# Mapping Mechanistic Pathways of Acute Oral Systemic Toxicity Using Chemical Structure and Bioactivity Measurements

**DOI:** 10.3389/ftox.2022.824094

**Published:** 2022-03-07

**Authors:** Stephen W. Edwards, Mark Nelms, Virginia K. Hench, Jessica Ponder, Kristie Sullivan

**Affiliations:** ^1^ GenOmics, Bioinformatics, and Translational Research Center, RTI International, Research Triangle Park, Durham, NC, United States; ^2^ Physicians Committee for Responsible Medicine, Washington, DC, United States

**Keywords:** acute oral systemic toxicity, adverse outcome pathway, new alternative methods, tiered testing, *in vitro* bioactivity assays, chemical structure-based clustering, tiered testing strategy

## Abstract

Regulatory agencies around the world have committed to reducing or eliminating animal testing for establishing chemical safety. Adverse outcome pathways can facilitate replacement by providing a mechanistic framework for identifying the appropriate non-animal methods and connecting them to apical adverse outcomes. This study separated 11,992 chemicals with curated rat oral acute toxicity information into clusters of structurally similar compounds. Each cluster was then assigned one or more ToxCast/Tox21 assays by looking for the minimum number of assays required to record at least one positive hit call below cytotoxicity for all acutely toxic chemicals in the cluster. When structural information is used to select assays for testing, none of the chemicals required more than four assays and 98% required two assays or less. Both the structure-based clusters and activity from the associated assays were significantly associated with the GHS toxicity classification of the chemicals, which suggests that a combination of bioactivity and structural information could be as reproducible as traditional *in vivo* studies. Predictivity is improved when the *in vitro* assay directly corresponds to the mechanism of toxicity, but many indirect assays showed promise as well. Given the lower cost of *in vitro* testing, a small assay battery including both general cytotoxicity assays and two or more orthogonal assays targeting the toxicological mechanism could be used to improve performance further. This approach illustrates the promise of combining existing in silico approaches, such as the Collaborative Acute Toxicity Modeling Suite (CATMoS), with structure-based bioactivity information as part of an efficient tiered testing strategy that can reduce or eliminate animal testing for acute oral toxicity.

## Introduction

An international workshop was held in 2015 to evaluate the state of the science supporting the replacement of animals for acute systemic toxicity testing (Hamm et al., 2017). As part of that workshop, the participants considered alternative test methods including *in vitro* methods and fish embryo models in addition to in silico options such as (Q)SAR (Table 1) and read-across. A tiered testing approach originally proposed by the National Academy of Sciences for the Department of Defense was evaluated as an organizing framework for this alternative testing strategy (National Academies of Sciences, 2015). The workshop concluded with eight general recommendations: 1) evaluate the suitability of *in vivo* reference data as the “gold standard” for validating NAMs, 2) continue mechanistic research to improve confidence in NAMs, 3) address the lack ADME of chemicals in non-animal tests, 4) develop IATA to better utilize all available evidence, 5) increase education and training to increase adoption of NAMs, 6) identify opportunities such as waivers under existing regulatory requirements and assemble the data to support policy changes where needed, 7) promote international harmonization of regulatory testing requirements, 8) encourage data curation and sharing.

**TABLE 1:** Summary of abbreviations and assay names used in the text.

Abbreviation	Definition
AC50	Active Concentration 50
ACE	Angiotensin converting enzyme
ADME	Absorption, distribution, metabolism, and excretion
AOP	Adverse Outcome Pathway
ATP	adenosine triphosphate
ATWG	Acute Toxicity Working Group
CASRN	Chemical Abstracts Service Registry Number
CATMoS	Collaborative Acute Toxicity Modeling Suite
DNA	deoxyribonucleic acid
DSSTox	Distributed Structure-Searchable Toxicity
DT40	chicken B cell line
EPA	Environmental Protection Agency
FDR	False Discovery Rate
FP, FN	False positive, False Negative
GABA	Gamma-aminobutyric acid
GHS	Globally Harmonized System
HTS	High Throughput Screening
IATA	Integrated Approach to Testing and Assessment
ICCVAM	Interagency Coordinating committee for the Validation of Alternative Methods
LD50	Lethal Dose 50
MCC	Matthew’s Correlation Coefficient
NAM	New Approach Methodology
NMDA	N-methyl-D-aspartate
OECD	Organisation for Economic Cooperation and Development
PPAR *δ*	Peroxisome Proliferator Activated Receptor Delta
PPAR *γ*	Peroxisome Proliferator Activated Receptor Gamma
(Q)SAR	(Quantitative) Structure Activity Relationship
SMILES	simplified molecular-input line-entry system
tcpl	ToxCast Analysis Pipeline
Tox21	Toxicology in the 21st Century
ToxCast	Toxicity Forecaster
TP, TN	True Positive, True Negative
Assay Abbreviation	Assay Name
ATG_ERa_TRANS_up	Attagene TRANS-FACTORIAL HepG2 Human Estrogen Receptor Alpha Activation Assay
ATG_NRF2_ARE_CIS_dn	Attagene HepG2 Human Nuclear factor-erythroid factor 2-related factor 2 antioxidant response element Inhibition Assay
ATG_VDR_TRANS_up	Attagene HepG2 Human Vitamin D Transactivation Assay
BSK_LPS_PGE2_down	Bioseek Human Primary Vascular Prostaglandin E_2_ Inhibition Assay
BSK_Sag_Eselectin_up	Bioseek Human Primary Vascular Superantigen E-Selectin Activation Assay
NCCT_TPO_AUR_dn	National Center for Computational Toxicology Thyroperoxidase Inhibition Assay
NHEERL_ZF_144hpf_TERATOSCORE_up	National Health and Environmental Effects Research Laboratory
NVS_ENZ_hAChE	Novascreen Human Acetylcholinesterase Enzyme Assay
NVS_ENZ_rAChE	Novascreen Rat Acetylcholinesterase Enzyme Assay
NVS_GPCR_gOpiateK	Novascreen Guinea Pig Cerebellar Membrane Opiod Receptor Assay
NVS_LGIC_rGlyRStrSens	Novascreen Norway Rat Spinal Cord Membrane Glycine Receptor Assay
TOX21_AR_LUC_MDAK_B2_Agonist_3uM_Nilutamide	Tox21 Human Breast Cancer Cell Line Androgen Receptor Agonist Assay
TOX21_DT40	Tox21Chicken B Cell Line Assay
TOX21_DT40_100	Tox21Chicken B Cell Line Gene Deletion Assay
TOX21_DT40_657	Tox21Chicken B Cell Line Gene Deletion Assay
TOX21_PPARg_BLA_Agonist_ch2	Tox21 Basolateral Human Kidney Cell Line PPAR gamma Agonism Assay
TOX21_PR_BLA_Agonist_ch1	Tox21 Basolateral Human Kidney Cell Line Progesterone Agonism Assay
TOX21_VDR_BLA_agonist_ch2	Tox21 Basolateral Human Kidney Cell Line Vitamin D Receptor Agonism Assay
UPITT_HCI_U2OS_AR_TIF2_Nucleoli_Agonist	University of Pittsburgh Human Bone Cell Line Androgen Receptor Agonism Assay

There has been considerable progress on all the goals set forth during that workshop including research efforts on inhalation toxicity (Clippinger et al., 2018) and oral toxicity (Sullivan et al., 2021) as well as regulatory policies focused on waiving acute dermal toxicity testing (EPA, 2016; Health Canada, 2017; EPA, 2020). For acute oral toxicity, an evaluation of the variability for the traditional *in vivo* tests found that repeat measurements from the same assay predict the same hazard category less than 80% of the time, though the two predictions differ by no more than one level more than 90% of the time (Karmaus et al., 2021). These results are consistent with previous evaluations of other guideline studies (Browne et al., 2015; Luechtefeld et al., 2016; Kleinstreuer et al., 2018a; Browne et al., 2018; Pham et al., 2020; Rooney et al., 2021). Research supporting the use of existing data and in silico approaches to predict acute oral toxicity of mixtures has been successful as well (Chushak et al., 2021; Hamm et al., 2021). Computational modeling was a key focus area (Kleinstreuer et al., 2018b) and a large international effort resulted in CATMoS (Mansouri et al., 2021). The Department of Defense has begun implementation of the tiered testing paradigm recommended by the National Academy (National Academies of Sciences, 2015; Sullivan et al., 2021). By coupling in silico predictions with *in vitro* measurements from tiered testing it should be possible to achieve reproducibility comparable to that seen with repeated animal studies. To support the development of the relevant *in vitro* assays, there have been several efforts to comprehensively identify mechanisms of acute toxicity (Wijeyesakere et al., 2018; Wilson et al., 2018; Prieto et al., 2019; Sullivan et al., 2021).

AOPs have been proposed as the ideal framework for organizing the mechanistic information (Hamm et al., 2017; Prieto et al., 2019; Sullivan et al., 2021) to support the use of new alternative methods for predicting acute toxicity. AOPs describe the toxicological mechanism as a series of key events that start from the initial interaction of a chemical with the biological system (molecular initiating event) and progresses through to an adverse outcome for an organism (Ankley et al., 2010; Villeneuve et al., 2014; Ankley and Edwards, 2018). The LD50 is the most common endpoint used to measure the adverse outcome in traditional oral acute toxicity studies. New alternative methods tend to target the molecular initiating event or other early key events as those are amenable to quantification by *in vitro* methods. In addition to their role in summarizing our understanding of the biological processes of particular pathways, a key feature of AOPs is that they provide a scaffold for the data to link these disparate endpoints that often occur in separate studies (Maxwell et al., 2014; Ankley and Edwards, 2018). This enables a variety of quantitative modeling approaches that can integrate data from in silico, *in vitro*, and *in vivo* testing (Jaworska et al., 2013; Foran et al., 2019; Perkins et al., 2019; Zgheib et al., 2019; Spinu et al., 2020; Paini et al., 2021).

Our work builds upon the previous efforts to create a comprehensive list of AOPs covering acute oral toxicity (Prieto et al., 2019; Sullivan et al., 2021) by mapping structural classes of chemicals and new alternative methods to these mechanisms. This process will identify data gaps in the existing catalog of acute oral toxicity AOPs and link in silico and *in vitro* data to AOPs related to acute toxicity. By linking the new alternative methods to specific AOPs, our work will facilitate the tiered testing paradigm proposed by the National Academy (National Academies of Sciences, 2015). This should enhance the ongoing work by the Department of Defense as well as other related efforts.

## Materials and Methods

The code for all analyses described below is available here: https://github.com/RTIInternational/acute-tox-aop-testing.

Abbreviations used throughout the paper are summarized in Table 1.

### Data Sources

#### Acute Toxicity Dataset

The rat acute oral systemic toxicity dataset assembled by the ICCVAM ATWG was used as the reference upon which the analyses in this study were conducted.

A detailed summary of the data compilation and curation process is available on the collaborative modelling page of the ATWG (NTP, 2020). Briefly, the U.S. National Toxicology Program Interagency Center for the Evaluation of Alternative Toxicological Methods and the U.S. EPA’s Center for Computational Toxicology and Exposure collated 21,200 LD50 values (both point estimate and limit test values) for 15,688 unique substances. For this curation effort, the LD50 values represent the dose that is lethal for 50% of the animals in a rat acute oral toxicity study (Karmaus et al., 2019). These data were collected from a variety of publicly available data sources, including, but not limited to ChemIDplus, the European Commission Joint Research Council’s Acutetoxbase, and OECD’s eChemPortal (Kleinstreuer et al., 2018b; NTP, 2018; Karmaus et al., 2019; Mansouri et al., 2021).

These data underwent further processing to remove duplicate study values, amend obvious transcription errors (e.g., limit test LD50 value of “20005000 mg/kg”), definition of a representative LD50 value for chemicals with 3 or more point estimate values, and the retrieval of chemical structure information from the EPA’s CompTox Chemicals Dashboard and other public resources. After all of the processing steps, the final acute toxicity dataset (herein termed the ATWG dataset) consisted of 11,992 unique substances with at least one toxicity outcome, of which 8,979 had a processed LD50 value.

#### ToxCast Data

*In vitro* bioactivity data were aggregated for the approximately 4,000 ATWG chemicals tested in at least one of the almost 1,600 high-throughput screening assays that comprise the EPA’s ToxCast/Tox21 program. These data were obtained from invitroDB v3.3 using the tcpl R package (v2.0) (Filer et al., 2016). The chemical-assay data that were extracted included: 1) whether a chemical was tested in a particular assay, 2) the AC50 values (i.e., the 50% of maximal activity concentration), and 3) the binary hit calls (i.e., whether a chemical was active [1] or inactive [0] within a given assay). Additionally, the chemical-specific cytotoxicity and lower bound of cytotoxicity values were extracted.

Subsequently, we compared the AC50 with the lower bound estimate of cytotoxicity defined by Judson et al. to develop a hit call matrix whereby the cytotoxicity-associated burst phenomenon was taken into account (Judson et al., 2016). To generate this burst hit call matrix, we compared chemical-assay AC50 values against the chemical-specific lower bound of cytotoxicity as calculated by the tcplCytoPt function in the tcpl R package (Filer et al., 2016). Chemical-assay combinations whereby the AC50 was below the lower bound of cytotoxicity for the chemical were considered active and assigned a value of 1. Meanwhile, chemical-assay combinations whereby the AC50 value was above the lower bound of cytotoxicity for the chemical were considered inactive and assigned a value of 0.

#### Chemical Structure Data

To perform the chemical clustering and subsequent data analyses in this study, defined chemical structure information was required. To ensure consistency and reliability in the chemical structures we utilized the EPA’s DSSTox database to retrieve QSAR-ready SMILES. In addition to the QSAR-ready SMILES, we extracted the DSSTox substance identifier, preferred chemical name, and regular SMILES for each substance.

To obtain this information, we performed a batch search of the EPA CompTox Chemicals Dashboard (www.comptox.epa.gov/dashboard, accessed June 2021) using the CASRN as the input. The search of the CompTox Chemicals Dashboard returned a total of 10,886 chemicals with QSAR-ready SMILES (Richard et al., 2016; Williams et al., 2017; Grulke et al., 2019; Richard et al., 2021).

### Structure-Based Clustering

Prior to clustering, we used the publicly available ChemoTyper software (https://chemotyper.org) and ToxPrint chemotype feature set (v2.0_r711, https://toxprint.org) to create a binary molecular fingerprint for each chemical with a QSAR-ready SMILES string. The ToxPrint fingerprints identify whether the 729 specific substructural features that comprise the ToxPrint feature set are present in a molecule.

The complete fingerprint matrix was subsequently utilized to calculate the pairwise Tanimoto distance between all 10,886 chemicals. The Tanimoto distance is computed as the inverse of the Tanimoto similarity coefficient. The Tanimoto similarity coefficient is calculated as the ratio of bits present in the molecular fingerprint of both chemicals (i.e., the size of the intersection) divided by the number of bits present in the molecular fingerprint of one or both chemicals (i.e., the size of the union). Next, the resulting distance matrix was used within a hierarchical clustering algorithm implementing Ward’s agglomerative clustering method. To create the final chemical clusters a cut height of 0.7 was chosen. The distance calculations and hierarchical clustering were undertaken using the philentropy and cluster R packages, respectively, as implemented in R version 4.0.3 (2020-10-10) (R Core Team, 2020).

### Enrichment Analysis

Three separate enrichment analyses were conducted in this study. The first involved investigating which, if any, ToxCast assays are enriched for activity below cytotoxicity in the acutely toxic chemicals (i.e., those not identified as nontoxic in the ATWG set, or those with rat oral LD50 ≤ 2,000 mg/kg) relative to the full set of ATWG chemicals. The second involved investigating which, if any, ToxCast assays are enriched for activity below cytotoxicity in the very acutely toxic chemicals relative to the combined set of very acutely toxic and nontoxic ATWG chemicals. The final enrichment analysis involved investigating which, if any, ToxCast assays are enriched for activity below cytotoxicity for the acutely toxic chemicals within a cluster relative to the acutely toxic chemicals in all clusters. For each analysis, chemicals identified as being active (in the case of ToxCast assays) or (very) acutely toxic were indicated by a value of 1, while chemicals identified as being inactive (in the case of ToxCast assays) or nontoxic were indicated by a value of 0.

A ToxCast assay was considered enriched if the computed MCC was ≥ 0.1, a *p*-value ≤ 0.05, and at least 3 chemicals were toxic/in the cluster and active in the ToxCast assay. The MCC was calculated based upon the following formula:

$$MCC\;=\;\frac{\left(TP\;\cdot \;TN\right)-\left(FP\;\cdot \;FN\right)}{\sqrt{\left(TP\;+\;FP\right)\left(TP\;+\;FN\right)\left(TN\;+\;FP\right)\left(TN\;+\;FN\right)}}$$

Where, TP = True Positive, i.e., the chemical is toxic/in the cluster and active in the ToxCast assay; TN = True Negative, i.e., the chemical is nontoxic/not in the cluster and inactive in the ToxCast assay; FP = False Positive, i.e., the chemical is toxic/in the cluster and inactive in the ToxCast assay; and FN = False Negative, i.e., the chemical is nontoxic/not in the cluster and active in the ToxCast assay. Additionally, the *p*-value was calculated using the Fisher’s exact test; thereby, indicating the level of significance of the enrichment.

### Identifying the Minimum Number of Assays to Identify all Toxic Chemicals

**FIGURE 1:**
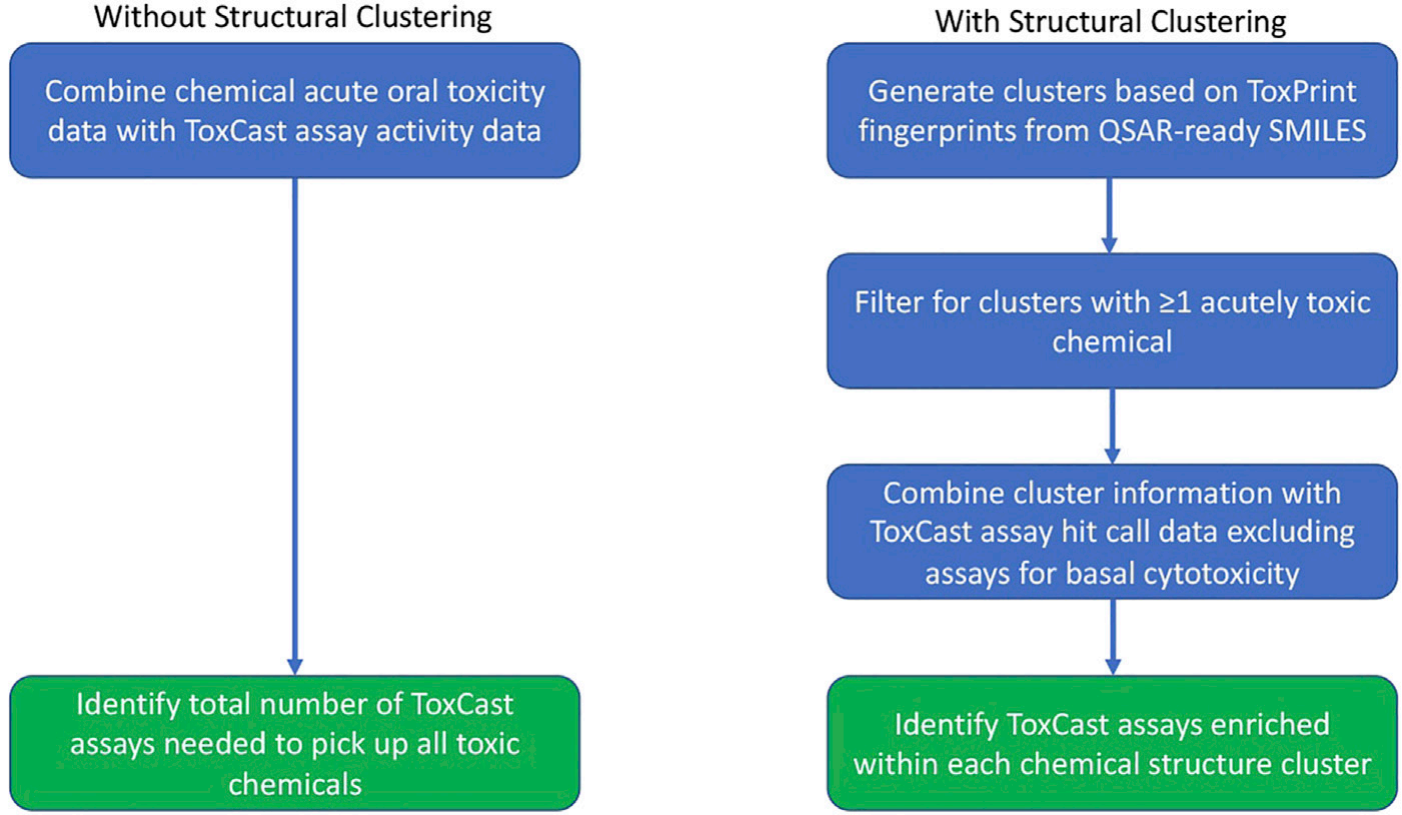
Workflow undertaken for the assay analysis both with and without use of the chemical clusters generated based upon structural similarity.

The activity of the toxic chemicals in the various *in vitro* assays was used to identify the minimal set of assays required to provide full coverage of the toxic chemicals within the set. This was performed on all toxic chemicals together and then repeated for the chemicals within each cluster as illustrated in Figure 1. The activity of a chemical was determined using the burst hit call matrix described above, which indicates activity below concentrations expected to cause cytotoxicity (Judson et al., 2016). When processing all chemicals, any assay with activity for at least one acutely toxic chemical was included. When performing the analysis for a single chemical cluster, the assays that were enriched for each cluster as defined above were used as the starting point. The assays were sorted by the number of acutely toxic chemicals identified by the assay minus the number of nontoxic chemicals with activity in the assay. In the case of a tie, the assays were then sorted by the total number of toxic chemicals with activity in the assay. If more than one chemical still had the same top value, then assays that were enriched for toxic chemicals in general (see previous section) were sorted by the false discovery rate of the assay in that analysis. For ties at this stage, the same process was applied using the enrichment for the very toxic chemicals. Once an assay was identified, all acutely toxic chemicals with activity in that assay were removed and the process was repeated until all chemicals in the set showed activity in at least one assay.

### Final Activity Assessment

All ATWG chemicals were assigned a composite AC50 value based on the activity of the chemical in the cluster-specific acute toxicity-relevant assay(s) and the ToxCast cytotoxicity value. The cluster-specific AC50 value was obtained by taking the minimum AC50 for each chemical based on the assays defined for the structural cluster containing that chemical. The cytotoxicity point defined by ToxCast using the activity from a set of 88 cytotoxicity assays was used to define the AC50 value for cytotoxicity (Judson et al., 2016). The final AC50 value was considered the minimum between the cluster-specific and cytotoxicity AC50 values. Chemicals having no activity in the cluster-specific assay(s) or for which no cluster-specific assays exist were assigned the cytotoxicity AC50. If a chemical had no assigned cytotoxicity value or cluster-specific activity, no AC50 value was assigned for that chemical.

The final activity of a chemical was then determined from the composite AC50 value. Chemicals with a composite AC50 below 1 mM were considered active, and the other chemicals that were tested in either the cluster-specific assay or with a ToxCast-defined cytotoxicity point were considered inactive. Out of 11,992 chemicals in the ATWG set, 7,997 chemicals had not been tested in ToxCast. An additional 38 chemicals were not tested in the assay corresponding to their structural cluster and had no assigned cytotoxicity point. The remaining 3,957 chemicals were used to evaluate how *in vitro* activity compares with the previously determined acute oral toxicity of those chemicals.

Two different measures of toxicity were compared against the ToxCast activity. The first was a binary determination of toxicity with chemicals having an LD50 > 2,000 mg/kg classified as nontoxic(18). The second measure used the five United Nations Globally Harmonized System (GHS) categories for acute oral toxicity (United Nations, 2021). For the binary analyses, 2 chemicals had no toxicity designation resulting in a total of 3,955 chemicals evaluated. For the GHS analysis, an additional 5 chemicals had no GHS classification resulting in a total of 3,950 chemicals. Statistical significance of the association between ToxCast composite activity or structural cluster and toxicity was determined using Fisher’s Exact Test as implemented in R version 4.0.3 (2020-10-10) (R Core Team, 2020). For the multiclass GHS comparisons, the *p*-value was estimated by Monte Carlo simulation based on 20,000 replicates.

## Results

This study demonstrates the utility of combining *in vitro* bioactivity data with chemical structure information to help improve oral acute systemic toxicity predictions. To ensure that the chemical activity used to identify the minimal assay sets was more likely to represent target-mediated effects, we used filters to remove chemical-assay results that could, potentially, be confounded by non-specific cytotoxicity. This filtering involved utilizing the chemical-specific lower bound cytotoxicity values. Whilst this is likely to have resulted in a relatively high false negative rate, it also increases our confidence that an active hit call is due to a target-mediated mechanism. For chemicals with no activity below the cytotoxicity range, the mechanism was considered to be cytotoxicity and the cytotoxicity point was used to determine the AC50 value for the chemical.

### Mapping ATWG Chemicals to ToxCast Assays

**FIGURE 2:**
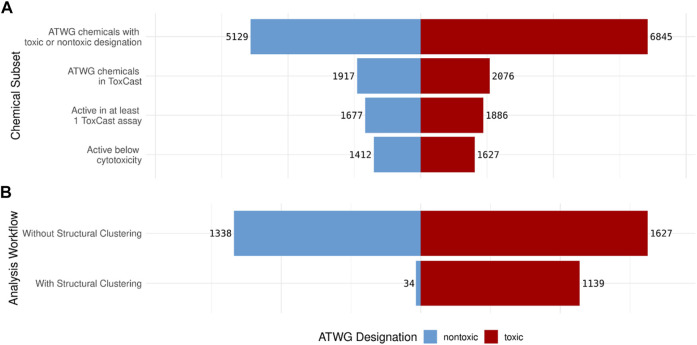
Pyramid plots showing: **(A)** The number of nontoxic (blue) and toxic(red) ATWG chemicals that meet each criterion and **(B)** The number of nontoxic and toxic chemicals identified by the minimum ToxCast assays generated by the assay analysis workflow both without and with using the structural clustering information. The number of nontoxic chemicals identified by the minimum assays is drastically reduced when the clustering information is used with only a slight loss in the number of toxic compounds.

As illustrated in Figure 2A, the vast majority (11,974, 99.8%) of the 11,992 chemicals in the ATWG dataset had a logical (i.e., true or false) designation of whether the chemical was identified as being nontoxic. Of these, 3,993 (33.3%) ATWG chemicals have been tested in at least one ToxCast assay. Of the 3,993 ATWG chemicals tested in ToxCast 3,563 were active in at least one assay, with 3,039 chemicals having activity below cytotoxicity in at least one assay. In this study, chemicals with a nontoxic designation of “false” (or those with rat oral LD50 ≤ 2,000 mg/kg) were considered to be toxic and will be referred to as such throughout. Approximately 43% (5,129) of the ATWG chemicals with a toxicity designation were identified as nontoxic with the remaining 57% (6,845) identified as toxic (Figure 2A). Of the chemicals with ToxCast/Tox21 data, the toxic and nontoxic chemicals were evenly split with anywhere from 52 to 54% of the chemicals classified as toxic at each stage.

The assay analysis conducted without considering structural clustering was able to identify all 1,627 acutely toxic chemicals with activity below cytotoxicity; however, it also captures 1,338 nontoxic chemicals (Figure 2B). This is almost the same number of nontoxic that have activity below cytotoxicity in at least one ToxCast assay; therefore, this approach performs only marginally better than using the ToxCast data for all assays directly. The assay analysis conducted with the structural clustering taken into consideration, meanwhile, performs drastically better in this respect: identifying only 34 nontoxic chemicals. However, it also identifies slightly fewer acutely toxic chemicals: 1,139 of the 1,627 acutely toxic chemicals with activity below cytotoxicity (Figure 2B).

**FIGURE 3:**
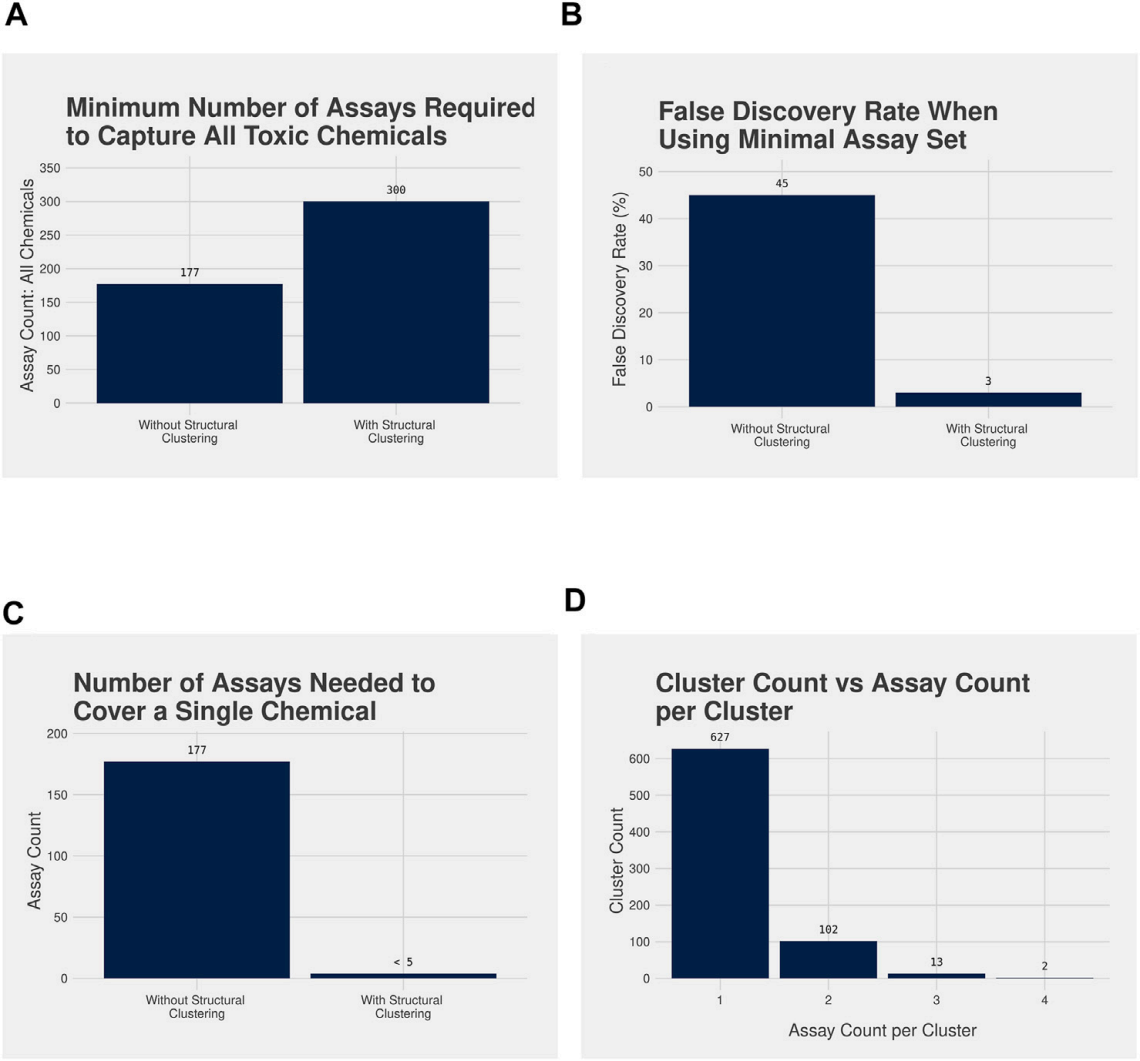
**(A)** Bar plot illustrating the minimum number of assays required to identify all acutely toxic chemicals after performing the assay analysis workflow without (177 assays) and with (300 assays) the structure-based clustering, and **(B)** bar plot illustrating the false discovery rate (FDR) associated with the minimal assay set without (45%) and with (3%) the structure-based clustering. A larger number of assays are required to capture all acutely toxic chemicals with the structure-based clustering, but the FDR is dramatically reduced when compared with not using the structure-based clusters. **(C)** Bar plot highlighting the number of assays required to assess the potential acute toxicity of a single chemical without using the structural clustering compared with when the structural clustering is used, and **(D)** Bar plot illustrating the number of chemical clusters requiring testing in 4 or fewer assays to cover all acutely toxic chemicals, with bioactivity data in ToxCast. Without structural clustering a novel chemical would be required to be tested in all 177 assays in the minimal assay set. With the cluster information fewer than 5 assays would be required: 98% of clusters would only need testing in 1 or 2 assays.

Using the analysis workflow without structure-based clustering, a minimal set of 177 unique assays were needed to cover all 1,627 acutely toxic chemicals; however, the false discovery rate (FDR) is quite high at 45% (Figures 3A,B). Meanwhile, when the assay data were combined with the structural clustering the minimum number of unique assays needed to cover the 1,139 acutely toxic chemicals increased to 300; however, this coincided with a considerable decrease in FDR to 3% (Figures 3A,B). It should be noted that the FDRs reported here are based on our analysis of the existing data and is not an indication of how predictive the assays are for unknown chemicals. Nevertheless, these results illustrate the value of combining bioactivity data with structural information.

A further benefit of the approach incorporating structural clustering can be observed when we consider the number of assays a novel chemical would be required to be tested in to be covered by both approaches (Figure 3C). Under the analysis workflow without structural clustering, a novel chemical would need to be tested in all 177 assays that comprise the minimal assay set to predict its acute oral toxicity. However, when using the workflow with structural clustering a novel chemical would need only be tested in, at most, 4 assays with the specific assays being determined by the structural cluster to which the novel chemical is assigned (Figure 3C). Furthermore, 98% of chemical clusters required only one or two assays to cover all acutely toxic chemicals within the cluster that had been tested within ToxCast, with the vast majority needing only one assay (Figure 3D).

### Evaluation of ToxCast Assay Mapping

The full list of the ATWG chemicals with their structural cluster designation is provided in Supplementary Table S1. When a ToxCast/Tox21 assay was assigned to the cluster, that information is provided as well. Not all chemicals in the cluster necessarily showed activity in the assay, however. The ToxCast/Tox21 activity of the individual chemicals is summarized in Supplementary Datasheets S2, S3. Supplementary Datasheet S2 shows only those assays that were enriched for the given chemical cluster along with all chemicals from the cluster that showed activity below the cytotoxicity range in at least one assay. Supplementary Datasheet S3 shows all assays for which at least one chemical in the cluster had activity and all chemicals from the cluster that were active in at least one assay. Five chemical clusters will be discussed in detail to evaluate the findings as well as to highlight several of the different situations we observed.

#### Cluster 678: Carbamate Pesticides

**FIGURE 4:**
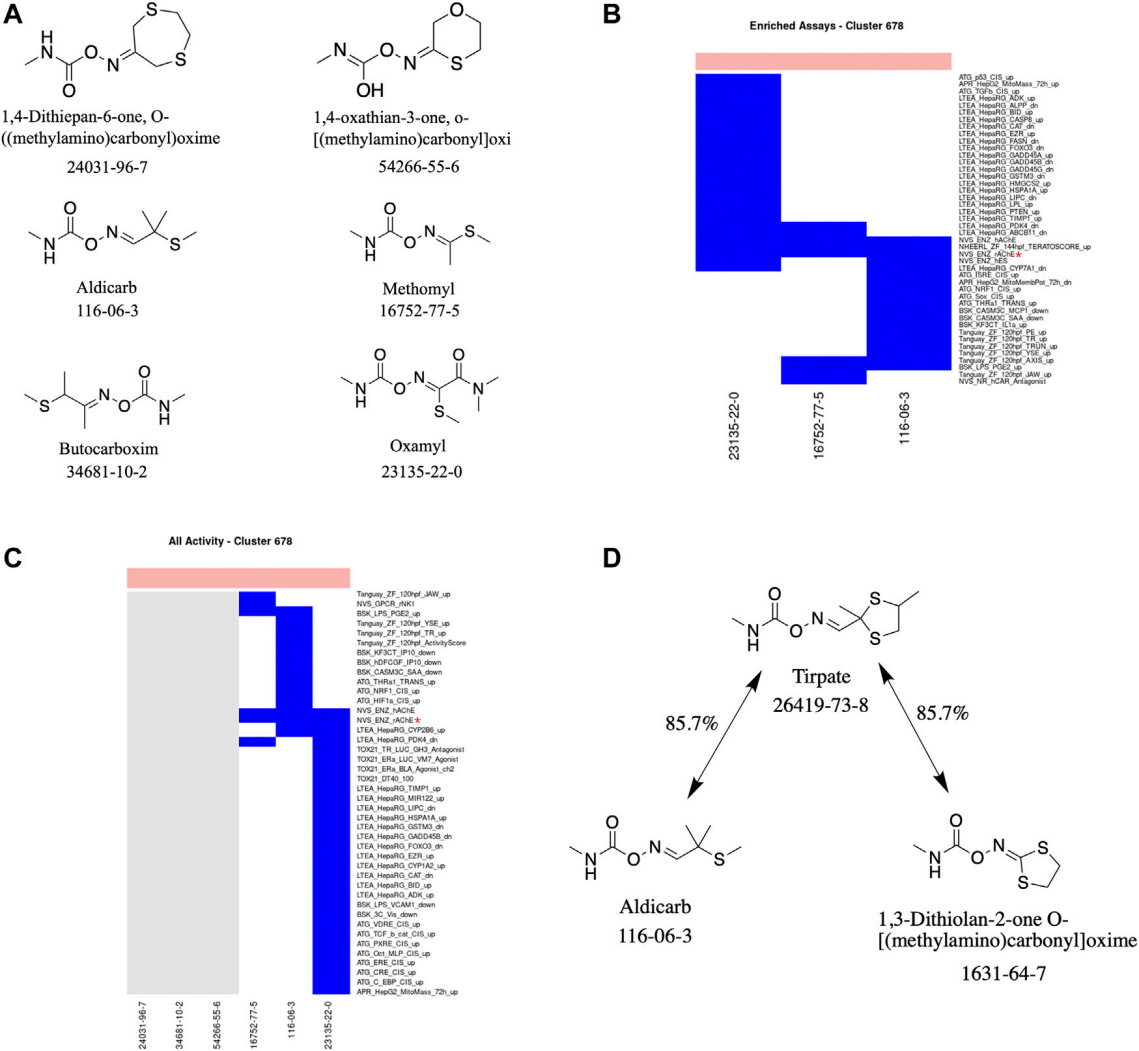
Cluster 678 structure and activity information. **(A)** Structures for chemicals in cluster 678. **(B)** Heatmap showing activity below the cytotoxicity range in the assays that were enriched for this cluster. Only chemicals with activity in at least one assay are shown. **(C)** Heatmap showing activity for all assays where at least one chemical from the cluster was active. Not all assay names are shown. **(D)** Structure of tirpate compared with chemicals from clusters 390 and 678. Heatmap colors: blue = active below cytotoxicity, light blue = active above cytotoxicity, white = inactive, grey = not tested. Top of heatmap: red = toxic, blue = nontoxic. Red asterisks identify the assay(s) selected for that cluster.

A total of six carbamate-containing chemicals were assigned to this cluster (Figures 4A–C). Four are pesticides, and the other two appear to be early-stage compounds with insecticidal properties that have not yet been developed into a commercial product. All the chemicals are acutely toxic with LD50 values ranging from 0.5 to 400 mg/kg. Three of the six chemicals (aldicarb, methomyl, and oxamyl) have been tested in at least 1,350 of the almost 1,600 ToxCast assays, with all three chemicals exhibiting activity below the level of cytotoxicity within three assays: namely, NVS_ENZ_hAChE, NVS_ENZ_rAChE, and NHEERL_ZF_144hpf_TERATOSCORE_up. Of the assays in which at least one chemical had been tested, 58 were enriched for activity compared to the acutely toxic chemicals not in this cluster. Two of the top three enriched assays, in terms of their MCC, for this cluster were the NVS_ENZ_hAChE and NVS_ENZ_rAChE assays: with MCC values of 0.396 and 0.352, respectively. In theory, any of the three assays in which all the chemicals were active could have been chosen; however, the minimal assay identified by the analysis workflow was the NVS_ENZ_rAChE assay. Together, these results suggest that the chemicals act *via* inhibition of acetylcholinesterase activity.

This is corroborated by the literature-reported mechanism for the four chemicals in the cluster for which information on a mechanism could be identified. Each of the four chemicals (aldicarb, butocarboxim, methomyl, and oxamyl) are recognized as acting by inhibiting the enzyme acetylcholinesterase. Acetylcholinesterase is responsible for the termination of impulse transmission at various nerve endings in the central and peripheral nervous systems by catalyzing the breakdown of the neurotransmitter acetylcholine into choline and acetic acid (Risher et al., 1987; U.S. EPA, 2007; National Advisory Committee, 2009; Costa et al., 2010; Colovic et al., 2013). These chemicals, along with other carbamate pesticides, prevent acetylcholinesterase from catalyzing this reaction; thereby, increasing the amount and/or duration acetylcholine is present causing a hyperstimulation of the cholinergic receptors and disrupted neurotransmission.

This cluster is an example of the ideal scenario where a consistent mechanism could be identified across multiple chemicals and where the biological target of the minimal assay identified by the analysis workflow corresponds to the literature-reported mechanism. It should be noted that *a priori* knowledge of the mechanism of action of the chemicals within a cluster could also be utilized to assist in prioritizing/identifying the most appropriate assay(s) for testing.

Additionally, we can use the literature-reported mechanism(s) and minimal assay(s) associated with a cluster to help guide the testing of a similar chemical (in terms of chemical structure and/or physicochemical properties) outside the cluster. For example, tirpate (Cluster 390) is approximately 86% similar to two chemicals (in terms of chemical structure): 1) 1,3-dithiolan-2-one O-[(methylamino)carbonyl]oxime, which is in Cluster 390 along with tirpate, 2) aldicarb from Cluster 678 (Figure 4D). As tirpate, along with the other chemicals in Cluster 390, has not been tested in ToxCast we can use its similarity to aldicarb to prioritize the assay(s) in which tirpate should be tested. In this case it would be the NVS_ENZ_rAChE assay.

#### Cluster 1860: Indole Alkaloids

**FIGURE 5:**
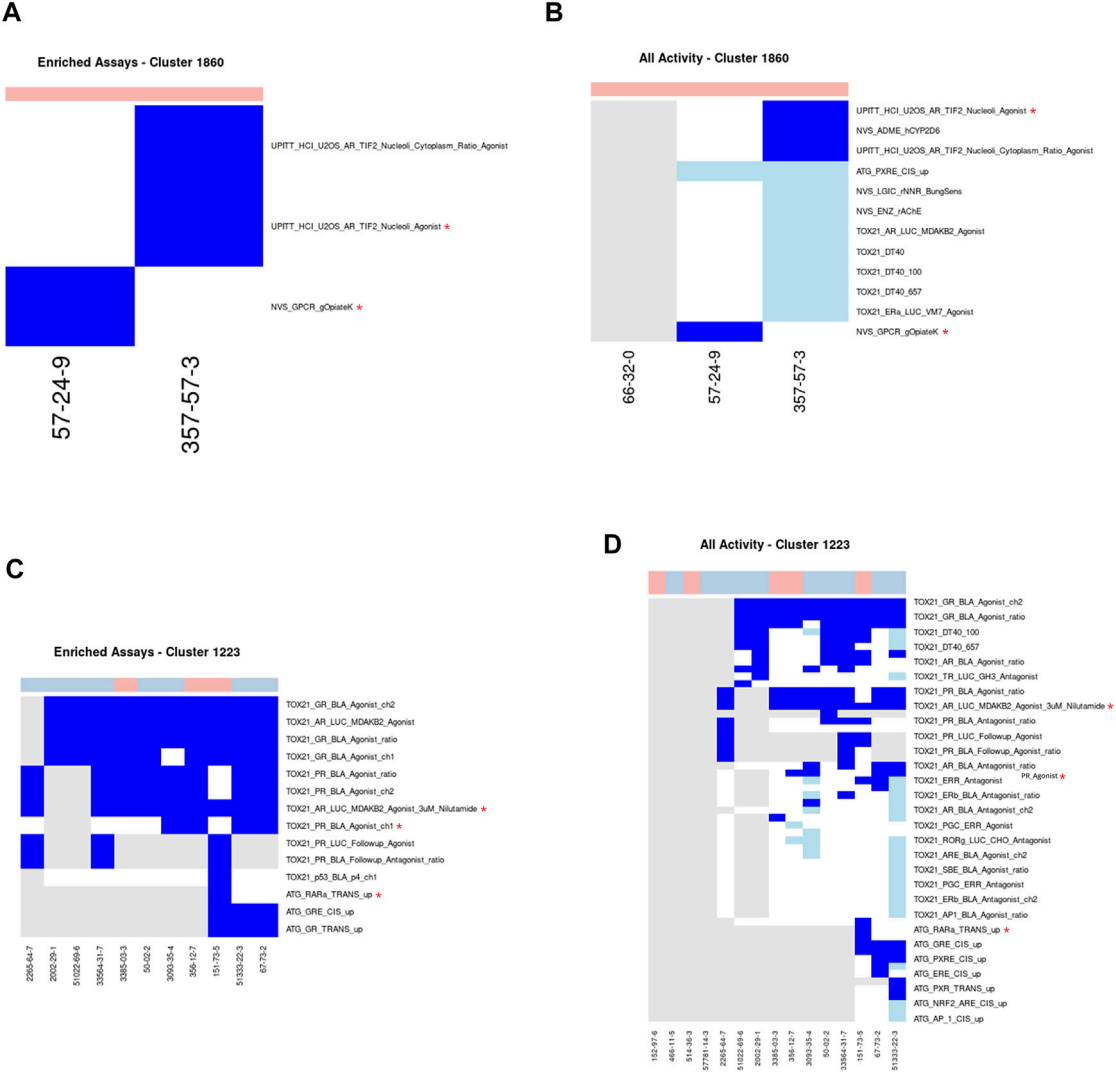
Clusters 1860 and 1223. A/C. Heatmap showing activity below the cytotoxicity range in the assays that were enriched for clusters 1860 **(A)** and 1223. **(C)** Only chemicals with activity in at least one assay are shown. B/D. Heatmap showing activity for all assays where at least one chemical from the clusters 1860 **(B)** or 1223 **(D)** was active. Heatmap colors: blue = active below the cytotoxicity point, light blue = active above the cytotoxicity point, white = inactive, grey = not tested. Top of heatmap: red = toxic, blue = nontoxic, aquamarine = undefined. Red asterisks identify the assay(s) selected for that cluster.

Cluster 1860 consists of three indole alkaloids (brucine, strychnine, and strychnine nitrate), all of which are very acutely toxic (Figures 5A,B). Brucine and strychnine have been seen to have pharmacological effects at a variety of receptors of neurotransmitters and as such have been used as medicines to treat a wide range of ailments (Jensen et al., 2006; Teske et al., 2011). However, these uses were discontinued due to the narrow therapeutic window. More recently, strychnine has been used as a rodenticide. Two of the three chemicals (strychnine and brucine) are well studied, having been tested in 312 and 470 ToxCast assays, respectively. Unlike the previous cluster, no one assay captures the activity across both chemicals. Therefore, the analysis workflow identified two minimal assays for this cluster: namely, the NVS_GPCR_gOpiateK and the UPITT_HCI_U2OS_AR_TIF2_Nucleoli_Agonist assays. There were a total of 4 assays enriched for activity in this cluster compared to the acutely toxic chemicals not in this cluster, with MCC values ranging from 0.097 to 0.212. Both of the assays identified as the minimal assays for this cluster have different biological targets: the target for the NVS_GPCR_gOpiateK assay being the kappa 1 opioid receptor and the target for the UPITT_HCI_U2OS_AR_TIF2_Nucleoli_Agonist assay being the androgen receptor.

Meanwhile, the predominant mechanisms for these chemicals identified in the literature are as antagonists of glycine receptor and cholinergic receptor signaling. The post-synaptic glycine receptor is a ligand-gated ion channel that is a member of the acetylcholine receptor family and is well known to function as an inhibitor of neurotransmission in the spinal cord and brain stem (Lynch, 2004). Strychnine is an extremely potent competitive antagonist of the glycine receptor with inhibitory constant values in the nanomolar region (Rajendra et al., 1997; Laube et al., 2002; Lynch, 2004; Jensen et al., 2006). Strychnine appears to bind to the glycine receptor at a similar, but not identical, site as glycine. Once bound, strychnine and brucine block glycine from binding to the glycine receptor and exhibiting its inhibitory effects on the post-synaptic neuron, leading to hyperexcitability and, subsequently, death by asphyxiation. Additionally, these chemicals can act on the nicotinic and muscarinic acetylcholine receptors as antagonists and allosteric modulators, respectively (Kuijpers et al., 1994; Jensen et al., 2006).

Even though the two minimal assays identified for this cluster and the literature reported mechanisms of action do not appear wholly related to one another, the reason for this discrepancy can likely be explained by the fact that none of the chemicals in this cluster have been tested in the glycine receptor-related assay in ToxCast: NVS_LGIC_rGlyRStrSens. However, strychnine nitrate is used as the positive control within the NVS_LGIC_rGlyRStrSens assay. As such, it is probably reasonable to hypothesize that if these chemicals were tested in the NVS_LGIC_rGlyRStrSens assay they would all likely be active. Therefore, this cluster is an example of how the minimal assay(s) chosen are a result of which assays the chemicals have been tested in and don’t necessarily correspond with the mechanism of acute toxicity.

#### Cluster 1223: Corticosteroids

Cluster 1223 consists of 15 corticosteroid chemicals, of which 5 are acutely toxic and 10 are not acutely toxic (Figures 5C,D). Eleven of the 15 chemicals have been tested in a large number of ToxCast assays ranging from 79 to 413 endpoints per chemical, with the vast majority being tested only in the Tox21 assays. All 10 of the chemicals tested in the glucocorticoid receptor assay exhibited activity below cytotoxicity, including three acutely toxic and seven nontoxic chemicals. As with Cluster 1860, more than one assay was chosen as part of the minimal assay set, in this instance three assays were required: ATG_NRF2_ARE_CIS_dn, TOX21_AR_LUC_MDAKB2_Agonist_3uM_Nilutamide, and TOX21_PR_BLA_Agonist_ch1. Even though each of the acutely toxic chemicals were active in the glucocorticoid receptor-related assays, these were not identified as part of the minimal assay set because of the high number of false positives (i.e., the high number of nontoxic chemicals that were also active in these assays). A total of 17 assays were enriched for activity in the acutely toxic chemicals in this cluster when compared against the remaining acutely toxic chemicals. As expected, six of the top seven enriched assays were related to the glucocorticoid receptor, which is the target mechanism of corticosteroids.

While the long-term use of glucocorticoid agonists can lead to a variety of adverse effects such as adrenal atrophy, immunosuppression, hypertension, and hyperglycaemia (Wallace et al., 2004); less appears to be known about the mechanism by which these chemicals elicit toxicity after acute exposure. Without this information it is difficult to verify whether the assays identified in the minimal assay set are mechanistically relevant. As such, this cluster is an example of how it can be difficult to ascertain whether the minimal assay(s) identified are relevant to the mechanism of acute toxicity. Additionally, this cluster demonstrates how information on a structural class, particularly one with pharmacological uses, can be integrated with bioactivity information to identify assays that are likely not informative for the acute toxicity mechanism.

#### Cluster 217: Methylphenols

**FIGURE 6:**
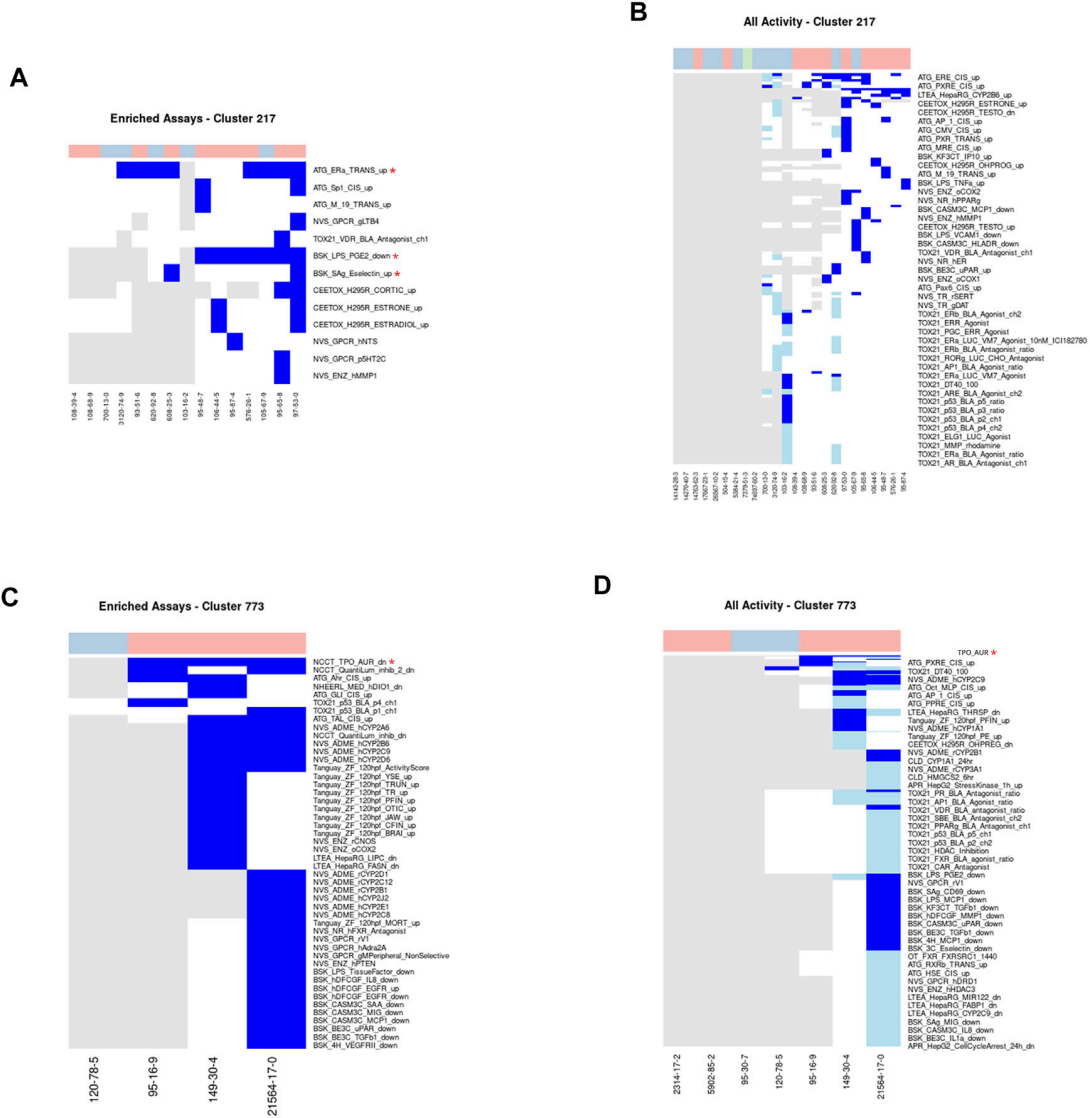
Clusters 217 and 773.A/C. Heatmap showing activity below the cytotoxicity range in the assays that were enriched for clusters 217 **(A)** and 773 **(C)**. Only chemicals with activity in at least one assay are shown. B/D. Heatmap showing activity for all assays where at least one chemical from the clusters 217 **(B)** or 773 **(D)** was active. Heatmap colors: blue = active below the cytotoxicity point, light blue = active above the cytotoxicity point, white = inactive, grey = not tested. Top of heatmap: red = toxic, blue = nontoxic, aquamarine = undefined. Red asterisks identify the assay(s) selected for that cluster.

A total of 24 methylphenol chemicals were assigned to Cluster 217, of which half were considered acutely toxic (Figures 6A,B). Methylphenols are commonly used as precursors or intermediates in the production of a variety of substances including antioxidants, pesticides, and pharmaceuticals (Thompson et al., 1996). Fifteen chemicals, ten acutely toxic and five nontoxic, have been tested in between 250 and 1,206 ToxCast assays, with a median of 638. Three assays were required to cover all acutely toxic chemicals within this cluster: namely, ATG_ERa_TRANS_up, BSK_LPS_PGE2_down, and BSK_Sag_Eselectin_up. Additionally, 34 assays were enriched for activity in the toxic chemicals in Cluster 217 compared to those not in Cluster 217 with MCC values ranging from 0.053 to 0.256. The enriched assays cover a wide variety of different biological endpoints, including but not limited to the arginine vasopressin receptor, the estrogen receptor, matrix metallopeptidase, and the change in the transcription factor activity of the SP1 gene.

While there is limited information about the potential toxic mechanism of methylphenols, effects have been observed in the liver, kidney, gastrointestinal tract, and central nervous system (Thompson et al., 1996; U.S. EPA, 2000; Agency for Toxic Substances and Disease Registry, 2008; National Center for Biotechnology Information, 2021). Several methylphenols (such as meta-, ortho-, and para-cresol present in this cluster) are known chemical irritants and corrosives at high concentrations that appear able to denature and precipitate proteins leading to coagulative necrosis (Agency for Toxic Substances and Disease Registry, 2008). After studying the hepatotoxic effects of p-cresol and other substituted cresols within rat liver slices, Thompson and colleagues (1996) hypothesized a cytotoxic mechanism whereby p-cresol is bioactivated to a quinone methide intermediate, which can covalently bind to cellular macromolecules *via* Michael addition (Thompson et al., 1993; Thompson et al., 1996; Agency for Toxic Substances and Disease Registry, 2008; Bolton, 2014). An alternative hypothesis was suggested by Kitagawa (2001) that cresol may exhibit their hepatotoxicity by inhibiting mitochondrial respiration and/or causing/accelerating mitochondrial swelling (Kitagawa, 2001). Additionally, quinone methides may alter the redox balance within cells through the depletion of glutathione (Bolton, 2014).

Given the sparsity and inconsistency in activity within the ToxCast assays and the diversity of hypothesized mechanisms, this cluster is an example of a cluster where there does not appear to be a clear, coherent mechanism across the chemicals. As these chemicals are corrosive irritants, they are likely to have a broad range of non-specific effects. Alternatively, it may be that the cause of the activity across different assays is due to assay interference *via* the hypothesized protein binding mechanism from the literature.

#### Cluster 773: Benzothiazoles

This cluster consists of seven benzothiazole-containing chemicals. Five of these seven chemicals are considered acutely toxic (Figures 6C,D). Benzothiazoles can occur naturally as constituents of tea leaves, but are mainly manufactured for a variety of industrial and consumer purposes, including as corrosion inhibitors, fungicides, and insecticides, as well as vulcanization accelerators in the production of rubber and within the paper and pharmaceutical industries (De Wever and Verachtert, 1997; Liao et al., 2018). Four chemicals [2,2′-dithiobisbenzothiazole, benzothiazole, 2-mercaptobenzothiazole, and 2-(thiocyanomethylthio) benzothiazole] have been tested in between 235 and 1,383 ToxCast assays. All three of the acutely toxic chemicals were active below cytotoxicity in the NCCT_TPO_AUR_dn assay, which measures a loss of activity in the thyroid peroxidase enzyme. Consequently, this assay was identified as the minimal assay for this cluster. Additionally, inhibition of activity in the thyroid peroxidase enzyme has been observed within other studies (Hornung et al., 2015). The mechanism of acute toxicity for benzothiazoles is unclear; however, it is unlikely that inhibition of thyroid peroxidase activity is the mechanism as this does not correspond to any known pathways associated with acute toxicity.

### Catalog of AOPs Associated With Acute Systemic Toxicity

We identified previously published mechanisms (Prieto et al., 2019; Sullivan et al., 2021) associated with our chemical clusters (Table 2) to evaluate the breadth of acute toxicity mechanisms across the clusters. A comprehensive evaluation of known mechanisms of action was performed for chemical clusters that included extremely toxic chemicals with an acute oral LD50 of 25 mg/kg or less and which had one or more ToxCast assay assigned to the cluster (108 clusters met these criteria). Importantly, given the emphasis on collecting known mechanisms associated with the chemicals in the clusters, the resulting list in Table 2 includes mechanisms that are not likely to lead to acute toxicity, such as endocrine system disruption and inflammation inhibition. The previous publications collectively covered 69% of the precise known mechanisms identified within the extremely toxic chemicals. In most cases where the mechanism wasn’t previously described, the broader mechanism was captured but the specific mechanism was missed. For example, Prieto and colleagues identified hemorrhage as a mechanism of acute toxicity but didn’t specify the inhibition of vitamin-K recycling as one of the upstream events leading to hemorrhage. There are also a number of acutely toxic chemicals for which the mechanism of toxicity is still unknown. As previously noted, traditional *in vivo* testing methods do not provide the information needed to define a mechanism for chemicals lacking this information from other sources (National Academies of Sciences, 2015).

**TABLE 2:** Summary of previously published mechanisms for selected chemical clusters. Extremely Toxic Chemicals: LD50≤25 mg/kg. Options for inclusion of the mechanism in the AOP-Wiki are as follows: AOP = An AOP exists that includes the key event corresponding to this mechanism and an adverse outcome of death (mortality). Key Event = A key event corresponding to this mechanism exists but no AOPs containing that key event have an adverse outcome of death (mortality). No = No key event was found that matches the mechanism.*Cardiac channel blocking includes several different channels, but the AOP is specifically focused on the Ether-a-go-go channels, which have not been definitively mapped to a cluster from our analysis at this time.

Known mechanisms Associated with Clusters including Extremely Toxic Chemicals	Associated with Extremely Toxic Chemicals	Reported in Sullivan et al	Included in the AOP-Wiki	Reported in Prieto et al
Adrenergic receptor interaction	X	X	Key Event	X
Cholinergic signaling	X	X	AOP	X
Histaminergic signaling	—	X	AOP	X
Dopamine receptor interaction	X	X	Key Event	X
GABA receptor signaling	X	—	AOP	X
Glycine receptor signaling	X	—	No	X
NMDA receptor signaling	X	X	Key Event	X
Norepinephrine reuptake inhibition	—	X	No	X
Opioid receptor interaction	X	X	Key Event	—
Serotonin reuptake inhibition	—	X	Key Event	X
Steroid receptor signaling	X	—	Key Event	—
Endocrine system disruption	X	—	Key Event	—
Cardiac ATPase inhibition	X	—	No	X
Cardiac channel blocking	X	X	AOP*	X
Alkali-associated toxicity	X	—	No	—
ACE inhibition	X	—	Key Event	—
Vitamin-K recycling inhibition	X	—	AOP	—
TRPA1 interaction	—	X	Key Event	—
Prostaglandin synthesis inhibition	X	—	Key Event	X
Inhibition of inflammation (anti-inflammatory agents)	X	—	Key Event	X
Vitamin D receptor inhibition	X	—	Key Event	—
Aconitase inhibition	X	X	No	—
Aldose Reductase inhibition	X	X	No	—
Alkylation of biomolecules (alkylating agents)	X	—	Key Event	X
Aryl hydrocarbon receptor activation	X	—	Key Event	—
Dihydrofolate reductase inhibition	—	X	No	—
DNA damage	X	—	AOP	X
Heme biosynthesis inhibition	—	X	Key Event	—
Oxidative phosphorylation inhibition	X	X	AOP	X
Oxidative phosphorylation inhibition via cytochrome-C oxidase	X	X	No	—
Ion balance disruption (ionophores)	X	—	No	—
Mitochondrial inhibitors	X	X	AOP	X
PPAR signaling inhibition	X	—	Key Event	—
Protein synthesis inhibition	—	X	No	X
Tubulin binding	—	X	Key Event	X
Voltage-gated ion channel interference	X	X	AOP	X

A comparison with key events in the AOP-Wiki (https://aopwiki.org/) showed similar results. While 75% of the mechanisms associated with extreme toxicity had a relevant key event in the AOP-Wiki, only 29% had the full AOP leading to acute mortality. Of those AOPs, the majority were applicable to fish and invertebrates and may not be relevant for mammalian toxicity. Of interest, there are only two cases (alkali-associated toxicity and ion balance disruption by ionophores) where no mechanistic information was present across all data sources. This suggests that a great deal of the needed information is available and just has not been formally assembled in the AOP-Wiki at this stage.

The effort to inventory mechanisms associated with extremely toxic chemicals helped us to evaluate the extent to which assays selected for a cluster align with what is already known about mechanisms of action for the chemicals in a cluster. The cluster-to-assay relationships can be organized into the following bins: 1) Cluster where the selected assay aligns with the best-known mechanisms associated with the chemicals in the cluster, 2) Cluster where the selected assay is indirectly associated with the best-known mechanisms for chemicals in the cluster, 3) Clusters that did not get assays assigned, and 4) Clusters where a relationship between the selected assay(s) and what is already known about the MOA for chemicals in the cluster was not readily apparent.

**FIGURE 7:**
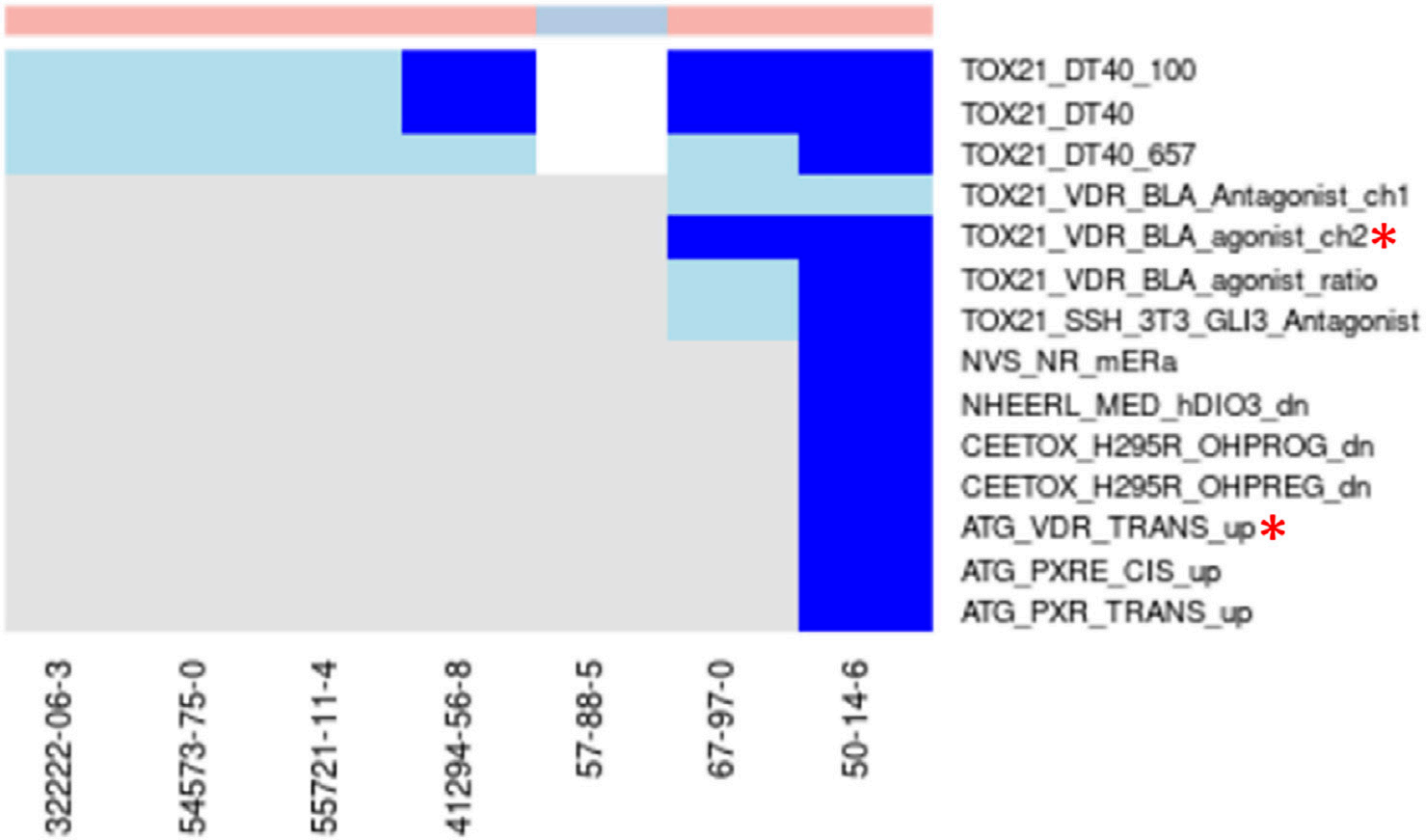
Vitamin-D analogs.Combined heatmap showing chemicals from clusters 1798 and 1964 containing vitamin-D analogs. All assays corresponding to the vitamin-D receptor and any other assay with activity below the cytotoxicity point for at least one chemical are shown. Heatmap colors: blue = active below the cytotoxicity point, light blue = active above the cytotoxicity point, white = inactive, grey = not tested. Top of heatmap: red = toxic, blue = nontoxic, aquamarine = undefined. Red asterisks identify the assay(s) selected for that cluster.

Example where a selected assay aligns with best-known Mechanisms for Chemicals in the Cluster. Vitamin-D analogs—Clusters 1798 and 1964 (Figure 7): Two clusters were enriched for chemicals known to be Vitamin-D analogs (1798 with 6 chemicals and 1964 with 2 chemicals) and each cluster had a single chemical (Vitamin D–Cluster 1798, Ergocalciferol–Cluster 1964) that was tested in the Tox21 Vitamin-D receptor assays. Ergocalciferol (CAS# 50-14-6) was also tested in the ToxCast Attagene assay. None of the other chemicals were tested in any of the vitamin-D receptor assays. Both chemicals showed activity in all vitamin-D receptor assays in which they were tested, but some activity was within the cytotoxicity range. The TOX21_VDR_BLA_agonist_ch2 endpoint was selected for cluster 1798 and showed activity for both of the tested chemicals below the cytotoxicity point. The ATG_VDR_TRANS_up assay was selected for cluster 1964. While it is encouraging that the correct assays were selected when only a single chemical was tested from each cluster, the algorithm is designed to use the aggregate signal across multiple chemicals in the cluster. For this reason, the results found in this study could be dramatically improved if the assay coverage for the chemicals was more extensive. We are also evaluating the clustering to determine why these chemicals were split into two clusters and whether other similar examples exist.

Another interesting observation from this pair of clusters is the activity of all toxic compounds in the Tox21 DT40 assays with no activity for the nontoxic compound (Figure 7). These assays all use the CellTiter-Glo (Promega) method to measure cytotoxicity (Yamamoto et al., 2011). Two of the assays (TOX21_DT40_657, TOX21_DT40_100) rely on DT40 cells with mutations that induce deficiency in DNA repair, and comparison of cytotoxicity between those cell lines and the isogenic wild-type cells (TOX21_DT40) is used to evaluate chemicals as DNA damaging agents. Our results suggest that these cells may also provide a sensitive assay for cytotoxicity, which has been previously shown as a good predictor of acute systemic toxicity (Prieto et al., 2013). This is consistent with previous characterization of these cell lines (Yamamoto et al., 2011). Activity in this highly sensitive cytotoxicity assay below the cytotoxicity range established by the aggregate signal across many assays was common across the different chemical clusters (examples in Figures 5, 6), but the specificity for acutely toxic chemicals is not universal. It could, however, represent an interesting addition to a multi-assay testing battery.

**FIGURE 8:**
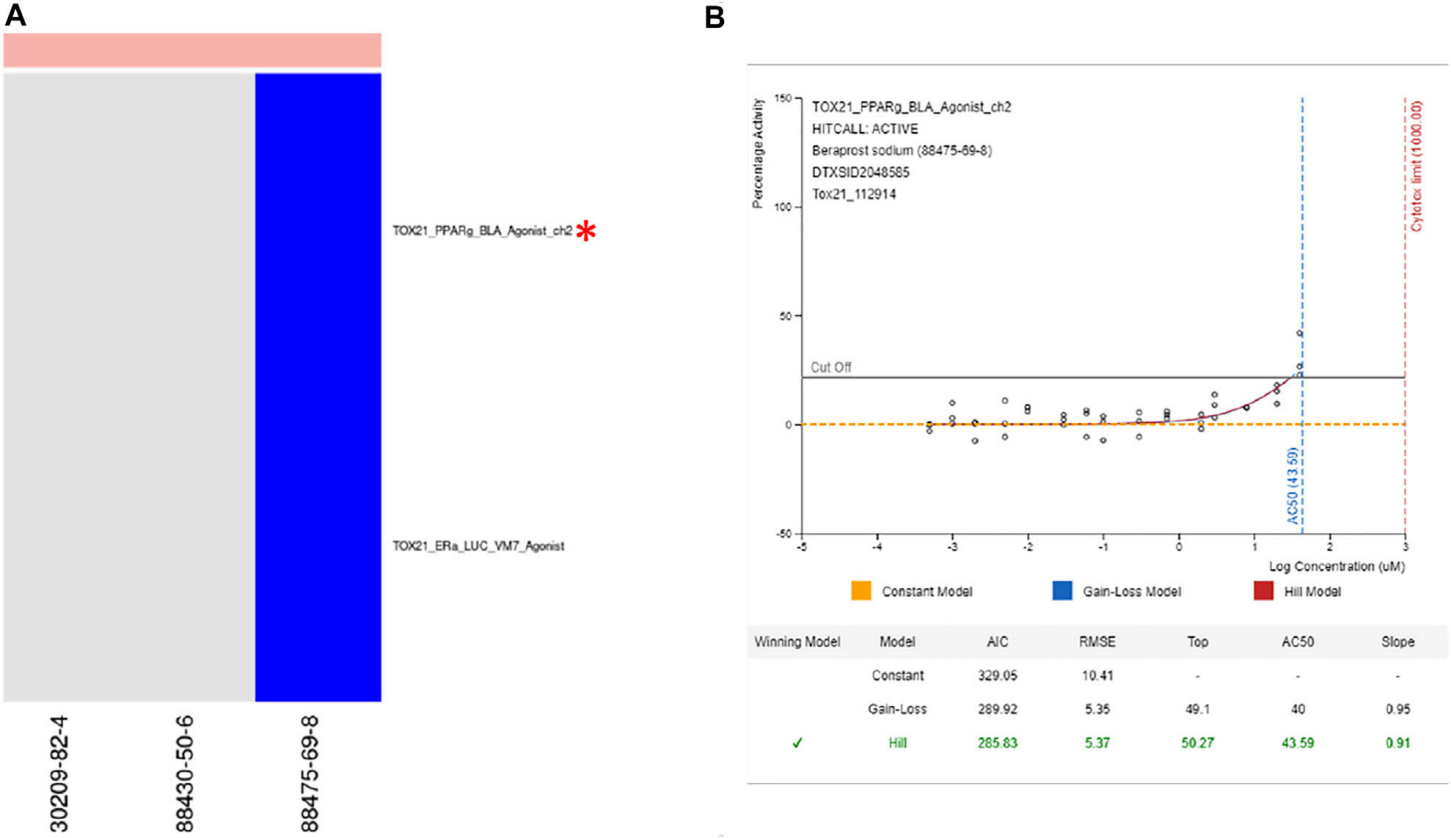
Prostaglandin receptor agonists. **(A)** Heatmap showing activity for all assays where at least one chemical from cluster 941 was active. **(B)** Dose response curve for beraprost sodium in selected assay showing modest activity. From https://comptox.epa.gov/dashboard/dsstoxdb/results?search=DTXSID2048585#invitrodb-bioassays-toxcast-tox21. Accessed 11/19/2021. Heatmap colors: blue = active below the cytotoxicity point, light blue = active above the cytotoxicity point, white = inactive, grey = not tested. Top of heatmap: red = toxic, blue = nontoxic, aquamarine = undefined. Red asterisks identify the assay(s) selected for that cluster.

Examples where the selected assay is indirectly linked with the best-known mechanisms for chemicals in the cluster. Prostaglandin Receptor Agonist—Cluster 941 (Figure 8): Cluster 941 drew our attention because all three chemicals are known to be prostaglandin receptor agonists (Beraprost sodium, Beraprost, and Latanoprost). None of the chemicals were tested in ToxCast, but Beraprost sodium had been tested in 79 Tox21 assays, none of which are specific for prostaglandin receptors. Based on this chemical, the assay selected for the cluster was TOX21_PPARg_BLA_Agonist_ch2. While PPAR *γ* is not a direct target of prostaglandins, there is documented bidirectional communication between PPAR *γ* and different prostaglandin signaling pathways (Koeffler, 2003; Fujimori, 2012; Evans et al., 2019; Lee et al., 2021) and 15-deoxy-Δ^12,14^-prostaglandin J_2_ is the endogenous ligand for this receptor (Kliewer et al., 1995; Lee et al., 2021). Beraprost is an analog for prostaglandin I or prostacyclin, which has been shown to modulate PPAR δ (Forman et al., 1997; Lim et al., 1999). Since prostacyclin has not shown activity for PPAR *γ* (Forman et al., 1997), the activity picked up in the ToxCast assay could be off-target effects of beraprost or a consequence of micromolar concentrations applied directly to the receptor. Either way, the expectation would be that activity in the PPAR δ assay would occur at lower concentrations and the activity for the prostaglandin I receptor at lower concentrations still. While the AC50 values used in our analysis are a good starting point, they are not indicative of what could be achieved with a tailored tiered testing strategy that uses mechanism-based targets.

**FIGURE 9:**
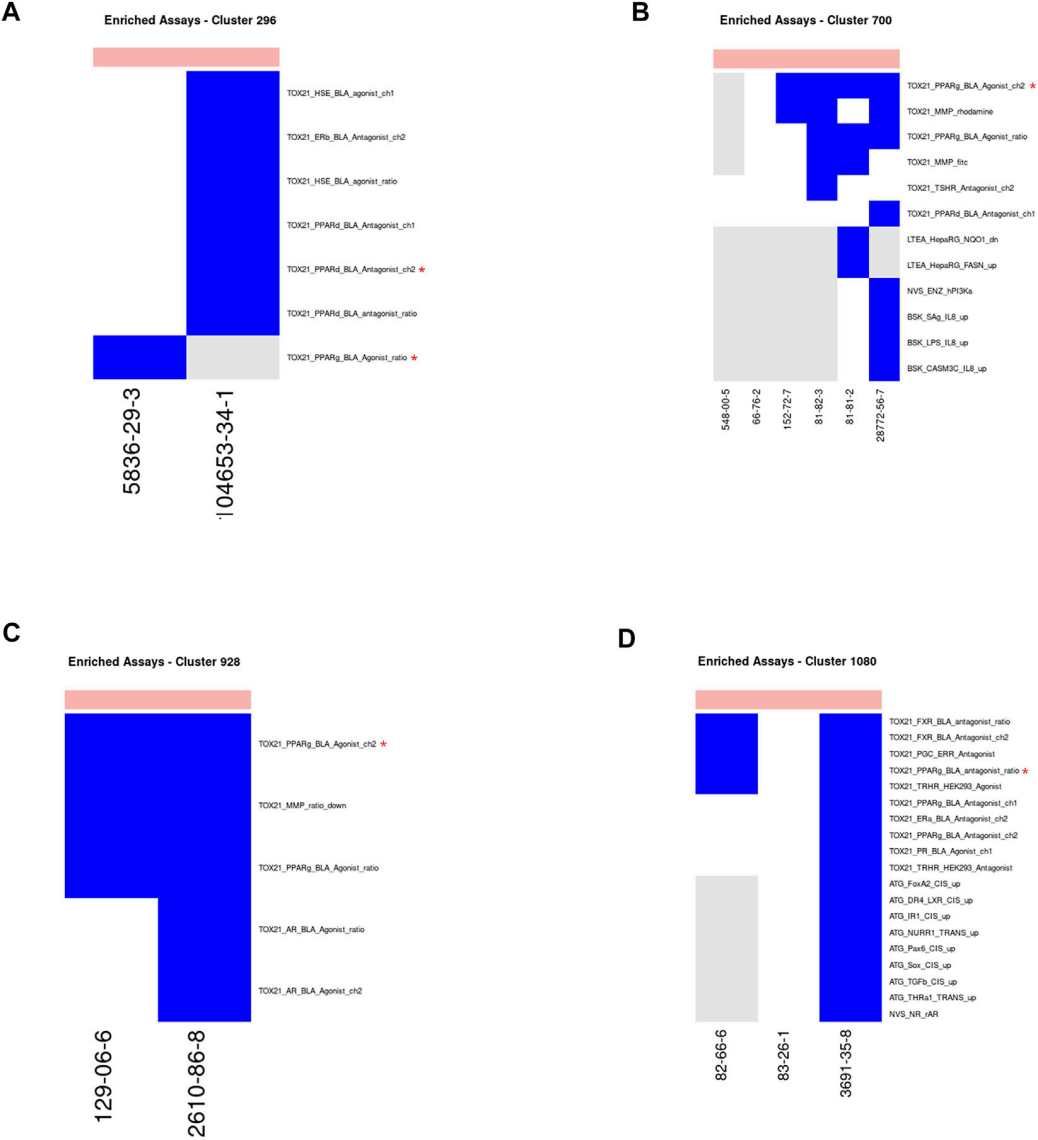
Vitamin-K epoxide reductase inhibitors. Heatmap showing activity below the cytotoxicity range in the assays that were enriched for clusters 296 **(A)**, 700 **(B)**, 928 **(C)** and 1080 **(D)**. Only chemicals with activity in at least one assay are shown. Heatmap colors: blue = active below the cytotoxicity point, light blue = active above the cytotoxicity point, white = inactive, grey = not tested. Top of heatmap: red = toxic, blue = nontoxic, aquamarine = undefined. Red asterisks identify the assay(s) selected for that cluster.

Vitamin-K Epoxide Reductase Inhibitor—Clusters 296, 700, 928, 1080 (Figure 9): Four clusters containing extremely toxic chemicals consisted mostly of rodenticides that work by inhibiting the recycling of vitamin K, which is required for synthesis of coagulation factors VII, IX, X, and thrombin. The target of these chemicals is vitamin K epoxide, which is not a target in any ToxCast or Tox21 assays. In the absence of the primary target for these chemicals, the assays identified for all clusters related to PPAR *γ* signaling (Figure 9). As with the prostaglandin receptor agonists, there is evidence of an off-target impact on PPAR *γ* signaling for this chemical class. Warfarin, which is the prototypical chemical for this class has been previously shown to inhibit PPAR *γ* signaling (Tew et al., 2017). In the case of the Tox21 results, we identified both PPAR *γ* agonist (clusters 296, 700, and 928) and antagonist (cluster 1080) activity. This could mean that members of this class of compounds are partial agonists for the PPAR *γ* receptor. This is consistent with a general observation of low efficacy in the agonist assays. There were two assays identified for cluster 296 based on two chemicals within that cluster with activity below cytotoxicity in ToxCast/Tox21. One assay targets PPAR *γ* signaling and the other targets PPAR δ signaling. The chemical for which PPAR δ was chosen was not tested in the PPAR *γ* assay, so it is possible that this cluster would have been exclusive for PPAR *γ* had the chemical been tested in that assay. Many of the chemicals across all four clusters had activity in both the PPAR *γ* and PPAR *δ* assays, however, suggesting that there is considerable crosstalk among those assays. This is consistent with our conclusions for the prostaglandin receptor agonist cluster above.

**FIGURE 10:**
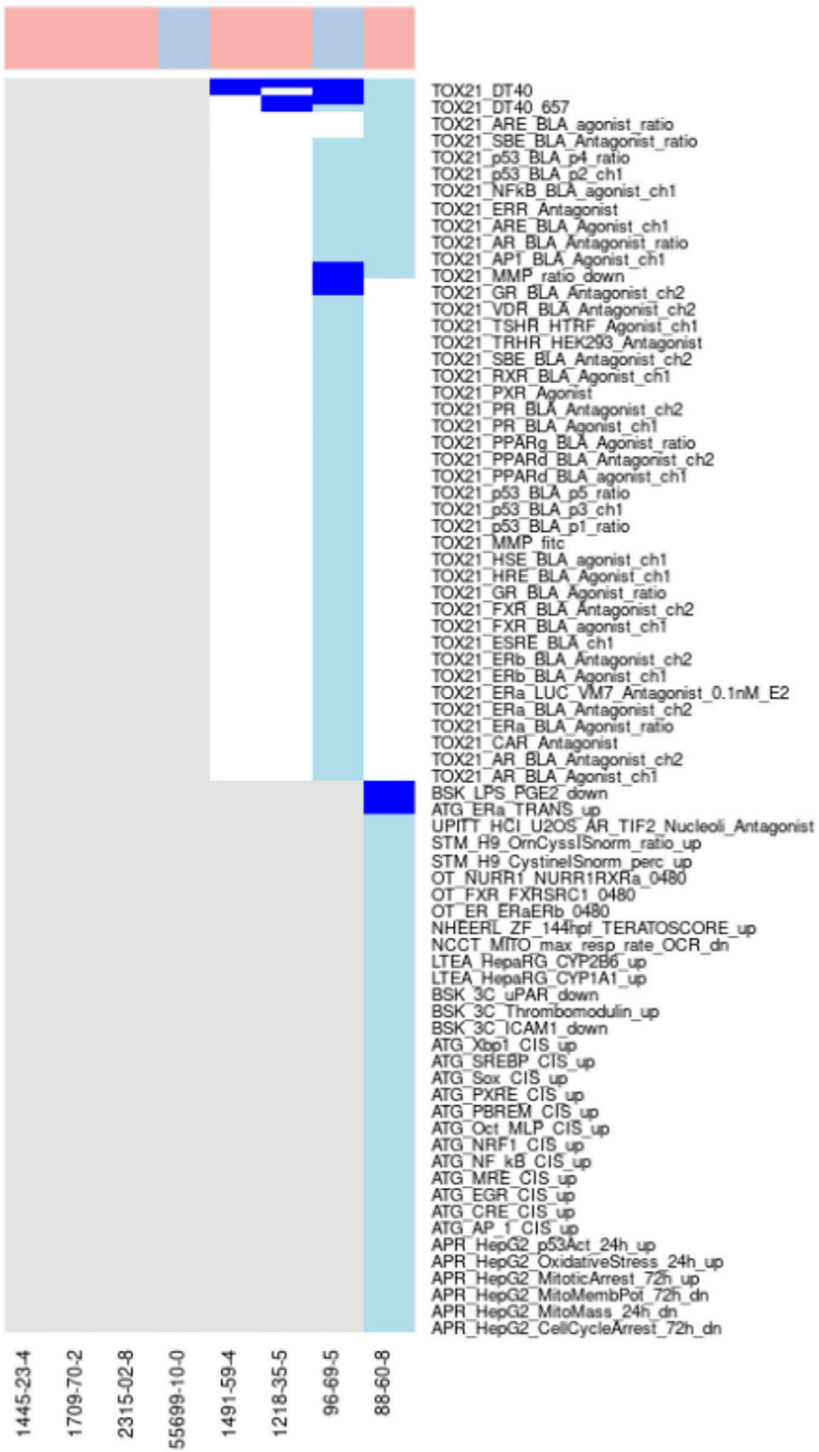
Adrenergic receptor agonists. Heatmap showing activity for all assays where at least one chemical from the cluster was active. Not all assay names are shown. Heatmap colors: blue = active below the cytotoxicity point, light blue = active above the cytotoxicity point, white = inactive, grey = not tested. Top of heatmap: red = toxic, blue = nontoxic, aquamarine = undefined.

Example of a cluster that did not get an assay assigned. Adrenergic Receptor—Cluster 803 (Figure 10): Cluster 803 came to our attention because it contained known alpha 1 adrenergic receptor agonists and “Adrenergic interaction” has been defined as a mechanism of acute lethality for which further assay development work is needed (Sullivan et al., 2021). When ingested orally or taken in too large a dose, certain imidazoline-containing chemicals, such as alpha adrenergic receptor agonists, can depress the central nervous system, reduce blood pressure, and induce bradycardia (Lowry and Brown, 2014; Karpushkina et al., 2021; Lionte et al., 2012; Norman and Nappe, 2021). This may be due to a loss of selectivity of the chemical towards the alpha-2 adrenergic receptor and/or *via* binding to the imidazoline receptor (Lowry and Brown, 2014; Karpushkina et al., 2021). It has been hypothesized that stimulation of either the alpha-2 adrenergic receptor or the imidazoline receptor can lead to activation of the other receptor (Lowry and Brown, 2014).

We wanted to understand why cluster 803 was not assigned an assay during our analysis despite having a previously defined mechanism for acute toxicity. There are three chemicals in the cluster known to impact adrenergic signaling (Oxymetazoline, Xylometazoline hydrochloride, and Oxymetazoline hydrochloride), but none of those chemicals were tested in any of the ToxCast assays for alpha adrenergic receptors. They were tested in the Tox21 assays, but those assays do not include measures of adrenergic signaling. Whereas the three known alpha1 adrenergic receptor agonists all have an imidazoline group, which is known to interact with the alpha adrenoceptor as well as the imidazoline receptors (Hong et al., 1994; Lowry and Brown, 2014), the other chemicals in this cluster lack that chemical feature and may act *via* a different mechanism. This is consistent with a lack of activity in adrenoreceptor assays for the one chemical from the cluster that was tested in the ToxCast assays. In this case, the original cluster should be split further based on the presence or absence of the imidazoline group. This cluster demonstrates how using the structure, bioactivity, and literature data can help to further refine the clustering. The Tox21 DT40 assays show activity for all chemicals tested in this cluster, but they are not eligible for a cluster-specific assay because they measure cytotoxicity. As noted previously, however, these assays do hold promise as a sensitive measure of cytotoxicity.

### Summary of Mechanistic Classification of ATWG Chemicals

The ToxCast activity for chemicals in the assays corresponding to their structural cluster was combined with the cytotoxicity activity from ToxCast to create a composite activity assessment and corresponding AC50 value for comparison with the previously defined toxicity classification for those chemicals. The cytotoxicity activity was included along with assays identified above because this readout has been previously determined to be informative regarding acute toxicity (Prieto et al., 2013; Prieto et al., 2019). Out of 11,992 chemicals in the ATWG set, 3957 chemicals had ToxCast data deemed relevant for acute toxicity evaluation. Of these, 2028 chemicals were classified as active and 1929 showed no activity. Comparison of the ToxCast activity assessment against a binary classification of toxicity (Mansouri et al., 2021) showed a highly significant association (Table 3).

**TABLE 3:** Comparison of ToxCast activity and acute oral toxicitySignificance based on Fisher’s Exact Test: *p*-value = 2.2 × 10^−16^, odds ratio = 4.46. Nontoxic chemicals are defined as LD50 > 2,000 mg/kg.

ToxCast Activity	ATWG Toxicity Classification	
	Toxic	Nontoxic
Active	1,406	621
Not Active	649	1,279

**FIGURE 11:**
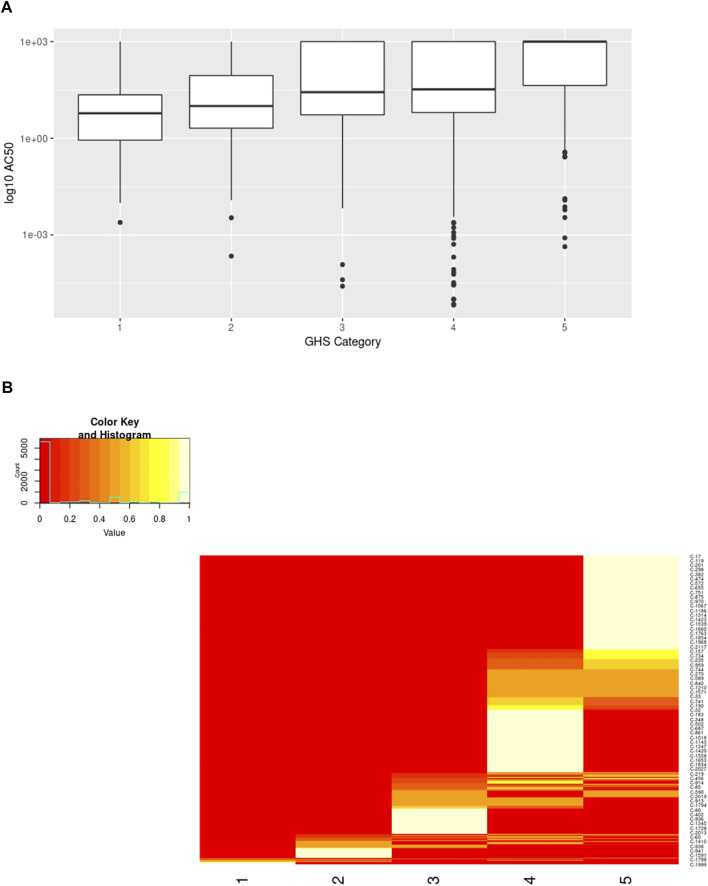
Comparison of ToxCast AC50 values and structural clusters with GHS Categories. **(A)** Boxplots show the median along with the first and third quartiles, and outliers are shown *via* dots and whiskers. Median AC50 values (micromolar) for each GHS category are as follows: 1 = 5.97, 2 = 10, 3 = 27, 4 = 33, 5 = 1000. **(B)** Heatmap shows the percentage of the chemicals within a cluster having the specified GHS category. The majority of clusters span 1-2 neighboring GHS categories.

A similar result was seen when comparing the activity in ToxCast vs the GHS categories for the chemicals. While the *p*-value from the Fisher’s Exact Test was higher than that for the binary classification, it was still significant (estimated *p*-value = 5 × 10^−4^). Comparison of the AC50 values across the different categories also shows a trend with higher AC50 values associated with less toxicity (Figure 11A). However, there is a high degree of overlap across the different categories making it impossible to predict the acute toxicity category from the ToxCast activity data alone.

The structural cluster to which a chemical belongs also provides valuable information regarding the potential acute toxicity potential of the chemical as expected (Figure 11B). This trend was also significant when evaluated using the Fisher’s Exact Test (estimated *p*-value = 5 × 10^−5^). A large number of structural clusters are unique to a single GHS category when considering only the ToxCast chemicals with activity data (corresponding to the AC50 values plotted in Figure 11A). Of the remainder, most clusters include chemicals from two neighboring categories. If chemicals that have not been tested in ToxCast are included, the distribution across the categories is broader but still shows a similar pattern. The use of in vitro-in vivo extrapolation to account for toxicokinetics would be expected to improve the correspondence between the bioactivity and in vivo-based toxicity classifications as discussed below (Honda et al., 2019; Ring et al., 2021).

## Discussion

Our results suggest that a combination of in silico and *in vitro* approaches can be sufficient for determining acute oral toxicity without the need for *in vivo* animal testing. We have identified a set of *in vitro* assays from the ToxCast program that can be used to evaluate toxicity and shown that coupling those assays with structural information can improve their predictive capability. Subsequent evaluation of the assays highlighted opportunities and challenges associated with the use of the existing *in vitro* assays for this purpose. The biggest issue was the lack of assay data for many of the chemicals. In many cases, the minimal assay chosen was based upon data availability rather than measured activity. There were also many cases where a single chemical from a cluster was solely responsible for the assay selection. The methods employed are intended to leverage the aggregate activity across the cluster and are not expected to perform well when a single chemical is used for the selection.

The *in vitro* assays identified herein are not intended as a definitive list but instead as a starting point for a mechanism-driven approach to identifying and cataloging assays that cover the acute oral toxicity space. Our approach looked for the best assays to identify toxic chemicals within a cluster, but it was not designed to evaluate the quality of those assays. Assays that only identify a small percentage of the toxic chemicals within the cluster or don’t discriminate between the toxic and nontoxic chemicals will not be useful in a tiered testing paradigm. On the other hand, assays that correspond to the known mechanism of toxicity and correctly identified all toxic chemicals within a cluster can be used with confidence. It is important to note that not all assays need to be demonstrably tied to a precise mechanism to be valuable. There are many reasons why an assay may provide an accurate readout of the potential toxicity due to indirect effects on the target of the assay. The key is to accurately catalog those assays and include the necessary caveats for use when interpreting the data from those assays.

The current results can be expanded in a variety of ways. This study focused on data from the ToxCast screening program, but this represents only a fraction of the available assay data. Previous studies have evaluated results from the ACuteTox project (Kinsner-Ovaskainen et al., 2013) and data collected in PubChem (Russo et al., 2019), and there is a wealth of information in the published literature outside of large individual datasets. Combining the information from these different approaches and applying our methods to additional datasets should expand our coverage of the chemical space and increase the number of clusters for which a relevant assay is identified. The current analysis was intentionally data-driven and relied exclusively on the chemical structure and bioactivity information. The rationale for this choice was to complement other recent efforts that attempted to approach the problem by defining the biological mechanisms (Prieto et al., 2019; Sullivan et al., 2021). There will be many cases where the ideal bioassay is readily available and can be easily identified purely by identifying the mechanism by which a given chemical cluster causes toxicity. Many of these assays may not be amenable to high throughput screening (HTS) and would therefore never be identified by analyzing the results from HTS efforts.

Integration of information regarding the mechanisms of toxicity should greatly improve our ability to identify and verify the ideal *in vitro* assays for different structural classes of chemicals. To this end, we catalogued known mechanisms of acute oral toxicity from three sources and compared them with the *in vitro* assays identified for the chemicals associated with those mechanisms. Finally, we evaluated how well the ToxCast *in vitro* activity and the structural clustering corresponded to the known acute oral toxicity potential of the chemicals. These results suggest that a combination of chemical structure and bioactivity can be predictive of *in vivo* toxicity. For example, 73% of the structural clusters containing at least one toxic chemical includes chemicals from at most two GHS categories. These findings are consistent with the previously published evaluations of the CATMoS model (Mansouri et al., 2021) and the related QSAR efforts (Alberga et al., 2019; Ballabio et al., 2019; Gadaleta et al., 2019). They also highlight the potential value of mechanism-based profilers (Wijeyesakere et al., 2018; Wilson et al., 2018). We’ve shown that 98% of the clusters containing chemicals tested as part of the ToxCast/Tox21 programs required two assays or less to identify the toxic chemicals within those clusters (Figure 3D). While the overlap in ac50 values (Figure 11A) is currently too great for accurate discrimination, there are several obvious improvements to be made: identifying assays more causally associated with the toxicological mechanism, accounting for toxicokinetics of the chemicals, testing chemicals in lower throughput assays to potentially increase accuracy and precision.

Our findings highlight the value of incorporating mechanistic information into any tiered testing strategy. Not only does the information about mechanisms of toxicity for a certain structural class aid in identifying the appropriate assay, but it also provides the information to interpret the results from those assays. Assays that focus directly on essential targets for the mechanism of toxicity give more confidence in a hazard assessment than assays that monitor tangential effects. Knowledge of the mechanisms of toxicity also provide a framework for integrating data from in silico, *in vitro*, and *in vivo* studies to more effectively leverage all available information when assessing toxicity (Tollefsen et al., 2014; Marshall et al., 2018). AOPs were specifically designed for this purpose and offer the ideal framework for assembling this type of information (Ankley and Edwards, 2018; Watford et al., 2019). Our results show that much of the relevant information is already included in the AOP-Wiki, but in many cases, the full AOPs relevant for acute oral toxicity have not been defined.

A common misconception is that an AOP must be exhaustively documented and reviewed in order to be used for IATA development or toxicity assessment, but many acute toxicity mechanisms are well known and would not require extensive documentation for use in a tiered testing environment. In fact, the assembly of the AOP often highlights data gaps and aids in identifying mechanisms where additional information is needed to increase the confidence. In particular, if AOPs are developed in concert with the assay identification and assembly of the tiered testing framework, the information requirements for the AOPs will be driven by the testing paradigm. This focuses the AOP development effort and reduces the time and effort required to create a fit for purpose AOP. Should those AOPs be needed for a different purpose down the road, they can be more comprehensively reviewed to meet those needs.

An AOP-driven integrated approach also reduces the reliance on any single source of toxicity information. By tailoring the bioactivity assays to the structural clusters, we can add an additional evidence layer to the information provided by the chemical structure. The additional information from a carefully selected bioactivity assay is critical when defining mechanism-based profilers (Wijeyesakere et al., 2018; Wilson et al., 2018) since small changes in structures could lead to drastic changes in bioactivity. This integrated approach can be used to support read-across and other related applications (Prieto et al., 2013; Floris et al., 2014; De Abrew et al., 2019; Russo et al., 2019; Karmaus et al., 2020). There are probabilistic approaches such as Bayesian networks that allow simultaneous consideration of all lines of evidence as well. By using AOPs as the organizing framework, these data can be jointly utilized to support decision making rather than considered individually within a tiered testing paradigm.

While more work is needed to comprehensively cover the entire chemical space associated with acute oral toxicity, there is pretty good coverage of known toxic chemicals today (Table 4). The structural clusters include 92% of the known toxic chemicals, and the structural information alone provides a good indication of the GHS category for most of the chemicals (Figure 11B). While Figure 11B is focused on those clusters that are linked to a ToxCast assay, the results are similar when evaluating all of the structural clusters. Looking at the toxic chemicals, 58% of the structural clusters contain chemicals that all fall within the same GHS category. Furthermore, 85% of the clusters include chemicals from no more than two neighboring categories, which means that information on the chemical cluster alone could narrow the possible GHS categories considerably when coupled with any other qualitative measure of toxicity.

**TABLE 4:** Coverage of the acute oral toxicity chemical spaceEvaluation of the percentage of toxic chemicals from the ATWG list covered by structural clusters and ToxCast activity.

	Number of Chemicals	Percentage (%)	Number of Clusters
Toxic ATWG Chemicals	6,845	100	N/A
Chemicals in clusters	6,299	92	1,810
Chemicals in clusters associated with ToxCast activity	3,723	54	990

Over half of the chemicals (54%) are in structural clusters for which ToxCast assays were identified as having the potential to provide a bioactivity readout. As discussed above, not all of the ToxCast assays identified will be appropriate for future screening, but in many of those cases the appropriate assay is readily identified by looking at the shared target for the chemicals in the cluster. Even where the existing assay is suboptimal, in many cases it may be sufficient for estimating the GHS category with a reasonable degree of accuracy.

The focus of this study was to better understand the options for evaluating acute oral toxicity not to design new predictive models. Because of this, all analyses performed were descriptive of the existing chemicals and do not provide any information on the actual performance of the approaches for new chemicals. However, these results provide the information needed to design such approaches with an emphasis on combining multiple sources of information to predict acute oral toxicity rather than reliance on a single measurement. The survey of the existing data is encouraging, however, and suggests that predictive models that combine structural and bioactivity information could be successful. A large percentage of the chemicals remain untested in the new alternative methods, which presents a perfect opportunity to test predictive models that combine in silico (e.g. QSAR) and *in vitro* data prospectively as they are developed.

The Department of Defense has previously developed a tiered approach to toxicity testing that could easily incorporate the results from our study (National Academies of Sciences, 2015; Sullivan et al., 2021). The first step is an in silico evaluation of chemicals, which is easily covered by the CATMoS modeling suite. Our structural clustering of over 11,000 chemicals builds upon this work and provides a bridge to the second tier of testing. Tier II of the testing paradigm is the use of high and medium throughput screening assays. By explicitly connecting the *in vitro* assays to the structural clusters from the in silico analysis, we are able to dramatically reduce the number of assays needed for the tier II screening. This not only saves time and money on unnecessary testing, but it also allows assays that are not amenable for high throughput screening to be used by limiting the number of assays for each single chemical. By further mapping our chemical clusters to biological mechanisms, we help guide the selection of the assays for each cluster and increase our confidence in the Tier II results. Utilizing the AOP framework for defining the biological mechanisms also provides a data integration platform to facilitate Tier III of the testing paradigm. The AOP allows alignment of all data (in silico, *in vitro*, *in vivo* data from read across) in a consistent manner for all chemicals. This increases the confidence in qualitative integration strategies and provides a framework for quantitative integration. By enhancing each of the first three tiers of the testing paradigm, we hope to reduce the number of chemicals for which Tier IV *in vivo* testing is required.

For tier II screening, the cluster assignments can be used to design a focused screening strategy with a small number of lower throughput assays per chemical to emphasize precise estimates of the dose response rather than screening large numbers of chemicals. This should reduce the variability in the predicted toxicity classification for chemicals within the cluster. This can be further improved by using multiple orthogonal assays to evaluate the toxicity. This can include both cluster-specific assays as well as general assays such as cytotoxicity. For chemicals with no existing assay, the knowledge of the chemical mechanism could be used to identify the appropriate assay. In many cases, the assay will already exist and has just not been adapted for high throughput testing.

In the short term, additional chemicals from the cluster could be run in parallel to further refine our knowledge regarding the bioactivity for that cluster. Longer term, the previously evaluated chemicals from the cluster could be used as positive controls for the screening of new chemicals to account for batch effects. Testing of 4-8 chemicals at the time would still allow for higher assay volumes and increased replicates to improve the precision of the results. Testing 8 chemicals across 4 or more *in vitro* assays would be considerably cheaper than a corresponding *in vivo* animal study for a single chemical and is likely to provide more accurate results when the assays are appropriately chosen.

There has been increased attention on the reproducibility of the “gold standard” *in vivo* toxicity tests traditionally used for both hazard assessment and the evaluation of new alternative methods. When comparing alternative methods for assessing endocrine disruption potential, it was found that models based on alternative methods could predict the results of an *in vivo* test with comparable accuracy to a secondary evaluation using the same *in vivo* assay (Browne et al., 2015; Kleinstreuer et al., 2018a; Browne et al., 2018). This led to an evaluation of the reproducibility of guideline *in vivo* studies in general (Pham et al., 2020) and of eye irritation (Luechtefeld et al., 2016), skin irritation (Rooney et al., 2021), and oral toxicity (Karmaus et al., 2021). These studies show a similar variability when comparing two repeated tests using a standard *in vivo* assay that is seen when evaluating in silico and *in vitro* methods based both on our current results and previous reports (Nelms et al., 2020; Mansouri et al., 2021).

A persistent limitation with non-animal testing is the ADME for the chemicals. This can result in substantial over or under prediction of toxicity depending on whether the active chemical is rapidly eliminated or is created during the metabolism of the parent chemical. While consideration of the structural features within each cluster coupled with computer-based metabolism predictions can provide some insights regarding the potential for this confounding effect, our work does not specifically address this outstanding issue. There has been considerable progress in recent years both with better in silico predictions (Pinto et al., 2016; Leonard et al., 2018; Ring et al., 2021) and *in vitro* approaches (DeGroot et al., 2018; Deisenroth et al., 2020; Franzosa et al., 2021). As these efforts continue to progress in parallel with efforts such as ours, the robustness of an *in vitro* testing paradigm should rapidly increase.

By using the AOP framework to assemble the in silico, *in vitro*, and existing *in vivo* data, the data integration tier can fully leverage our knowledge of the biological mechanism when integrating all toxicity data available for the chemical in question and related chemicals. This will improve the resulting decisions by providing qualitative evidence to support the evaluation of the results and by providing a framework on which to build quantitative models that better account for the known biological mechanism. Integrated models that include all existing information relevant for assessing the toxicity of a chemical are necessarily better than any single measure of toxicity. The AOP framework provides a scientifically sound, biologically based way in which to achieve this integration.

## Data Availability

All datasets used for this work are publicly available as noted in the Methods. The full results from our analysis are provided as supplemental tables with this article.
