# Supplementary material for: Mapping Mechanistic Pathways of Acute Oral Systemic Toxicity Using Chemical Structure and Bioactivity Measurements
Source: Front Toxicol. 2022 Mar 7;4:824094. doi: 10.3389/ftox.2022.824094 (PMC8915918; doi:10.3389/ftox.2022.824094)

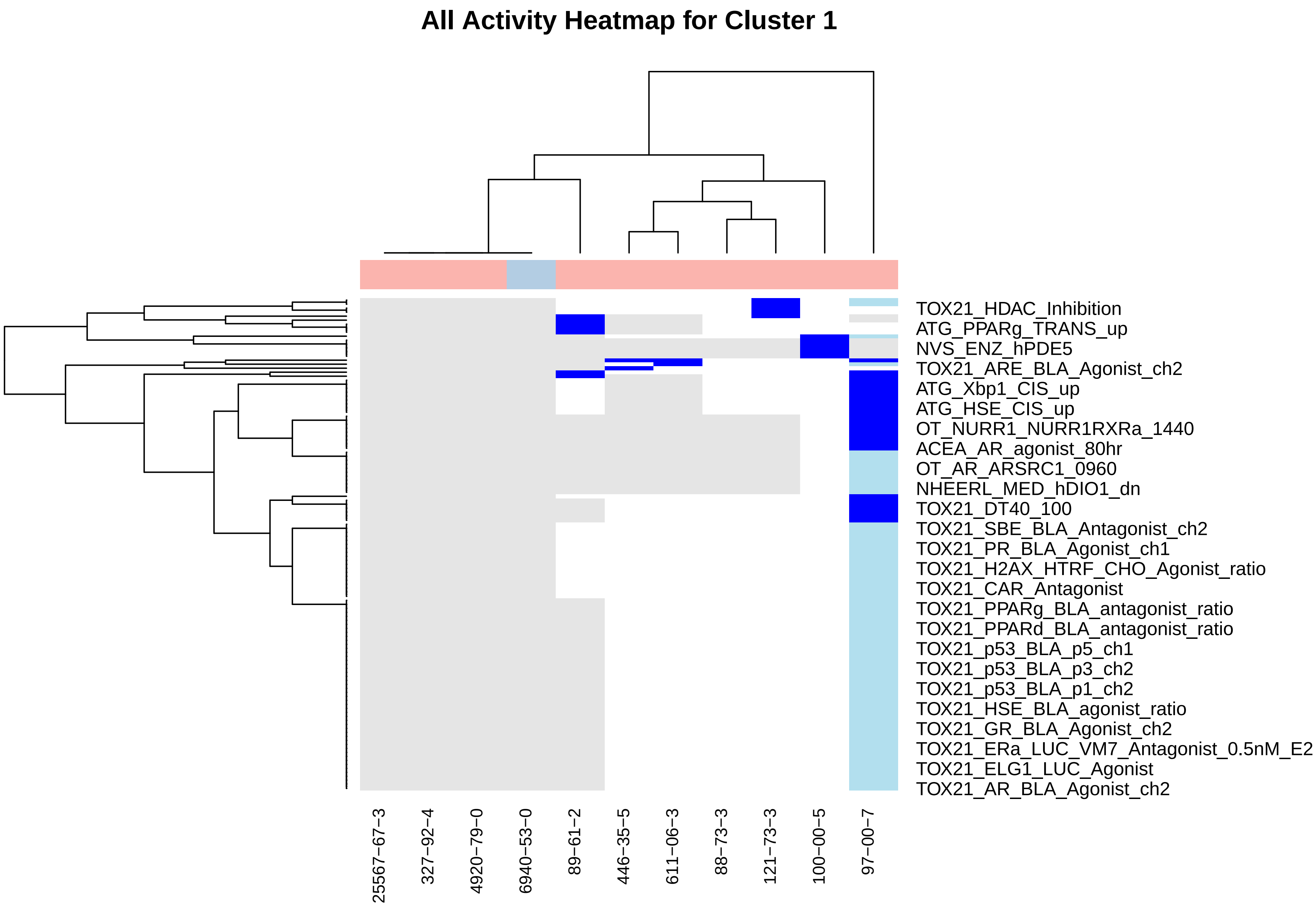
All Activity Heatmap for Cluster 1
TOX21_HDAC_Inhibition
ATG_PPARg_TRANS_up
NVS_ENZ_hPDE5
TOX21_ARE_BLA_Agonist_ch2
ATG_Xbp1_CIS_up
ATG_HSE_CIS_up
OT_NURR1_NURR1RXRa_1440
ACEA_AR_agonist_80hr
OT_AR_ARSRC1_0960
NHEERL_MED_hDIO1_dn
TOX21_DT40_100
TOX21_SBE_BLA_Antagonist_ch2
TOX21_PR_BLA_Agonist_ch1
TOX21_H2AX_HTRF_CHO_Agonist_ratio
TOX21_CAR_Antagonist
TOX21_PPARg_BLA_antagonist_ratio
TOX21_PPARd_BLA_antagonist_ratio
TOX21_p53_BLA_p5_ch1
TOX21_p53_BLA_p3_ch2
TOX21_p53_BLA_p1_ch2
TOX21_HSE_BLA_agonist_ratio
TOX21_GR_BLA_Agonist_ch2
TOX21_ERa_LUC_VM7_Antagonist_0.5nM_E2
TOX21_ELG1_LUC_Agonist
TOX21_AR_BLA_Agonist_ch2
25567−67−3
327−92−4
4920−79−0
6940−53−0
89−61−2
446−35−5
611−06−3
88−73−3
121−73−3
100−00−5
97−00−7

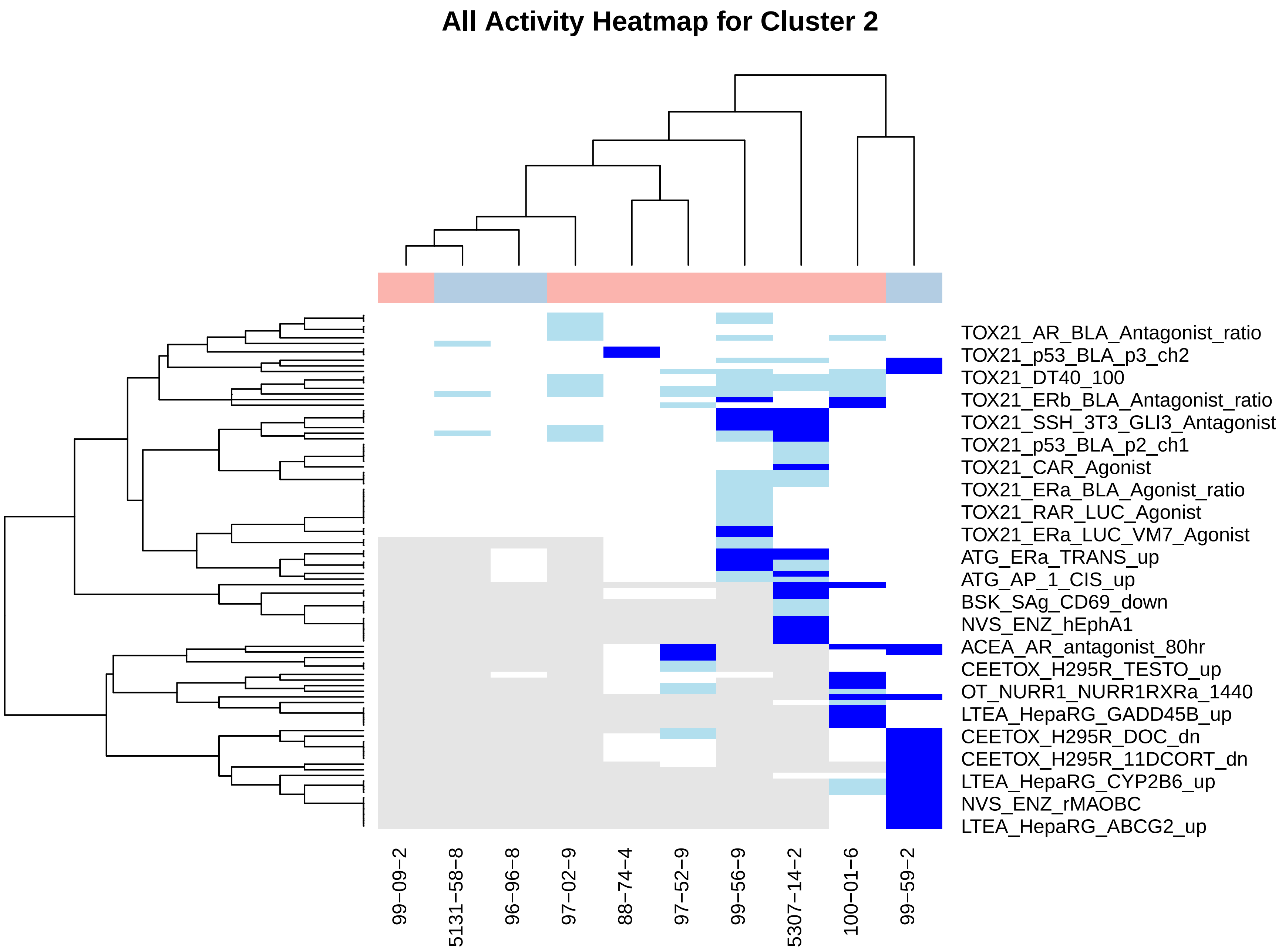
All Activity Heatmap for Cluster 2
TOX21_AR_BLA_Antagonist_ratio
TOX21_p53_BLA_p3_ch2
TOX21_DT40_100
TOX21_ERb_BLA_Antagonist_ratio
TOX21_SSH_3T3_GLI3_Antagonist
TOX21_p53_BLA_p2_ch1
TOX21_CAR_Agonist
TOX21_ERa_BLA_Agonist_ratio
TOX21_RAR_LUC_Agonist
TOX21_ERa_LUC_VM7_Agonist
ATG_ERa_TRANS_up
ATG_AP_1_CIS_up
BSK_SAg_CD69_down
NVS_ENZ_hEphA1
ACEA_AR_antagonist_80hr
CEETOX_H295R_TESTO_up
OT_NURR1_NURR1RXRa_1440
LTEA_HepaRG_GADD45B_up
CEETOX_H295R_DOC_dn
CEETOX_H295R_11DCORT_dn
LTEA_HepaRG_CYP2B6_up
NVS_ENZ_rMAOBC
LTEA_HepaRG_ABCG2_up
99−09−2
5131−58−8
96−96−8
97−02−9
88−74−4
97−52−9
99−56−9
5307−14−2
100−01−6
99−59−2

All Activity Heatmap for Cluster 3

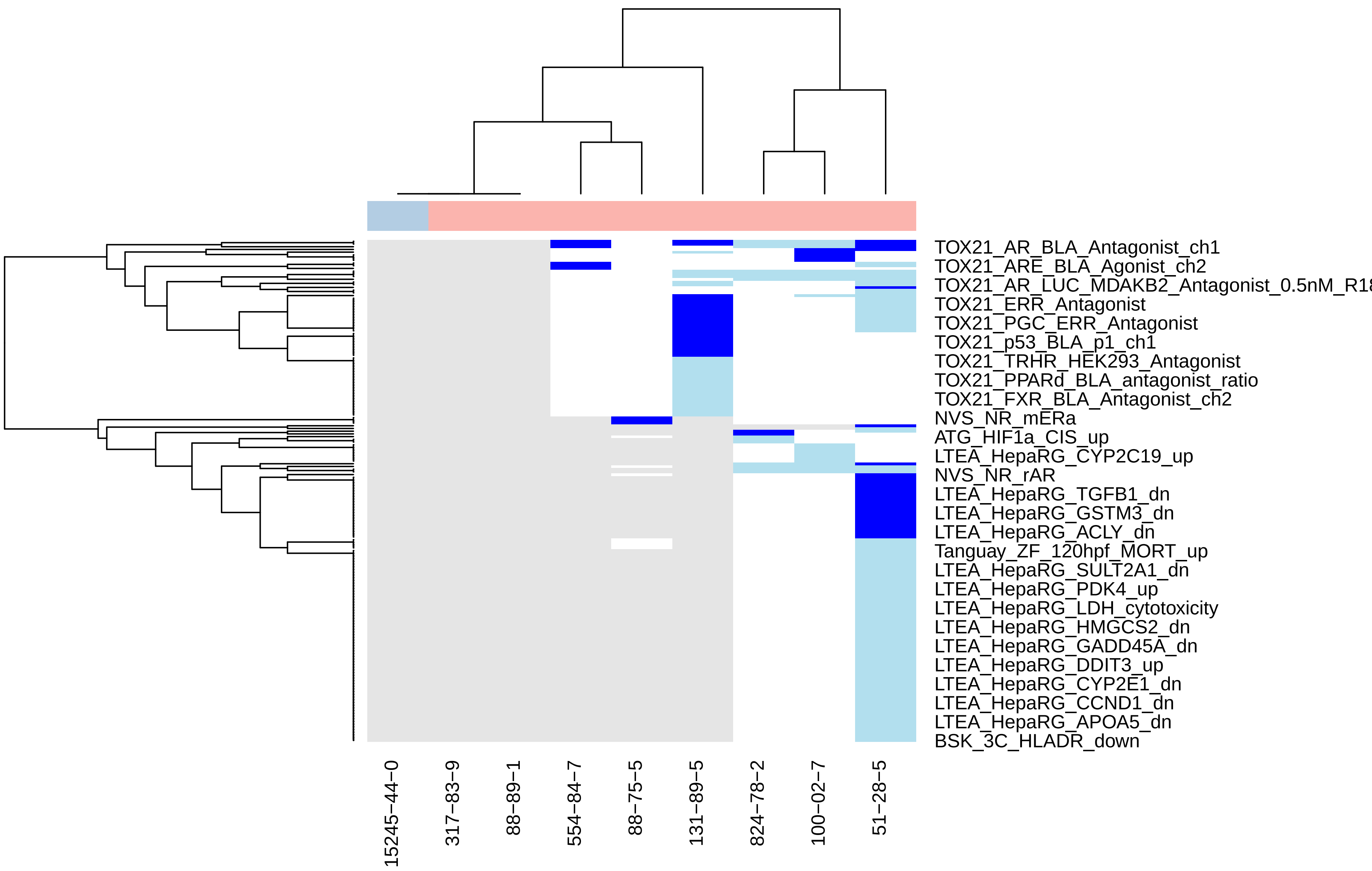

All Activity Heatmap for Cluster 4

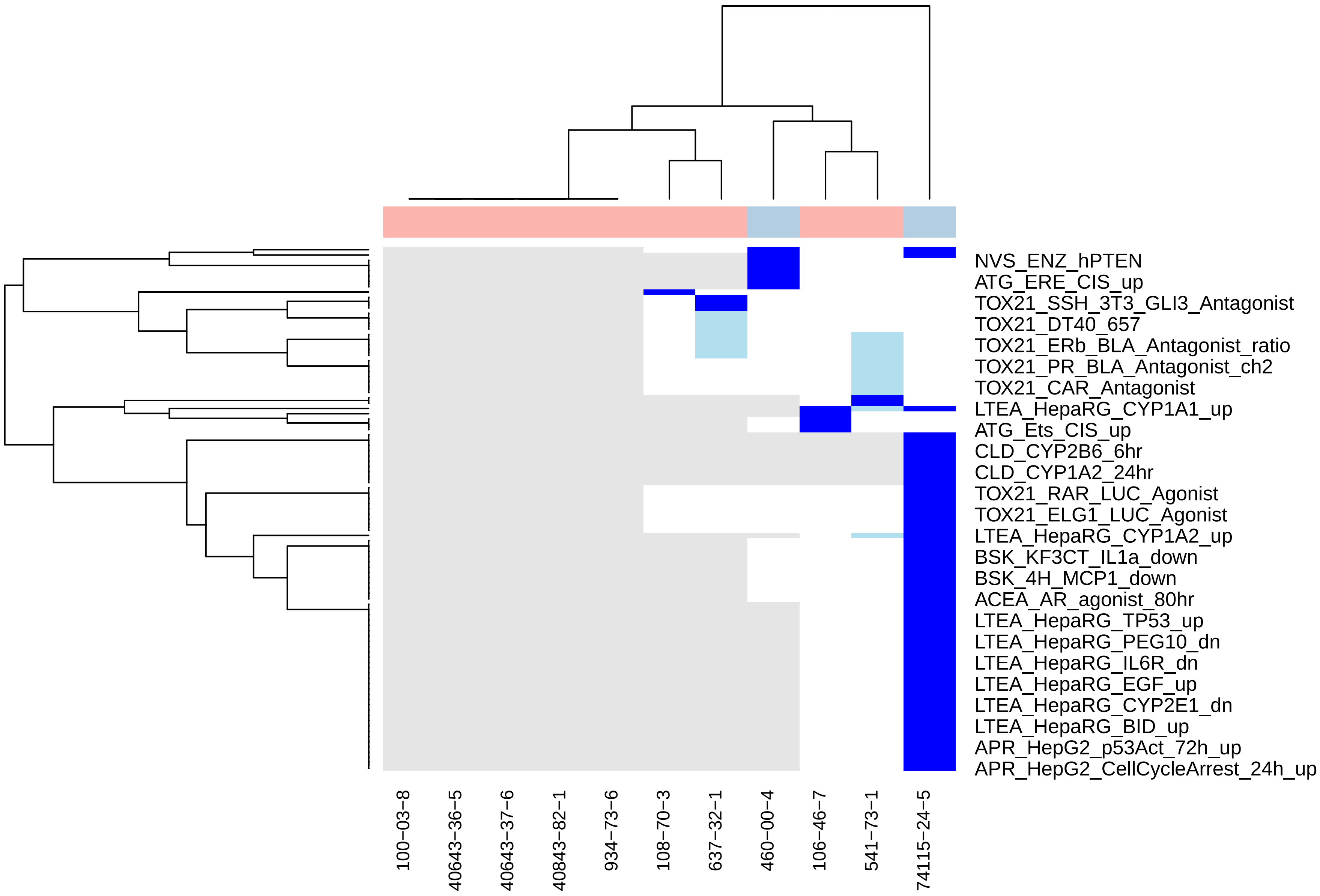

All Activity Heatmap for Cluster 5

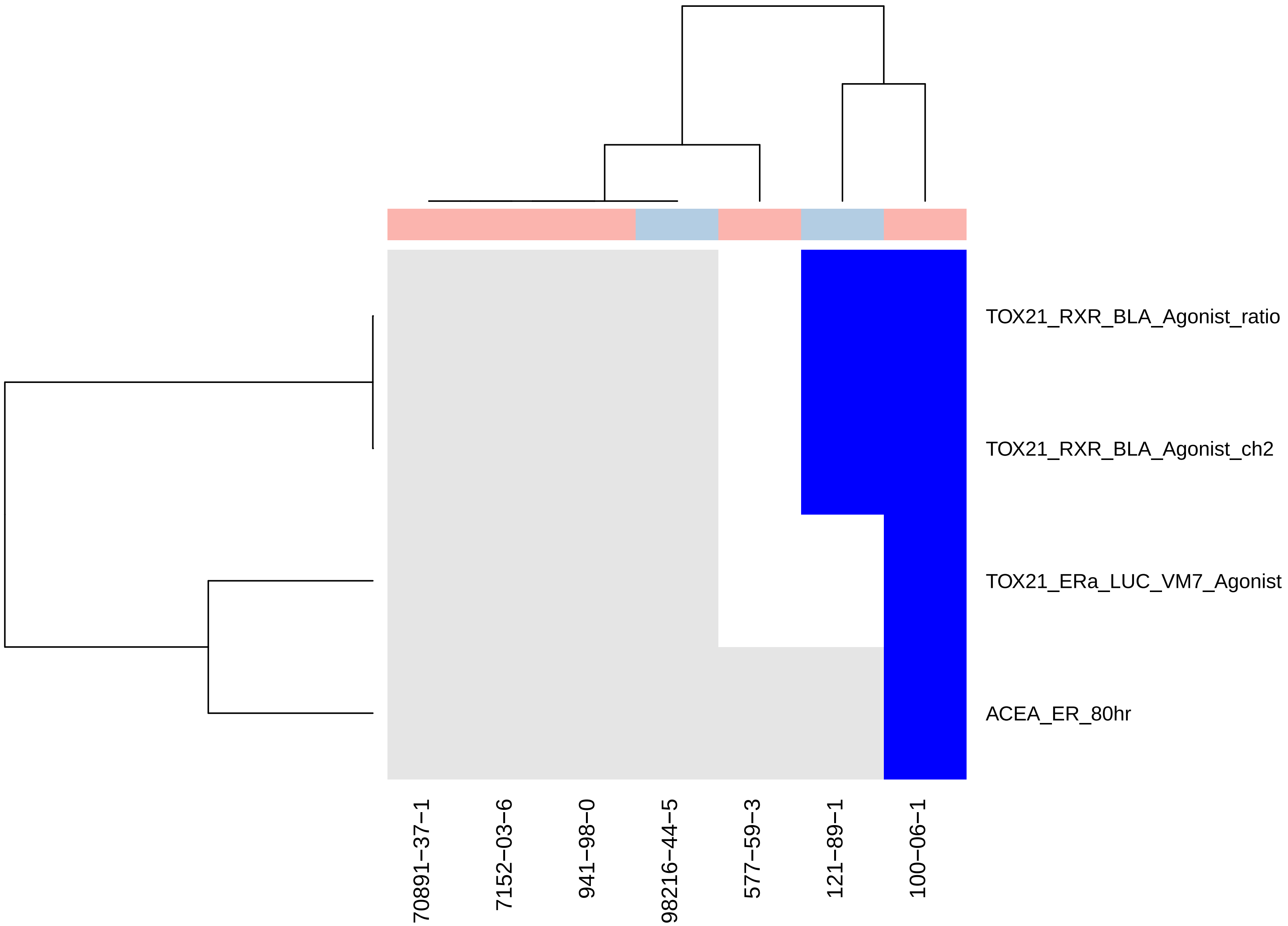

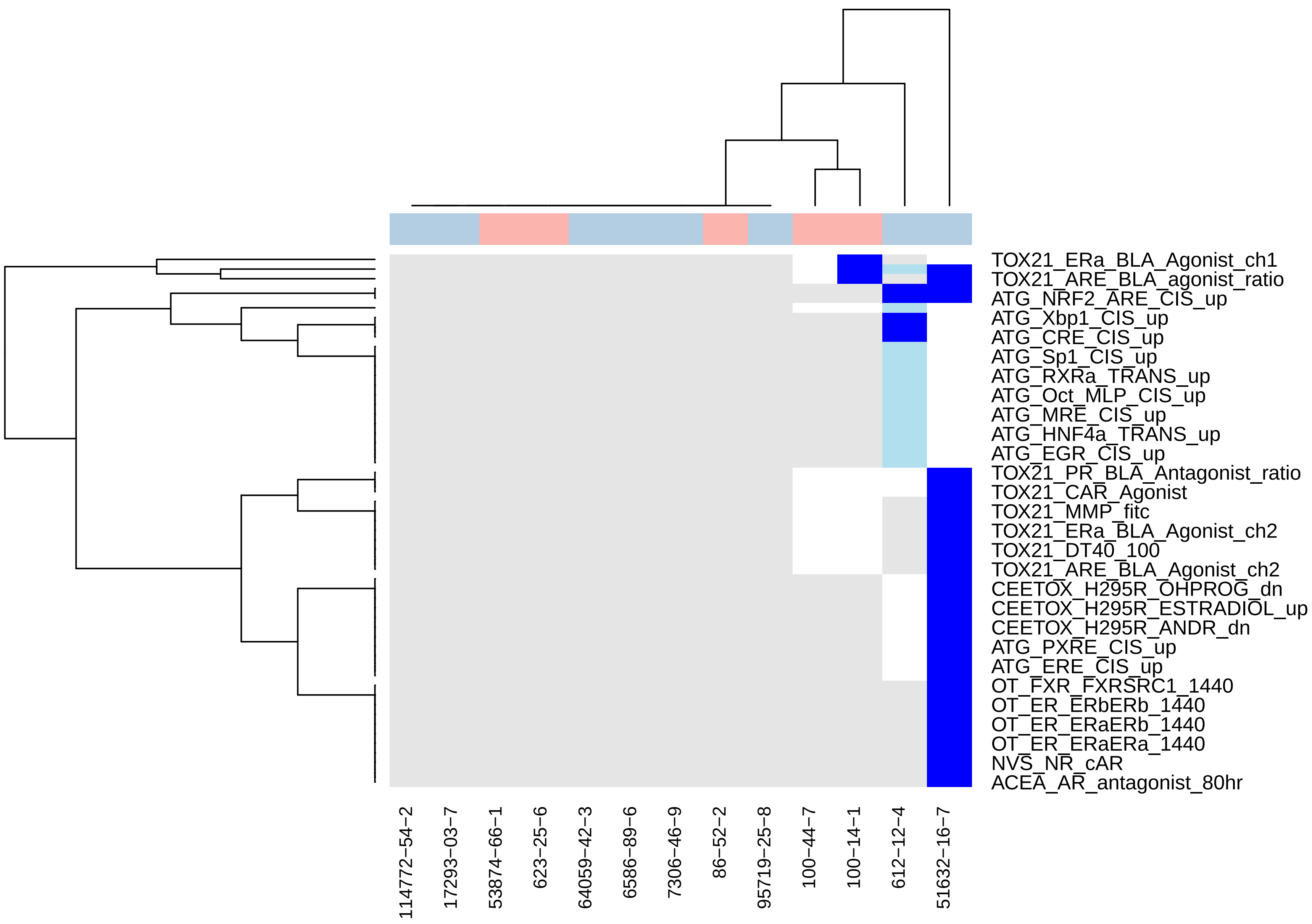

All Activity Heatmap for Cluster 6
TOX21_ERa_BLA_Agonist_ch1
TOX21_ARE_BLA_agonist_ratio
ATG_NRF2_ARE_CIS_up
ATG_Xbp1_CIS_up
ATG_CRE_CIS_up
ATG_Sp1_CIS_up
ATG_RXRa_TRANS_up
ATG_Oct_MLP_CIS_up
ATG_MRE_CIS_up
ATG_HNF4a_TRANS_up
ATG_EGR_CIS_up
TOX21_PR_BLA_Antagonist_ratio
TOX21_CAR_Agonist
TOX21_MMP_fitc
TOX21_ERa_BLA_Agonist_ch2
TOX21_DT40_100
TOX21_ARE_BLA_Agonist_ch2
CEETOX_H295R_OHPROG_dn
CEETOX_H295R_ESTRADIOL_up
CEETOX_H295R_ANDR_dn
ATG_PXRE_CIS_up
ATG_ERE_CIS_up
OT_FXR_FXRSRC1_1440
OT_ER_ERbERb_1440
OT_ER_ERaERb_1440
OT_ER_ERaERa_1440
NVS_NR_cAR
ACEA_AR_antagonist_80hr
114772−54−2
17293−03−7
53874−66−1
623−25−6
64059−42−3
6586−89−6
7306−46−9
86−52−2
95719−25−8
100−44−7
100−14−1
612−12−4
51632−16−7

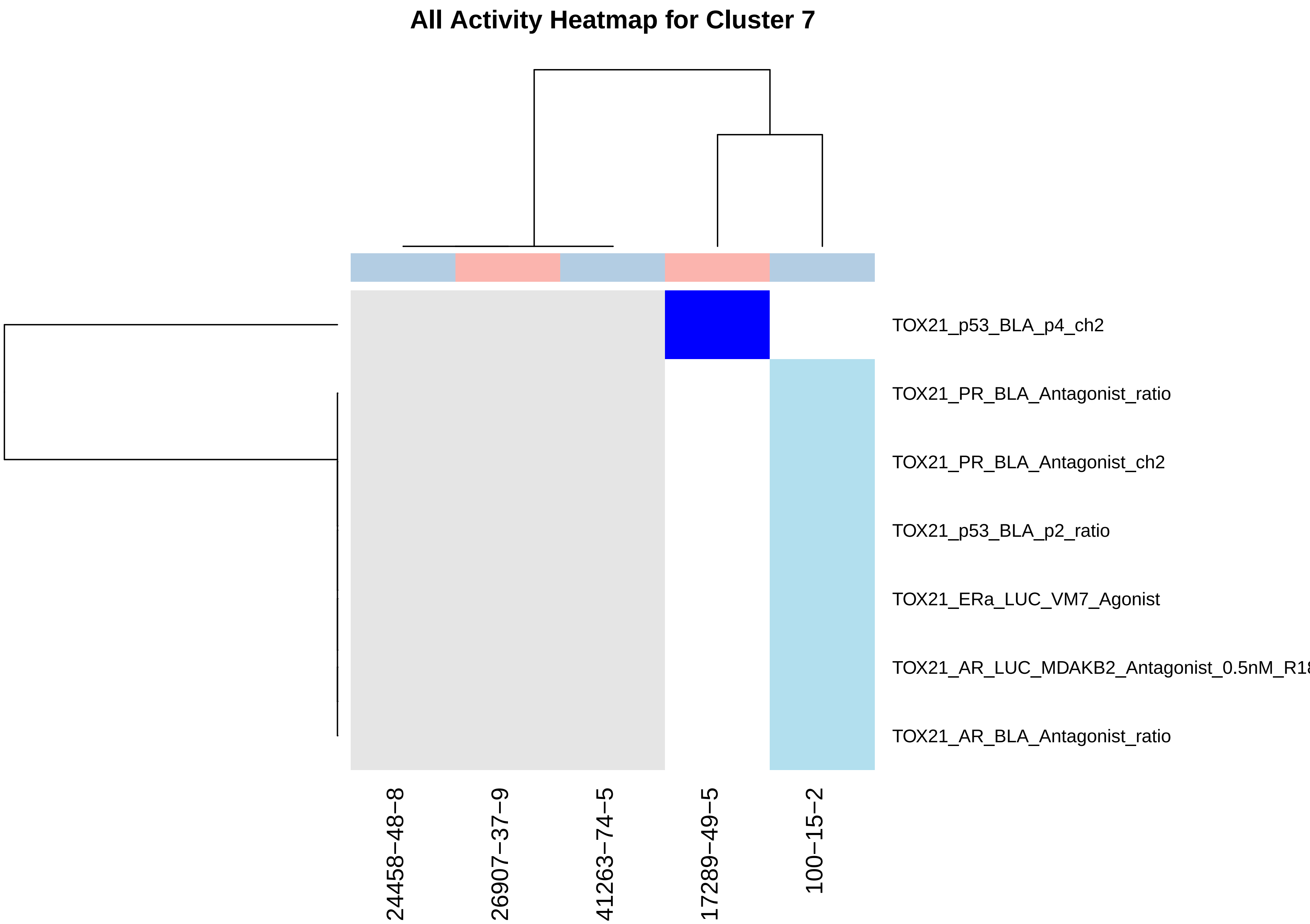

All Activity Heatmap for Cluster 7
TOX21_p53_BLA_p4_ch2
TOX21_PR_BLA_Antagonist_ratio
TOX21_PR_BLA_Antagonist_ch2
TOX21_p53_BLA_p2_ratio
TOX21_ERa_LUC_VM7_Agonist
TOX21_AR_LUC_MDAKB2_Antagonist_0.5nM_R1
TOX21_AR_BLA_Antagonist_ratio
24458−48−8
26907−37−9
41263−74−5
17289−49−5
100−15−2

All Activity Heatmap for Cluster 8

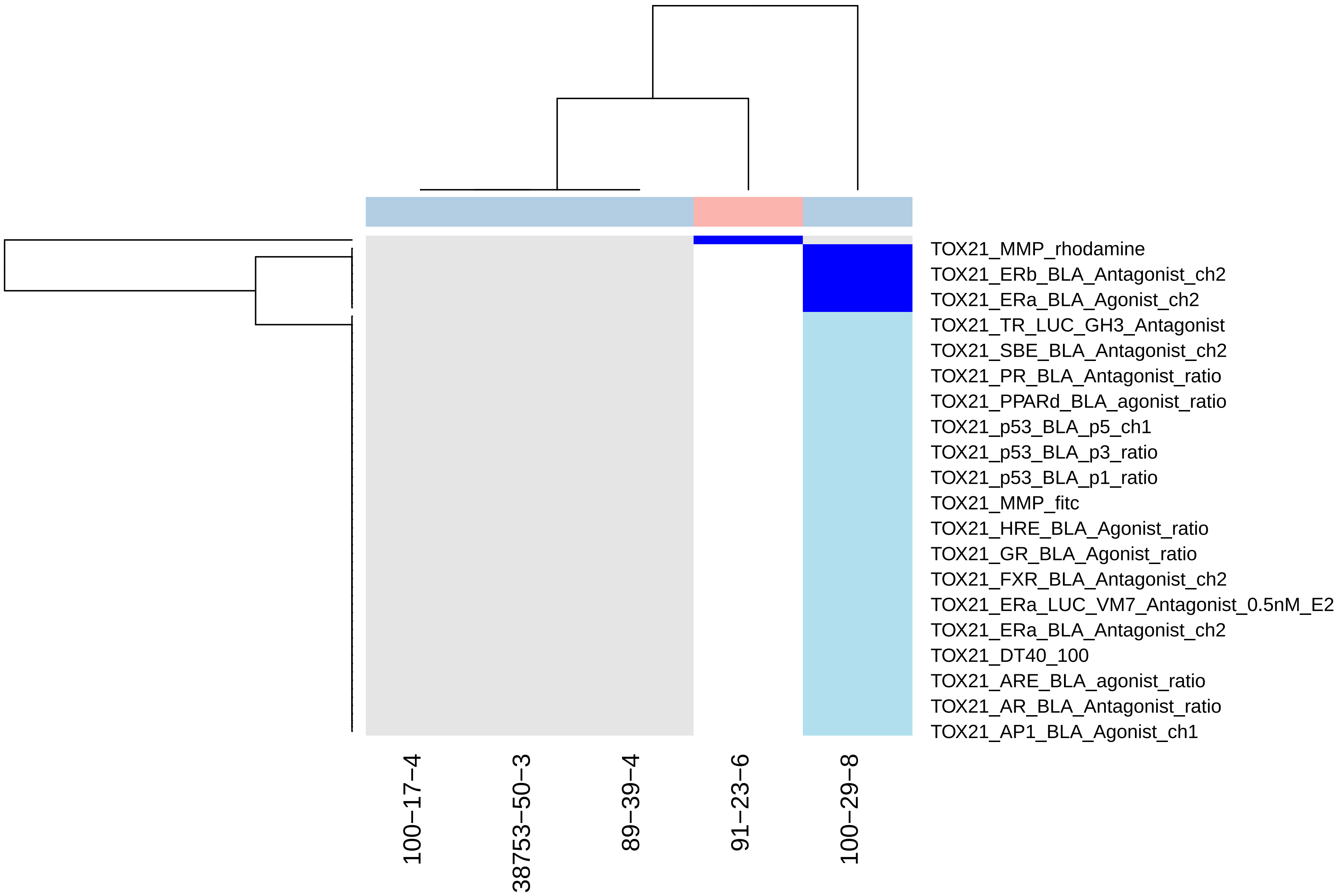

All Activity Heatmap for Cluster 9

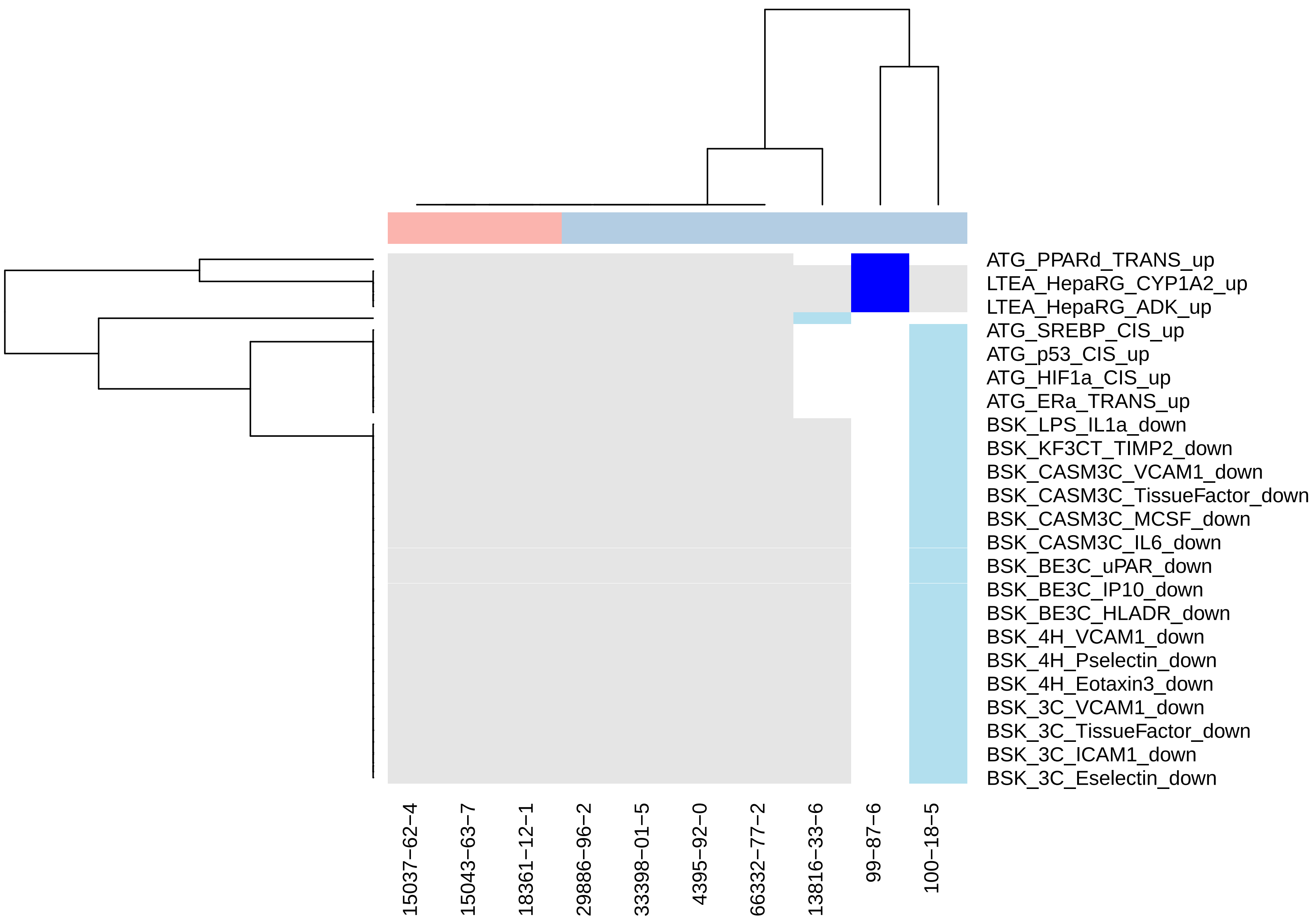

All Activity Heatmap for Cluster 10

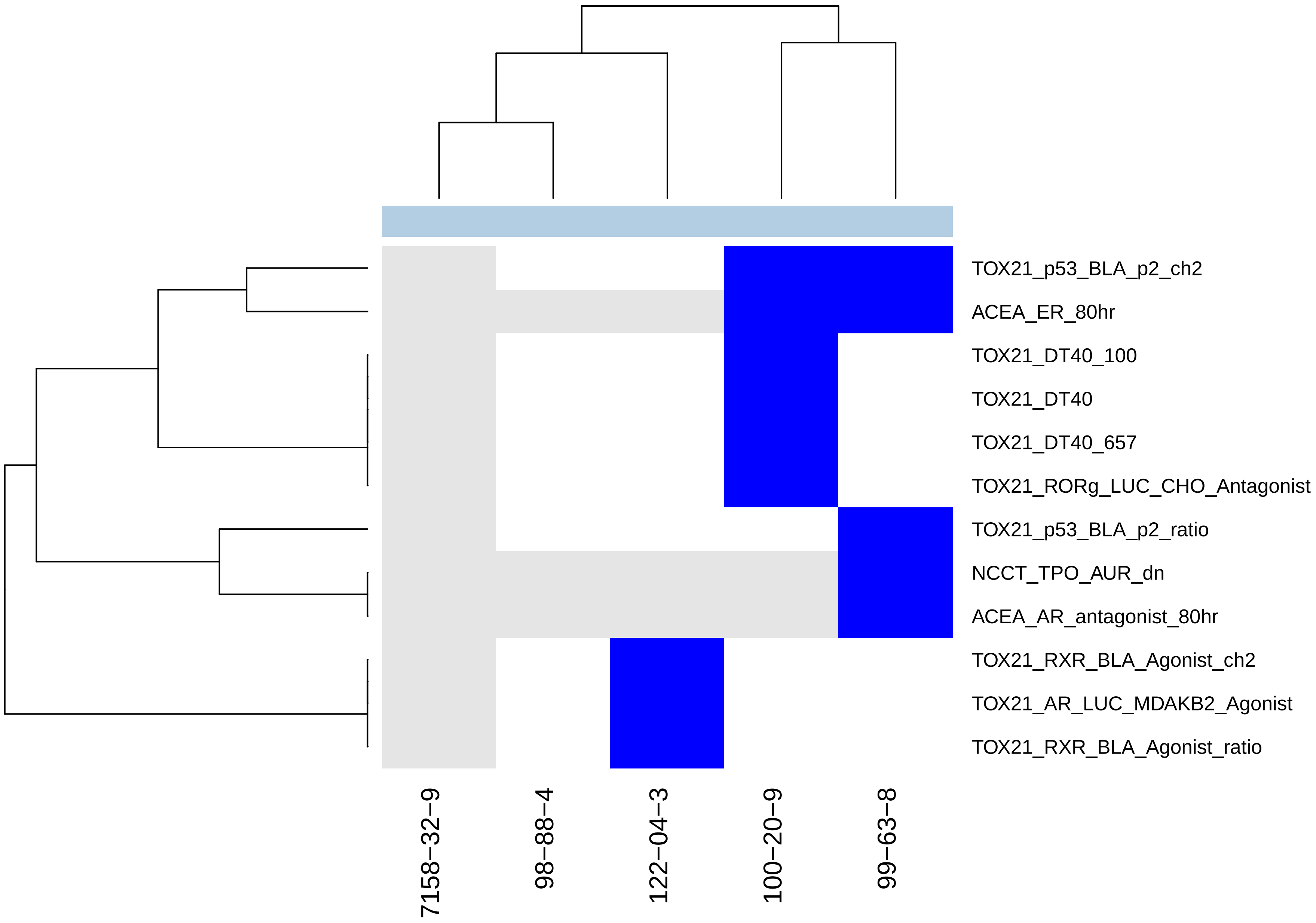

All Activity Heatmap for Cluster 11

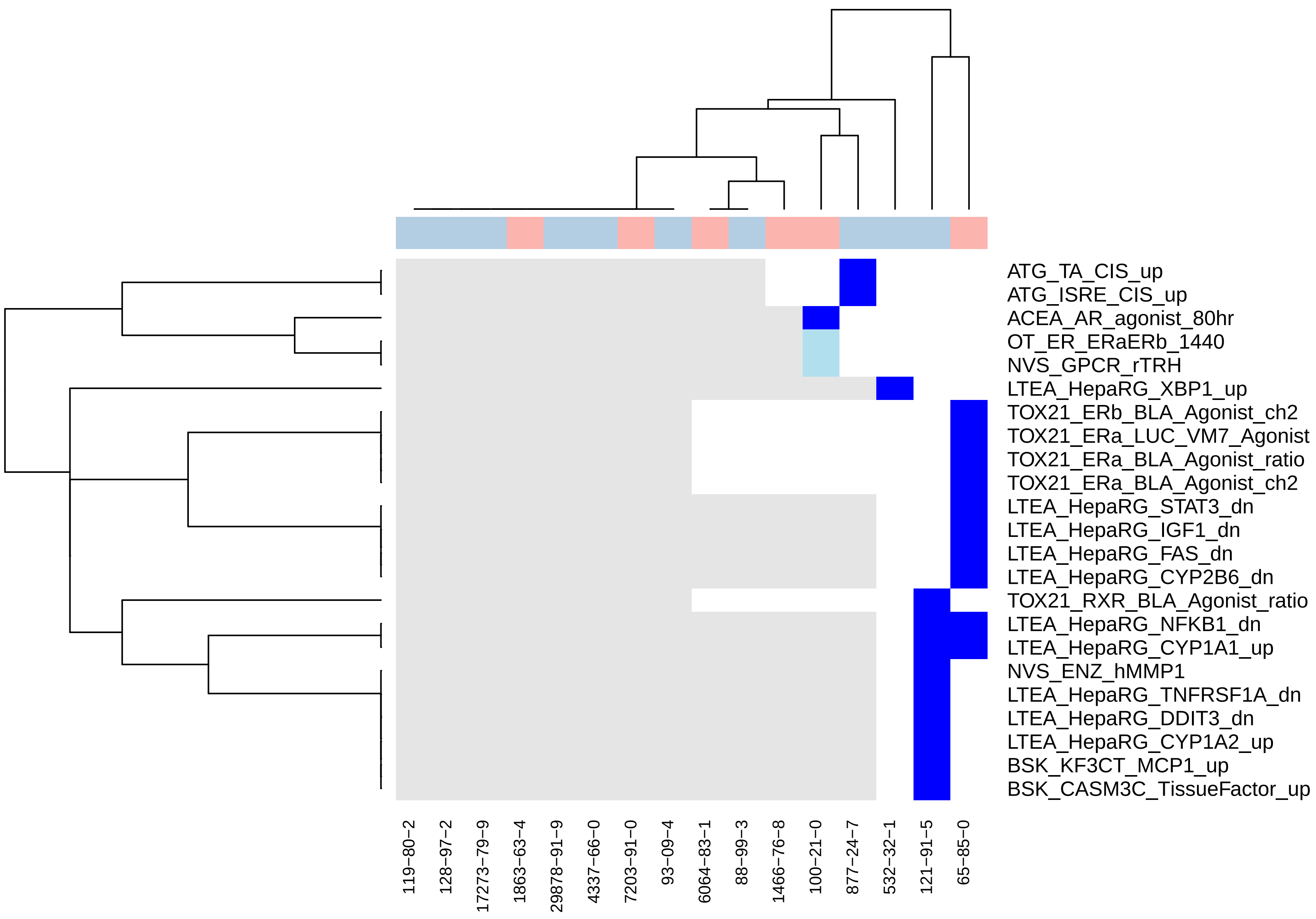

All Activity Heatmap for Cluster 12

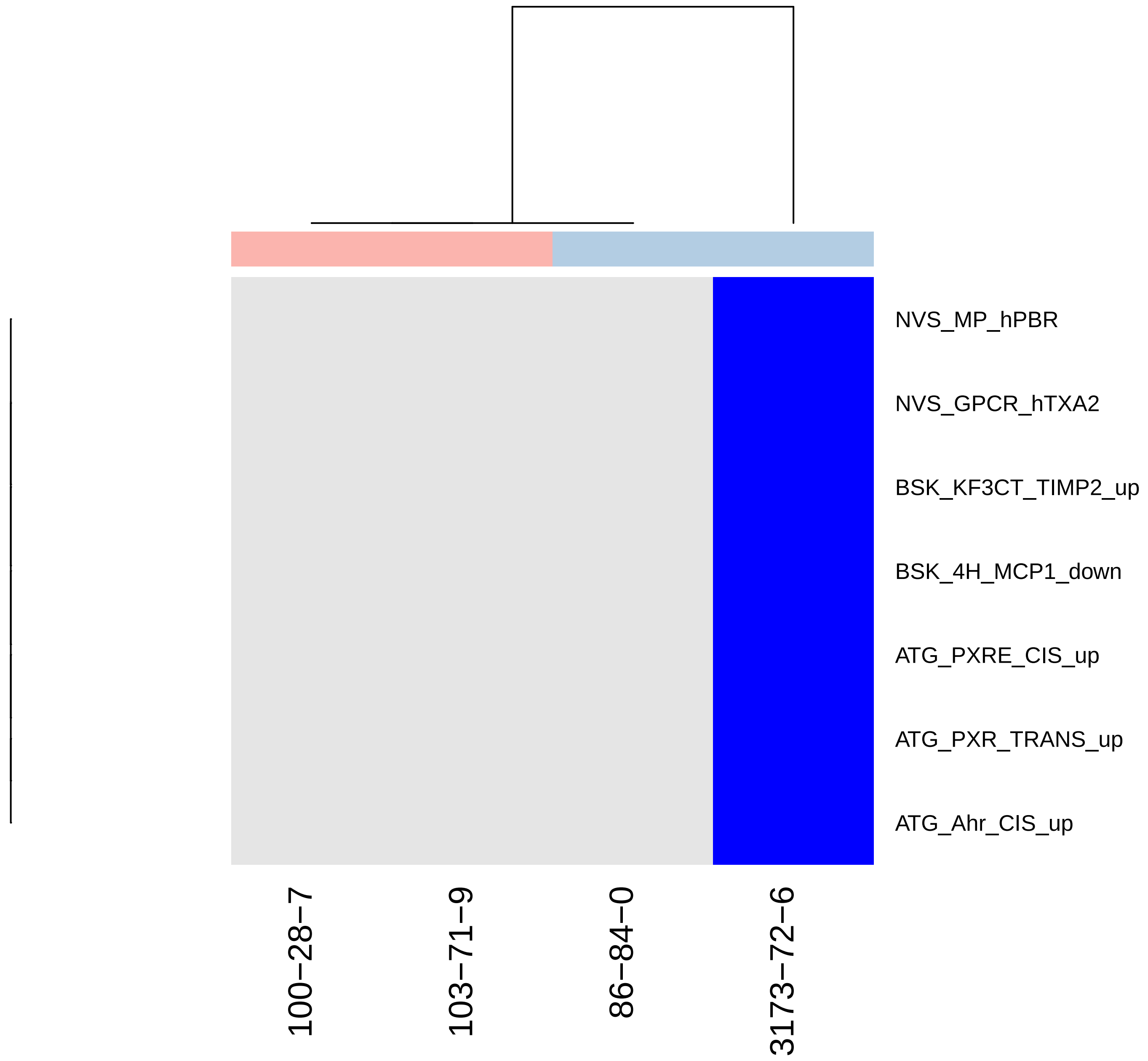

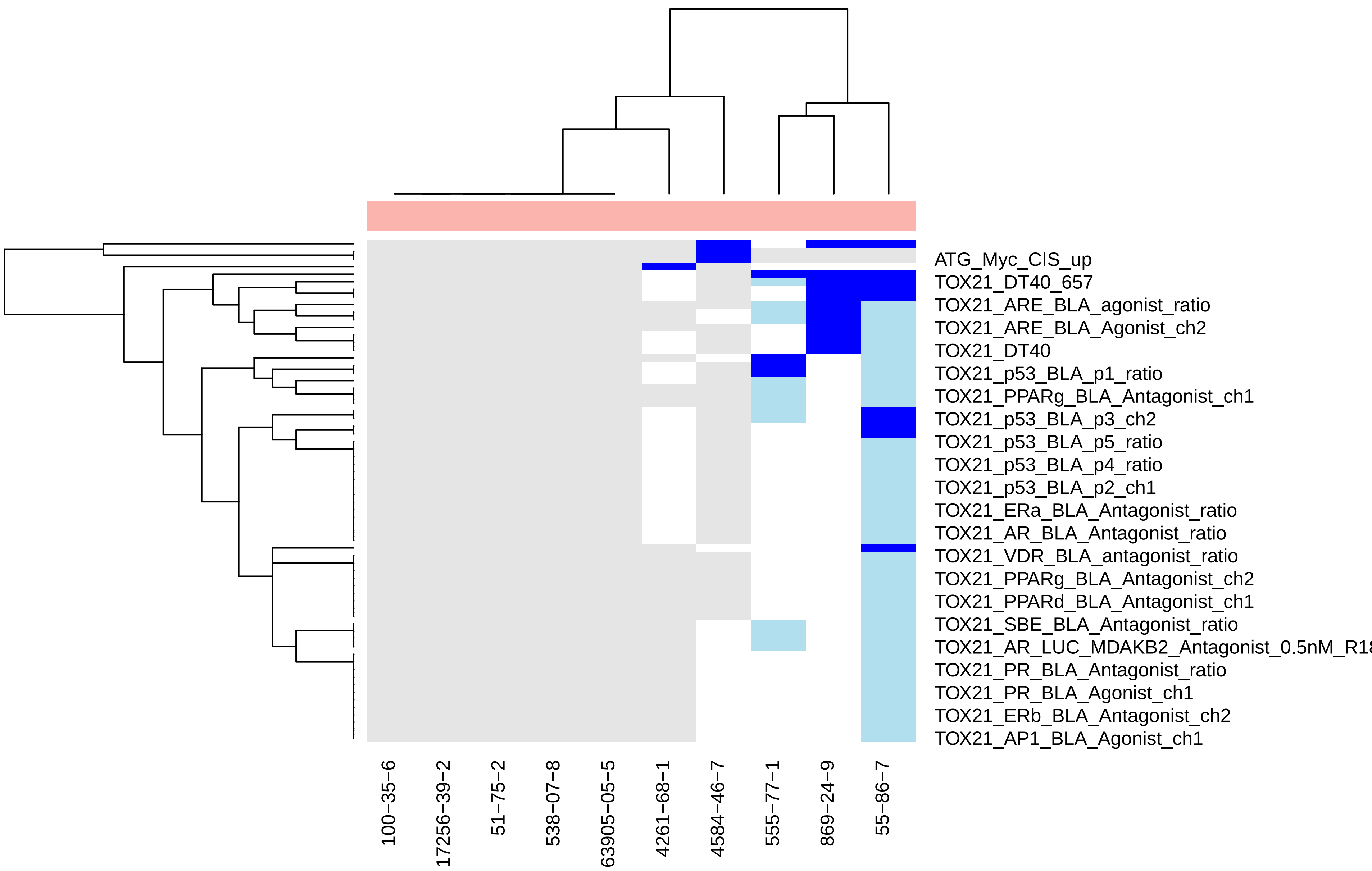

All Activity Heatmap for Cluster 13
ATG_Myc_CIS_up
TOX21_DT40_657
TOX21_ARE_BLA_agonist_ratio
TOX21_ARE_BLA_Agonist_ch2
TOX21_DT40
TOX21_p53_BLA_p1_ratio
TOX21_PPARg_BLA_Antagonist_ch1
TOX21_p53_BLA_p3_ch2
TOX21_p53_BLA_p5_ratio
TOX21_p53_BLA_p4_ratio
TOX21_p53_BLA_p2_ch1
TOX21_ERa_BLA_Antagonist_ratio
TOX21_AR_BLA_Antagonist_ratio
TOX21_VDR_BLA_antagonist_ratio
TOX21_PPARg_BLA_Antagonist_ch2
TOX21_PPARd_BLA_Antagonist_ch1
TOX21_SBE_BLA_Antagonist_ratio
TOX21_AR_LUC_MDAKB2_Antagonist_0.5nM_R18
TOX21_PR_BLA_Antagonist_ratio
TOX21_PR_BLA_Agonist_ch1
TOX21_ERb_BLA_Antagonist_ch2
TOX21_AP1_BLA_Agonist_ch1
100−35−6
17256−39−2
51−75−2
538−07−8
63905−05−5
4261−68−1
4584−46−7
555−77−1
869−24−9
55−86−7

All Activity Heatmap for Cluster 15

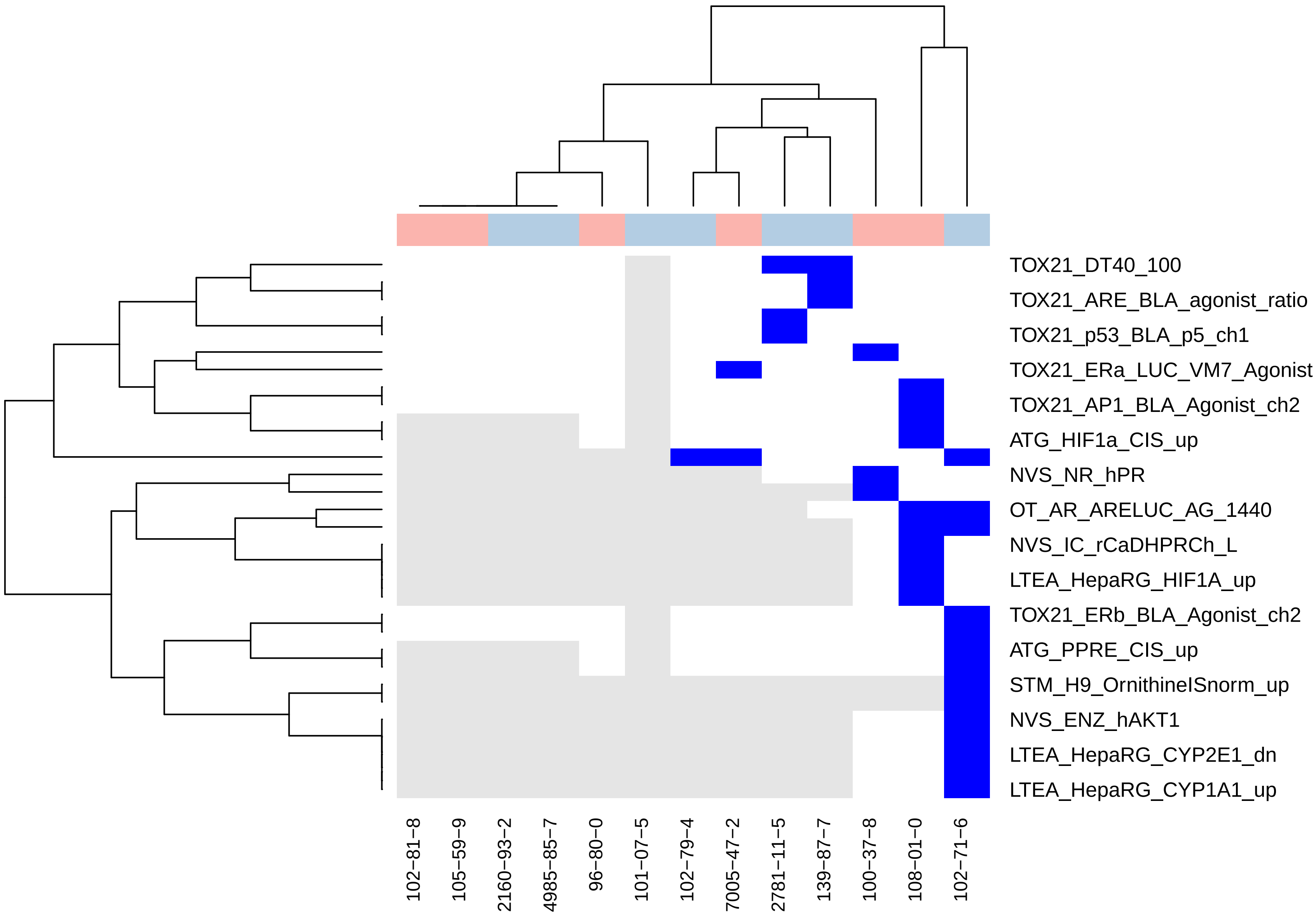

All Activity Heatmap for Cluster 16

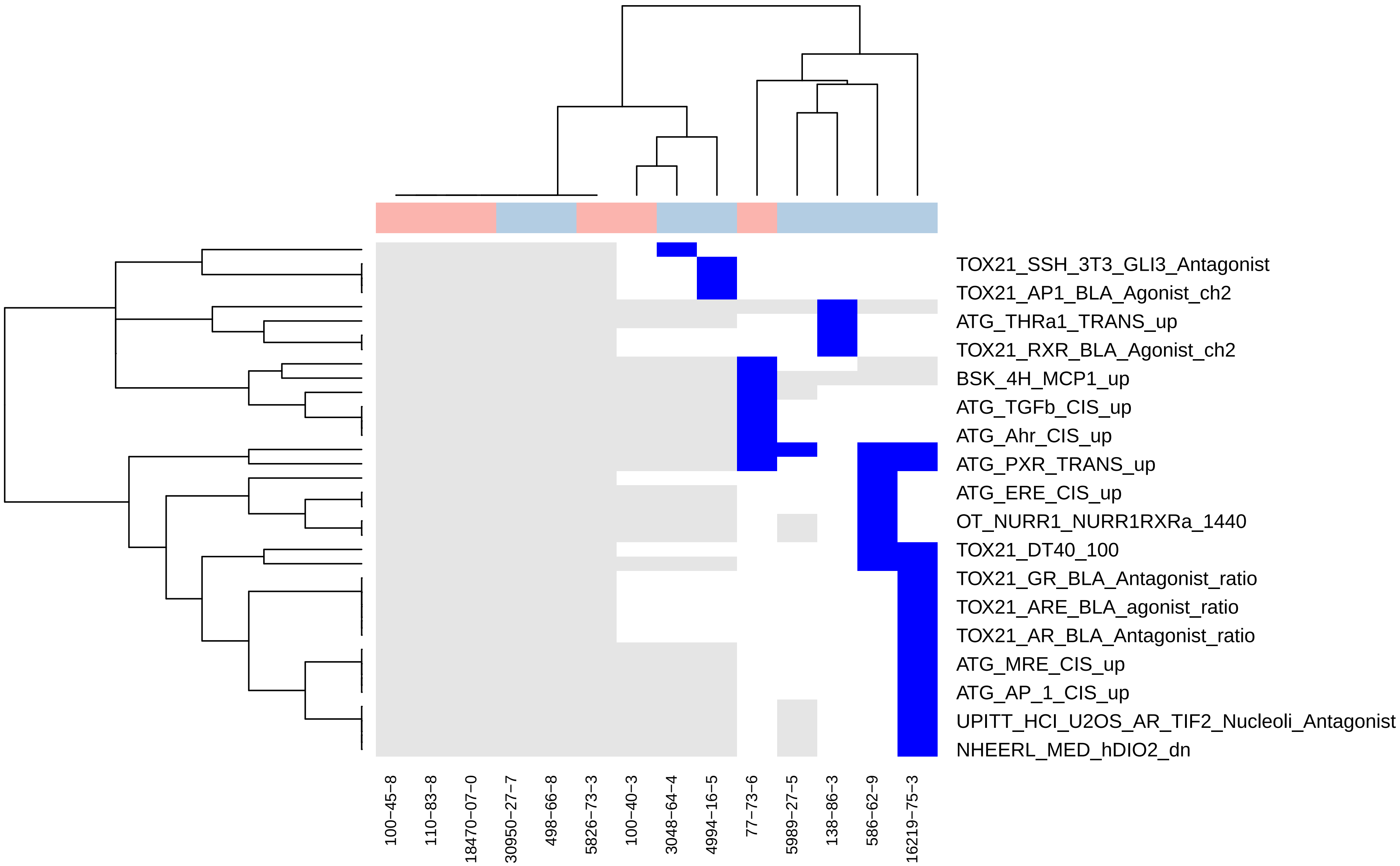

All Activity Heatmap for Cluster 17

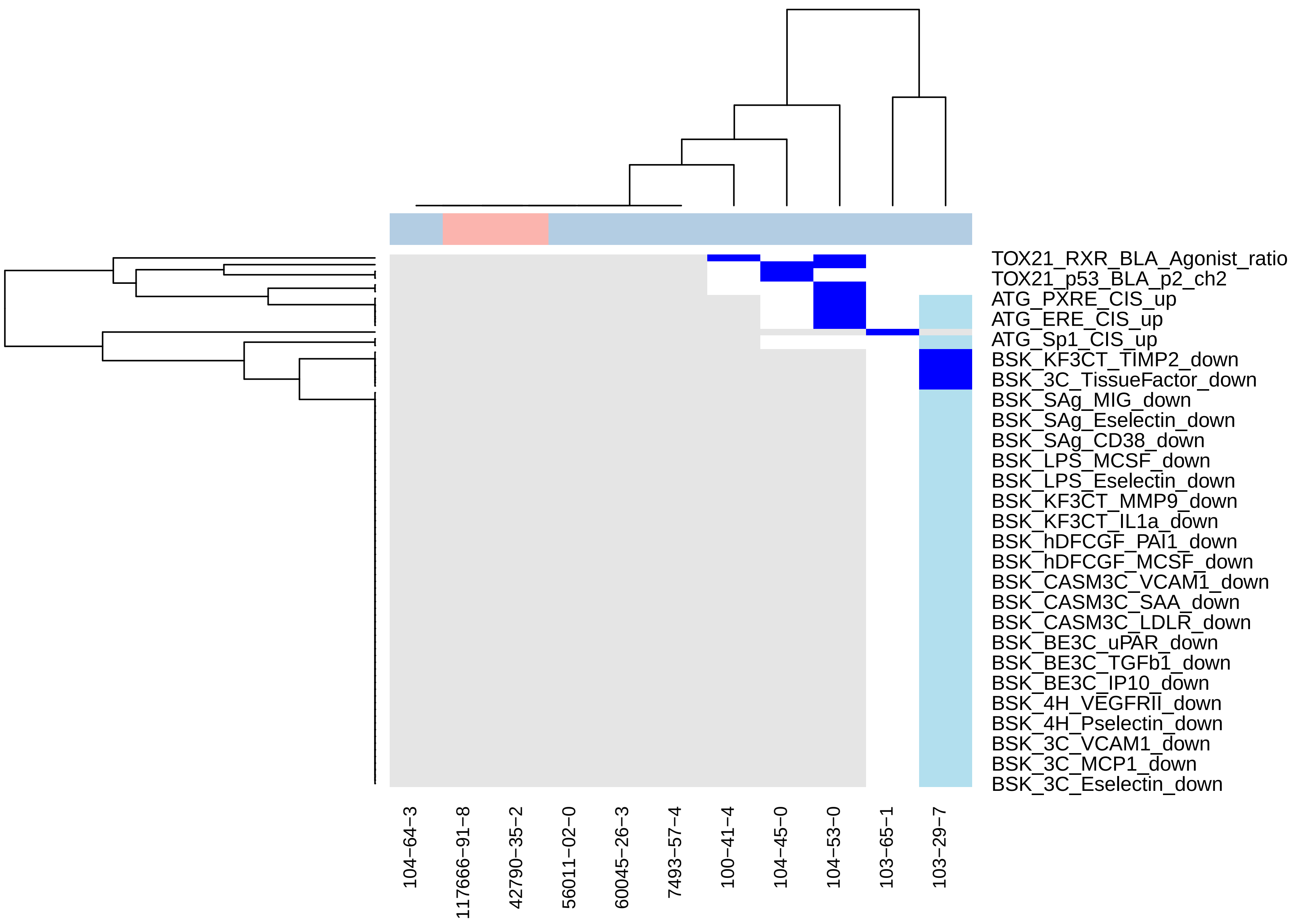

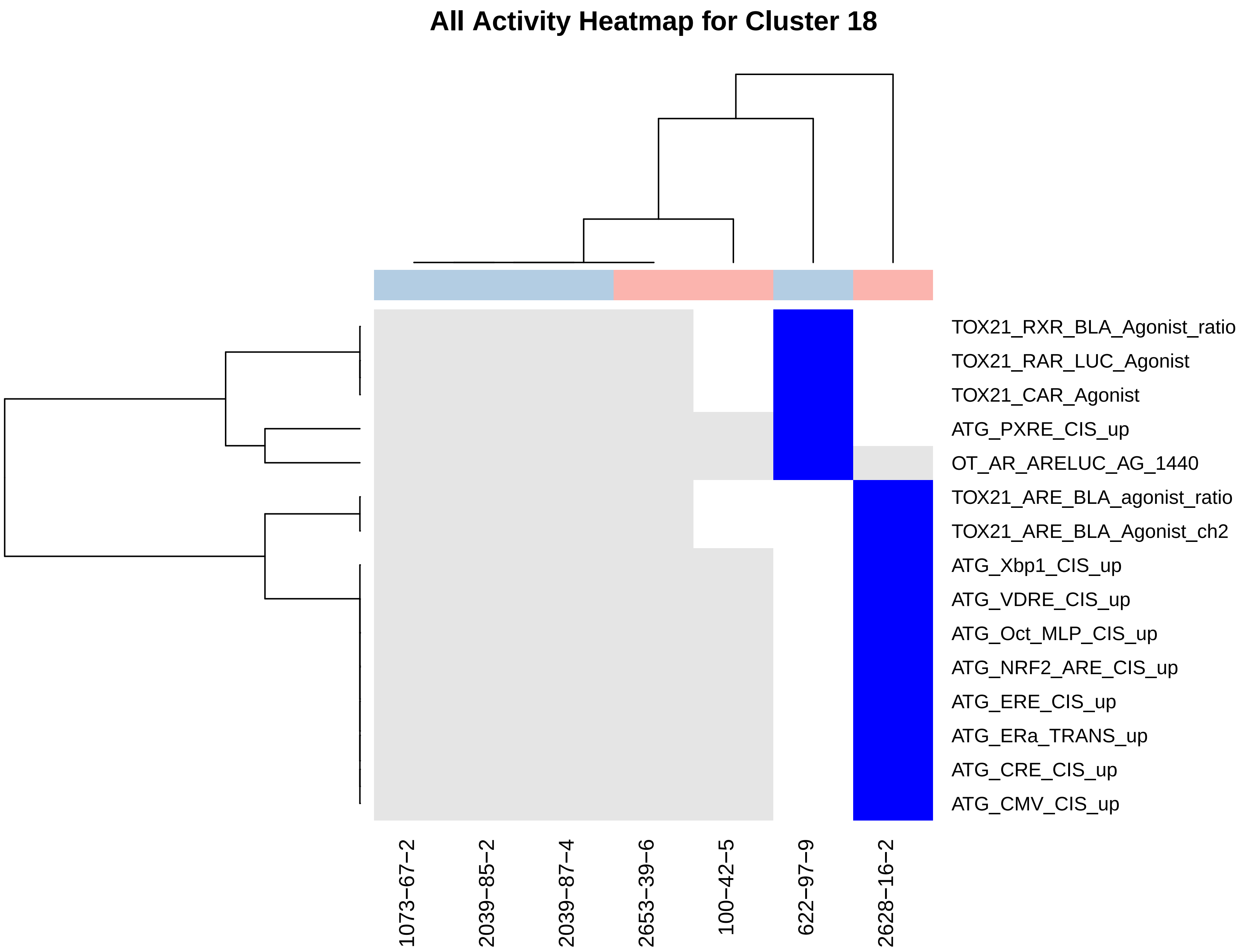
All Activity Heatmap for Cluster 18
TOX21_RXR_BLA_Agonist_ratio
TOX21_RAR_LUC_Agonist
TOX21_CAR_Agonist
ATG_PXRE_CIS_up
OT_AR_ARELUC_AG_1440
TOX21_ARE_BLA_agonist_ratio
TOX21_ARE_BLA_Agonist_ch2
ATG_Xbp1_CIS_up
ATG_VDRE_CIS_up
ATG_Oct_MLP_CIS_up
ATG_NRF2_ARE_CIS_up
ATG_ERE_CIS_up
ATG_ERa_TRANS_up
ATG_CRE_CIS_up
ATG_CMV_CIS_up
1073−67−2
2039−85−2
2039−87−4
2653−39−6
100−42−5
622−97−9
2628−16−2

All Activity Heatmap for Cluster 19

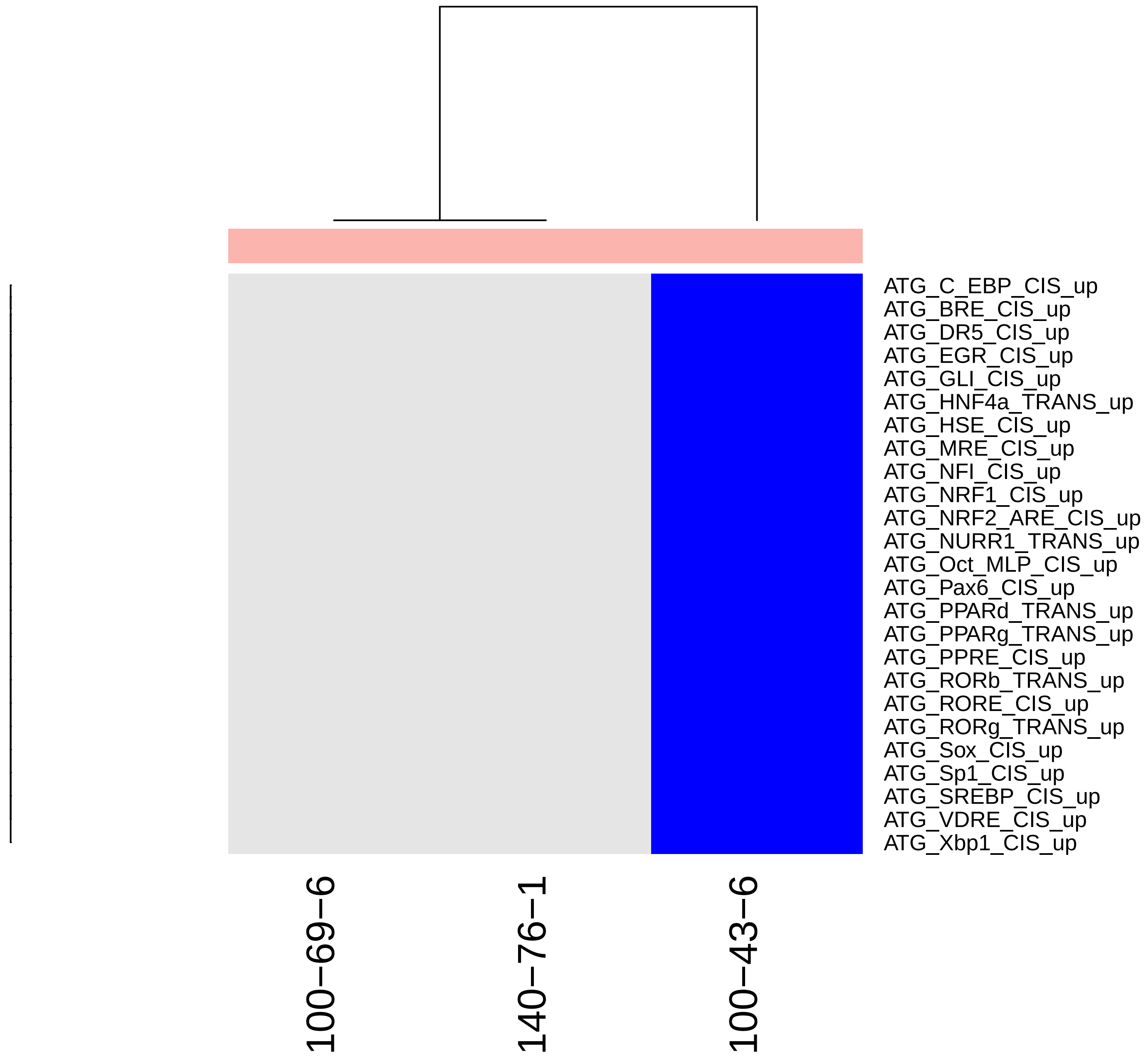

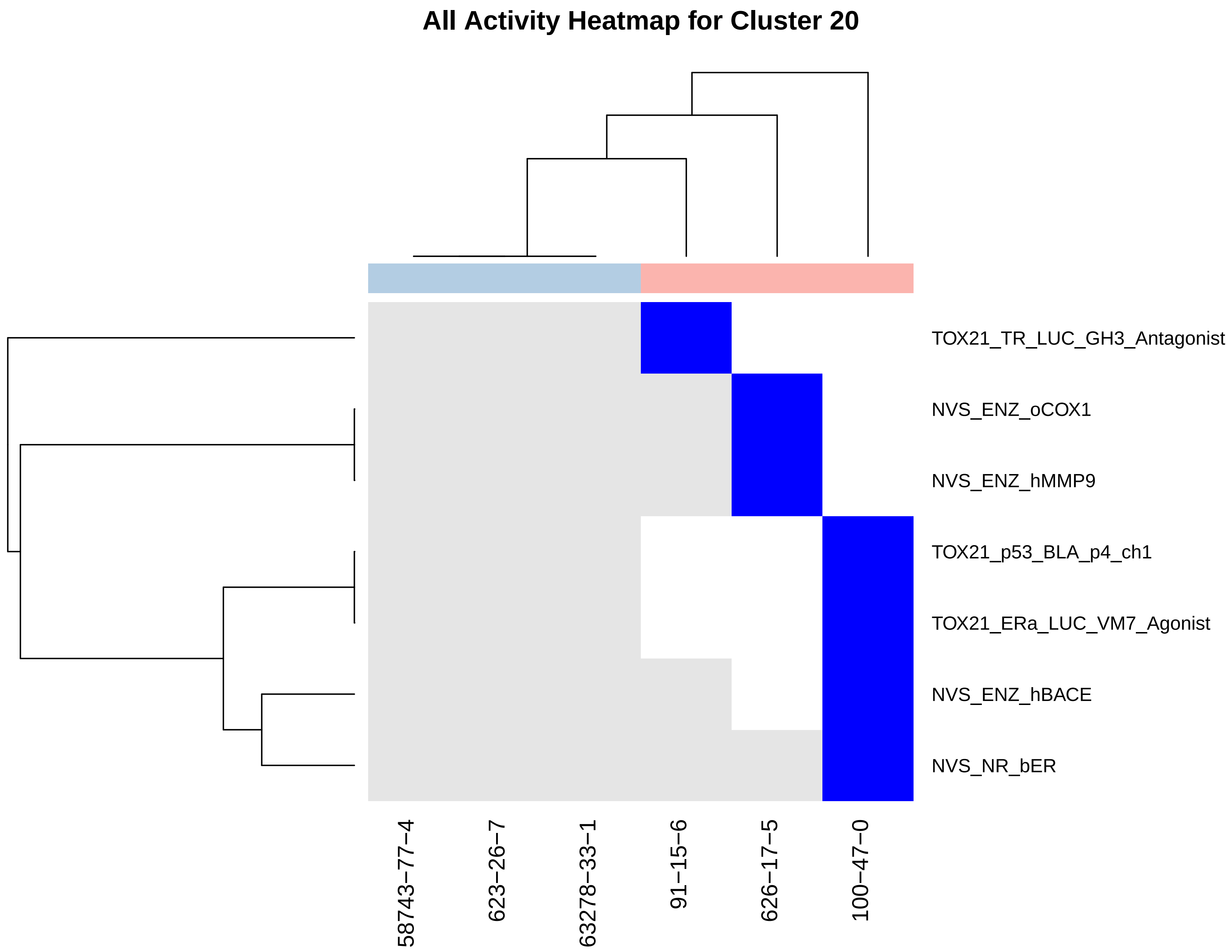
All Activity Heatmap for Cluster 20
TOX21_TR_LUC_GH3_Antagonist
NVS_ENZ_oCOX1
NVS_ENZ_hMMP9
TOX21_p53_BLA_p4_ch1
TOX21_ERa_LUC_VM7_Agonist
NVS_ENZ_hBACE
NVS_NR_bER
58743−77−4
623−26−7
63278−33−1
91−15−6
626−17−5
100−47−0

All Activity Heatmap for Cluster 21

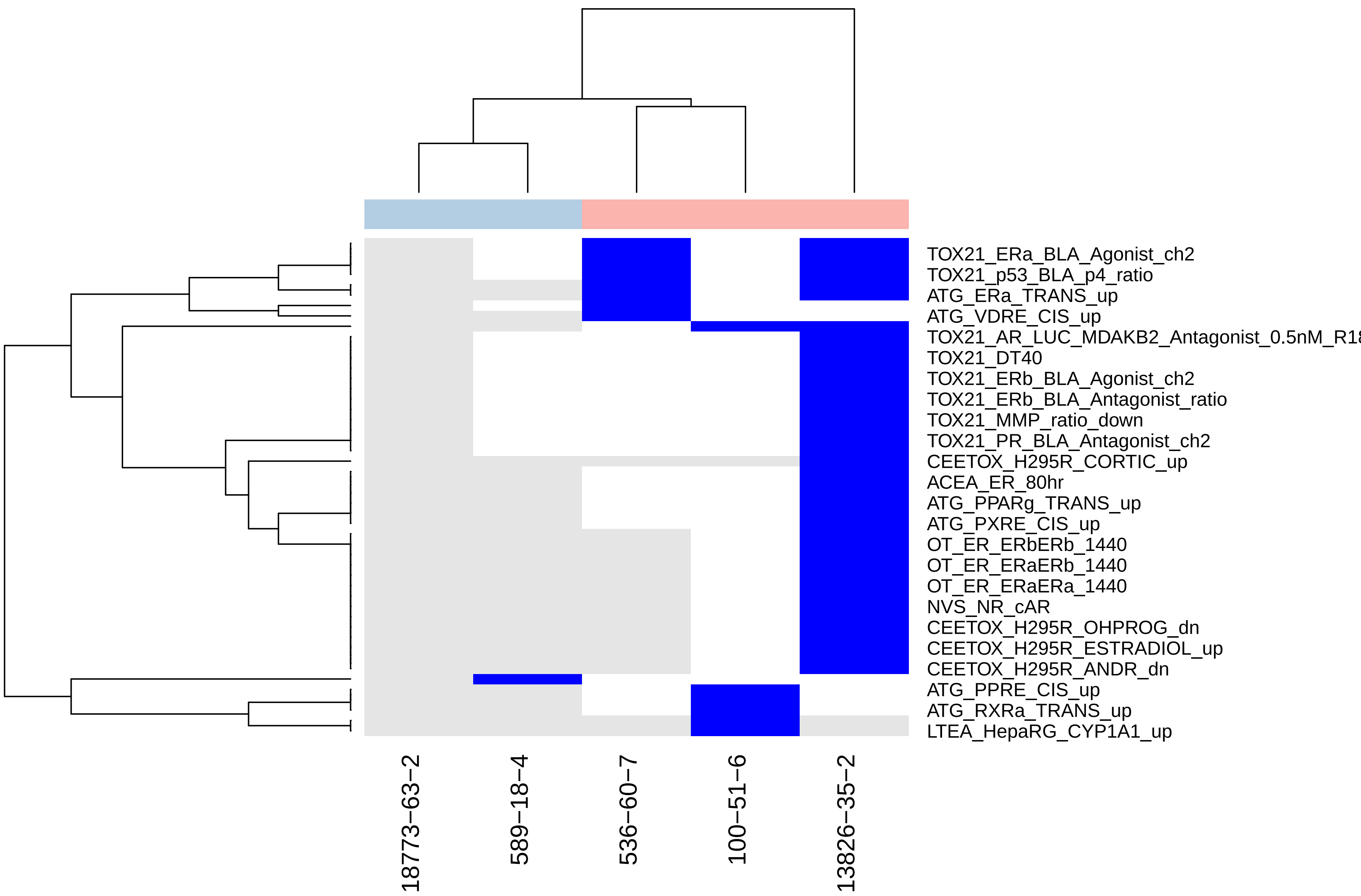

All Activity Heatmap for Cluster 22

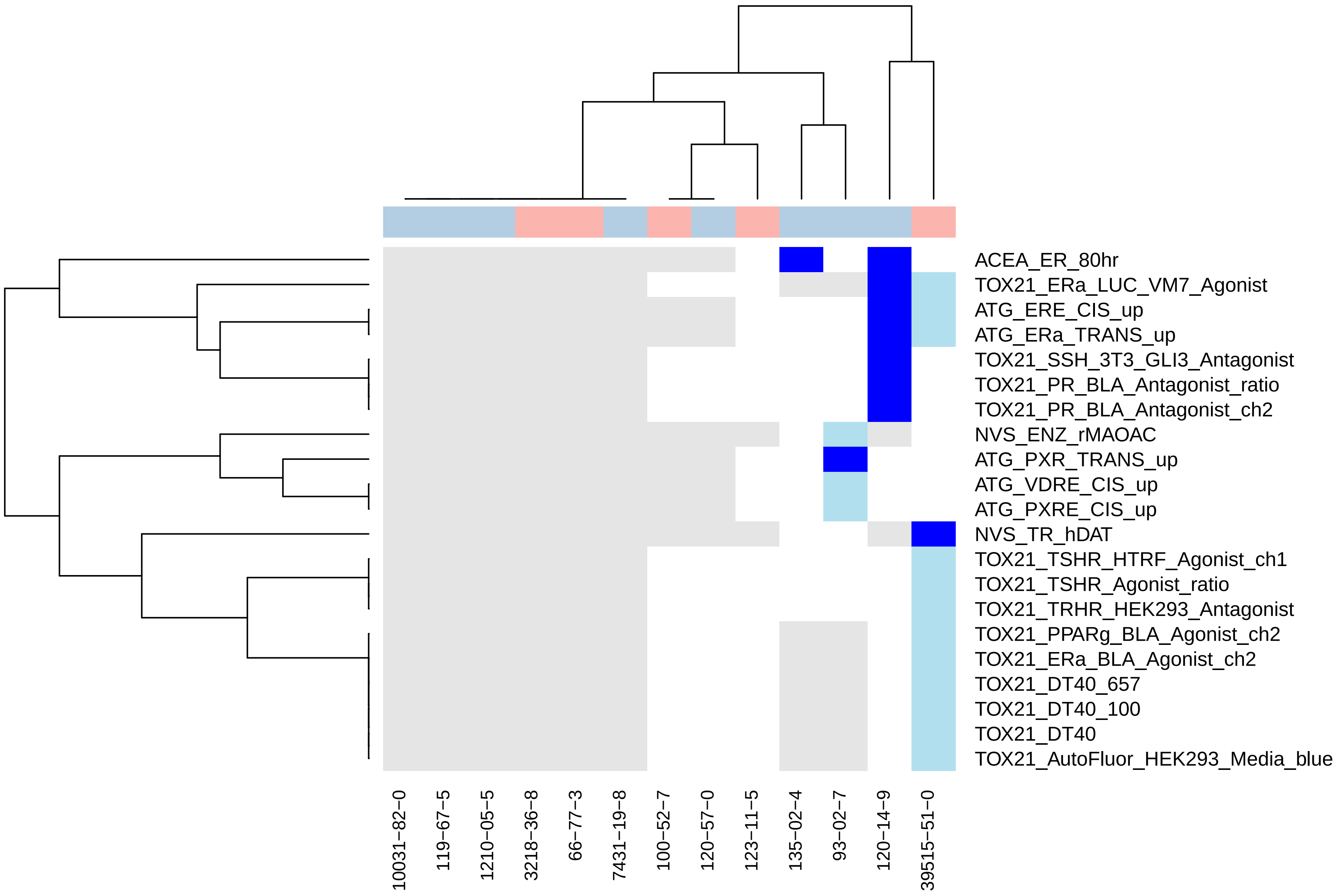

All Activity Heatmap for Cluster 23

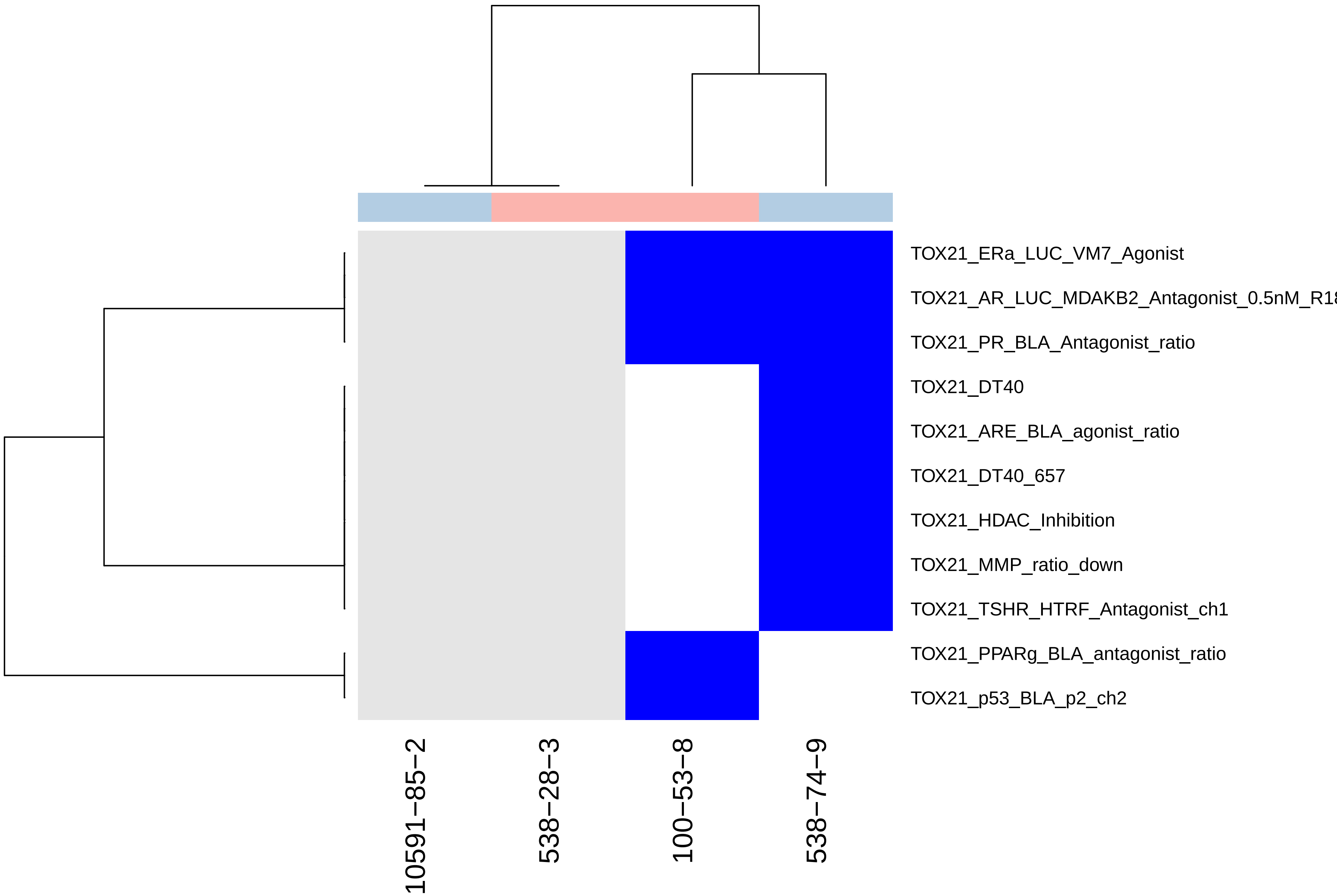

All Activity Heatmap for Cluster 24

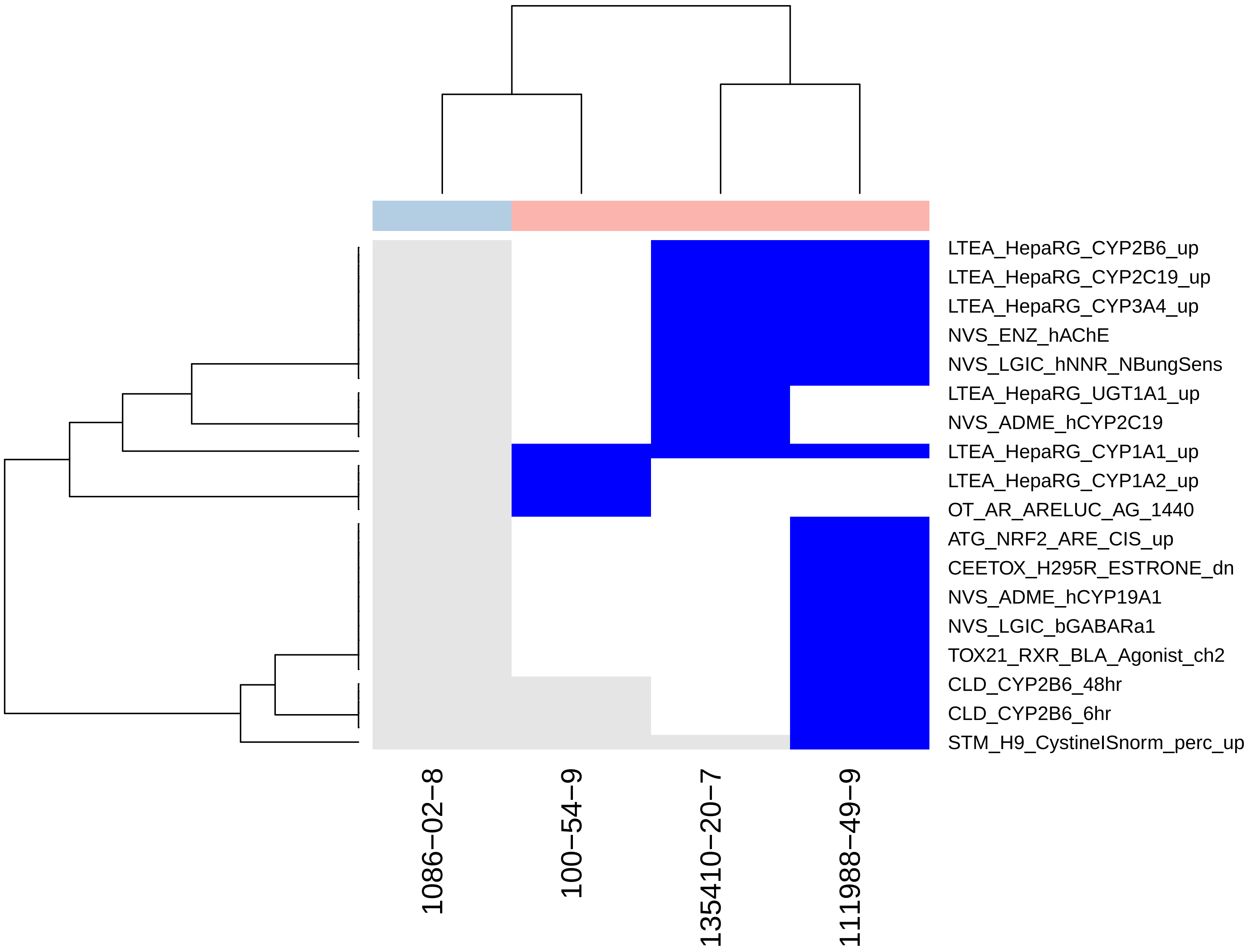

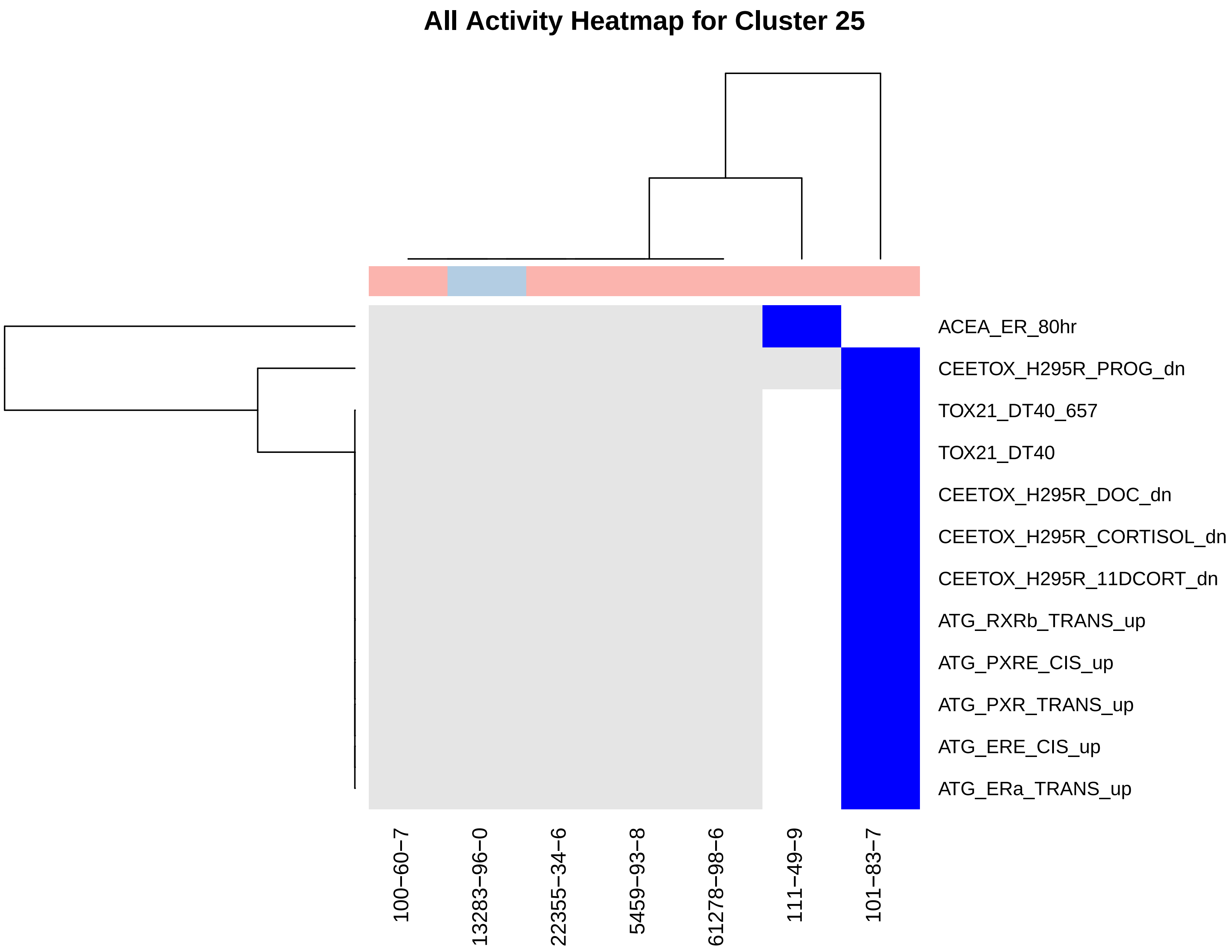
All Activity Heatmap for Cluster 25
ACEA_ER_80hr
CEETOX_H295R_PROG_dn
TOX21_DT40_657
TOX21_DT40
CEETOX_H295R_DOC_dn
CEETOX_H295R_CORTISOL_dn
CEETOX_H295R_11DCORT_dn
ATG_RXRb_TRANS_up
ATG_PXRE_CIS_up
ATG_PXR_TRANS_up
ATG_ERE_CIS_up
ATG_ERa_TRANS_up
100−60−7
13283−96−0
22355−34−6
5459−93−8
61278−98−6
111−49−9
101−83−7

All Activity Heatmap for Cluster 26

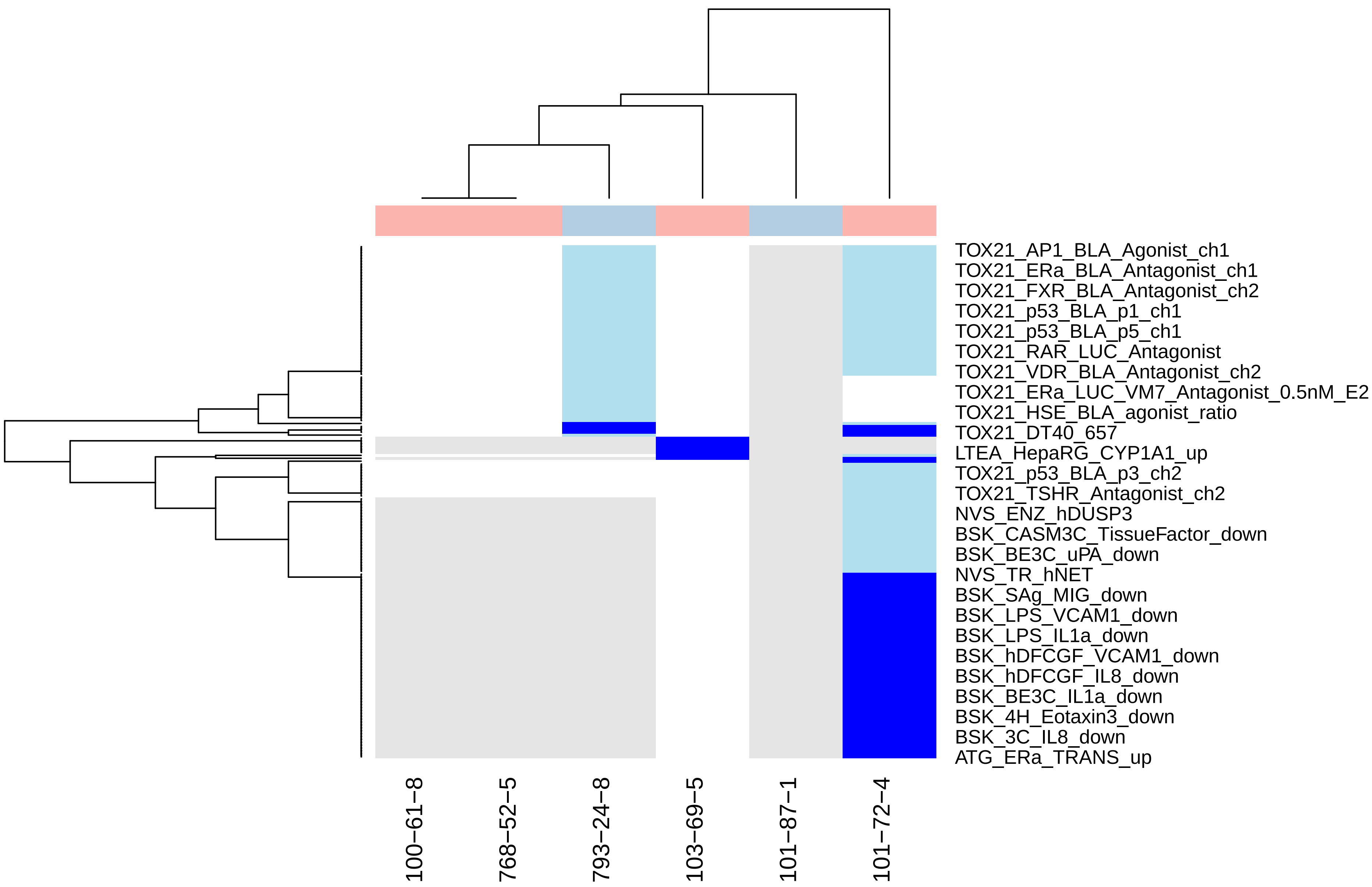

All Activity Heatmap for Cluster 27

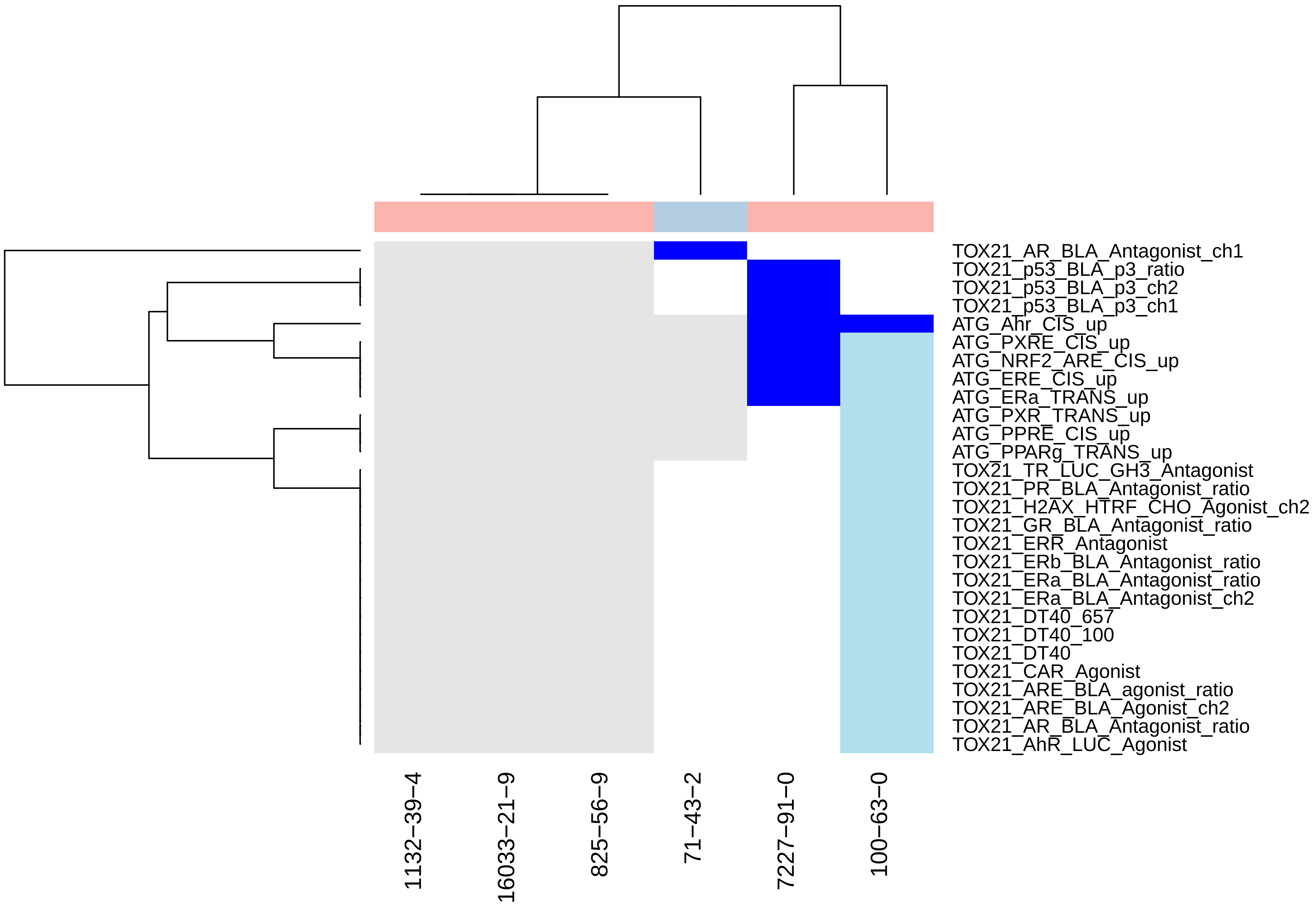

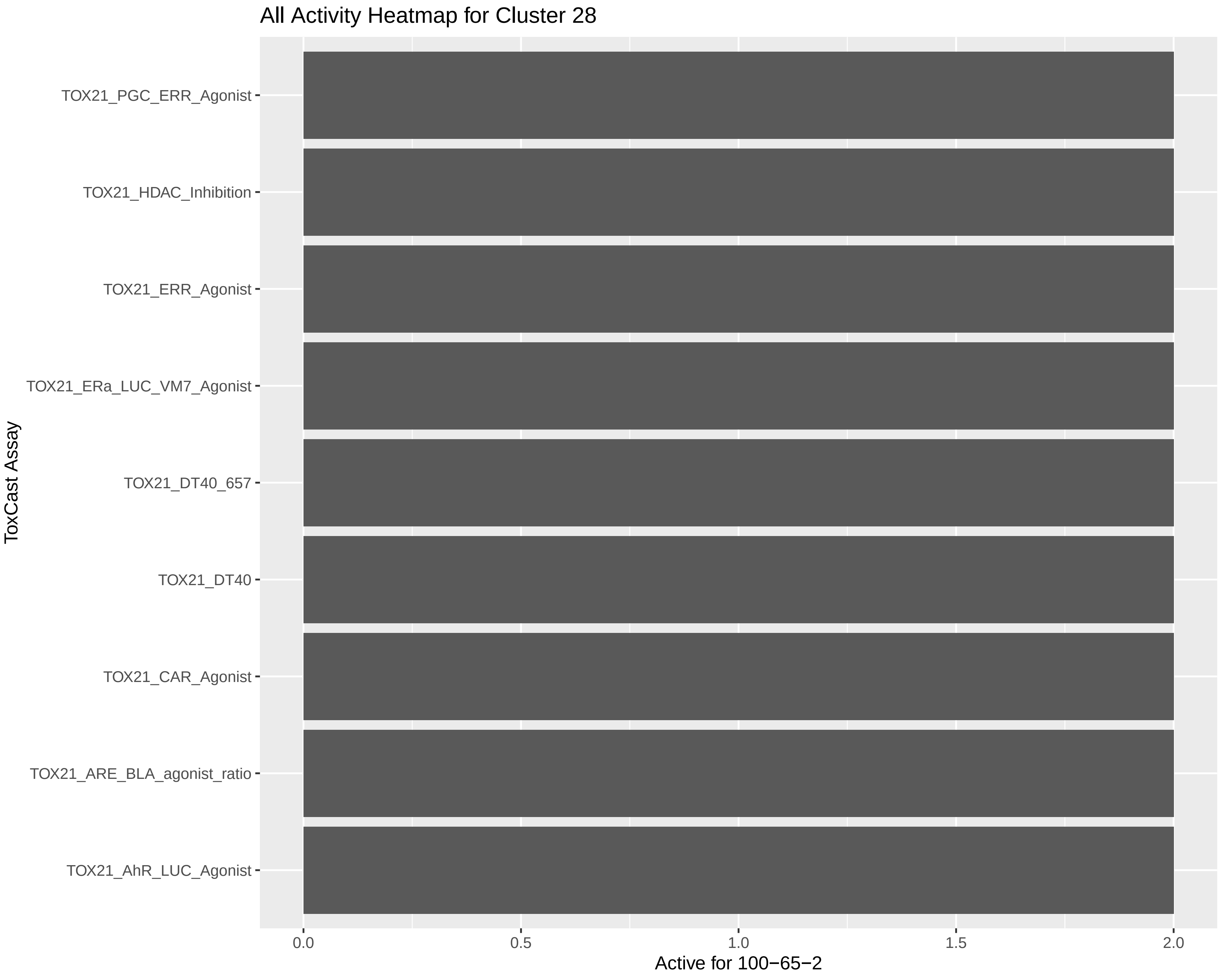
All Activity Heatmap for Cluster 28
ToxCast Assay
TOX21_PGC_ERR_Agonist
TOX21_HDAC_Inhibition
TOX21_ERR_Agonist
TOX21_ERa_LUC_VM7_Agonist
TOX21_DT40_657
TOX21_DT40
TOX21_CAR_Agonist
TOX21_ARE_BLA_agonist_ratio
TOX21_AhR_LUC_Agonist
0.0
0.5
1.0
1.5
2.0
Active for 100−65−2

All Activity Heatmap for Cluster 31

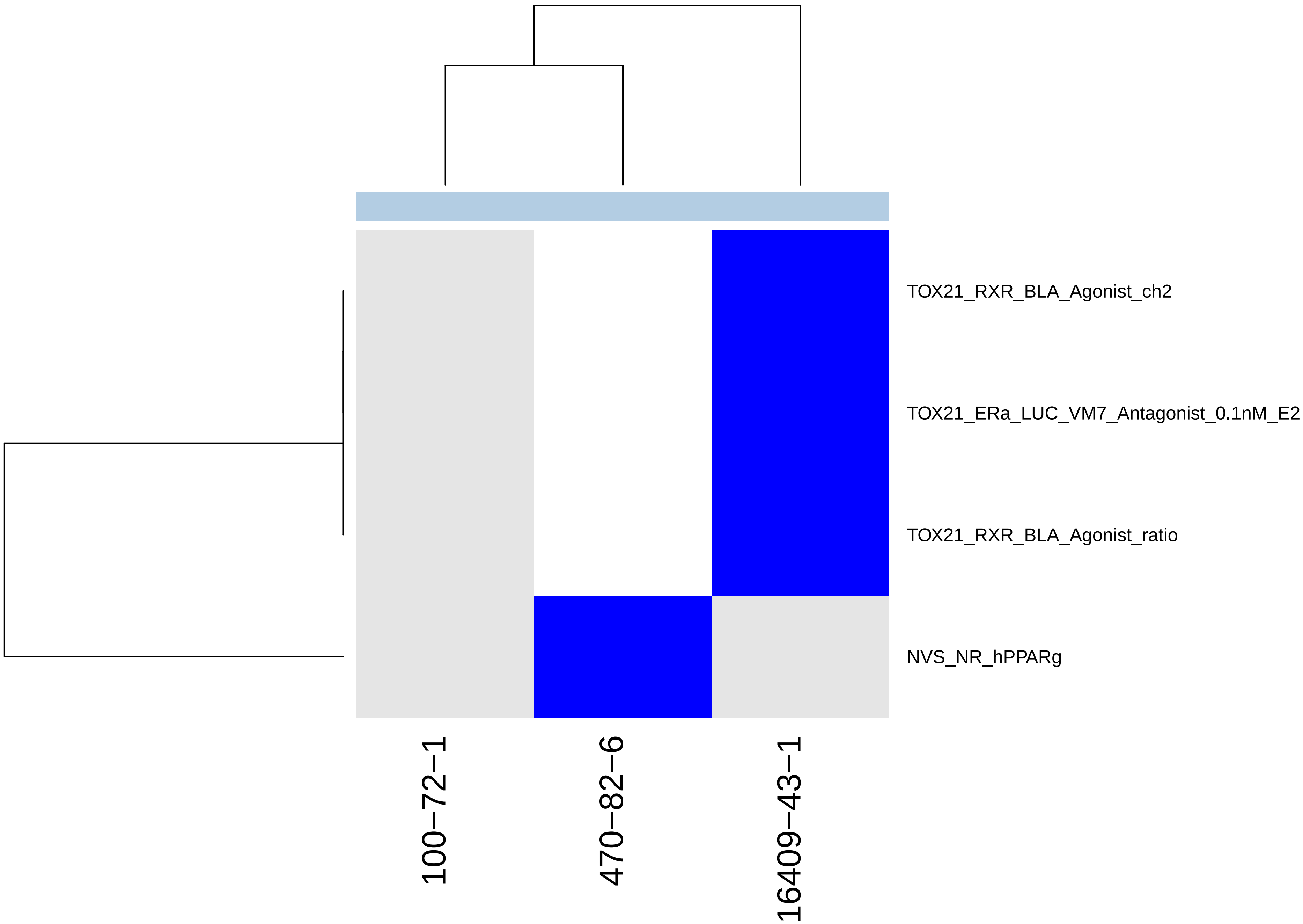

All Activity Heatmap for Cluster 32

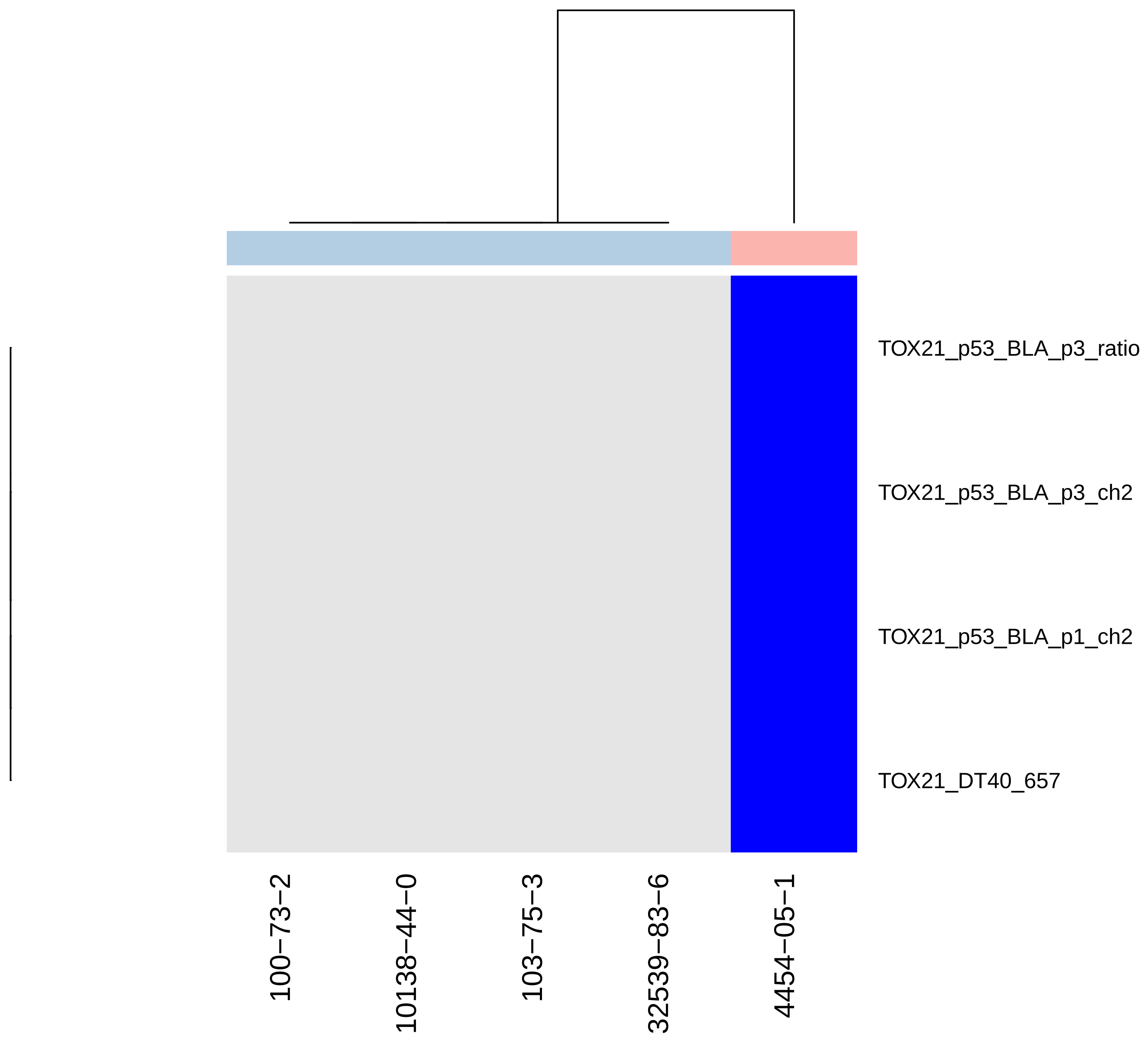

All Activity Heatmap for Cluster 33

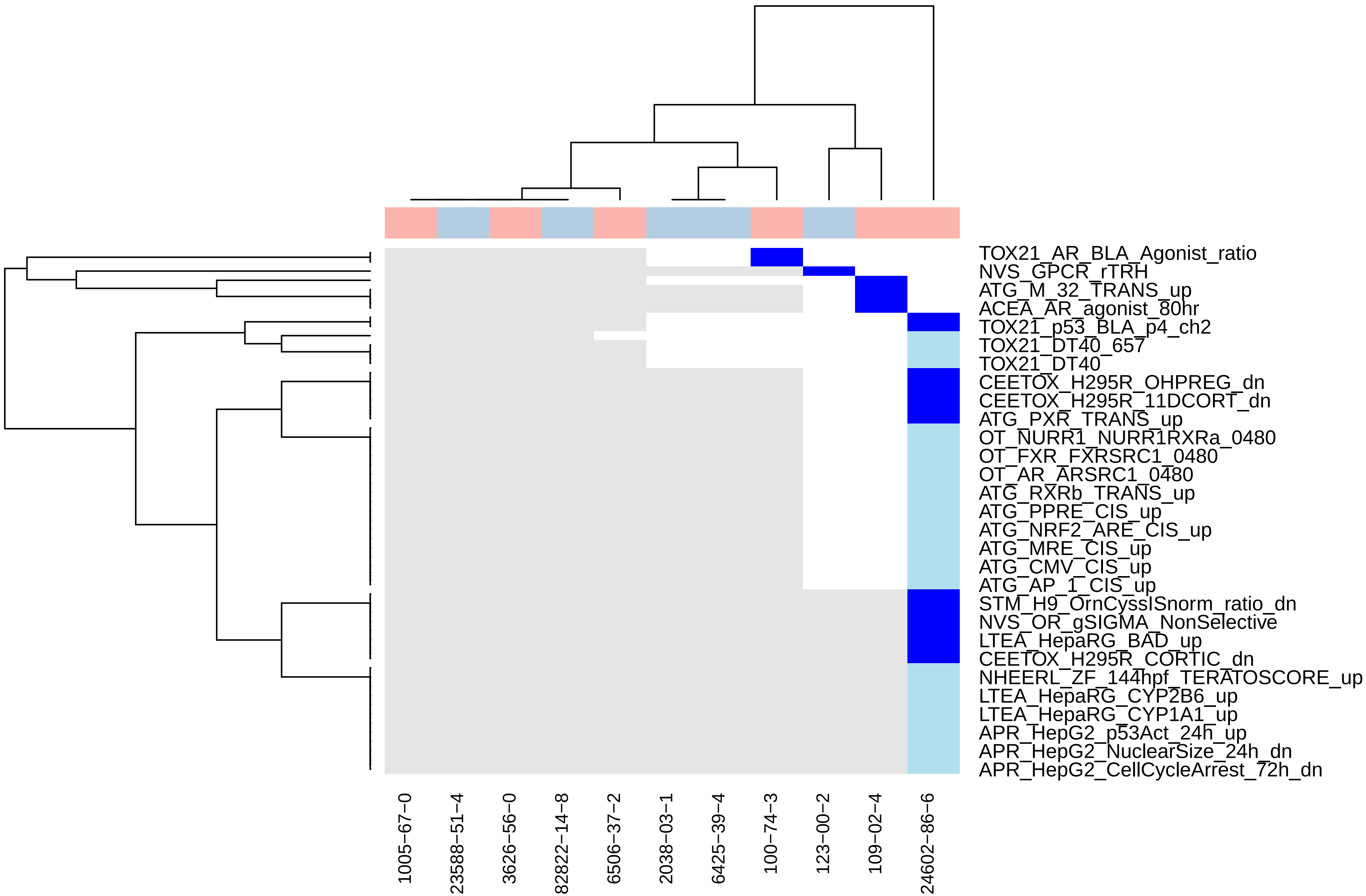

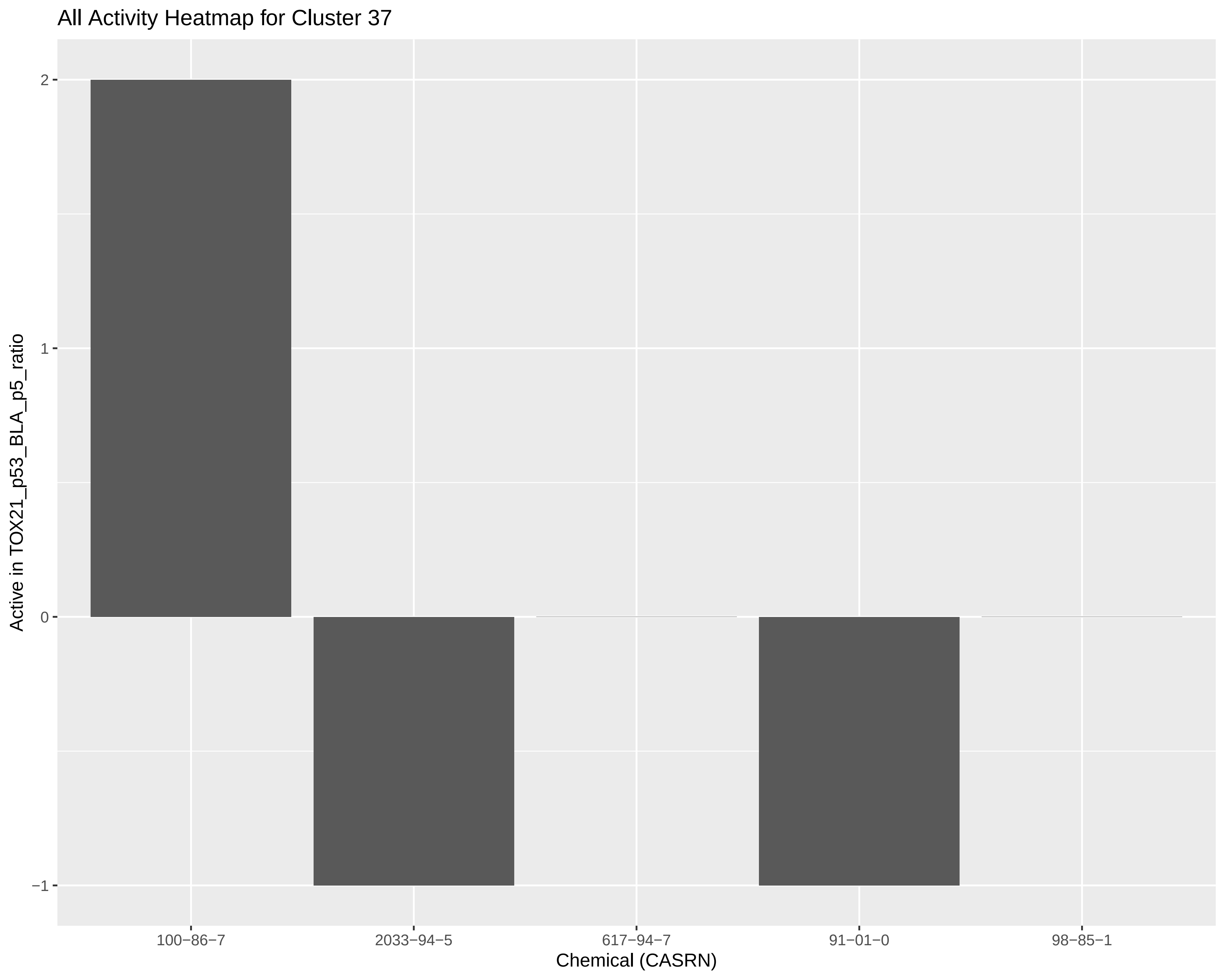

All Activity Heatmap for Cluster 37
Active in TOX21_p53_BLA_p5_ratio
2
1
0
−1
100−86−7
2033−94−5
617−94−7
91−01−0
98−85−1
Chemical (CASRN)

All Activity Heatmap for Cluster 38

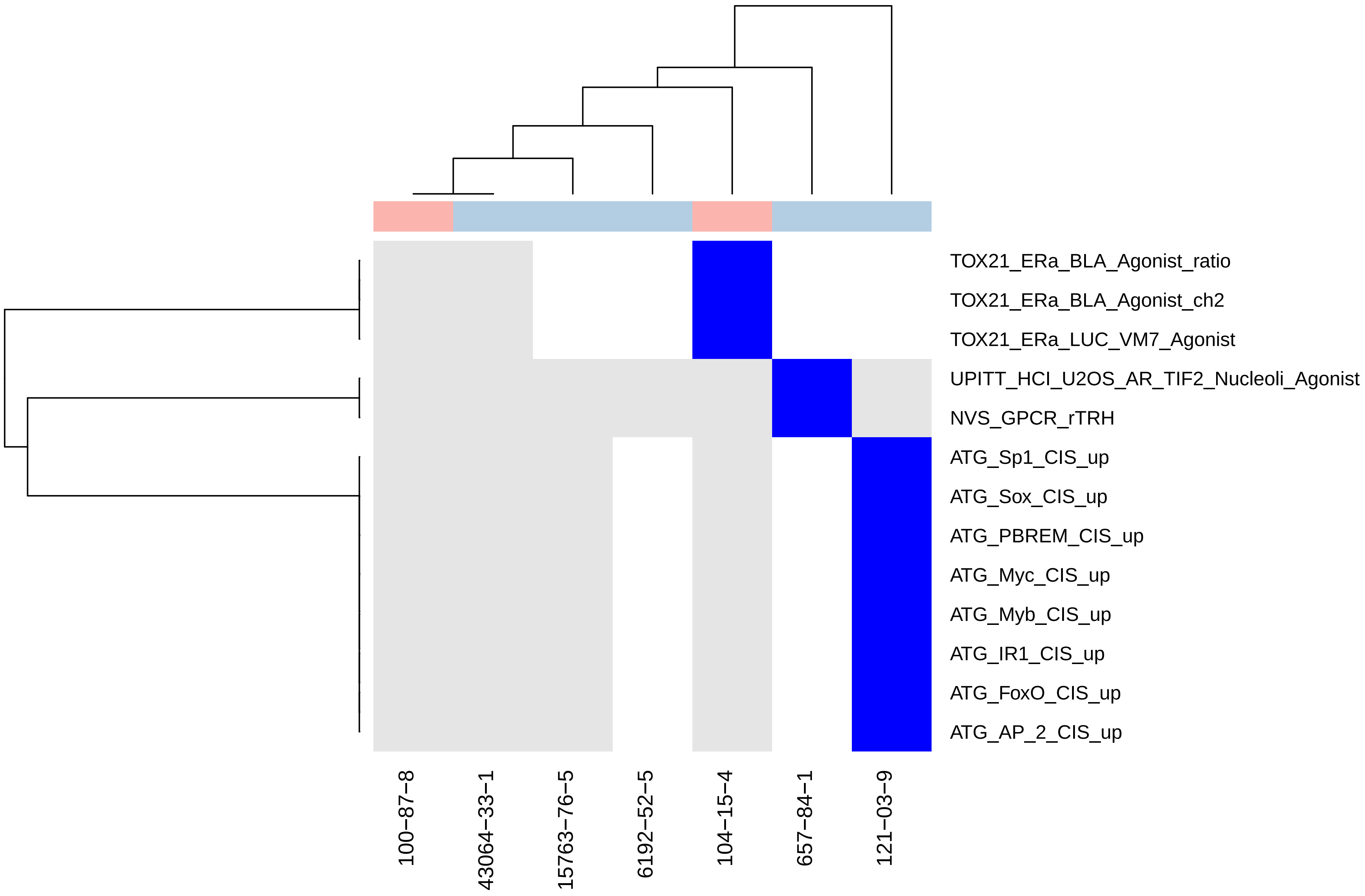

All Activity Heatmap for Cluster 39

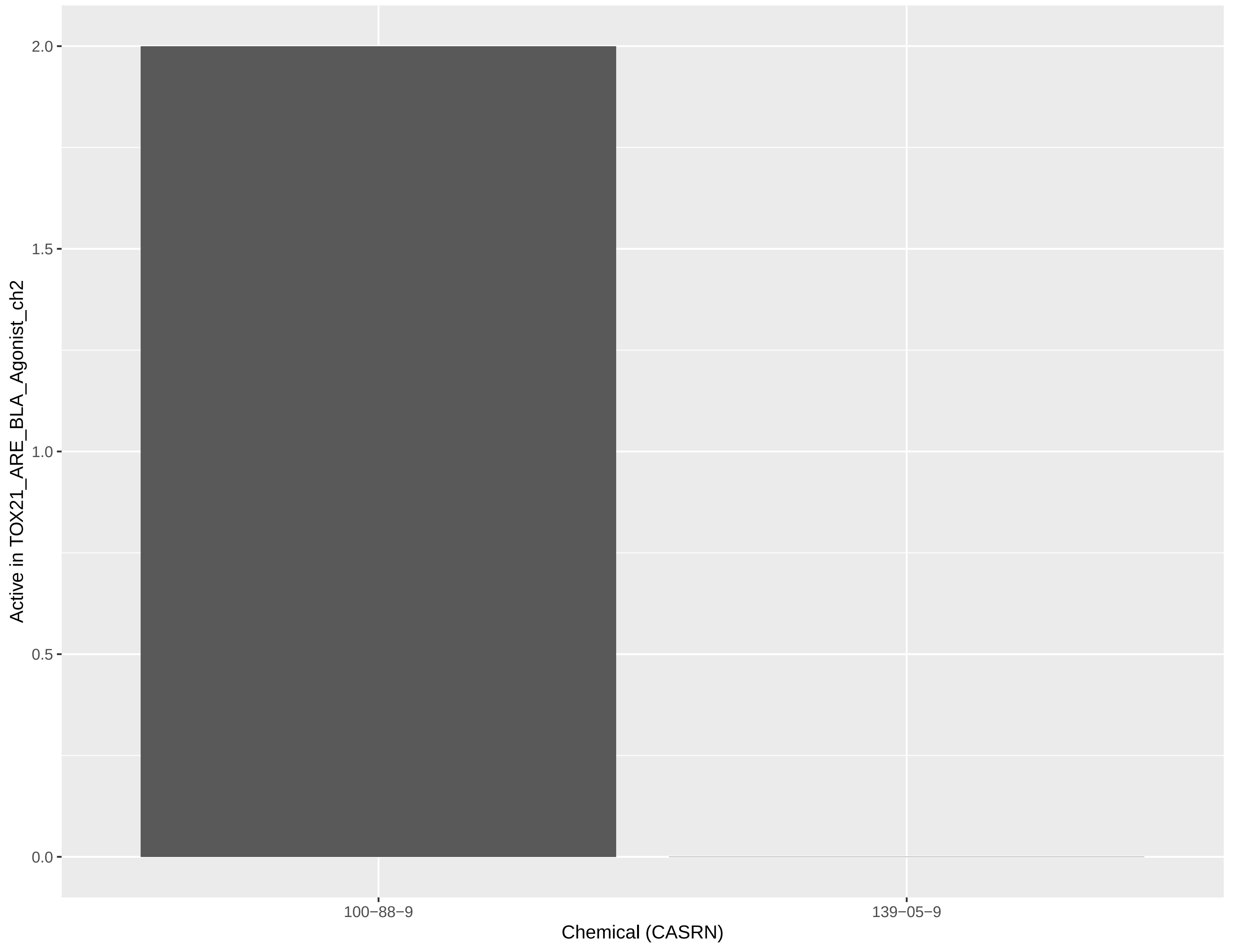

All Activity Heatmap for Cluster 41

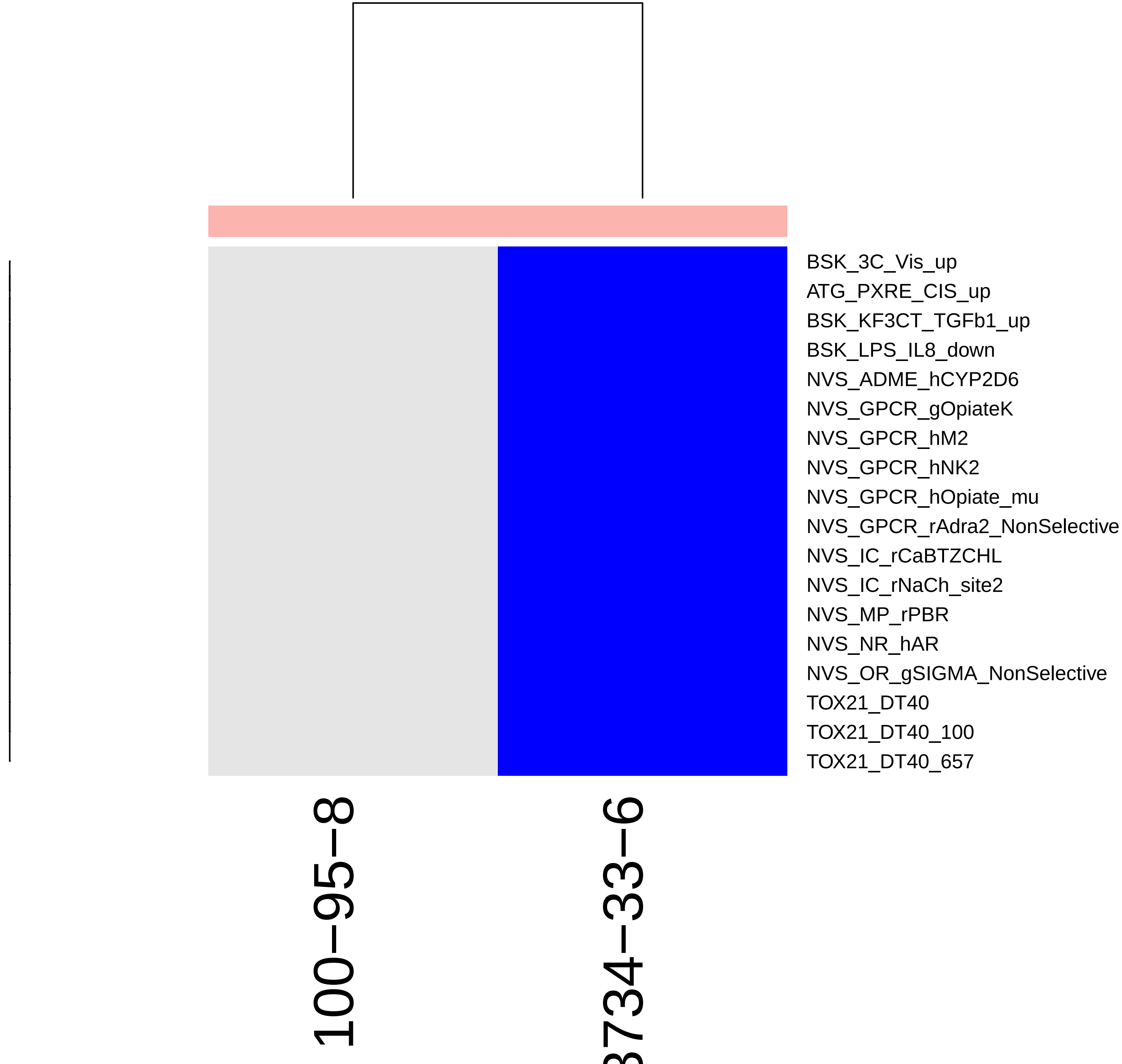

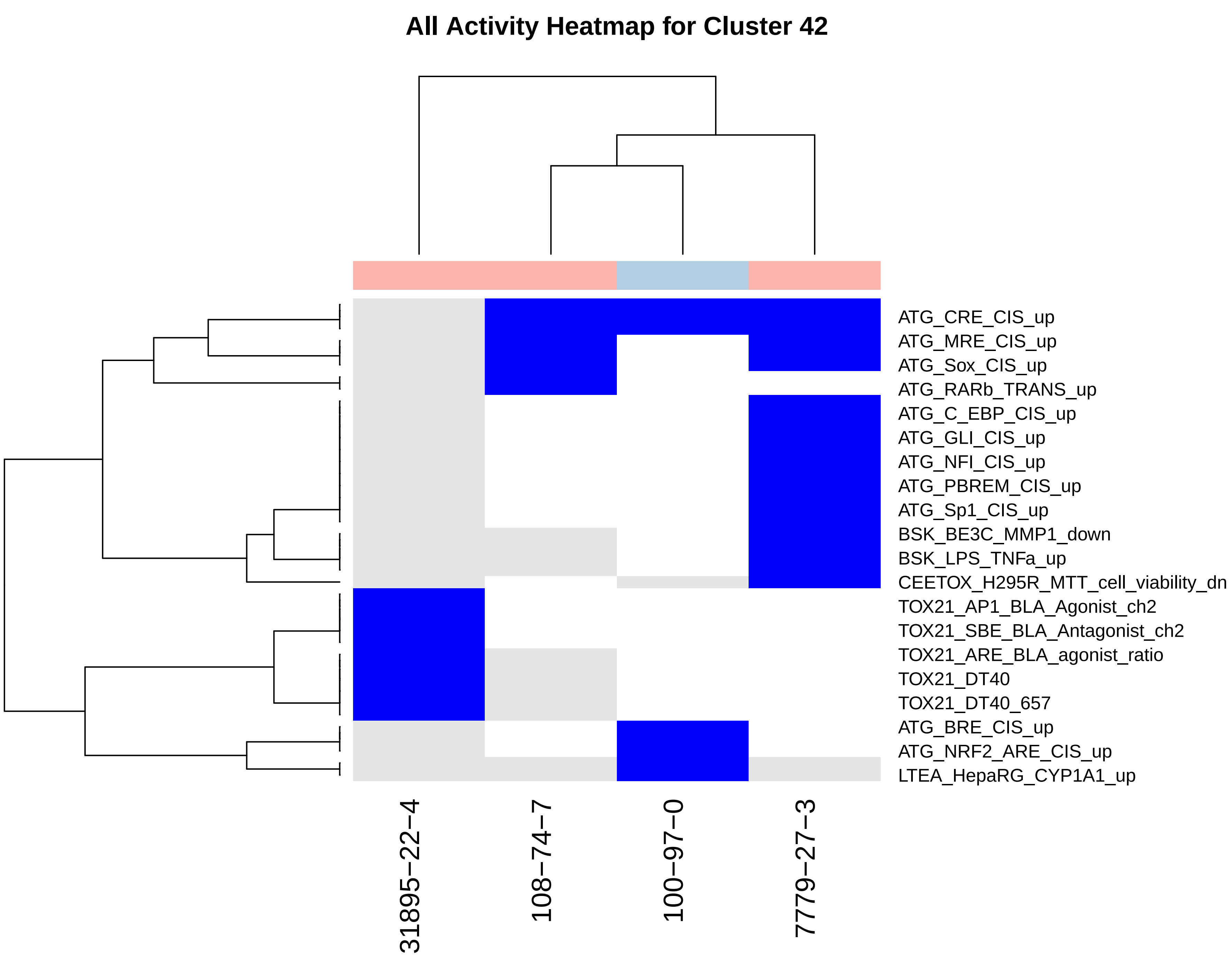
All Activity Heatmap for Cluster 42
ATG_CRE_CIS_up
ATG_MRE_CIS_up
ATG_Sox_CIS_up
ATG_RARb_TRANS_up
ATG_C_EBP_CIS_up
ATG_GLI_CIS_up
ATG_NFI_CIS_up
ATG_PBREM_CIS_up
ATG_Sp1_CIS_up
BSK_BE3C_MMP1_down
BSK_LPS_TNFa_up
CEETOX_H295R_MTT_cell_viability_dn
TOX21_AP1_BLA_Agonist_ch2
TOX21_SBE_BLA_Antagonist_ch2
TOX21_ARE_BLA_agonist_ratio
TOX21_DT40
TOX21_DT40_657
ATG_BRE_CIS_up
ATG_NRF2_ARE_CIS_up
LTEA_HepaRG_CYP1A1_up
31895−22−4
108−74−7
100−97−0
7779−27−3

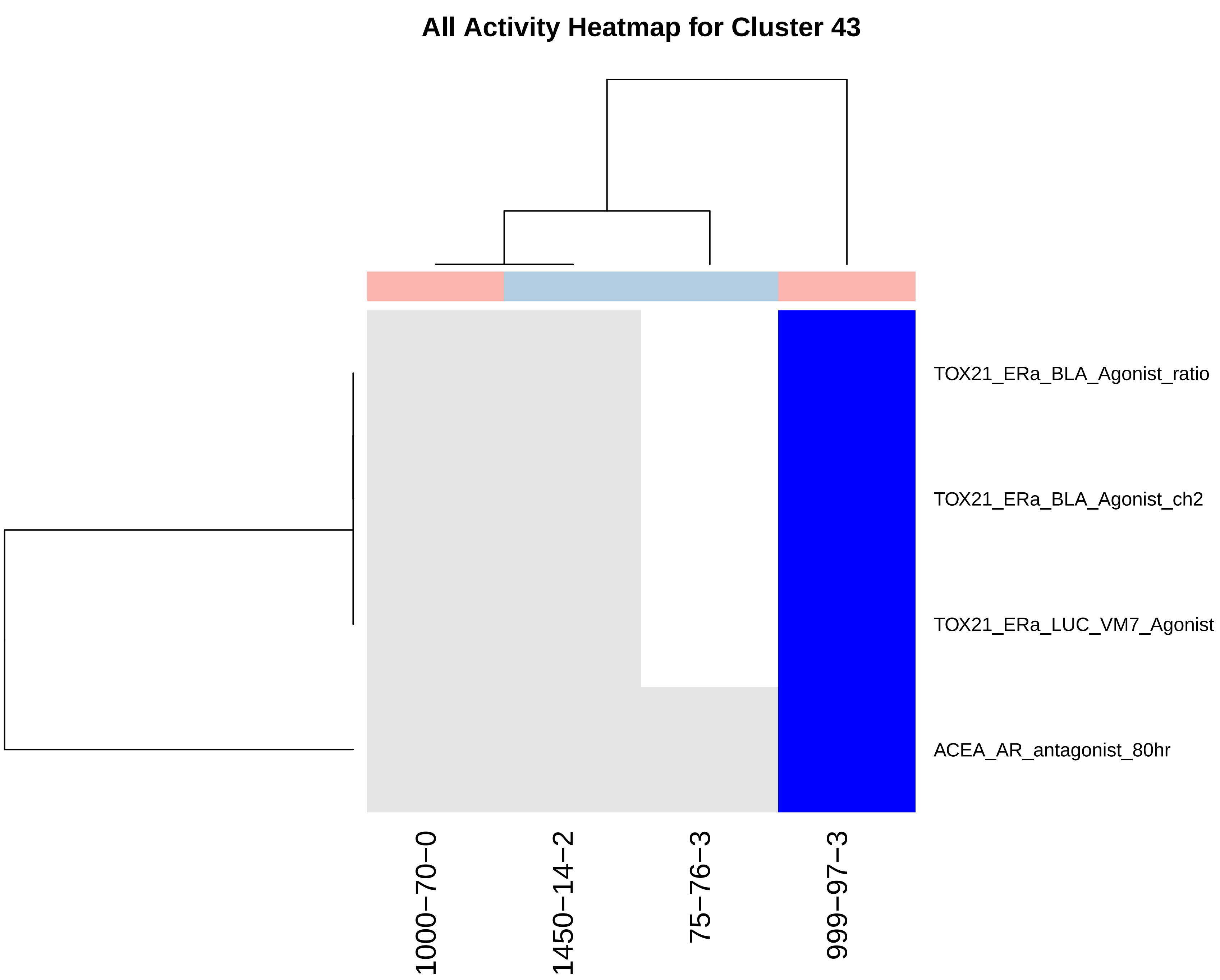
All Activity Heatmap for Cluster 43
TOX21_ERa_BLA_Agonist_ratio
TOX21_ERa_BLA_Agonist_ch2
TOX21_ERa_LUC_VM7_Agonist
ACEA_AR_antagonist_80hr
1000−70−0
1450−14−2
75−76−3
999−97−3

All Activity Heatmap for Cluster 44

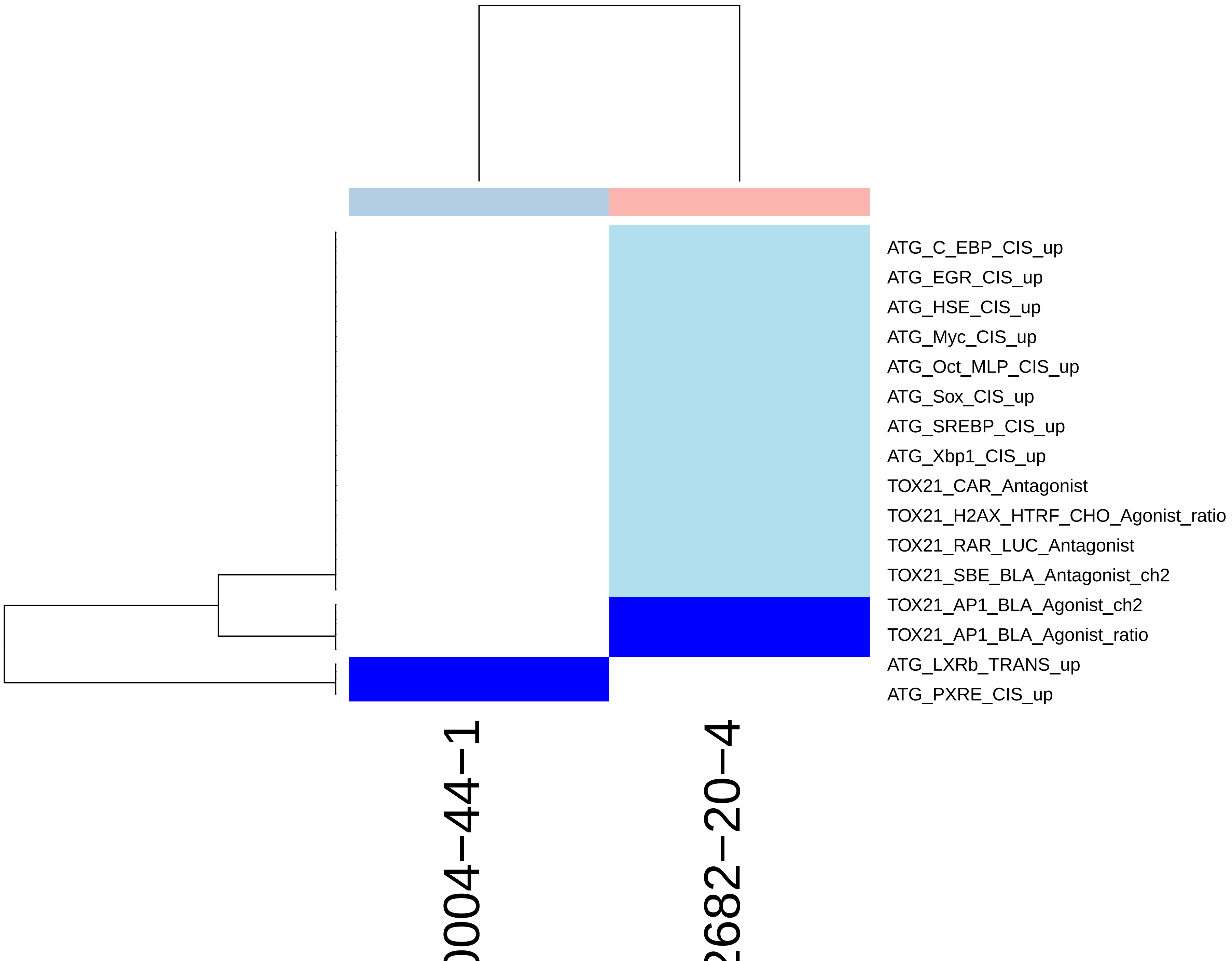

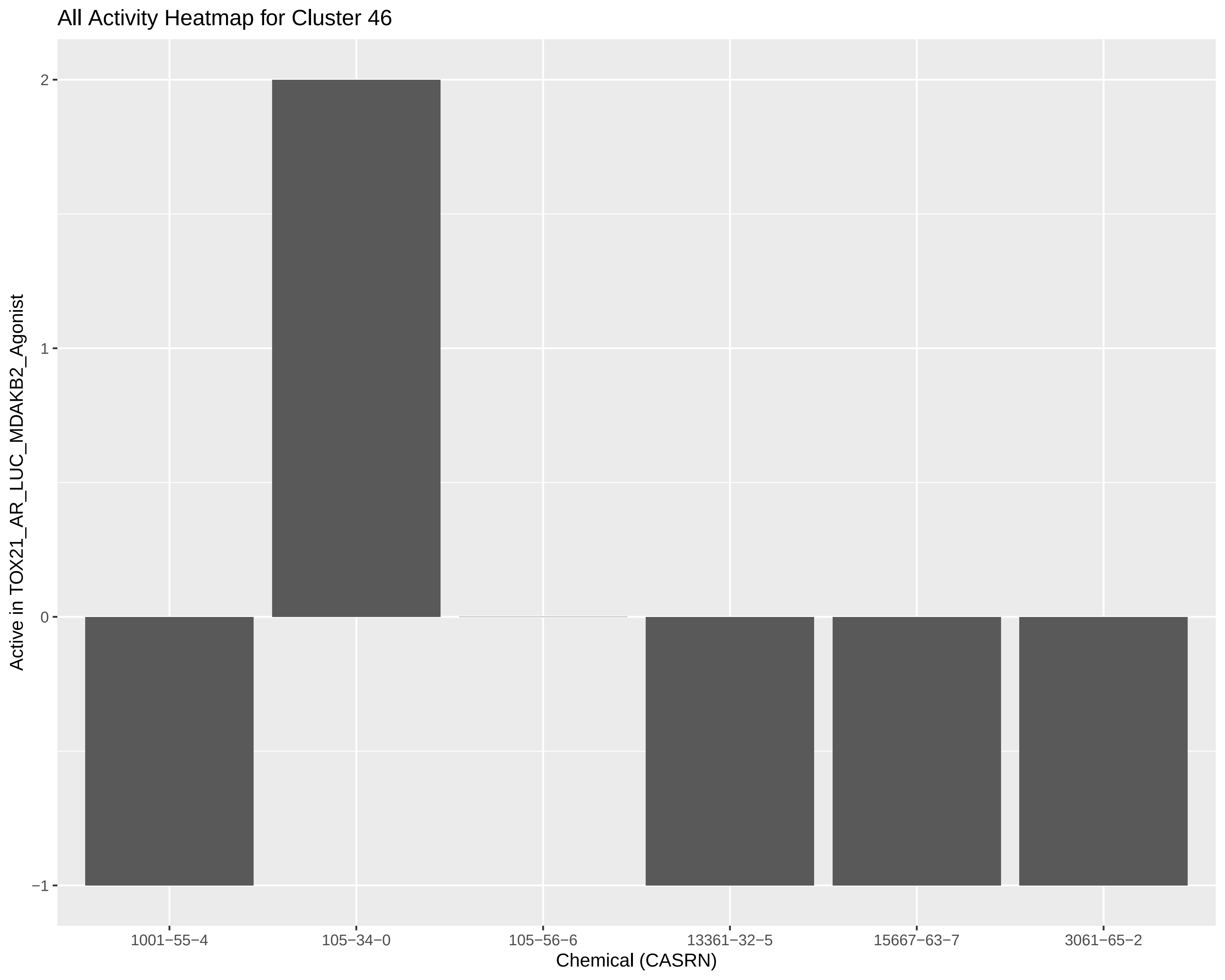
All Activity Heatmap for Cluster 46
Active in TOX21_AR_LUC_MDAKB2_Agonist
2
1
0
−1
1001−55−4
105−34−0
105−56−6
13361−32−5
15667−63−7
3061−65−2
Chemical (CASRN)

All Activity Heatmap for Cluster 52

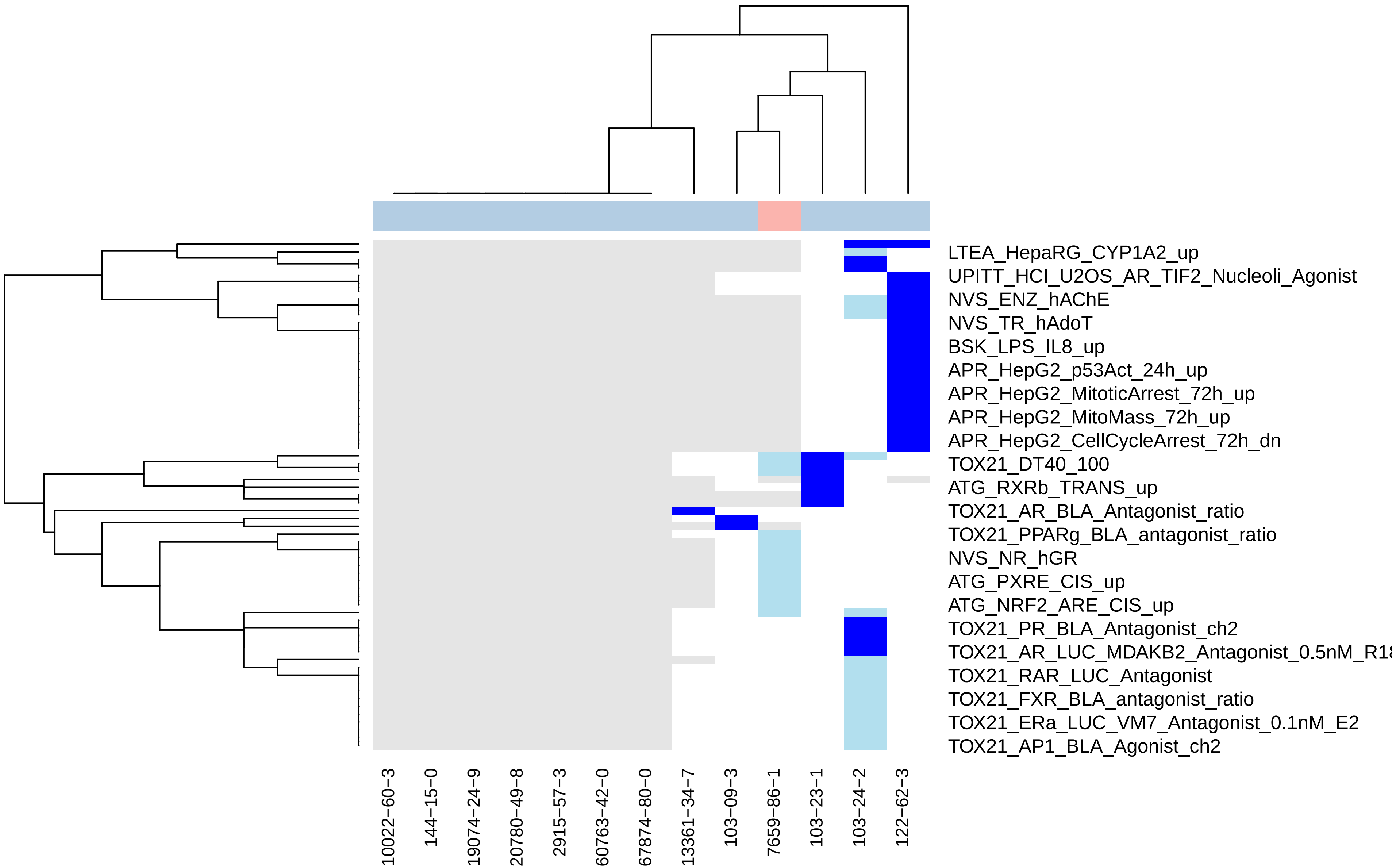

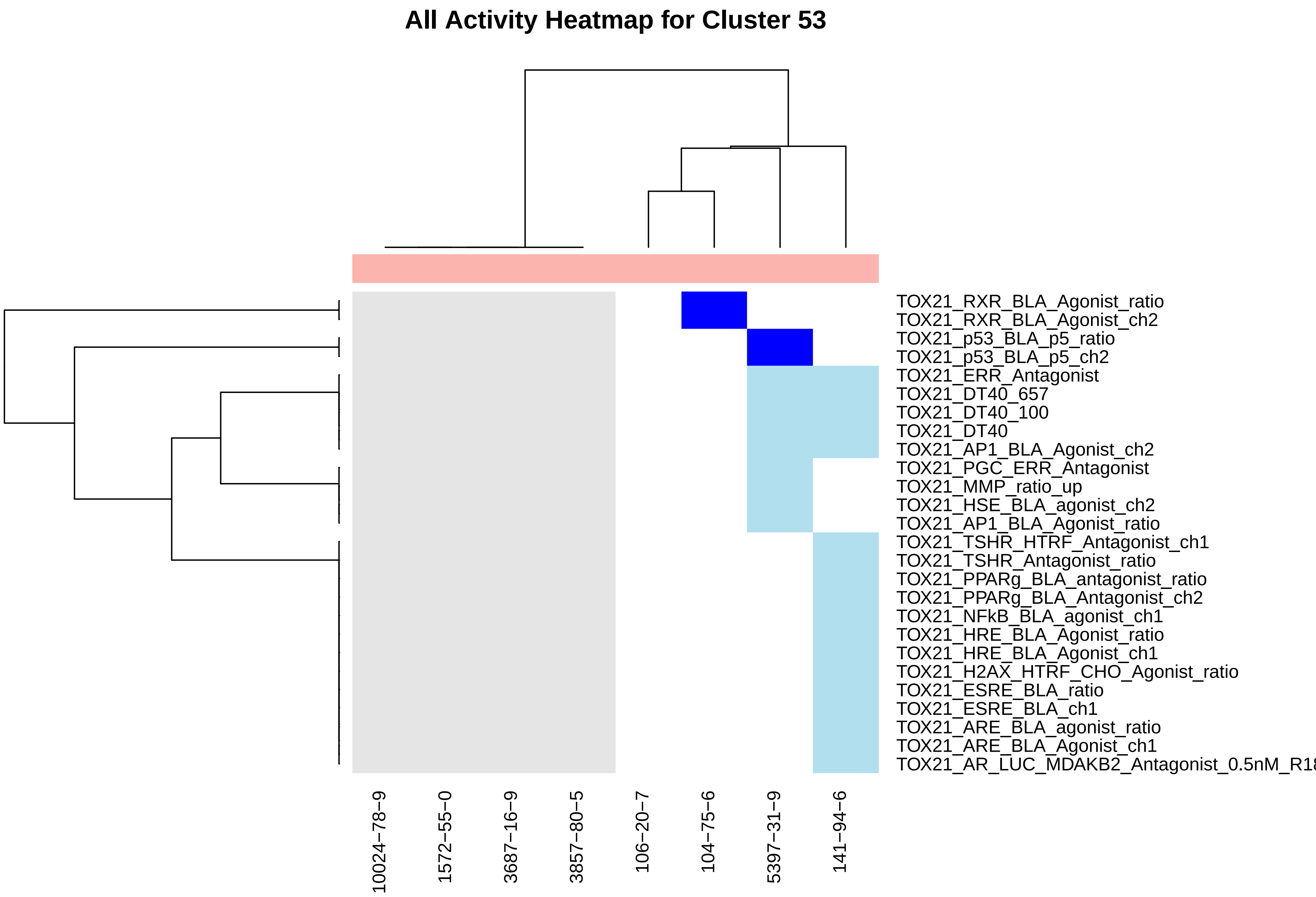
All Activity Heatmap for Cluster 53
TOX21_RXR_BLA_Agonist_ratio
TOX21_RXR_BLA_Agonist_ch2
TOX21_p53_BLA_p5_ratio
TOX21_p53_BLA_p5_ch2
TOX21_ERR_Antagonist
TOX21_DT40_657
TOX21_DT40_100
TOX21_DT40
TOX21_AP1_BLA_Agonist_ch2
TOX21_PGC_ERR_Antagonist
TOX21_MMP_ratio_up
TOX21_HSE_BLA_agonist_ch2
TOX21_AP1_BLA_Agonist_ratio
TOX21_TSHR_HTRF_Antagonist_ch1
TOX21_TSHR_Antagonist_ratio
TOX21_PPARg_BLA_antagonist_ratio
TOX21_PPARg_BLA_Antagonist_ch2
TOX21_NFkB_BLA_agonist_ch1
TOX21_HRE_BLA_Agonist_ratio
TOX21_HRE_BLA_Agonist_ch1
TOX21_H2AX_HTRF_CHO_Agonist_ratio
TOX21_ESRE_BLA_ratio
TOX21_ESRE_BLA_ch1
TOX21_ARE_BLA_agonist_ratio
TOX21_ARE_BLA_Agonist_ch1
TOX21_AR_LUC_MDAKB2_Antagonist_0.5nM_R18
10024−78−9
1572−55−0
3687−16−9
3857−80−5
106−20−7
104−75−6
5397−31−9
141−94−6

All Activity Heatmap for Cluster 54

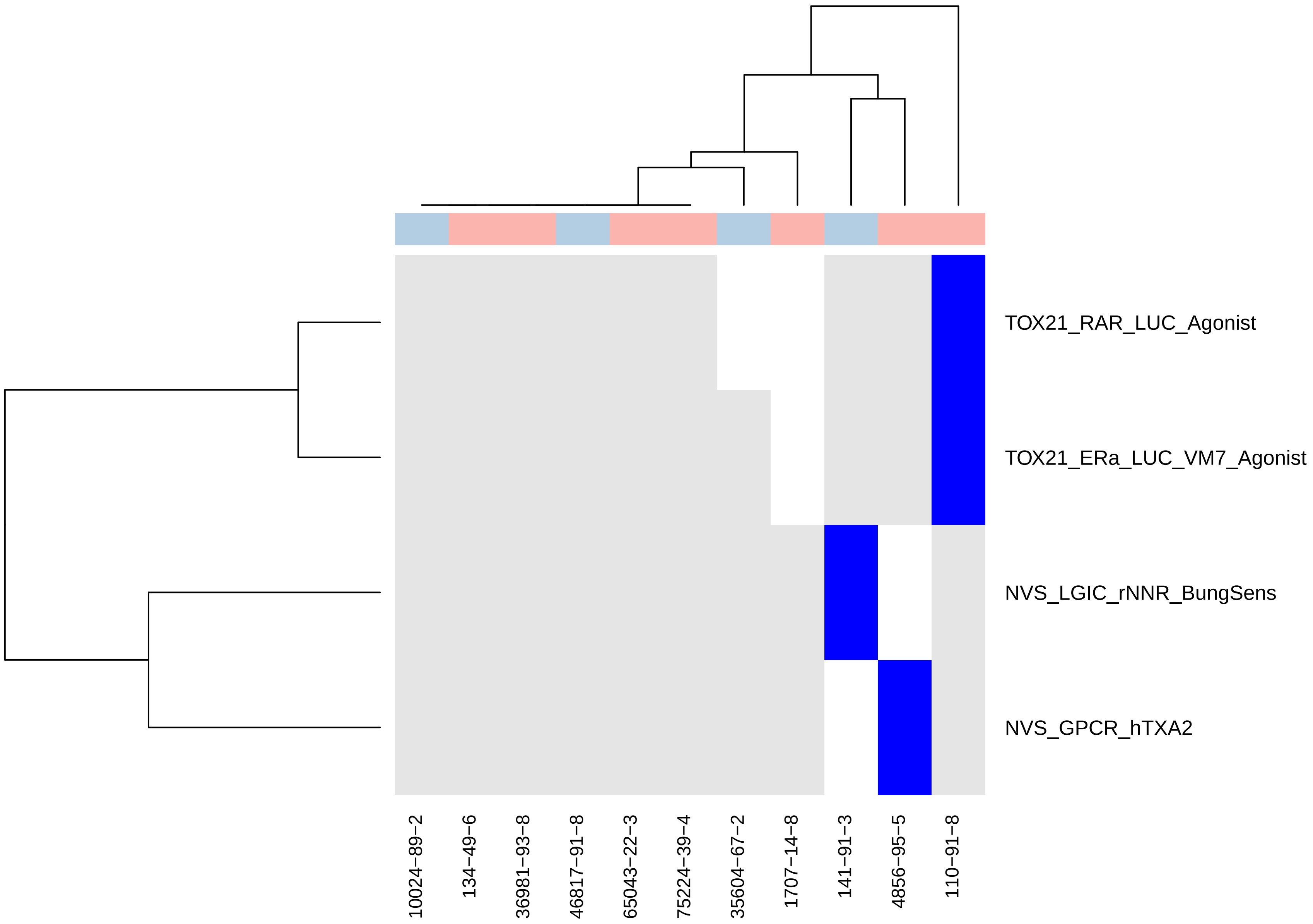

All Activity Heatmap for Cluster 55

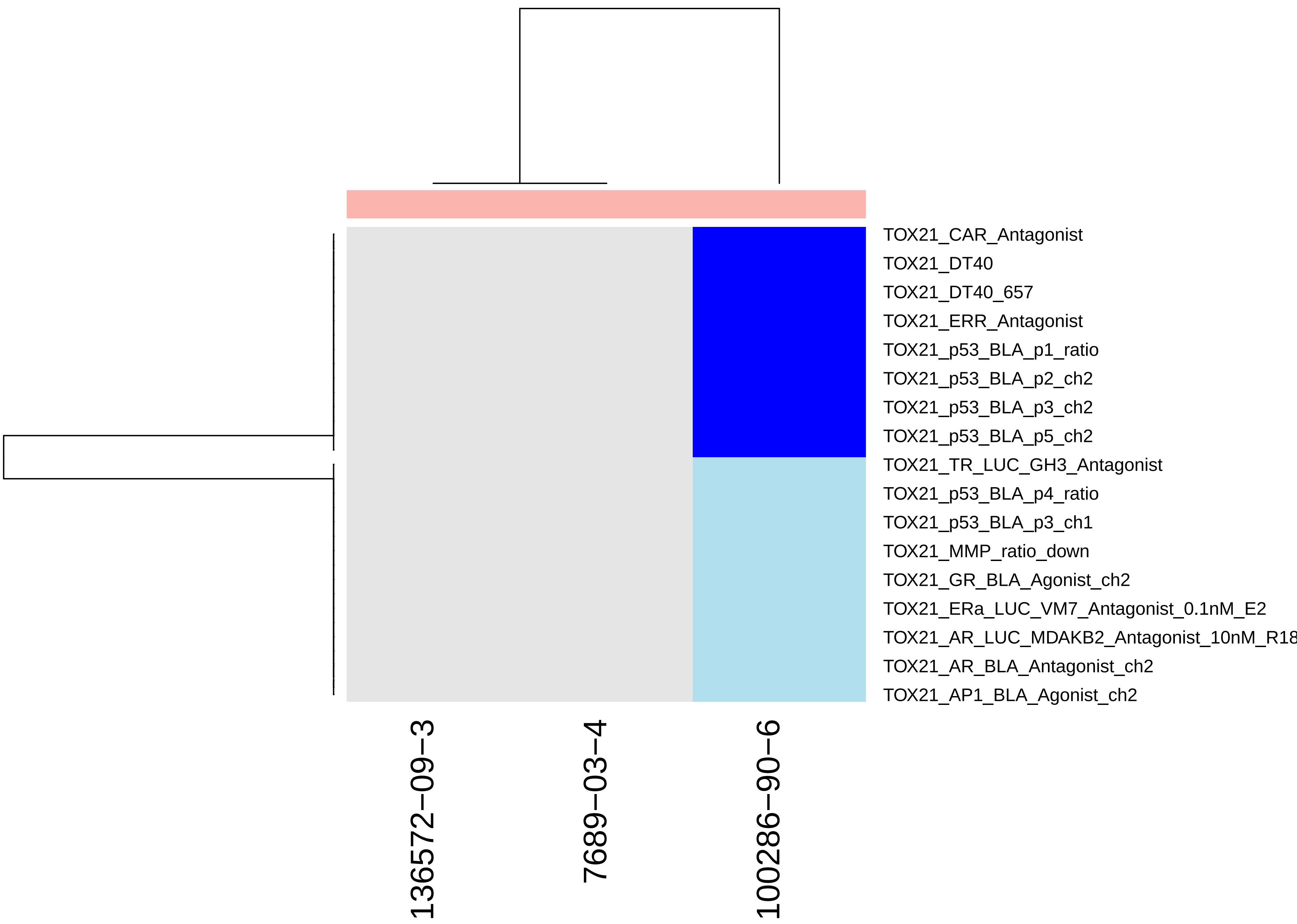

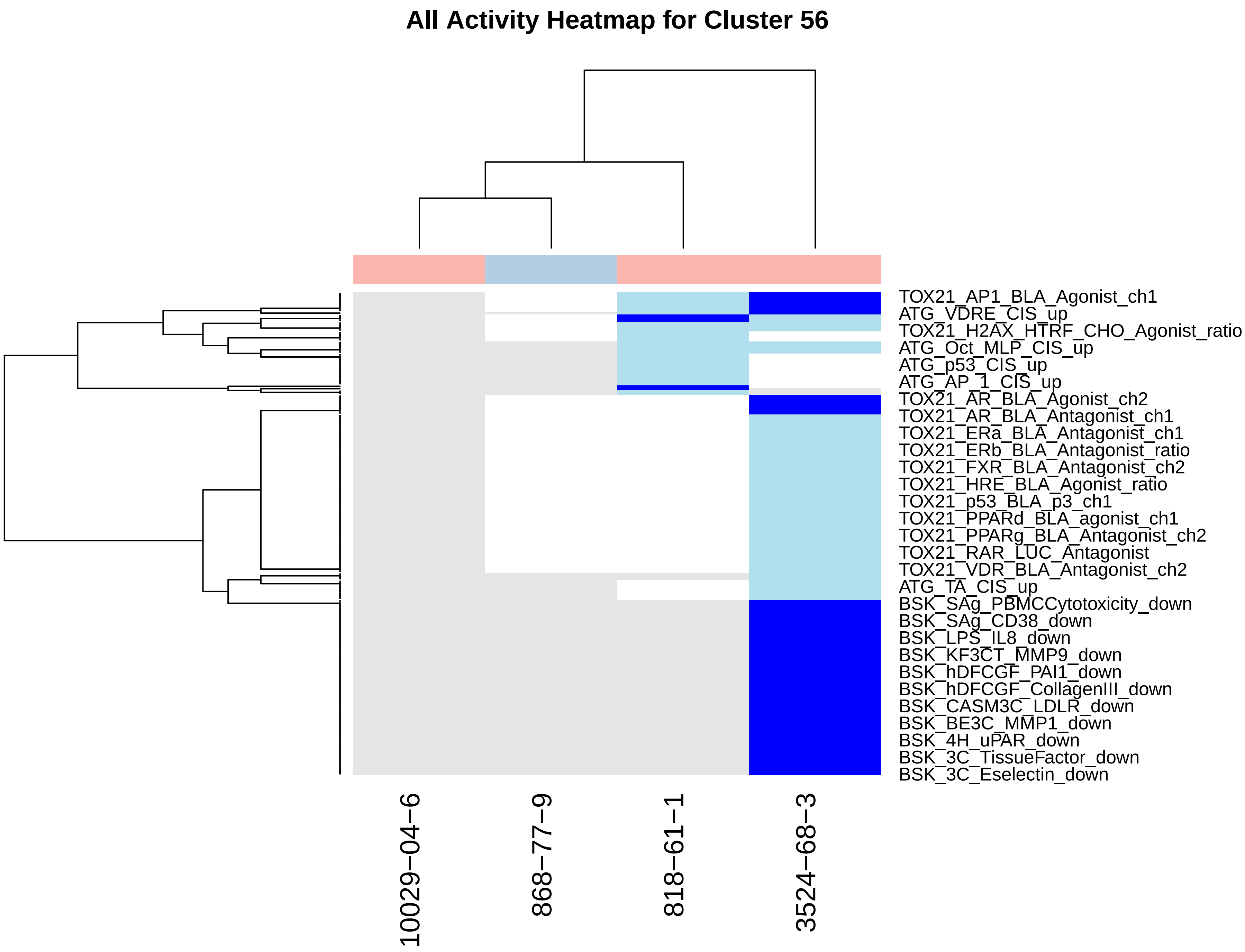
All Activity Heatmap for Cluster 56
TOX21_AP1_BLA_Agonist_ch1
ATG_VDRE_CIS_up
TOX21_H2AX_HTRF_CHO_Agonist_ratio
ATG_Oct_MLP_CIS_up
ATG_p53_CIS_up
ATG_AP_1_CIS_up
TOX21_AR_BLA_Agonist_ch2
TOX21_AR_BLA_Antagonist_ch1
TOX21_ERa_BLA_Antagonist_ch1
TOX21_ERb_BLA_Antagonist_ratio
TOX21_FXR_BLA_Antagonist_ch2
TOX21_HRE_BLA_Agonist_ratio
TOX21_p53_BLA_p3_ch1
TOX21_PPARd_BLA_agonist_ch1
TOX21_PPARg_BLA_Antagonist_ch2
TOX21_RAR_LUC_Antagonist
TOX21_VDR_BLA_Antagonist_ch2
ATG_TA_CIS_up
BSK_SAg_PBMCCytotoxicity_down
BSK_SAg_CD38_down
BSK_LPS_IL8_down
BSK_KF3CT_MMP9_down
BSK_hDFCGF_PAI1_down
BSK_hDFCGF_CollagenIII_down
BSK_CASM3C_LDLR_down
BSK_BE3C_MMP1_down
BSK_4H_uPAR_down
BSK_3C_TissueFactor_down
BSK_3C_Eselectin_down
10029−04−6
868−77−9
818−61−1
3524−68−3

All Activity Heatmap for Cluster 58

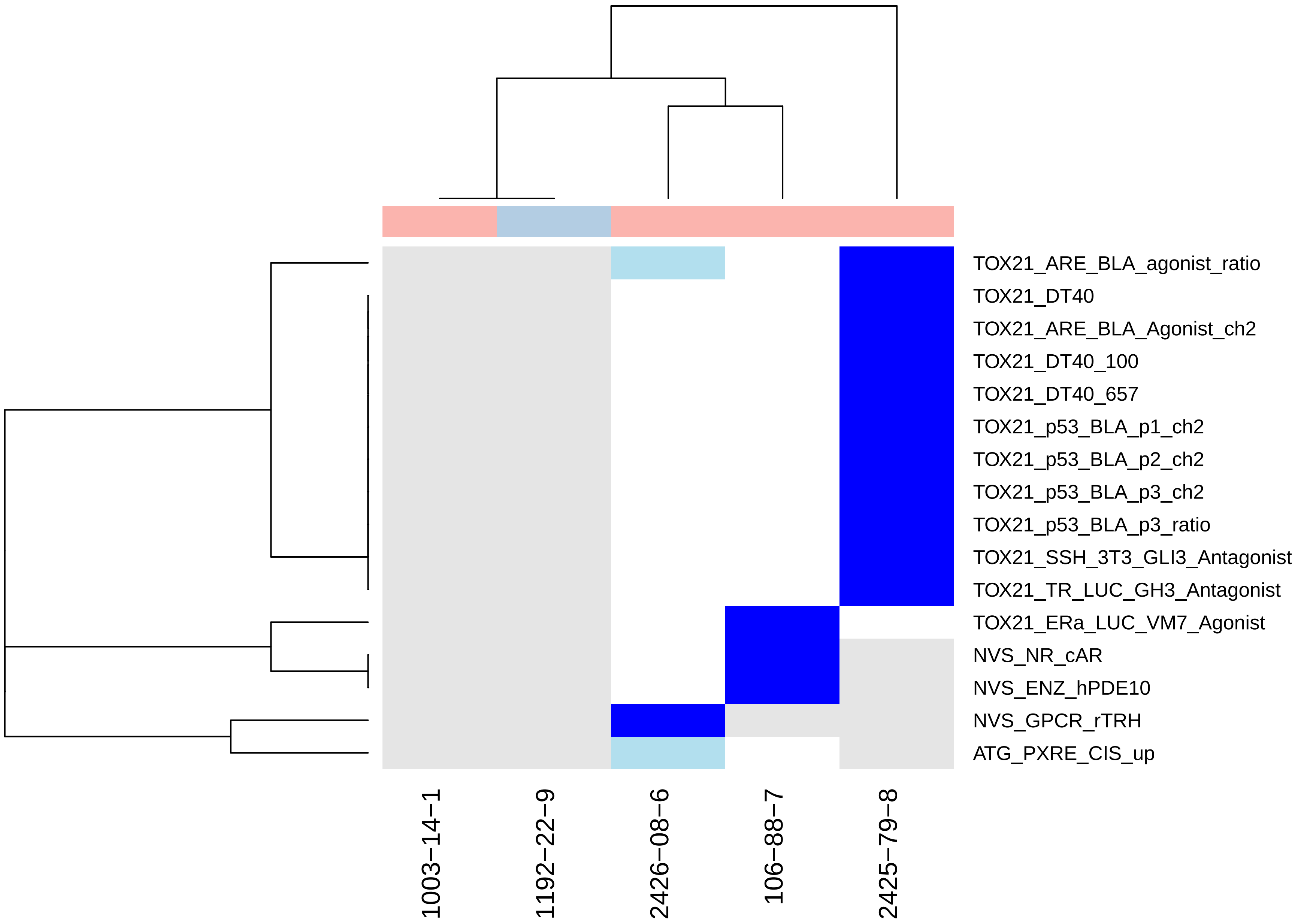

All Activity Heatmap for Cluster 59

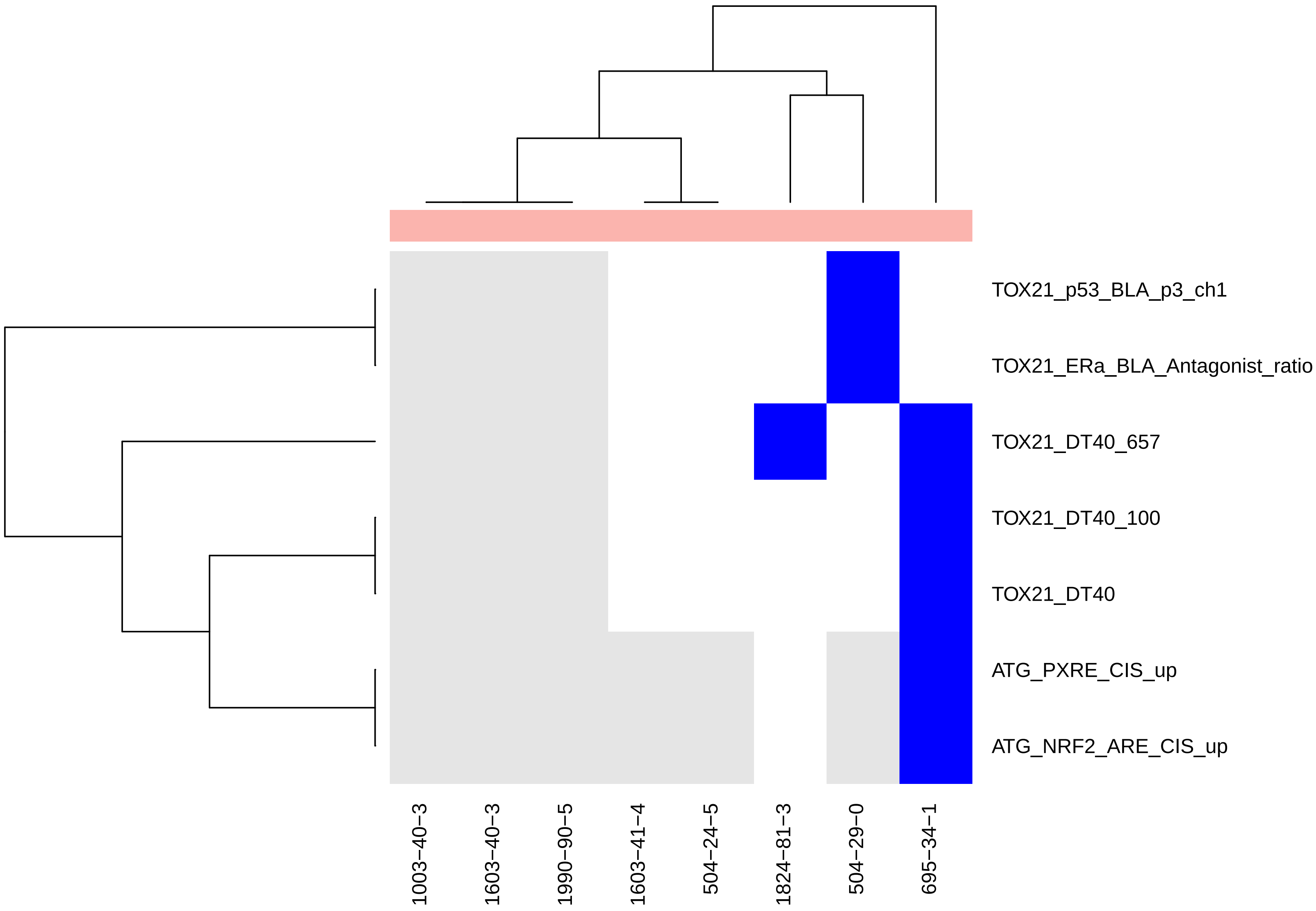

All Activity Heatmap for Cluster 60

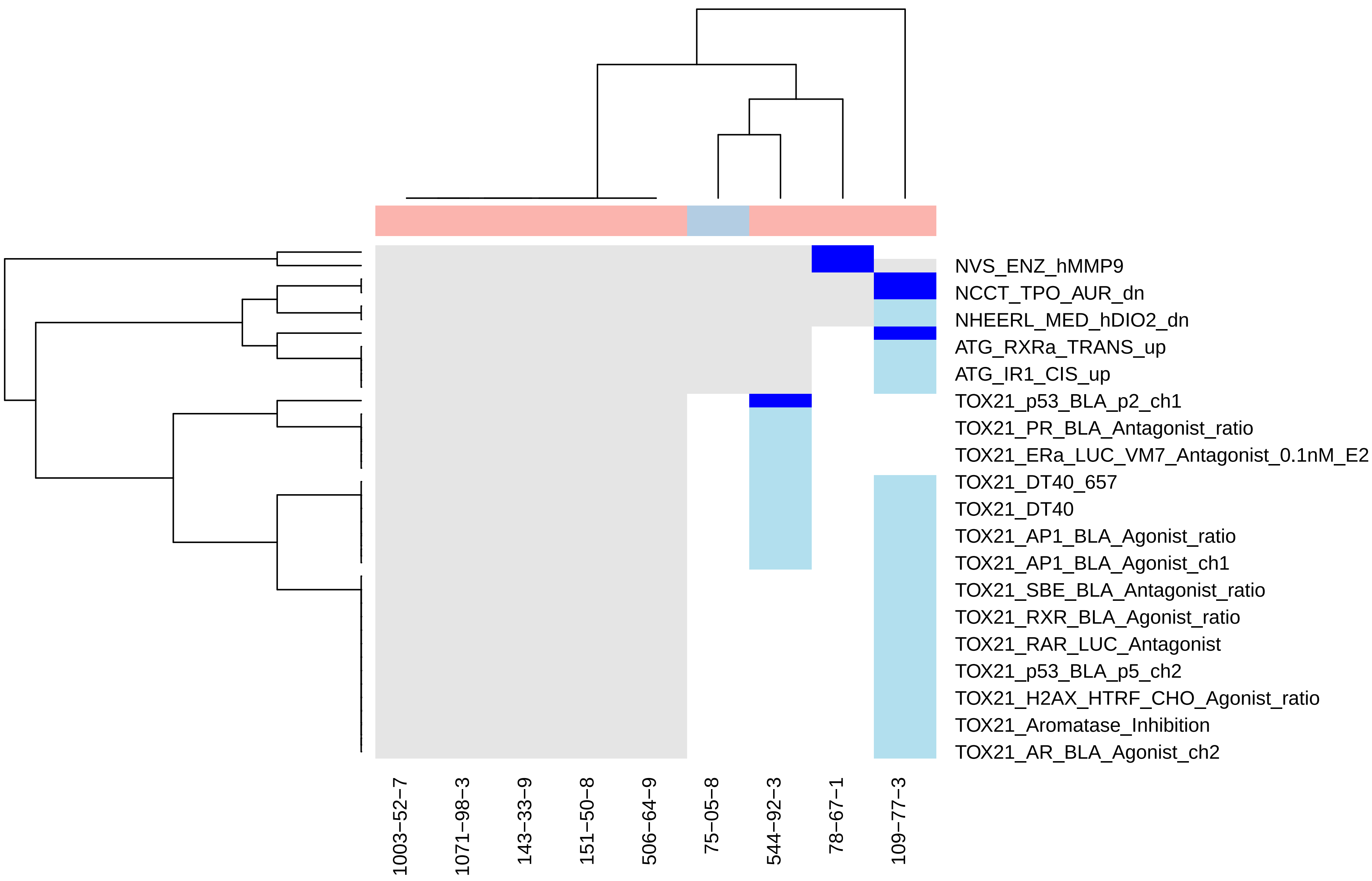

All Activity Heatmap for Cluster 62

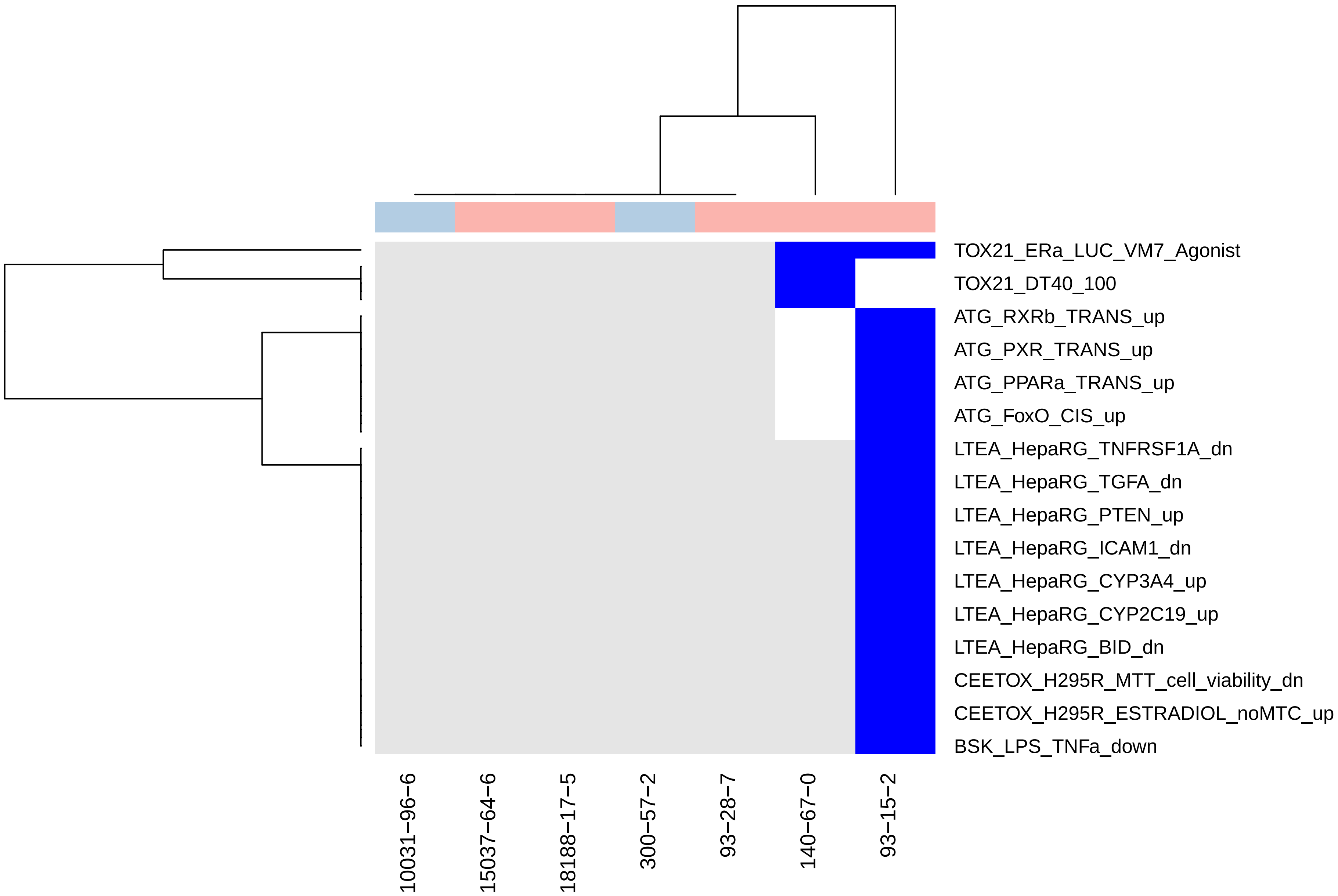

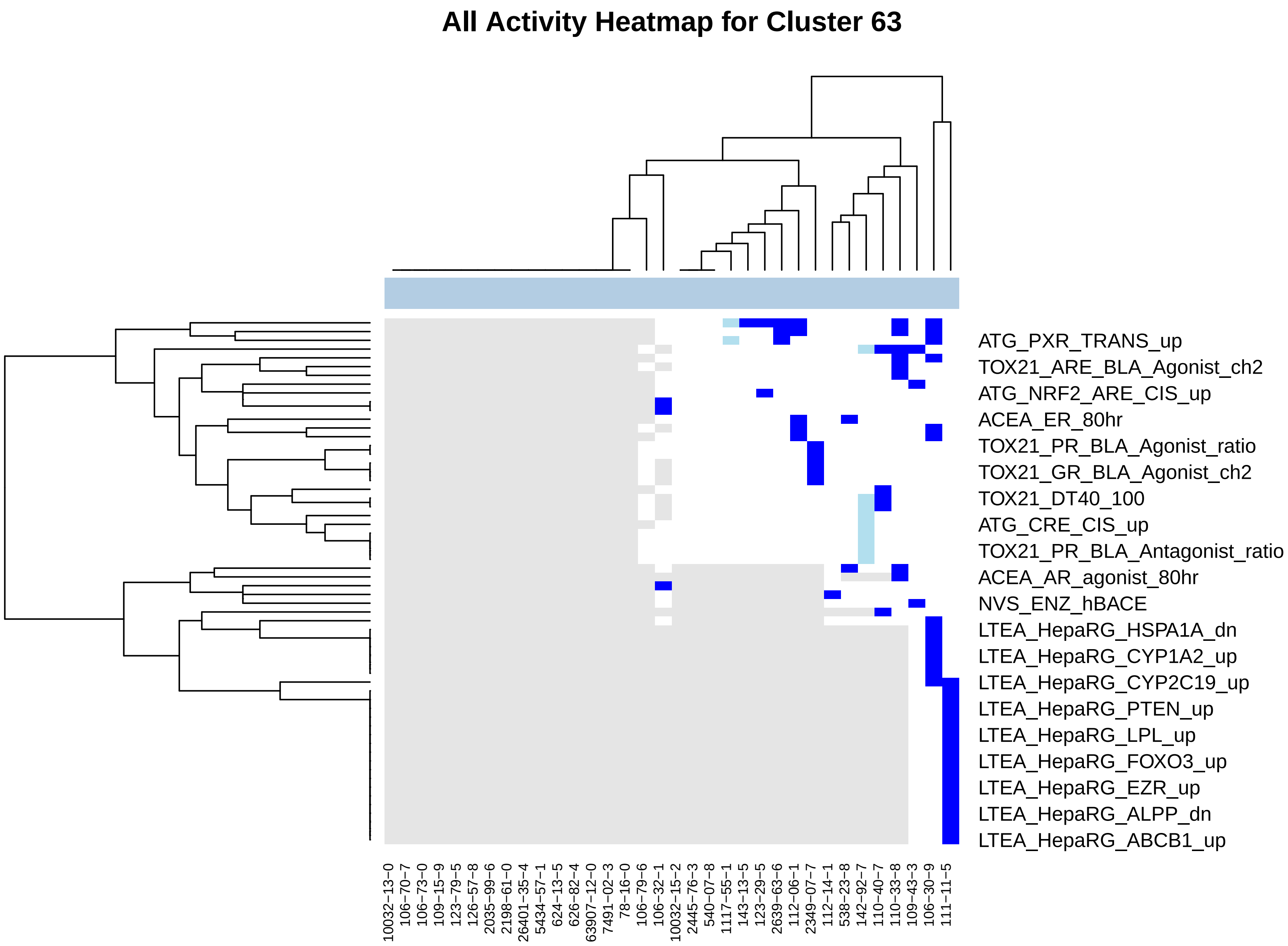

All Activity Heatmap for Cluster 63
ATG_PXR_TRANS_up
TOX21_ARE_BLA_Agonist_ch2
ATG_NRF2_ARE_CIS_up
ACEA_ER_80hr
TOX21_PR_BLA_Agonist_ratio
TOX21_GR_BLA_Agonist_ch2
TOX21_DT40_100
ATG_CRE_CIS_up
TOX21_PR_BLA_Antagonist_ratio
ACEA_AR_agonist_80hr
NVS_ENZ_hBACE
LTEA_HepaRG_HSPA1A_dn
LTEA_HepaRG_CYP1A2_up
LTEA_HepaRG_CYP2C19_up
LTEA_HepaRG_PTEN_up
LTEA_HepaRG_LPL_up
LTEA_HepaRG_FOXO3_up
LTEA_HepaRG_EZR_up
LTEA_HepaRG_ALPP_dn
LTEA_HepaRG_ABCB1_up
10032−13−0
106−70−7
106−73−0
109−15−9
123−79−5
126−57−8
2035−99−6
2198−61−0
26401−35−4
5434−57−1
624−13−5
626−82−4
63907−12−0
7491−02−3
78−16−0
106−79−6
106−32−1
10032−15−2
2445−76−3
540−07−8
1117−55−1
143−13−5
123−29−5
2639−63−6
112−06−1
2349−07−7
112−14−1
538−23−8
142−92−7
110−40−7
110−33−8
109−43−3
106−30−9
111−11−5

All Activity Heatmap for Cluster 64

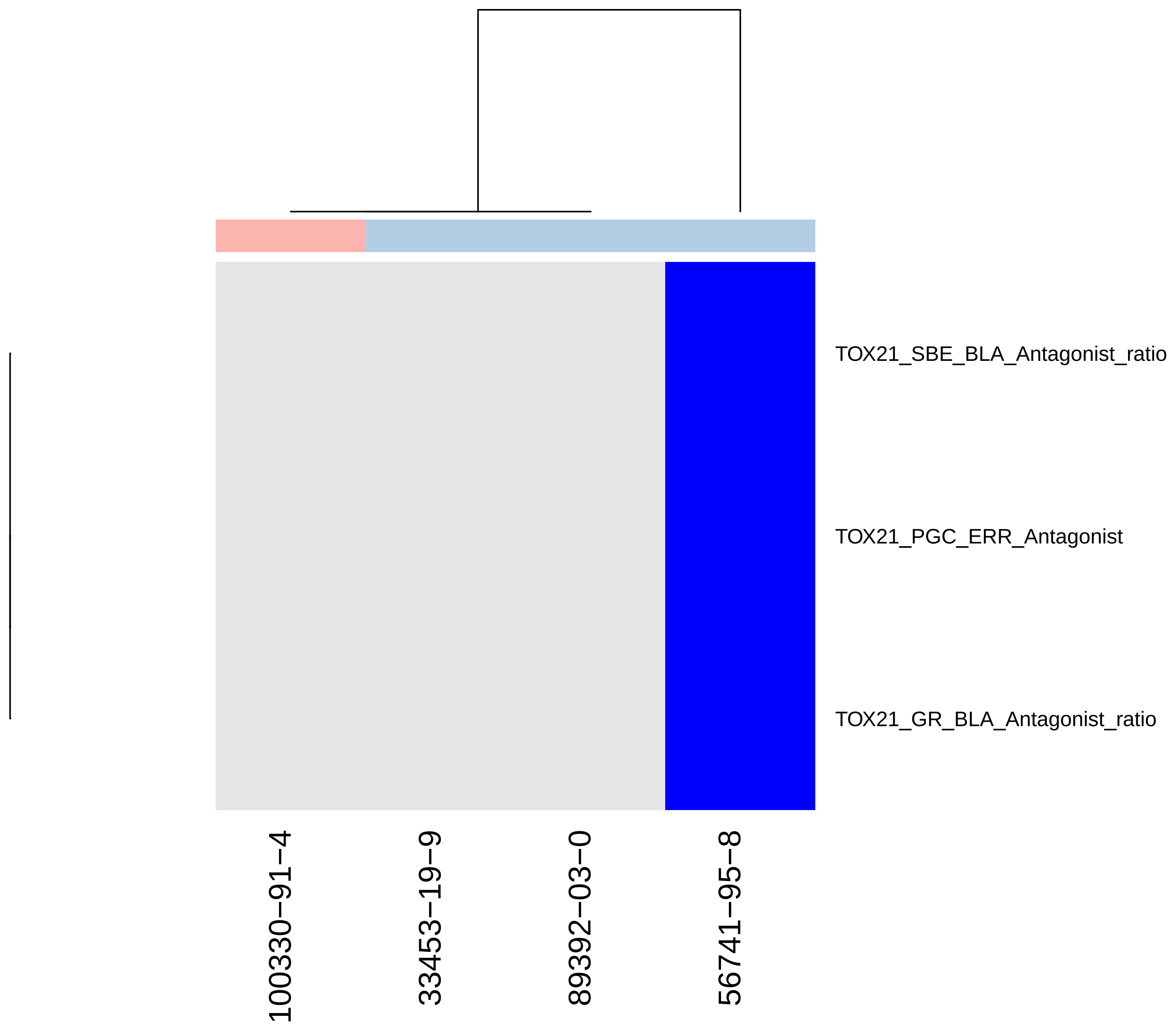

All Activity Heatmap for Cluster 67

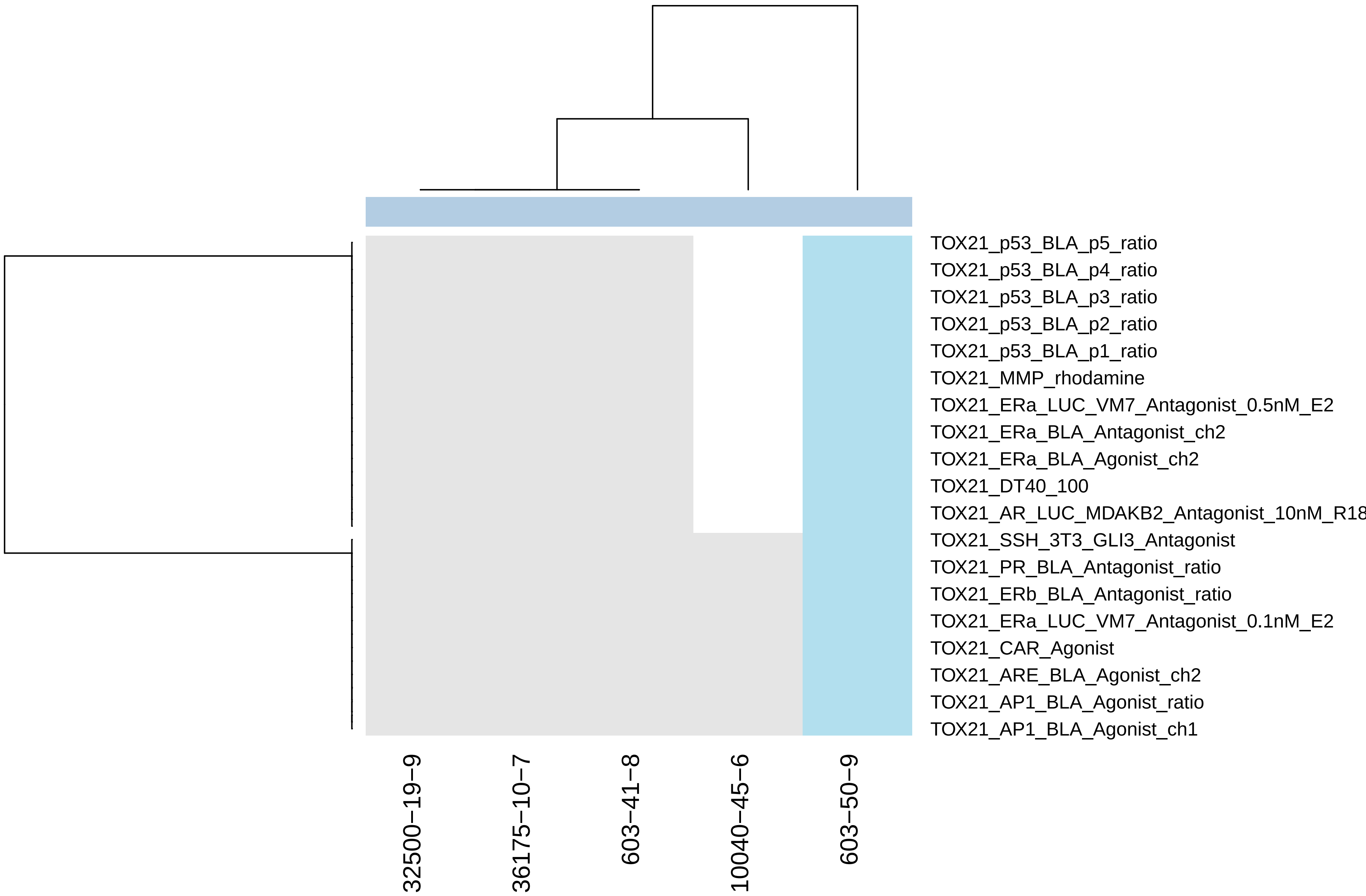

All Activity Heatmap for Cluster 68

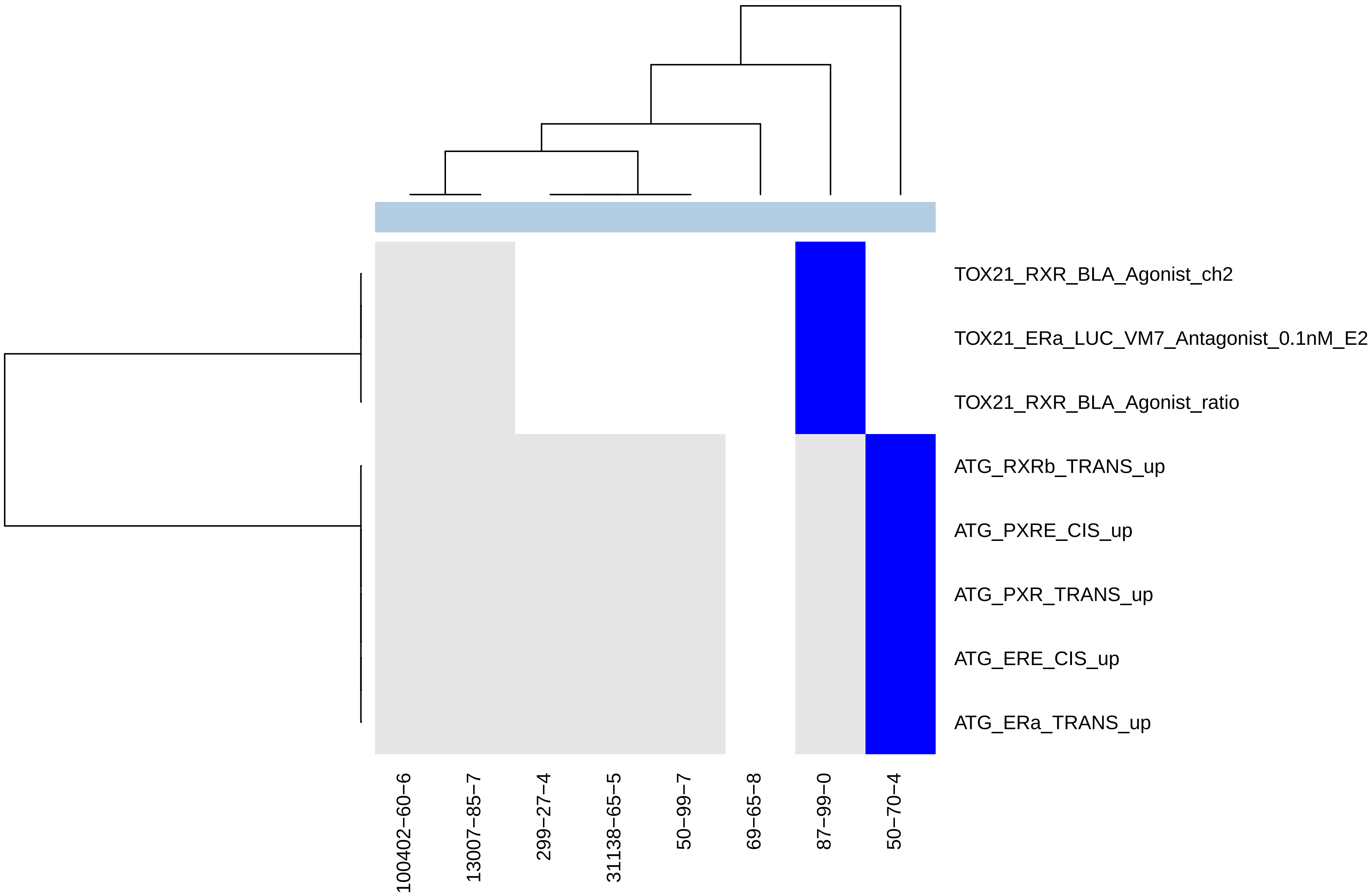

All Activity Heatmap for Cluster 70

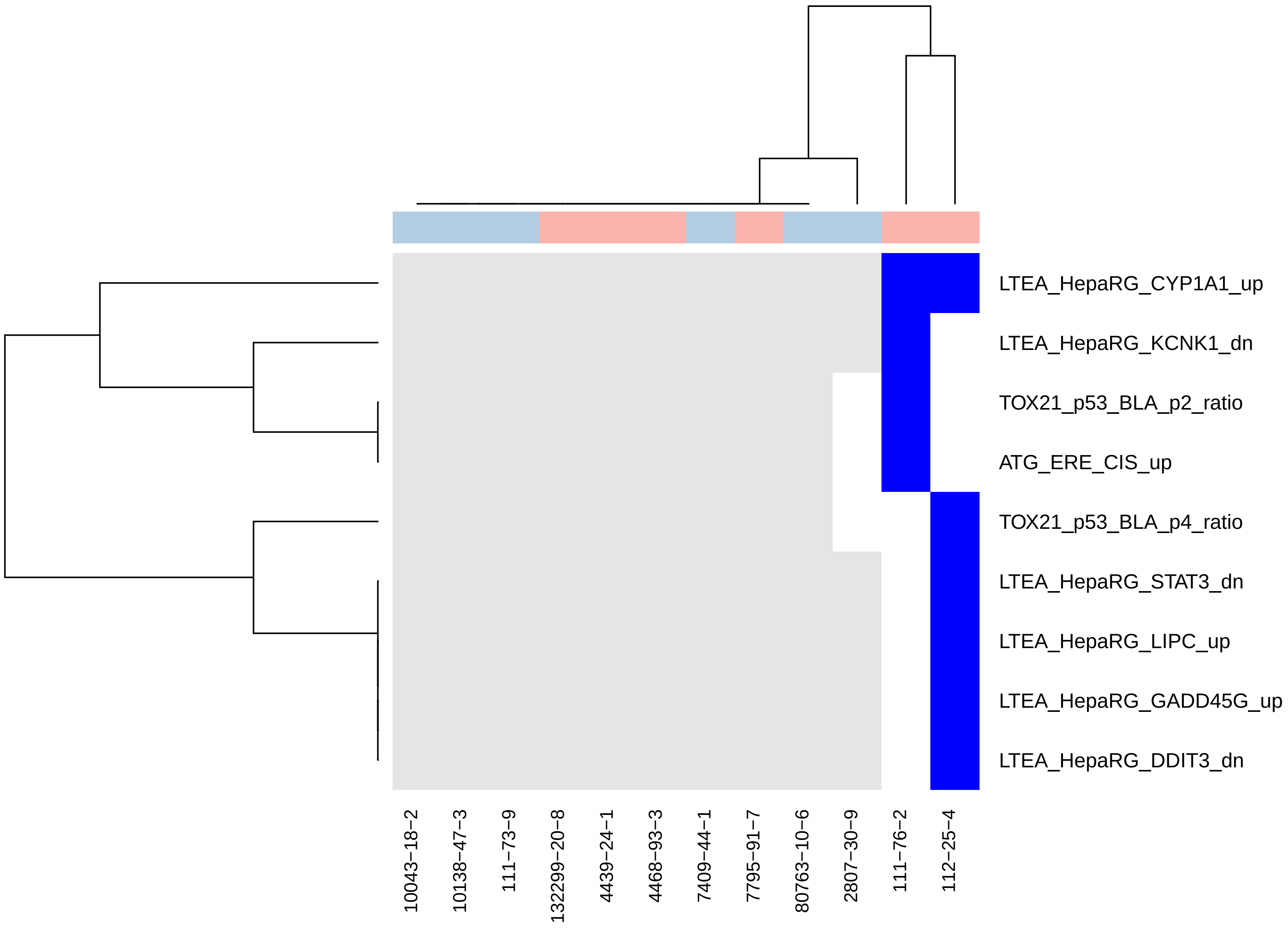

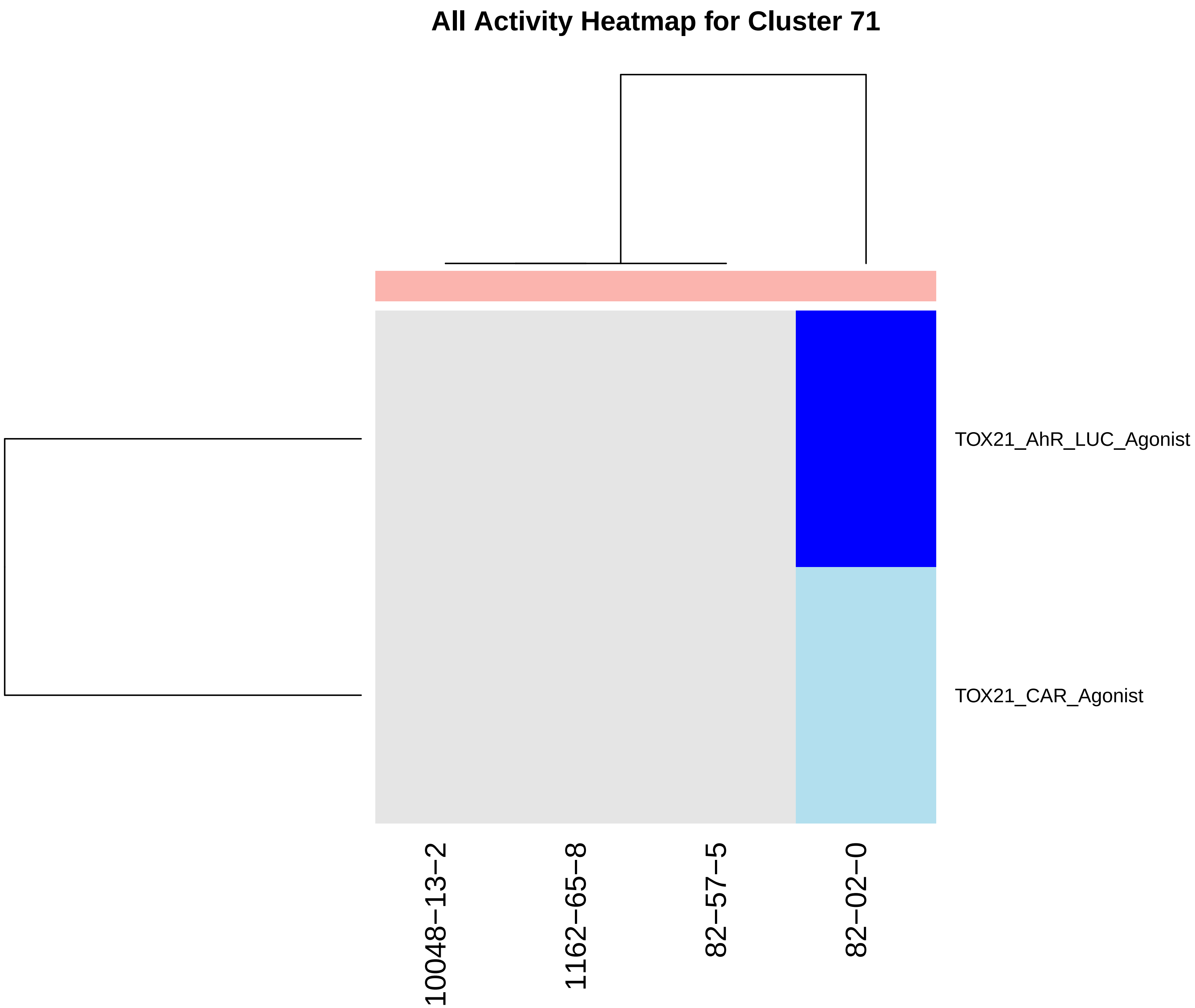
All Activity Heatmap for Cluster 71
TOX21_AhR_LUC_Agonist
TOX21_CAR_Agonist
10048−13−2
1162−65−8
82−57−5
82−02−0

All Activity Heatmap for Cluster 72

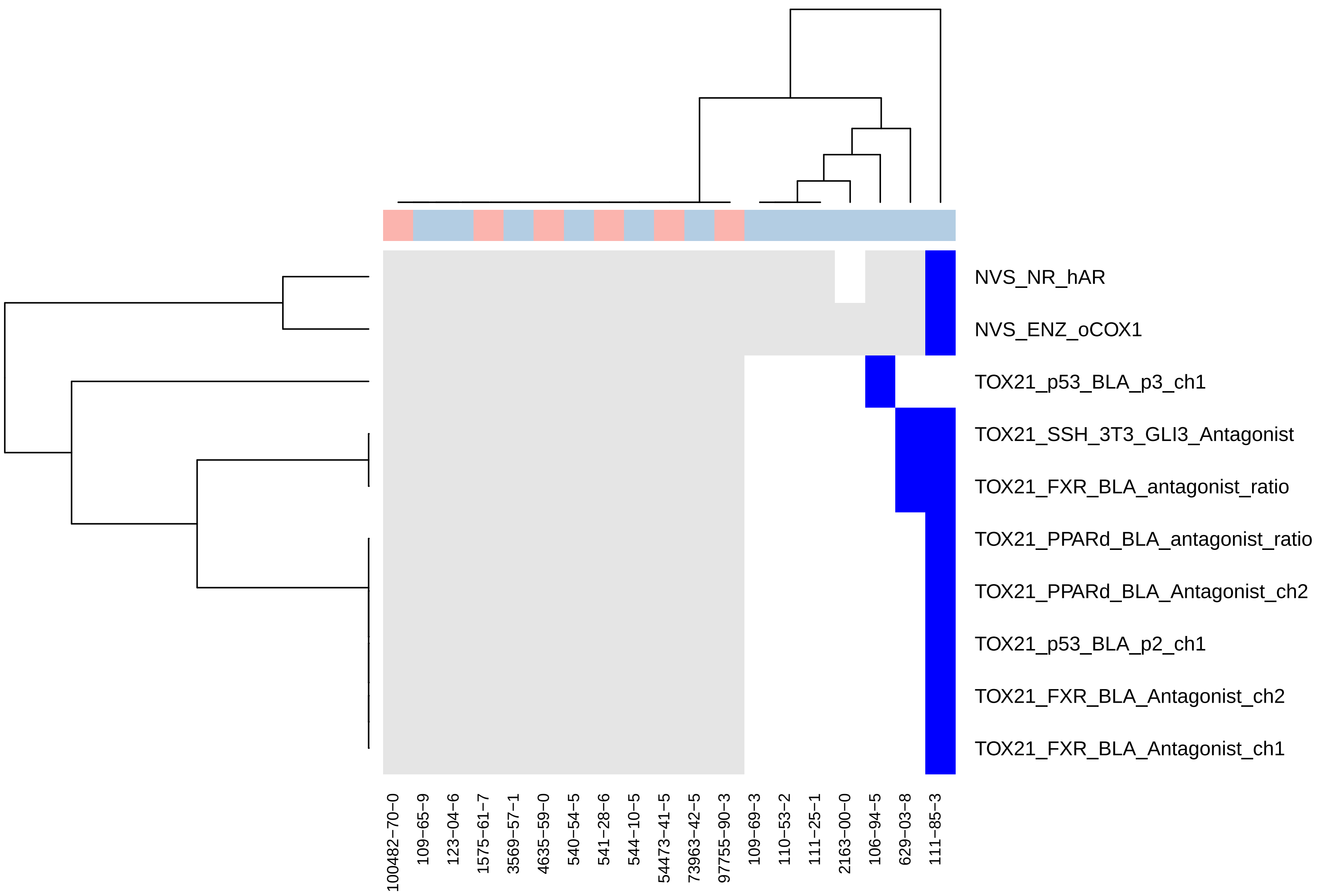

All Activity Heatmap for Cluster 74

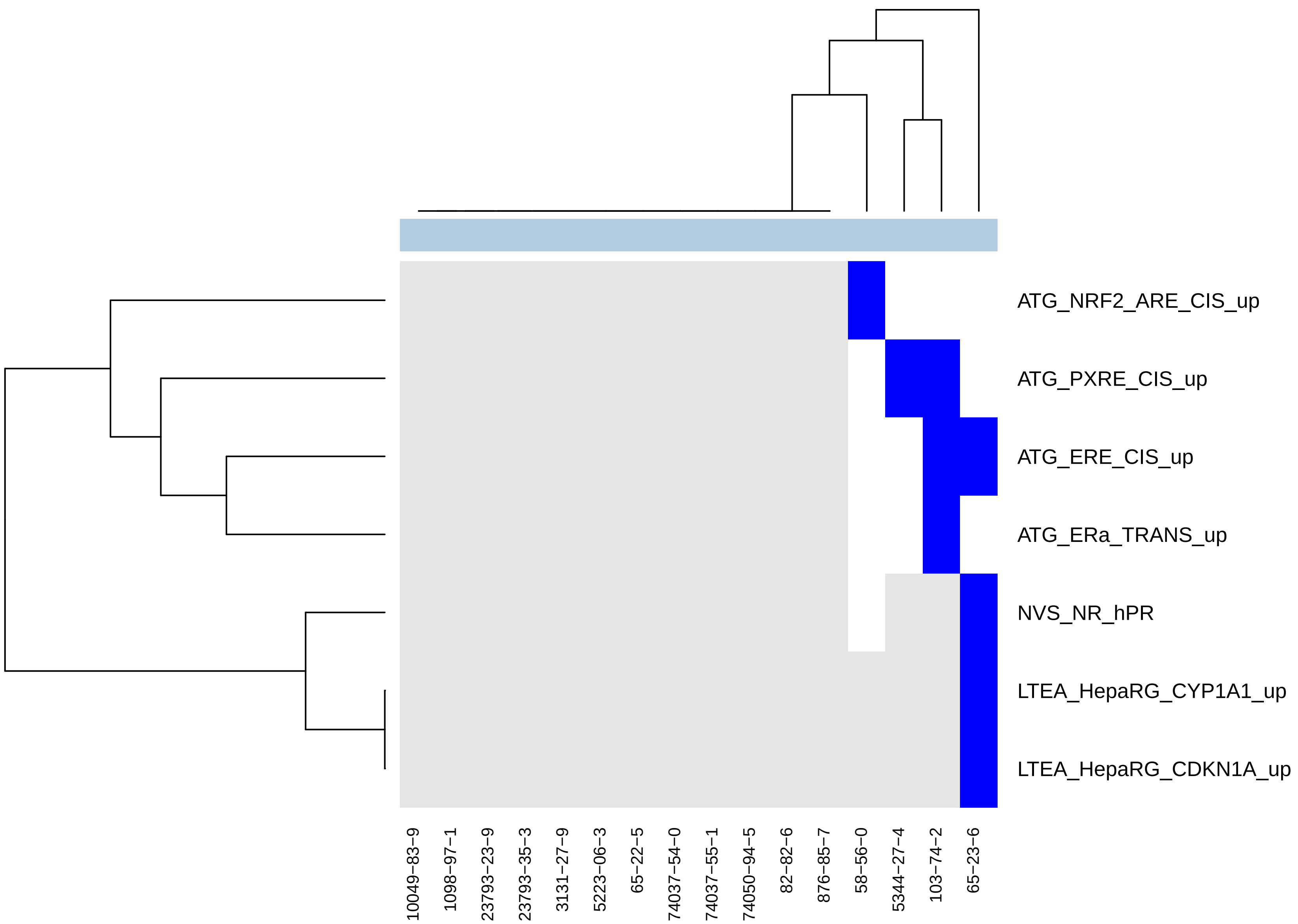

All Activity Heatmap for Cluster 76

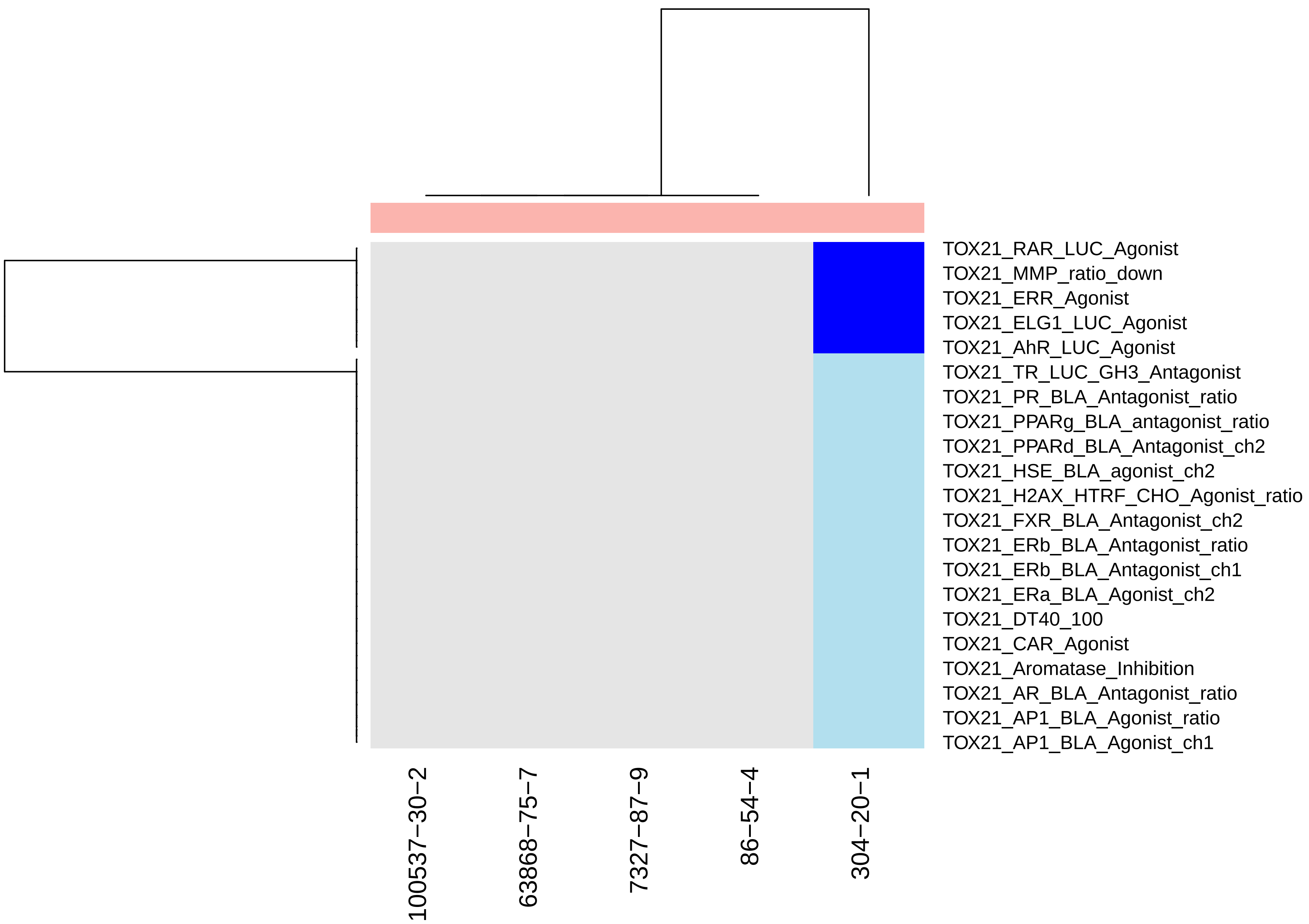

All Activity Heatmap for Cluster 77

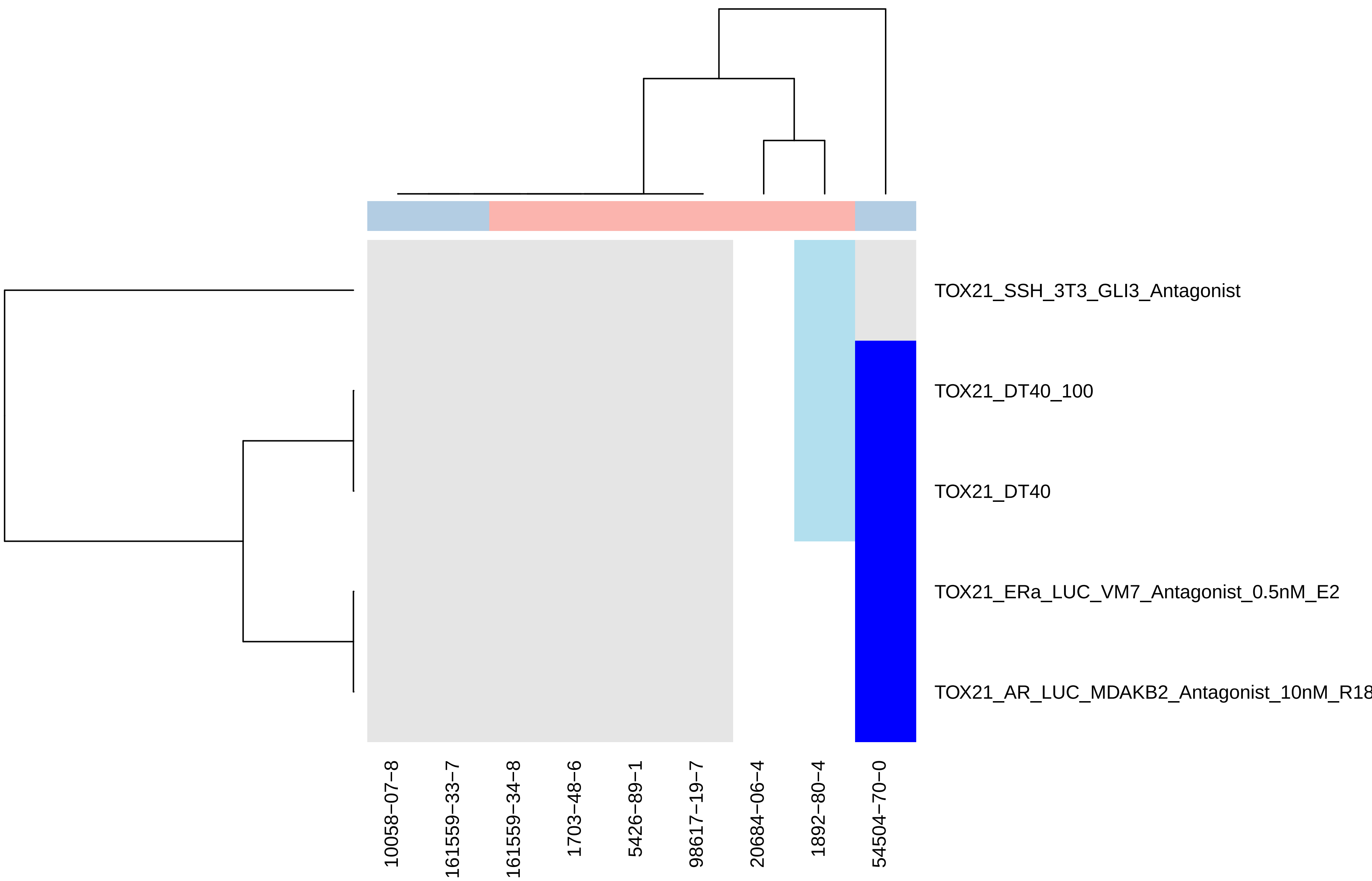

All Activity Heatmap for Cluster 78

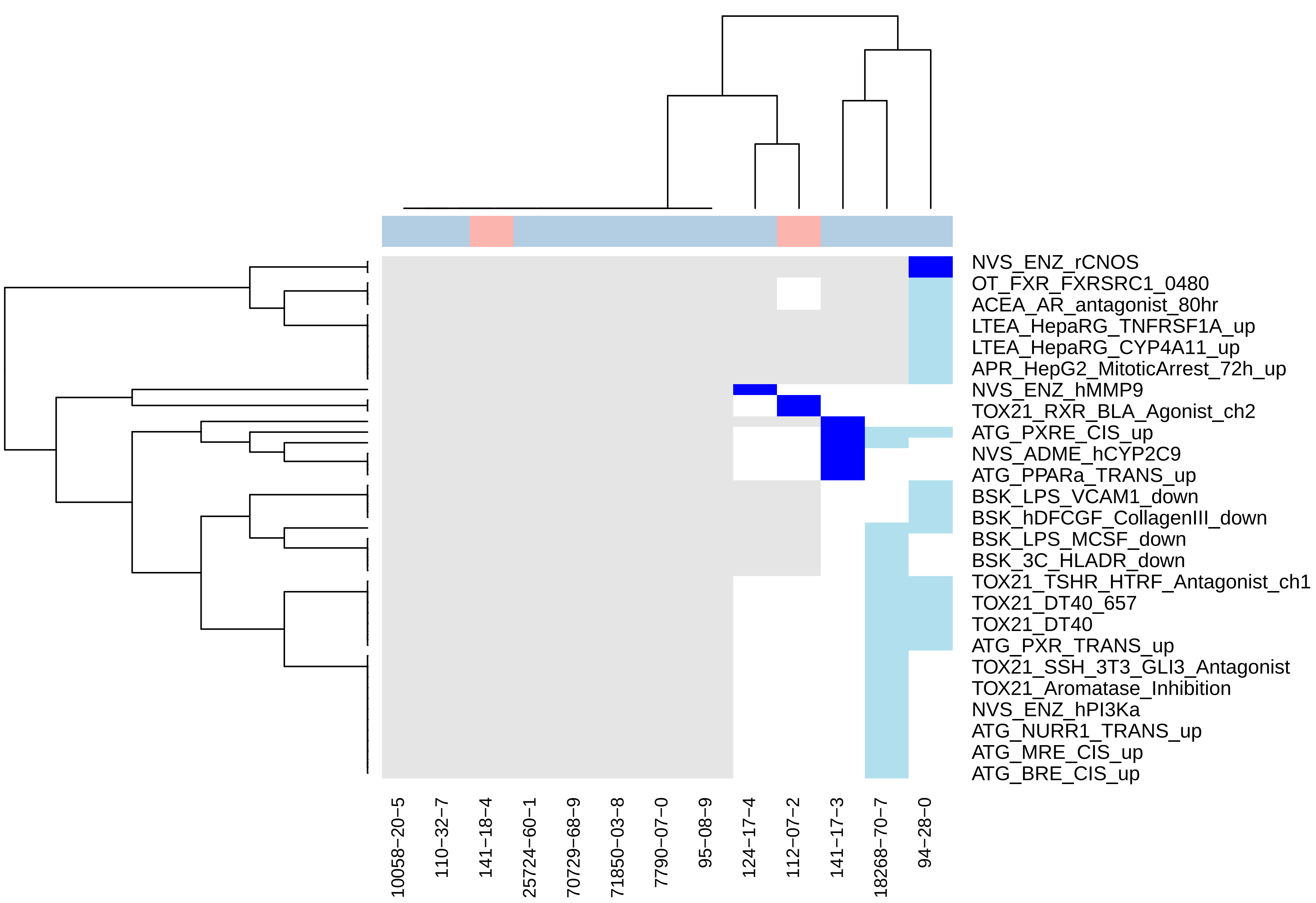

All Activity Heatmap for Cluster 79

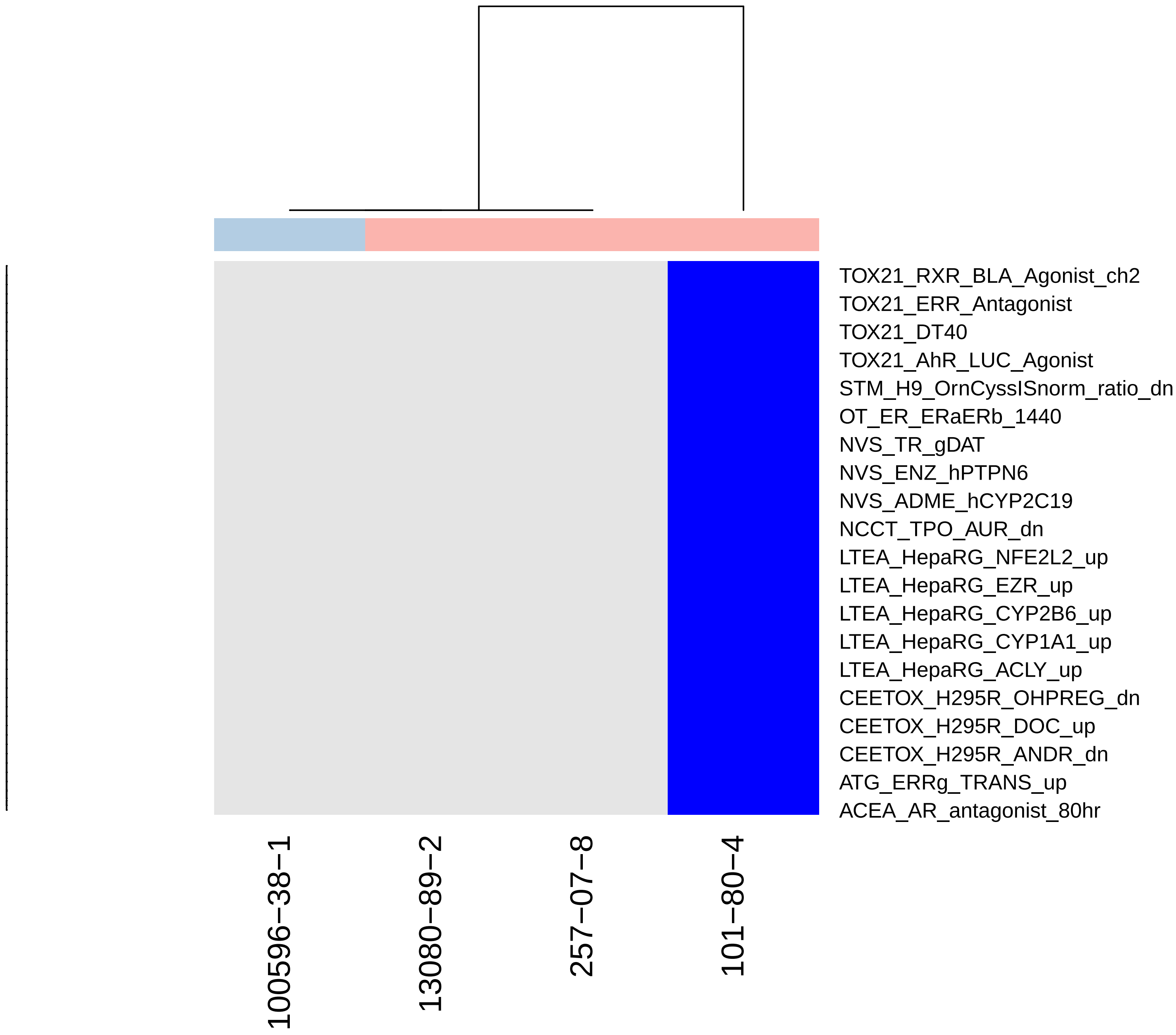

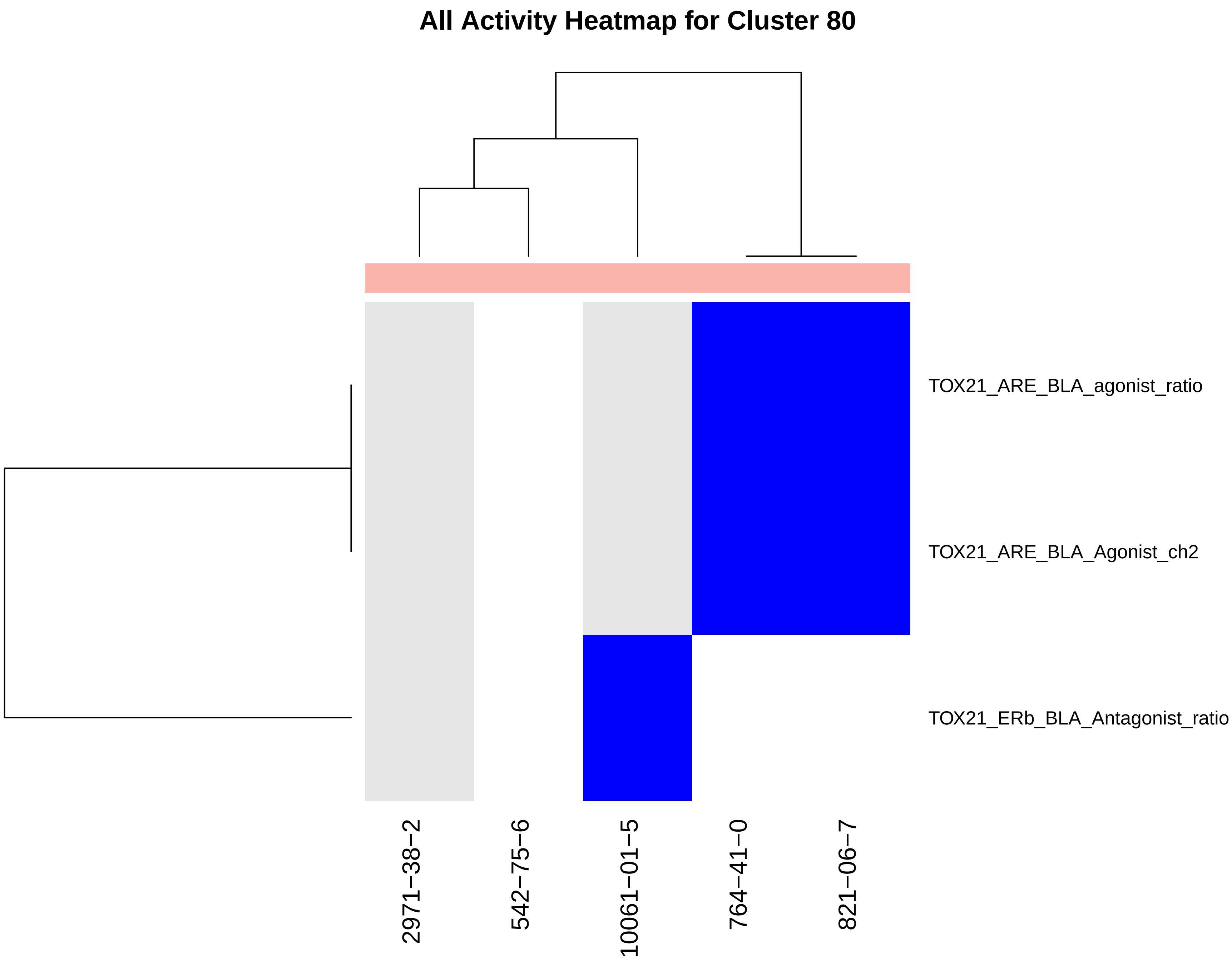

All Activity Heatmap for Cluster 80
TOX21_ARE_BLA_agonist_ratio
TOX21_ARE_BLA_Agonist_ch2
TOX21_ERb_BLA_Antagonist_ratio
2971−38−2
542−75−6
10061−01−5
764−41−0
821−06−7

All Activity Heatmap for Cluster 81

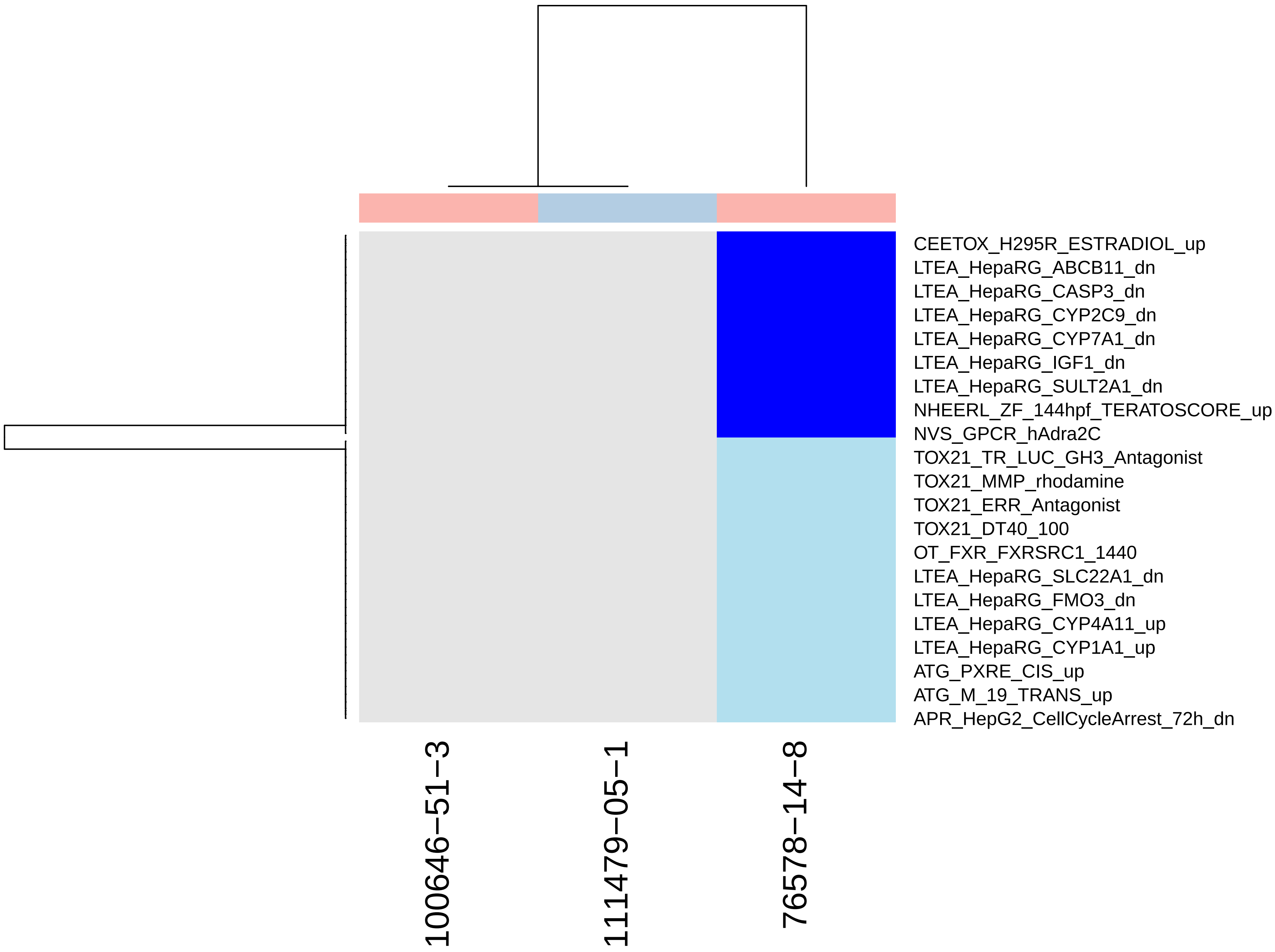

All Activity Heatmap for Cluster 82

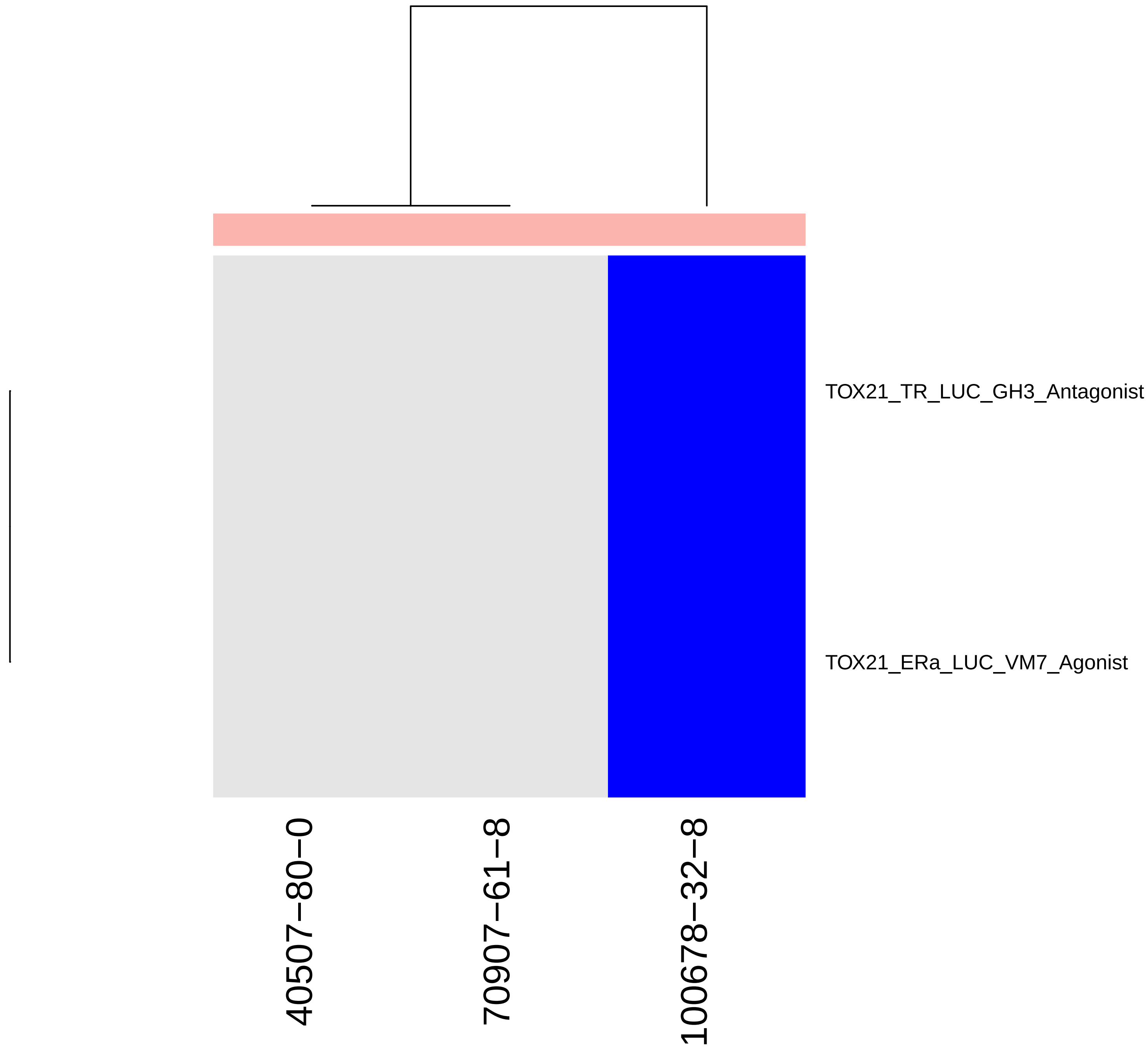

All Activity Heatmap for Cluster 83

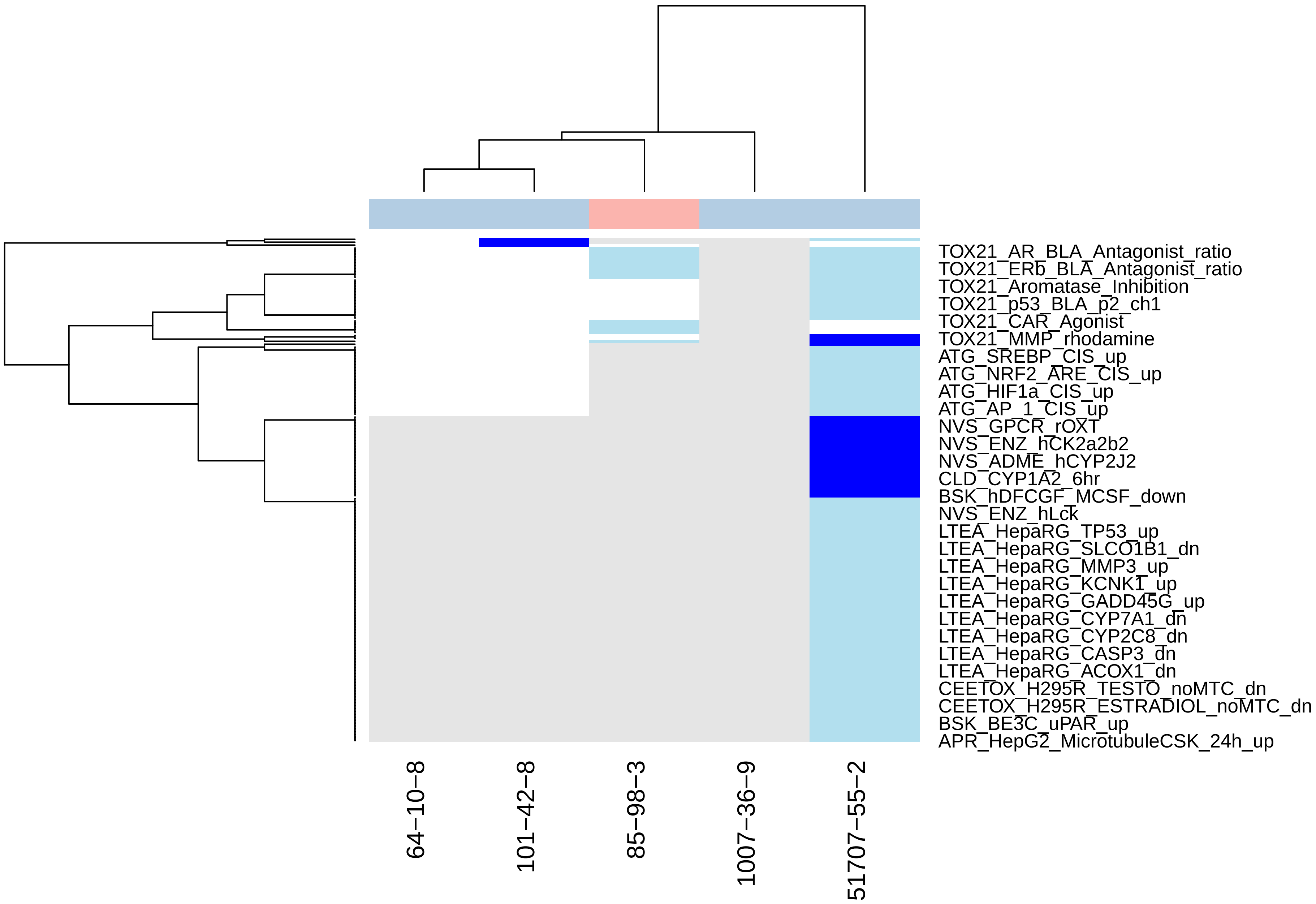

All Activity Heatmap for Cluster 84

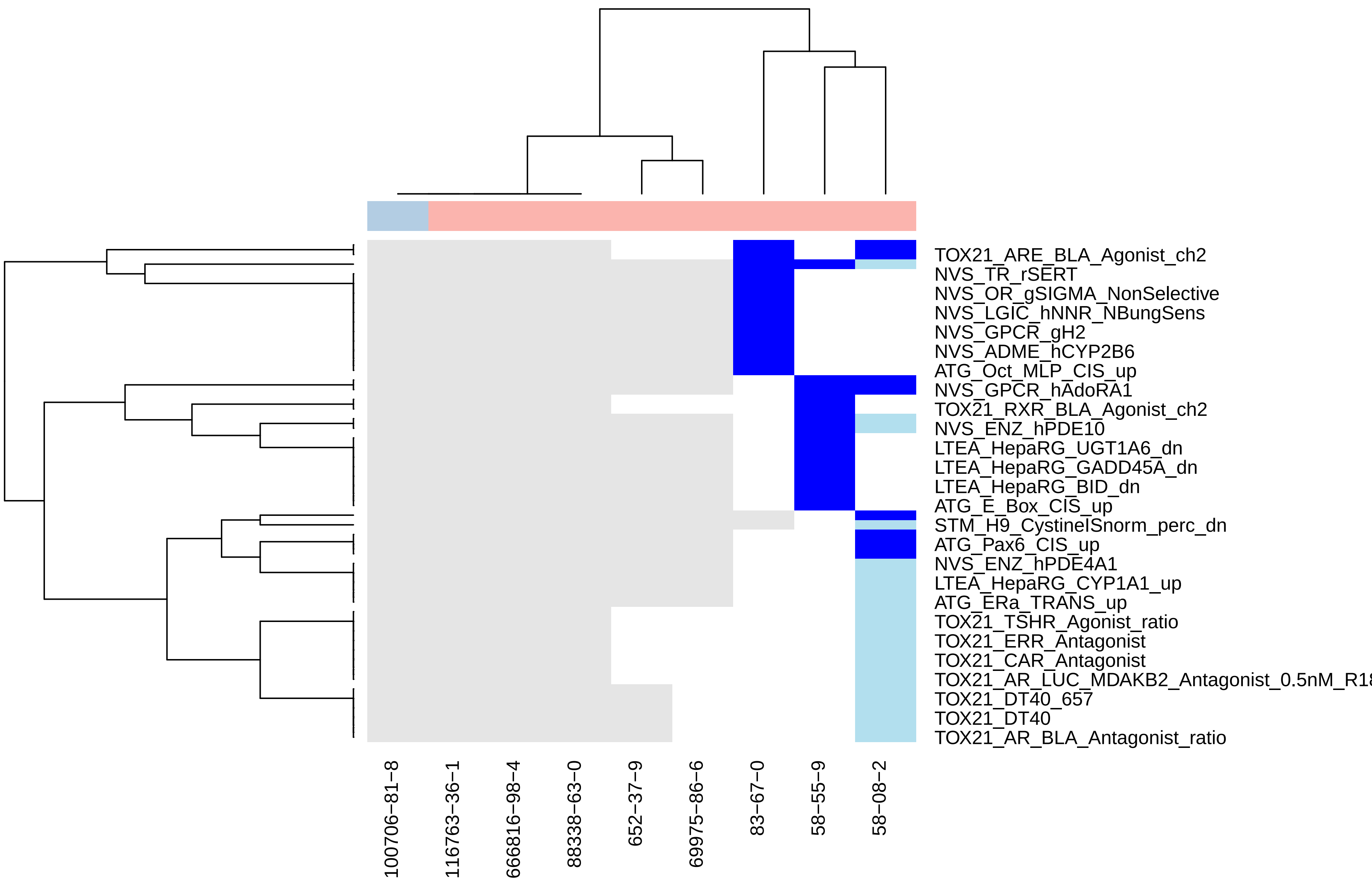

All Activity Heatmap for Cluster 85

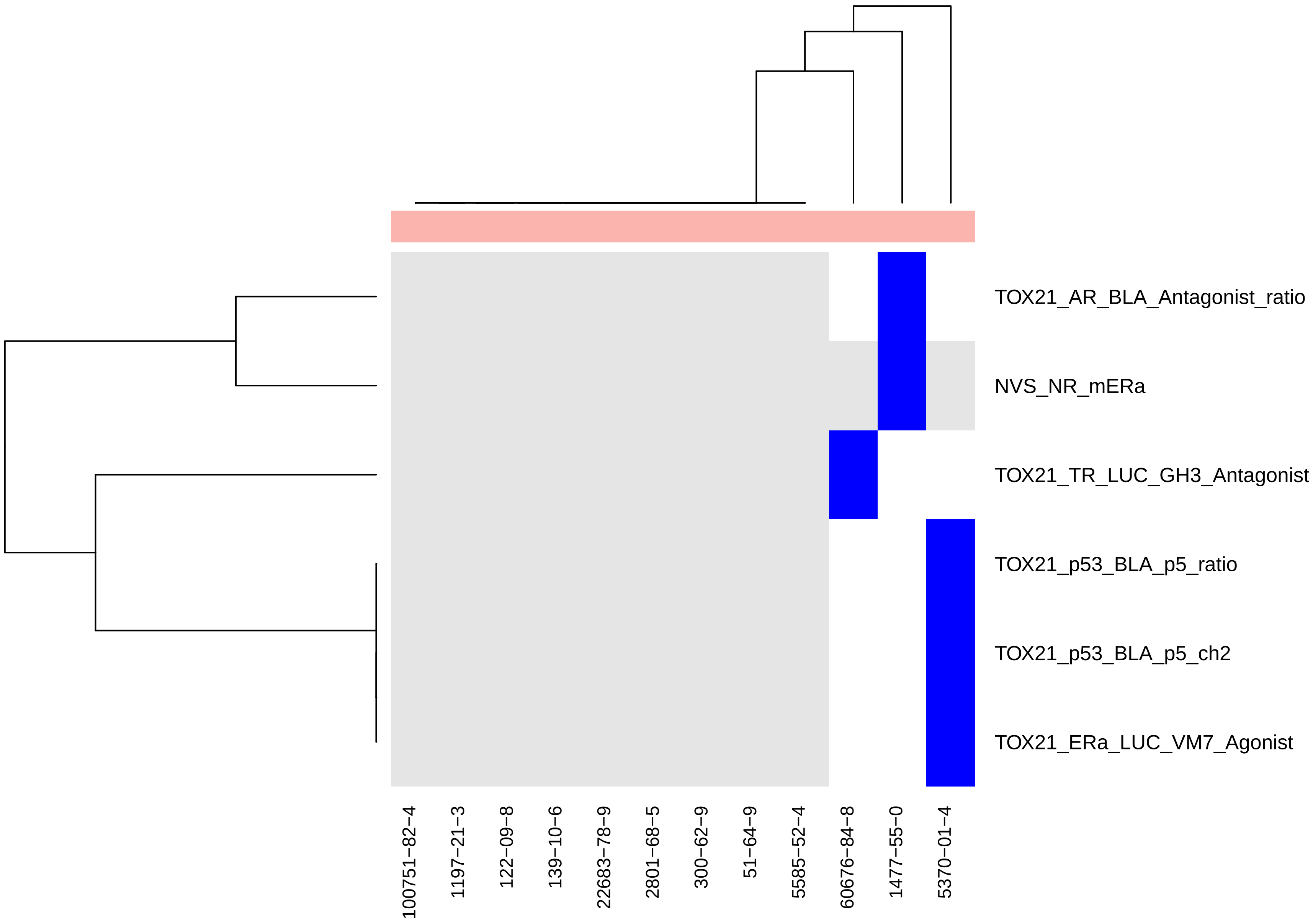

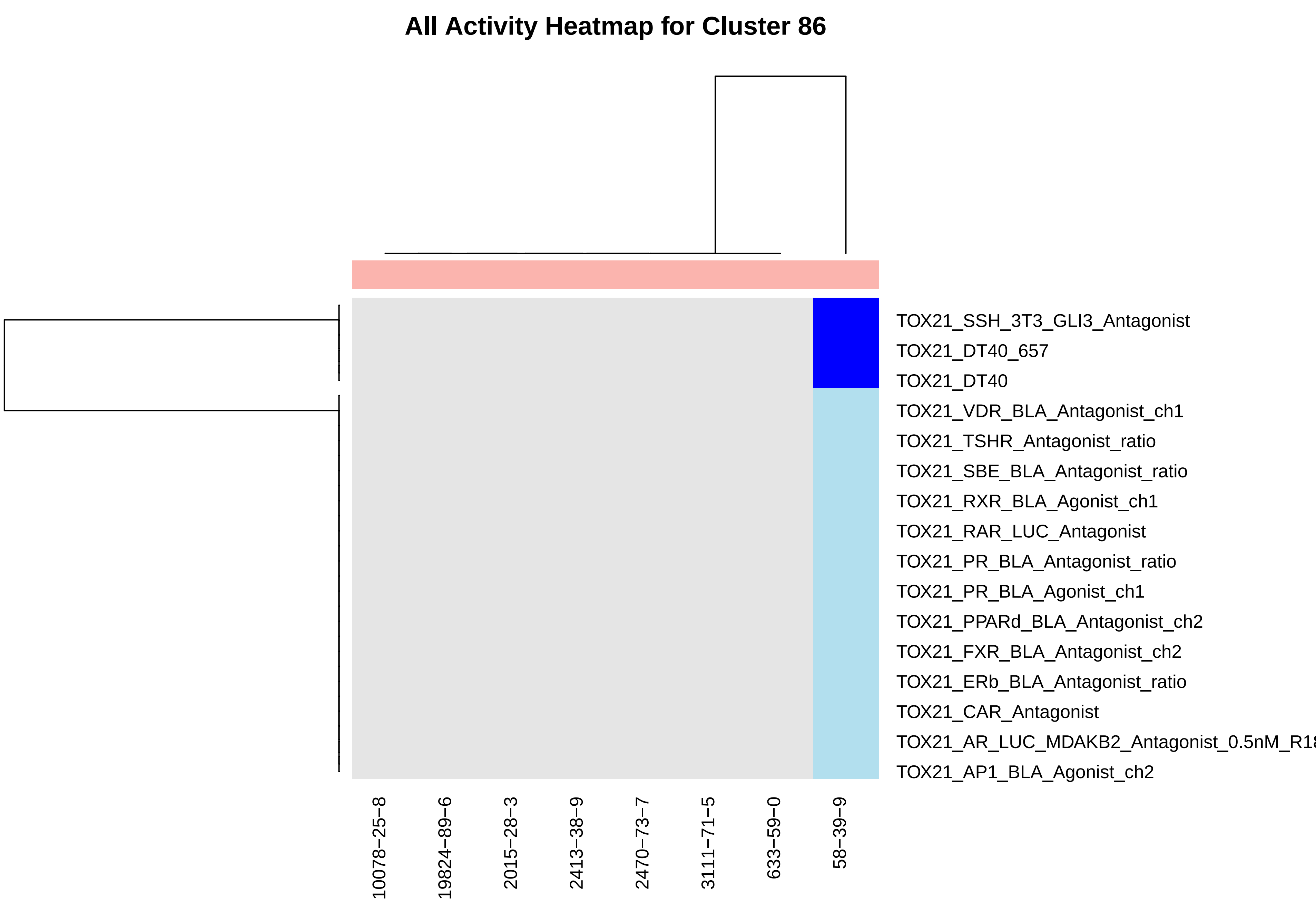
All Activity Heatmap for Cluster 86
TOX21_SSH_3T3_GLI3_Antagonist
TOX21_DT40_657
TOX21_DT40
TOX21_VDR_BLA_Antagonist_ch1
TOX21_TSHR_Antagonist_ratio
TOX21_SBE_BLA_Antagonist_ratio
TOX21_RXR_BLA_Agonist_ch1
TOX21_RAR_LUC_Antagonist
TOX21_PR_BLA_Antagonist_ratio
TOX21_PR_BLA_Agonist_ch1
TOX21_PPARd_BLA_Antagonist_ch2
TOX21_FXR_BLA_Antagonist_ch2
TOX21_ERb_BLA_Antagonist_ratio
TOX21_CAR_Antagonist
TOX21_AR_LUC_MDAKB2_Antagonist_0.5nM_R18
TOX21_AP1_BLA_Agonist_ch2
10078−25−8
19824−89−6
2015−28−3
2413−38−9
2470−73−7
3111−71−5
633−59−0
58−39−9

All Activity Heatmap for Cluster 87

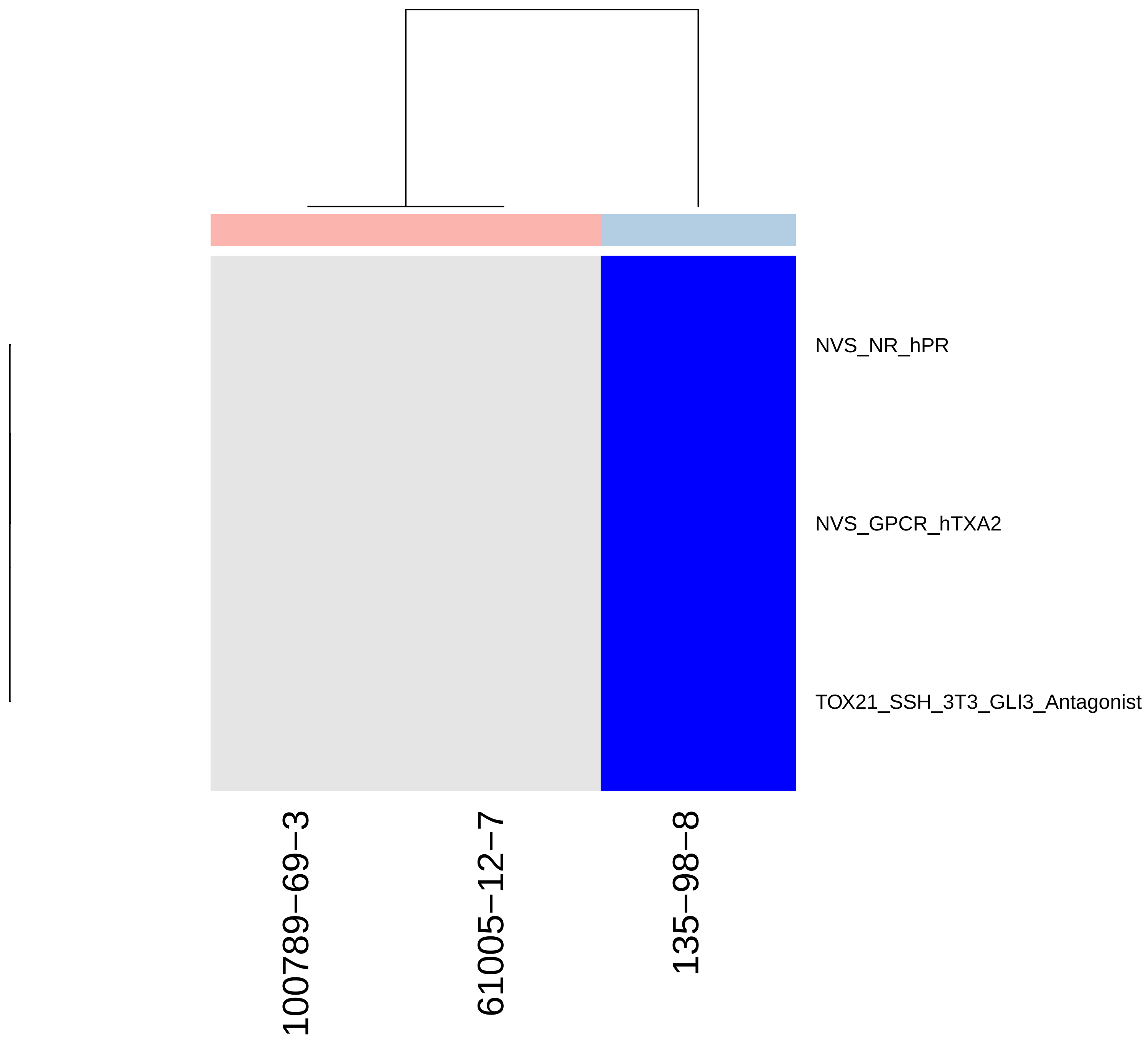

All Activity Heatmap for Cluster 88

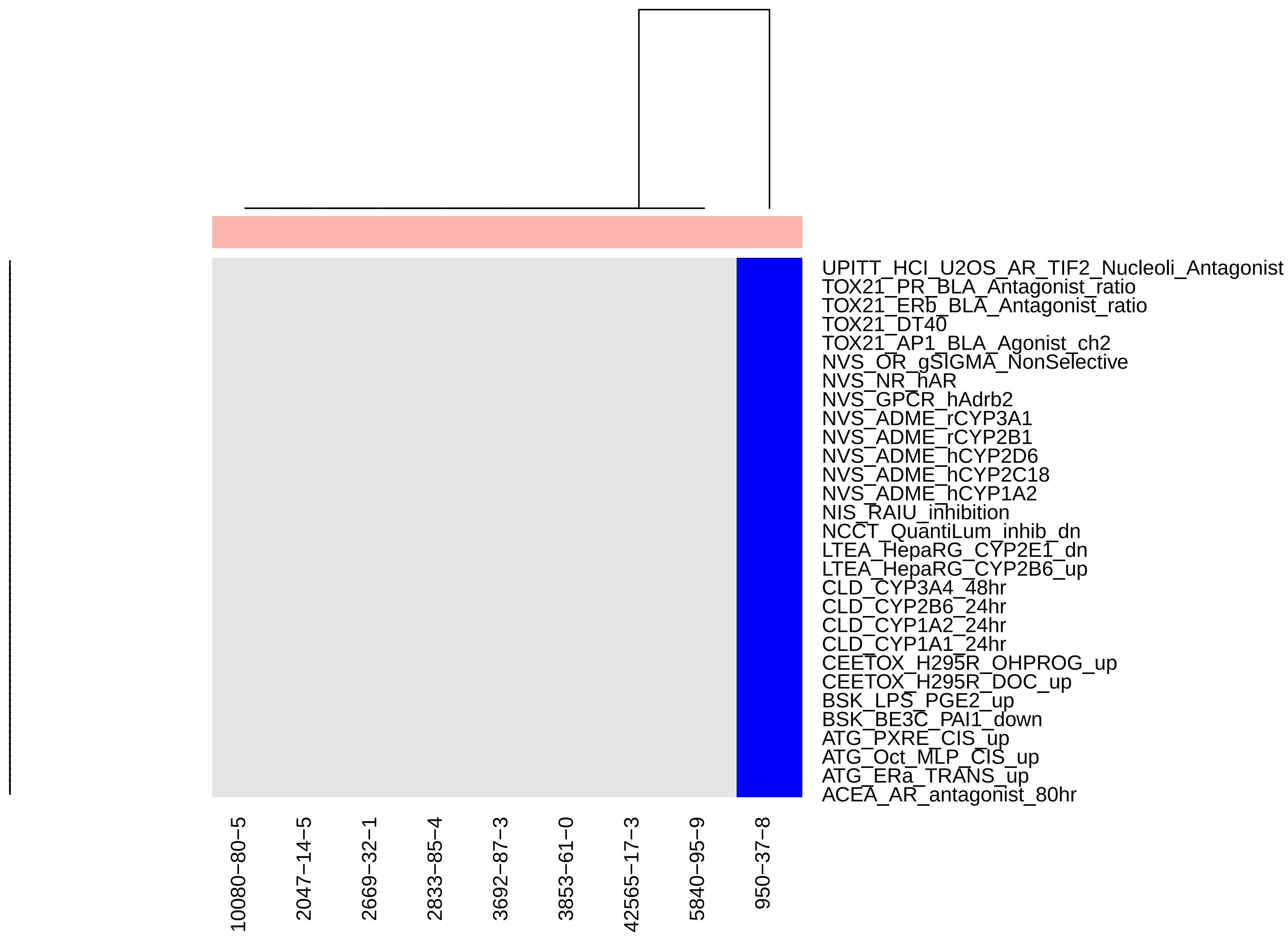

All Activity Heatmap for Cluster 89

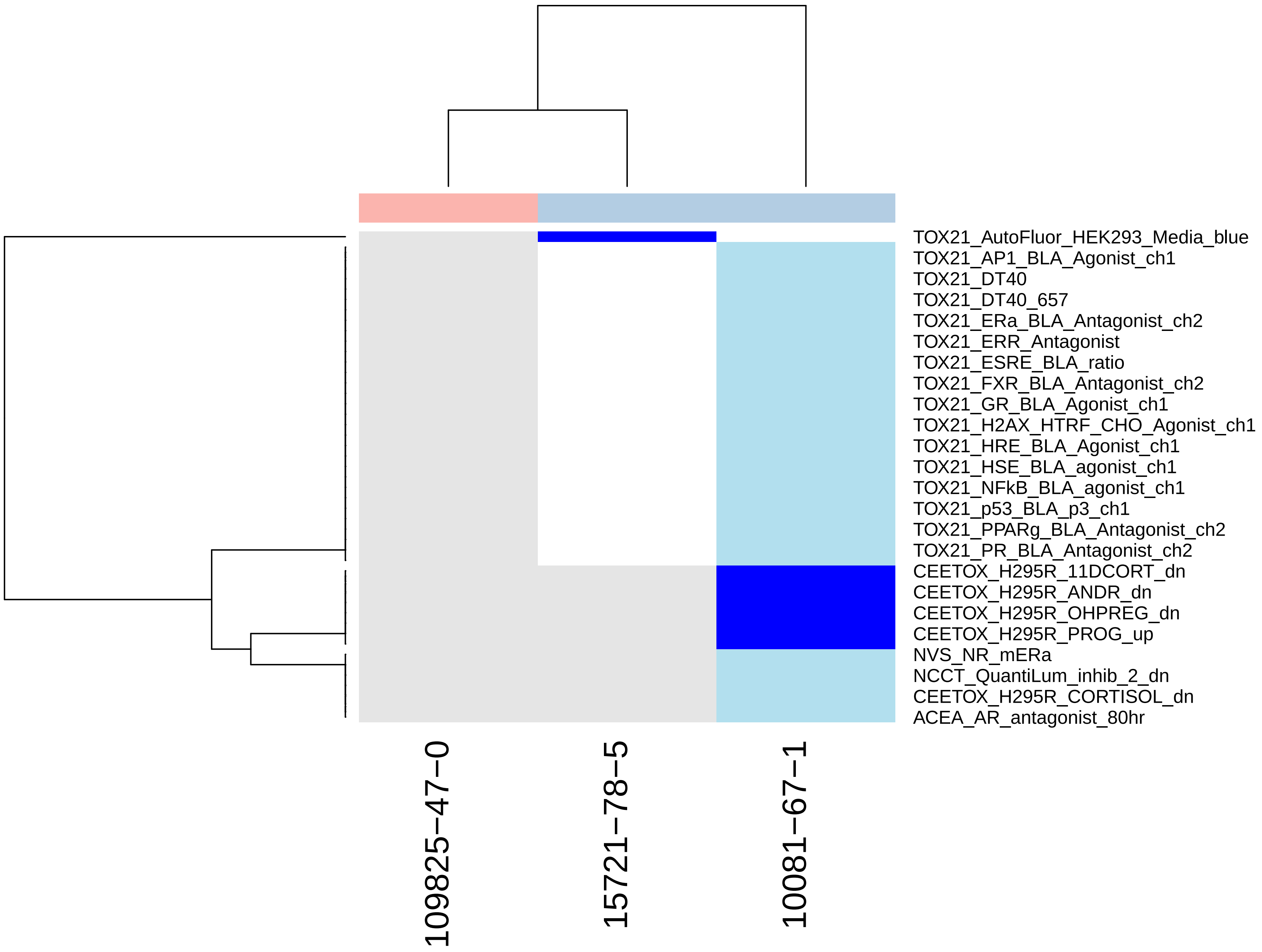

All Activity Heatmap for Cluster 90

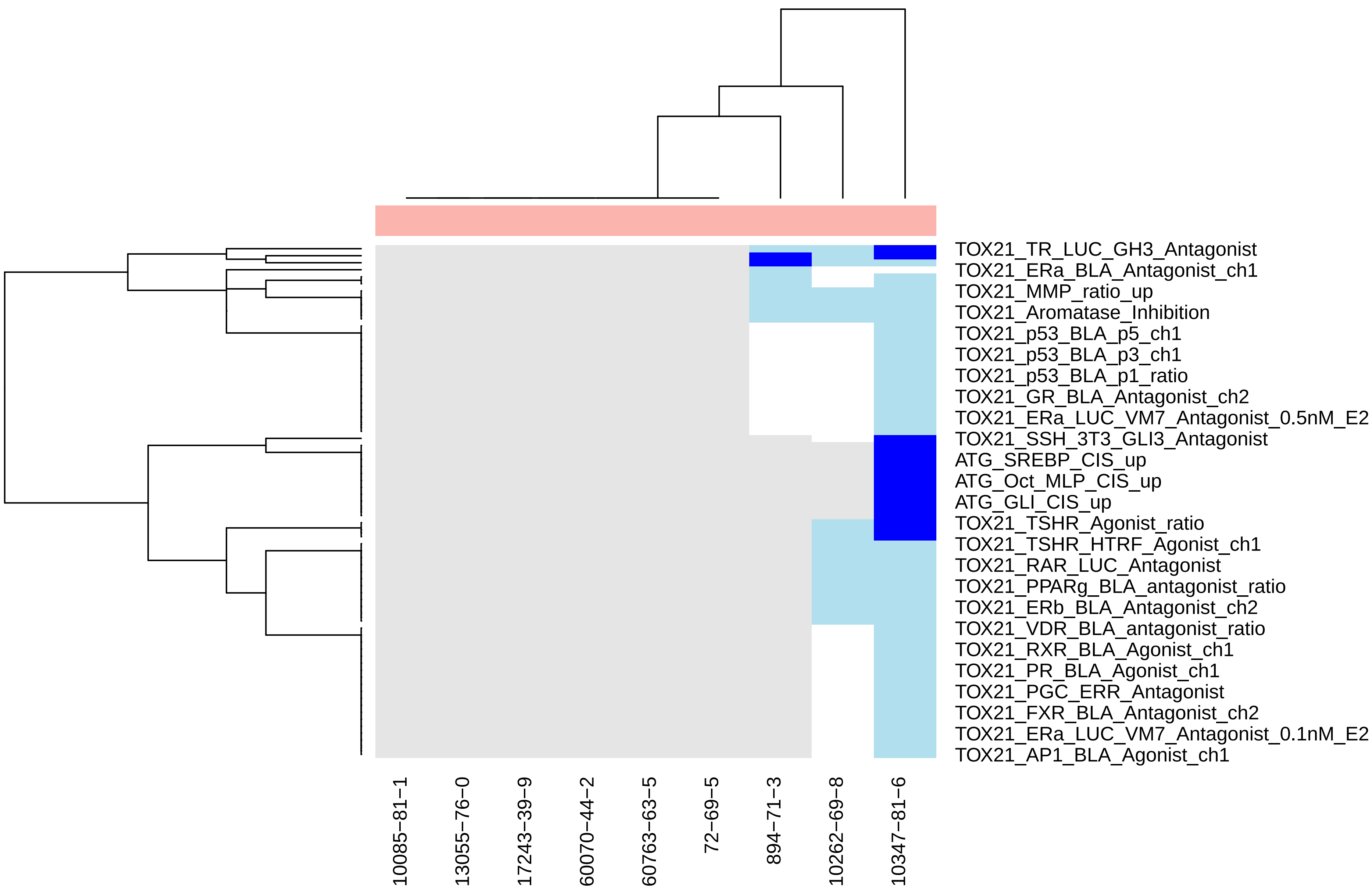

All Activity Heatmap for Cluster 91

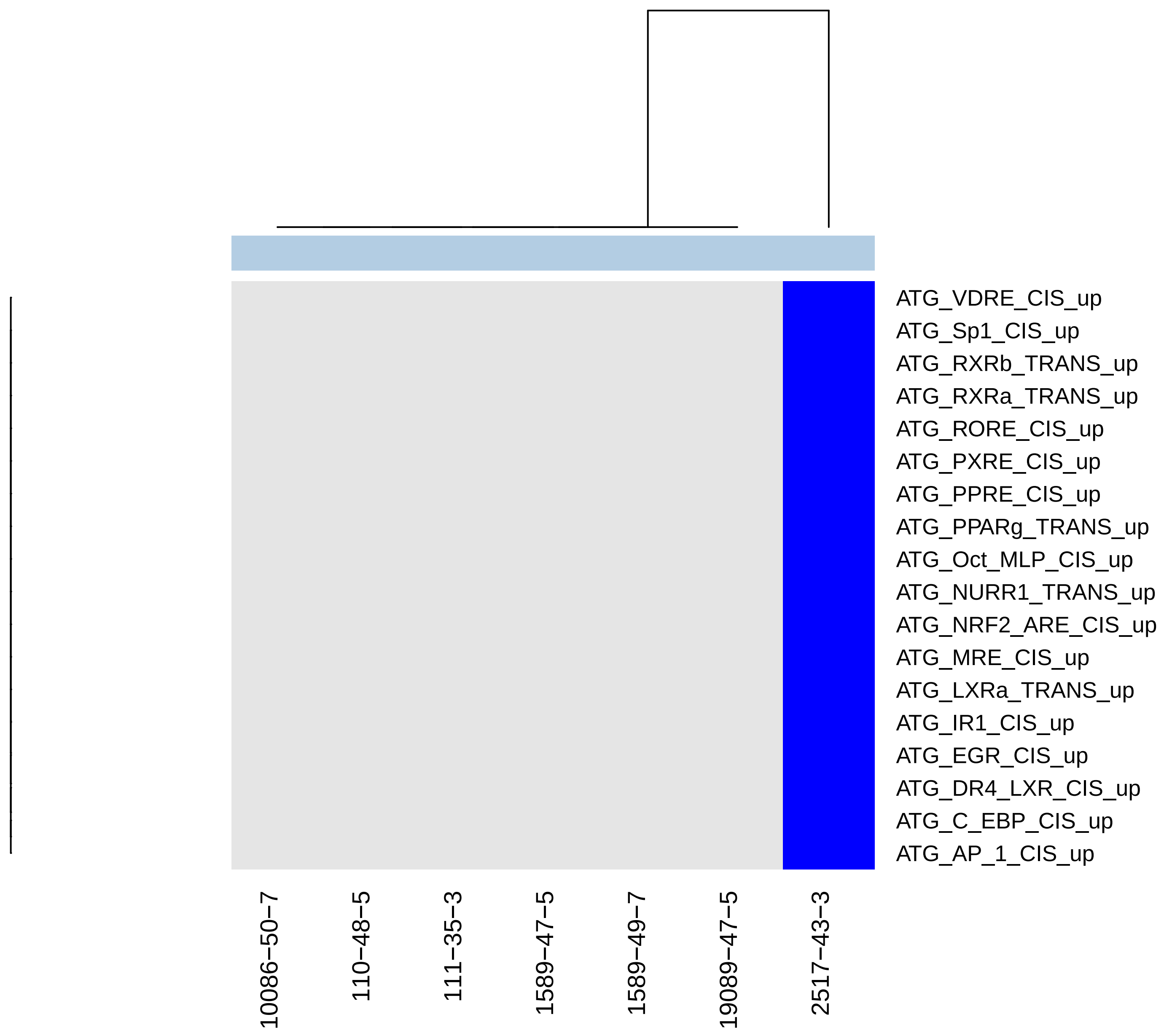

All Activity Heatmap for Cluster 92

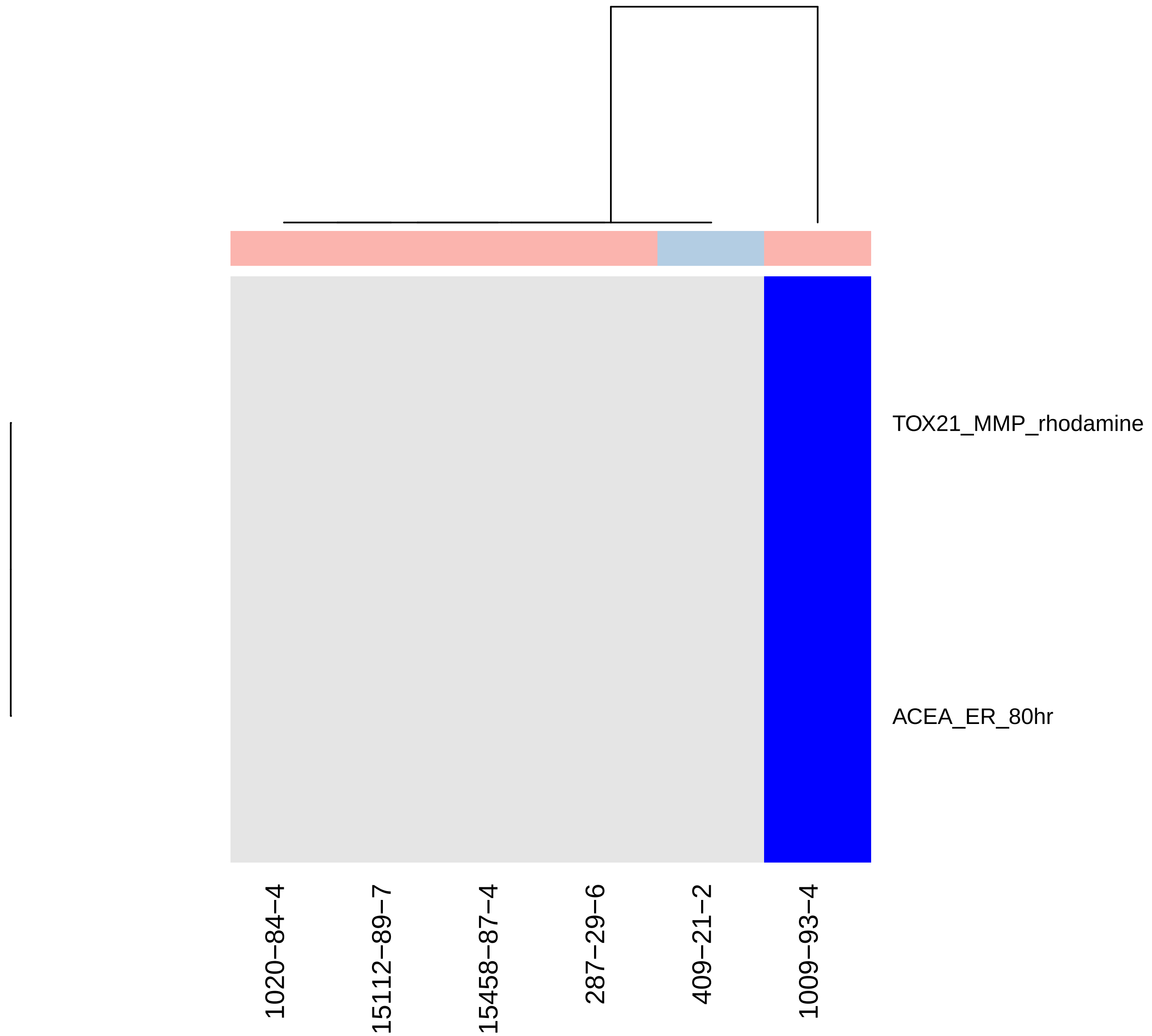

All Activity Heatmap for Cluster 93

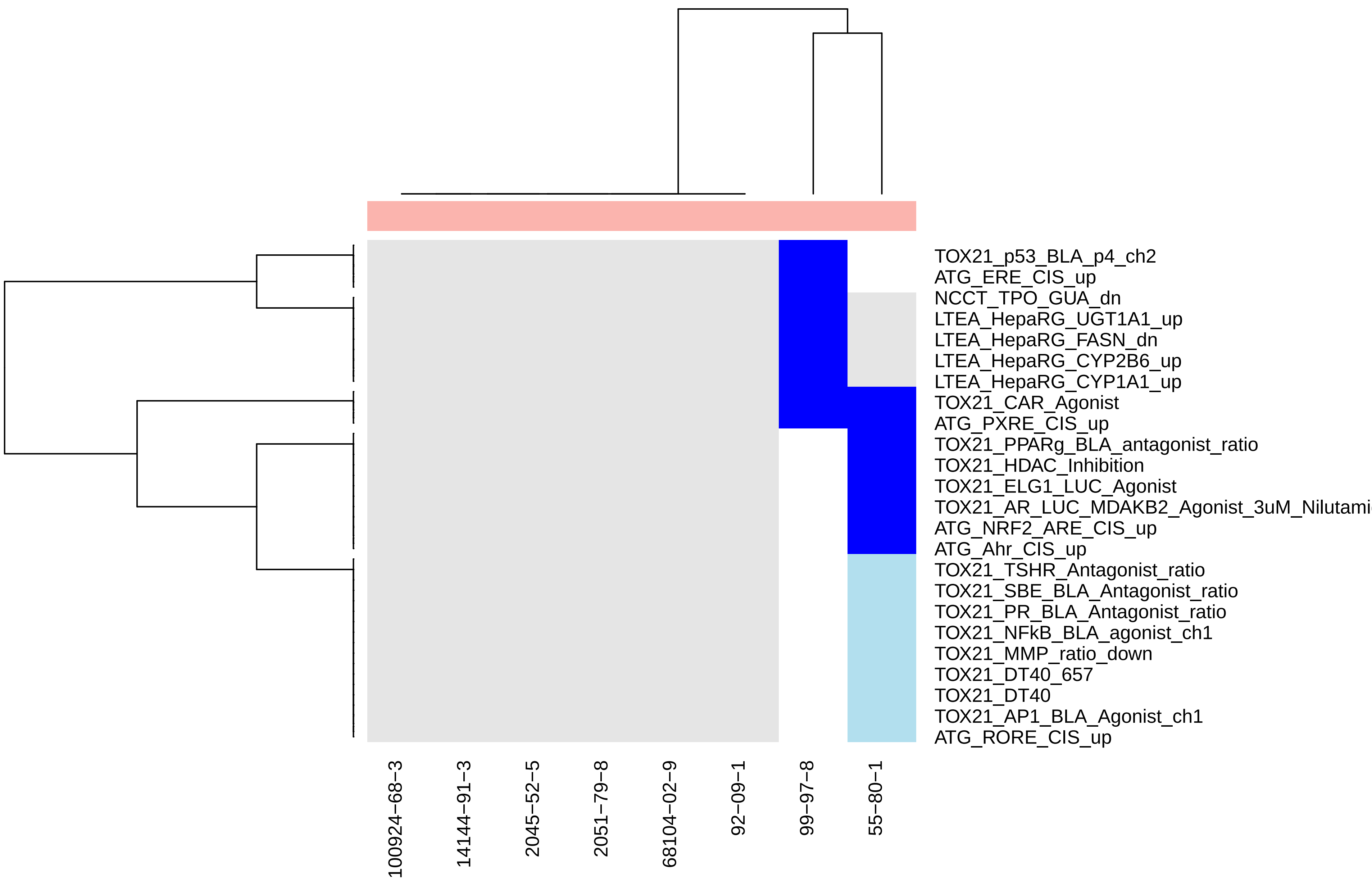

All Activity Heatmap for Cluster 94

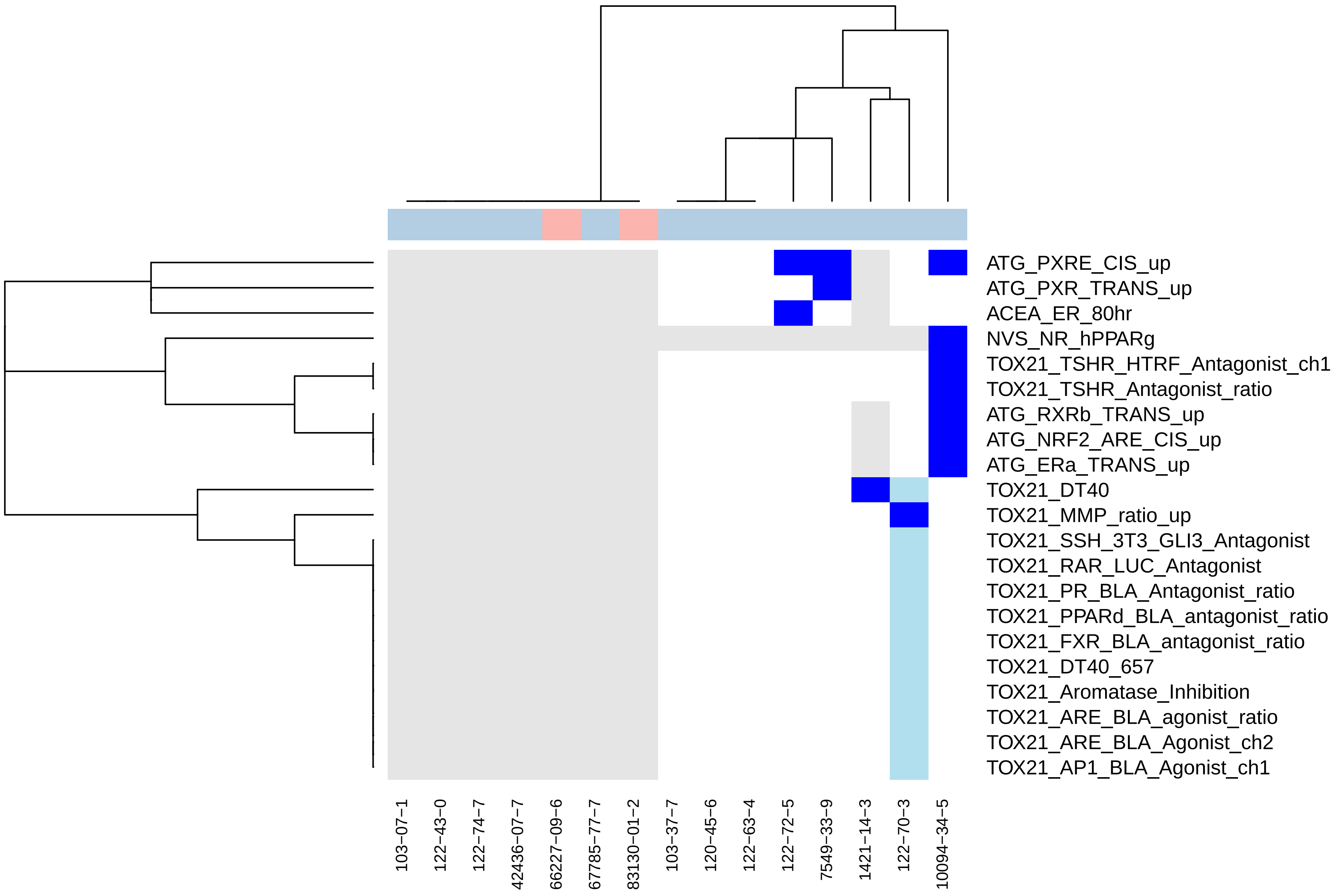

All Activity Heatmap for Cluster 96

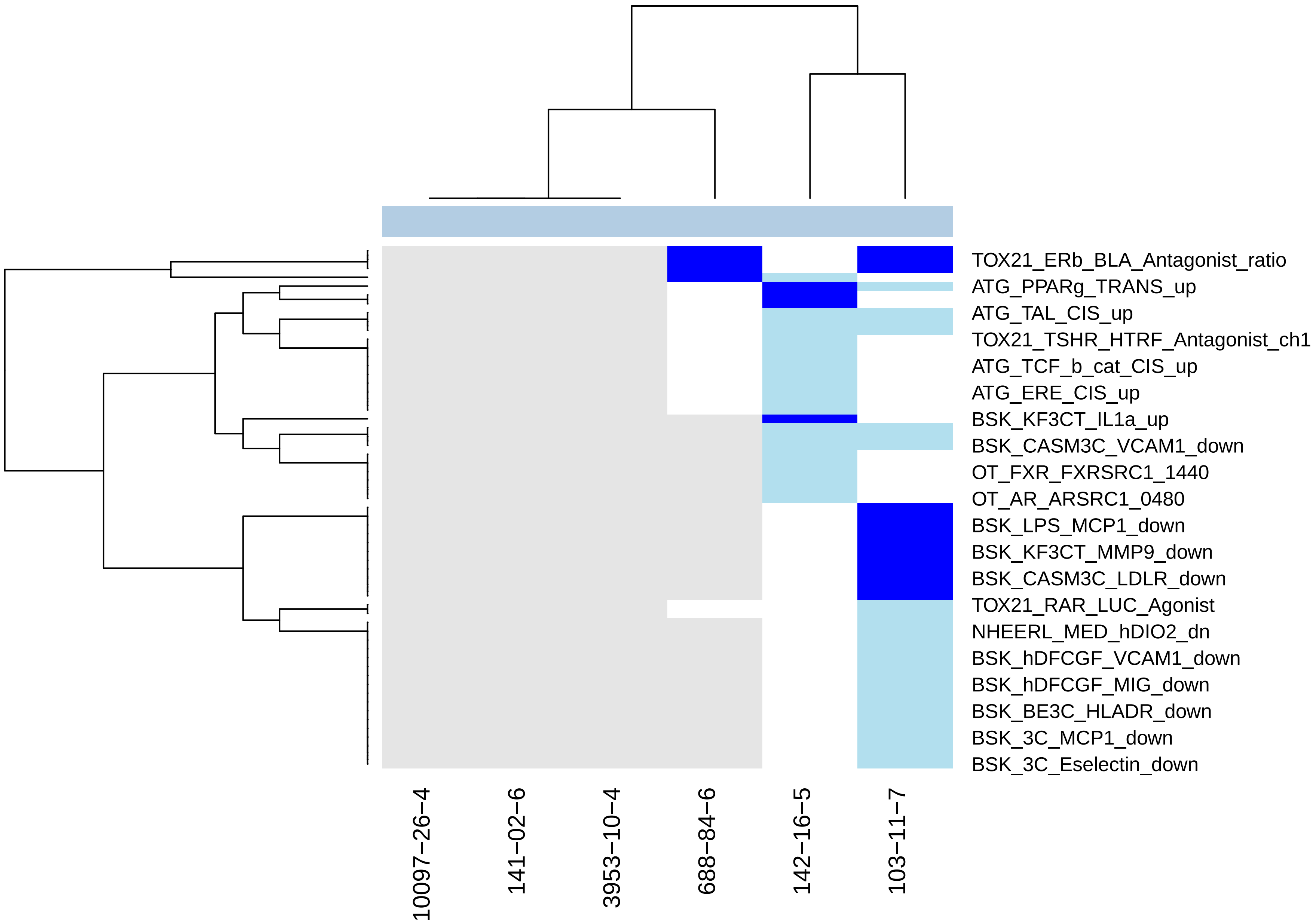

All Activity Heatmap for Cluster 97

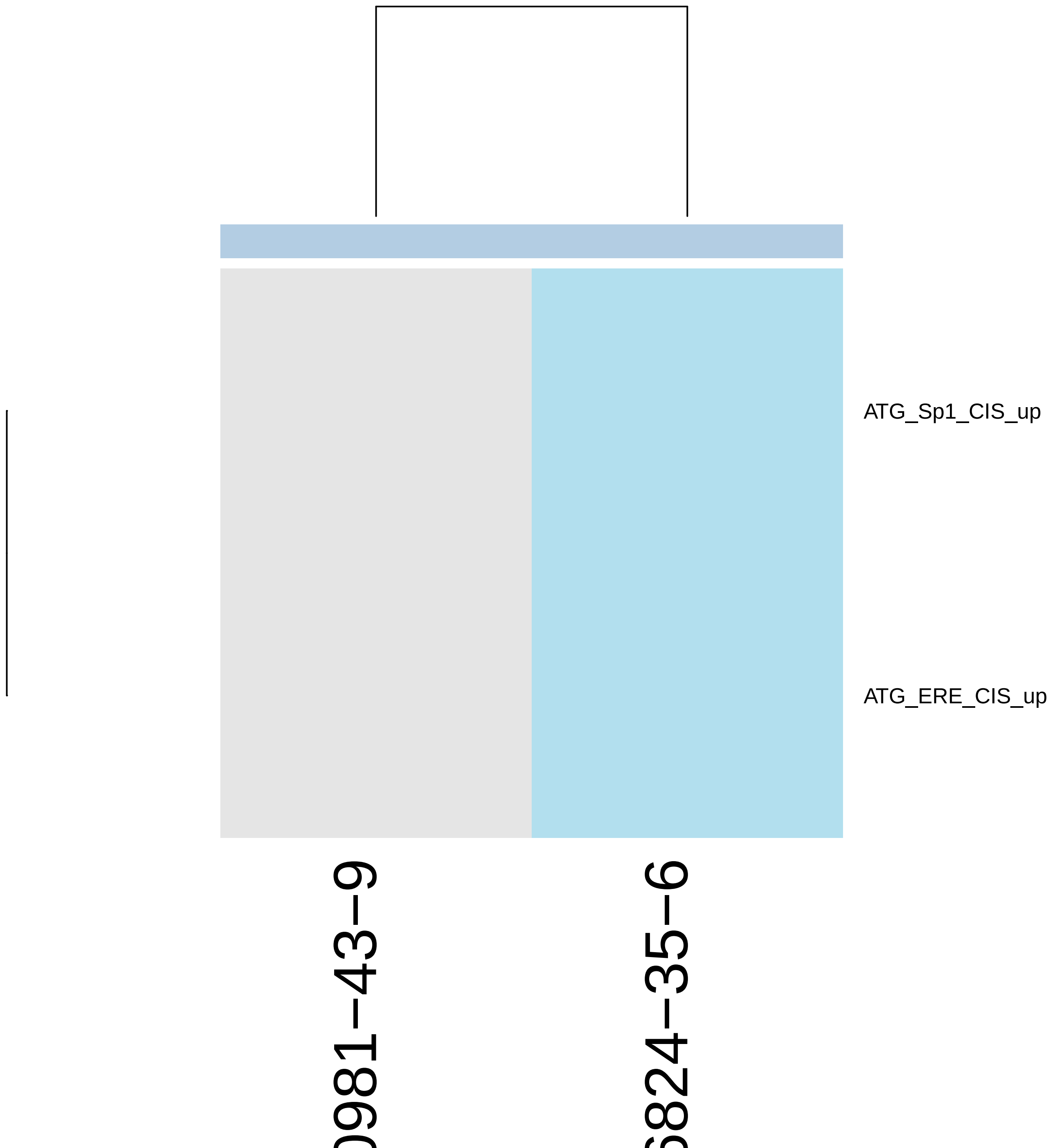

All Activity Heatmap for Cluster 98

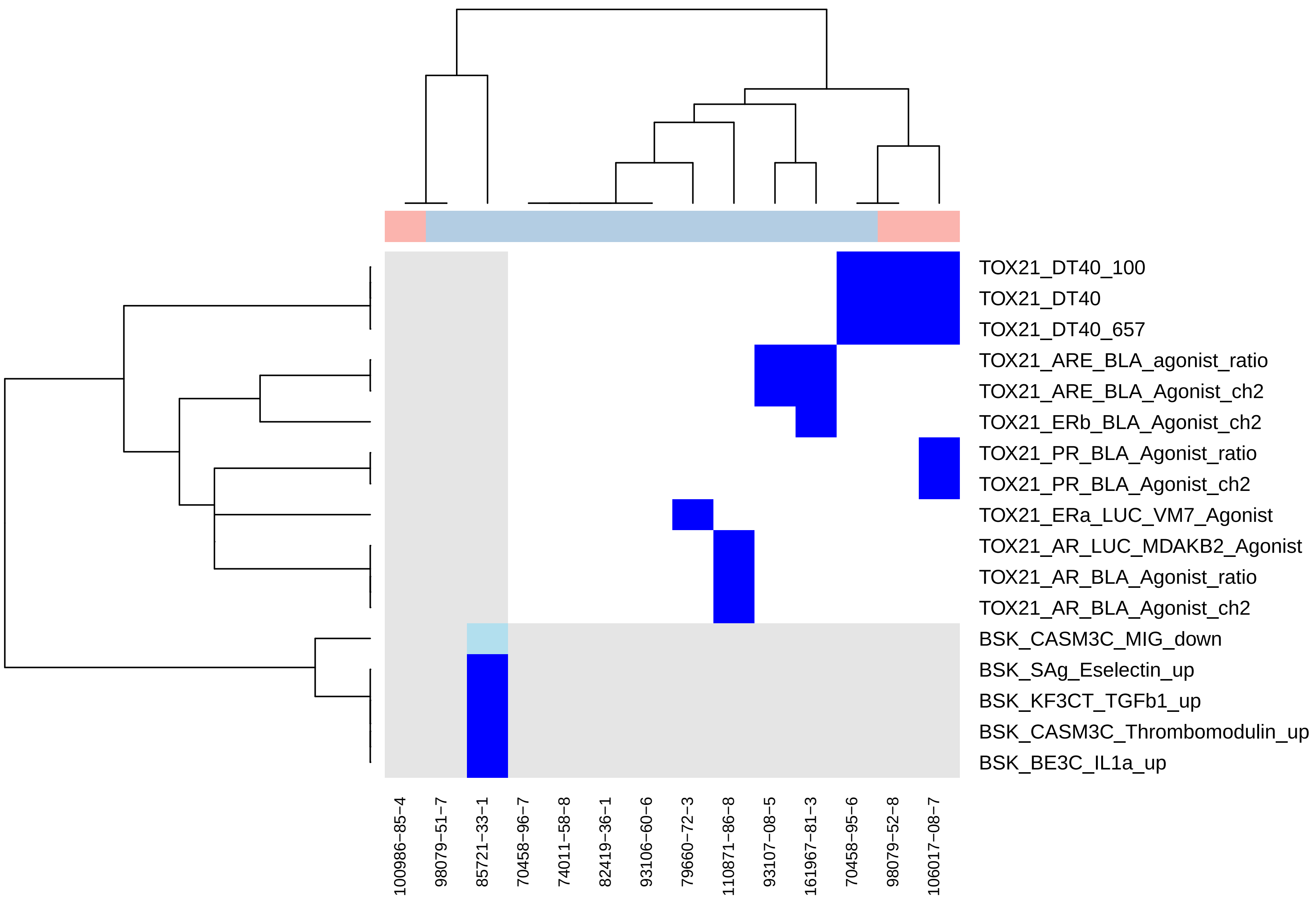

All Activity Heatmap for Cluster 99

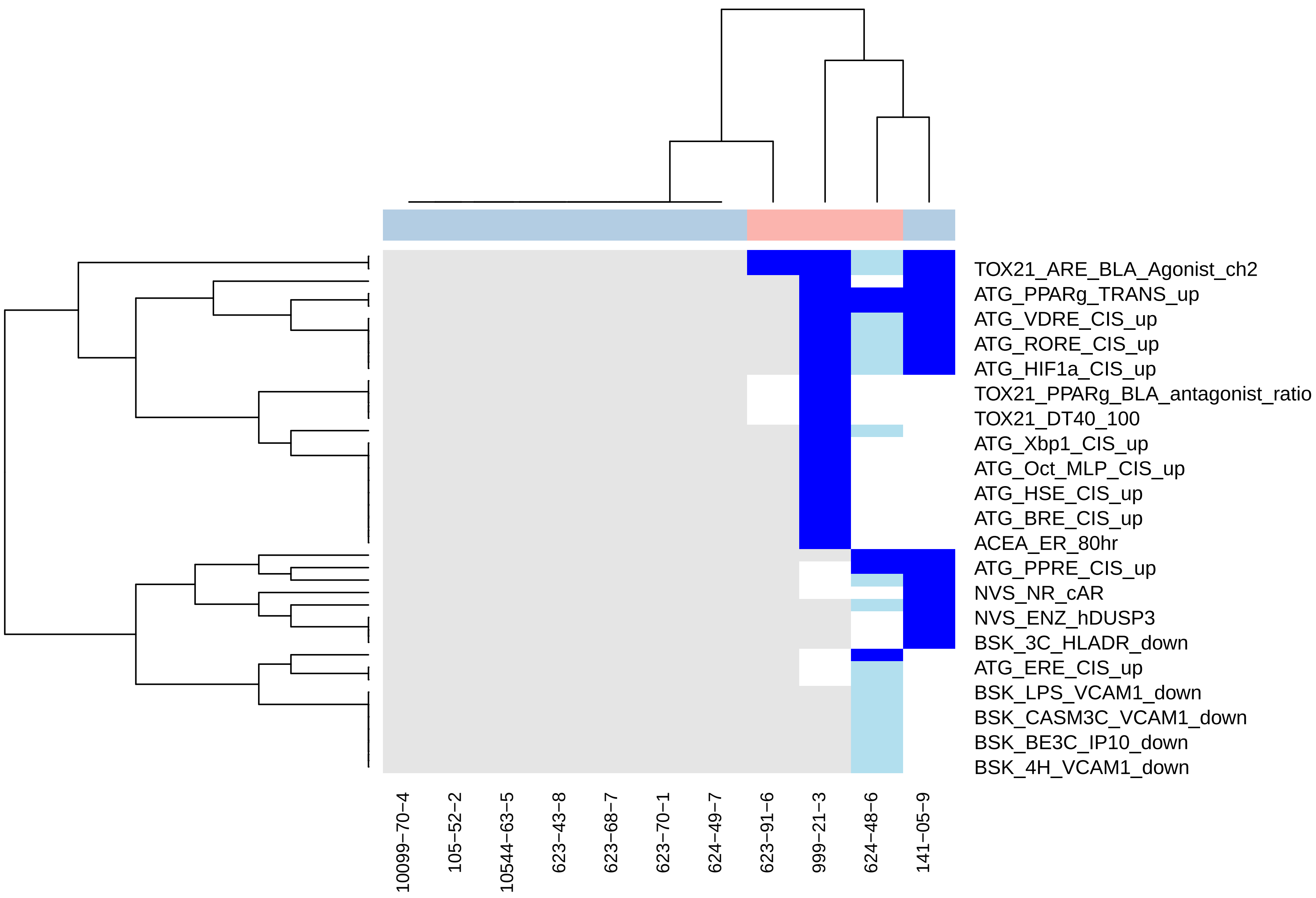

All Activity Heatmap for Cluster 100

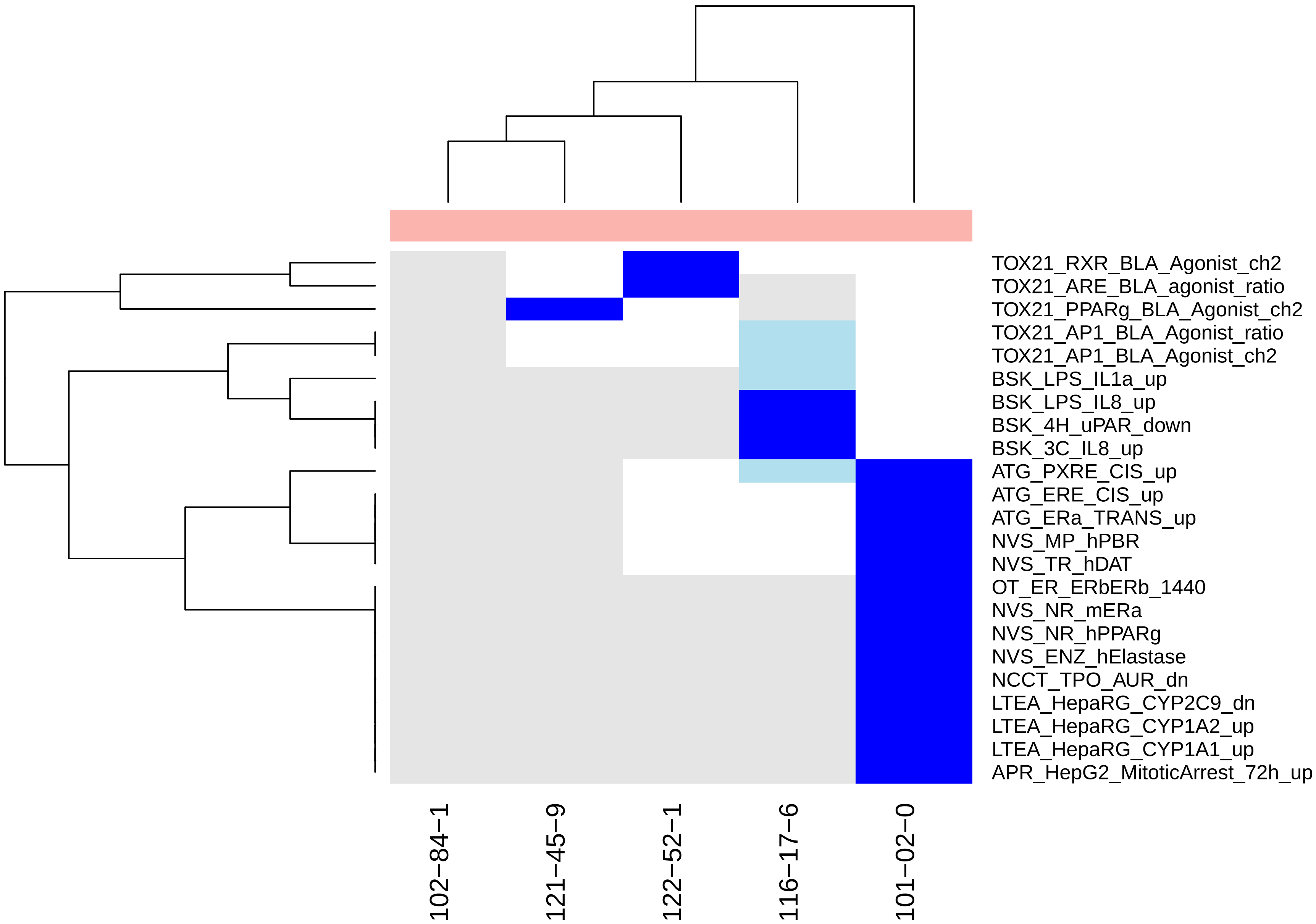

All Activity Heatmap for Cluster 101

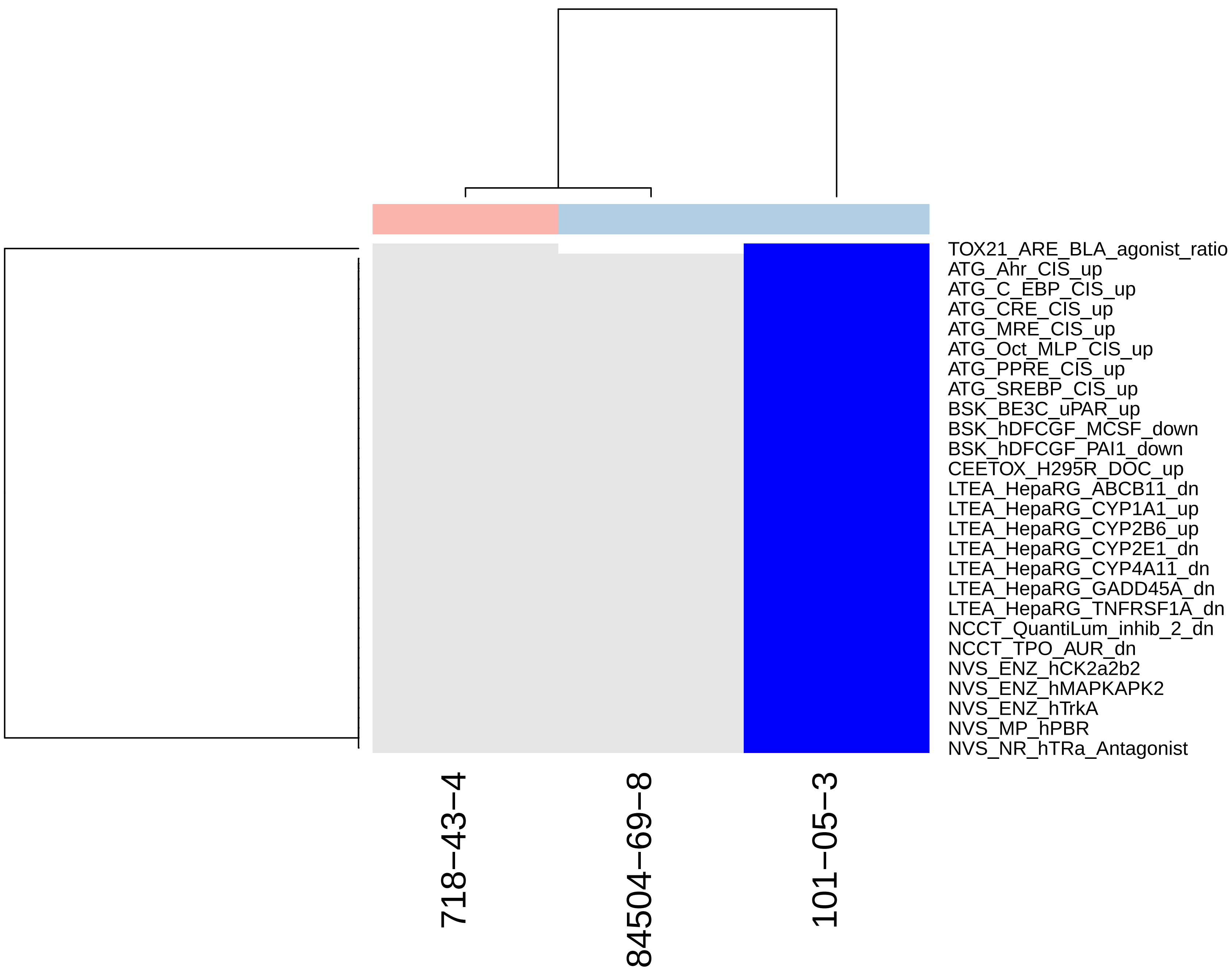

All Activity Heatmap for Cluster 102

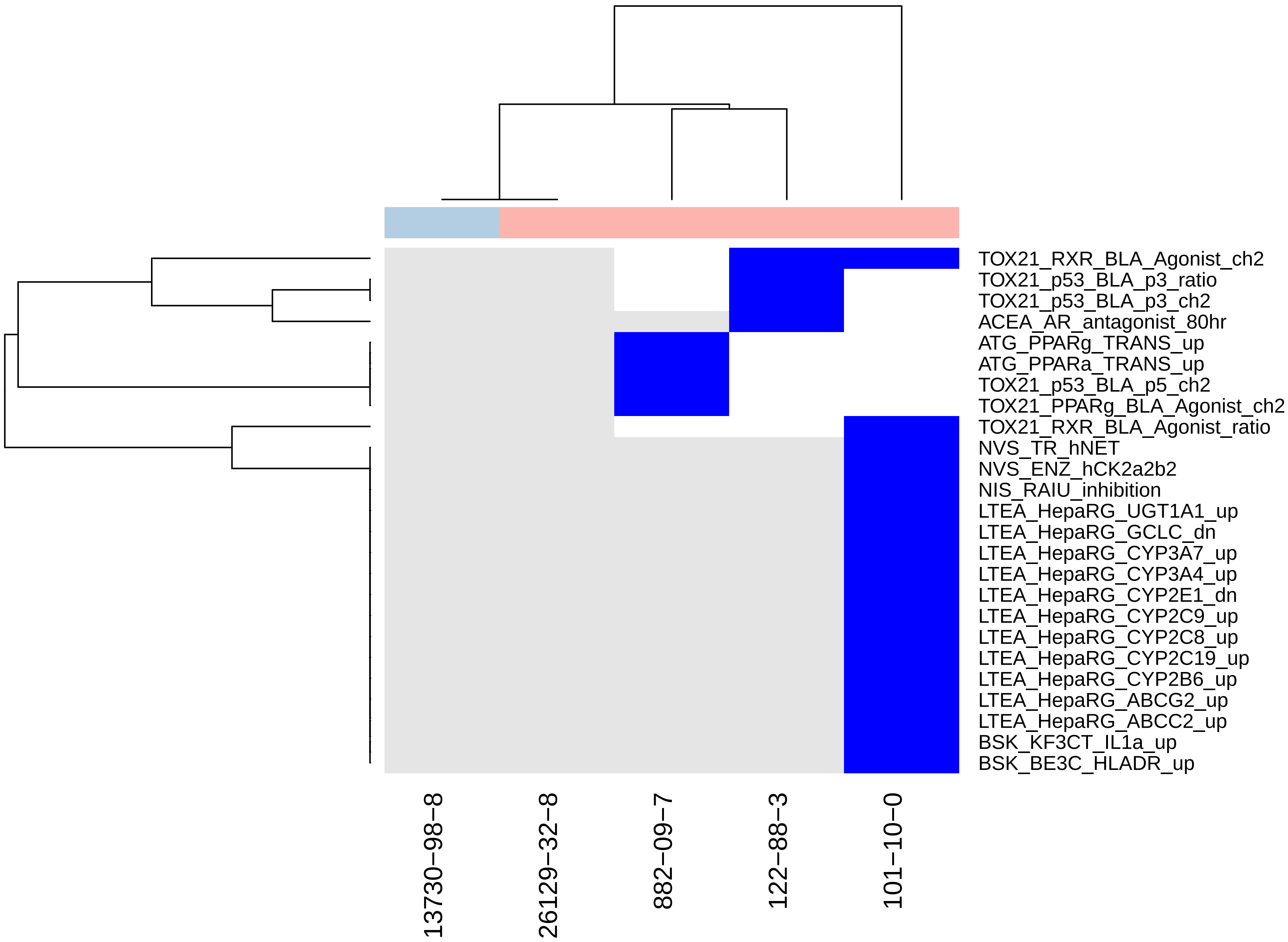

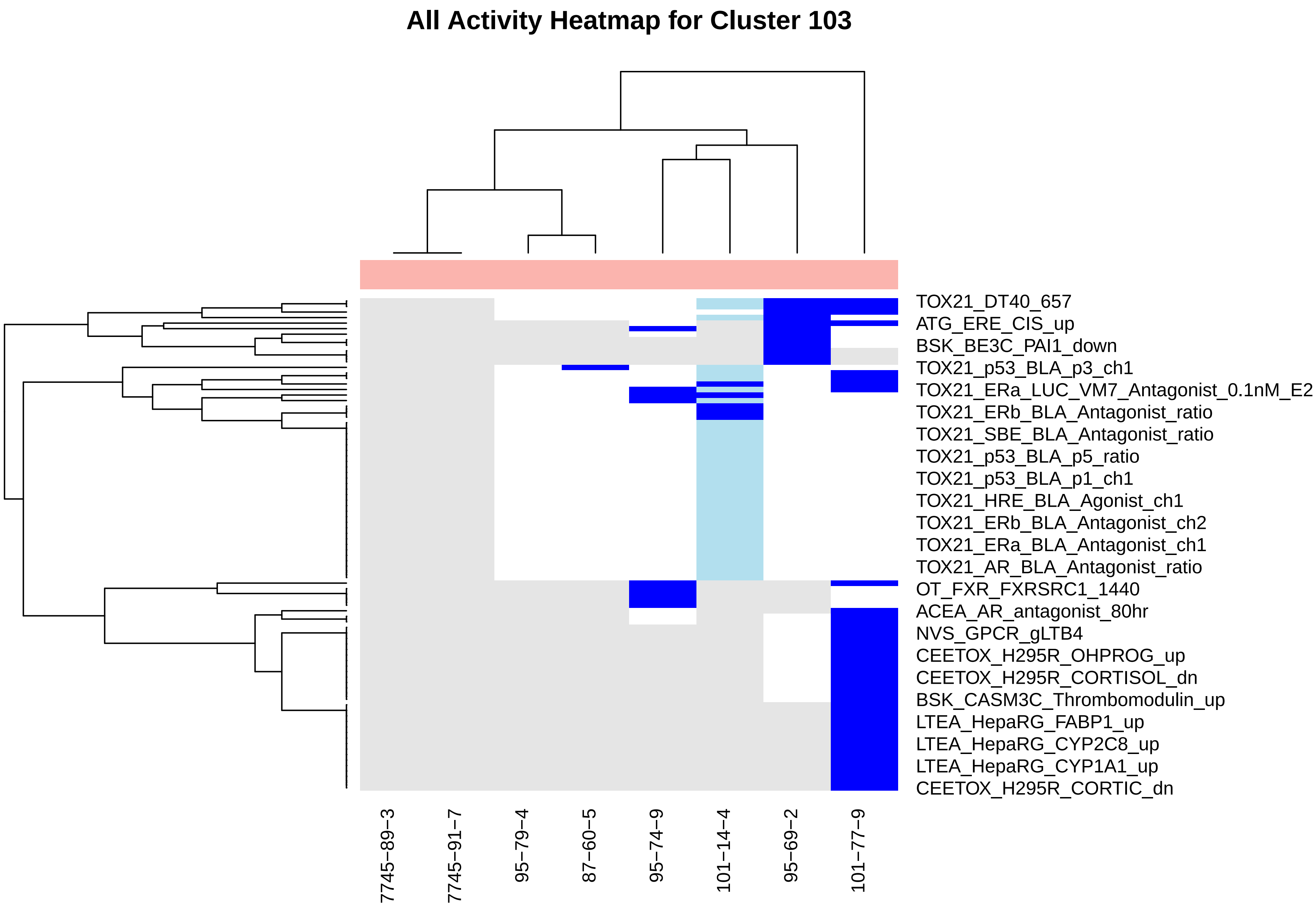
All Activity Heatmap for Cluster 103
TOX21_DT40_657
ATG_ERE_CIS_up
BSK_BE3C_PAI1_down
TOX21_p53_BLA_p3_ch1
TOX21_ERa_LUC_VM7_Antagonist_0.1nM_E2
TOX21_ERb_BLA_Antagonist_ratio
TOX21_SBE_BLA_Antagonist_ratio
TOX21_p53_BLA_p5_ratio
TOX21_p53_BLA_p1_ch1
TOX21_HRE_BLA_Agonist_ch1
TOX21_ERb_BLA_Antagonist_ch2
TOX21_ERa_BLA_Antagonist_ch1
TOX21_AR_BLA_Antagonist_ratio
OT_FXR_FXRSRC1_1440
ACEA_AR_antagonist_80hr
NVS_GPCR_gLTB4
CEETOX_H295R_OHPROG_up
CEETOX_H295R_CORTISOL_dn
BSK_CASM3C_Thrombomodulin_up
LTEA_HepaRG_FABP1_up
LTEA_HepaRG_CYP2C8_up
LTEA_HepaRG_CYP1A1_up
CEETOX_H295R_CORTIC_dn
7745−89−3
7745−91−7
95−79−4
87−60−5
95−74−9
101−14−4
95−69−2
101−77−9

All Activity Heatmap for Cluster 104

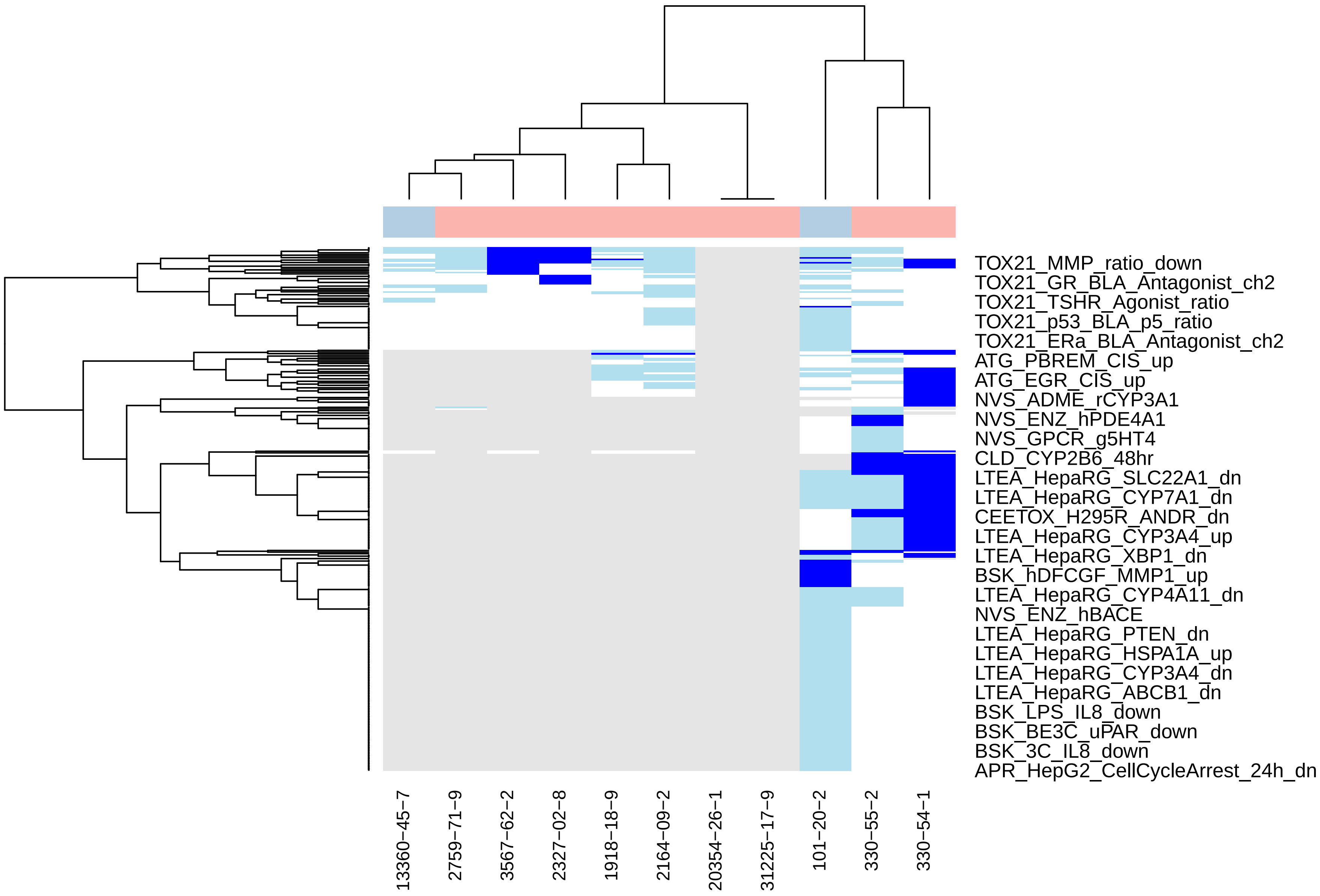

All Activity Heatmap for Cluster 105

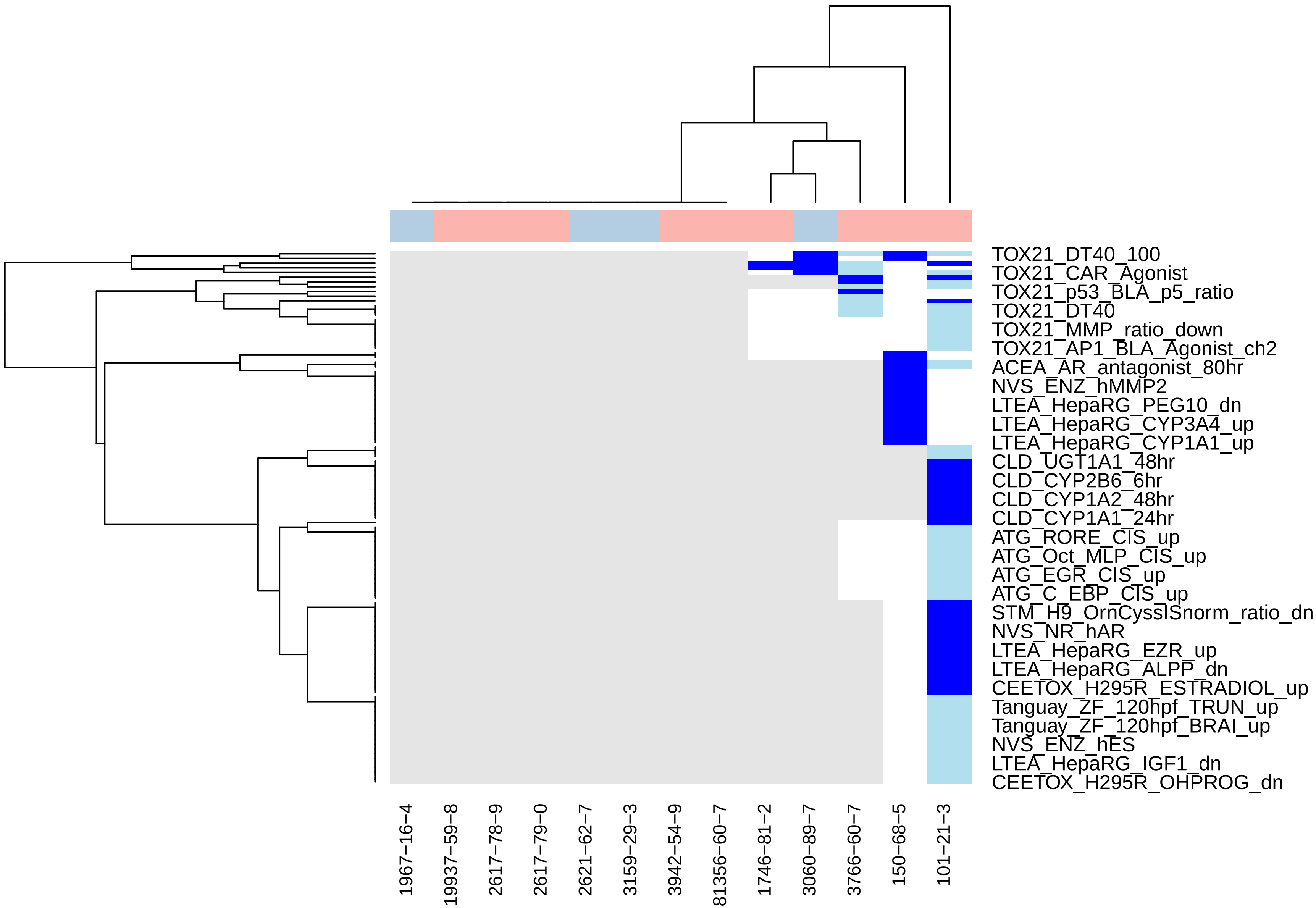

All Activity Heatmap for Cluster 107

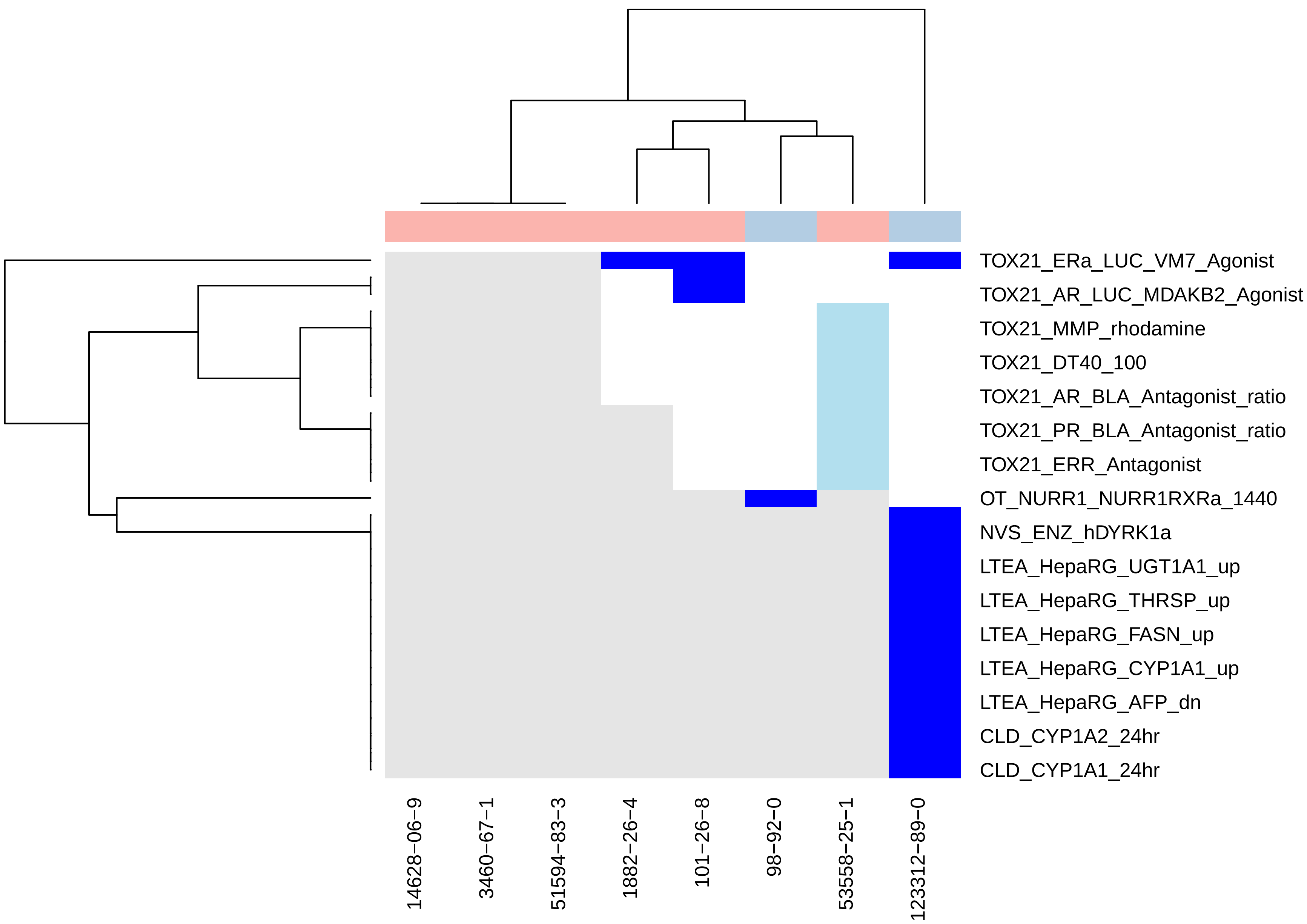

All Activity Heatmap for Cluster 108

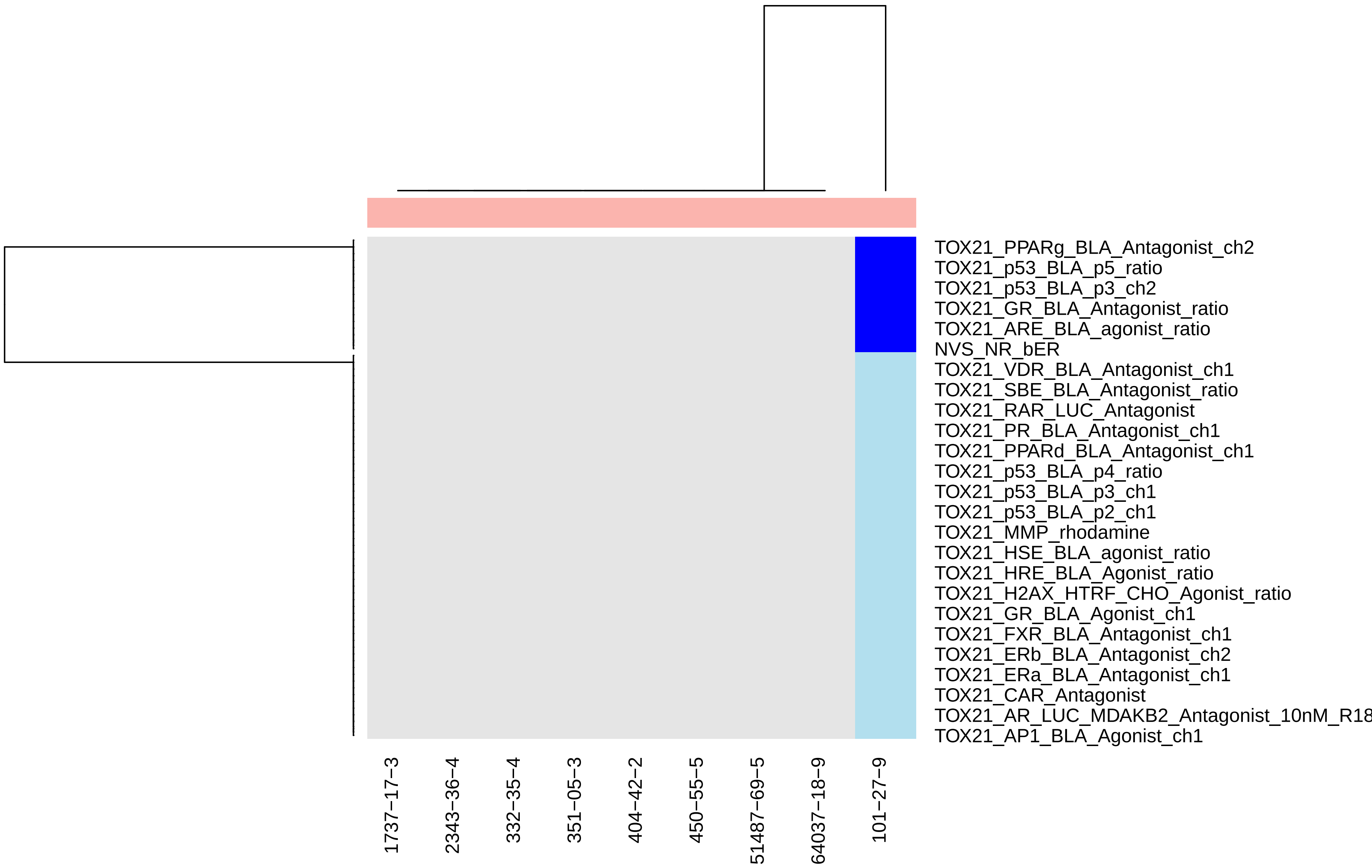

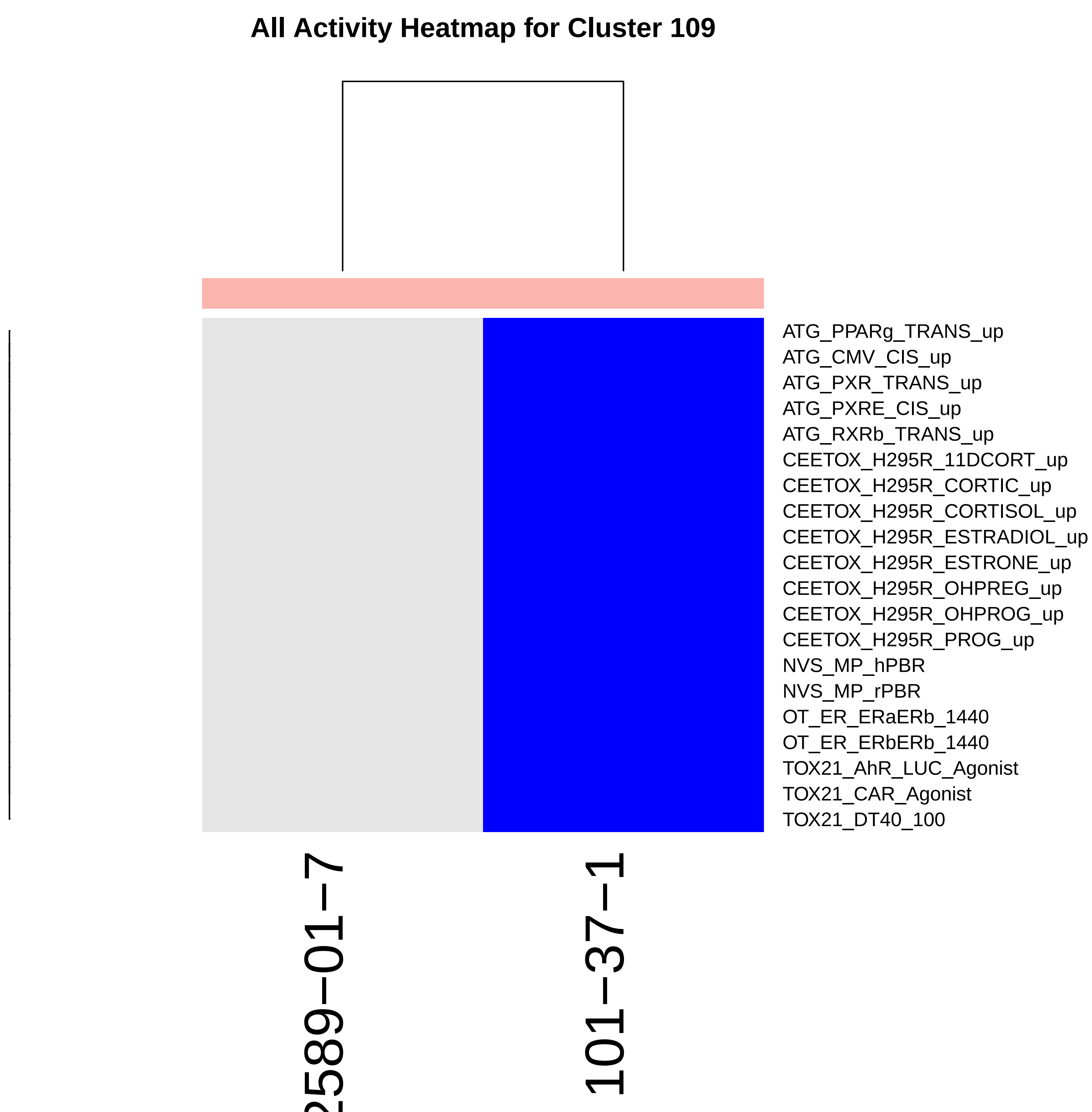
All Activity Heatmap for Cluster 109
ATG_PPARg_TRANS_up
ATG_CMV_CIS_up
ATG_PXR_TRANS_up
ATG_PXRE_CIS_up
ATG_RXRb_TRANS_up
CEETOX_H295R_11DCORT_up
CEETOX_H295R_CORTIC_up
CEETOX_H295R_CORTISOL_up
CEETOX_H295R_ESTRADIOL_up
CEETOX_H295R_ESTRONE_up
CEETOX_H295R_OHPREG_up
CEETOX_H295R_OHPROG_up
CEETOX_H295R_PROG_up
NVS_MP_hPBR
NVS_MP_rPBR
OT_ER_ERaERb_1440
OT_ER_ERbERb_1440
TOX21_AhR_LUC_Agonist
TOX21_CAR_Agonist
TOX21_DT40_100
2589−01−7
101−37−1

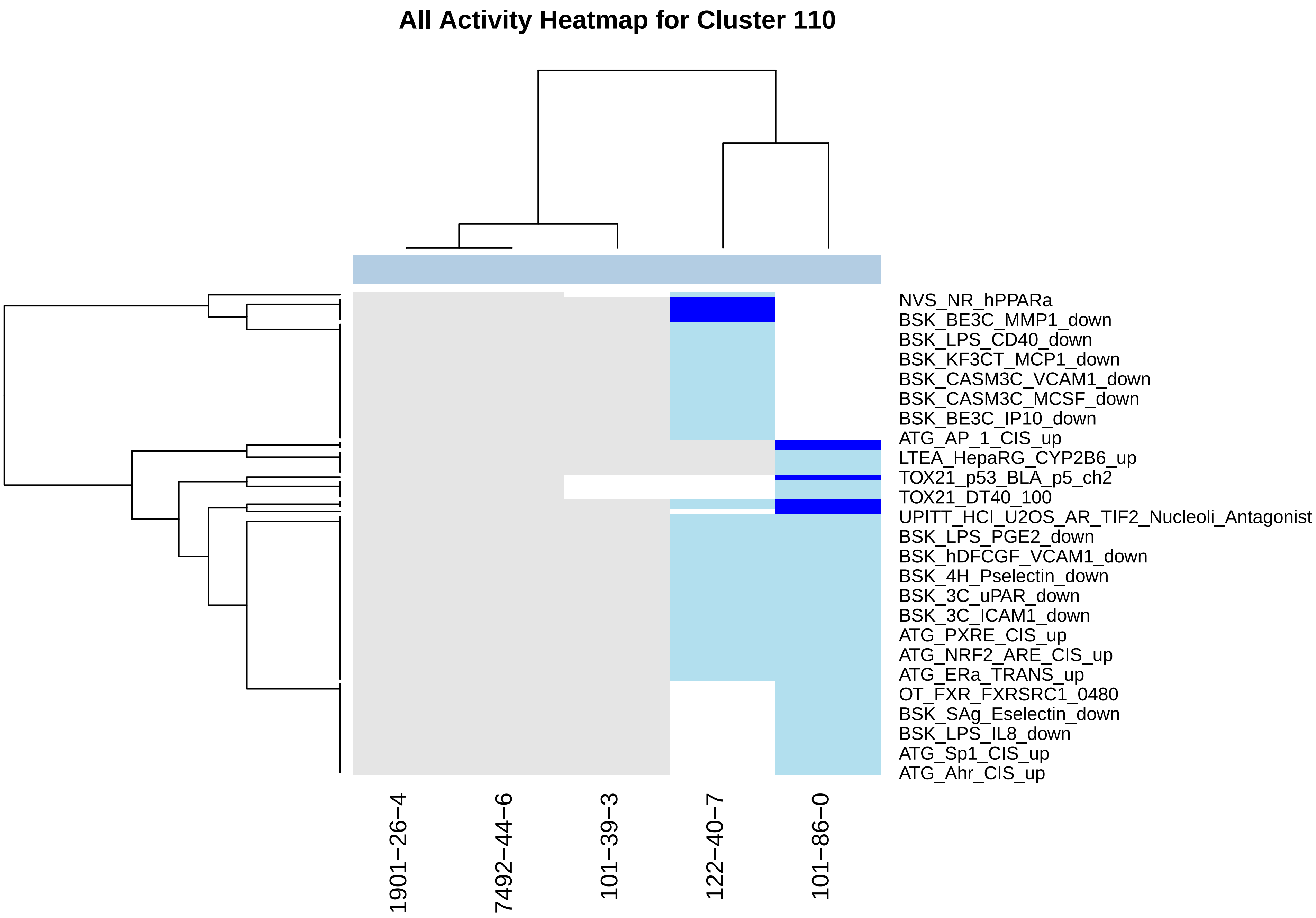
All Activity Heatmap for Cluster 110
NVS_NR_hPPARa
BSK_BE3C_MMP1_down
BSK_LPS_CD40_down
BSK_KF3CT_MCP1_down
BSK_CASM3C_VCAM1_down
BSK_CASM3C_MCSF_down
BSK_BE3C_IP10_down
ATG_AP_1_CIS_up
LTEA_HepaRG_CYP2B6_up
TOX21_p53_BLA_p5_ch2
TOX21_DT40_100
UPITT_HCI_U2OS_AR_TIF2_Nucleoli_Antagonist
BSK_LPS_PGE2_down
BSK_hDFCGF_VCAM1_down
BSK_4H_Pselectin_down
BSK_3C_uPAR_down
BSK_3C_ICAM1_down
ATG_PXRE_CIS_up
ATG_NRF2_ARE_CIS_up
ATG_ERa_TRANS_up
OT_FXR_FXRSRC1_0480
BSK_SAg_Eselectin_down
BSK_LPS_IL8_down
ATG_Sp1_CIS_up
ATG_Ahr_CIS_up
1901−26−4
7492−44−6
101−39−3
122−40−7
101−86−0

All Activity Heatmap for Cluster 111

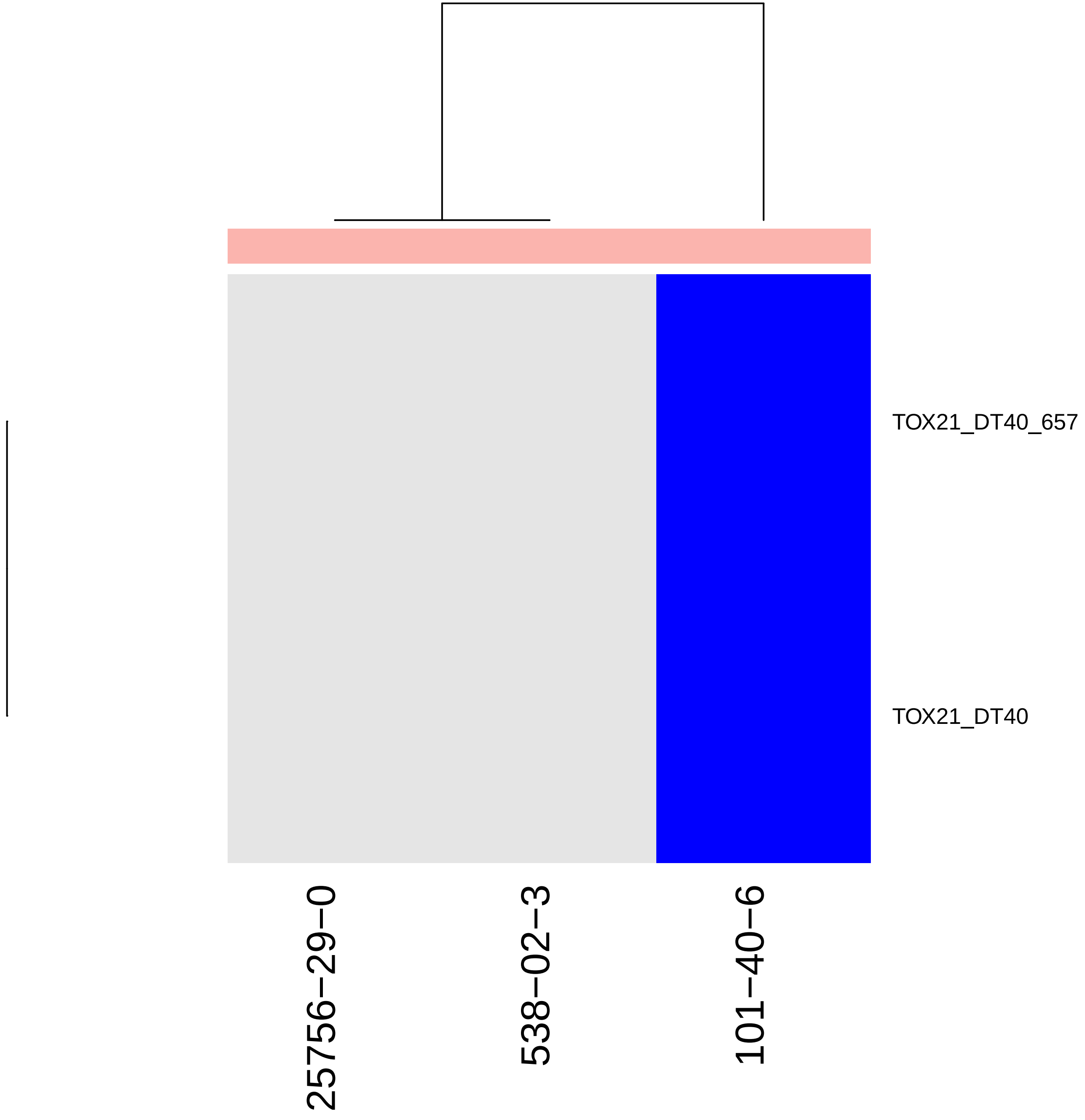

All Activity Heatmap for Cluster 112

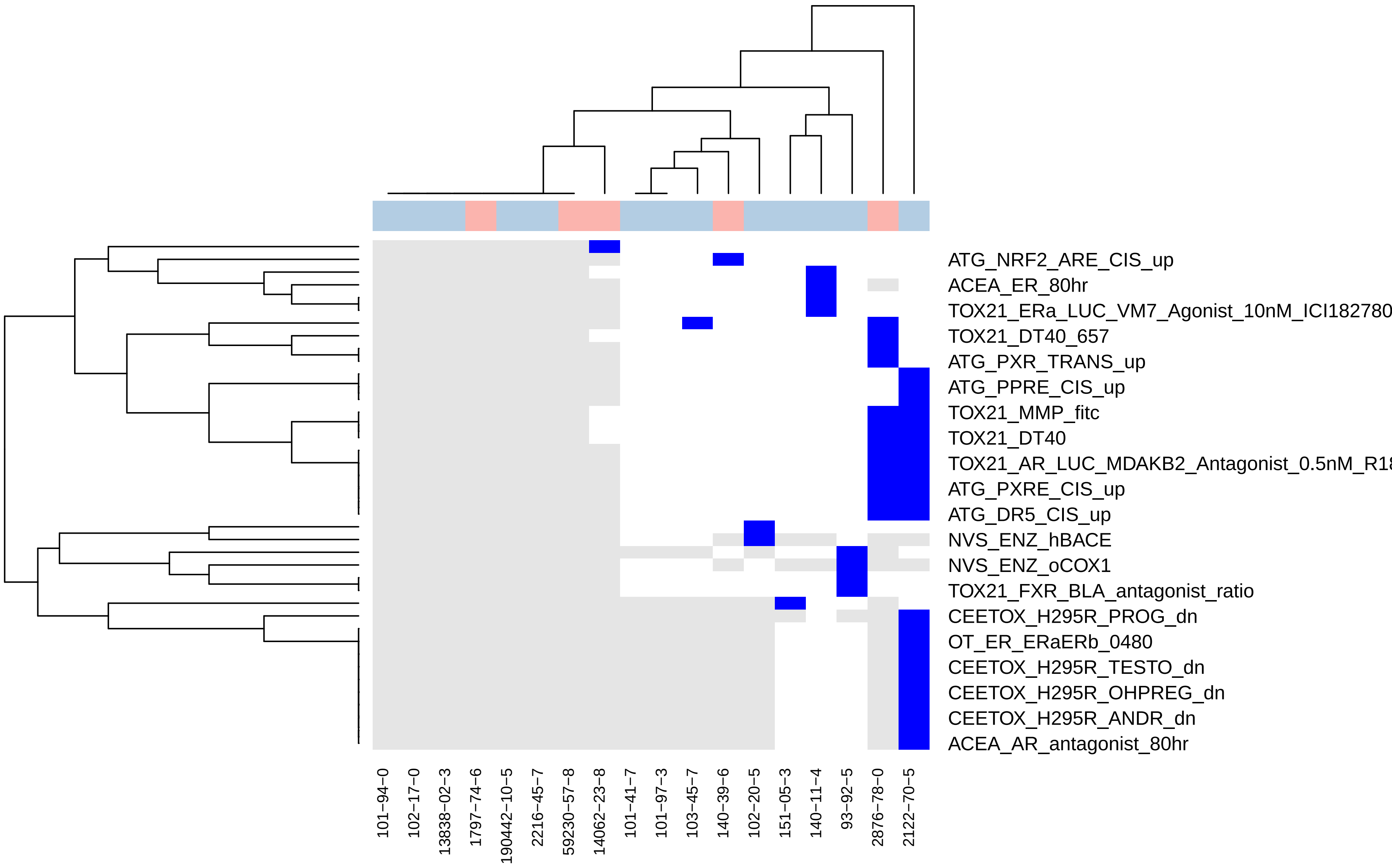

All Activity Heatmap for Cluster 114

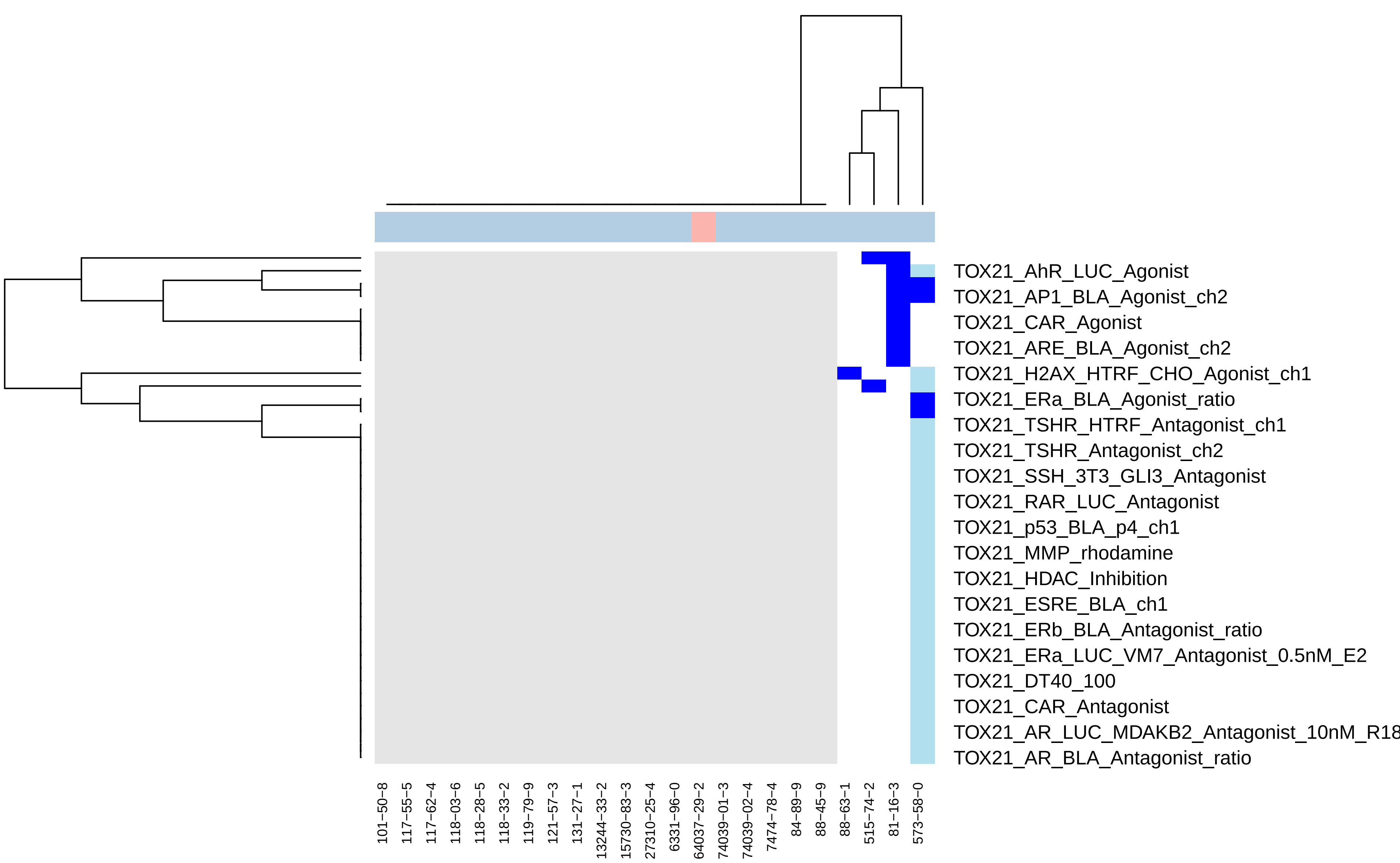

All Activity Heatmap for Cluster 115

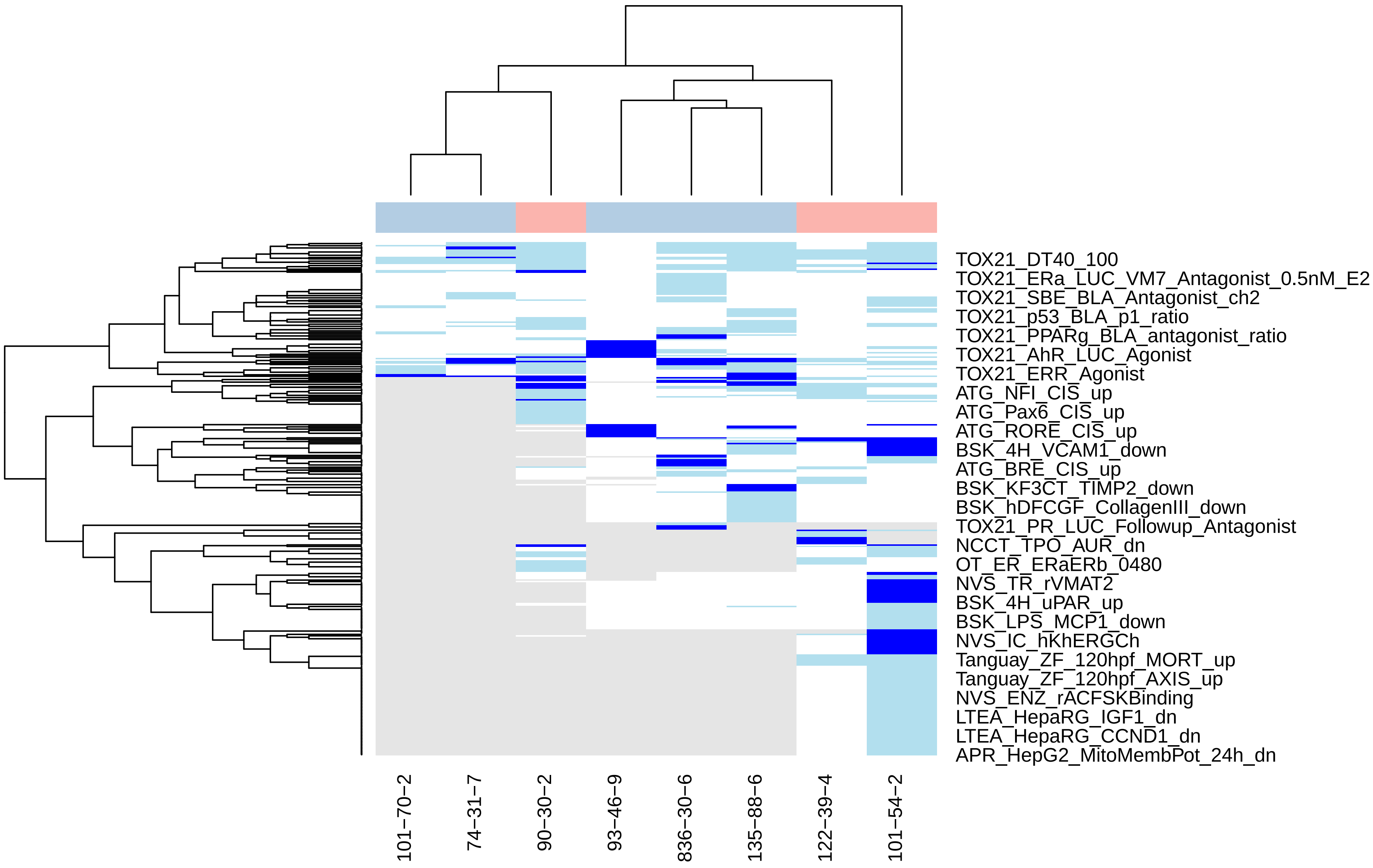

All Activity Heatmap for Cluster 116

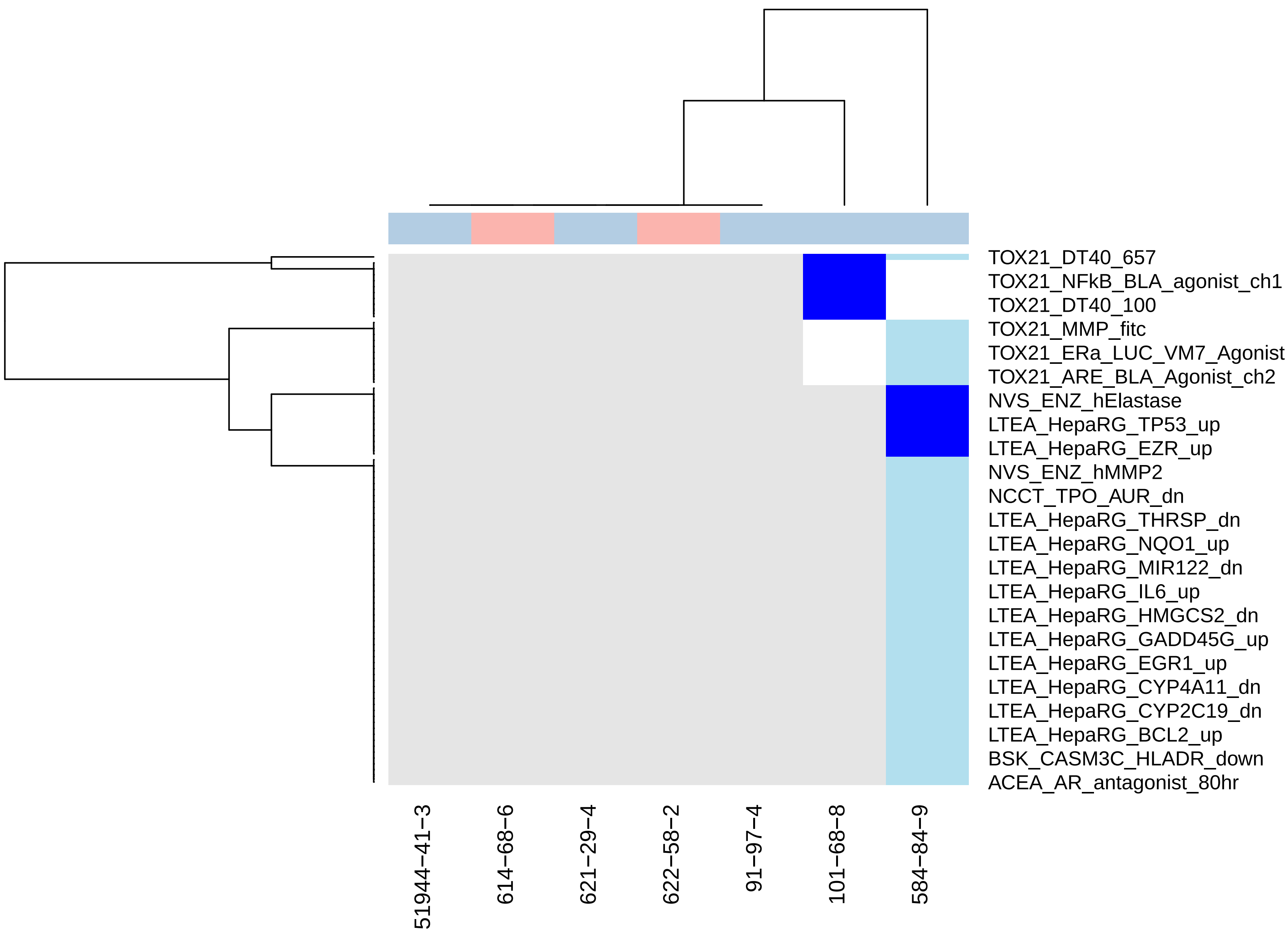

All Activity Heatmap for Cluster 118

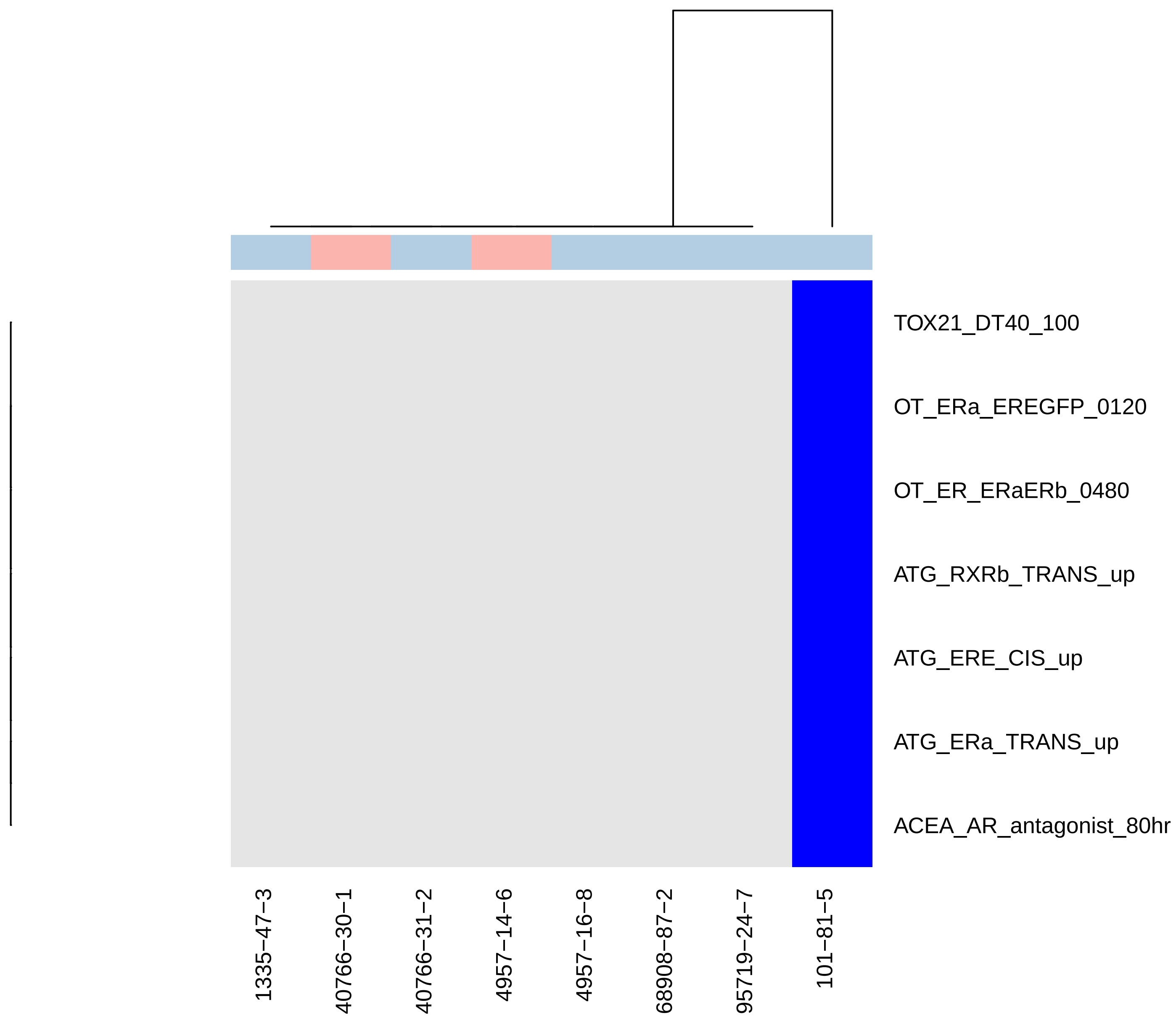

All Activity Heatmap for Cluster 119

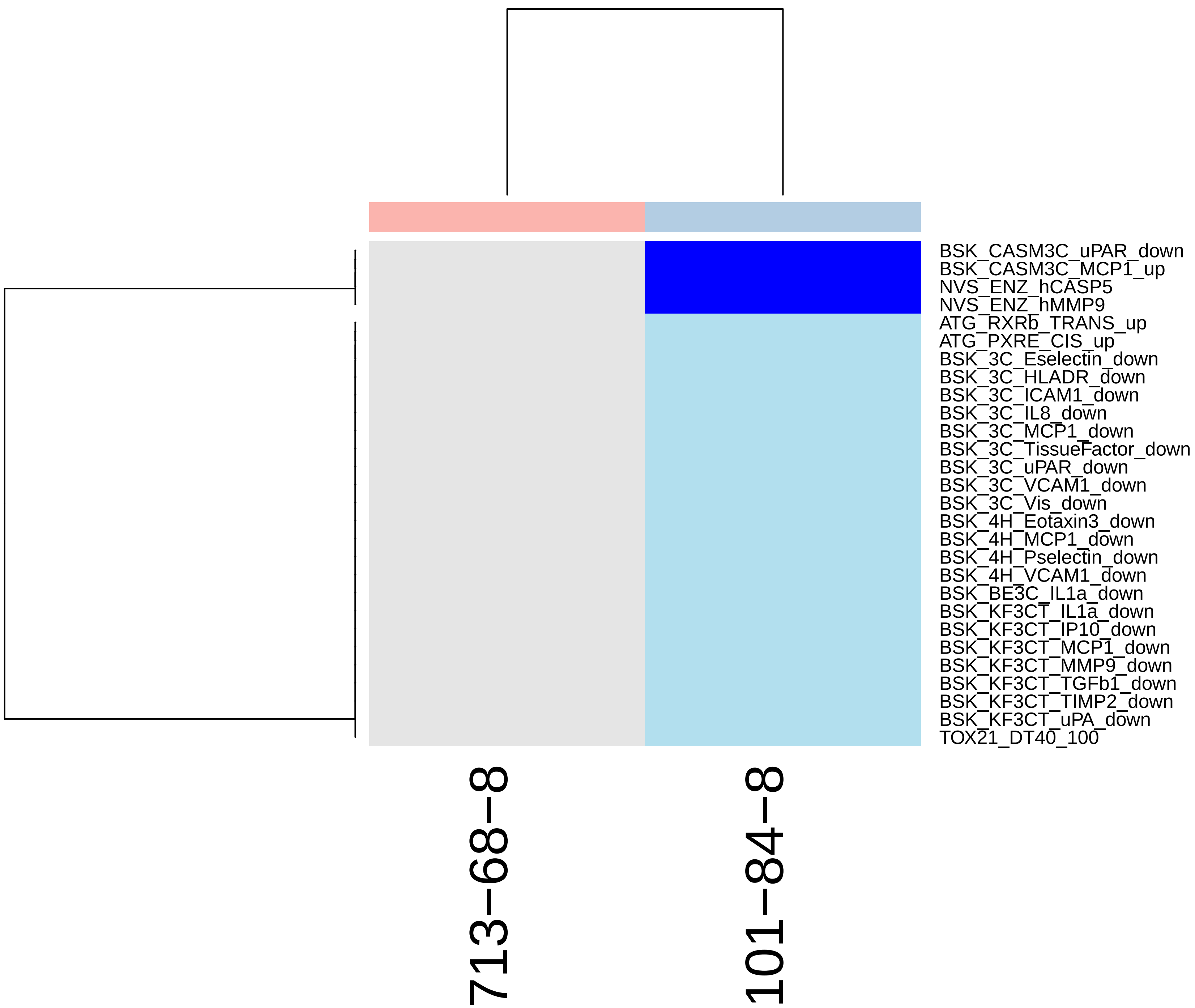

All Activity Heatmap for Cluster 120

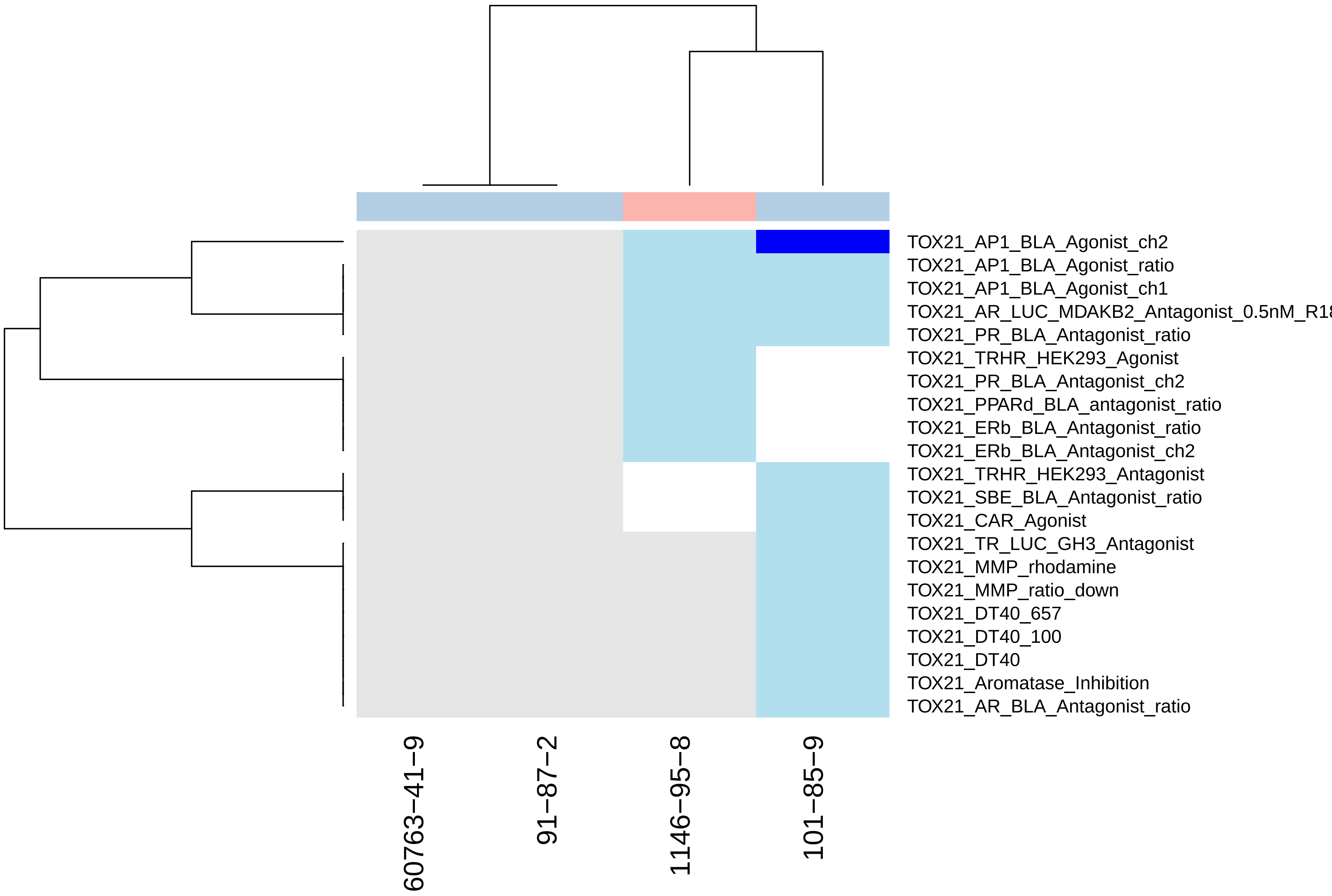

All Activity Heatmap for Cluster 121

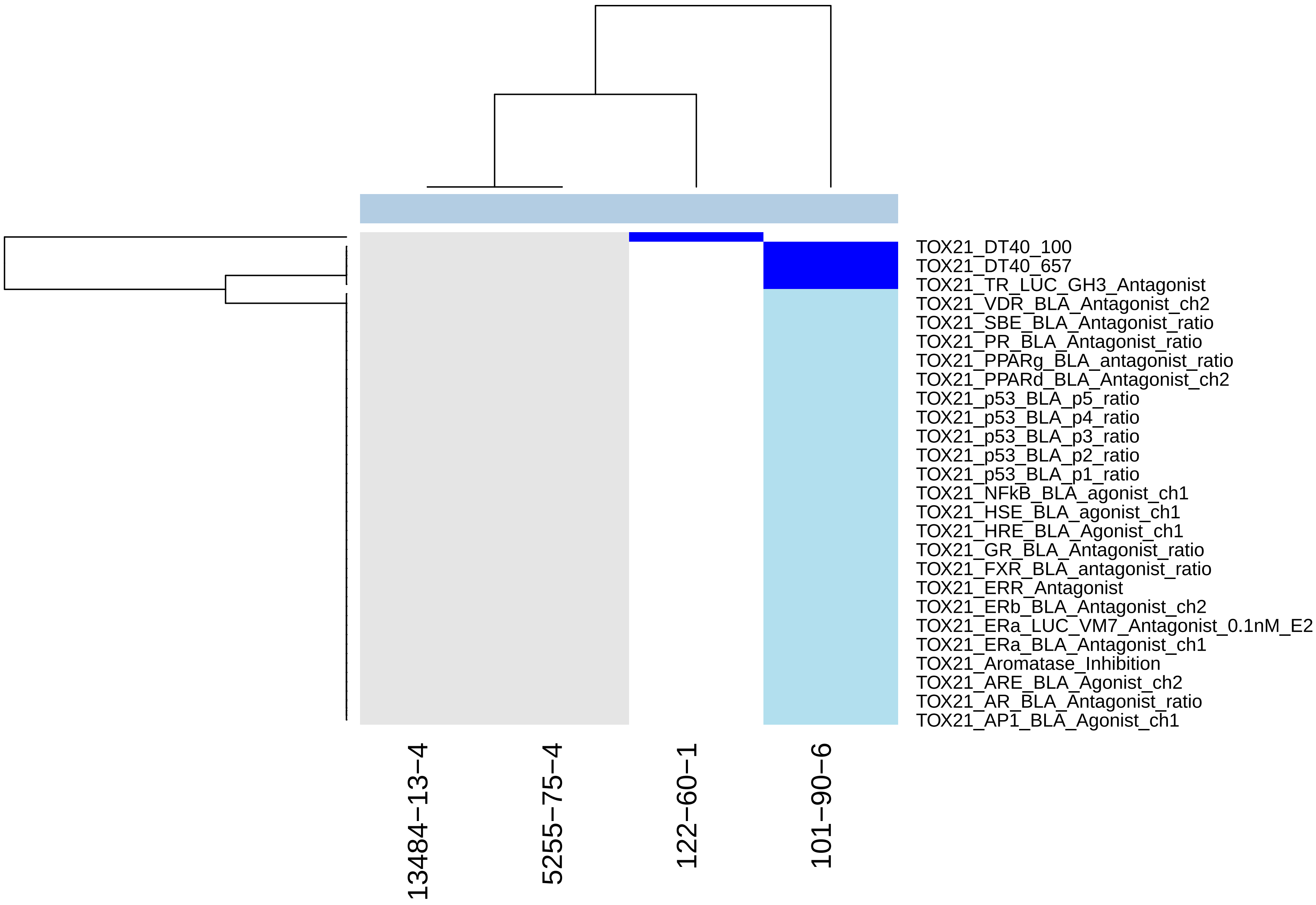

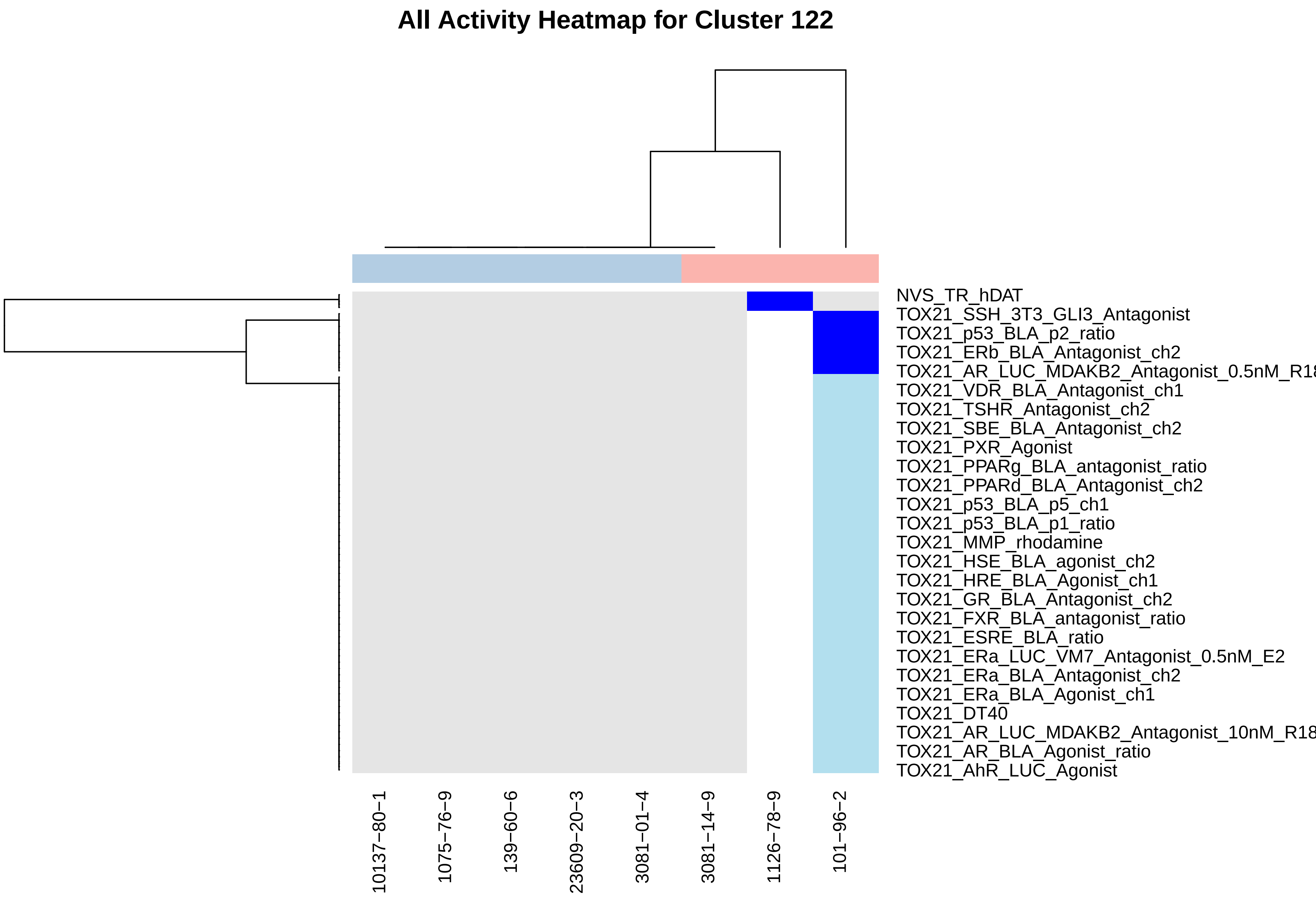
All Activity Heatmap for Cluster 122
NVS_TR_hDAT
TOX21_SSH_3T3_GLI3_Antagonist
TOX21_p53_BLA_p2_ratio
TOX21_ERb_BLA_Antagonist_ch2
TOX21_AR_LUC_MDAKB2_Antagonist_0.5nM_R18
TOX21_VDR_BLA_Antagonist_ch1
TOX21_TSHR_Antagonist_ch2
TOX21_SBE_BLA_Antagonist_ch2
TOX21_PXR_Agonist
TOX21_PPARg_BLA_antagonist_ratio
TOX21_PPARd_BLA_Antagonist_ch2
TOX21_p53_BLA_p5_ch1
TOX21_p53_BLA_p1_ratio
TOX21_MMP_rhodamine
TOX21_HSE_BLA_agonist_ch2
TOX21_HRE_BLA_Agonist_ch1
TOX21_GR_BLA_Antagonist_ch2
TOX21_FXR_BLA_antagonist_ratio
TOX21_ESRE_BLA_ratio
TOX21_ERa_LUC_VM7_Antagonist_0.5nM_E2
TOX21_ERa_BLA_Antagonist_ch2
TOX21_ERa_BLA_Agonist_ch1
TOX21_DT40
TOX21_AR_LUC_MDAKB2_Antagonist_10nM_R18
TOX21_AR_BLA_Agonist_ratio
TOX21_AhR_LUC_Agonist
10137−80−1
1075−76−9
139−60−6
23609−20−3
3081−01−4
3081−14−9
1126−78−9
101−96−2

All Activity Heatmap for Cluster 123

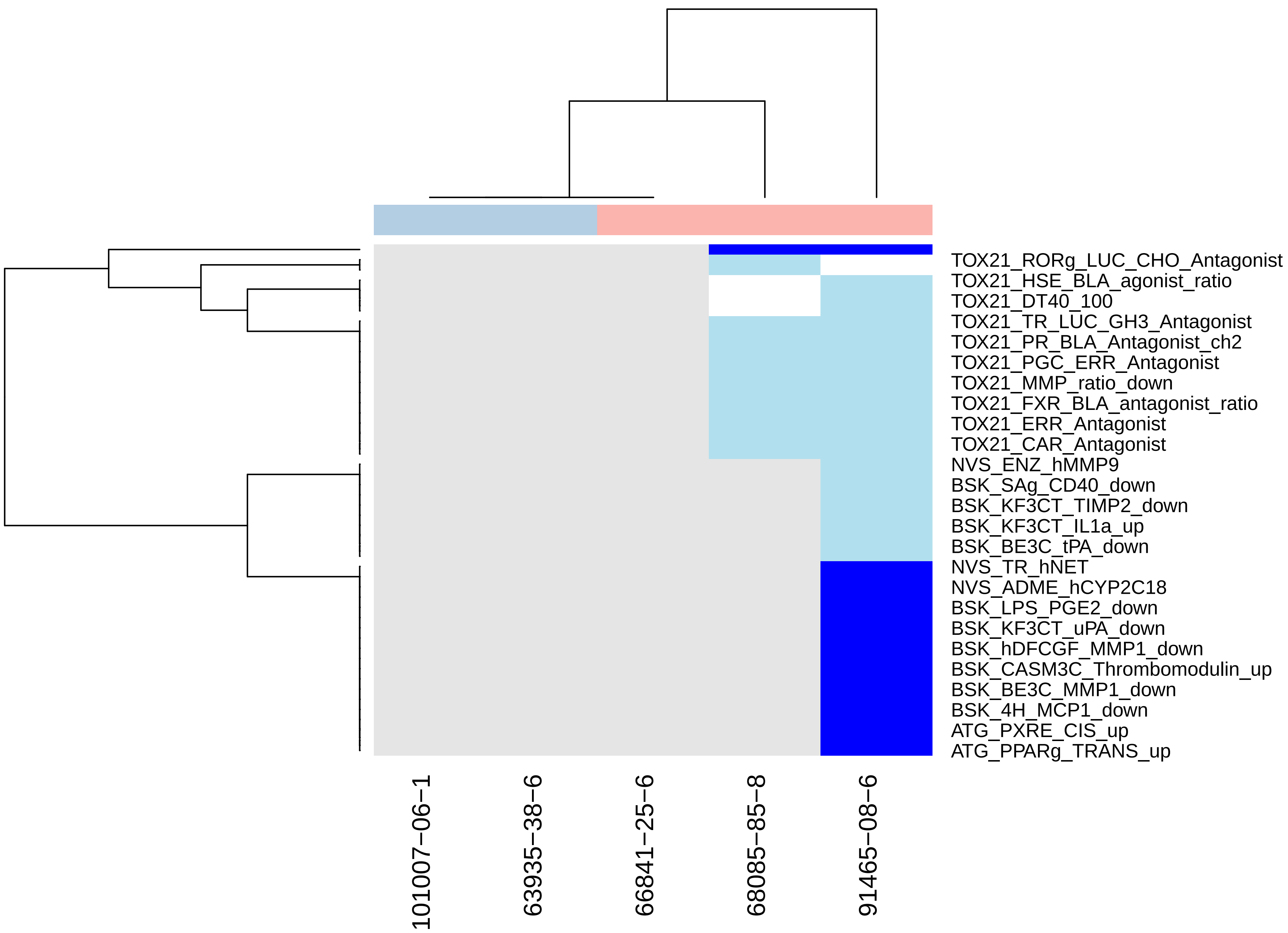

All Activity Heatmap for Cluster 124

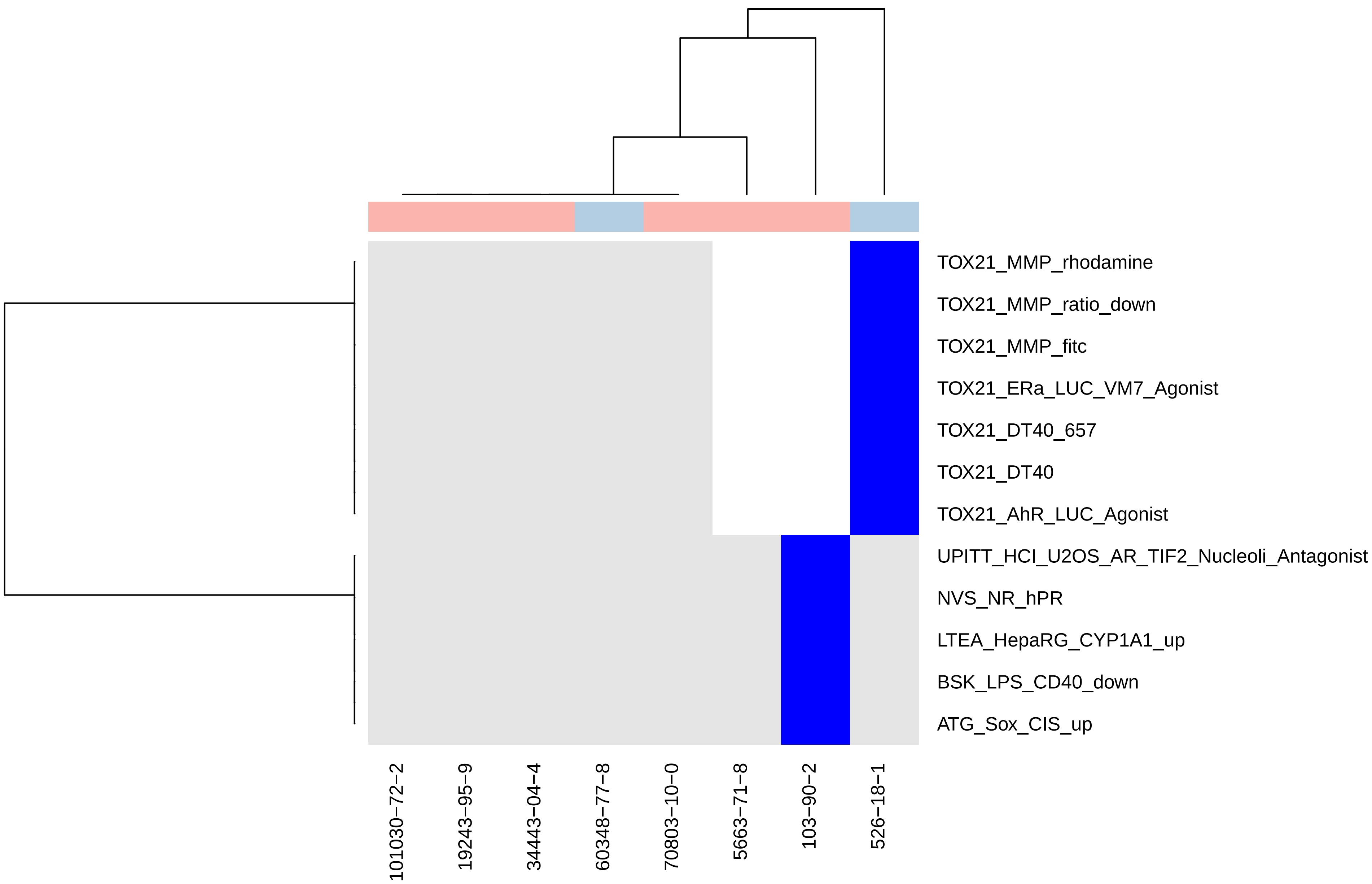

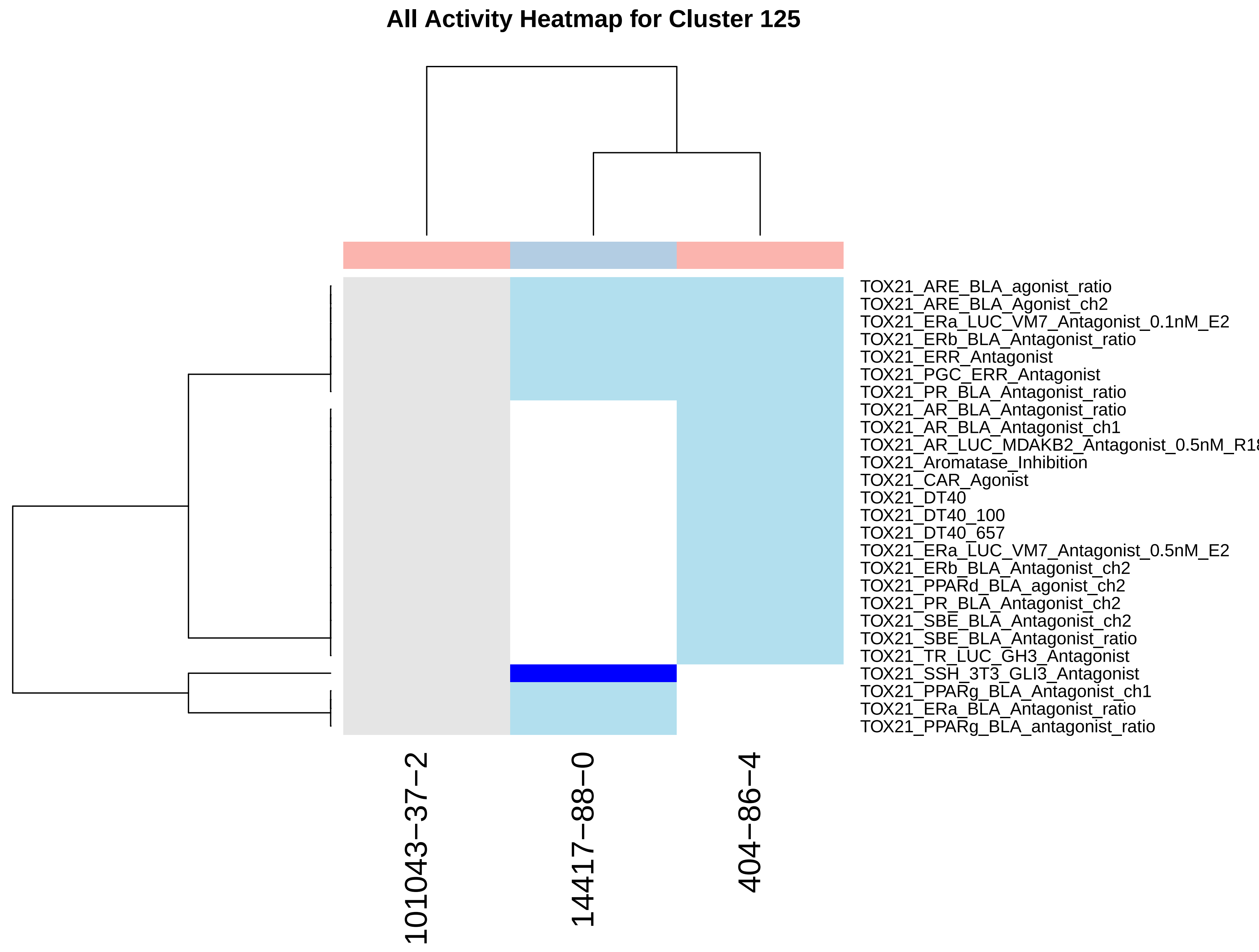
All Activity Heatmap for Cluster 125
TOX21_ARE_BLA_agonist_ratio
TOX21_ARE_BLA_Agonist_ch2
TOX21_ERa_LUC_VM7_Antagonist_0.1nM_E2
TOX21_ERb_BLA_Antagonist_ratio
TOX21_ERR_Antagonist
TOX21_PGC_ERR_Antagonist
TOX21_PR_BLA_Antagonist_ratio
TOX21_AR_BLA_Antagonist_ratio
TOX21_AR_BLA_Antagonist_ch1
TOX21_AR_LUC_MDAKB2_Antagonist_0.5nM_R18
TOX21_Aromatase_Inhibition
TOX21_CAR_Agonist
TOX21_DT40
TOX21_DT40_100
TOX21_DT40_657
TOX21_ERa_LUC_VM7_Antagonist_0.5nM_E2
TOX21_ERb_BLA_Antagonist_ch2
TOX21_PPARd_BLA_agonist_ch2
TOX21_PR_BLA_Antagonist_ch2
TOX21_SBE_BLA_Antagonist_ch2
TOX21_SBE_BLA_Antagonist_ratio
TOX21_TR_LUC_GH3_Antagonist
TOX21_SSH_3T3_GLI3_Antagonist
TOX21_PPARg_BLA_Antagonist_ch1
TOX21_ERa_BLA_Antagonist_ratio
TOX21_PPARg_BLA_antagonist_ratio
101043−37−2
14417−88−0
404−86−4

All Activity Heatmap for Cluster 127

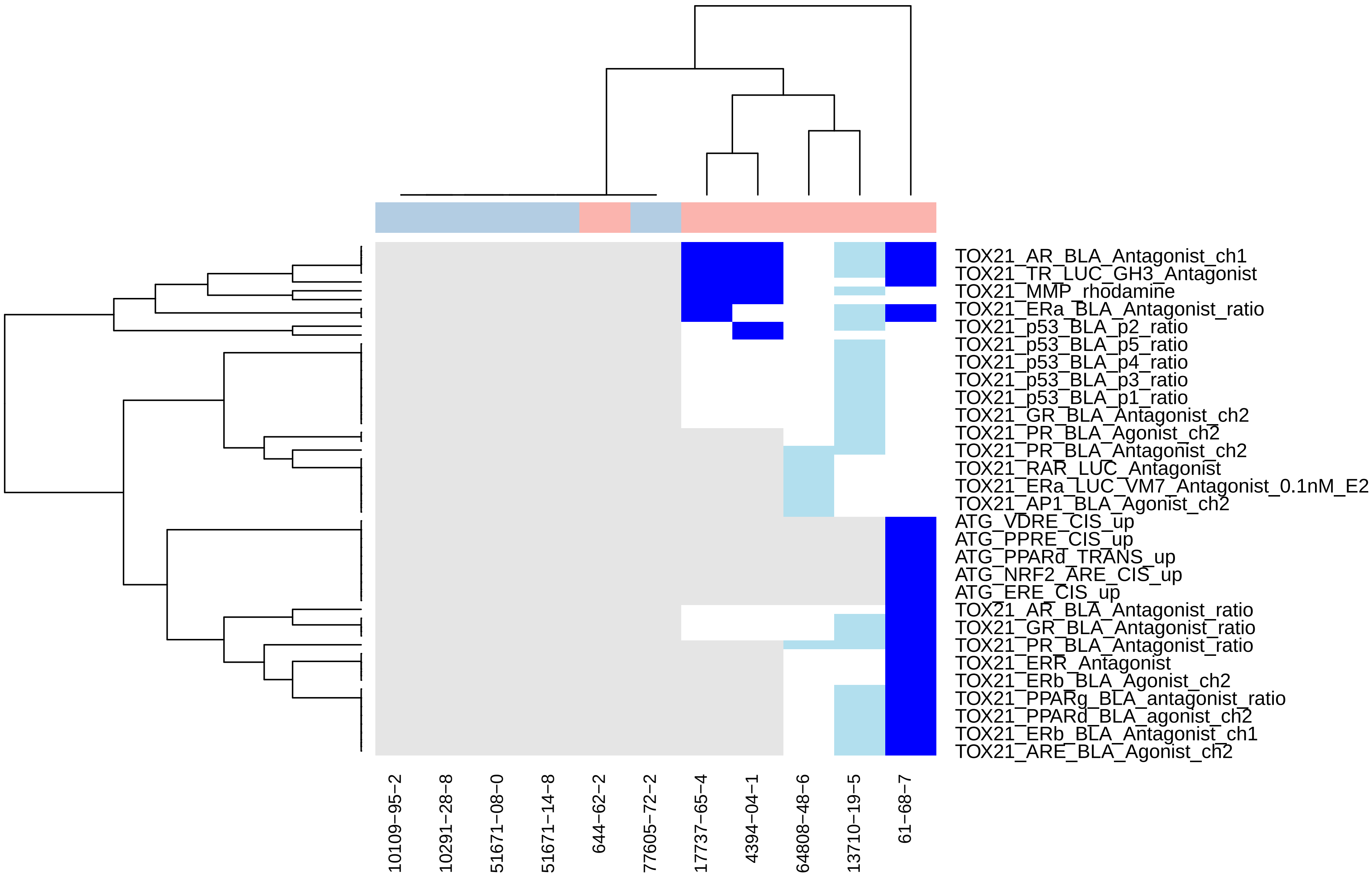

All Activity Heatmap for Cluster 129

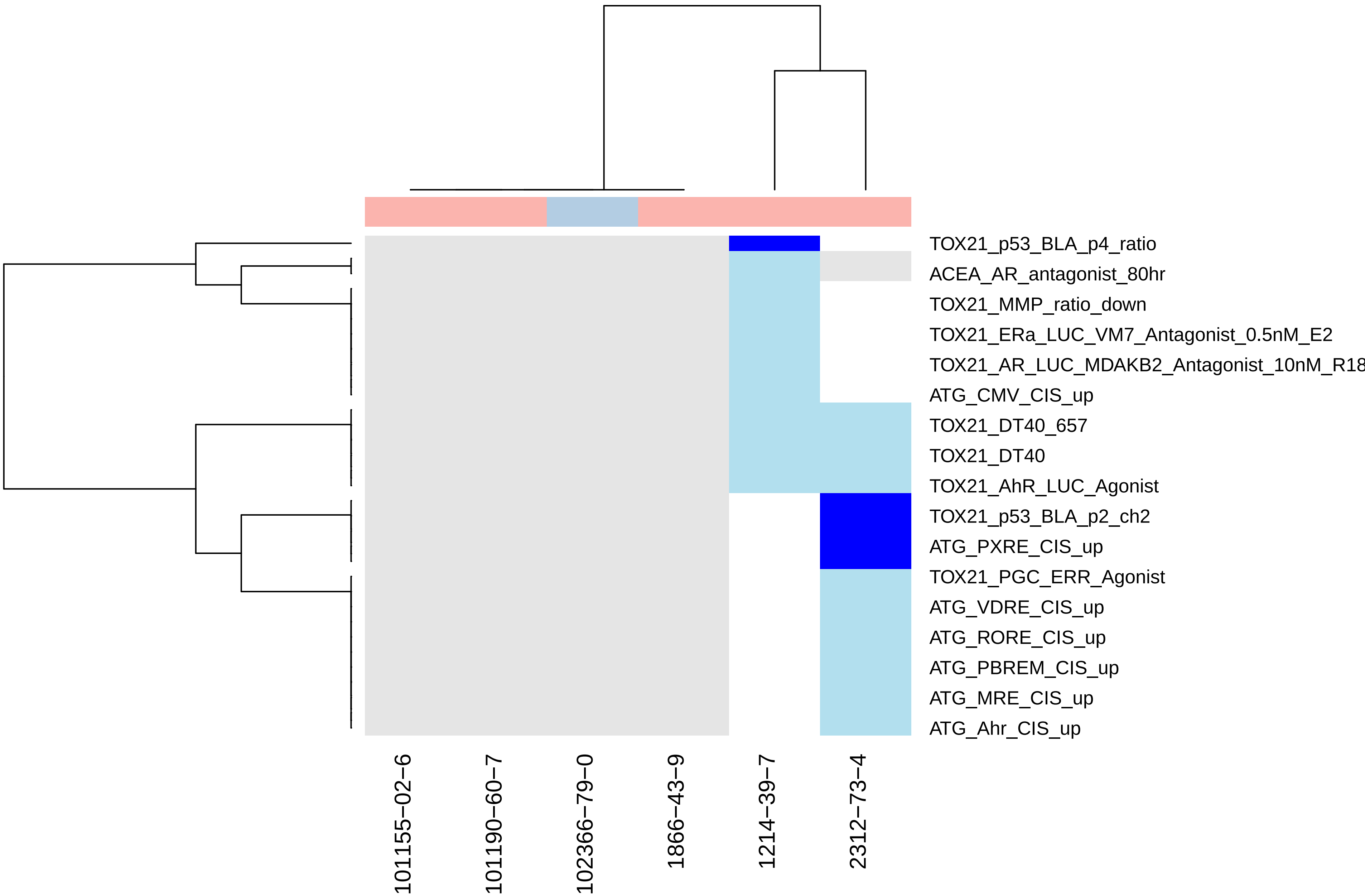

All Activity Heatmap for Cluster 130

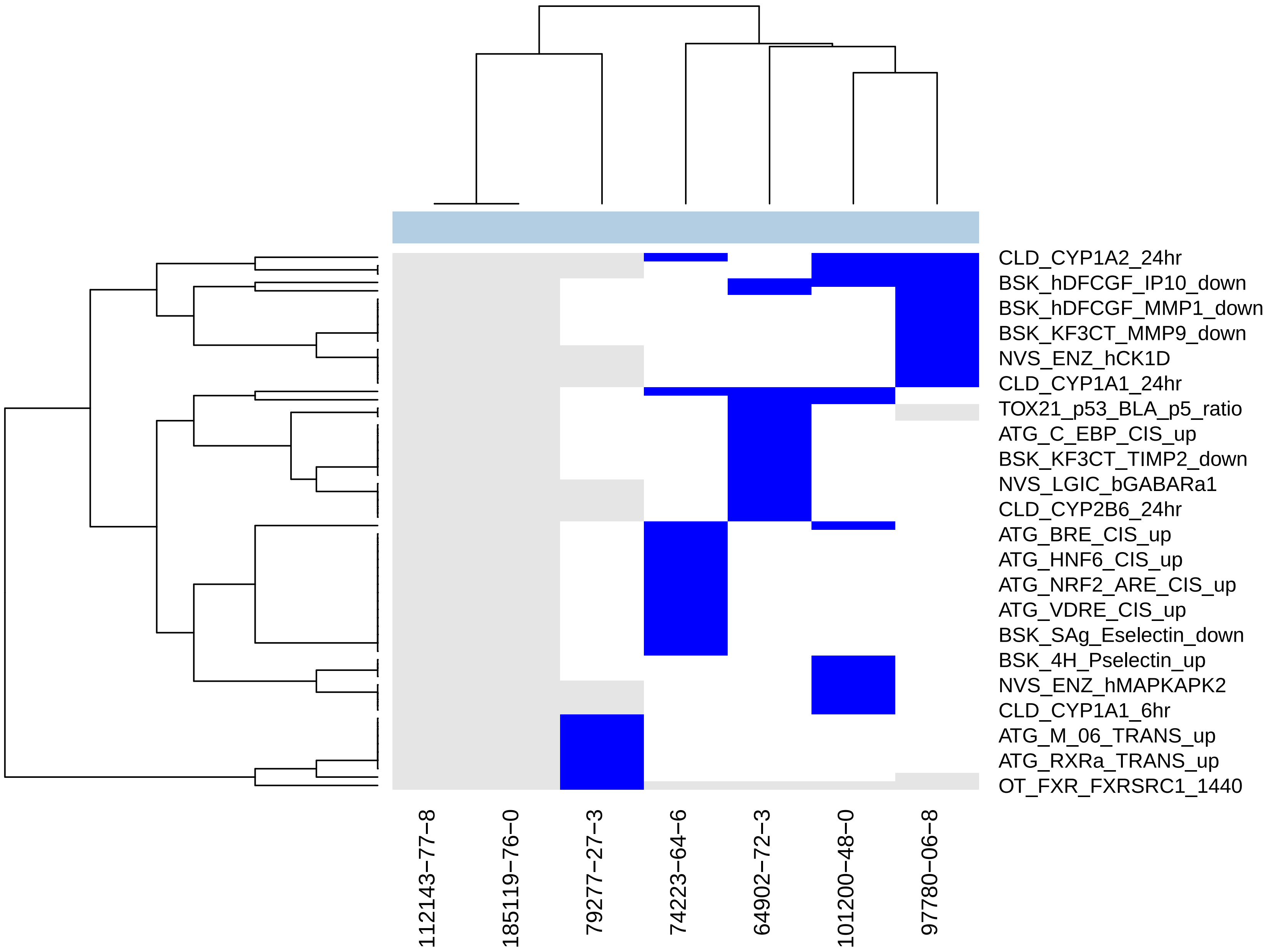

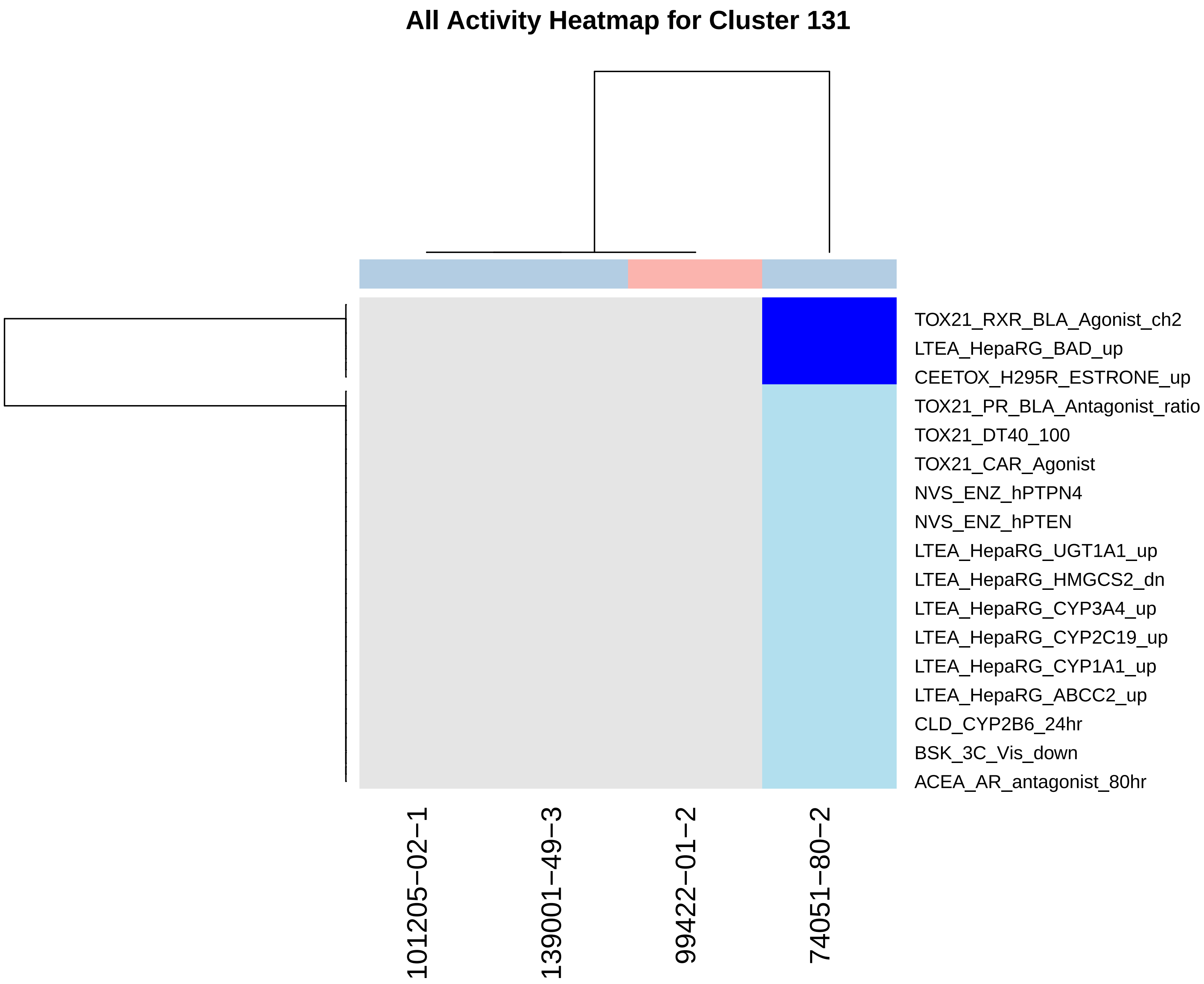
All Activity Heatmap for Cluster 131
TOX21_RXR_BLA_Agonist_ch2
LTEA_HepaRG_BAD_up
CEETOX_H295R_ESTRONE_up
TOX21_PR_BLA_Antagonist_ratio
TOX21_DT40_100
TOX21_CAR_Agonist
NVS_ENZ_hPTPN4
NVS_ENZ_hPTEN
LTEA_HepaRG_UGT1A1_up
LTEA_HepaRG_HMGCS2_dn
LTEA_HepaRG_CYP3A4_up
LTEA_HepaRG_CYP2C19_up
LTEA_HepaRG_CYP1A1_up
LTEA_HepaRG_ABCC2_up
CLD_CYP2B6_24hr
BSK_3C_Vis_down
ACEA_AR_antagonist_80hr
101205−02−1
139001−49−3
99422−01−2
74051−80−2

All Activity Heatmap for Cluster 132

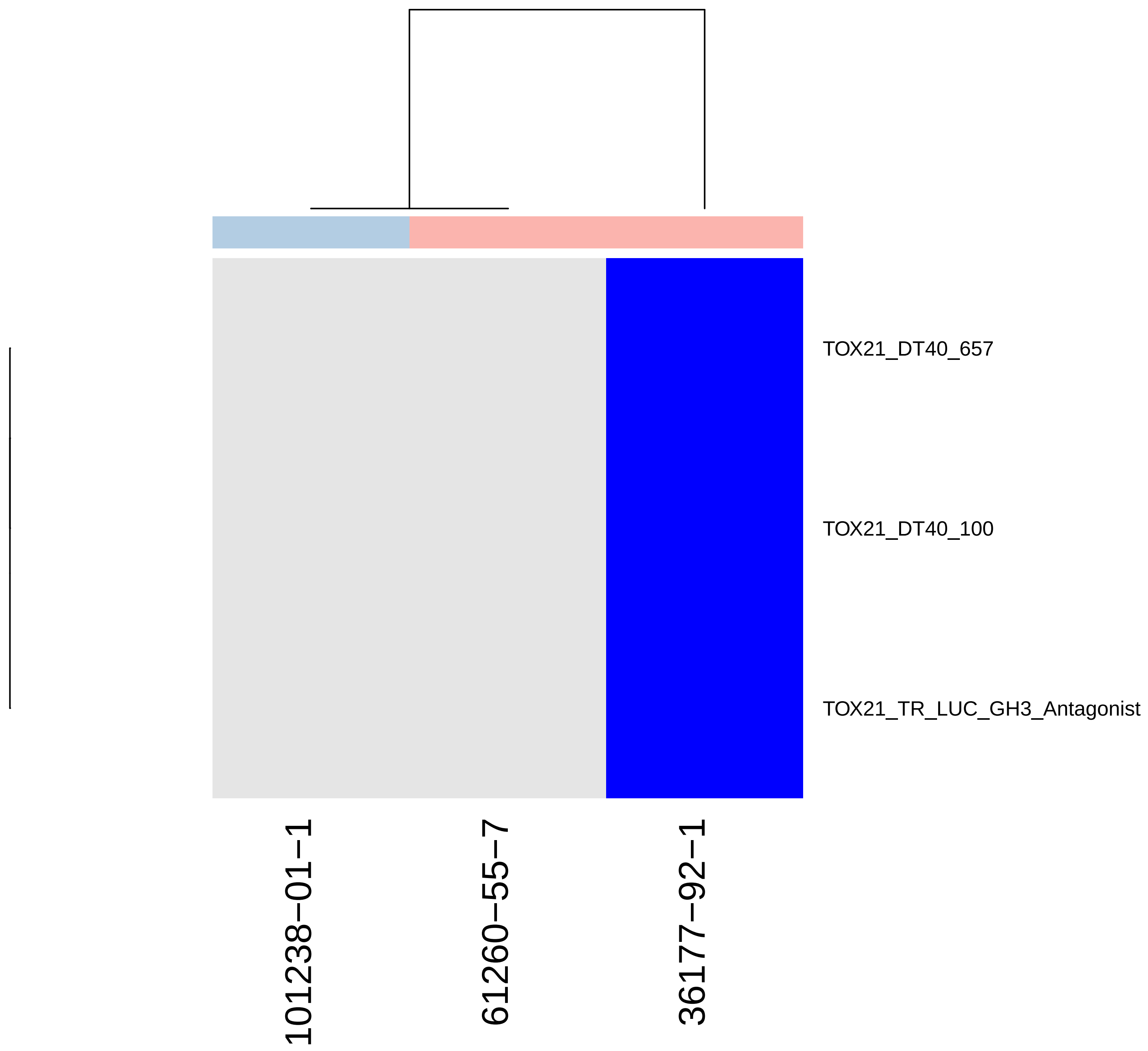

All Activity Heatmap for Cluster 133

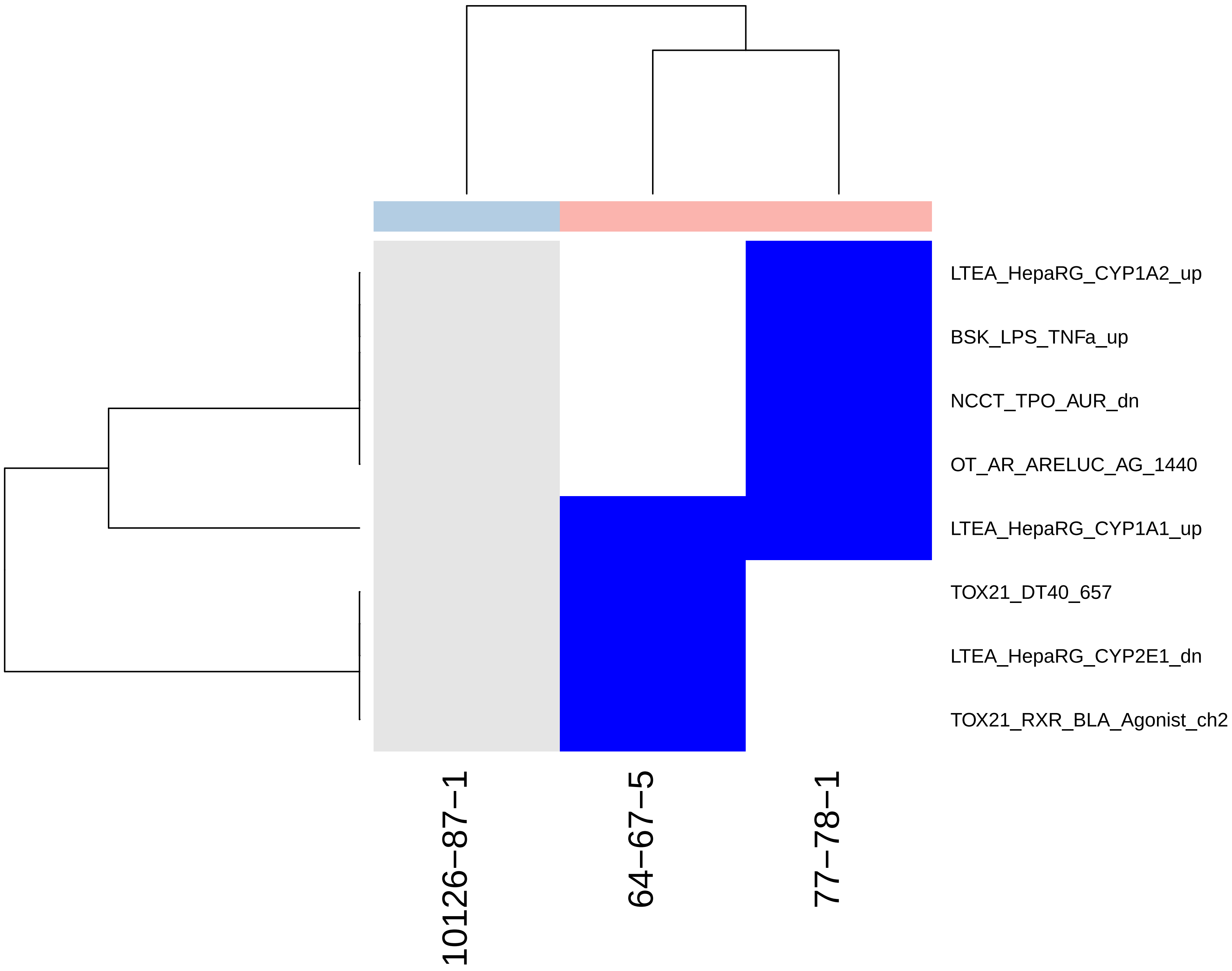

All Activity Heatmap for Cluster 134

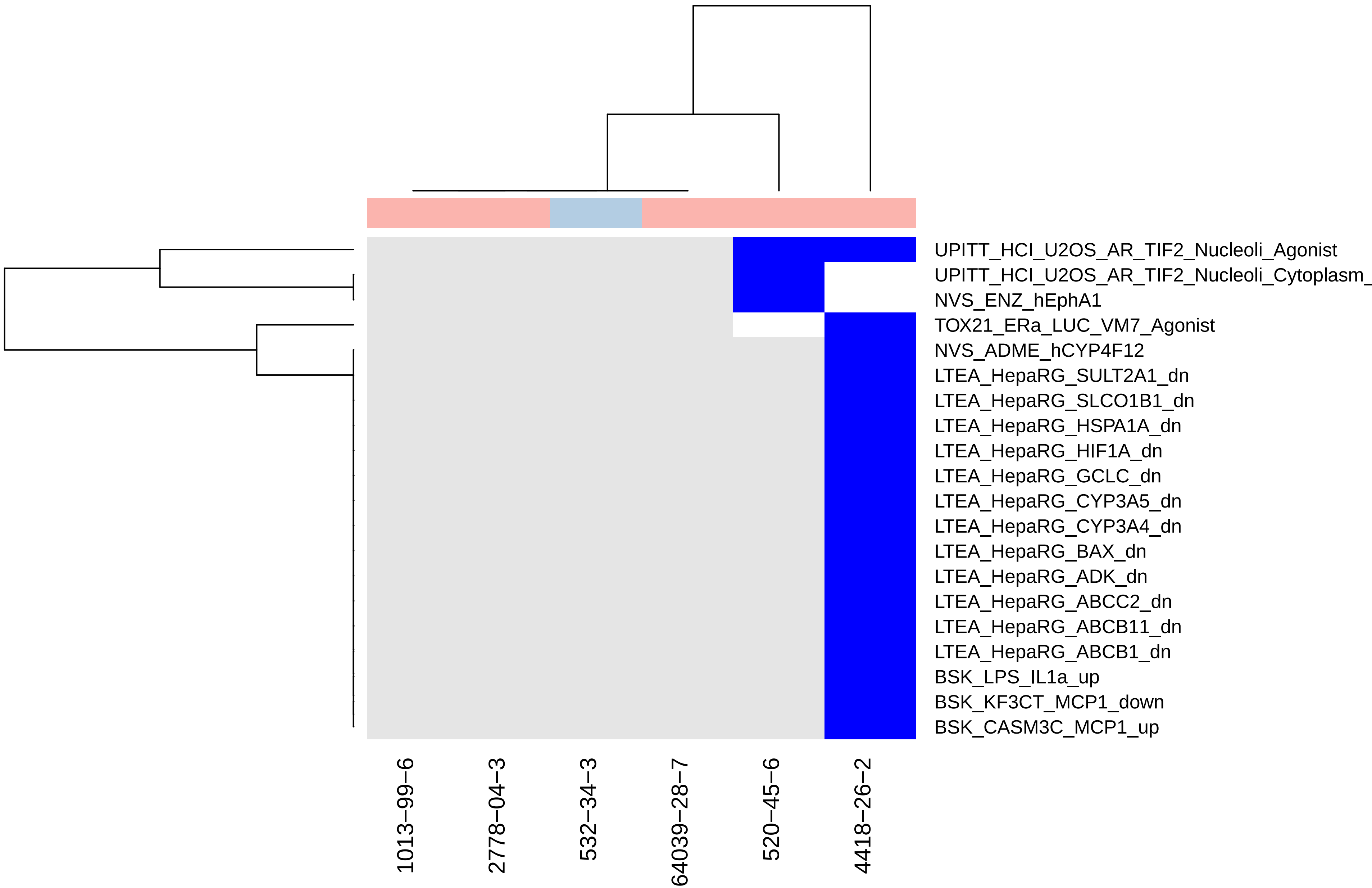

All Activity Heatmap for Cluster 138

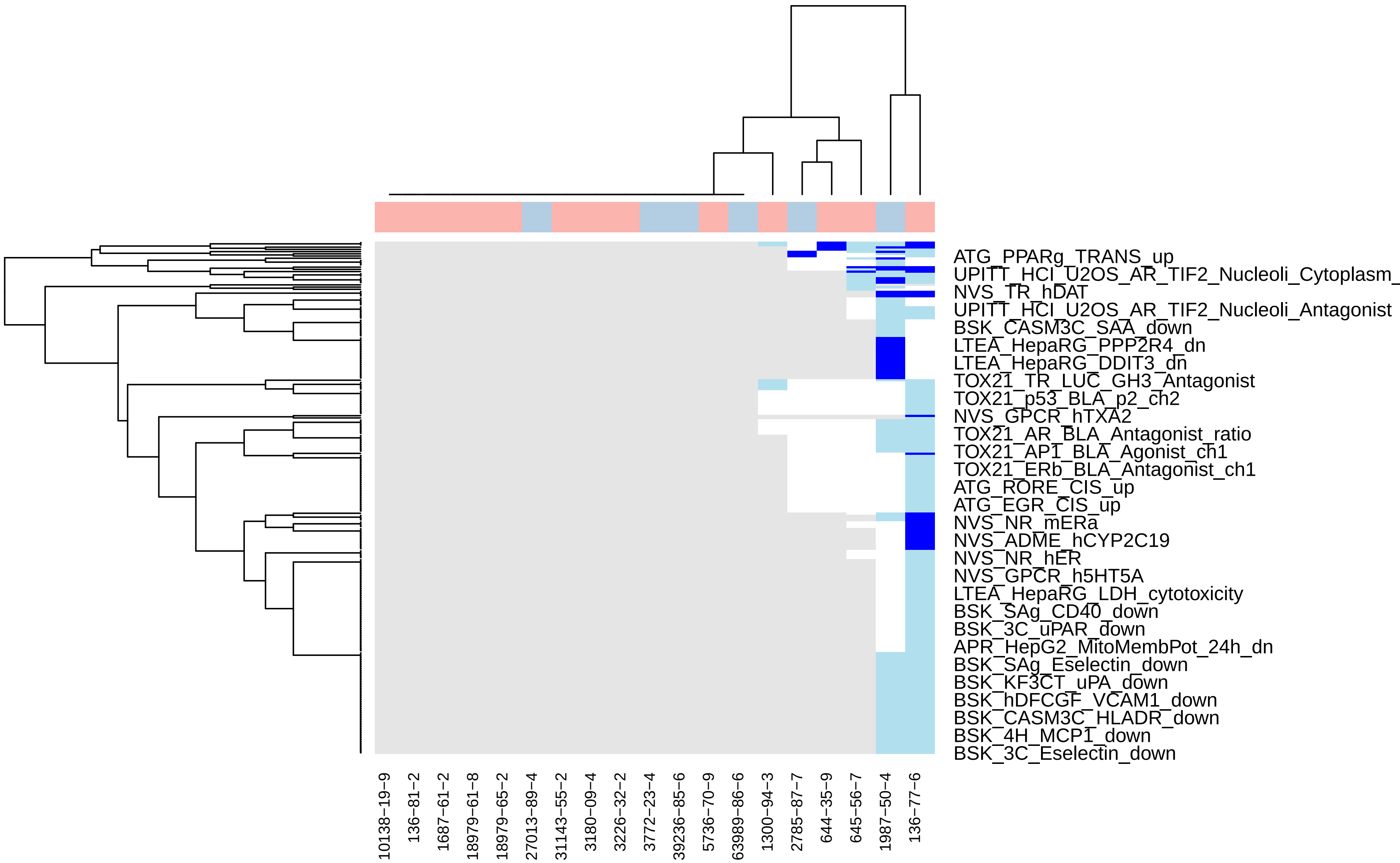

All Activity Heatmap for Cluster 139

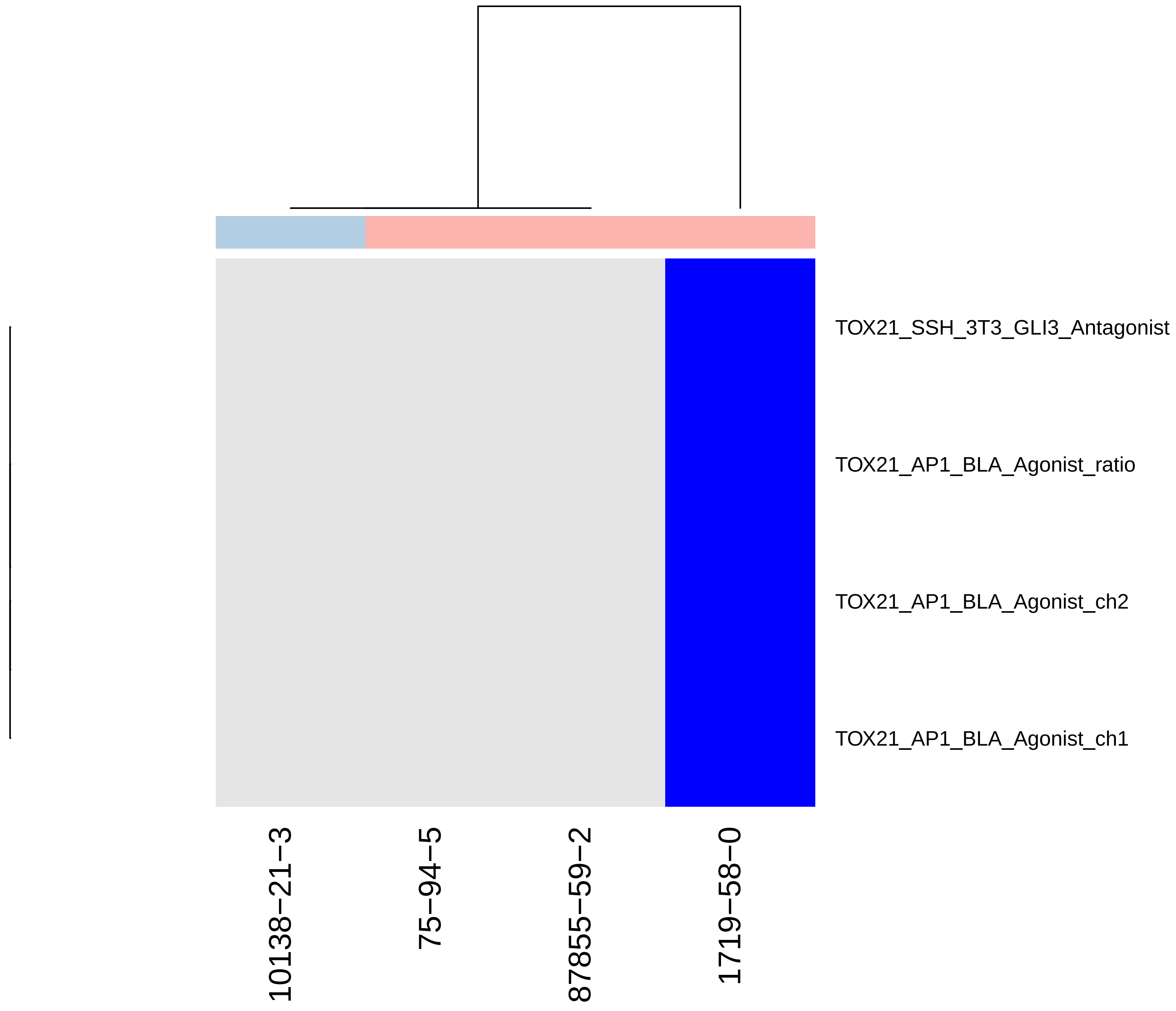

All Activity Heatmap for Cluster 140

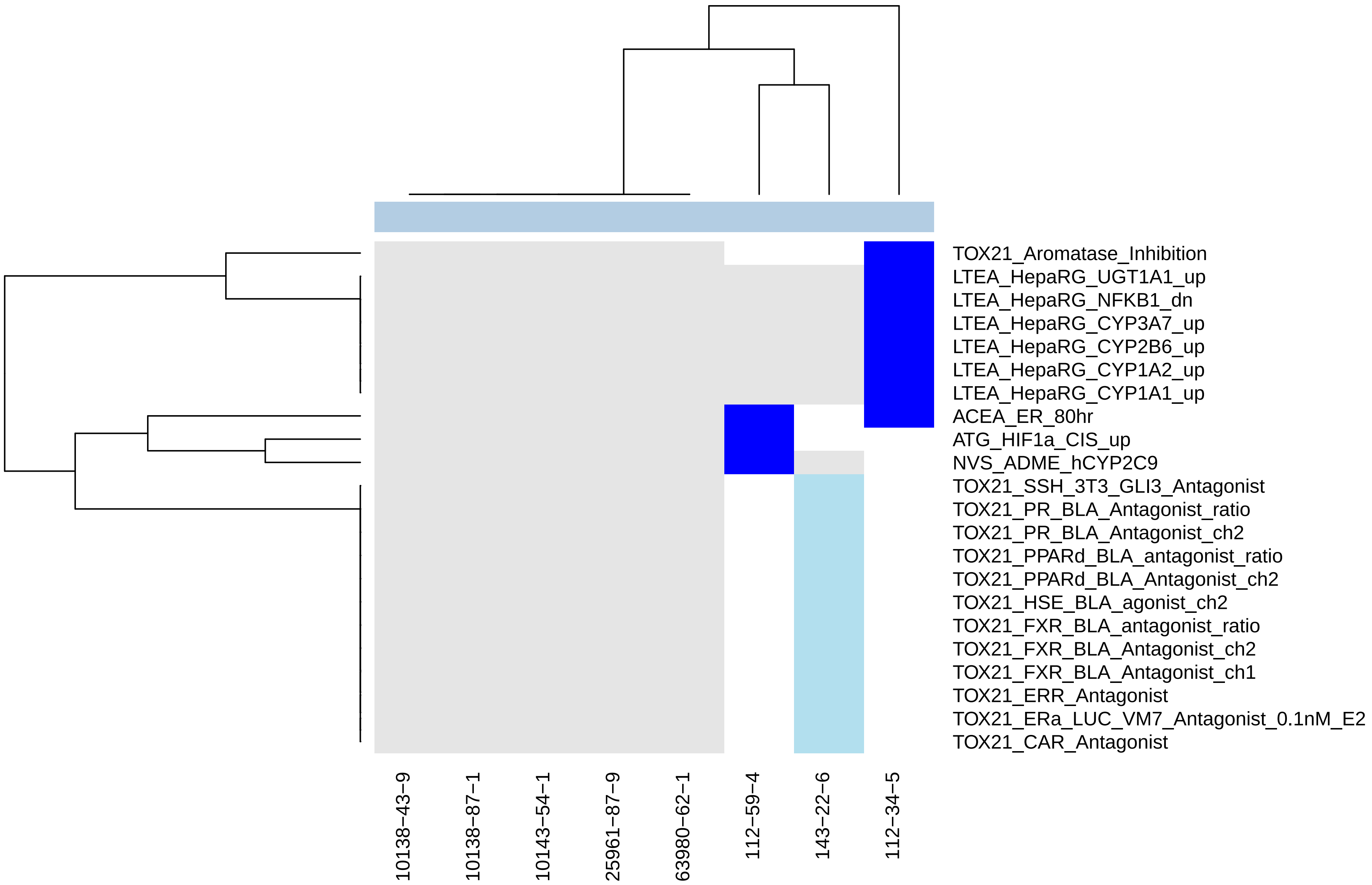

All Activity Heatmap for Cluster 141

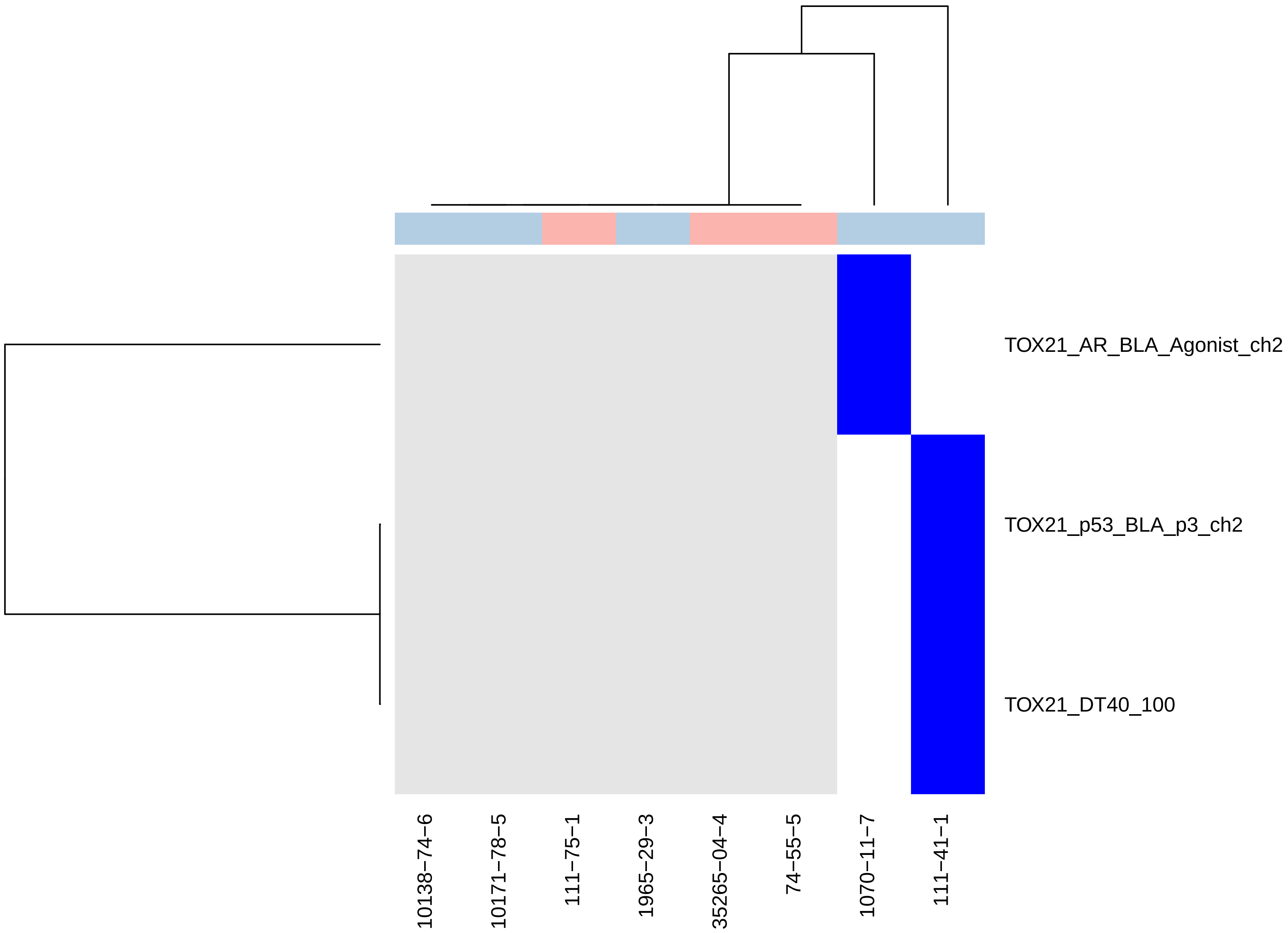

All Activity Heatmap for Cluster 142

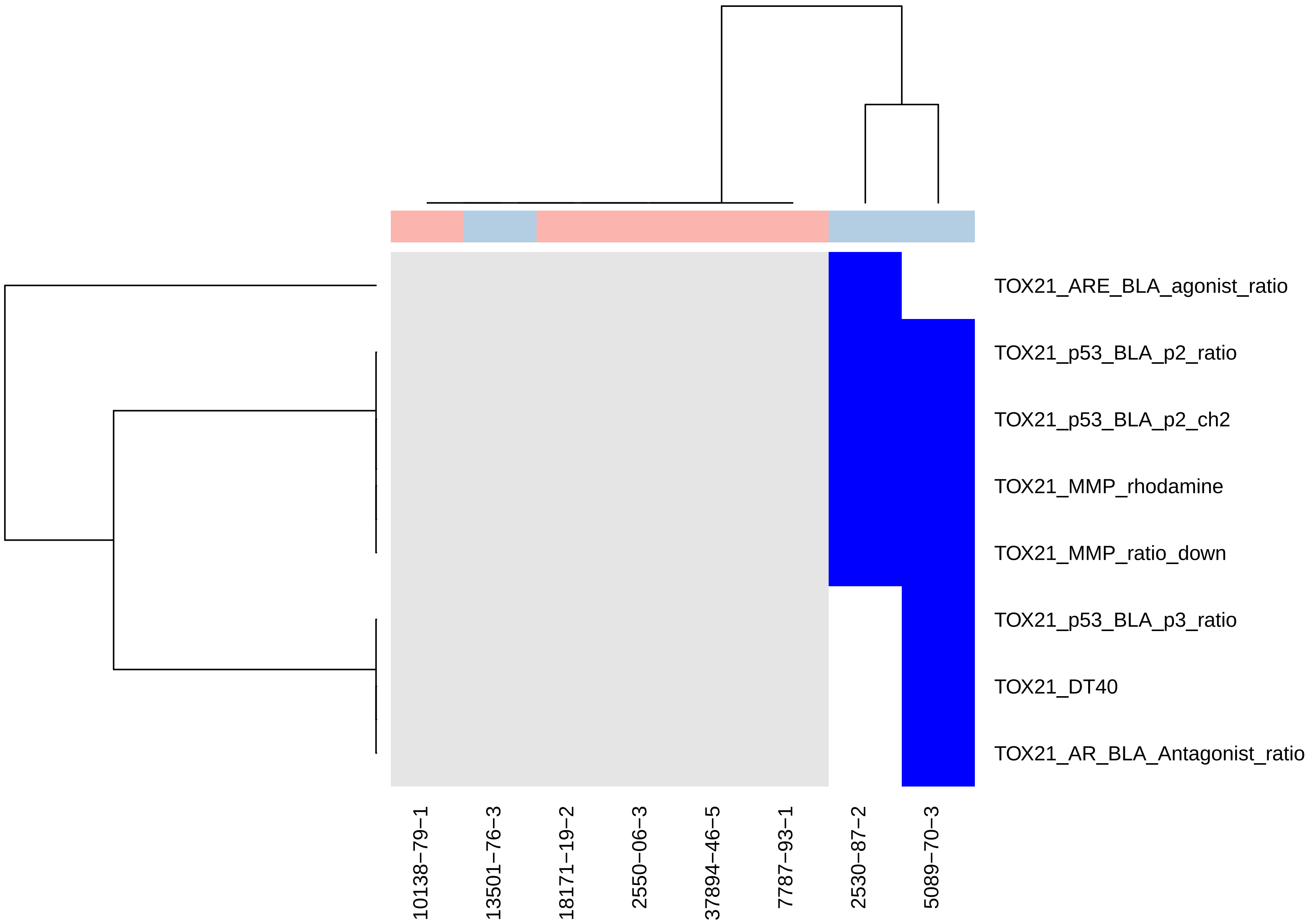

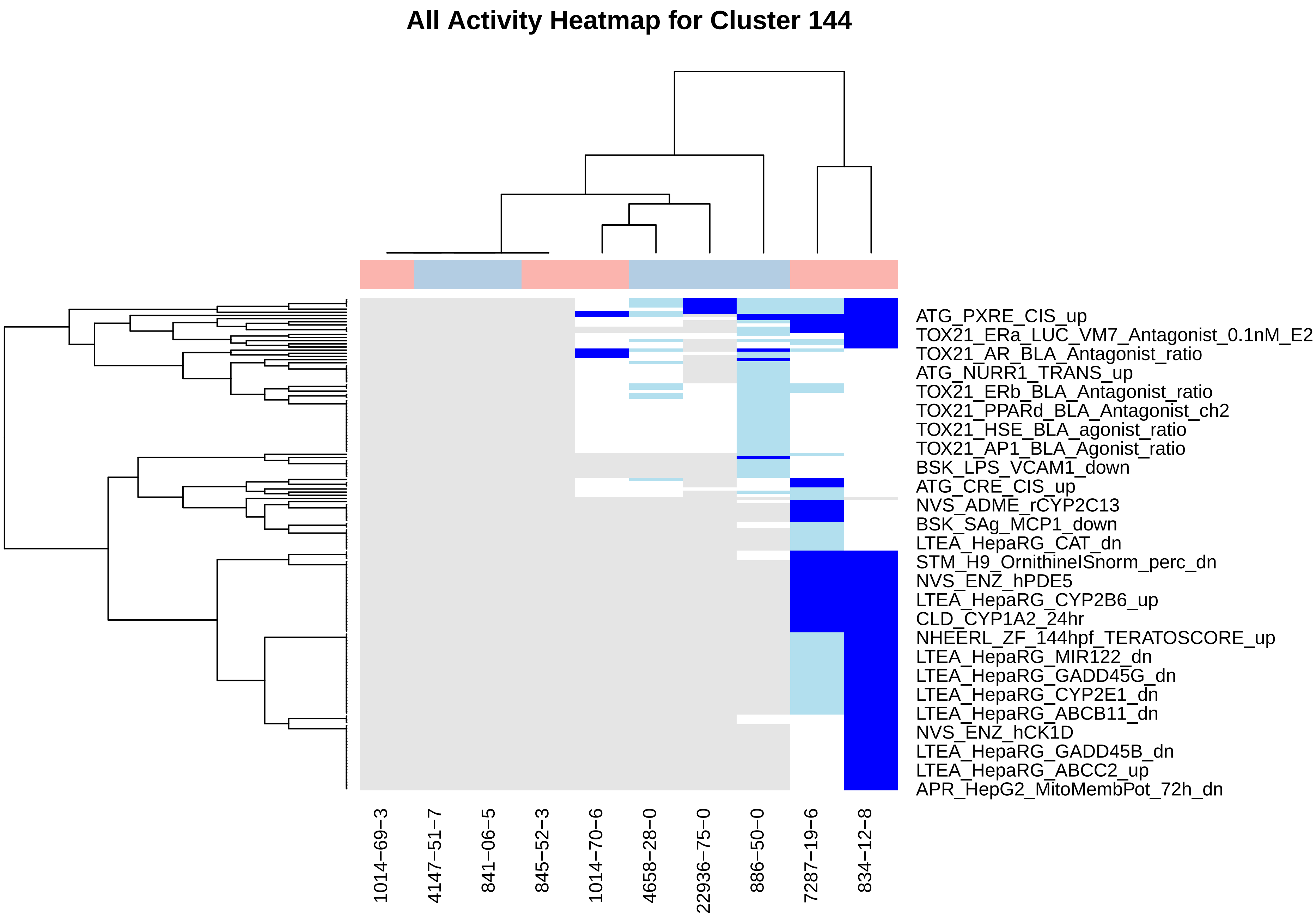
All Activity Heatmap for Cluster 144
ATG_PXRE_CIS_up
TOX21_ERa_LUC_VM7_Antagonist_0.1nM_E2
TOX21_AR_BLA_Antagonist_ratio
ATG_NURR1_TRANS_up
TOX21_ERb_BLA_Antagonist_ratio
TOX21_PPARd_BLA_Antagonist_ch2
TOX21_HSE_BLA_agonist_ratio
TOX21_AP1_BLA_Agonist_ratio
BSK_LPS_VCAM1_down
ATG_CRE_CIS_up
NVS_ADME_rCYP2C13
BSK_SAg_MCP1_down
LTEA_HepaRG_CAT_dn
STM_H9_OrnithineISnorm_perc_dn
NVS_ENZ_hPDE5
LTEA_HepaRG_CYP2B6_up
CLD_CYP1A2_24hr
NHEERL_ZF_144hpf_TERATOSCORE_up
LTEA_HepaRG_MIR122_dn
LTEA_HepaRG_GADD45G_dn
LTEA_HepaRG_CYP2E1_dn
LTEA_HepaRG_ABCB11_dn
NVS_ENZ_hCK1D
LTEA_HepaRG_GADD45B_dn
LTEA_HepaRG_ABCC2_up
APR_HepG2_MitoMembPot_72h_dn
1014−69−3
4147−51−7
841−06−5
845−52−3
1014−70−6
4658−28−0
22936−75−0
886−50−0
7287−19−6
834−12−8

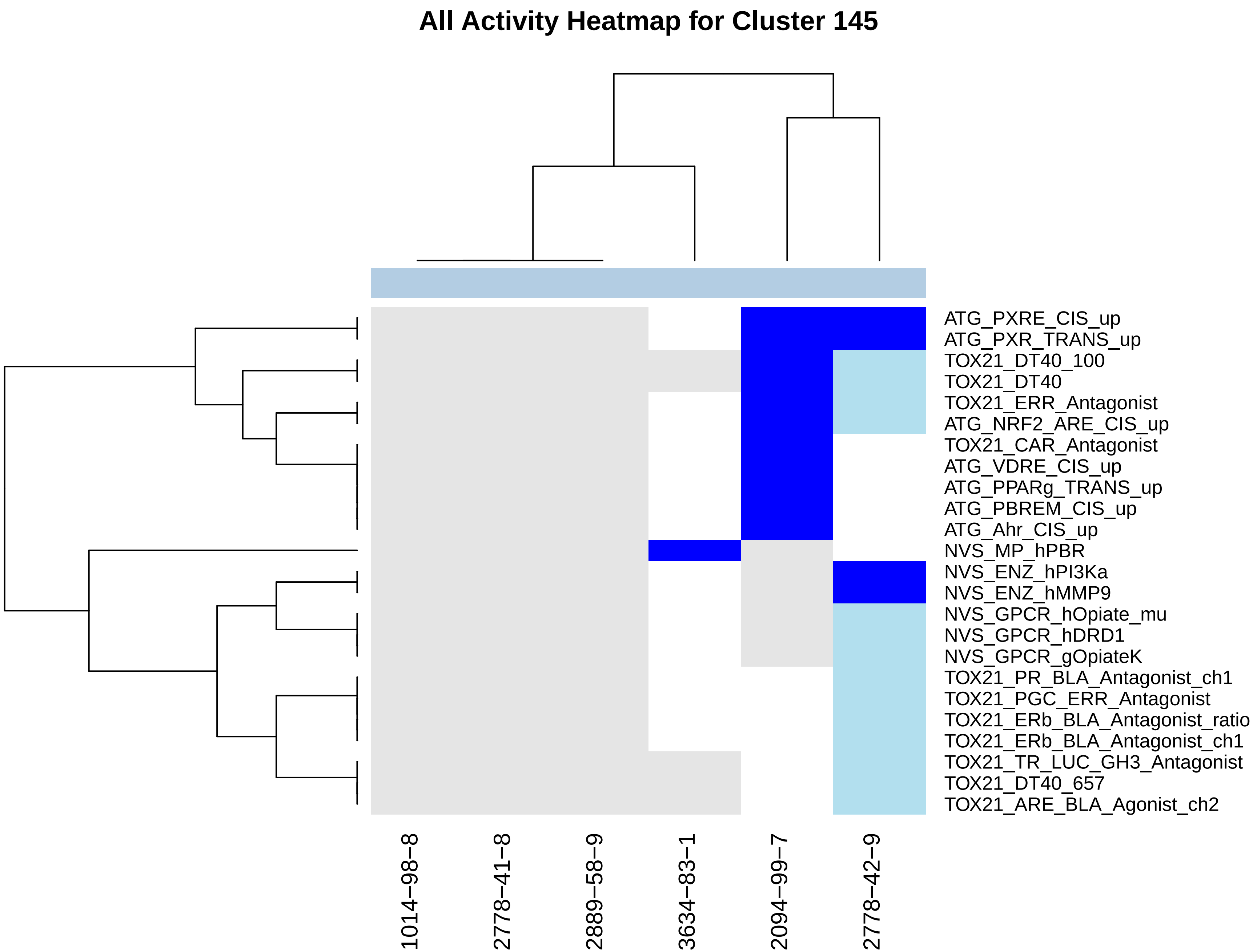
All Activity Heatmap for Cluster 145
ATG_PXRE_CIS_up
ATG_PXR_TRANS_up
TOX21_DT40_100
TOX21_DT40
TOX21_ERR_Antagonist
ATG_NRF2_ARE_CIS_up
TOX21_CAR_Antagonist
ATG_VDRE_CIS_up
ATG_PPARg_TRANS_up
ATG_PBREM_CIS_up
ATG_Ahr_CIS_up
NVS_MP_hPBR
NVS_ENZ_hPI3Ka
NVS_ENZ_hMMP9
NVS_GPCR_hOpiate_mu
NVS_GPCR_hDRD1
NVS_GPCR_gOpiateK
TOX21_PR_BLA_Antagonist_ch1
TOX21_PGC_ERR_Antagonist
TOX21_ERb_BLA_Antagonist_ratio
TOX21_ERb_BLA_Antagonist_ch1
TOX21_TR_LUC_GH3_Antagonist
TOX21_DT40_657
TOX21_ARE_BLA_Agonist_ch2
1014−98−8
2778−41−8
2889−58−9
3634−83−1
2094−99−7
2778−42−9

All Activity Heatmap for Cluster 147

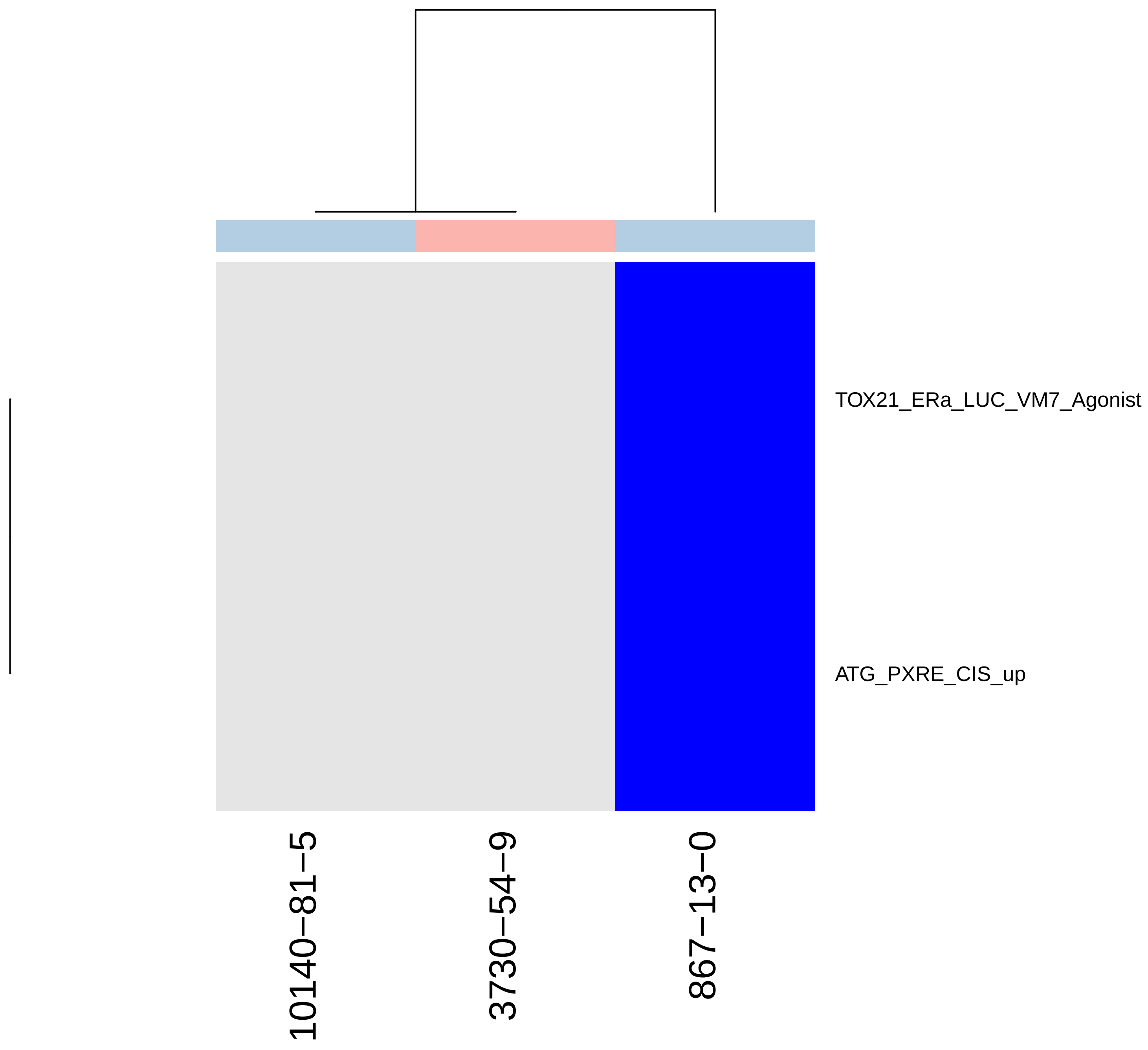

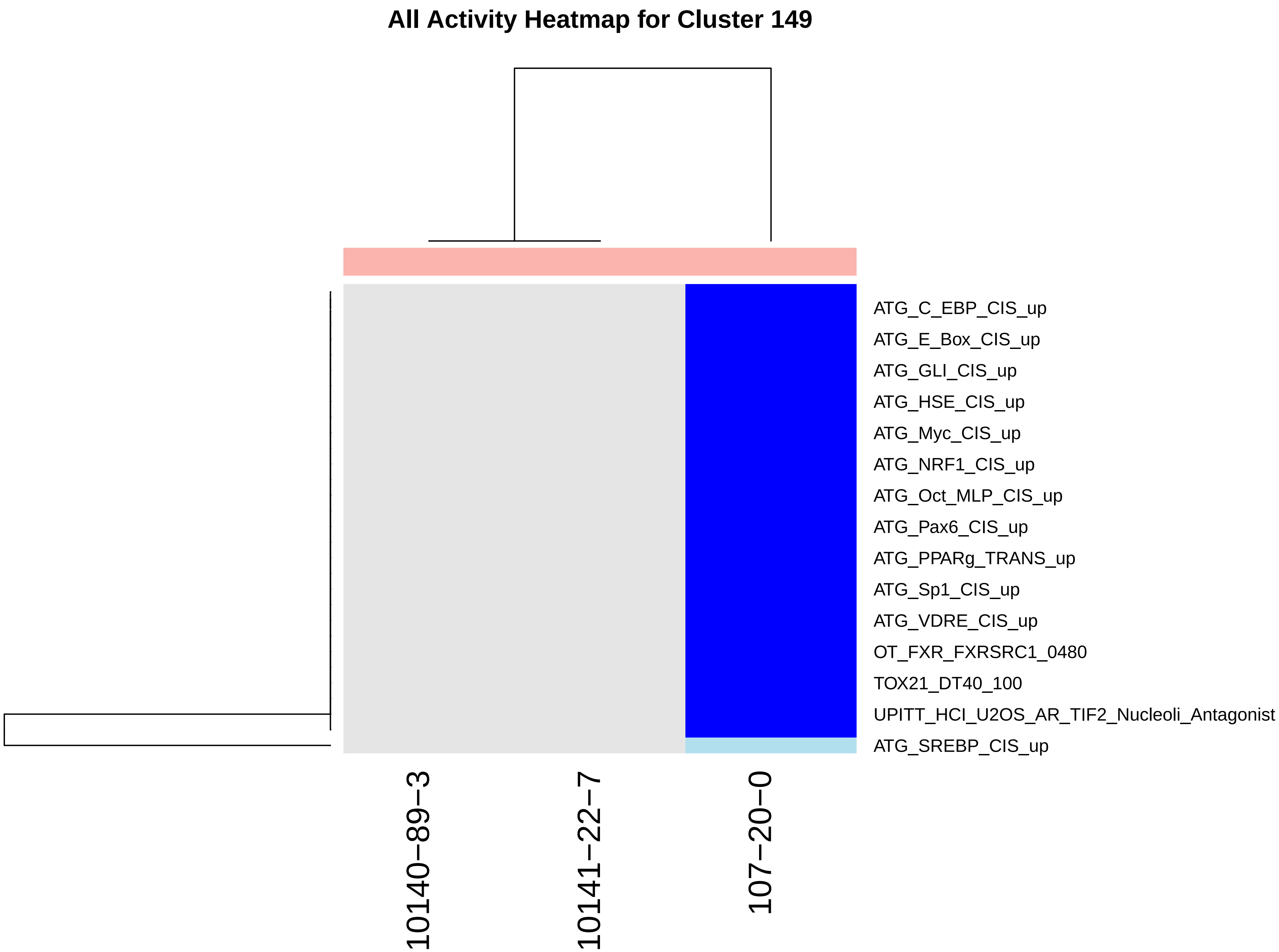
All Activity Heatmap for Cluster 149
ATG_C_EBP_CIS_up
ATG_E_Box_CIS_up
ATG_GLI_CIS_up
ATG_HSE_CIS_up
ATG_Myc_CIS_up
ATG_NRF1_CIS_up
ATG_Oct_MLP_CIS_up
ATG_Pax6_CIS_up
ATG_PPARg_TRANS_up
ATG_Sp1_CIS_up
ATG_VDRE_CIS_up
OT_FXR_FXRSRC1_0480
TOX21_DT40_100
UPITT_HCI_U2OS_AR_TIF2_Nucleoli_Antagonist
ATG_SREBP_CIS_up
10140−89−3
10141−22−7
107−20−0

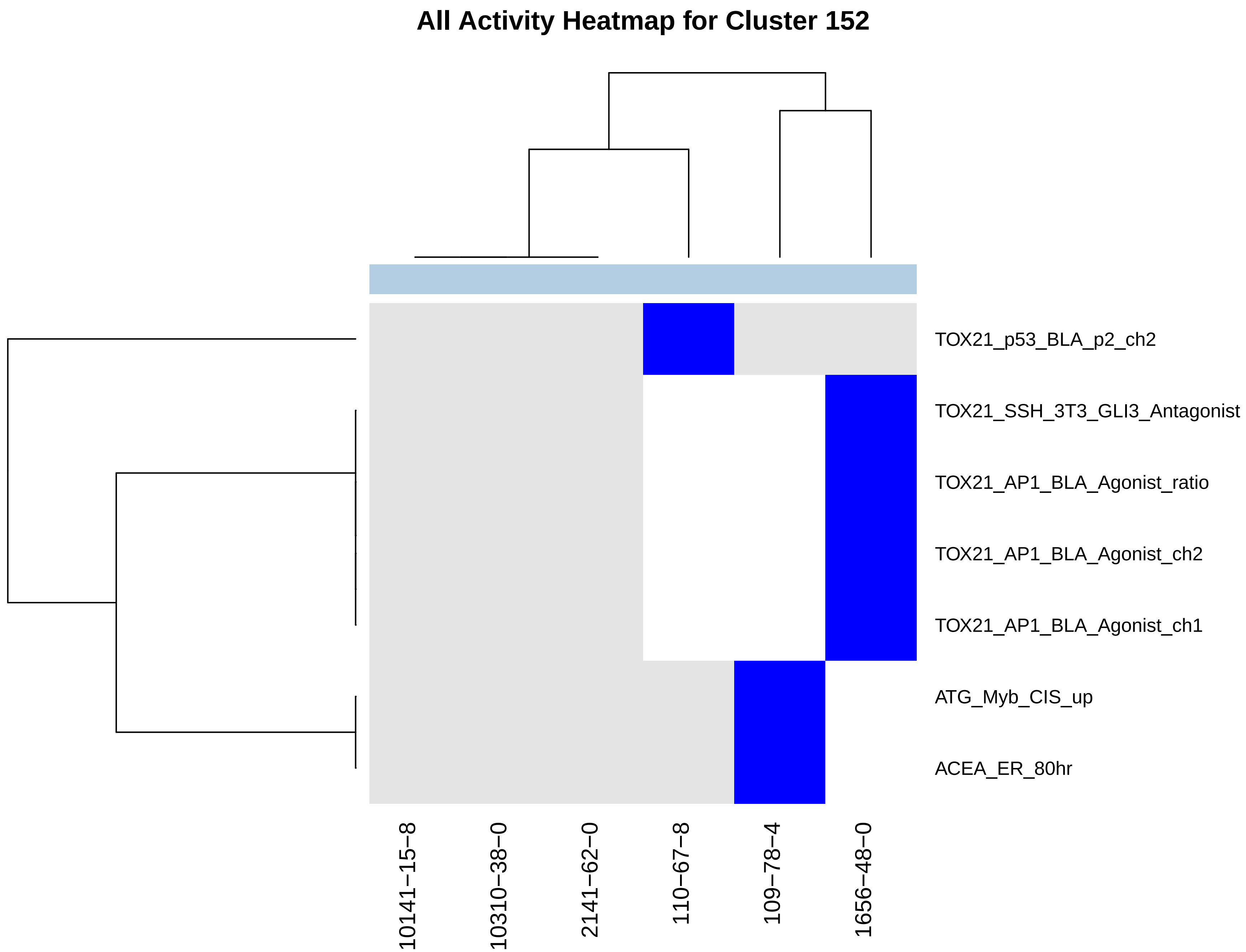
All Activity Heatmap for Cluster 152
TOX21_p53_BLA_p2_ch2
TOX21_SSH_3T3_GLI3_Antagonist
TOX21_AP1_BLA_Agonist_ratio
TOX21_AP1_BLA_Agonist_ch2
TOX21_AP1_BLA_Agonist_ch1
ATG_Myb_CIS_up
ACEA_ER_80hr
10141−15−8
10310−38−0
2141−62−0
110−67−8
109−78−4
1656−48−0

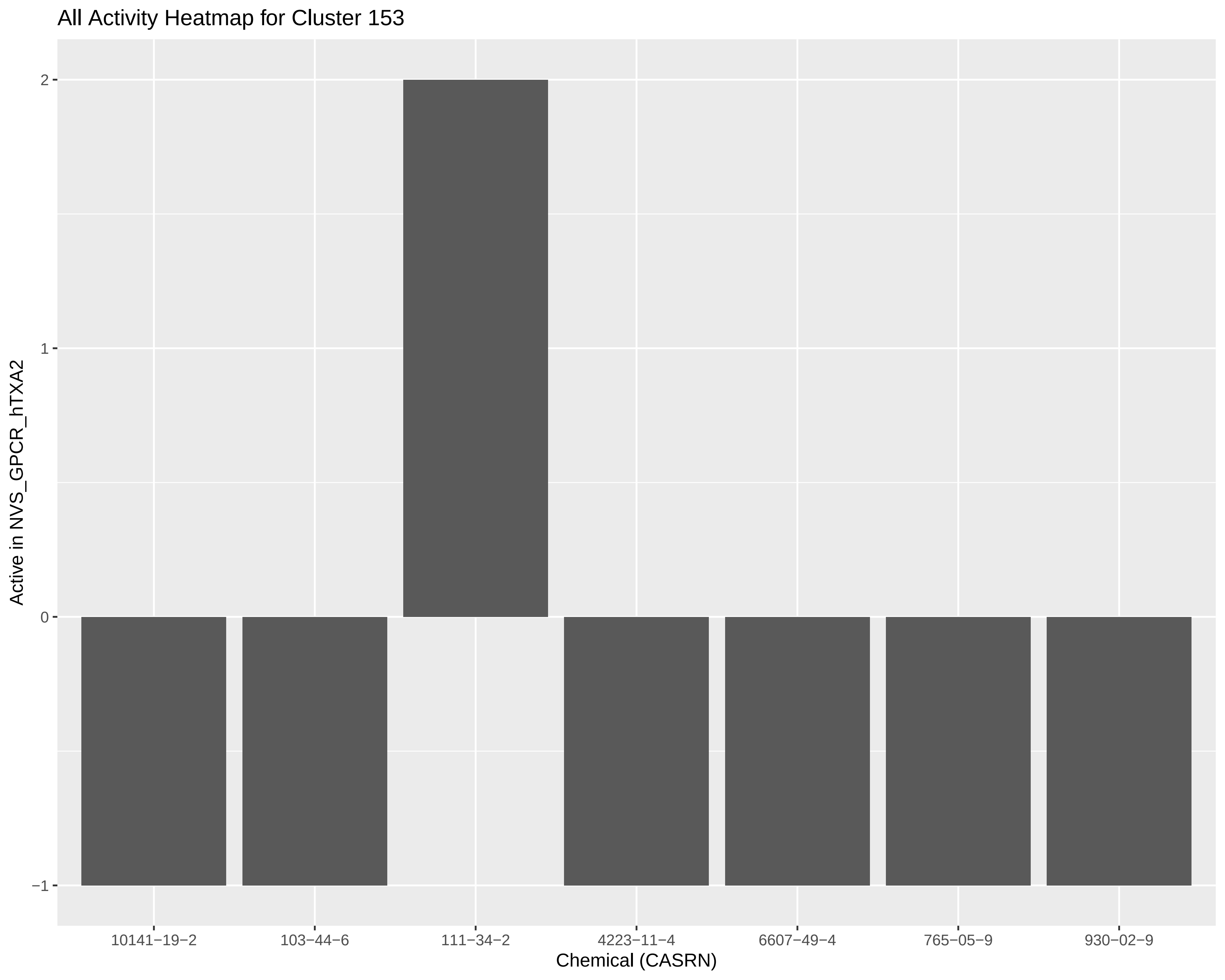
All Activity Heatmap for Cluster 153
Active in NVS_GPCR_hTXA2
2
1
0
−1
10141−19−2
103−44−6
111−34−2
4223−11−4
6607−49−4
765−05−9
930−02−9
Chemical (CASRN)

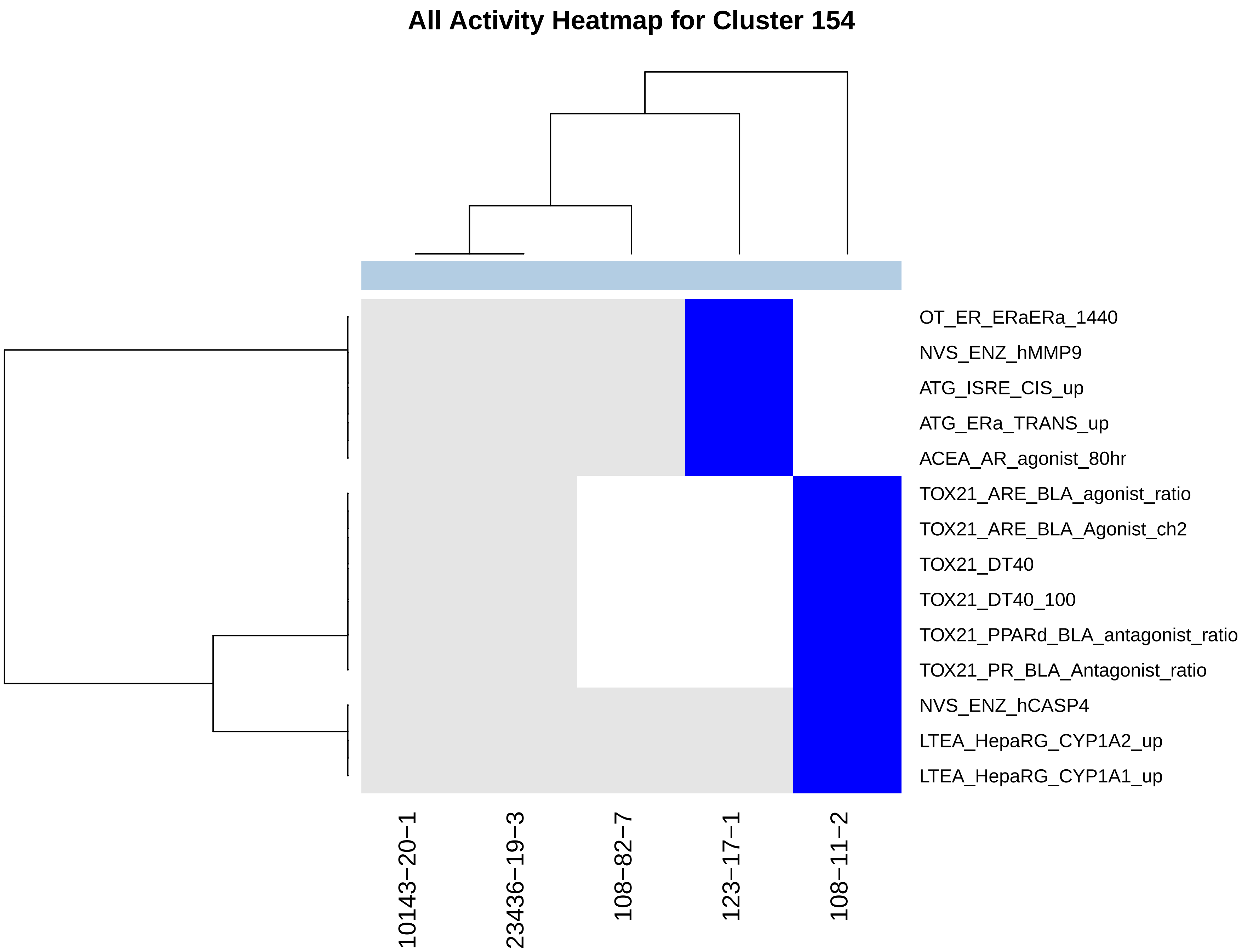

All Activity Heatmap for Cluster 154
OT_ER_ERaERa_1440
NVS_ENZ_hMMP9
ATG_ISRE_CIS_up
ATG_ERa_TRANS_up
ACEA_AR_agonist_80hr
TOX21_ARE_BLA_agonist_ratio
TOX21_ARE_BLA_Agonist_ch2
TOX21_DT40
TOX21_DT40_100
TOX21_PPARd_BLA_antagonist_ratio
TOX21_PR_BLA_Antagonist_ratio
NVS_ENZ_hCASP4
LTEA_HepaRG_CYP1A2_up
LTEA_HepaRG_CYP1A1_up
10143−20−1
23436−19−3
108−82−7
123−17−1
108−11−2

All Activity Heatmap for Cluster 155

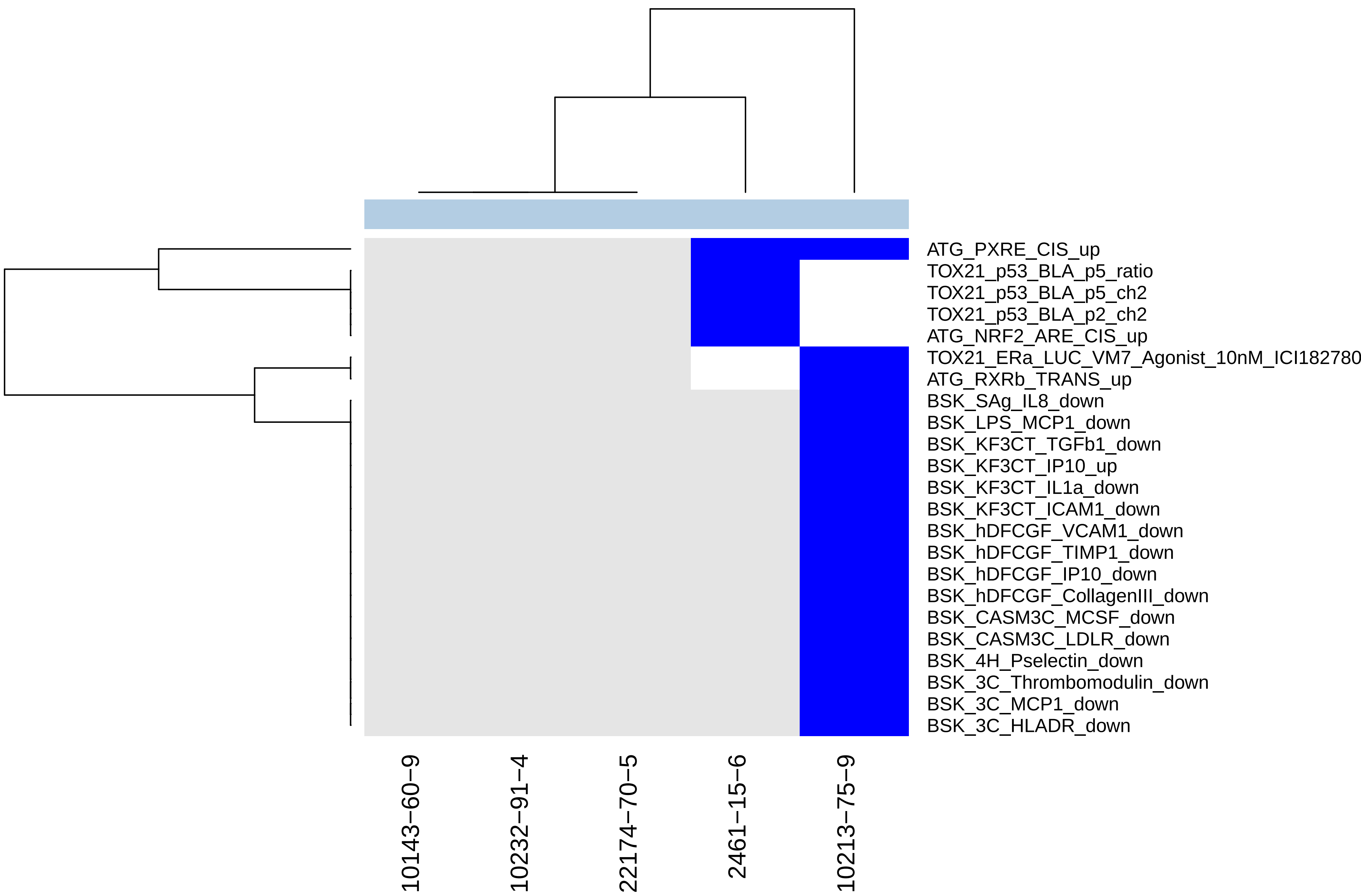

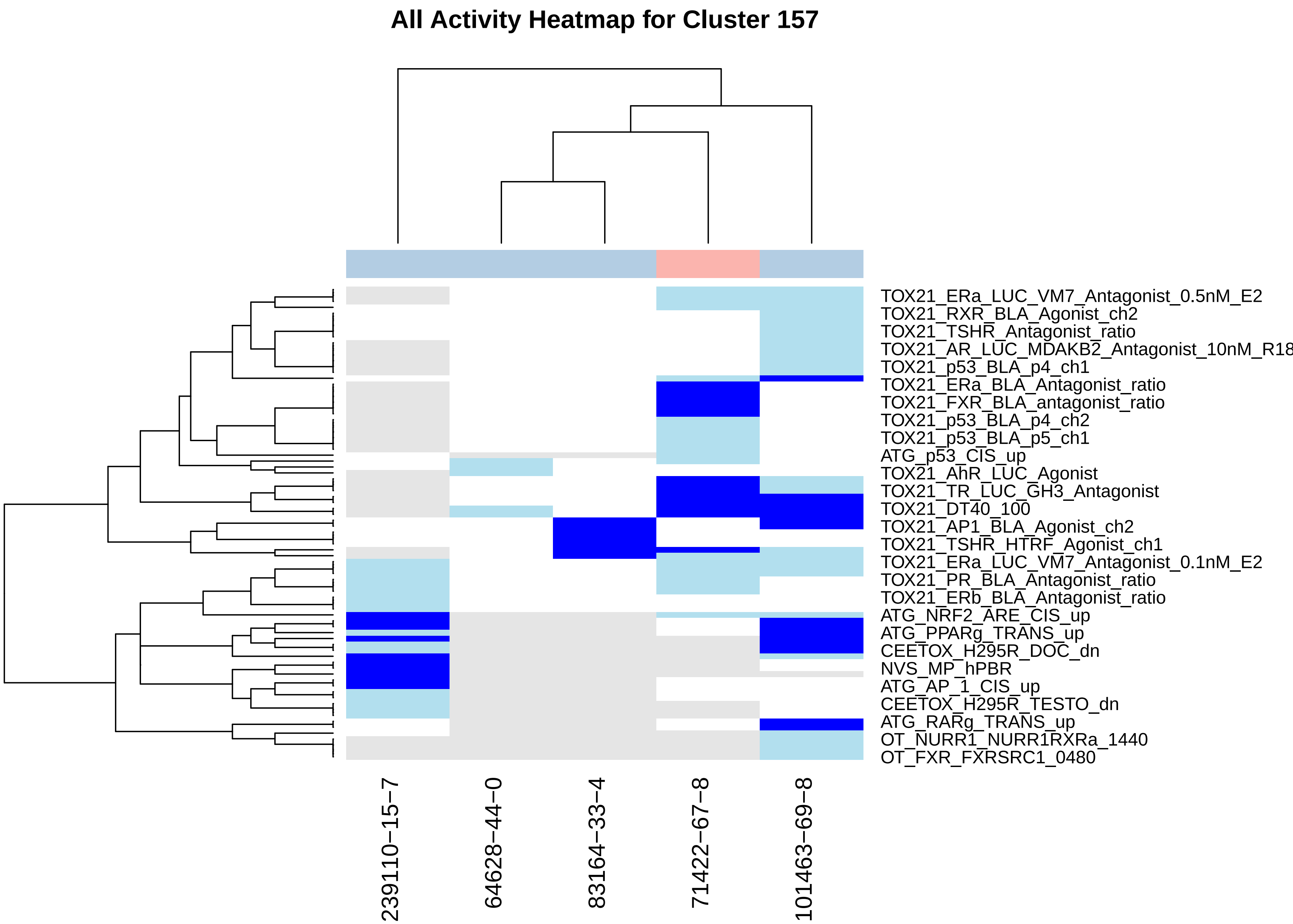
All Activity Heatmap for Cluster 157
TOX21_ERa_LUC_VM7_Antagonist_0.5nM_E2
TOX21_RXR_BLA_Agonist_ch2
TOX21_TSHR_Antagonist_ratio
TOX21_AR_LUC_MDAKB2_Antagonist_10nM_R18
TOX21_p53_BLA_p4_ch1
TOX21_ERa_BLA_Antagonist_ratio
TOX21_FXR_BLA_antagonist_ratio
TOX21_p53_BLA_p4_ch2
TOX21_p53_BLA_p5_ch1
ATG_p53_CIS_up
TOX21_AhR_LUC_Agonist
TOX21_TR_LUC_GH3_Antagonist
TOX21_DT40_100
TOX21_AP1_BLA_Agonist_ch2
TOX21_TSHR_HTRF_Agonist_ch1
TOX21_ERa_LUC_VM7_Antagonist_0.1nM_E2
TOX21_PR_BLA_Antagonist_ratio
TOX21_ERb_BLA_Antagonist_ratio
ATG_NRF2_ARE_CIS_up
ATG_PPARg_TRANS_up
CEETOX_H295R_DOC_dn
NVS_MP_hPBR
ATG_AP_1_CIS_up
CEETOX_H295R_TESTO_dn
ATG_RARg_TRANS_up
OT_NURR1_NURR1RXRa_1440
OT_FXR_FXRSRC1_0480
239110−15−7
64628−44−0
83164−33−4
71422−67−8
101463−69−8

All Activity Heatmap for Cluster 158

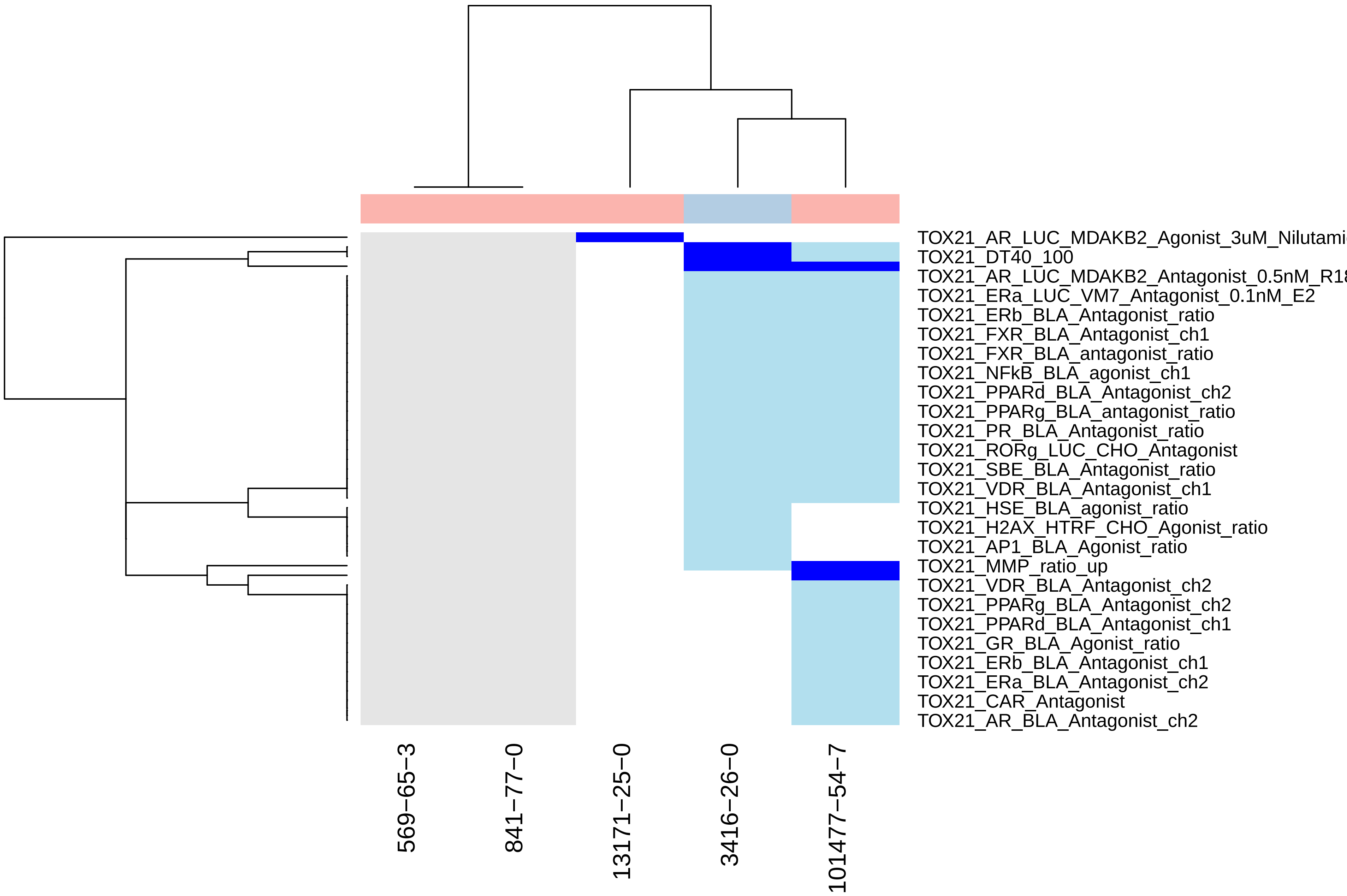

All Activity Heatmap for Cluster 159

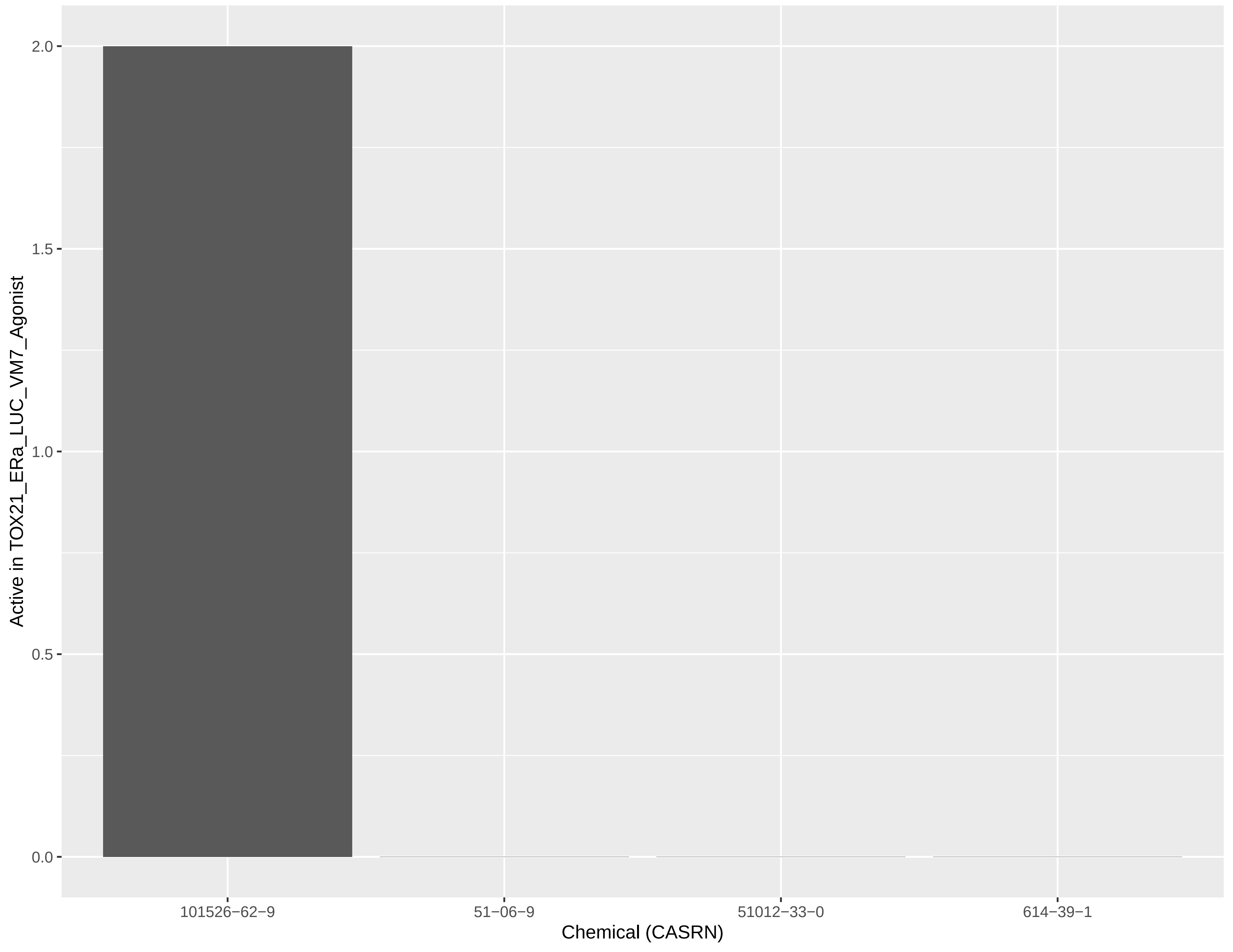

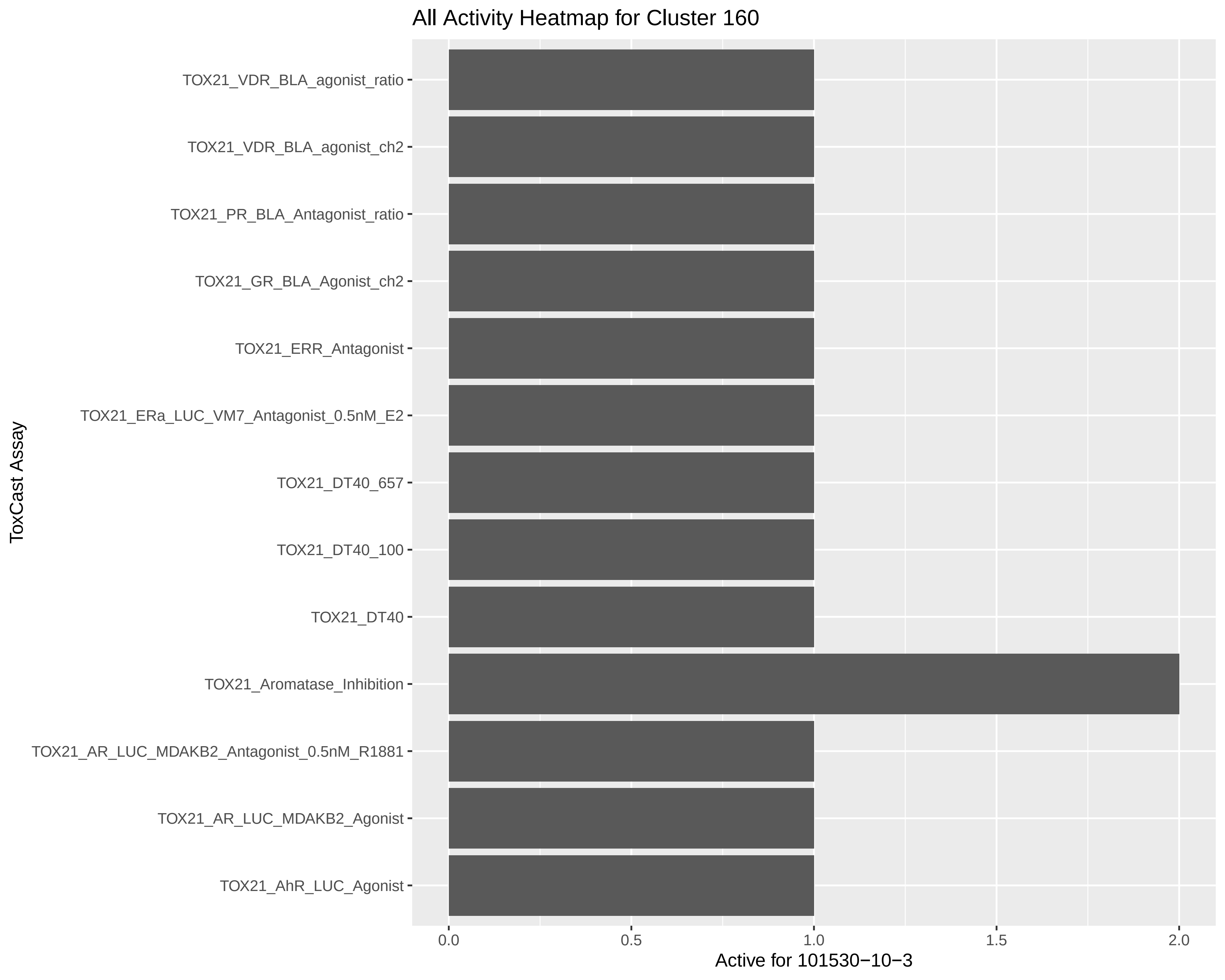

All Activity Heatmap for Cluster 160
ToxCast Assay
TOX21_VDR_BLA_agonist_ratio
TOX21_VDR_BLA_agonist_ch2
TOX21_PR_BLA_Antagonist_ratio
TOX21_GR_BLA_Agonist_ch2
TOX21_ERR_Antagonist
TOX21_ERa_LUC_VM7_Antagonist_0.5nM_E2
TOX21_DT40_657
TOX21_DT40_100
TOX21_DT40
TOX21_Aromatase_Inhibition
TOX21_AR_LUC_MDAKB2_Antagonist_0.5nM_R1881
TOX21_AR_LUC_MDAKB2_Agonist
TOX21_AhR_LUC_Agonist
0.0
0.5
1.0
1.5
2.0
Active for 101530−10−3

All Activity Heatmap for Cluster 162

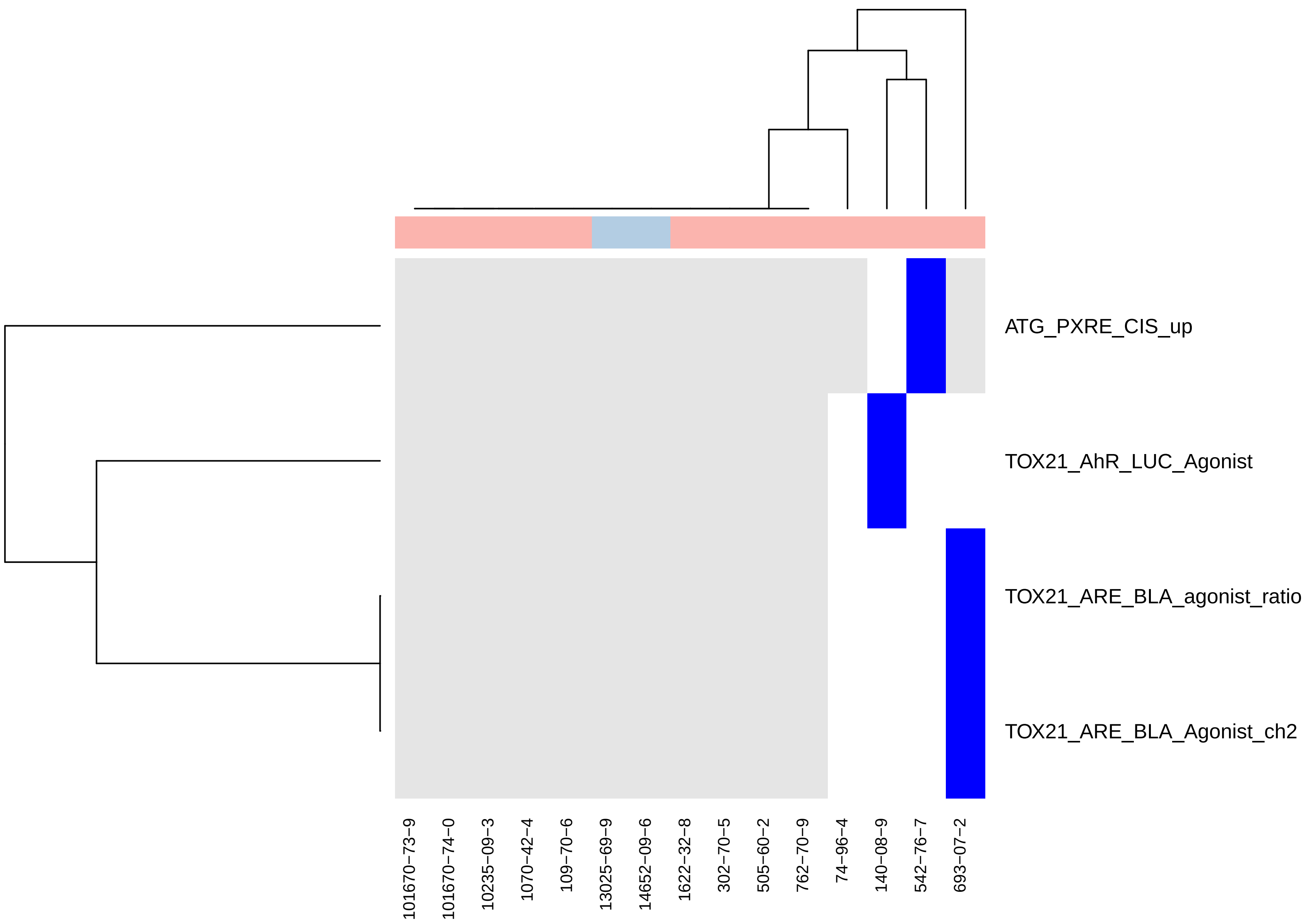

All Activity Heatmap for Cluster 165

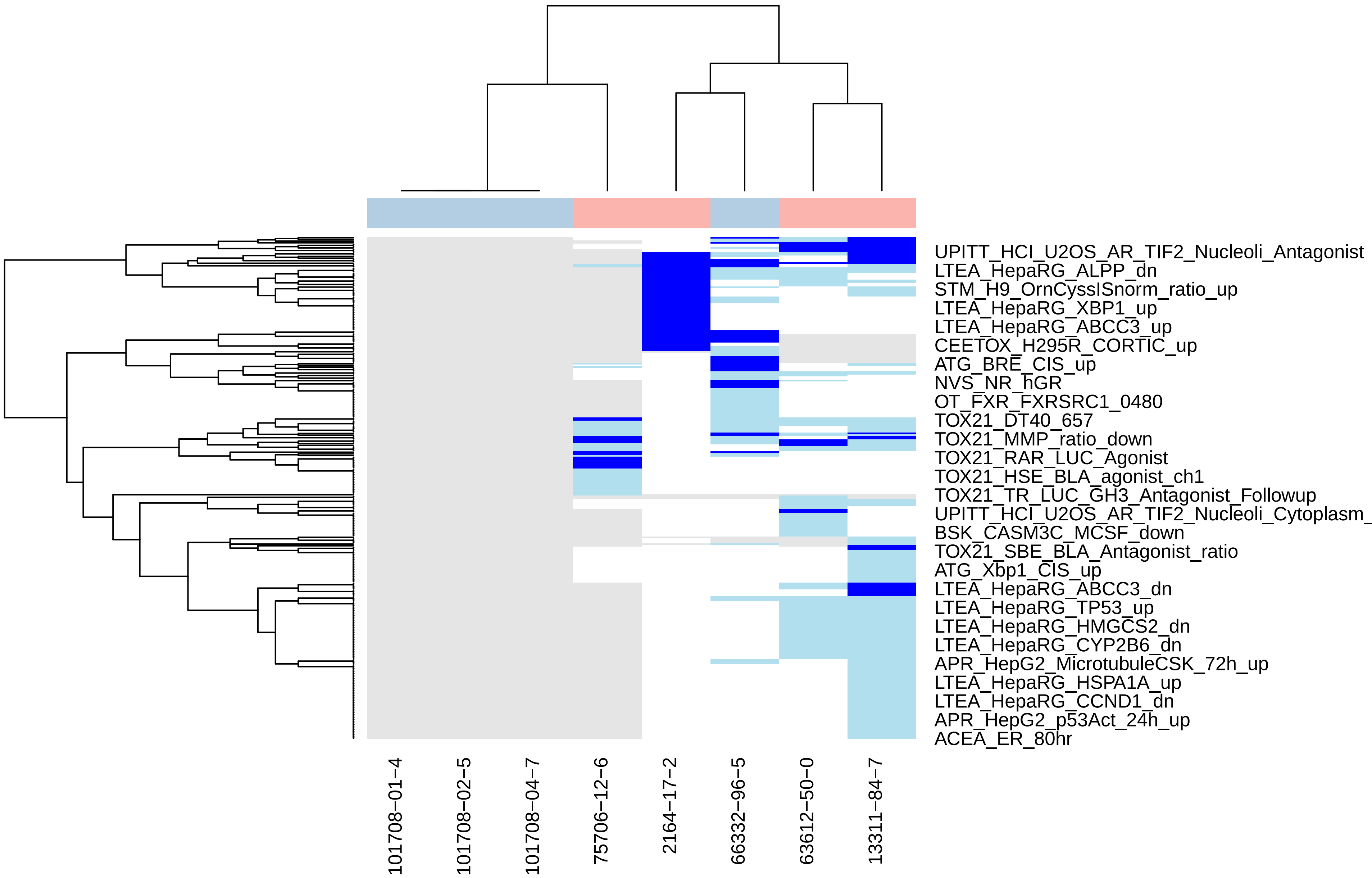

All Activity Heatmap for Cluster 166

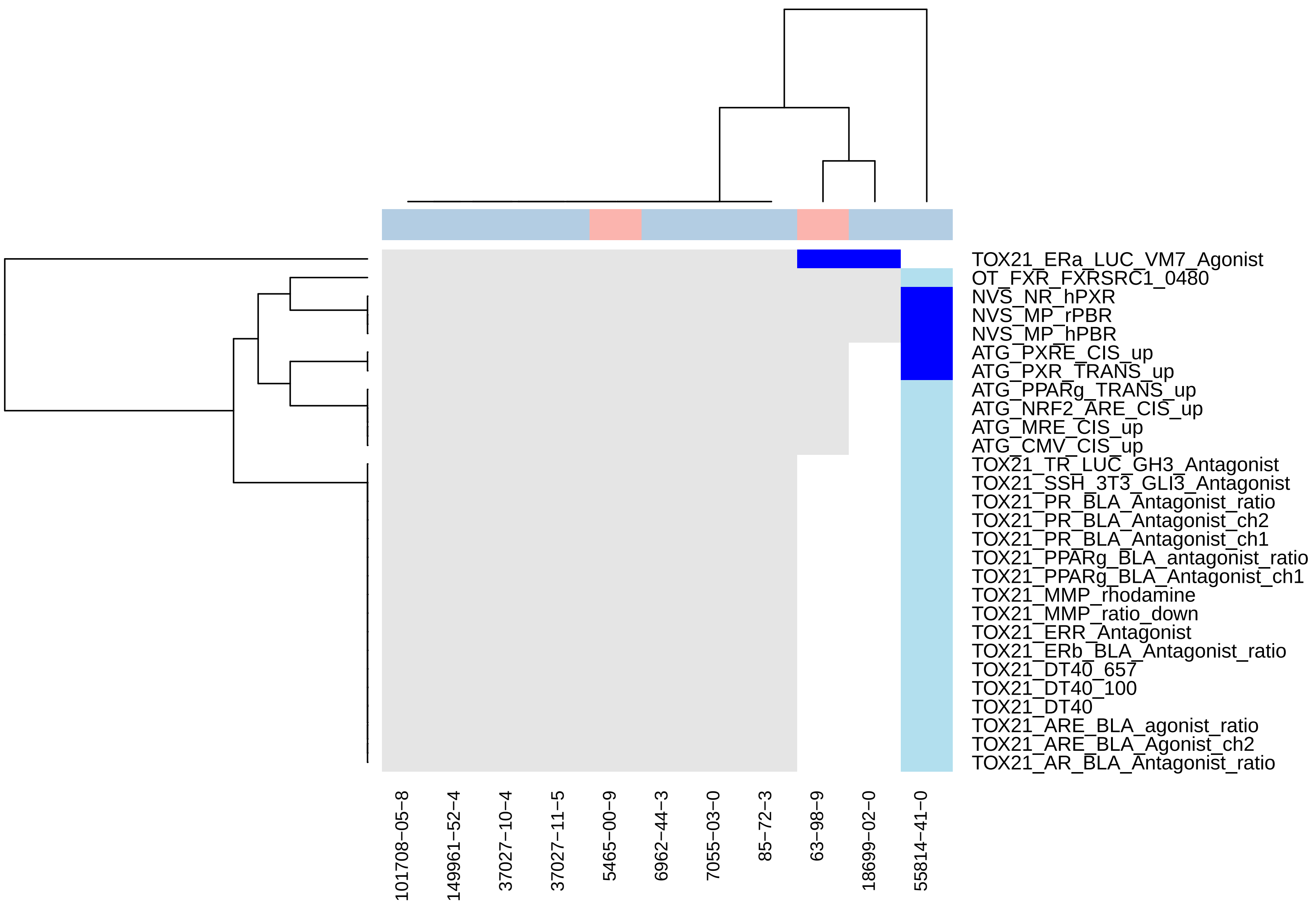

All Activity Heatmap for Cluster 167

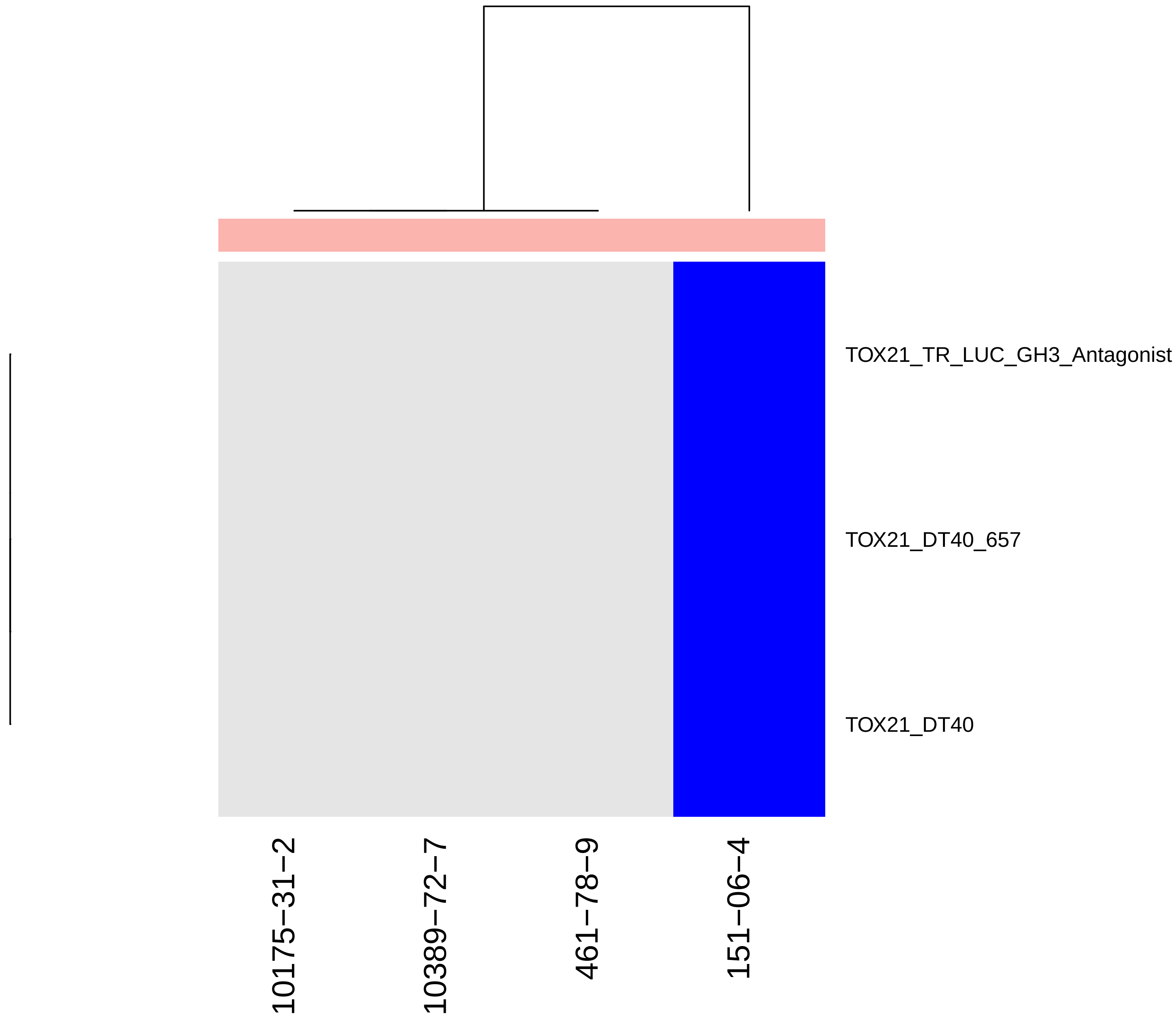

All Activity Heatmap for Cluster 168

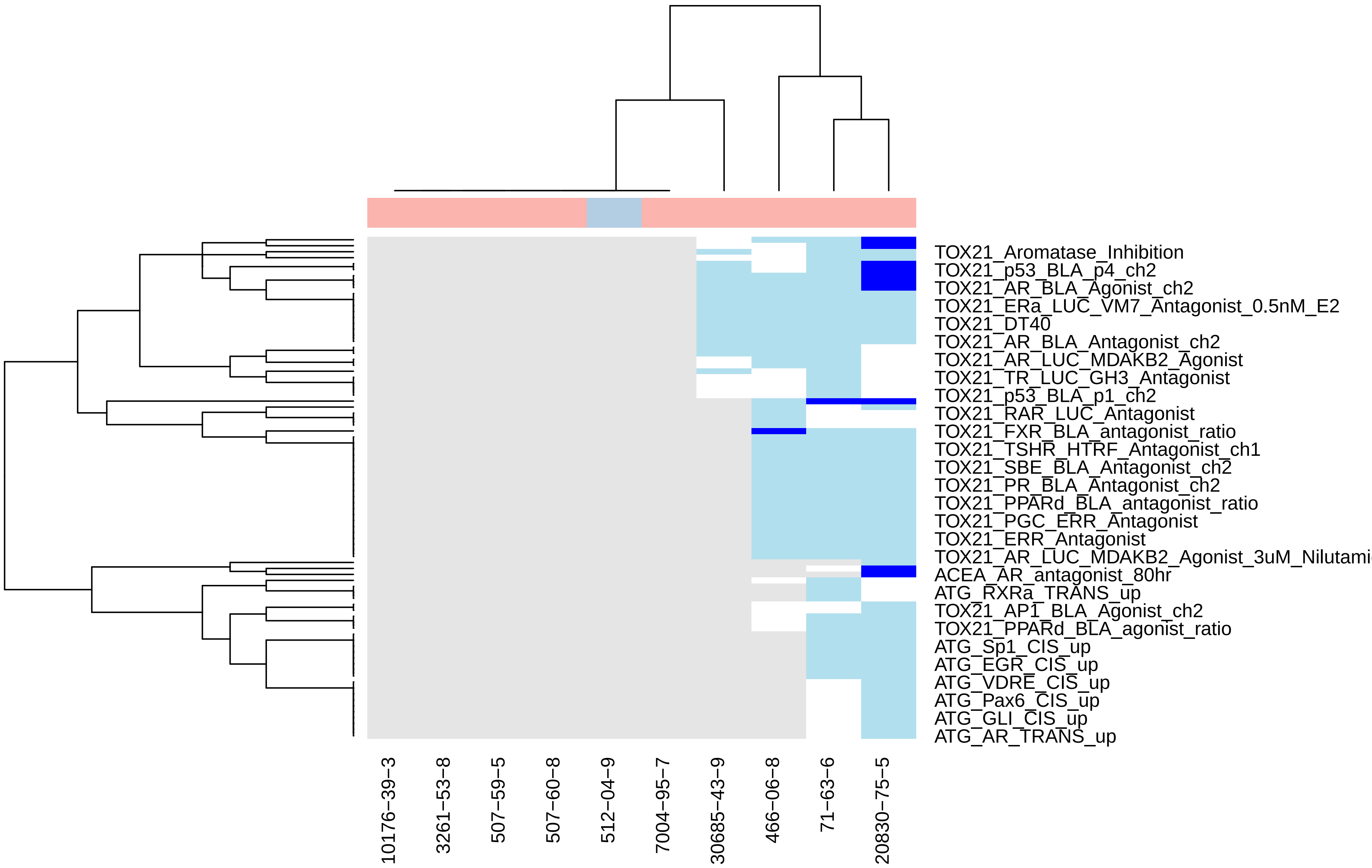

All Activity Heatmap for Cluster 169

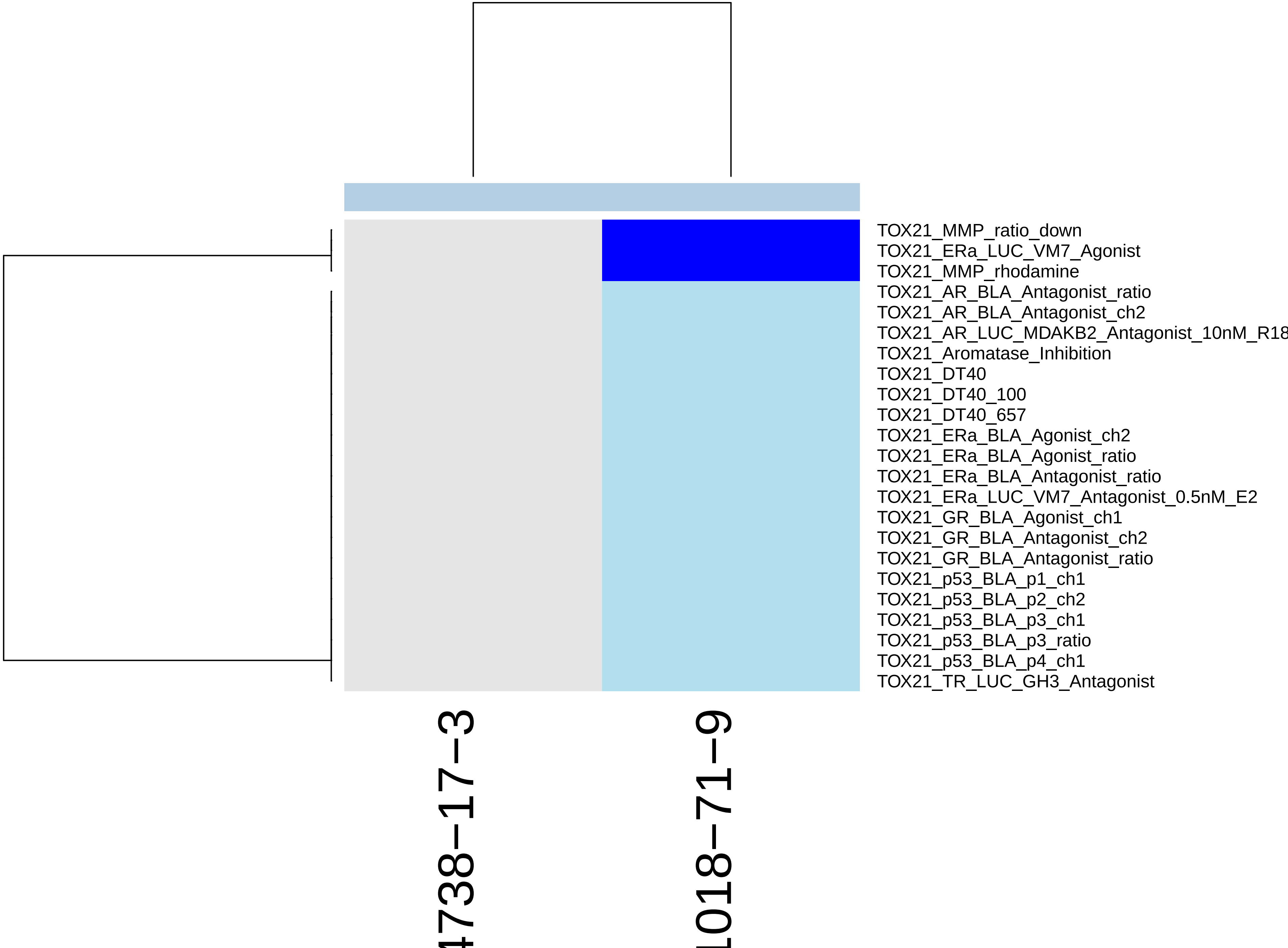

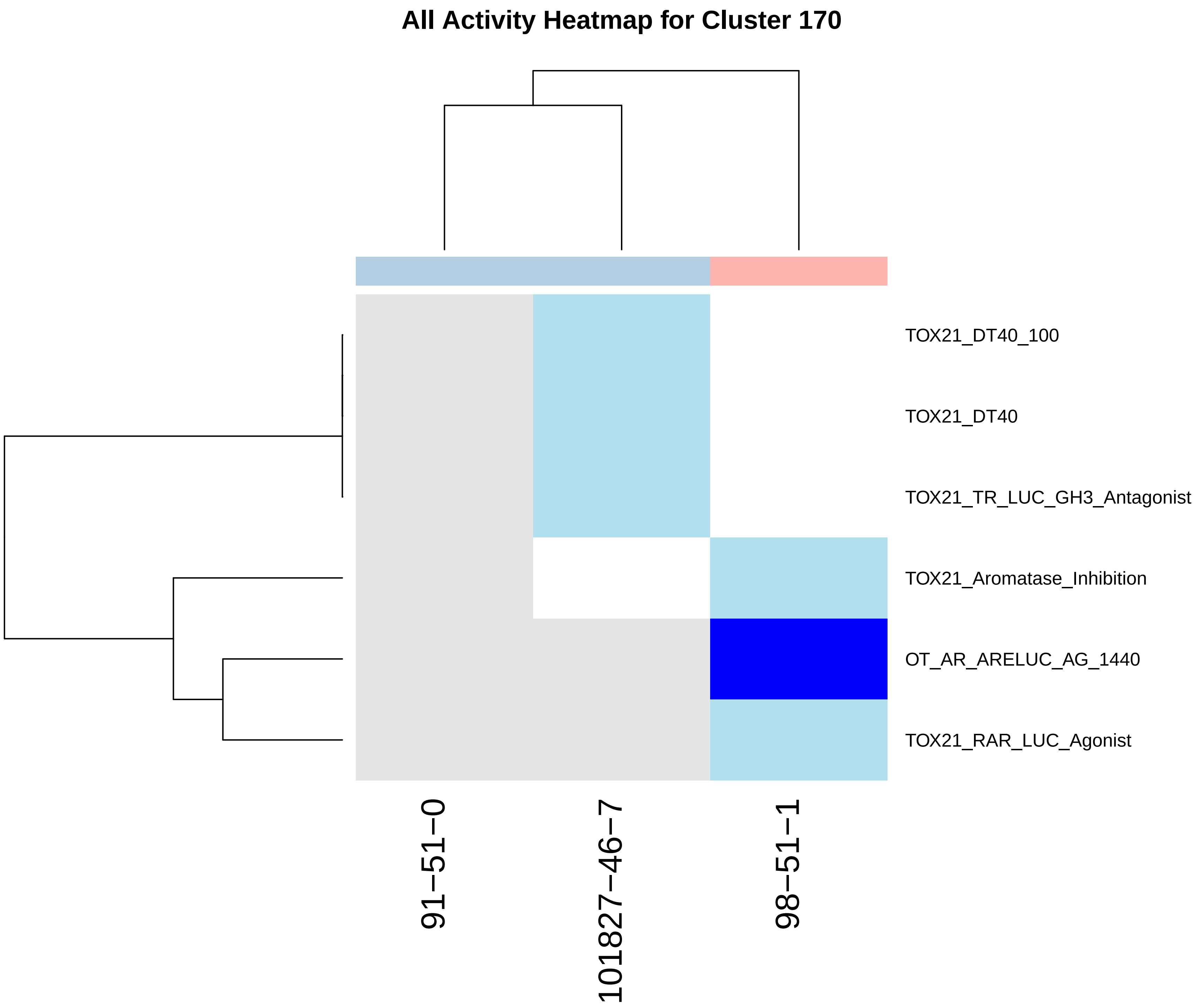

All Activity Heatmap for Cluster 170
TOX21_DT40_100
TOX21_DT40
TOX21_TR_LUC_GH3_Antagonist
TOX21_Aromatase_Inhibition
OT_AR_ARELUC_AG_1440
TOX21_RAR_LUC_Agonist
91−51−0
101827−46−7
98−51−1

All Activity Heatmap for Cluster 173

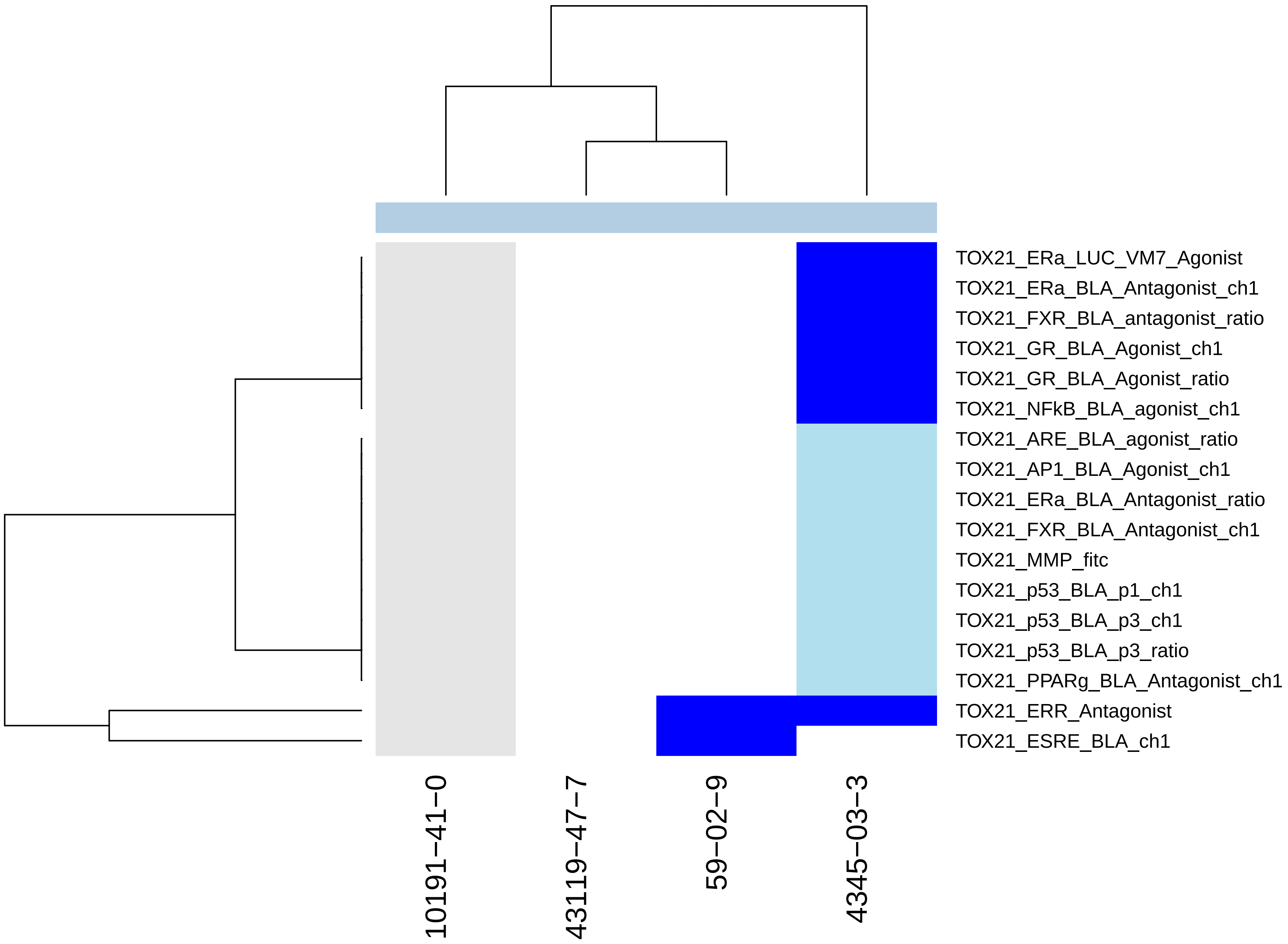

All Activity Heatmap for Cluster 174

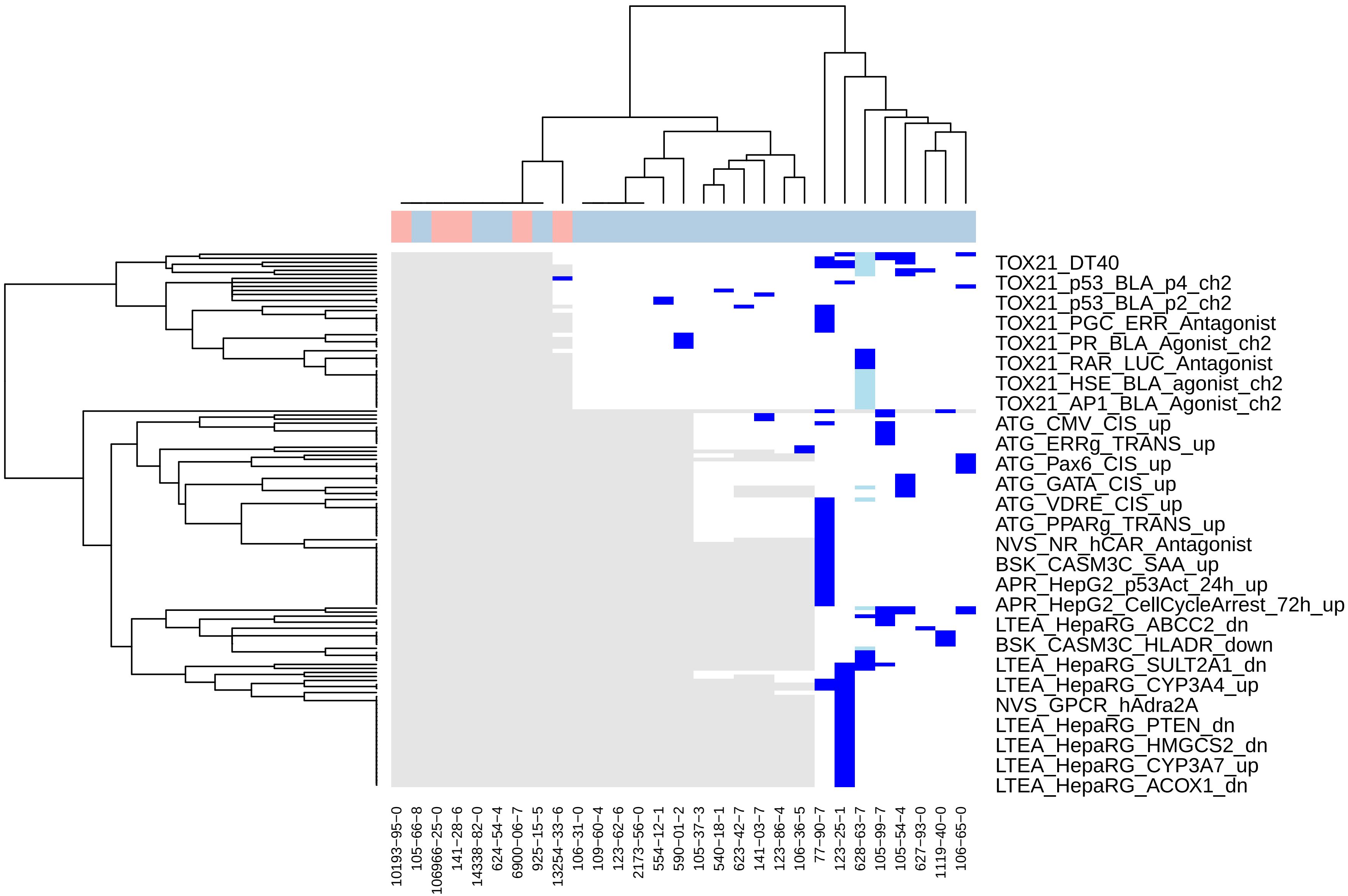

All Activity Heatmap for Cluster 175

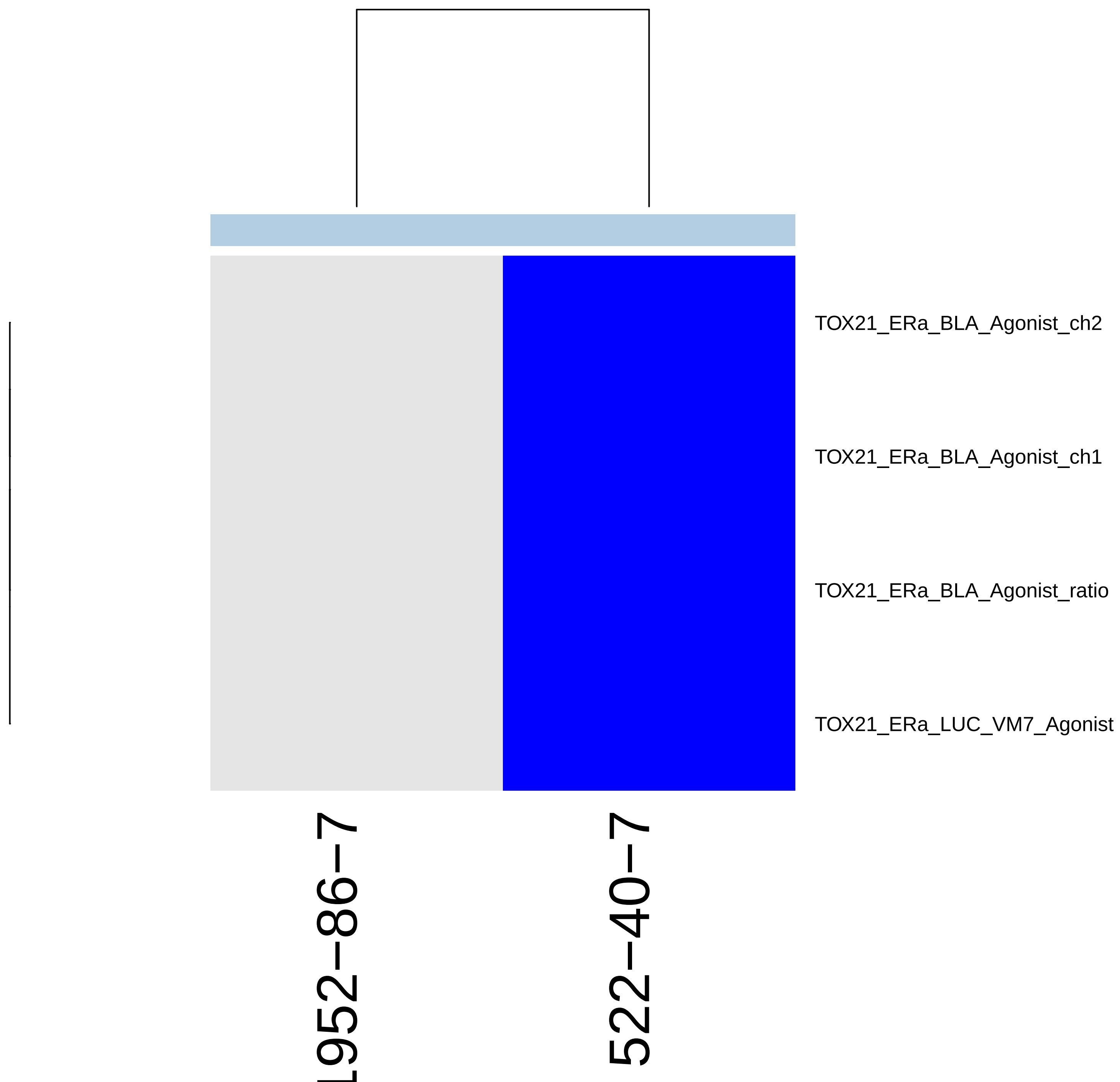

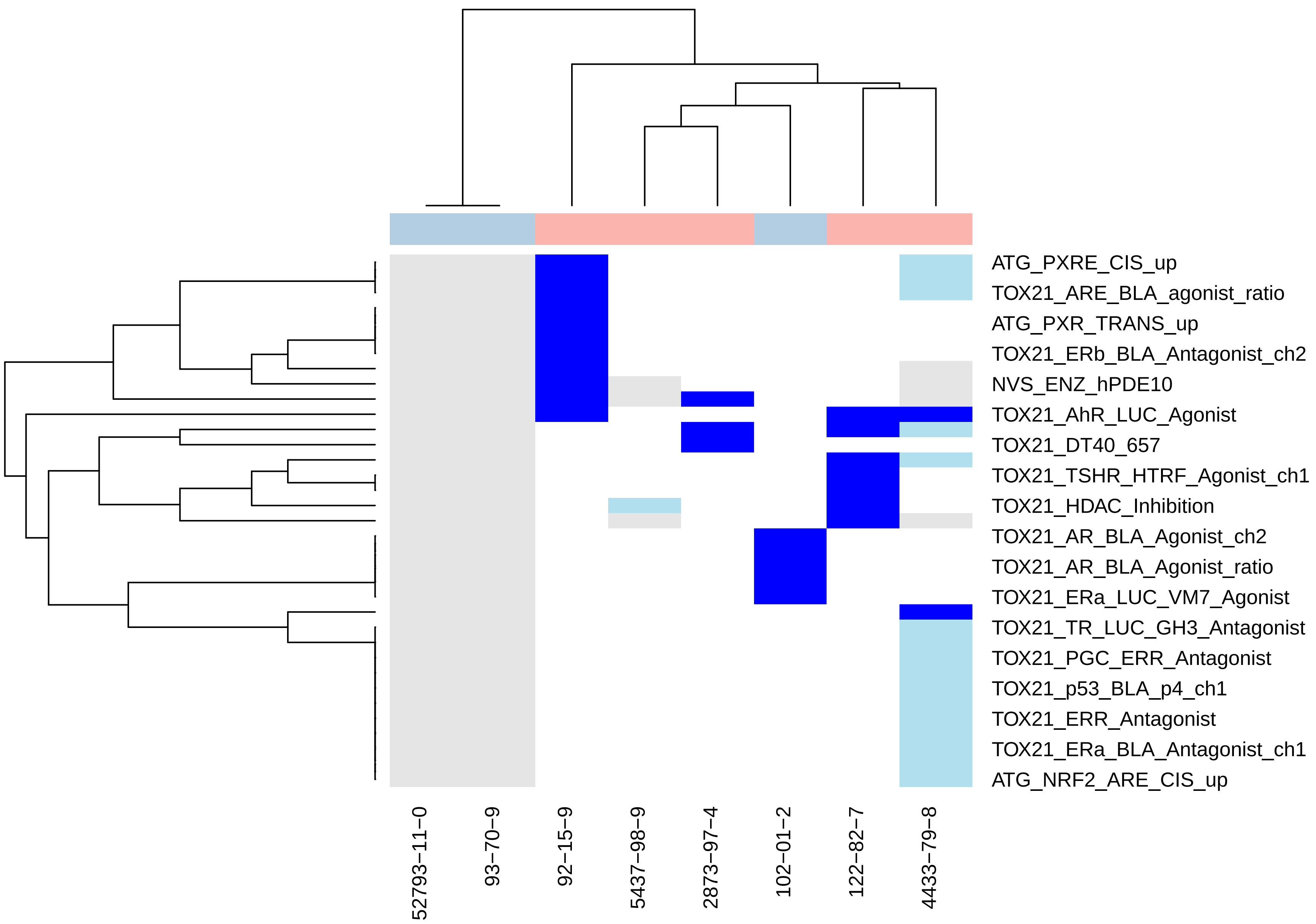
All Activity Heatmap for Cluster 176
ATG_PXRE_CIS_up
TOX21_ARE_BLA_agonist_ratio
ATG_PXR_TRANS_up
TOX21_ERb_BLA_Antagonist_ch2
NVS_ENZ_hPDE10
TOX21_AhR_LUC_Agonist
TOX21_DT40_657
TOX21_TSHR_HTRF_Agonist_ch1
TOX21_HDAC_Inhibition
TOX21_AR_BLA_Agonist_ch2
TOX21_AR_BLA_Agonist_ratio
TOX21_ERa_LUC_VM7_Agonist
TOX21_TR_LUC_GH3_Antagonist
TOX21_PGC_ERR_Antagonist
TOX21_p53_BLA_p4_ch1
TOX21_ERR_Antagonist
TOX21_ERa_BLA_Antagonist_ch1
ATG_NRF2_ARE_CIS_up
52793−11−0
93−70−9
92−15−9
5437−98−9
2873−97−4
102−01−2
122−82−7
4433−79−8

All Activity Heatmap for Cluster 178

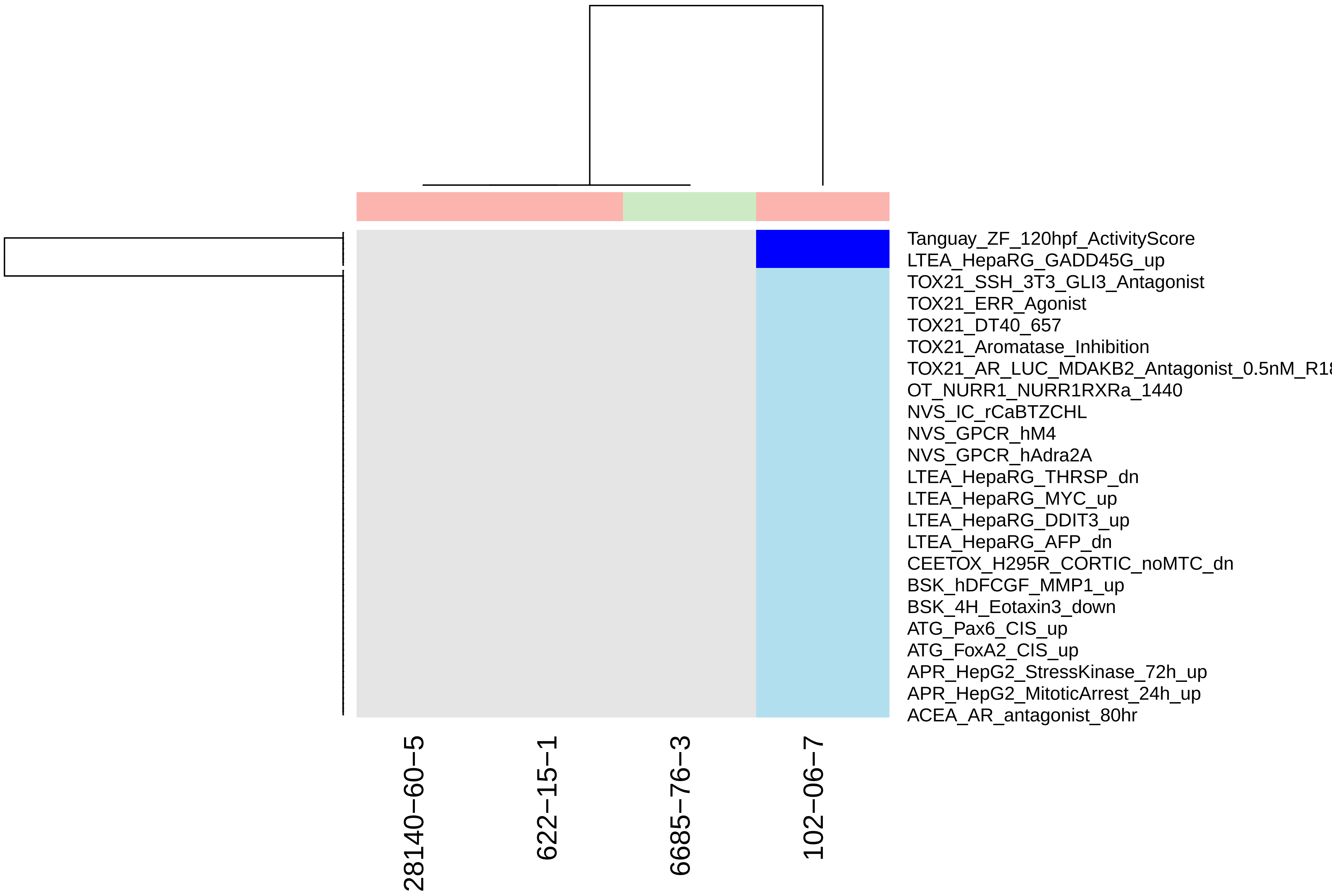

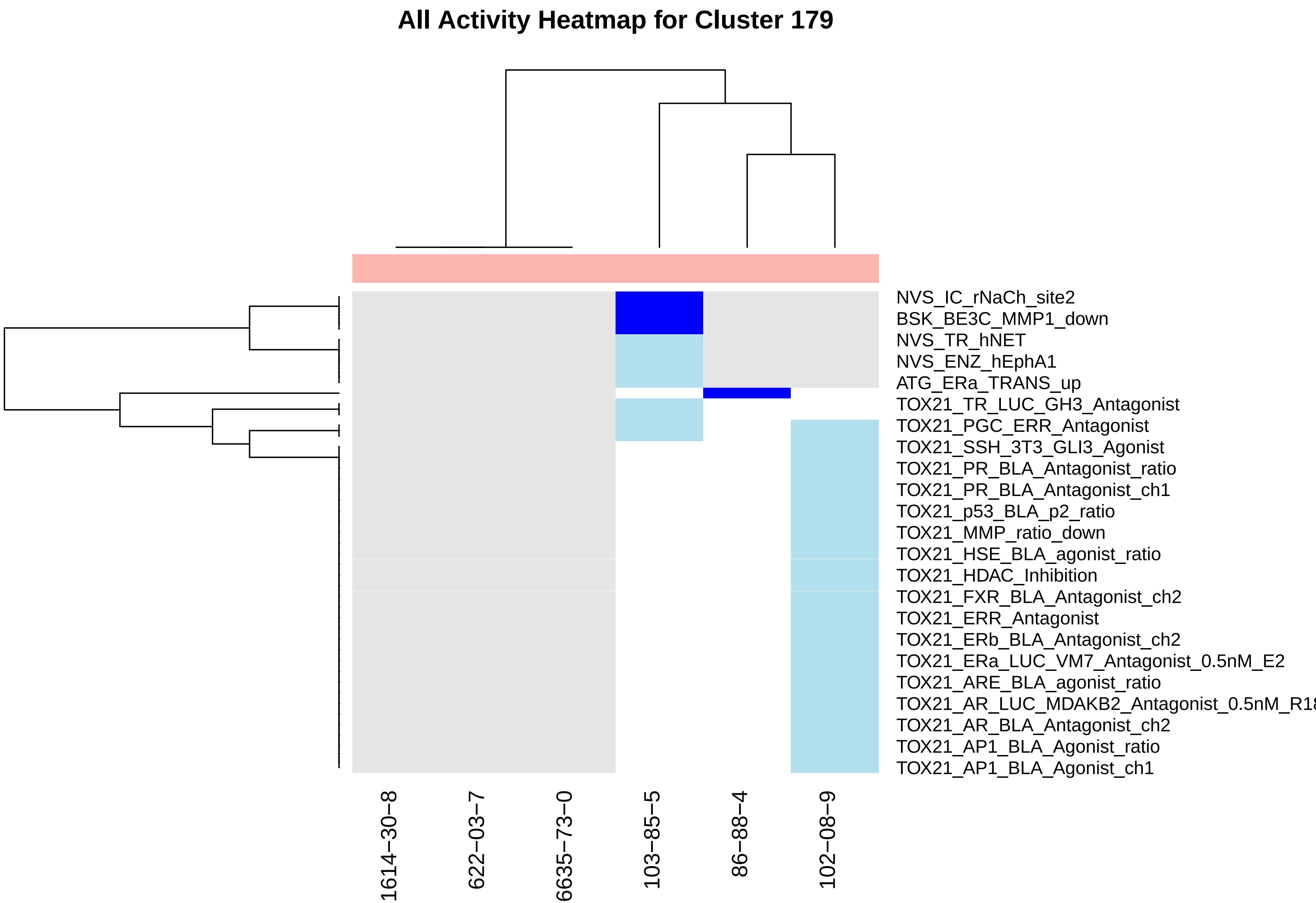
All Activity Heatmap for Cluster 179
NVS_IC_rNaCh_site2
BSK_BE3C_MMP1_down
NVS_TR_hNET
NVS_ENZ_hEphA1
ATG_ERa_TRANS_up
TOX21_TR_LUC_GH3_Antagonist
TOX21_PGC_ERR_Antagonist
TOX21_SSH_3T3_GLI3_Agonist
TOX21_PR_BLA_Antagonist_ratio
TOX21_PR_BLA_Antagonist_ch1
TOX21_p53_BLA_p2_ratio
TOX21_MMP_ratio_down
TOX21_HSE_BLA_agonist_ratio
TOX21_HDAC_Inhibition
TOX21_FXR_BLA_Antagonist_ch2
TOX21_ERR_Antagonist
TOX21_ERb_BLA_Antagonist_ch2
TOX21_ERa_LUC_VM7_Antagonist_0.5nM_E2
TOX21_ARE_BLA_agonist_ratio
TOX21_AR_LUC_MDAKB2_Antagonist_0.5nM_R1
TOX21_AR_BLA_Antagonist_ch2
TOX21_AP1_BLA_Agonist_ratio
TOX21_AP1_BLA_Agonist_ch1
1614−30−8
622−03−7
6635−73−0
103−85−5
86−88−4
102−08−9

All Activity Heatmap for Cluster 180

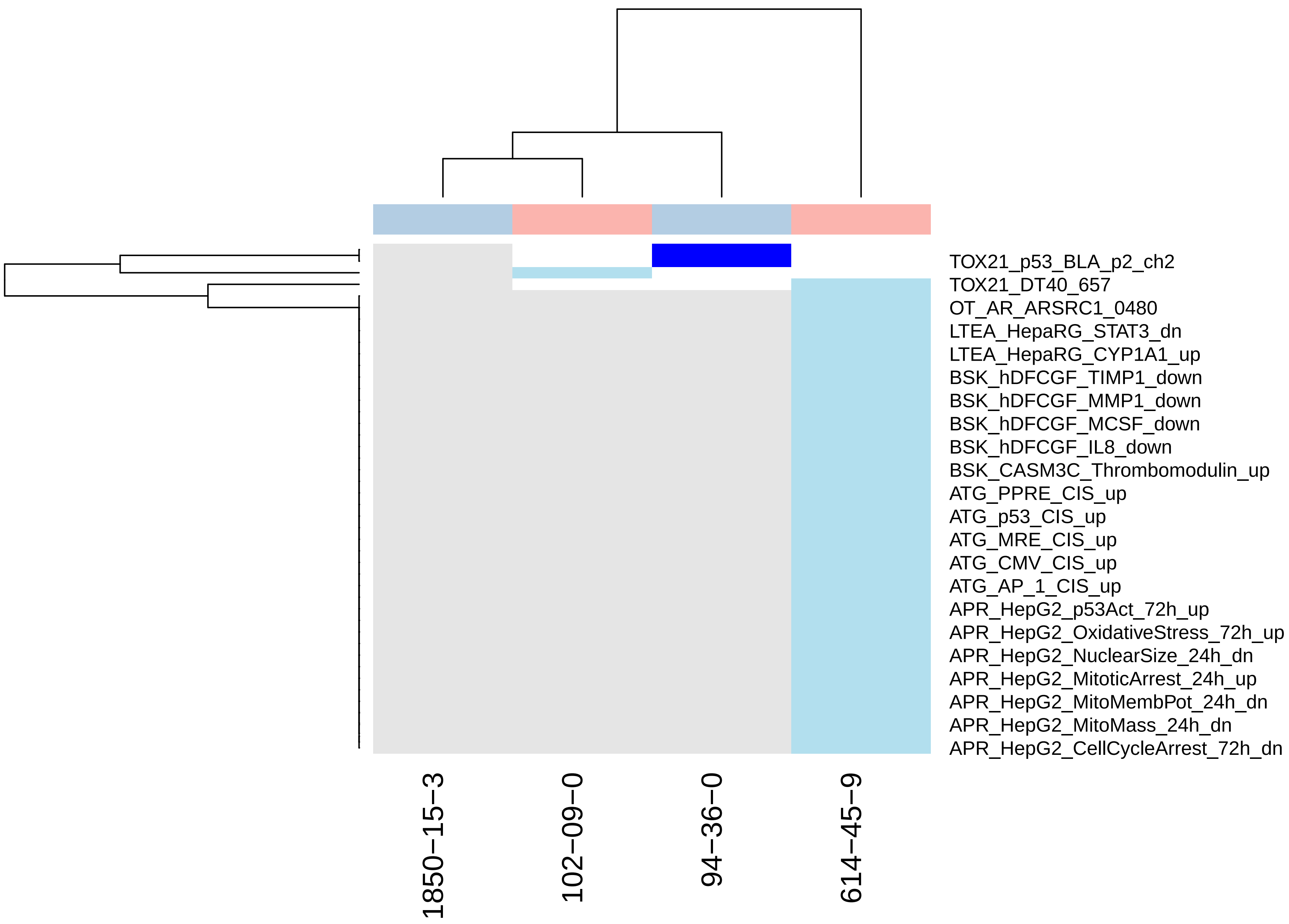

All Activity Heatmap for Cluster 181

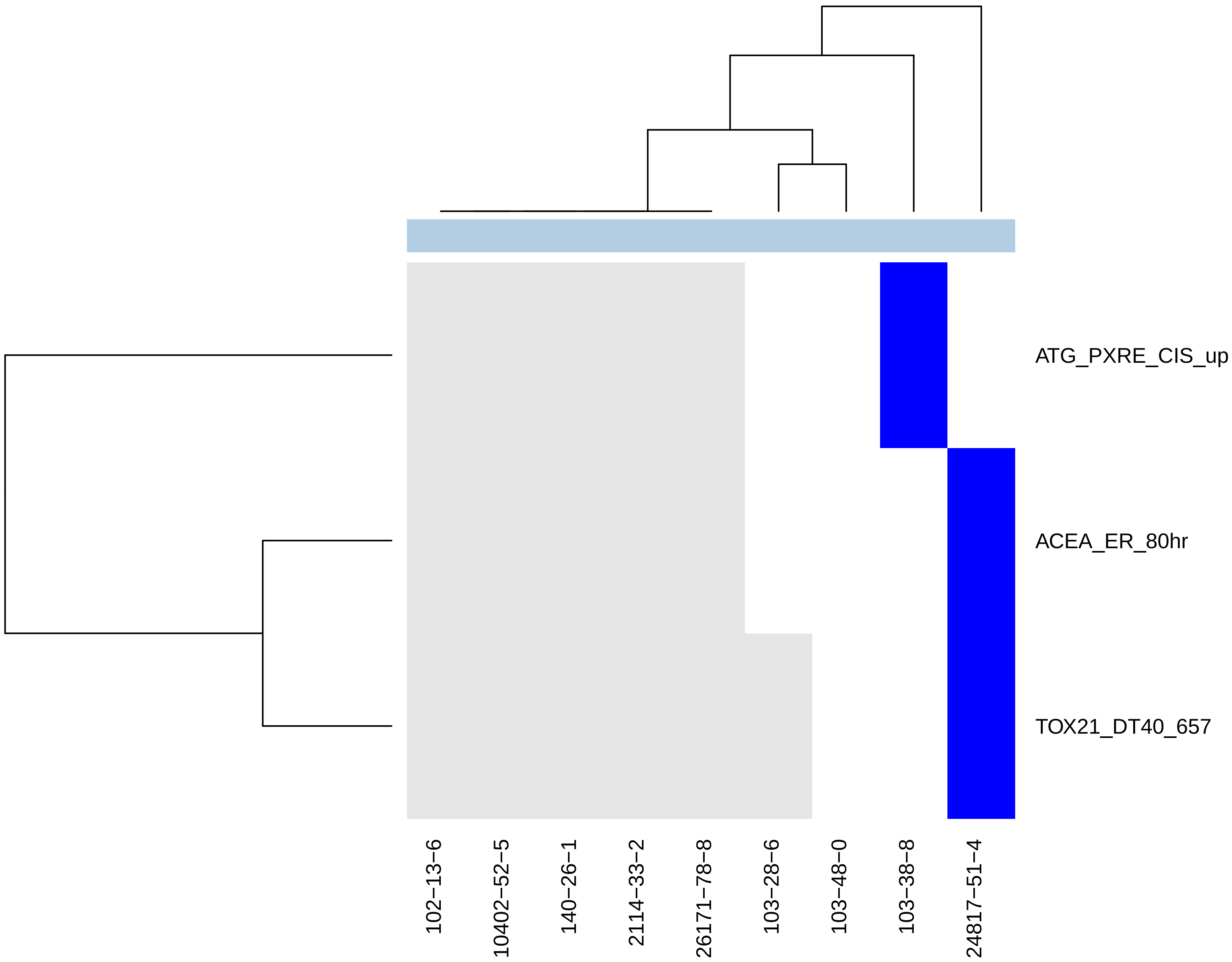

All Activity Heatmap for Cluster 183

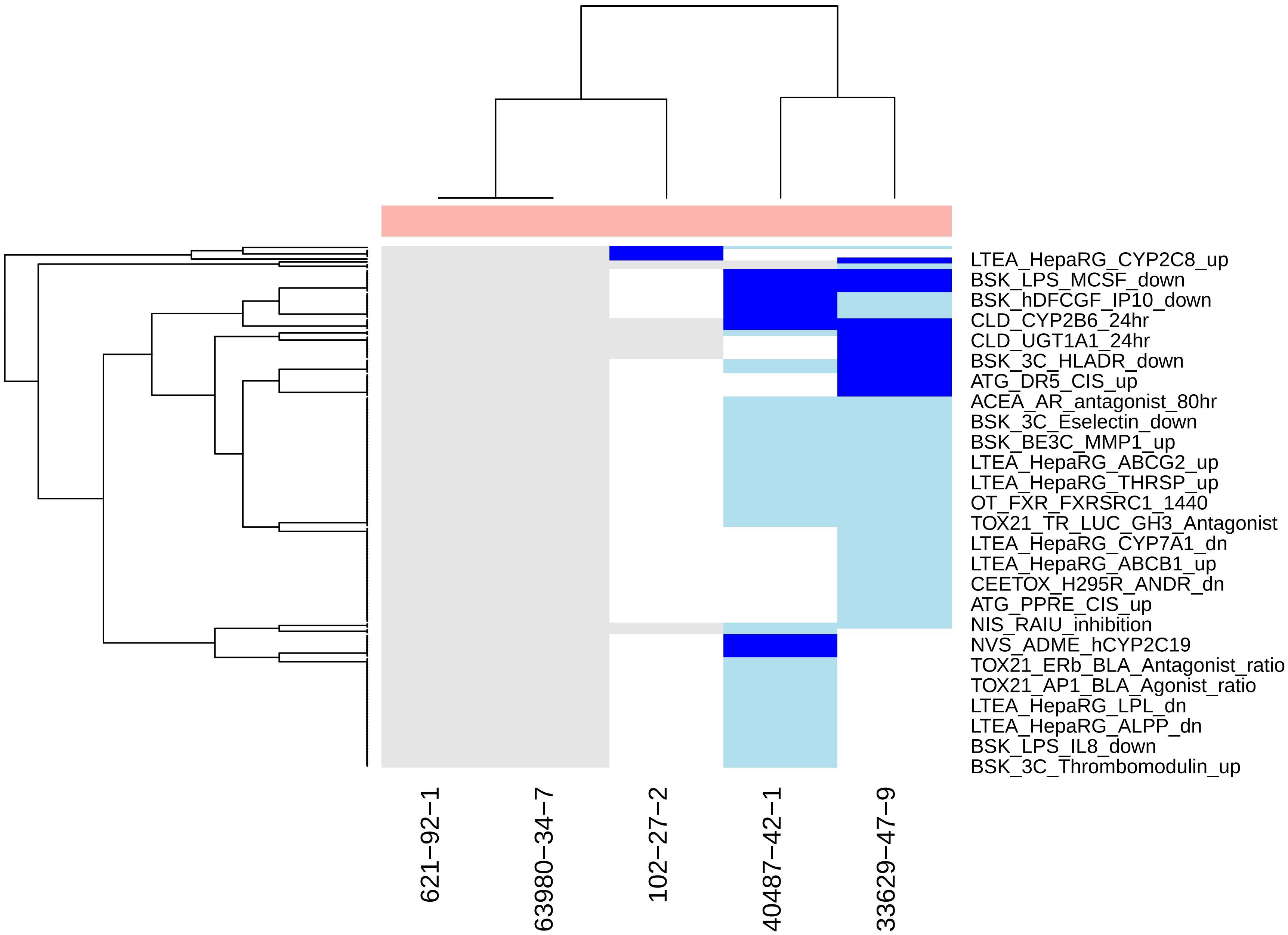

All Activity Heatmap for Cluster 184

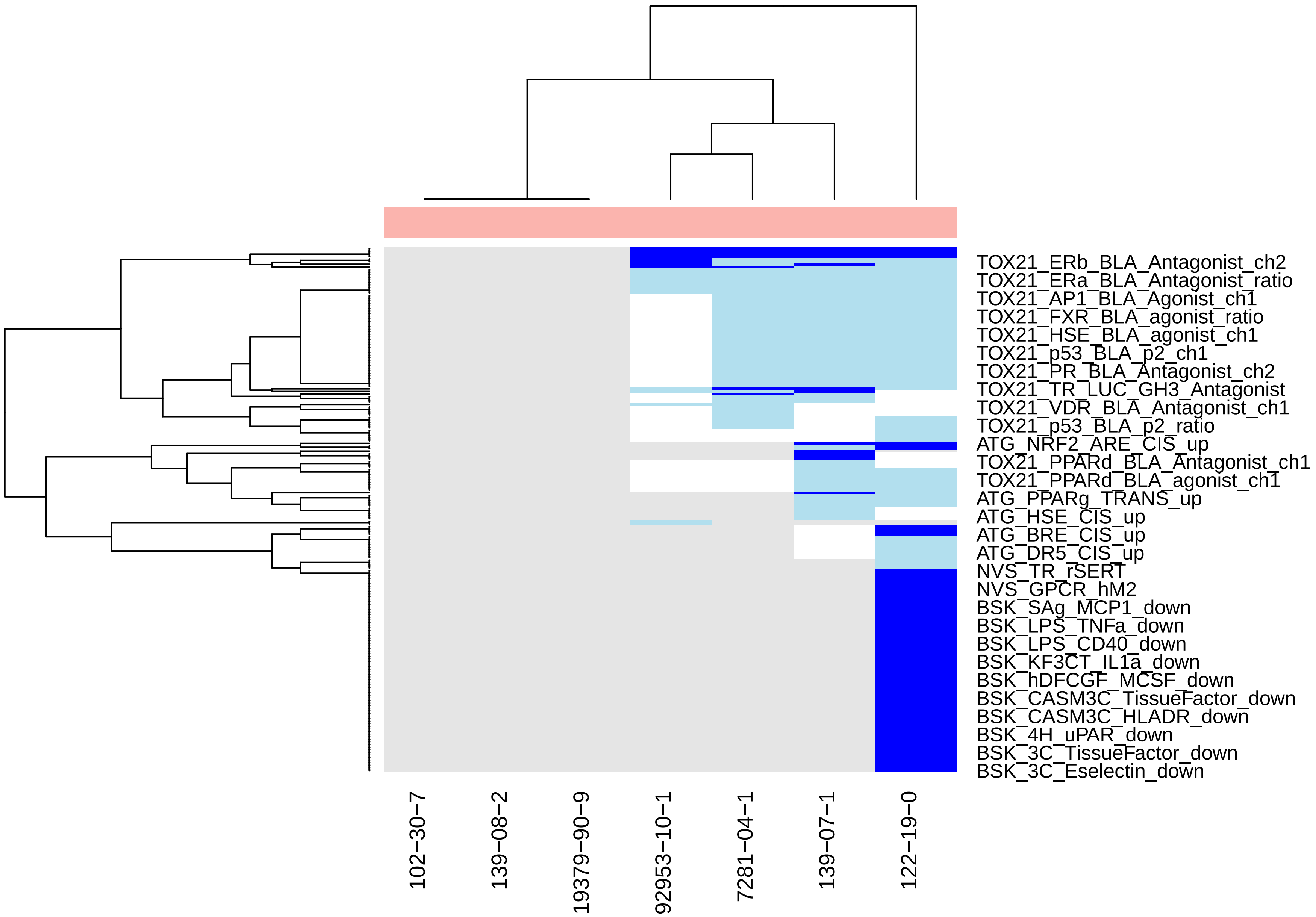

All Activity Heatmap for Cluster 185

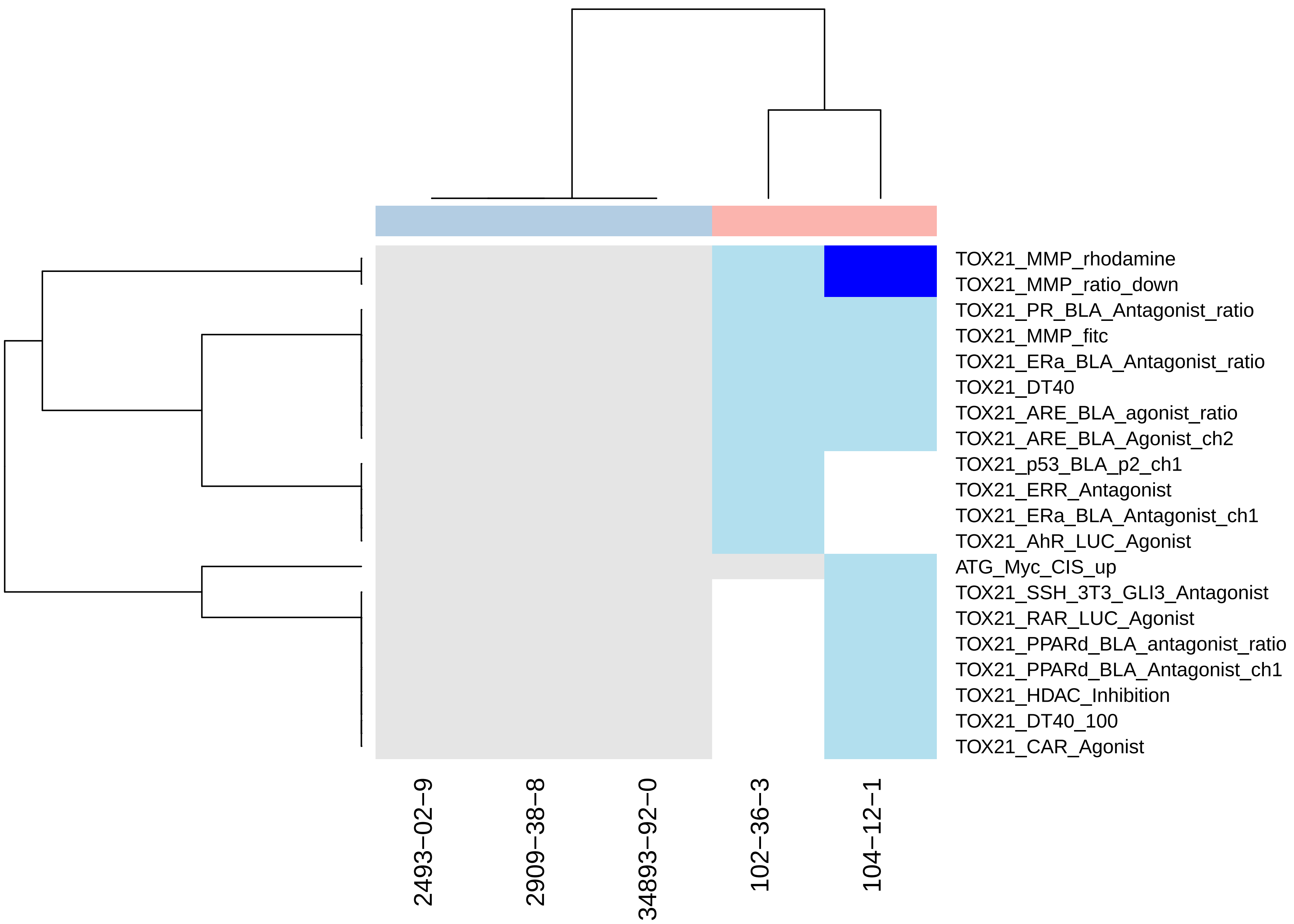

All Activity Heatmap for Cluster 186

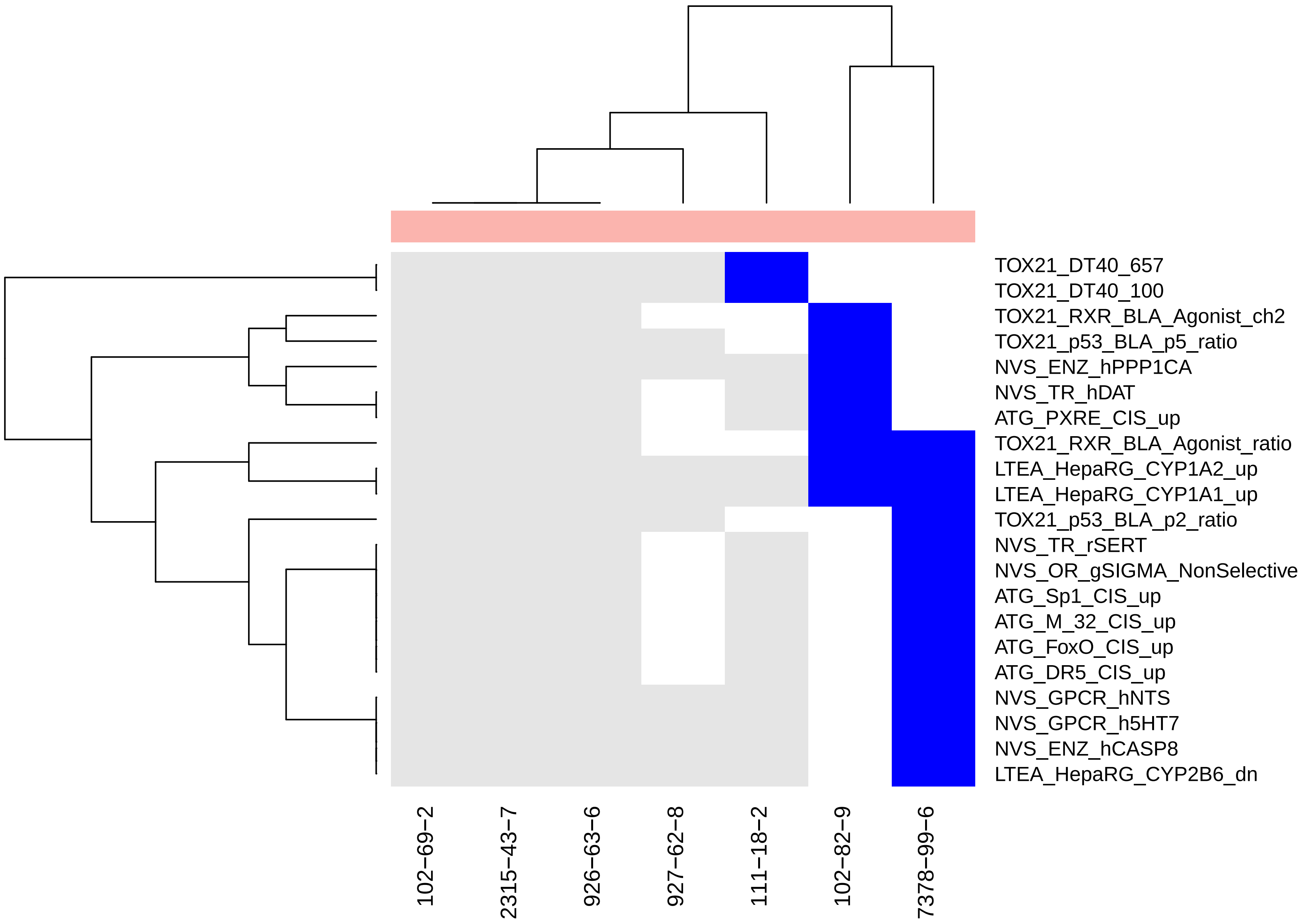

All Activity Heatmap for Cluster 187

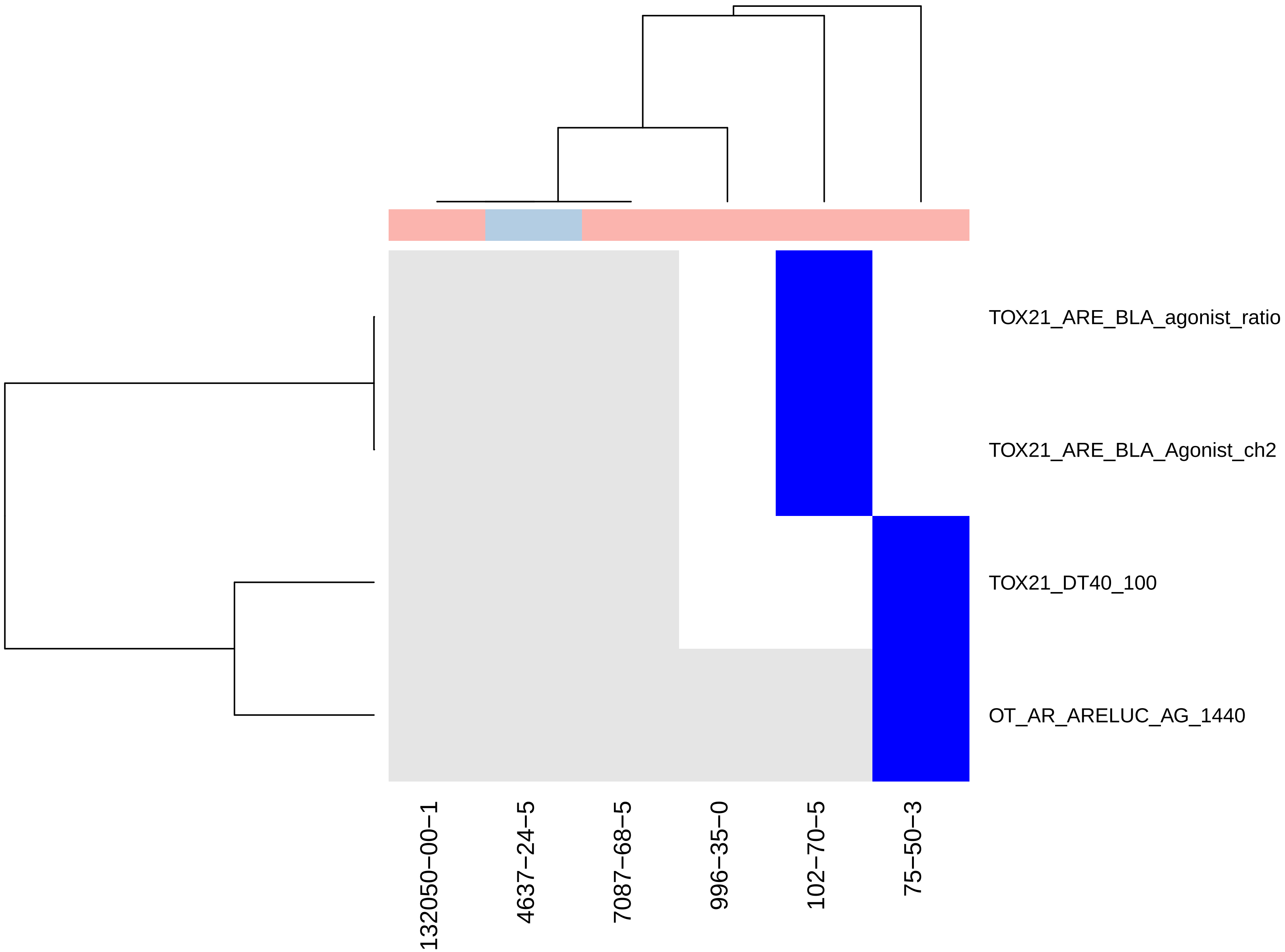

All Activity Heatmap for Cluster 188

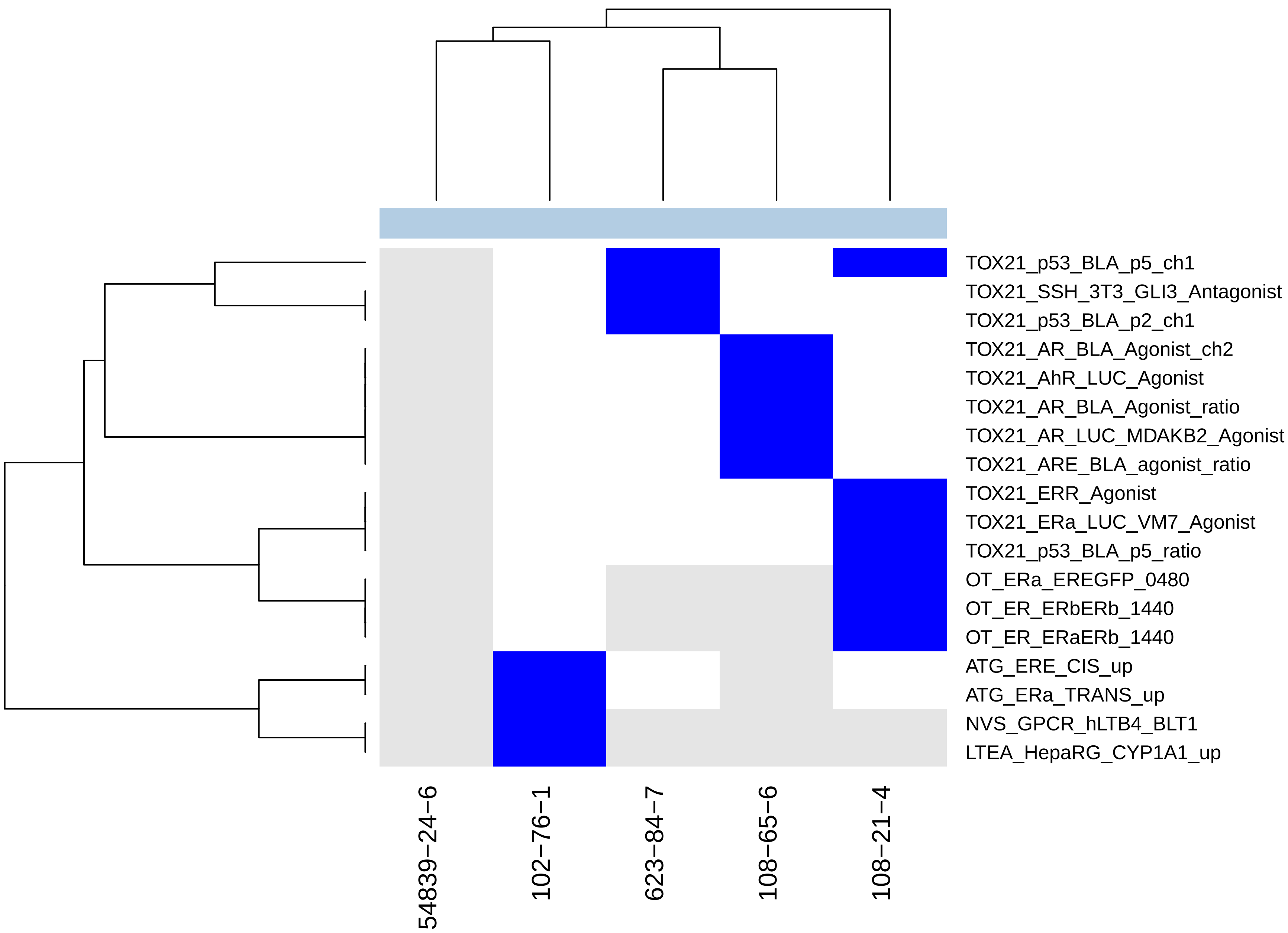

All Activity Heatmap for Cluster 189

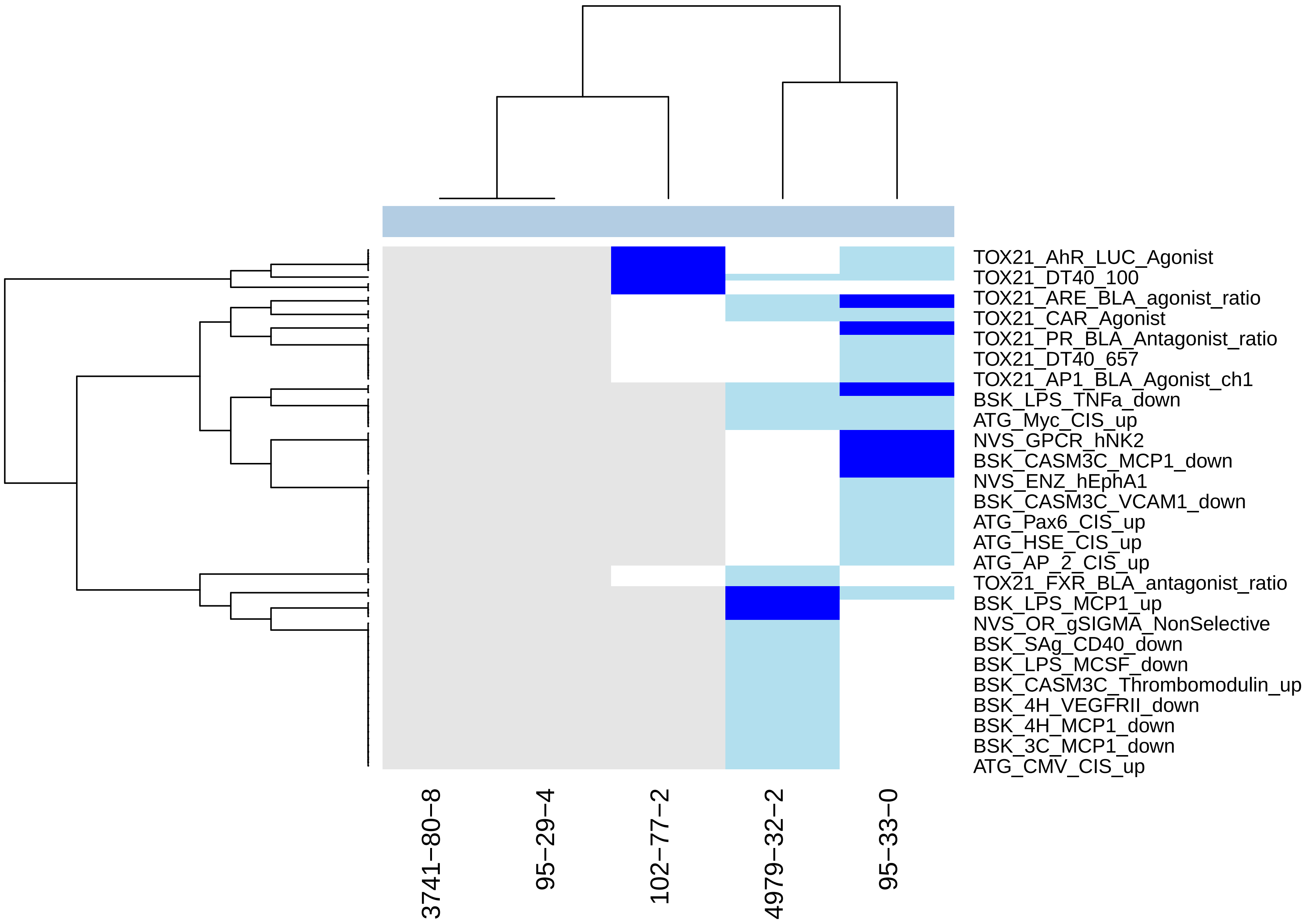

All Activity Heatmap for Cluster 190

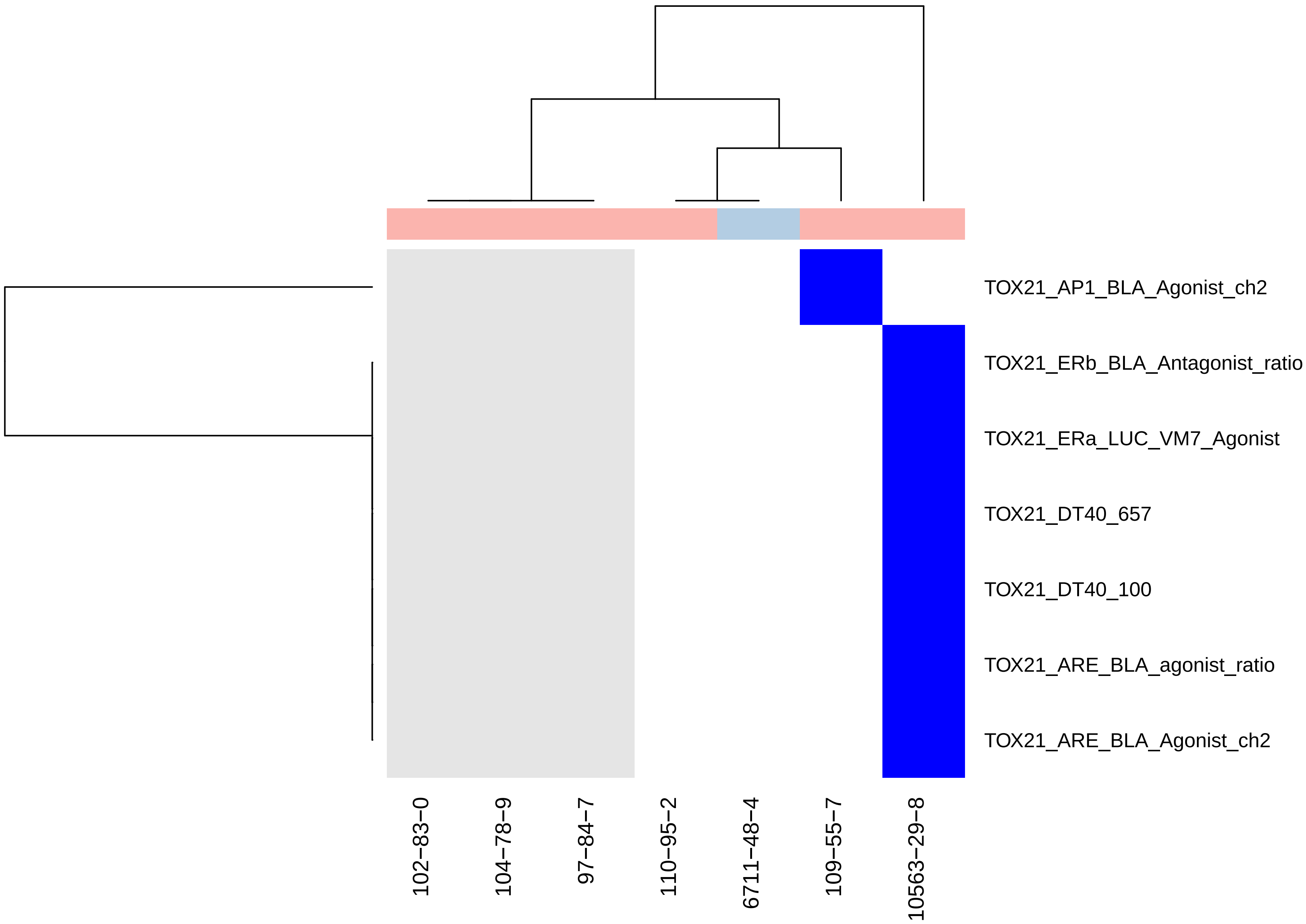

All Activity Heatmap for Cluster 191

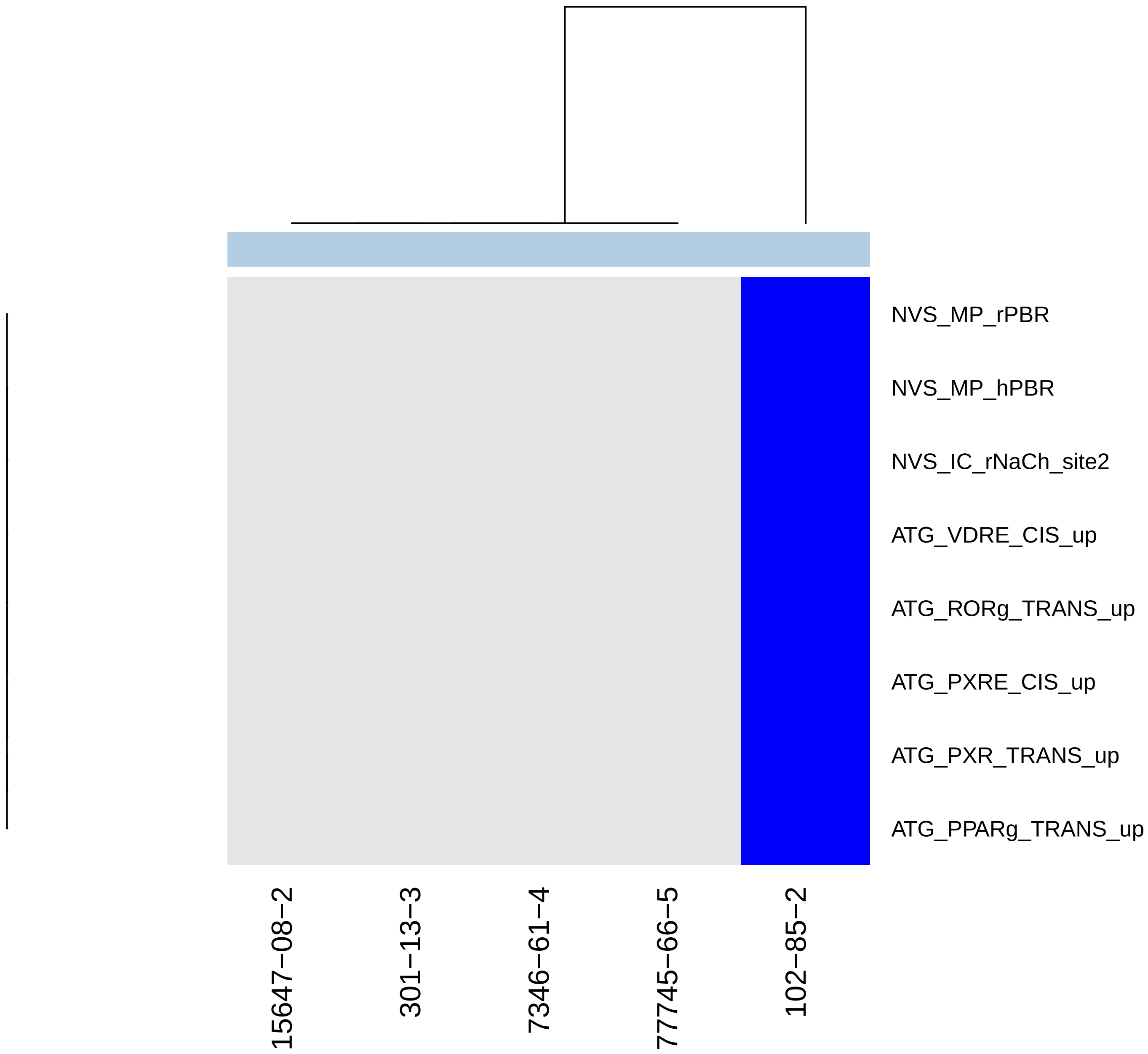

All Activity Heatmap for Cluster 192

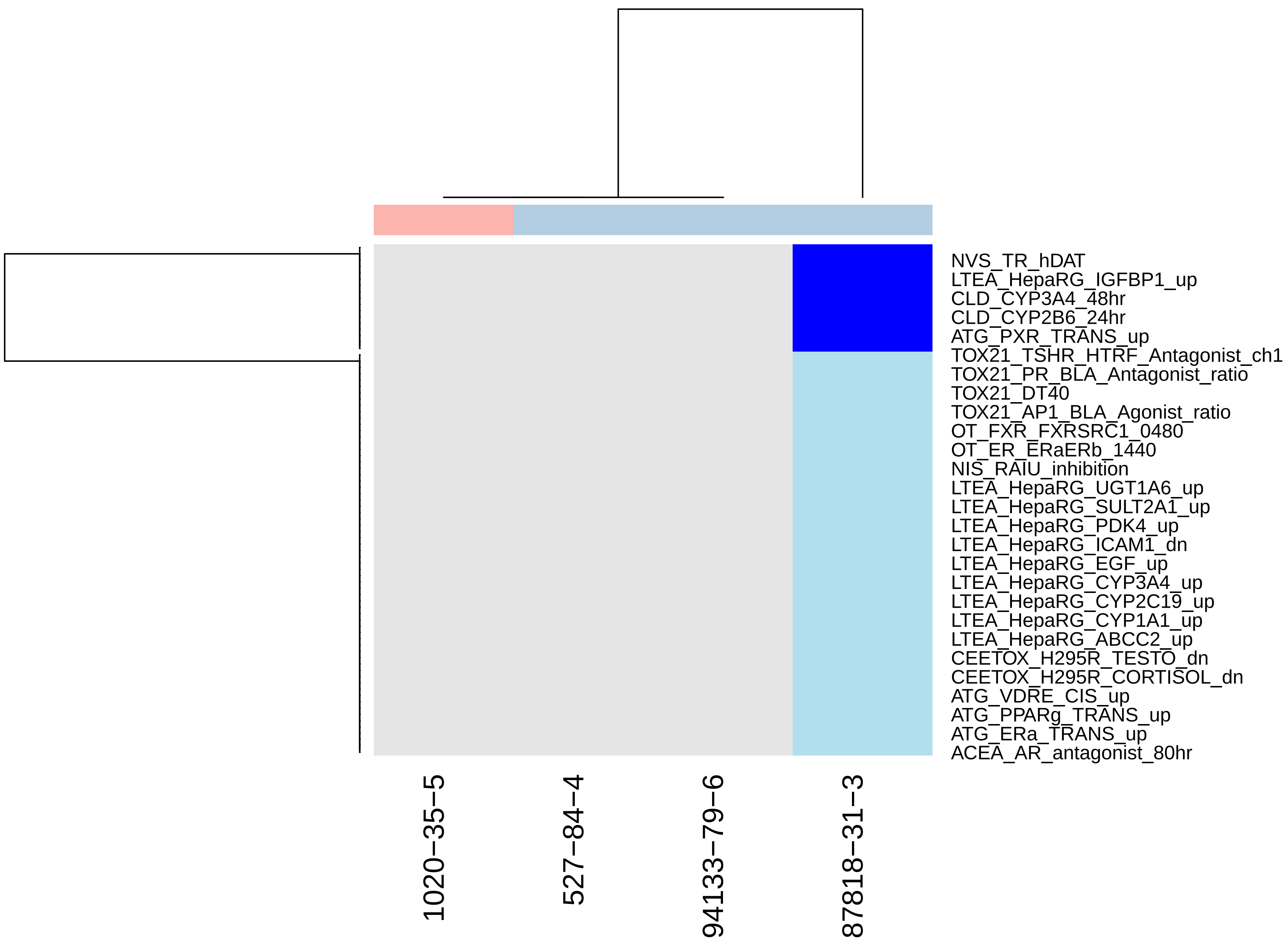

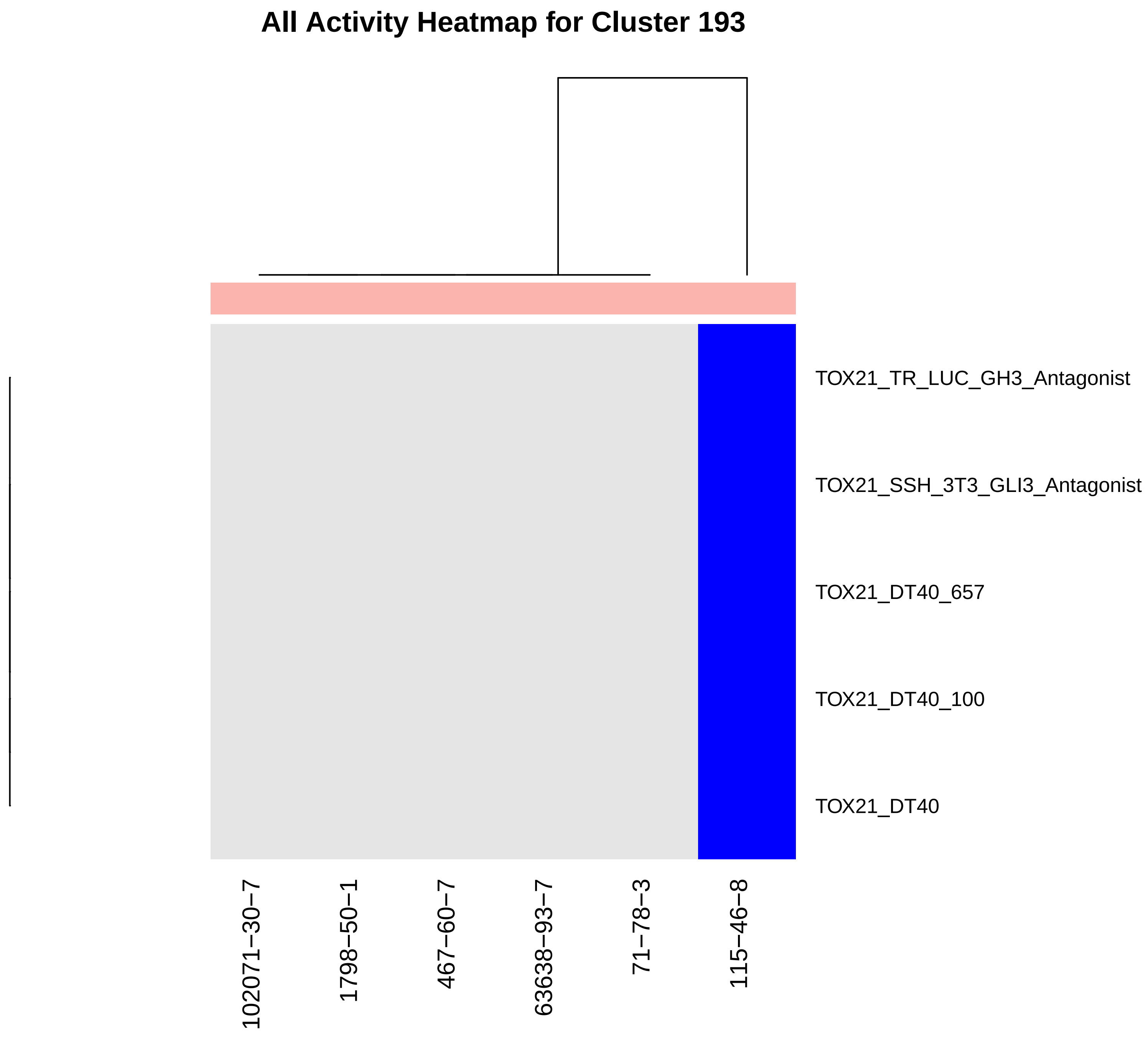
All Activity Heatmap for Cluster 193
TOX21_TR_LUC_GH3_Antagonist
TOX21_SSH_3T3_GLI3_Antagonist
TOX21_DT40_657
TOX21_DT40_100
TOX21_DT40
102071−30−7
1798−50−1
467−60−7
63638−93−7
71−78−3
115−46−8

All Activity Heatmap for Cluster 194

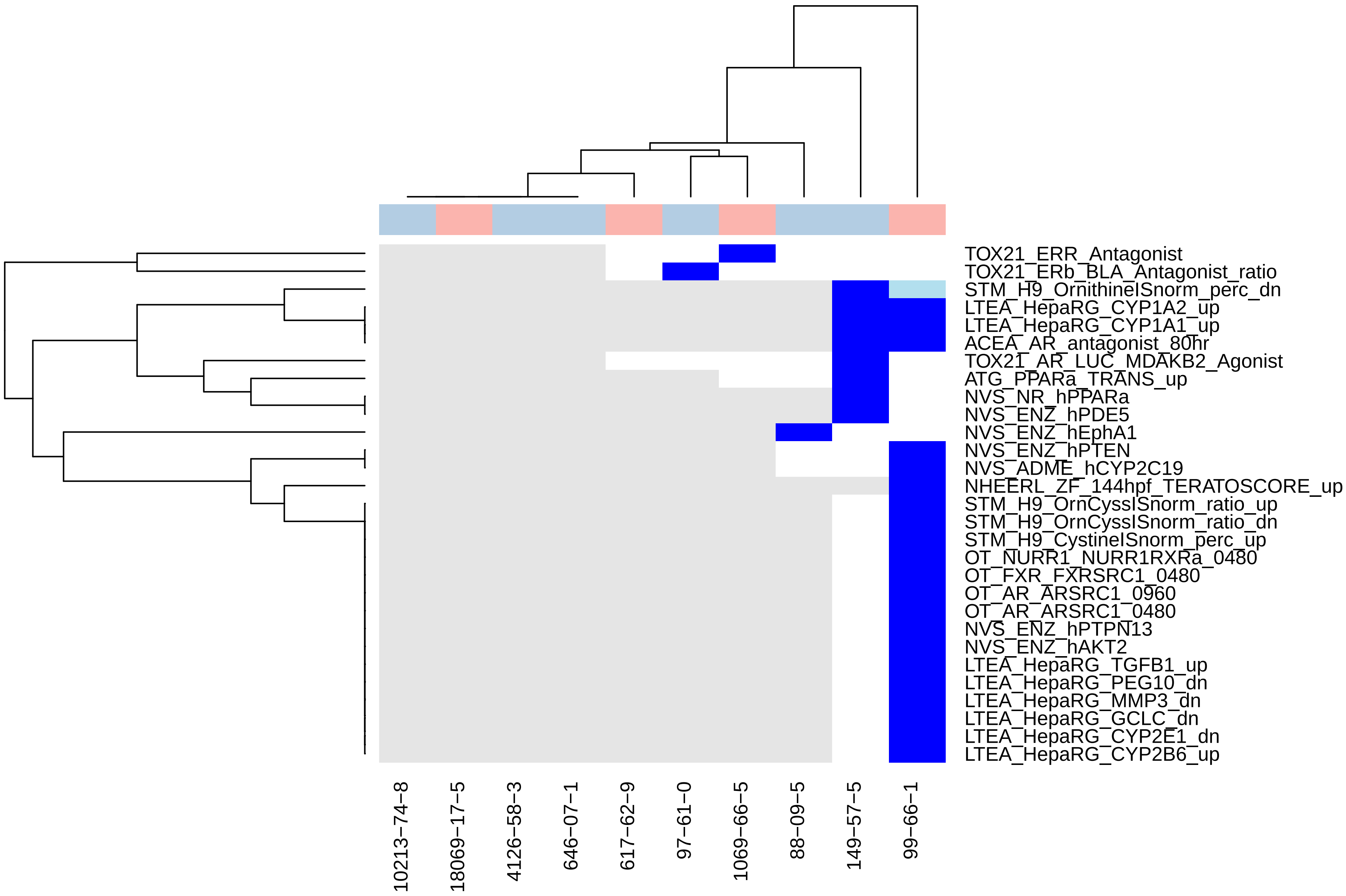

All Activity Heatmap for Cluster 195

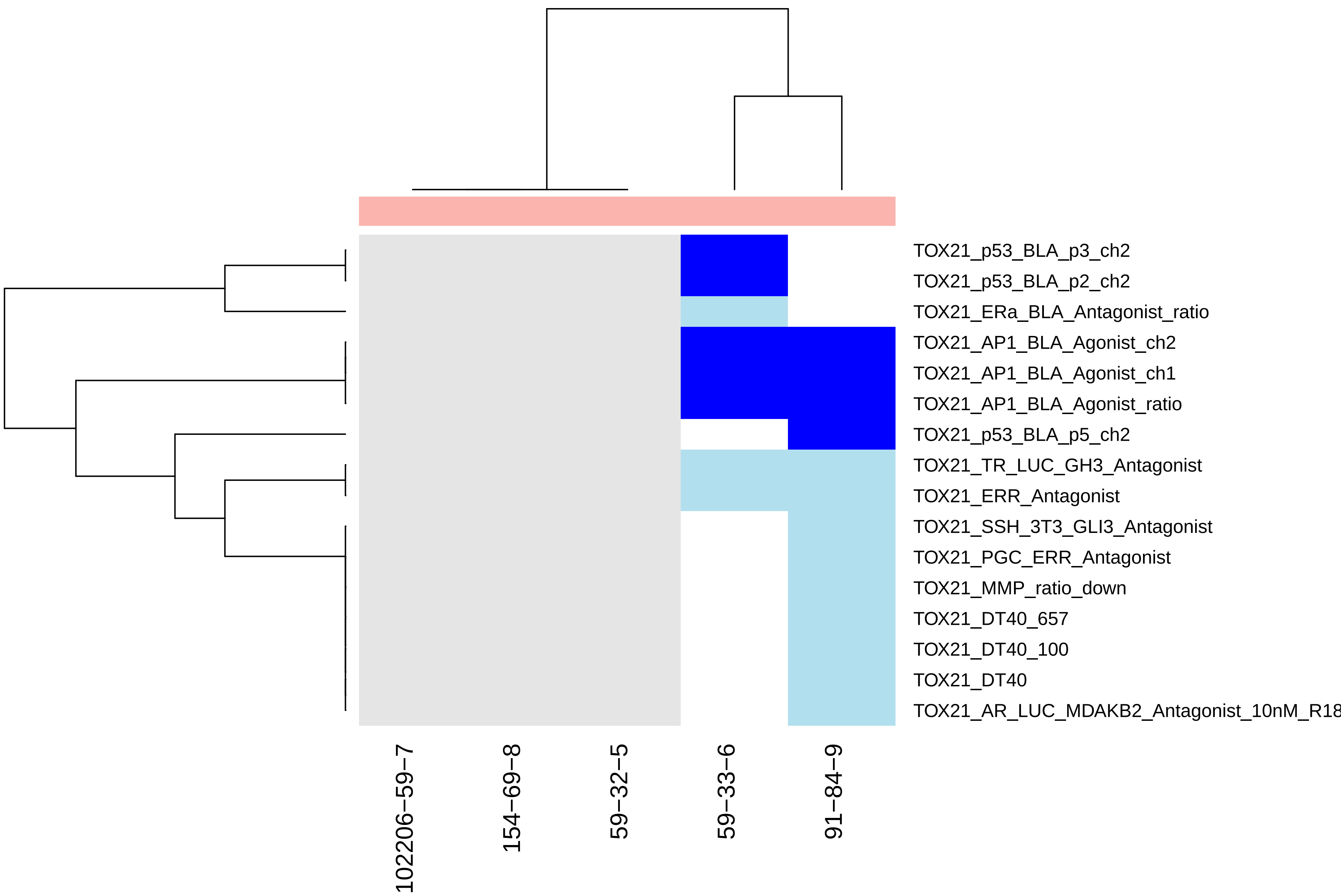

All Activity Heatmap for Cluster 197

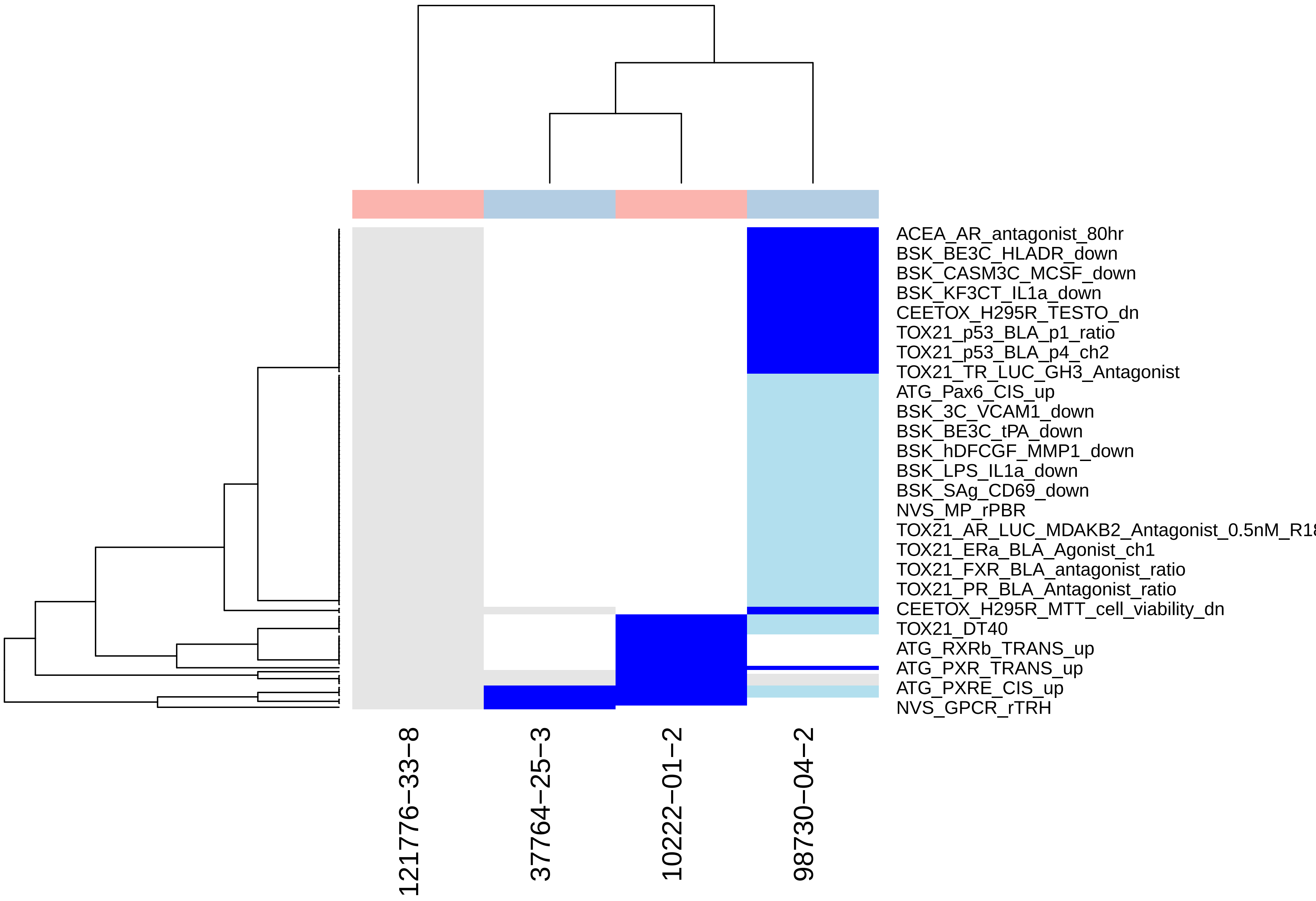

All Activity Heatmap for Cluster 199

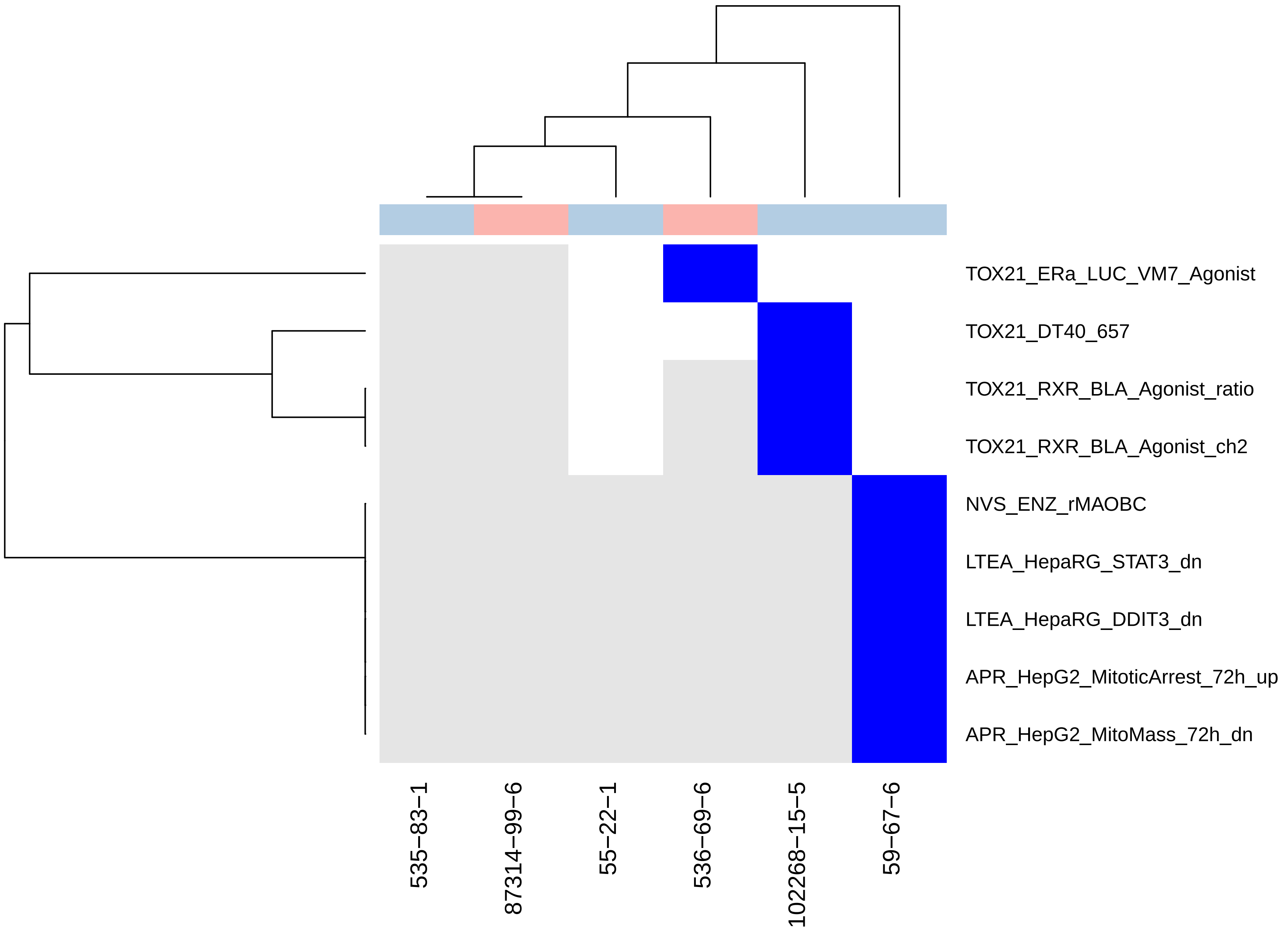

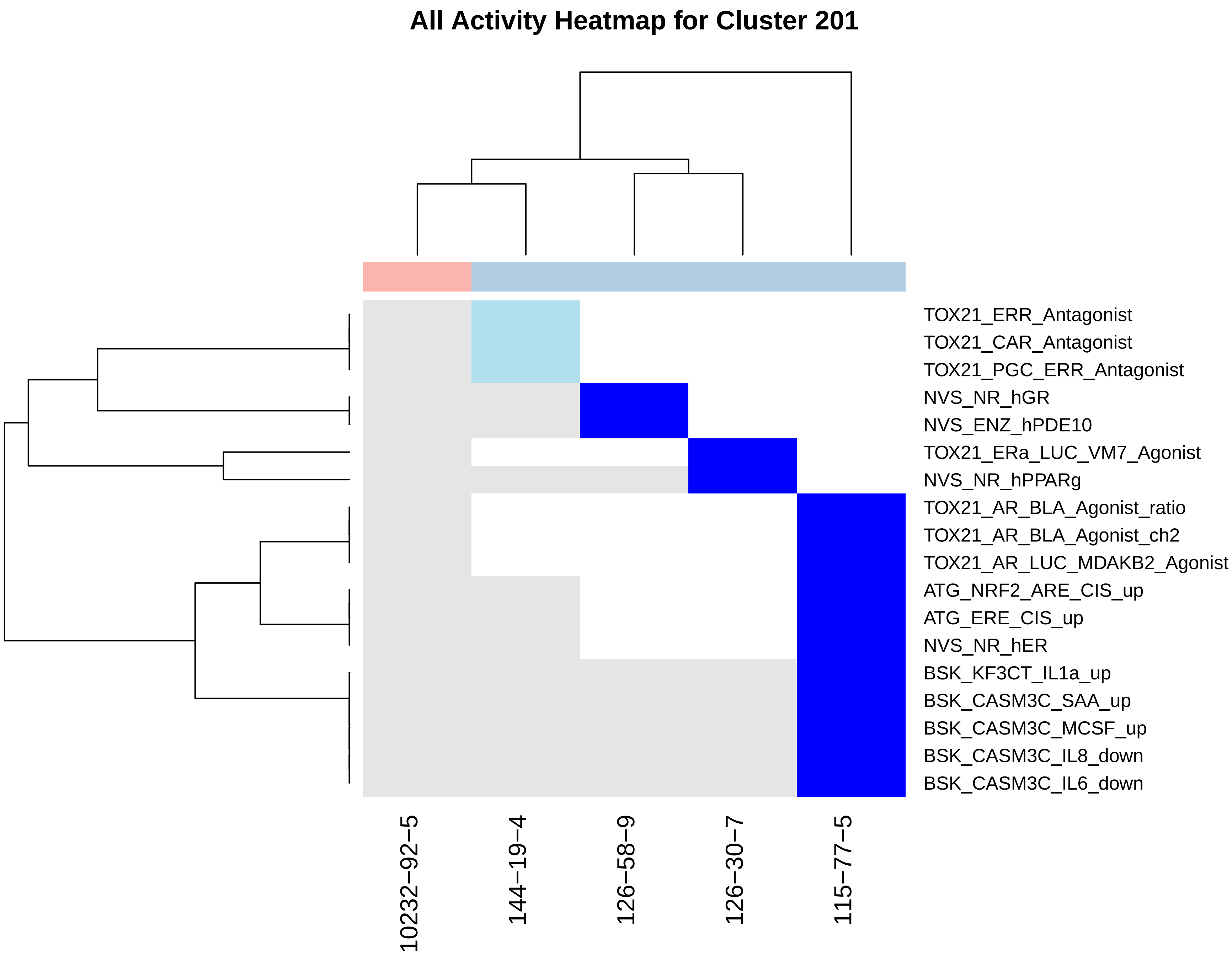
All Activity Heatmap for Cluster 201
TOX21_ERR_Antagonist
TOX21_CAR_Antagonist
TOX21_PGC_ERR_Antagonist
NVS_NR_hGR
NVS_ENZ_hPDE10
TOX21_ERa_LUC_VM7_Agonist
NVS_NR_hPPARg
TOX21_AR_BLA_Agonist_ratio
TOX21_AR_BLA_Agonist_ch2
TOX21_AR_LUC_MDAKB2_Agonist
ATG_NRF2_ARE_CIS_up
ATG_ERE_CIS_up
NVS_NR_hER
BSK_KF3CT_IL1a_up
BSK_CASM3C_SAA_up
BSK_CASM3C_MCSF_up
BSK_CASM3C_IL8_down
BSK_CASM3C_IL6_down
10232−92−5
144−19−4
126−58−9
126−30−7
115−77−5

All Activity Heatmap for Cluster 202

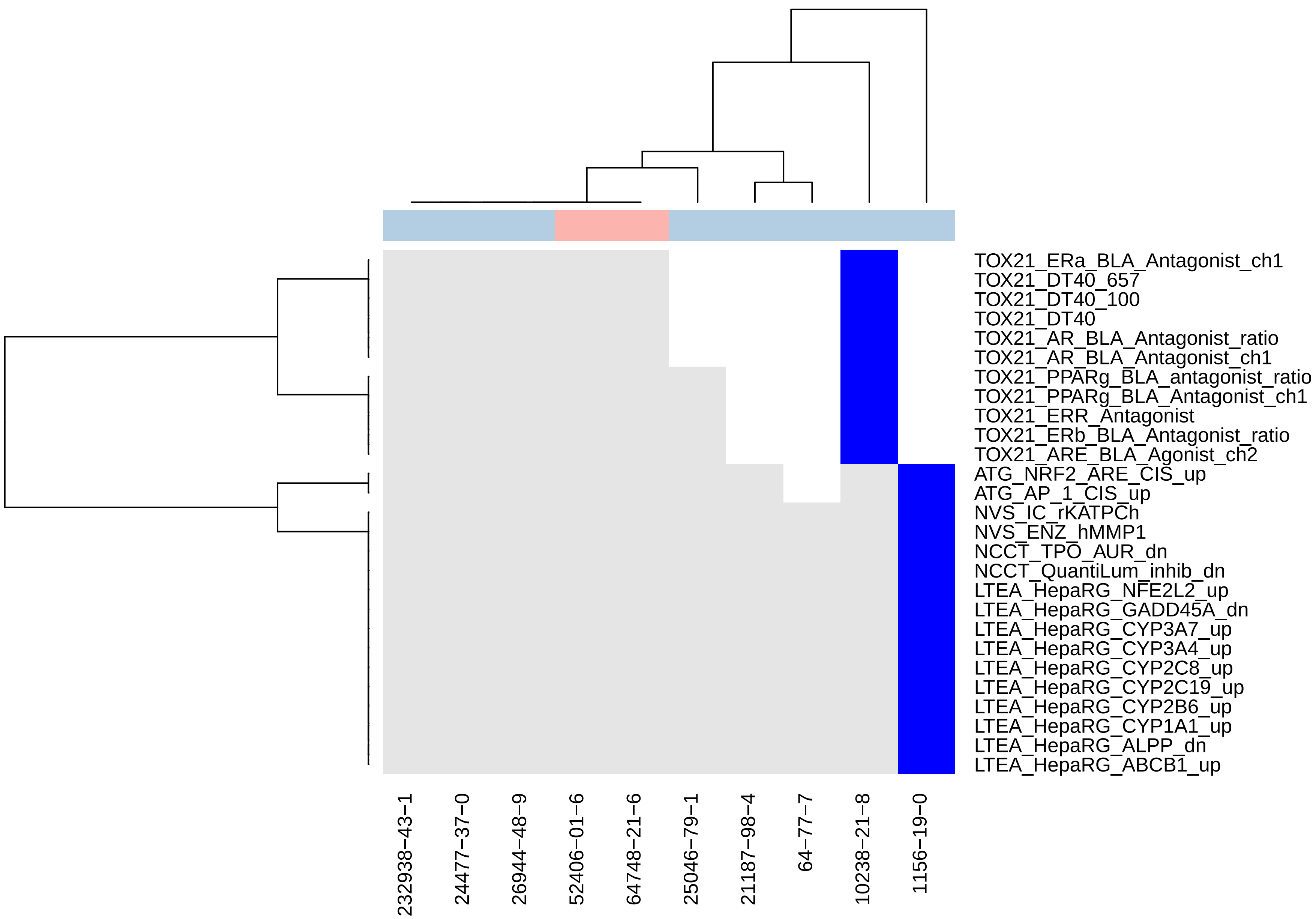

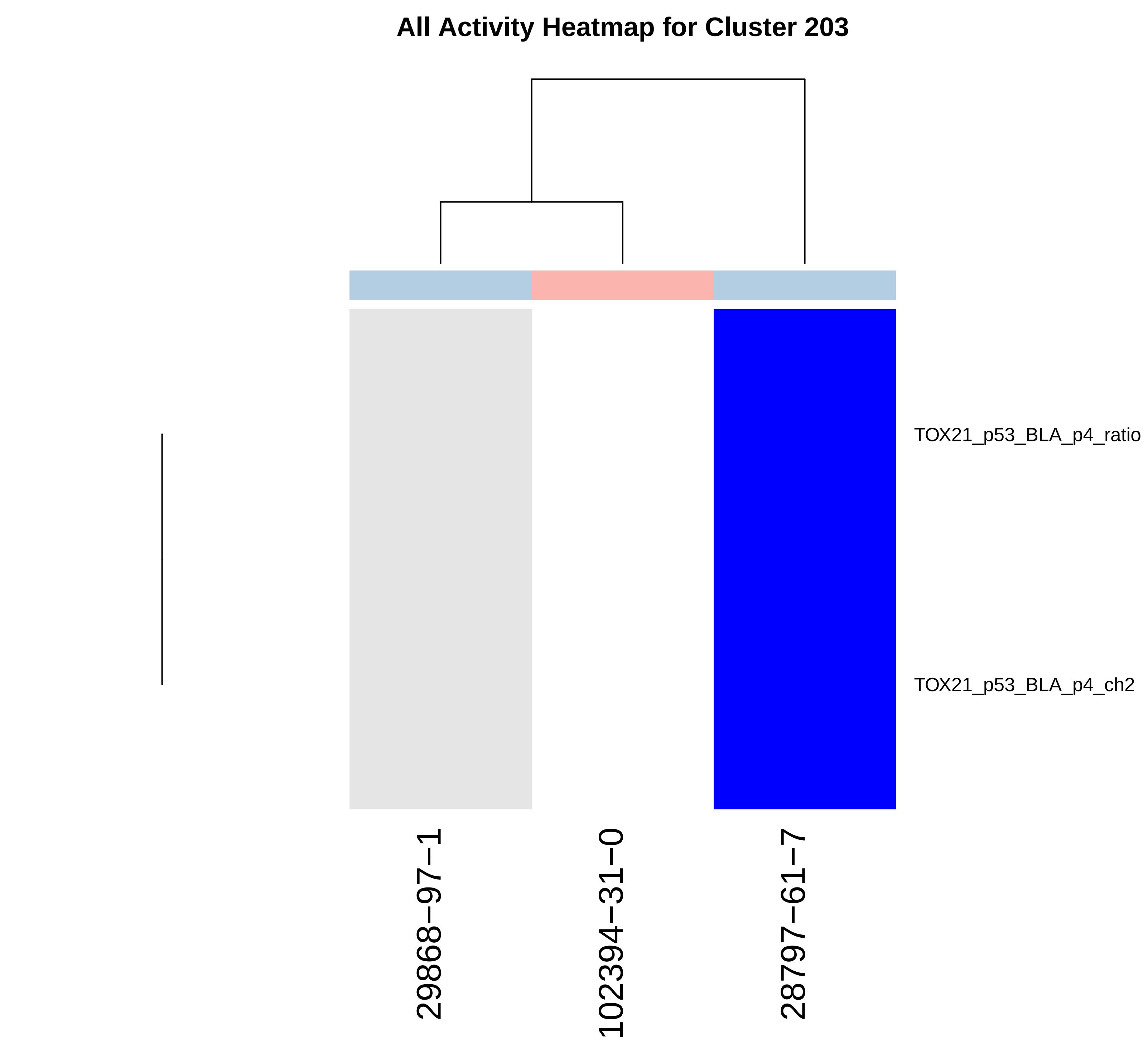
All Activity Heatmap for Cluster 203
TOX21_p53_BLA_p4_ratio
TOX21_p53_BLA_p4_ch2
29868−97−1
102394−31−0
28797−61−7

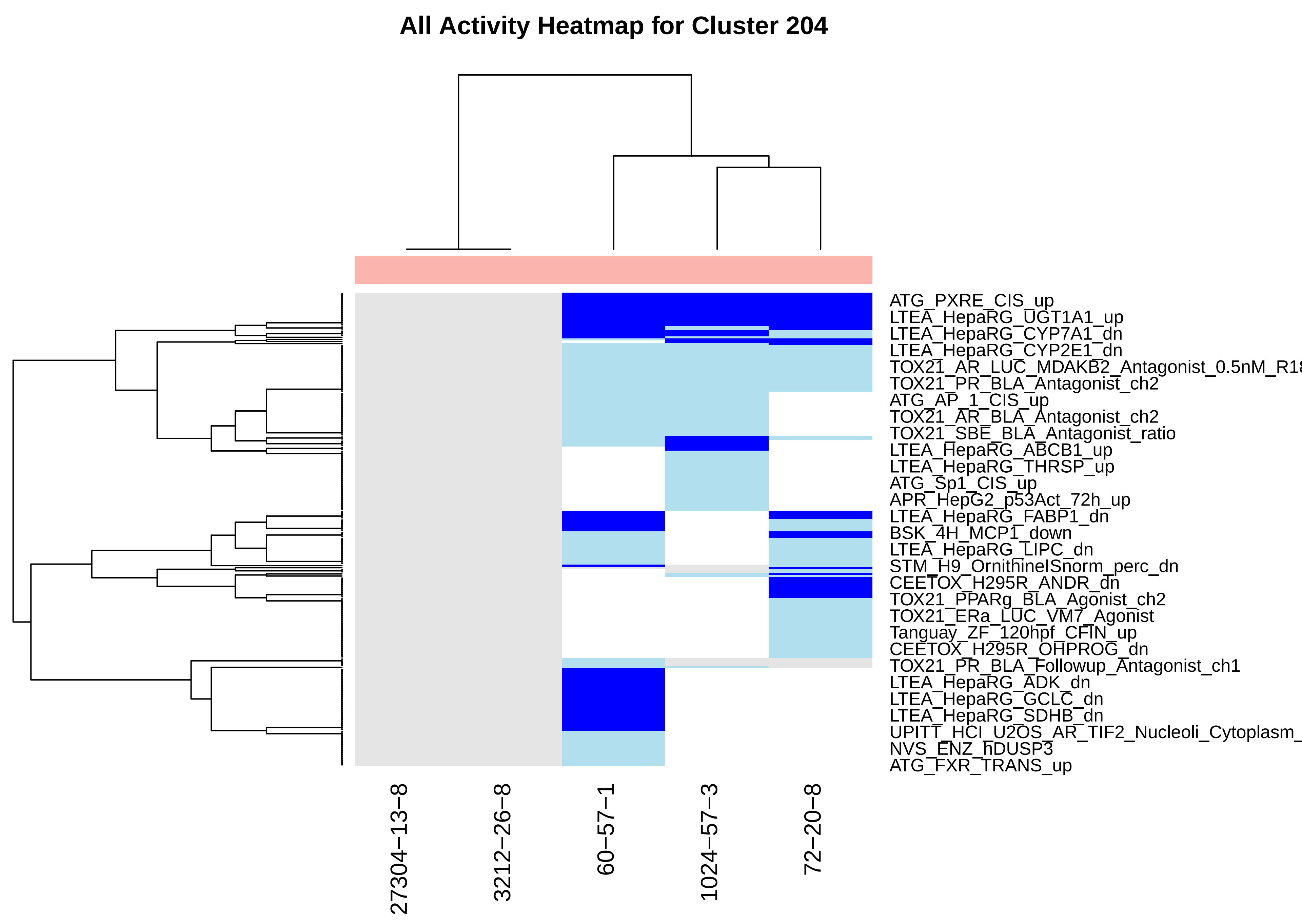
All Activity Heatmap for Cluster 204
ATG_PXRE_CIS_up
LTEA_HepaRG_UGT1A1_up
LTEA_HepaRG_CYP7A1_dn
LTEA_HepaRG_CYP2E1_dn
TOX21_AR_LUC_MDAKB2_Antagonist_0.5nM_R18
TOX21_PR_BLA_Antagonist_ch2
ATG_AP_1_CIS_up
TOX21_AR_BLA_Antagonist_ch2
TOX21_SBE_BLA_Antagonist_ratio
LTEA_HepaRG_ABCB1_up
LTEA_HepaRG_THRSP_up
ATG_Sp1_CIS_up
APR_HepG2_p53Act_72h_up
LTEA_HepaRG_FABP1_dn
BSK_4H_MCP1_down
LTEA_HepaRG_LIPC_dn
STM_H9_OrnithineISnorm_perc_dn
CEETOX_H295R_ANDR_dn
TOX21_PPARg_BLA_Agonist_ch2
TOX21_ERa_LUC_VM7_Agonist
Tanguay_ZF_120hpf_CFIN_up
CEETOX_H295R_OHPROG_dn
TOX21_PR_BLA_Followup_Antagonist_ch1
LTEA_HepaRG_ADK_dn
LTEA_HepaRG_GCLC_dn
LTEA_HepaRG_SDHB_dn
UPITT_HCI_U2OS_AR_TIF2_Nucleoli_Cytoplasm_
NVS_ENZ_hDUSP3
ATG_FXR_TRANS_up
27304−13−8
3212−26−8
60−57−1
1024−57−3
72−20−8

All Activity Heatmap for Cluster 206

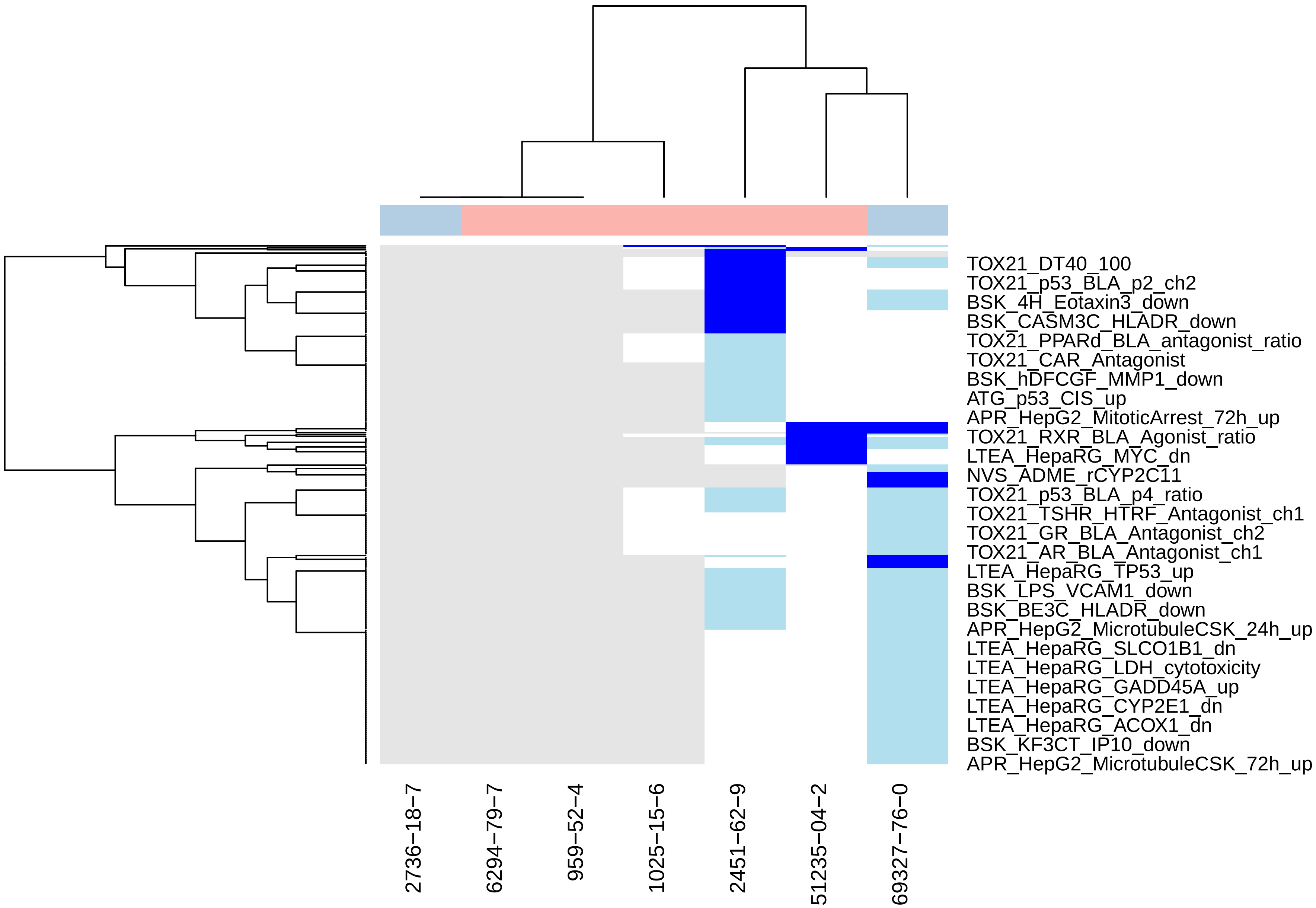

All Activity Heatmap for Cluster 207

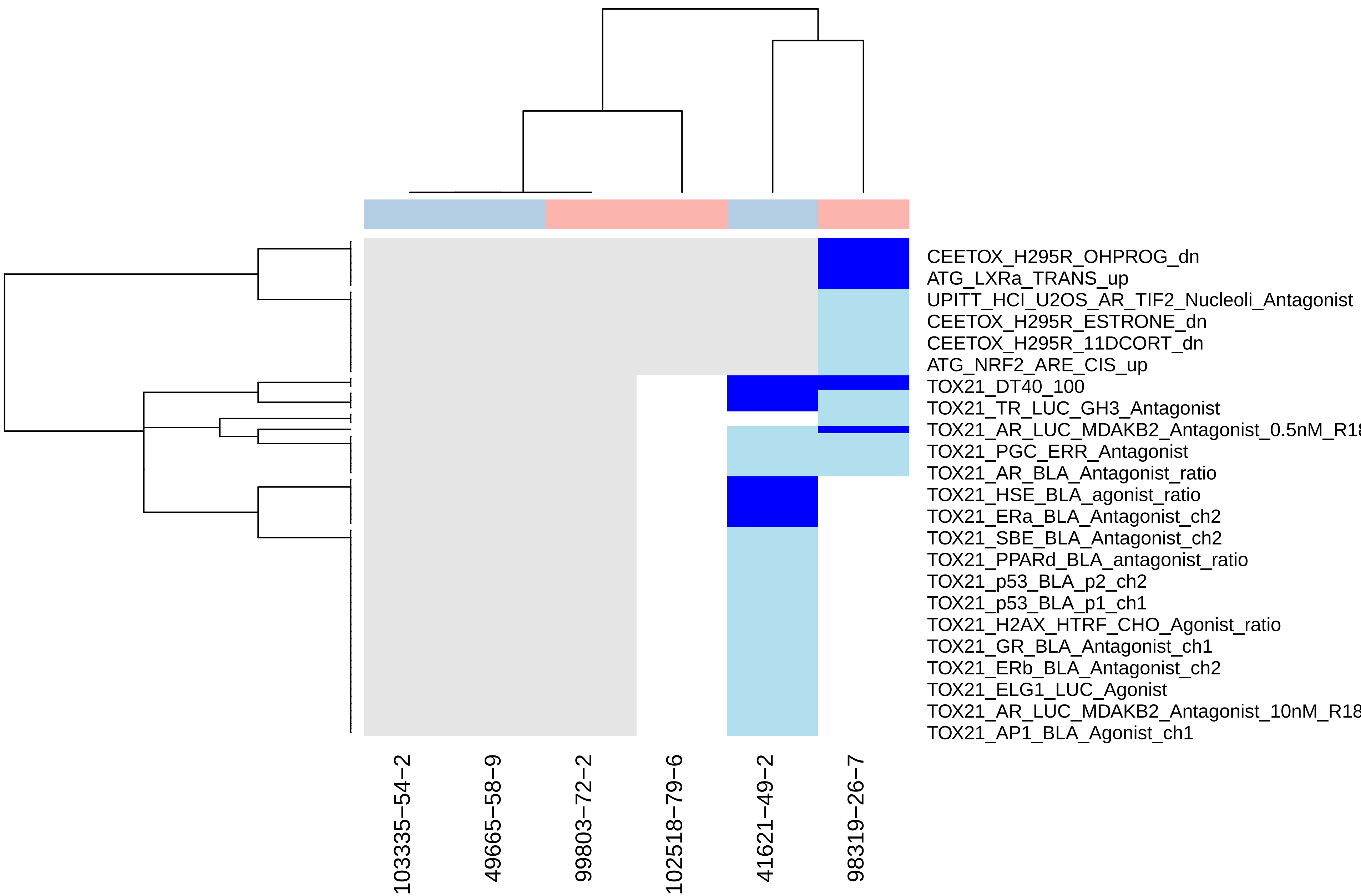

All Activity Heatmap for Cluster 208

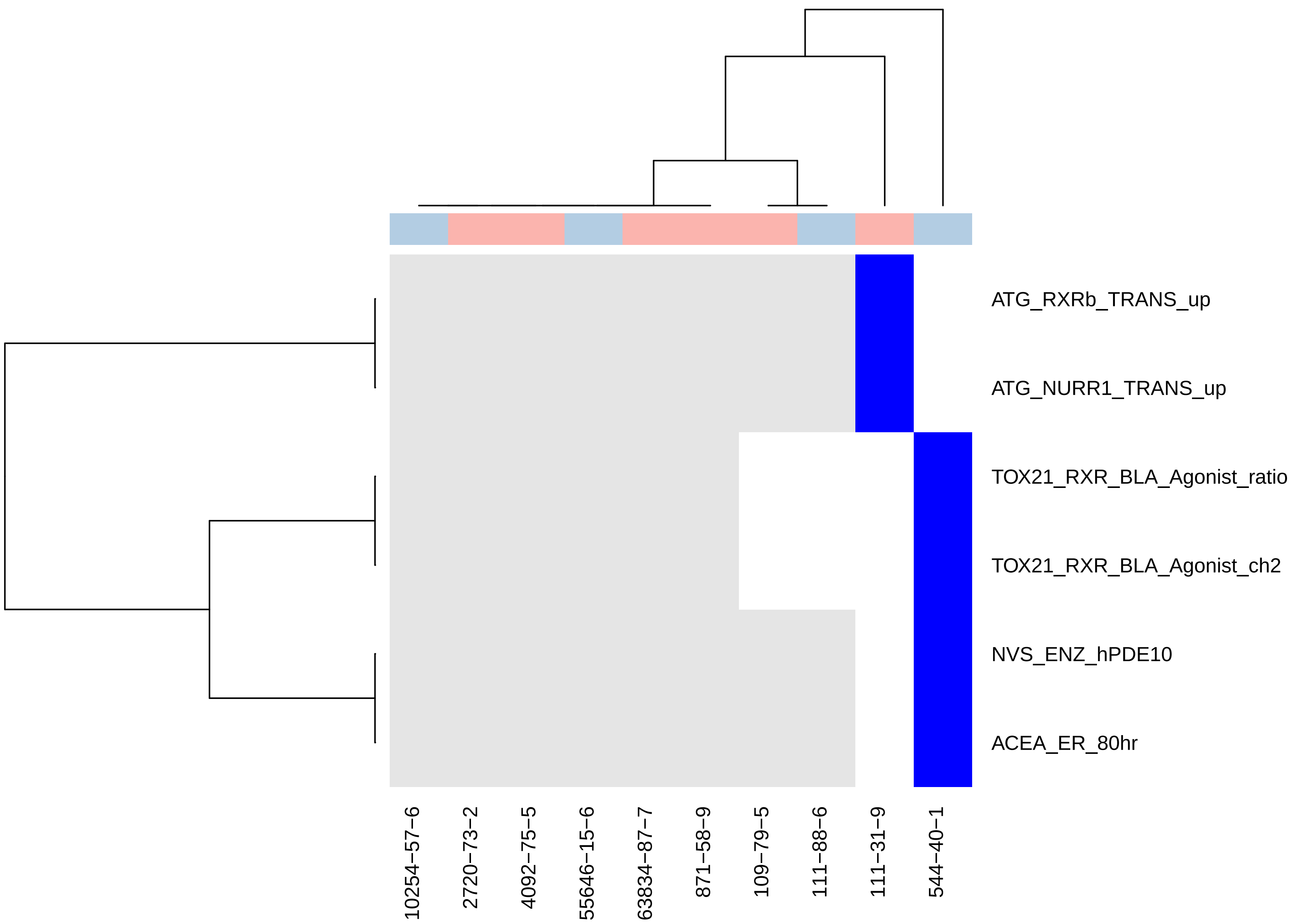

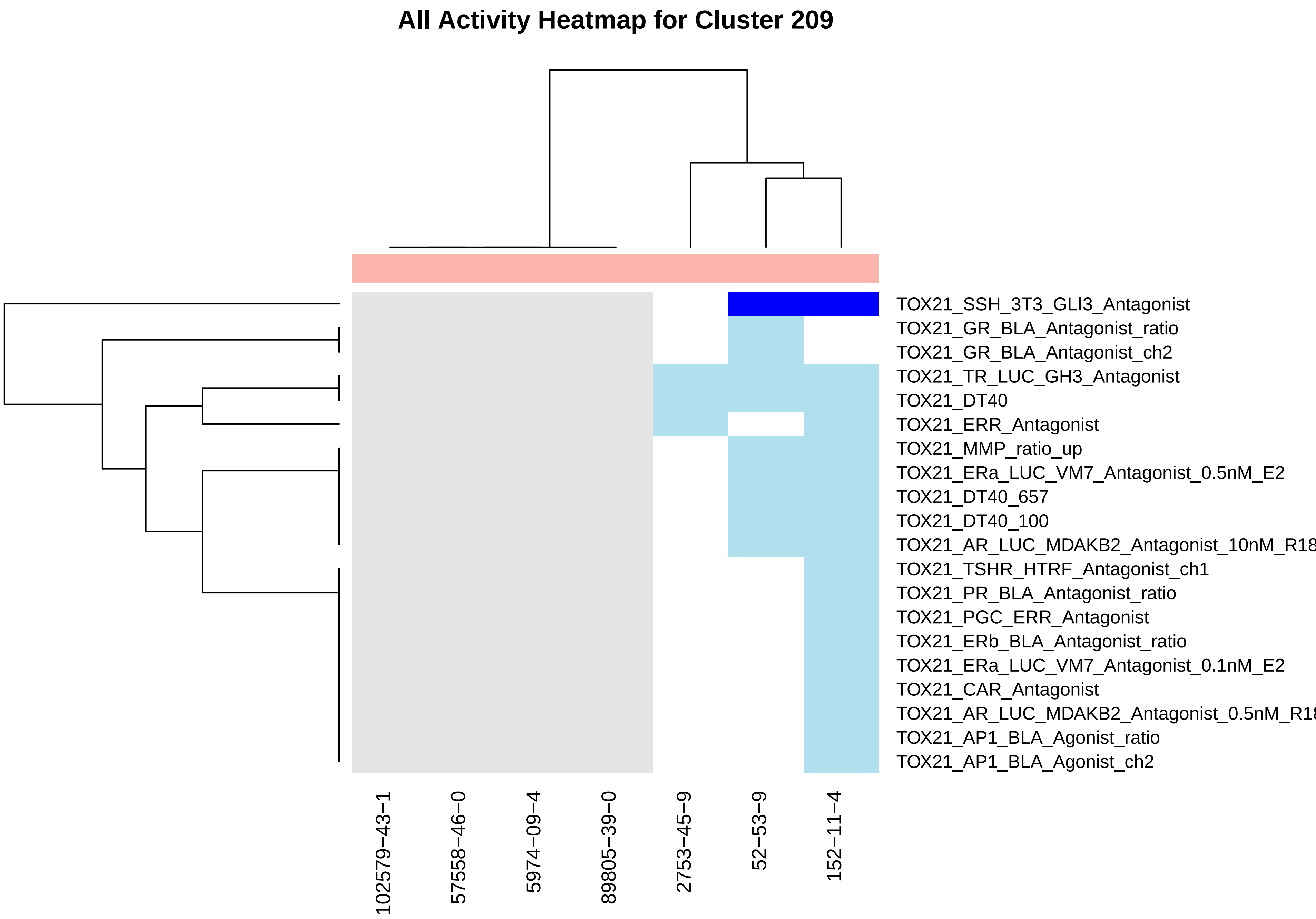

All Activity Heatmap for Cluster 209
TOX21_SSH_3T3_GLI3_Antagonist
TOX21_GR_BLA_Antagonist_ratio
TOX21_GR_BLA_Antagonist_ch2
TOX21_TR_LUC_GH3_Antagonist
TOX21_DT40
TOX21_ERR_Antagonist
TOX21_MMP_ratio_up
TOX21_ERa_LUC_VM7_Antagonist_0.5nM_E2
TOX21_DT40_657
TOX21_DT40_100
TOX21_AR_LUC_MDAKB2_Antagonist_10nM_R18
TOX21_TSHR_HTRF_Antagonist_ch1
TOX21_PR_BLA_Antagonist_ratio
TOX21_PGC_ERR_Antagonist
TOX21_ERb_BLA_Antagonist_ratio
TOX21_ERa_LUC_VM7_Antagonist_0.1nM_E2
TOX21_CAR_Antagonist
TOX21_AR_LUC_MDAKB2_Antagonist_0.5nM_R18
TOX21_AP1_BLA_Agonist_ratio
TOX21_AP1_BLA_Agonist_ch2
102579−43−1
57558−46−0
5974−09−4
89805−39−0
2753−45−9
52−53−9
152−11−4

All Activity Heatmap for Cluster 210

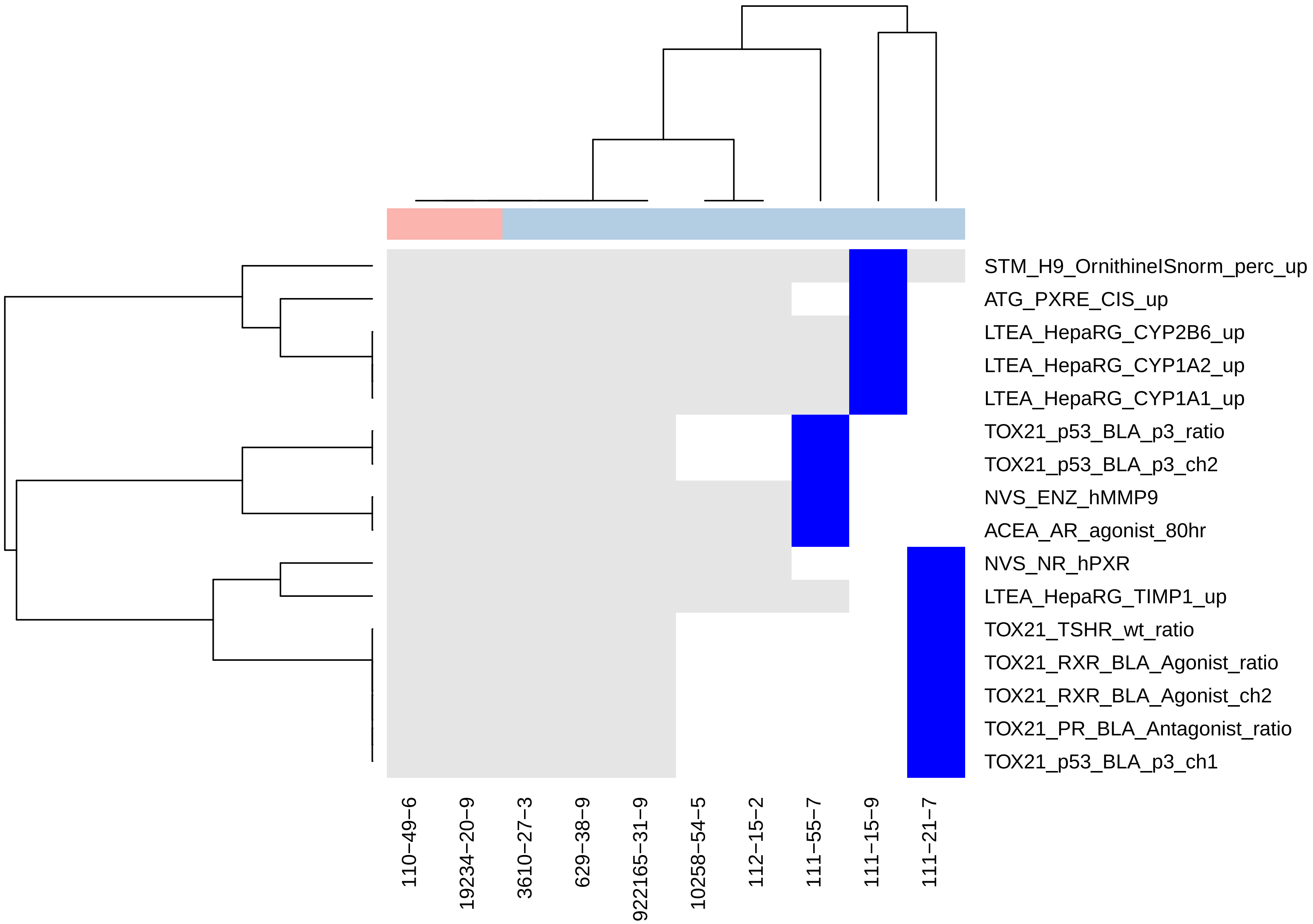

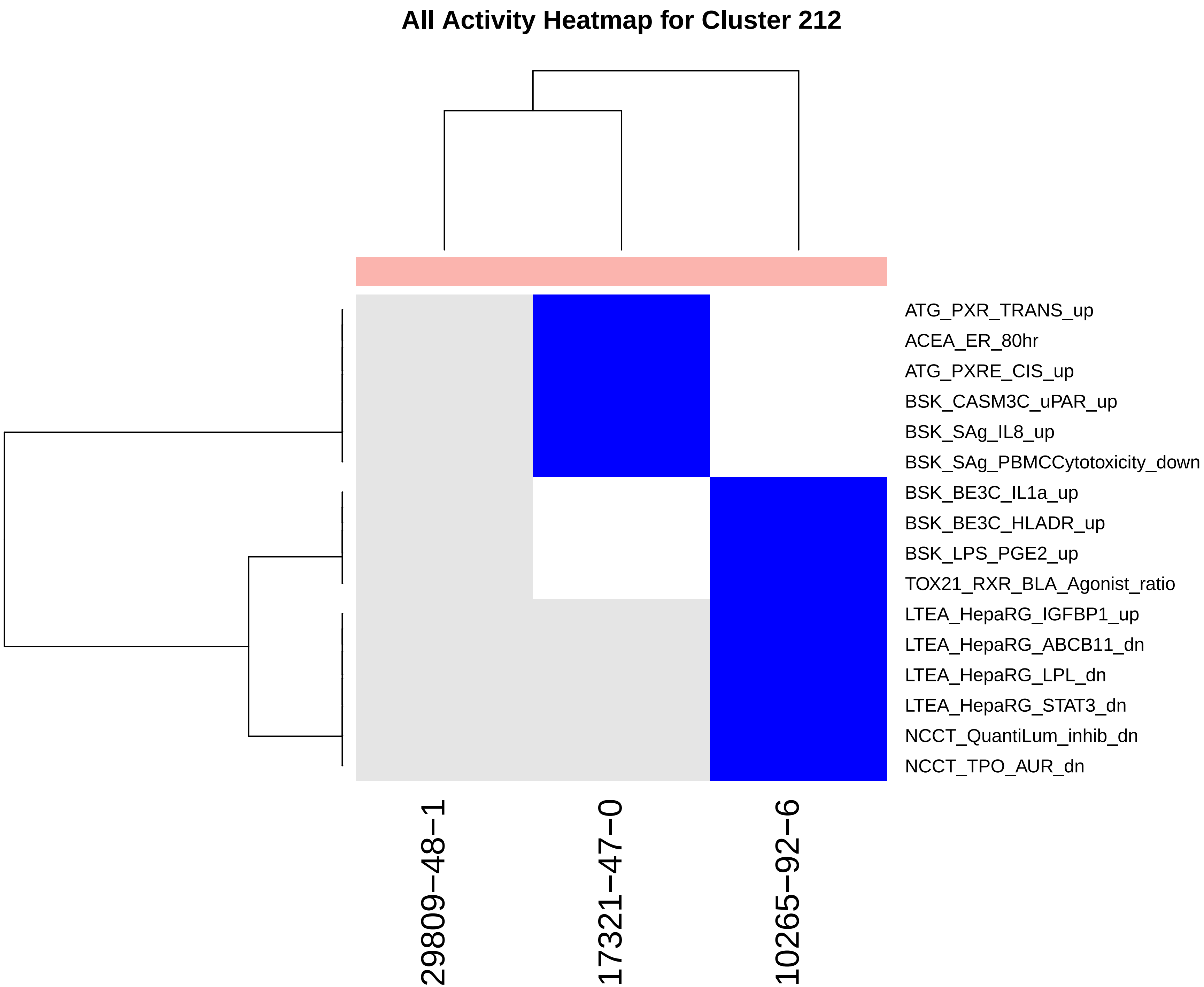
All Activity Heatmap for Cluster 212
ATG_PXR_TRANS_up
ACEA_ER_80hr
ATG_PXRE_CIS_up
BSK_CASM3C_uPAR_up
BSK_SAg_IL8_up
BSK_SAg_PBMCCytotoxicity_down
BSK_BE3C_IL1a_up
BSK_BE3C_HLADR_up
BSK_LPS_PGE2_up
TOX21_RXR_BLA_Agonist_ratio
LTEA_HepaRG_IGFBP1_up
LTEA_HepaRG_ABCB11_dn
LTEA_HepaRG_LPL_dn
LTEA_HepaRG_STAT3_dn
NCCT_QuantiLum_inhib_dn
NCCT_TPO_AUR_dn
29809−48−1
17321−47−0
10265−92−6

All Activity Heatmap for Cluster 213

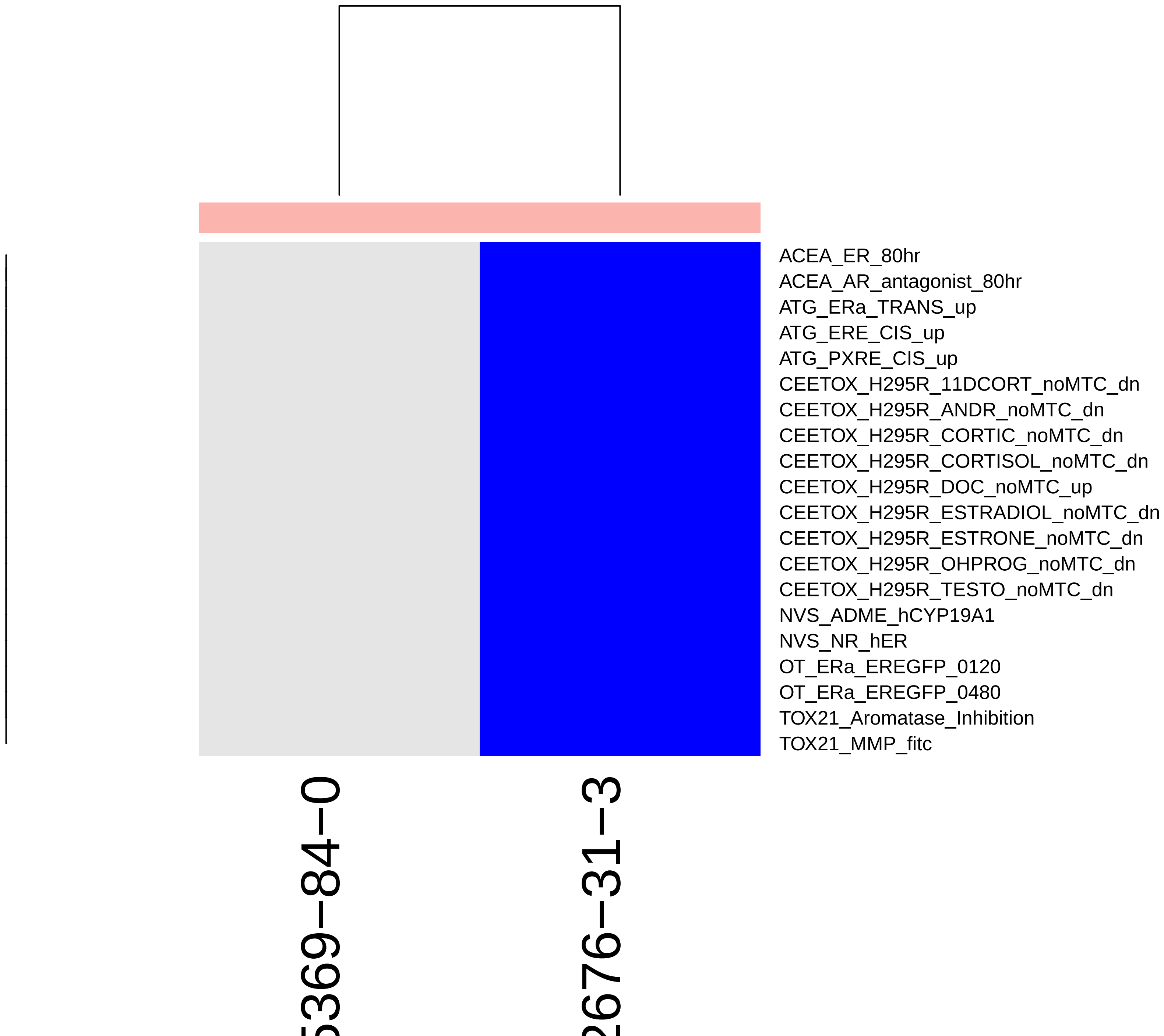

All Activity Heatmap for Cluster 215

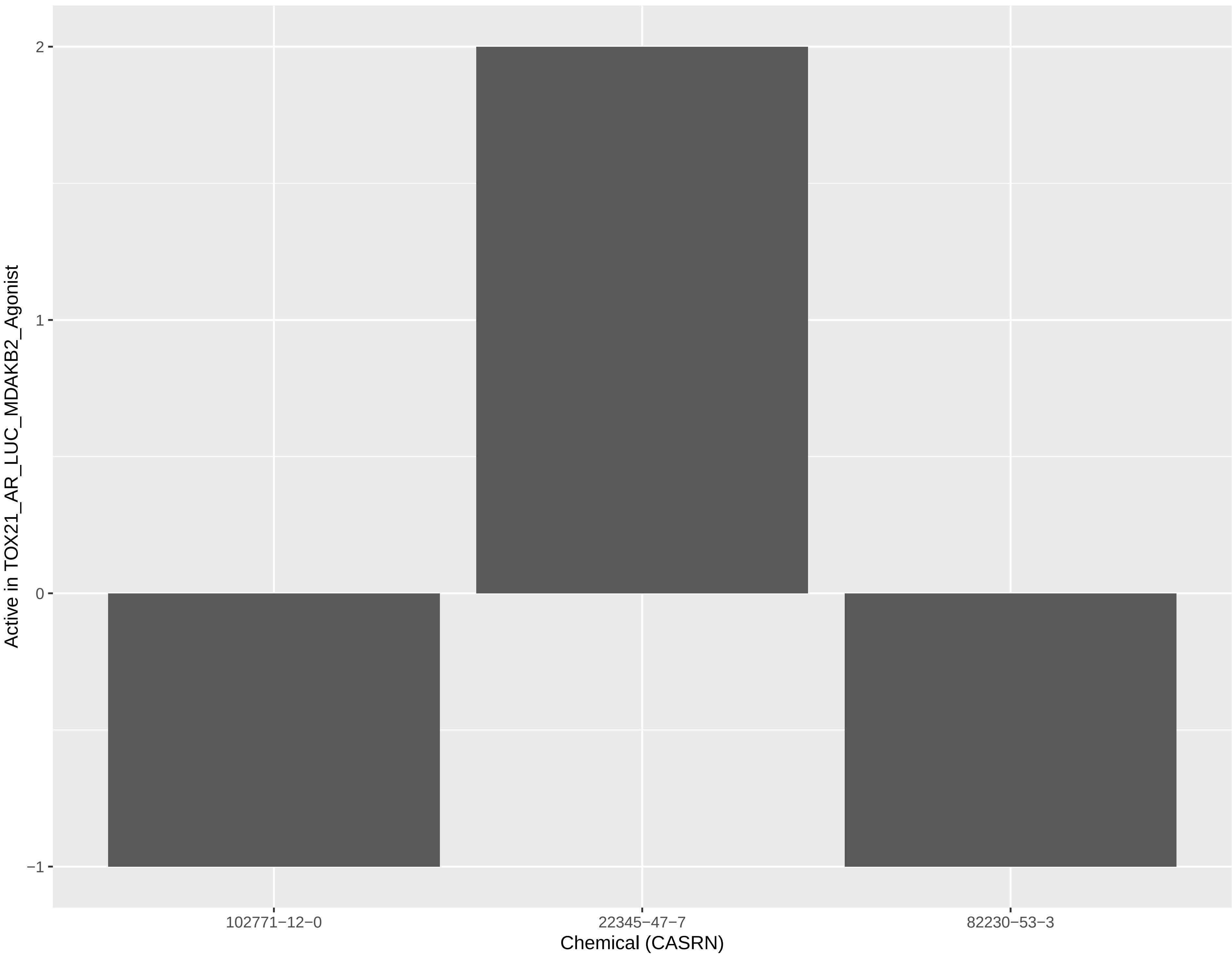

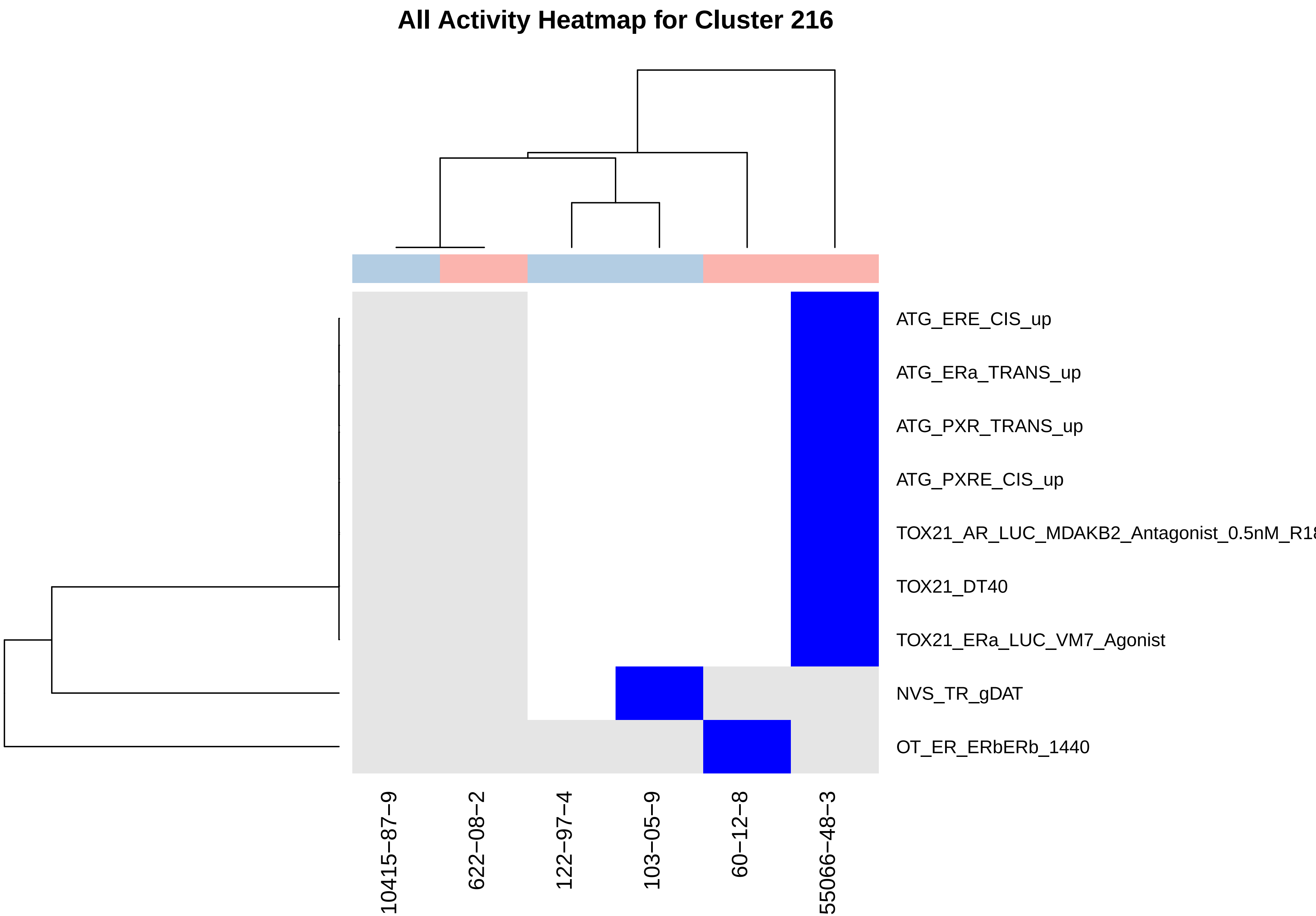
All Activity Heatmap for Cluster 216
ATG_ERE_CIS_up
ATG_ERa_TRANS_up
ATG_PXR_TRANS_up
ATG_PXRE_CIS_up
TOX21_AR_LUC_MDAKB2_Antagonist_0.5nM_R1
TOX21_DT40
TOX21_ERa_LUC_VM7_Agonist
NVS_TR_gDAT
OT_ER_ERbERb_1440
10415−87−9
622−08−2
122−97−4
103−05−9
60−12−8
55066−48−3

All Activity Heatmap for Cluster 217

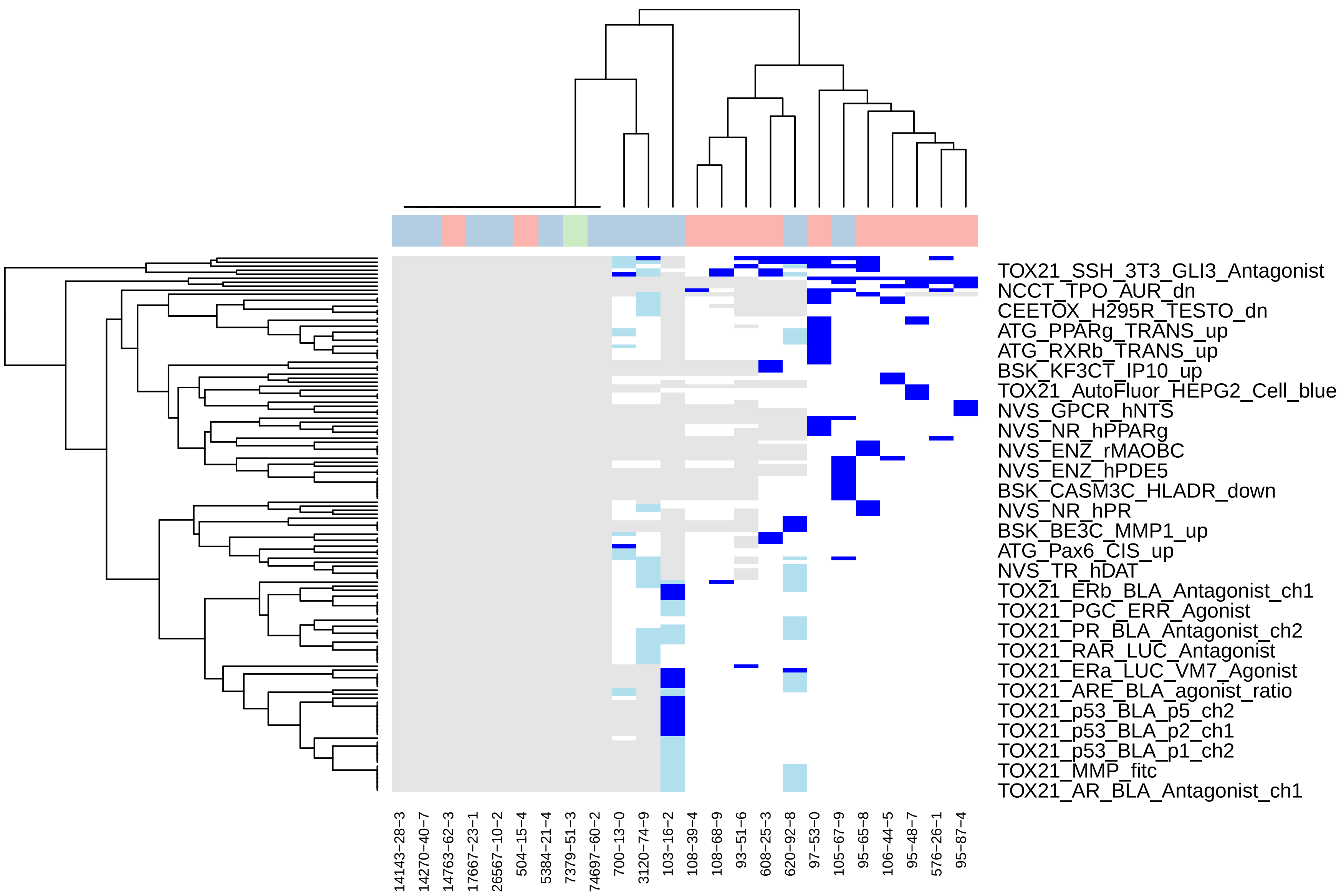

All Activity Heatmap for Cluster 218

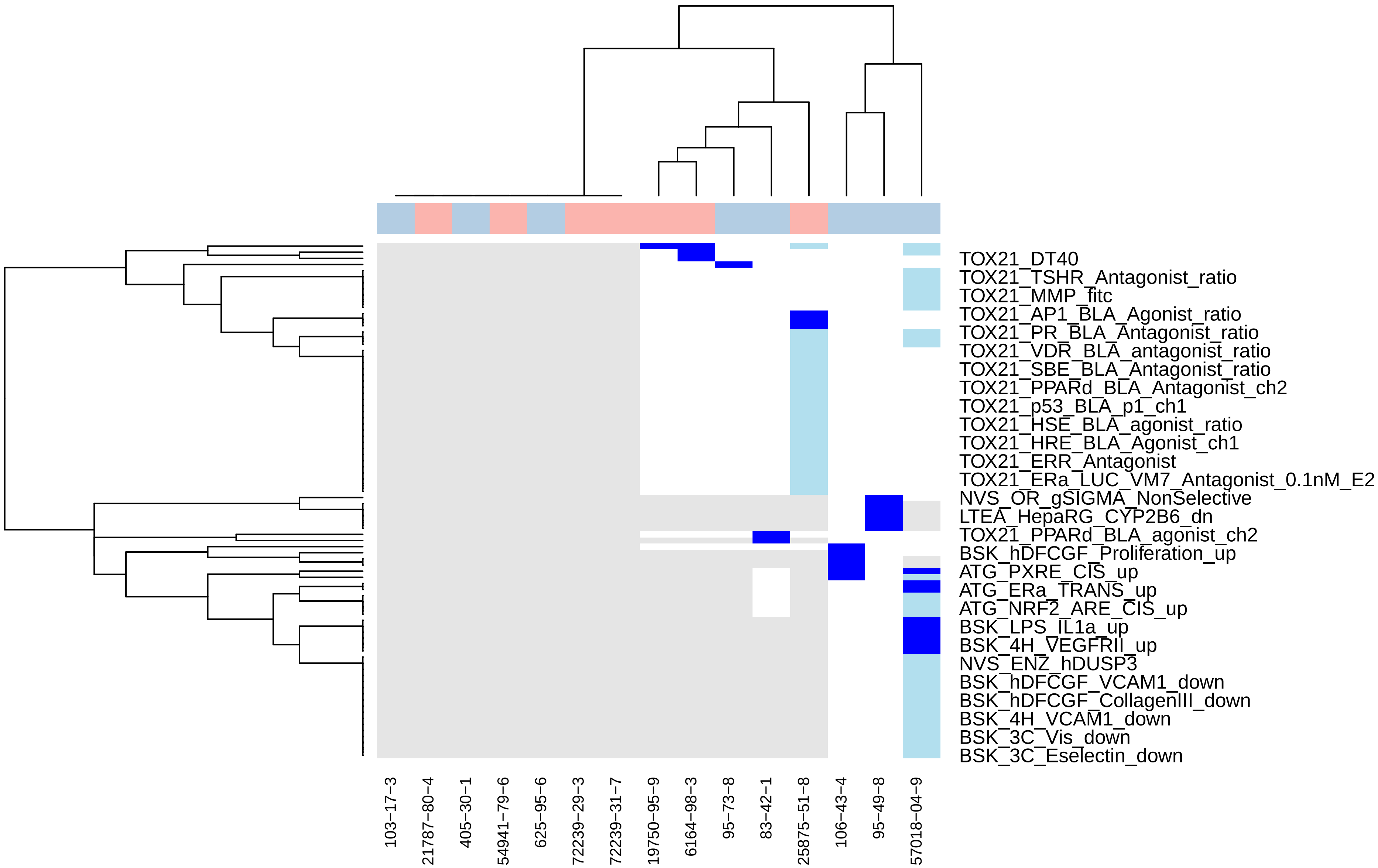

All Activity Heatmap for Cluster 219

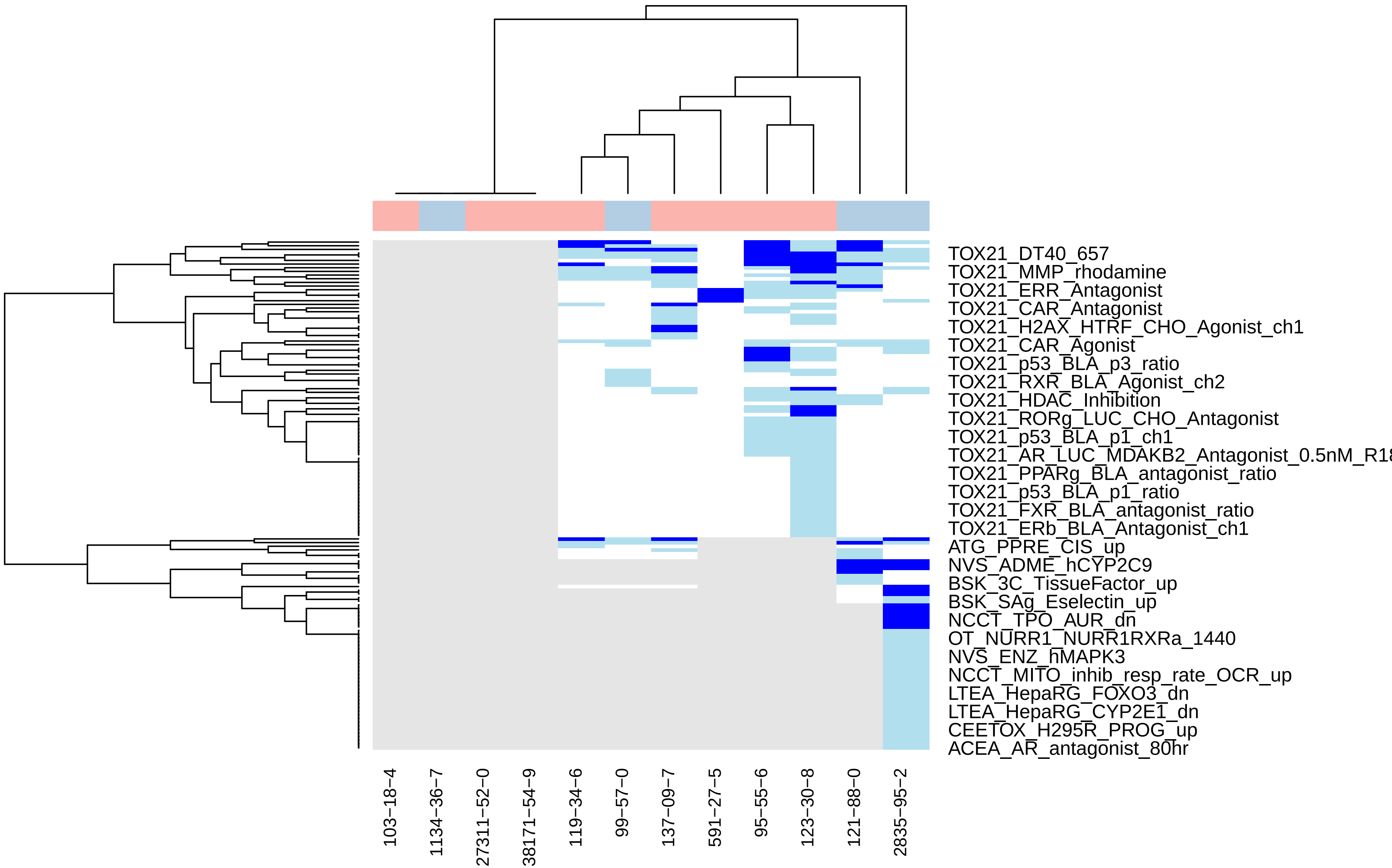

All Activity Heatmap for Cluster 220

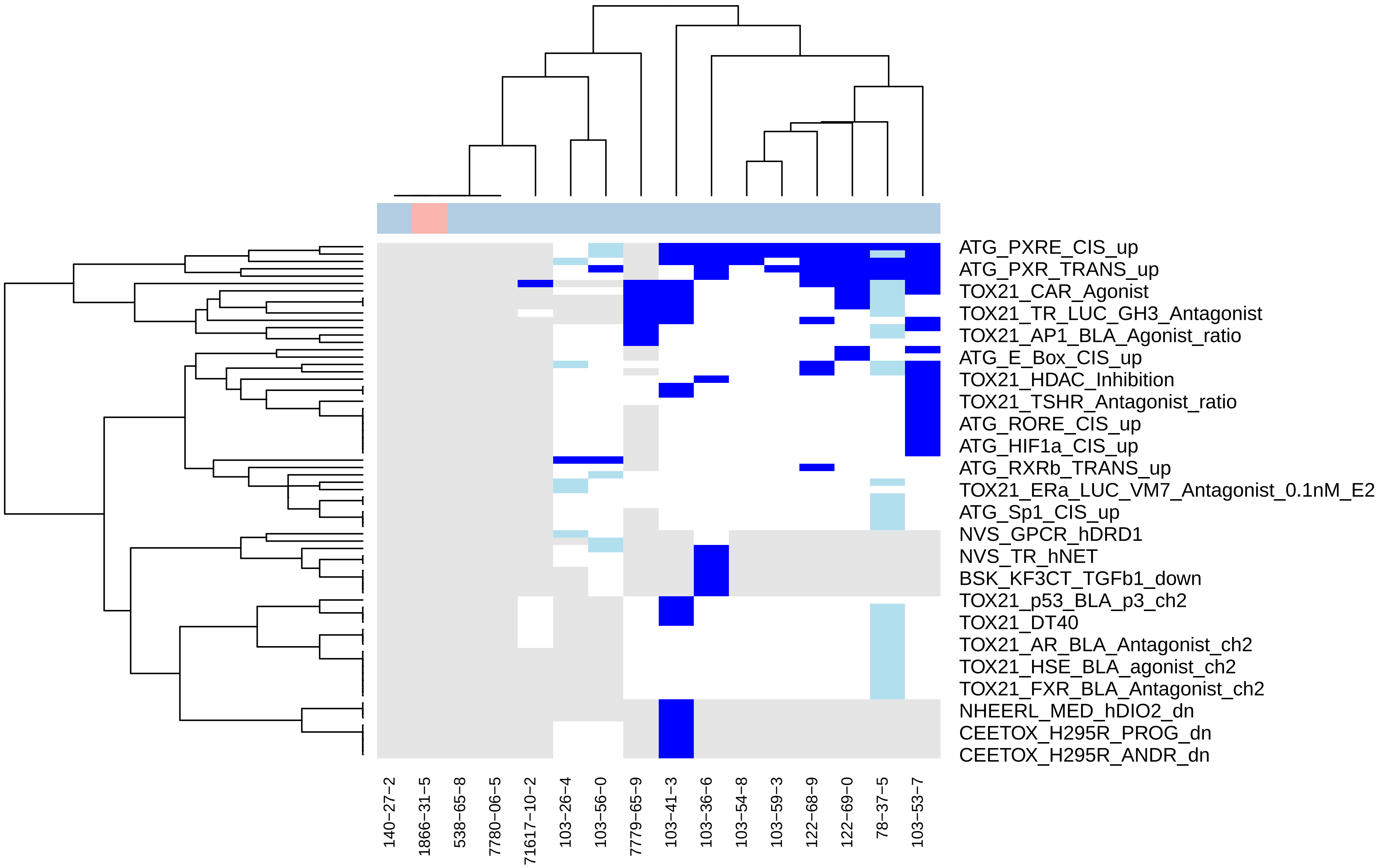

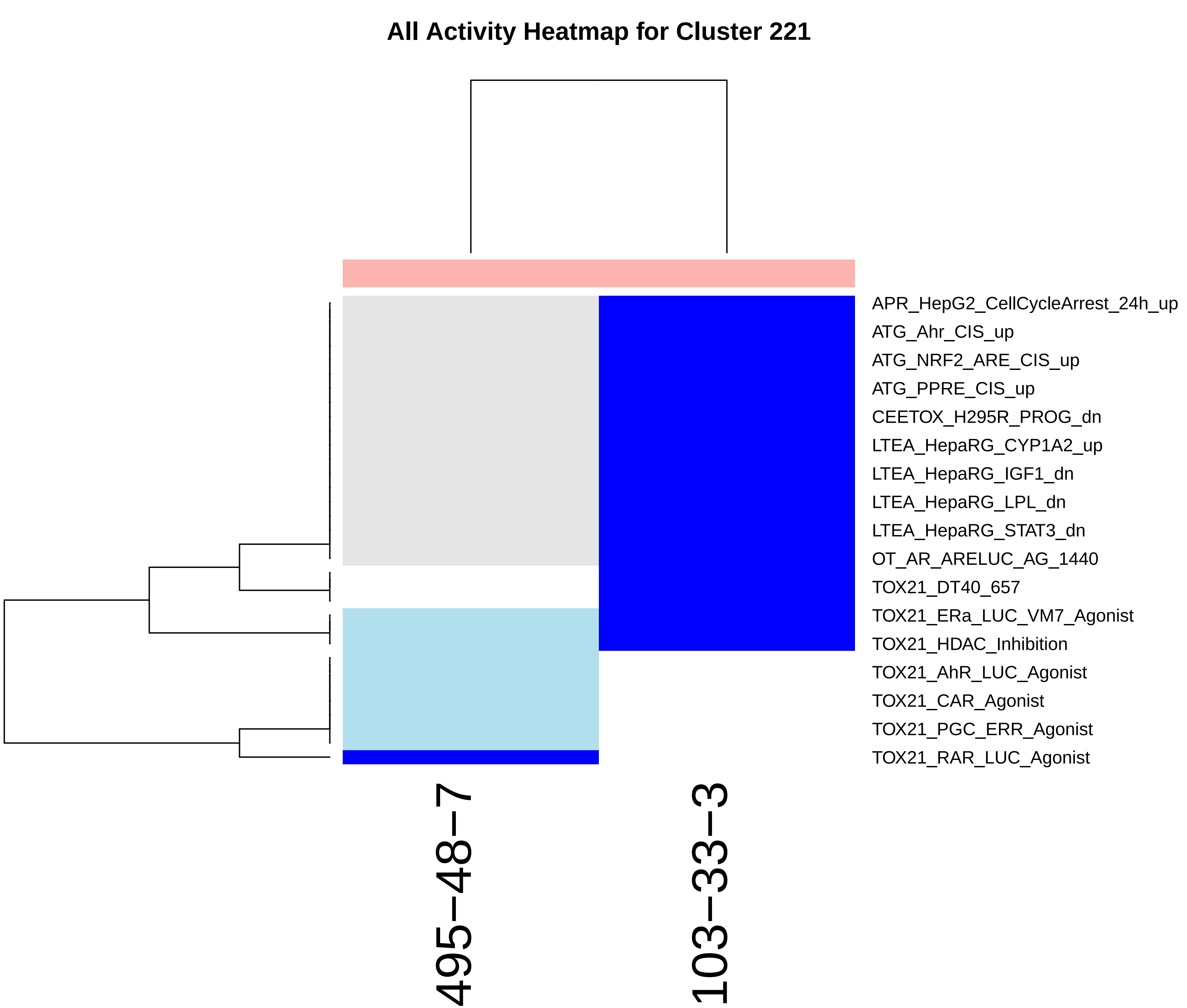
All Activity Heatmap for Cluster 221
APR_HepG2_CellCycleArrest_24h_up
ATG_Ahr_CIS_up
ATG_NRF2_ARE_CIS_up
ATG_PPRE_CIS_up
CEETOX_H295R_PROG_dn
LTEA_HepaRG_CYP1A2_up
LTEA_HepaRG_IGF1_dn
LTEA_HepaRG_LPL_dn
LTEA_HepaRG_STAT3_dn
OT_AR_ARELUC_AG_1440
TOX21_DT40_657
TOX21_ERa_LUC_VM7_Agonist
TOX21_HDAC_Inhibition
TOX21_AhR_LUC_Agonist
TOX21_CAR_Agonist
TOX21_PGC_ERR_Agonist
TOX21_RAR_LUC_Agonist
495−48−7
103−33−3

All Activity Heatmap for Cluster 222

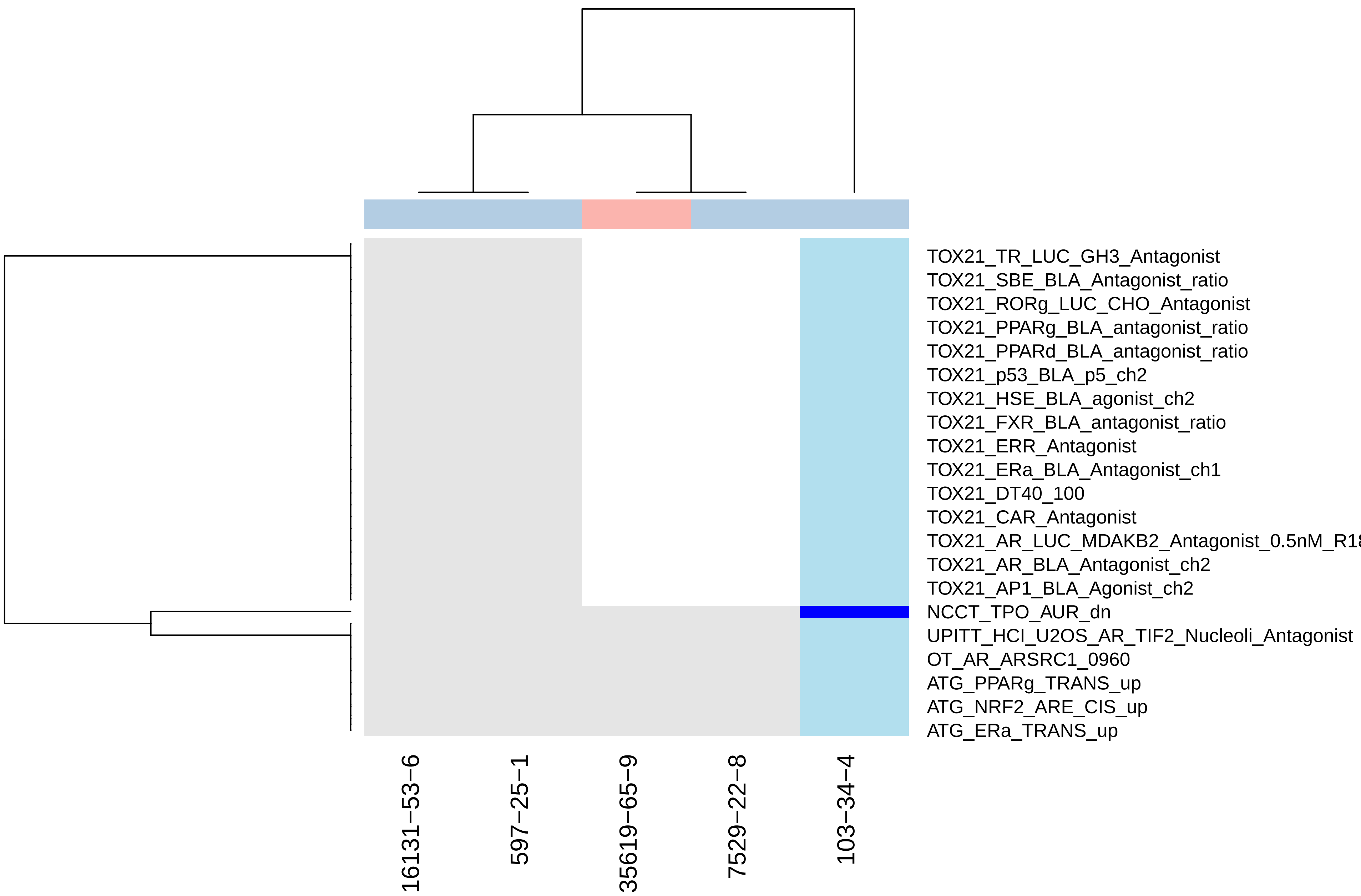

All Activity Heatmap for Cluster 223

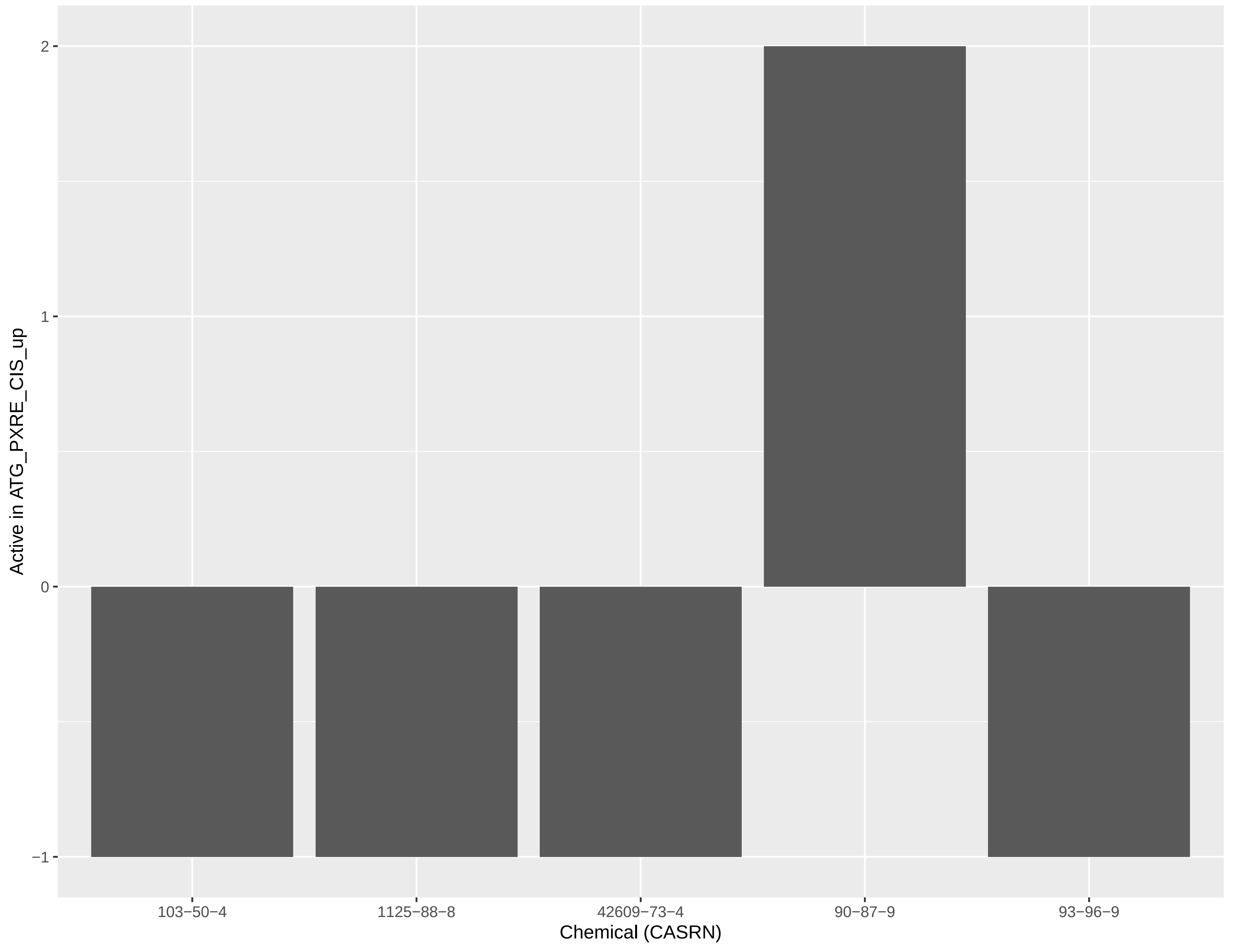

All Activity Heatmap for Cluster 224

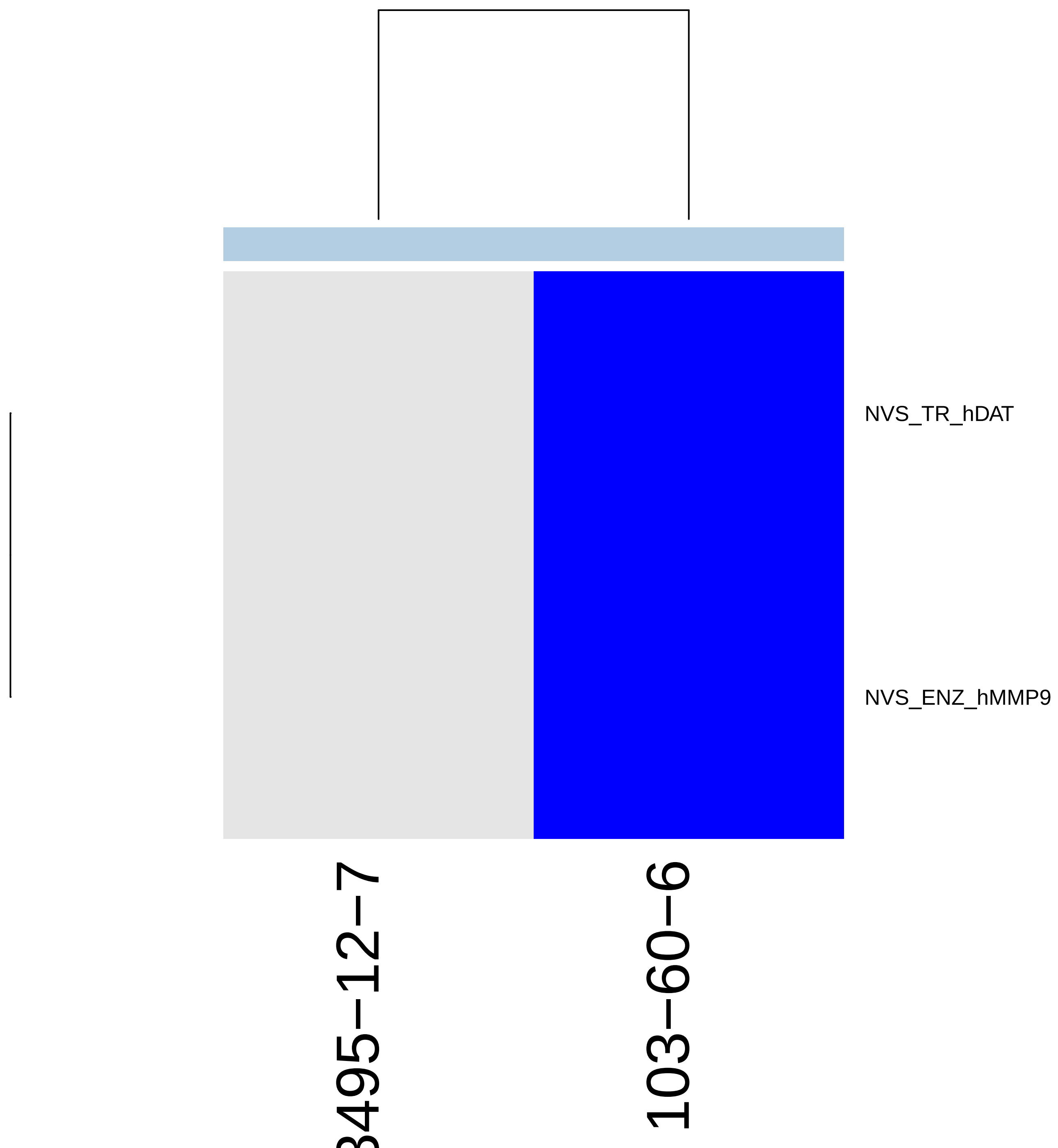

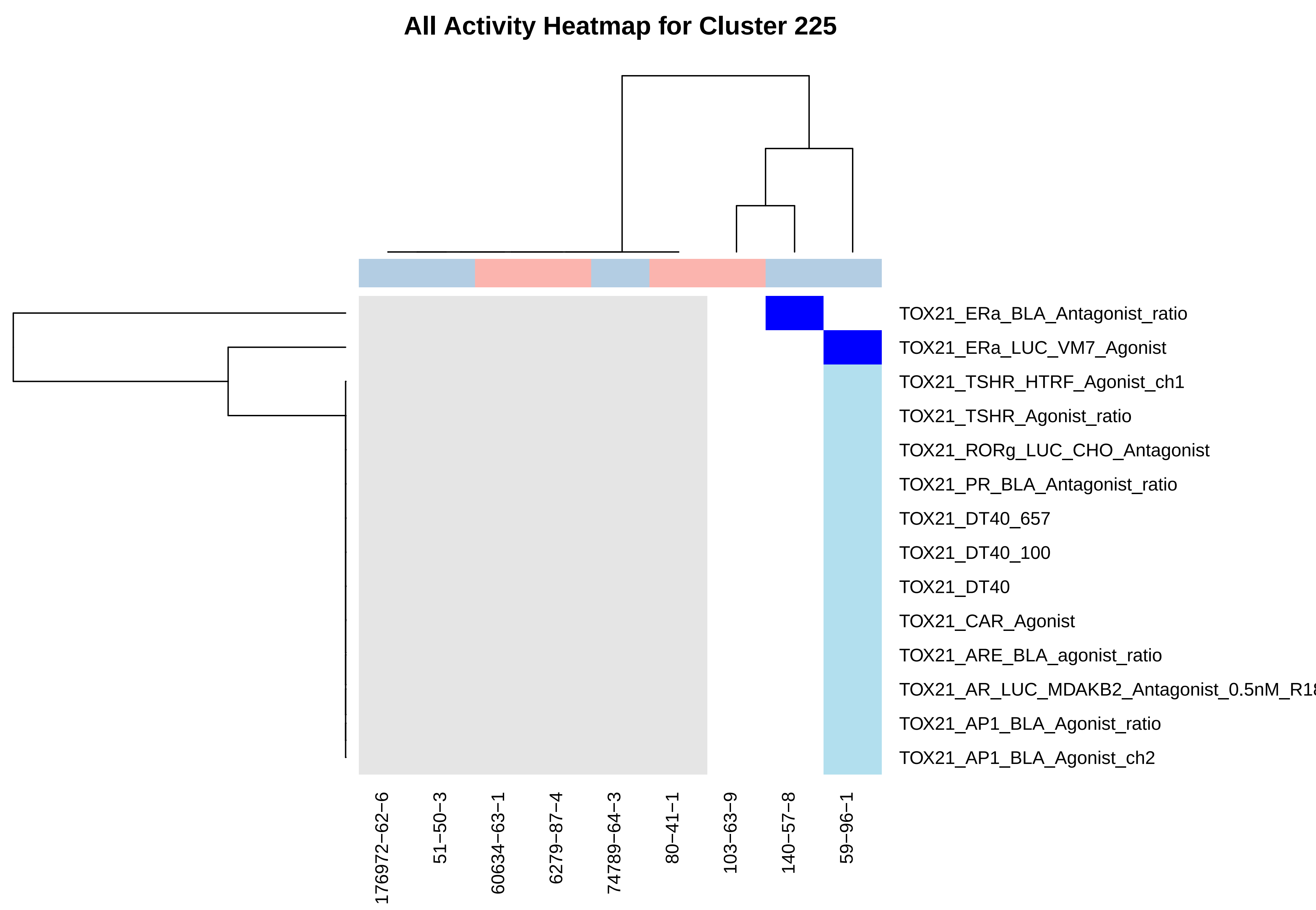
All Activity Heatmap for Cluster 225
TOX21_ERa_BLA_Antagonist_ratio
TOX21_ERa_LUC_VM7_Agonist
TOX21_TSHR_HTRF_Agonist_ch1
TOX21_TSHR_Agonist_ratio
TOX21_RORg_LUC_CHO_Antagonist
TOX21_PR_BLA_Antagonist_ratio
TOX21_DT40_657
TOX21_DT40_100
TOX21_DT40
TOX21_CAR_Agonist
TOX21_ARE_BLA_agonist_ratio
TOX21_AR_LUC_MDAKB2_Antagonist_0.5nM_R1
TOX21_AP1_BLA_Agonist_ratio
TOX21_AP1_BLA_Agonist_ch2
176972−62−6
51−50−3
60634−63−1
6279−87−4
74789−64−3
80−41−1
103−63−9
140−57−8
59−96−1

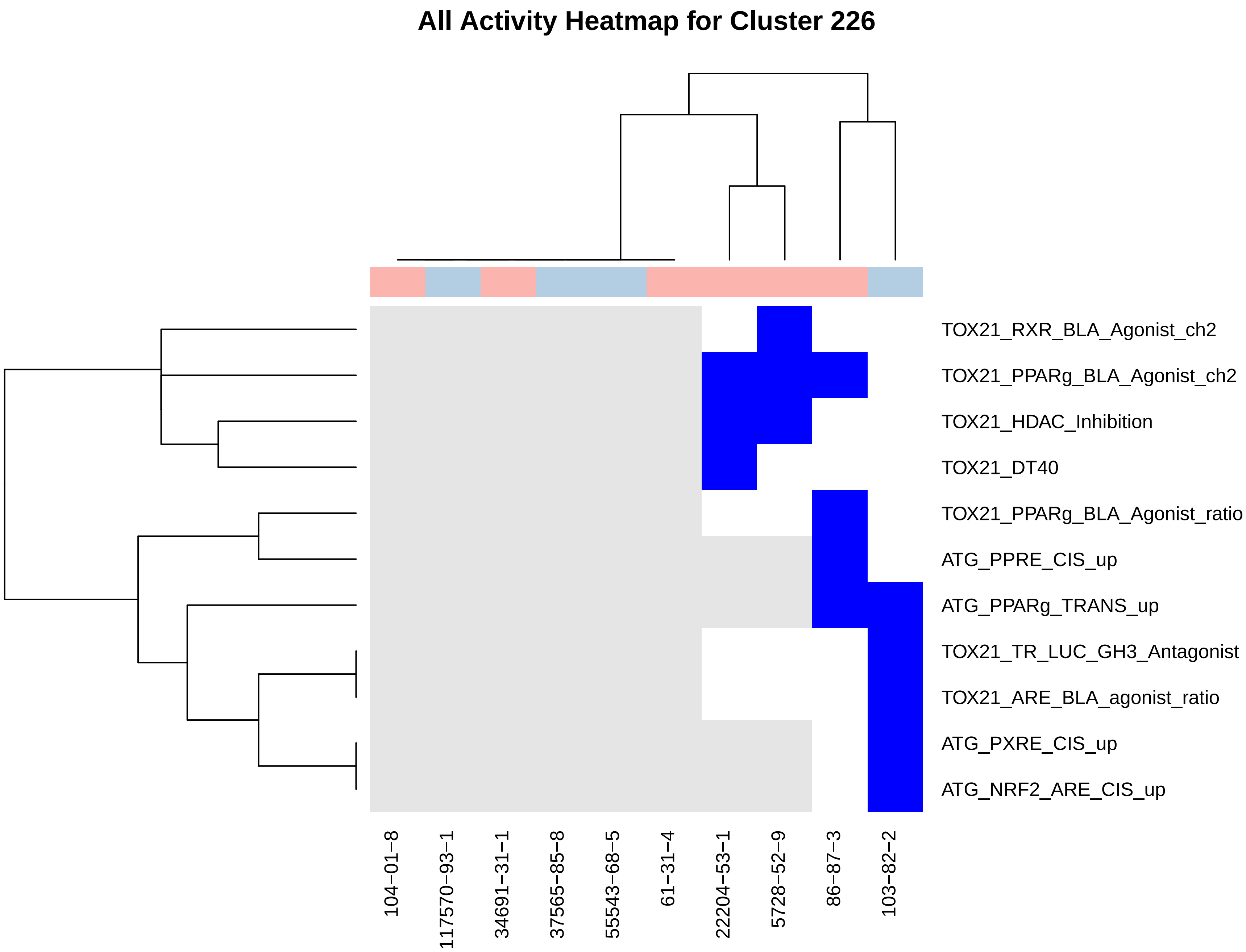

All Activity Heatmap for Cluster 226
TOX21_RXR_BLA_Agonist_ch2
TOX21_PPARg_BLA_Agonist_ch2
TOX21_HDAC_Inhibition
TOX21_DT40
TOX21_PPARg_BLA_Agonist_ratio
ATG_PPRE_CIS_up
ATG_PPARg_TRANS_up
TOX21_TR_LUC_GH3_Antagonist
TOX21_ARE_BLA_agonist_ratio
ATG_PXRE_CIS_up
ATG_NRF2_ARE_CIS_up
104−01−8
117570−93−1
34691−31−1
37565−85−8
55543−68−5
61−31−4
22204−53−1
5728−52−9
86−87−3
103−82−2

All Activity Heatmap for Cluster 227

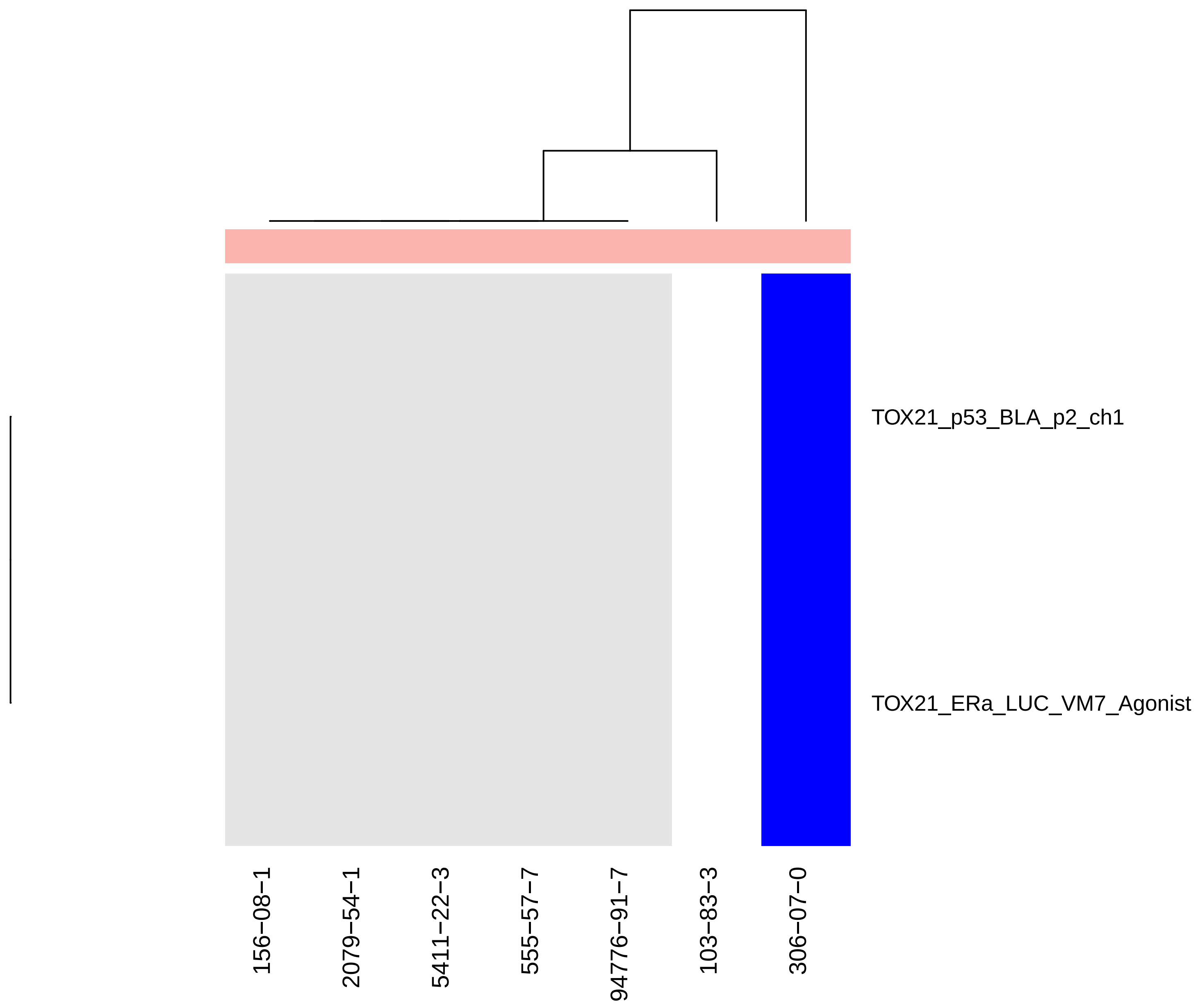

All Activity Heatmap for Cluster 228

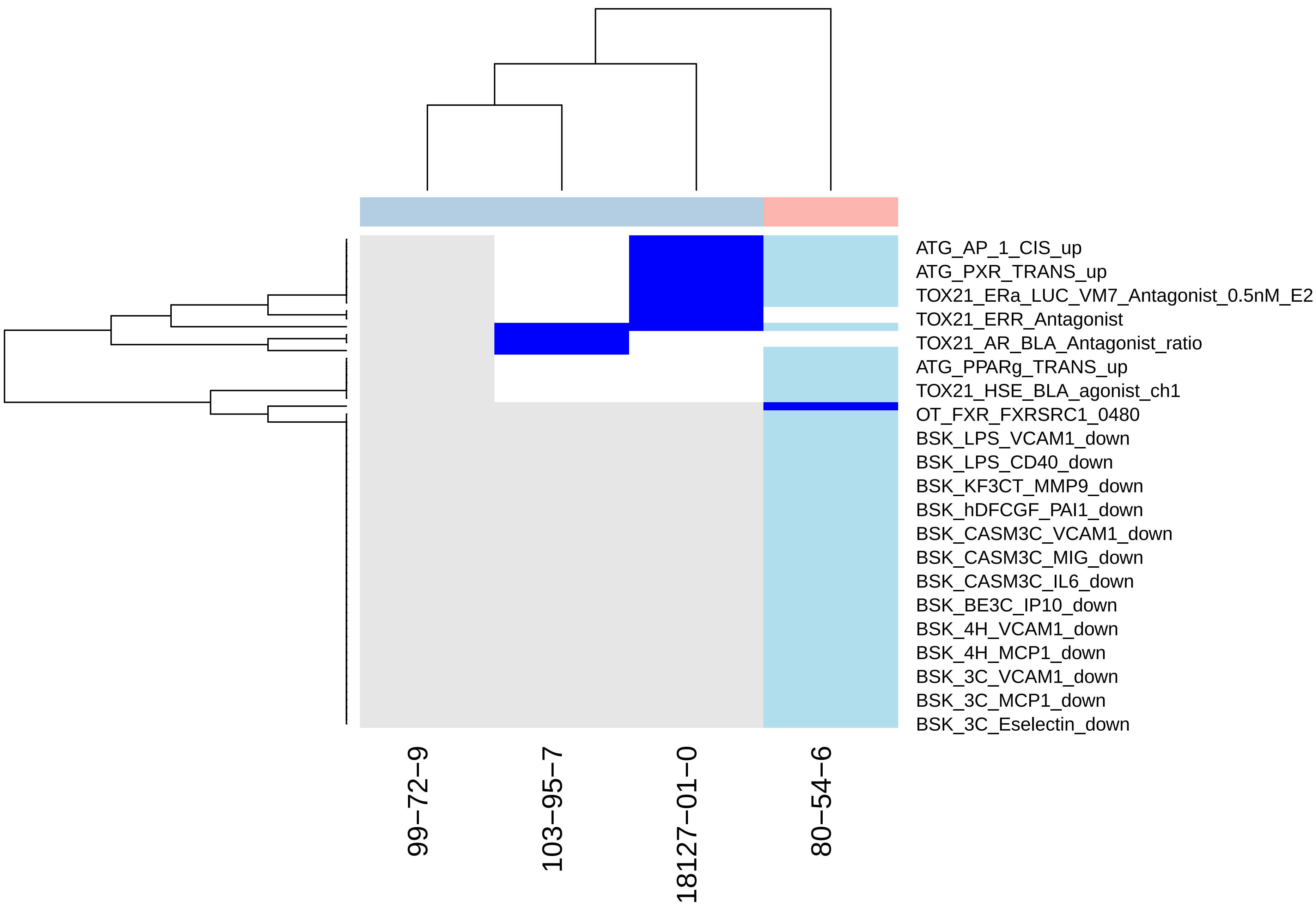

All Activity Heatmap for Cluster 230

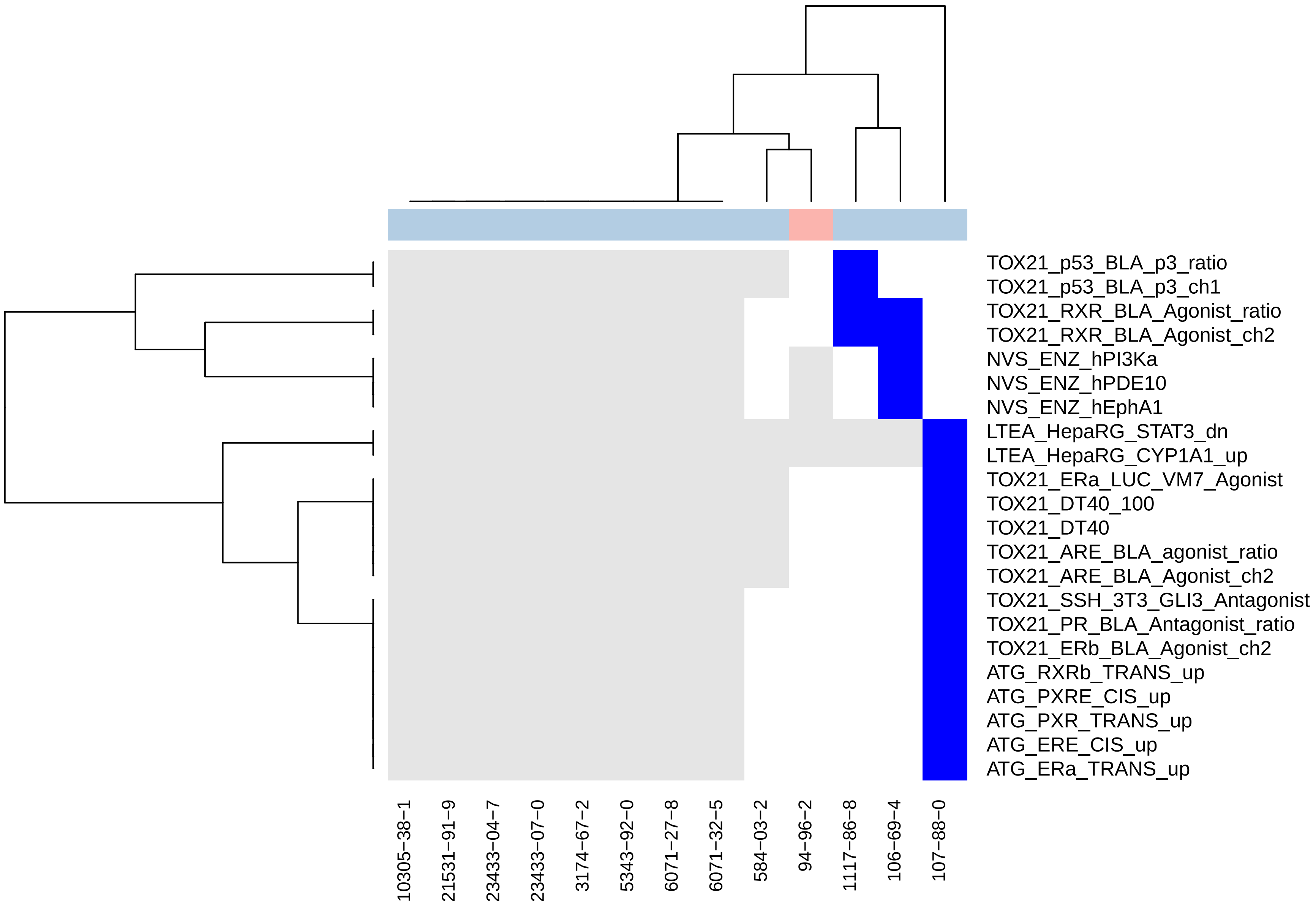

All Activity Heatmap for Cluster 231

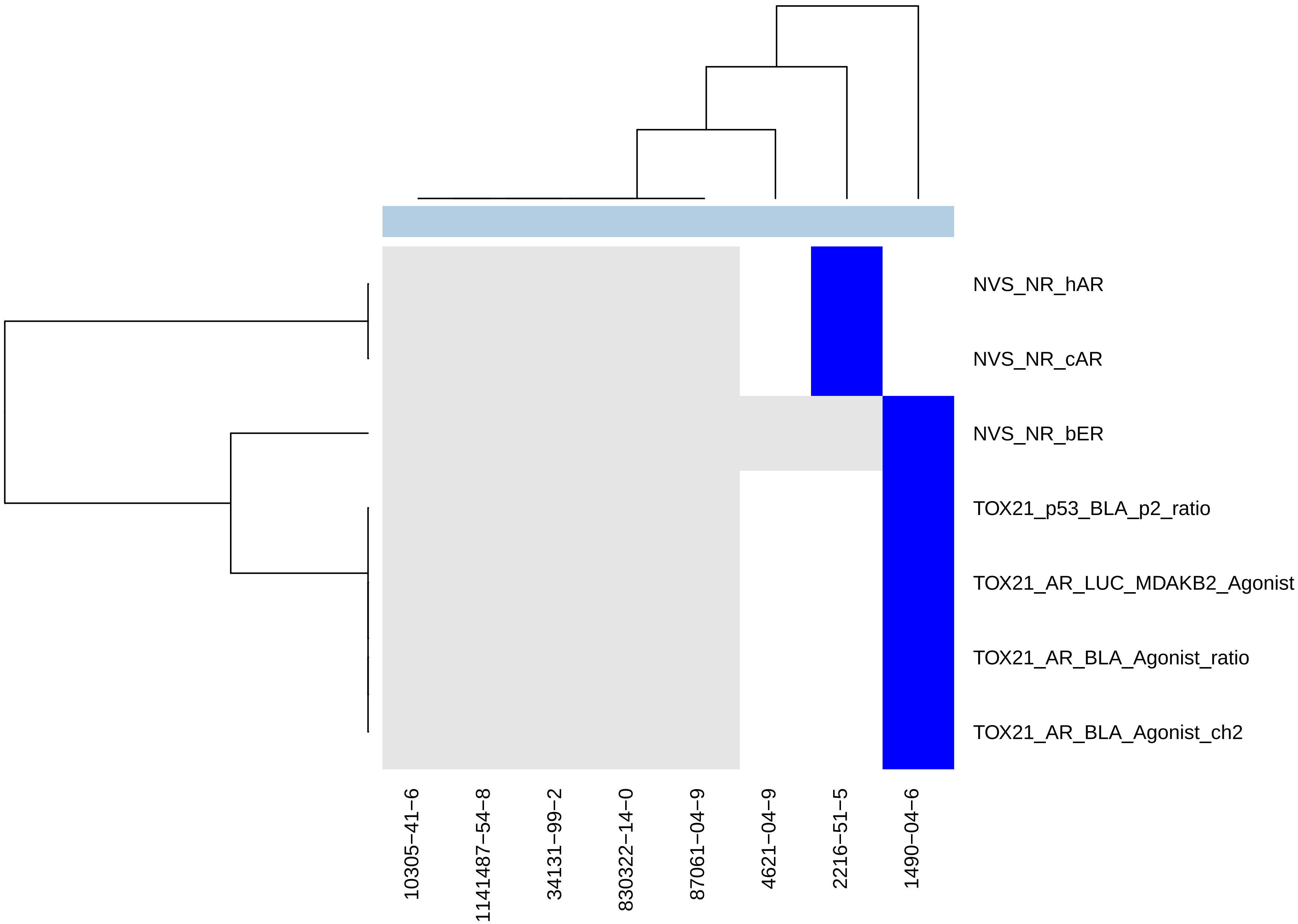

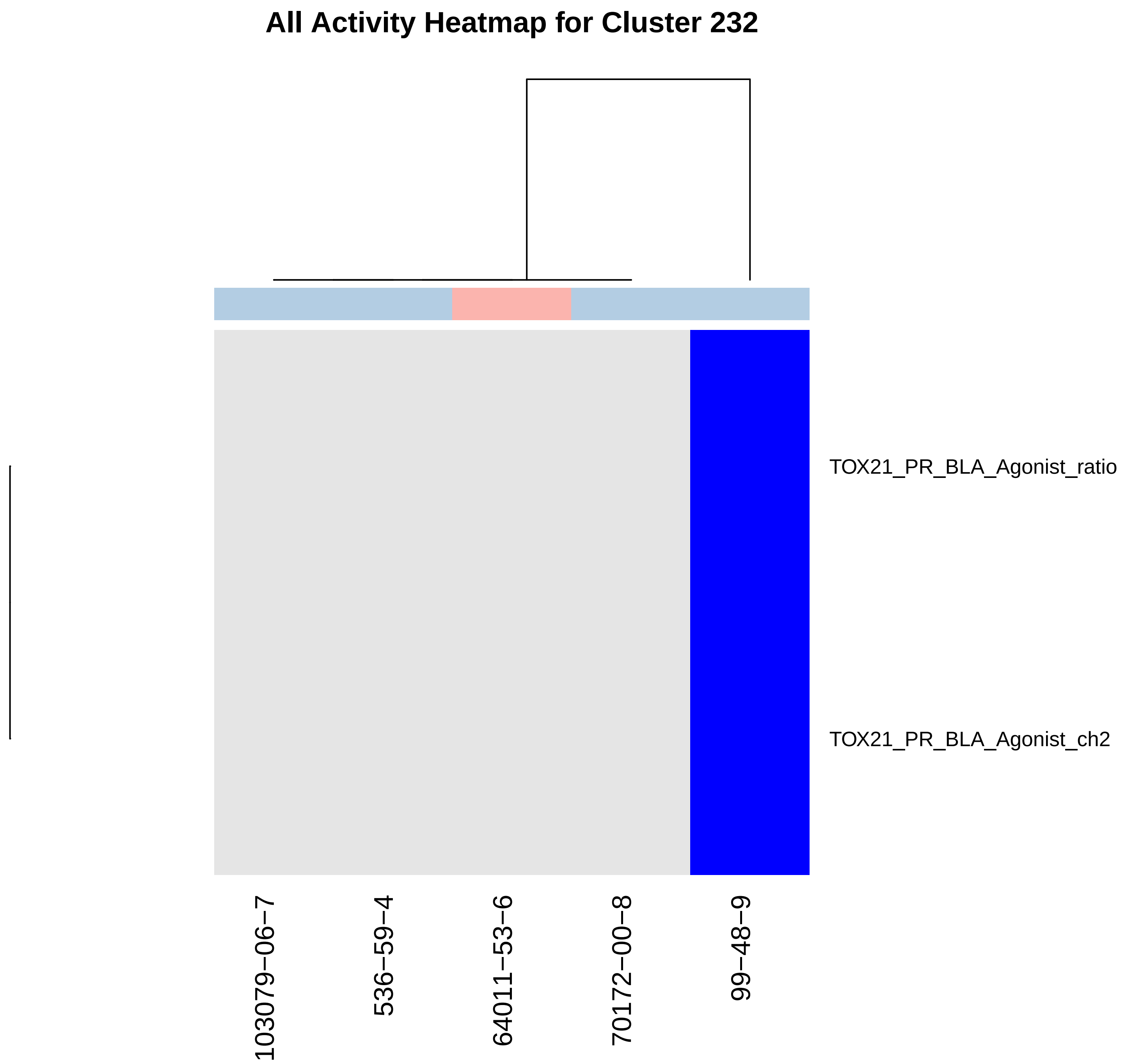
All Activity Heatmap for Cluster 232
TOX21_PR_BLA_Agonist_ratio
TOX21_PR_BLA_Agonist_ch2
103079−06−7
536−59−4
64011−53−6
70172−00−8
99−48−9

All Activity Heatmap for Cluster 233

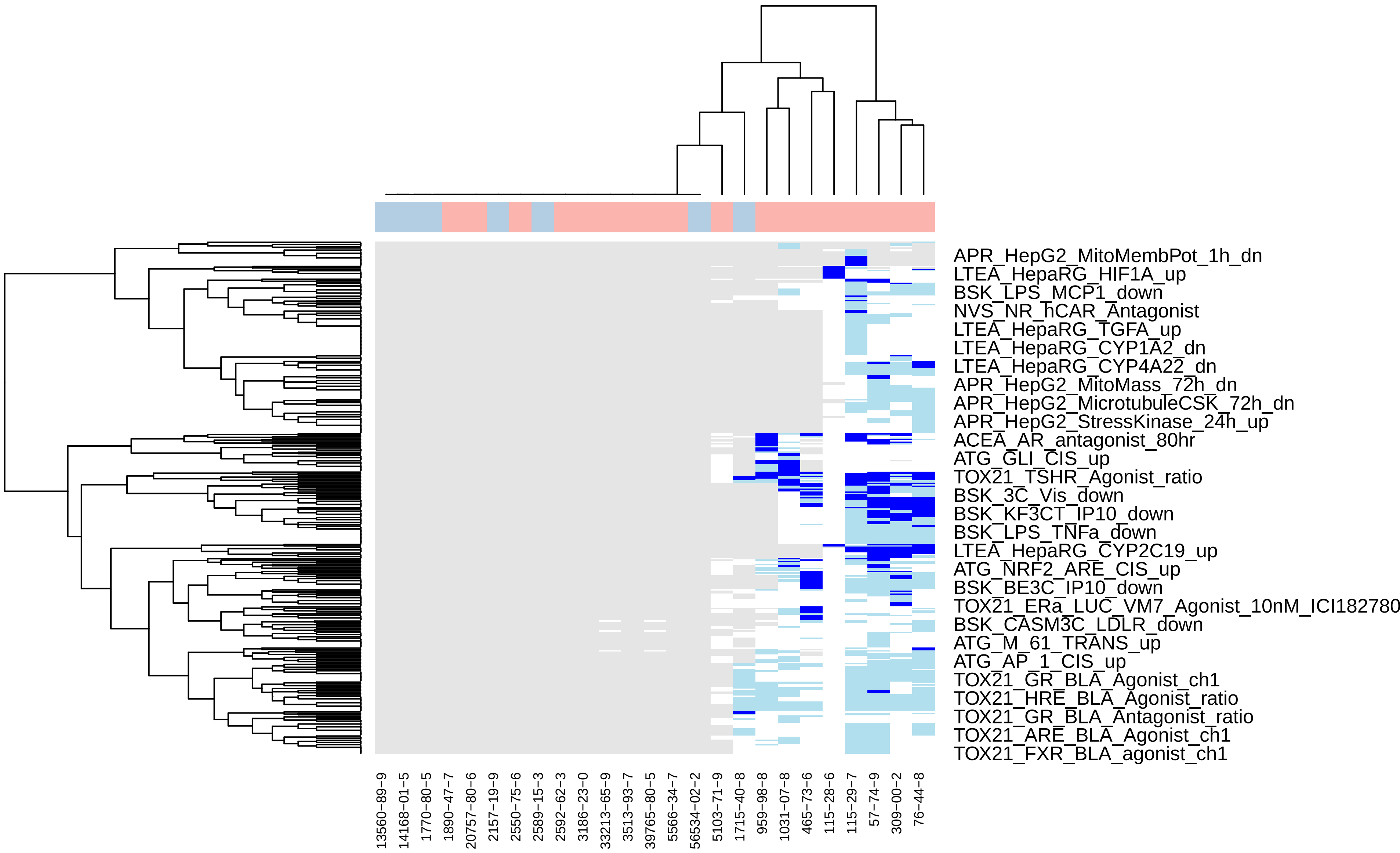

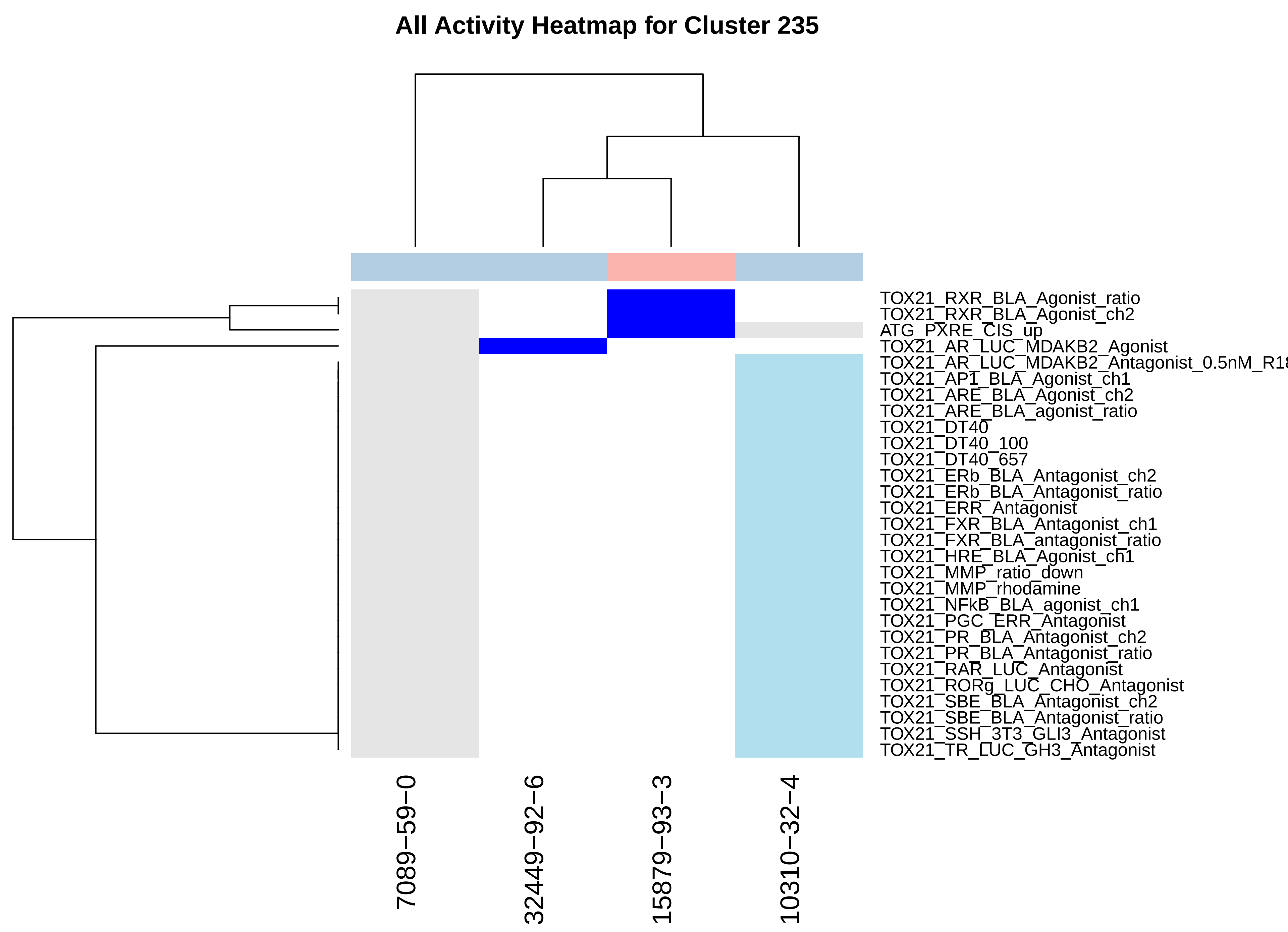

All Activity Heatmap for Cluster 235
TOX21_RXR_BLA_Agonist_ratio
TOX21_RXR_BLA_Agonist_ch2
ATG_PXRE_CIS_up
TOX21_AR_LUC_MDAKB2_Agonist
TOX21_AR_LUC_MDAKB2_Antagonist_0.5nM_R18
TOX21_AP1_BLA_Agonist_ch1
TOX21_ARE_BLA_Agonist_ch2
TOX21_ARE_BLA_agonist_ratio
TOX21_DT40
TOX21_DT40_100
TOX21_DT40_657
TOX21_ERb_BLA_Antagonist_ch2
TOX21_ERb_BLA_Antagonist_ratio
TOX21_ERR_Antagonist
TOX21_FXR_BLA_Antagonist_ch1
TOX21_FXR_BLA_antagonist_ratio
TOX21_HRE_BLA_Agonist_ch1
TOX21_MMP_ratio_down
TOX21_MMP_rhodamine
TOX21_NFkB_BLA_agonist_ch1
TOX21_PGC_ERR_Antagonist
TOX21_PR_BLA_Antagonist_ch2
TOX21_PR_BLA_Antagonist_ratio
TOX21_RAR_LUC_Antagonist
TOX21_RORg_LUC_CHO_Antagonist
TOX21_SBE_BLA_Antagonist_ch2
TOX21_SBE_BLA_Antagonist_ratio
TOX21_SSH_3T3_GLI3_Antagonist
TOX21_TR_LUC_GH3_Antagonist
7089−59−0
32449−92−6
15879−93−3
10310−32−4

All Activity Heatmap for Cluster 236

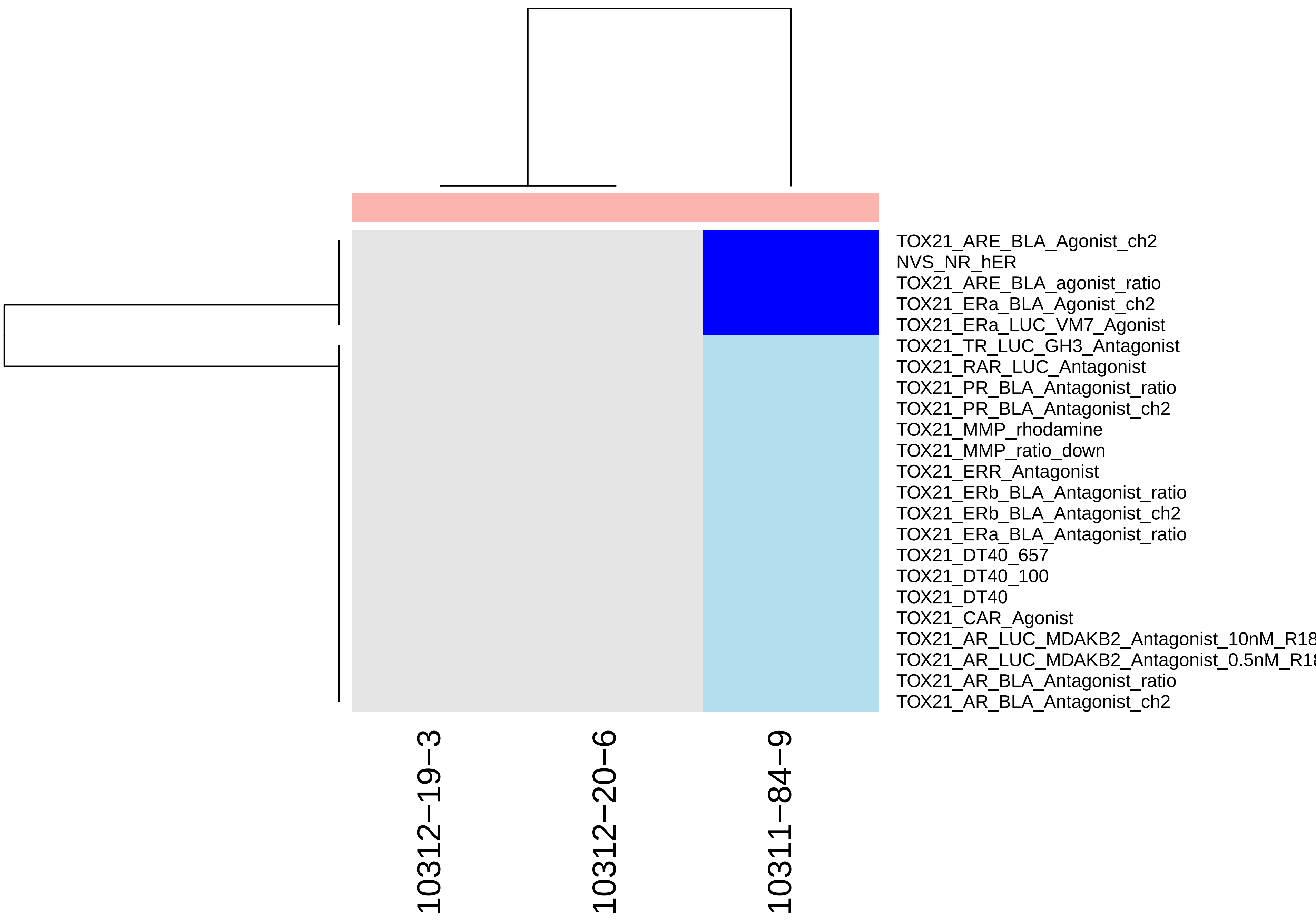

All Activity Heatmap for Cluster 237

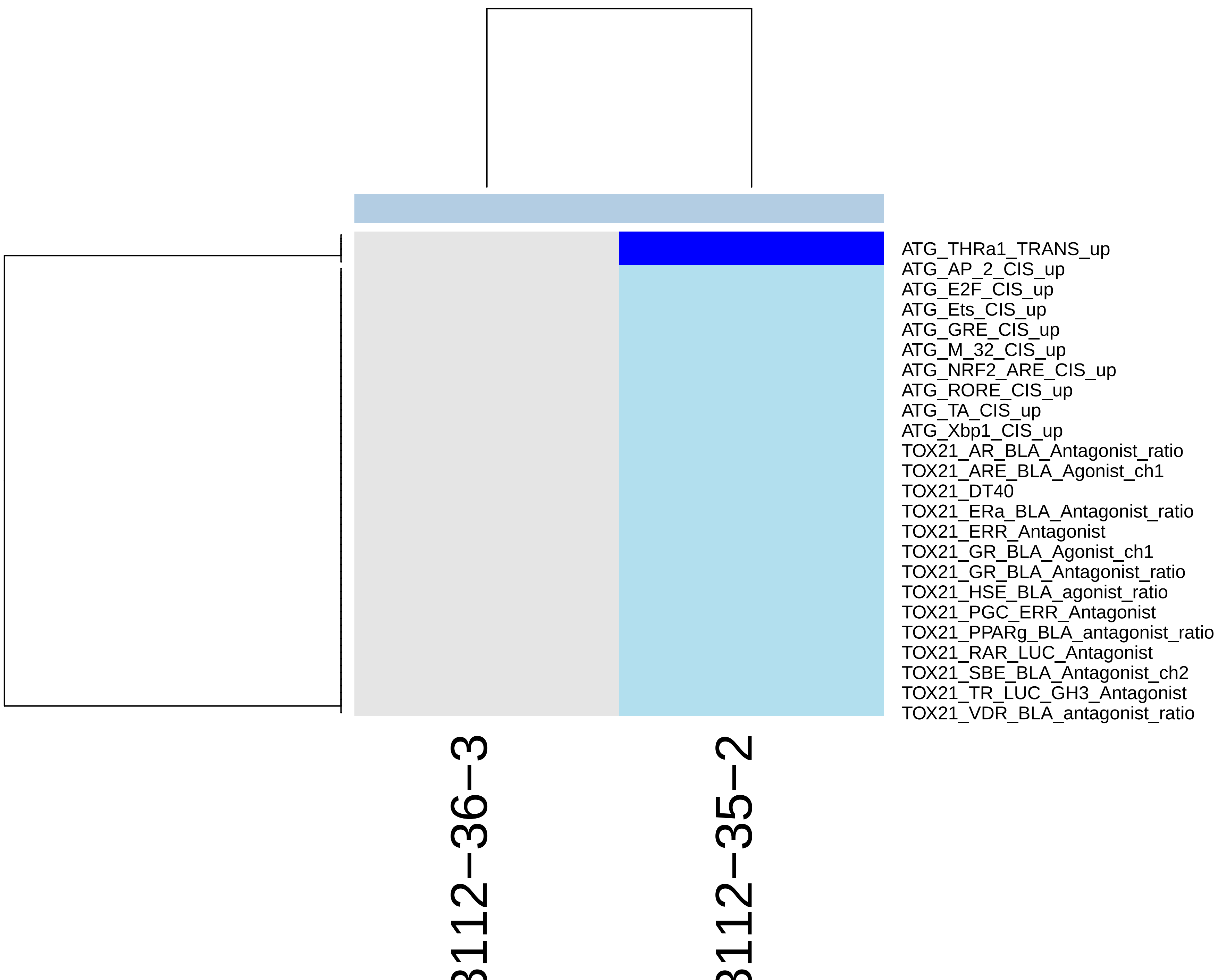

All Activity Heatmap for Cluster 238

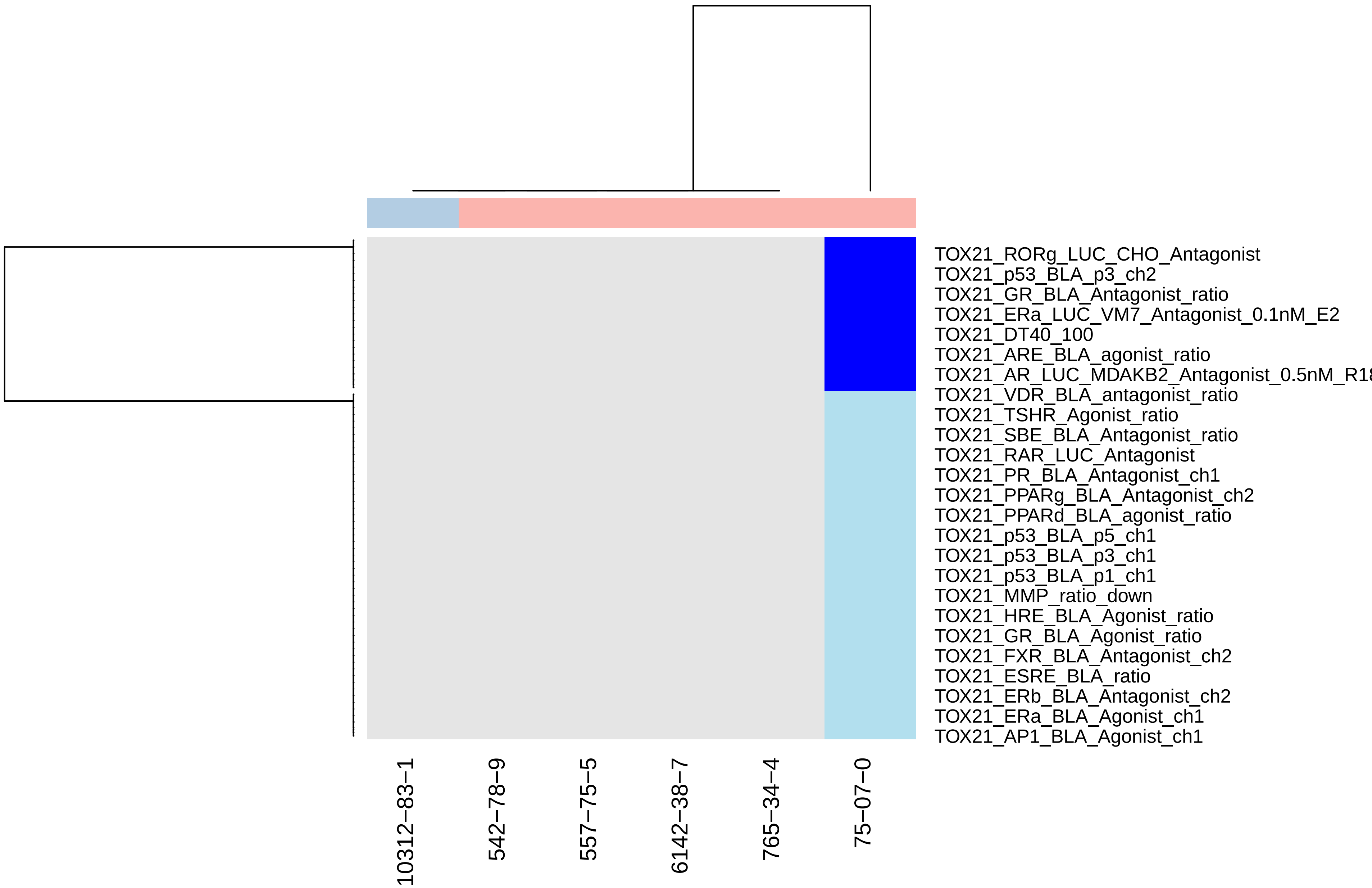

All Activity Heatmap for Cluster 240

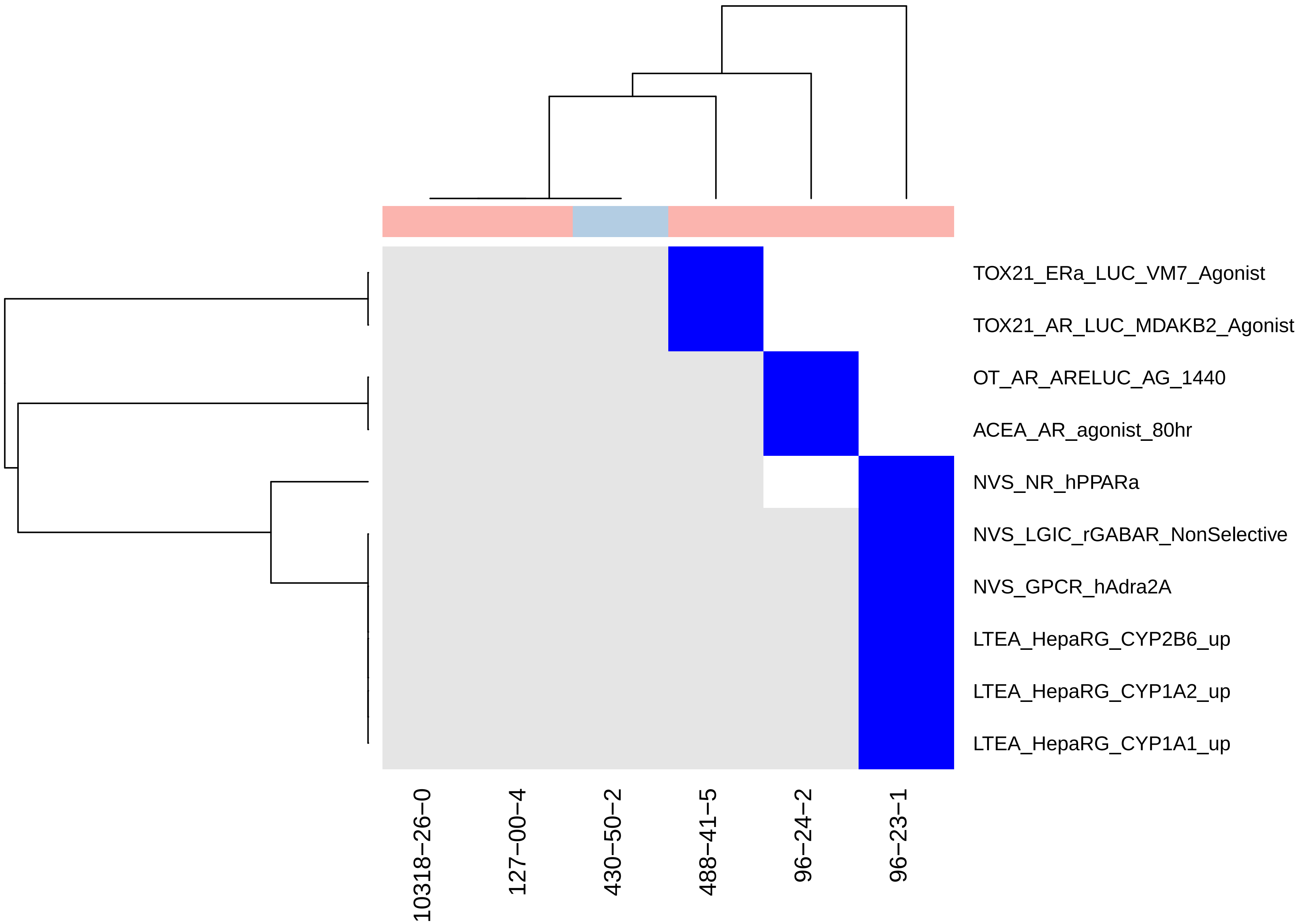

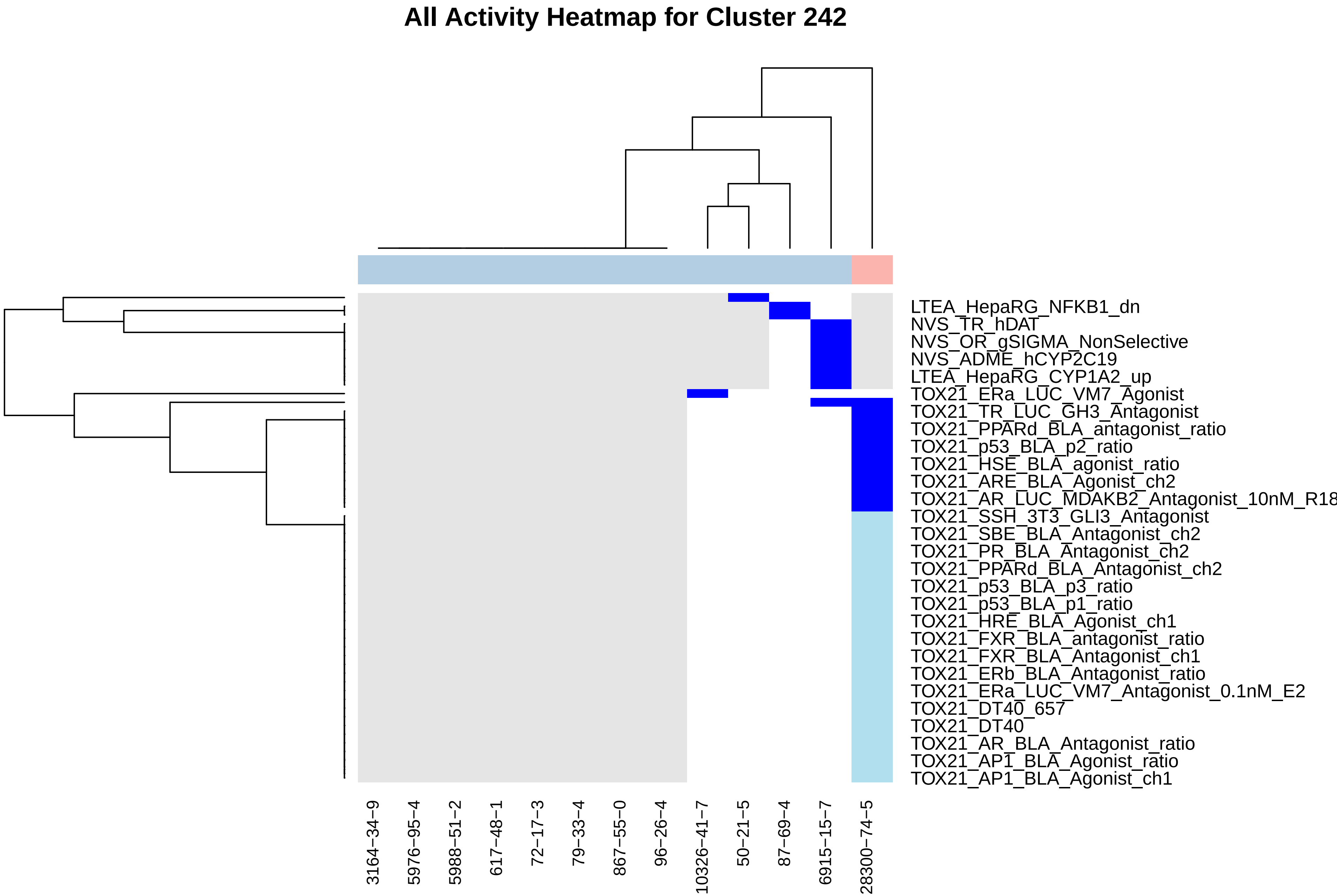
All Activity Heatmap for Cluster 242
LTEA_HepaRG_NFKB1_dn
NVS_TR_hDAT
NVS_OR_gSIGMA_NonSelective
NVS_ADME_hCYP2C19
LTEA_HepaRG_CYP1A2_up
TOX21_ERa_LUC_VM7_Agonist
TOX21_TR_LUC_GH3_Antagonist
TOX21_PPARd_BLA_antagonist_ratio
TOX21_p53_BLA_p2_ratio
TOX21_HSE_BLA_agonist_ratio
TOX21_ARE_BLA_Agonist_ch2
TOX21_AR_LUC_MDAKB2_Antagonist_10nM_R18
TOX21_SSH_3T3_GLI3_Antagonist
TOX21_SBE_BLA_Antagonist_ch2
TOX21_PR_BLA_Antagonist_ch2
TOX21_PPARd_BLA_Antagonist_ch2
TOX21_p53_BLA_p3_ratio
TOX21_p53_BLA_p1_ratio
TOX21_HRE_BLA_Agonist_ch1
TOX21_FXR_BLA_antagonist_ratio
TOX21_FXR_BLA_Antagonist_ch1
TOX21_ERb_BLA_Antagonist_ratio
TOX21_ERa_LUC_VM7_Antagonist_0.1nM_E2
TOX21_DT40_657
TOX21_DT40
TOX21_AR_BLA_Antagonist_ratio
TOX21_AP1_BLA_Agonist_ratio
TOX21_AP1_BLA_Agonist_ch1
3164−34−9
5976−95−4
5988−51−2
617−48−1
72−17−3
79−33−4
867−55−0
96−26−4
10326−41−7
50−21−5
87−69−4
6915−15−7
28300−74−5

All Activity Heatmap for Cluster 243

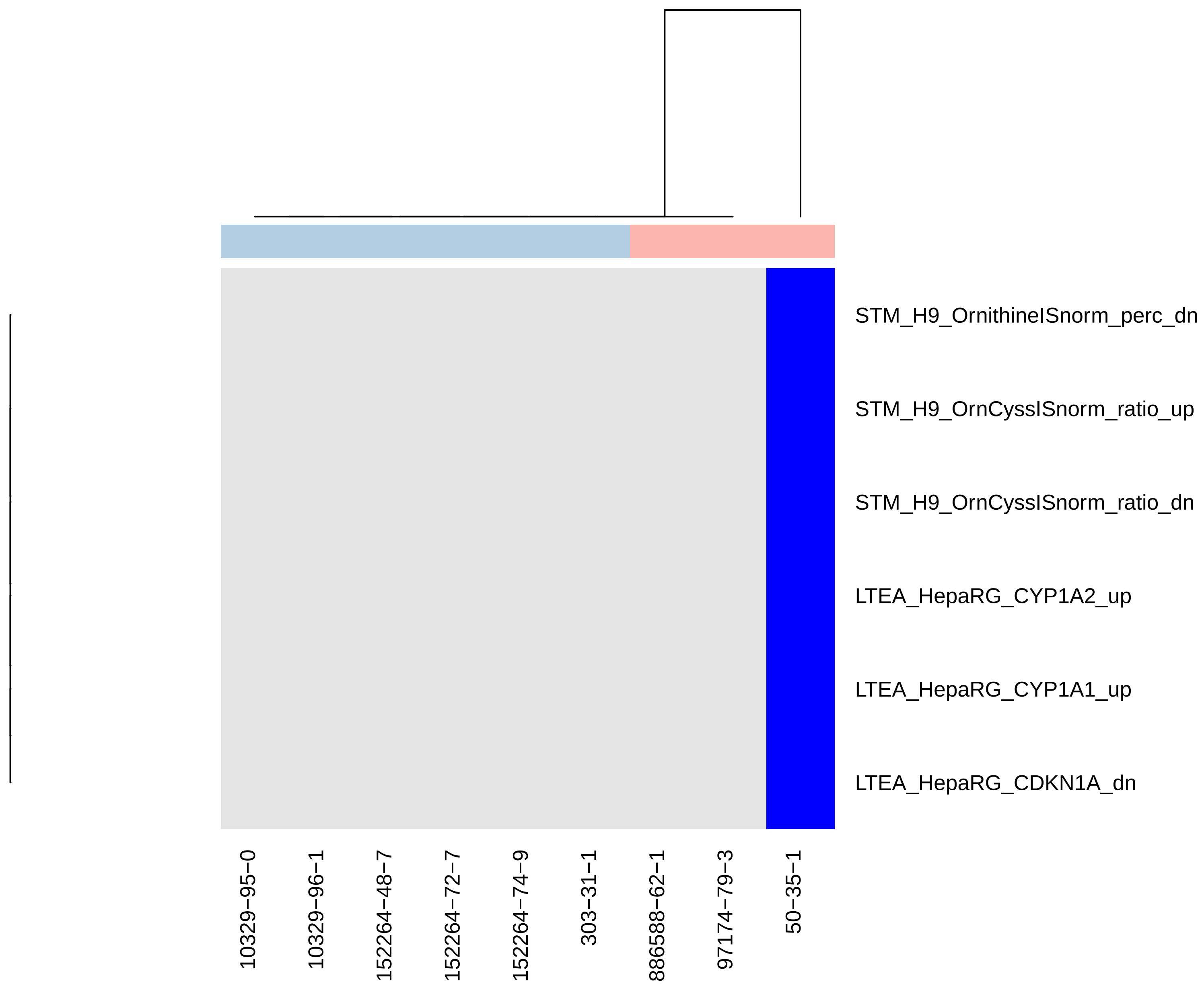

All Activity Heatmap for Cluster 244

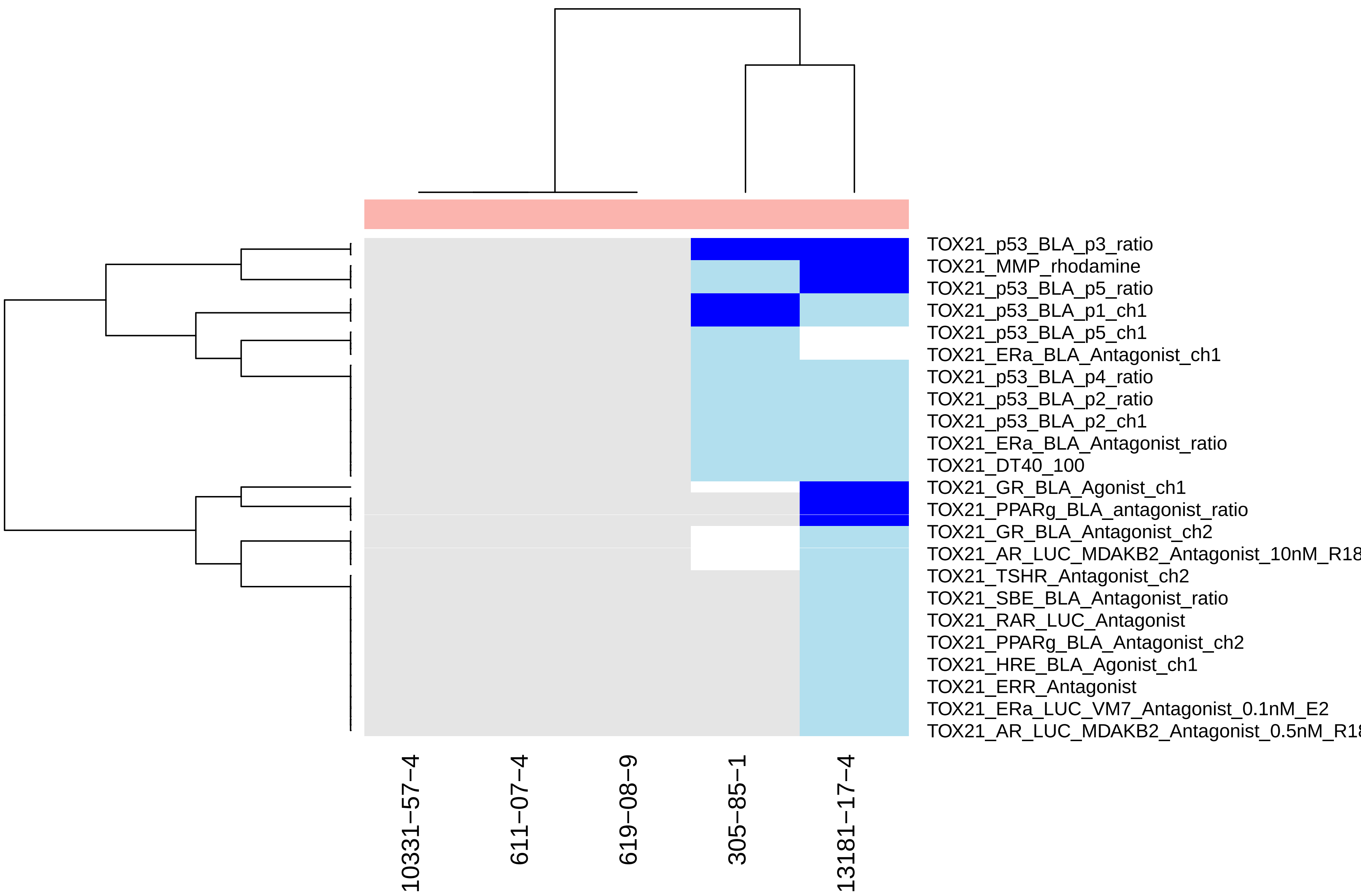

All Activity Heatmap for Cluster 245

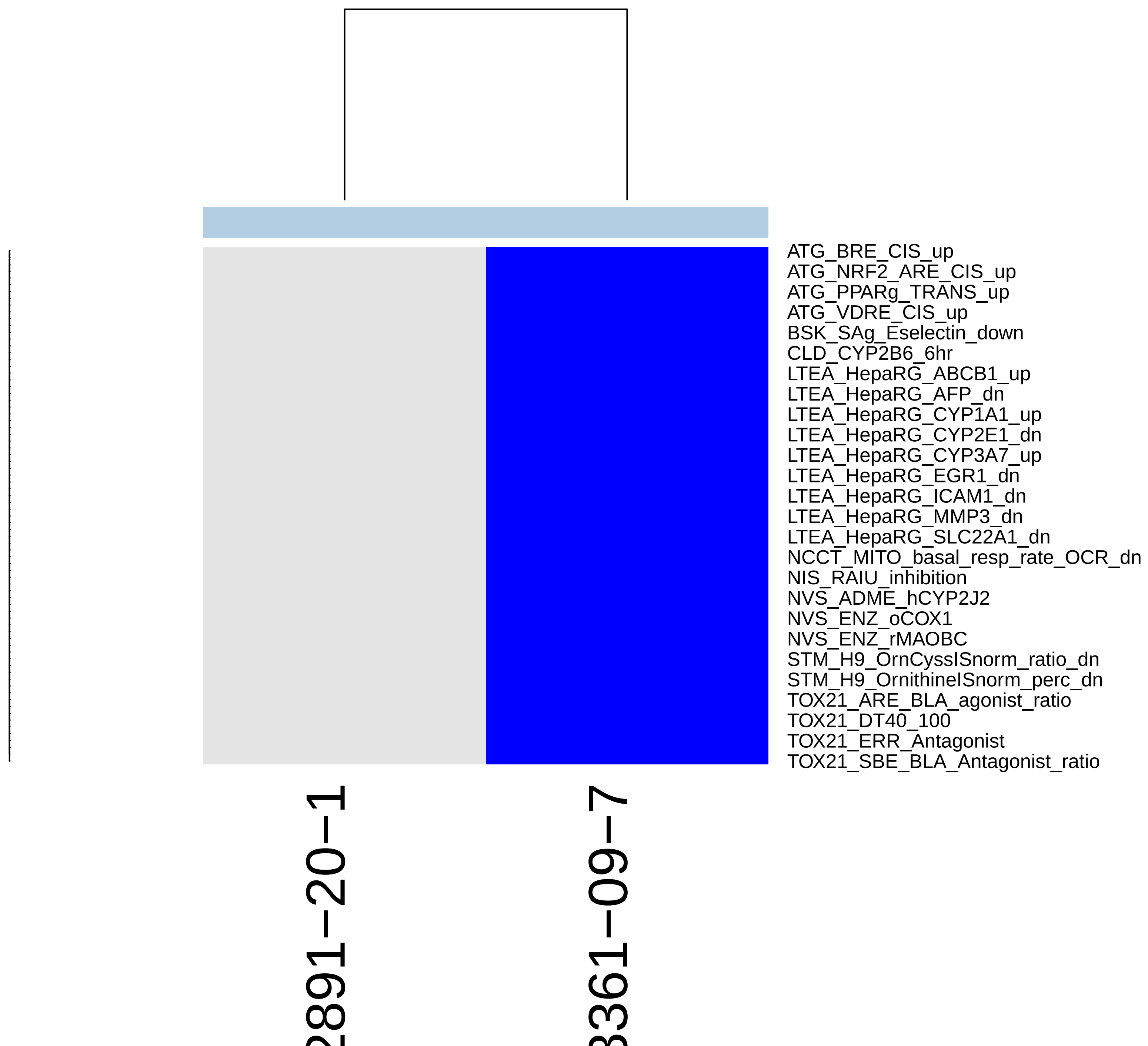

All Activity Heatmap for Cluster 246

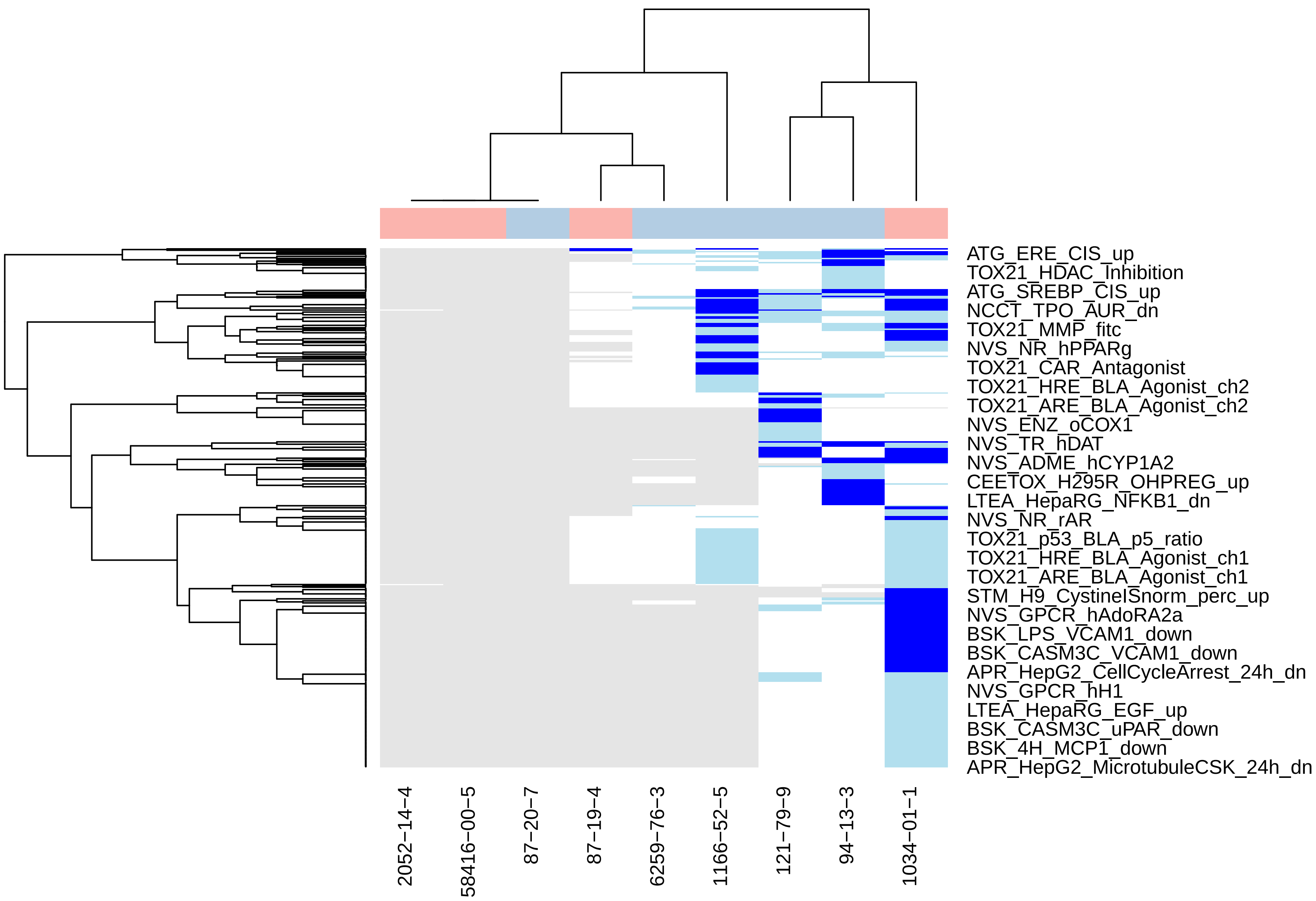

All Activity Heatmap for Cluster 247

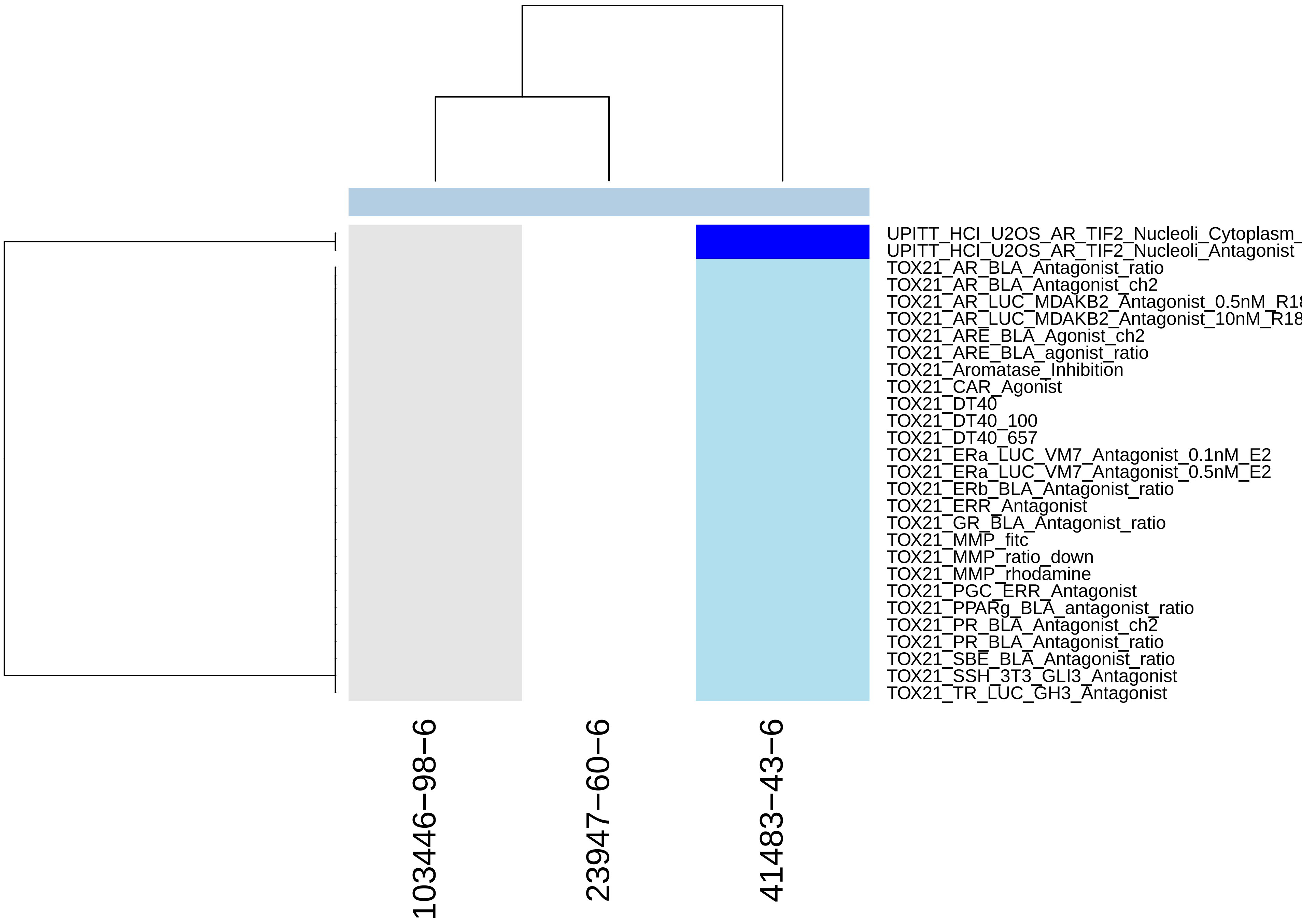

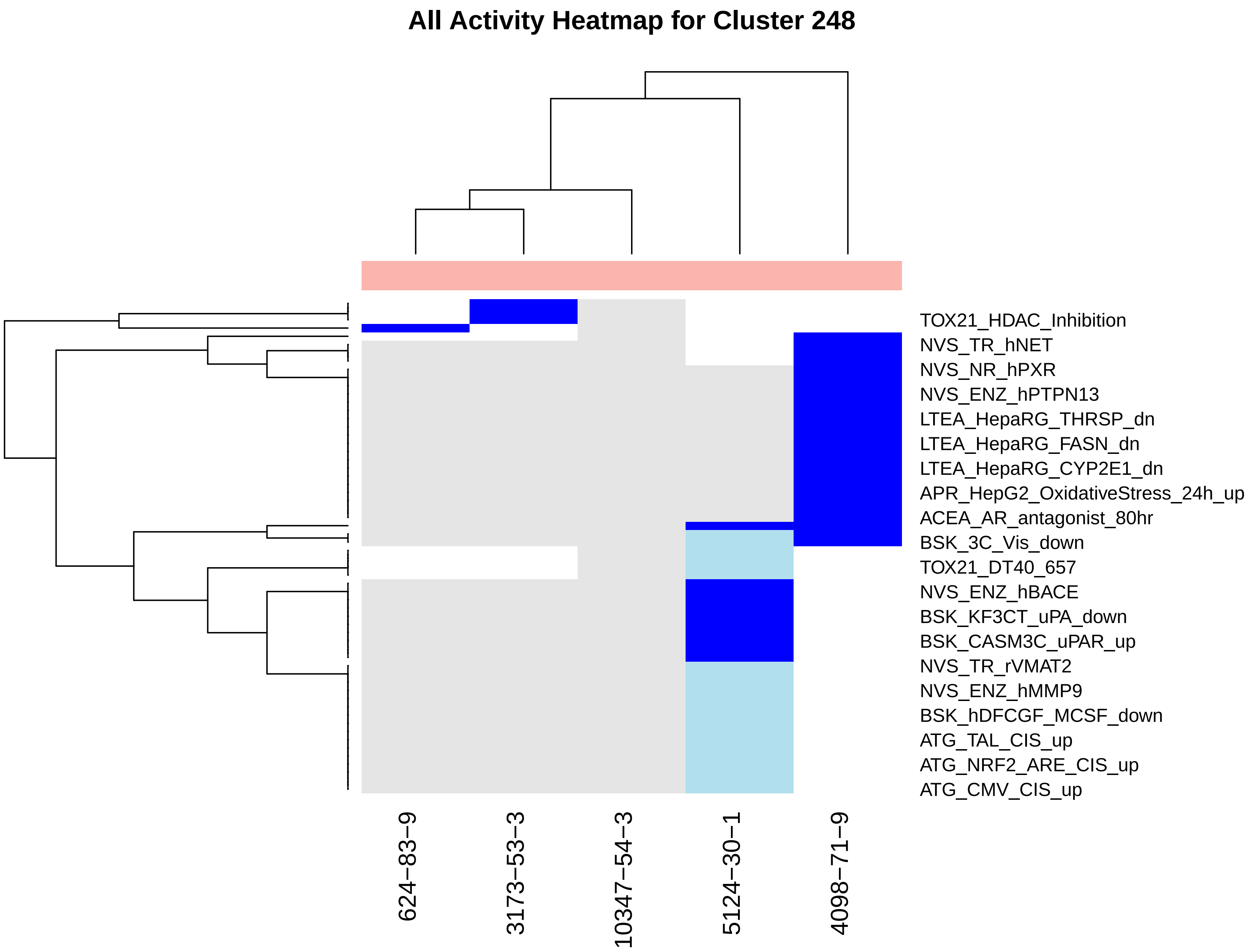
All Activity Heatmap for Cluster 248
TOX21_HDAC_Inhibition
NVS_TR_hNET
NVS_NR_hPXR
NVS_ENZ_hPTPN13
LTEA_HepaRG_THRSP_dn
LTEA_HepaRG_FASN_dn
LTEA_HepaRG_CYP2E1_dn
APR_HepG2_OxidativeStress_24h_up
ACEA_AR_antagonist_80hr
BSK_3C_Vis_down
TOX21_DT40_657
NVS_ENZ_hBACE
BSK_KF3CT_uPA_down
BSK_CASM3C_uPAR_up
NVS_TR_rVMAT2
NVS_ENZ_hMMP9
BSK_hDFCGF_MCSF_down
ATG_TAL_CIS_up
ATG_NRF2_ARE_CIS_up
ATG_CMV_CIS_up
624−83−9
3173−53−3
10347−54−3
5124−30−1
4098−71−9

All Activity Heatmap for Cluster 249

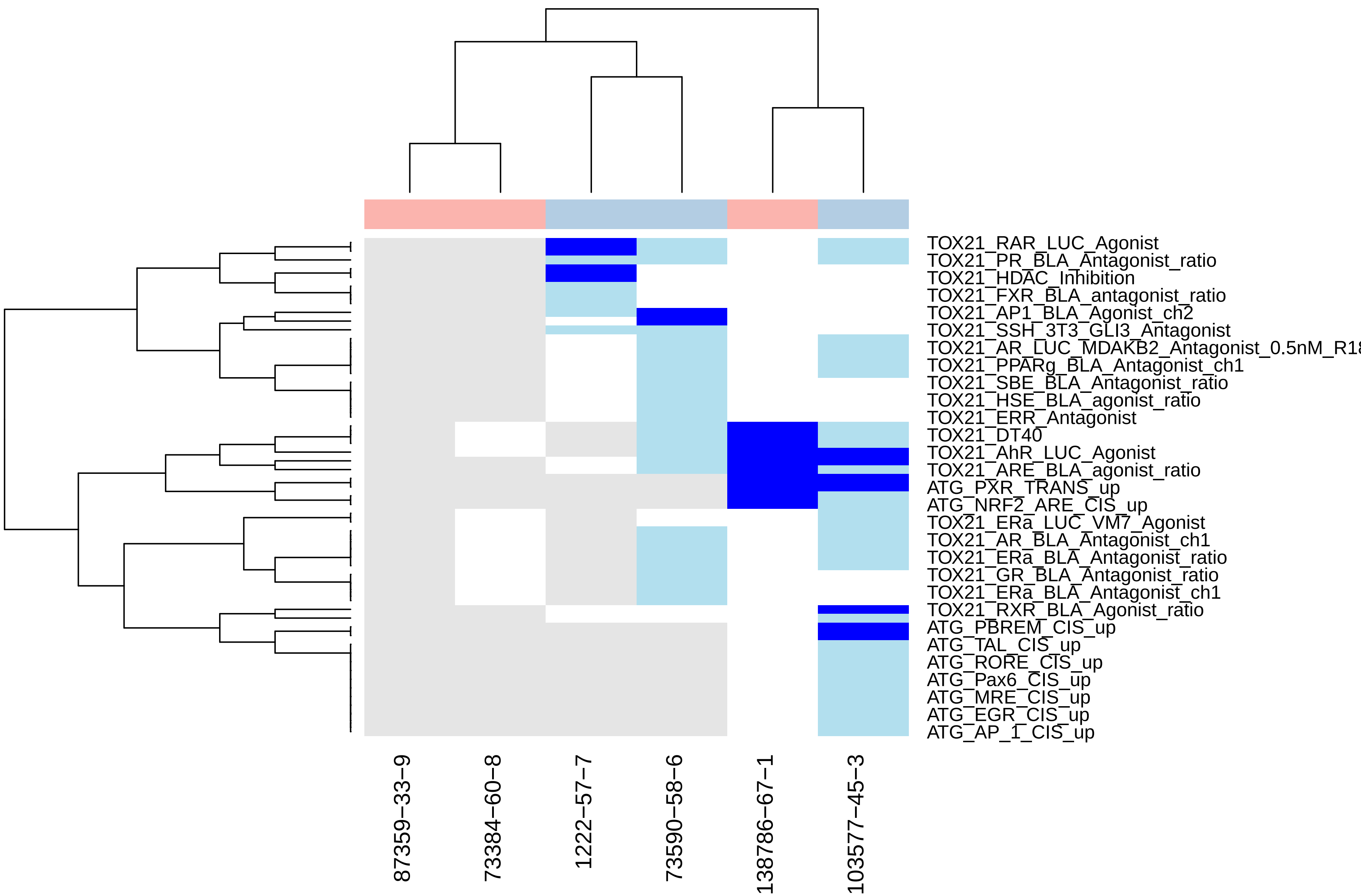

All Activity Heatmap for Cluster 251

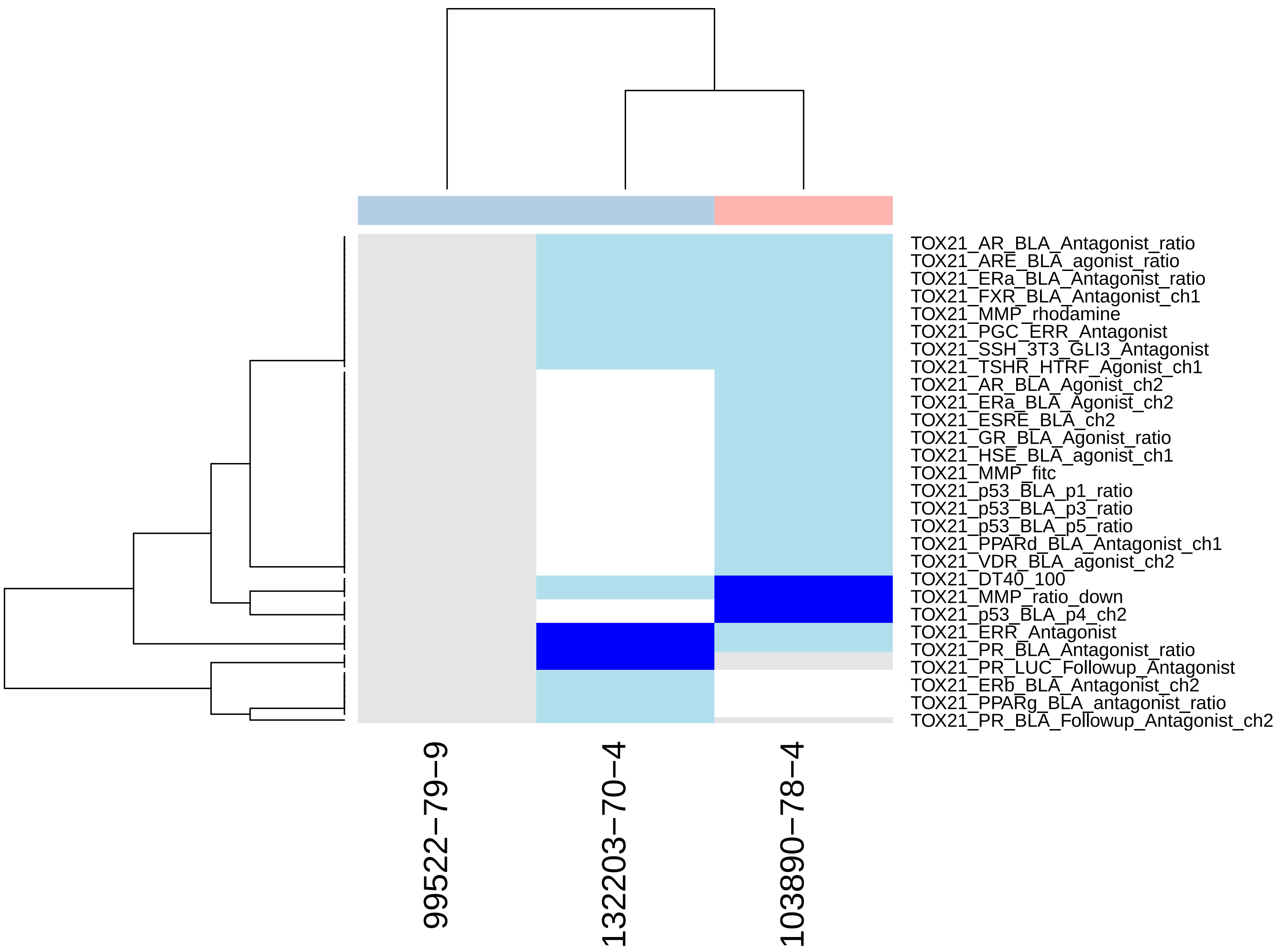

All Activity Heatmap for Cluster 252

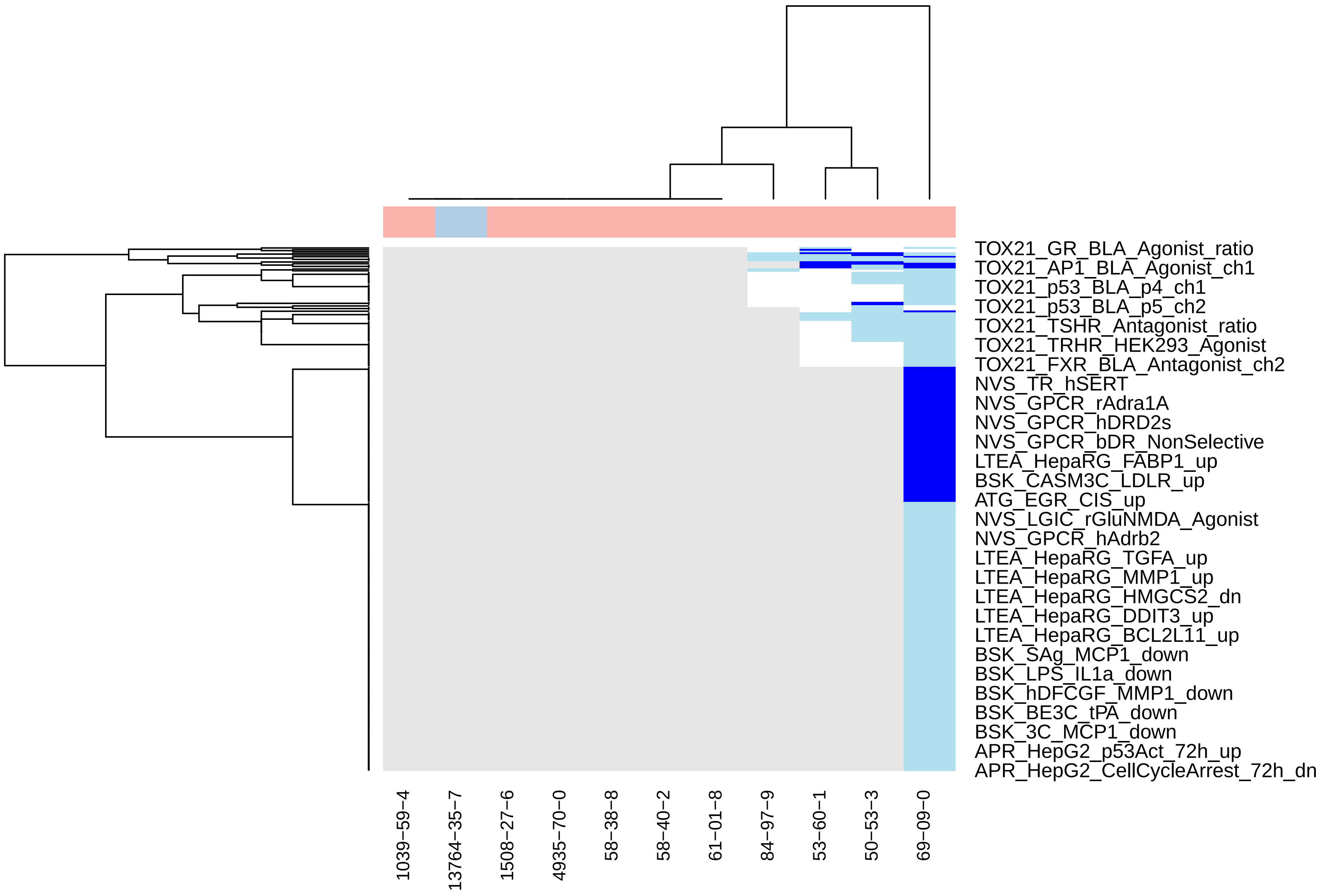

All Activity Heatmap for Cluster 253

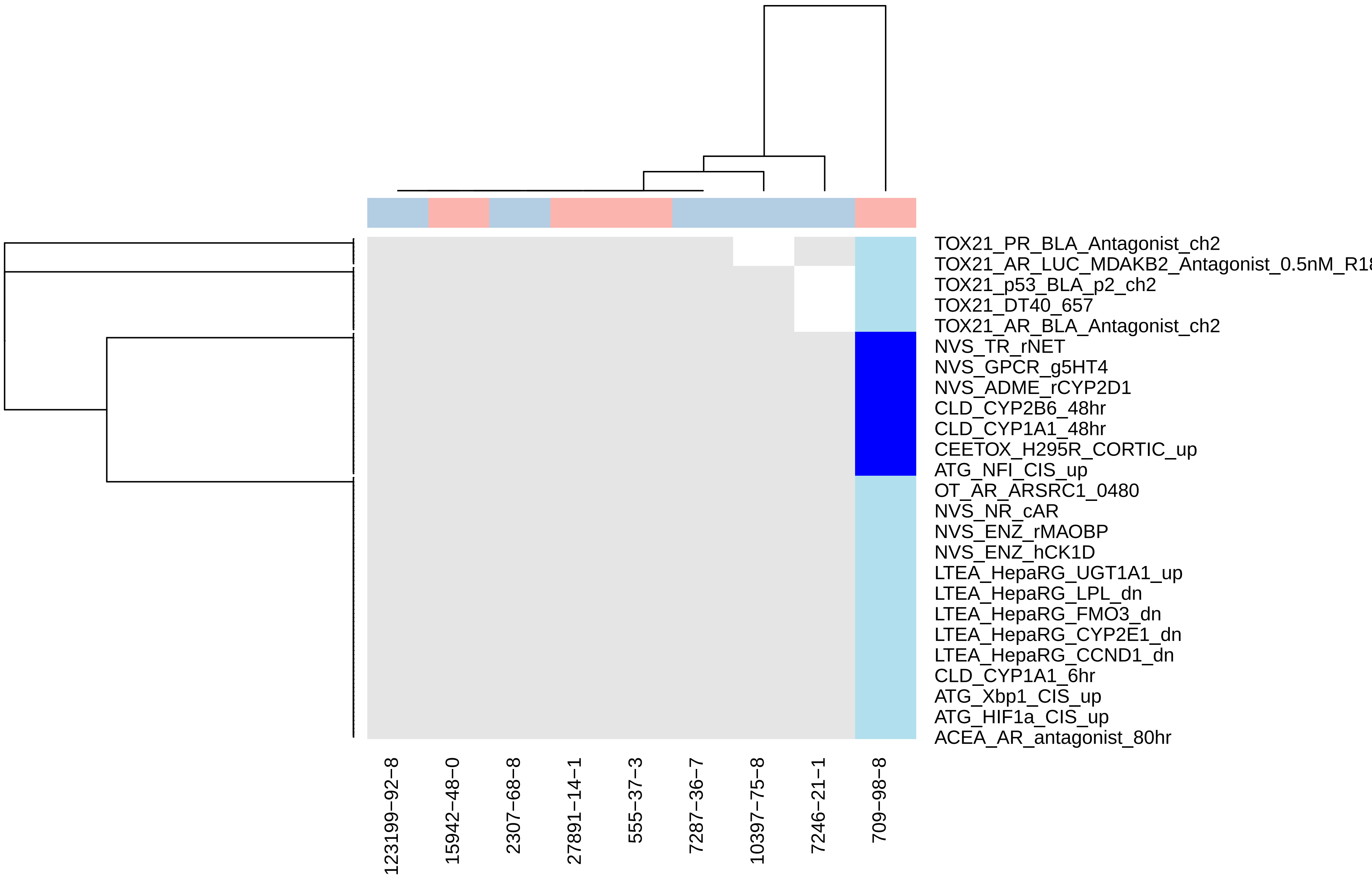

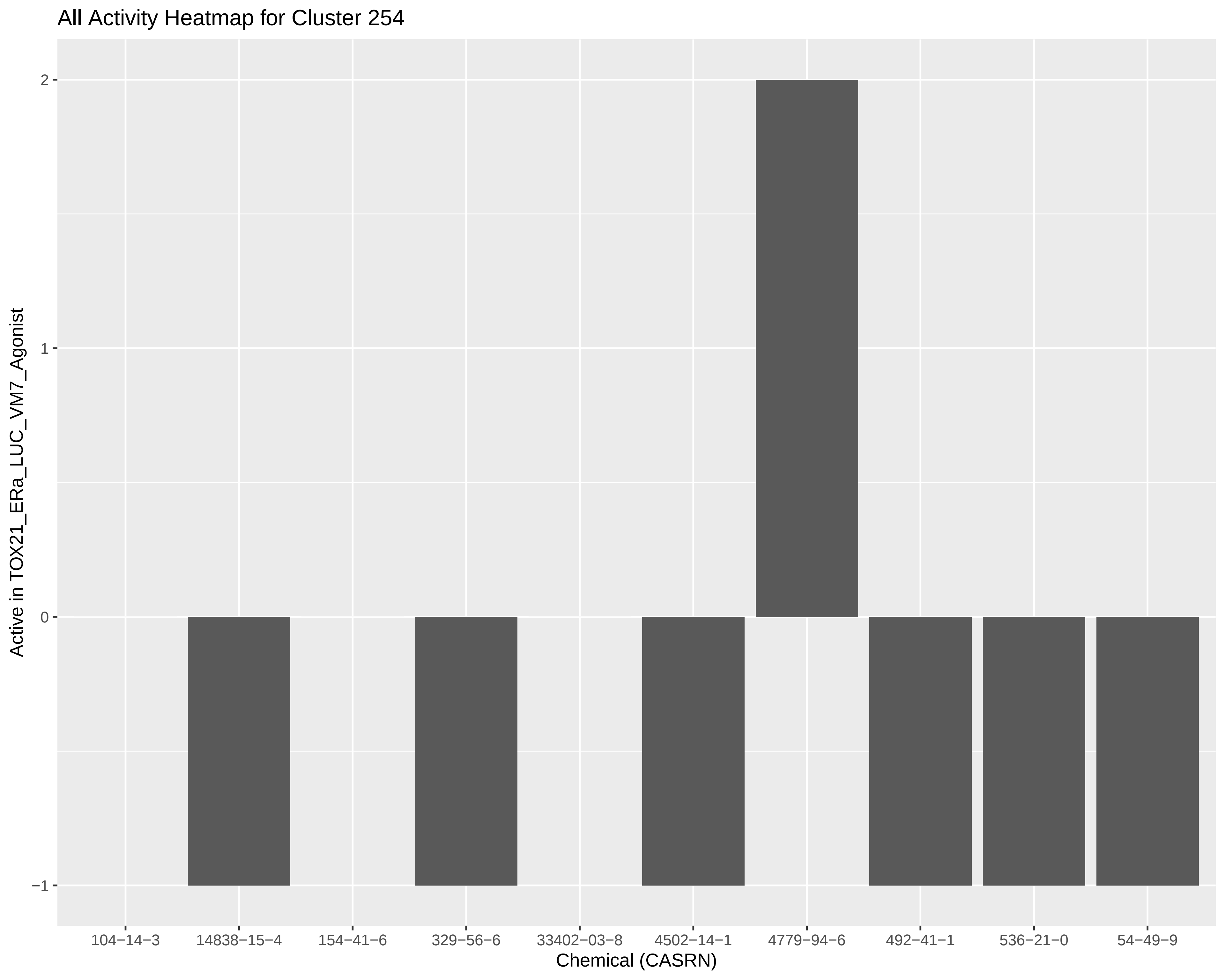

All Activity Heatmap for Cluster 254
Active in TOX21_ERa_LUC_VM7_Agonist
2
1
0
−1
104−14−3
14838−15−4
154−41−6
329−56−6
33402−03−8
4502−14−1
4779−94−6
492−41−1
536−21−0
54−49−9
Chemical (CASRN)

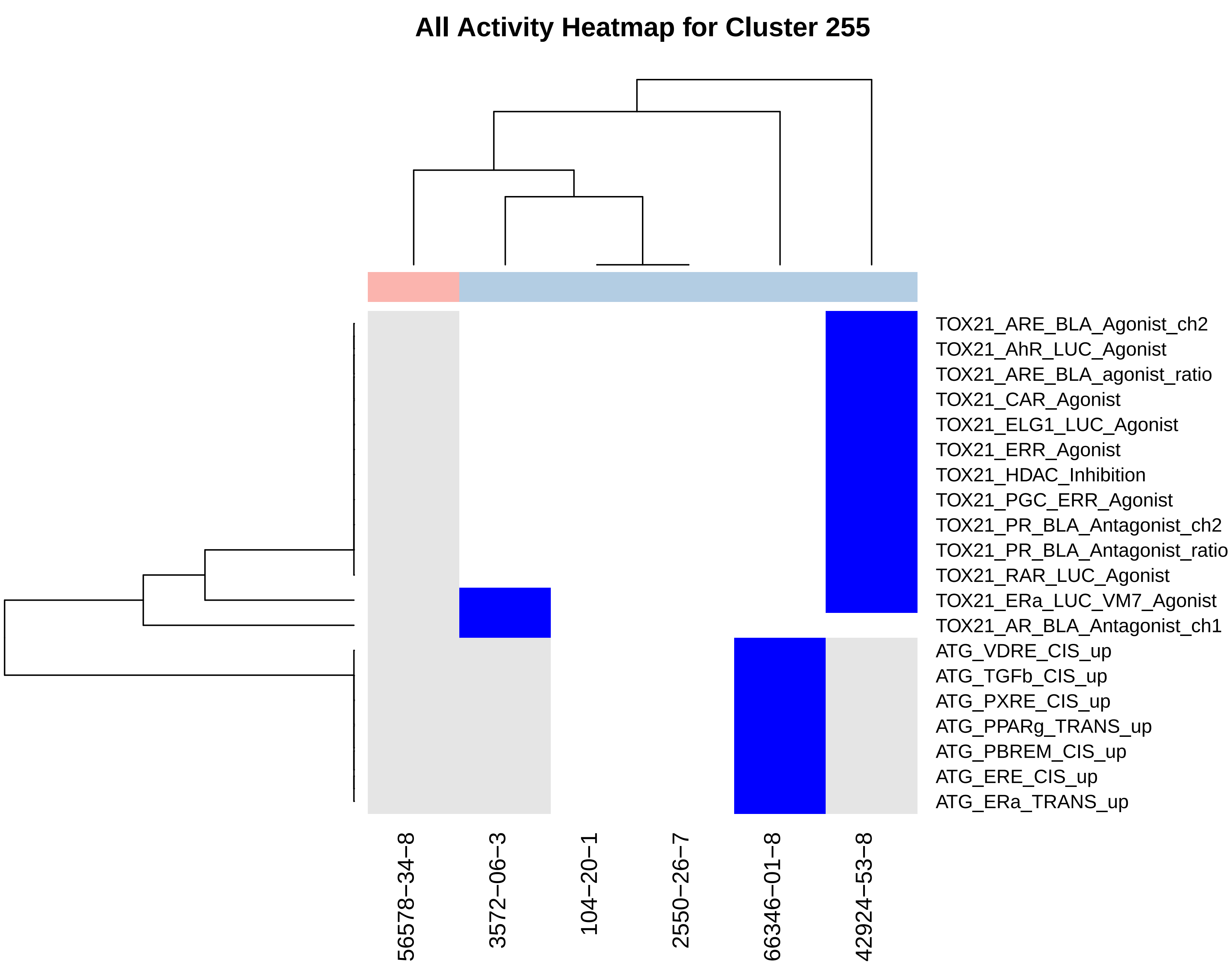

All Activity Heatmap for Cluster 255
TOX21_ARE_BLA_Agonist_ch2
TOX21_AhR_LUC_Agonist
TOX21_ARE_BLA_agonist_ratio
TOX21_CAR_Agonist
TOX21_ELG1_LUC_Agonist
TOX21_ERR_Agonist
TOX21_HDAC_Inhibition
TOX21_PGC_ERR_Agonist
TOX21_PR_BLA_Antagonist_ch2
TOX21_PR_BLA_Antagonist_ratio
TOX21_RAR_LUC_Agonist
TOX21_ERa_LUC_VM7_Agonist
TOX21_AR_BLA_Antagonist_ch1
ATG_VDRE_CIS_up
ATG_TGFb_CIS_up
ATG_PXRE_CIS_up
ATG_PPARg_TRANS_up
ATG_PBREM_CIS_up
ATG_ERE_CIS_up
ATG_ERa_TRANS_up
56578−34−8
3572−06−3
104−20−1
2550−26−7
66346−01−8
42924−53−8

All Activity Heatmap for Cluster 256

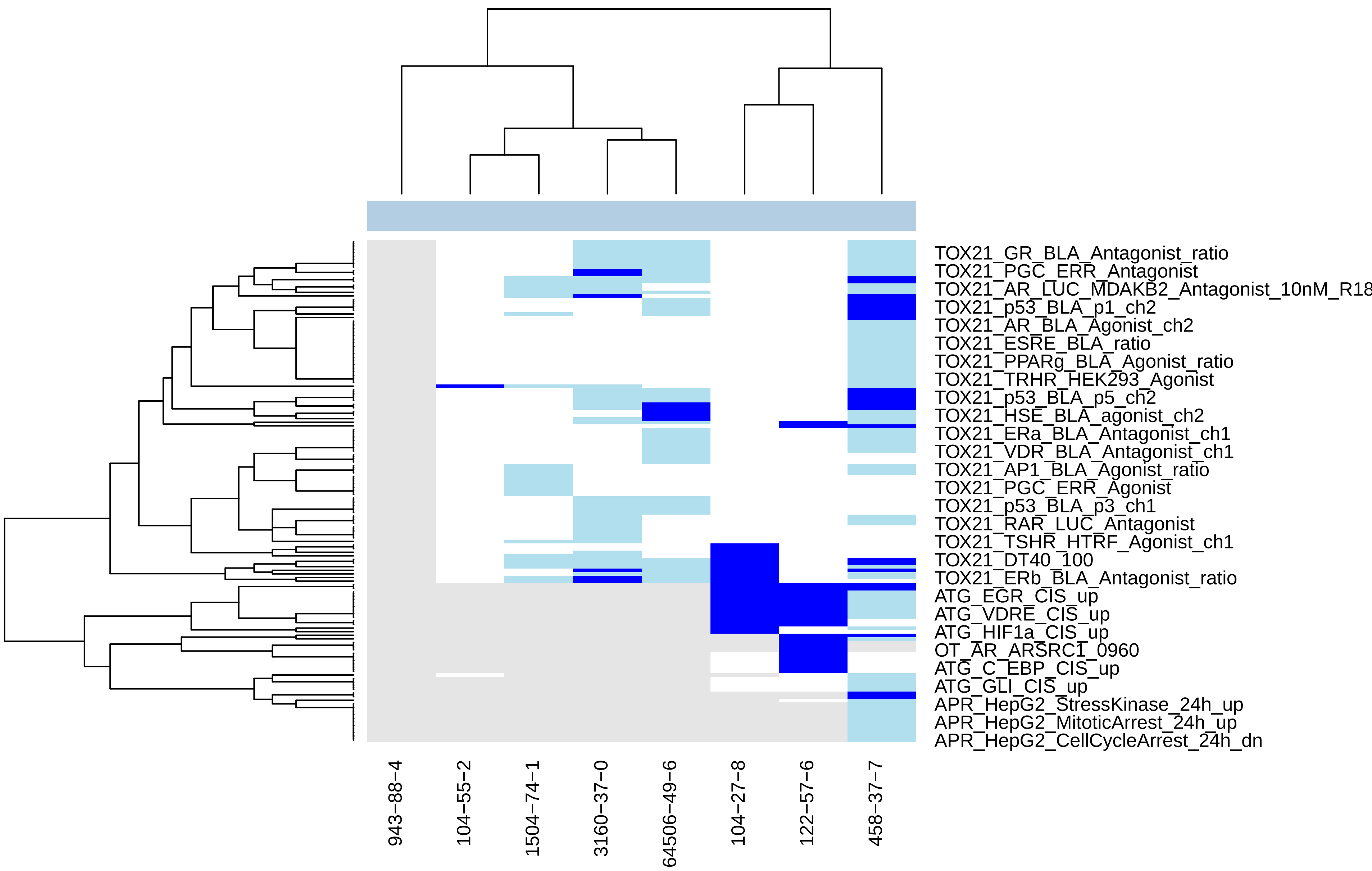

All Activity Heatmap for Cluster 257

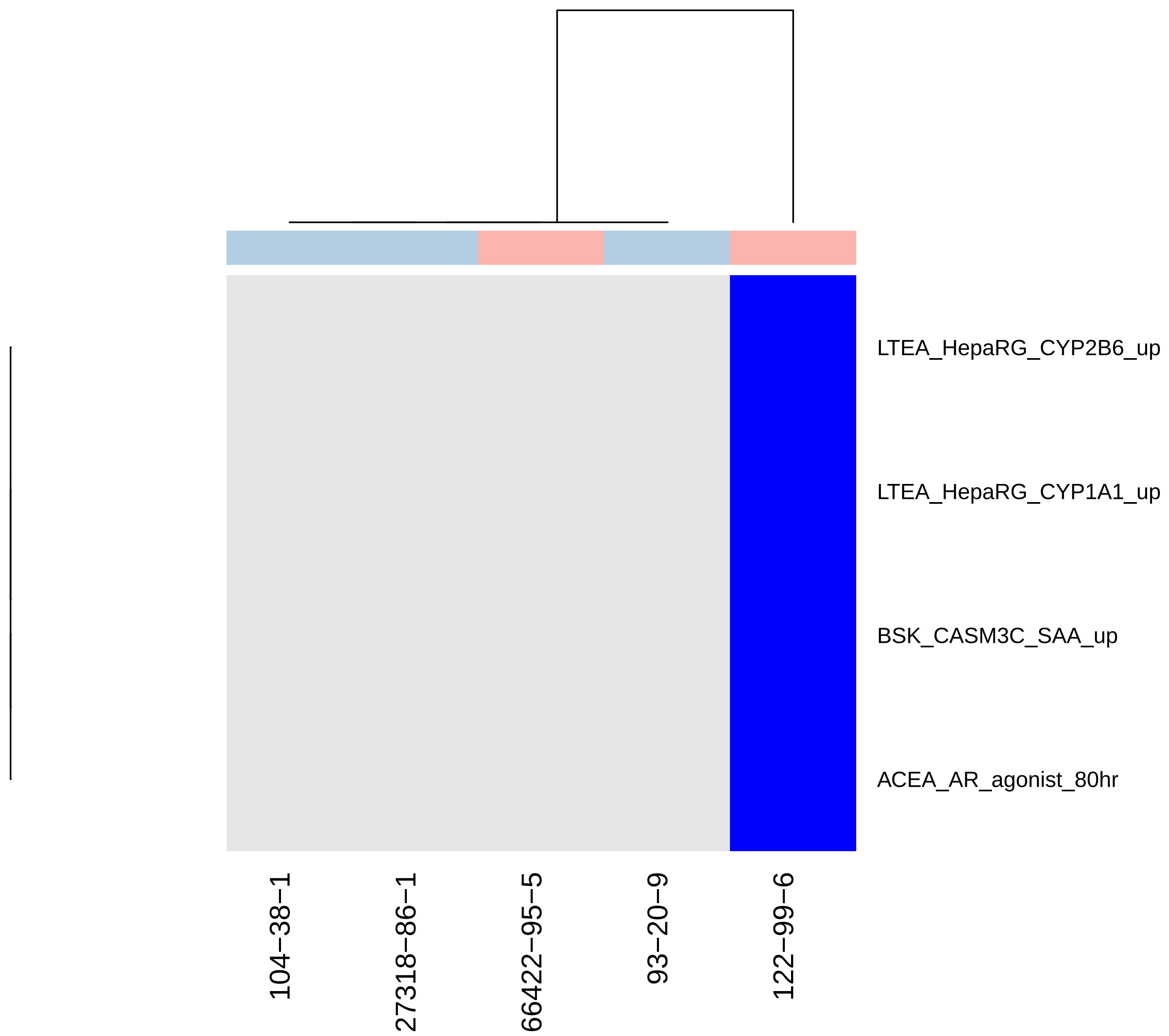

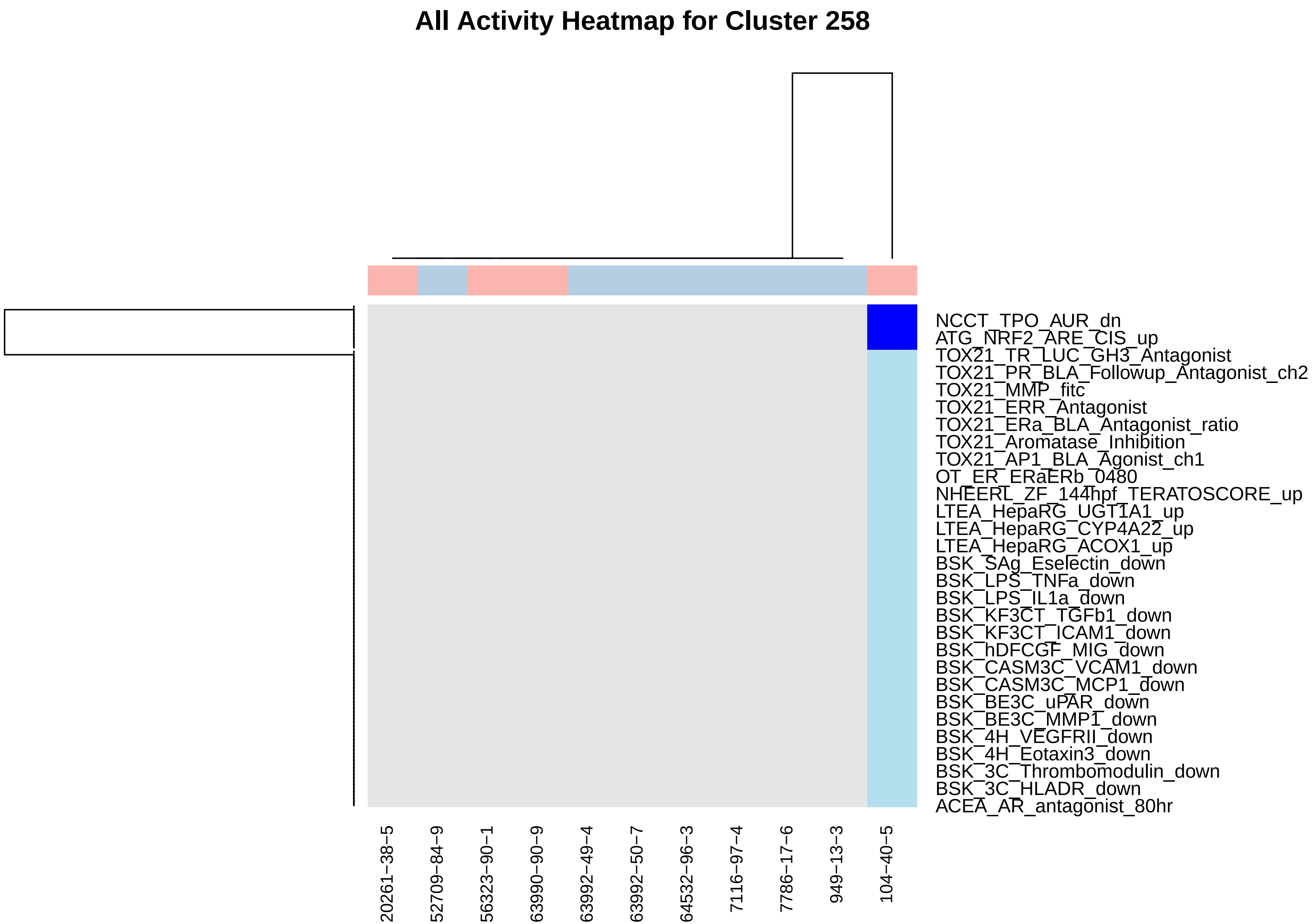
All Activity Heatmap for Cluster 258
NCCT_TPO_AUR_dn
ATG_NRF2_ARE_CIS_up
TOX21_TR_LUC_GH3_Antagonist
TOX21_PR_BLA_Followup_Antagonist_ch2
TOX21_MMP_fitc
TOX21_ERR_Antagonist
TOX21_ERa_BLA_Antagonist_ratio
TOX21_Aromatase_Inhibition
TOX21_AP1_BLA_Agonist_ch1
OT_ER_ERaERb_0480
NHEERL_ZF_144hpf_TERATOSCORE_up
LTEA_HepaRG_UGT1A1_up
LTEA_HepaRG_CYP4A22_up
LTEA_HepaRG_ACOX1_up
BSK_SAg_Eselectin_down
BSK_LPS_TNFa_down
BSK_LPS_IL1a_down
BSK_KF3CT_TGFb1_down
BSK_KF3CT_ICAM1_down
BSK_hDFCGF_MIG_down
BSK_CASM3C_VCAM1_down
BSK_CASM3C_MCP1_down
BSK_BE3C_uPAR_down
BSK_BE3C_MMP1_down
BSK_4H_VEGFRII_down
BSK_4H_Eotaxin3_down
BSK_3C_Thrombomodulin_down
BSK_3C_HLADR_down
ACEA_AR_antagonist_80hr
20261−38−5
52709−84−9
56323−90−1
63990−90−9
63992−49−4
63992−50−7
64532−96−3
7116−97−4
7786−17−6
949−13−3
104−40−5

All Activity Heatmap for Cluster 259

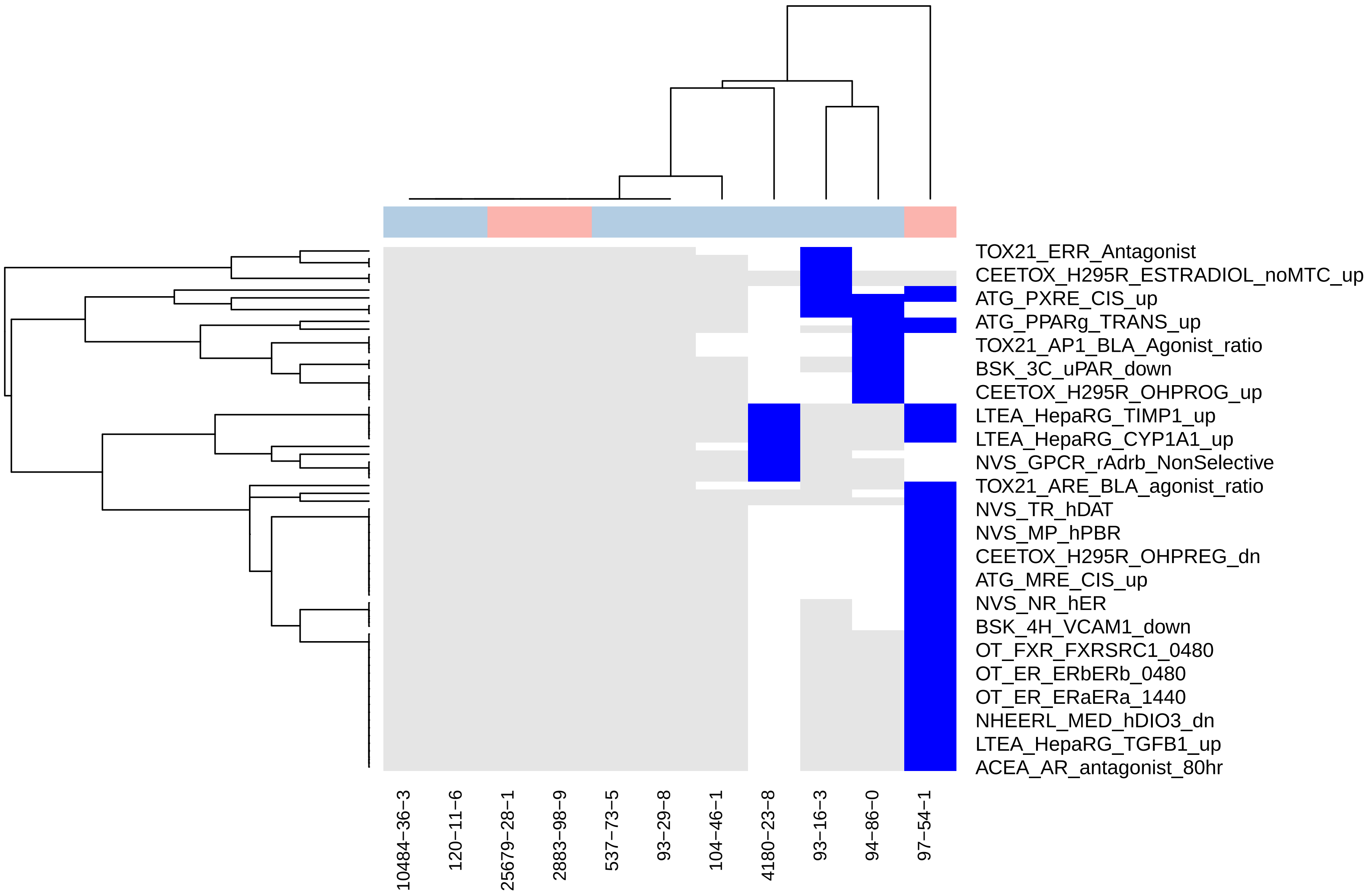

All Activity Heatmap for Cluster 260

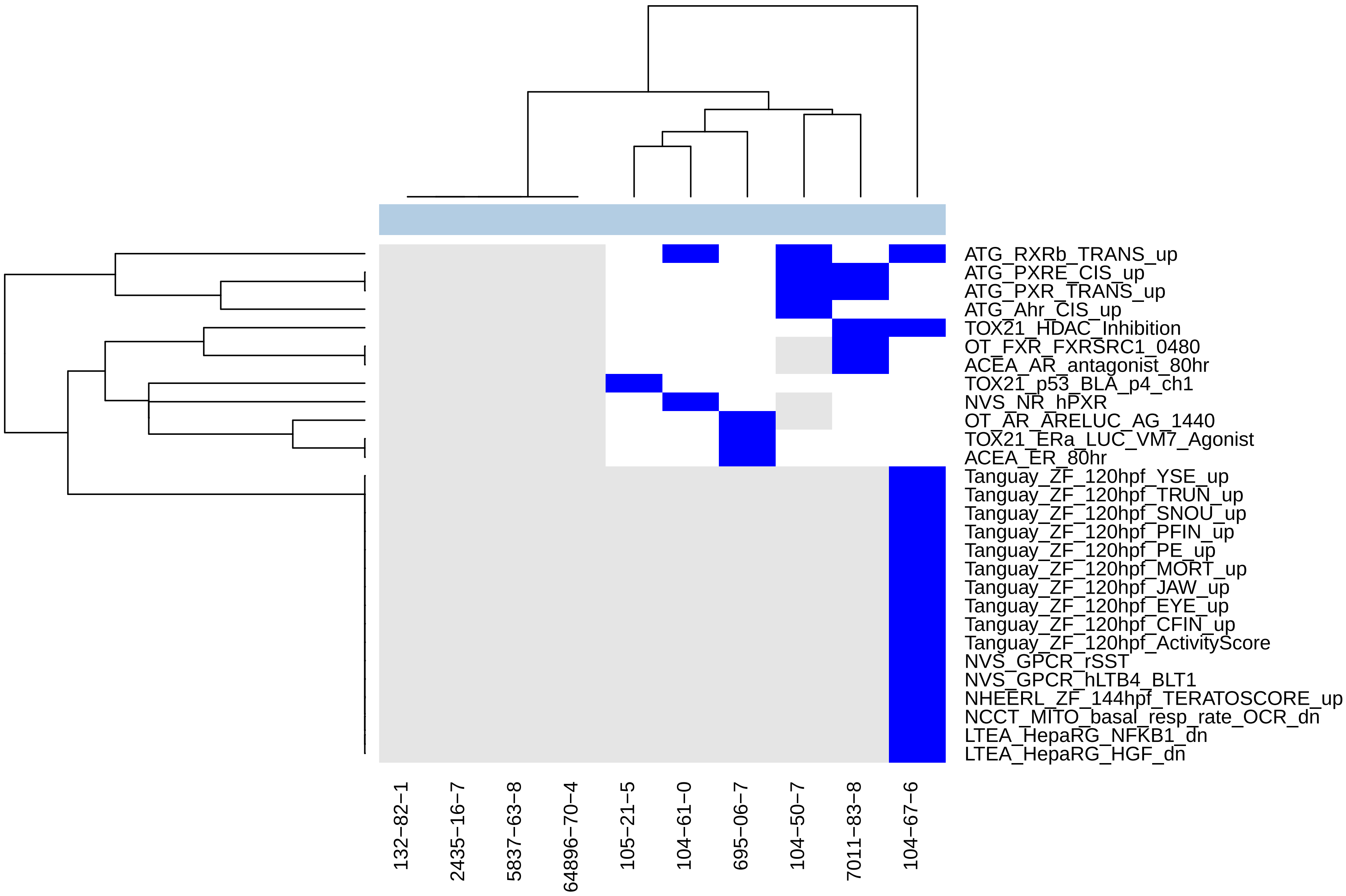

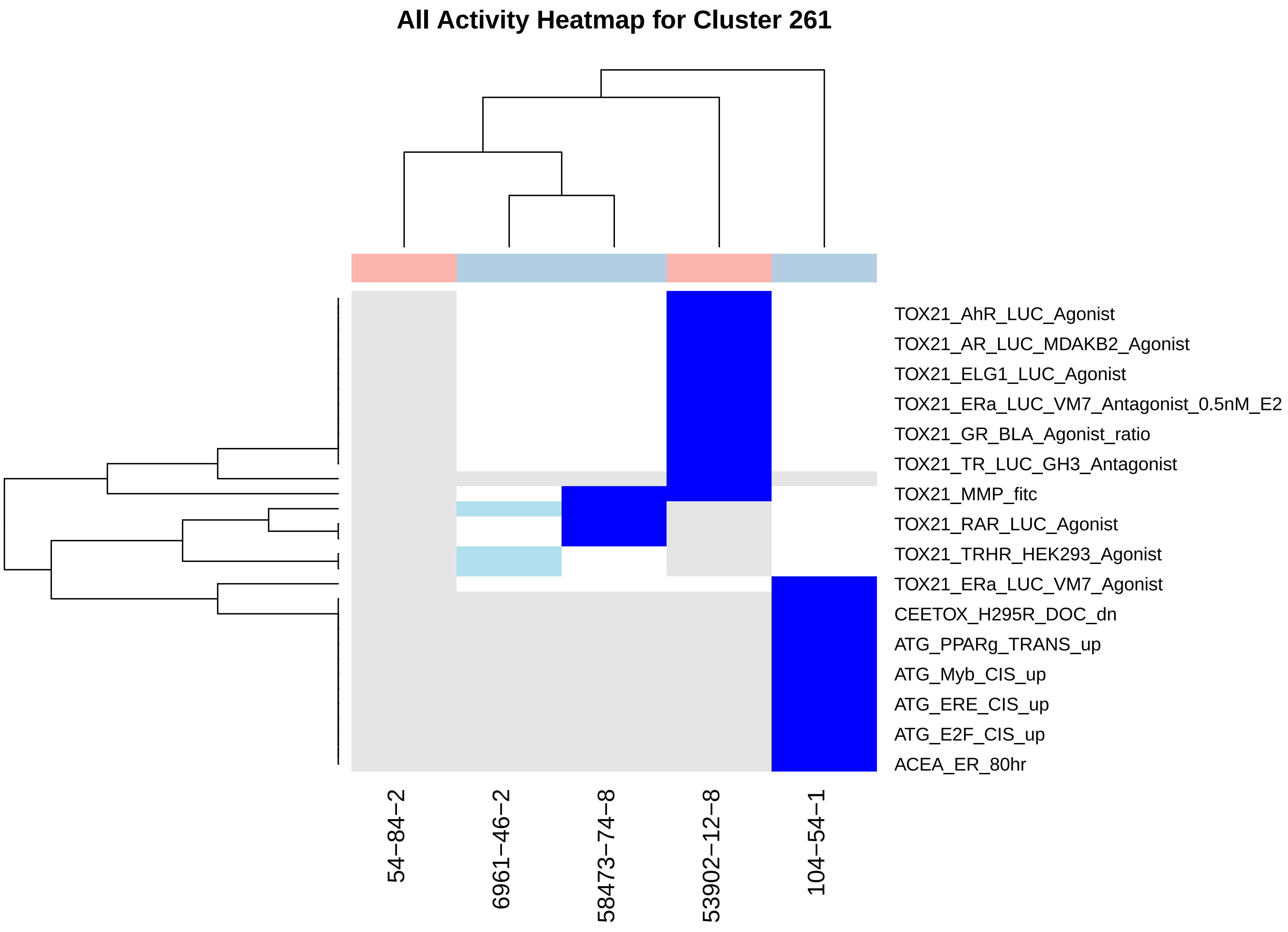
All Activity Heatmap for Cluster 261
TOX21_AhR_LUC_Agonist
TOX21_AR_LUC_MDAKB2_Agonist
TOX21_ELG1_LUC_Agonist
TOX21_ERa_LUC_VM7_Antagonist_0.5nM_E2
TOX21_GR_BLA_Agonist_ratio
TOX21_TR_LUC_GH3_Antagonist
TOX21_MMP_fitc
TOX21_RAR_LUC_Agonist
TOX21_TRHR_HEK293_Agonist
TOX21_ERa_LUC_VM7_Agonist
CEETOX_H295R_DOC_dn
ATG_PPARg_TRANS_up
ATG_Myb_CIS_up
ATG_ERE_CIS_up
ATG_E2F_CIS_up
ACEA_ER_80hr
54−84−2
6961−46−2
58473−74−8
53902−12−8
104−54−1

All Activity Heatmap for Cluster 262

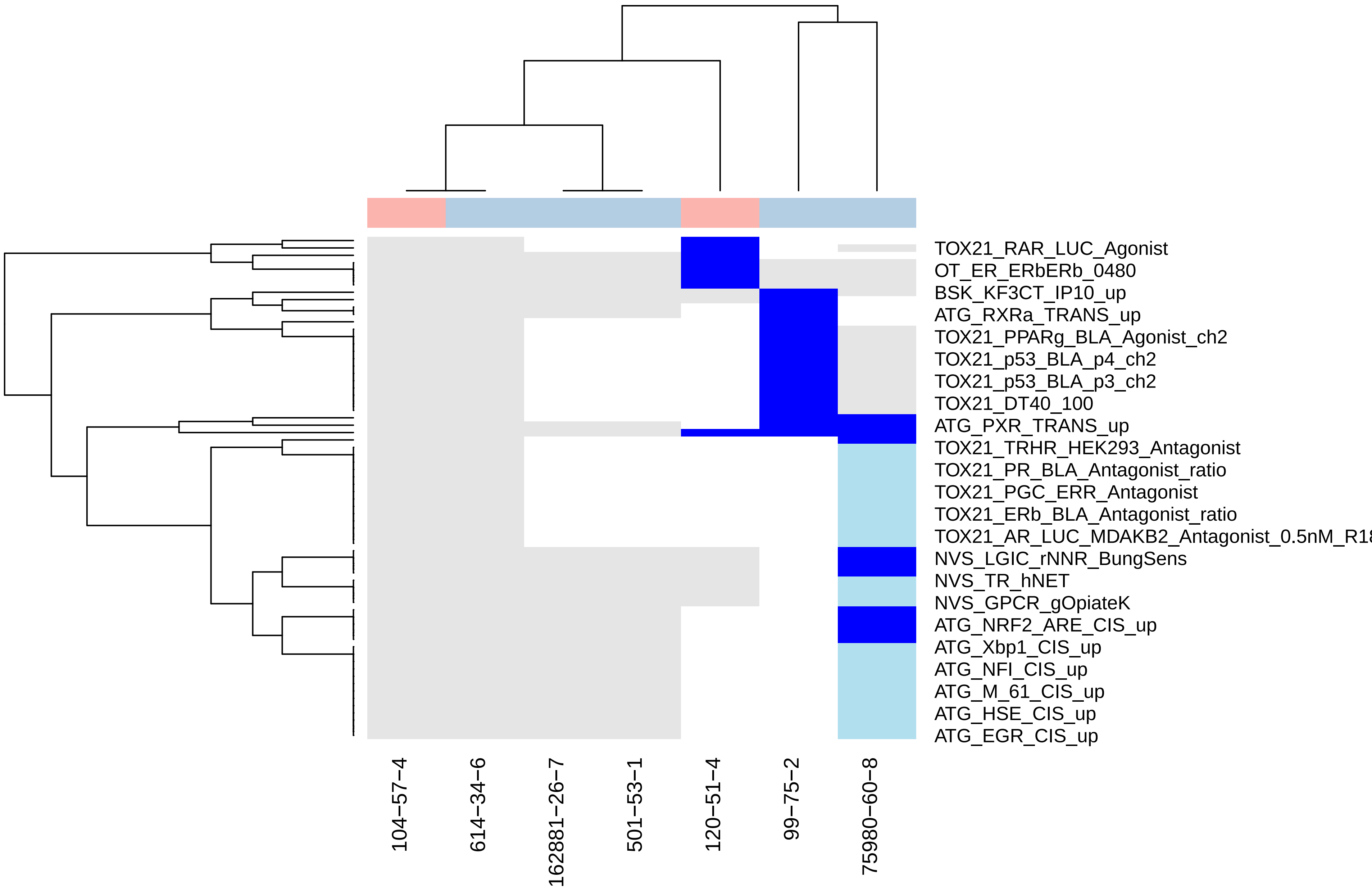

All Activity Heatmap for Cluster 263

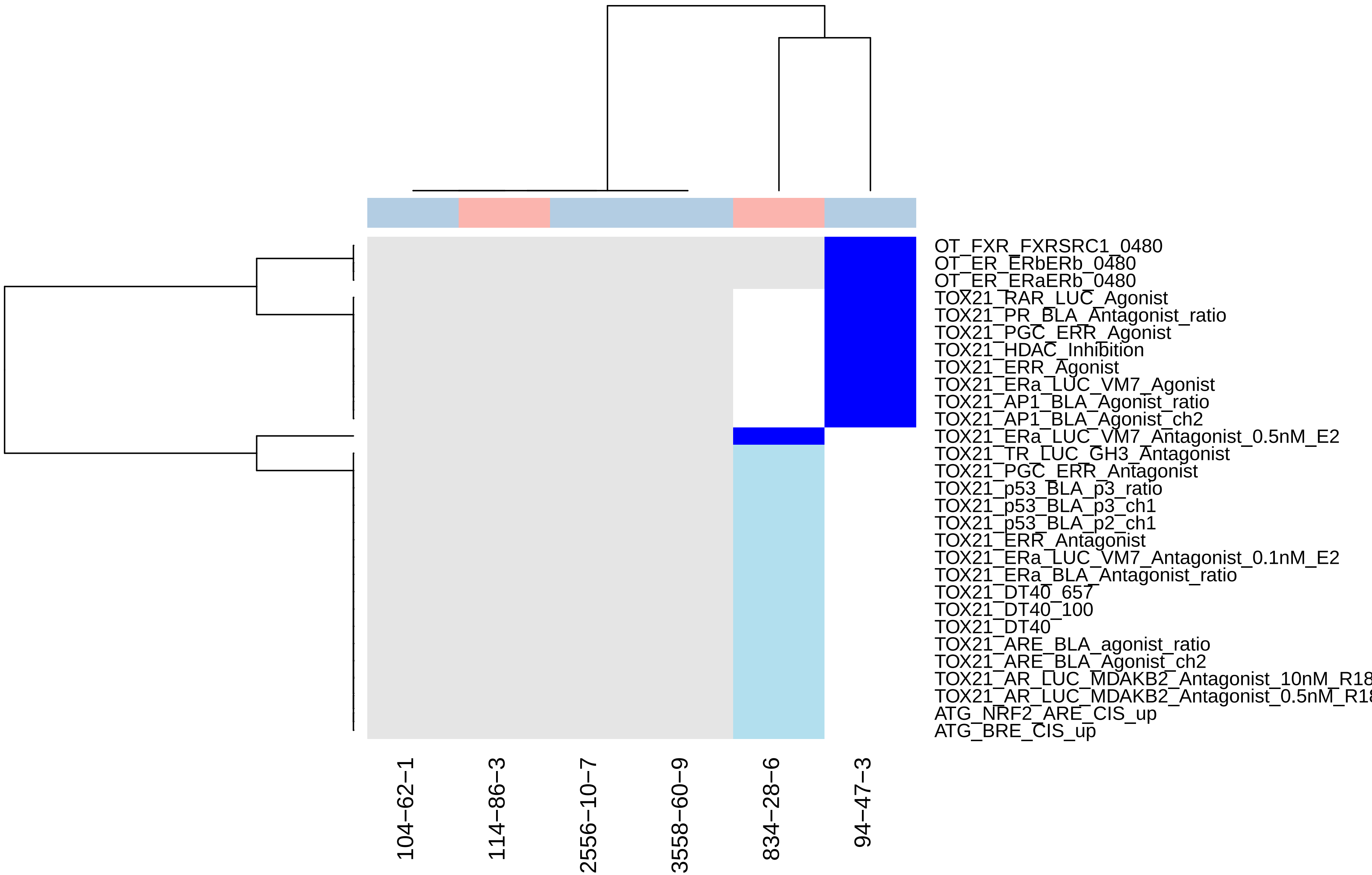

All Activity Heatmap for Cluster 264

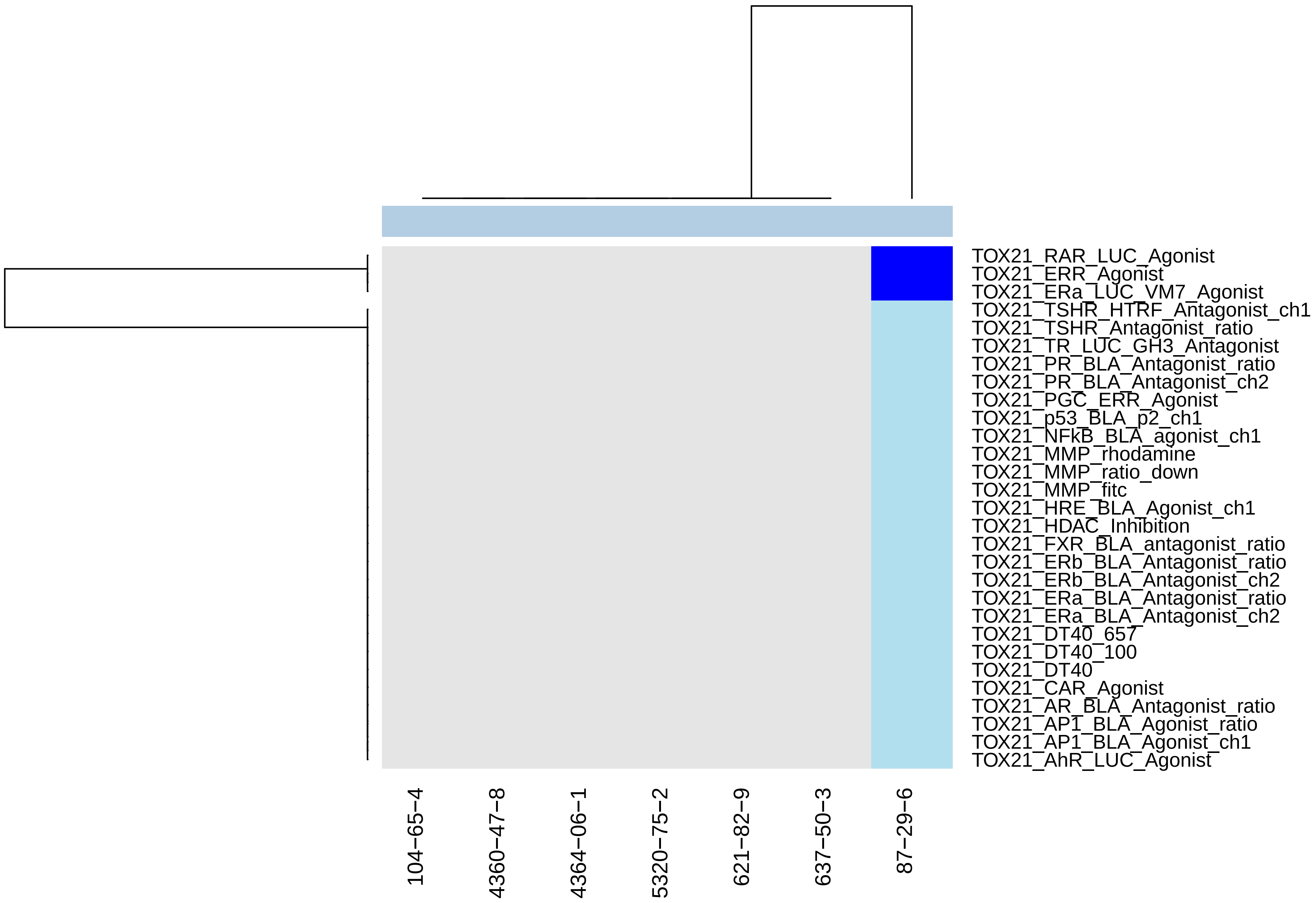

All Activity Heatmap for Cluster 265

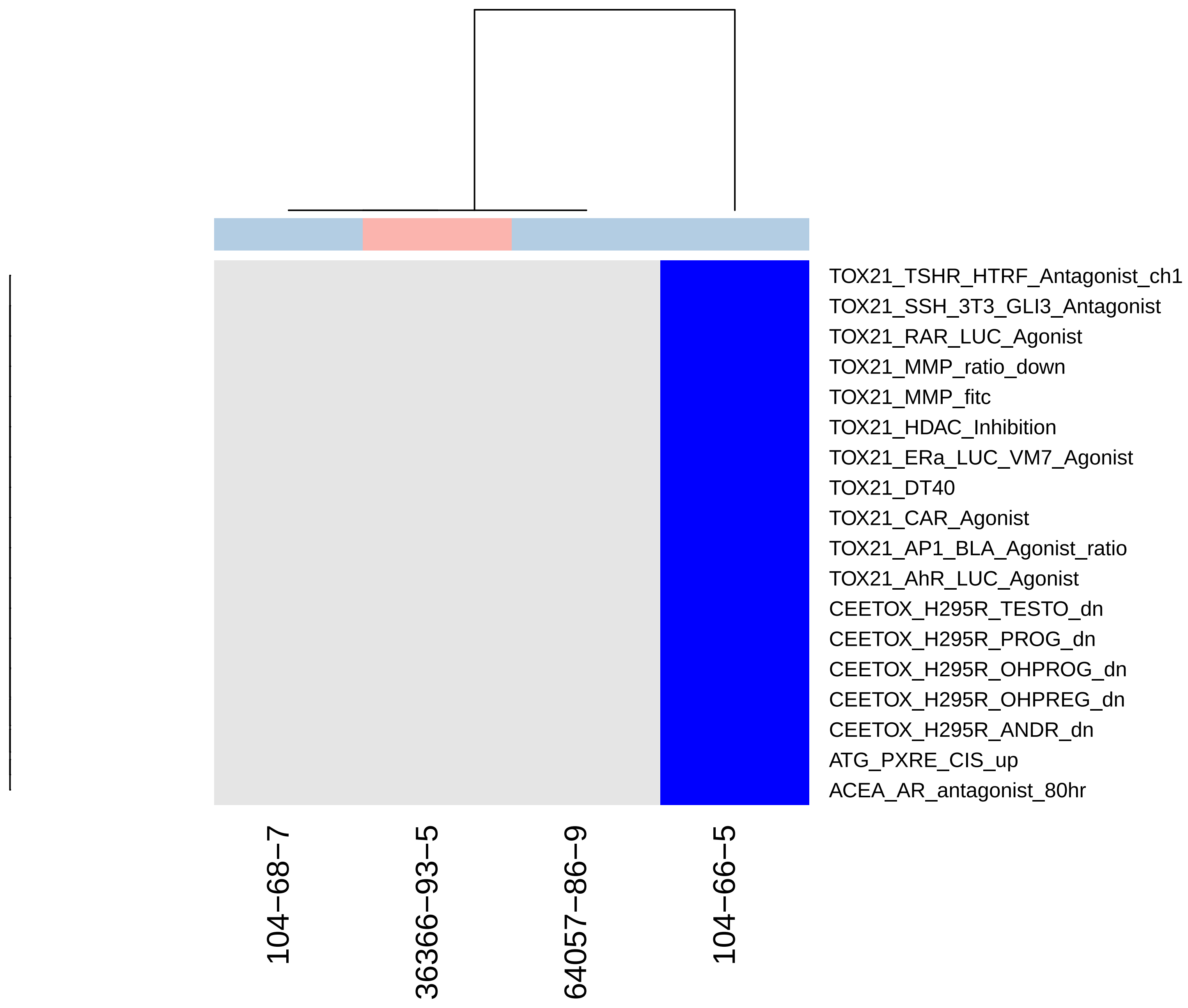

All Activity Heatmap for Cluster 266

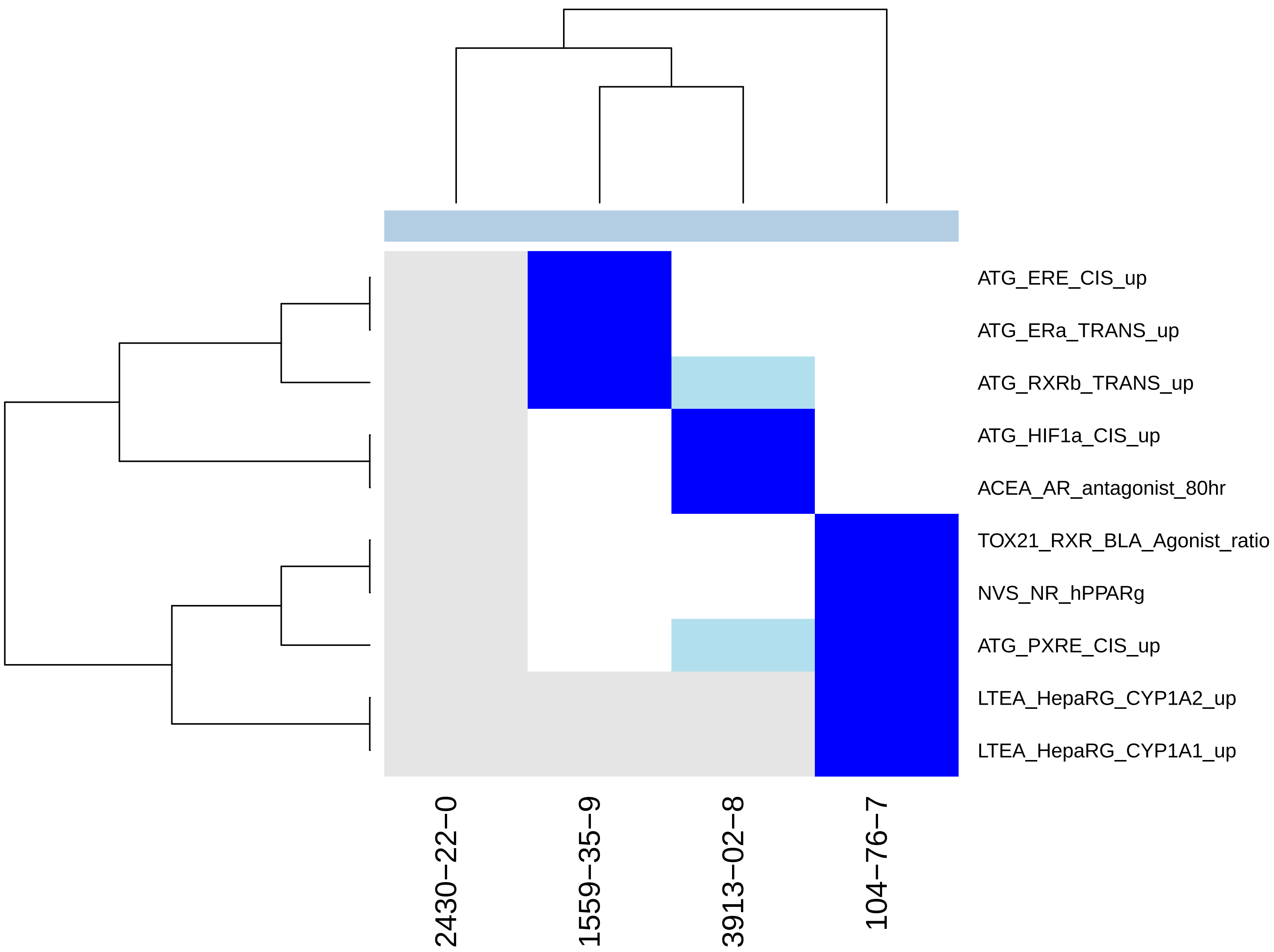

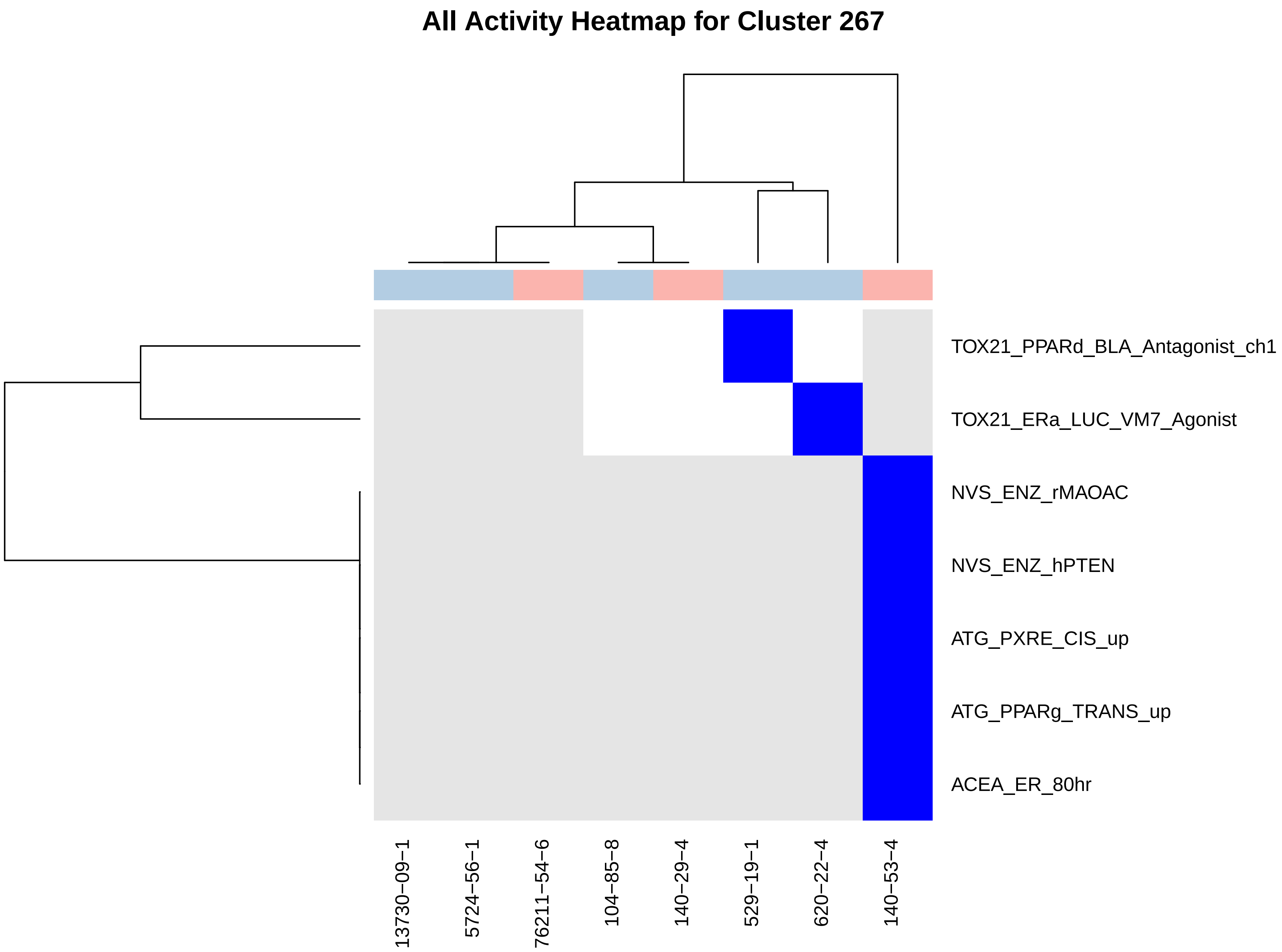
All Activity Heatmap for Cluster 267
TOX21_PPARd_BLA_Antagonist_ch1
TOX21_ERa_LUC_VM7_Agonist
NVS_ENZ_rMAOAC
NVS_ENZ_hPTEN
ATG_PXRE_CIS_up
ATG_PPARg_TRANS_up
ACEA_ER_80hr
13730−09−1
5724−56−1
76211−54−6
104−85−8
140−29−4
529−19−1
620−22−4
140−53−4

All Activity Heatmap for Cluster 268

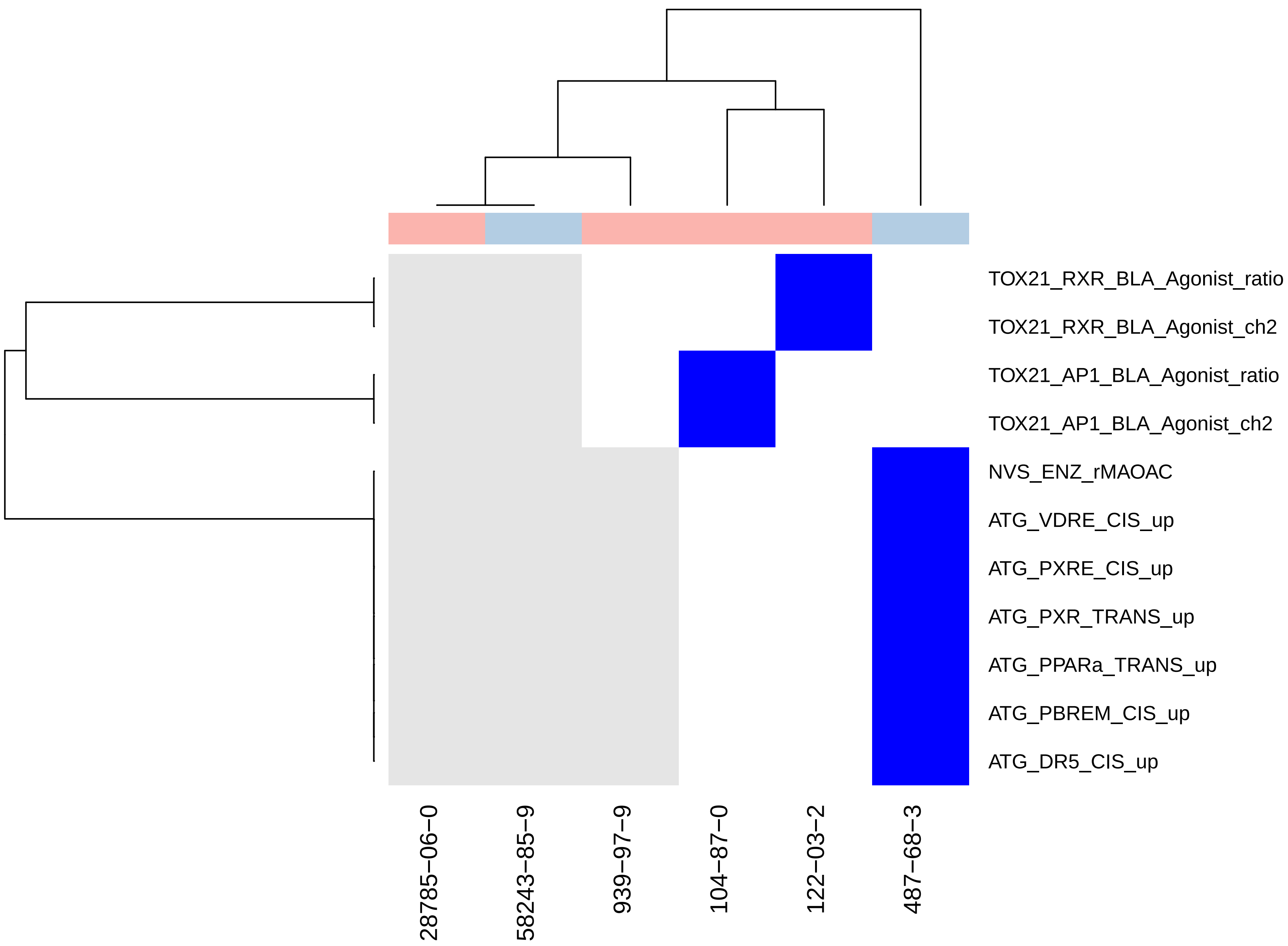

All Activity Heatmap for Cluster 269

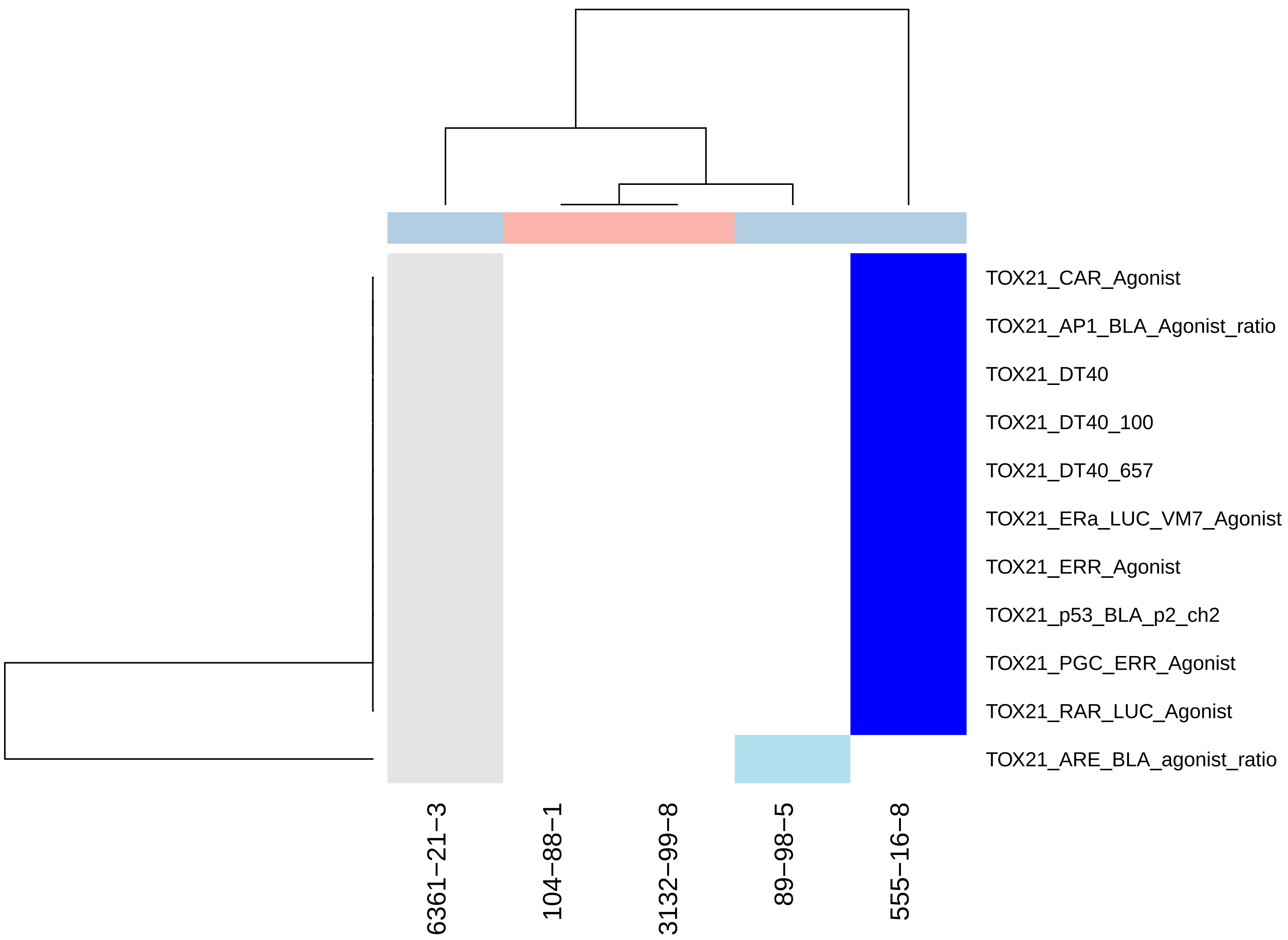

All Activity Heatmap for Cluster 270

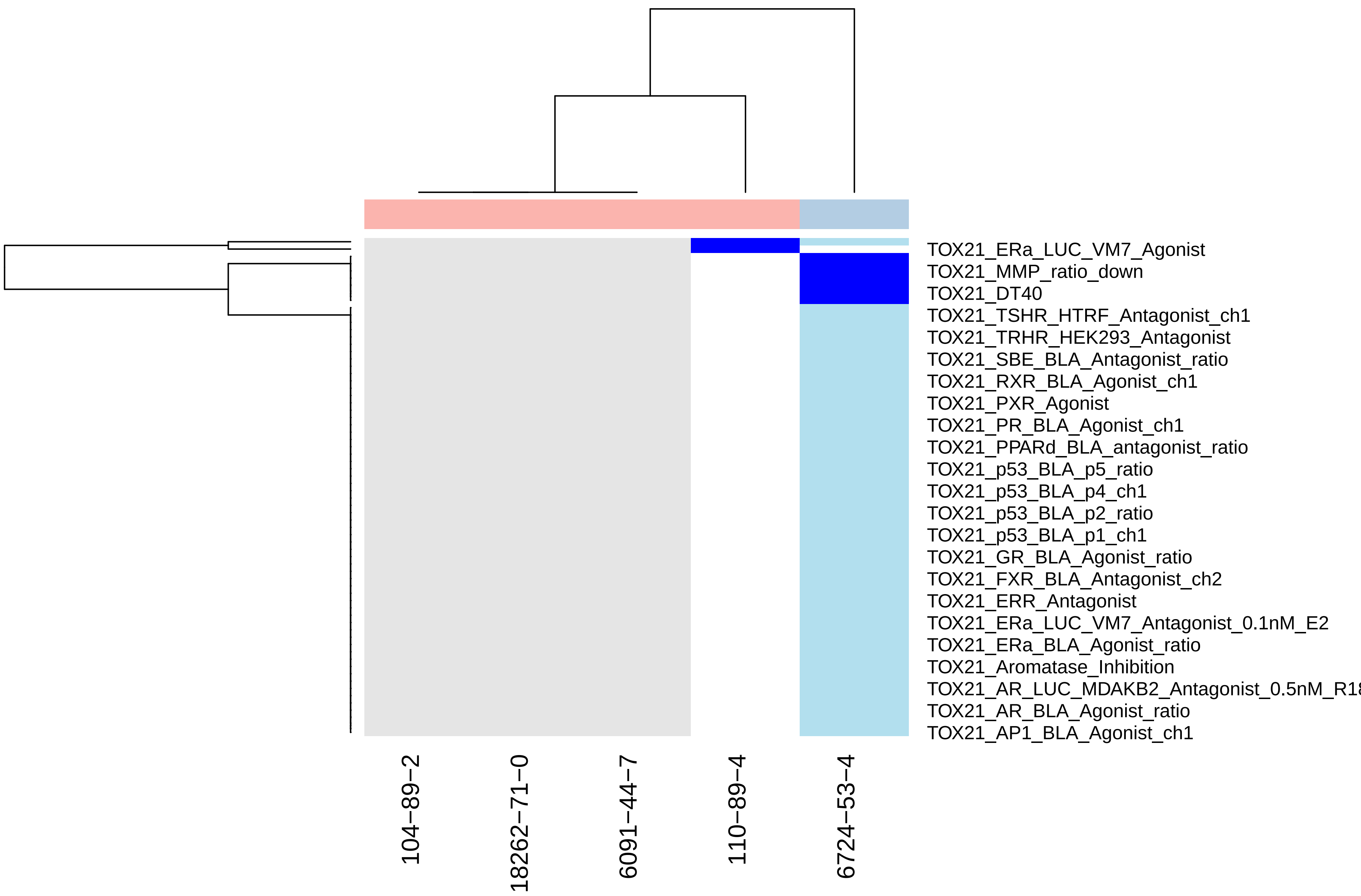

All Activity Heatmap for Cluster 271

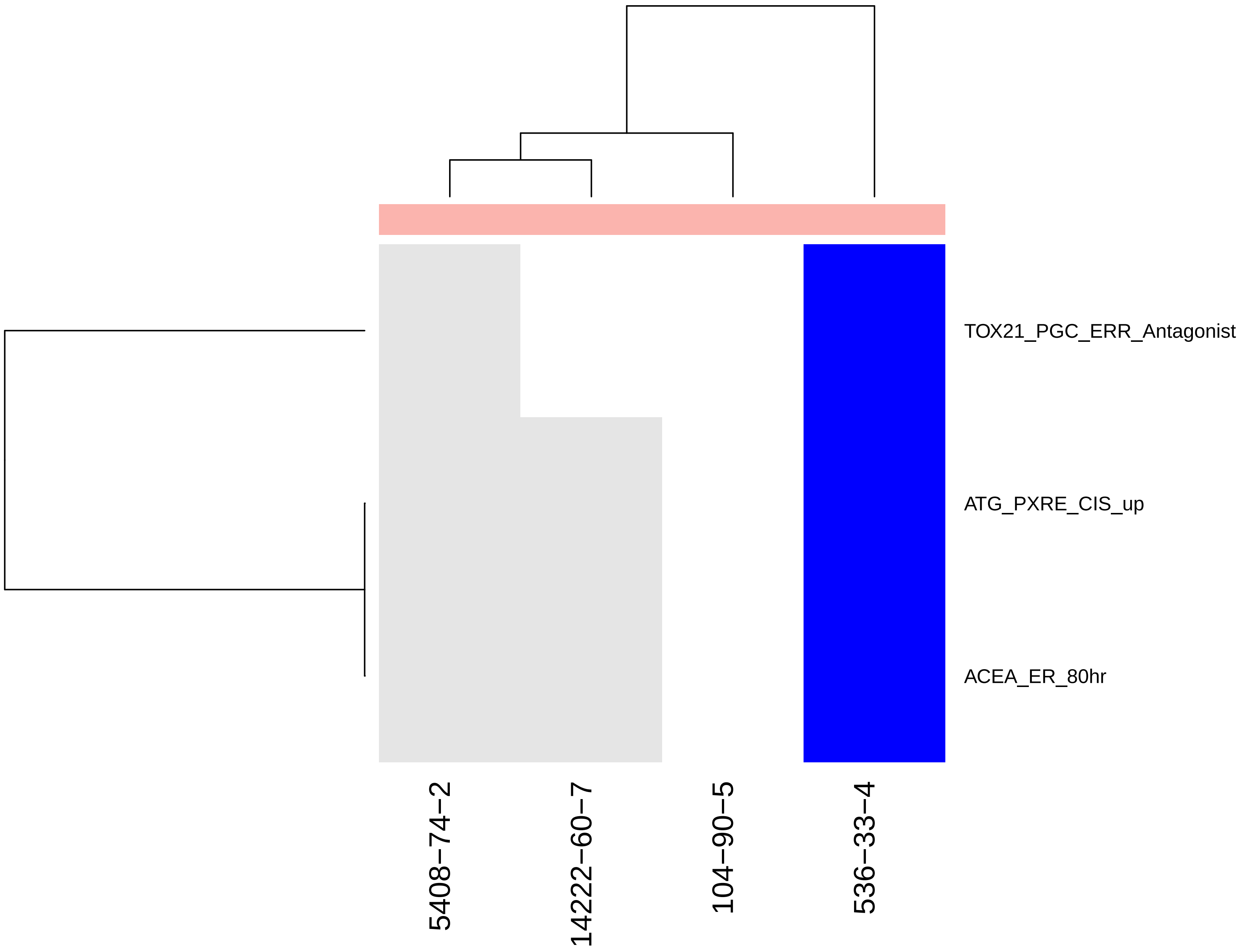

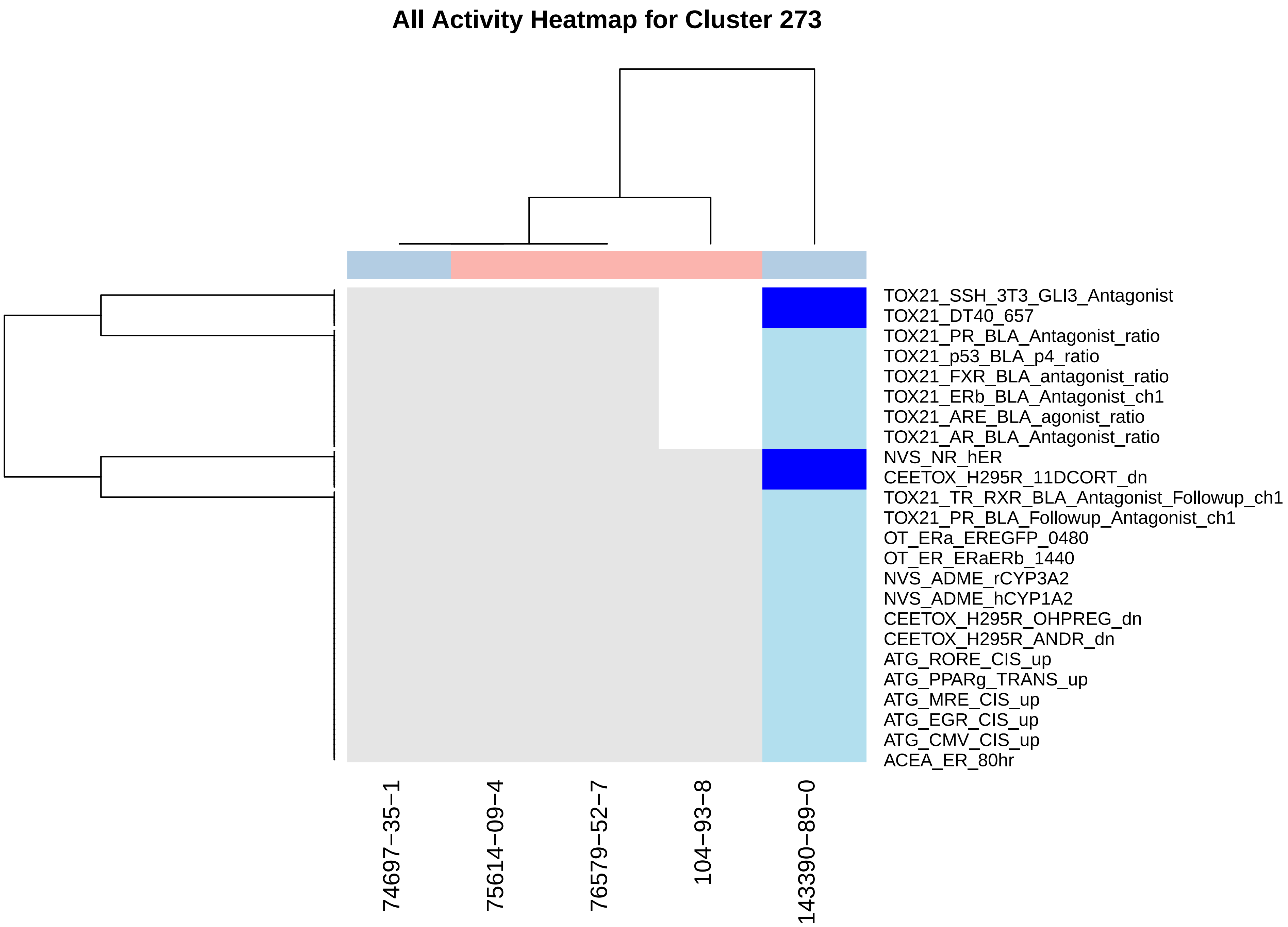
All Activity Heatmap for Cluster 273
TOX21_SSH_3T3_GLI3_Antagonist
TOX21_DT40_657
TOX21_PR_BLA_Antagonist_ratio
TOX21_p53_BLA_p4_ratio
TOX21_FXR_BLA_antagonist_ratio
TOX21_ERb_BLA_Antagonist_ch1
TOX21_ARE_BLA_agonist_ratio
TOX21_AR_BLA_Antagonist_ratio
NVS_NR_hER
CEETOX_H295R_11DCORT_dn
TOX21_TR_RXR_BLA_Antagonist_Followup_ch1
TOX21_PR_BLA_Followup_Antagonist_ch1
OT_ERa_EREGFP_0480
OT_ER_ERaERb_1440
NVS_ADME_rCYP3A2
NVS_ADME_hCYP1A2
CEETOX_H295R_OHPREG_dn
CEETOX_H295R_ANDR_dn
ATG_RORE_CIS_up
ATG_PPARg_TRANS_up
ATG_MRE_CIS_up
ATG_EGR_CIS_up
ATG_CMV_CIS_up
ACEA_ER_80hr
74697−35−1
75614−09−4
76579−52−7
104−93−8
143390−89−0

All Activity Heatmap for Cluster 274

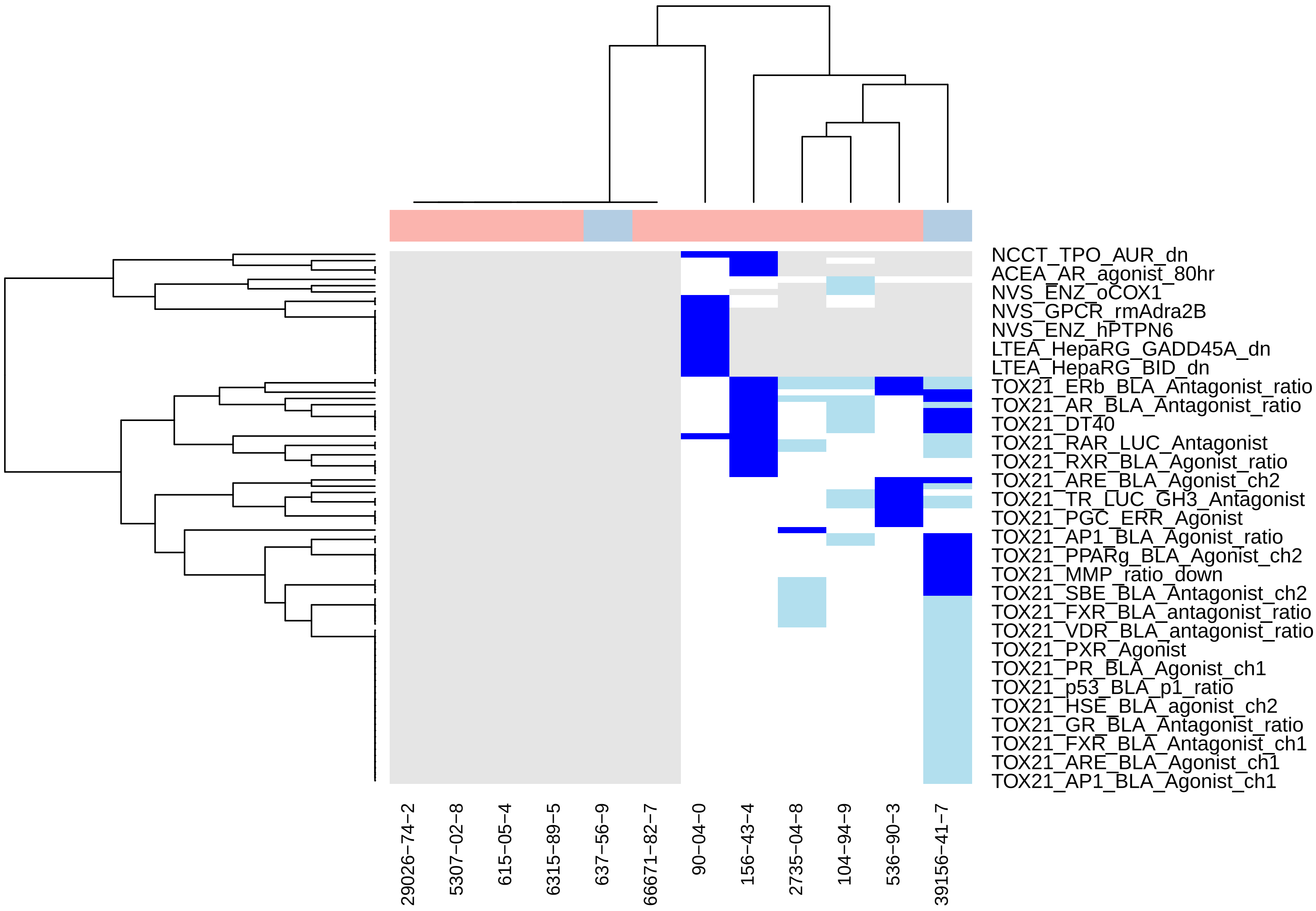

All Activity Heatmap for Cluster 275

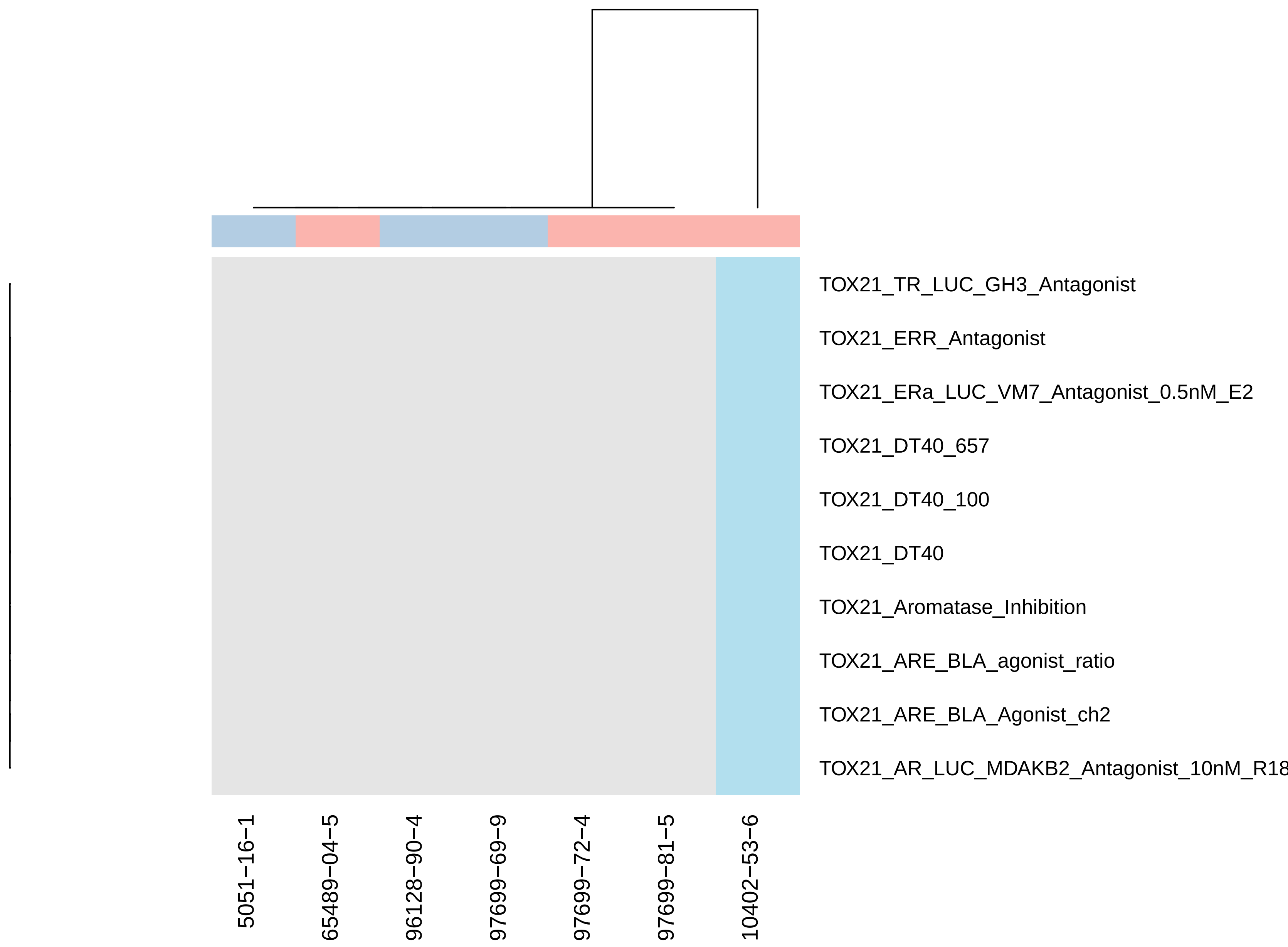

All Activity Heatmap for Cluster 276

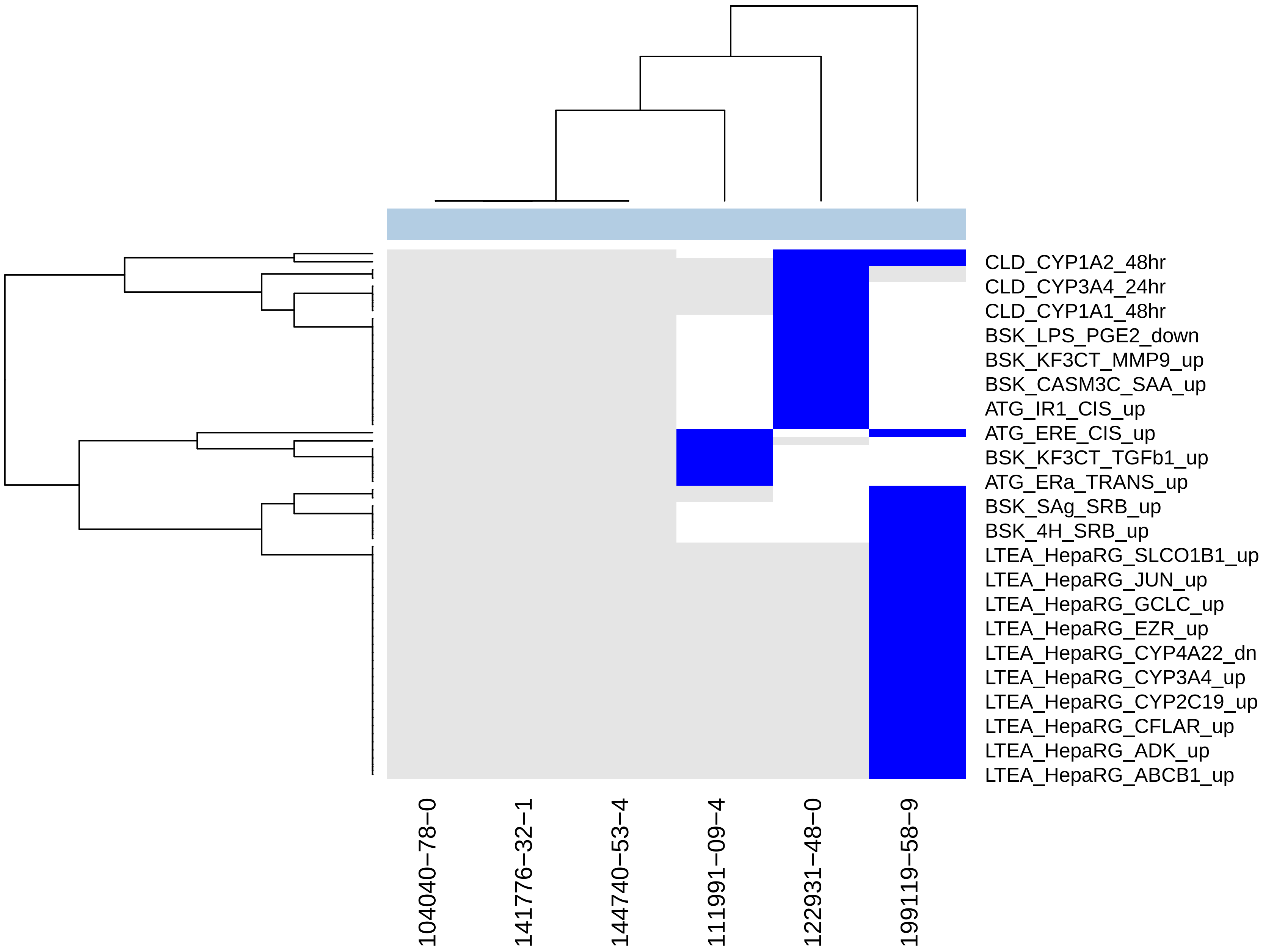

All Activity Heatmap for Cluster 277

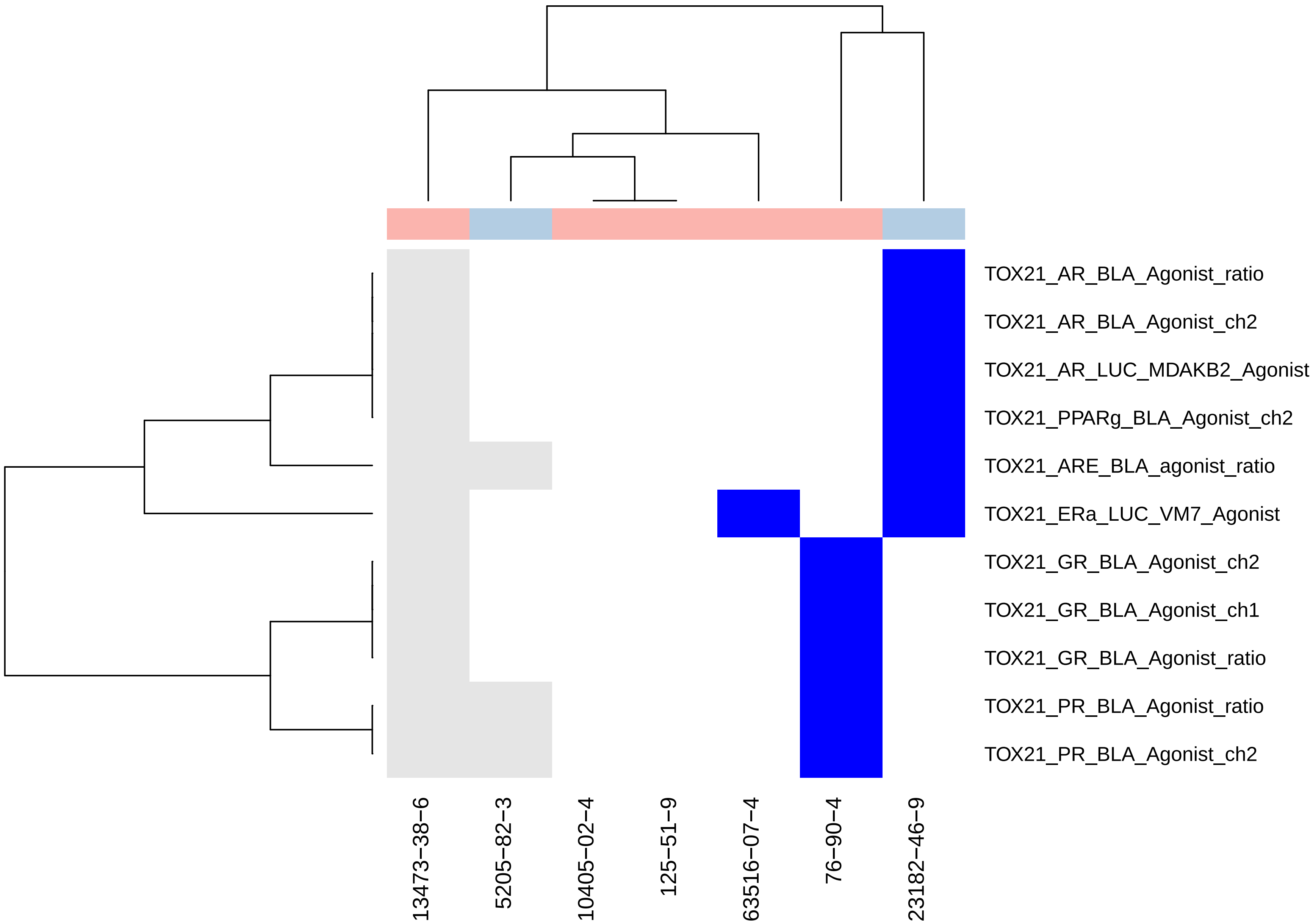

All Activity Heatmap for Cluster 278

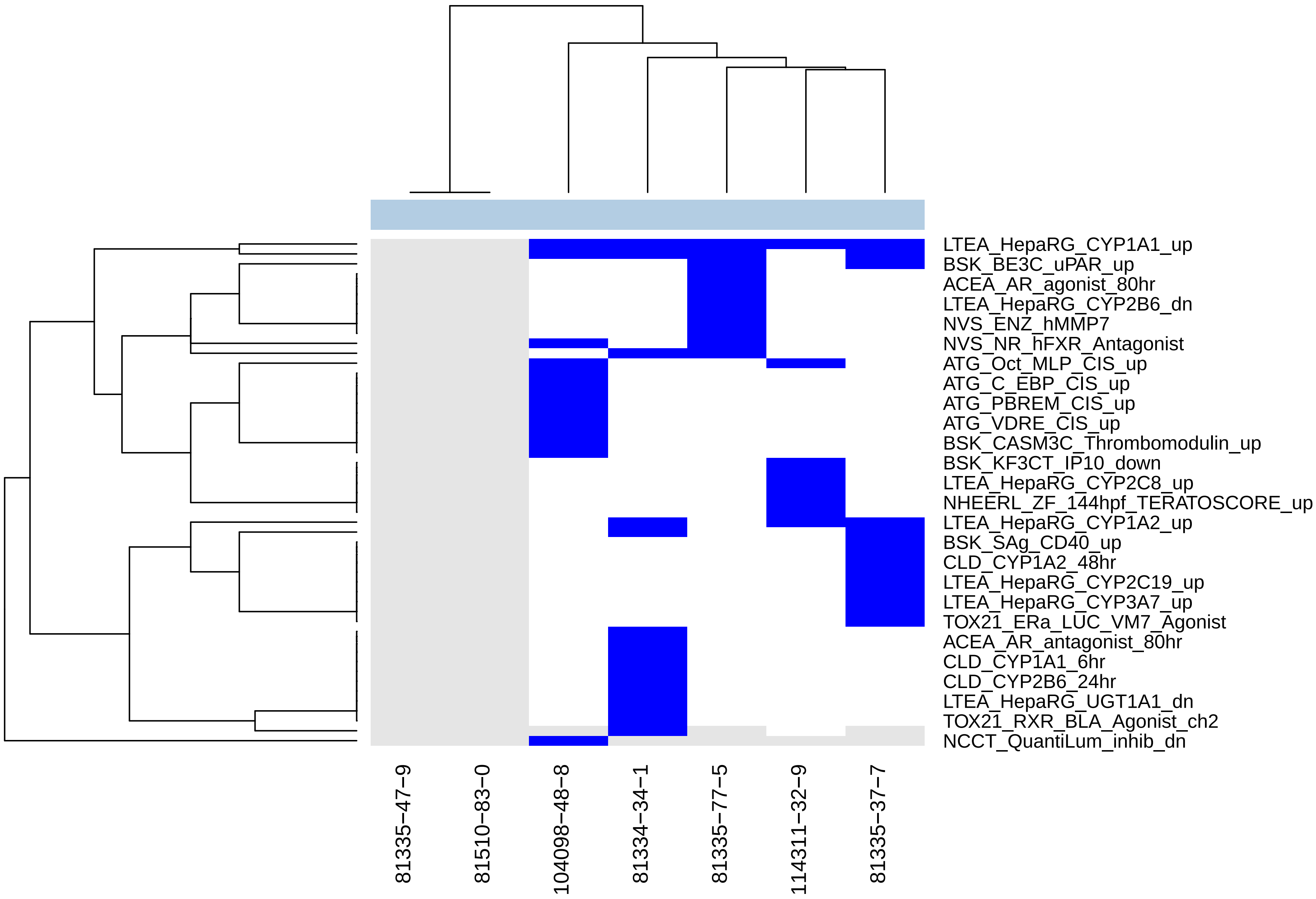

All Activity Heatmap for Cluster 279

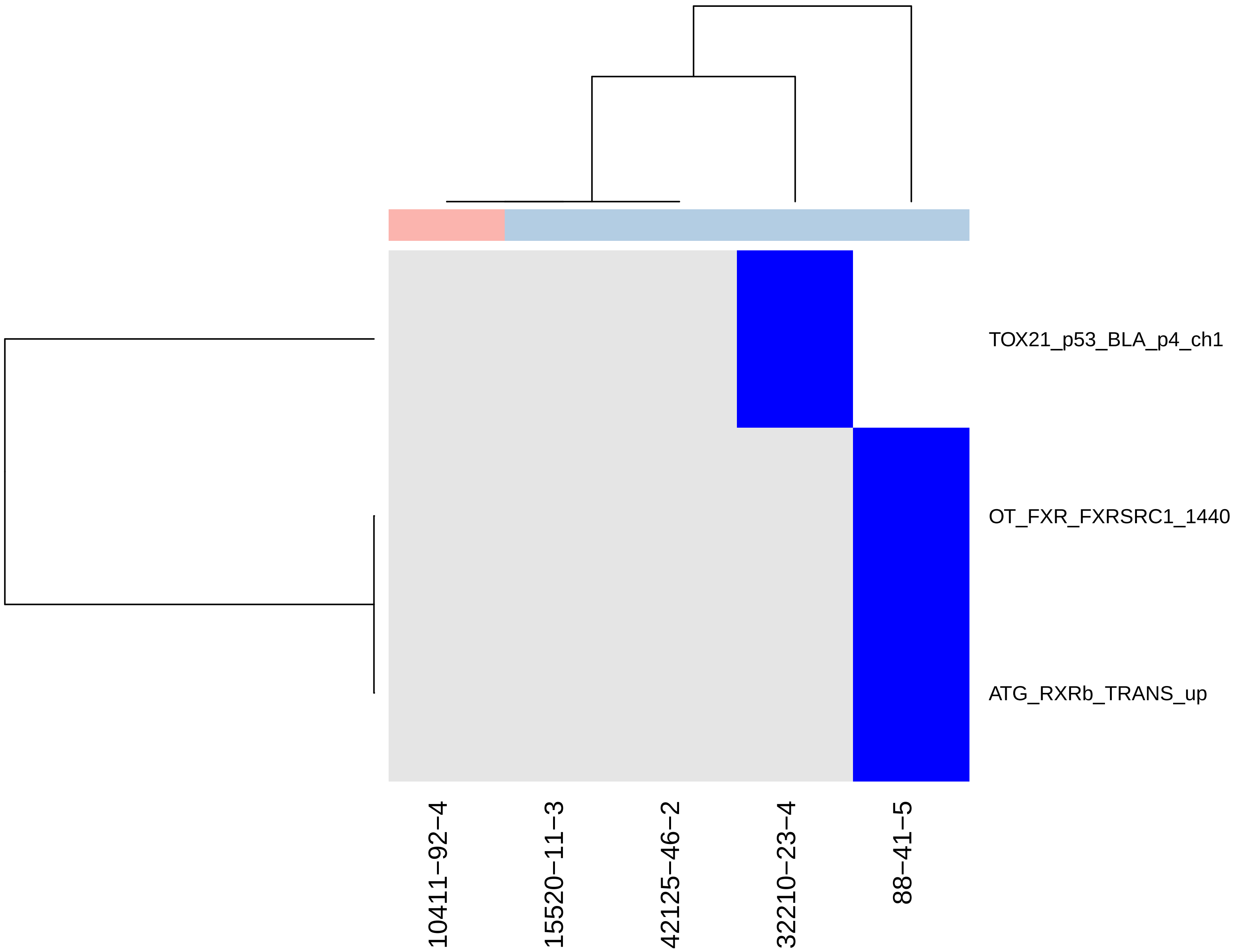

All Activity Heatmap for Cluster 280

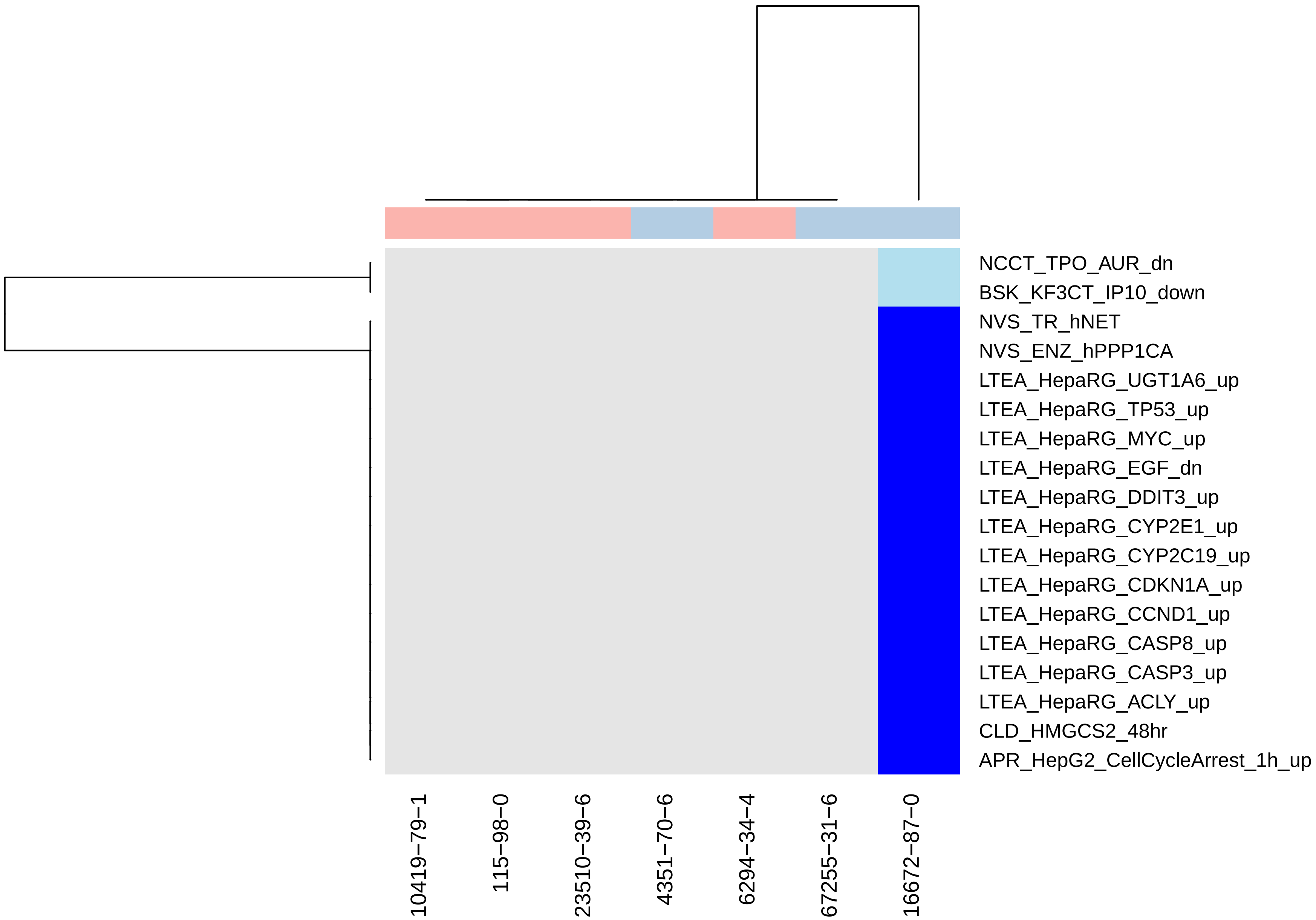

All Activity Heatmap for Cluster 282

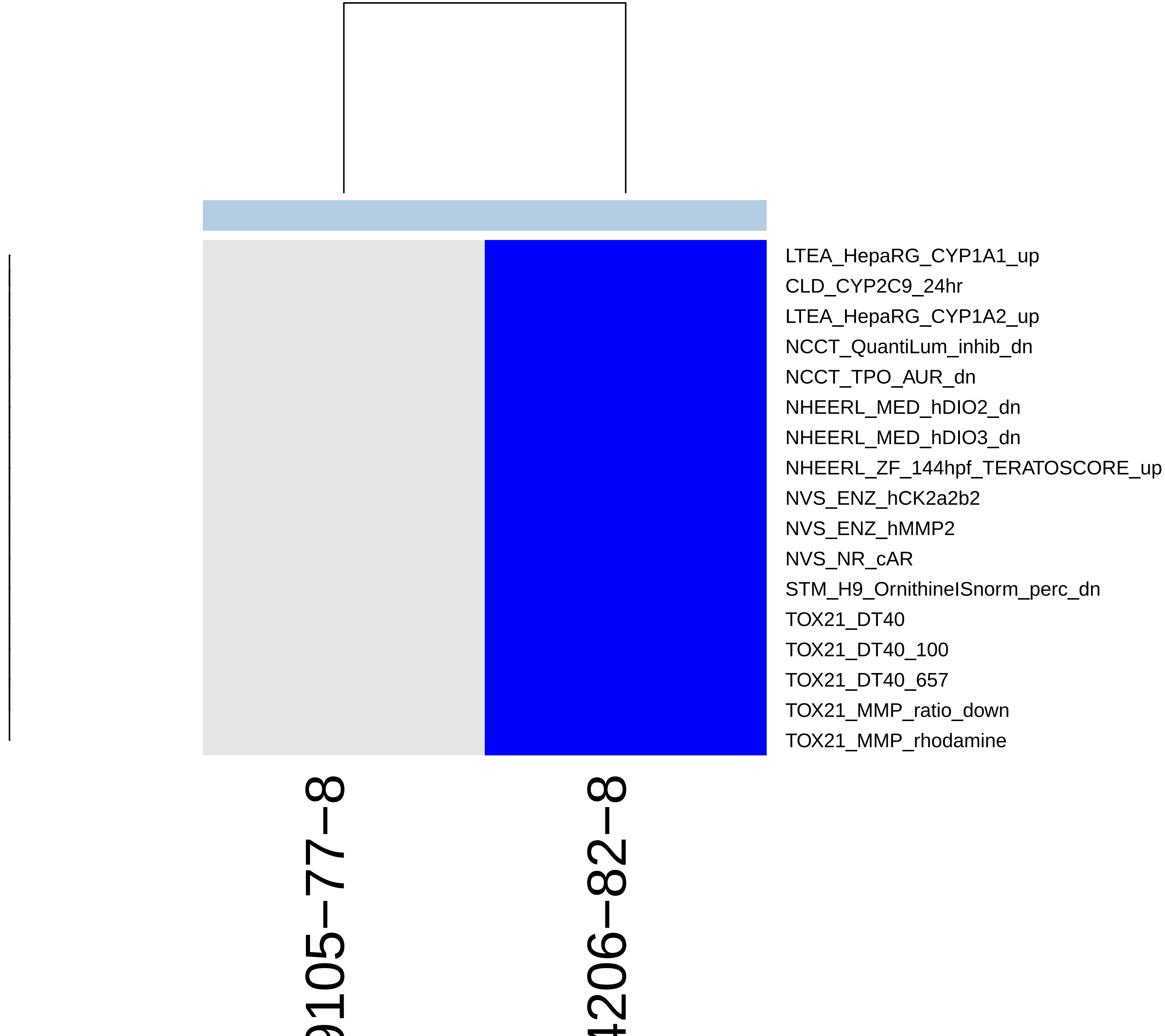

All Activity Heatmap for Cluster 283

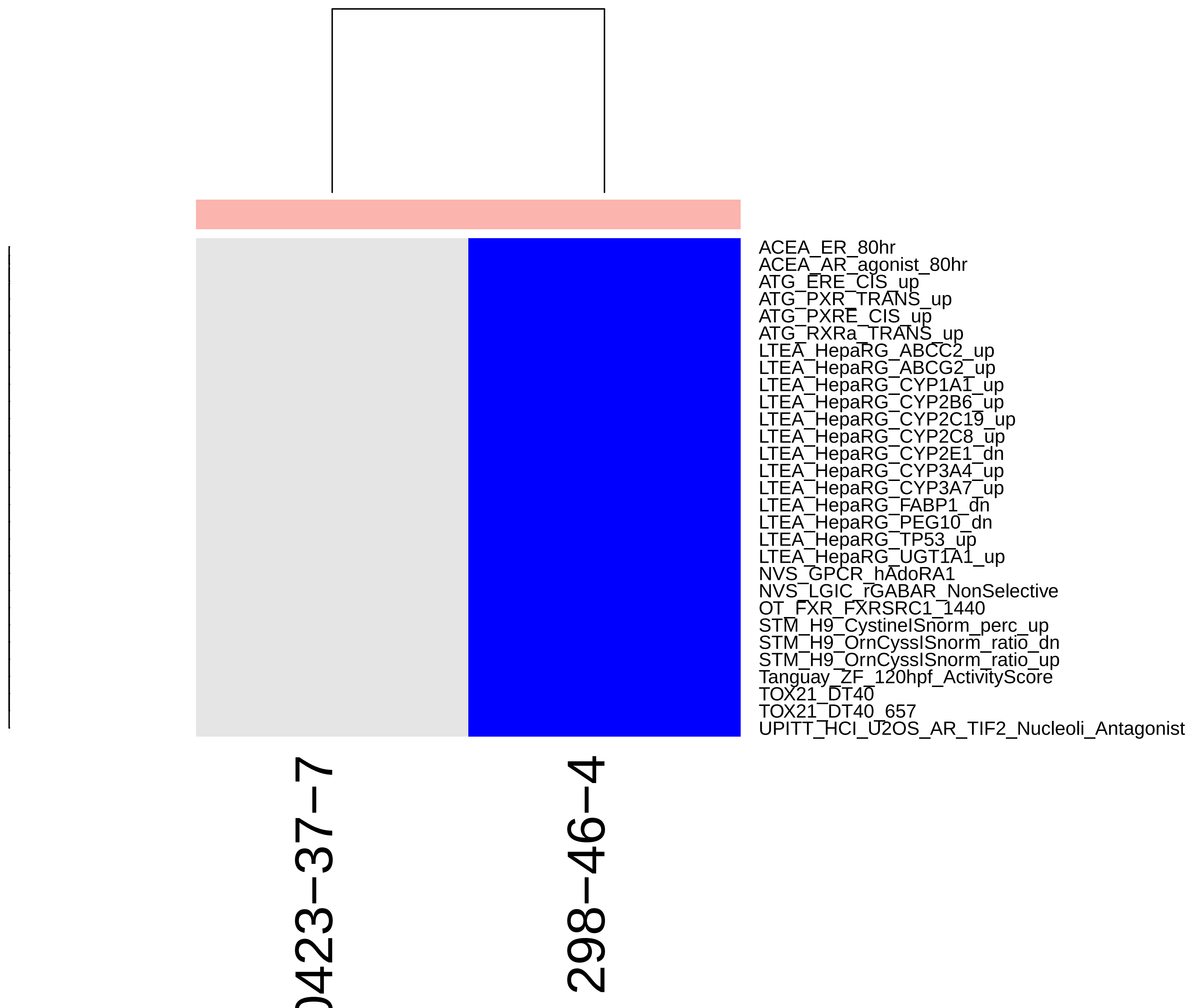

All Activity Heatmap for Cluster 284

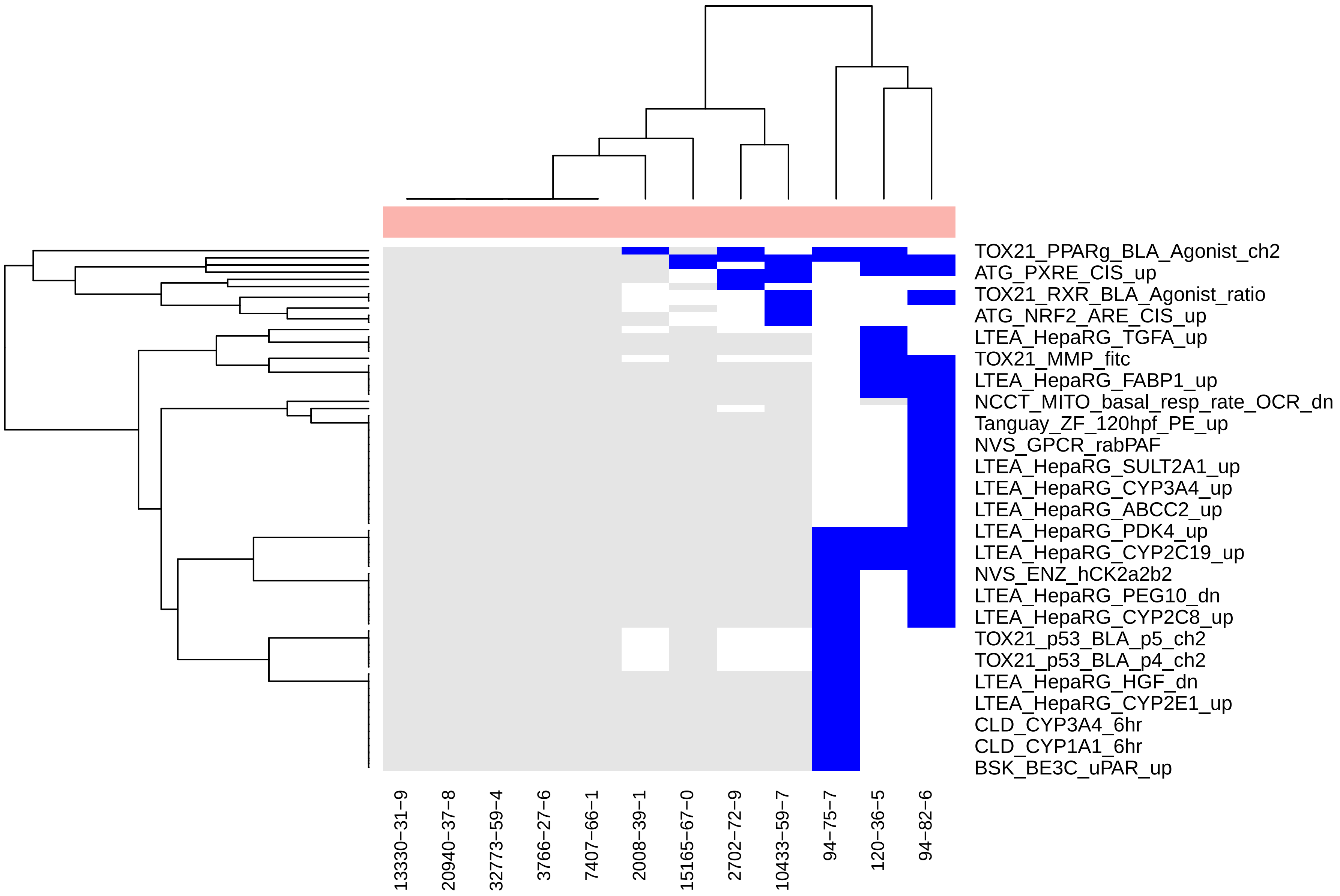

All Activity Heatmap for Cluster 285

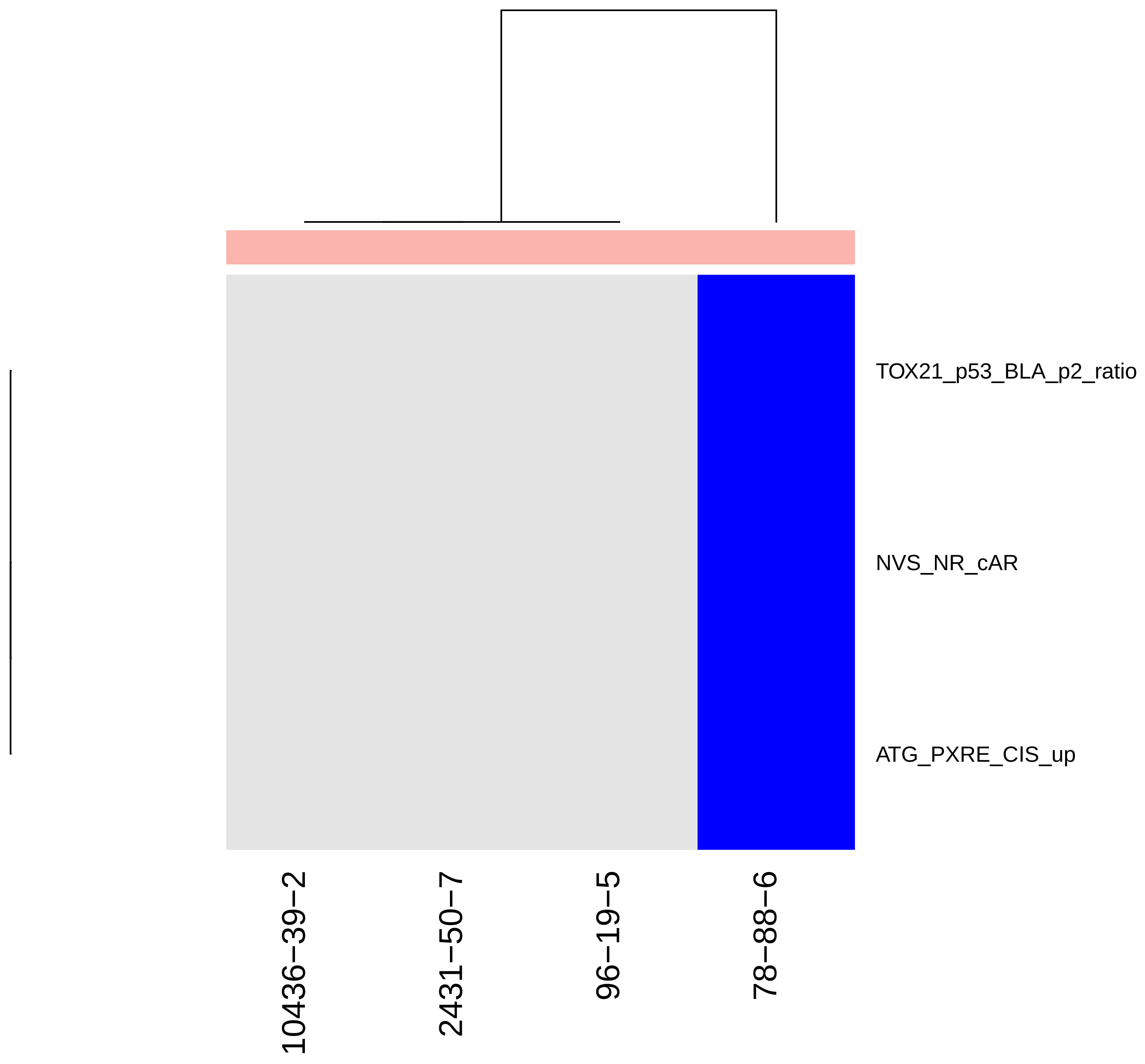

All Activity Heatmap for Cluster 286

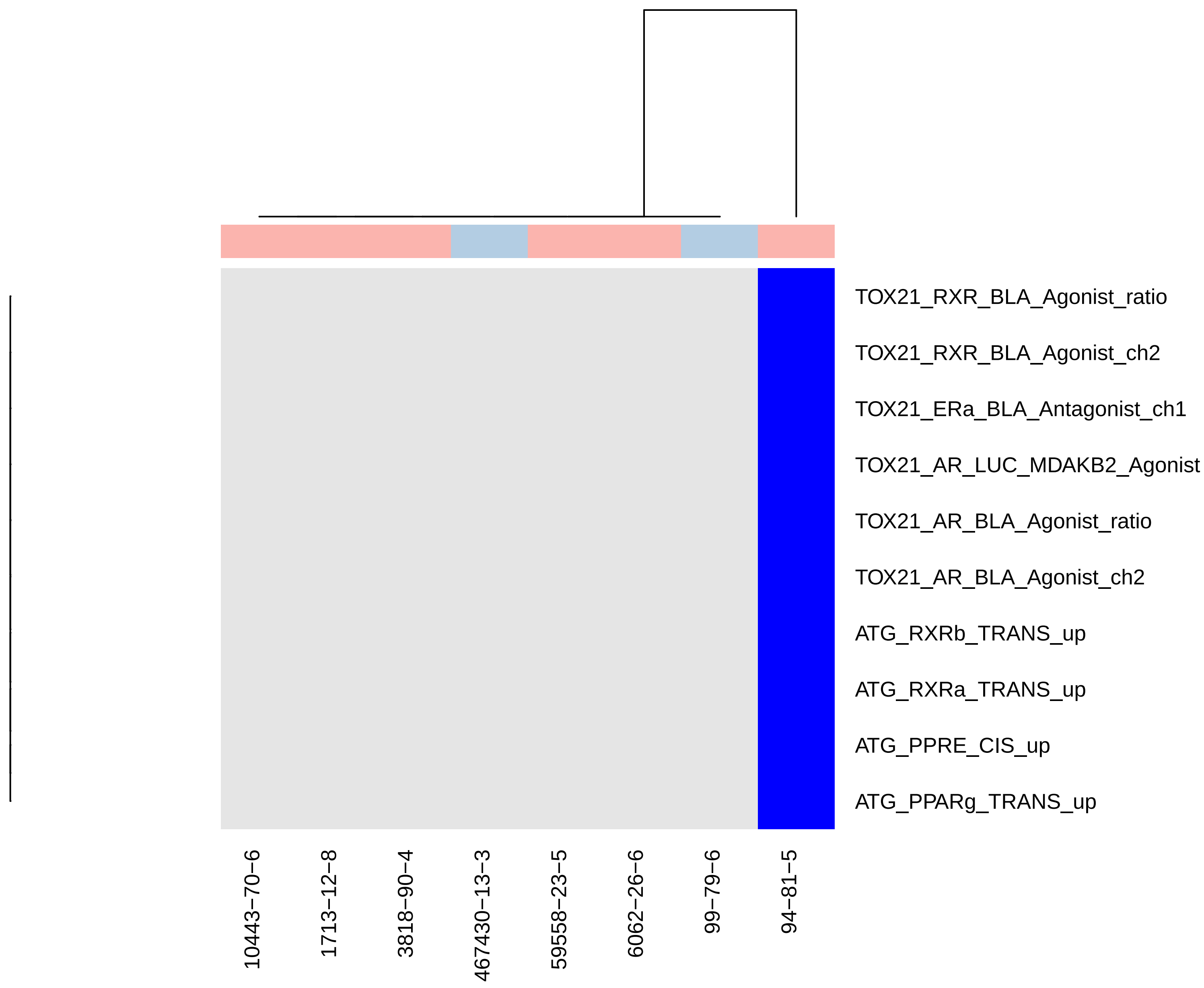

All Activity Heatmap for Cluster 287

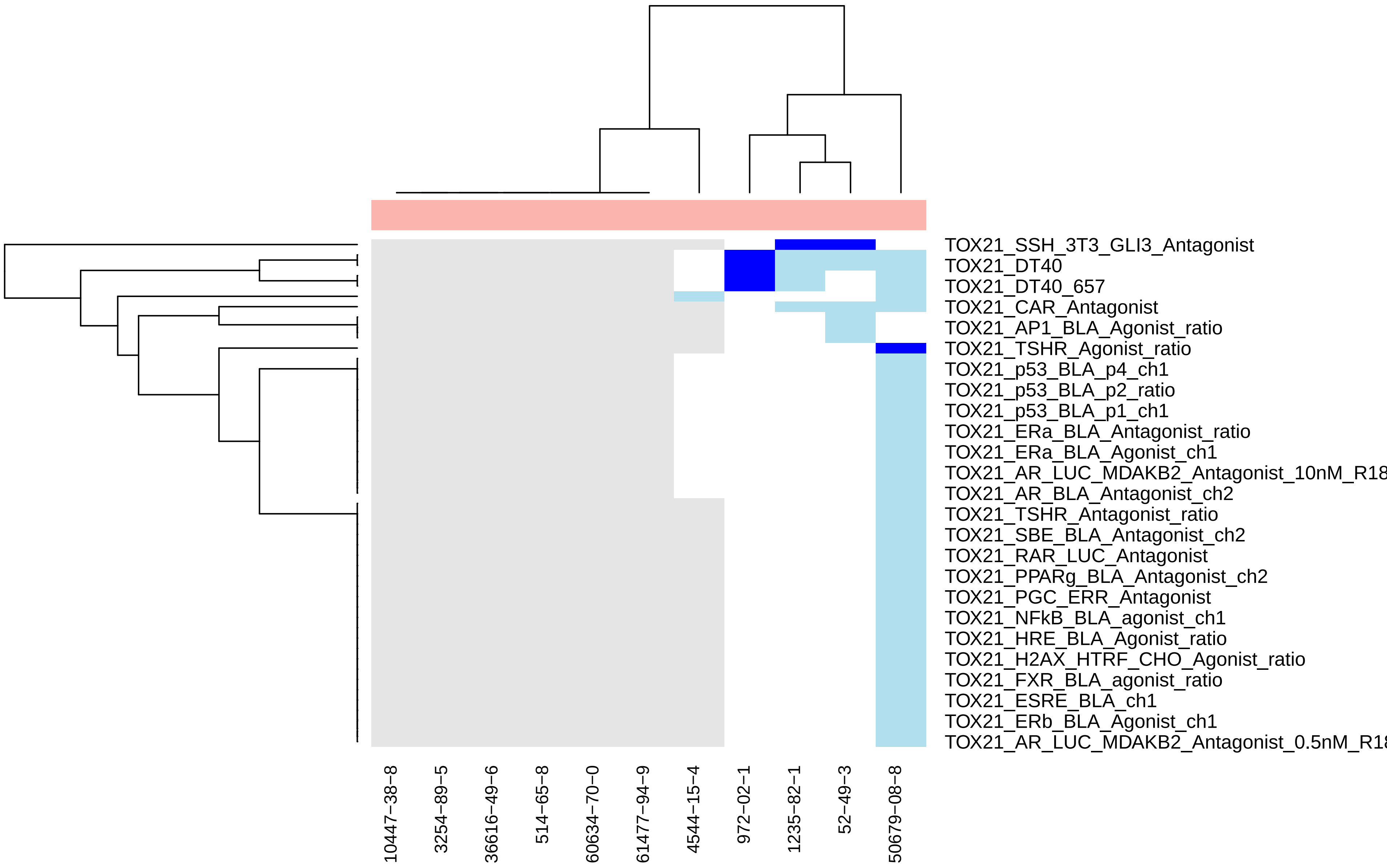

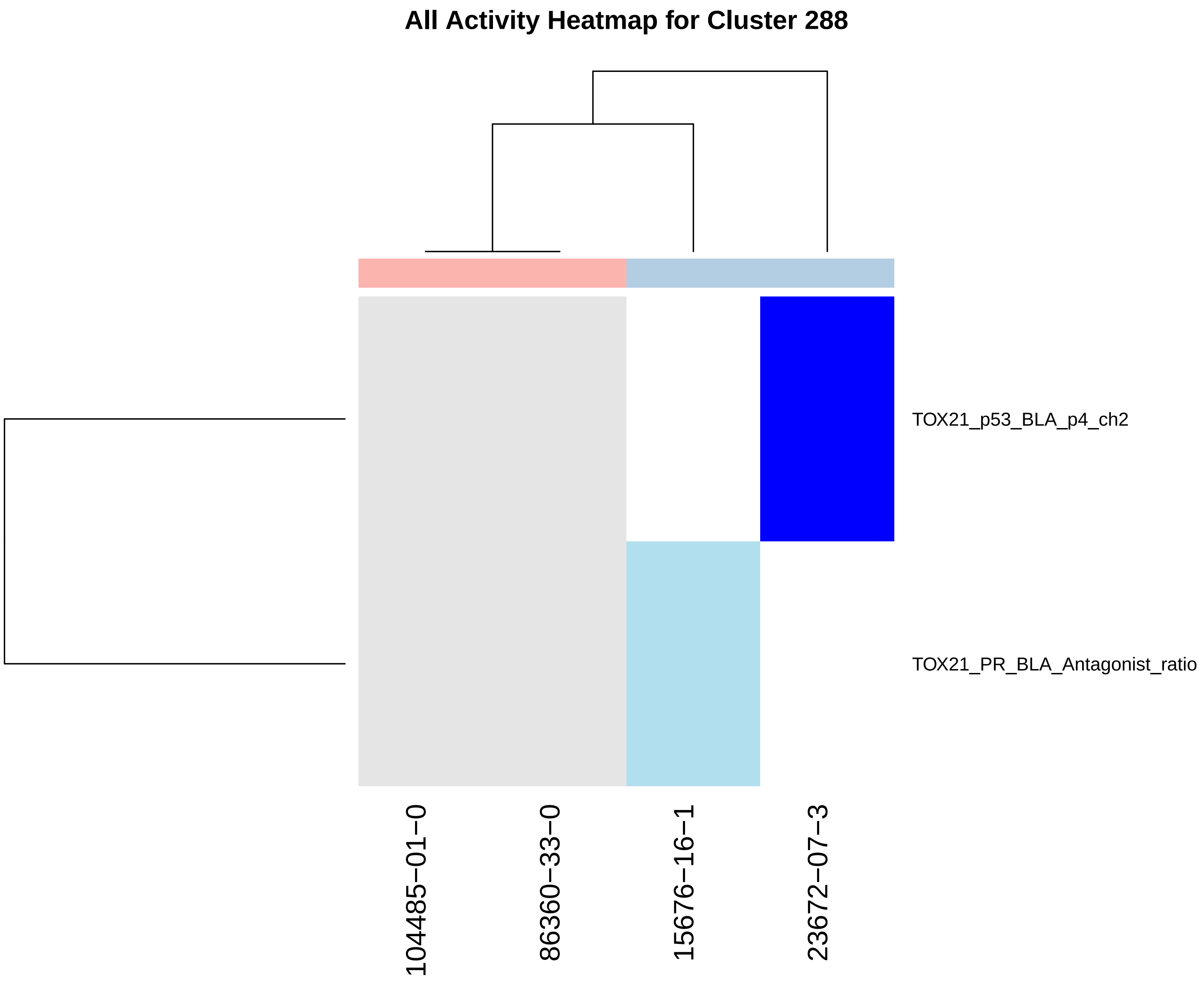

All Activity Heatmap for Cluster 288
TOX21_p53_BLA_p4_ch2
TOX21_PR_BLA_Antagonist_ratio
104485−01−0
86360−33−0
15676−16−1
23672−07−3

All Activity Heatmap for Cluster 289

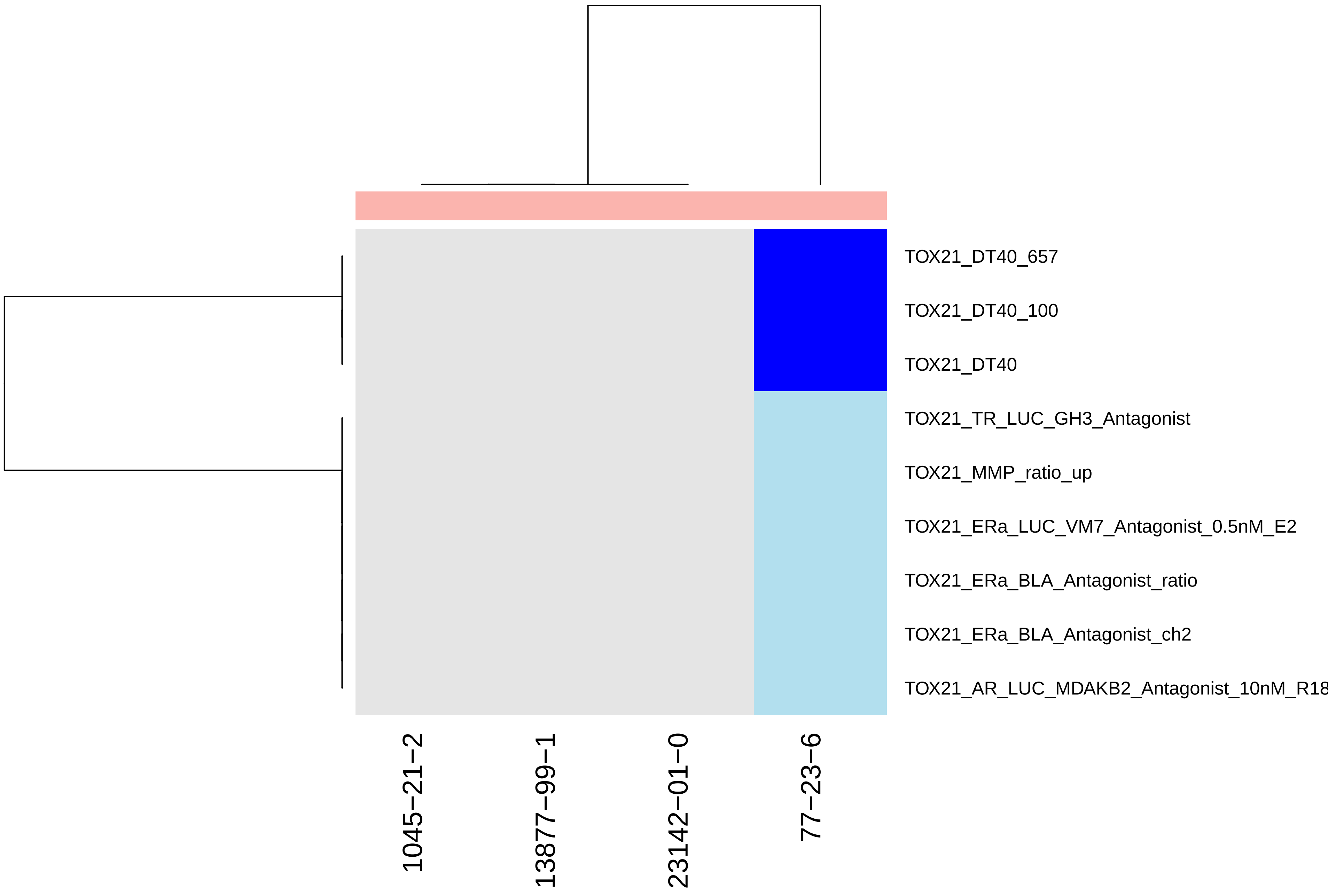

All Activity Heatmap for Cluster 290

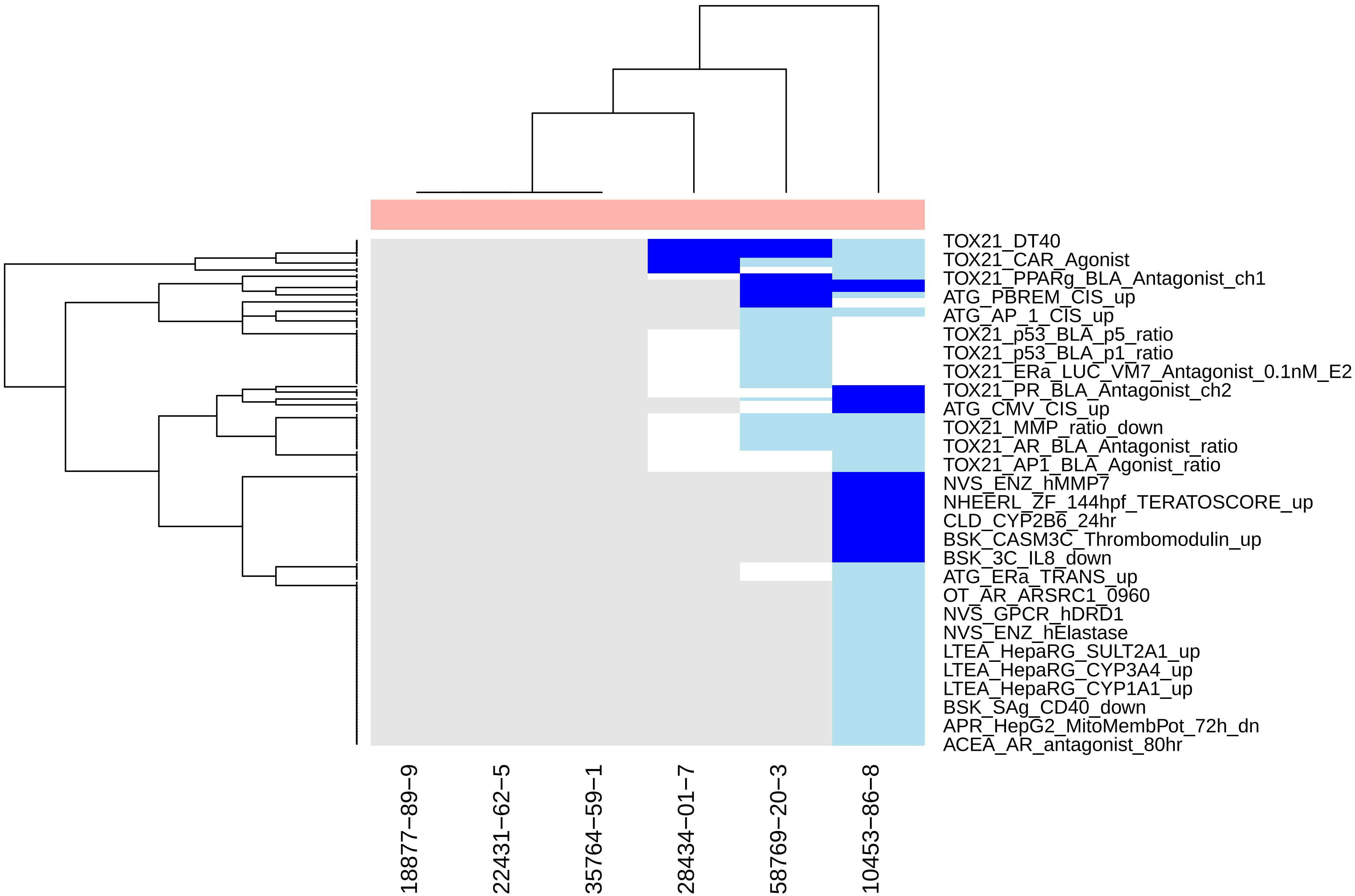

All Activity Heatmap for Cluster 292

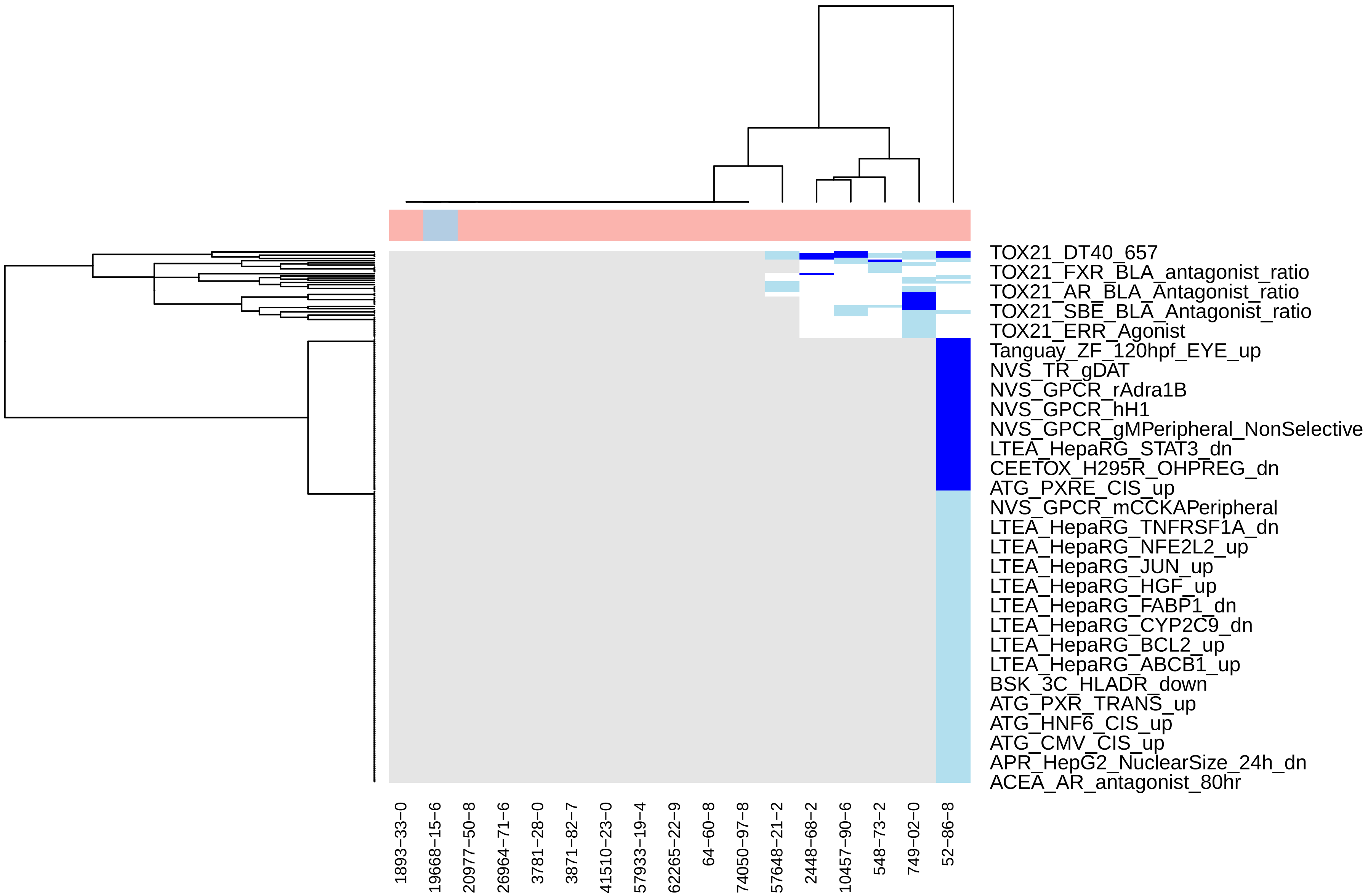

All Activity Heatmap for Cluster 293

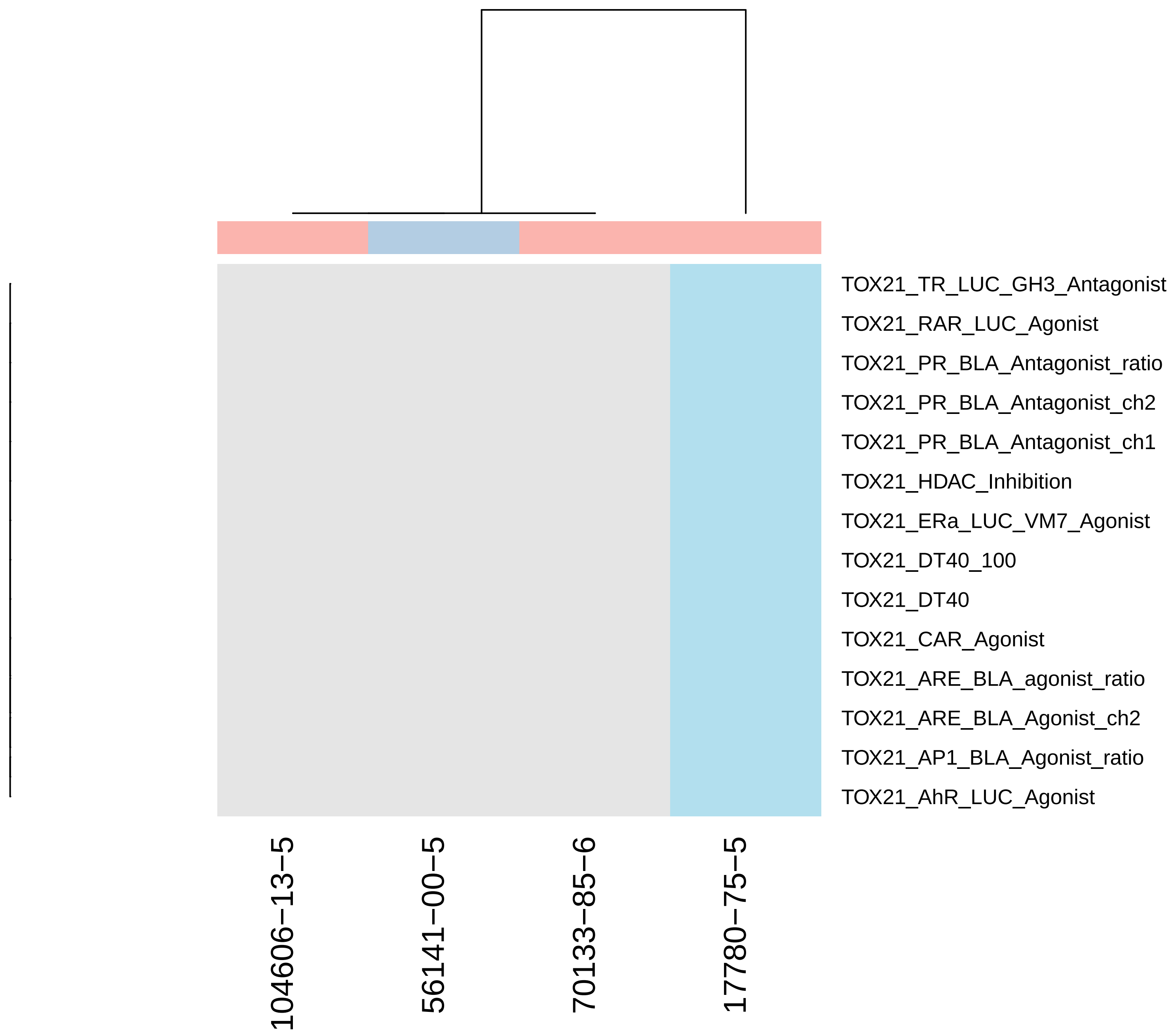

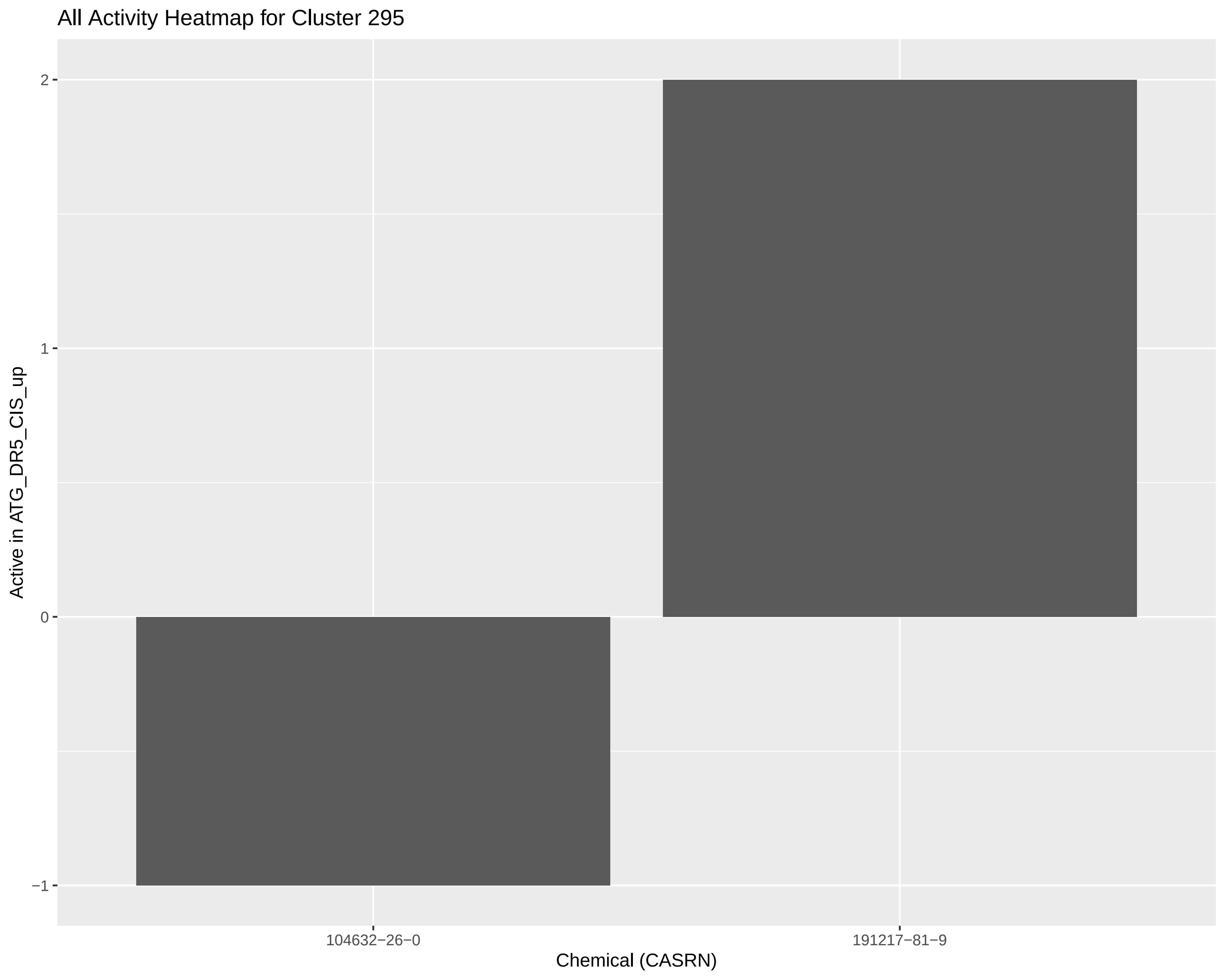

All Activity Heatmap for Cluster 295
Active in ATG_DR5_CIS_up
2
1
0
−1
104632−26−0
191217−81−9
Chemical (CASRN)

All Activity Heatmap for Cluster 296

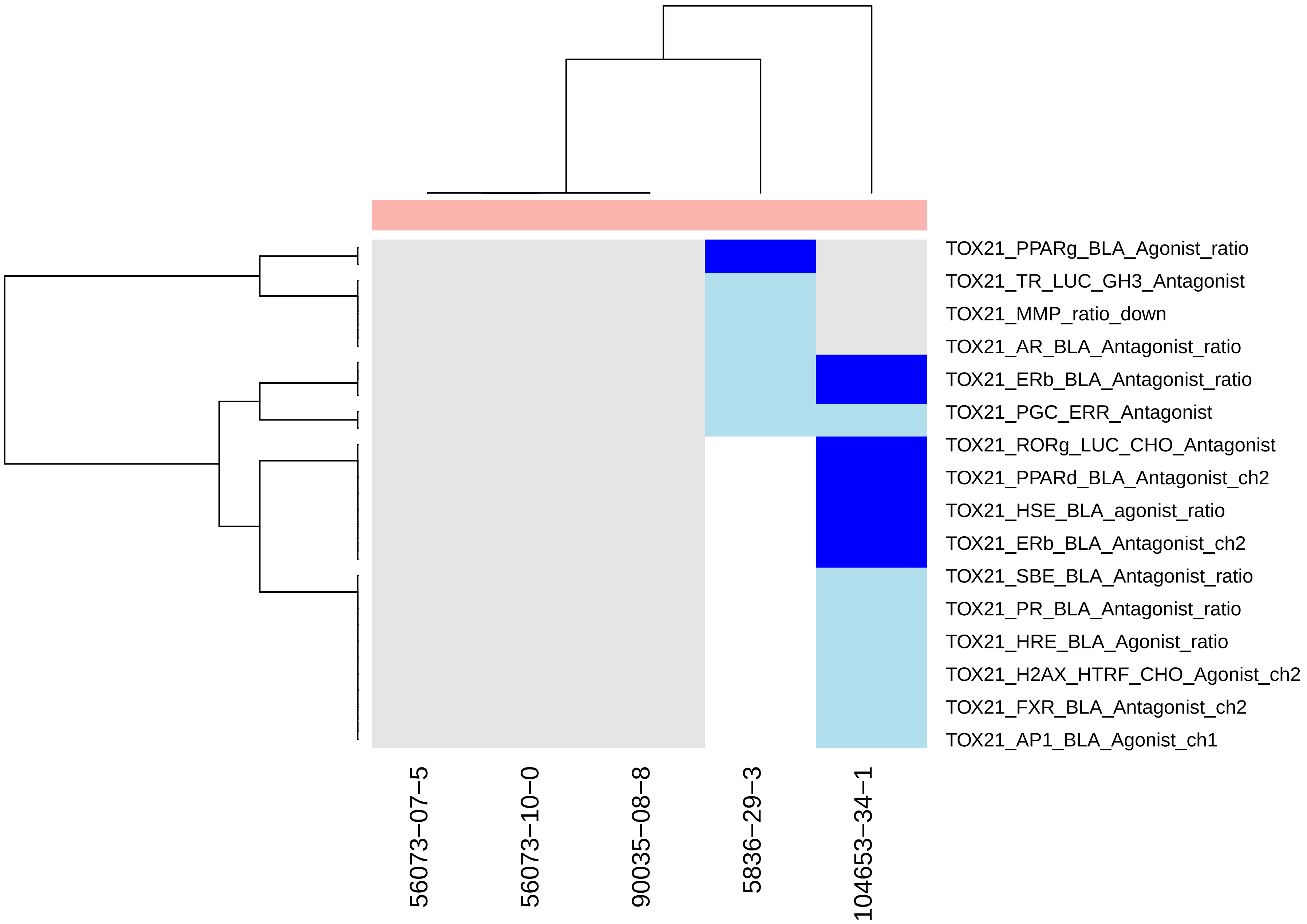

All Activity Heatmap for Cluster 298

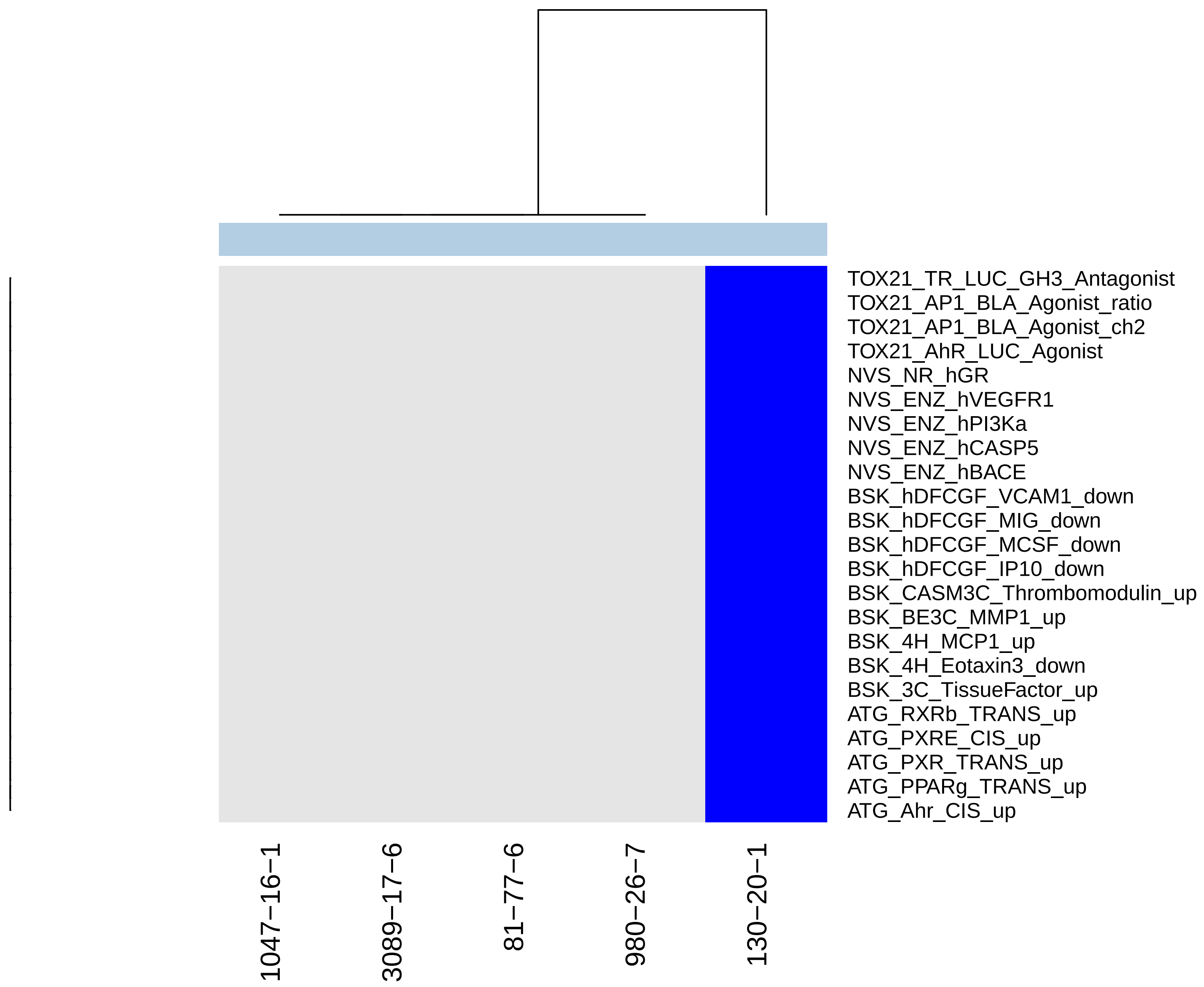

All Activity Heatmap for Cluster 299

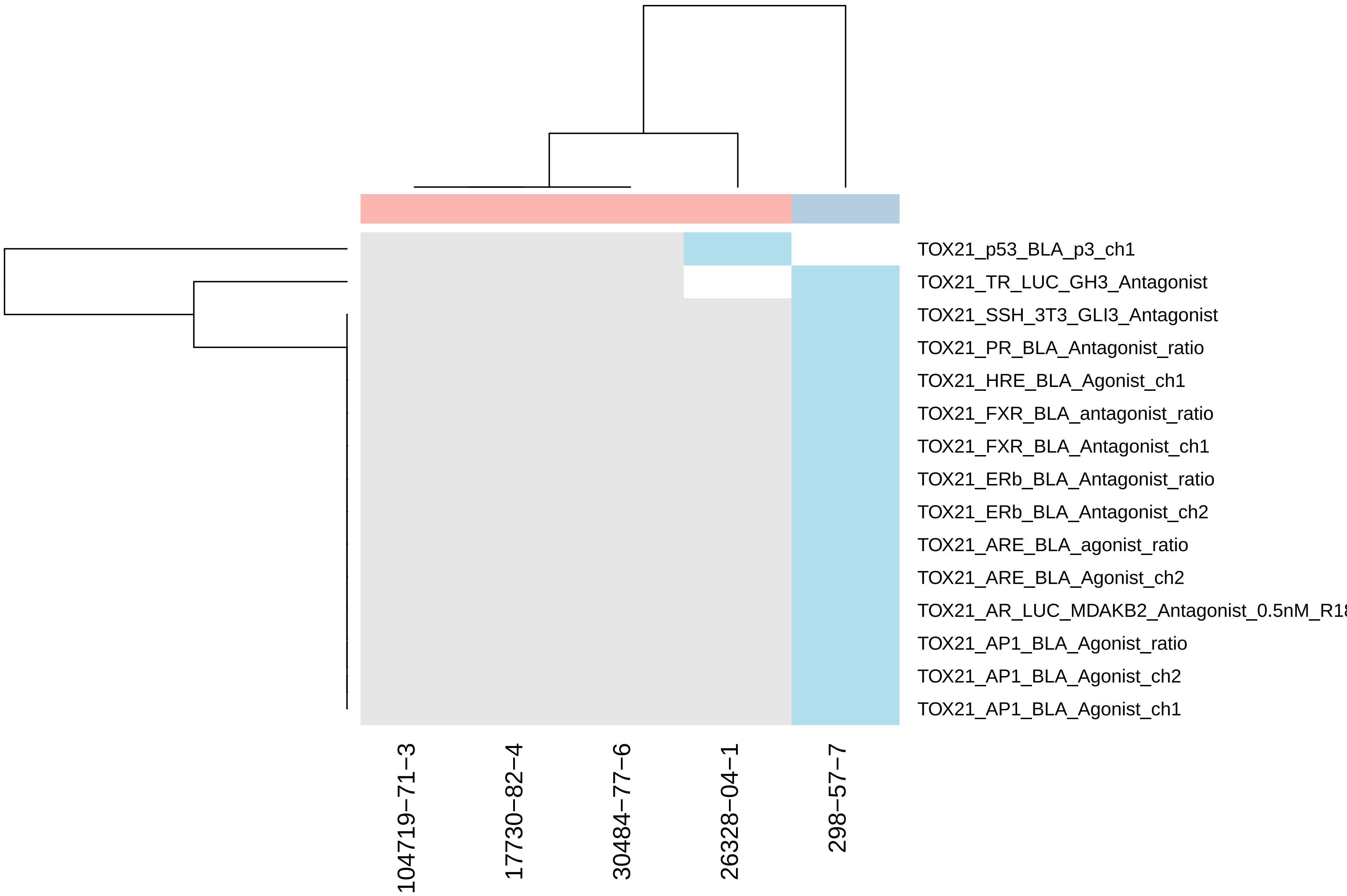

All Activity Heatmap for Cluster 300

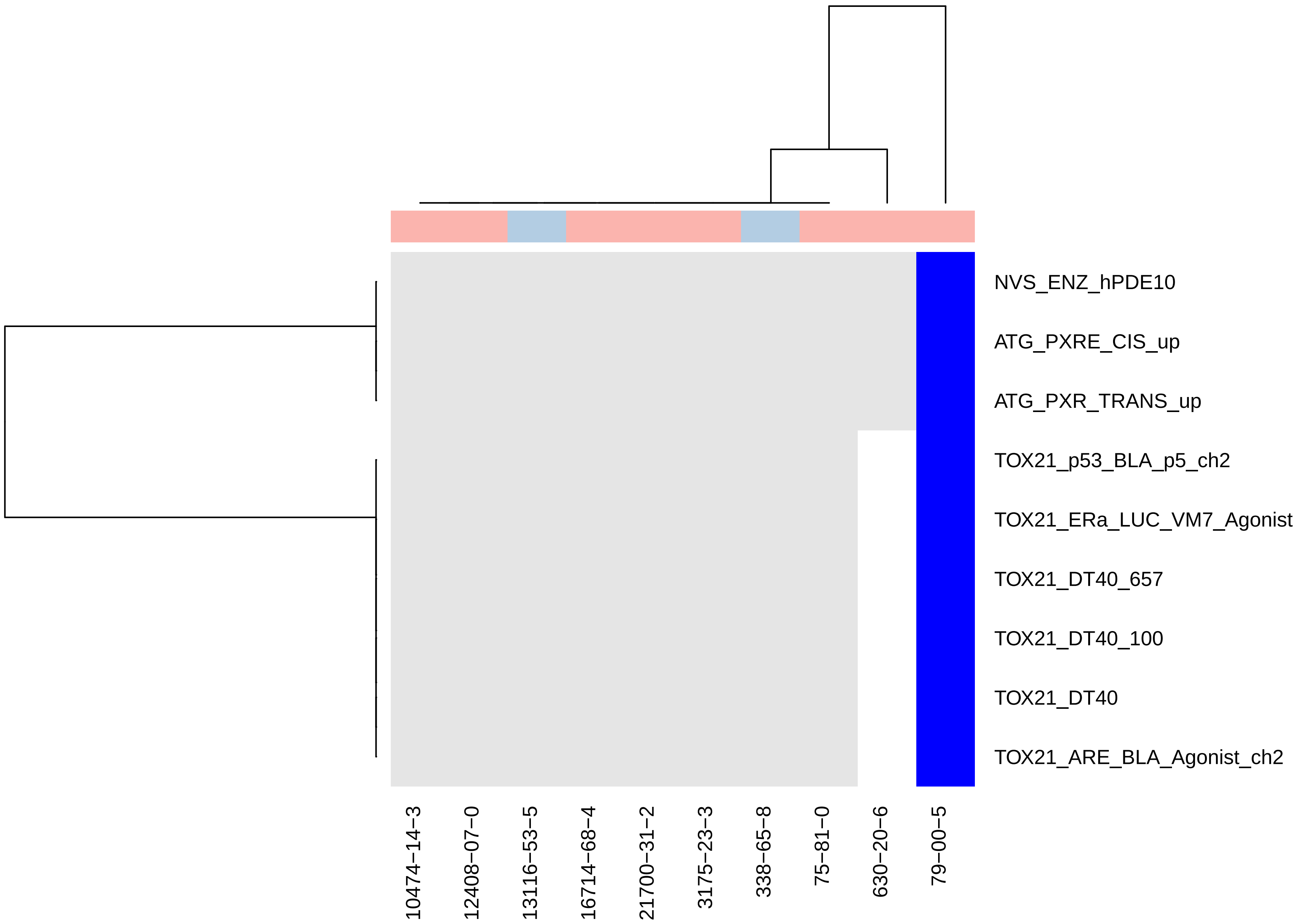

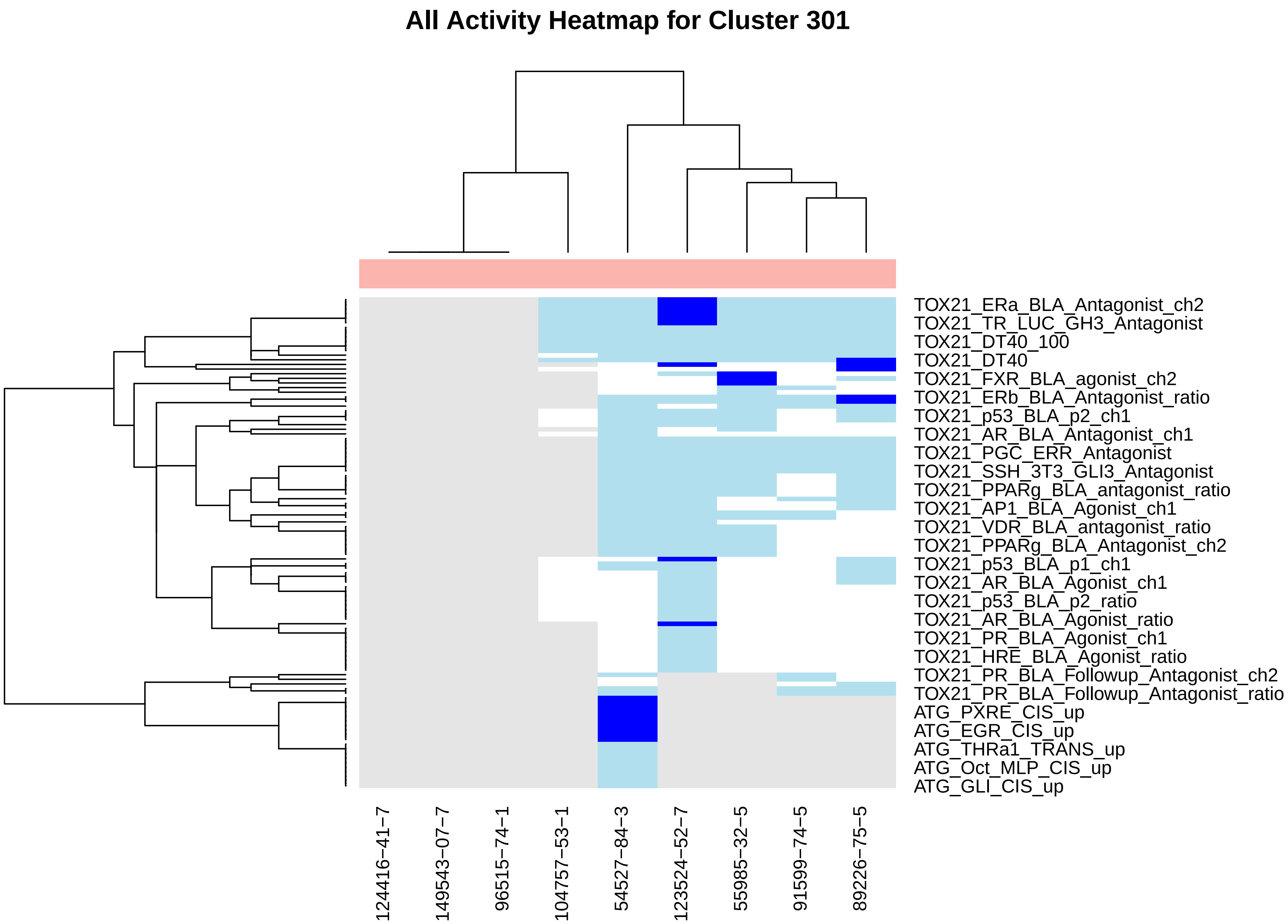
All Activity Heatmap for Cluster 301
TOX21_ERa_BLA_Antagonist_ch2
TOX21_TR_LUC_GH3_Antagonist
TOX21_DT40_100
TOX21_DT40
TOX21_FXR_BLA_agonist_ch2
TOX21_ERb_BLA_Antagonist_ratio
TOX21_p53_BLA_p2_ch1
TOX21_AR_BLA_Antagonist_ch1
TOX21_PGC_ERR_Antagonist
TOX21_SSH_3T3_GLI3_Antagonist
TOX21_PPARg_BLA_antagonist_ratio
TOX21_AP1_BLA_Agonist_ch1
TOX21_VDR_BLA_antagonist_ratio
TOX21_PPARg_BLA_Antagonist_ch2
TOX21_p53_BLA_p1_ch1
TOX21_AR_BLA_Agonist_ch1
TOX21_p53_BLA_p2_ratio
TOX21_AR_BLA_Agonist_ratio
TOX21_PR_BLA_Agonist_ch1
TOX21_HRE_BLA_Agonist_ratio
TOX21_PR_BLA_Followup_Antagonist_ch2
TOX21_PR_BLA_Followup_Antagonist_ratio
ATG_PXRE_CIS_up
ATG_EGR_CIS_up
ATG_THRa1_TRANS_up
ATG_Oct_MLP_CIS_up
ATG_GLI_CIS_up
124416−41−7
149543−07−7
96515−74−1
104757−53−1
54527−84−3
123524−52−7
55985−32−5
91599−74−5
89226−75−5

All Activity Heatmap for Cluster 303

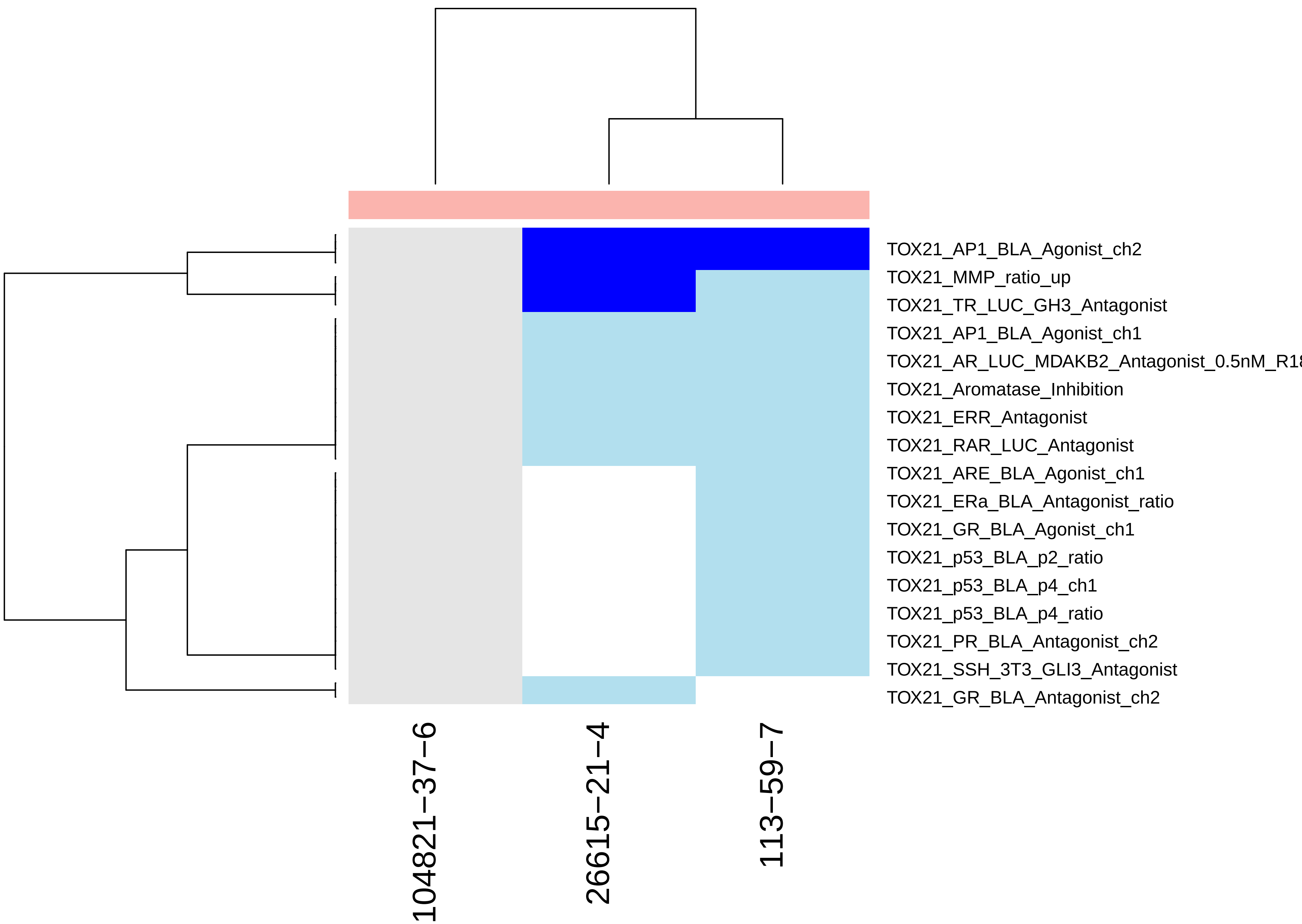

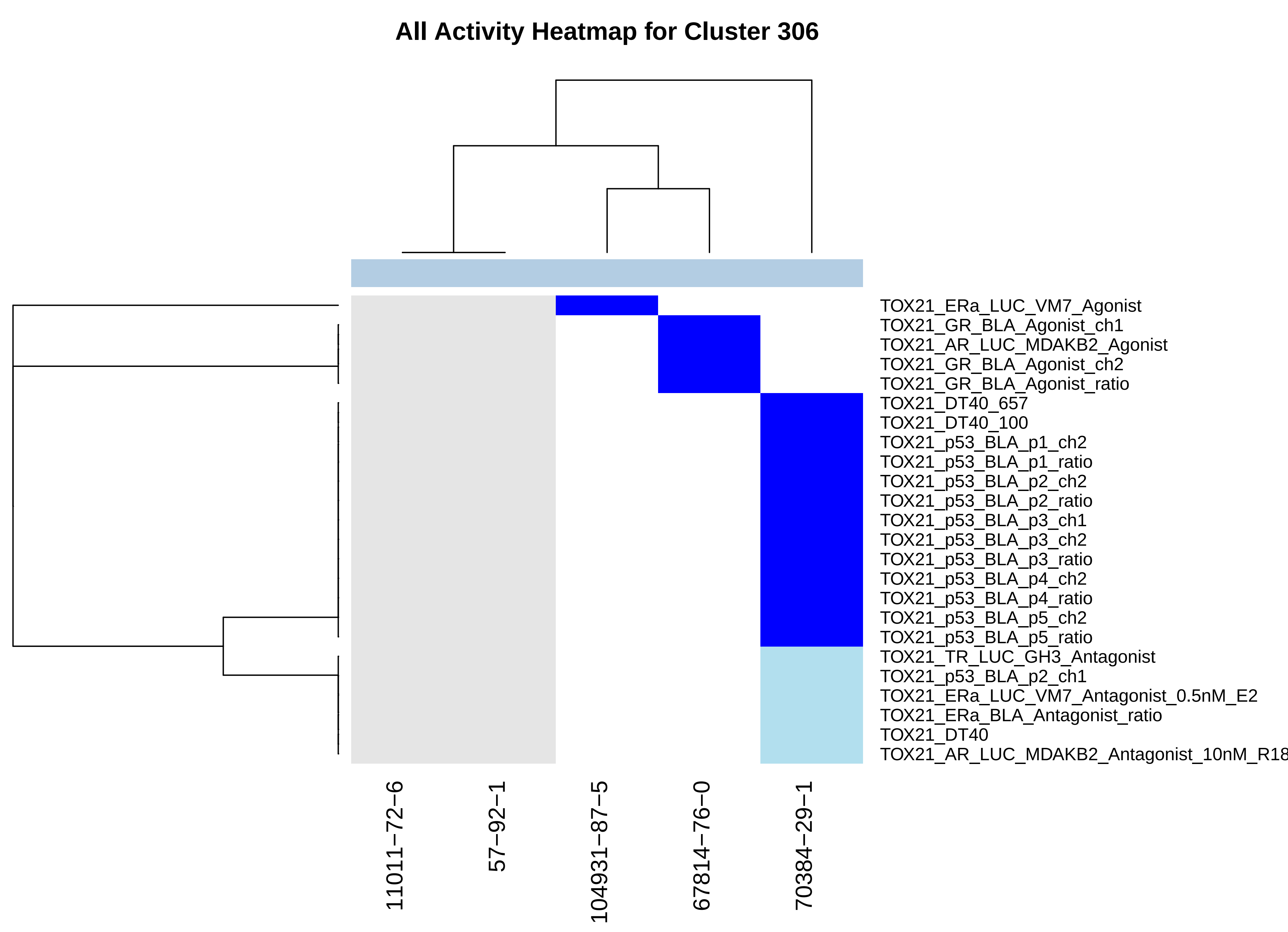

All Activity Heatmap for Cluster 306
TOX21_ERa_LUC_VM7_Agonist
TOX21_GR_BLA_Agonist_ch1
TOX21_AR_LUC_MDAKB2_Agonist
TOX21_GR_BLA_Agonist_ch2
TOX21_GR_BLA_Agonist_ratio
TOX21_DT40_657
TOX21_DT40_100
TOX21_p53_BLA_p1_ch2
TOX21_p53_BLA_p1_ratio
TOX21_p53_BLA_p2_ch2
TOX21_p53_BLA_p2_ratio
TOX21_p53_BLA_p3_ch1
TOX21_p53_BLA_p3_ch2
TOX21_p53_BLA_p3_ratio
TOX21_p53_BLA_p4_ch2
TOX21_p53_BLA_p4_ratio
TOX21_p53_BLA_p5_ch2
TOX21_p53_BLA_p5_ratio
TOX21_TR_LUC_GH3_Antagonist
TOX21_p53_BLA_p2_ch1
TOX21_ERa_LUC_VM7_Antagonist_0.5nM_E2
TOX21_ERa_BLA_Antagonist_ratio
TOX21_DT40
TOX21_AR_LUC_MDAKB2_Antagonist_10nM_R18
11011−72−6
57−92−1
104931−87−5
67814−76−0
70384−29−1

All Activity Heatmap for Cluster 307

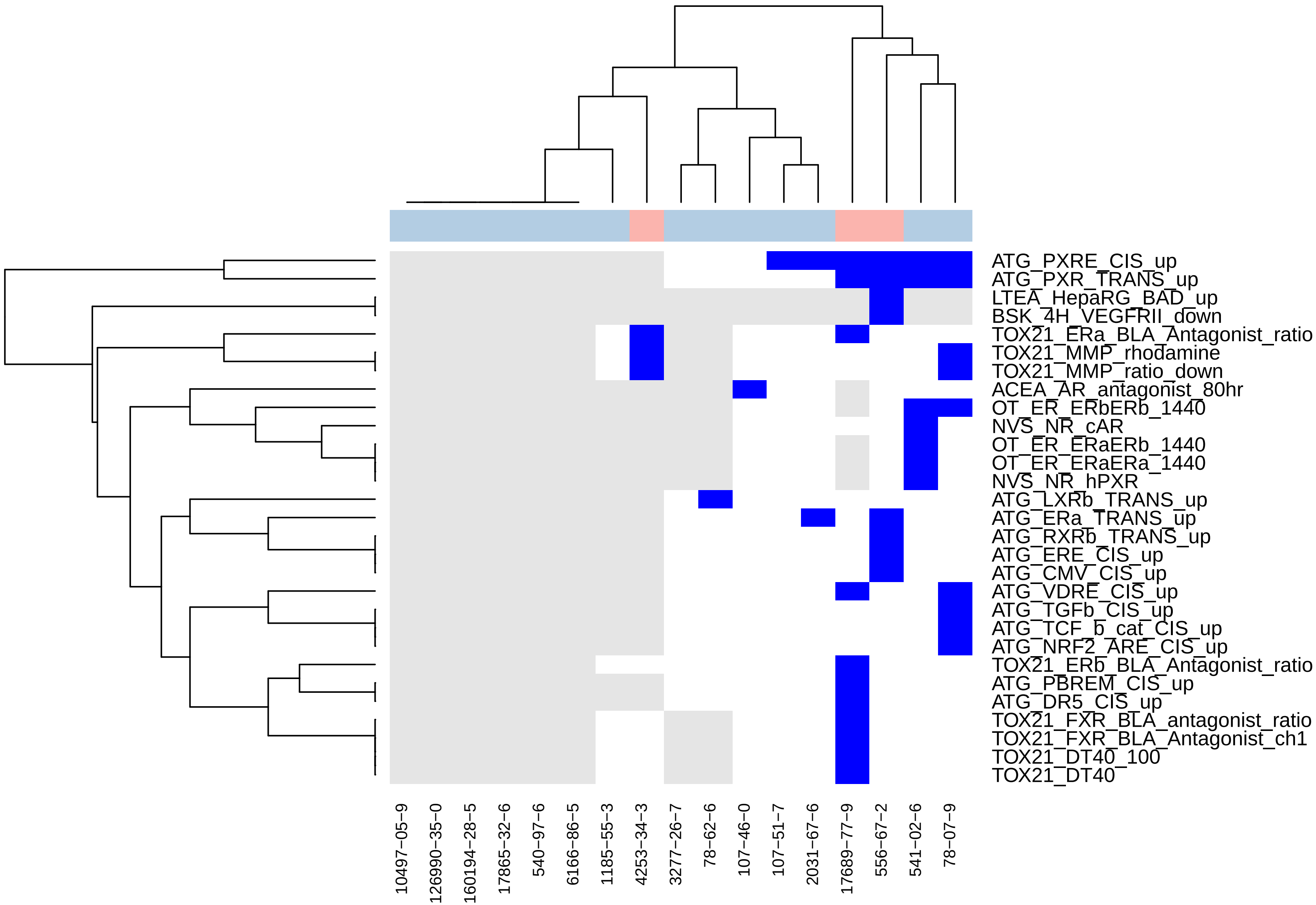

All Activity Heatmap for Cluster 308

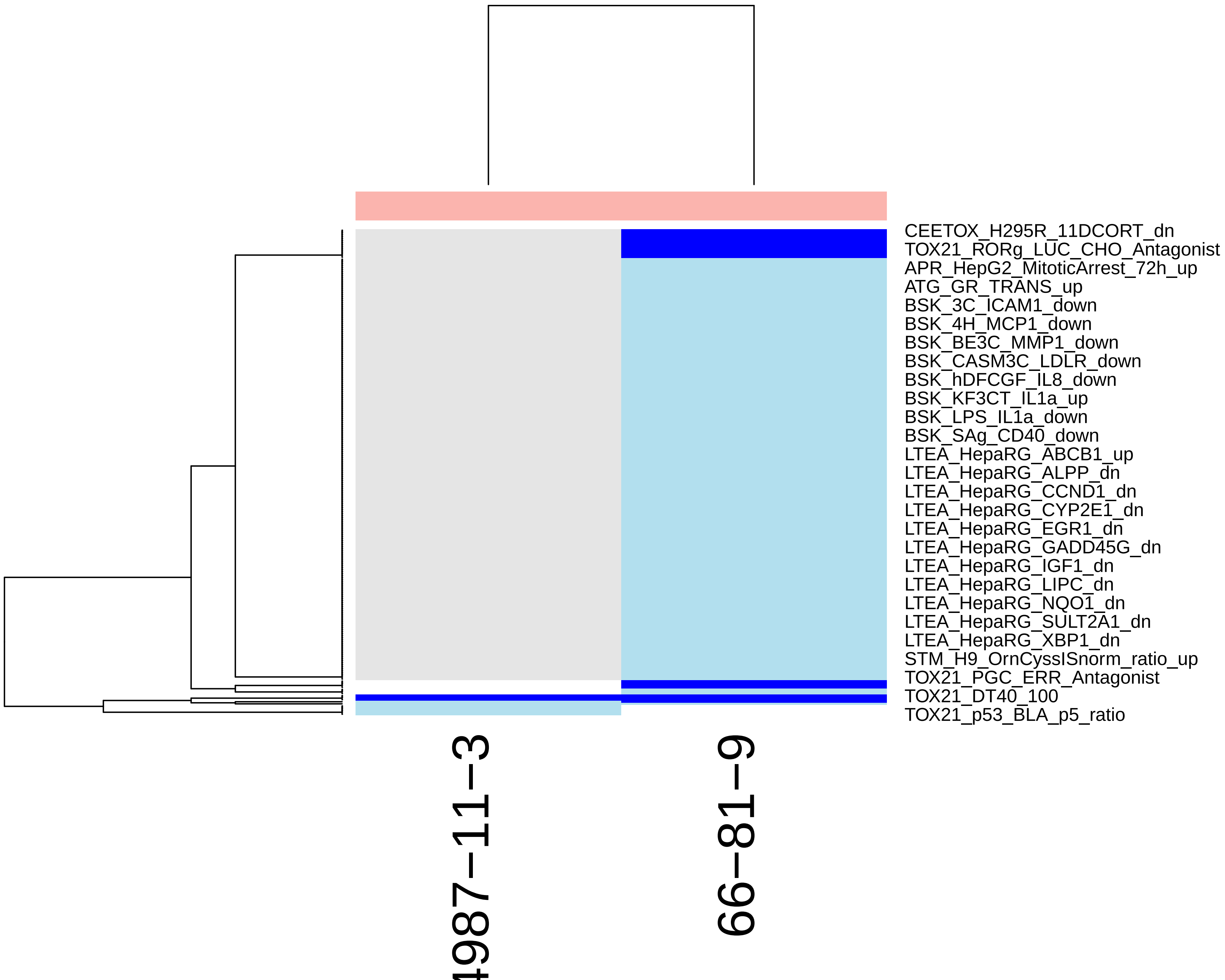

All Activity Heatmap for Cluster 309

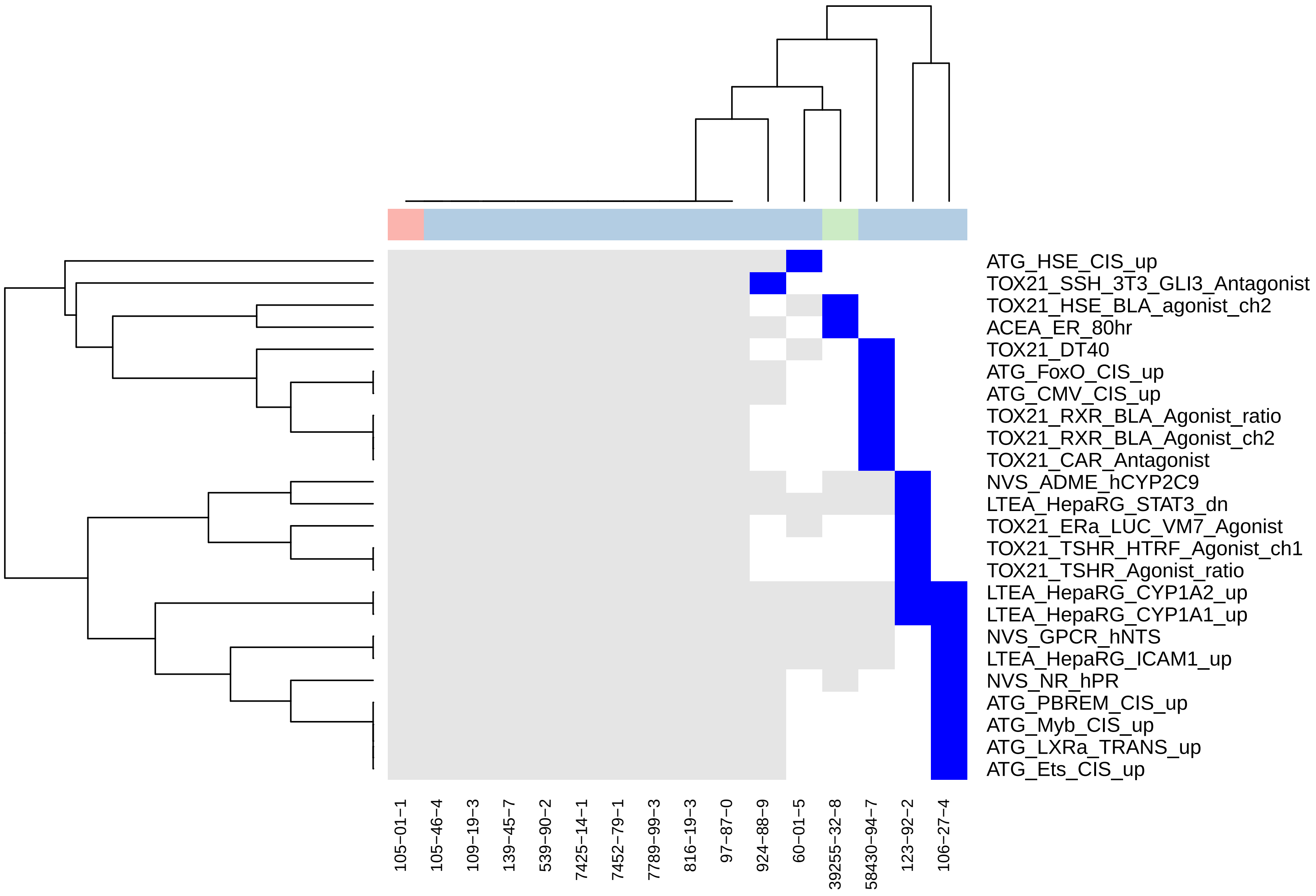

All Activity Heatmap for Cluster 311

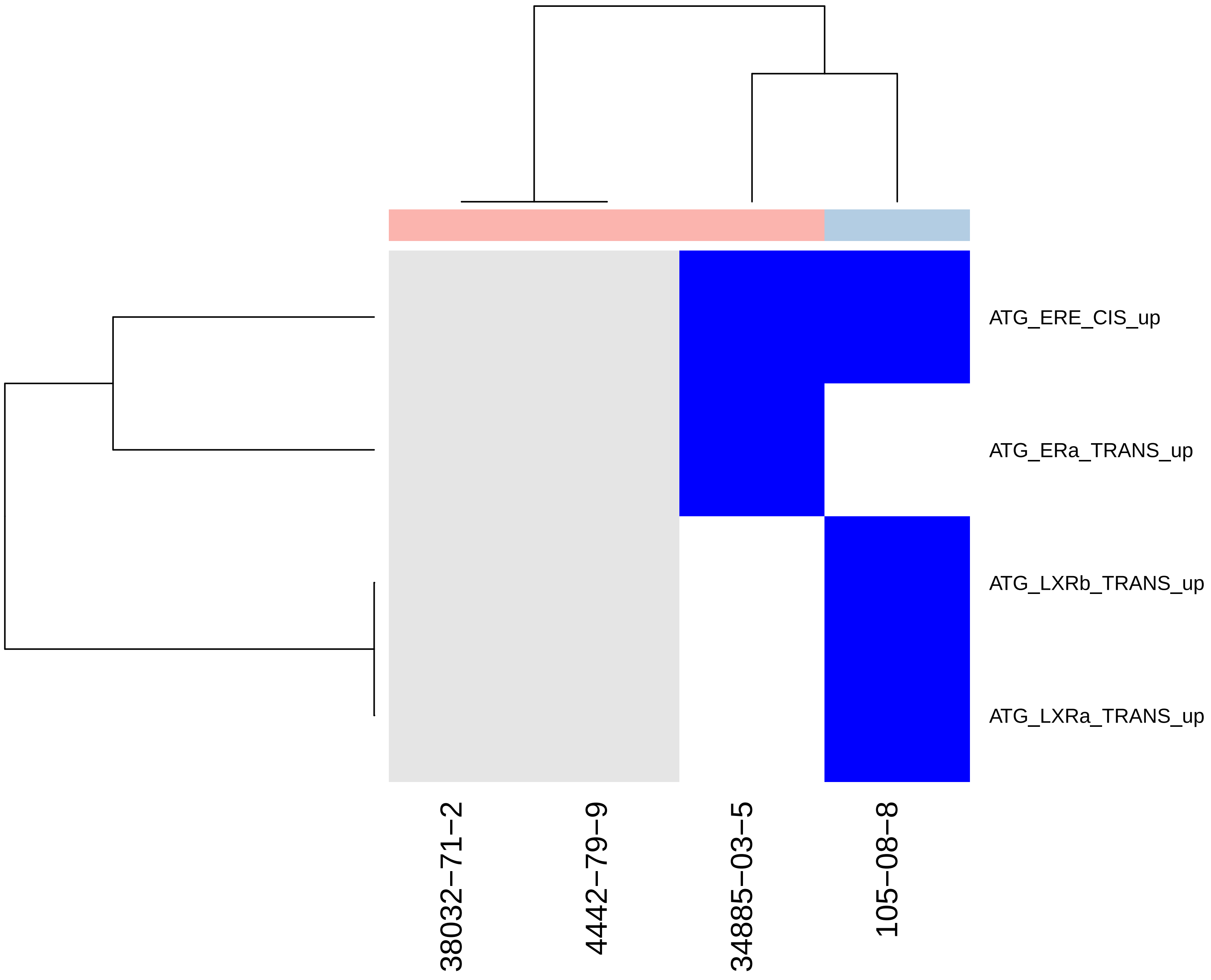

All Activity Heatmap for Cluster 312

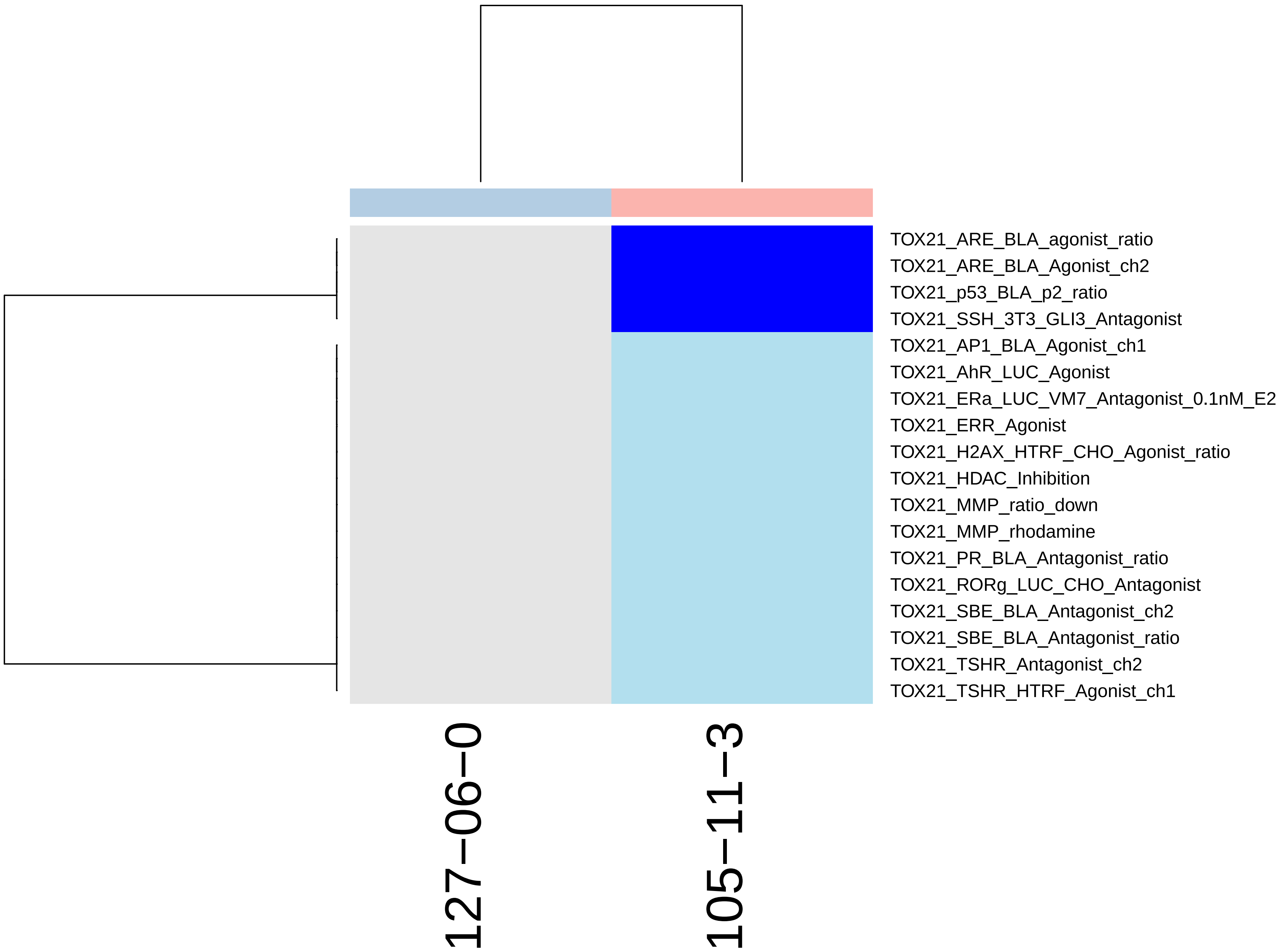

All Activity Heatmap for Cluster 314

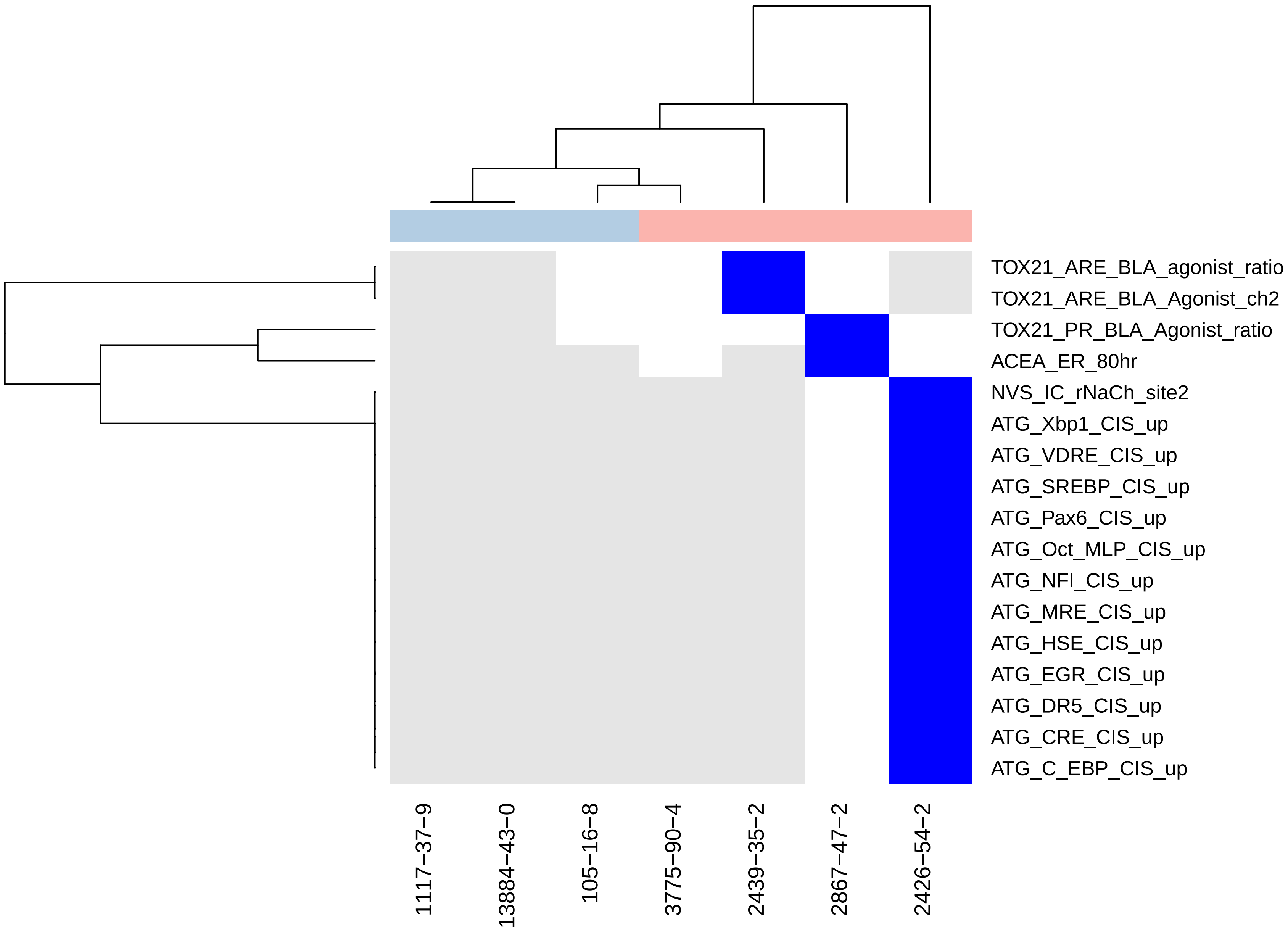

All Activity Heatmap for Cluster 315

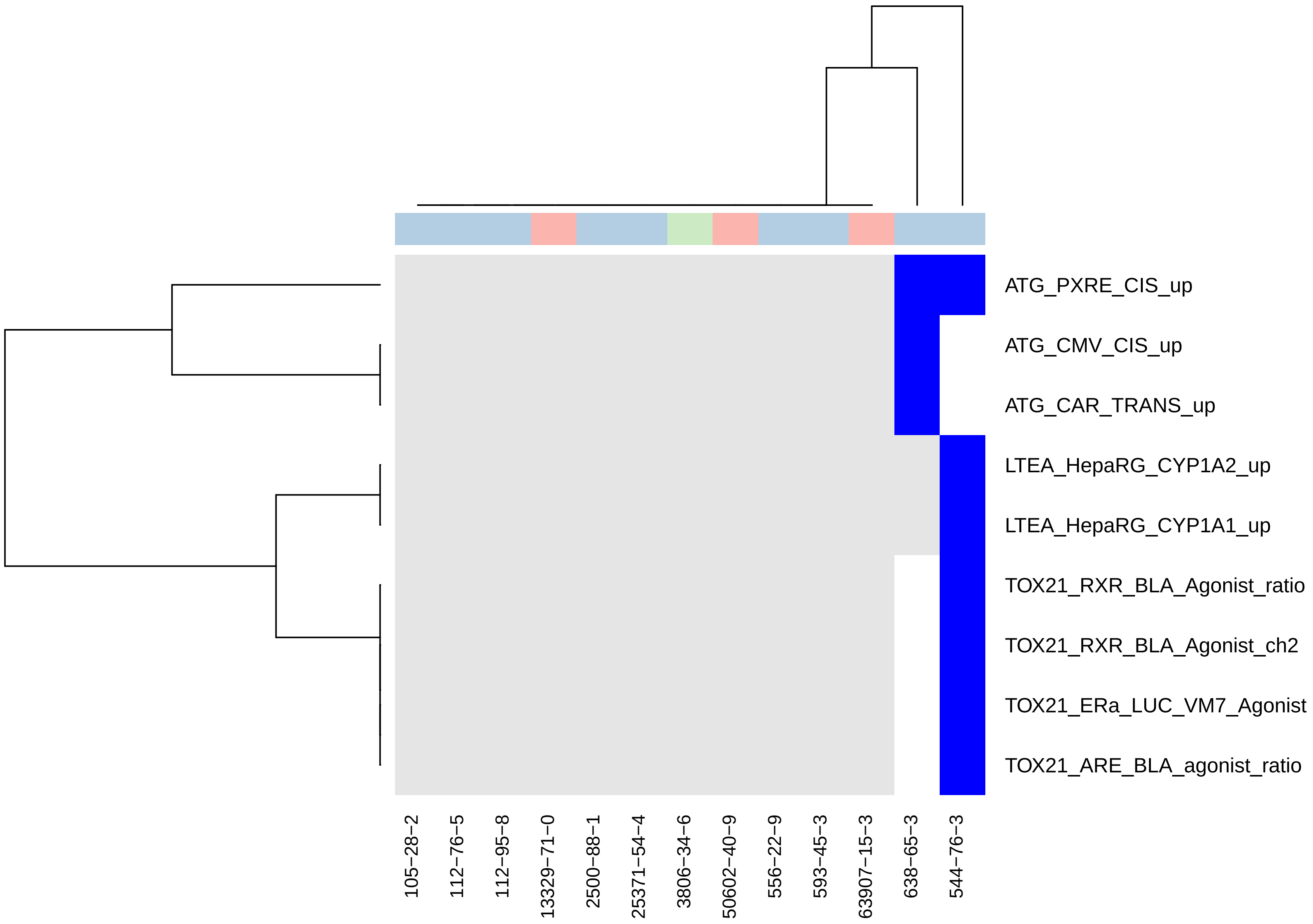

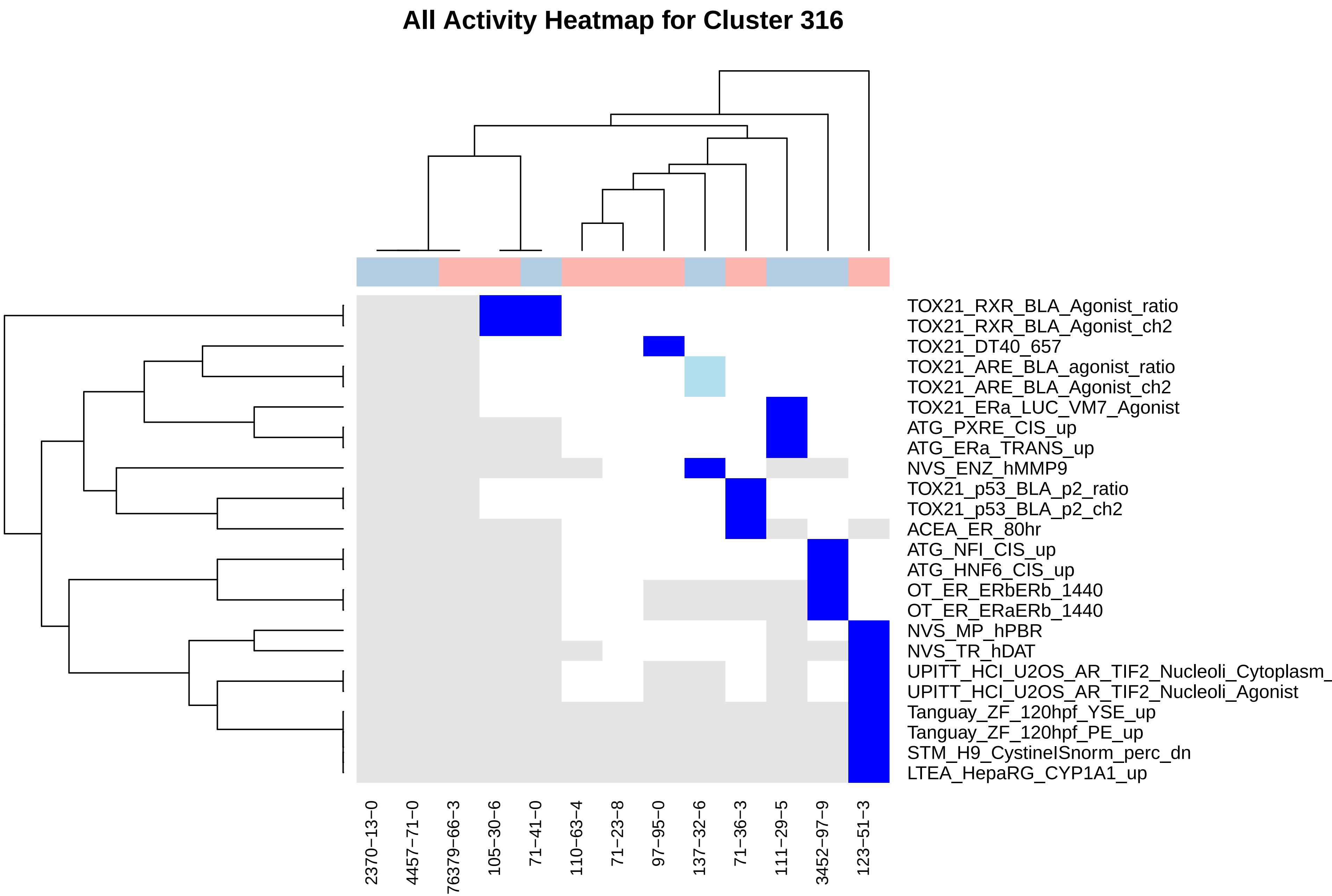
All Activity Heatmap for Cluster 316
TOX21_RXR_BLA_Agonist_ratio
TOX21_RXR_BLA_Agonist_ch2
TOX21_DT40_657
TOX21_ARE_BLA_agonist_ratio
TOX21_ARE_BLA_Agonist_ch2
TOX21_ERa_LUC_VM7_Agonist
ATG_PXRE_CIS_up
ATG_ERa_TRANS_up
NVS_ENZ_hMMP9
TOX21_p53_BLA_p2_ratio
TOX21_p53_BLA_p2_ch2
ACEA_ER_80hr
ATG_NFI_CIS_up
ATG_HNF6_CIS_up
OT_ER_ERbERb_1440
OT_ER_ERaERb_1440
NVS_MP_hPBR
NVS_TR_hDAT
UPITT_HCI_U2OS_AR_TIF2_Nucleoli_Cytoplasm_
UPITT_HCI_U2OS_AR_TIF2_Nucleoli_Agonist
Tanguay_ZF_120hpf_YSE_up
Tanguay_ZF_120hpf_PE_up
STM_H9_CystineISnorm_perc_dn
LTEA_HepaRG_CYP1A1_up
2370−13−0
4457−71−0
76379−66−3
105−30−6
71−41−0
110−63−4
71−23−8
97−95−0
137−32−6
71−36−3
111−29−5
3452−97−9
123−51−3

All Activity Heatmap for Cluster 317

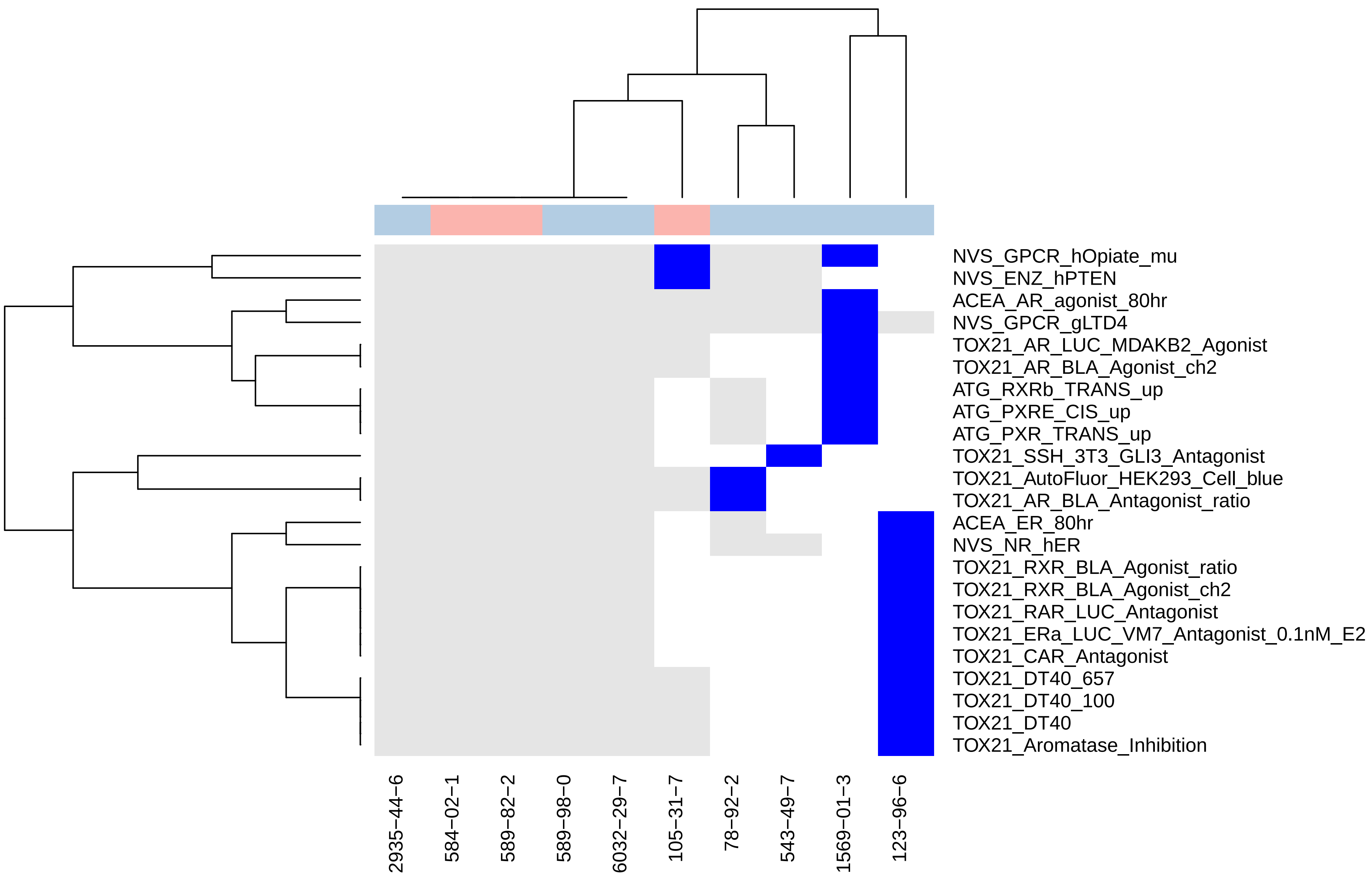

All Activity Heatmap for Cluster 318

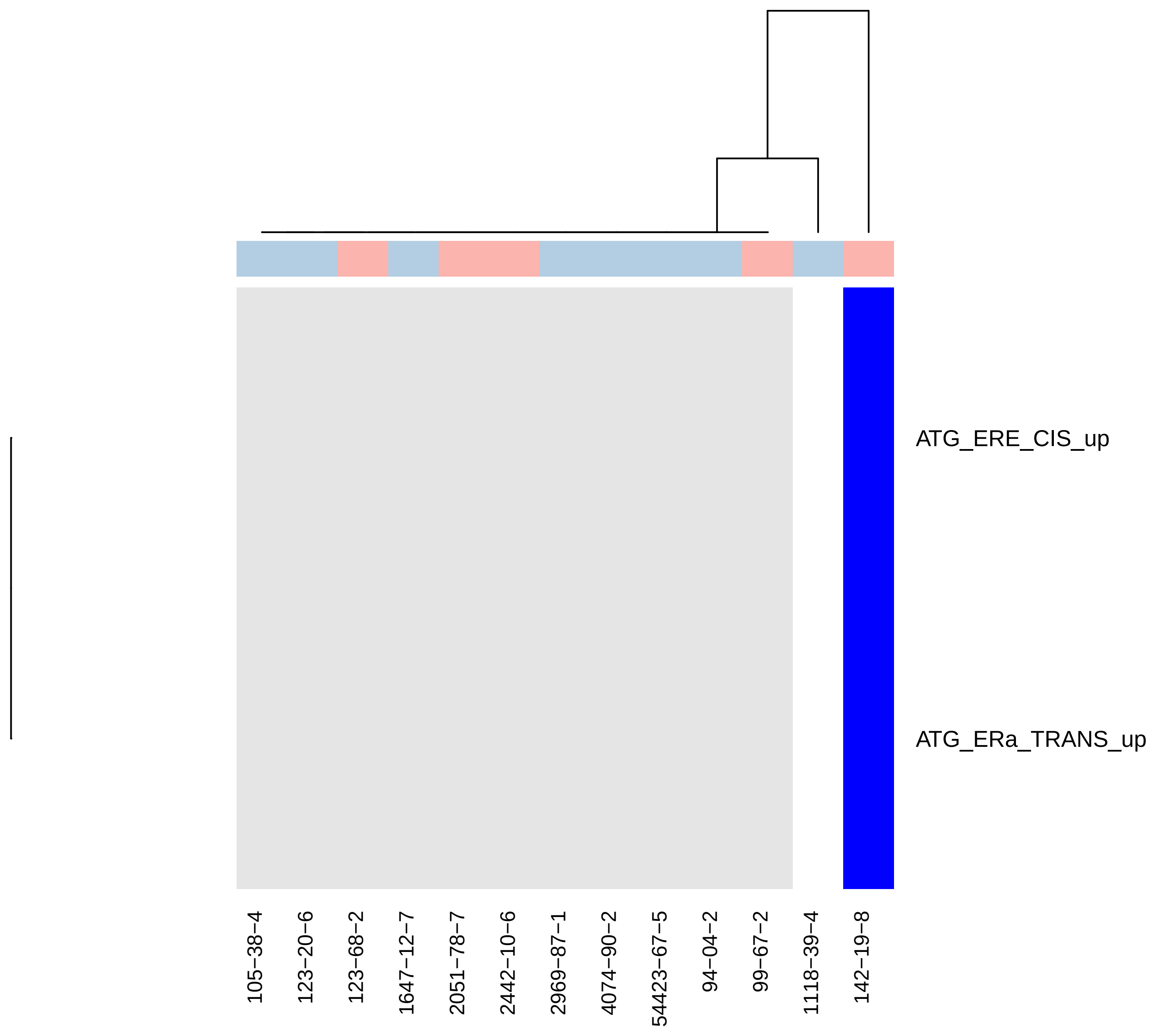

All Activity Heatmap for Cluster 319

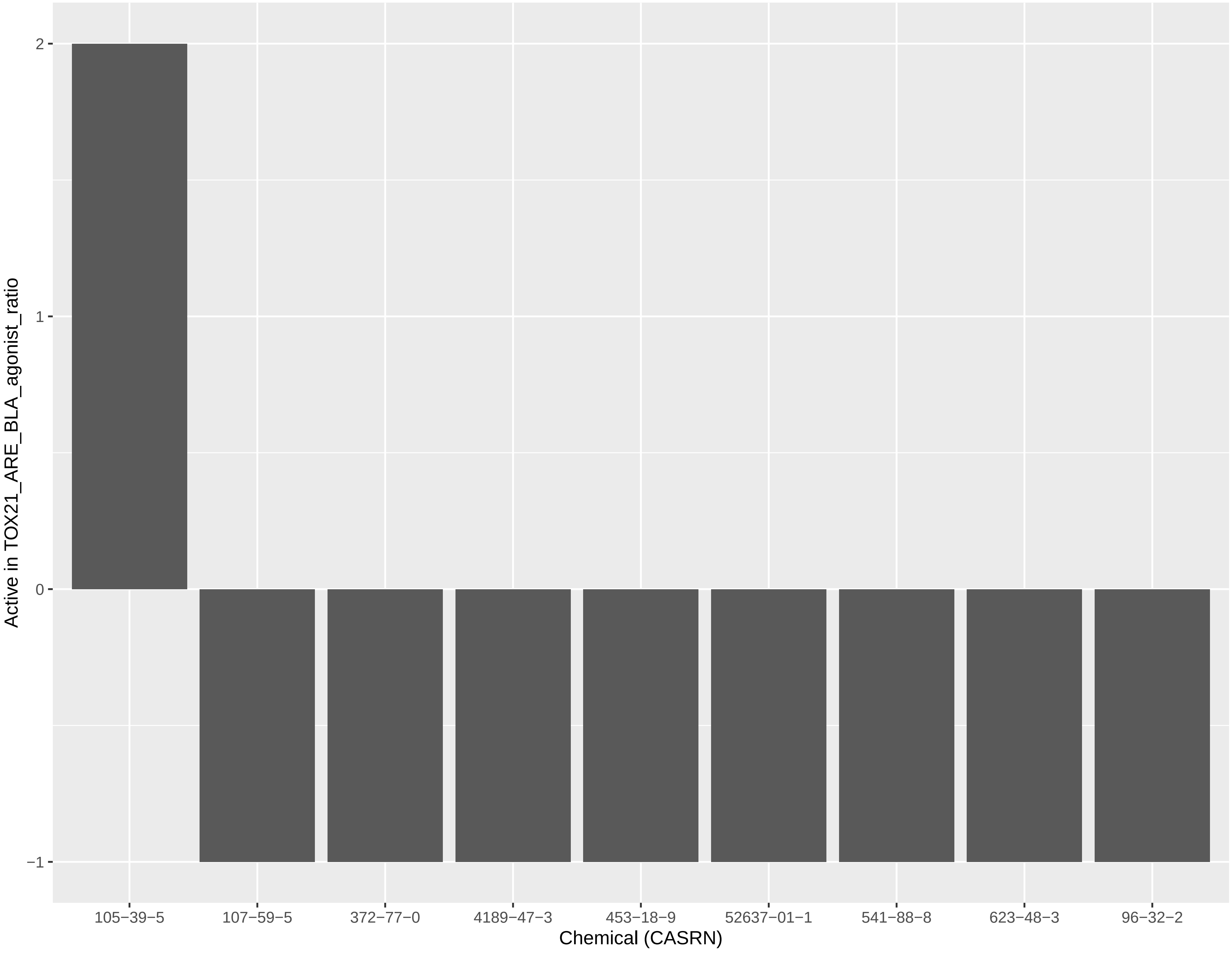

All Activity Heatmap for Cluster 320

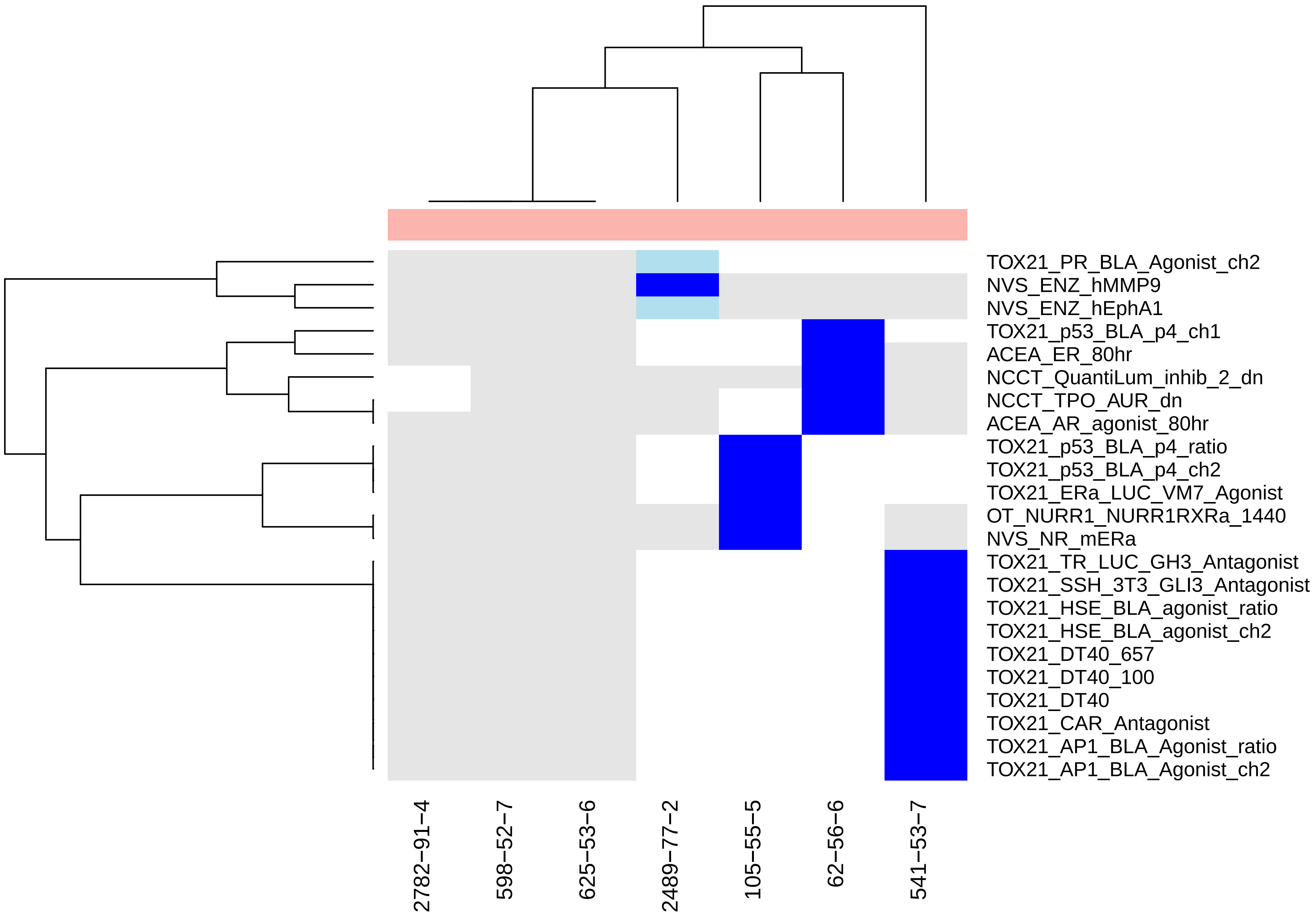

All Activity Heatmap for Cluster 321

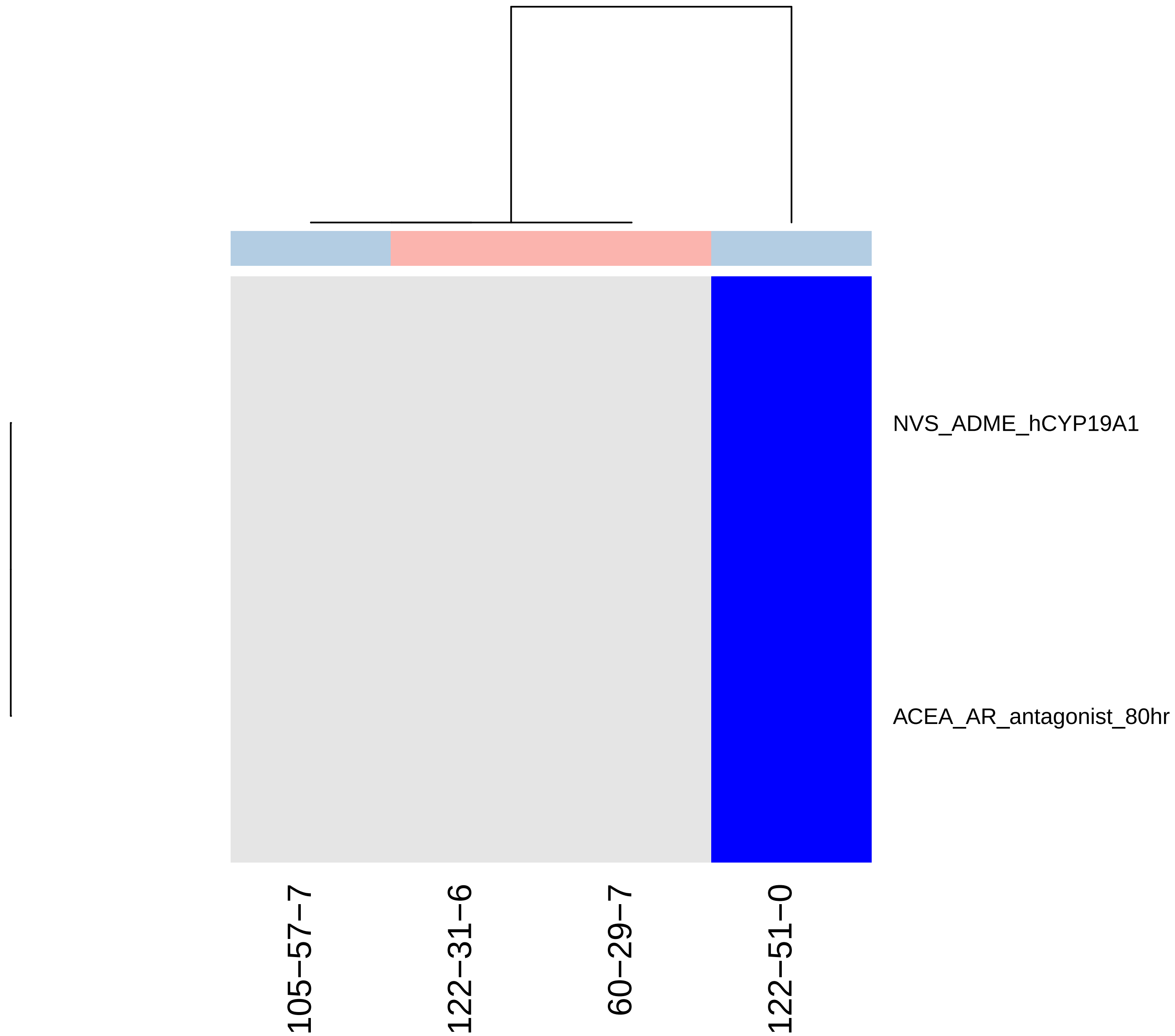

All Activity Heatmap for Cluster 322

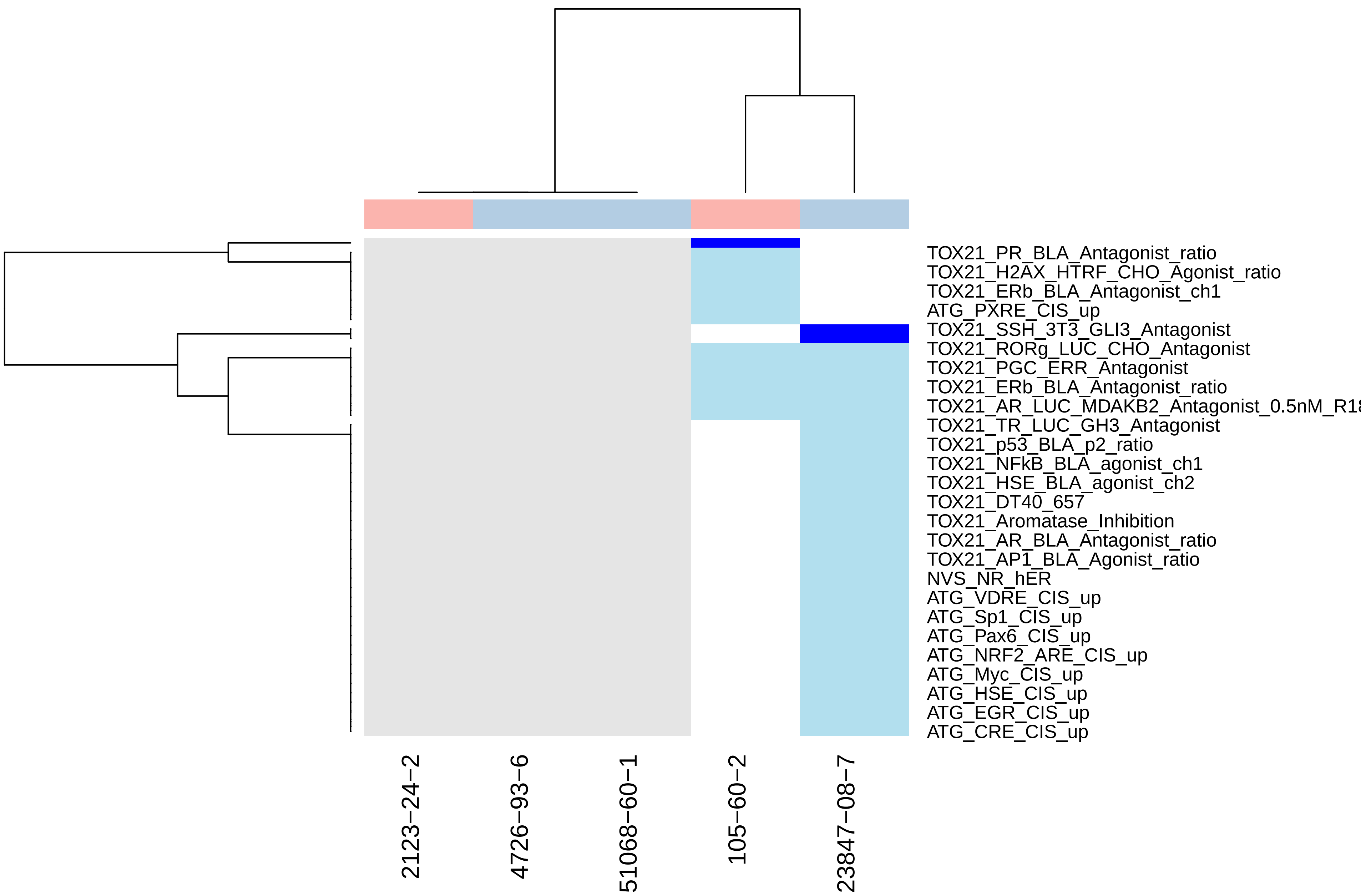

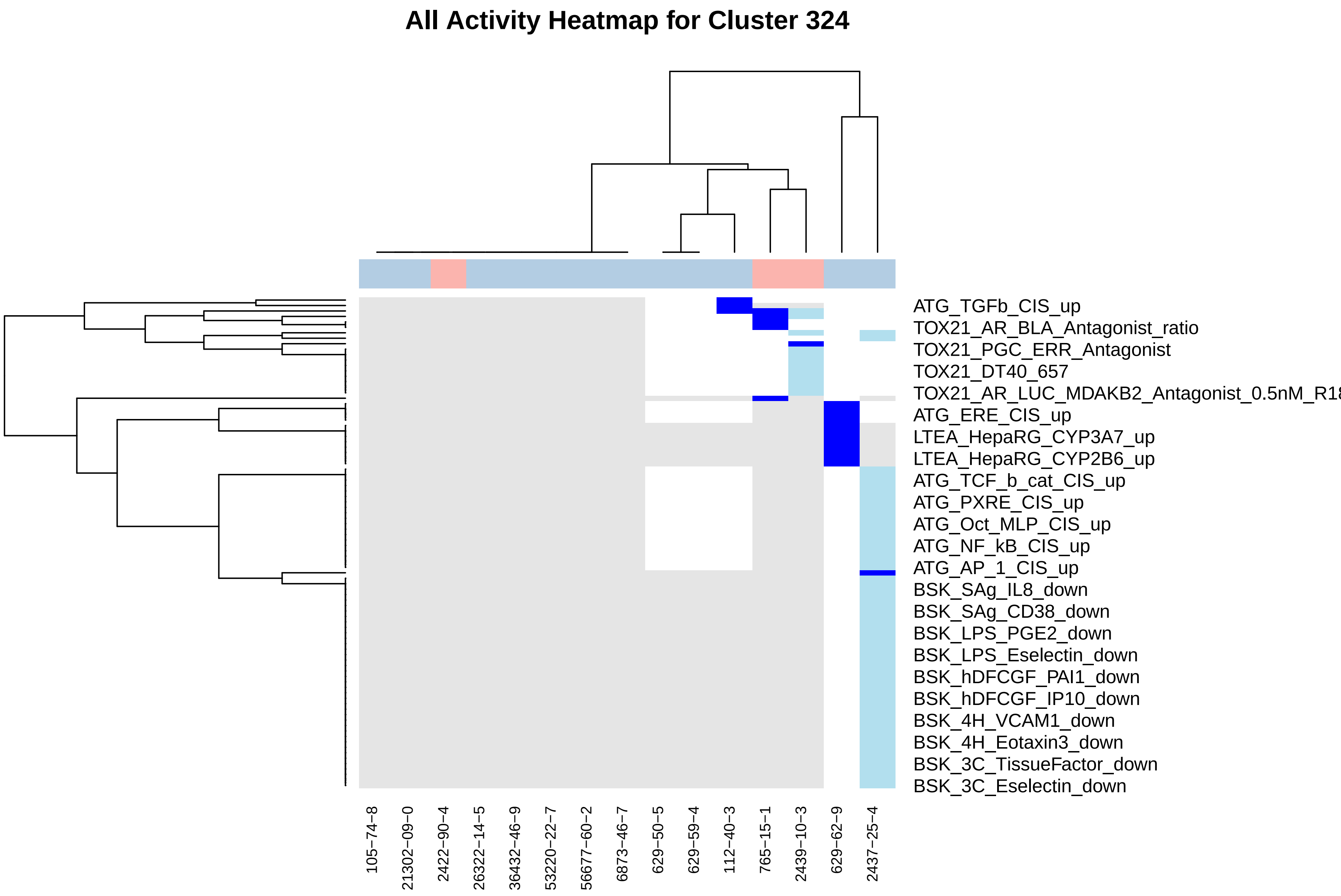
All Activity Heatmap for Cluster 324
ATG_TGFb_CIS_up
TOX21_AR_BLA_Antagonist_ratio
TOX21_PGC_ERR_Antagonist
TOX21_DT40_657
ATG_ERE_CIS_up
LTEA_HepaRG_CYP3A7_up
LTEA_HepaRG_CYP2B6_up
ATG_TCF_b_cat_CIS_up
ATG_PXRE_CIS_up
ATG_Oct_MLP_CIS_up
ATG_NF_kB_CIS_up
ATG_AP_1_CIS_up
BSK_SAg_IL8_down
BSK_SAg_CD38_down
BSK_LPS_PGE2_down
BSK_LPS_Eselectin_down
BSK_hDFCGF_PAI1_down
BSK_hDFCGF_IP10_down
BSK_4H_VCAM1_down
BSK_4H_Eotaxin3_down
BSK_3C_TissueFactor_down
BSK_3C_Eselectin_down
105−74−8
21302−09−0
2422−90−4
26322−14−5
36432−46−9
53220−22−7
56677−60−2
6873−46−7
629−50−5
629−59−4
112−40−3
765−15−1
2439−10−3
629−62−9
2437−25−4

All Activity Heatmap for Cluster 325

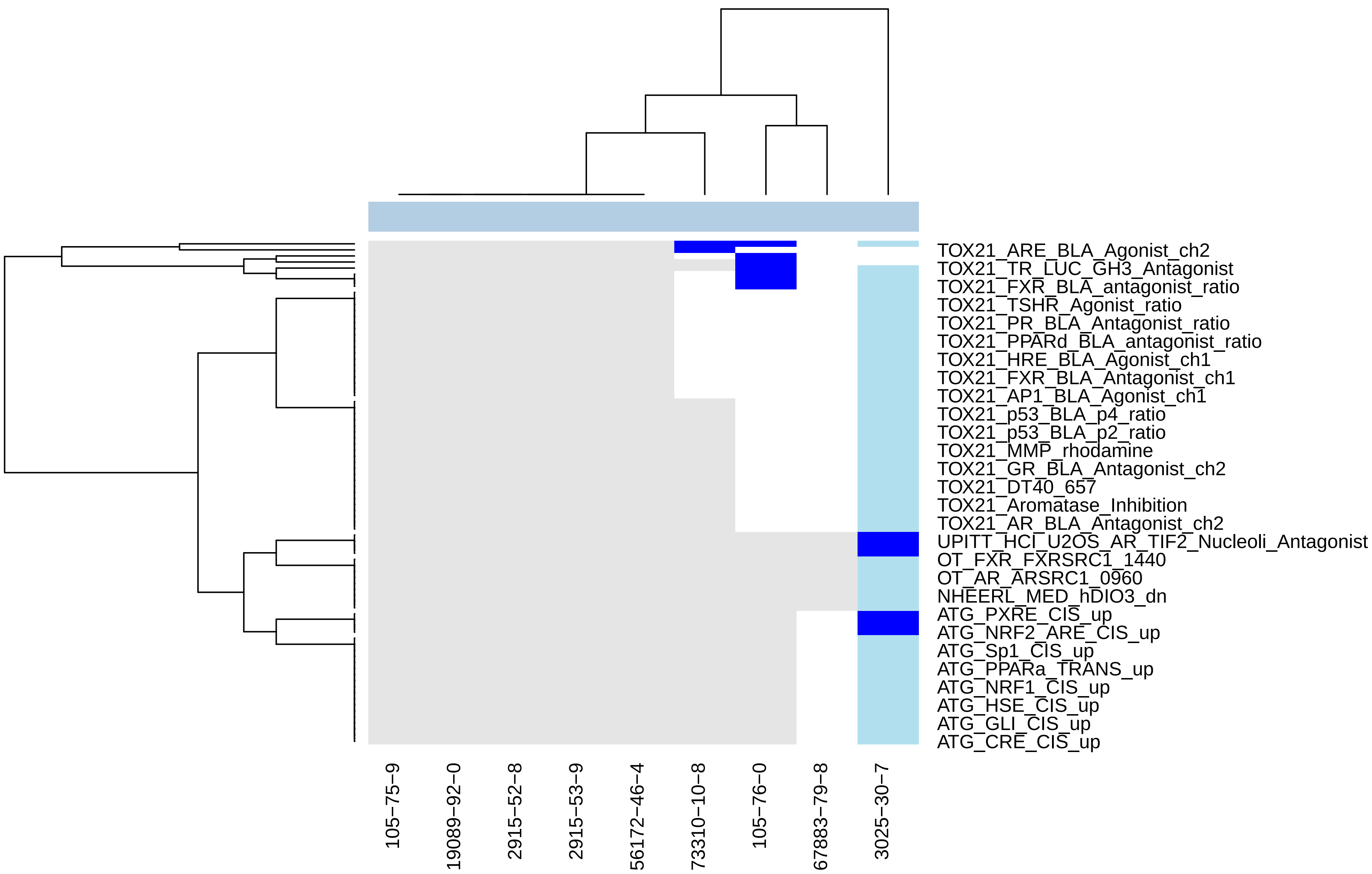

All Activity Heatmap for Cluster 326

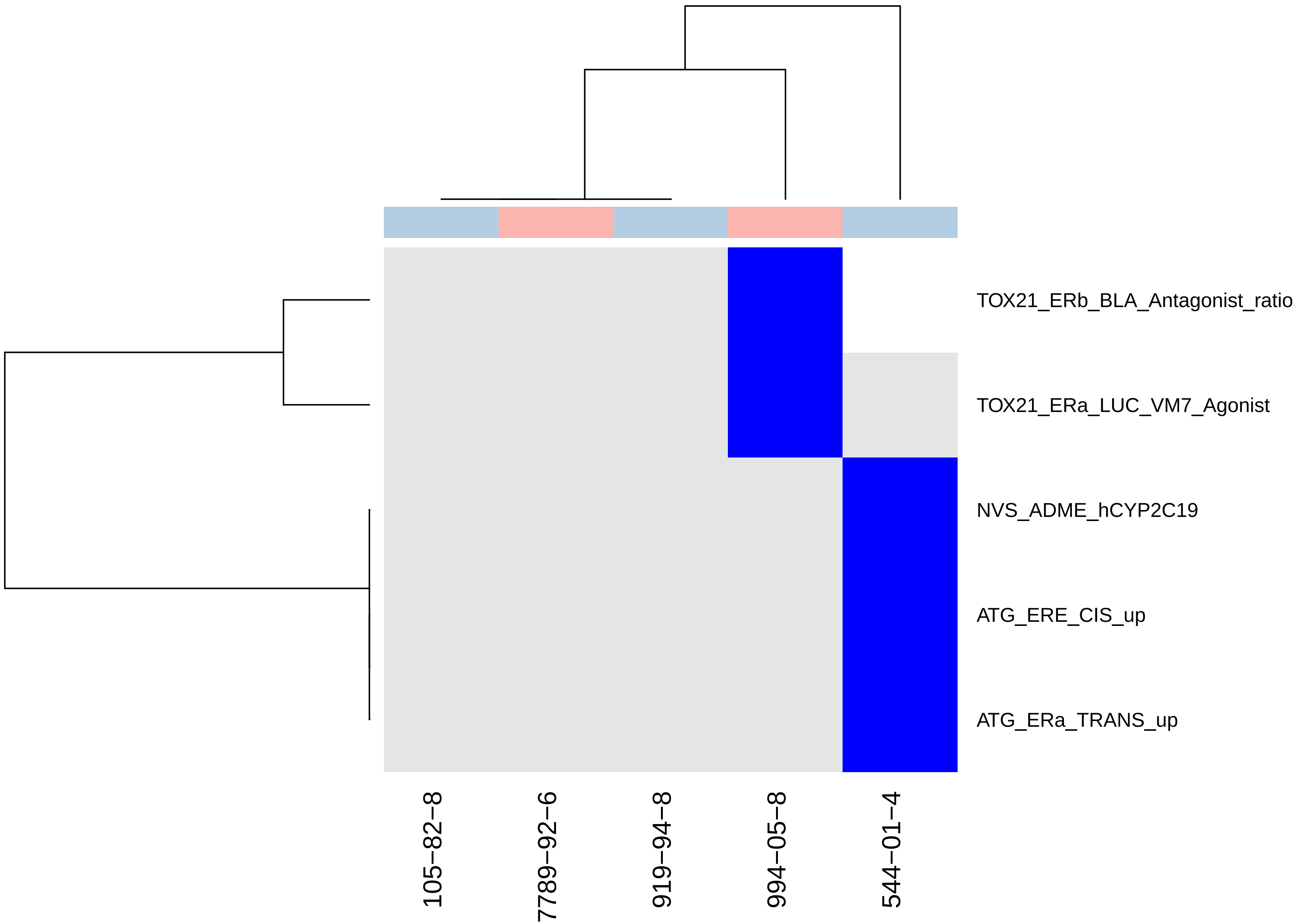

All Activity Heatmap for Cluster 327

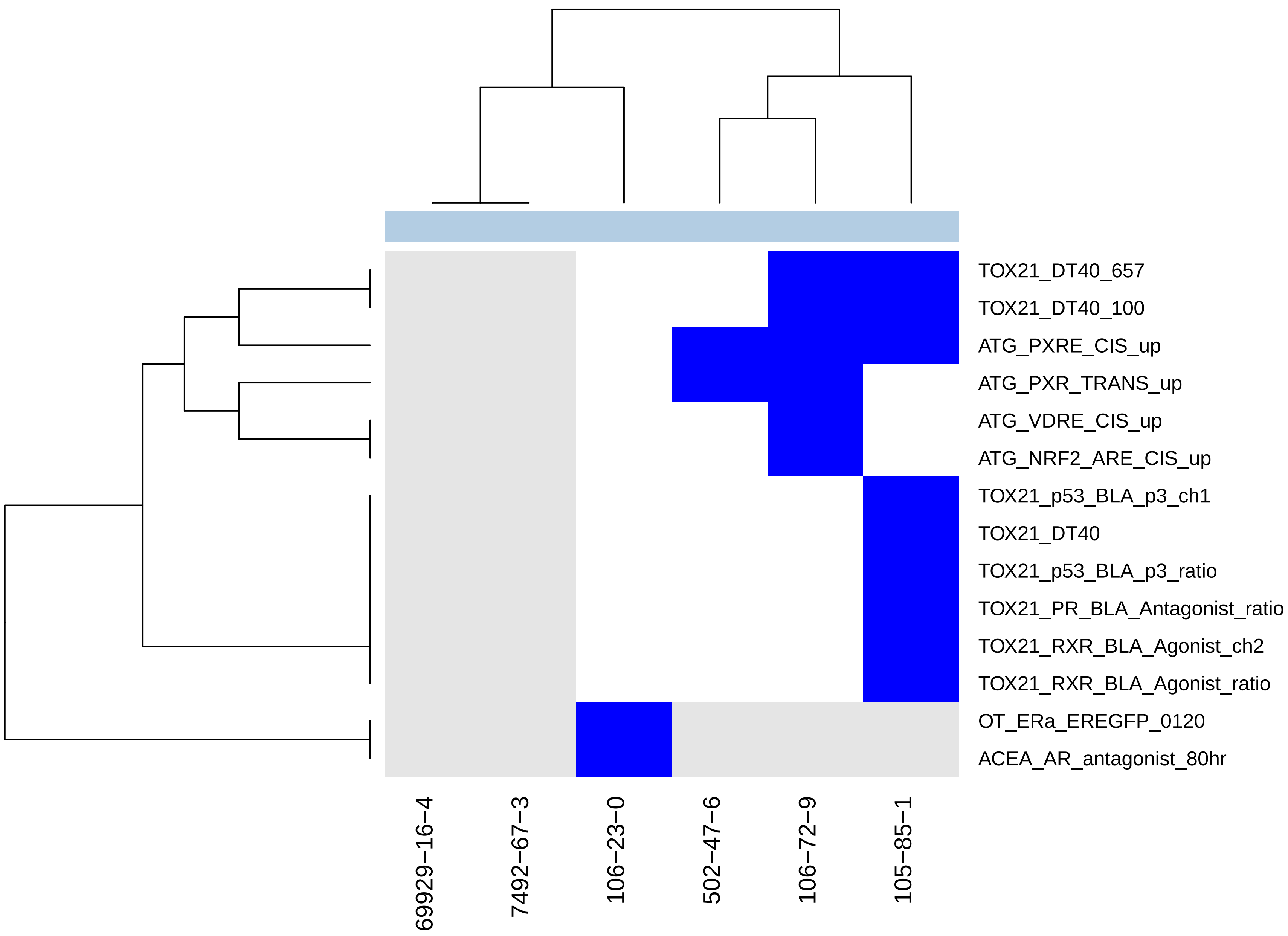

All Activity Heatmap for Cluster 328

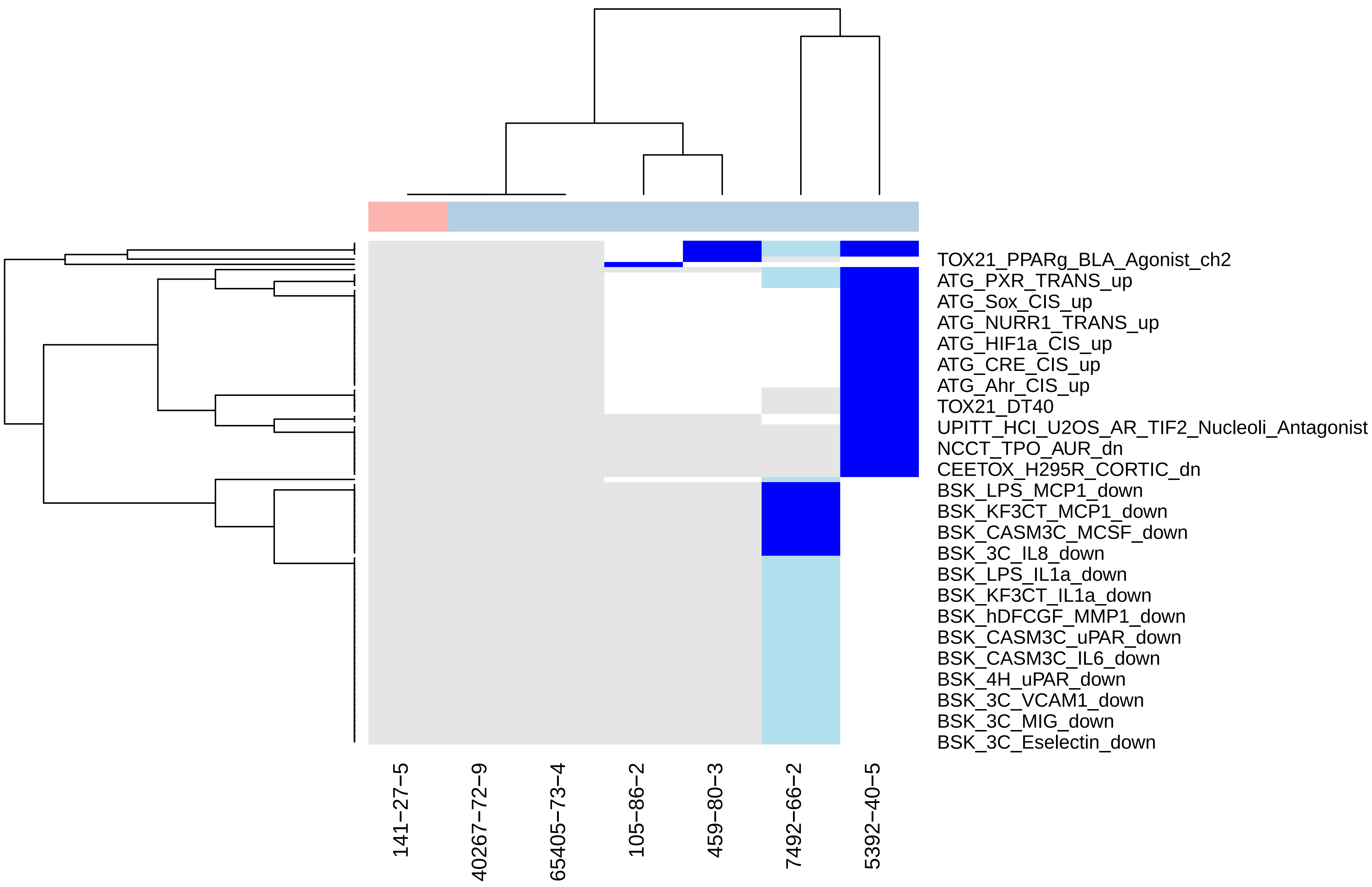

All Activity Heatmap for Cluster 329

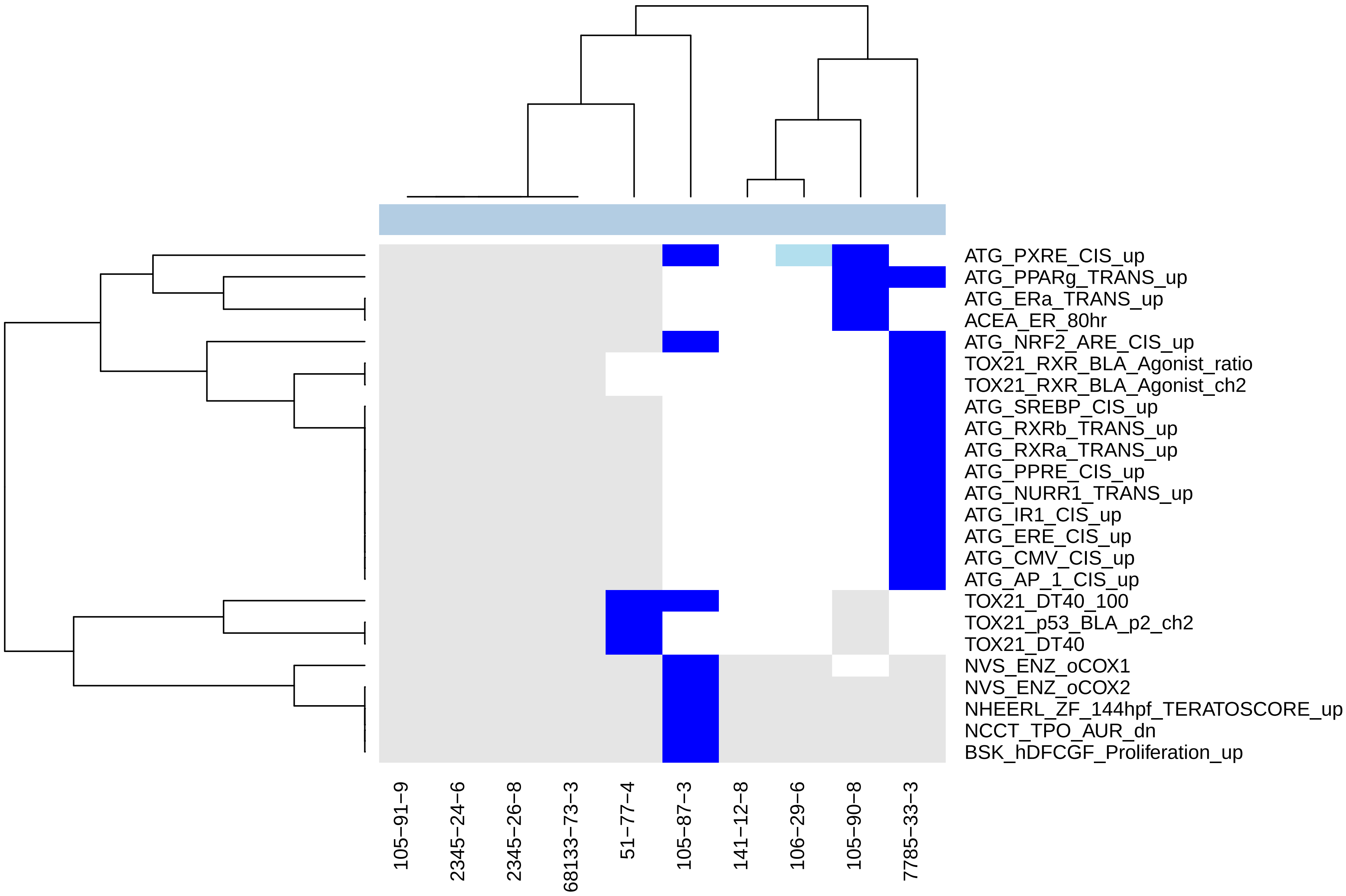

All Activity Heatmap for Cluster 330

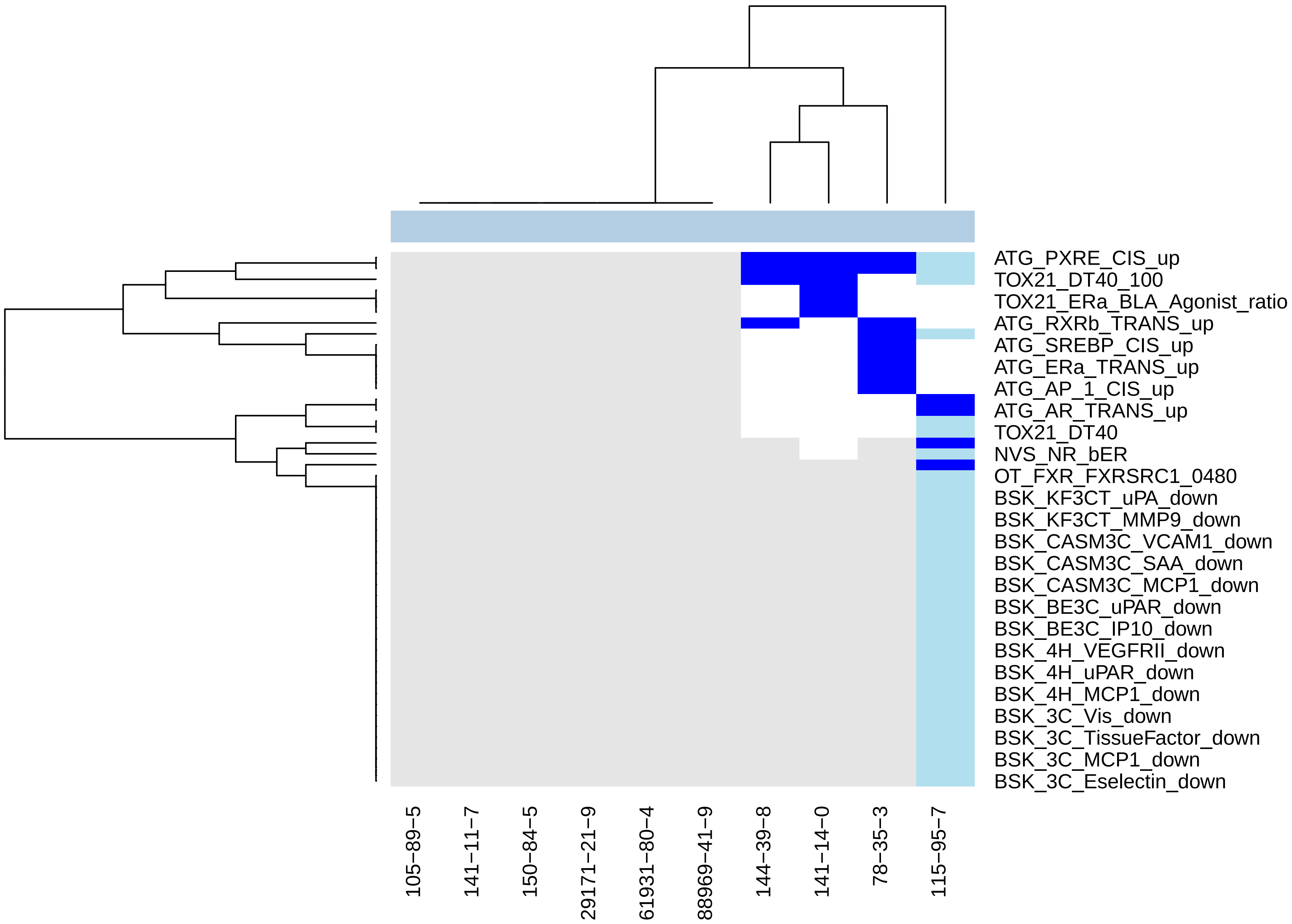

All Activity Heatmap for Cluster 331

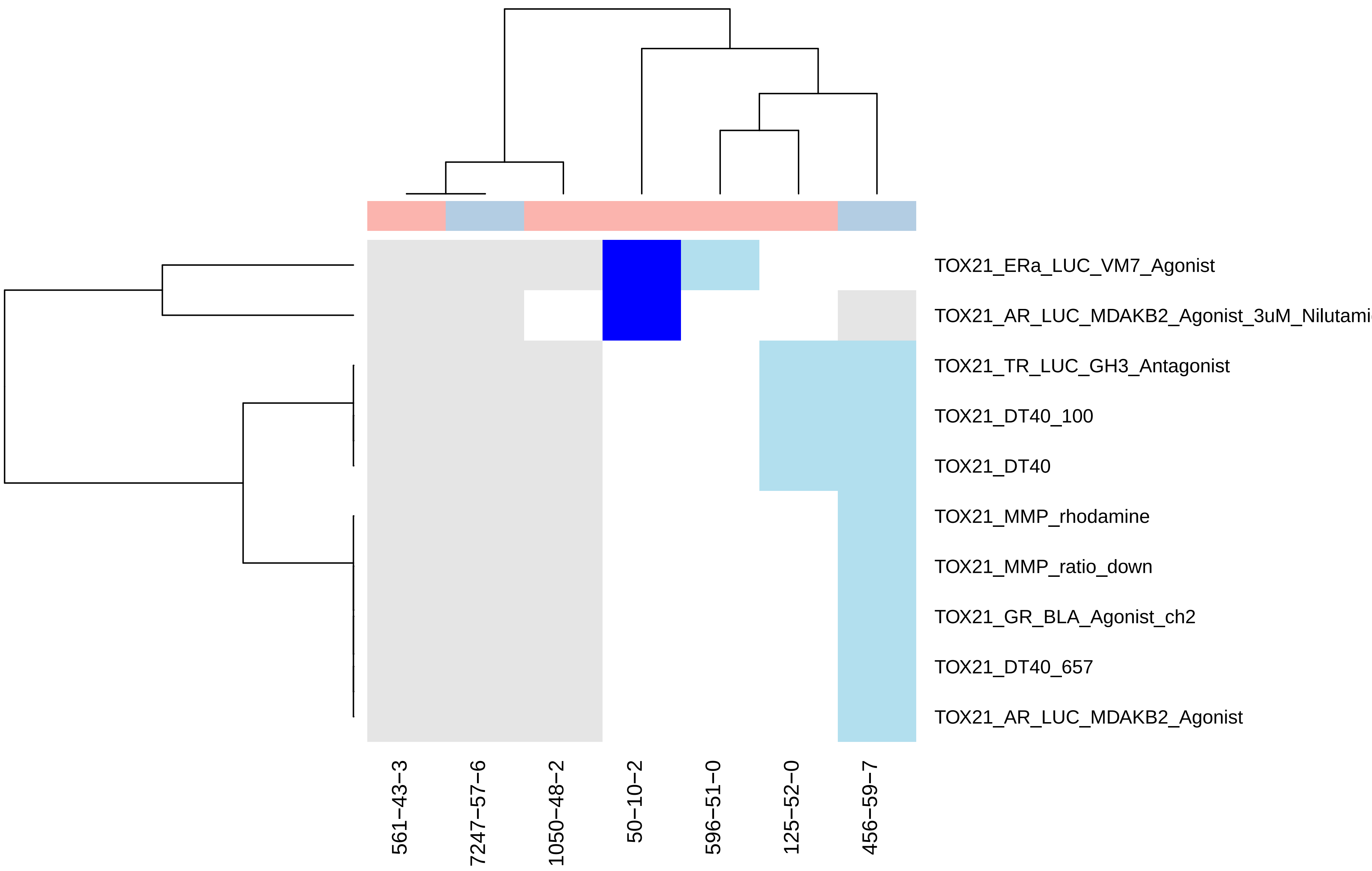

All Activity Heatmap for Cluster 332

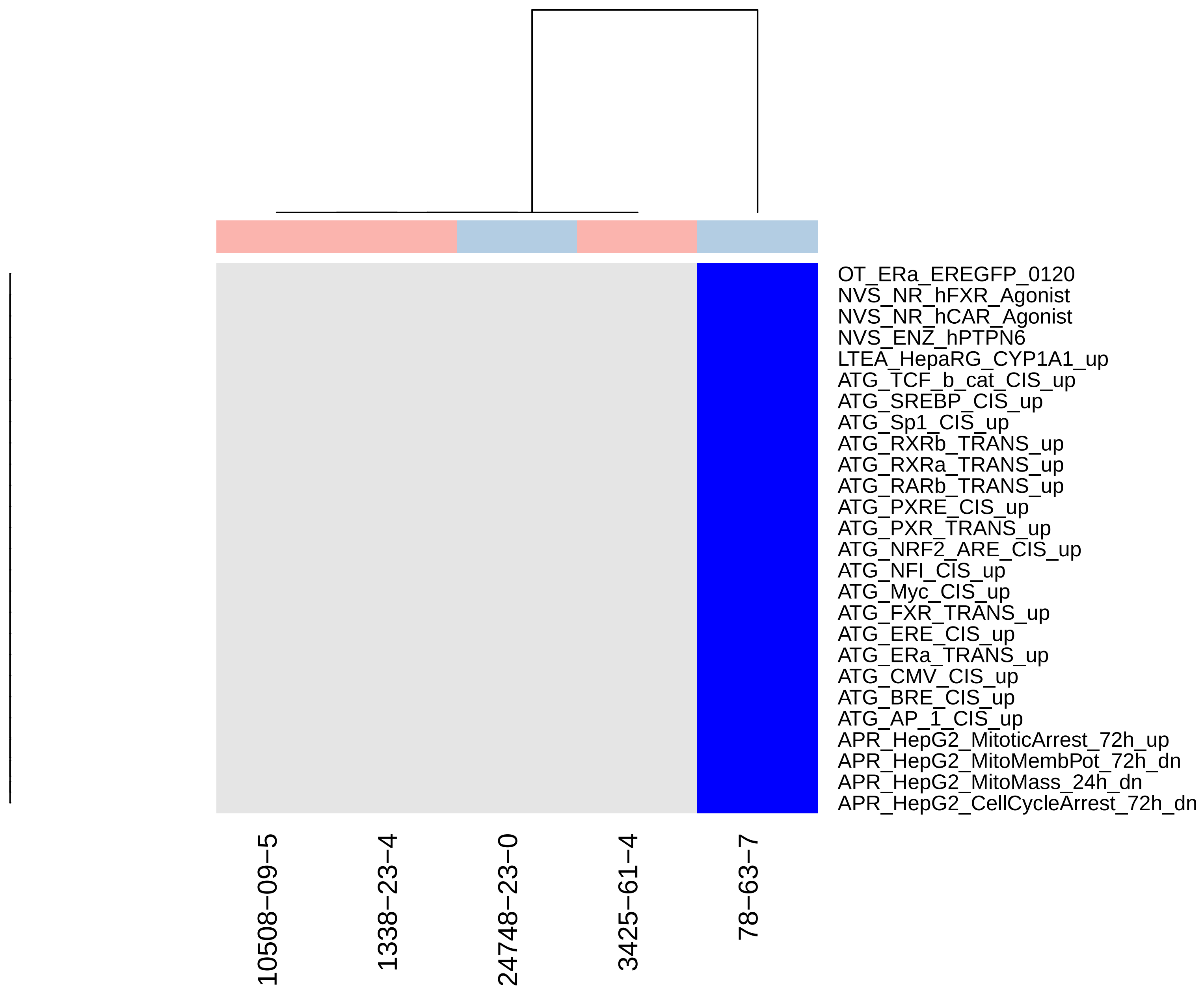

All Activity Heatmap for Cluster 333

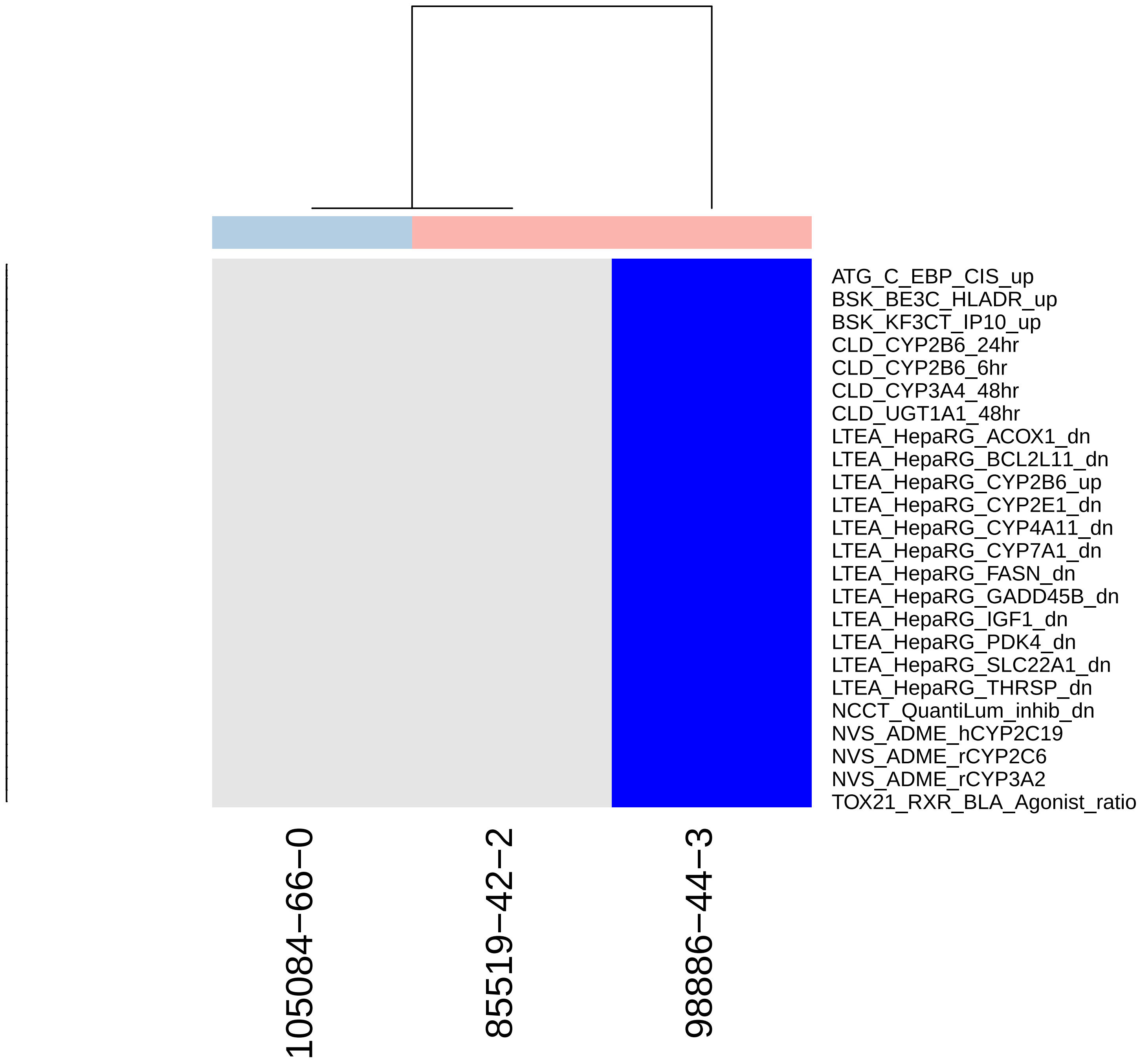

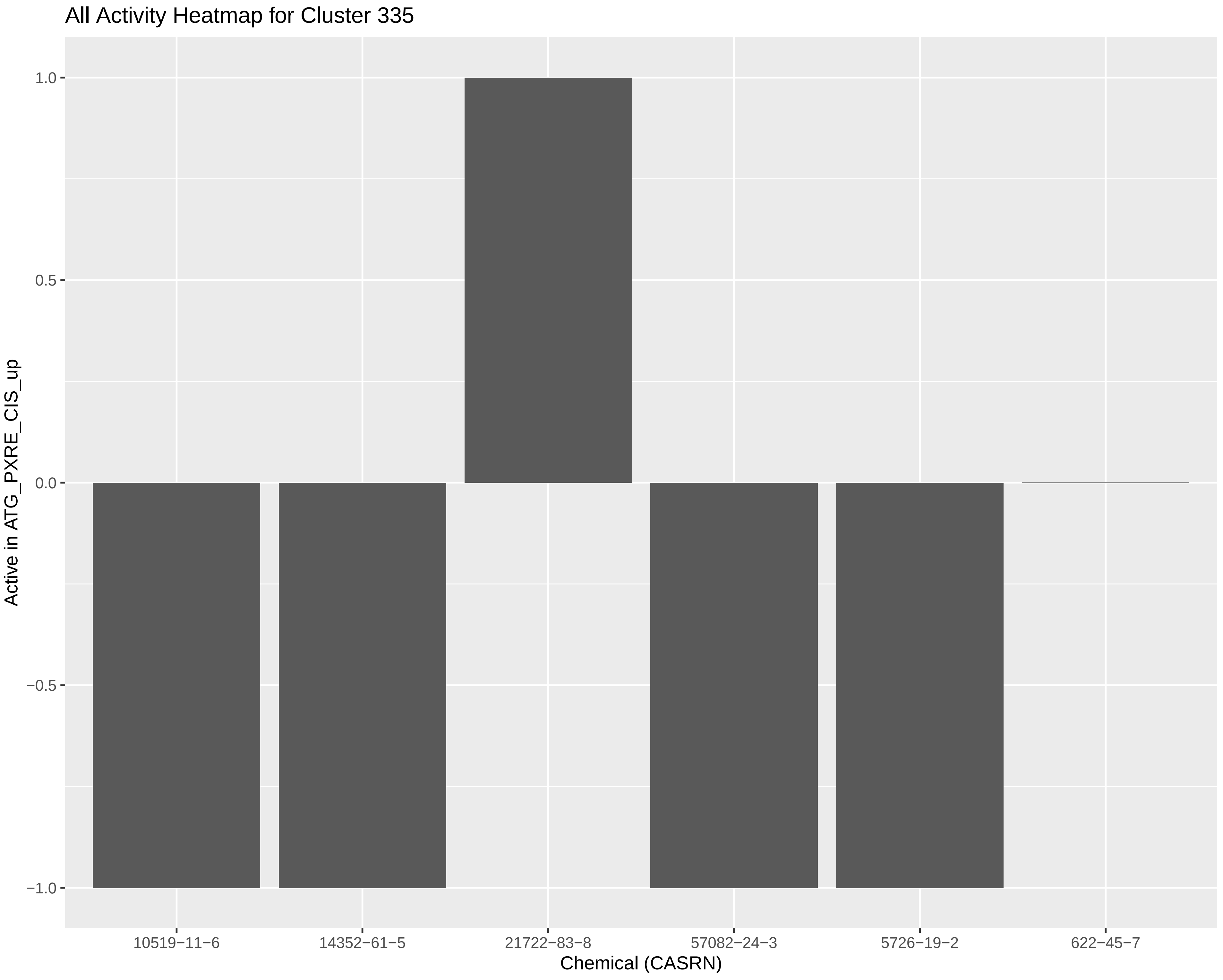

All Activity Heatmap for Cluster 335
Active in ATG_PXRE_CIS_up
1.0
0.5
0.0
−0.5
−1.0
10519−11−6
14352−61−5
21722−83−8
57082−24−3
5726−19−2
622−45−7
Chemical (CASRN)

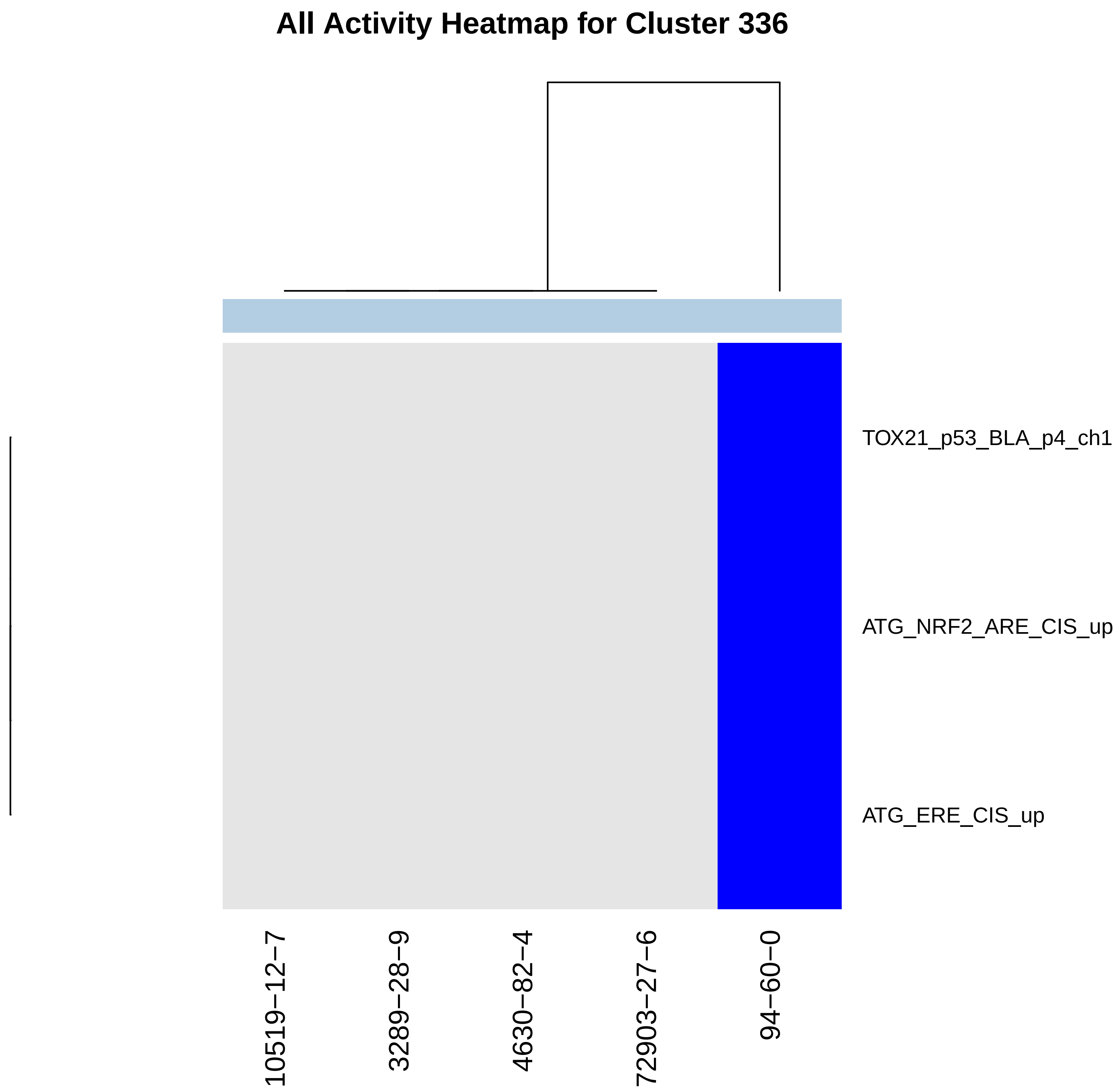

All Activity Heatmap for Cluster 336
TOX21_p53_BLA_p4_ch1
ATG_NRF2_ARE_CIS_up
ATG_ERE_CIS_up
10519−12−7
3289−28−9
4630−82−4
72903−27−6
94−60−0

All Activity Heatmap for Cluster 337

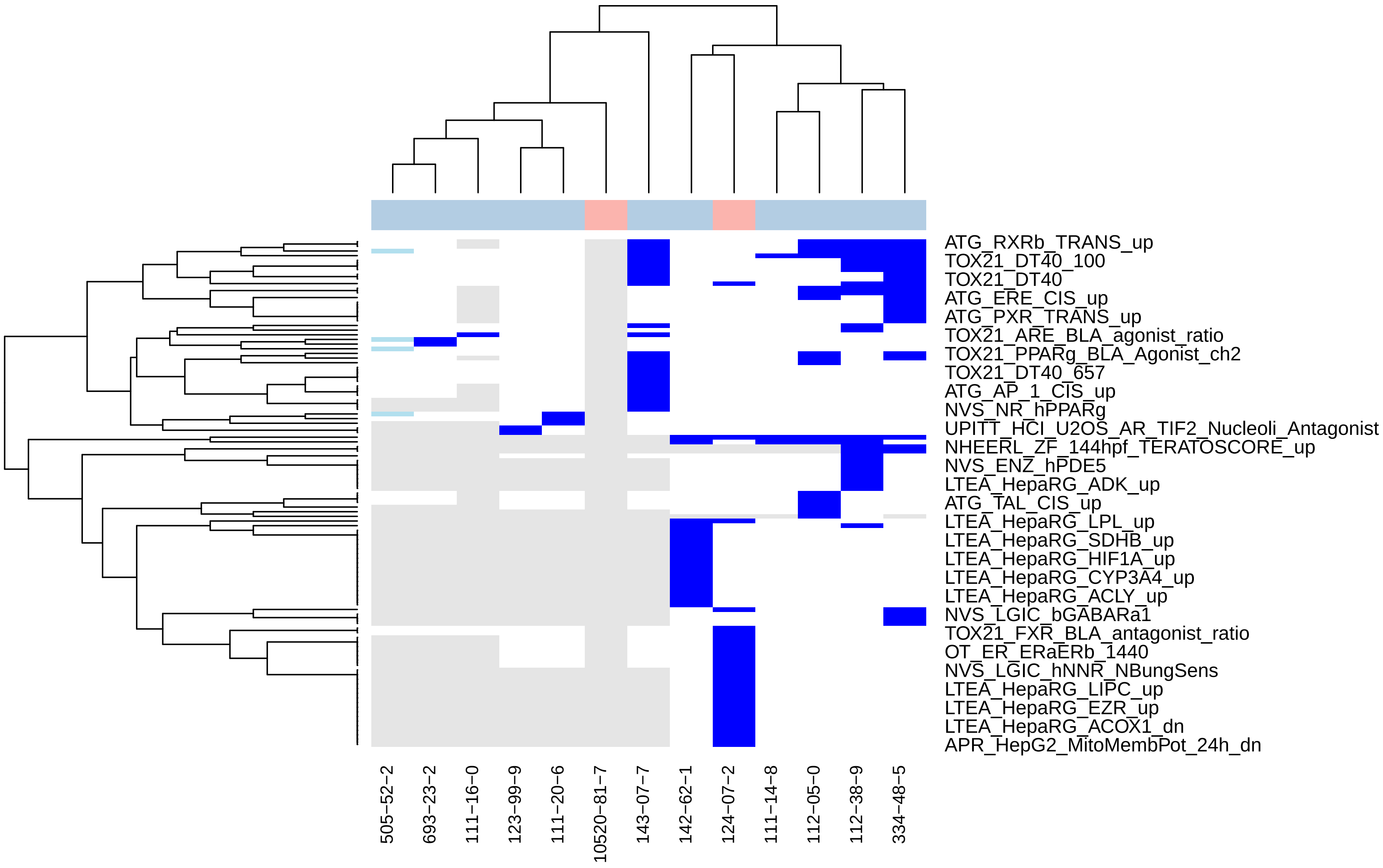

All Activity Heatmap for Cluster 338

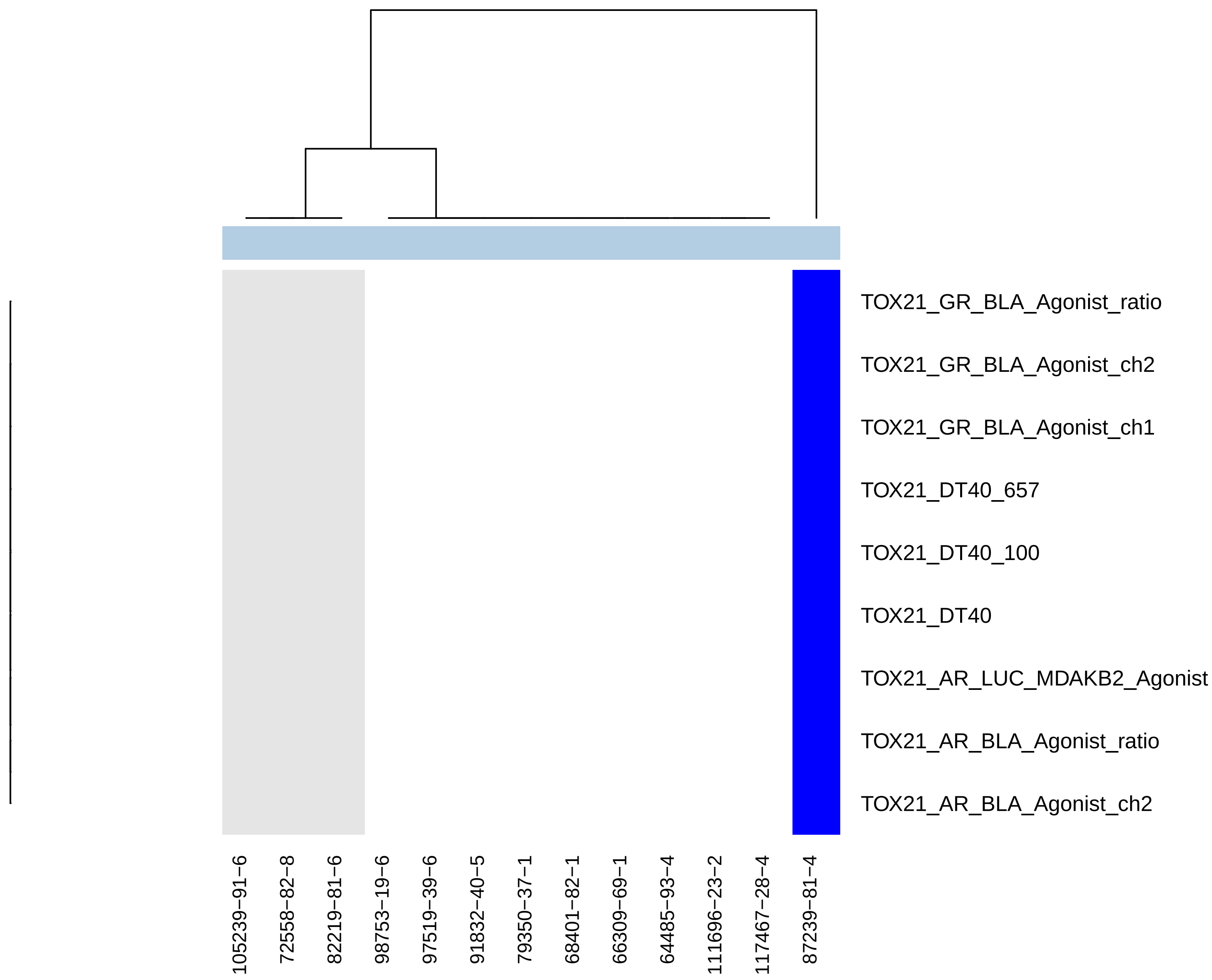

All Activity Heatmap for Cluster 339

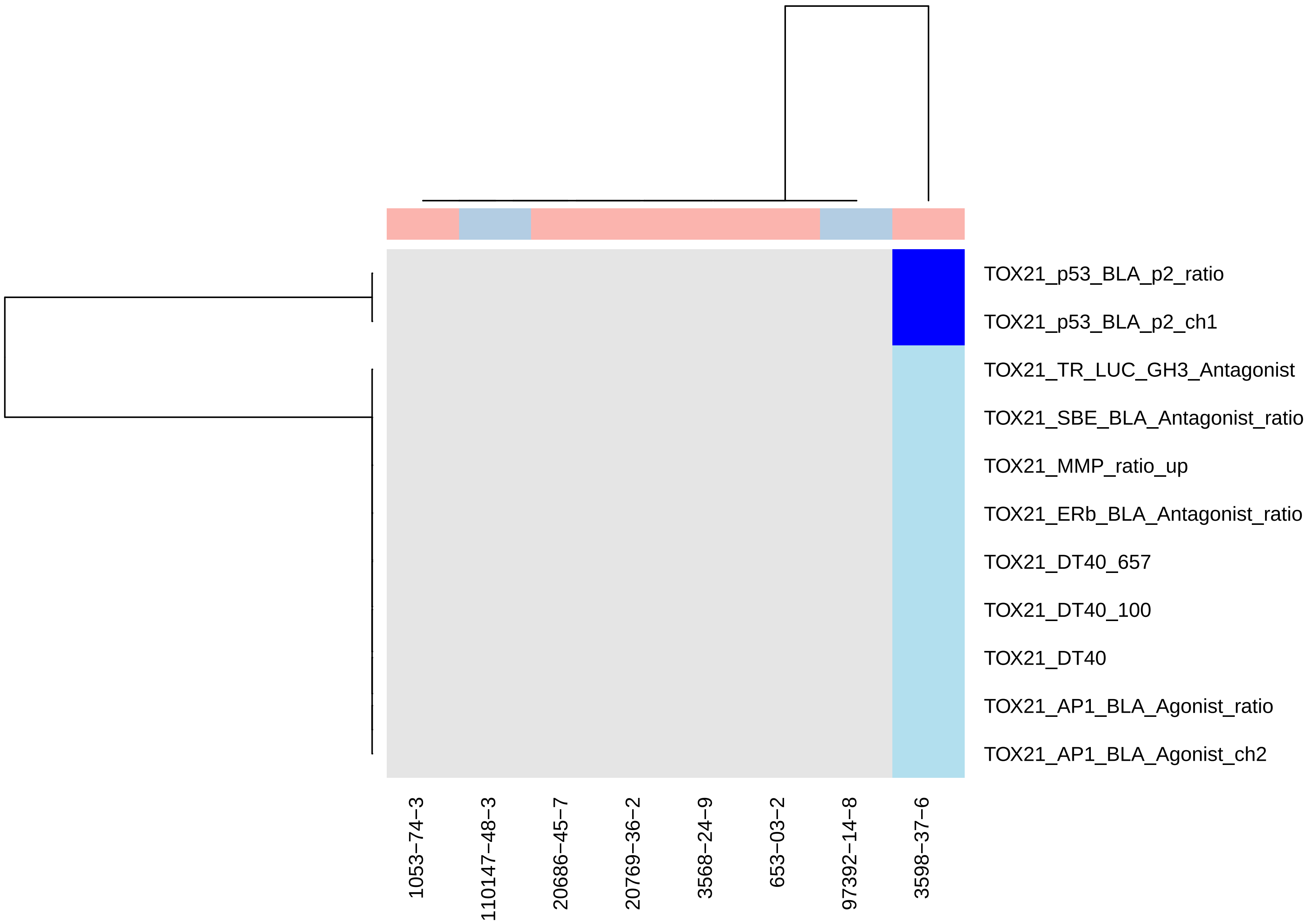

All Activity Heatmap for Cluster 341

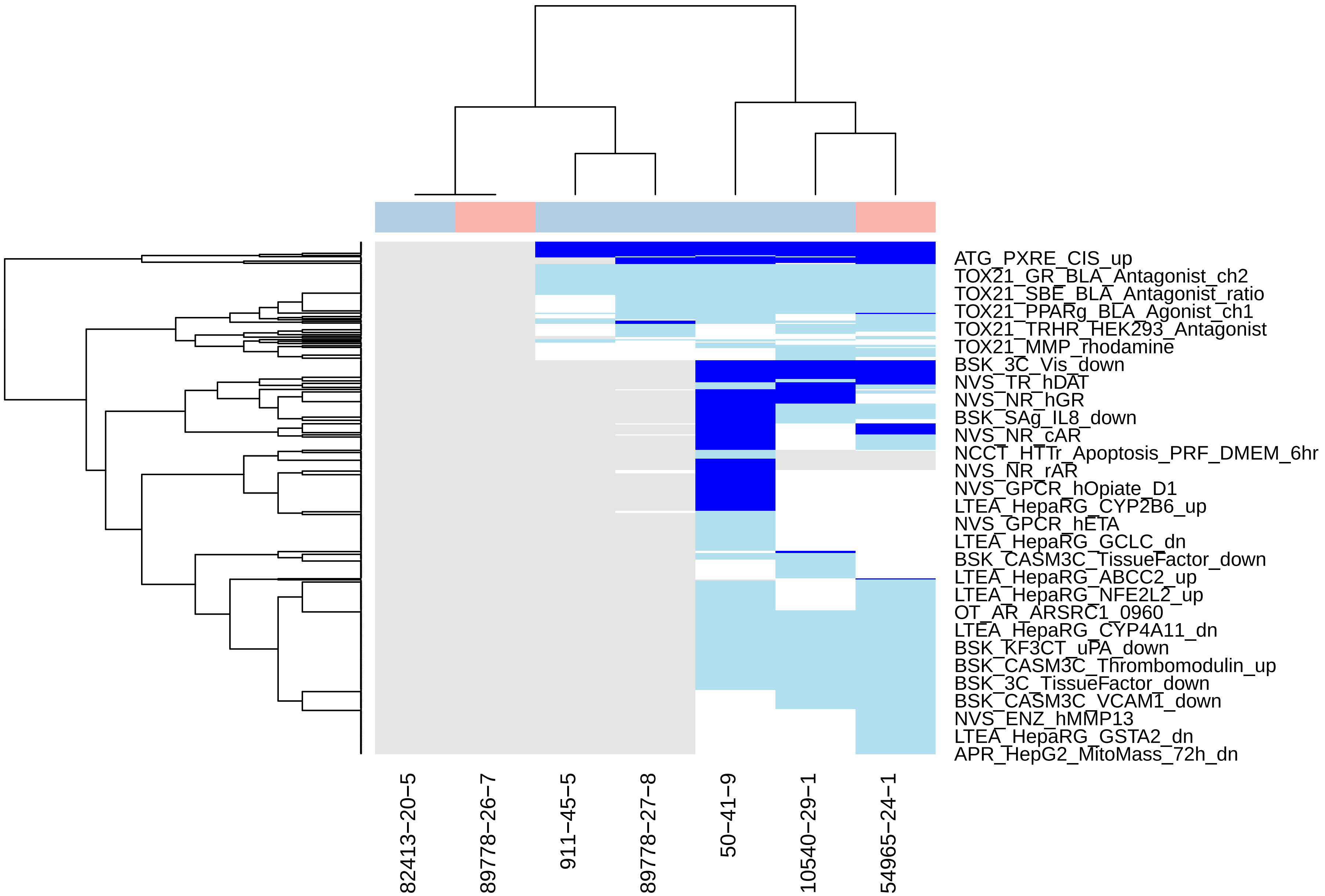

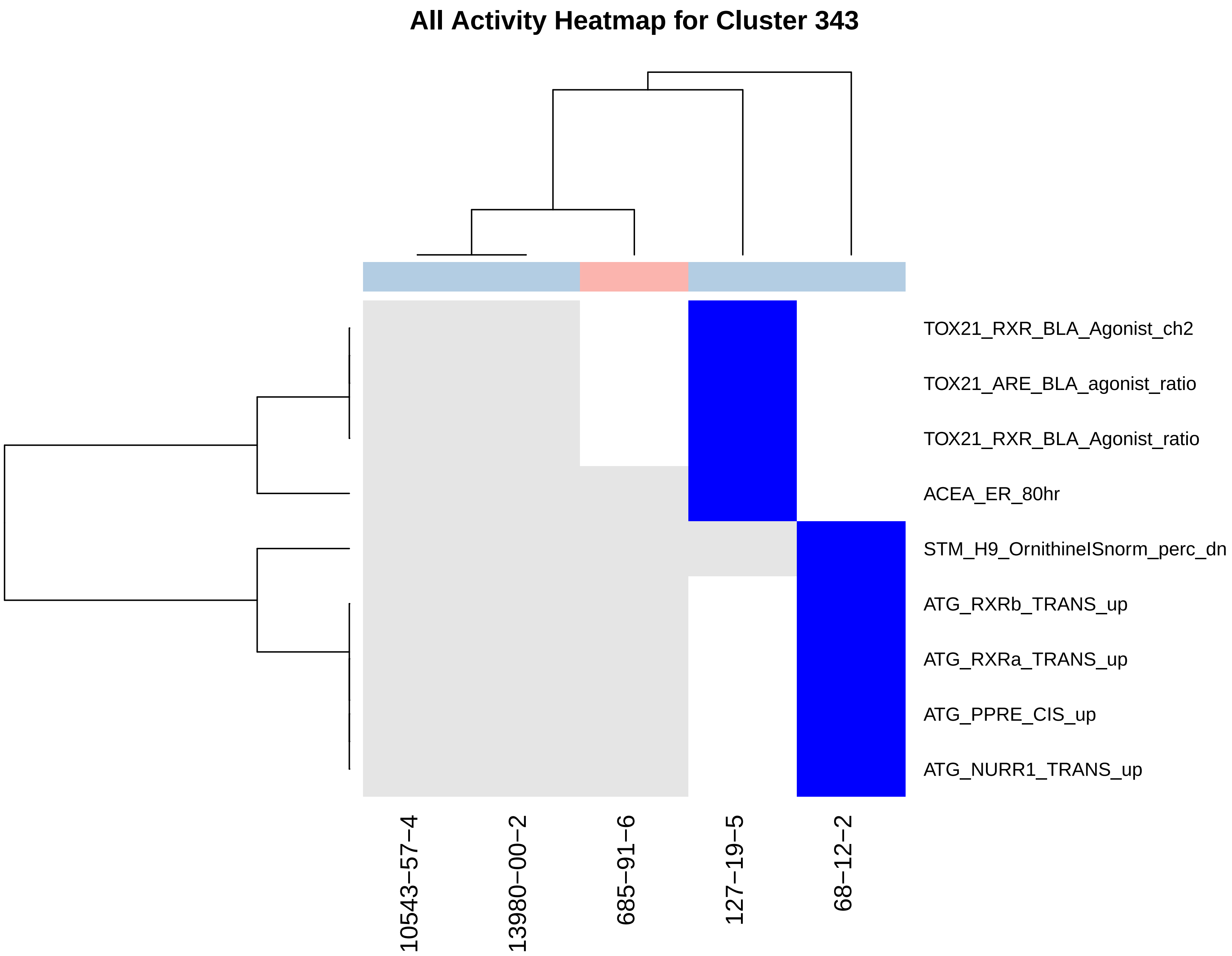

All Activity Heatmap for Cluster 343
TOX21_RXR_BLA_Agonist_ch2
TOX21_ARE_BLA_agonist_ratio
TOX21_RXR_BLA_Agonist_ratio
ACEA_ER_80hr
STM_H9_OrnithineISnorm_perc_dn
ATG_RXRb_TRANS_up
ATG_RXRa_TRANS_up
ATG_PPRE_CIS_up
ATG_NURR1_TRANS_up
10543−57−4
13980−00−2
685−91−6
127−19−5
68−12−2

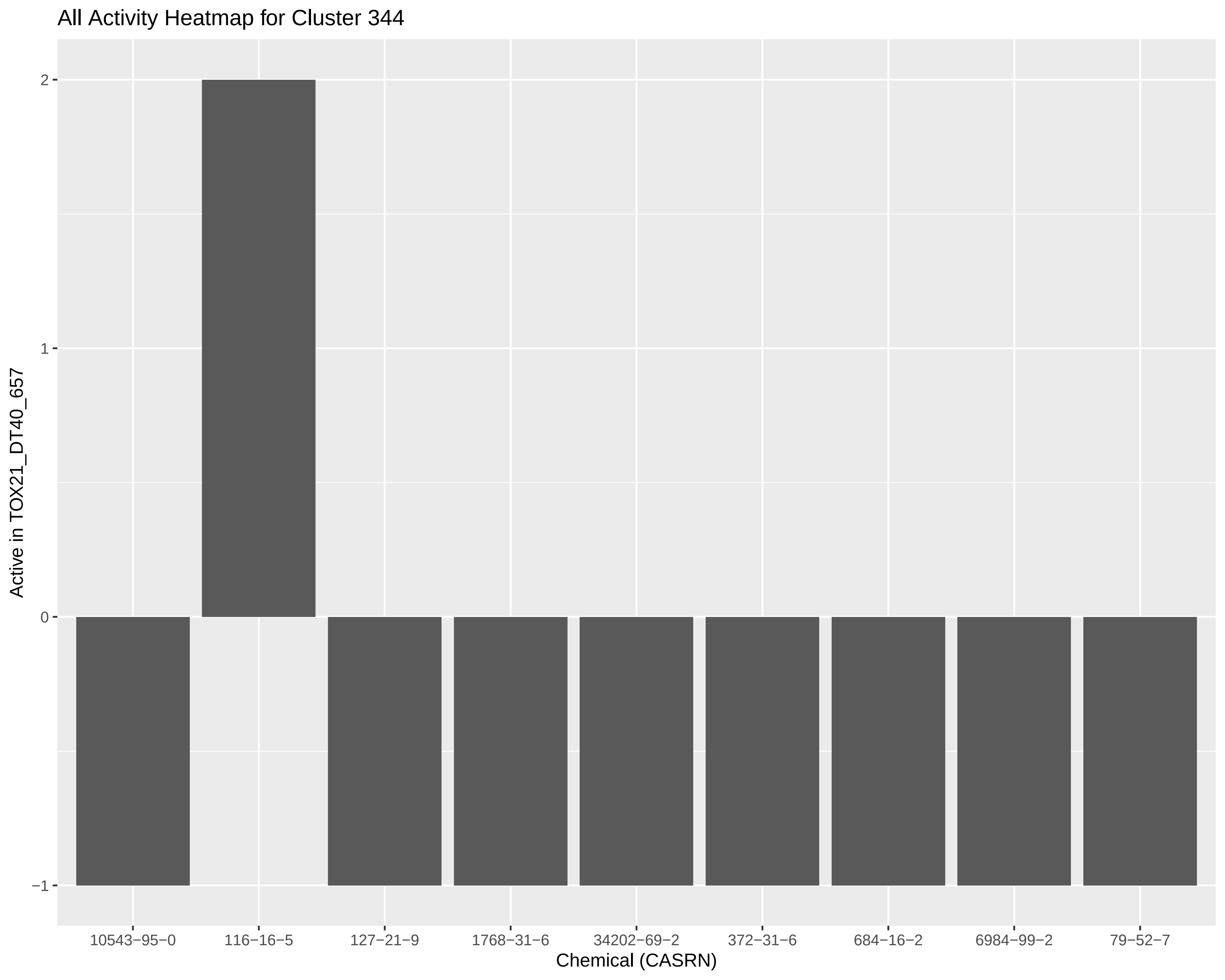

All Activity Heatmap for Cluster 344
Active in TOX21_DT40_657
Chemical (CASRN)
2
1
0
−1
10543−95−0
116−16−5
127−21−9
1768−31−6
34202−69−2
372−31−6
684−16−2
6984−99−2
79−52−7

All Activity Heatmap for Cluster 345

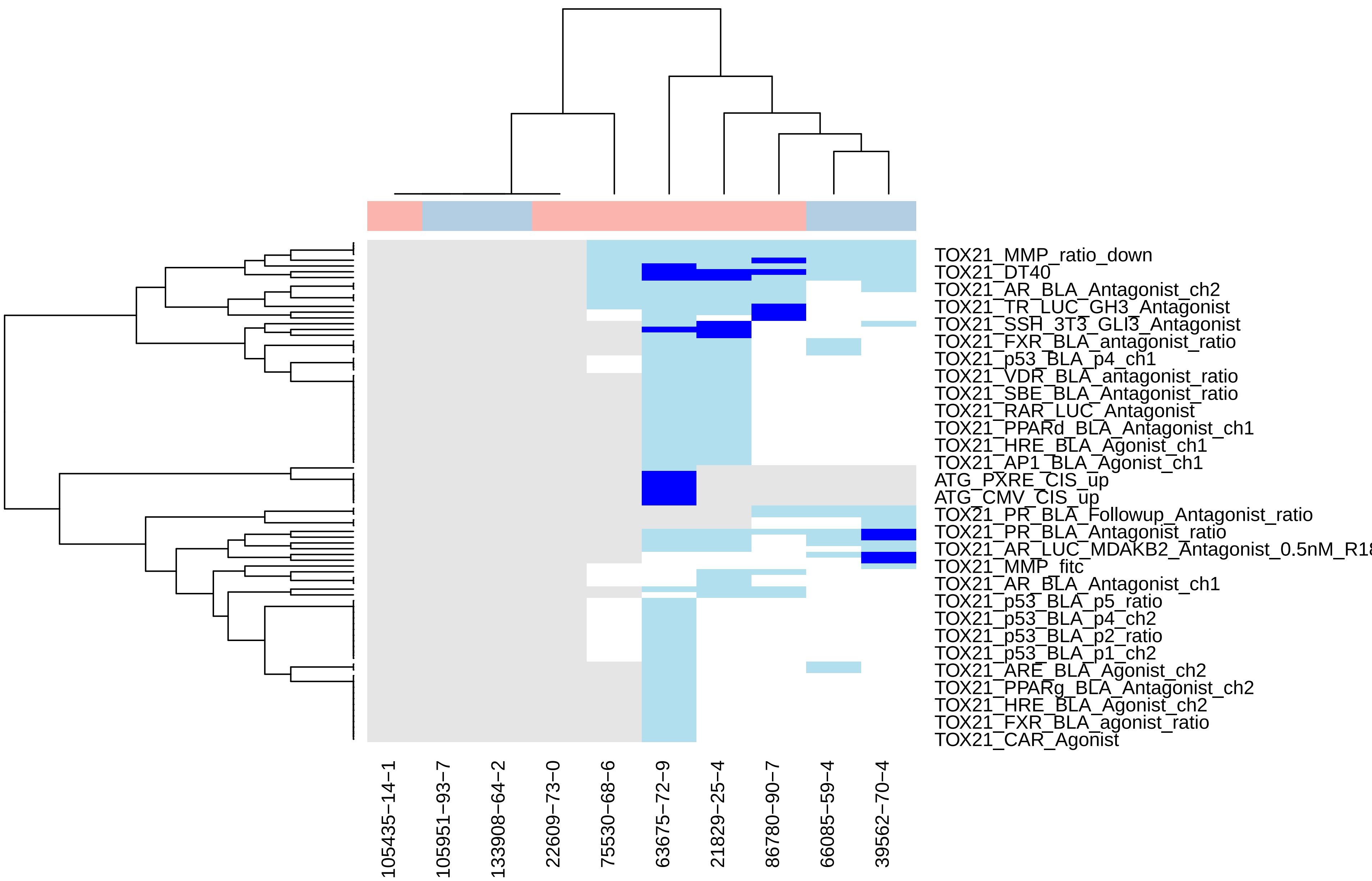

All Activity Heatmap for Cluster 346

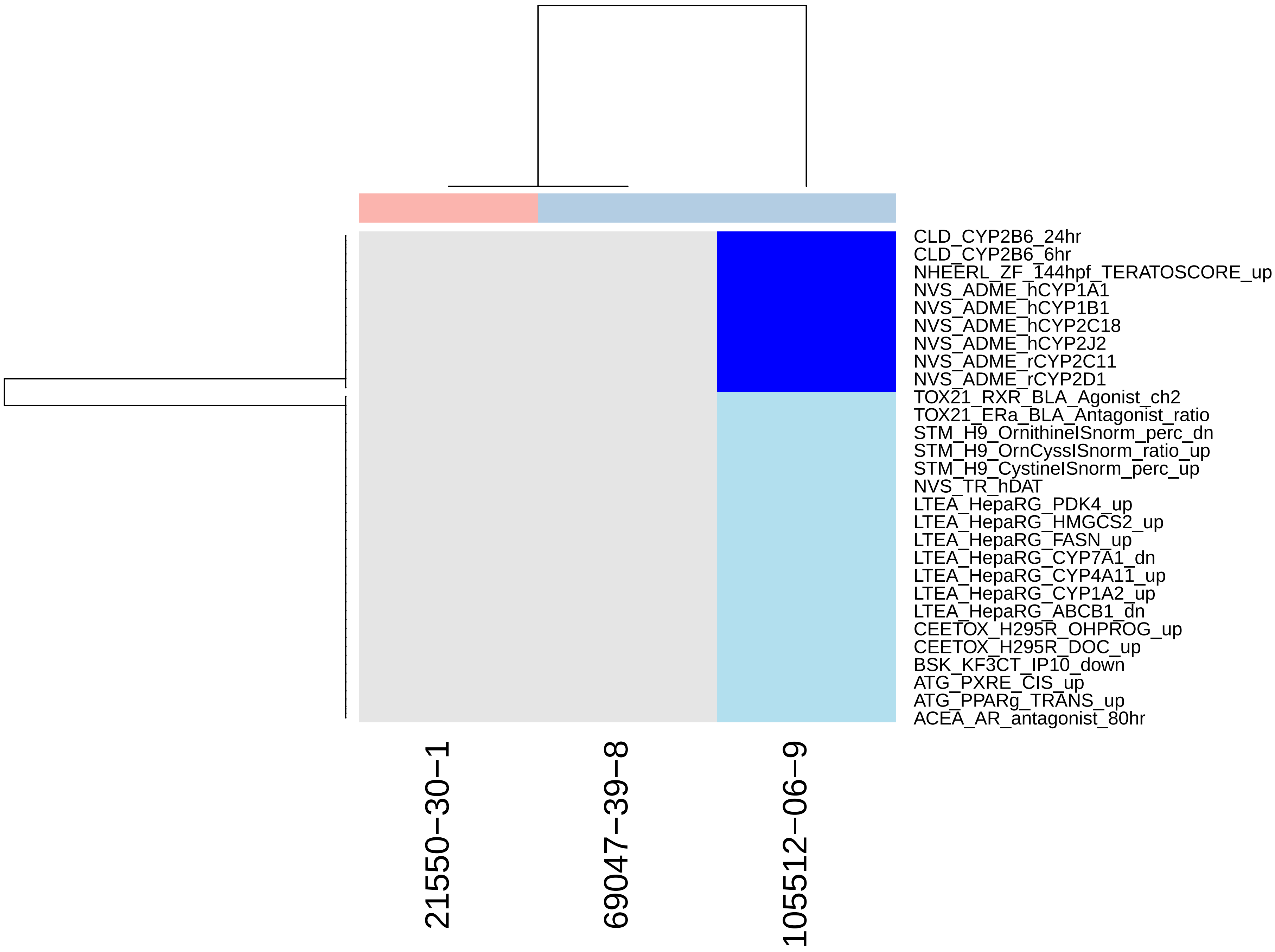

All Activity Heatmap for Cluster 347

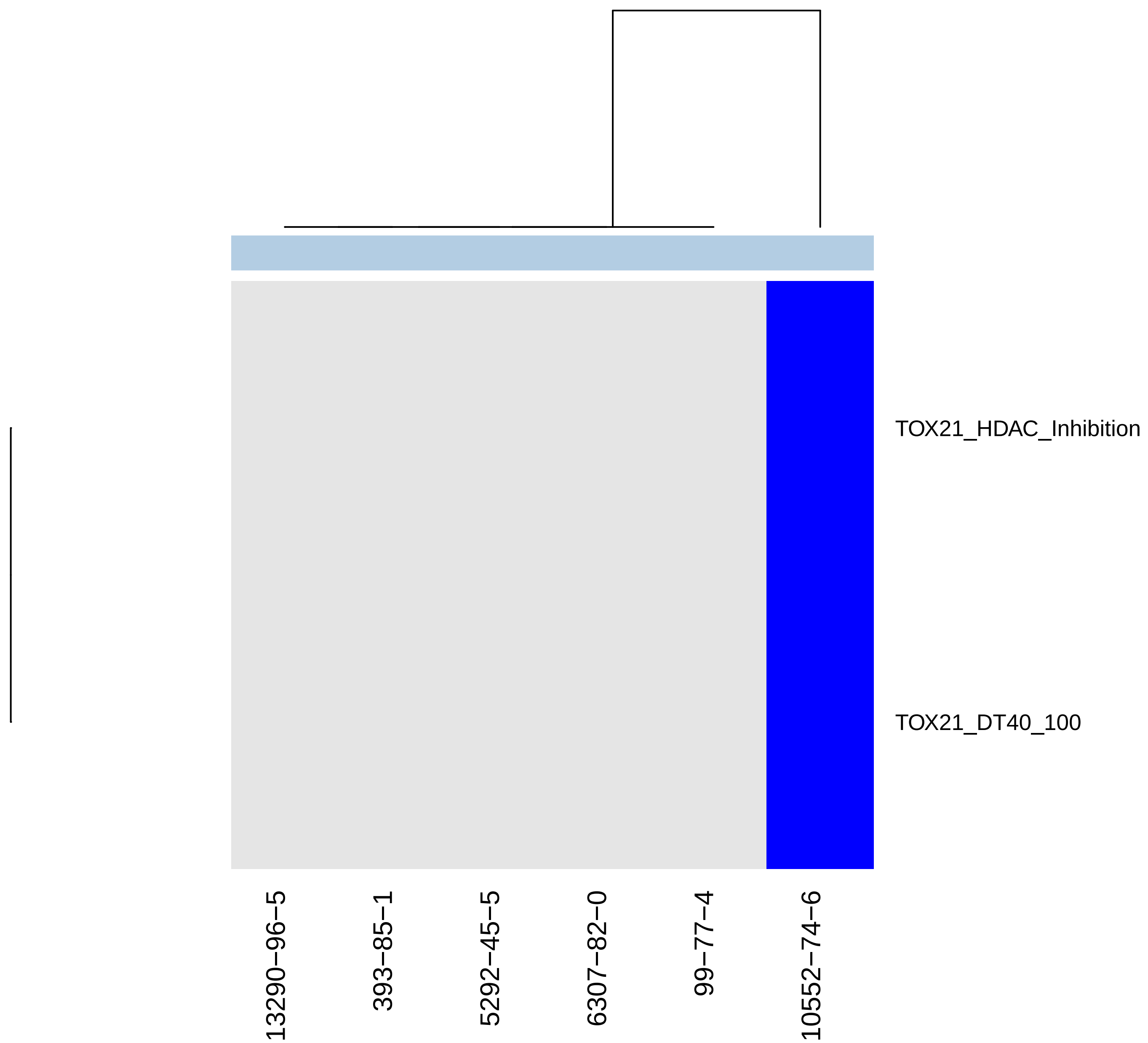

All Activity Heatmap for Cluster 348

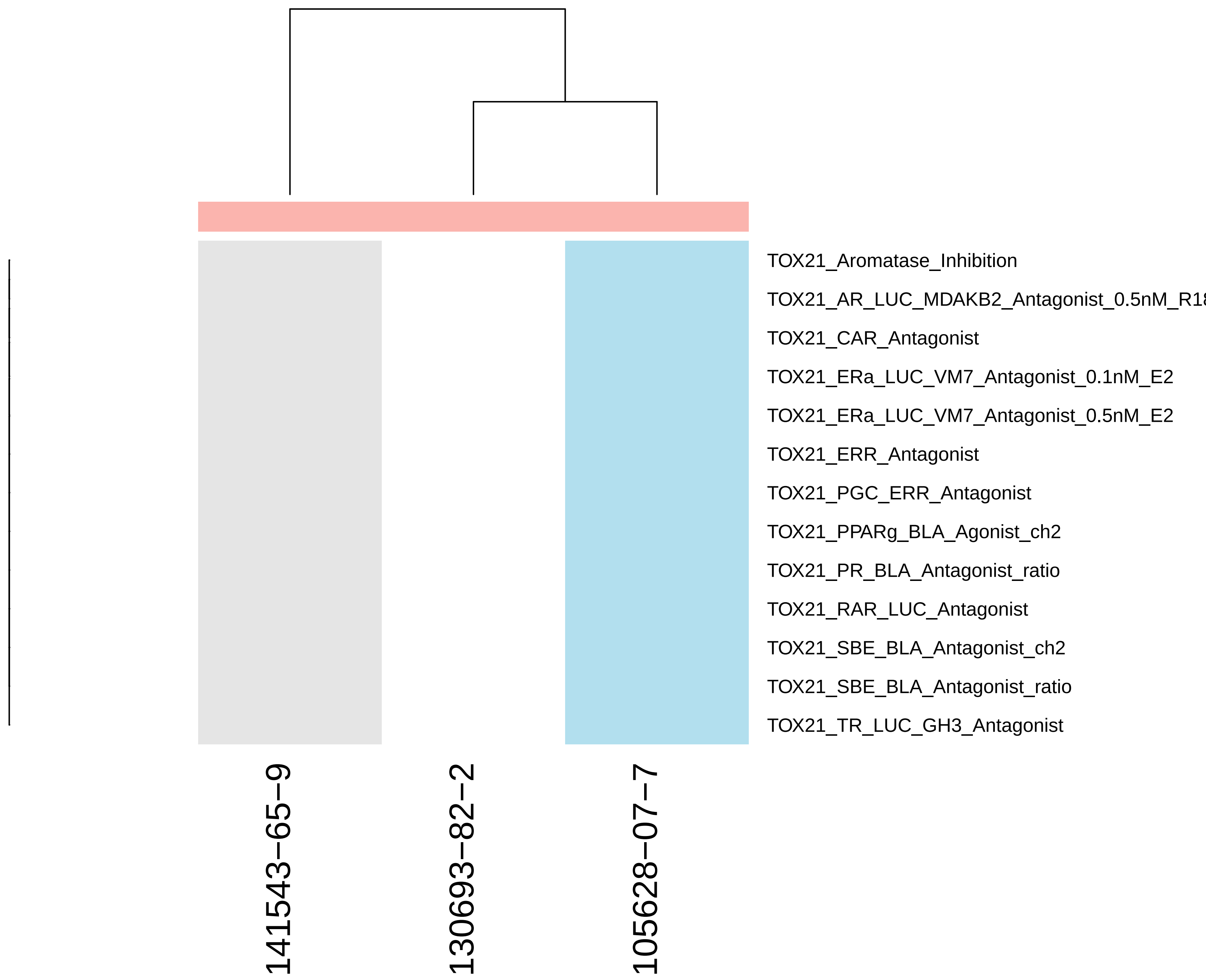

All Activity Heatmap for Cluster 349

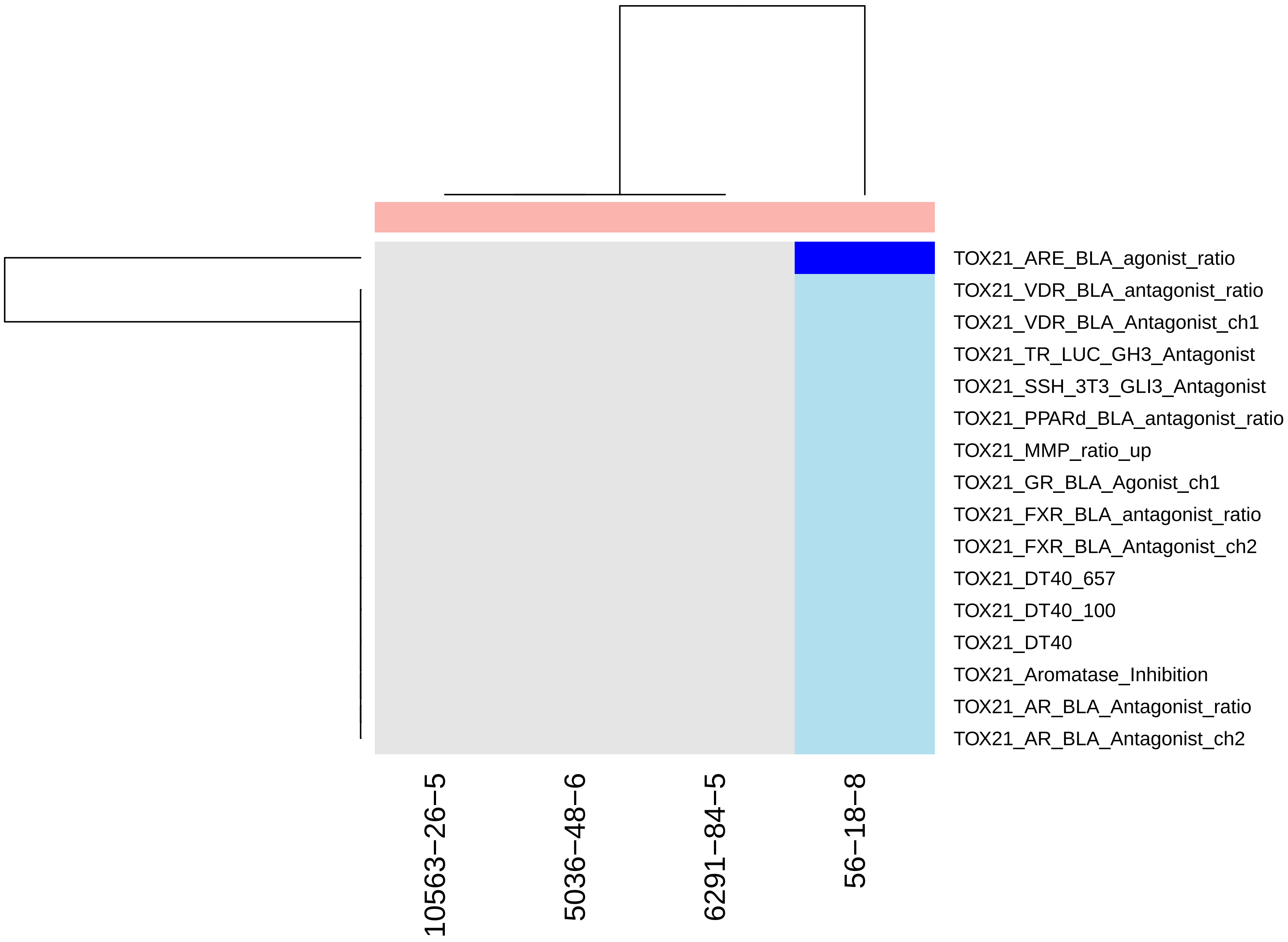

All Activity Heatmap for Cluster 350

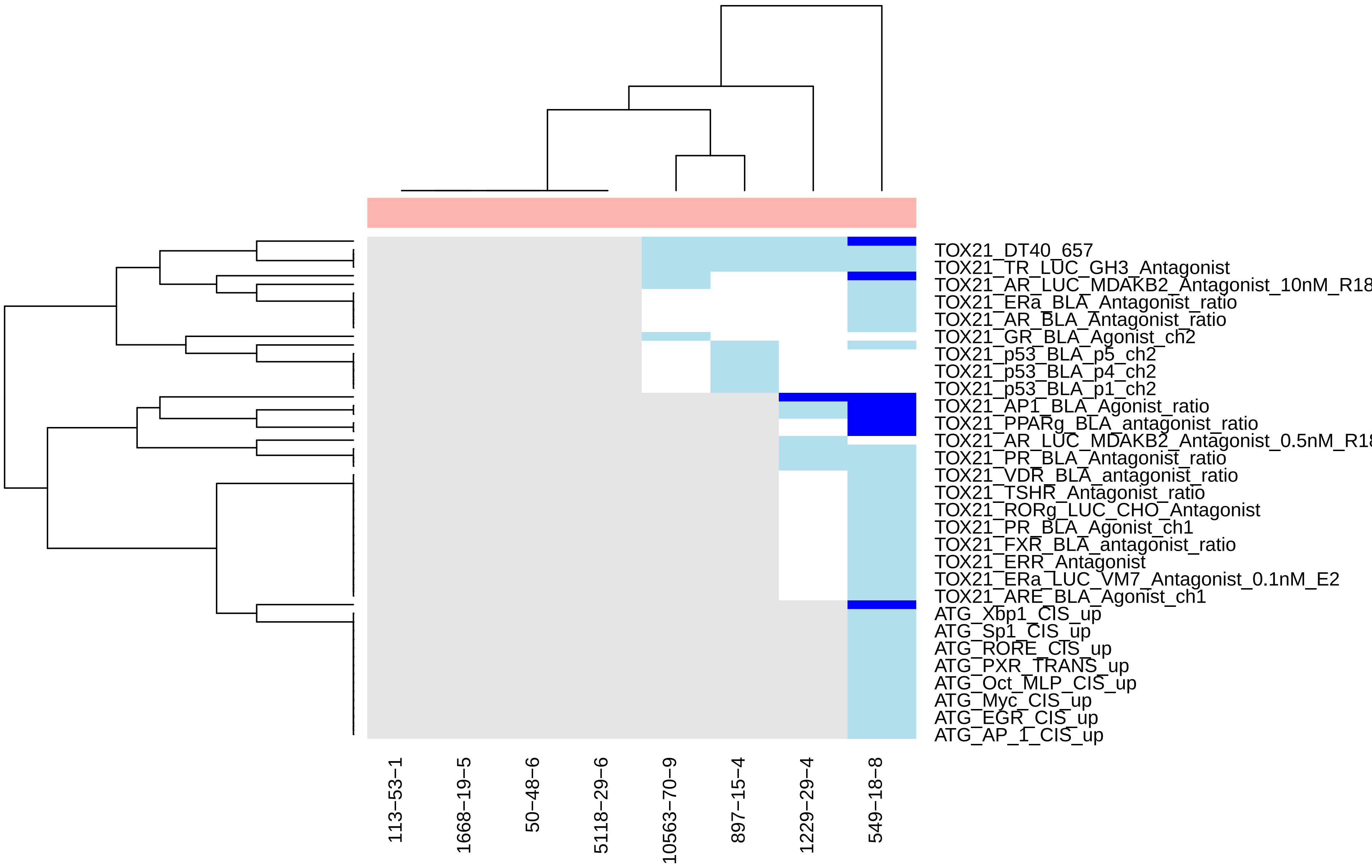

All Activity Heatmap for Cluster 351

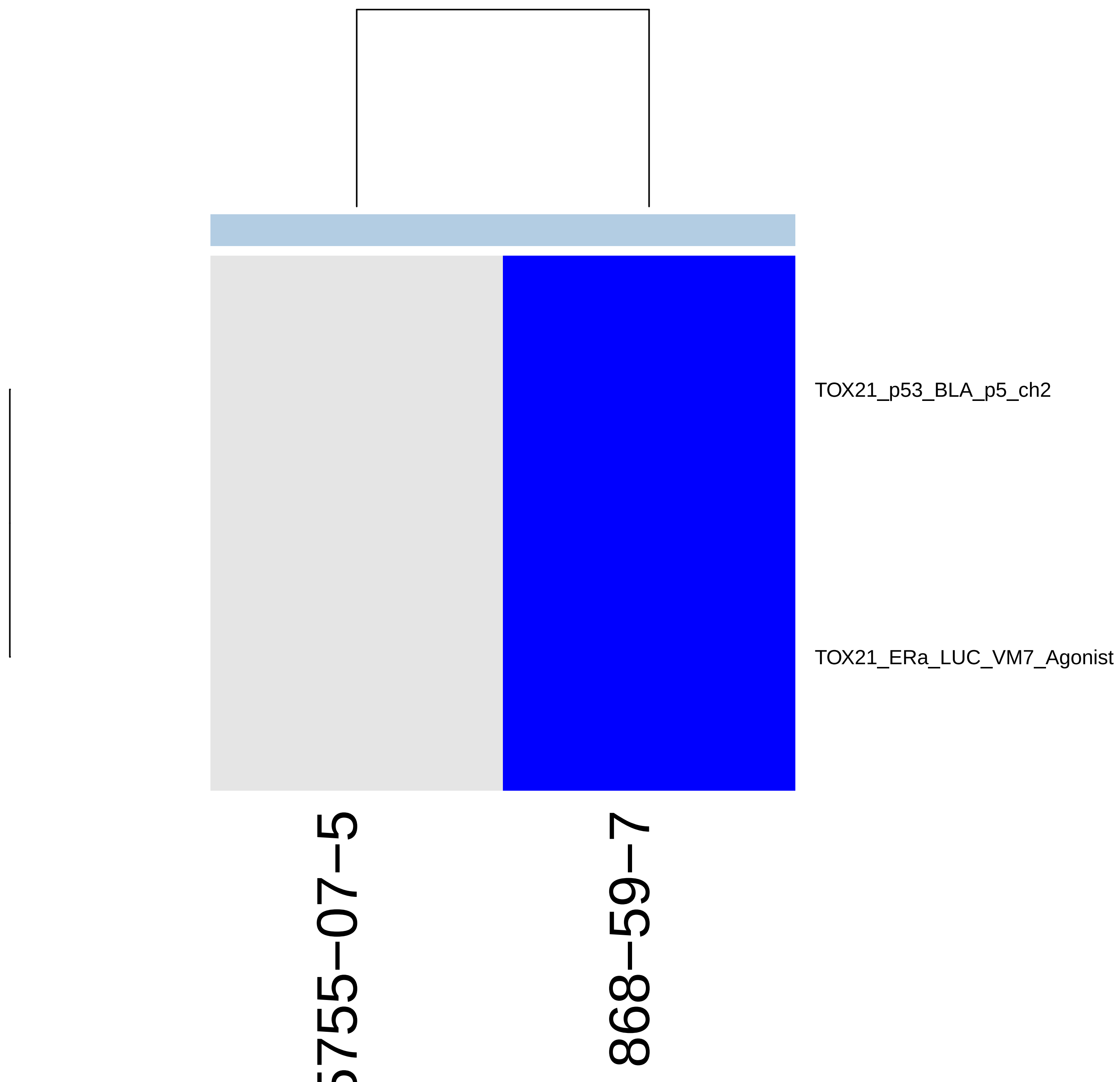

All Activity Heatmap for Cluster 352

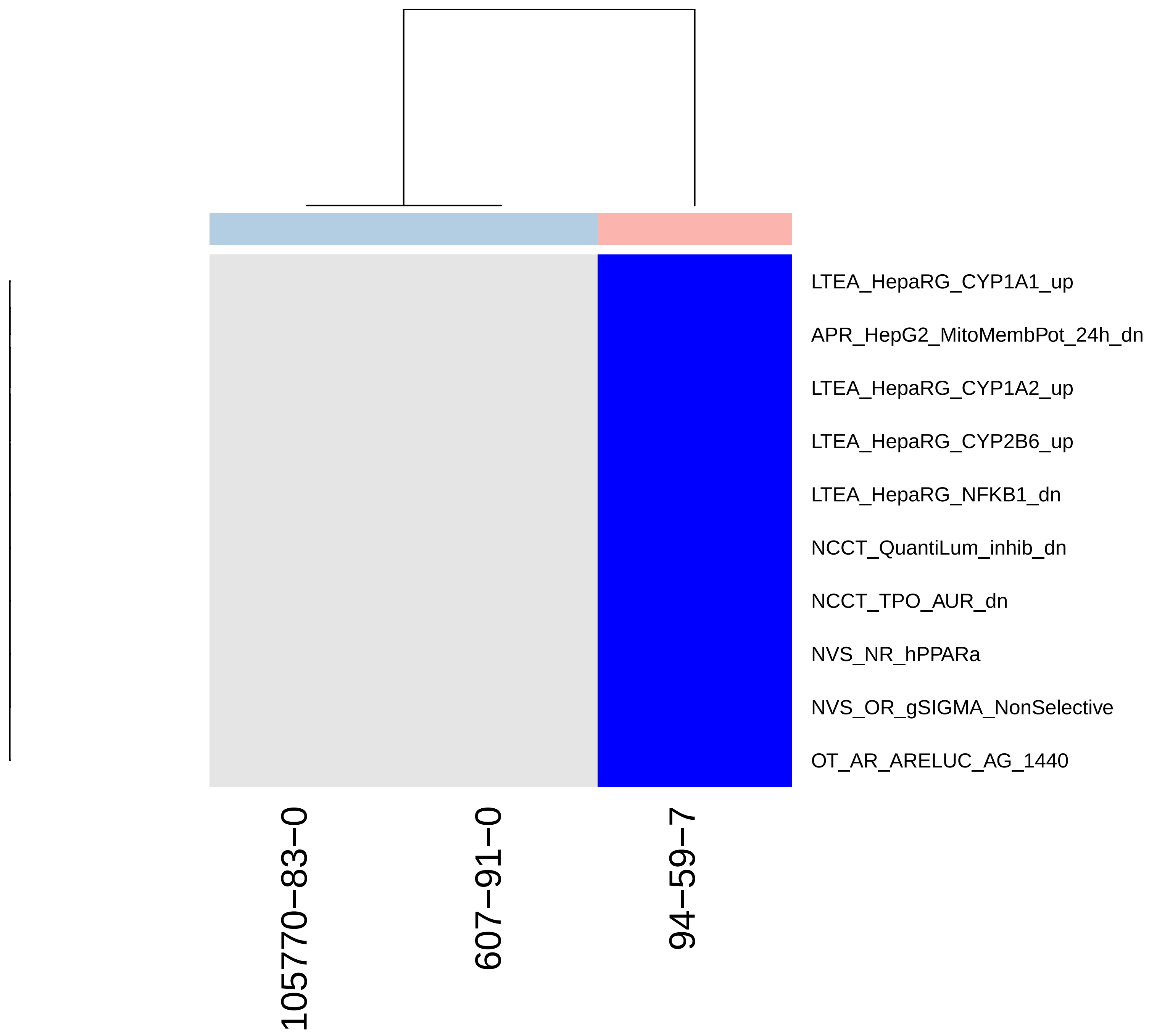

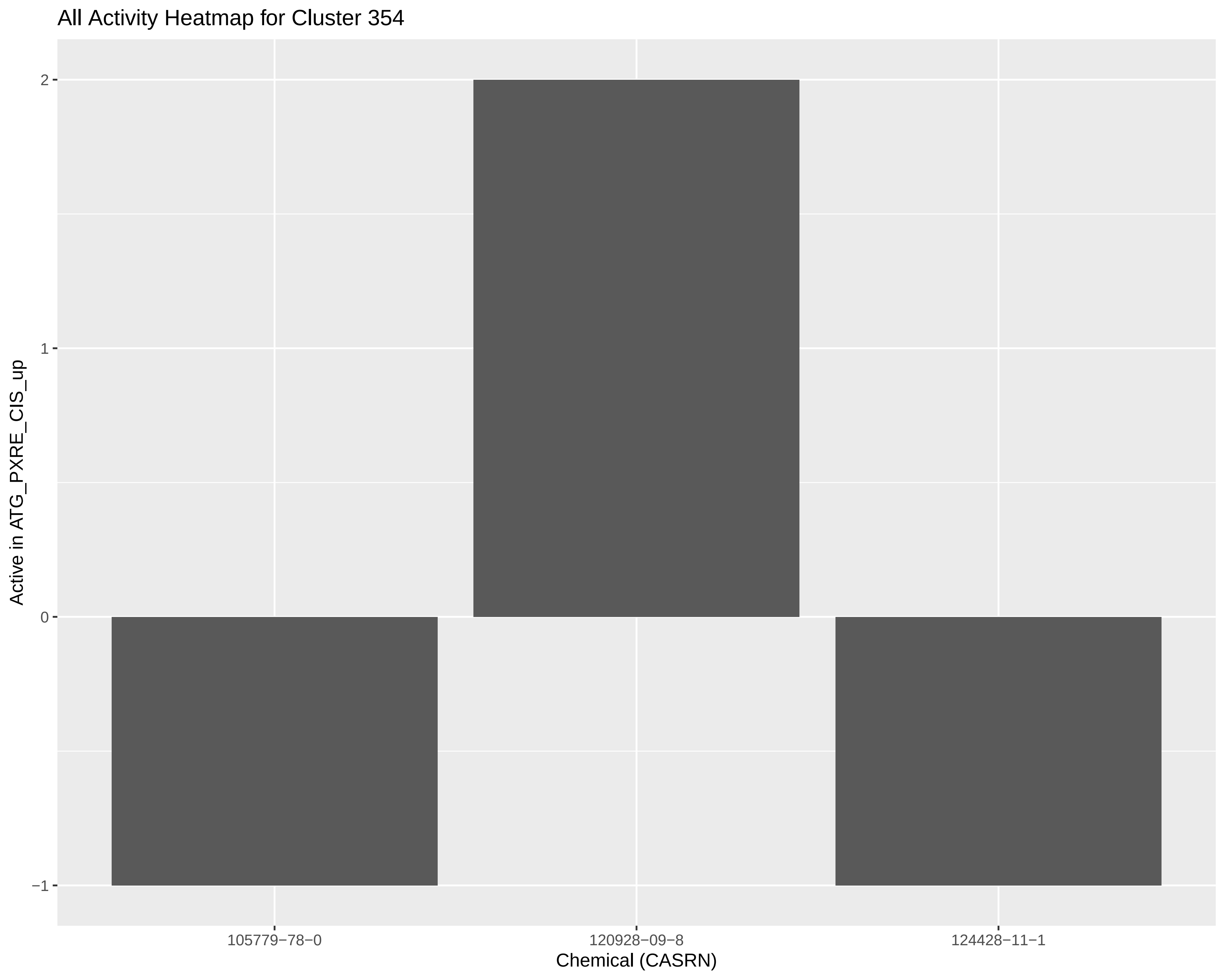

All Activity Heatmap for Cluster 354
Active in ATG_PXRE_CIS_up
2
1
0
−1
105779−78−0
120928−09−8
124428−11−1
Chemical (CASRN)

All Activity Heatmap for Cluster 356

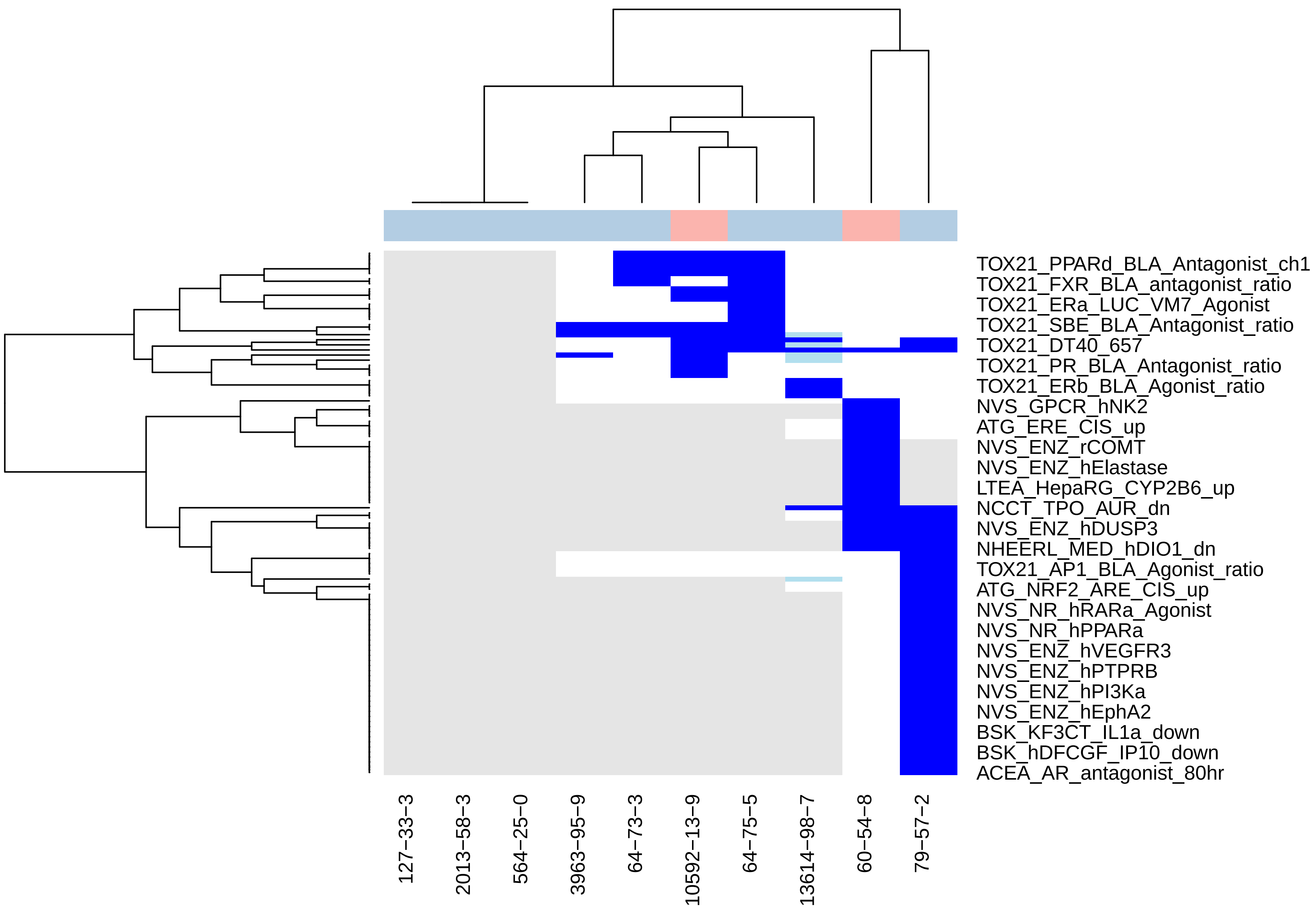

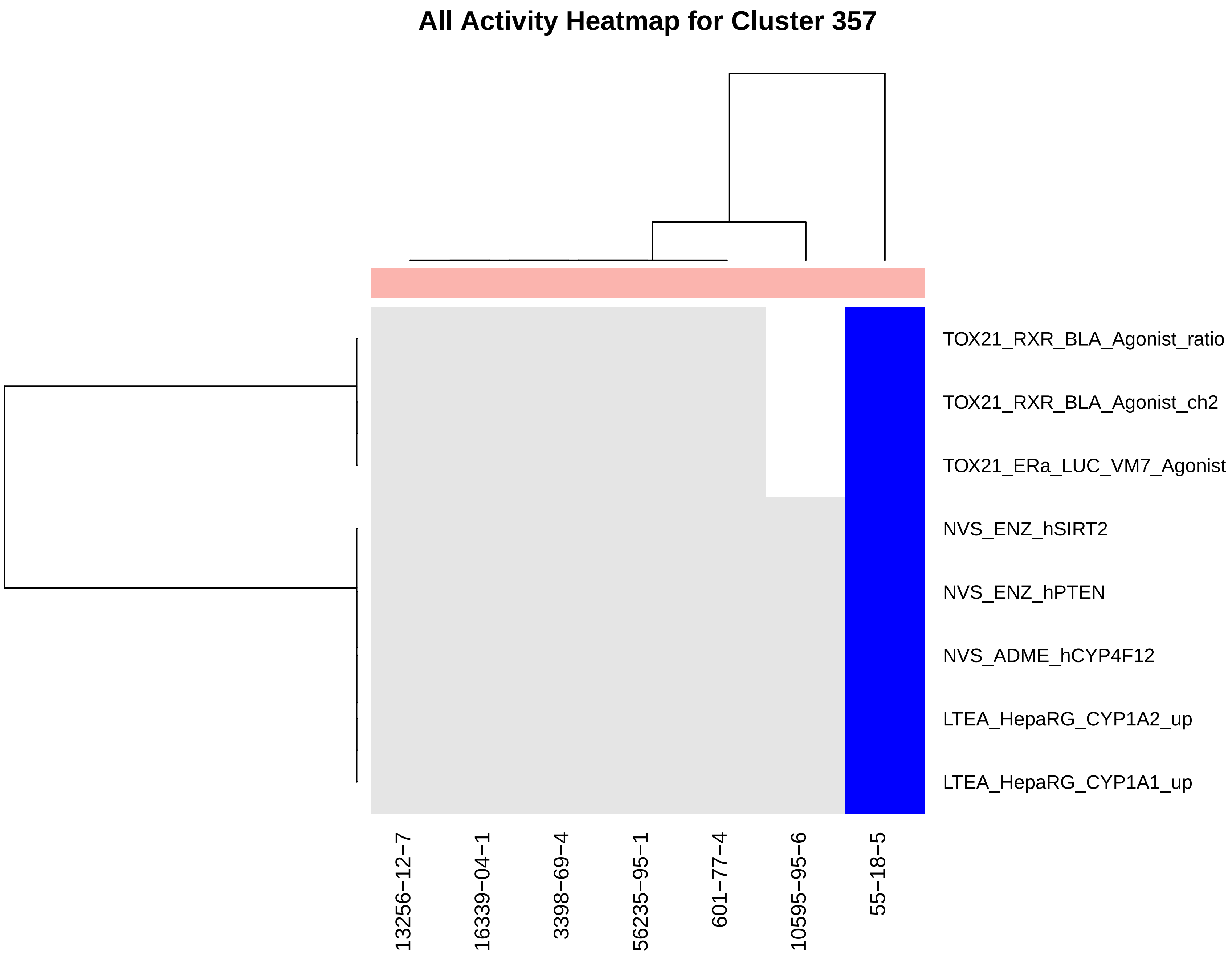
All Activity Heatmap for Cluster 357
TOX21_RXR_BLA_Agonist_ratio
TOX21_RXR_BLA_Agonist_ch2
TOX21_ERa_LUC_VM7_Agonist
NVS_ENZ_hSIRT2
NVS_ENZ_hPTEN
NVS_ADME_hCYP4F12
LTEA_HepaRG_CYP1A2_up
LTEA_HepaRG_CYP1A1_up
13256−12−7
16339−04−1
3398−69−4
56235−95−1
601−77−4
10595−95−6
55−18−5

All Activity Heatmap for Cluster 358

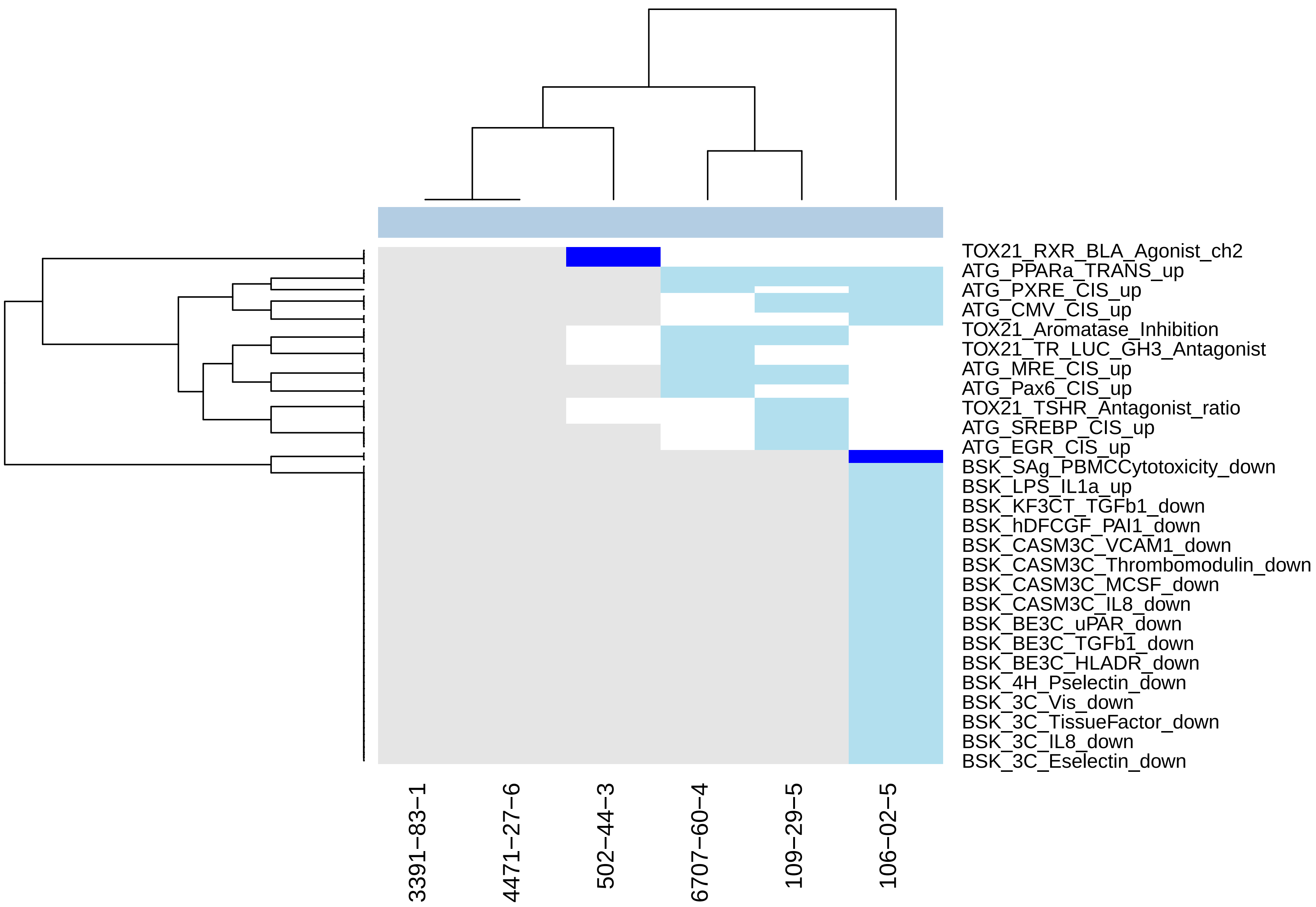

All Activity Heatmap for Cluster 360

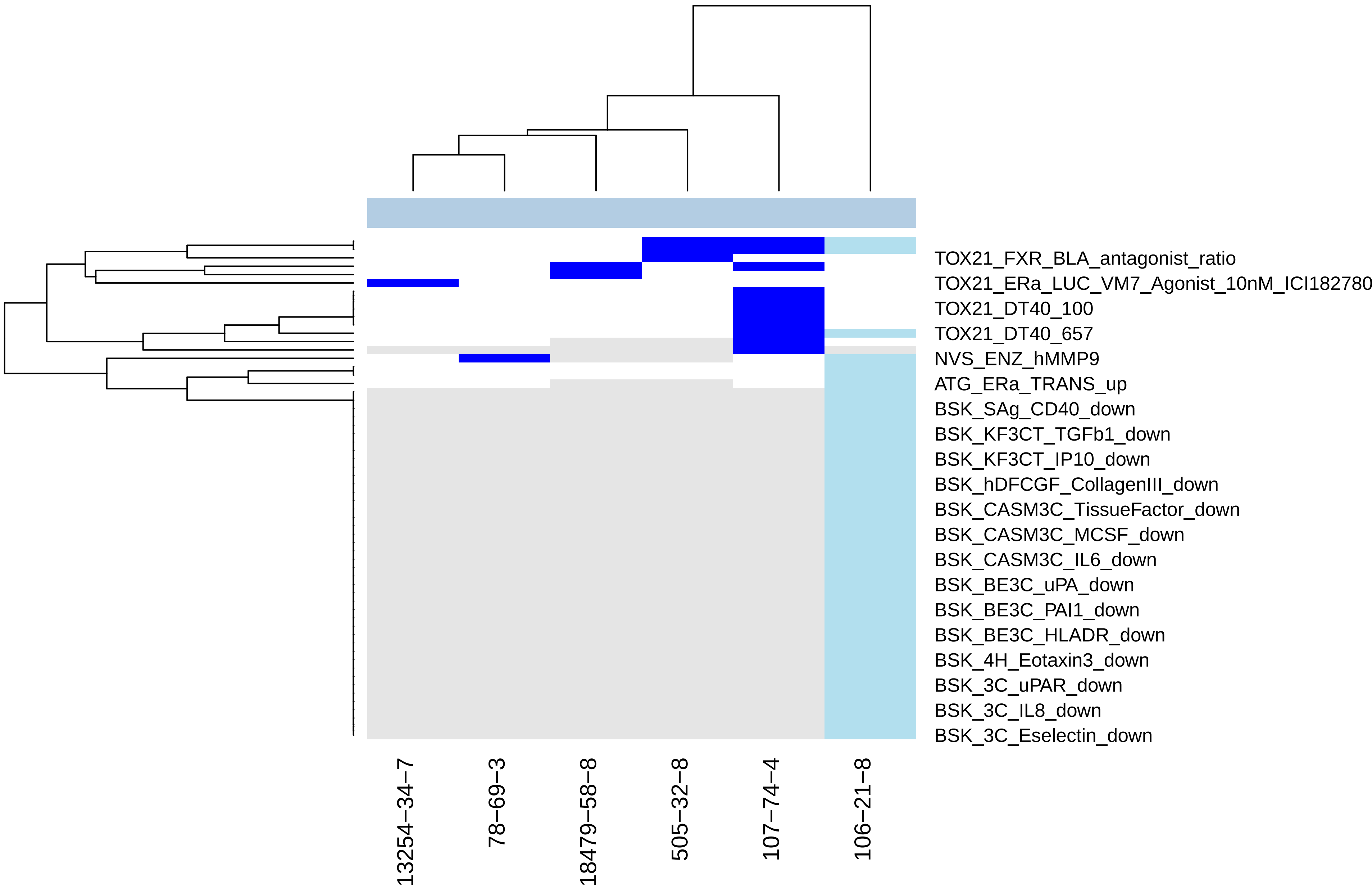

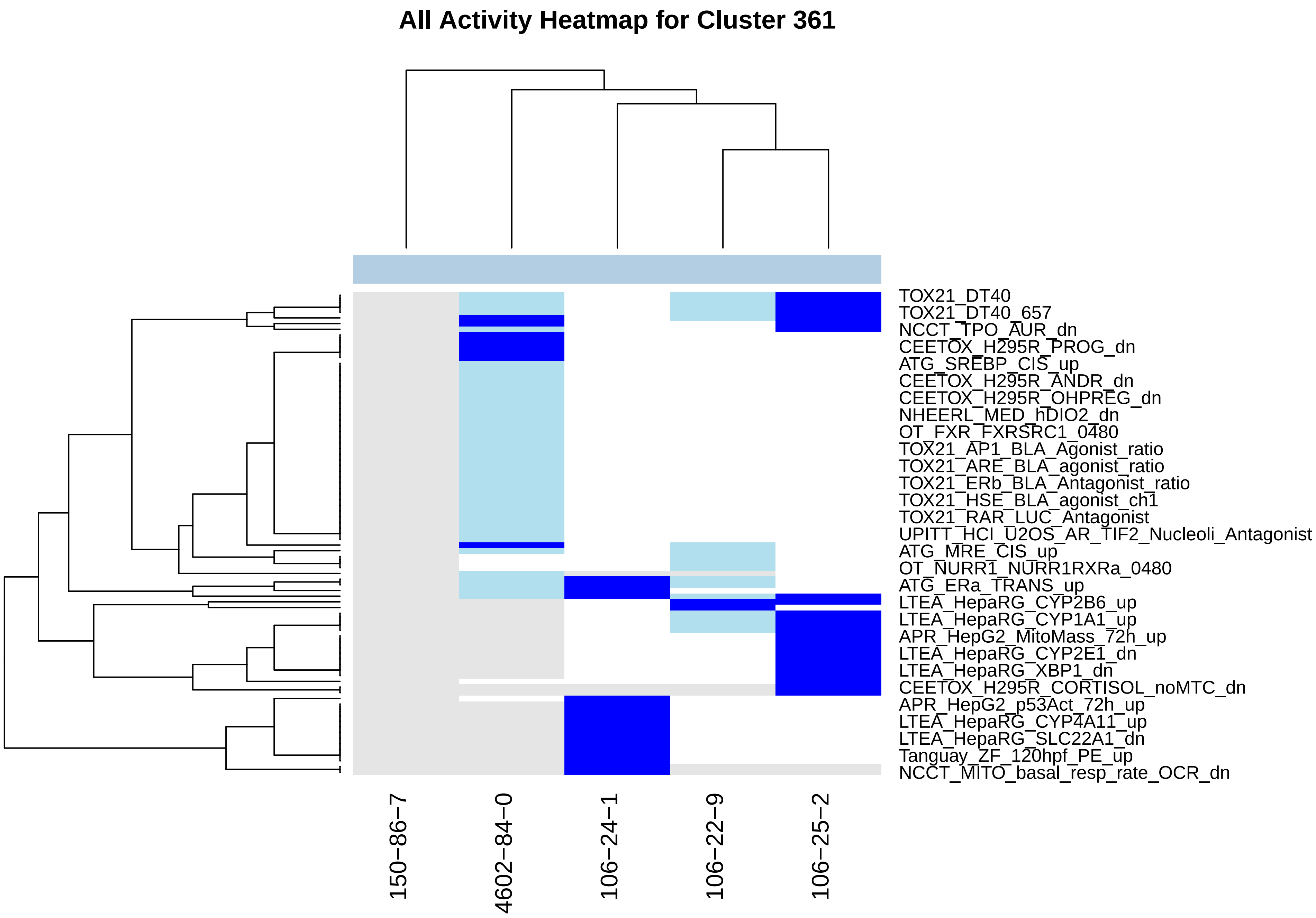
All Activity Heatmap for Cluster 361
TOX21_DT40
TOX21_DT40_657
NCCT_TPO_AUR_dn
CEETOX_H295R_PROG_dn
ATG_SREBP_CIS_up
CEETOX_H295R_ANDR_dn
CEETOX_H295R_OHPREG_dn
NHEERL_MED_hDIO2_dn
OT_FXR_FXRSRC1_0480
TOX21_AP1_BLA_Agonist_ratio
TOX21_ARE_BLA_agonist_ratio
TOX21_ERb_BLA_Antagonist_ratio
TOX21_HSE_BLA_agonist_ch1
TOX21_RAR_LUC_Antagonist
UPITT_HCI_U2OS_AR_TIF2_Nucleoli_Antagonist
ATG_MRE_CIS_up
OT_NURR1_NURR1RXRa_0480
ATG_ERa_TRANS_up
LTEA_HepaRG_CYP2B6_up
LTEA_HepaRG_CYP1A1_up
APR_HepG2_MitoMass_72h_up
LTEA_HepaRG_CYP2E1_dn
LTEA_HepaRG_XBP1_dn
CEETOX_H295R_CORTISOL_noMTC_dn
APR_HepG2_p53Act_72h_up
LTEA_HepaRG_CYP4A11_up
LTEA_HepaRG_SLC22A1_dn
Tanguay_ZF_120hpf_PE_up
NCCT_MITO_basal_resp_rate_OCR_dn
150−86−7
4602−84−0
106−24−1
106−22−9
106−25−2

All Activity Heatmap for Cluster 362

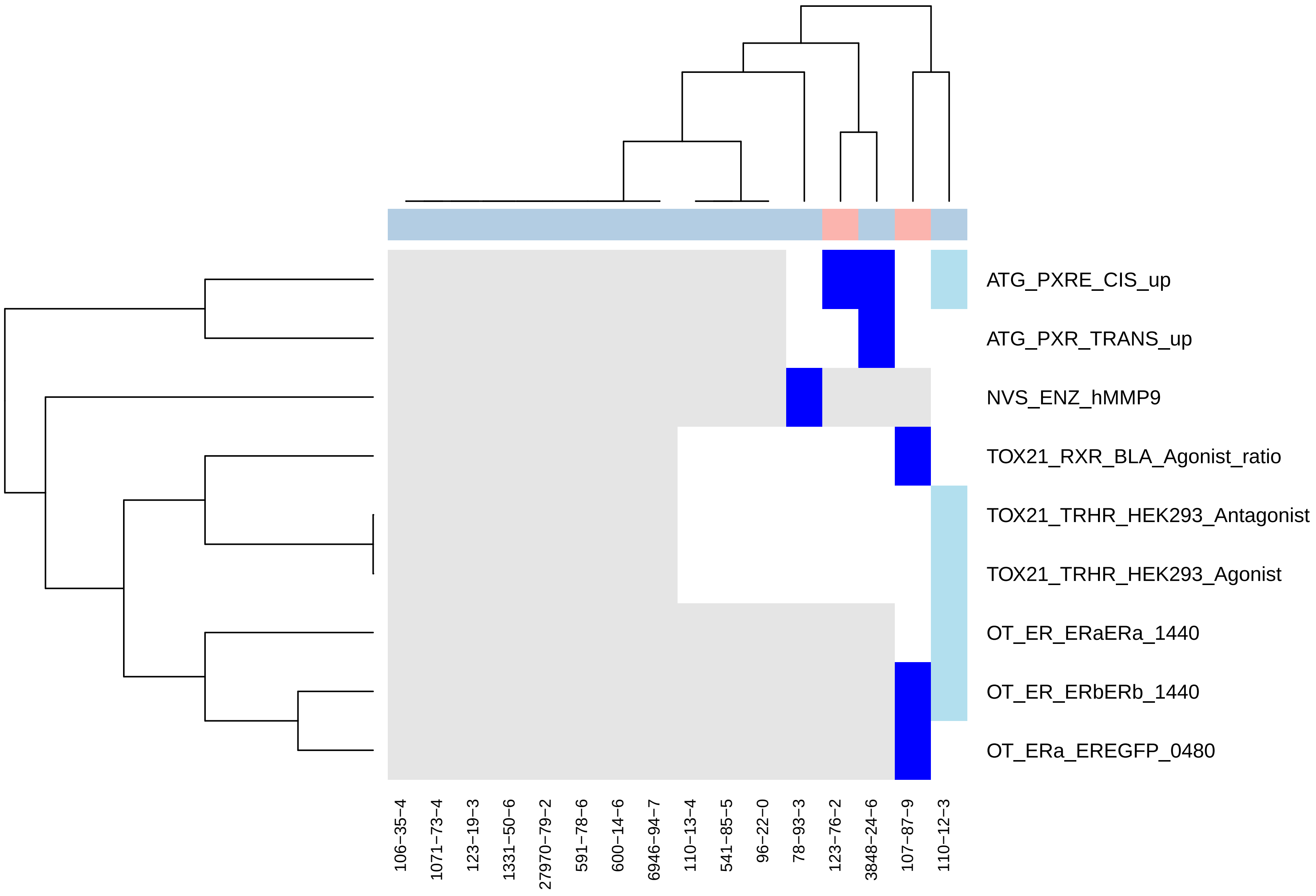

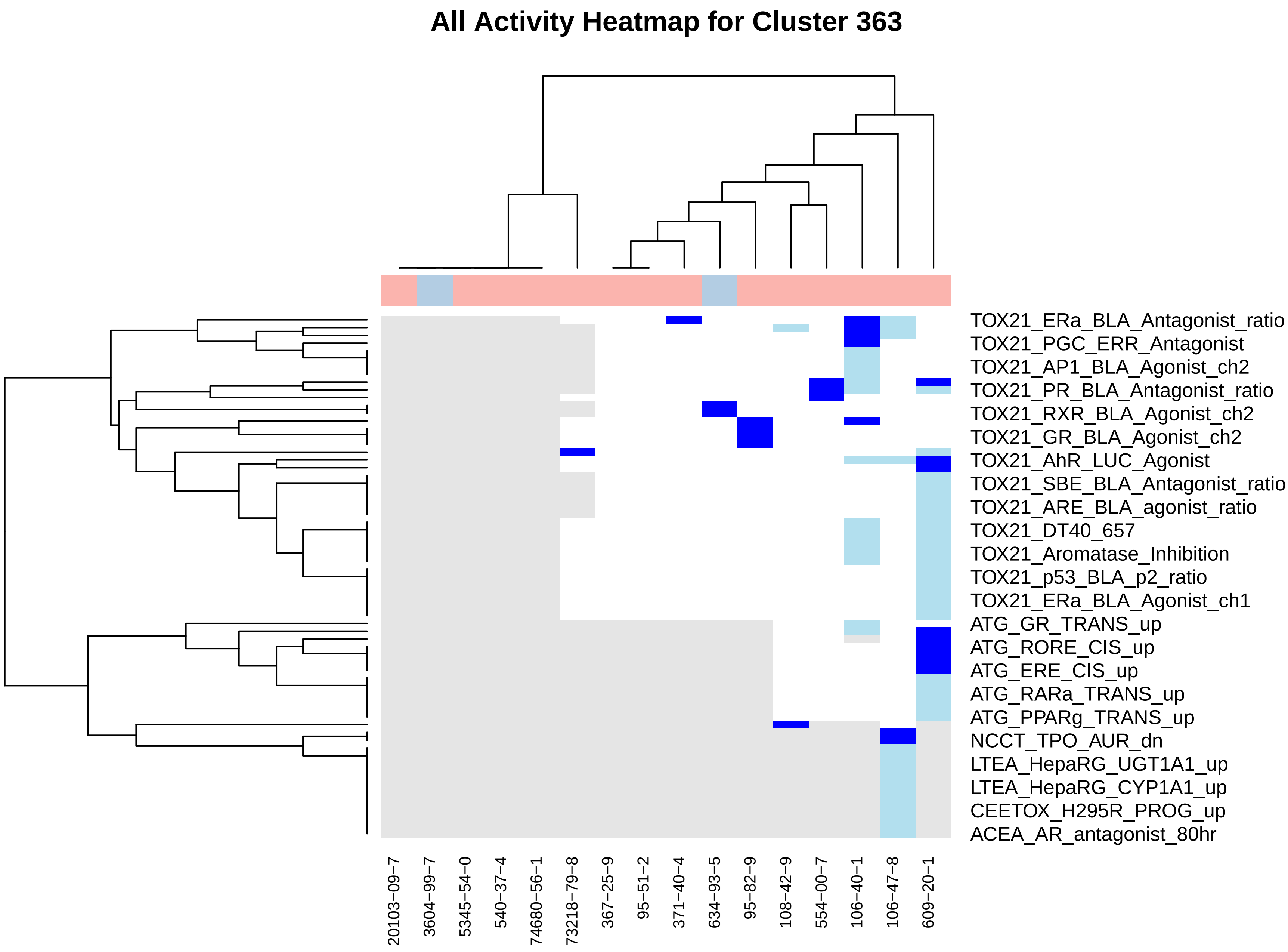
All Activity Heatmap for Cluster 363
TOX21_ERa_BLA_Antagonist_ratio
TOX21_PGC_ERR_Antagonist
TOX21_AP1_BLA_Agonist_ch2
TOX21_PR_BLA_Antagonist_ratio
TOX21_RXR_BLA_Agonist_ch2
TOX21_GR_BLA_Agonist_ch2
TOX21_AhR_LUC_Agonist
TOX21_SBE_BLA_Antagonist_ratio
TOX21_ARE_BLA_agonist_ratio
TOX21_DT40_657
TOX21_Aromatase_Inhibition
TOX21_p53_BLA_p2_ratio
TOX21_ERa_BLA_Agonist_ch1
ATG_GR_TRANS_up
ATG_RORE_CIS_up
ATG_ERE_CIS_up
ATG_RARa_TRANS_up
ATG_PPARg_TRANS_up
NCCT_TPO_AUR_dn
LTEA_HepaRG_UGT1A1_up
LTEA_HepaRG_CYP1A1_up
CEETOX_H295R_PROG_up
ACEA_AR_antagonist_80hr
20103−09−7
3604−99−7
5345−54−0
540−37−4
74680−56−1
73218−79−8
367−25−9
95−51−2
371−40−4
634−93−5
95−82−9
108−42−9
554−00−7
106−40−1
106−47−8
609−20−1

All Activity Heatmap for Cluster 364

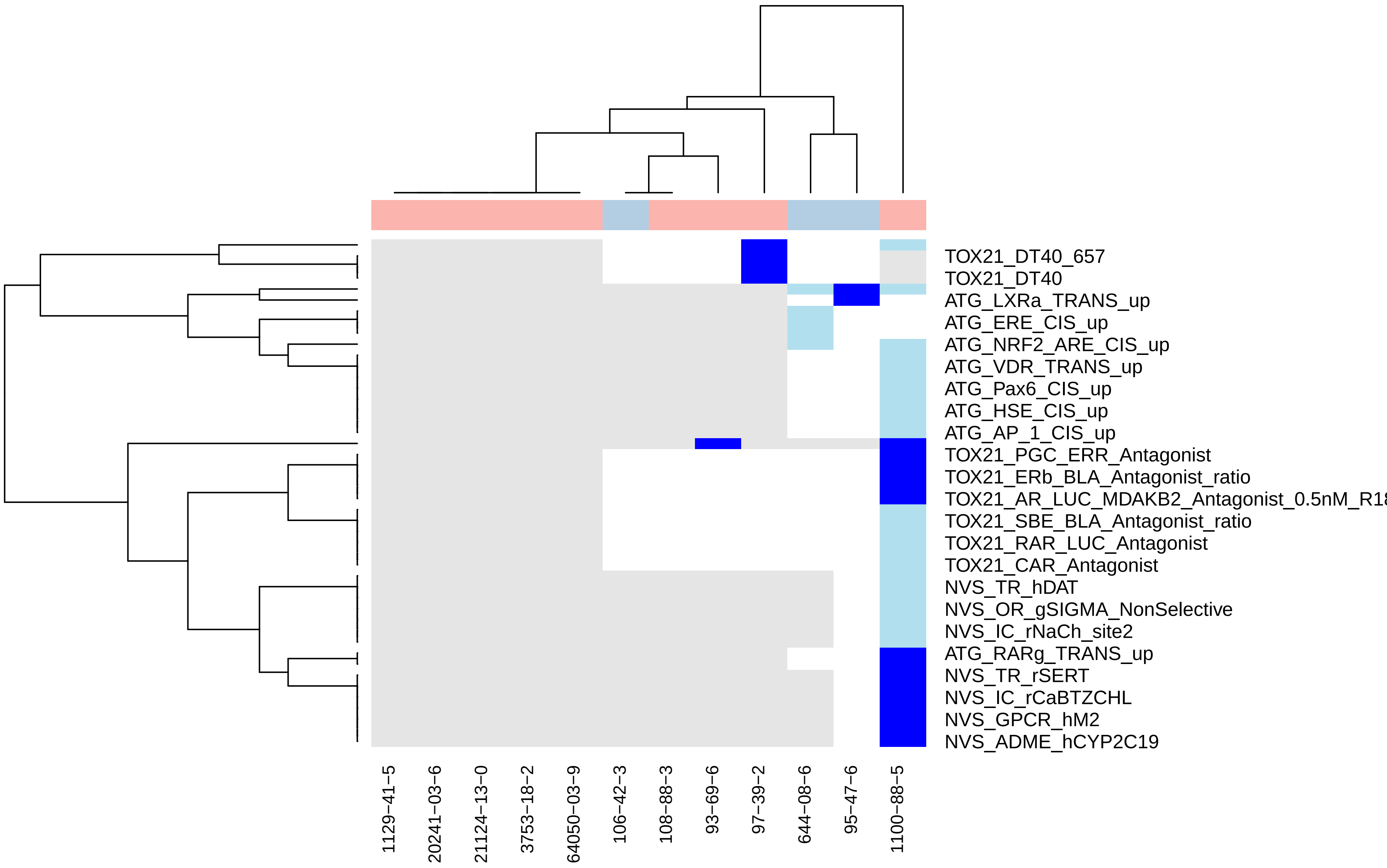

All Activity Heatmap for Cluster 365

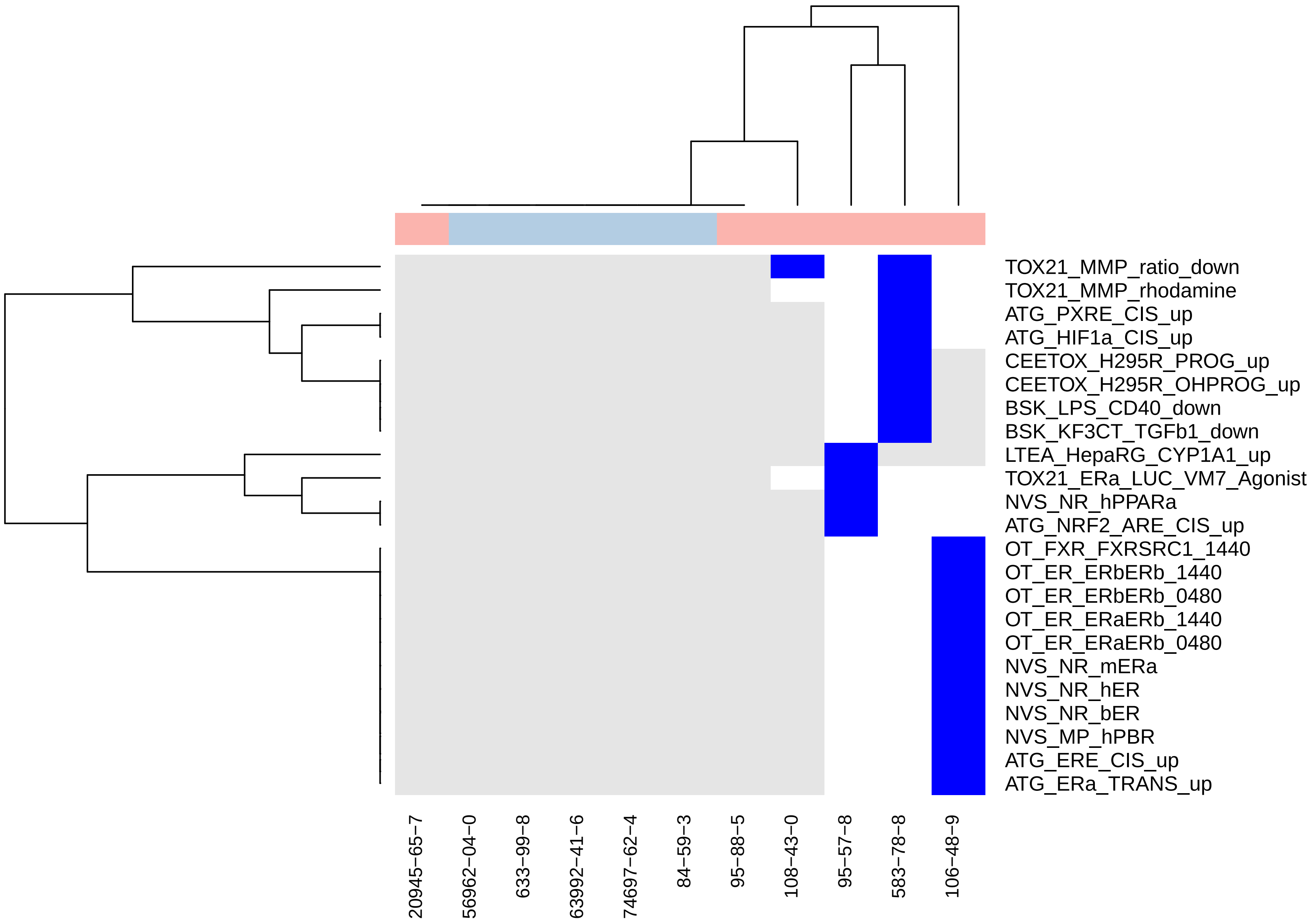

All Activity Heatmap for Cluster 366

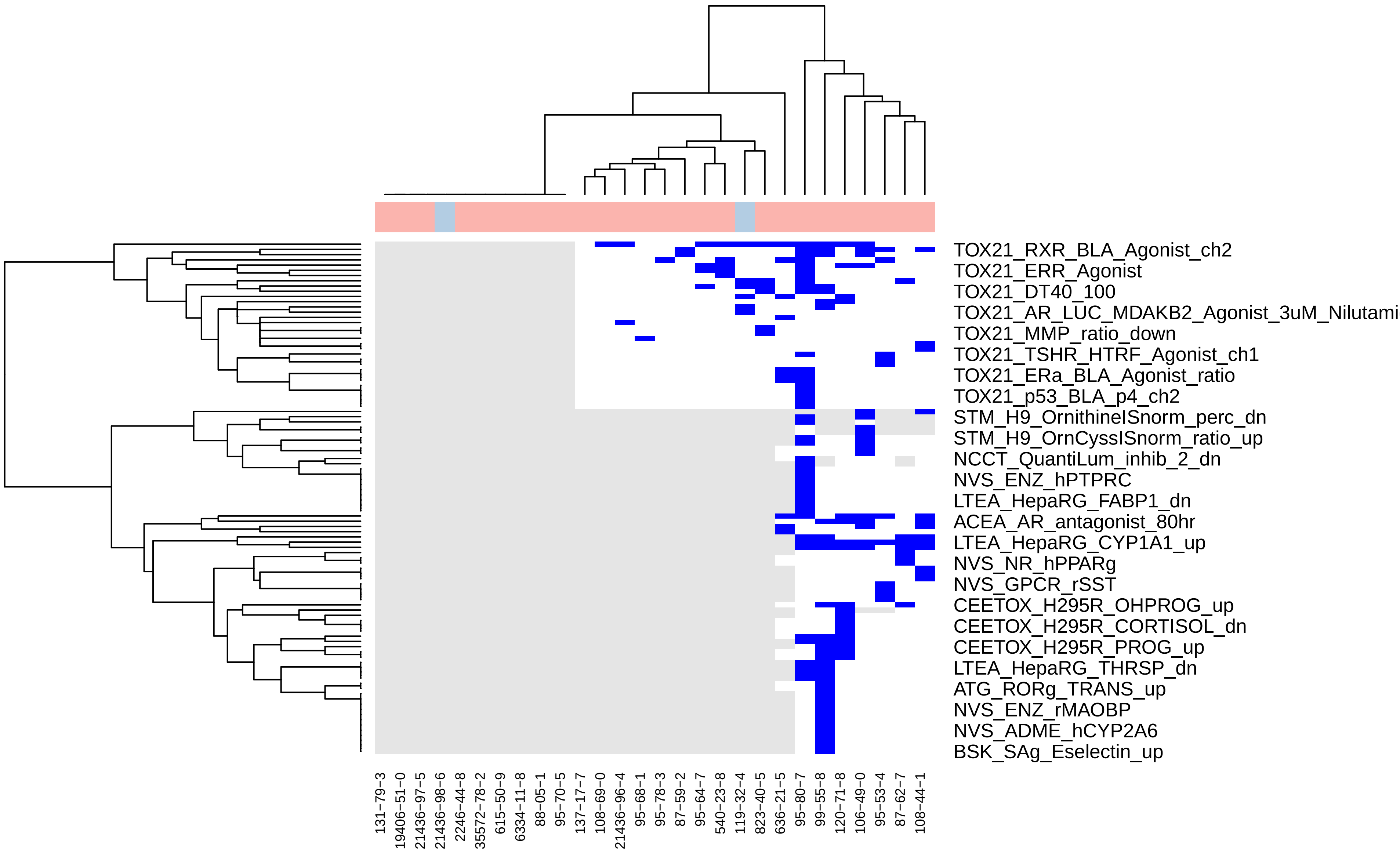

All Activity Heatmap for Cluster 367

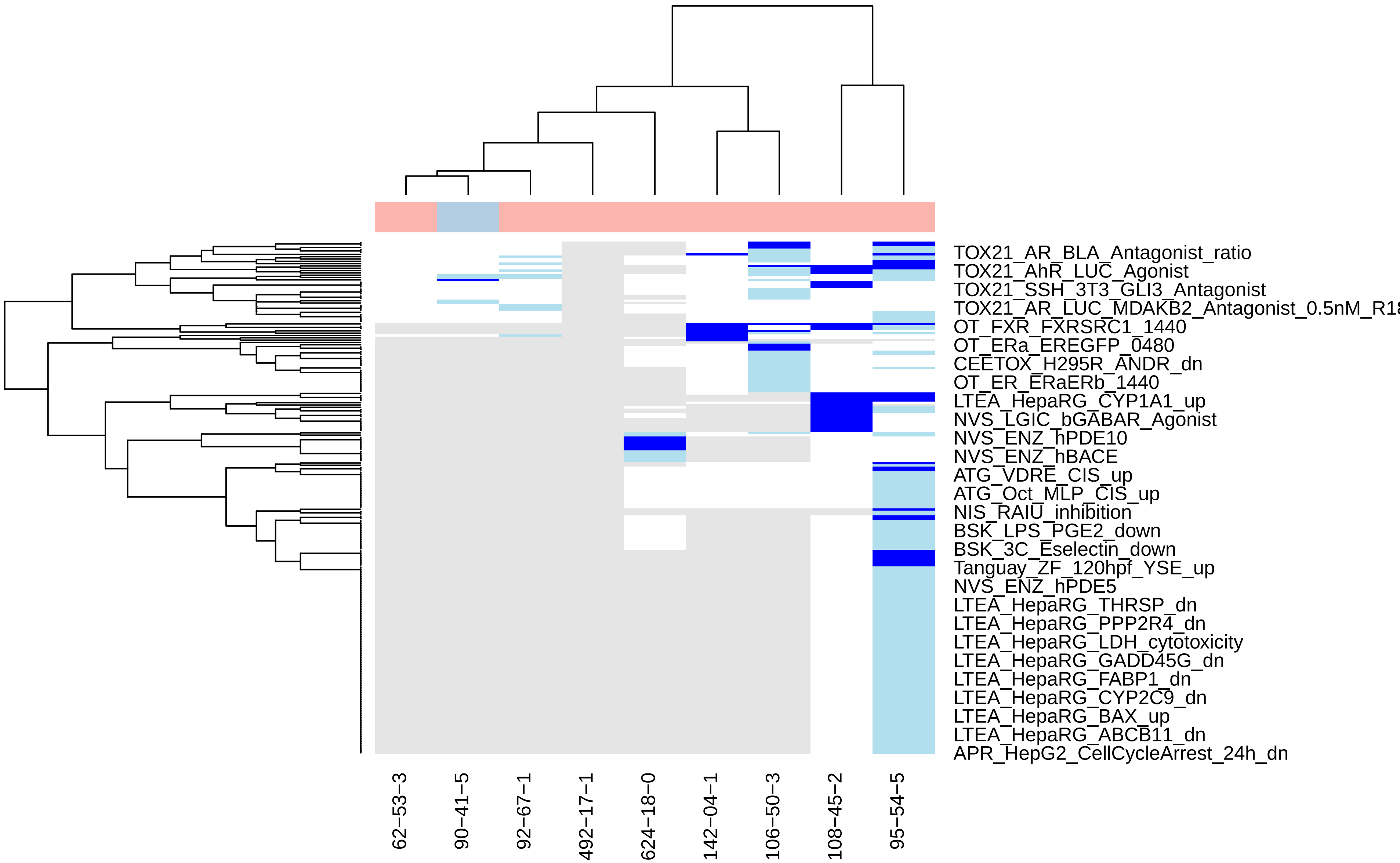

All Activity Heatmap for Cluster 368

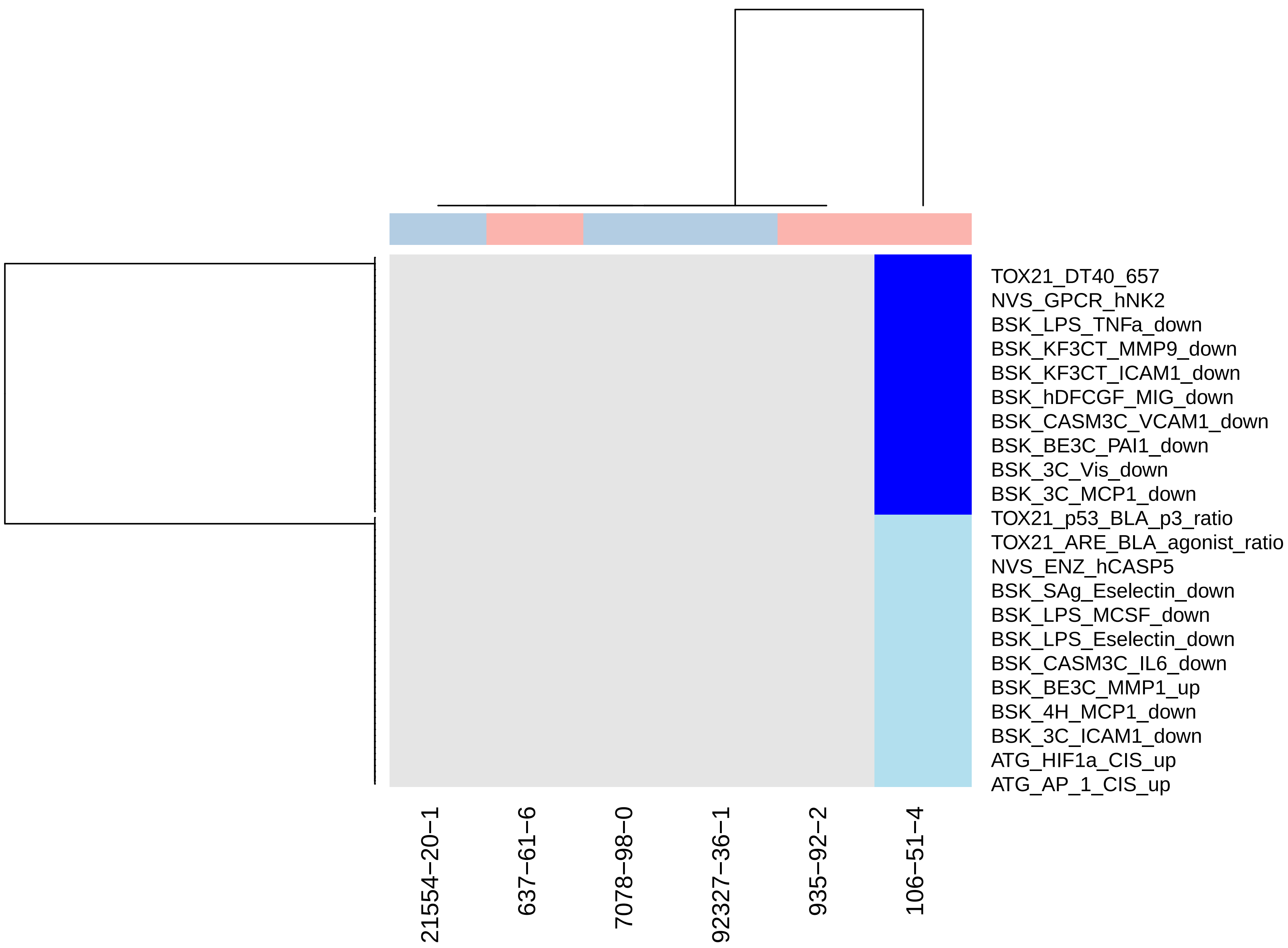

All Activity Heatmap for Cluster 369

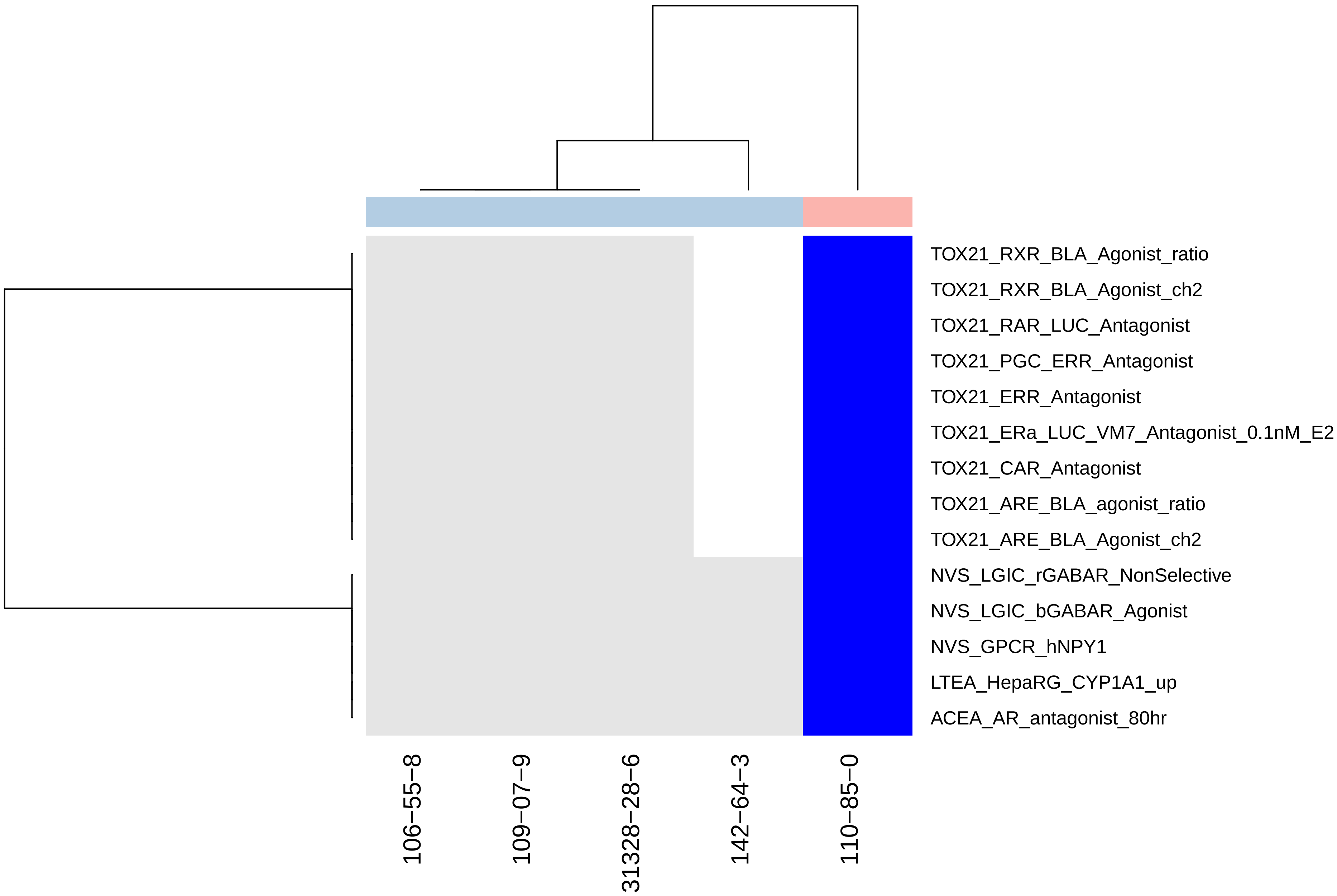

All Activity Heatmap for Cluster 370

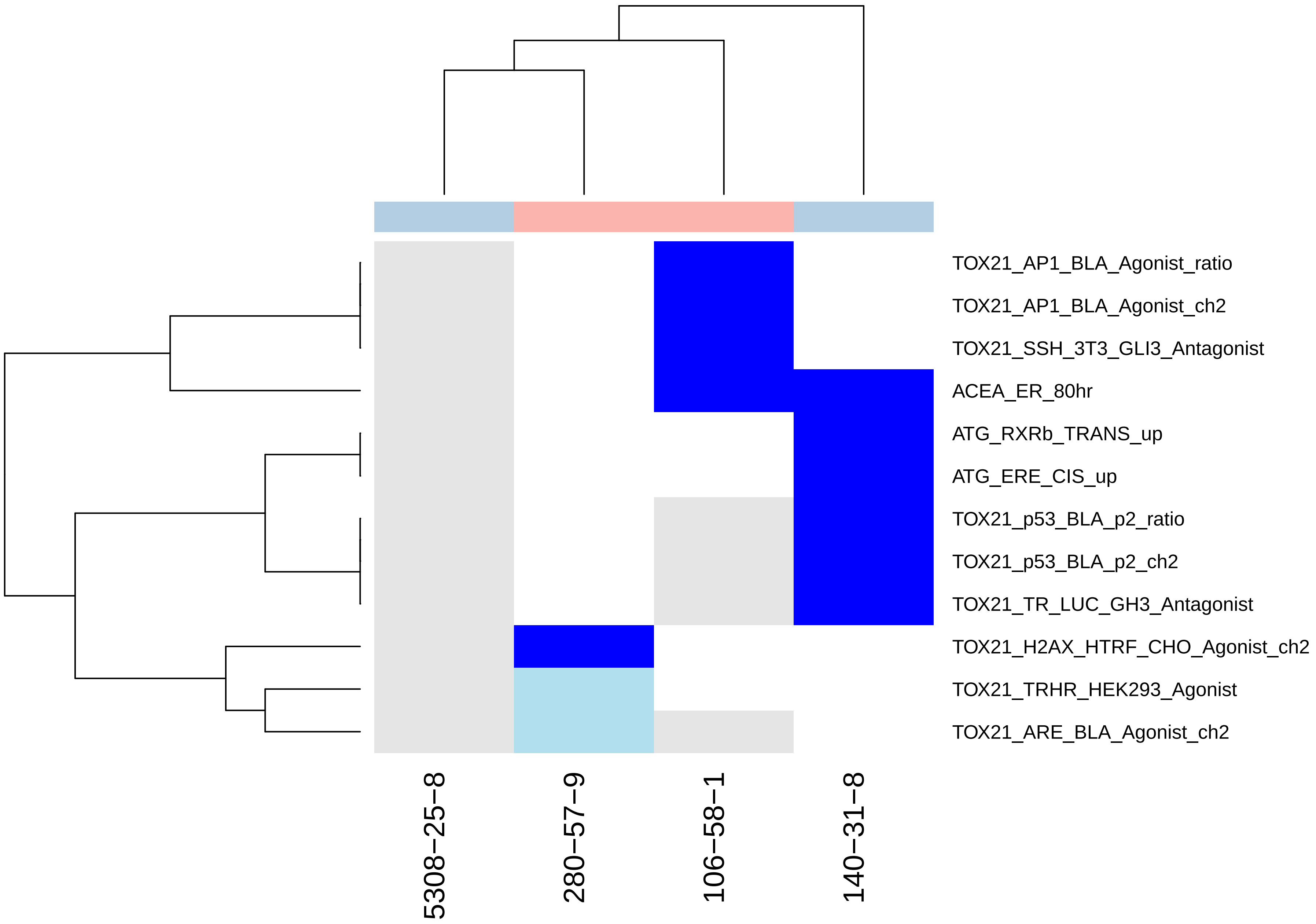

All Activity Heatmap for Cluster 371

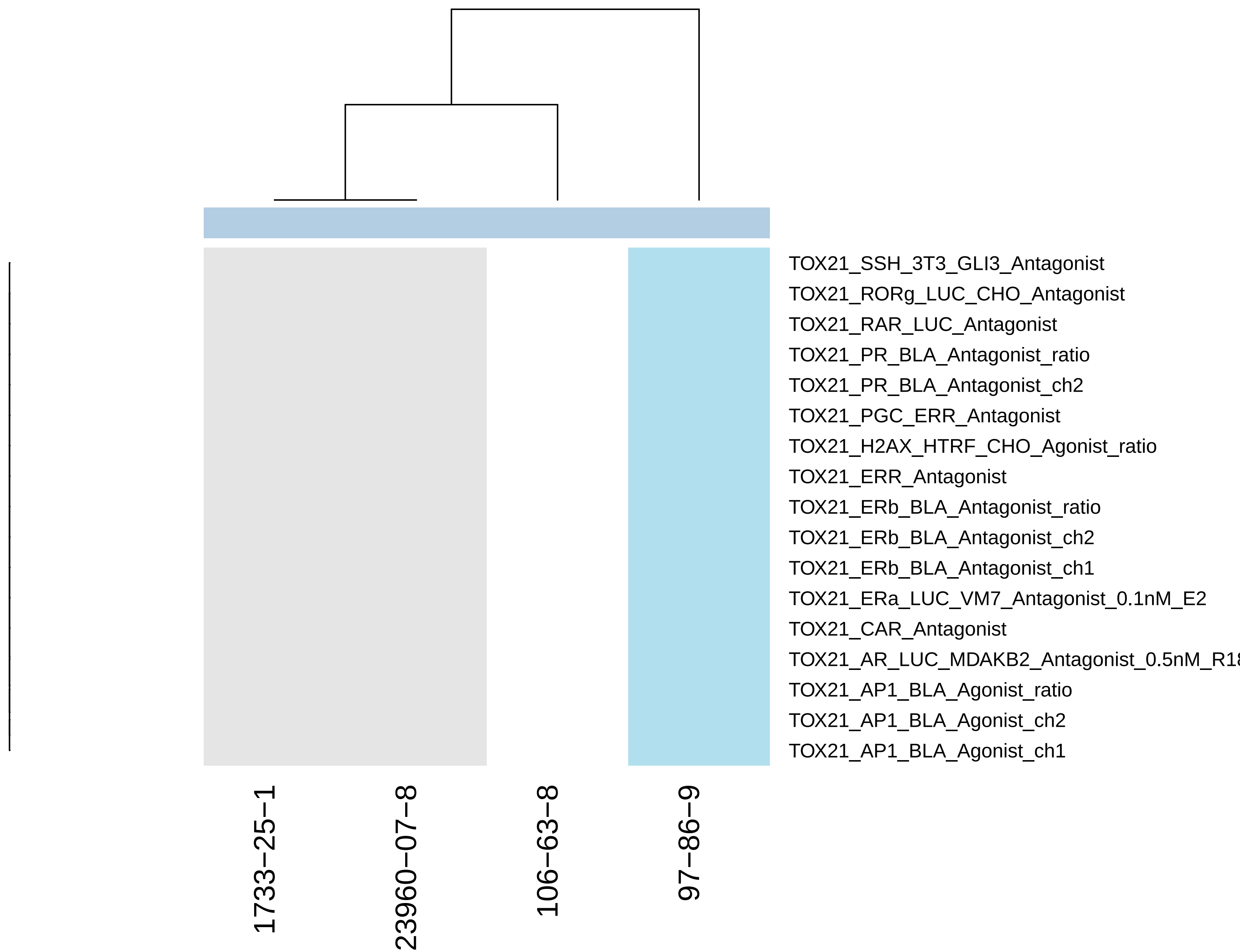

All Activity Heatmap for Cluster 372

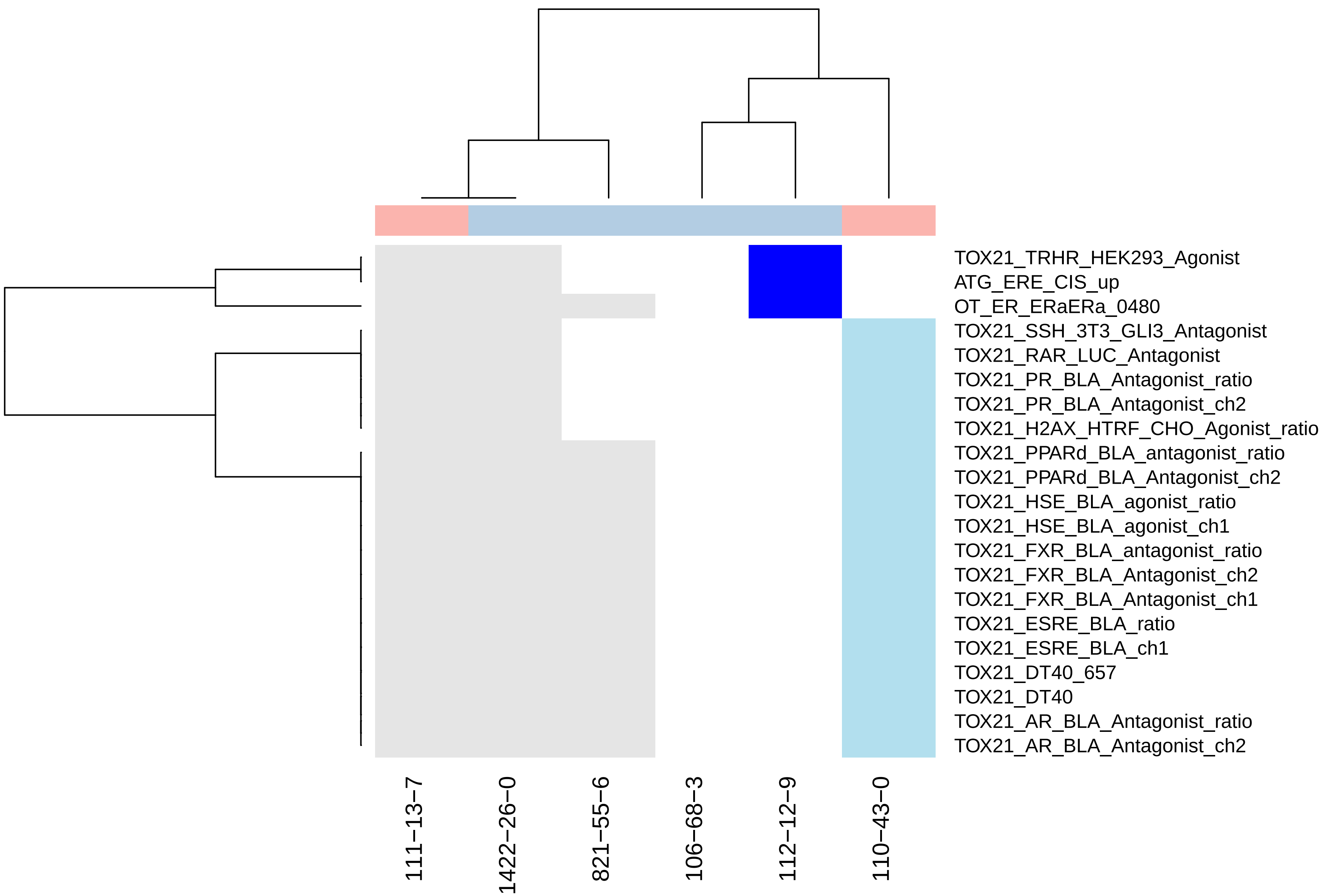

All Activity Heatmap for Cluster 373

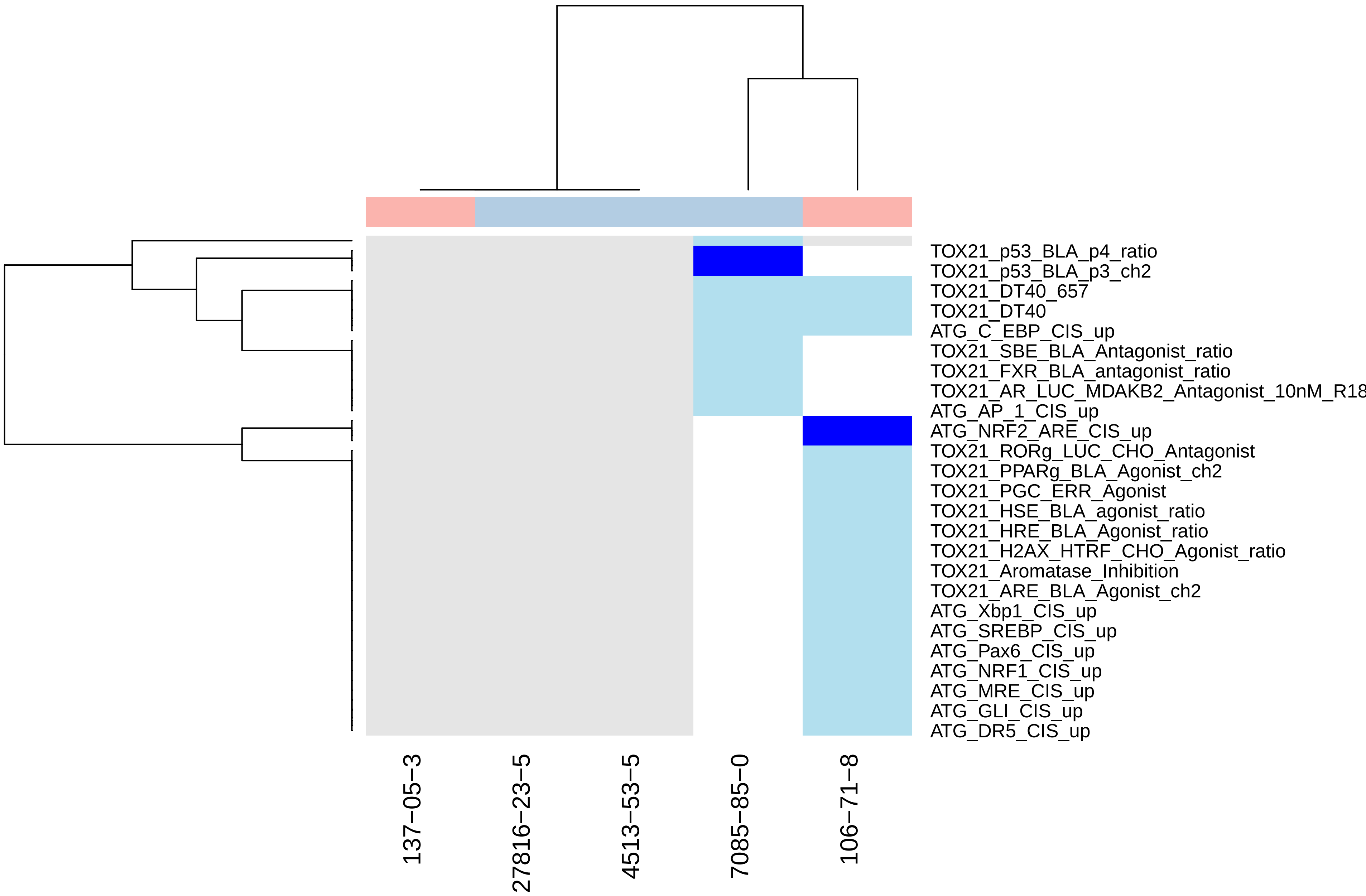

All Activity Heatmap for Cluster 374

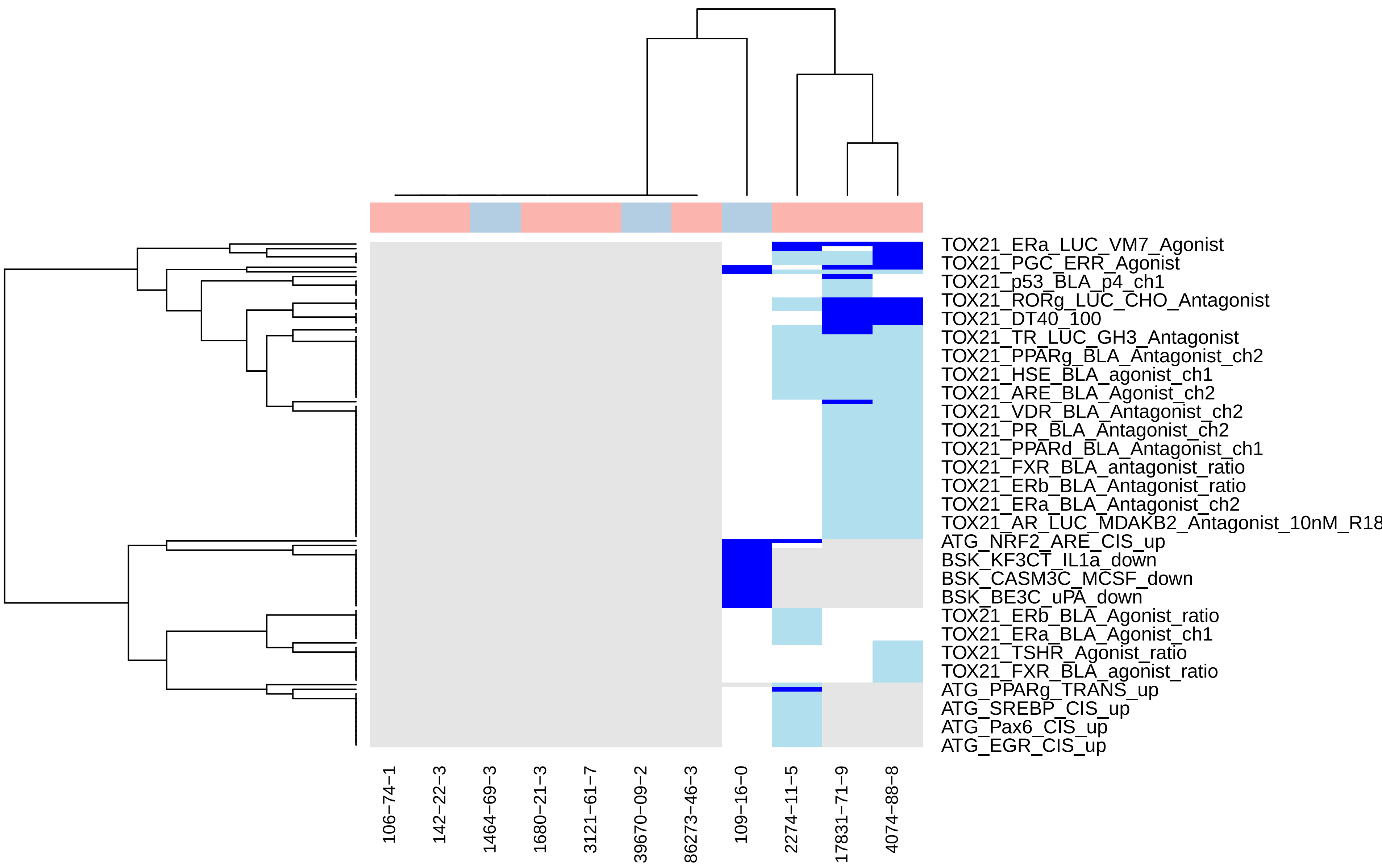

All Activity Heatmap for Cluster 375

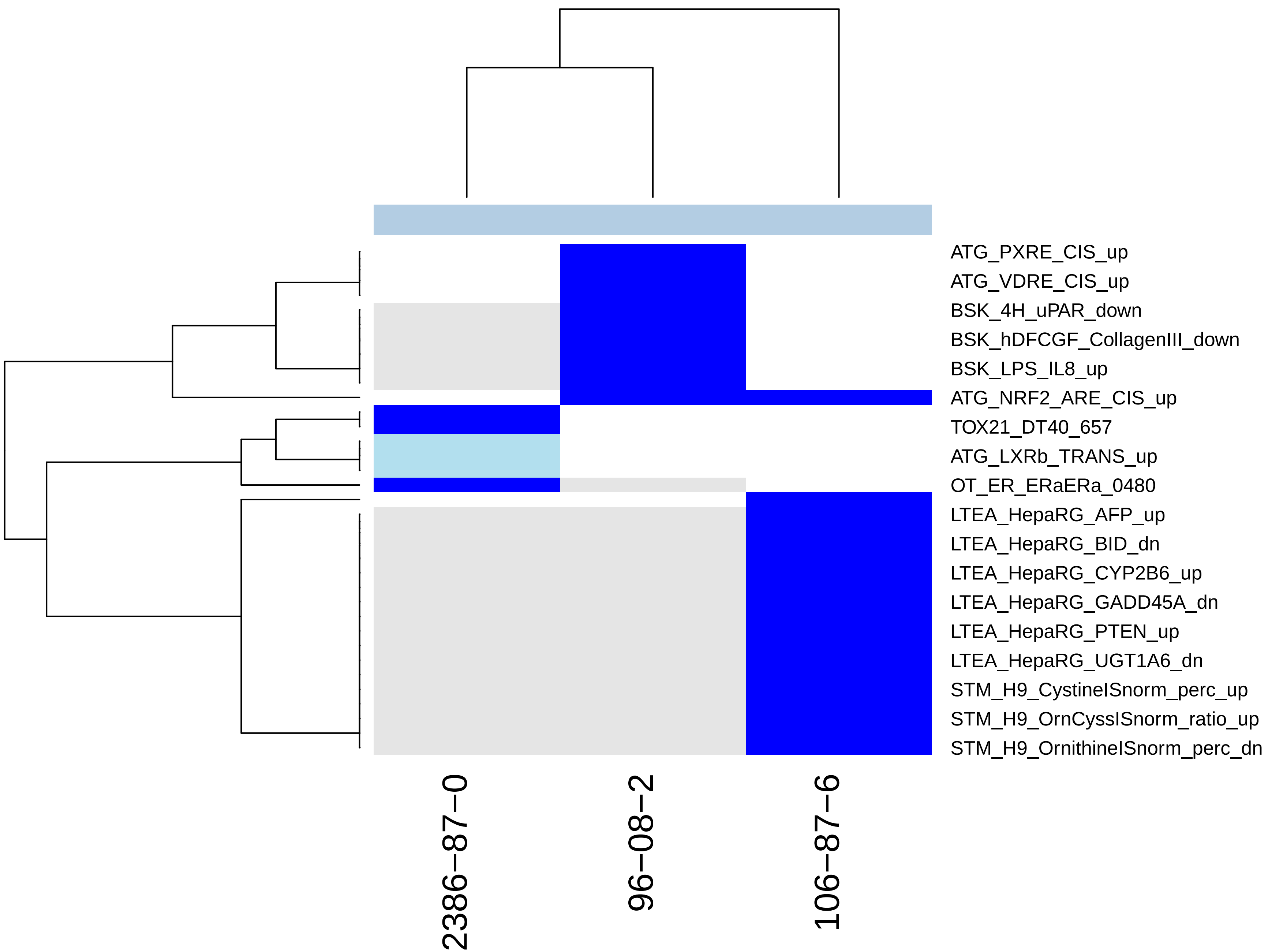

All Activity Heatmap for Cluster 376

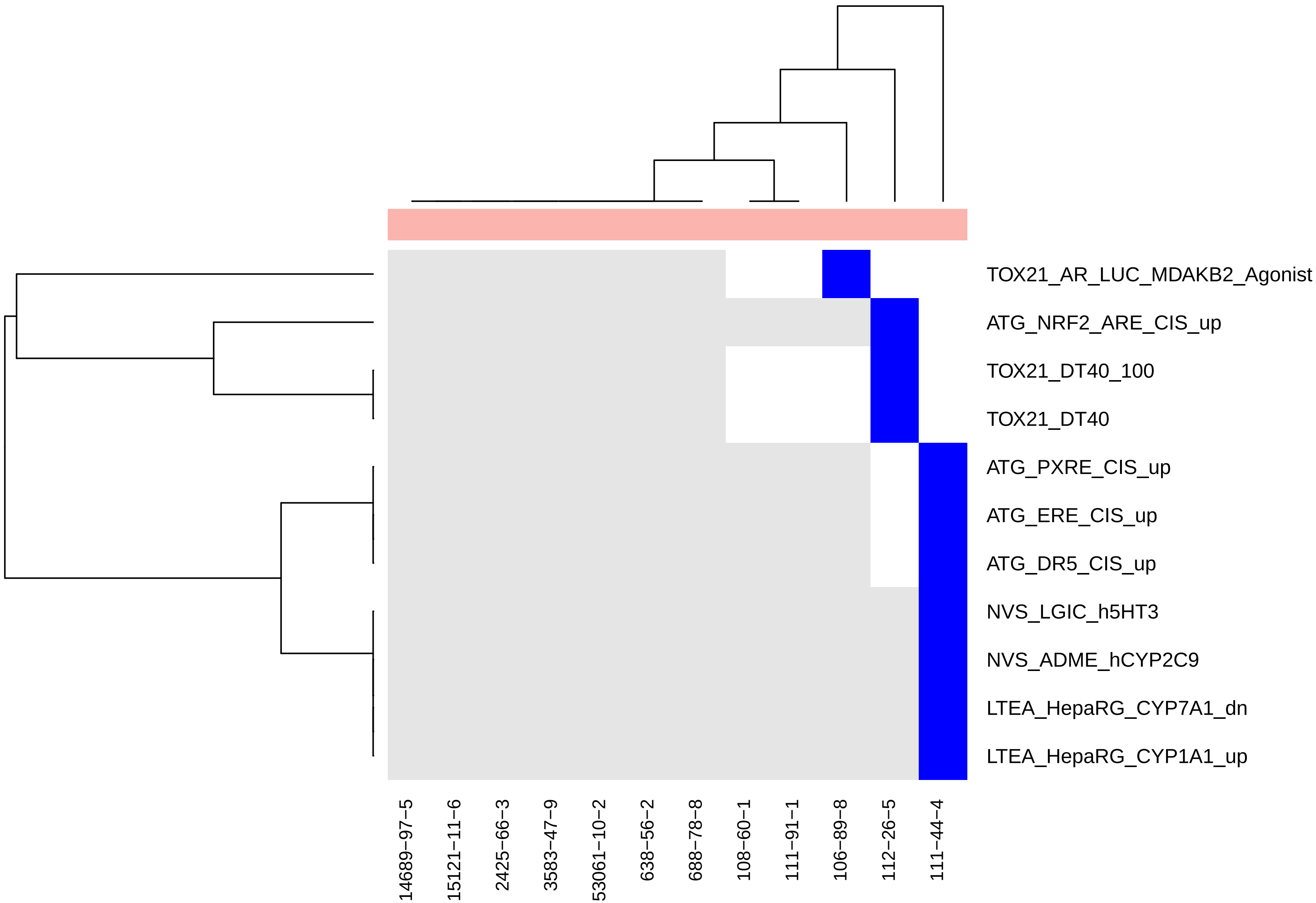

All Activity Heatmap for Cluster 377

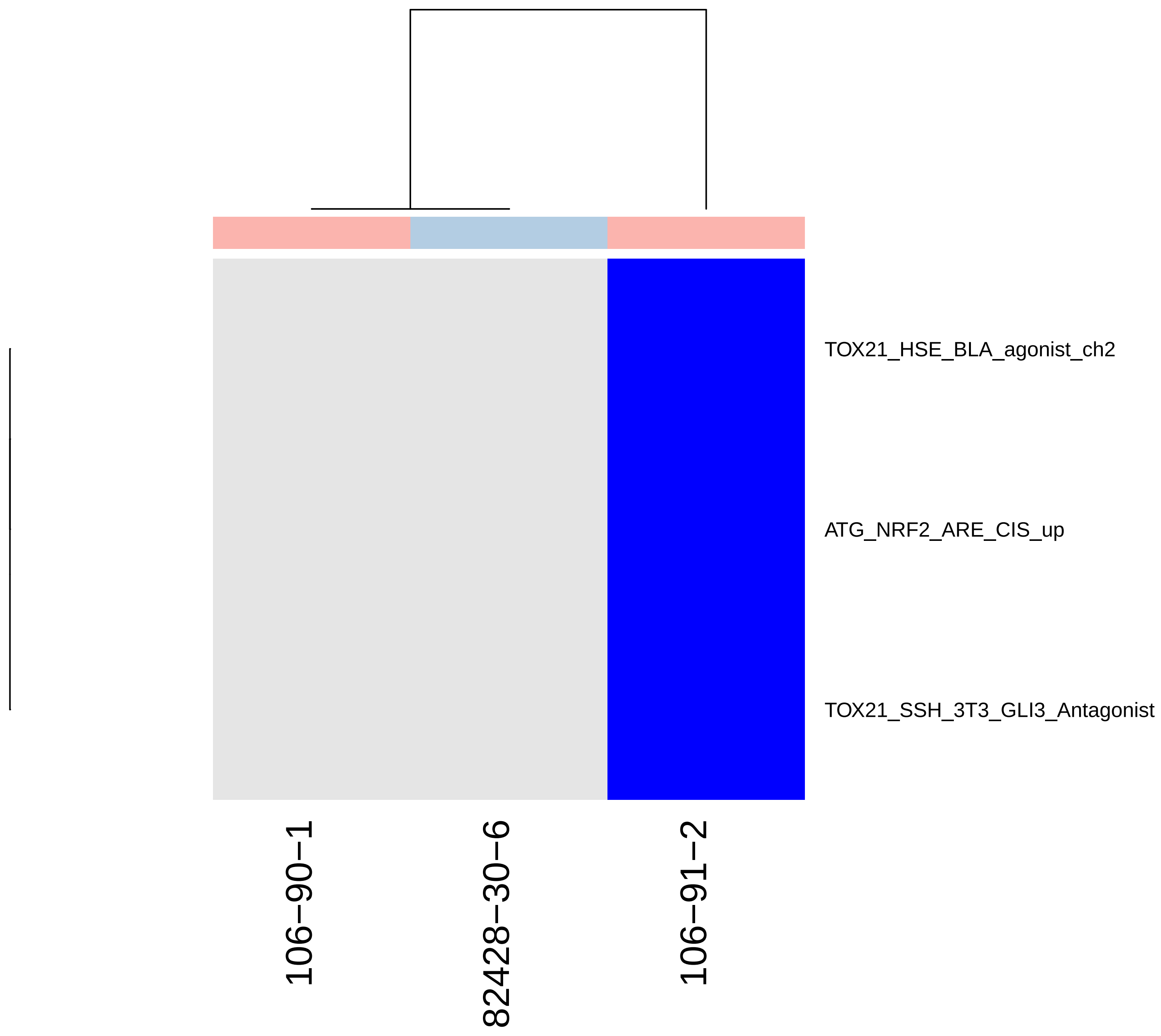

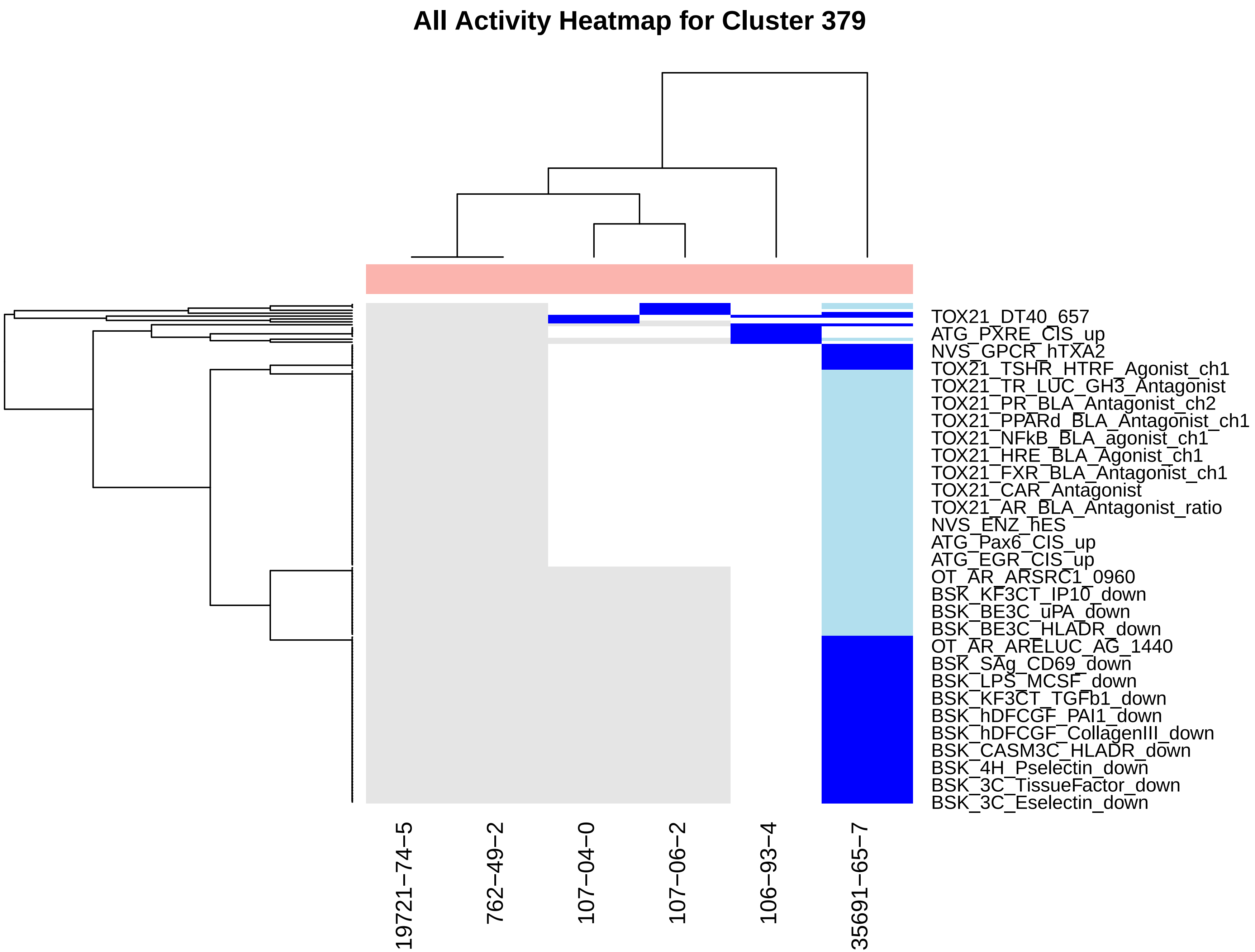
All Activity Heatmap for Cluster 379
TOX21_DT40_657
ATG_PXRE_CIS_up
NVS_GPCR_hTXA2
TOX21_TSHR_HTRF_Agonist_ch1
TOX21_TR_LUC_GH3_Antagonist
TOX21_PR_BLA_Antagonist_ch2
TOX21_PPARd_BLA_Antagonist_ch1
TOX21_NFkB_BLA_agonist_ch1
TOX21_HRE_BLA_Agonist_ch1
TOX21_FXR_BLA_Antagonist_ch1
TOX21_CAR_Antagonist
TOX21_AR_BLA_Antagonist_ratio
NVS_ENZ_hES
ATG_Pax6_CIS_up
ATG_EGR_CIS_up
OT_AR_ARSRC1_0960
BSK_KF3CT_IP10_down
BSK_BE3C_uPA_down
BSK_BE3C_HLADR_down
OT_AR_ARELUC_AG_1440
BSK_SAg_CD69_down
BSK_LPS_MCSF_down
BSK_KF3CT_TGFb1_down
BSK_hDFCGF_PAI1_down
BSK_hDFCGF_CollagenIII_down
BSK_CASM3C_HLADR_down
BSK_4H_Pselectin_down
BSK_3C_TissueFactor_down
BSK_3C_Eselectin_down
19721−74−5
762−49−2
107−04−0
107−06−2
106−93−4
35691−65−7

All Activity Heatmap for Cluster 380

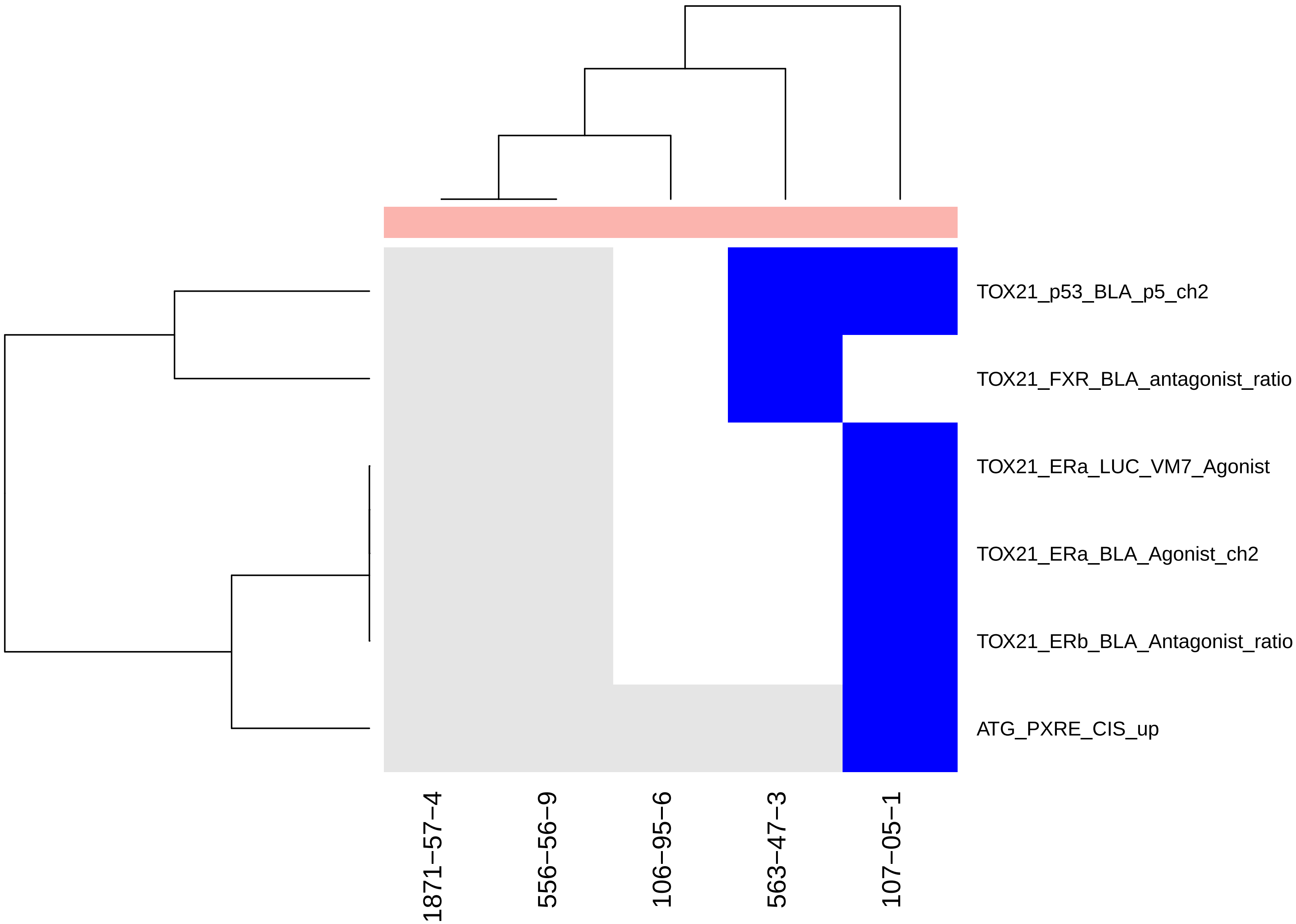

All Activity Heatmap for Cluster 381

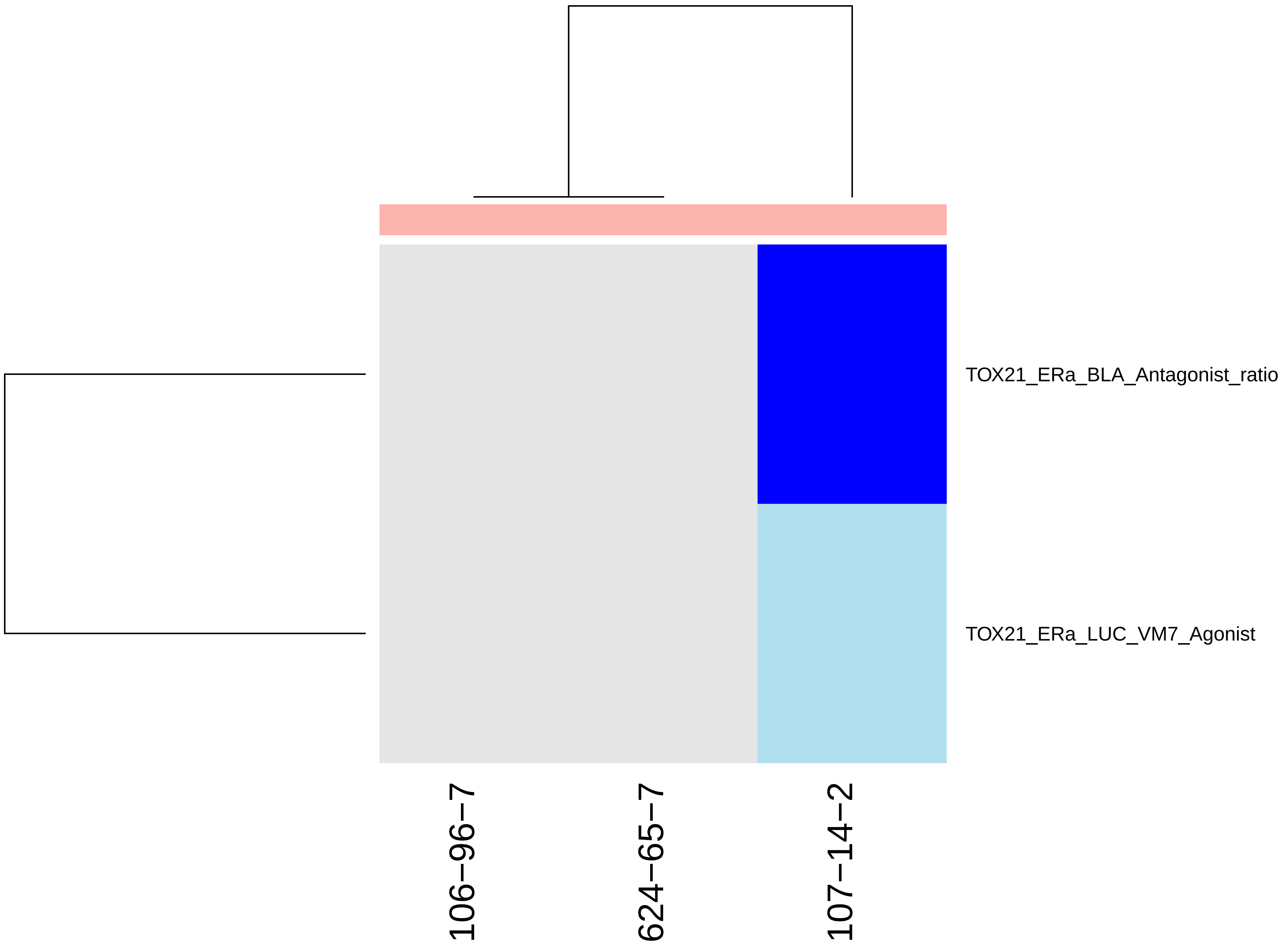

All Activity Heatmap for Cluster 383

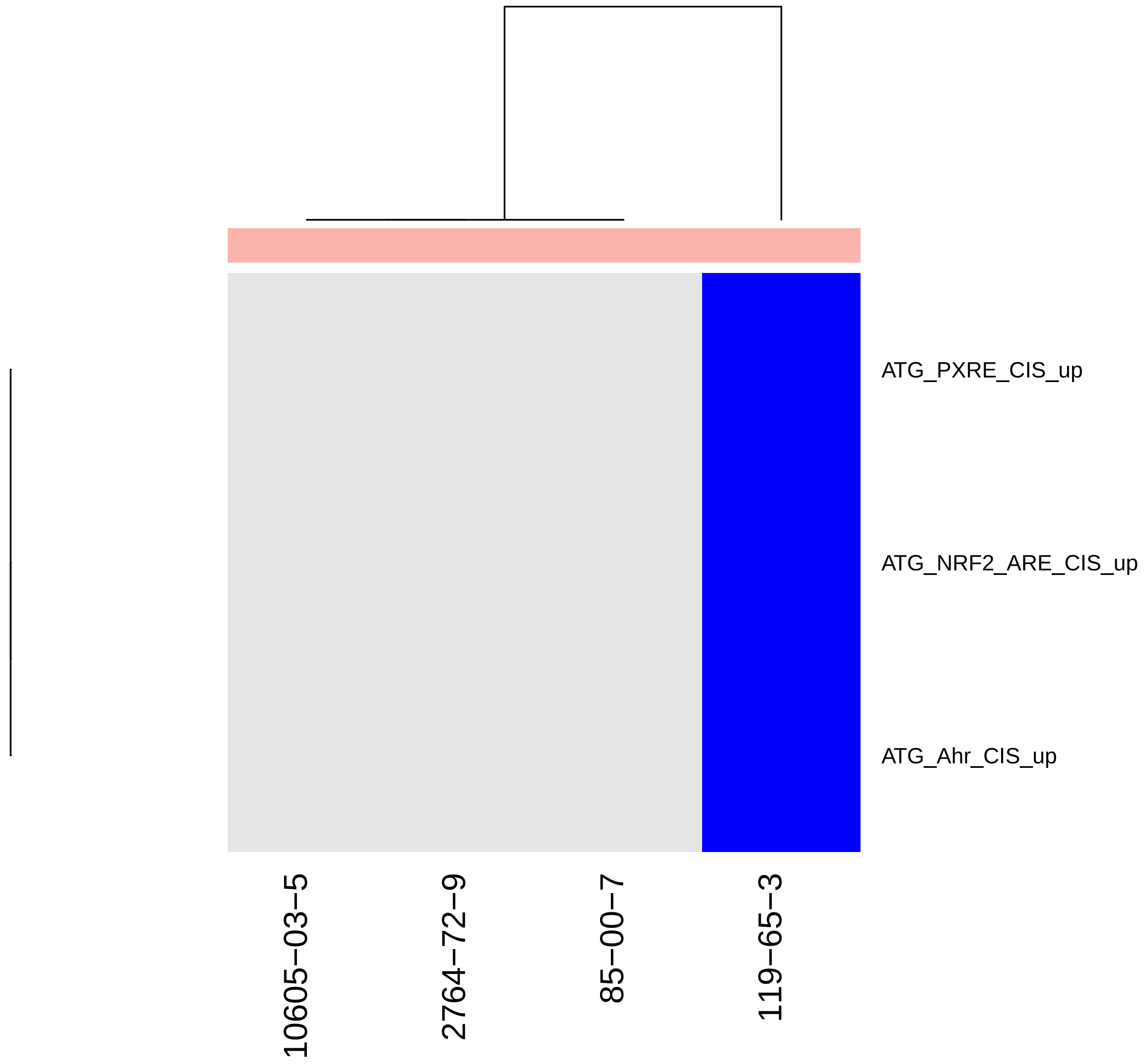

All Activity Heatmap for Cluster 384

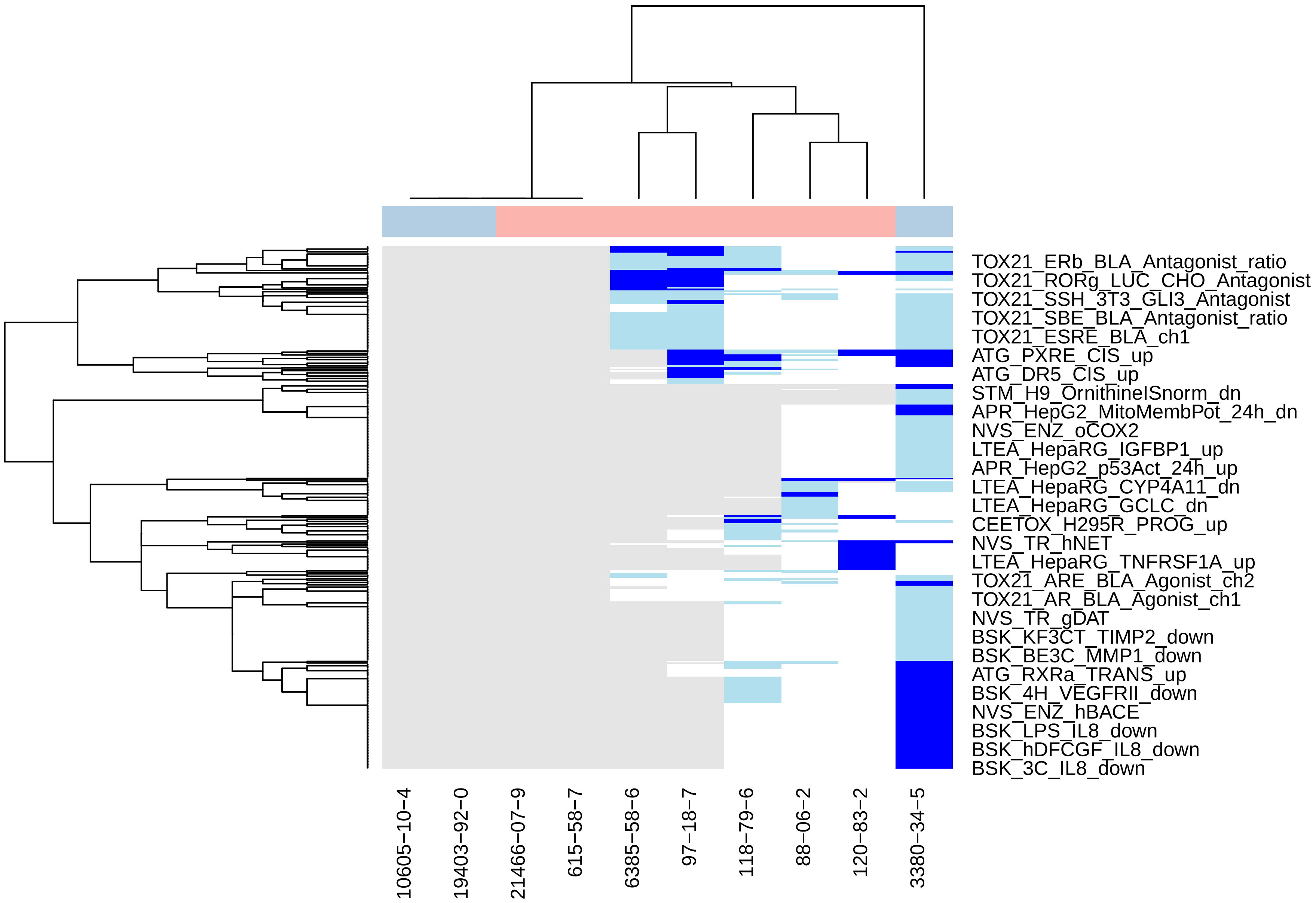

All Activity Heatmap for Cluster 385

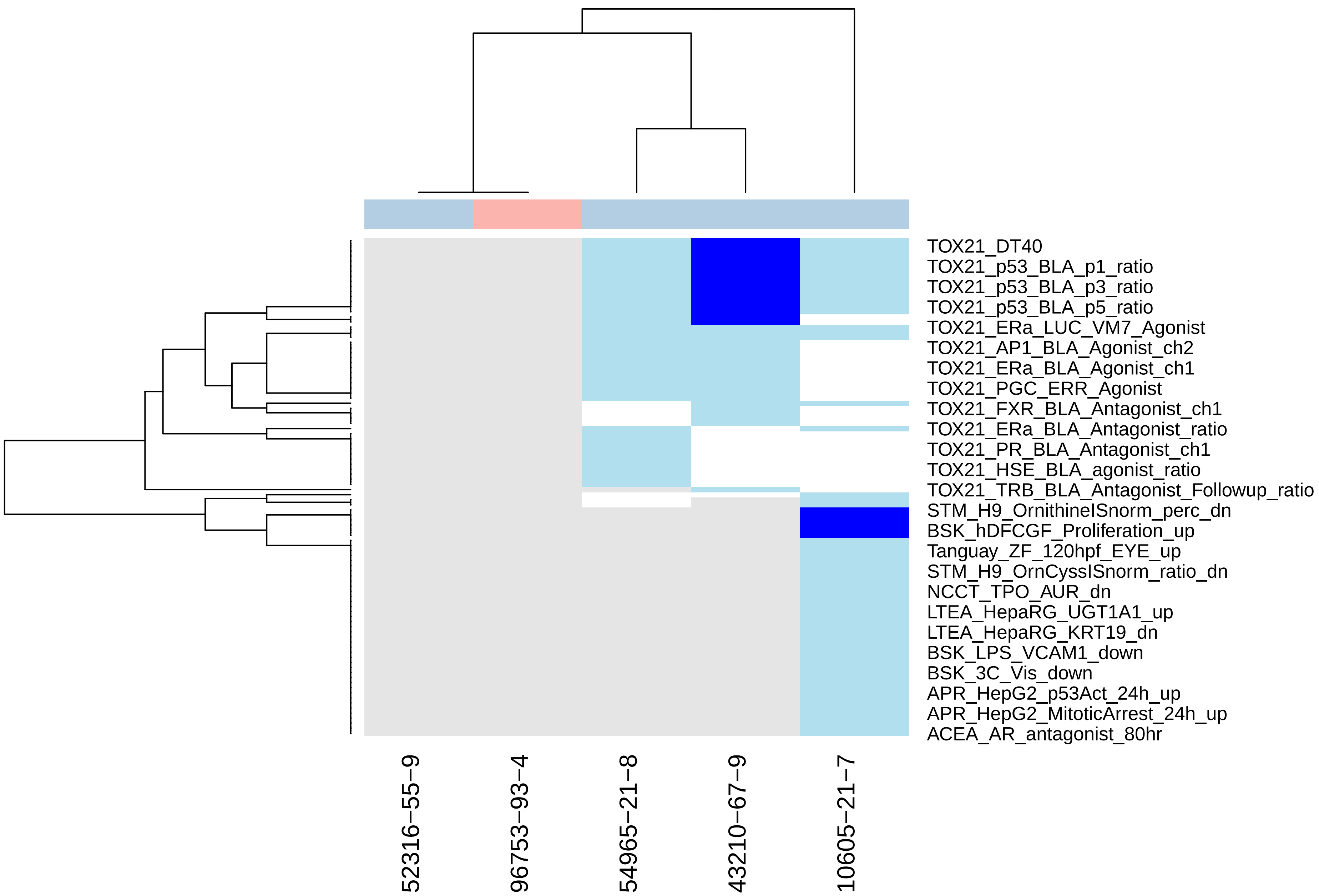

All Activity Heatmap for Cluster 386

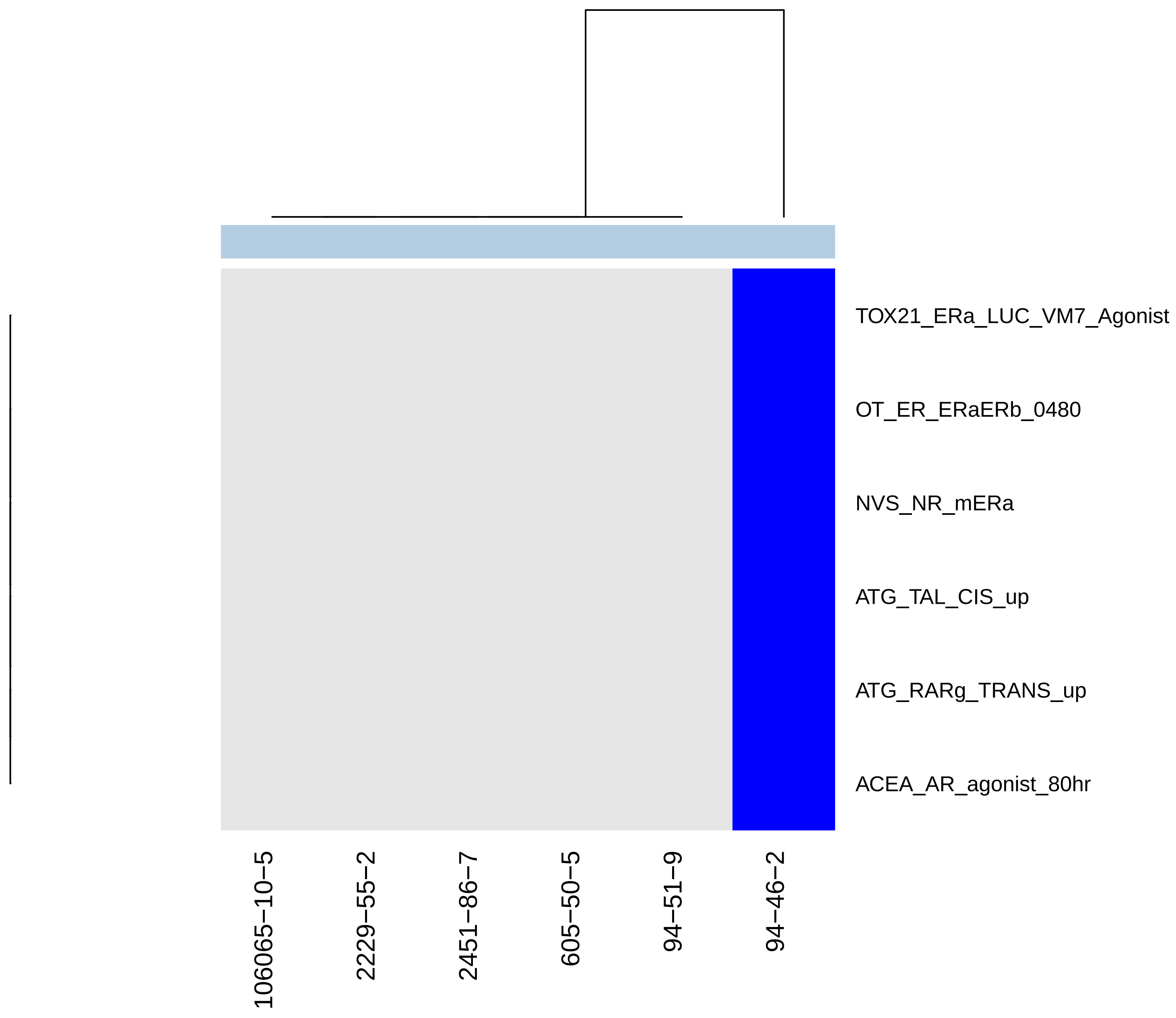

All Activity Heatmap for Cluster 388

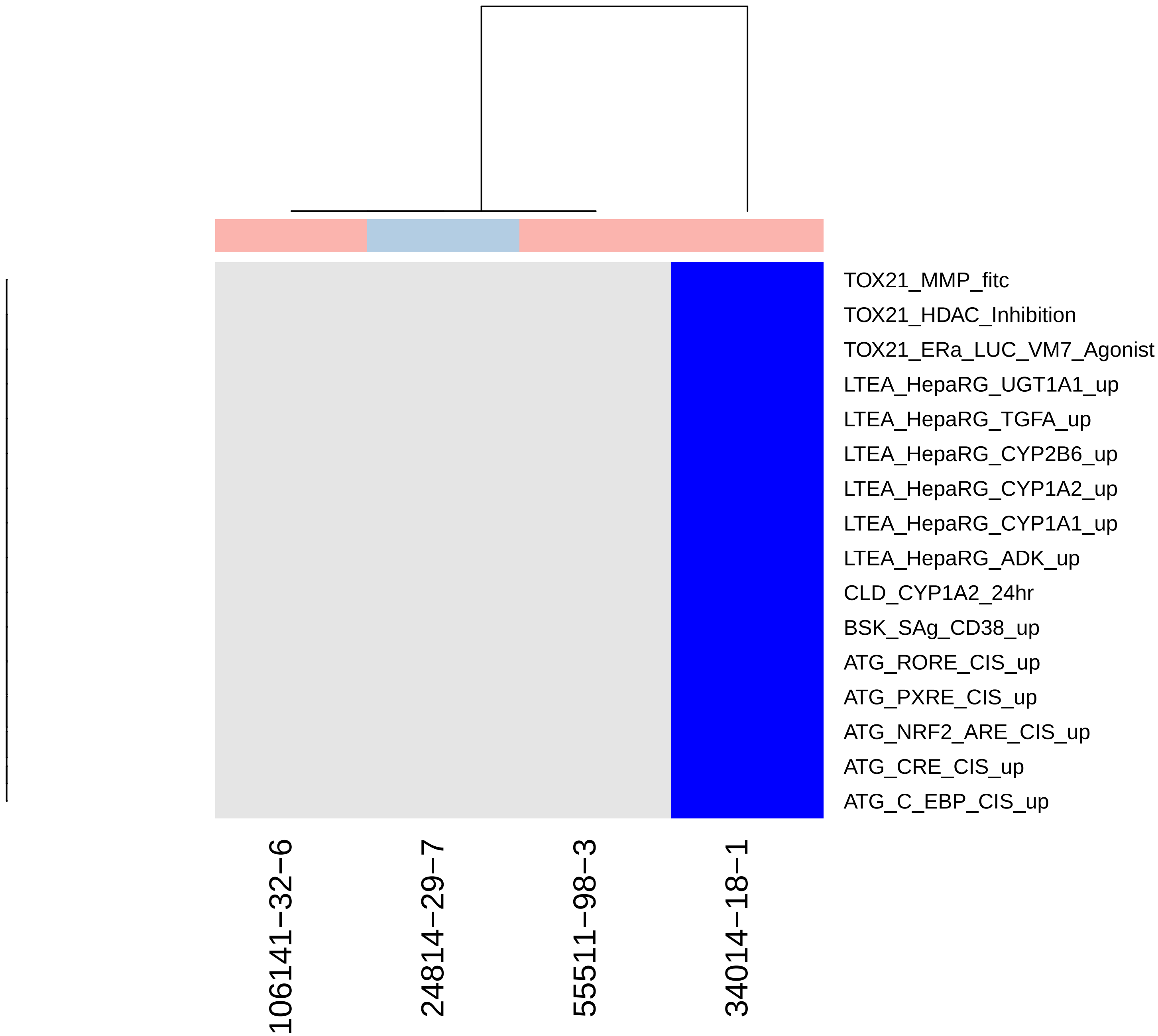

All Activity Heatmap for Cluster 389

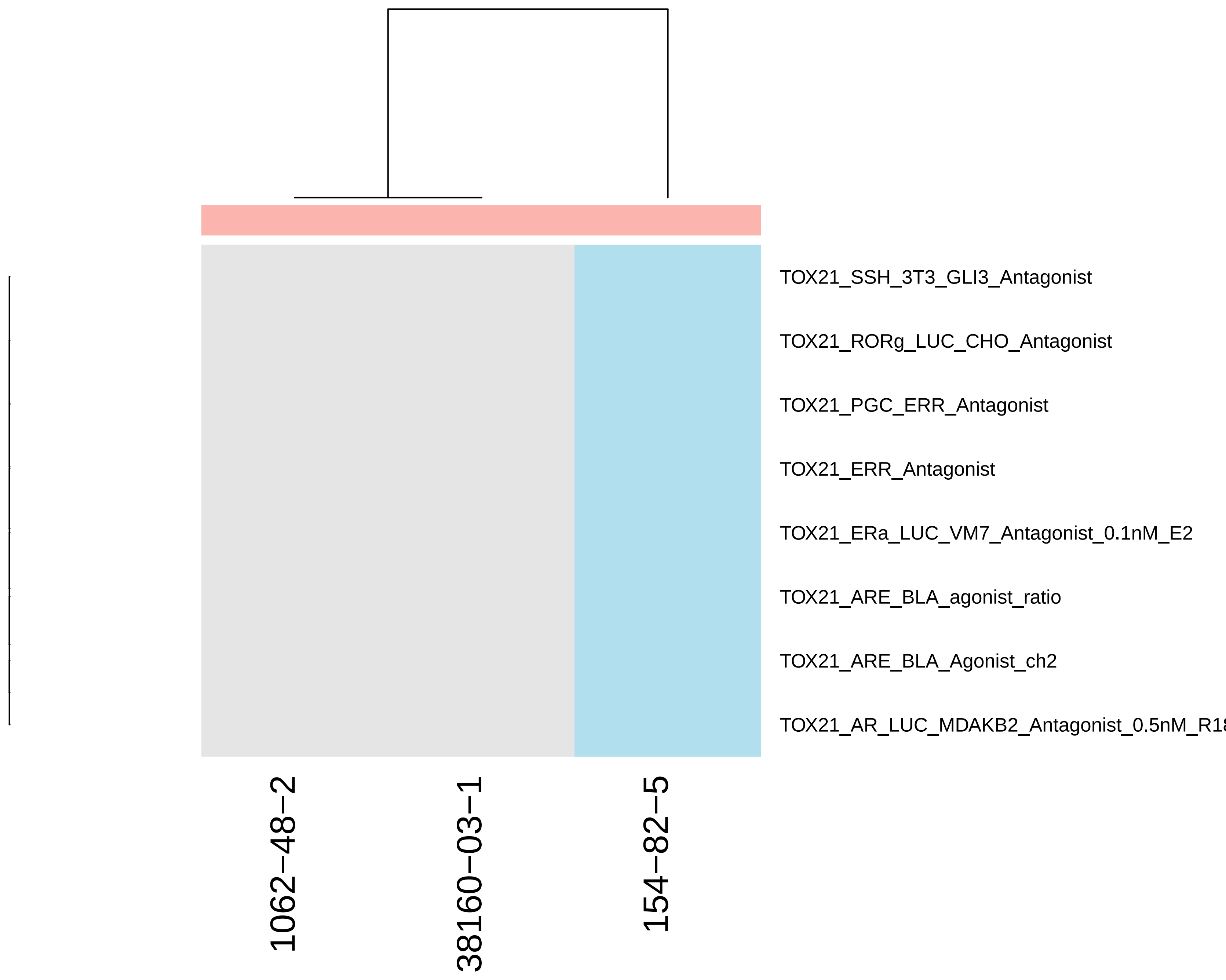

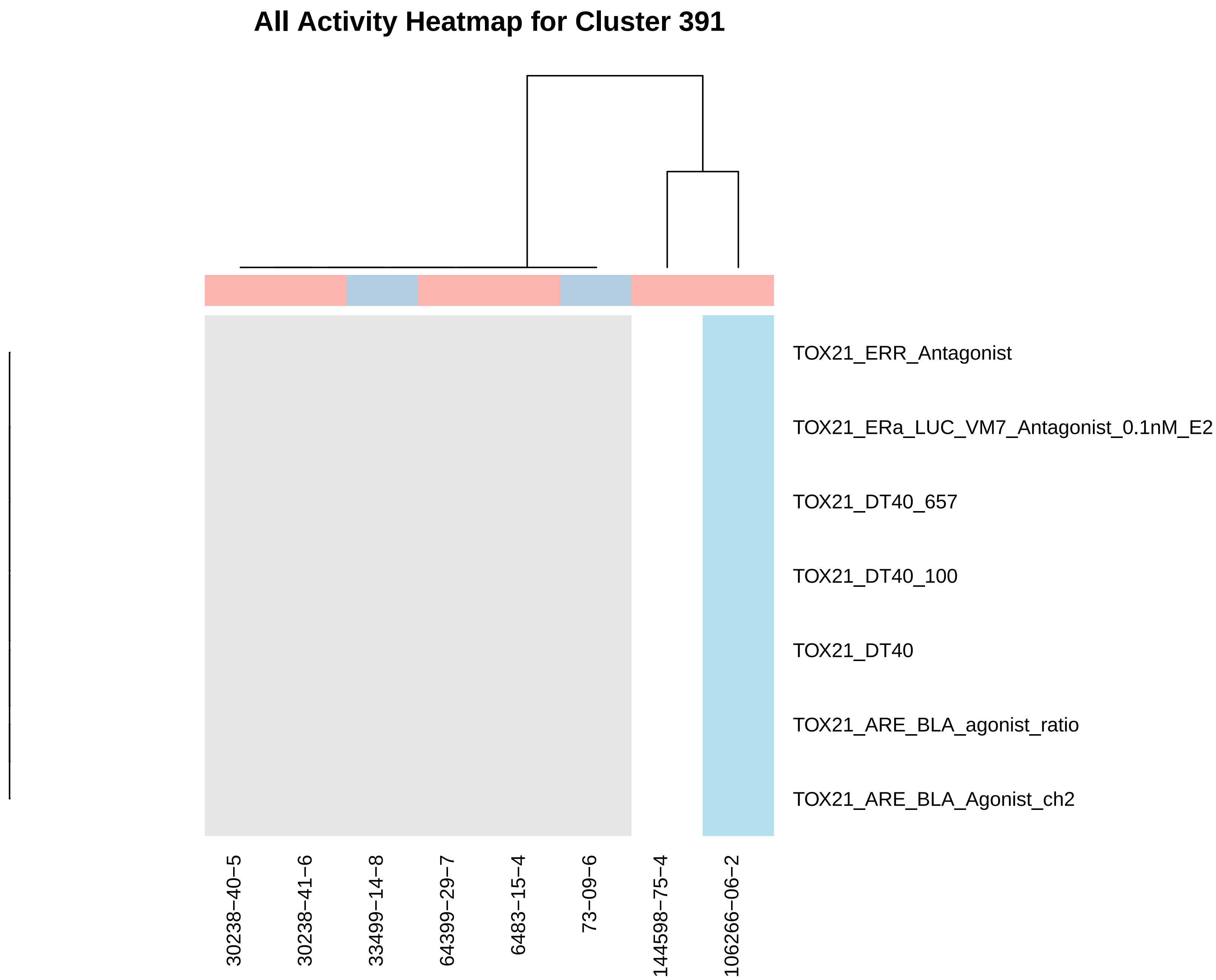
All Activity Heatmap for Cluster 391
TOX21_ERR_Antagonist
TOX21_ERa_LUC_VM7_Antagonist_0.1nM_E2
TOX21_DT40_657
TOX21_DT40_100
TOX21_DT40
TOX21_ARE_BLA_agonist_ratio
TOX21_ARE_BLA_Agonist_ch2
30238−40−5
30238−41−6
33499−14−8
64399−29−7
6483−15−4
73−09−6
144598−75−4
106266−06−2

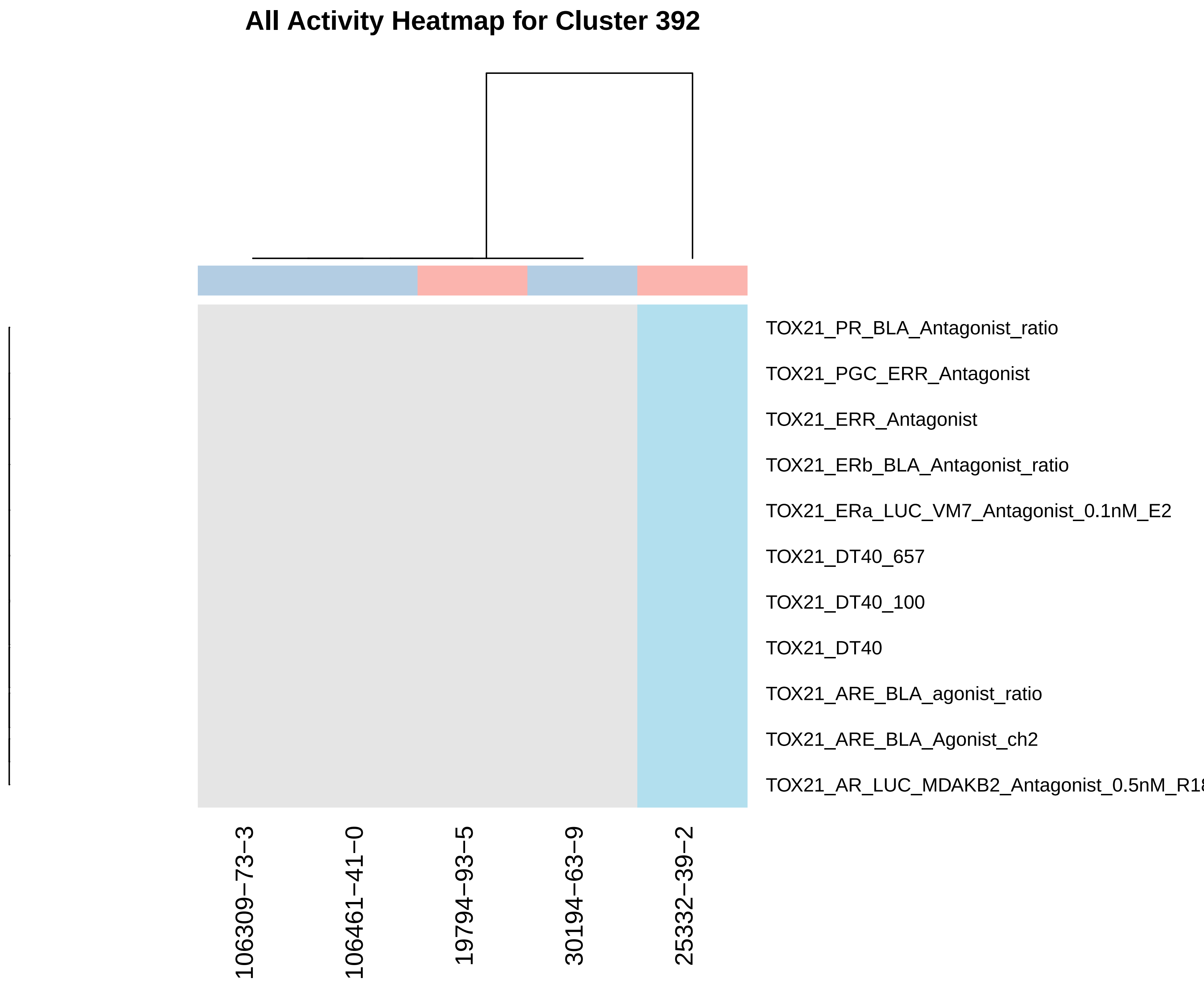
All Activity Heatmap for Cluster 392
TOX21_PR_BLA_Antagonist_ratio
TOX21_PGC_ERR_Antagonist
TOX21_ERR_Antagonist
TOX21_ERb_BLA_Antagonist_ratio
TOX21_ERa_LUC_VM7_Antagonist_0.1nM_E2
TOX21_DT40_657
TOX21_DT40_100
TOX21_DT40
TOX21_ARE_BLA_agonist_ratio
TOX21_ARE_BLA_Agonist_ch2
TOX21_AR_LUC_MDAKB2_Antagonist_0.5nM_R18
106309−73−3
106461−41−0
19794−93−5
30194−63−9
25332−39−2

All Activity Heatmap for Cluster 393

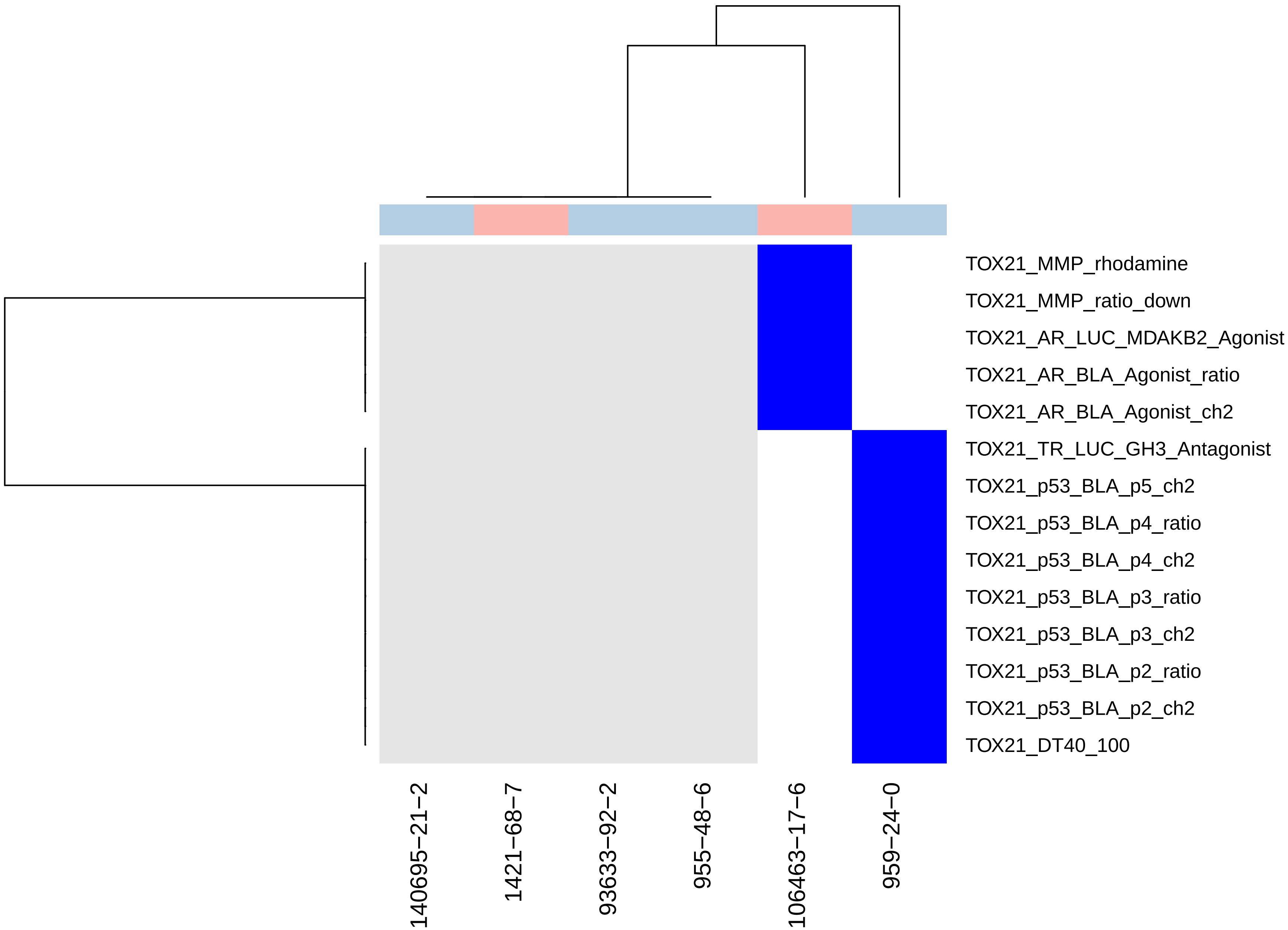

All Activity Heatmap for Cluster 395

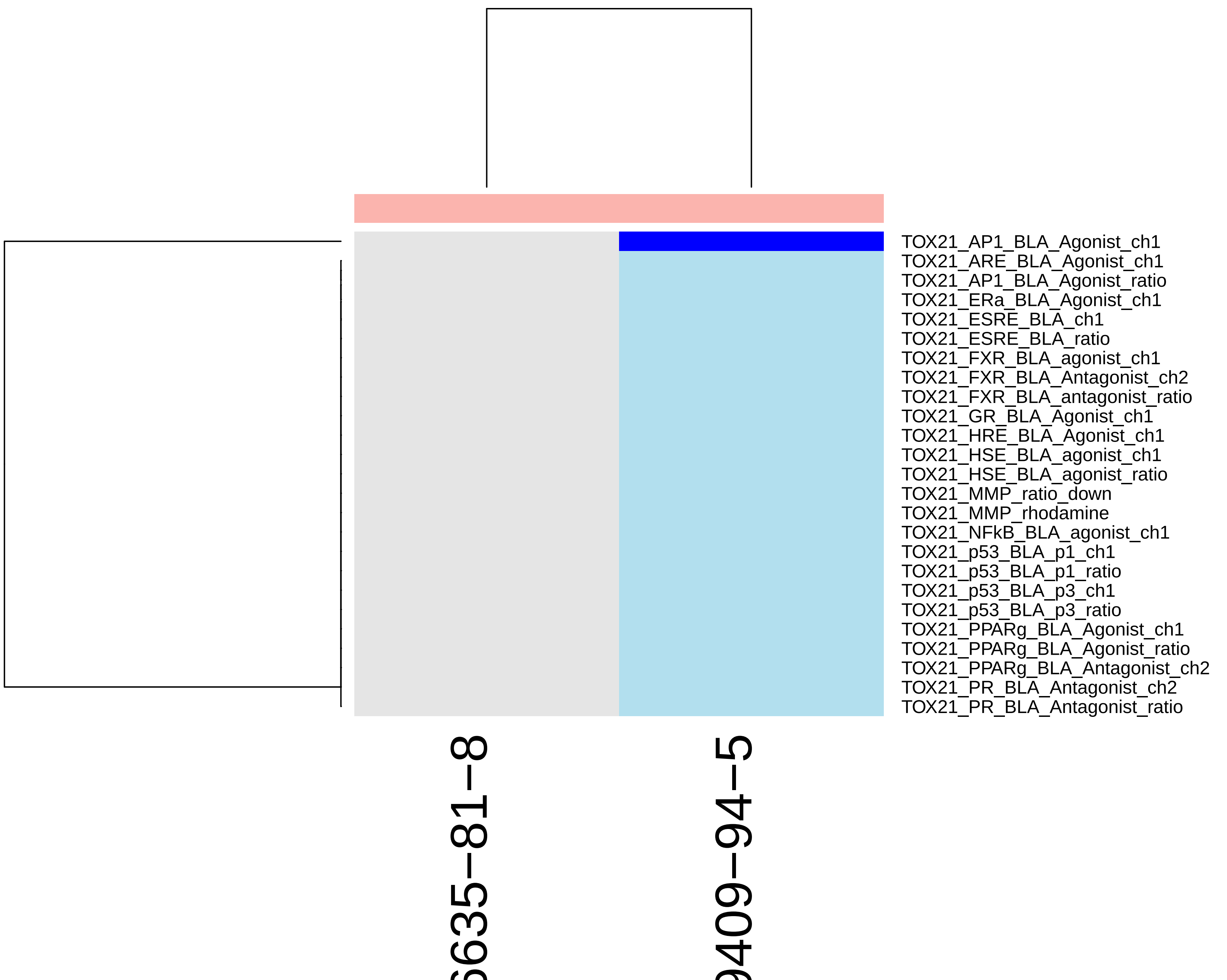

All Activity Heatmap for Cluster 396

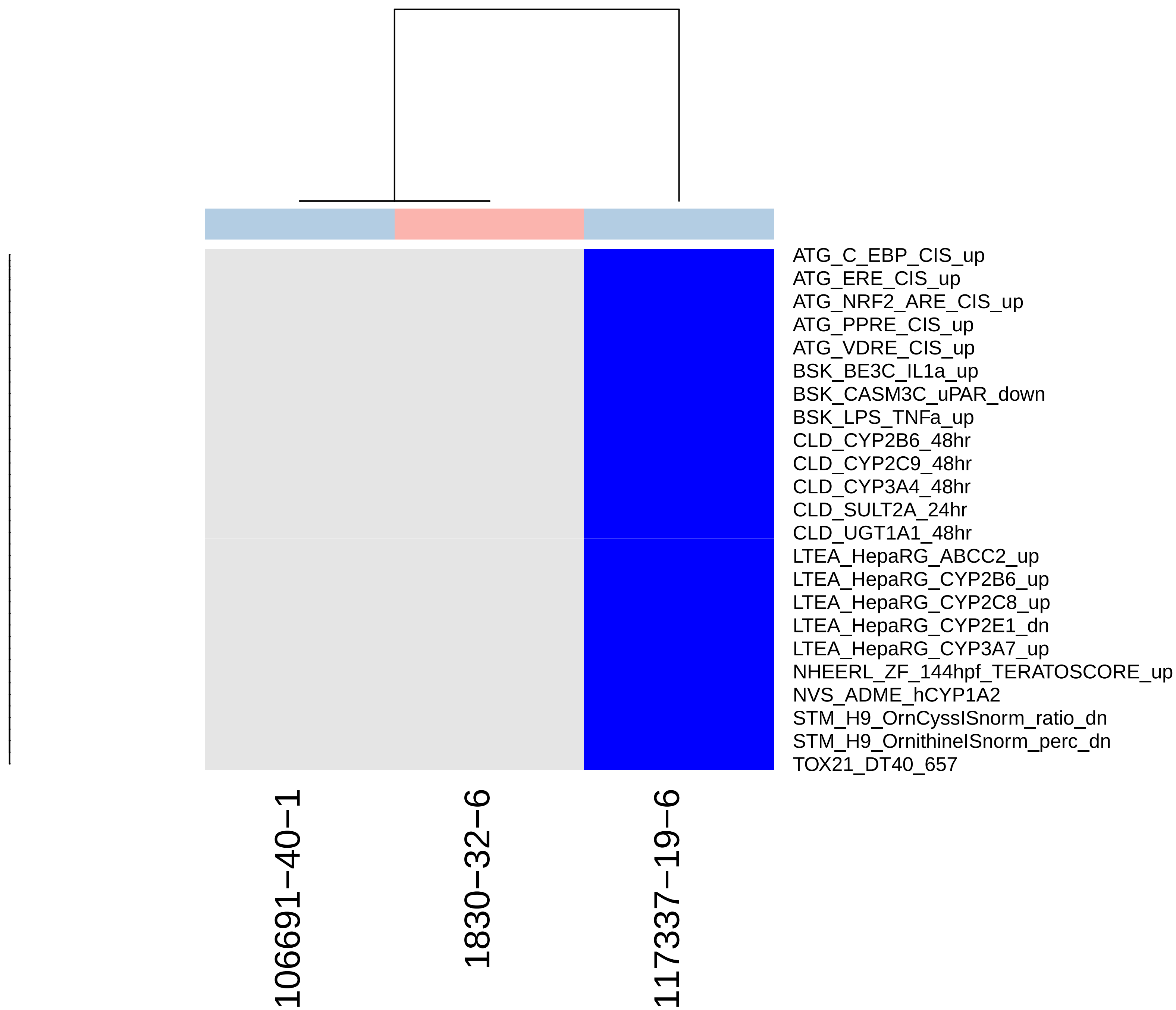

All Activity Heatmap for Cluster 397

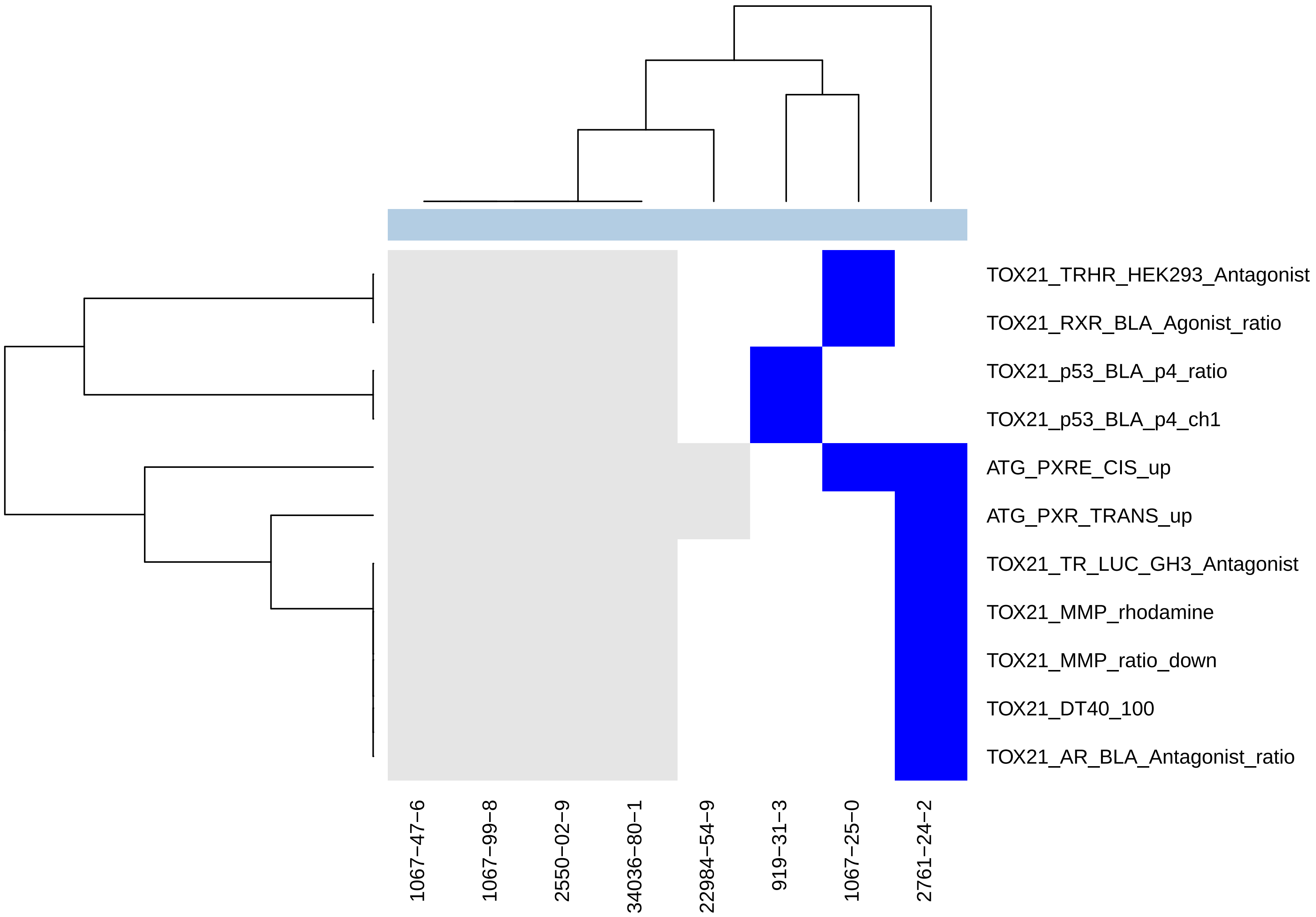

All Activity Heatmap for Cluster 398

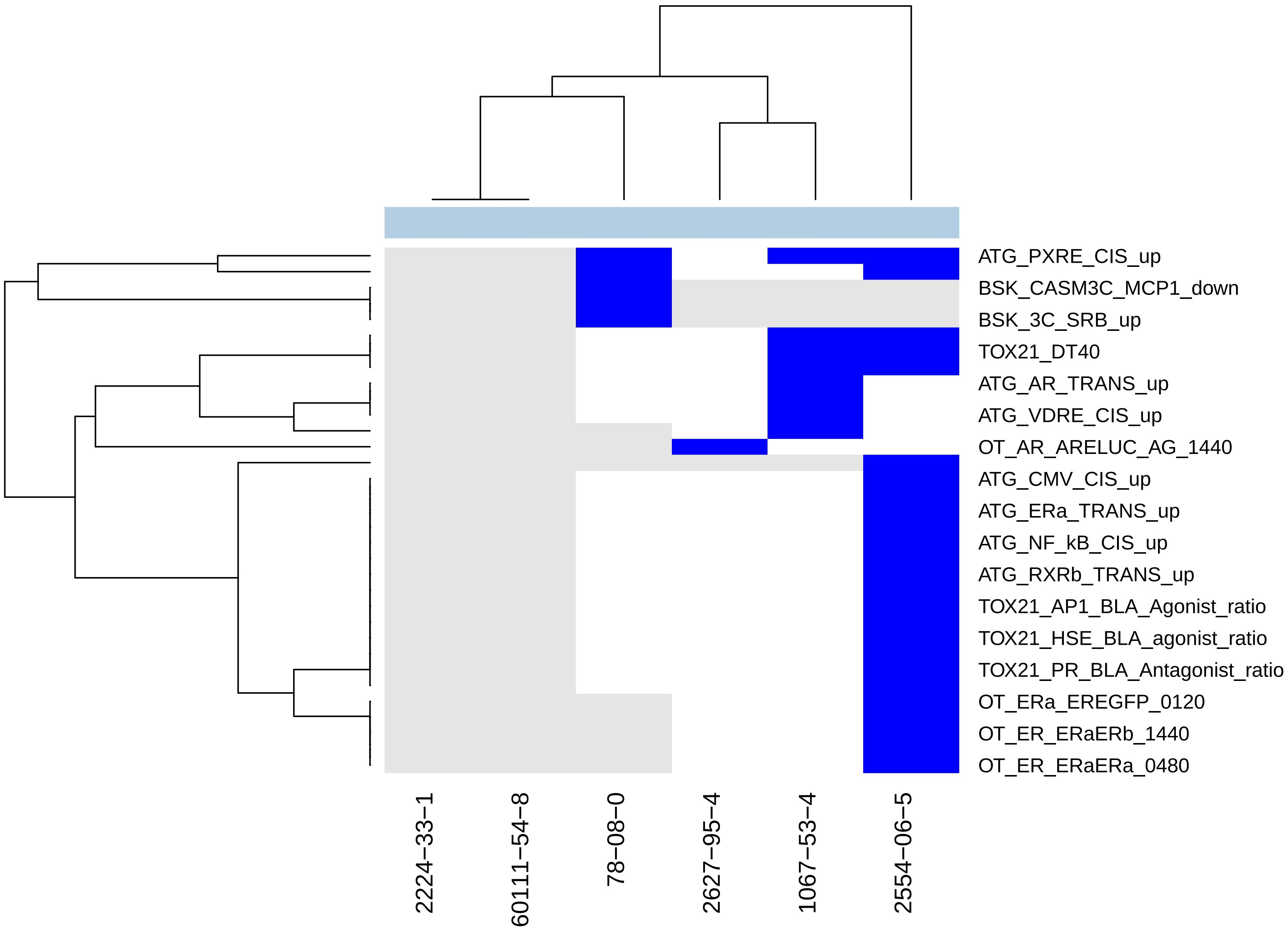

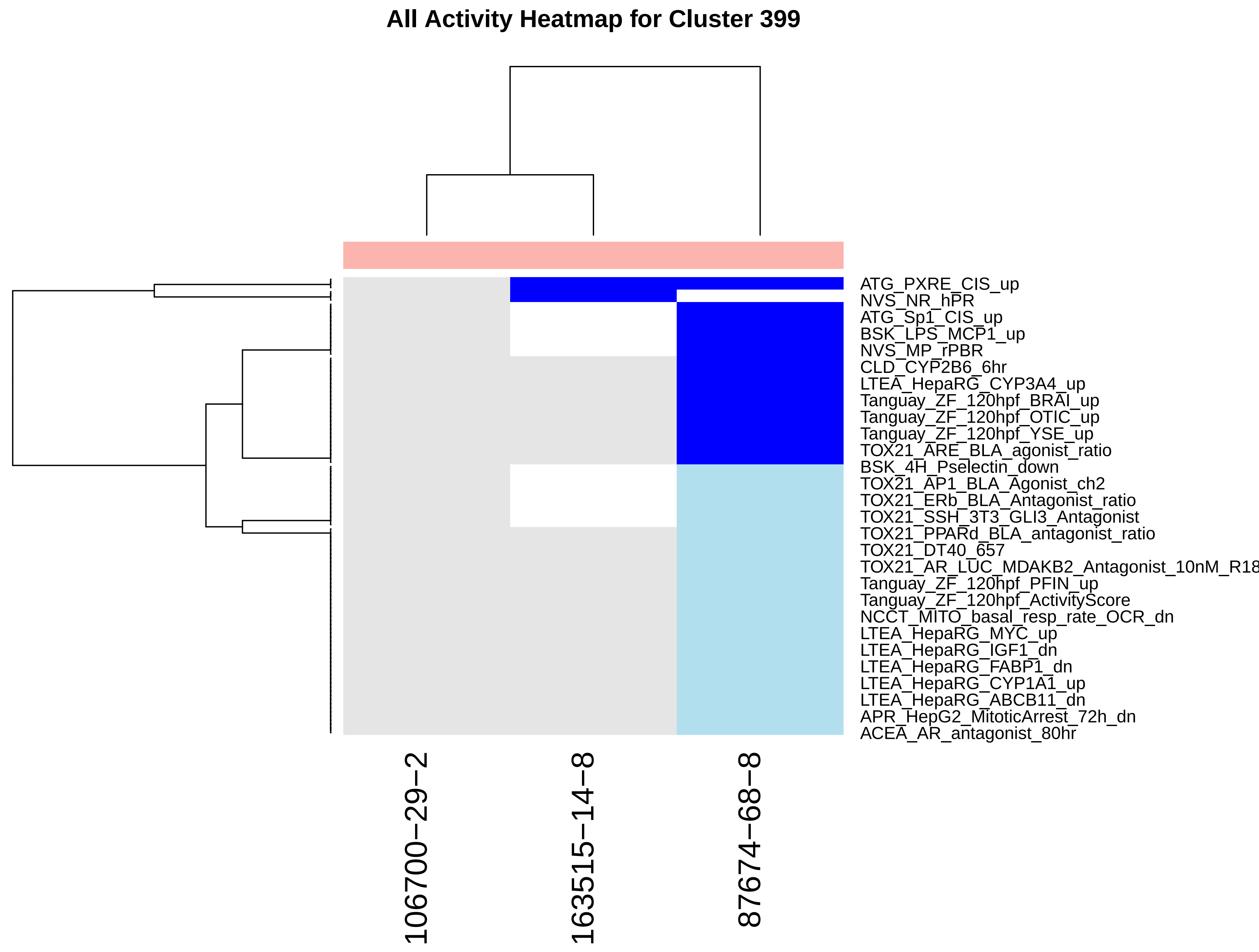

All Activity Heatmap for Cluster 399
ATG_PXRE_CIS_up
NVS_NR_hPR
ATG_Sp1_CIS_up
BSK_LPS_MCP1_up
NVS_MP_rPBR
CLD_CYP2B6_6hr
LTEA_HepaRG_CYP3A4_up
Tanguay_ZF_120hpf_BRAI_up
Tanguay_ZF_120hpf_OTIC_up
Tanguay_ZF_120hpf_YSE_up
TOX21_ARE_BLA_agonist_ratio
BSK_4H_Pselectin_down
TOX21_AP1_BLA_Agonist_ch2
TOX21_ERb_BLA_Antagonist_ratio
TOX21_SSH_3T3_GLI3_Antagonist
TOX21_PPARd_BLA_antagonist_ratio
TOX21_DT40_657
TOX21_AR_LUC_MDAKB2_Antagonist_10nM_R18
Tanguay_ZF_120hpf_PFIN_up
Tanguay_ZF_120hpf_ActivityScore
NCCT_MITO_basal_resp_rate_OCR_dn
LTEA_HepaRG_MYC_up
LTEA_HepaRG_IGF1_dn
LTEA_HepaRG_FABP1_dn
LTEA_HepaRG_CYP1A1_up
LTEA_HepaRG_ABCB11_dn
APR_HepG2_MitoticArrest_72h_dn
ACEA_AR_antagonist_80hr
106700−29−2
163515−14−8
87674−68−8

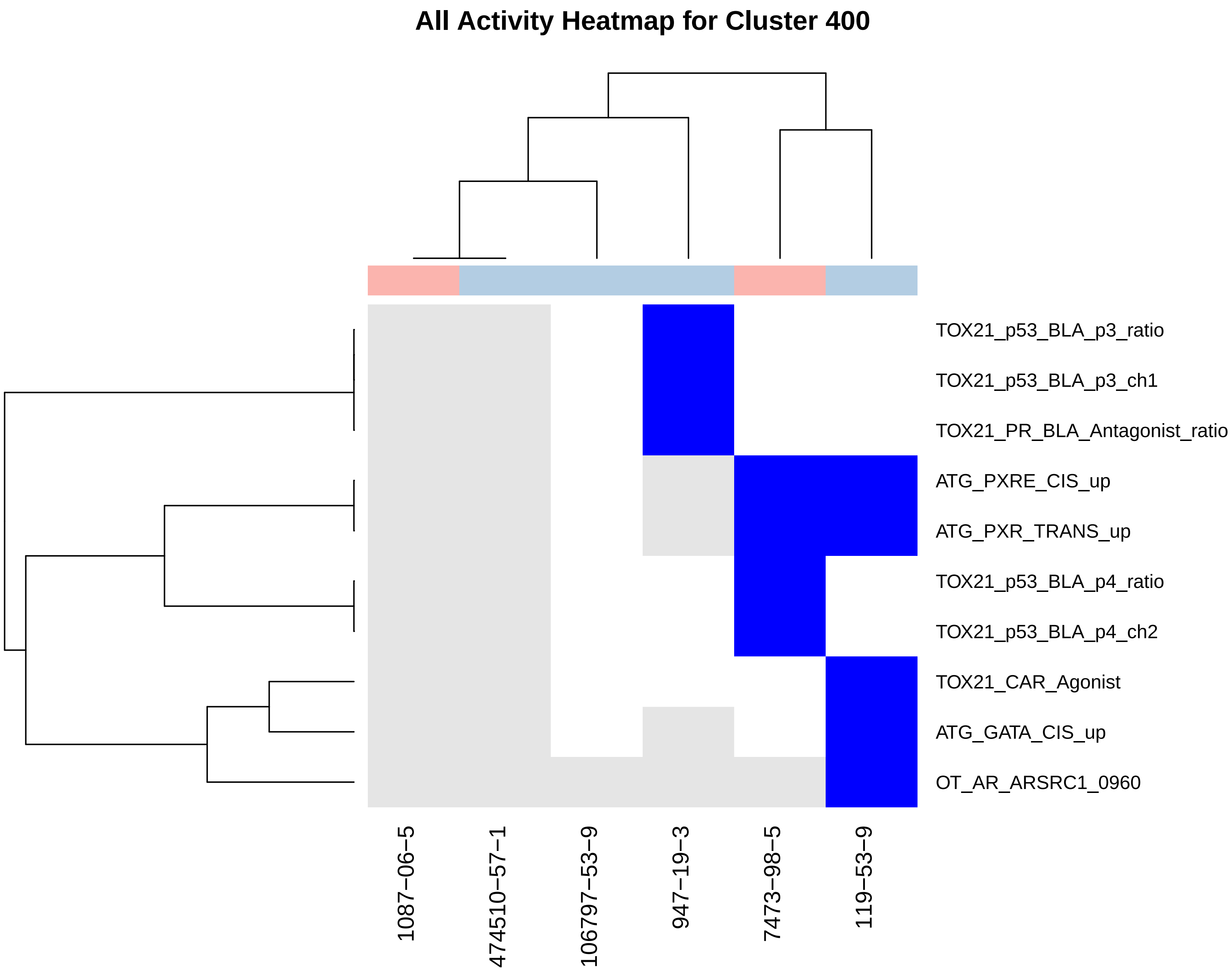
All Activity Heatmap for Cluster 400
TOX21_p53_BLA_p3_ratio
TOX21_p53_BLA_p3_ch1
TOX21_PR_BLA_Antagonist_ratio
ATG_PXRE_CIS_up
ATG_PXR_TRANS_up
TOX21_p53_BLA_p4_ratio
TOX21_p53_BLA_p4_ch2
TOX21_CAR_Agonist
ATG_GATA_CIS_up
OT_AR_ARSRC1_0960
1087−06−5
474510−57−1
106797−53−9
947−19−3
7473−98−5
119−53−9

All Activity Heatmap for Cluster 401

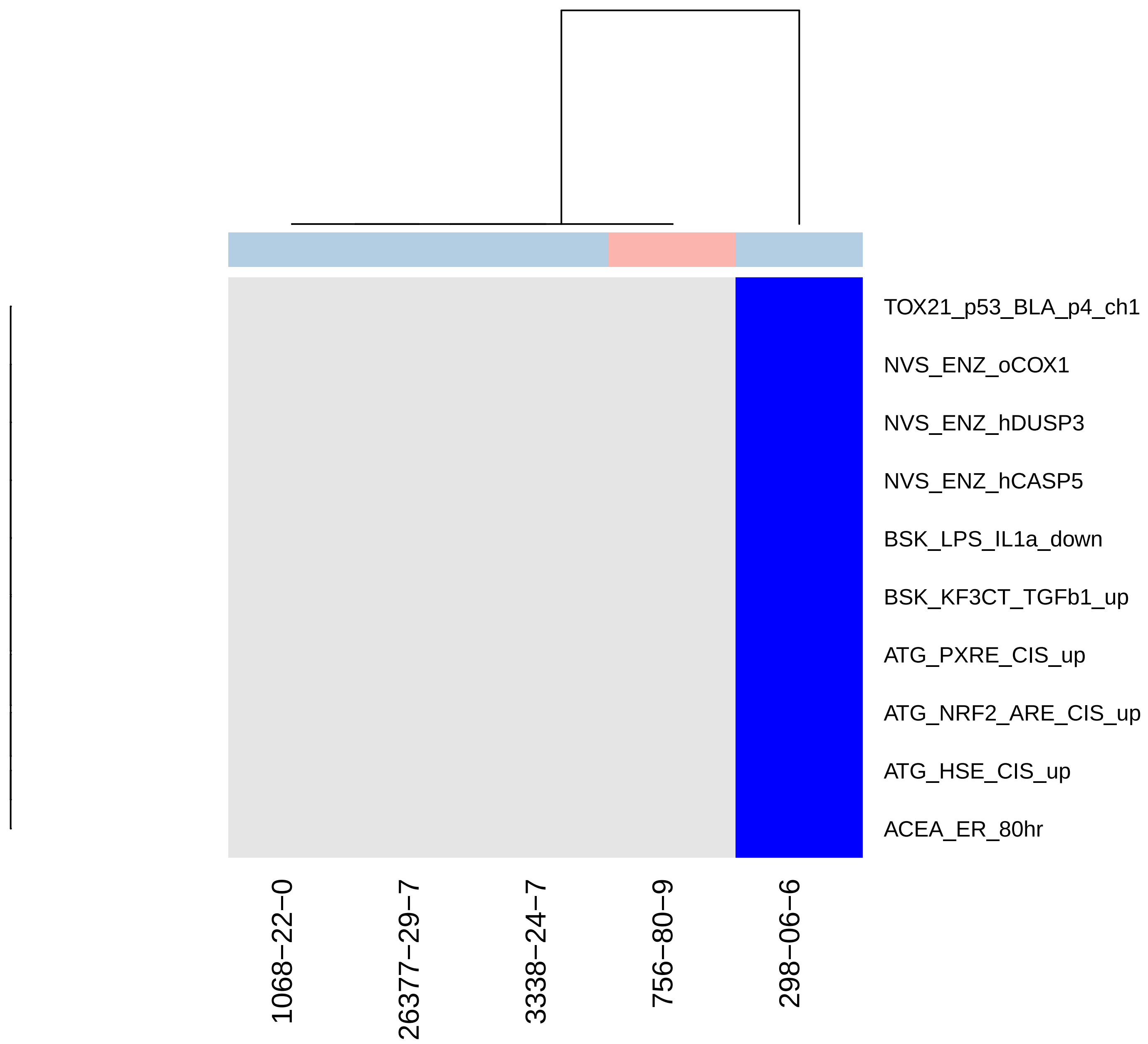

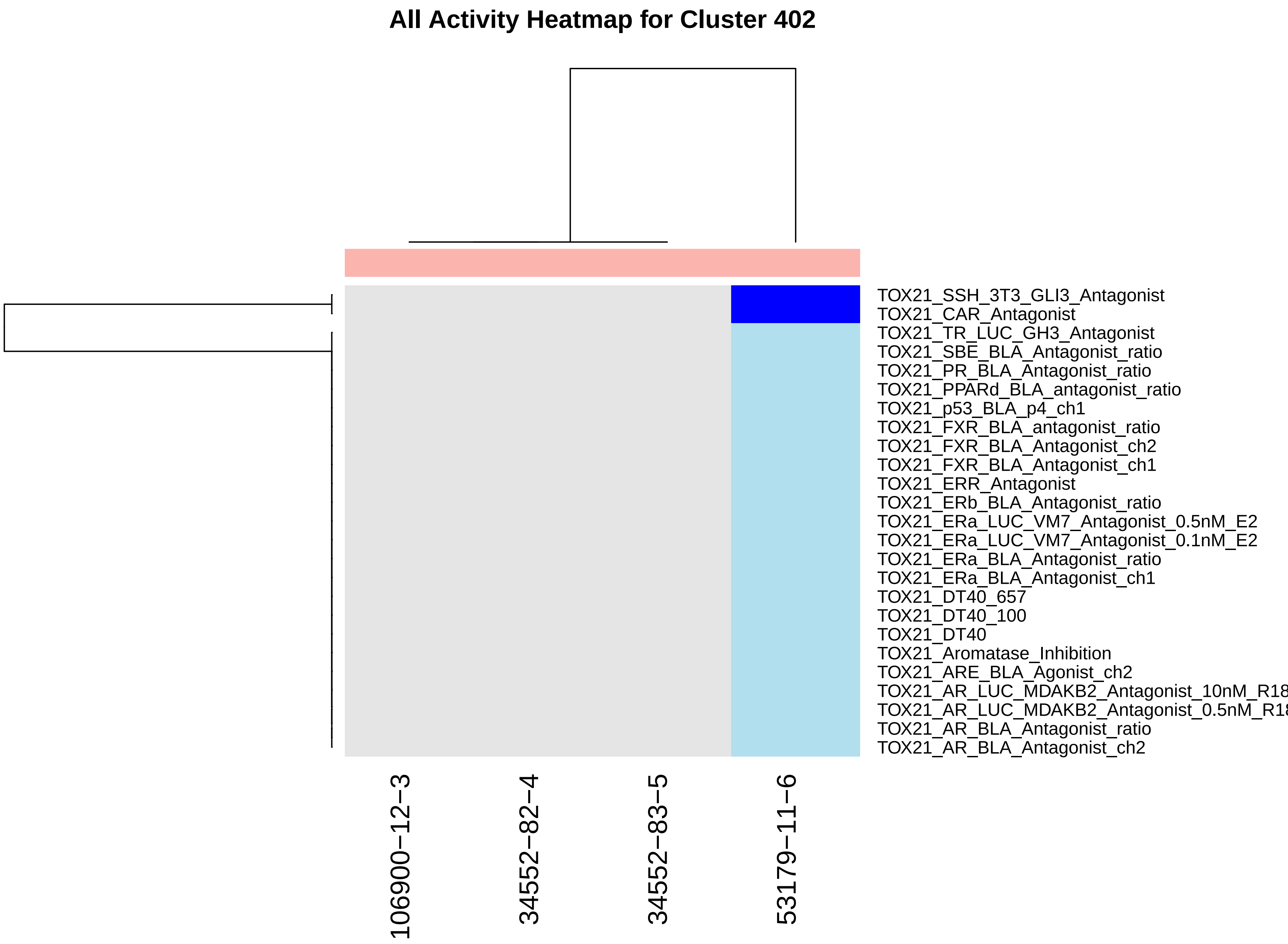
All Activity Heatmap for Cluster 402
TOX21_SSH_3T3_GLI3_Antagonist
TOX21_CAR_Antagonist
TOX21_TR_LUC_GH3_Antagonist
TOX21_SBE_BLA_Antagonist_ratio
TOX21_PR_BLA_Antagonist_ratio
TOX21_PPARd_BLA_antagonist_ratio
TOX21_p53_BLA_p4_ch1
TOX21_FXR_BLA_antagonist_ratio
TOX21_FXR_BLA_Antagonist_ch2
TOX21_FXR_BLA_Antagonist_ch1
TOX21_ERR_Antagonist
TOX21_ERb_BLA_Antagonist_ratio
TOX21_ERa_LUC_VM7_Antagonist_0.5nM_E2
TOX21_ERa_LUC_VM7_Antagonist_0.1nM_E2
TOX21_ERa_BLA_Antagonist_ratio
TOX21_ERa_BLA_Antagonist_ch1
TOX21_DT40_657
TOX21_DT40_100
TOX21_DT40
TOX21_Aromatase_Inhibition
TOX21_ARE_BLA_Agonist_ch2
TOX21_AR_LUC_MDAKB2_Antagonist_10nM_R18
TOX21_AR_LUC_MDAKB2_Antagonist_0.5nM_R18
TOX21_AR_BLA_Antagonist_ratio
TOX21_AR_BLA_Antagonist_ch2
106900−12−3
34552−82−4
34552−83−5
53179−11−6

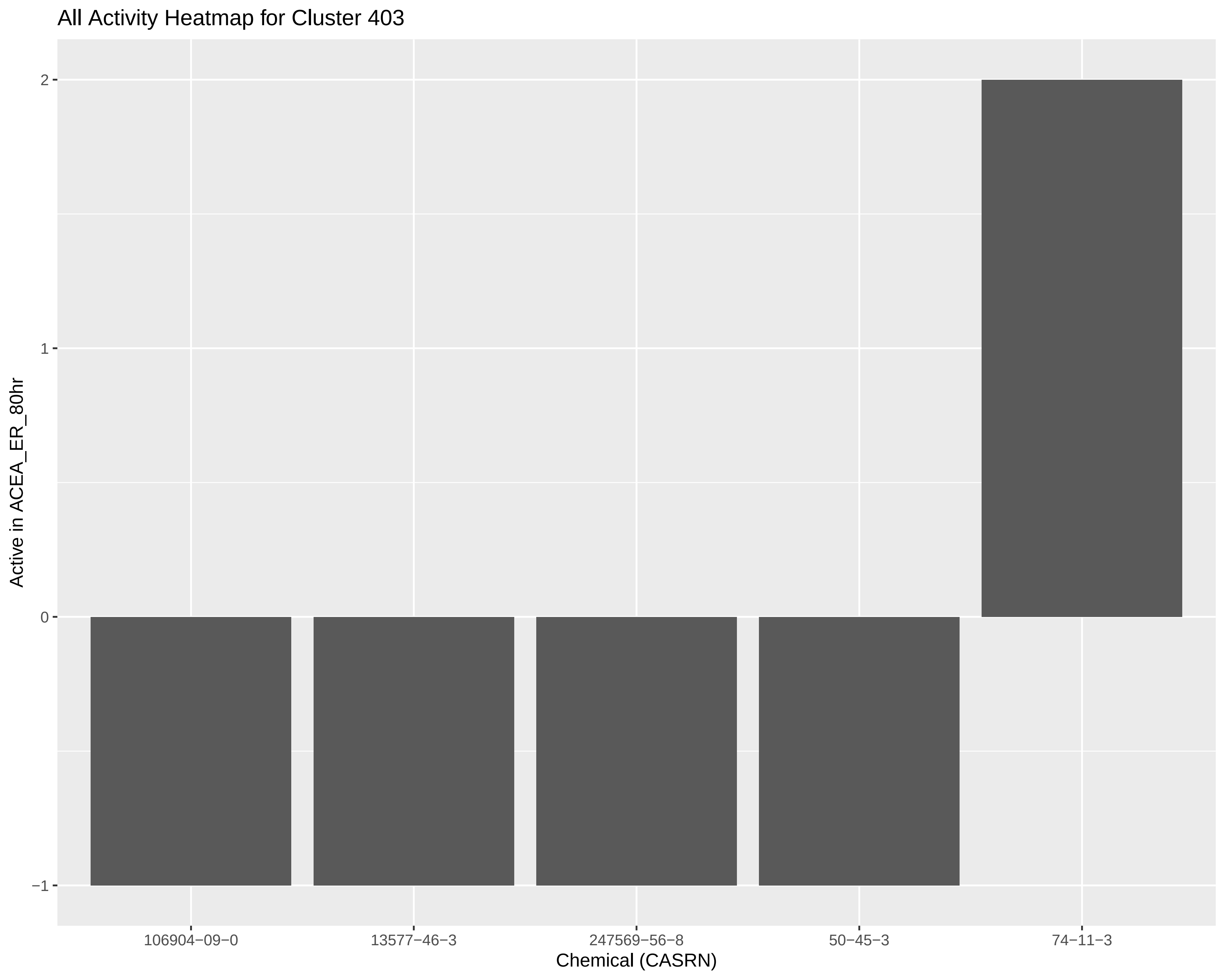

All Activity Heatmap for Cluster 403
Active in ACEA_ER_80hr
2
1
0
−1
106904−09−0
13577−46−3
247569−56−8
50−45−3
74−11−3
Chemical (CASRN)

All Activity Heatmap for Cluster 405

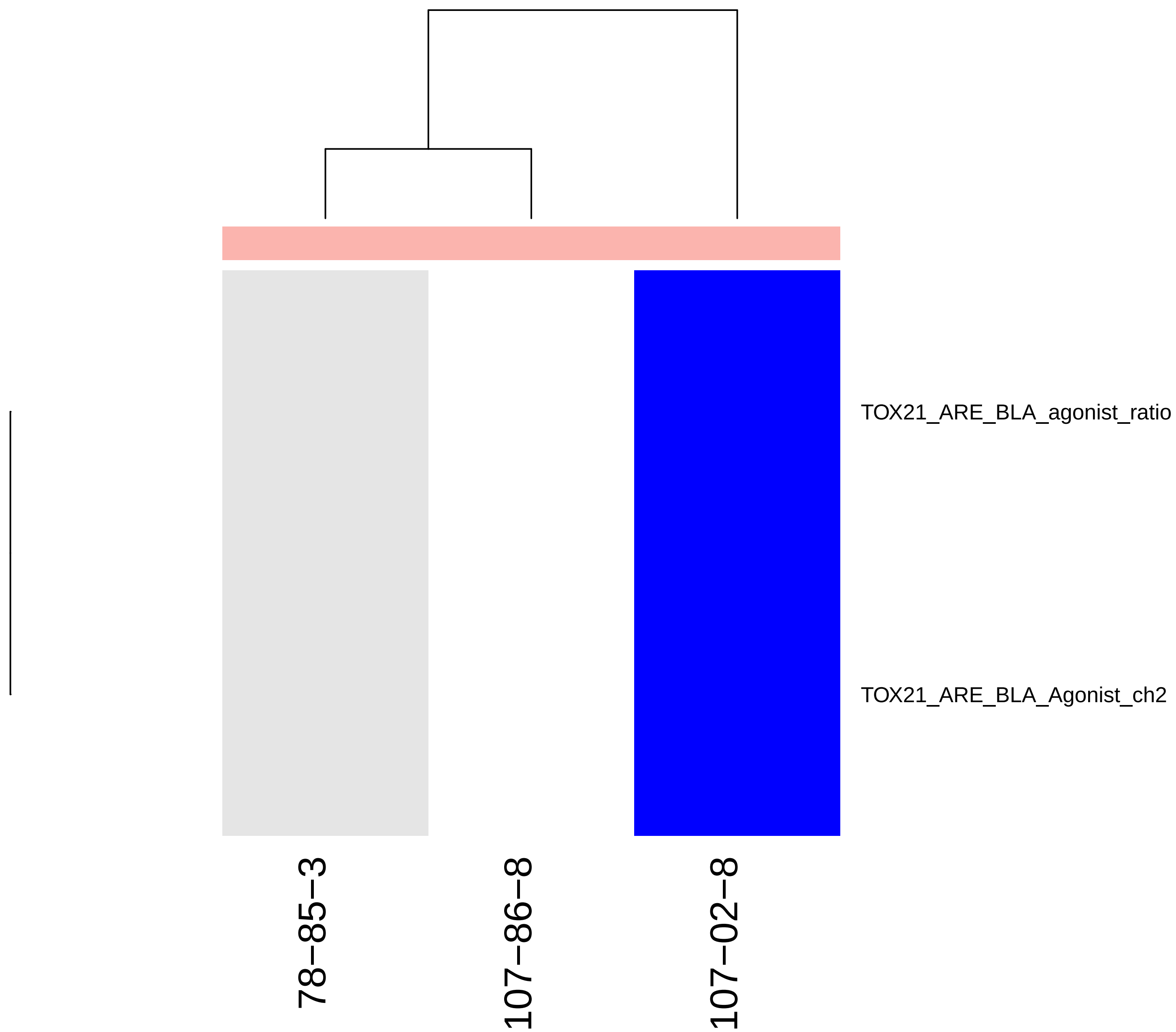

All Activity Heatmap for Cluster 407

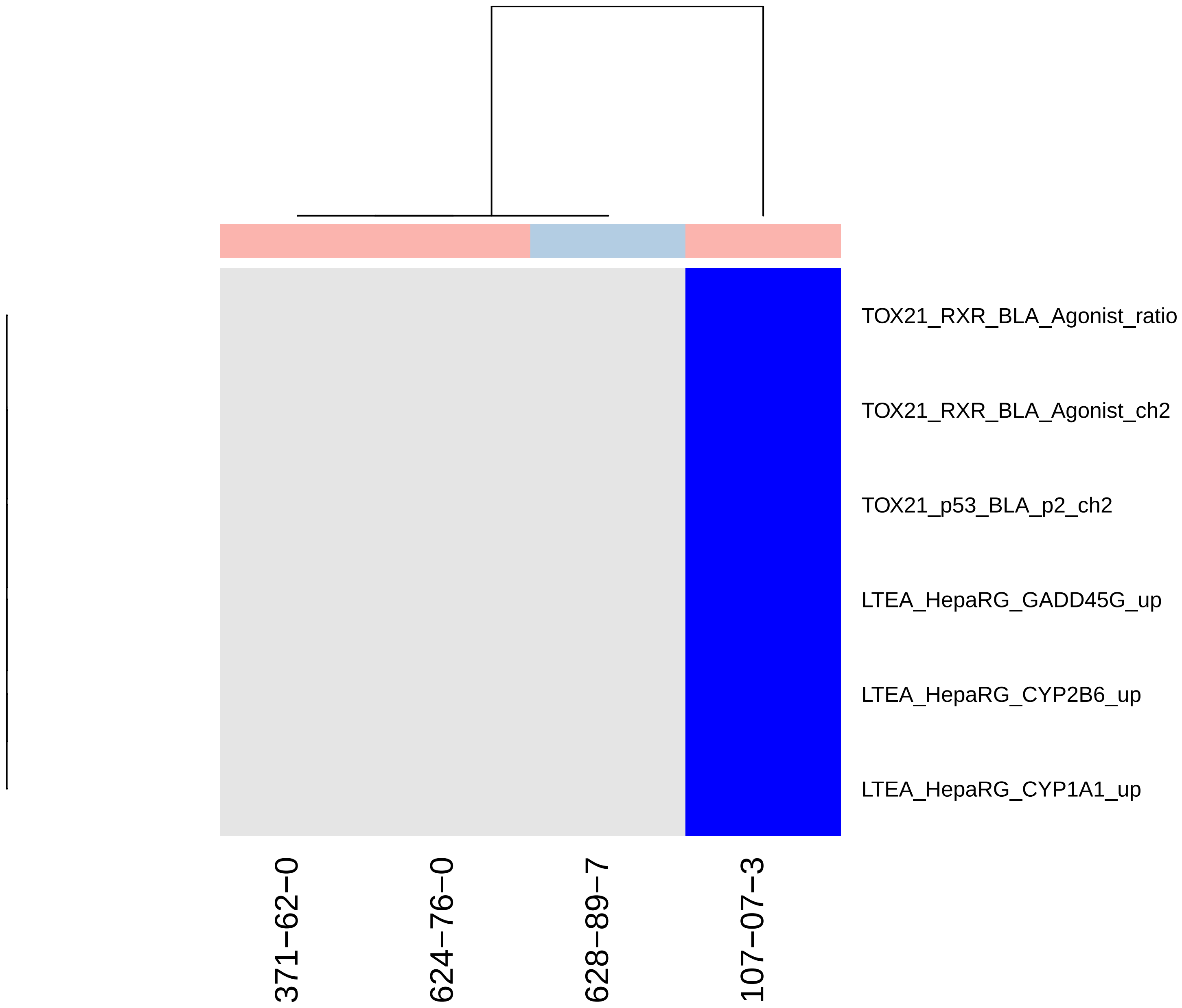

All Activity Heatmap for Cluster 408

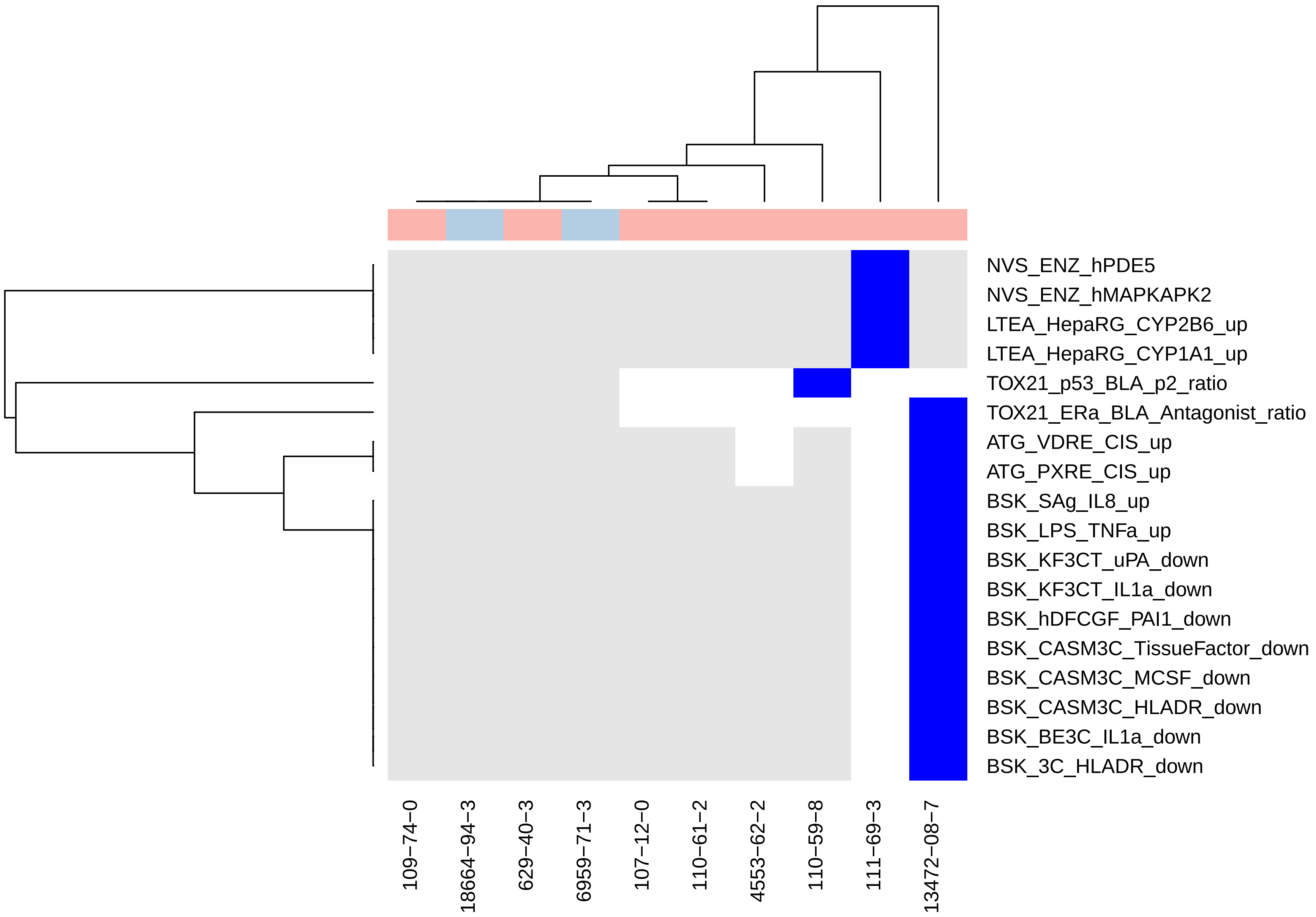

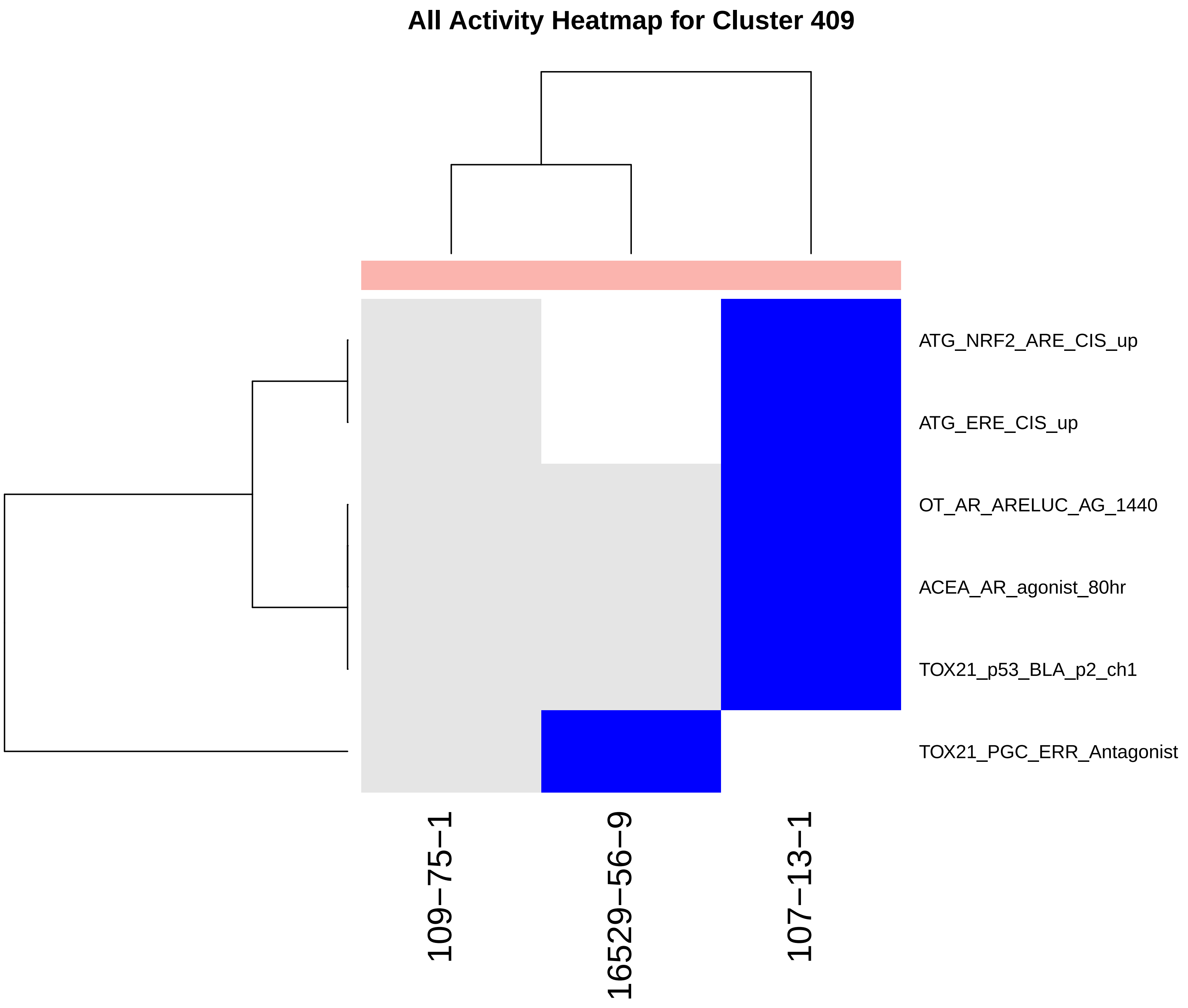
All Activity Heatmap for Cluster 409
ATG_NRF2_ARE_CIS_up
ATG_ERE_CIS_up
OT_AR_ARELUC_AG_1440
ACEA_AR_agonist_80hr
TOX21_p53_BLA_p2_ch1
TOX21_PGC_ERR_Antagonist
109−75−1
16529−56−9
107−13−1

All Activity Heatmap for Cluster 410

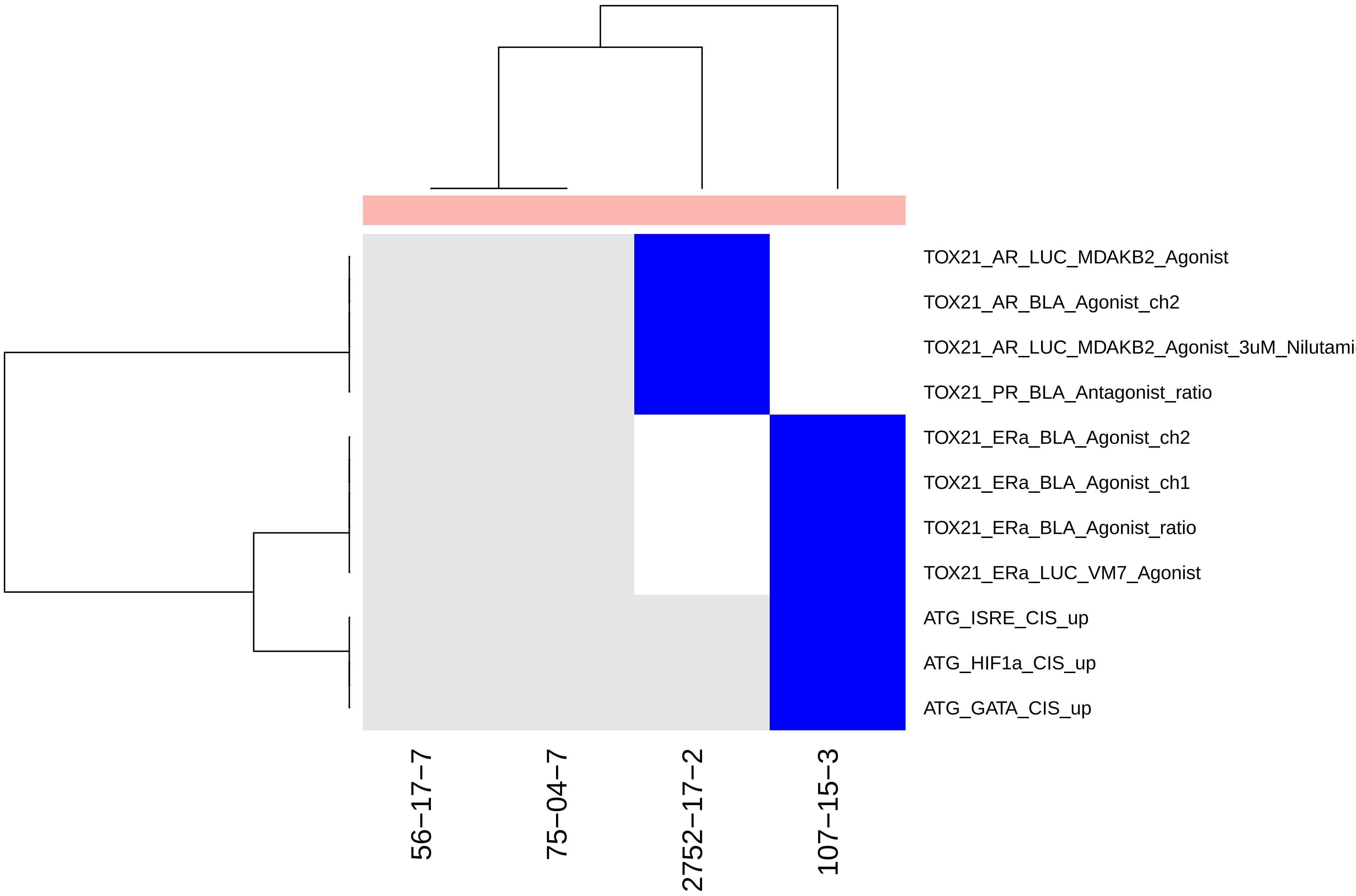

All Activity Heatmap for Cluster 411

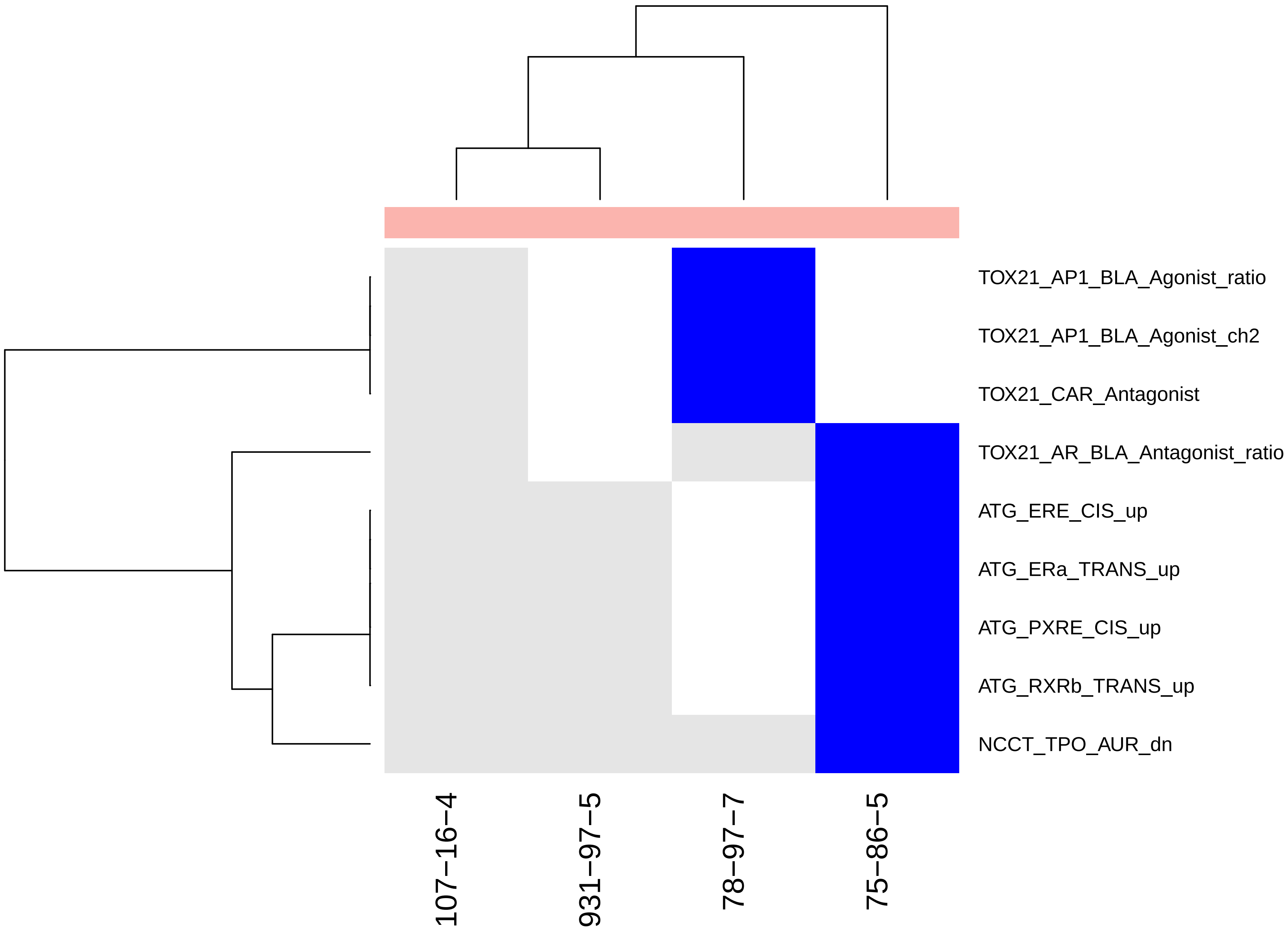

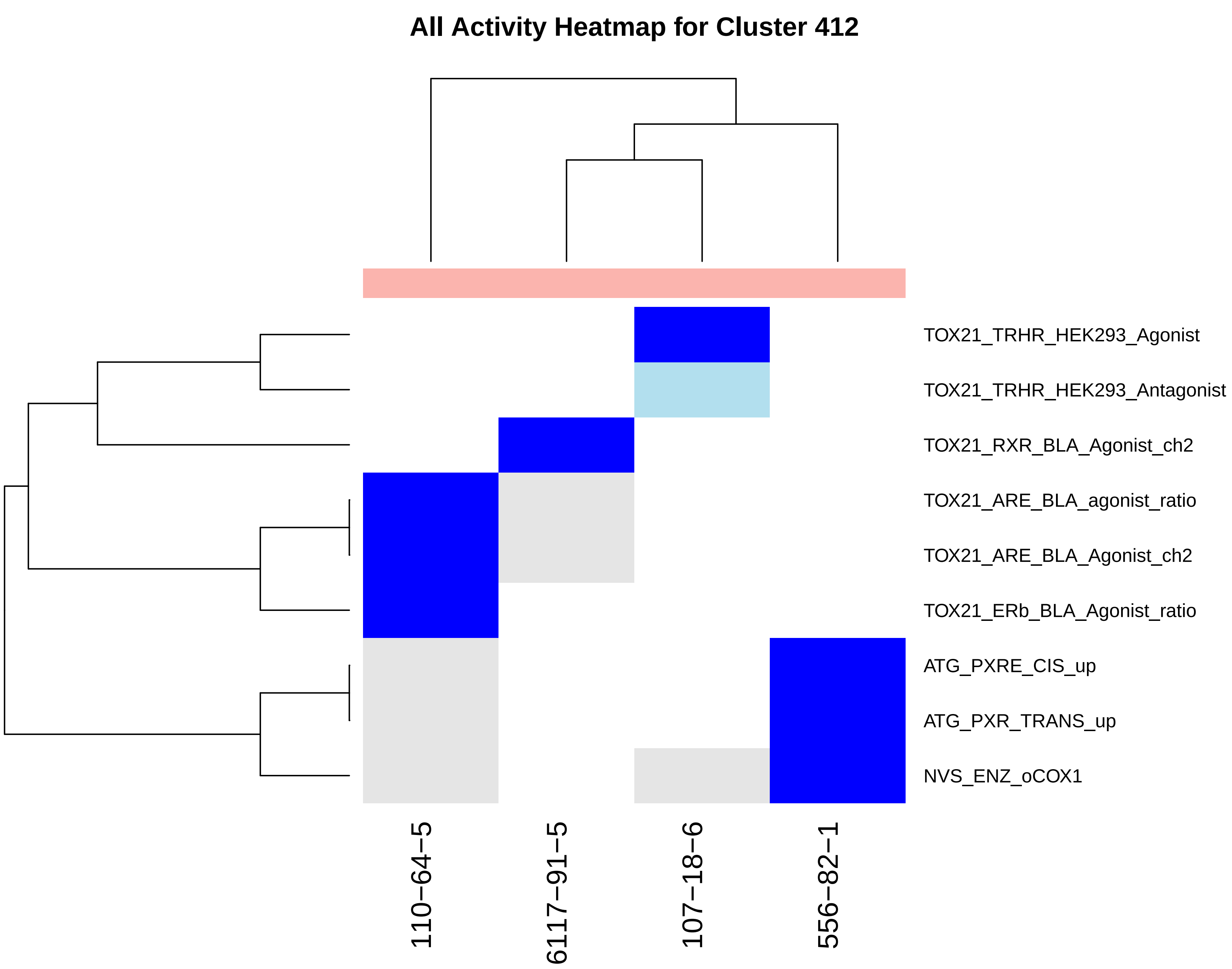
All Activity Heatmap for Cluster 412
TOX21_TRHR_HEK293_Agonist
TOX21_TRHR_HEK293_Antagonist
TOX21_RXR_BLA_Agonist_ch2
TOX21_ARE_BLA_agonist_ratio
TOX21_ARE_BLA_Agonist_ch2
TOX21_ERb_BLA_Agonist_ratio
ATG_PXRE_CIS_up
ATG_PXR_TRANS_up
NVS_ENZ_oCOX1
110−64−5
6117−91−5
107−18−6
556−82−1

All Activity Heatmap for Cluster 413

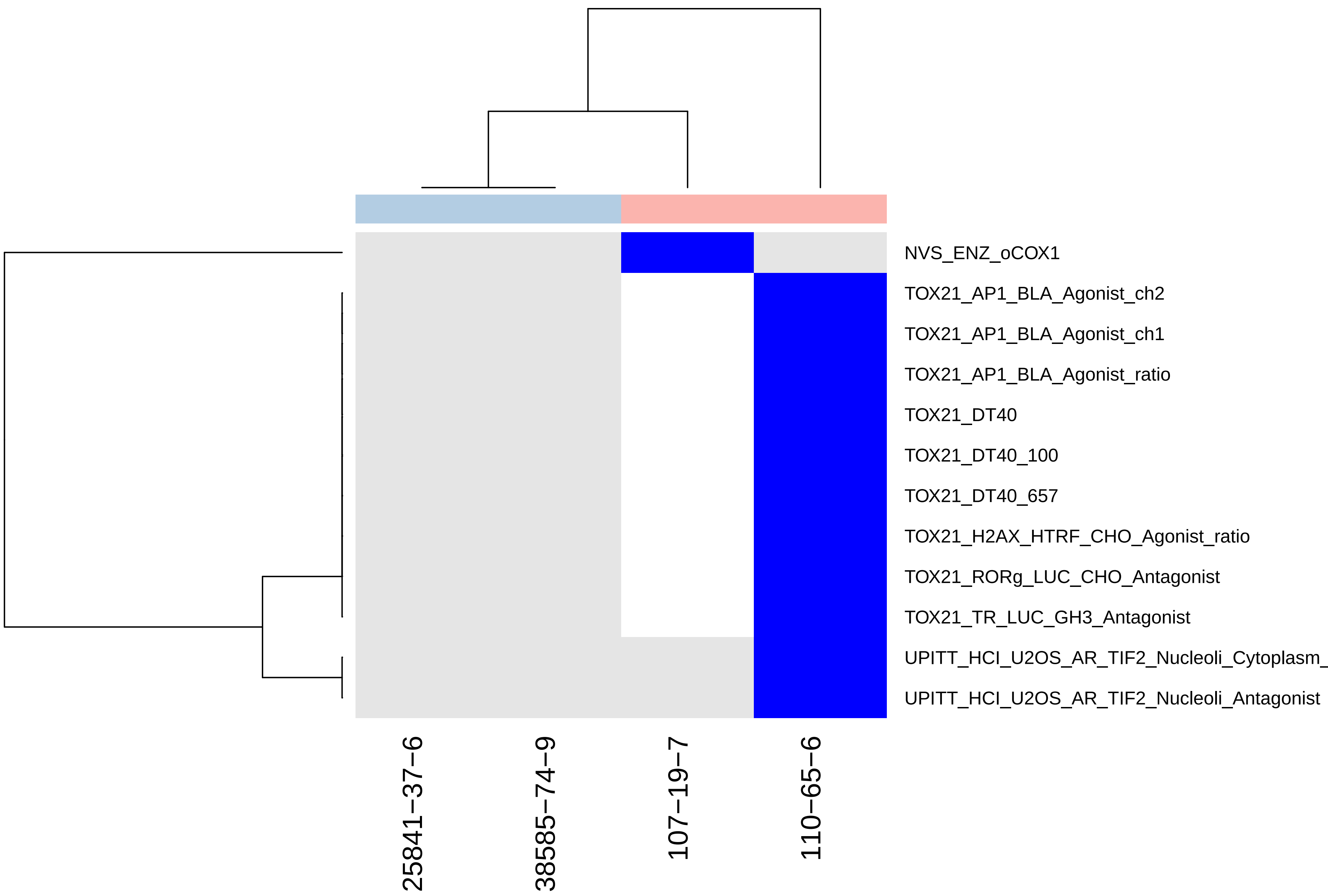

All Activity Heatmap for Cluster 414

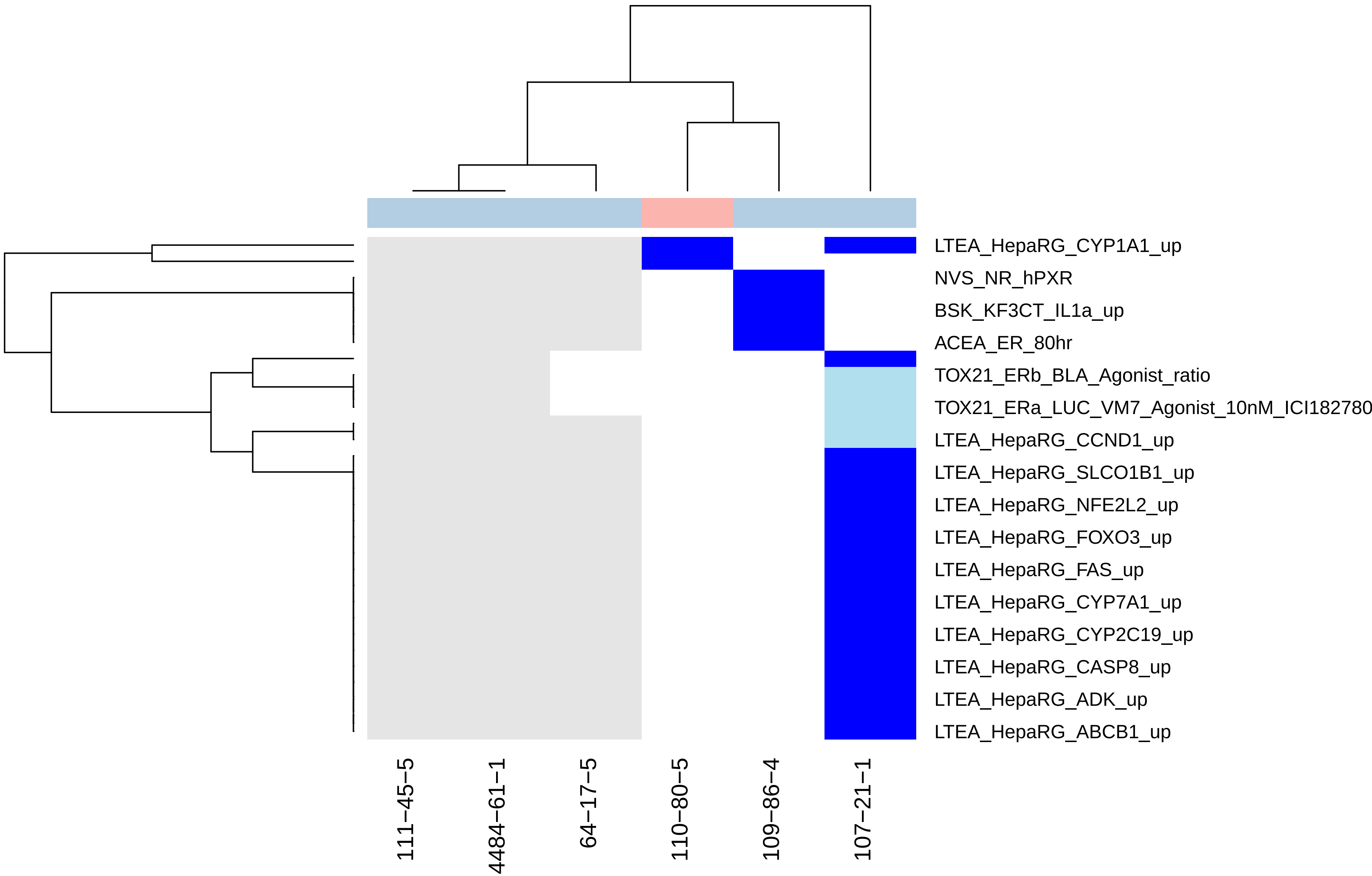

All Activity Heatmap for Cluster 415

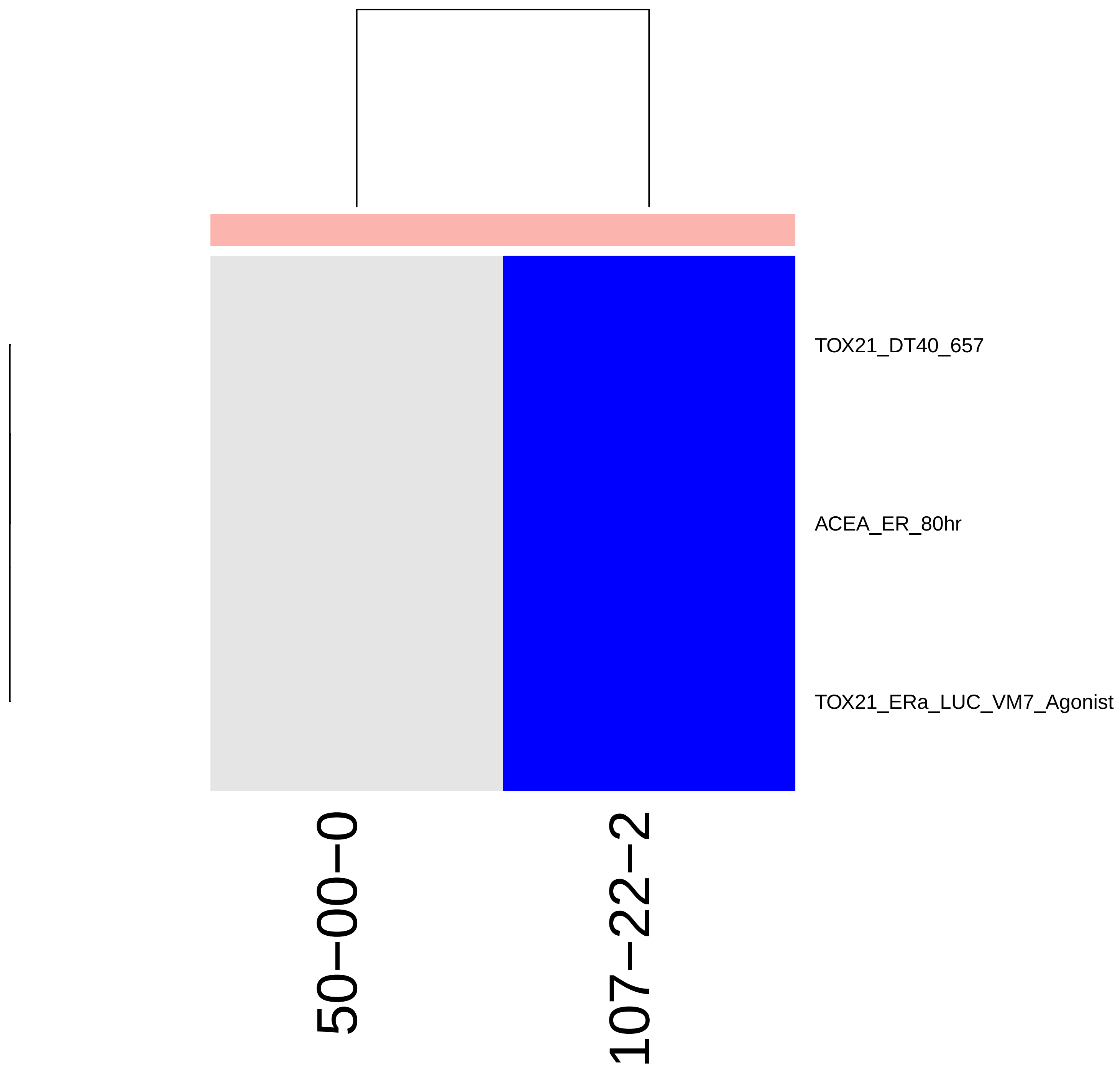

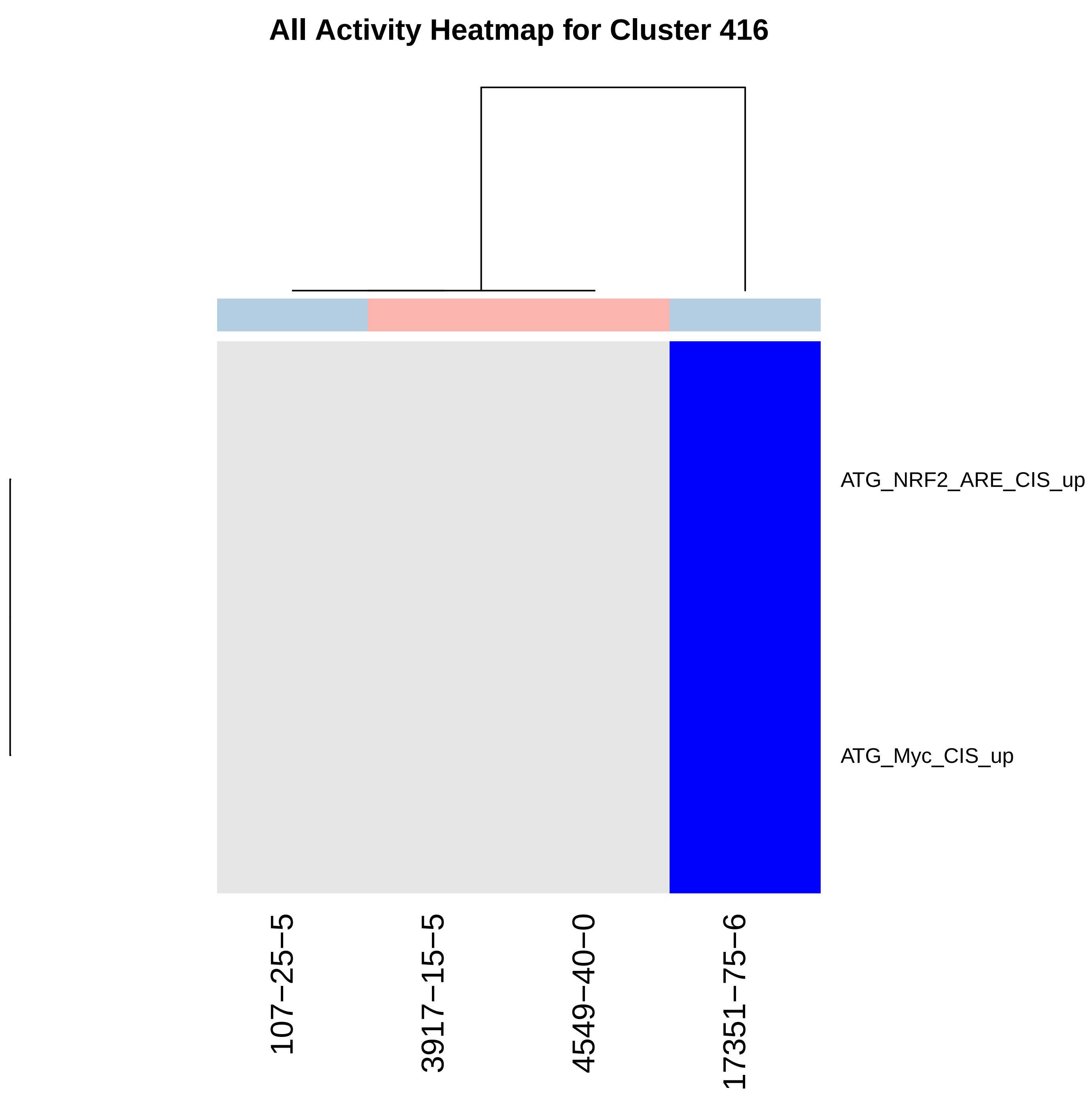

All Activity Heatmap for Cluster 416
ATG_NRF2_ARE_CIS_up
ATG_Myc_CIS_up
107−25−5
3917−15−5
4549−40−0
17351−75−6

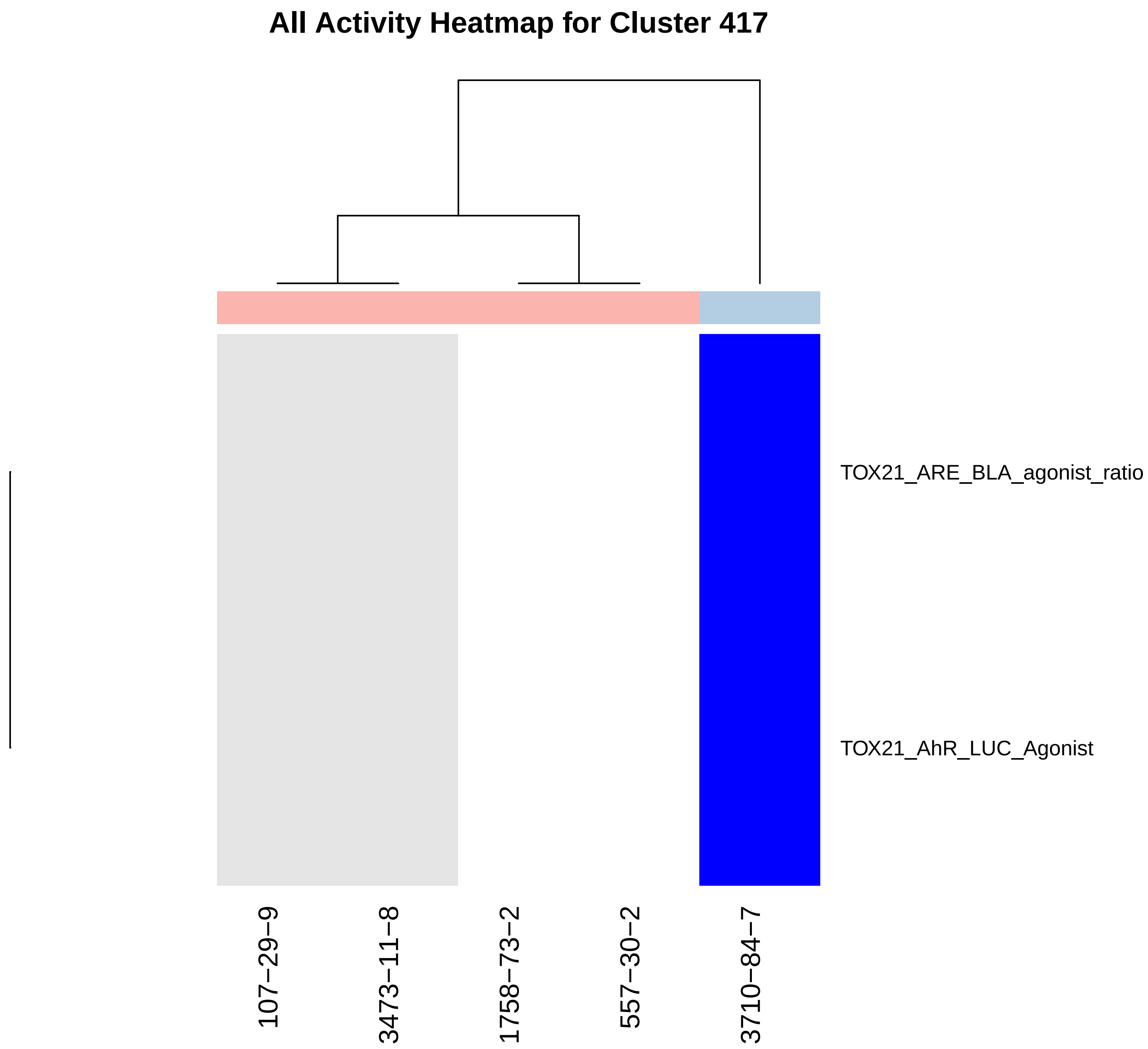
All Activity Heatmap for Cluster 417
TOX21_ARE_BLA_agonist_ratio
TOX21_AhR_LUC_Agonist
107−29−9
3473−11−8
1758−73−2
557−30−2
3710−84−7

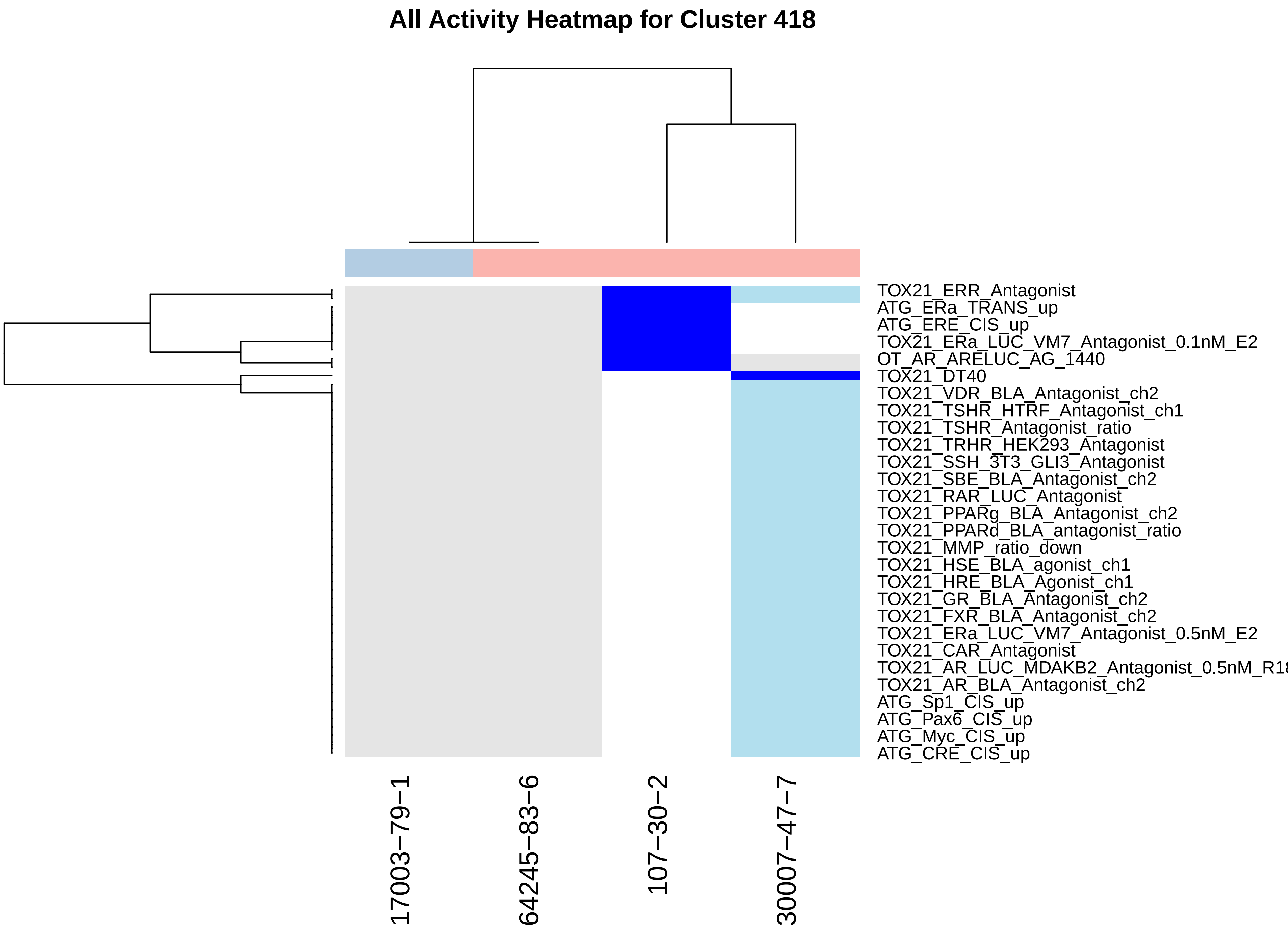
All Activity Heatmap for Cluster 418
TOX21_ERR_Antagonist
ATG_ERa_TRANS_up
ATG_ERE_CIS_up
TOX21_ERa_LUC_VM7_Antagonist_0.1nM_E2
OT_AR_ARELUC_AG_1440
TOX21_DT40
TOX21_VDR_BLA_Antagonist_ch2
TOX21_TSHR_HTRF_Antagonist_ch1
TOX21_TSHR_Antagonist_ratio
TOX21_TRHR_HEK293_Antagonist
TOX21_SSH_3T3_GLI3_Antagonist
TOX21_SBE_BLA_Antagonist_ch2
TOX21_RAR_LUC_Antagonist
TOX21_PPARg_BLA_Antagonist_ch2
TOX21_PPARd_BLA_antagonist_ratio
TOX21_MMP_ratio_down
TOX21_HSE_BLA_agonist_ch1
TOX21_HRE_BLA_Agonist_ch1
TOX21_GR_BLA_Antagonist_ch2
TOX21_FXR_BLA_Antagonist_ch2
TOX21_ERa_LUC_VM7_Antagonist_0.5nM_E2
TOX21_CAR_Antagonist
TOX21_AR_LUC_MDAKB2_Antagonist_0.5nM_R18
TOX21_AR_BLA_Antagonist_ch2
ATG_Sp1_CIS_up
ATG_Pax6_CIS_up
ATG_Myc_CIS_up
ATG_CRE_CIS_up
17003−79−1
64245−83−6
107−30−2
30007−47−7

All Activity Heatmap for Cluster 419

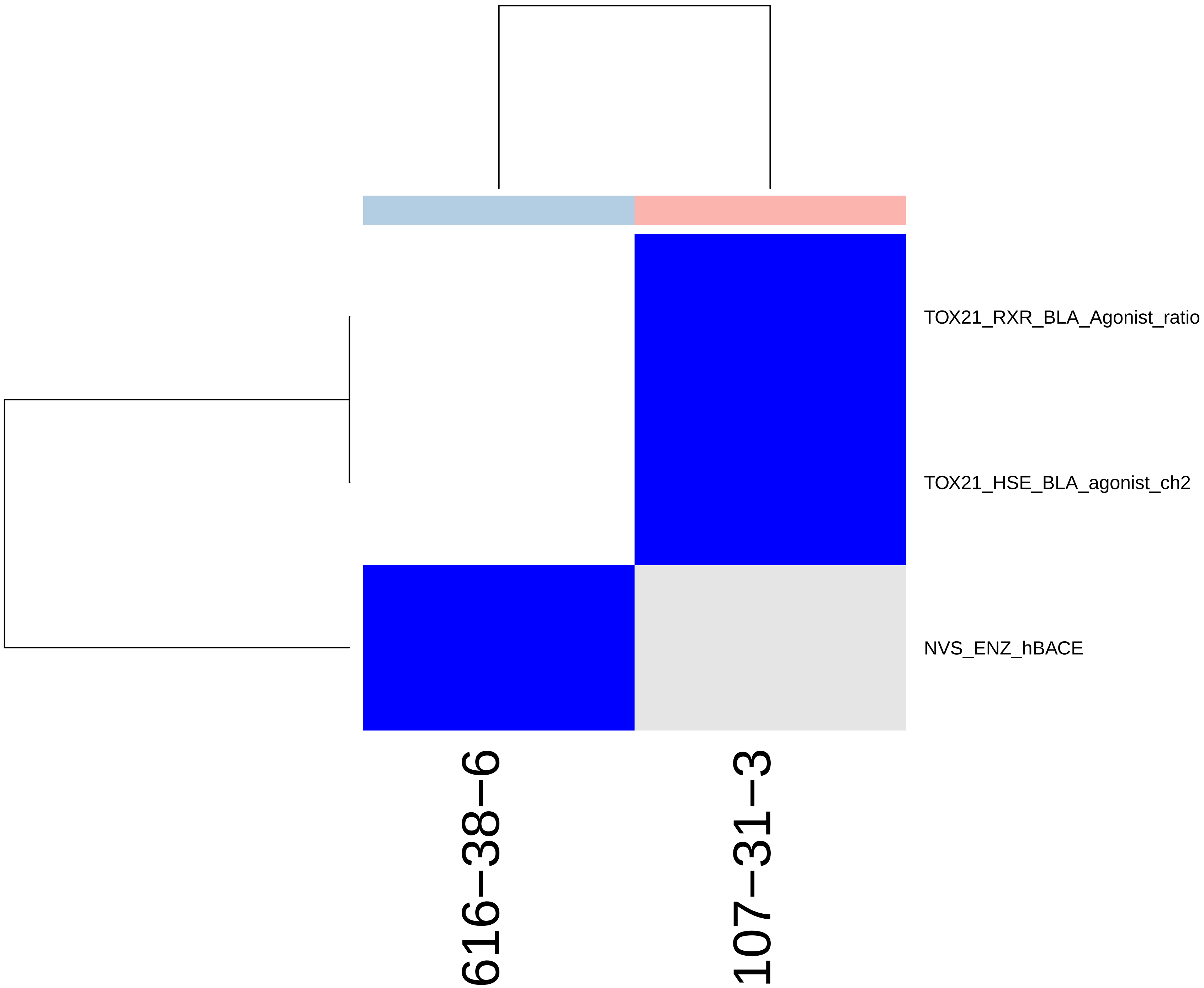

All Activity Heatmap for Cluster 420

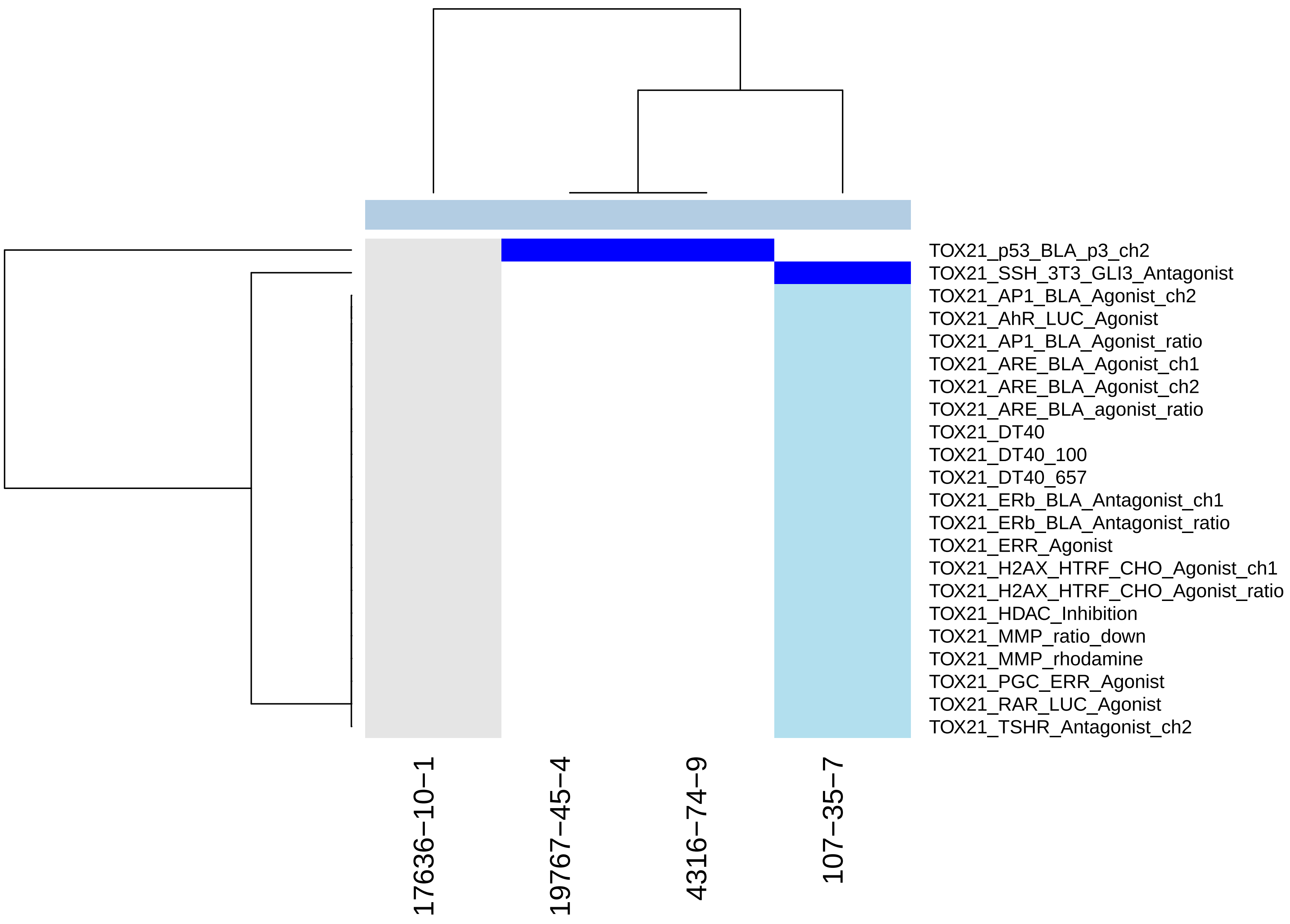

All Activity Heatmap for Cluster 421

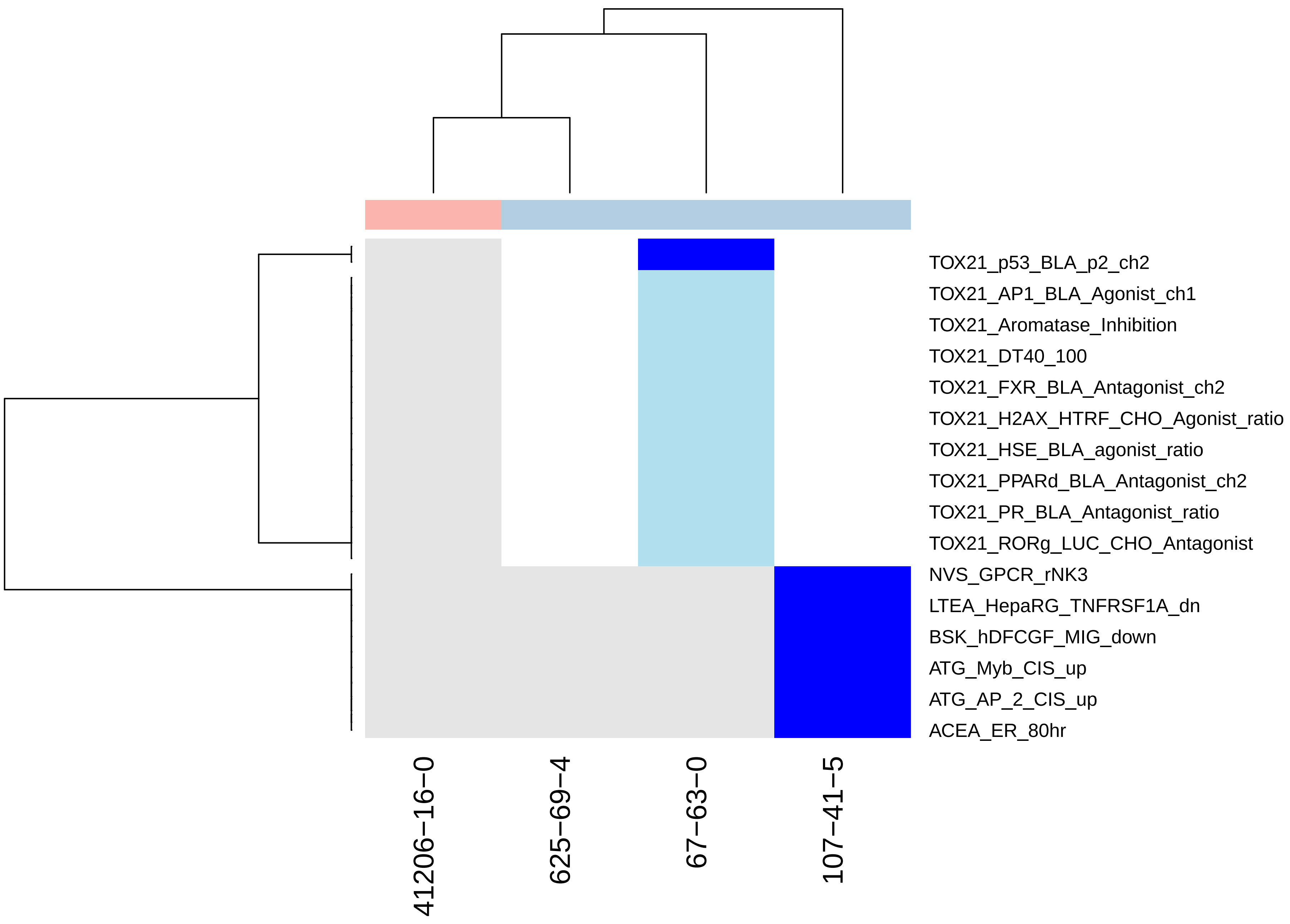

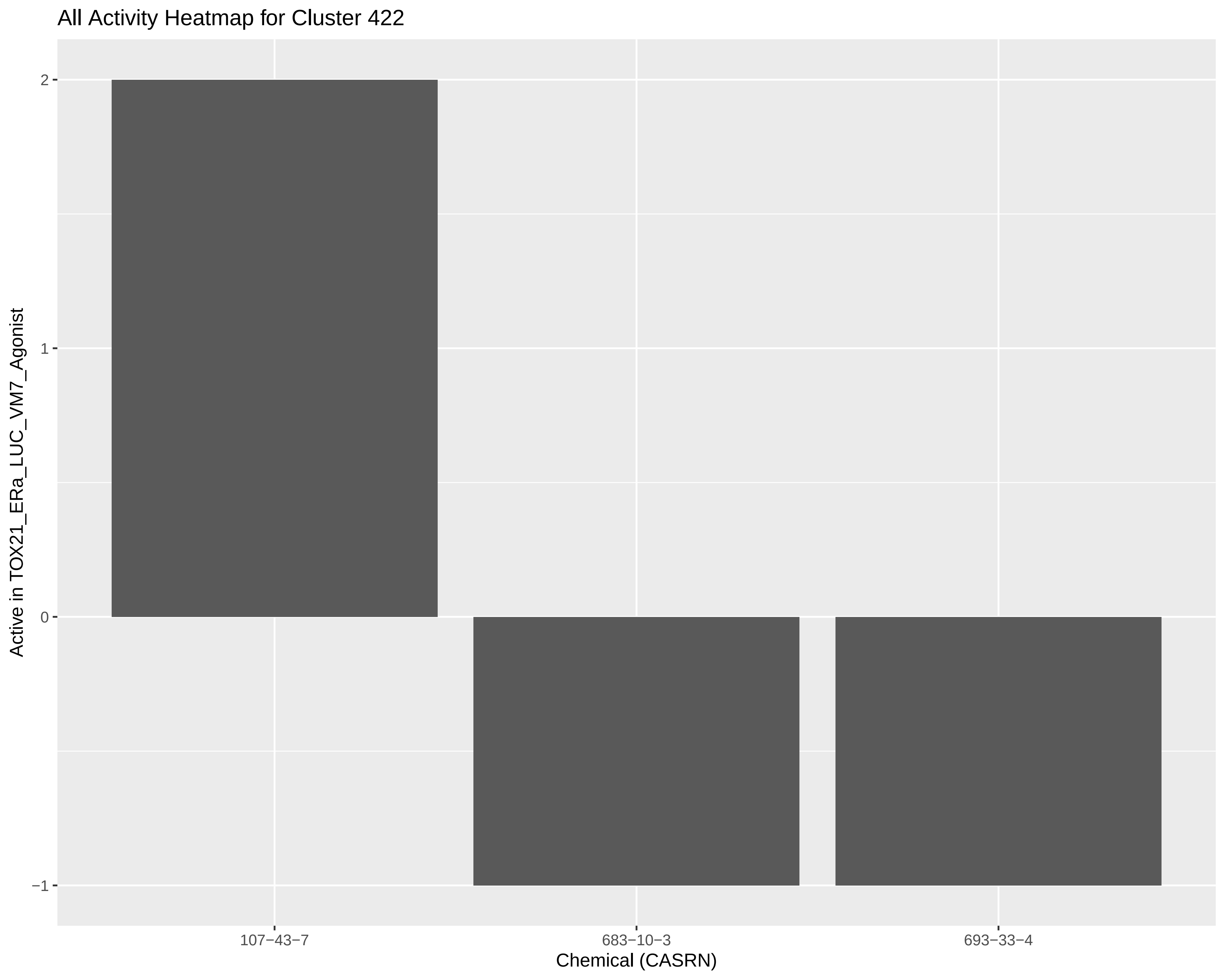
All Activity Heatmap for Cluster 422
Active in TOX21_ERa_LUC_VM7_Agonist
2
1
0
−1
107−43−7
683−10−3
693−33−4
Chemical (CASRN)

All Activity Heatmap for Cluster 423

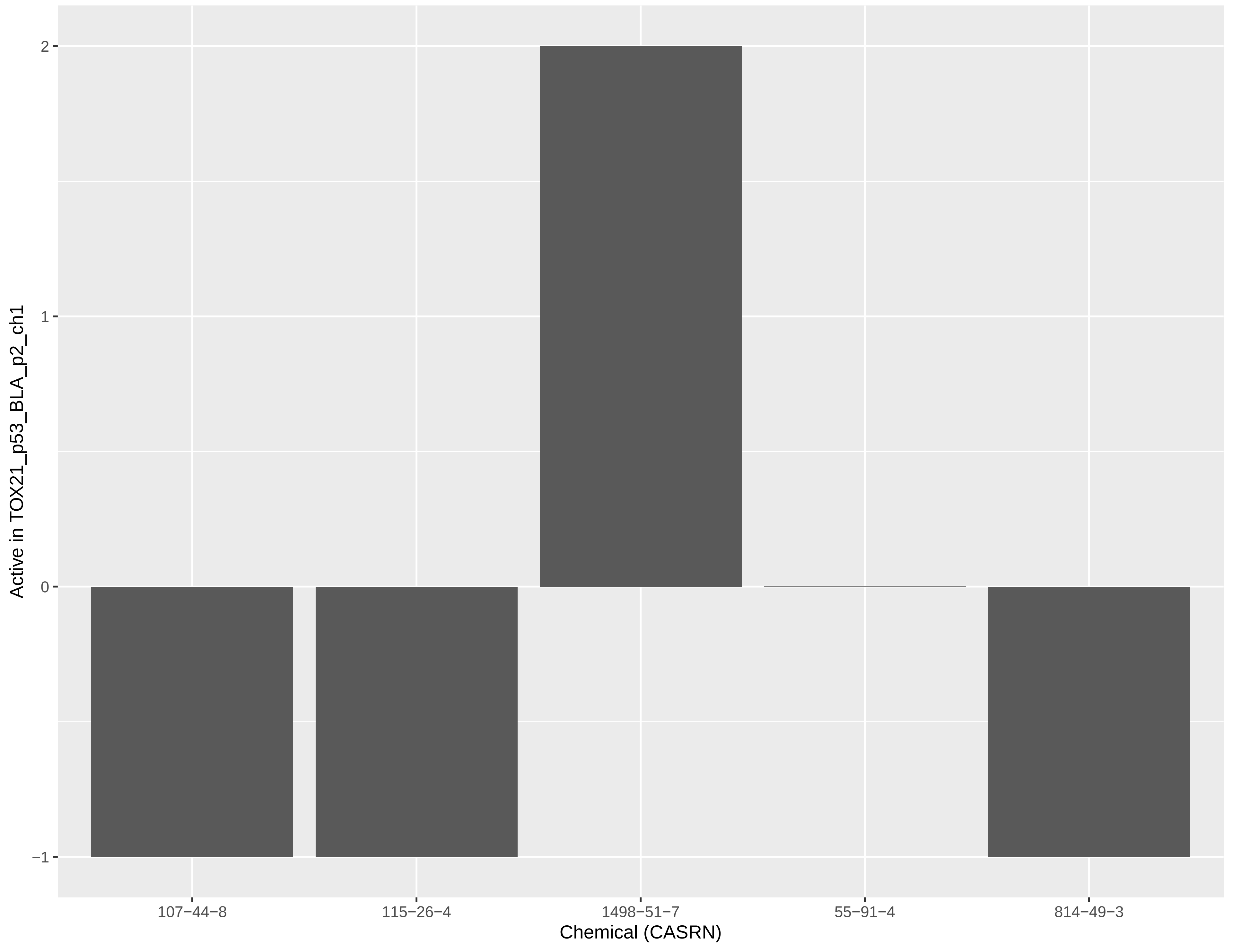

All Activity Heatmap for Cluster 424

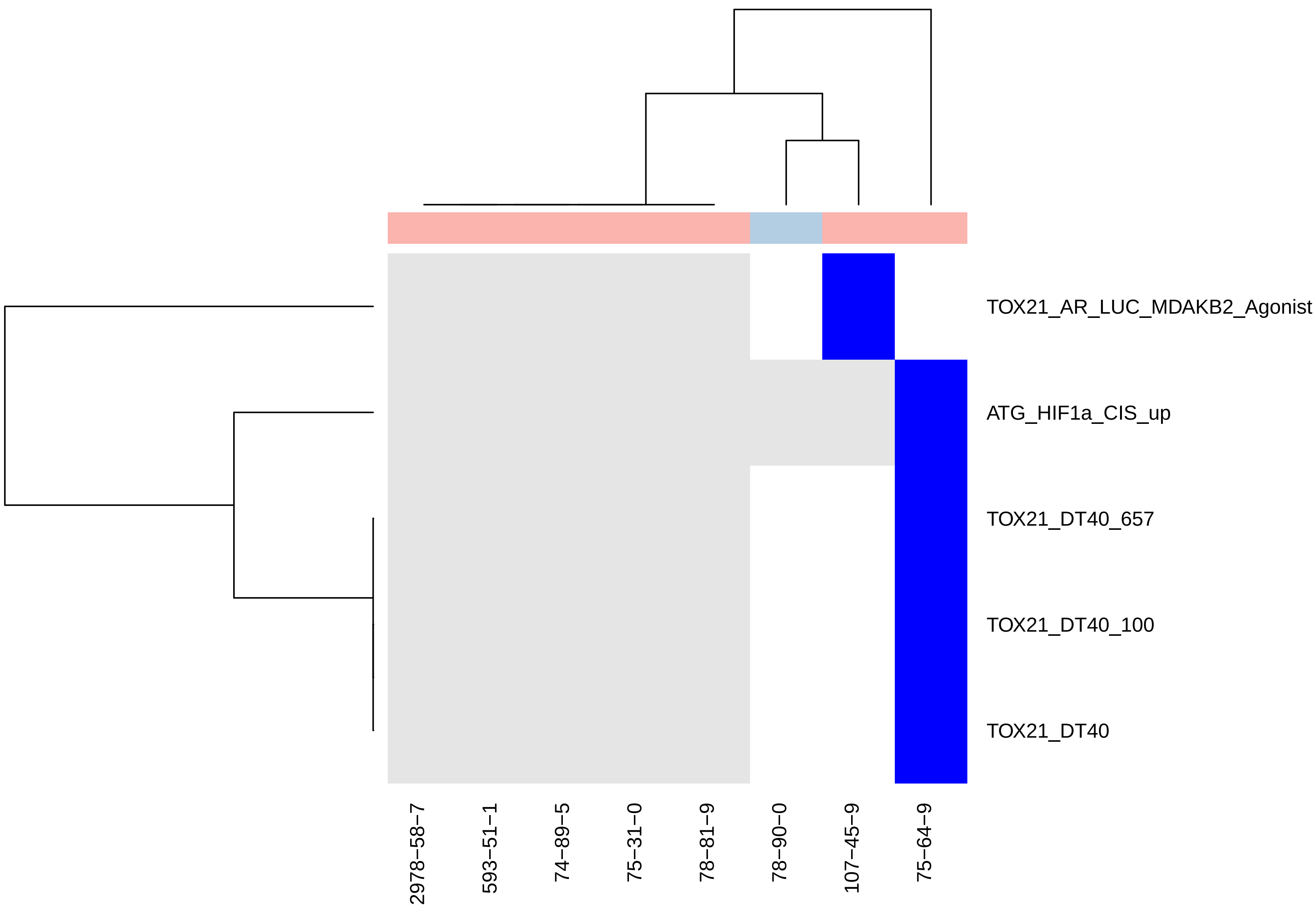

All Activity Heatmap for Cluster 425

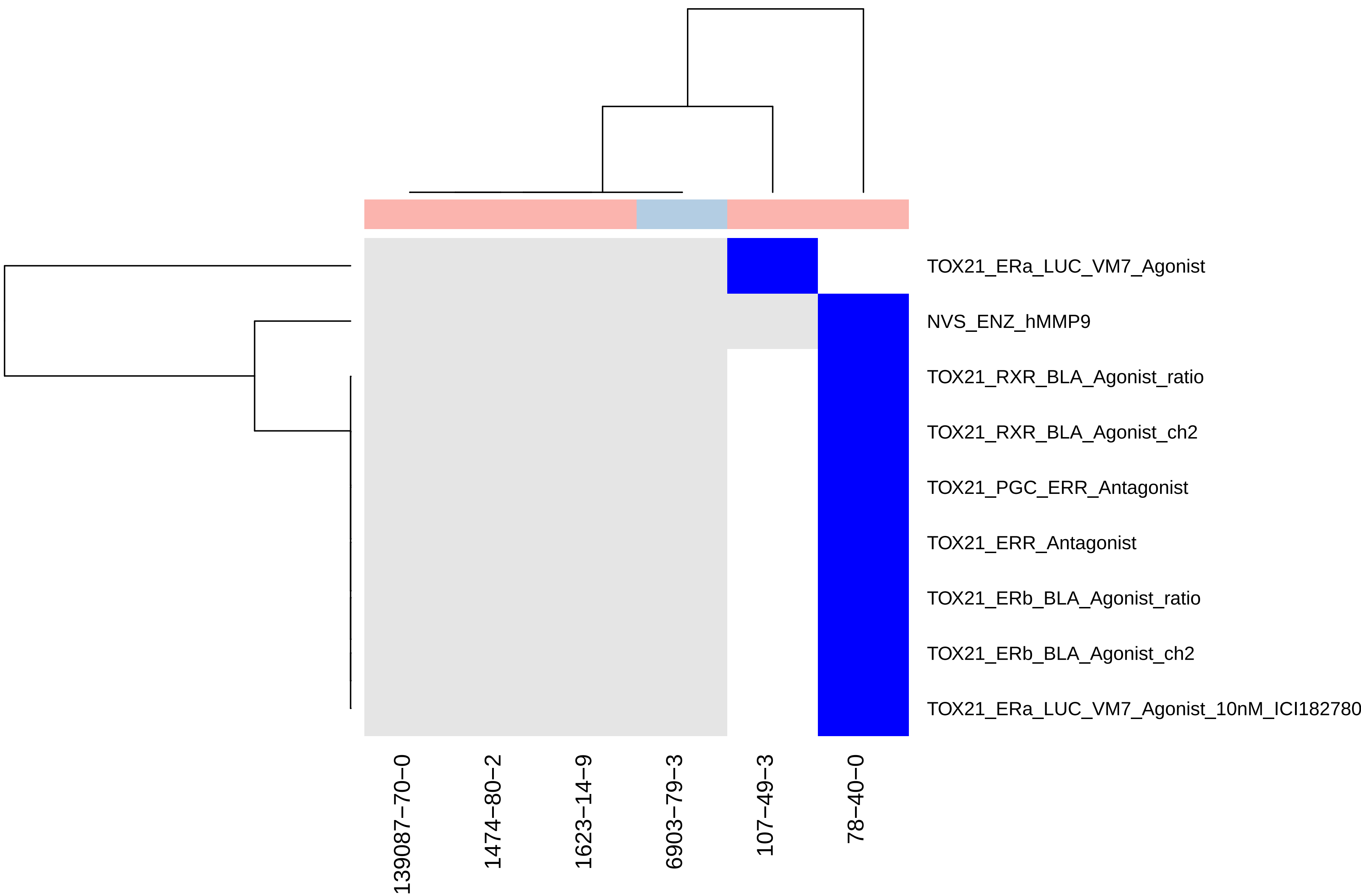

All Activity Heatmap for Cluster 426

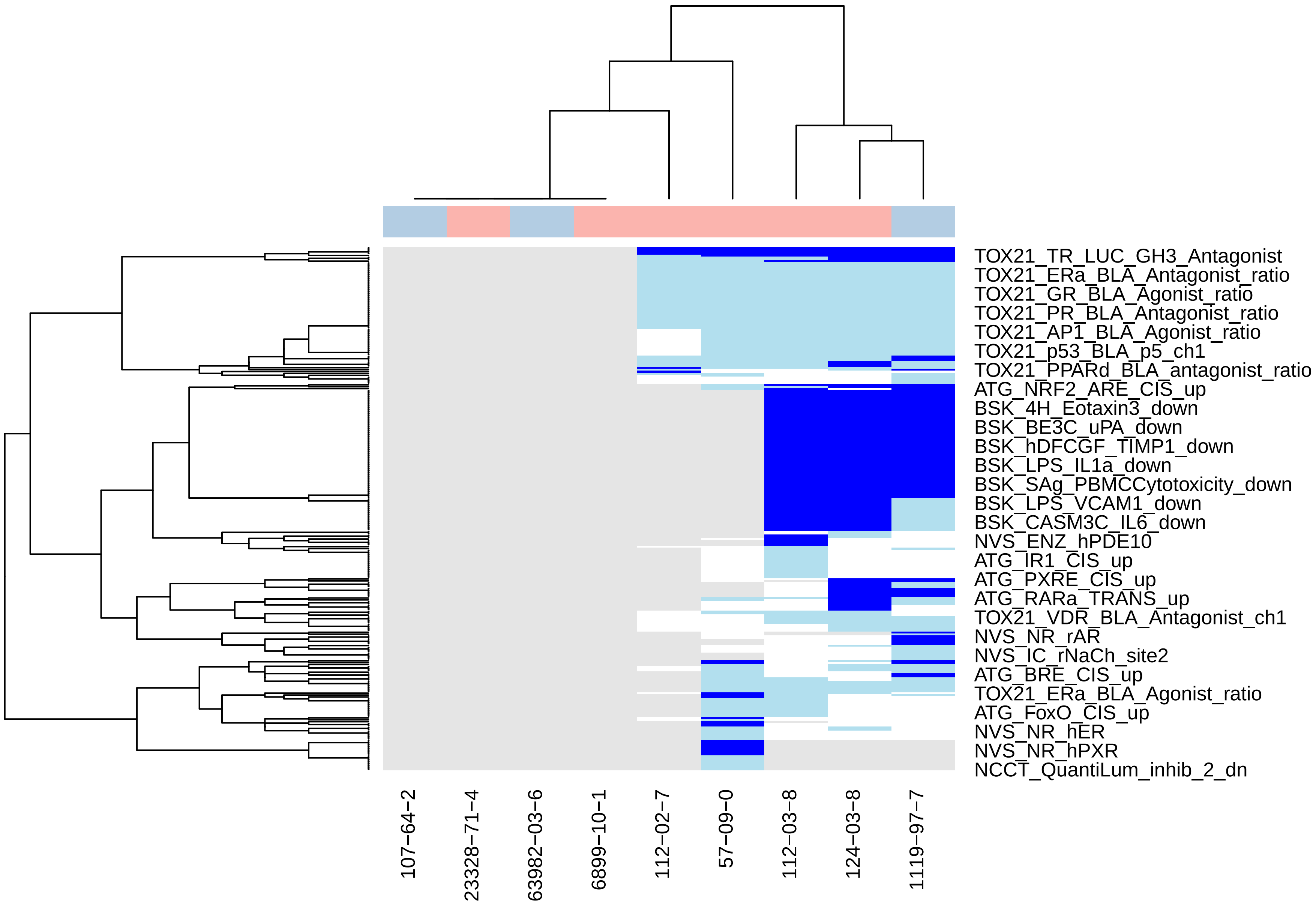

All Activity Heatmap for Cluster 427

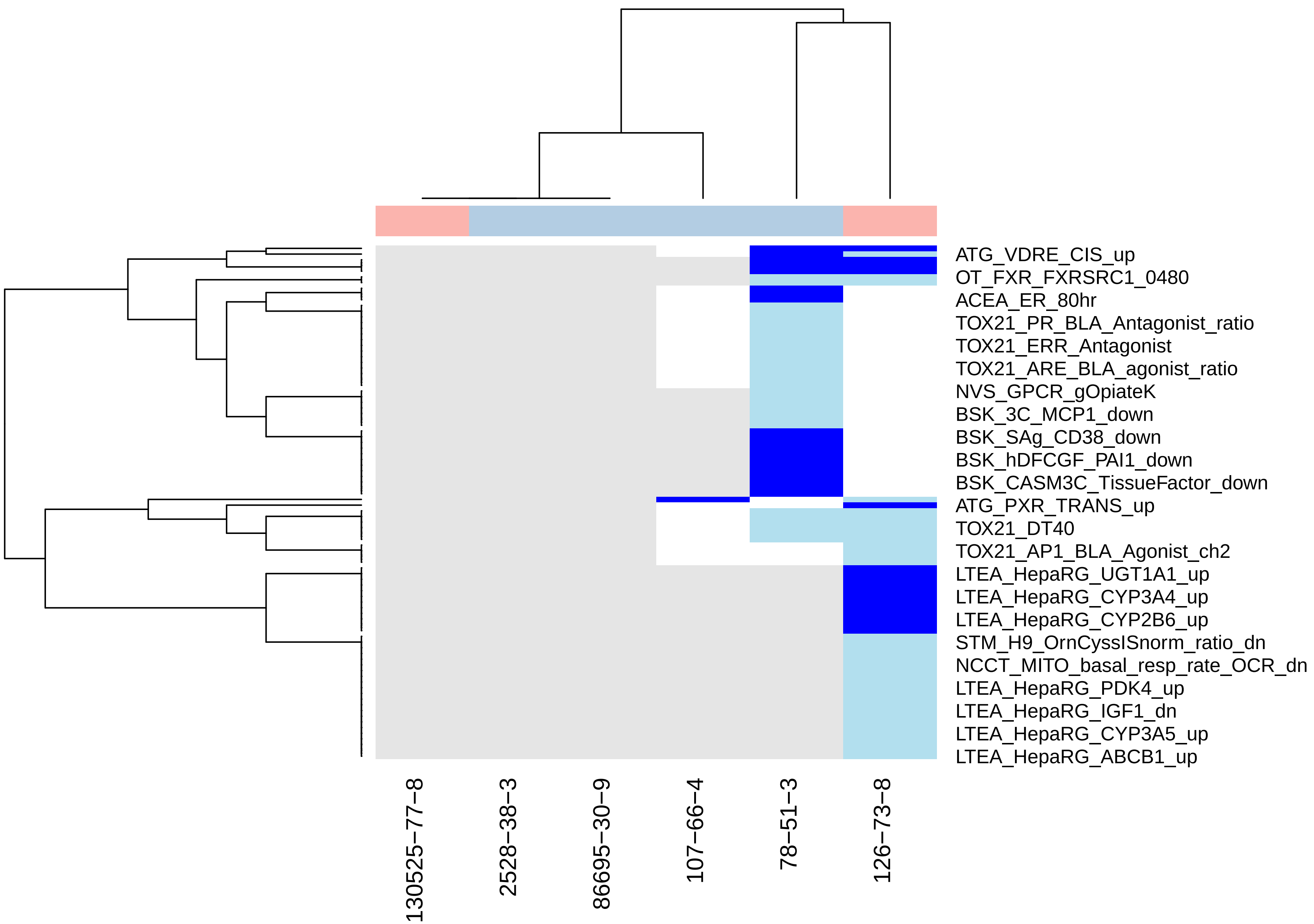

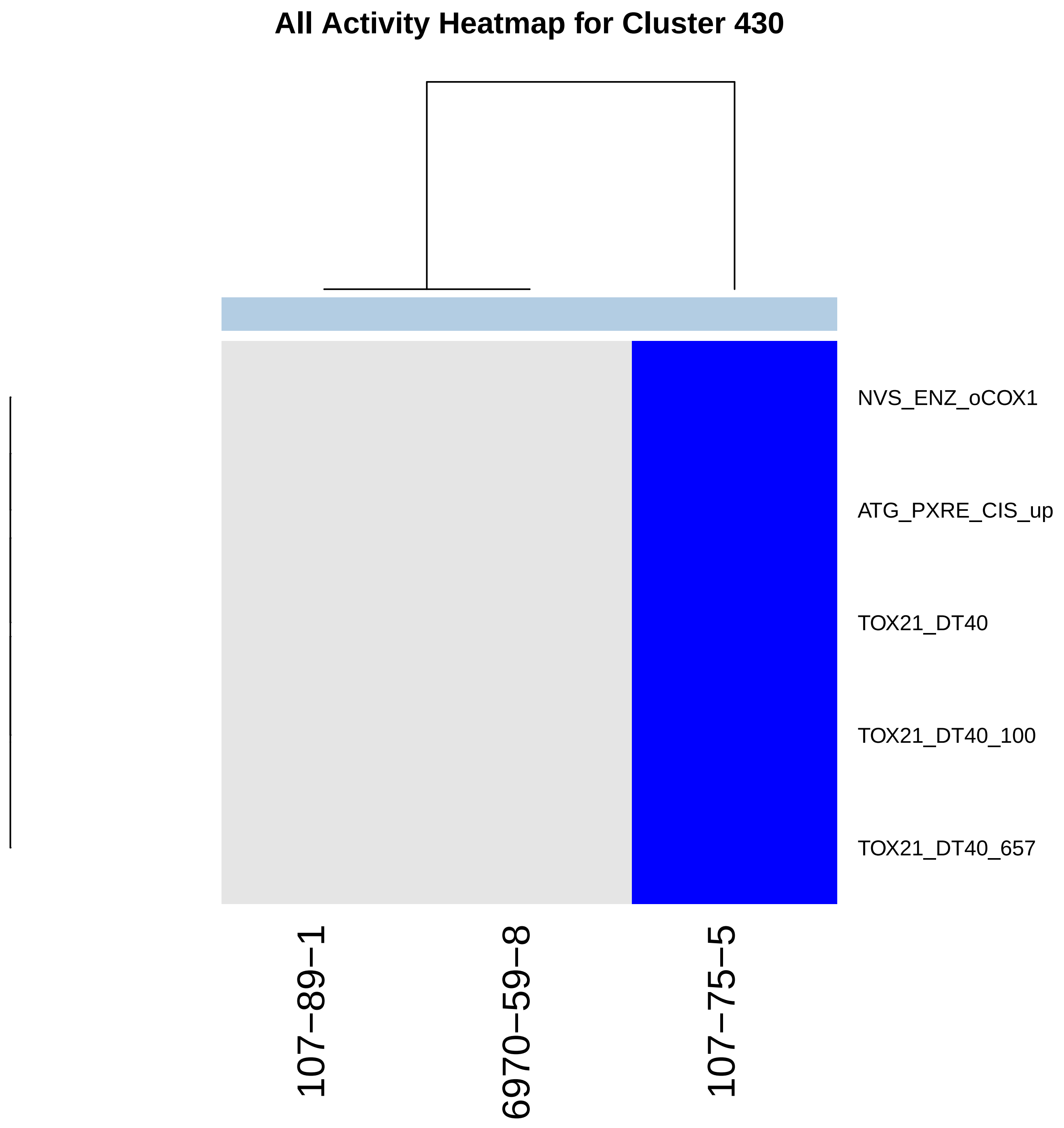
All Activity Heatmap for Cluster 430
NVS_ENZ_oCOX1
ATG_PXRE_CIS_up
TOX21_DT40
TOX21_DT40_100
TOX21_DT40_657
107−89−1
6970−59−8
107−75−5

All Activity Heatmap for Cluster 431

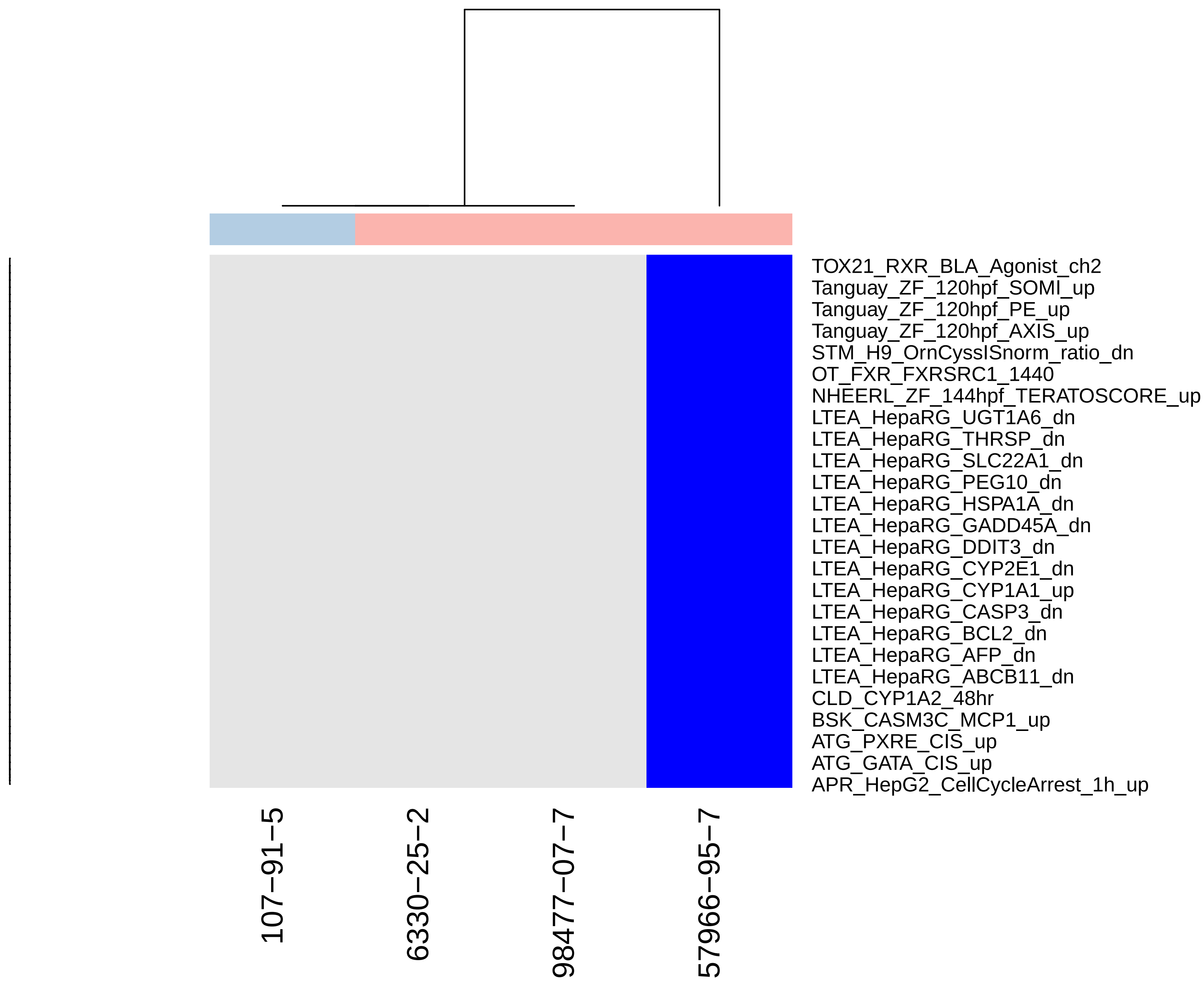

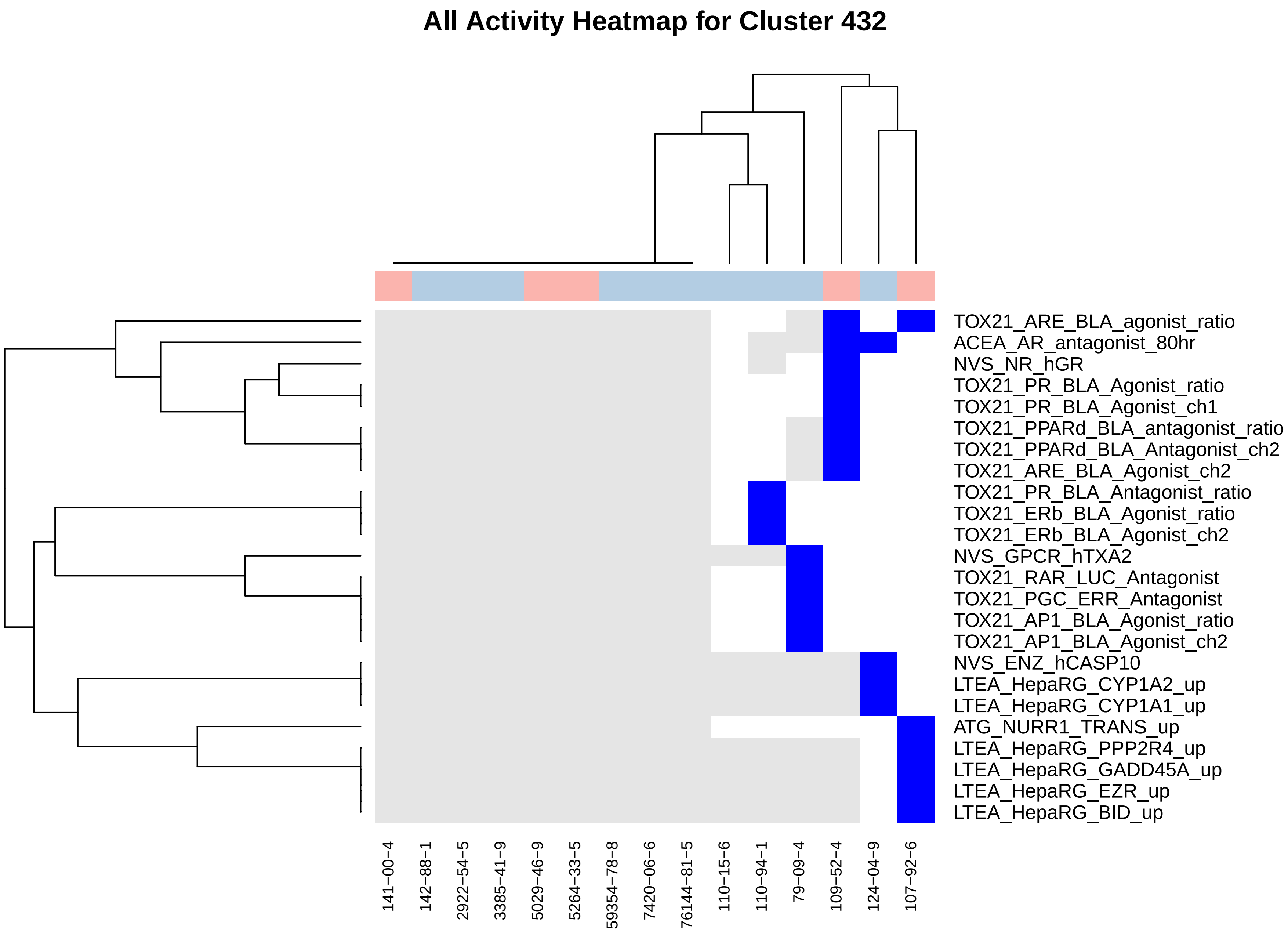

All Activity Heatmap for Cluster 432
TOX21_ARE_BLA_agonist_ratio
ACEA_AR_antagonist_80hr
NVS_NR_hGR
TOX21_PR_BLA_Agonist_ratio
TOX21_PR_BLA_Agonist_ch1
TOX21_PPARd_BLA_antagonist_ratio
TOX21_PPARd_BLA_Antagonist_ch2
TOX21_ARE_BLA_Agonist_ch2
TOX21_PR_BLA_Antagonist_ratio
TOX21_ERb_BLA_Agonist_ratio
TOX21_ERb_BLA_Agonist_ch2
NVS_GPCR_hTXA2
TOX21_RAR_LUC_Antagonist
TOX21_PGC_ERR_Antagonist
TOX21_AP1_BLA_Agonist_ratio
TOX21_AP1_BLA_Agonist_ch2
NVS_ENZ_hCASP10
LTEA_HepaRG_CYP1A2_up
LTEA_HepaRG_CYP1A1_up
ATG_NURR1_TRANS_up
LTEA_HepaRG_PPP2R4_up
LTEA_HepaRG_GADD45A_up
LTEA_HepaRG_EZR_up
LTEA_HepaRG_BID_up
141−00−4
142−88−1
2922−54−5
3385−41−9
5029−46−9
5264−33−5
59354−78−8
7420−06−6
76144−81−5
110−15−6
110−94−1
79−09−4
109−52−4
124−04−9
107−92−6

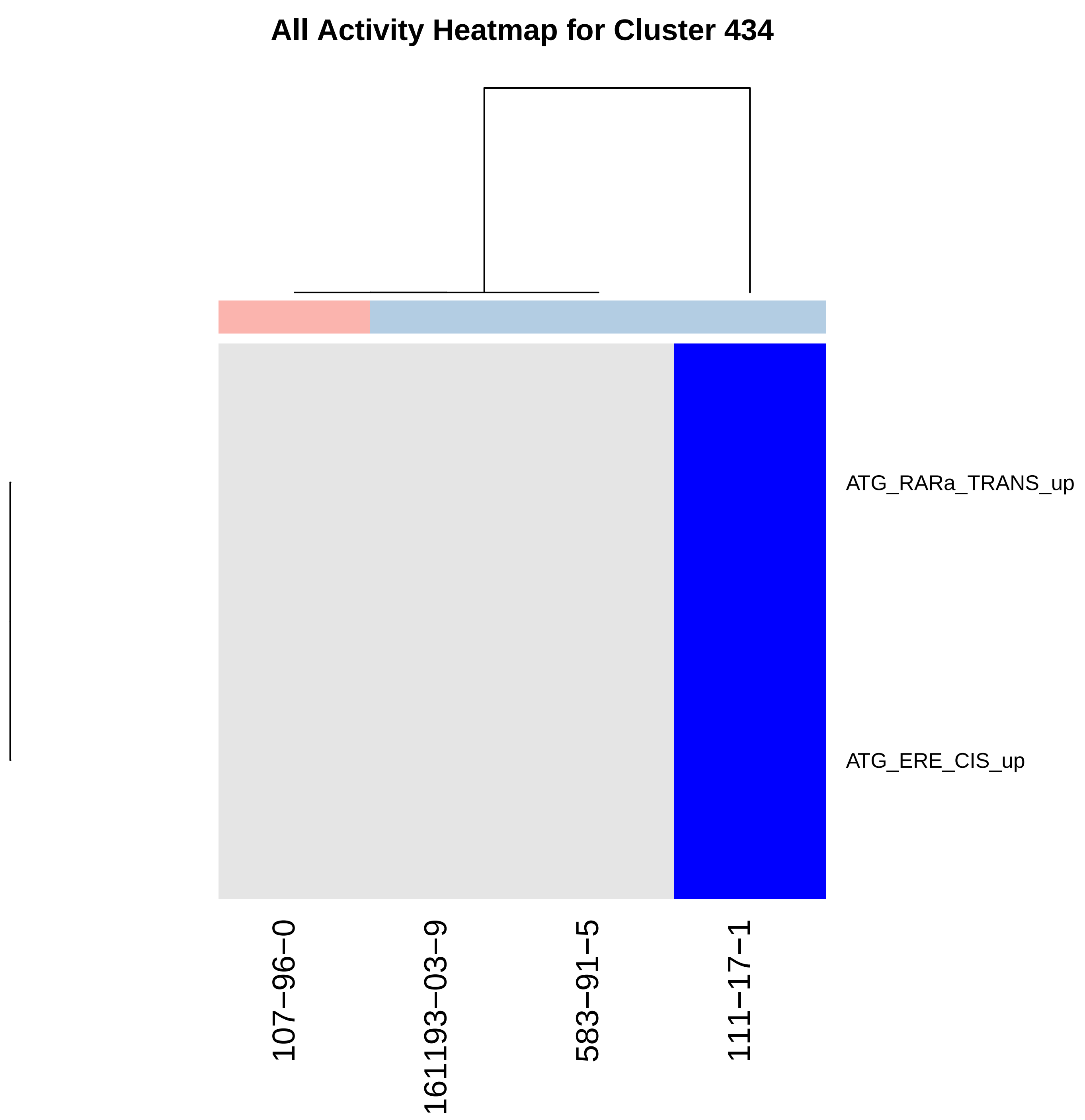
All Activity Heatmap for Cluster 434
ATG_RARa_TRANS_up
ATG_ERE_CIS_up
107−96−0
161193−03−9
583−91−5
111−17−1

All Activity Heatmap for Cluster 435

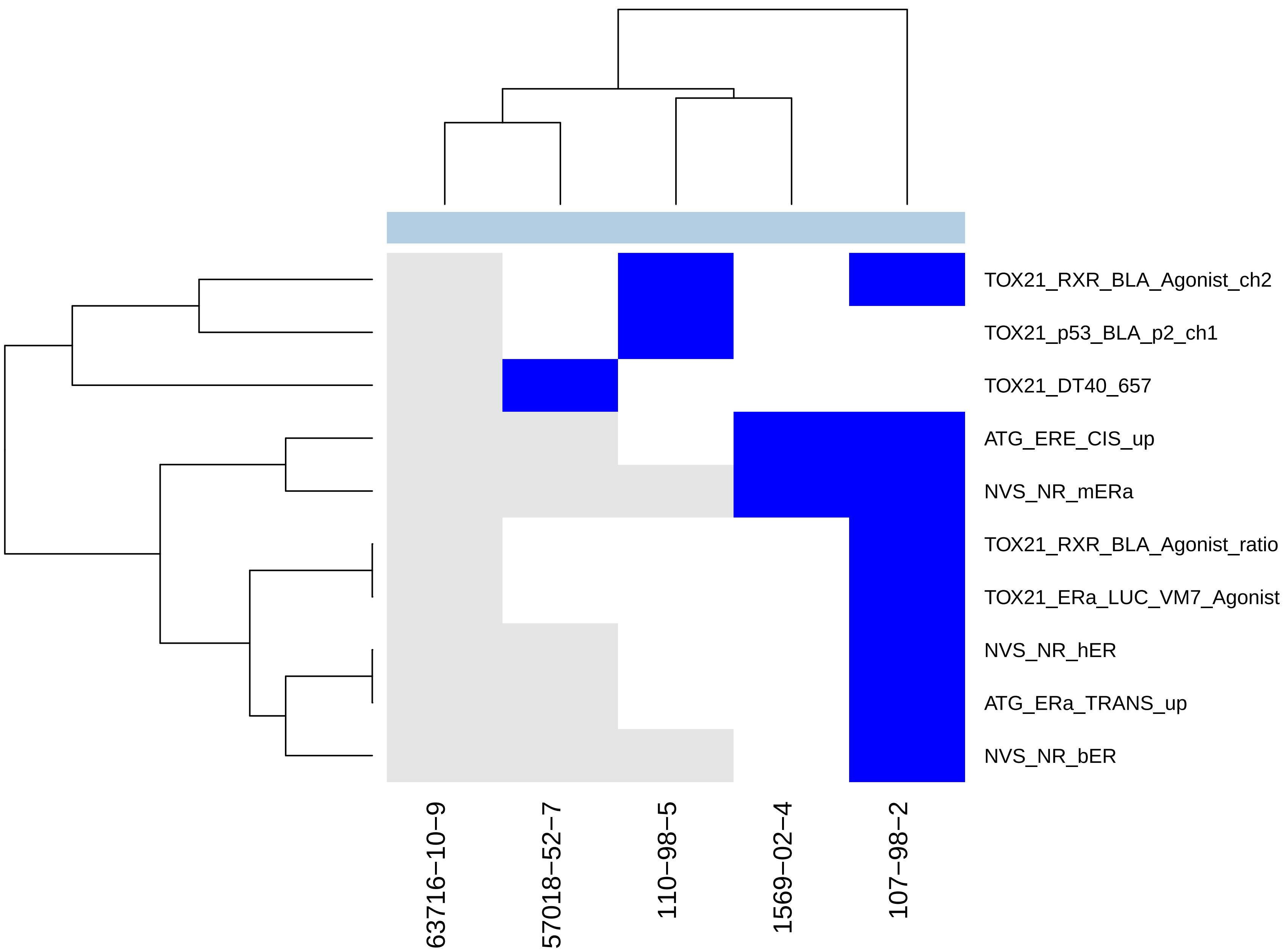

All Activity Heatmap for Cluster 436

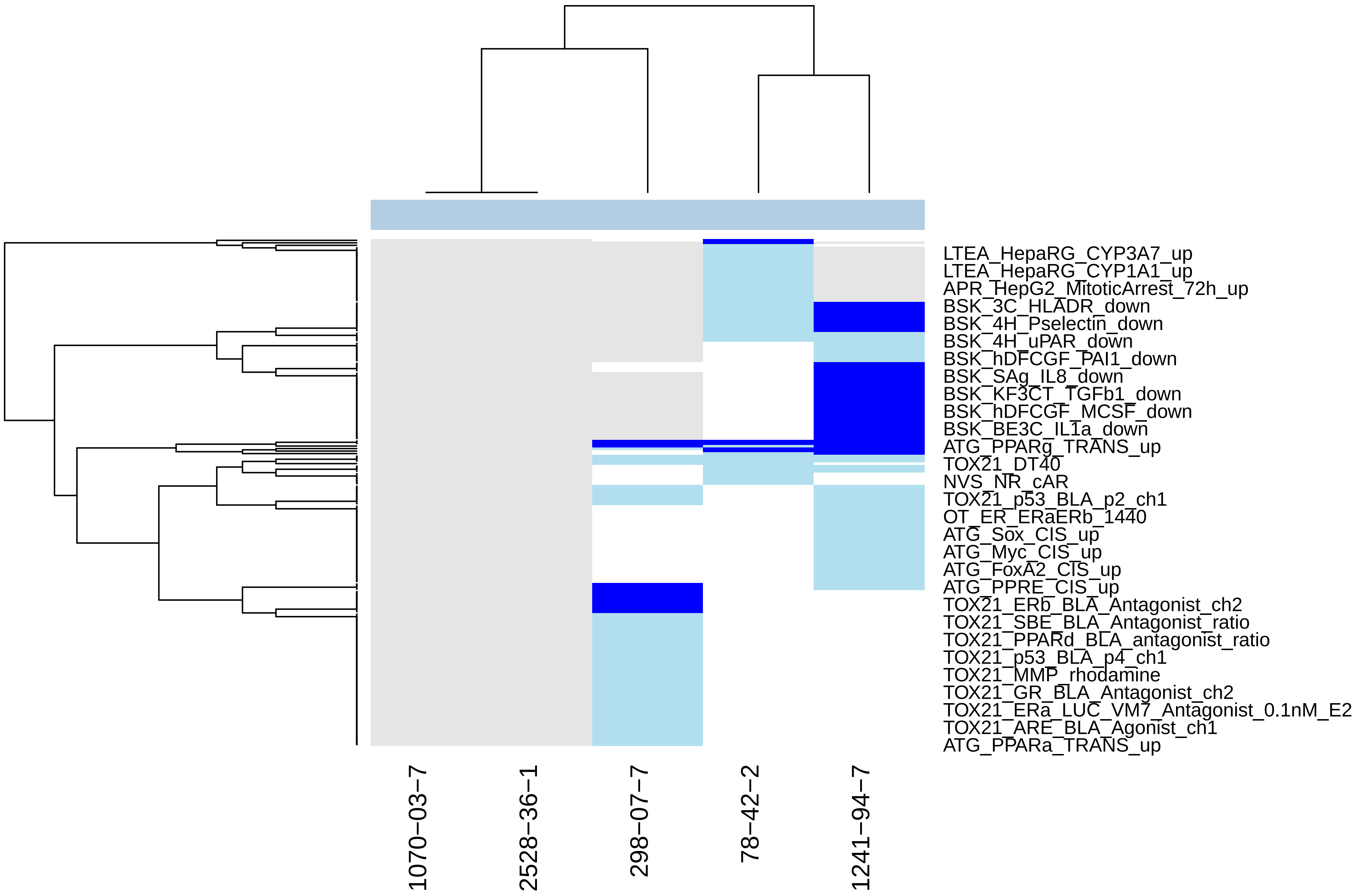

All Activity Heatmap for Cluster 437

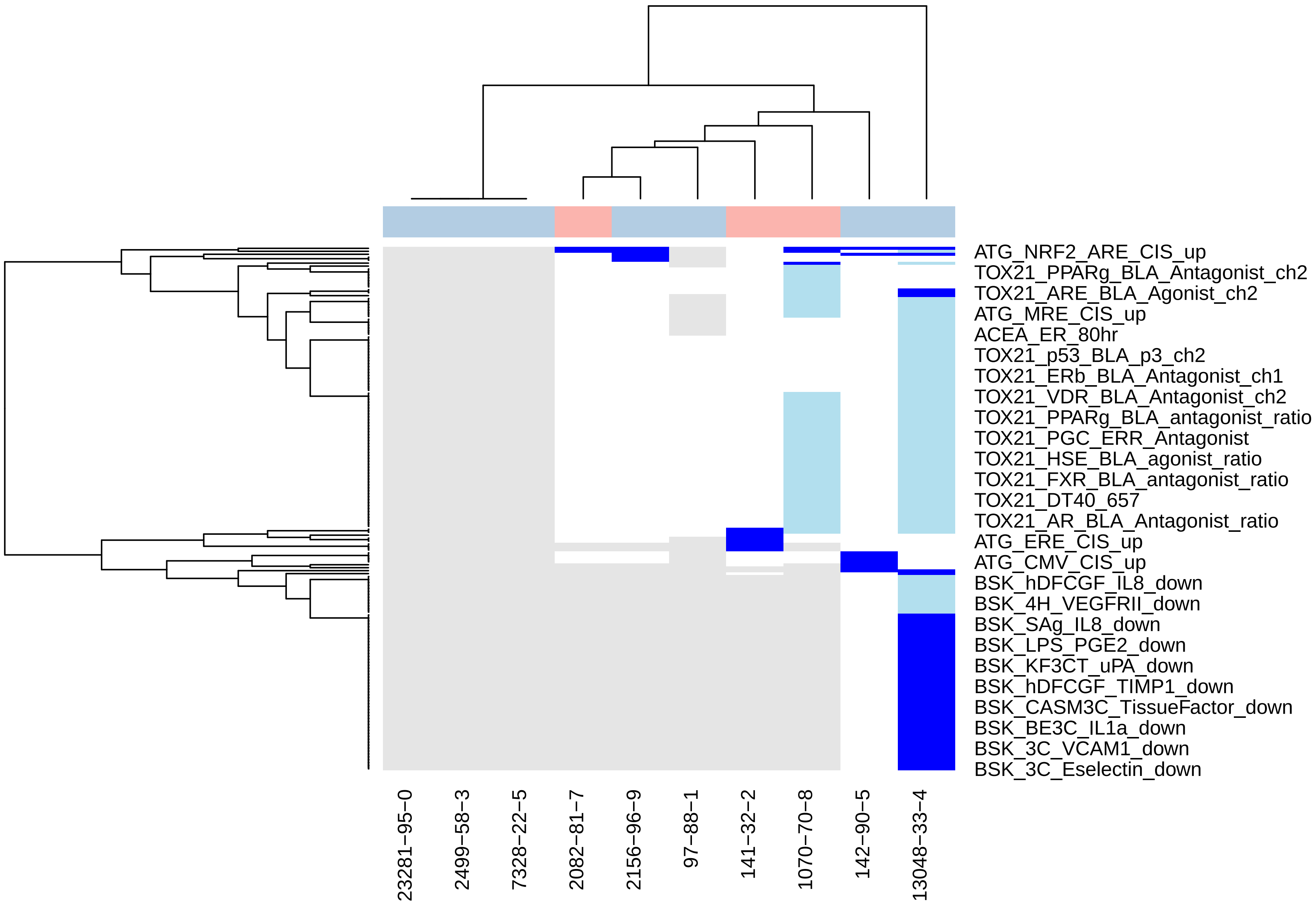

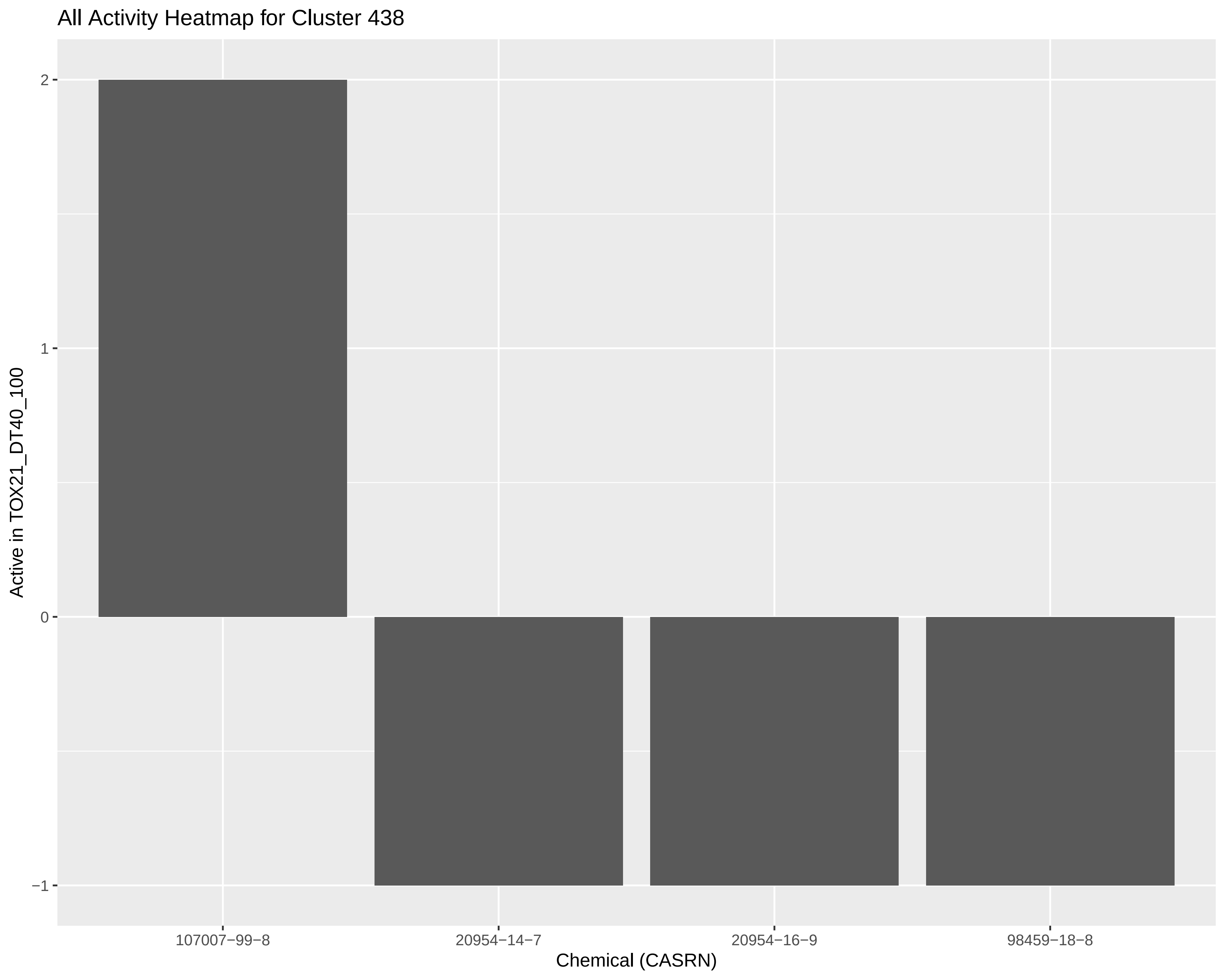
All Activity Heatmap for Cluster 438
Active in TOX21_DT40_100
2
1
0
−1
107007−99−8
20954−14−7
20954−16−9
98459−18−8
Chemical (CASRN)

All Activity Heatmap for Cluster 441

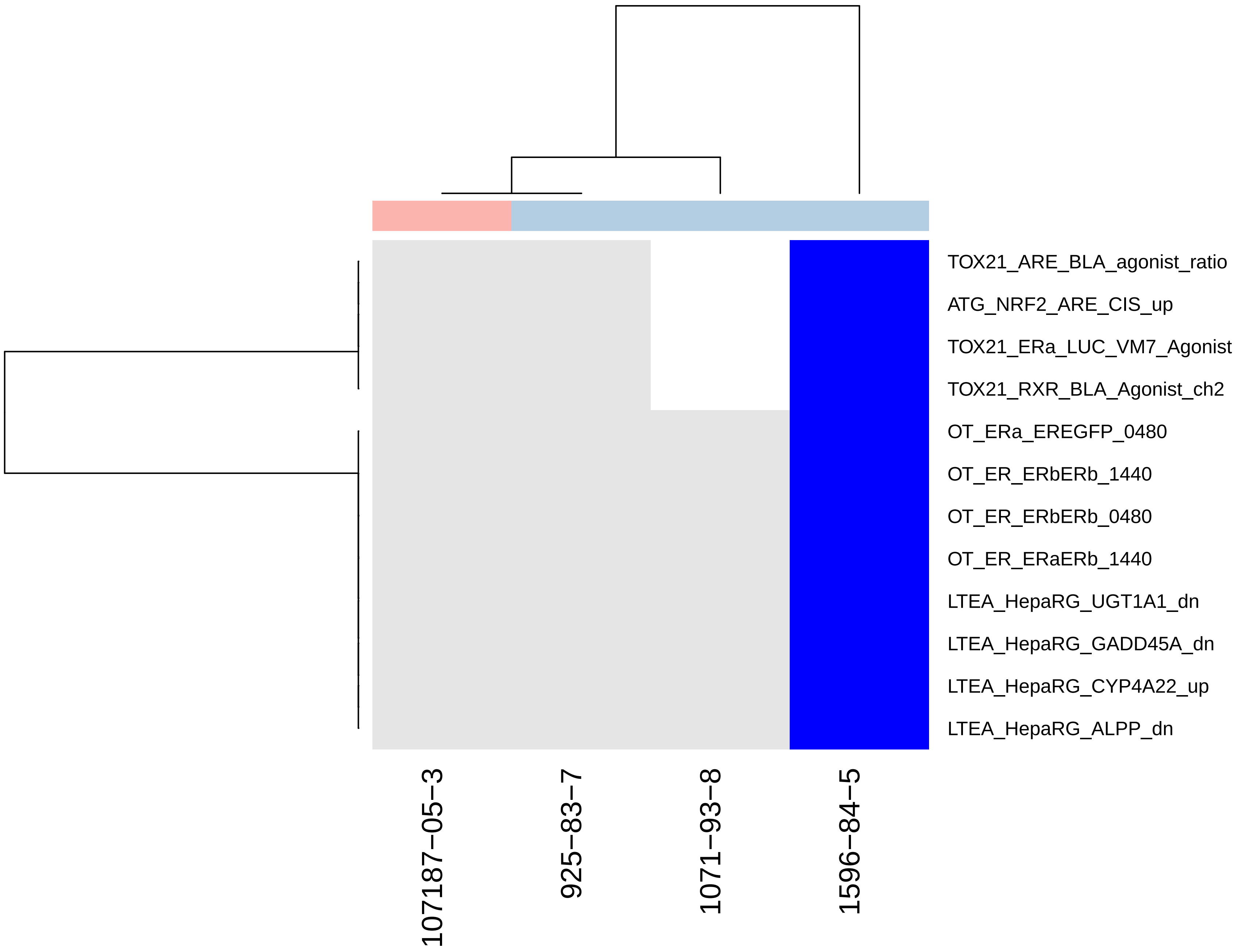

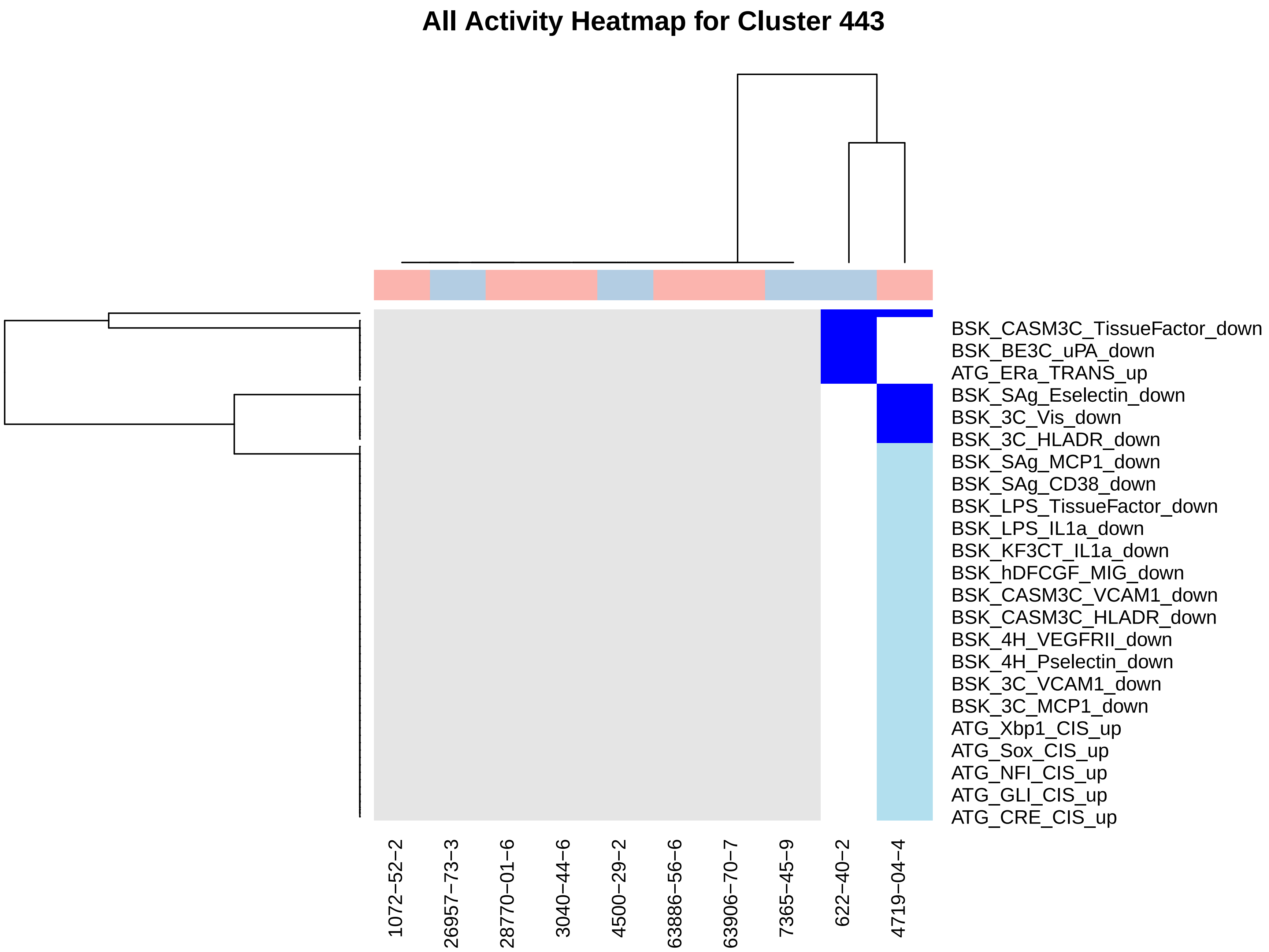
All Activity Heatmap for Cluster 443
BSK_CASM3C_TissueFactor_down
BSK_BE3C_uPA_down
ATG_ERa_TRANS_up
BSK_SAg_Eselectin_down
BSK_3C_Vis_down
BSK_3C_HLADR_down
BSK_SAg_MCP1_down
BSK_SAg_CD38_down
BSK_LPS_TissueFactor_down
BSK_LPS_IL1a_down
BSK_KF3CT_IL1a_down
BSK_hDFCGF_MIG_down
BSK_CASM3C_VCAM1_down
BSK_CASM3C_HLADR_down
BSK_4H_VEGFRII_down
BSK_4H_Pselectin_down
BSK_3C_VCAM1_down
BSK_3C_MCP1_down
ATG_Xbp1_CIS_up
ATG_Sox_CIS_up
ATG_NFI_CIS_up
ATG_GLI_CIS_up
ATG_CRE_CIS_up
1072−52−2
26957−73−3
28770−01−6
3040−44−6
4500−29−2
63886−56−6
63906−70−7
7365−45−9
622−40−2
4719−04−4

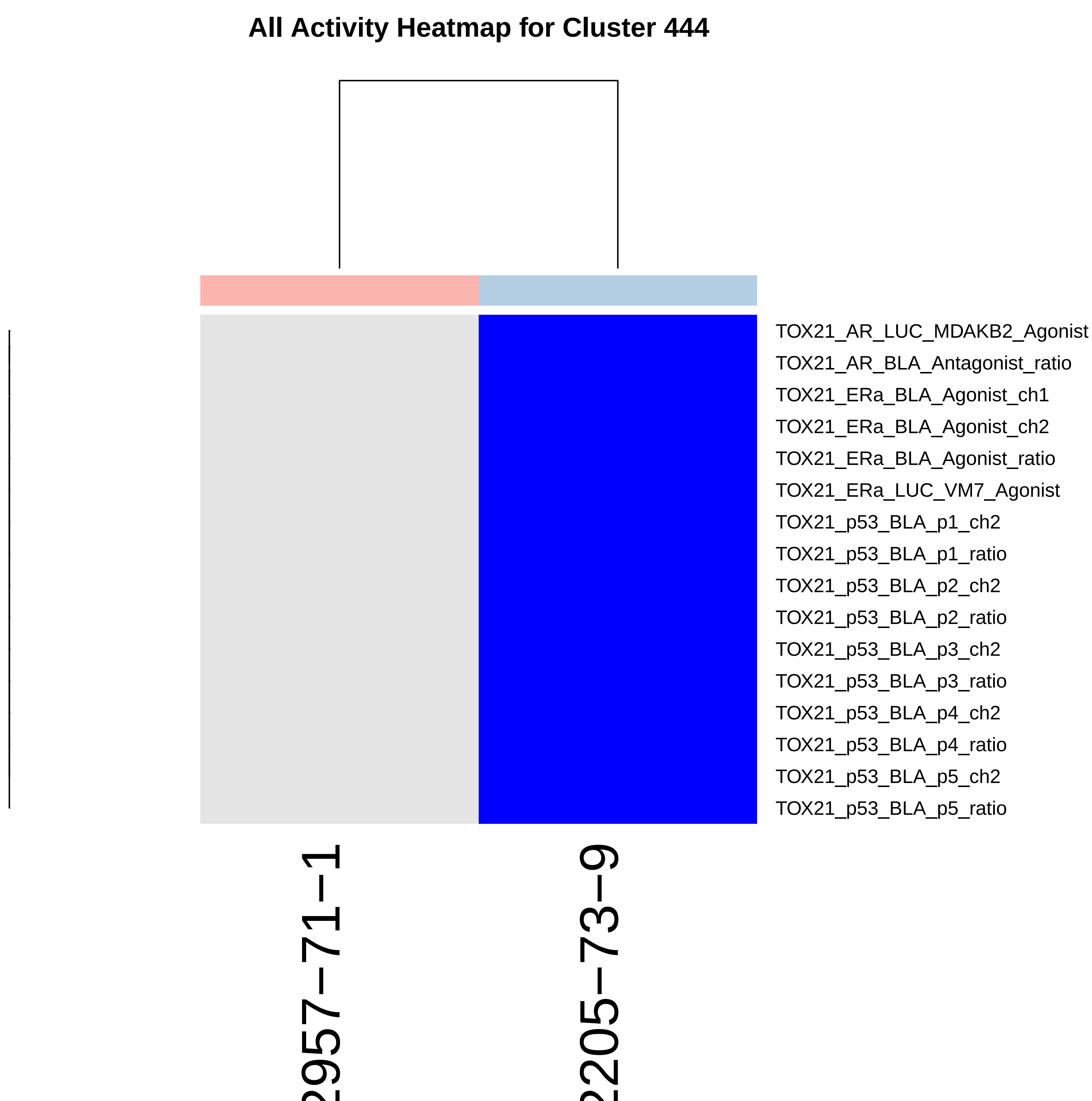
All Activity Heatmap for Cluster 444
TOX21_AR_LUC_MDAKB2_Agonist
TOX21_AR_BLA_Antagonist_ratio
TOX21_ERa_BLA_Agonist_ch1
TOX21_ERa_BLA_Agonist_ch2
TOX21_ERa_BLA_Agonist_ratio
TOX21_ERa_LUC_VM7_Agonist
TOX21_p53_BLA_p1_ch2
TOX21_p53_BLA_p1_ratio
TOX21_p53_BLA_p2_ch2
TOX21_p53_BLA_p2_ratio
TOX21_p53_BLA_p3_ch2
TOX21_p53_BLA_p3_ratio
TOX21_p53_BLA_p4_ch2
TOX21_p53_BLA_p4_ratio
TOX21_p53_BLA_p5_ch2
TOX21_p53_BLA_p5_ratio
2957−71−1
2205−73−9

All Activity Heatmap for Cluster 446

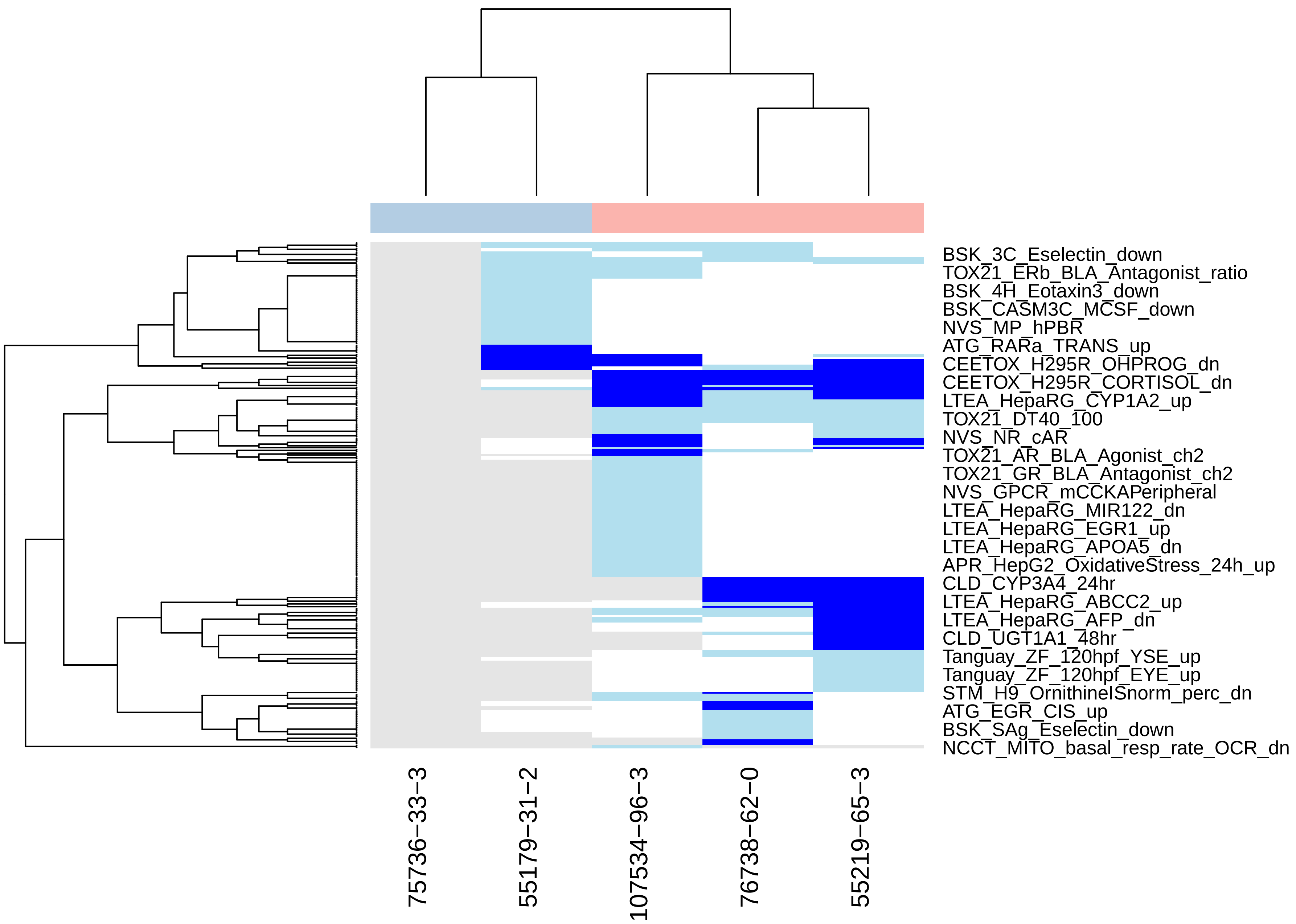

All Activity Heatmap for Cluster 447

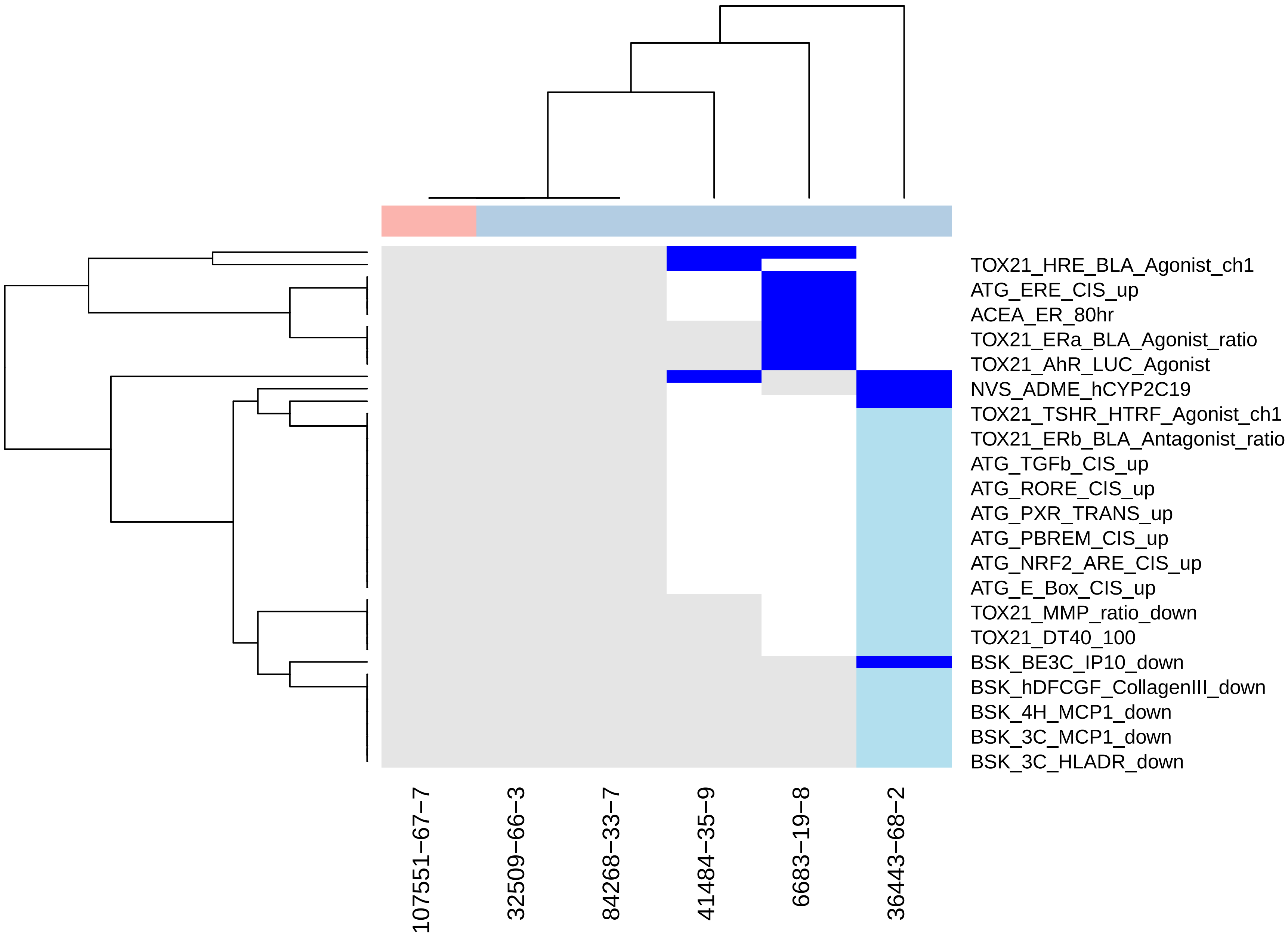

All Activity Heatmap for Cluster 448

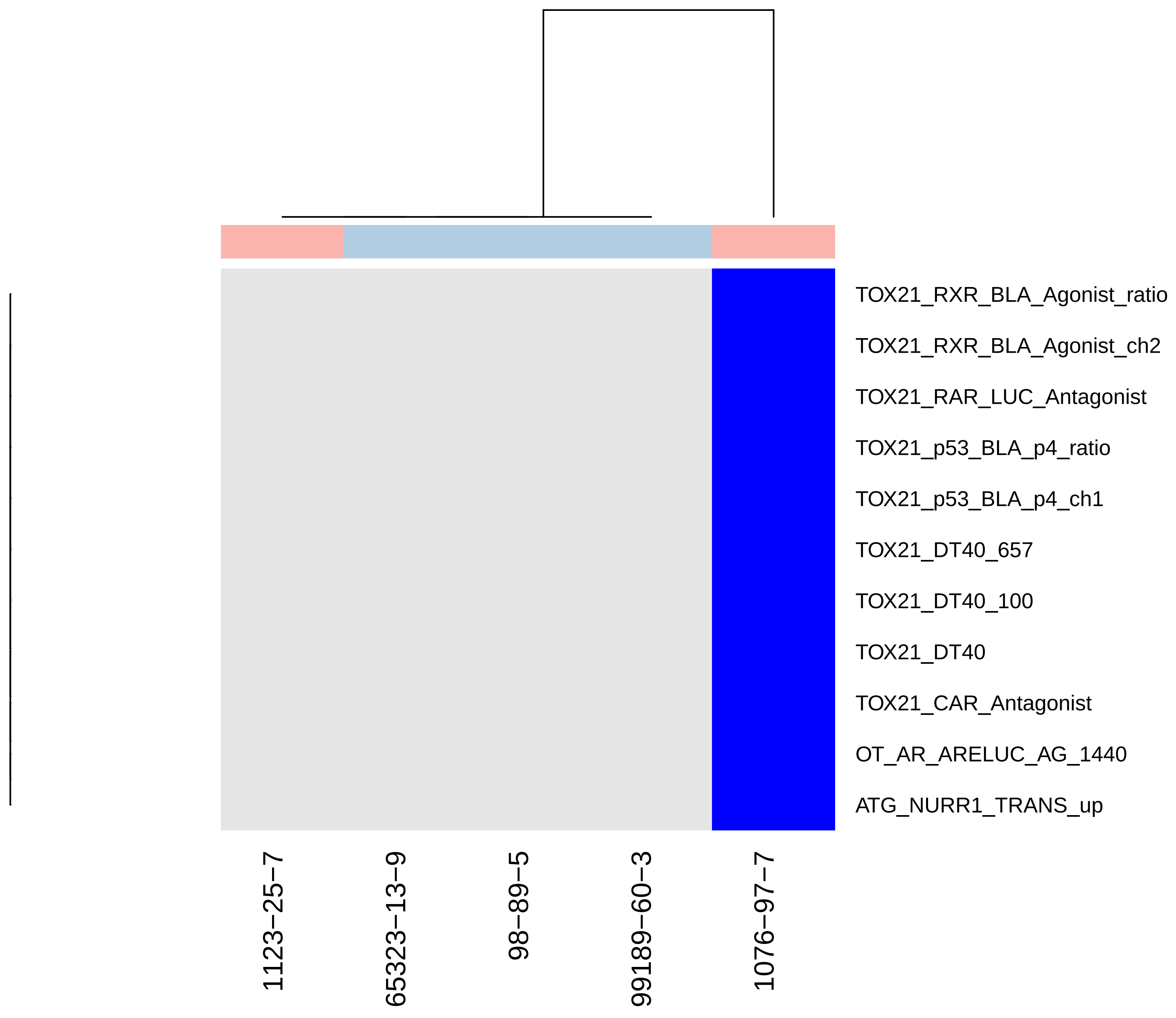

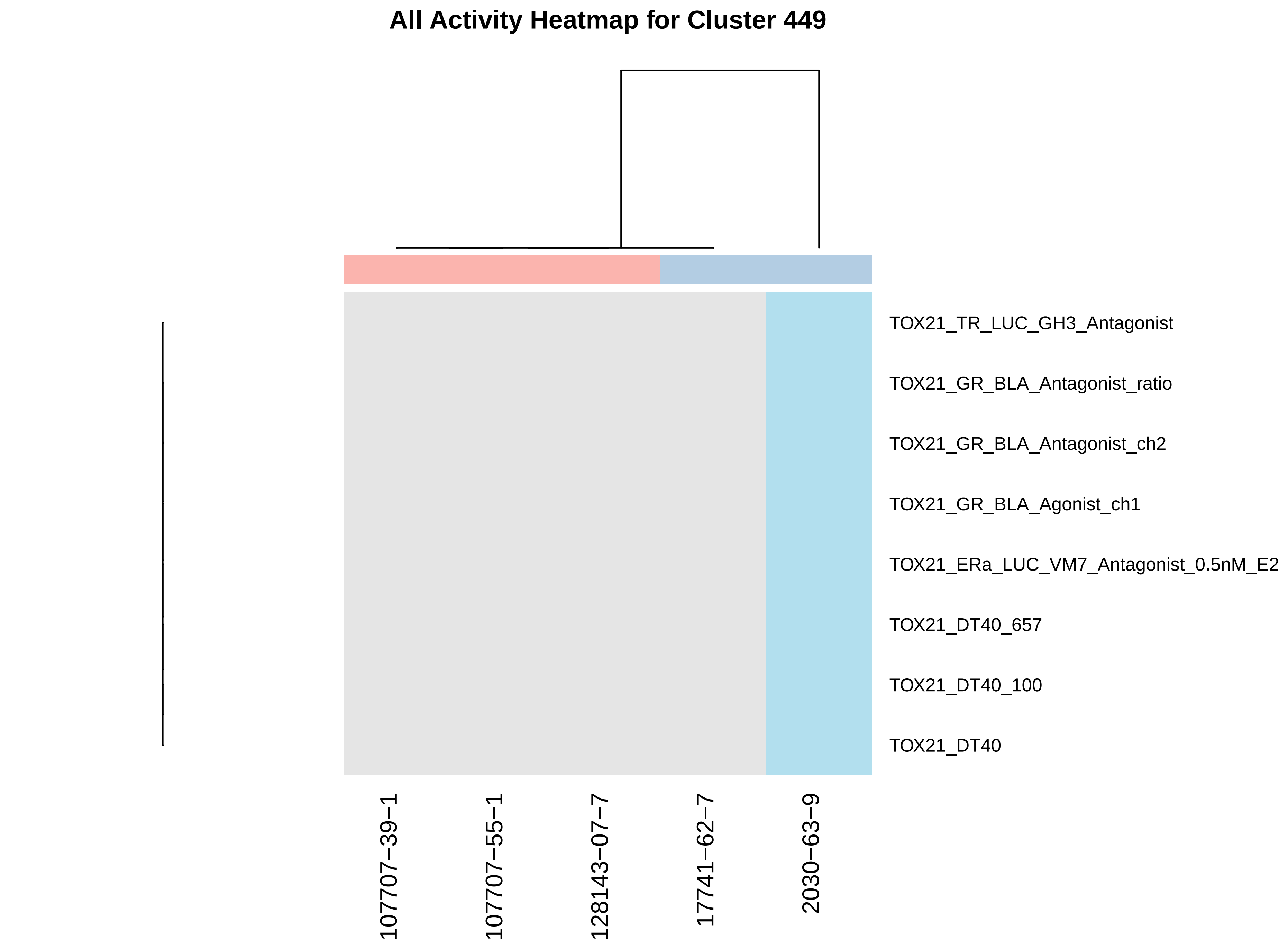

All Activity Heatmap for Cluster 449
TOX21_TR_LUC_GH3_Antagonist
TOX21_GR_BLA_Antagonist_ratio
TOX21_GR_BLA_Antagonist_ch2
TOX21_GR_BLA_Agonist_ch1
TOX21_ERa_LUC_VM7_Antagonist_0.5nM_E2
TOX21_DT40_657
TOX21_DT40_100
TOX21_DT40
107707−39−1
107707−55−1
128143−07−7
17741−62−7
2030−63−9

All Activity Heatmap for Cluster 450

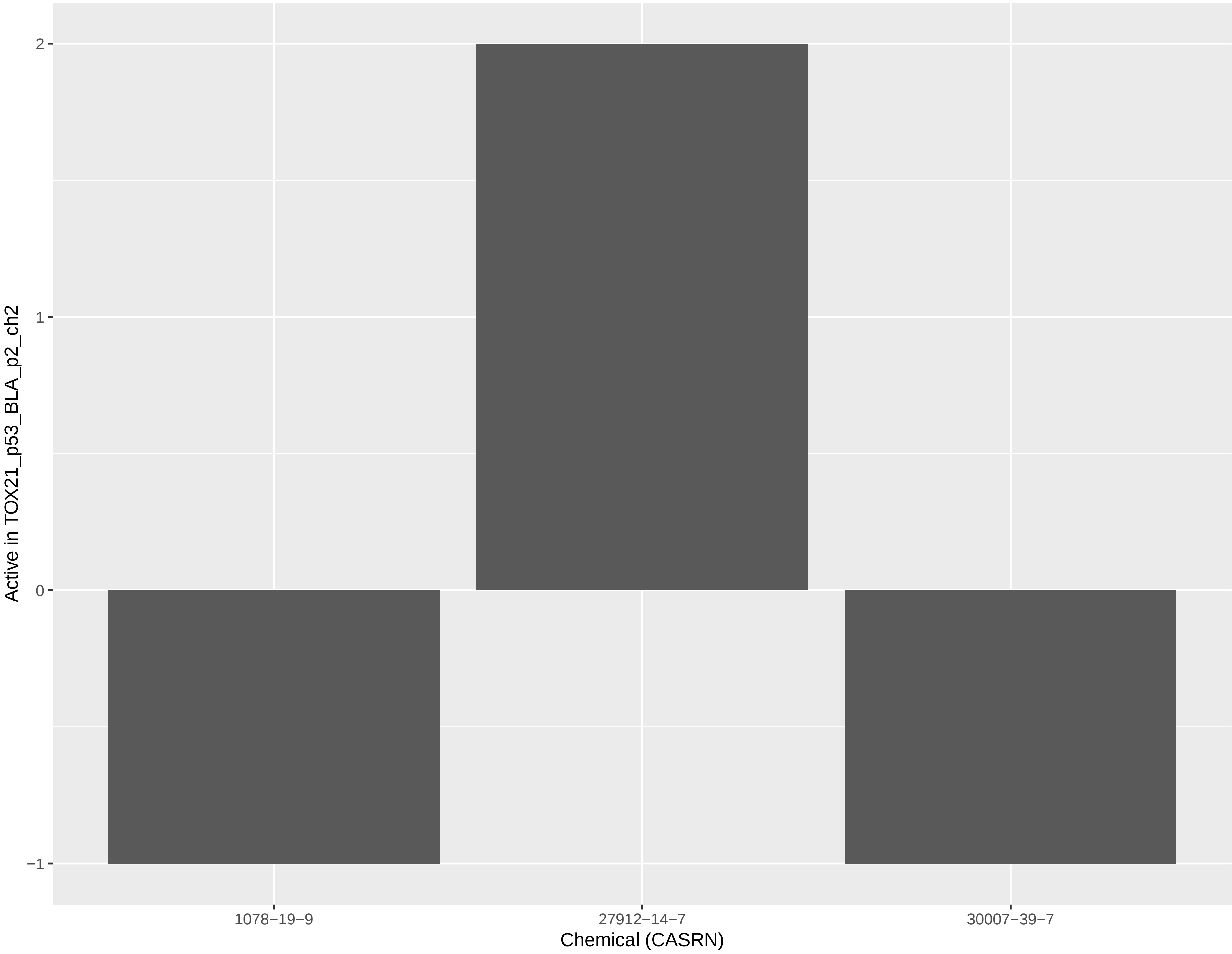

All Activity Heatmap for Cluster 452

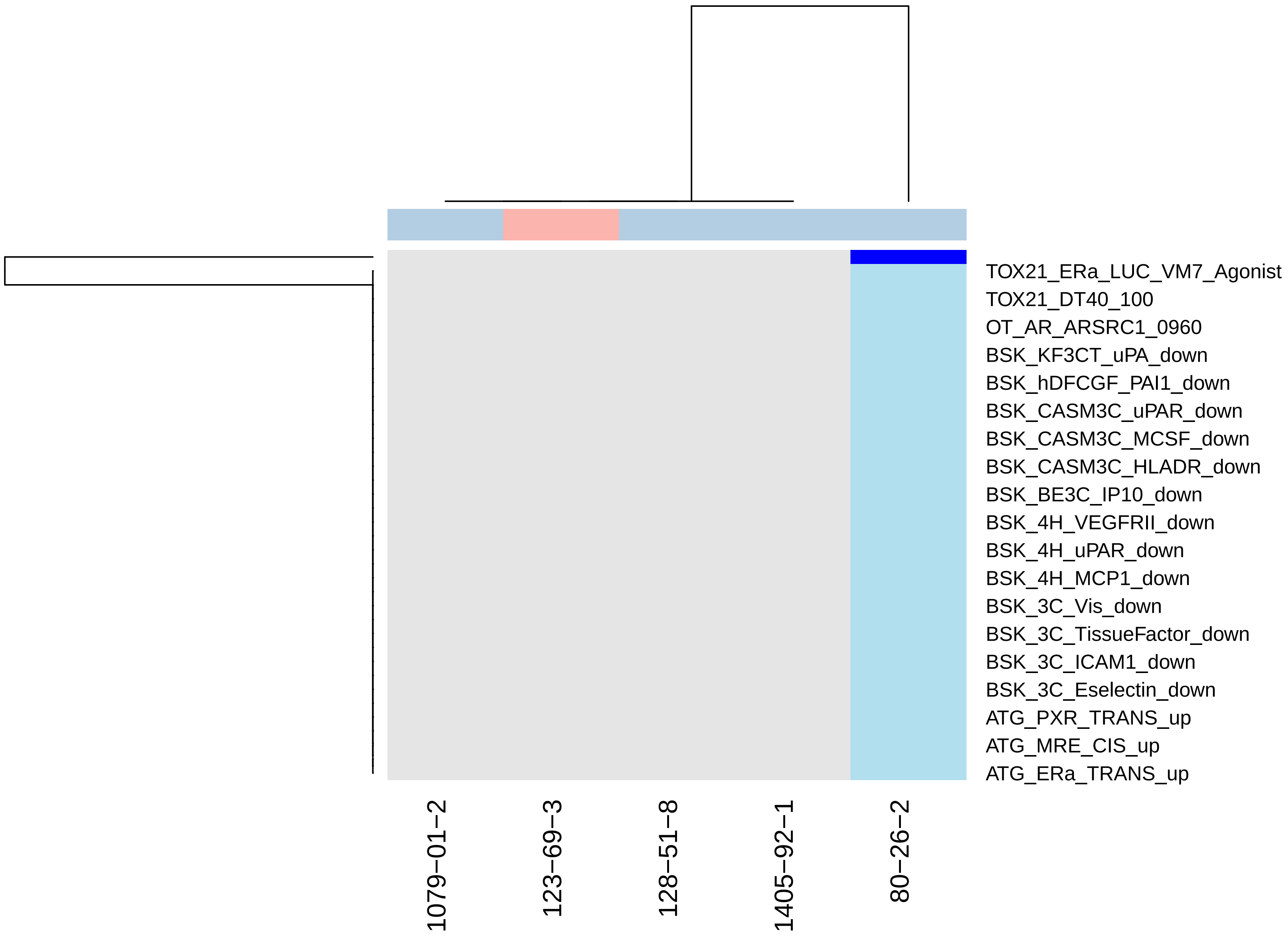

All Activity Heatmap for Cluster 455

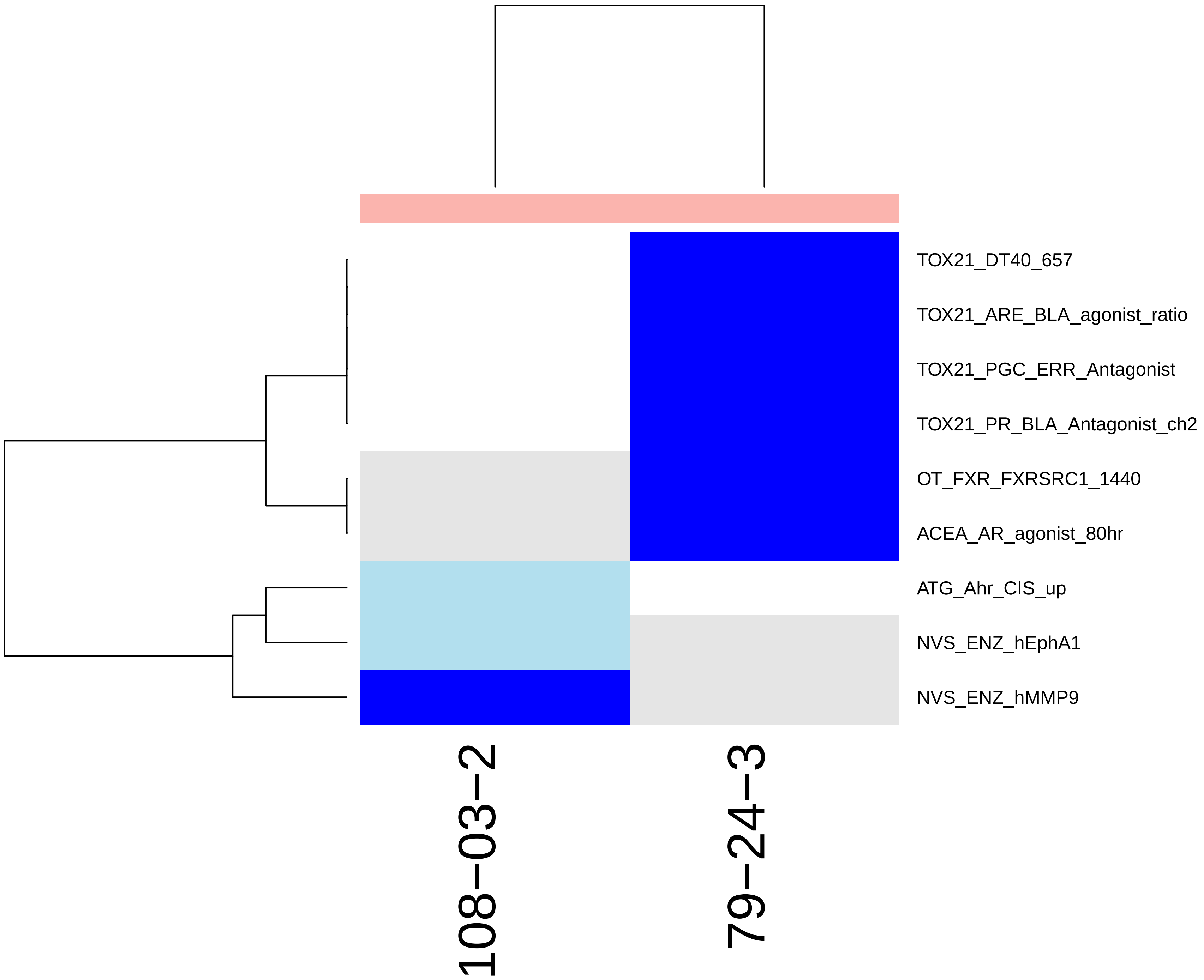

All Activity Heatmap for Cluster 456

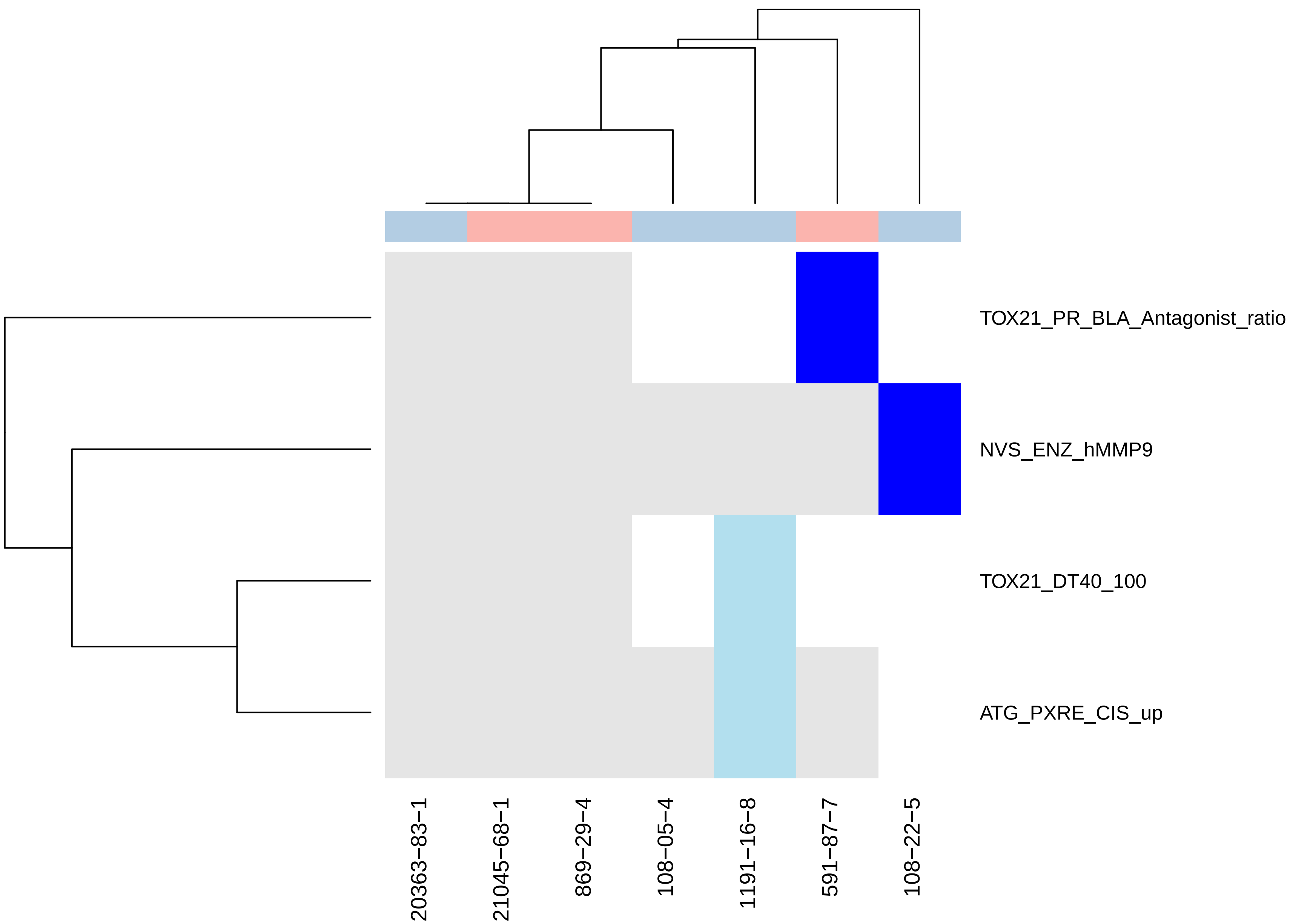

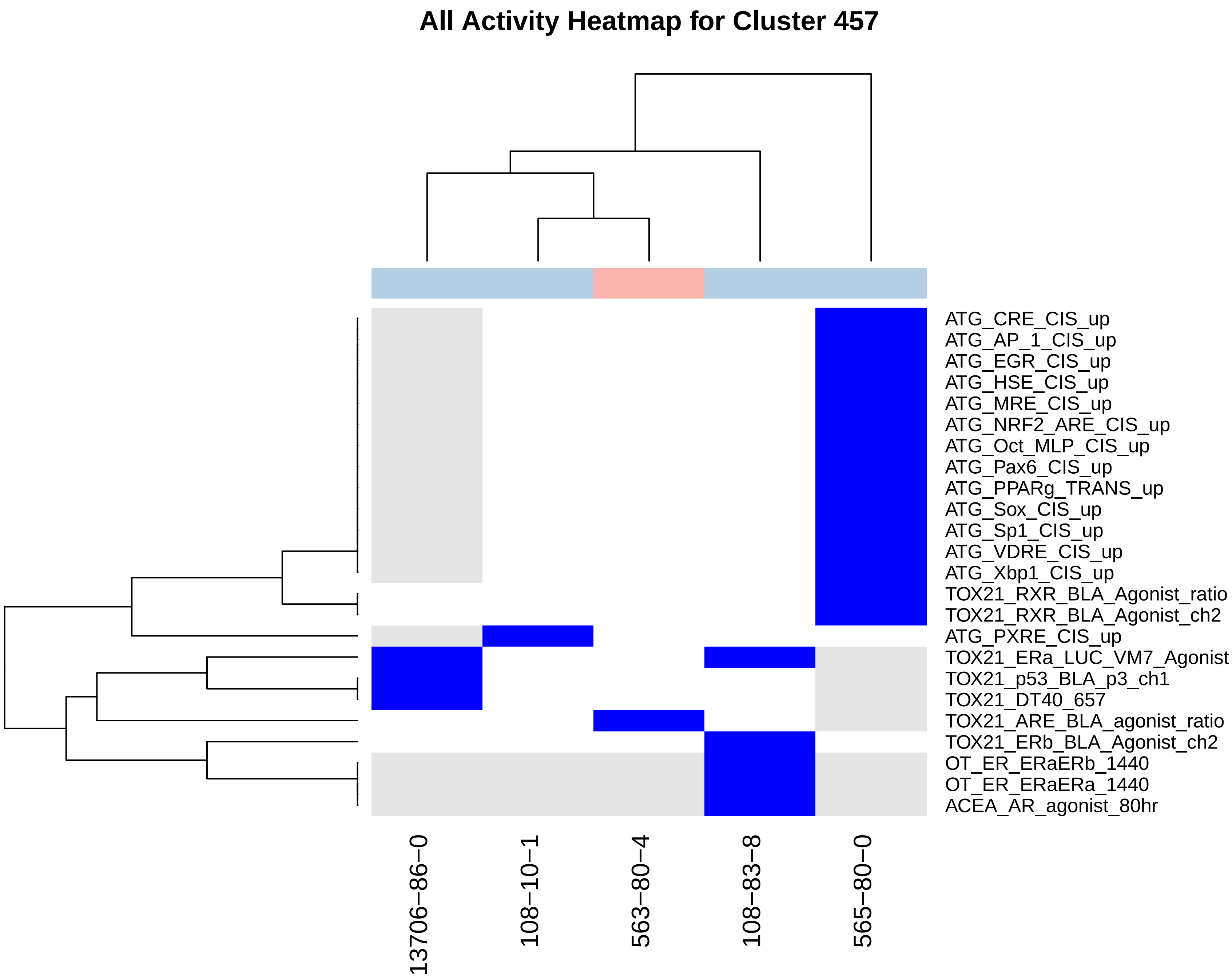
All Activity Heatmap for Cluster 457
ATG_CRE_CIS_up
ATG_AP_1_CIS_up
ATG_EGR_CIS_up
ATG_HSE_CIS_up
ATG_MRE_CIS_up
ATG_NRF2_ARE_CIS_up
ATG_Oct_MLP_CIS_up
ATG_Pax6_CIS_up
ATG_PPARg_TRANS_up
ATG_Sox_CIS_up
ATG_Sp1_CIS_up
ATG_VDRE_CIS_up
ATG_Xbp1_CIS_up
TOX21_RXR_BLA_Agonist_ratio
TOX21_RXR_BLA_Agonist_ch2
ATG_PXRE_CIS_up
TOX21_ERa_LUC_VM7_Agonist
TOX21_p53_BLA_p3_ch1
TOX21_DT40_657
TOX21_ARE_BLA_agonist_ratio
TOX21_ERb_BLA_Agonist_ch2
OT_ER_ERaERb_1440
OT_ER_ERaERa_1440
ACEA_AR_agonist_80hr
13706−86−0
108−10−1
563−80−4
108−83−8
565−80−0

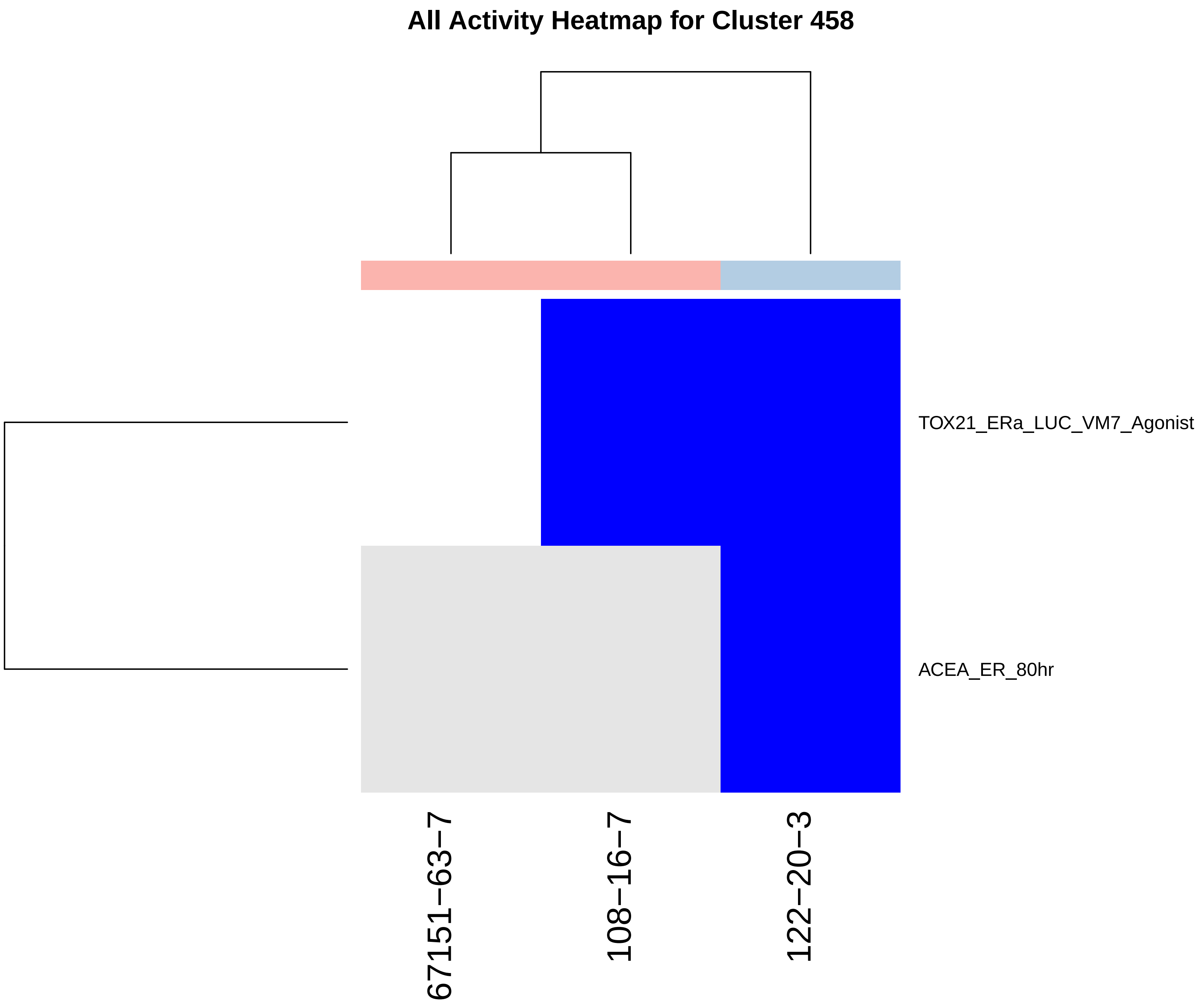
All Activity Heatmap for Cluster 458
TOX21_ERa_LUC_VM7_Agonist
ACEA_ER_80hr
67151−63−7
108−16−7
122−20−3

All Activity Heatmap for Cluster 459

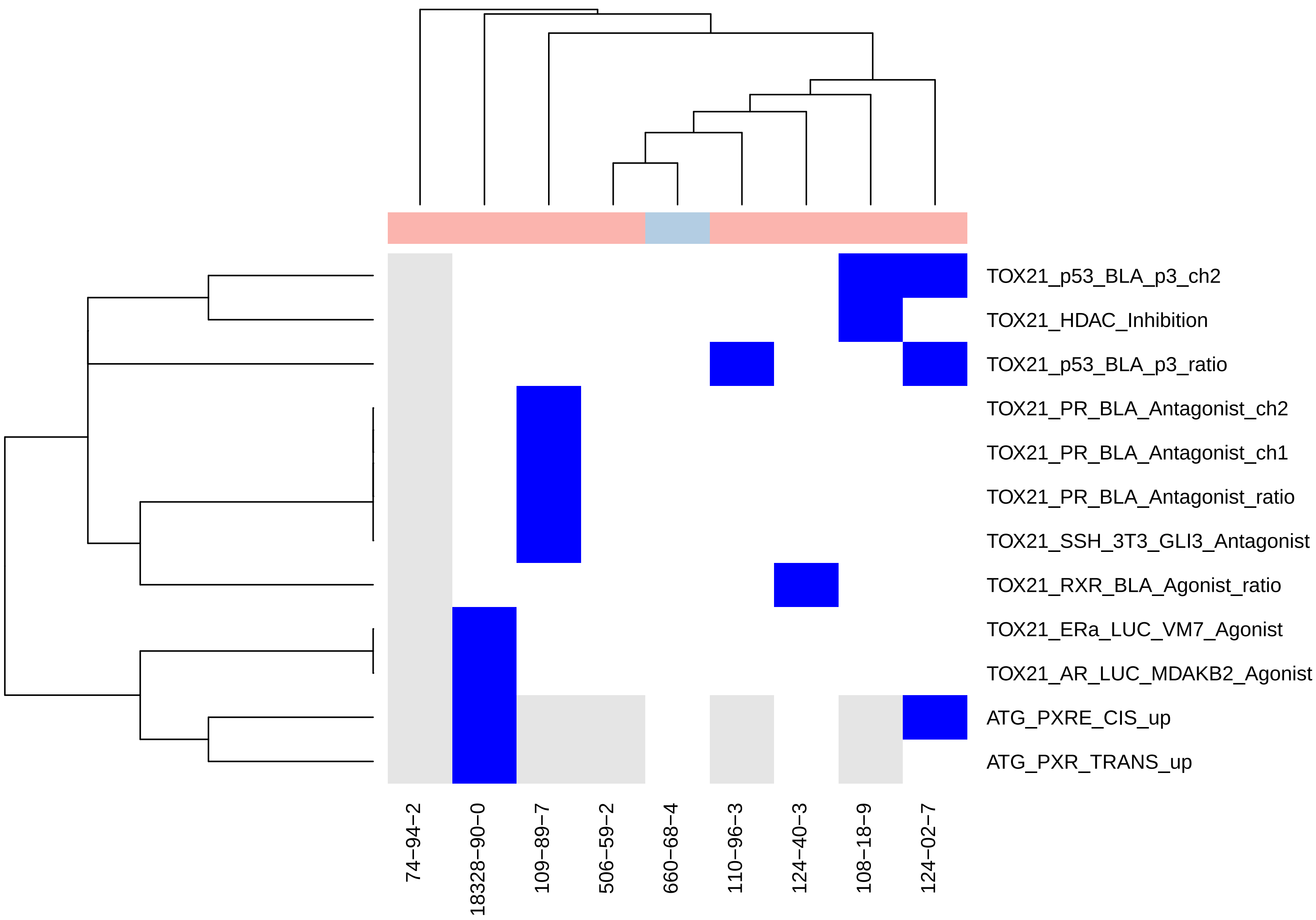

All Activity Heatmap for Cluster 461

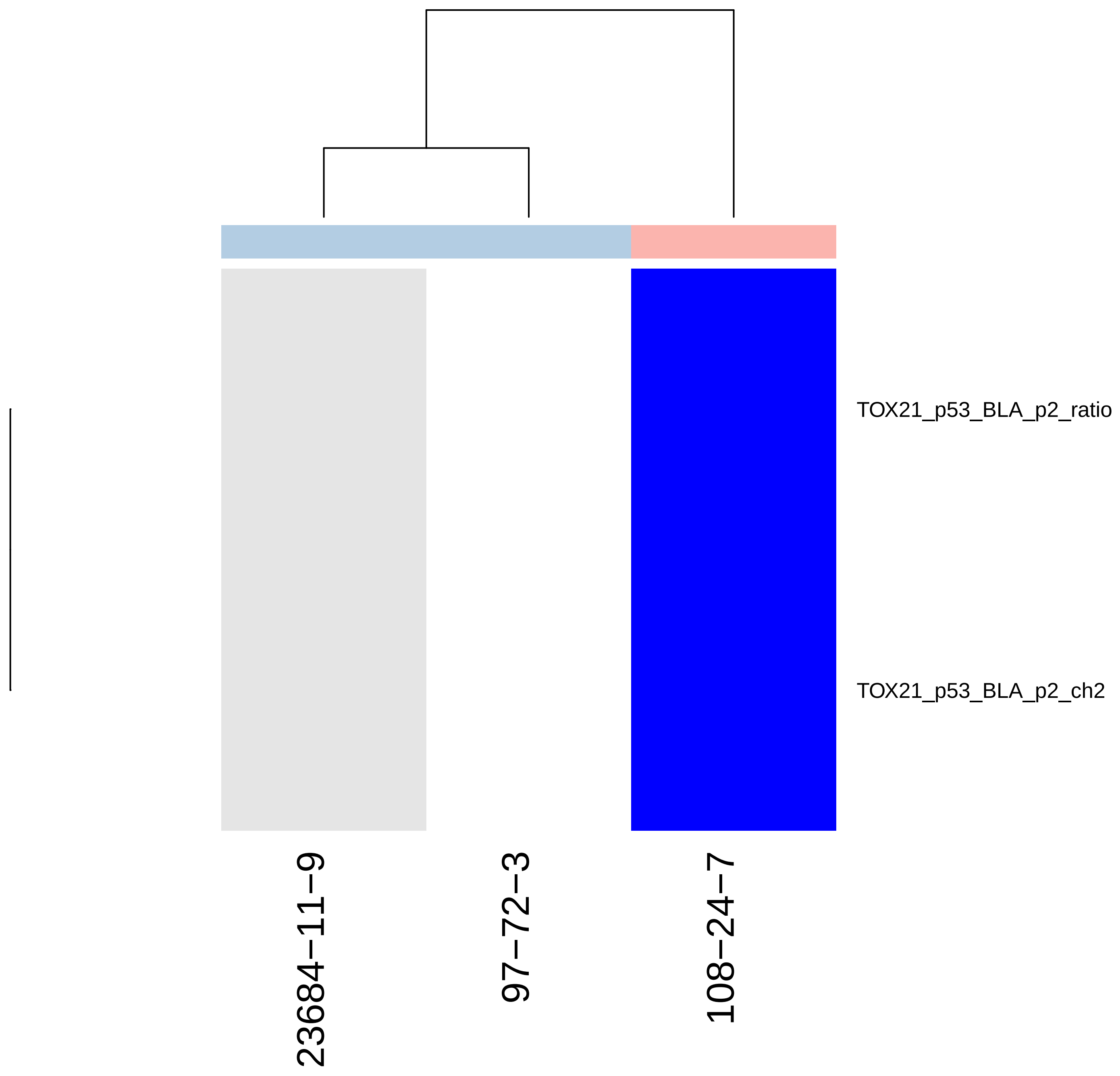

All Activity Heatmap for Cluster 462

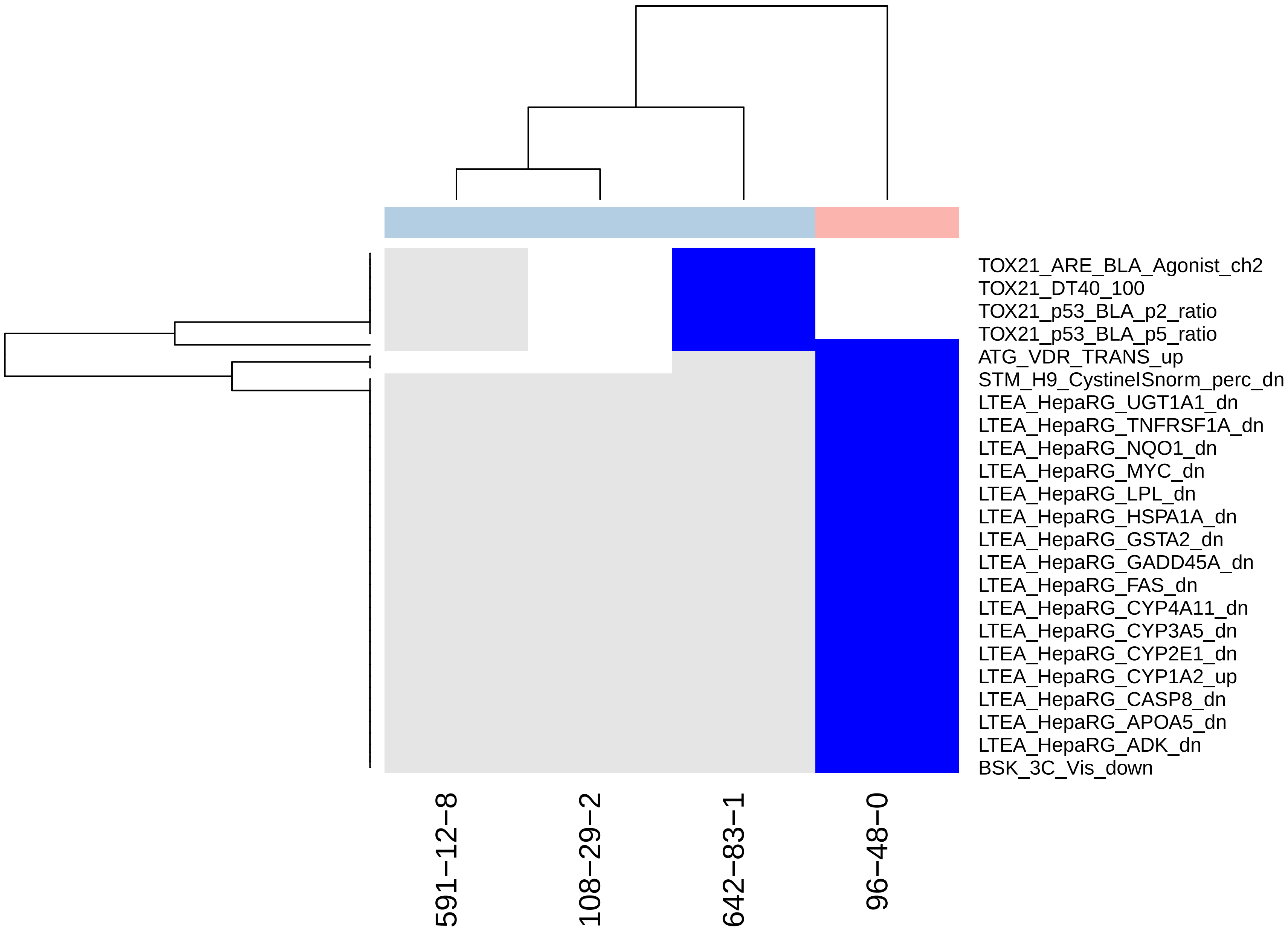

All Activity Heatmap for Cluster 463

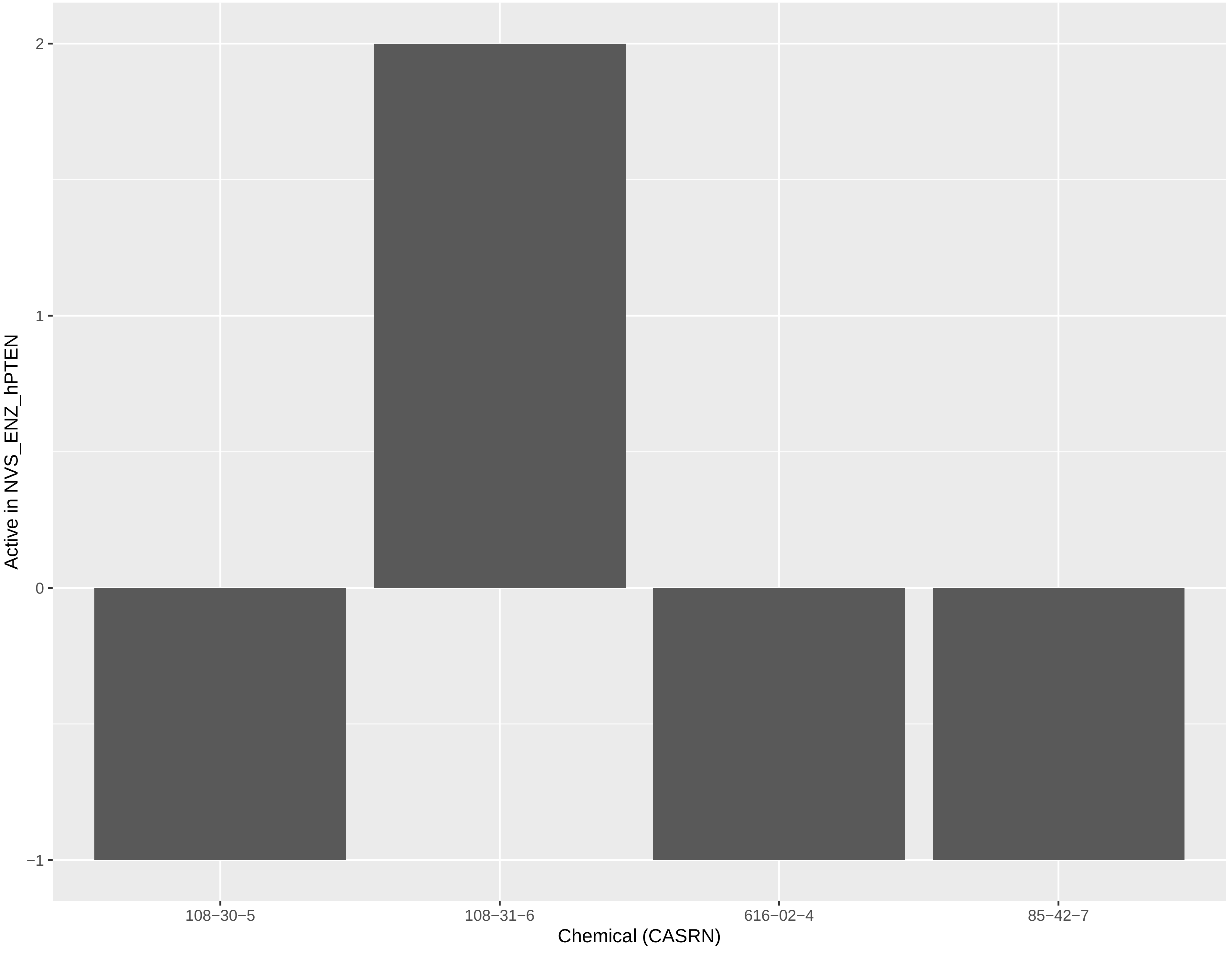

All Activity Heatmap for Cluster 464

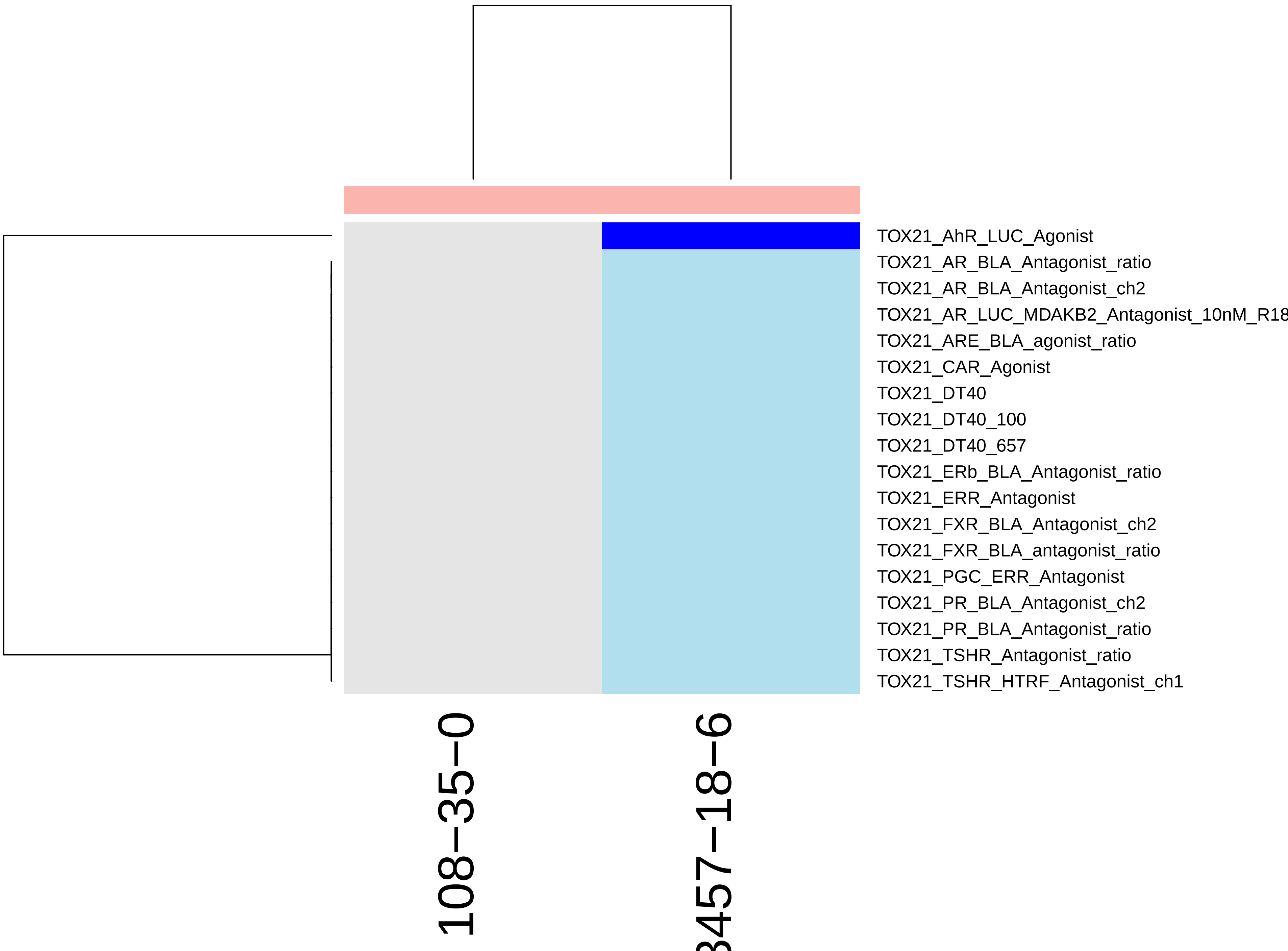

All Activity Heatmap for Cluster 465

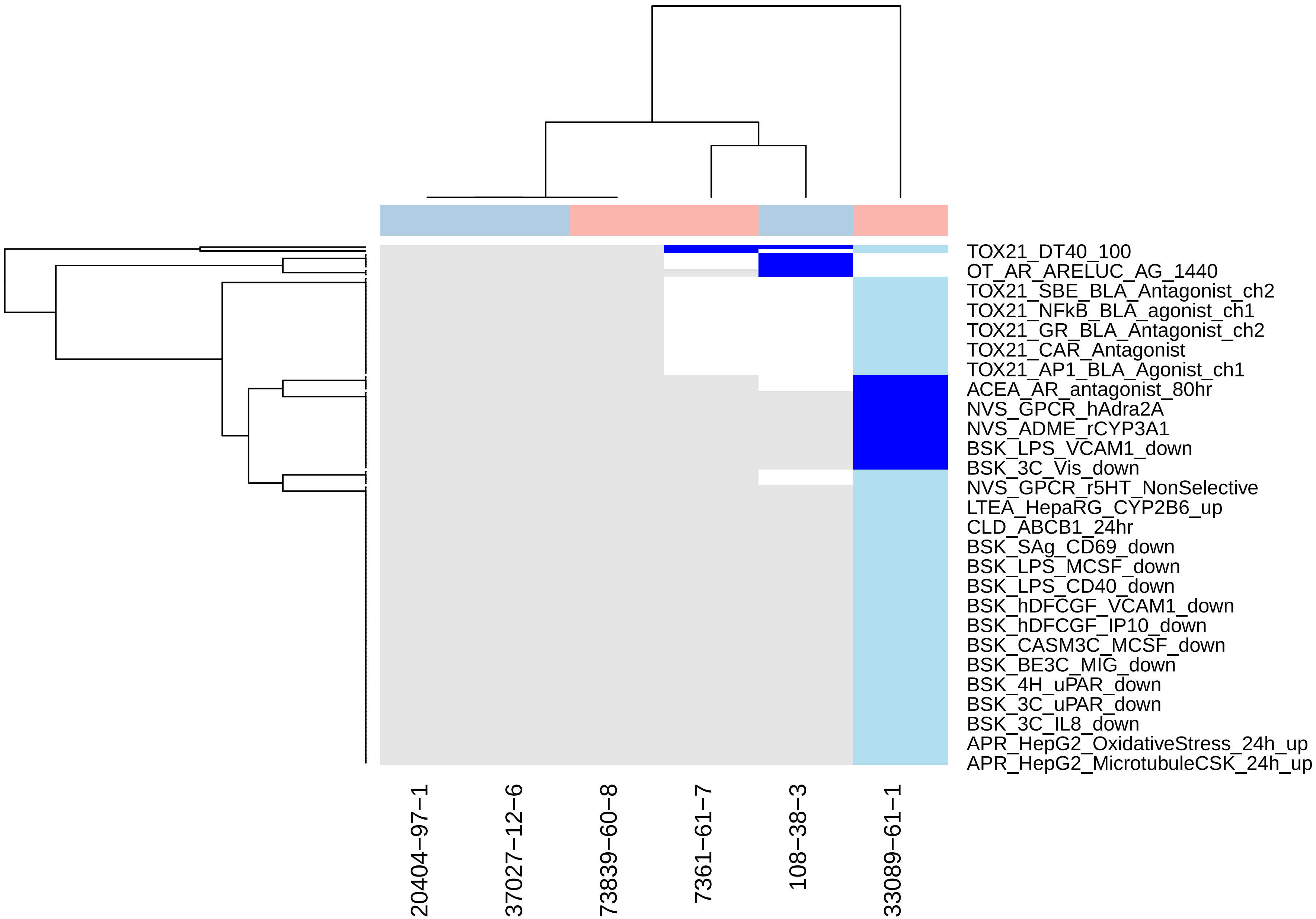

All Activity Heatmap for Cluster 466

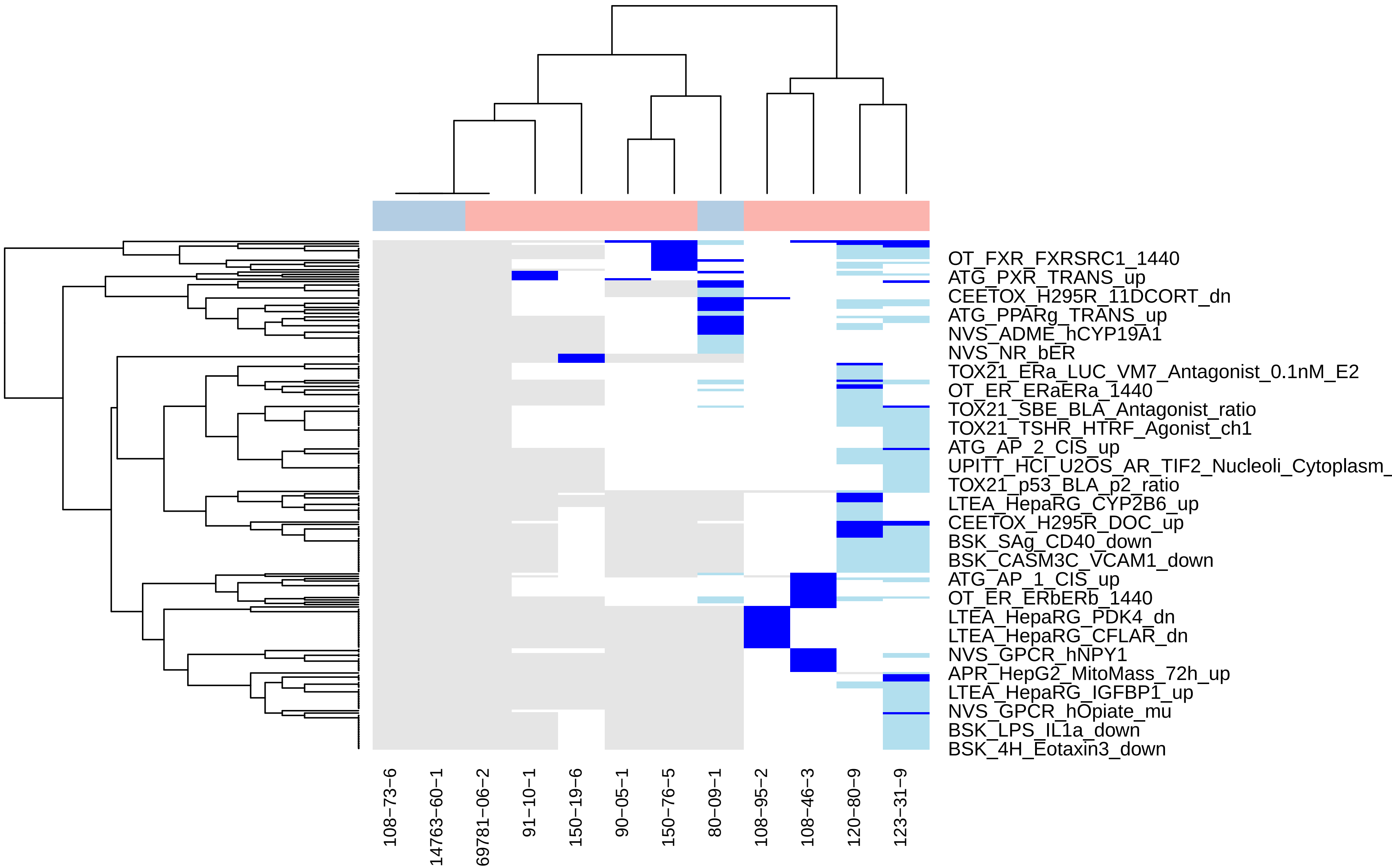

All Activity Heatmap for Cluster 467

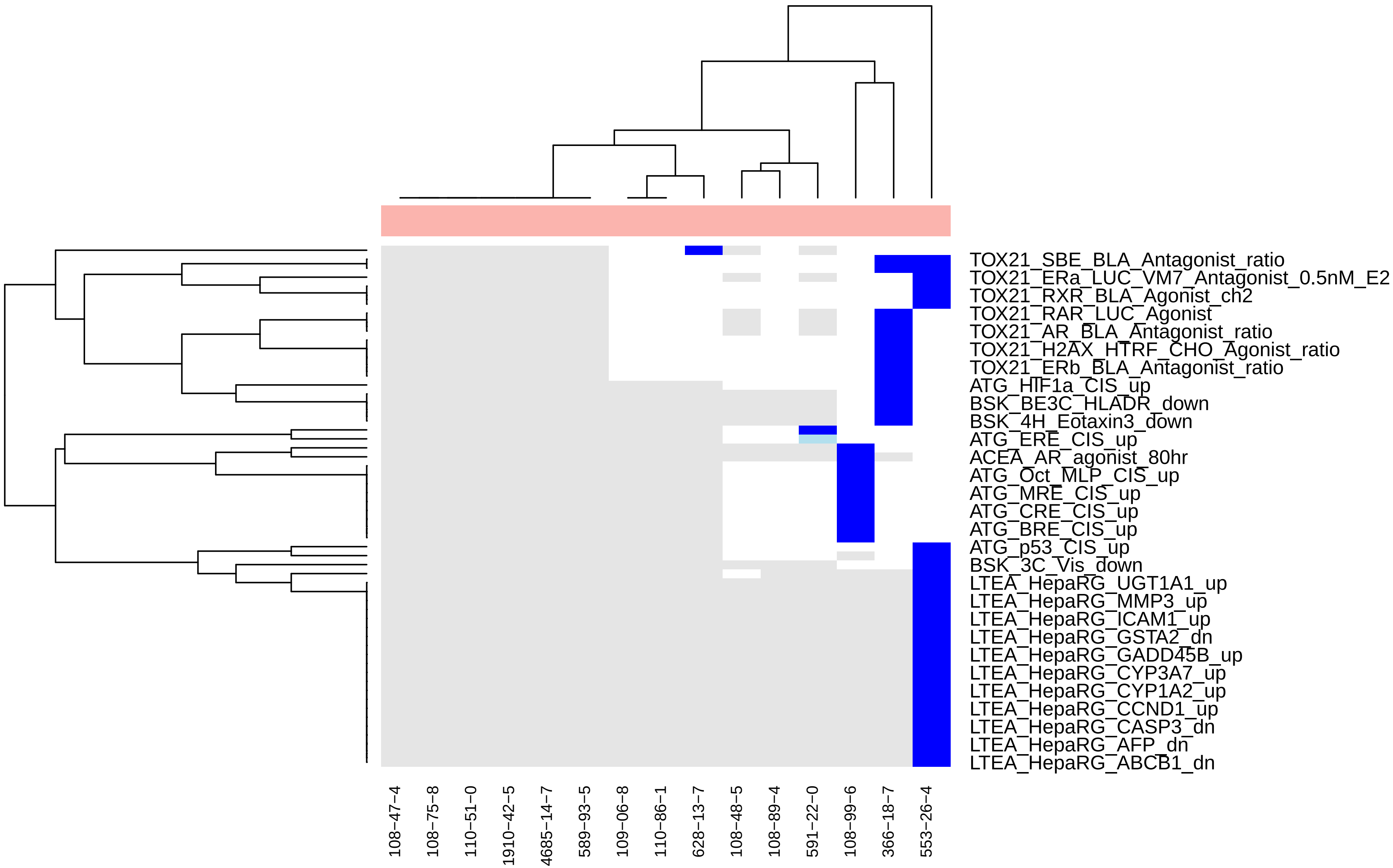

All Activity Heatmap for Cluster 468

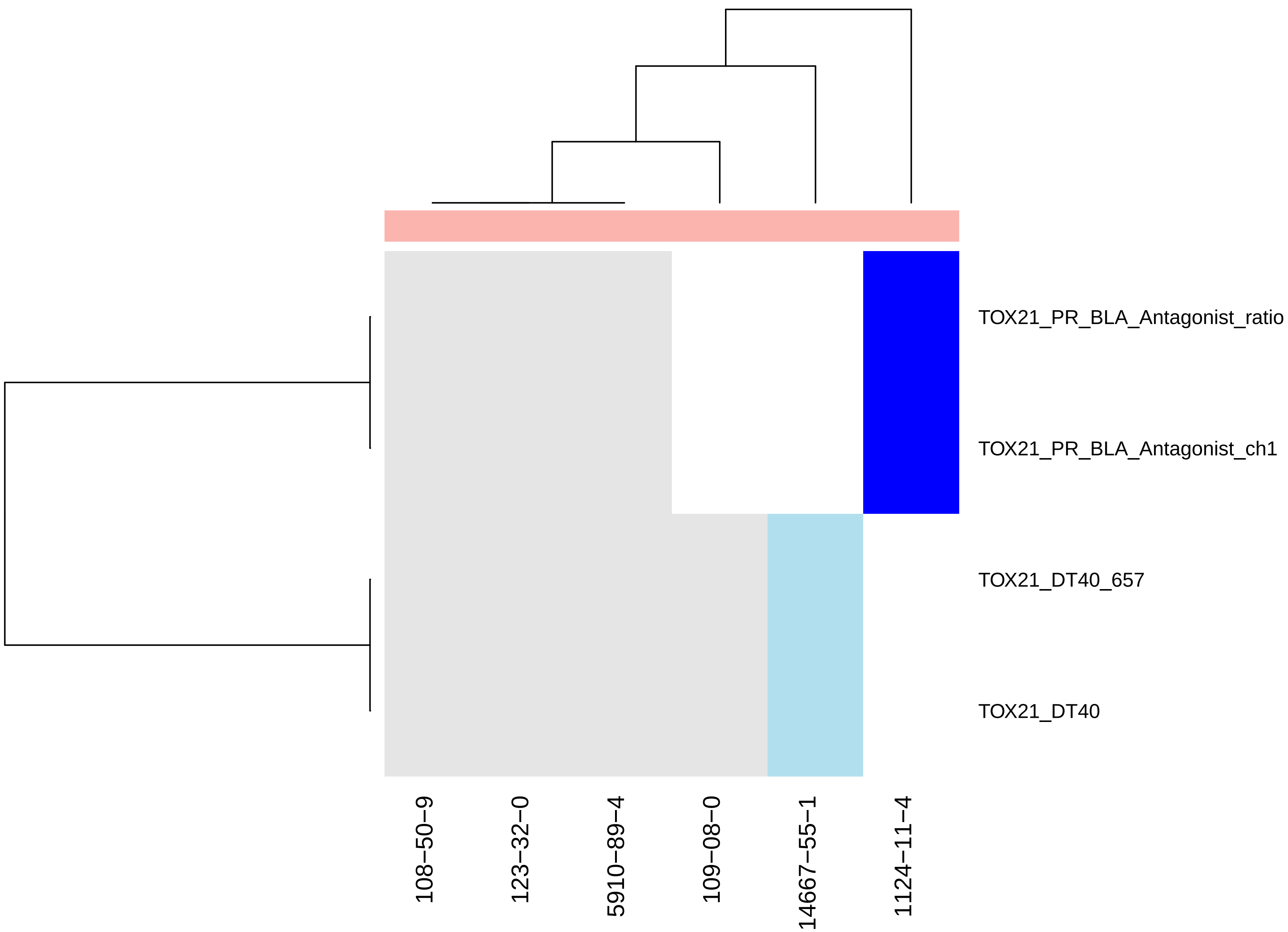

All Activity Heatmap for Cluster 469

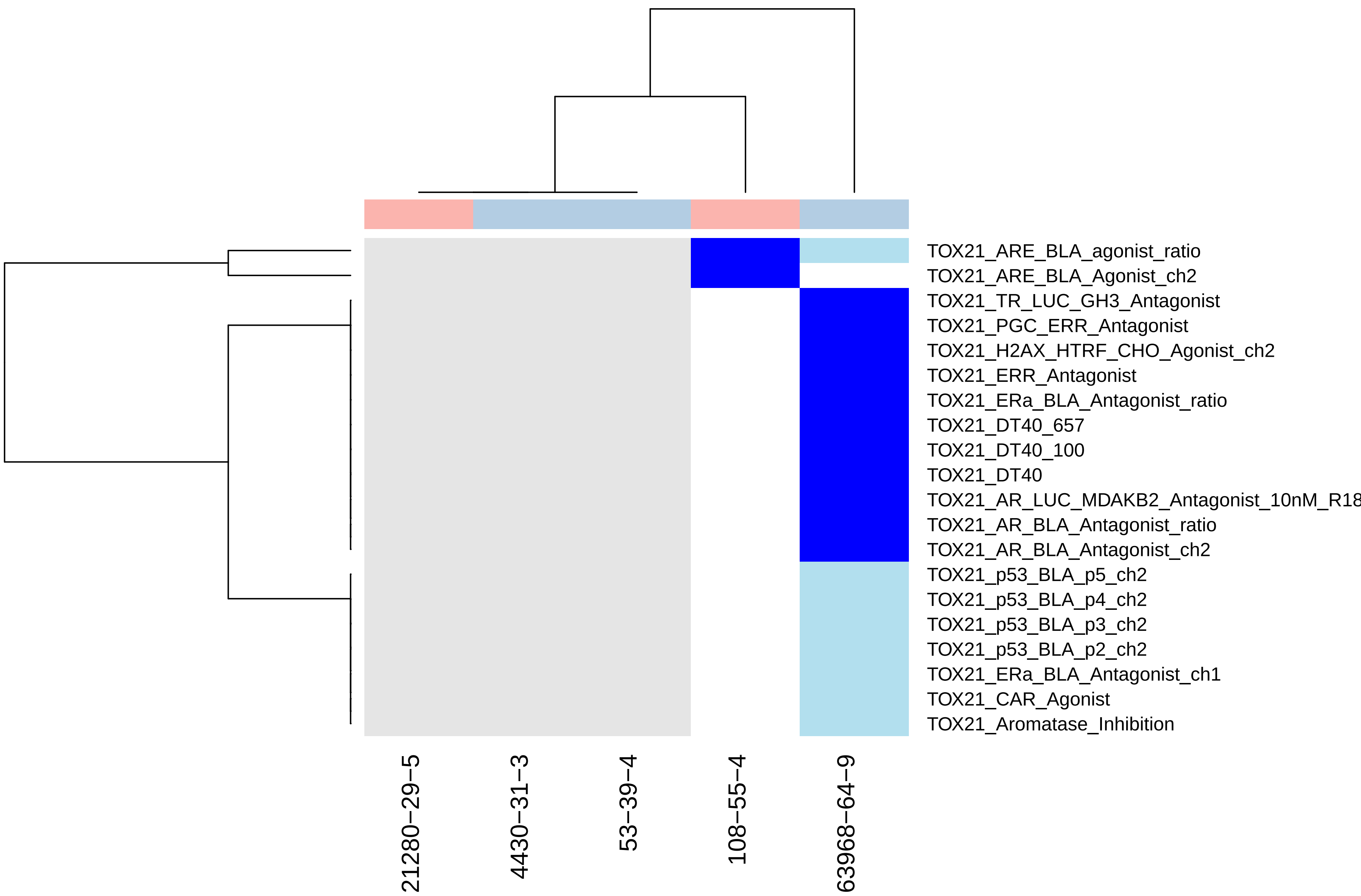

All Activity Heatmap for Cluster 470

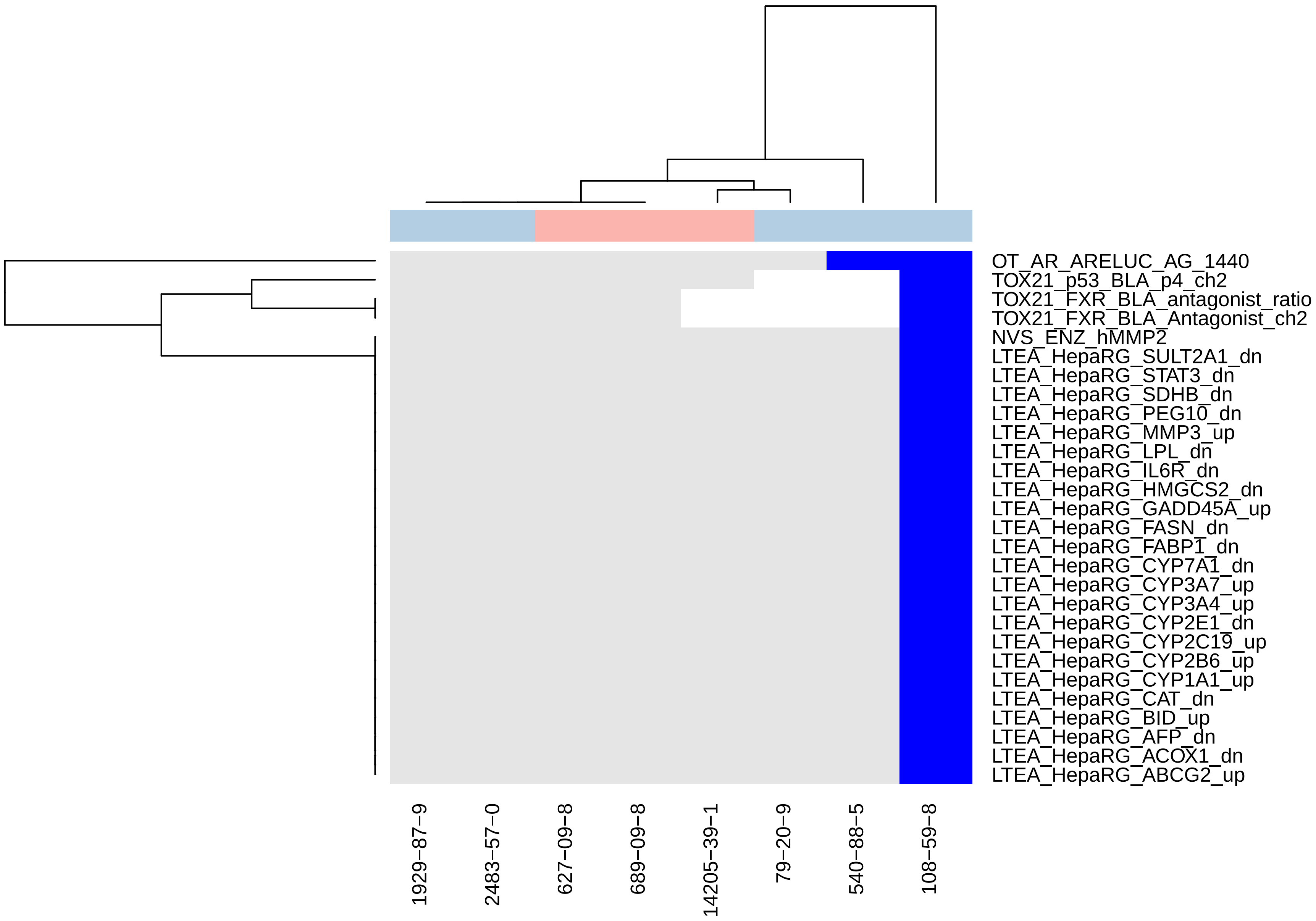

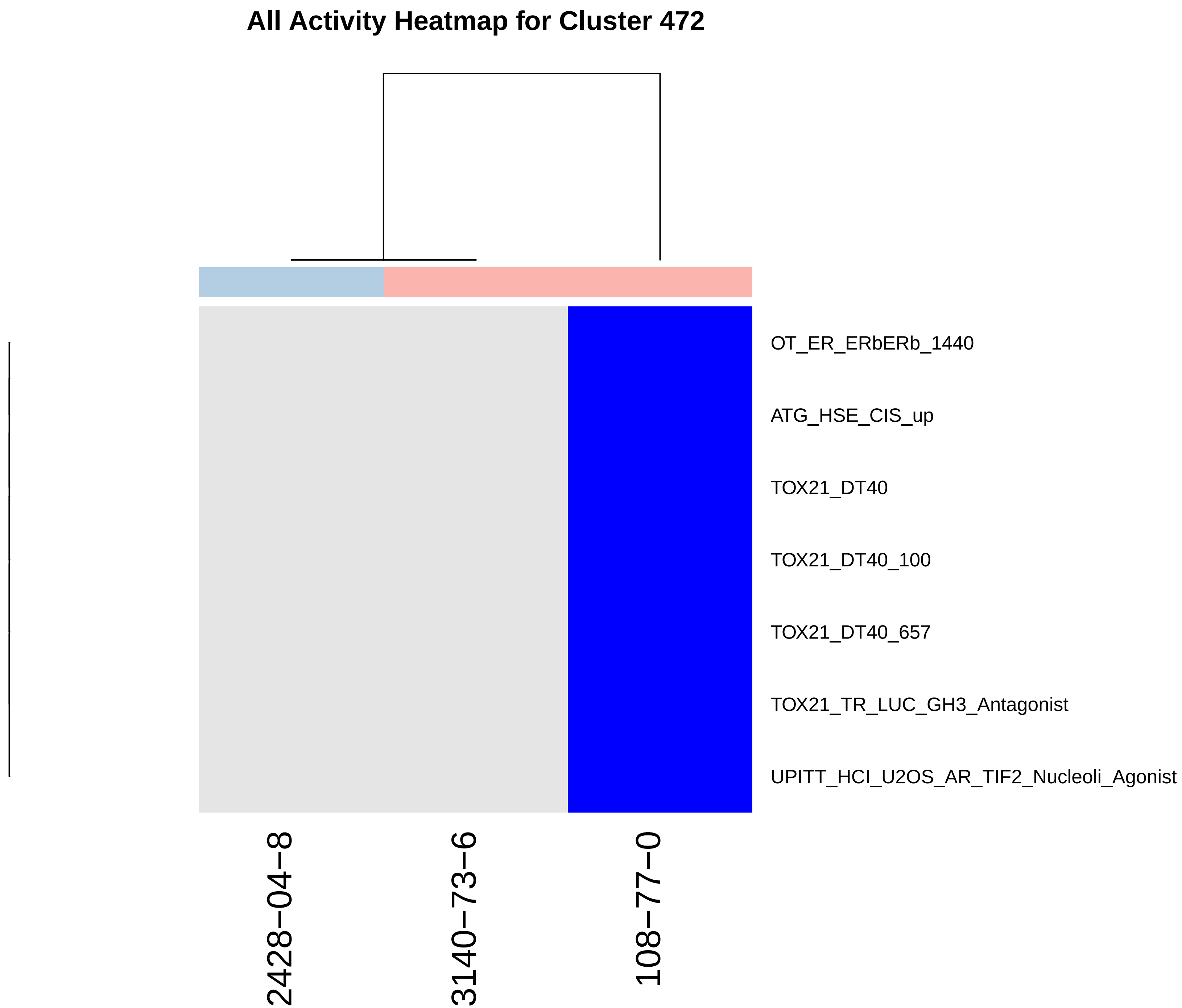
All Activity Heatmap for Cluster 472
OT_ER_ERbERb_1440
ATG_HSE_CIS_up
TOX21_DT40
TOX21_DT40_100
TOX21_DT40_657
TOX21_TR_LUC_GH3_Antagonist
UPITT_HCI_U2OS_AR_TIF2_Nucleoli_Agonist
2428−04−8
3140−73−6
108−77−0

All Activity Heatmap for Cluster 473

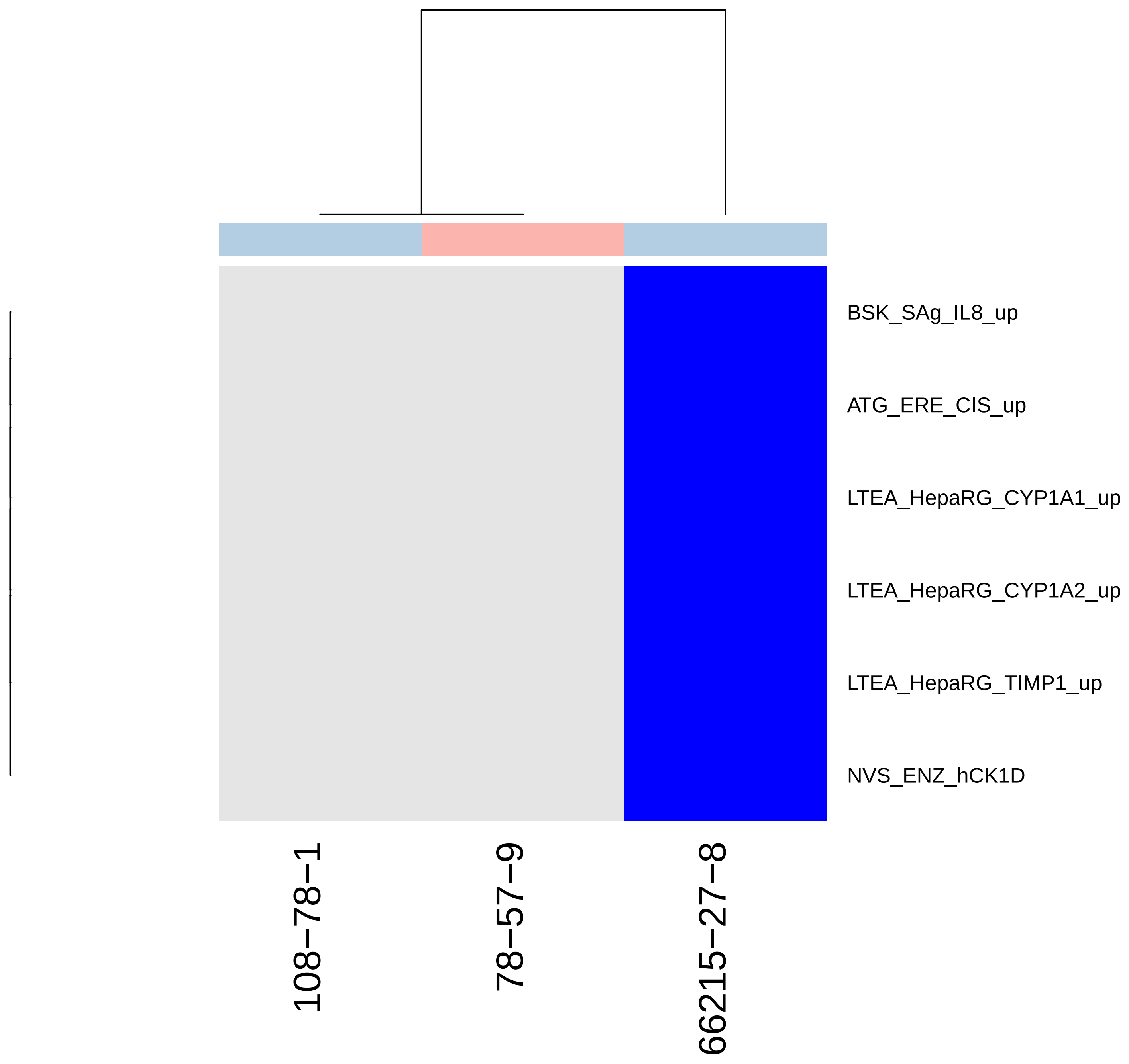

All Activity Heatmap for Cluster 474

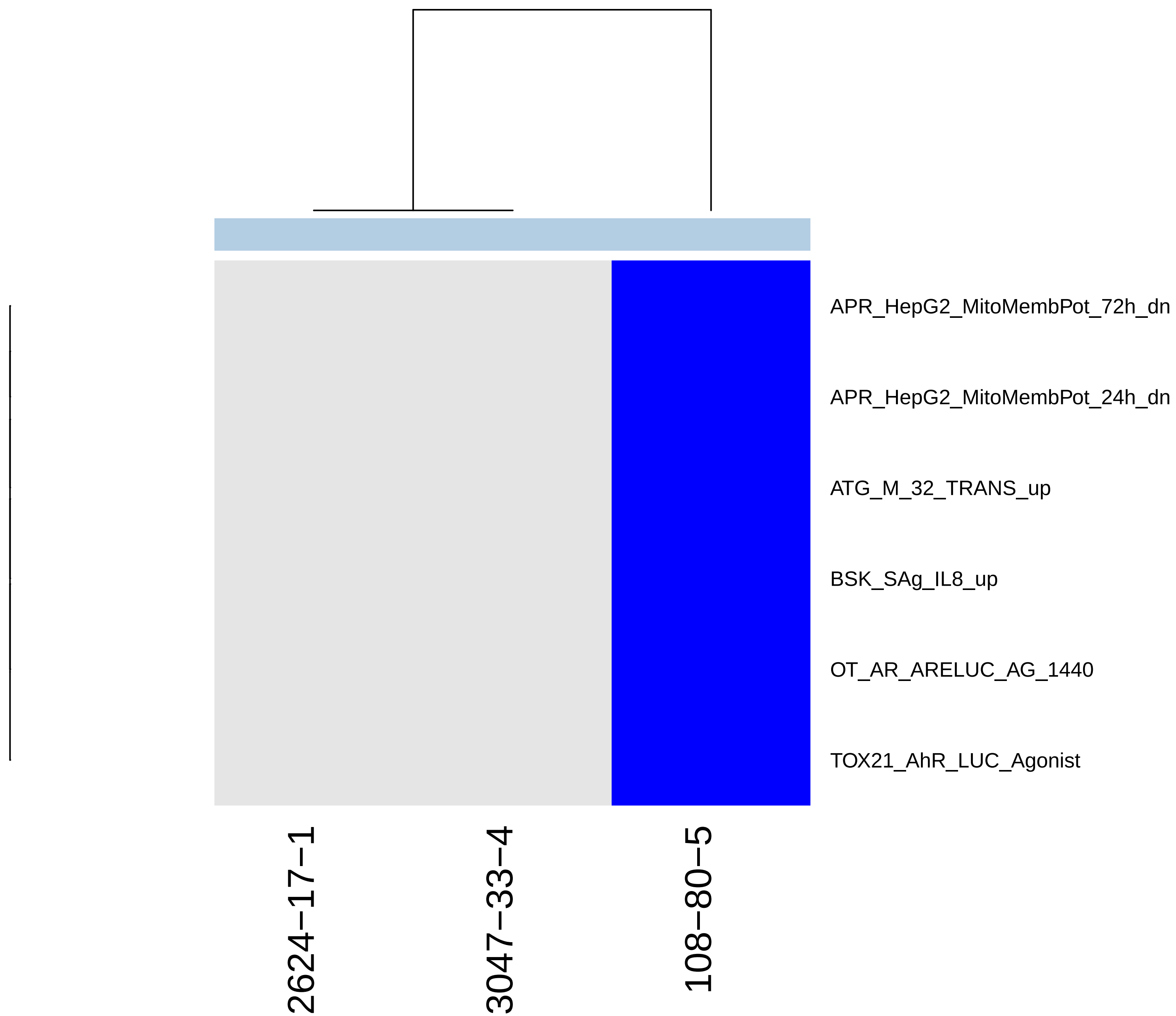

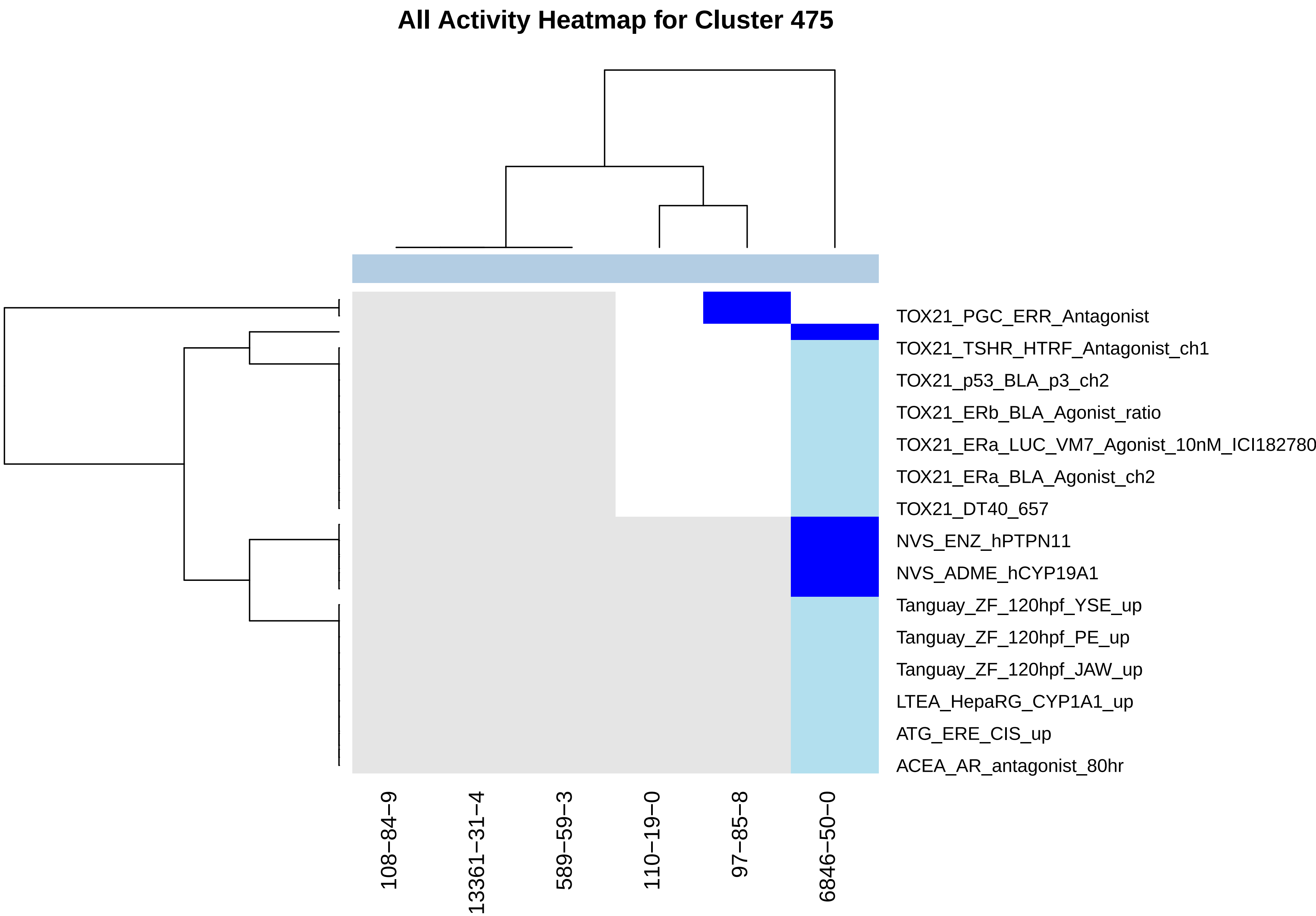
All Activity Heatmap for Cluster 475
TOX21_PGC_ERR_Antagonist
TOX21_TSHR_HTRF_Antagonist_ch1
TOX21_p53_BLA_p3_ch2
TOX21_ERb_BLA_Agonist_ratio
TOX21_ERa_LUC_VM7_Agonist_10nM_ICI182780
TOX21_ERa_BLA_Agonist_ch2
TOX21_DT40_657
NVS_ENZ_hPTPN11
NVS_ADME_hCYP19A1
Tanguay_ZF_120hpf_YSE_up
Tanguay_ZF_120hpf_PE_up
Tanguay_ZF_120hpf_JAW_up
LTEA_HepaRG_CYP1A1_up
ATG_ERE_CIS_up
ACEA_AR_antagonist_80hr
108−84−9
13361−31−4
589−59−3
110−19−0
97−85−8
6846−50−0

All Activity Heatmap for Cluster 476

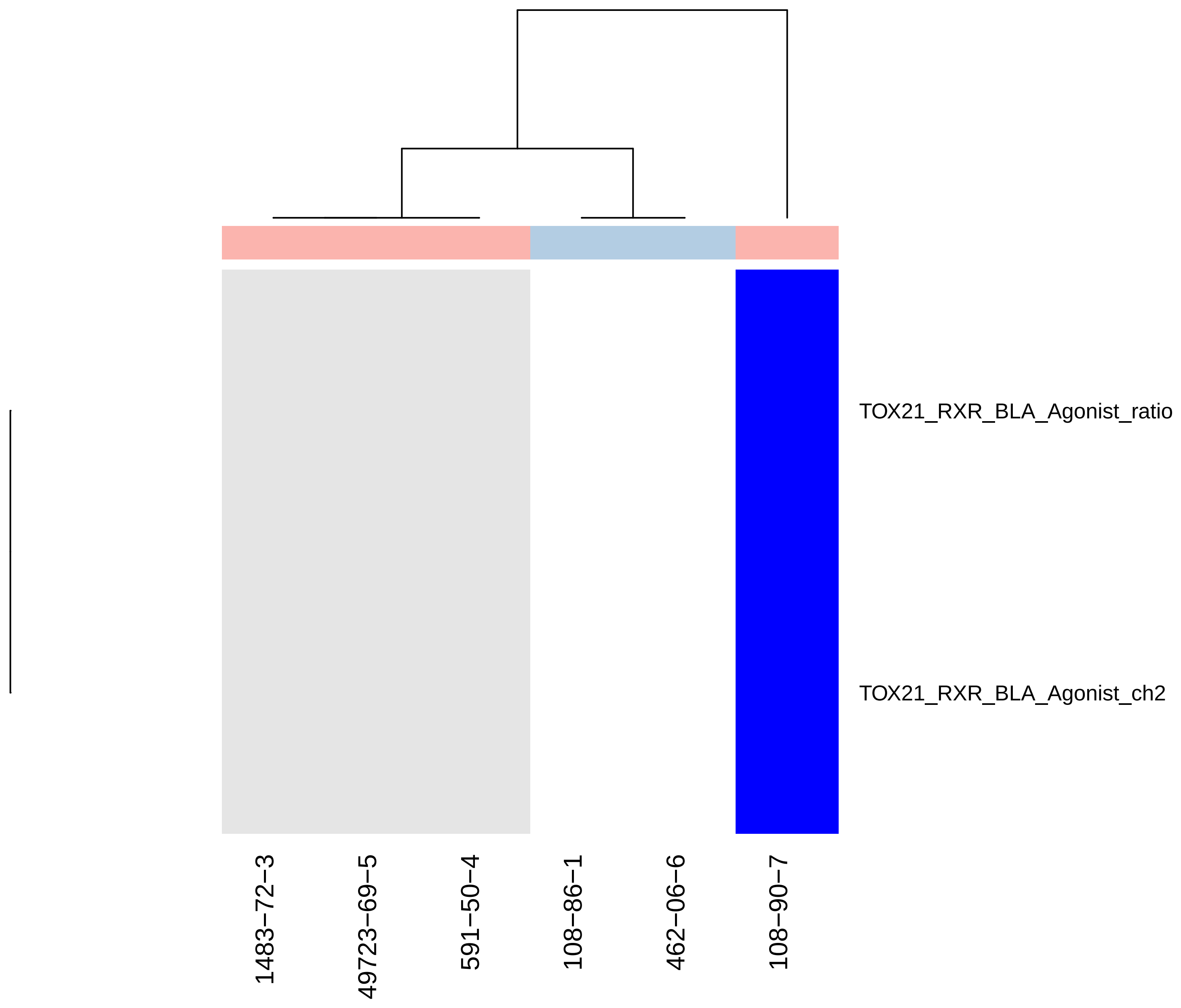

All Activity Heatmap for Cluster 477

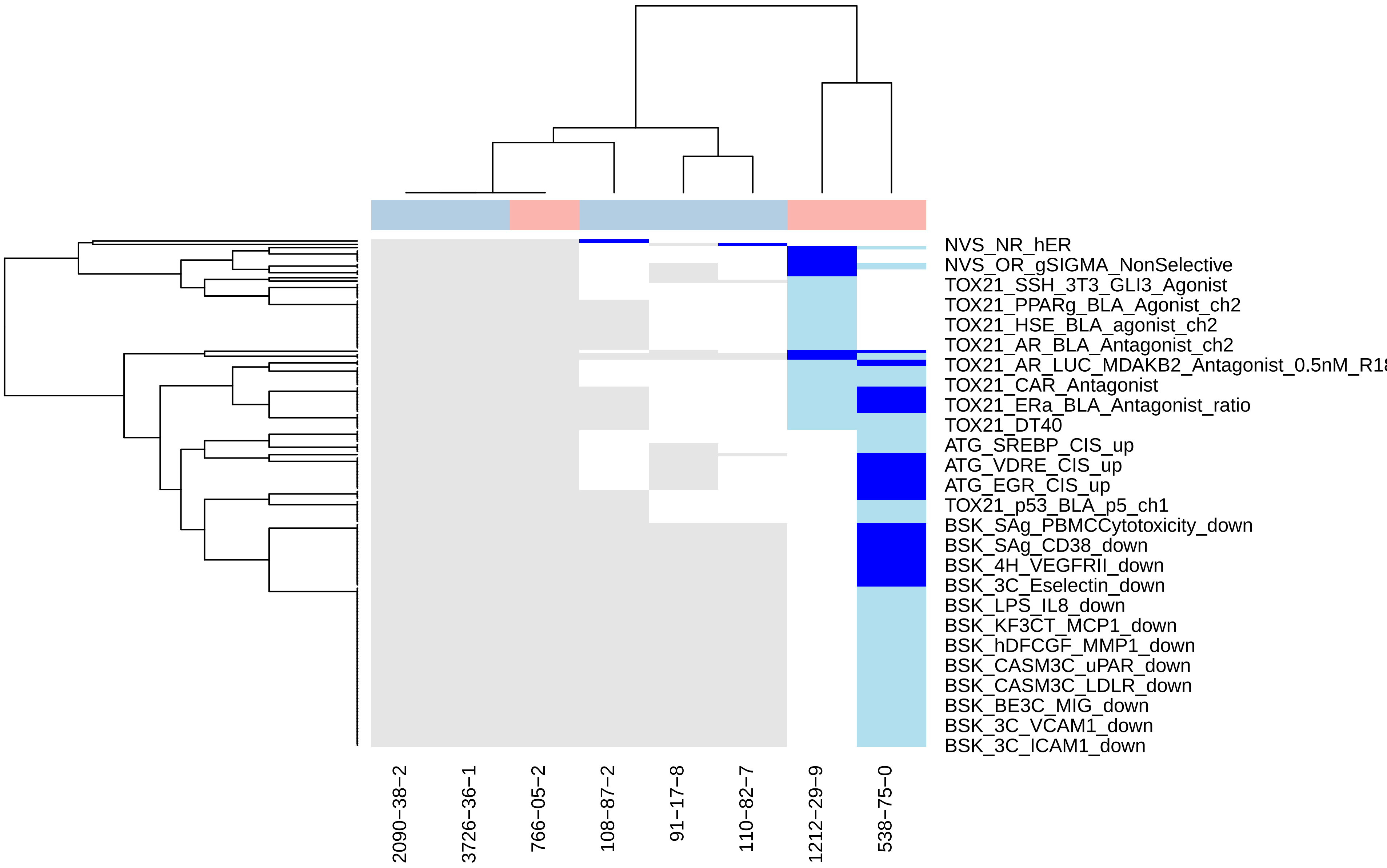

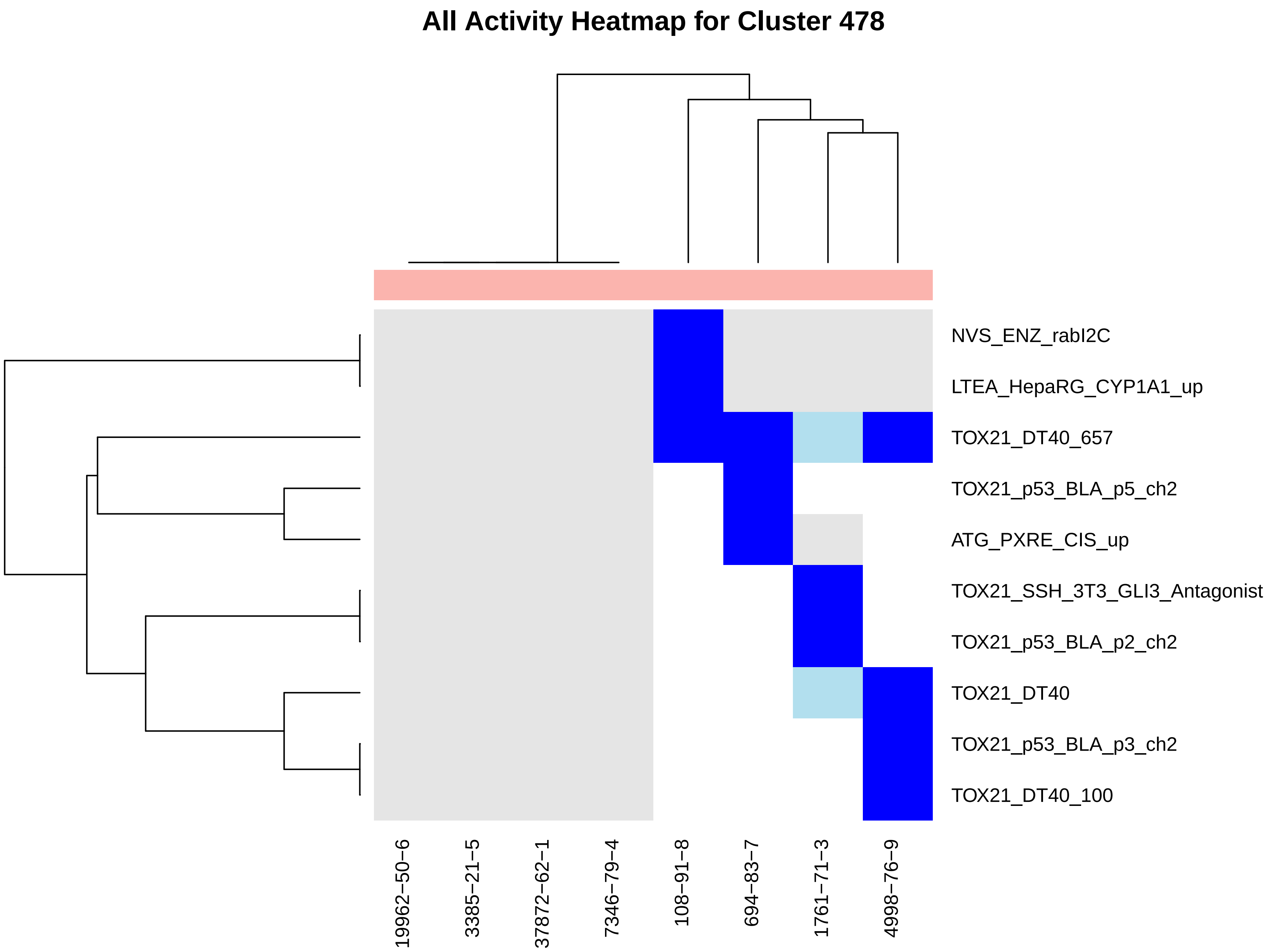
All Activity Heatmap for Cluster 478
NVS_ENZ_rabI2C
LTEA_HepaRG_CYP1A1_up
TOX21_DT40_657
TOX21_p53_BLA_p5_ch2
ATG_PXRE_CIS_up
TOX21_SSH_3T3_GLI3_Antagonist
TOX21_p53_BLA_p2_ch2
TOX21_DT40
TOX21_p53_BLA_p3_ch2
TOX21_DT40_100
19962−50−6
3385−21−5
37872−62−1
7346−79−4
108−91−8
694−83−7
1761−71−3
4998−76−9

All Activity Heatmap for Cluster 479

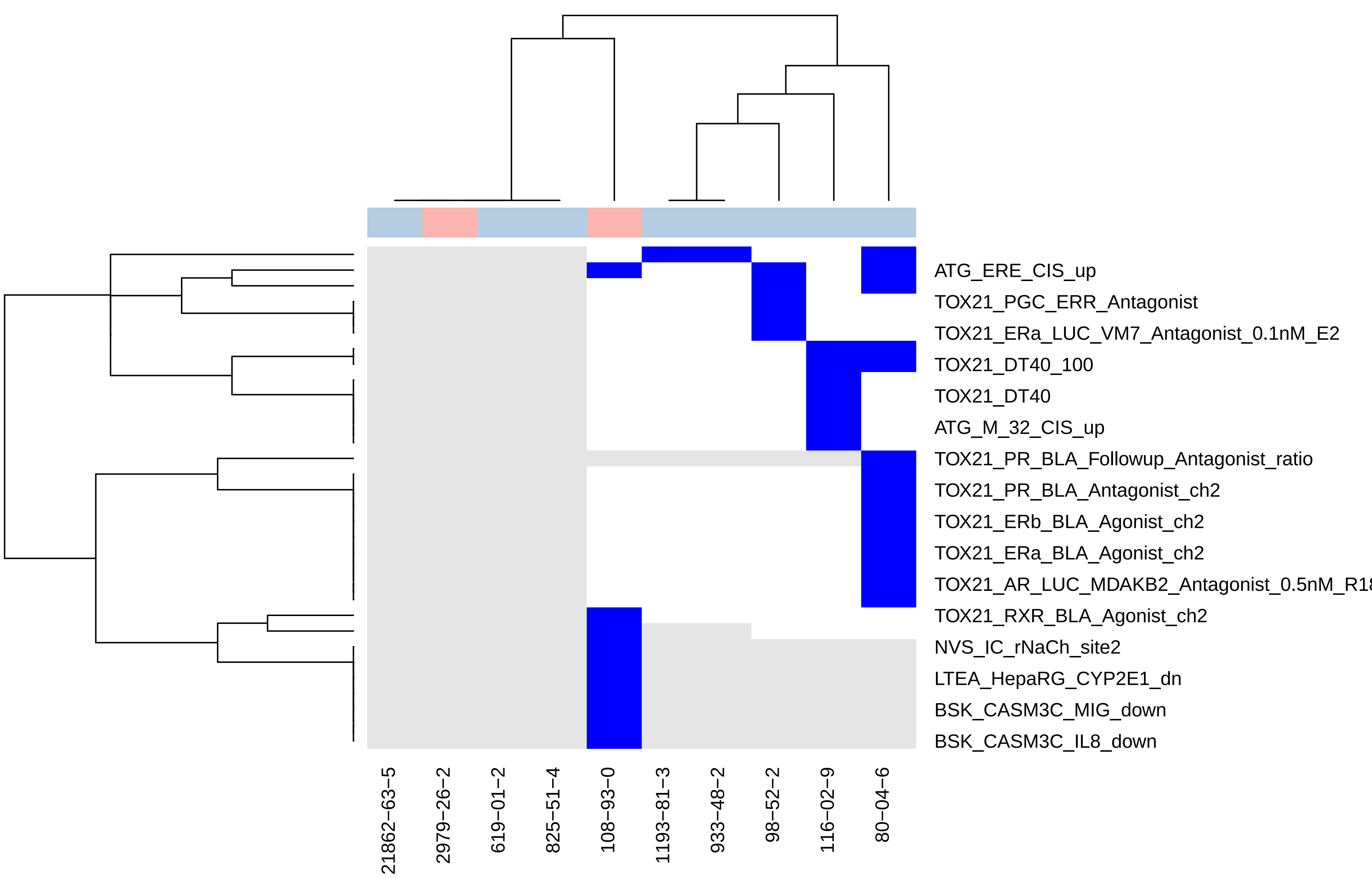

All Activity Heatmap for Cluster 480

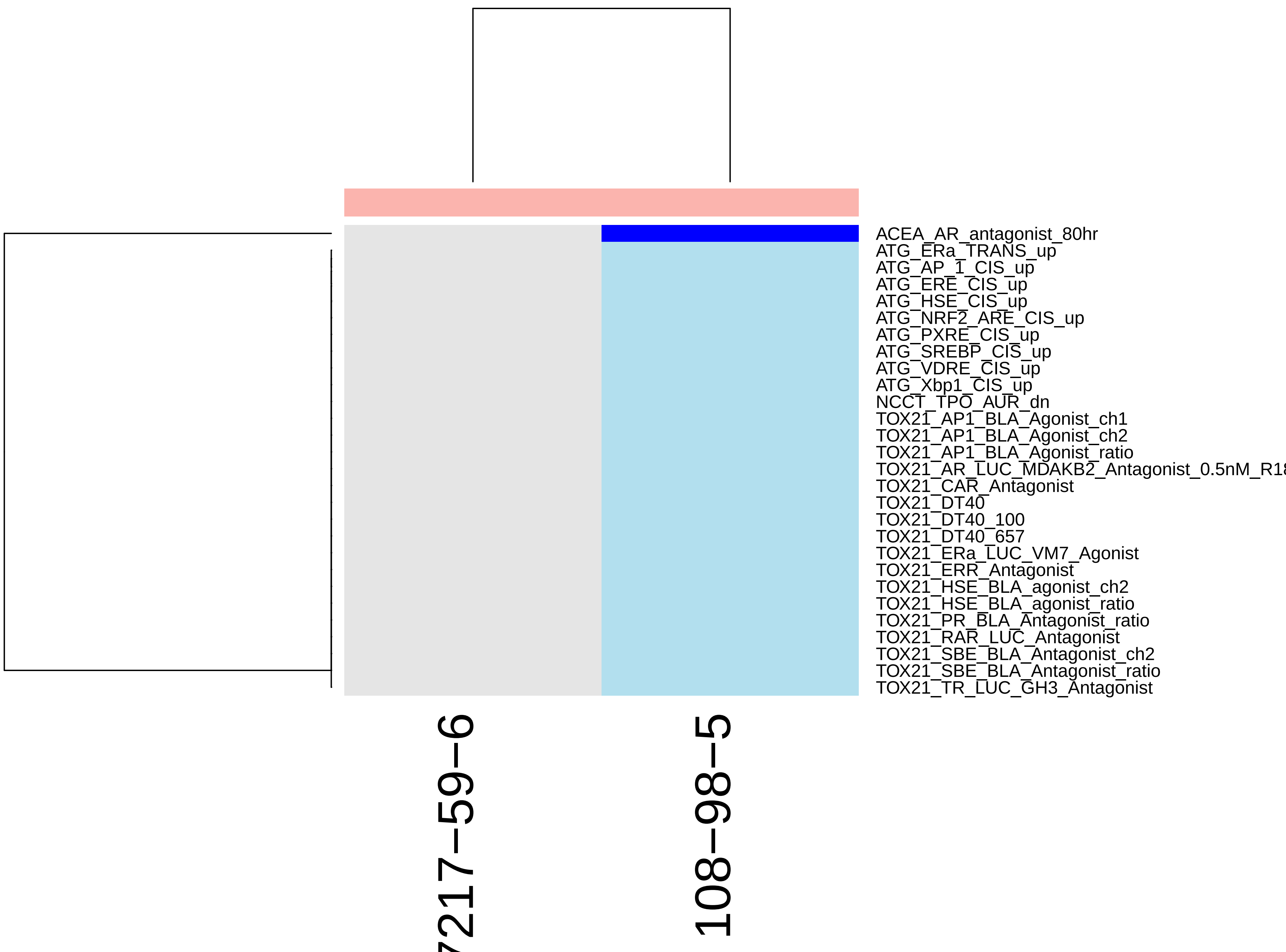

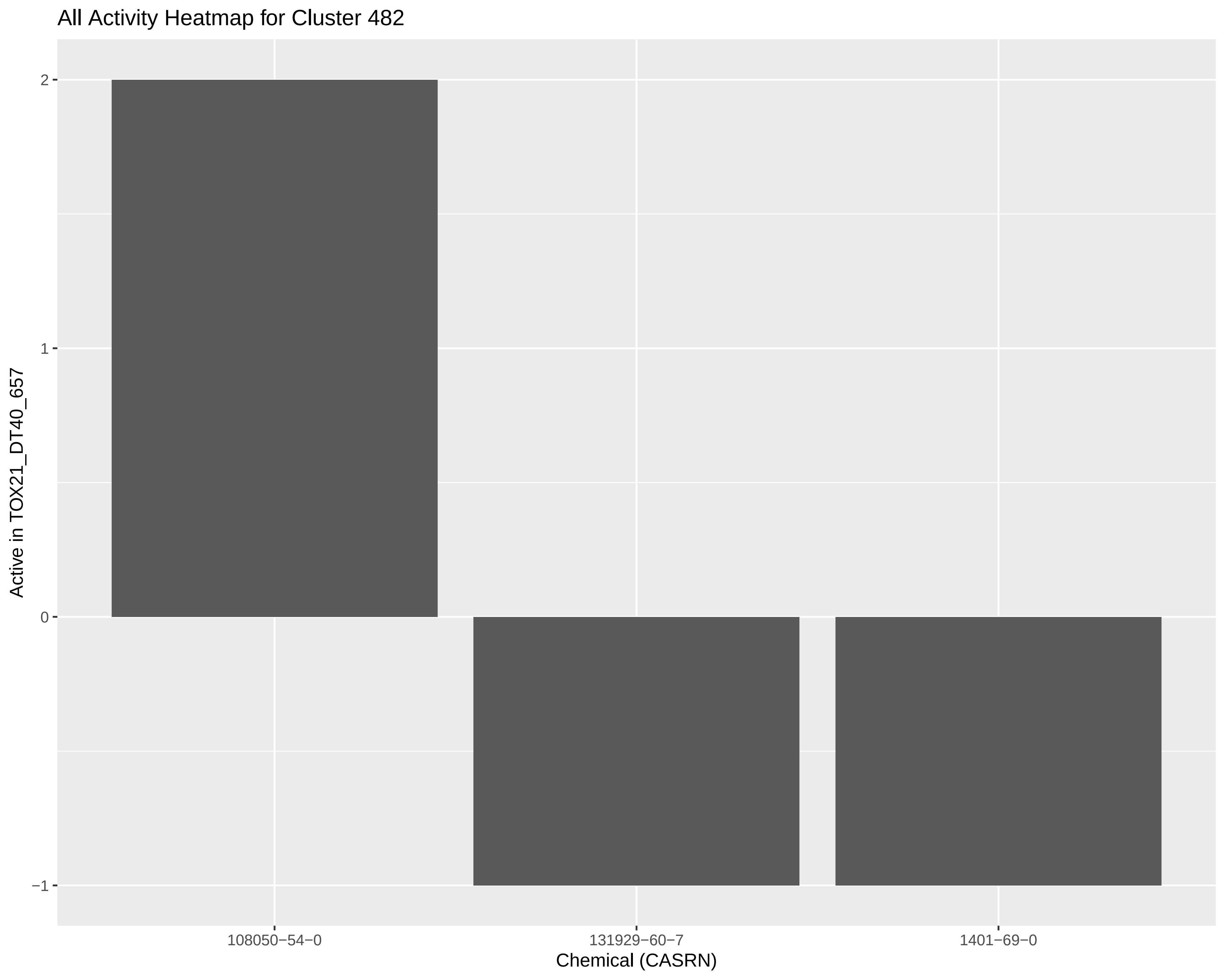

All Activity Heatmap for Cluster 482
Active in TOX21_DT40_657
2
1
0
−1
108050−54−0
131929−60−7
1401−69−0
Chemical (CASRN)

All Activity Heatmap for Cluster 483

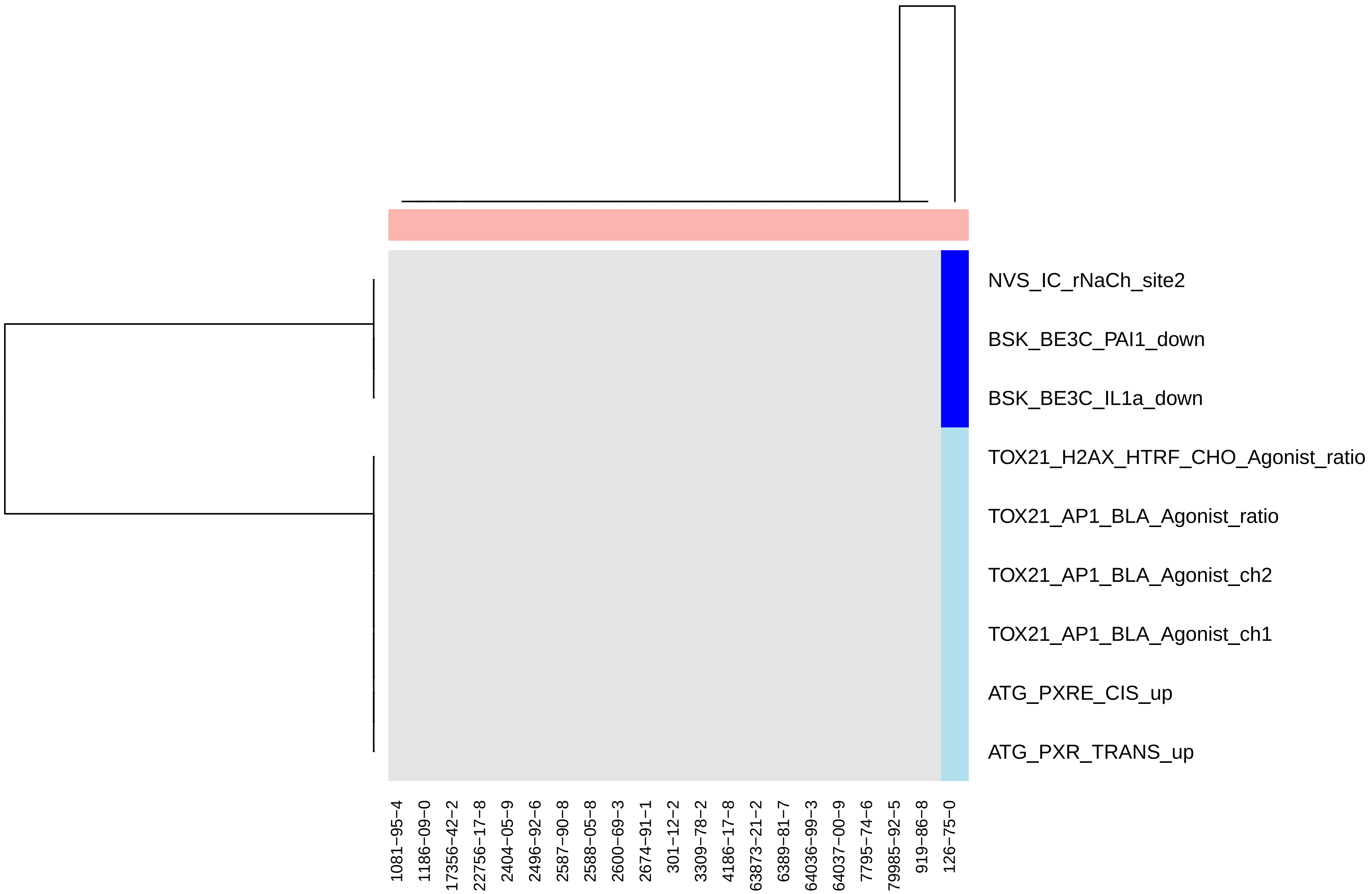

All Activity Heatmap for Cluster 485

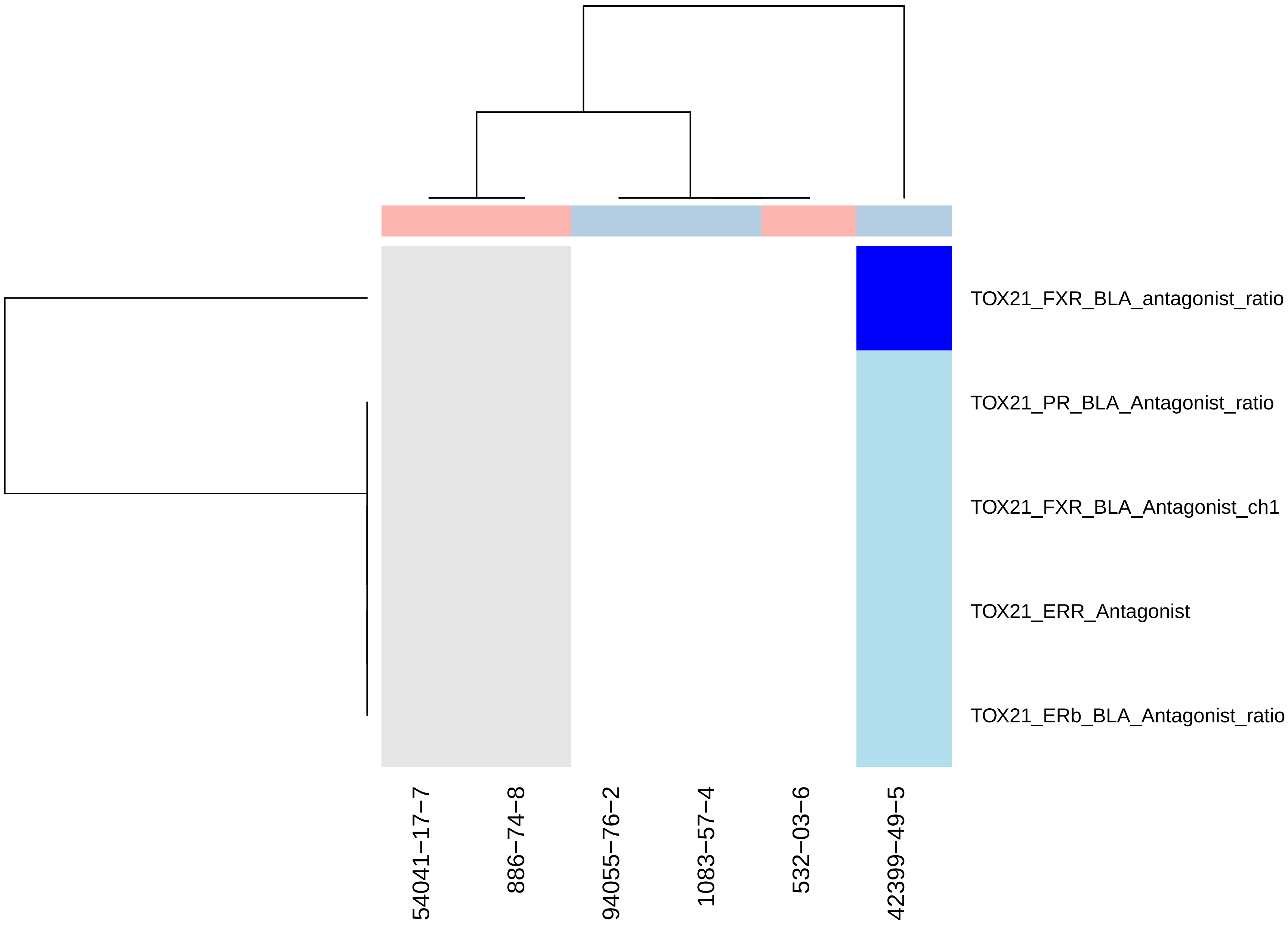

All Activity Heatmap for Cluster 486

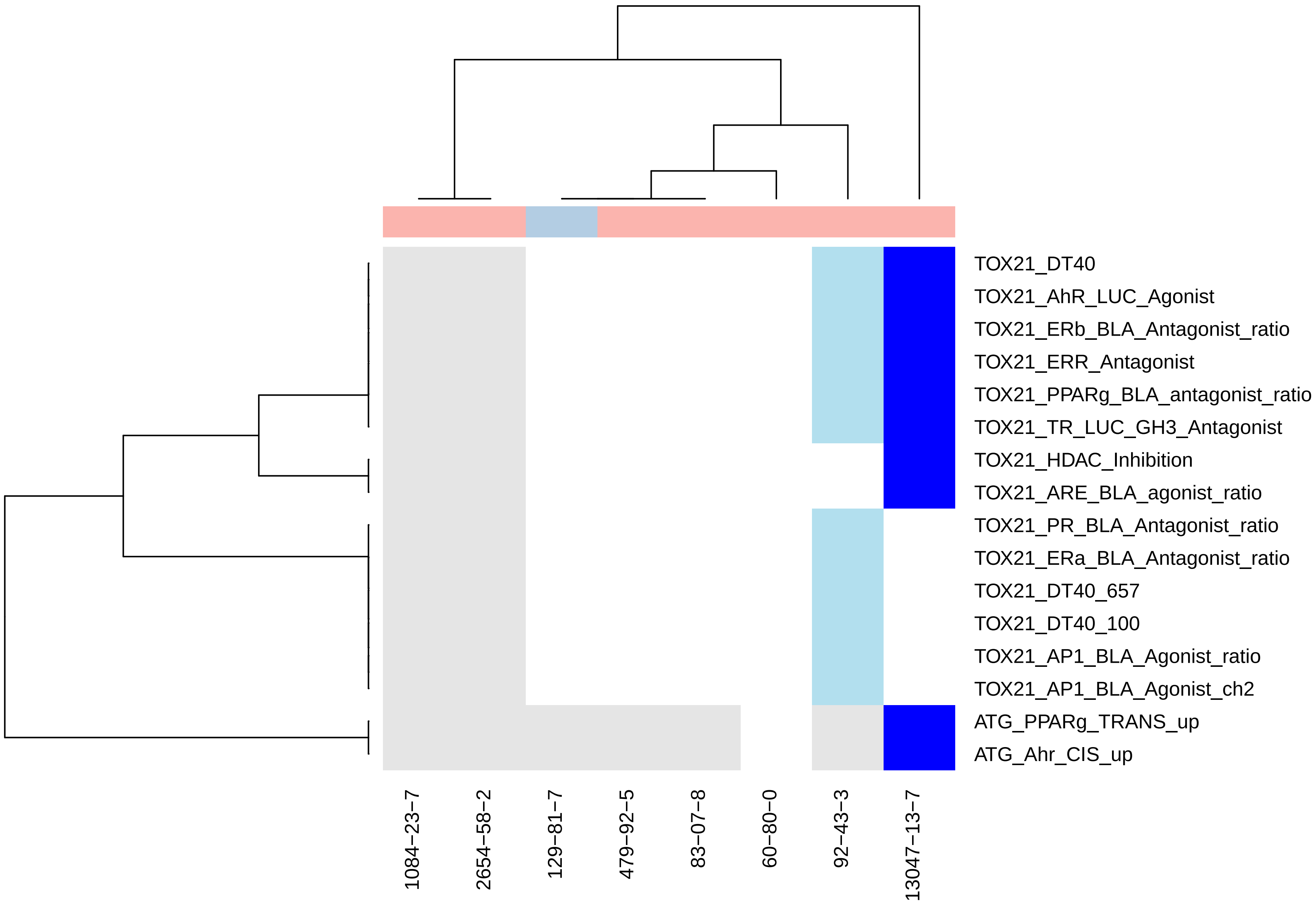

All Activity Heatmap for Cluster 487

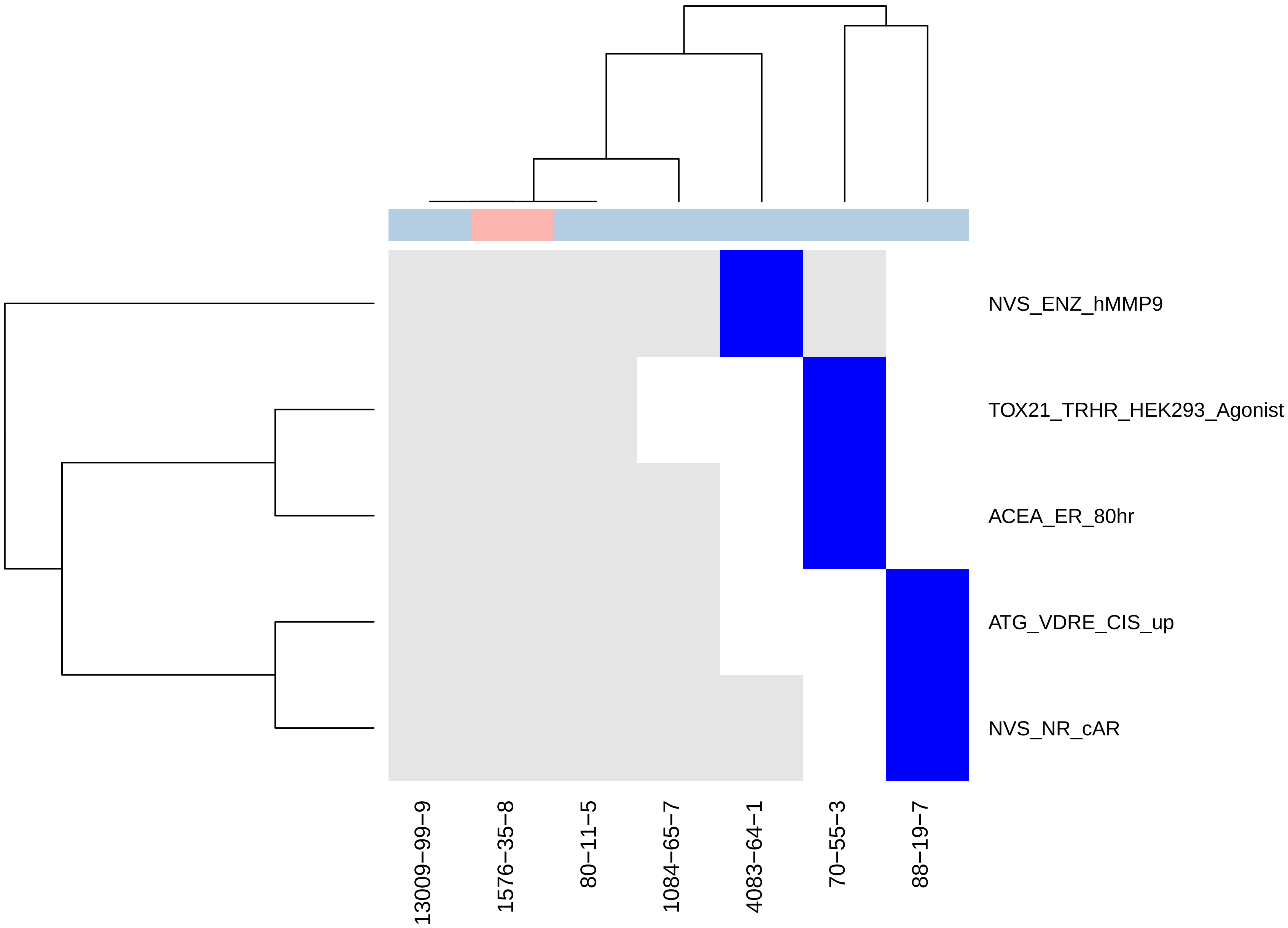

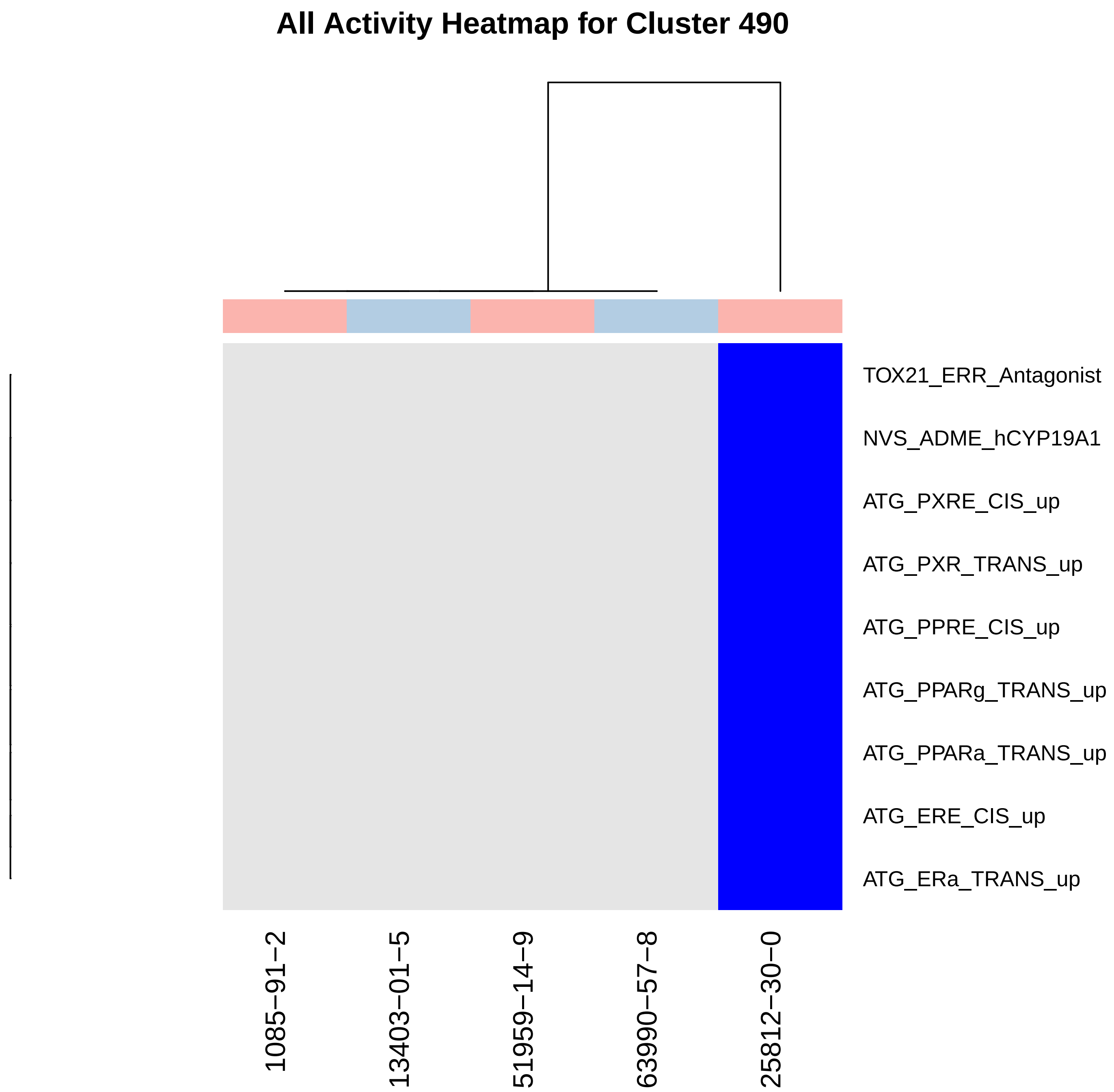
All Activity Heatmap for Cluster 490
TOX21_ERR_Antagonist
NVS_ADME_hCYP19A1
ATG_PXRE_CIS_up
ATG_PXR_TRANS_up
ATG_PPRE_CIS_up
ATG_PPARg_TRANS_up
ATG_PPARa_TRANS_up
ATG_ERE_CIS_up
ATG_ERa_TRANS_up
1085–91–2
13403–01–5
51959–14–9
63990–57–8
25812–30–0

All Activity Heatmap for Cluster 491

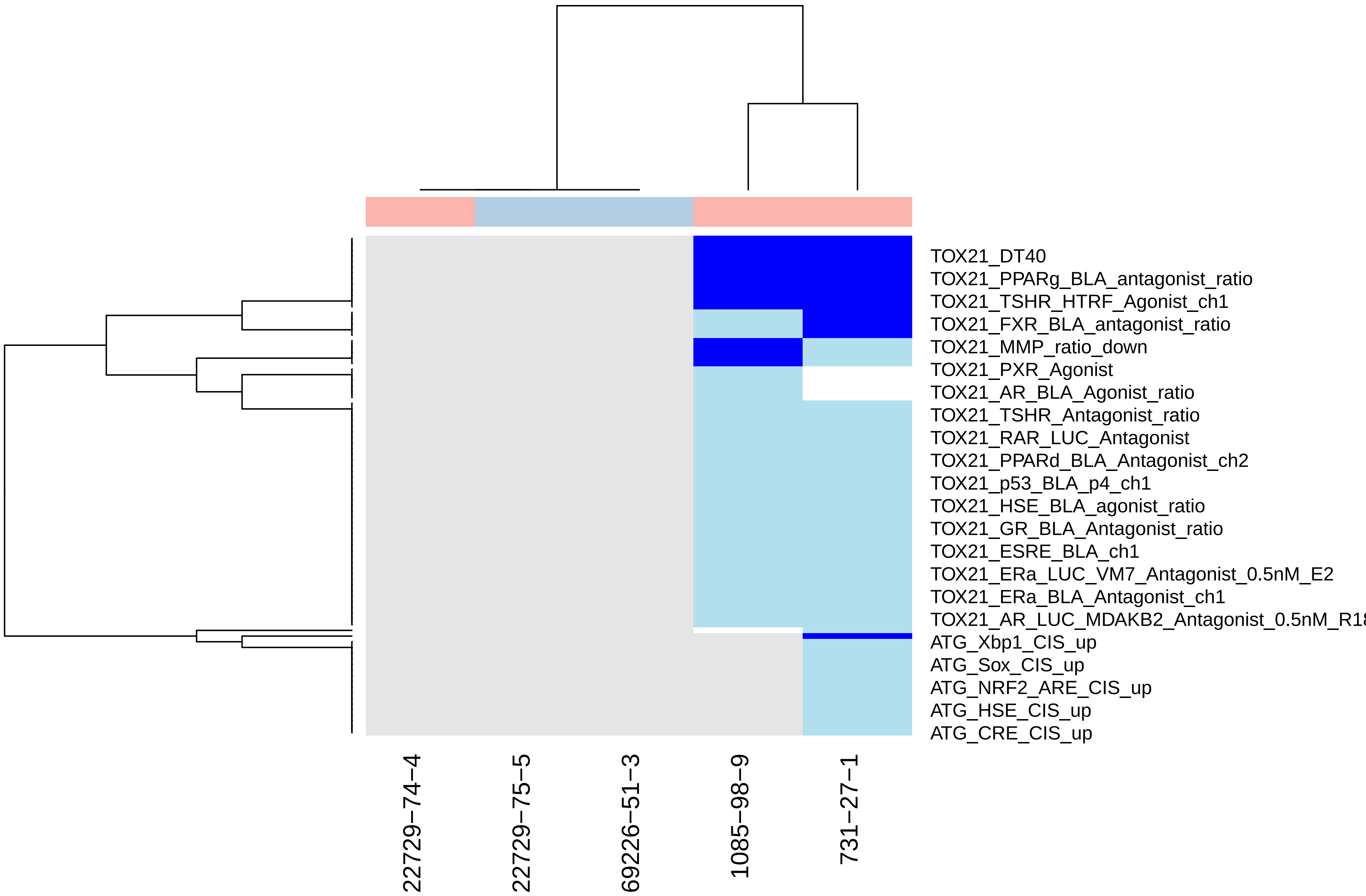

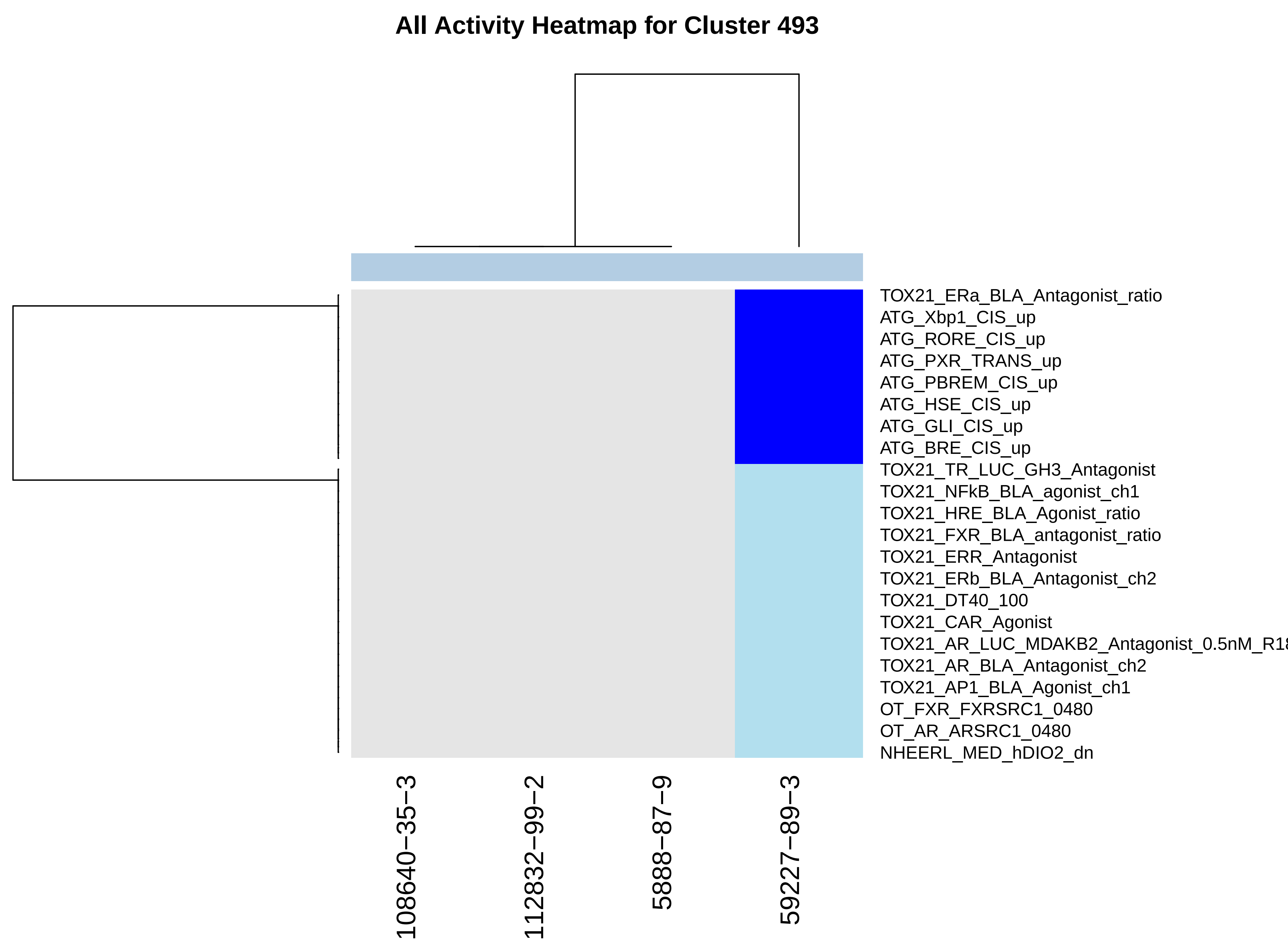
All Activity Heatmap for Cluster 493
TOX21_ERa_BLA_Antagonist_ratio
ATG_Xbp1_CIS_up
ATG_RORE_CIS_up
ATG_PXR_TRANS_up
ATG_PBREM_CIS_up
ATG_HSE_CIS_up
ATG_GLI_CIS_up
ATG_BRE_CIS_up
TOX21_TR_LUC_GH3_Antagonist
TOX21_NFkB_BLA_agonist_ch1
TOX21_HRE_BLA_Agonist_ratio
TOX21_FXR_BLA_antagonist_ratio
TOX21_ERR_Antagonist
TOX21_ERb_BLA_Antagonist_ch2
TOX21_DT40_100
TOX21_CAR_Agonist
TOX21_AR_LUC_MDAKB2_Antagonist_0.5nM_R1
TOX21_AR_BLA_Antagonist_ch2
TOX21_AP1_BLA_Agonist_ch1
OT_FXR_FXRSRC1_0480
OT_AR_ARSRC1_0480
NHEERL_MED_hDIO2_dn
108640−35−3
112832−99−2
5888−87−9
59227−89−3

All Activity Heatmap for Cluster 494

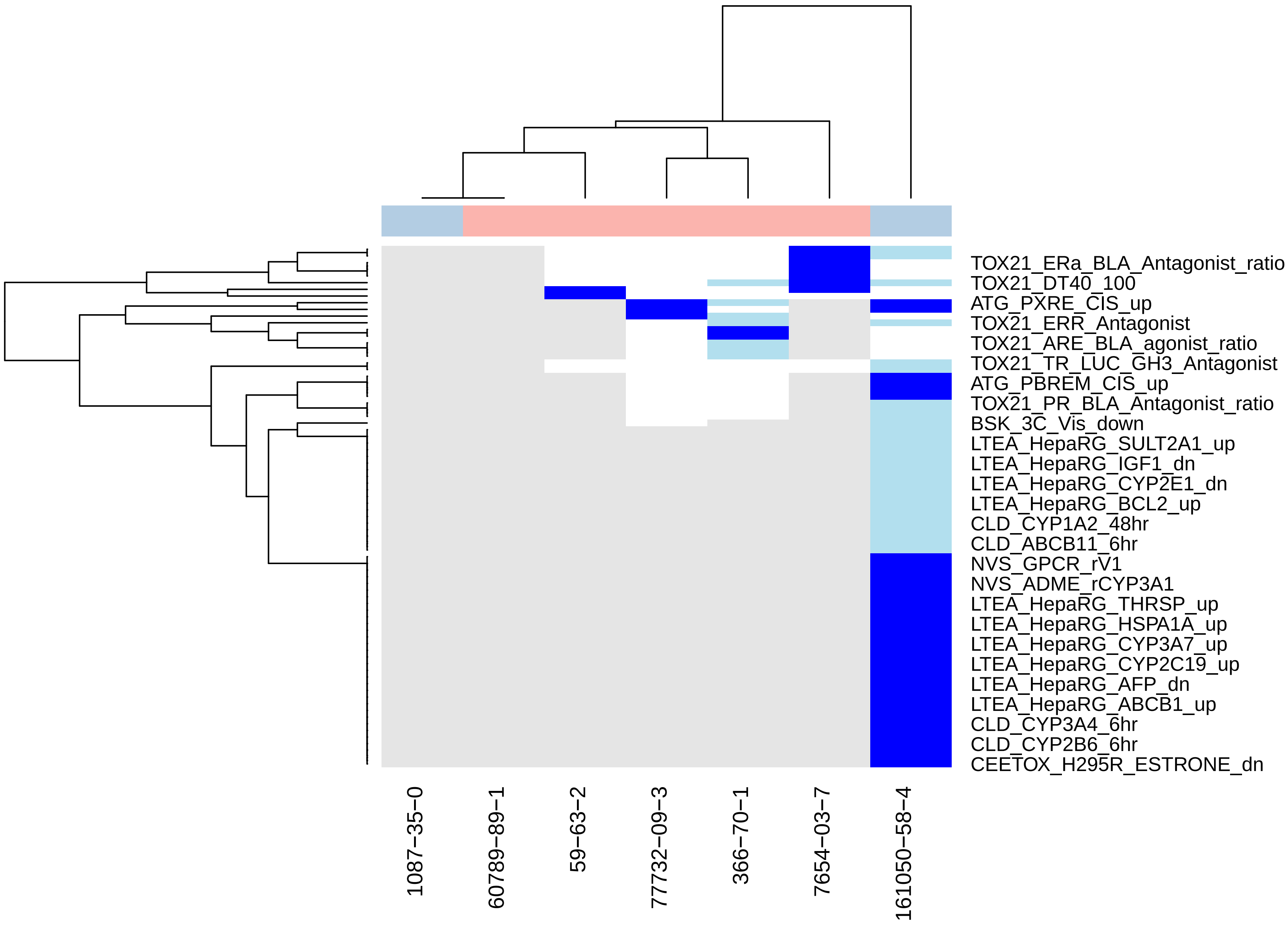

All Activity Heatmap for Cluster 495

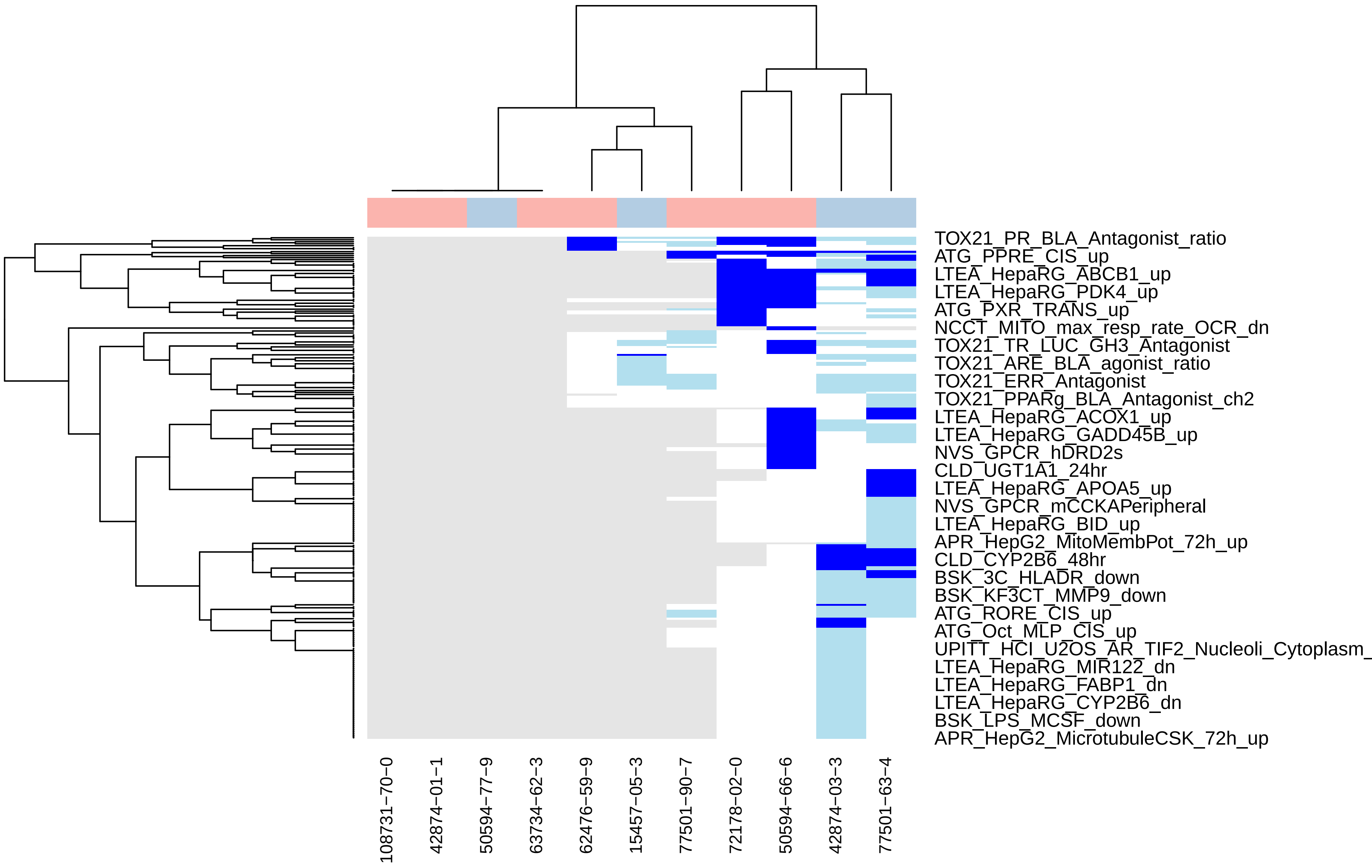

All Activity Heatmap for Cluster 497

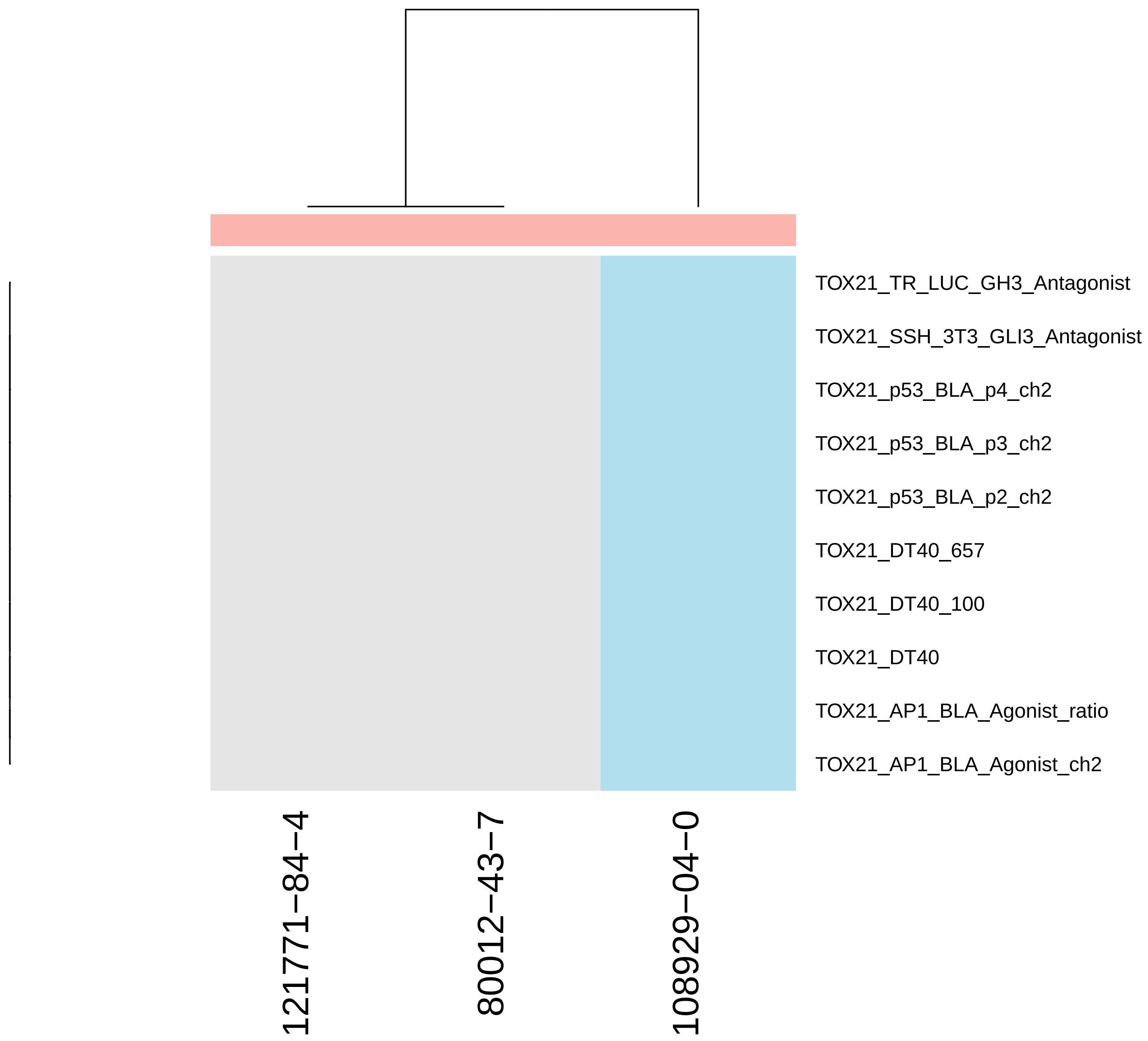

All Activity Heatmap for Cluster 499

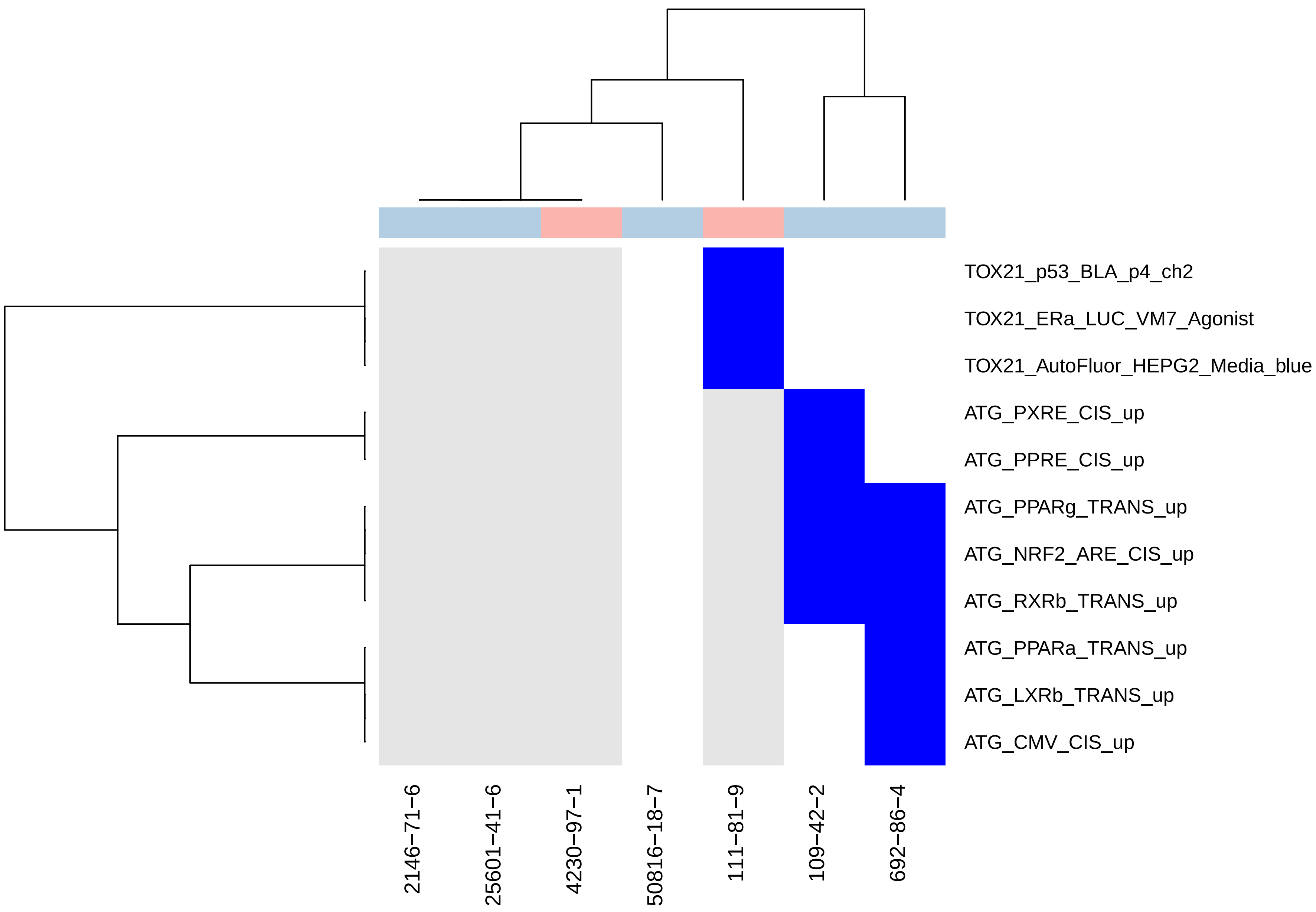

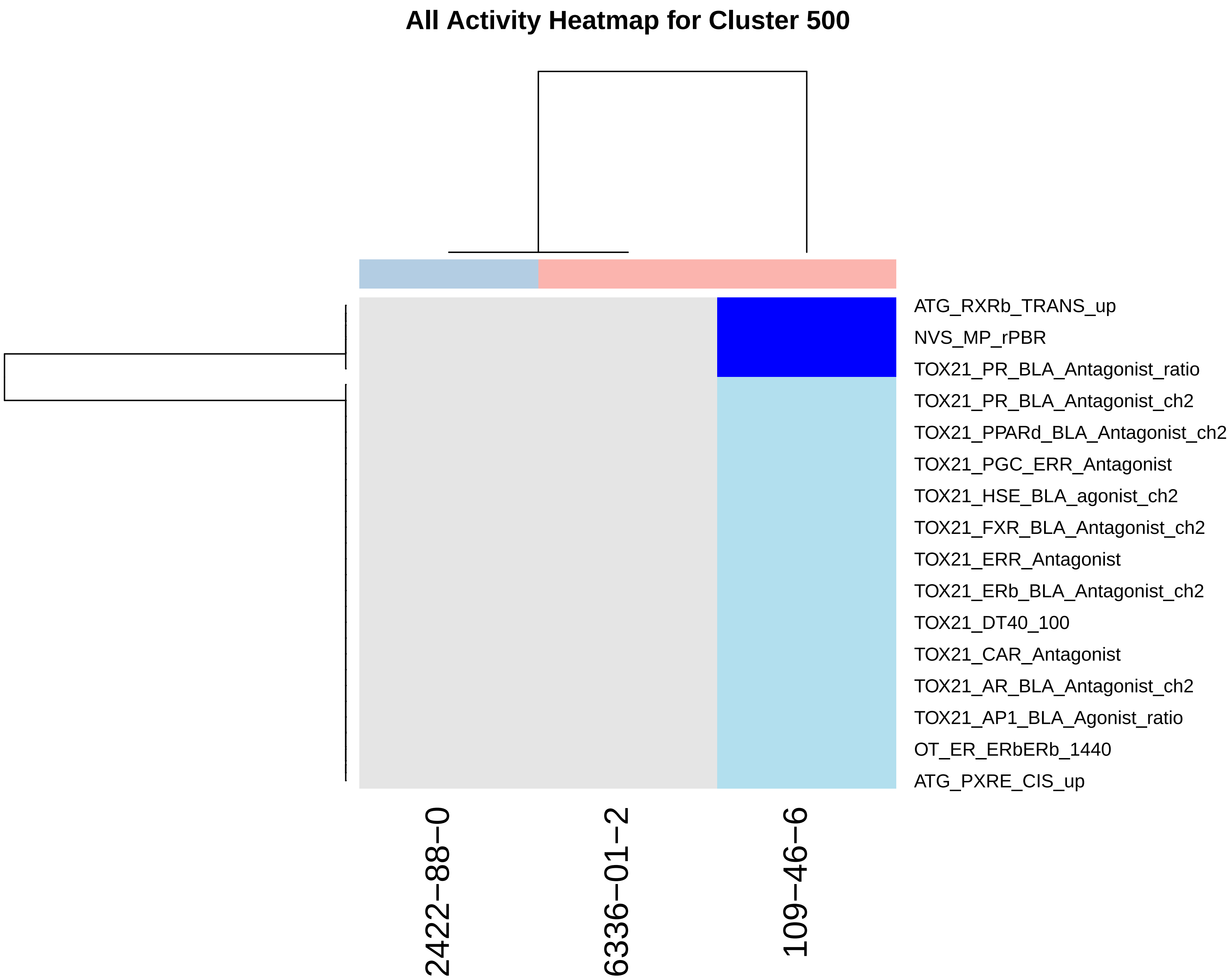
All Activity Heatmap for Cluster 500
ATG_RXRb_TRANS_up
NVS_MP_rPBR
TOX21_PR_BLA_Antagonist_ratio
TOX21_PR_BLA_Antagonist_ch2
TOX21_PPARd_BLA_Antagonist_ch2
TOX21_PGC_ERR_Antagonist
TOX21_HSE_BLA_agonist_ch2
TOX21_FXR_BLA_Antagonist_ch2
TOX21_ERR_Antagonist
TOX21_ERb_BLA_Antagonist_ch2
TOX21_DT40_100
TOX21_CAR_Antagonist
TOX21_AR_BLA_Antagonist_ch2
TOX21_AP1_BLA_Agonist_ratio
OT_ER_ERbERb_1440
ATG_PXRE_CIS_up
2422−88−0
6336−01−2
109−46−6

All Activity Heatmap for Cluster 501

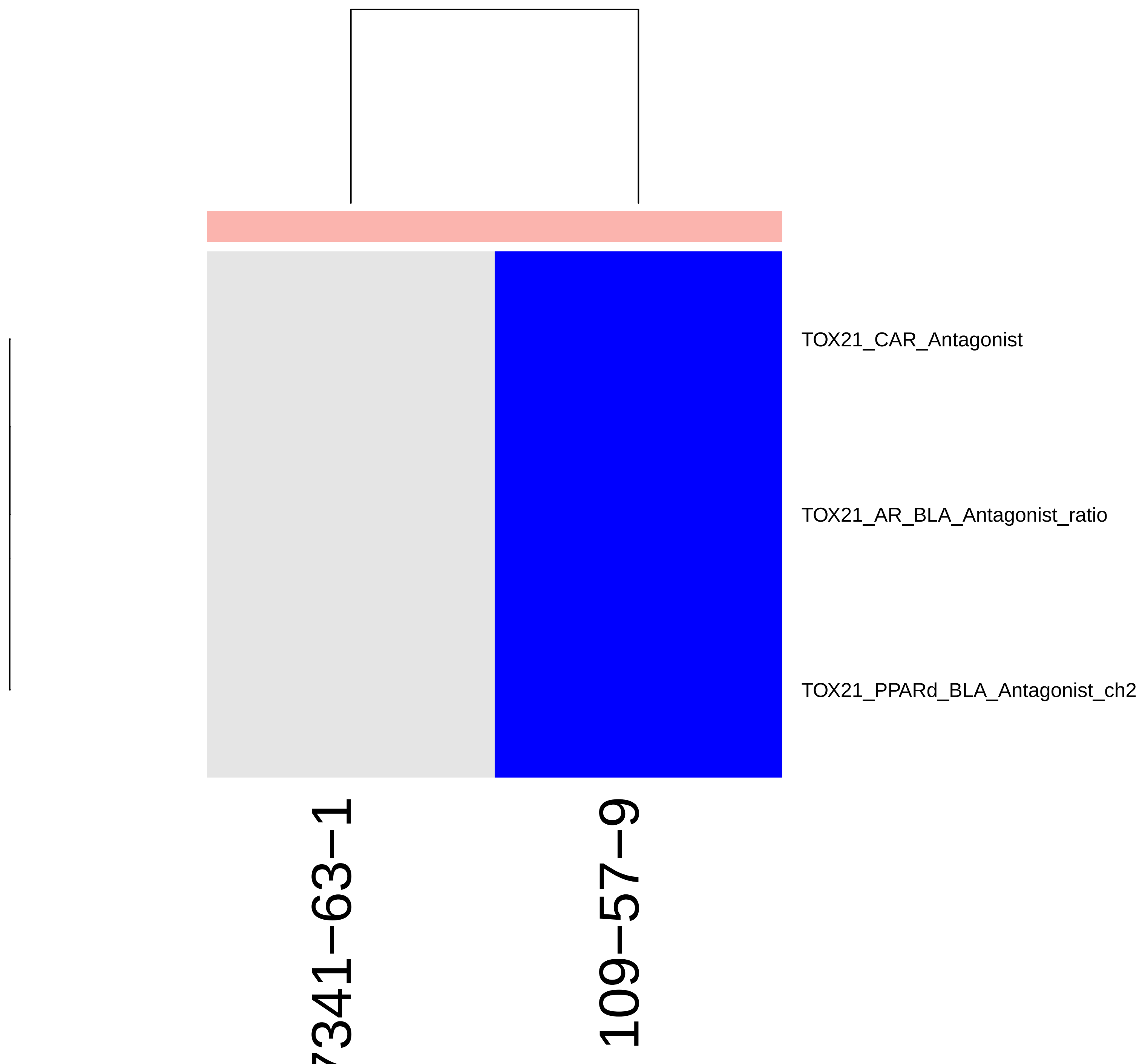

All Activity Heatmap for Cluster 502

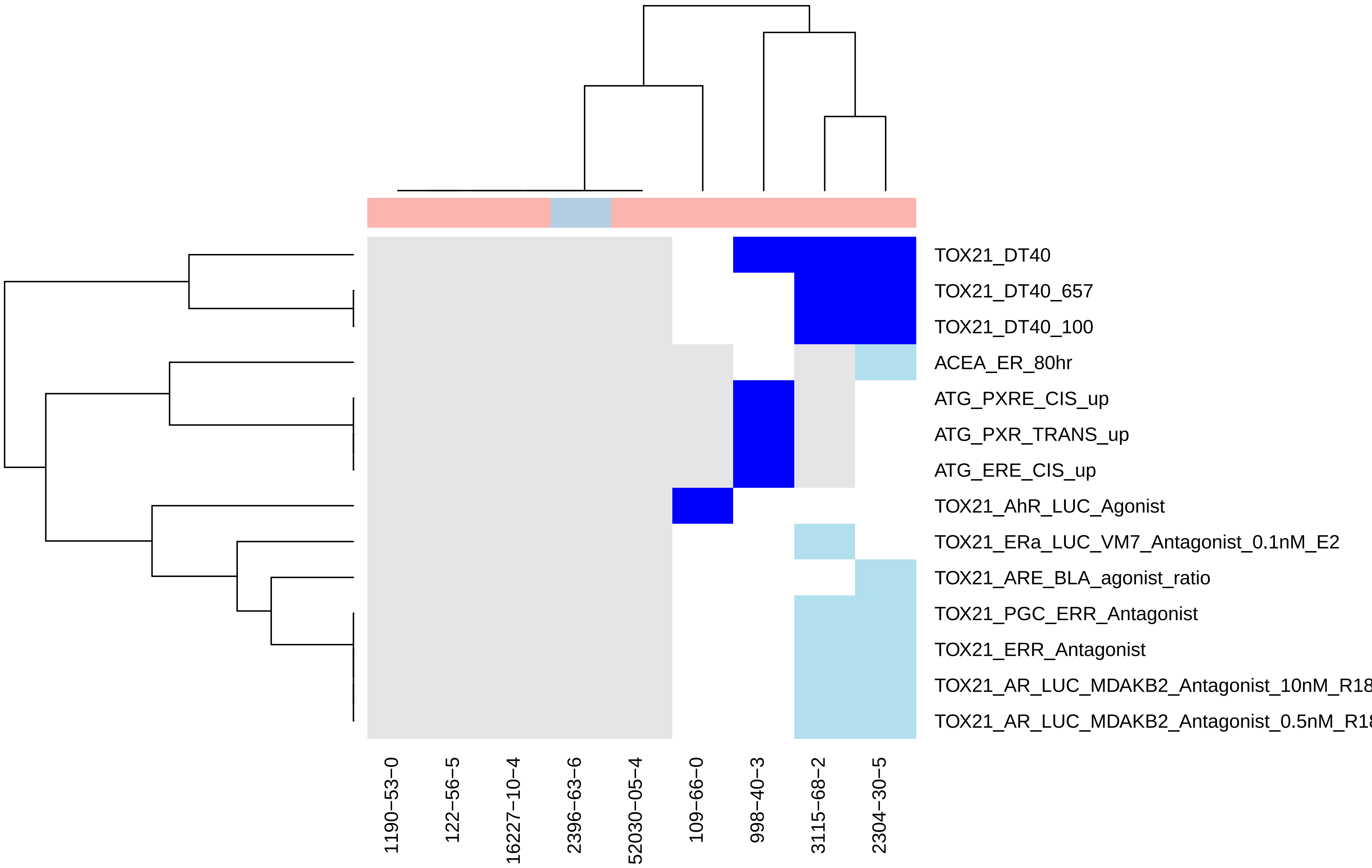

All Activity Heatmap for Cluster 504

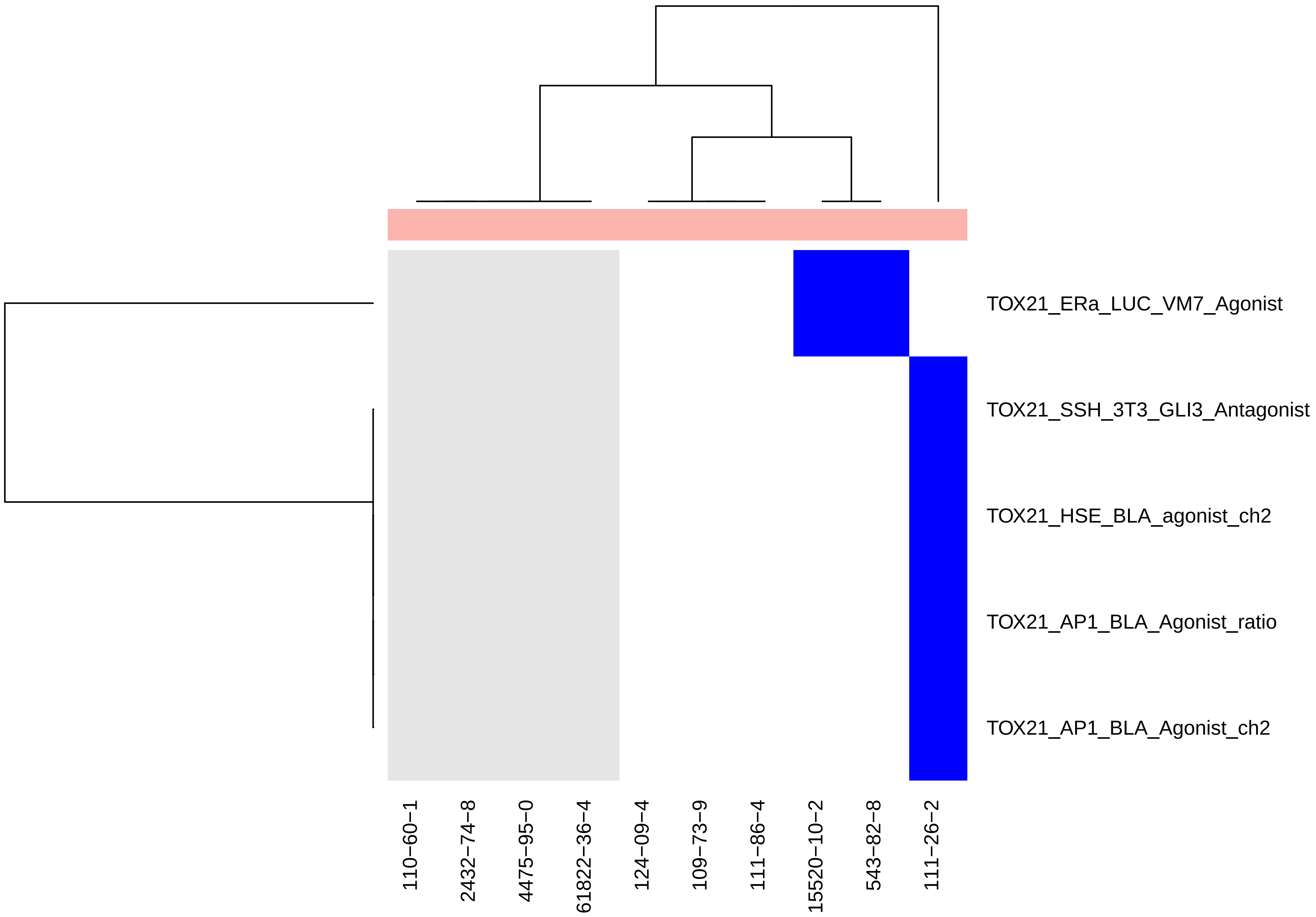

All Activity Heatmap for Cluster 505

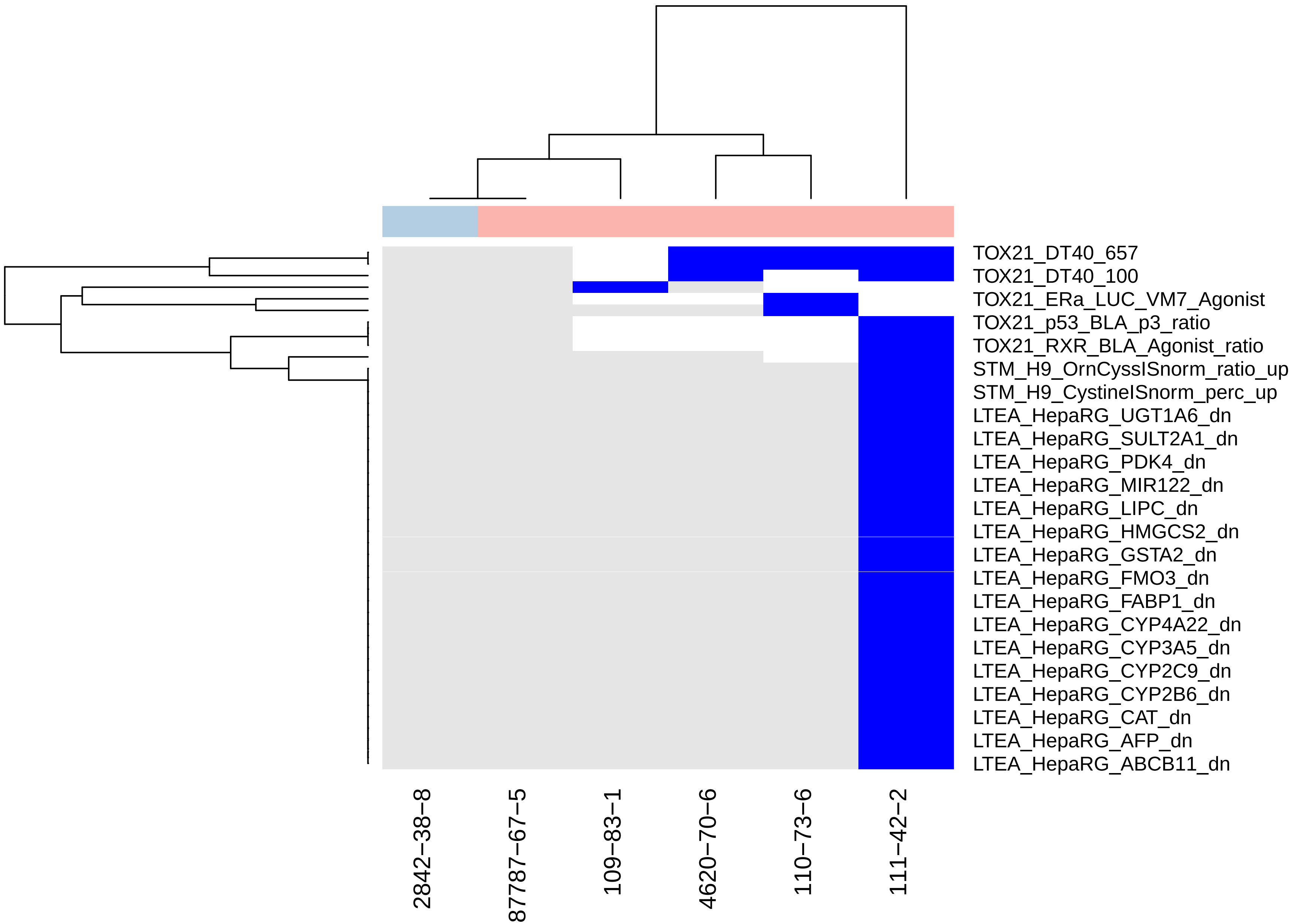

All Activity Heatmap for Cluster 507

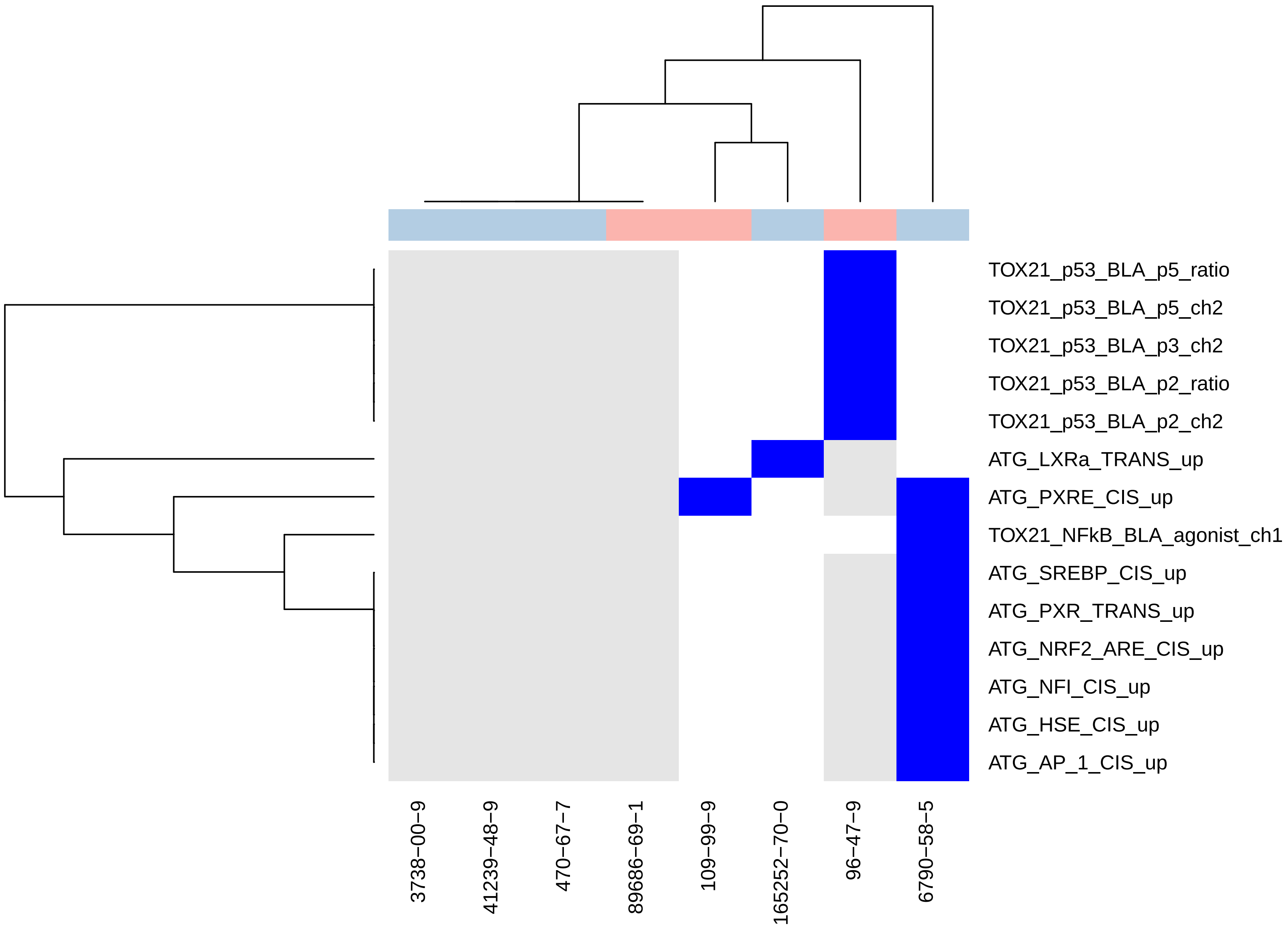

All Activity Heatmap for Cluster 508

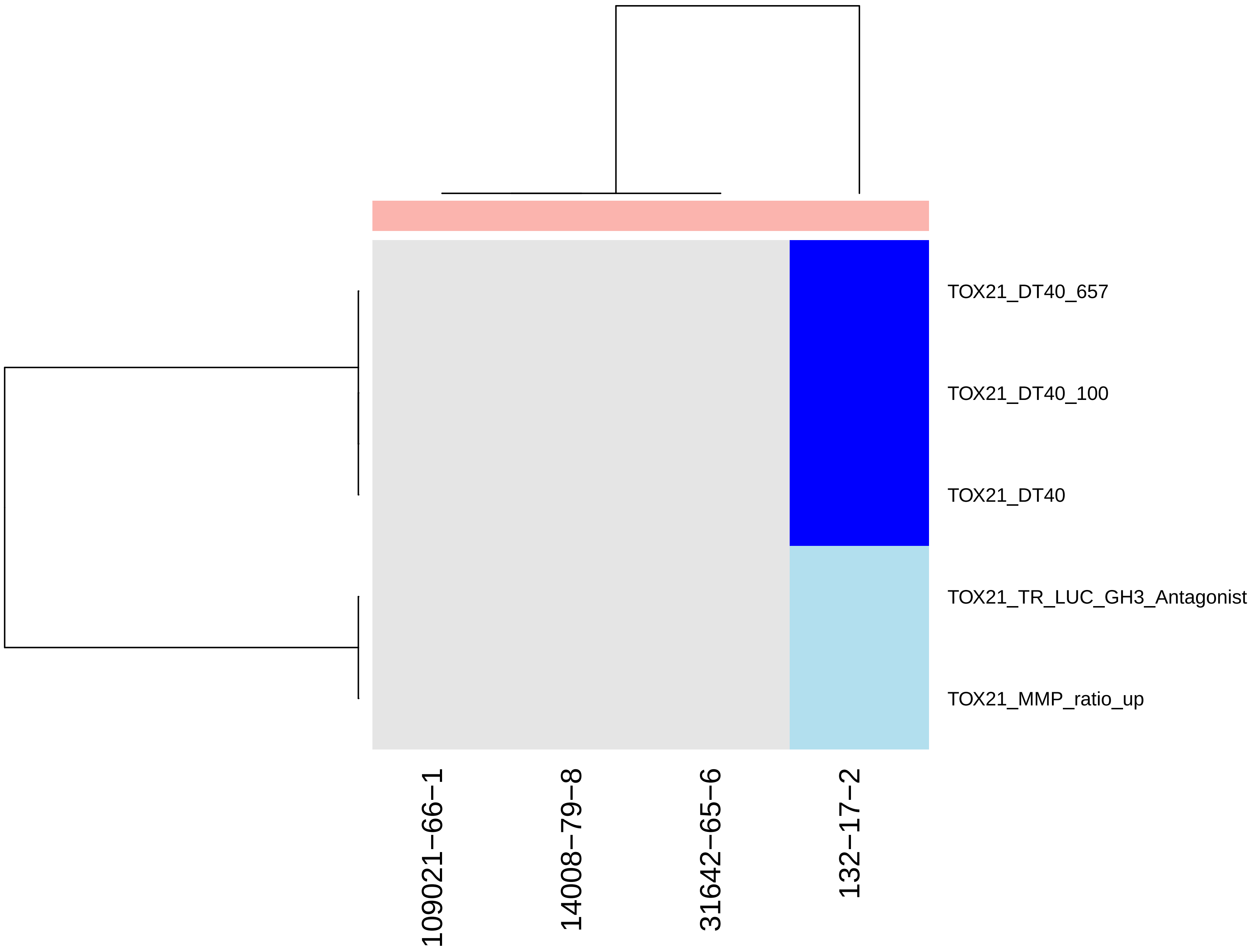

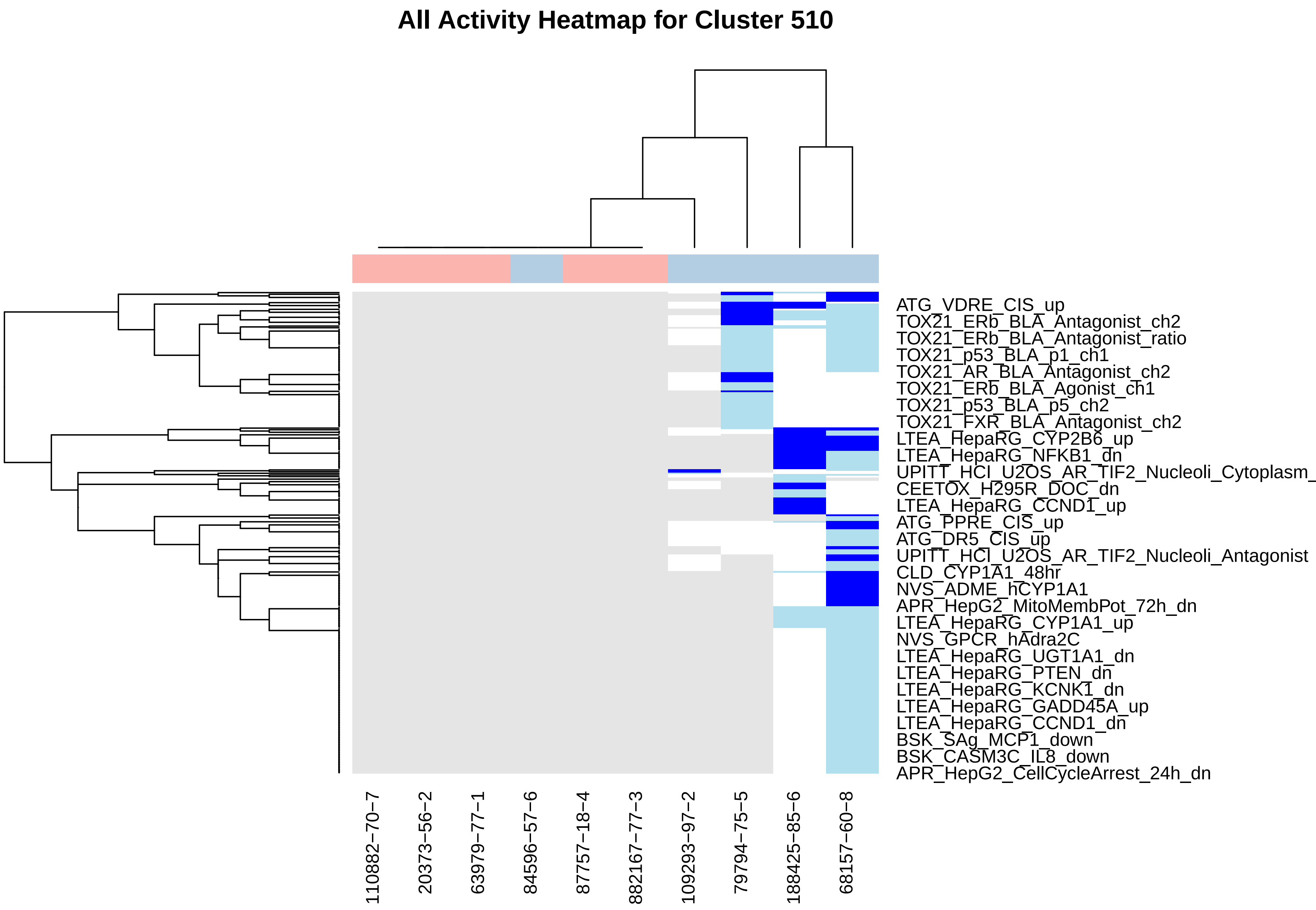

All Activity Heatmap for Cluster 510
ATG_VDRE_CIS_up
TOX21_ERb_BLA_Antagonist_ch2
TOX21_ERb_BLA_Antagonist_ratio
TOX21_p53_BLA_p1_ch1
TOX21_AR_BLA_Antagonist_ch2
TOX21_ERb_BLA_Agonist_ch1
TOX21_p53_BLA_p5_ch2
TOX21_FXR_BLA_Antagonist_ch2
LTEA_HepaRG_CYP2B6_up
LTEA_HepaRG_NFKB1_dn
UPITT_HCI_U2OS_AR_TIF2_Nucleoli_Cytoplasm_
CEETOX_H295R_DOC_dn
LTEA_HepaRG_CCND1_up
ATG_PPRE_CIS_up
ATG_DR5_CIS_up
UPITT_HCI_U2OS_AR_TIF2_Nucleoli_Antagonist
CLD_CYP1A1_48hr
NVS_ADME_hCYP1A1
APR_HepG2_MitoMembPot_72h_dn
LTEA_HepaRG_CYP1A1_up
NVS_GPCR_hAdra2C
LTEA_HepaRG_UGT1A1_dn
LTEA_HepaRG_PTEN_dn
LTEA_HepaRG_KCNK1_dn
LTEA_HepaRG_GADD45A_up
LTEA_HepaRG_CCND1_dn
BSK_SAg_MCP1_down
BSK_CASM3C_IL8_down
APR_HepG2_CellCycleArrest_24h_dn
110882−70−7
20373−56−2
63979−77−1
84596−57−6
87757−18−4
882167−77−3
109293−97−2
79794−75−5
188425−85−6
68157−60−8

All Activity Heatmap for Cluster 511

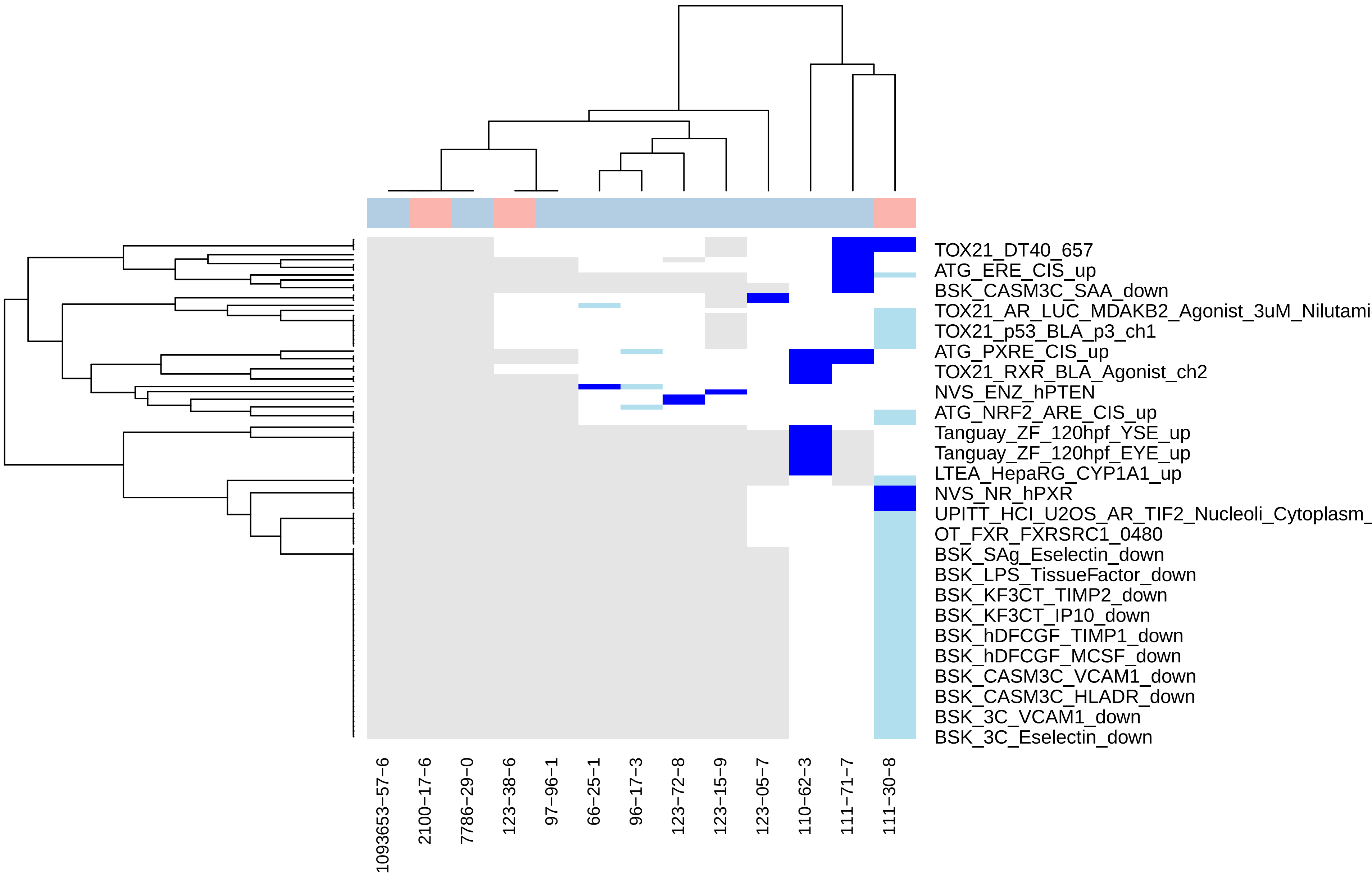

All Activity Heatmap for Cluster 512

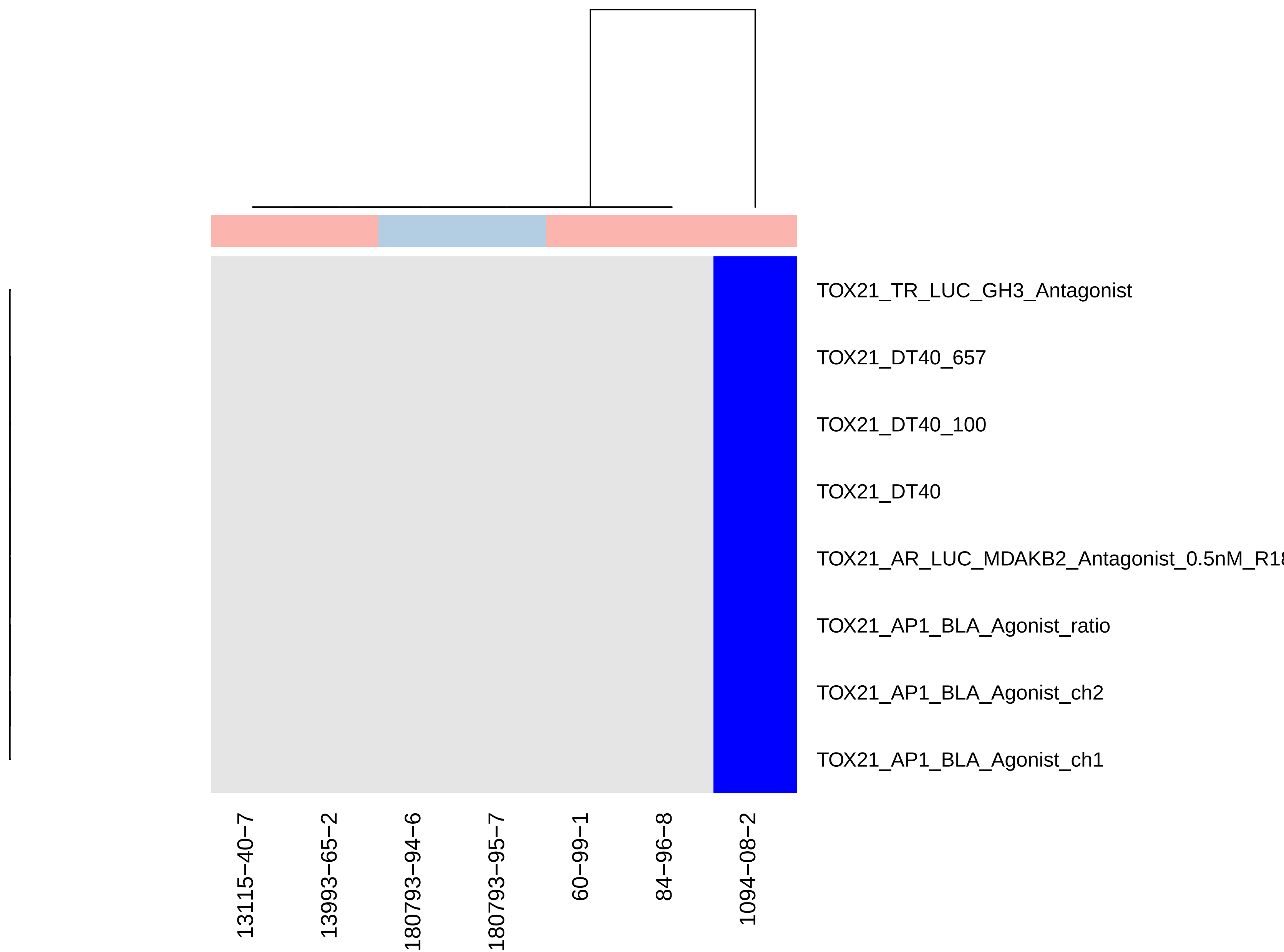

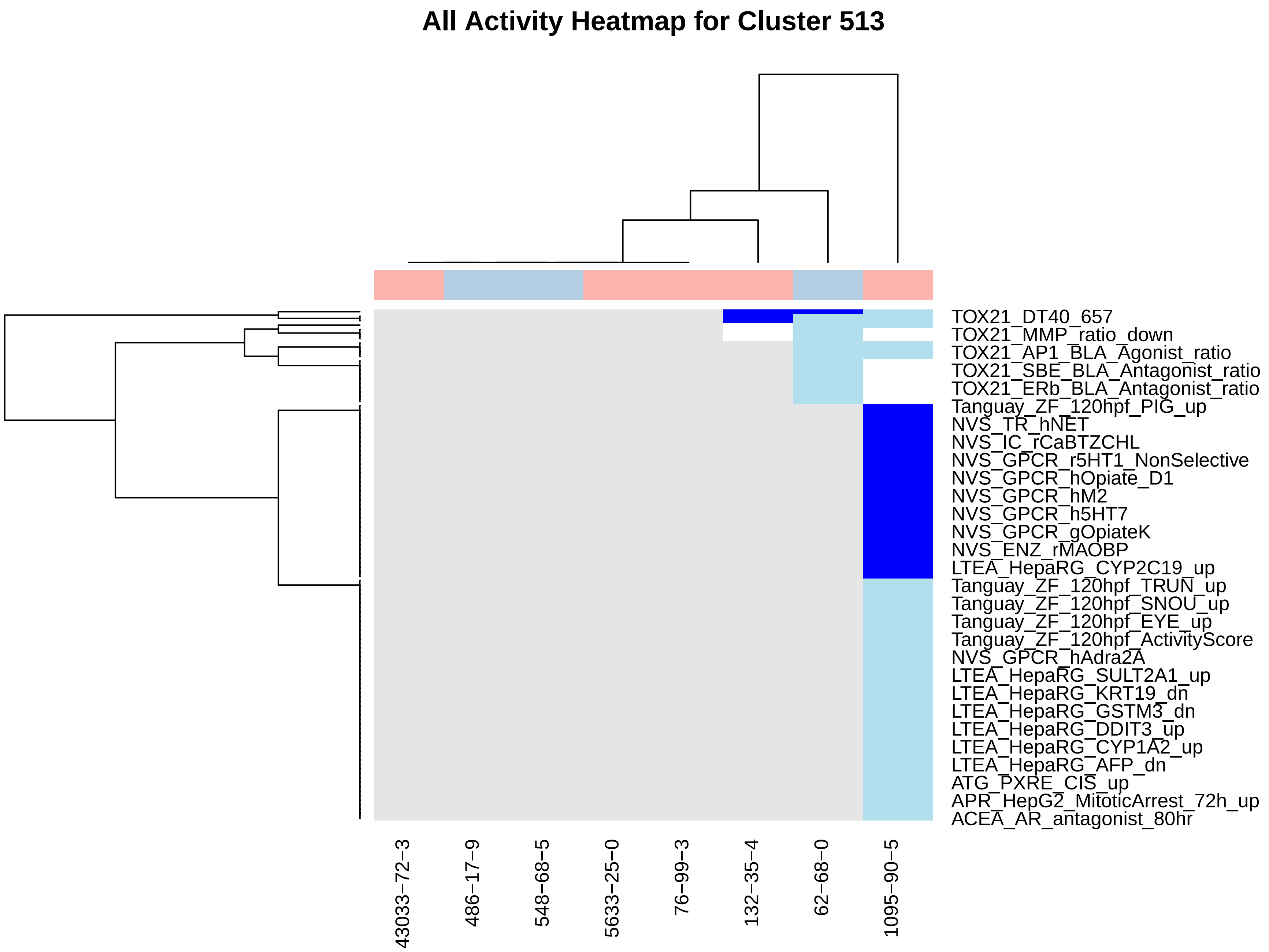

All Activity Heatmap for Cluster 513
TOX21_DT40_657
TOX21_MMP_ratio_down
TOX21_AP1_BLA_Agonist_ratio
TOX21_SBE_BLA_Antagonist_ratio
TOX21_ERb_BLA_Antagonist_ratio
Tanguay_ZF_120hpf_PIG_up
NVS_TR_hNET
NVS_IC_rCaBTZCHL
NVS_GPCR_r5HT1_NonSelective
NVS_GPCR_hOpiate_D1
NVS_GPCR_hM2
NVS_GPCR_h5HT7
NVS_GPCR_gOpiateK
NVS_ENZ_rMAOBP
LTEA_HepaRG_CYP2C19_up
Tanguay_ZF_120hpf_TRUN_up
Tanguay_ZF_120hpf_SNOU_up
Tanguay_ZF_120hpf_EYE_up
Tanguay_ZF_120hpf_ActivityScore
NVS_GPCR_hAdra2A
LTEA_HepaRG_SULT2A1_up
LTEA_HepaRG_KRT19_dn
LTEA_HepaRG_GSTM3_dn
LTEA_HepaRG_DDIT3_up
LTEA_HepaRG_CYP1A2_up
LTEA_HepaRG_AFP_dn
ATG_PXRE_CIS_up
APR_HepG2_MitoticArrest_72h_up
ACEA_AR_antagonist_80hr
43033−72−3
486−17−9
548−68−5
5633−25−0
76−99−3
132−35−4
62−68−0
1095−90−5

All Activity Heatmap for Cluster 515

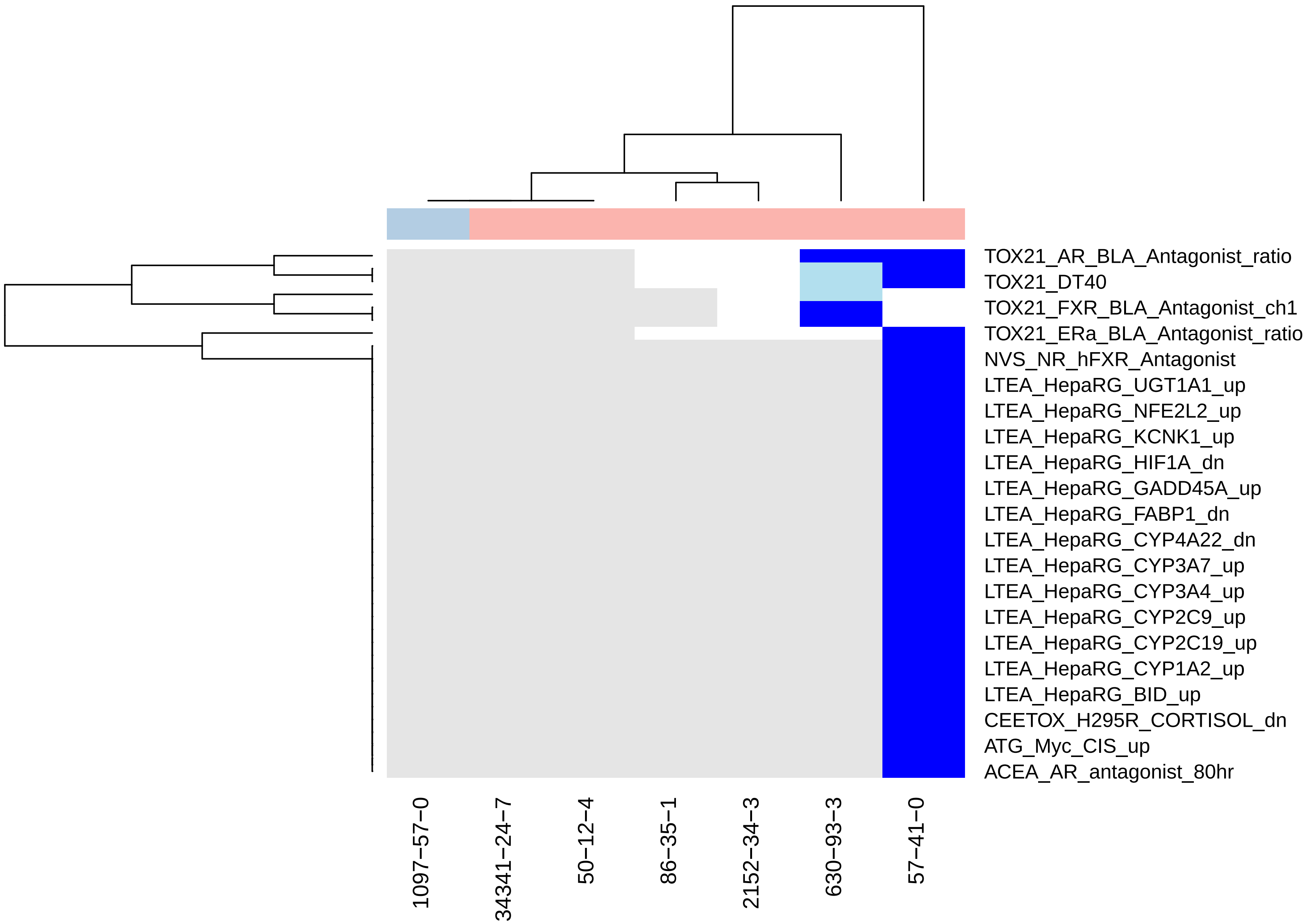

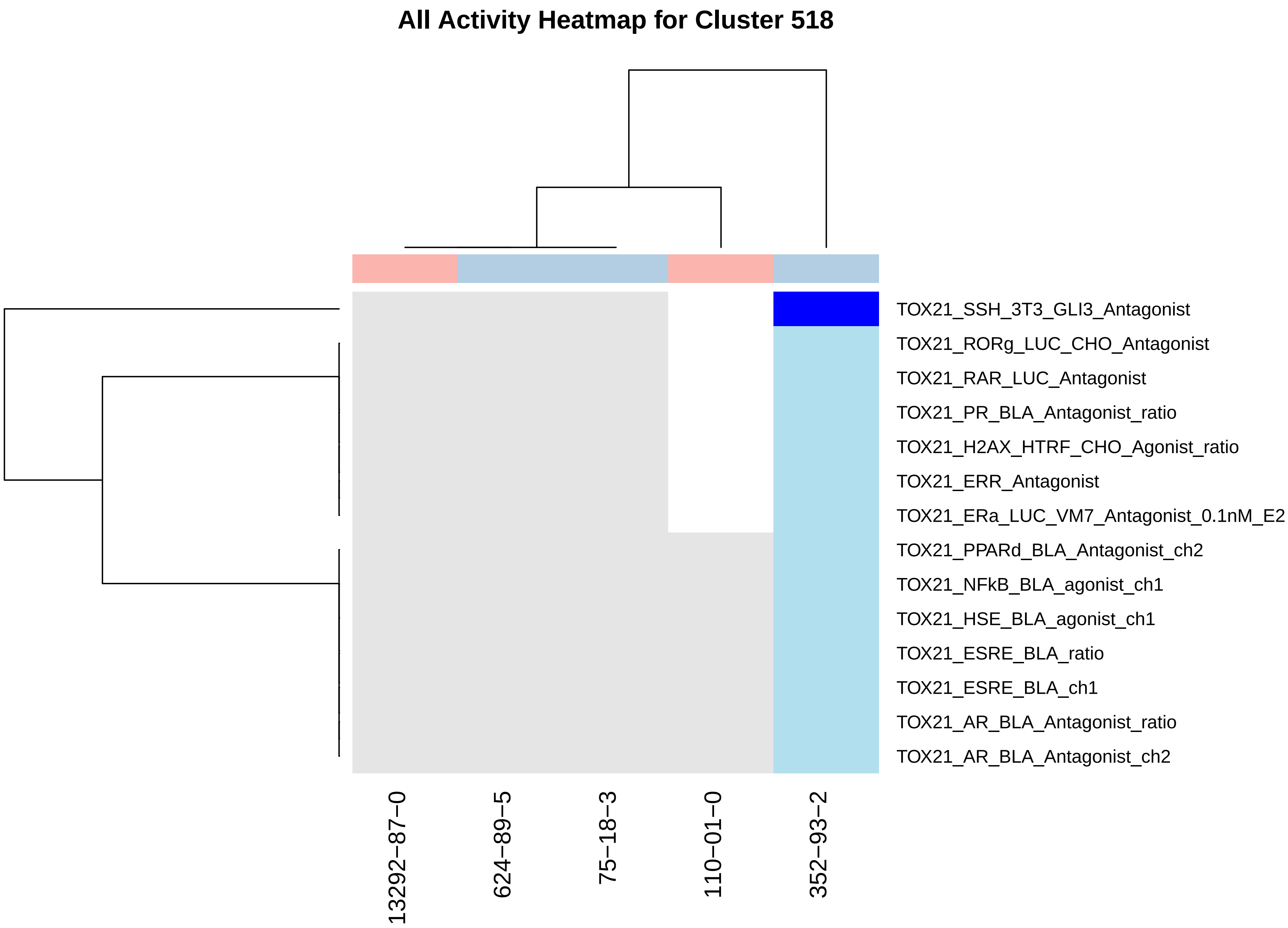
All Activity Heatmap for Cluster 518
TOX21_SSH_3T3_GLI3_Antagonist
TOX21_RORg_LUC_CHO_Antagonist
TOX21_RAR_LUC_Antagonist
TOX21_PR_BLA_Antagonist_ratio
TOX21_H2AX_HTRF_CHO_Agonist_ratio
TOX21_ERR_Antagonist
TOX21_ERa_LUC_VM7_Antagonist_0.1nM_E2
TOX21_PPARd_BLA_Antagonist_ch2
TOX21_NFkB_BLA_agonist_ch1
TOX21_HSE_BLA_agonist_ch1
TOX21_ESRE_BLA_ratio
TOX21_ESRE_BLA_ch1
TOX21_AR_BLA_Antagonist_ratio
TOX21_AR_BLA_Antagonist_ch2
13292−87−0
624−89−5
75−18−3
110−01−0
352−93−2

All Activity Heatmap for Cluster 521

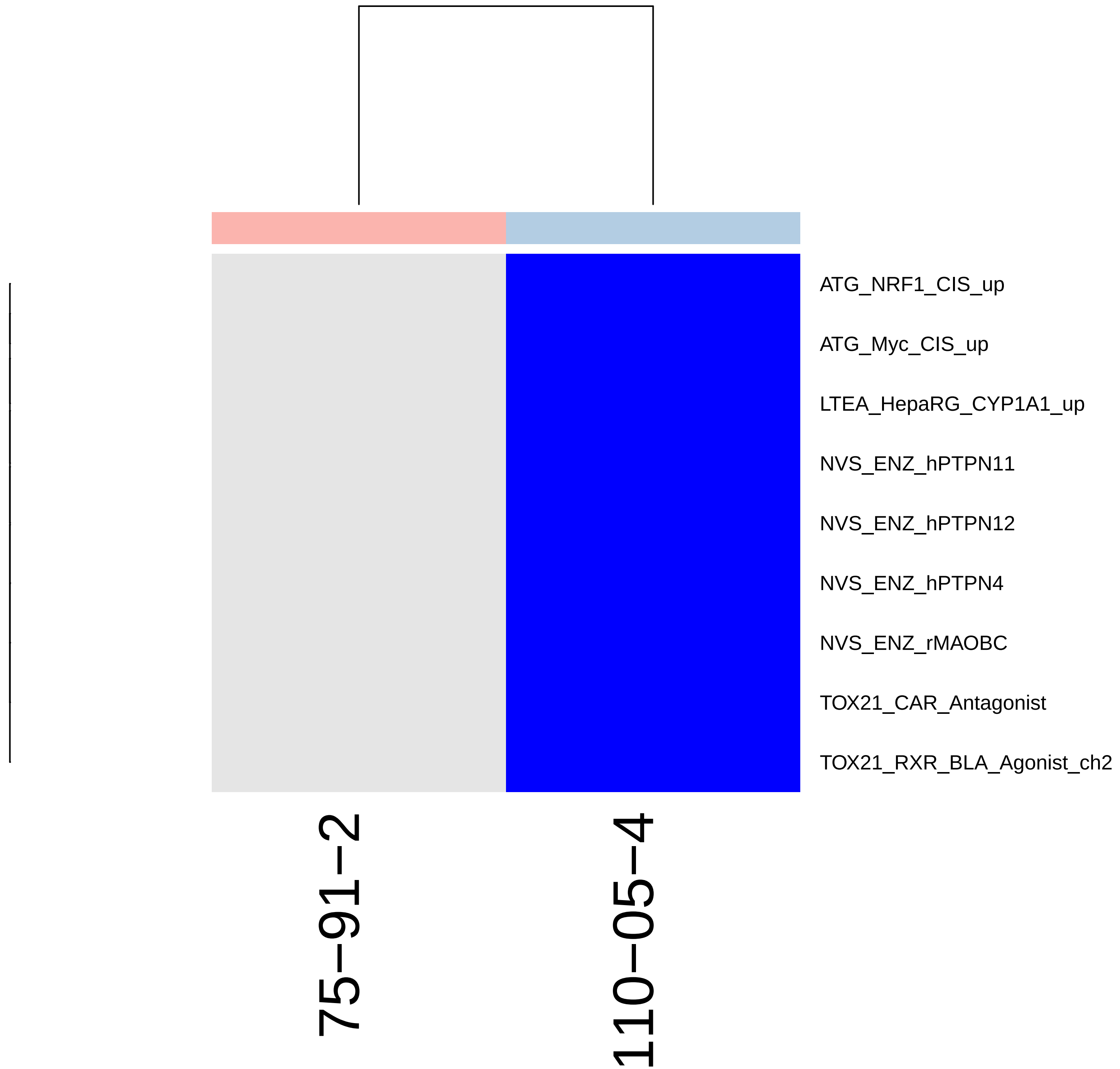

All Activity Heatmap for Cluster 522

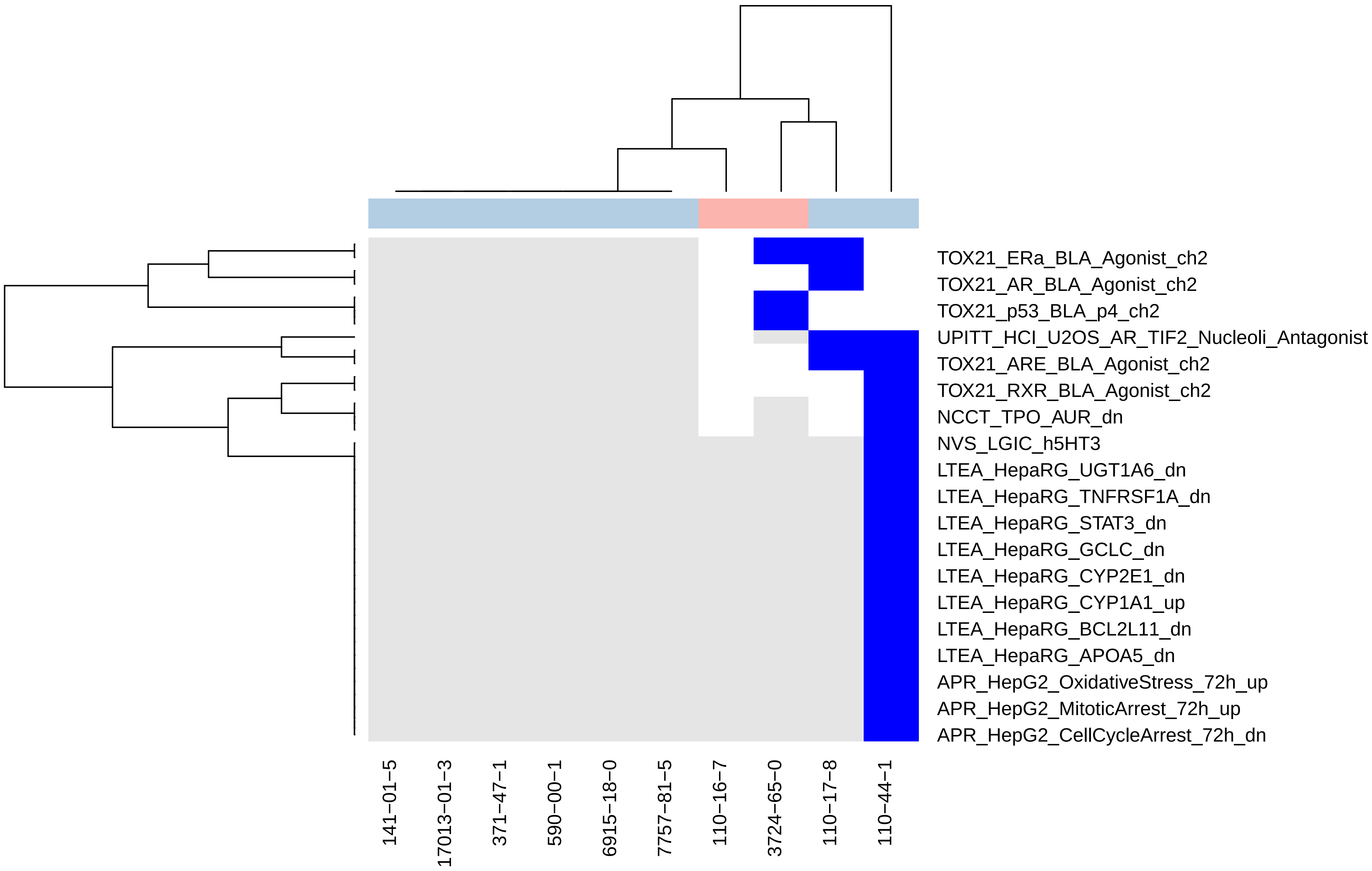

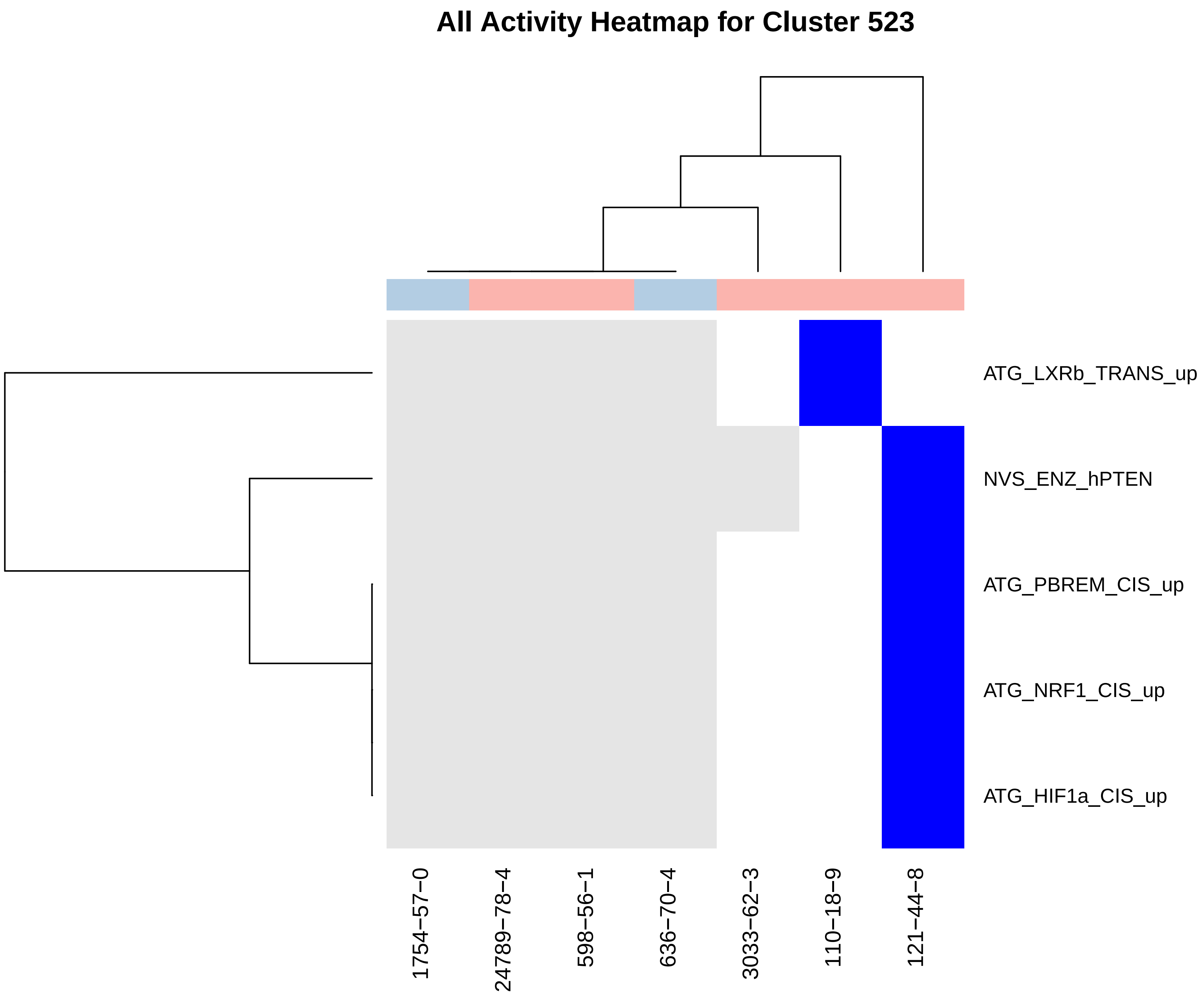
All Activity Heatmap for Cluster 523
ATG_LXRb_TRANS_up
NVS_ENZ_hPTEN
ATG_PBREM_CIS_up
ATG_NRF1_CIS_up
ATG_HIF1a_CIS_up
1754−57−0
24789−78−4
598−56−1
636−70−4
3033−62−3
110−18−9
121−44−8

All Activity Heatmap for Cluster 525

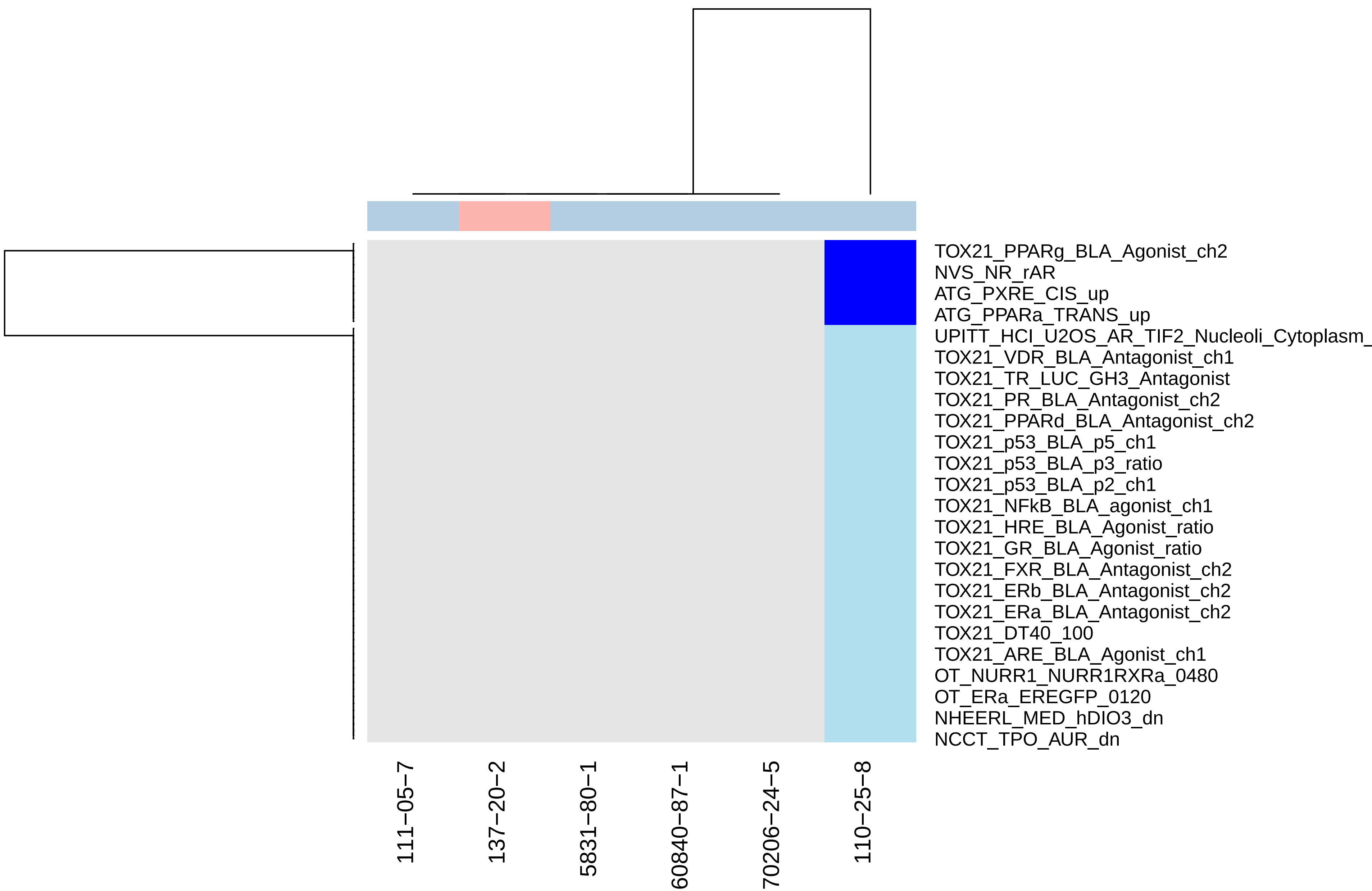

All Activity Heatmap for Cluster 526

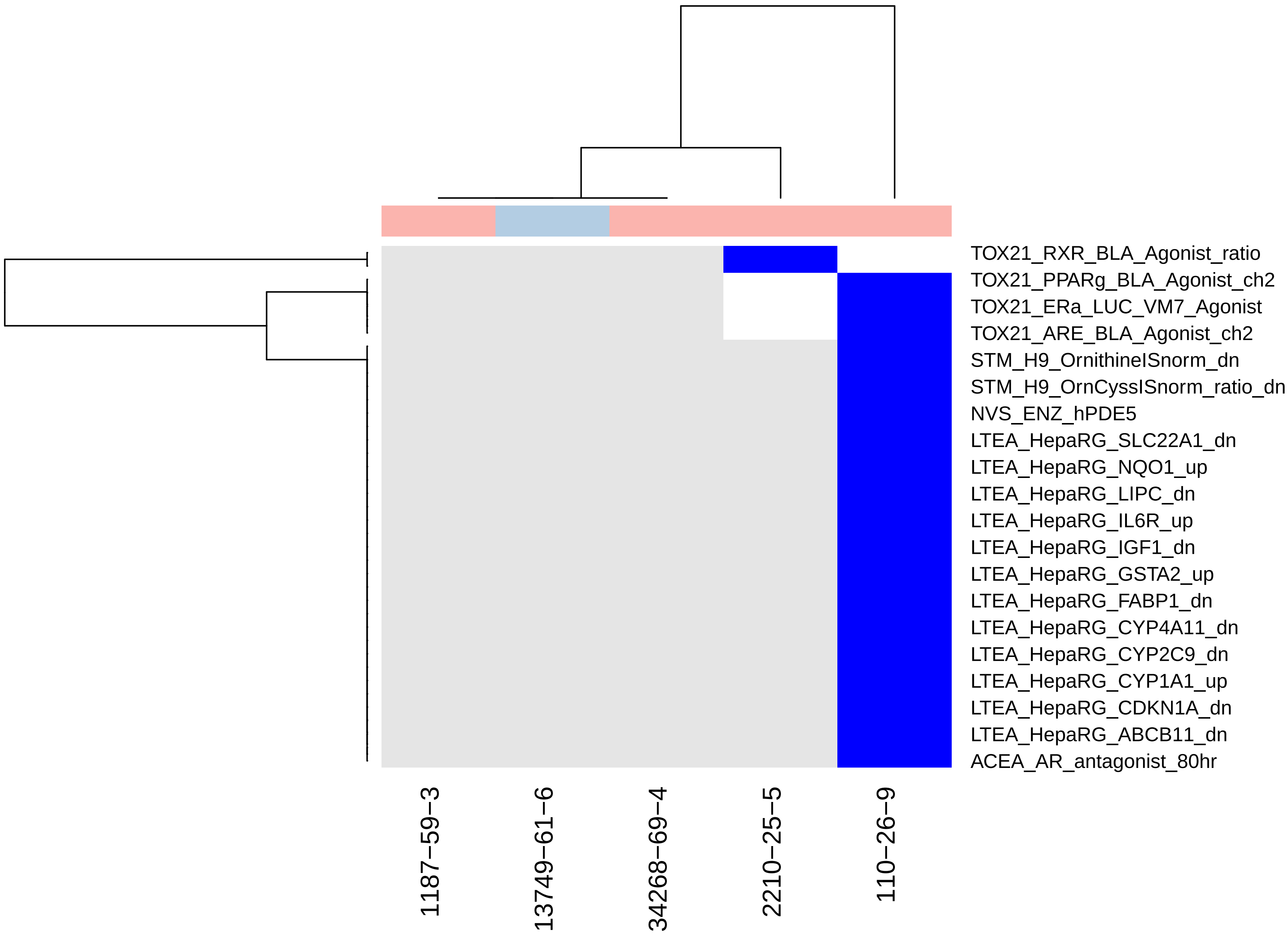

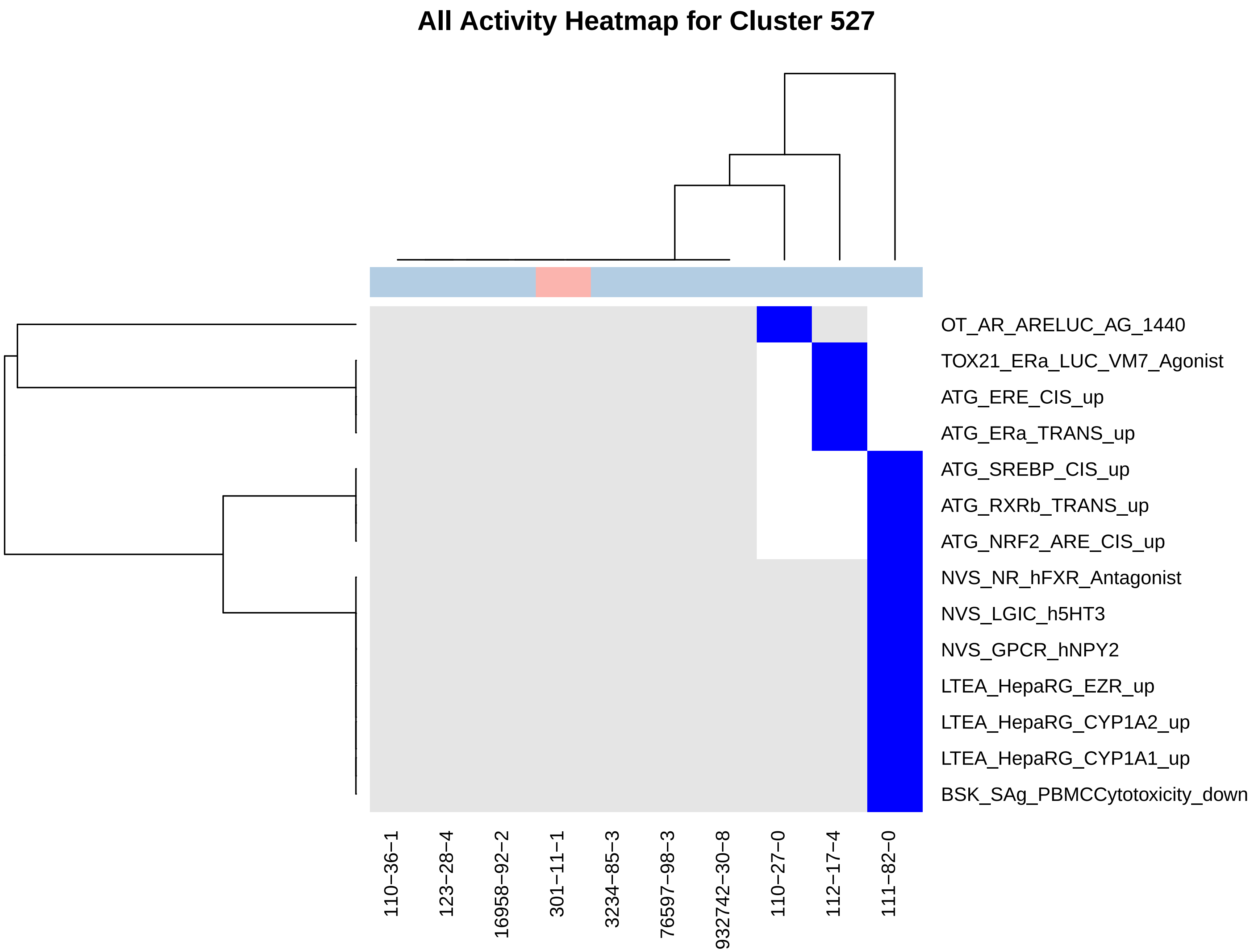
All Activity Heatmap for Cluster 527
OT_AR_ARELUC_AG_1440
TOX21_ERa_LUC_VM7_Agonist
ATG_ERE_CIS_up
ATG_ERa_TRANS_up
ATG_SREBP_CIS_up
ATG_RXRb_TRANS_up
ATG_NRF2_ARE_CIS_up
NVS_NR_hFXR_Antagonist
NVS_LGIC_h5HT3
NVS_GPCR_hNPY2
LTEA_HepaRG_EZR_up
LTEA_HepaRG_CYP1A2_up
LTEA_HepaRG_CYP1A1_up
BSK_SAg_PBMCCytotoxicity_down
110−36−1
123−28−4
16958−92−2
301−11−1
3234−85−3
76597−98−3
932742−30−8
110−27−0
112−17−4
111−82−0

All Activity Heatmap for Cluster 528

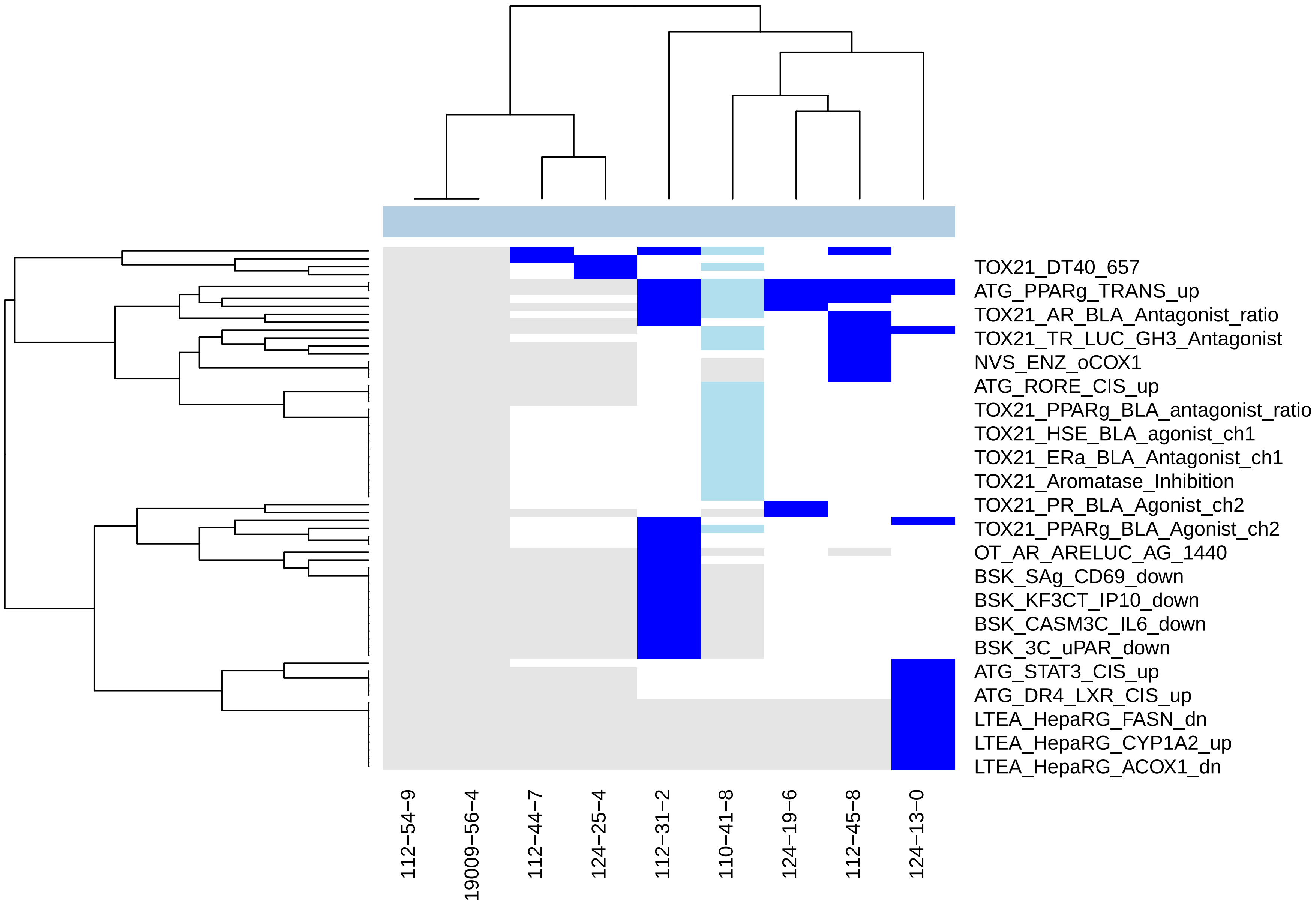

All Activity Heatmap for Cluster 529

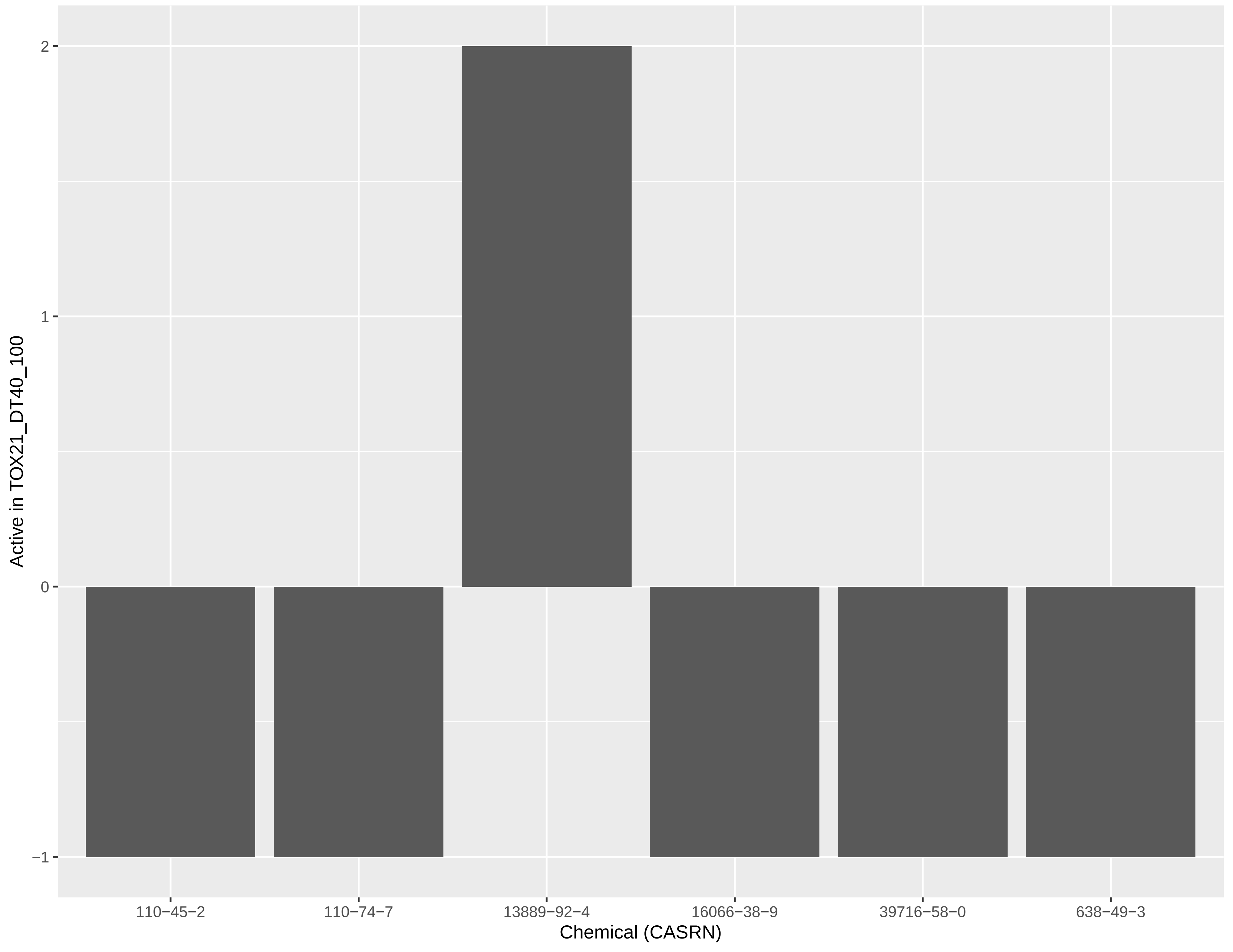

All Activity Heatmap for Cluster 531

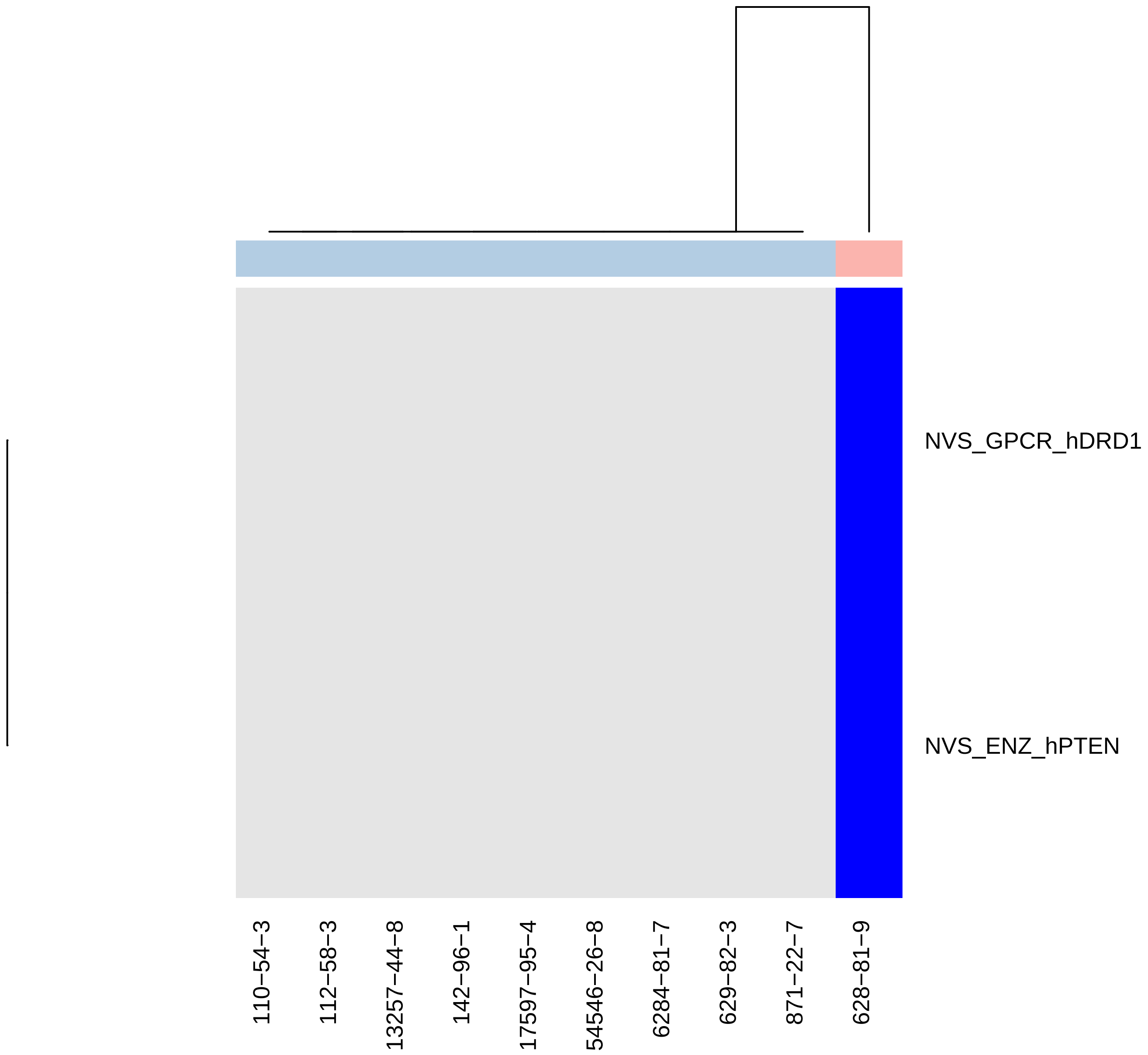

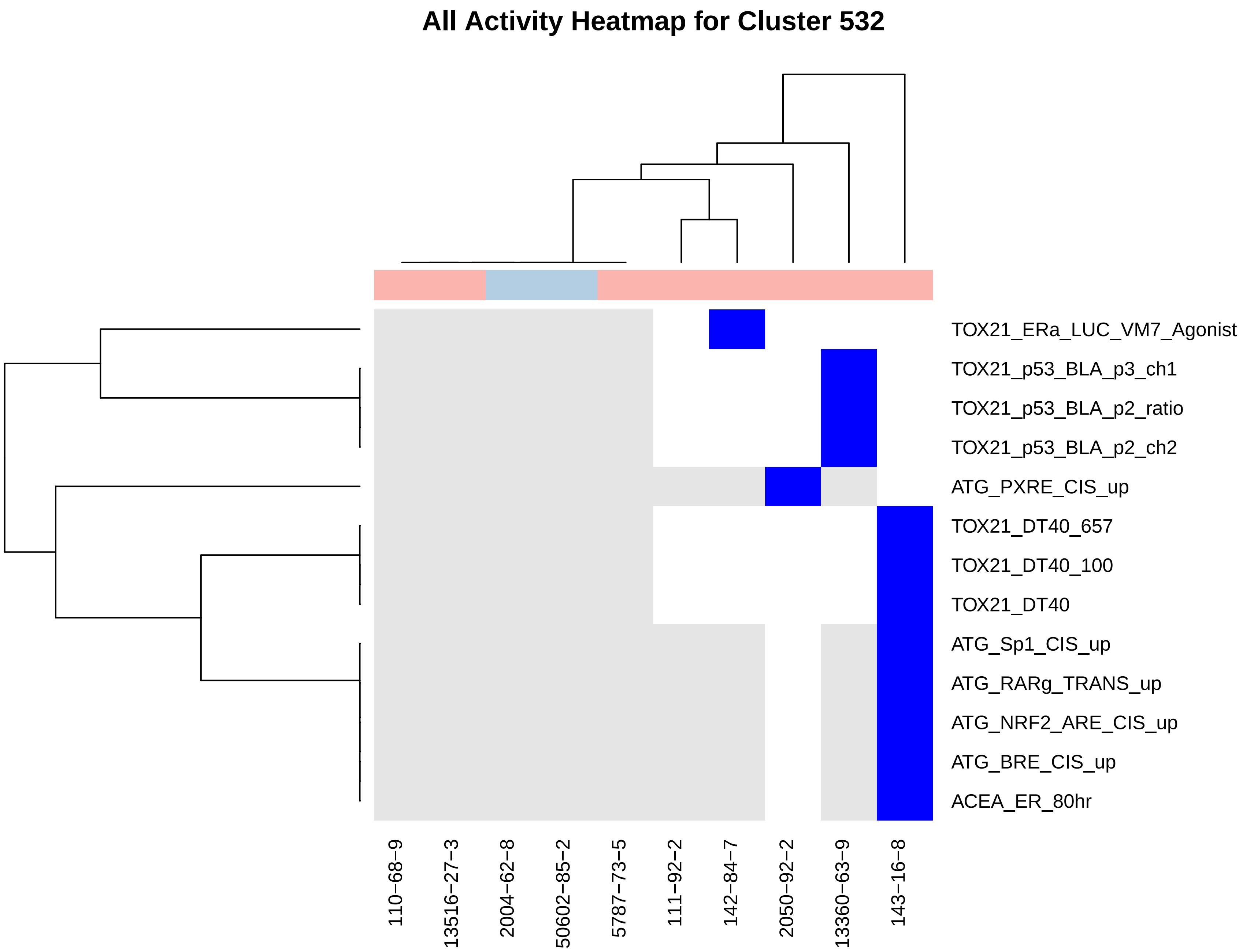
All Activity Heatmap for Cluster 532
TOX21_ERa_LUC_VM7_Agonist
TOX21_p53_BLA_p3_ch1
TOX21_p53_BLA_p2_ratio
TOX21_p53_BLA_p2_ch2
ATG_PXRE_CIS_up
TOX21_DT40_657
TOX21_DT40_100
TOX21_DT40
ATG_Sp1_CIS_up
ATG_RARg_TRANS_up
ATG_NRF2_ARE_CIS_up
ATG_BRE_CIS_up
ACEA_ER_80hr
110−68−9
13516−27−3
2004−62−8
50602−85−2
5787−73−5
111−92−2
142−84−7
2050−92−2
13360−63−9
143−16−8

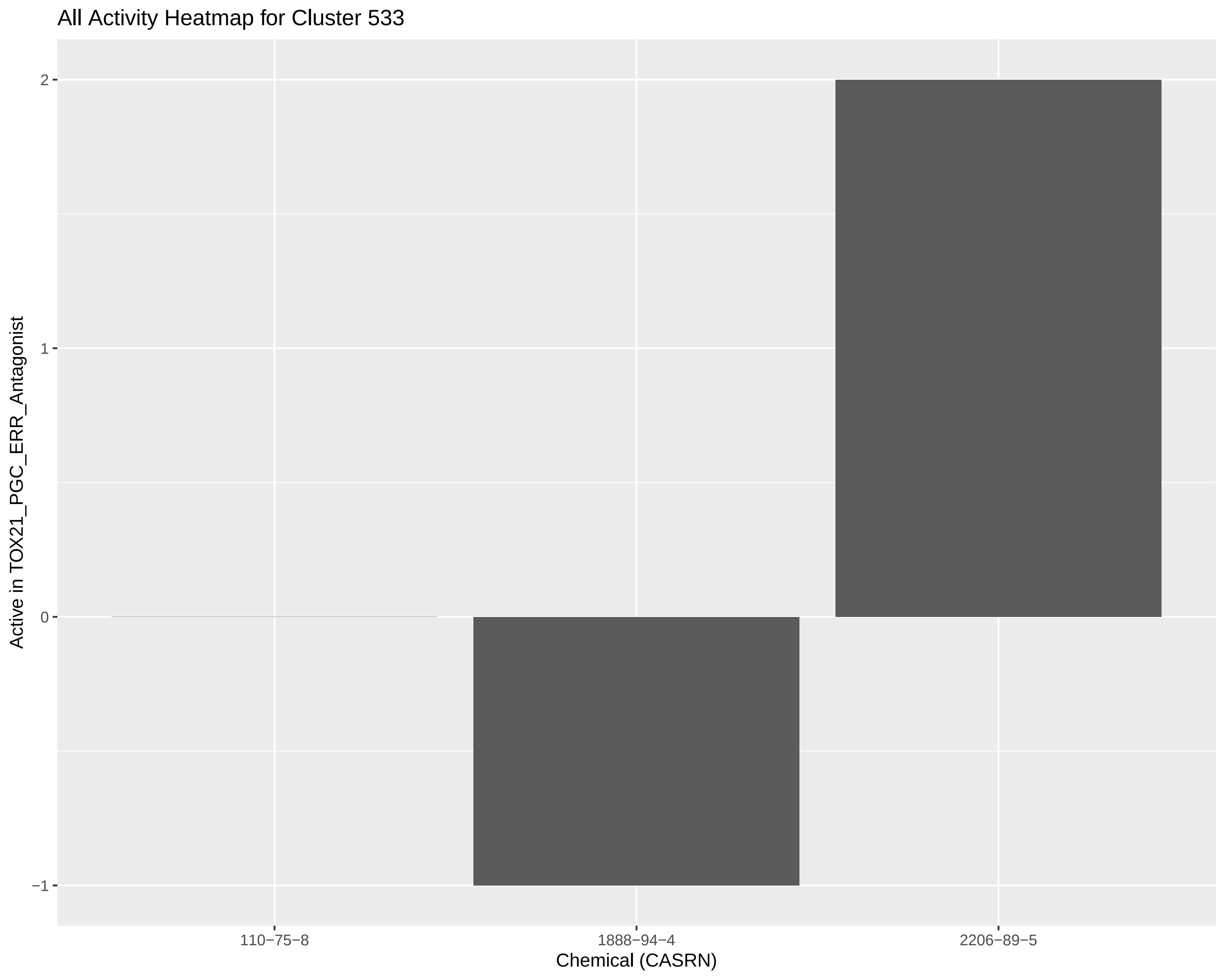
All Activity Heatmap for Cluster 533
Active in TOX21_PGC_ERR_Antagonist
2
1
0
−1
110−75−8
1888−94−4
2206−89−5
Chemical (CASRN)

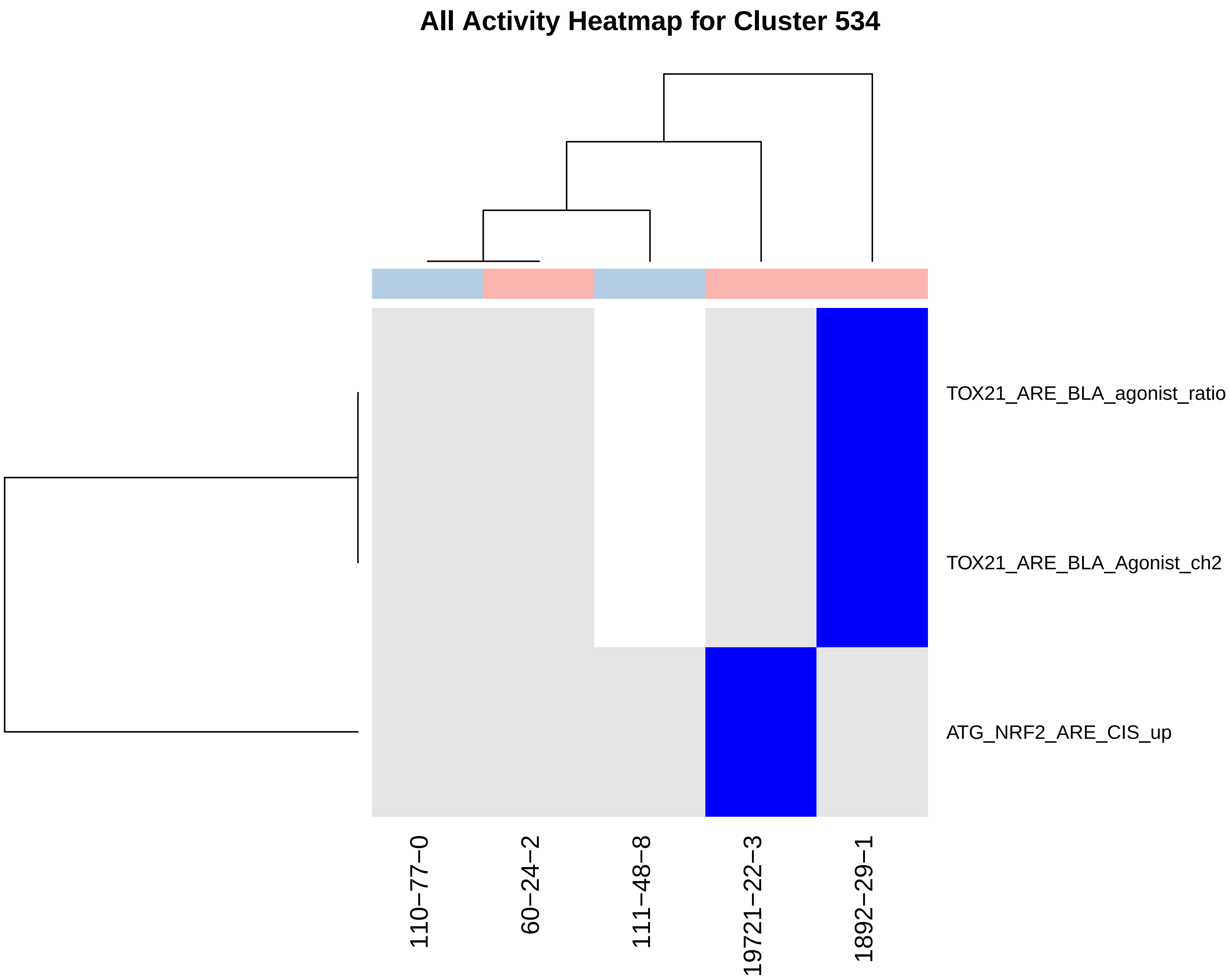
All Activity Heatmap for Cluster 534
TOX21_ARE_BLA_agonist_ratio
TOX21_ARE_BLA_Agonist_ch2
ATG_NRF2_ARE_CIS_up
110−77−0
60−24−2
111−48−8
19721−22−3
1892−29−1

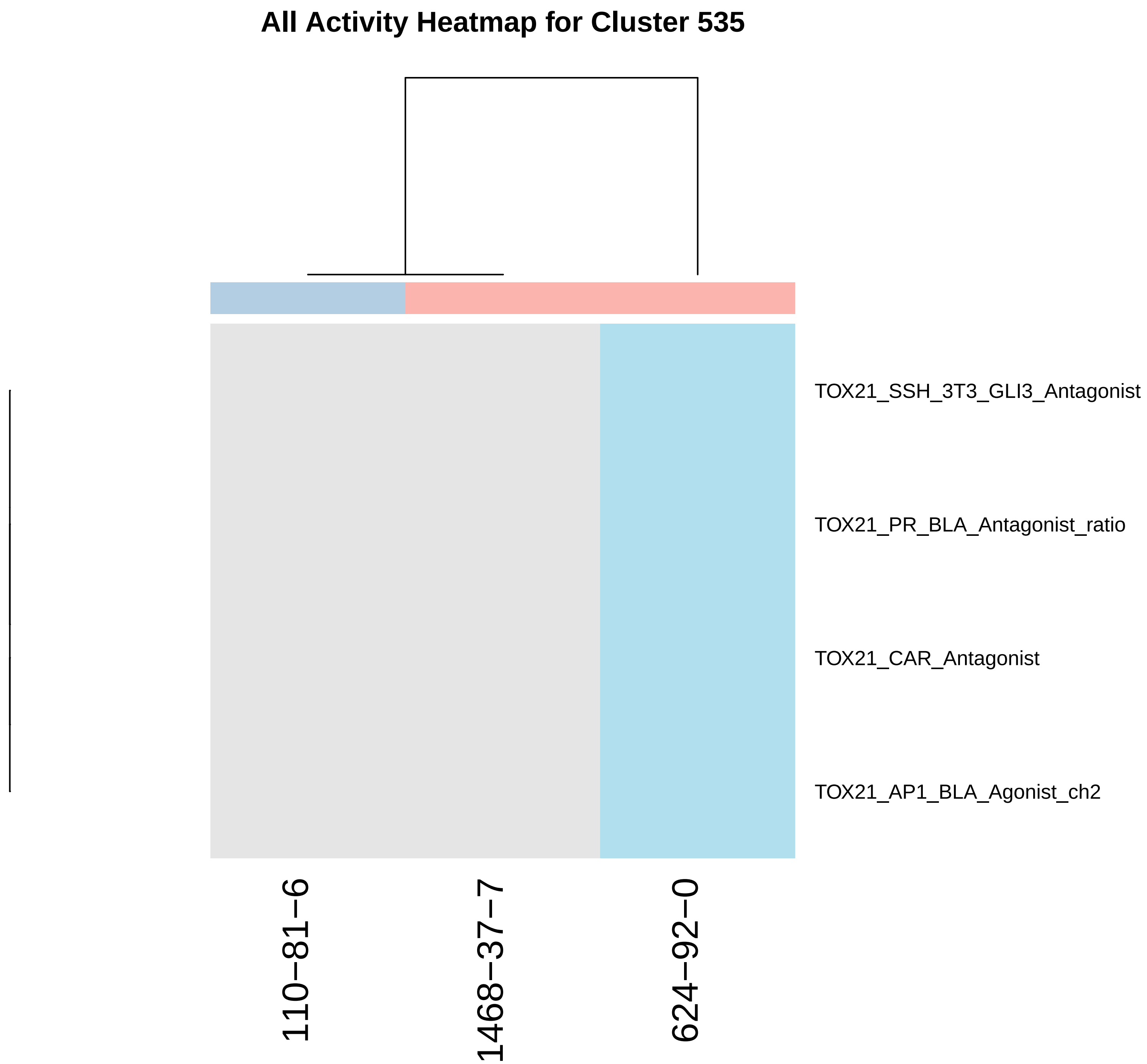
All Activity Heatmap for Cluster 535
TOX21_SSH_3T3_GLI3_Antagonist
TOX21_PR_BLA_Antagonist_ratio
TOX21_CAR_Antagonist
TOX21_AP1_BLA_Agonist_ch2
110−81−6
1468−37−7
624−92−0

All Activity Heatmap for Cluster 536

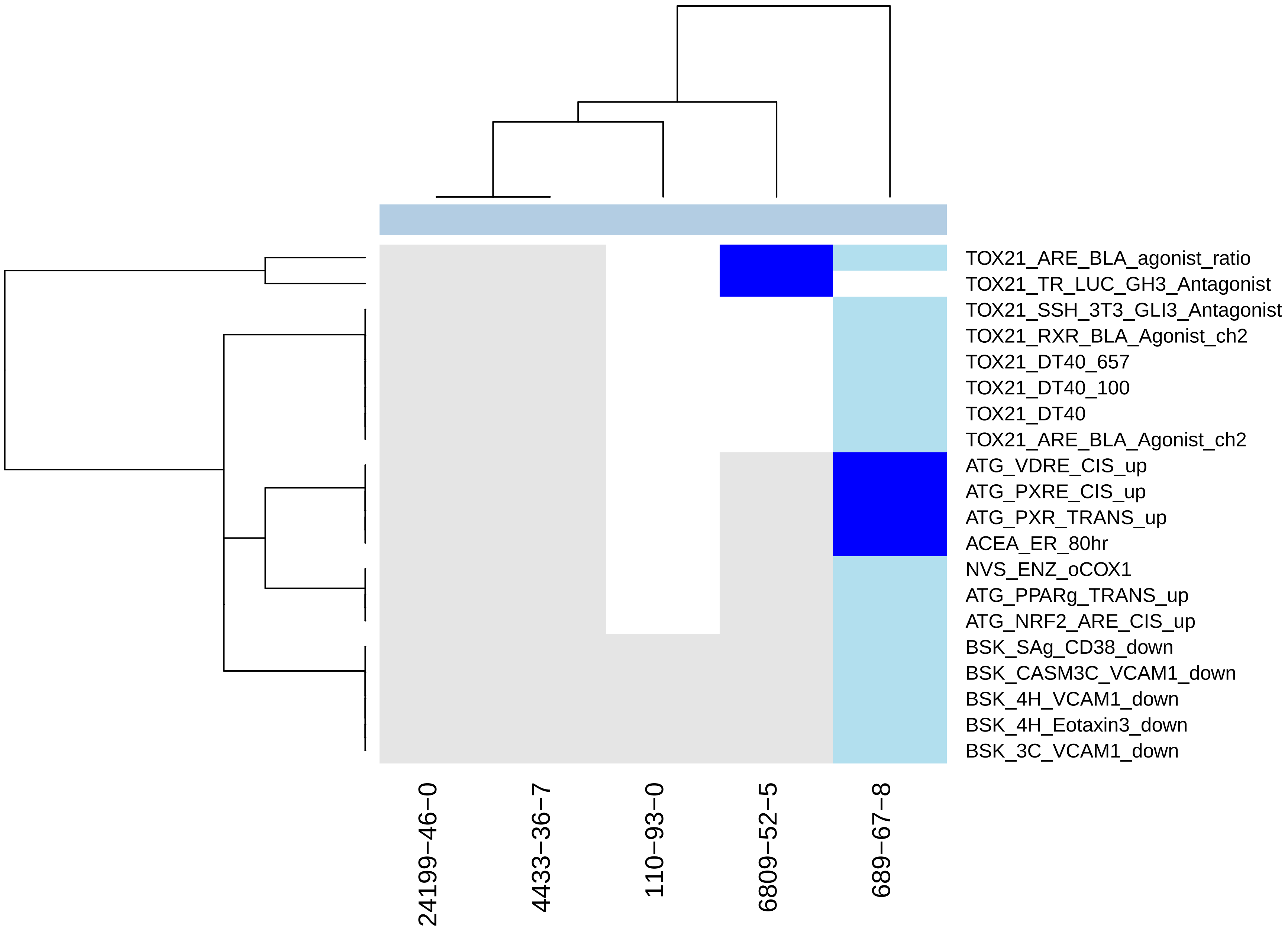

All Activity Heatmap for Cluster 537

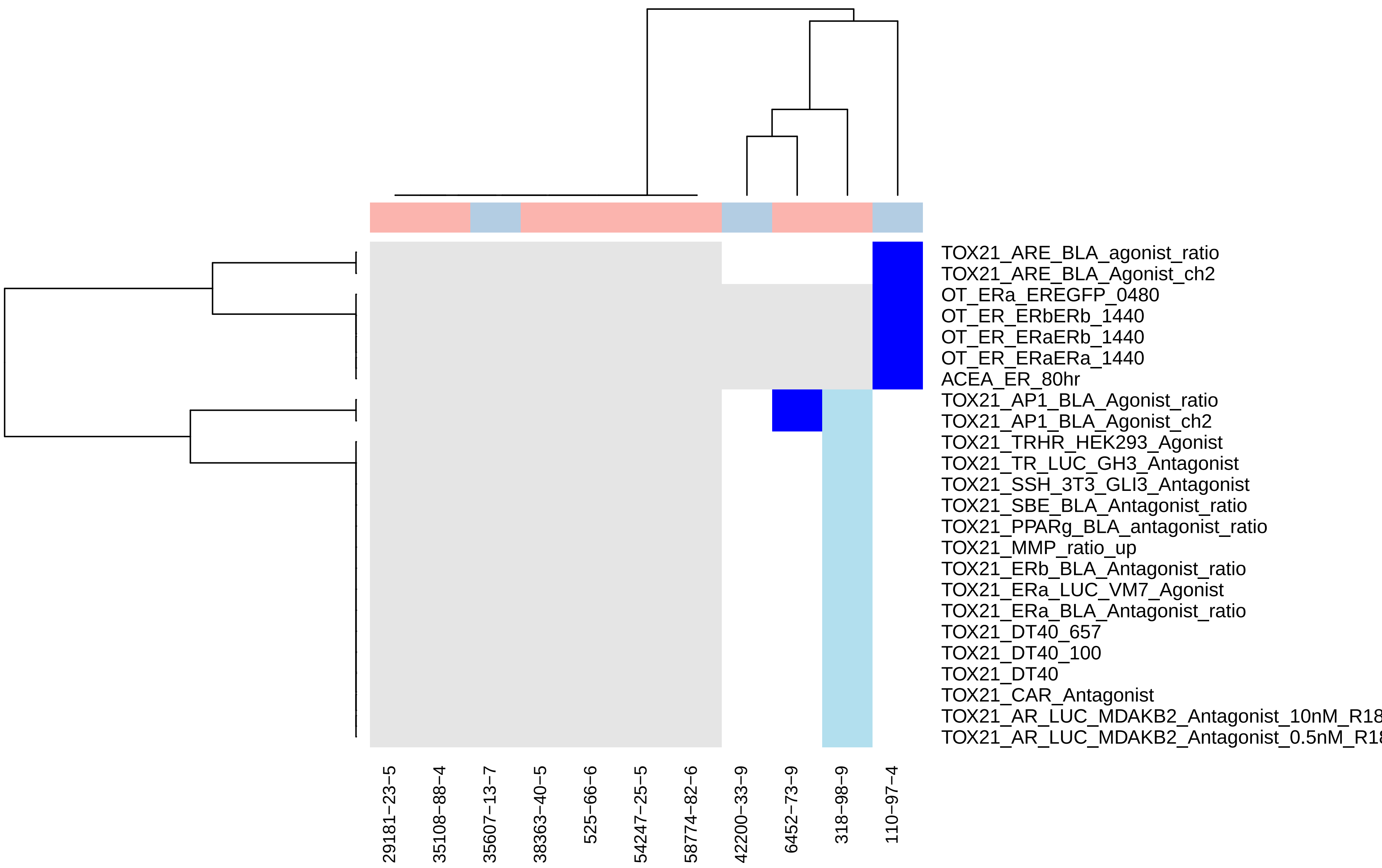

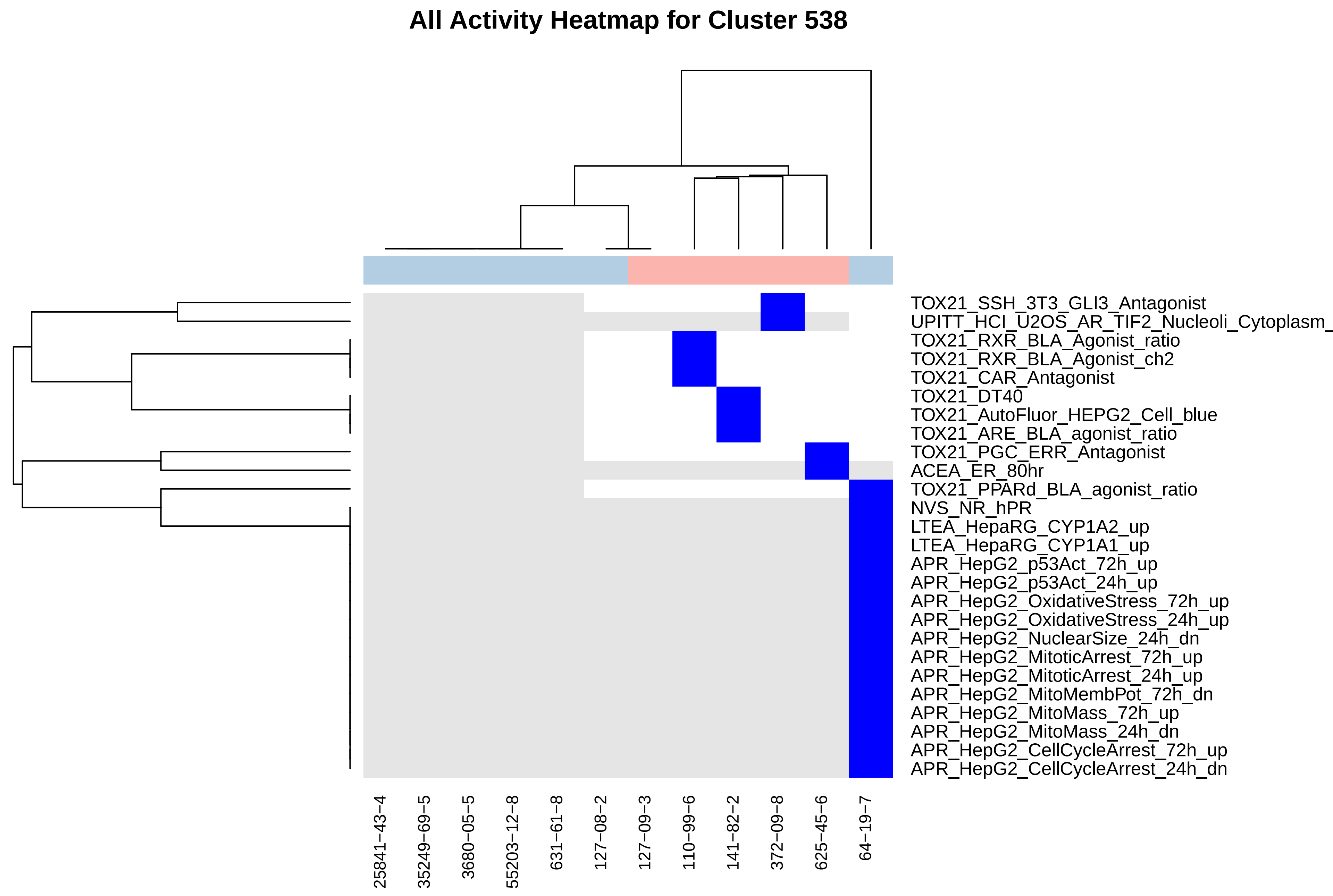
All Activity Heatmap for Cluster 538
TOX21_SSH_3T3_GLI3_Antagonist
UPITT_HCI_U2OS_AR_TIF2_Nucleoli_Cytoplasm_
TOX21_RXR_BLA_Agonist_ratio
TOX21_RXR_BLA_Agonist_ch2
TOX21_CAR_Antagonist
TOX21_DT40
TOX21_AutoFluor_HEPG2_Cell_blue
TOX21_ARE_BLA_agonist_ratio
TOX21_PGC_ERR_Antagonist
ACEA_ER_80hr
TOX21_PPARd_BLA_agonist_ratio
NVS_NR_hPR
LTEA_HepaRG_CYP1A2_up
LTEA_HepaRG_CYP1A1_up
APR_HepG2_p53Act_72h_up
APR_HepG2_p53Act_24h_up
APR_HepG2_OxidativeStress_72h_up
APR_HepG2_OxidativeStress_24h_up
APR_HepG2_NuclearSize_24h_dn
APR_HepG2_MitoticArrest_72h_up
APR_HepG2_MitoticArrest_24h_up
APR_HepG2_MitoMembPot_72h_dn
APR_HepG2_MitoMass_72h_up
APR_HepG2_MitoMass_24h_dn
APR_HepG2_CellCycleArrest_72h_up
APR_HepG2_CellCycleArrest_24h_dn
25841−43−4
35249−69−5
3680−05−5
55203−12−8
631−61−8
127−08−2
127−09−3
110−99−6
141−82−2
372−09−8
625−45−6
64−19−7

All Activity Heatmap for Cluster 539

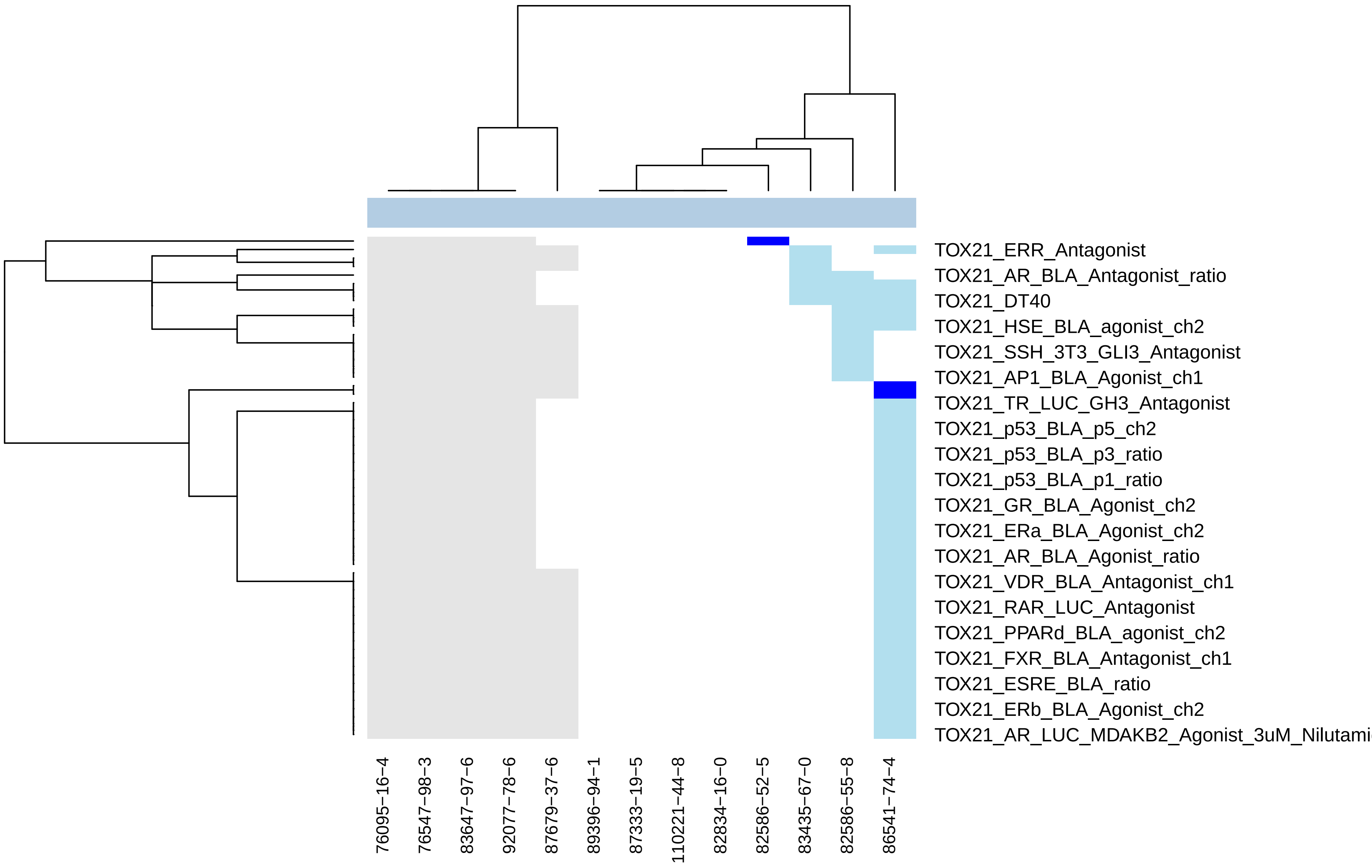

All Activity Heatmap for Cluster 540

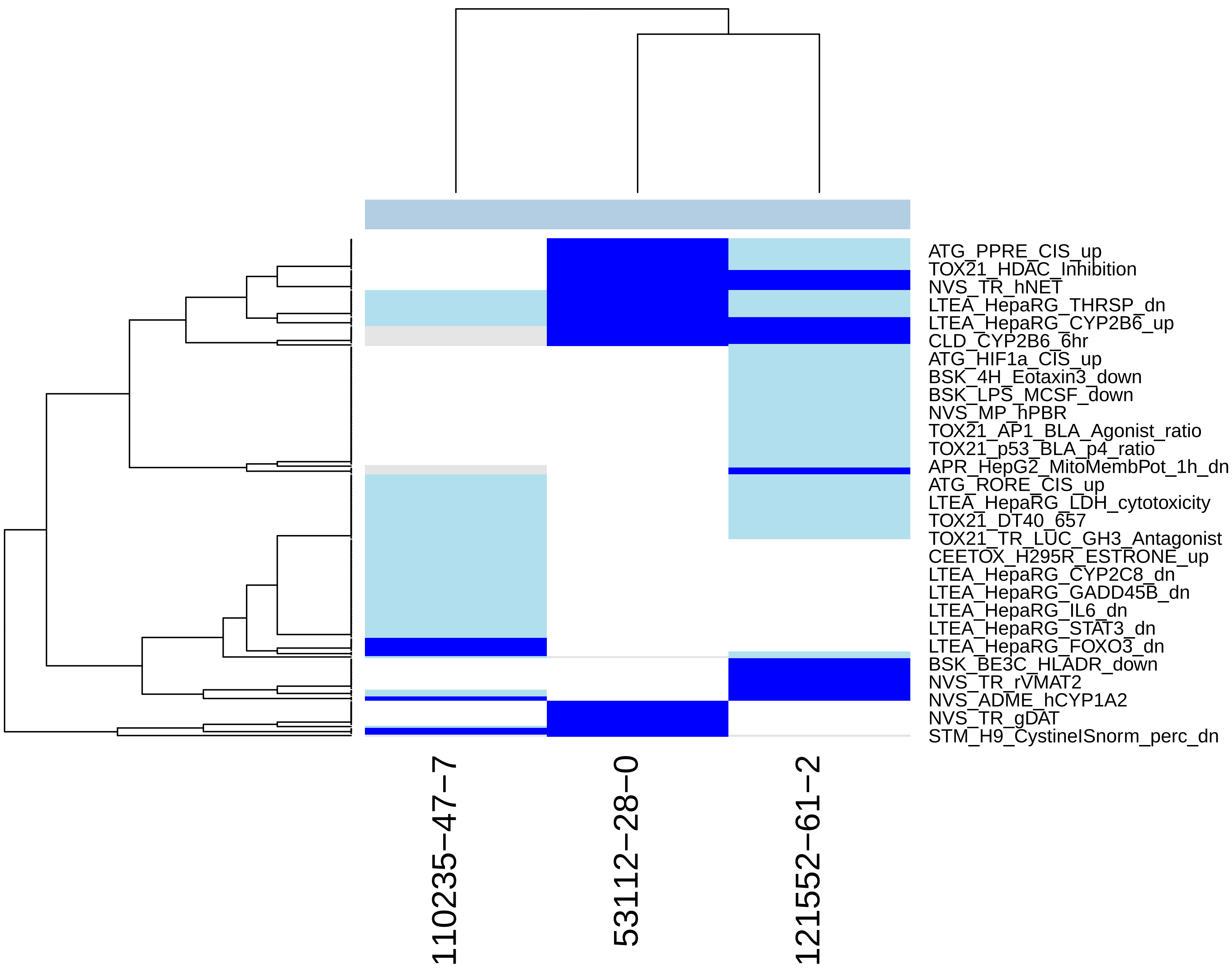

All Activity Heatmap for Cluster 541

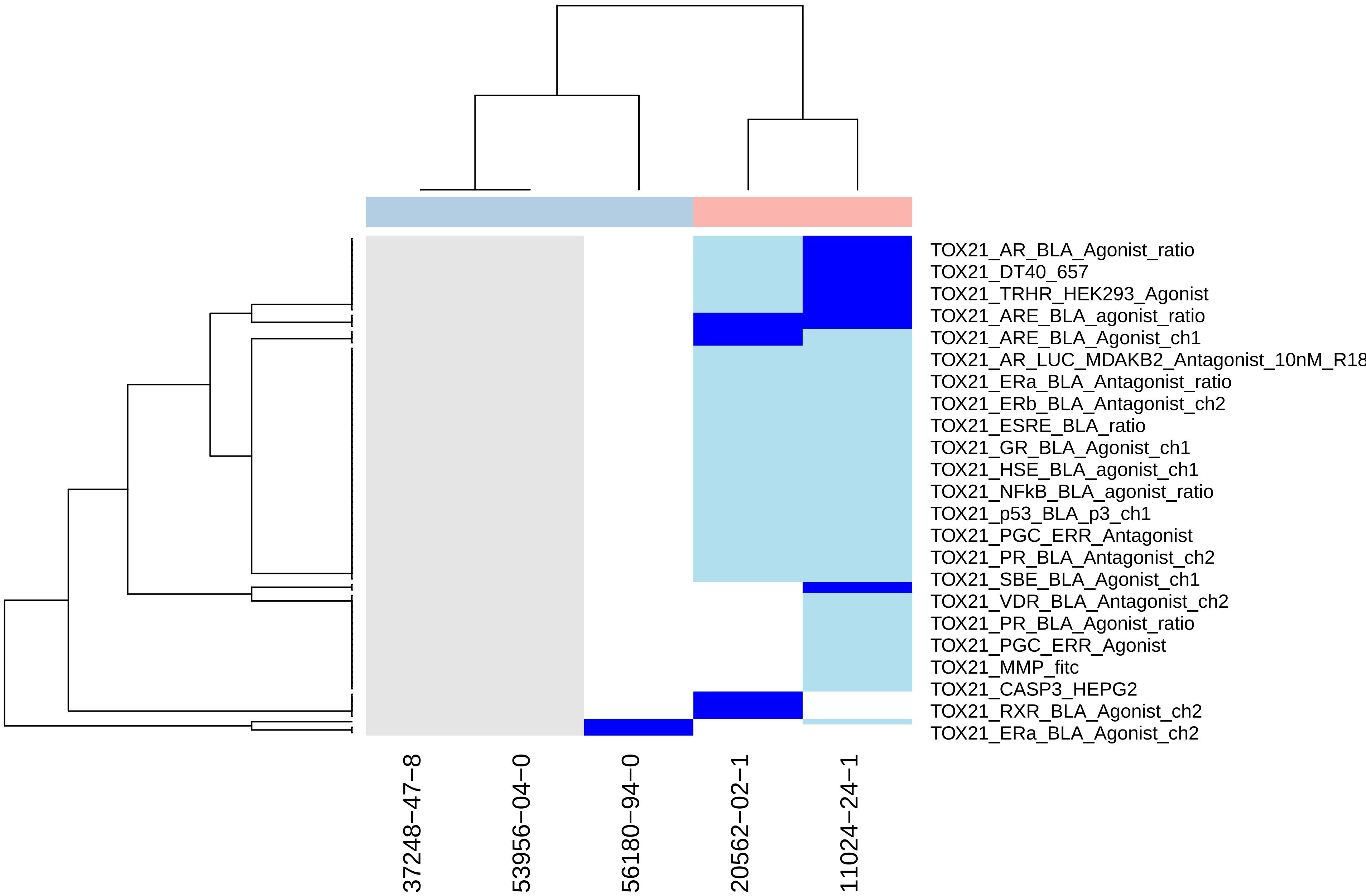

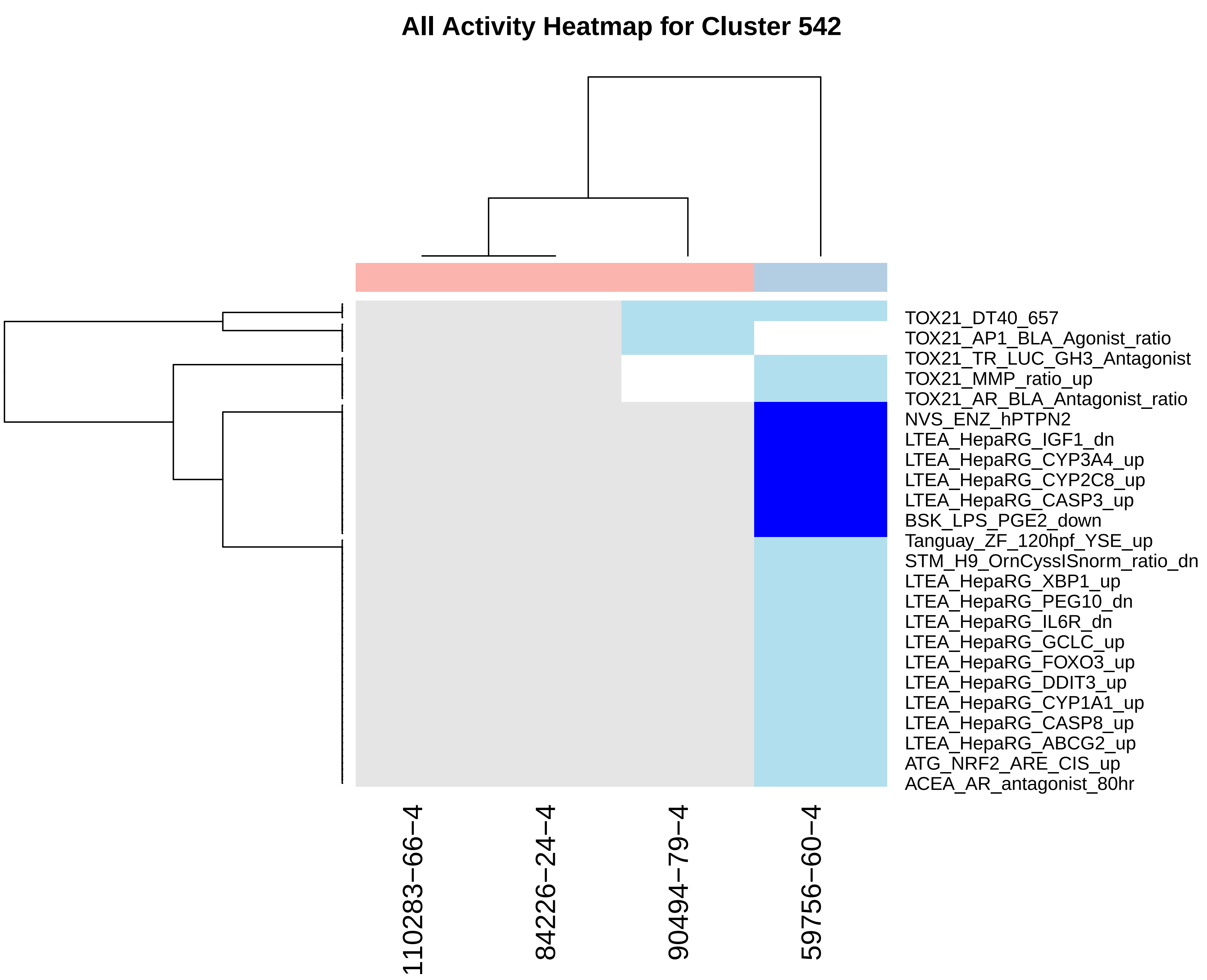
All Activity Heatmap for Cluster 542
TOX21_DT40_657
TOX21_AP1_BLA_Agonist_ratio
TOX21_TR_LUC_GH3_Antagonist
TOX21_MMP_ratio_up
TOX21_AR_BLA_Antagonist_ratio
NVS_ENZ_hPTPN2
LTEA_HepaRG_IGF1_dn
LTEA_HepaRG_CYP3A4_up
LTEA_HepaRG_CYP2C8_up
LTEA_HepaRG_CASP3_up
BSK_LPS_PGE2_down
Tanguay_ZF_120hpf_YSE_up
STM_H9_OrnCyssISnorm_ratio_dn
LTEA_HepaRG_XBP1_up
LTEA_HepaRG_PEG10_dn
LTEA_HepaRG_IL6R_dn
LTEA_HepaRG_GCLC_up
LTEA_HepaRG_FOXO3_up
LTEA_HepaRG_DDIT3_up
LTEA_HepaRG_CYP1A1_up
LTEA_HepaRG_CASP8_up
LTEA_HepaRG_ABCG2_up
ATG_NRF2_ARE_CIS_up
ACEA_AR_antagonist_80hr
110283−66−4
84226−24−4
90494−79−4
59756−60−4

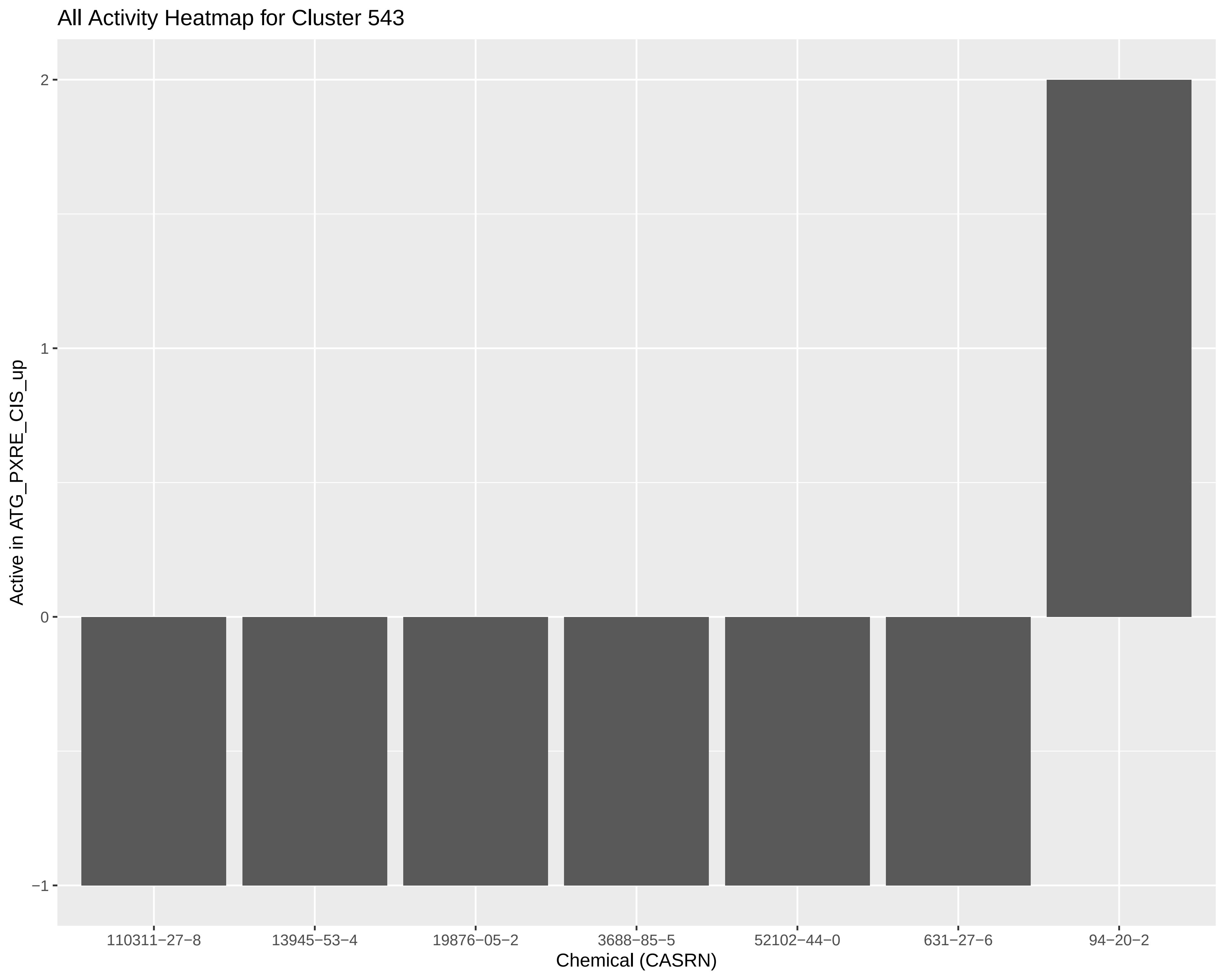

All Activity Heatmap for Cluster 543
Active in ATG_PXRE_CIS_up
2
1
0
−1
110311−27−8
13945−53−4
19876−05−2
3688−85−5
52102−44−0
631−27−6
94−20−2
Chemical (CASRN)

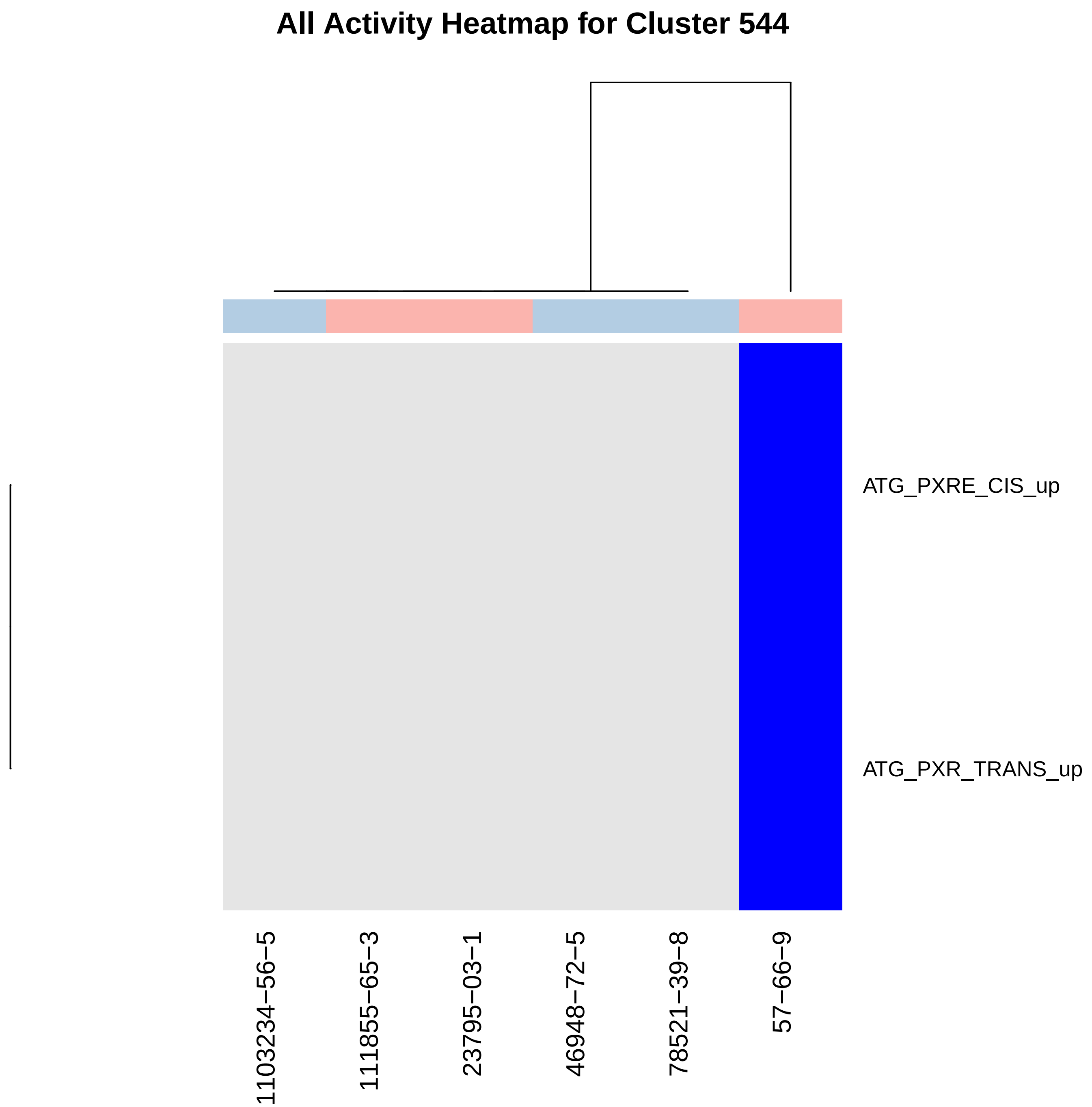

All Activity Heatmap for Cluster 544
ATG_PXRE_CIS_up
ATG_PXR_TRANS_up
1103234−56−5
111855−65−3
23795−03−1
46948−72−5
78521−39−8
57−66−9

All Activity Heatmap for Cluster 545

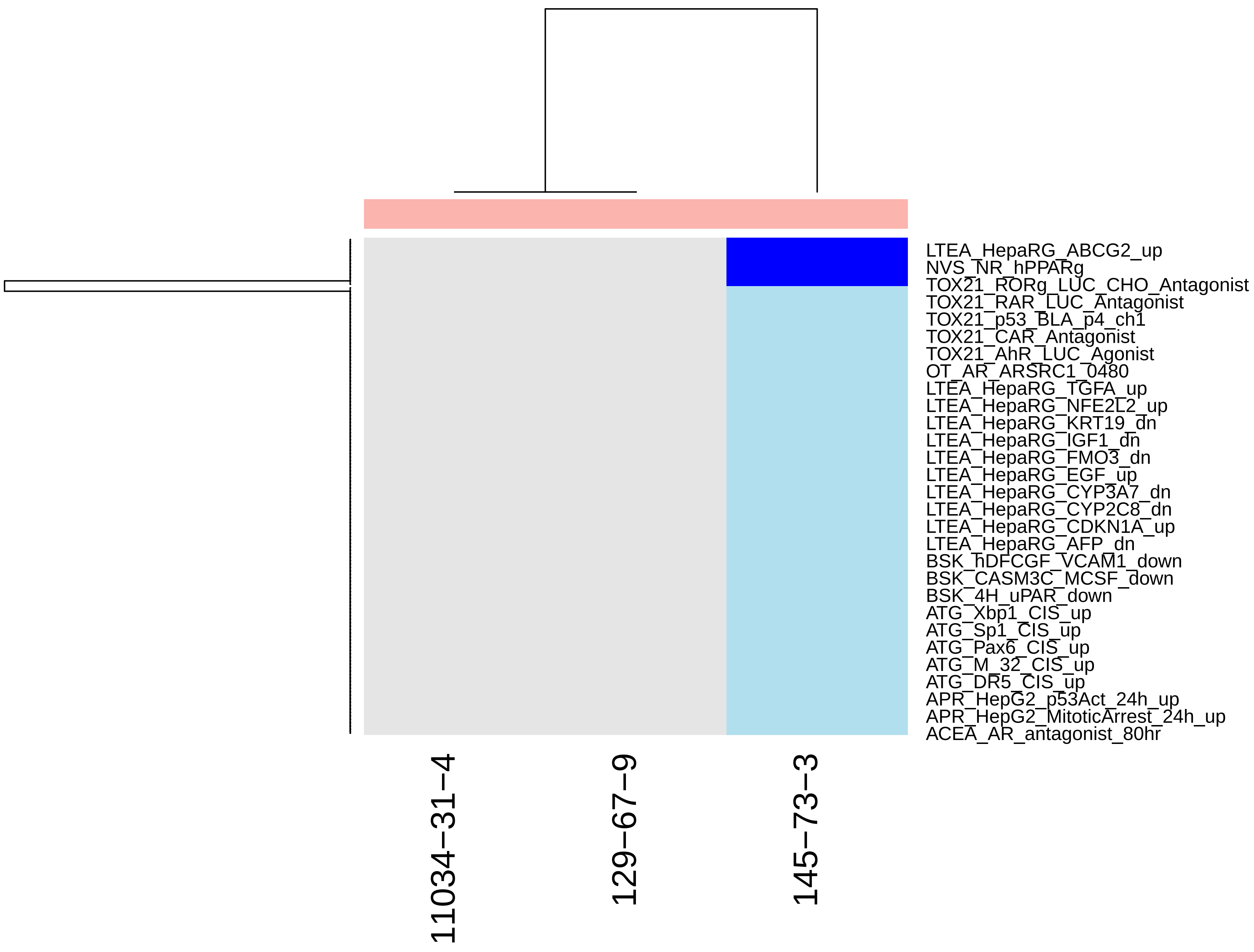

All Activity Heatmap for Cluster 546

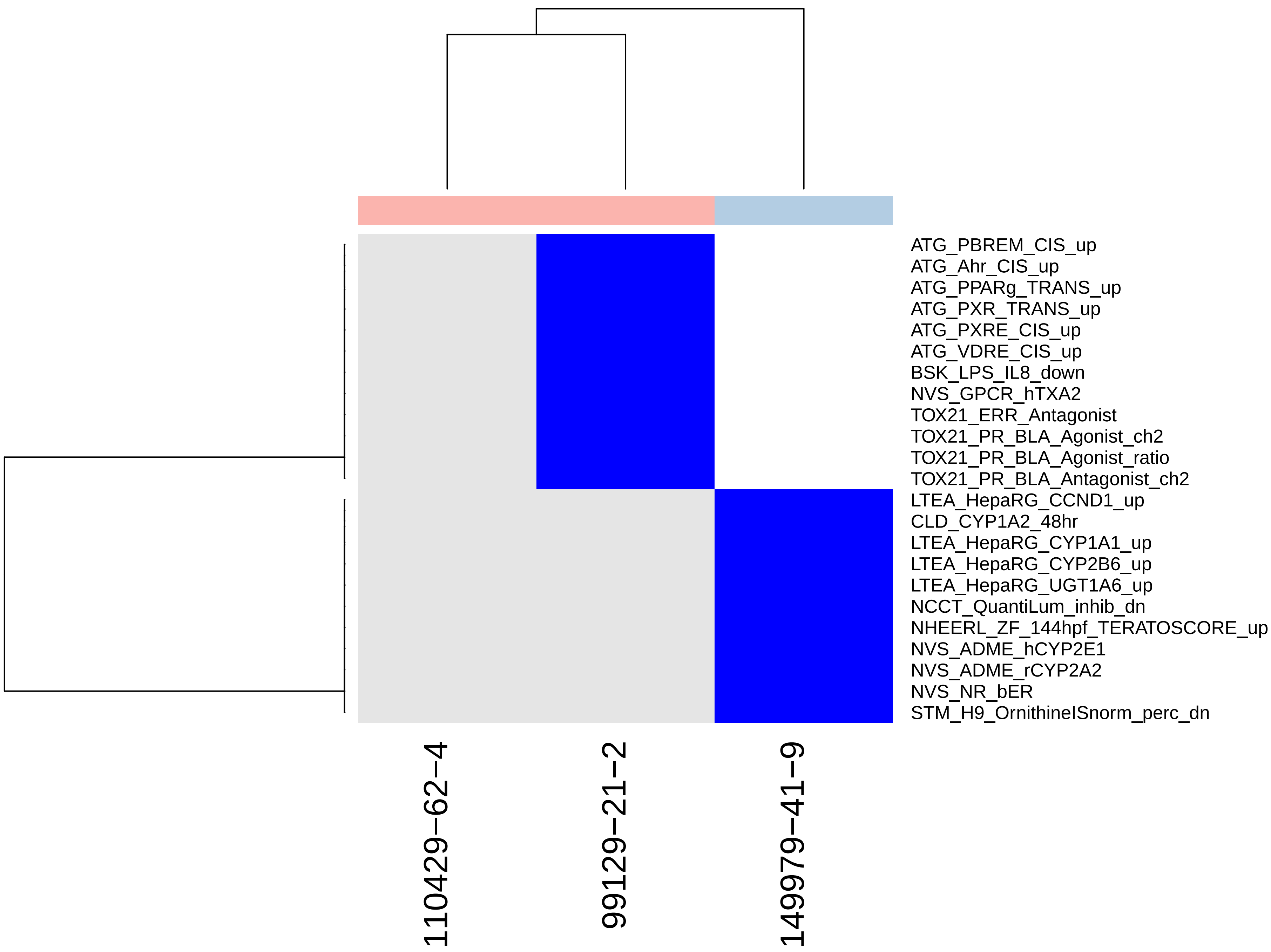

All Activity Heatmap for Cluster 547

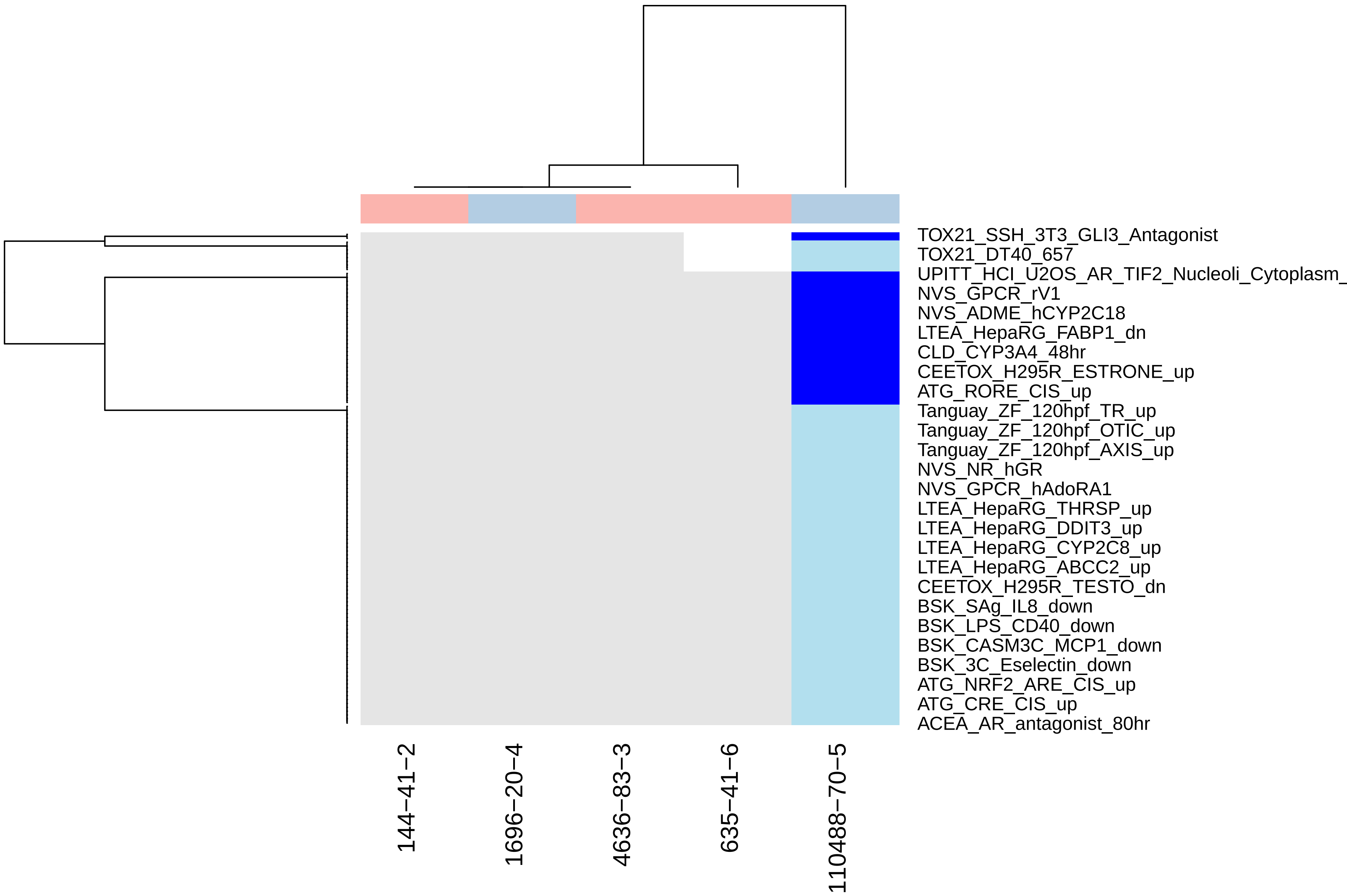

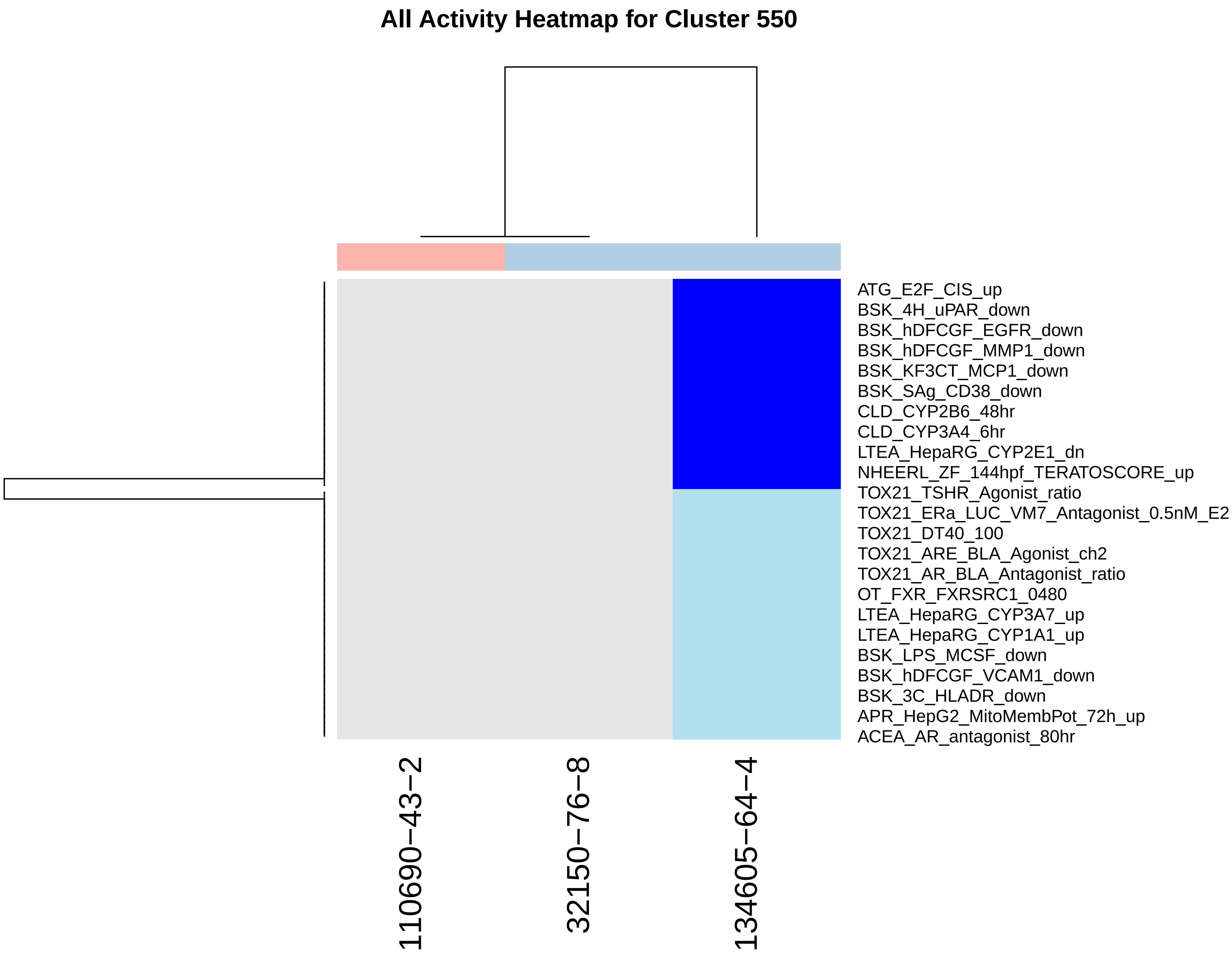
All Activity Heatmap for Cluster 550
ATG_E2F_CIS_up
BSK_4H_uPAR_down
BSK_hDFCGF_EGFR_down
BSK_hDFCGF_MMP1_down
BSK_KF3CT_MCP1_down
BSK_SAg_CD38_down
CLD_CYP2B6_48hr
CLD_CYP3A4_6hr
LTEA_HepaRG_CYP2E1_dn
NHEERL_ZF_144hpf_TERATOSCORE_up
TOX21_TSHR_Agonist_ratio
TOX21_ERa_LUC_VM7_Antagonist_0.5nM_E2
TOX21_DT40_100
TOX21_ARE_BLA_Agonist_ch2
TOX21_AR_BLA_Antagonist_ratio
OT_FXR_FXRSRC1_0480
LTEA_HepaRG_CYP3A7_up
LTEA_HepaRG_CYP1A1_up
BSK_LPS_MCSF_down
BSK_hDFCGF_VCAM1_down
BSK_3C_HLADR_down
APR_HepG2_MitoMembPot_72h_up
ACEA_AR_antagonist_80hr
110690−43−2
32150−76−8
134605−64−4

All Activity Heatmap for Cluster 552

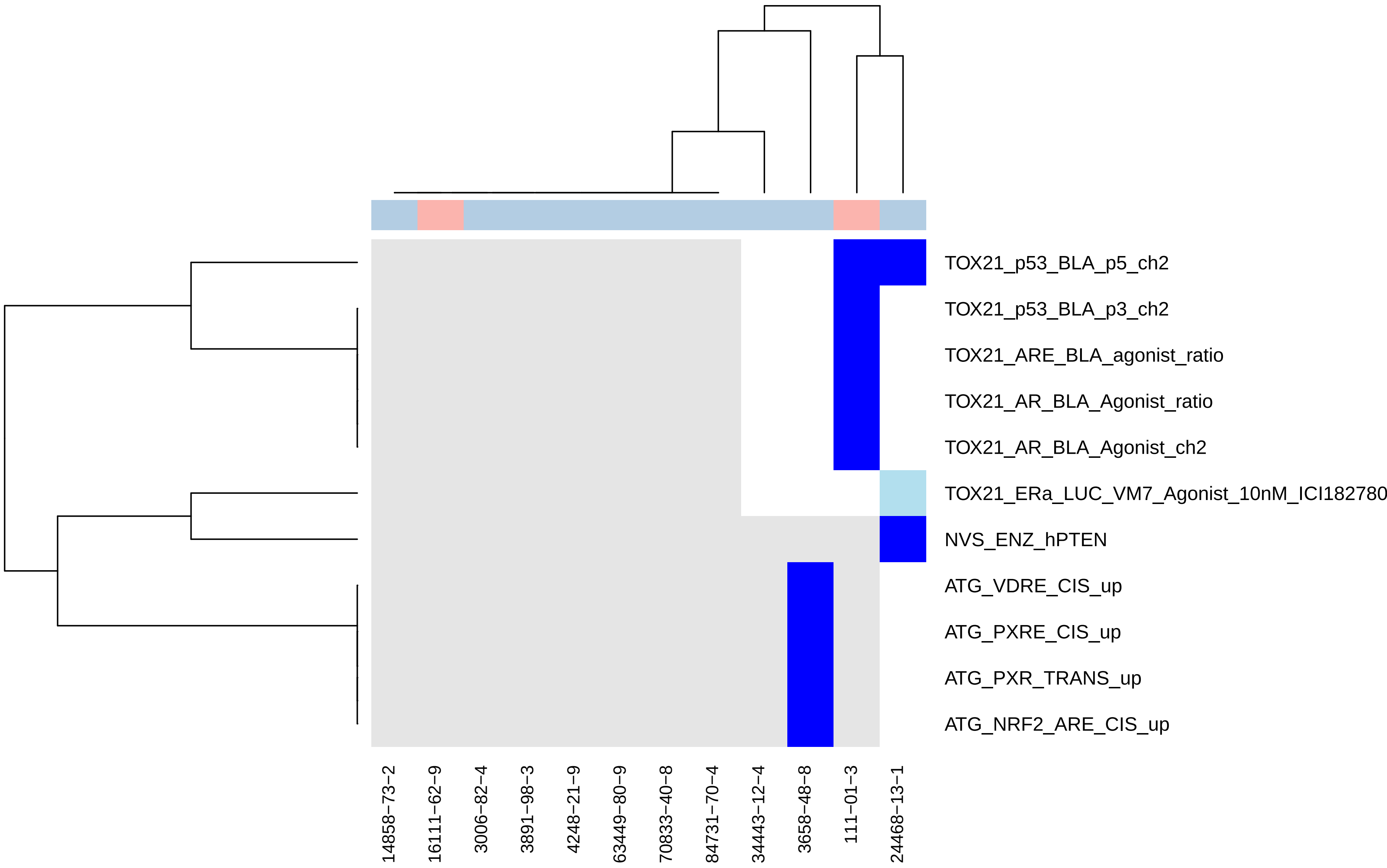

All Activity Heatmap for Cluster 553

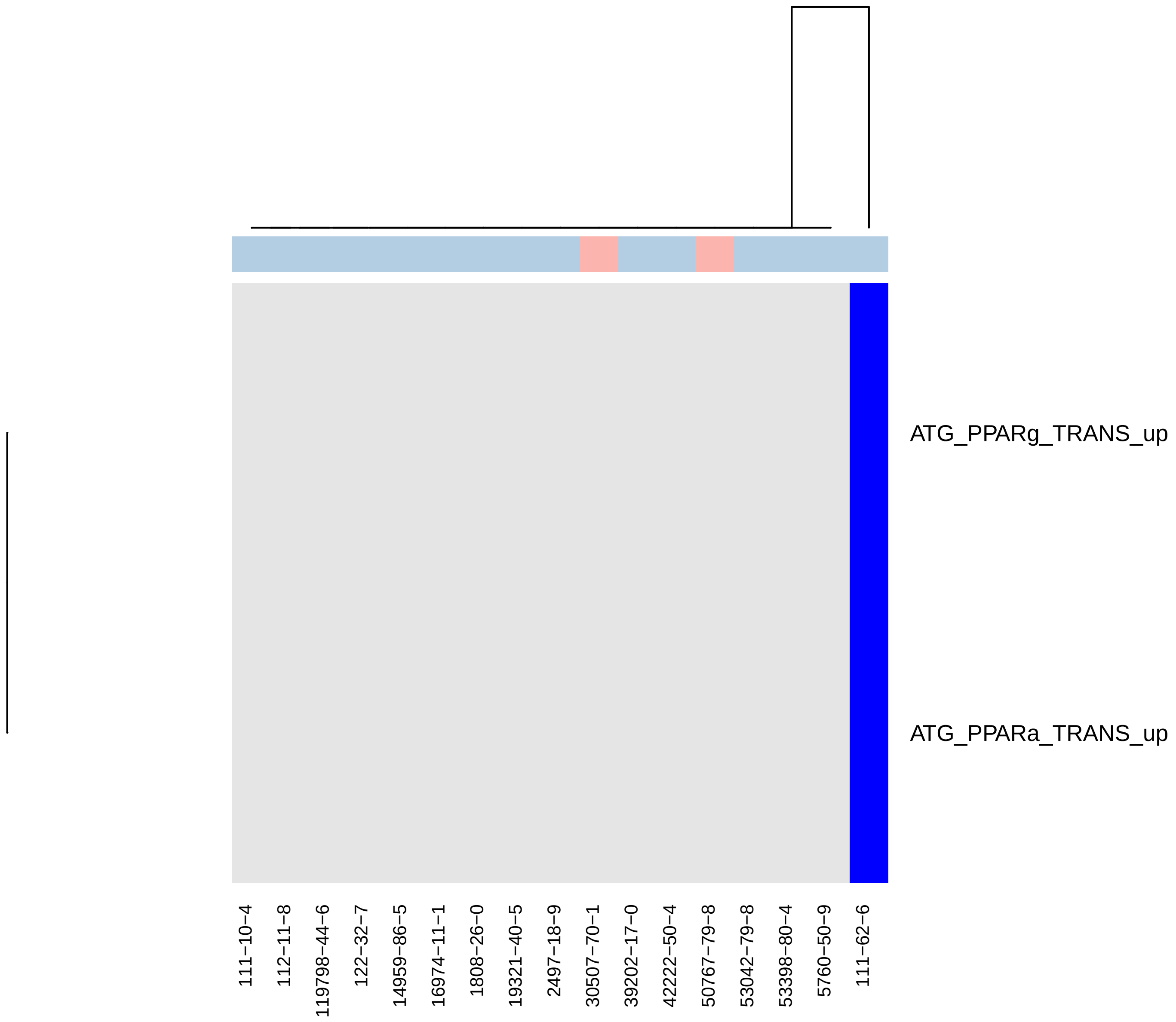

All Activity Heatmap for Cluster 554

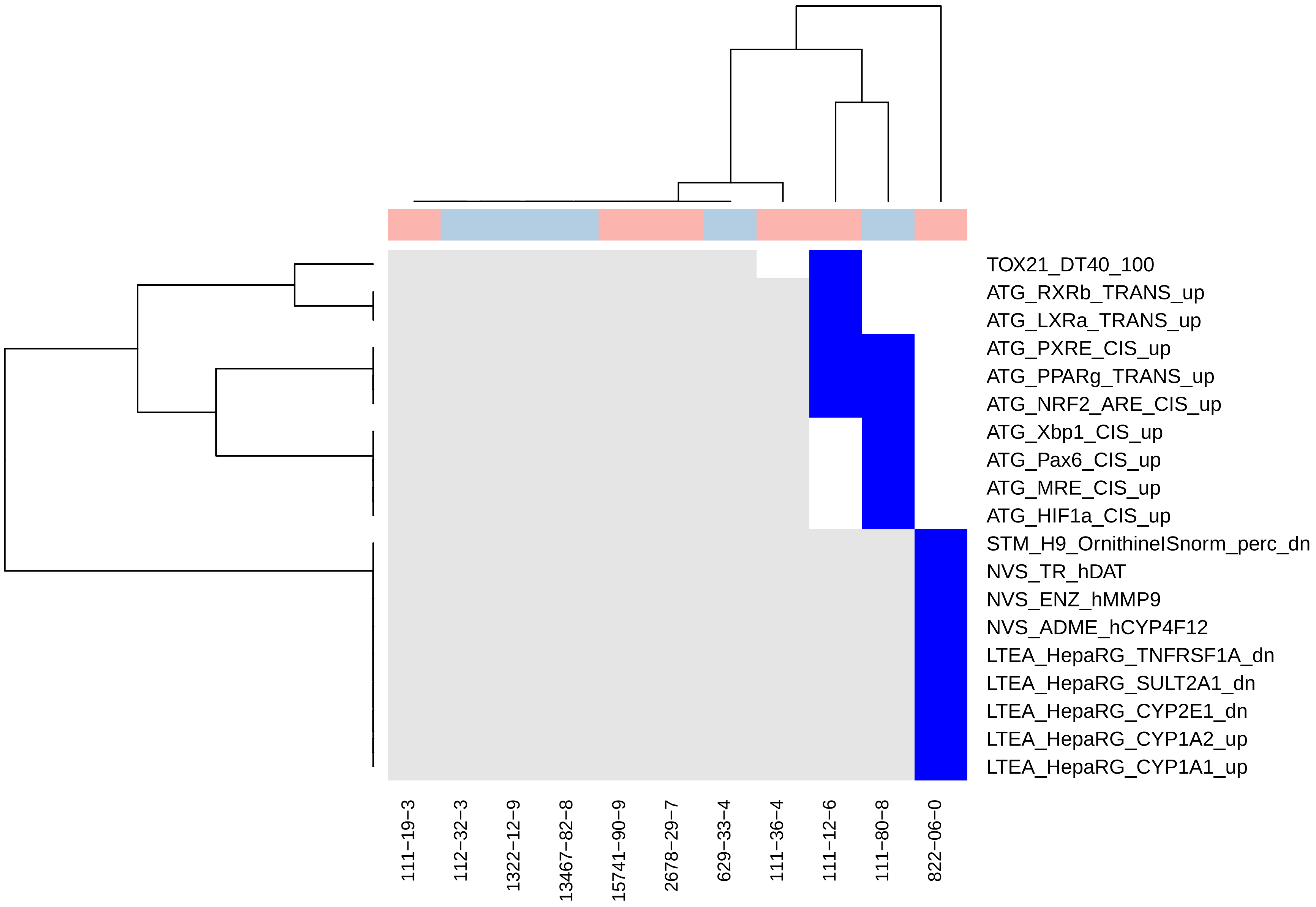

All Activity Heatmap for Cluster 555

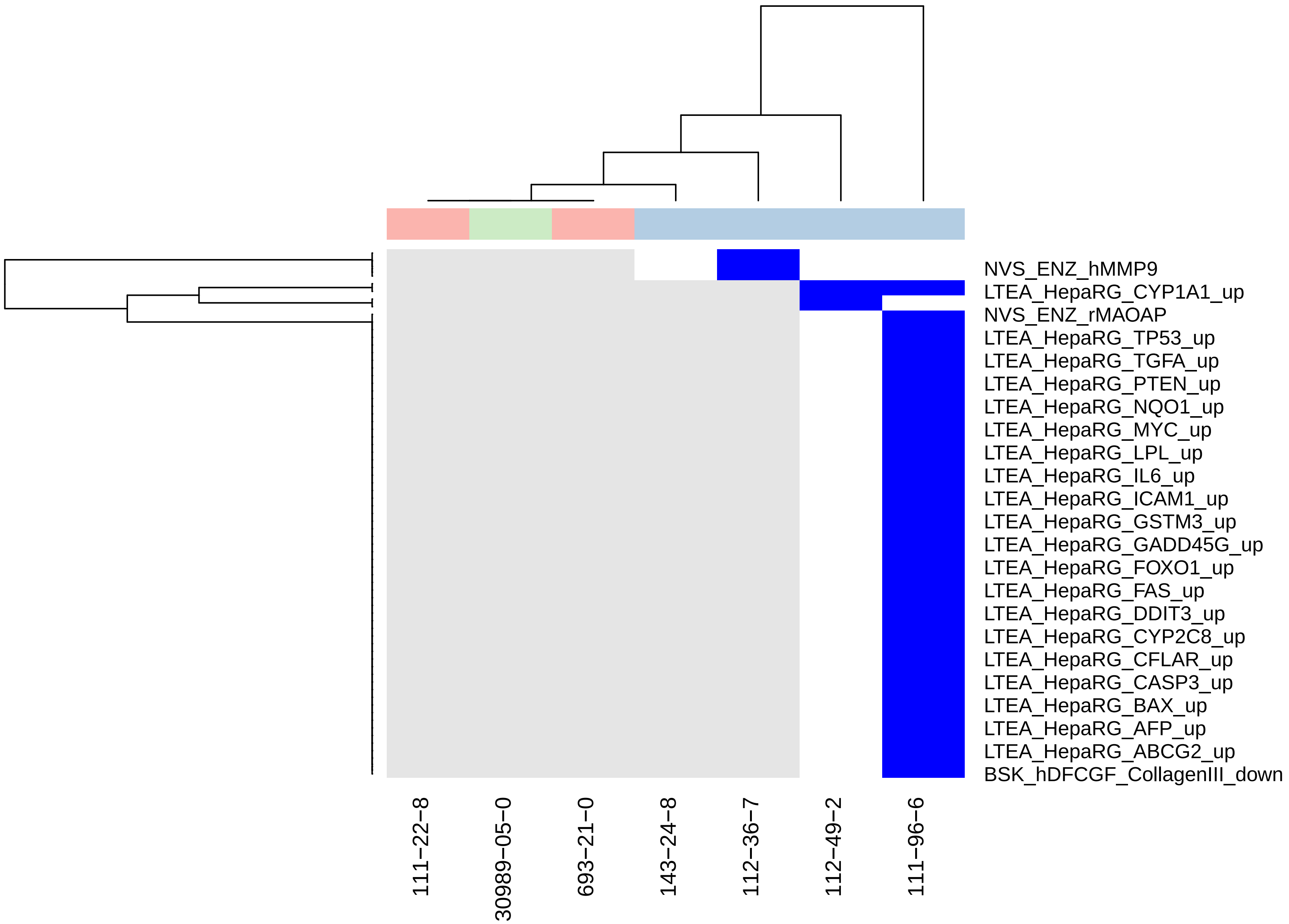

All Activity Heatmap for Cluster 556

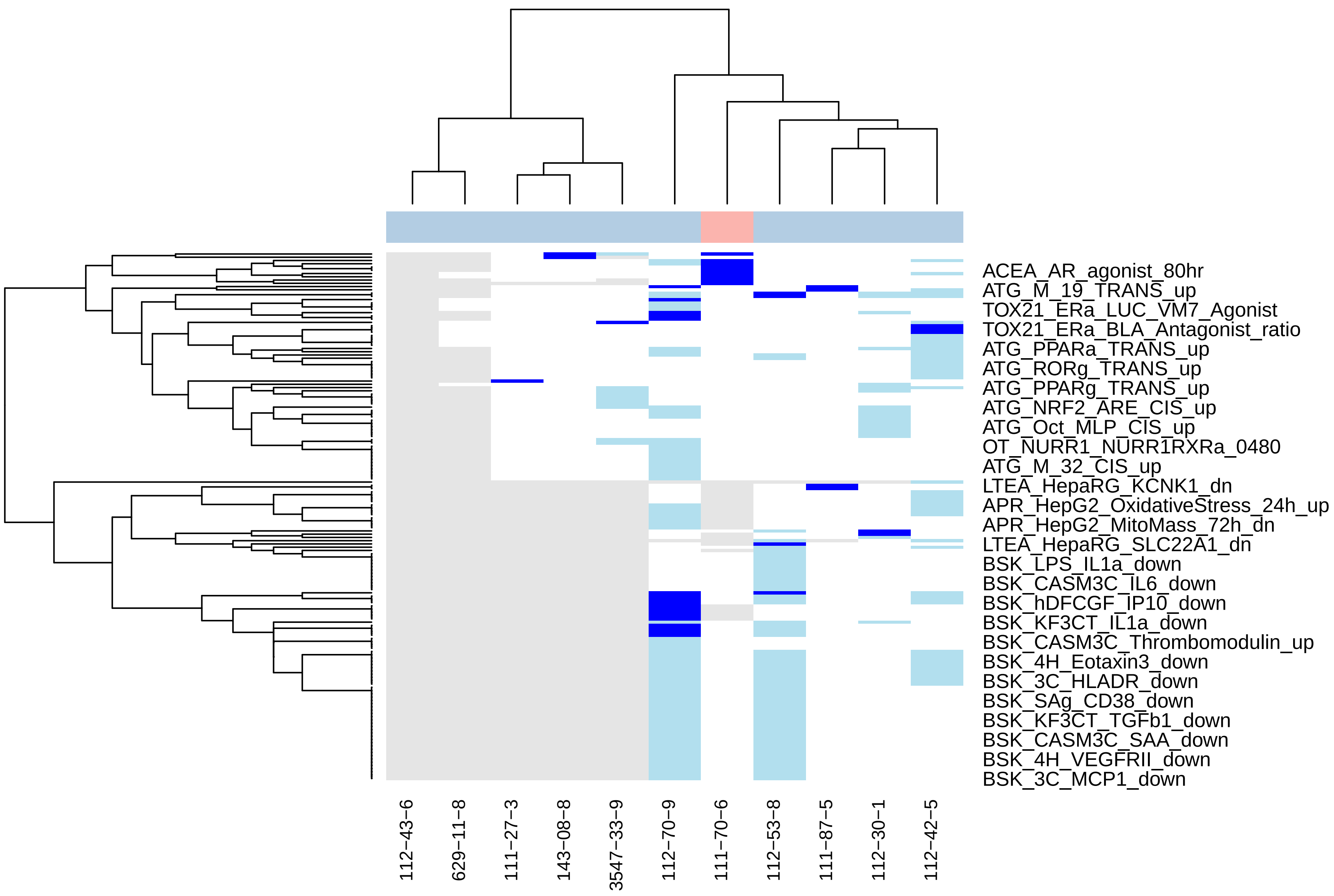

All Activity Heatmap for Cluster 557

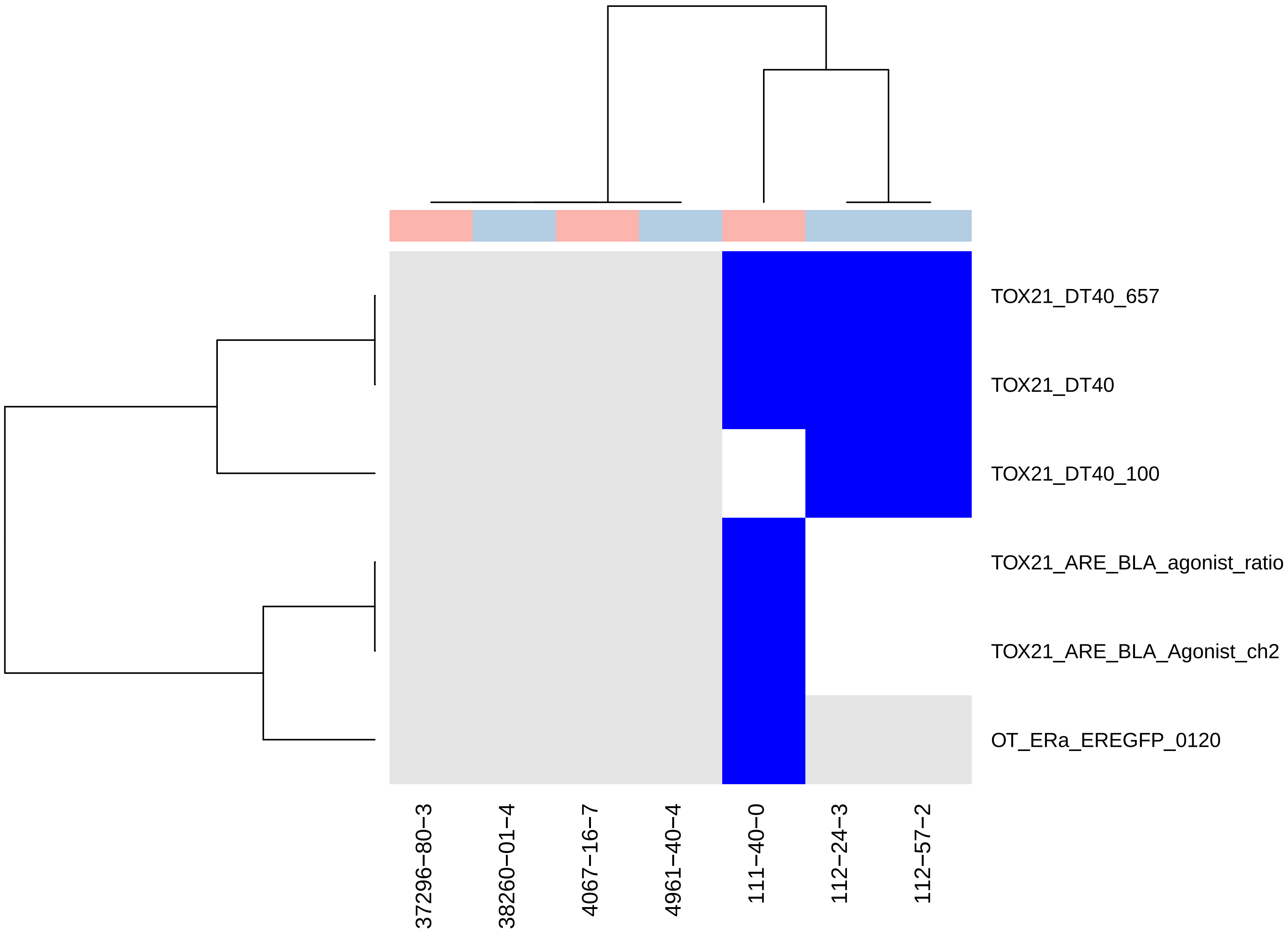

All Activity Heatmap for Cluster 558

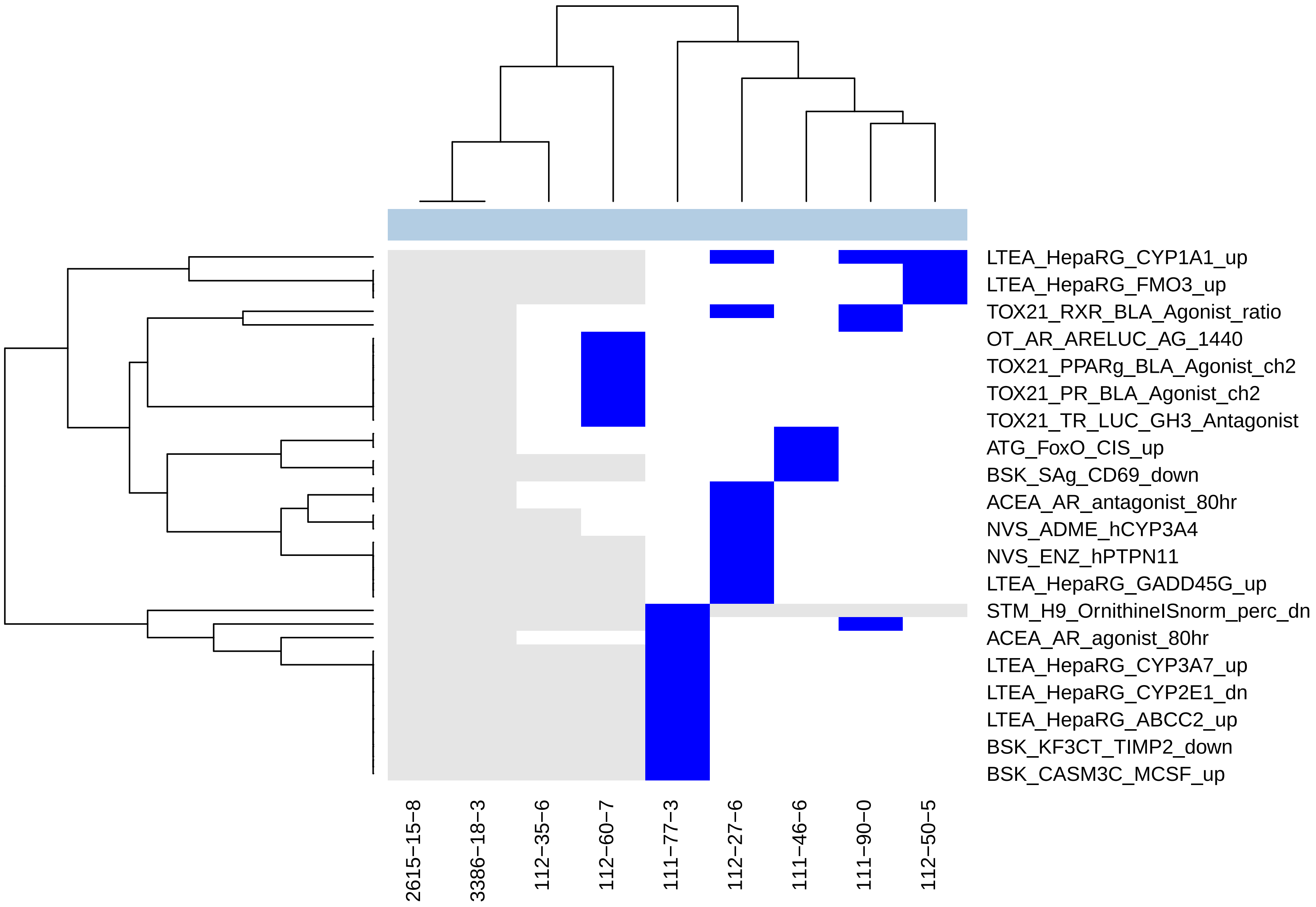

All Activity Heatmap for Cluster 559

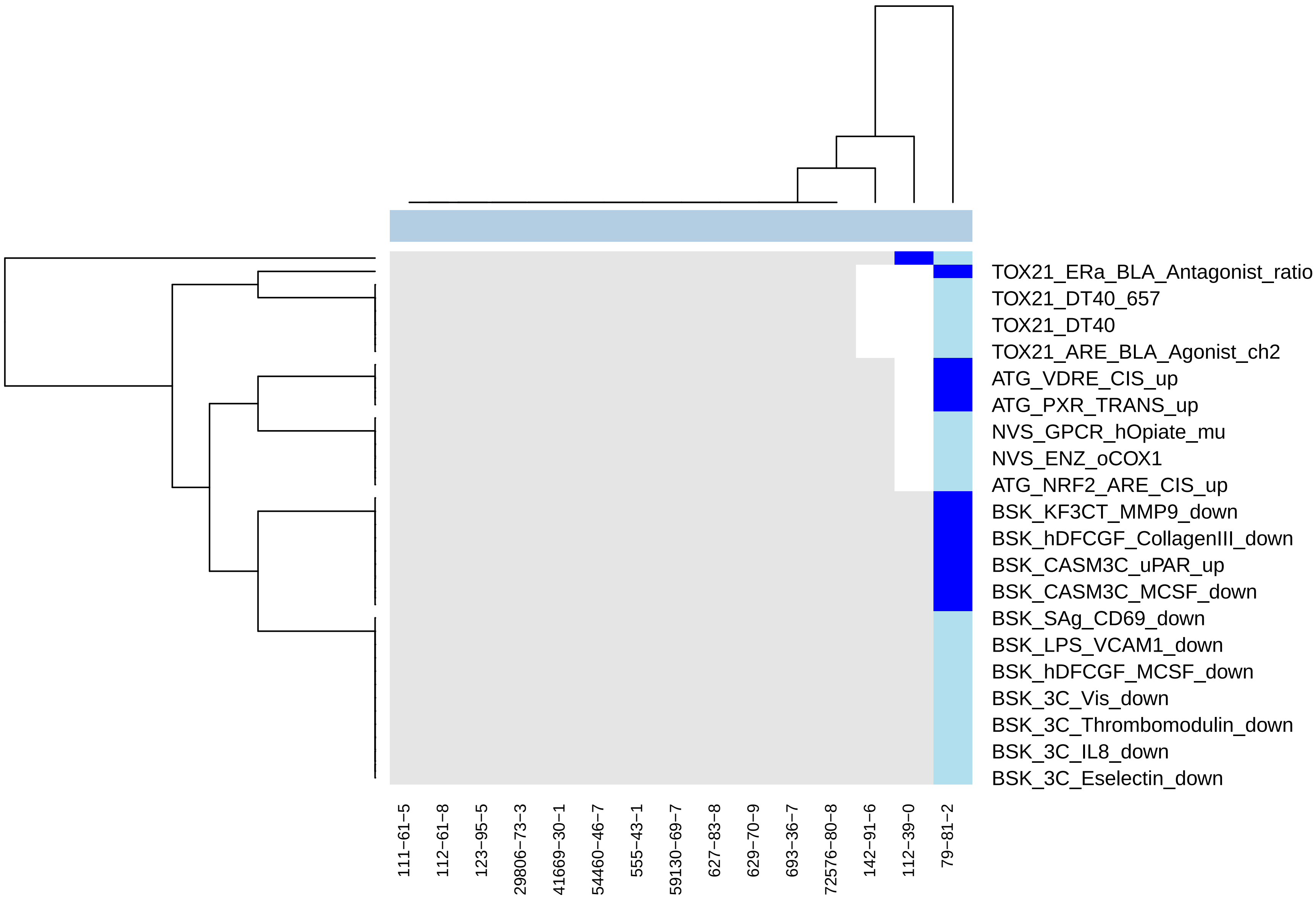

All Activity Heatmap for Cluster 560

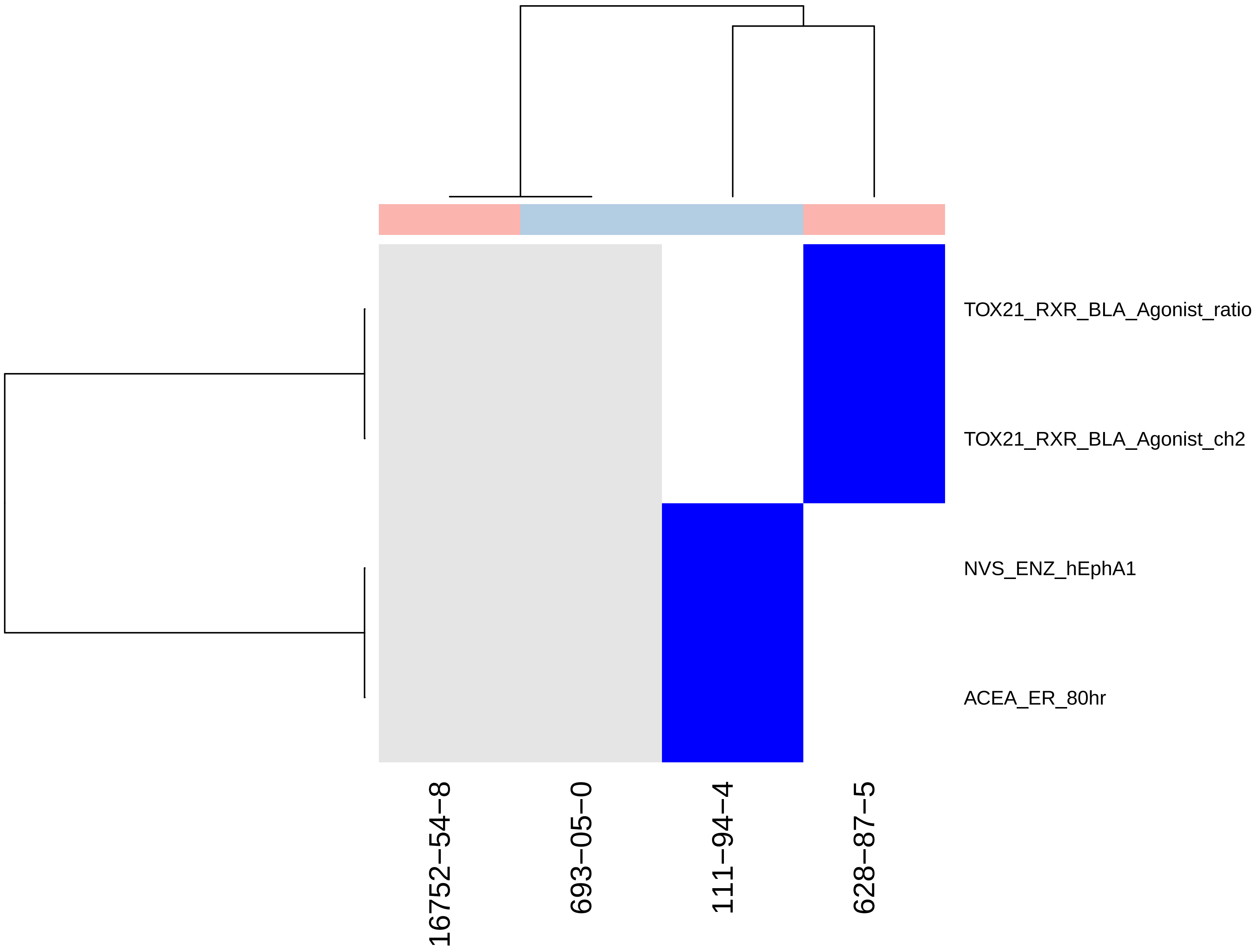

All Activity Heatmap for Cluster 561

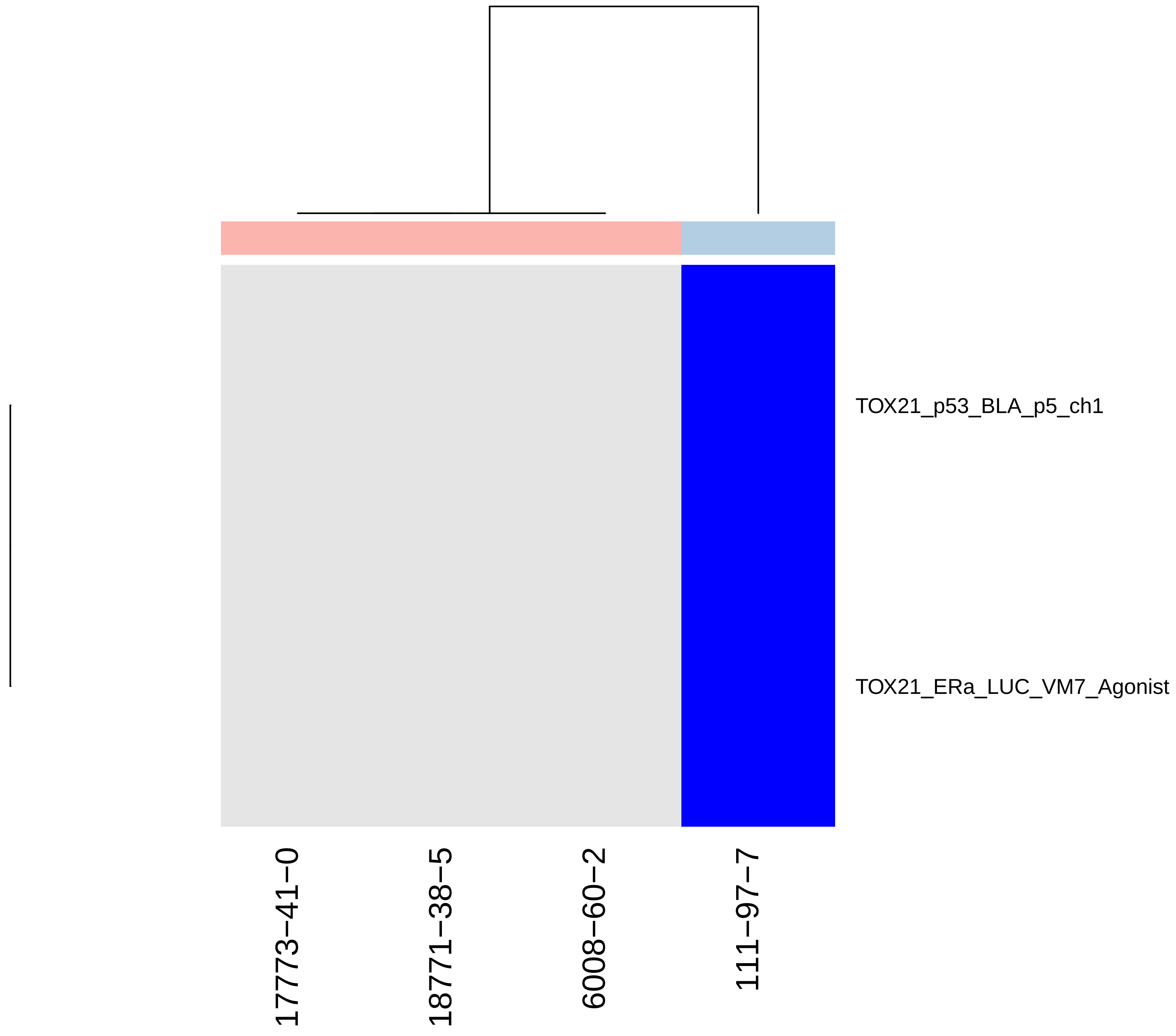

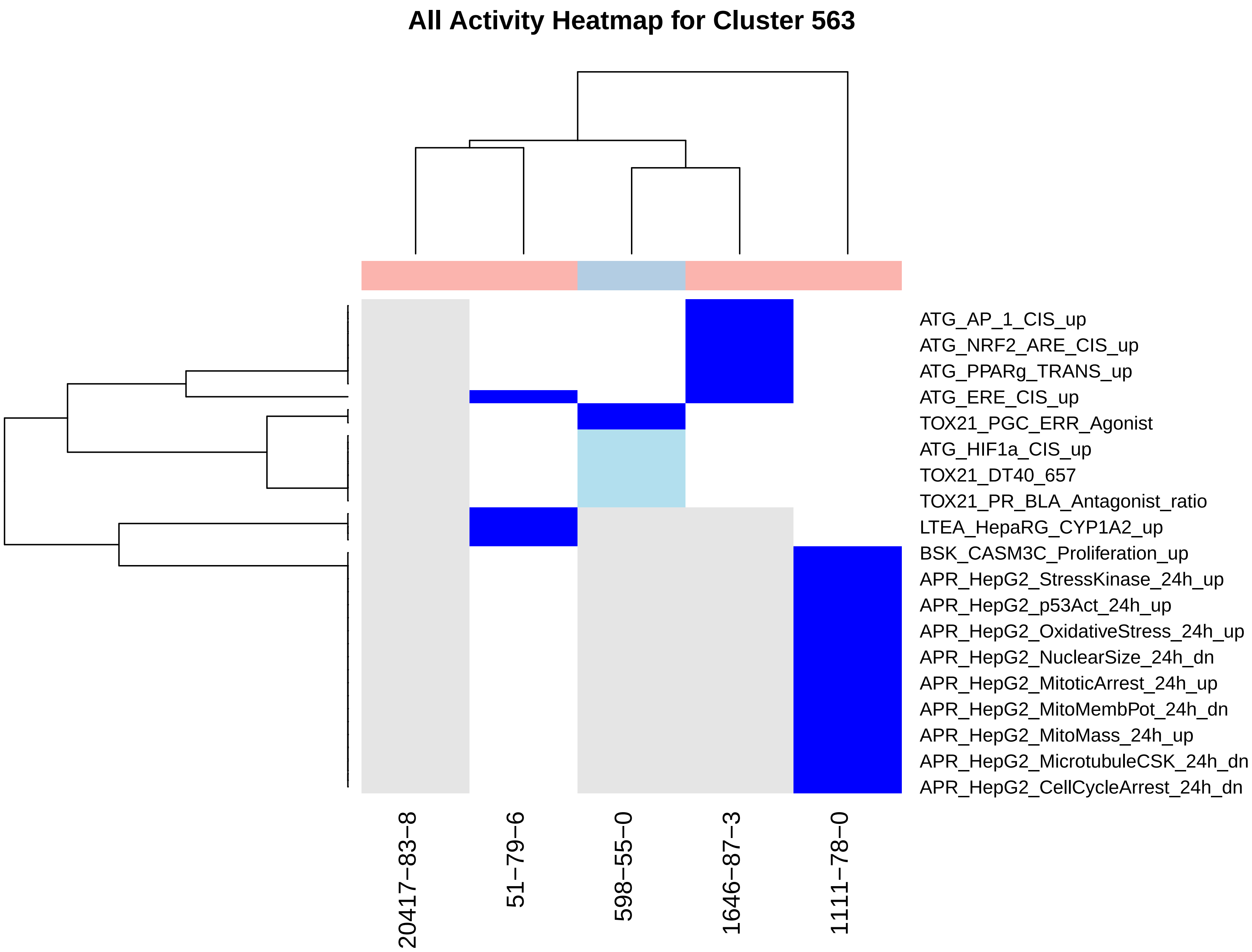

All Activity Heatmap for Cluster 563
ATG_AP_1_CIS_up
ATG_NRF2_ARE_CIS_up
ATG_PPARg_TRANS_up
ATG_ERE_CIS_up
TOX21_PGC_ERR_Agonist
ATG_HIF1a_CIS_up
TOX21_DT40_657
TOX21_PR_BLA_Antagonist_ratio
LTEA_HepaRG_CYP1A2_up
BSK_CASM3C_Proliferation_up
APR_HepG2_StressKinase_24h_up
APR_HepG2_p53Act_24h_up
APR_HepG2_OxidativeStress_24h_up
APR_HepG2_NuclearSize_24h_dn
APR_HepG2_MitoticArrest_24h_up
APR_HepG2_MitoMembPot_24h_dn
APR_HepG2_MitoMass_24h_up
APR_HepG2_MicrotubuleCSK_24h_dn
APR_HepG2_CellCycleArrest_24h_dn
20417−83−8
51−79−6
598−55−0
1646−87−3
1111−78−0

All Activity Heatmap for Cluster 565

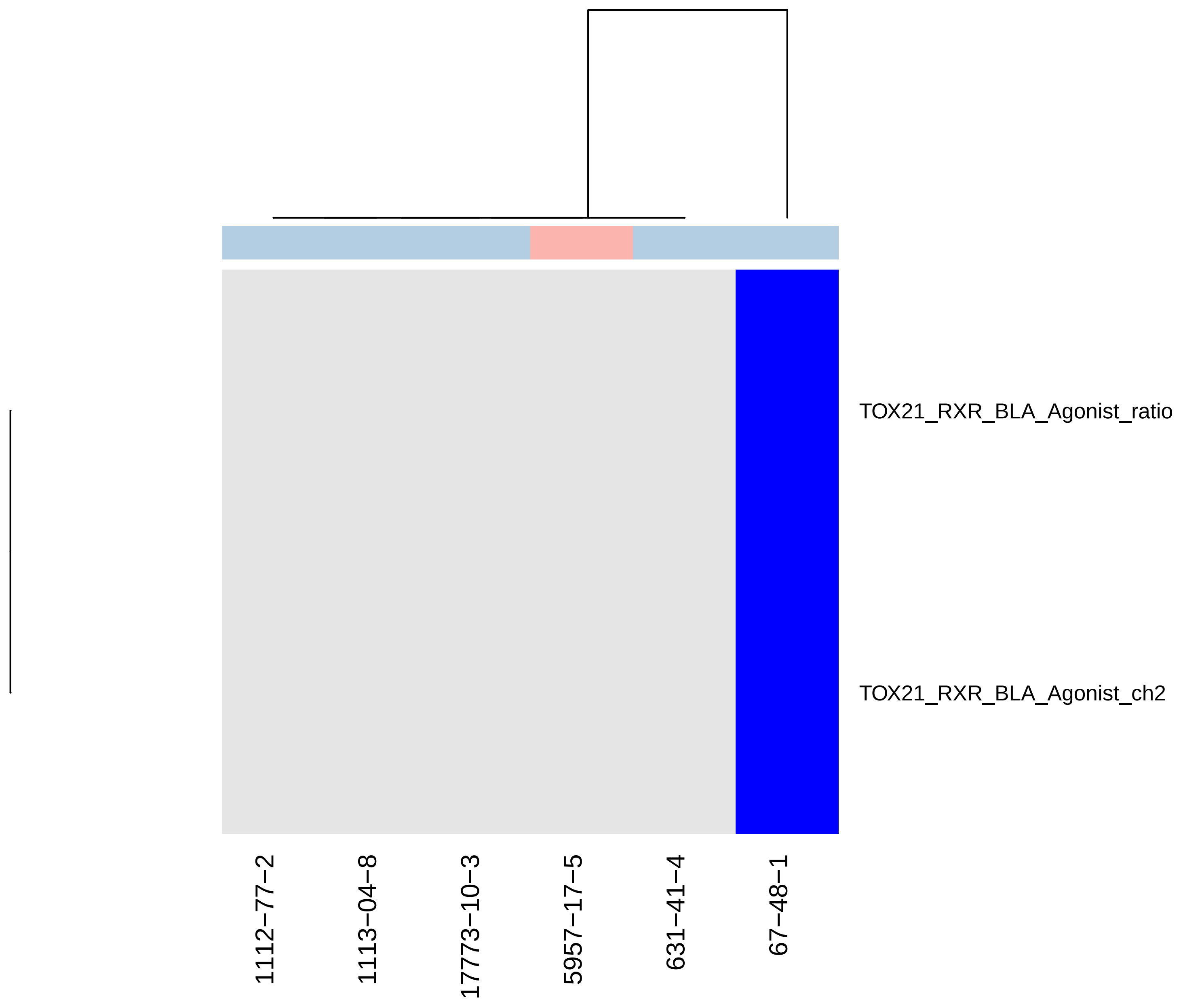

All Activity Heatmap for Cluster 566

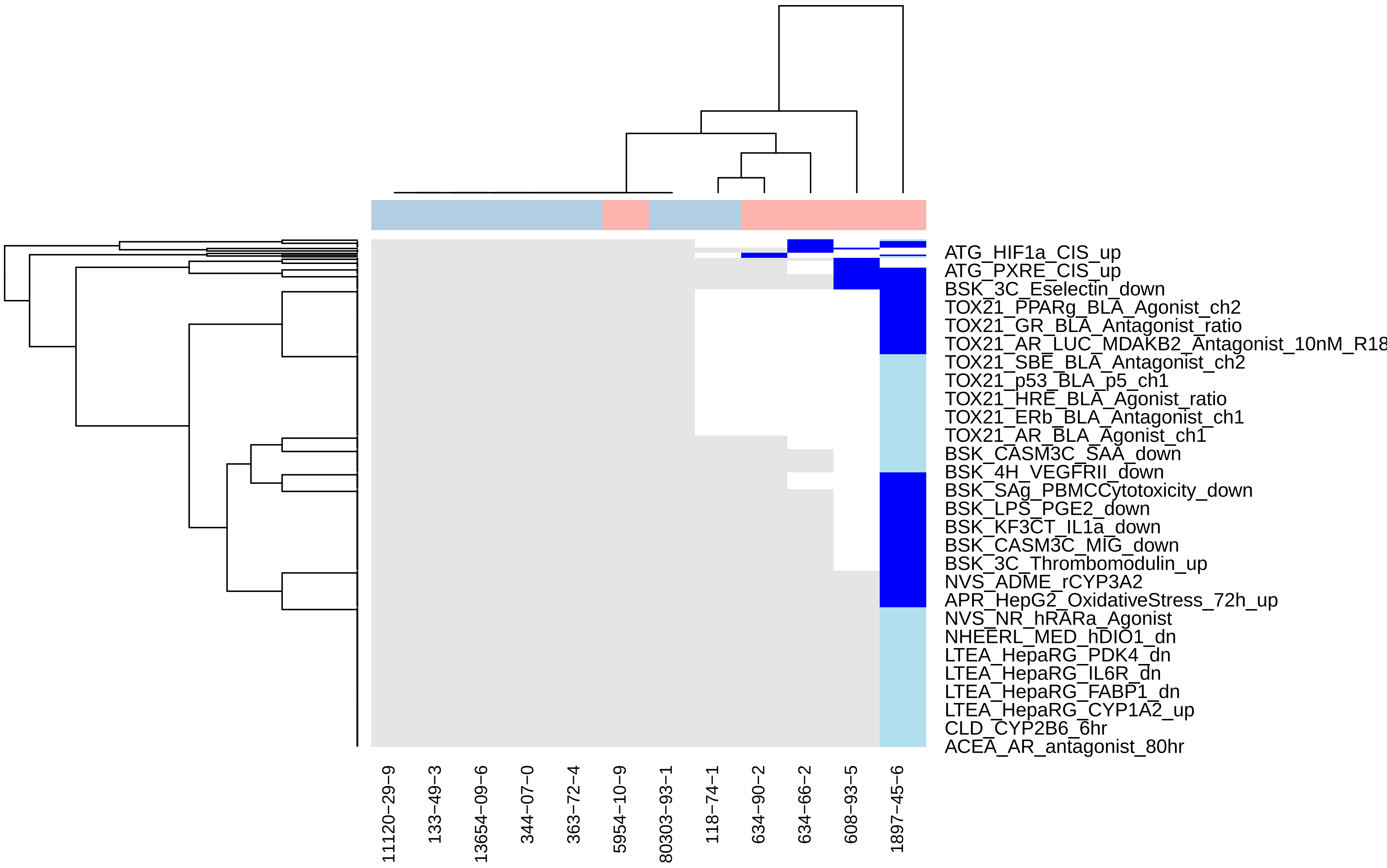

All Activity Heatmap for Cluster 568

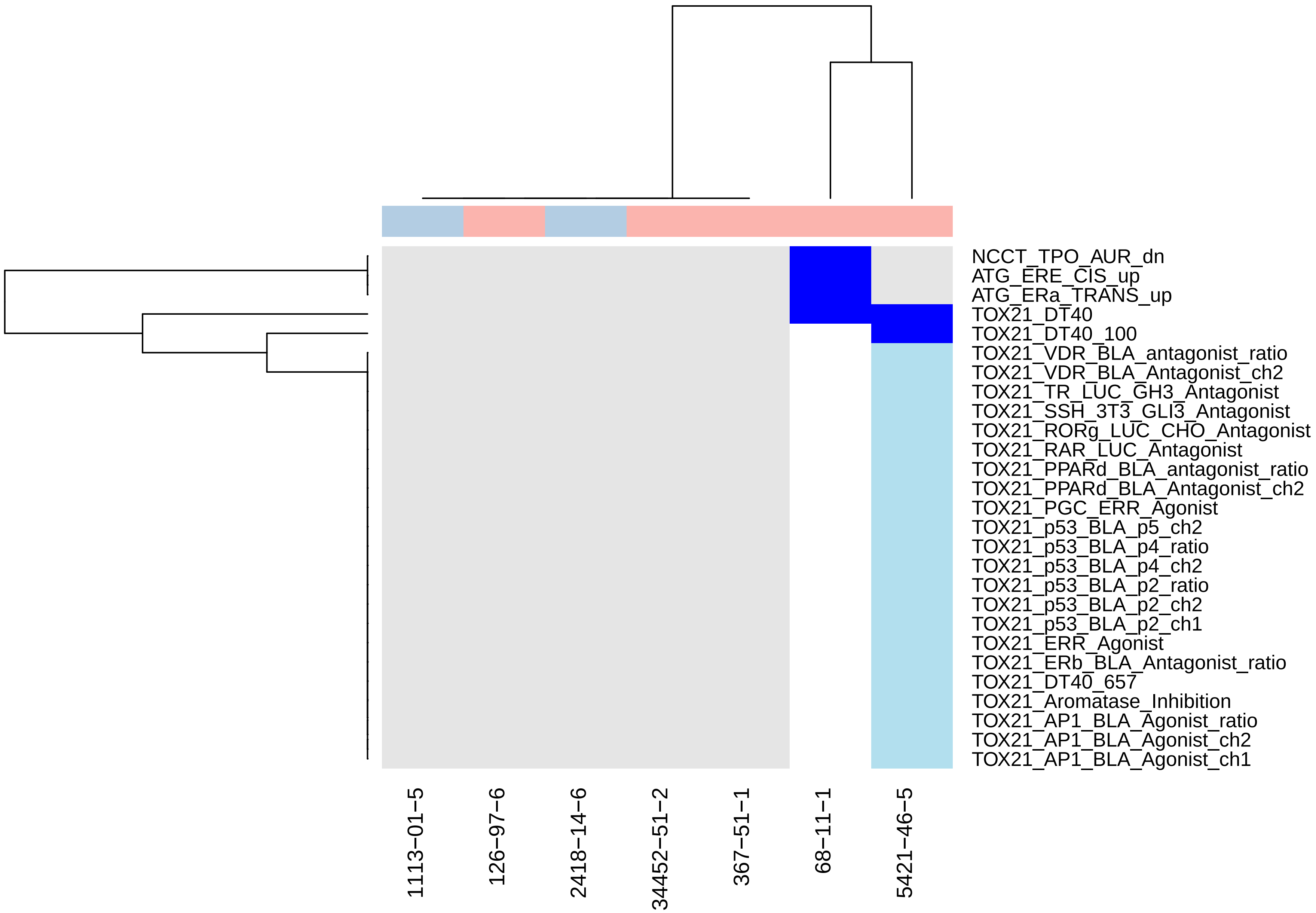

All Activity Heatmap for Cluster 569

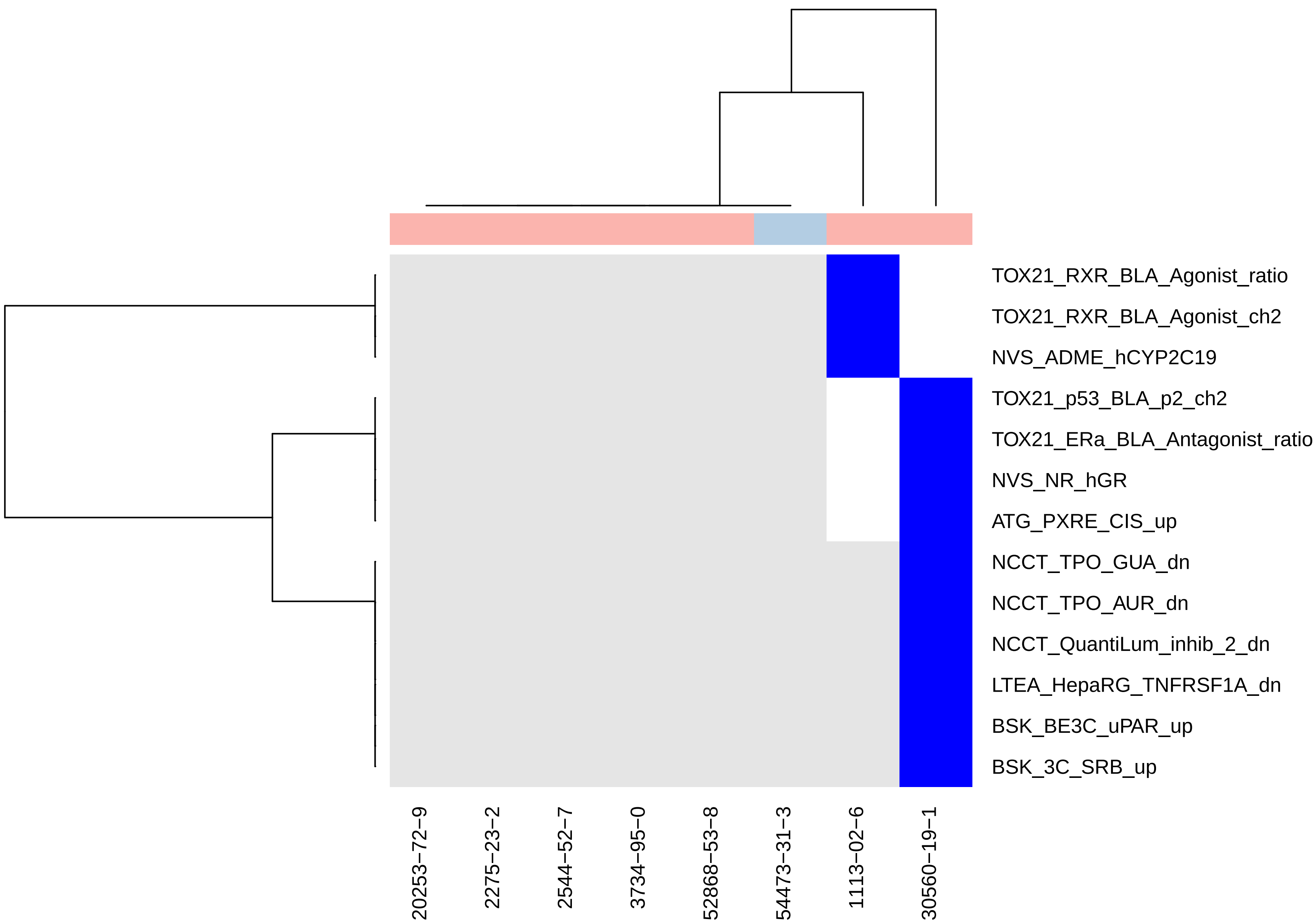

All Activity Heatmap for Cluster 572

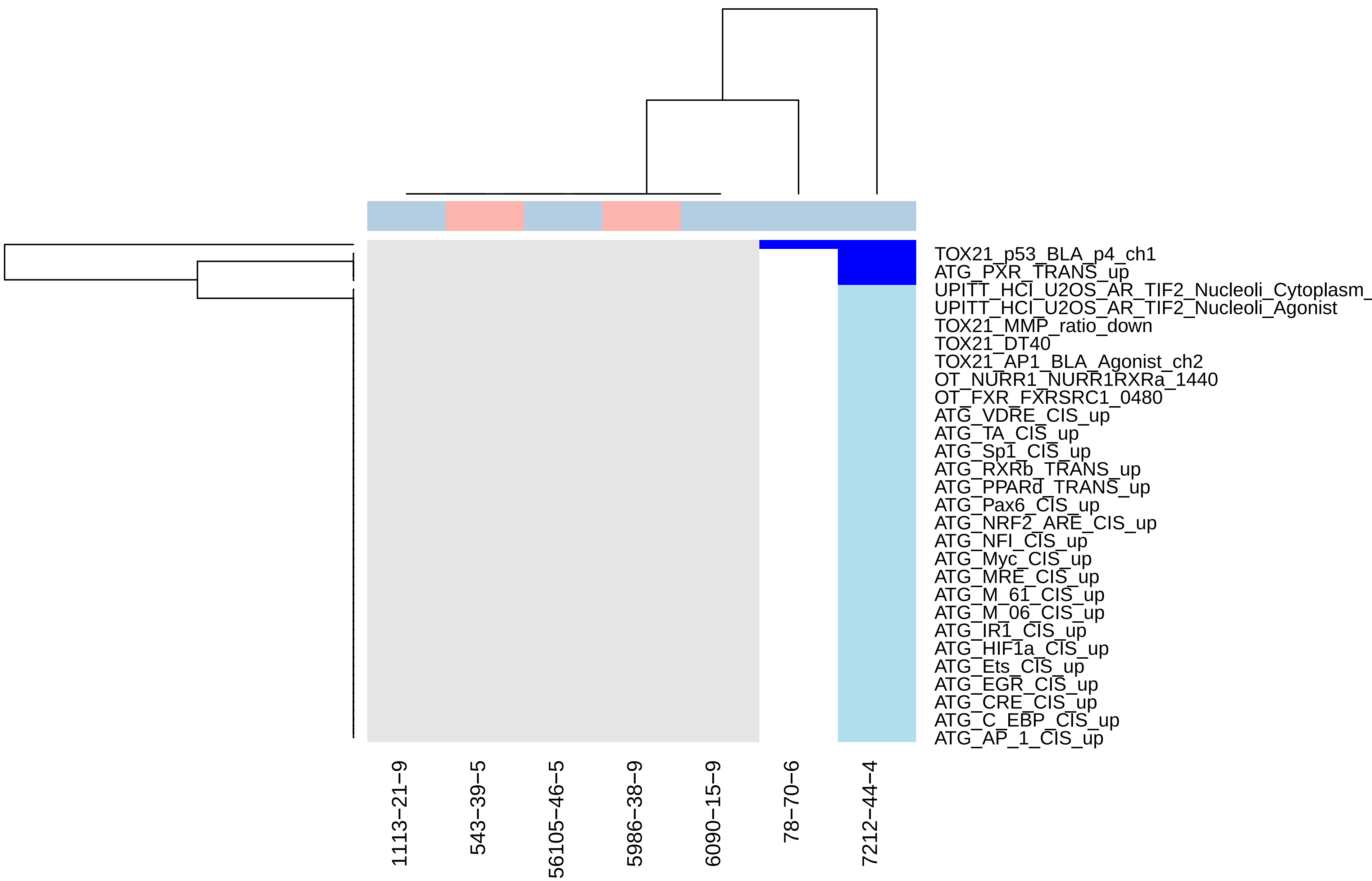

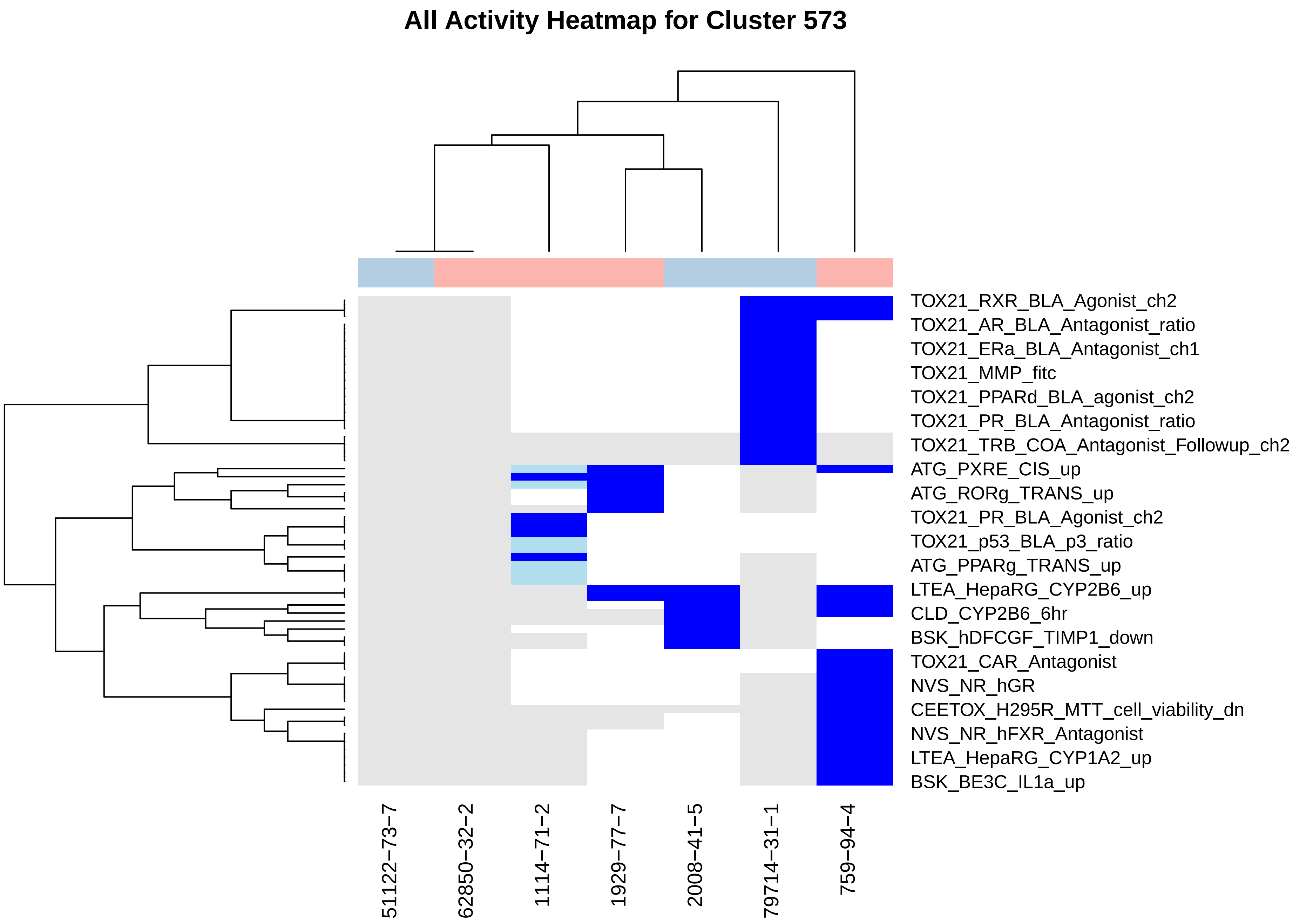
All Activity Heatmap for Cluster 573
TOX21_RXR_BLA_Agonist_ch2
TOX21_AR_BLA_Antagonist_ratio
TOX21_ERa_BLA_Antagonist_ch1
TOX21_MMP_fitc
TOX21_PPARd_BLA_agonist_ch2
TOX21_PR_BLA_Antagonist_ratio
TOX21_TRB_COA_Antagonist_Followup_ch2
ATG_PXRE_CIS_up
ATG_RORg_TRANS_up
TOX21_PR_BLA_Agonist_ch2
TOX21_p53_BLA_p3_ratio
ATG_PPARg_TRANS_up
LTEA_HepaRG_CYP2B6_up
CLD_CYP2B6_6hr
BSK_hDFCGF_TIMP1_down
TOX21_CAR_Antagonist
NVS_NR_hGR
CEETOX_H295R_MTT_cell_viability_dn
NVS_NR_hFXR_Antagonist
LTEA_HepaRG_CYP1A2_up
BSK_BE3C_IL1a_up
51122−73−7
62850−32−2
1114−71−2
1929−77−7
2008−41−5
79714−31−1
759−94−4

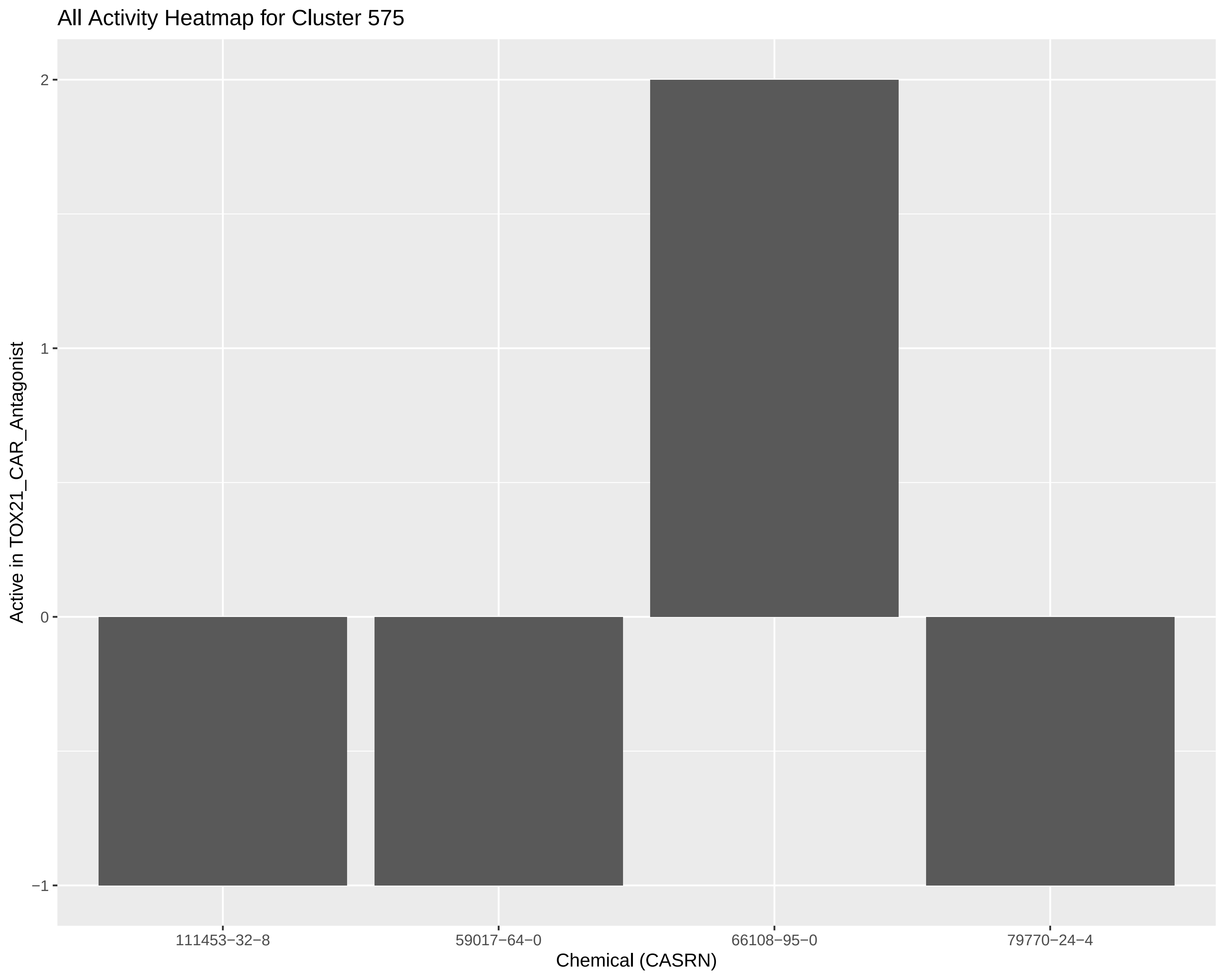

All Activity Heatmap for Cluster 575
Active in TOX21_CAR_Antagonist
2
1
0
−1
111453−32−8
59017−64−0
66108−95−0
79770−24−4
Chemical (CASRN)

All Activity Heatmap for Cluster 576

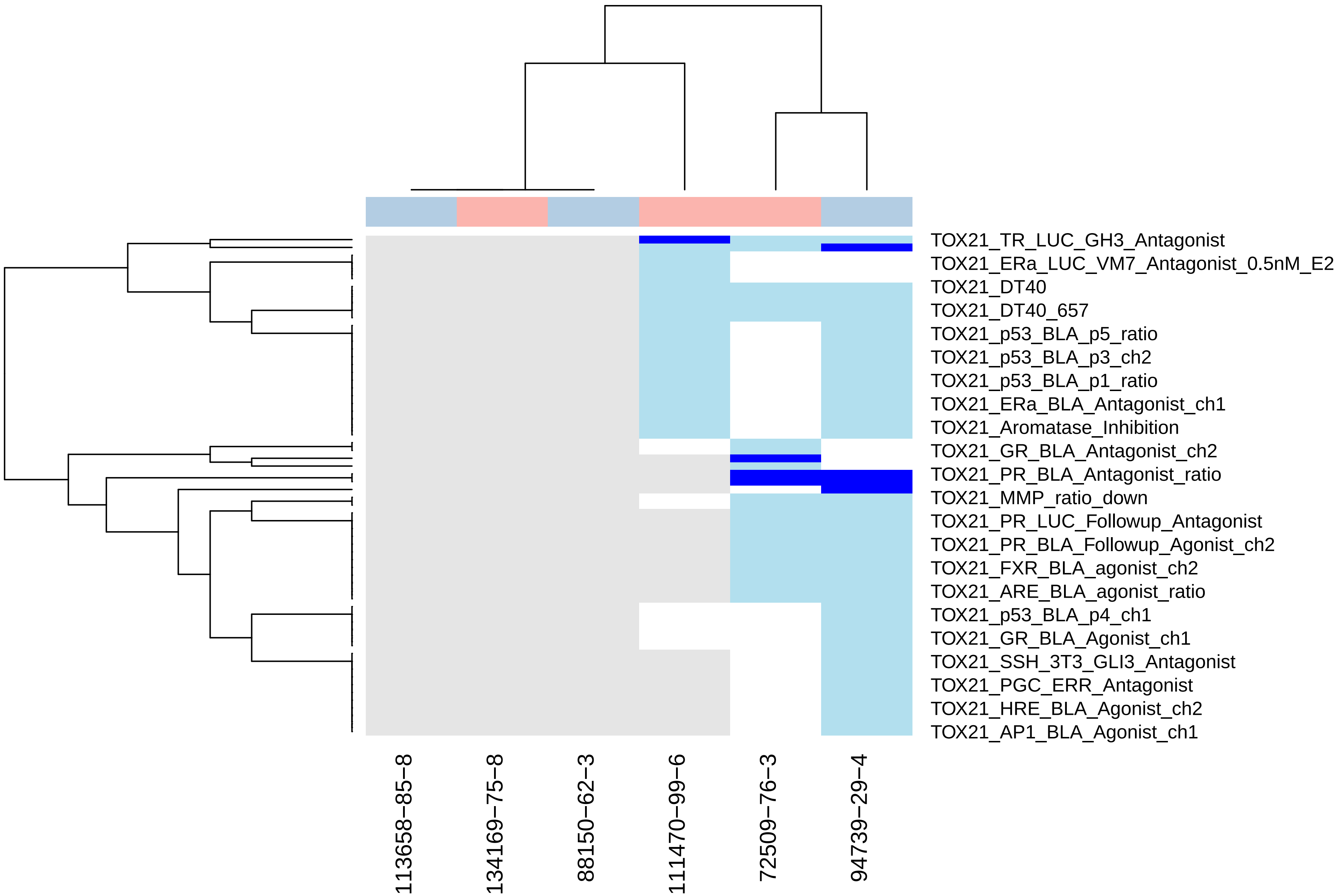

All Activity Heatmap for Cluster 577

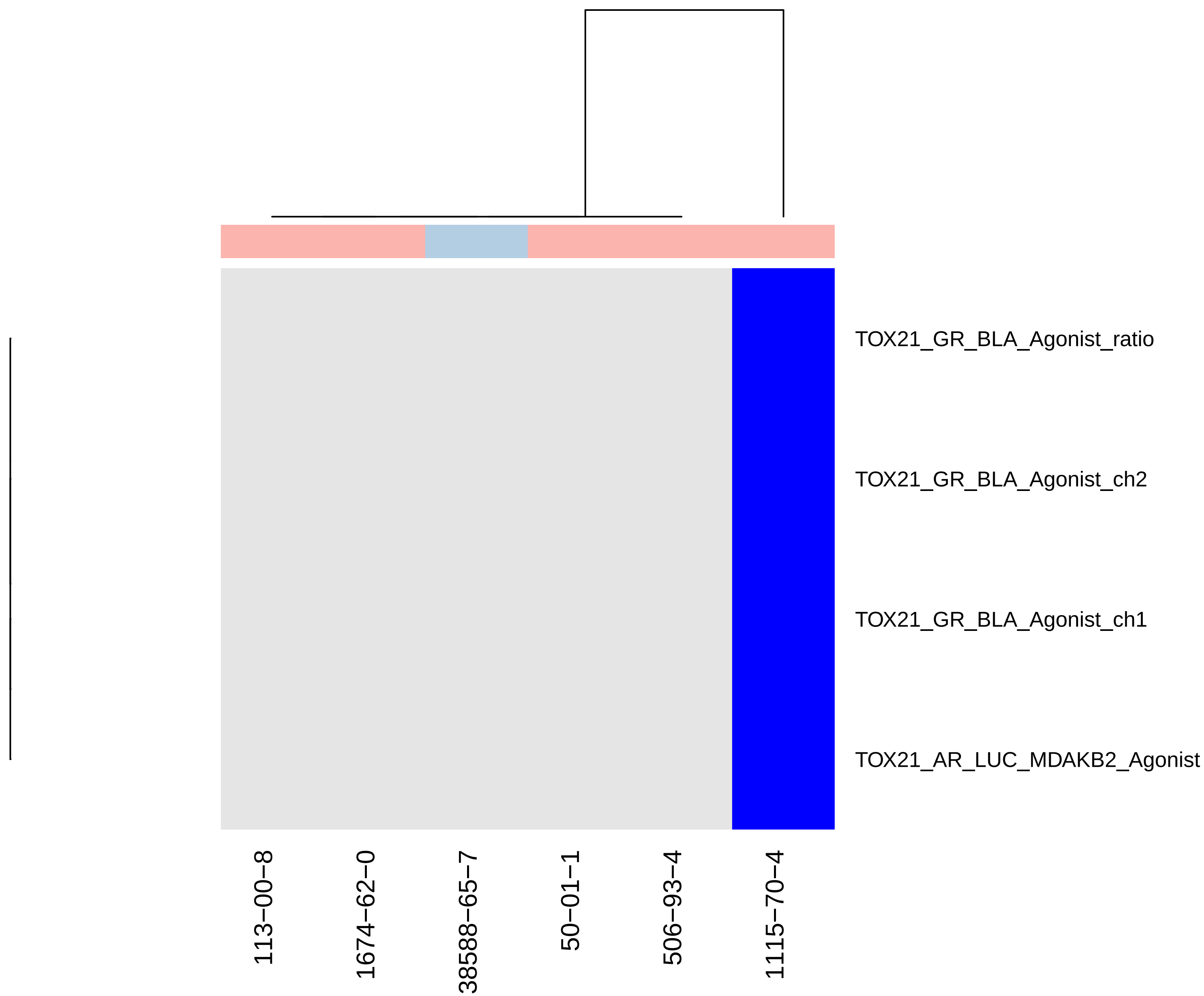

All Activity Heatmap for Cluster 578

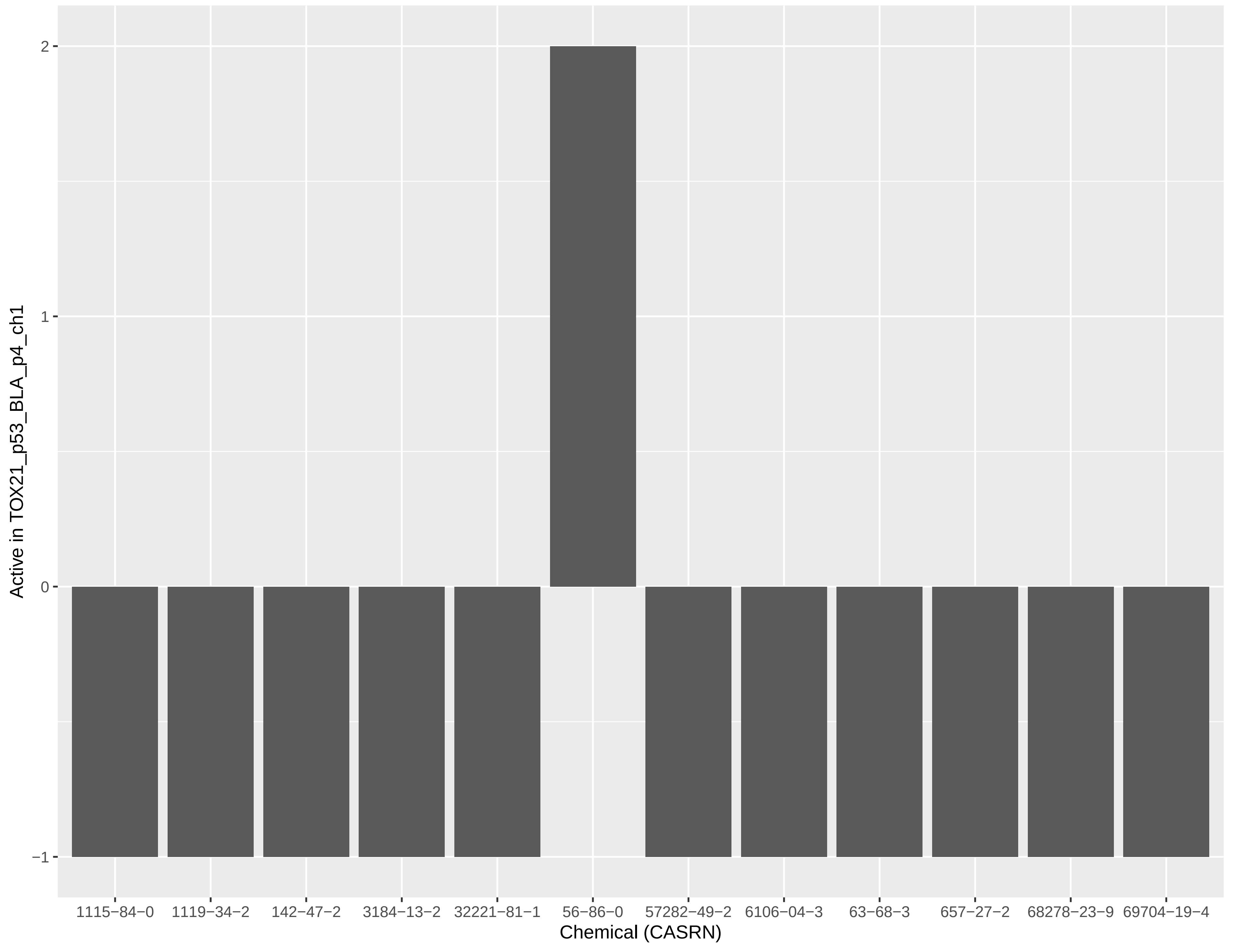

All Activity Heatmap for Cluster 580

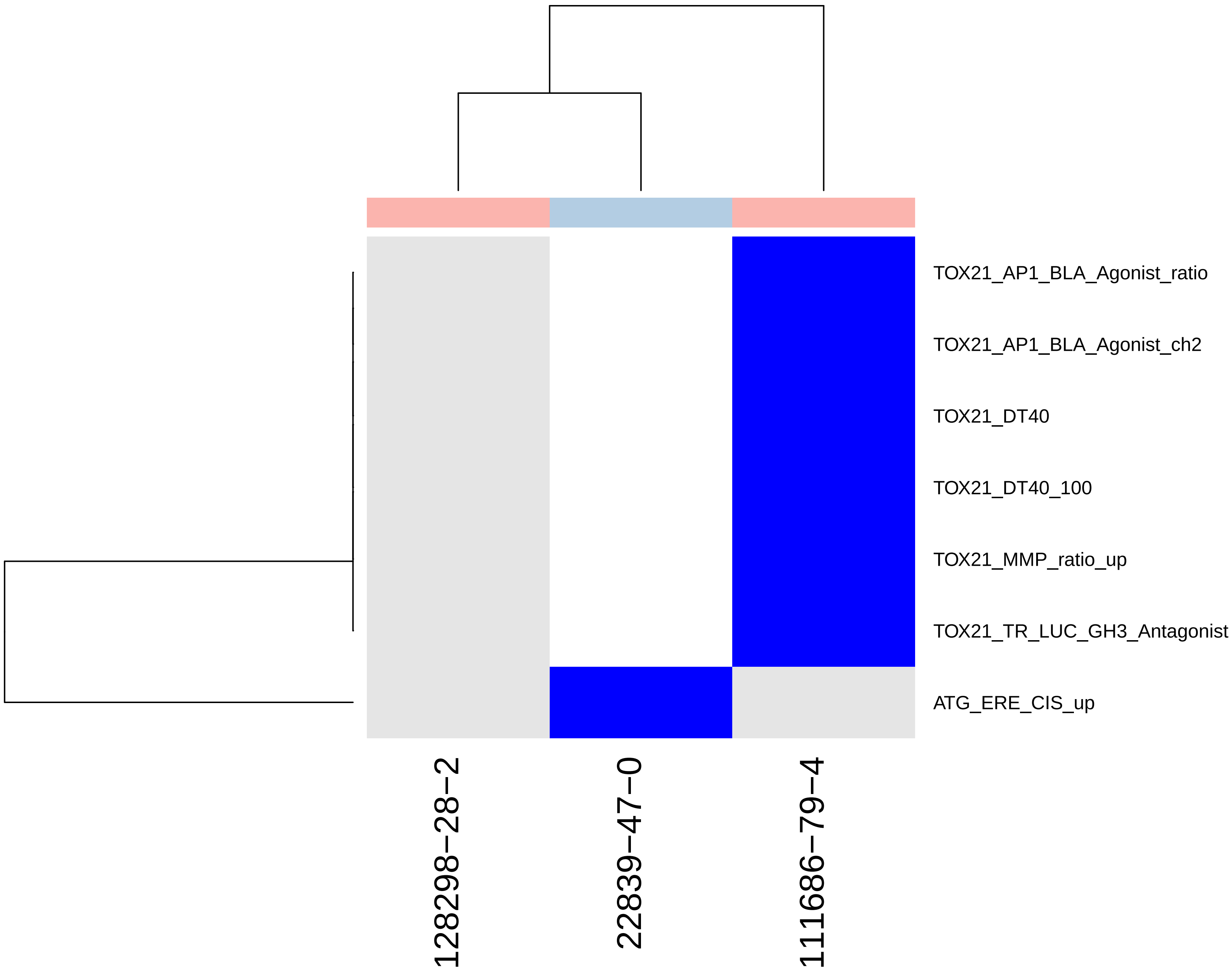

All Activity Heatmap for Cluster 583

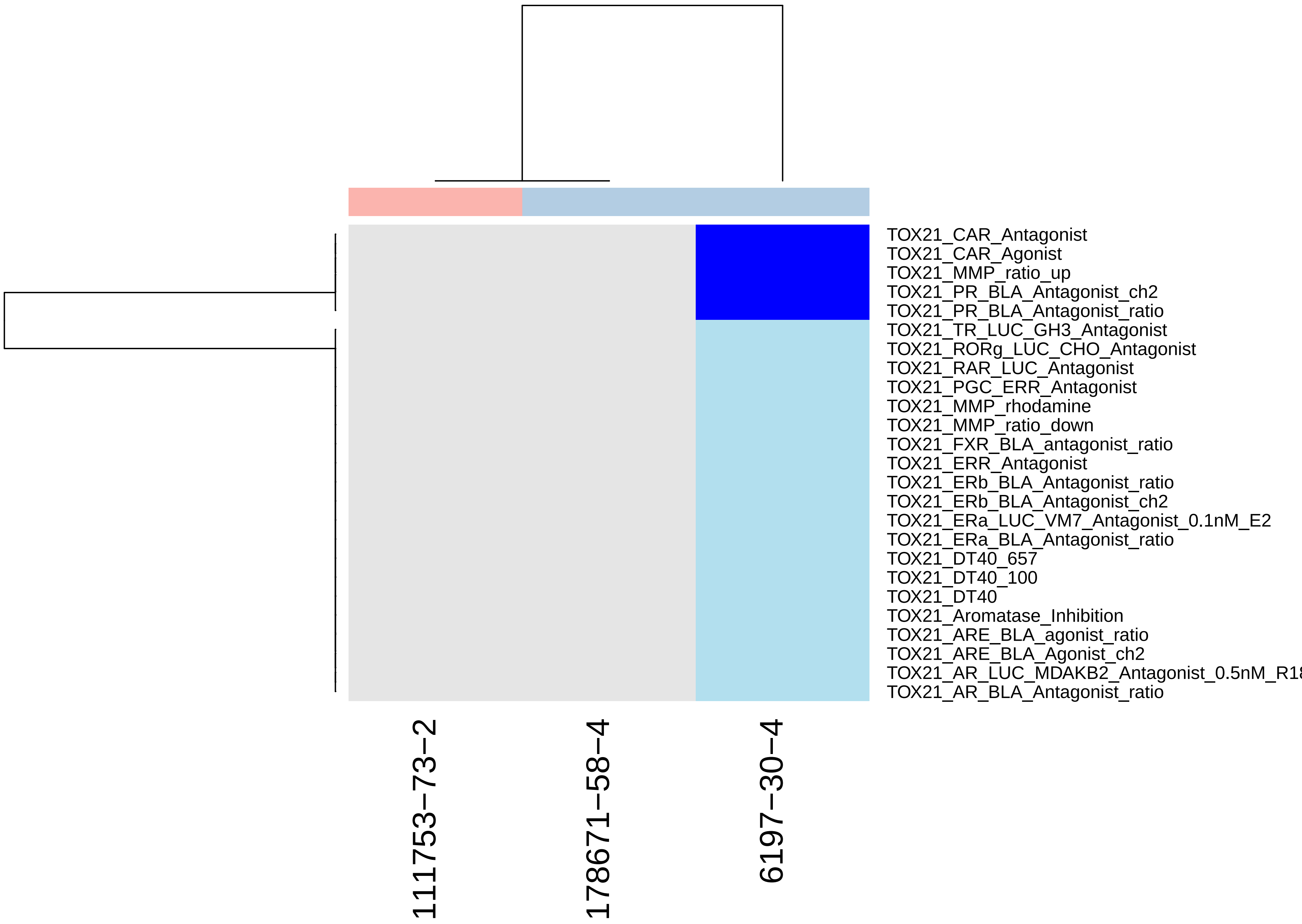

All Activity Heatmap for Cluster 584

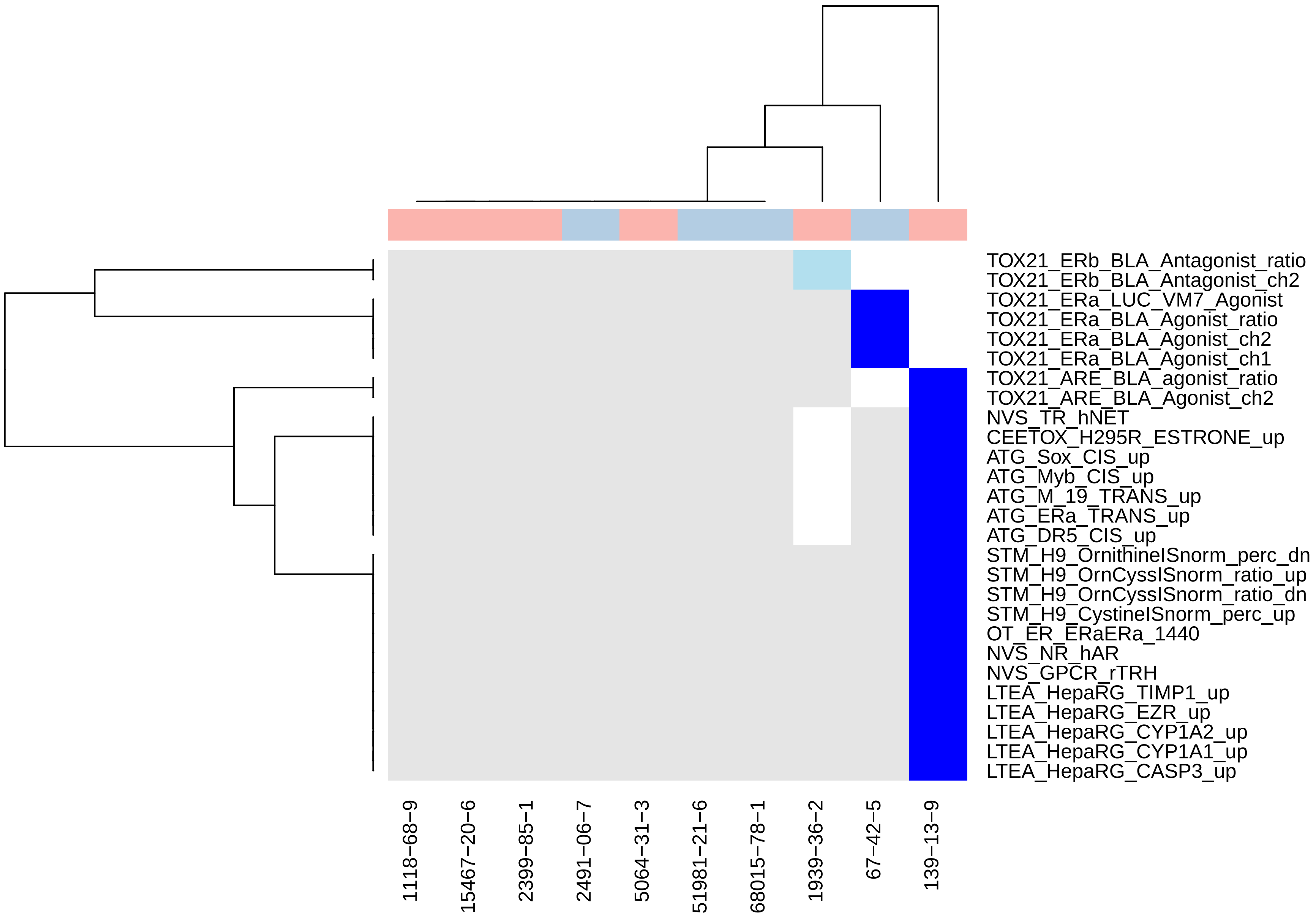

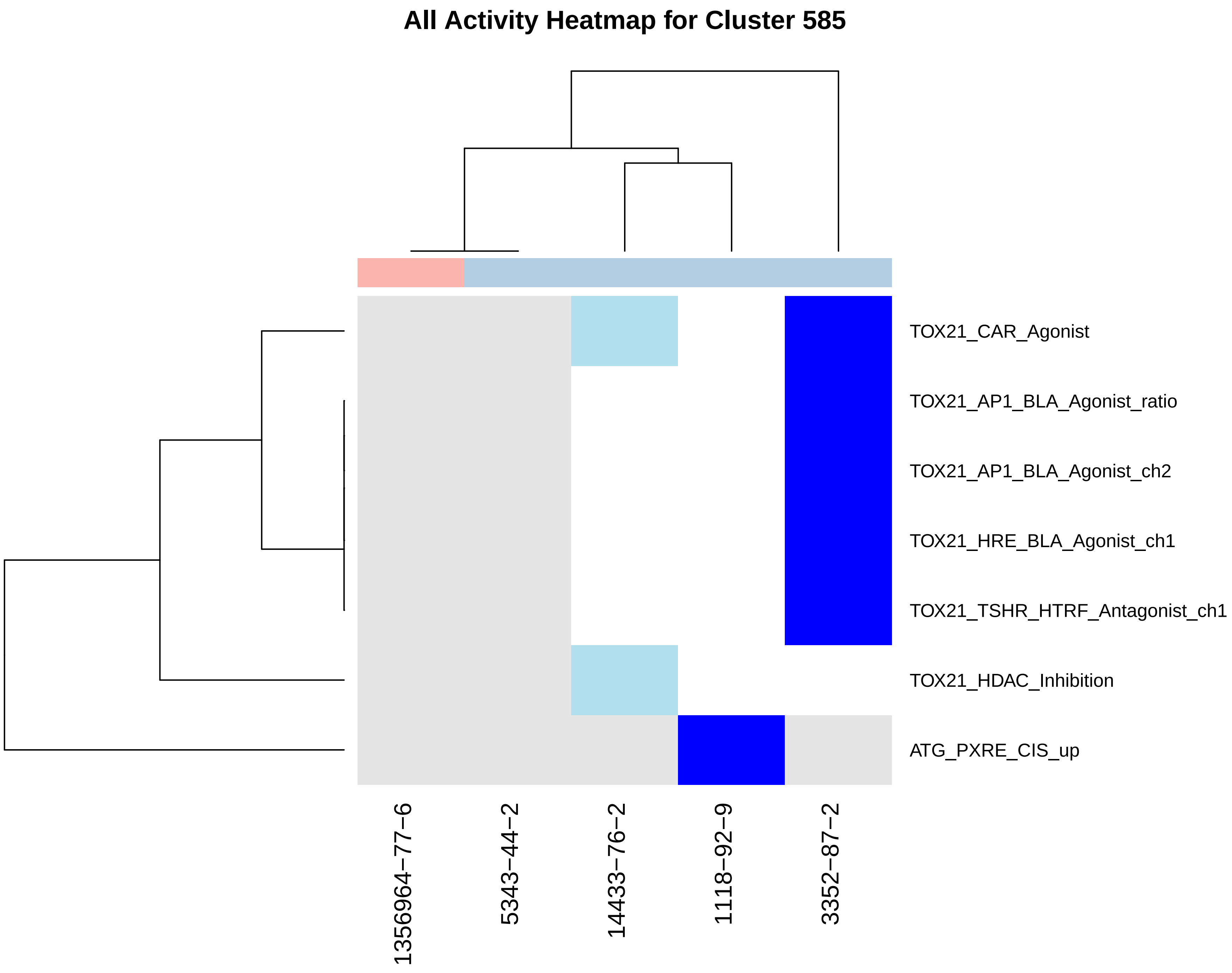
All Activity Heatmap for Cluster 585
TOX21_CAR_Agonist
TOX21_AP1_BLA_Agonist_ratio
TOX21_AP1_BLA_Agonist_ch2
TOX21_HRE_BLA_Agonist_ch1
TOX21_TSHR_HTRF_Antagonist_ch1
TOX21_HDAC_Inhibition
ATG_PXRE_CIS_up
1356964−77−6
5343−44−2
14433−76−2
1118−92−9
3352−87−2

All Activity Heatmap for Cluster 586

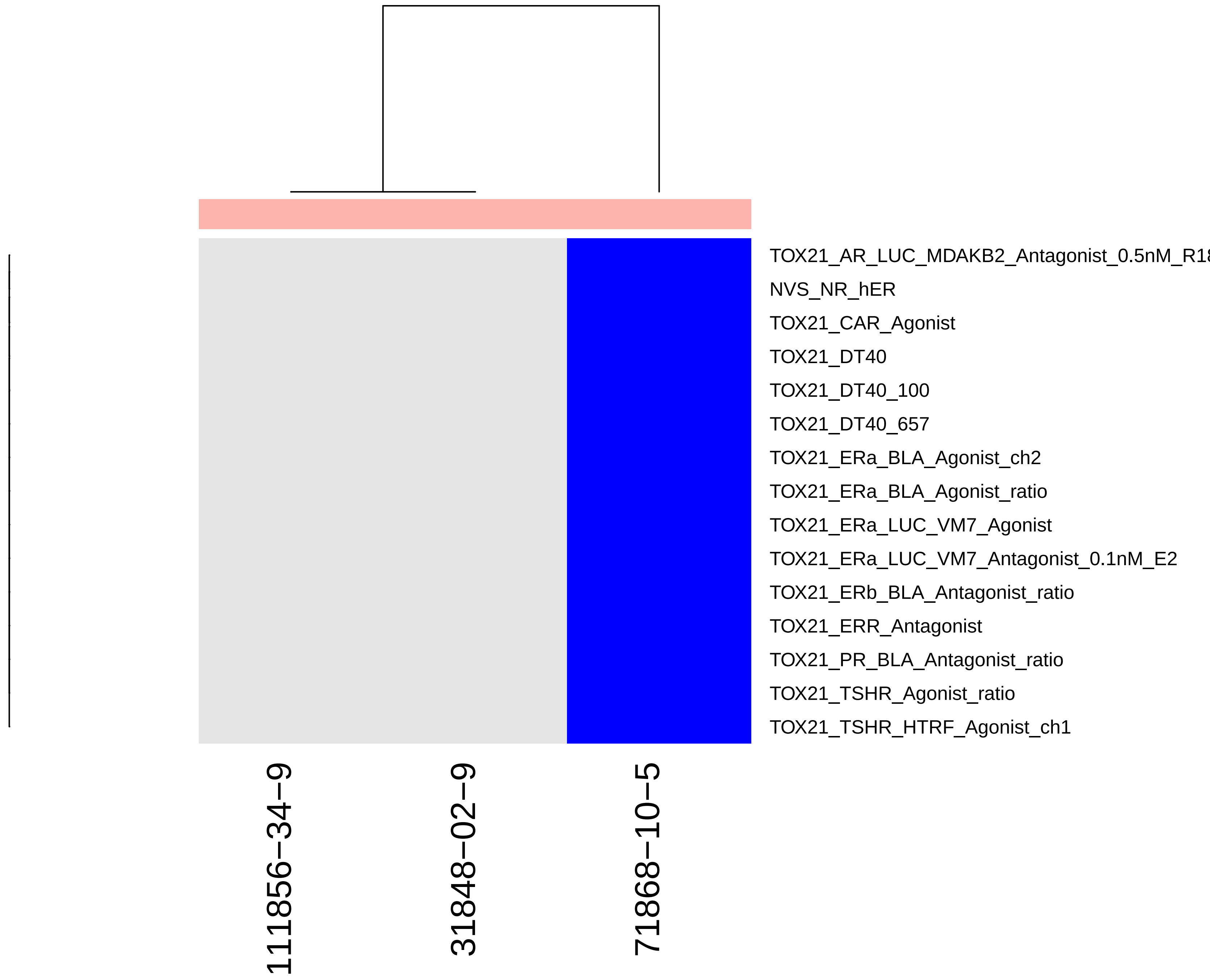

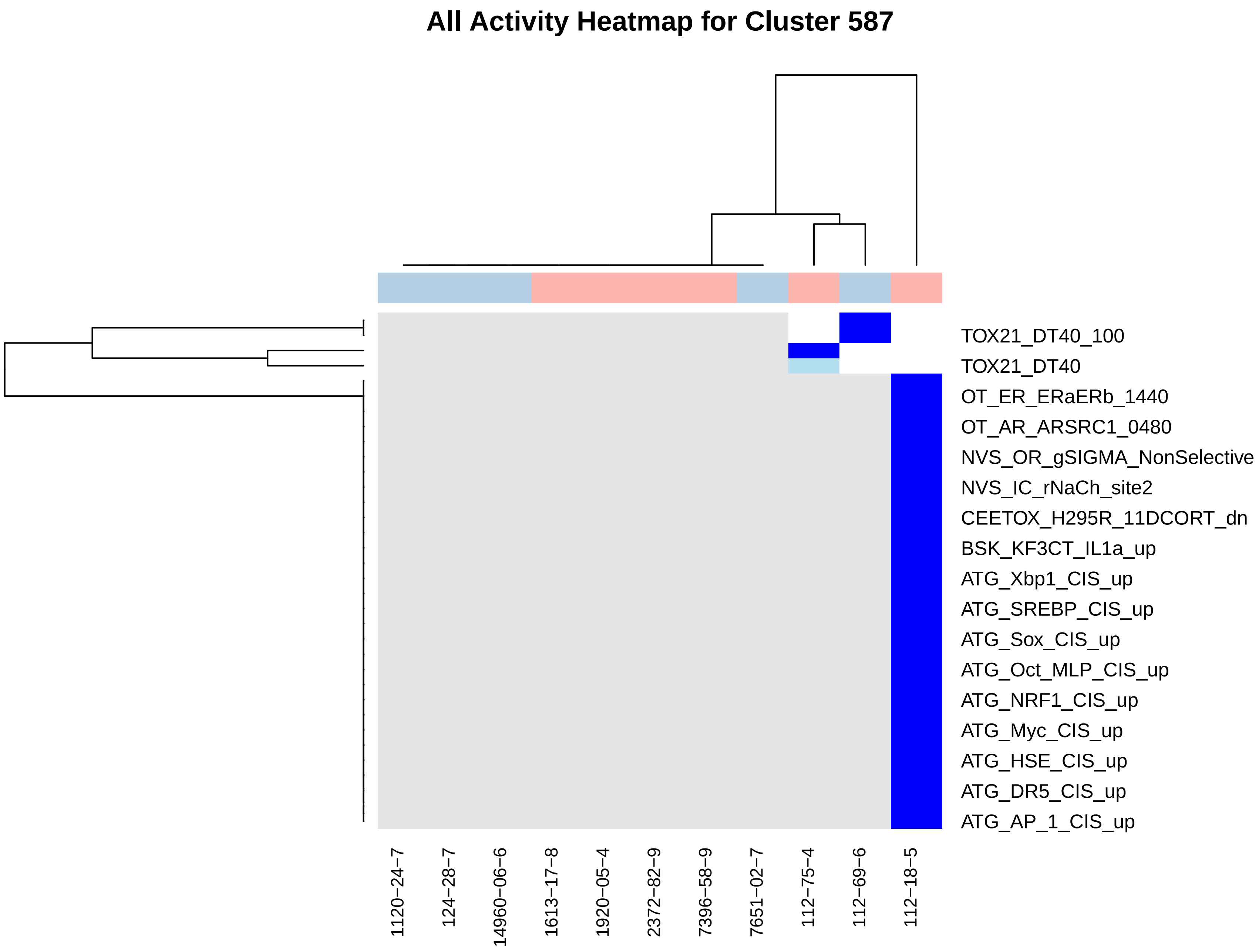

All Activity Heatmap for Cluster 587
TOX21_DT40_100
TOX21_DT40
OT_ER_ERaERb_1440
OT_AR_ARSRC1_0480
NVS_OR_gSIGMA_NonSelective
NVS_IC_rNaCh_site2
CEETOX_H295R_11DCORT_dn
BSK_KF3CT_IL1a_up
ATG_Xbp1_CIS_up
ATG_SREBP_CIS_up
ATG_Sox_CIS_up
ATG_Oct_MLP_CIS_up
ATG_NRF1_CIS_up
ATG_Myc_CIS_up
ATG_HSE_CIS_up
ATG_DR5_CIS_up
ATG_AP_1_CIS_up
1120−24−7
124−28−7
14960−06−6
1613−17−8
1920−05−4
2372−82−9
7396−58−9
7651−02−7
112−75−4
112−69−6
112−18−5

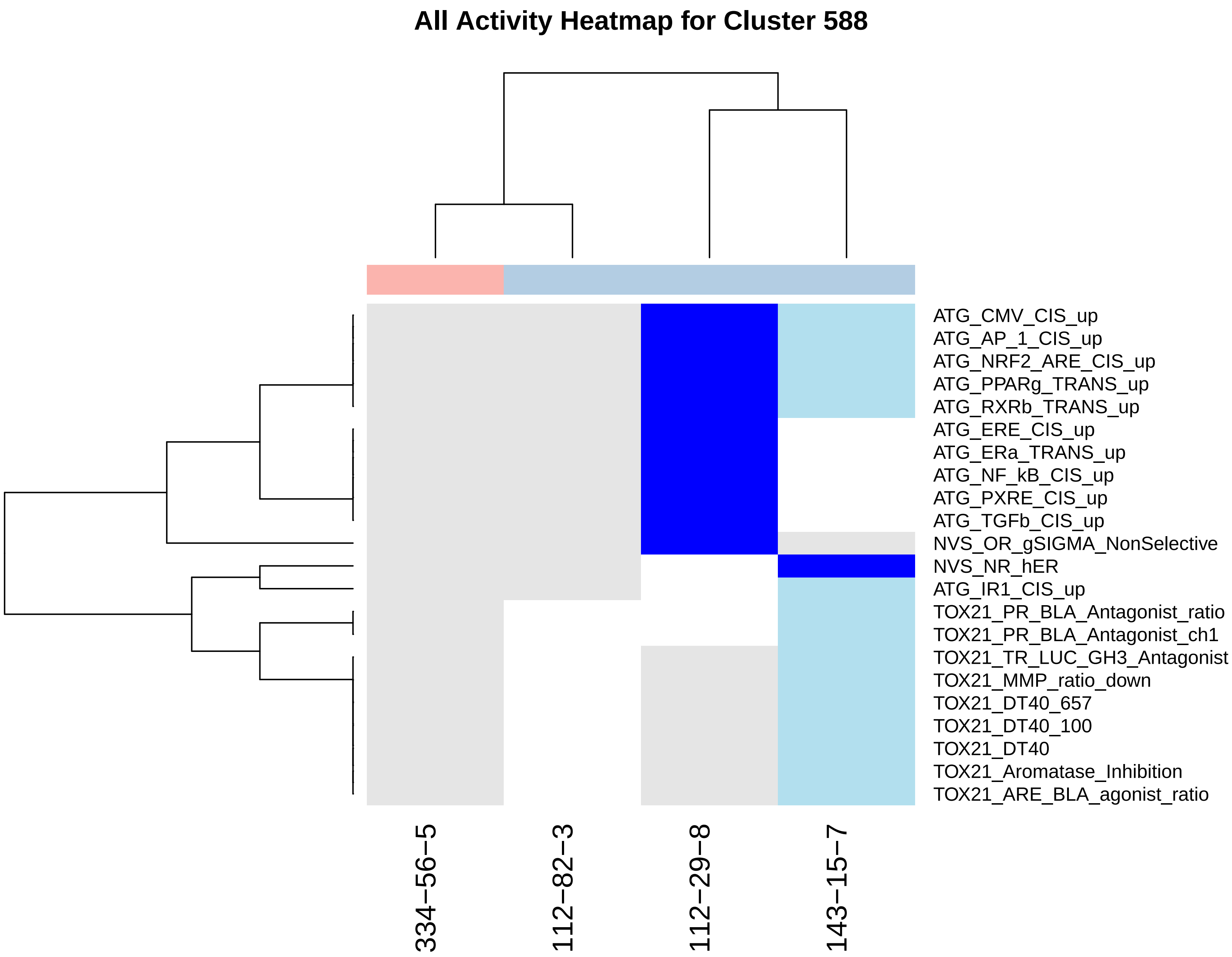
All Activity Heatmap for Cluster 588
ATG_CMV_CIS_up
ATG_AP_1_CIS_up
ATG_NRF2_ARE_CIS_up
ATG_PPARg_TRANS_up
ATG_RXRb_TRANS_up
ATG_ERE_CIS_up
ATG_ERa_TRANS_up
ATG_NF_kB_CIS_up
ATG_PXRE_CIS_up
ATG_TGFb_CIS_up
NVS_OR_gSIGMA_NonSelective
NVS_NR_hER
ATG_IR1_CIS_up
TOX21_PR_BLA_Antagonist_ratio
TOX21_PR_BLA_Antagonist_ch1
TOX21_TR_LUC_GH3_Antagonist
TOX21_MMP_ratio_down
TOX21_DT40_657
TOX21_DT40_100
TOX21_DT40
TOX21_Aromatase_Inhibition
TOX21_ARE_BLA_agonist_ratio
334−56−5
112−82−3
112−29−8
143−15−7

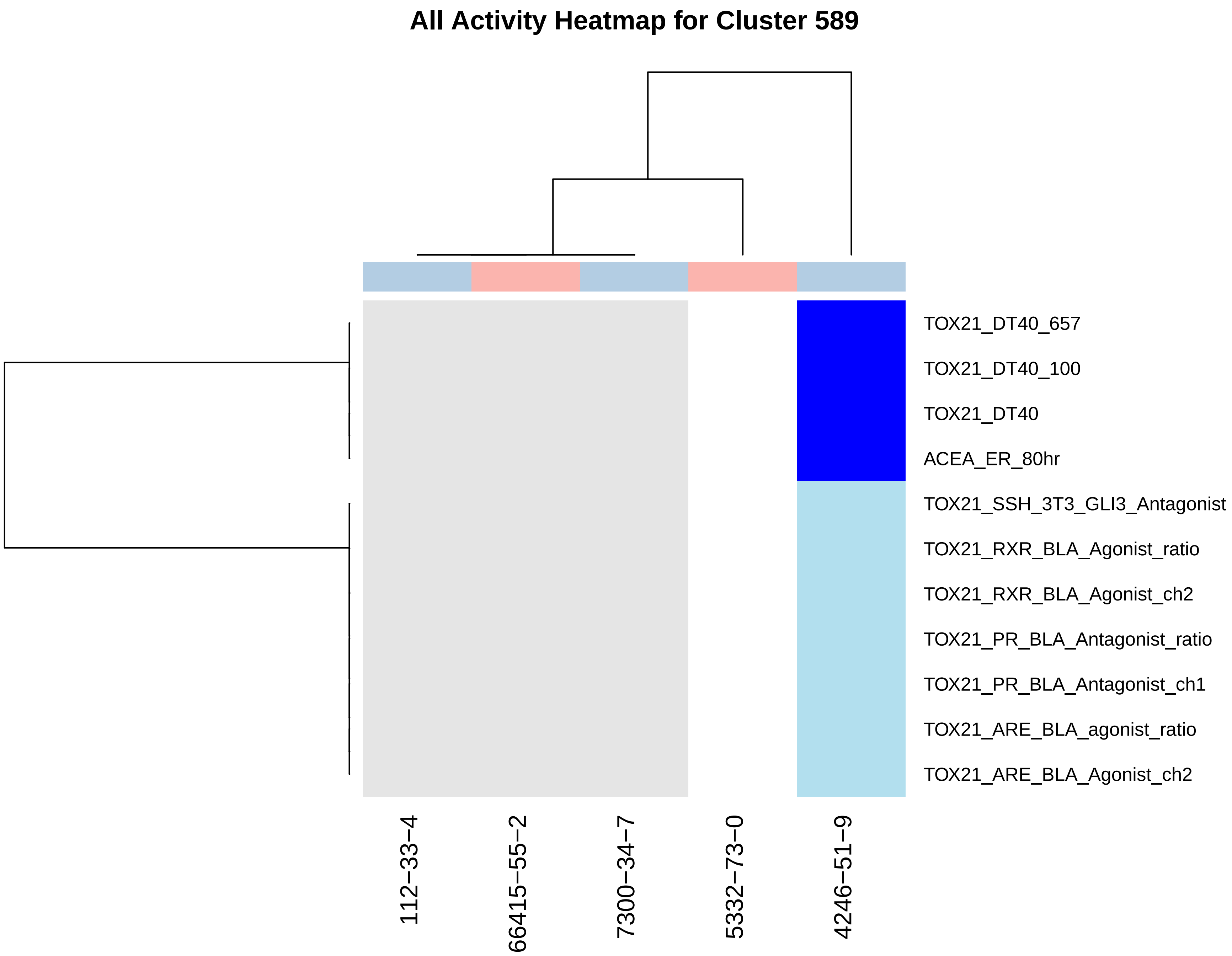
All Activity Heatmap for Cluster 589
TOX21_DT40_657
TOX21_DT40_100
TOX21_DT40
ACEA_ER_80hr
TOX21_SSH_3T3_GLI3_Antagonist
TOX21_RXR_BLA_Agonist_ratio
TOX21_RXR_BLA_Agonist_ch2
TOX21_PR_BLA_Antagonist_ratio
TOX21_PR_BLA_Antagonist_ch1
TOX21_ARE_BLA_agonist_ratio
TOX21_ARE_BLA_Agonist_ch2
112−33−4
66415−55−2
7300−34−7
5332−73−0
4246−51−9

All Activity Heatmap for Cluster 590

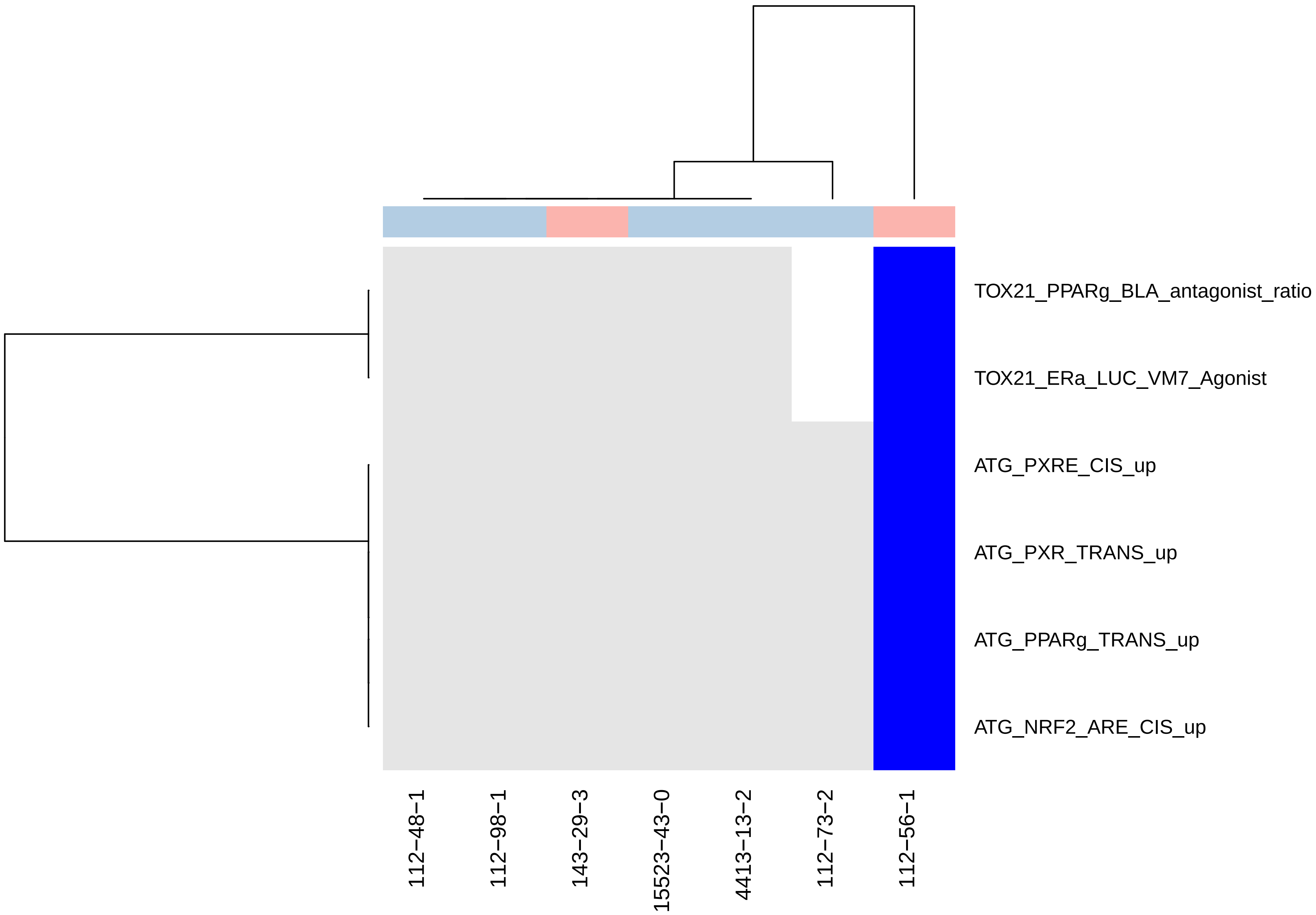

All Activity Heatmap for Cluster 591

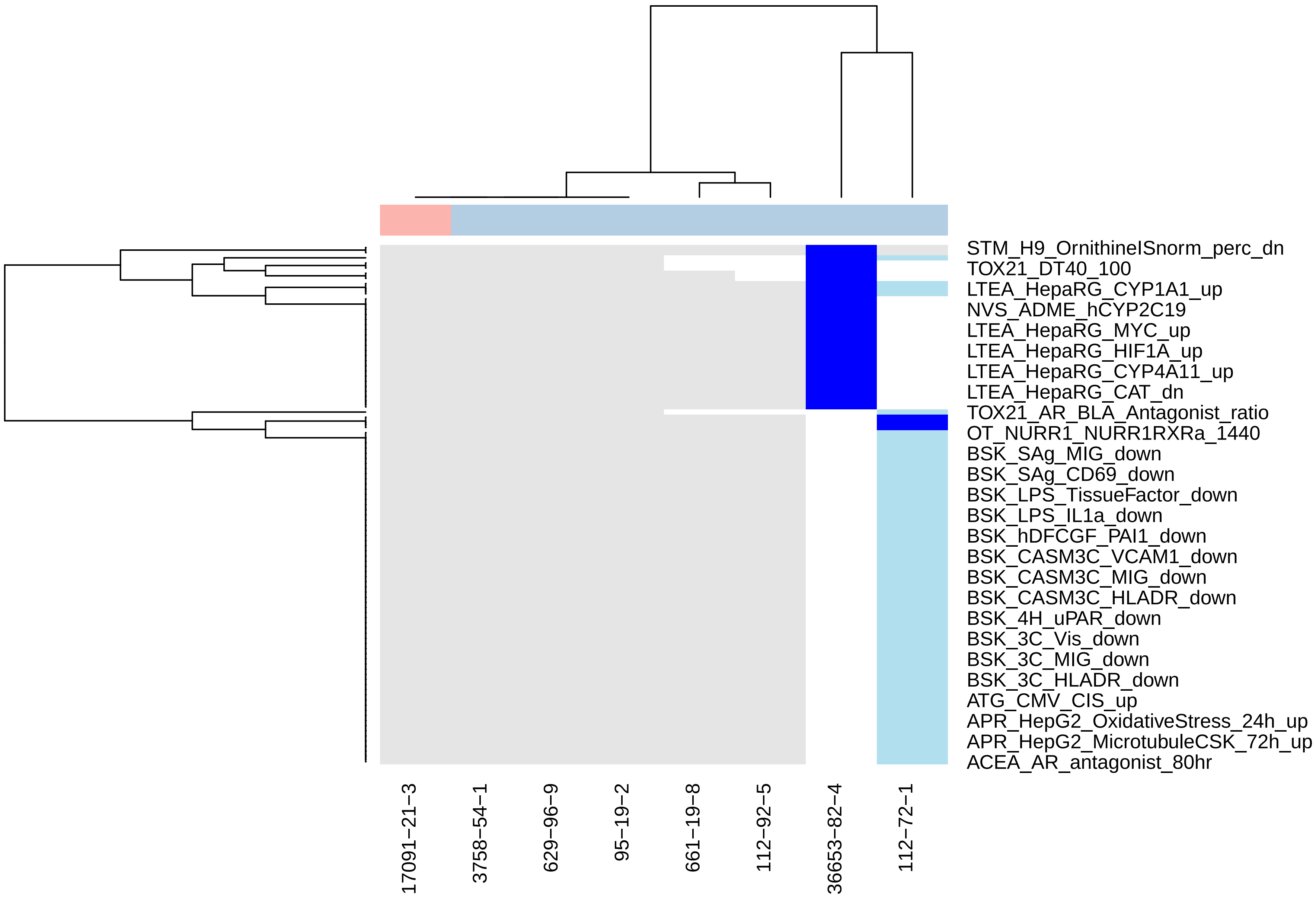

All Activity Heatmap for Cluster 592

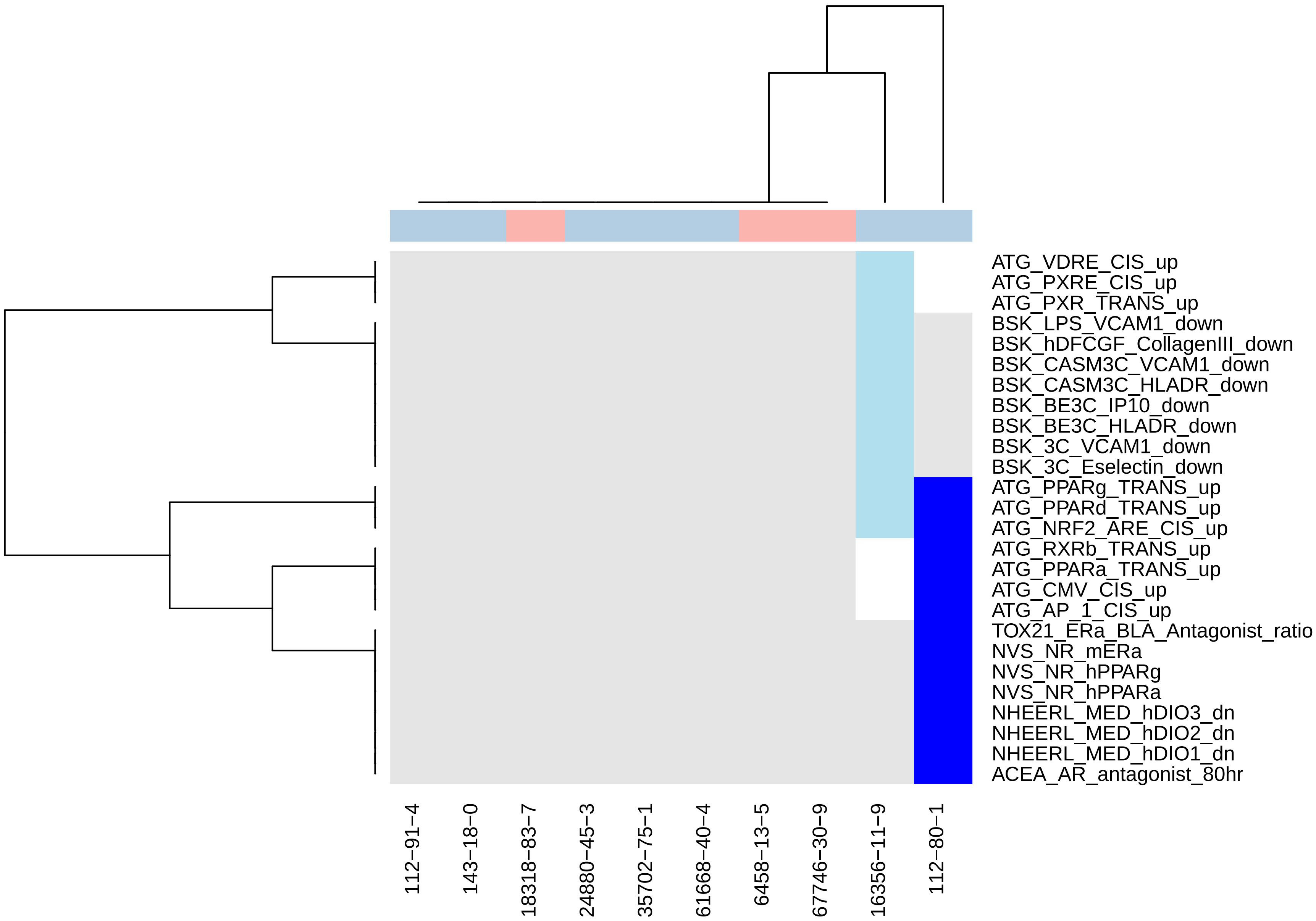

All Activity Heatmap for Cluster 594

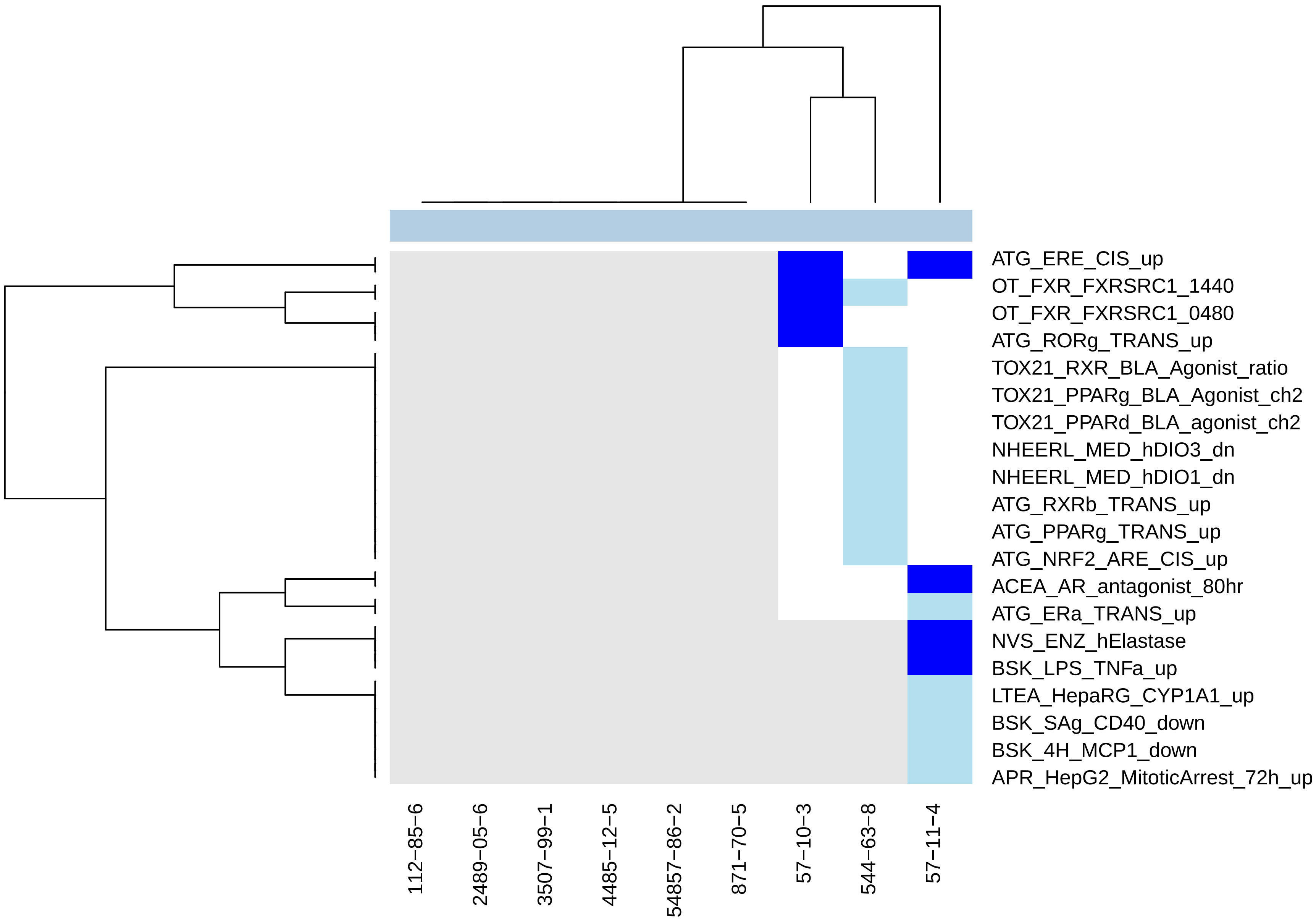

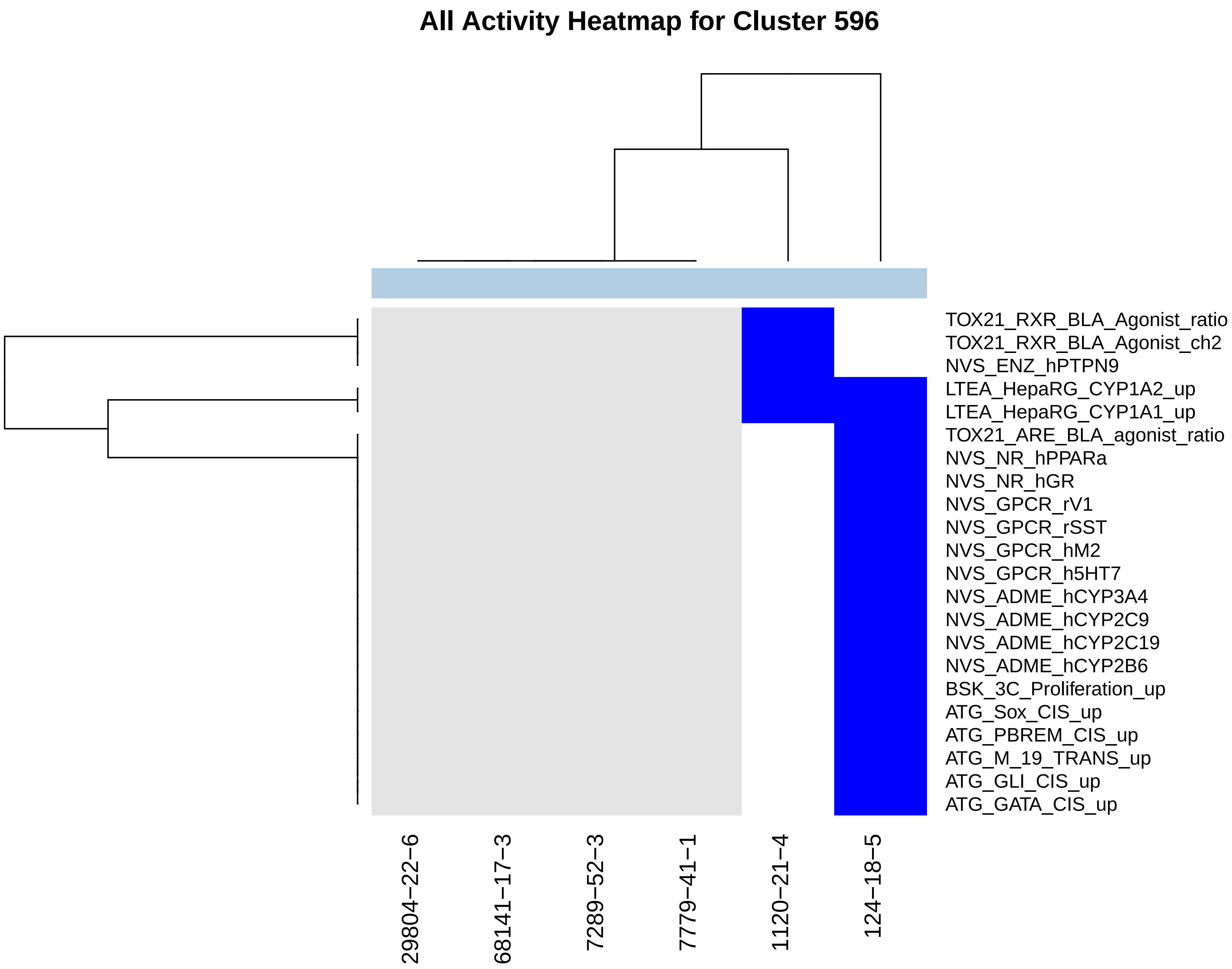
All Activity Heatmap for Cluster 596
TOX21_RXR_BLA_Agonist_ratio
TOX21_RXR_BLA_Agonist_ch2
NVS_ENZ_hPTPN9
LTEA_HepaRG_CYP1A2_up
LTEA_HepaRG_CYP1A1_up
TOX21_ARE_BLA_agonist_ratio
NVS_NR_hPPARa
NVS_NR_hGR
NVS_GPCR_rV1
NVS_GPCR_rSST
NVS_GPCR_hM2
NVS_GPCR_h5HT7
NVS_ADME_hCYP3A4
NVS_ADME_hCYP2C9
NVS_ADME_hCYP2C19
NVS_ADME_hCYP2B6
BSK_3C_Proliferation_up
ATG_Sox_CIS_up
ATG_PBREM_CIS_up
ATG_M_19_TRANS_up
ATG_GLI_CIS_up
ATG_GATA_CIS_up
29804−22−6
68141−17−3
7289−52−3
7779−41−1
1120−21−4
124−18−5

All Activity Heatmap for Cluster 597

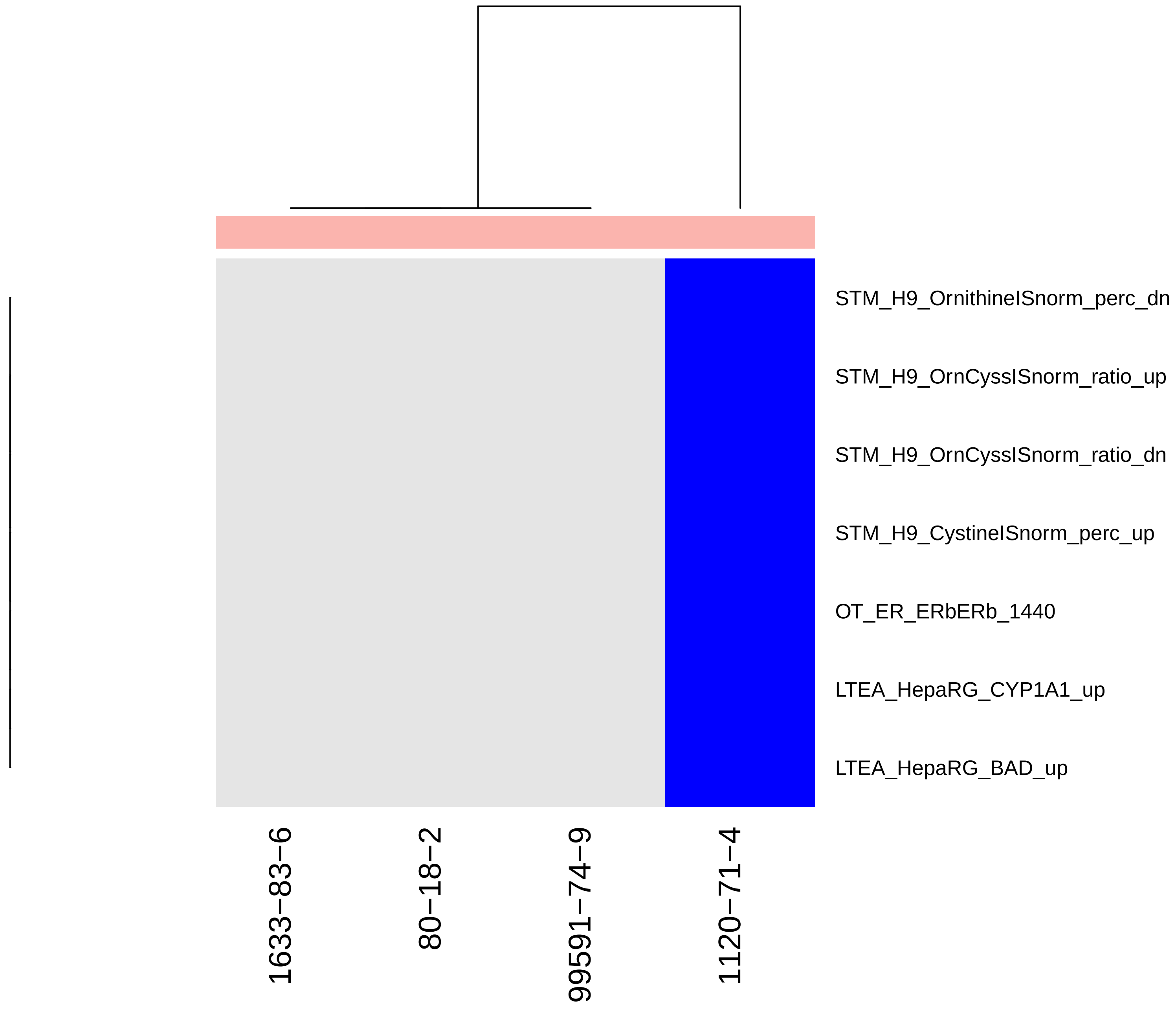

All Activity Heatmap for Cluster 598

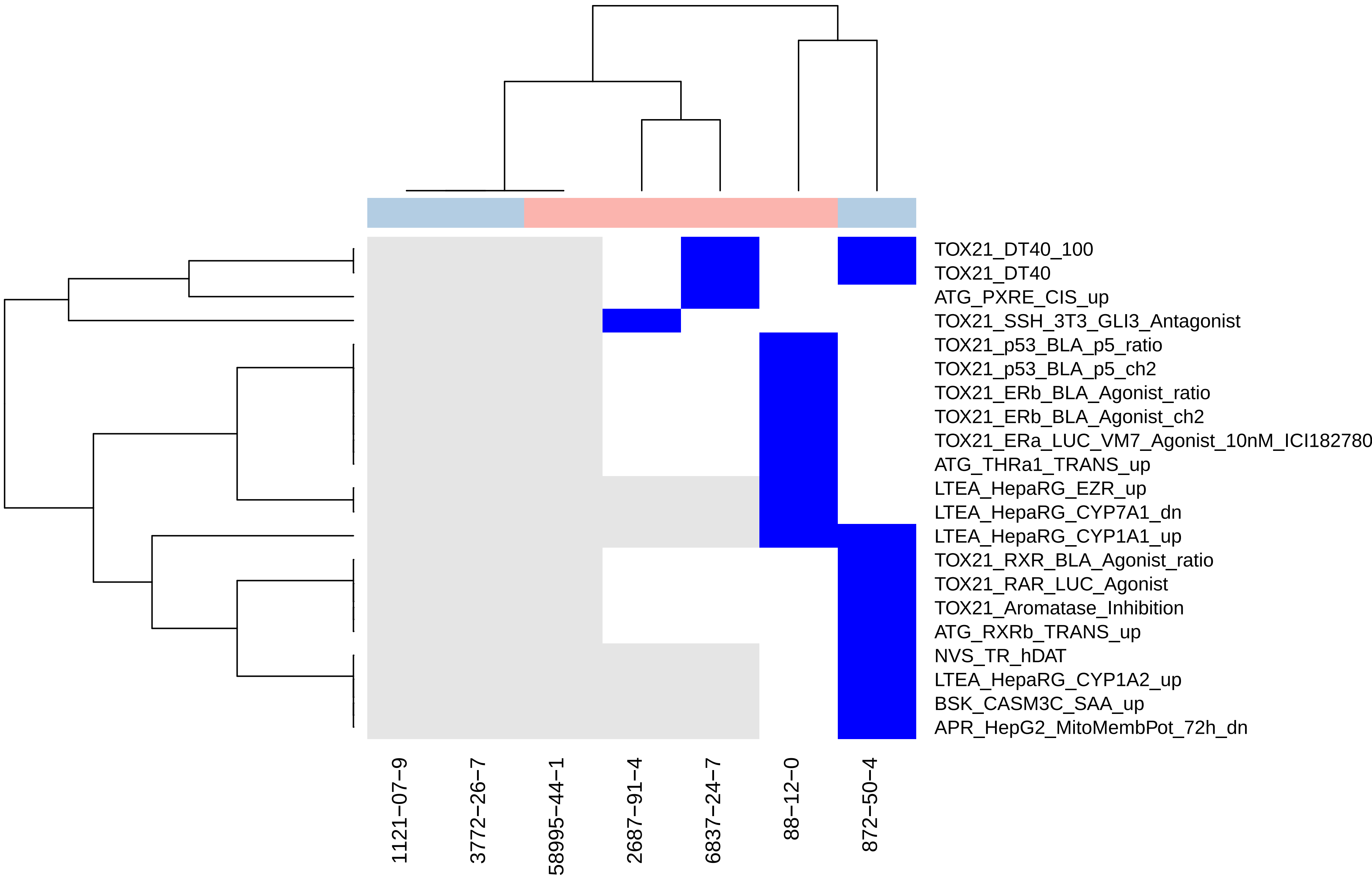

All Activity Heatmap for Cluster 599

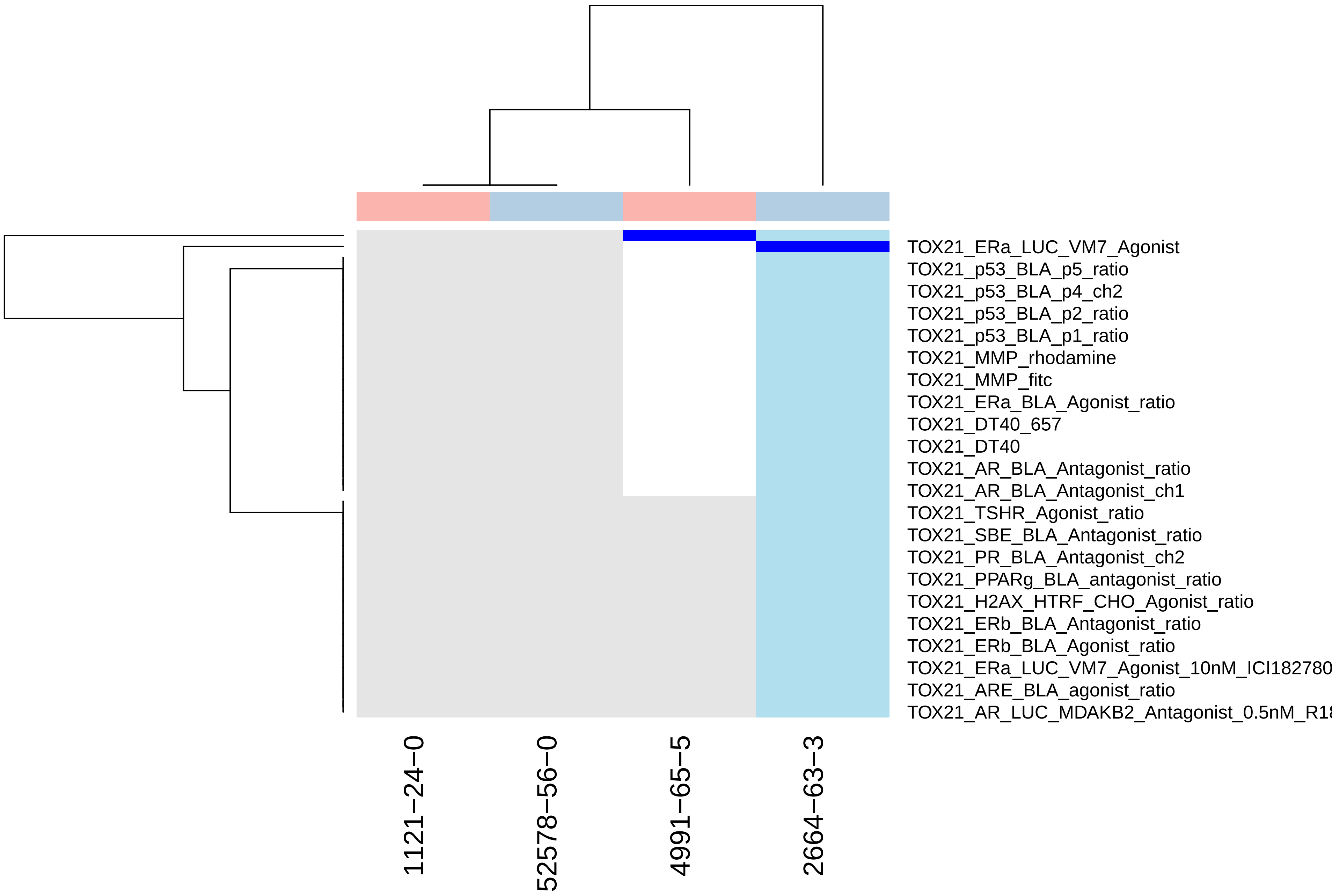

All Activity Heatmap for Cluster 602

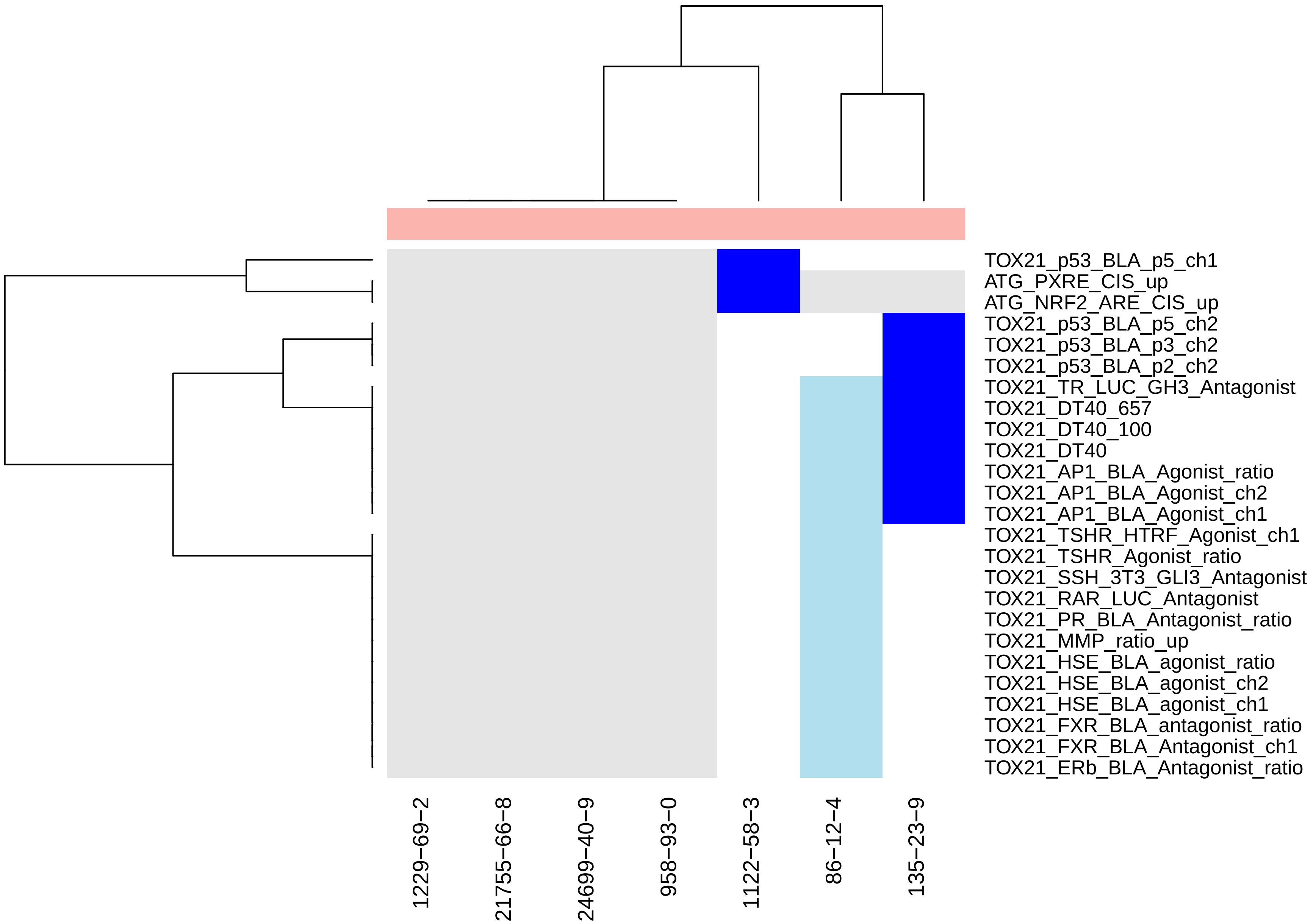

All Activity Heatmap for Cluster 603

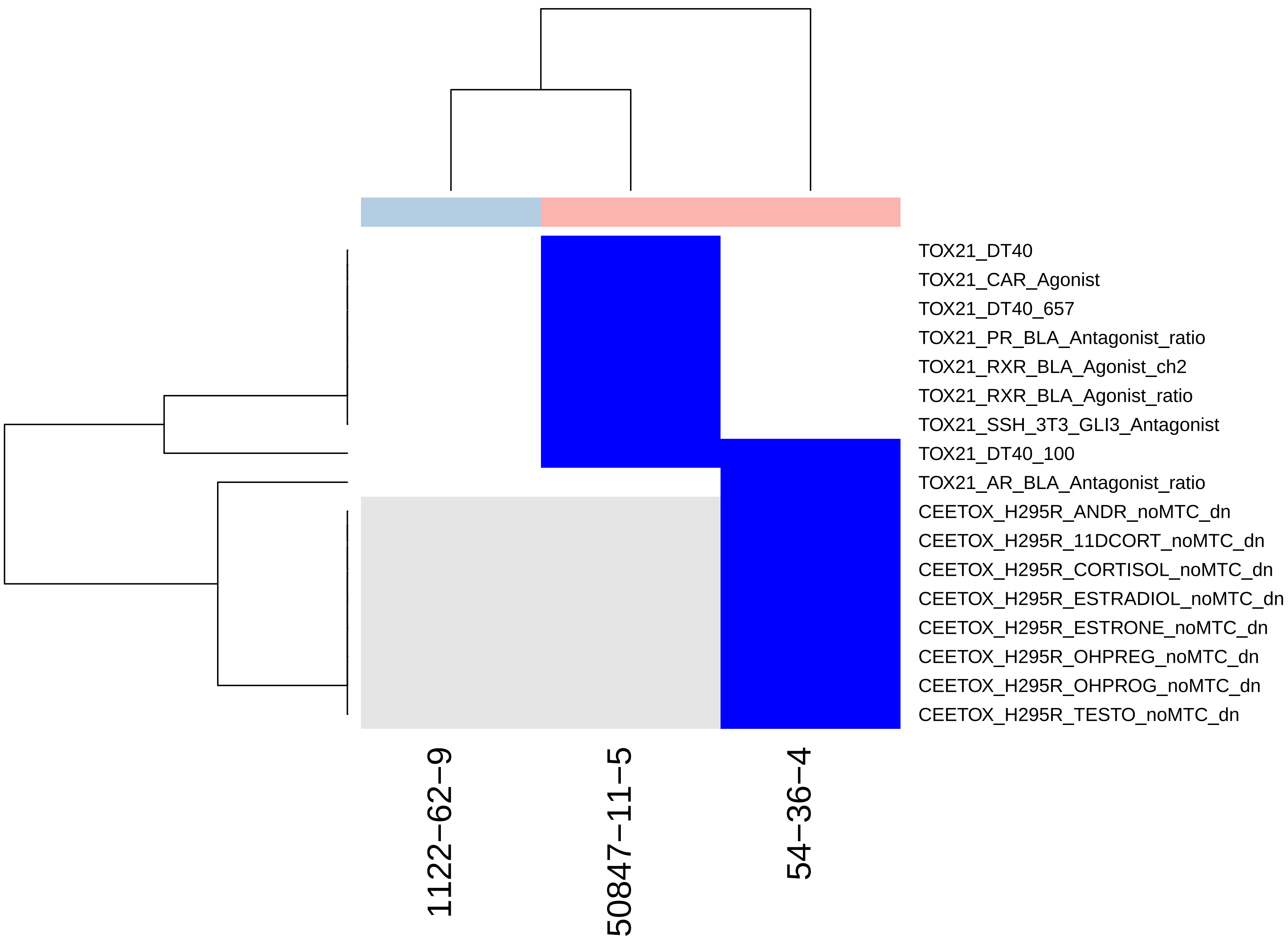

All Activity Heatmap for Cluster 604

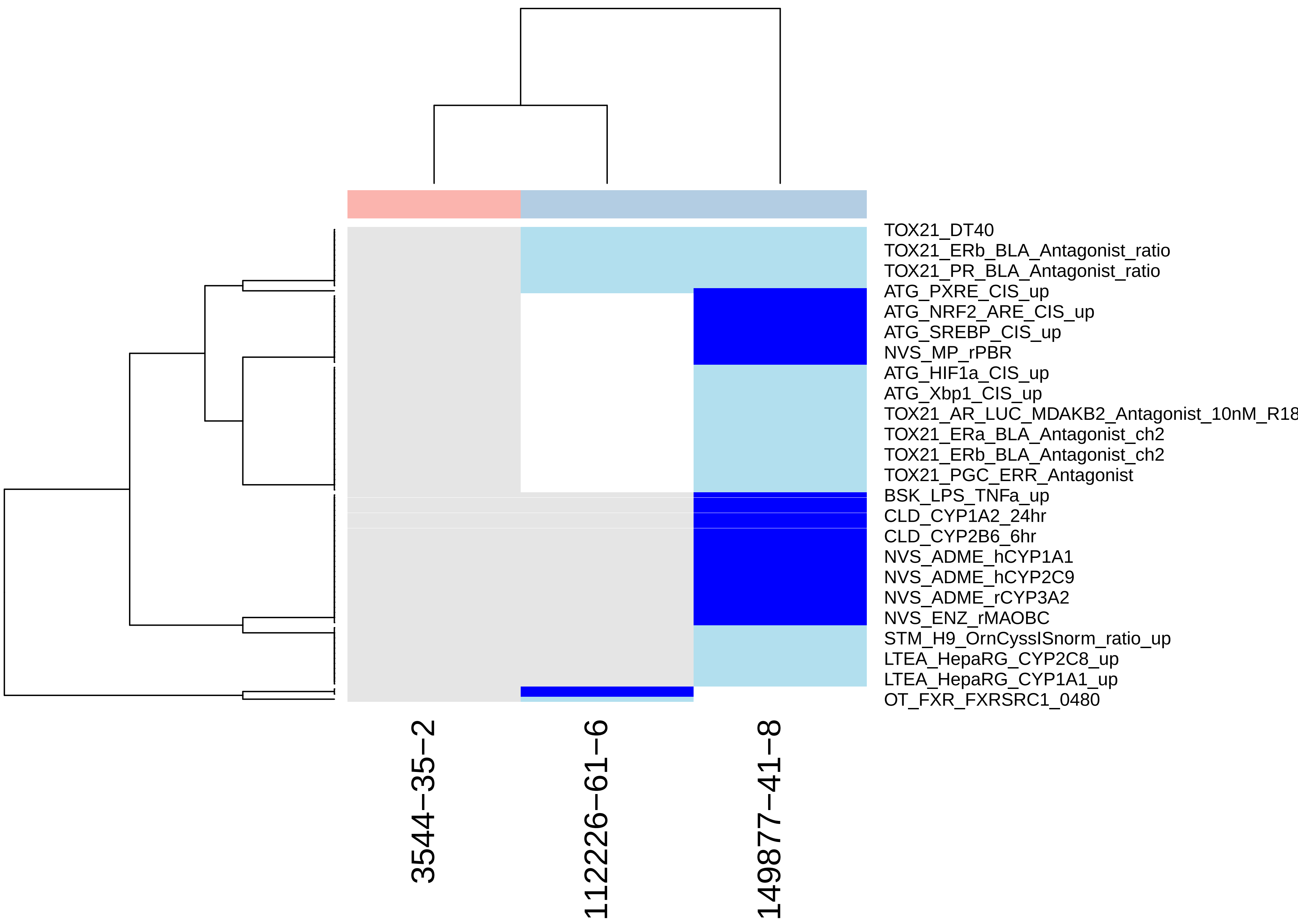

All Activity Heatmap for Cluster 605

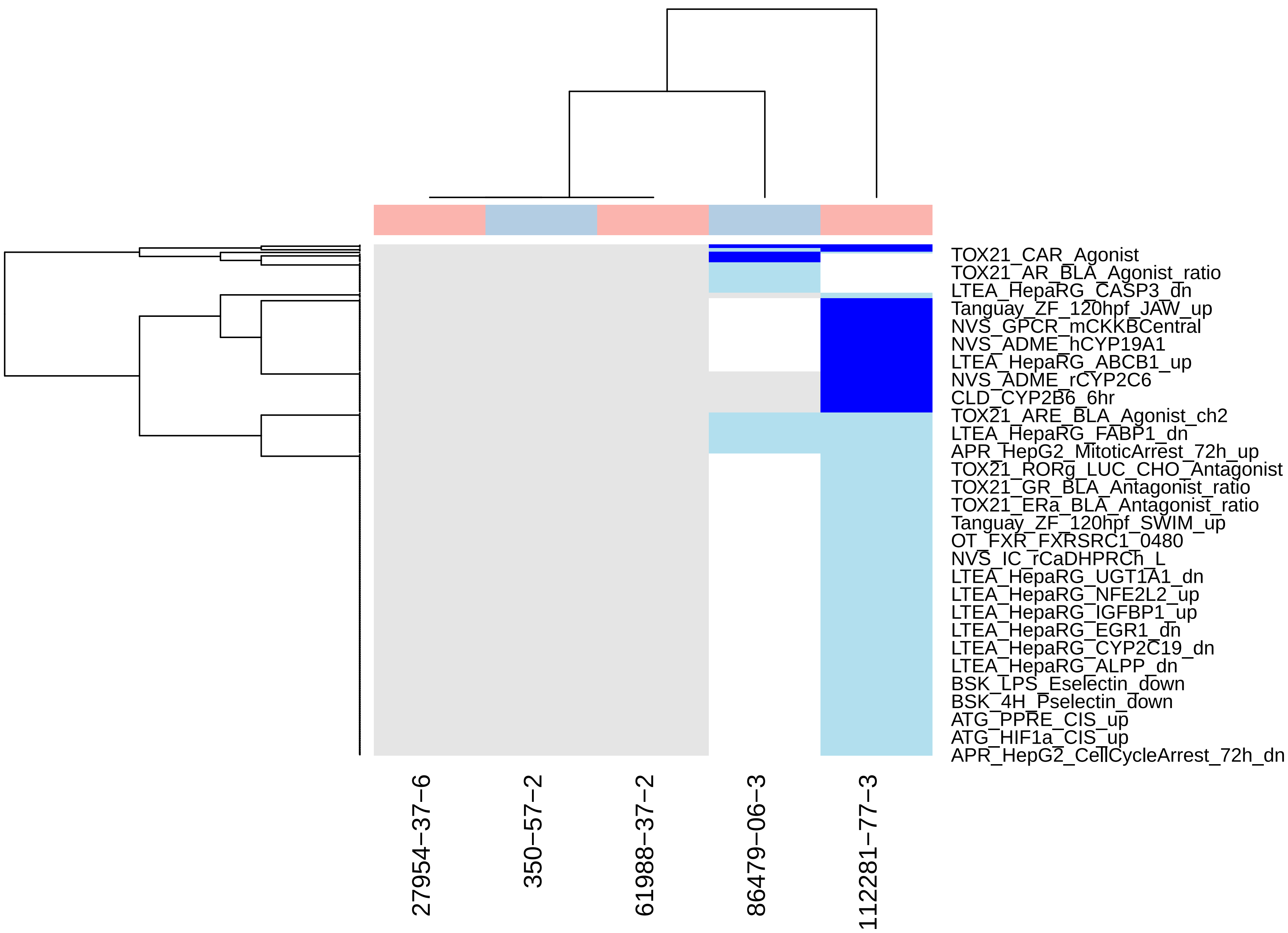

All Activity Heatmap for Cluster 606

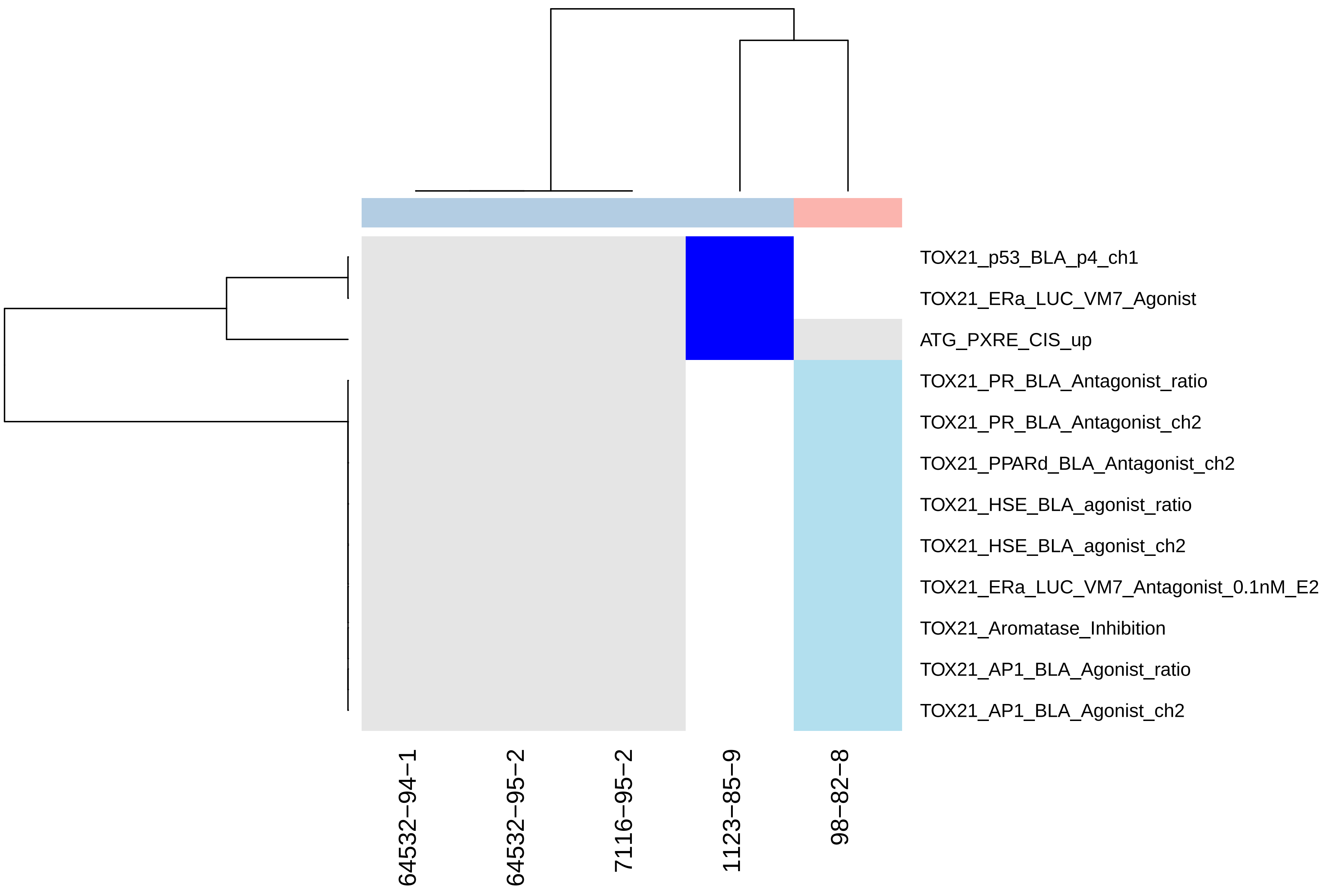

All Activity Heatmap for Cluster 607

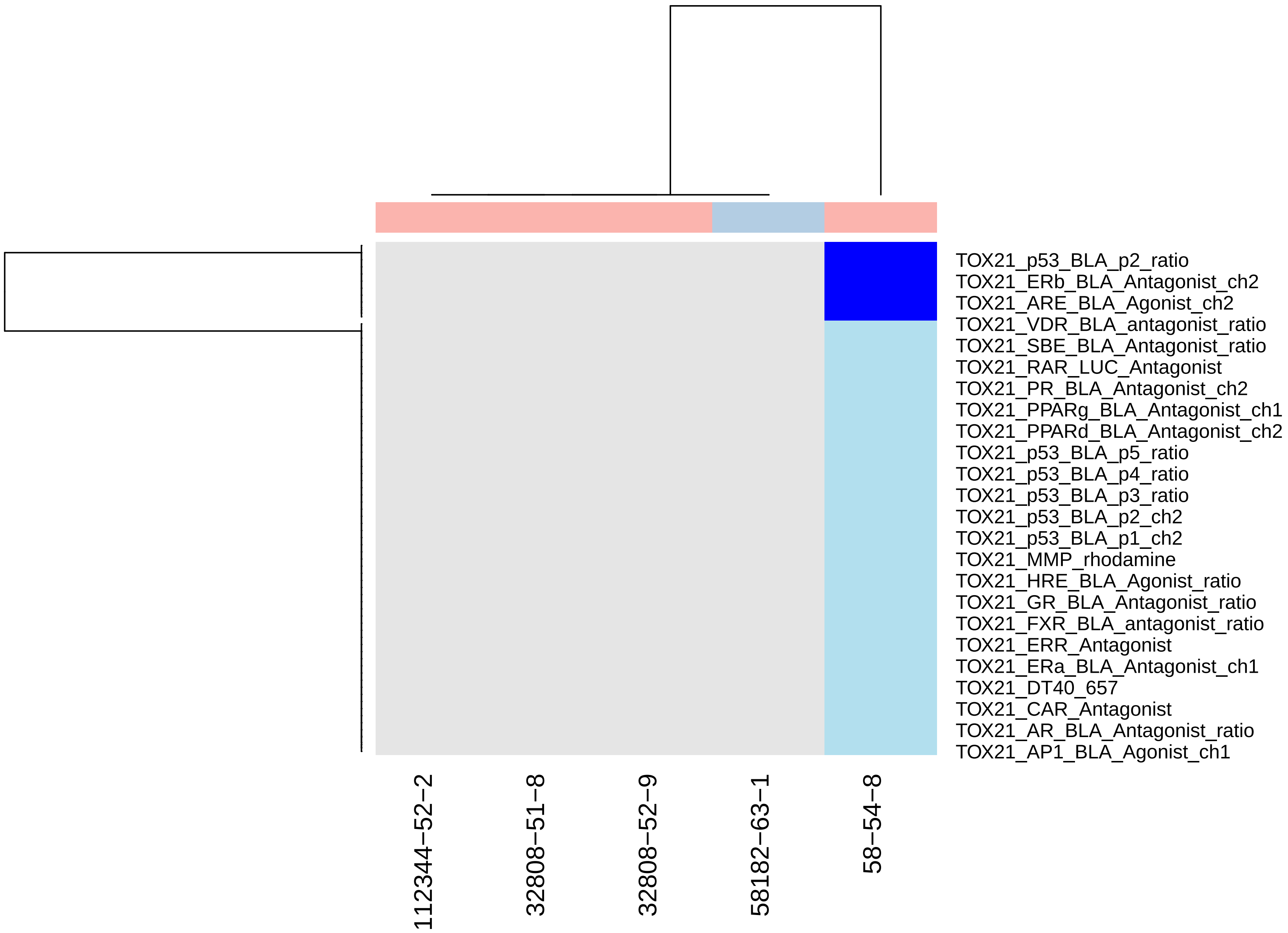

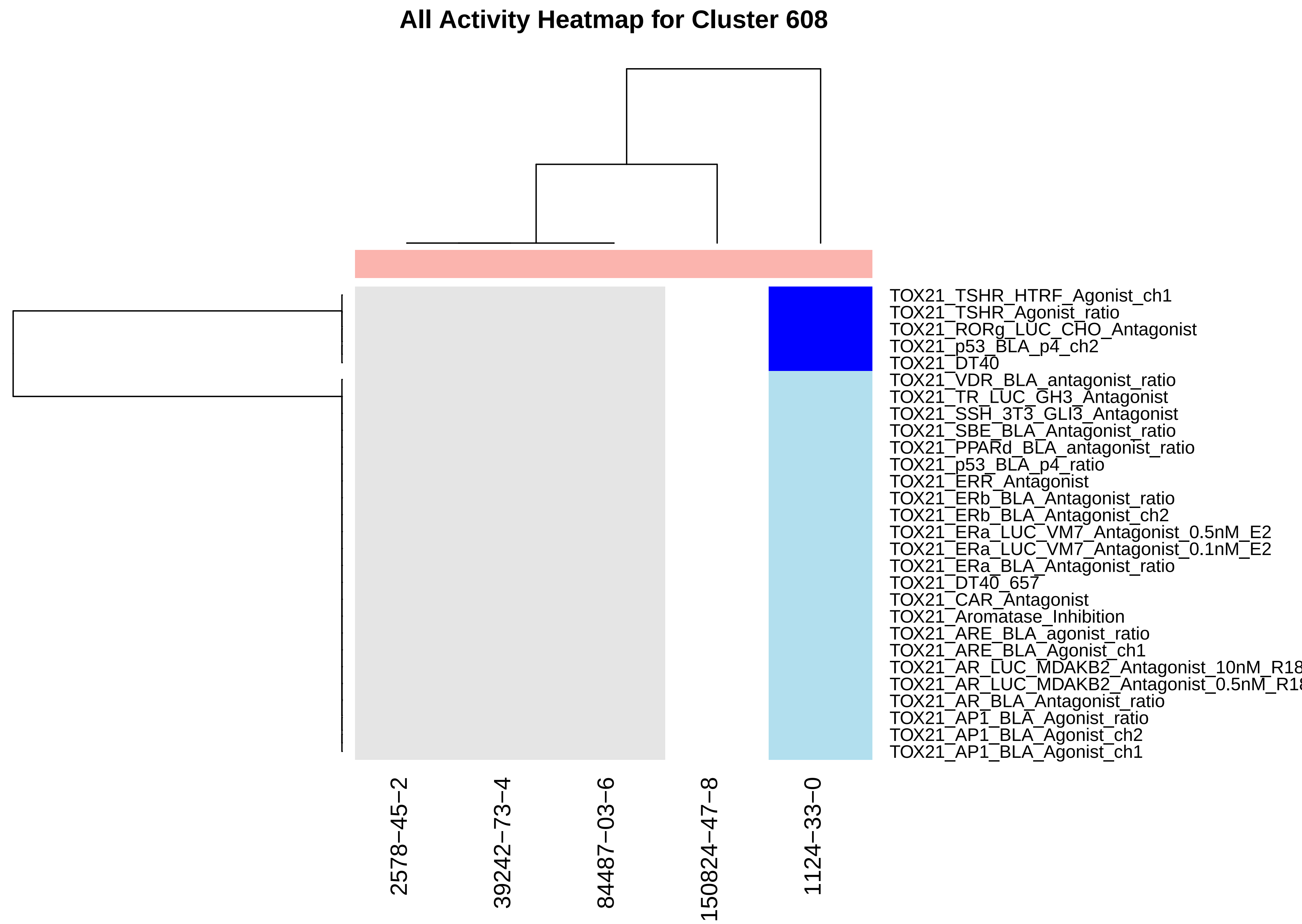
All Activity Heatmap for Cluster 608
TOX21_TSHR_HTRF_Agonist_ch1
TOX21_TSHR_Agonist_ratio
TOX21_RORg_LUC_CHO_Antagonist
TOX21_p53_BLA_p4_ch2
TOX21_DT40
TOX21_VDR_BLA_antagonist_ratio
TOX21_TR_LUC_GH3_Antagonist
TOX21_SSH_3T3_GLI3_Antagonist
TOX21_SBE_BLA_Antagonist_ratio
TOX21_PPARd_BLA_antagonist_ratio
TOX21_p53_BLA_p4_ratio
TOX21_ERR_Antagonist
TOX21_ERb_BLA_Antagonist_ratio
TOX21_ERb_BLA_Antagonist_ch2
TOX21_ERa_LUC_VM7_Antagonist_0.5nM_E2
TOX21_ERa_LUC_VM7_Antagonist_0.1nM_E2
TOX21_ERa_BLA_Antagonist_ratio
TOX21_DT40_657
TOX21_CAR_Antagonist
TOX21_Aromatase_Inhibition
TOX21_ARE_BLA_agonist_ratio
TOX21_ARE_BLA_Agonist_ch1
TOX21_AR_LUC_MDAKB2_Antagonist_10nM_R18
TOX21_AR_LUC_MDAKB2_Antagonist_0.5nM_R18
TOX21_AR_BLA_Antagonist_ratio
TOX21_AP1_BLA_Agonist_ratio
TOX21_AP1_BLA_Agonist_ch2
TOX21_AP1_BLA_Agonist_ch1
2578−45−2
39242−73−4
84487−03−6
150824−47−8
1124−33−0

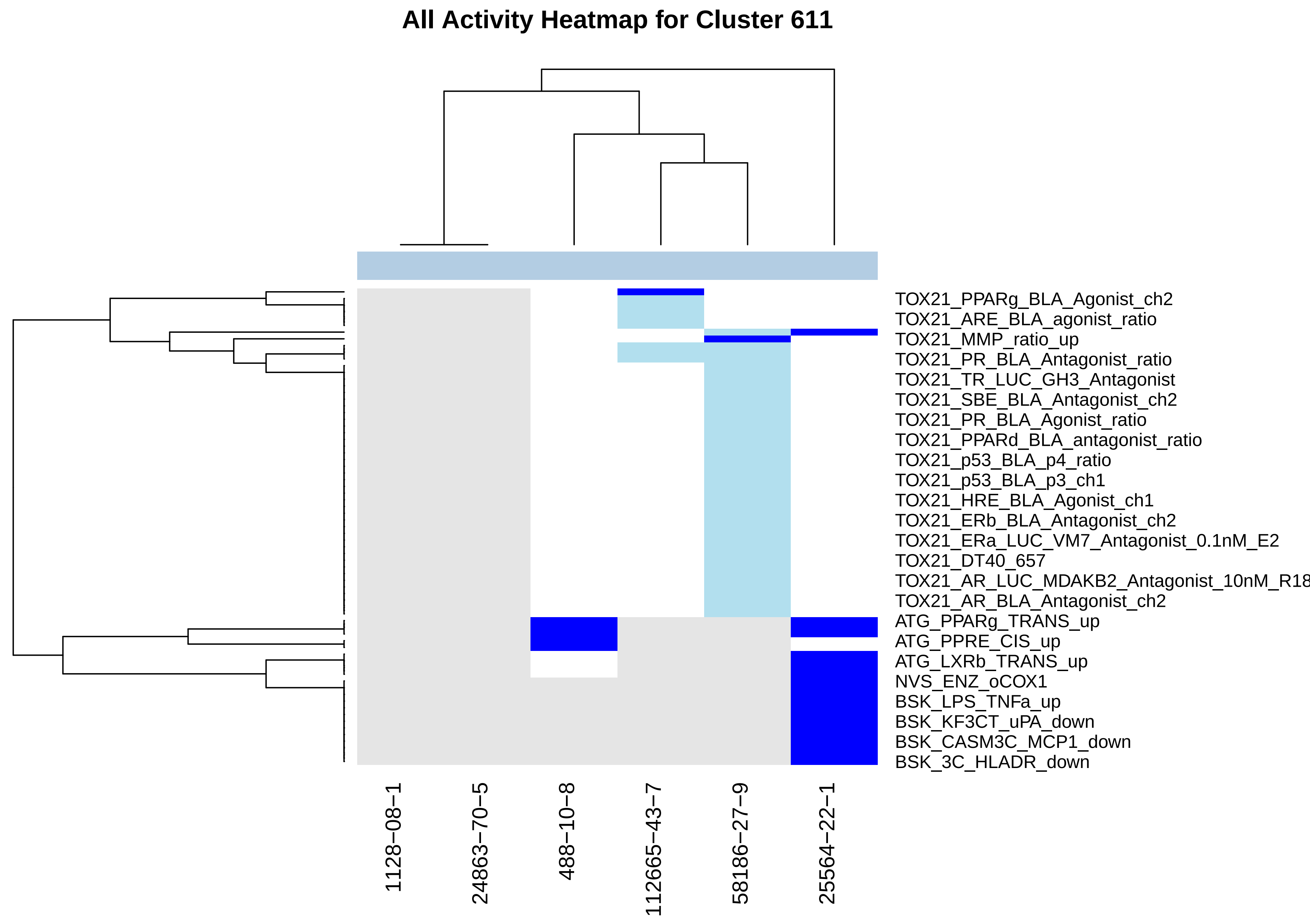

All Activity Heatmap for Cluster 611
TOX21_PPARg_BLA_Agonist_ch2
TOX21_ARE_BLA_agonist_ratio
TOX21_MMP_ratio_up
TOX21_PR_BLA_Antagonist_ratio
TOX21_TR_LUC_GH3_Antagonist
TOX21_SBE_BLA_Antagonist_ch2
TOX21_PR_BLA_Agonist_ratio
TOX21_PPARd_BLA_antagonist_ratio
TOX21_p53_BLA_p4_ratio
TOX21_p53_BLA_p3_ch1
TOX21_HRE_BLA_Agonist_ch1
TOX21_ERb_BLA_Antagonist_ch2
TOX21_ERa_LUC_VM7_Antagonist_0.1nM_E2
TOX21_DT40_657
TOX21_AR_LUC_MDAKB2_Antagonist_10nM_R18
TOX21_AR_BLA_Antagonist_ch2
ATG_PPARg_TRANS_up
ATG_PPRE_CIS_up
ATG_LXRb_TRANS_up
NVS_ENZ_oCOX1
BSK_LPS_TNFa_up
BSK_KF3CT_uPA_down
BSK_CASM3C_MCP1_down
BSK_3C_HLADR_down
1128−08−1
24863−70−5
488−10−8
112665−43−7
58186−27−9
25564−22−1

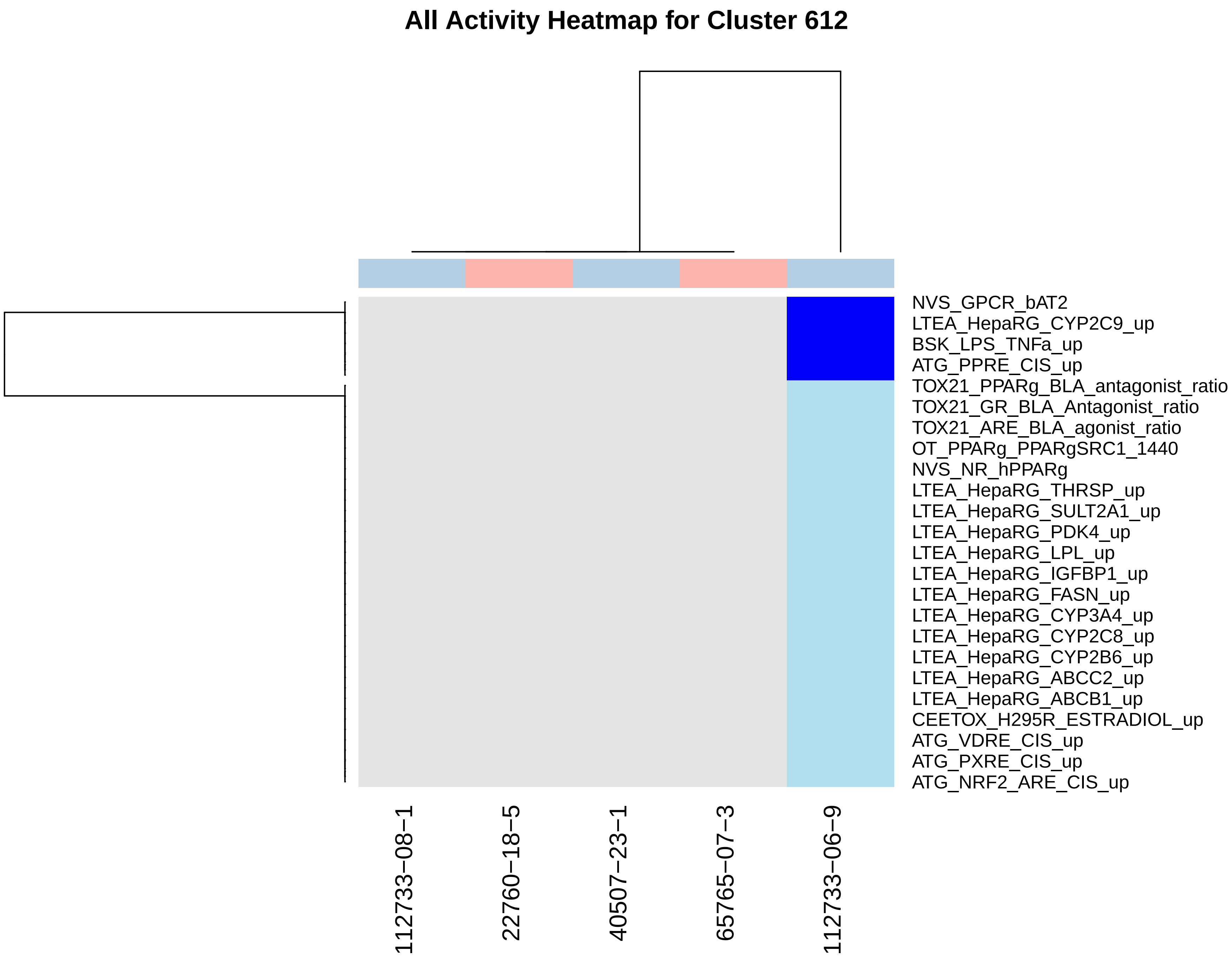
All Activity Heatmap for Cluster 612
NVS_GPCR_bAT2
LTEA_HepaRG_CYP2C9_up
BSK_LPS_TNFa_up
ATG_PPRE_CIS_up
TOX21_PPARg_BLA_antagonist_ratio
TOX21_GR_BLA_Antagonist_ratio
TOX21_ARE_BLA_agonist_ratio
OT_PPARg_PPARgSRC1_1440
NVS_NR_hPPARg
LTEA_HepaRG_THRSP_up
LTEA_HepaRG_SULT2A1_up
LTEA_HepaRG_PDK4_up
LTEA_HepaRG_LPL_up
LTEA_HepaRG_IGFBP1_up
LTEA_HepaRG_FASN_up
LTEA_HepaRG_CYP3A4_up
LTEA_HepaRG_CYP2C8_up
LTEA_HepaRG_CYP2B6_up
LTEA_HepaRG_ABCC2_up
LTEA_HepaRG_ABCB1_up
CEETOX_H295R_ESTRADIOL_up
ATG_VDRE_CIS_up
ATG_PXRE_CIS_up
ATG_NRF2_ARE_CIS_up
112733−08−1
22760−18−5
40507−23−1
65765−07−3
112733−06−9

All Activity Heatmap for Cluster 613

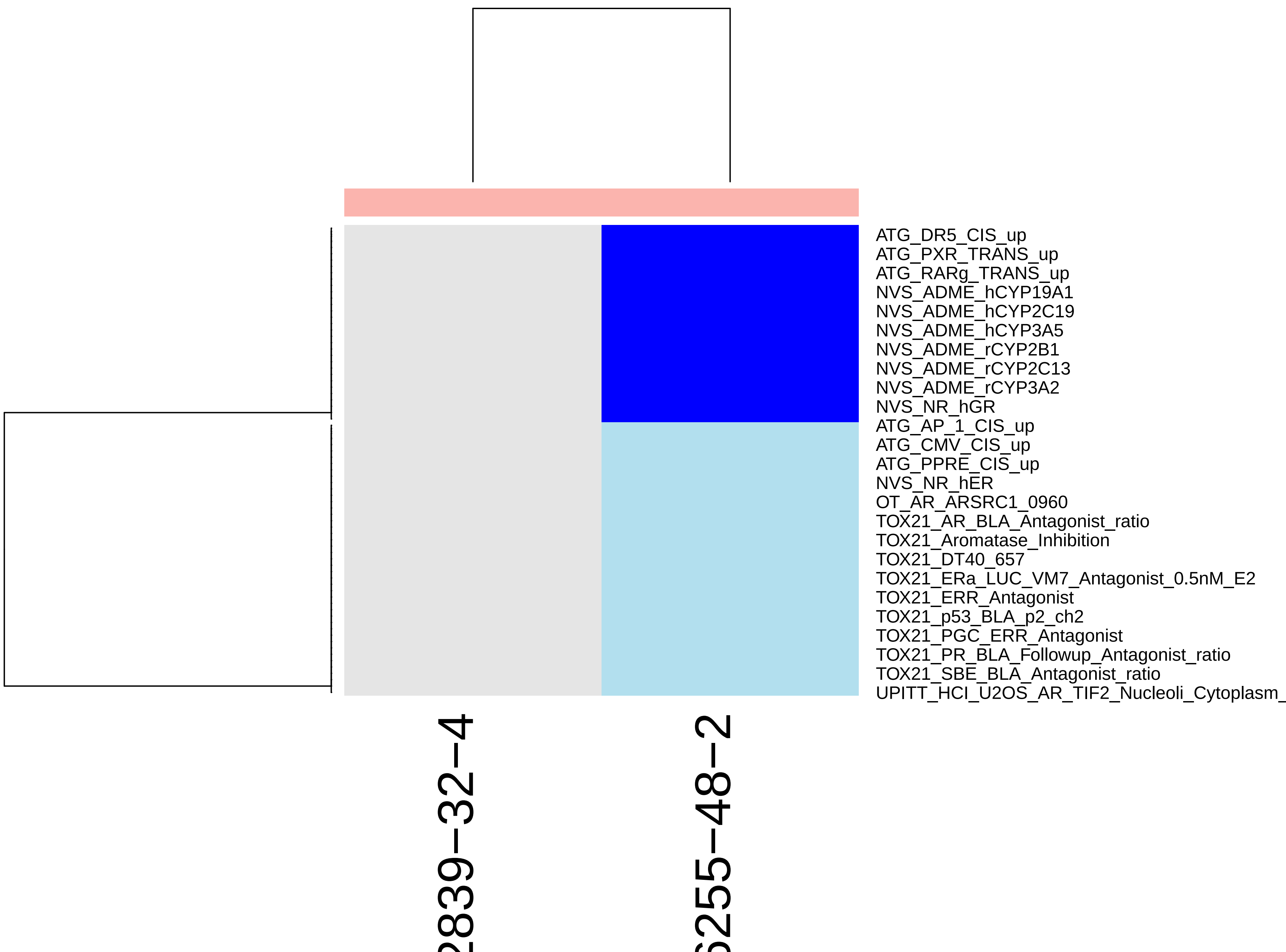

All Activity Heatmap for Cluster 614

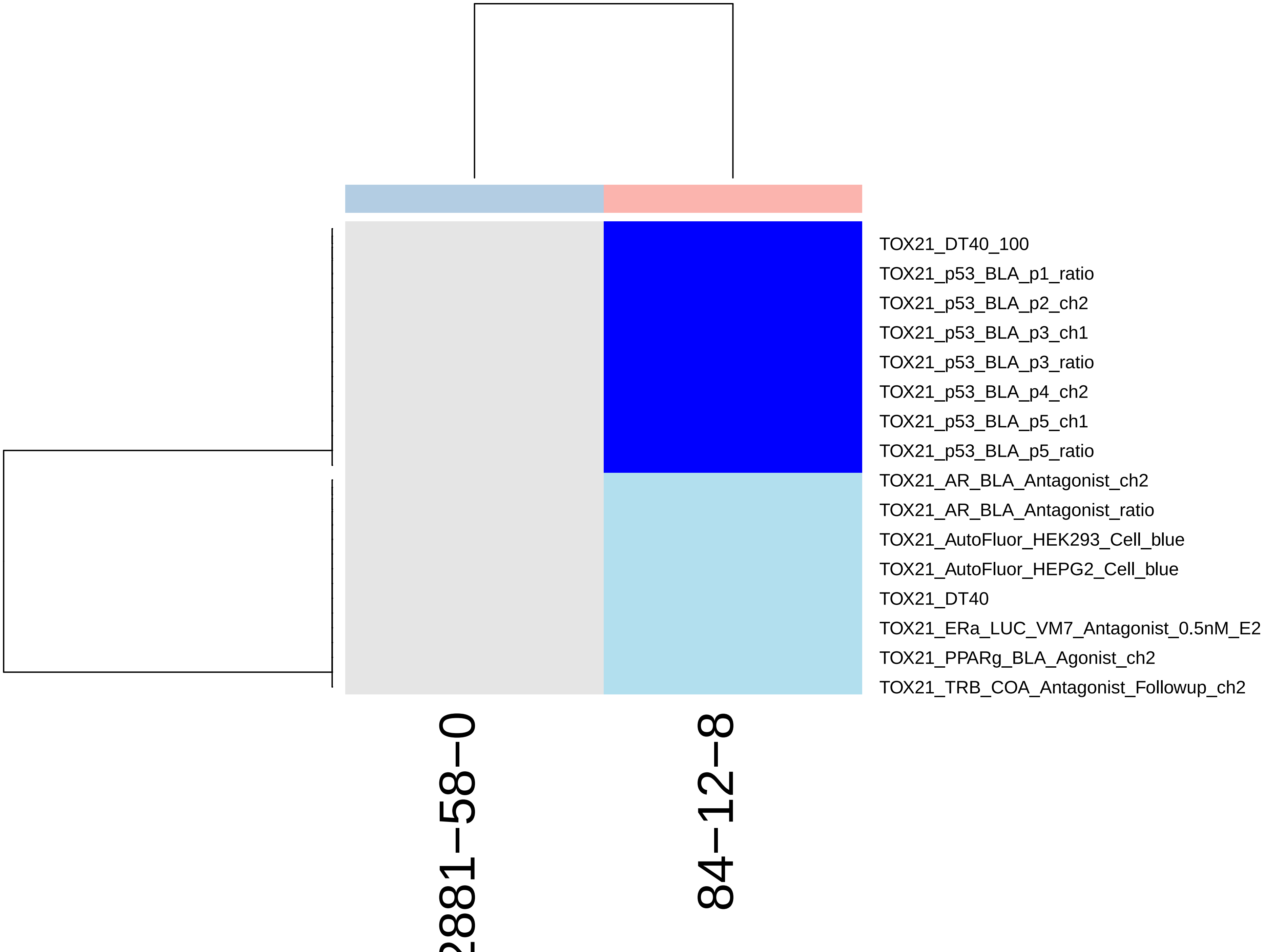

All Activity Heatmap for Cluster 615

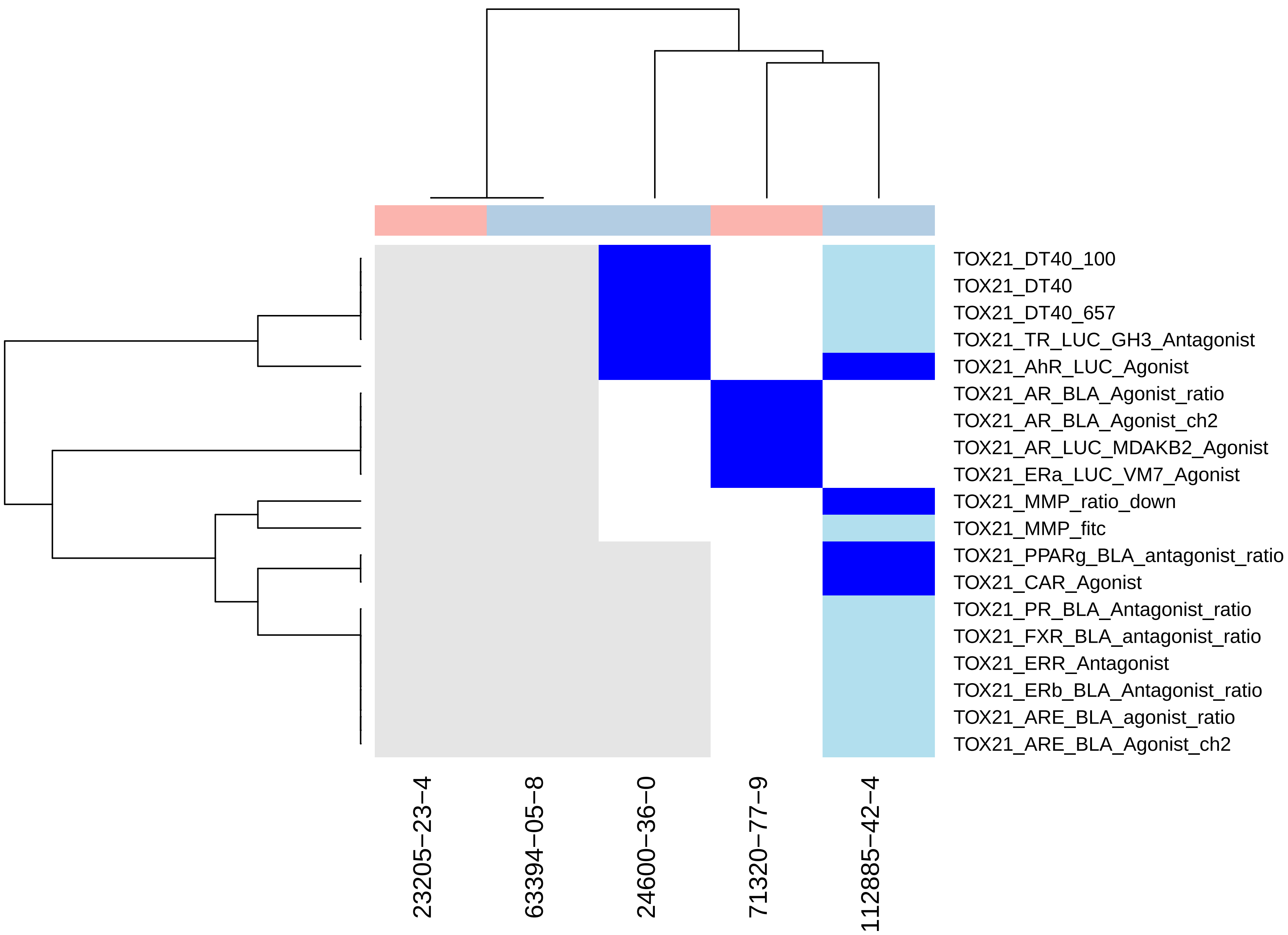

All Activity Heatmap for Cluster 616

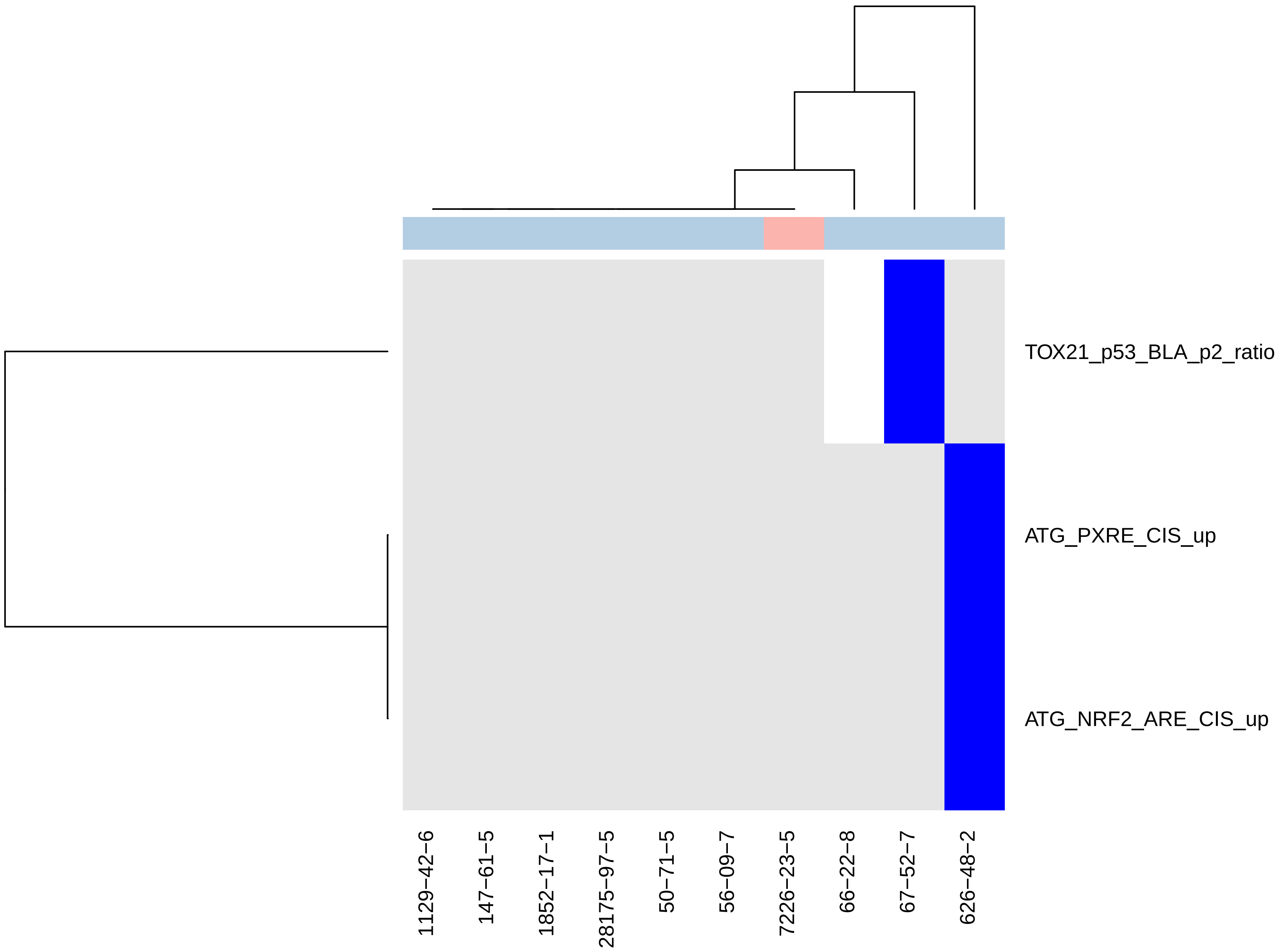

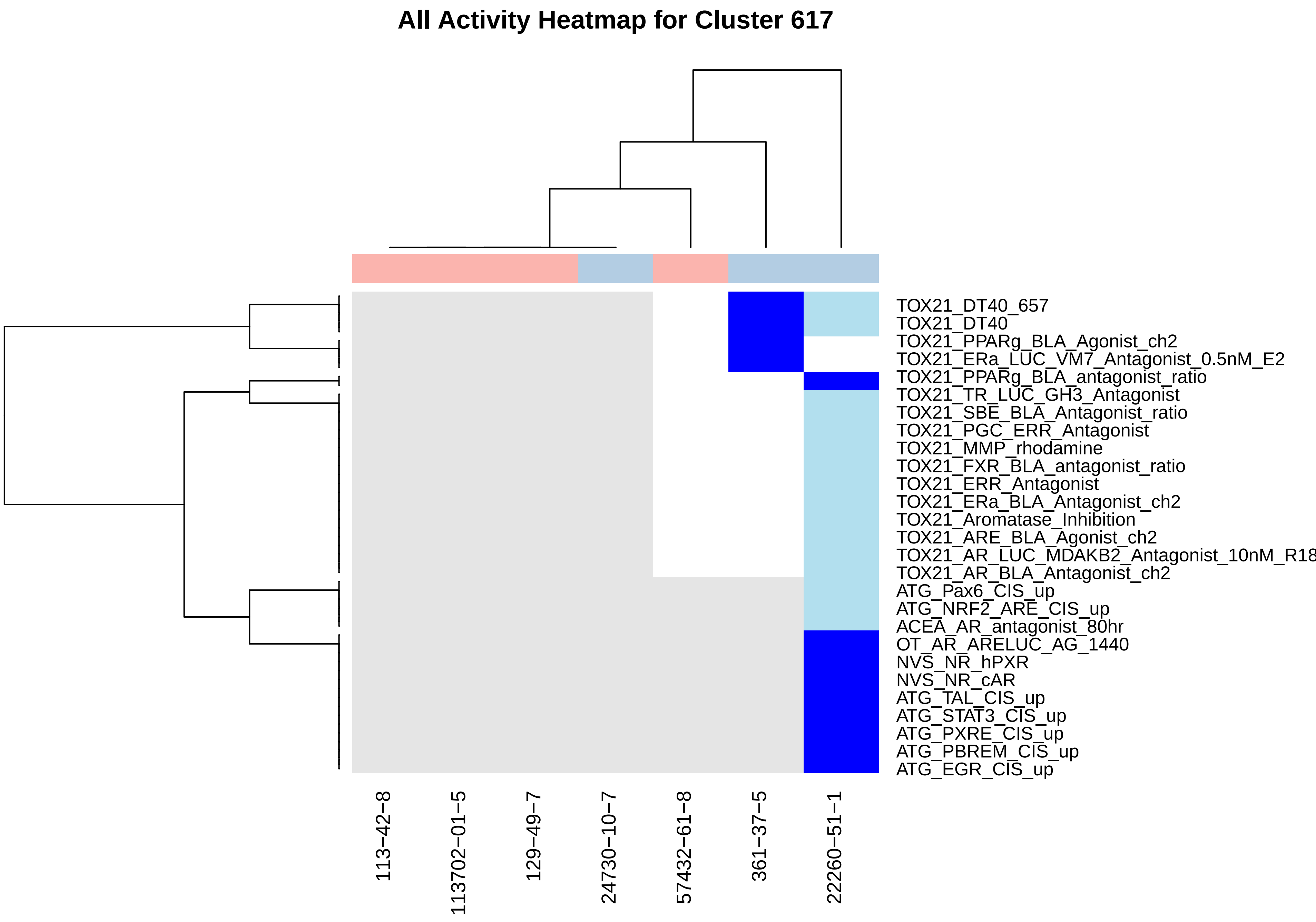
All Activity Heatmap for Cluster 617
TOX21_DT40_657
TOX21_DT40
TOX21_PPARg_BLA_Agonist_ch2
TOX21_ERa_LUC_VM7_Antagonist_0.5nM_E2
TOX21_PPARg_BLA_antagonist_ratio
TOX21_TR_LUC_GH3_Antagonist
TOX21_SBE_BLA_Antagonist_ratio
TOX21_PGC_ERR_Antagonist
TOX21_MMP_rhodamine
TOX21_FXR_BLA_antagonist_ratio
TOX21_ERR_Antagonist
TOX21_ERa_BLA_Antagonist_ch2
TOX21_Aromatase_Inhibition
TOX21_ARE_BLA_Agonist_ch2
TOX21_AR_LUC_MDAKB2_Antagonist_10nM_R18
TOX21_AR_BLA_Antagonist_ch2
ATG_Pax6_CIS_up
ATG_NRF2_ARE_CIS_up
ACEA_AR_antagonist_80hr
OT_AR_ARELUC_AG_1440
NVS_NR_hPXR
NVS_NR_cAR
ATG_TAL_CIS_up
ATG_STAT3_CIS_up
ATG_PXRE_CIS_up
ATG_PBREM_CIS_up
ATG_EGR_CIS_up
113−42−8
113702−01−5
129−49−7
24730−10−7
57432−61−8
361−37−5
22260−51−1

All Activity Heatmap for Cluster 619

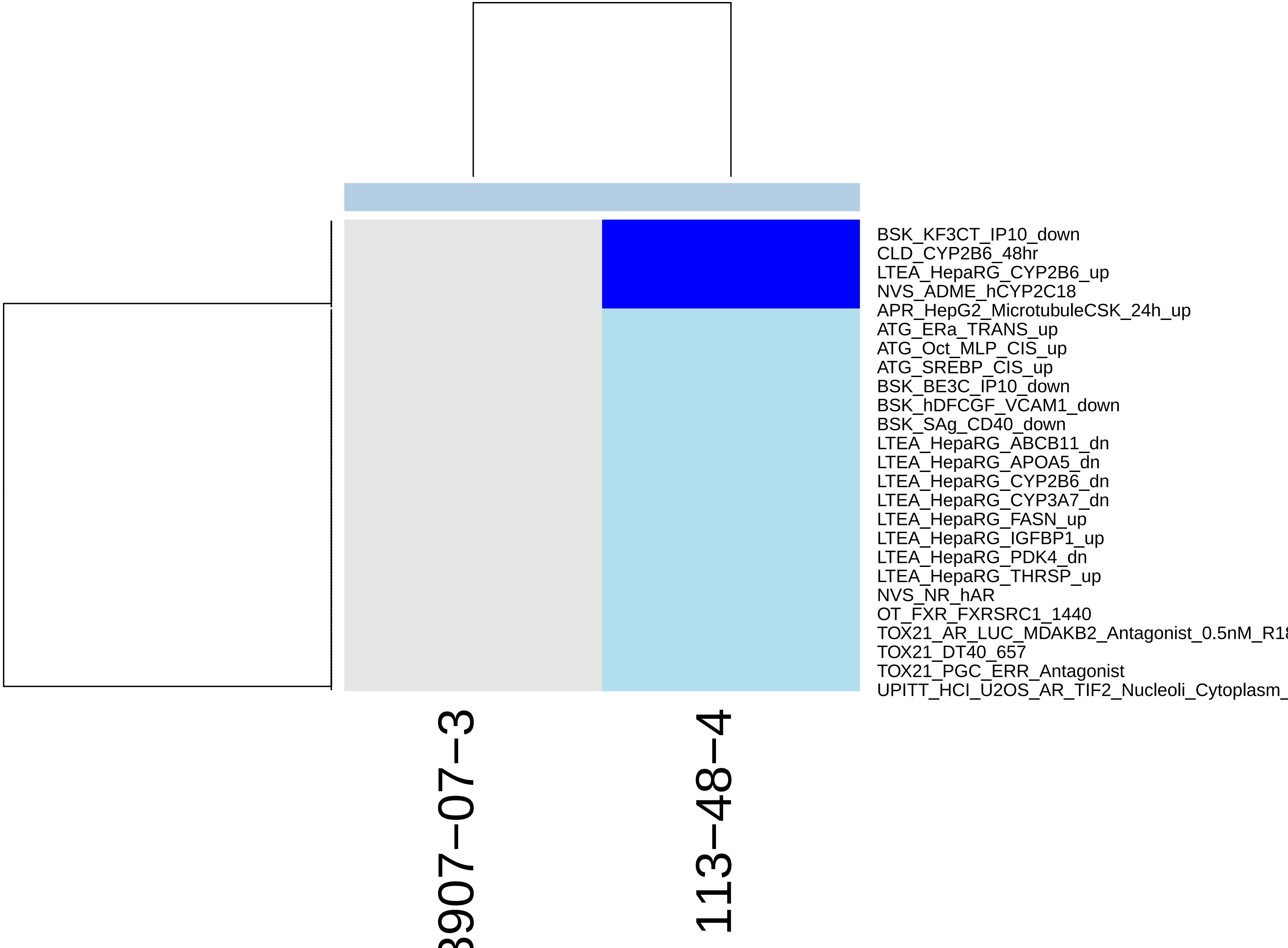

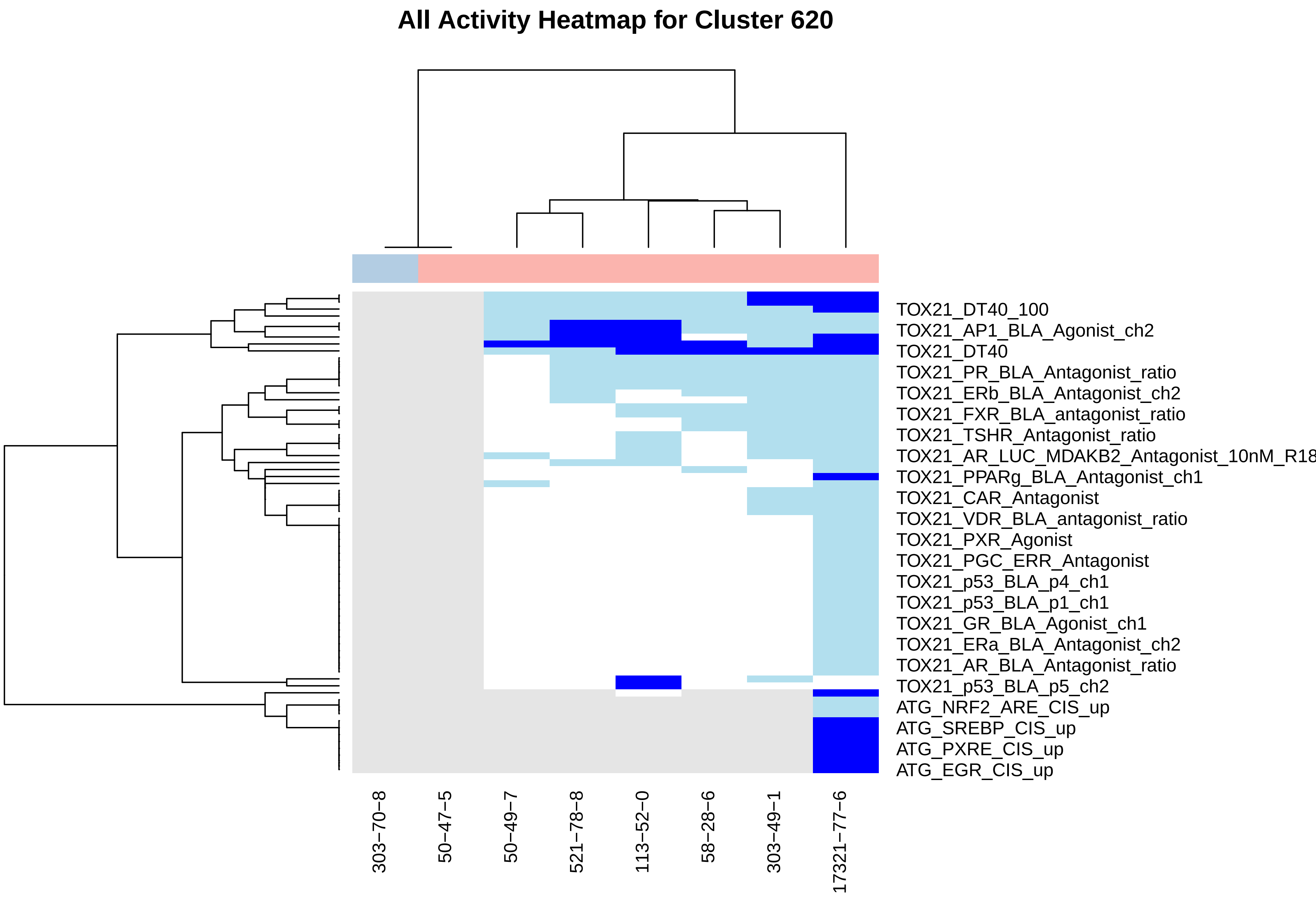
All Activity Heatmap for Cluster 620
TOX21_DT40_100
TOX21_AP1_BLA_Agonist_ch2
TOX21_DT40
TOX21_PR_BLA_Antagonist_ratio
TOX21_ERb_BLA_Antagonist_ch2
TOX21_FXR_BLA_antagonist_ratio
TOX21_TSHR_Antagonist_ratio
TOX21_AR_LUC_MDAKB2_Antagonist_10nM_R18
TOX21_PPARg_BLA_Antagonist_ch1
TOX21_CAR_Antagonist
TOX21_VDR_BLA_antagonist_ratio
TOX21_PXR_Agonist
TOX21_PGC_ERR_Antagonist
TOX21_p53_BLA_p4_ch1
TOX21_p53_BLA_p1_ch1
TOX21_GR_BLA_Agonist_ch1
TOX21_ERa_BLA_Antagonist_ch2
TOX21_AR_BLA_Antagonist_ratio
TOX21_p53_BLA_p5_ch2
ATG_NRF2_ARE_CIS_up
ATG_SREBP_CIS_up
ATG_PXRE_CIS_up
ATG_EGR_CIS_up
303−70−8
50−47−5
50−49−7
521−78−8
113−52−0
58−28−6
303−49−1
17321−77−6

All Activity Heatmap for Cluster 621

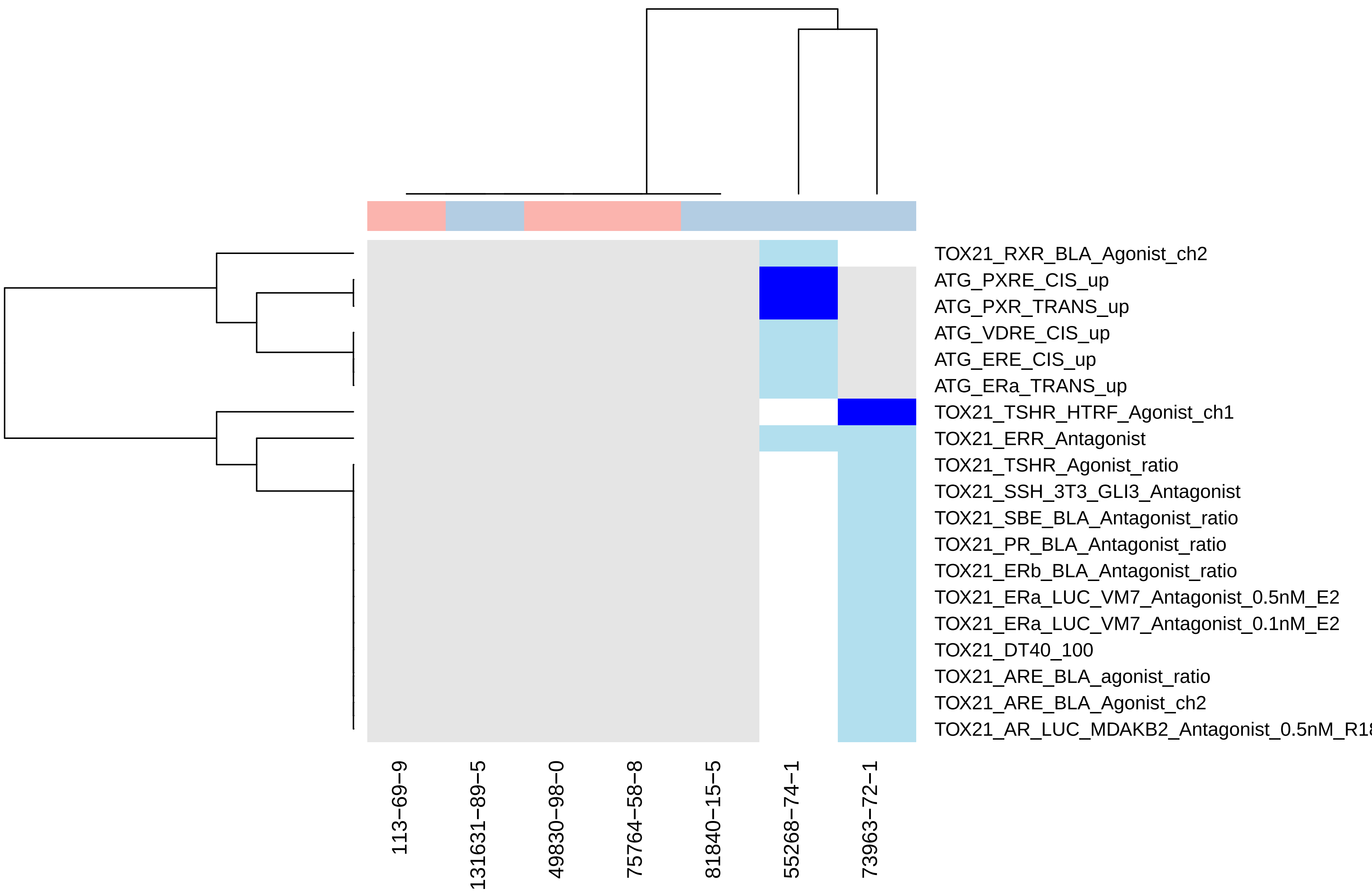

All Activity Heatmap for Cluster 622

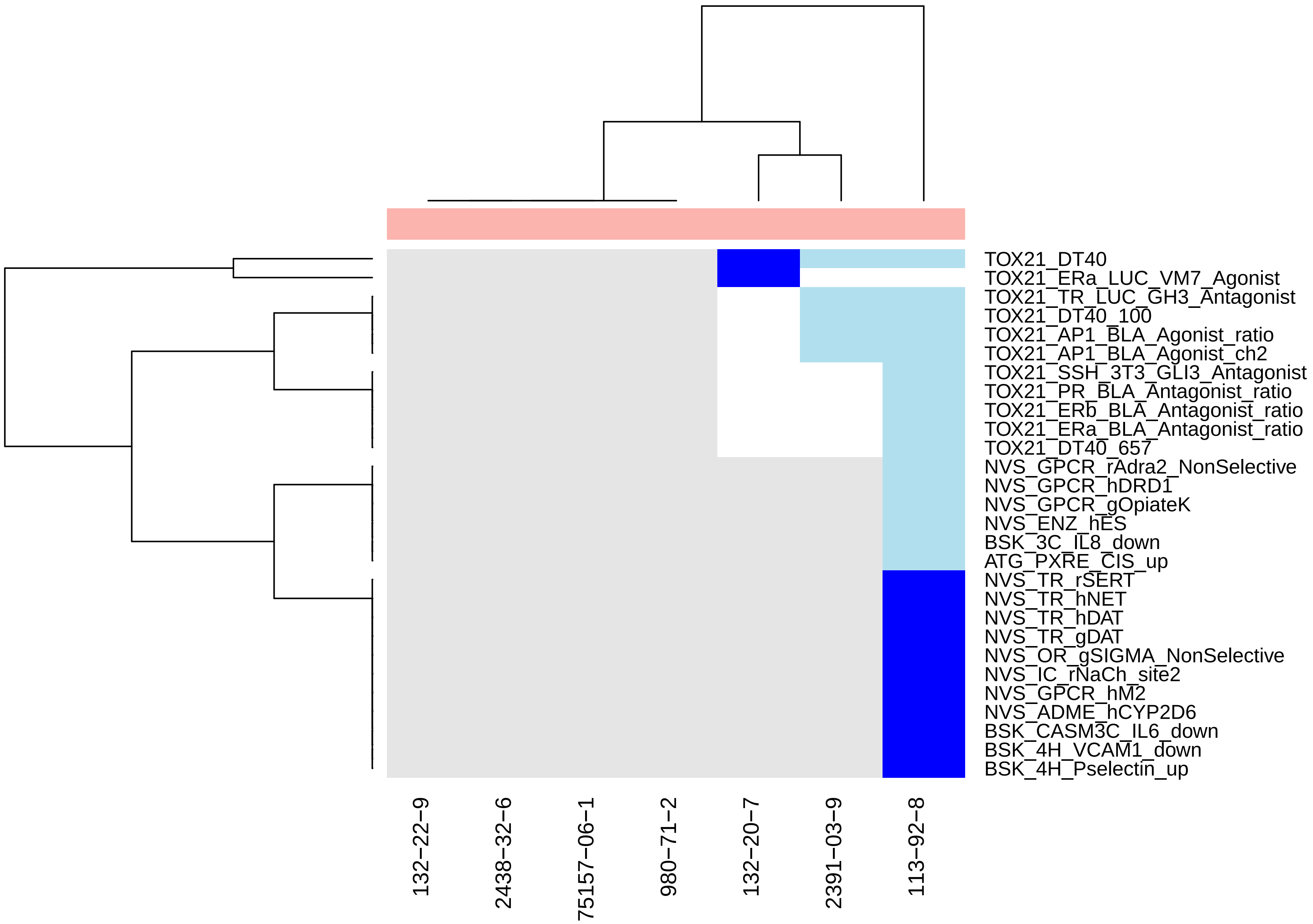

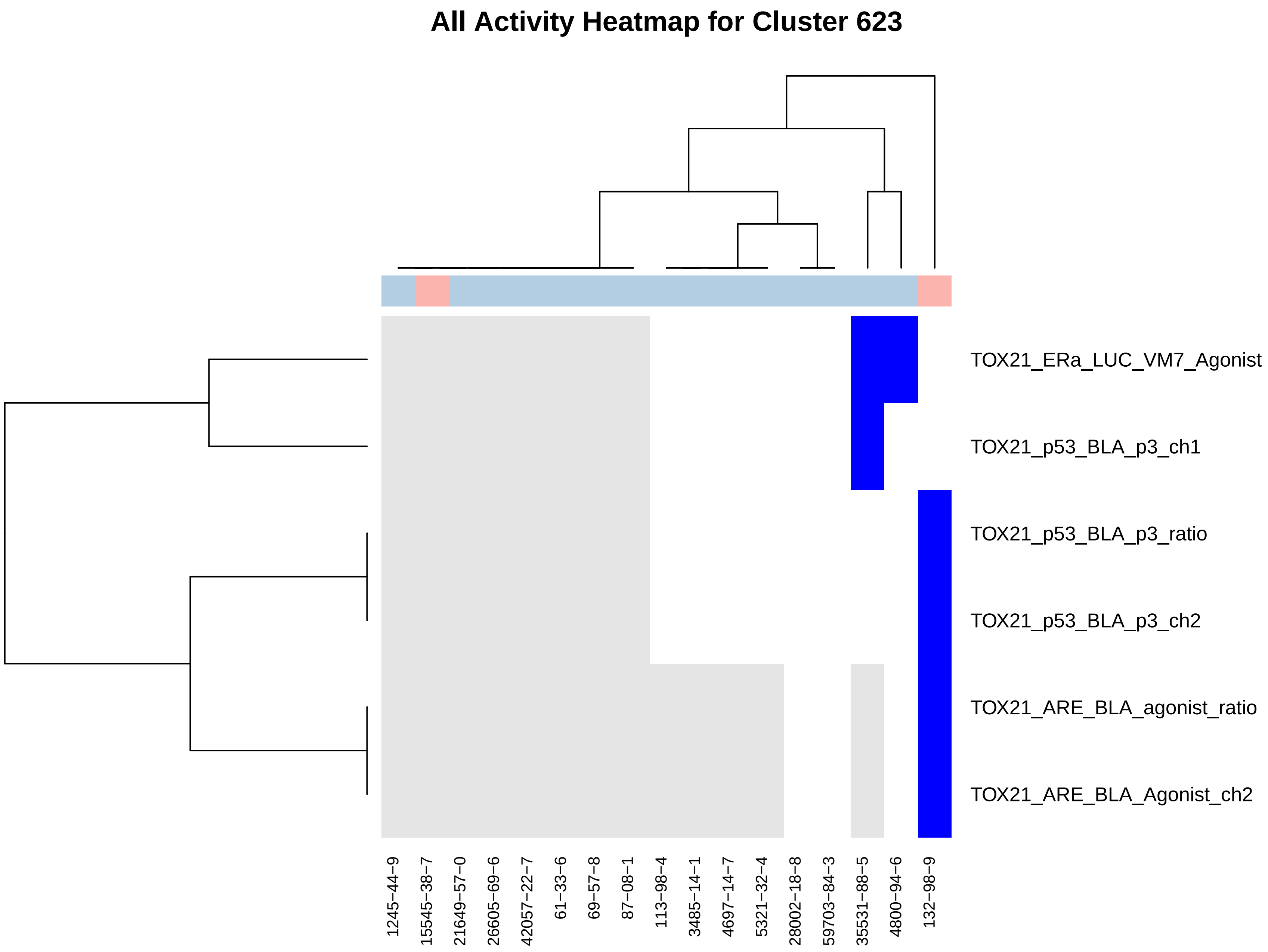
All Activity Heatmap for Cluster 623
TOX21_ERa_LUC_VM7_Agonist
TOX21_p53_BLA_p3_ch1
TOX21_p53_BLA_p3_ratio
TOX21_p53_BLA_p3_ch2
TOX21_ARE_BLA_agonist_ratio
TOX21_ARE_BLA_Agonist_ch2
1245−44−9
15545−38−7
21649−57−0
26605−69−6
42057−22−7
61−33−6
69−57−8
87−08−1
113−98−4
3485−14−1
4697−14−7
5321−32−4
28002−18−8
59703−84−3
35531−88−5
4800−94−6
132−98−9

All Activity Heatmap for Cluster 624

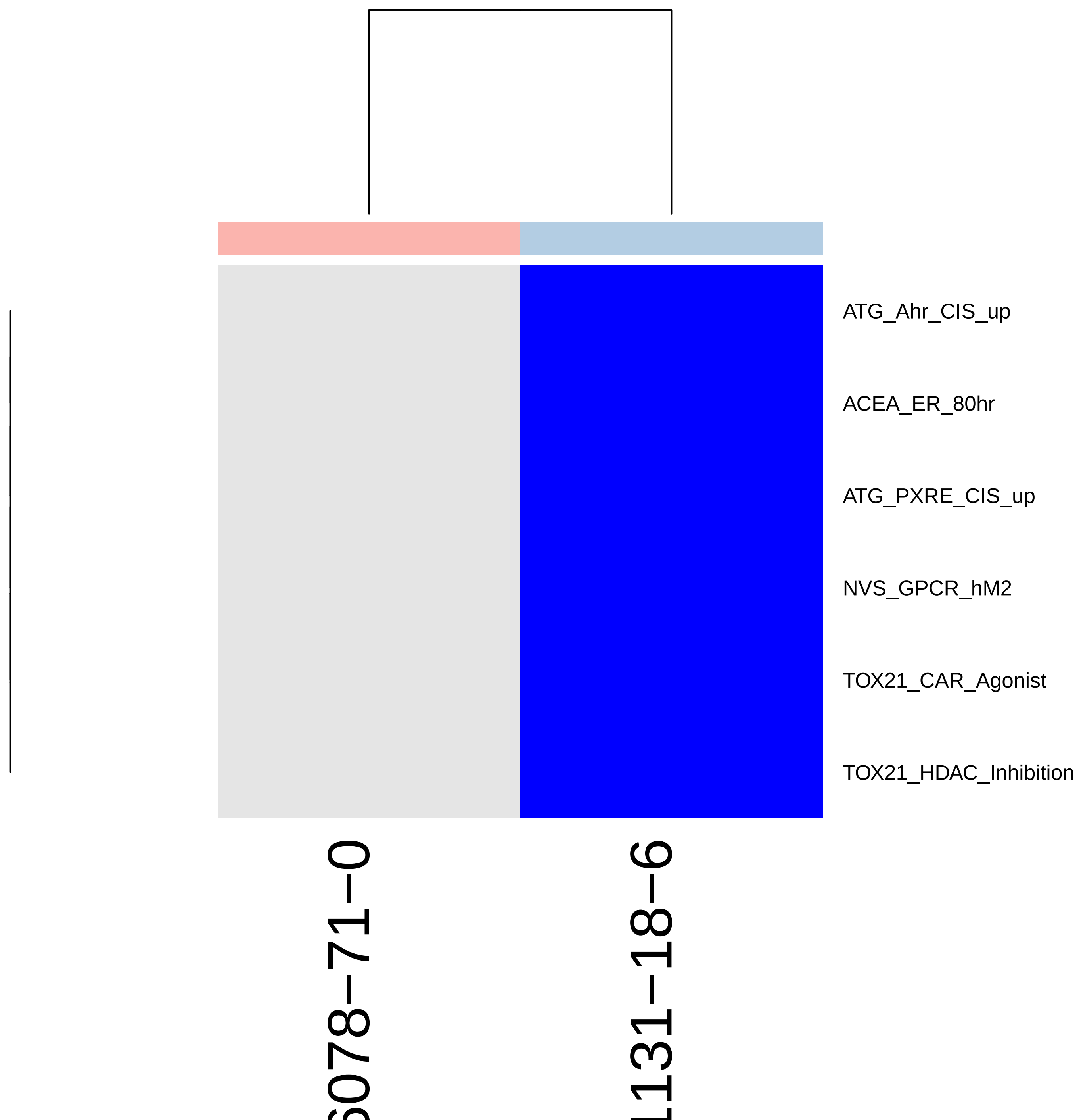

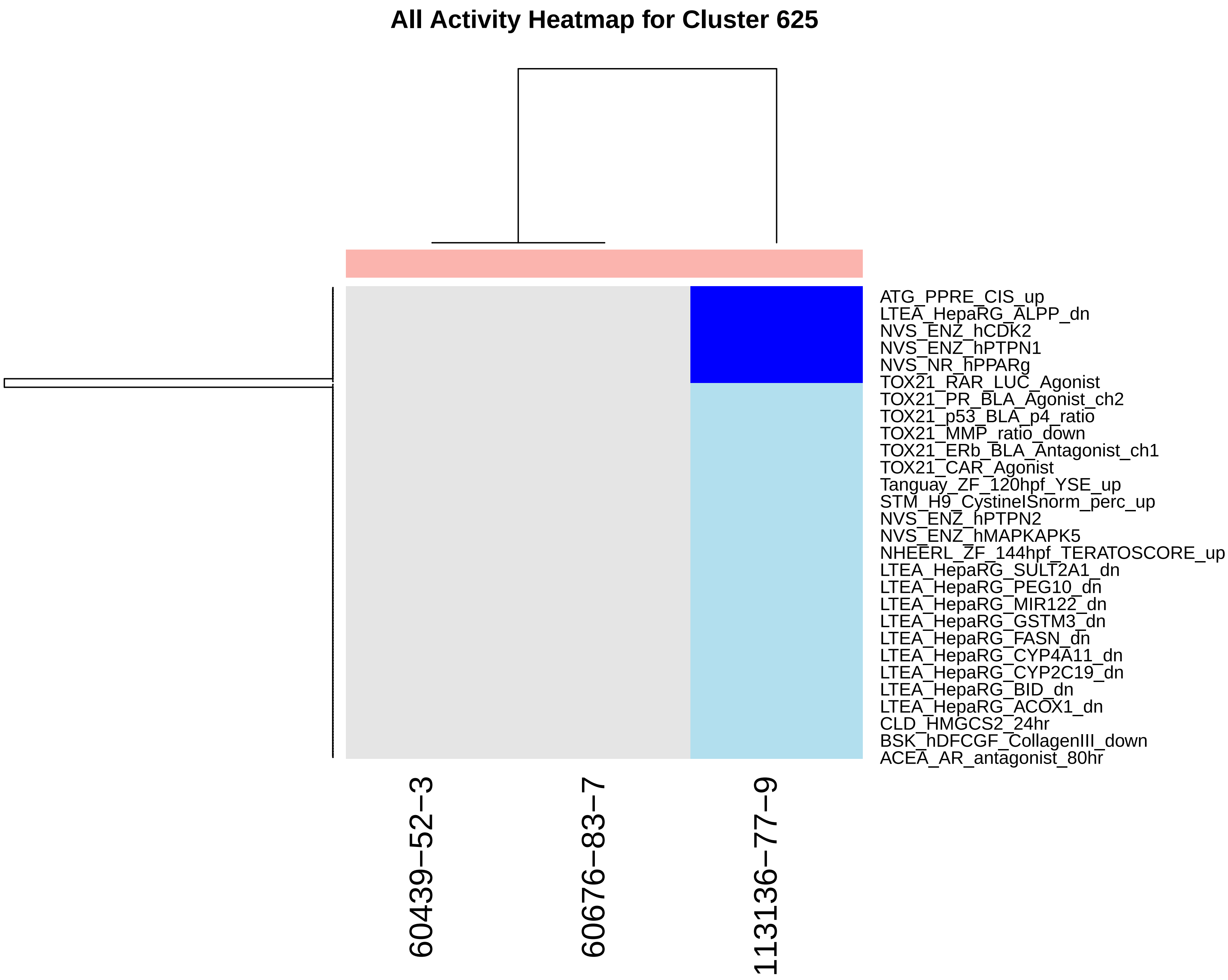
All Activity Heatmap for Cluster 625
ATG_PPRE_CIS_up
LTEA_HepaRG_ALPP_dn
NVS_ENZ_hCDK2
NVS_ENZ_hPTPN1
NVS_NR_hPPARg
TOX21_RAR_LUC_Agonist
TOX21_PR_BLA_Agonist_ch2
TOX21_p53_BLA_p4_ratio
TOX21_MMP_ratio_down
TOX21_ERb_BLA_Antagonist_ch1
TOX21_CAR_Agonist
Tanguay_ZF_120hpf_YSE_up
STM_H9_CystineISnorm_perc_up
NVS_ENZ_hPTPN2
NVS_ENZ_hMAPKAPK5
NHEERL_ZF_144hpf_TERATOSCORE_up
LTEA_HepaRG_SULT2A1_dn
LTEA_HepaRG_PEG10_dn
LTEA_HepaRG_MIR122_dn
LTEA_HepaRG_GSTM3_dn
LTEA_HepaRG_FASN_dn
LTEA_HepaRG_CYP4A11_dn
LTEA_HepaRG_CYP2C19_dn
LTEA_HepaRG_BID_dn
LTEA_HepaRG_ACOX1_dn
CLD_HMGCS2_24hr
BSK_hDFCGF_CollagenIII_down
ACEA_AR_antagonist_80hr
60439−52−3
60676−83−7
113136−77−9

All Activity Heatmap for Cluster 626

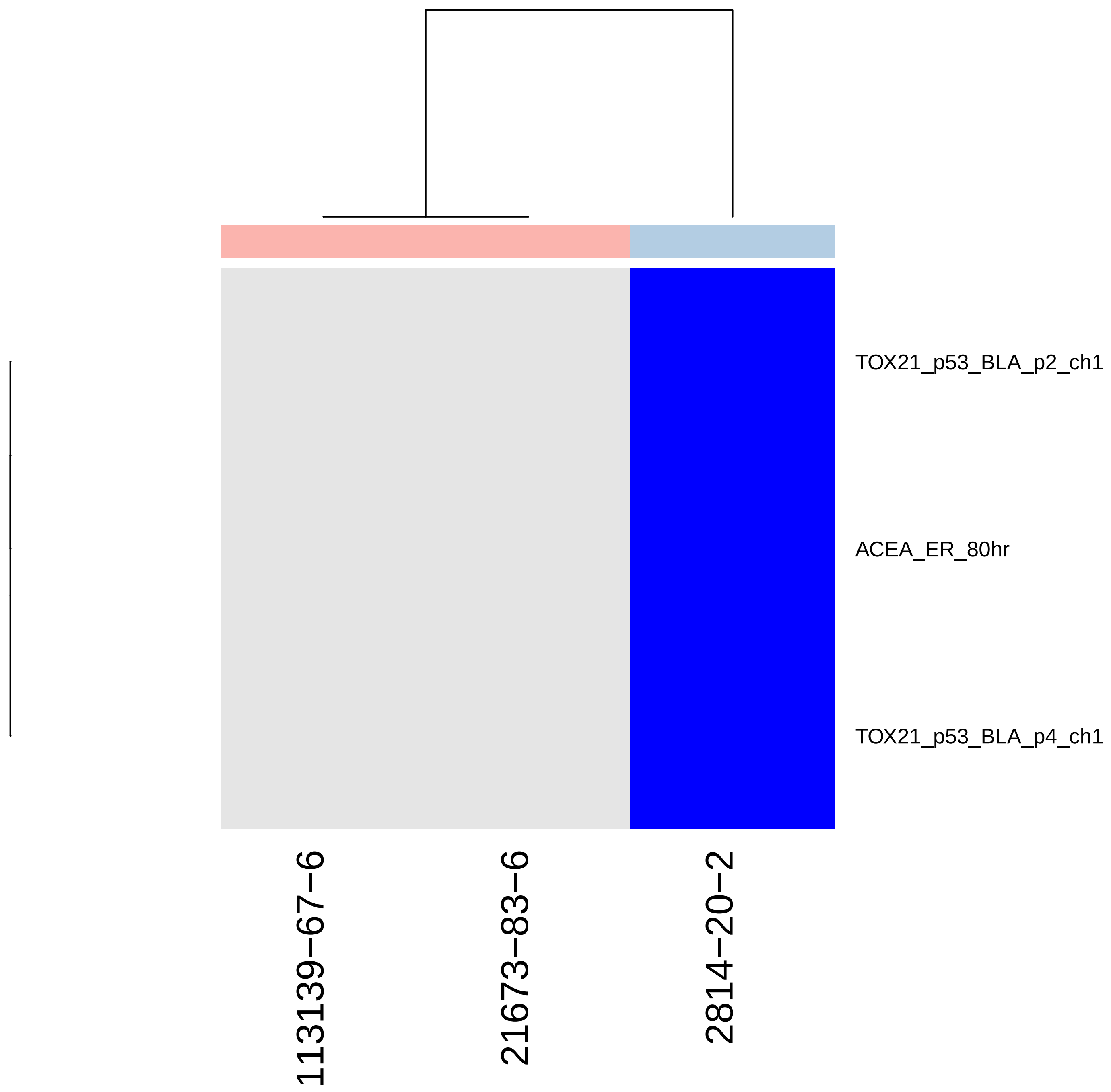

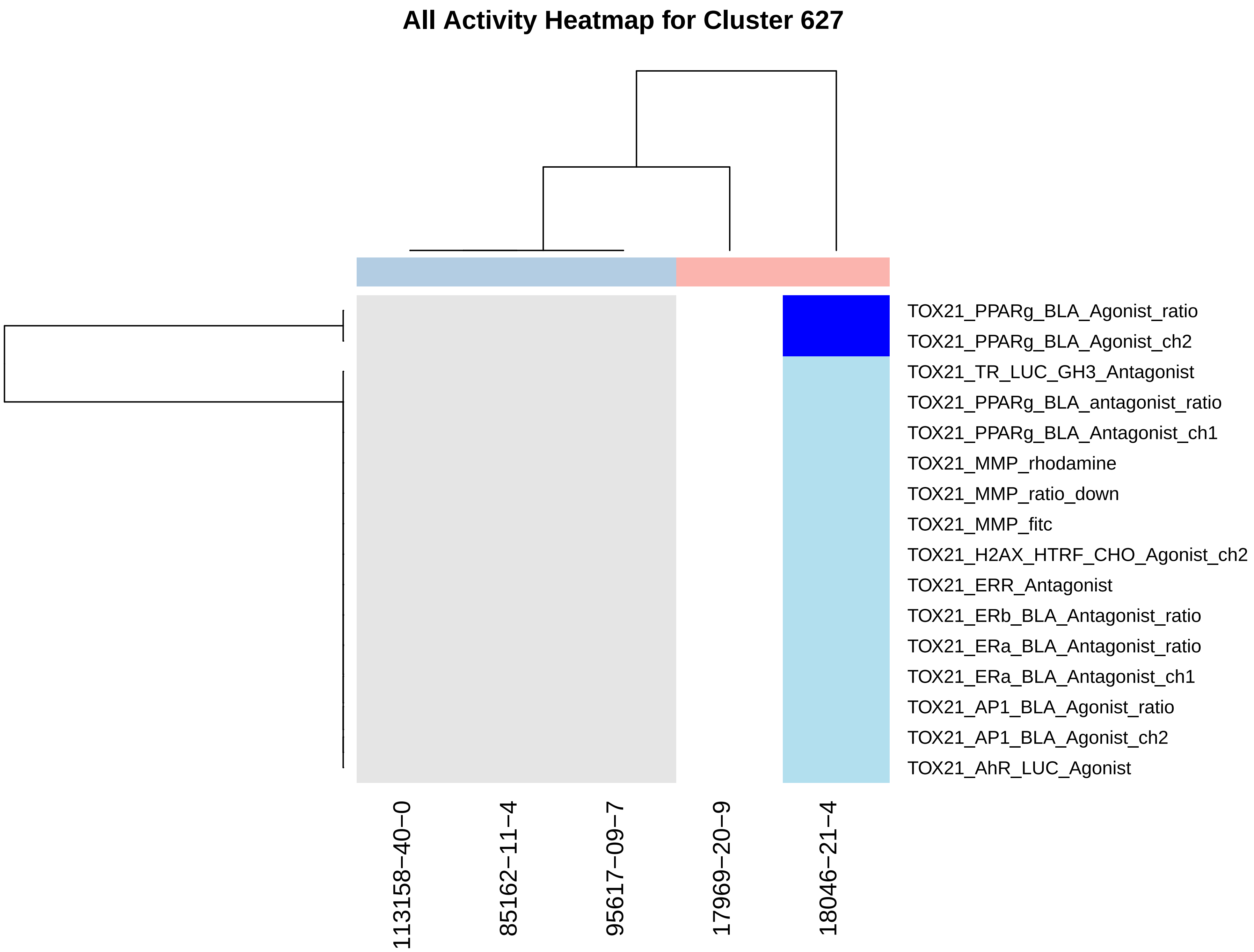
All Activity Heatmap for Cluster 627
TOX21_PPARg_BLA_Agonist_ratio
TOX21_PPARg_BLA_Agonist_ch2
TOX21_TR_LUC_GH3_Antagonist
TOX21_PPARg_BLA_antagonist_ratio
TOX21_PPARg_BLA_Antagonist_ch1
TOX21_MMP_rhodamine
TOX21_MMP_ratio_down
TOX21_MMP_fitc
TOX21_H2AX_HTRF_CHO_Agonist_ch2
TOX21_ERR_Antagonist
TOX21_ERb_BLA_Antagonist_ratio
TOX21_ERa_BLA_Antagonist_ratio
TOX21_ERa_BLA_Antagonist_ch1
TOX21_AP1_BLA_Agonist_ratio
TOX21_AP1_BLA_Agonist_ch2
TOX21_AhR_LUC_Agonist
113158−40−0
85162−11−4
95617−09−7
17969−20−9
18046−21−4

All Activity Heatmap for Cluster 629

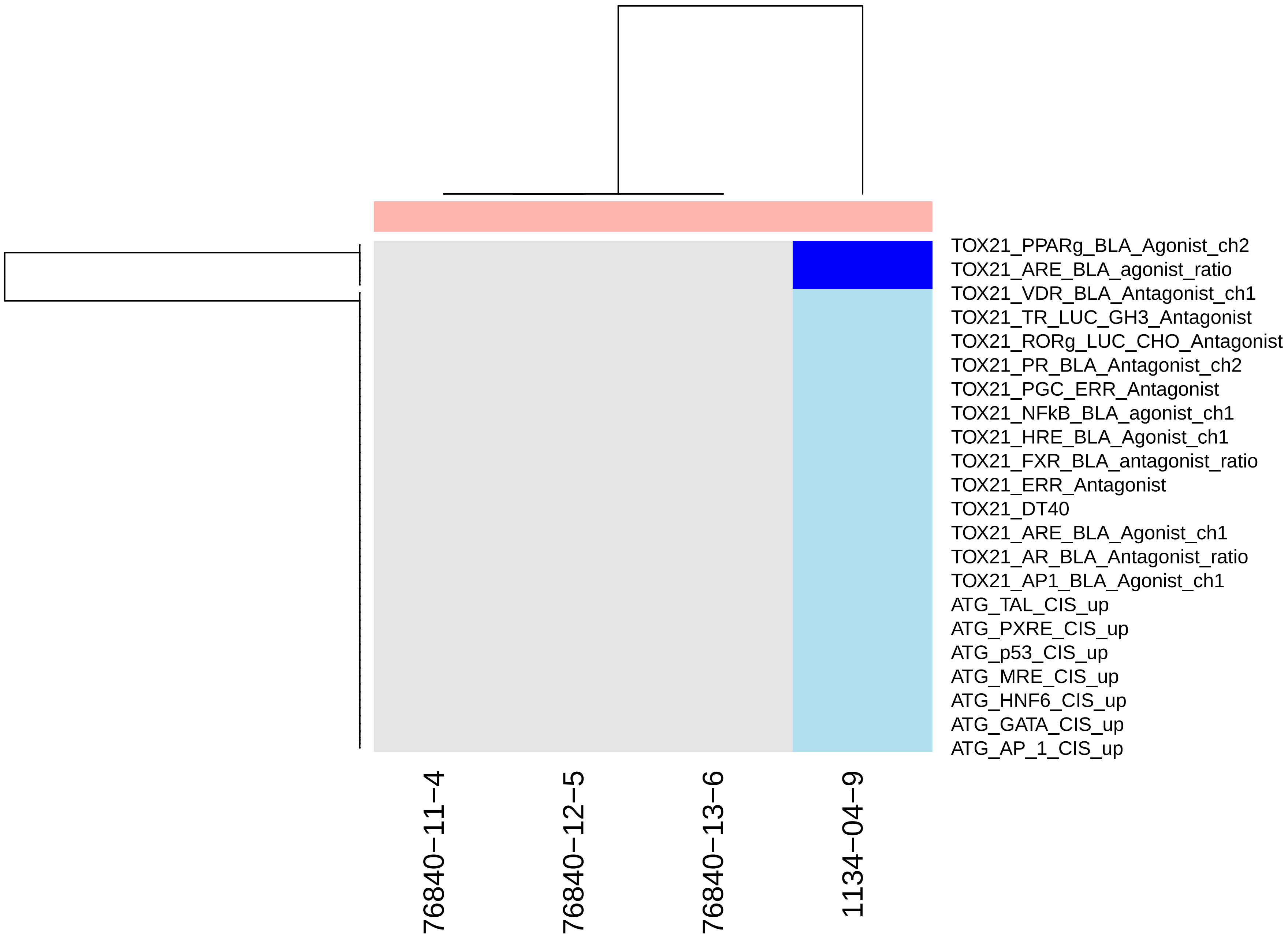

All Activity Heatmap for Cluster 630

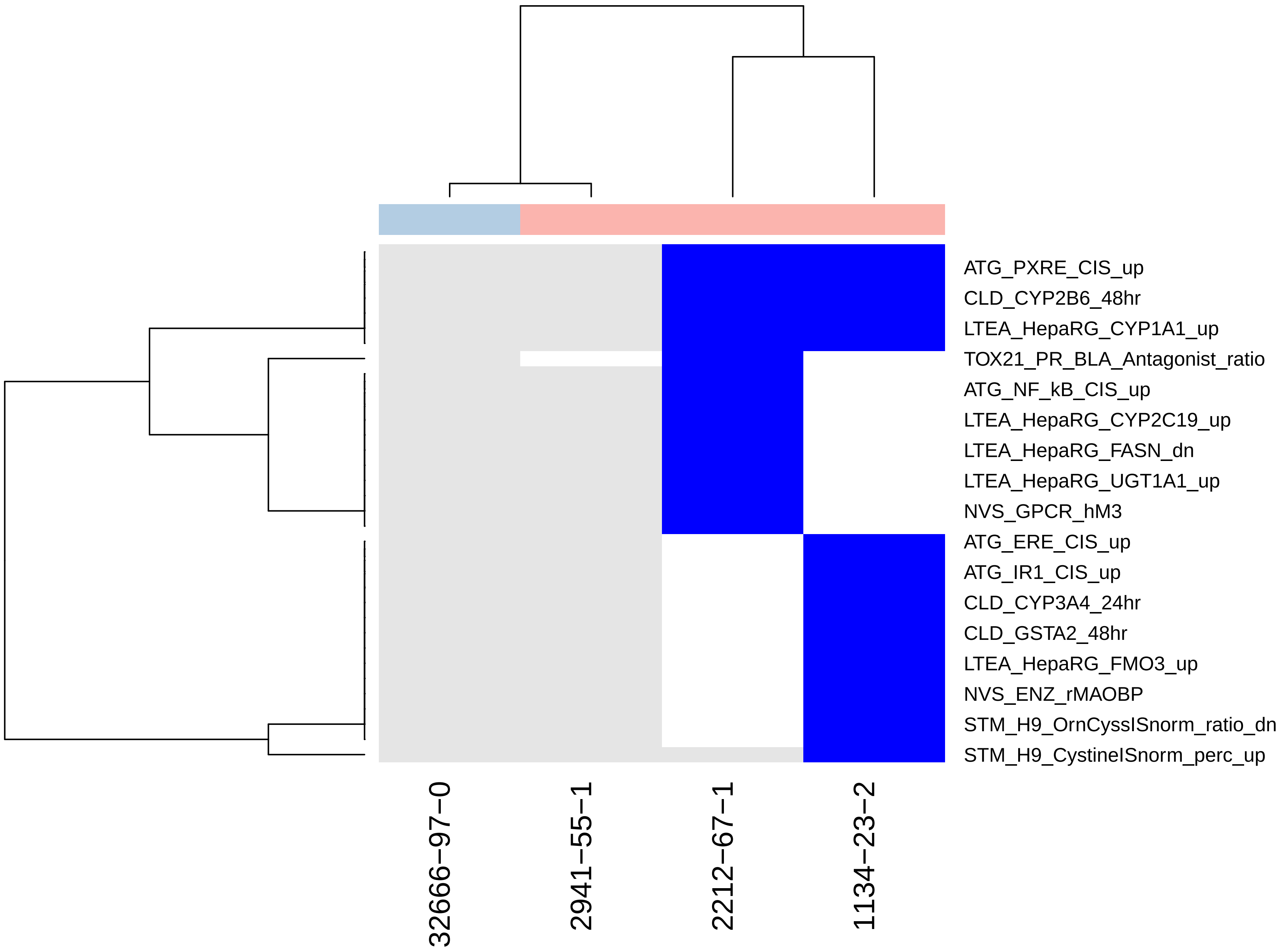

All Activity Heatmap for Cluster 631

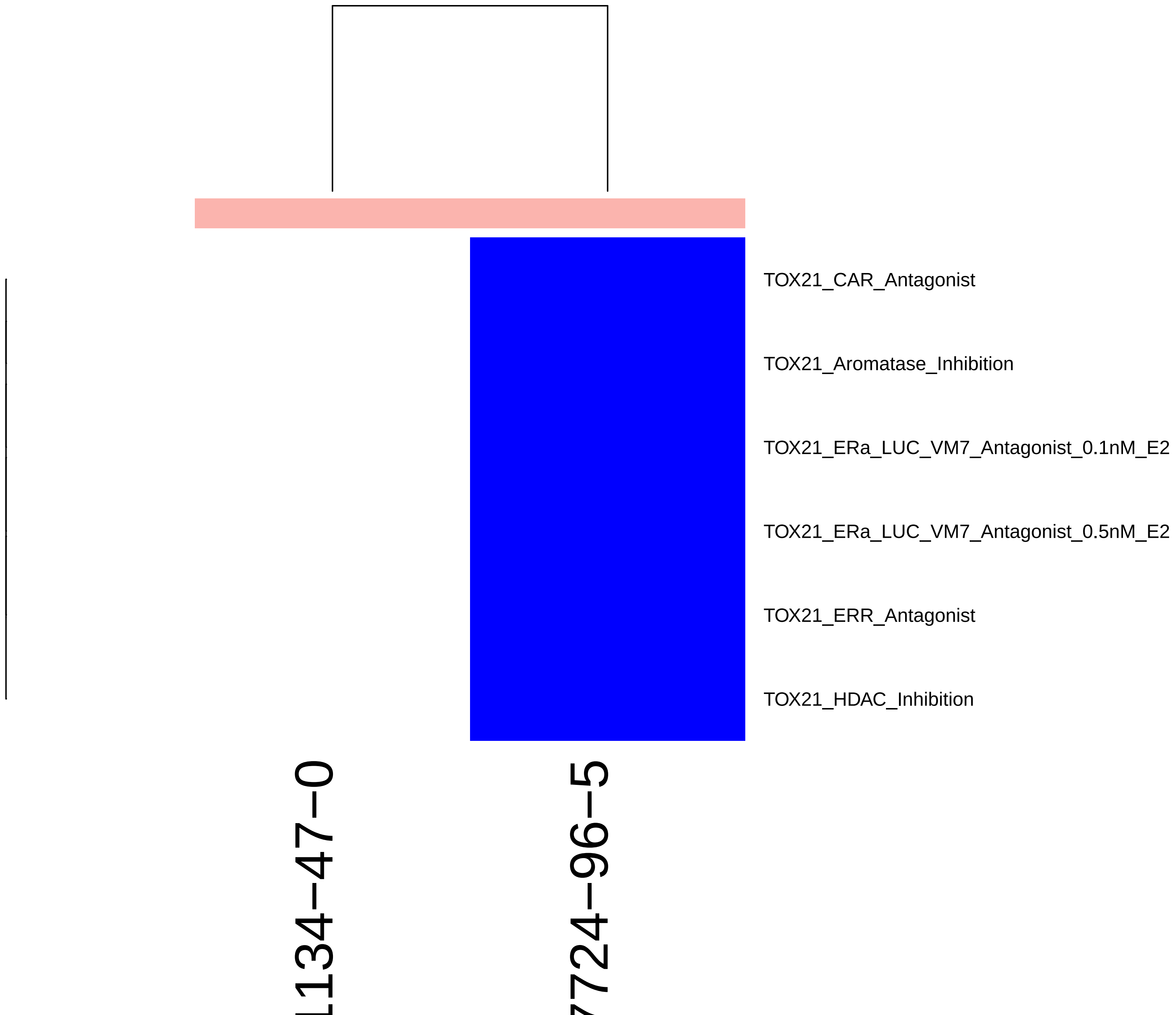

All Activity Heatmap for Cluster 632

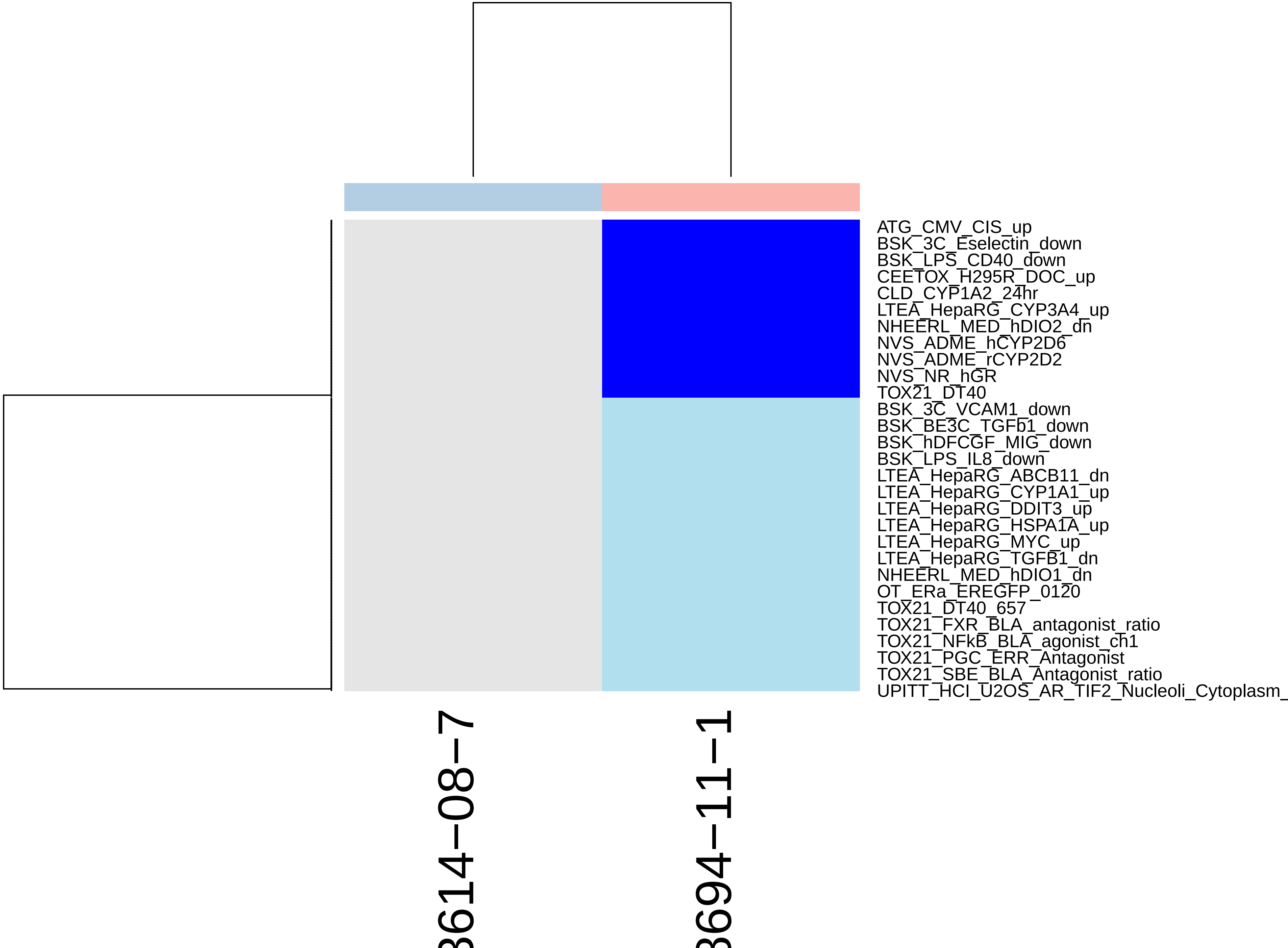

All Activity Heatmap for Cluster 633

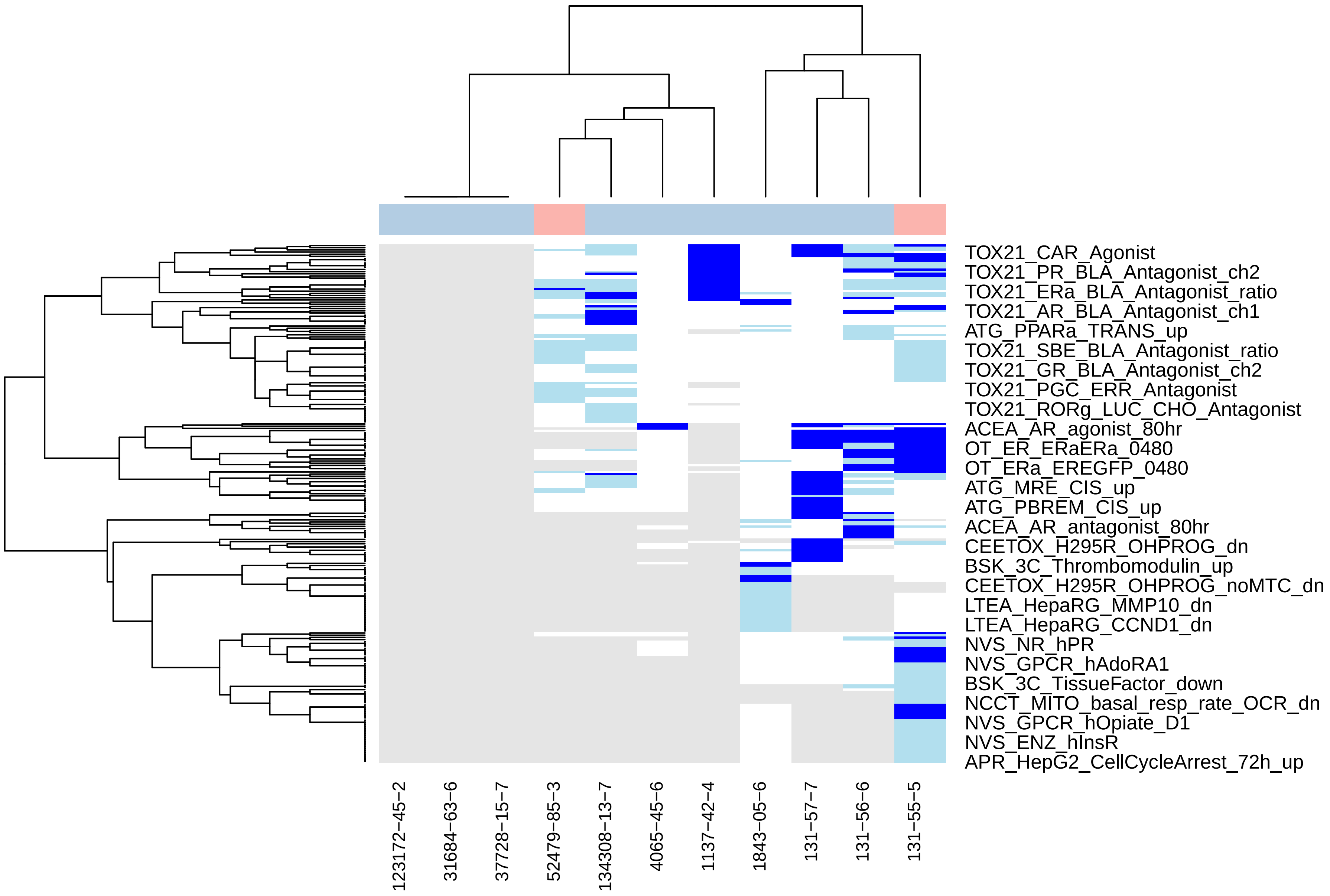

All Activity Heatmap for Cluster 634

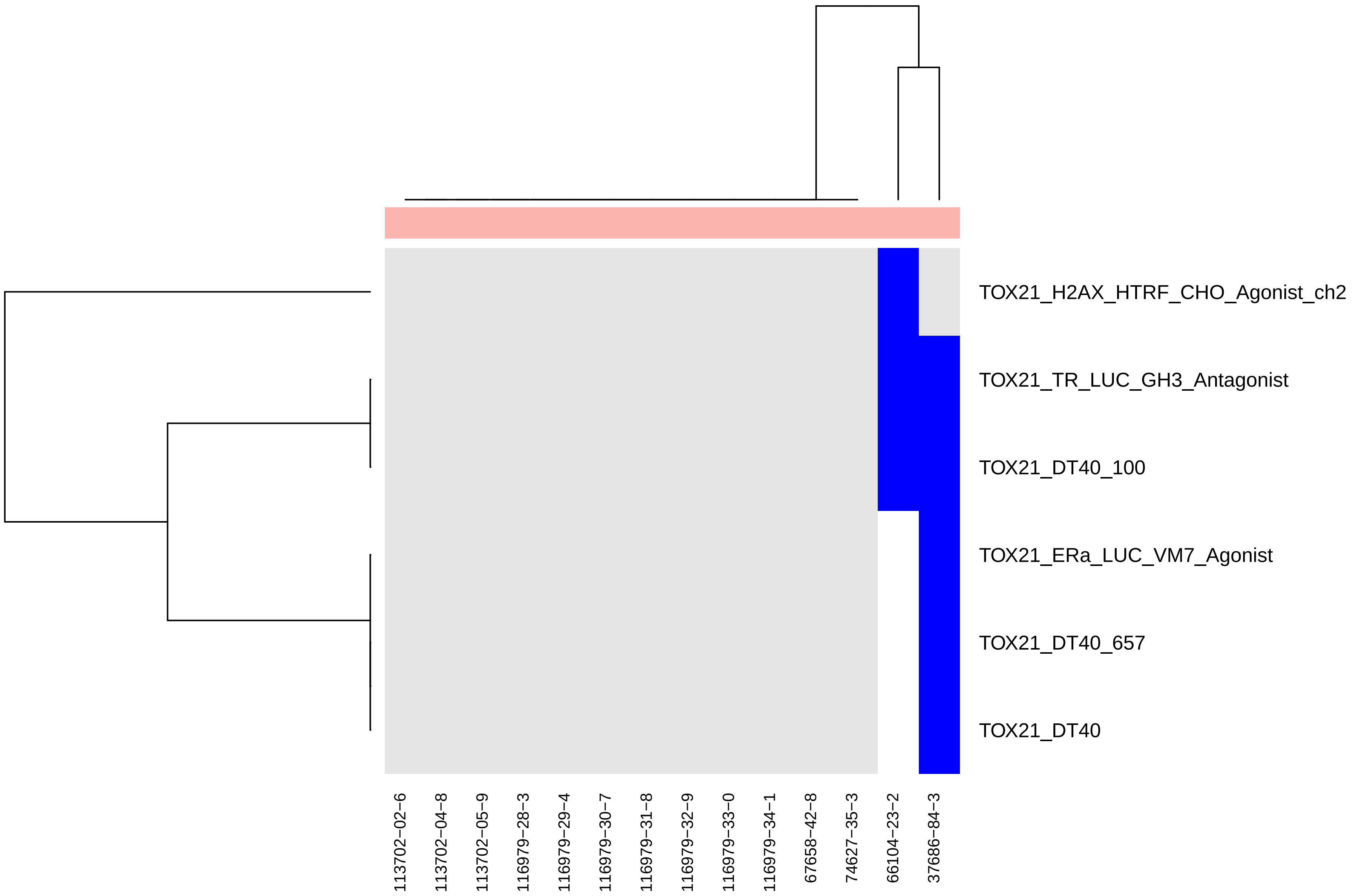

All Activity Heatmap for Cluster 635

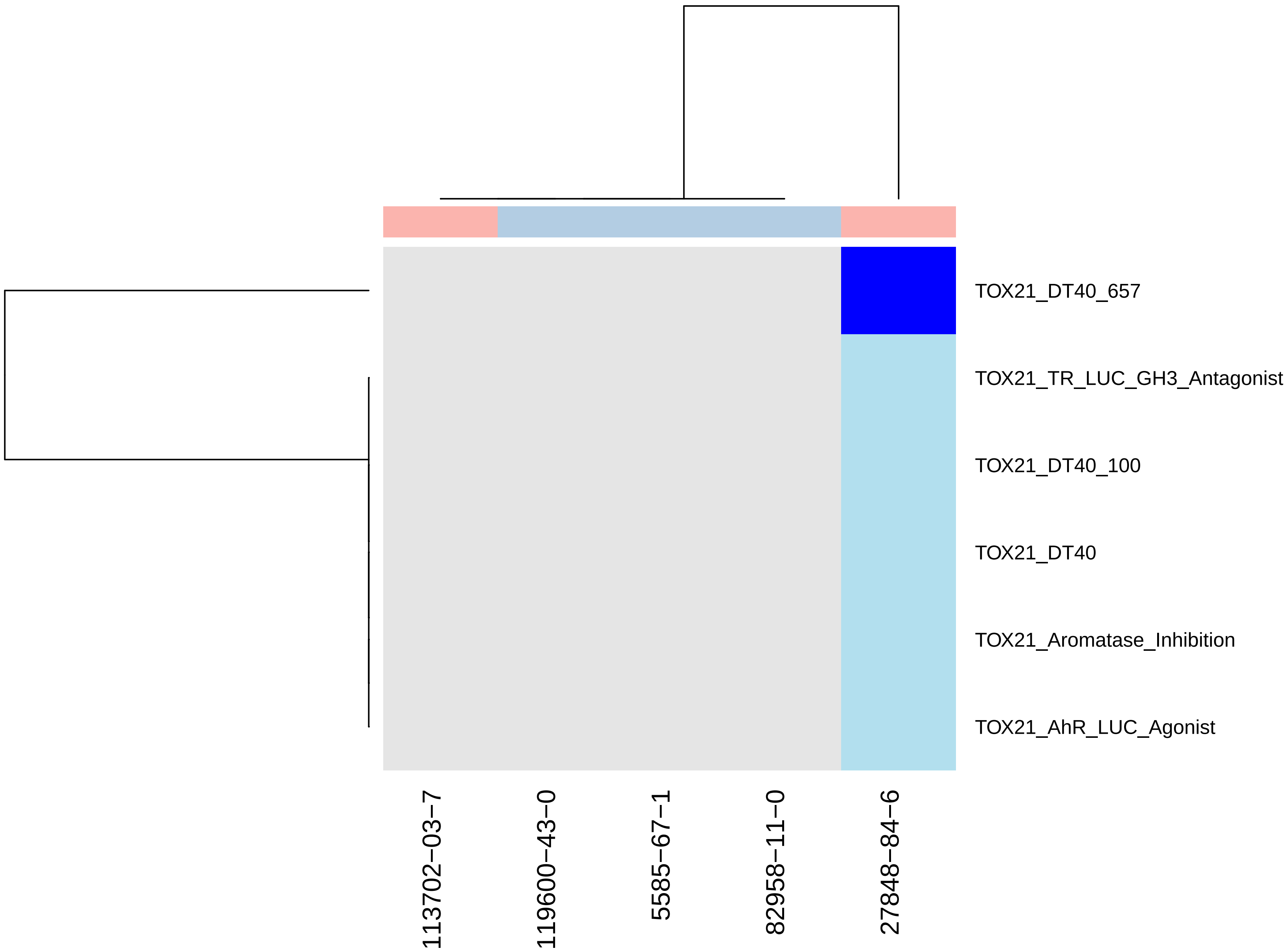

All Activity Heatmap for Cluster 636

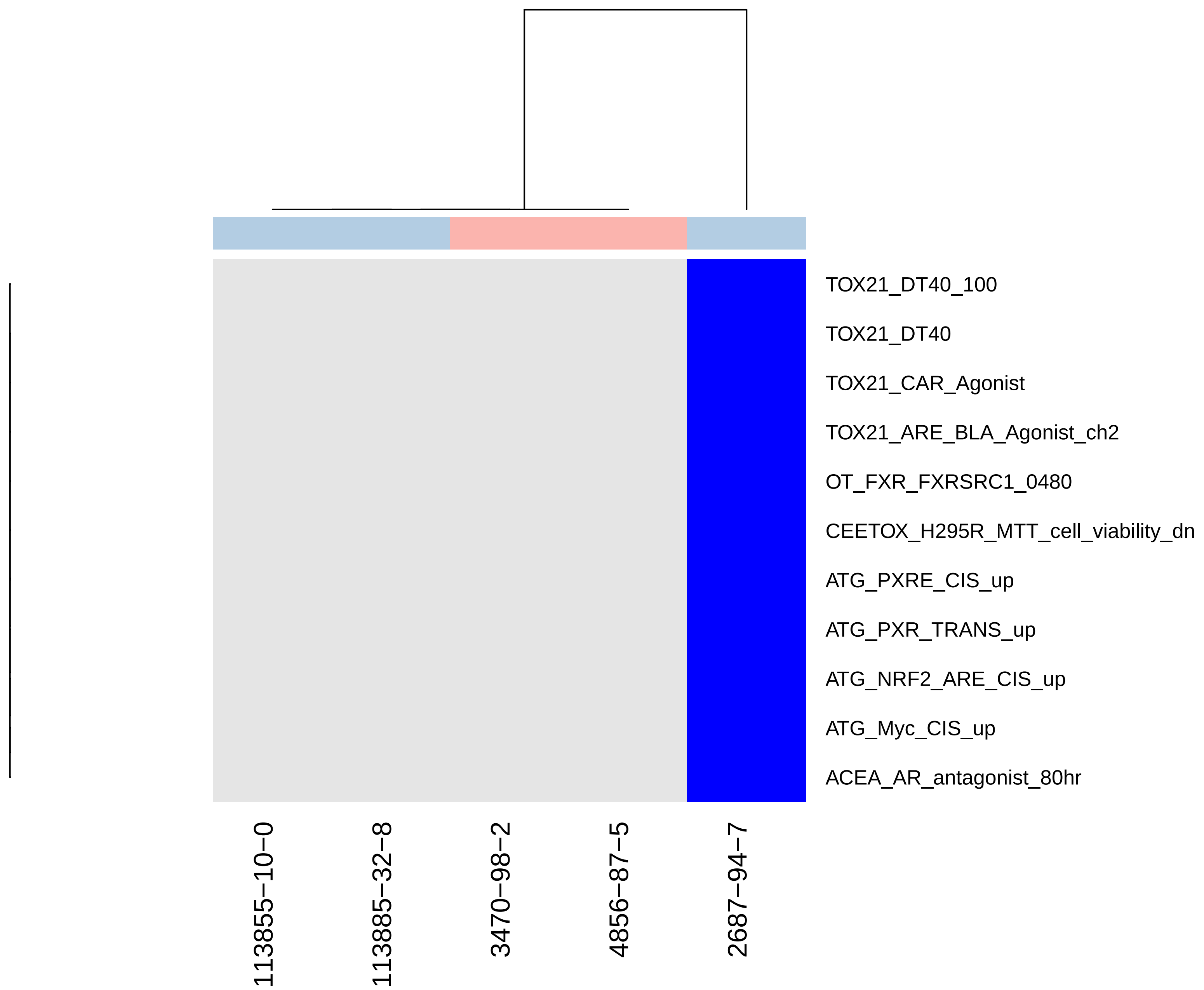

All Activity Heatmap for Cluster 637

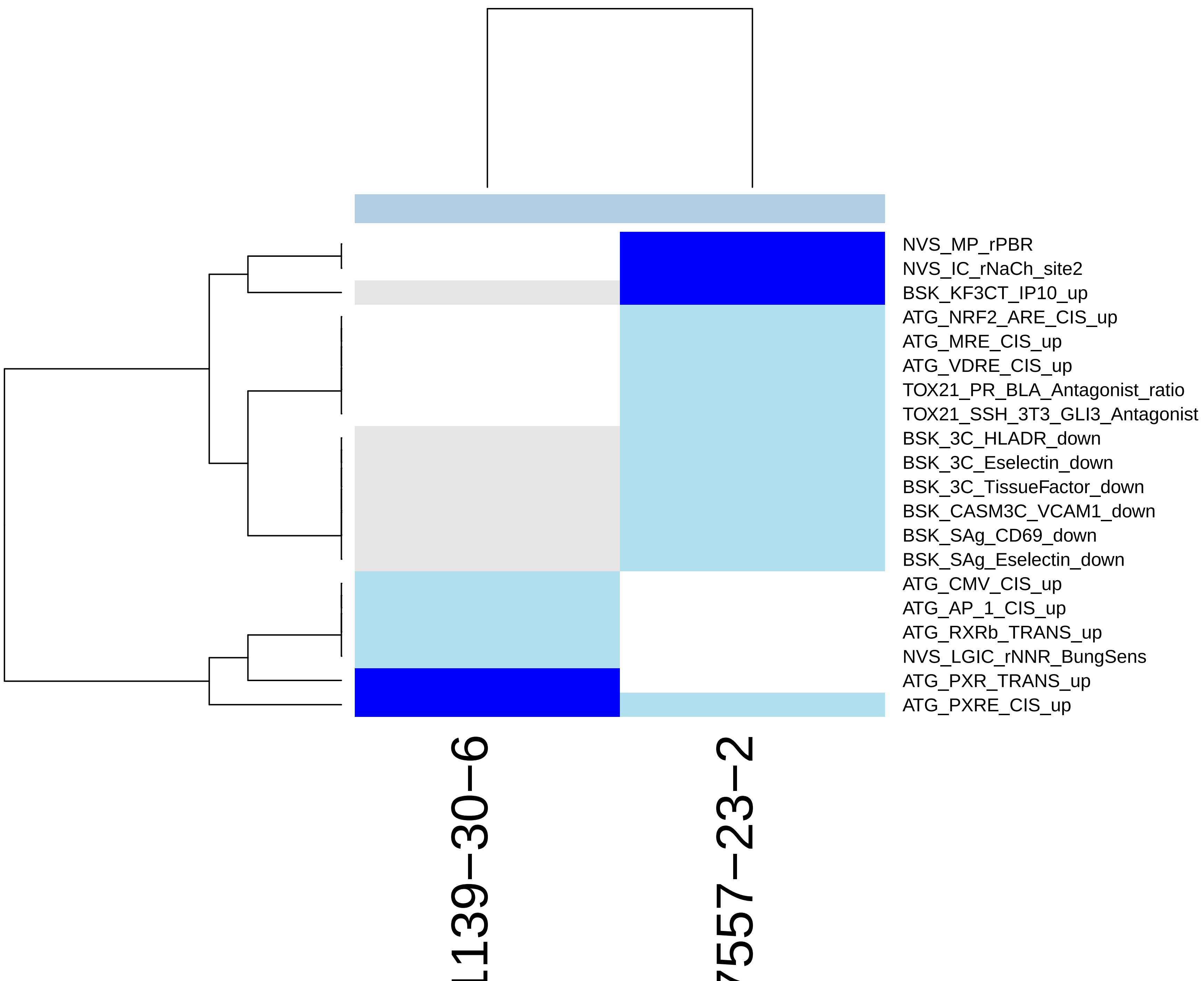

All Activity Heatmap for Cluster 638

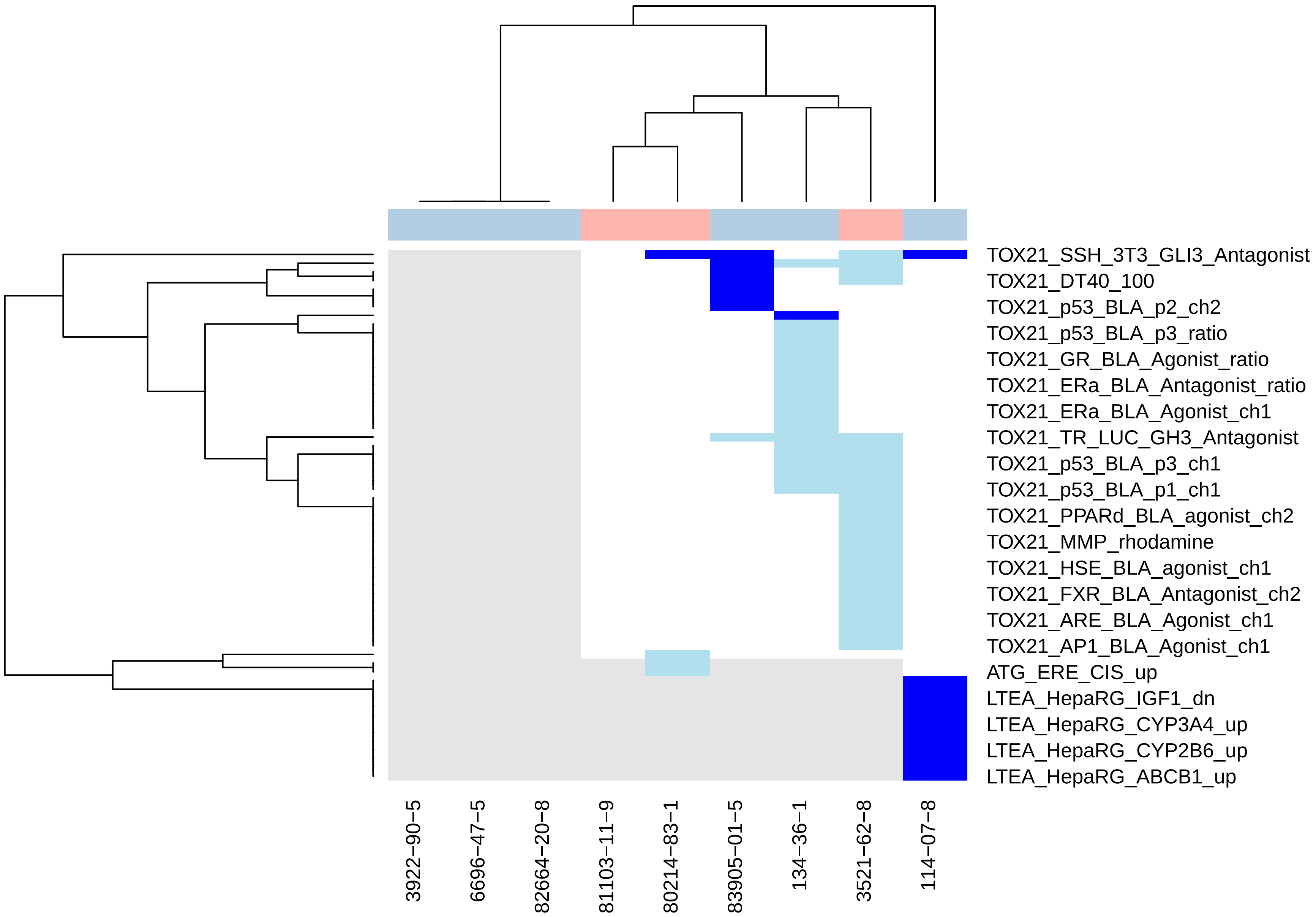

All Activity Heatmap for Cluster 639

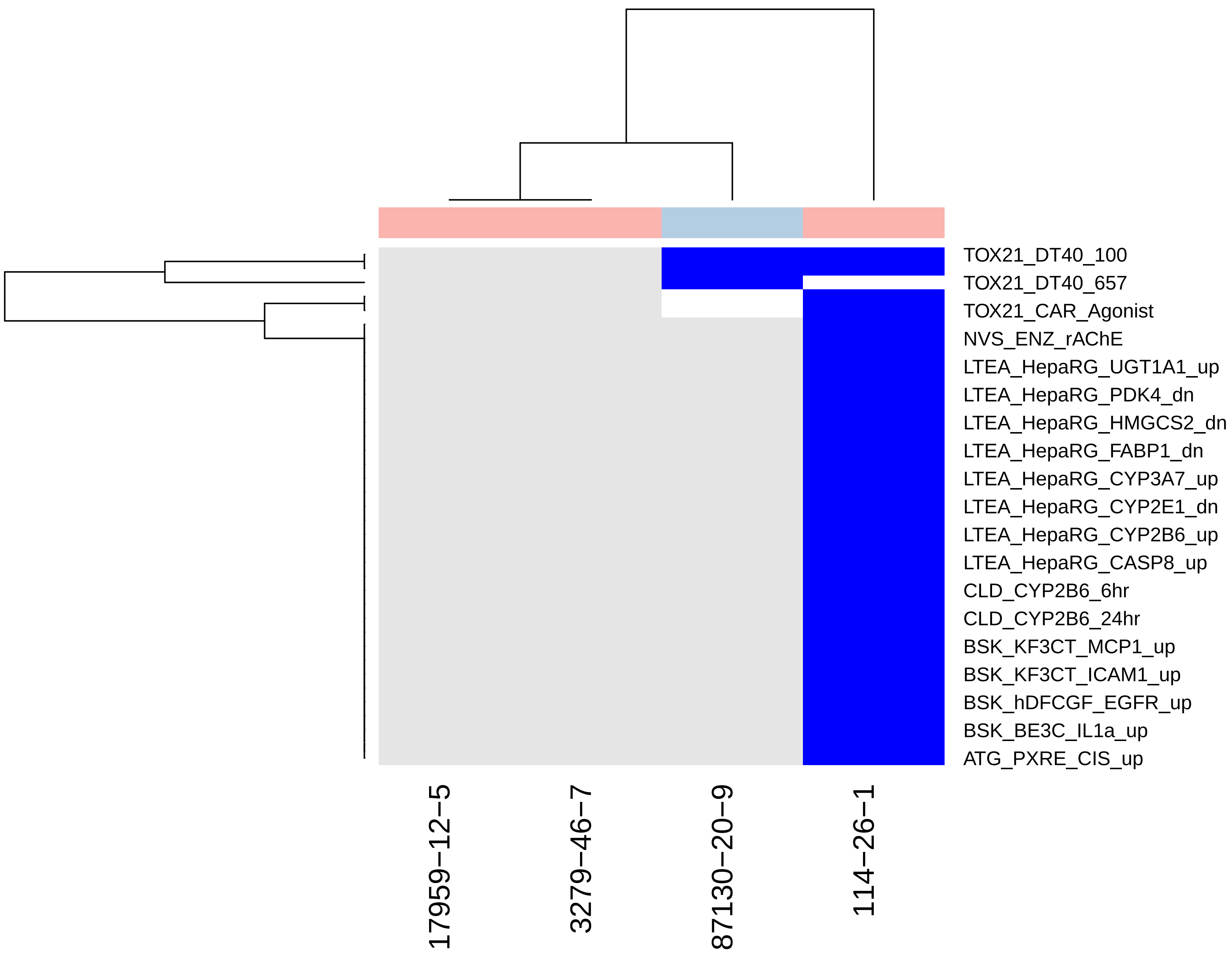

All Activity Heatmap for Cluster 640

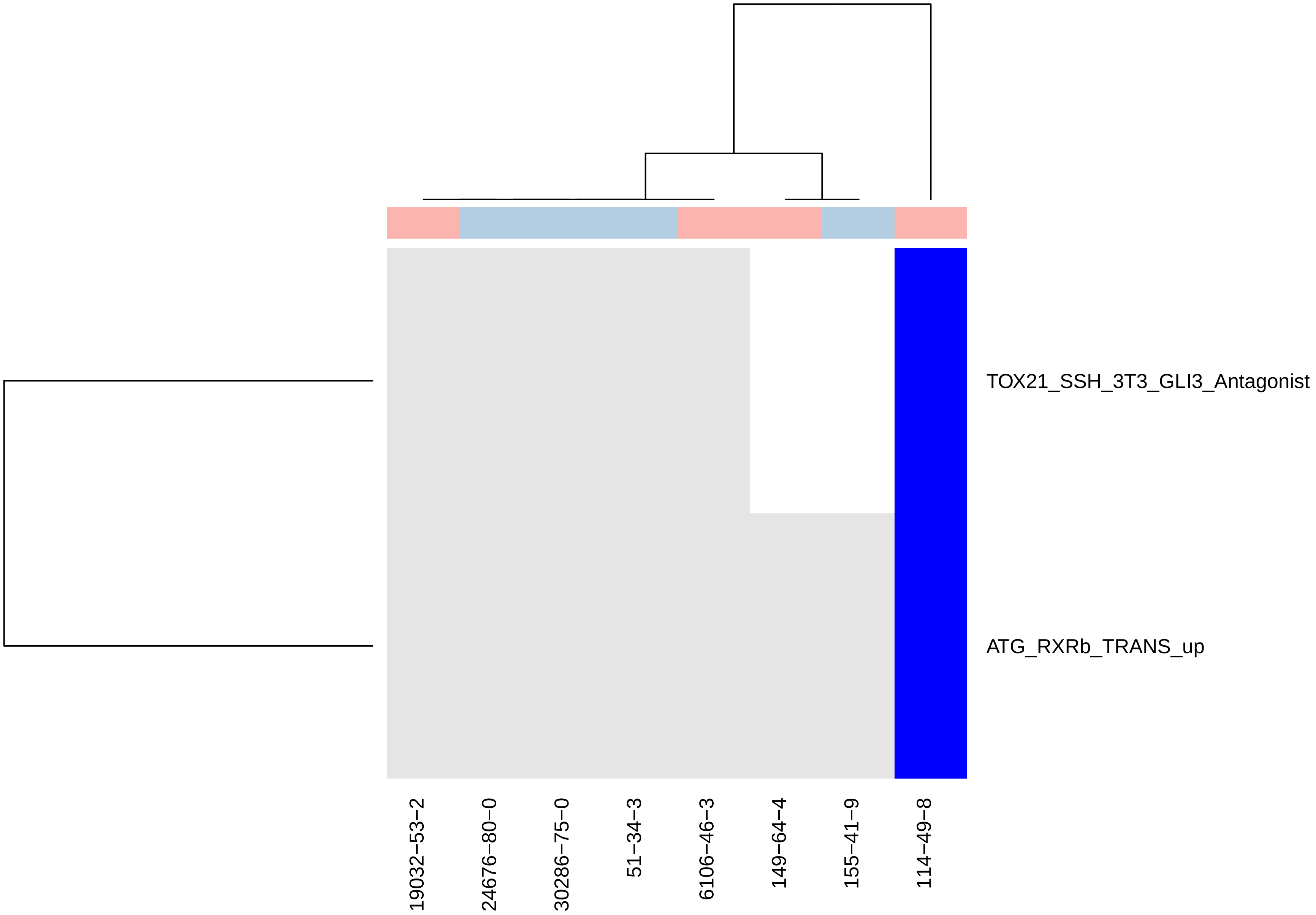

All Activity Heatmap for Cluster 641

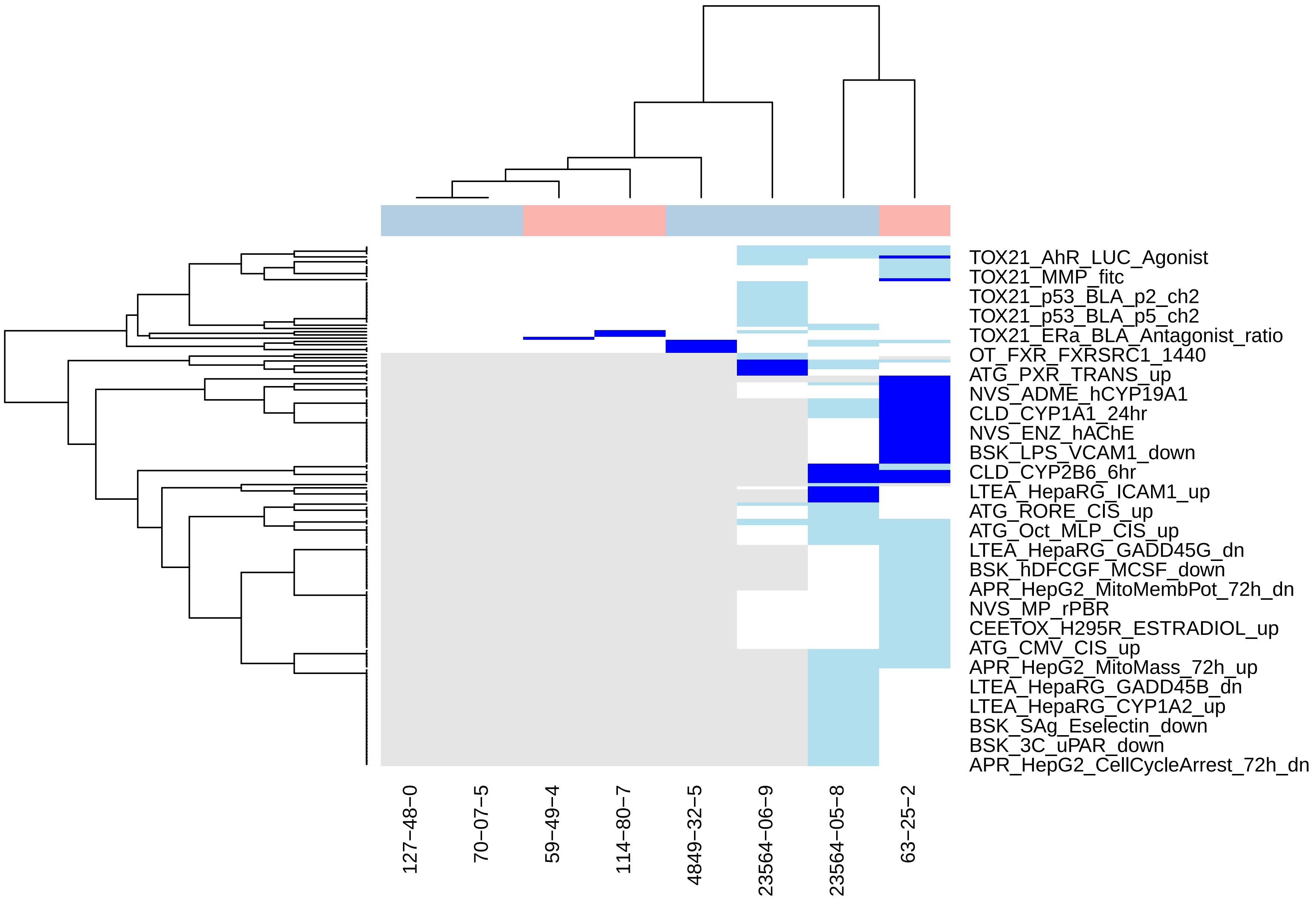

All Activity Heatmap for Cluster 642

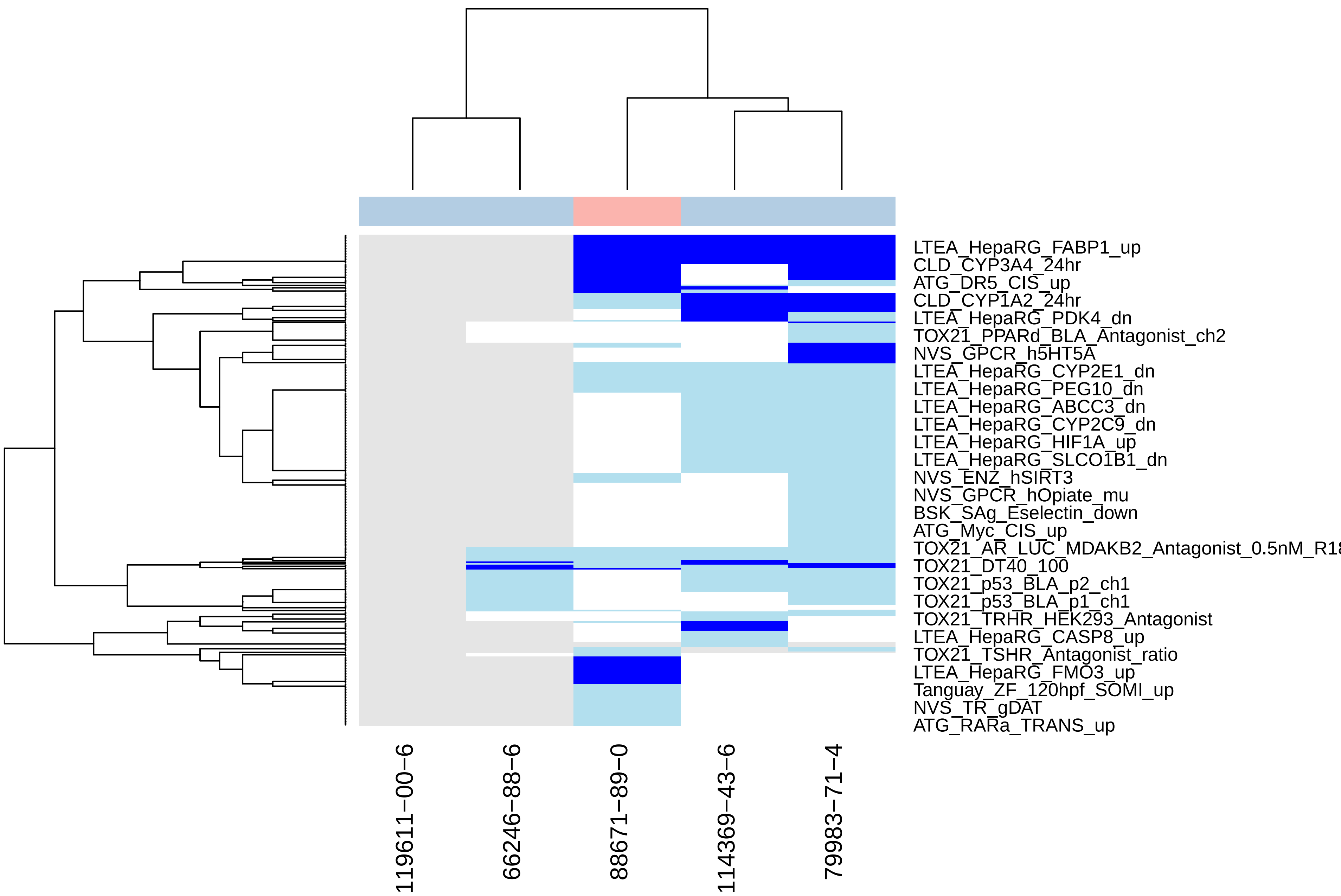

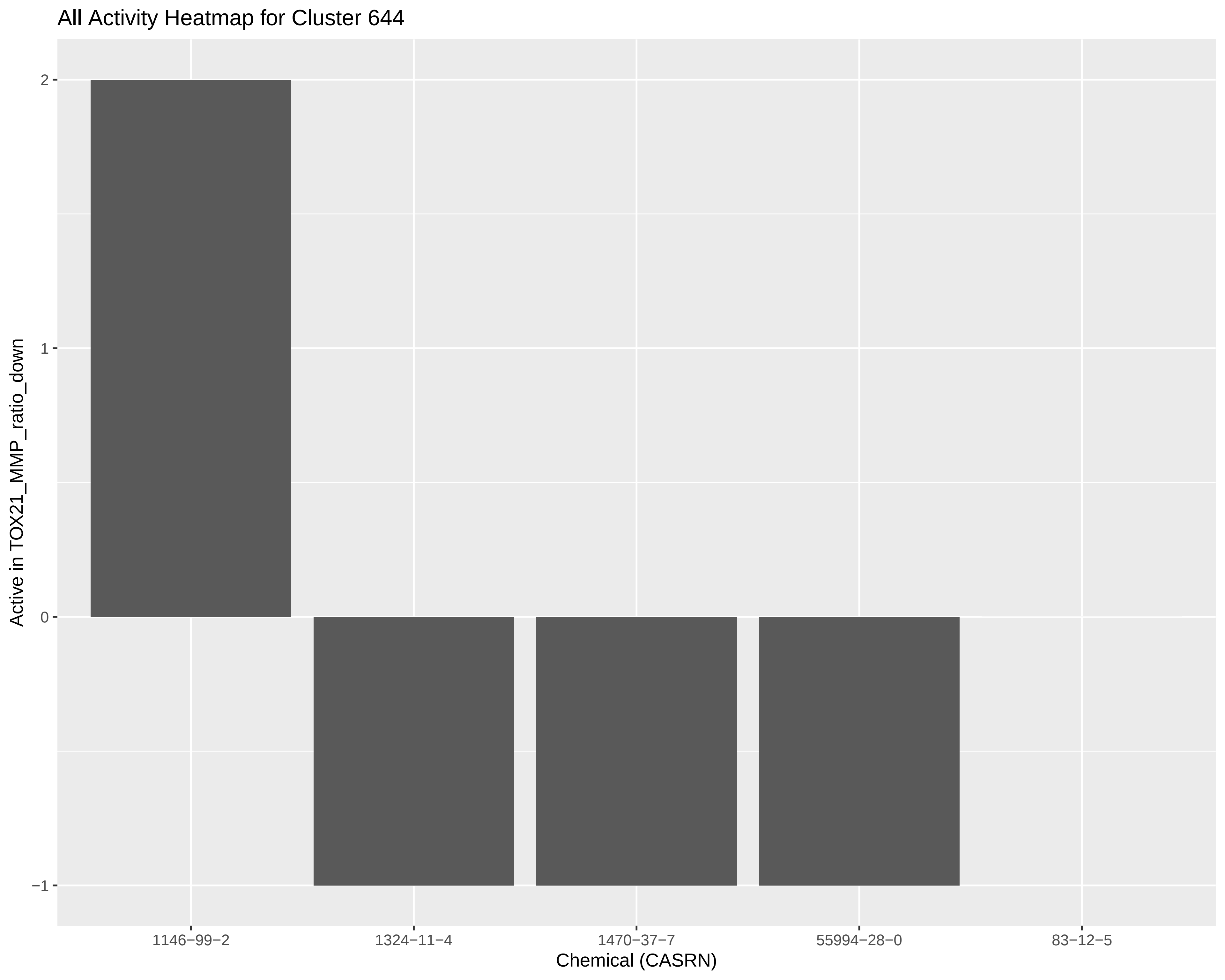

All Activity Heatmap for Cluster 644
Active in TOX21_MMP_ratio_down
2
1
0
−1
1146−99−2
1324−11−4
1470−37−7
55994−28−0
83−12−5
Chemical (CASRN)

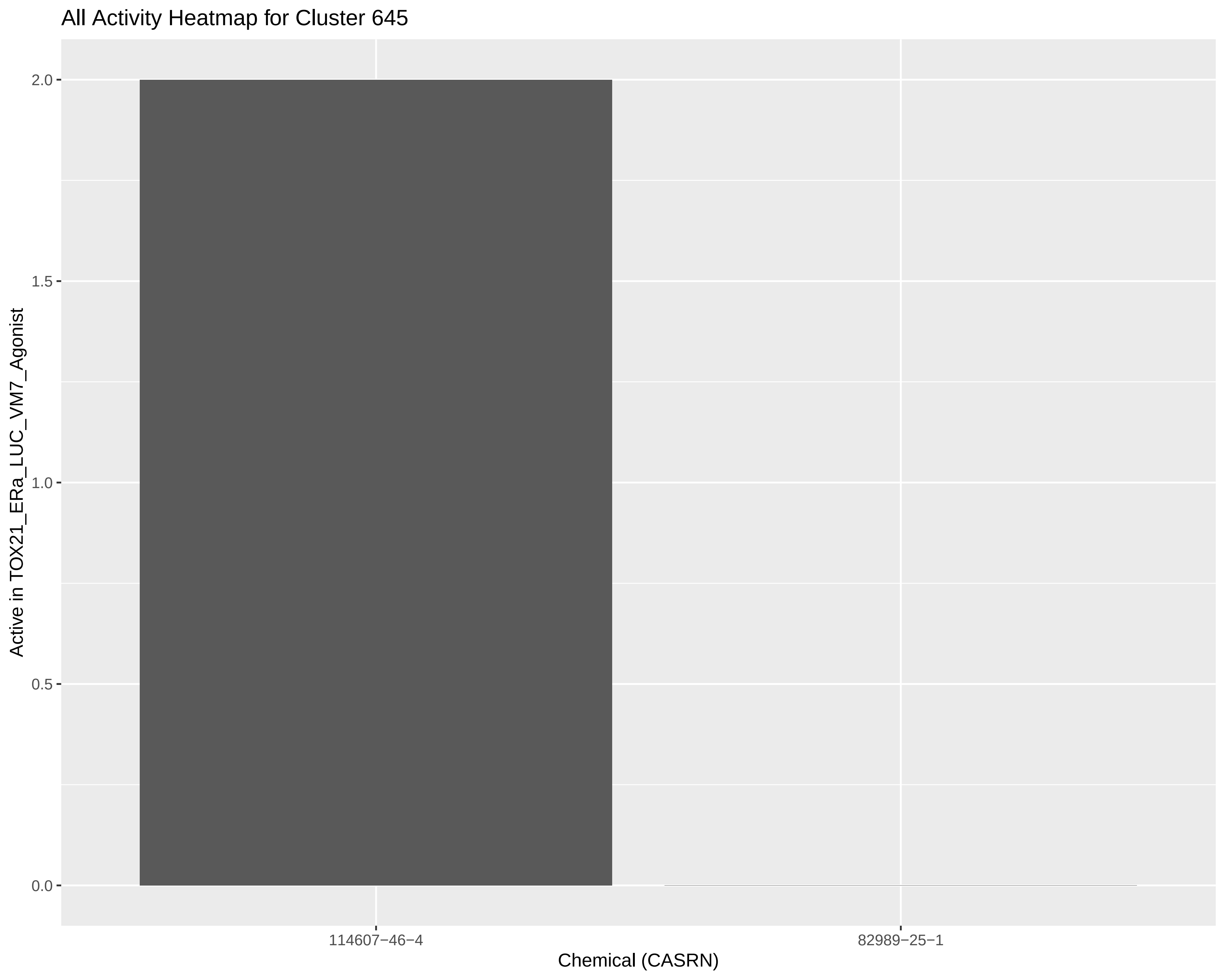

All Activity Heatmap for Cluster 645
Active in TOX21_ERa_LUC_VM7_Agonist
2.0
1.5
1.0
0.5
0.0
114607−46−4
82989−25−1
Chemical (CASRN)

All Activity Heatmap for Cluster 646

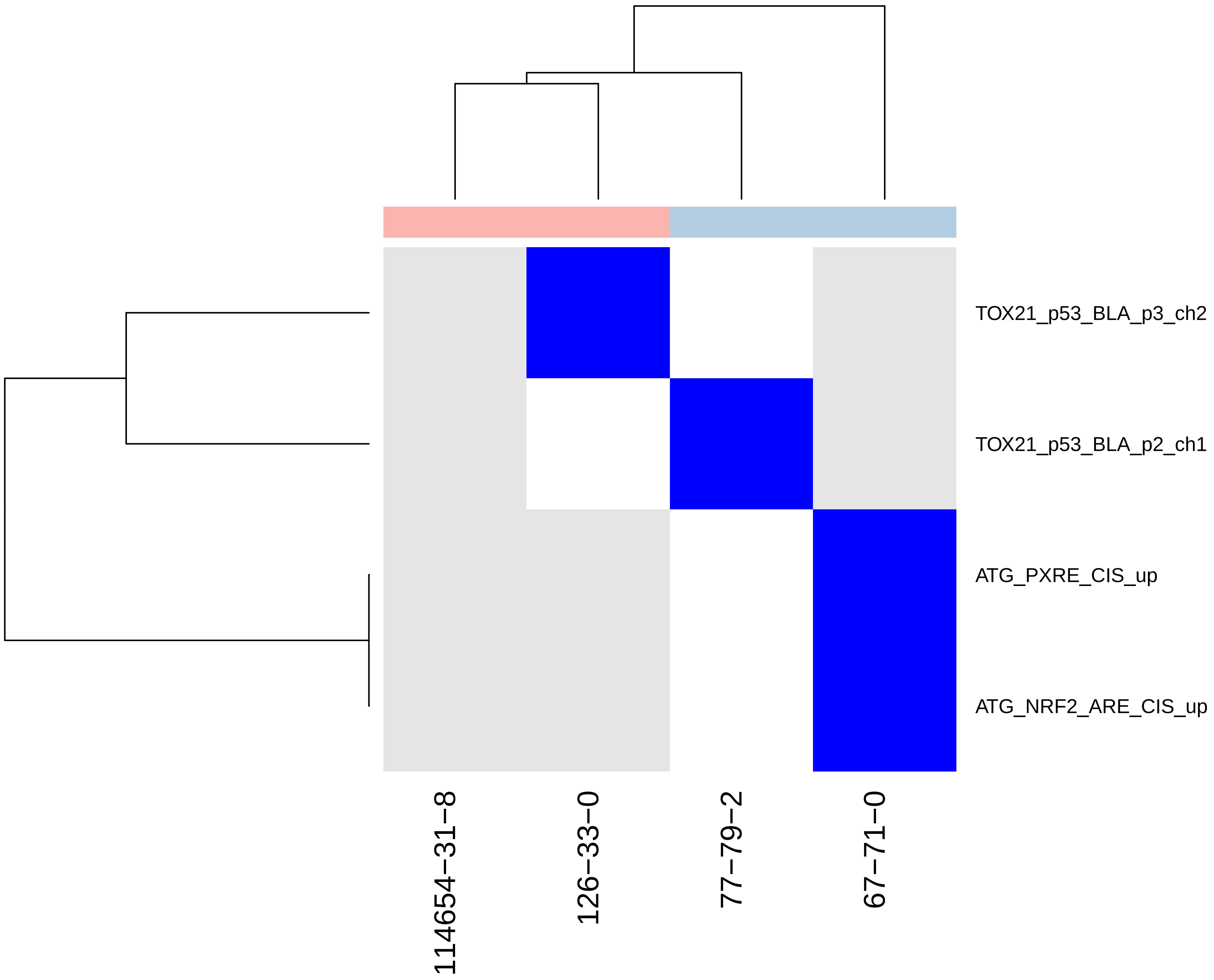

All Activity Heatmap for Cluster 648

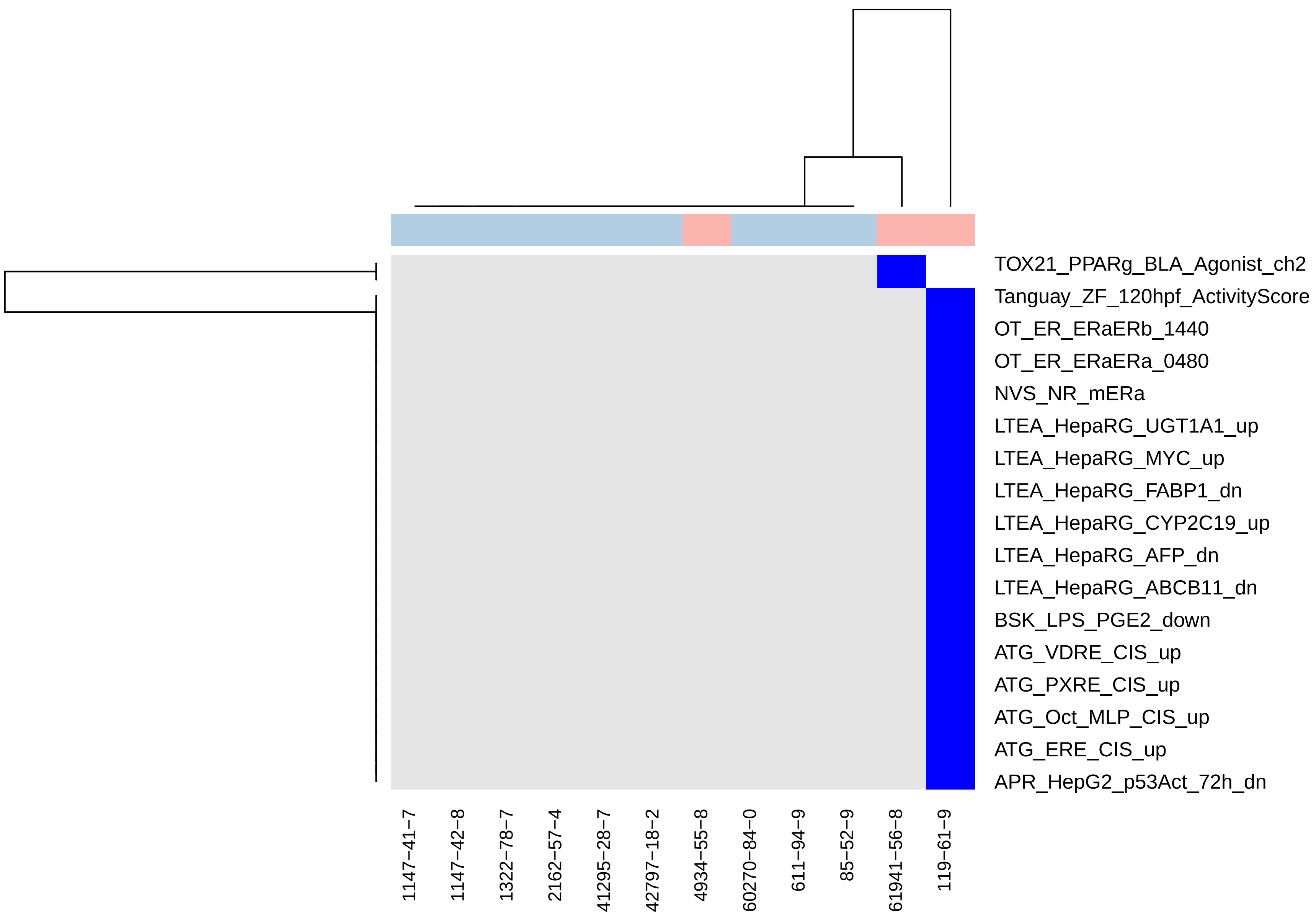

All Activity Heatmap for Cluster 649

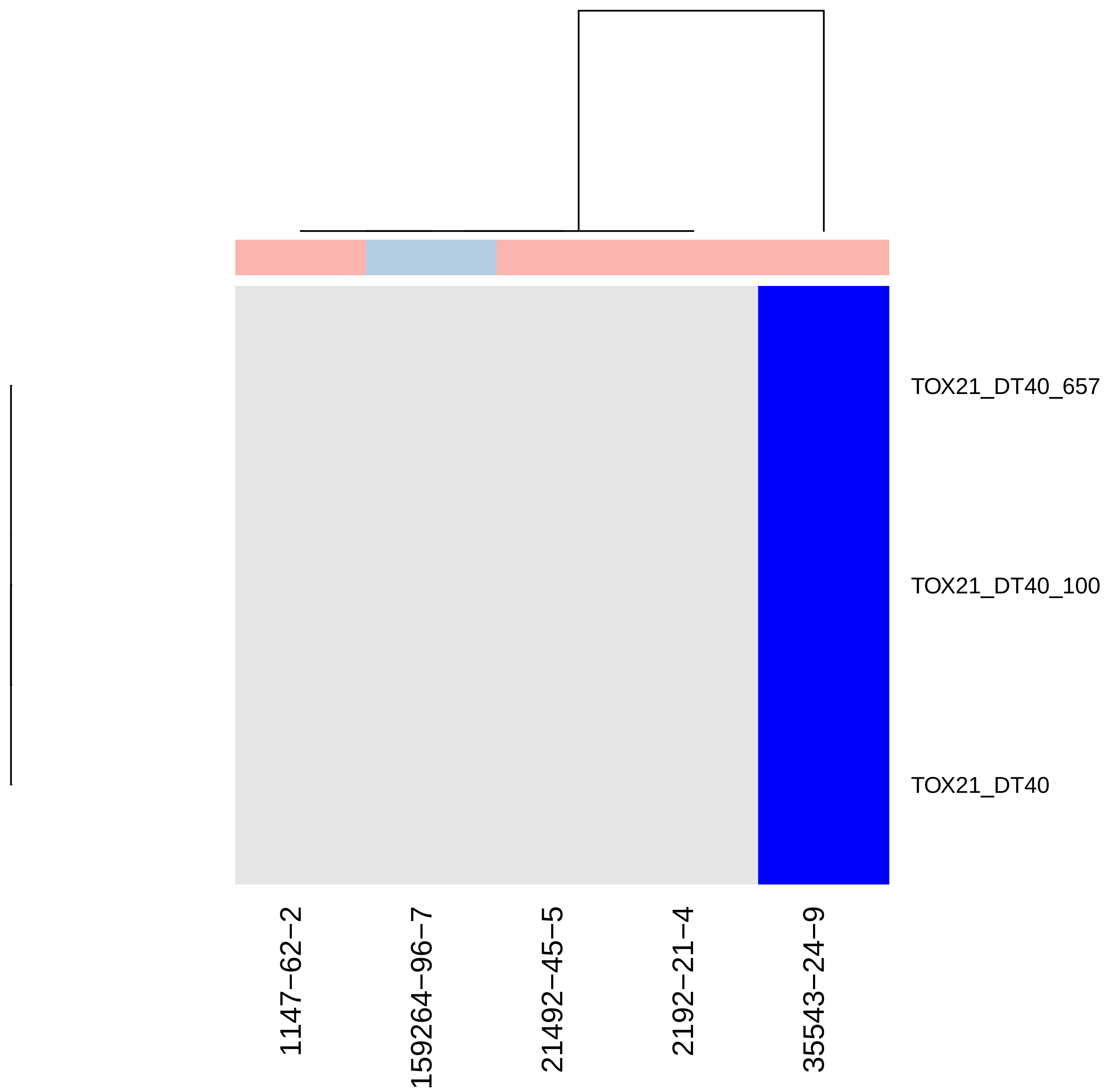

All Activity Heatmap for Cluster 650

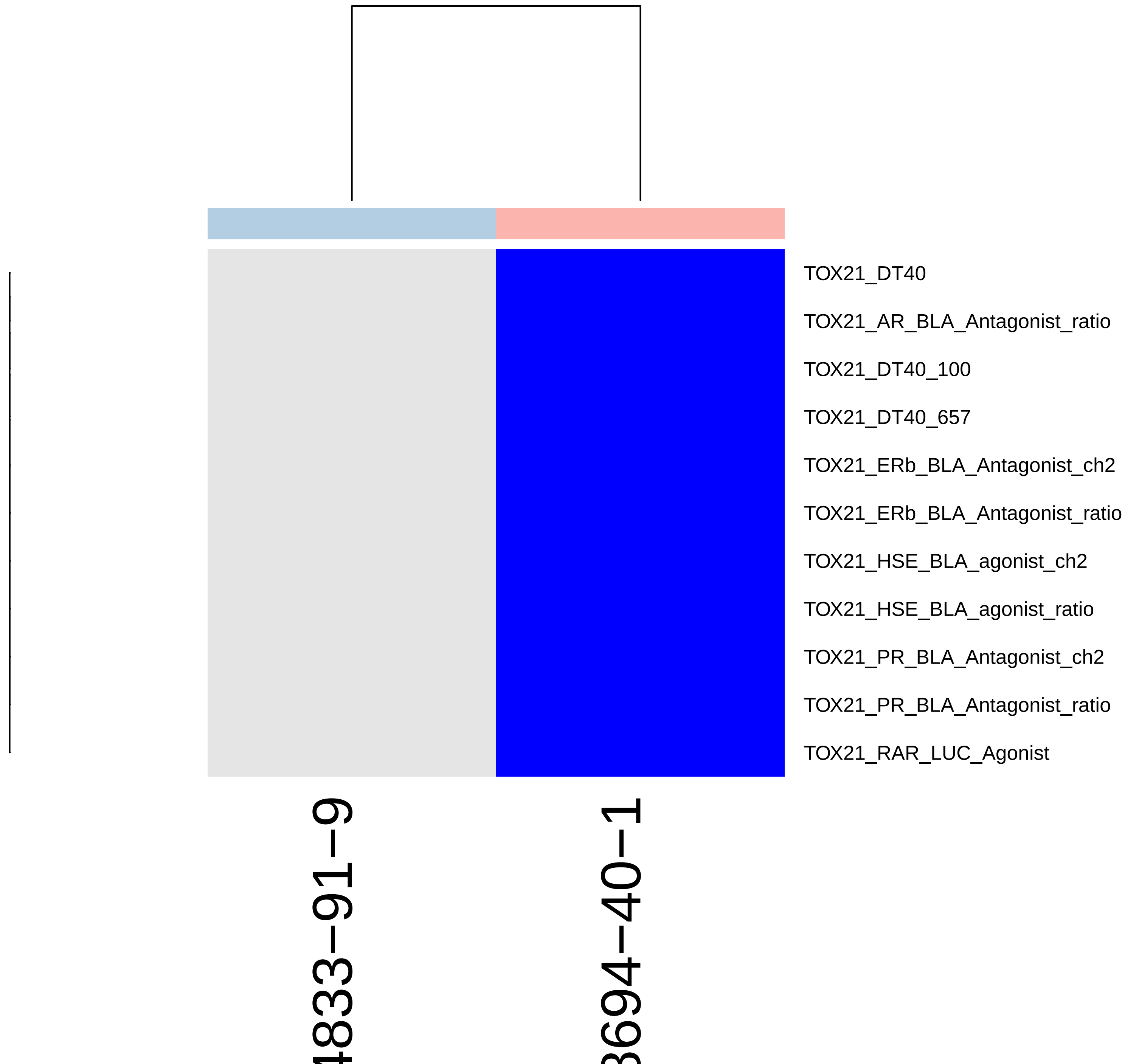

All Activity Heatmap for Cluster 651

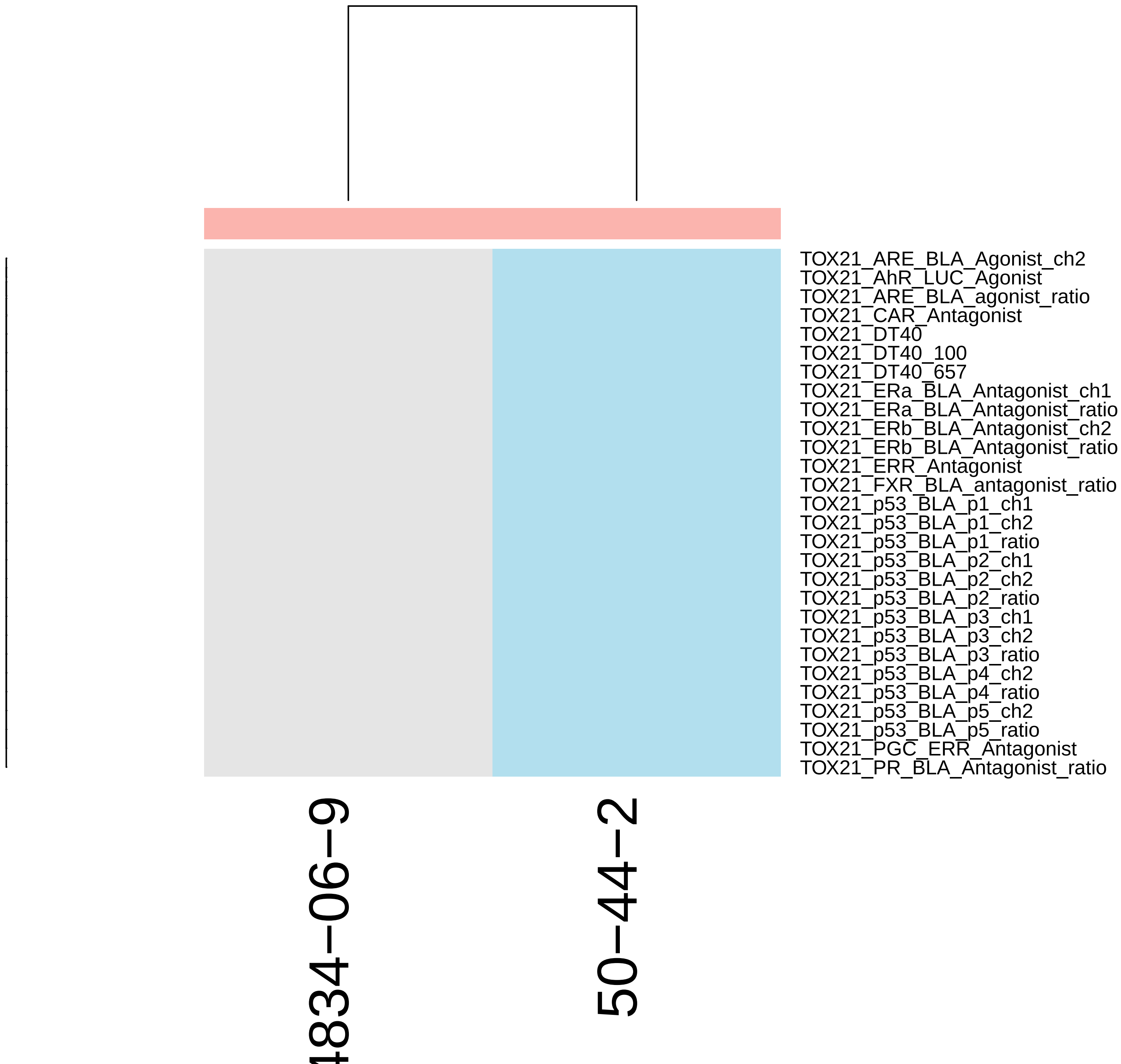

All Activity Heatmap for Cluster 652

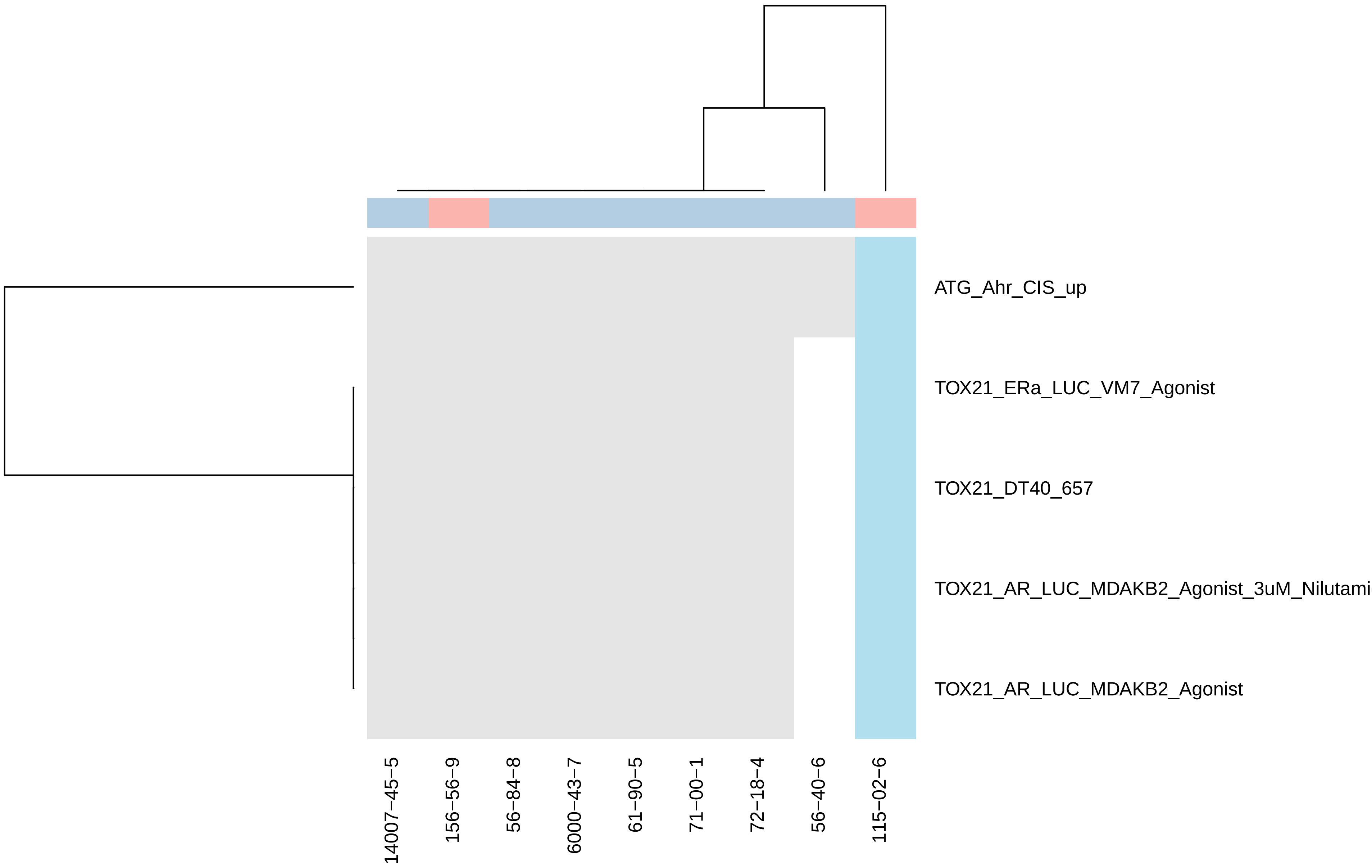

All Activity Heatmap for Cluster 653

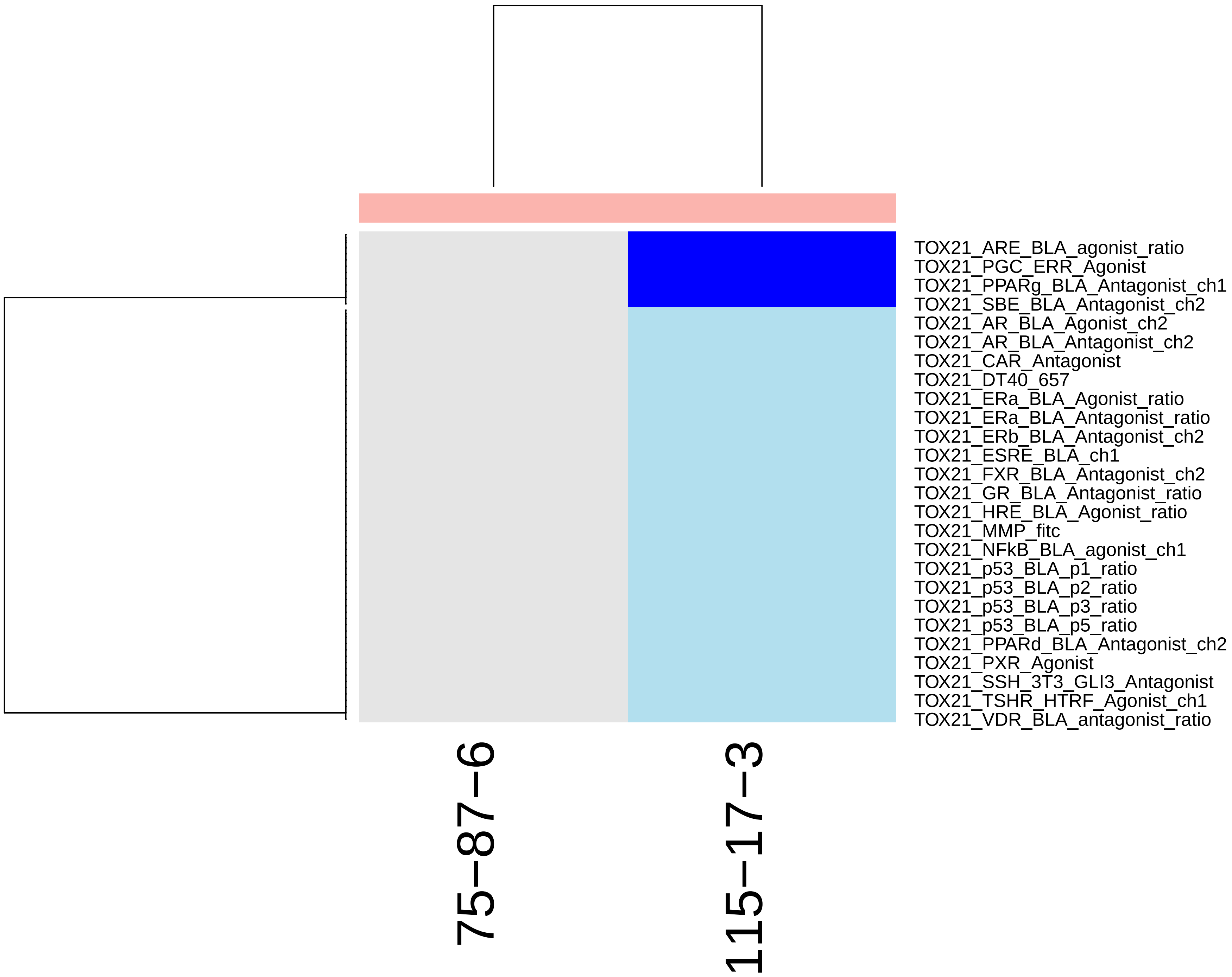

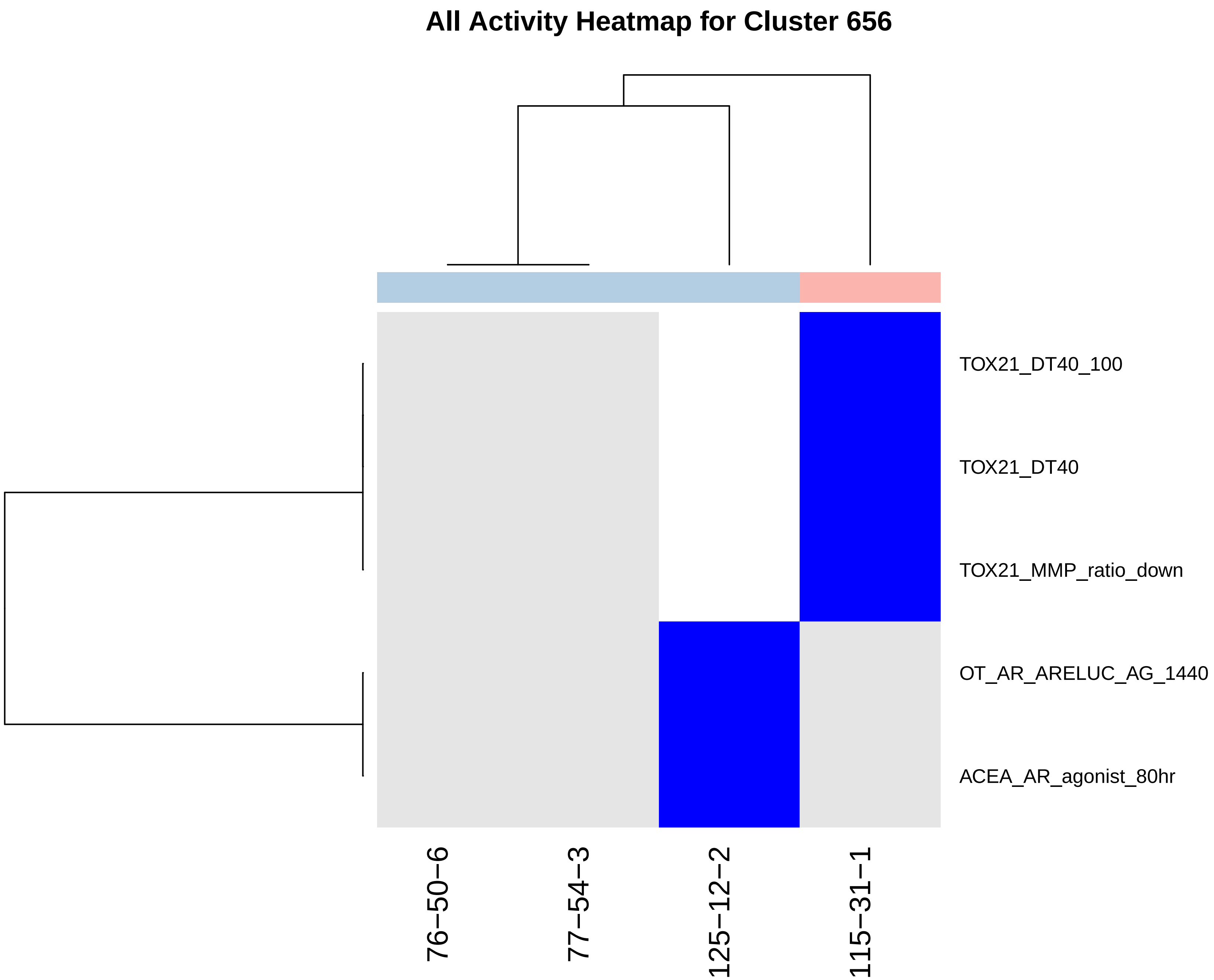

All Activity Heatmap for Cluster 656
TOX21_DT40_100
TOX21_DT40
TOX21_MMP_ratio_down
OT_AR_ARELUC_AG_1440
ACEA_AR_agonist_80hr
76−50−6
77−54−3
125−12−2
115−31−1

All Activity Heatmap for Cluster 657

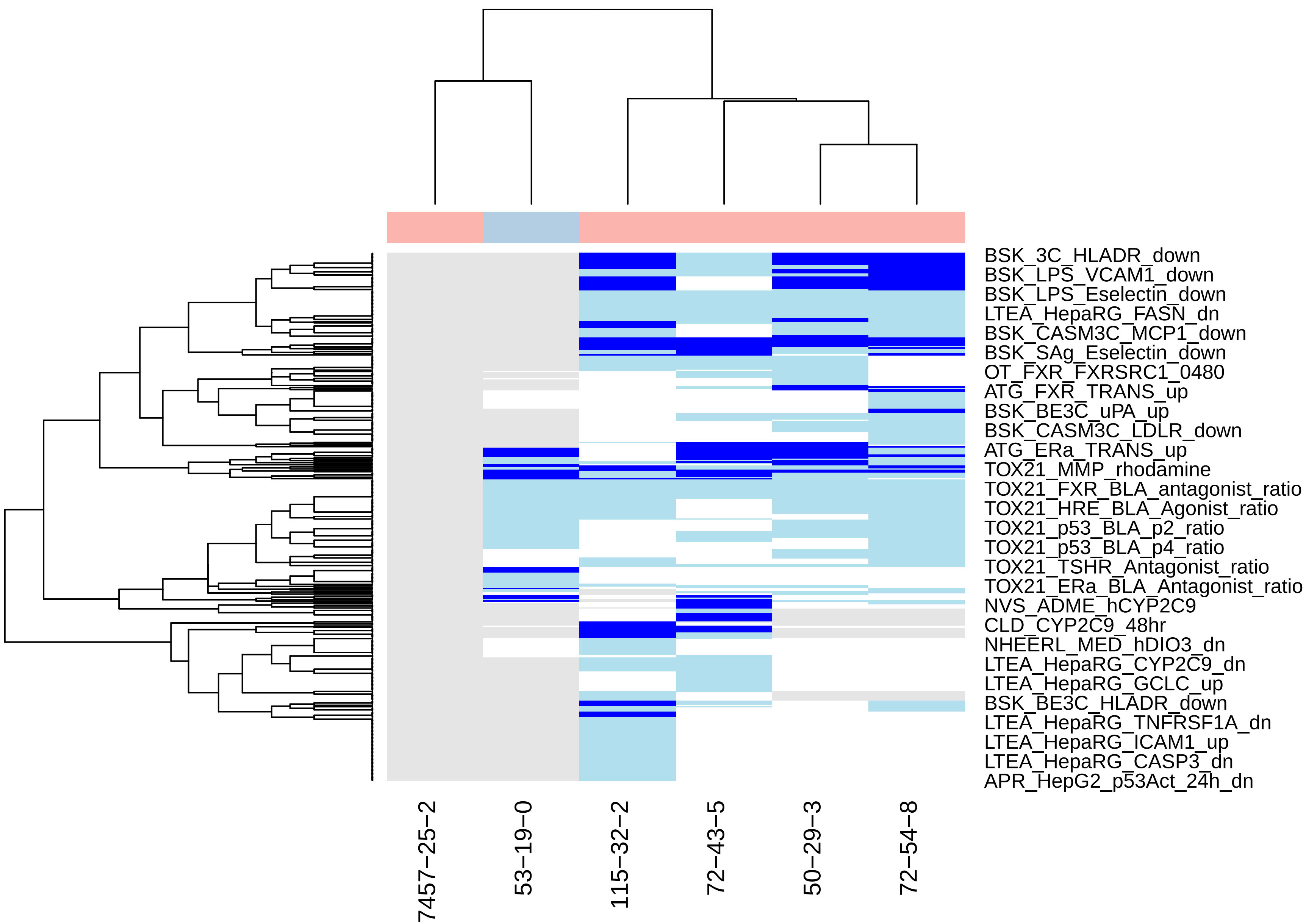

All Activity Heatmap for Cluster 658

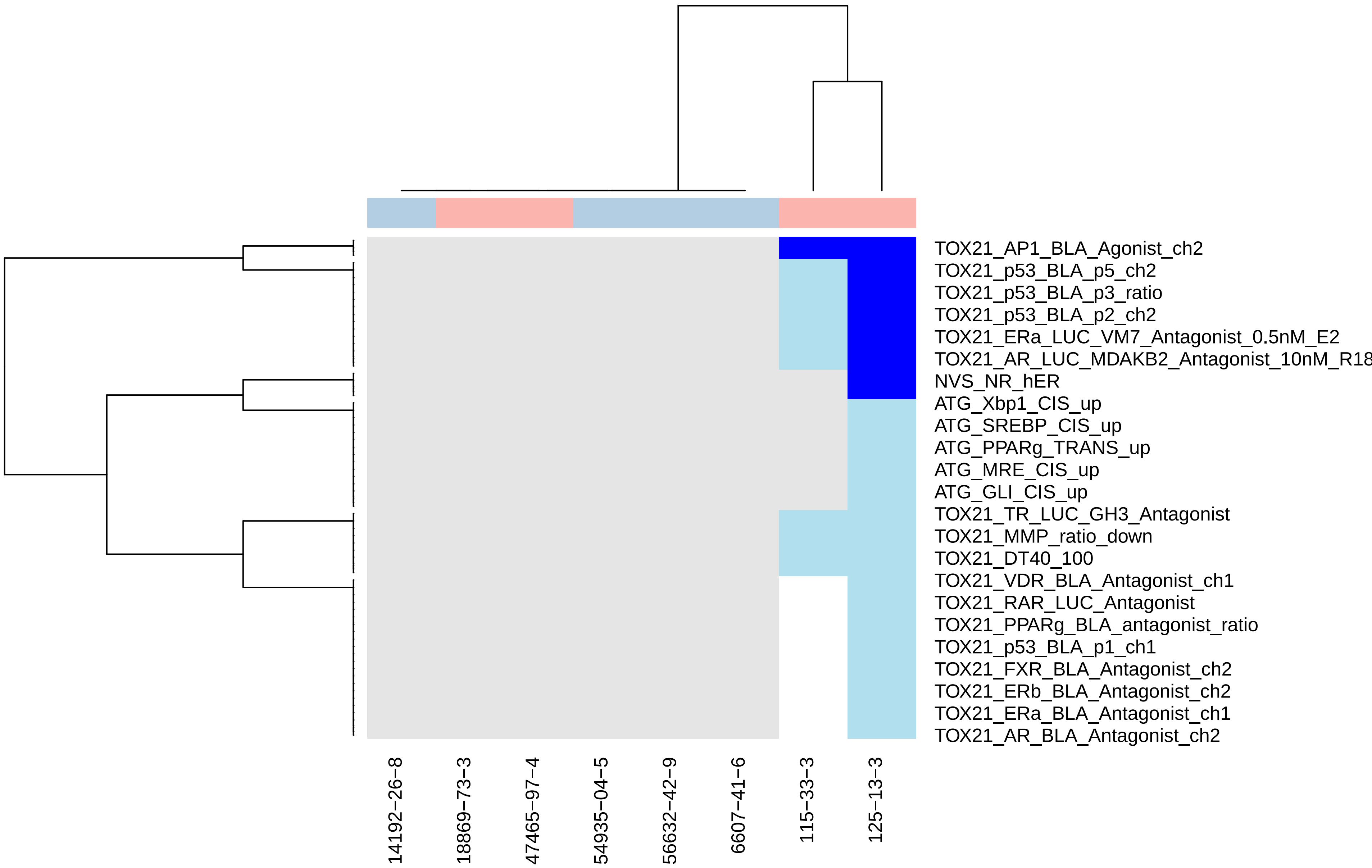

All Activity Heatmap for Cluster 659

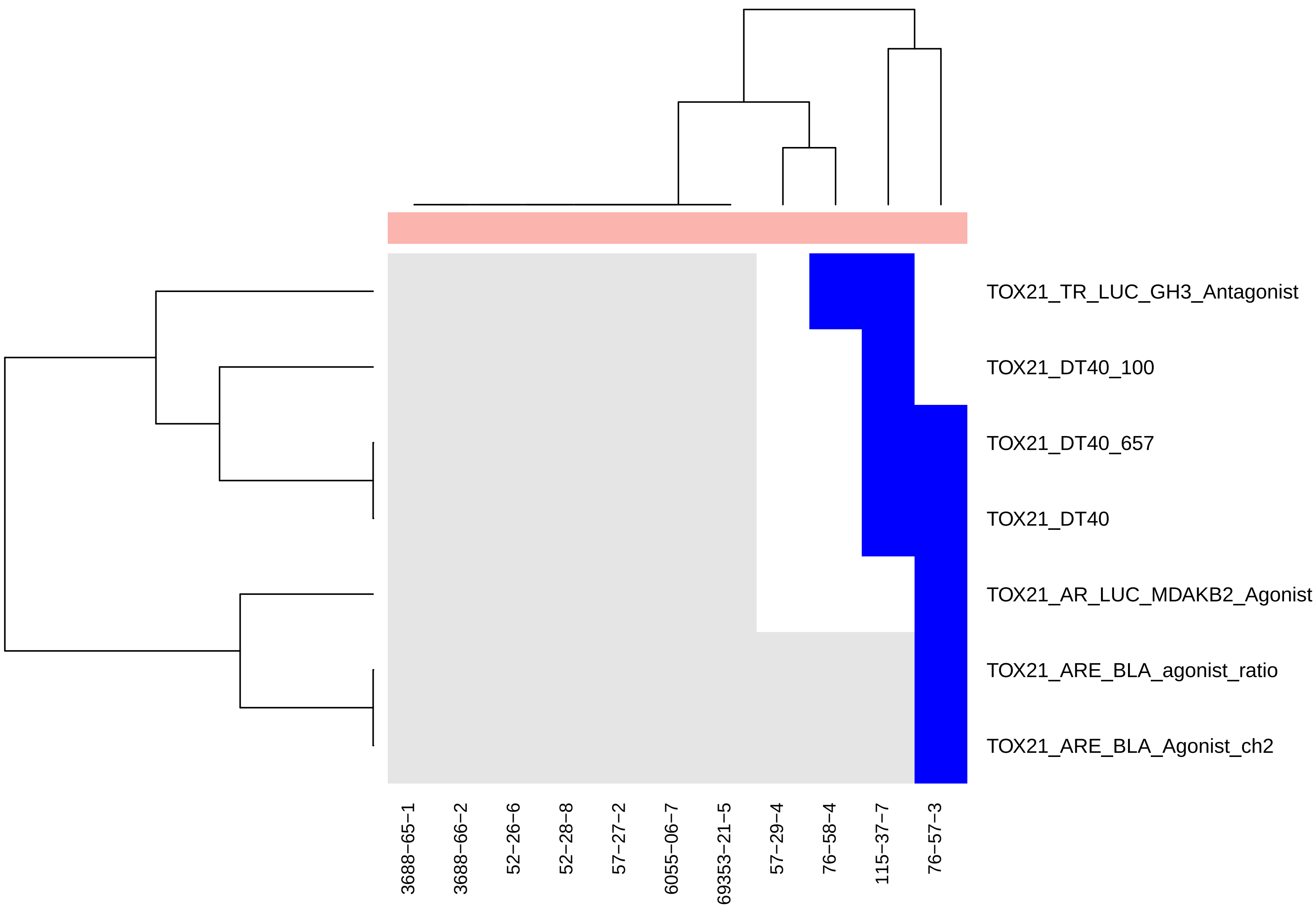

All Activity Heatmap for Cluster 660

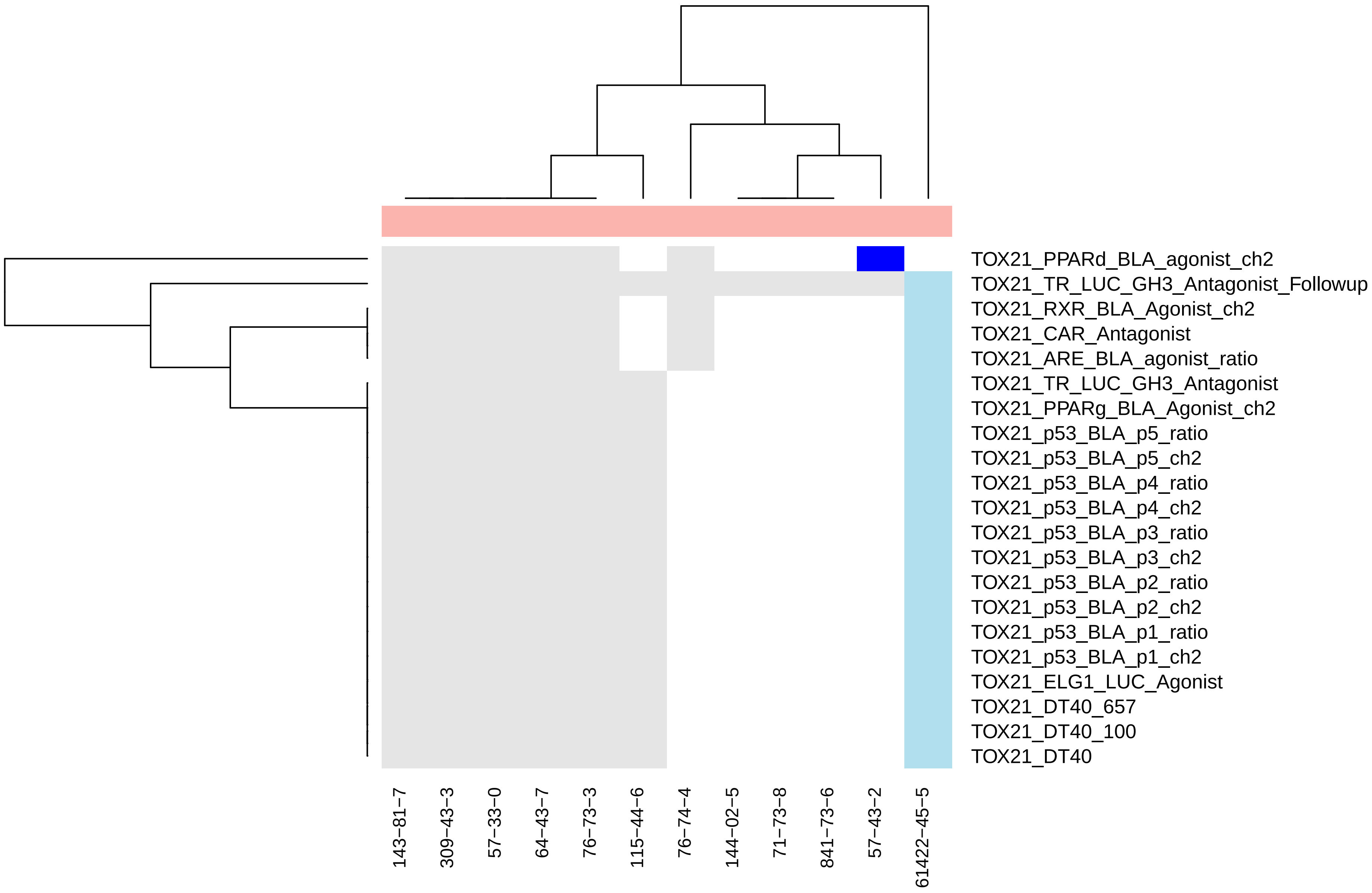

All Activity Heatmap for Cluster 661

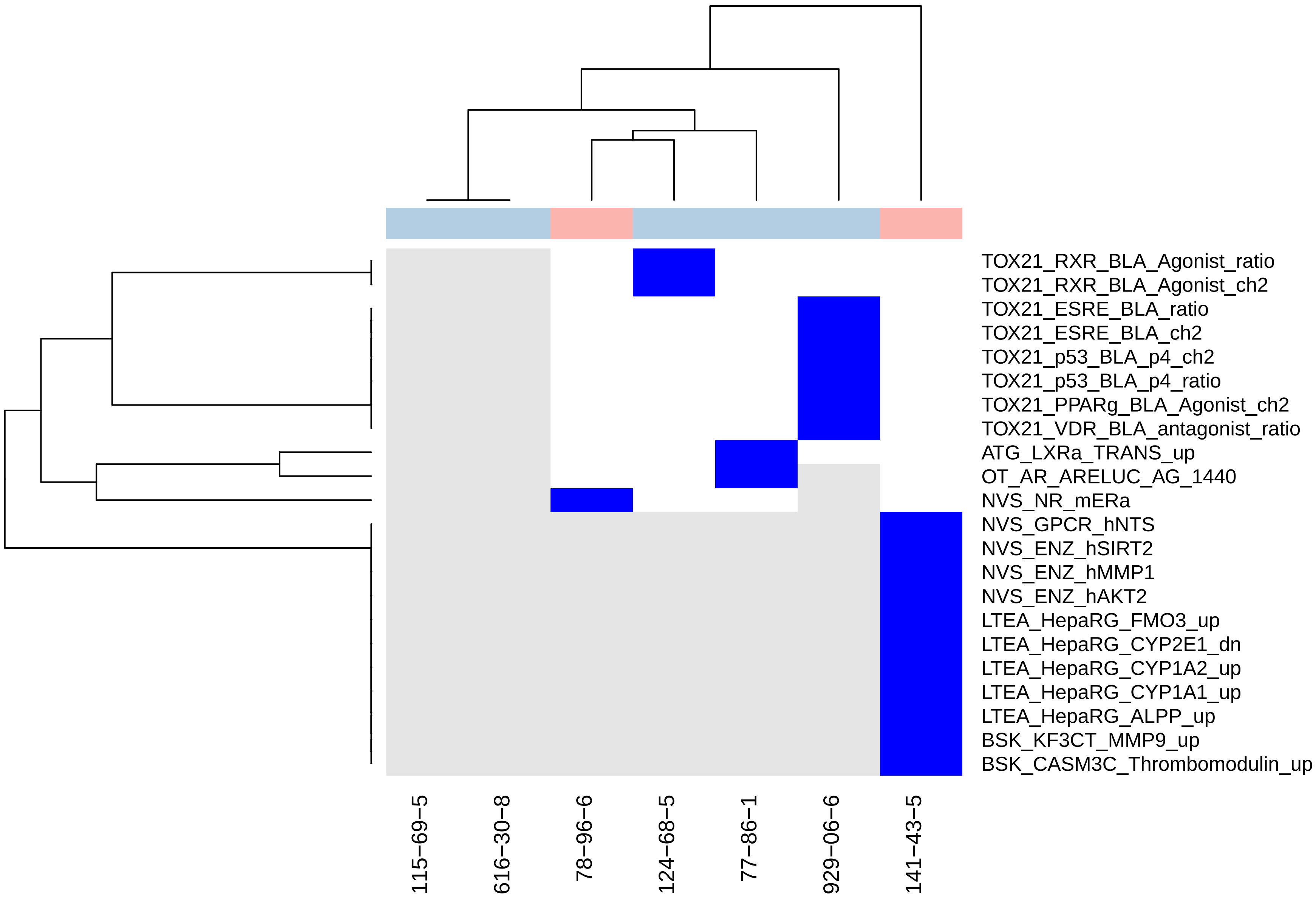

All Activity Heatmap for Cluster 662

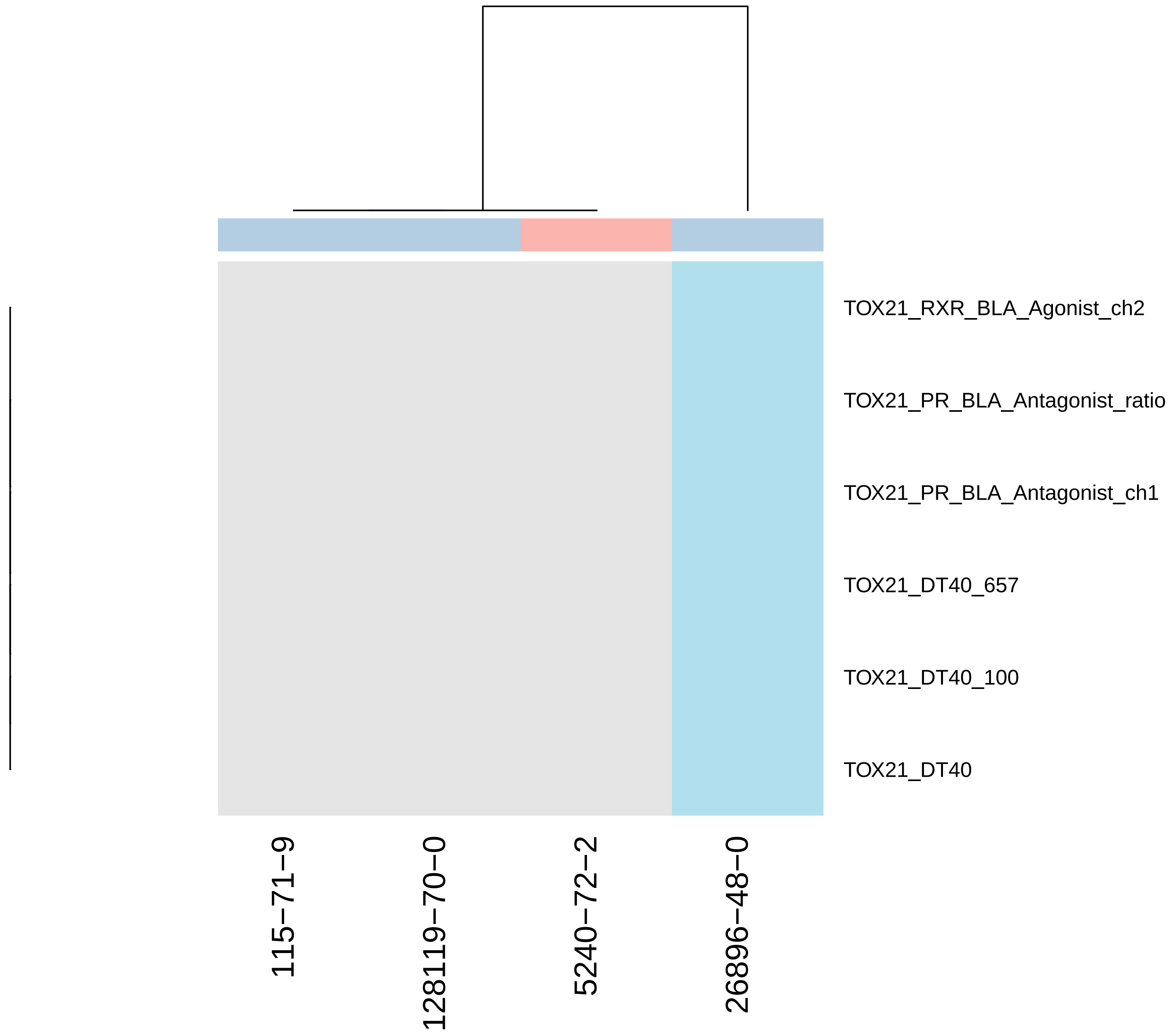

All Activity Heatmap for Cluster 663

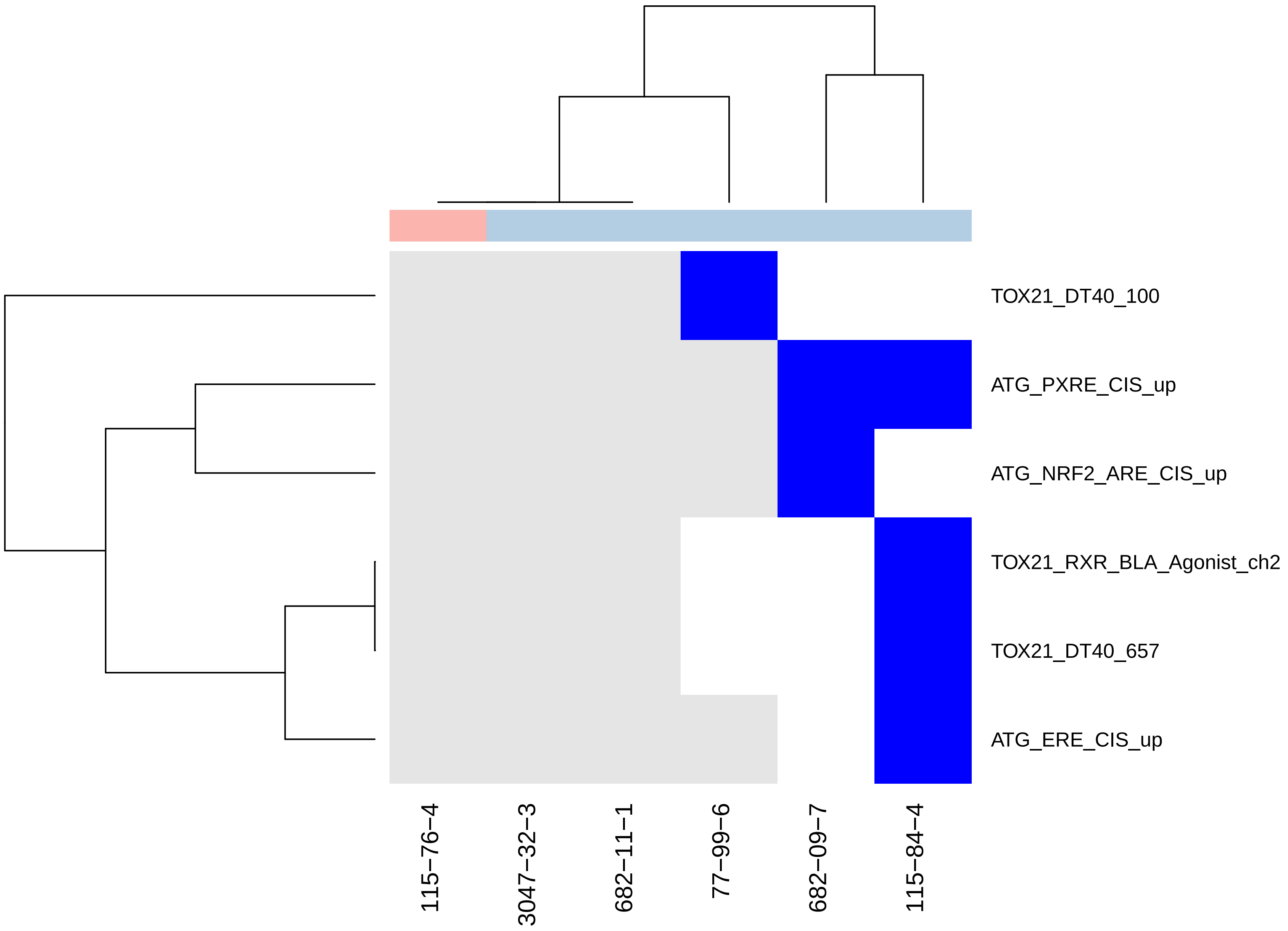

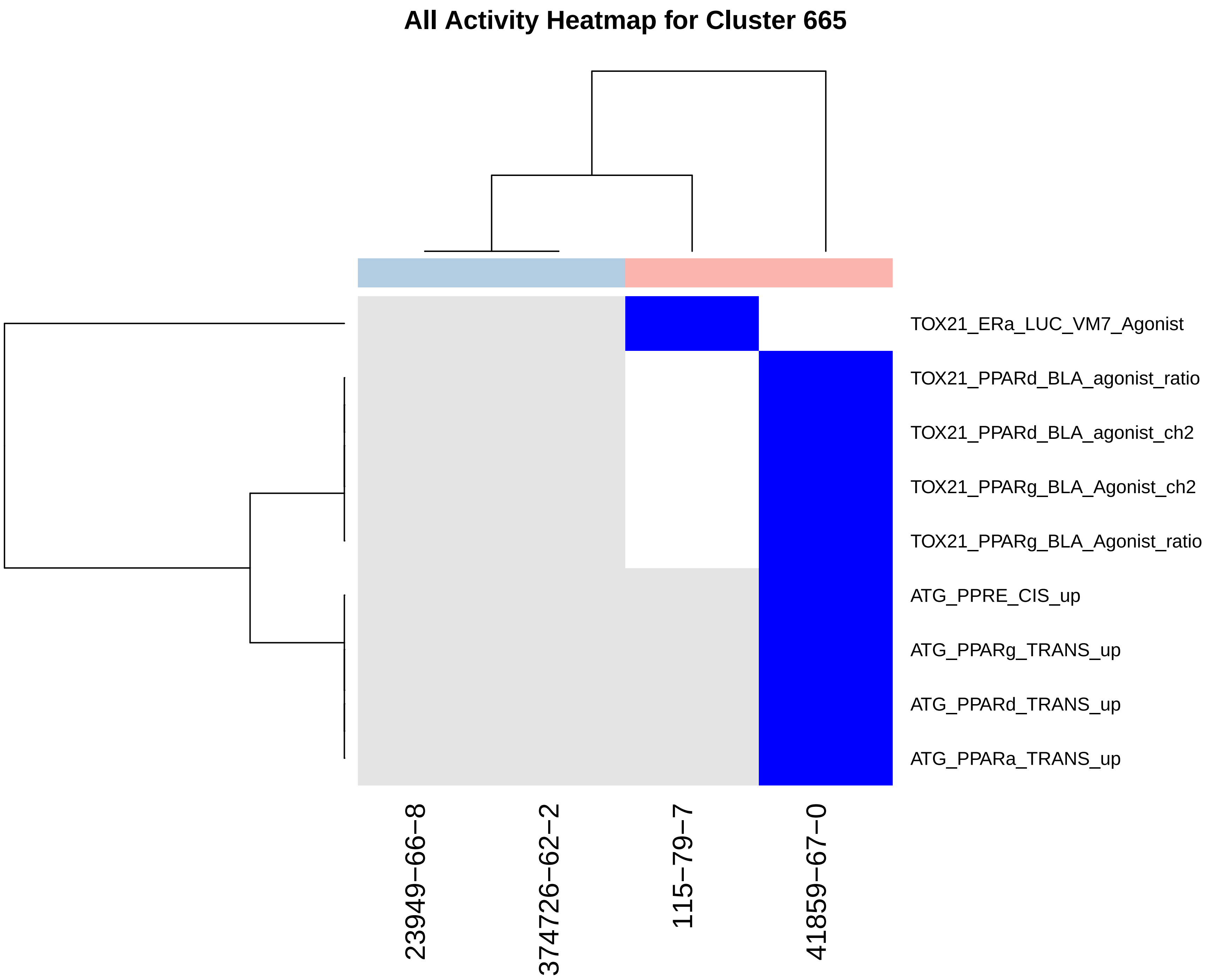
All Activity Heatmap for Cluster 665
TOX21_ERa_LUC_VM7_Agonist
TOX21_PPARd_BLA_agonist_ratio
TOX21_PPARd_BLA_agonist_ch2
TOX21_PPARg_BLA_Agonist_ch2
TOX21_PPARg_BLA_Agonist_ratio
ATG_PPRE_CIS_up
ATG_PPARg_TRANS_up
ATG_PPARd_TRANS_up
ATG_PPARa_TRANS_up
23949−66−8
374726−62−2
115−79−7
41859−67−0

All Activity Heatmap for Cluster 666

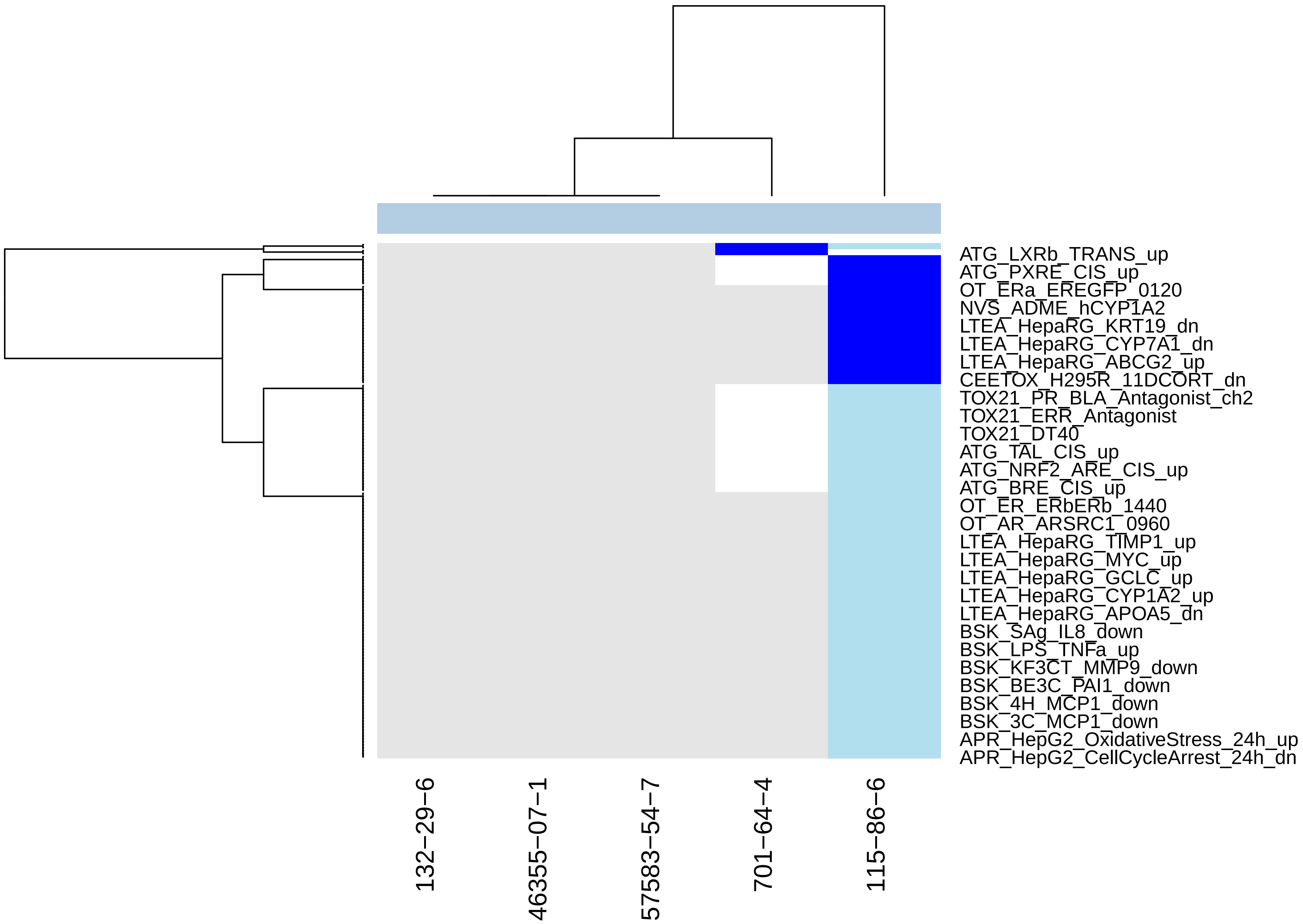

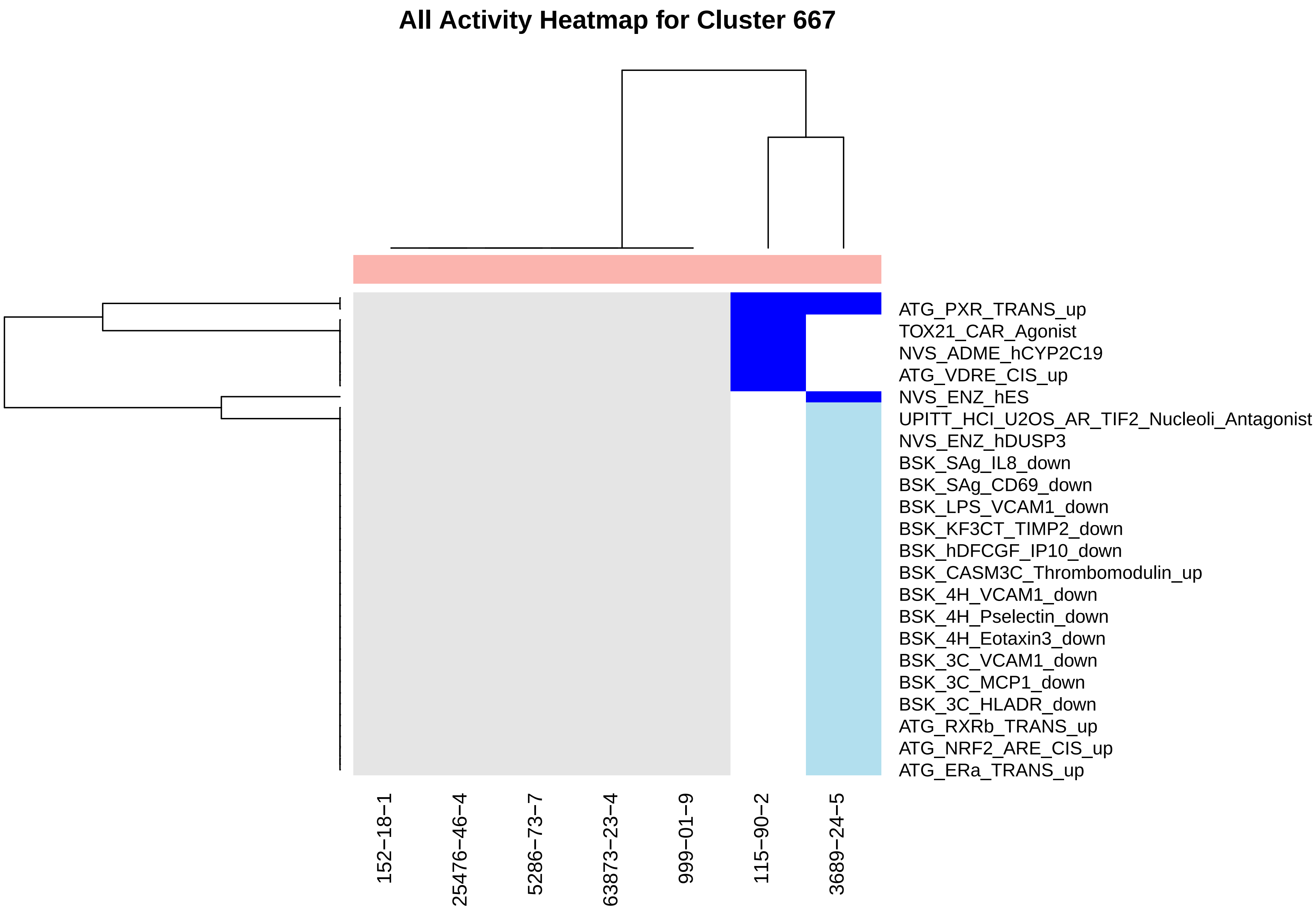
All Activity Heatmap for Cluster 667
ATG_PXR_TRANS_up
TOX21_CAR_Agonist
NVS_ADME_hCYP2C19
ATG_VDRE_CIS_up
NVS_ENZ_hES
UPITT_HCI_U2OS_AR_TIF2_Nucleoli_Antagonist
NVS_ENZ_hDUSP3
BSK_SAg_IL8_down
BSK_SAg_CD69_down
BSK_LPS_VCAM1_down
BSK_KF3CT_TIMP2_down
BSK_hDFCGF_IP10_down
BSK_CASM3C_Thrombomodulin_up
BSK_4H_VCAM1_down
BSK_4H_Pselectin_down
BSK_4H_Eotaxin3_down
BSK_3C_VCAM1_down
BSK_3C_MCP1_down
BSK_3C_HLADR_down
ATG_RXRb_TRANS_up
ATG_NRF2_ARE_CIS_up
ATG_ERa_TRANS_up
152−18−1
25476−46−4
5286−73−7
63873−23−4
999−01−9
115−90−2
3689−24−5

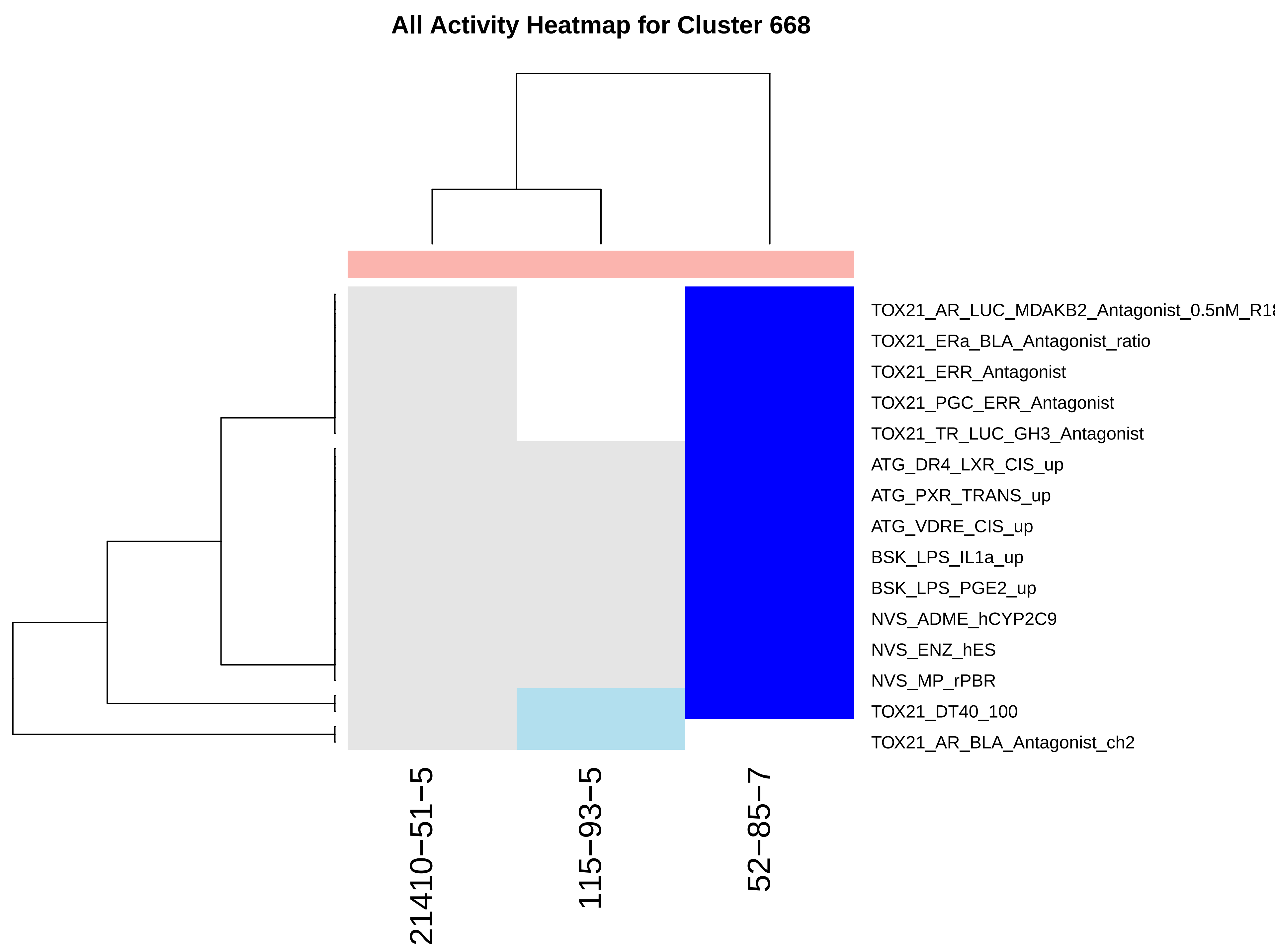
All Activity Heatmap for Cluster 668
TOX21_AR_LUC_MDAKB2_Antagonist_0.5nM_R18
TOX21_ERa_BLA_Antagonist_ratio
TOX21_ERR_Antagonist
TOX21_PGC_ERR_Antagonist
TOX21_TR_LUC_GH3_Antagonist
ATG_DR4_LXR_CIS_up
ATG_PXR_TRANS_up
ATG_VDRE_CIS_up
BSK_LPS_IL1a_up
BSK_LPS_PGE2_up
NVS_ADME_hCYP2C9
NVS_ENZ_hES
NVS_MP_rPBR
TOX21_DT40_100
TOX21_AR_BLA_Antagonist_ch2
21410−51−5
115−93−5
52−85−7

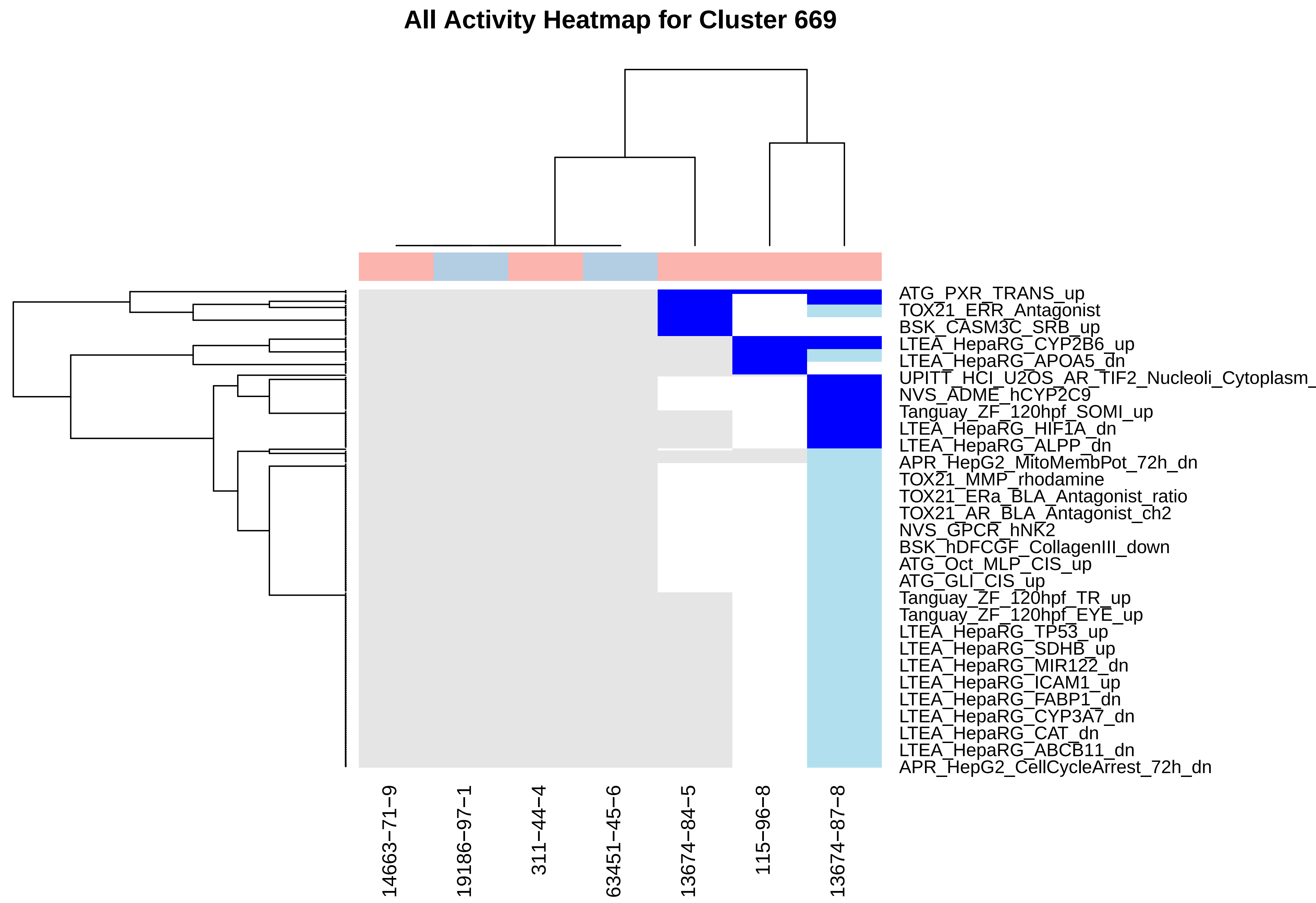
All Activity Heatmap for Cluster 669
ATG_PXR_TRANS_up
TOX21_ERR_Antagonist
BSK_CASM3C_SRB_up
LTEA_HepaRG_CYP2B6_up
LTEA_HepaRG_APOA5_dn
UPITT_HCI_U2OS_AR_TIF2_Nucleoli_Cytoplasm_
NVS_ADME_hCYP2C9
Tanguay_ZF_120hpf_SOMI_up
LTEA_HepaRG_HIF1A_dn
LTEA_HepaRG_ALPP_dn
APR_HepG2_MitoMembPot_72h_dn
TOX21_MMP_rhodamine
TOX21_ERa_BLA_Antagonist_ratio
TOX21_AR_BLA_Antagonist_ch2
NVS_GPCR_hNK2
BSK_hDFCGF_CollagenIII_down
ATG_Oct_MLP_CIS_up
ATG_GLI_CIS_up
Tanguay_ZF_120hpf_TR_up
Tanguay_ZF_120hpf_EYE_up
LTEA_HepaRG_TP53_up
LTEA_HepaRG_SDHB_up
LTEA_HepaRG_MIR122_dn
LTEA_HepaRG_ICAM1_up
LTEA_HepaRG_FABP1_dn
LTEA_HepaRG_CYP3A7_dn
LTEA_HepaRG_CAT_dn
LTEA_HepaRG_ABCB11_dn
APR_HepG2_CellCycleArrest_72h_dn
14663−71−9
19186−97−1
311−44−4
63451−45−6
13674−84−5
115−96−8
13674−87−8

All Activity Heatmap for Cluster 670

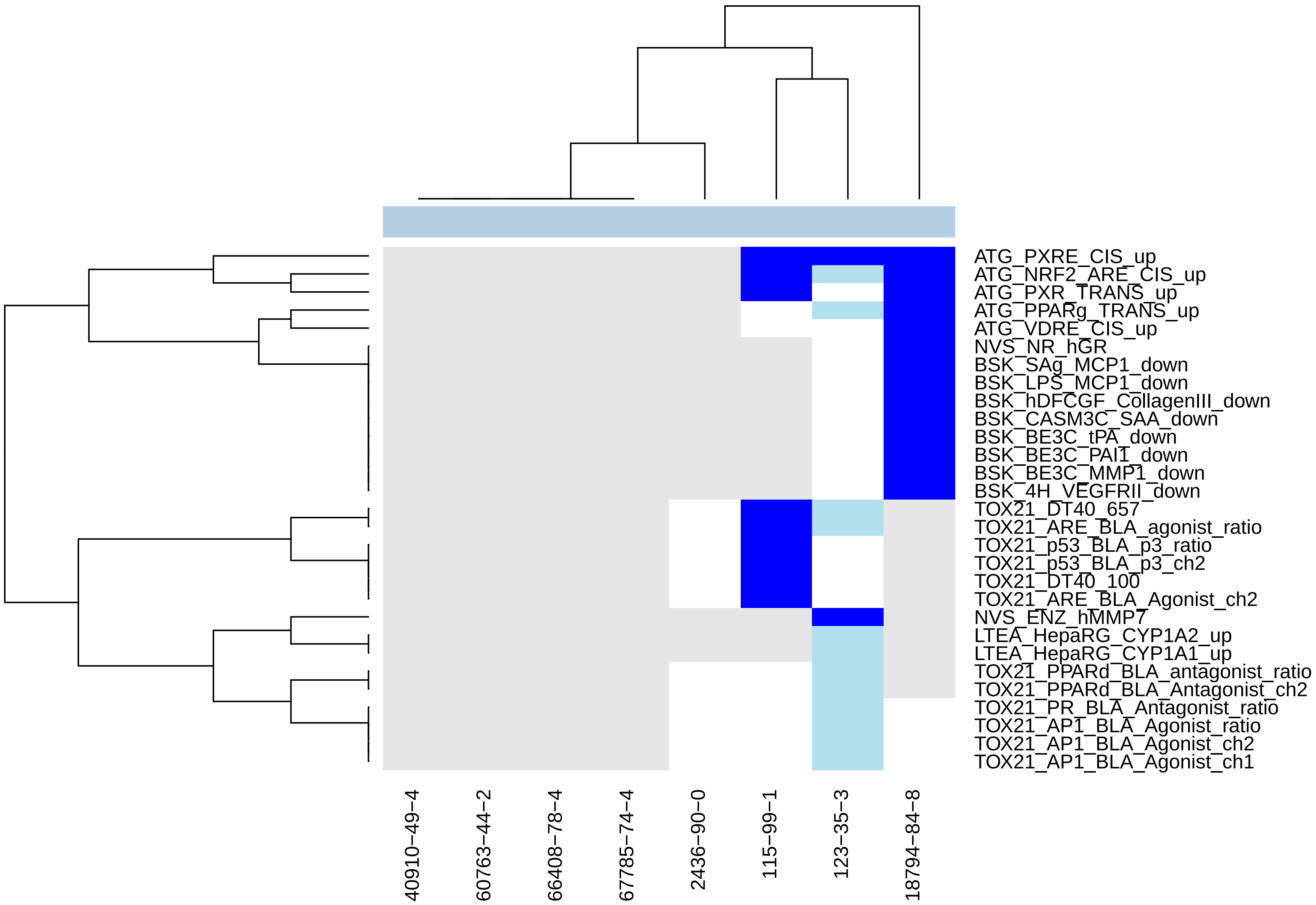

All Activity Heatmap for Cluster 671

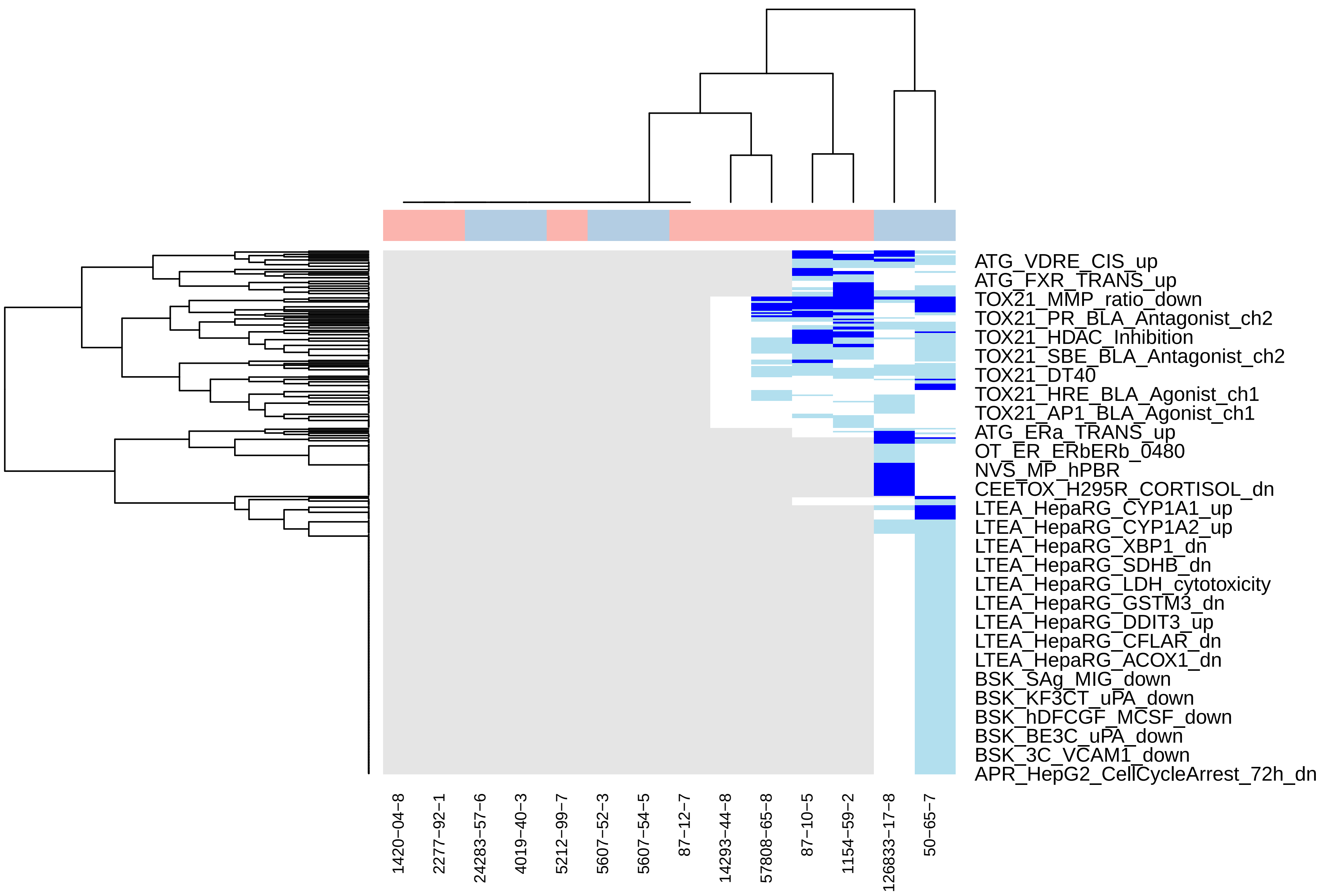

All Activity Heatmap for Cluster 674

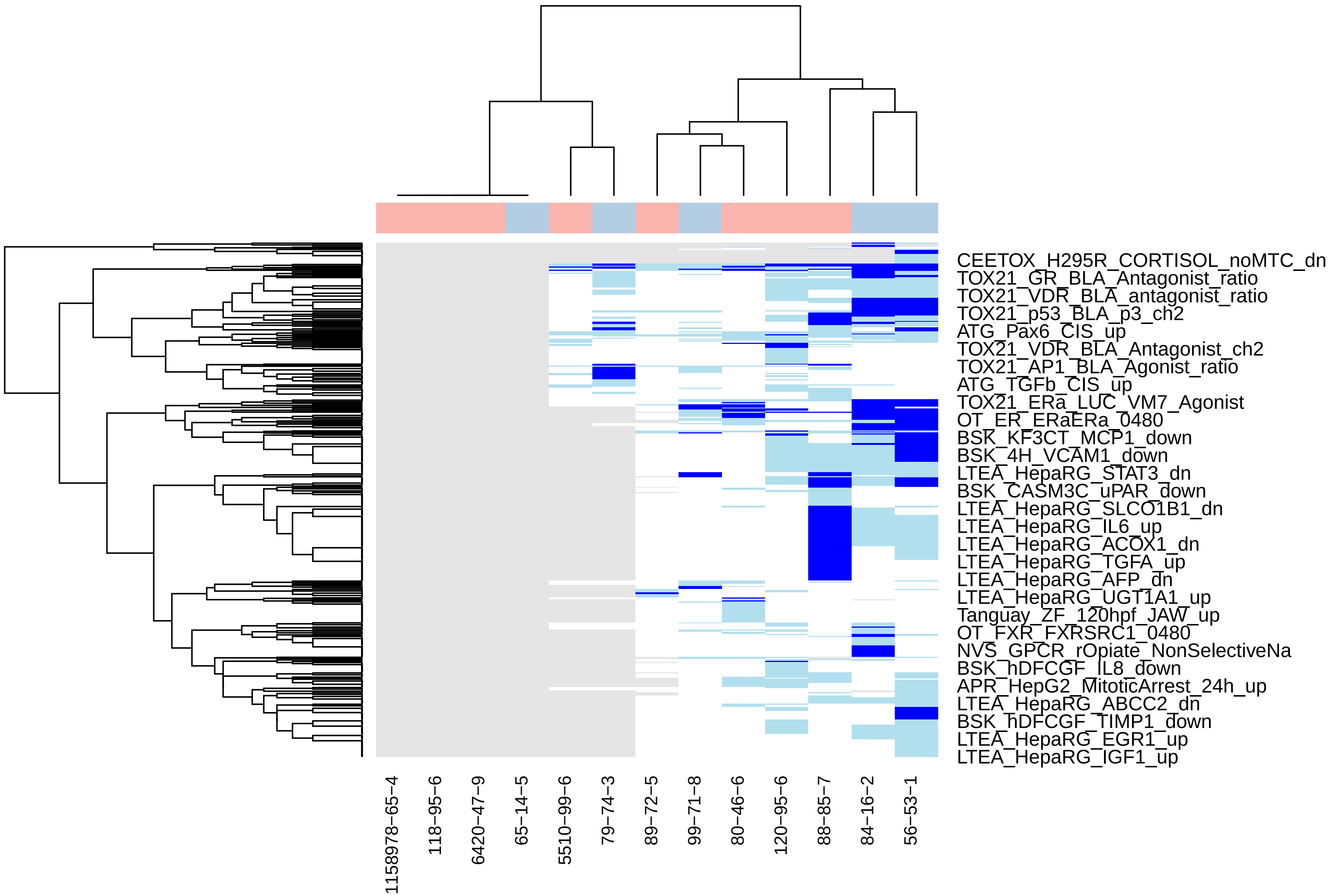

All Activity Heatmap for Cluster 675

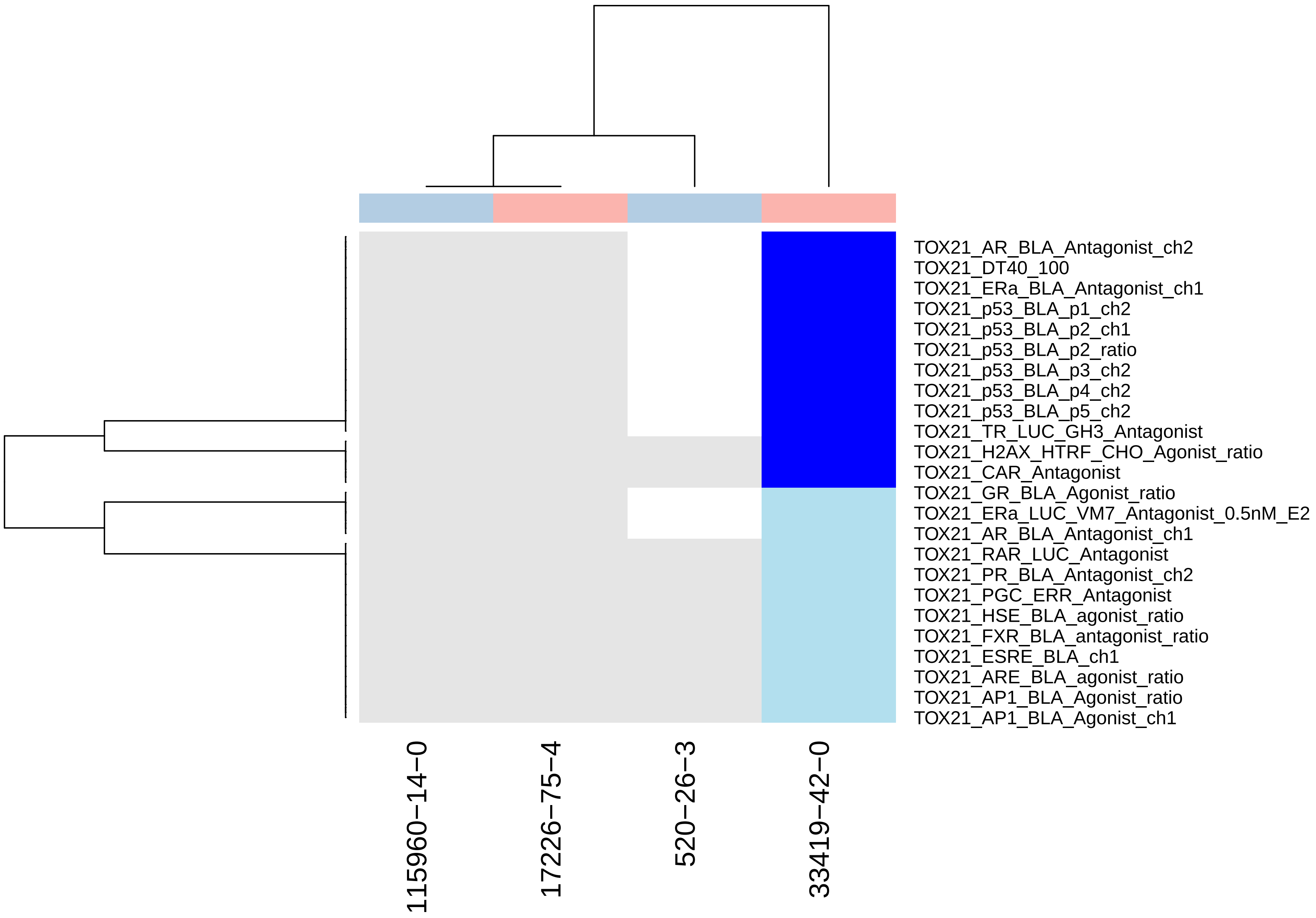

All Activity Heatmap for Cluster 676

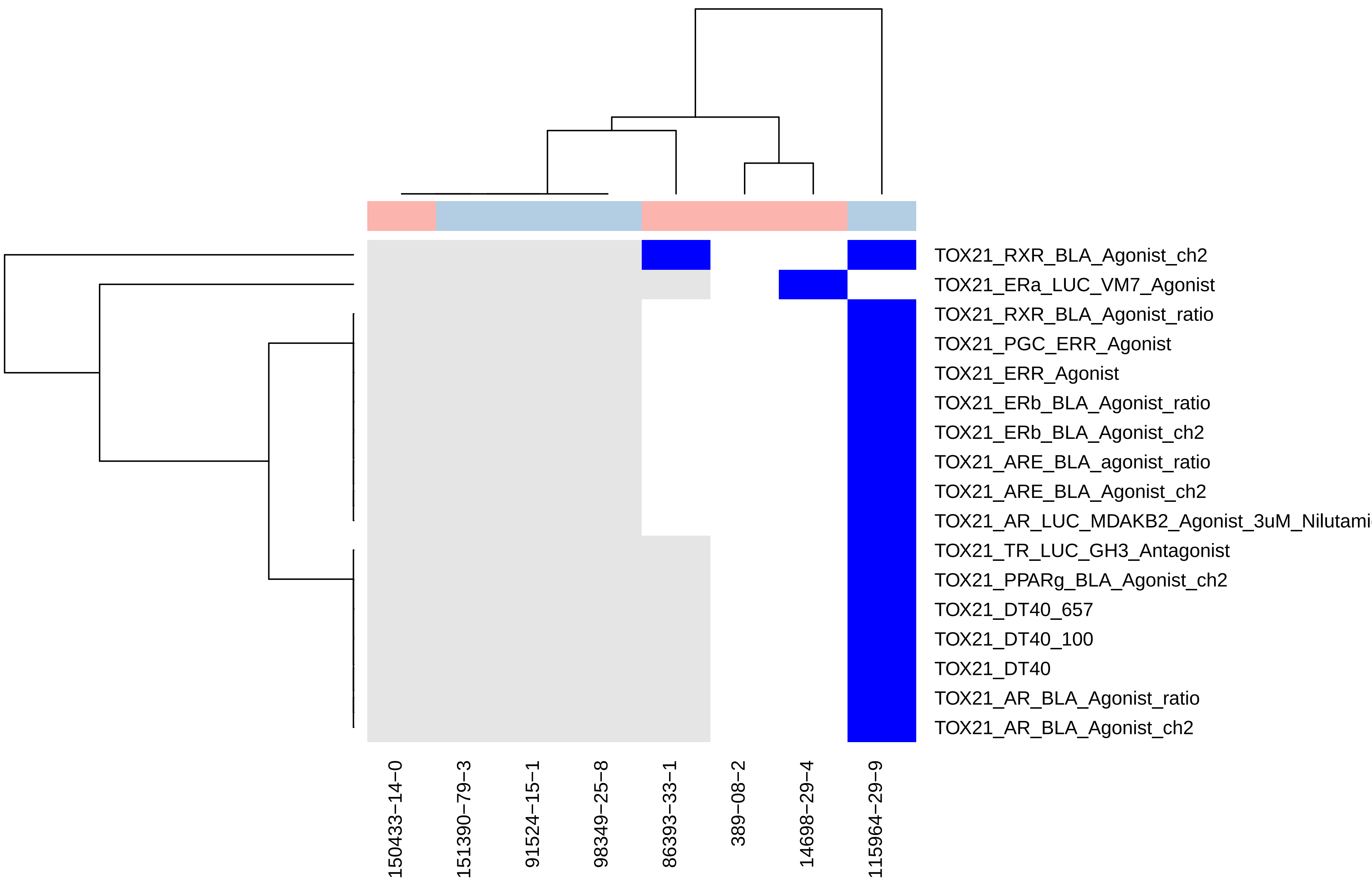

All Activity Heatmap for Cluster 677

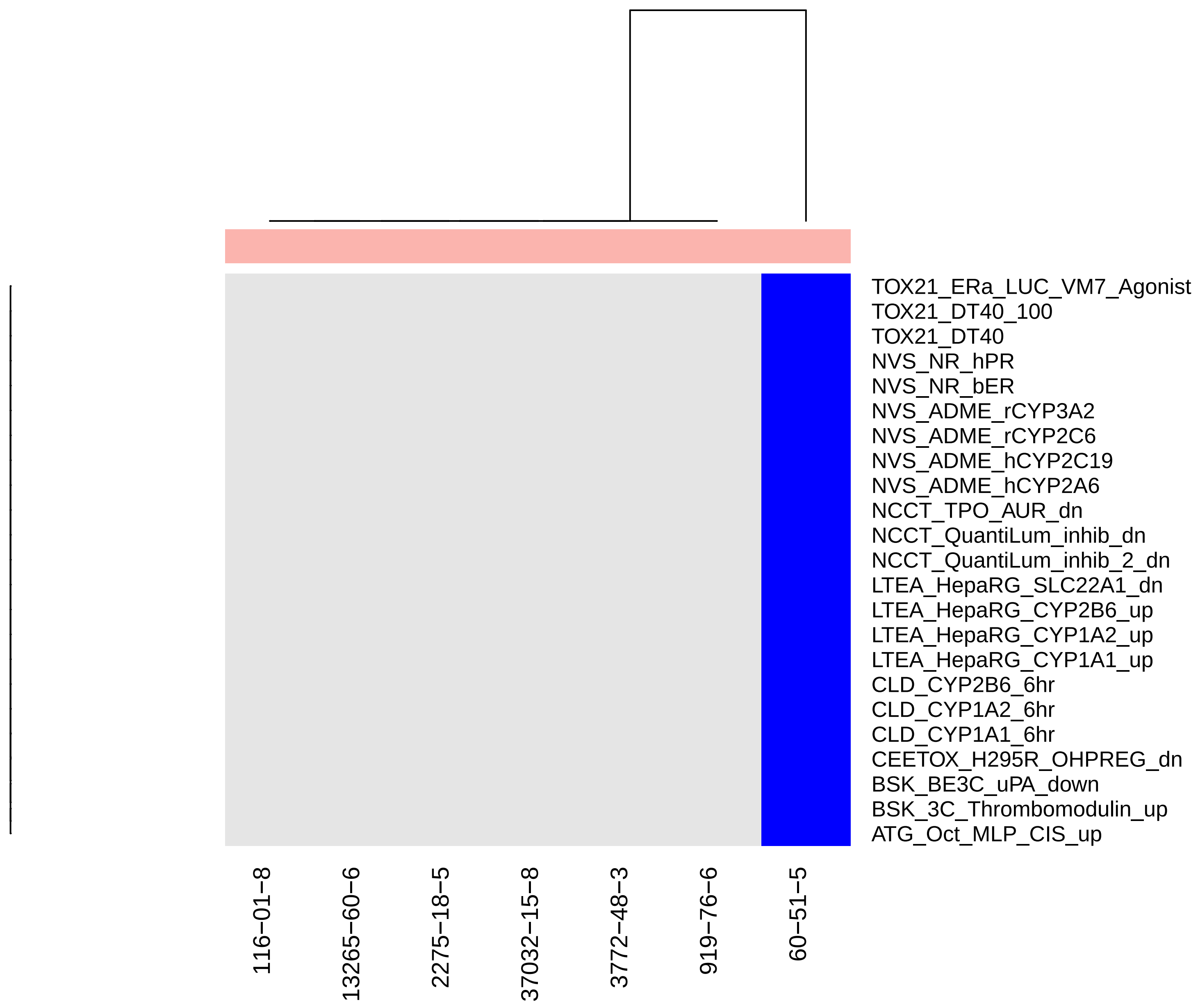

All Activity Heatmap for Cluster 678

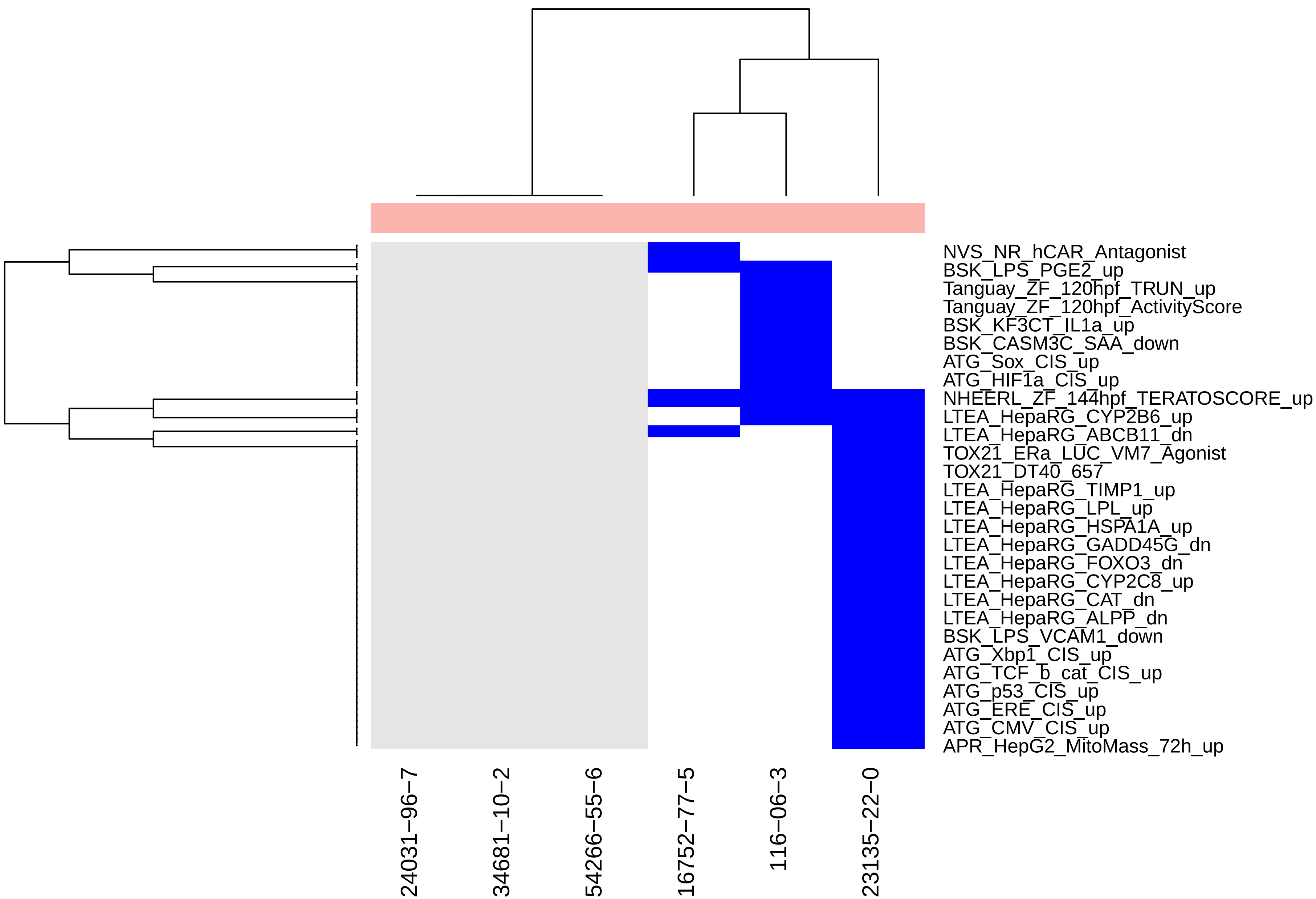

All Activity Heatmap for Cluster 679

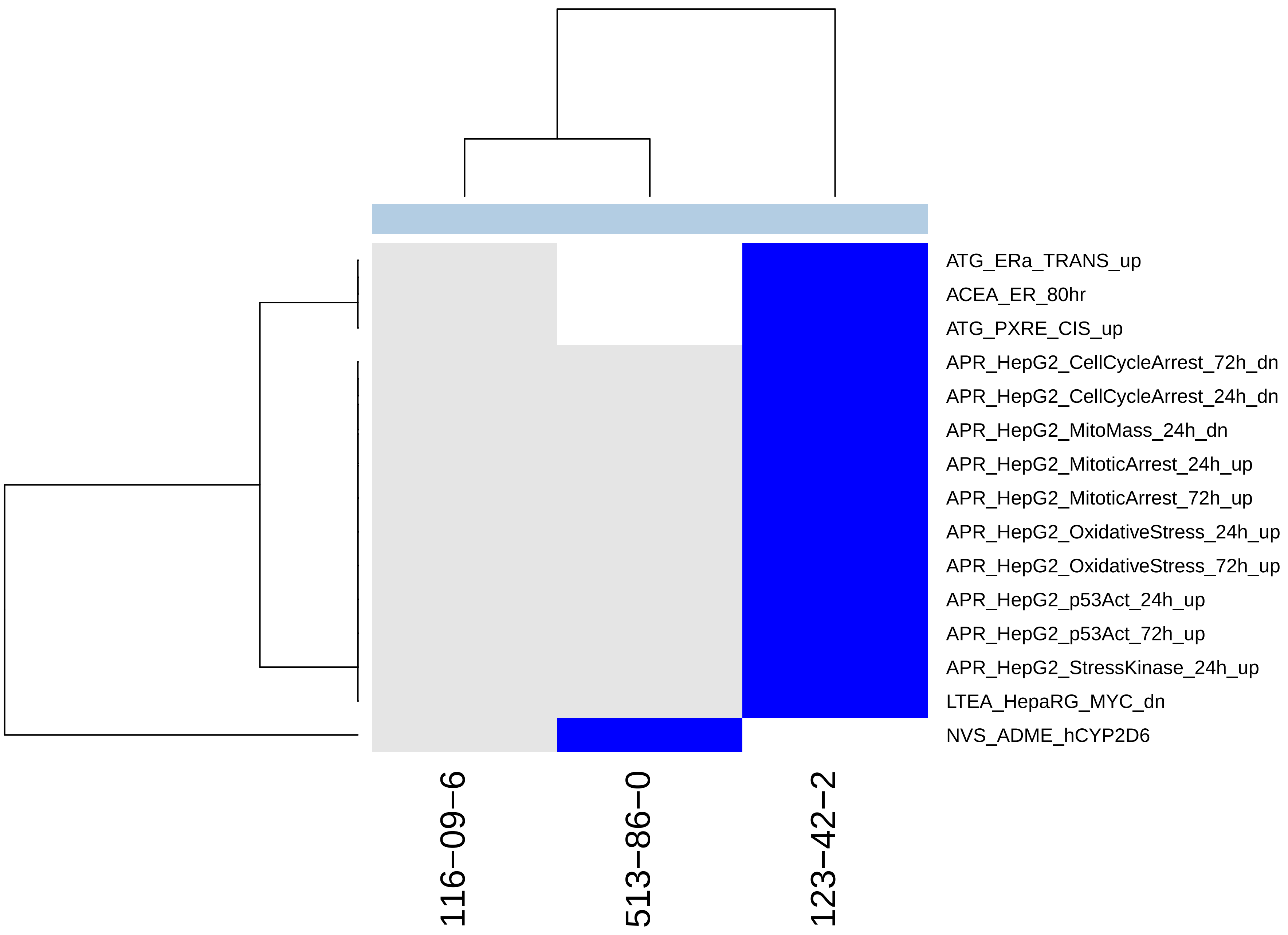

All Activity Heatmap for Cluster 680

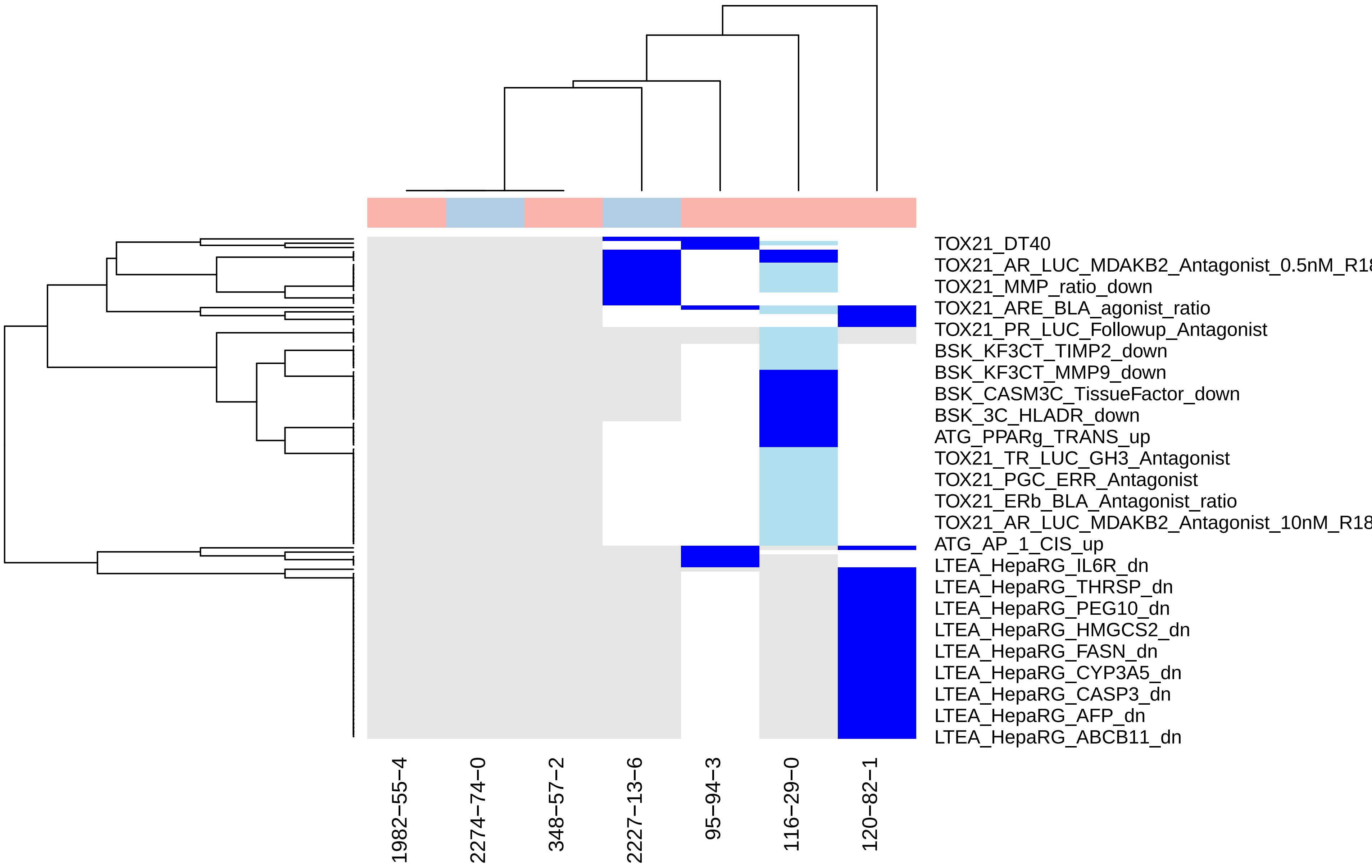

All Activity Heatmap for Cluster 681

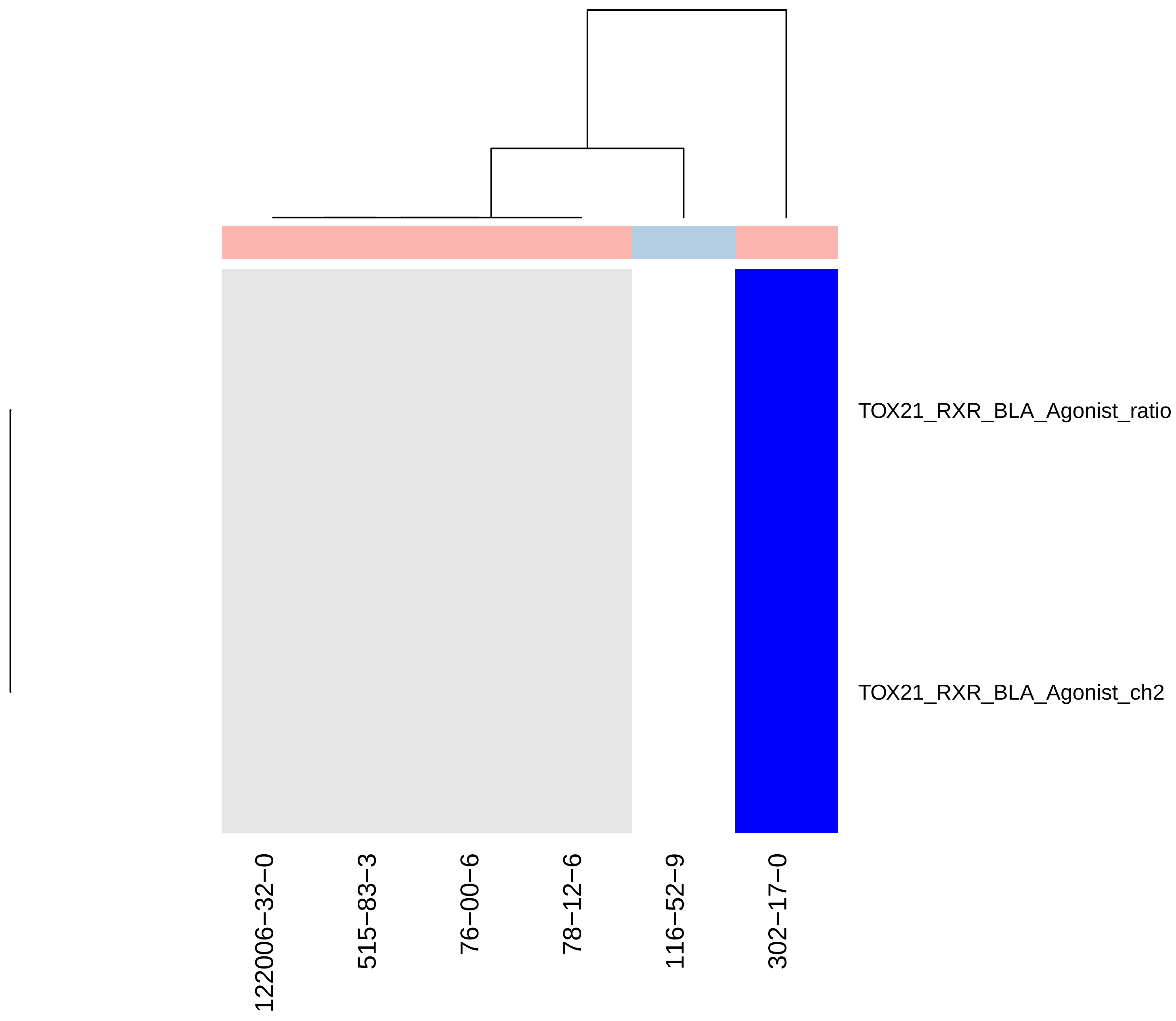

All Activity Heatmap for Cluster 682

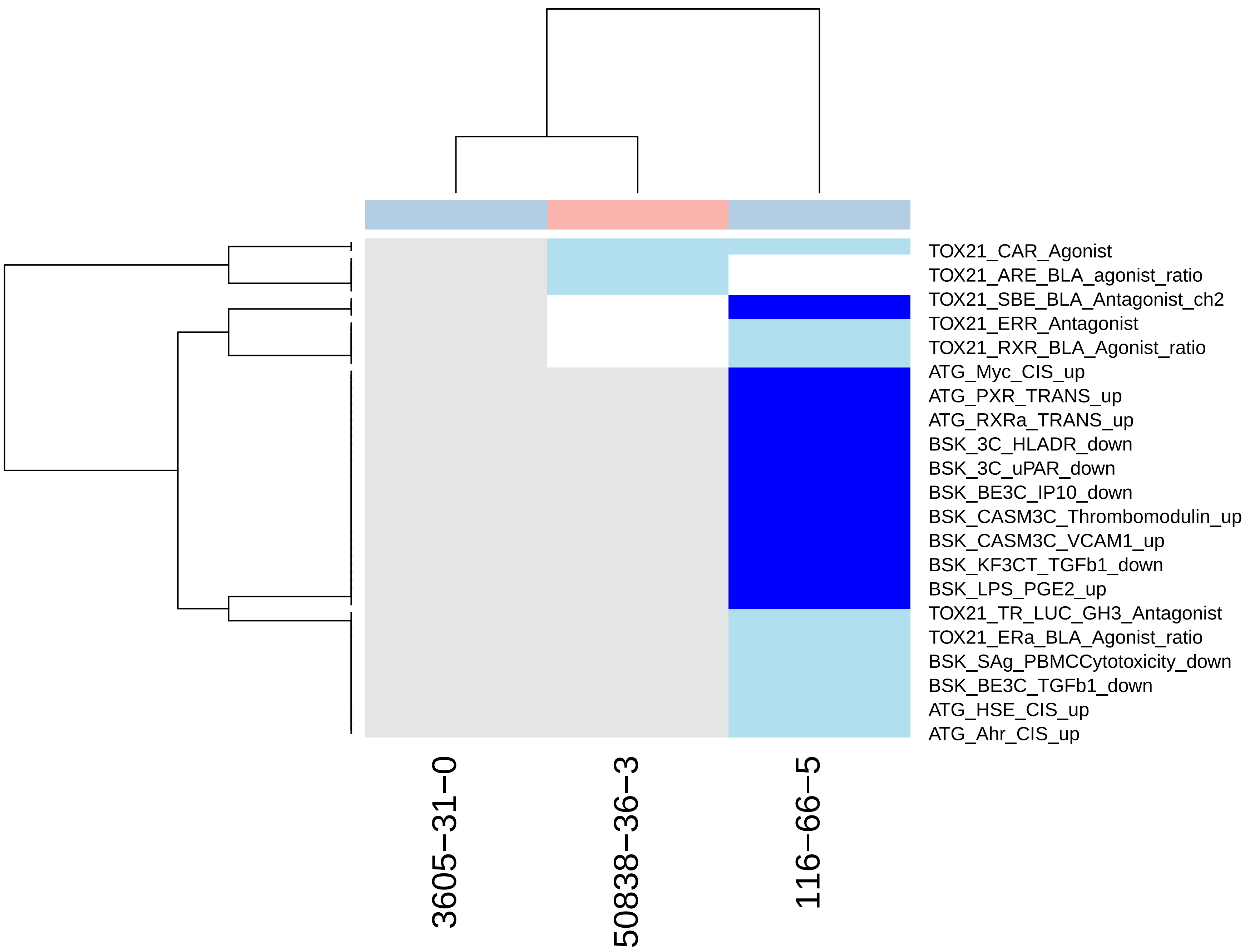

All Activity Heatmap for Cluster 683

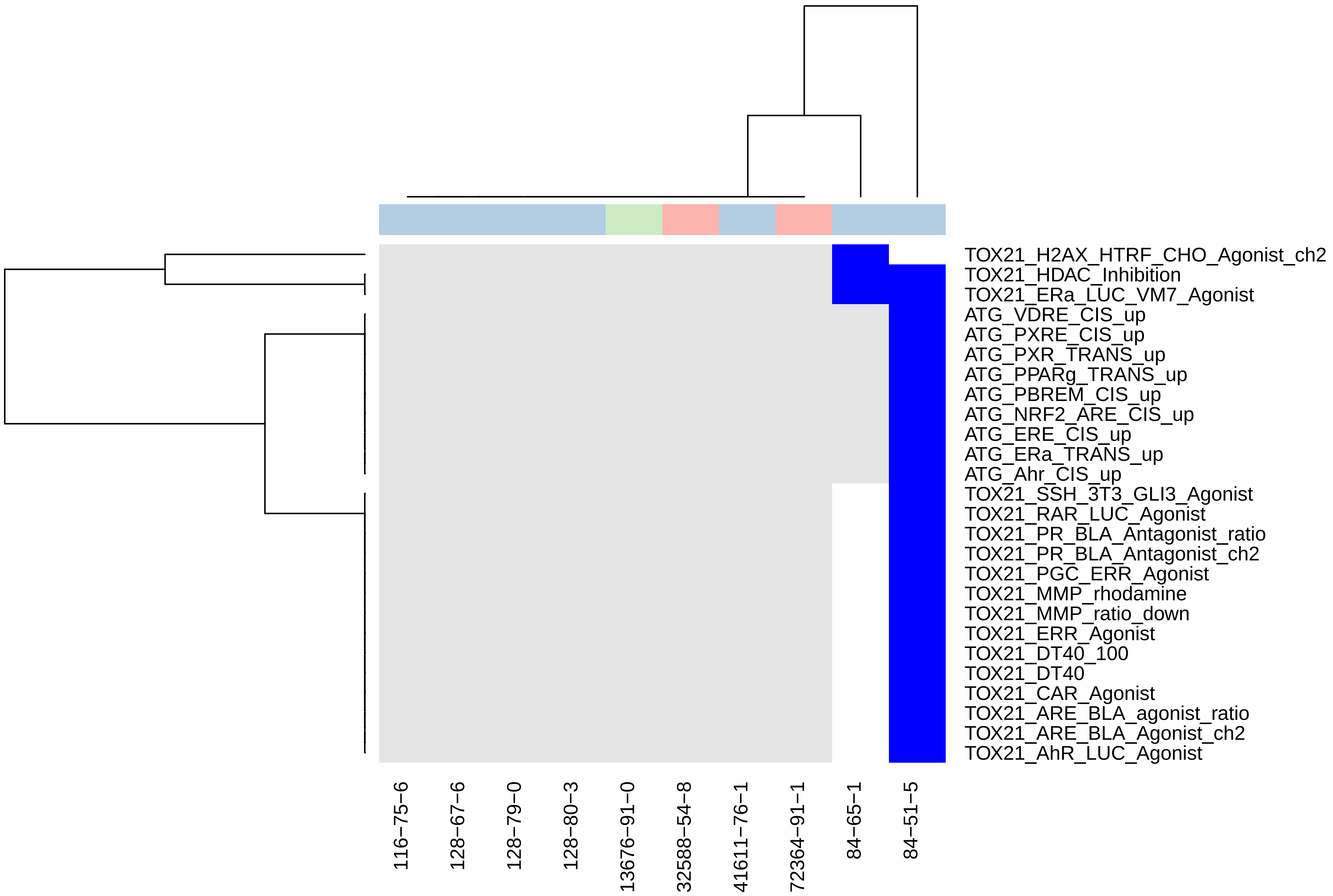

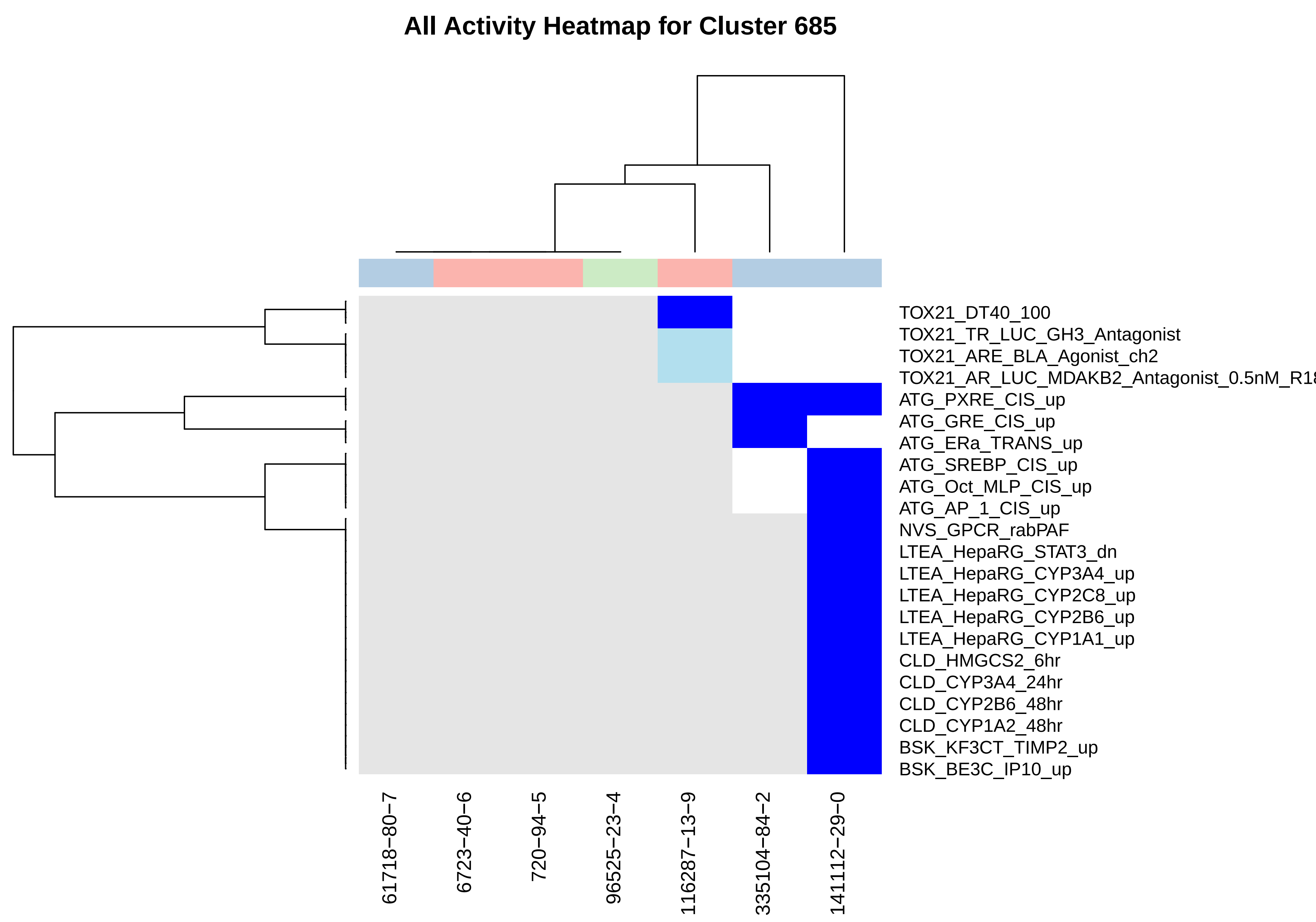
All Activity Heatmap for Cluster 685
TOX21_DT40_100
TOX21_TR_LUC_GH3_Antagonist
TOX21_ARE_BLA_Agonist_ch2
TOX21_AR_LUC_MDAKB2_Antagonist_0.5nM_R18
ATG_PXRE_CIS_up
ATG_GRE_CIS_up
ATG_ERa_TRANS_up
ATG_SREBP_CIS_up
ATG_Oct_MLP_CIS_up
ATG_AP_1_CIS_up
NVS_GPCR_rabPAF
LTEA_HepaRG_STAT3_dn
LTEA_HepaRG_CYP3A4_up
LTEA_HepaRG_CYP2C8_up
LTEA_HepaRG_CYP2B6_up
LTEA_HepaRG_CYP1A1_up
CLD_HMGCS2_6hr
CLD_CYP3A4_24hr
CLD_CYP2B6_48hr
CLD_CYP1A2_48hr
BSK_KF3CT_TIMP2_up
BSK_BE3C_IP10_up
61718−80−7
6723−40−6
720−94−5
96525−23−4
116287−13−9
335104−84−2
141112−29−0

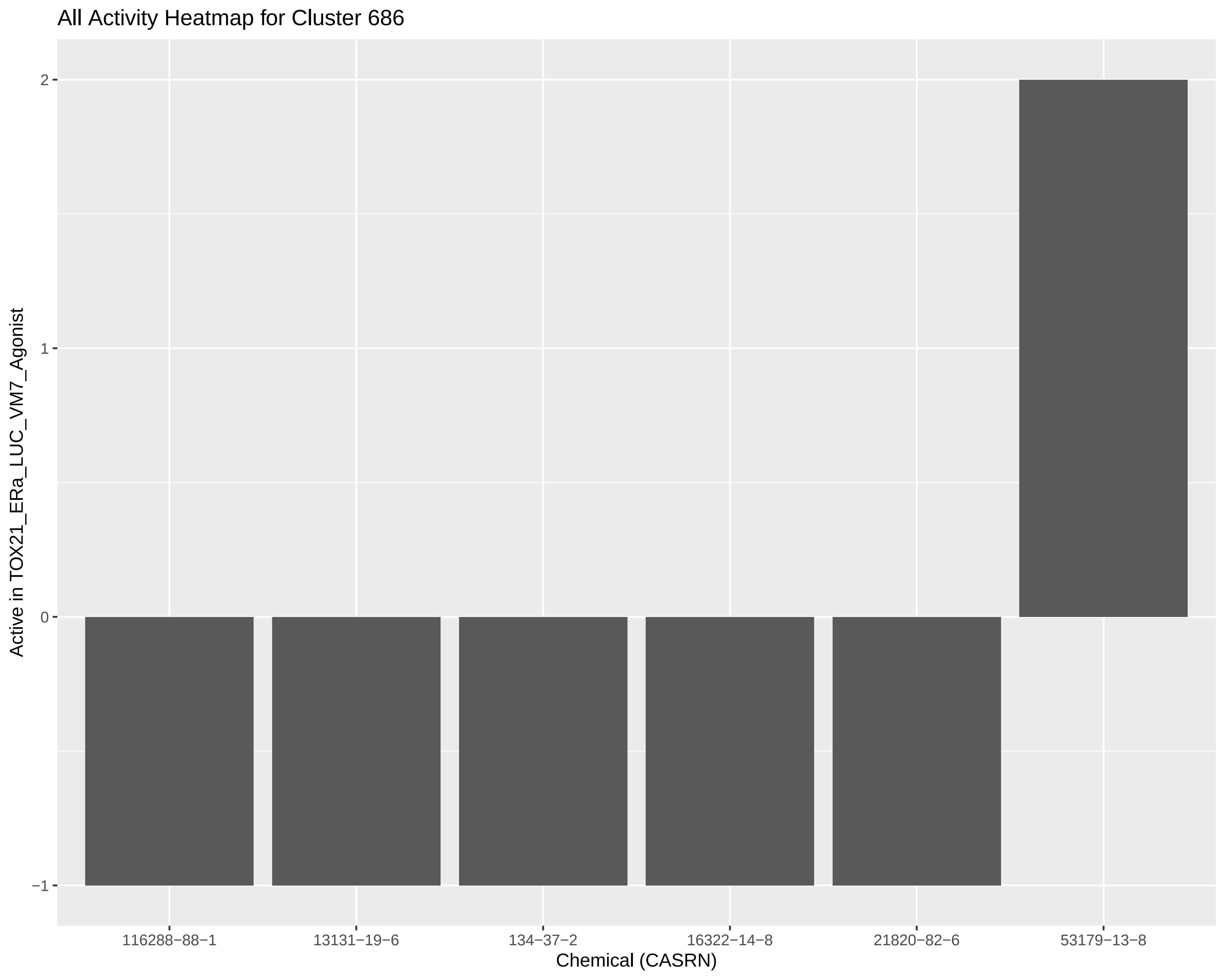

All Activity Heatmap for Cluster 686
Active in TOX21_ERa_LUC_VM7_Agonist
2
1
0
−1
116288−88−1
13131−19−6
134−37−2
16322−14−8
21820−82−6
53179−13−8
Chemical (CASRN)

All Activity Heatmap for Cluster 687

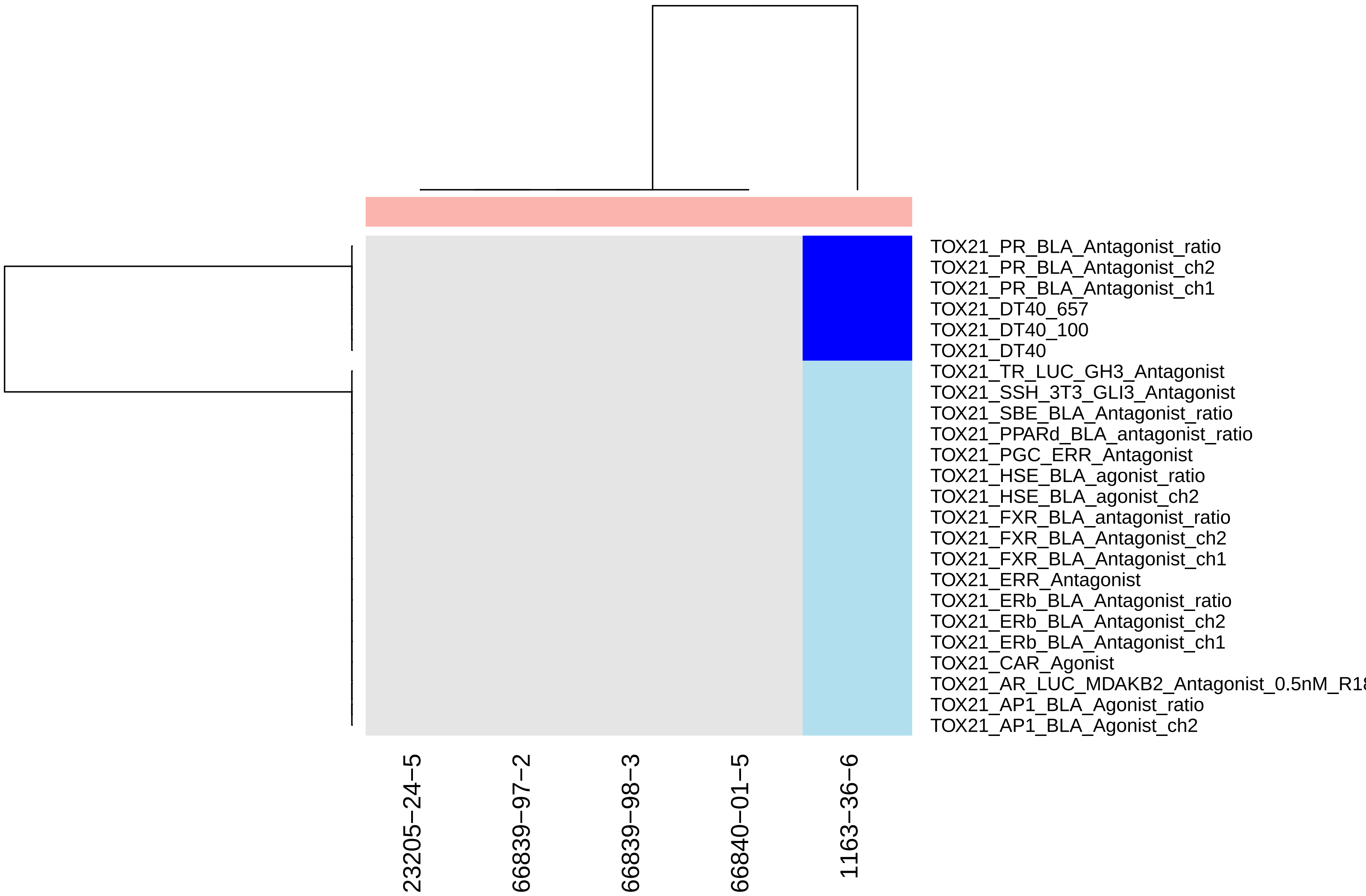

All Activity Heatmap for Cluster 690

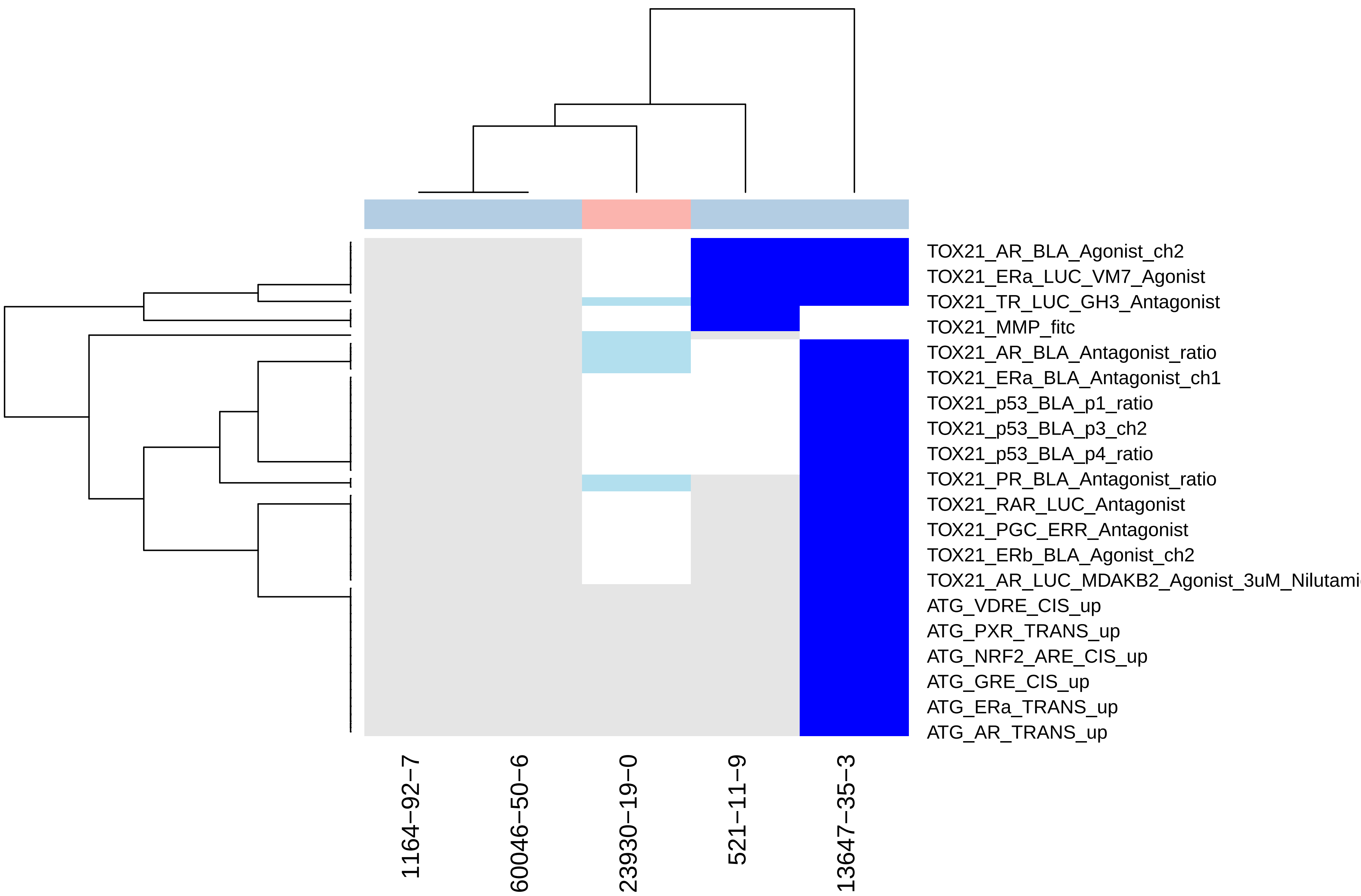

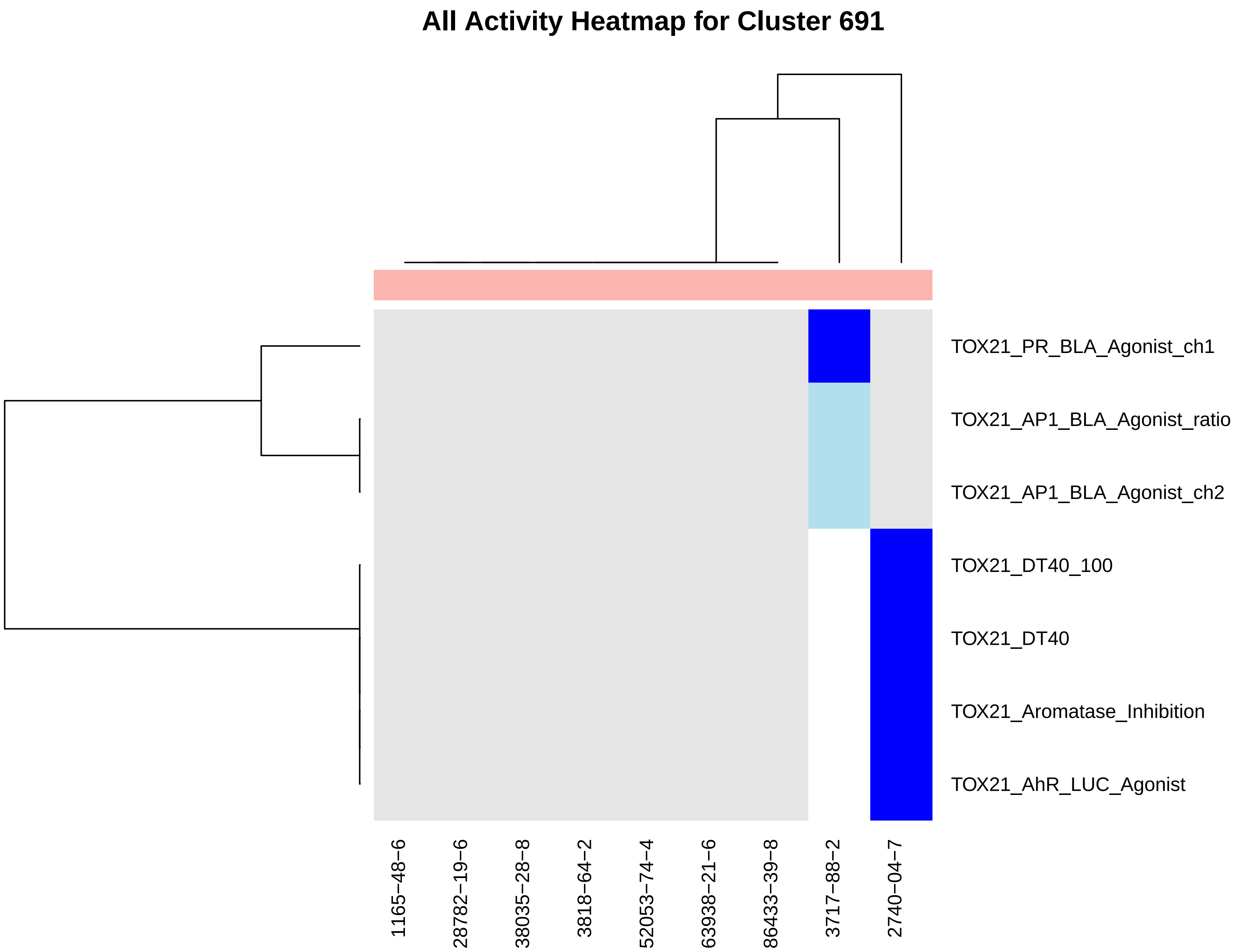
All Activity Heatmap for Cluster 691
TOX21_PR_BLA_Agonist_ch1
TOX21_AP1_BLA_Agonist_ratio
TOX21_AP1_BLA_Agonist_ch2
TOX21_DT40_100
TOX21_DT40
TOX21_Aromatase_Inhibition
TOX21_AhR_LUC_Agonist
1165−48−6
28782−19−6
38035−28−8
3818−64−2
52053−74−4
63938−21−6
86433−39−8
3717−88−2
2740−04−7

All Activity Heatmap for Cluster 692

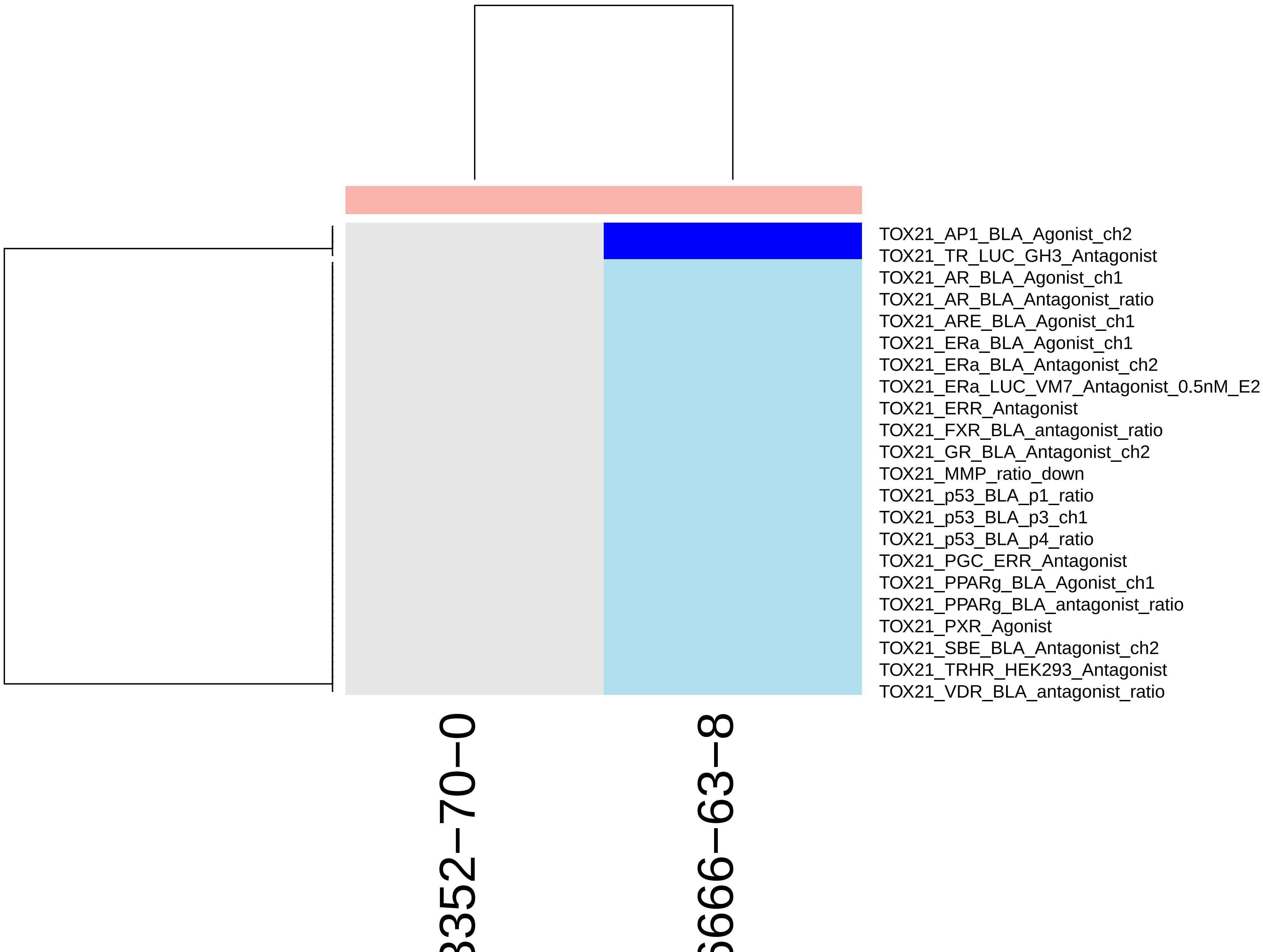

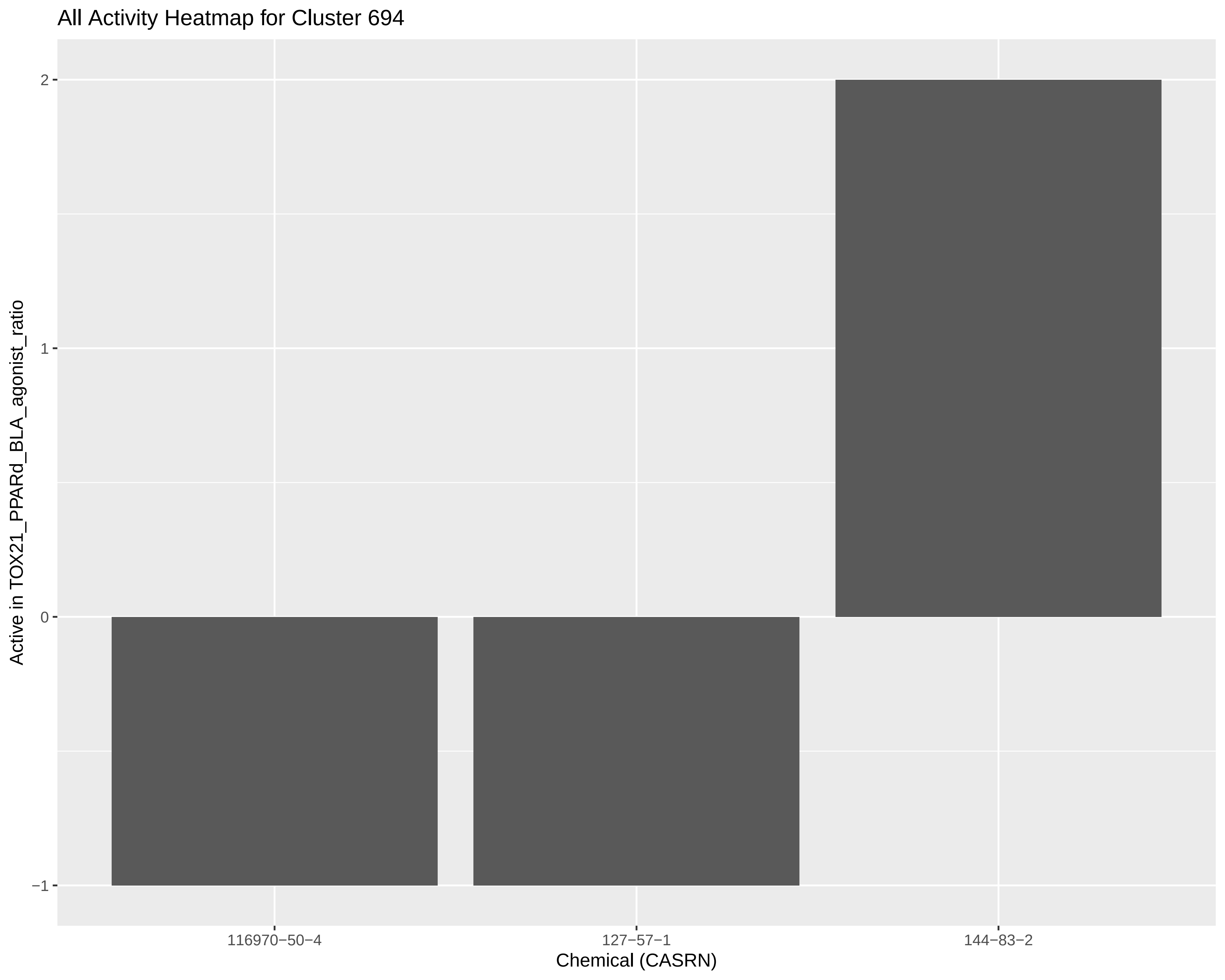
All Activity Heatmap for Cluster 694
Active in TOX21_PPARd_BLA_agonist_ratio
2
1
0
−1
116970−50−4
127−57−1
144−83−2
Chemical (CASRN)

All Activity Heatmap for Cluster 695

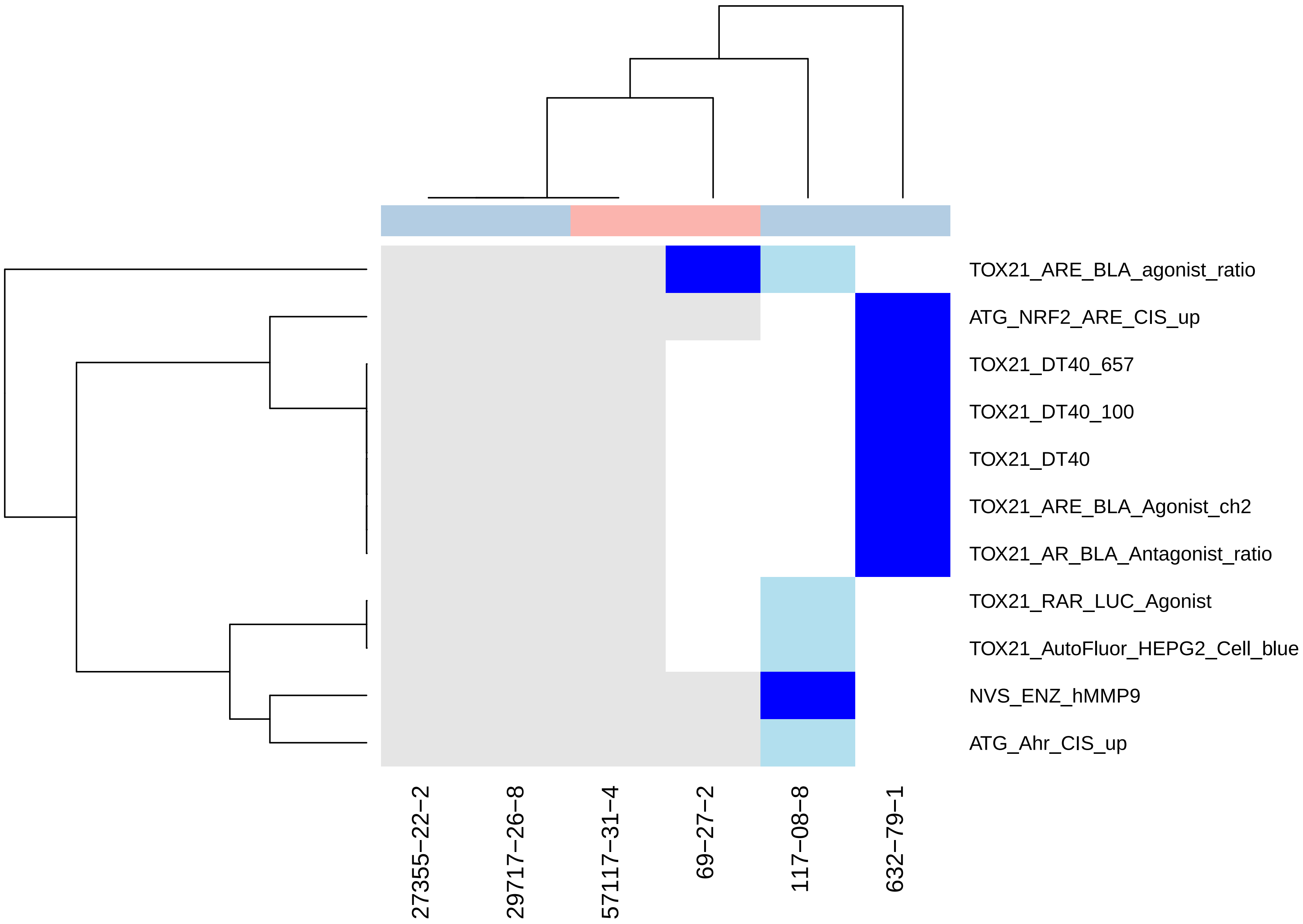

All Activity Heatmap for Cluster 697

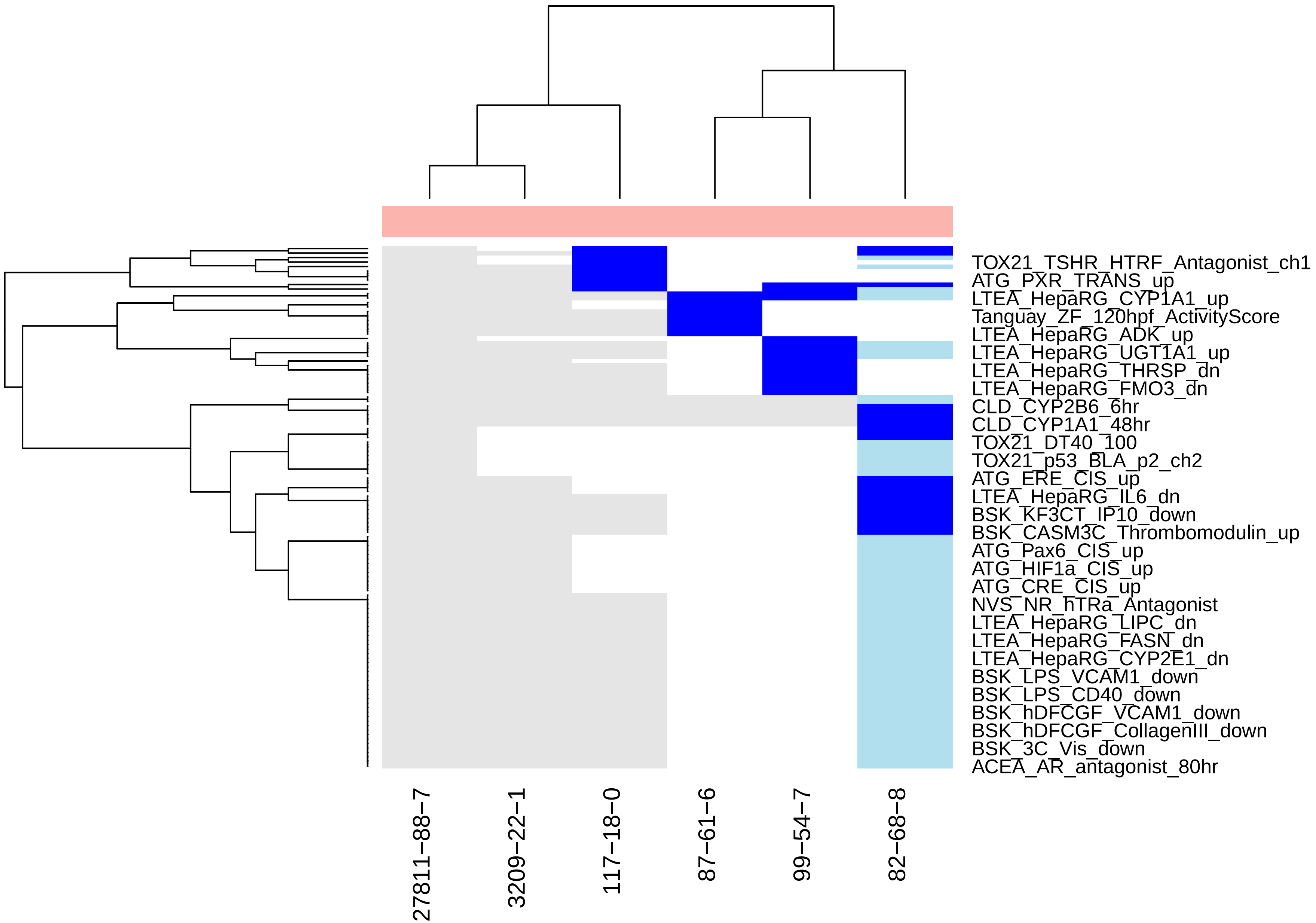

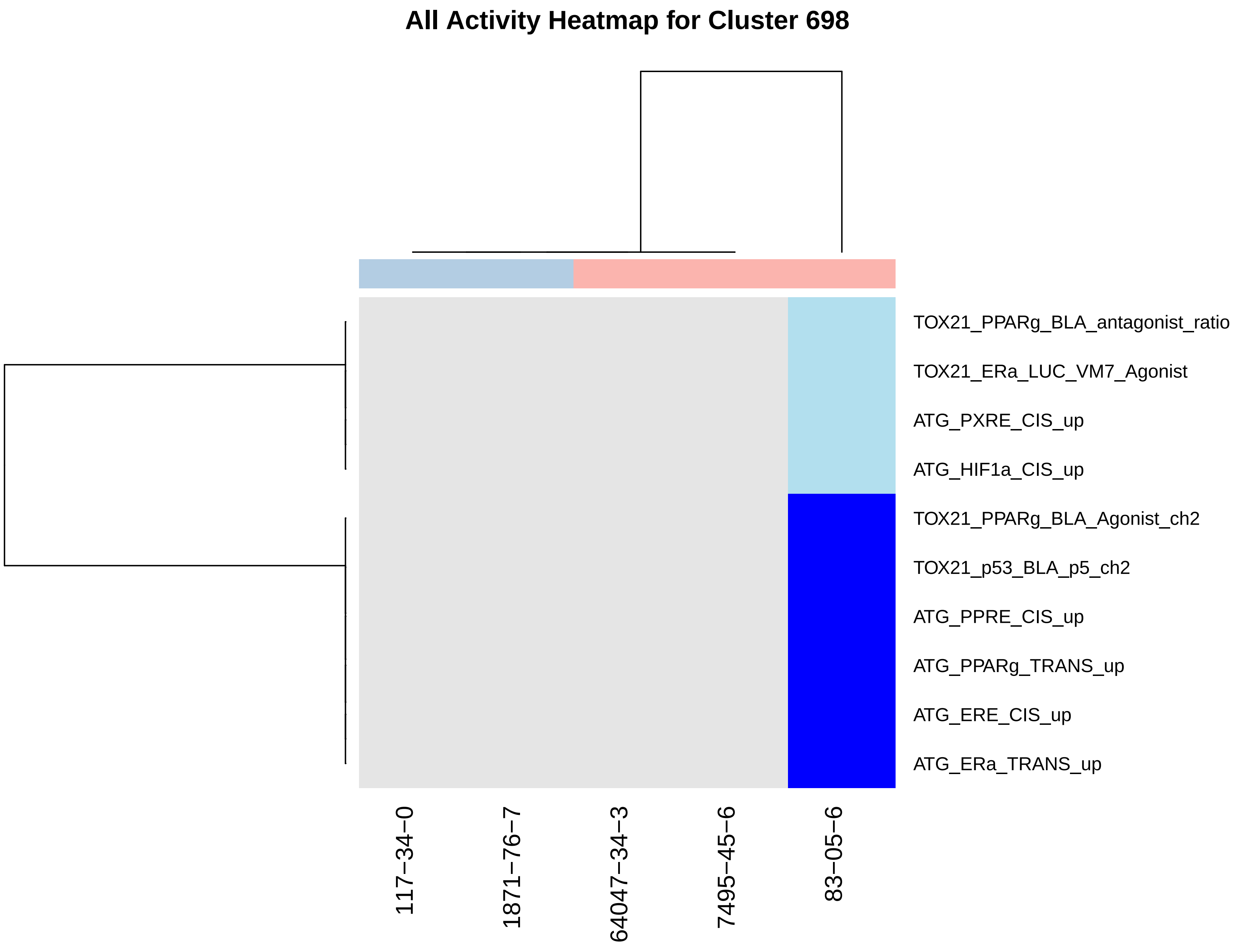
All Activity Heatmap for Cluster 698
TOX21_PPARg_BLA_antagonist_ratio
TOX21_ERa_LUC_VM7_Agonist
ATG_PXRE_CIS_up
ATG_HIF1a_CIS_up
TOX21_PPARg_BLA_Agonist_ch2
TOX21_p53_BLA_p5_ch2
ATG_PPRE_CIS_up
ATG_PPARg_TRANS_up
ATG_ERE_CIS_up
ATG_ERa_TRANS_up
117−34−0
1871−76−7
64047−34−3
7495−45−6
83−05−6

All Activity Heatmap for Cluster 699

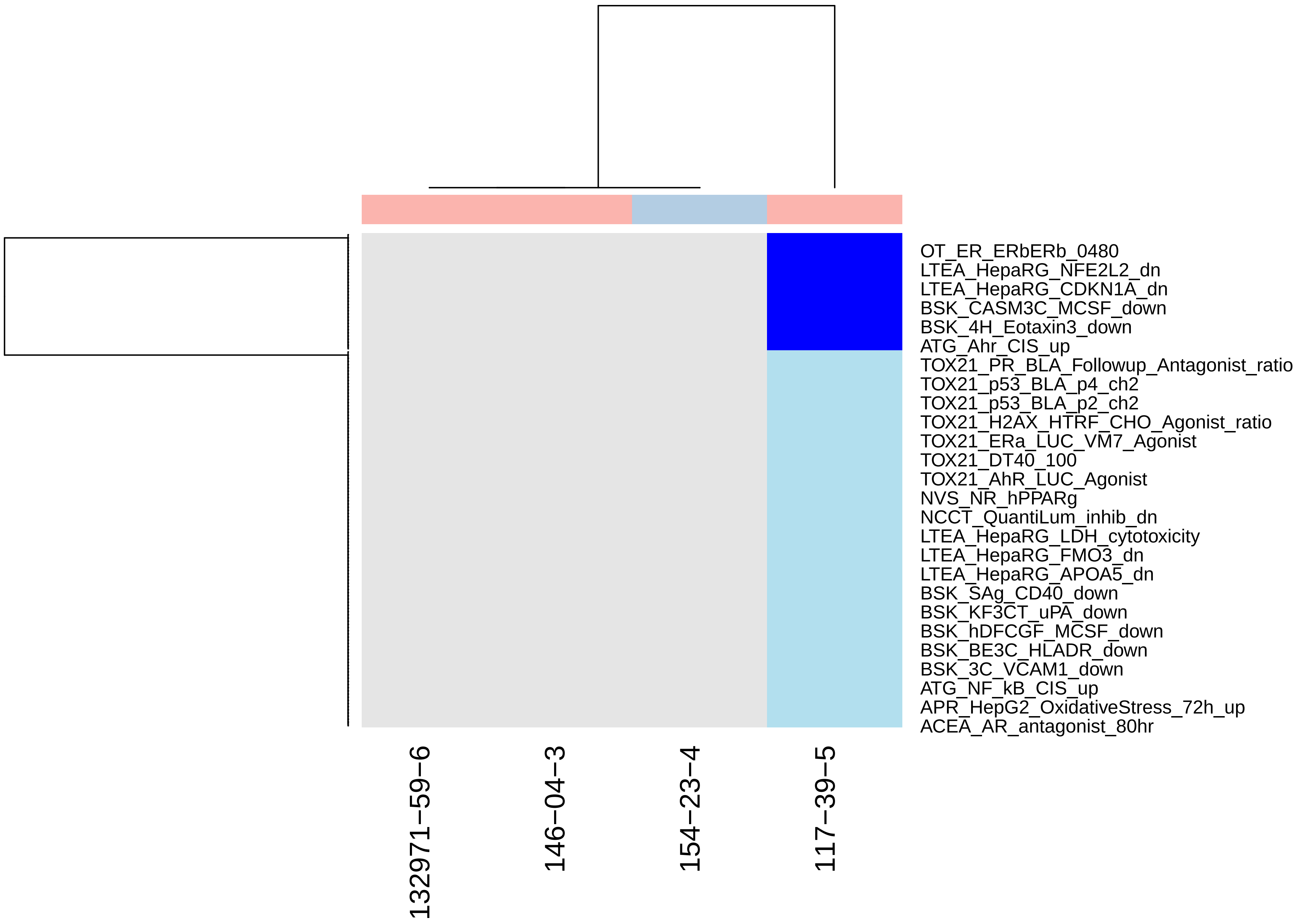

All Activity Heatmap for Cluster 700

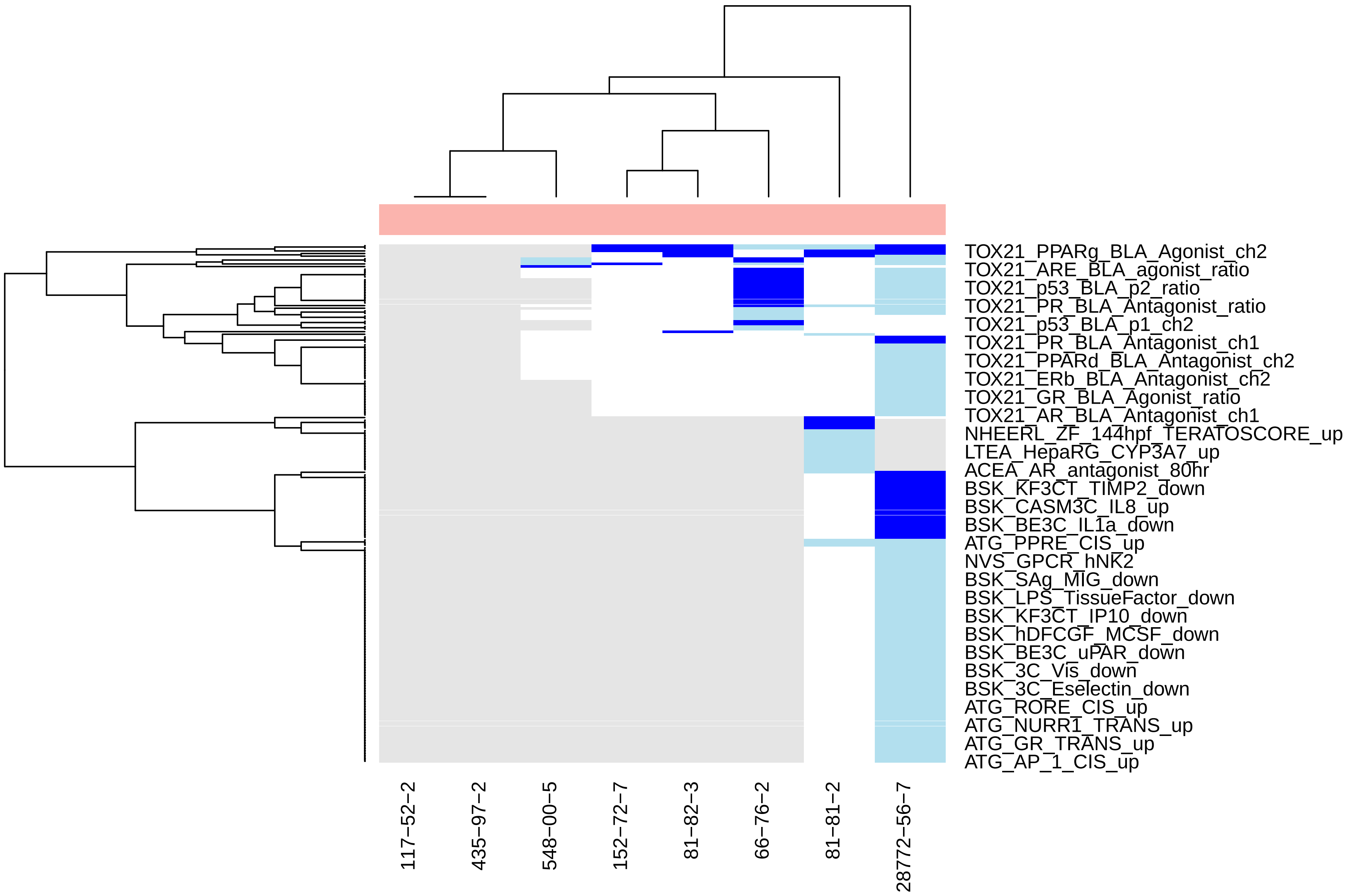

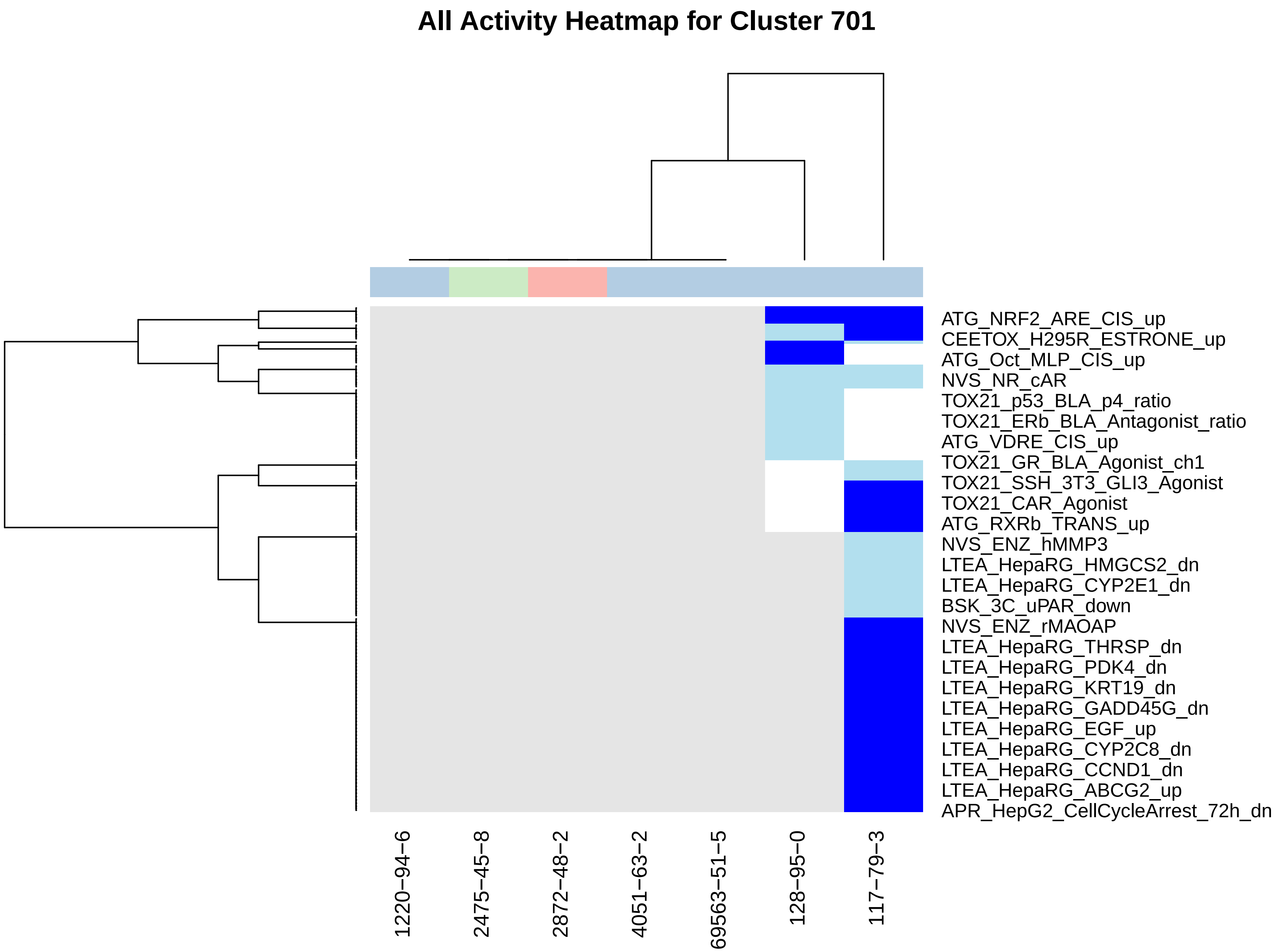

All Activity Heatmap for Cluster 701
ATG_NRF2_ARE_CIS_up
CEETOX_H295R_ESTRONE_up
ATG_Oct_MLP_CIS_up
NVS_NR_cAR
TOX21_p53_BLA_p4_ratio
TOX21_ERb_BLA_Antagonist_ratio
ATG_VDRE_CIS_up
TOX21_GR_BLA_Agonist_ch1
TOX21_SSH_3T3_GLI3_Agonist
TOX21_CAR_Agonist
ATG_RXRb_TRANS_up
NVS_ENZ_hMMP3
LTEA_HepaRG_HMGCS2_dn
LTEA_HepaRG_CYP2E1_dn
BSK_3C_uPAR_down
NVS_ENZ_rMAOAP
LTEA_HepaRG_THRSP_dn
LTEA_HepaRG_PDK4_dn
LTEA_HepaRG_KRT19_dn
LTEA_HepaRG_GADD45G_dn
LTEA_HepaRG_EGF_up
LTEA_HepaRG_CYP2C8_dn
LTEA_HepaRG_CCND1_dn
LTEA_HepaRG_ABCG2_up
APR_HepG2_CellCycleArrest_72h_dn
1220−94−6
2475−45−8
2872−48−2
4051−63−2
69563−51−5
128−95−0
117−79−3

All Activity Heatmap for Cluster 702

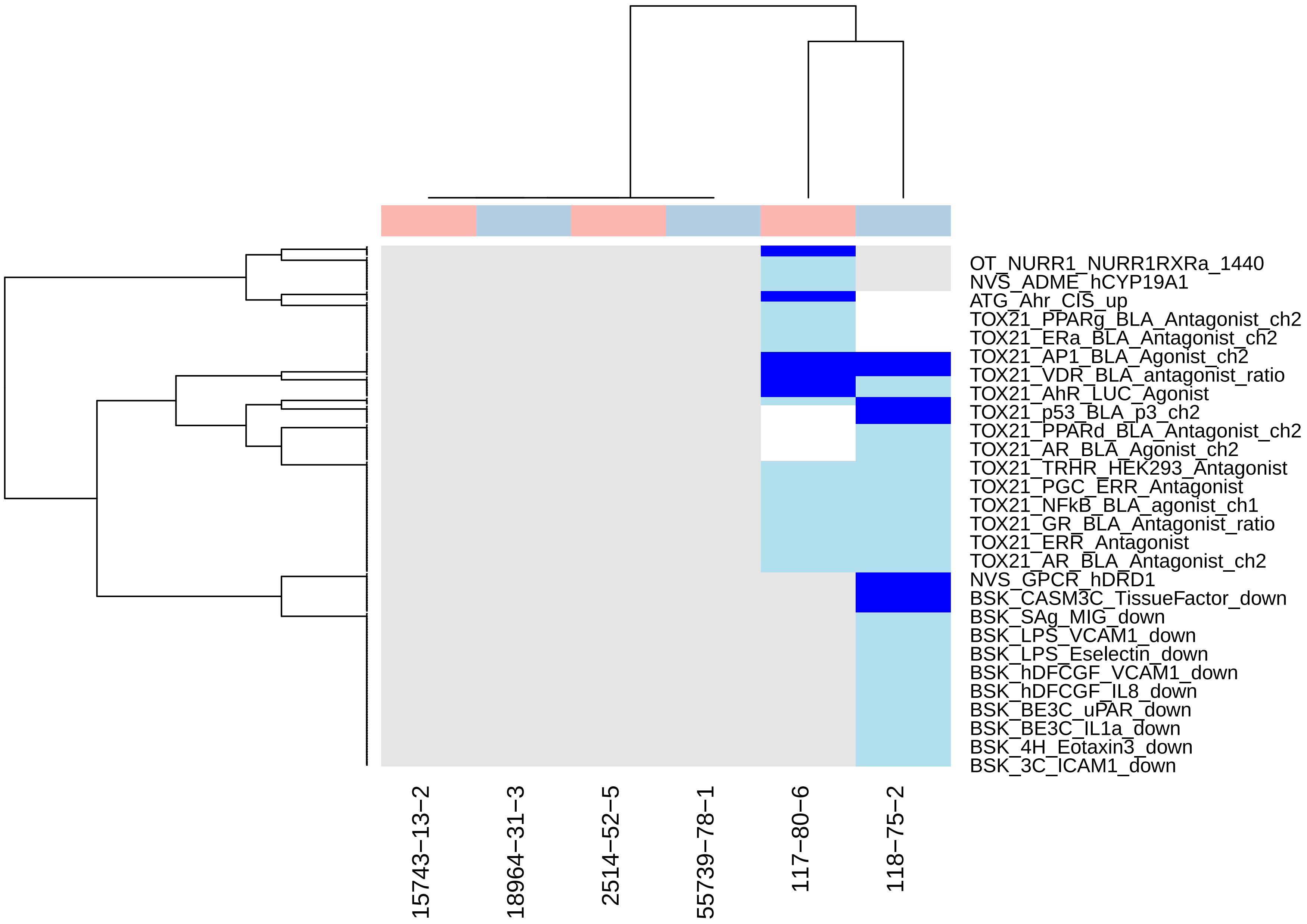

All Activity Heatmap for Cluster 703

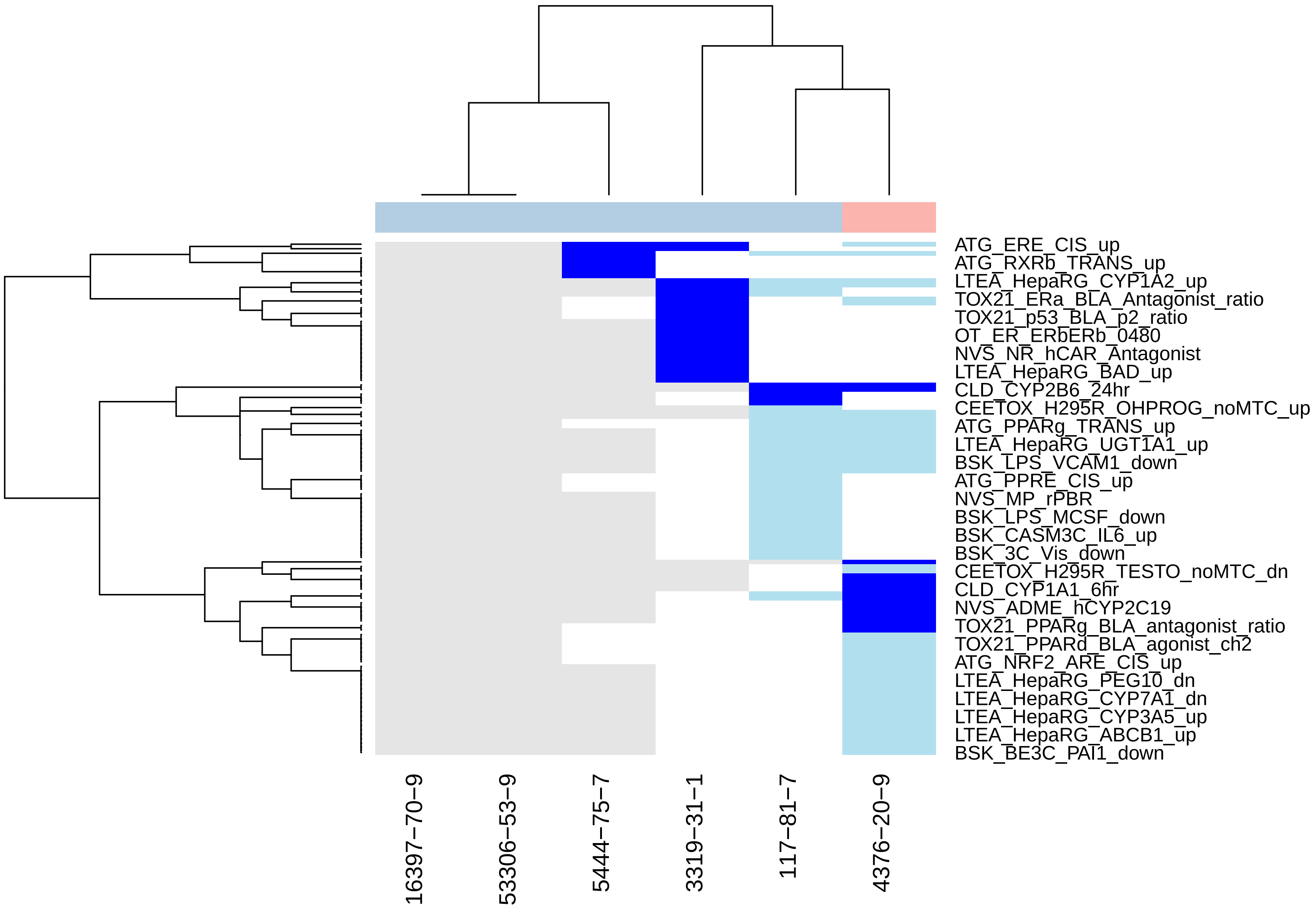

All Activity Heatmap for Cluster 704

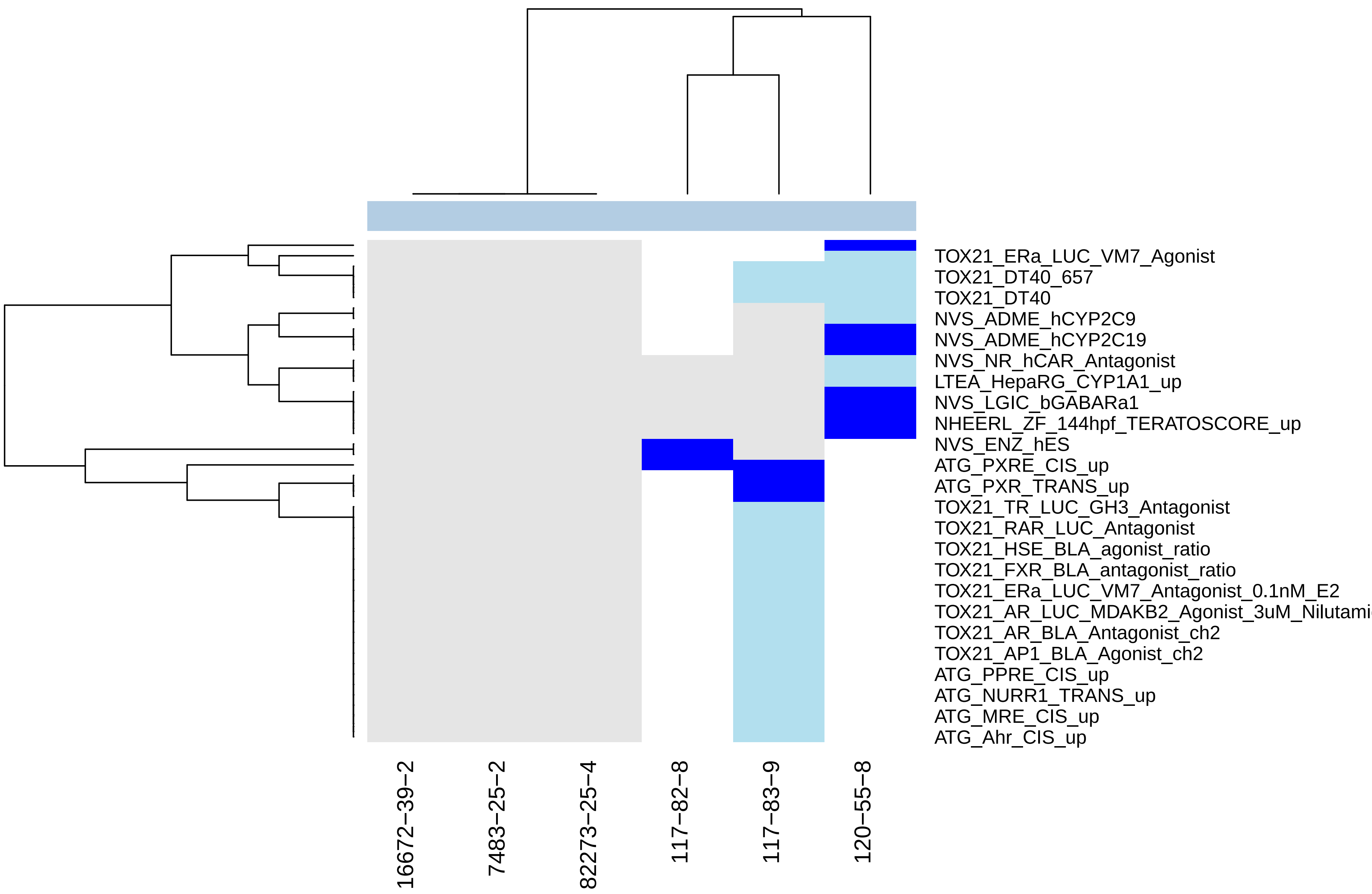

All Activity Heatmap for Cluster 705

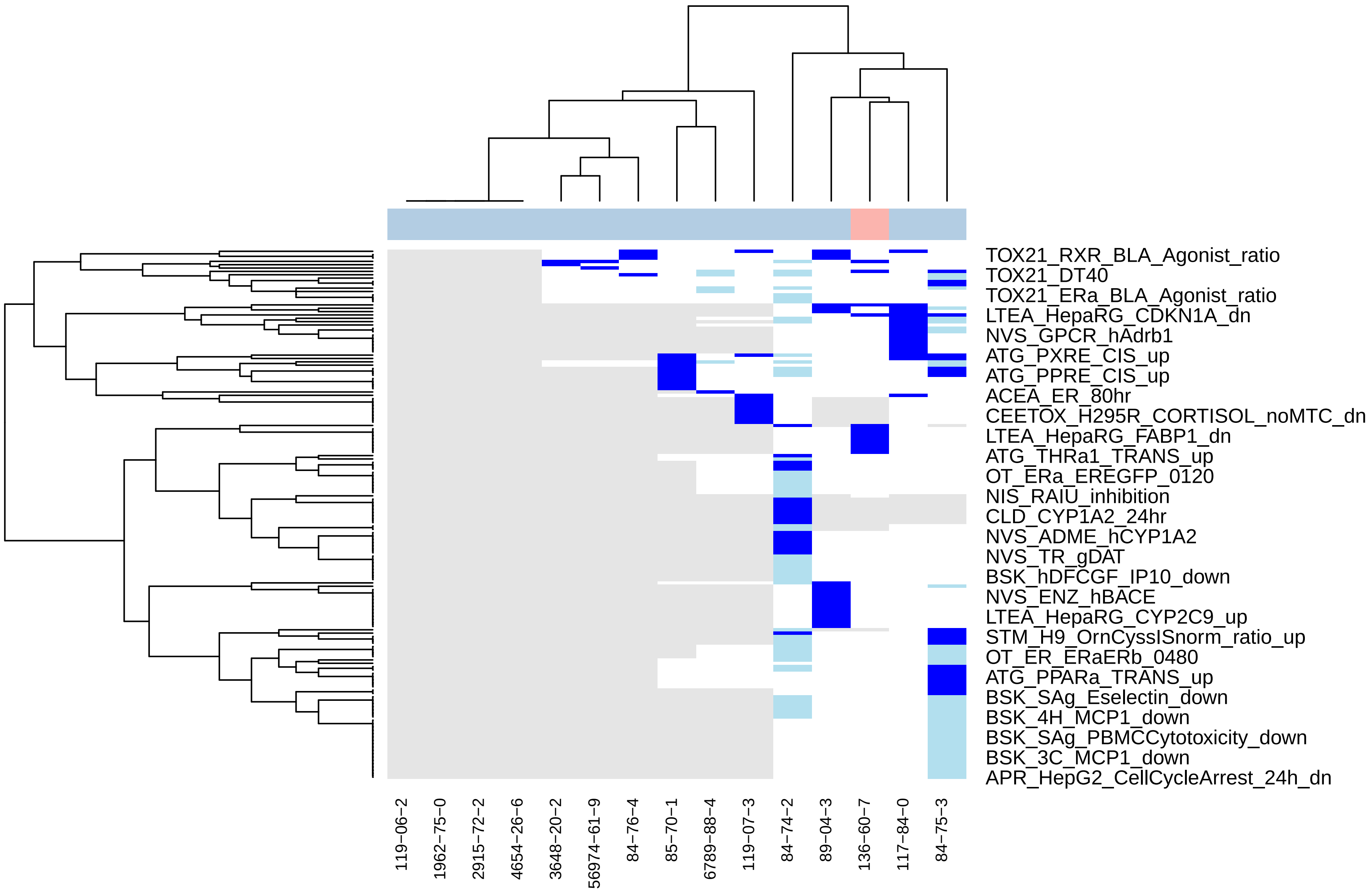

All Activity Heatmap for Cluster 706

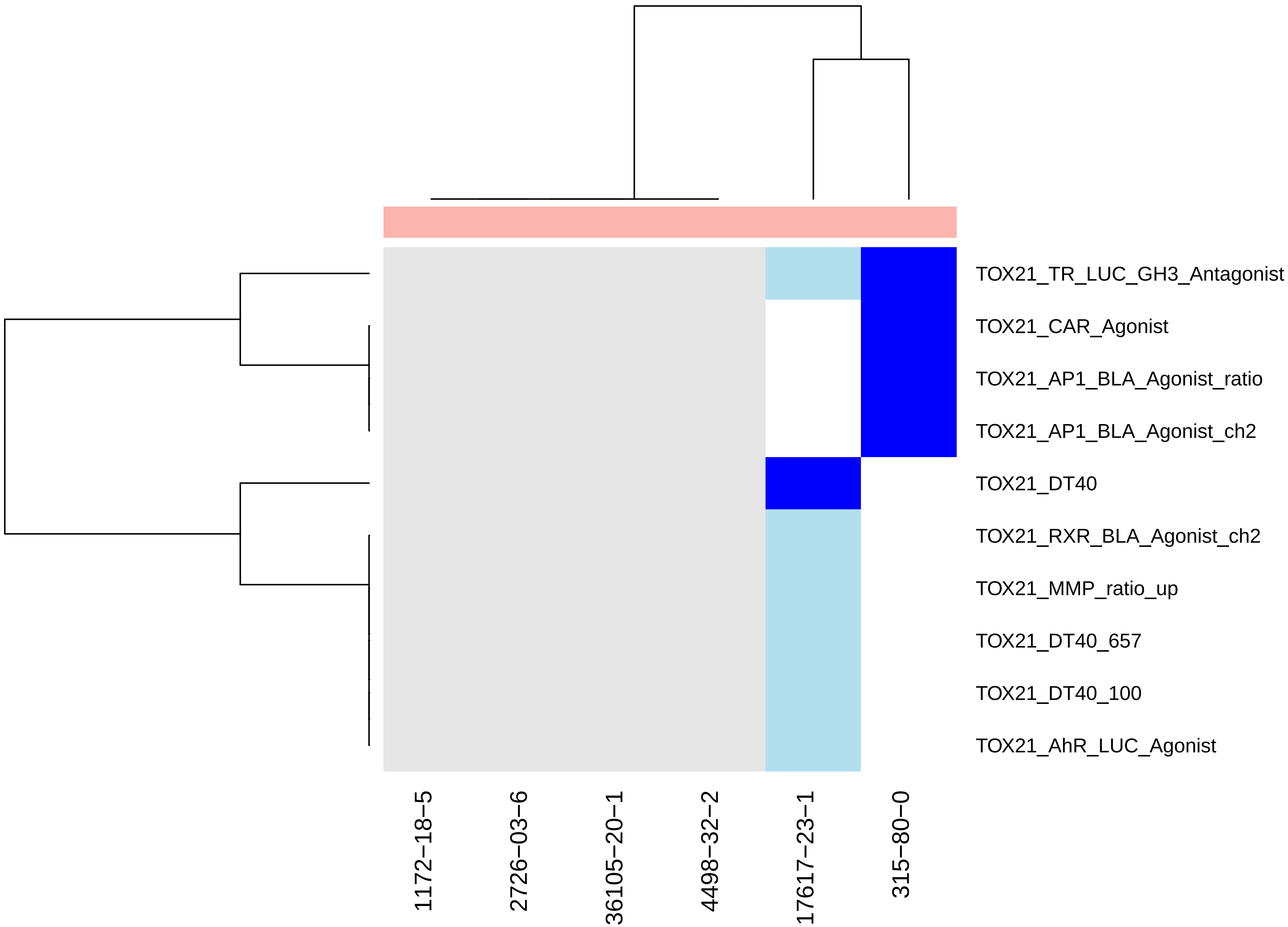

All Activity Heatmap for Cluster 707

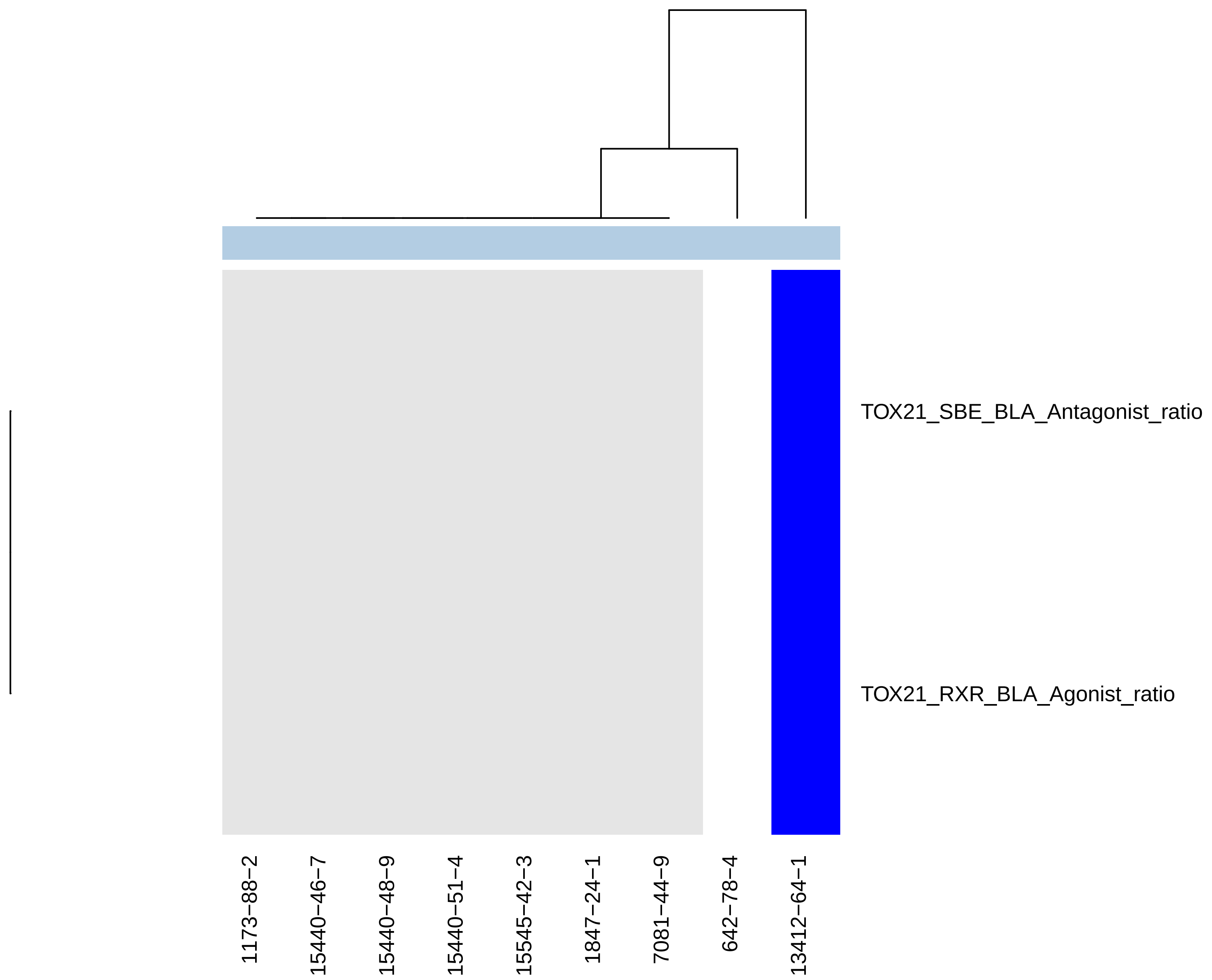

All Activity Heatmap for Cluster 710

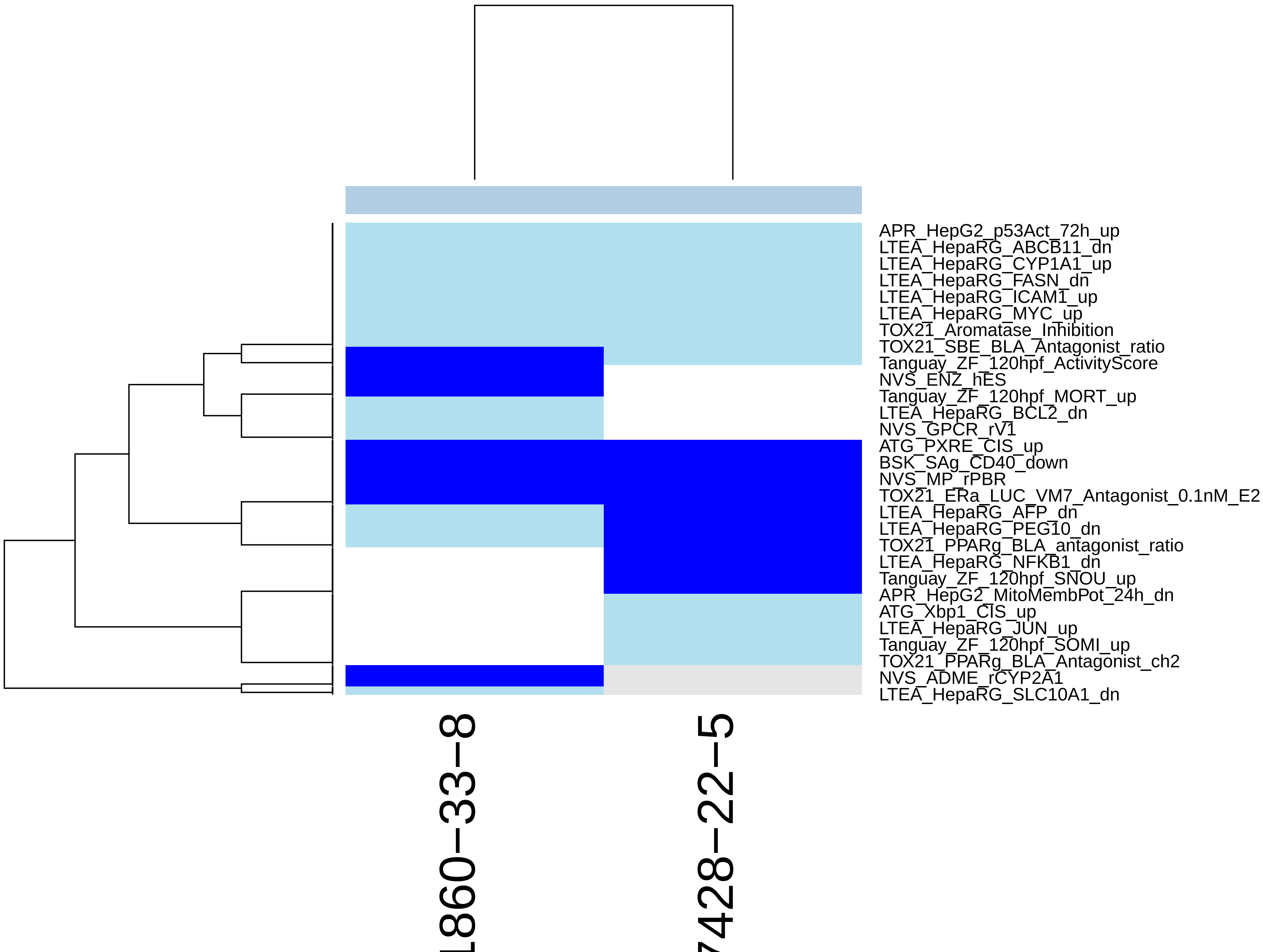

All Activity Heatmap for Cluster 711

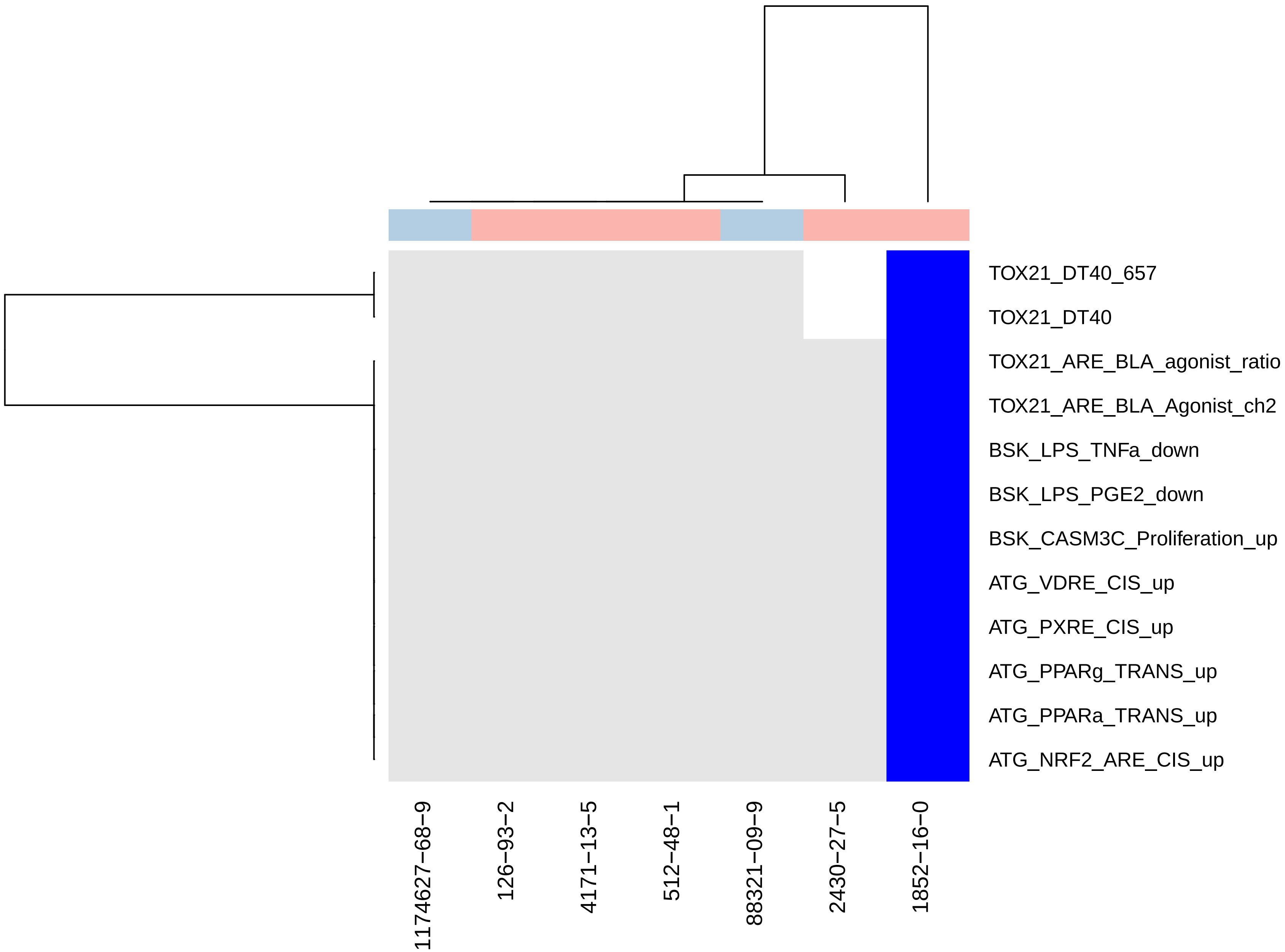

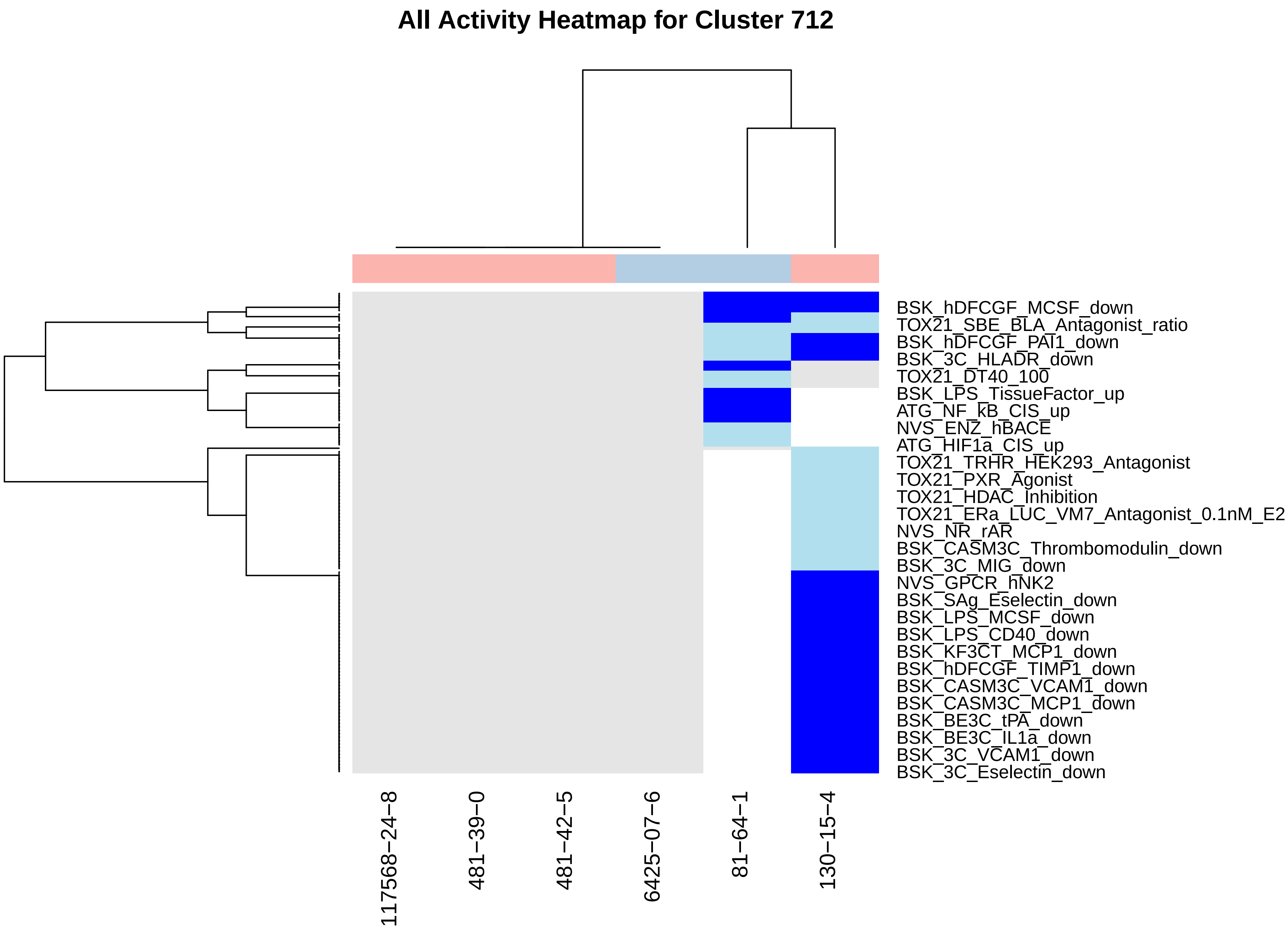
All Activity Heatmap for Cluster 712
BSK_hDFCGF_MCSF_down
TOX21_SBE_BLA_Antagonist_ratio
BSK_hDFCGF_PAI1_down
BSK_3C_HLADR_down
TOX21_DT40_100
BSK_LPS_TissueFactor_up
ATG_NF_kB_CIS_up
NVS_ENZ_hBACE
ATG_HIF1a_CIS_up
TOX21_TRHR_HEK293_Antagonist
TOX21_PXR_Agonist
TOX21_HDAC_Inhibition
TOX21_ERa_LUC_VM7_Antagonist_0.1nM_E2
NVS_NR_rAR
BSK_CASM3C_Thrombomodulin_down
BSK_3C_MIG_down
NVS_GPCR_hNK2
BSK_SAg_Eselectin_down
BSK_LPS_MCSF_down
BSK_LPS_CD40_down
BSK_KF3CT_MCP1_down
BSK_hDFCGF_TIMP1_down
BSK_CASM3C_VCAM1_down
BSK_CASM3C_MCP1_down
BSK_BE3C_tPA_down
BSK_BE3C_IL1a_down
BSK_3C_VCAM1_down
BSK_3C_Eselectin_down
117568−24−8
481−39−0
481−42−5
6425−07−6
81−64−1
130−15−4

All Activity Heatmap for Cluster 713

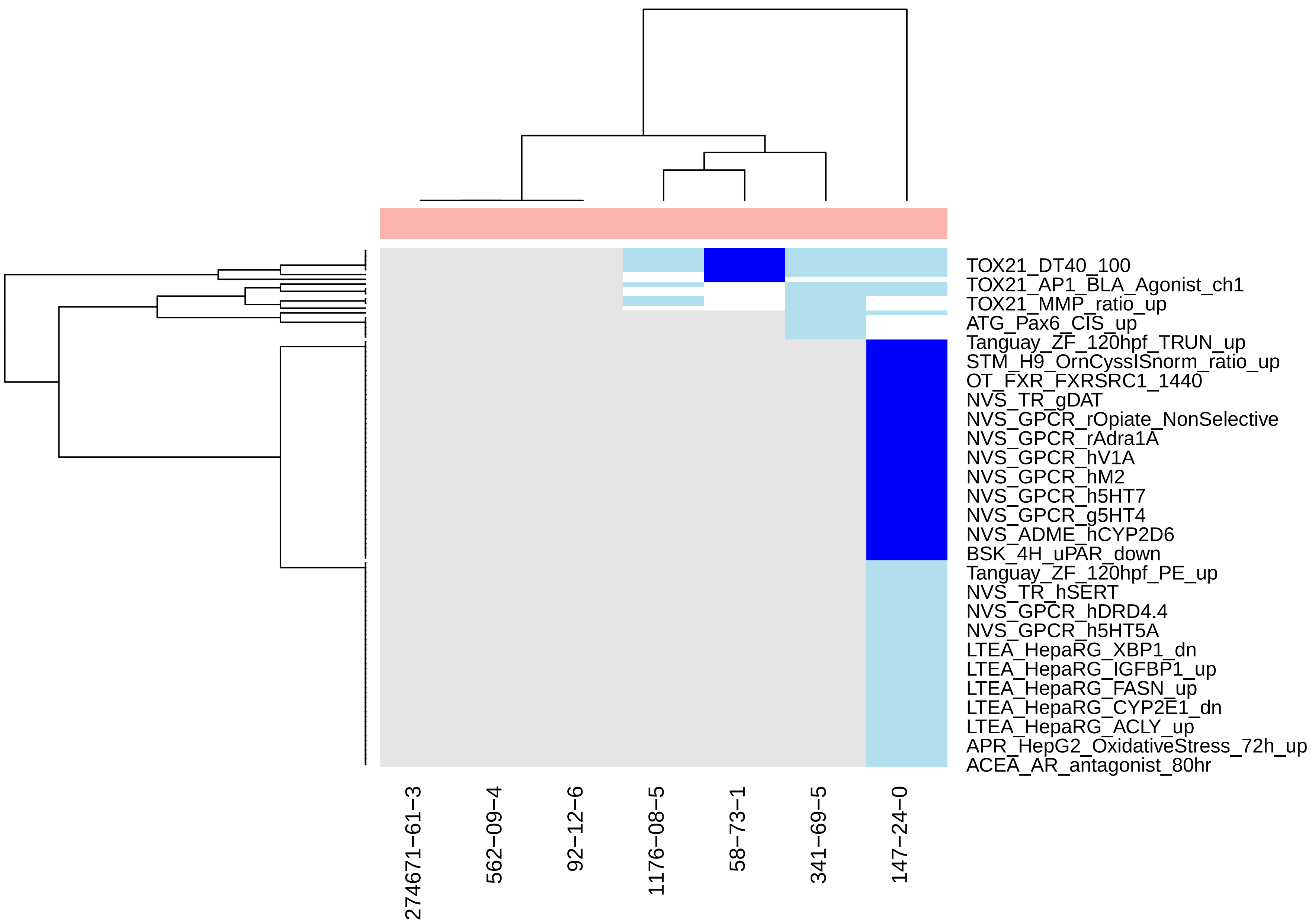

All Activity Heatmap for Cluster 716

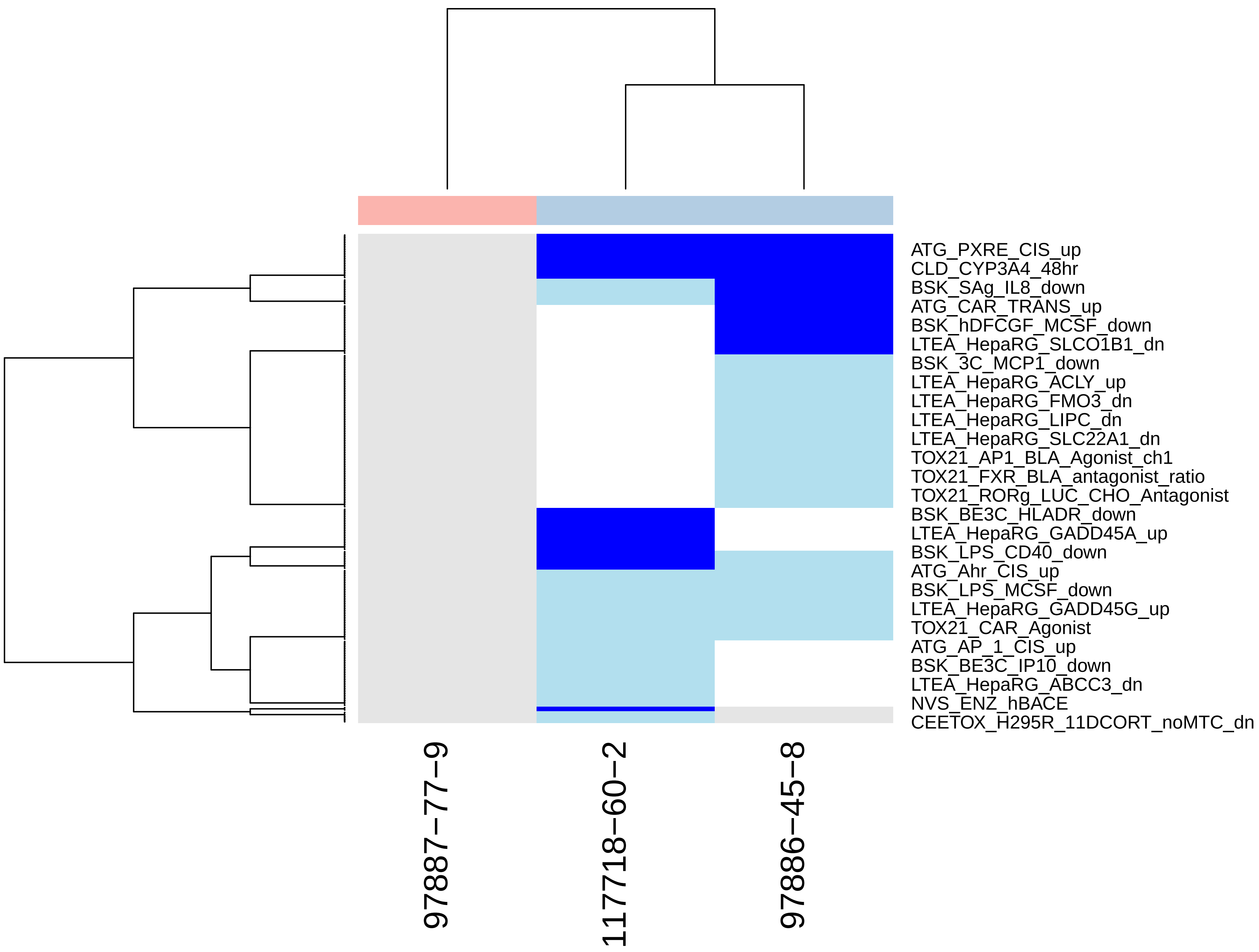

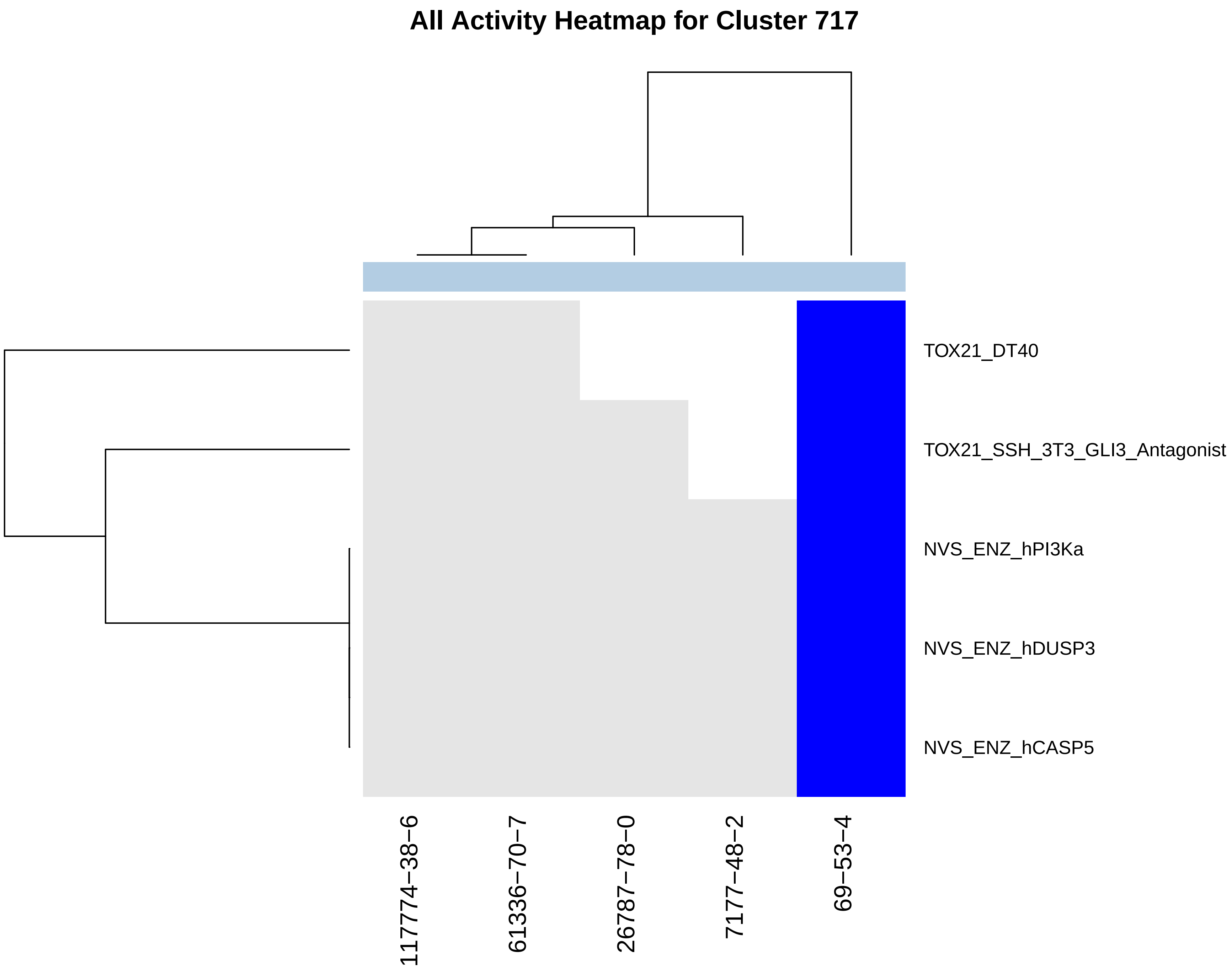
All Activity Heatmap for Cluster 717
TOX21_DT40
TOX21_SSH_3T3_GLI3_Antagonist
NVS_ENZ_hPI3Ka
NVS_ENZ_hDUSP3
NVS_ENZ_hCASP5
117774−38−6
61336−70−7
26787−78−0
7177−48−2
69−53−4

All Activity Heatmap for Cluster 718

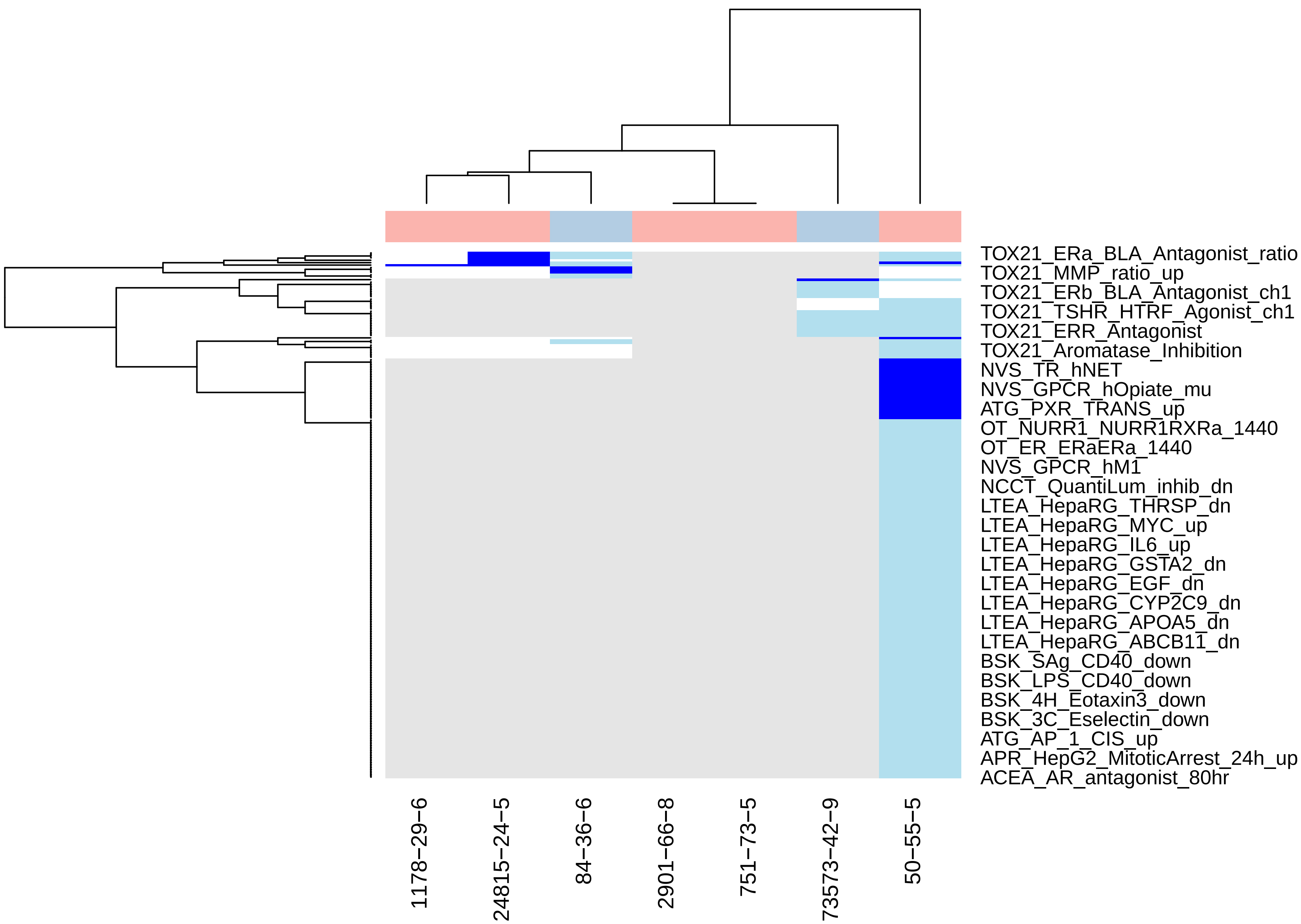

All Activity Heatmap for Cluster 720

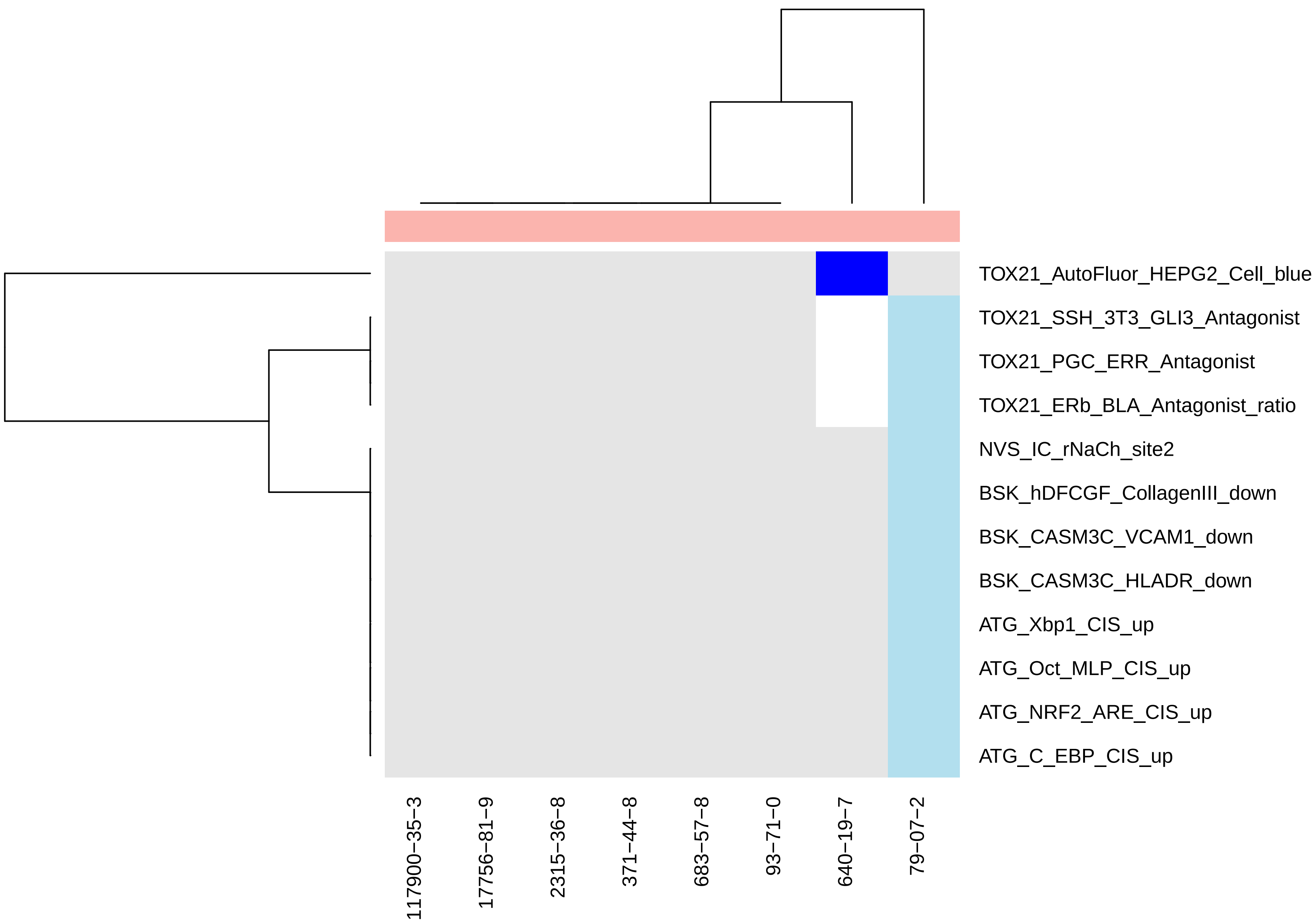

All Activity Heatmap for Cluster 721

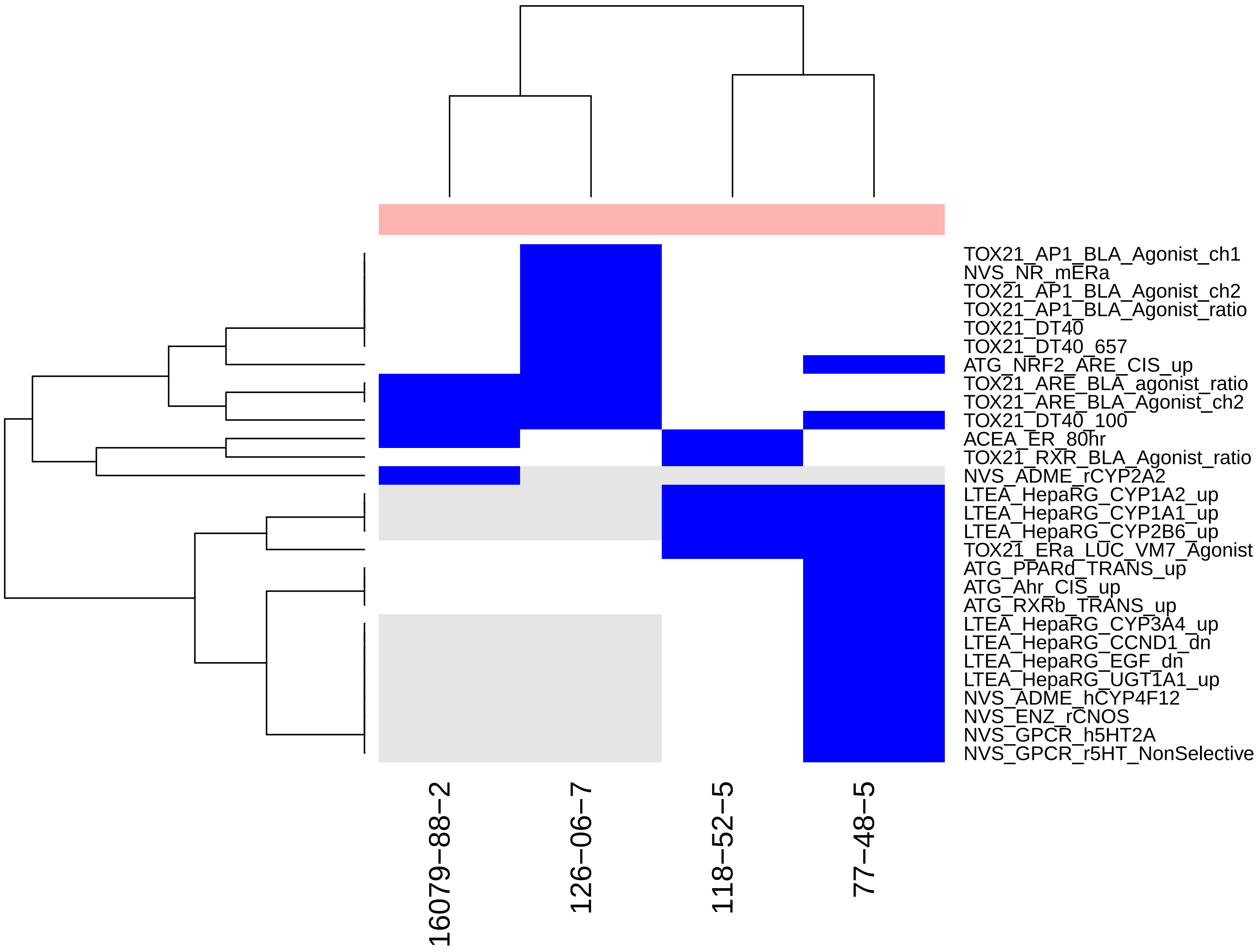

All Activity Heatmap for Cluster 722

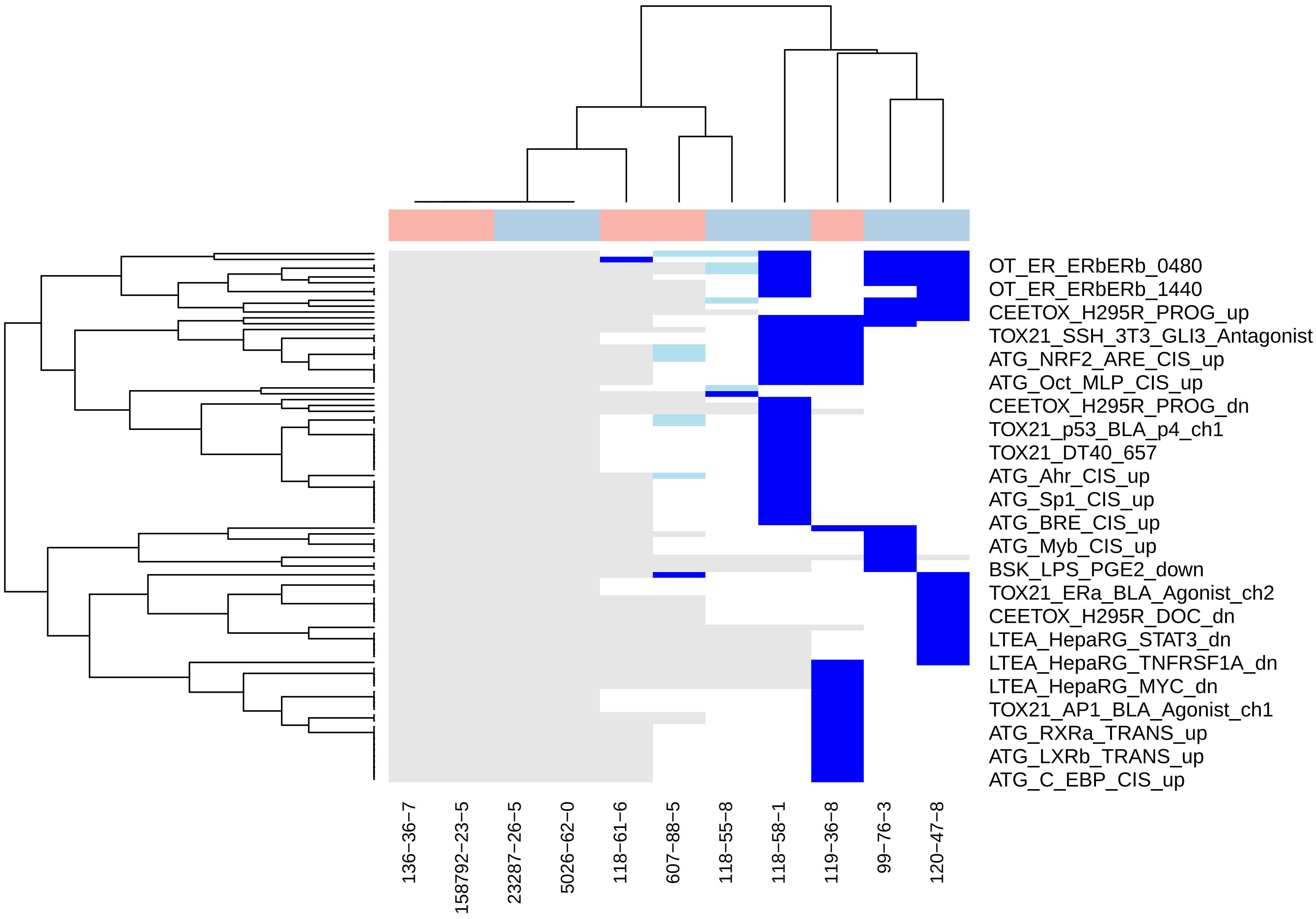

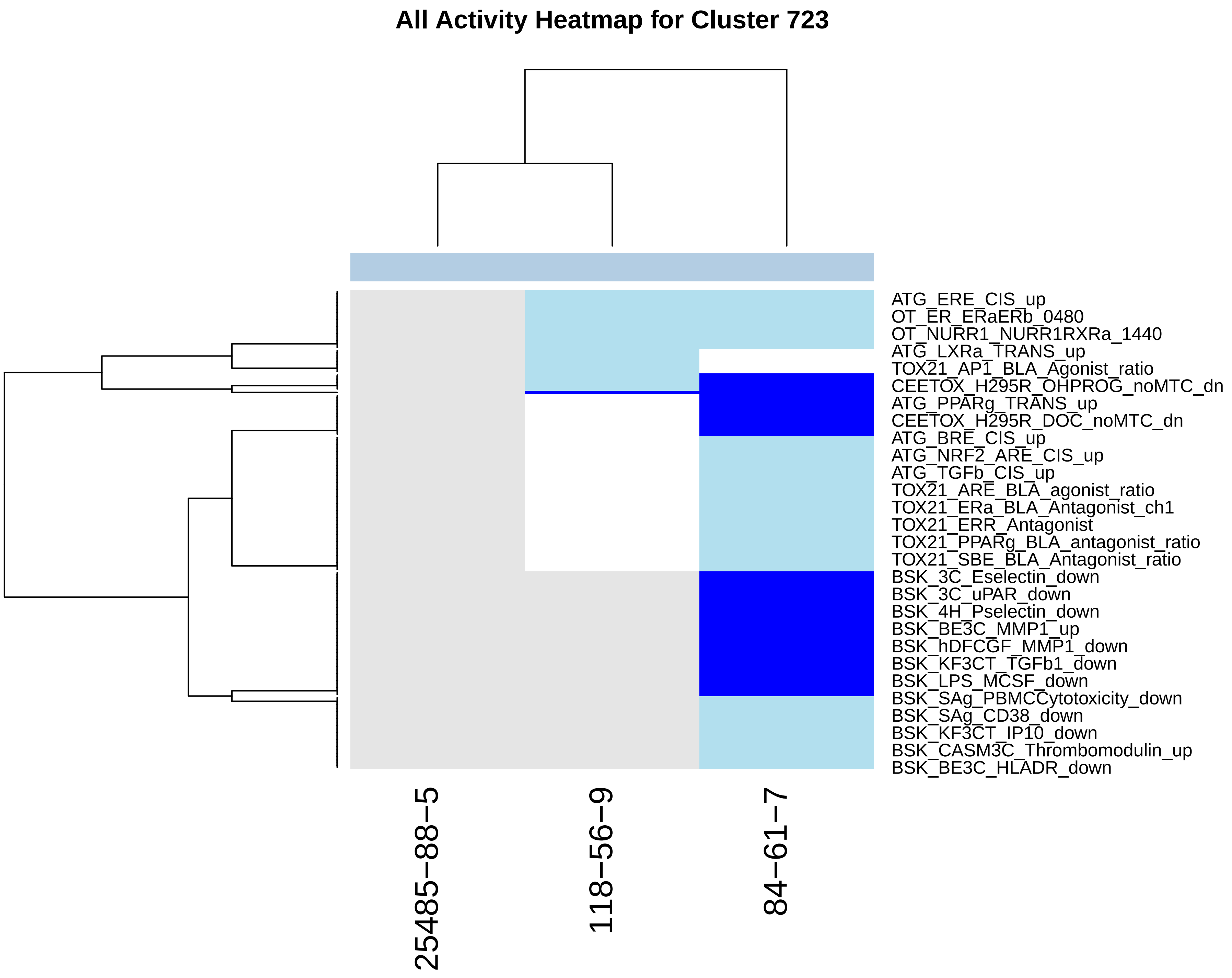

All Activity Heatmap for Cluster 723
ATG_ERE_CIS_up
OT_ER_ERaERb_0480
OT_NURR1_NURR1RXRa_1440
ATG_LXRa_TRANS_up
TOX21_AP1_BLA_Agonist_ratio
CEETOX_H295R_OHPROG_noMTC_dn
ATG_PPARg_TRANS_up
CEETOX_H295R_DOC_noMTC_dn
ATG_BRE_CIS_up
ATG_NRF2_ARE_CIS_up
ATG_TGFb_CIS_up
TOX21_ARE_BLA_agonist_ratio
TOX21_ERa_BLA_Antagonist_ch1
TOX21_ERR_Antagonist
TOX21_PPARg_BLA_antagonist_ratio
TOX21_SBE_BLA_Antagonist_ratio
BSK_3C_Eselectin_down
BSK_3C_uPAR_down
BSK_4H_Pselectin_down
BSK_BE3C_MMP1_up
BSK_hDFCGF_MMP1_down
BSK_KF3CT_TGFb1_down
BSK_LPS_MCSF_down
BSK_SAg_PBMCCytotoxicity_down
BSK_SAg_CD38_down
BSK_KF3CT_IP10_down
BSK_CASM3C_Thrombomodulin_up
BSK_BE3C_HLADR_down
25485−88−5
118−56−9
84−61−7

All Activity Heatmap for Cluster 724

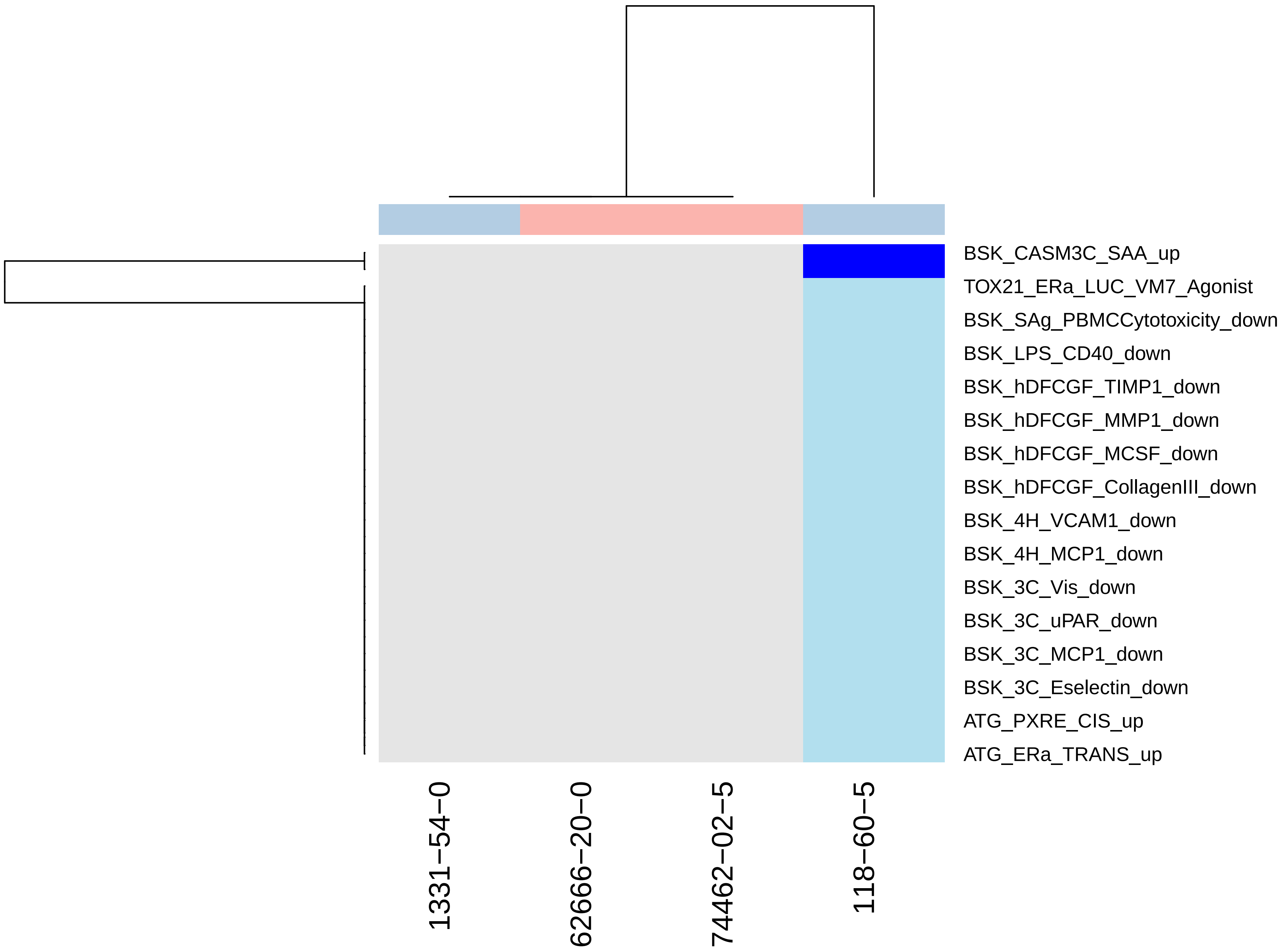

All Activity Heatmap for Cluster 725

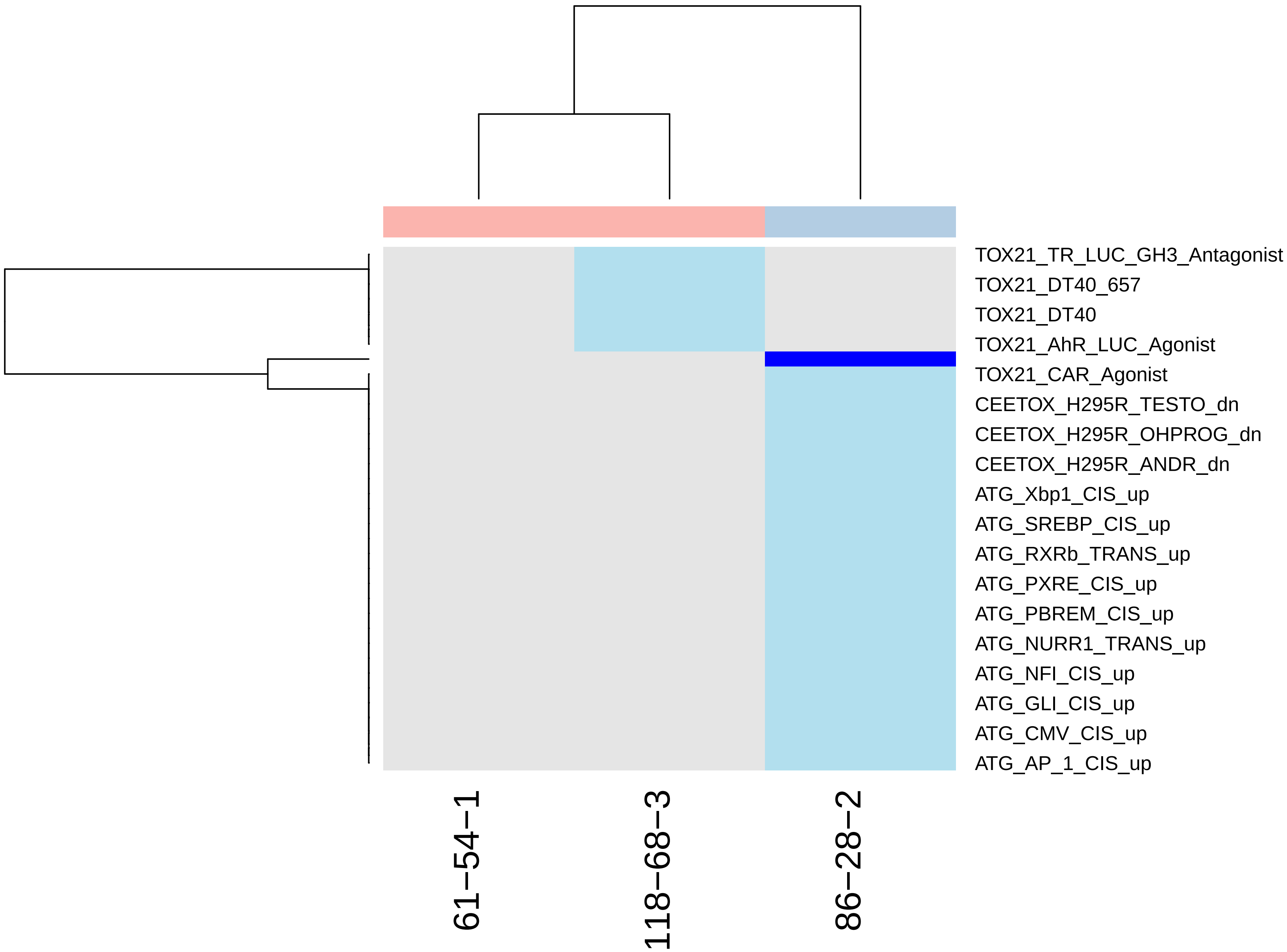

All Activity Heatmap for Cluster 726

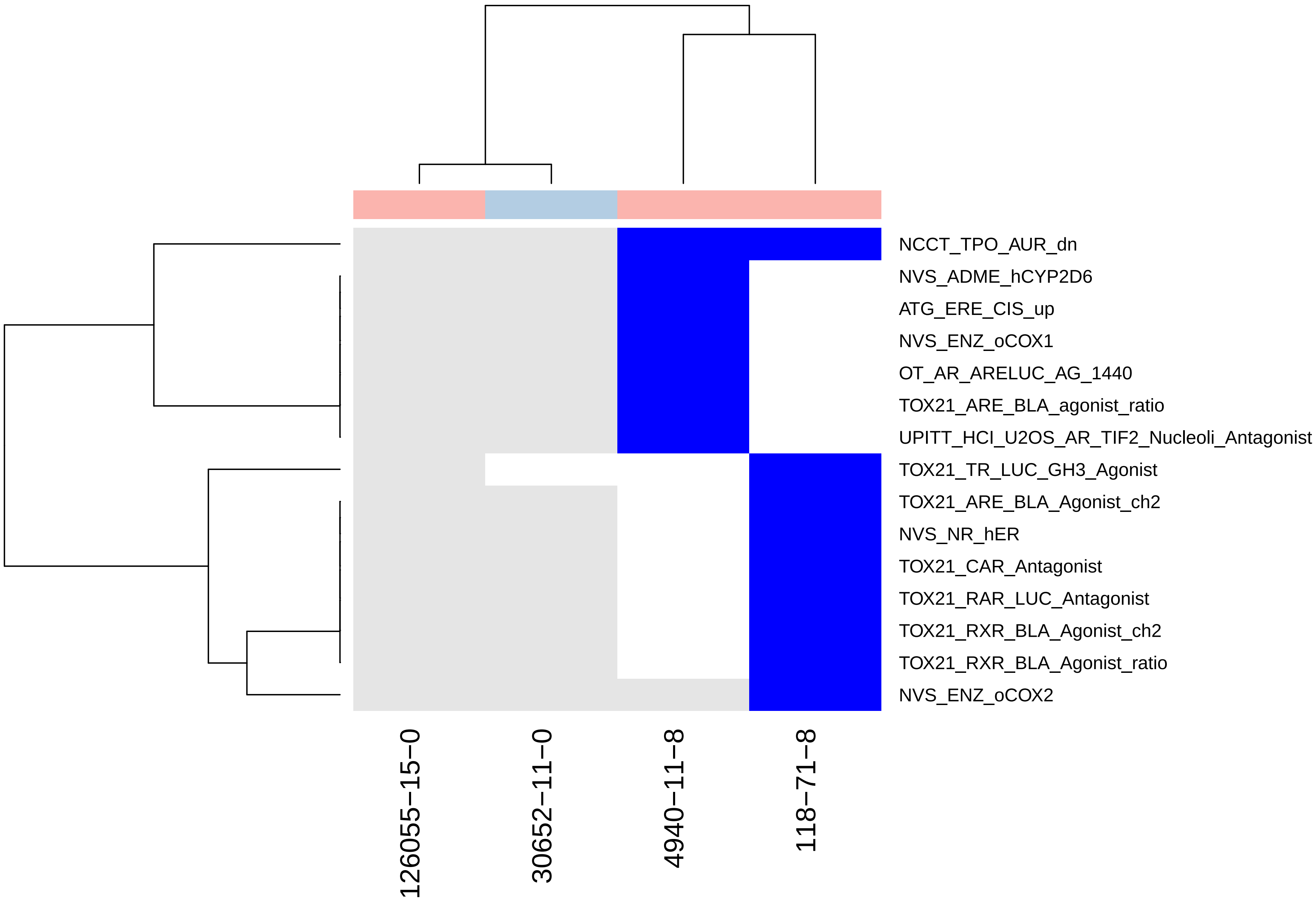

All Activity Heatmap for Cluster 727

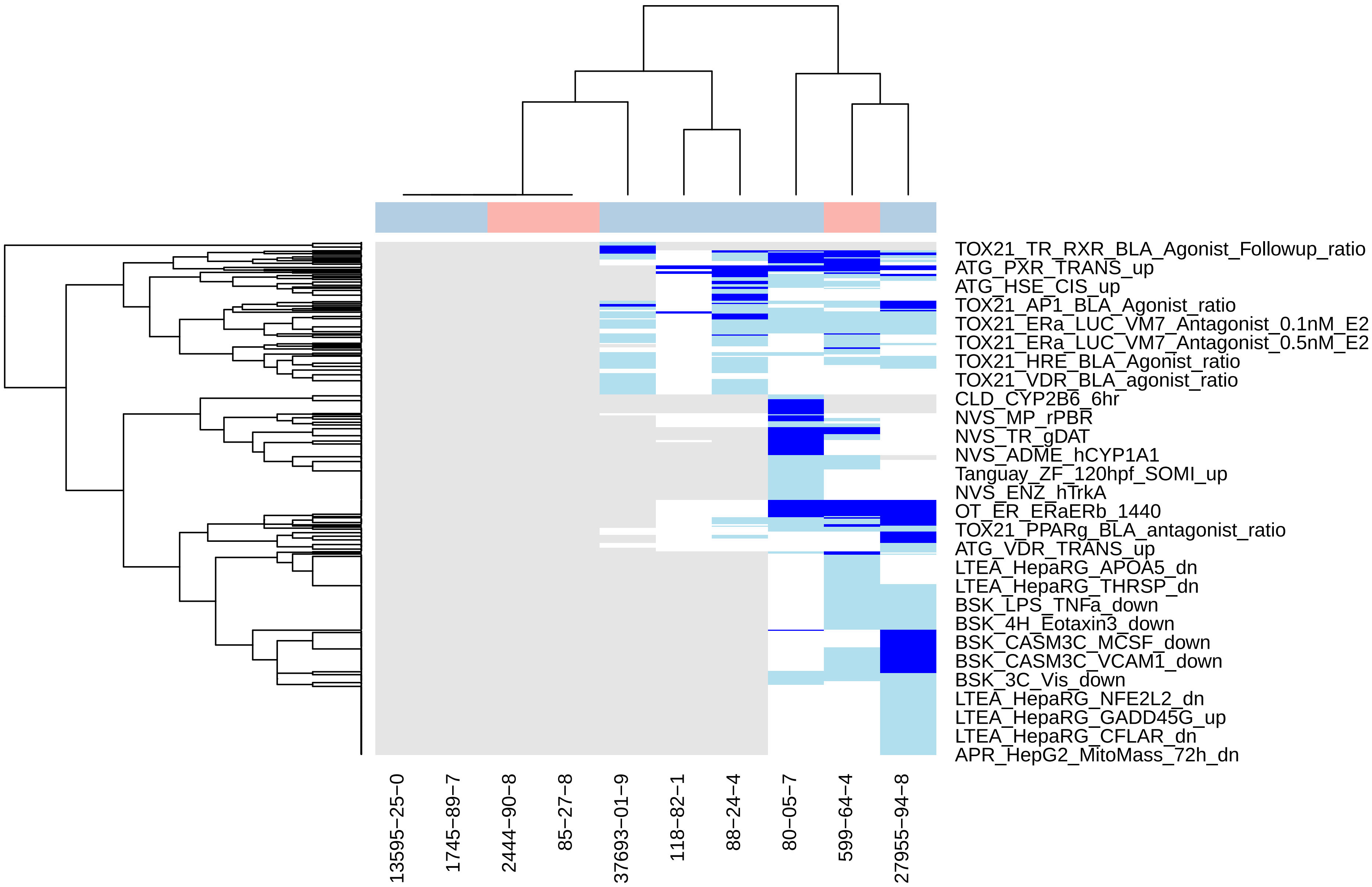

All Activity Heatmap for Cluster 728

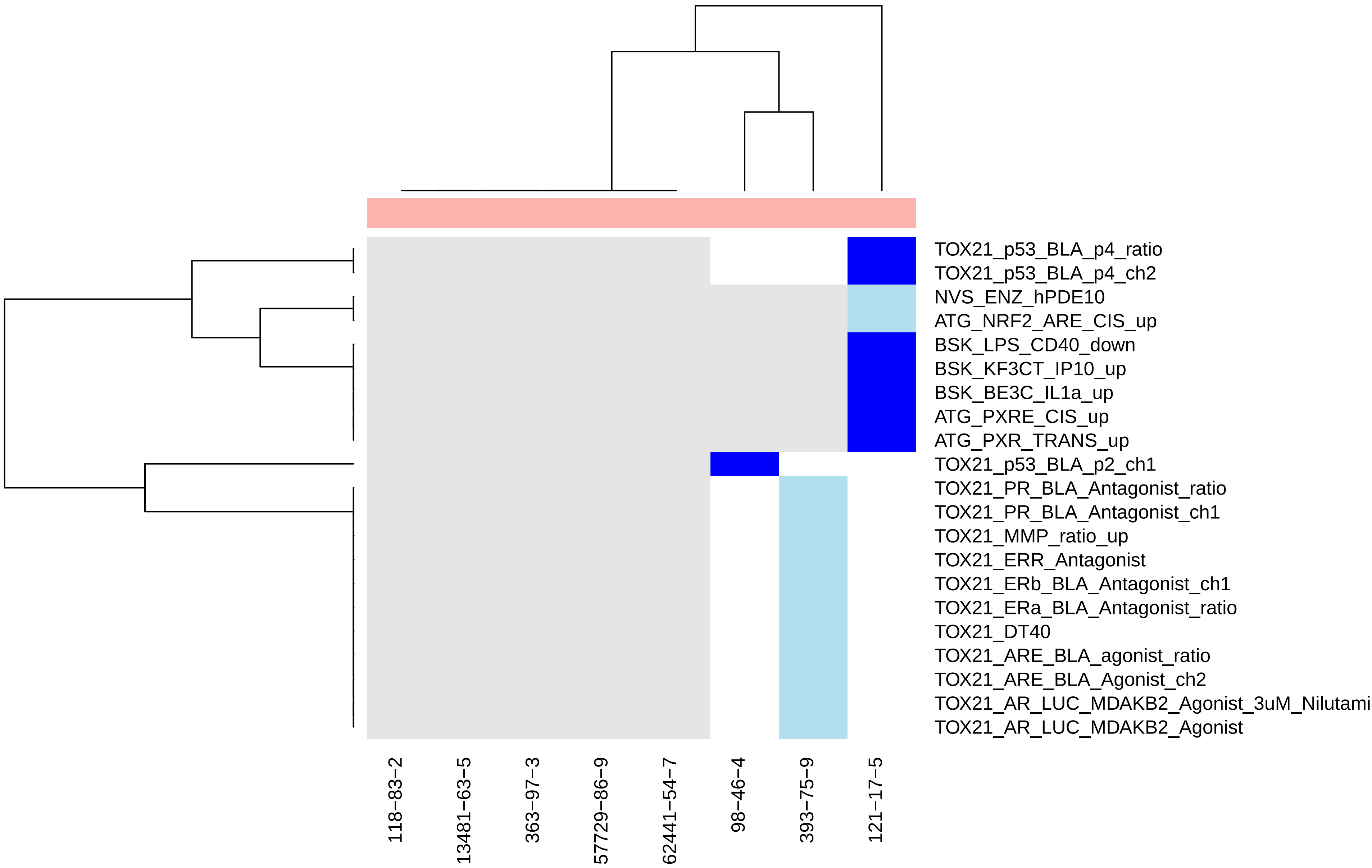

All Activity Heatmap for Cluster 729

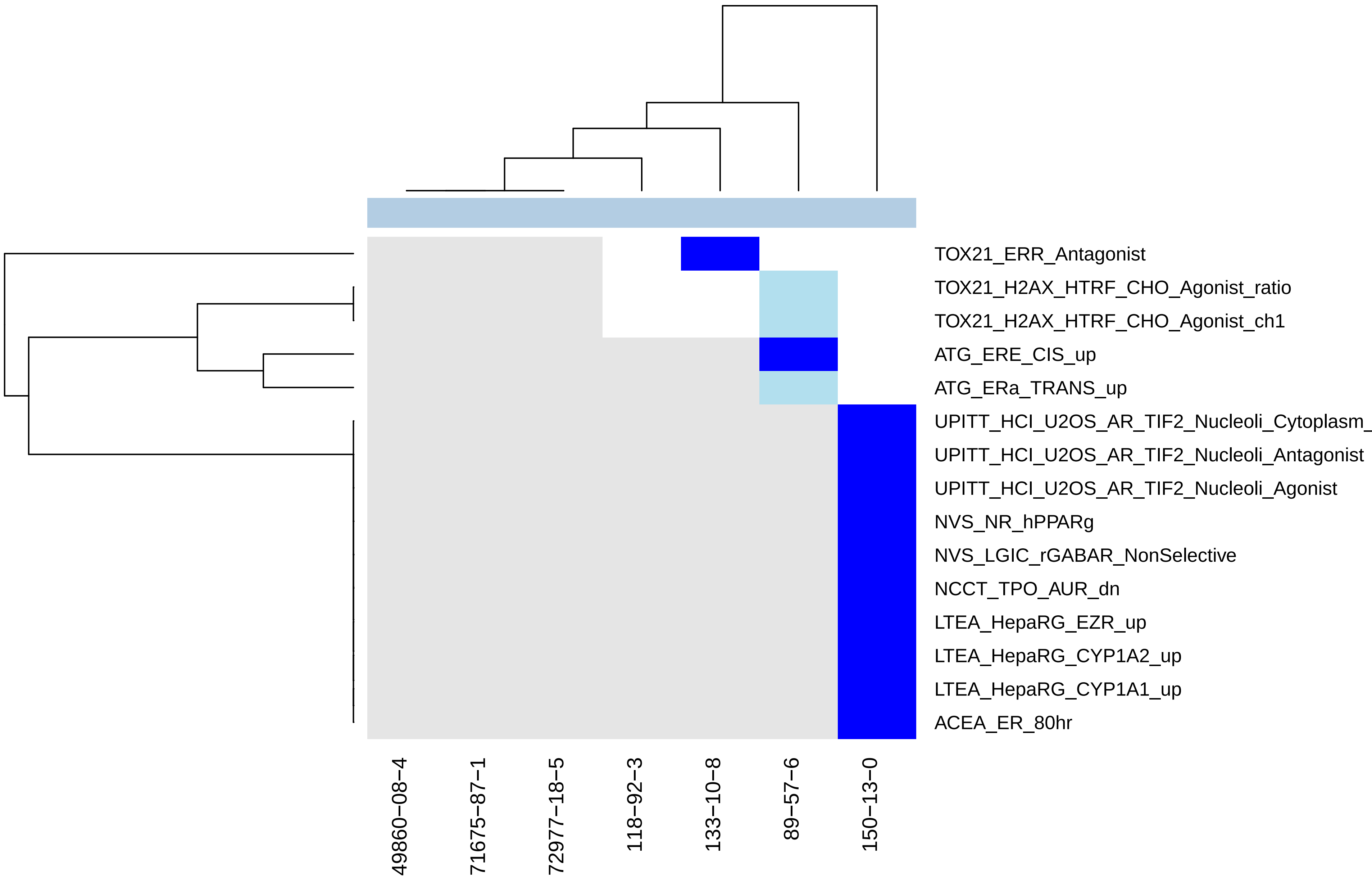

All Activity Heatmap for Cluster 730

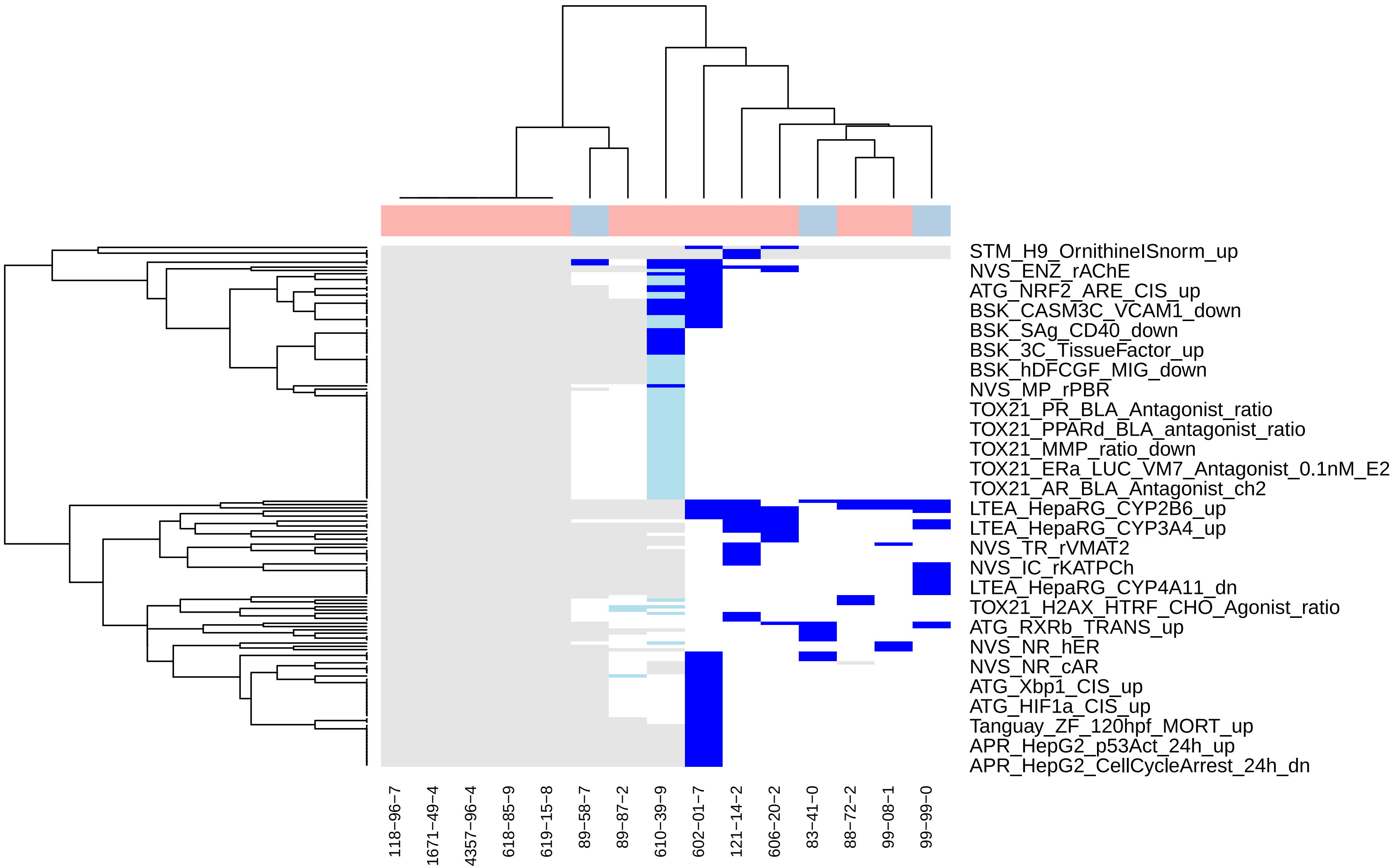

All Activity Heatmap for Cluster 731

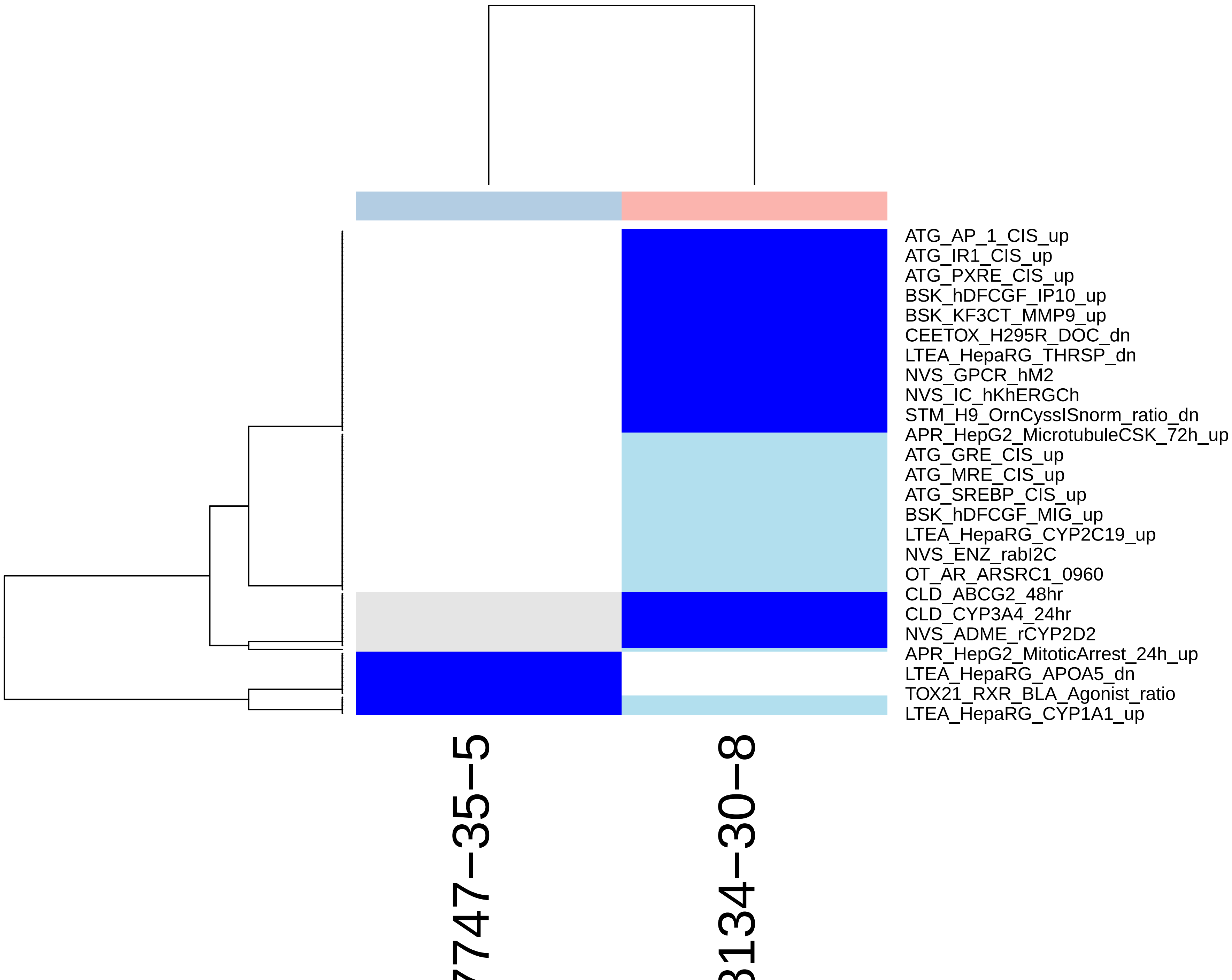

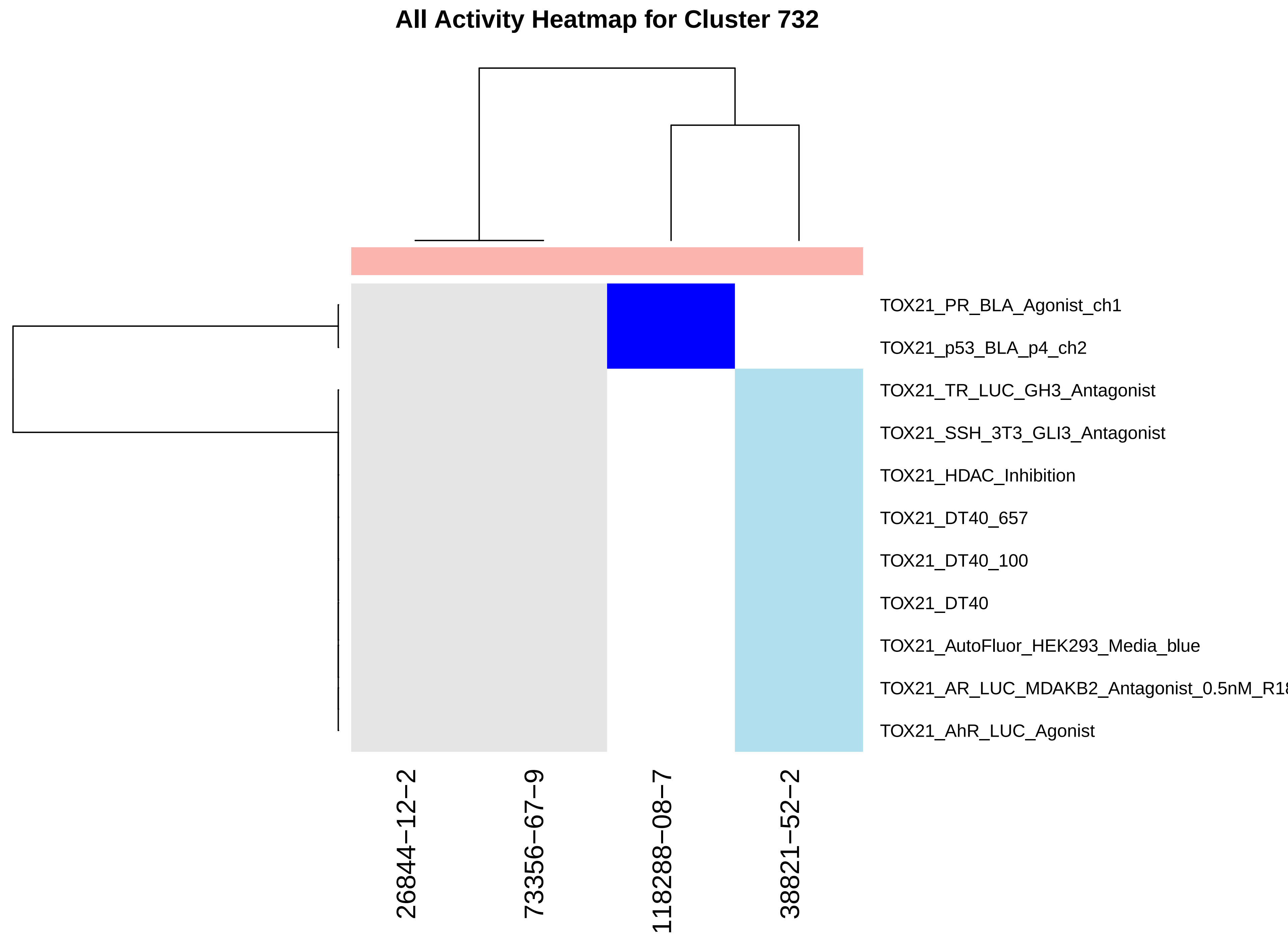
All Activity Heatmap for Cluster 732
TOX21_PR_BLA_Agonist_ch1
TOX21_p53_BLA_p4_ch2
TOX21_TR_LUC_GH3_Antagonist
TOX21_SSH_3T3_GLI3_Antagonist
TOX21_HDAC_Inhibition
TOX21_DT40_657
TOX21_DT40_100
TOX21_DT40
TOX21_AutoFluor_HEK293_Media_blue
TOX21_AR_LUC_MDAKB2_Antagonist_0.5nM_R1
TOX21_AhR_LUC_Agonist
26844−12−2
73356−67−9
118288−08−7
38821−52−2

All Activity Heatmap for Cluster 734

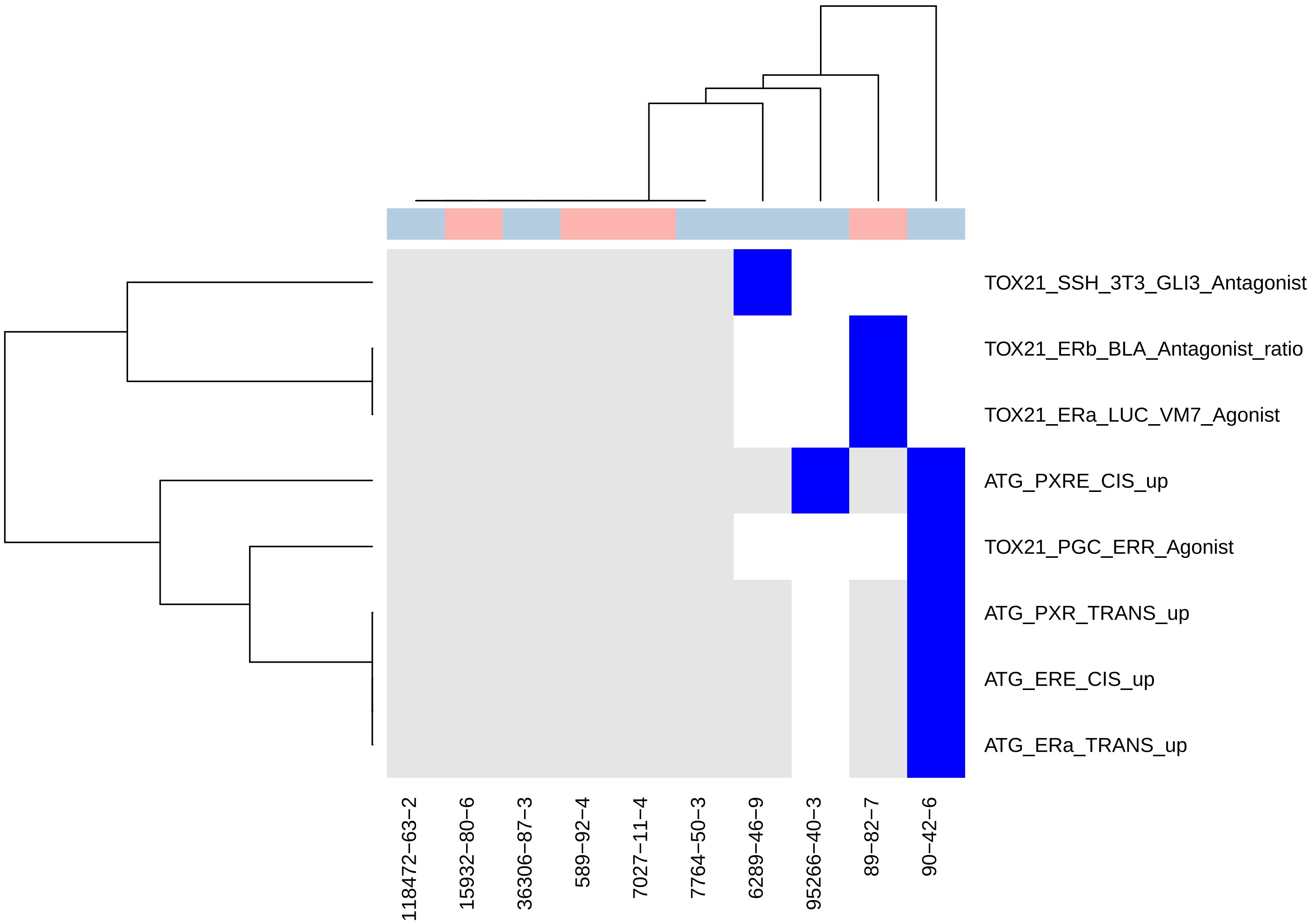

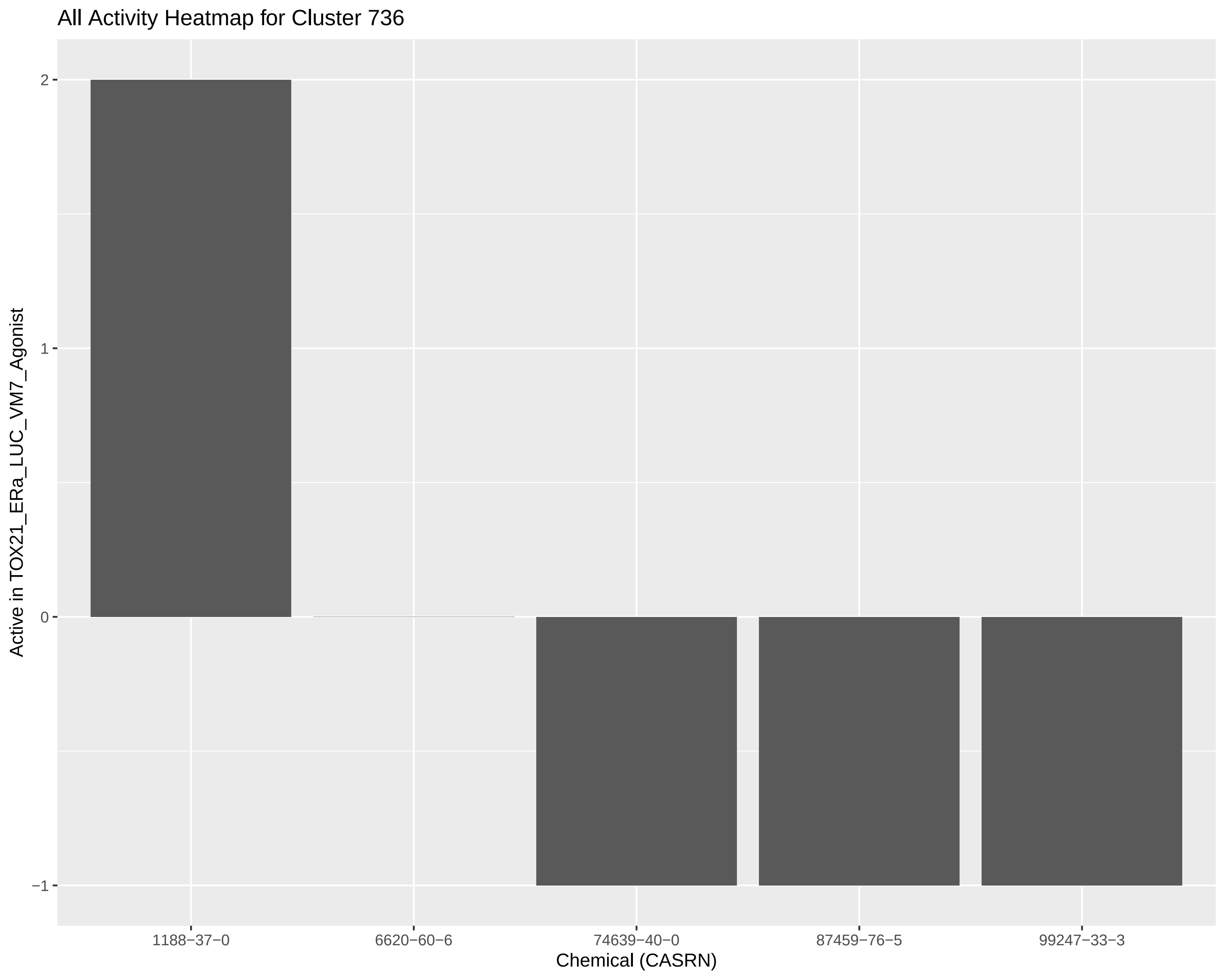
All Activity Heatmap for Cluster 736
Active in TOX21_ERa_LUC_VM7_Agonist
2
1
0
−1
1188−37−0
6620−60−6
74639−40−0
87459−76−5
99247−33−3
Chemical (CASRN)

All Activity Heatmap for Cluster 738

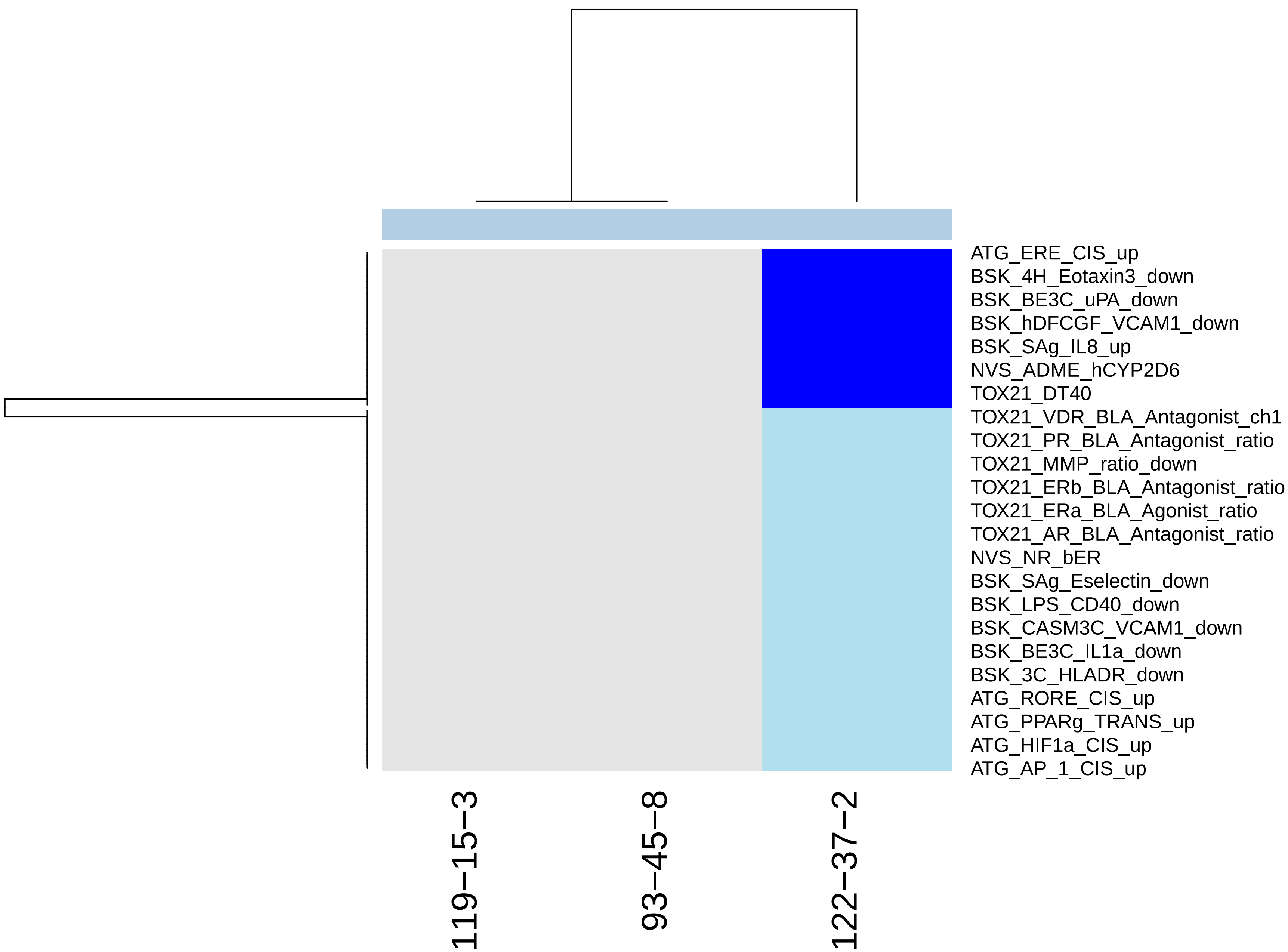

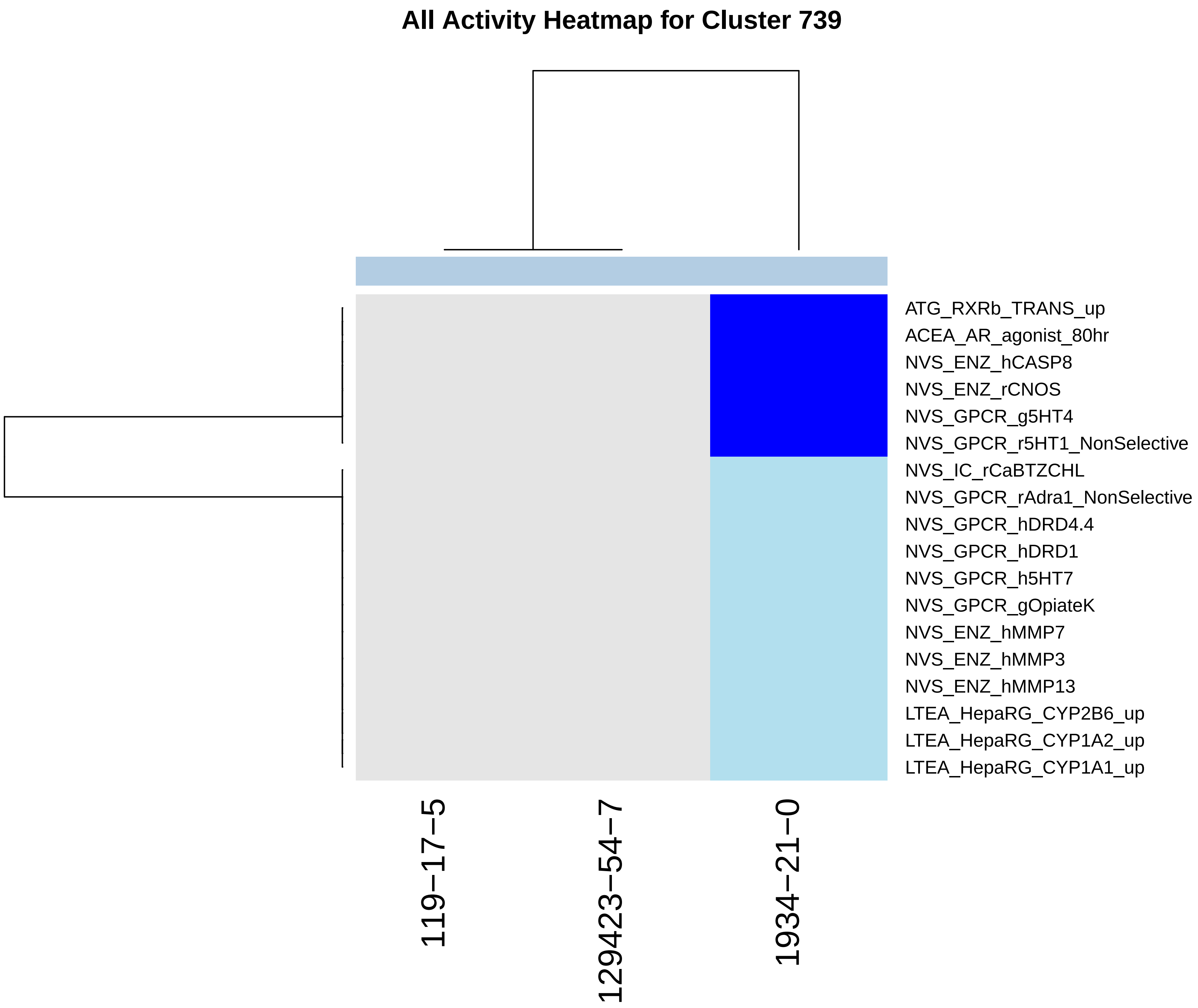
All Activity Heatmap for Cluster 739
ATG_RXRb_TRANS_up
ACEA_AR_agonist_80hr
NVS_ENZ_hCASP8
NVS_ENZ_rCNOS
NVS_GPCR_g5HT4
NVS_GPCR_r5HT1_NonSelective
NVS_IC_rCaBTZCHL
NVS_GPCR_rAdra1_NonSelective
NVS_GPCR_hDRD4.4
NVS_GPCR_hDRD1
NVS_GPCR_h5HT7
NVS_GPCR_gOpiateK
NVS_ENZ_hMMP7
NVS_ENZ_hMMP3
NVS_ENZ_hMMP13
LTEA_HepaRG_CYP2B6_up
LTEA_HepaRG_CYP1A2_up
LTEA_HepaRG_CYP1A1_up
119−17−5
129423−54−7
1934−21−0

All Activity Heatmap for Cluster 740

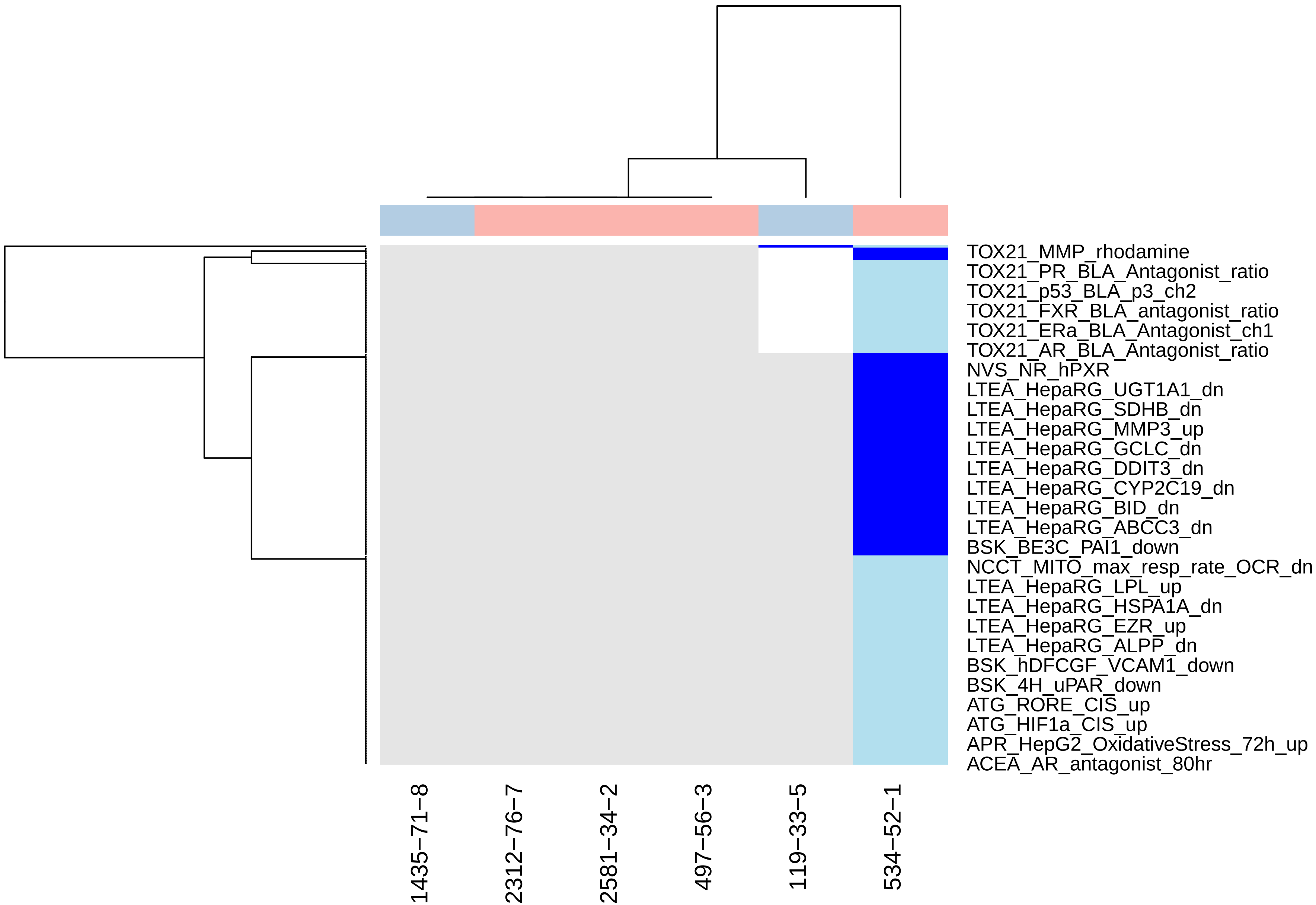

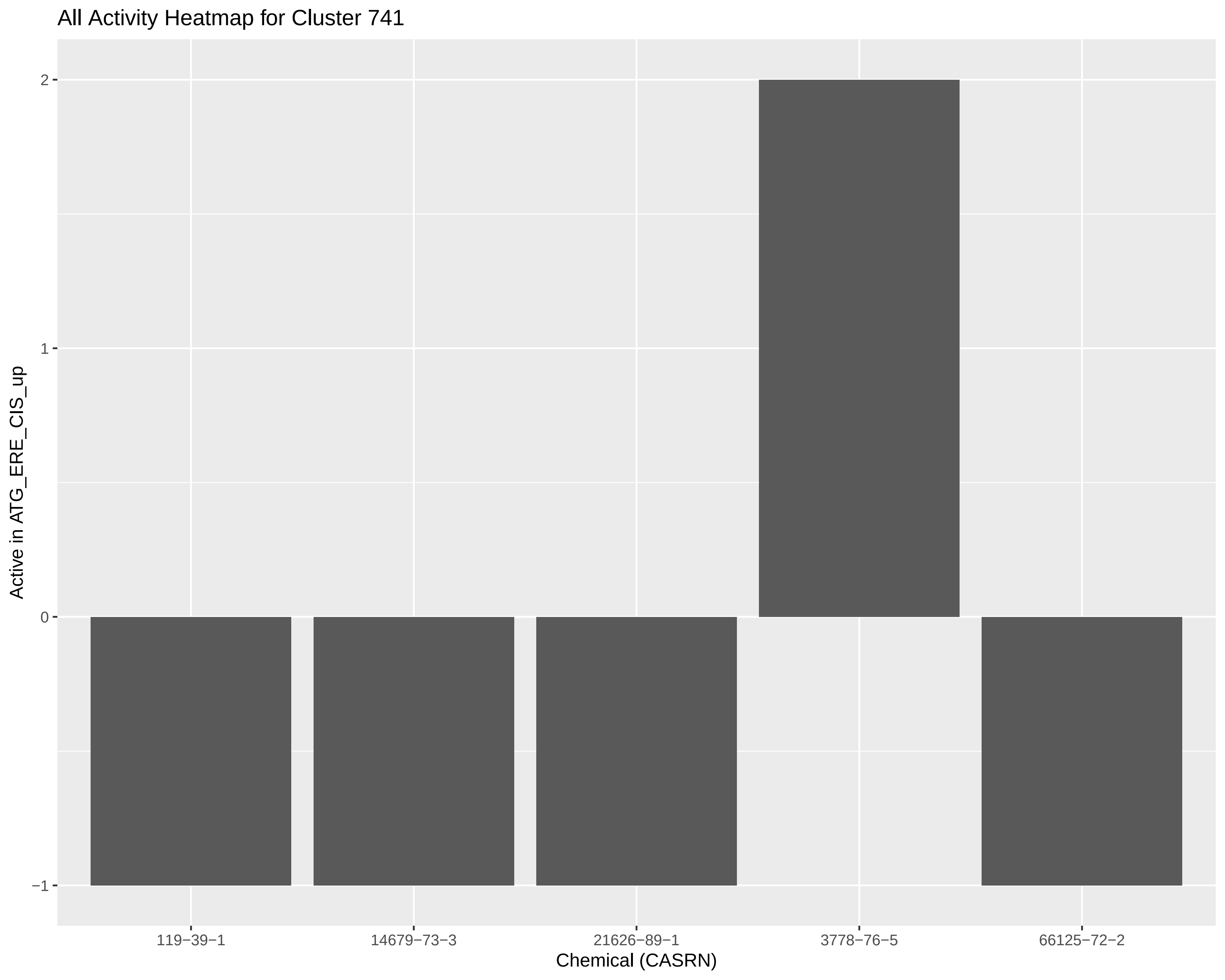

All Activity Heatmap for Cluster 741
Active in ATG_ERE_CIS_up
2
1
0
−1
119−39−1
14679−73−3
21626−89−1
3778−76−5
66125−72−2
Chemical (CASRN)

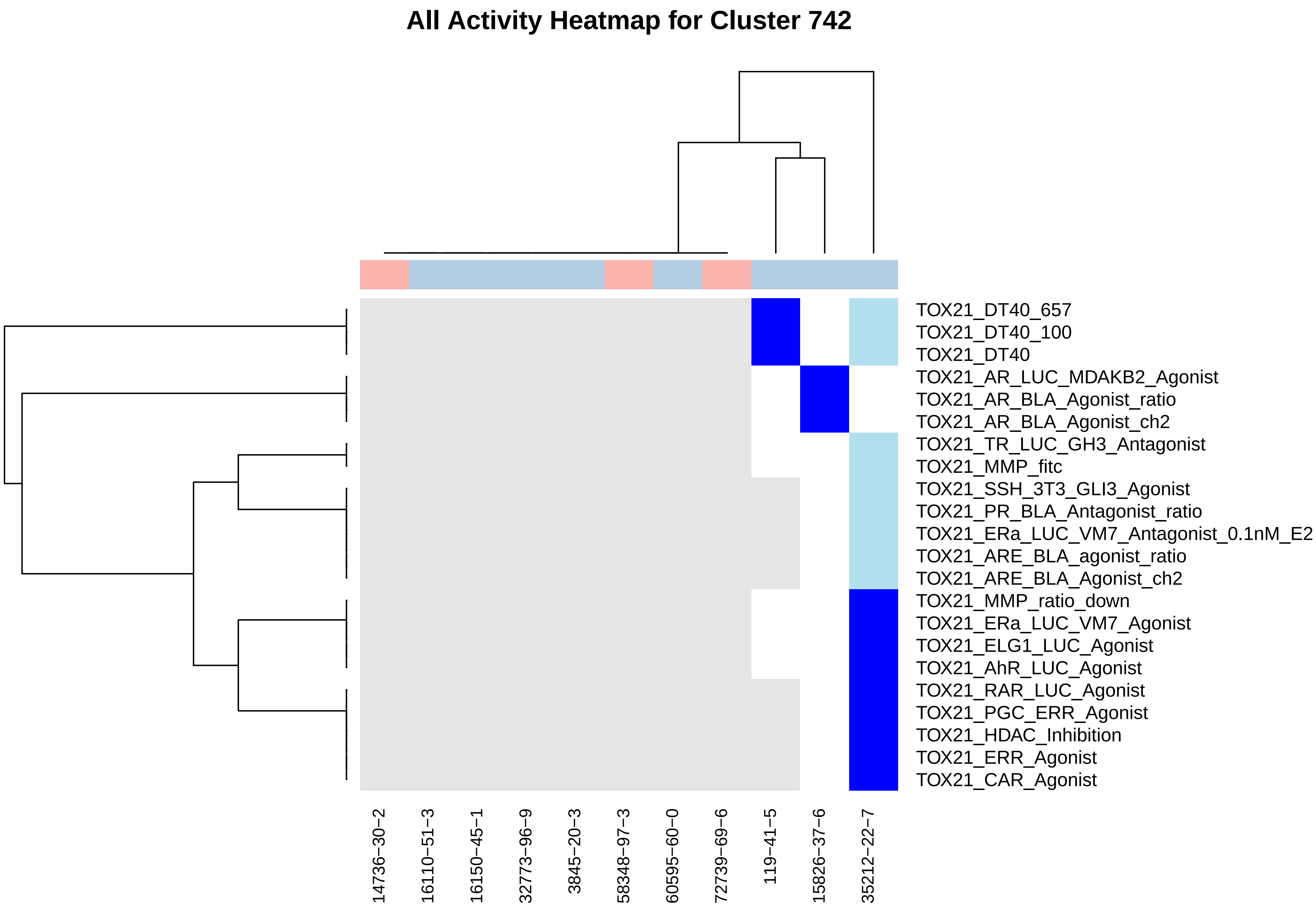

All Activity Heatmap for Cluster 742
TOX21_DT40_657
TOX21_DT40_100
TOX21_DT40
TOX21_AR_LUC_MDAKB2_Agonist
TOX21_AR_BLA_Agonist_ratio
TOX21_AR_BLA_Agonist_ch2
TOX21_TR_LUC_GH3_Antagonist
TOX21_MMP_fitc
TOX21_SSH_3T3_GLI3_Agonist
TOX21_PR_BLA_Antagonist_ratio
TOX21_ERa_LUC_VM7_Antagonist_0.1nM_E2
TOX21_ARE_BLA_agonist_ratio
TOX21_ARE_BLA_Agonist_ch2
TOX21_MMP_ratio_down
TOX21_ERa_LUC_VM7_Agonist
TOX21_ELG1_LUC_Agonist
TOX21_AhR_LUC_Agonist
TOX21_RAR_LUC_Agonist
TOX21_PGC_ERR_Agonist
TOX21_HDAC_Inhibition
TOX21_ERR_Agonist
TOX21_CAR_Agonist
14736−30−2
16110−51−3
16150−45−1
32773−96−9
3845−20−3
58348−97−3
60595−60−0
72739−69−6
119−41−5
15826−37−6
35212−22−7

All Activity Heatmap for Cluster 743

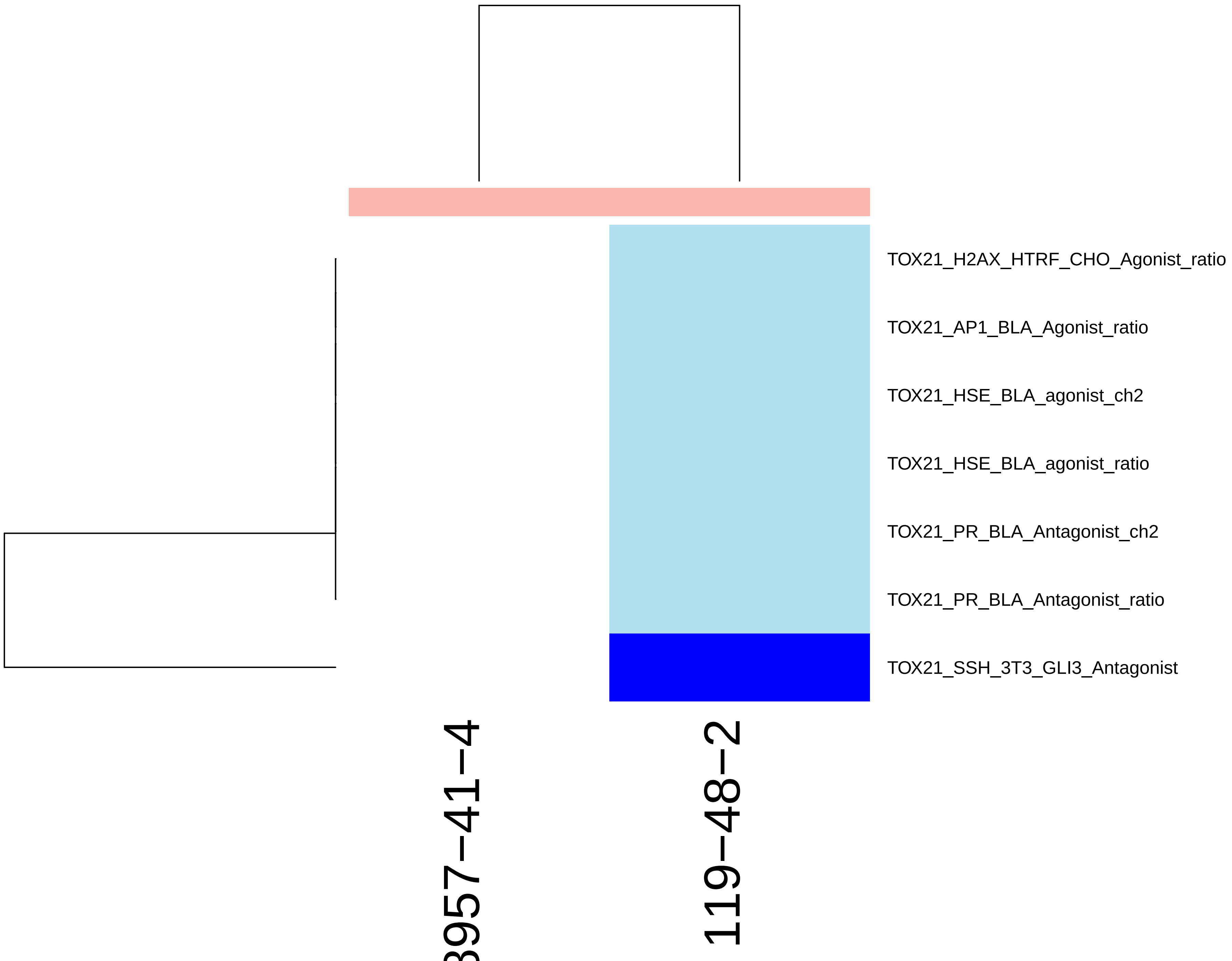

All Activity Heatmap for Cluster 744

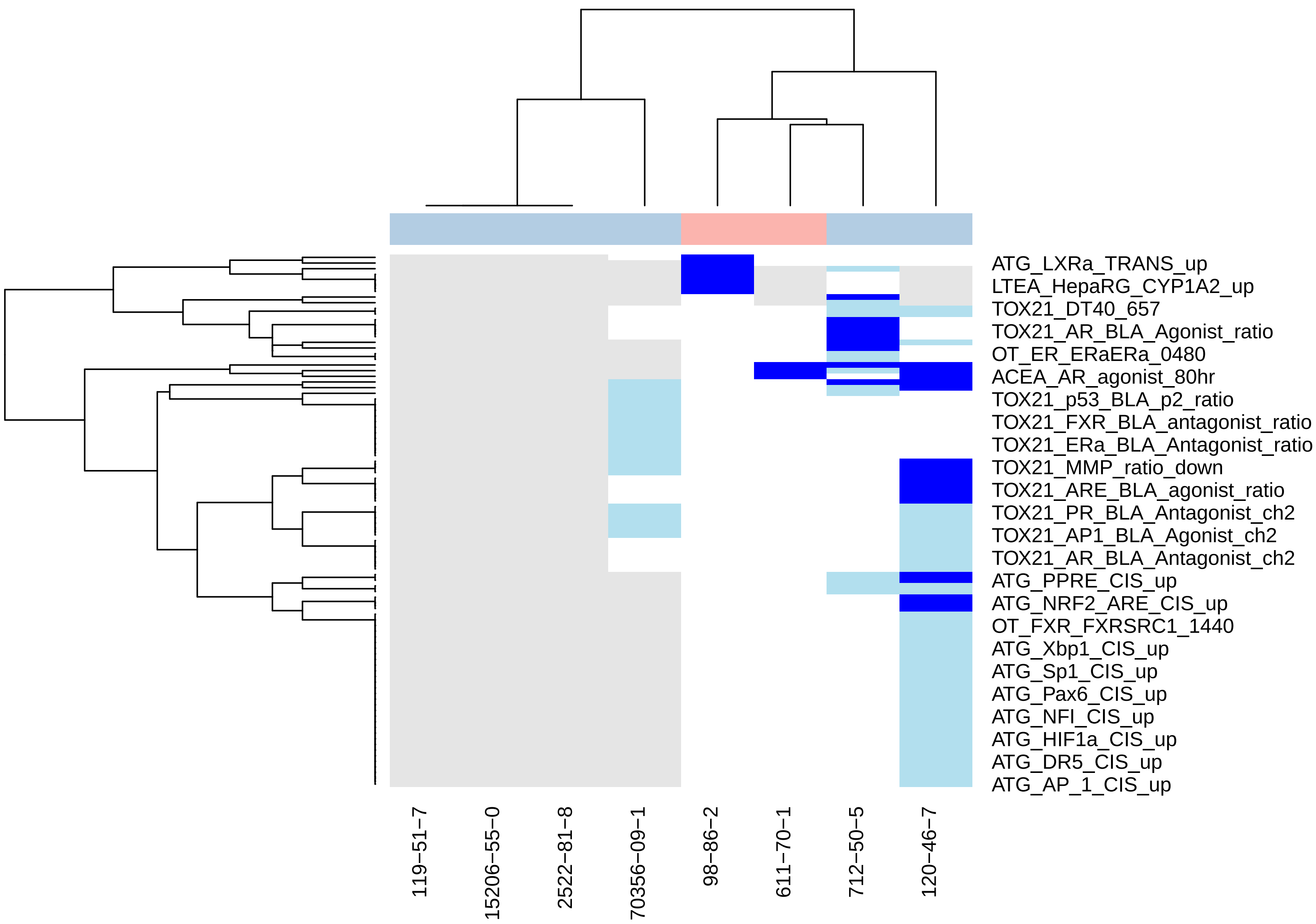

All Activity Heatmap for Cluster 745

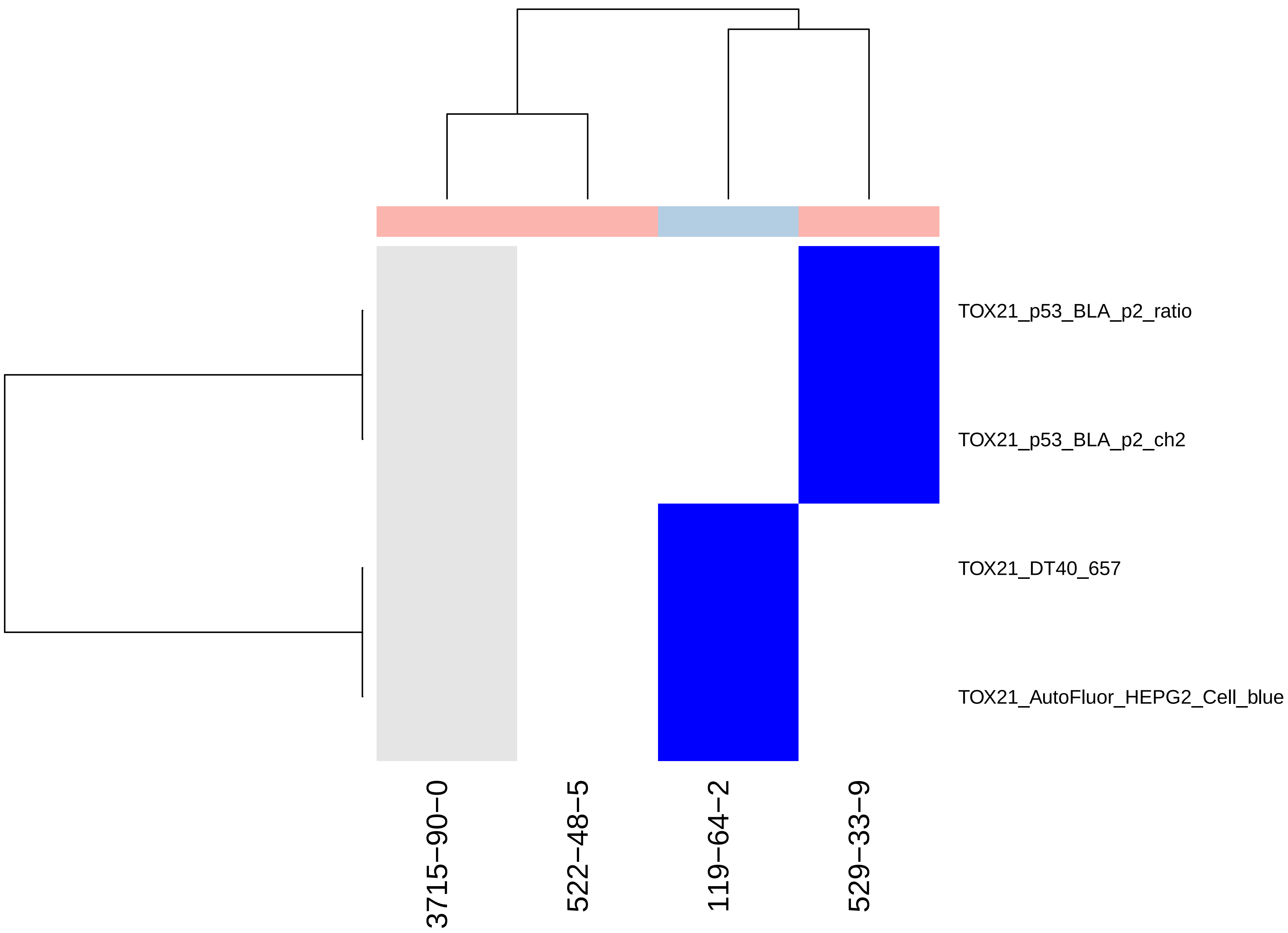

All Activity Heatmap for Cluster 746

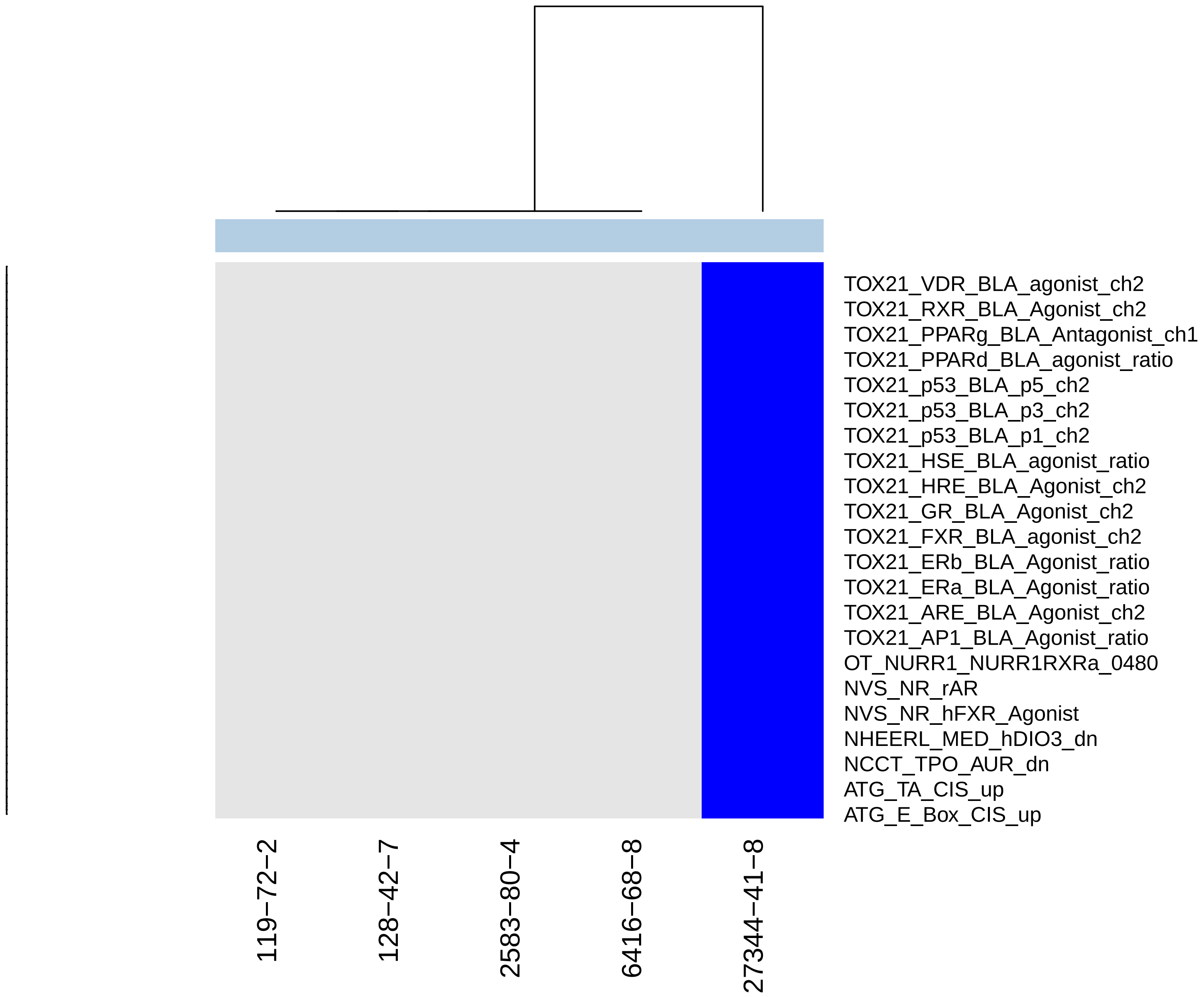

All Activity Heatmap for Cluster 747

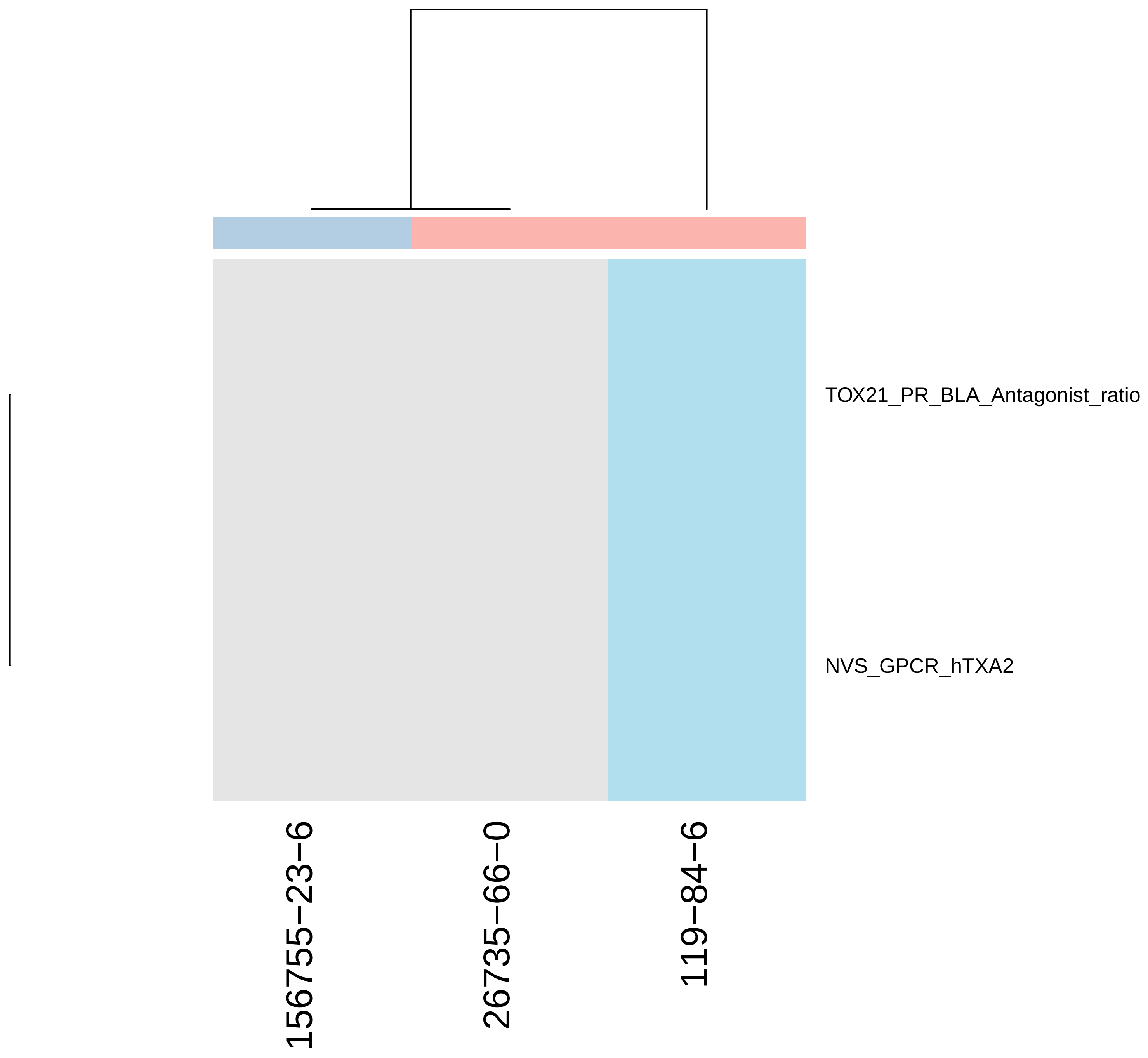

All Activity Heatmap for Cluster 749

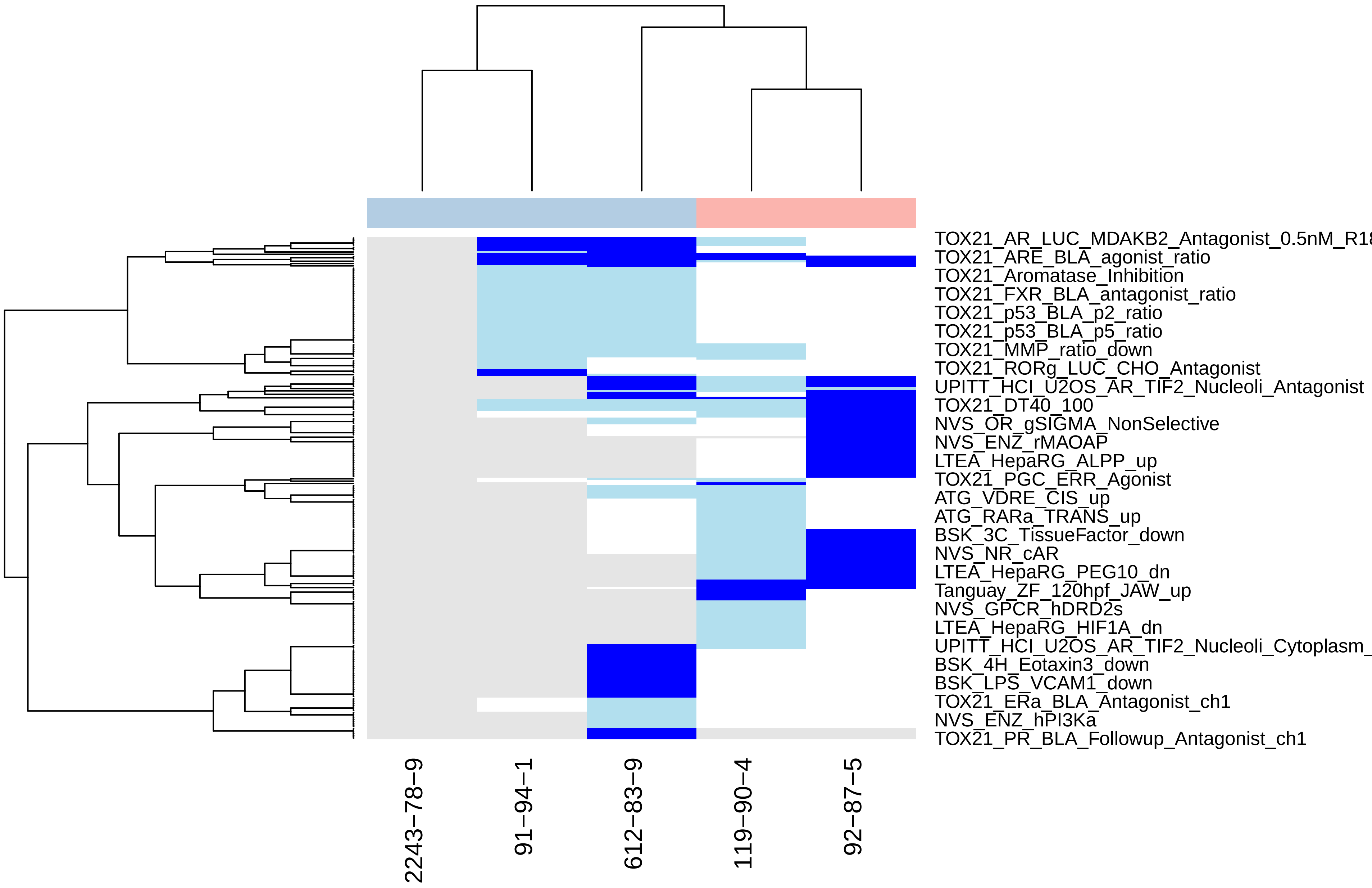

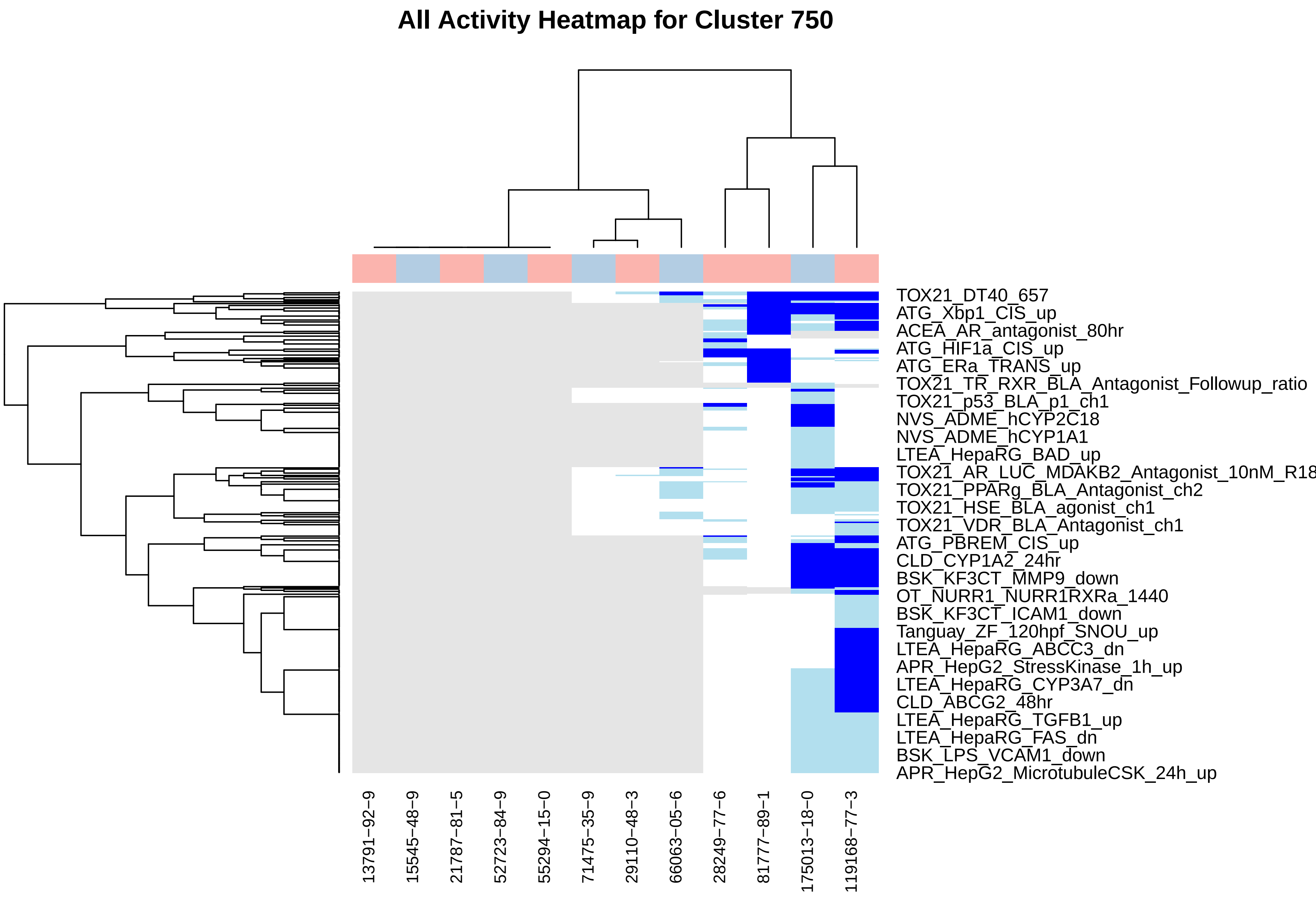
All Activity Heatmap for Cluster 750
TOX21_DT40_657
ATG_Xbp1_CIS_up
ACEA_AR_antagonist_80hr
ATG_HIF1a_CIS_up
ATG_ERa_TRANS_up
TOX21_TR_RXR_BLA_Antagonist_Followup_ratio
TOX21_p53_BLA_p1_ch1
NVS_ADME_hCYP2C18
NVS_ADME_hCYP1A1
LTEA_HepaRG_BAD_up
TOX21_AR_LUC_MDAKB2_Antagonist_10nM_R18
TOX21_PPARg_BLA_Antagonist_ch2
TOX21_HSE_BLA_agonist_ch1
TOX21_VDR_BLA_Antagonist_ch1
ATG_PBREM_CIS_up
CLD_CYP1A2_24hr
BSK_KF3CT_MMP9_down
OT_NURR1_NURR1RXRa_1440
BSK_KF3CT_ICAM1_down
Tanguay_ZF_120hpf_SNOU_up
LTEA_HepaRG_ABCC3_dn
APR_HepG2_StressKinase_1h_up
LTEA_HepaRG_CYP3A7_dn
CLD_ABCG2_48hr
LTEA_HepaRG_TGFB1_up
LTEA_HepaRG_FAS_dn
BSK_LPS_VCAM1_down
APR_HepG2_MicrotubuleCSK_24h_up
13791−92−9
15545−48−9
21787−81−5
52723−84−9
55294−15−0
71475−35−9
29110−48−3
66063−05−6
28249−77−6
81777−89−1
175013−18−0
119168−77−3

All Activity Heatmap for Cluster 751

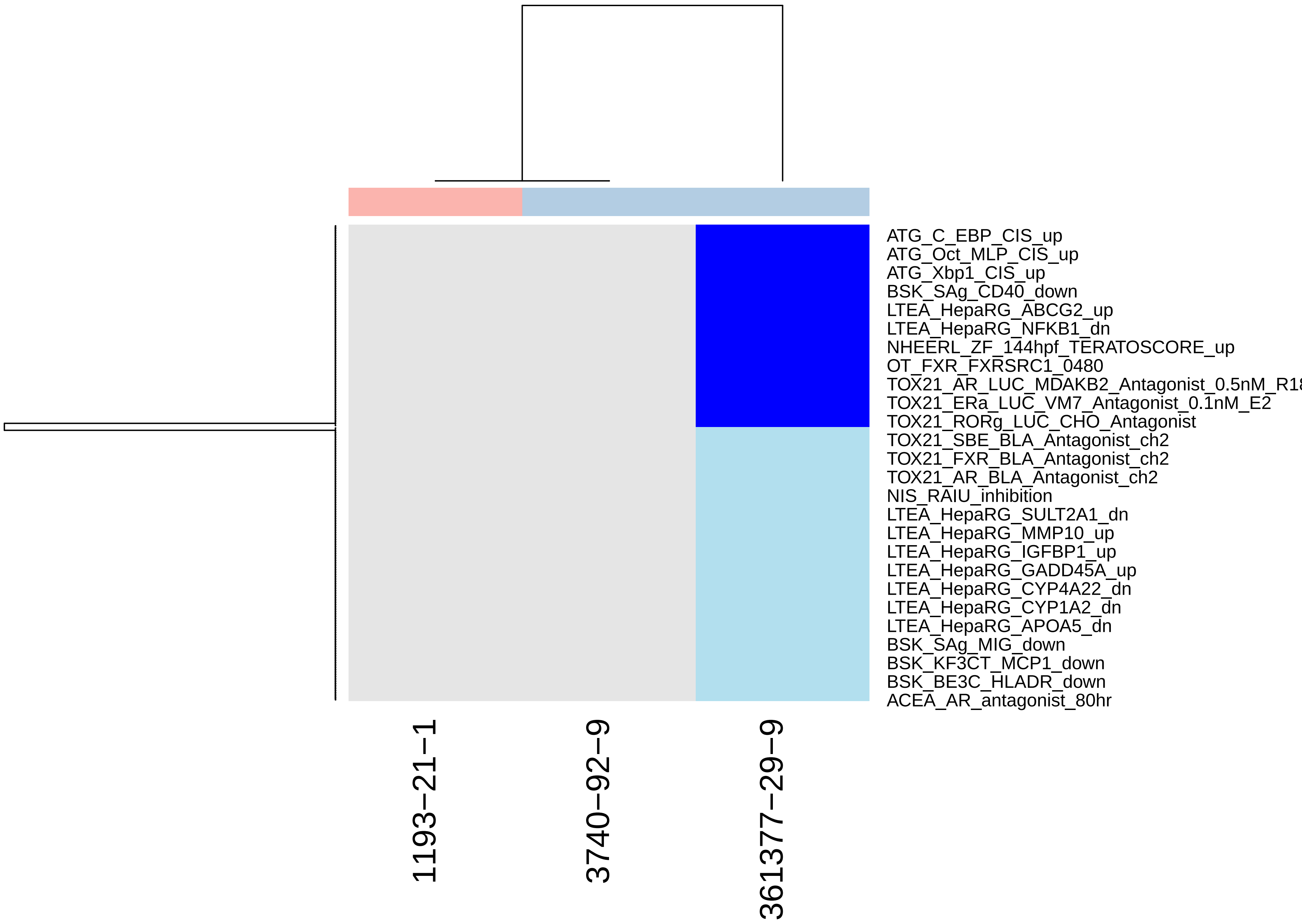

All Activity Heatmap for Cluster 753

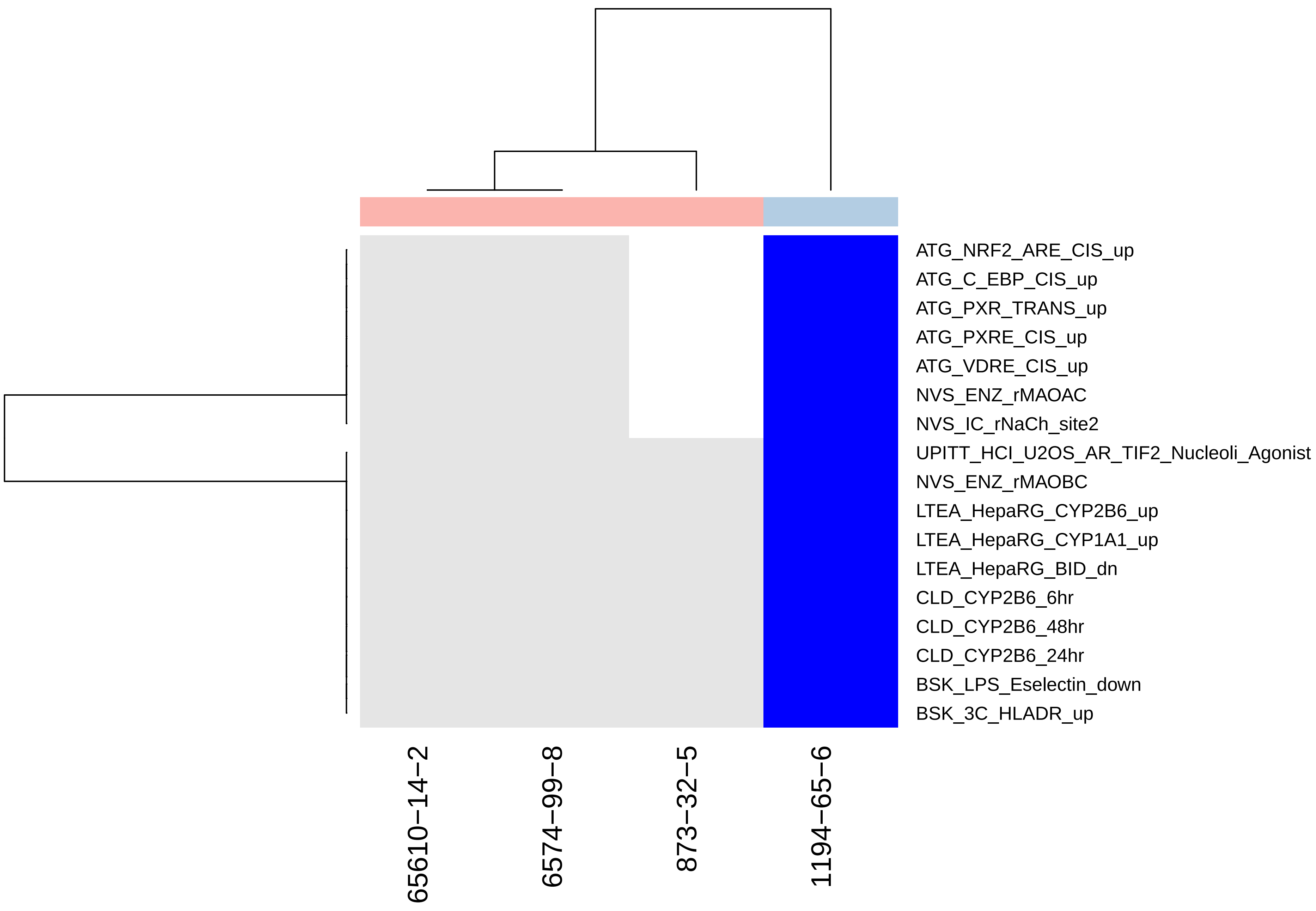

All Activity Heatmap for Cluster 754

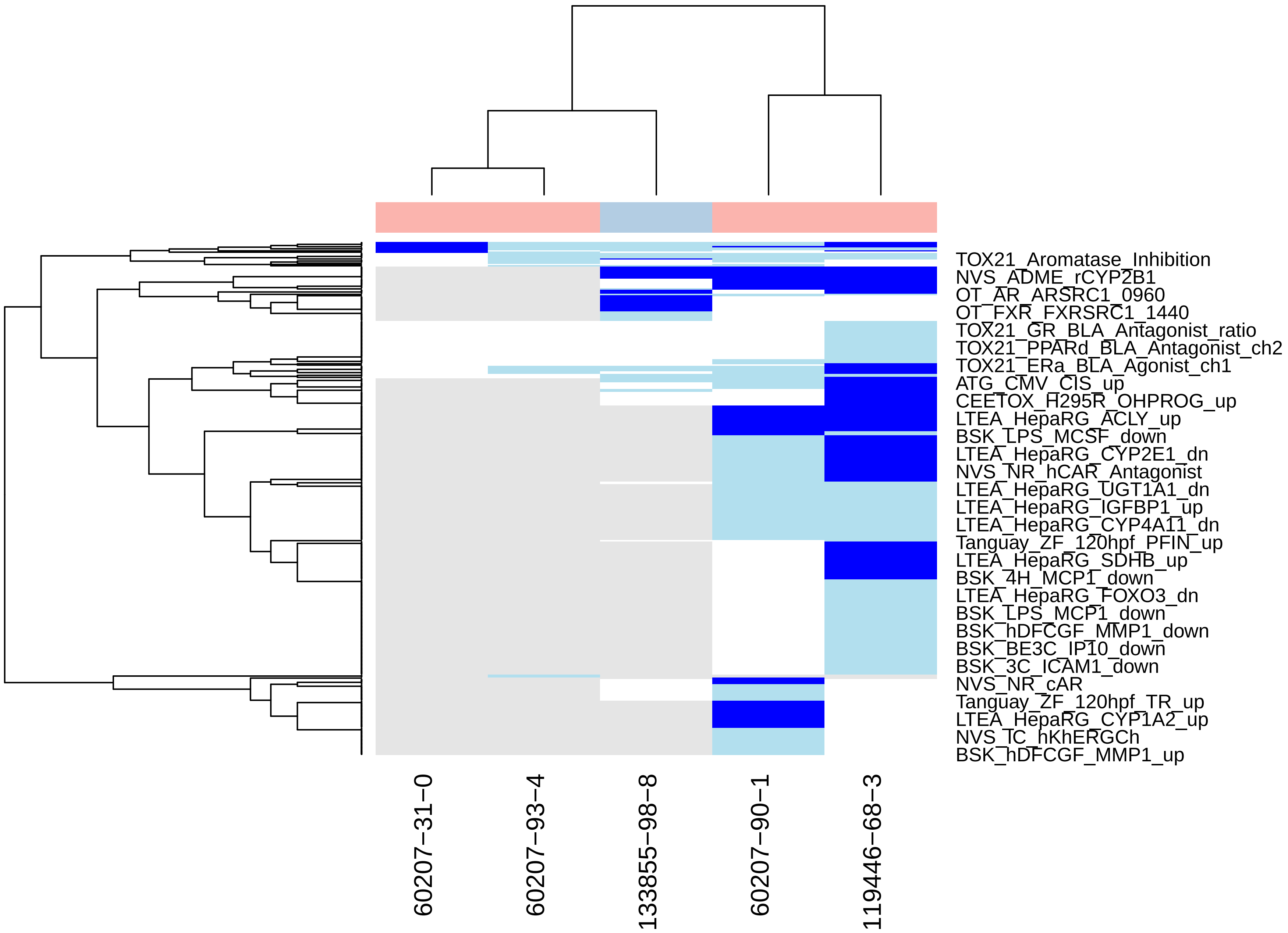

All Activity Heatmap for Cluster 755

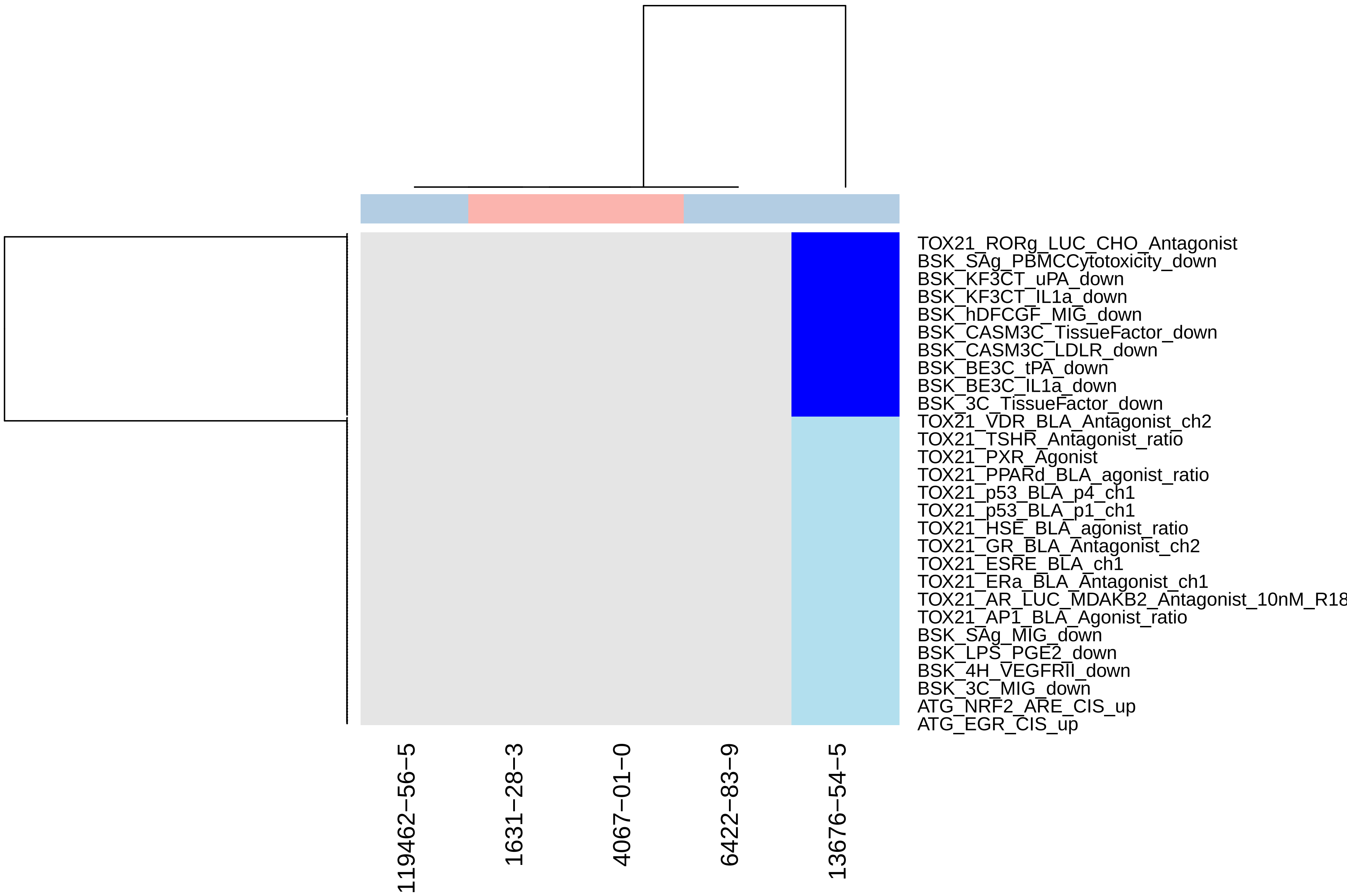

All Activity Heatmap for Cluster 756

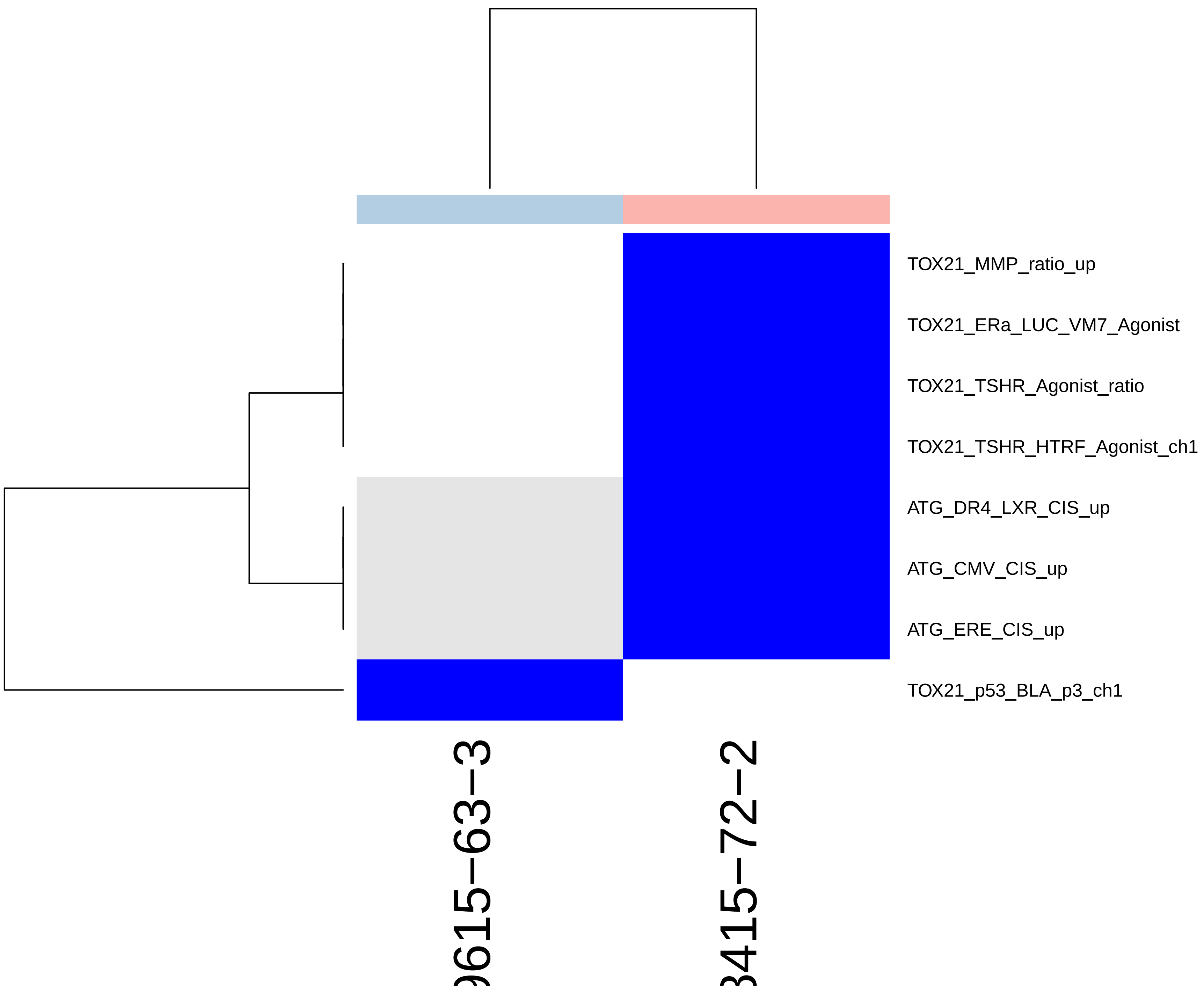

All Activity Heatmap for Cluster 757

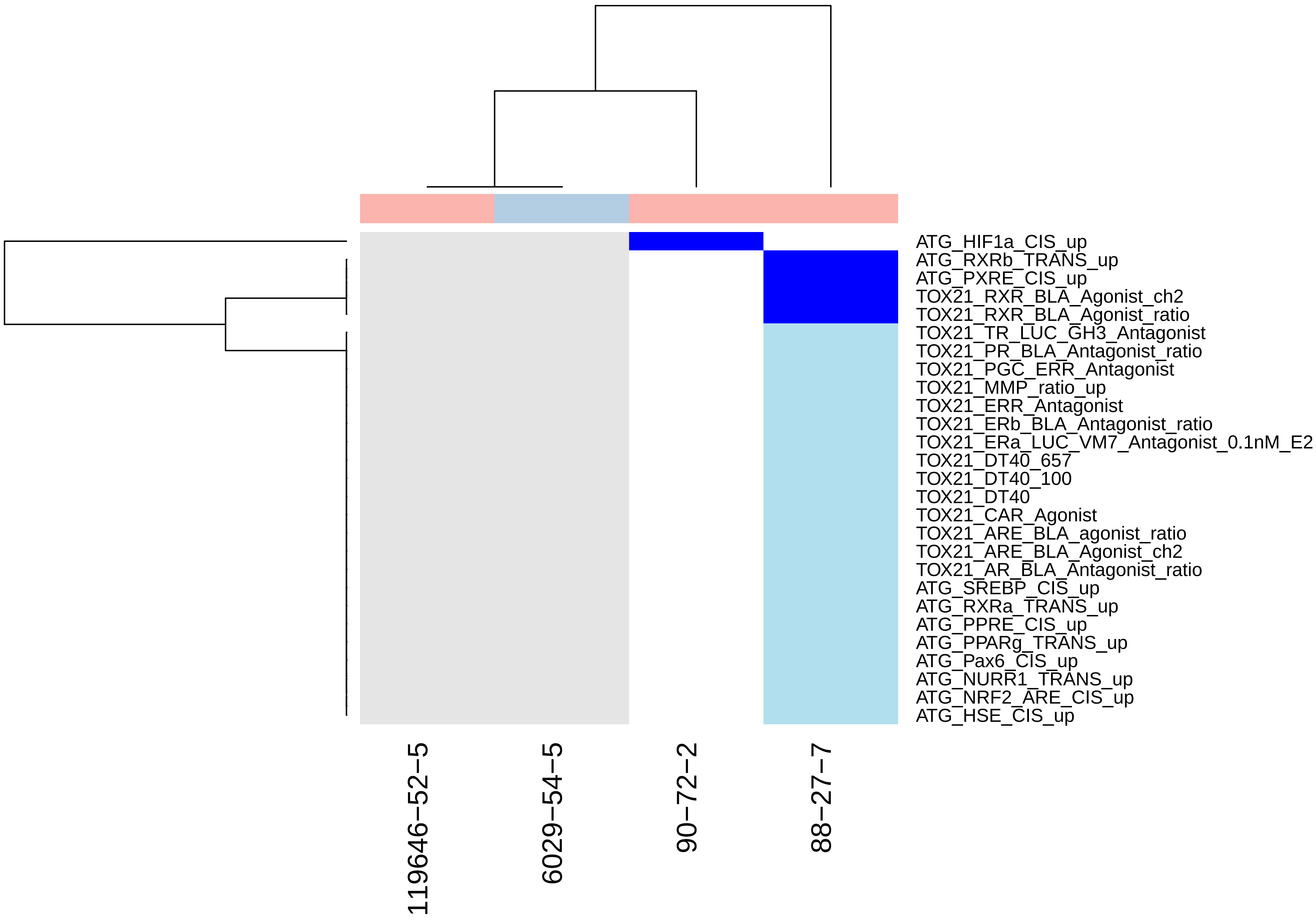

All Activity Heatmap for Cluster 759

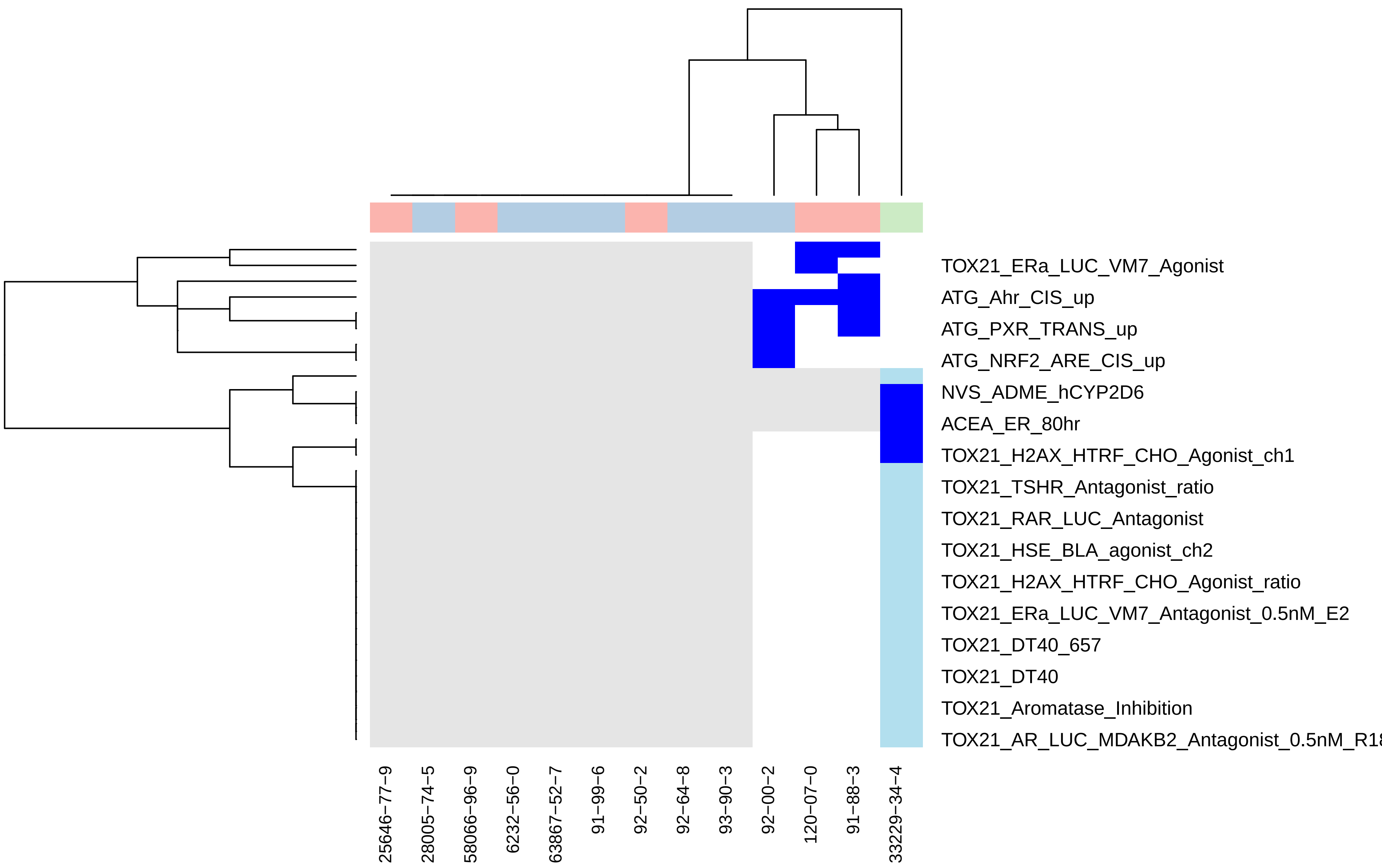

All Activity Heatmap for Cluster 760

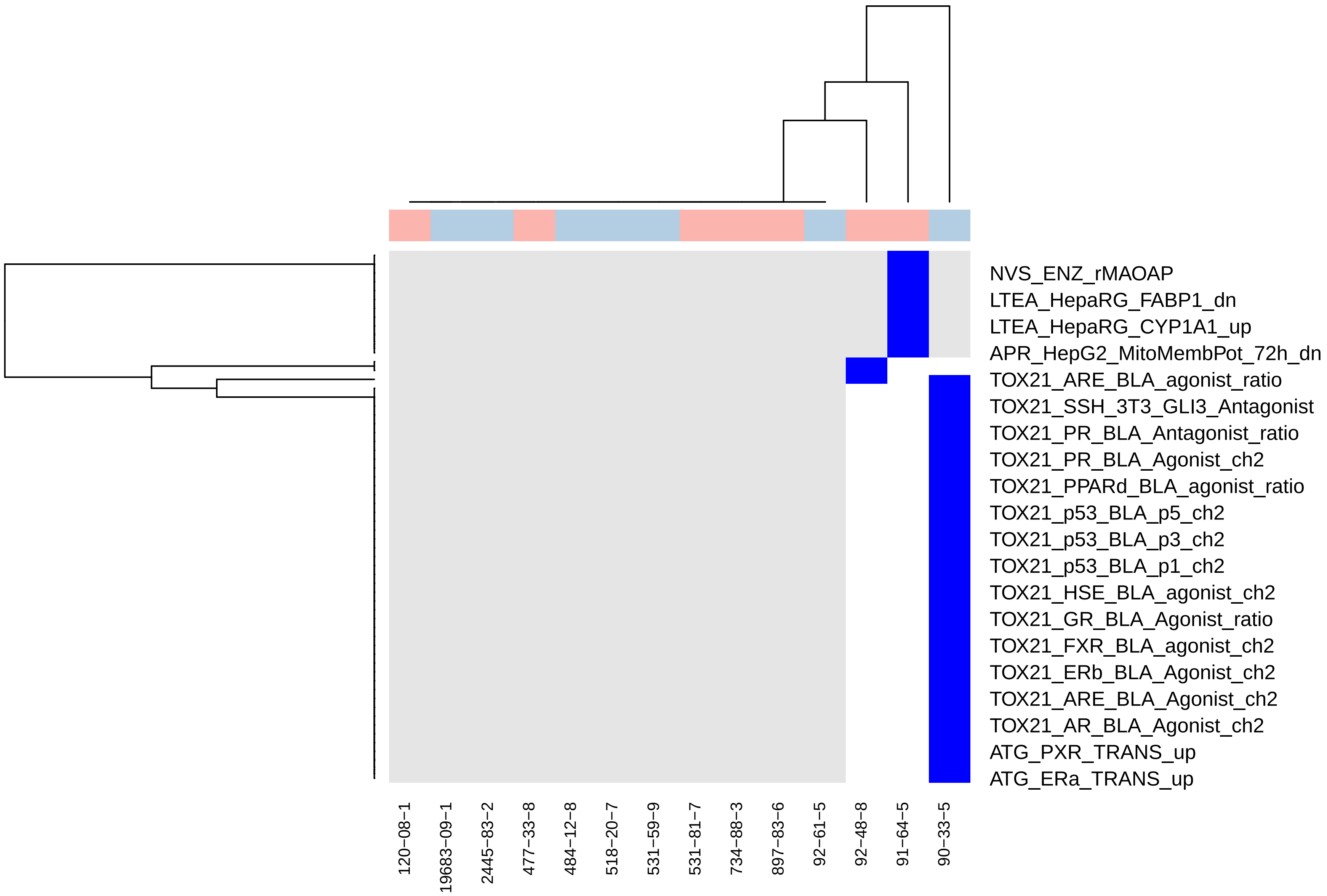

All Activity Heatmap for Cluster 761

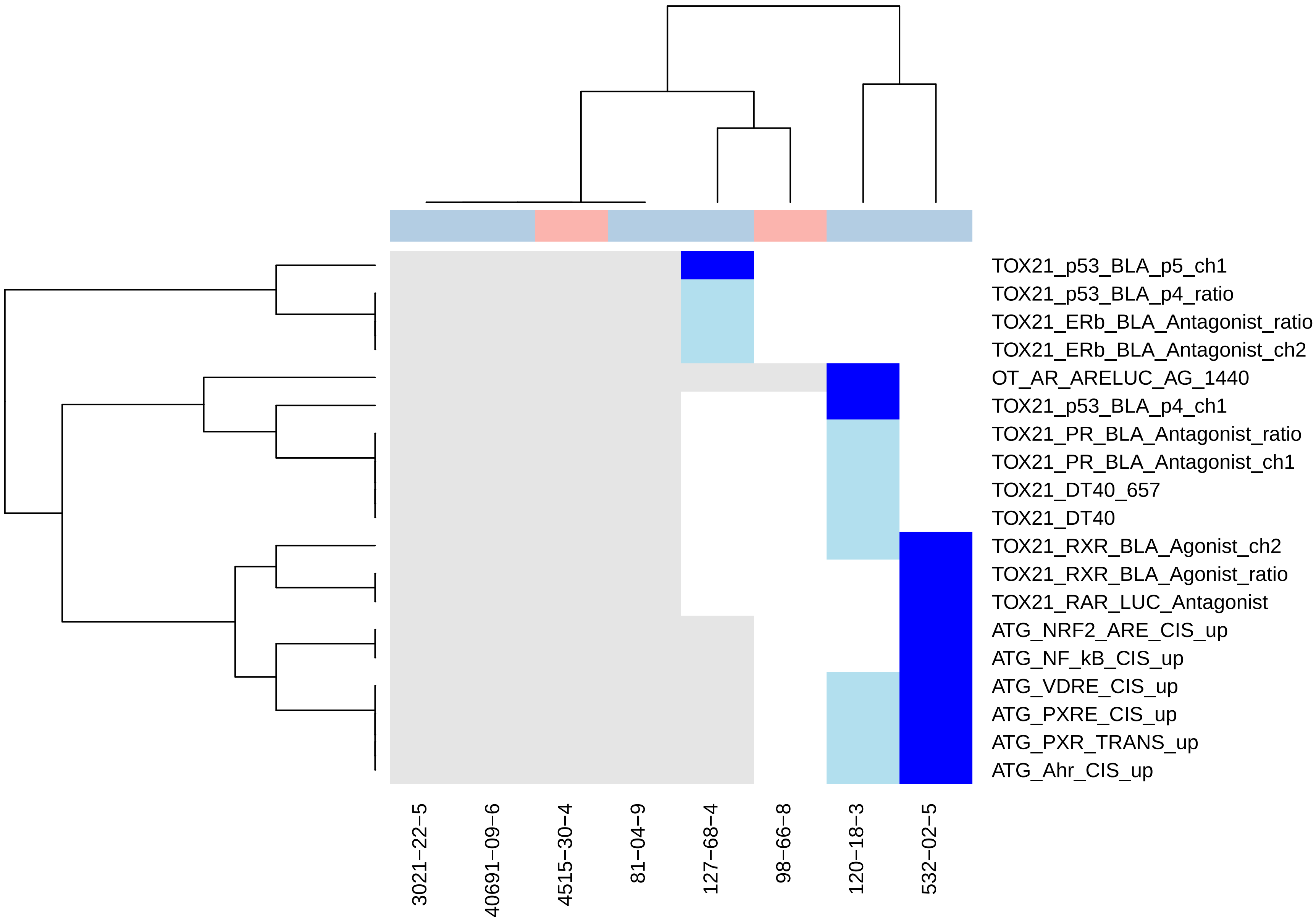

All Activity Heatmap for Cluster 762

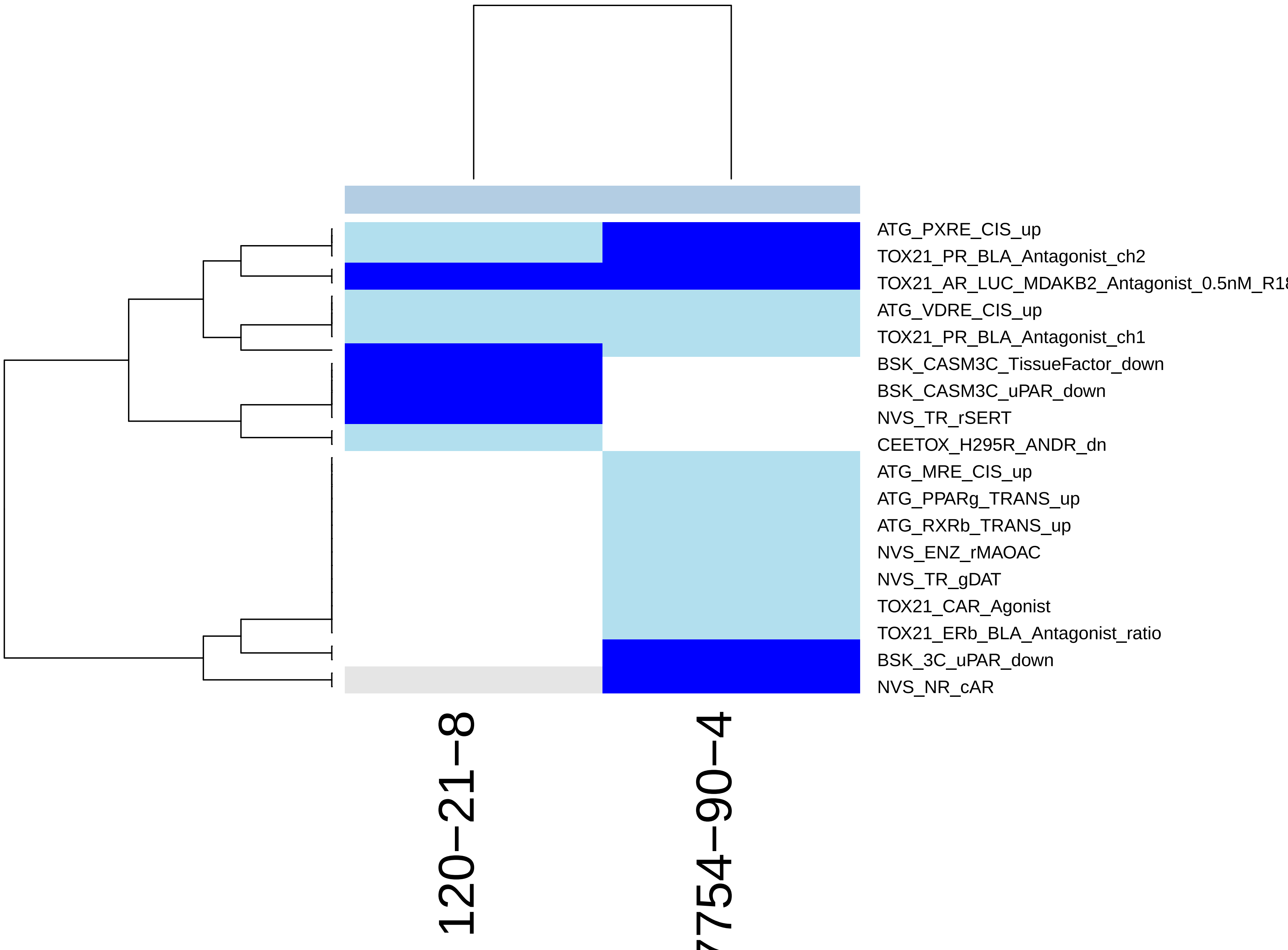

All Activity Heatmap for Cluster 763

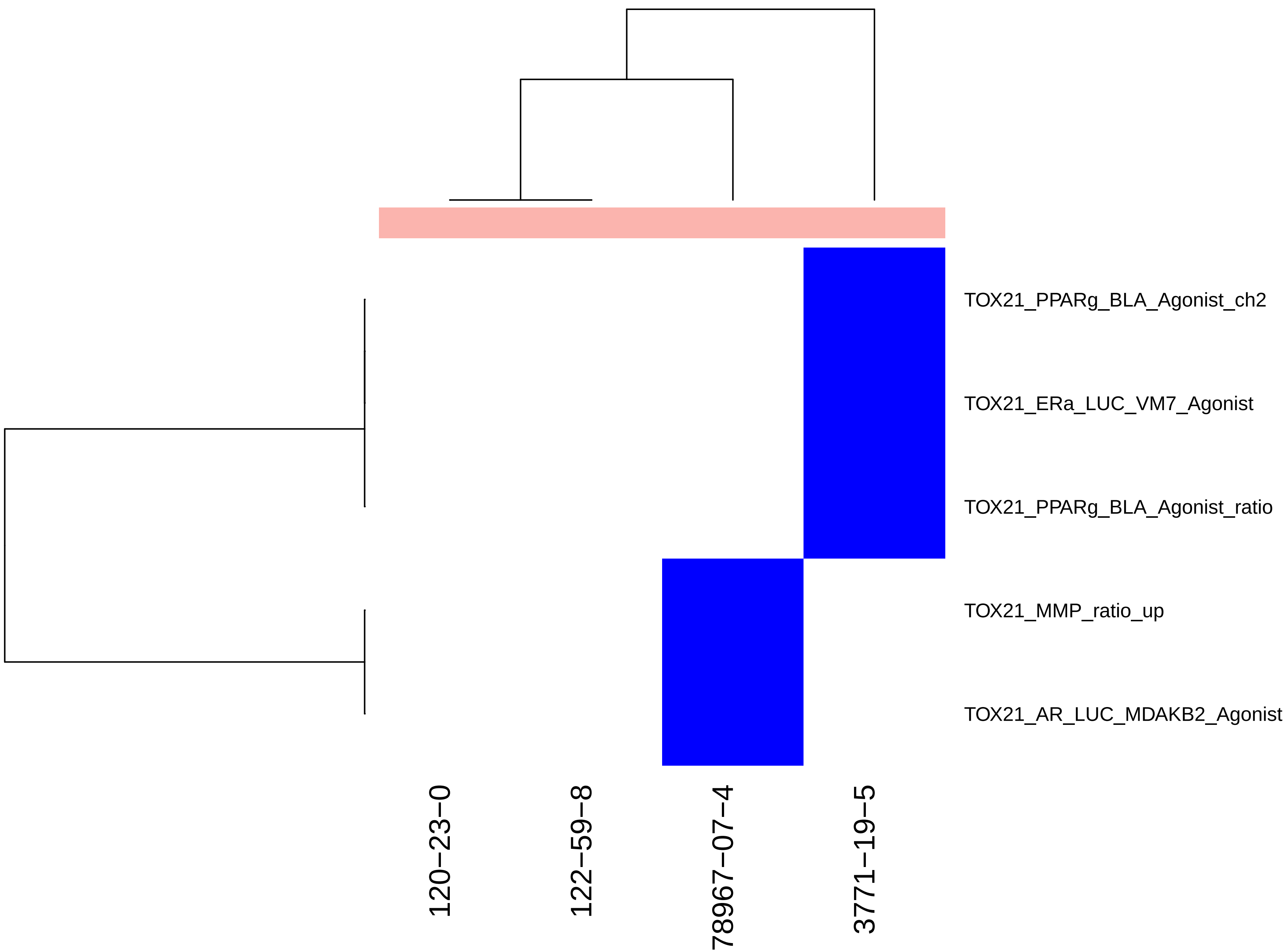

All Activity Heatmap for Cluster 764

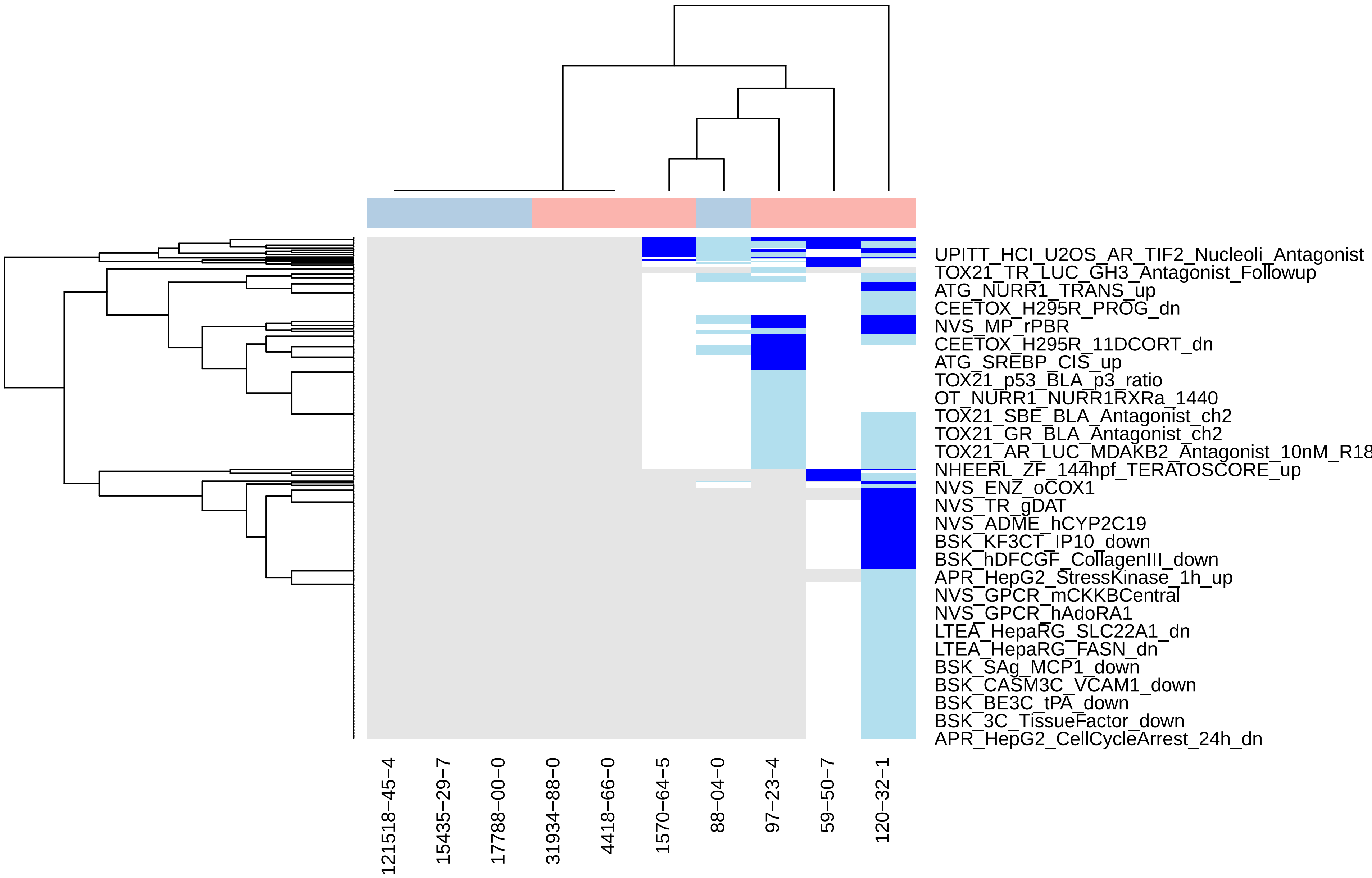

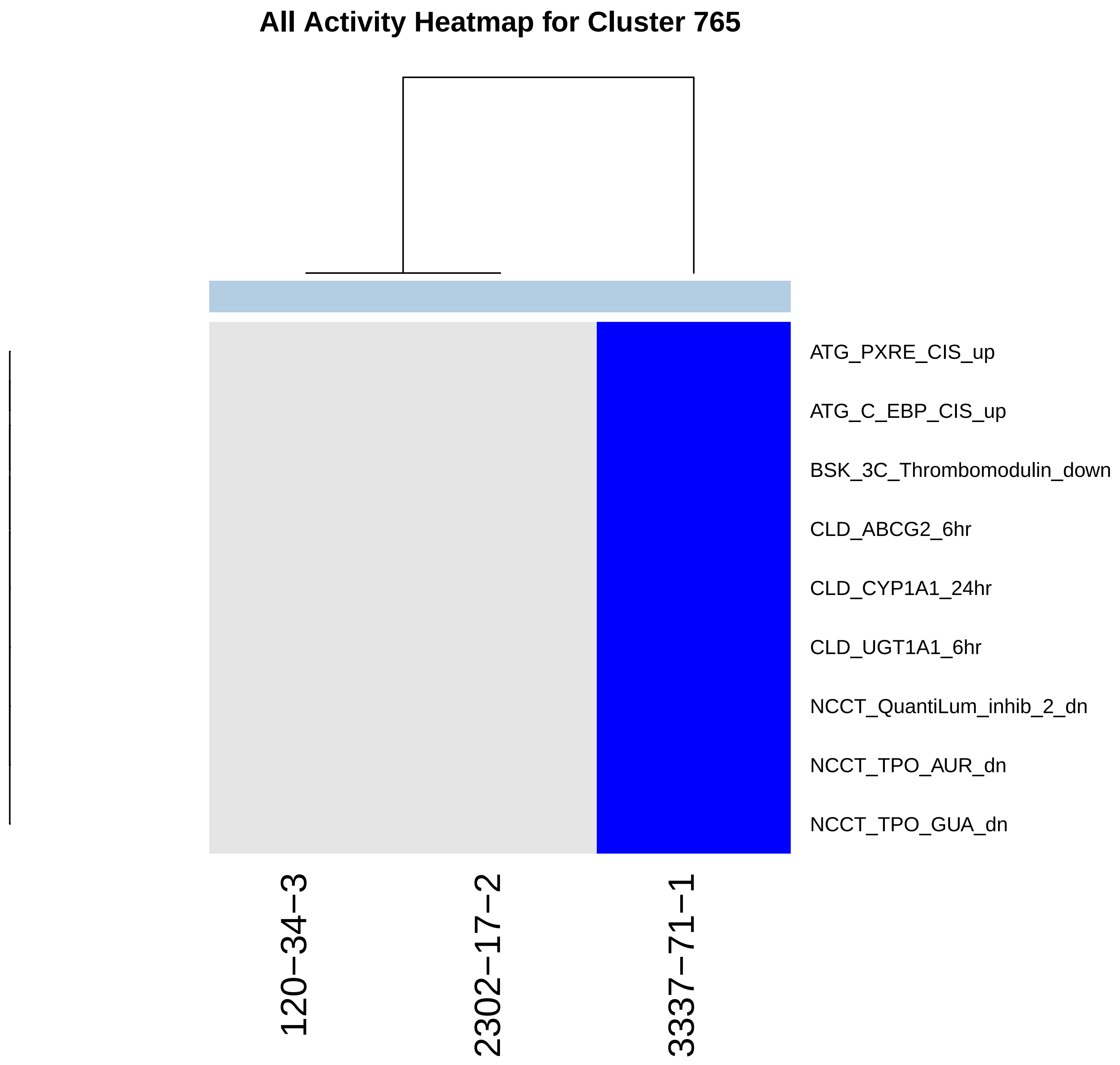

All Activity Heatmap for Cluster 765
ATG_PXRE_CIS_up
ATG_C_EBP_CIS_up
BSK_3C_Thrombomodulin_down
CLD_ABCG2_6hr
CLD_CYP1A1_24hr
CLD_UGT1A1_6hr
NCCT_QuantiLum_inhib_2_dn
NCCT_TPO_AUR_dn
NCCT_TPO_GUA_dn
120−34−3
2302−17−2
3337−71−1

All Activity Heatmap for Cluster 766

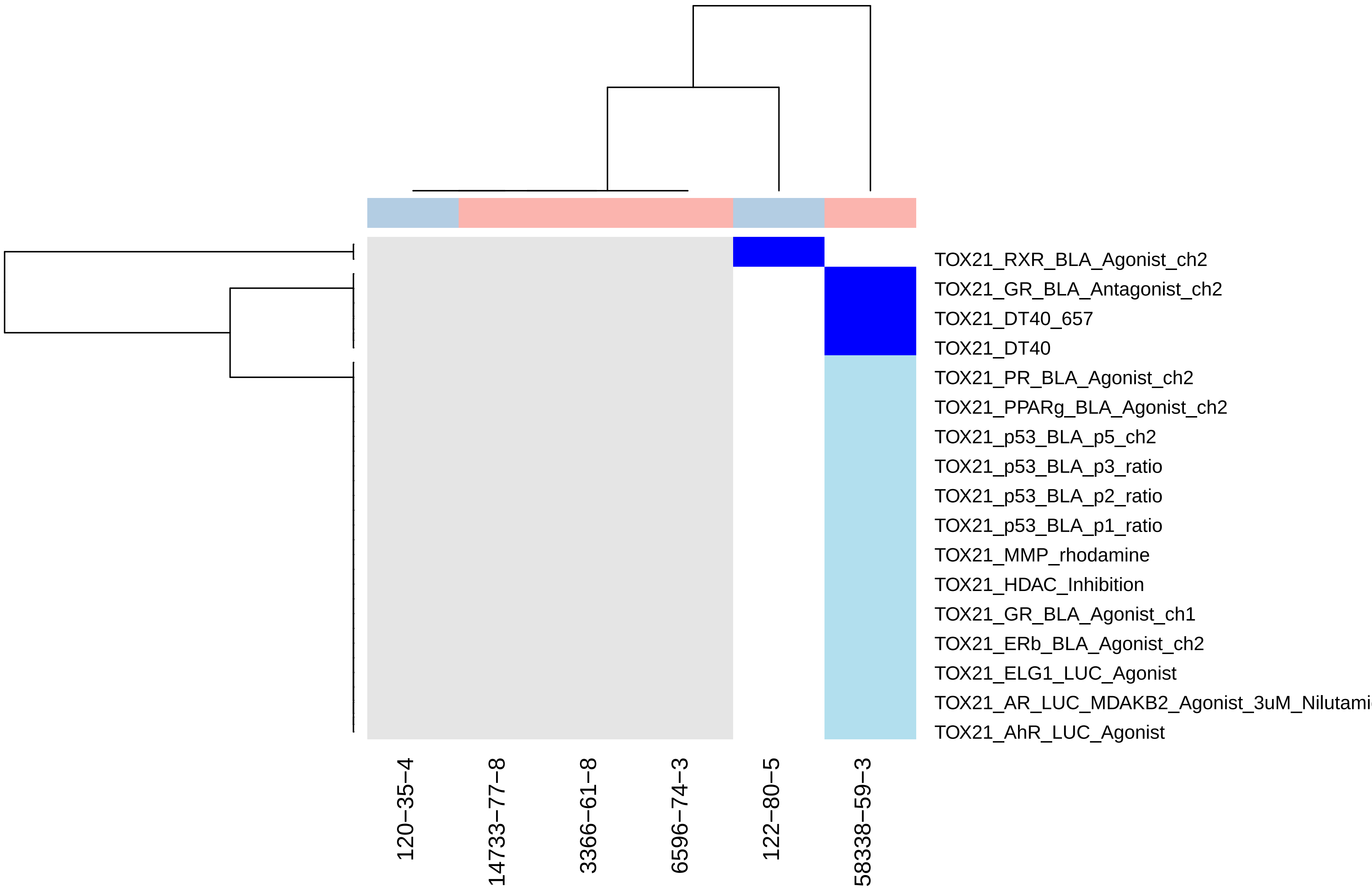

All Activity Heatmap for Cluster 767

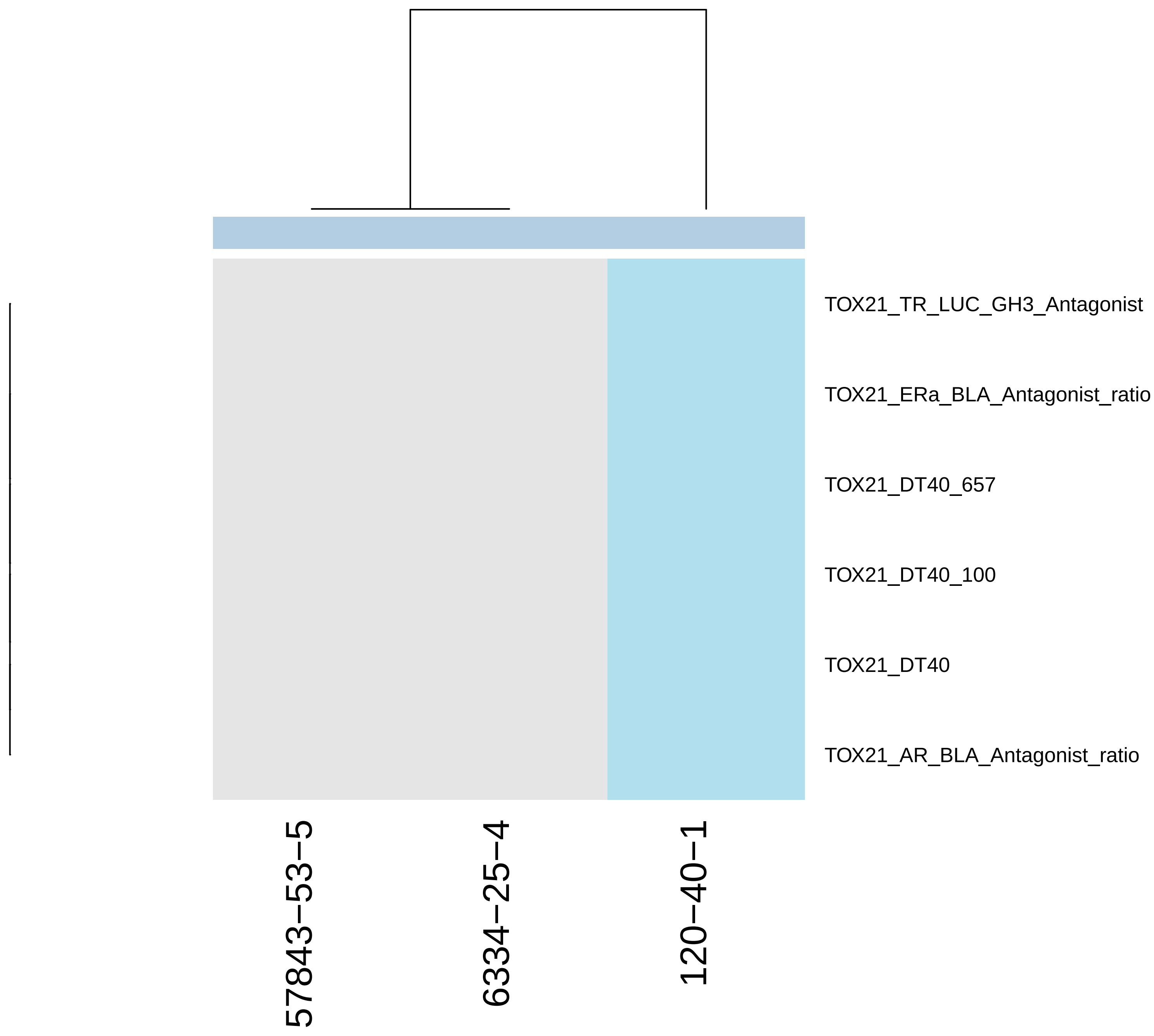

All Activity Heatmap for Cluster 768

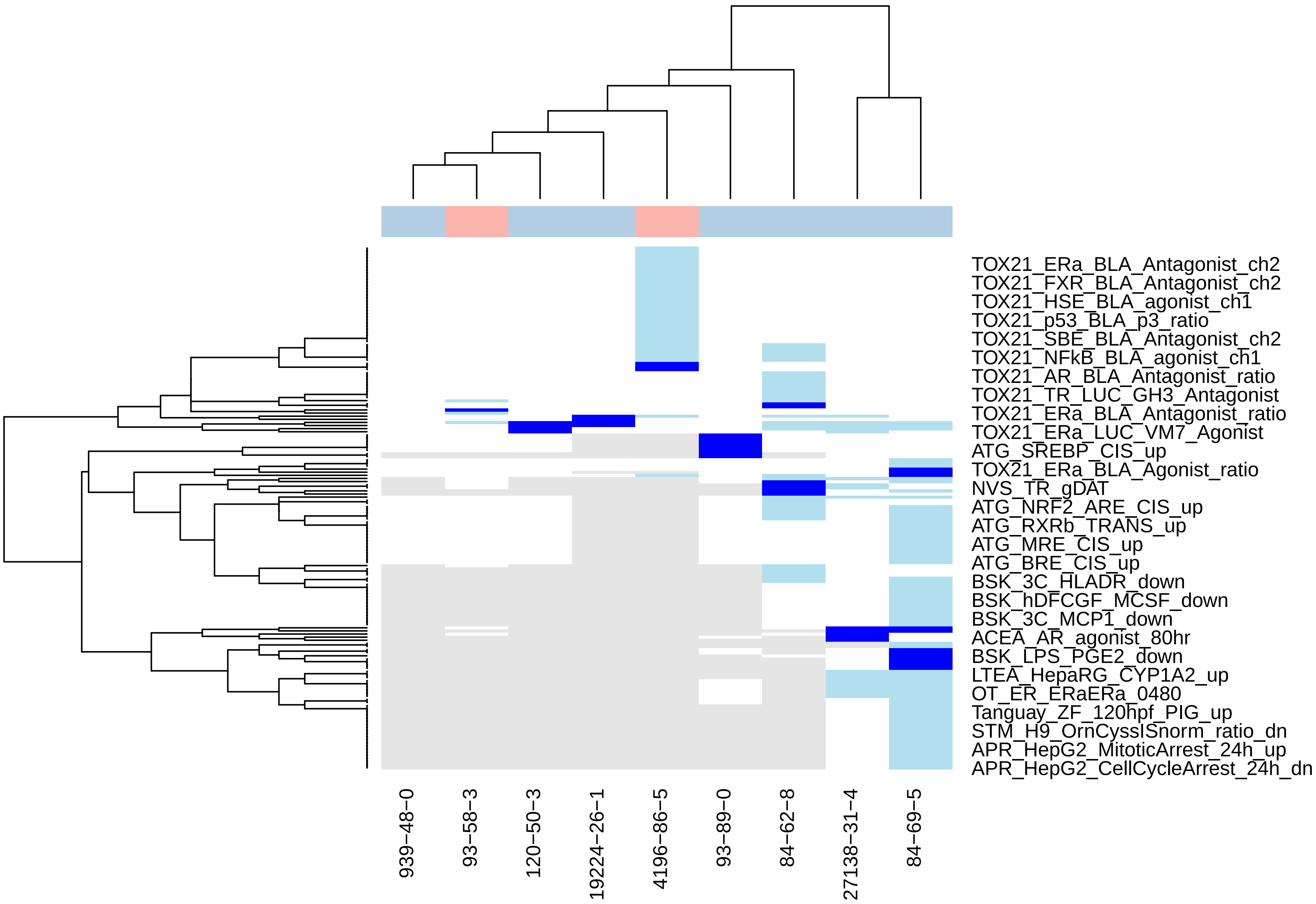

All Activity Heatmap for Cluster 769

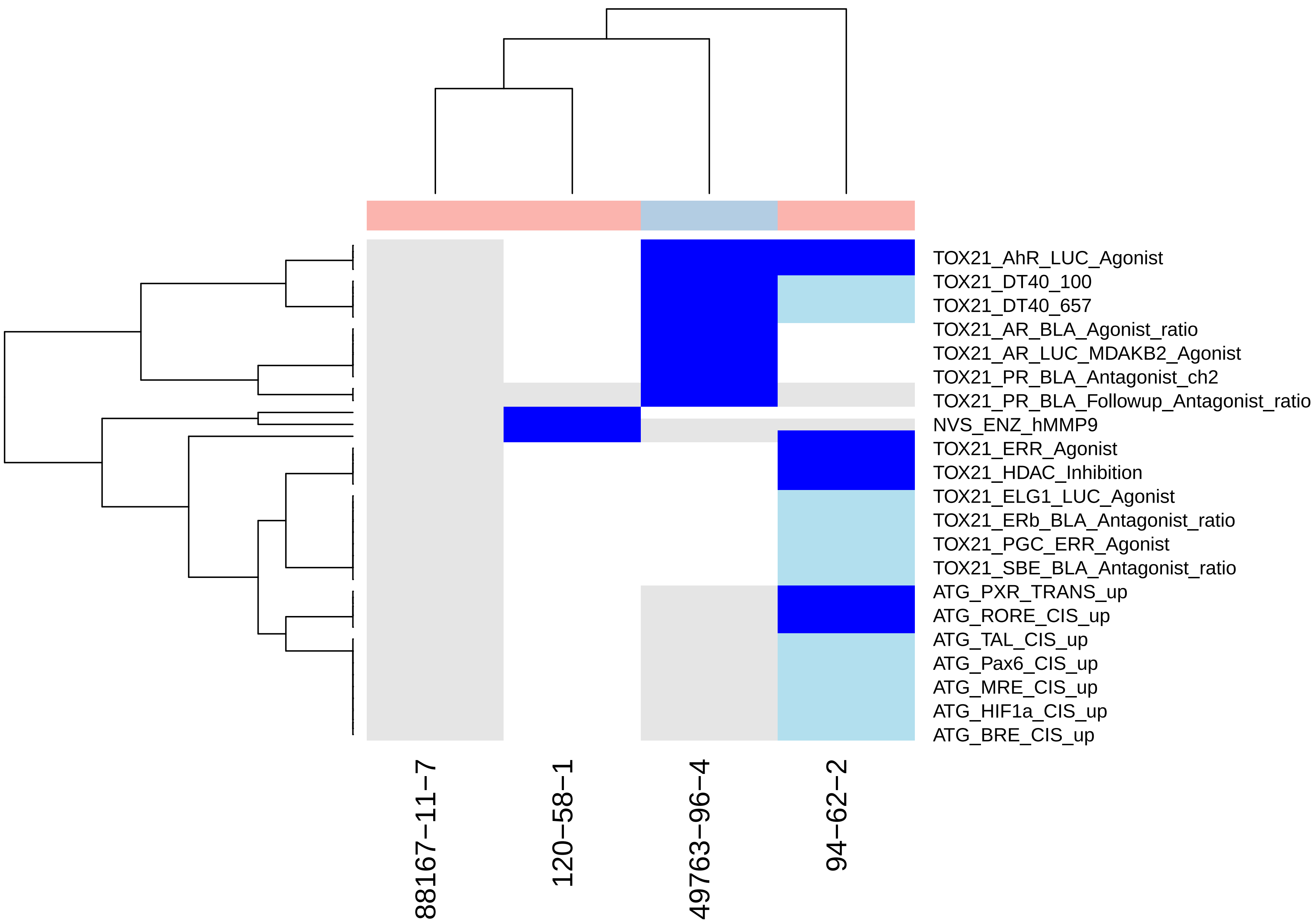

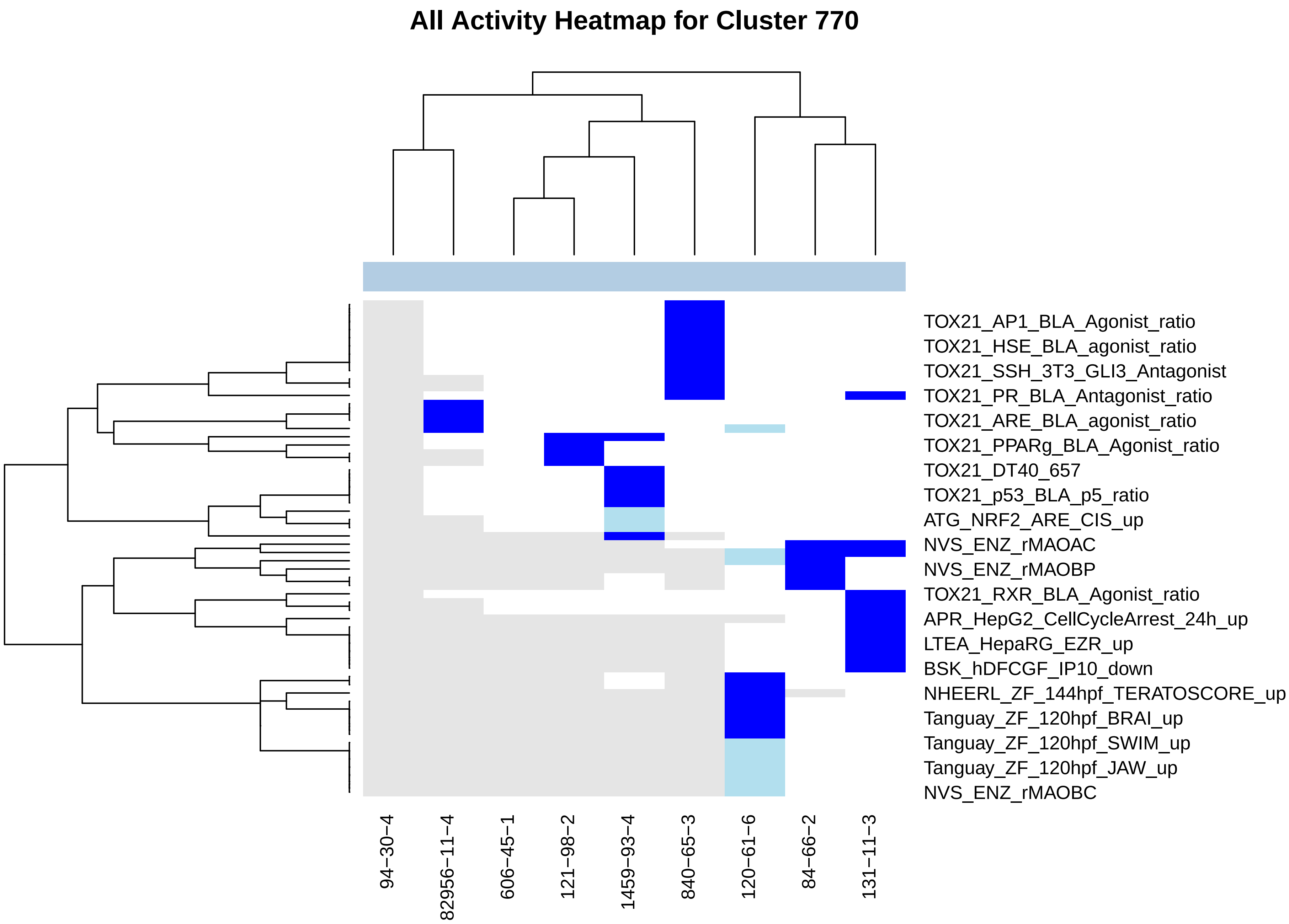
All Activity Heatmap for Cluster 770
TOX21_AP1_BLA_Agonist_ratio
TOX21_HSE_BLA_agonist_ratio
TOX21_SSH_3T3_GLI3_Antagonist
TOX21_PR_BLA_Antagonist_ratio
TOX21_ARE_BLA_agonist_ratio
TOX21_PPARg_BLA_Agonist_ratio
TOX21_DT40_657
TOX21_p53_BLA_p5_ratio
ATG_NRF2_ARE_CIS_up
NVS_ENZ_rMAOAC
NVS_ENZ_rMAOBP
TOX21_RXR_BLA_Agonist_ratio
APR_HepG2_CellCycleArrest_24h_up
LTEA_HepaRG_EZR_up
BSK_hDFCGF_IP10_down
NHEERL_ZF_144hpf_TERATOSCORE_up
Tanguay_ZF_120hpf_BRAI_up
Tanguay_ZF_120hpf_SWIM_up
Tanguay_ZF_120hpf_JAW_up
NVS_ENZ_rMAOBC
94−30−4
82956−11−4
606−45−1
121−98−2
1459−93−4
840−65−3
120−61−6
84−66−2
131−11−3

All Activity Heatmap for Cluster 771

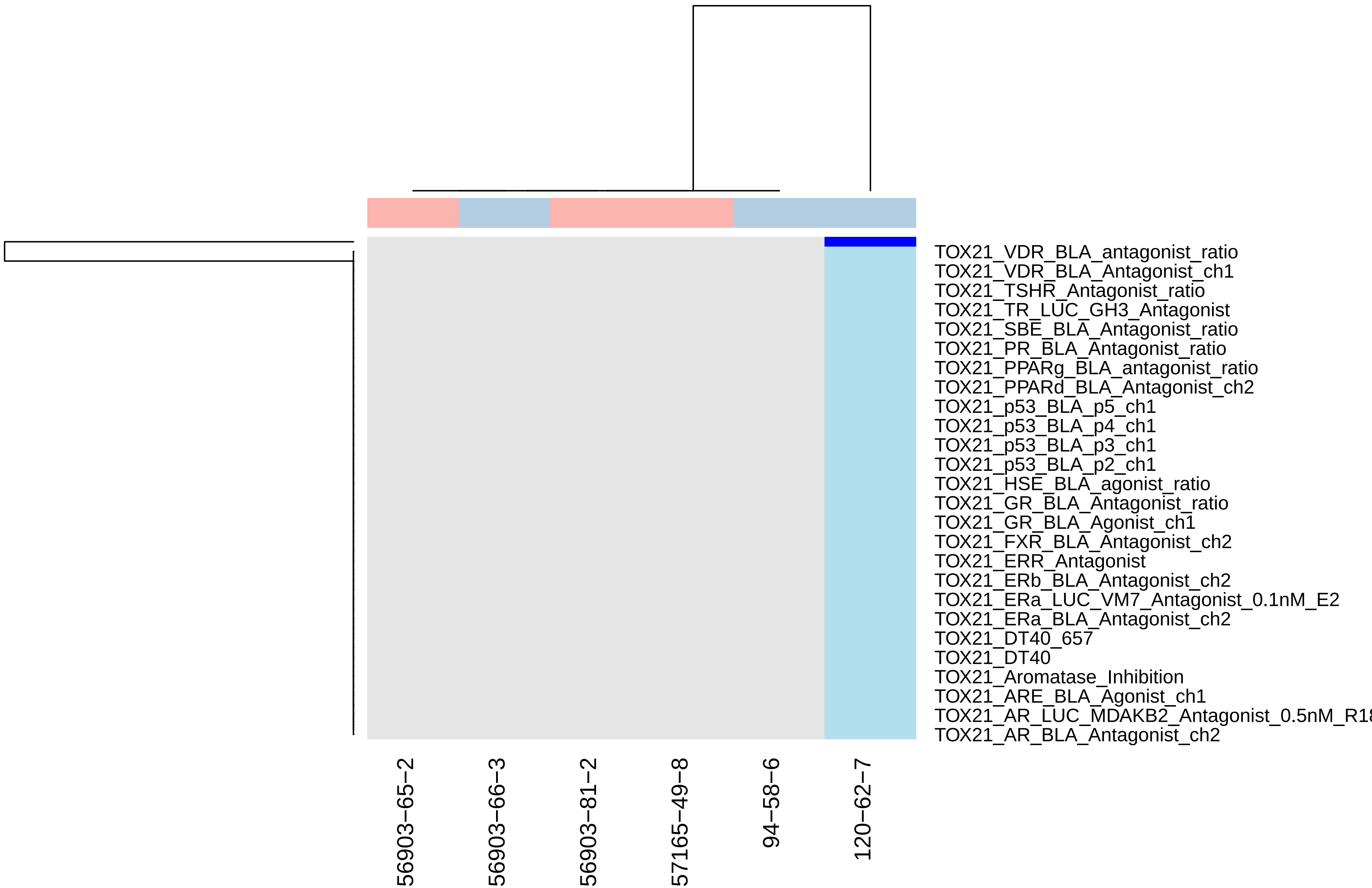

All Activity Heatmap for Cluster 772

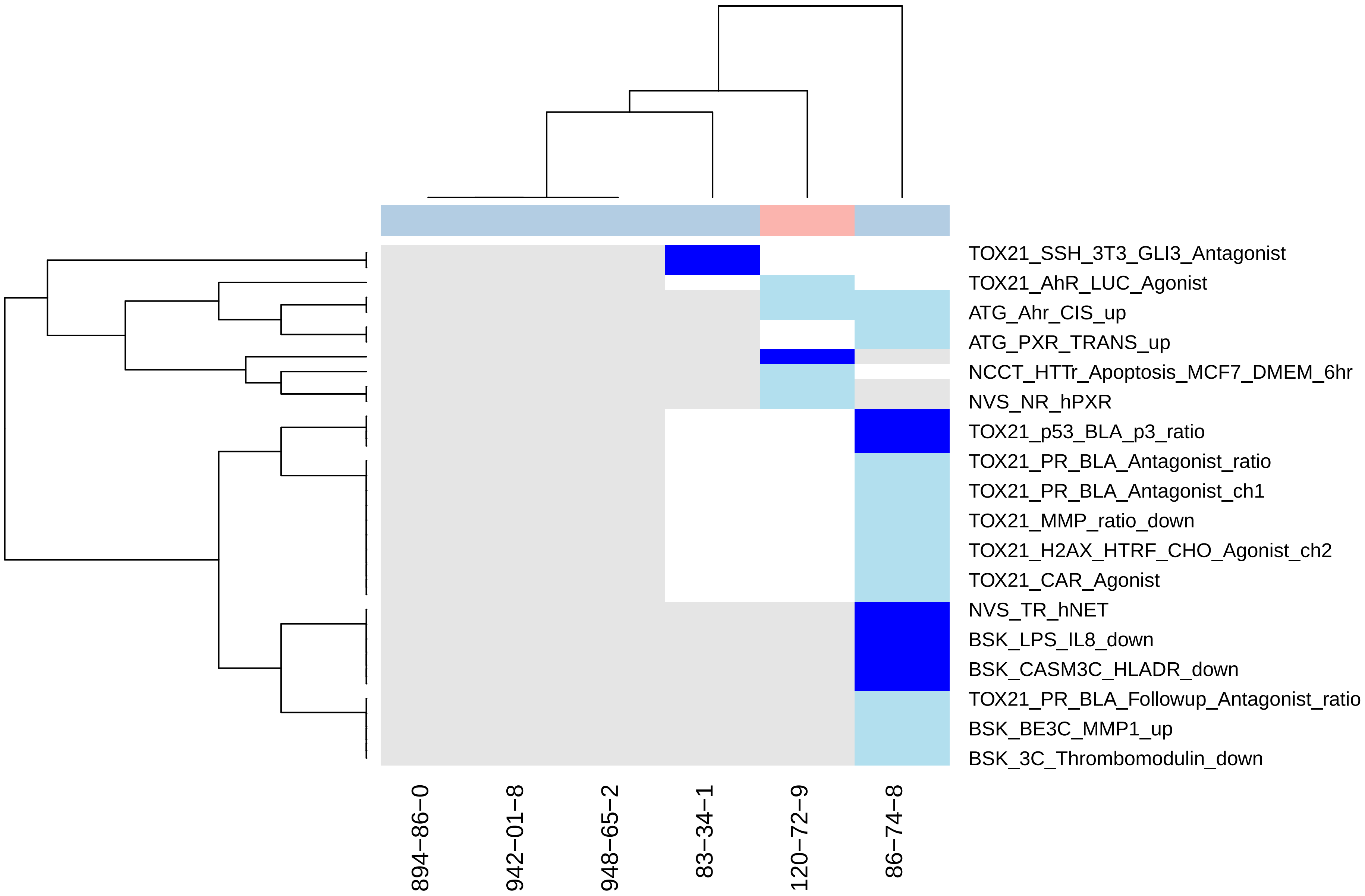

All Activity Heatmap for Cluster 773

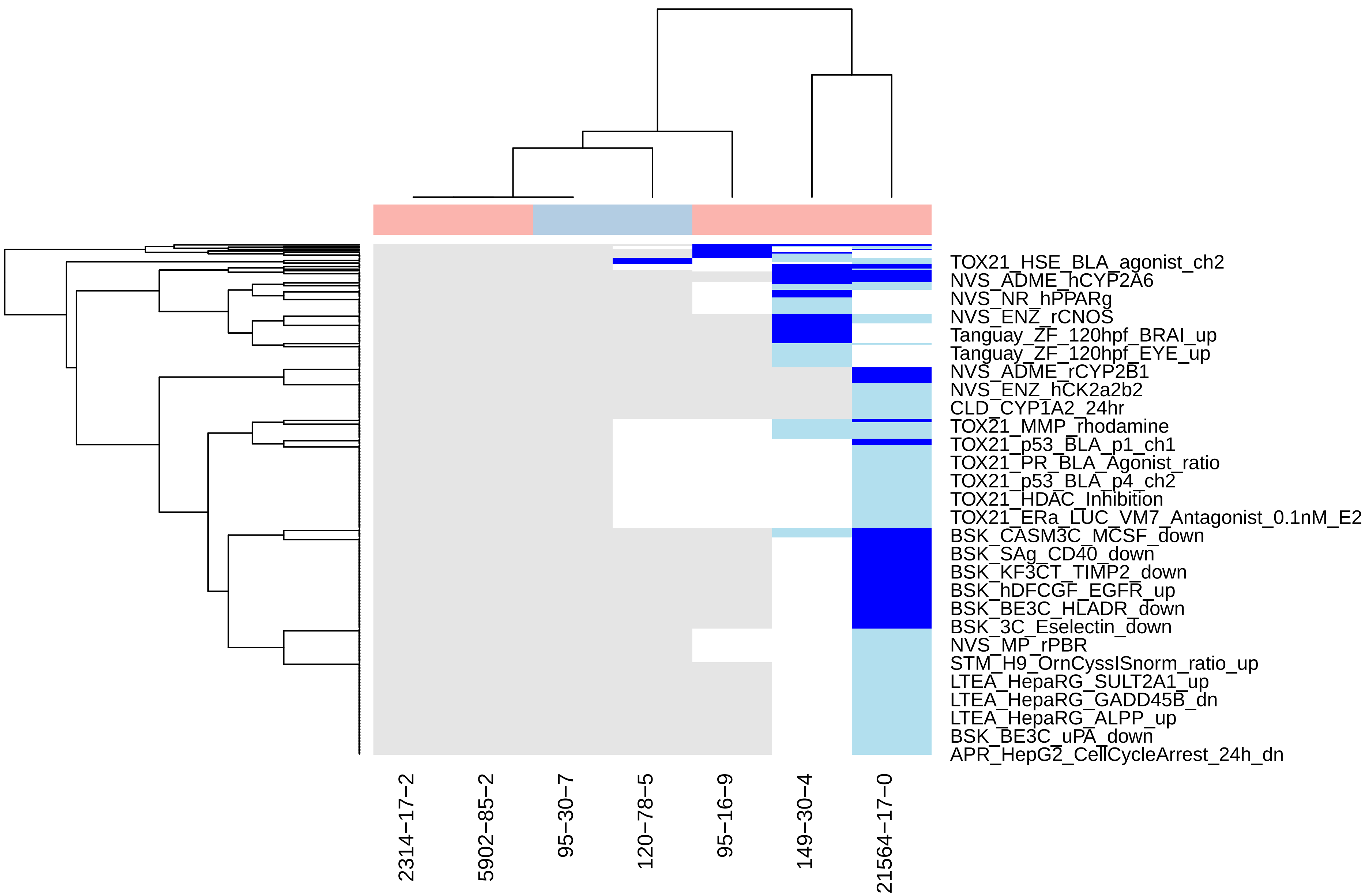

All Activity Heatmap for Cluster 774

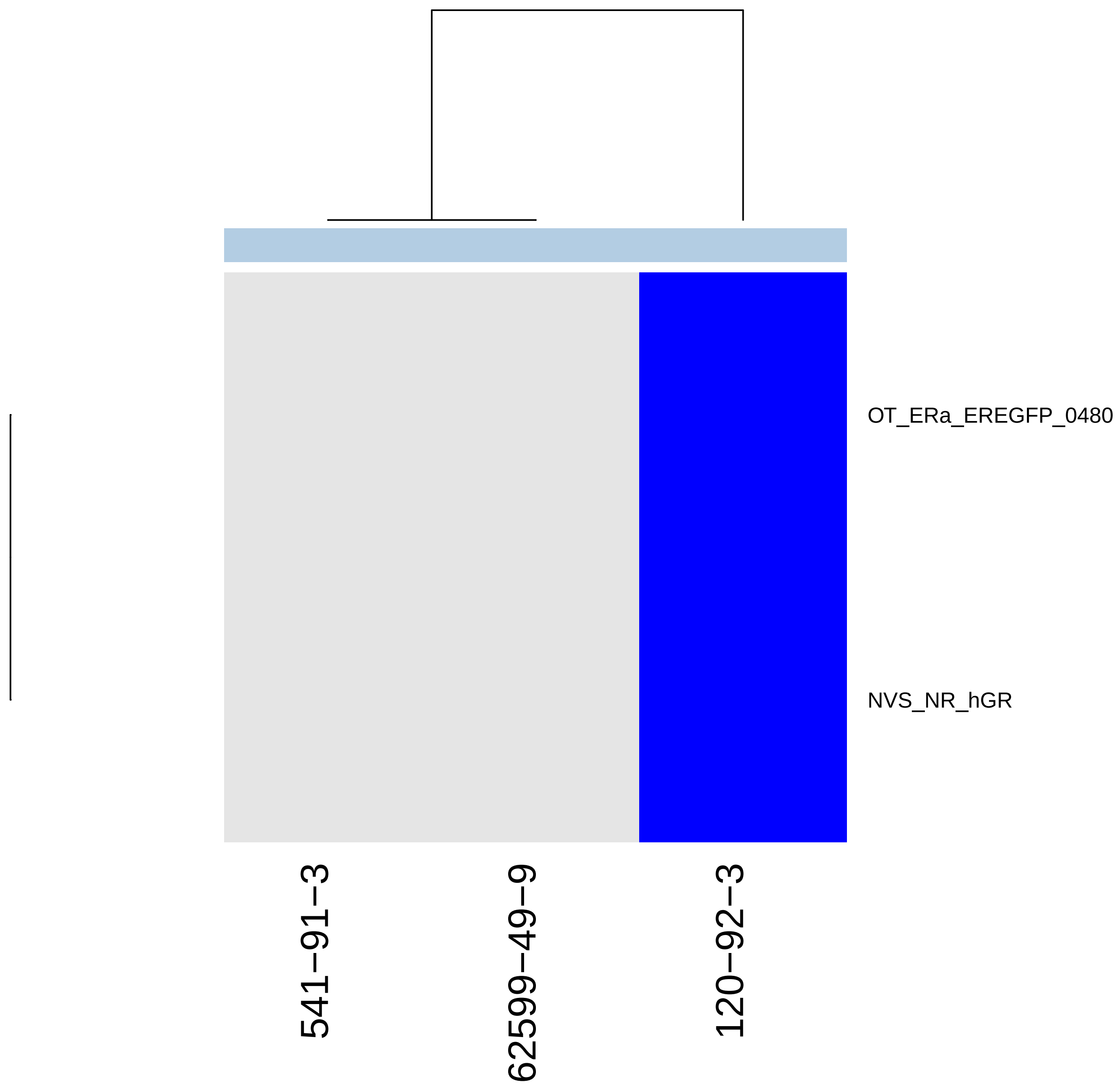

All Activity Heatmap for Cluster 775

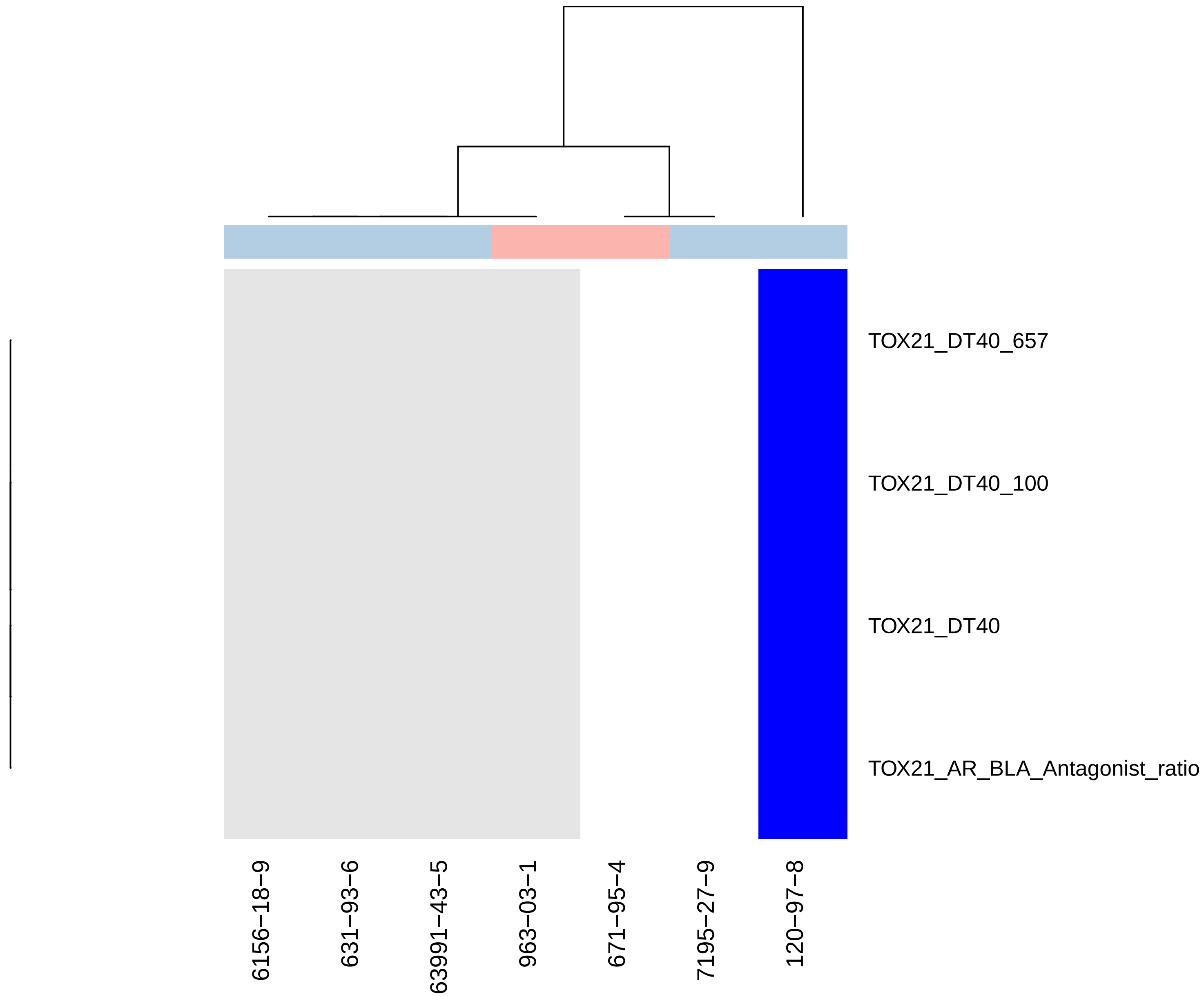

All Activity Heatmap for Cluster 776

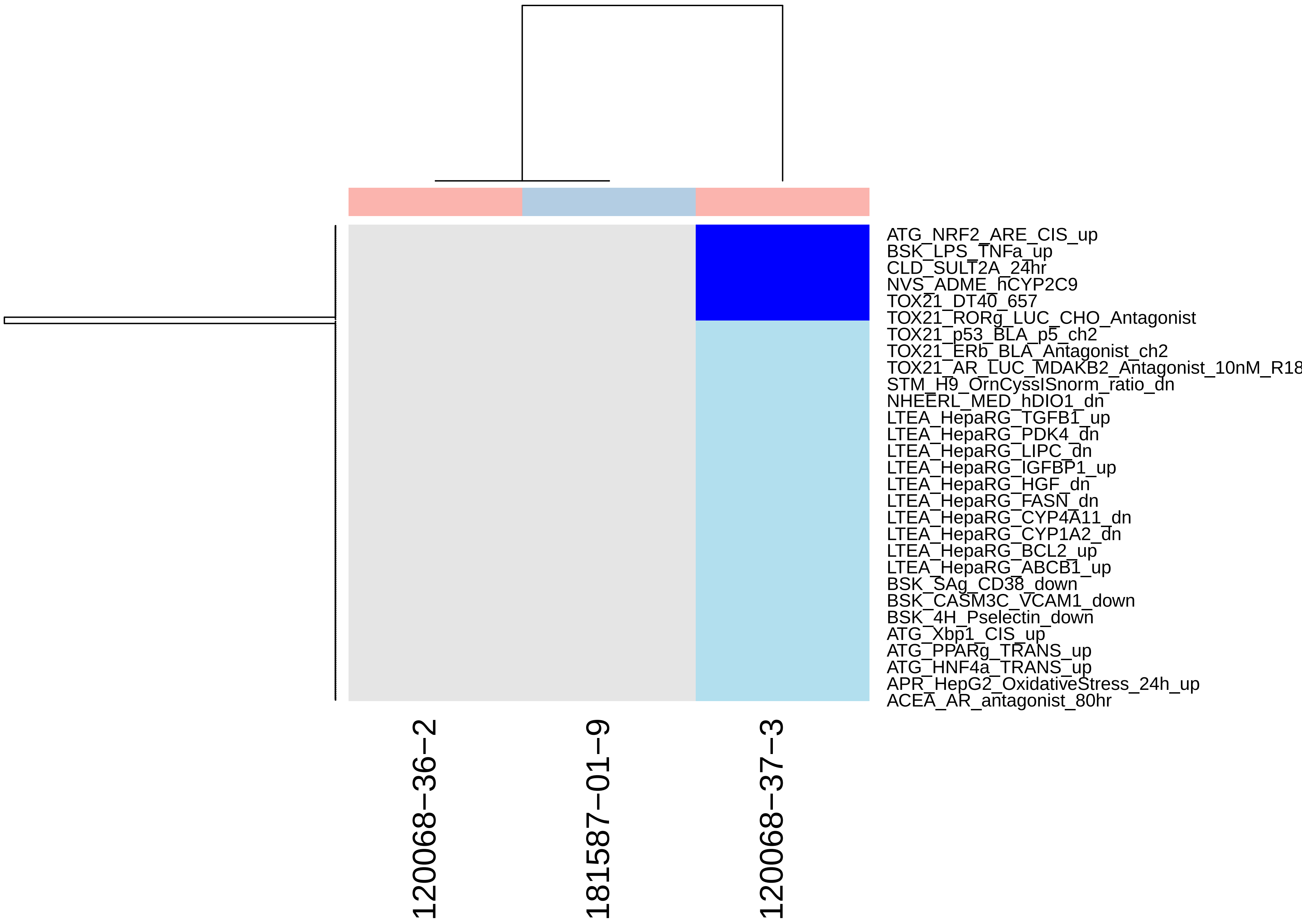

All Activity Heatmap for Cluster 777

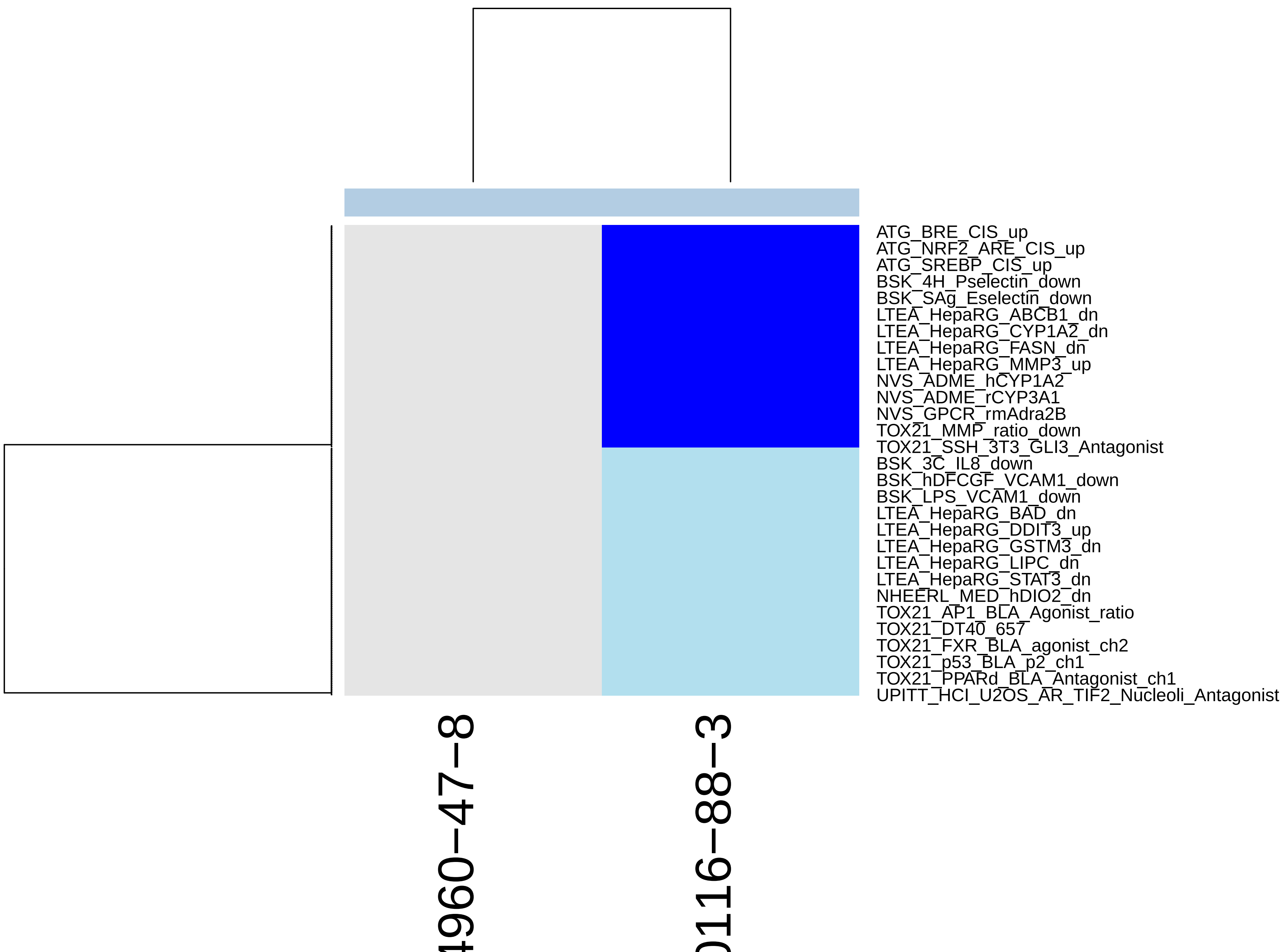

All Activity Heatmap for Cluster 779

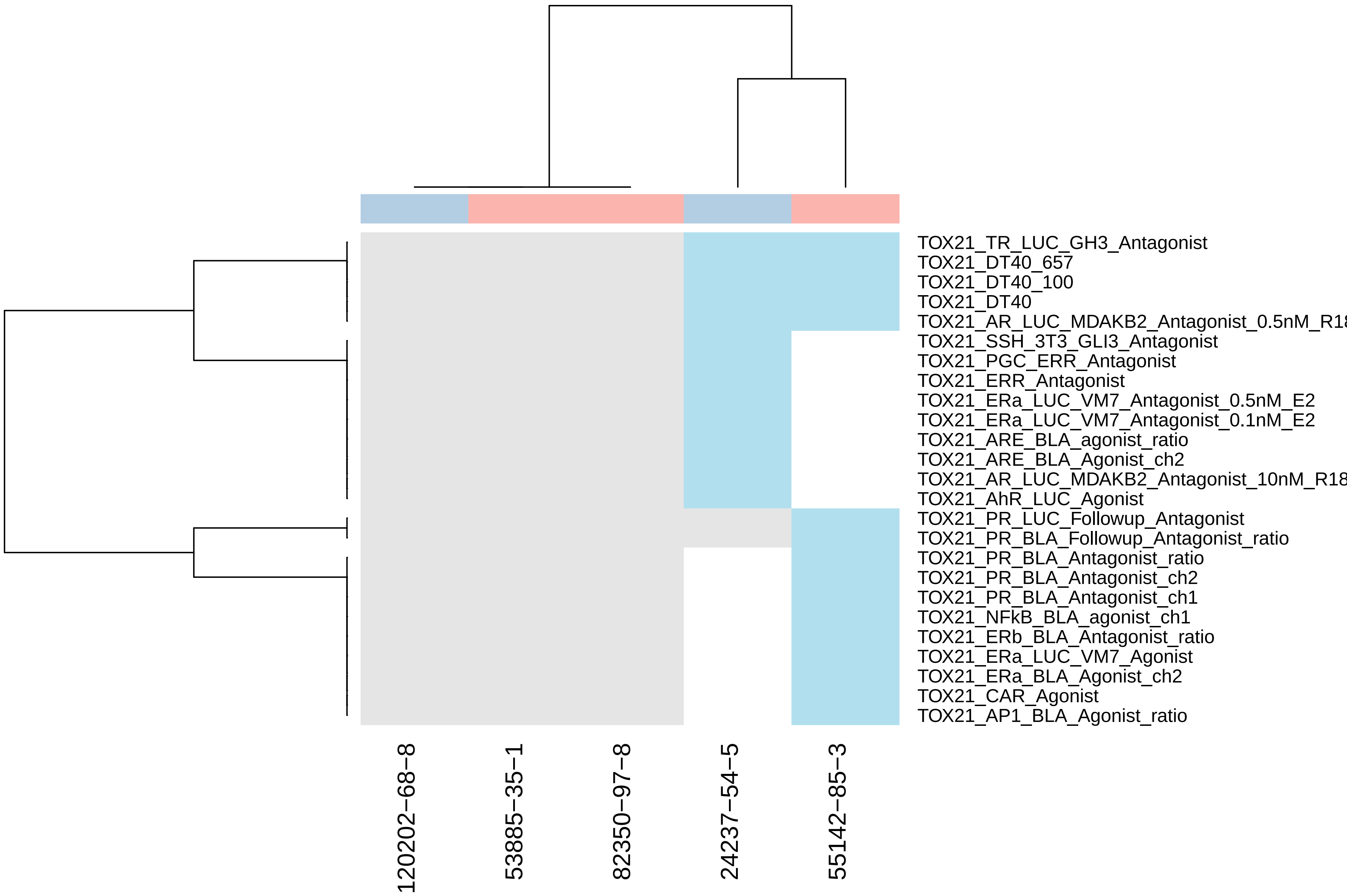

All Activity Heatmap for Cluster 783

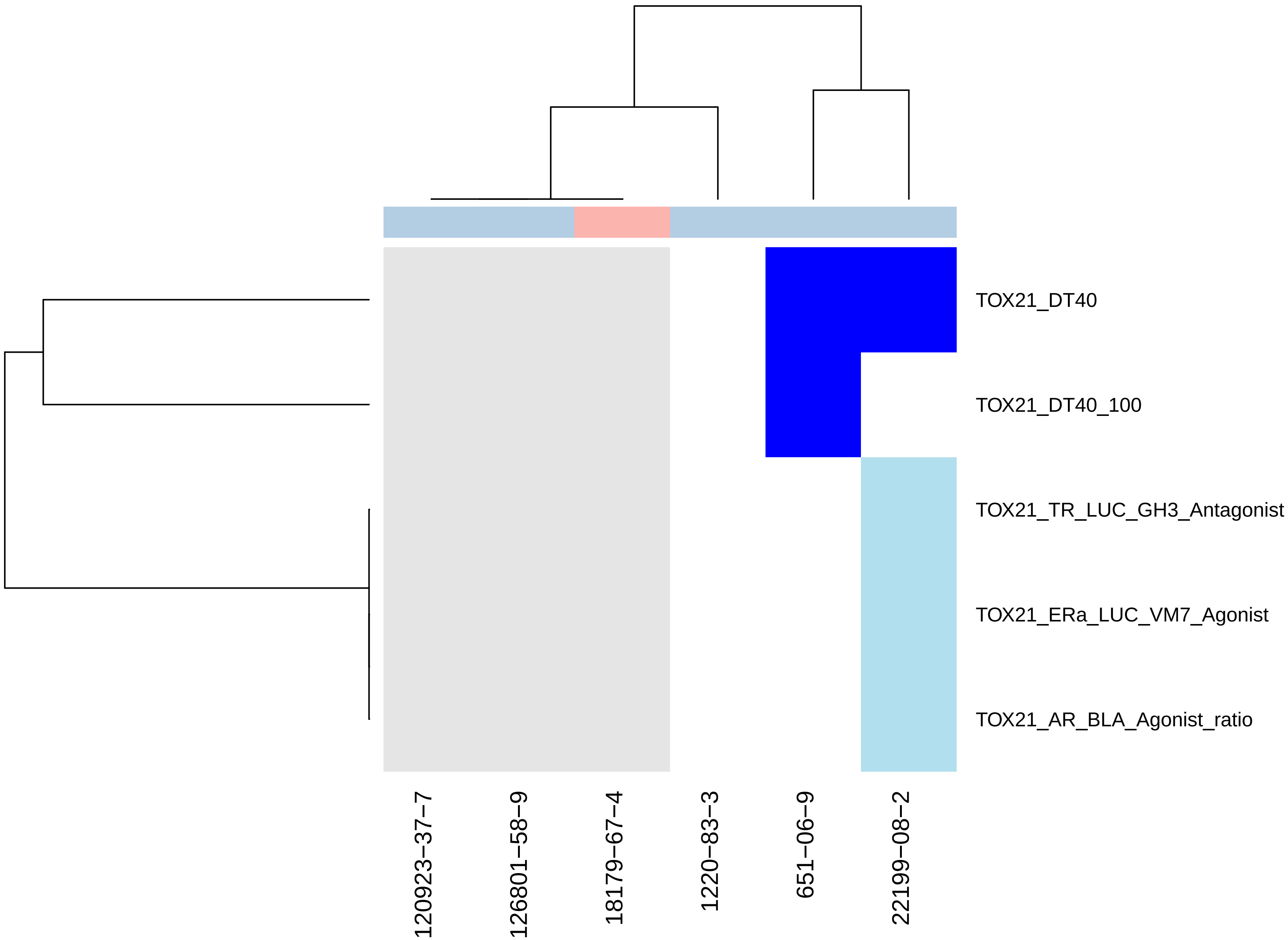

All Activity Heatmap for Cluster 784

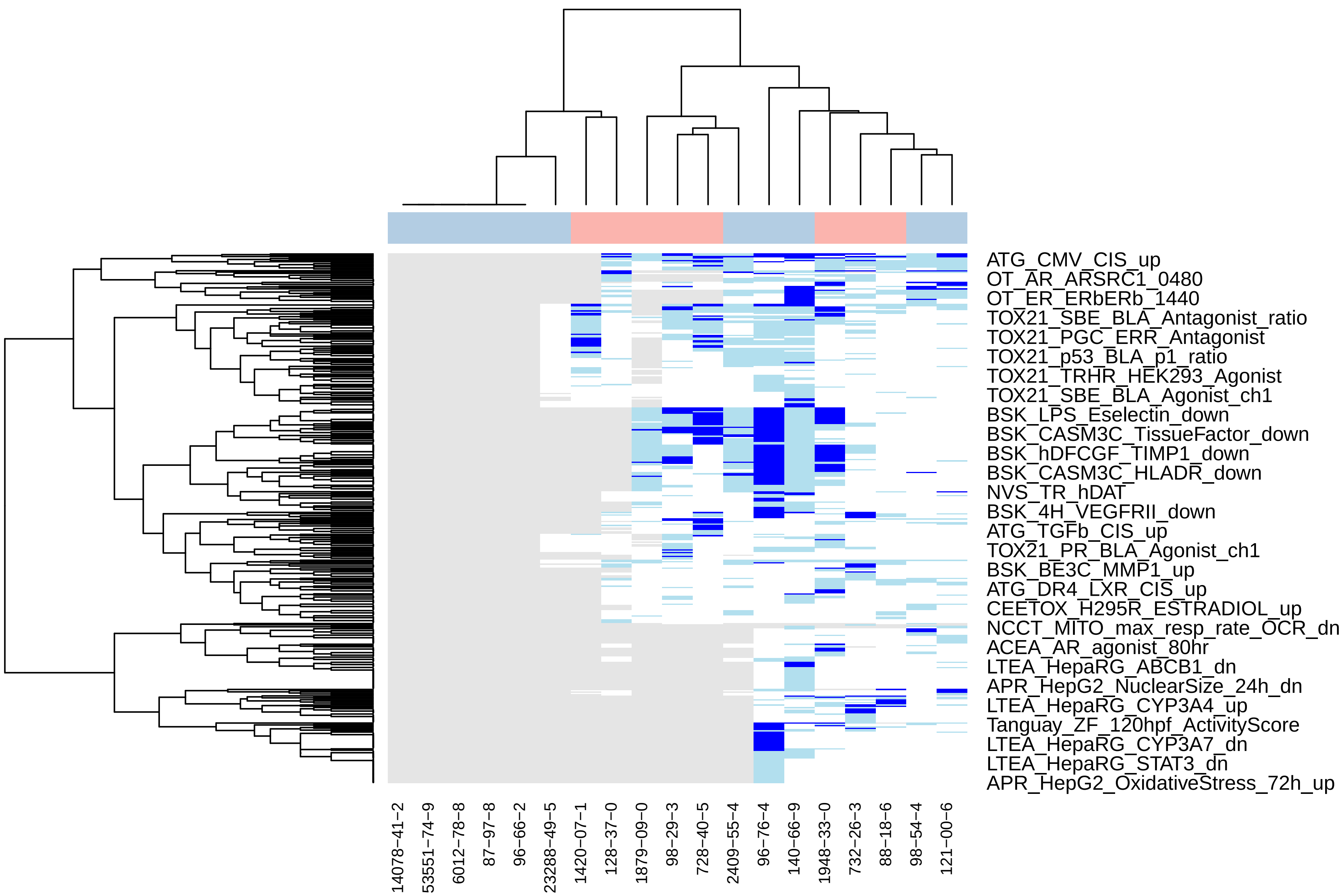

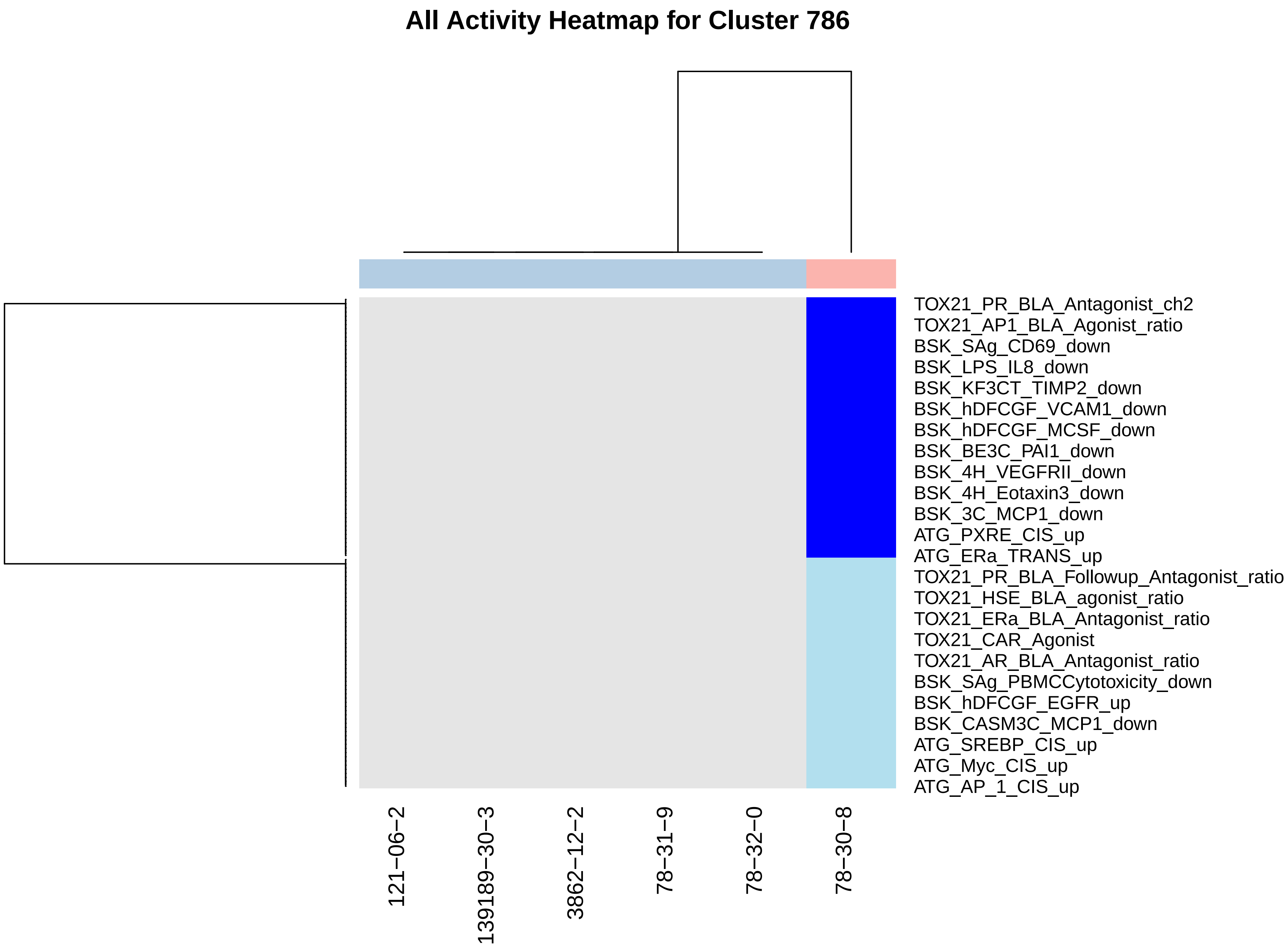
All Activity Heatmap for Cluster 786
TOX21_PR_BLA_Antagonist_ch2
TOX21_AP1_BLA_Agonist_ratio
BSK_SAg_CD69_down
BSK_LPS_IL8_down
BSK_KF3CT_TIMP2_down
BSK_hDFCGF_VCAM1_down
BSK_hDFCGF_MCSF_down
BSK_BE3C_PAI1_down
BSK_4H_VEGFRII_down
BSK_4H_Eotaxin3_down
BSK_3C_MCP1_down
ATG_PXRE_CIS_up
ATG_ERa_TRANS_up
TOX21_PR_BLA_Followup_Antagonist_ratio
TOX21_HSE_BLA_agonist_ratio
TOX21_ERa_BLA_Antagonist_ratio
TOX21_CAR_Agonist
TOX21_AR_BLA_Antagonist_ratio
BSK_SAg_PBMCCytotoxicity_down
BSK_hDFCGF_EGFR_up
BSK_CASM3C_MCP1_down
ATG_SREBP_CIS_up
ATG_Myc_CIS_up
ATG_AP_1_CIS_up
121−06−2
139189−30−3
3862−12−2
78−31−9
78−32−0
78−30−8

All Activity Heatmap for Cluster 787

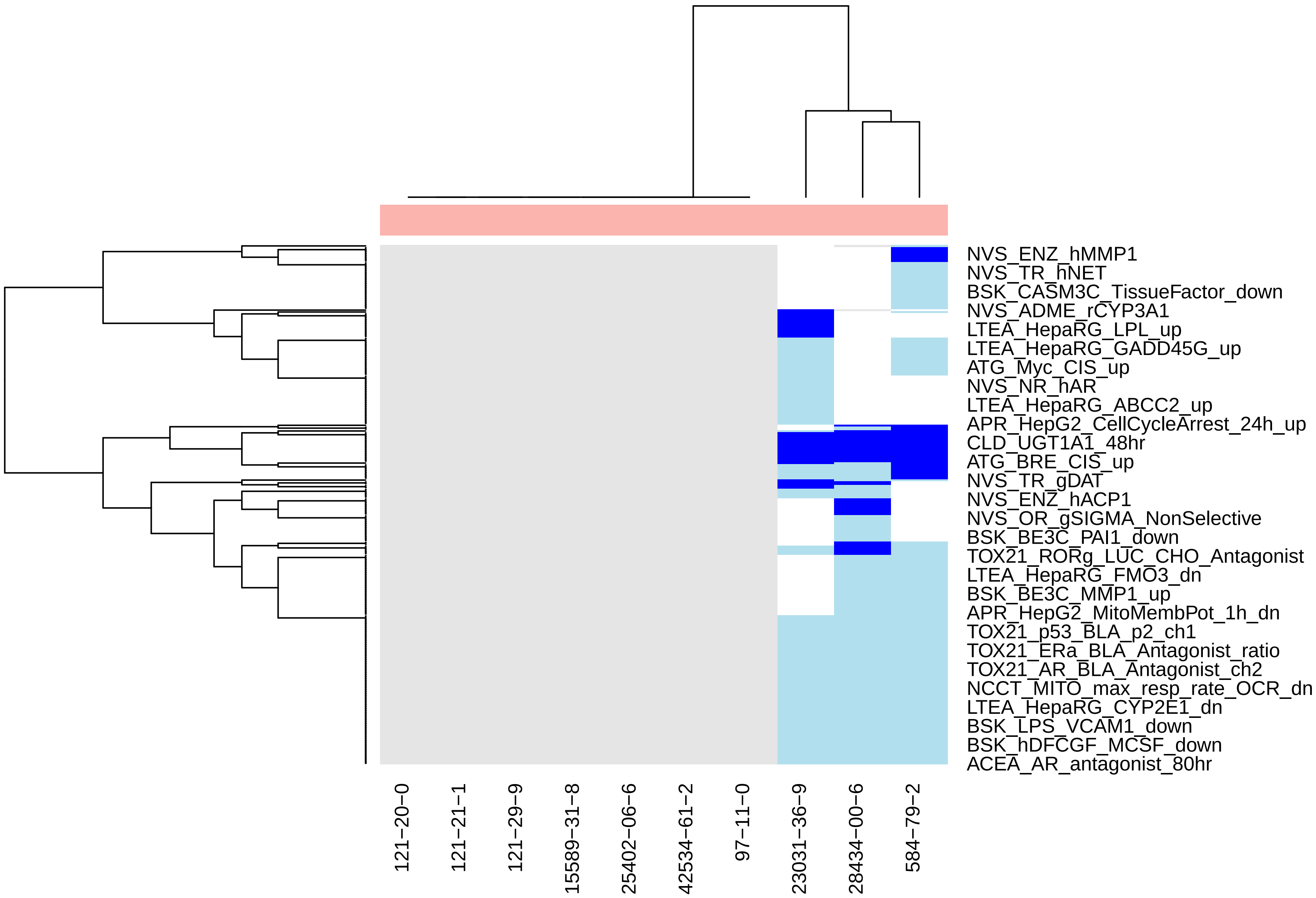

All Activity Heatmap for Cluster 788

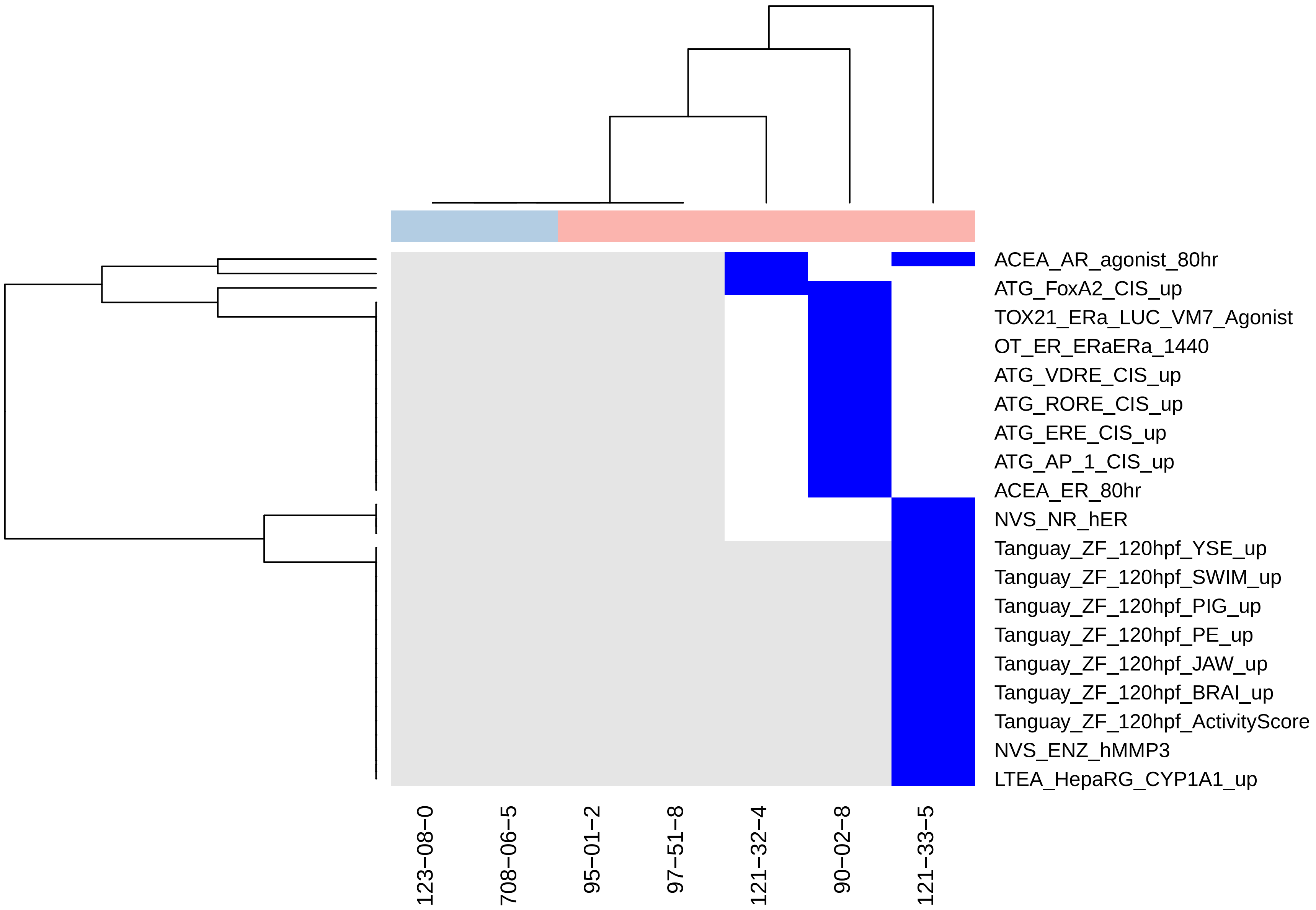

All Activity Heatmap for Cluster 789

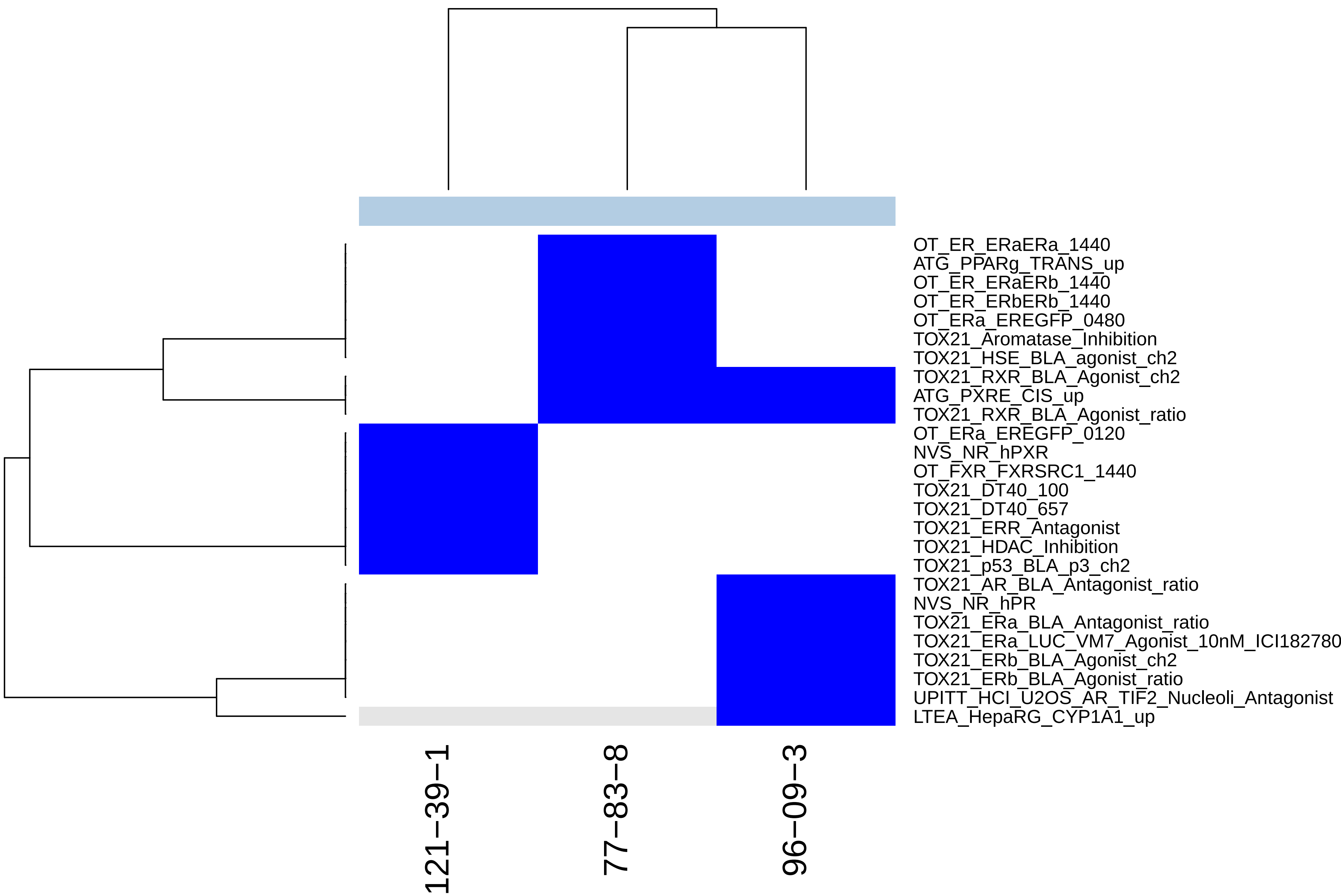

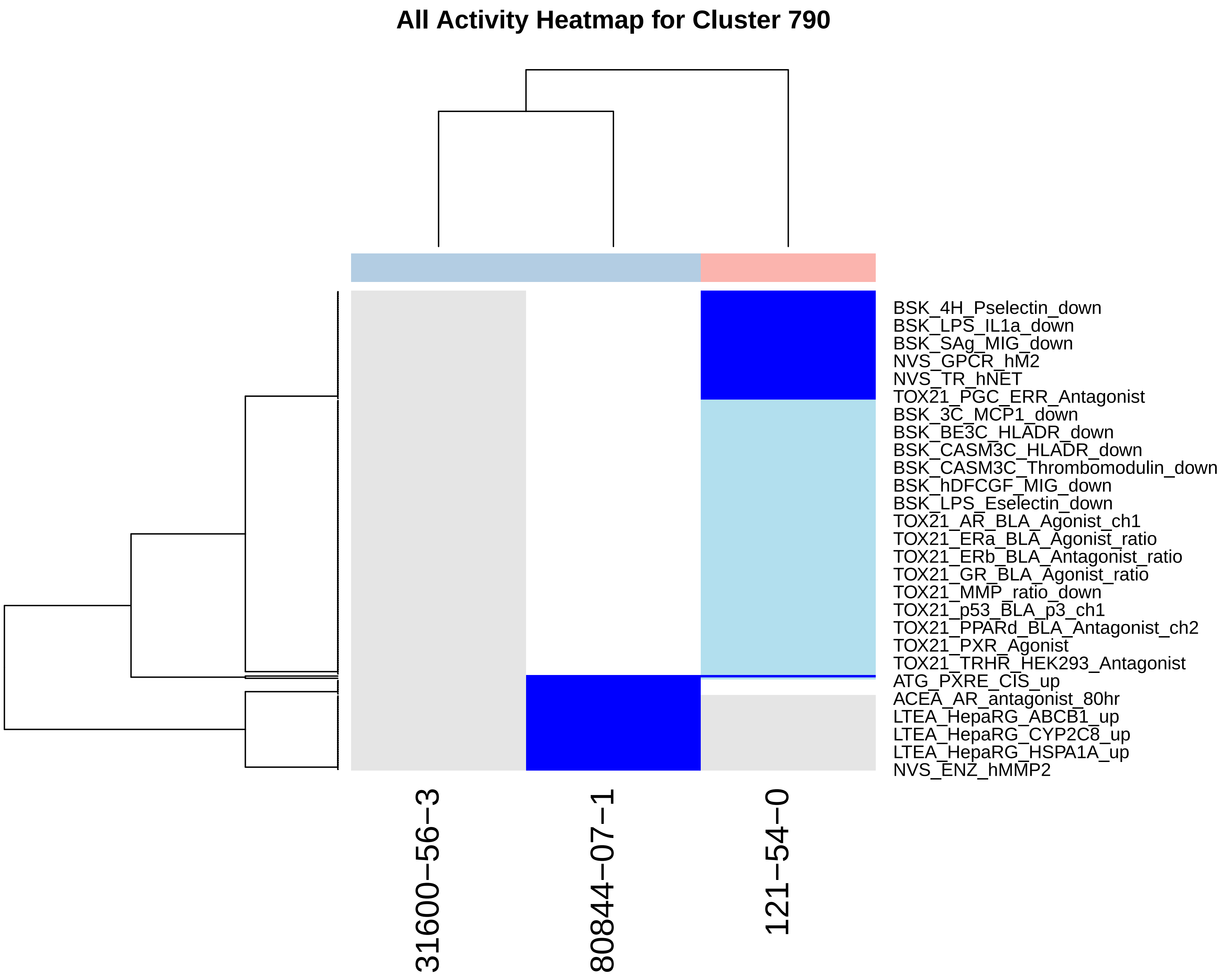

All Activity Heatmap for Cluster 790
BSK_4H_Pselectin_down
BSK_LPS_IL1a_down
BSK_SAg_MIG_down
NVS_GPCR_hM2
NVS_TR_hNET
TOX21_PGC_ERR_Antagonist
BSK_3C_MCP1_down
BSK_BE3C_HLADR_down
BSK_CASM3C_HLADR_down
BSK_CASM3C_Thrombomodulin_down
BSK_hDFCGF_MIG_down
BSK_LPS_Eselectin_down
TOX21_AR_BLA_Agonist_ch1
TOX21_ERa_BLA_Agonist_ratio
TOX21_ERb_BLA_Antagonist_ratio
TOX21_GR_BLA_Agonist_ratio
TOX21_MMP_ratio_down
TOX21_p53_BLA_p3_ch1
TOX21_PPARd_BLA_Antagonist_ch2
TOX21_PXR_Agonist
TOX21_TRHR_HEK293_Antagonist
ATG_PXRE_CIS_up
ACEA_AR_antagonist_80hr
LTEA_HepaRG_ABCB1_up
LTEA_HepaRG_CYP2C8_up
LTEA_HepaRG_HSPA1A_up
NVS_ENZ_hMMP2
31600−56−3
80844−07−1
121−54−0

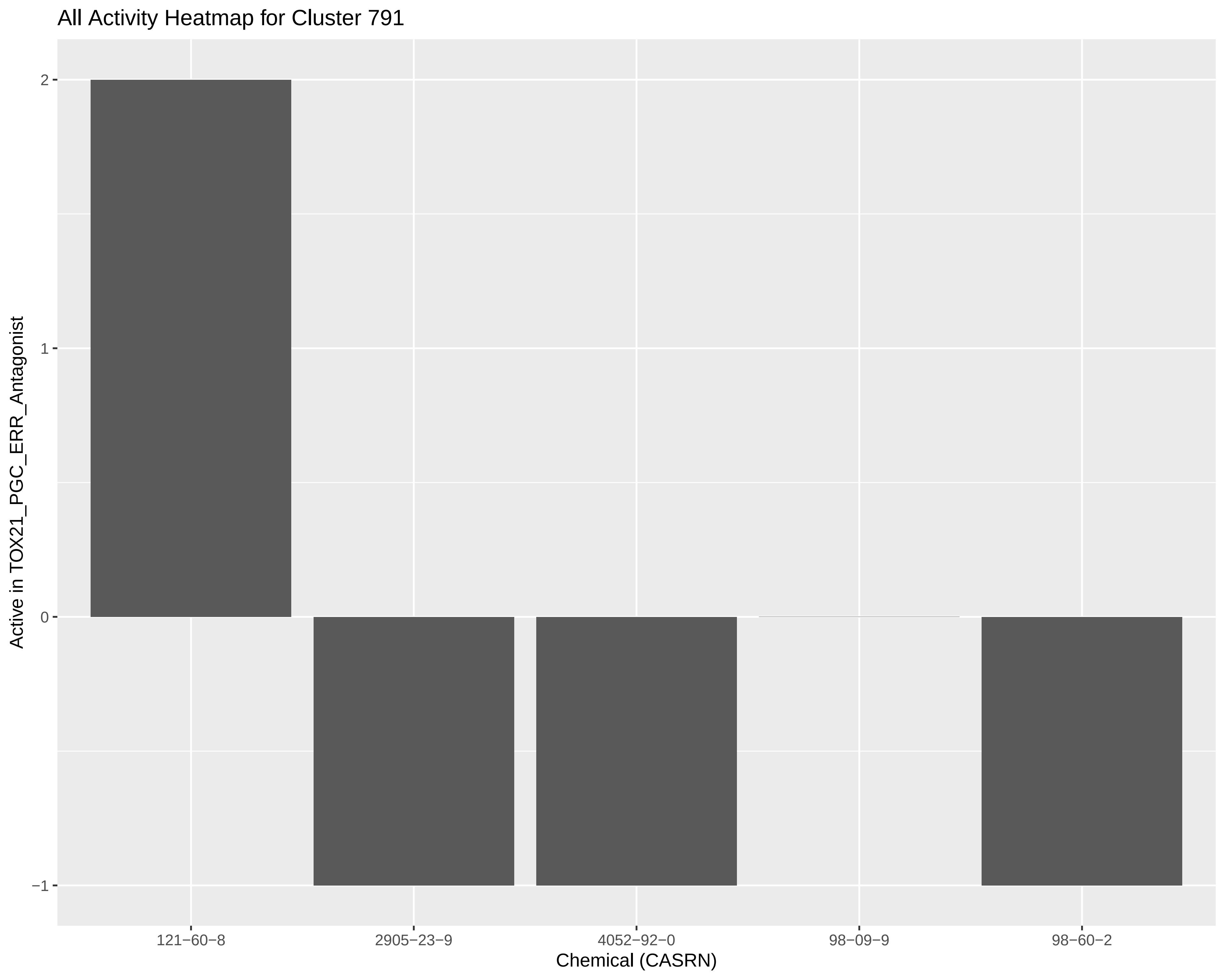

All Activity Heatmap for Cluster 791
Active in TOX21_PGC_ERR_Antagonist
2
1
0
−1
121−60−8
2905−23−9
4052−92−0
98−09−9
98−60−2
Chemical (CASRN)

All Activity Heatmap for Cluster 792

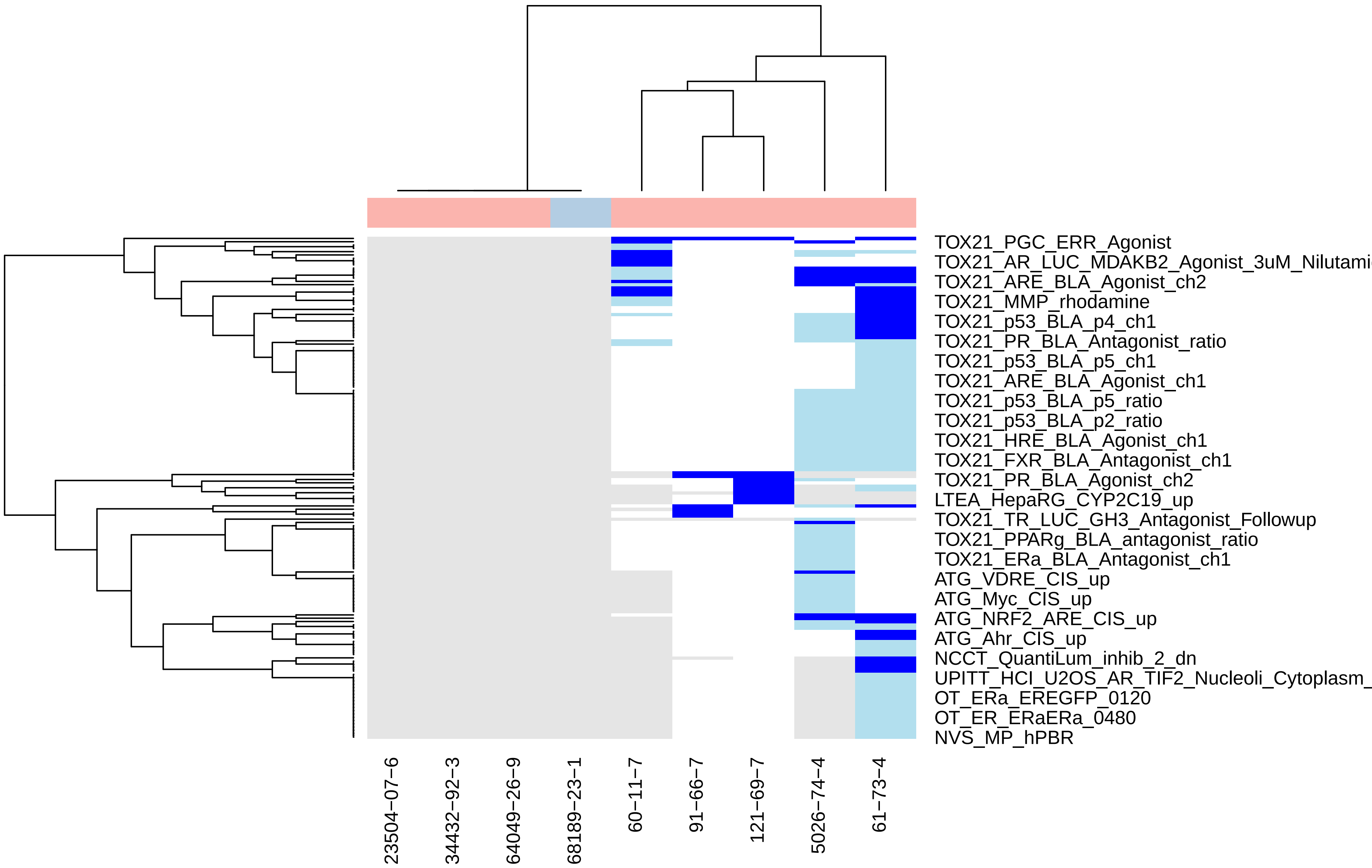

All Activity Heatmap for Cluster 793

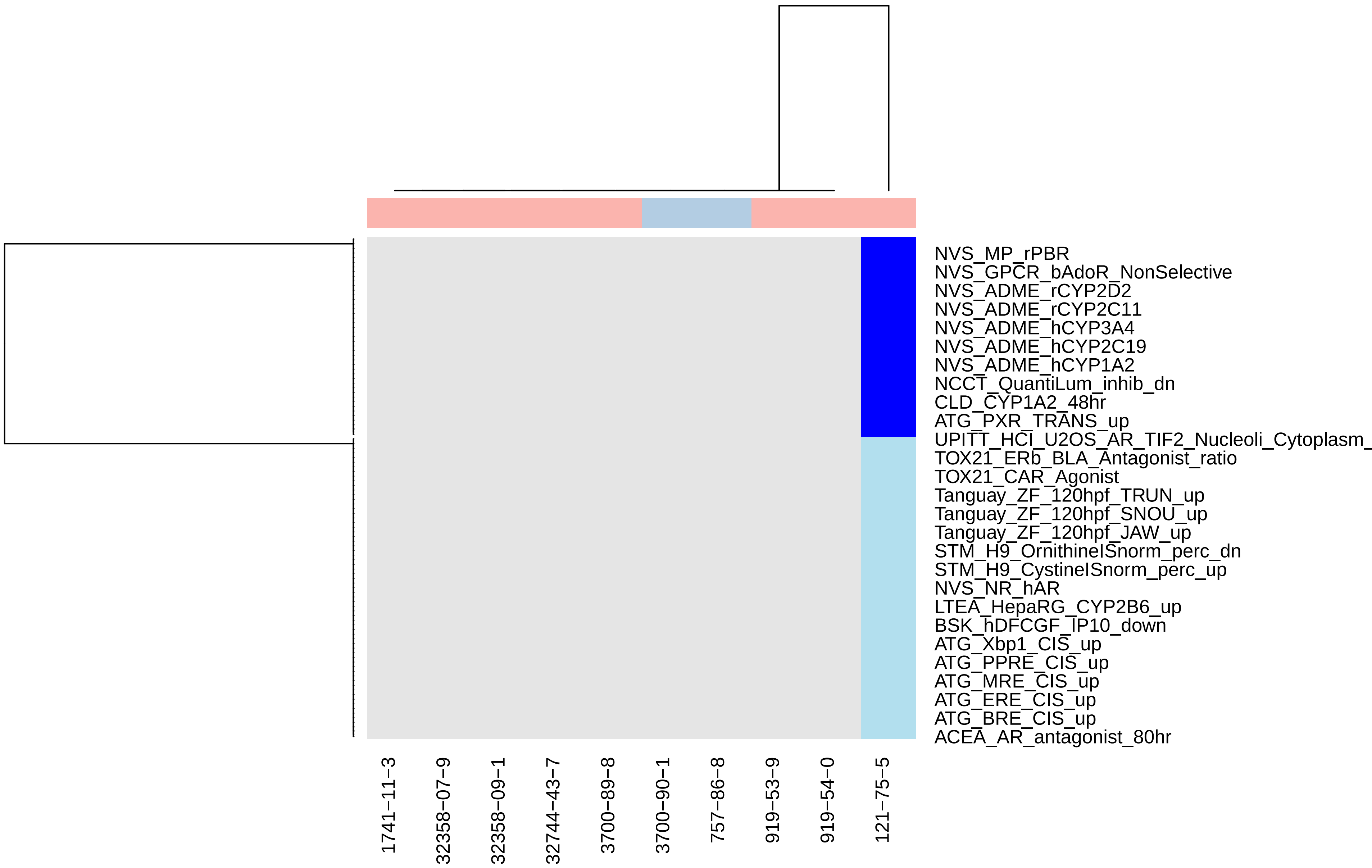

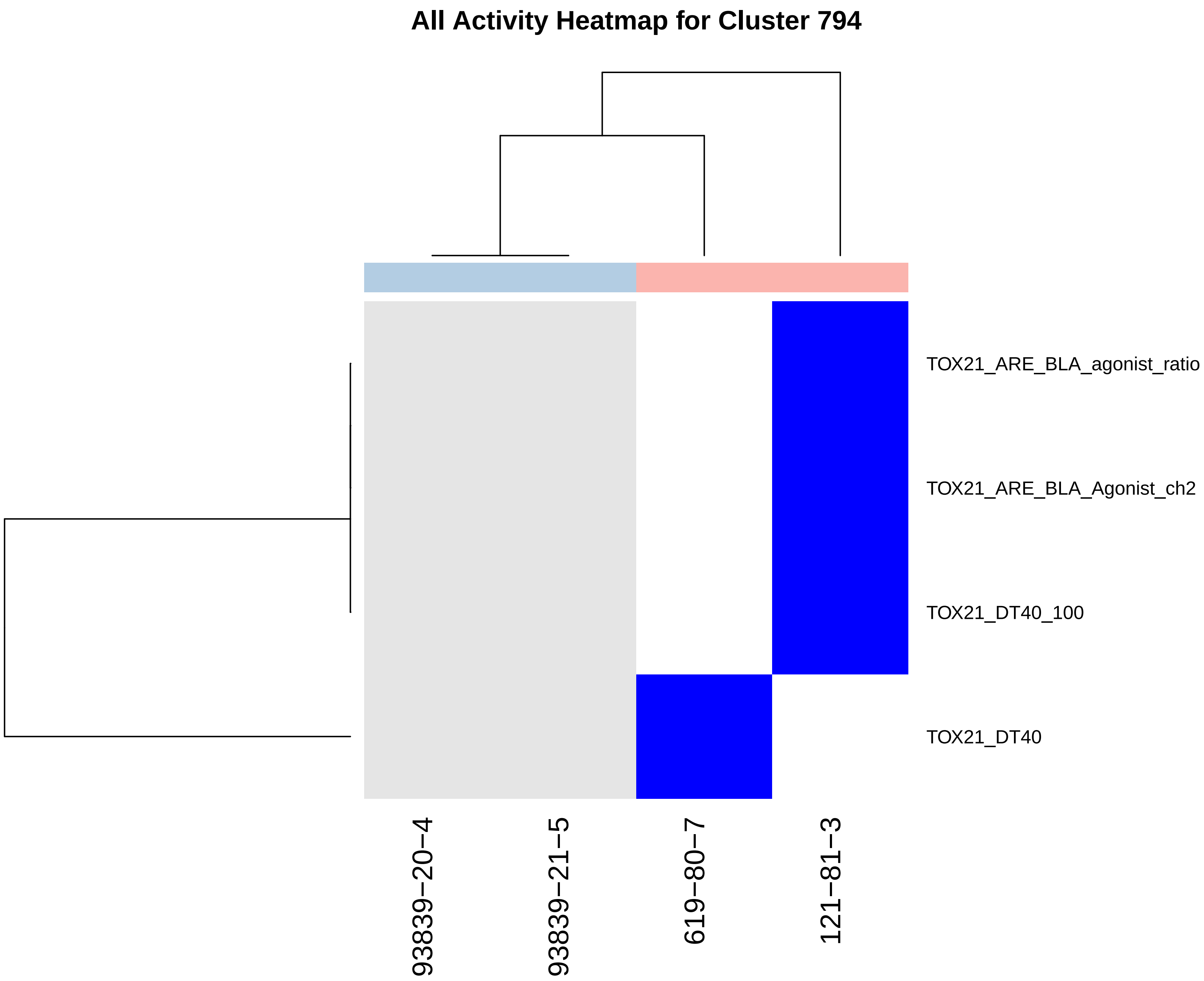
All Activity Heatmap for Cluster 794
TOX21_ARE_BLA_agonist_ratio
TOX21_ARE_BLA_Agonist_ch2
TOX21_DT40_100
TOX21_DT40
93839−20−4
93839−21−5
619−80−7
121−81−3

All Activity Heatmap for Cluster 795

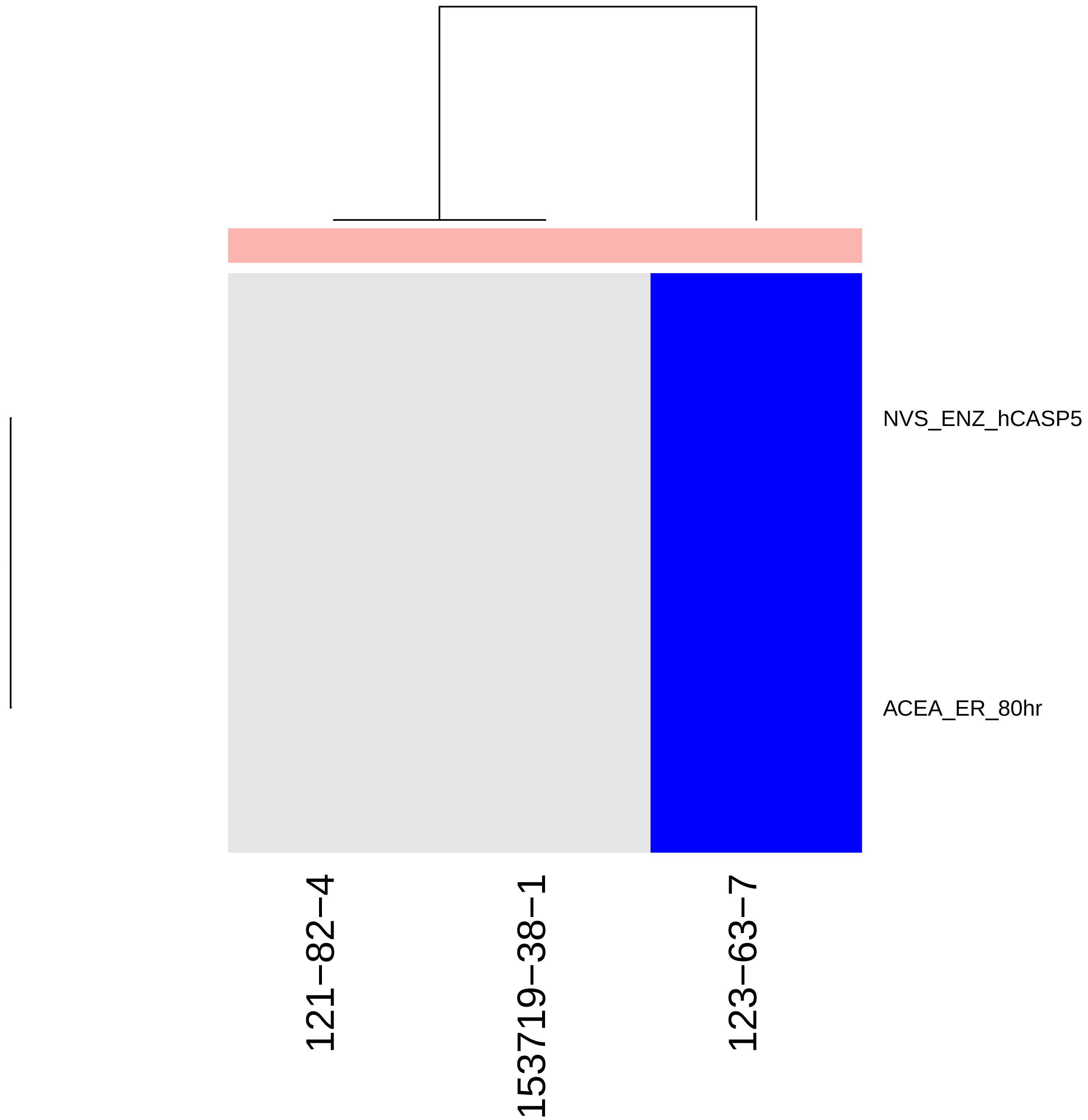

All Activity Heatmap for Cluster 796

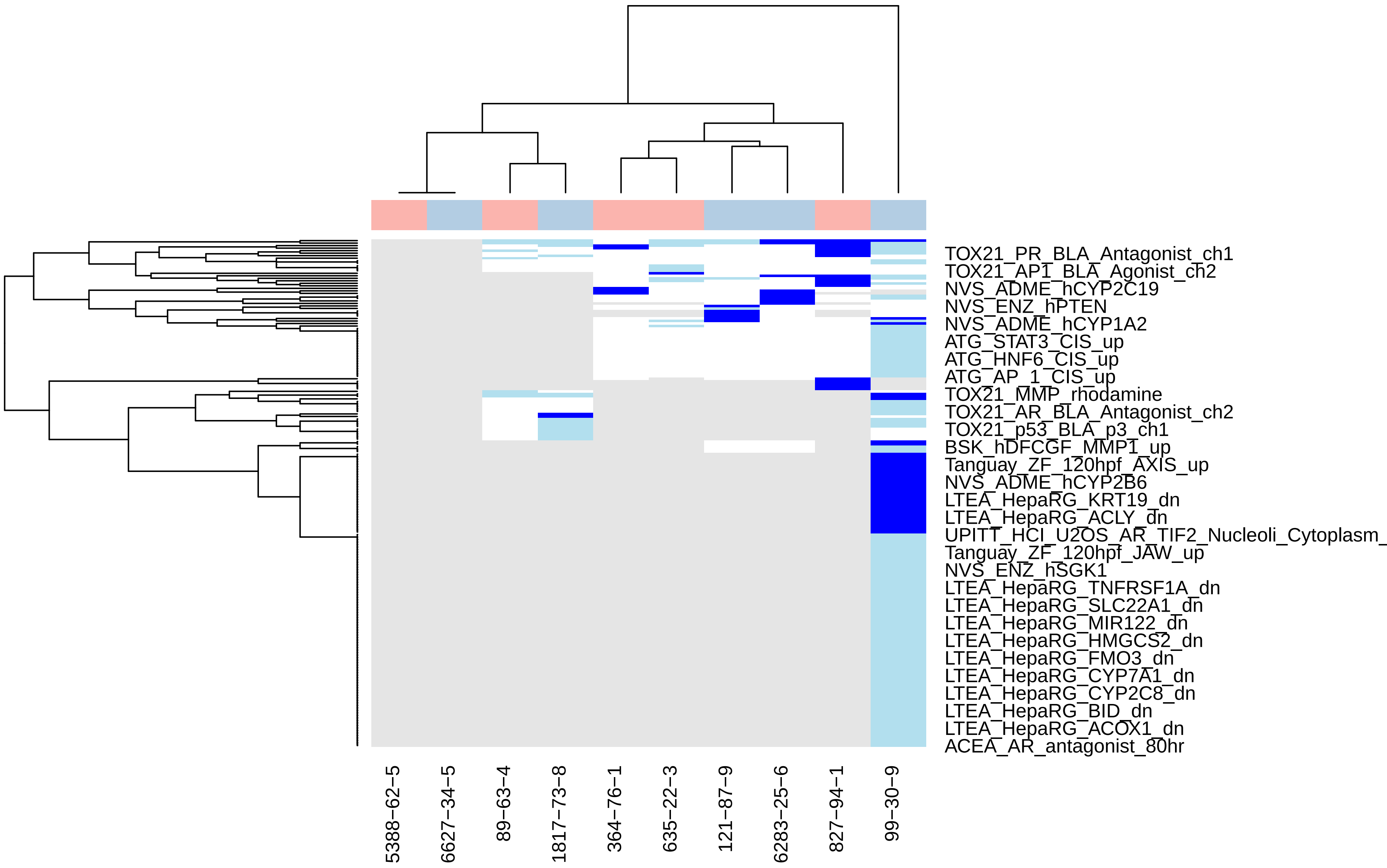

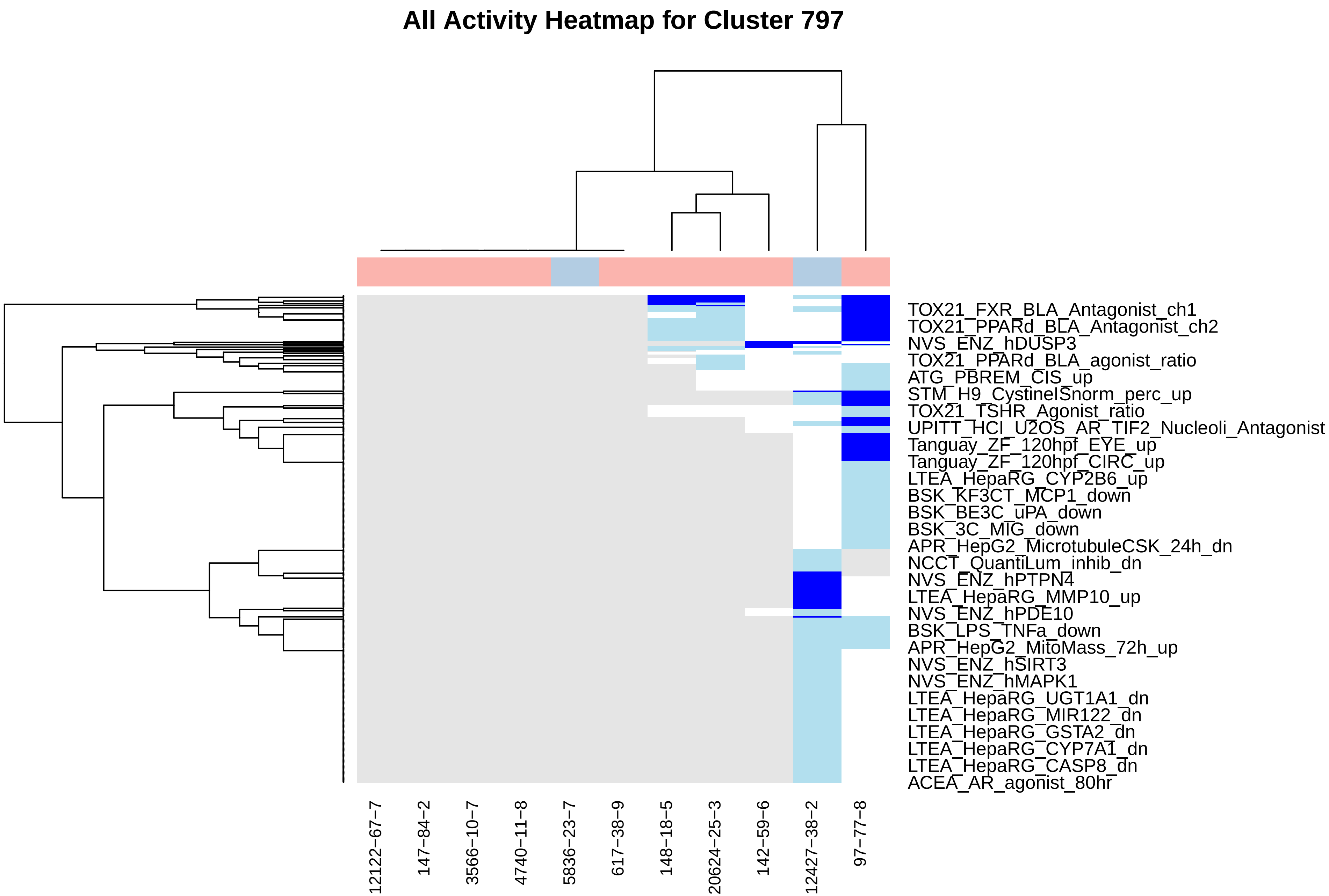
All Activity Heatmap for Cluster 797
TOX21_FXR_BLA_Antagonist_ch1
TOX21_PPARd_BLA_Antagonist_ch2
NVS_ENZ_hDUSP3
TOX21_PPARd_BLA_agonist_ratio
ATG_PBREM_CIS_up
STM_H9_CystineISnorm_perc_up
TOX21_TSHR_Agonist_ratio
UPITT_HCI_U2OS_AR_TIF2_Nucleoli_Antagonist
Tanguay_ZF_120hpf_EYE_up
Tanguay_ZF_120hpf_CIRC_up
LTEA_HepaRG_CYP2B6_up
BSK_KF3CT_MCP1_down
BSK_BE3C_uPA_down
BSK_3C_MIG_down
APR_HepG2_MicrotubuleCSK_24h_dn
NCCT_QuantiLum_inhib_dn
NVS_ENZ_hPTPN4
LTEA_HepaRG_MMP10_up
NVS_ENZ_hPDE10
BSK_LPS_TNFa_down
APR_HepG2_MitoMass_72h_up
NVS_ENZ_hSIRT3
NVS_ENZ_hMAPK1
LTEA_HepaRG_UGT1A1_dn
LTEA_HepaRG_MIR122_dn
LTEA_HepaRG_GSTA2_dn
LTEA_HepaRG_CYP7A1_dn
LTEA_HepaRG_CASP8_dn
ACEA_AR_agonist_80hr
12122−67−7
147−84−2
3566−10−7
4740−11−8
5836−23−7
617−38−9
148−18−5
20624−25−3
142−59−6
12427−38−2
97−77−8

All Activity Heatmap for Cluster 798

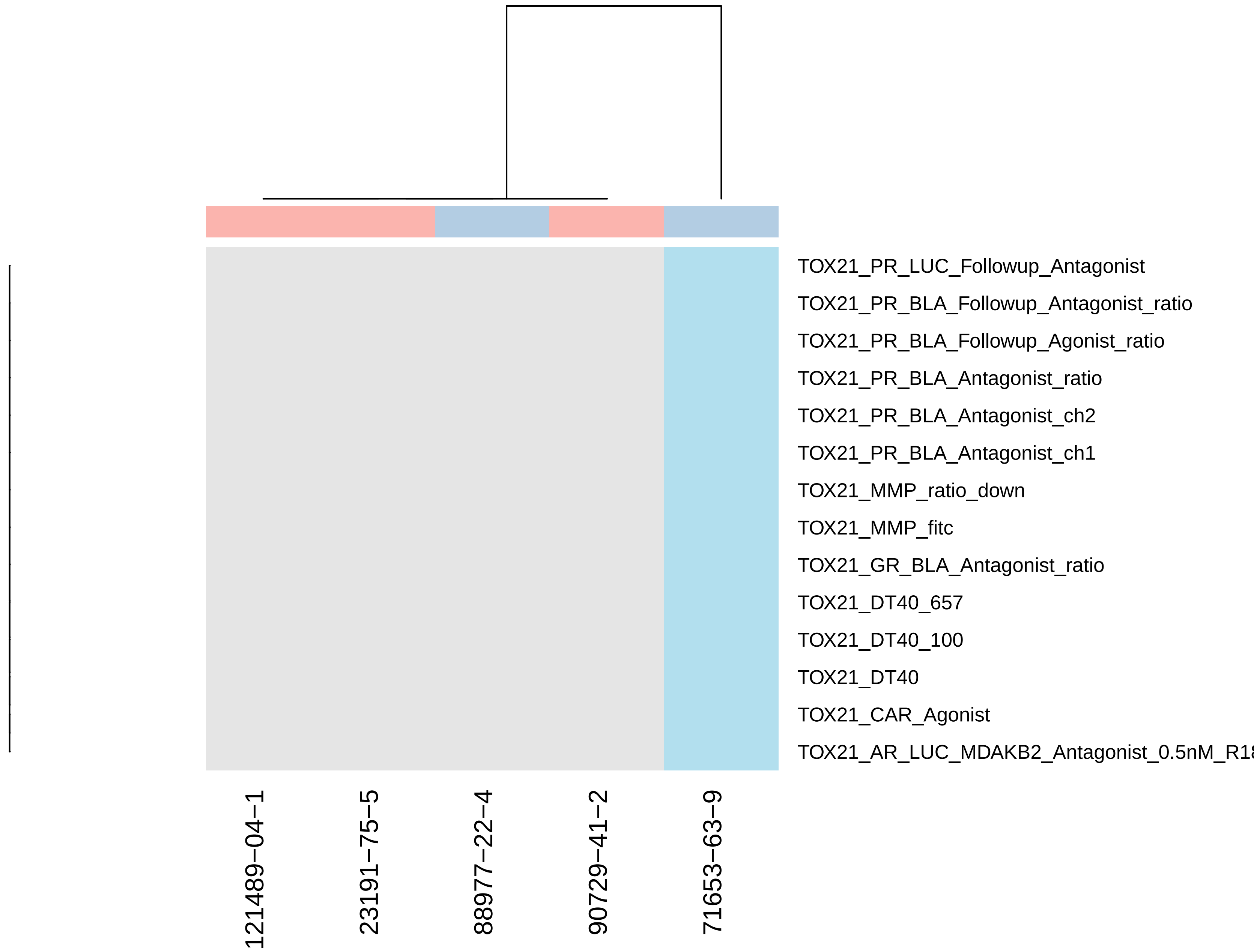

All Activity Heatmap for Cluster 799

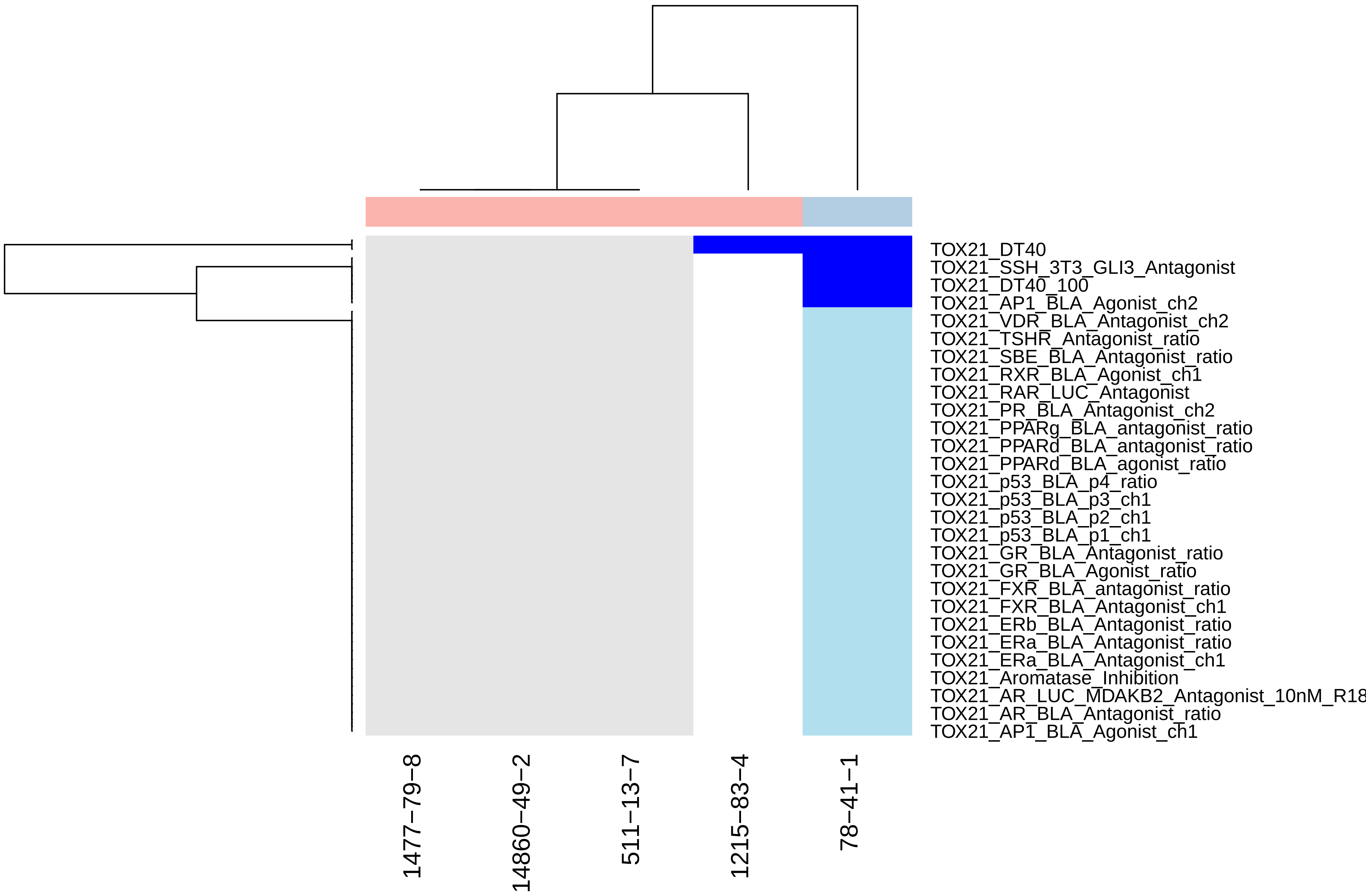

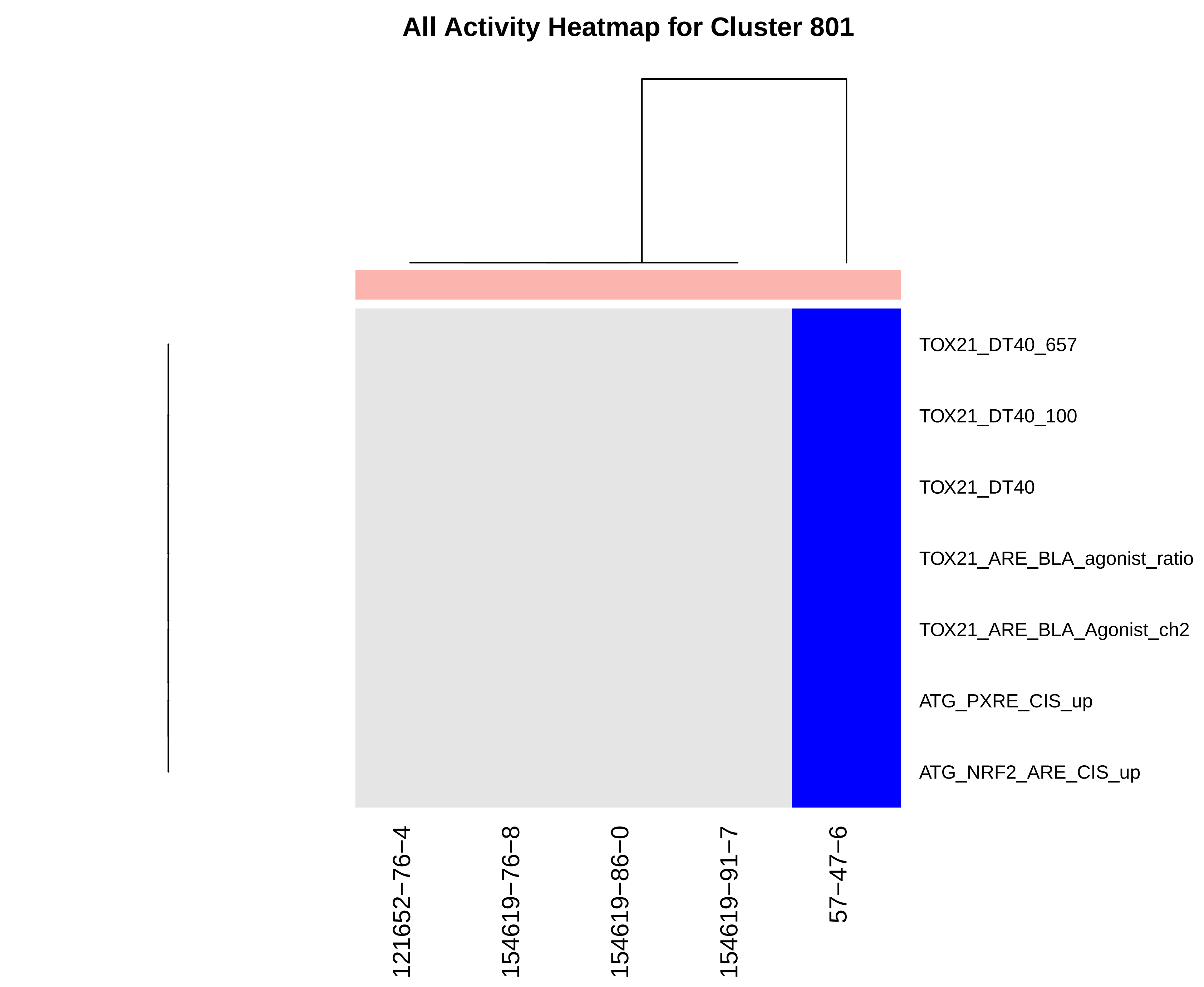

All Activity Heatmap for Cluster 801
TOX21_DT40_657
TOX21_DT40_100
TOX21_DT40
TOX21_ARE_BLA_agonist_ratio
TOX21_ARE_BLA_Agonist_ch2
ATG_PXRE_CIS_up
ATG_NRF2_ARE_CIS_up
121652−76−4
154619−76−8
154619−86−0
154619−91−7
57−47−6

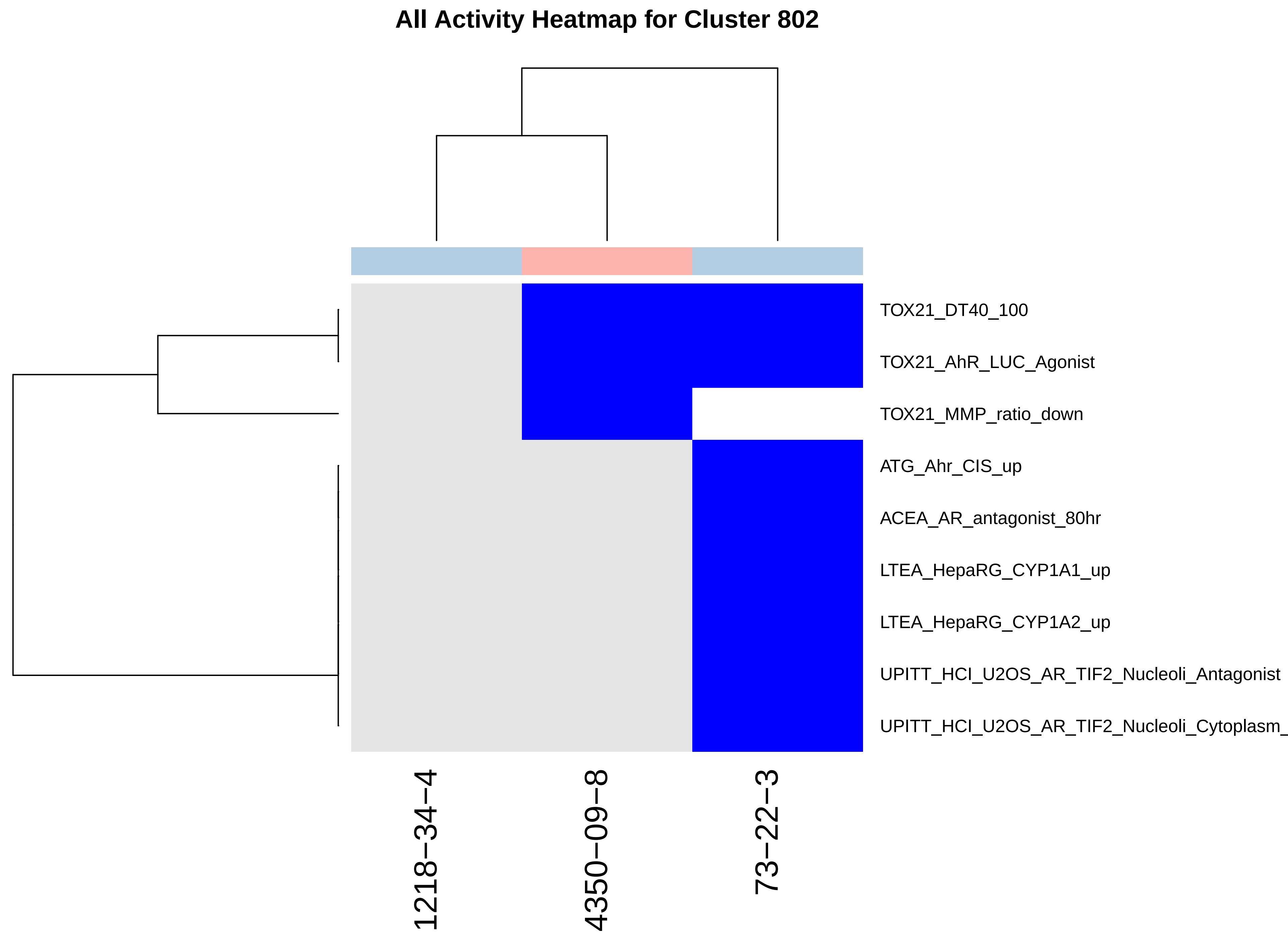

All Activity Heatmap for Cluster 802
TOX21_DT40_100
TOX21_AhR_LUC_Agonist
TOX21_MMP_ratio_down
ATG_Ahr_CIS_up
ACEA_AR_antagonist_80hr
LTEA_HepaRG_CYP1A1_up
LTEA_HepaRG_CYP1A2_up
UPITT_HCI_U2OS_AR_TIF2_Nucleoli_Antagonist
UPITT_HCI_U2OS_AR_TIF2_Nucleoli_Cytoplasm_
1218−34−4
4350−09−8
73−22−3

All Activity Heatmap for Cluster 803

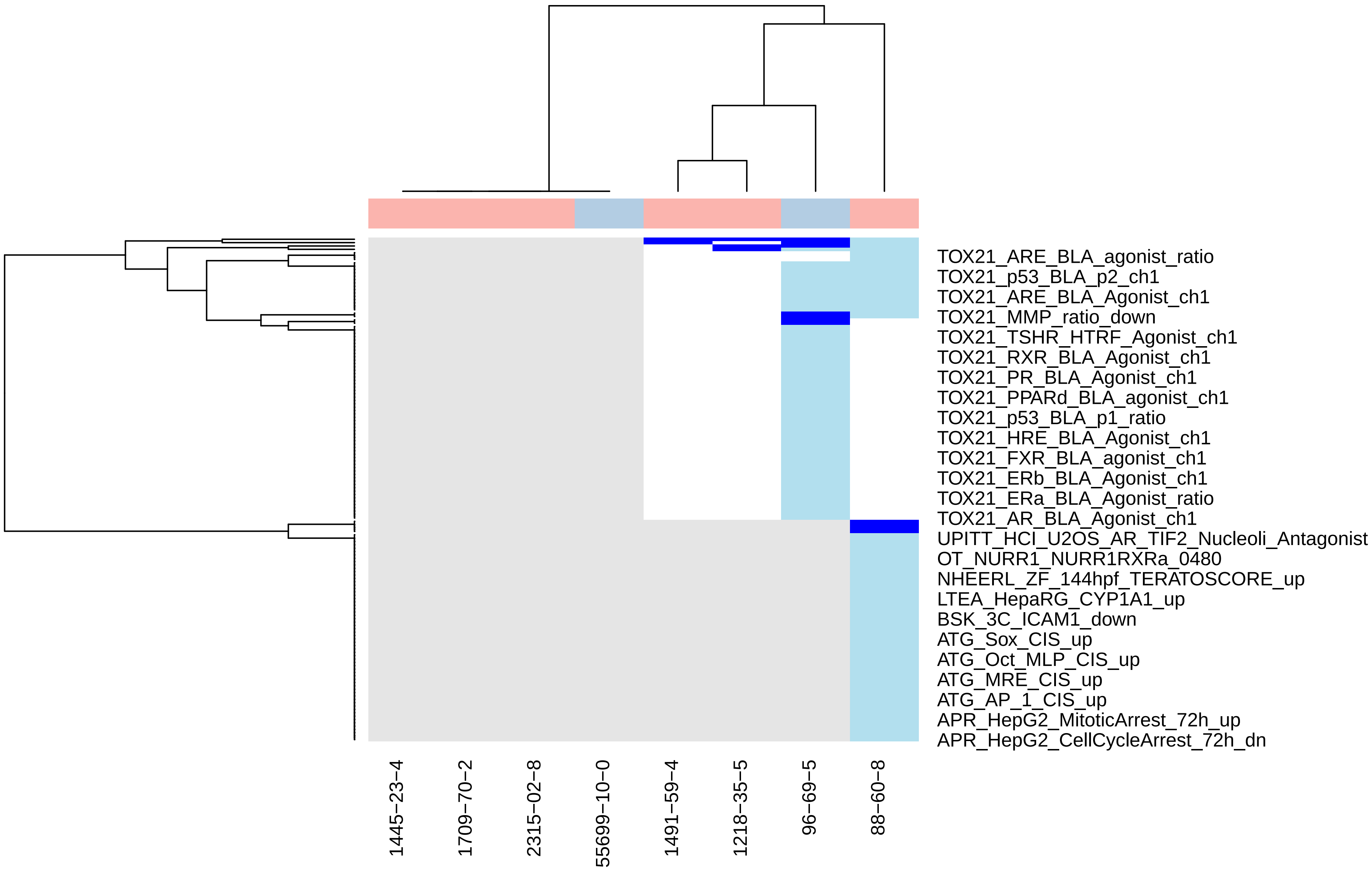

All Activity Heatmap for Cluster 805

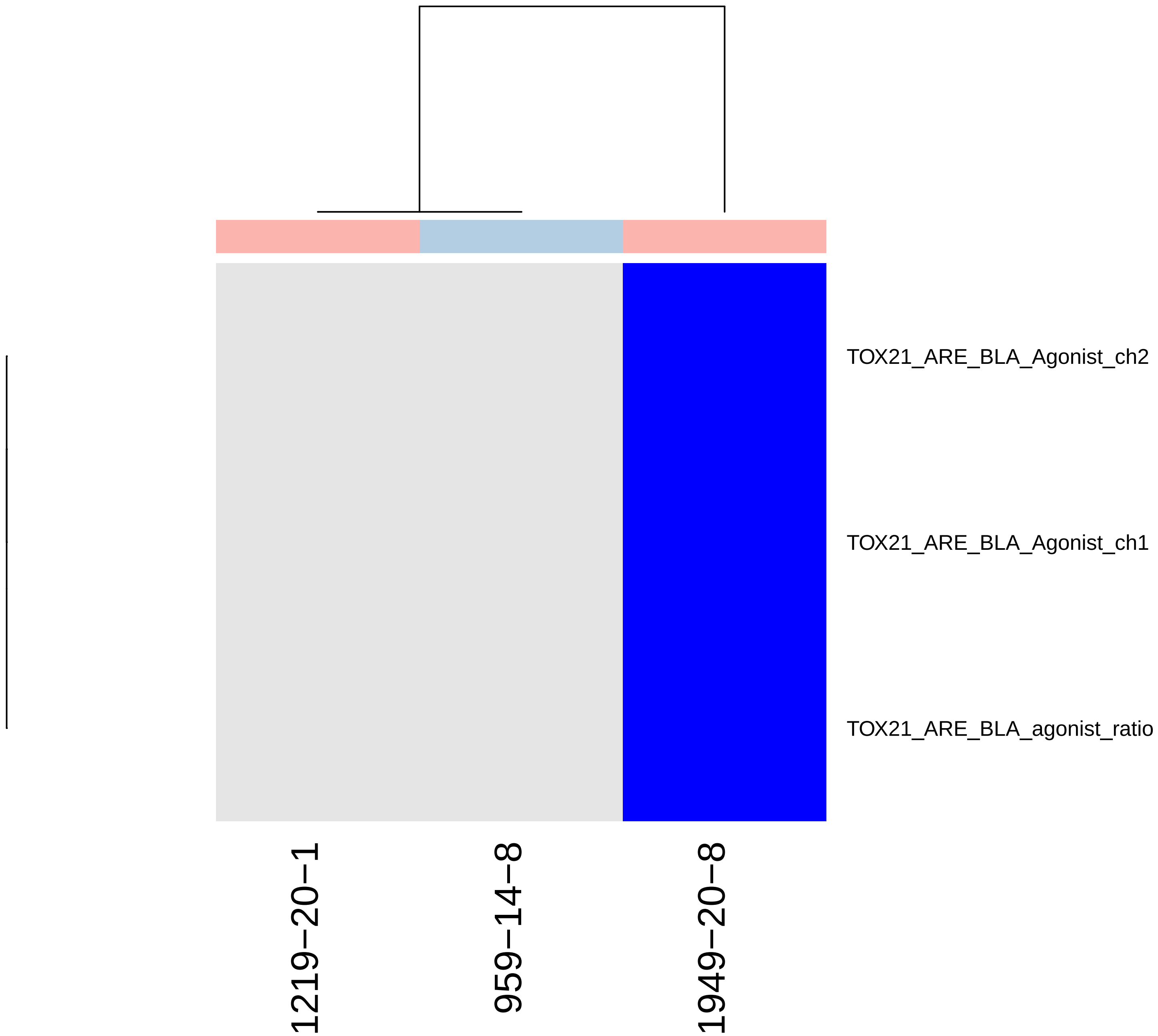

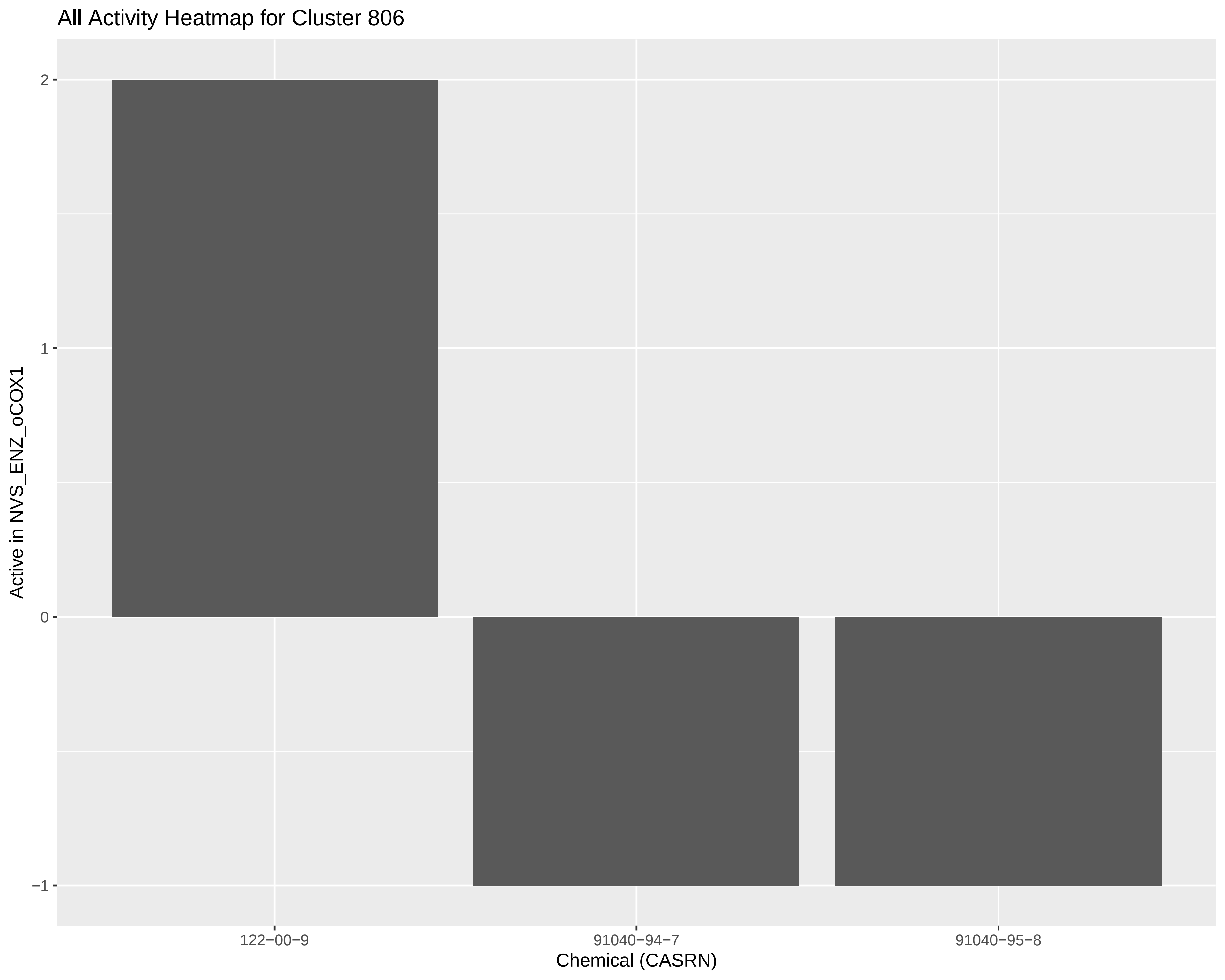
All Activity Heatmap for Cluster 806
Active in NVS_ENZ_oCOX1
2
1
0
−1
122−00−9
91040−94−7
91040−95−8
Chemical (CASRN)

All Activity Heatmap for Cluster 807

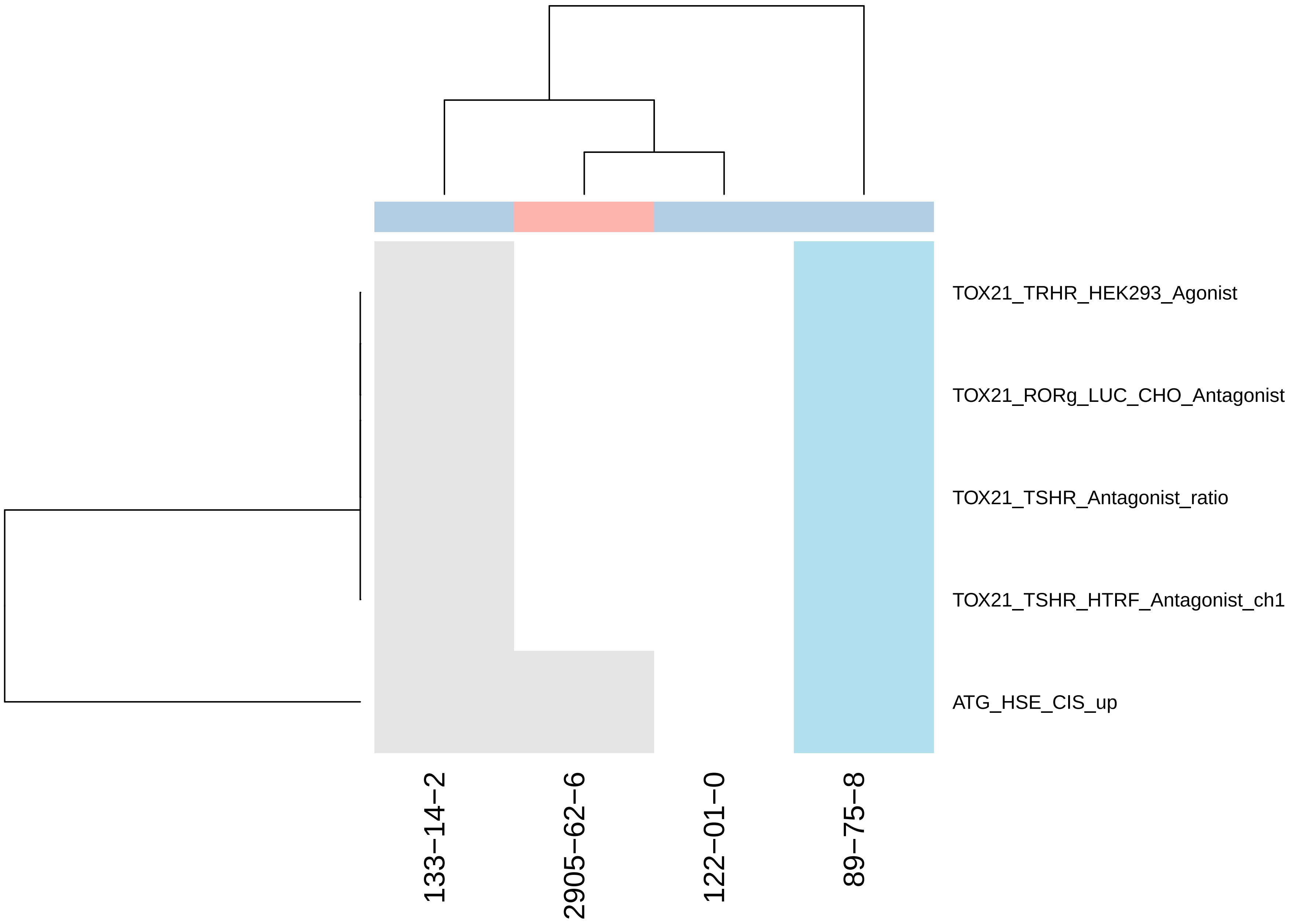

All Activity Heatmap for Cluster 808

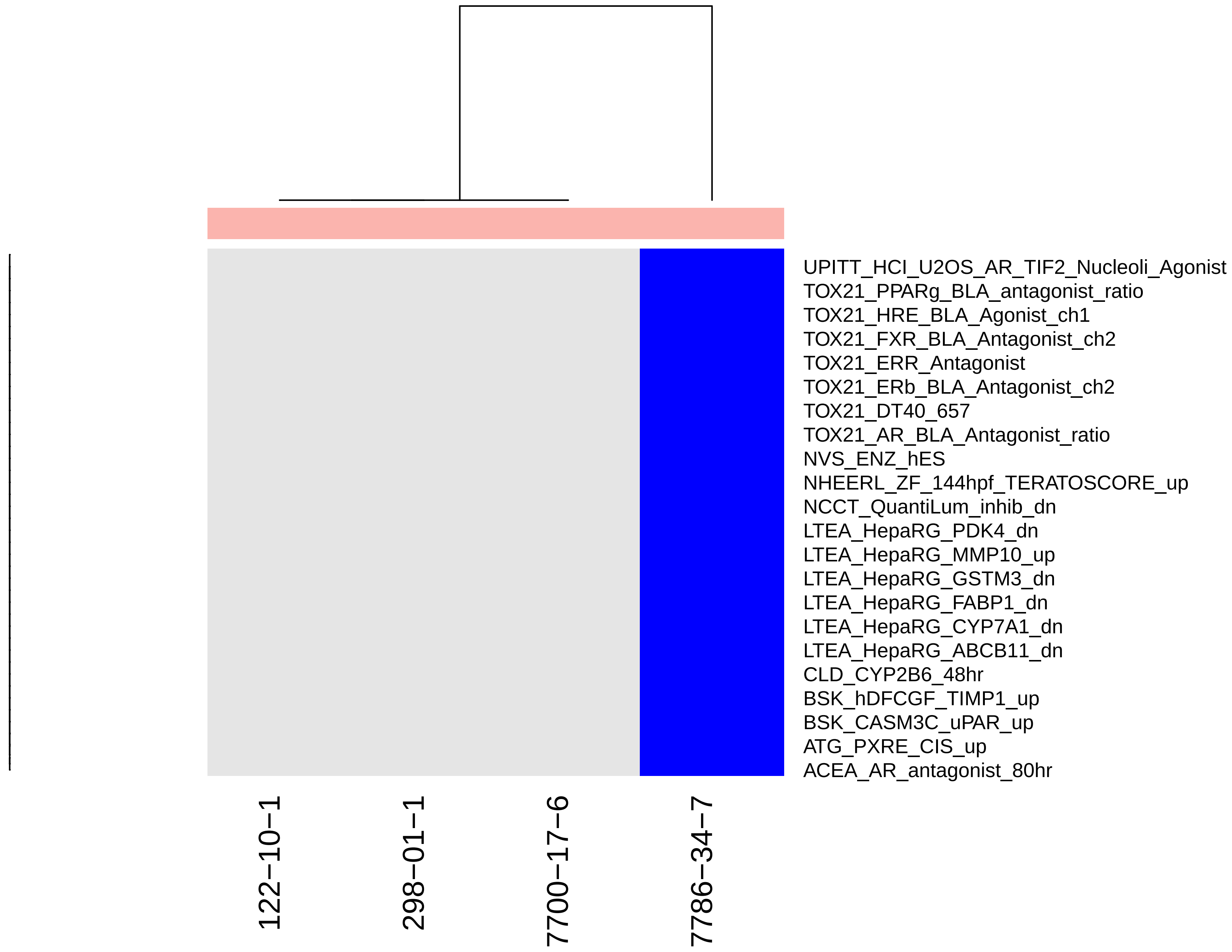

All Activity Heatmap for Cluster 809

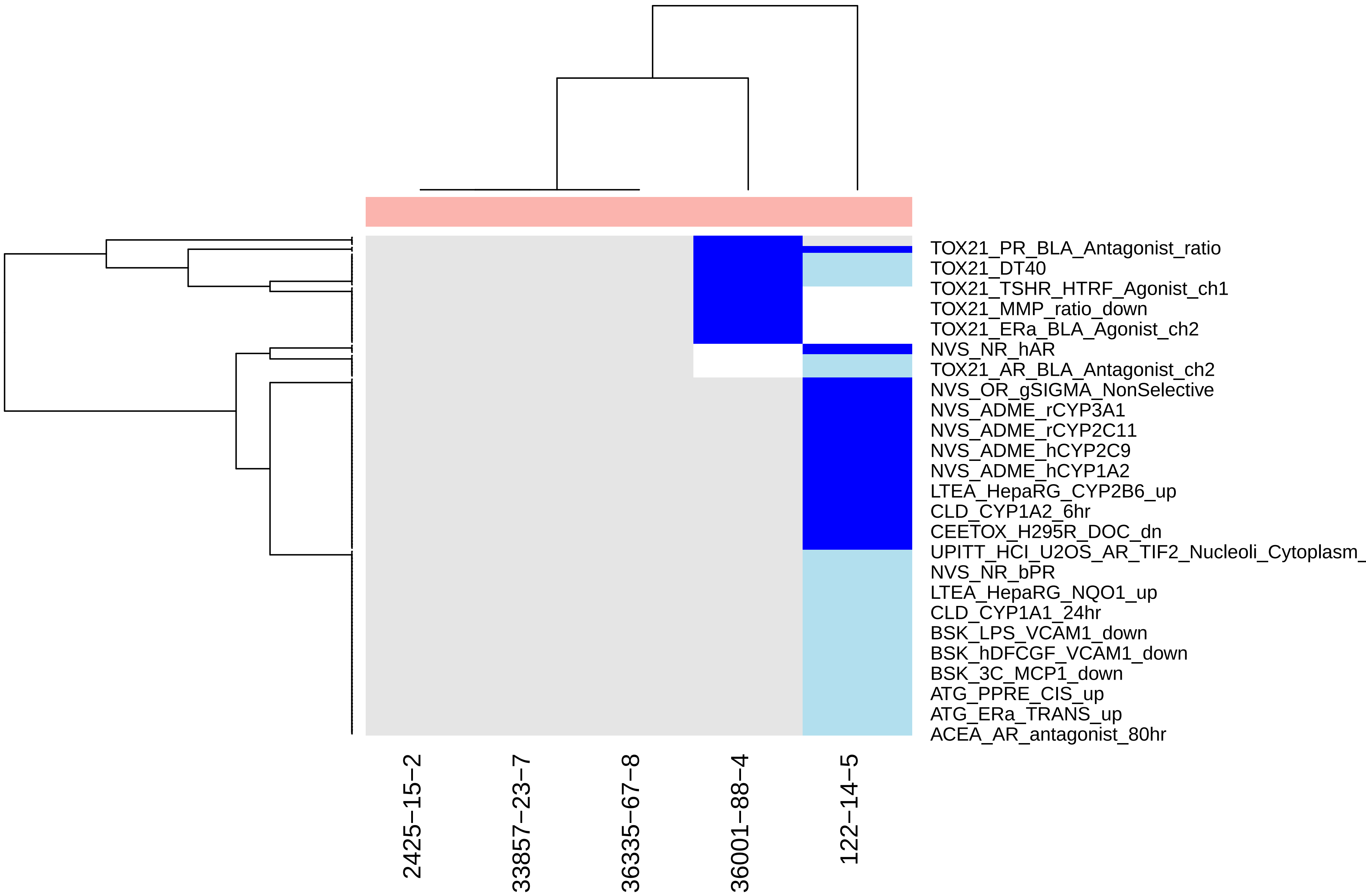

All Activity Heatmap for Cluster 810

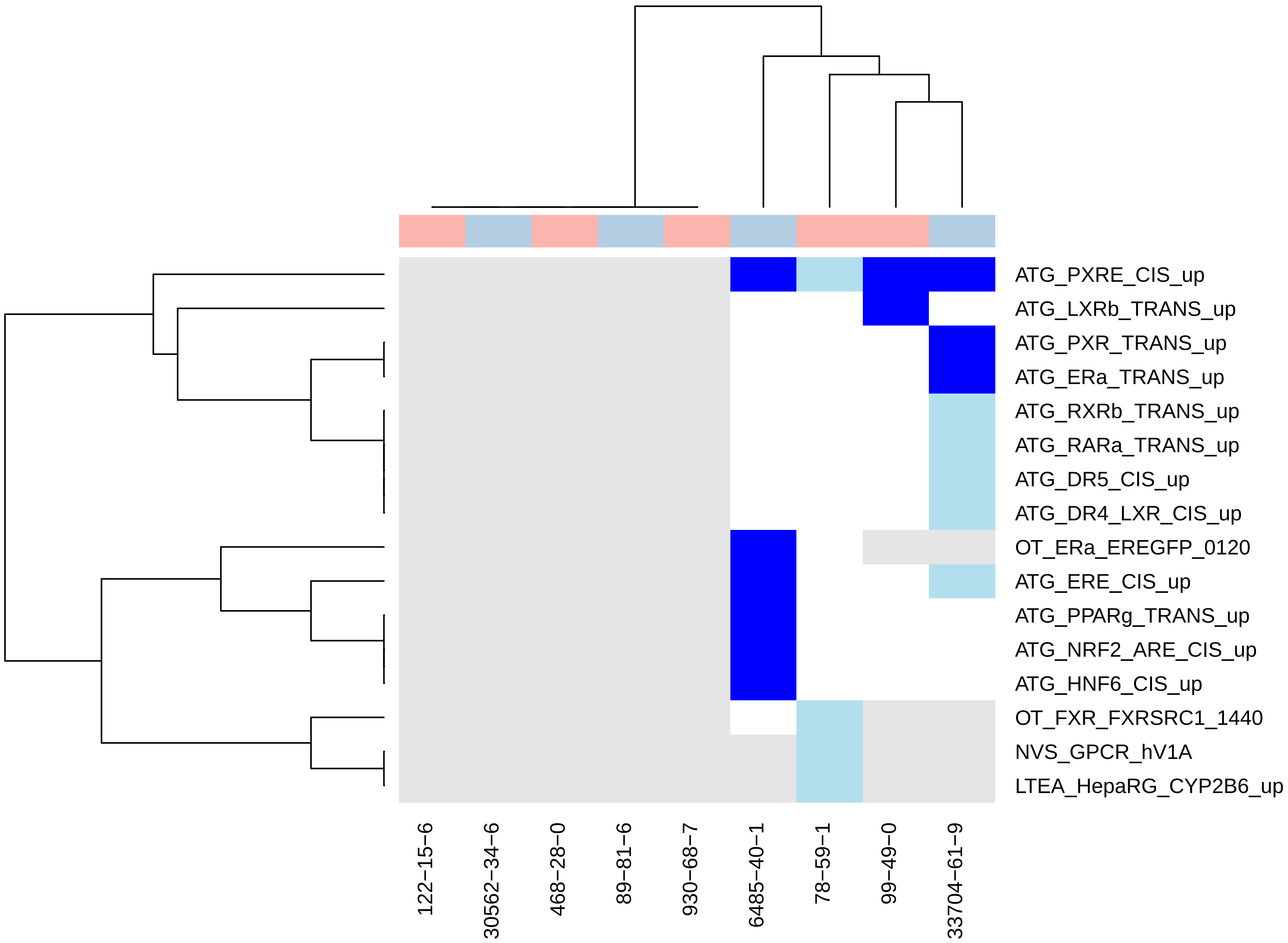

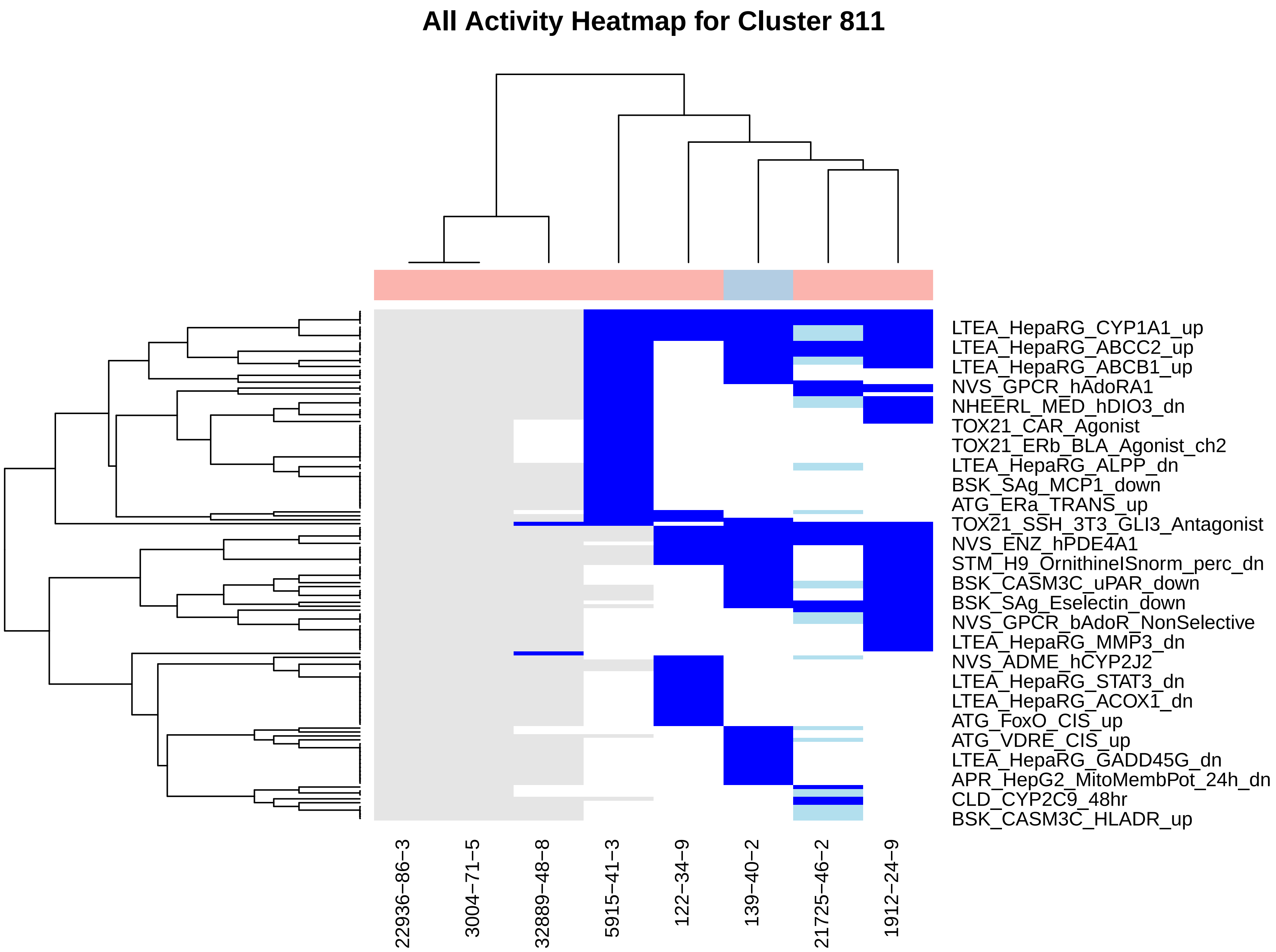
All Activity Heatmap for Cluster 811
LTEA_HepaRG_CYP1A1_up
LTEA_HepaRG_ABCC2_up
LTEA_HepaRG_ABCB1_up
NVS_GPCR_hAdoRA1
NHEERL_MED_hDIO3_dn
TOX21_CAR_Agonist
TOX21_ERb_BLA_Agonist_ch2
LTEA_HepaRG_ALPP_dn
BSK_SAg_MCP1_down
ATG_ERa_TRANS_up
TOX21_SSH_3T3_GLI3_Antagonist
NVS_ENZ_hPDE4A1
STM_H9_OrnithineISnorm_perc_dn
BSK_CASM3C_uPAR_down
BSK_SAg_Eselectin_down
NVS_GPCR_bAdoR_NonSelective
LTEA_HepaRG_MMP3_dn
NVS_ADME_hCYP2J2
LTEA_HepaRG_STAT3_dn
LTEA_HepaRG_ACOX1_dn
ATG_FoxO_CIS_up
ATG_VDRE_CIS_up
LTEA_HepaRG_GADD45G_dn
APR_HepG2_MitoMembPot_24h_dn
CLD_CYP2C9_48hr
BSK_CASM3C_HLADR_up
22936−86−3
3004−71−5
32889−48−8
5915−41−3
122−34−9
139−40−2
21725−46−2
1912−24−9

All Activity Heatmap for Cluster 812

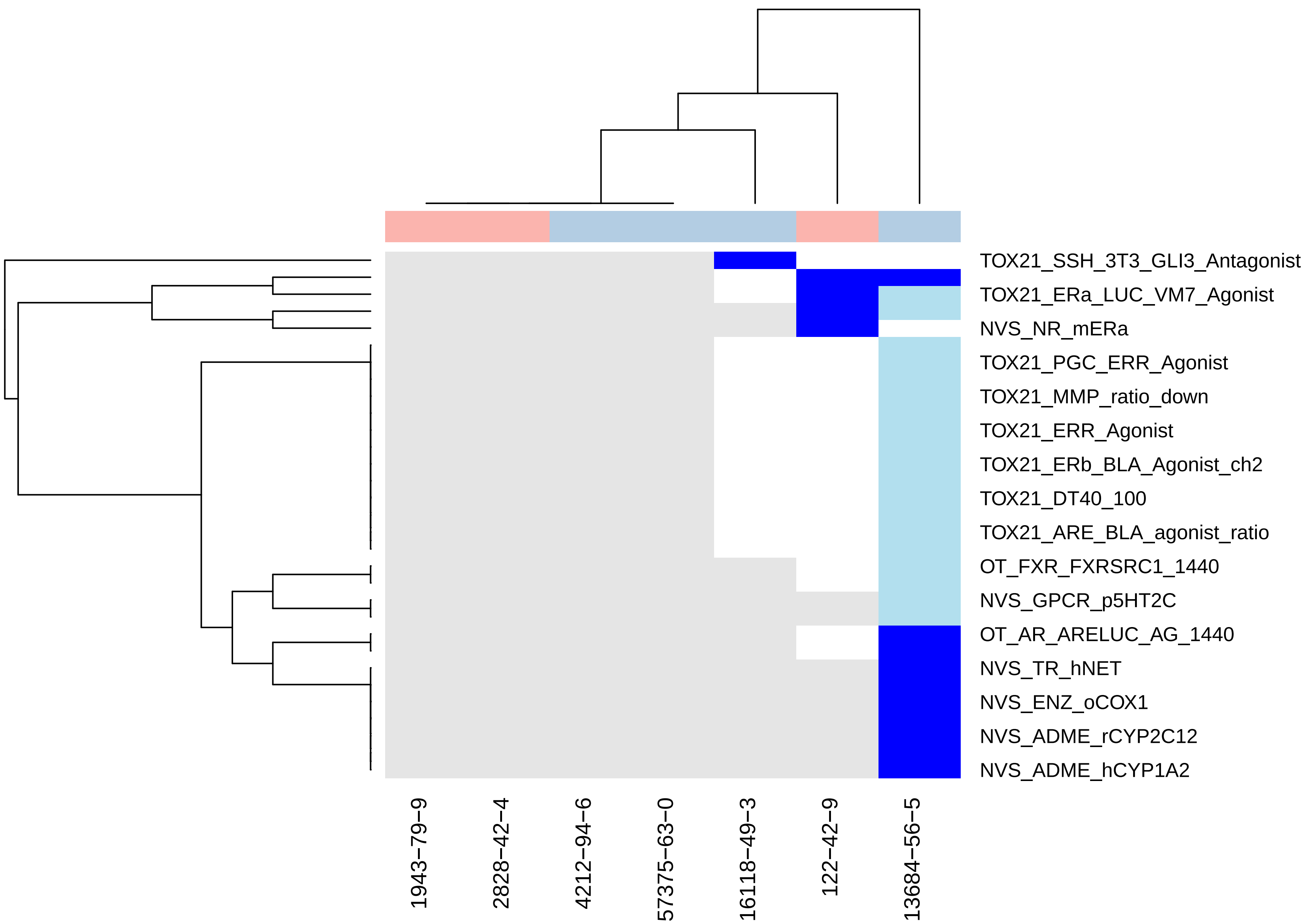

All Activity Heatmap for Cluster 813

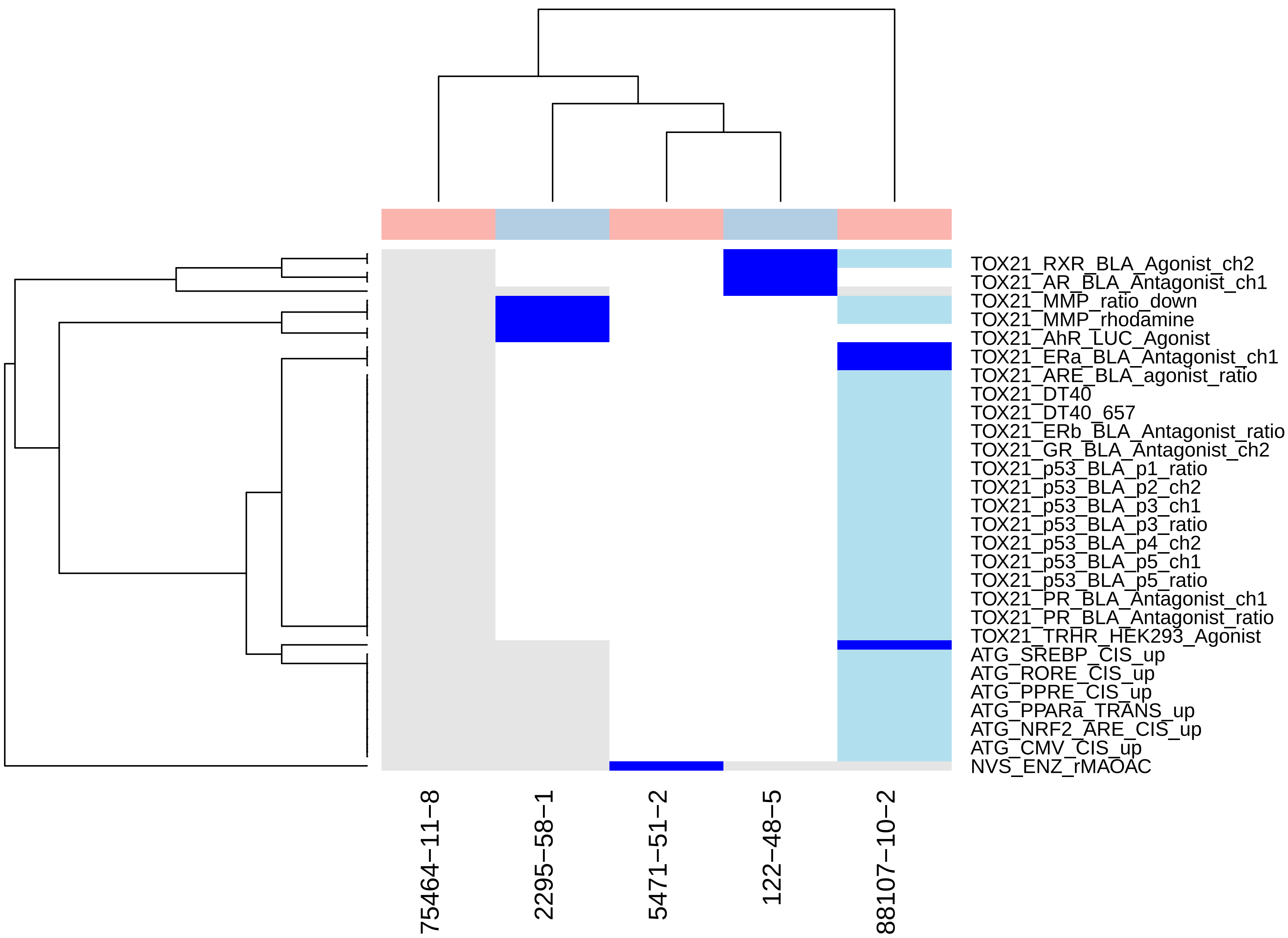

All Activity Heatmap for Cluster 814

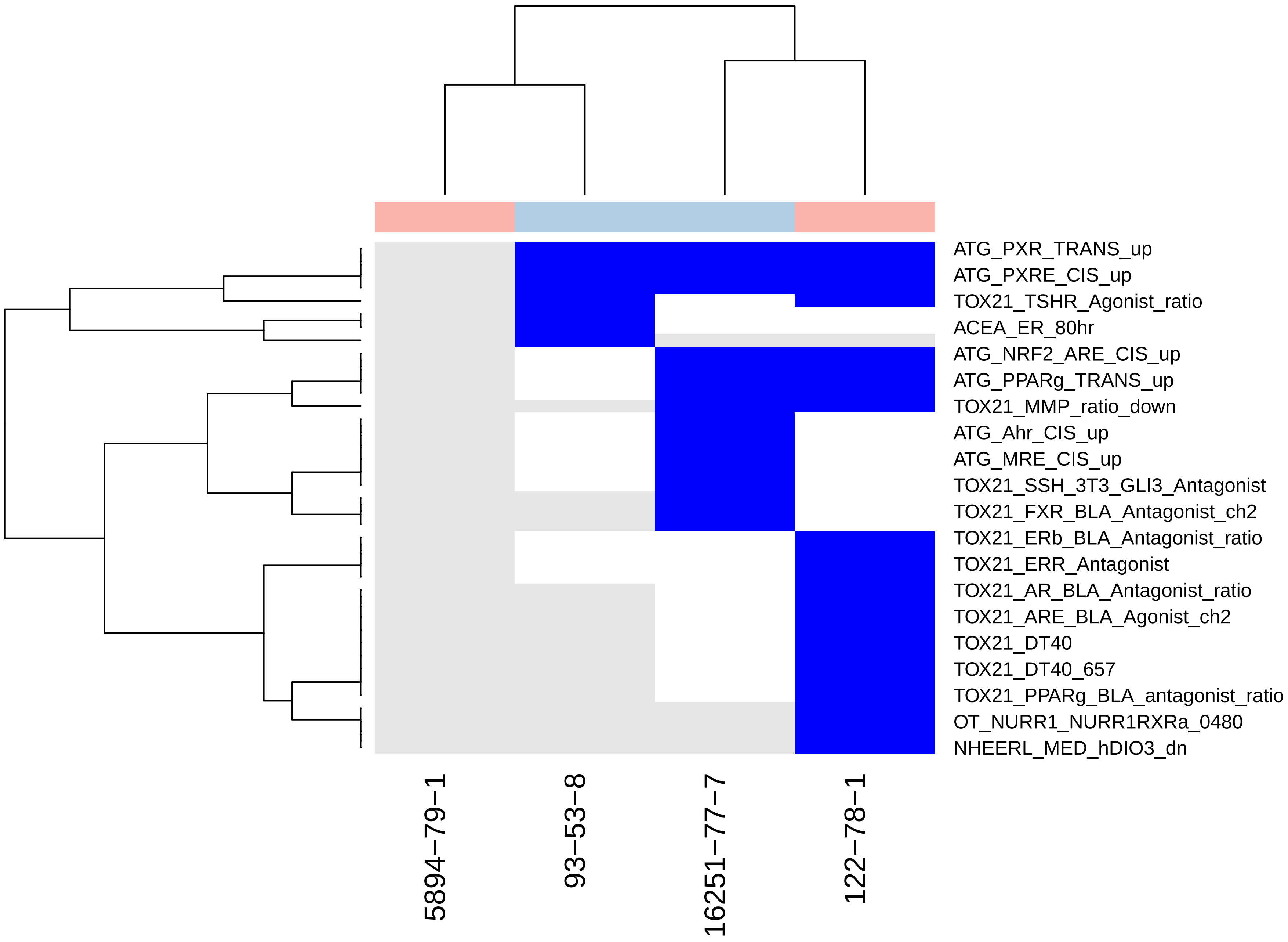

All Activity Heatmap for Cluster 815

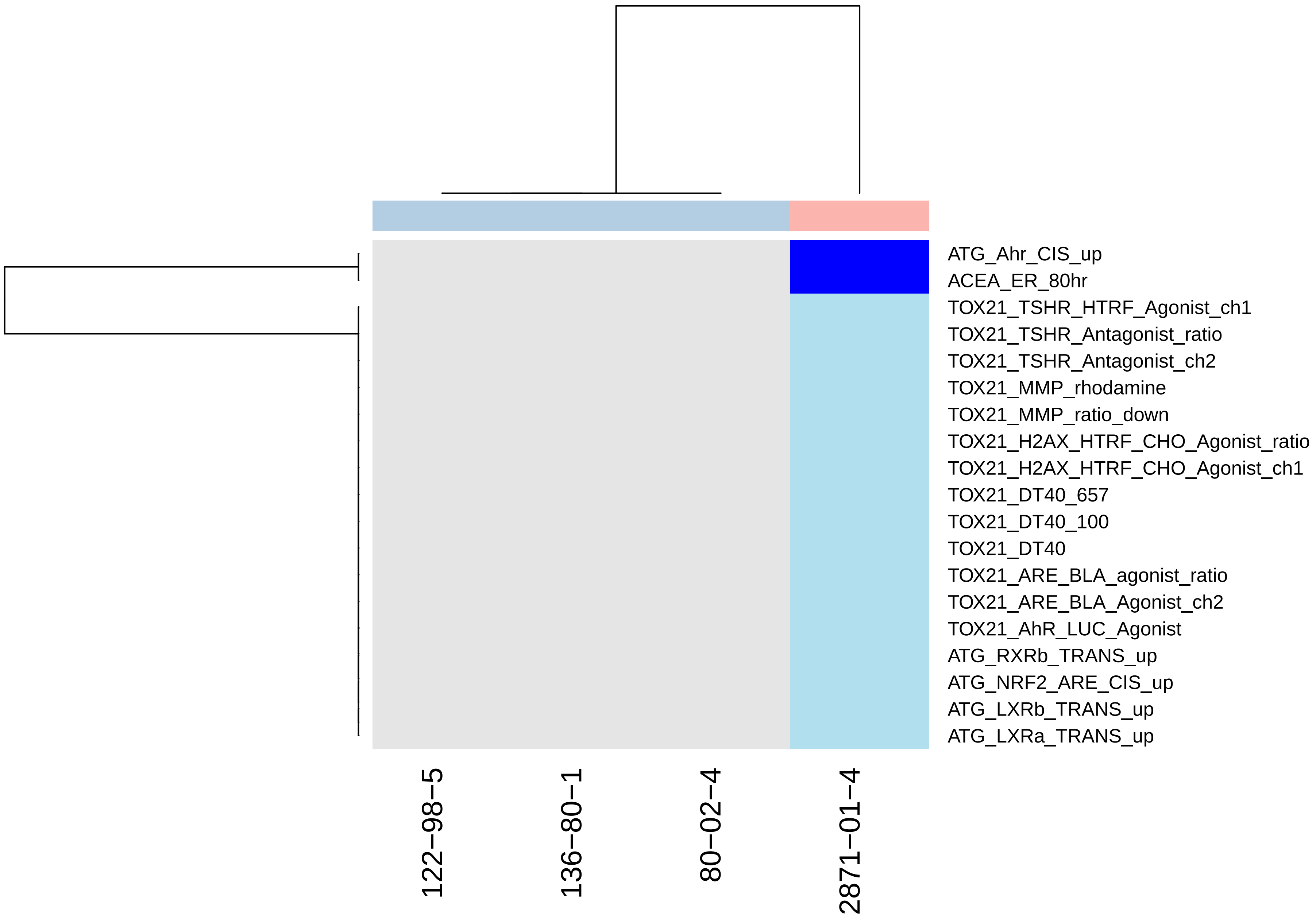

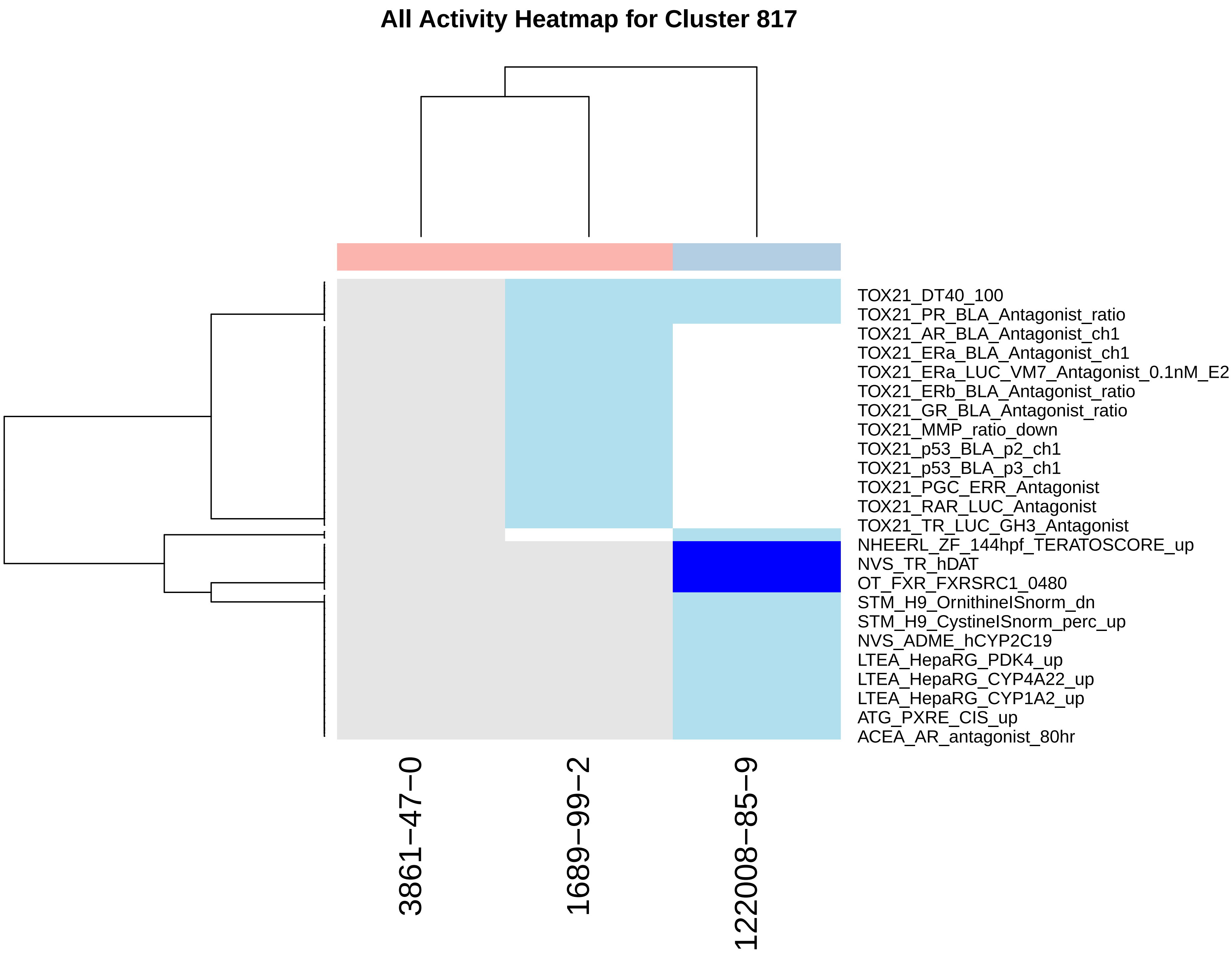
All Activity Heatmap for Cluster 817
TOX21_DT40_100
TOX21_PR_BLA_Antagonist_ratio
TOX21_AR_BLA_Antagonist_ch1
TOX21_ERa_BLA_Antagonist_ch1
TOX21_ERa_LUC_VM7_Antagonist_0.1nM_E2
TOX21_ERb_BLA_Antagonist_ratio
TOX21_GR_BLA_Antagonist_ratio
TOX21_MMP_ratio_down
TOX21_p53_BLA_p2_ch1
TOX21_p53_BLA_p3_ch1
TOX21_PGC_ERR_Antagonist
TOX21_RAR_LUC_Antagonist
TOX21_TR_LUC_GH3_Antagonist
NHEERL_ZF_144hpf_TERATOSCORE_up
NVS_TR_hDAT
OT_FXR_FXRSRC1_0480
STM_H9_OrnithineISnorm_dn
STM_H9_CystineISnorm_perc_up
NVS_ADME_hCYP2C19
LTEA_HepaRG_PDK4_up
LTEA_HepaRG_CYP4A22_up
LTEA_HepaRG_CYP1A2_up
ATG_PXRE_CIS_up
ACEA_AR_antagonist_80hr
3861−47−0
1689−99−2
122008−85−9

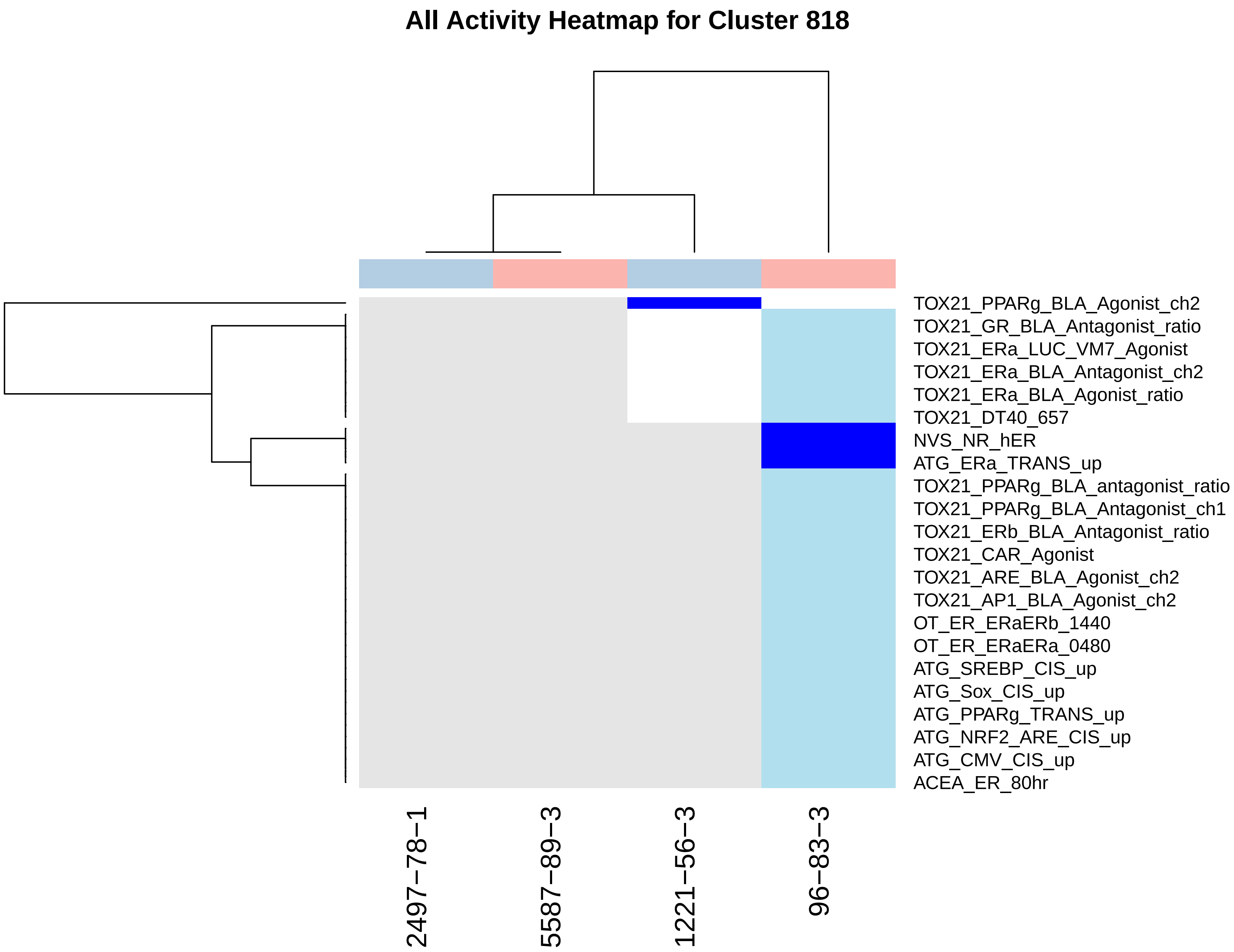
All Activity Heatmap for Cluster 818
TOX21_PPARg_BLA_Agonist_ch2
TOX21_GR_BLA_Antagonist_ratio
TOX21_ERa_LUC_VM7_Agonist
TOX21_ERa_BLA_Antagonist_ch2
TOX21_ERa_BLA_Agonist_ratio
TOX21_DT40_657
NVS_NR_hER
ATG_ERa_TRANS_up
TOX21_PPARg_BLA_antagonist_ratio
TOX21_PPARg_BLA_Antagonist_ch1
TOX21_ERb_BLA_Antagonist_ratio
TOX21_CAR_Agonist
TOX21_ARE_BLA_Agonist_ch2
TOX21_AP1_BLA_Agonist_ch2
OT_ER_ERaERb_1440
OT_ER_ERaERa_0480
ATG_SREBP_CIS_up
ATG_Sox_CIS_up
ATG_PPARg_TRANS_up
ATG_NRF2_ARE_CIS_up
ATG_CMV_CIS_up
ACEA_ER_80hr
2497−78−1
5587−89−3
1221−56−3
96−83−3

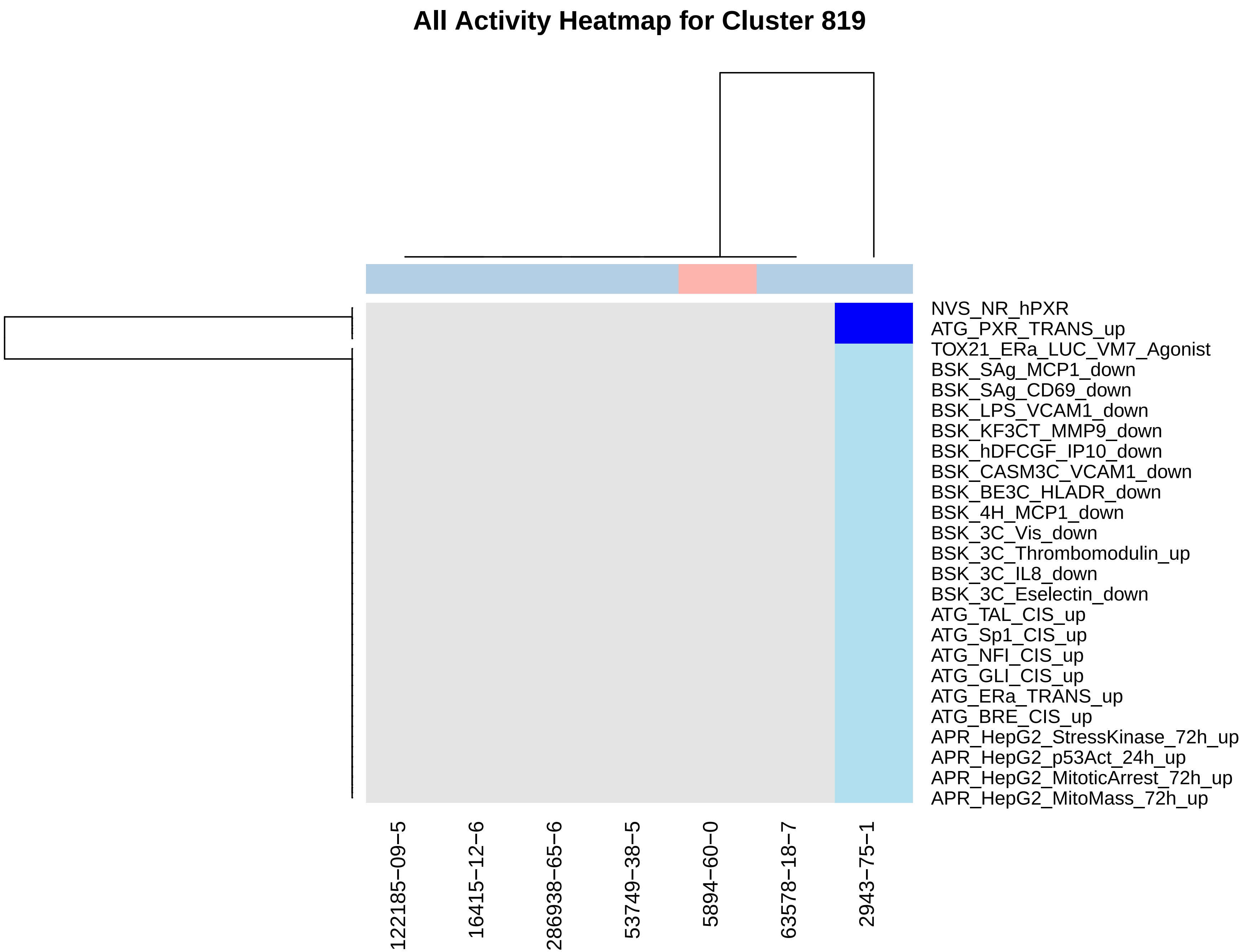
All Activity Heatmap for Cluster 819
NVS_NR_hPXR
ATG_PXR_TRANS_up
TOX21_ERa_LUC_VM7_Agonist
BSK_SAg_MCP1_down
BSK_SAg_CD69_down
BSK_LPS_VCAM1_down
BSK_KF3CT_MMP9_down
BSK_hDFCGF_IP10_down
BSK_CASM3C_VCAM1_down
BSK_BE3C_HLADR_down
BSK_4H_MCP1_down
BSK_3C_Vis_down
BSK_3C_Thrombomodulin_up
BSK_3C_IL8_down
BSK_3C_Eselectin_down
ATG_TAL_CIS_up
ATG_Sp1_CIS_up
ATG_NFI_CIS_up
ATG_GLI_CIS_up
ATG_ERa_TRANS_up
ATG_BRE_CIS_up
APR_HepG2_StressKinase_72h_up
APR_HepG2_p53Act_24h_up
APR_HepG2_MitoticArrest_72h_up
APR_HepG2_MitoMass_72h_up
122185–09–5
16415–12–6
286938–65–6
53749–38–5
5894–60–0
63578–18–7
2943–75–1

All Activity Heatmap for Cluster 820

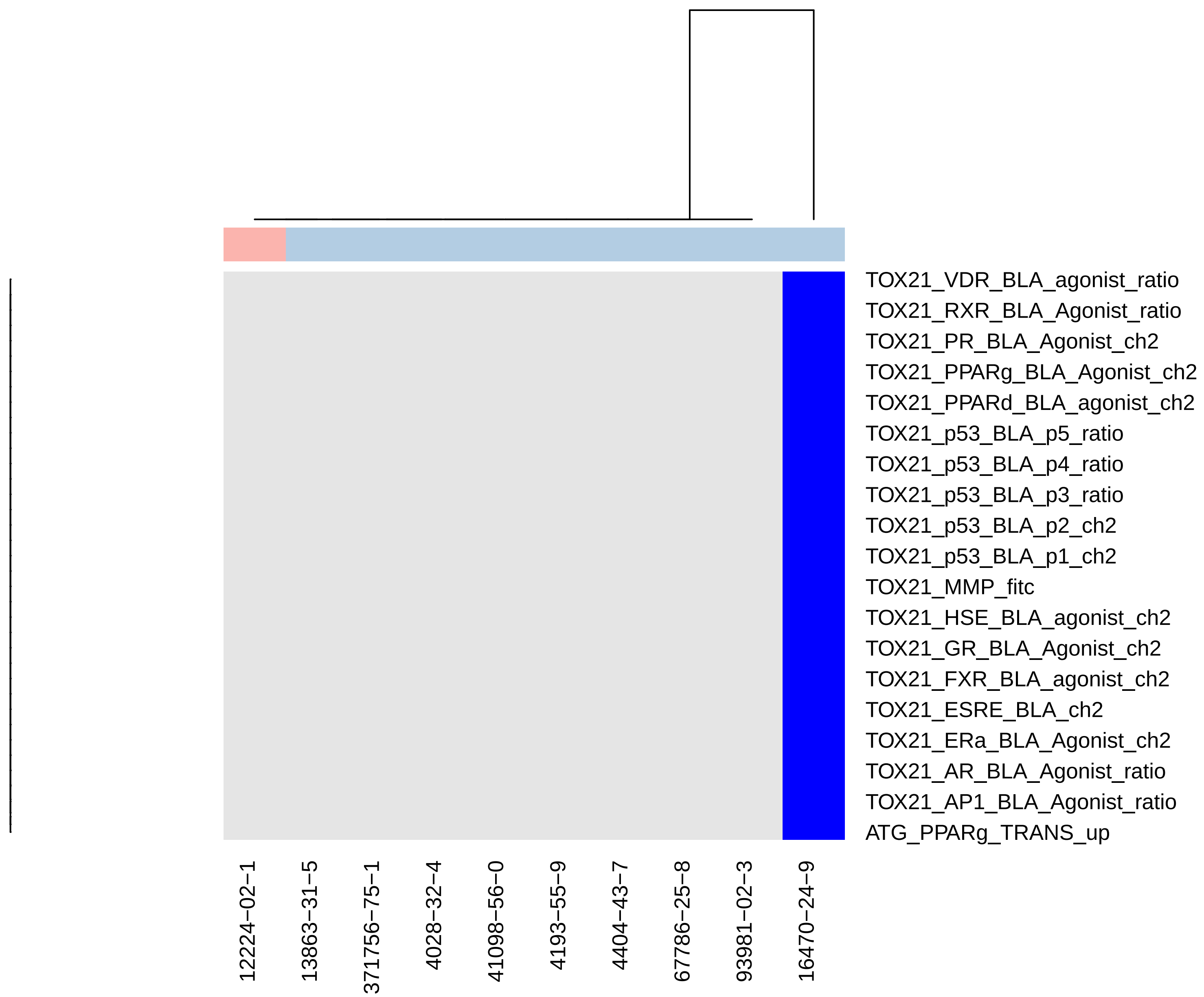

All Activity Heatmap for Cluster 822

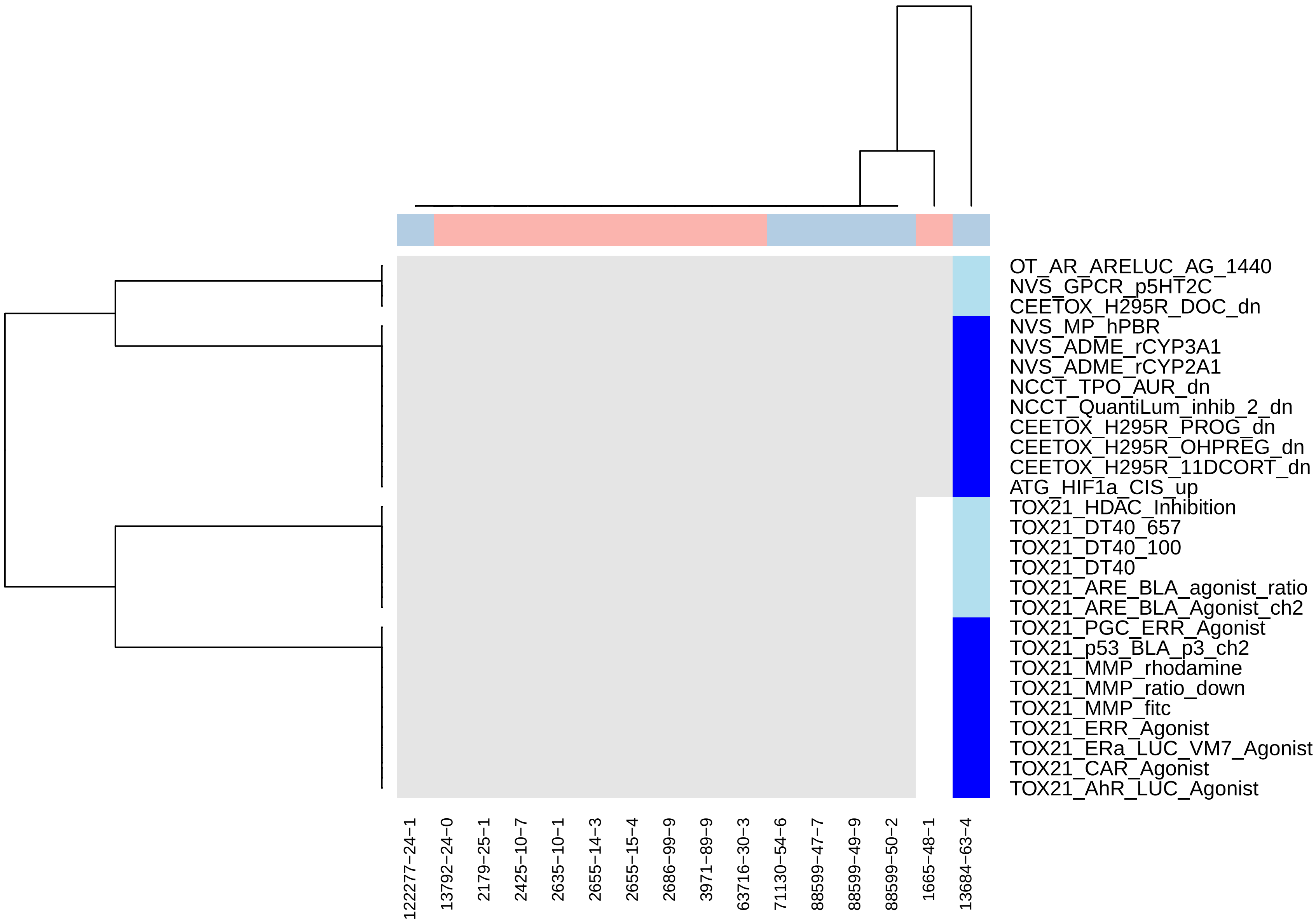

All Activity Heatmap for Cluster 825

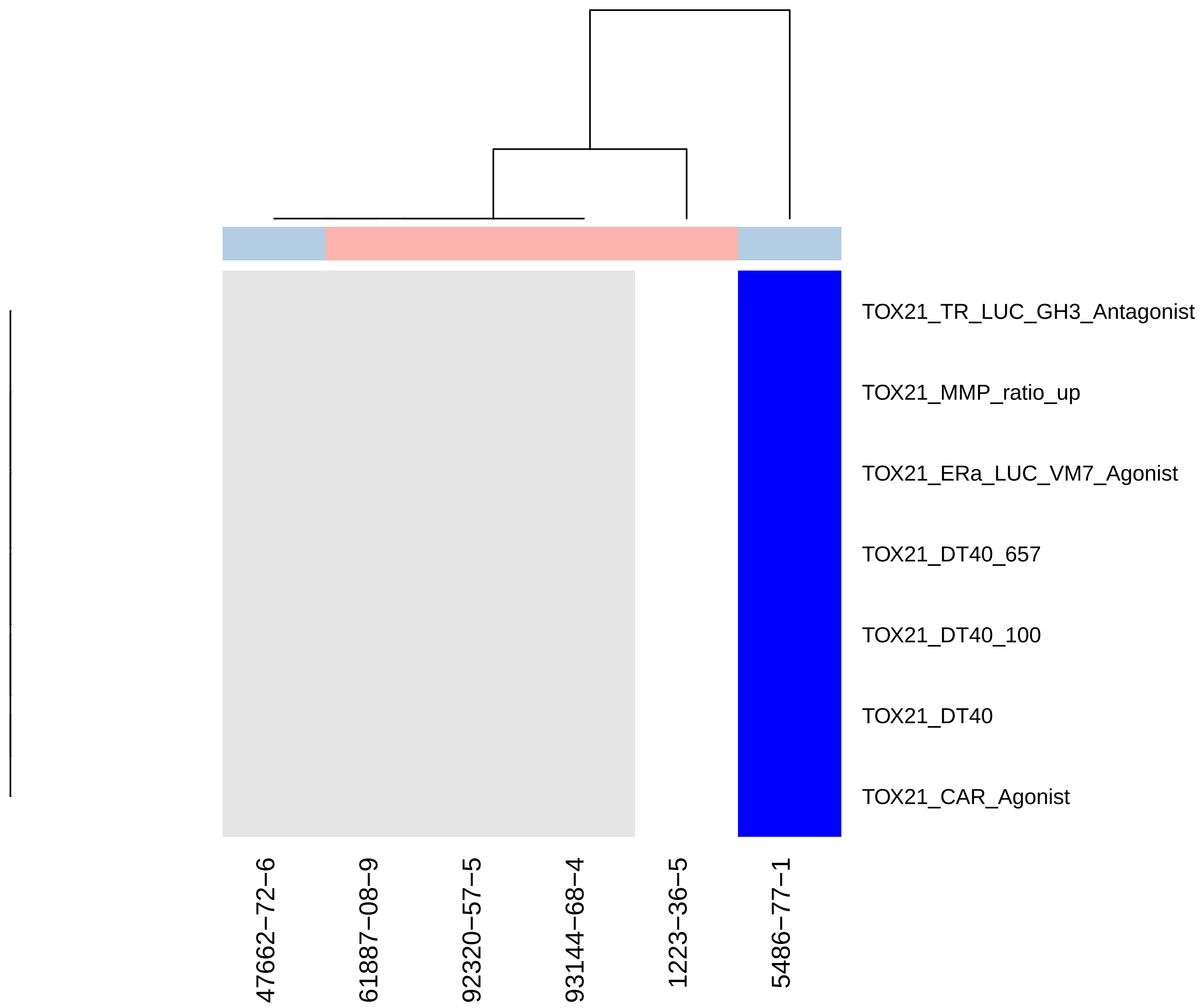

All Activity Heatmap for Cluster 827

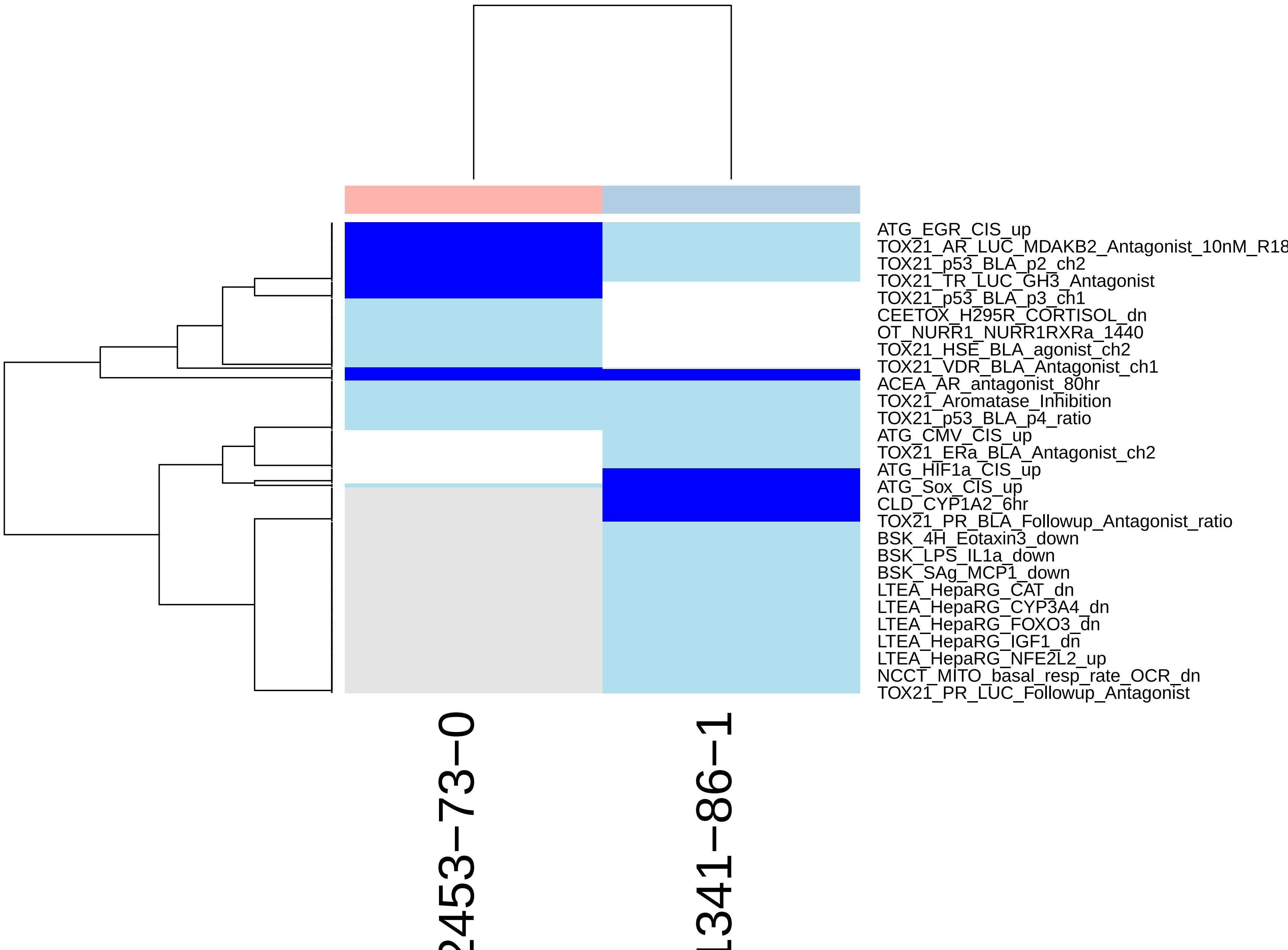

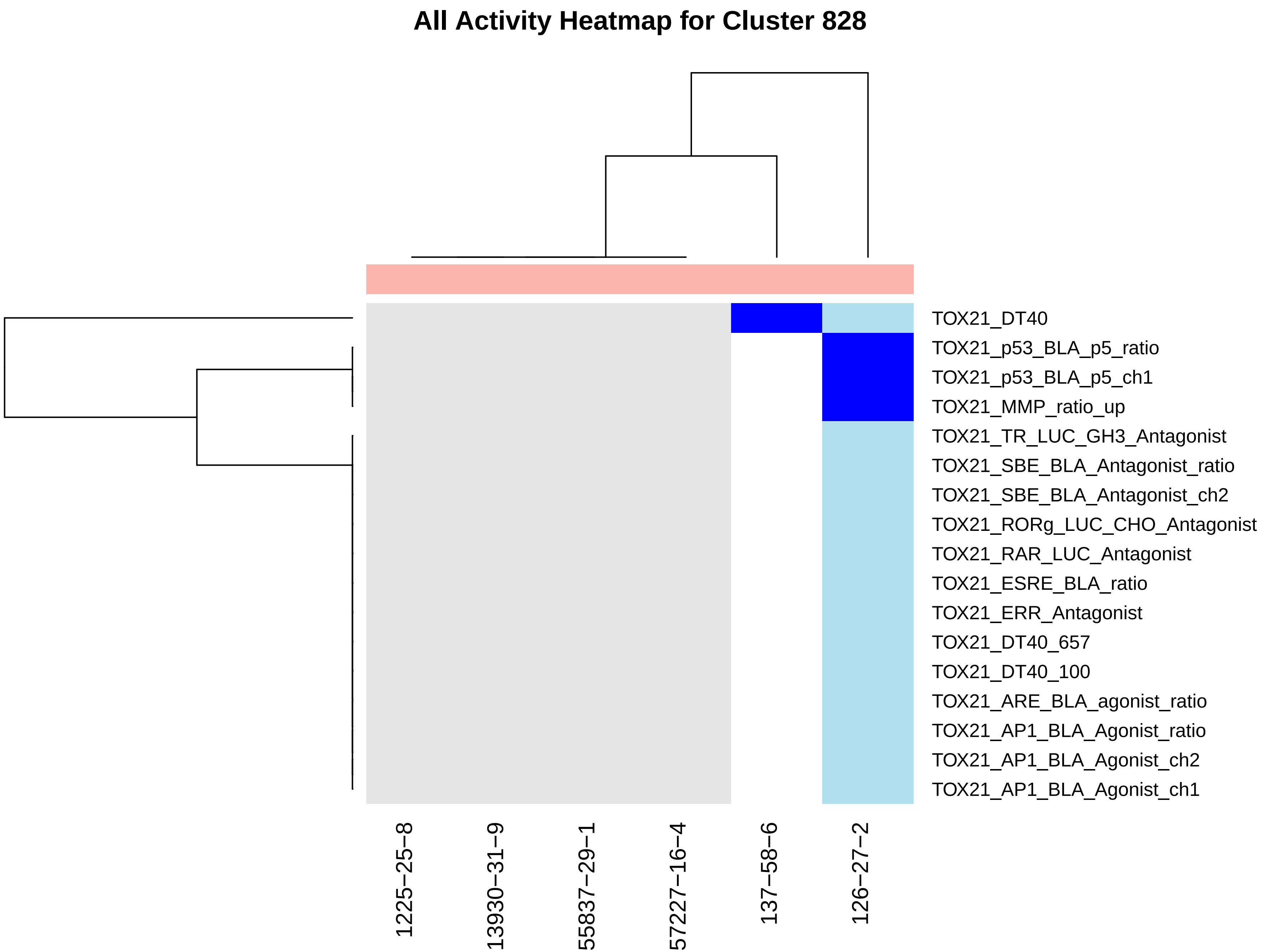
All Activity Heatmap for Cluster 828
TOX21_DT40
TOX21_p53_BLA_p5_ratio
TOX21_p53_BLA_p5_ch1
TOX21_MMP_ratio_up
TOX21_TR_LUC_GH3_Antagonist
TOX21_SBE_BLA_Antagonist_ratio
TOX21_SBE_BLA_Antagonist_ch2
TOX21_RORg_LUC_CHO_Antagonist
TOX21_RAR_LUC_Antagonist
TOX21_ESRE_BLA_ratio
TOX21_ERR_Antagonist
TOX21_DT40_657
TOX21_DT40_100
TOX21_ARE_BLA_agonist_ratio
TOX21_AP1_BLA_Agonist_ratio
TOX21_AP1_BLA_Agonist_ch2
TOX21_AP1_BLA_Agonist_ch1
1225−25−8
13930−31−9
55837−29−1
57227−16−4
137−58−6
126−27−2

All Activity Heatmap for Cluster 829

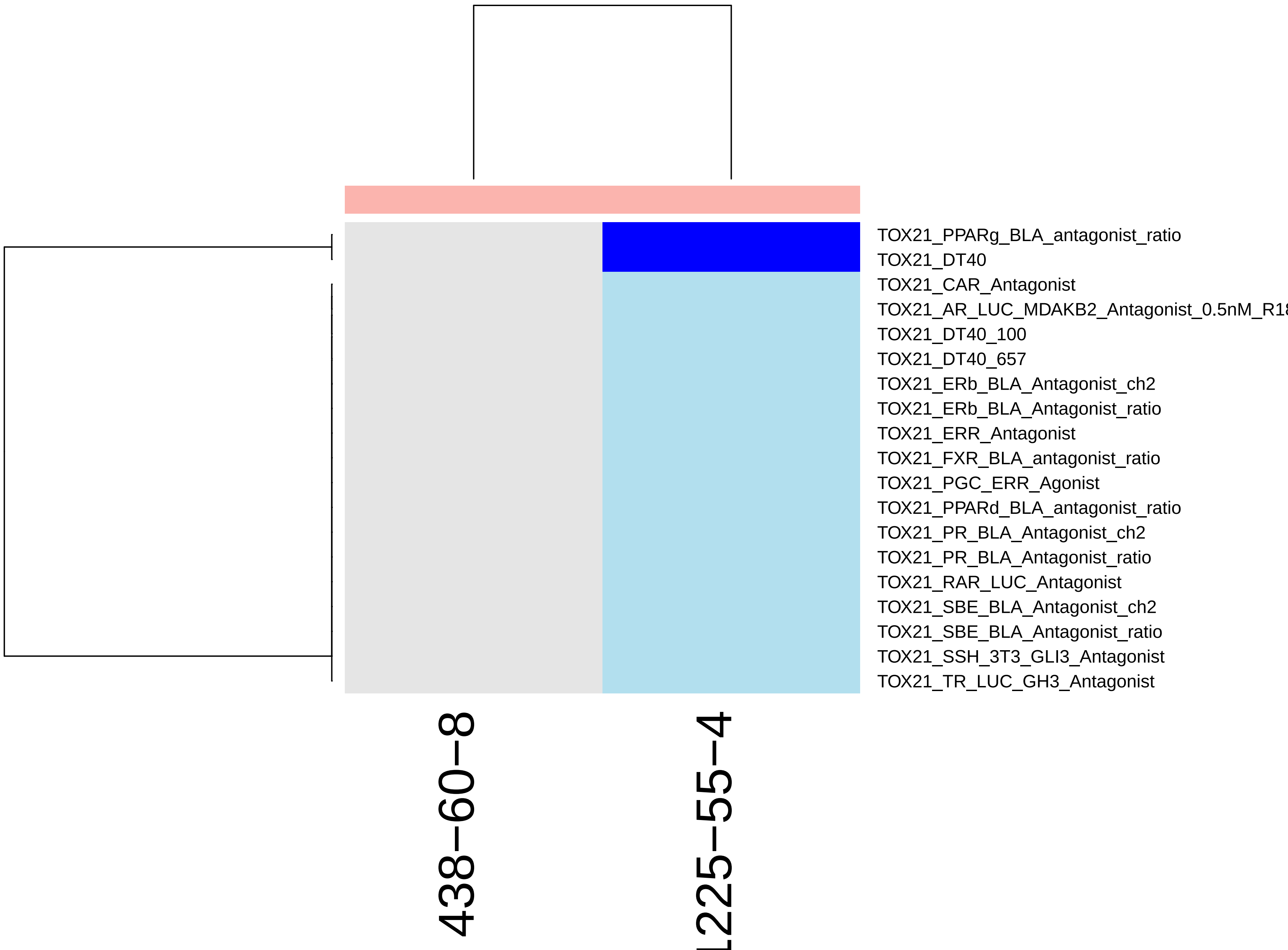

All Activity Heatmap for Cluster 830

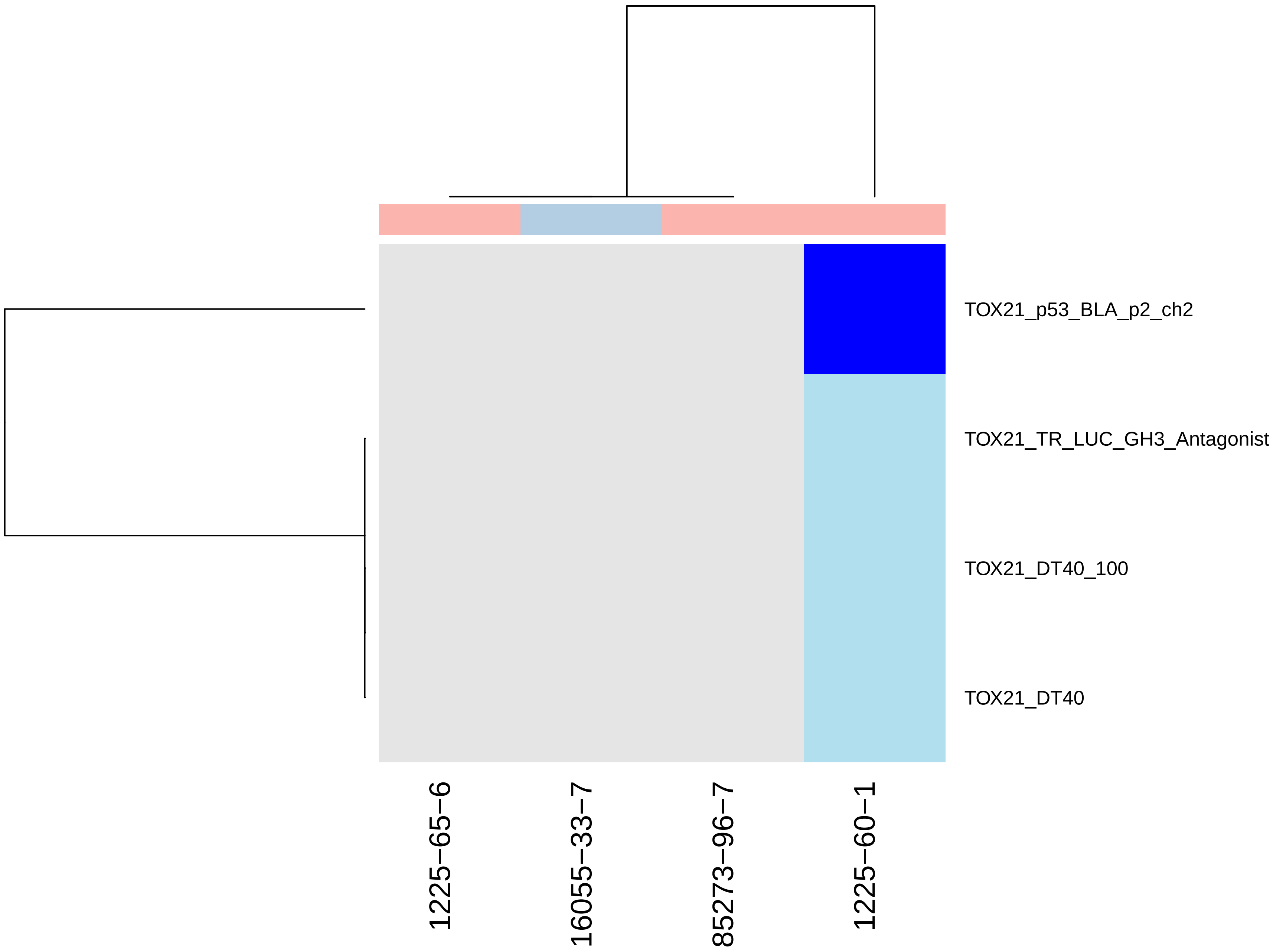

All Activity Heatmap for Cluster 831

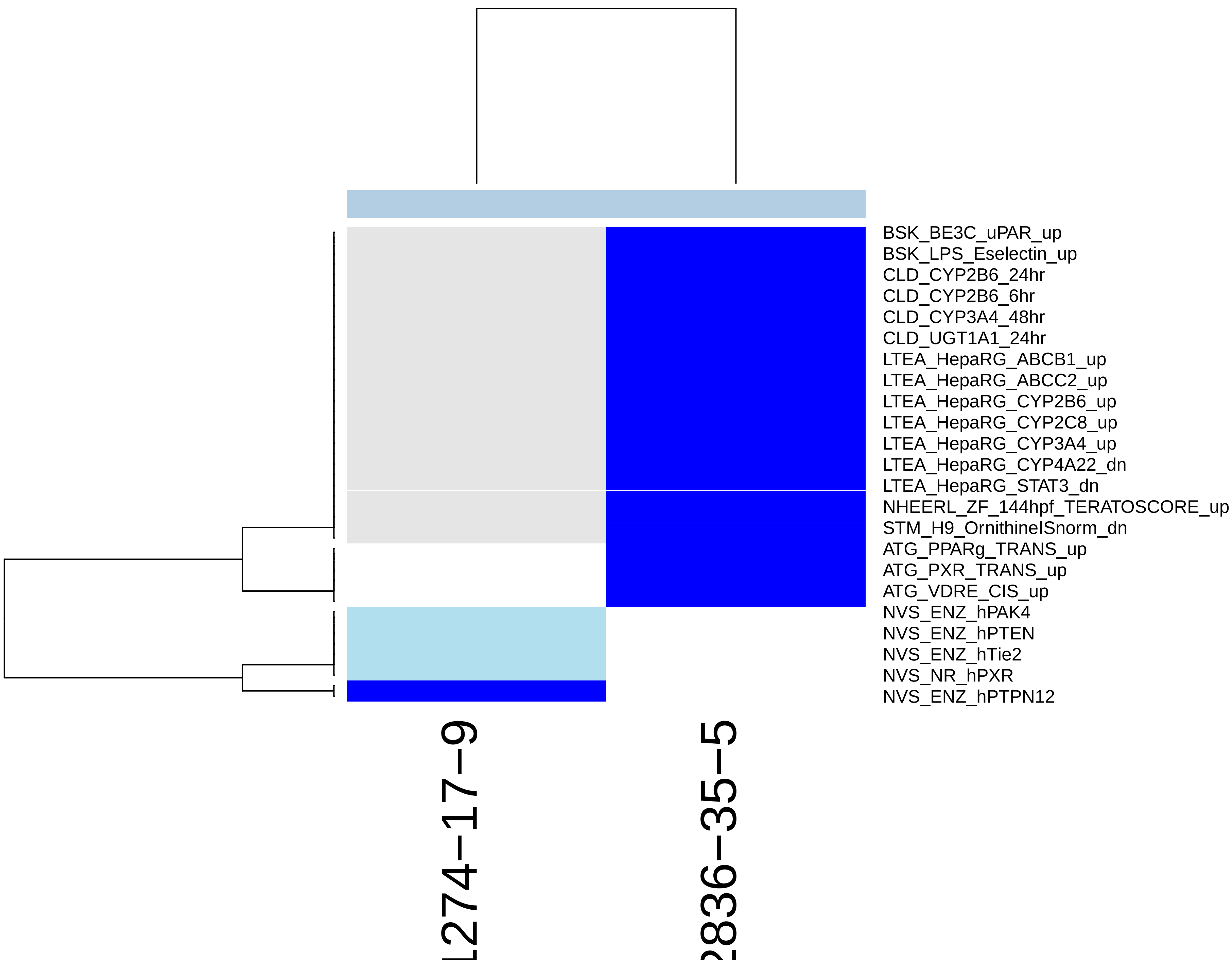

All Activity Heatmap for Cluster 832

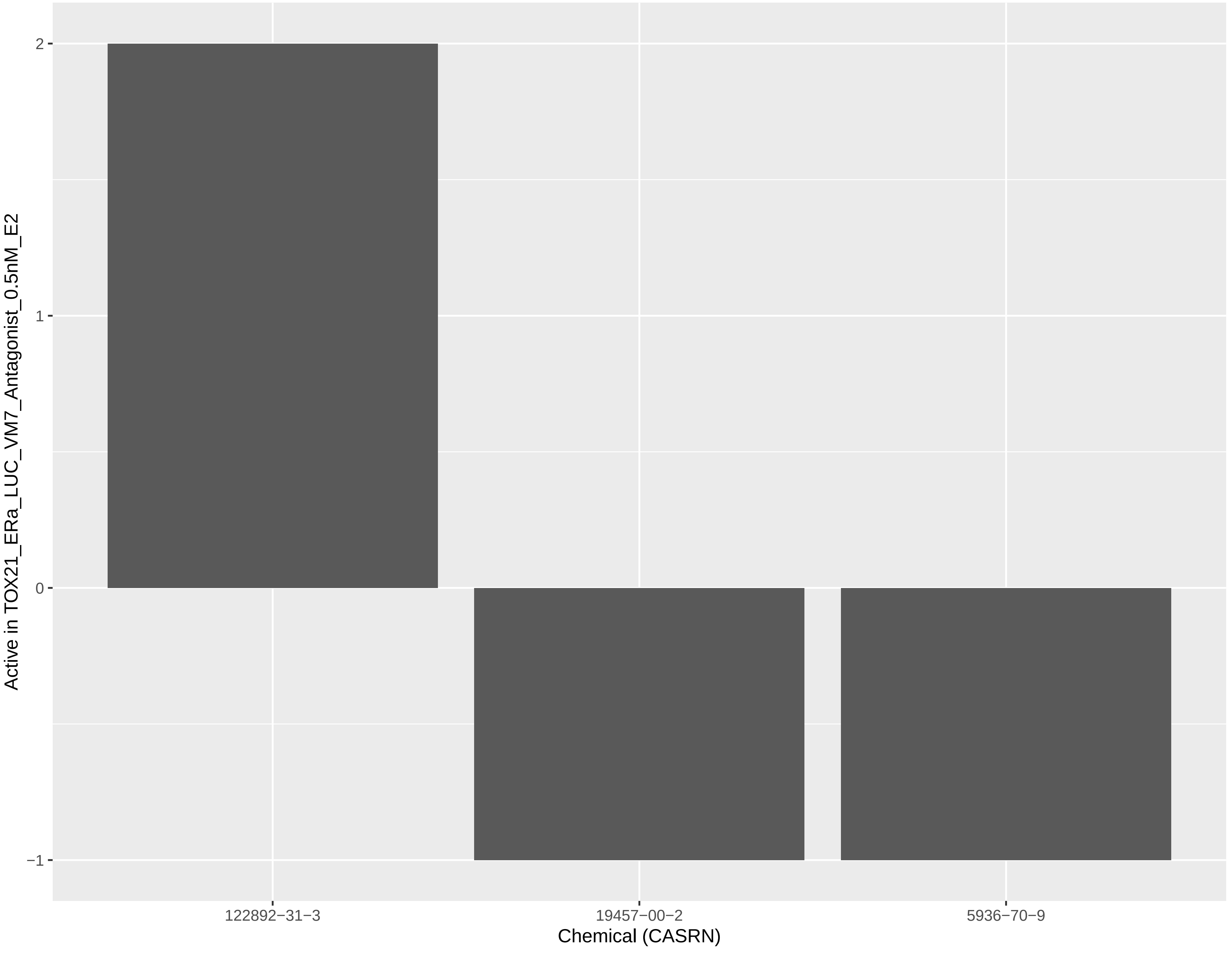

All Activity Heatmap for Cluster 833

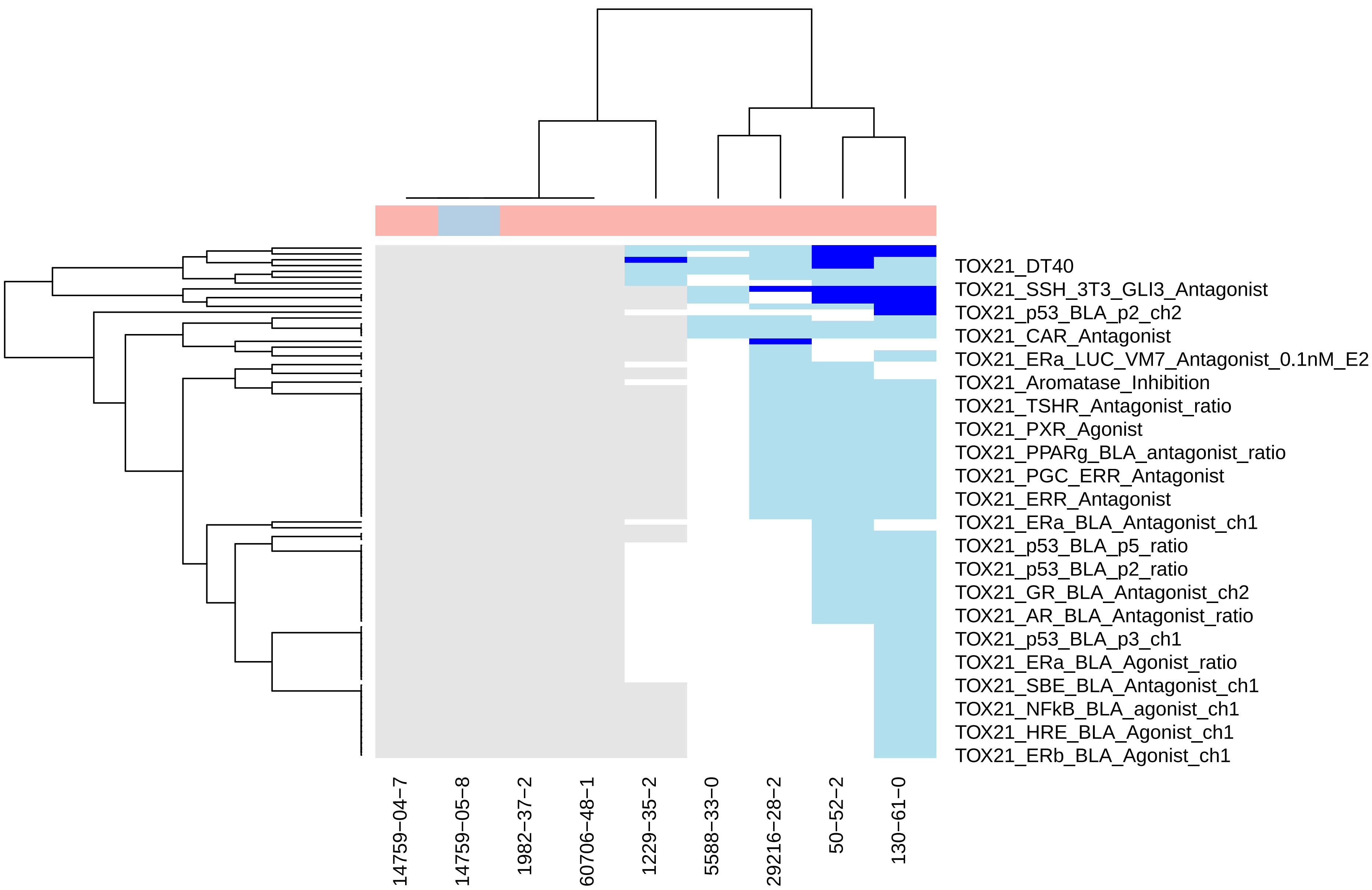

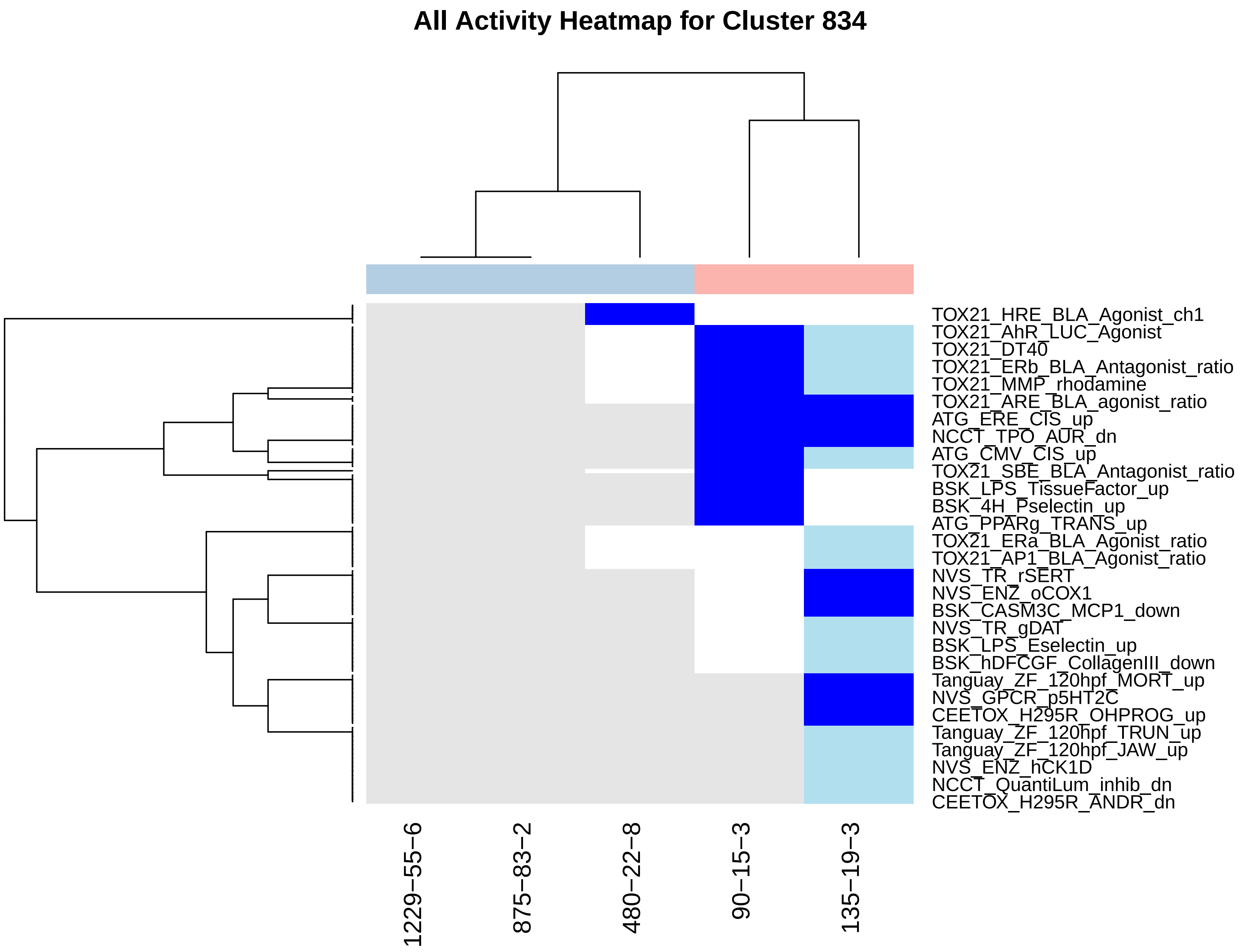
All Activity Heatmap for Cluster 834
TOX21_HRE_BLA_Agonist_ch1
TOX21_AhR_LUC_Agonist
TOX21_DT40
TOX21_ERb_BLA_Antagonist_ratio
TOX21_MMP_rhodamine
TOX21_ARE_BLA_agonist_ratio
ATG_ERE_CIS_up
NCCT_TPO_AUR_dn
ATG_CMV_CIS_up
TOX21_SBE_BLA_Antagonist_ratio
BSK_LPS_TissueFactor_up
BSK_4H_Pselectin_up
ATG_PPARg_TRANS_up
TOX21_ERa_BLA_Agonist_ratio
TOX21_AP1_BLA_Agonist_ratio
NVS_TR_rSERT
NVS_ENZ_oCOX1
BSK_CASM3C_MCP1_down
NVS_TR_gDAT
BSK_LPS_Eselectin_up
BSK_hDFCGF_CollagenIII_down
Tanguay_ZF_120hpf_MORT_up
NVS_GPCR_p5HT2C
CEETOX_H295R_OHPROG_up
Tanguay_ZF_120hpf_TRUN_up
Tanguay_ZF_120hpf_JAW_up
NVS_ENZ_hCK1D
NCCT_QuantiLum_inhib_dn
CEETOX_H295R_ANDR_dn
1229–55–6
875–83–2
480–22–8
90–15–3
135–19–3

All Activity Heatmap for Cluster 835

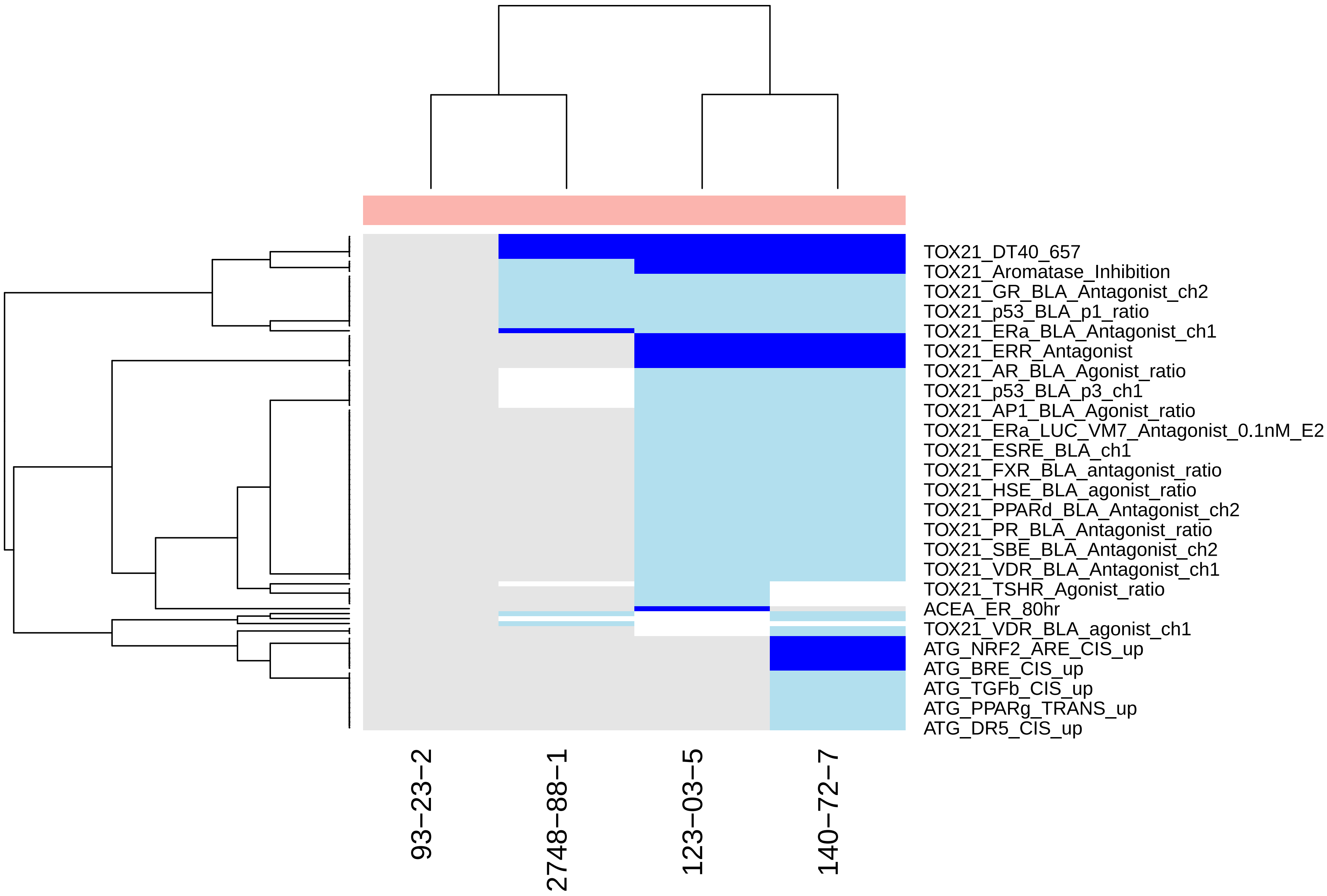

All Activity Heatmap for Cluster 837

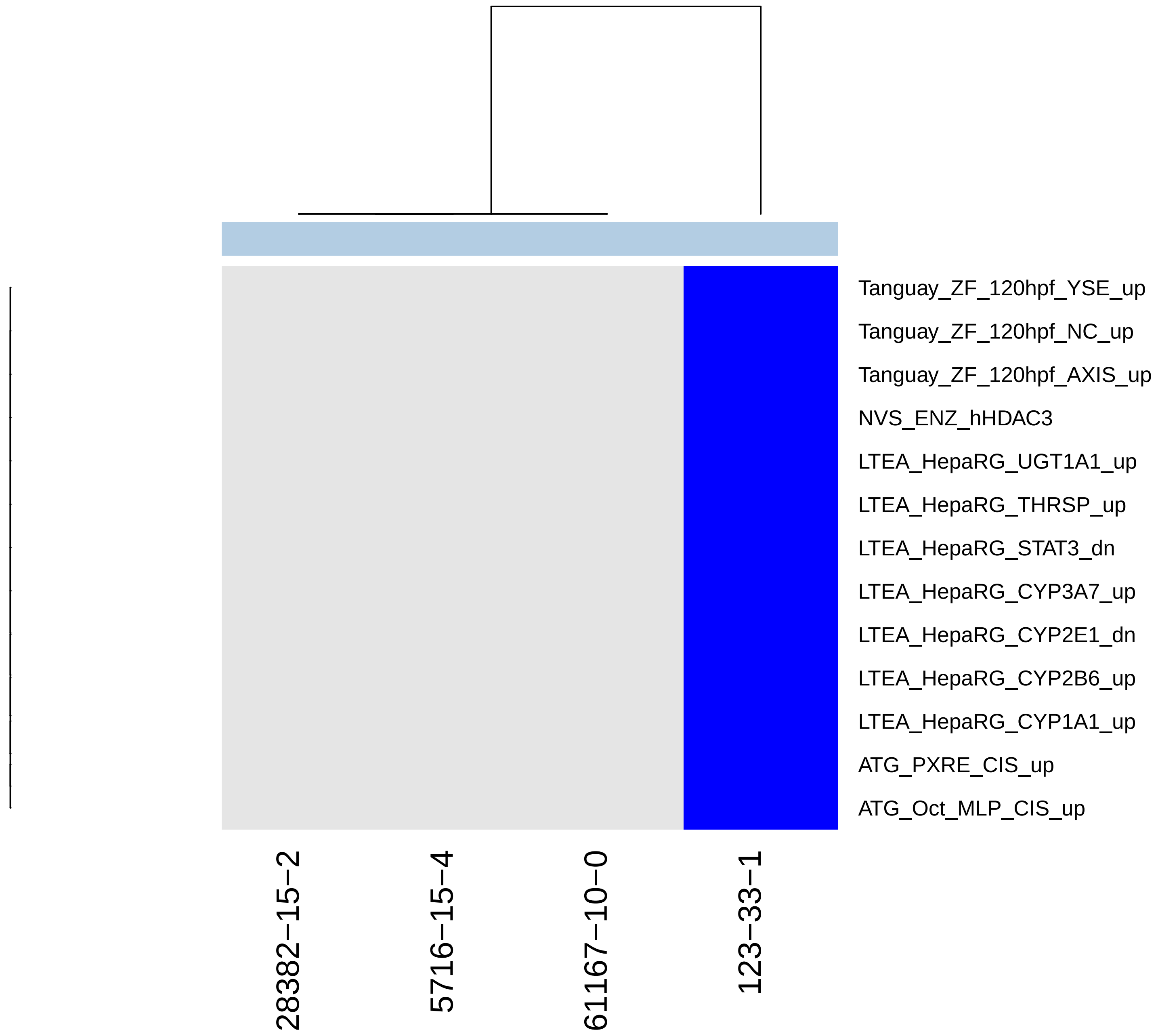

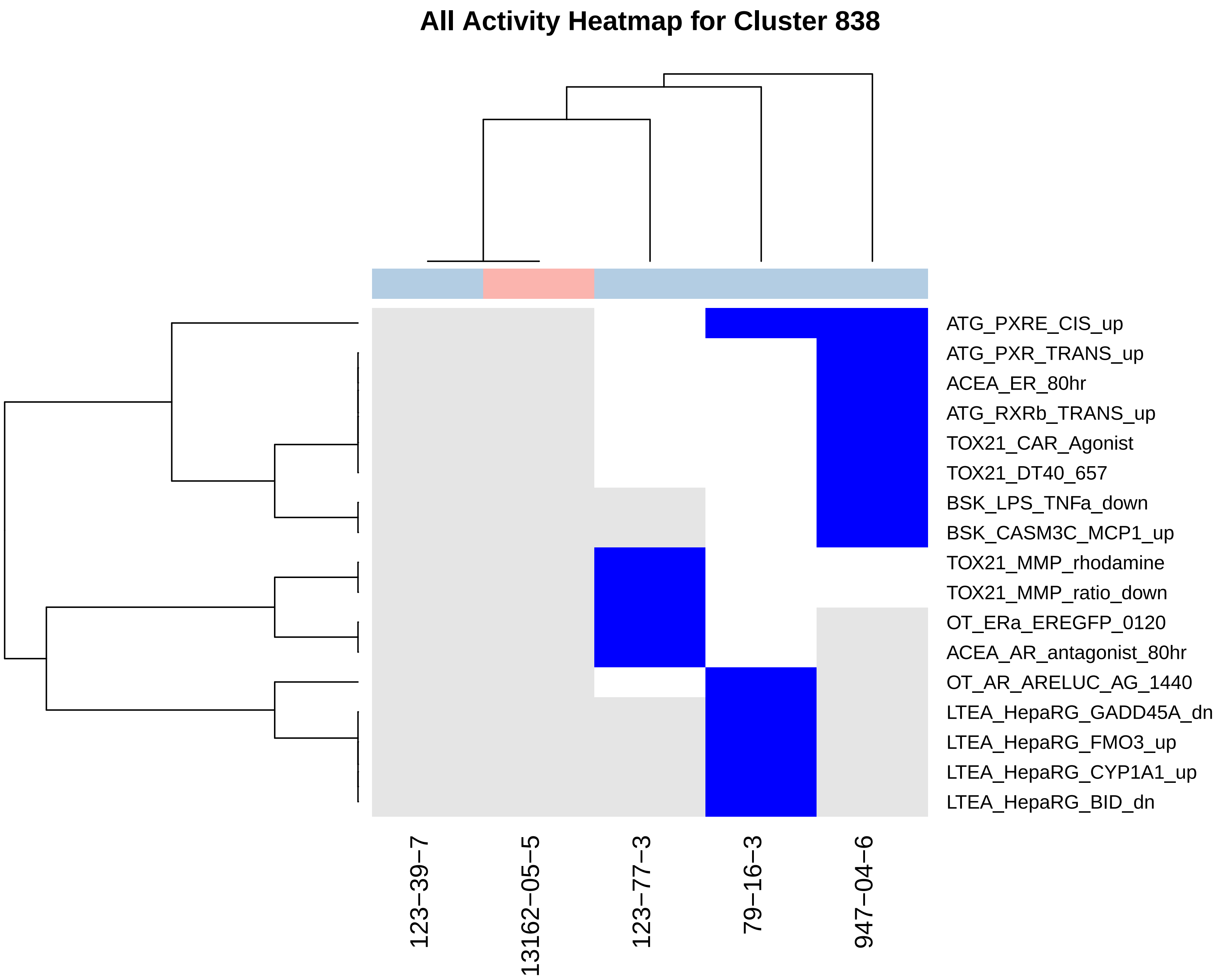
All Activity Heatmap for Cluster 838
ATG_PXRE_CIS_up
ATG_PXR_TRANS_up
ACEA_ER_80hr
ATG_RXRb_TRANS_up
TOX21_CAR_Agonist
TOX21_DT40_657
BSK_LPS_TNFa_down
BSK_CASM3C_MCP1_up
TOX21_MMP_rhodamine
TOX21_MMP_ratio_down
OT_ERa_EREGFP_0120
ACEA_AR_antagonist_80hr
OT_AR_ARELUC_AG_1440
LTEA_HepaRG_GADD45A_dn
LTEA_HepaRG_FMO3_up
LTEA_HepaRG_CYP1A1_up
LTEA_HepaRG_BID_dn
123−39−7
13162−05−5
123−77−3
79−16−3
947−04−6

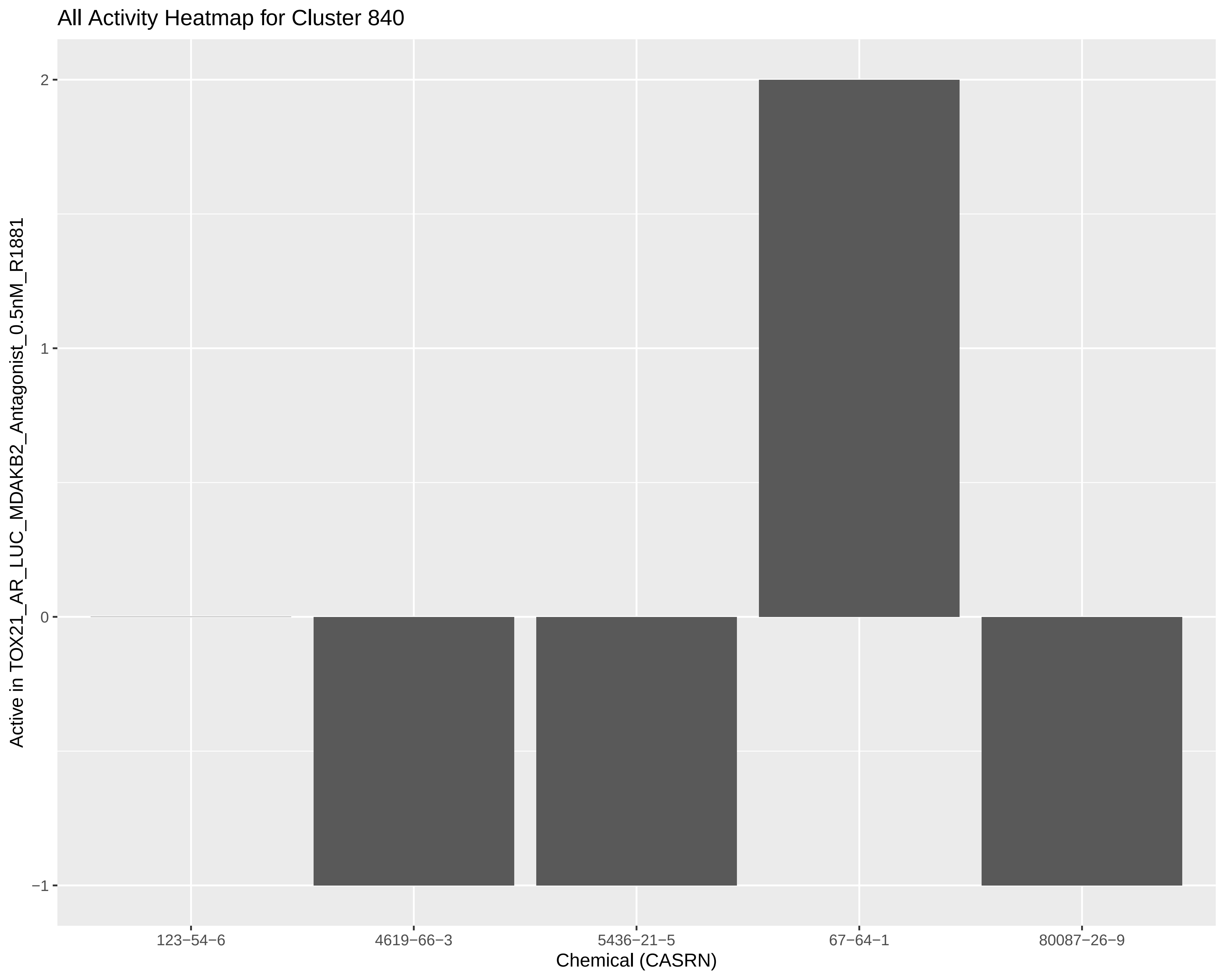

All Activity Heatmap for Cluster 840
Active in TOX21_AR_LUC_MDAKB2_Antagonist_0.5nM_R1881
2
1
0
−1
123−54−6
4619−66−3
5436−21−5
67−64−1
80087−26−9
Chemical (CASRN)

All Activity Heatmap for Cluster 841

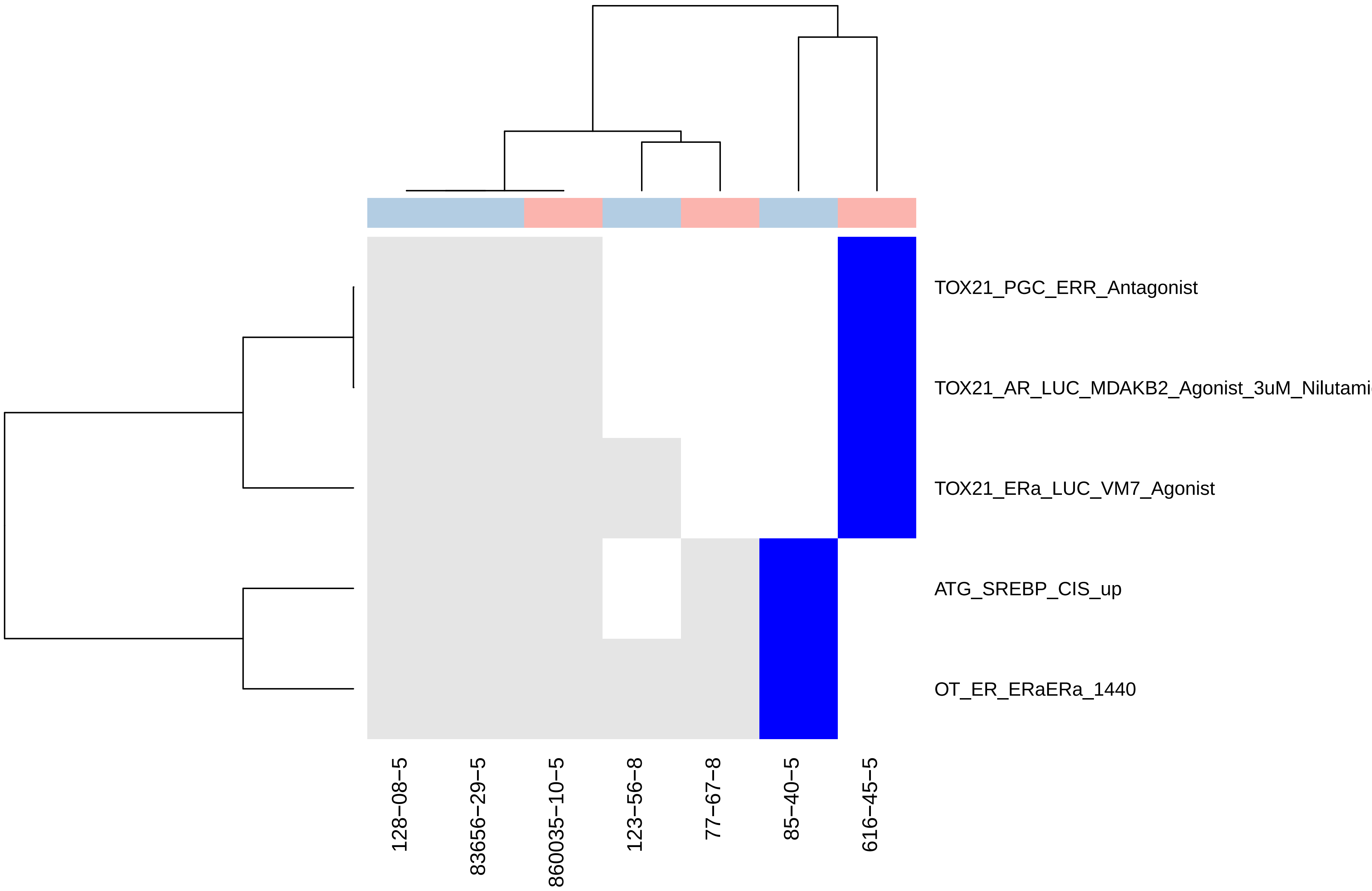

All Activity Heatmap for Cluster 842

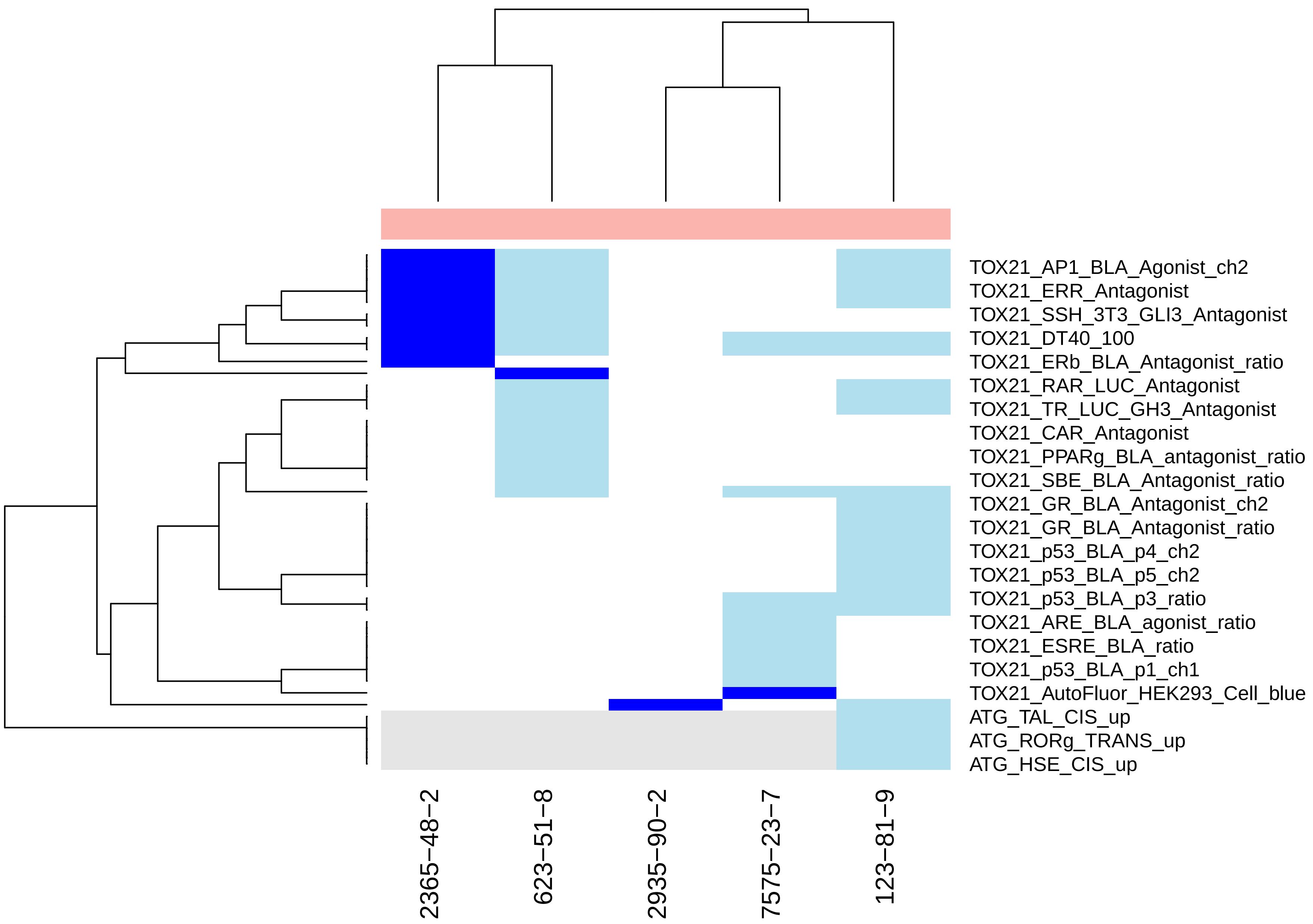

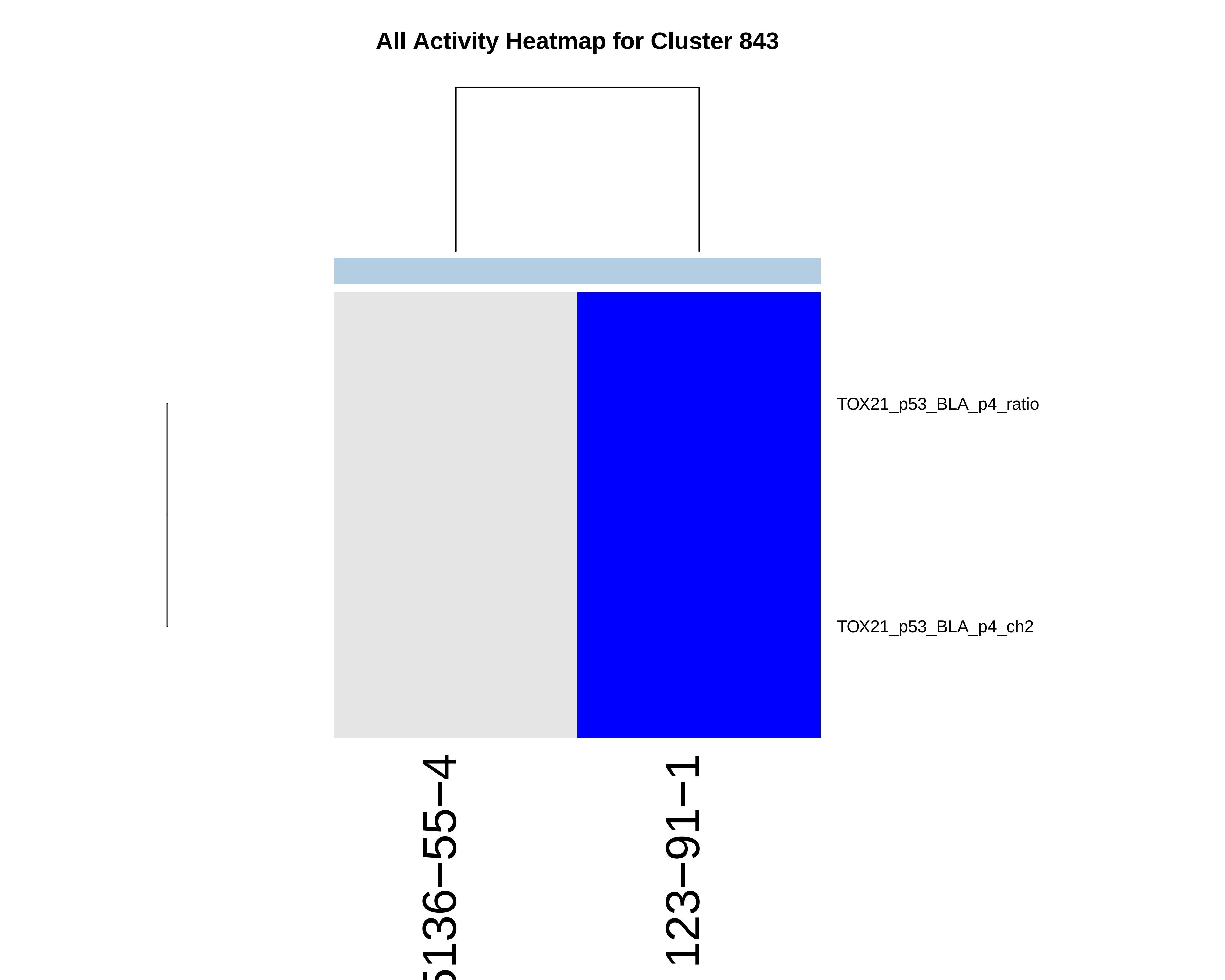
All Activity Heatmap for Cluster 843
TOX21_p53_BLA_p4_ratio
TOX21_p53_BLA_p4_ch2
5136−55−4
123−91−1

All Activity Heatmap for Cluster 844

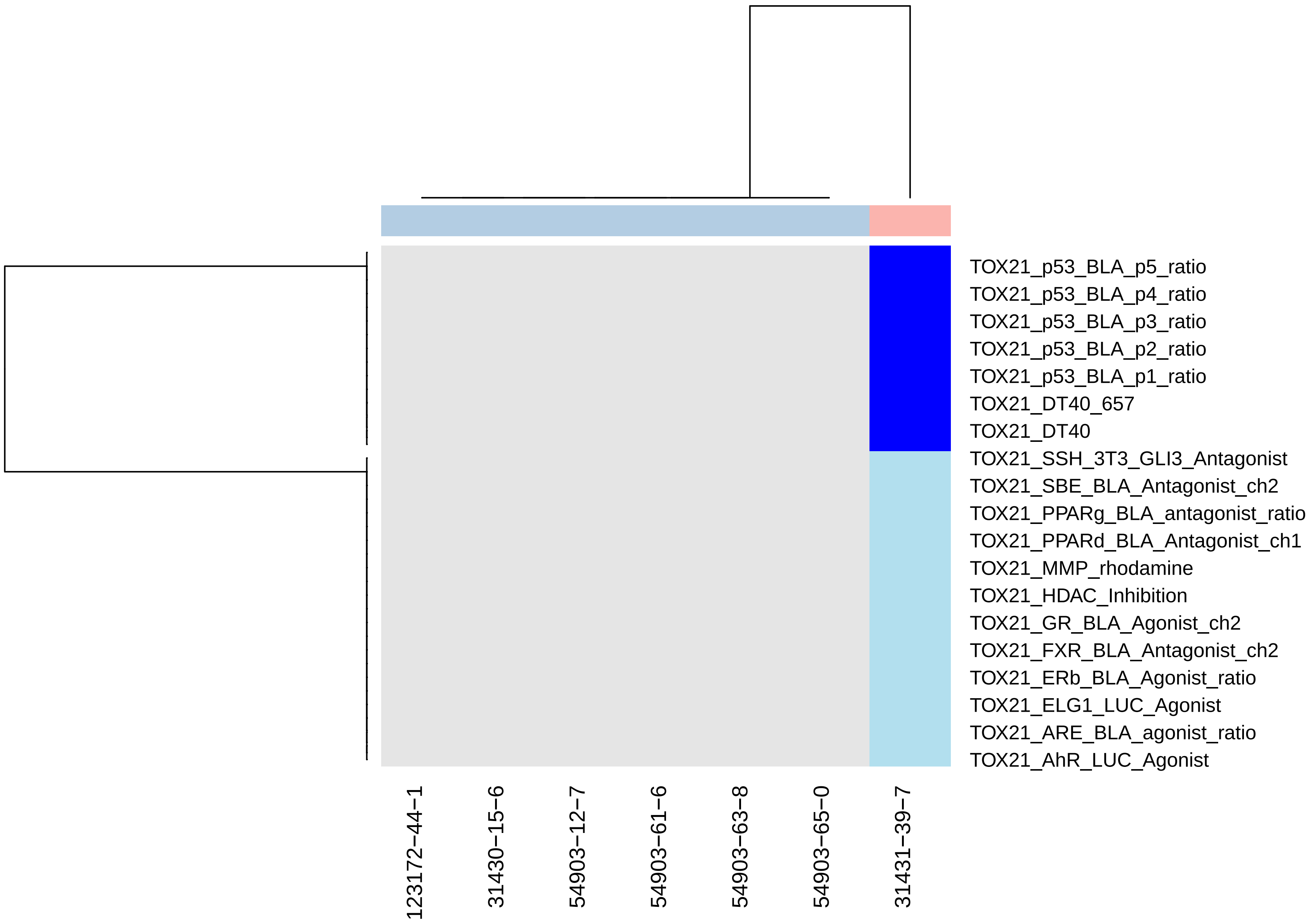

All Activity Heatmap for Cluster 850

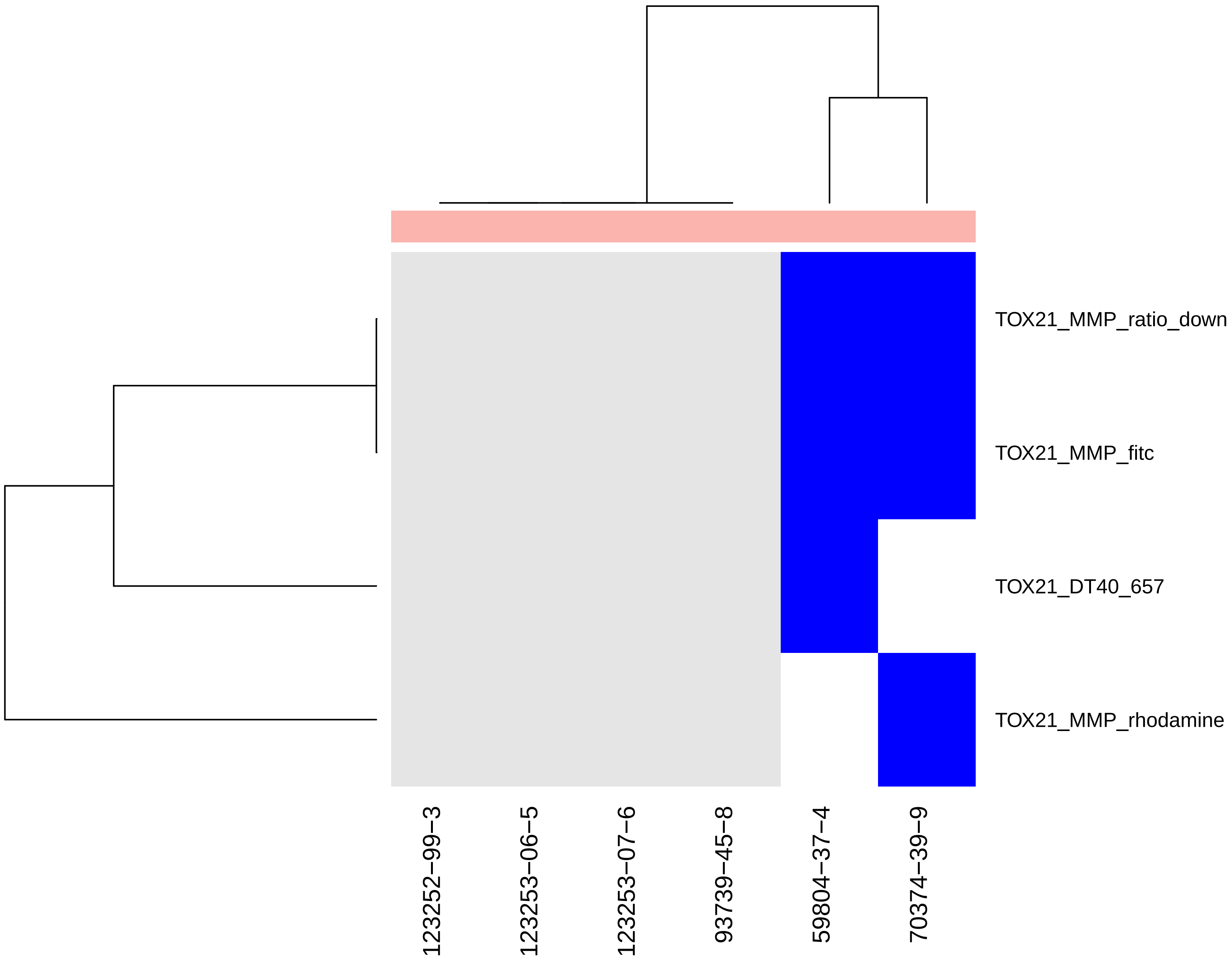

All Activity Heatmap for Cluster 851

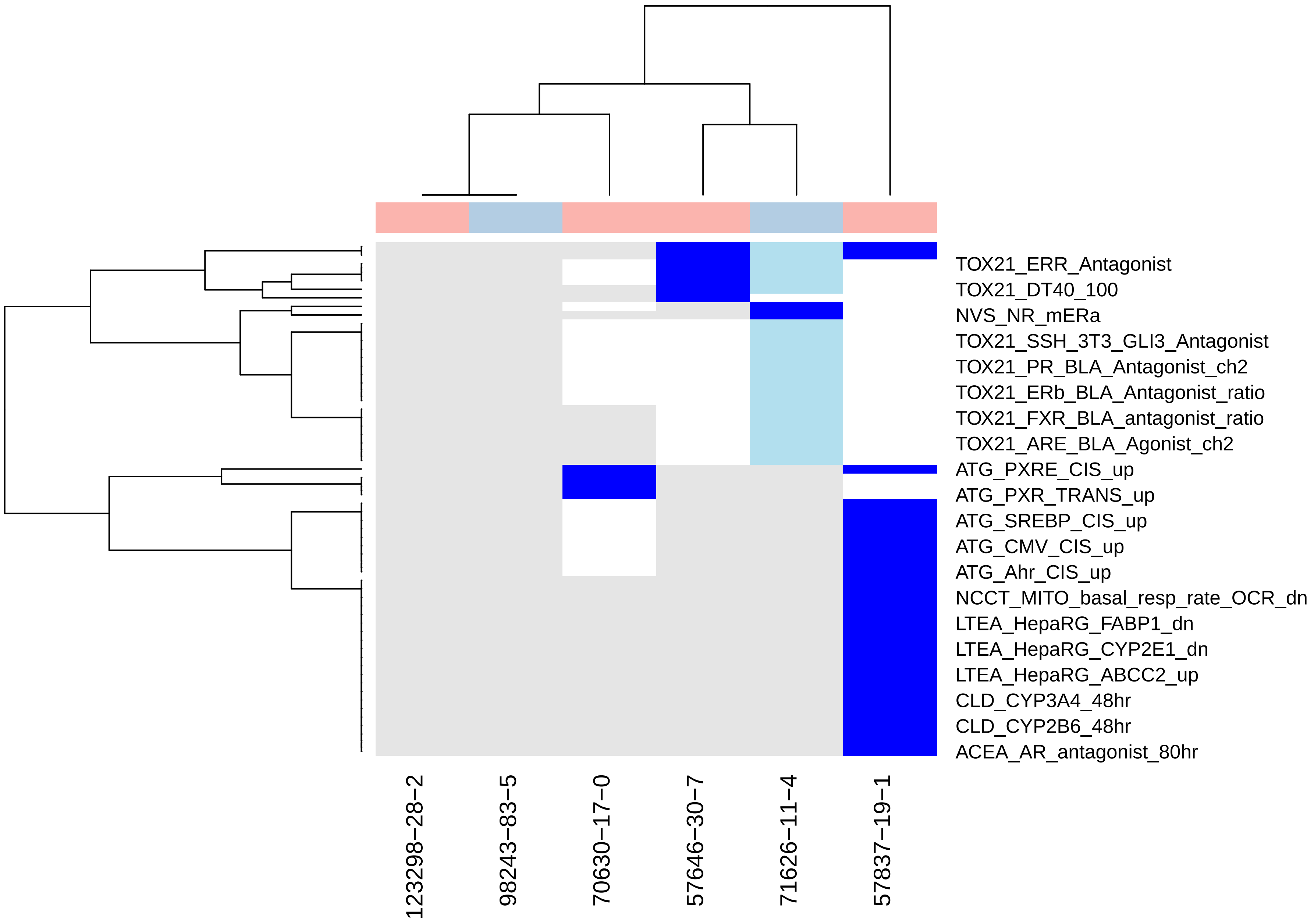

All Activity Heatmap for Cluster 853

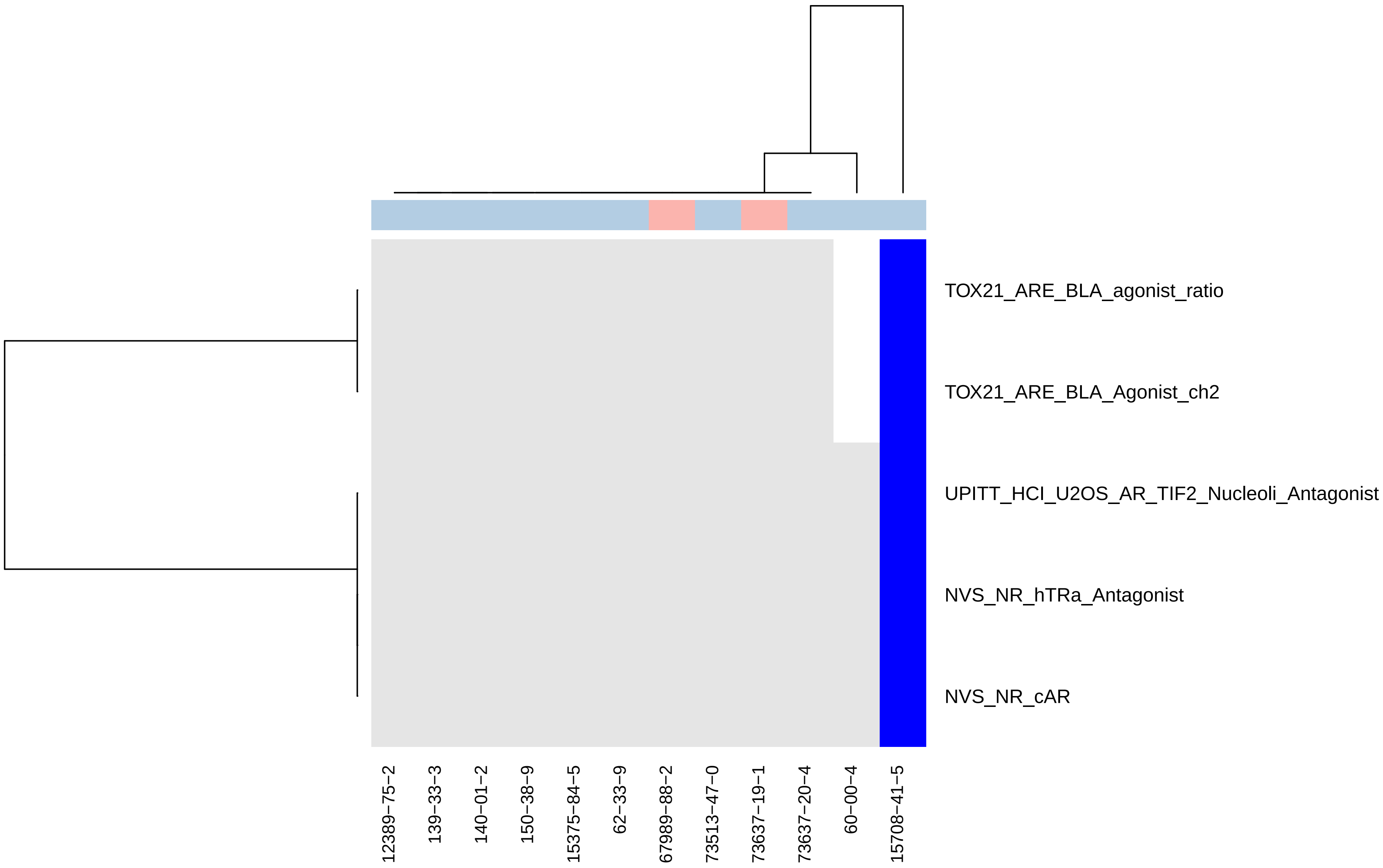

All Activity Heatmap for Cluster 854

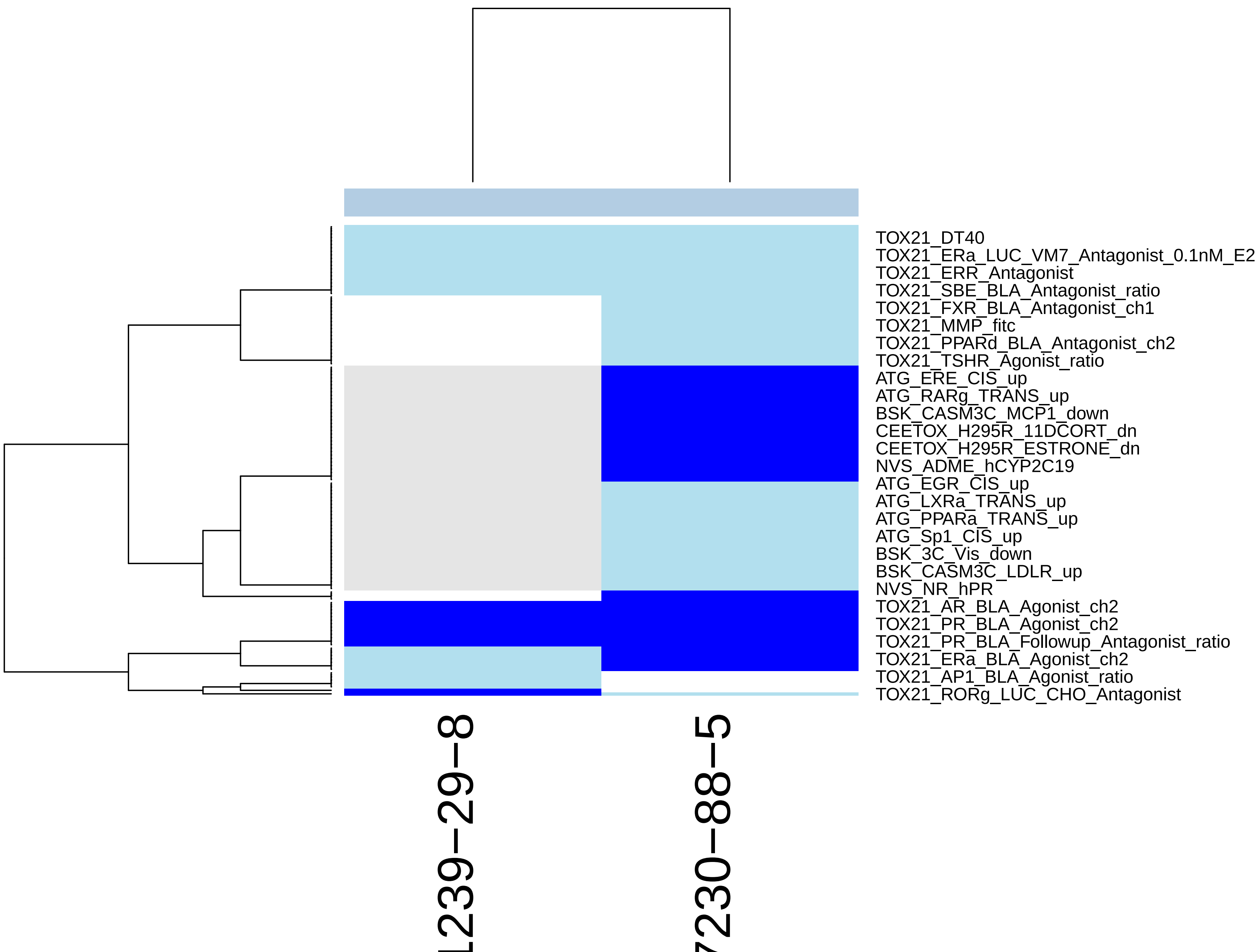

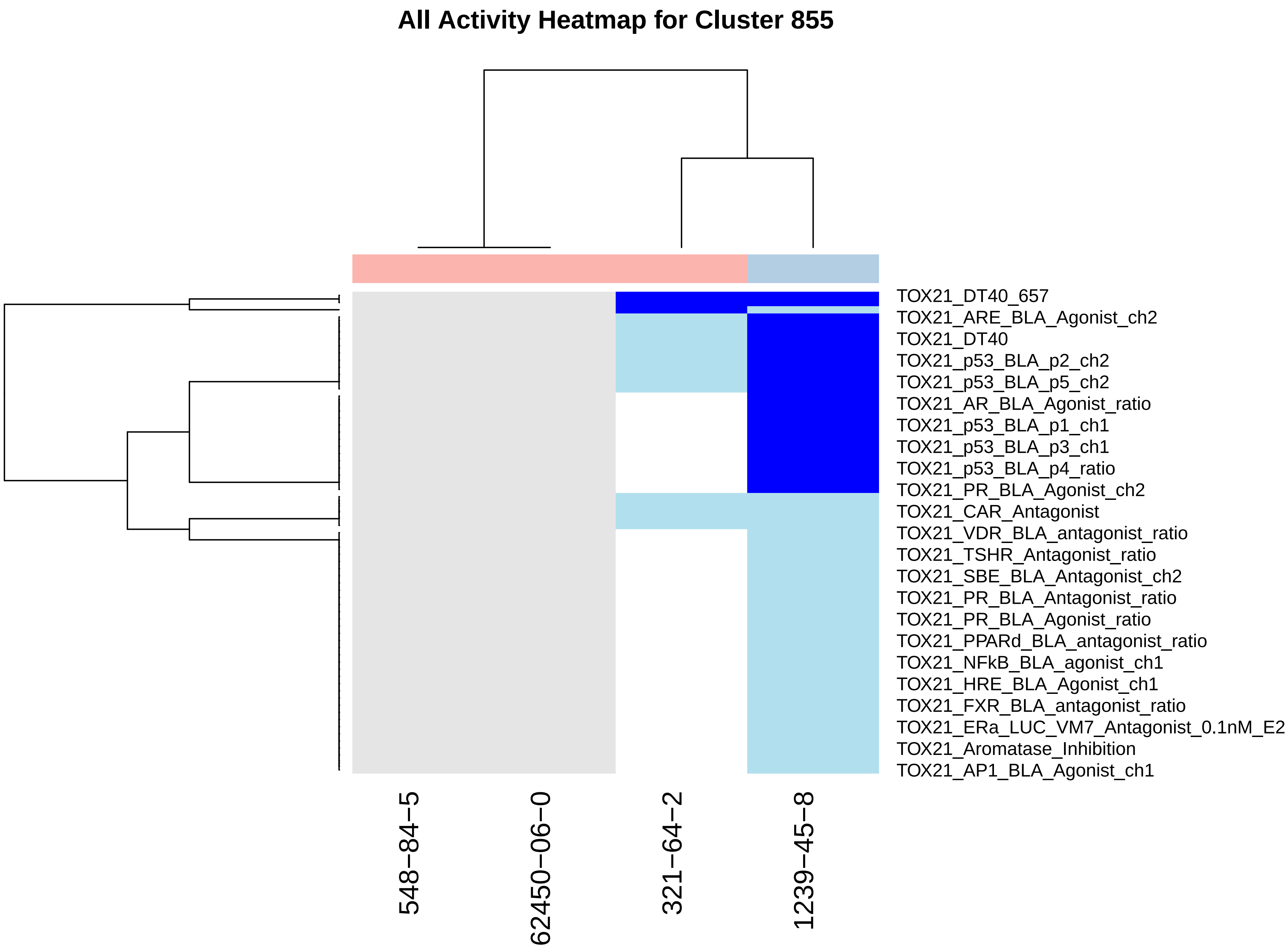

All Activity Heatmap for Cluster 855
TOX21_DT40_657
TOX21_ARE_BLA_Agonist_ch2
TOX21_DT40
TOX21_p53_BLA_p2_ch2
TOX21_p53_BLA_p5_ch2
TOX21_AR_BLA_Agonist_ratio
TOX21_p53_BLA_p1_ch1
TOX21_p53_BLA_p3_ch1
TOX21_p53_BLA_p4_ratio
TOX21_PR_BLA_Agonist_ch2
TOX21_CAR_Antagonist
TOX21_VDR_BLA_antagonist_ratio
TOX21_TSHR_Antagonist_ratio
TOX21_SBE_BLA_Antagonist_ch2
TOX21_PR_BLA_Antagonist_ratio
TOX21_PR_BLA_Agonist_ratio
TOX21_PPARd_BLA_antagonist_ratio
TOX21_NFkB_BLA_agonist_ch1
TOX21_HRE_BLA_Agonist_ch1
TOX21_FXR_BLA_antagonist_ratio
TOX21_ERa_LUC_VM7_Antagonist_0.1nM_E2
TOX21_Aromatase_Inhibition
TOX21_AP1_BLA_Agonist_ch1
548−84−5
62450−06−0
321−64−2
1239−45−8

All Activity Heatmap for Cluster 856

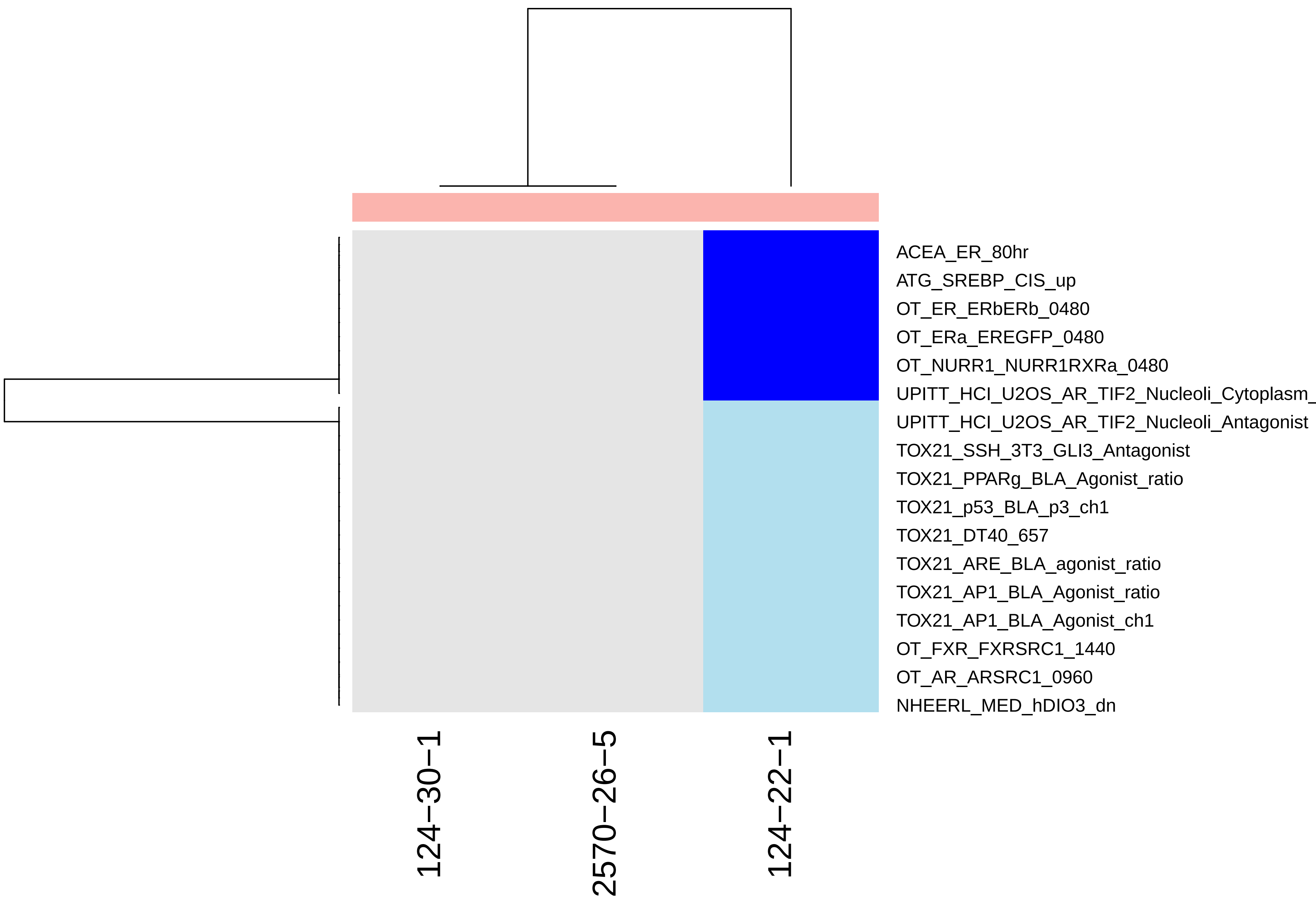

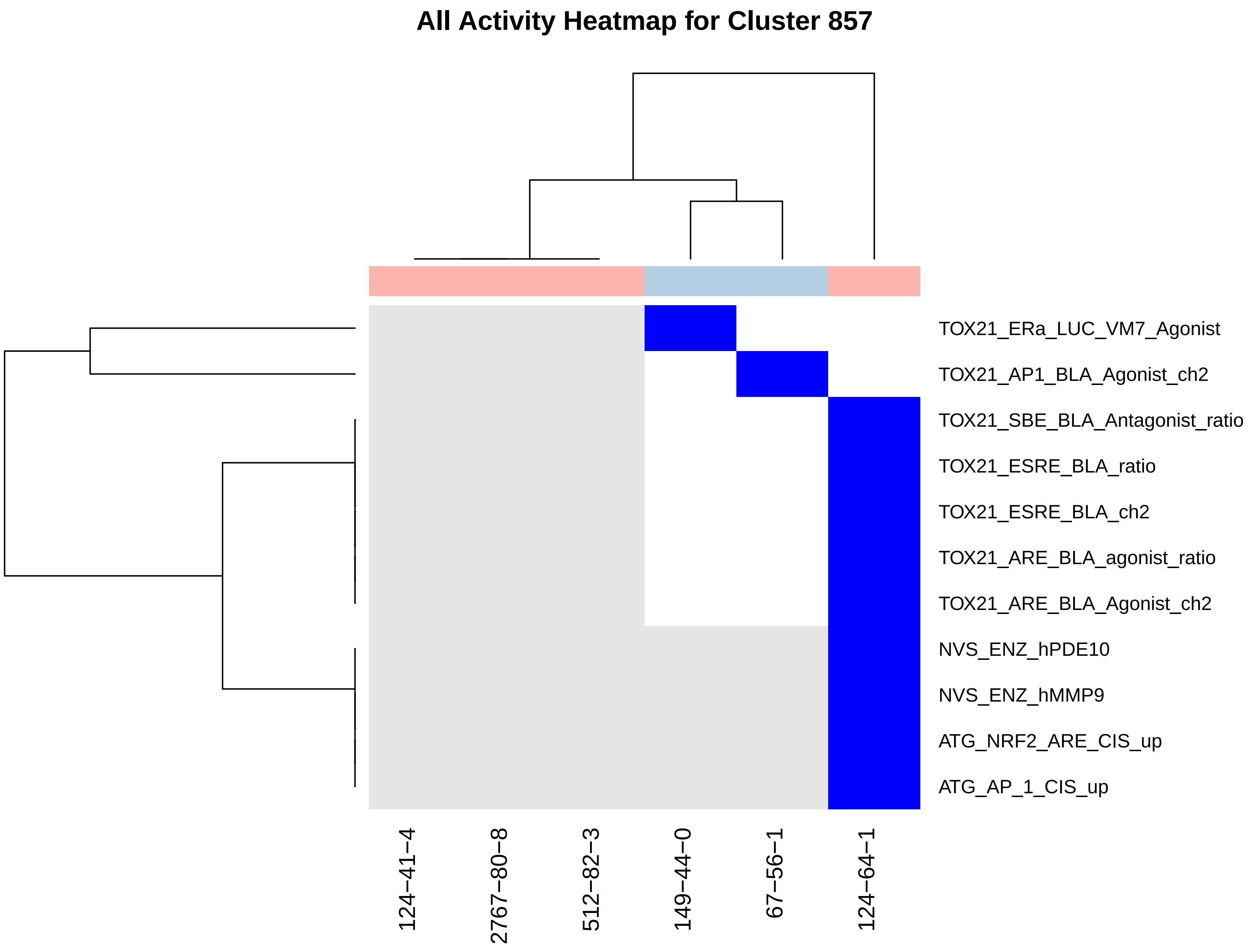
All Activity Heatmap for Cluster 857
TOX21_ERa_LUC_VM7_Agonist
TOX21_AP1_BLA_Agonist_ch2
TOX21_SBE_BLA_Antagonist_ratio
TOX21_ESRE_BLA_ratio
TOX21_ESRE_BLA_ch2
TOX21_ARE_BLA_agonist_ratio
TOX21_ARE_BLA_Agonist_ch2
NVS_ENZ_hPDE10
NVS_ENZ_hMMP9
ATG_NRF2_ARE_CIS_up
ATG_AP_1_CIS_up
124−41−4
2767−80−8
512−82−3
149−44−0
67−56−1
124−64−1

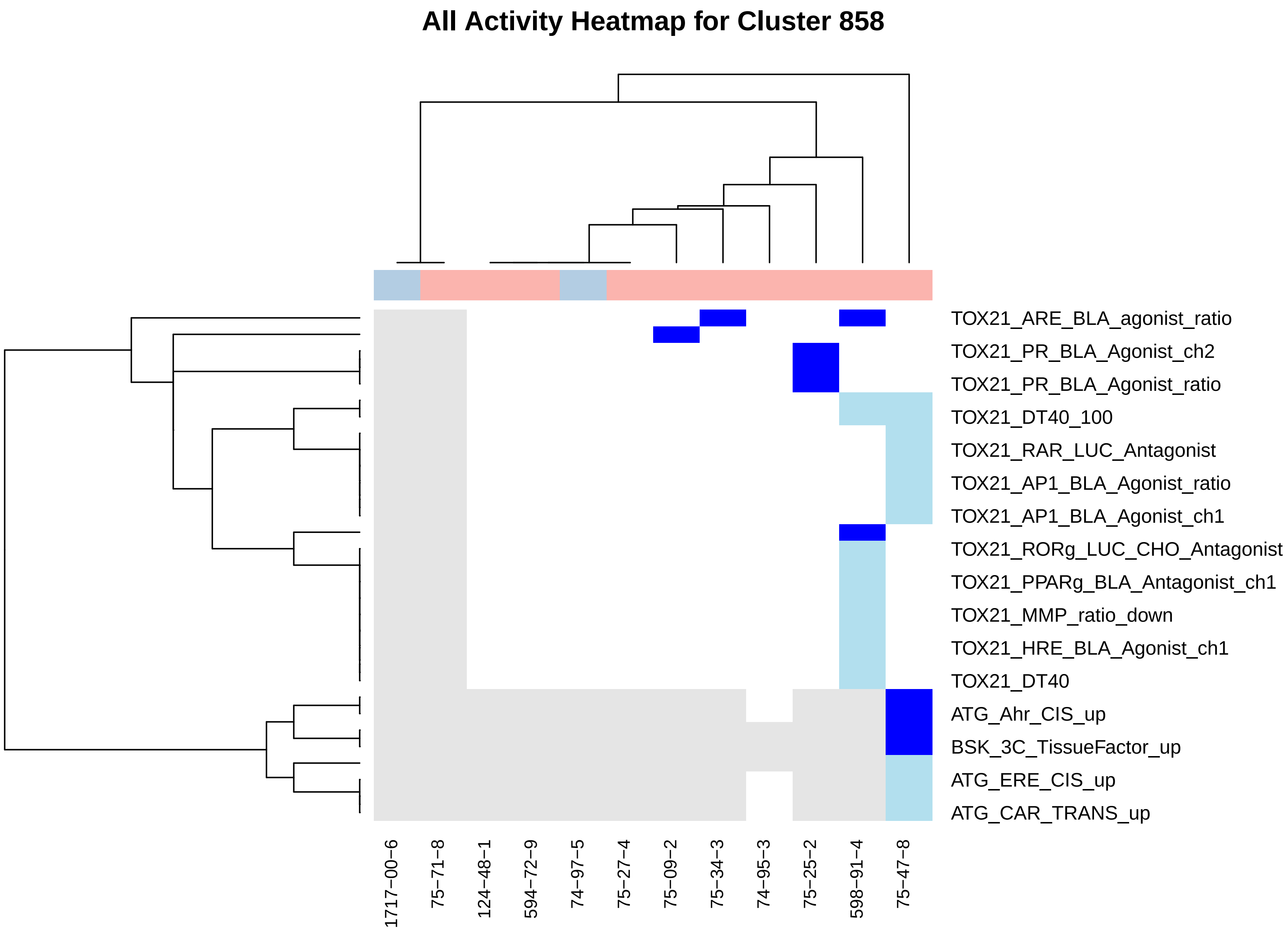
All Activity Heatmap for Cluster 858
TOX21_ARE_BLA_agonist_ratio
TOX21_PR_BLA_Agonist_ch2
TOX21_PR_BLA_Agonist_ratio
TOX21_DT40_100
TOX21_RAR_LUC_Antagonist
TOX21_AP1_BLA_Agonist_ratio
TOX21_AP1_BLA_Agonist_ch1
TOX21_RORg_LUC_CHO_Antagonist
TOX21_PPARg_BLA_Antagonist_ch1
TOX21_MMP_ratio_down
TOX21_HRE_BLA_Agonist_ch1
TOX21_DT40
ATG_Ahr_CIS_up
BSK_3C_TissueFactor_up
ATG_ERE_CIS_up
ATG_CAR_TRANS_up
1717−00−6
75−71−8
124−48−1
594−72−9
74−97−5
75−27−4
75−09−2
75−34−3
74−95−3
75−25−2
598−91−4
75−47−8

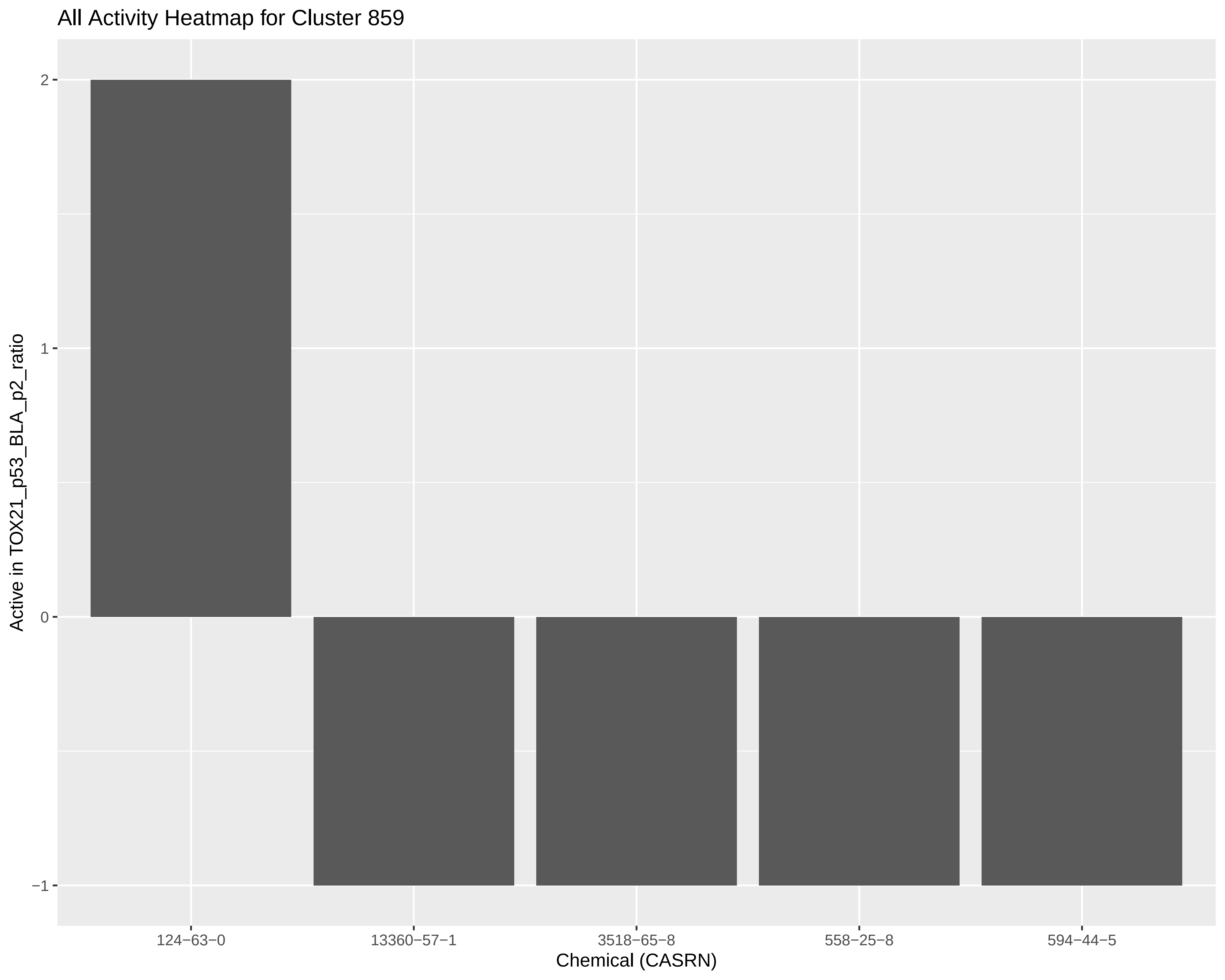

All Activity Heatmap for Cluster 859
Active in TOX21_p53_BLA_p2_ratio
2
1
0
−1
124−63−0
13360−57−1
3518−65−8
558−25−8
594−44−5
Chemical (CASRN)

All Activity Heatmap for Cluster 860

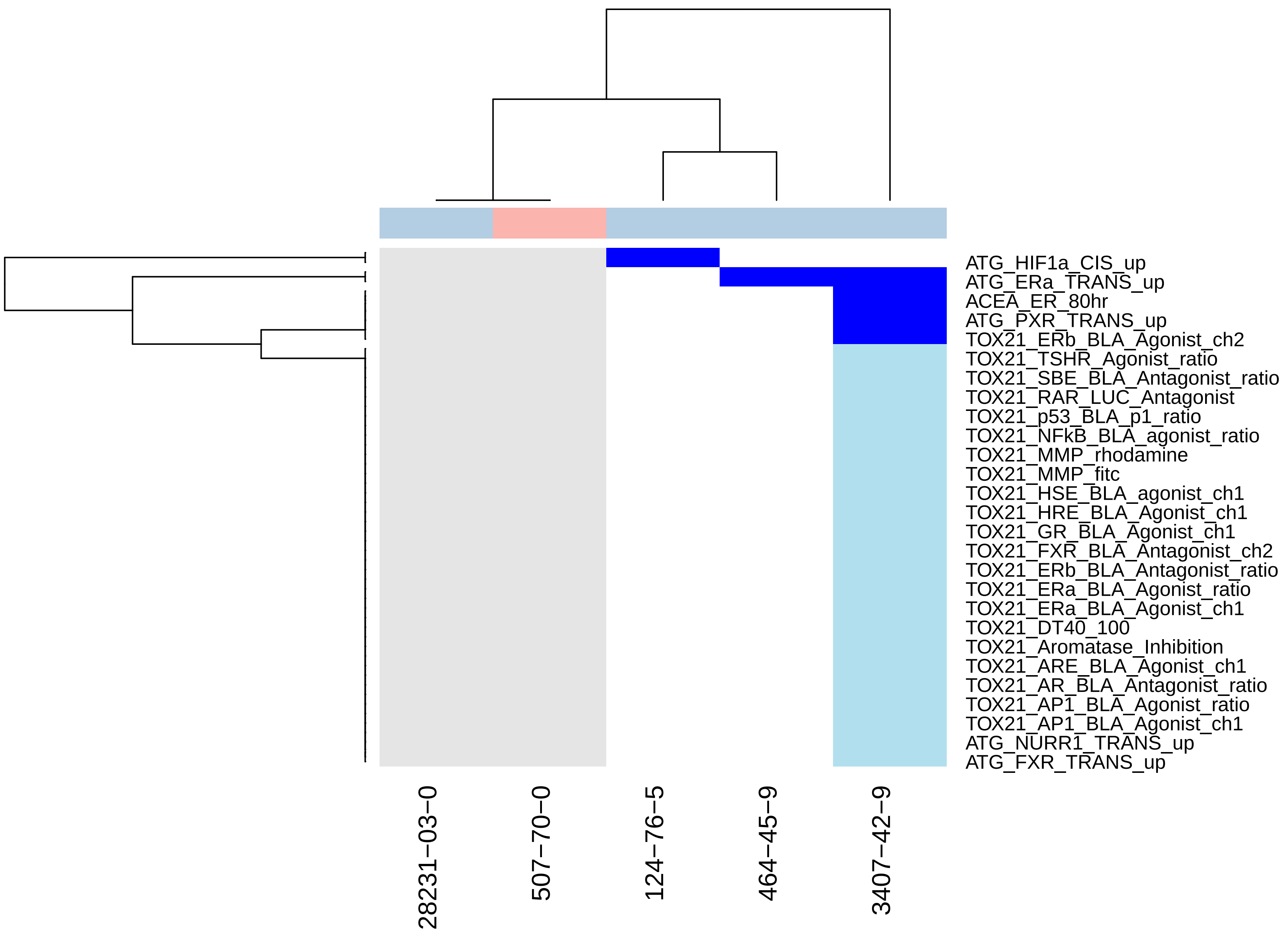

All Activity Heatmap for Cluster 861

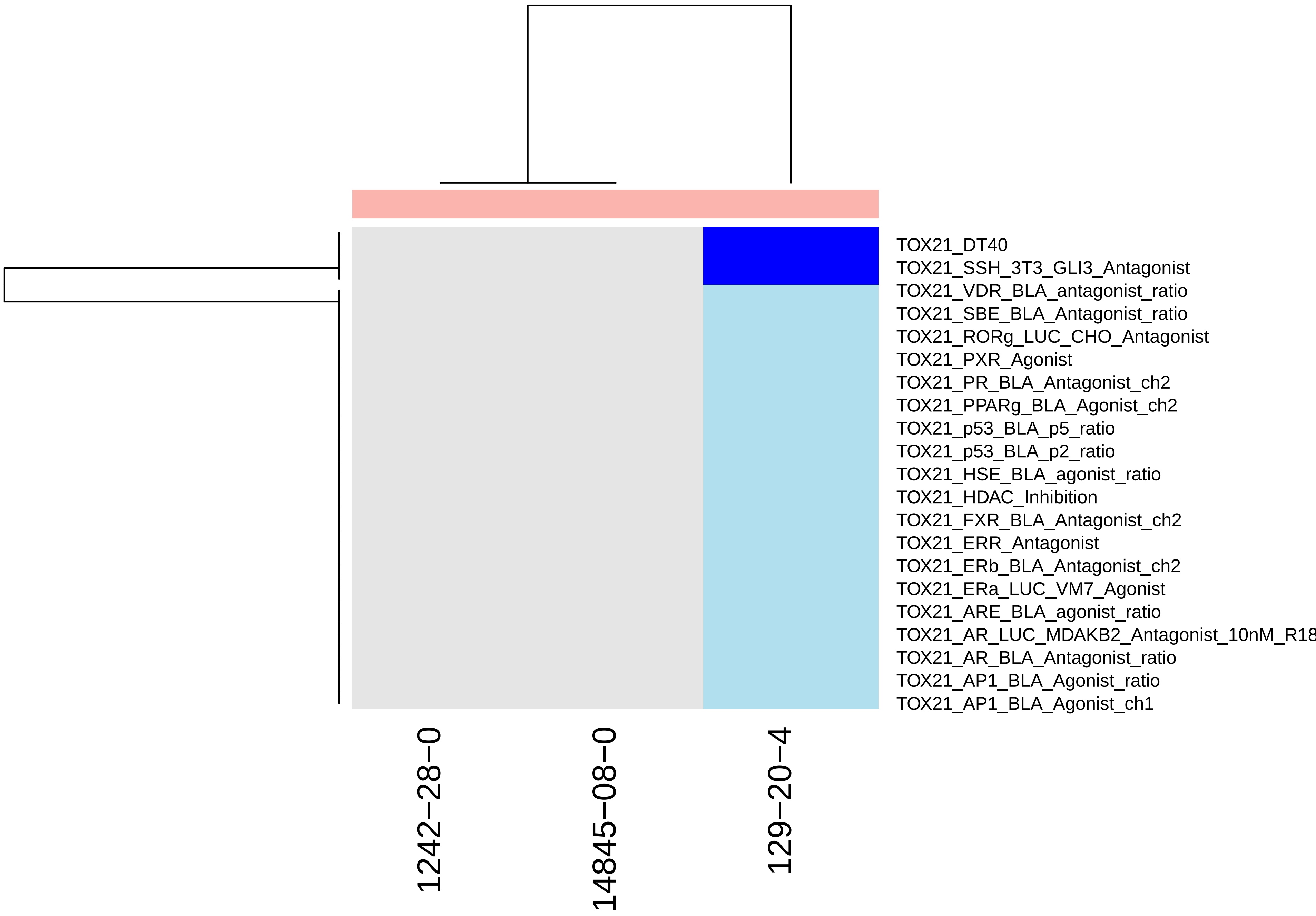

All Activity Heatmap for Cluster 863

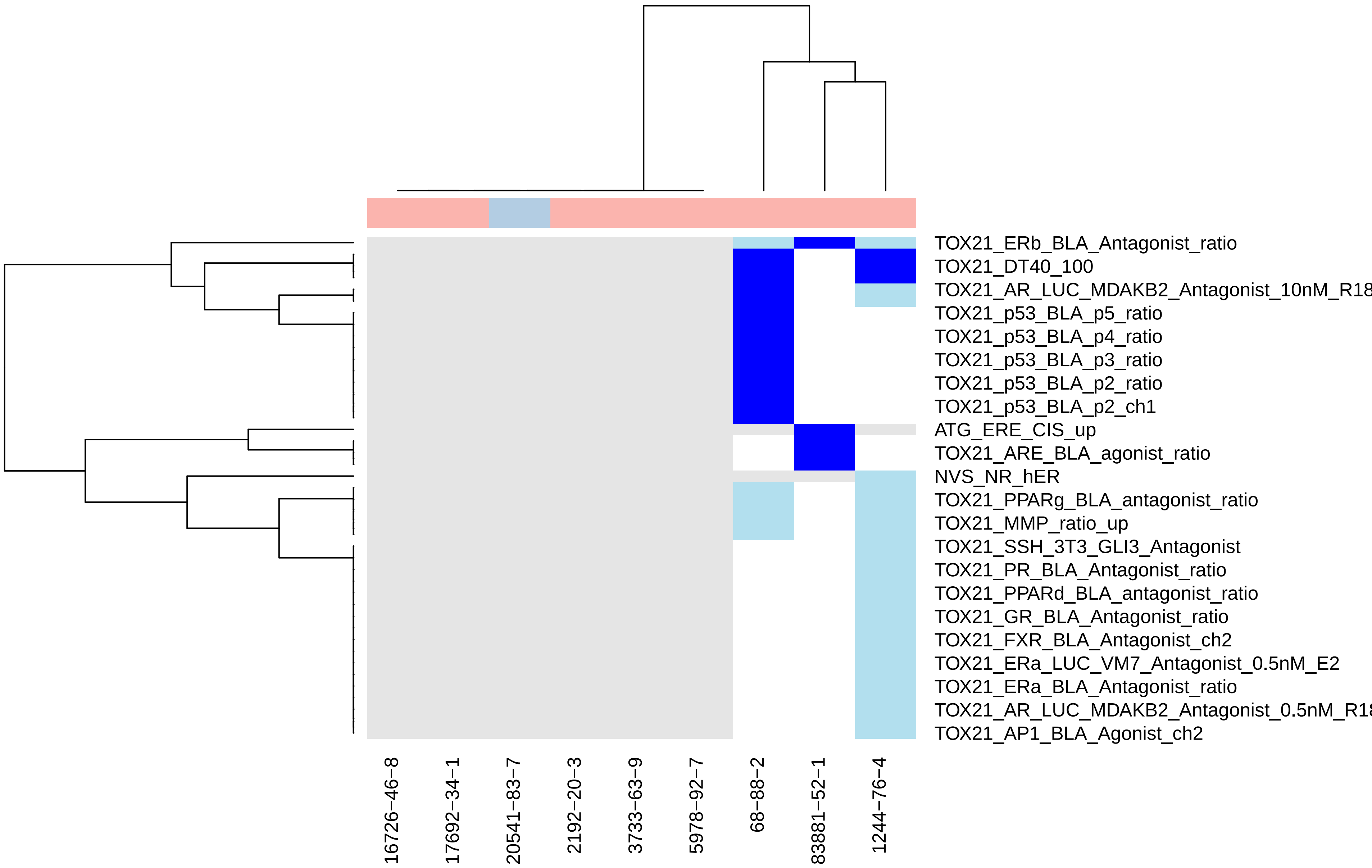

All Activity Heatmap for Cluster 864

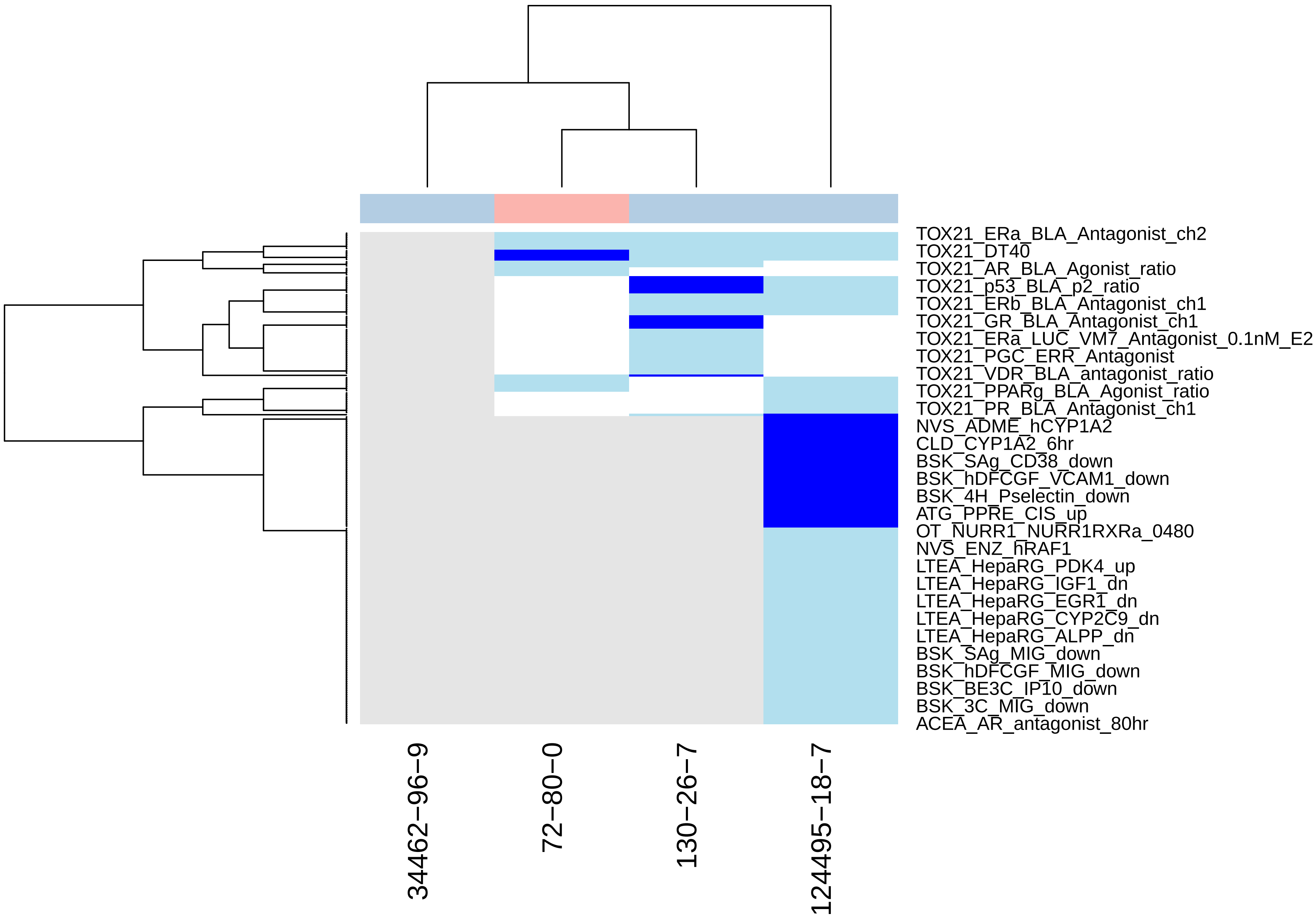

All Activity Heatmap for Cluster 865

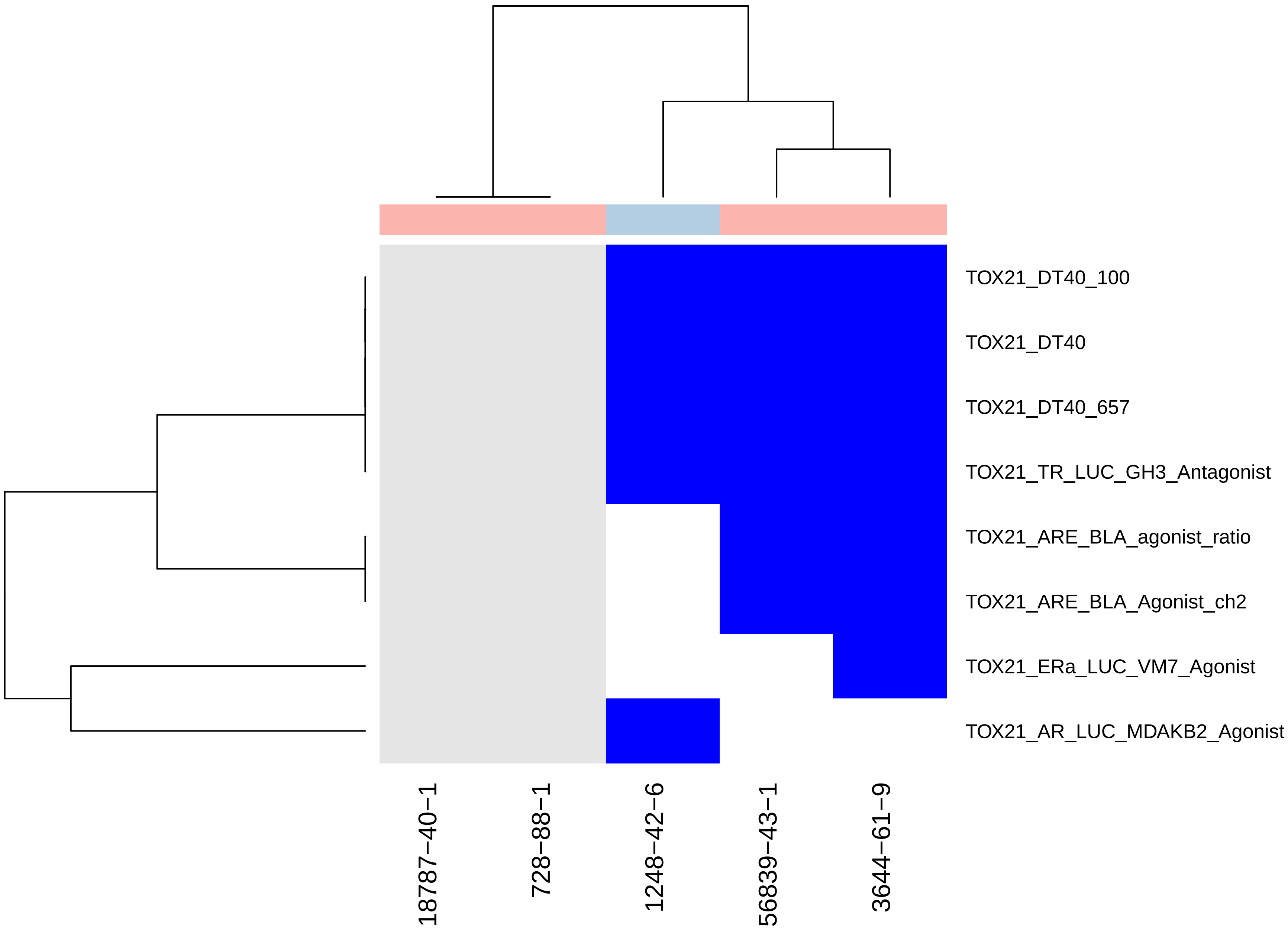

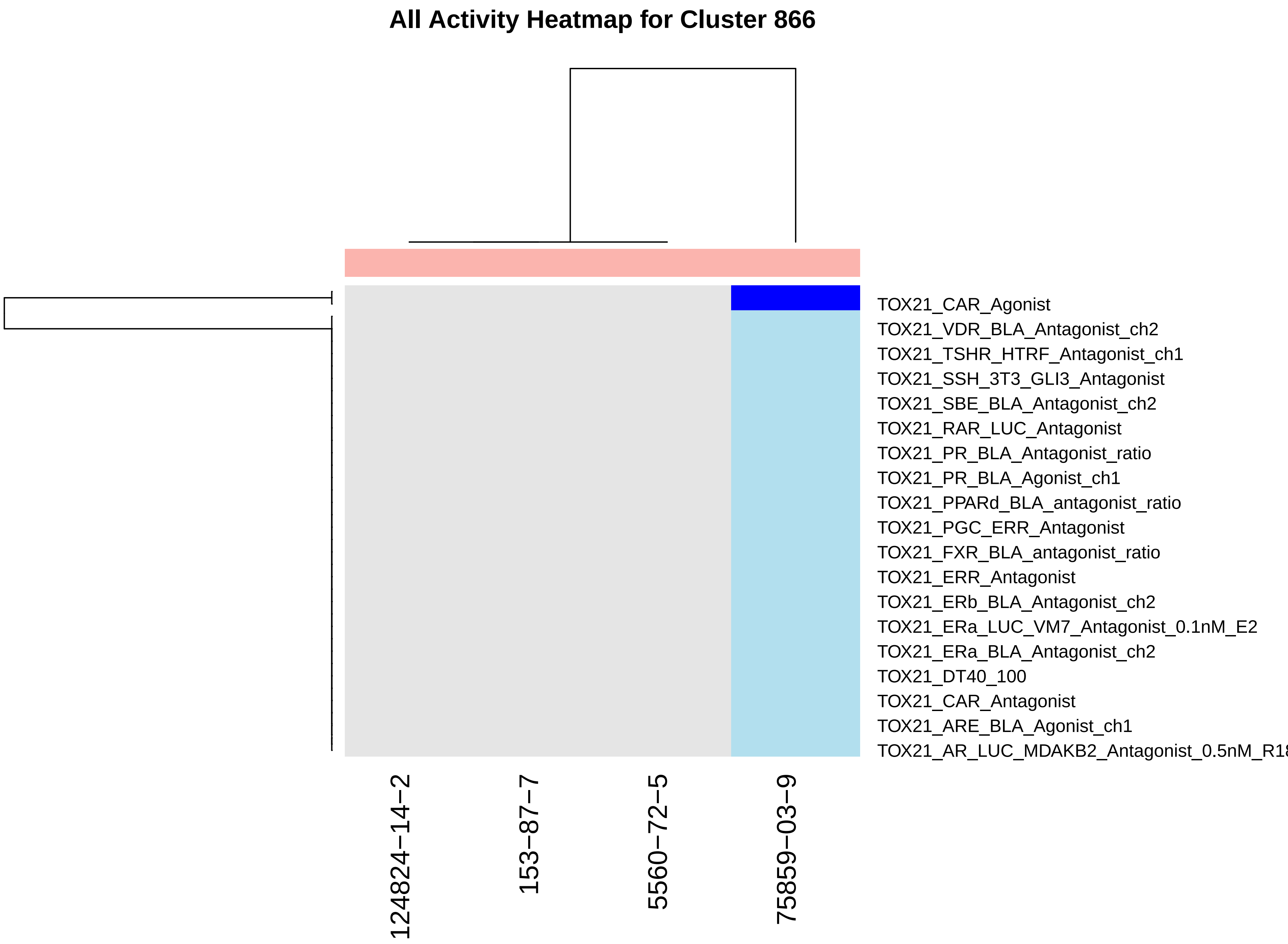

All Activity Heatmap for Cluster 866
TOX21_CAR_Agonist
TOX21_VDR_BLA_Antagonist_ch2
TOX21_TSHR_HTRF_Antagonist_ch1
TOX21_SSH_3T3_GLI3_Antagonist
TOX21_SBE_BLA_Antagonist_ch2
TOX21_RAR_LUC_Antagonist
TOX21_PR_BLA_Antagonist_ratio
TOX21_PR_BLA_Agonist_ch1
TOX21_PPARd_BLA_antagonist_ratio
TOX21_PGC_ERR_Antagonist
TOX21_FXR_BLA_antagonist_ratio
TOX21_ERR_Antagonist
TOX21_ERb_BLA_Antagonist_ch2
TOX21_ERa_LUC_VM7_Antagonist_0.1nM_E2
TOX21_ERa_BLA_Antagonist_ch2
TOX21_DT40_100
TOX21_CAR_Antagonist
TOX21_ARE_BLA_Agonist_ch1
TOX21_AR_LUC_MDAKB2_Antagonist_0.5nM_R1
124824−14−2
153−87−7
5560−72−5
75859−03−9

All Activity Heatmap for Cluster 869

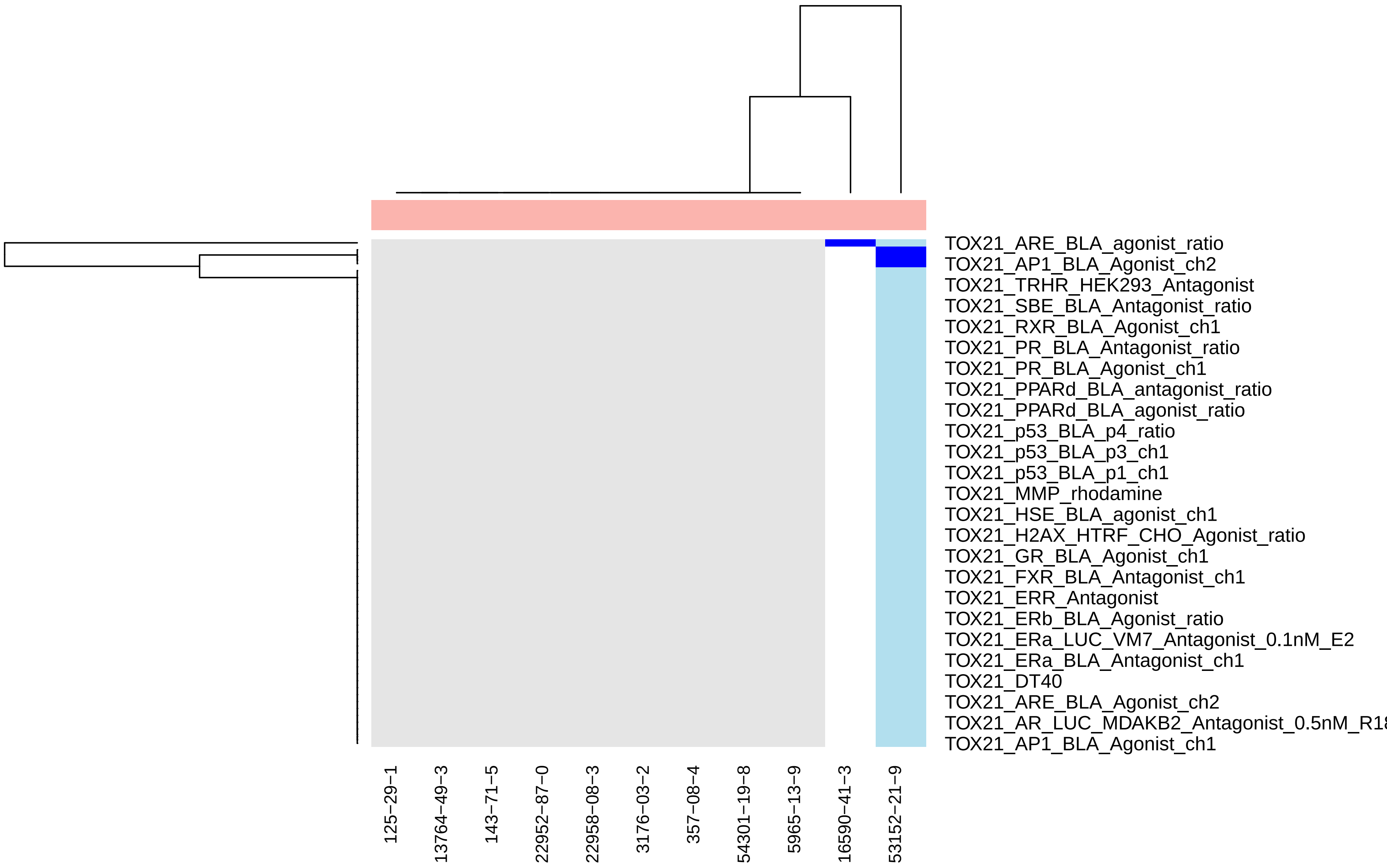

All Activity Heatmap for Cluster 870

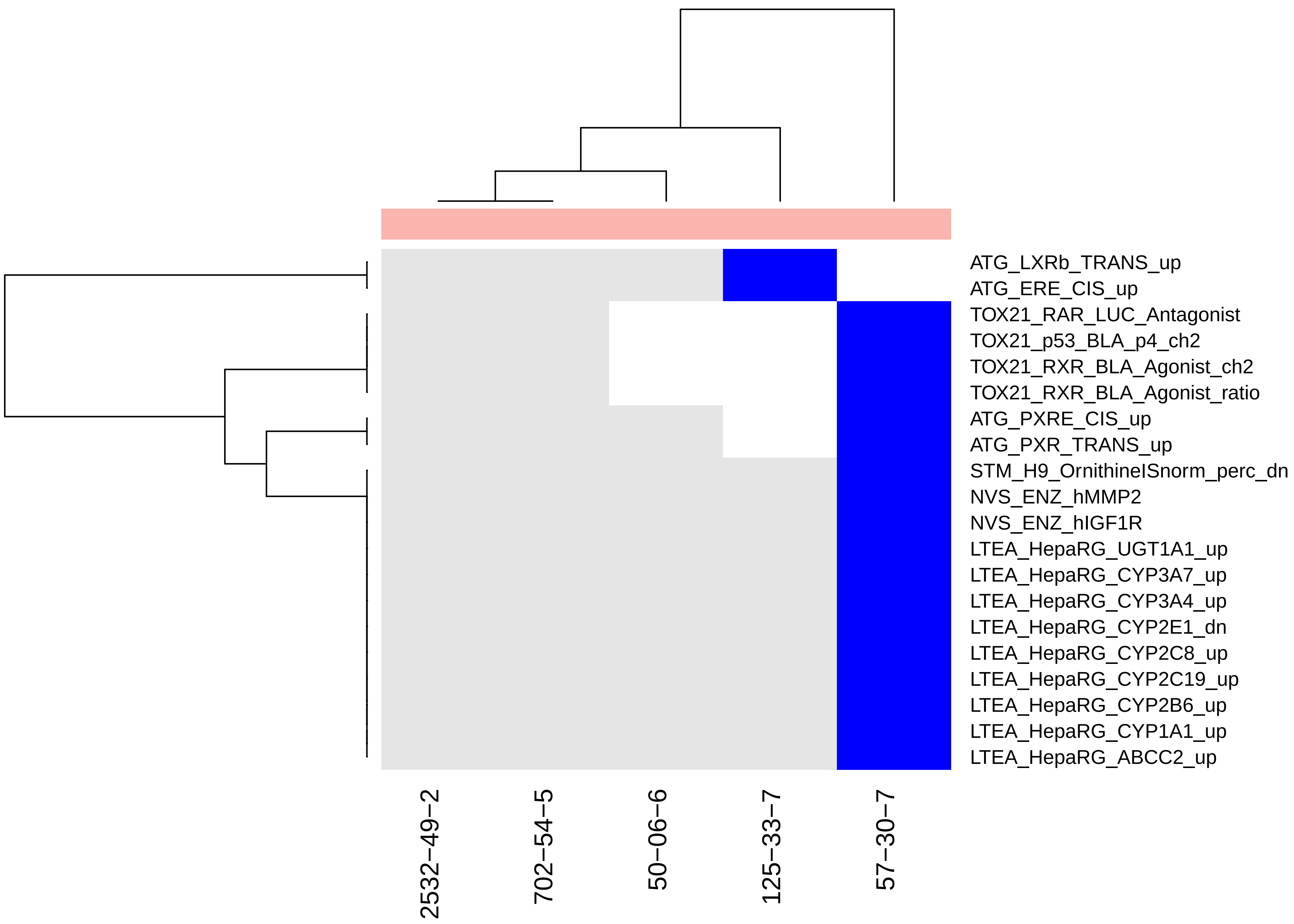

All Activity Heatmap for Cluster 872

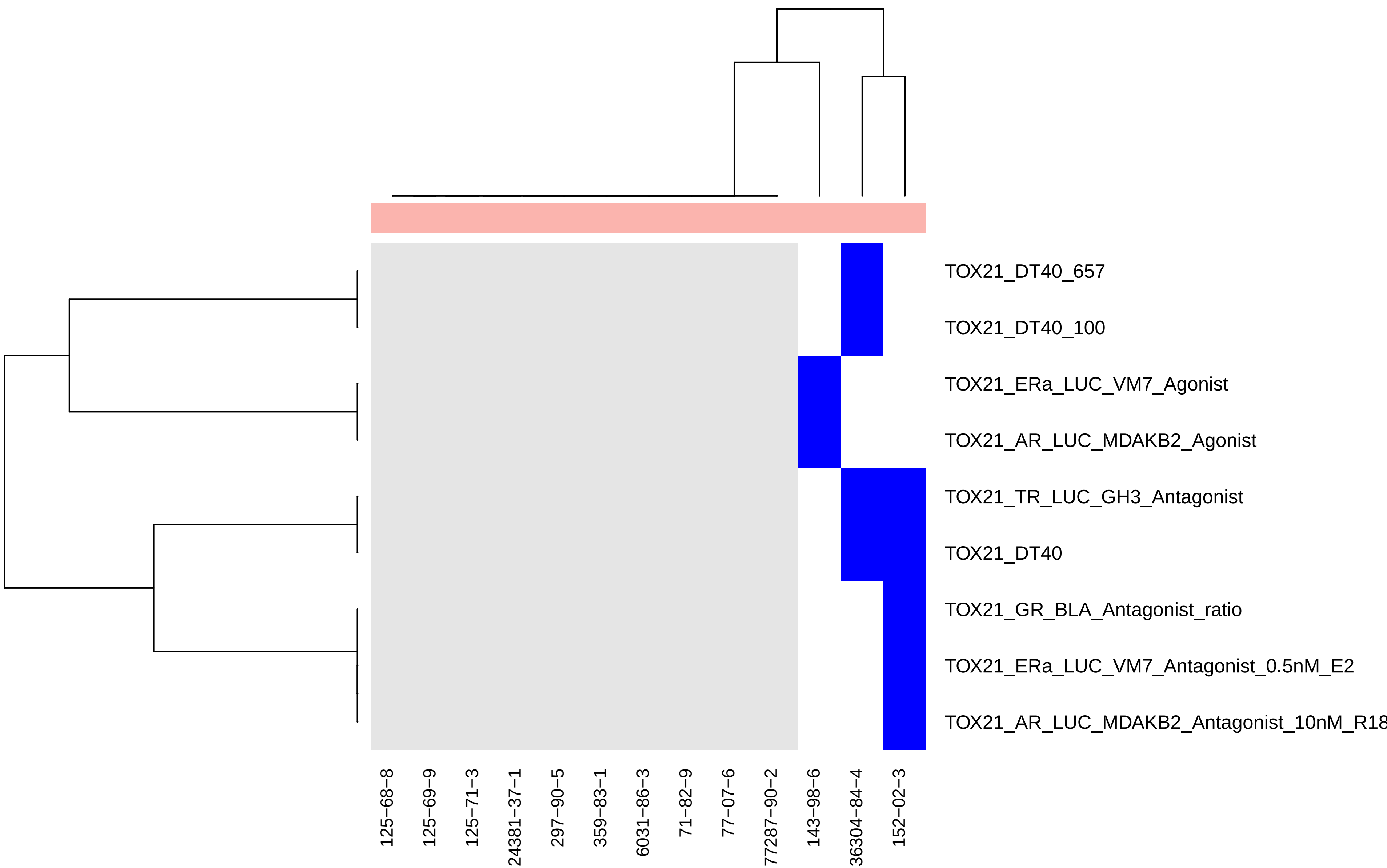

All Activity Heatmap for Cluster 875

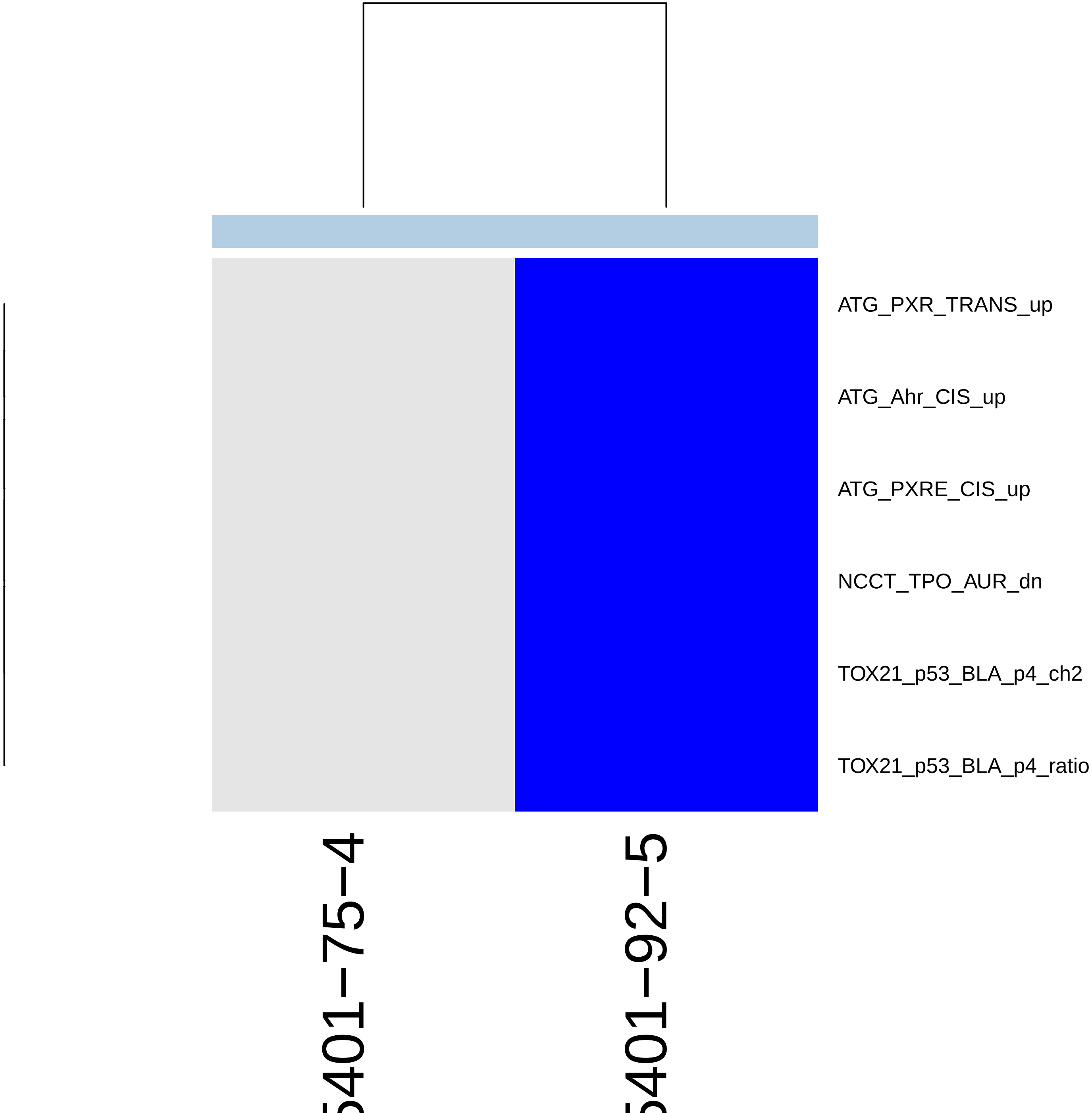

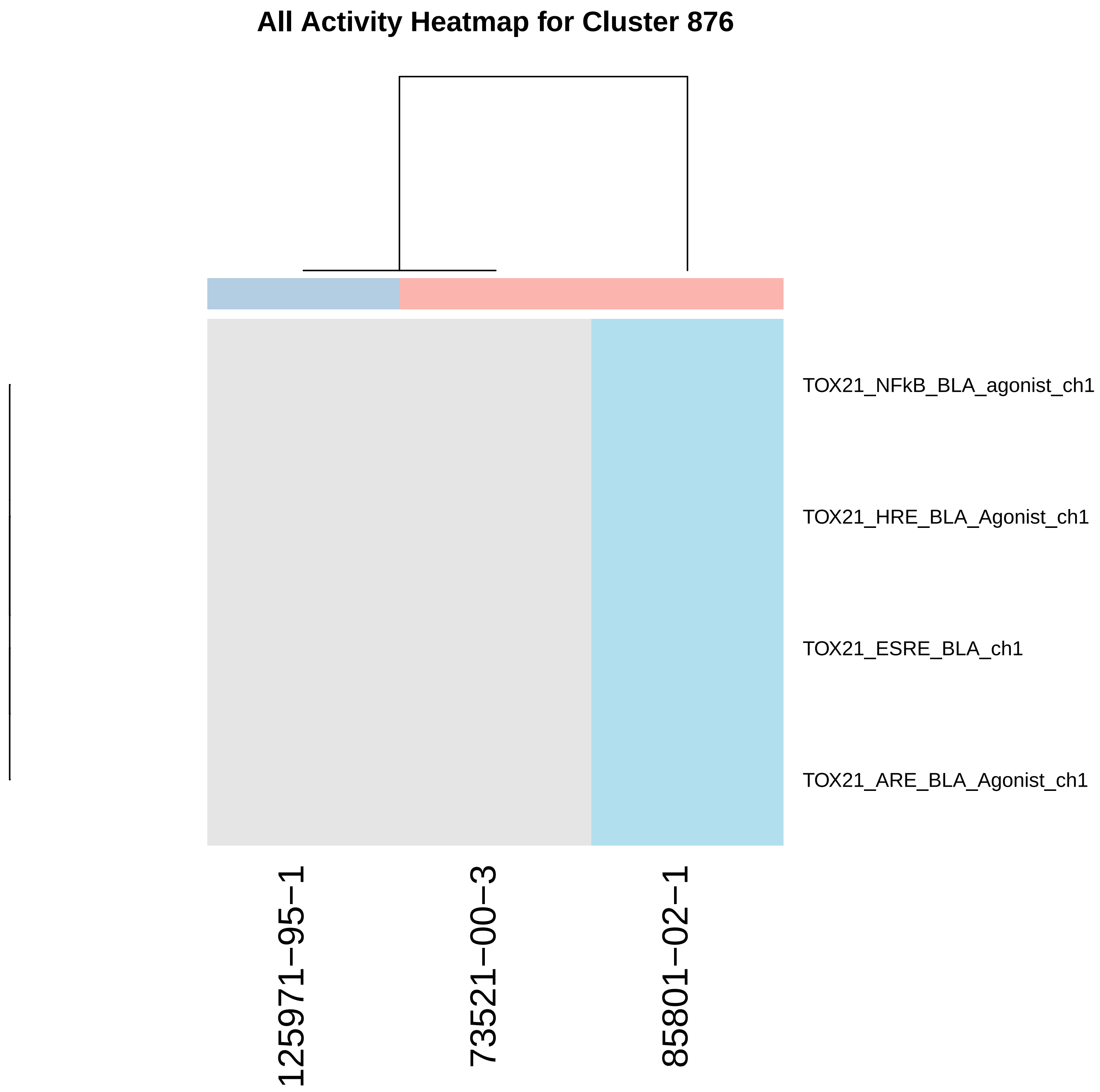
All Activity Heatmap for Cluster 876
TOX21_NFkB_BLA_agonist_ch1
TOX21_HRE_BLA_Agonist_ch1
TOX21_ESRE_BLA_ch1
TOX21_ARE_BLA_Agonist_ch1
125971−95−1
73521−00−3
85801−02−1

All Activity Heatmap for Cluster 877

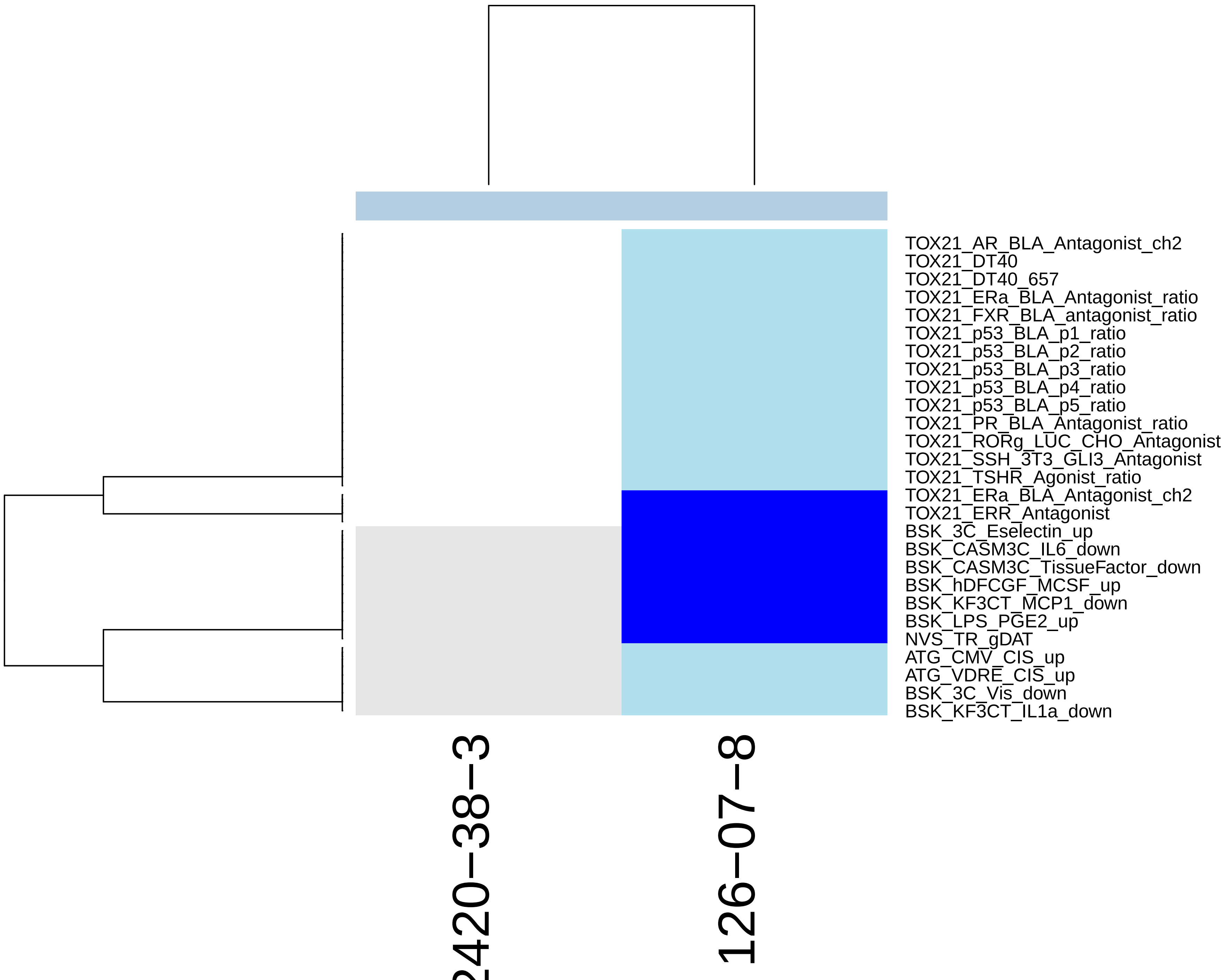

All Activity Heatmap for Cluster 878

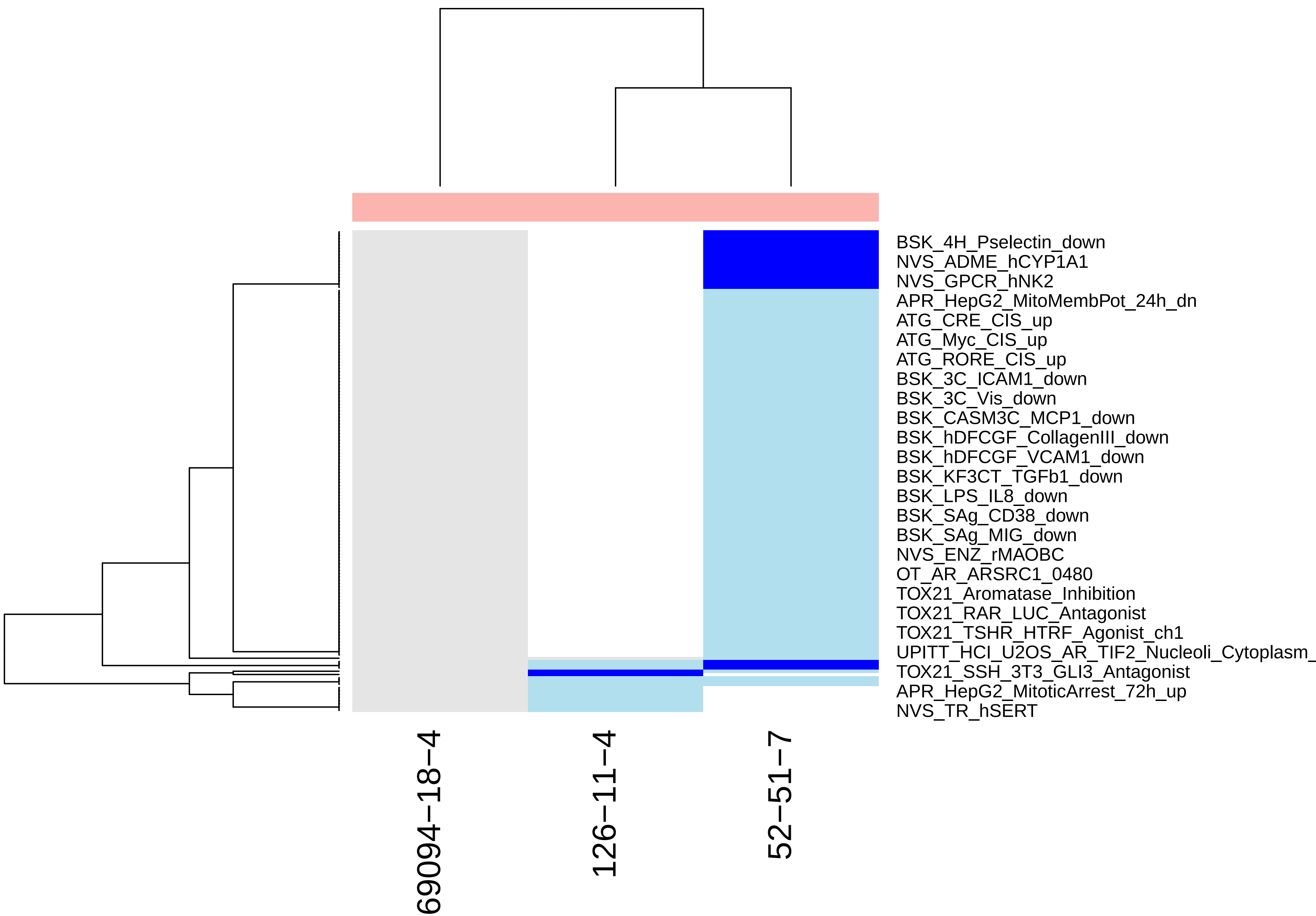

All Activity Heatmap for Cluster 880

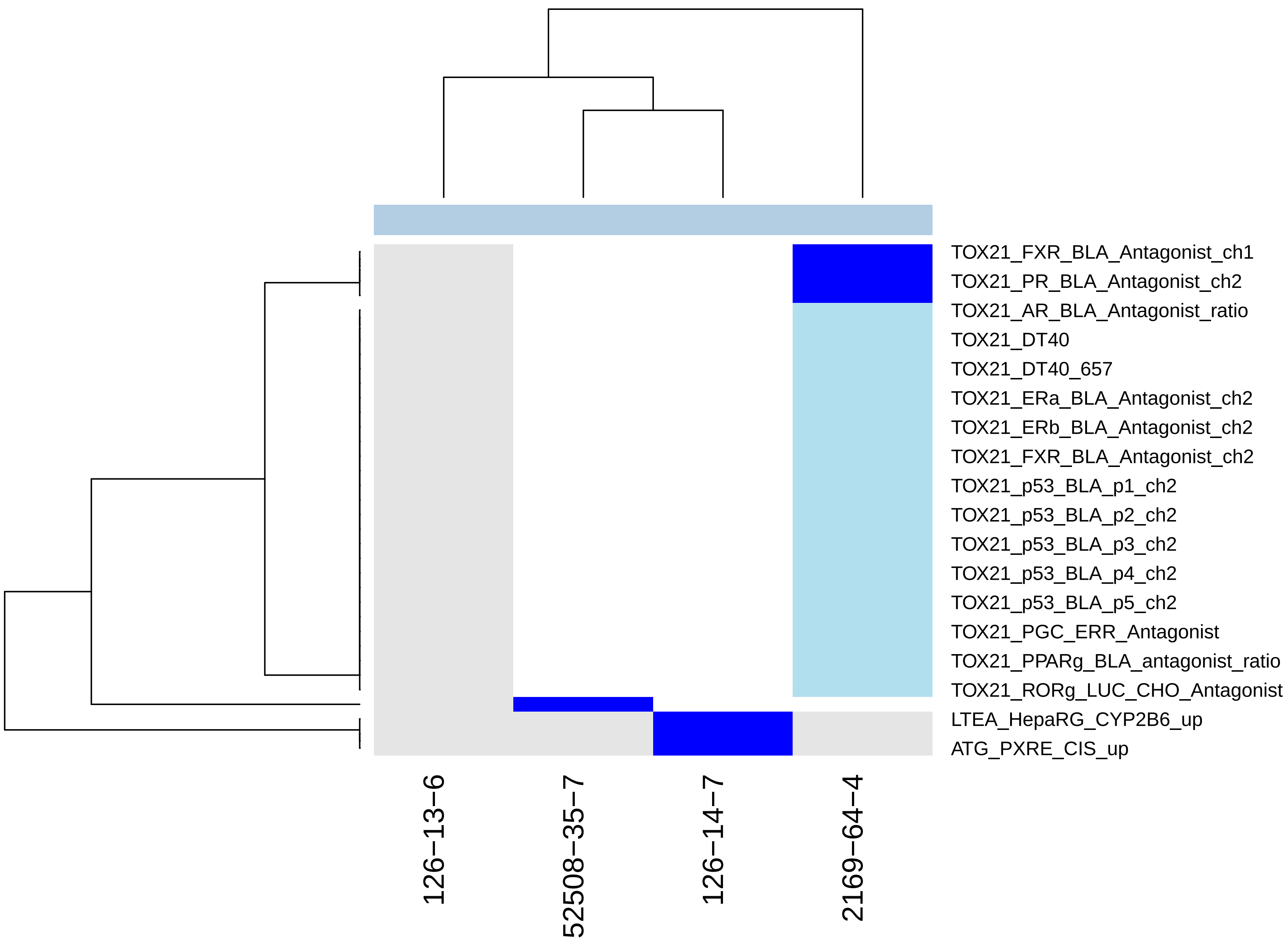

All Activity Heatmap for Cluster 882

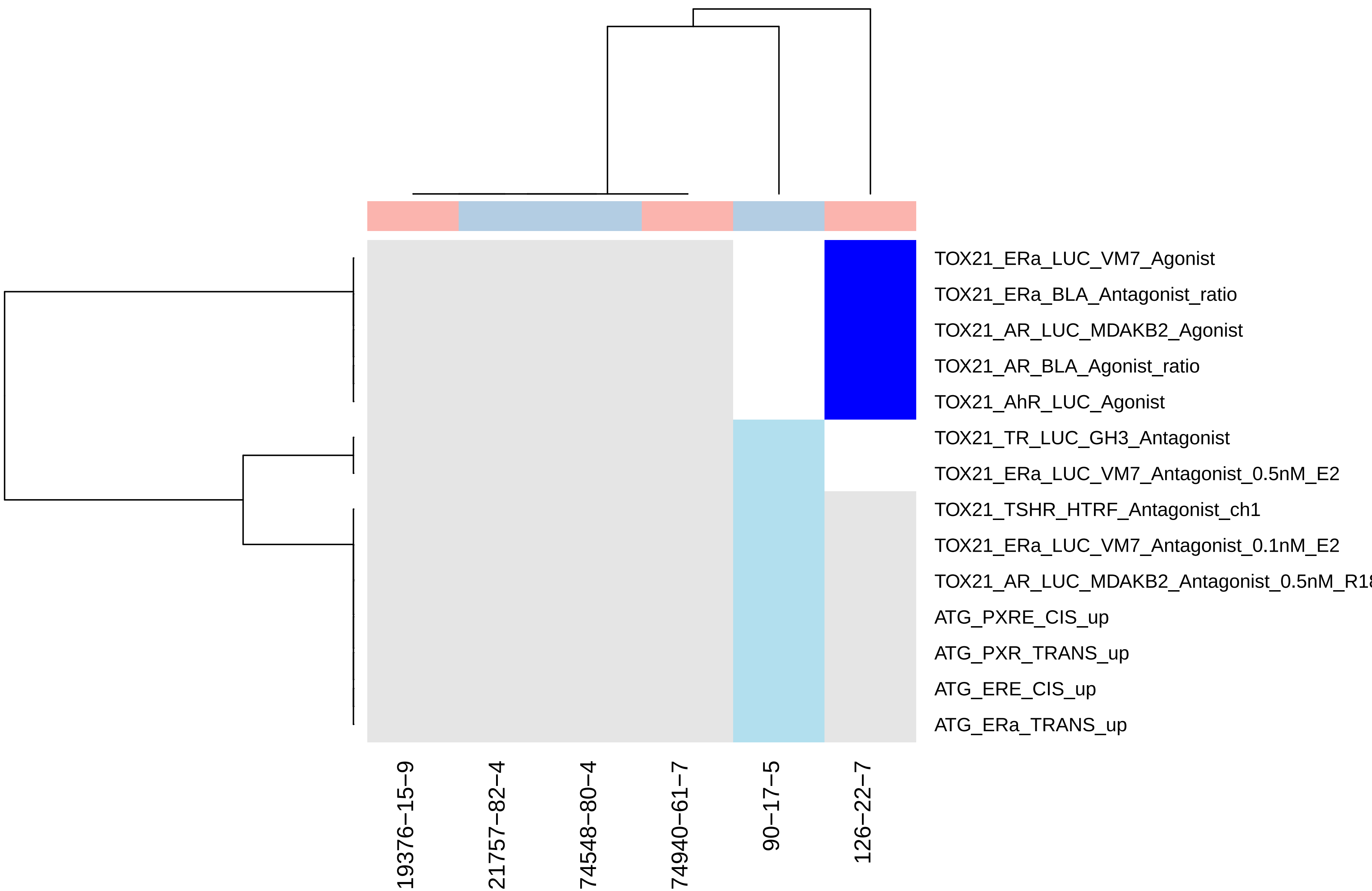

All Activity Heatmap for Cluster 883

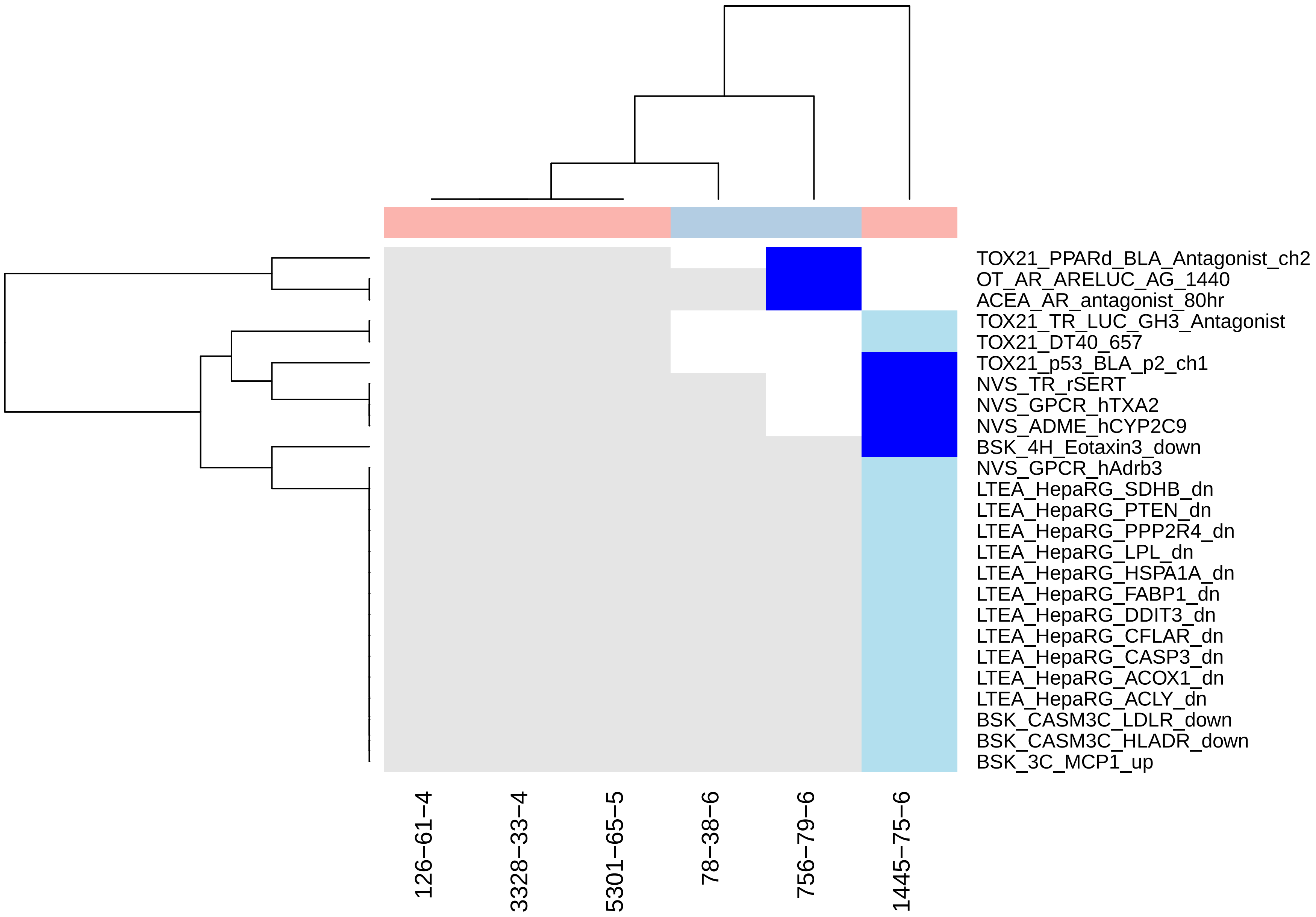

All Activity Heatmap for Cluster 884

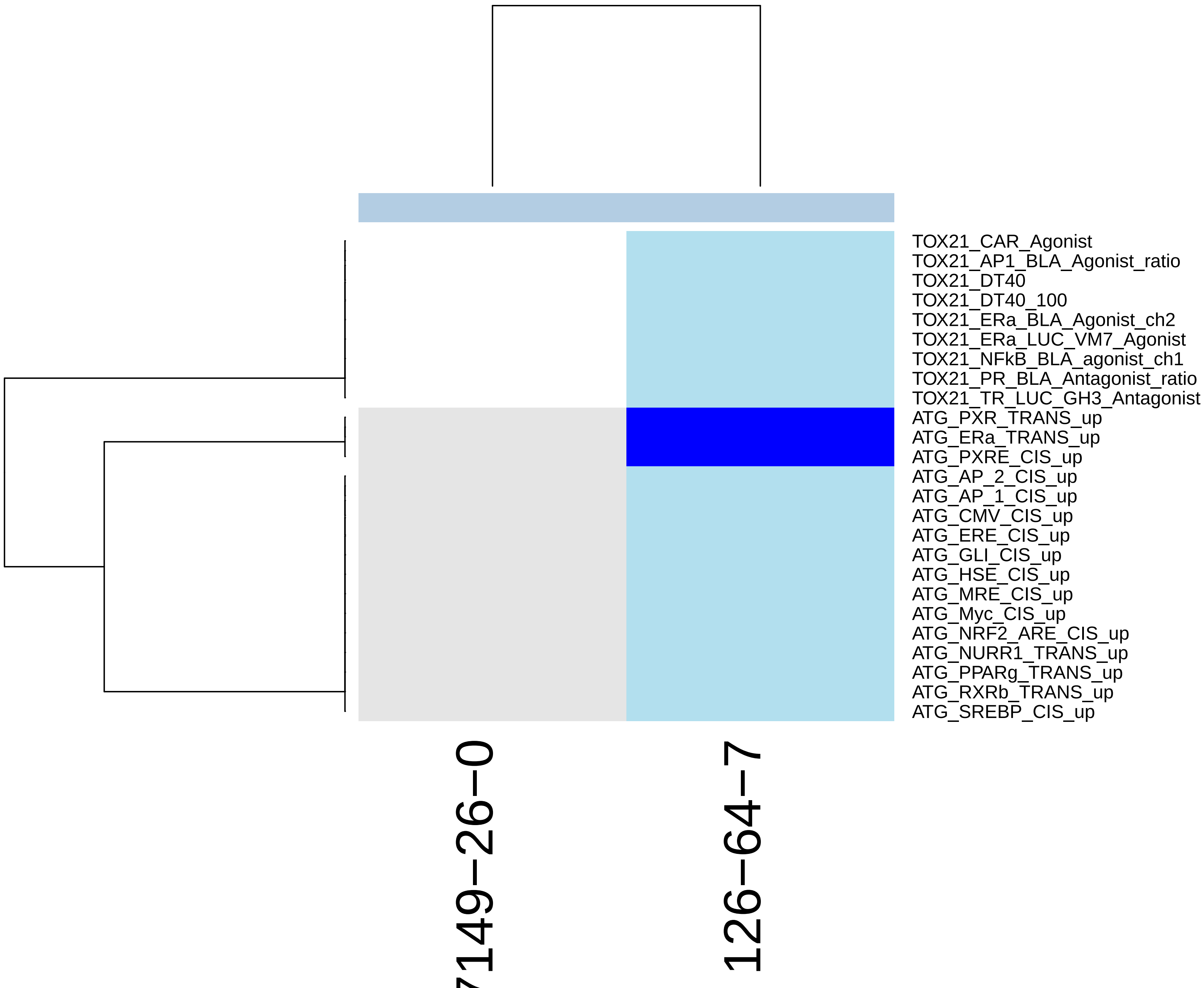

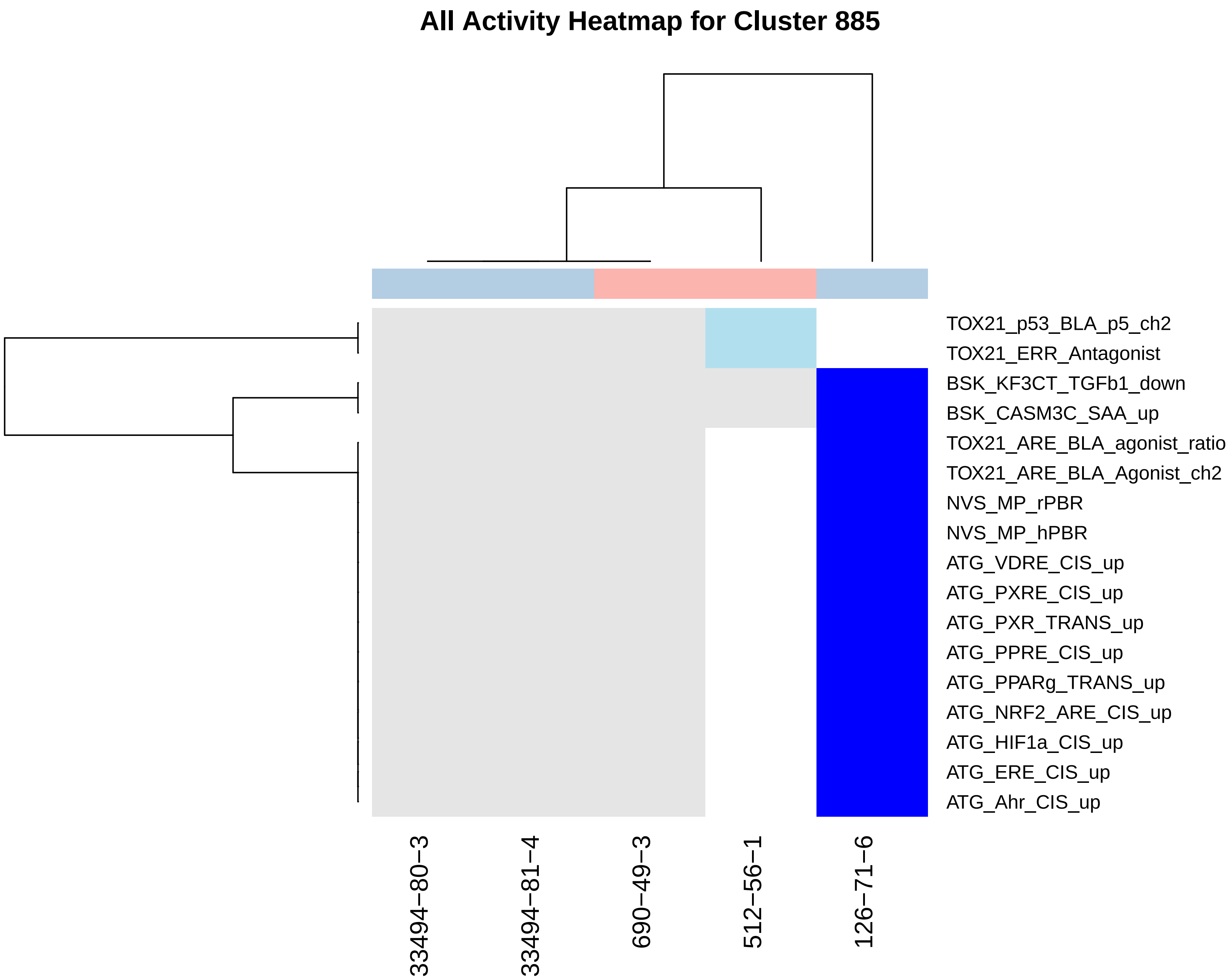
All Activity Heatmap for Cluster 885
TOX21_p53_BLA_p5_ch2
TOX21_ERR_Antagonist
BSK_KF3CT_TGFb1_down
BSK_CASM3C_SAA_up
TOX21_ARE_BLA_agonist_ratio
TOX21_ARE_BLA_Agonist_ch2
NVS_MP_rPBR
NVS_MP_hPBR
ATG_VDRE_CIS_up
ATG_PXRE_CIS_up
ATG_PXR_TRANS_up
ATG_PPRE_CIS_up
ATG_PPARg_TRANS_up
ATG_NRF2_ARE_CIS_up
ATG_HIF1a_CIS_up
ATG_ERE_CIS_up
ATG_Ahr_CIS_up
33494−80−3
33494−81−4
690−49−3
512−56−1
126−71−6

All Activity Heatmap for Cluster 886

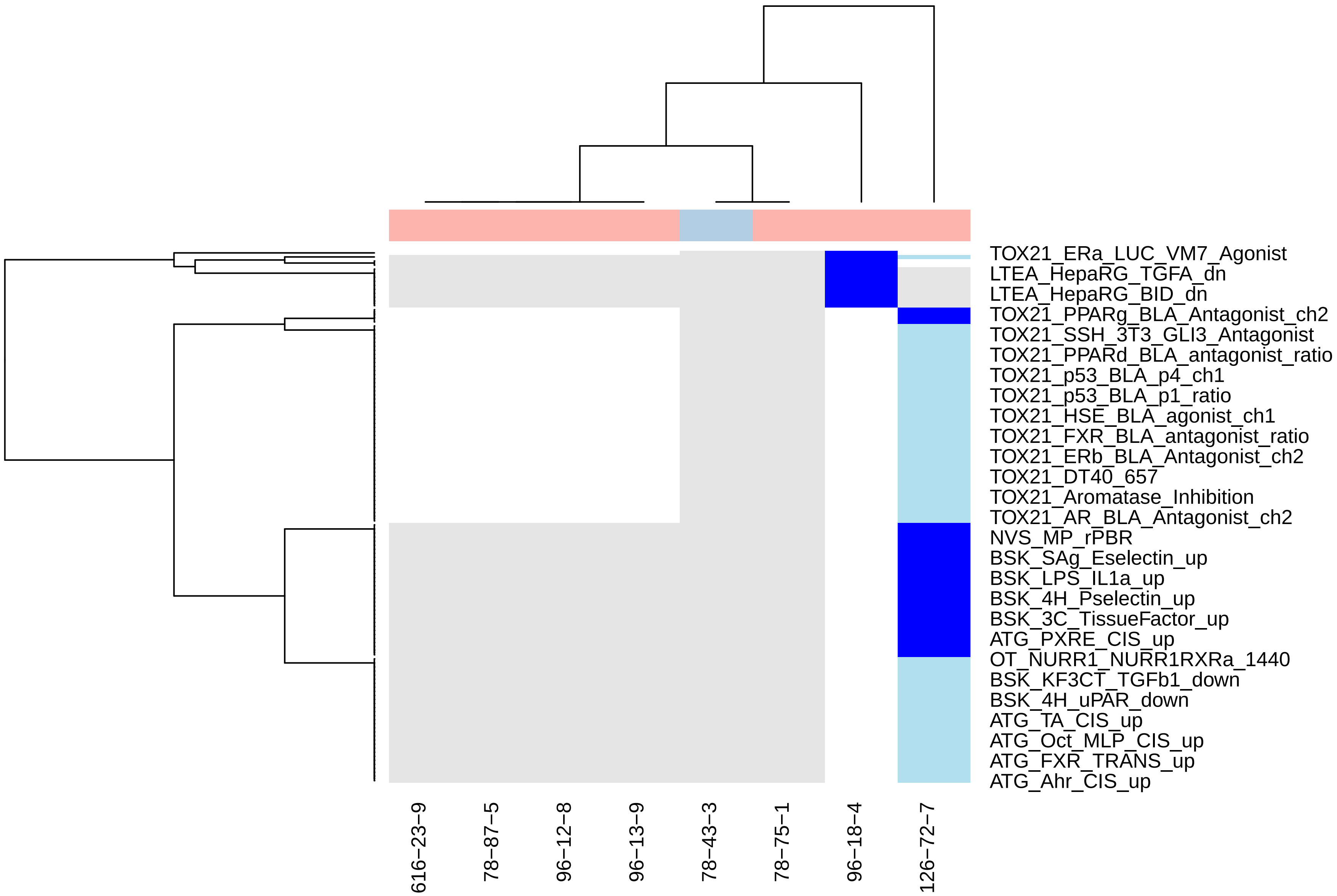

All Activity Heatmap for Cluster 887

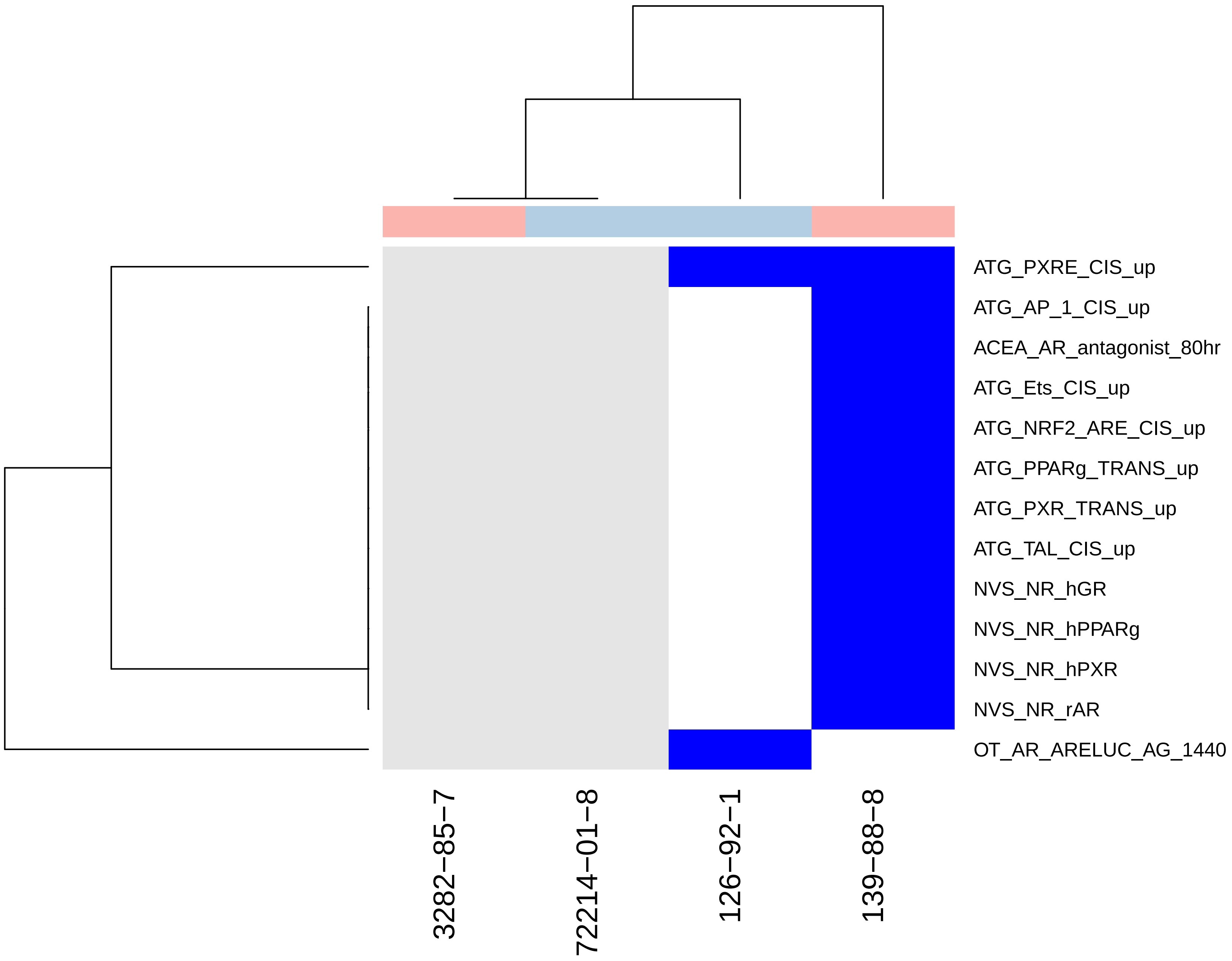

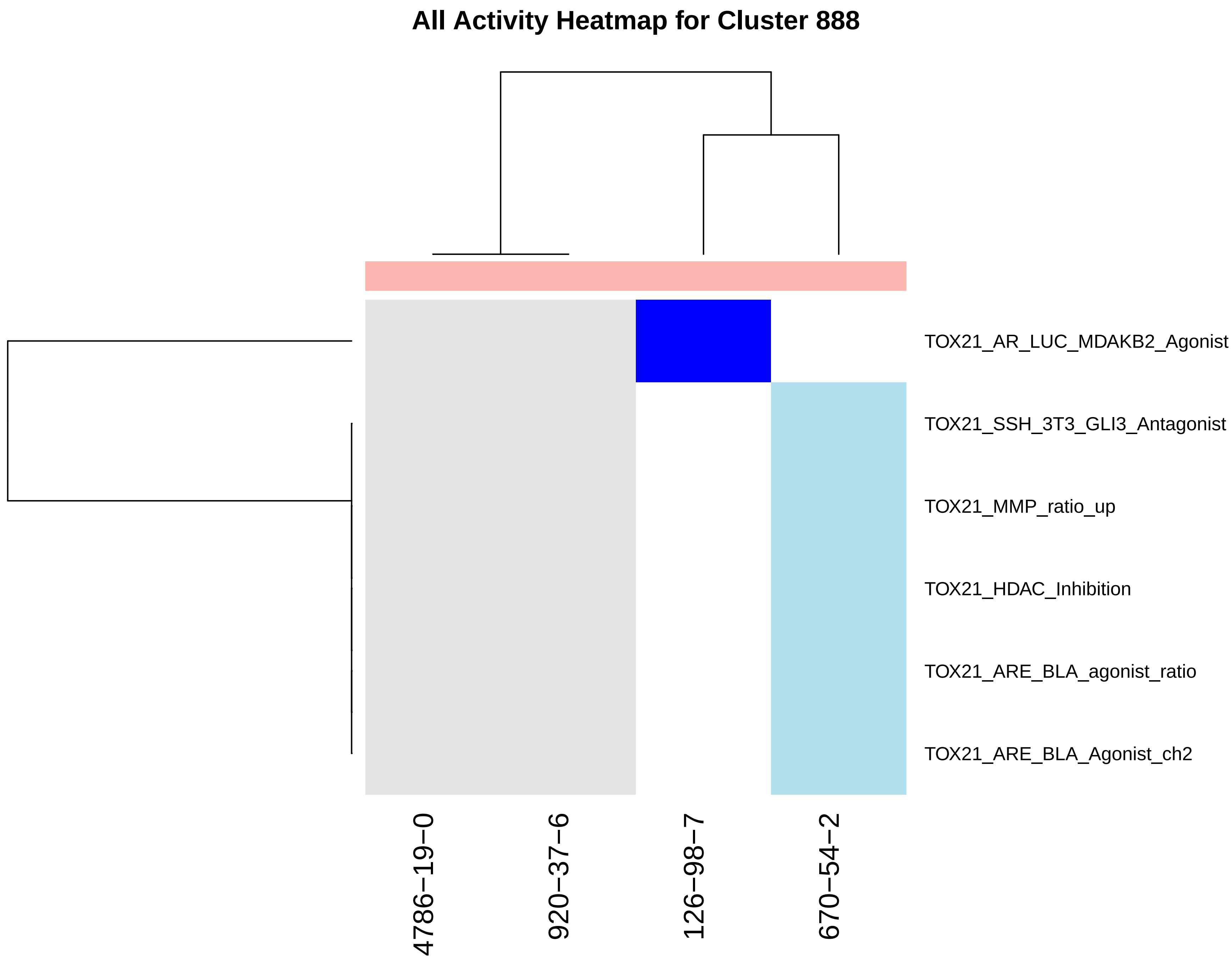
All Activity Heatmap for Cluster 888
TOX21_AR_LUC_MDAKB2_Agonist
TOX21_SSH_3T3_GLI3_Antagonist
TOX21_MMP_ratio_up
TOX21_HDAC_Inhibition
TOX21_ARE_BLA_agonist_ratio
TOX21_ARE_BLA_Agonist_ch2
4786−19−0
920−37−6
126−98−7
670−54−2

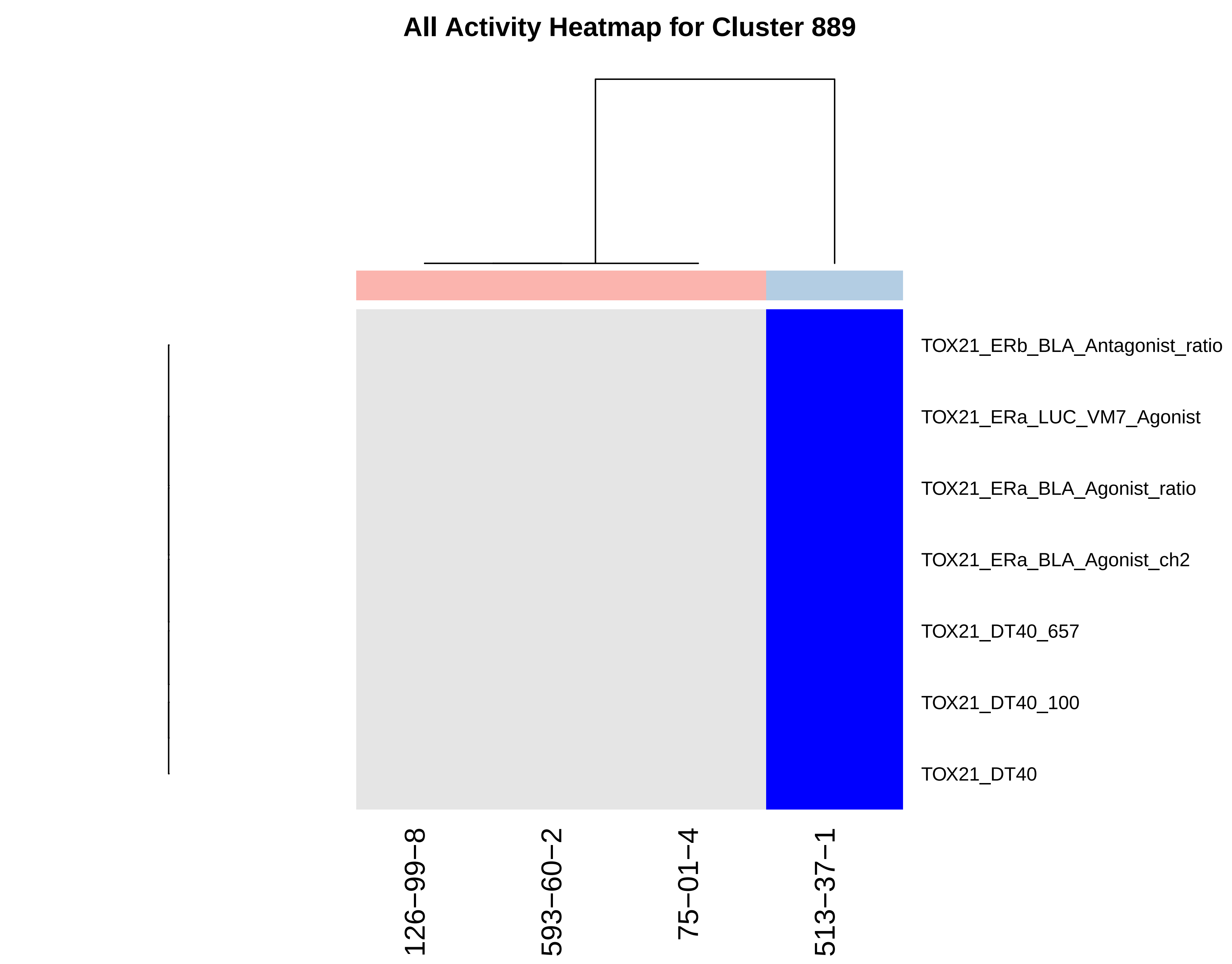
All Activity Heatmap for Cluster 889
TOX21_ERb_BLA_Antagonist_ratio
TOX21_ERa_LUC_VM7_Agonist
TOX21_ERa_BLA_Agonist_ratio
TOX21_ERa_BLA_Agonist_ch2
TOX21_DT40_657
TOX21_DT40_100
TOX21_DT40
126−99−8
593−60−2
75−01−4
513−37−1

All Activity Heatmap for Cluster 892

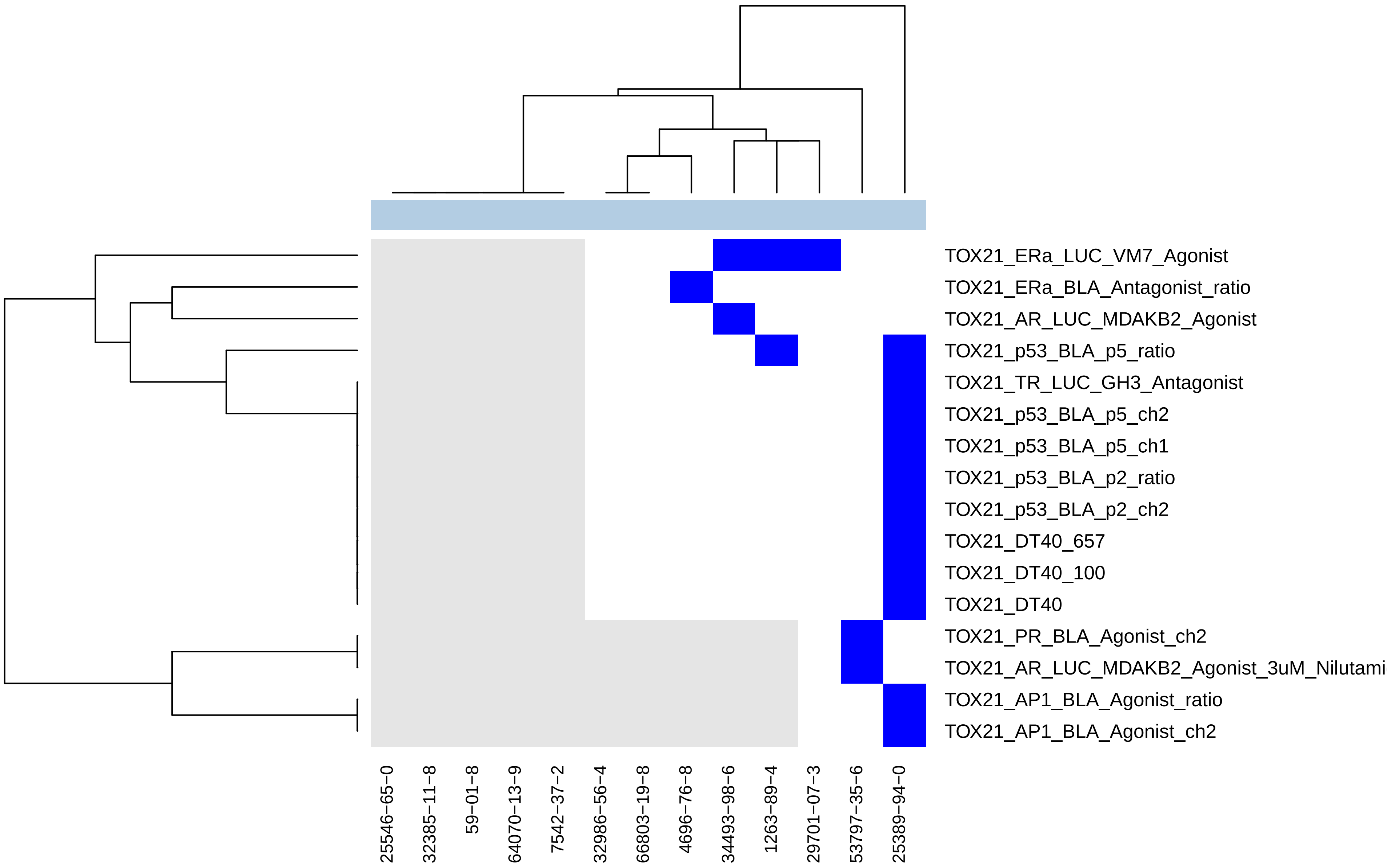

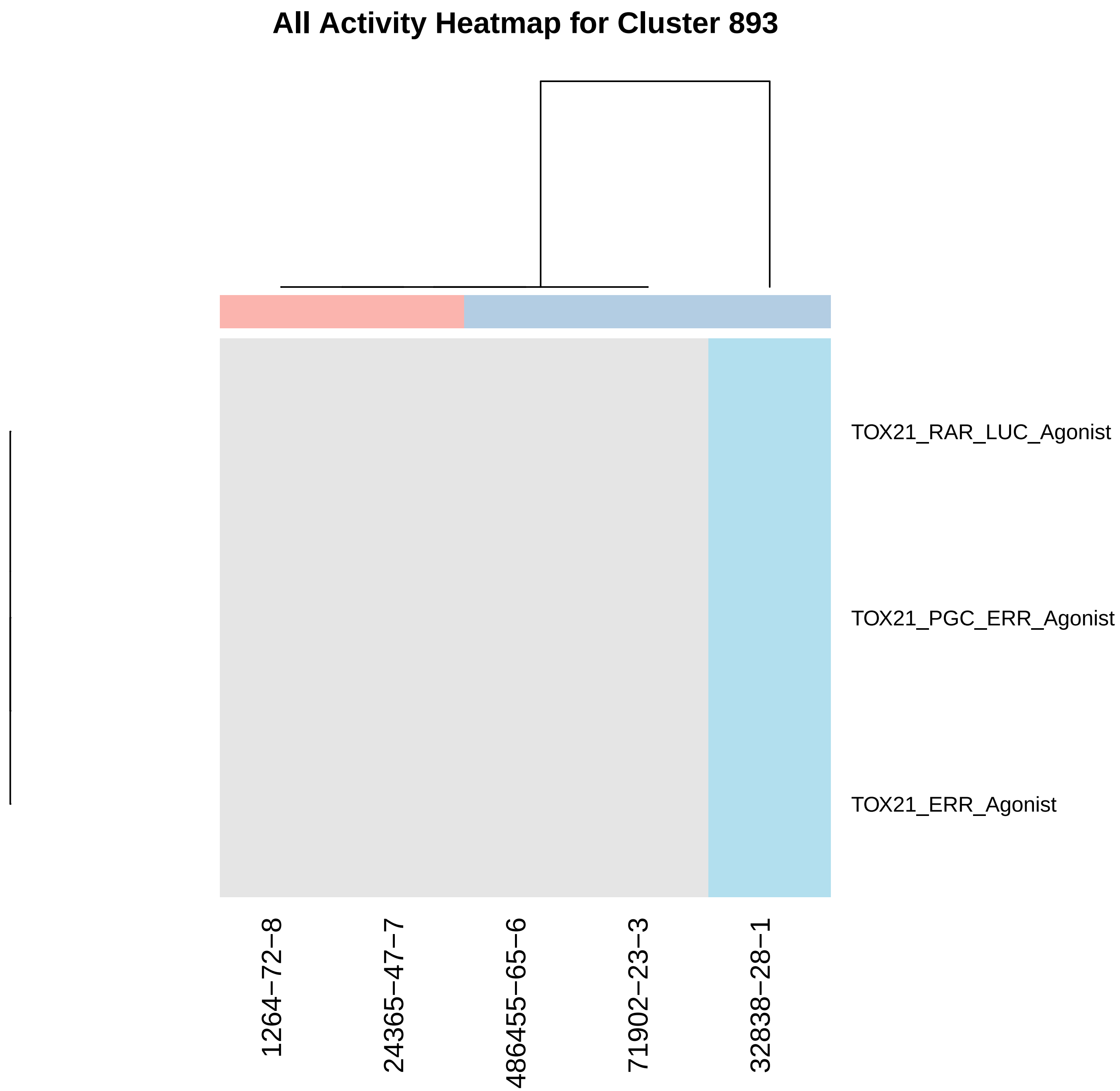
All Activity Heatmap for Cluster 893
TOX21_RAR_LUC_Agonist
TOX21_PGC_ERR_Agonist
TOX21_ERR_Agonist
1264−72−8
24365−47−7
486455−65−6
71902−23−3
32838−28−1

All Activity Heatmap for Cluster 894

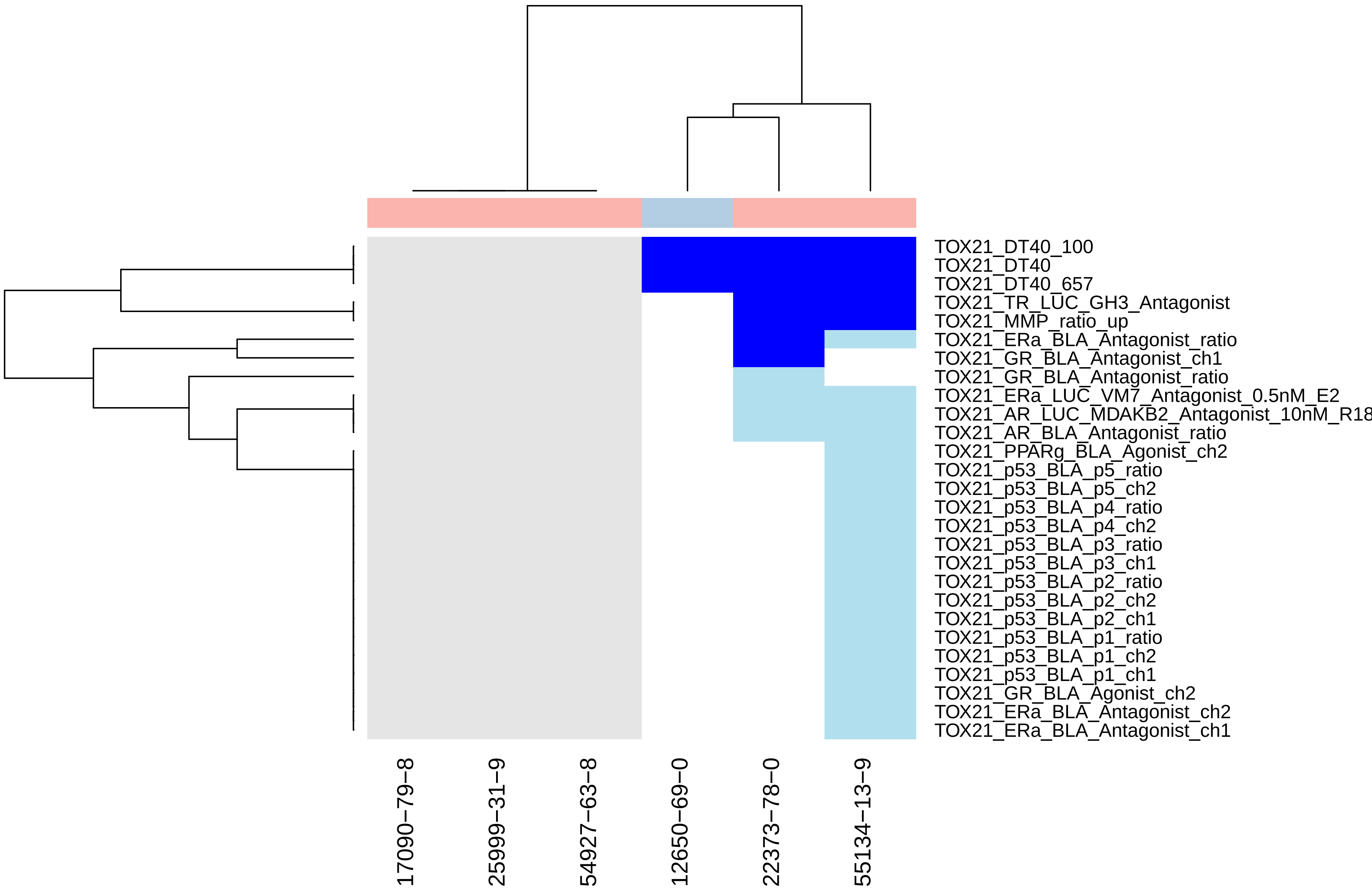

All Activity Heatmap for Cluster 897

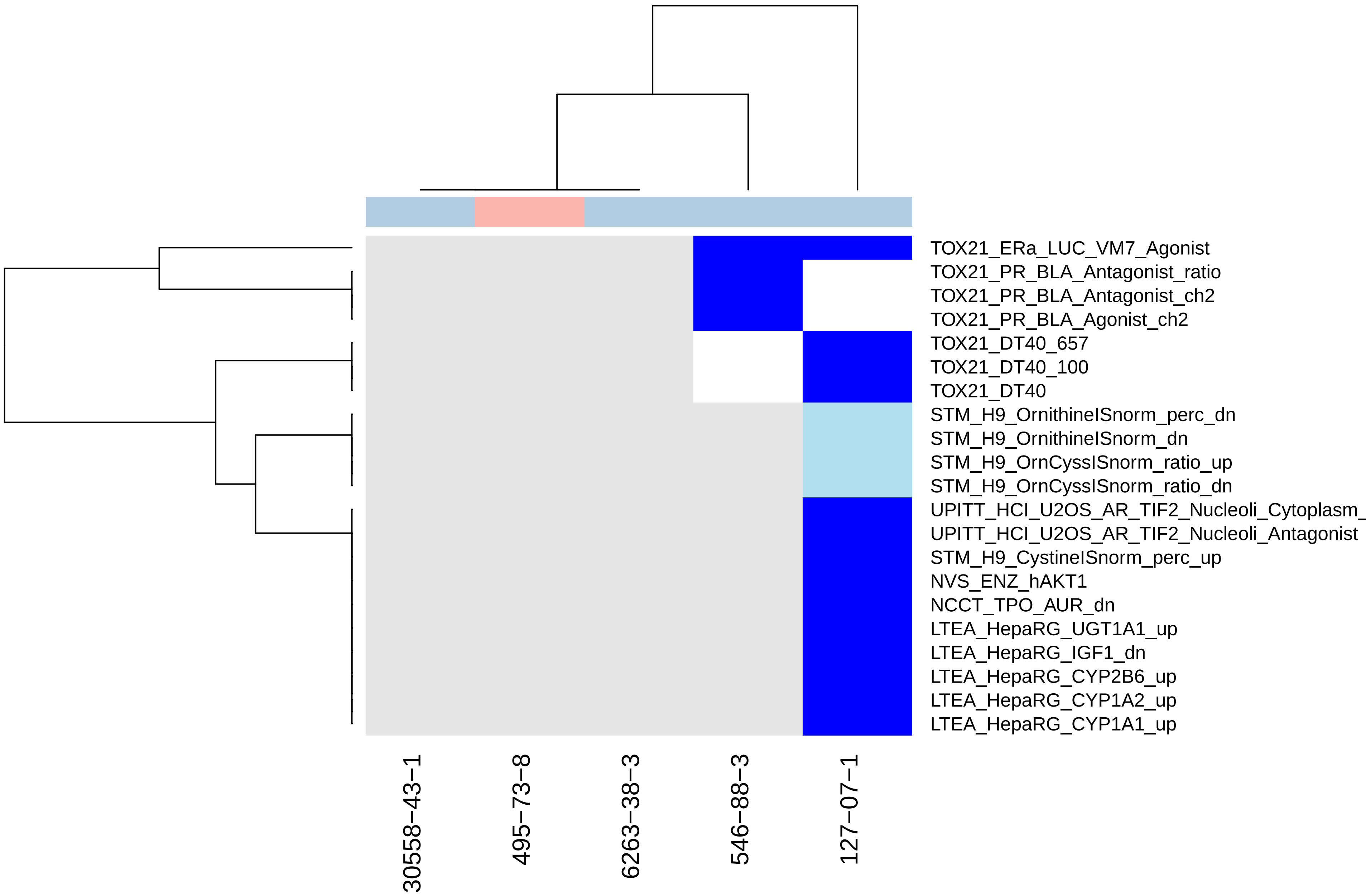

All Activity Heatmap for Cluster 898

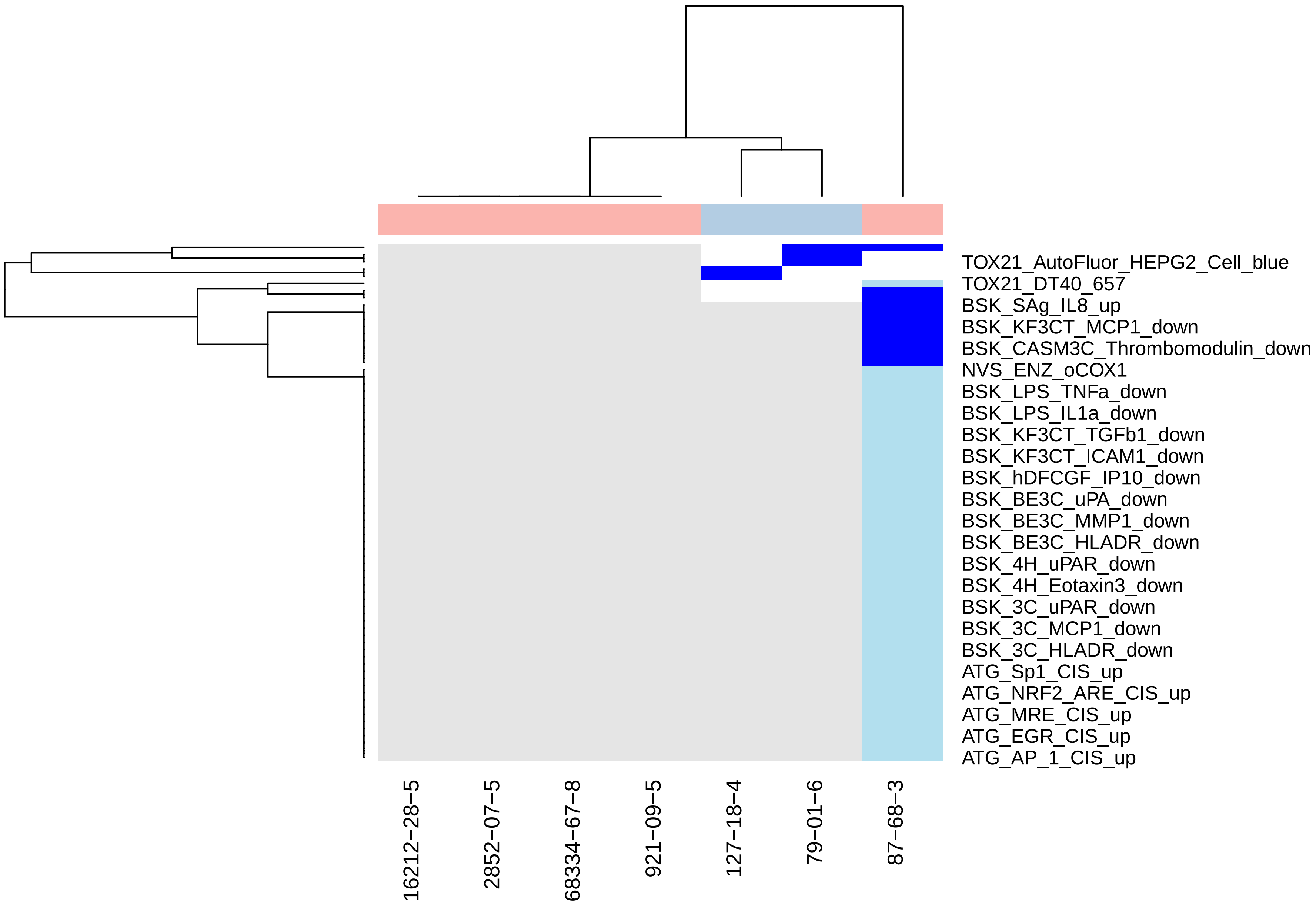

All Activity Heatmap for Cluster 899

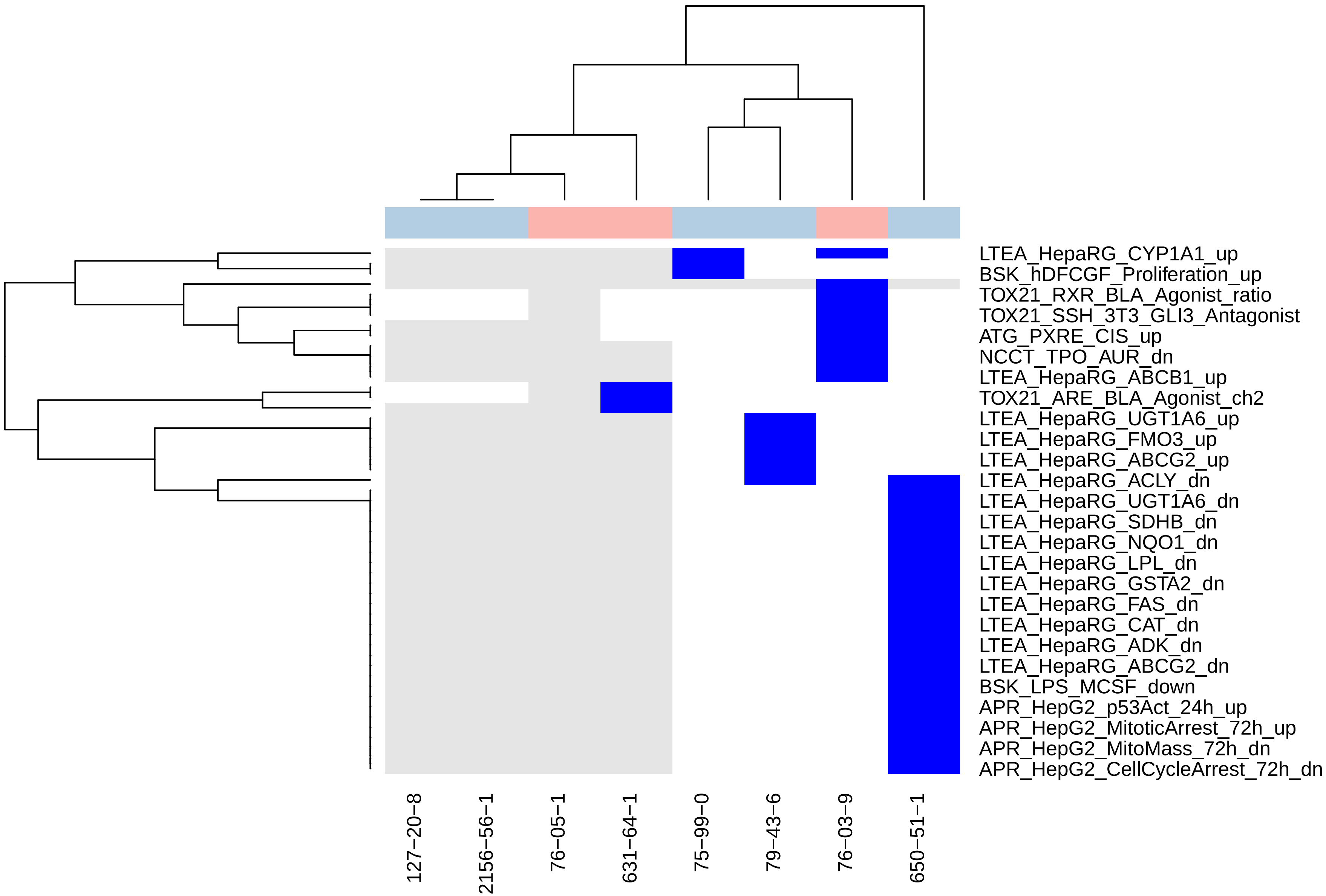

All Activity Heatmap for Cluster 900

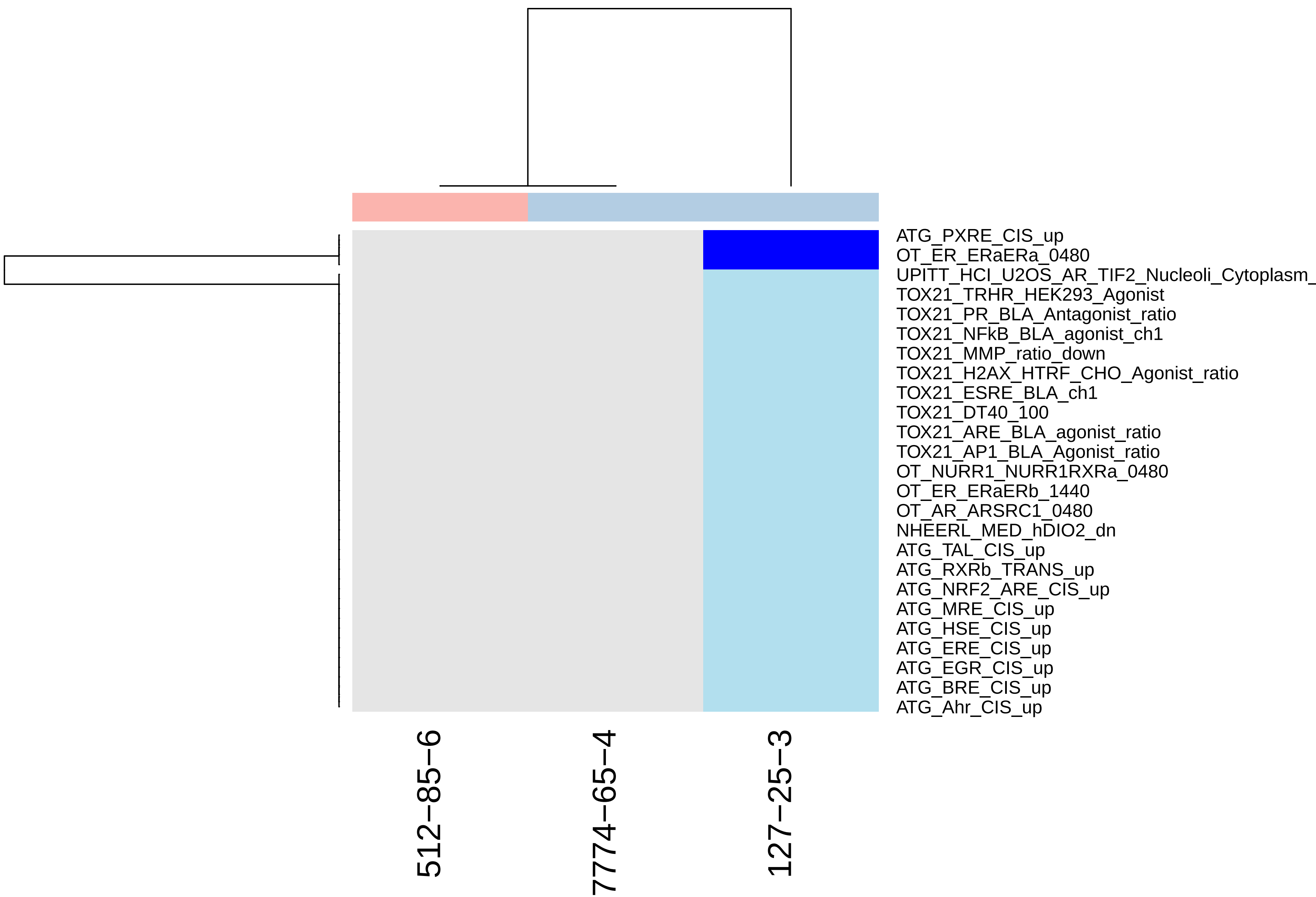

All Activity Heatmap for Cluster 901

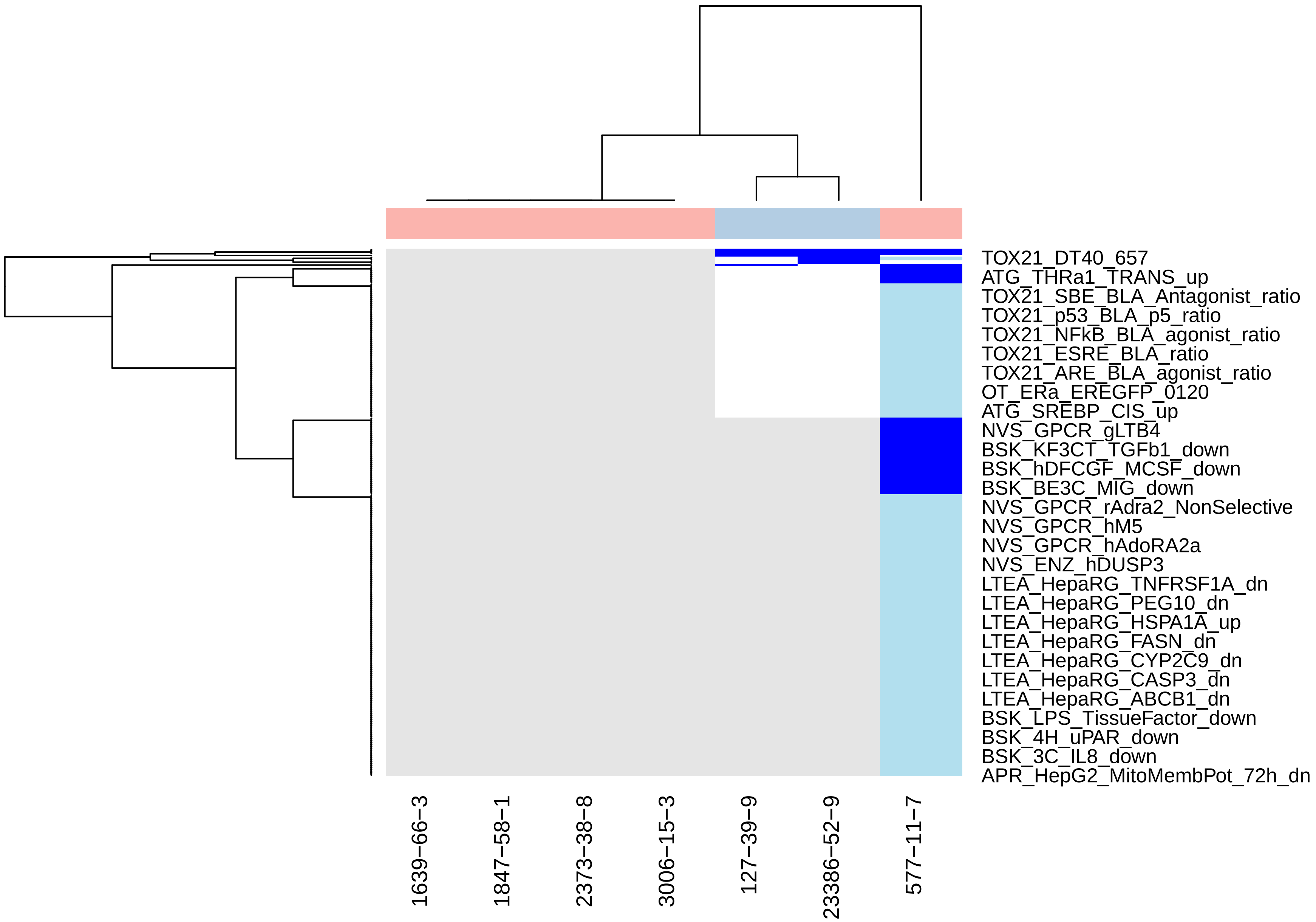

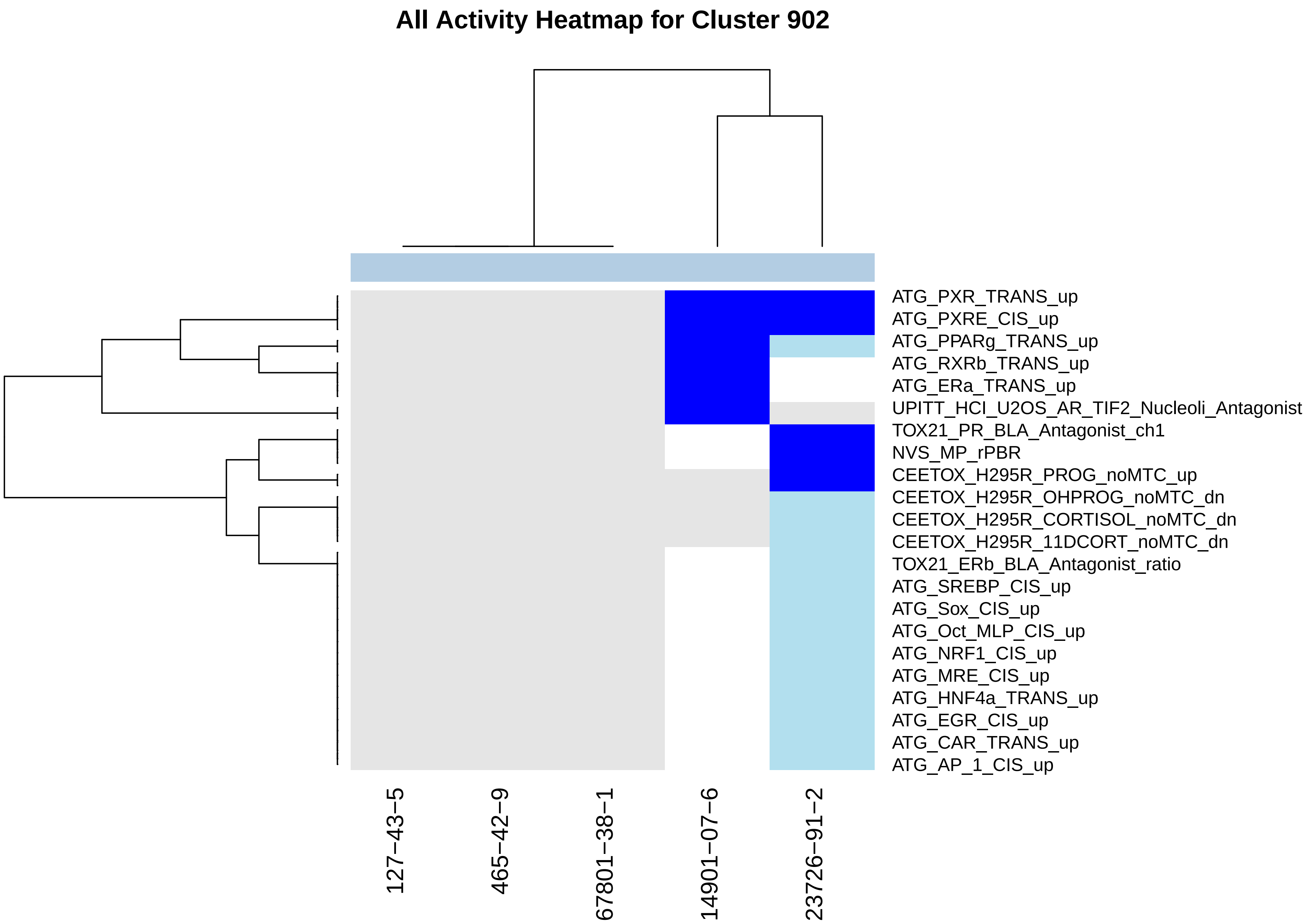
All Activity Heatmap for Cluster 902
ATG_PXR_TRANS_up
ATG_PXRE_CIS_up
ATG_PPARg_TRANS_up
ATG_RXRb_TRANS_up
ATG_ERa_TRANS_up
UPITT_HCI_U2OS_AR_TIF2_Nucleoli_Antagonist
TOX21_PR_BLA_Antagonist_ch1
NVS_MP_rPBR
CEETOX_H295R_PROG_noMTC_up
CEETOX_H295R_OHPROG_noMTC_dn
CEETOX_H295R_CORTISOL_noMTC_dn
CEETOX_H295R_11DCORT_noMTC_dn
TOX21_ERb_BLA_Antagonist_ratio
ATG_SREBP_CIS_up
ATG_Sox_CIS_up
ATG_Oct_MLP_CIS_up
ATG_NRF1_CIS_up
ATG_MRE_CIS_up
ATG_HNF4a_TRANS_up
ATG_EGR_CIS_up
ATG_CAR_TRANS_up
ATG_AP_1_CIS_up
127−43−5
465−42−9
67801−38−1
14901−07−6
23726−91−2

All Activity Heatmap for Cluster 904

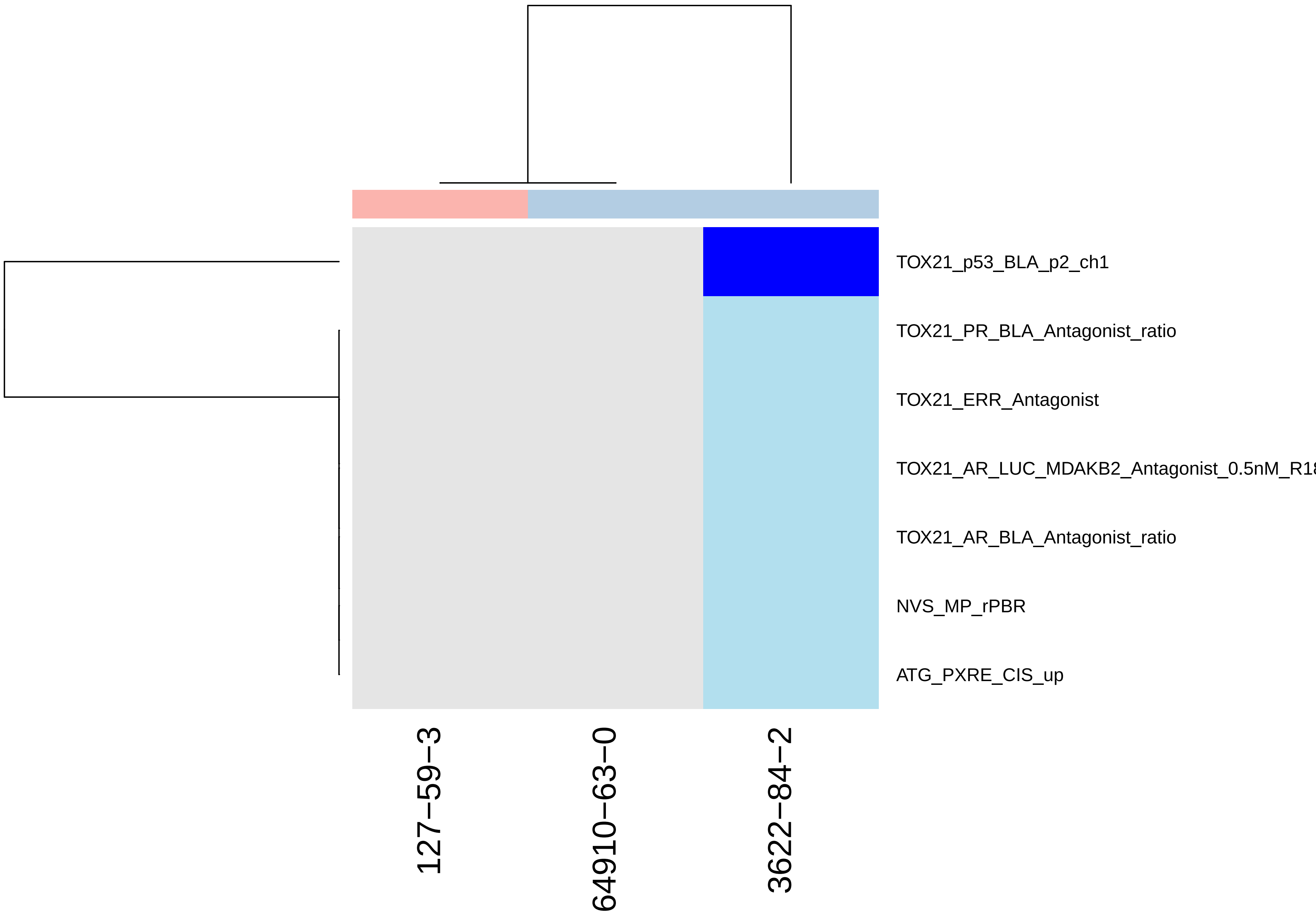

All Activity Heatmap for Cluster 905

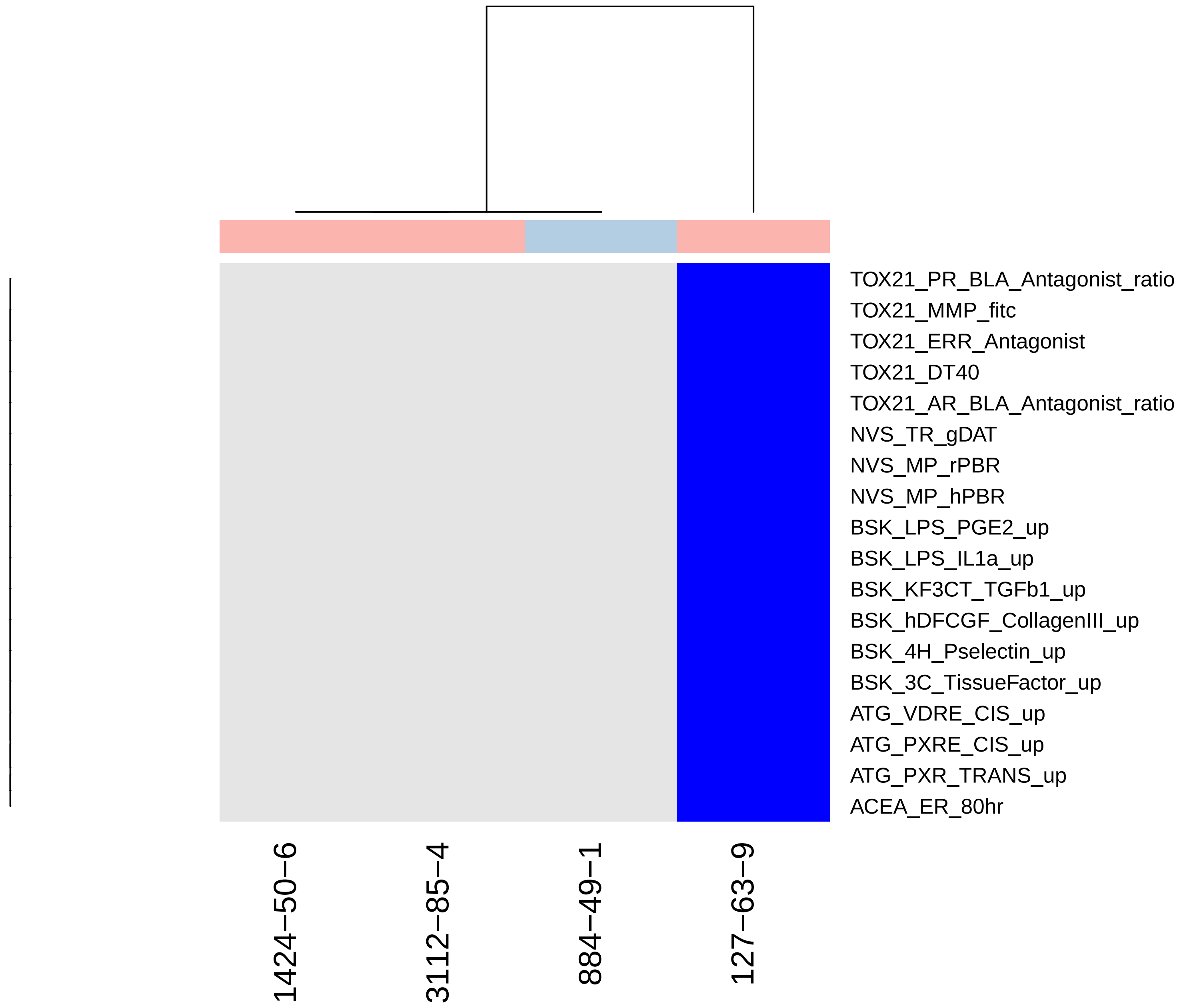

All Activity Heatmap for Cluster 906

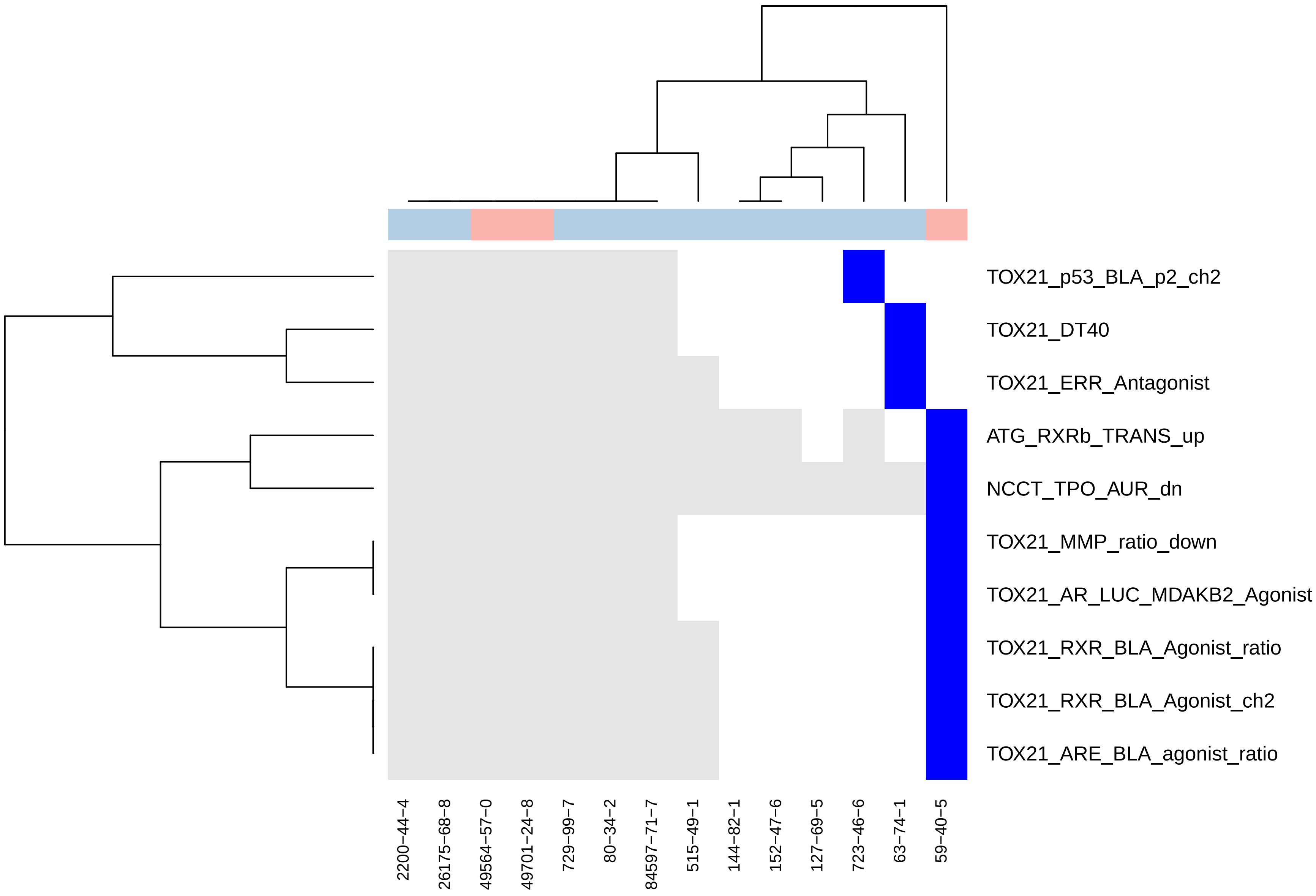

All Activity Heatmap for Cluster 907

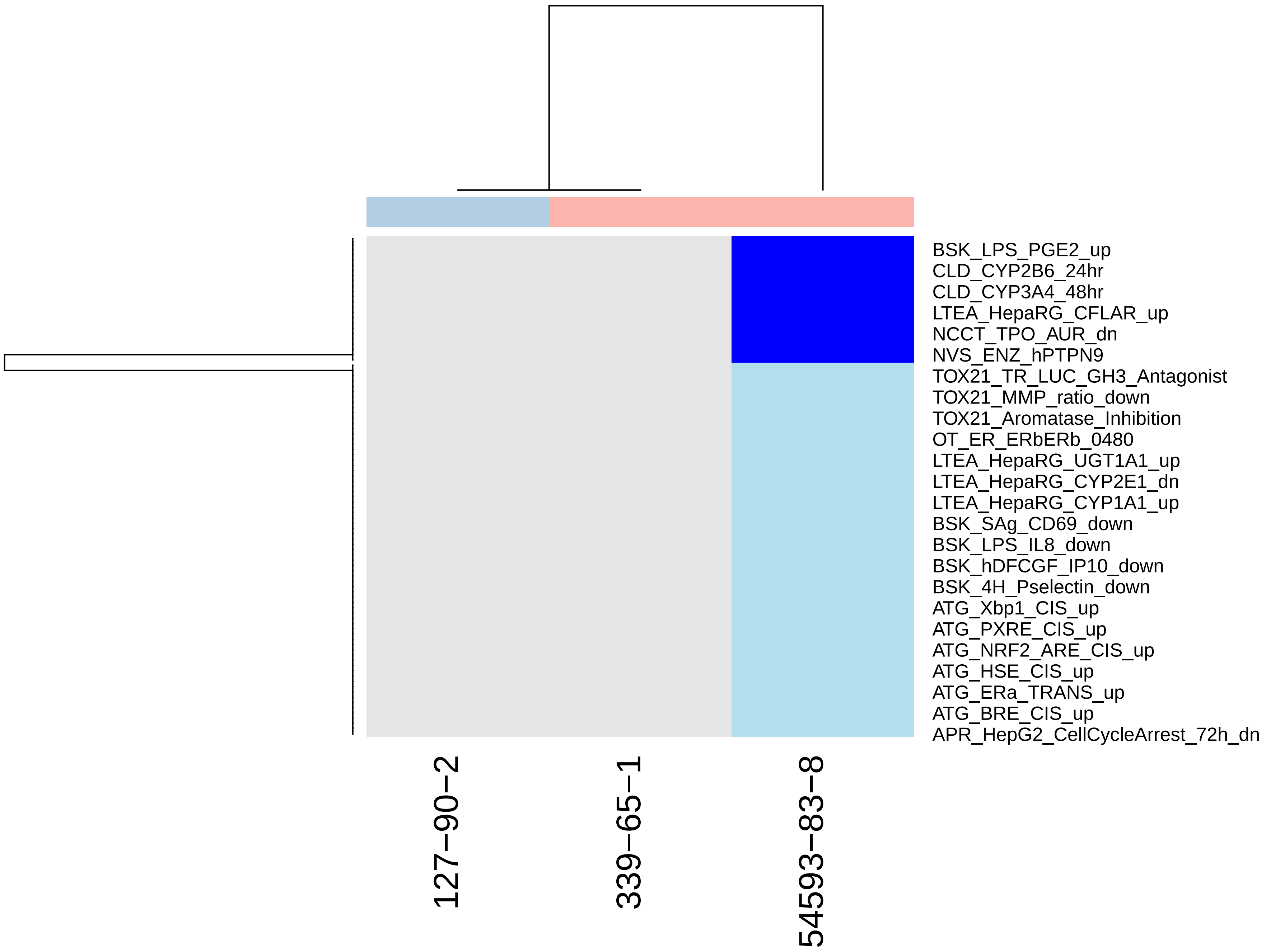

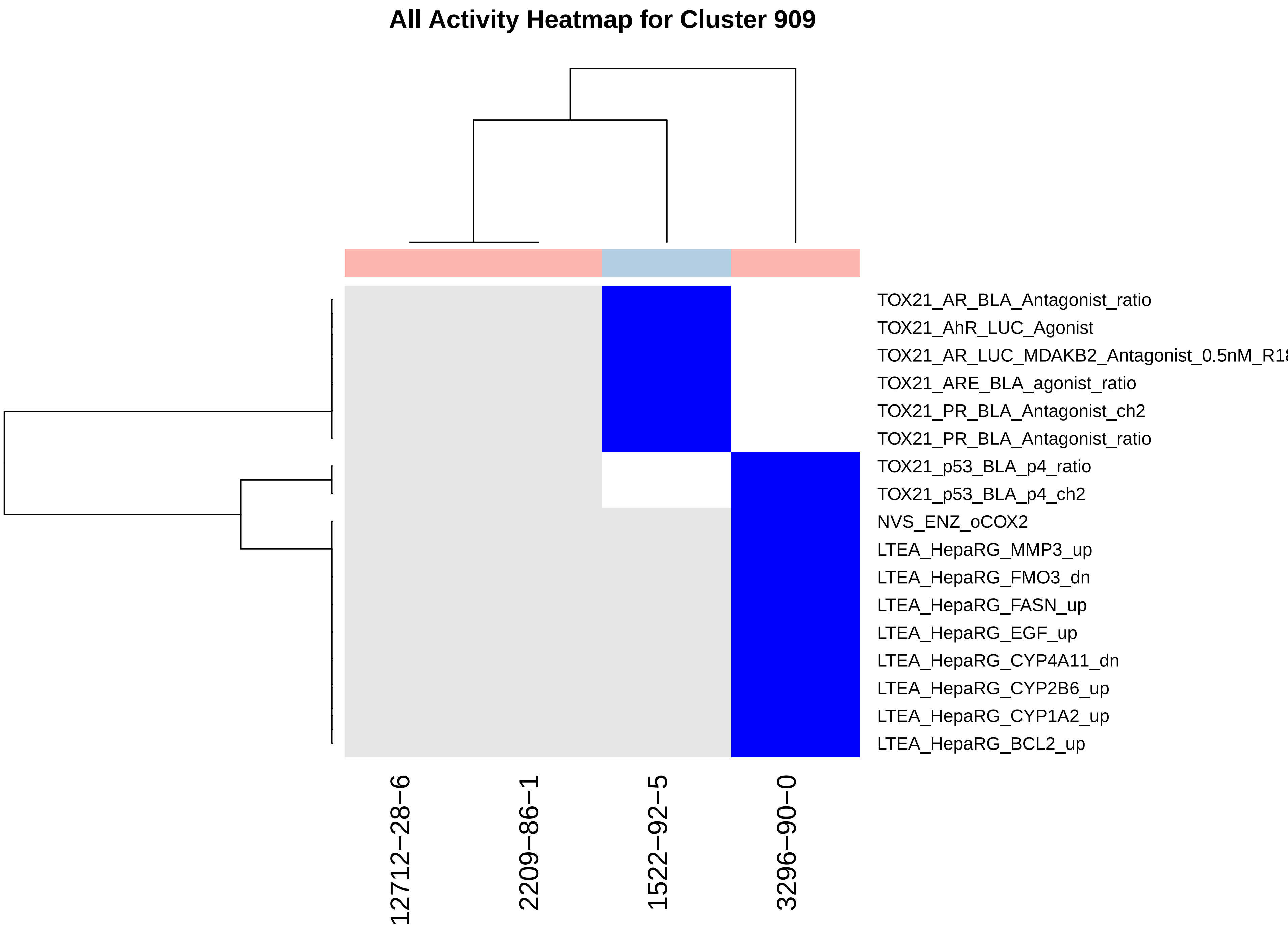

All Activity Heatmap for Cluster 909
TOX21_AR_BLA_Antagonist_ratio
TOX21_AhR_LUC_Agonist
TOX21_AR_LUC_MDAKB2_Antagonist_0.5nM_R18
TOX21_ARE_BLA_agonist_ratio
TOX21_PR_BLA_Antagonist_ch2
TOX21_PR_BLA_Antagonist_ratio
TOX21_p53_BLA_p4_ratio
TOX21_p53_BLA_p4_ch2
NVS_ENZ_oCOX2
LTEA_HepaRG_MMP3_up
LTEA_HepaRG_FMO3_dn
LTEA_HepaRG_FASN_up
LTEA_HepaRG_EGF_up
LTEA_HepaRG_CYP4A11_dn
LTEA_HepaRG_CYP2B6_up
LTEA_HepaRG_CYP1A2_up
LTEA_HepaRG_BCL2_up
12712−28−6
2209−86−1
1522−92−5
3296−90−0

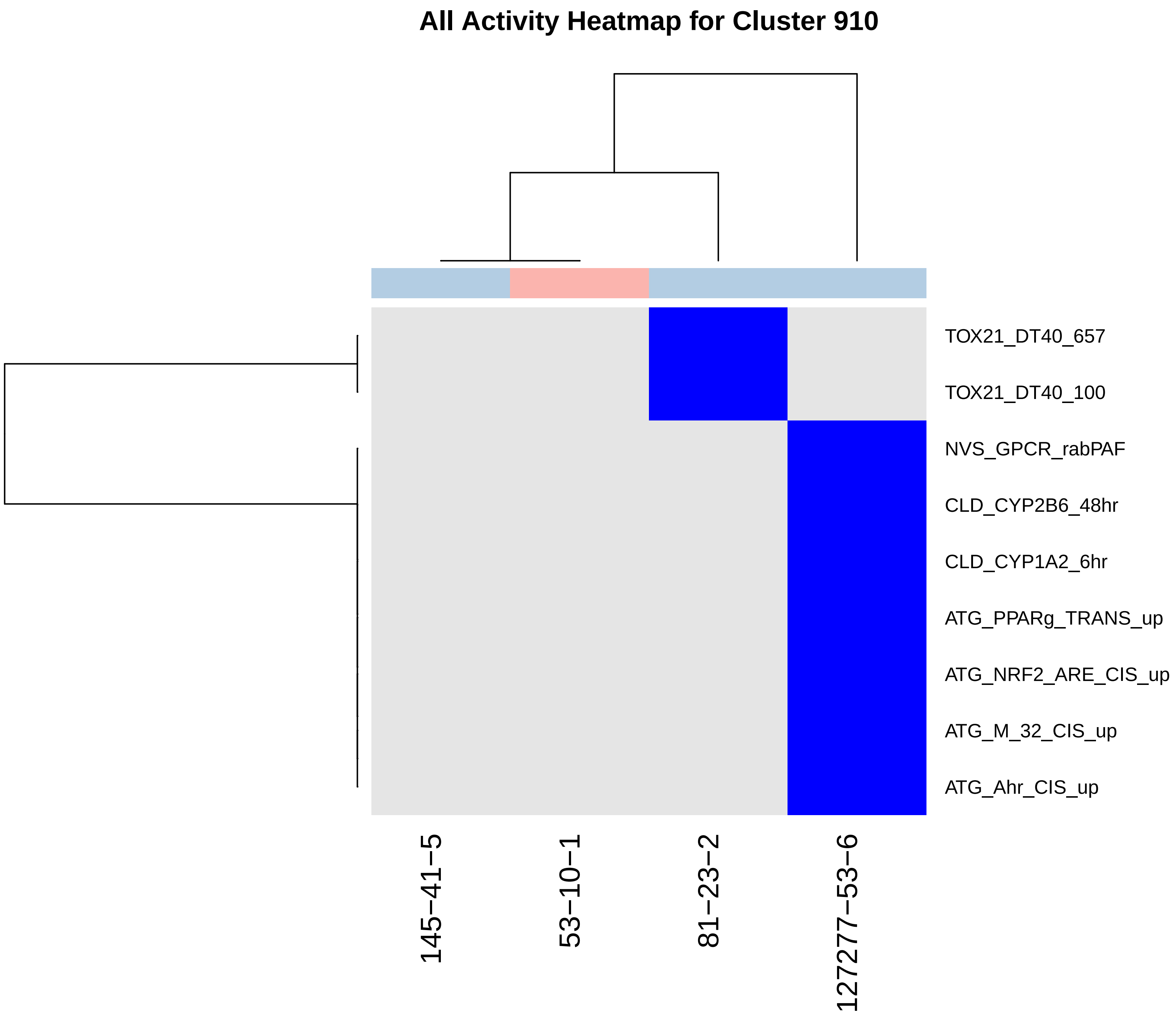

All Activity Heatmap for Cluster 910
TOX21_DT40_657
TOX21_DT40_100
NVS_GPCR_rabPAF
CLD_CYP2B6_48hr
CLD_CYP1A2_6hr
ATG_PPARg_TRANS_up
ATG_NRF2_ARE_CIS_up
ATG_M_32_CIS_up
ATG_Ahr_CIS_up
145−41−5
53−10−1
81−23−2
127277−53−6

All Activity Heatmap for Cluster 911

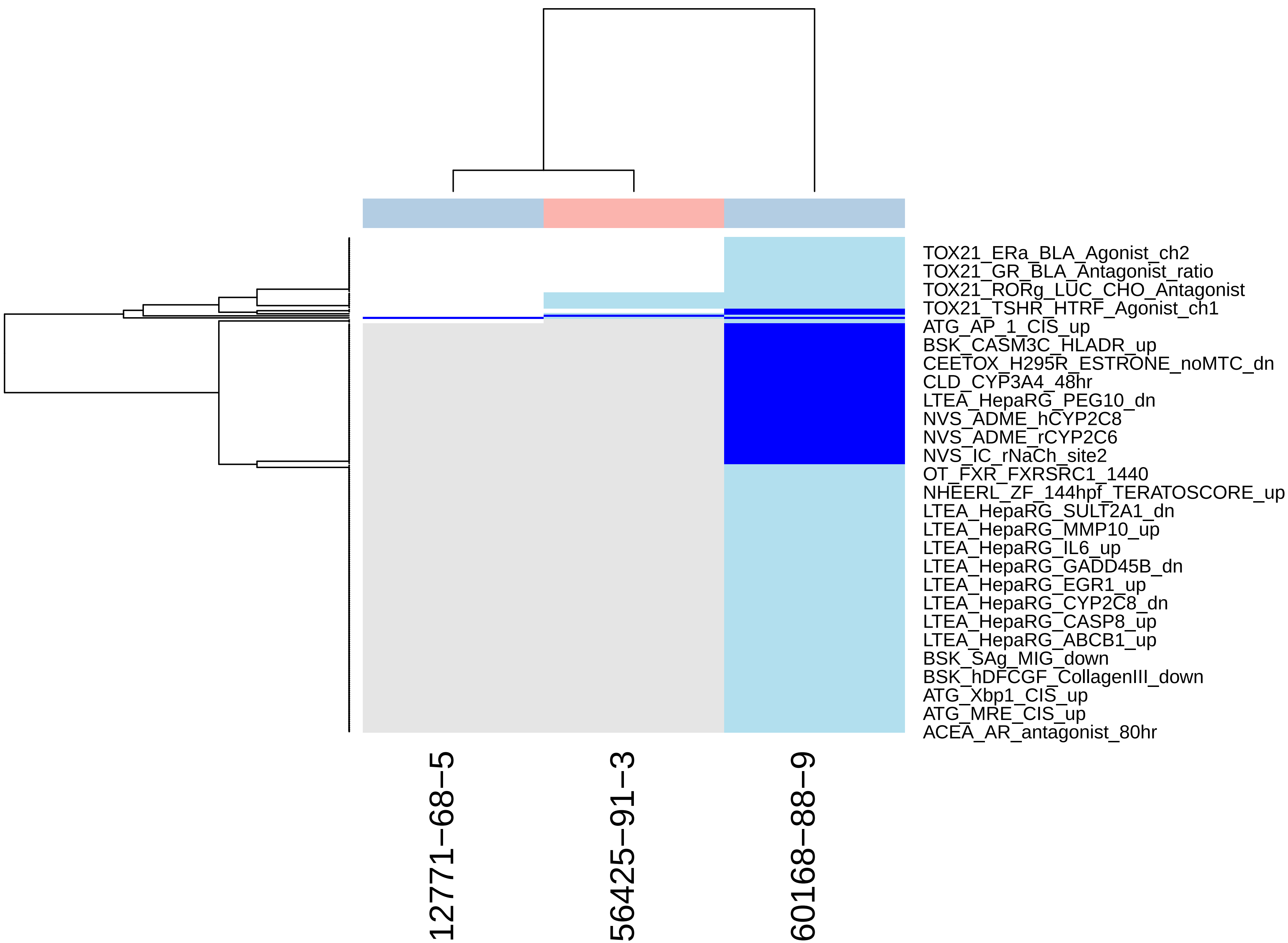

All Activity Heatmap for Cluster 913

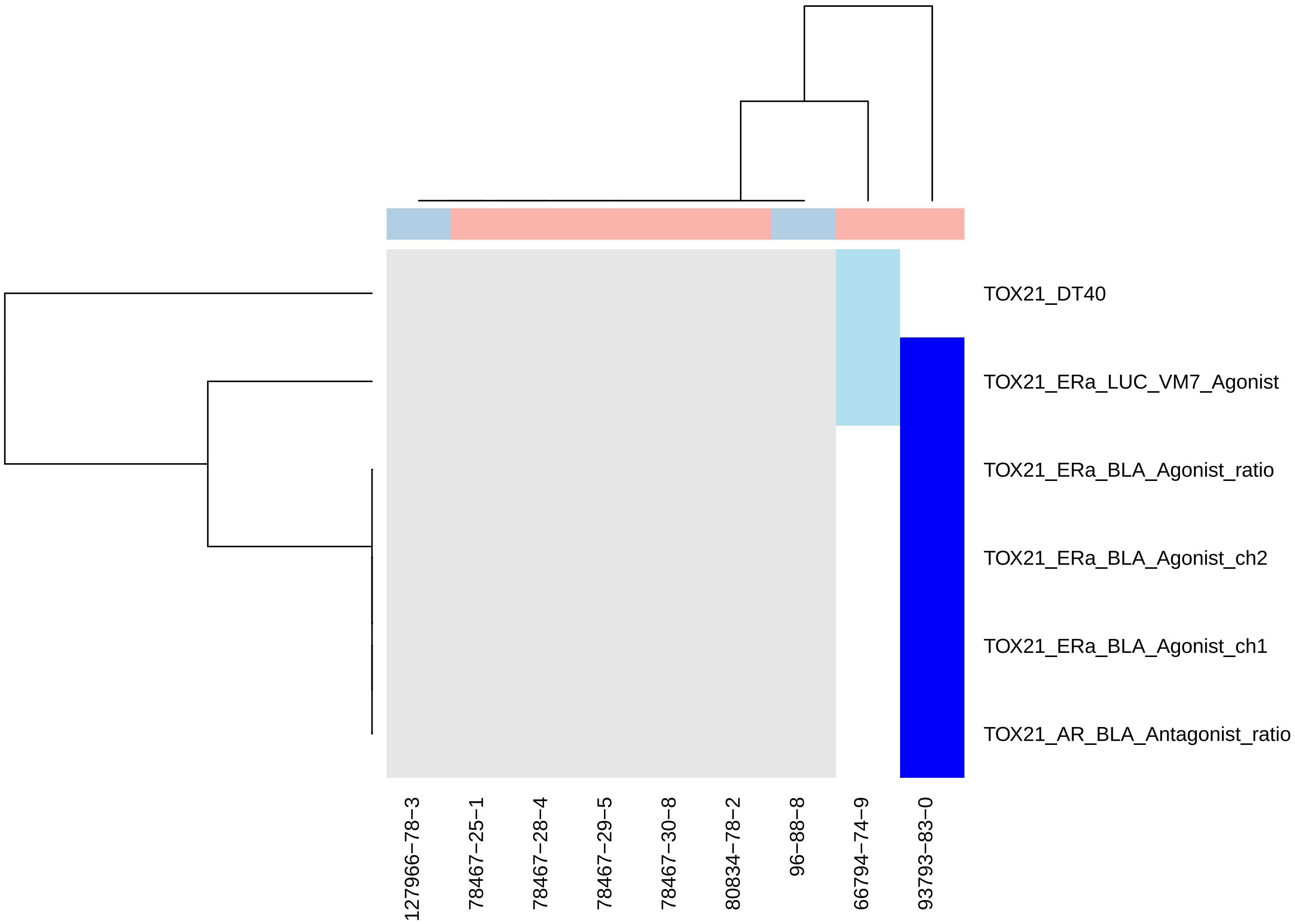

All Activity Heatmap for Cluster 914

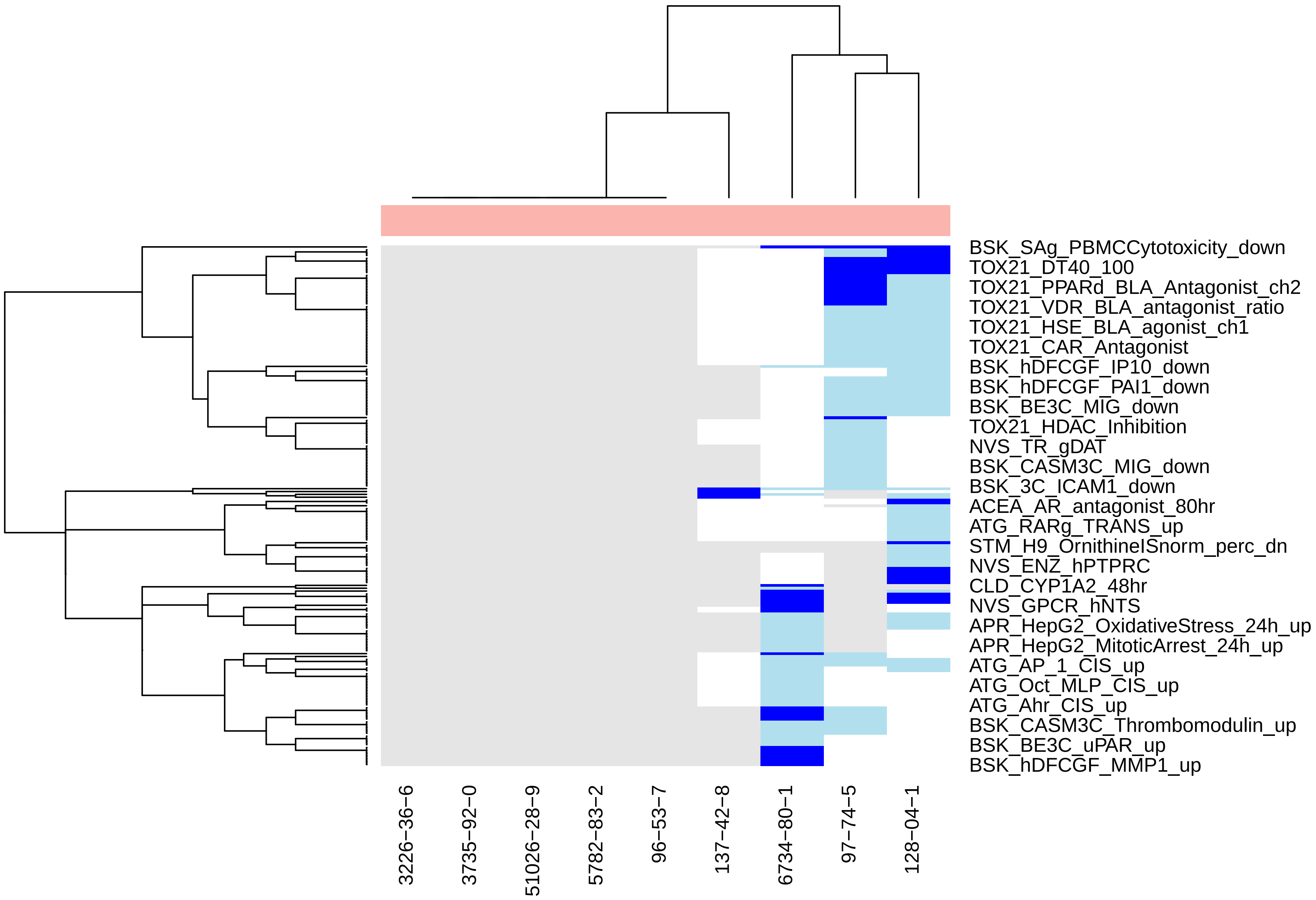

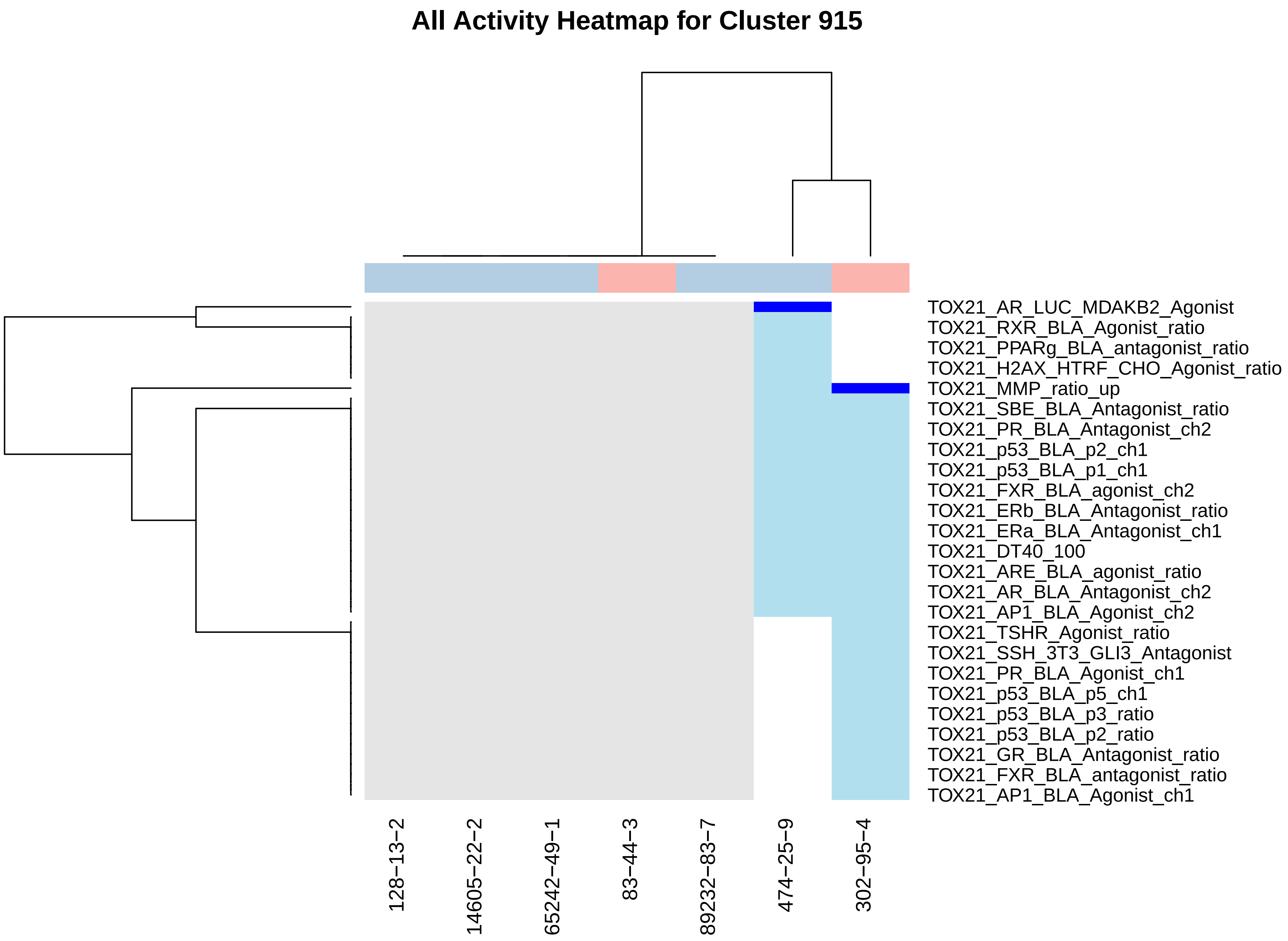
All Activity Heatmap for Cluster 915
TOX21_AR_LUC_MDAKB2_Agonist
TOX21_RXR_BLA_Agonist_ratio
TOX21_PPARg_BLA_antagonist_ratio
TOX21_H2AX_HTRF_CHO_Agonist_ratio
TOX21_MMP_ratio_up
TOX21_SBE_BLA_Antagonist_ratio
TOX21_PR_BLA_Antagonist_ch2
TOX21_p53_BLA_p2_ch1
TOX21_p53_BLA_p1_ch1
TOX21_FXR_BLA_agonist_ch2
TOX21_ERb_BLA_Antagonist_ratio
TOX21_ERa_BLA_Antagonist_ch1
TOX21_DT40_100
TOX21_ARE_BLA_agonist_ratio
TOX21_AR_BLA_Antagonist_ch2
TOX21_AP1_BLA_Agonist_ch2
TOX21_TSHR_Agonist_ratio
TOX21_SSH_3T3_GLI3_Antagonist
TOX21_PR_BLA_Agonist_ch1
TOX21_p53_BLA_p5_ch1
TOX21_p53_BLA_p3_ratio
TOX21_p53_BLA_p2_ratio
TOX21_GR_BLA_Antagonist_ratio
TOX21_FXR_BLA_antagonist_ratio
TOX21_AP1_BLA_Agonist_ch1
128−13−2
14605−22−2
65242−49−1
83−44−3
89232−83−7
474−25−9
302−95−4

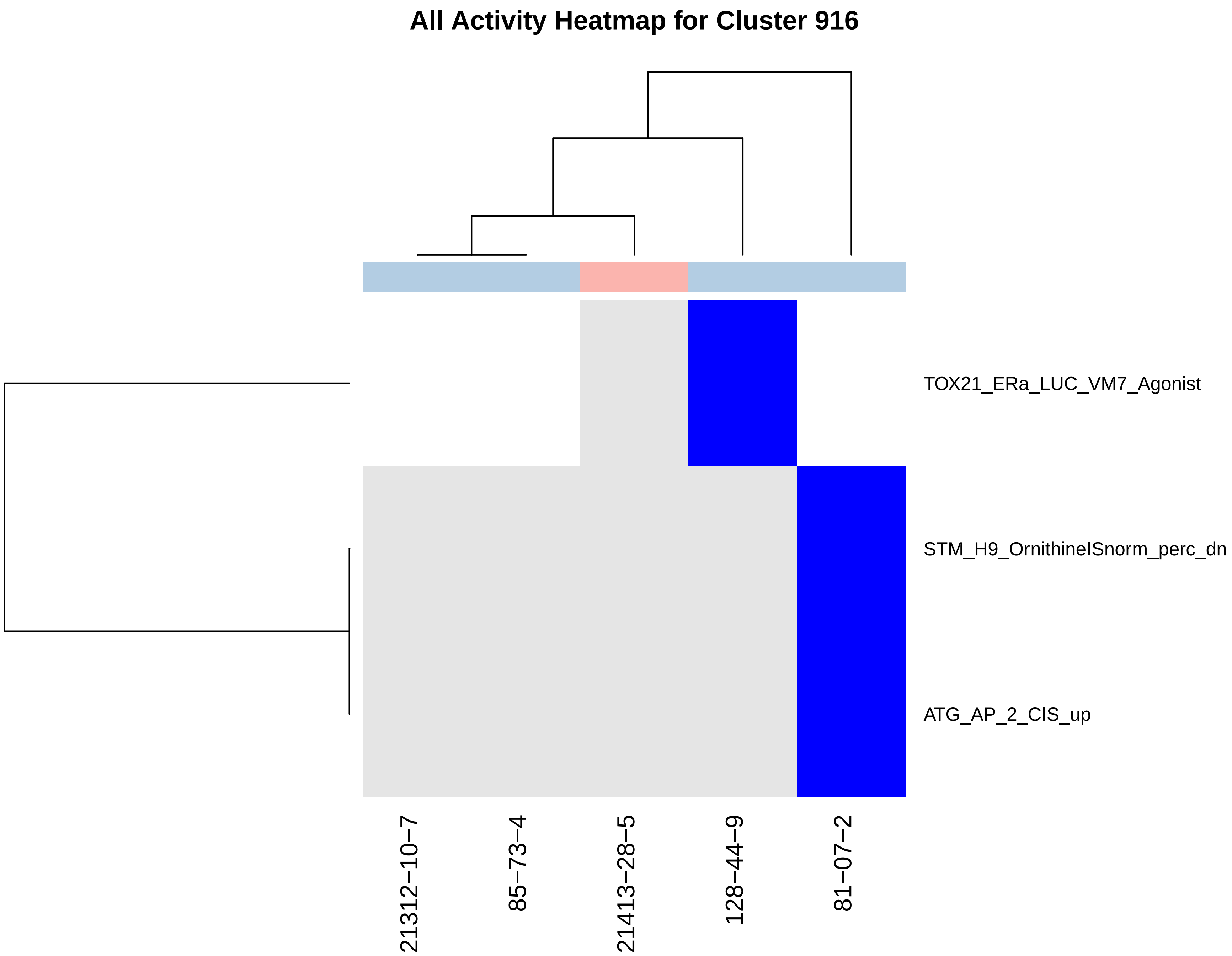

All Activity Heatmap for Cluster 916
TOX21_ERa_LUC_VM7_Agonist
STM_H9_OrnithineISnorm_perc_dn
ATG_AP_2_CIS_up
21312−10−7
85−73−4
21413−28−5
128−44−9
81−07−2

All Activity Heatmap for Cluster 917

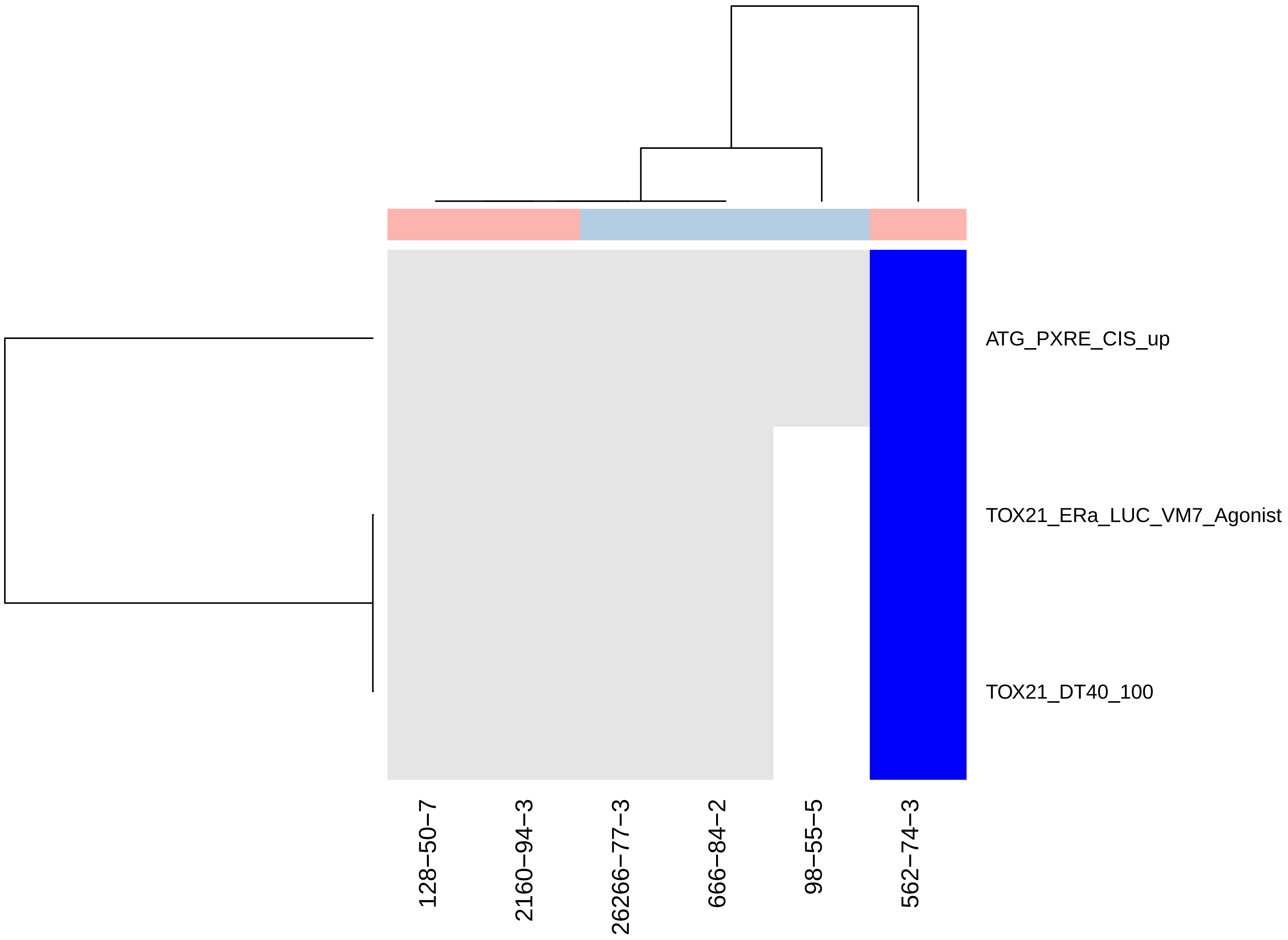

All Activity Heatmap for Cluster 918

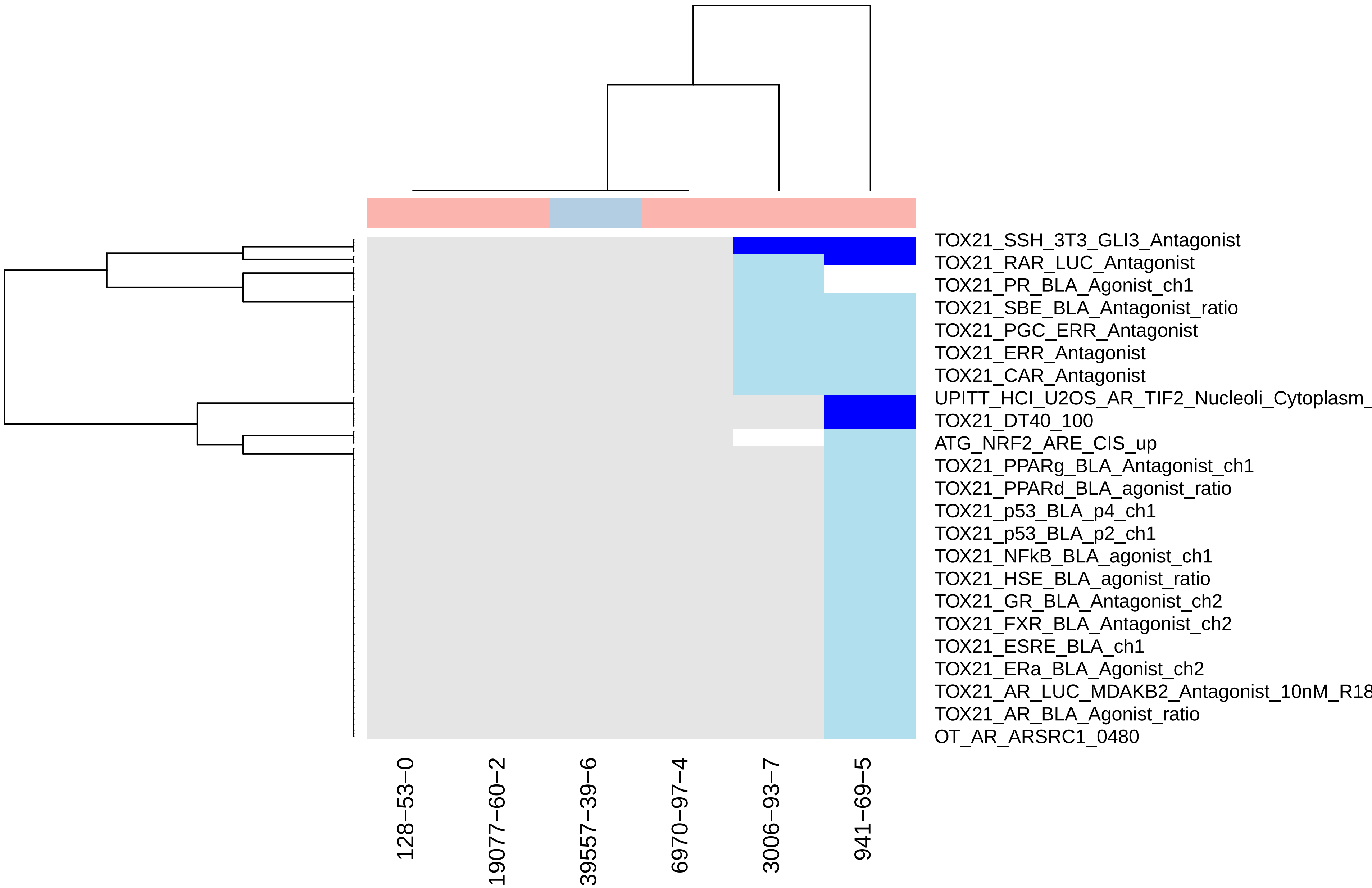

All Activity Heatmap for Cluster 919

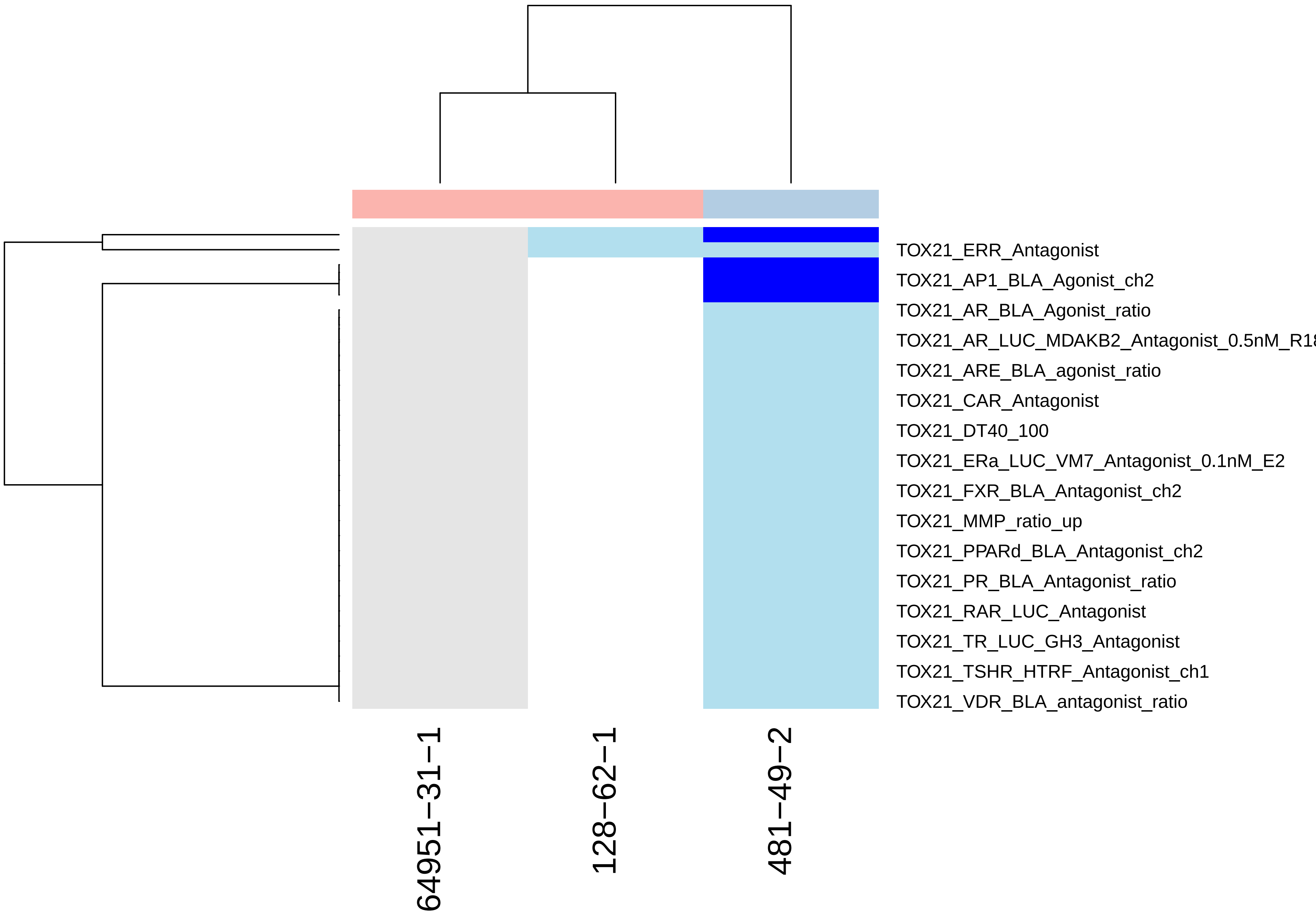

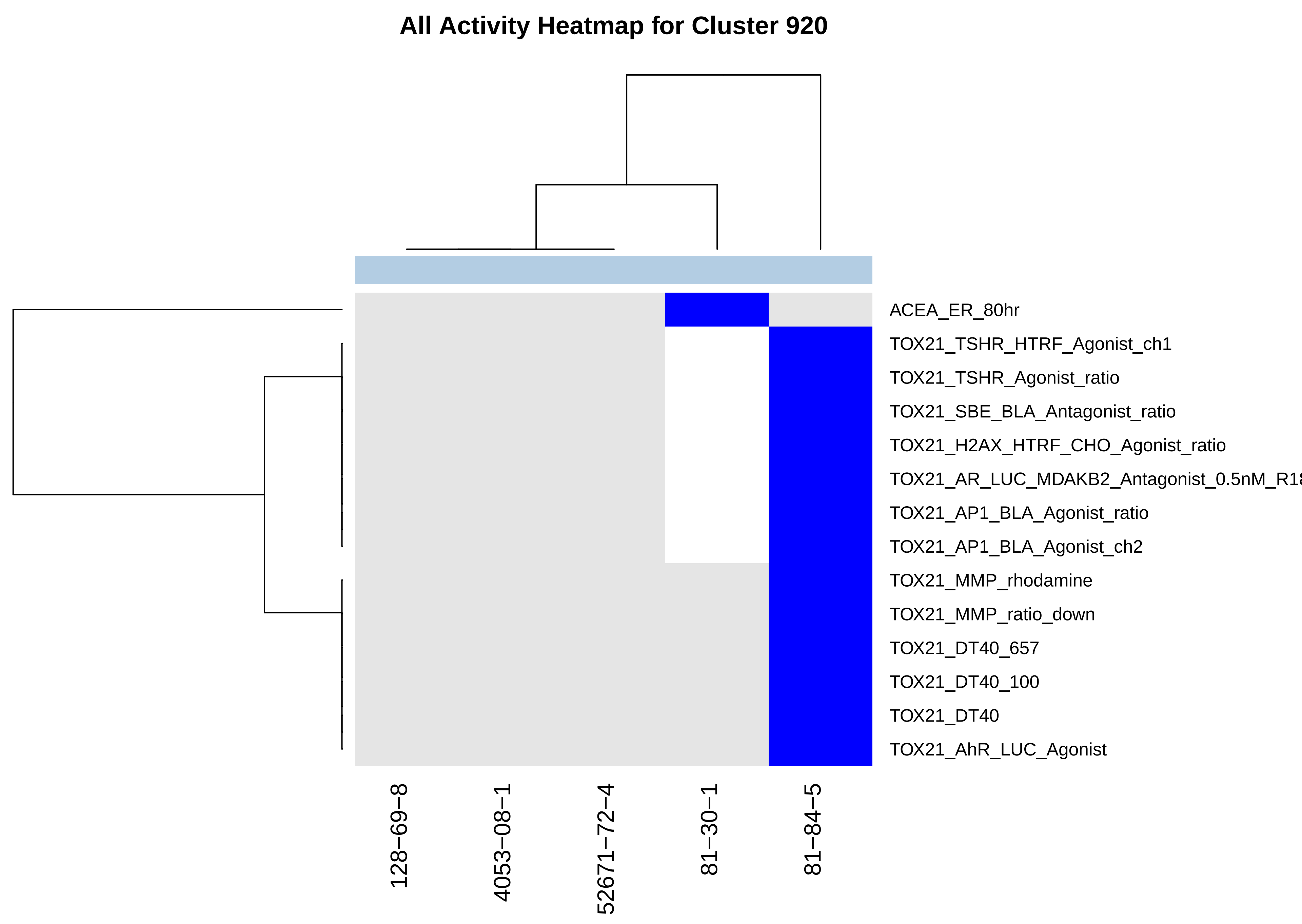

All Activity Heatmap for Cluster 920
ACEA_ER_80hr
TOX21_TSHR_HTRF_Agonist_ch1
TOX21_TSHR_Agonist_ratio
TOX21_SBE_BLA_Antagonist_ratio
TOX21_H2AX_HTRF_CHO_Agonist_ratio
TOX21_AR_LUC_MDAKB2_Antagonist_0.5nM_R18
TOX21_AP1_BLA_Agonist_ratio
TOX21_AP1_BLA_Agonist_ch2
TOX21_MMP_rhodamine
TOX21_MMP_ratio_down
TOX21_DT40_657
TOX21_DT40_100
TOX21_DT40
TOX21_AhR_LUC_Agonist
128−69−8
4053−08−1
52671−72−4
81−30−1
81−84−5

All Activity Heatmap for Cluster 921

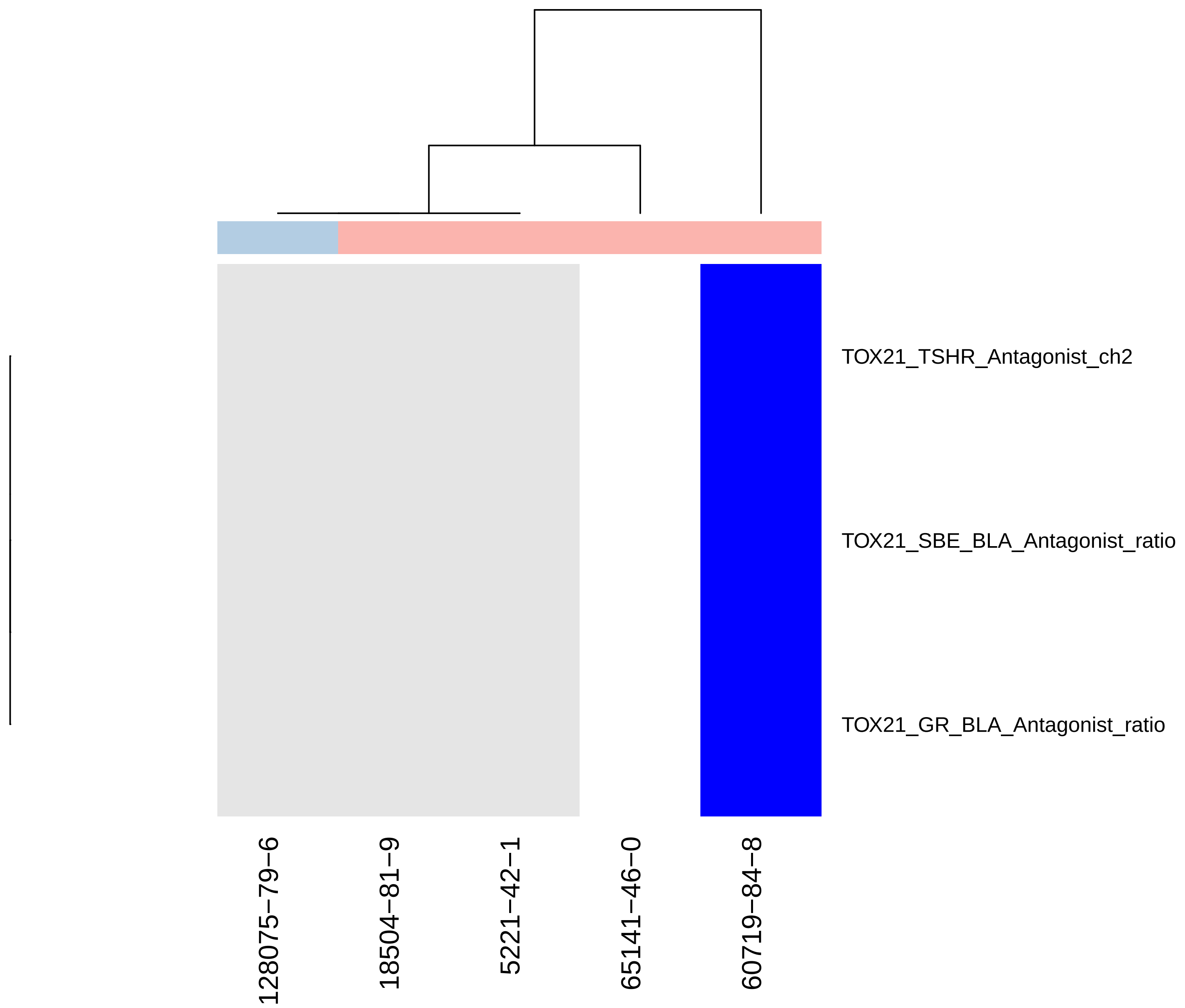

All Activity Heatmap for Cluster 922

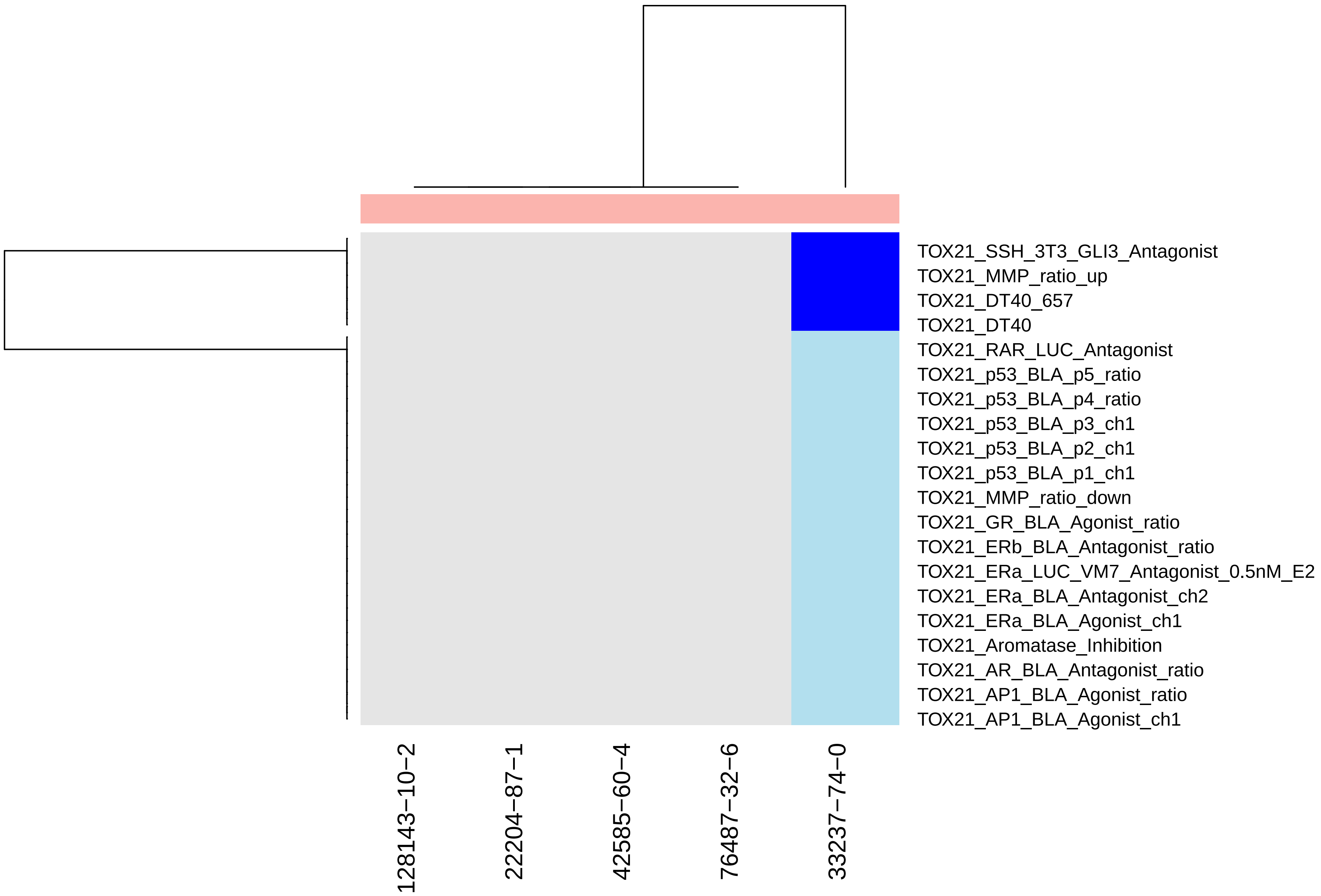

All Activity Heatmap for Cluster 924

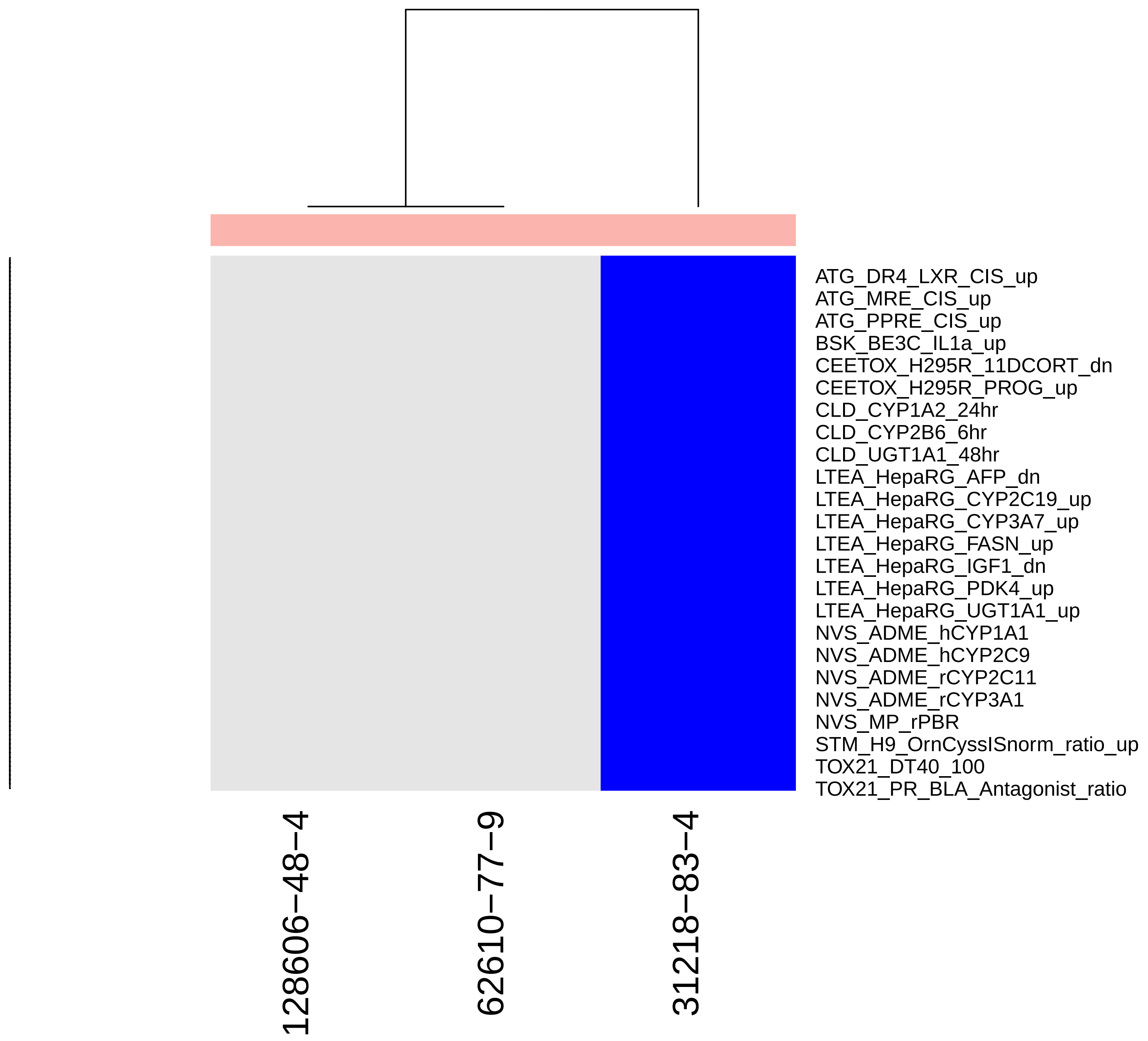

All Activity Heatmap for Cluster 925

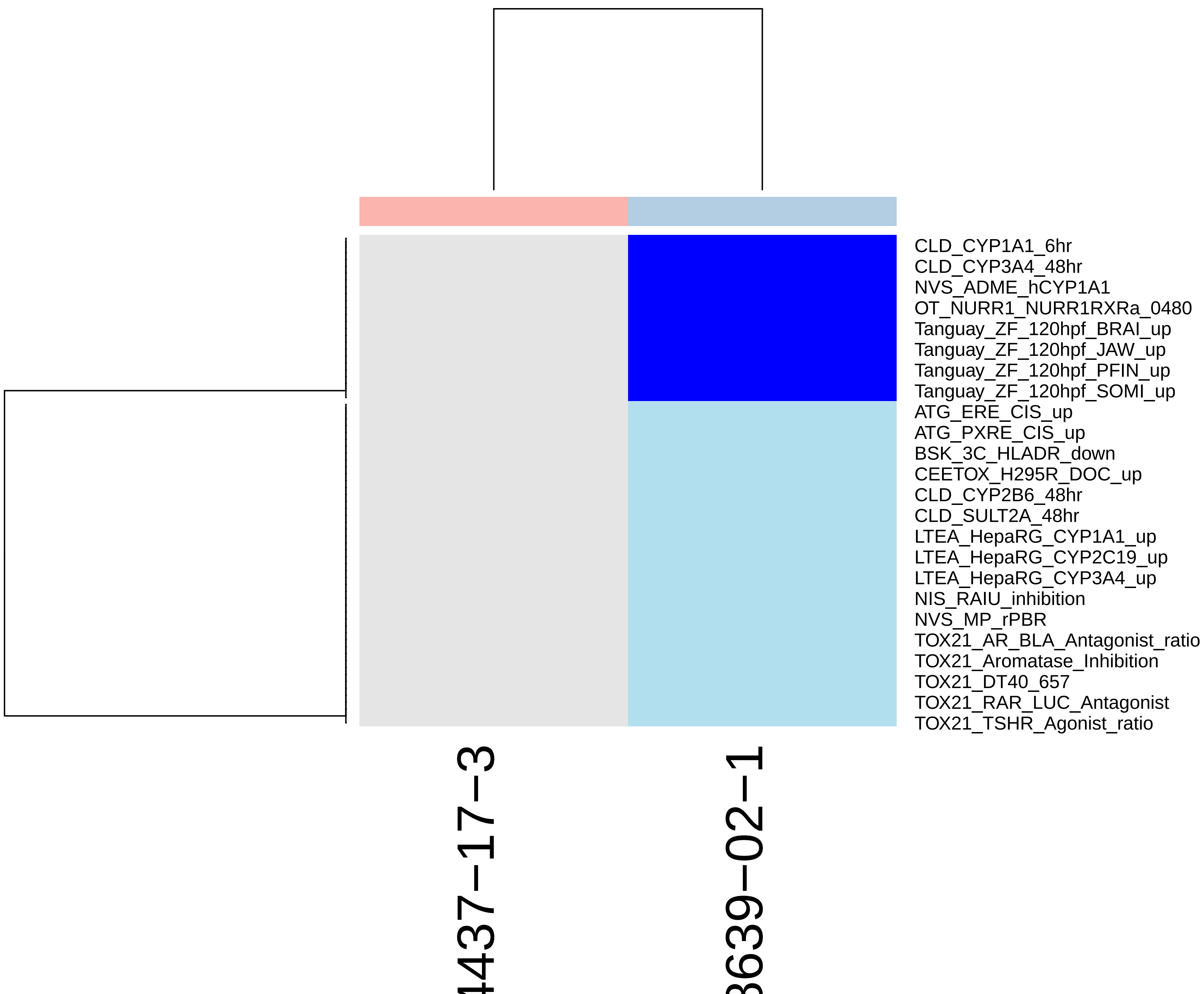

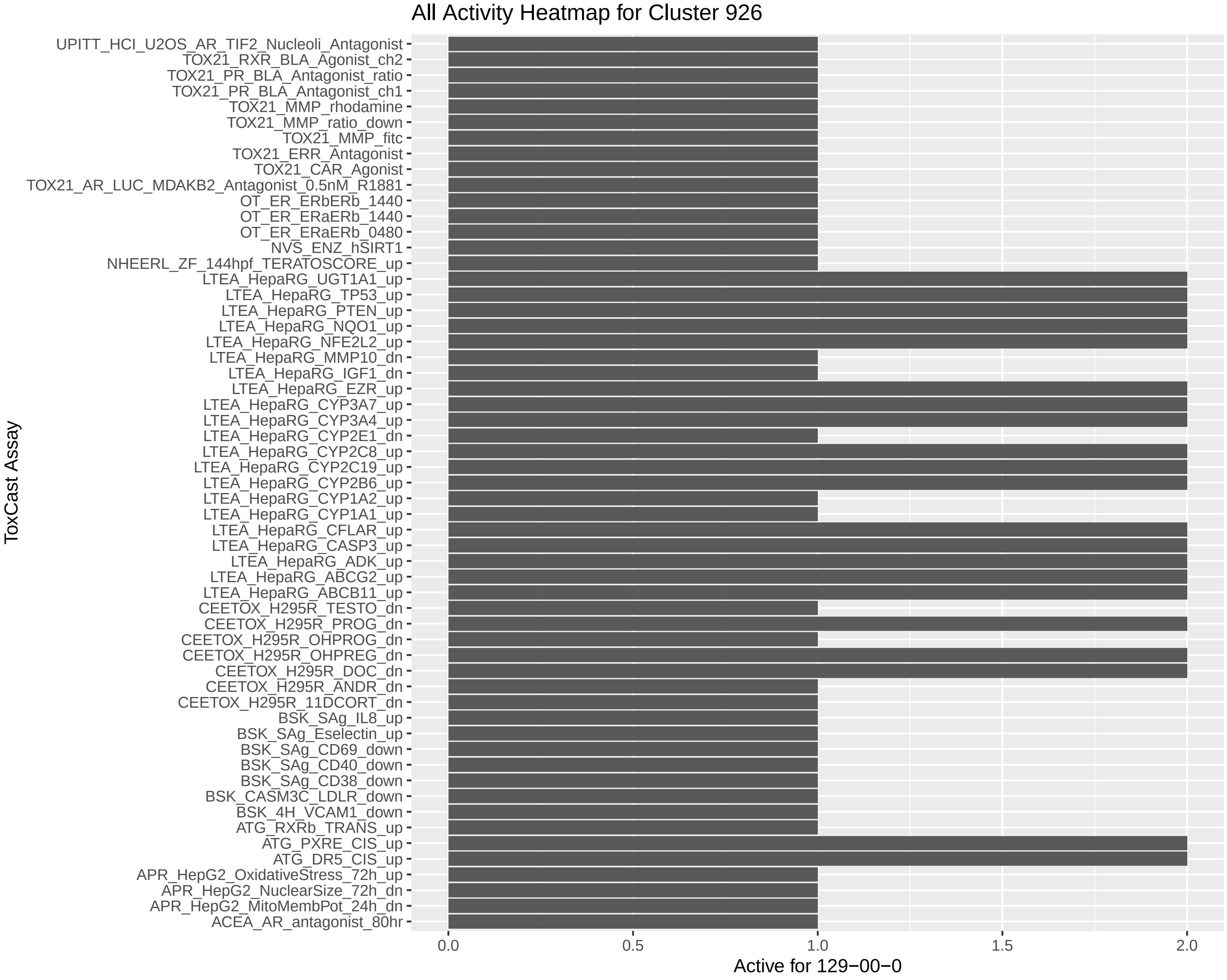
All Activity Heatmap for Cluster 926
ToxCast Assay
Active for 129−00−0
UPITT_HCI_U2OS_AR_TIF2_Nucleoli_Antagonist
TOX21_RXR_BLA_Agonist_ch2
TOX21_PR_BLA_Antagonist_ratio
TOX21_PR_BLA_Antagonist_ch1
TOX21_MMP_rhodamine
TOX21_MMP_ratio_down
TOX21_MMP_fitc
TOX21_ERR_Antagonist
TOX21_CAR_Agonist
TOX21_AR_LUC_MDAKB2_Antagonist_0.5nM_R1881
OT_ER_ERbERb_1440
OT_ER_ERaERb_1440
OT_ER_ERaERb_0480
NVS_ENZ_hSIRT1
NHEERL_ZF_144hpf_TERATOSCORE_up
LTEA_HepaRG_UGT1A1_up
LTEA_HepaRG_TP53_up
LTEA_HepaRG_PTEN_up
LTEA_HepaRG_NQO1_up
LTEA_HepaRG_NFE2L2_up
LTEA_HepaRG_MMP10_dn
LTEA_HepaRG_IGF1_dn
LTEA_HepaRG_EZR_up
LTEA_HepaRG_CYP3A7_up
LTEA_HepaRG_CYP3A4_up
LTEA_HepaRG_CYP2E1_dn
LTEA_HepaRG_CYP2C8_up
LTEA_HepaRG_CYP2C19_up
LTEA_HepaRG_CYP2B6_up
LTEA_HepaRG_CYP1A2_up
LTEA_HepaRG_CYP1A1_up
LTEA_HepaRG_CFLAR_up
LTEA_HepaRG_CASP3_up
LTEA_HepaRG_ADK_up
LTEA_HepaRG_ABCG2_up
LTEA_HepaRG_ABCB11_up
CEETOX_H295R_TESTO_dn
CEETOX_H295R_PROG_dn
CEETOX_H295R_OHPROG_dn
CEETOX_H295R_OHPREG_dn
CEETOX_H295R_DOC_dn
CEETOX_H295R_ANDR_dn
CEETOX_H295R_11DCORT_dn
BSK_SAg_IL8_up
BSK_SAg_Eselectin_up
BSK_SAg_CD69_down
BSK_SAg_CD40_down
BSK_SAg_CD38_down
BSK_CASM3C_LDLR_down
BSK_4H_VCAM1_down
ATG_RXRb_TRANS_up
ATG_PXRE_CIS_up
ATG_DR5_CIS_up
APR_HepG2_OxidativeStress_72h_up
APR_HepG2_NuclearSize_72h_dn
APR_HepG2_MitoMembPot_24h_dn
ACEA_AR_antagonist_80hr
0.0
0.5
1.0
1.5
2.0

All Activity Heatmap for Cluster 927

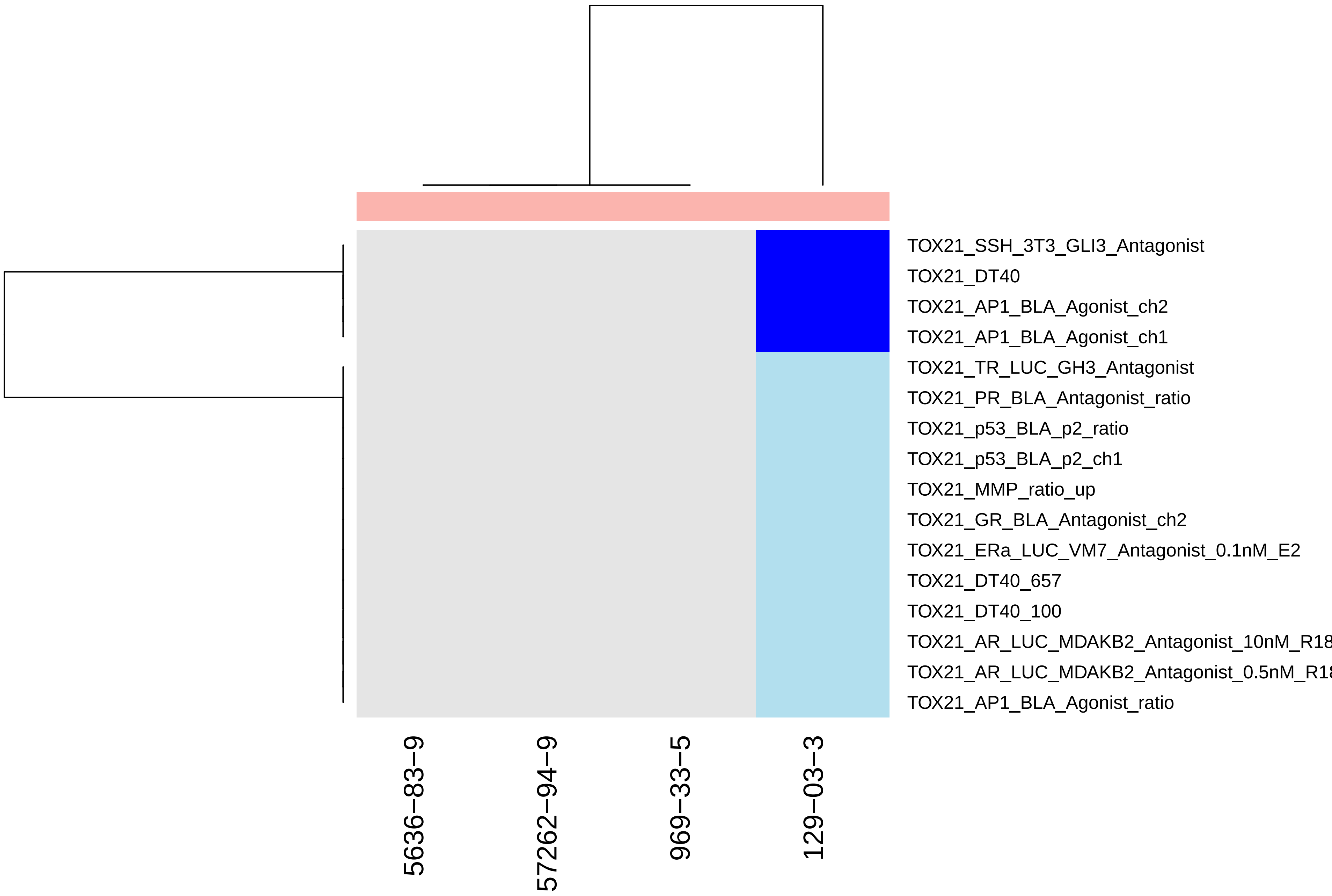

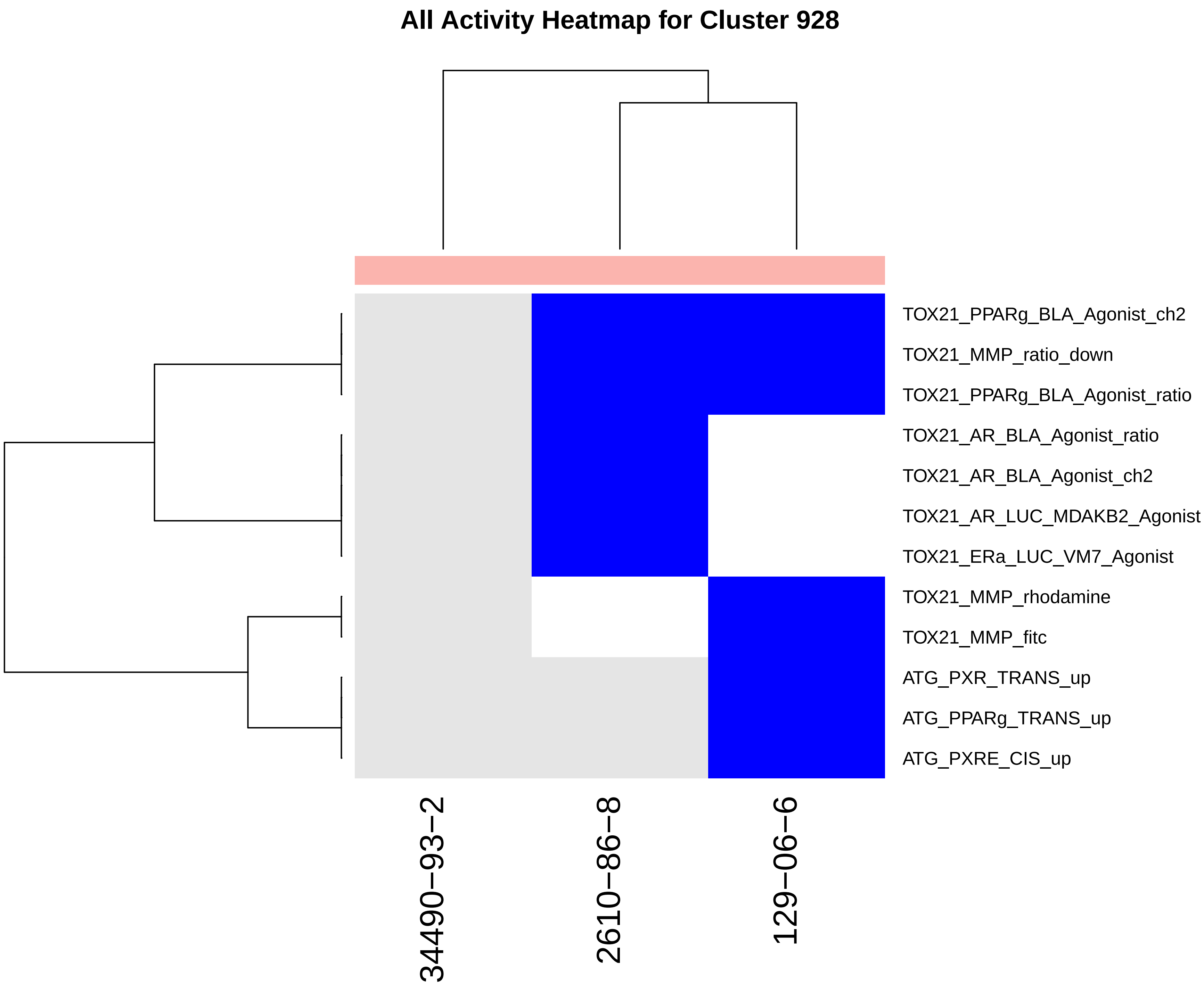

All Activity Heatmap for Cluster 928
TOX21_PPARg_BLA_Agonist_ch2
TOX21_MMP_ratio_down
TOX21_PPARg_BLA_Agonist_ratio
TOX21_AR_BLA_Agonist_ratio
TOX21_AR_BLA_Agonist_ch2
TOX21_AR_LUC_MDAKB2_Agonist
TOX21_ERa_LUC_VM7_Agonist
TOX21_MMP_rhodamine
TOX21_MMP_fitc
ATG_PXR_TRANS_up
ATG_PPARg_TRANS_up
ATG_PXRE_CIS_up
34490−93−2
2610−86−8
129−06−6

All Activity Heatmap for Cluster 930

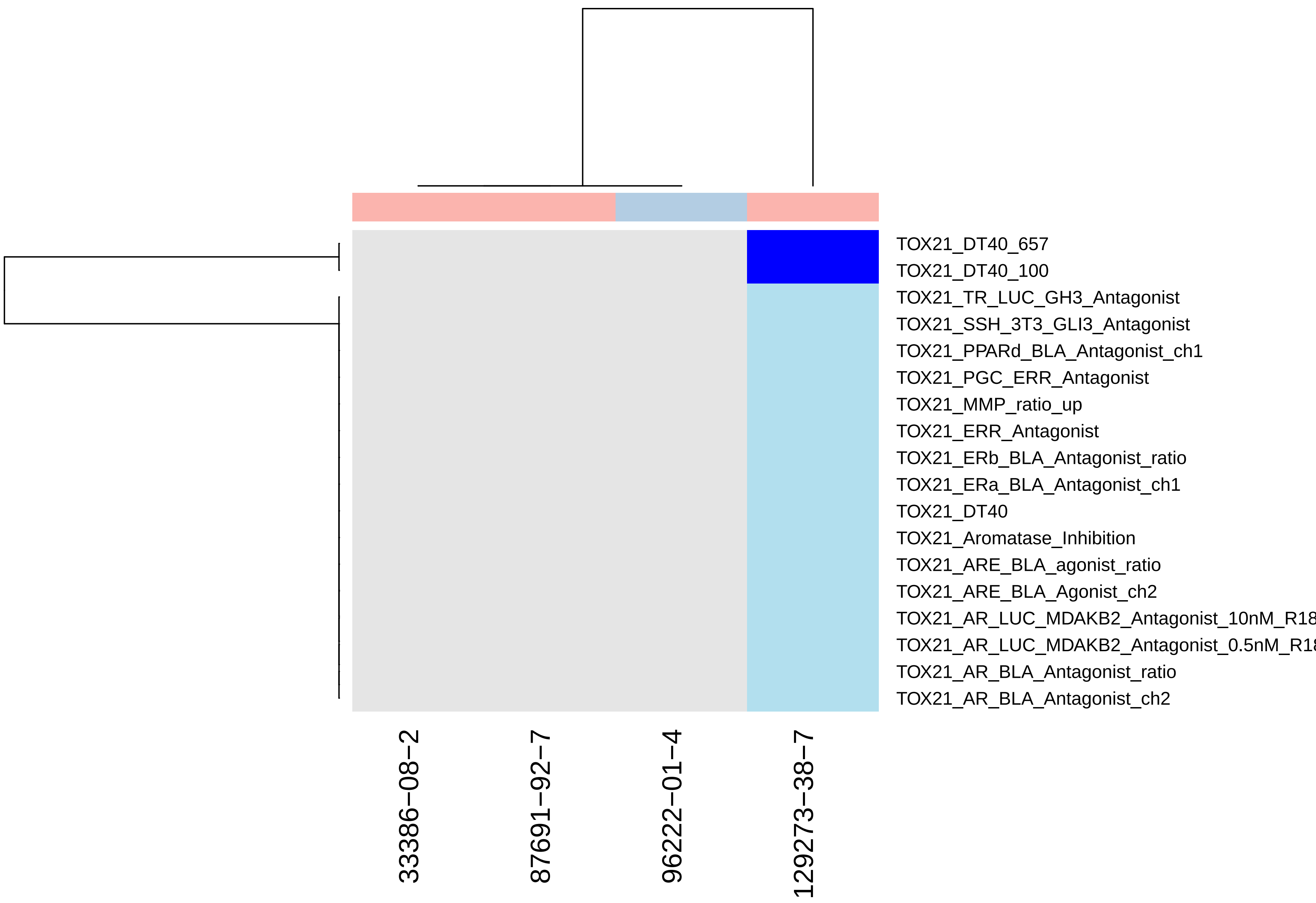

All Activity Heatmap for Cluster 931

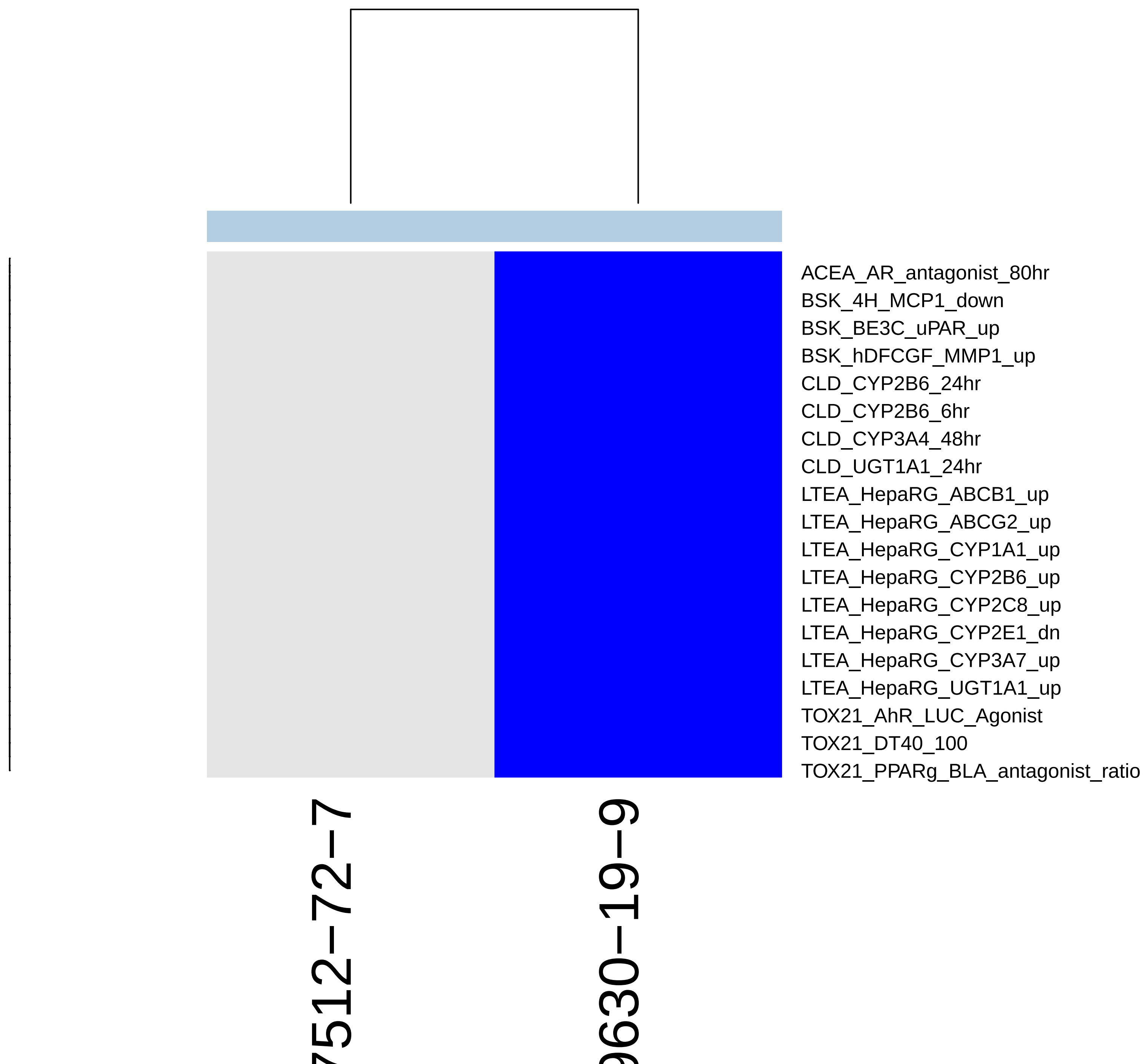

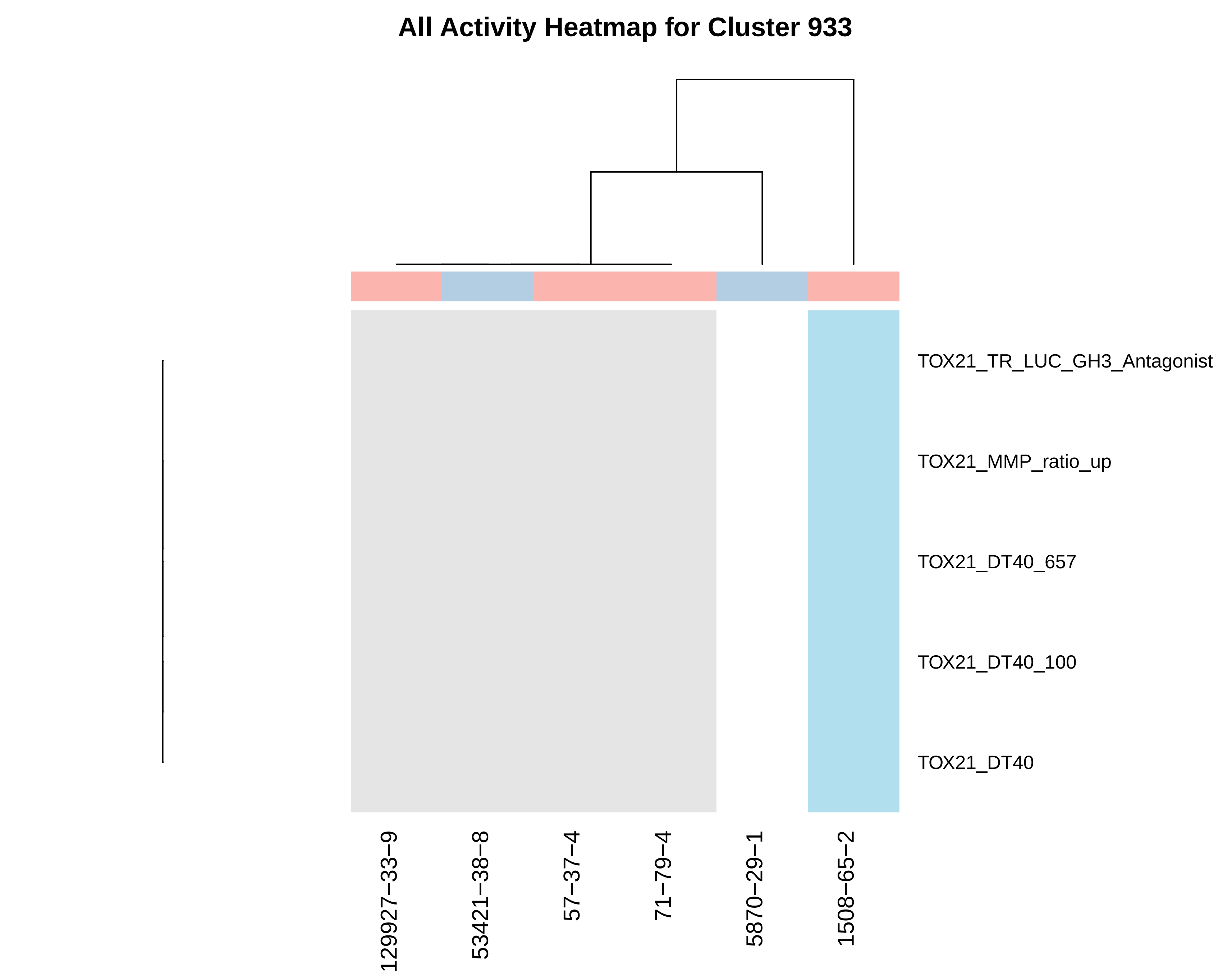
All Activity Heatmap for Cluster 933
TOX21_TR_LUC_GH3_Antagonist
TOX21_MMP_ratio_up
TOX21_DT40_657
TOX21_DT40_100
TOX21_DT40
129927−33−9
53421−38−8
57−37−4
71−79−4
5870−29−1
1508−65−2

All Activity Heatmap for Cluster 934

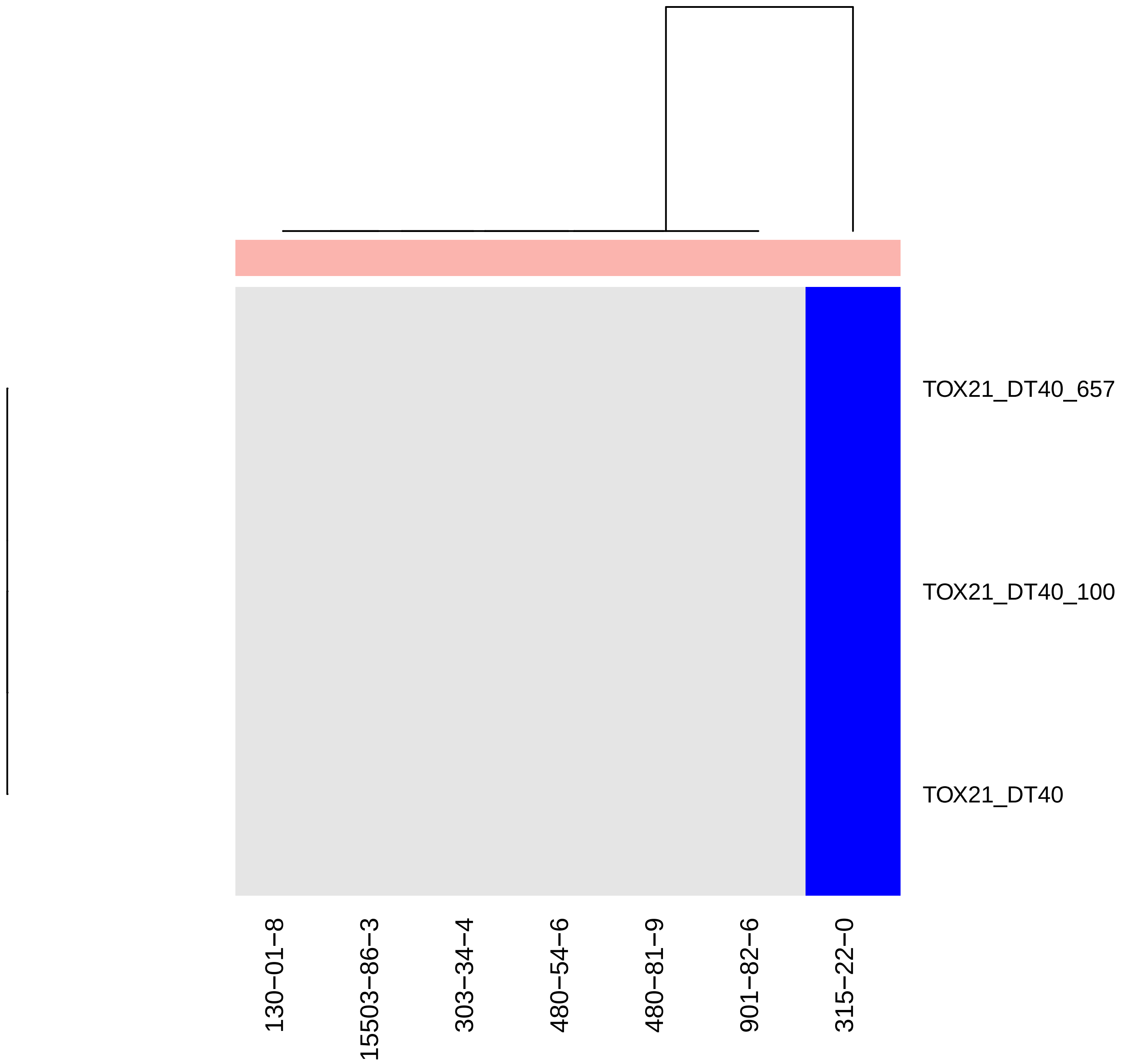

All Activity Heatmap for Cluster 935

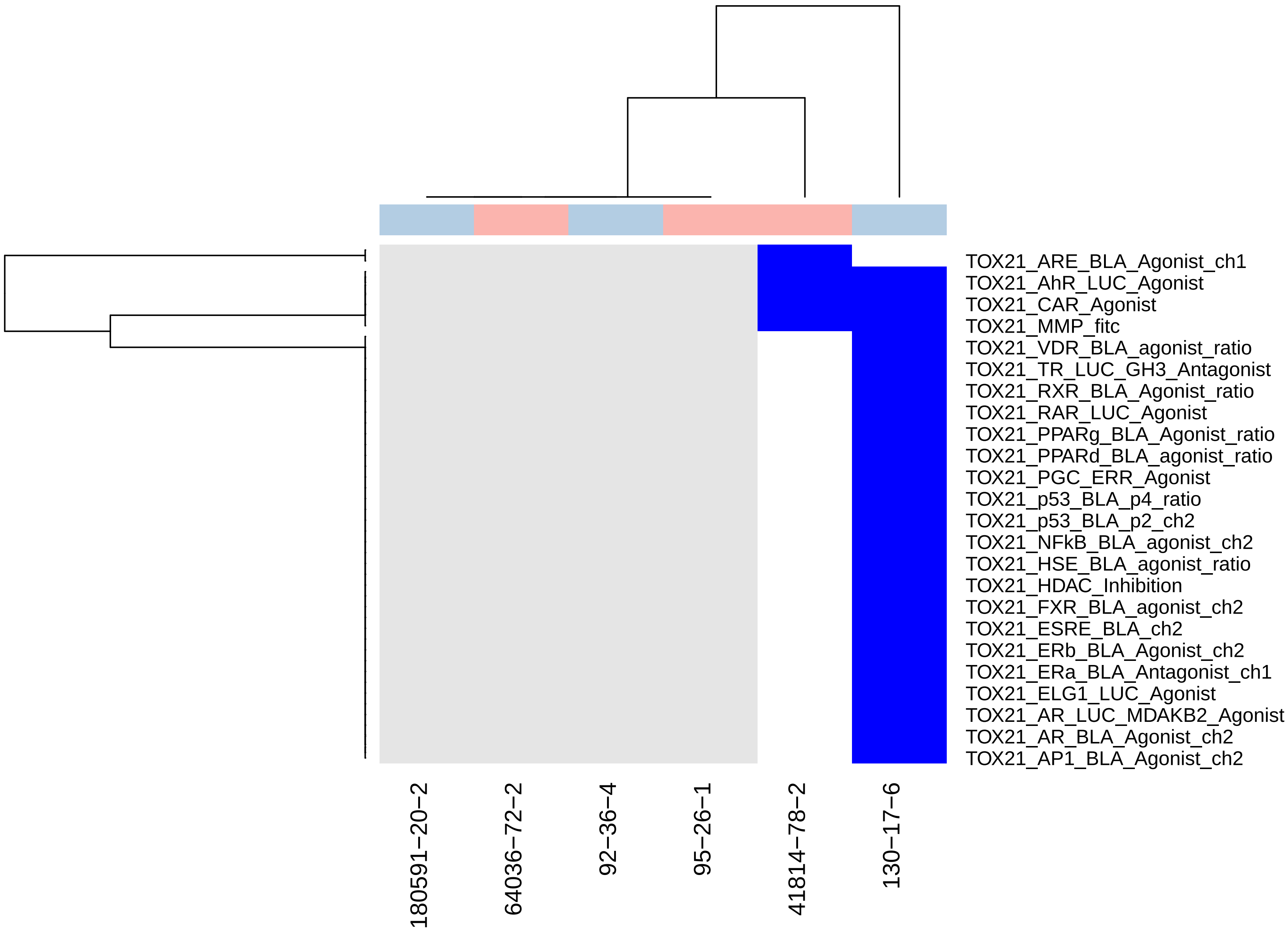

All Activity Heatmap for Cluster 936

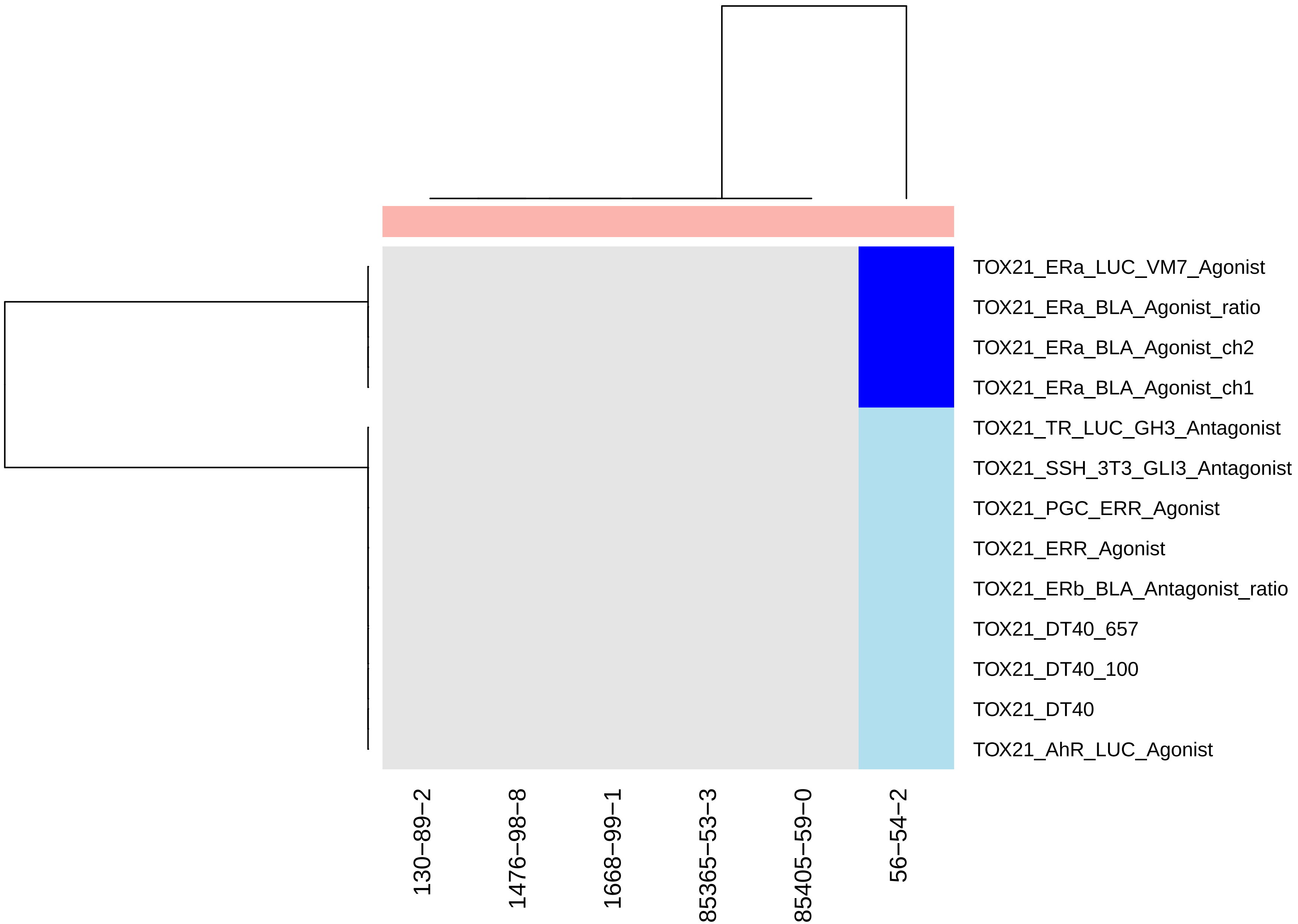

All Activity Heatmap for Cluster 937

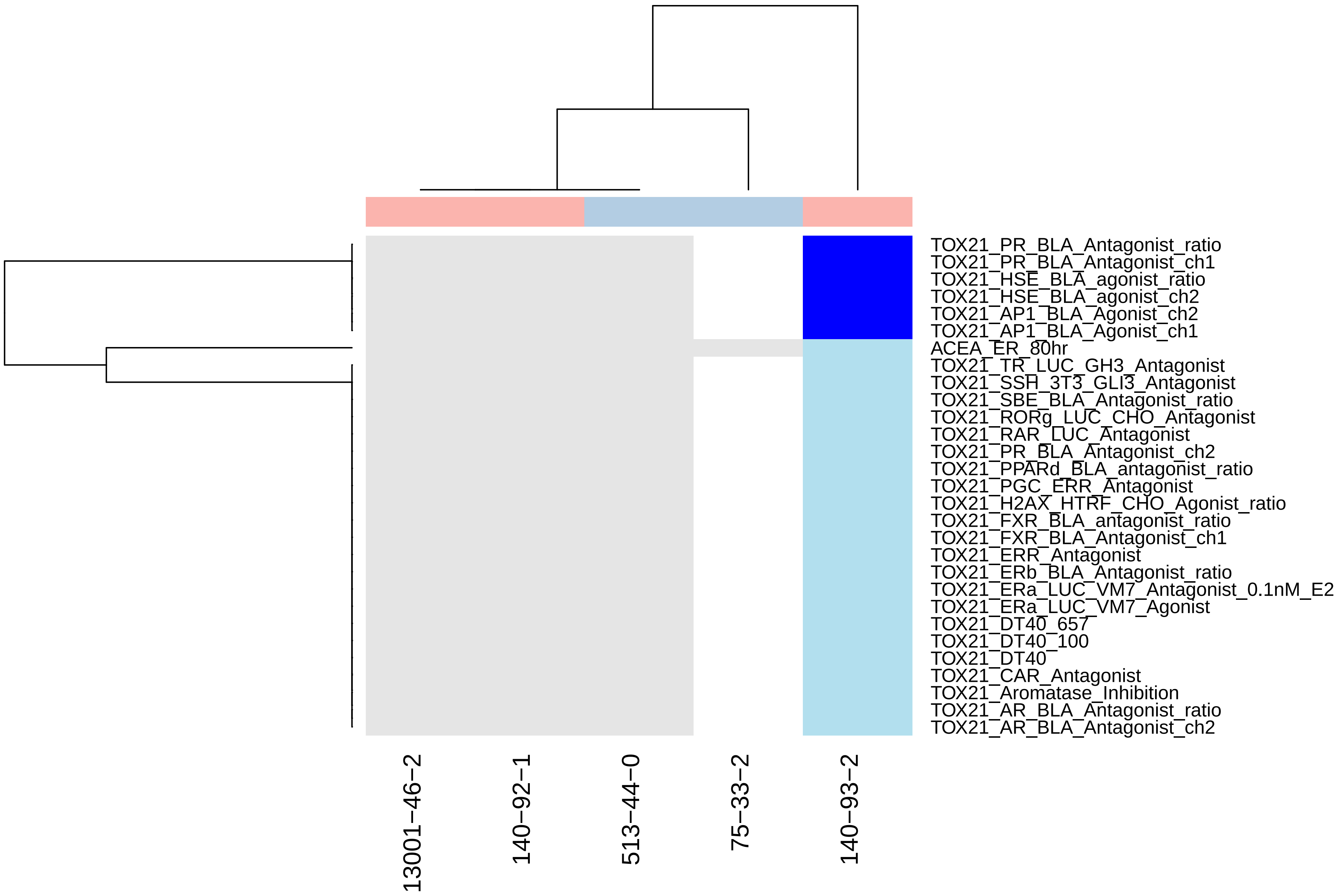

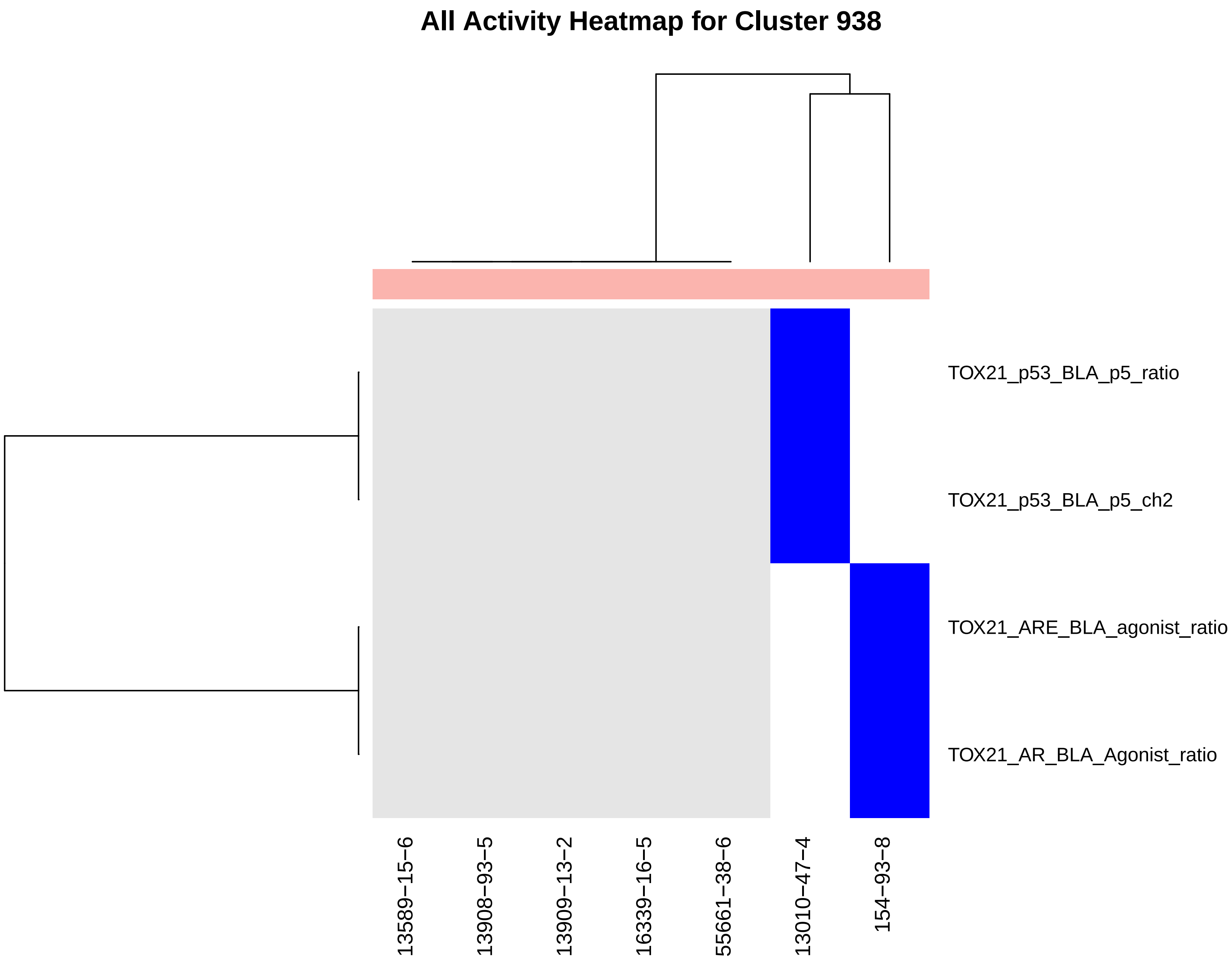
All Activity Heatmap for Cluster 938
TOX21_p53_BLA_p5_ratio
TOX21_p53_BLA_p5_ch2
TOX21_ARE_BLA_agonist_ratio
TOX21_AR_BLA_Agonist_ratio
13589−15−6
13908−93−5
13909−13−2
16339−16−5
55661−38−6
13010−47−4
154−93−8

All Activity Heatmap for Cluster 939

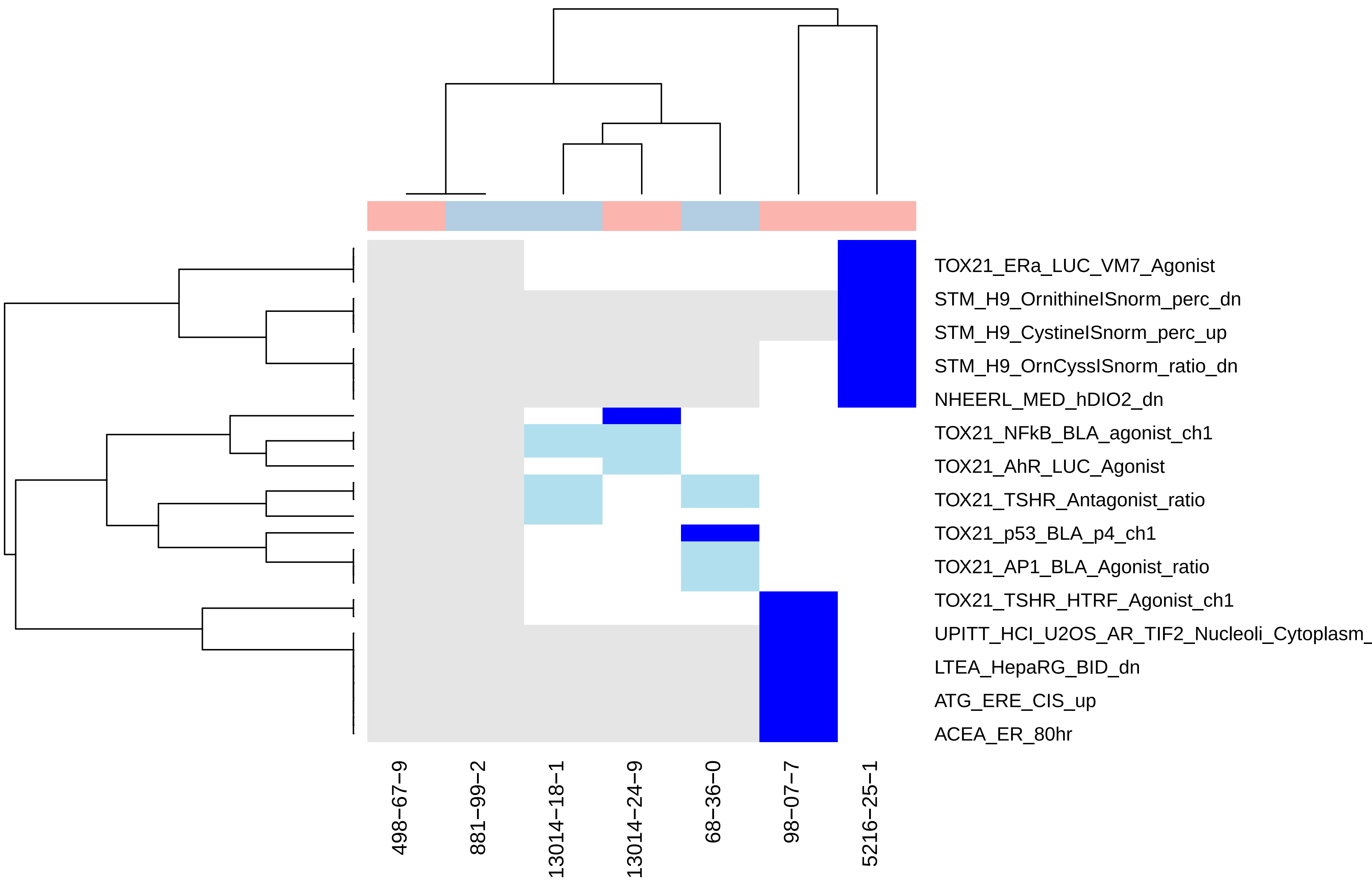

All Activity Heatmap for Cluster 941

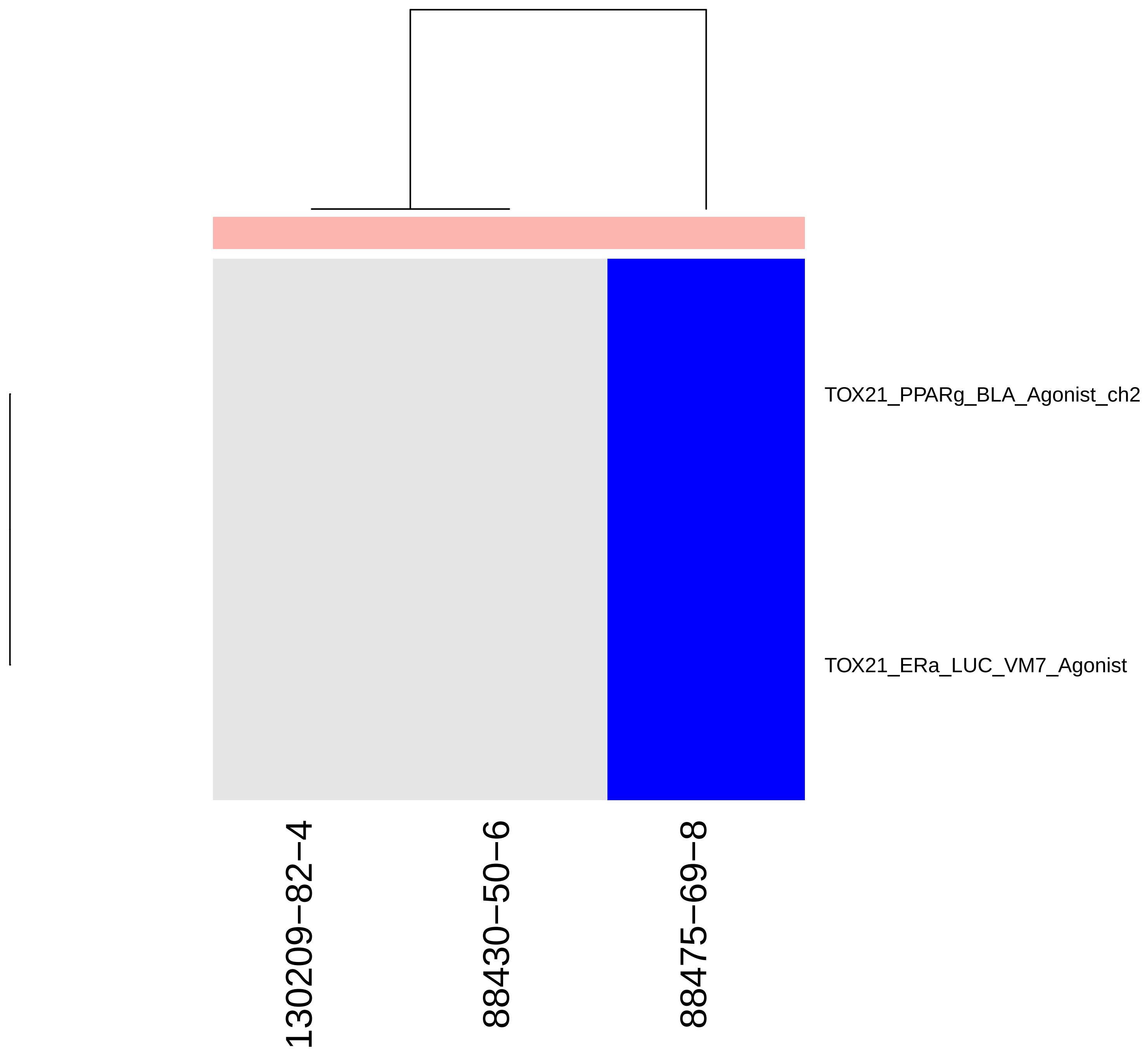

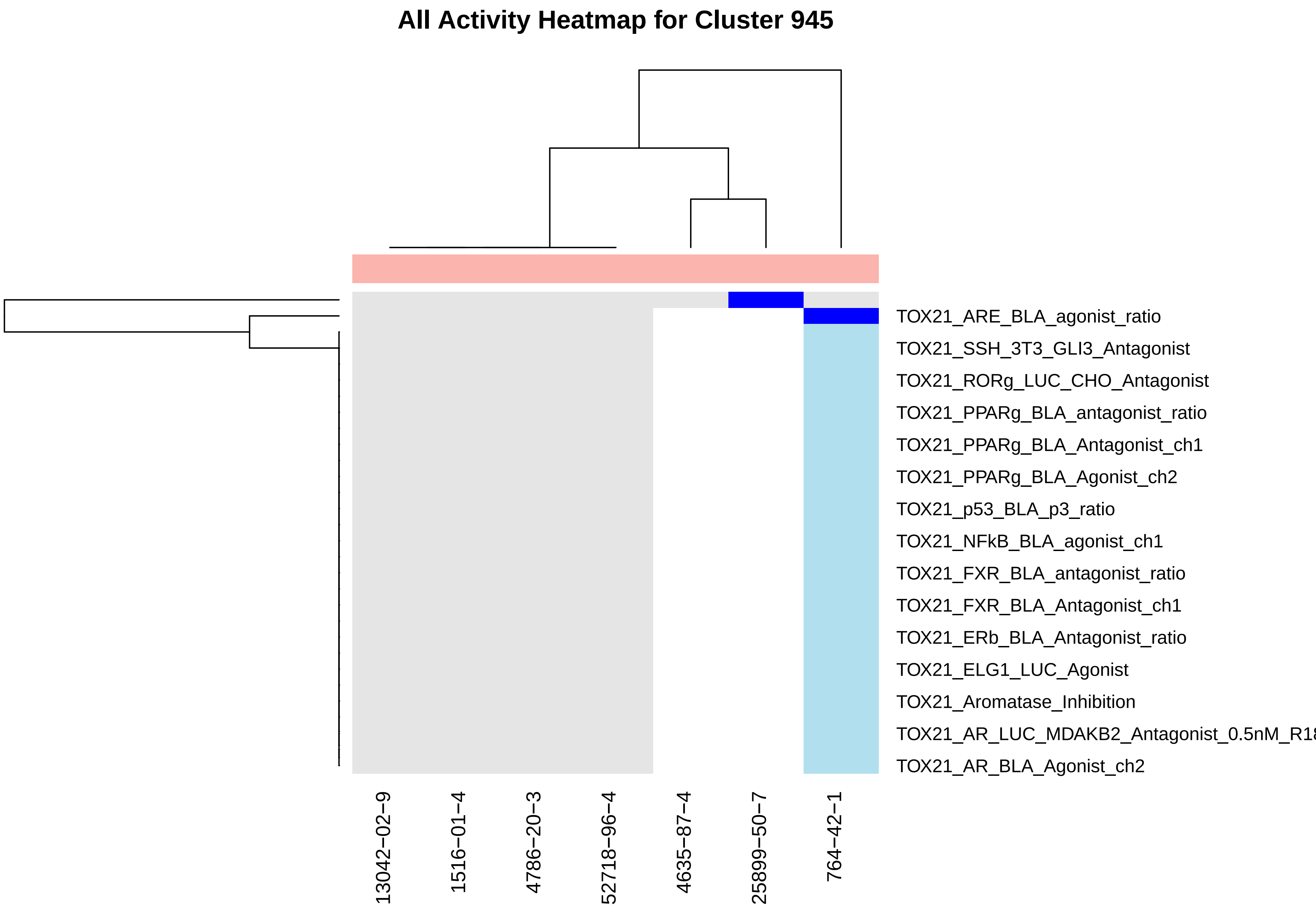
All Activity Heatmap for Cluster 945
TOX21_ARE_BLA_agonist_ratio
TOX21_SSH_3T3_GLI3_Antagonist
TOX21_RORg_LUC_CHO_Antagonist
TOX21_PPARg_BLA_antagonist_ratio
TOX21_PPARg_BLA_Antagonist_ch1
TOX21_PPARg_BLA_Agonist_ch2
TOX21_p53_BLA_p3_ratio
TOX21_NFkB_BLA_agonist_ch1
TOX21_FXR_BLA_antagonist_ratio
TOX21_FXR_BLA_Antagonist_ch1
TOX21_ERb_BLA_Antagonist_ratio
TOX21_ELG1_LUC_Agonist
TOX21_Aromatase_Inhibition
TOX21_AR_LUC_MDAKB2_Antagonist_0.5nM_R1
TOX21_AR_BLA_Agonist_ch2
13042−02−9
1516−01−4
4786−20−3
52718−96−4
4635−87−4
25899−50−7
764−42−1

All Activity Heatmap for Cluster 947

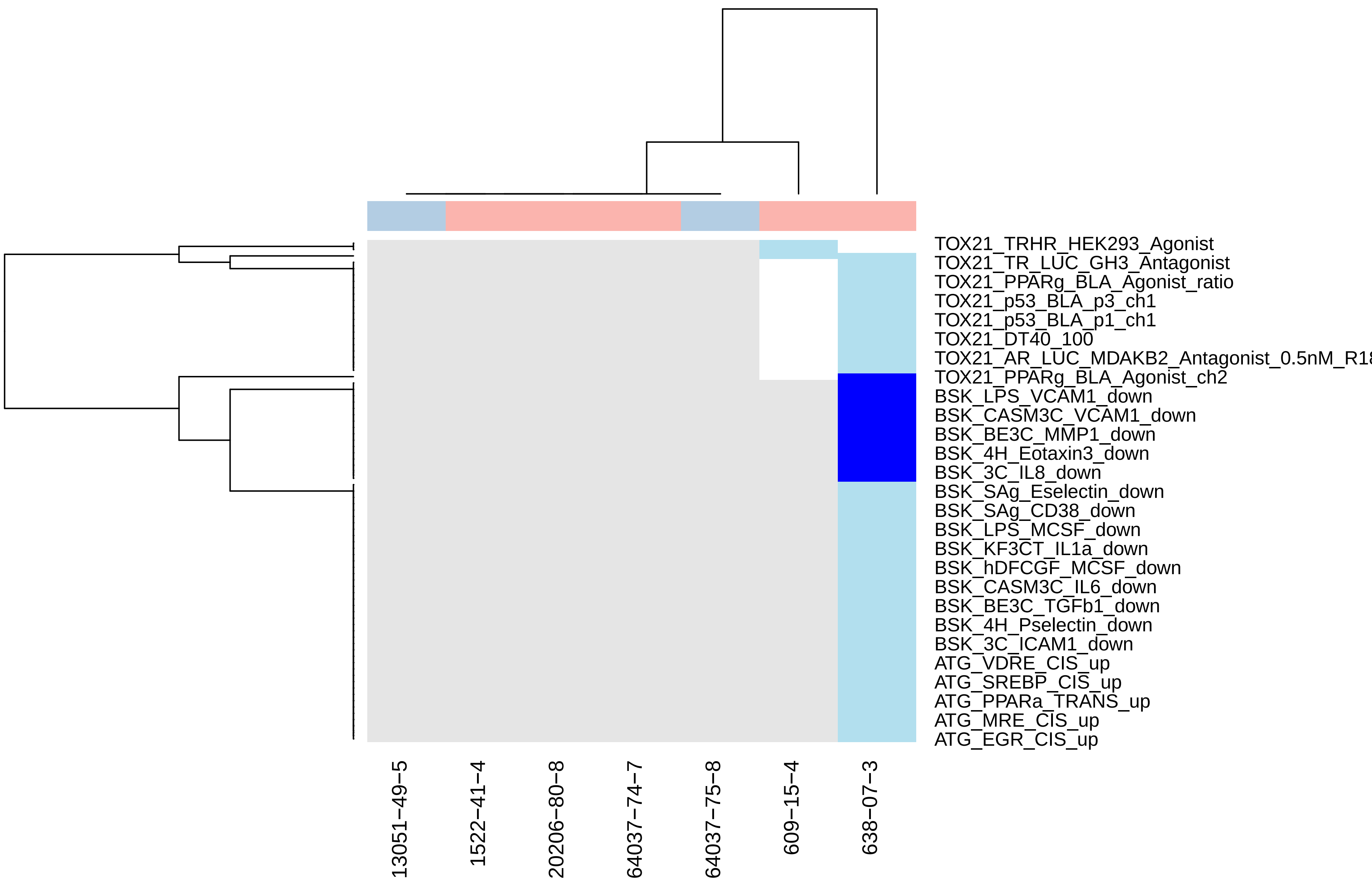

All Activity Heatmap for Cluster 948

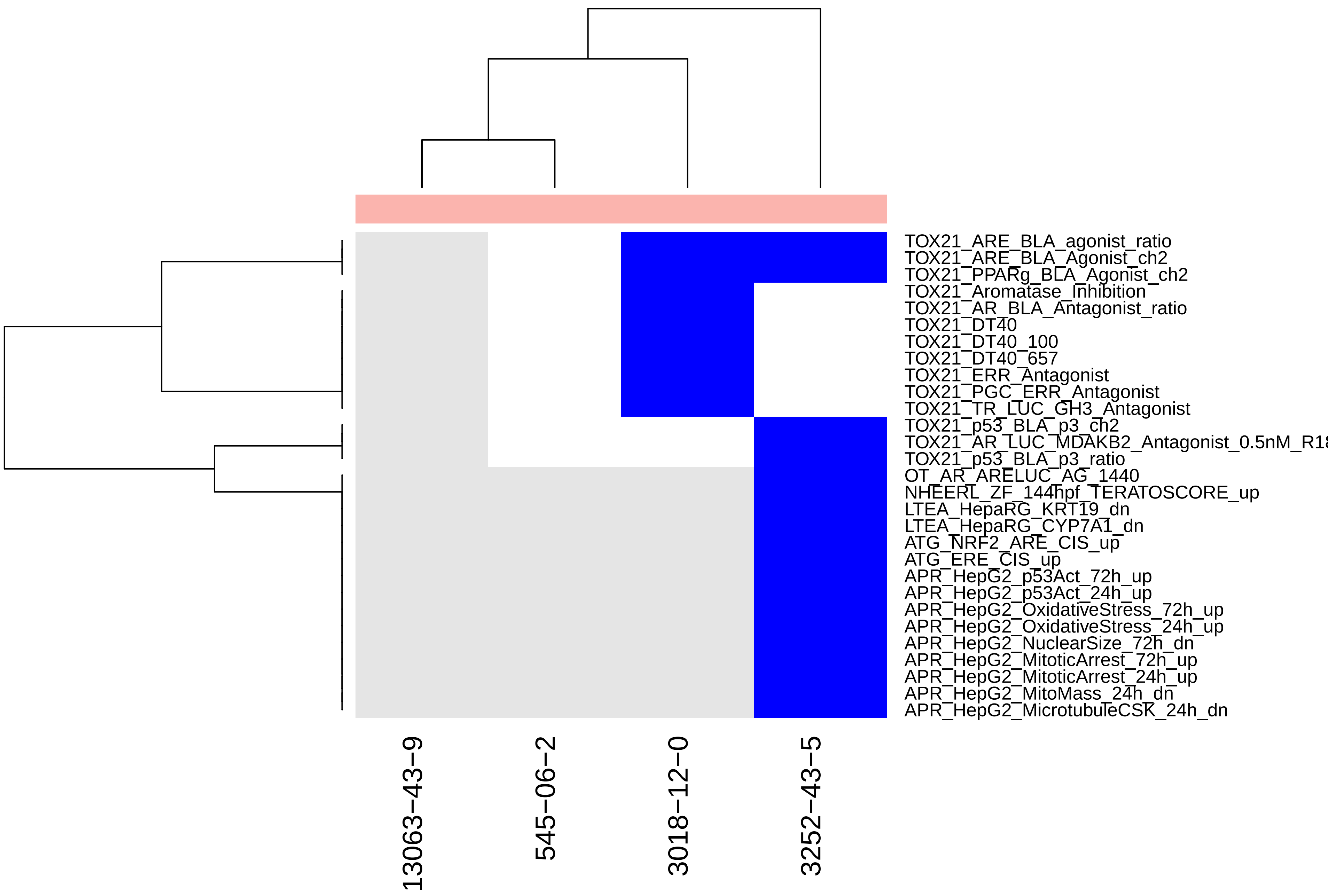

All Activity Heatmap for Cluster 949

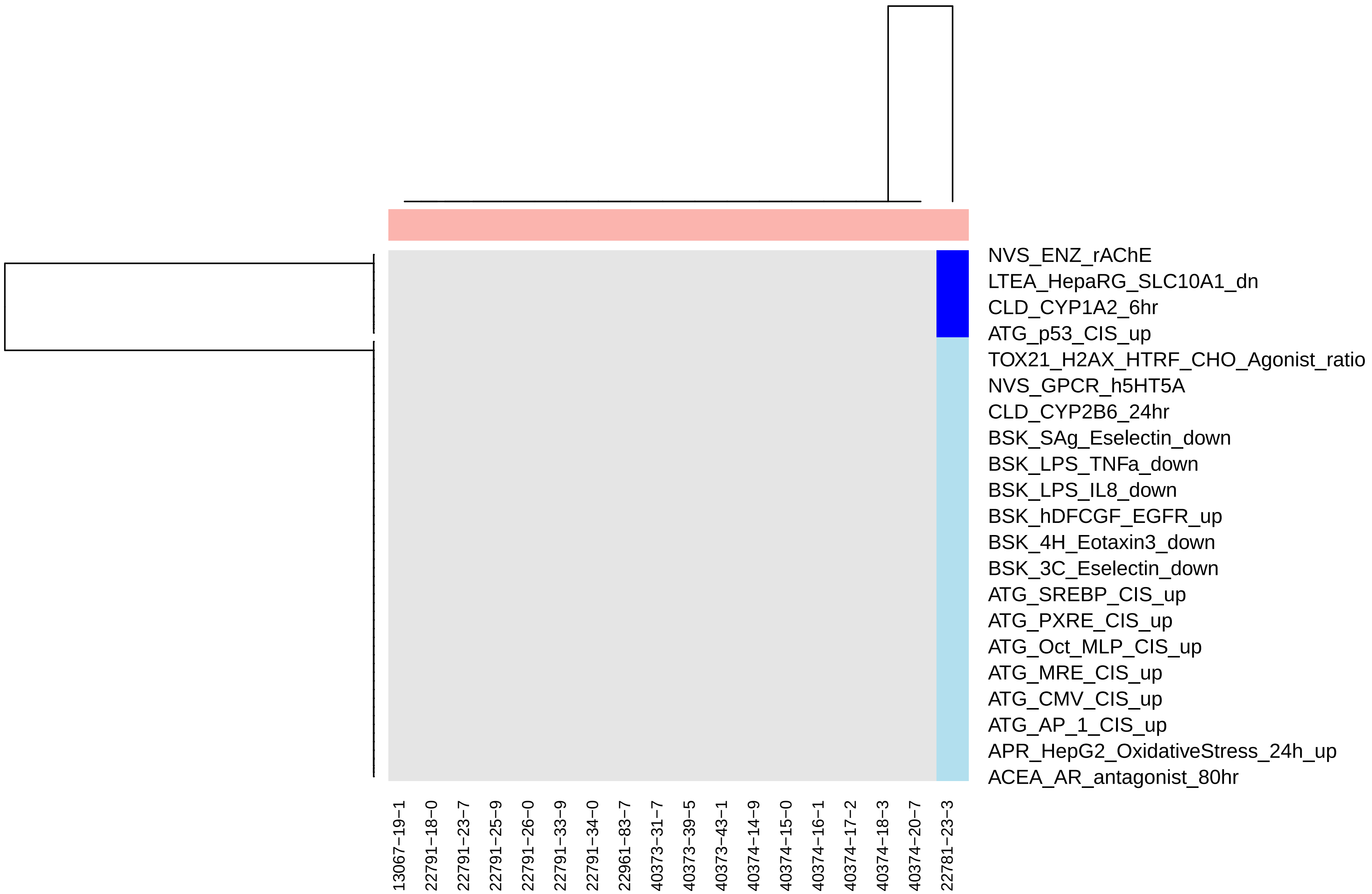

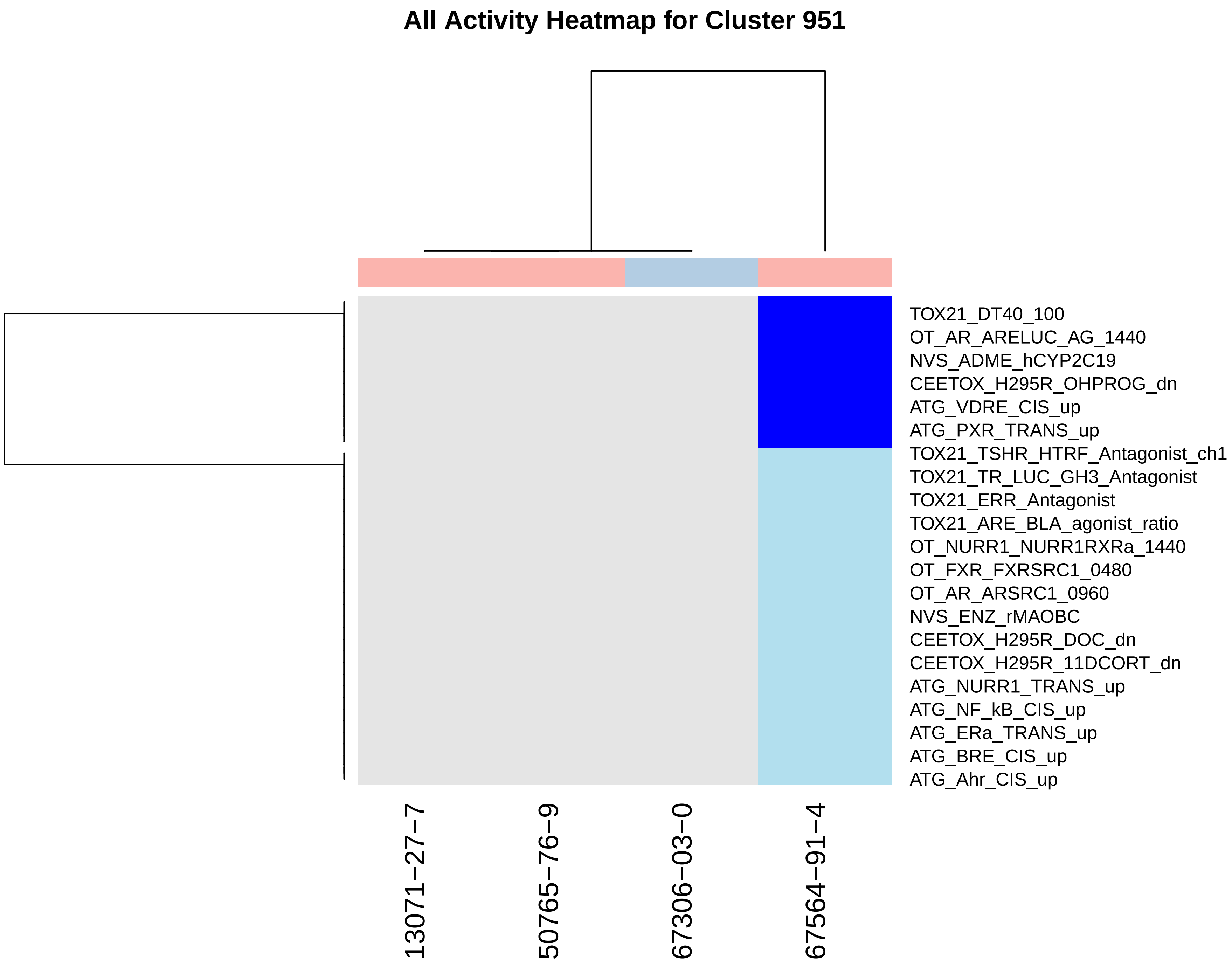
All Activity Heatmap for Cluster 951
TOX21_DT40_100
OT_AR_ARELUC_AG_1440
NVS_ADME_hCYP2C19
CEETOX_H295R_OHPROG_dn
ATG_VDRE_CIS_up
ATG_PXR_TRANS_up
TOX21_TSHR_HTRF_Antagonist_ch1
TOX21_TR_LUC_GH3_Antagonist
TOX21_ERR_Antagonist
TOX21_ARE_BLA_agonist_ratio
OT_NURR1_NURR1RXRa_1440
OT_FXR_FXRSRC1_0480
OT_AR_ARSRC1_0960
NVS_ENZ_rMAOBC
CEETOX_H295R_DOC_dn
CEETOX_H295R_11DCORT_dn
ATG_NURR1_TRANS_up
ATG_NF_kB_CIS_up
ATG_ERa_TRANS_up
ATG_BRE_CIS_up
ATG_Ahr_CIS_up
13071−27−7
50765−76−9
67306−03−0
67564−91−4

All Activity Heatmap for Cluster 952

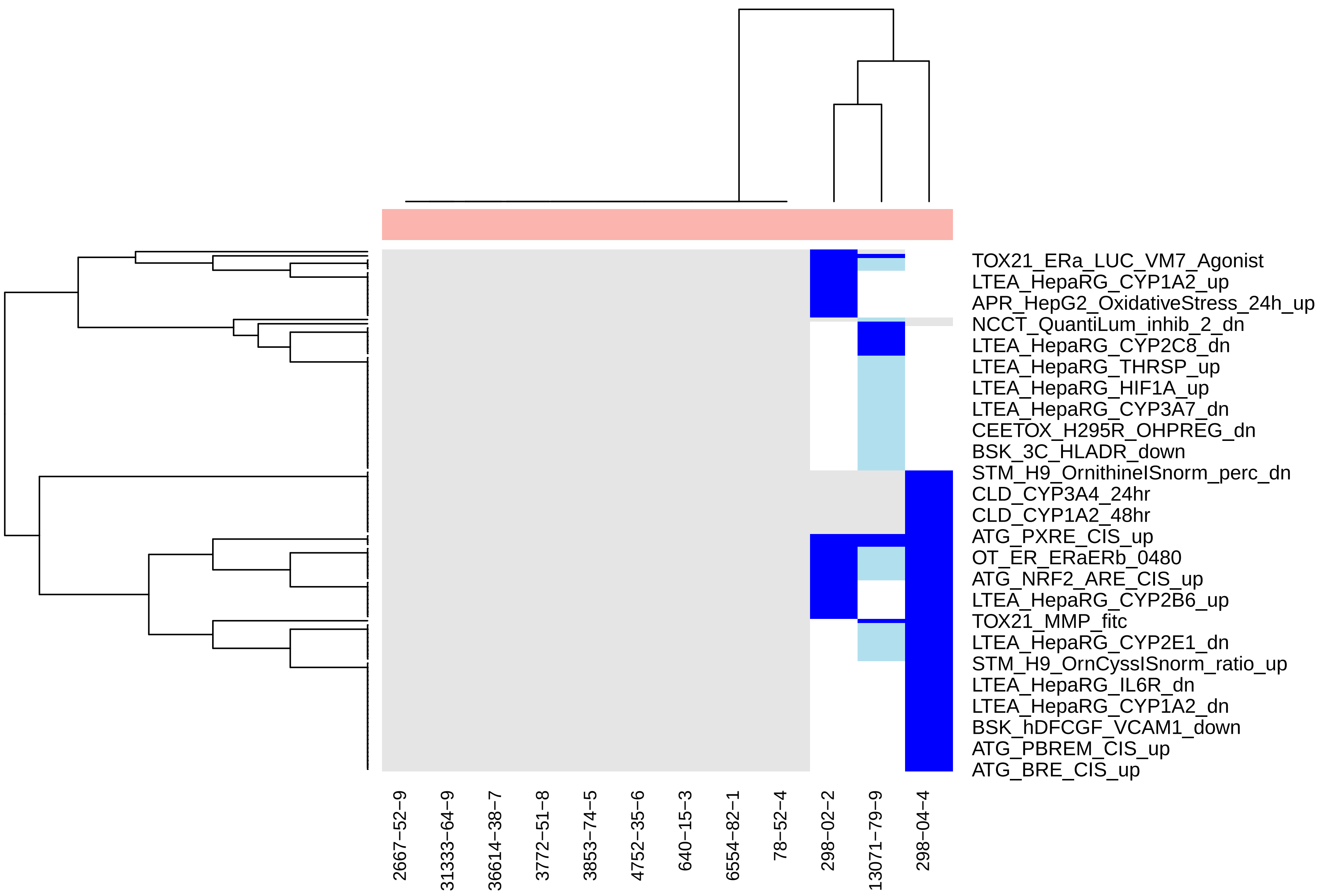

All Activity Heatmap for Cluster 953

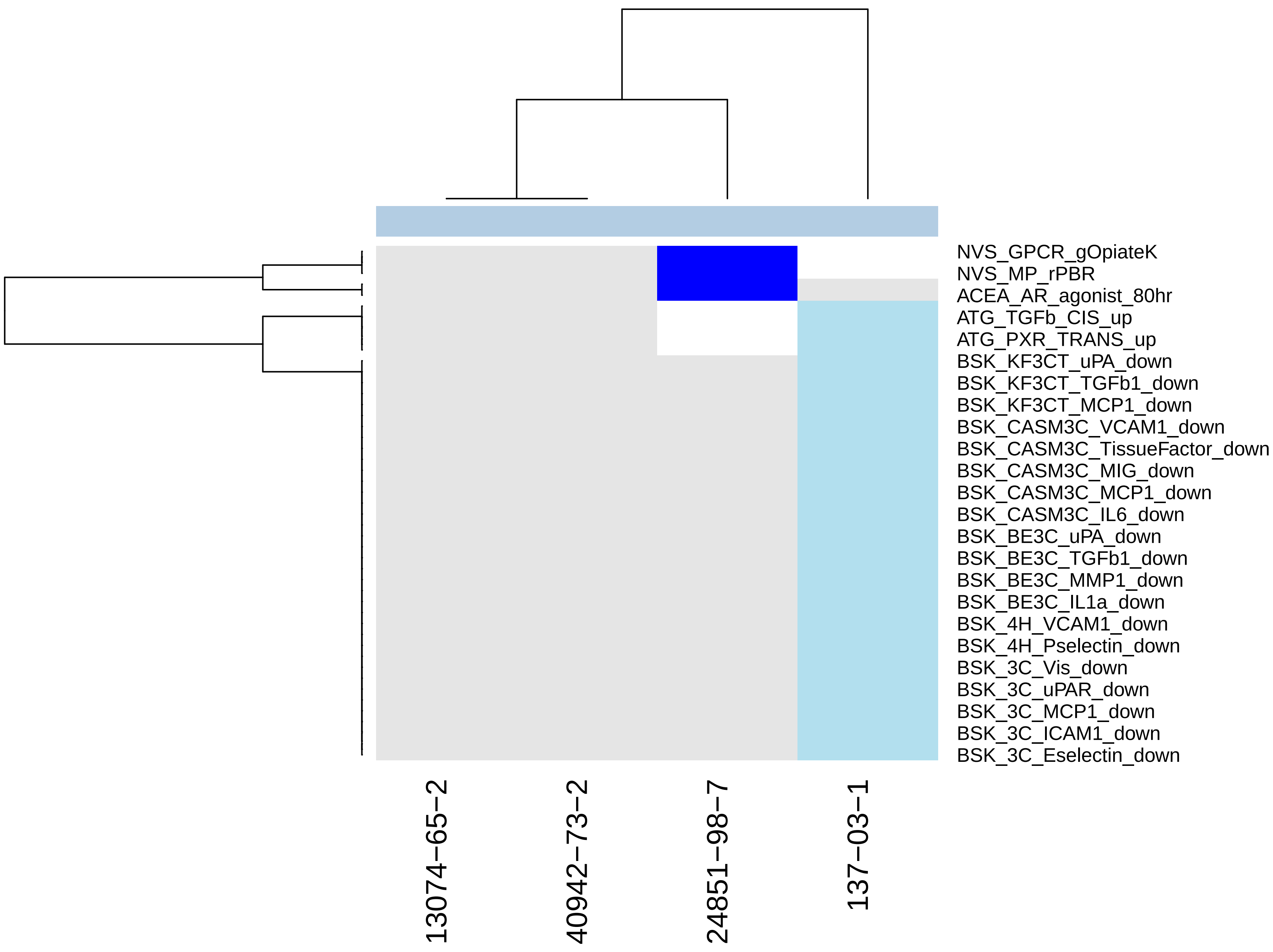

All Activity Heatmap for Cluster 954

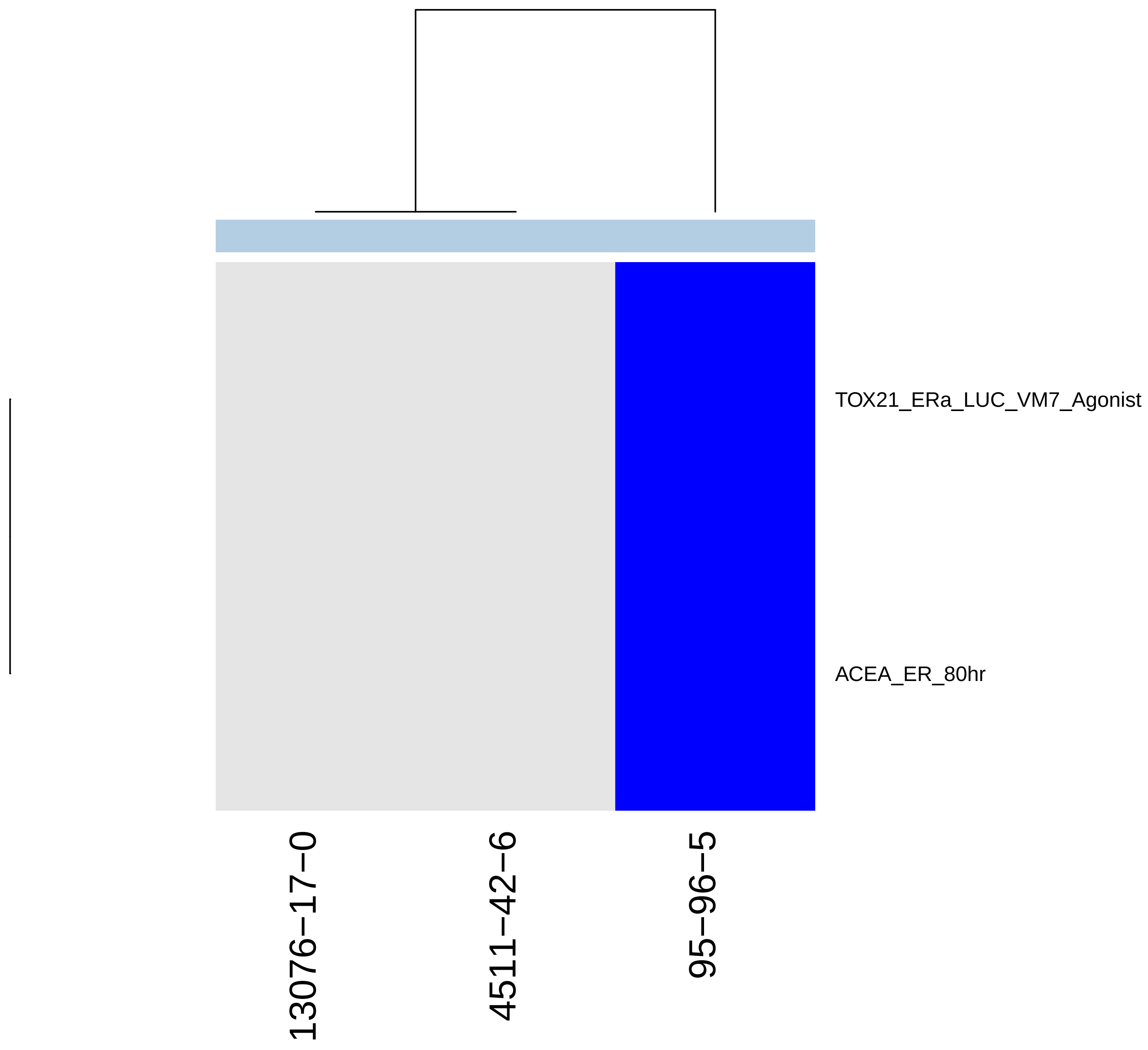

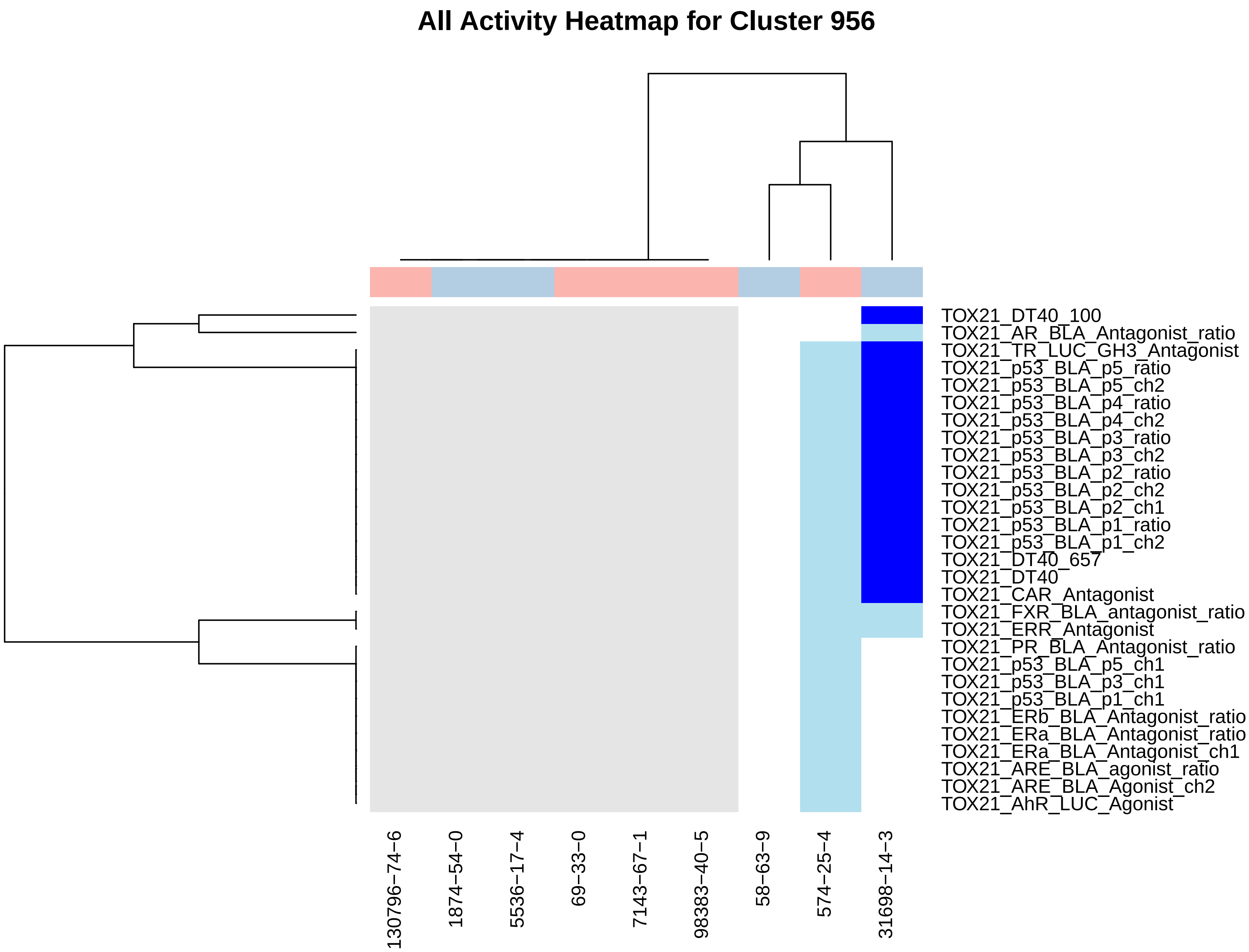

All Activity Heatmap for Cluster 956
TOX21_DT40_100
TOX21_AR_BLA_Antagonist_ratio
TOX21_TR_LUC_GH3_Antagonist
TOX21_p53_BLA_p5_ratio
TOX21_p53_BLA_p5_ch2
TOX21_p53_BLA_p4_ratio
TOX21_p53_BLA_p4_ch2
TOX21_p53_BLA_p3_ratio
TOX21_p53_BLA_p3_ch2
TOX21_p53_BLA_p2_ratio
TOX21_p53_BLA_p2_ch2
TOX21_p53_BLA_p2_ch1
TOX21_p53_BLA_p1_ratio
TOX21_p53_BLA_p1_ch2
TOX21_DT40_657
TOX21_DT40
TOX21_CAR_Antagonist
TOX21_FXR_BLA_antagonist_ratio
TOX21_ERR_Antagonist
TOX21_PR_BLA_Antagonist_ratio
TOX21_p53_BLA_p5_ch1
TOX21_p53_BLA_p3_ch1
TOX21_p53_BLA_p1_ch1
TOX21_ERb_BLA_Antagonist_ratio
TOX21_ERa_BLA_Antagonist_ratio
TOX21_ERa_BLA_Antagonist_ch1
TOX21_ARE_BLA_agonist_ratio
TOX21_ARE_BLA_Agonist_ch2
TOX21_AhR_LUC_Agonist
130796−74−6
1874−54−0
5536−17−4
69−33−0
7143−67−1
98383−40−5
58−63−9
574−25−4
31698−14−3

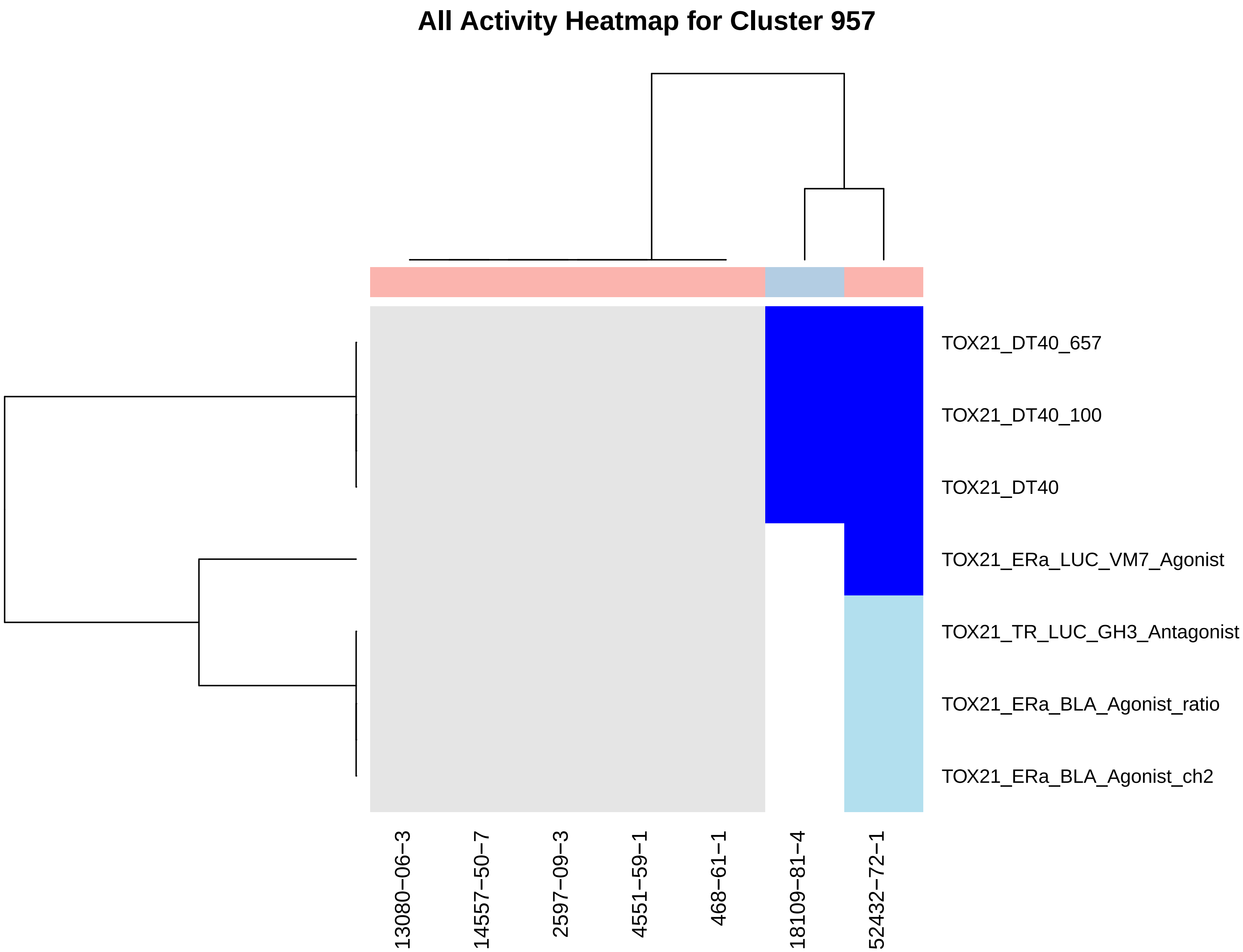

All Activity Heatmap for Cluster 957
TOX21_DT40_657
TOX21_DT40_100
TOX21_DT40
TOX21_ERa_LUC_VM7_Agonist
TOX21_TR_LUC_GH3_Antagonist
TOX21_ERa_BLA_Agonist_ratio
TOX21_ERa_BLA_Agonist_ch2
13080−06−3
14557−50−7
2597−09−3
4551−59−1
468−61−1
18109−81−4
52432−72−1

All Activity Heatmap for Cluster 958

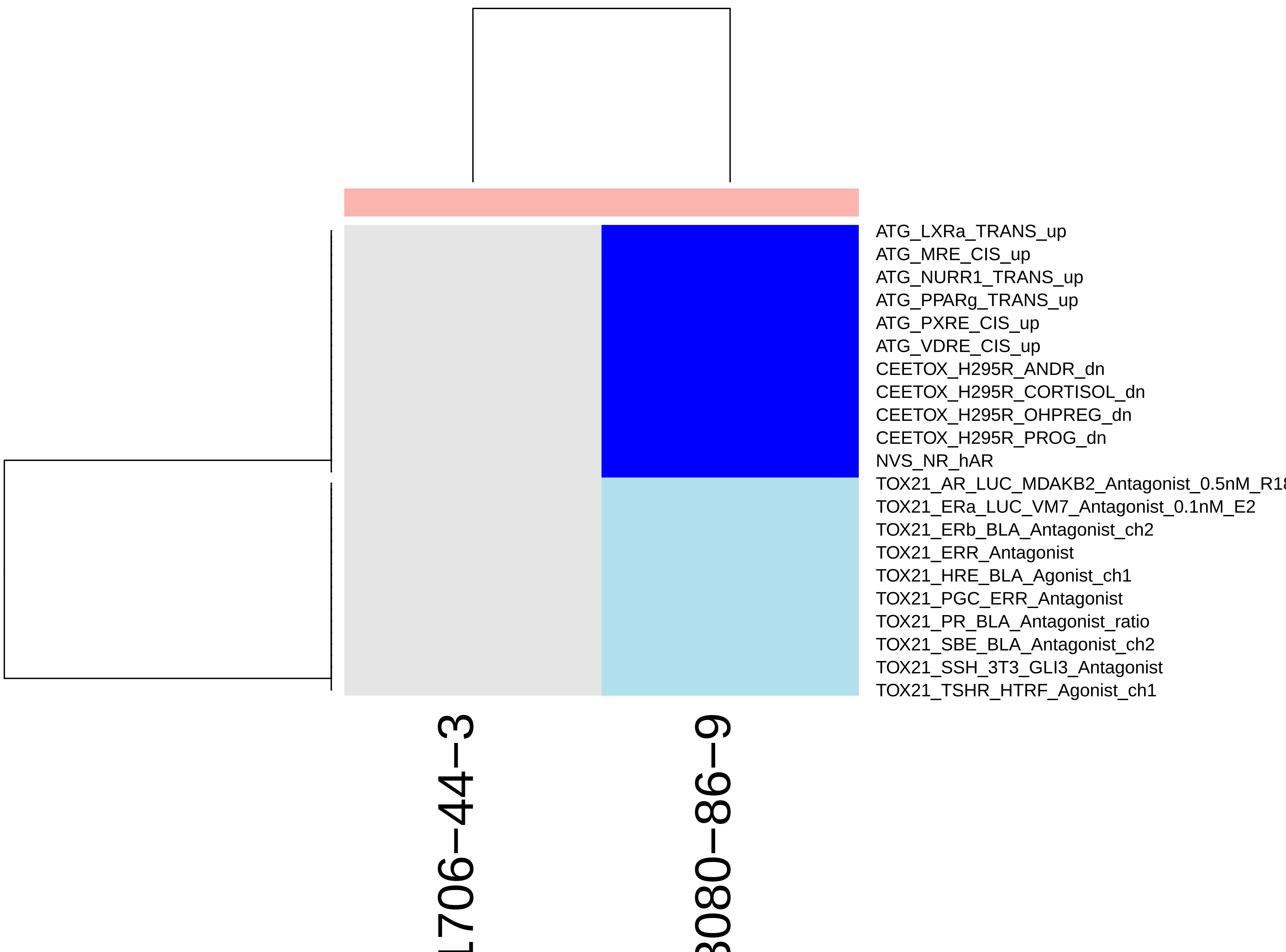

All Activity Heatmap for Cluster 959

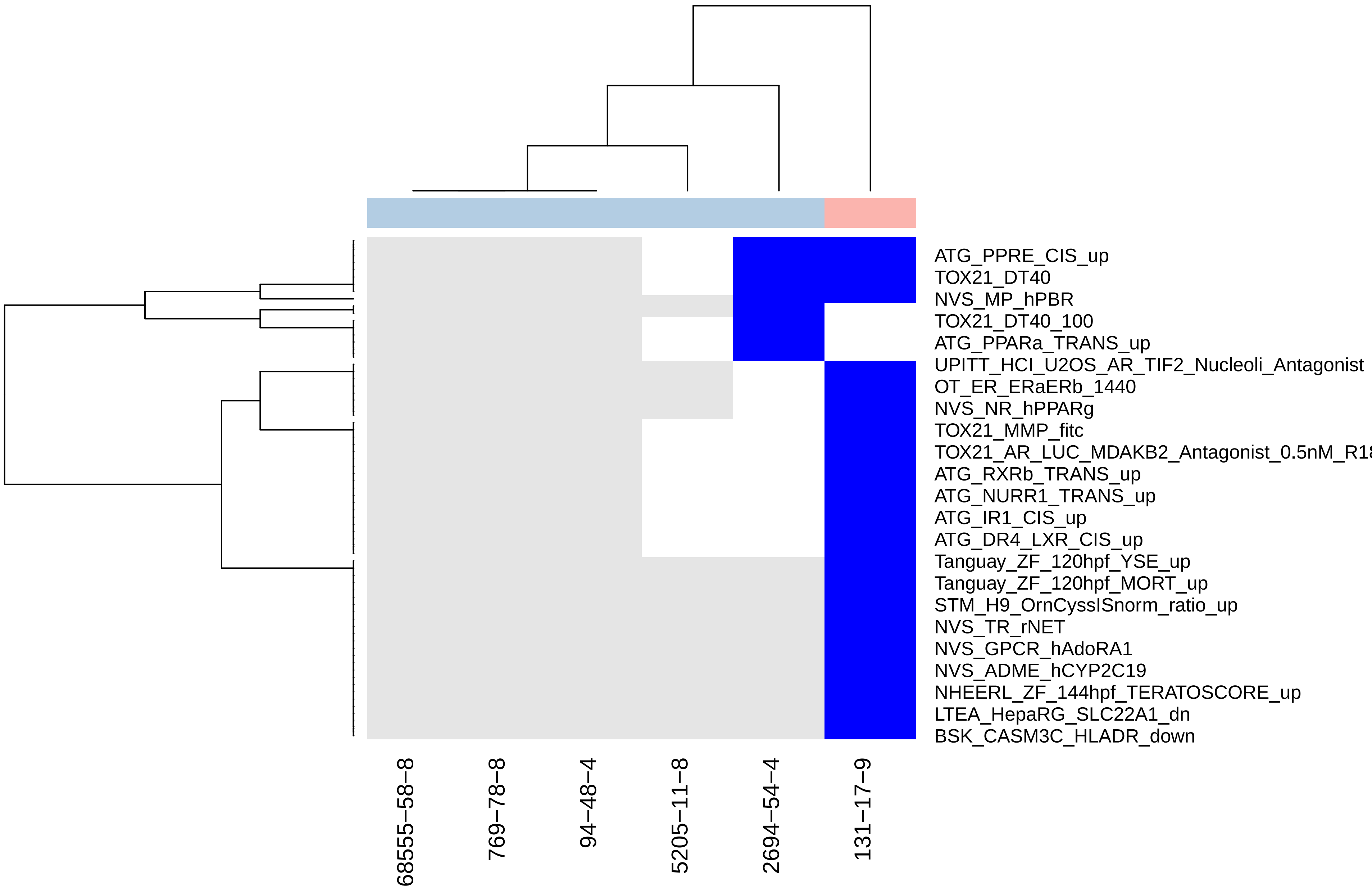

All Activity Heatmap for Cluster 960

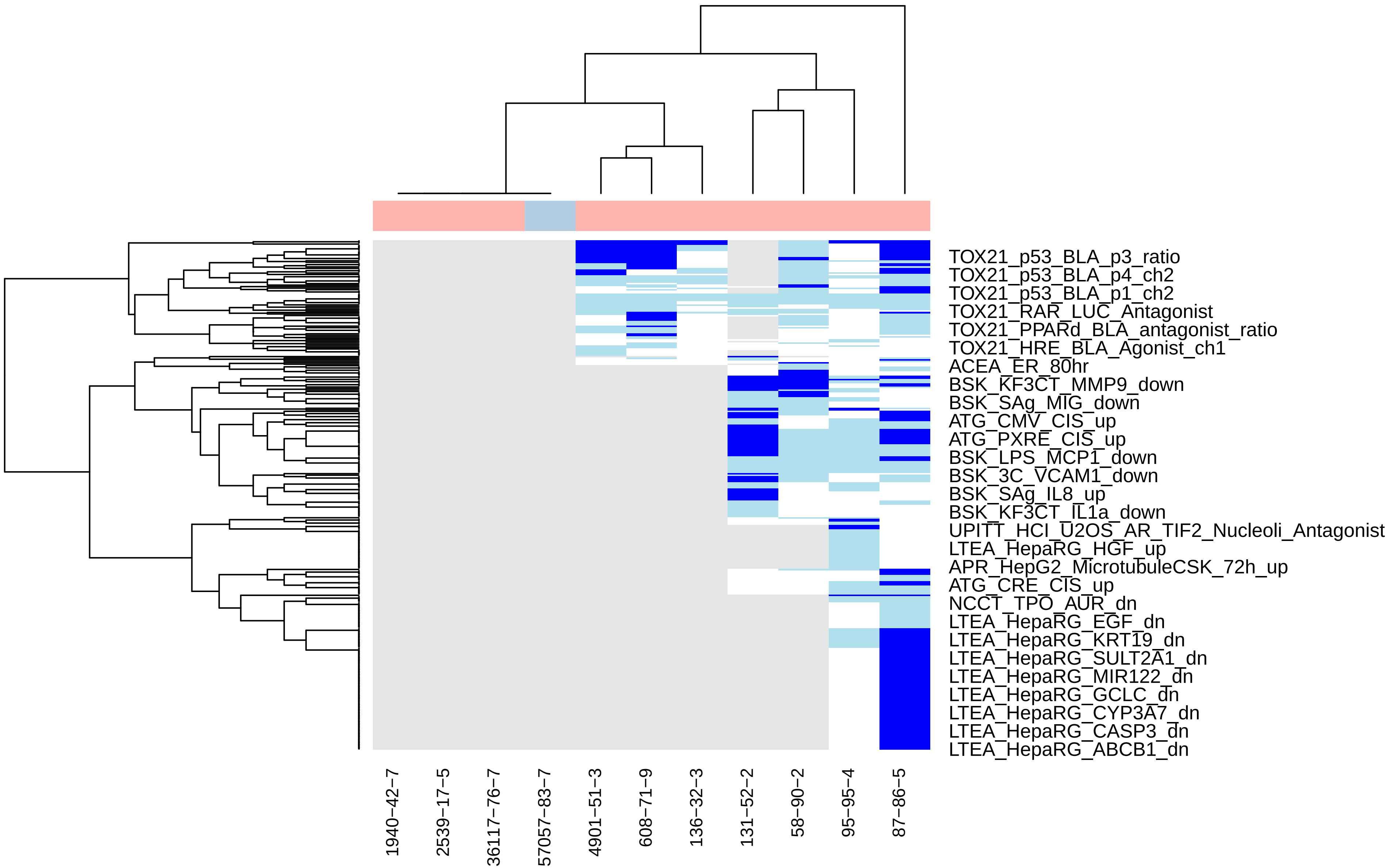

All Activity Heatmap for Cluster 961

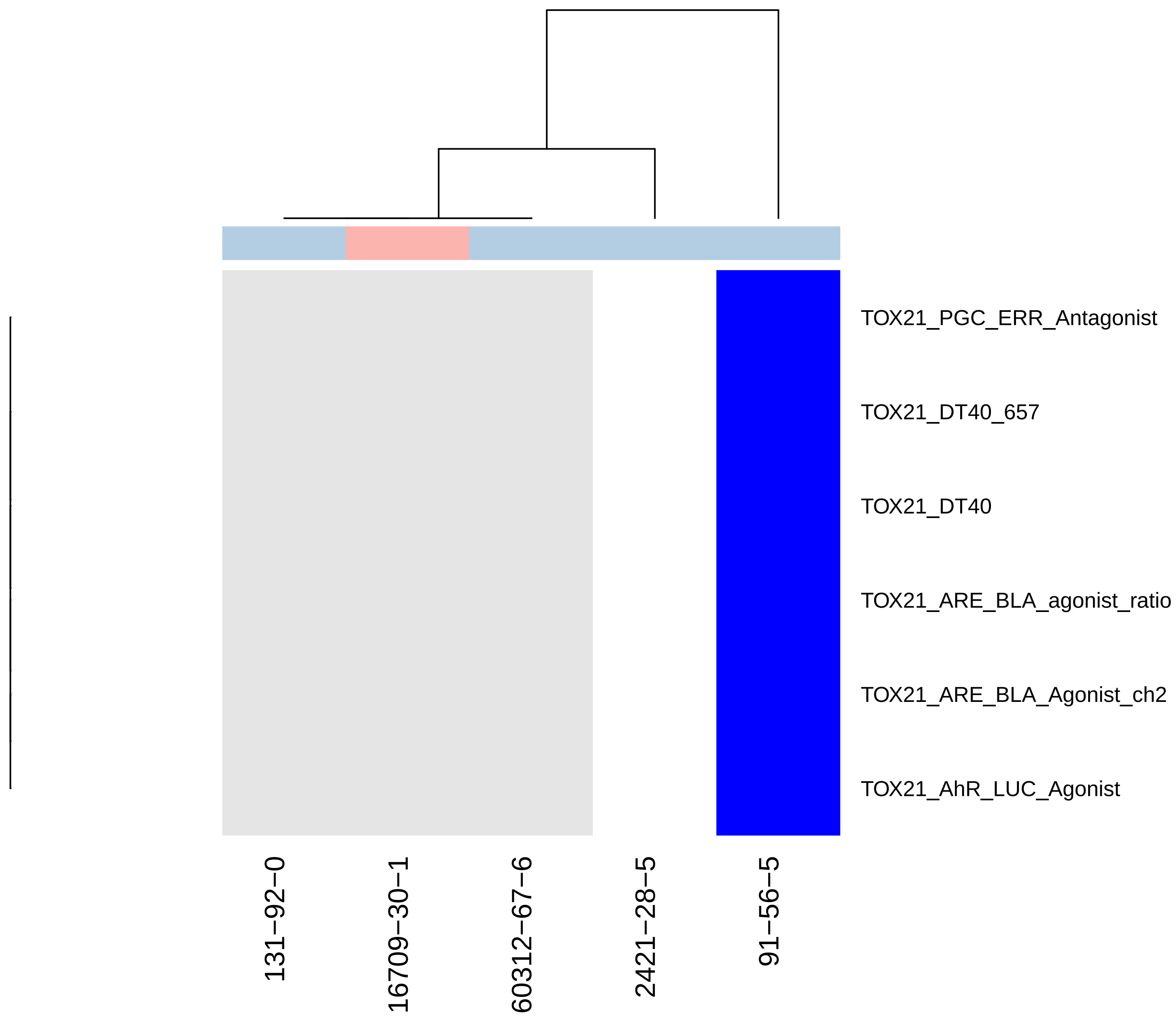

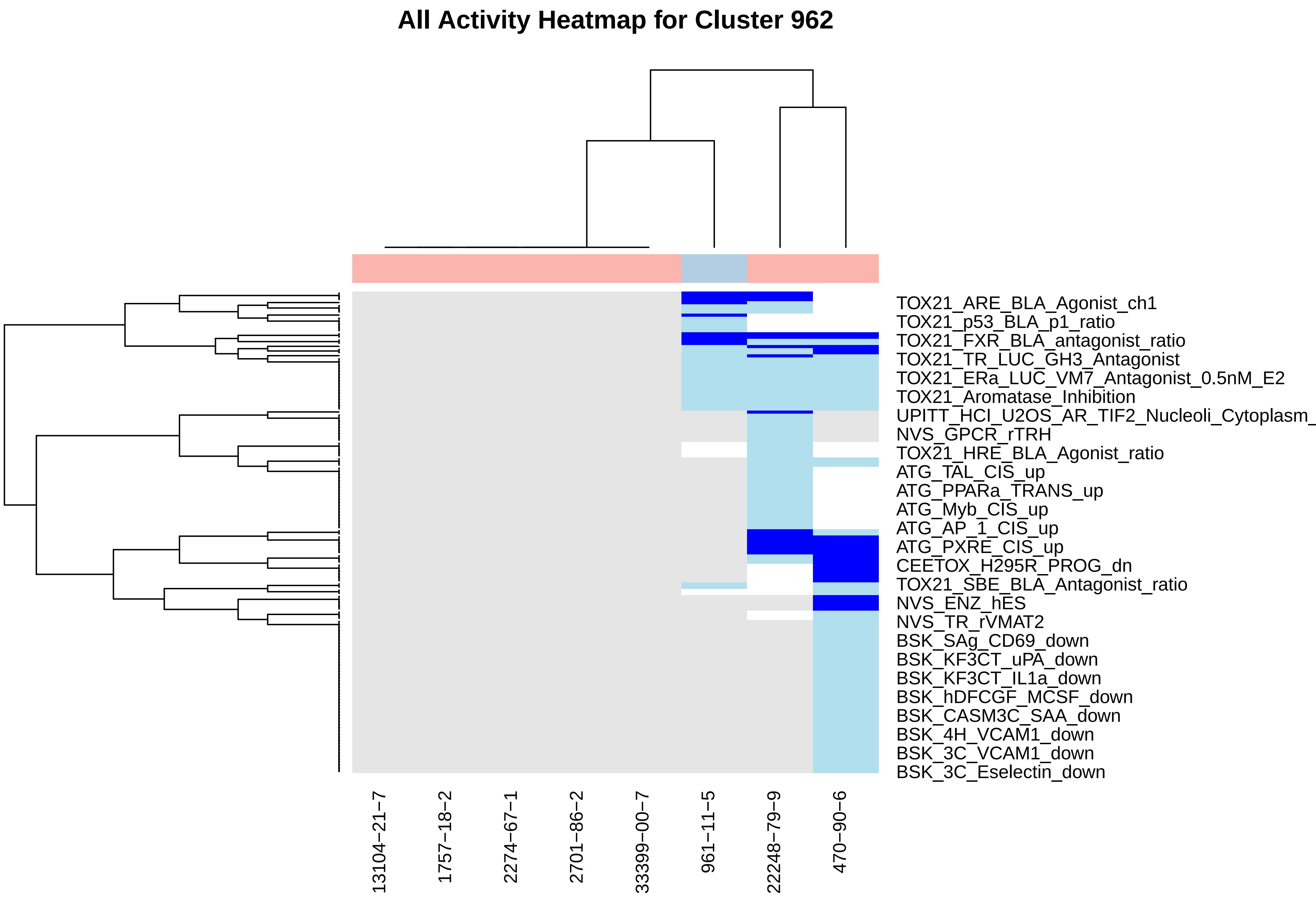
All Activity Heatmap for Cluster 962
TOX21_ARE_BLA_Agonist_ch1
TOX21_p53_BLA_p1_ratio
TOX21_FXR_BLA_antagonist_ratio
TOX21_TR_LUC_GH3_Antagonist
TOX21_ERa_LUC_VM7_Antagonist_0.5nM_E2
TOX21_Aromatase_Inhibition
UPITT_HCI_U2OS_AR_TIF2_Nucleoli_Cytoplasm_
NVS_GPCR_rTRH
TOX21_HRE_BLA_Agonist_ratio
ATG_TAL_CIS_up
ATG_PPARa_TRANS_up
ATG_Myb_CIS_up
ATG_AP_1_CIS_up
ATG_PXRE_CIS_up
CEETOX_H295R_PROG_dn
TOX21_SBE_BLA_Antagonist_ratio
NVS_ENZ_hES
NVS_TR_rVMAT2
BSK_SAg_CD69_down
BSK_KF3CT_uPA_down
BSK_KF3CT_IL1a_down
BSK_hDFCGF_MCSF_down
BSK_CASM3C_SAA_down
BSK_4H_VCAM1_down
BSK_3C_VCAM1_down
BSK_3C_Eselectin_down
13104−21−7
1757−18−2
2274−67−1
2701−86−2
33399−00−7
961−11−5
22248−79−9
470−90−6

All Activity Heatmap for Cluster 963

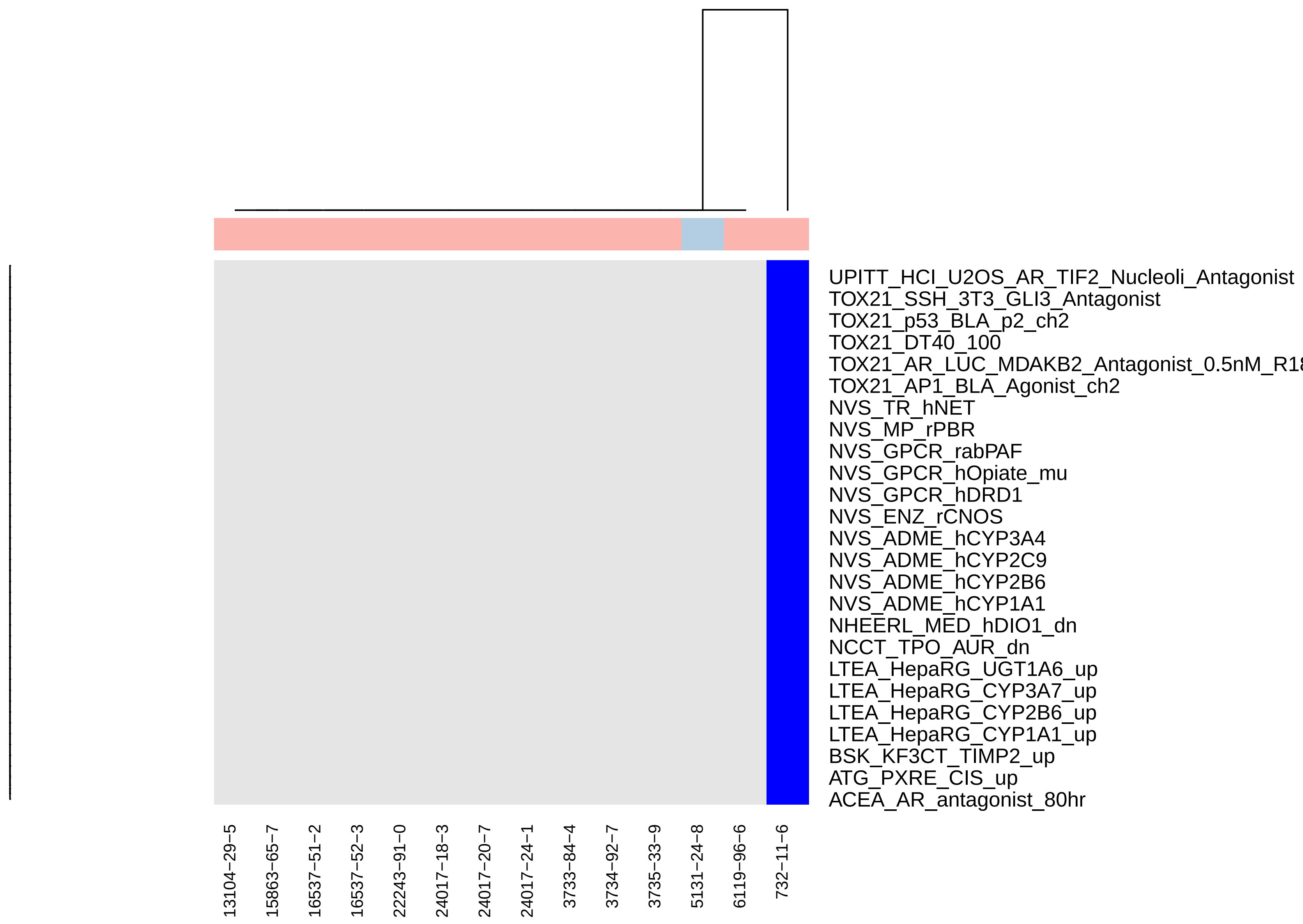

All Activity Heatmap for Cluster 964

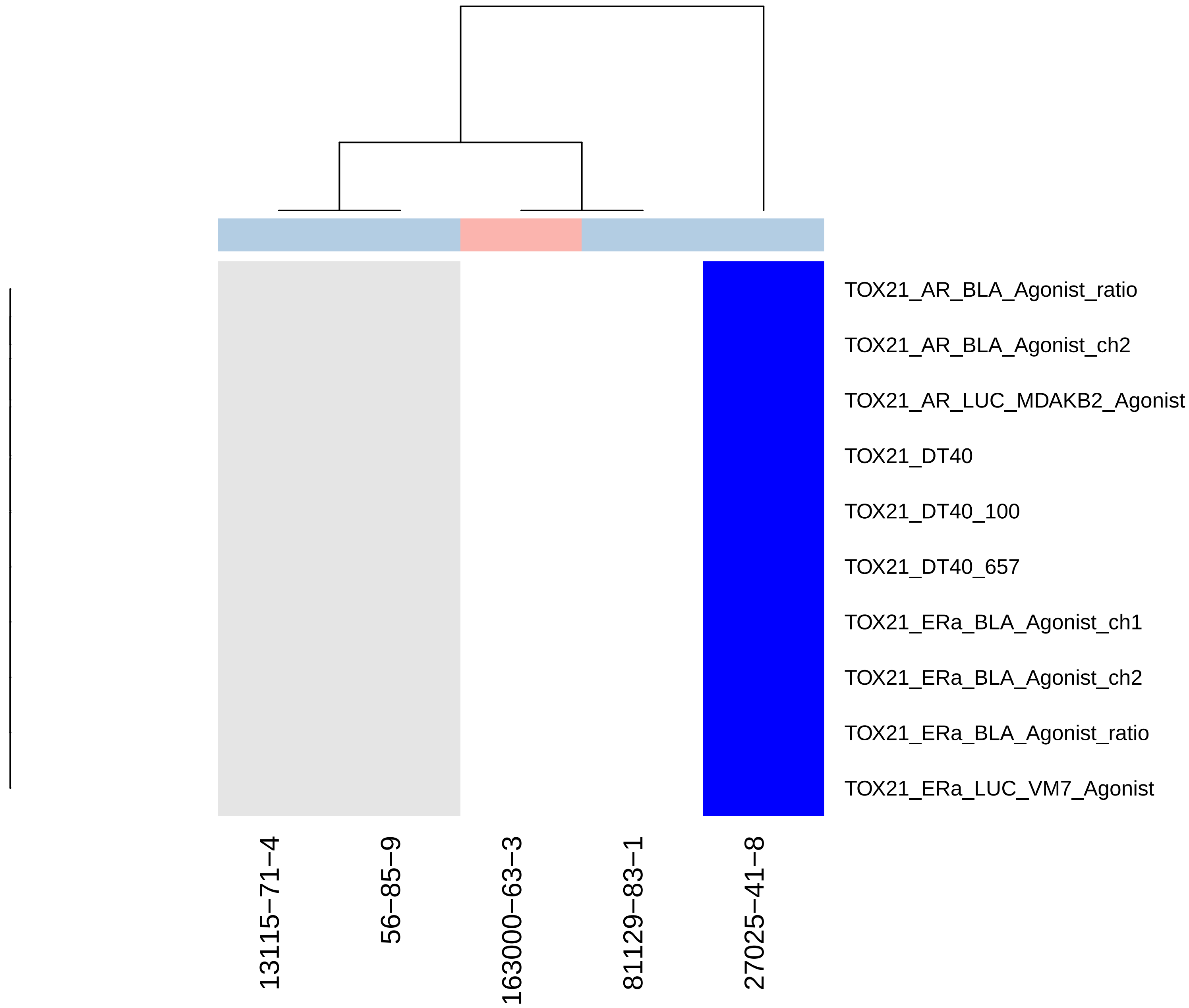

All Activity Heatmap for Cluster 965

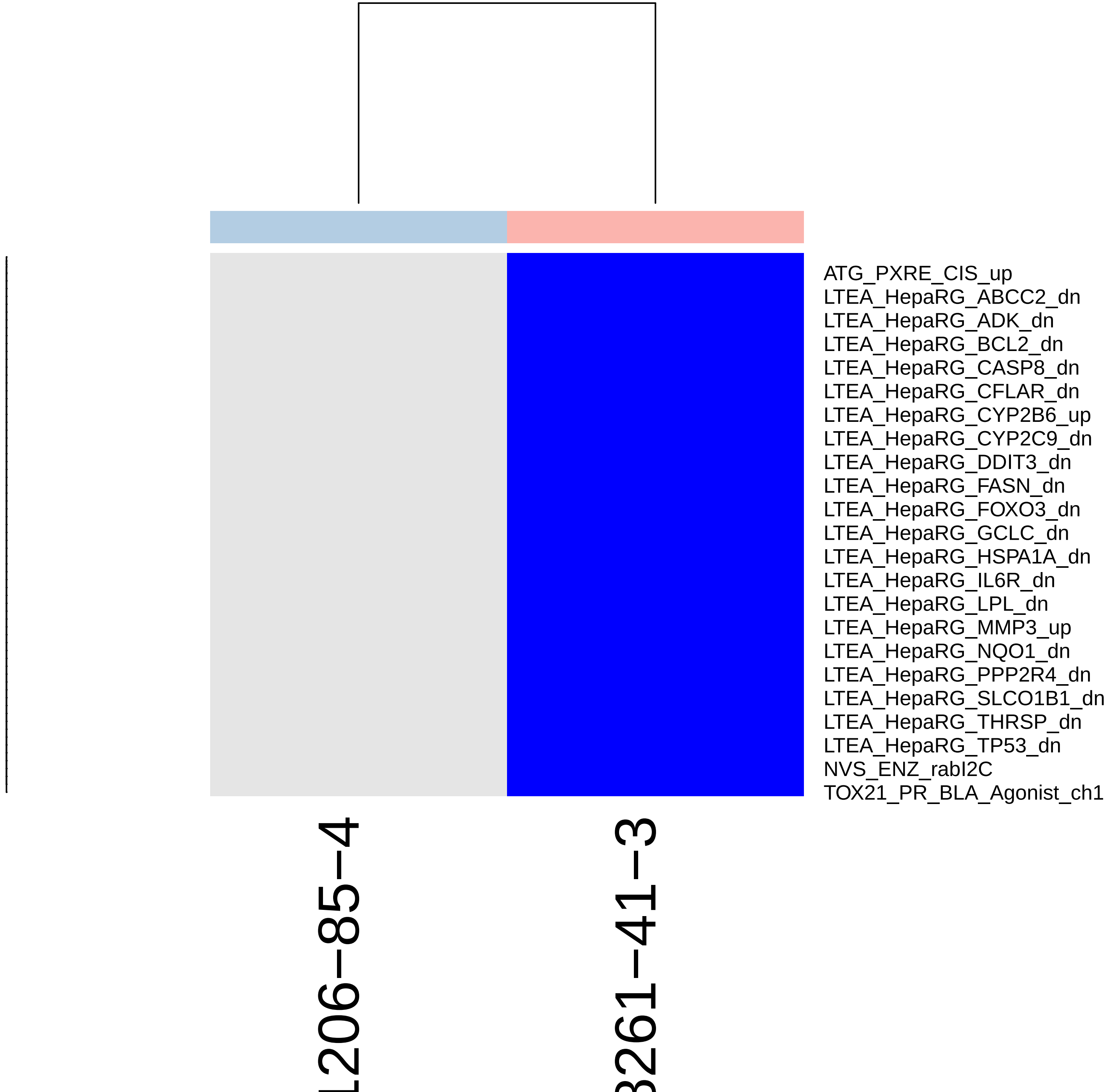

All Activity Heatmap for Cluster 966

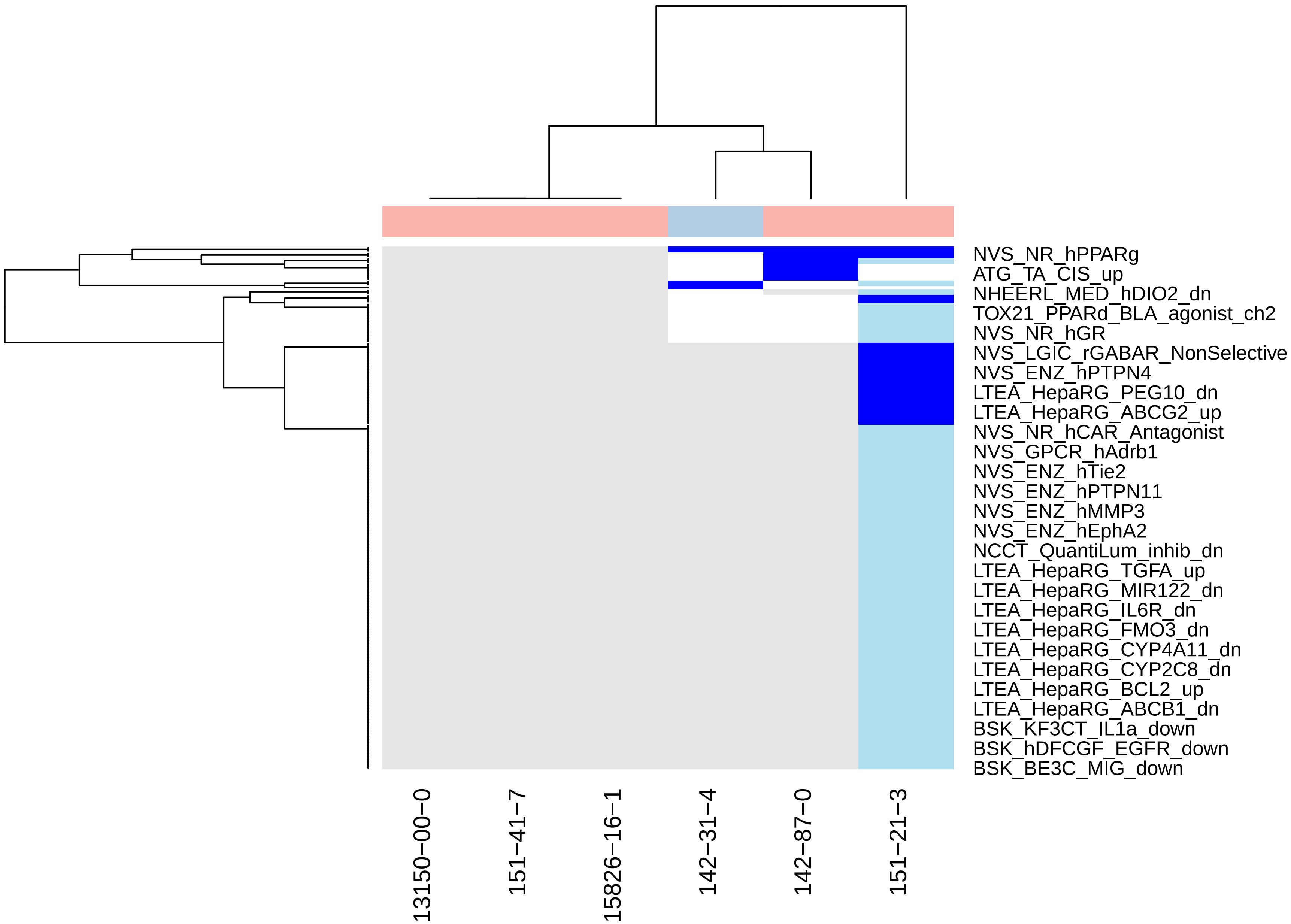

All Activity Heatmap for Cluster 968

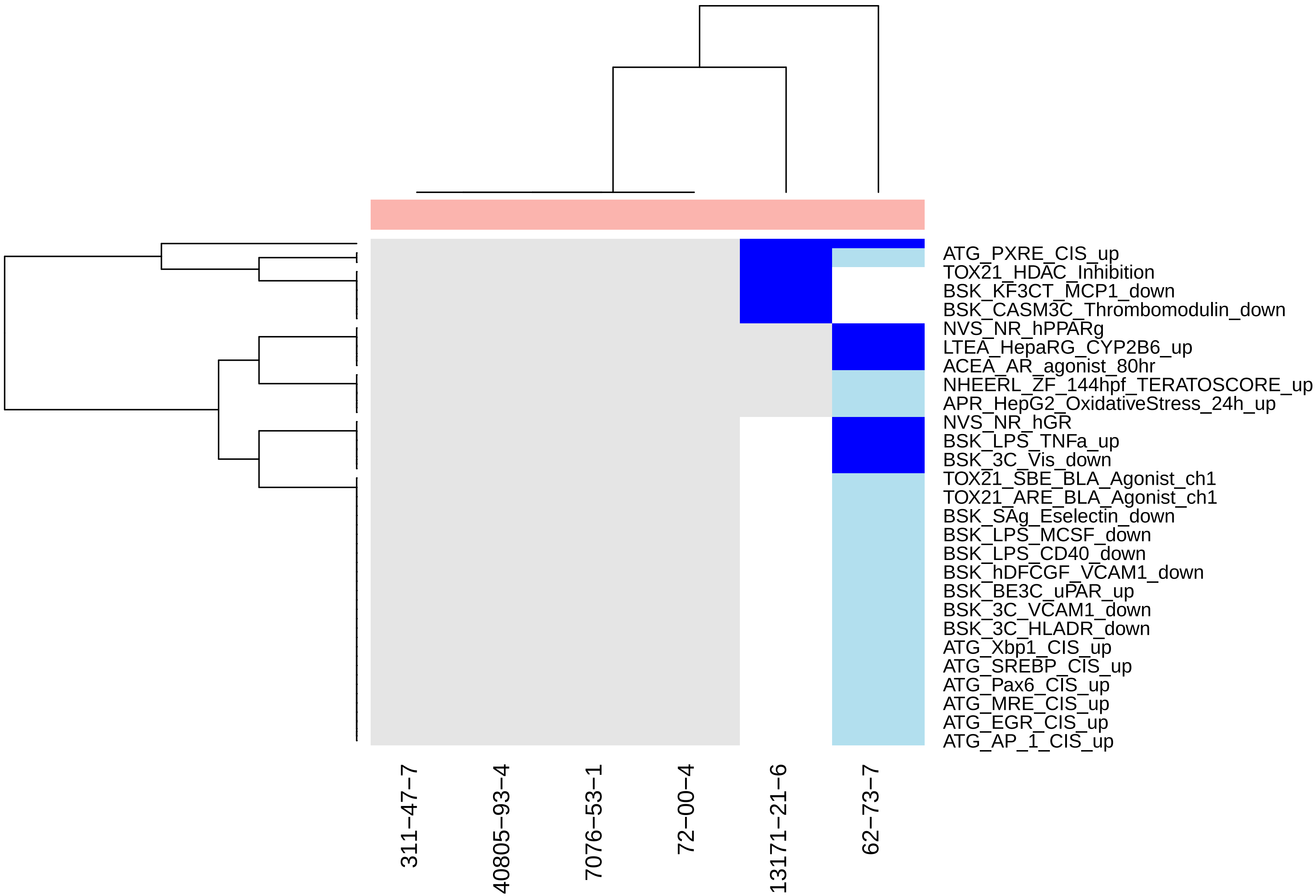

All Activity Heatmap for Cluster 969

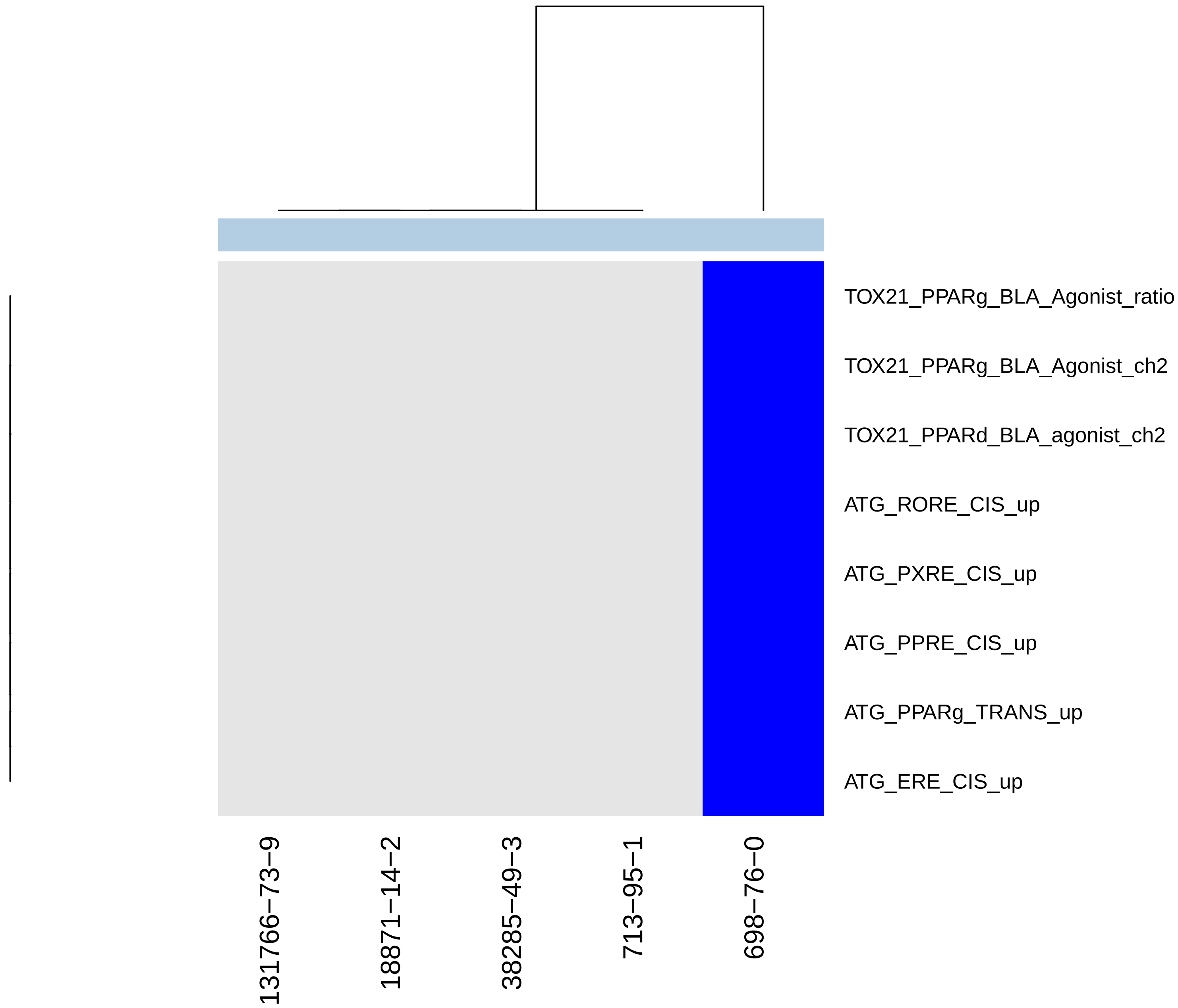

All Activity Heatmap for Cluster 970

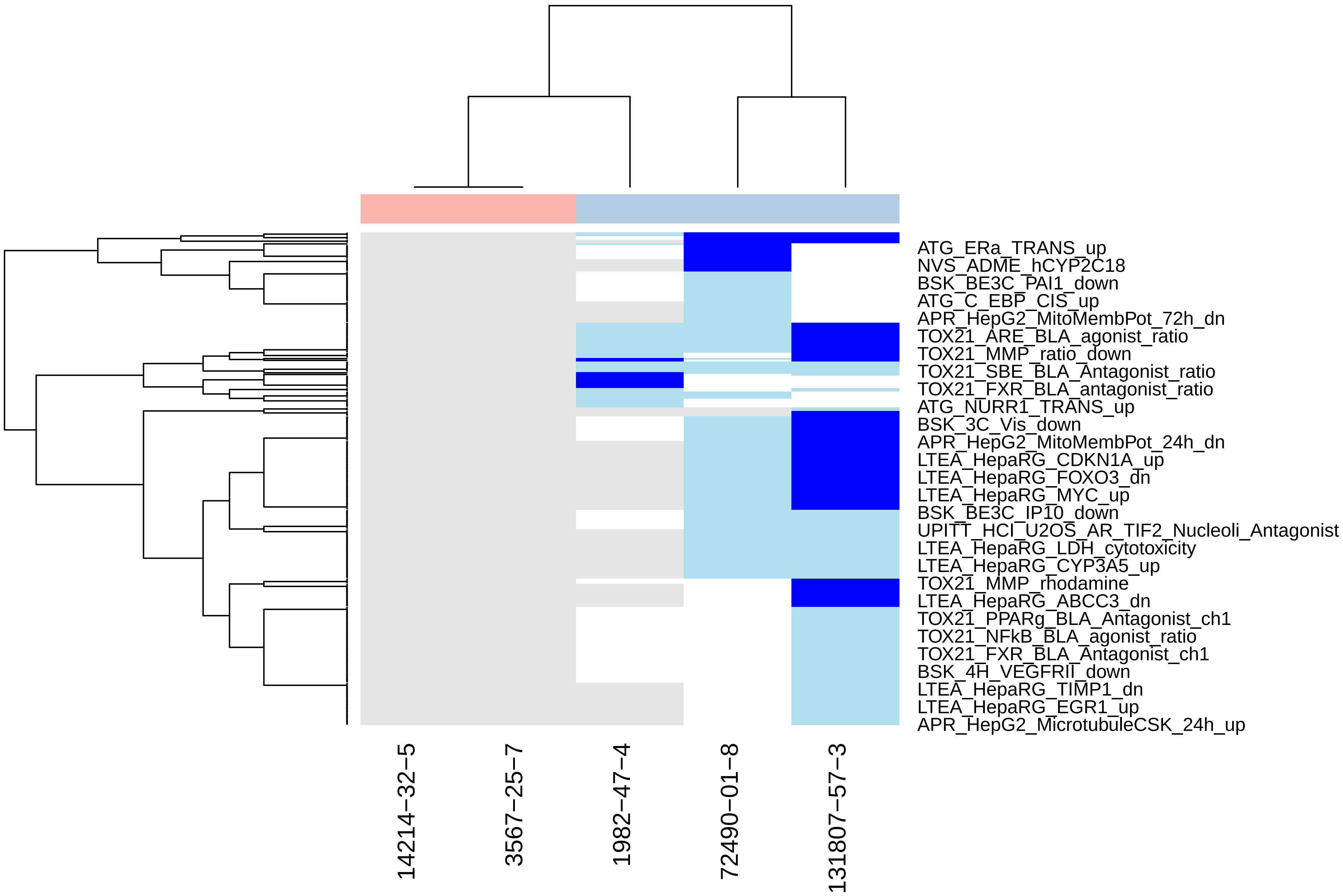

All Activity Heatmap for Cluster 971

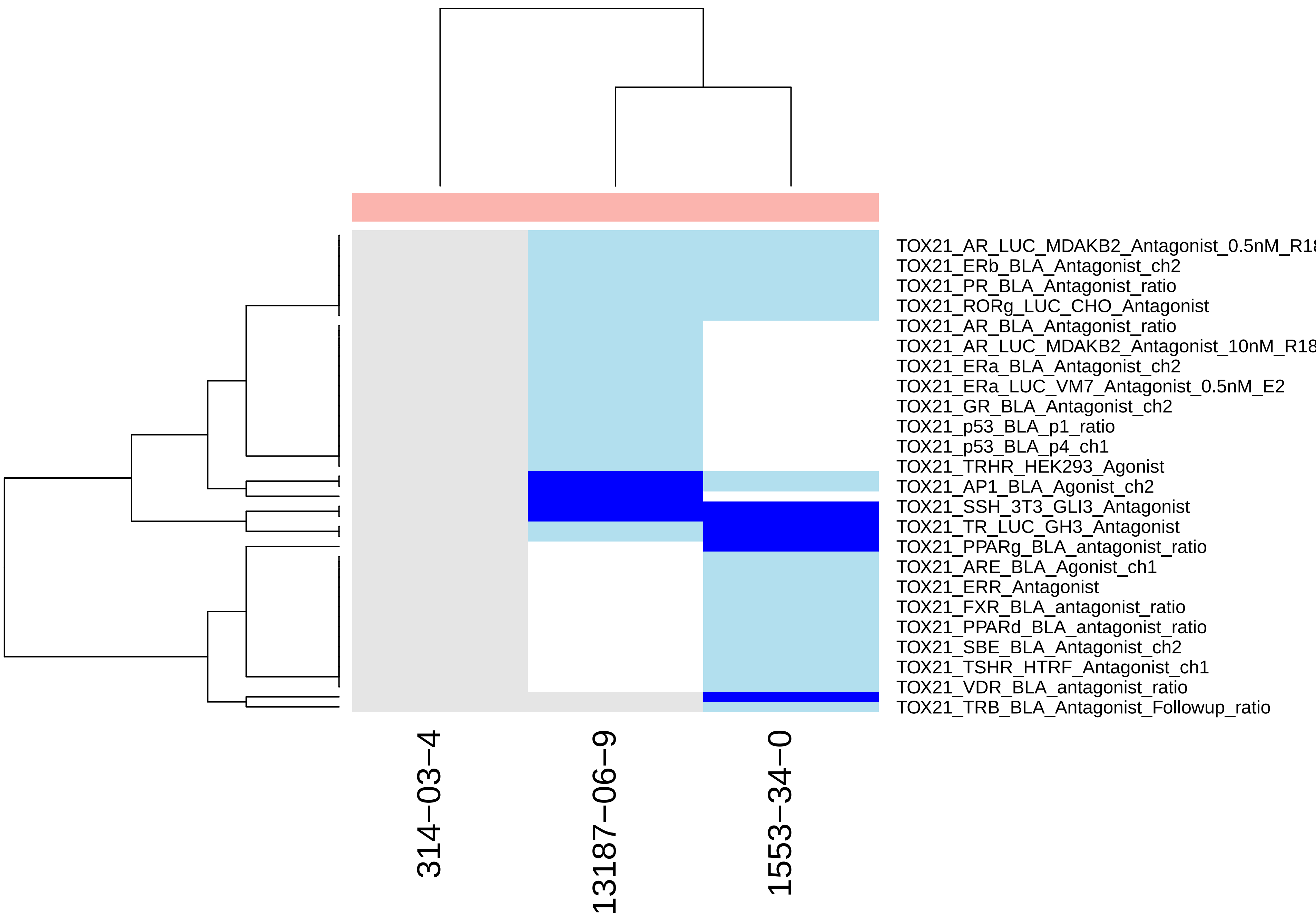

All Activity Heatmap for Cluster 972

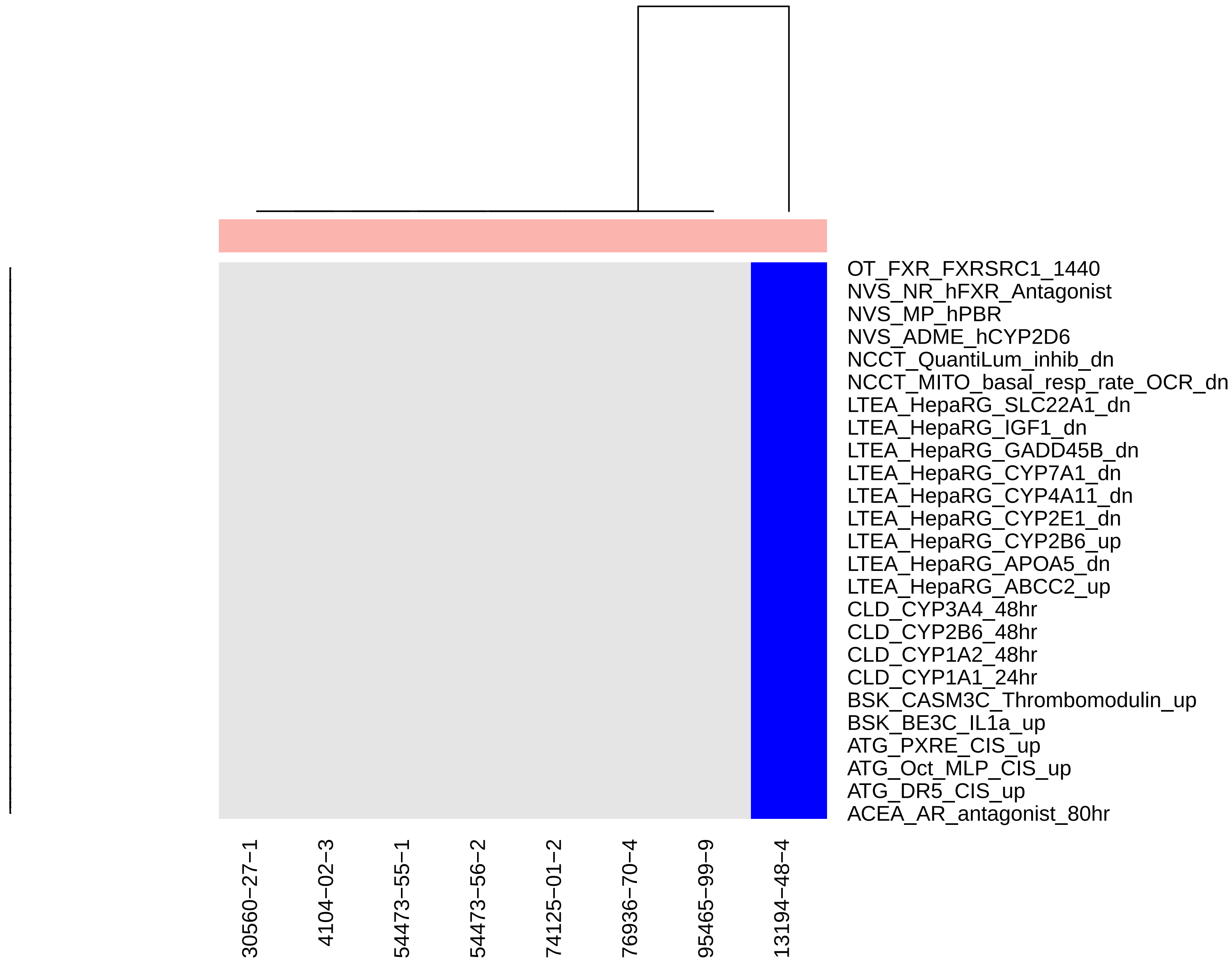

All Activity Heatmap for Cluster 973

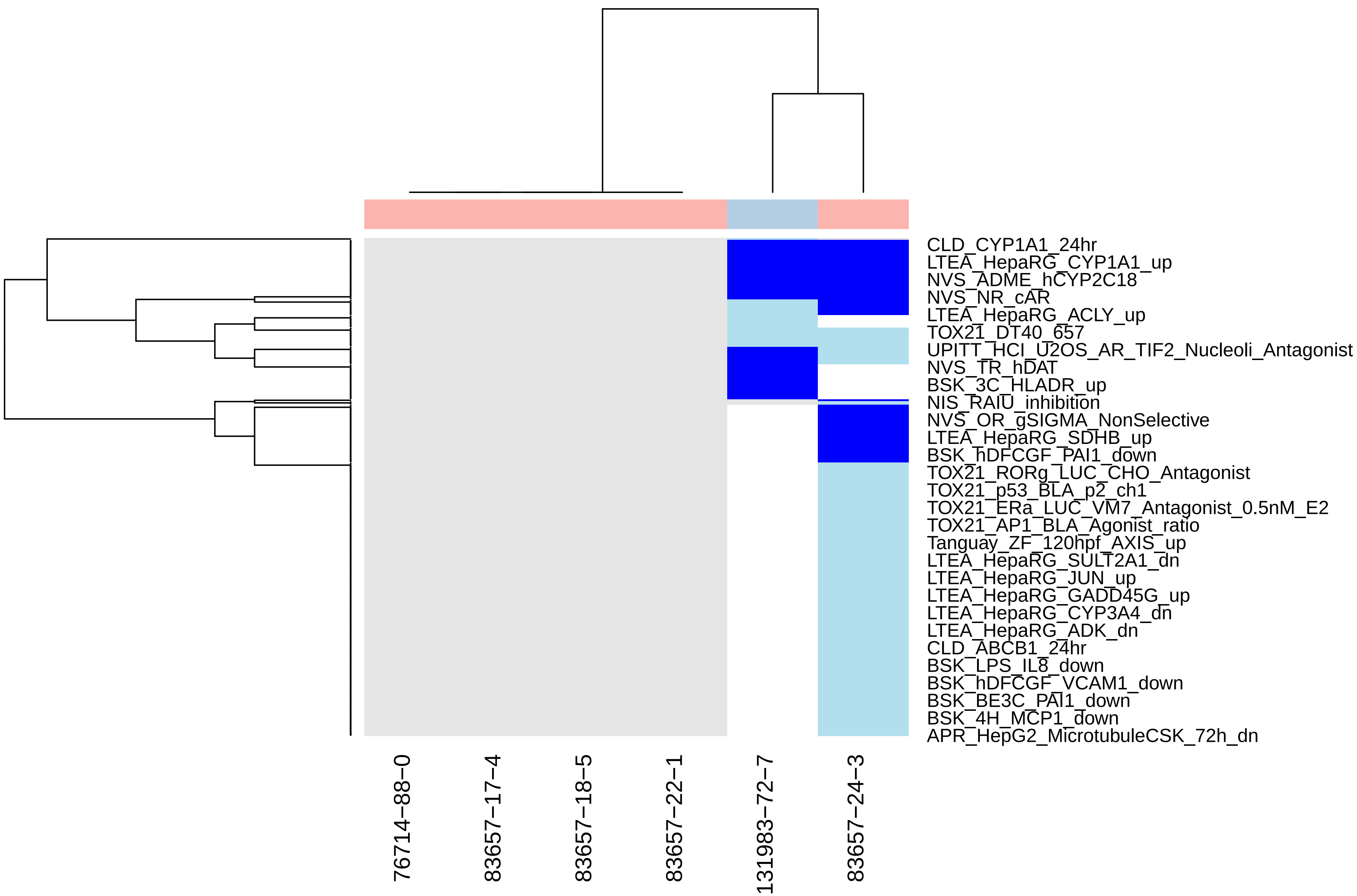

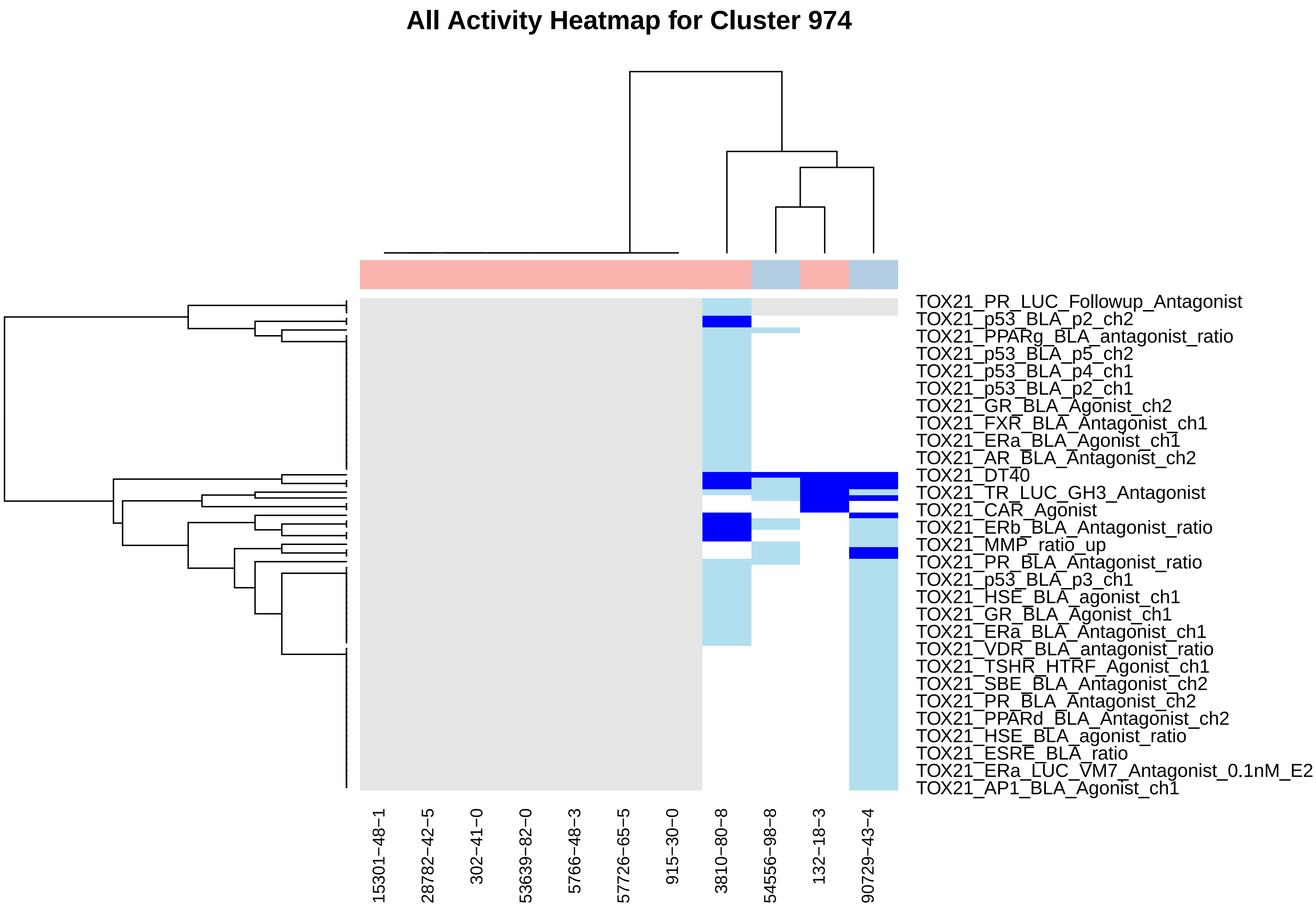
All Activity Heatmap for Cluster 974
TOX21_PR_LUC_Followup_Antagonist
TOX21_p53_BLA_p2_ch2
TOX21_PPARg_BLA_antagonist_ratio
TOX21_p53_BLA_p5_ch2
TOX21_p53_BLA_p4_ch1
TOX21_p53_BLA_p2_ch1
TOX21_GR_BLA_Agonist_ch2
TOX21_FXR_BLA_Antagonist_ch1
TOX21_ERa_BLA_Agonist_ch1
TOX21_AR_BLA_Antagonist_ch2
TOX21_DT40
TOX21_TR_LUC_GH3_Antagonist
TOX21_CAR_Agonist
TOX21_ERb_BLA_Antagonist_ratio
TOX21_MMP_ratio_up
TOX21_PR_BLA_Antagonist_ratio
TOX21_p53_BLA_p3_ch1
TOX21_HSE_BLA_agonist_ch1
TOX21_GR_BLA_Agonist_ch1
TOX21_ERa_BLA_Antagonist_ch1
TOX21_VDR_BLA_antagonist_ratio
TOX21_TSHR_HTRF_Agonist_ch1
TOX21_SBE_BLA_Antagonist_ch2
TOX21_PR_BLA_Antagonist_ch2
TOX21_PPARd_BLA_Antagonist_ch2
TOX21_HSE_BLA_agonist_ratio
TOX21_ESRE_BLA_ratio
TOX21_ERa_LUC_VM7_Antagonist_0.1nM_E2
TOX21_AP1_BLA_Agonist_ch1
15301−48−1
28782−42−5
302−41−0
53639−82−0
5766−48−3
57726−65−5
915−30−0
3810−80−8
54556−98−8
132−18−3
90729−43−4

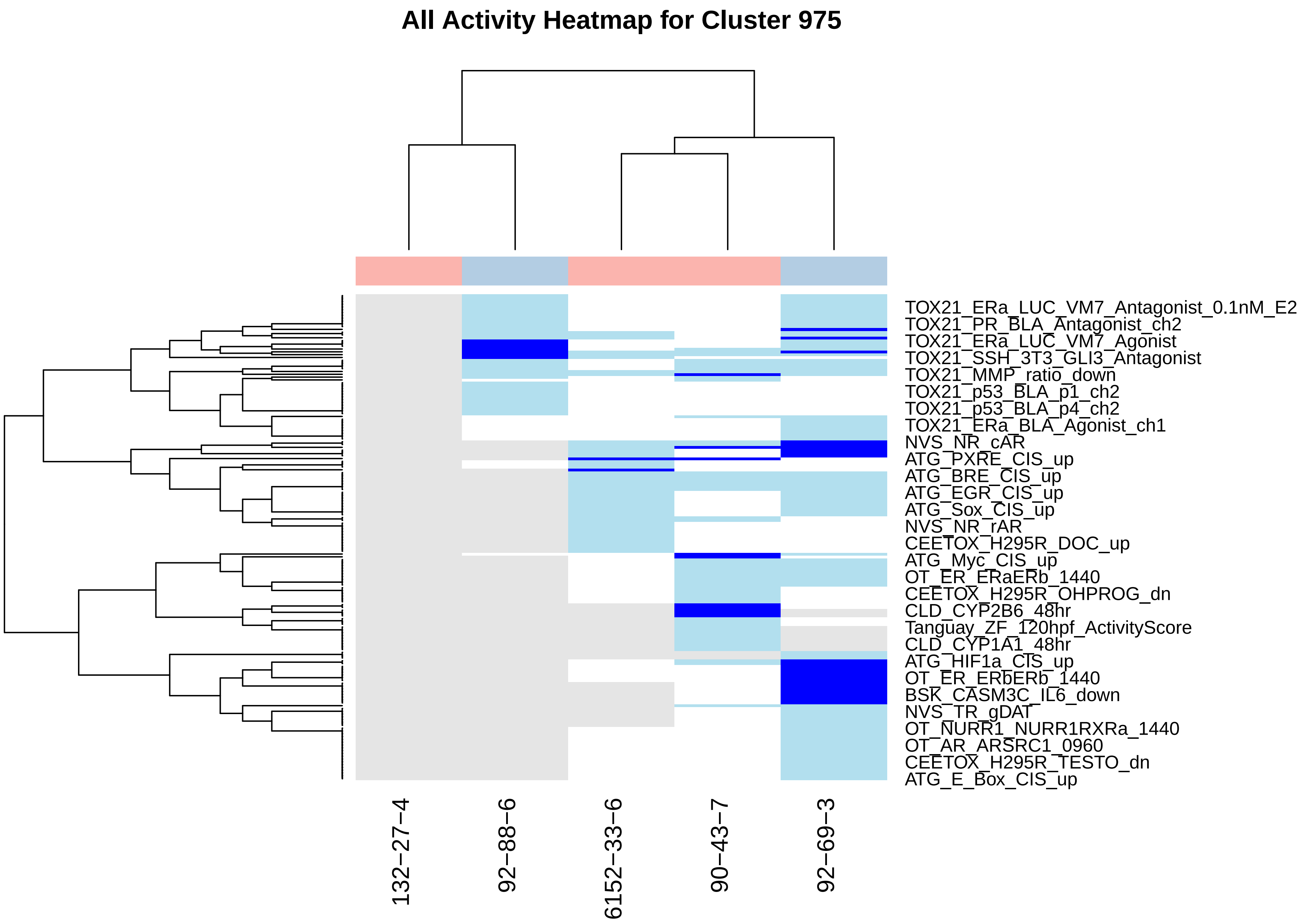
All Activity Heatmap for Cluster 975
TOX21_ERa_LUC_VM7_Antagonist_0.1nM_E2
TOX21_PR_BLA_Antagonist_ch2
TOX21_ERa_LUC_VM7_Agonist
TOX21_SSH_3T3_GLI3_Antagonist
TOX21_MMP_ratio_down
TOX21_p53_BLA_p1_ch2
TOX21_p53_BLA_p4_ch2
TOX21_ERa_BLA_Agonist_ch1
NVS_NR_cAR
ATG_PXRE_CIS_up
ATG_BRE_CIS_up
ATG_EGR_CIS_up
ATG_Sox_CIS_up
NVS_NR_rAR
CEETOX_H295R_DOC_up
ATG_Myc_CIS_up
OT_ER_ERaERb_1440
CEETOX_H295R_OHPROG_dn
CLD_CYP2B6_48hr
Tanguay_ZF_120hpf_ActivityScore
CLD_CYP1A1_48hr
ATG_HIF1a_CIS_up
OT_ER_ERbERb_1440
BSK_CASM3C_IL6_down
NVS_TR_gDAT
OT_NURR1_NURR1RXRa_1440
OT_AR_ARSRC1_0960
CEETOX_H295R_TESTO_dn
ATG_E_Box_CIS_up
132−27−4
92−88−6
6152−33−6
90−43−7
92−69−3

All Activity Heatmap for Cluster 976

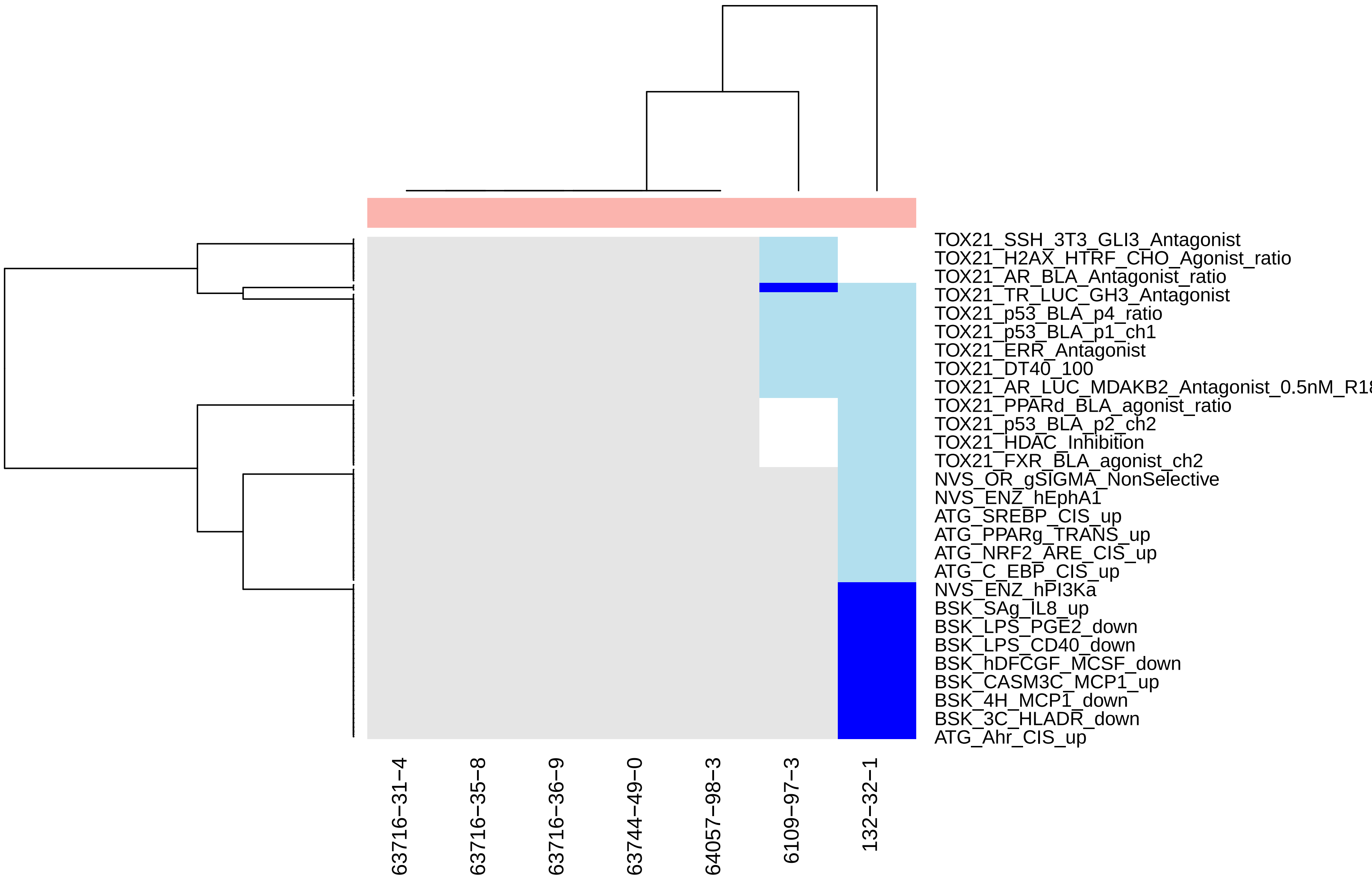

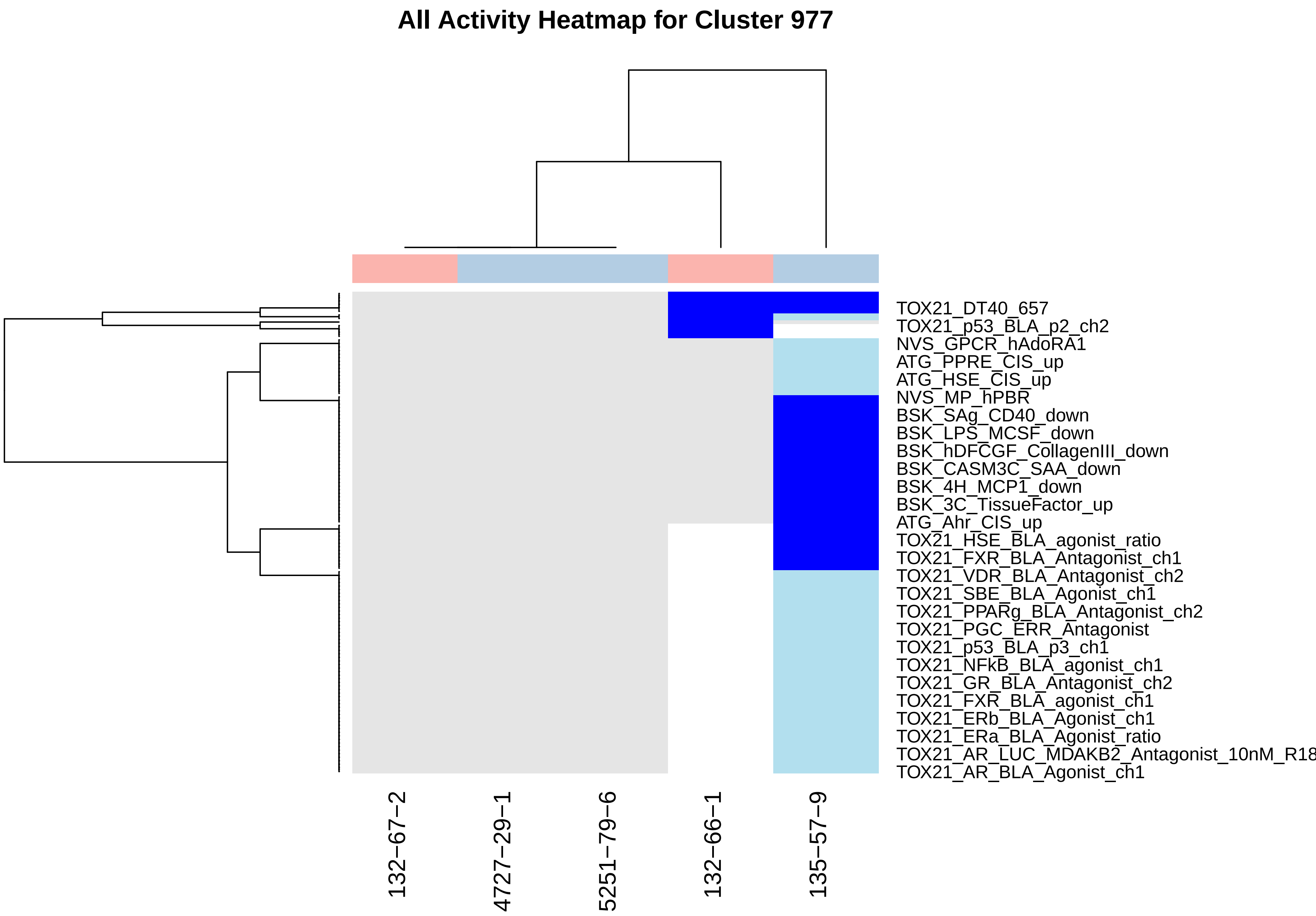
All Activity Heatmap for Cluster 977
TOX21_DT40_657
TOX21_p53_BLA_p2_ch2
NVS_GPCR_hAdoRA1
ATG_PPRE_CIS_up
ATG_HSE_CIS_up
NVS_MP_hPBR
BSK_SAg_CD40_down
BSK_LPS_MCSF_down
BSK_hDFCGF_CollagenIII_down
BSK_CASM3C_SAA_down
BSK_4H_MCP1_down
BSK_3C_TissueFactor_up
ATG_Ahr_CIS_up
TOX21_HSE_BLA_agonist_ratio
TOX21_FXR_BLA_Antagonist_ch1
TOX21_VDR_BLA_Antagonist_ch2
TOX21_SBE_BLA_Agonist_ch1
TOX21_PPARg_BLA_Antagonist_ch2
TOX21_PGC_ERR_Antagonist
TOX21_p53_BLA_p3_ch1
TOX21_NFkB_BLA_agonist_ch1
TOX21_GR_BLA_Antagonist_ch2
TOX21_FXR_BLA_agonist_ch1
TOX21_ERb_BLA_Agonist_ch1
TOX21_ERa_BLA_Agonist_ratio
TOX21_AR_LUC_MDAKB2_Antagonist_10nM_R18
TOX21_AR_BLA_Agonist_ch1
132−67−2
4727−29−1
5251−79−6
132−66−1
135−57−9

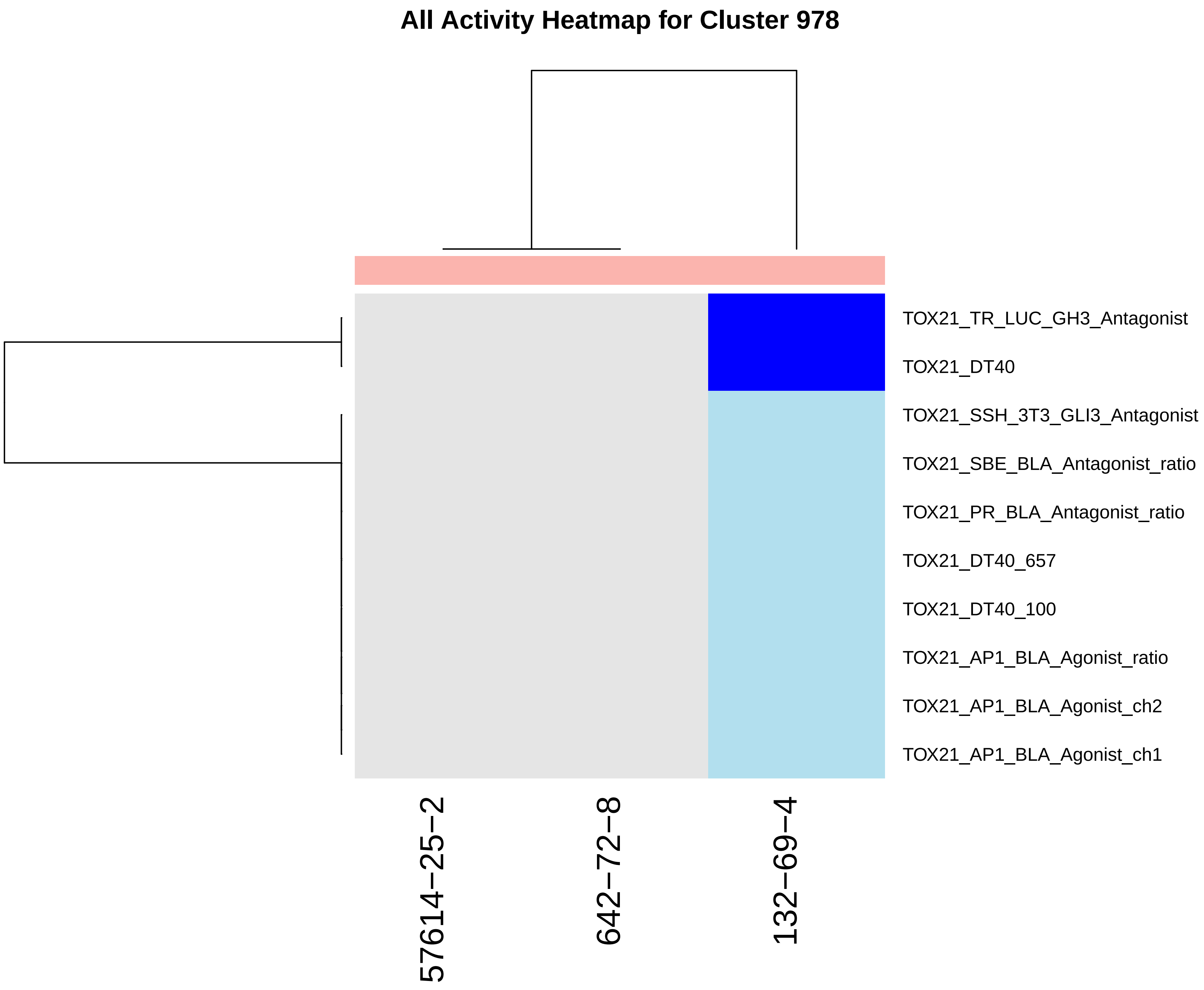
All Activity Heatmap for Cluster 978
TOX21_TR_LUC_GH3_Antagonist
TOX21_DT40
TOX21_SSH_3T3_GLI3_Antagonist
TOX21_SBE_BLA_Antagonist_ratio
TOX21_PR_BLA_Antagonist_ratio
TOX21_DT40_657
TOX21_DT40_100
TOX21_AP1_BLA_Agonist_ratio
TOX21_AP1_BLA_Agonist_ch2
TOX21_AP1_BLA_Agonist_ch1
57614−25−2
642−72−8
132−69−4

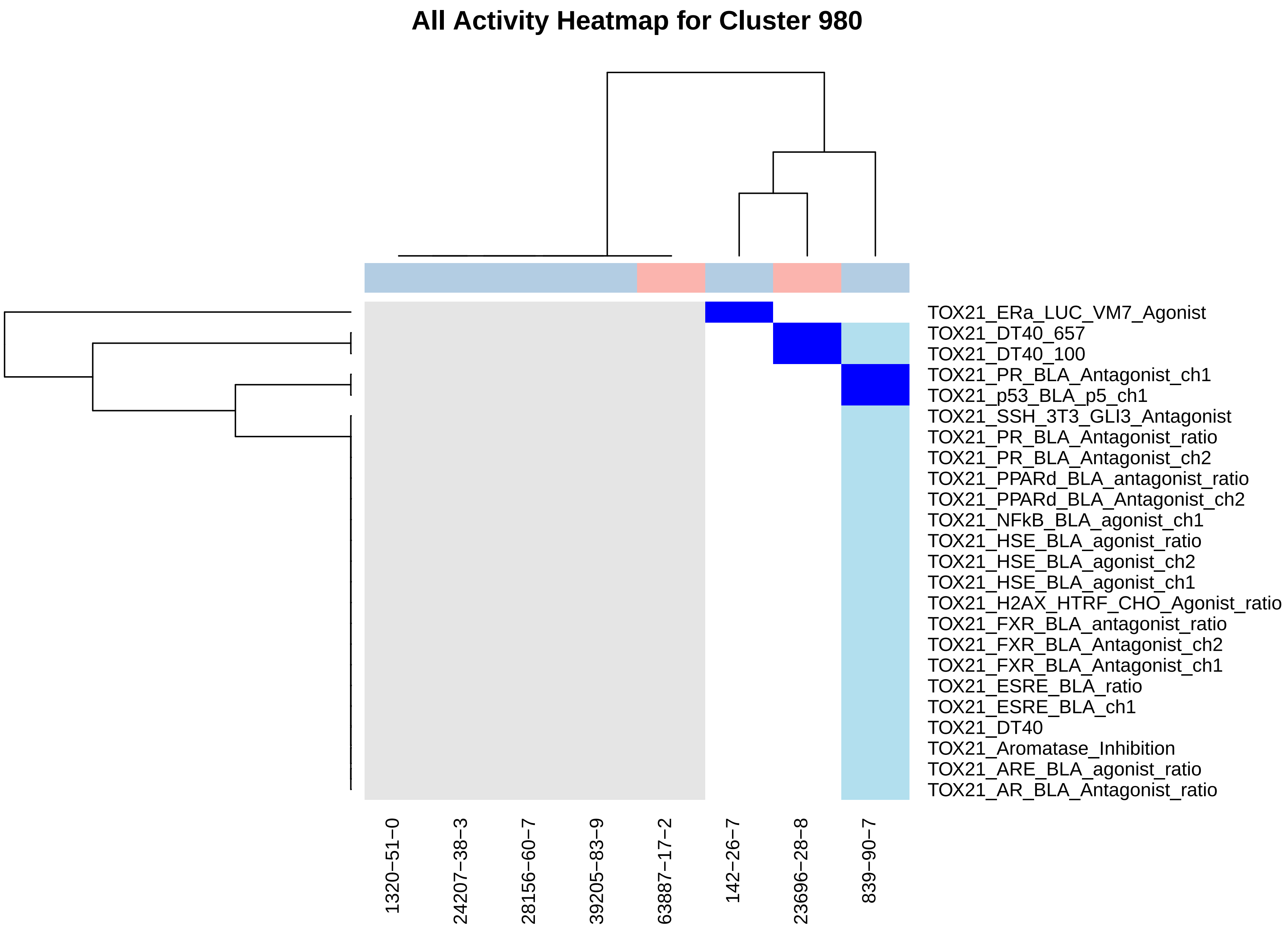
All Activity Heatmap for Cluster 980
TOX21_ERa_LUC_VM7_Agonist
TOX21_DT40_657
TOX21_DT40_100
TOX21_PR_BLA_Antagonist_ch1
TOX21_p53_BLA_p5_ch1
TOX21_SSH_3T3_GLI3_Antagonist
TOX21_PR_BLA_Antagonist_ratio
TOX21_PR_BLA_Antagonist_ch2
TOX21_PPARd_BLA_antagonist_ratio
TOX21_PPARd_BLA_Antagonist_ch2
TOX21_NFkB_BLA_agonist_ch1
TOX21_HSE_BLA_agonist_ratio
TOX21_HSE_BLA_agonist_ch2
TOX21_HSE_BLA_agonist_ch1
TOX21_H2AX_HTRF_CHO_Agonist_ratio
TOX21_FXR_BLA_antagonist_ratio
TOX21_FXR_BLA_Antagonist_ch2
TOX21_FXR_BLA_Antagonist_ch1
TOX21_ESRE_BLA_ratio
TOX21_ESRE_BLA_ch1
TOX21_DT40
TOX21_Aromatase_Inhibition
TOX21_ARE_BLA_agonist_ratio
TOX21_AR_BLA_Antagonist_ratio
1320−51−0
24207−38−3
28156−60−7
39205−83−9
63887−17−2
142−26−7
23696−28−8
839−90−7

All Activity Heatmap for Cluster 981

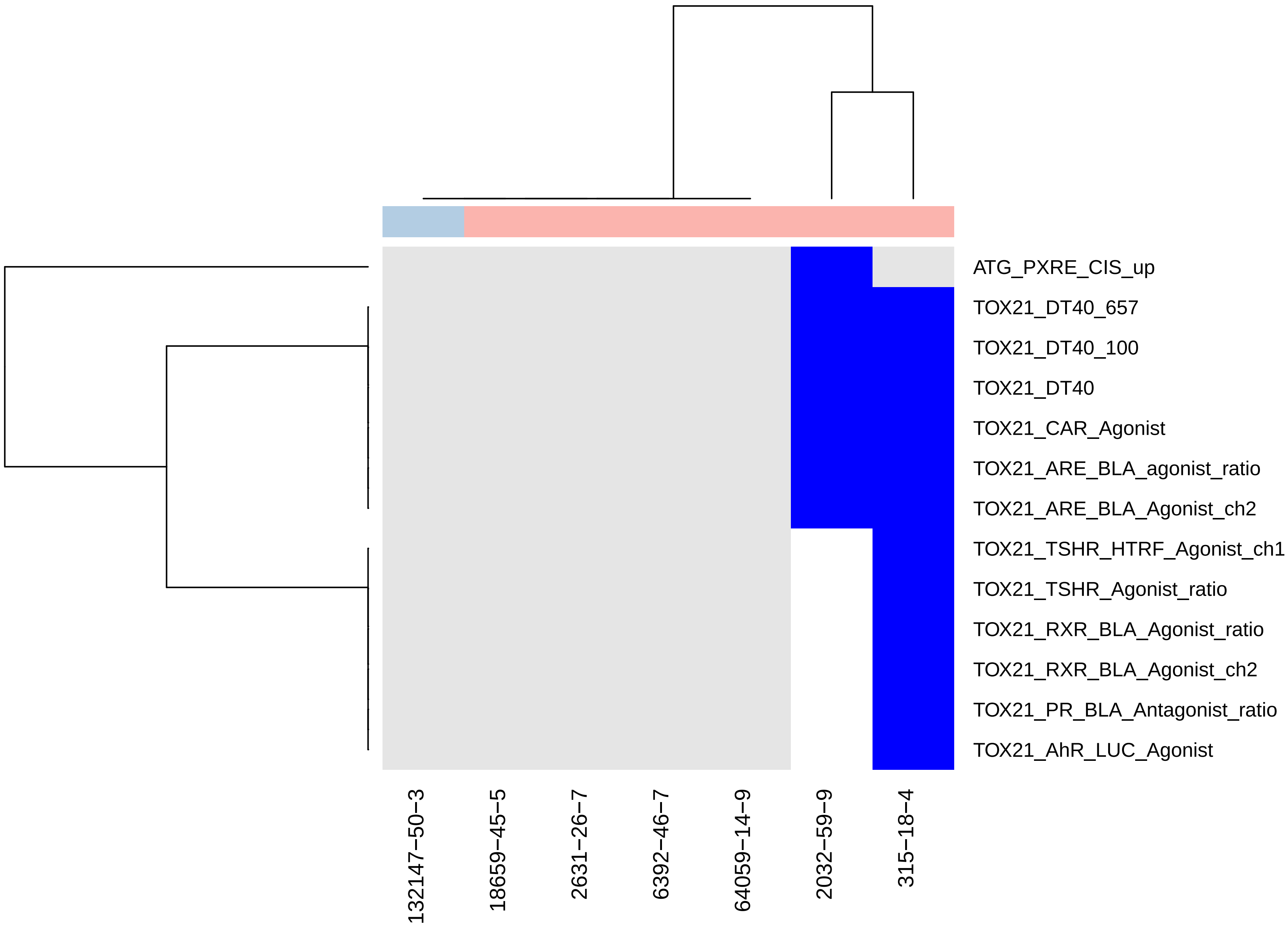

All Activity Heatmap for Cluster 985

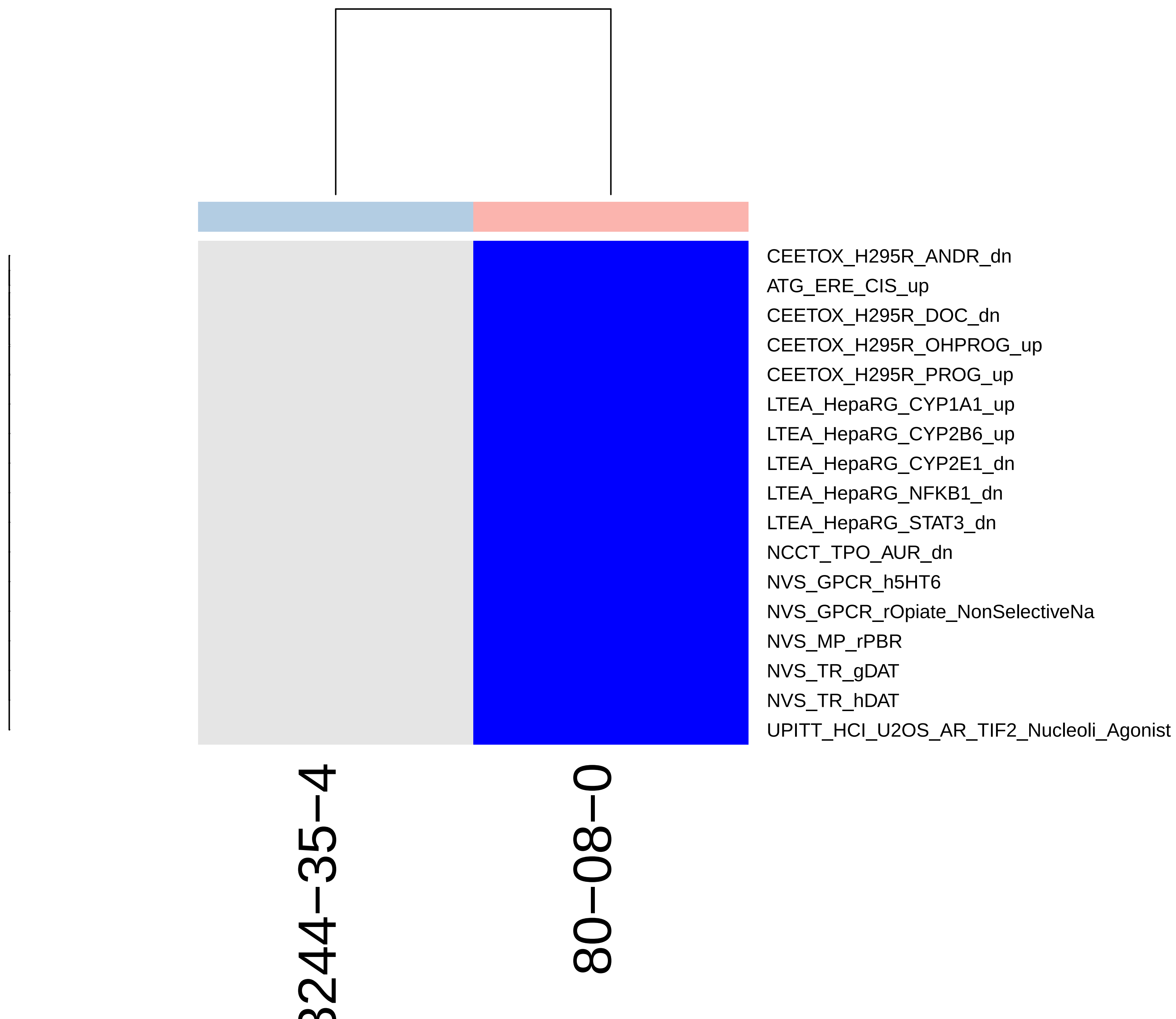

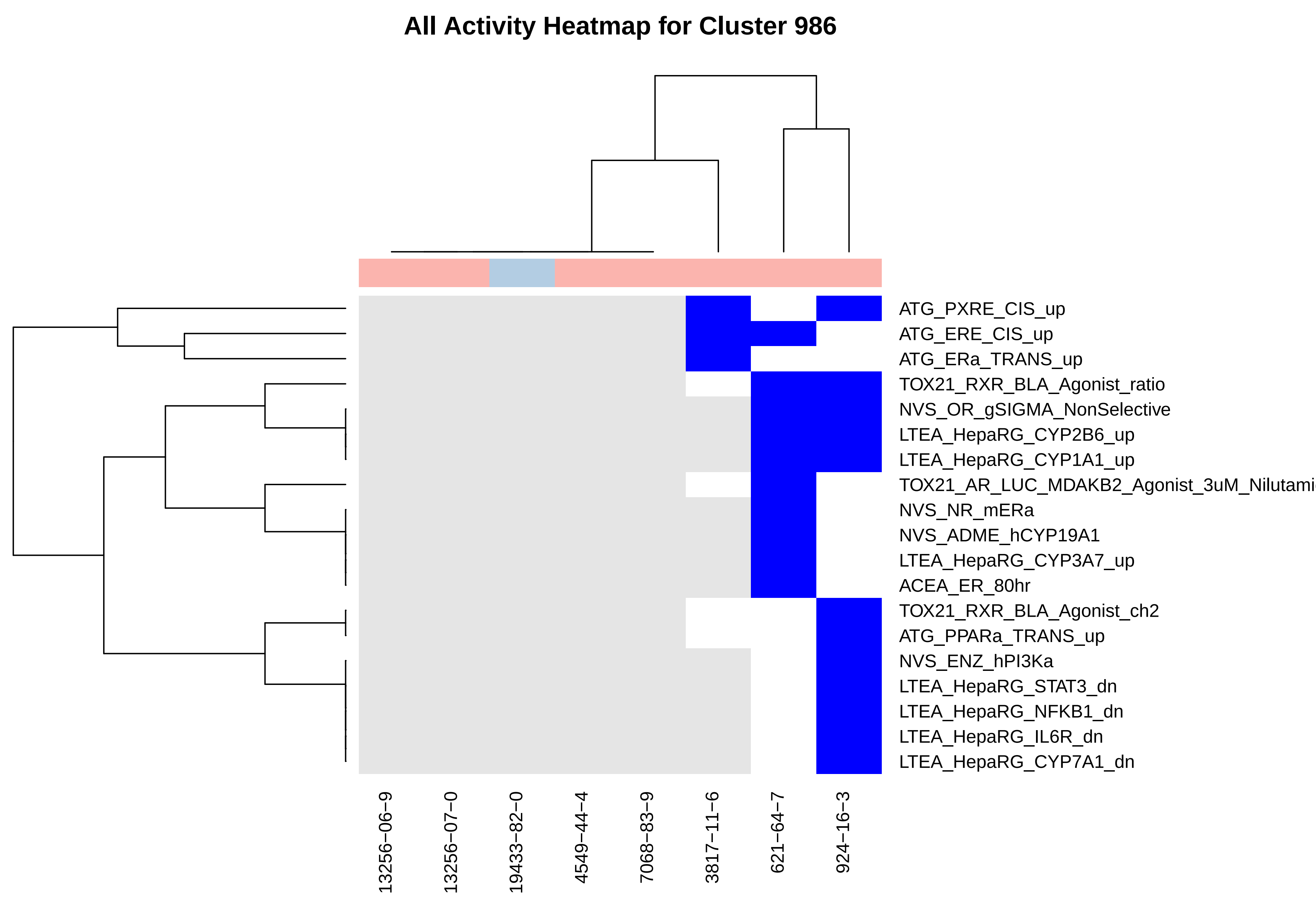

All Activity Heatmap for Cluster 986
ATG_PXRE_CIS_up
ATG_ERE_CIS_up
ATG_ERa_TRANS_up
TOX21_RXR_BLA_Agonist_ratio
NVS_OR_gSIGMA_NonSelective
LTEA_HepaRG_CYP2B6_up
LTEA_HepaRG_CYP1A1_up
TOX21_AR_LUC_MDAKB2_Agonist_3uM_Nilutami
NVS_NR_mERa
NVS_ADME_hCYP19A1
LTEA_HepaRG_CYP3A7_up
ACEA_ER_80hr
TOX21_RXR_BLA_Agonist_ch2
ATG_PPARa_TRANS_up
NVS_ENZ_hPI3Ka
LTEA_HepaRG_STAT3_dn
LTEA_HepaRG_NFKB1_dn
LTEA_HepaRG_IL6R_dn
LTEA_HepaRG_CYP7A1_dn
13256−06−9
13256−07−0
19433−82−0
4549−44−4
7068−83−9
3817−11−6
621−64−7
924−16−3

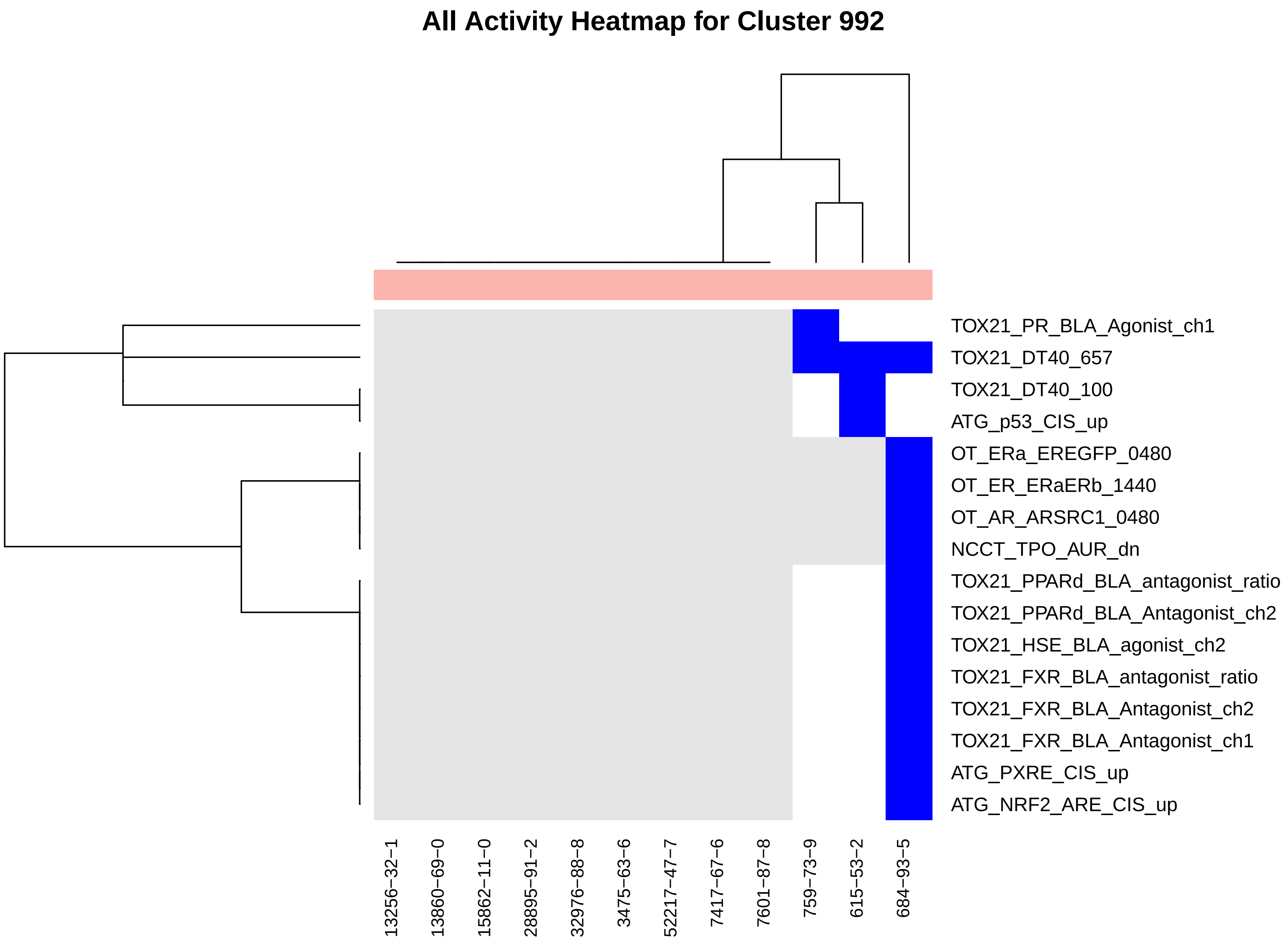

All Activity Heatmap for Cluster 992
TOX21_PR_BLA_Agonist_ch1
TOX21_DT40_657
TOX21_DT40_100
ATG_p53_CIS_up
OT_ERa_EREGFP_0480
OT_ER_ERaERb_1440
OT_AR_ARSRC1_0480
NCCT_TPO_AUR_dn
TOX21_PPARd_BLA_antagonist_ratio
TOX21_PPARd_BLA_Antagonist_ch2
TOX21_HSE_BLA_agonist_ch2
TOX21_FXR_BLA_antagonist_ratio
TOX21_FXR_BLA_Antagonist_ch2
TOX21_FXR_BLA_Antagonist_ch1
ATG_PXRE_CIS_up
ATG_NRF2_ARE_CIS_up
13256−32−1
13860−69−0
15862−11−0
28895−91−2
32976−88−8
3475−63−6
52217−47−7
7417−67−6
7601−87−8
759−73−9
615−53−2
684−93−5

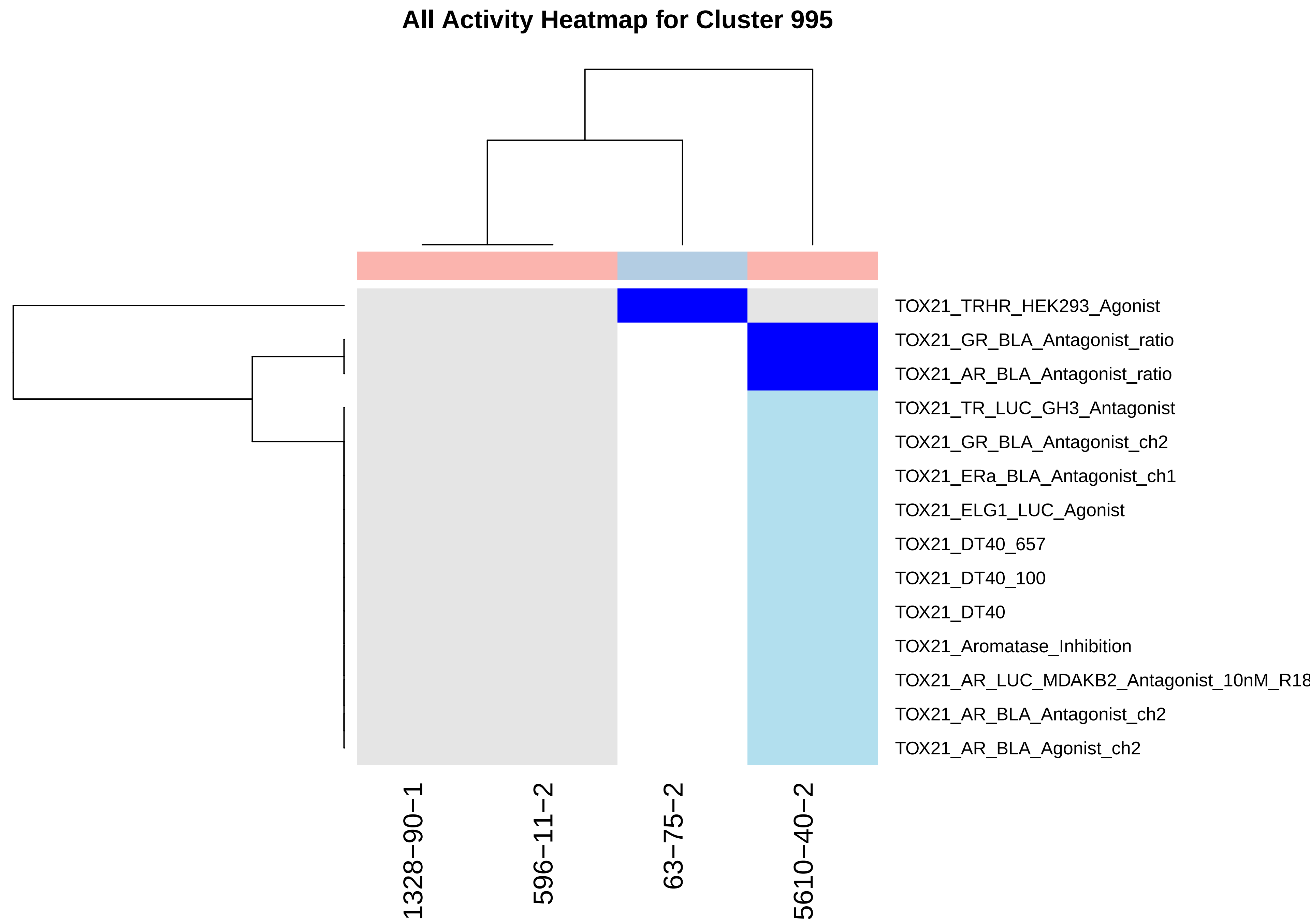

All Activity Heatmap for Cluster 995
TOX21_TRHR_HEK293_Agonist
TOX21_GR_BLA_Antagonist_ratio
TOX21_AR_BLA_Antagonist_ratio
TOX21_TR_LUC_GH3_Antagonist
TOX21_GR_BLA_Antagonist_ch2
TOX21_ERa_BLA_Antagonist_ch1
TOX21_ELG1_LUC_Agonist
TOX21_DT40_657
TOX21_DT40_100
TOX21_DT40
TOX21_Aromatase_Inhibition
TOX21_AR_LUC_MDAKB2_Antagonist_10nM_R18
TOX21_AR_BLA_Antagonist_ch2
TOX21_AR_BLA_Agonist_ch2
1328−90−1
596−11−2
63−75−2
5610−40−2

All Activity Heatmap for Cluster 998

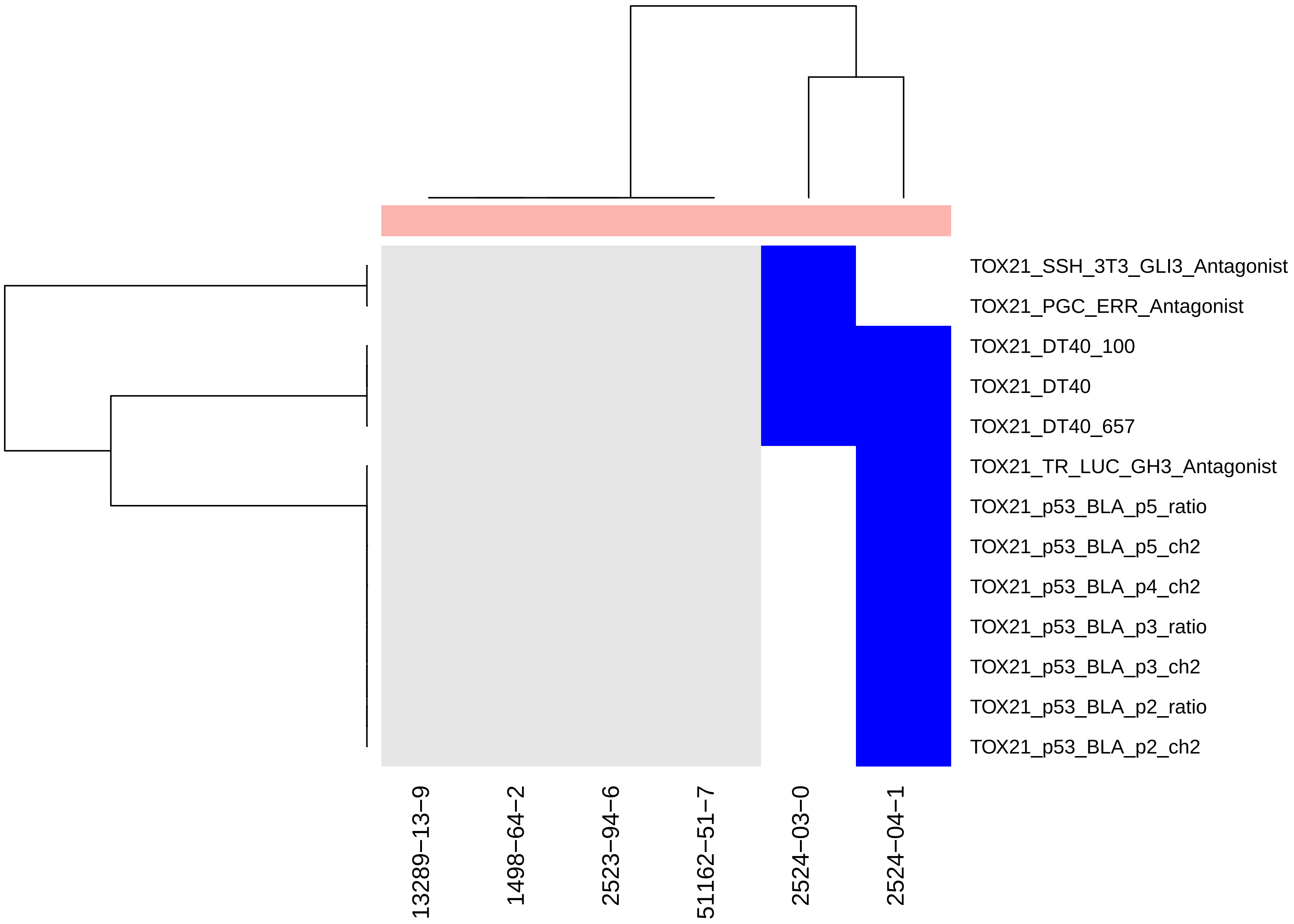

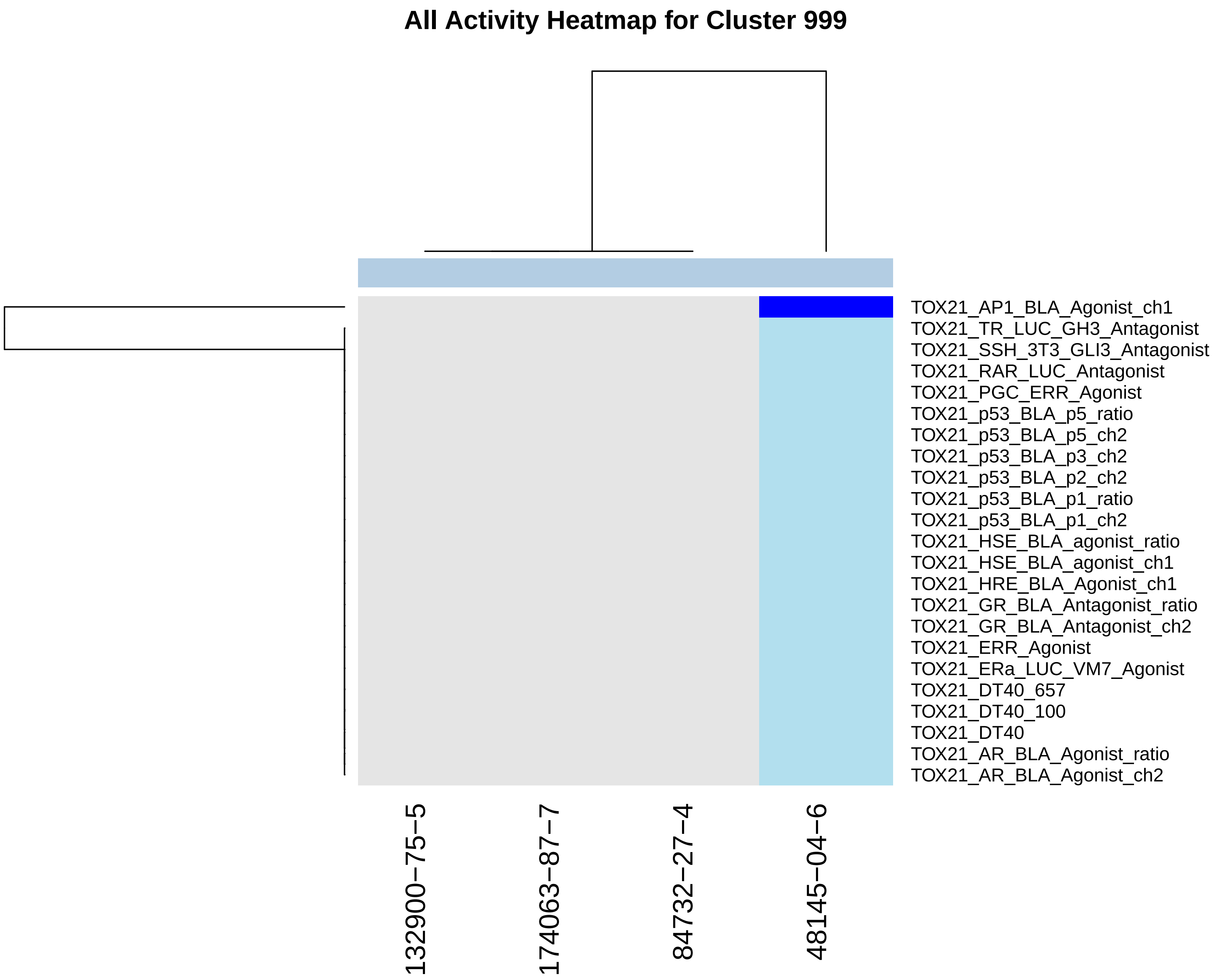

All Activity Heatmap for Cluster 999
TOX21_AP1_BLA_Agonist_ch1
TOX21_TR_LUC_GH3_Antagonist
TOX21_SSH_3T3_GLI3_Antagonist
TOX21_RAR_LUC_Antagonist
TOX21_PGC_ERR_Agonist
TOX21_p53_BLA_p5_ratio
TOX21_p53_BLA_p5_ch2
TOX21_p53_BLA_p3_ch2
TOX21_p53_BLA_p2_ch2
TOX21_p53_BLA_p1_ratio
TOX21_p53_BLA_p1_ch2
TOX21_HSE_BLA_agonist_ratio
TOX21_HSE_BLA_agonist_ch1
TOX21_HRE_BLA_Agonist_ch1
TOX21_GR_BLA_Antagonist_ratio
TOX21_GR_BLA_Antagonist_ch2
TOX21_ERR_Agonist
TOX21_ERa_LUC_VM7_Agonist
TOX21_DT40_657
TOX21_DT40_100
TOX21_DT40
TOX21_AR_BLA_Agonist_ratio
TOX21_AR_BLA_Agonist_ch2
132900−75−5
174063−87−7
84732−27−4
48145−04−6

All Activity Heatmap for Cluster 1000

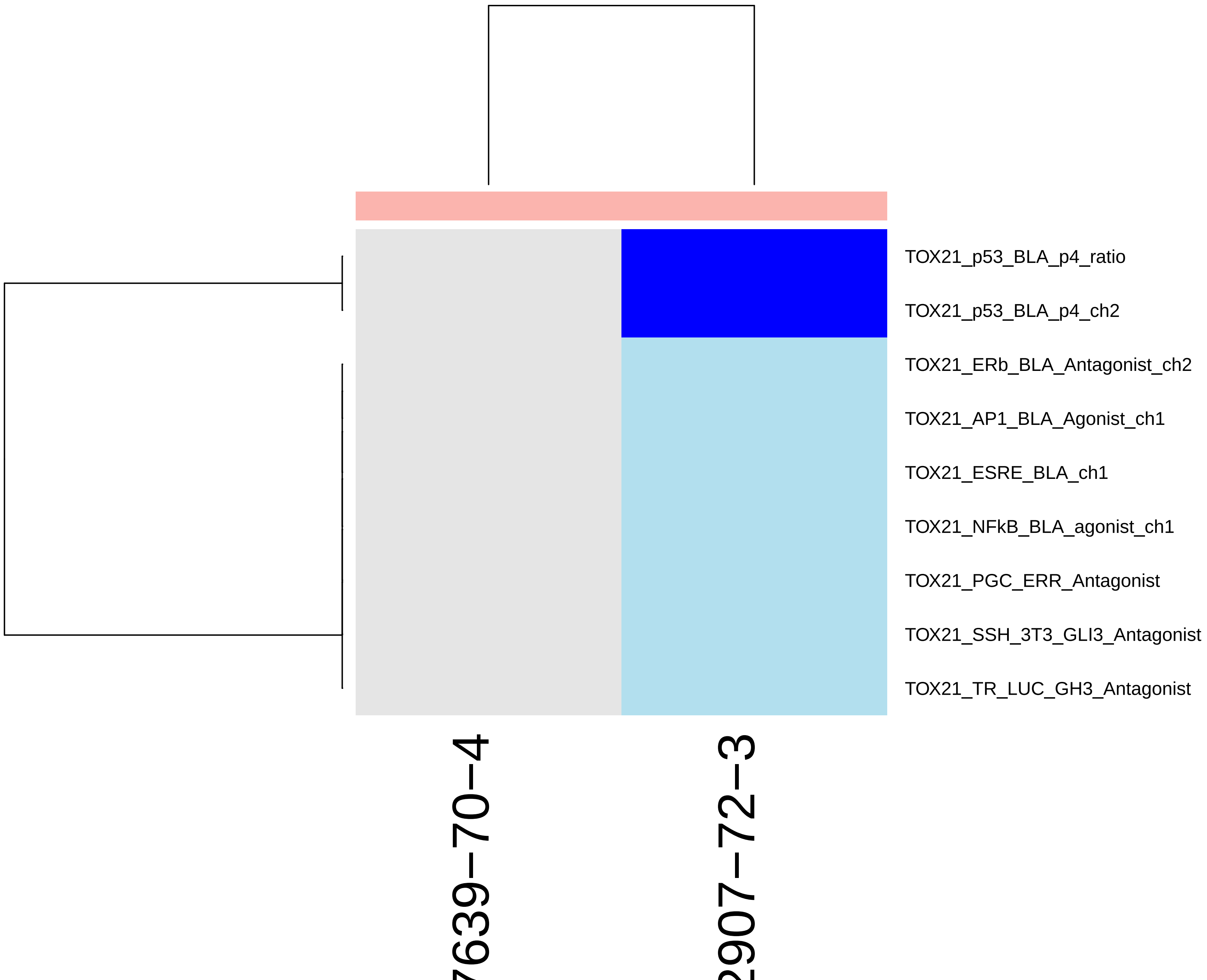

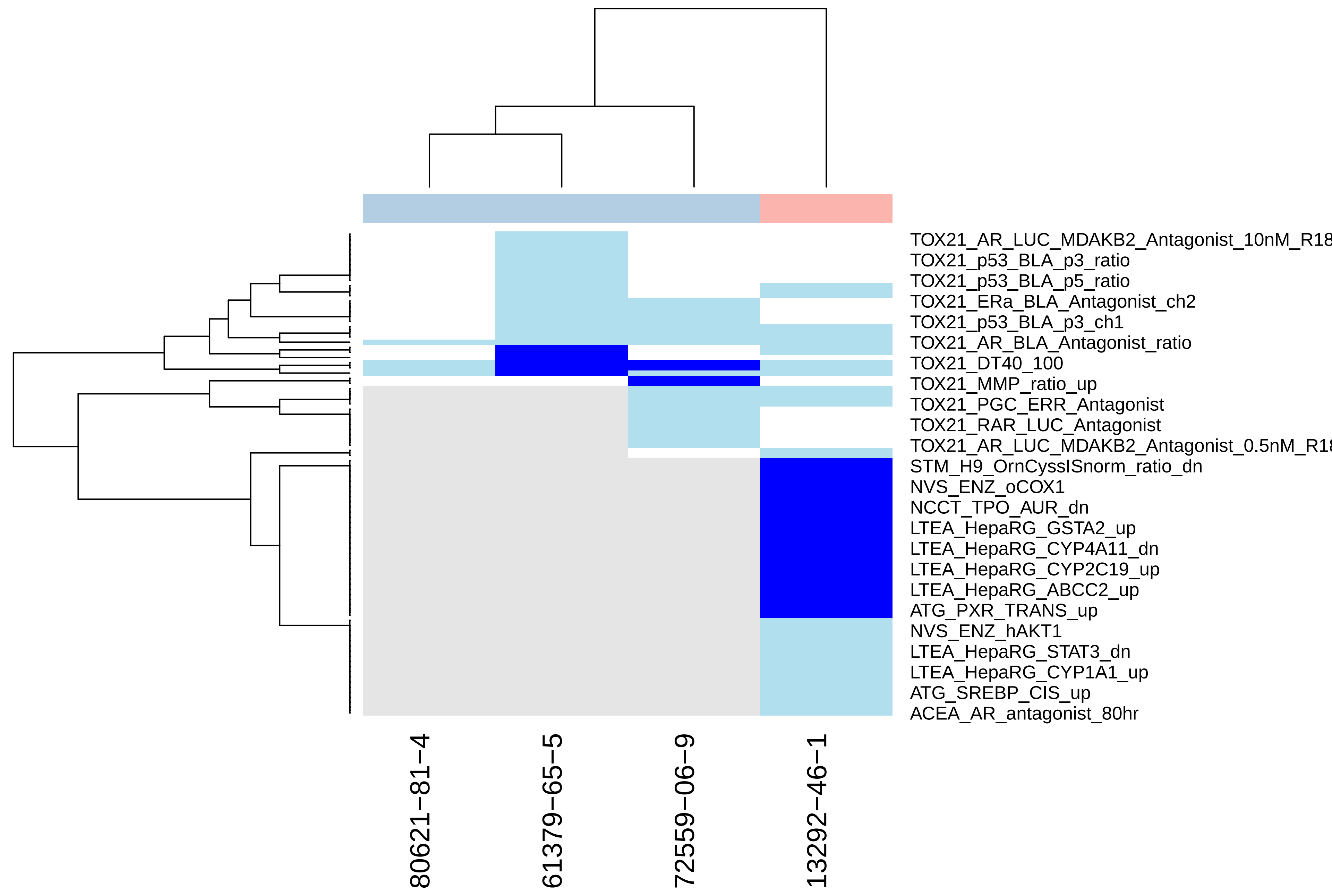
All Activity Heatmap for Cluster 1001
TOX21_AR_LUC_MDAKB2_Antagonist_10nM_R18
TOX21_p53_BLA_p3_ratio
TOX21_p53_BLA_p5_ratio
TOX21_ERa_BLA_Antagonist_ch2
TOX21_p53_BLA_p3_ch1
TOX21_AR_BLA_Antagonist_ratio
TOX21_DT40_100
TOX21_MMP_ratio_up
TOX21_PGC_ERR_Antagonist
TOX21_RAR_LUC_Antagonist
TOX21_AR_LUC_MDAKB2_Antagonist_0.5nM_R18
STM_H9_OrnCyssISnorm_ratio_dn
NVS_ENZ_oCOX1
NCCT_TPO_AUR_dn
LTEA_HepaRG_GSTA2_up
LTEA_HepaRG_CYP4A11_dn
LTEA_HepaRG_CYP2C19_up
LTEA_HepaRG_ABCC2_up
ATG_PXR_TRANS_up
NVS_ENZ_hAKT1
LTEA_HepaRG_STAT3_dn
LTEA_HepaRG_CYP1A1_up
ATG_SREBP_CIS_up
ACEA_AR_antagonist_80hr
80621−81−4
61379−65−5
72559−06−9
13292−46−1

All Activity Heatmap for Cluster 1002

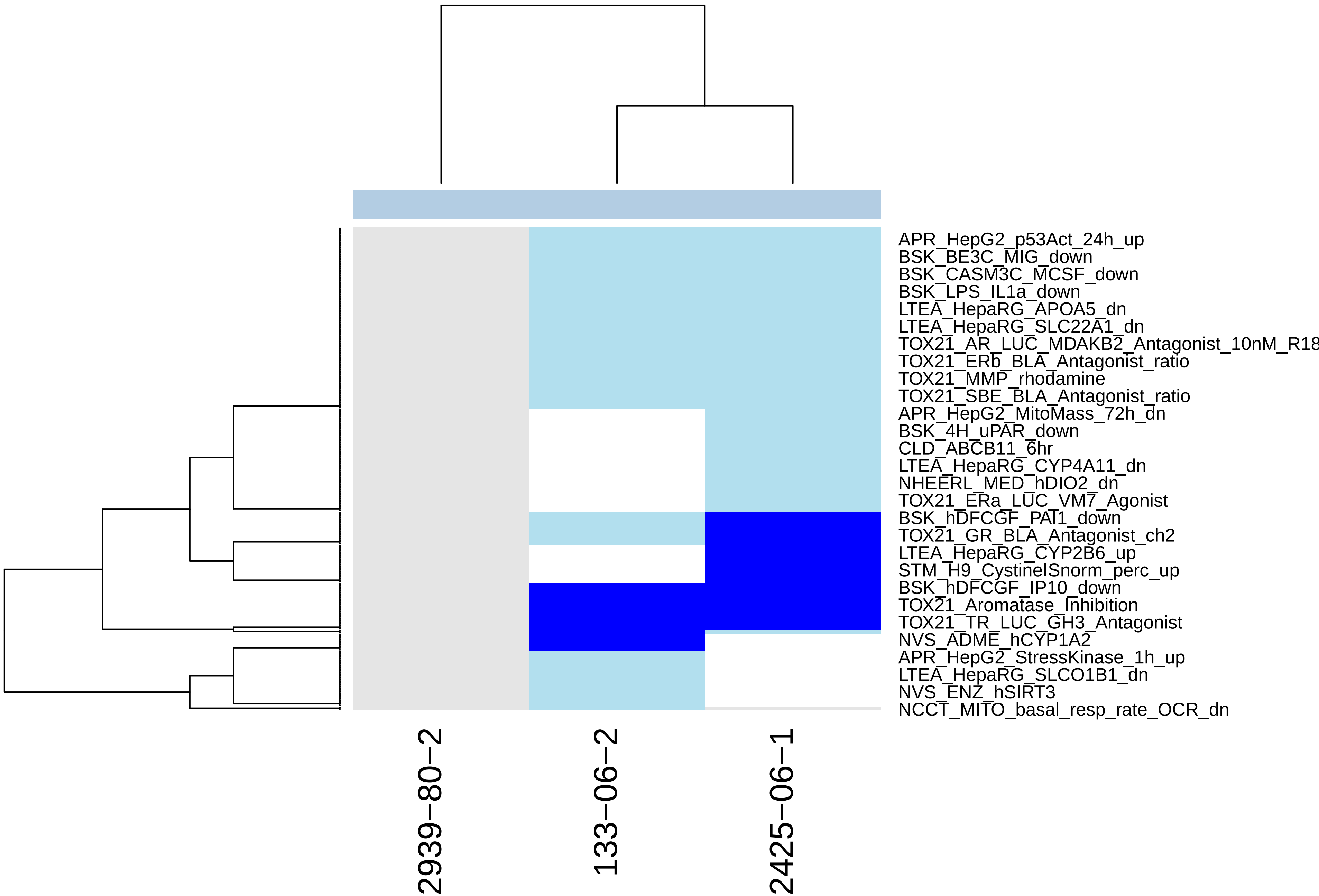

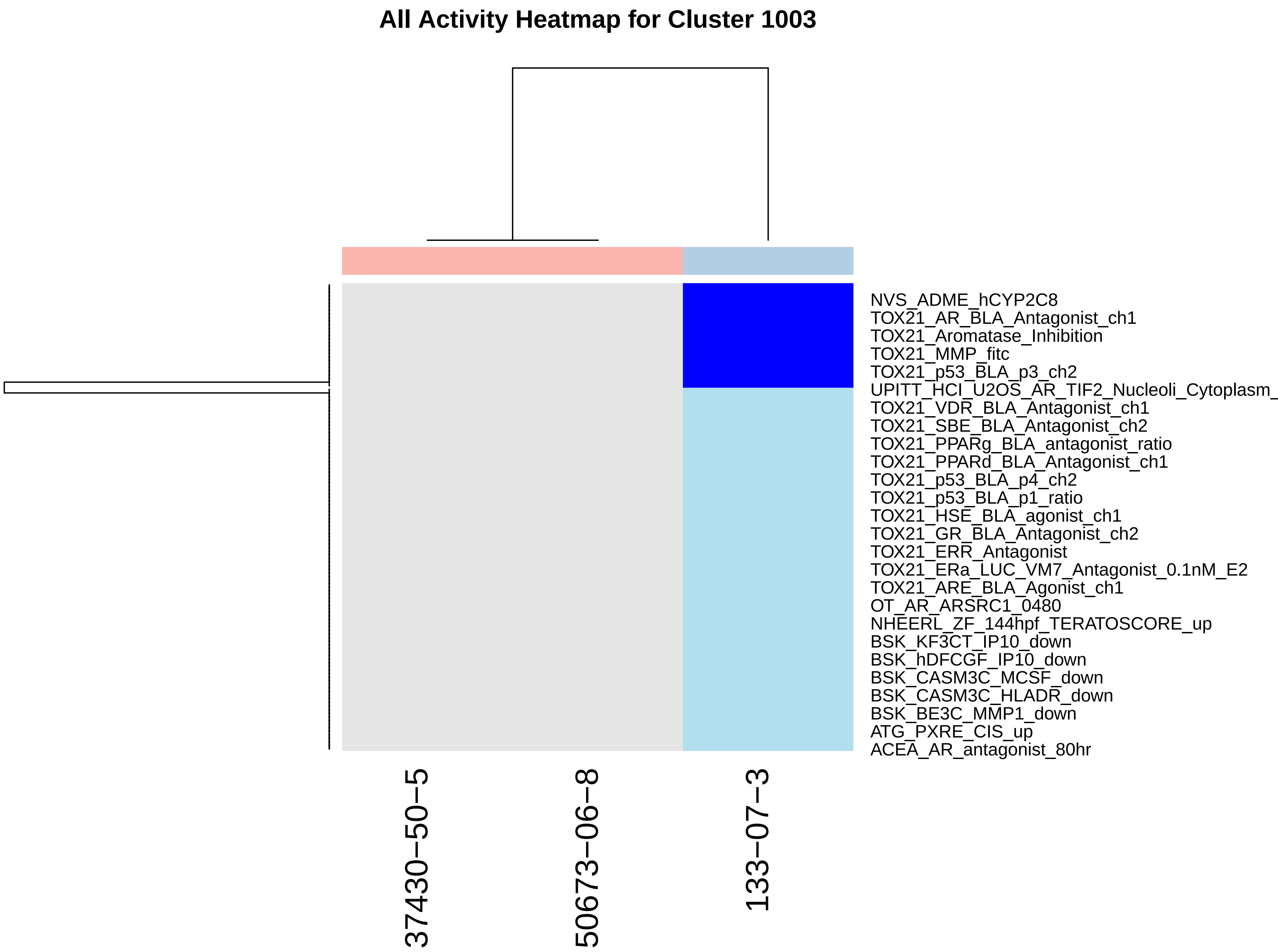
All Activity Heatmap for Cluster 1003
NVS_ADME_hCYP2C8
TOX21_AR_BLA_Antagonist_ch1
TOX21_Aromatase_Inhibition
TOX21_MMP_fitc
TOX21_p53_BLA_p3_ch2
UPITT_HCI_U2OS_AR_TIF2_Nucleoli_Cytoplasm_
TOX21_VDR_BLA_Antagonist_ch1
TOX21_SBE_BLA_Antagonist_ch2
TOX21_PPARg_BLA_antagonist_ratio
TOX21_PPARd_BLA_Antagonist_ch1
TOX21_p53_BLA_p4_ch2
TOX21_p53_BLA_p1_ratio
TOX21_HSE_BLA_agonist_ch1
TOX21_GR_BLA_Antagonist_ch2
TOX21_ERR_Antagonist
TOX21_ERa_LUC_VM7_Antagonist_0.1nM_E2
TOX21_ARE_BLA_Agonist_ch1
OT_AR_ARSRC1_0480
NHEERL_ZF_144hpf_TERATOSCORE_up
BSK_KF3CT_IP10_down
BSK_hDFCGF_IP10_down
BSK_CASM3C_MCSF_down
BSK_CASM3C_HLADR_down
BSK_BE3C_MMP1_down
ATG_PXRE_CIS_up
ACEA_AR_antagonist_80hr
37430−50−5
50673−06−8
133−07−3

All Activity Heatmap for Cluster 1005

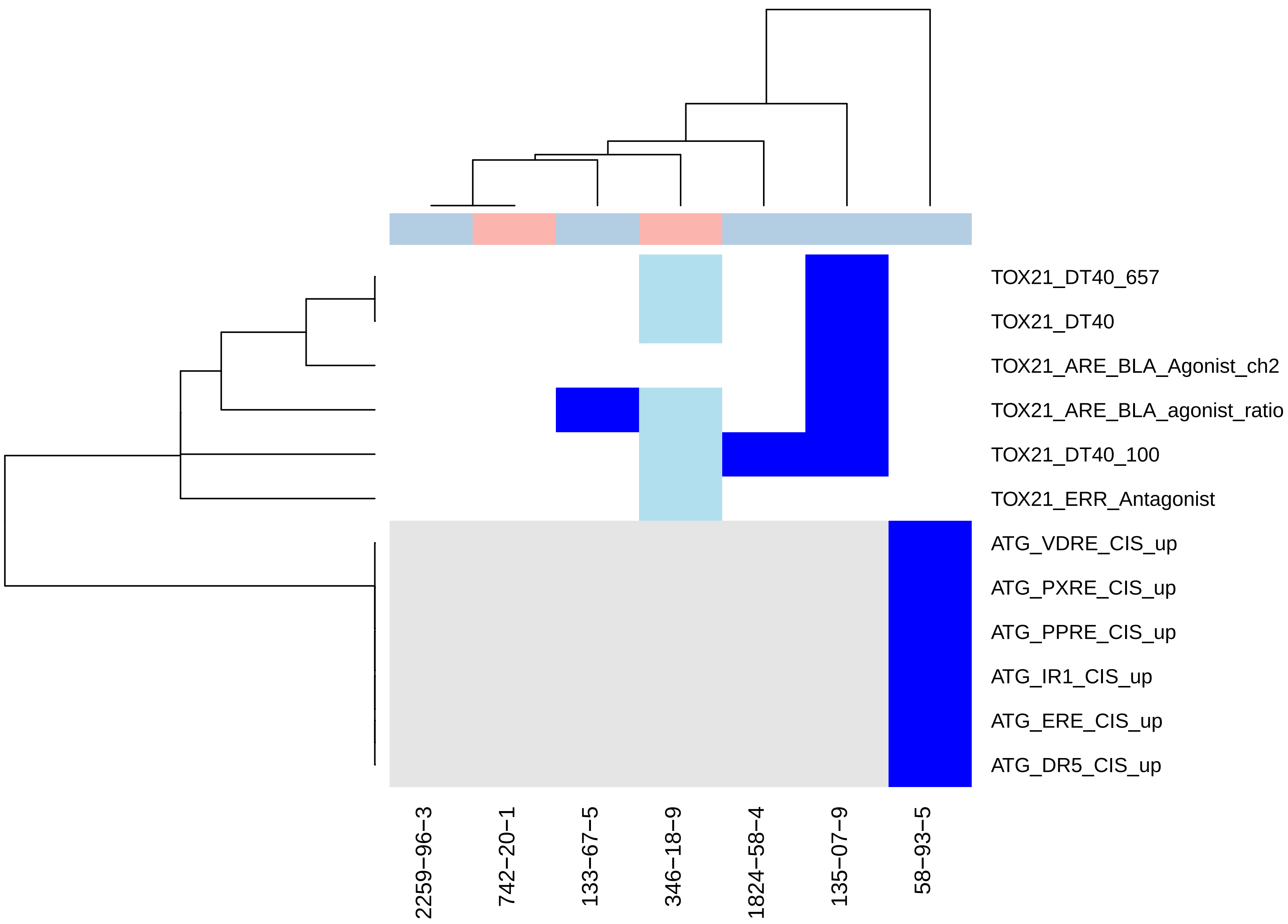

All Activity Heatmap for Cluster 1006

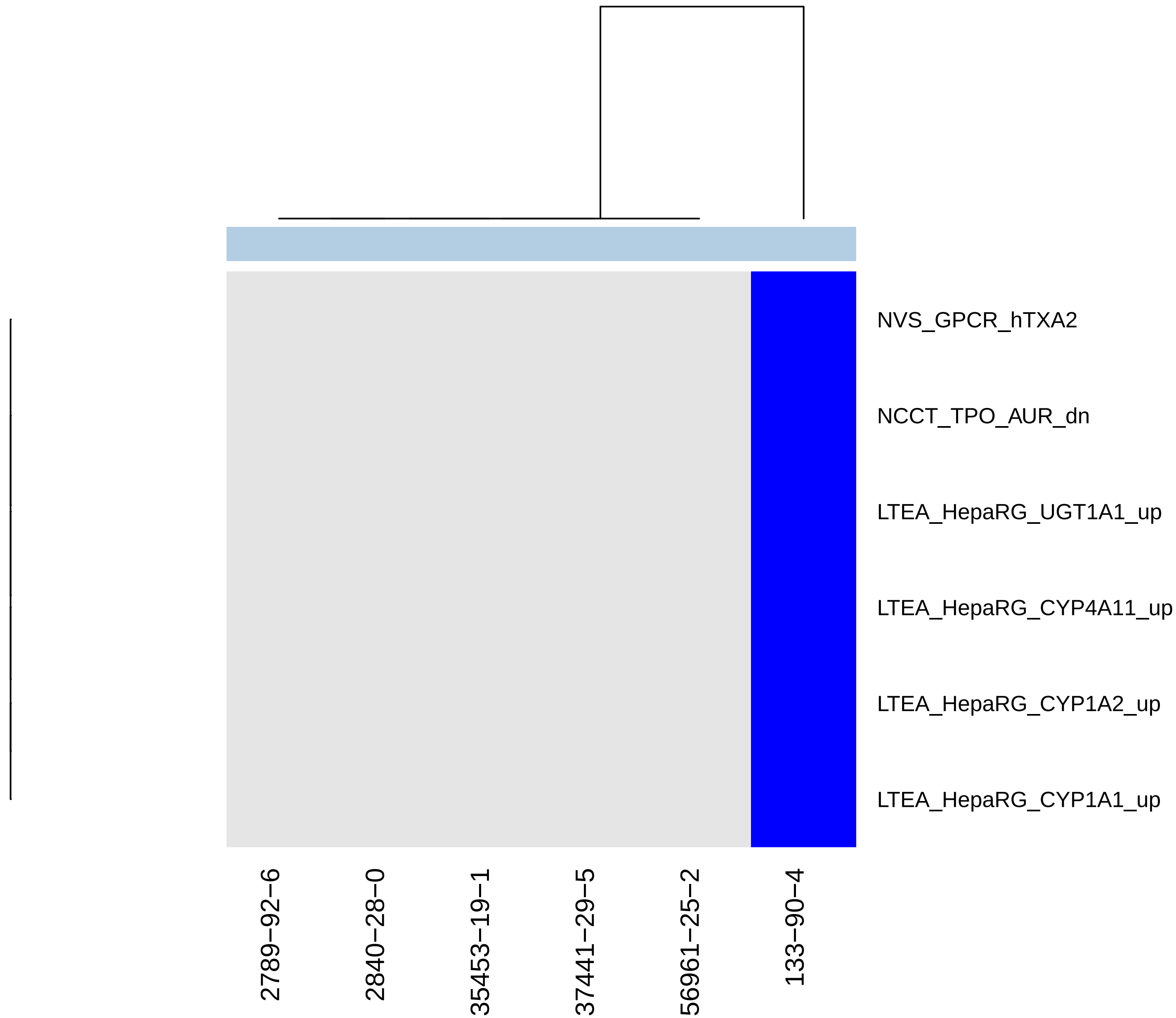

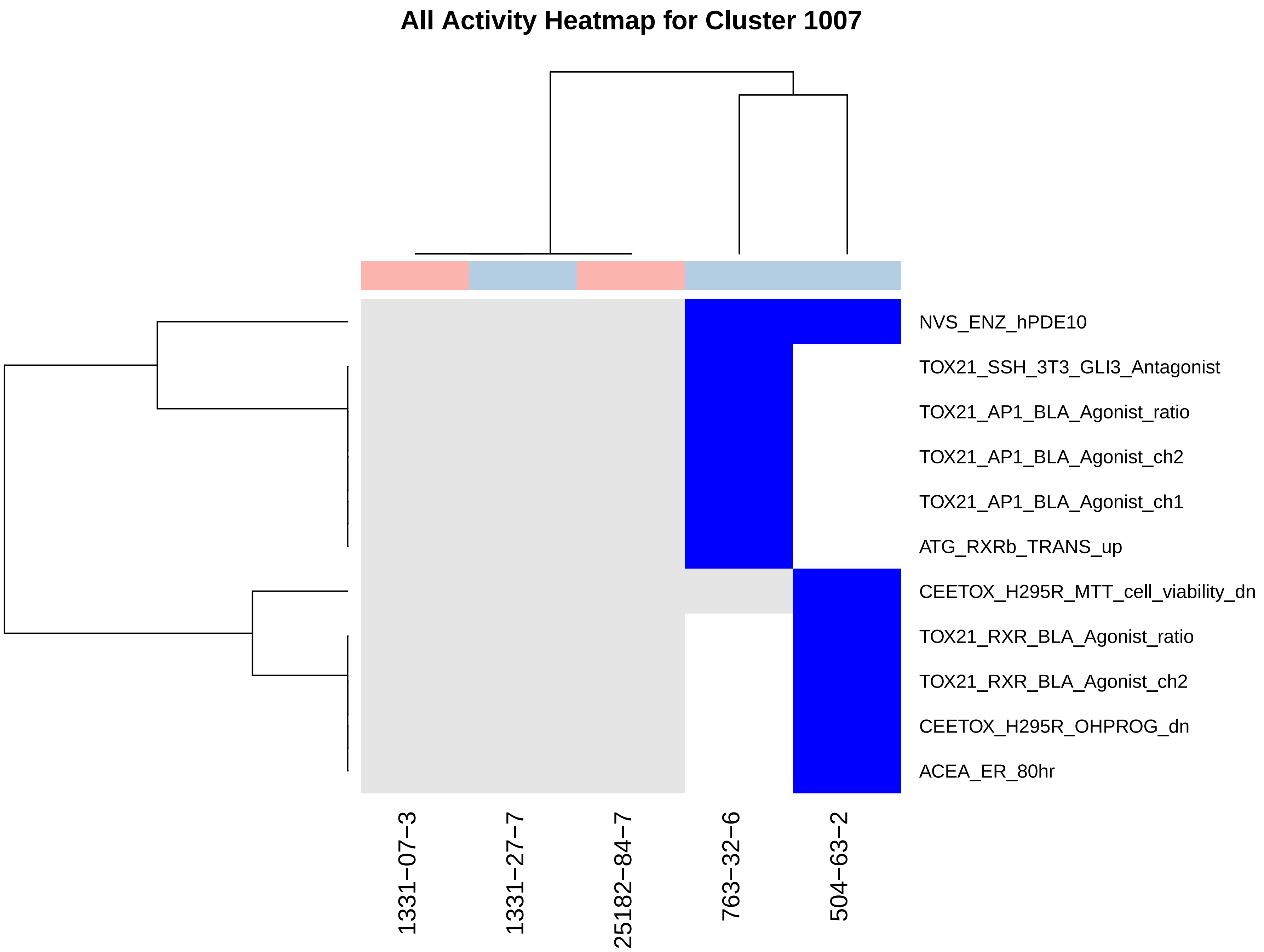
All Activity Heatmap for Cluster 1007
NVS_ENZ_hPDE10
TOX21_SSH_3T3_GLI3_Antagonist
TOX21_AP1_BLA_Agonist_ratio
TOX21_AP1_BLA_Agonist_ch2
TOX21_AP1_BLA_Agonist_ch1
ATG_RXRb_TRANS_up
CEETOX_H295R_MTT_cell_viability_dn
TOX21_RXR_BLA_Agonist_ratio
TOX21_RXR_BLA_Agonist_ch2
CEETOX_H295R_OHPROG_dn
ACEA_ER_80hr
1331−07−3
1331−27−7
25182−84−7
763−32−6
504−63−2

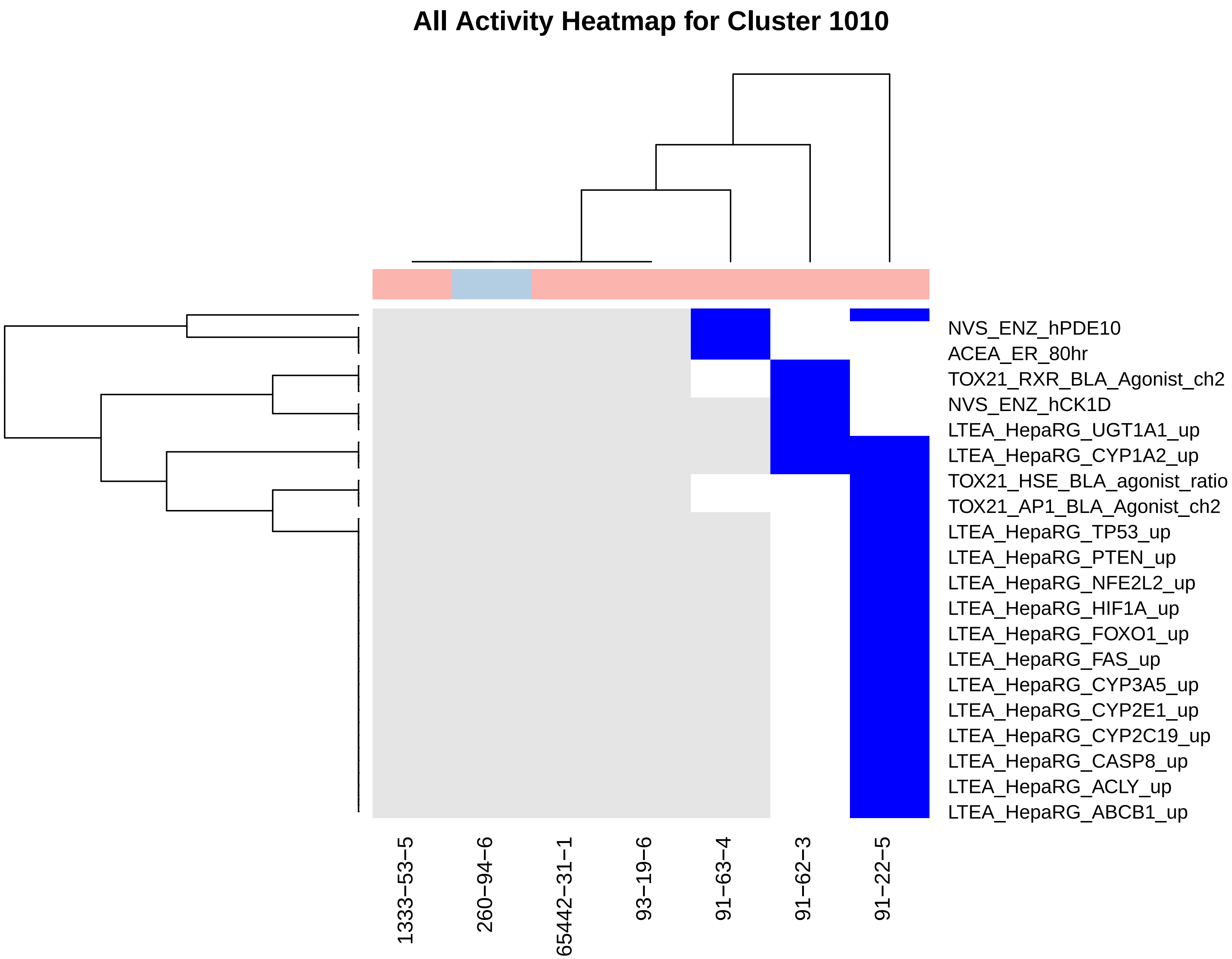
All Activity Heatmap for Cluster 1010
NVS_ENZ_hPDE10
ACEA_ER_80hr
TOX21_RXR_BLA_Agonist_ch2
NVS_ENZ_hCK1D
LTEA_HepaRG_UGT1A1_up
LTEA_HepaRG_CYP1A2_up
TOX21_HSE_BLA_agonist_ratio
TOX21_AP1_BLA_Agonist_ch2
LTEA_HepaRG_TP53_up
LTEA_HepaRG_PTEN_up
LTEA_HepaRG_NFE2L2_up
LTEA_HepaRG_HIF1A_up
LTEA_HepaRG_FOXO1_up
LTEA_HepaRG_FAS_up
LTEA_HepaRG_CYP3A5_up
LTEA_HepaRG_CYP2E1_up
LTEA_HepaRG_CYP2C19_up
LTEA_HepaRG_CASP8_up
LTEA_HepaRG_ACLY_up
LTEA_HepaRG_ABCB1_up
1333−53−5
260−94−6
65442−31−1
93−19−6
91−63−4
91−62−3
91−22−5

All Activity Heatmap for Cluster 1012

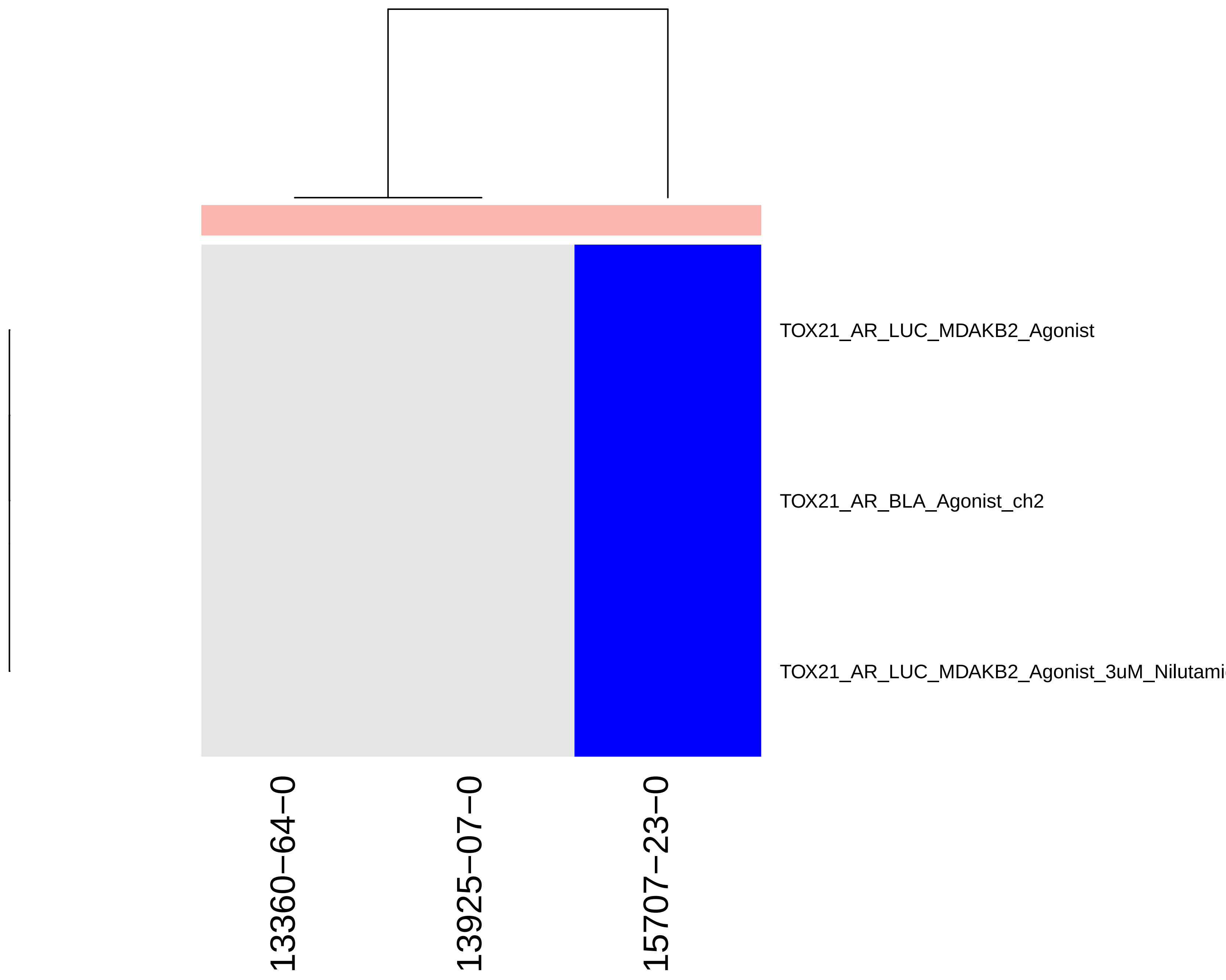

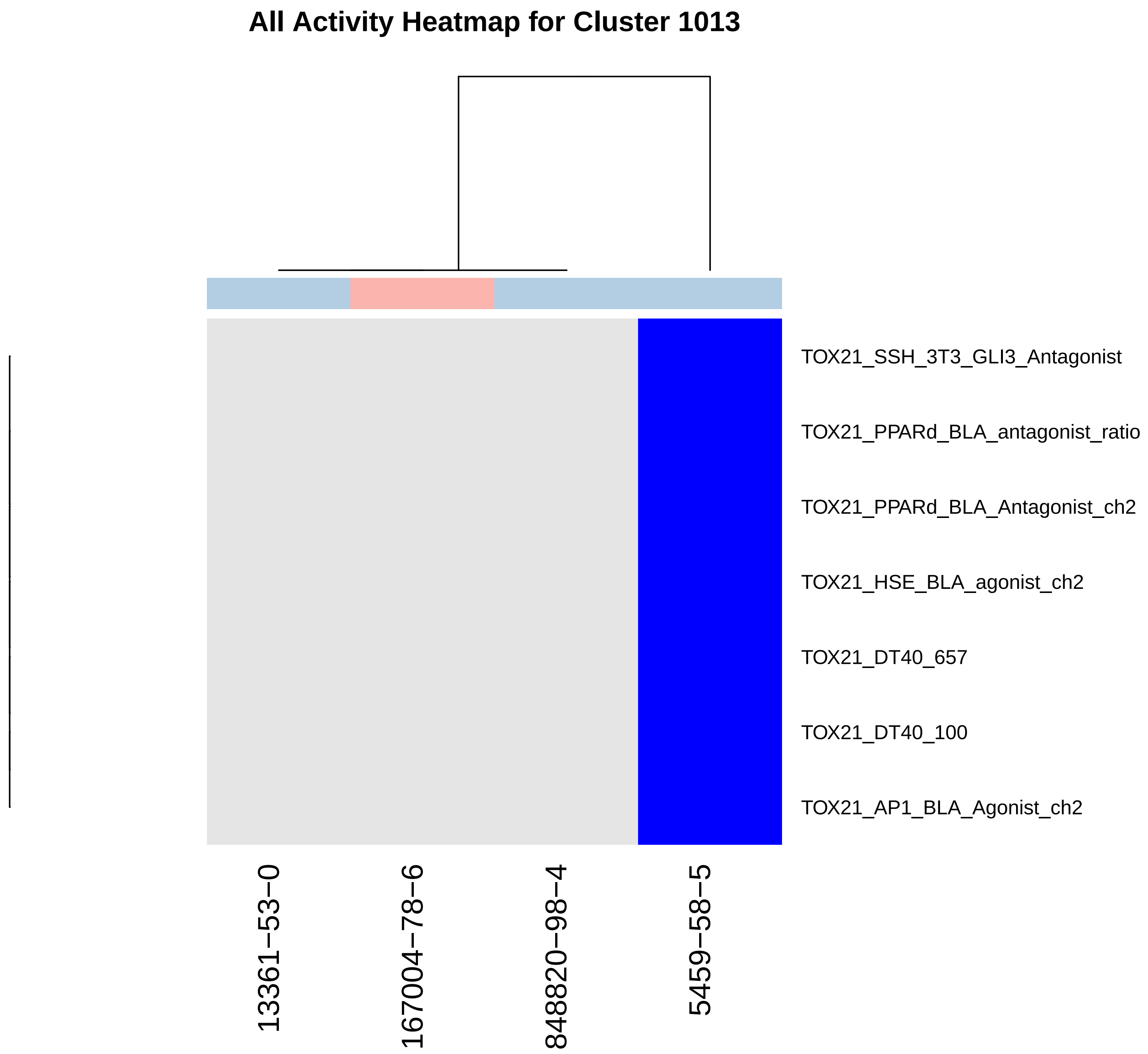
All Activity Heatmap for Cluster 1013
TOX21_SSH_3T3_GLI3_Antagonist
TOX21_PPARd_BLA_antagonist_ratio
TOX21_PPARd_BLA_Antagonist_ch2
TOX21_HSE_BLA_agonist_ch2
TOX21_DT40_657
TOX21_DT40_100
TOX21_AP1_BLA_Agonist_ch2
13361−53−0
167004−78−6
848820−98−4
5459−58−5

All Activity Heatmap for Cluster 1016

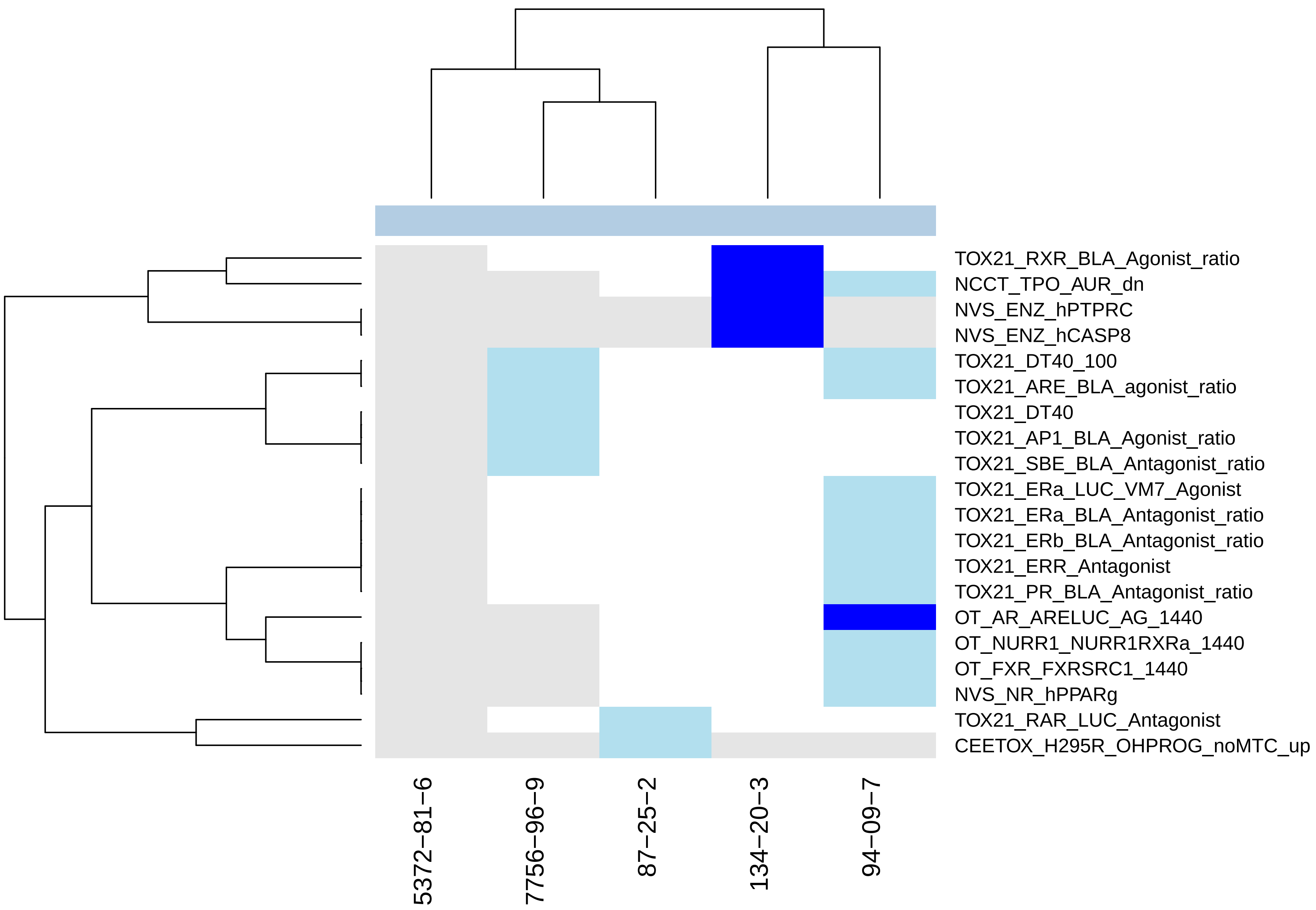

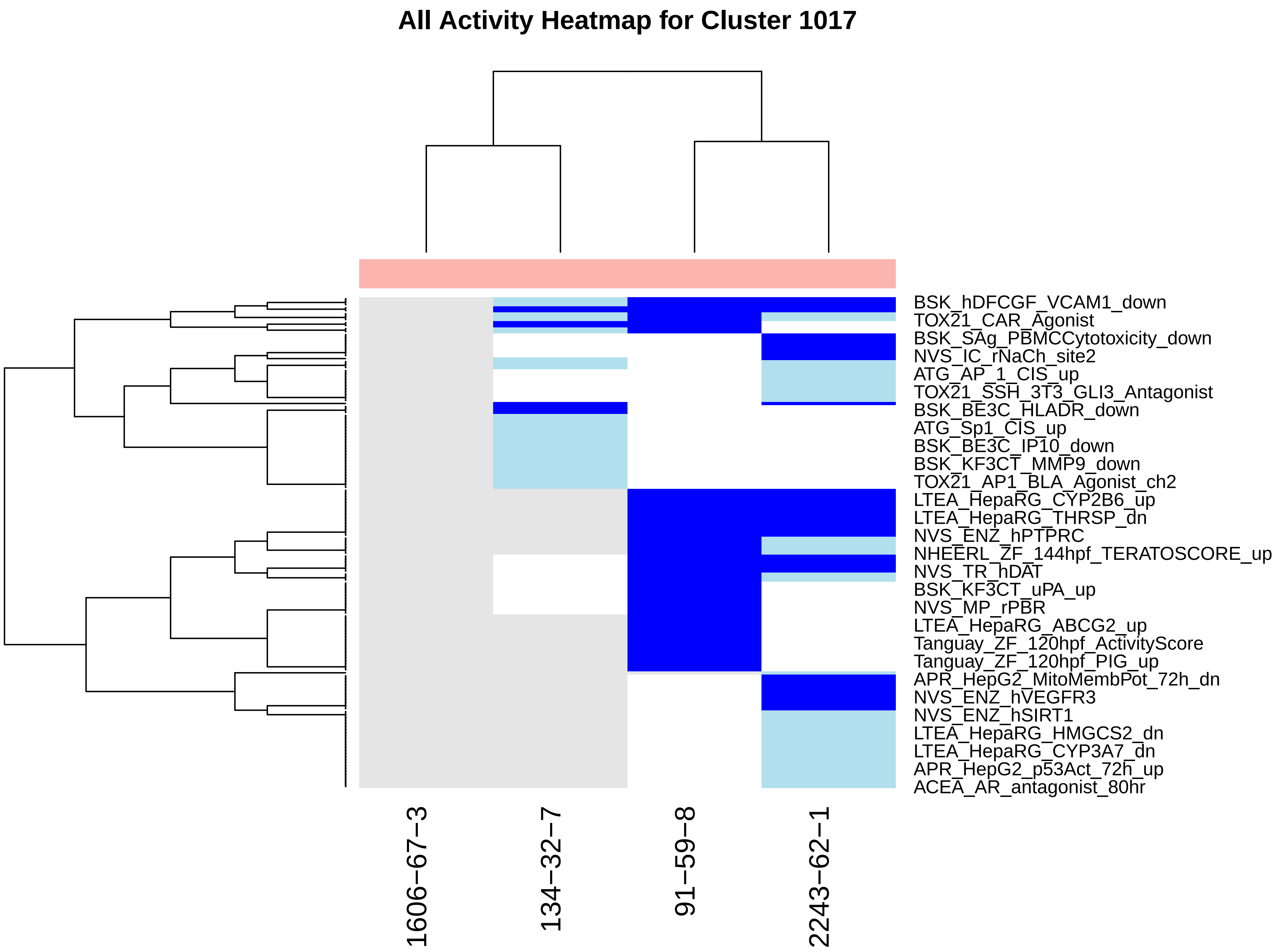
All Activity Heatmap for Cluster 1017
BSK_hDFCGF_VCAM1_down
TOX21_CAR_Agonist
BSK_SAg_PBMCCytotoxicity_down
NVS_IC_rNaCh_site2
ATG_AP_1_CIS_up
TOX21_SSH_3T3_GLI3_Antagonist
BSK_BE3C_HLADR_down
ATG_Sp1_CIS_up
BSK_BE3C_IP10_down
BSK_KF3CT_MMP9_down
TOX21_AP1_BLA_Agonist_ch2
LTEA_HepaRG_CYP2B6_up
LTEA_HepaRG_THRSP_dn
NVS_ENZ_hPTPRC
NHEERL_ZF_144hpf_TERATOSCORE_up
NVS_TR_hDAT
BSK_KF3CT_uPA_up
NVS_MP_rPBR
LTEA_HepaRG_ABCG2_up
Tanguay_ZF_120hpf_ActivityScore
Tanguay_ZF_120hpf_PIG_up
APR_HepG2_MitoMembPot_72h_dn
NVS_ENZ_hVEGFR3
NVS_ENZ_hSIRT1
LTEA_HepaRG_HMGCS2_dn
LTEA_HepaRG_CYP3A7_dn
APR_HepG2_p53Act_72h_up
ACEA_AR_antagonist_80hr
1606−67−3
134−32−7
91−59−8
2243−62−1

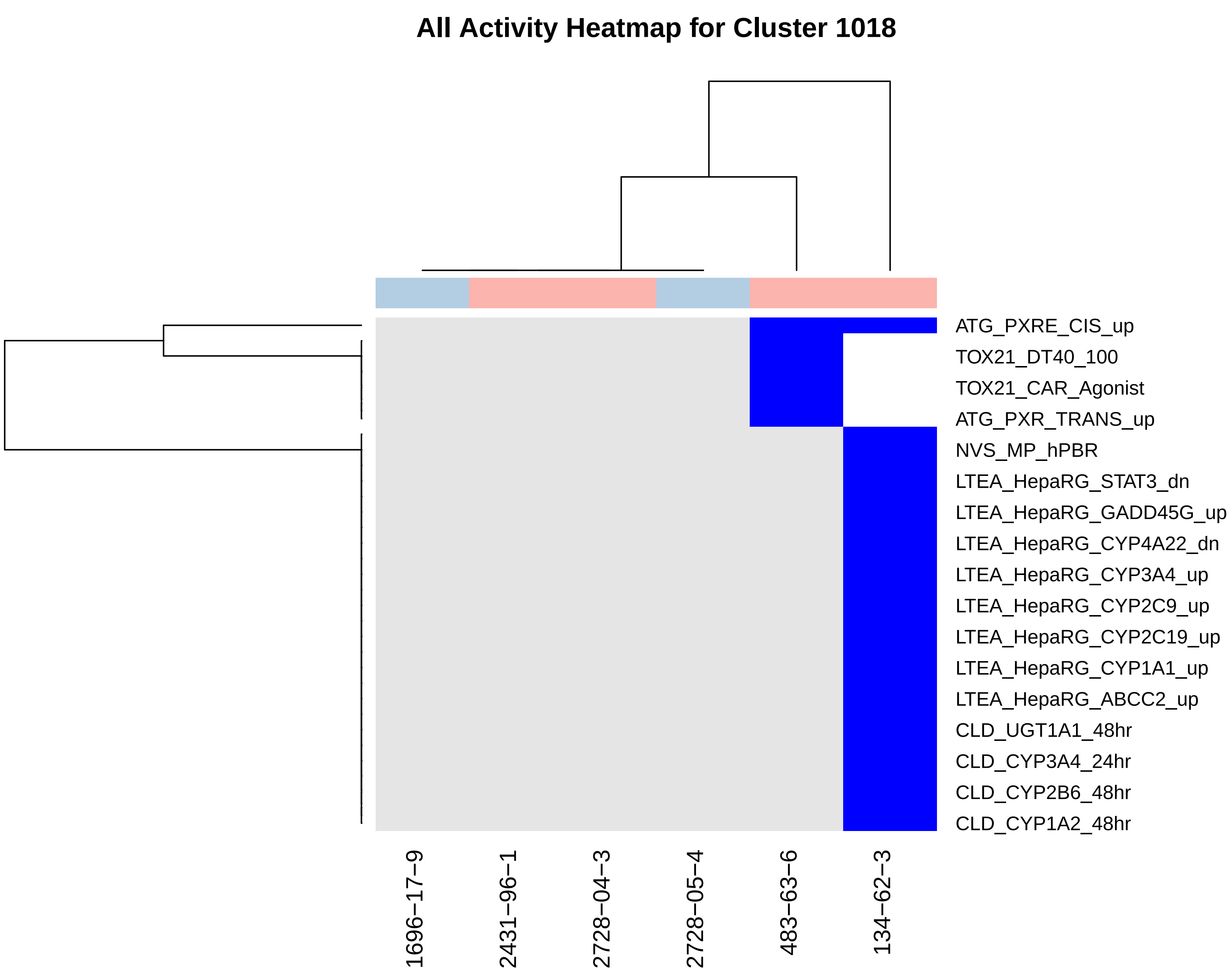
All Activity Heatmap for Cluster 1018
ATG_PXRE_CIS_up
TOX21_DT40_100
TOX21_CAR_Agonist
ATG_PXR_TRANS_up
NVS_MP_hPBR
LTEA_HepaRG_STAT3_dn
LTEA_HepaRG_GADD45G_up
LTEA_HepaRG_CYP4A22_dn
LTEA_HepaRG_CYP3A4_up
LTEA_HepaRG_CYP2C9_up
LTEA_HepaRG_CYP2C19_up
LTEA_HepaRG_CYP1A1_up
LTEA_HepaRG_ABCC2_up
CLD_UGT1A1_48hr
CLD_CYP3A4_24hr
CLD_CYP2B6_48hr
CLD_CYP1A2_48hr
1696−17−9
2431−96−1
2728−04−3
2728−05−4
483−63−6
134−62−3

All Activity Heatmap for Cluster 1020

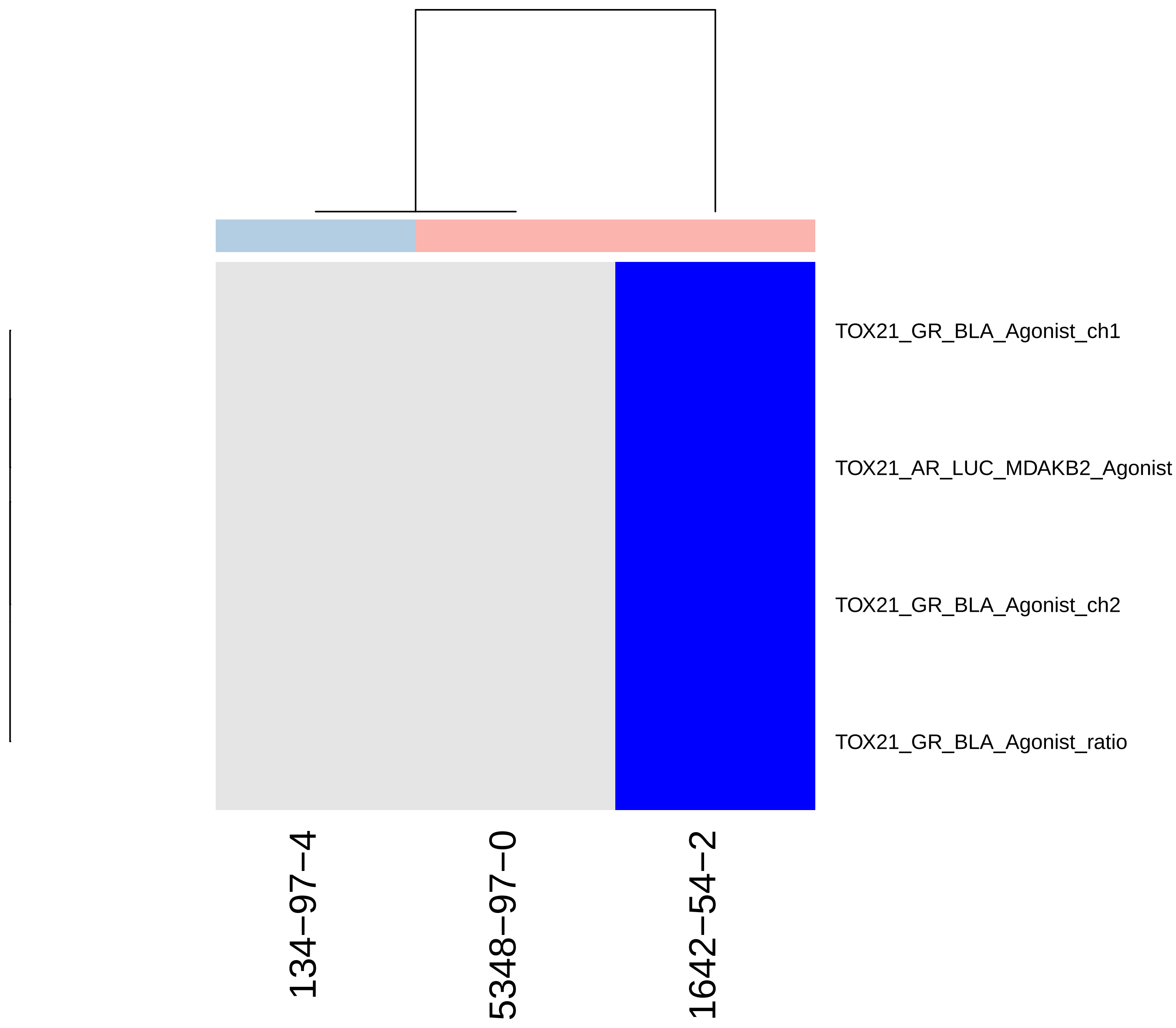

All Activity Heatmap for Cluster 1023

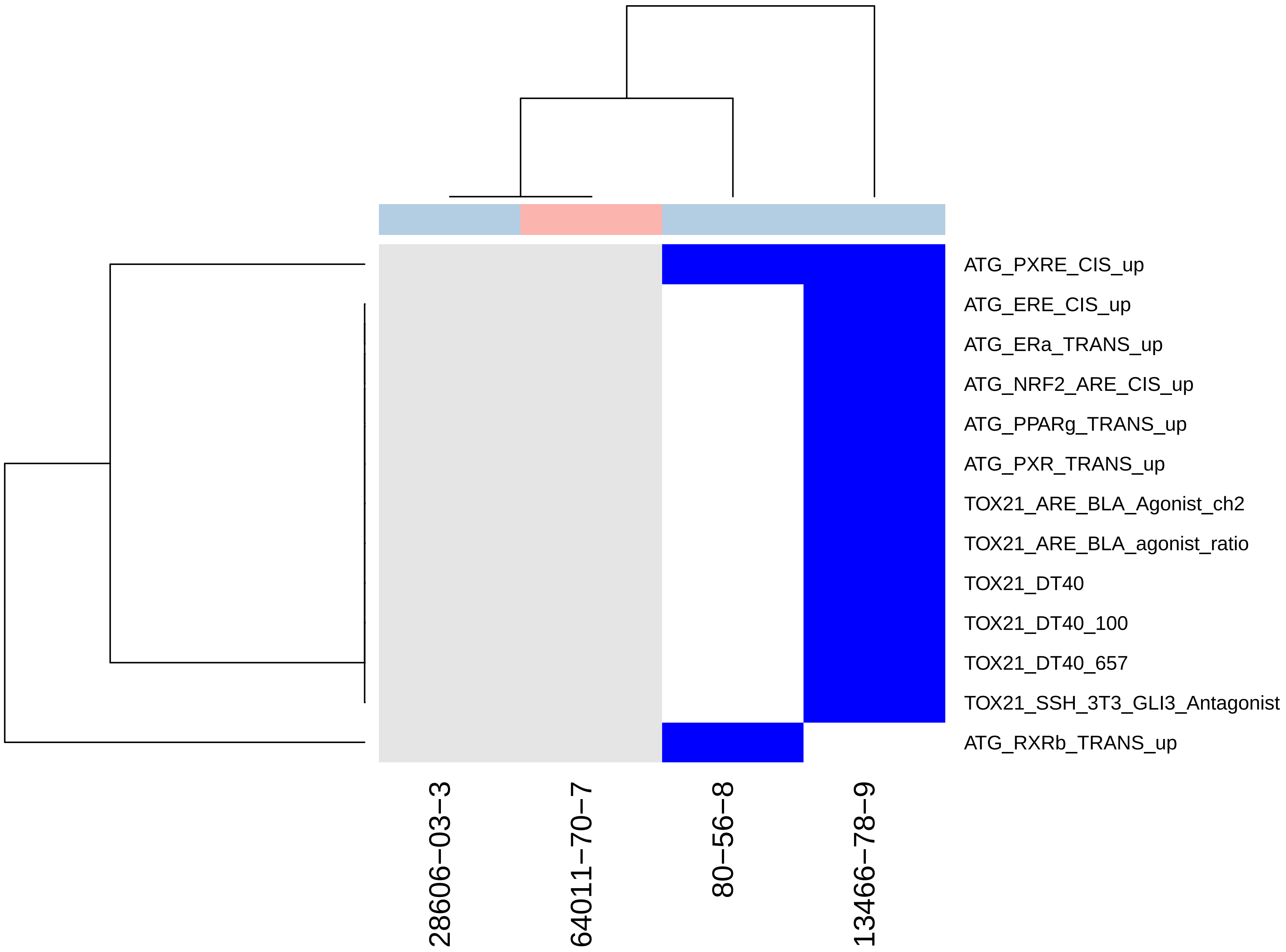

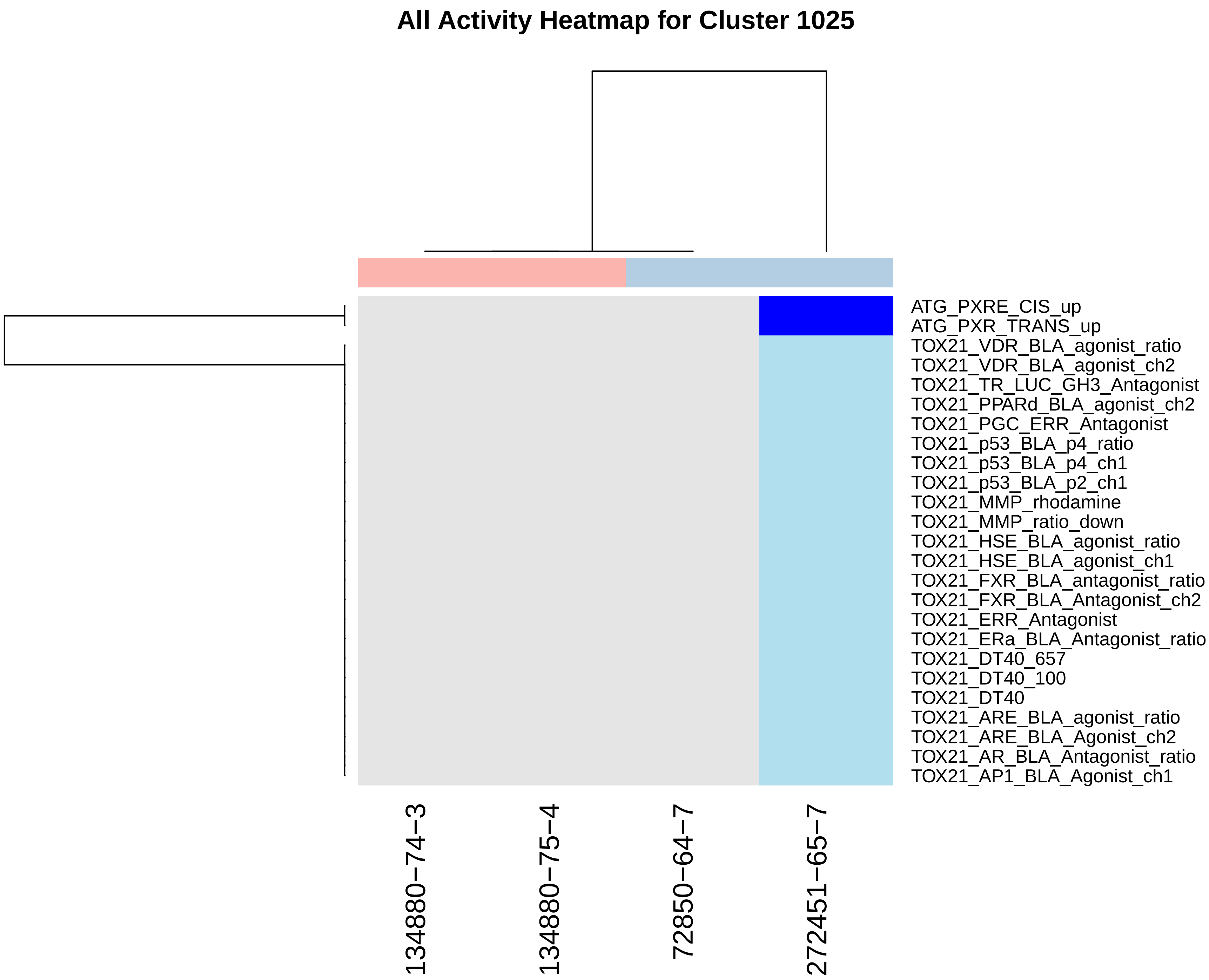
All Activity Heatmap for Cluster 1025
ATG_PXRE_CIS_up
ATG_PXR_TRANS_up
TOX21_VDR_BLA_agonist_ratio
TOX21_VDR_BLA_agonist_ch2
TOX21_TR_LUC_GH3_Antagonist
TOX21_PPARd_BLA_agonist_ch2
TOX21_PGC_ERR_Antagonist
TOX21_p53_BLA_p4_ratio
TOX21_p53_BLA_p4_ch1
TOX21_p53_BLA_p2_ch1
TOX21_MMP_rhodamine
TOX21_MMP_ratio_down
TOX21_HSE_BLA_agonist_ratio
TOX21_HSE_BLA_agonist_ch1
TOX21_FXR_BLA_antagonist_ratio
TOX21_FXR_BLA_Antagonist_ch2
TOX21_ERR_Antagonist
TOX21_ERa_BLA_Antagonist_ratio
TOX21_DT40_657
TOX21_DT40_100
TOX21_DT40
TOX21_ARE_BLA_agonist_ratio
TOX21_ARE_BLA_Agonist_ch2
TOX21_AR_BLA_Antagonist_ratio
TOX21_AP1_BLA_Agonist_ch1
134880−74−3
134880−75−4
72850−64−7
272451−65−7

All Activity Heatmap for Cluster 1026

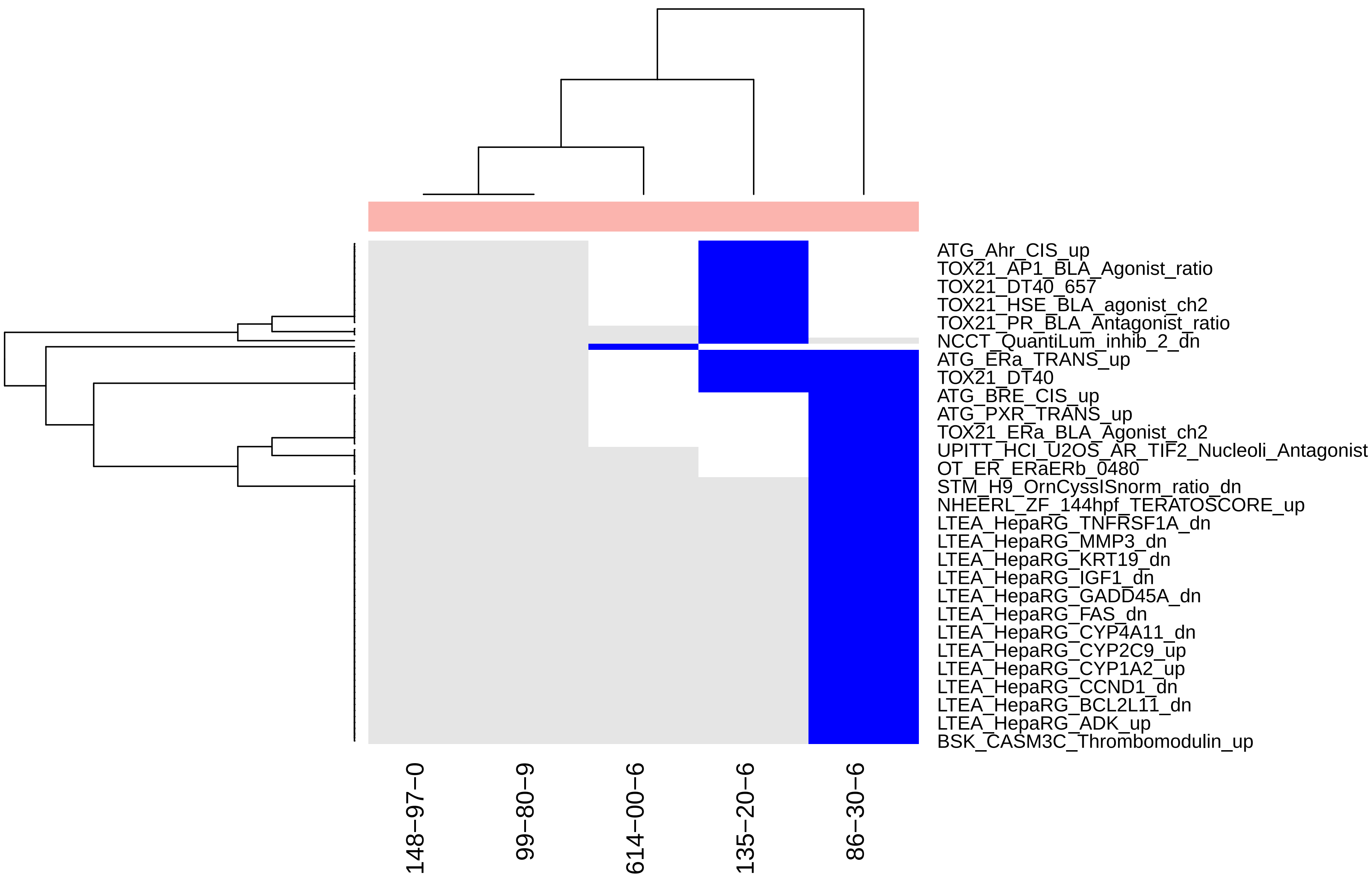

All Activity Heatmap for Cluster 1027

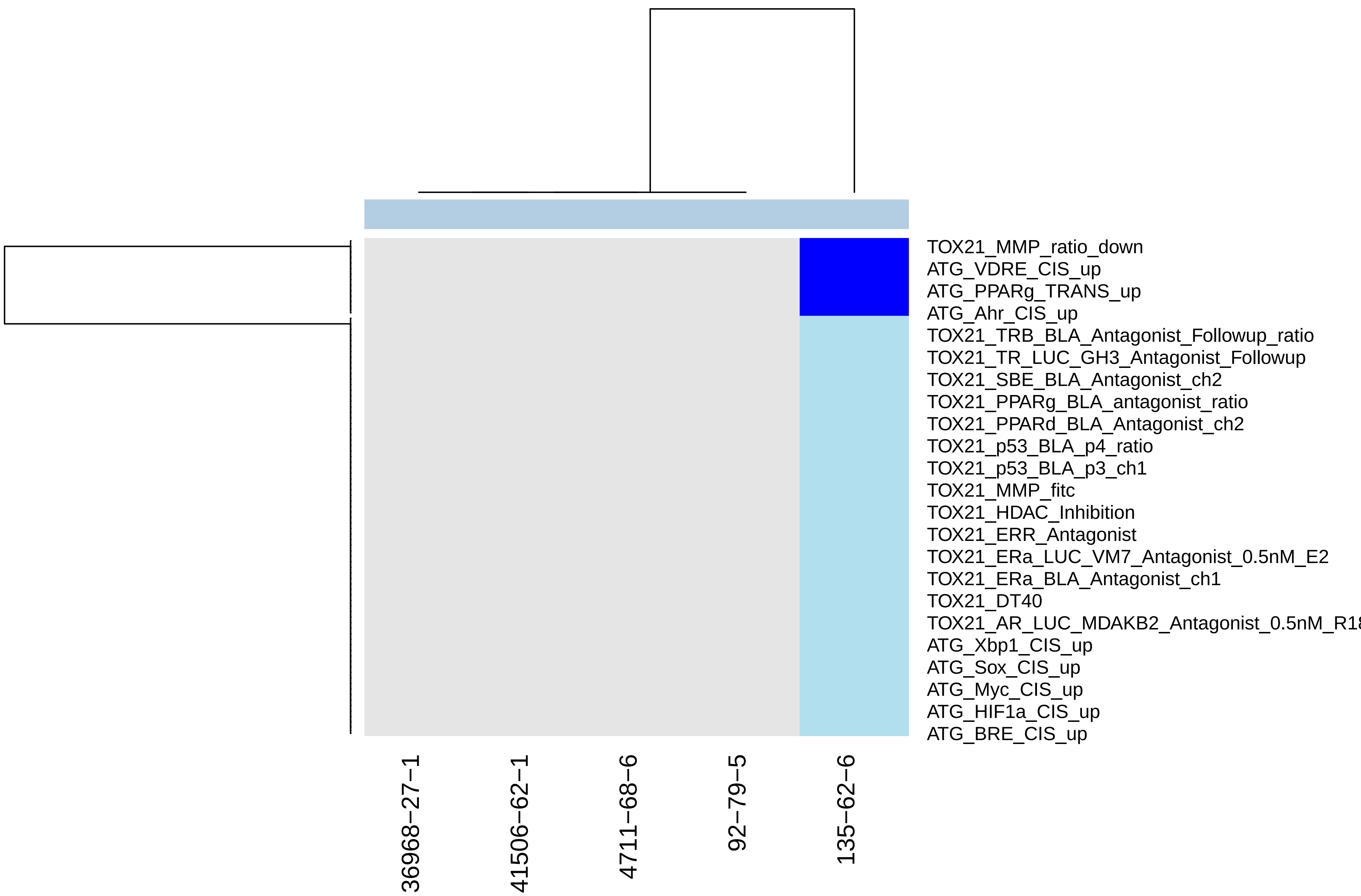

All Activity Heatmap for Cluster 1030

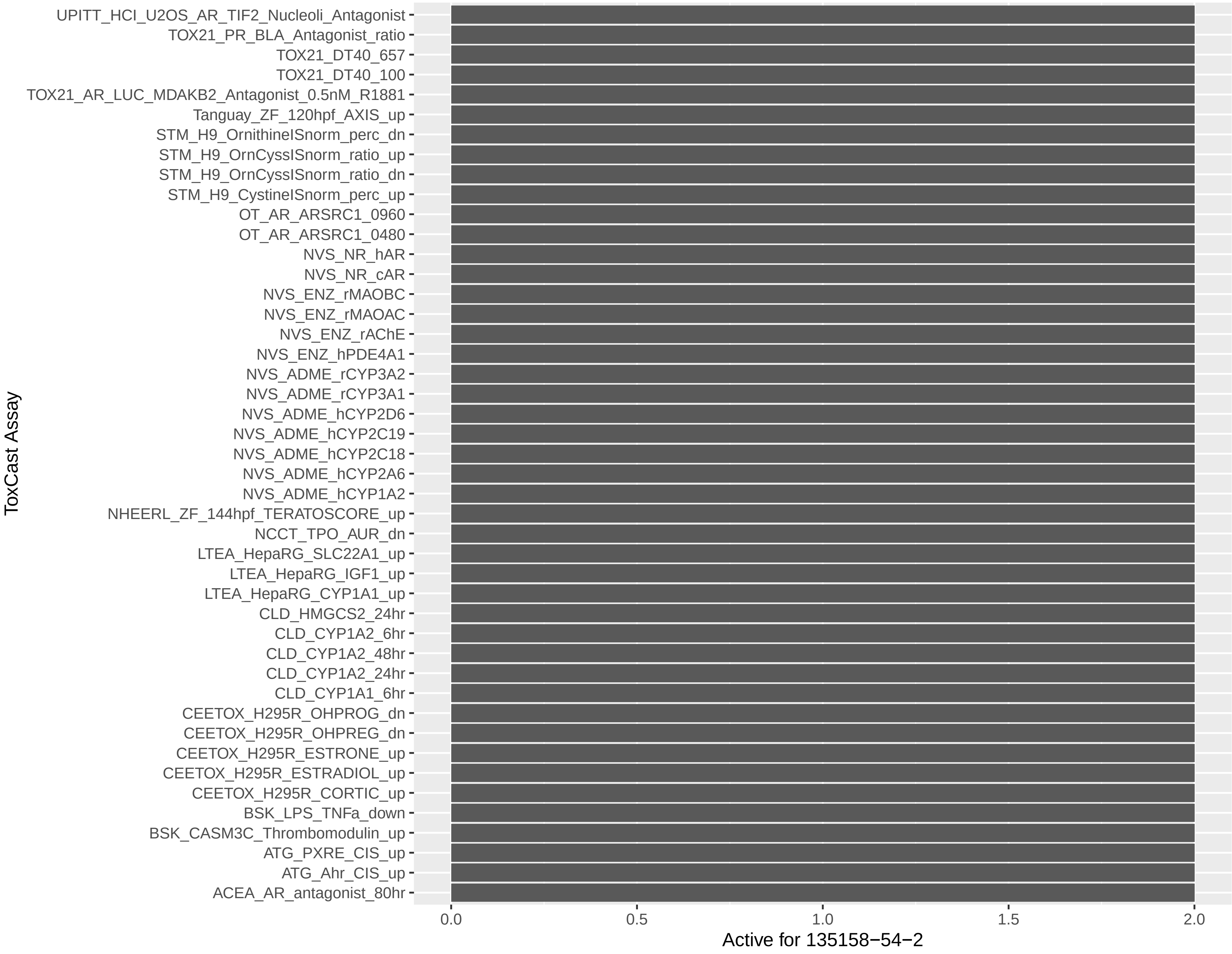

All Activity Heatmap for Cluster 1031

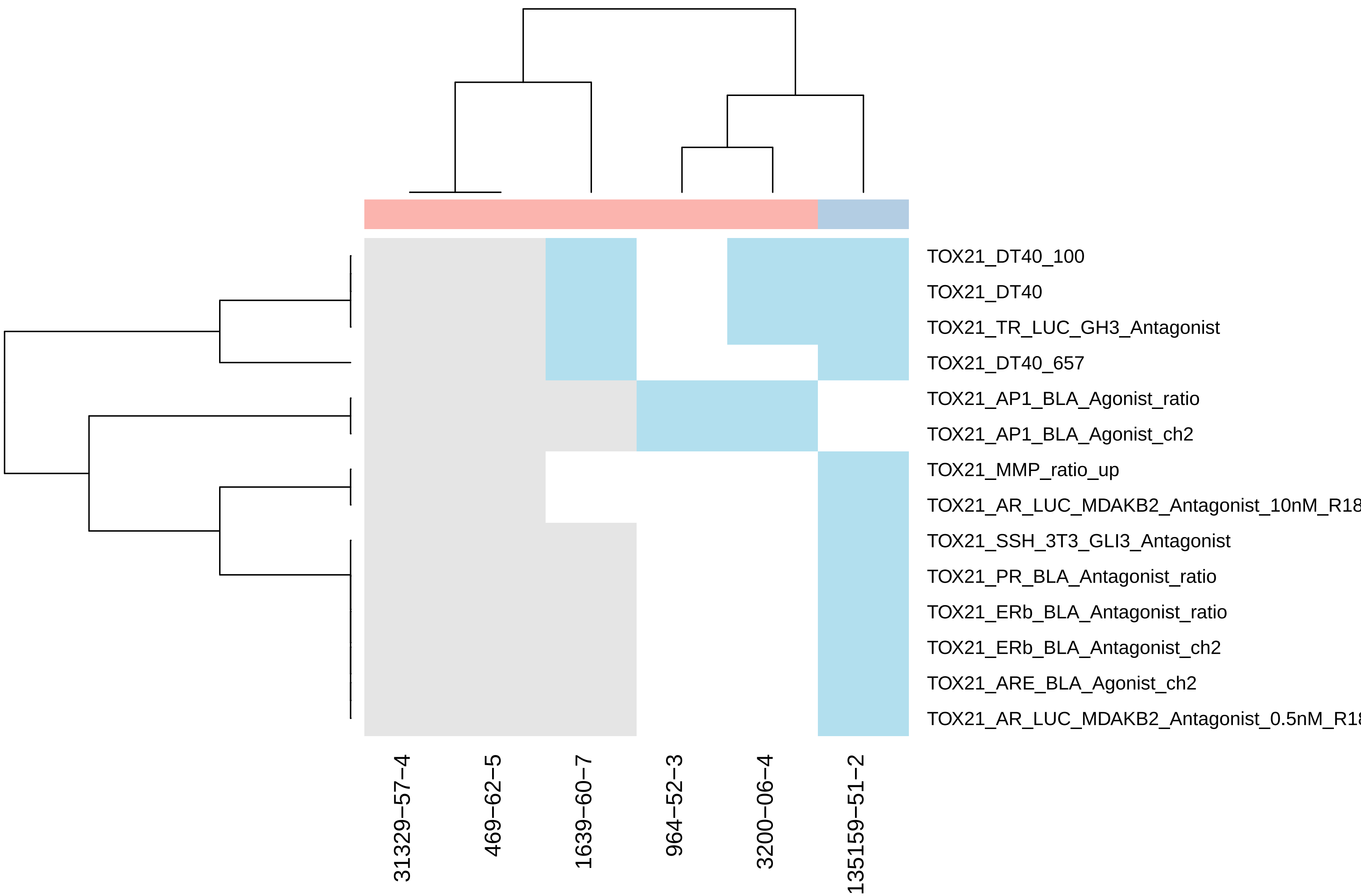

All Activity Heatmap for Cluster 1033

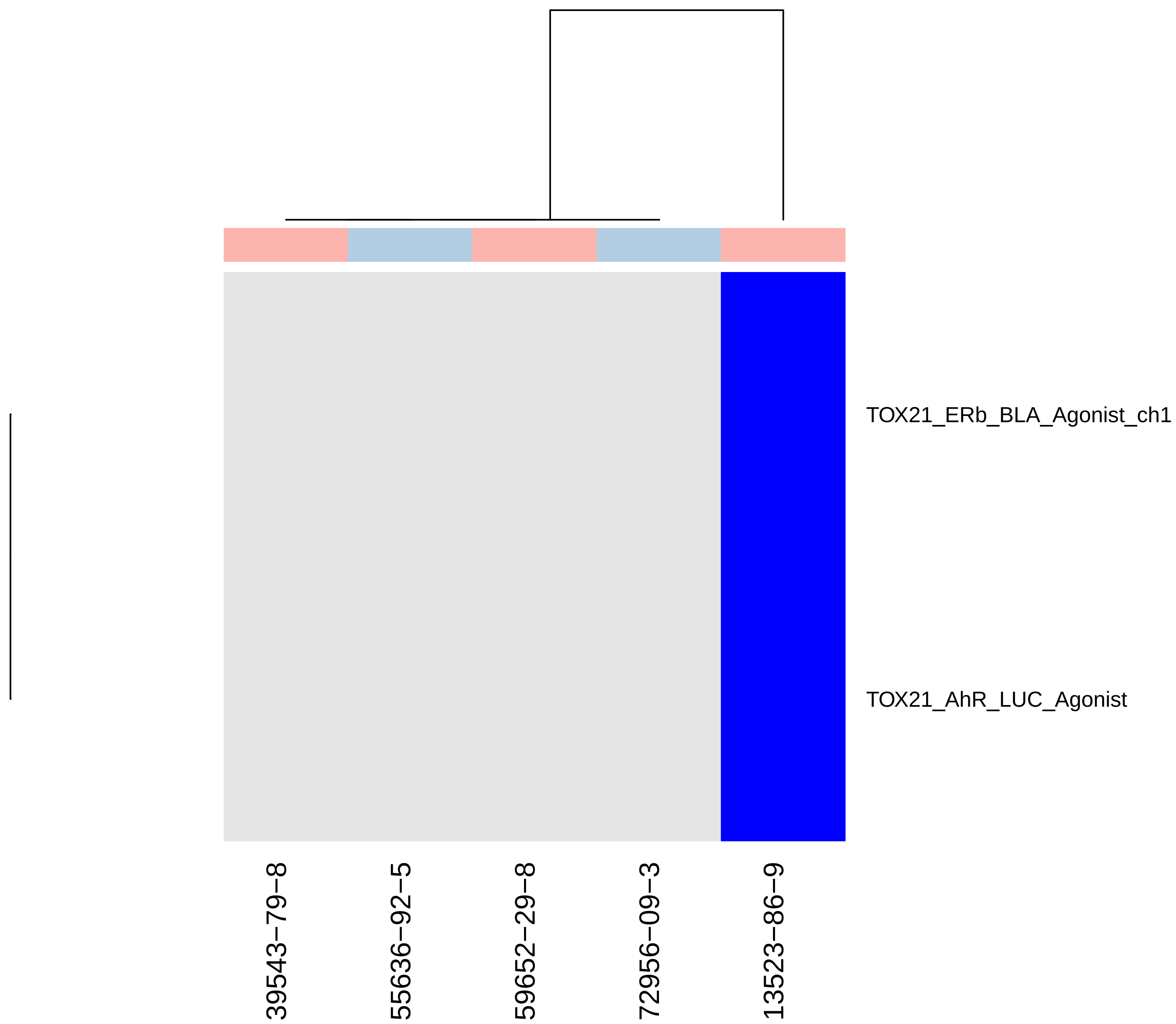

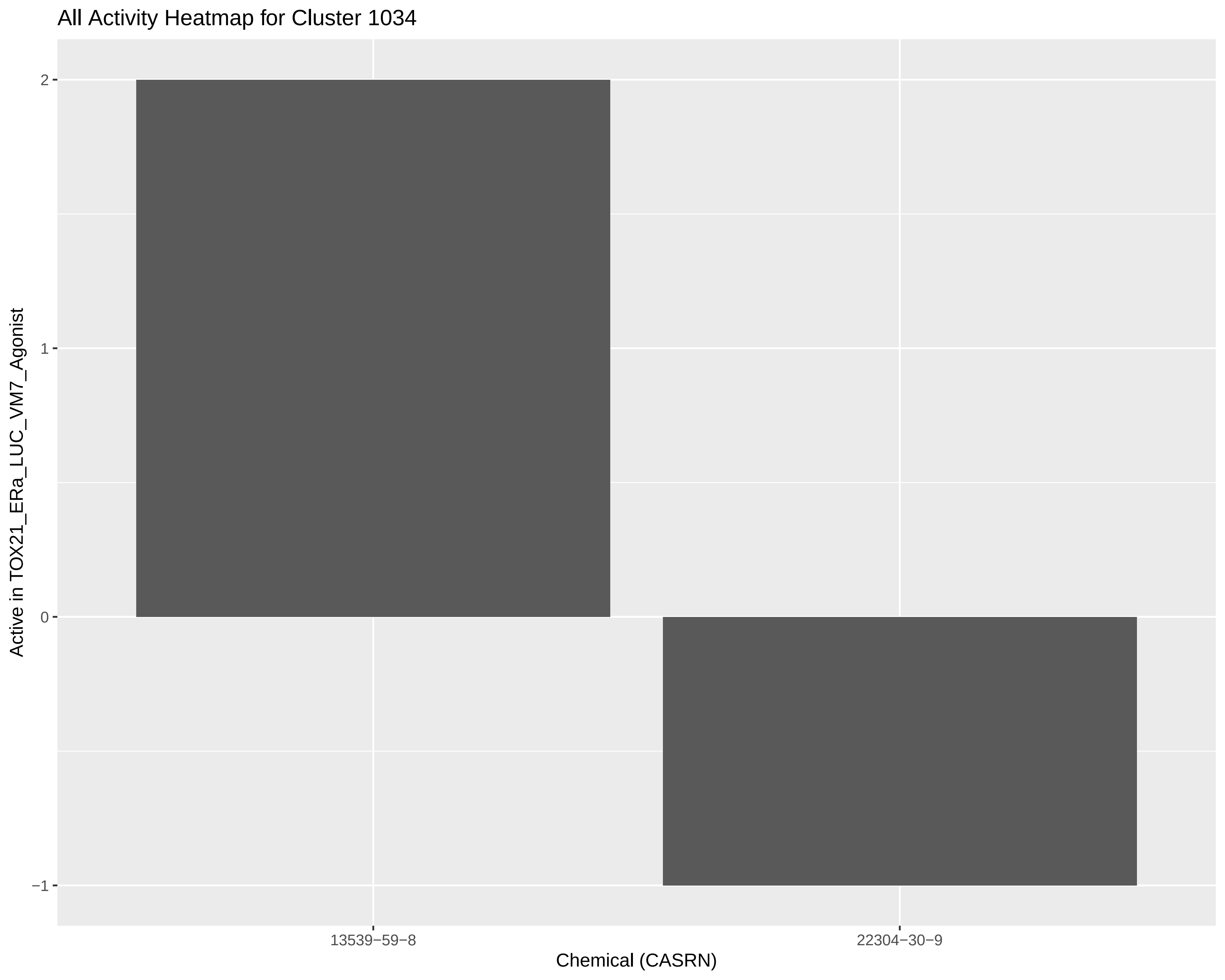
All Activity Heatmap for Cluster 1034
Active in TOX21_ERa_LUC_VM7_Agonist
2
1
0
−1
13539−59−8
22304−30−9
Chemical (CASRN)

All Activity Heatmap for Cluster 1035

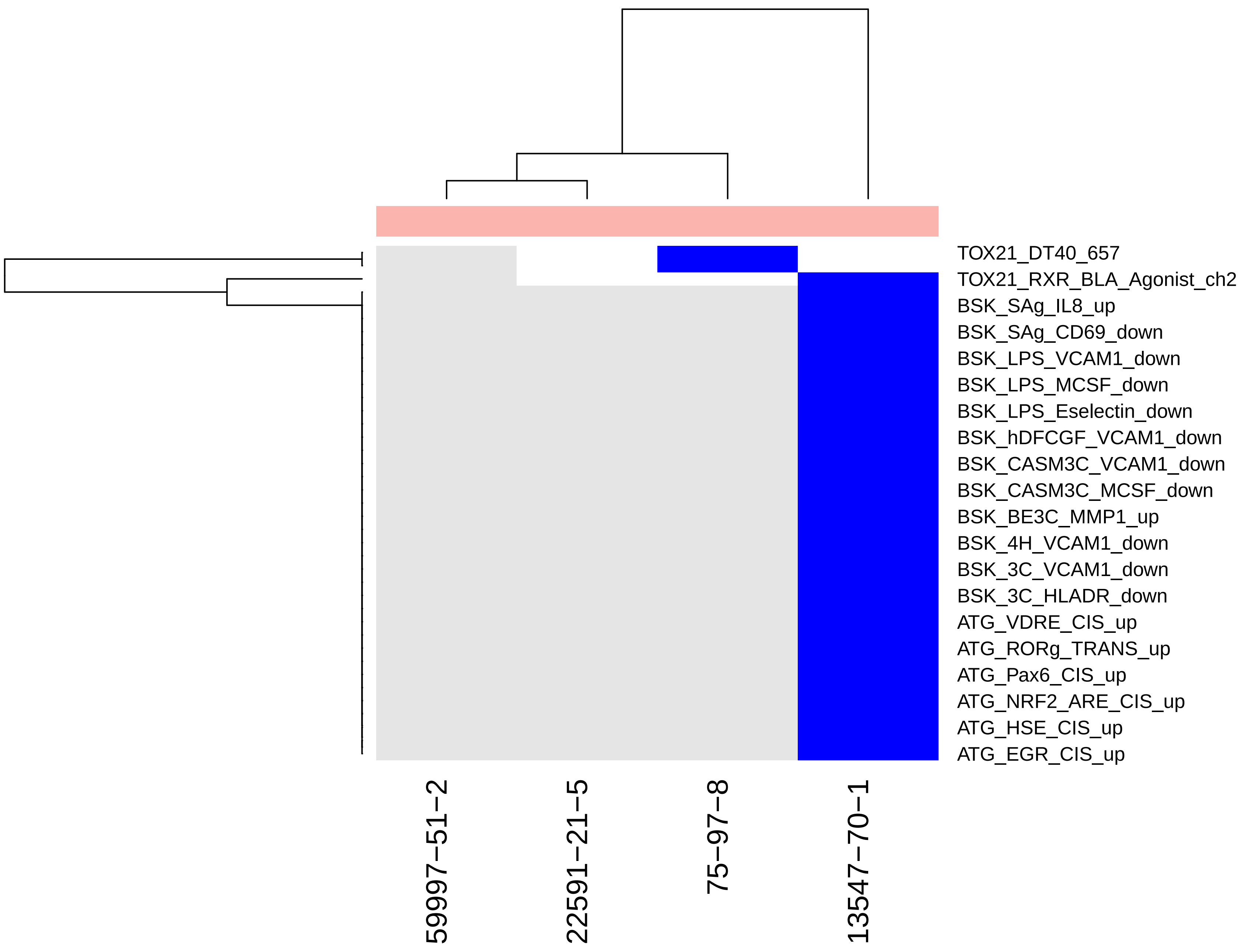

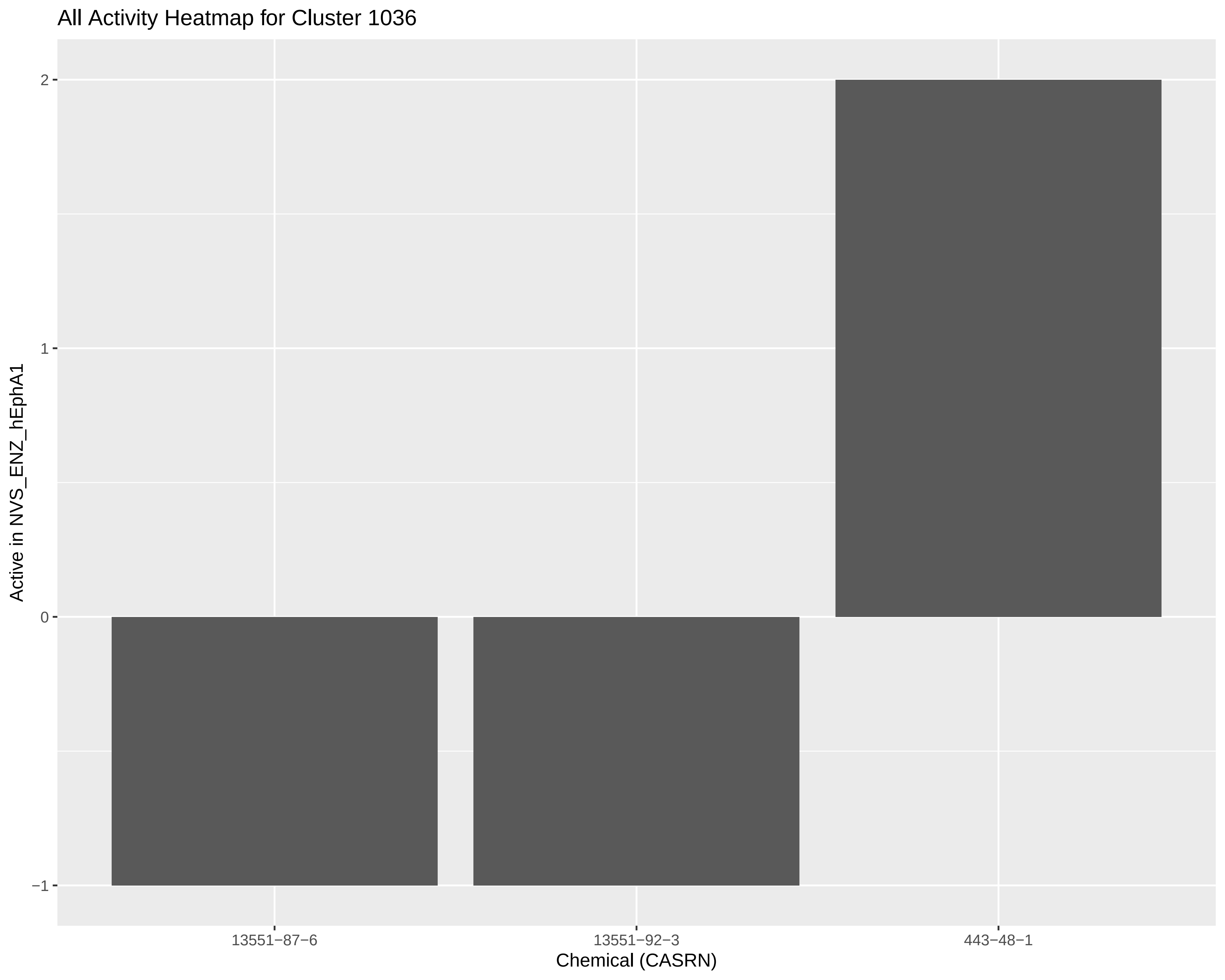

All Activity Heatmap for Cluster 1036
Active in NVS_ENZ_hEphA1
2
1
0
−1
13551−87−6
13551−92−3
443−48−1
Chemical (CASRN)

All Activity Heatmap for Cluster 1038

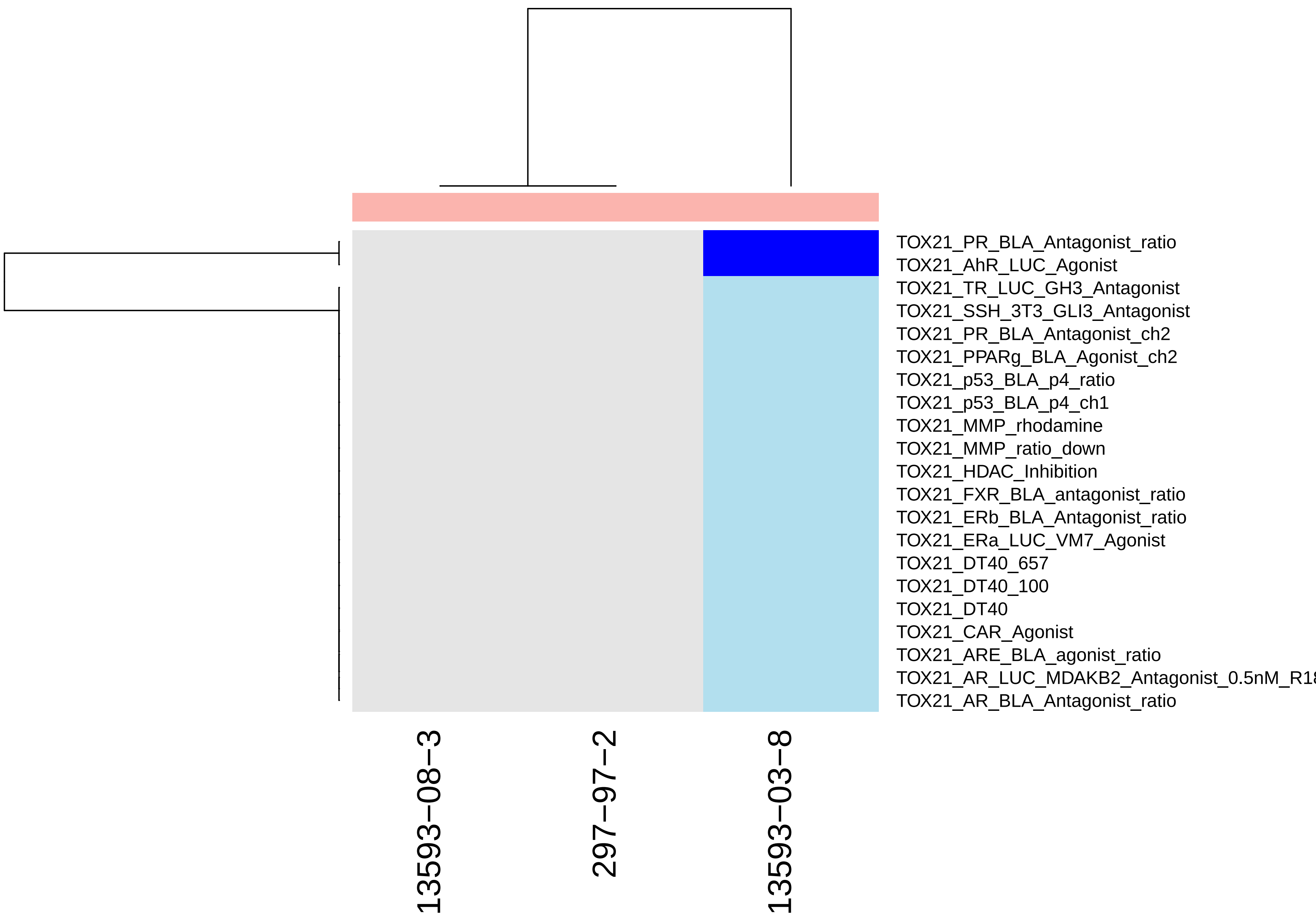

All Activity Heatmap for Cluster 1040

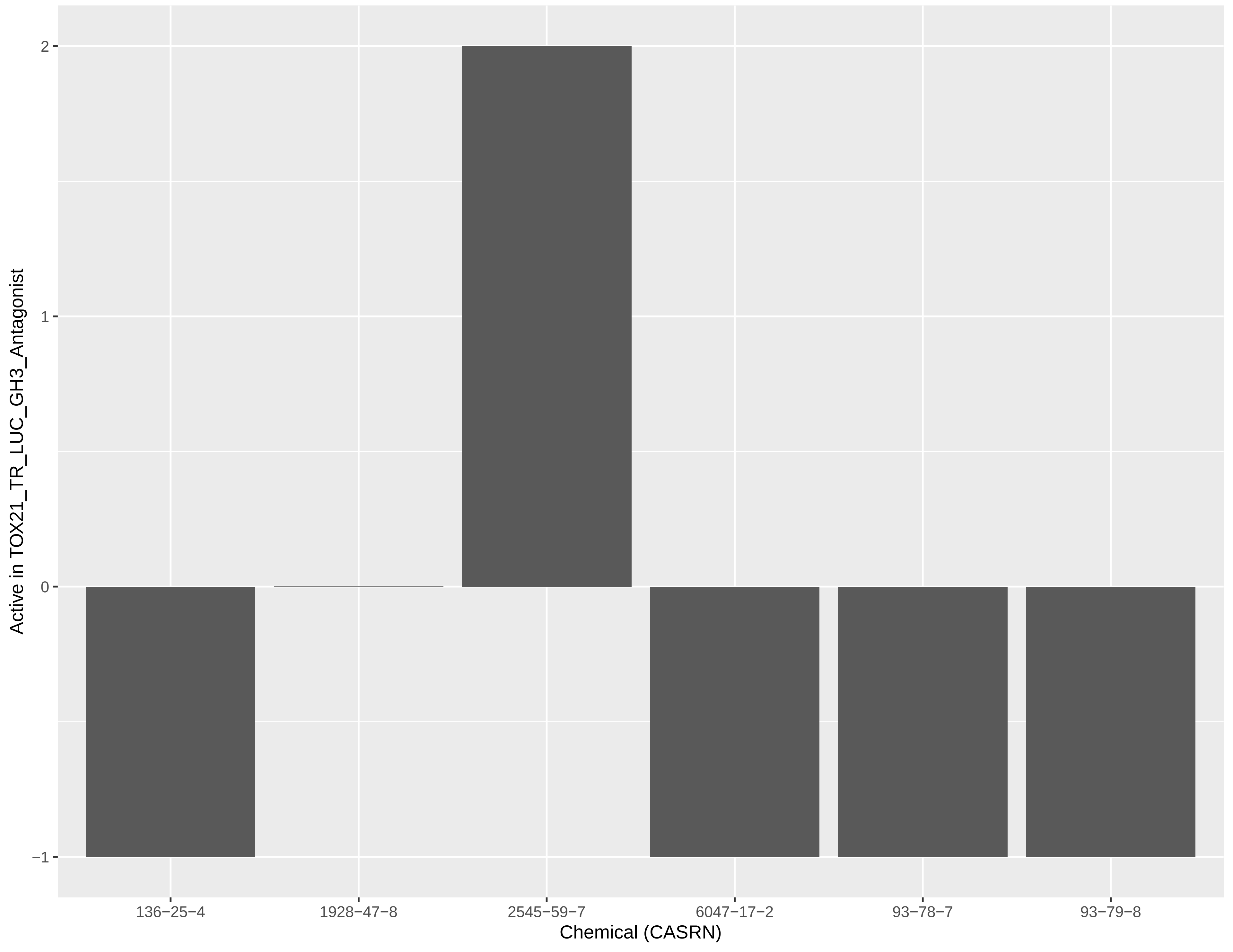

All Activity Heatmap for Cluster 1041

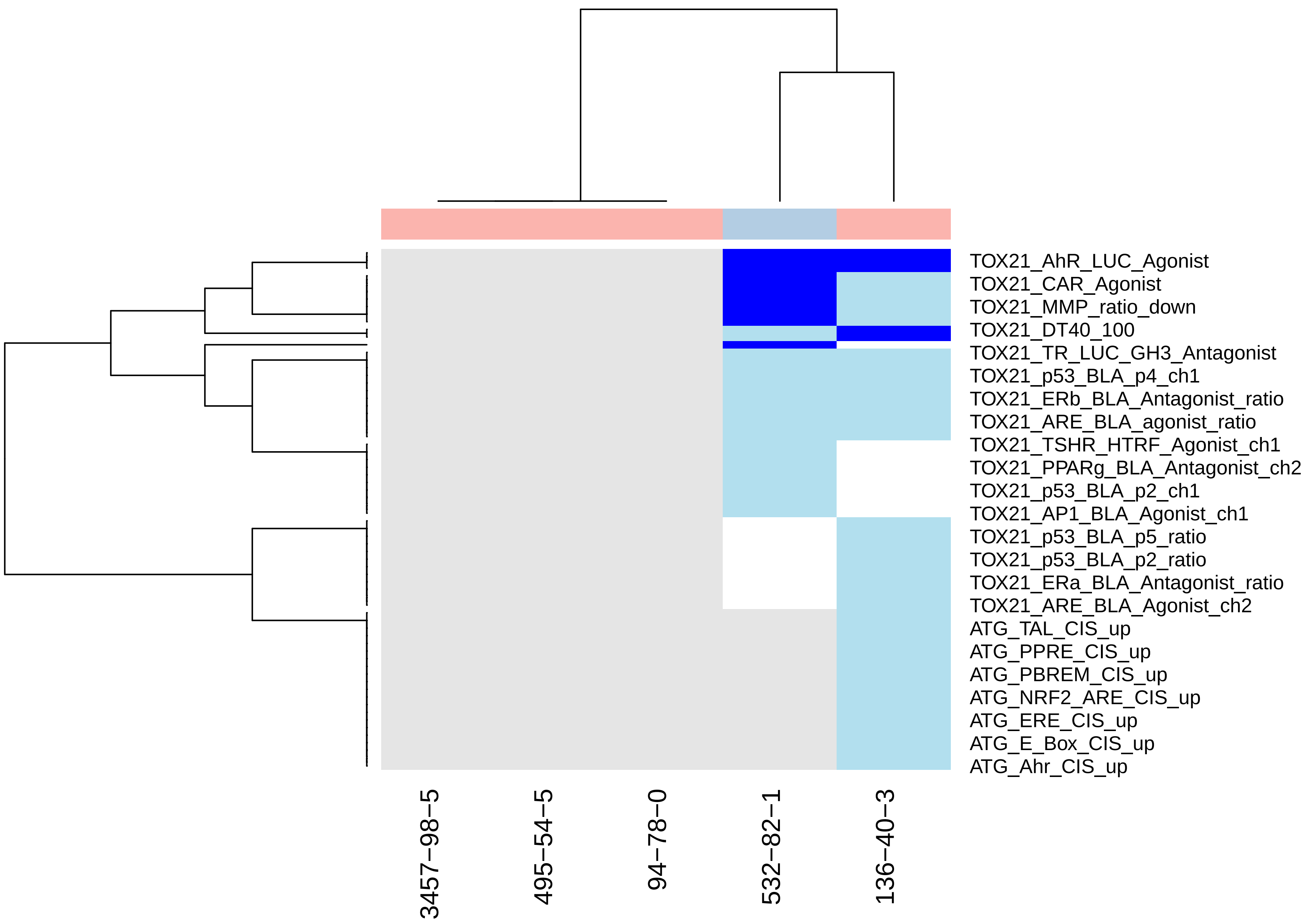

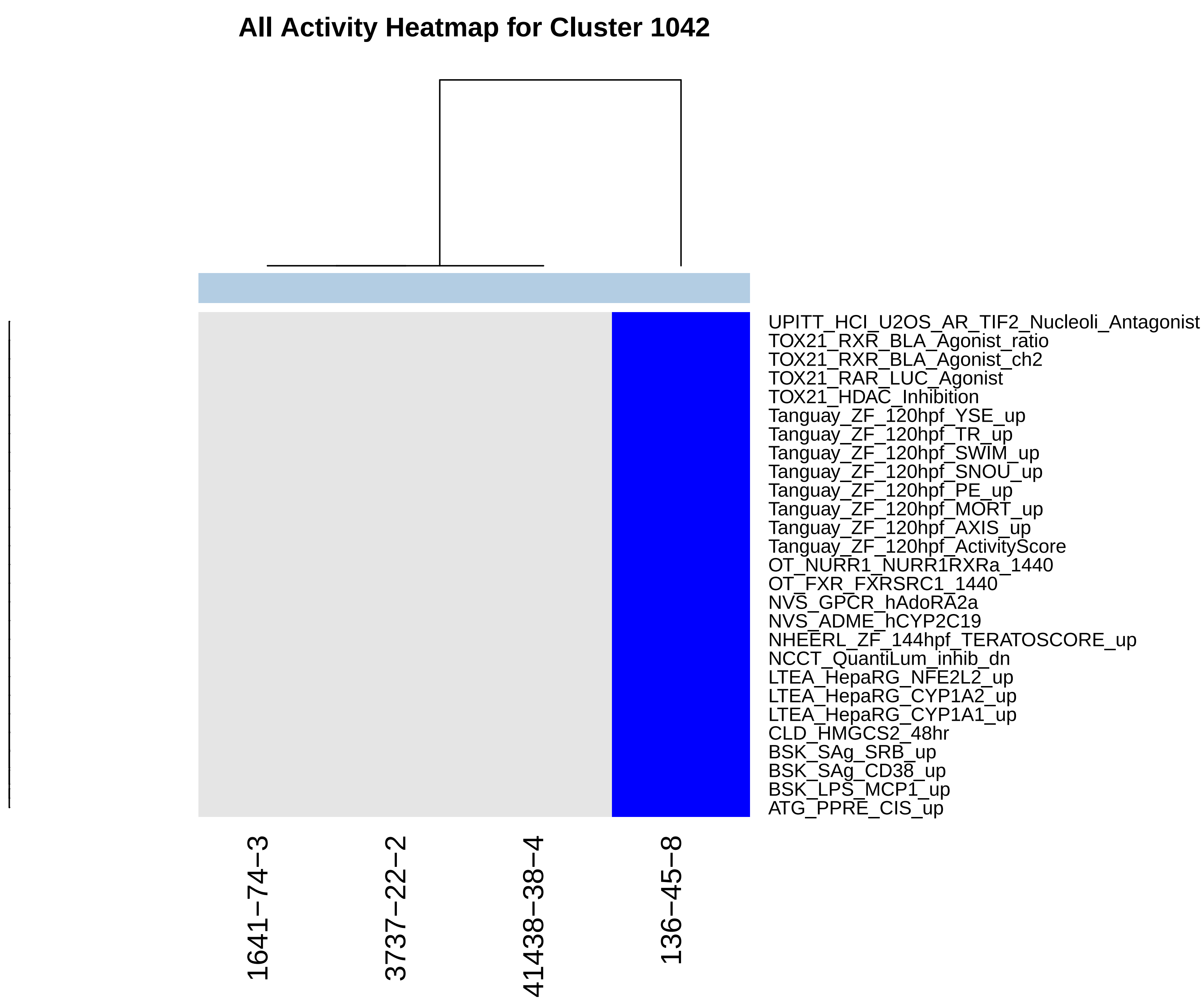
All Activity Heatmap for Cluster 1042
UPITT_HCI_U2OS_AR_TIF2_Nucleoli_Antagonist
TOX21_RXR_BLA_Agonist_ratio
TOX21_RXR_BLA_Agonist_ch2
TOX21_RAR_LUC_Agonist
TOX21_HDAC_Inhibition
Tanguay_ZF_120hpf_YSE_up
Tanguay_ZF_120hpf_TR_up
Tanguay_ZF_120hpf_SWIM_up
Tanguay_ZF_120hpf_SNOU_up
Tanguay_ZF_120hpf_PE_up
Tanguay_ZF_120hpf_MORT_up
Tanguay_ZF_120hpf_AXIS_up
Tanguay_ZF_120hpf_ActivityScore
OT_NURR1_NURR1RXRa_1440
OT_FXR_FXRSRC1_1440
NVS_GPCR_hAdoRA2a
NVS_ADME_hCYP2C19
NHEERL_ZF_144hpf_TERATOSCORE_up
NCCT_QuantiLum_inhib_dn
LTEA_HepaRG_NFE2L2_up
LTEA_HepaRG_CYP1A2_up
LTEA_HepaRG_CYP1A1_up
CLD_HMGCS2_48hr
BSK_SAg_SRB_up
BSK_SAg_CD38_up
BSK_LPS_MCP1_up
ATG_PPRE_CIS_up
1641−74−3
3737−22−2
41438−38−4
136−45−8

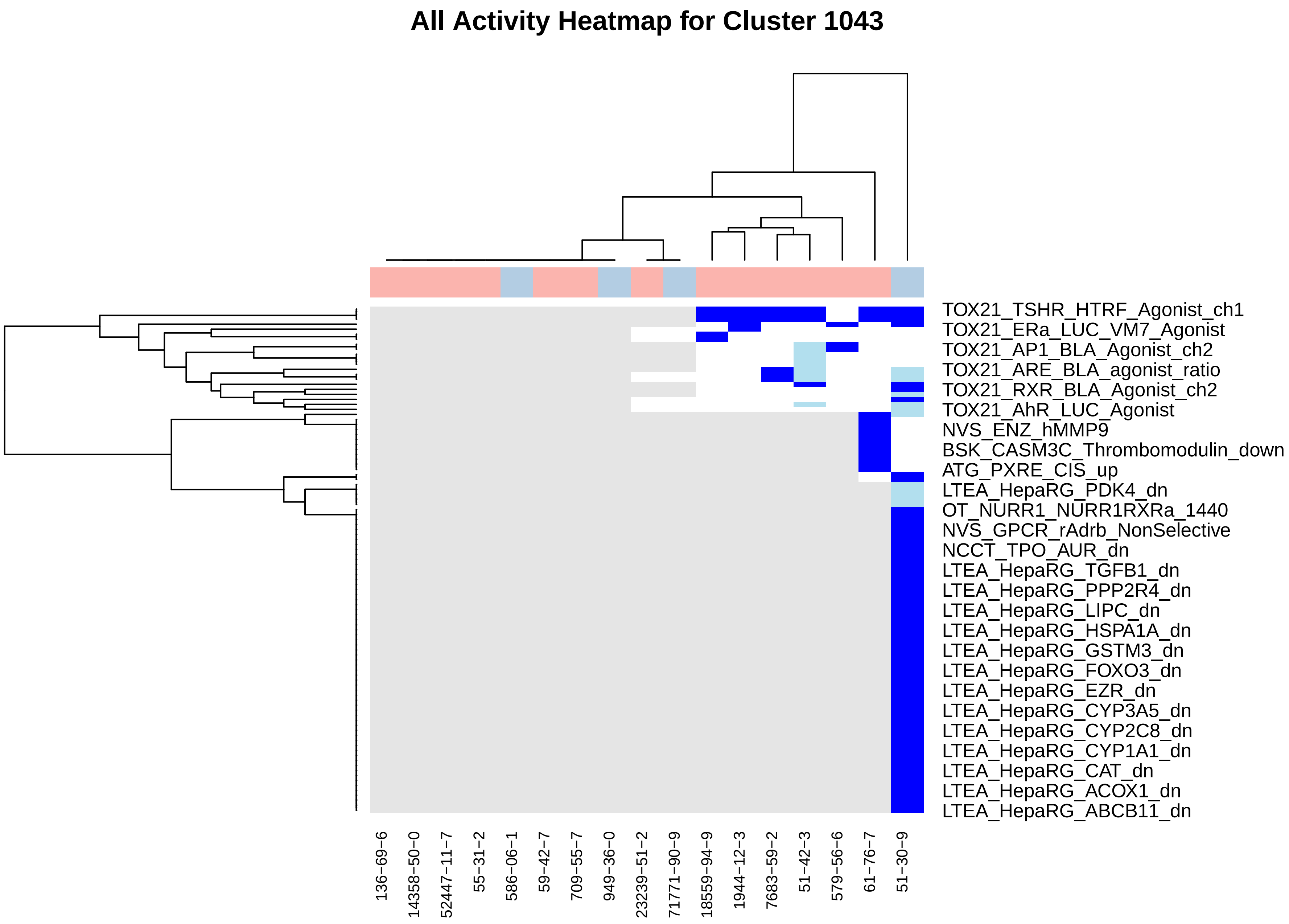
All Activity Heatmap for Cluster 1043
TOX21_TSHR_HTRF_Agonist_ch1
TOX21_ERa_LUC_VM7_Agonist
TOX21_AP1_BLA_Agonist_ch2
TOX21_ARE_BLA_agonist_ratio
TOX21_RXR_BLA_Agonist_ch2
TOX21_AhR_LUC_Agonist
NVS_ENZ_hMMP9
BSK_CASM3C_Thrombomodulin_down
ATG_PXRE_CIS_up
LTEA_HepaRG_PDK4_dn
OT_NURR1_NURR1RXRa_1440
NVS_GPCR_rAdrb_NonSelective
NCCT_TPO_AUR_dn
LTEA_HepaRG_TGFB1_dn
LTEA_HepaRG_PPP2R4_dn
LTEA_HepaRG_LIPC_dn
LTEA_HepaRG_HSPA1A_dn
LTEA_HepaRG_GSTM3_dn
LTEA_HepaRG_FOXO3_dn
LTEA_HepaRG_EZR_dn
LTEA_HepaRG_CYP3A5_dn
LTEA_HepaRG_CYP2C8_dn
LTEA_HepaRG_CYP1A1_dn
LTEA_HepaRG_CAT_dn
LTEA_HepaRG_ACOX1_dn
LTEA_HepaRG_ABCB11_dn
136−69−6
14358−50−0
52447−11−7
55−31−2
586−06−1
59−42−7
709−55−7
949−36−0
23239−51−2
71771−90−9
18559−94−9
1944−12−3
7683−59−2
51−42−3
579−56−6
61−76−7
51−30−9

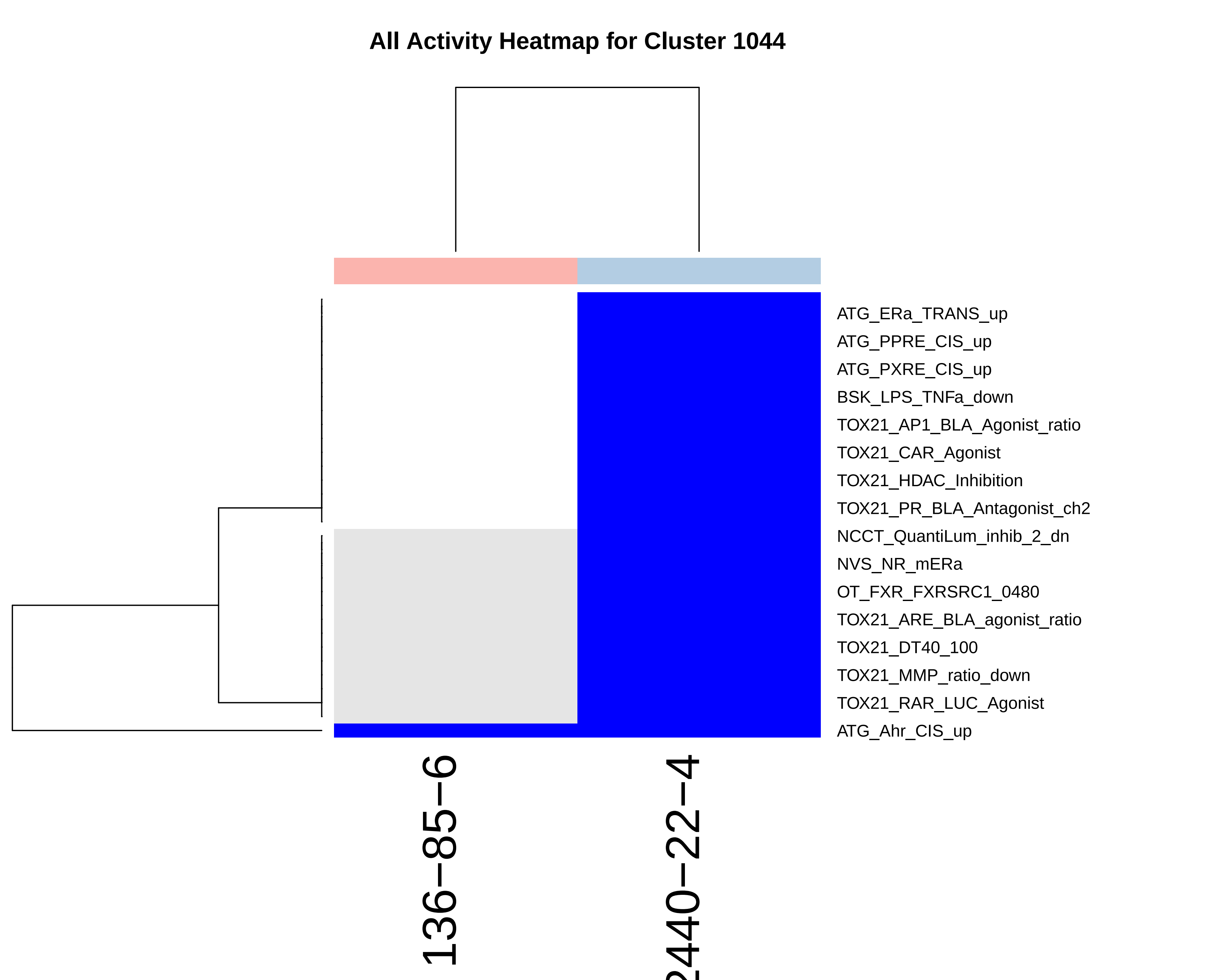
All Activity Heatmap for Cluster 1044
ATG_ERa_TRANS_up
ATG_PPRE_CIS_up
ATG_PXRE_CIS_up
BSK_LPS_TNFa_down
TOX21_AP1_BLA_Agonist_ratio
TOX21_CAR_Agonist
TOX21_HDAC_Inhibition
TOX21_PR_BLA_Antagonist_ch2
NCCT_QuantiLum_inhib_2_dn
NVS_NR_mERa
OT_FXR_FXRSRC1_0480
TOX21_ARE_BLA_agonist_ratio
TOX21_DT40_100
TOX21_MMP_ratio_down
TOX21_RAR_LUC_Agonist
ATG_Ahr_CIS_up
136−85−6

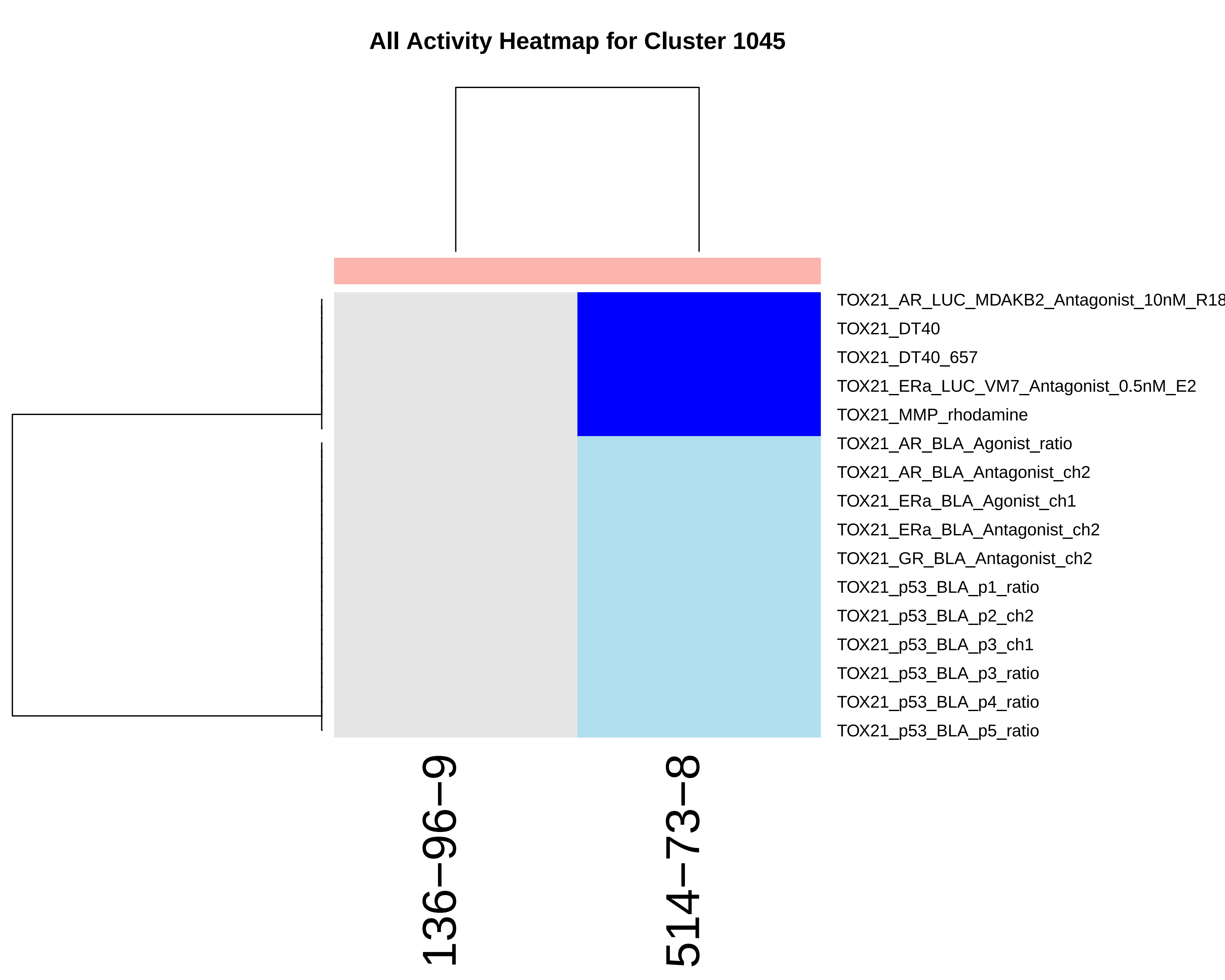
All Activity Heatmap for Cluster 1045
TOX21_AR_LUC_MDAKB2_Antagonist_10nM_R18
TOX21_DT40
TOX21_DT40_657
TOX21_ERa_LUC_VM7_Antagonist_0.5nM_E2
TOX21_MMP_rhodamine
TOX21_AR_BLA_Agonist_ratio
TOX21_AR_BLA_Antagonist_ch2
TOX21_ERa_BLA_Agonist_ch1
TOX21_ERa_BLA_Antagonist_ch2
TOX21_GR_BLA_Antagonist_ch2
TOX21_p53_BLA_p1_ratio
TOX21_p53_BLA_p2_ch2
TOX21_p53_BLA_p3_ch1
TOX21_p53_BLA_p3_ratio
TOX21_p53_BLA_p4_ratio
TOX21_p53_BLA_p5_ratio
136−96−9
514−73−8

All Activity Heatmap for Cluster 1046

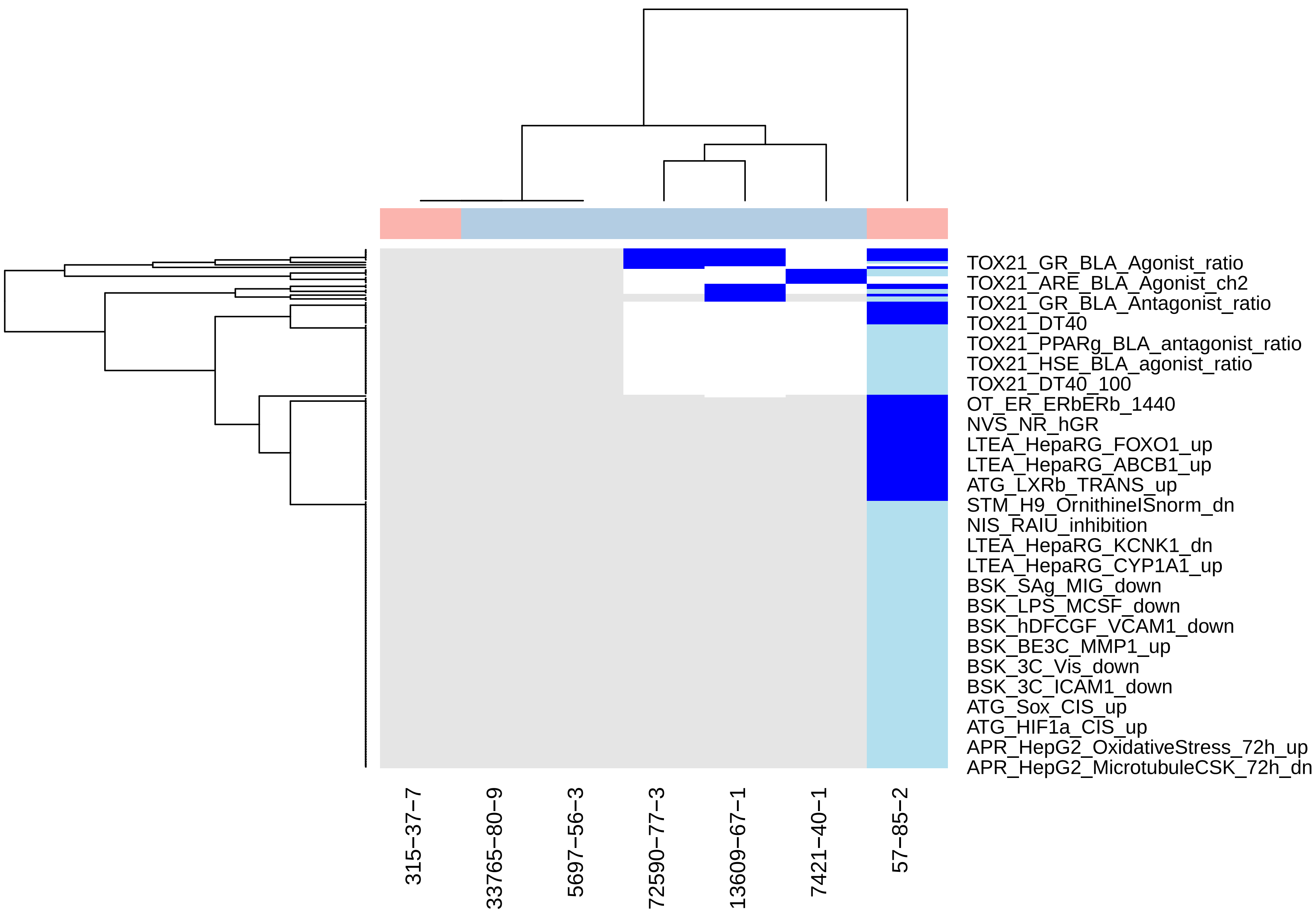

All Activity Heatmap for Cluster 1047

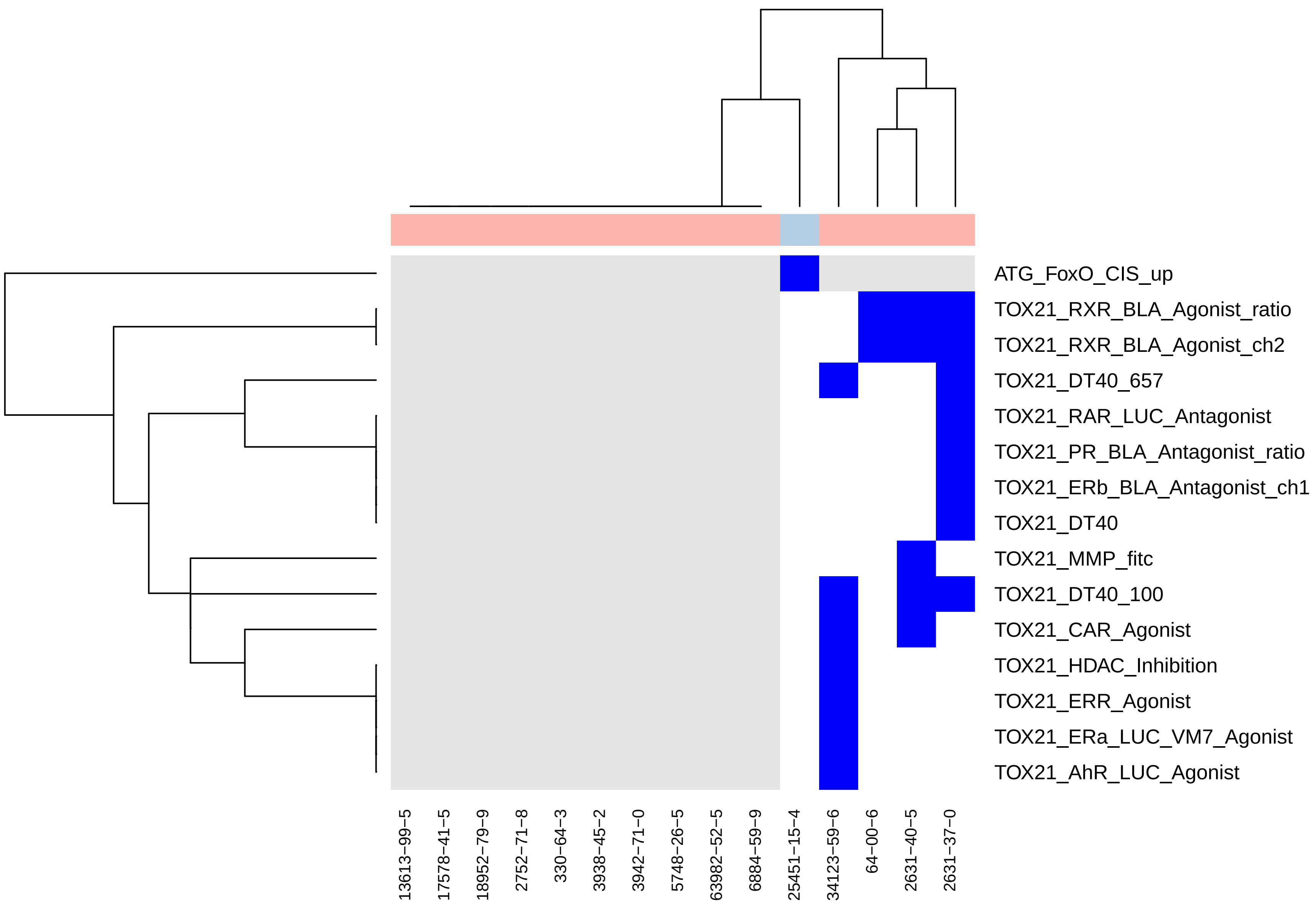

All Activity Heatmap for Cluster 1049

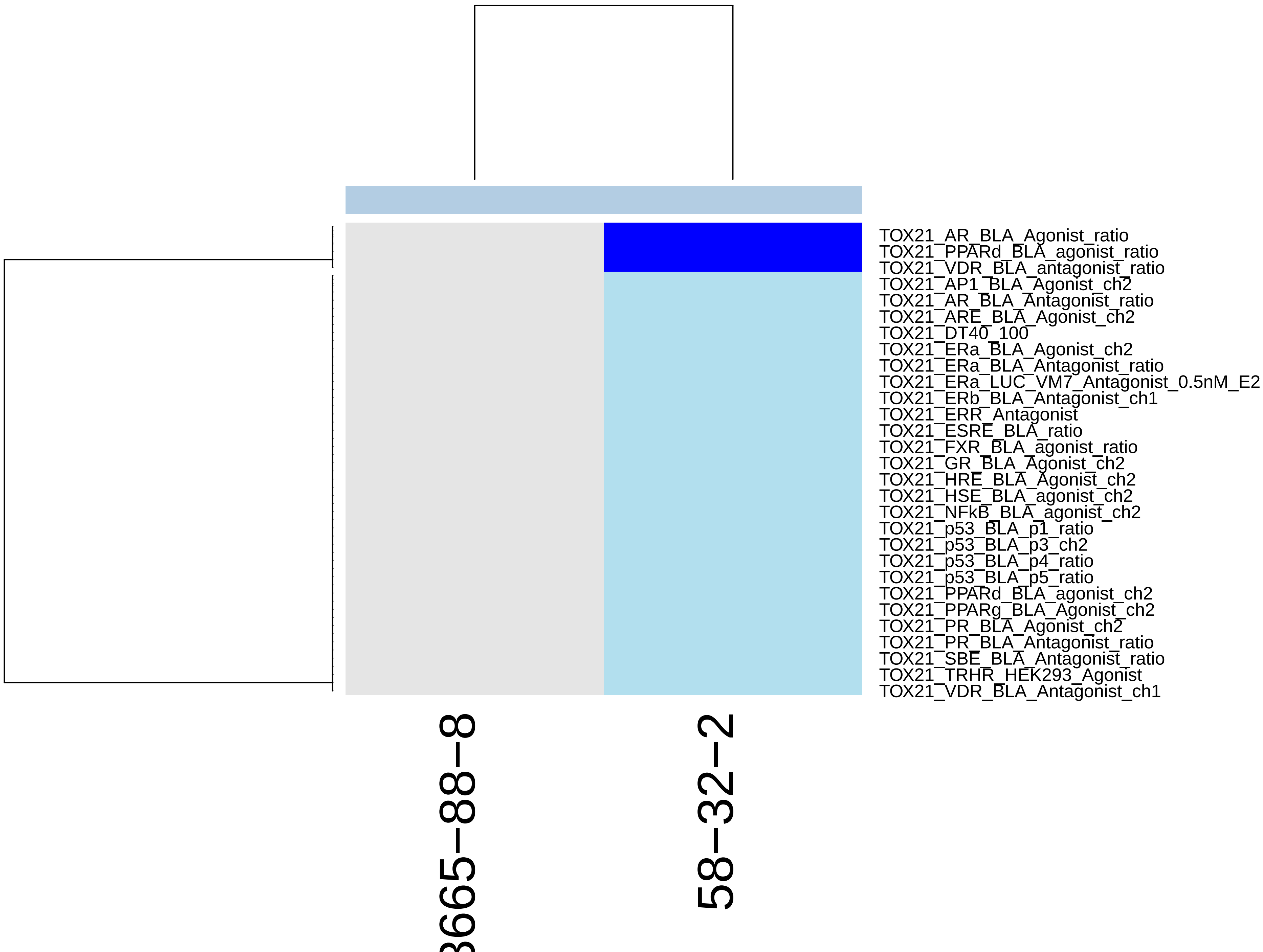

All Activity Heatmap for Cluster 1051

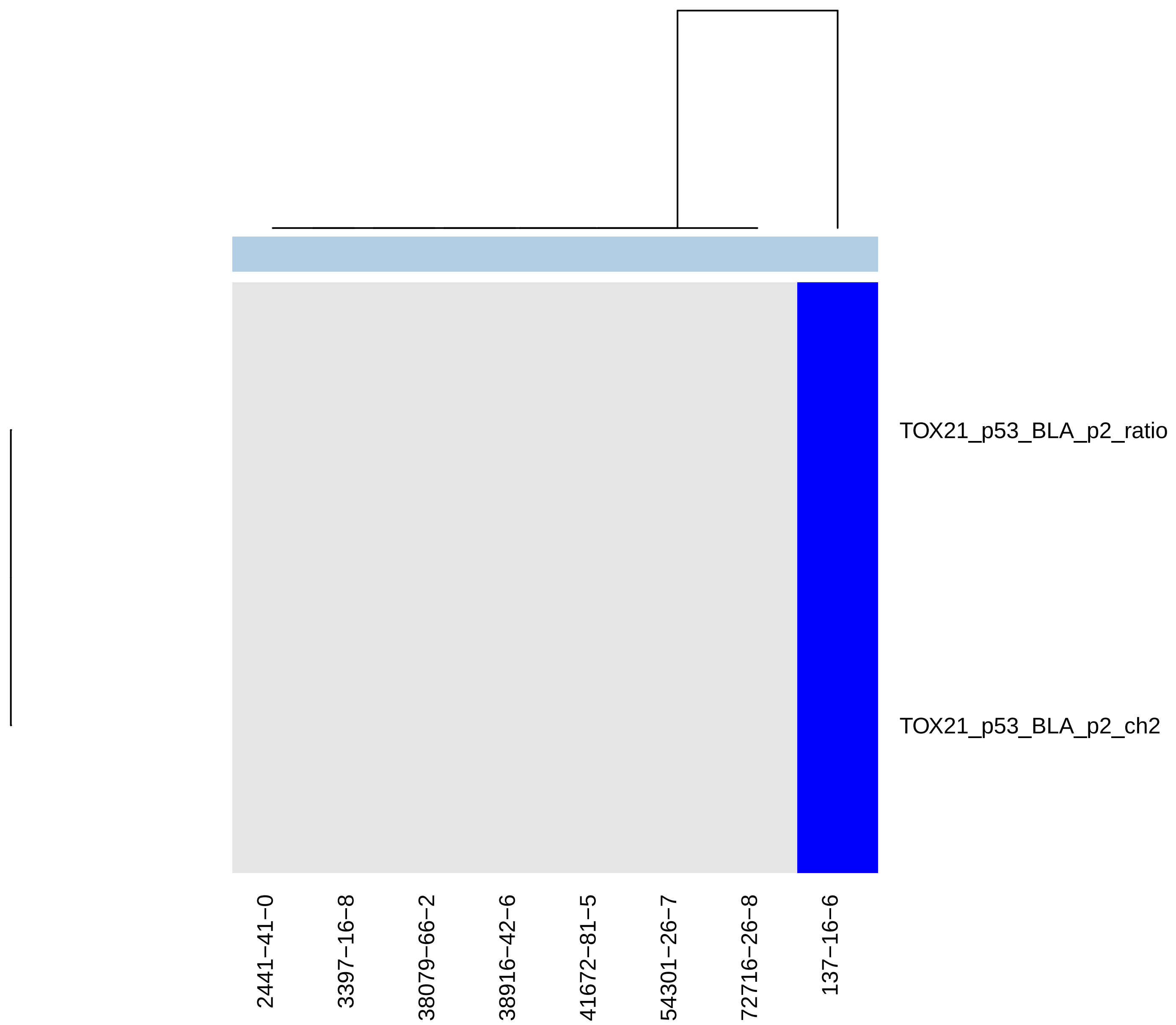

All Activity Heatmap for Cluster 1052

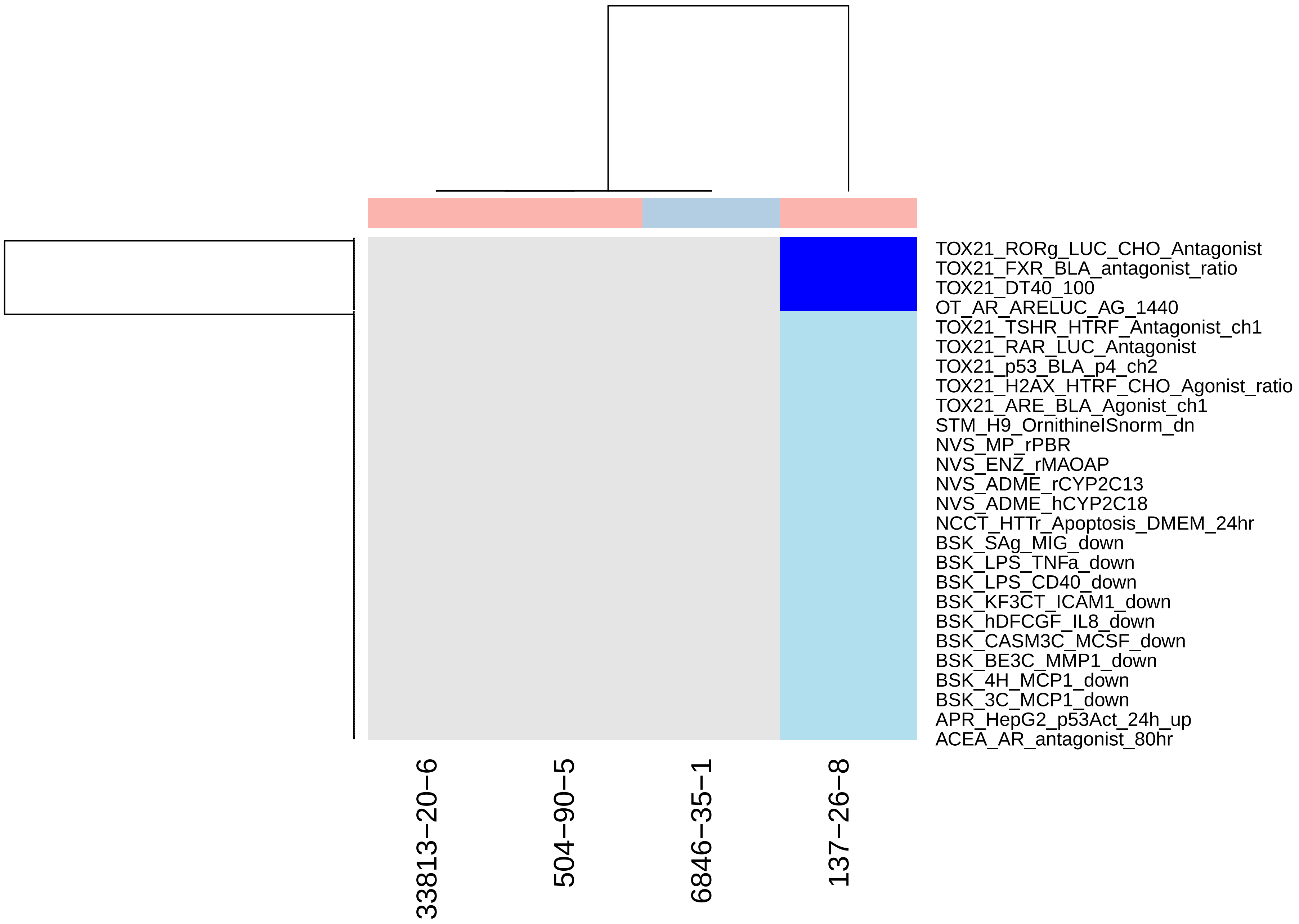

All Activity Heatmap for Cluster 1053

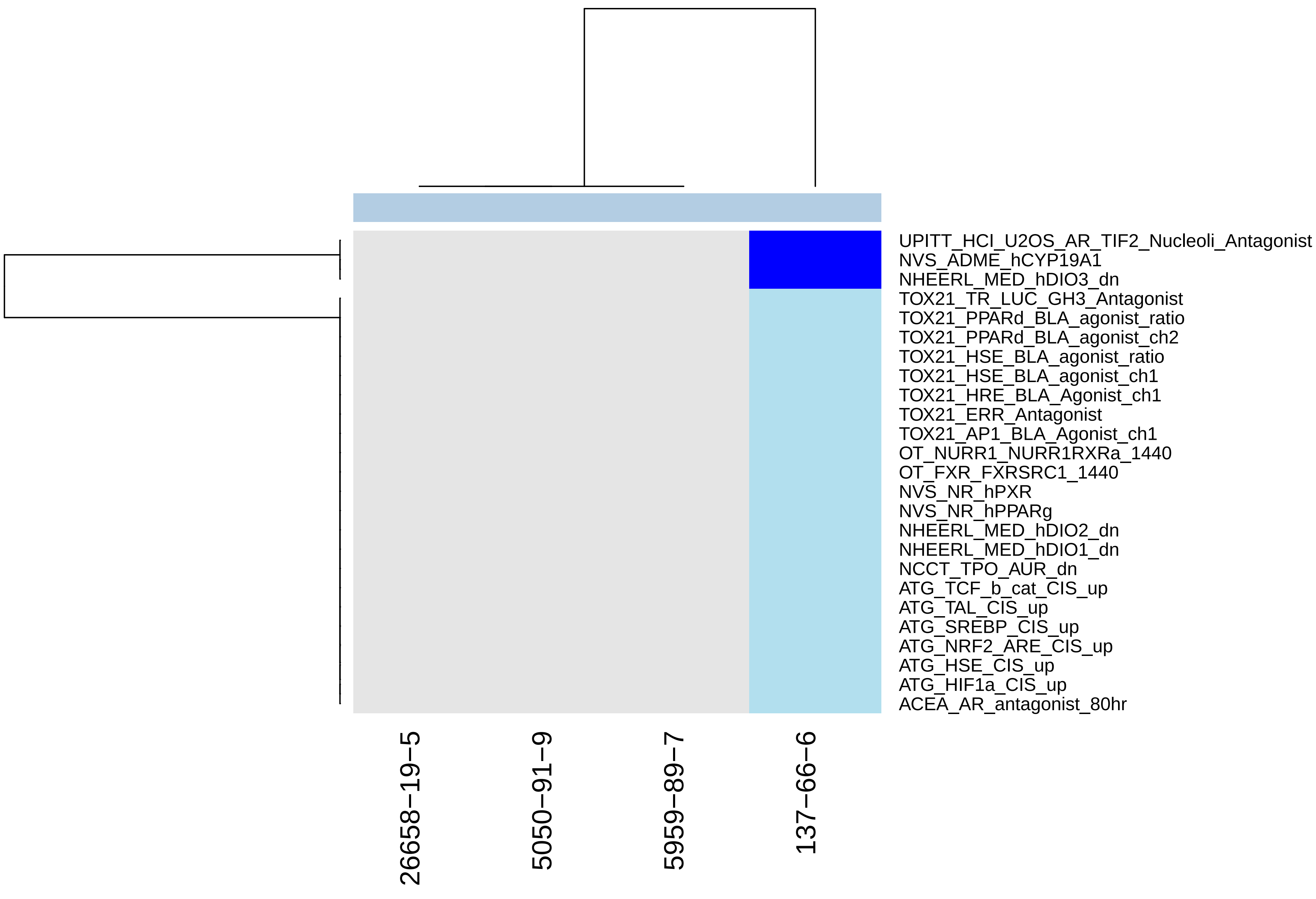

All Activity Heatmap for Cluster 1054

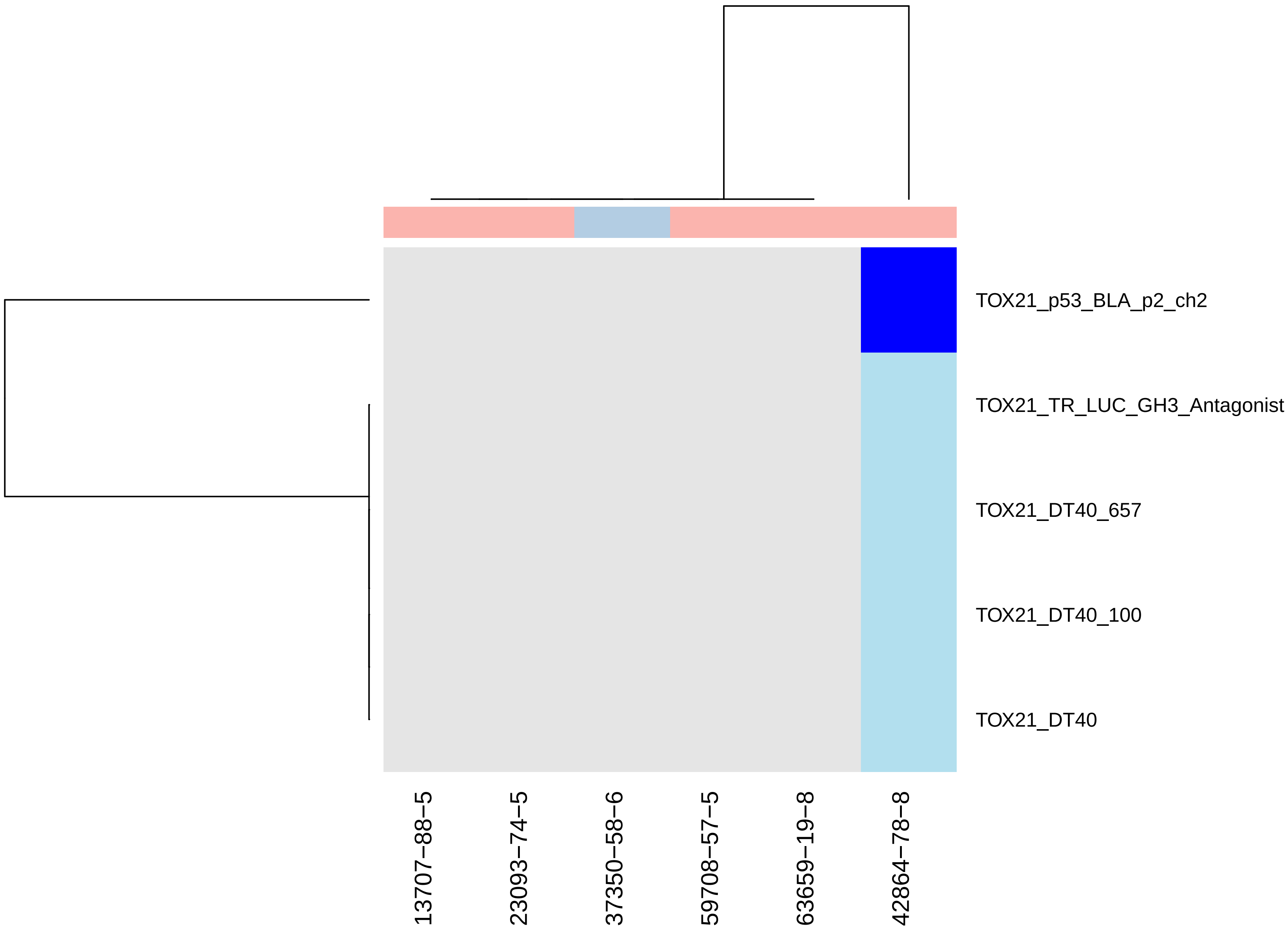

All Activity Heatmap for Cluster 1056

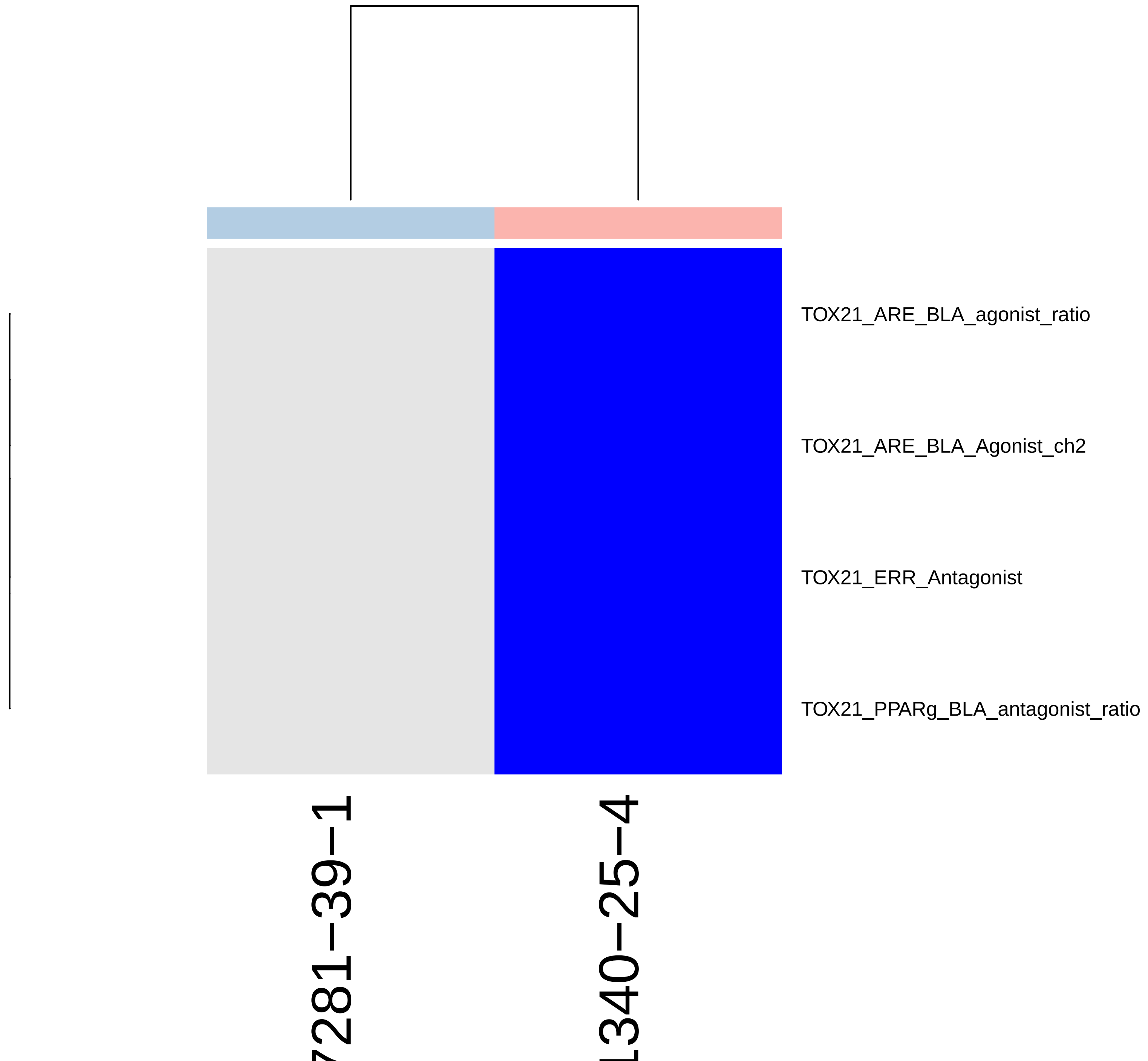

All Activity Heatmap for Cluster 1057

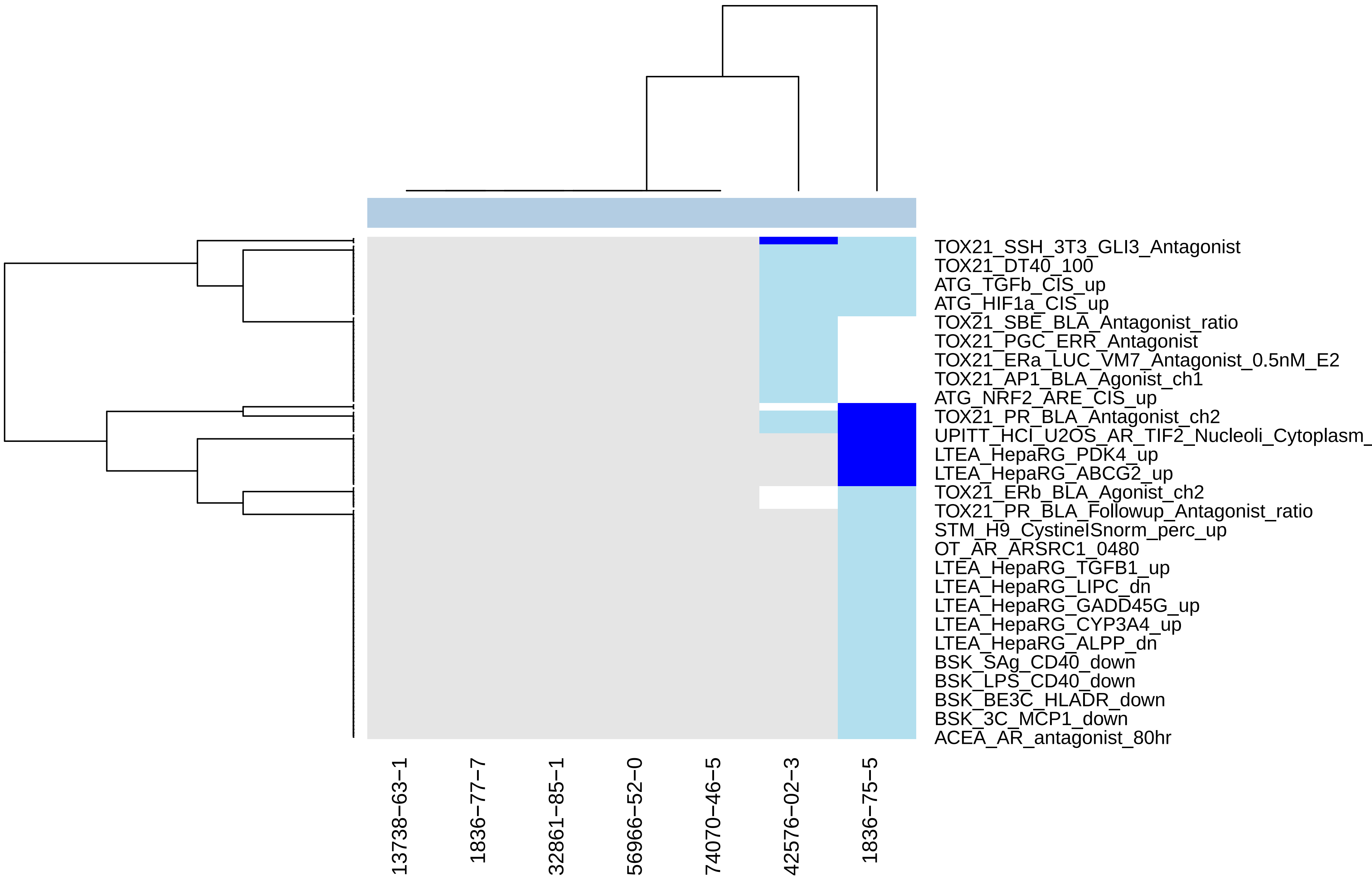

All Activity Heatmap for Cluster 1059

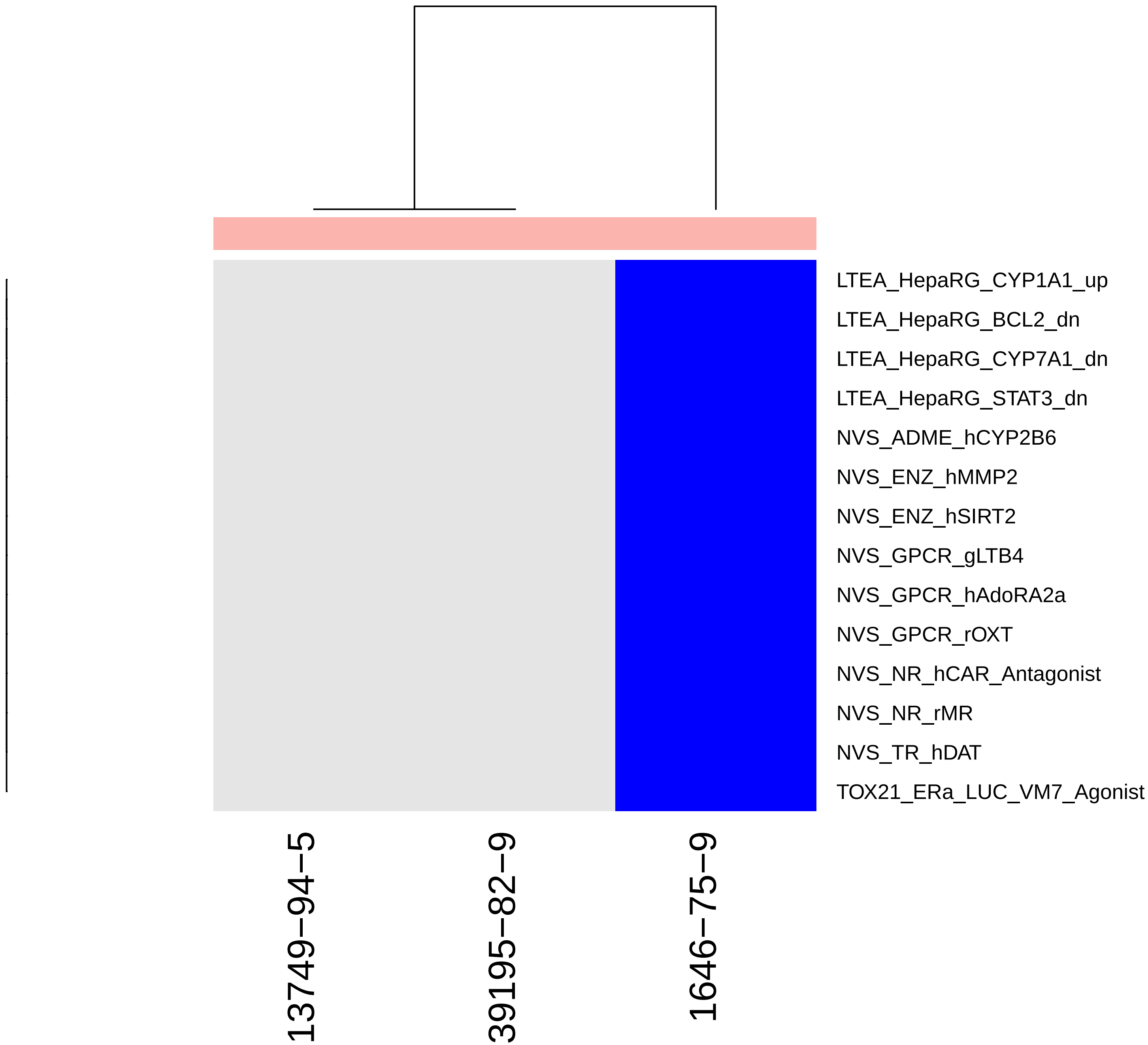

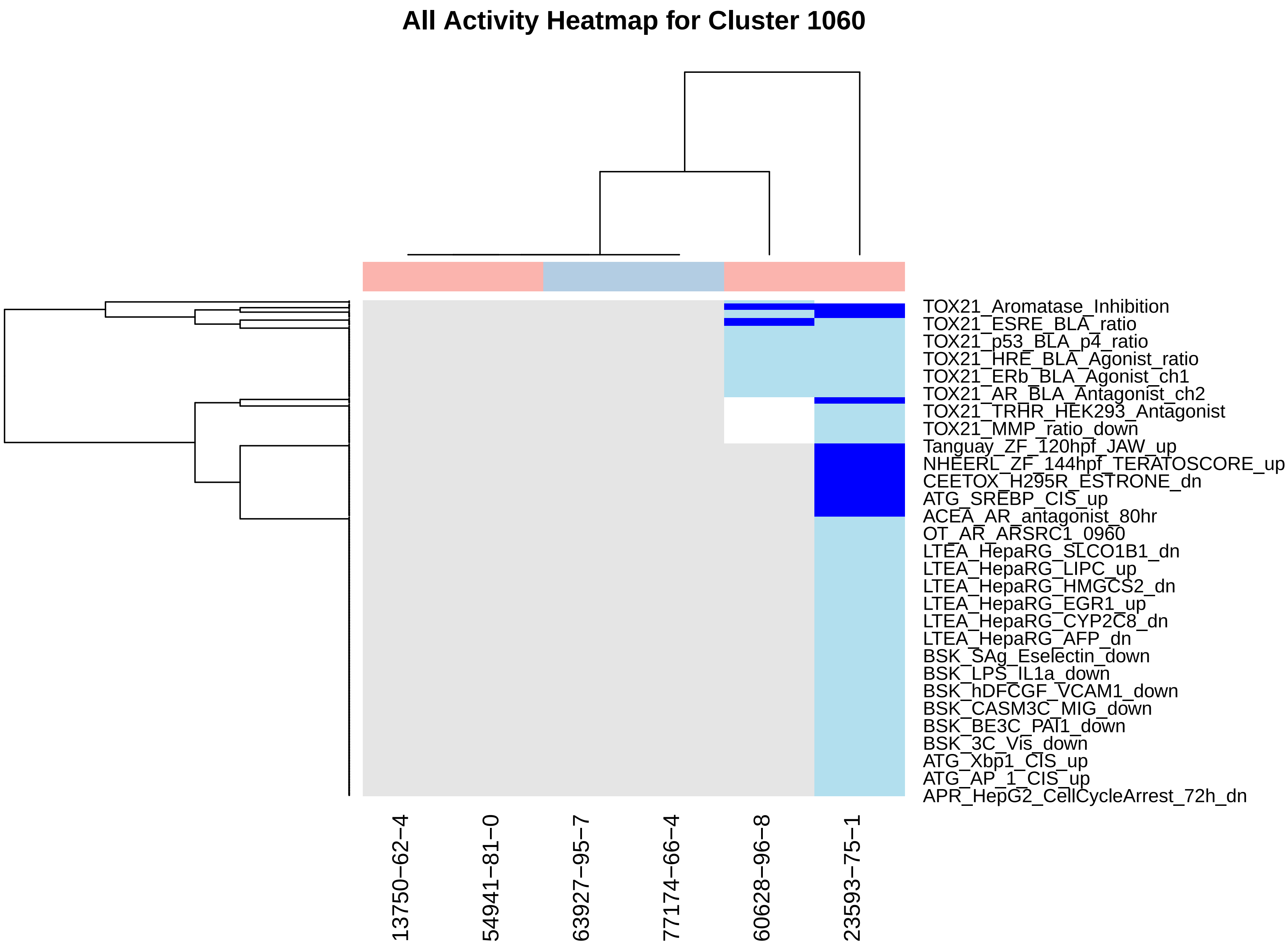
All Activity Heatmap for Cluster 1060
TOX21_Aromatase_Inhibition
TOX21_ESRE_BLA_ratio
TOX21_p53_BLA_p4_ratio
TOX21_HRE_BLA_Agonist_ratio
TOX21_ERb_BLA_Agonist_ch1
TOX21_AR_BLA_Antagonist_ch2
TOX21_TRHR_HEK293_Antagonist
TOX21_MMP_ratio_down
Tanguay_ZF_120hpf_JAW_up
NHEERL_ZF_144hpf_TERATOSCORE_up
CEETOX_H295R_ESTRONE_dn
ATG_SREBP_CIS_up
ACEA_AR_antagonist_80hr
OT_AR_ARSRC1_0960
LTEA_HepaRG_SLCO1B1_dn
LTEA_HepaRG_LIPC_up
LTEA_HepaRG_HMGCS2_dn
LTEA_HepaRG_EGR1_up
LTEA_HepaRG_CYP2C8_dn
LTEA_HepaRG_AFP_dn
BSK_SAg_Eselectin_down
BSK_LPS_IL1a_down
BSK_hDFCGF_VCAM1_down
BSK_CASM3C_MIG_down
BSK_BE3C_PAI1_down
BSK_3C_Vis_down
ATG_Xbp1_CIS_up
ATG_AP_1_CIS_up
APR_HepG2_CellCycleArrest_72h_dn
13750−62−4
54941−81−0
63927−95−7
77174−66−4
60628−96−8
23593−75−1

All Activity Heatmap for Cluster 1062

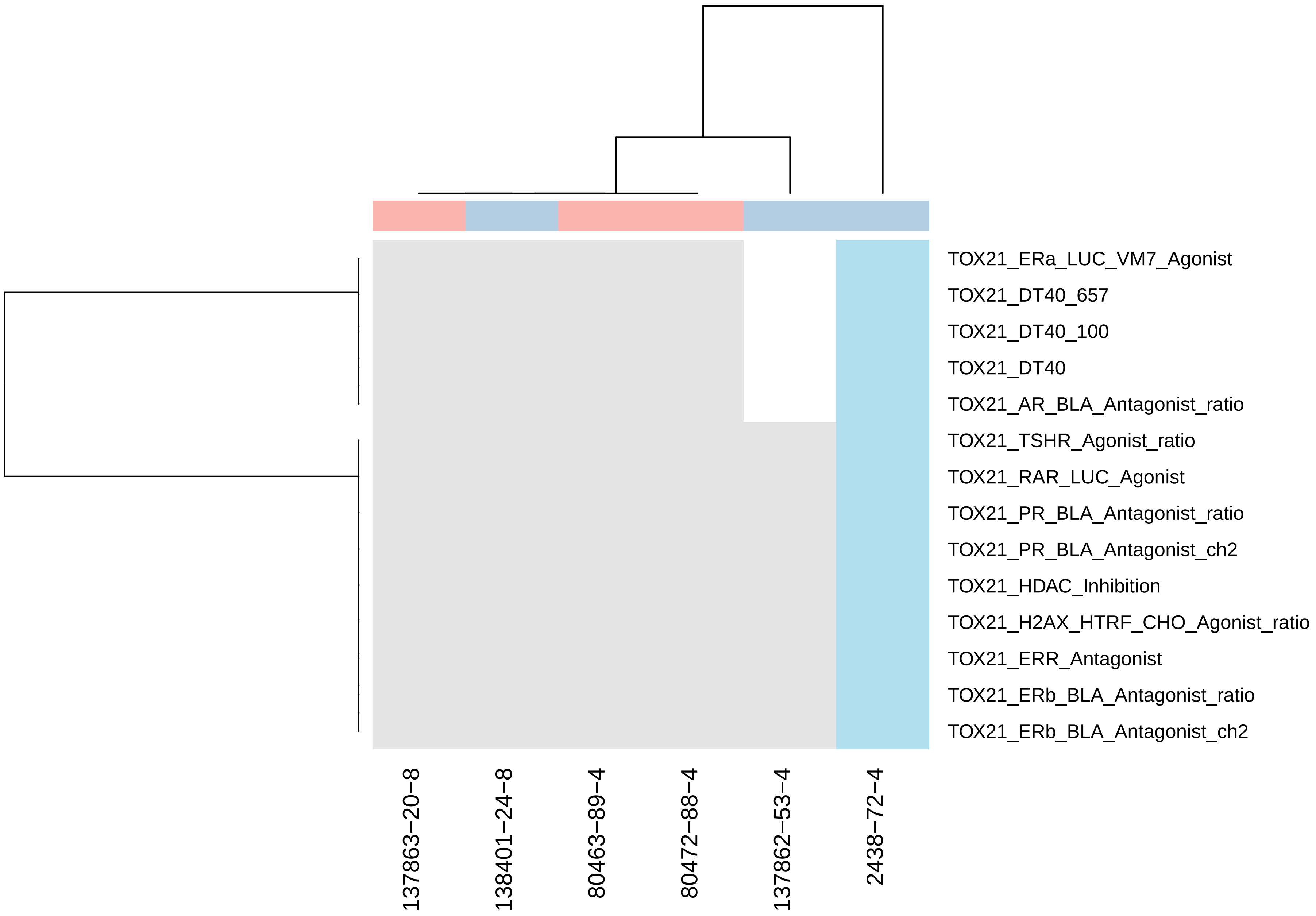

All Activity Heatmap for Cluster 1063

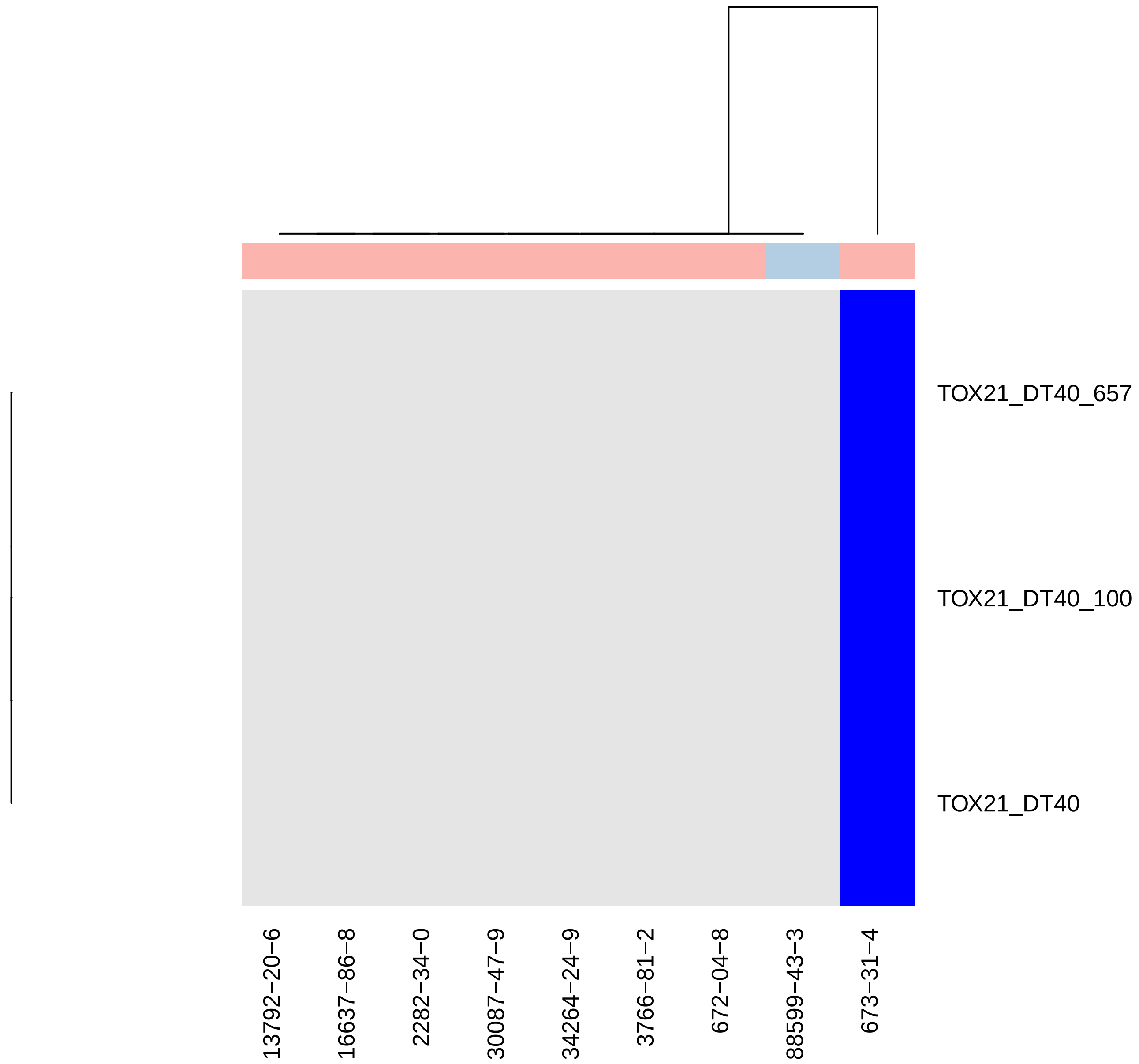

All Activity Heatmap for Cluster 1064

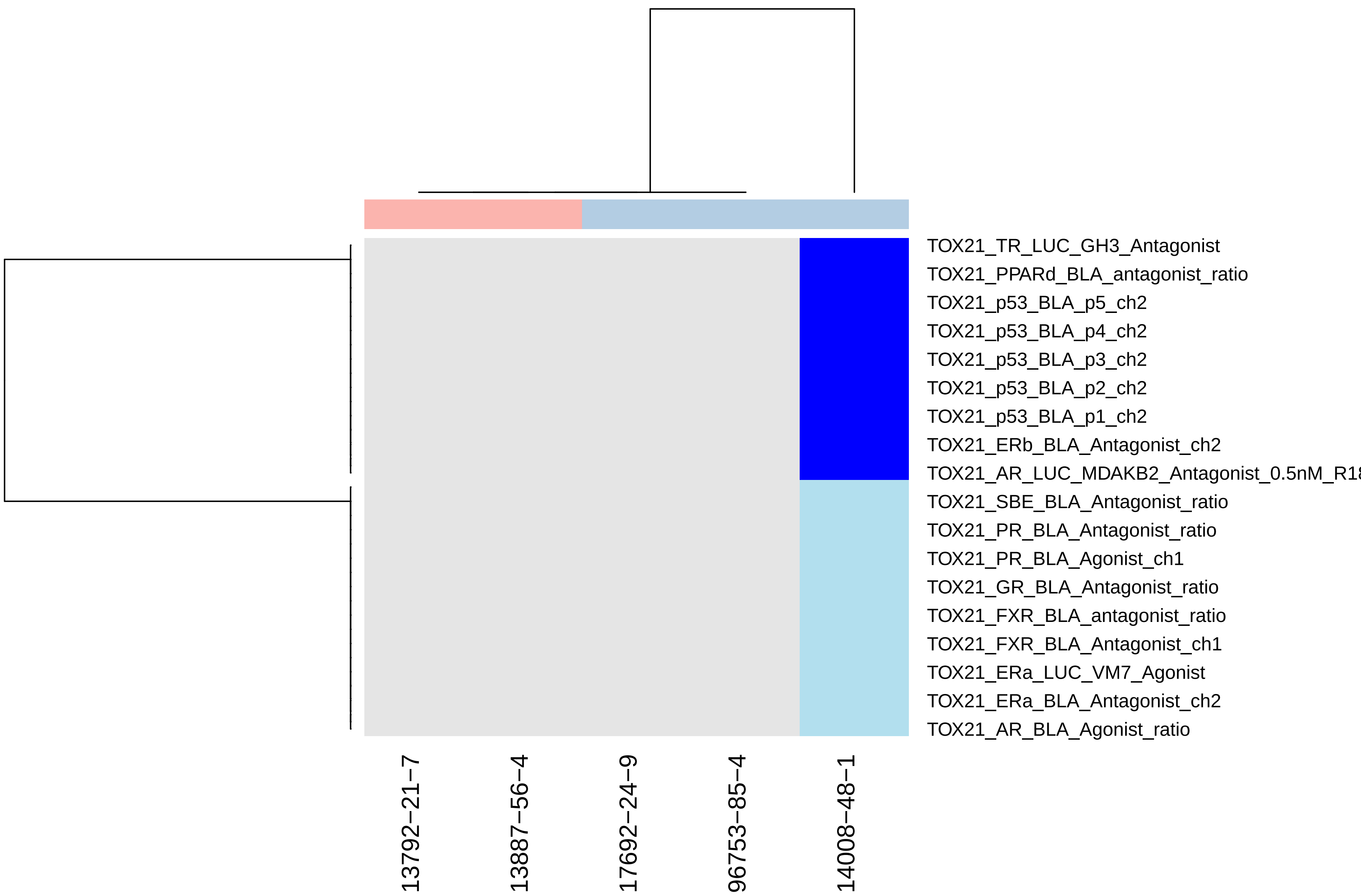

All Activity Heatmap for Cluster 1066

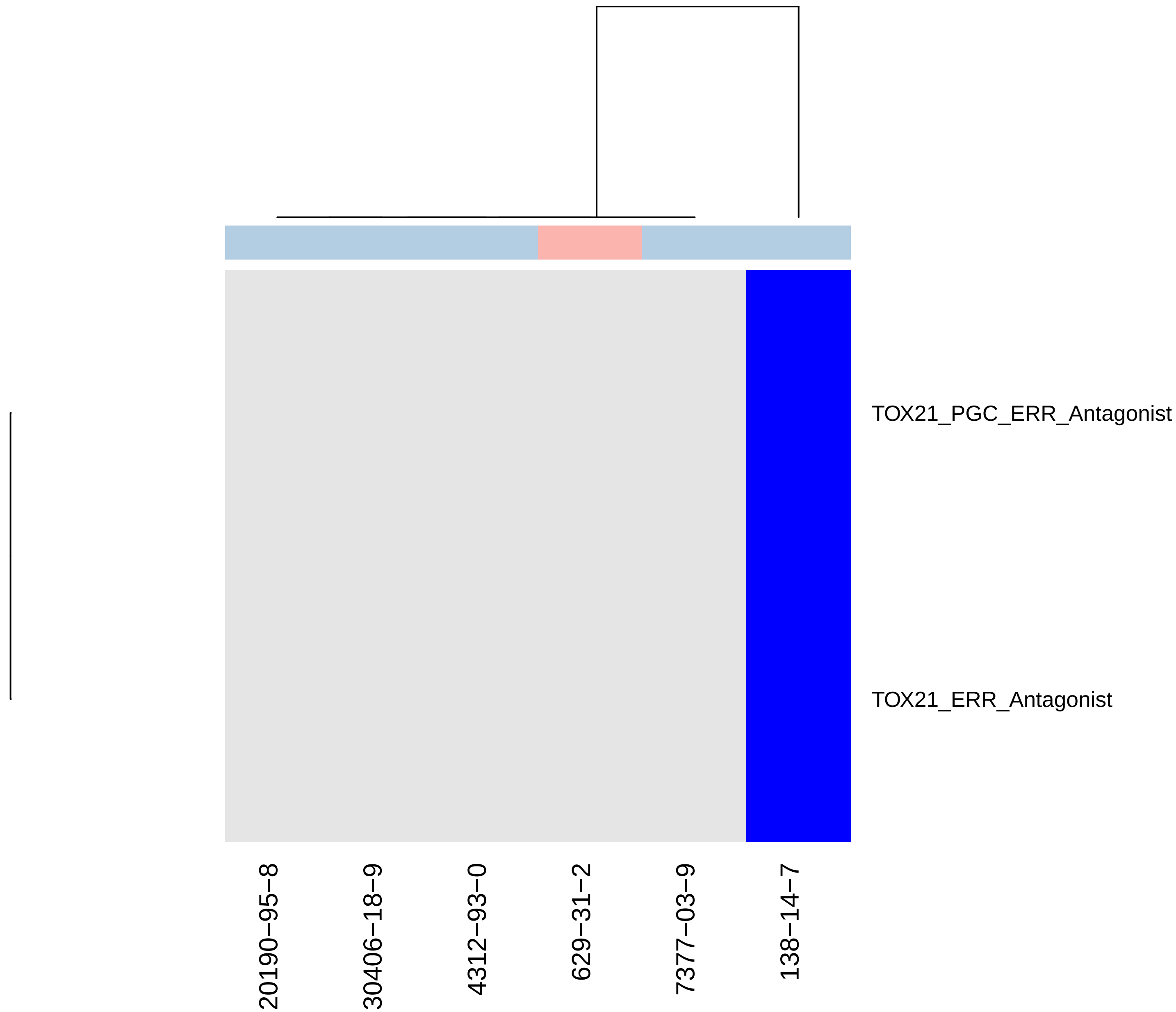

All Activity Heatmap for Cluster 1067

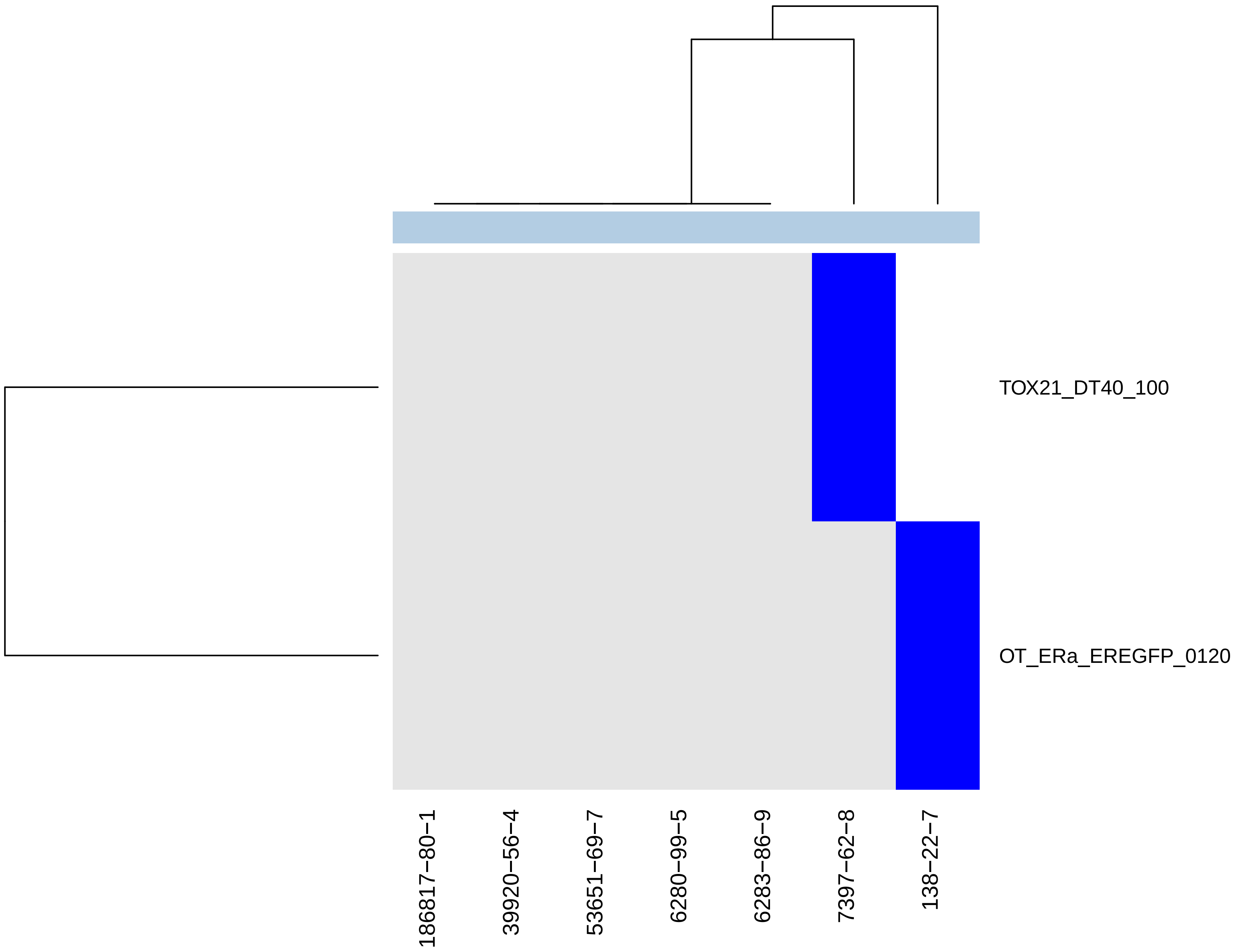

All Activity Heatmap for Cluster 1068

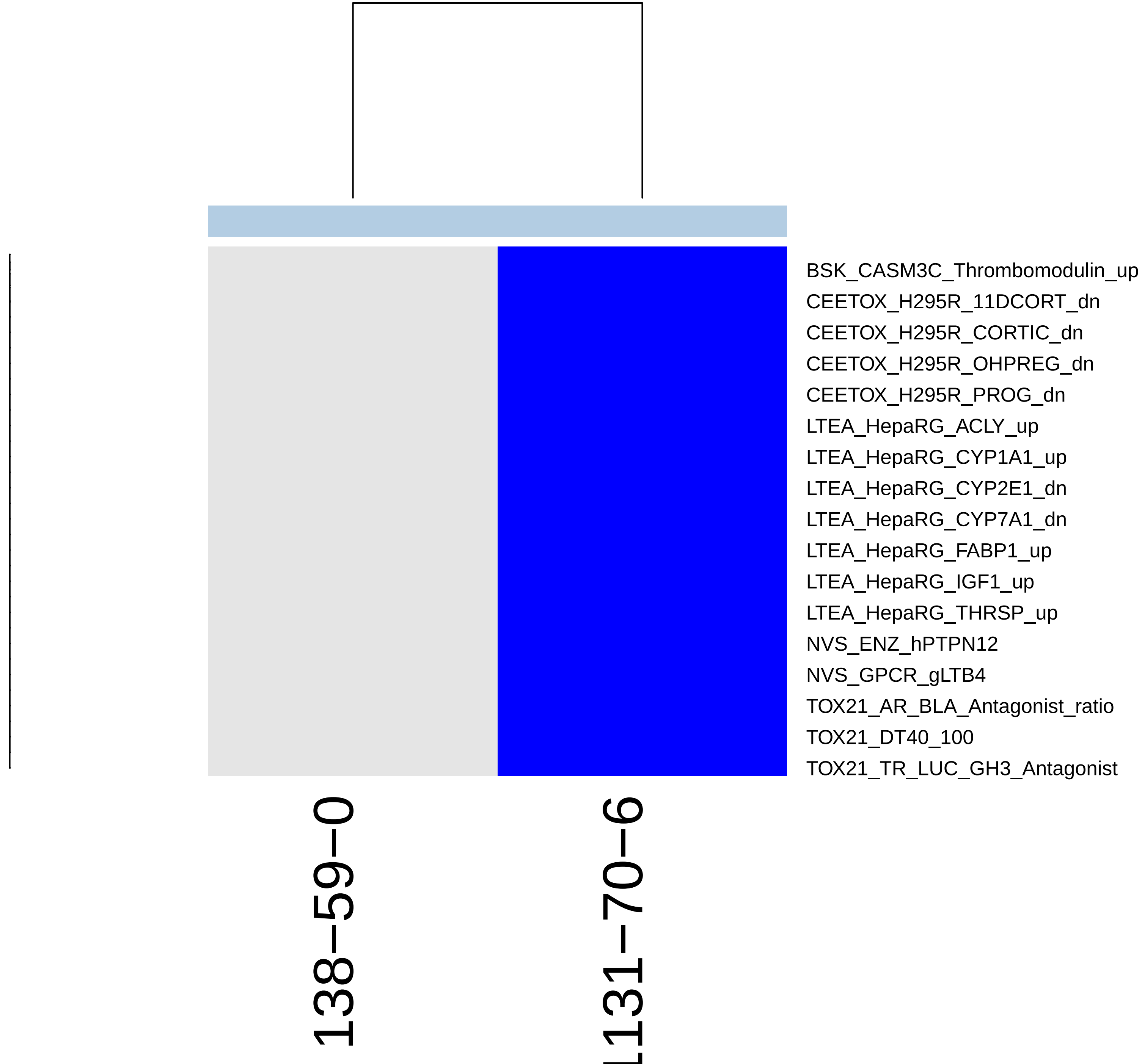

All Activity Heatmap for Cluster 1071

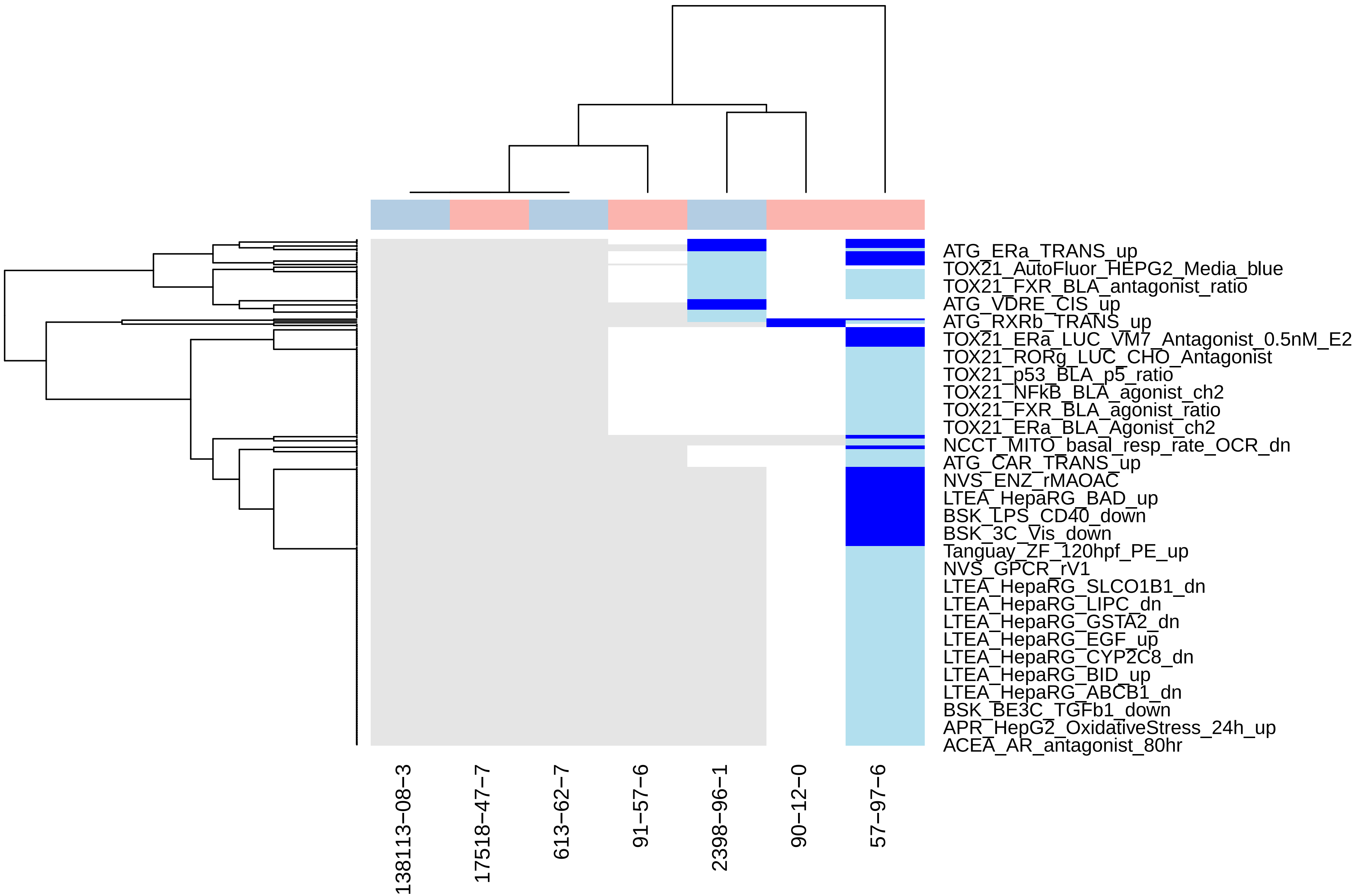

All Activity Heatmap for Cluster 1074

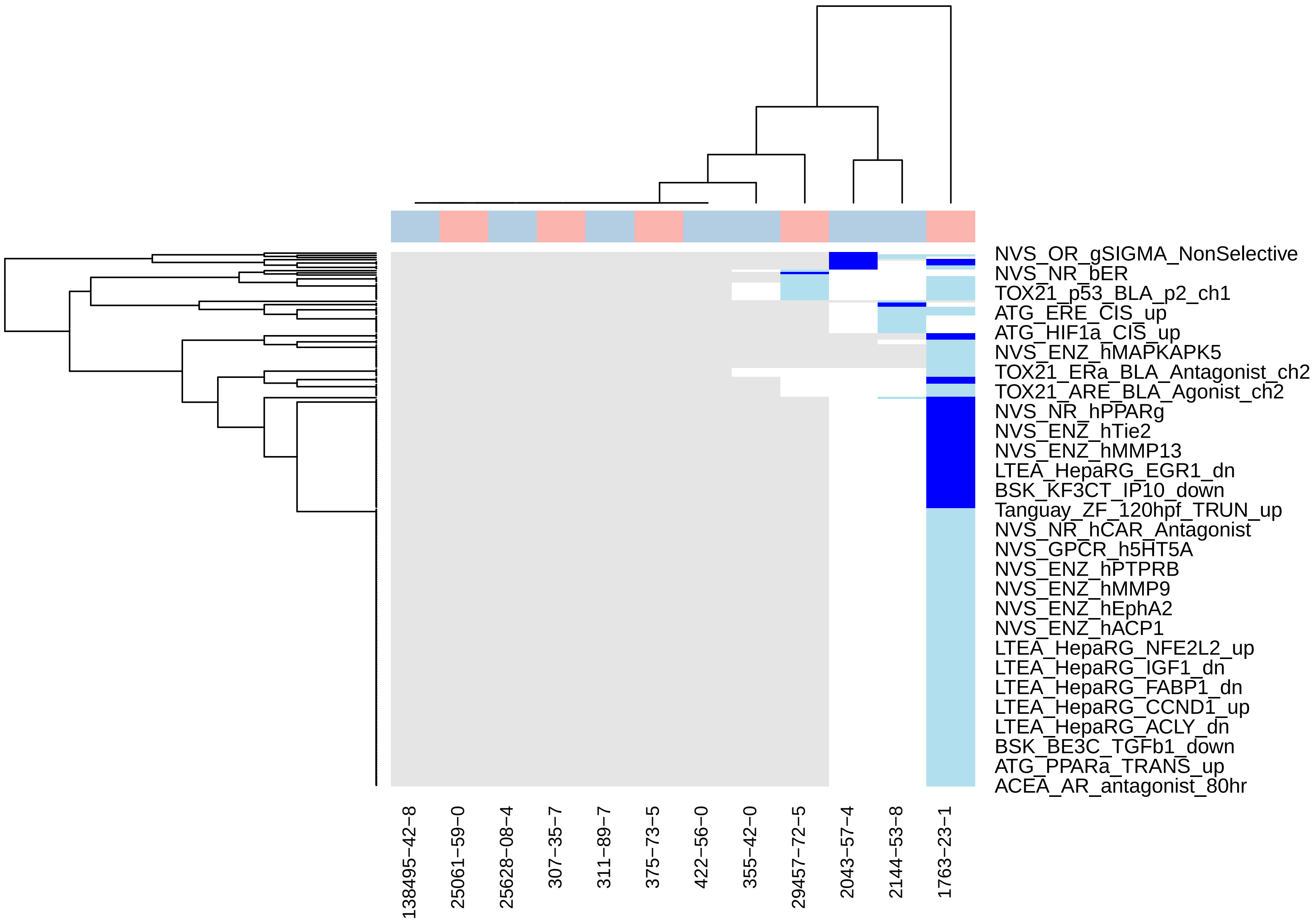

All Activity Heatmap for Cluster 1076

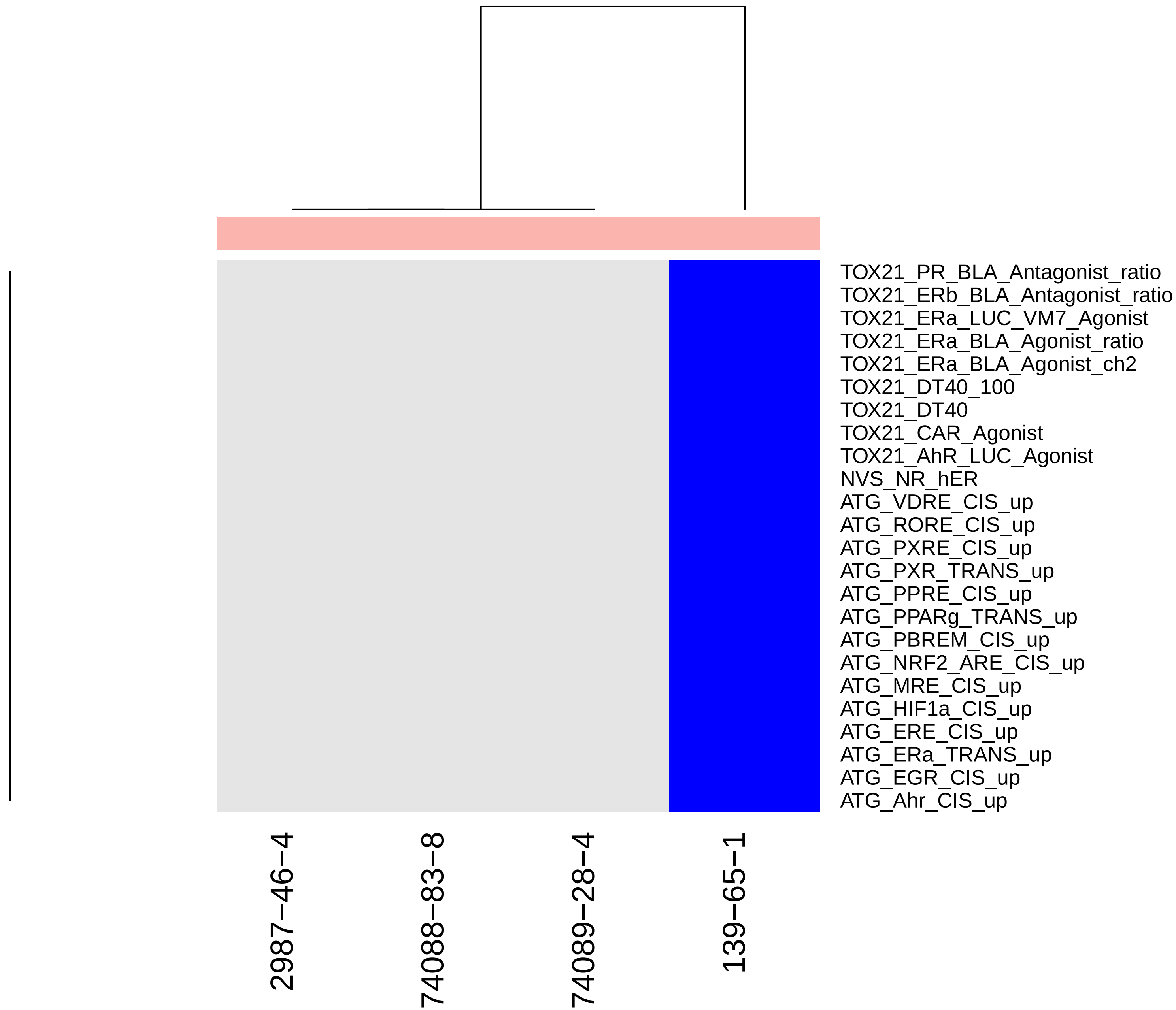

All Activity Heatmap for Cluster 1078

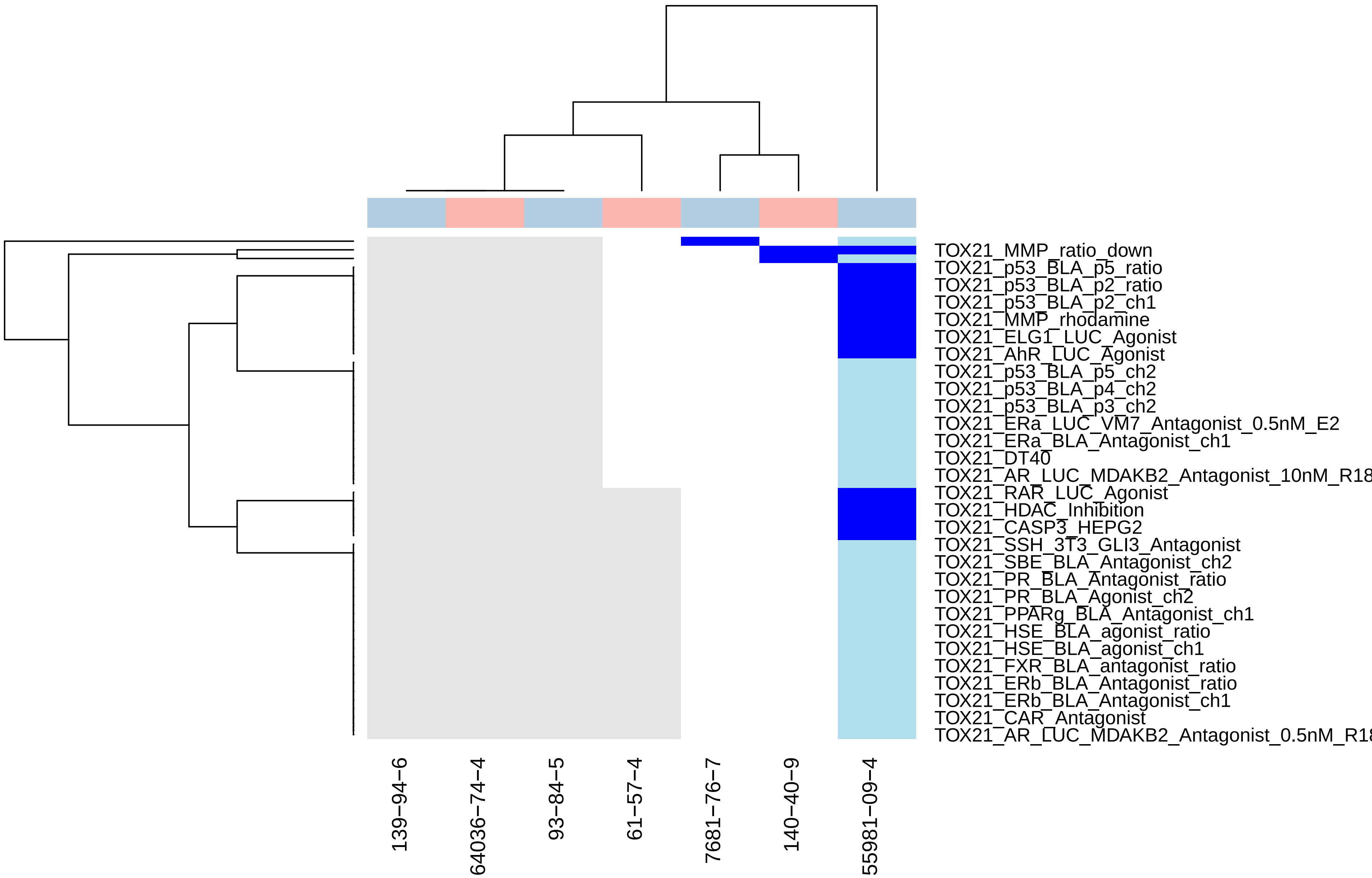

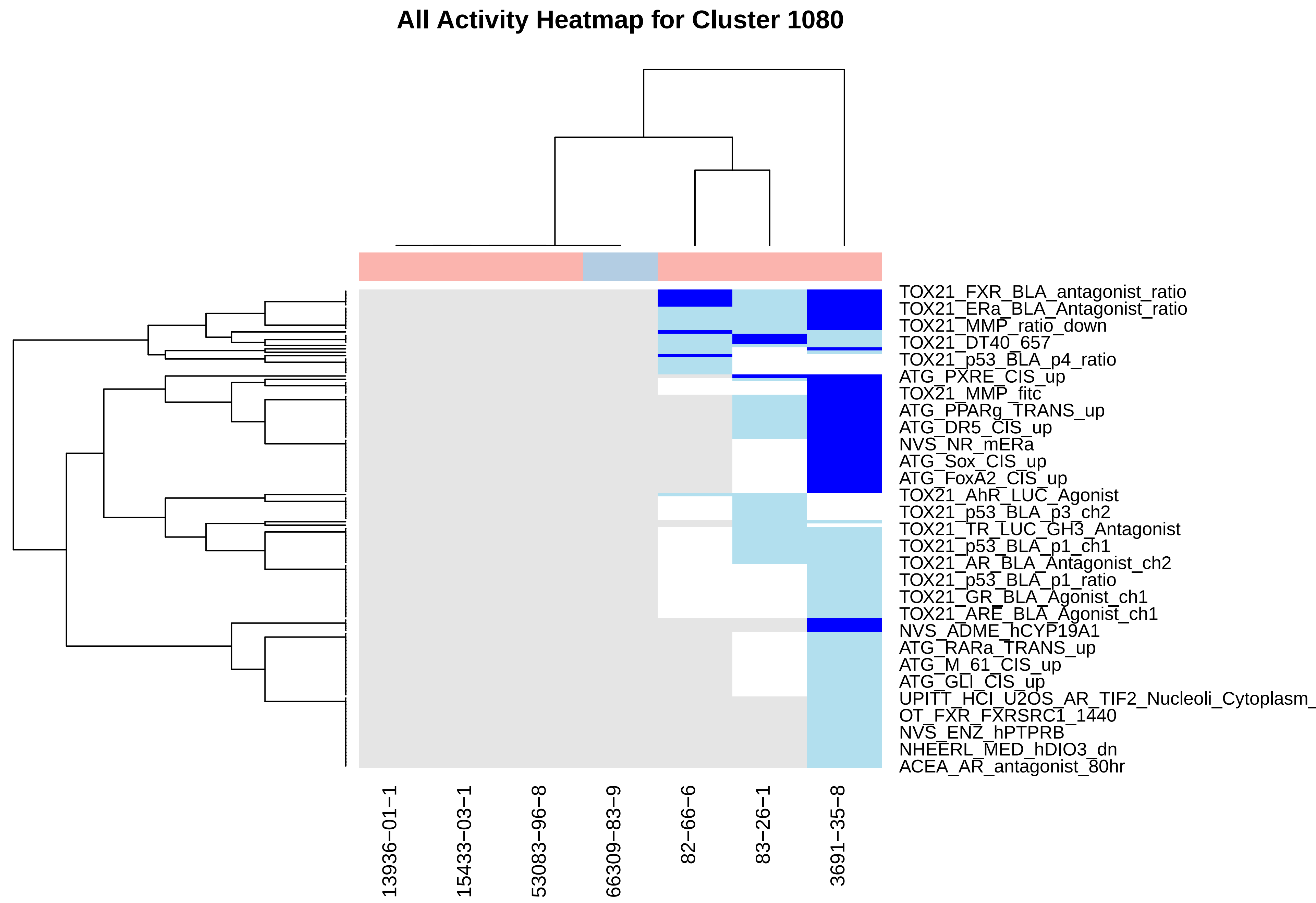
All Activity Heatmap for Cluster 1080
TOX21_FXR_BLA_antagonist_ratio
TOX21_ERa_BLA_Antagonist_ratio
TOX21_MMP_ratio_down
TOX21_DT40_657
TOX21_p53_BLA_p4_ratio
ATG_PXRE_CIS_up
TOX21_MMP_fitc
ATG_PPARg_TRANS_up
ATG_DR5_CIS_up
NVS_NR_mERa
ATG_Sox_CIS_up
ATG_FoxA2_CIS_up
TOX21_AhR_LUC_Agonist
TOX21_p53_BLA_p3_ch2
TOX21_TR_LUC_GH3_Antagonist
TOX21_p53_BLA_p1_ch1
TOX21_AR_BLA_Antagonist_ch2
TOX21_p53_BLA_p1_ratio
TOX21_GR_BLA_Agonist_ch1
TOX21_ARE_BLA_Agonist_ch1
NVS_ADME_hCYP19A1
ATG_RARa_TRANS_up
ATG_M_61_CIS_up
ATG_GLI_CIS_up
UPITT_HCI_U2OS_AR_TIF2_Nucleoli_Cytoplasm_
OT_FXR_FXRSRC1_1440
NVS_ENZ_hPTPRB
NHEERL_MED_hDIO3_dn
ACEA_AR_antagonist_80hr
13936-01-1
15433-03-1
53083-96-8
66309-83-9
82-66-6
83-26-1
3691-35-8

All Activity Heatmap for Cluster 1081

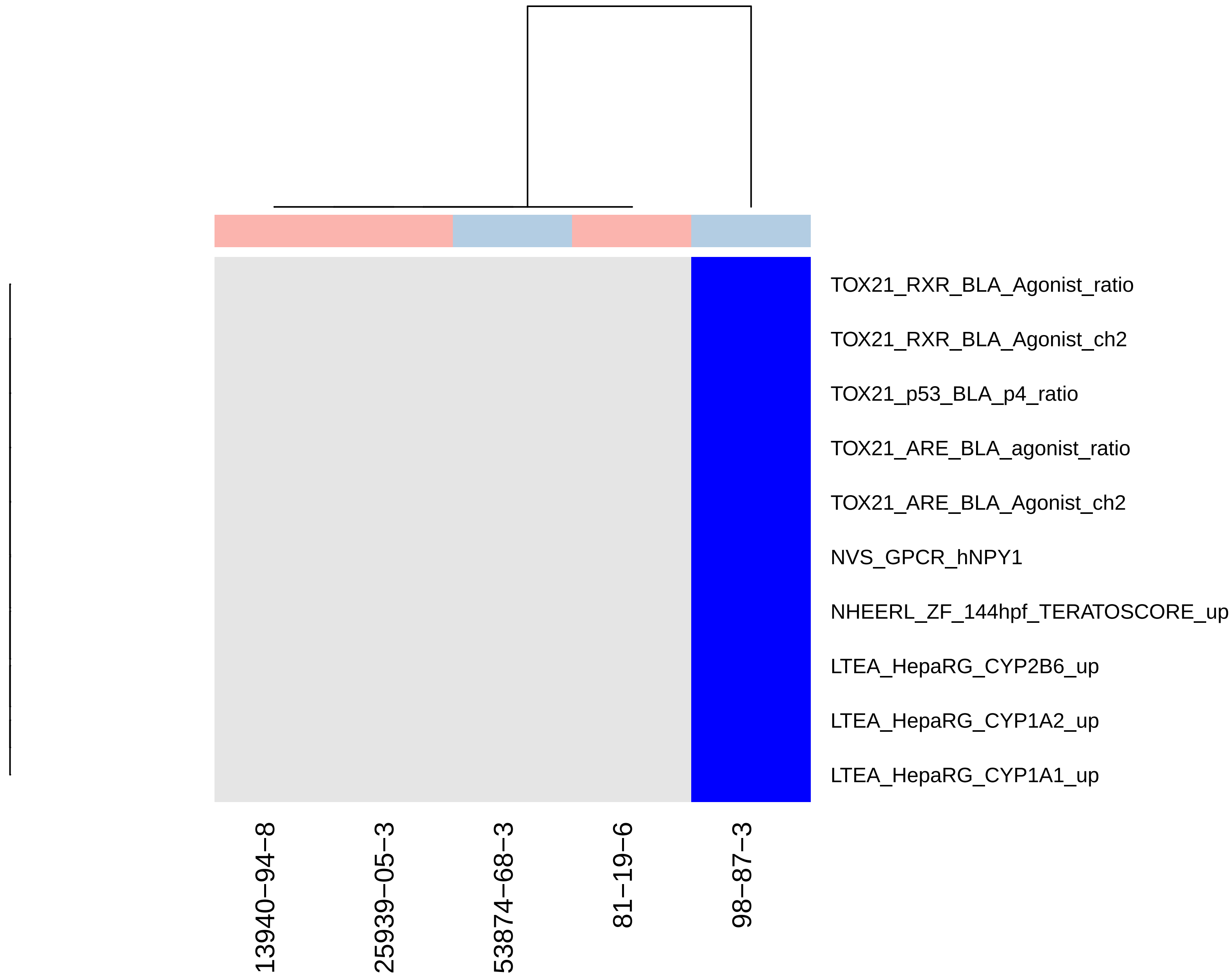

All Activity Heatmap for Cluster 1082

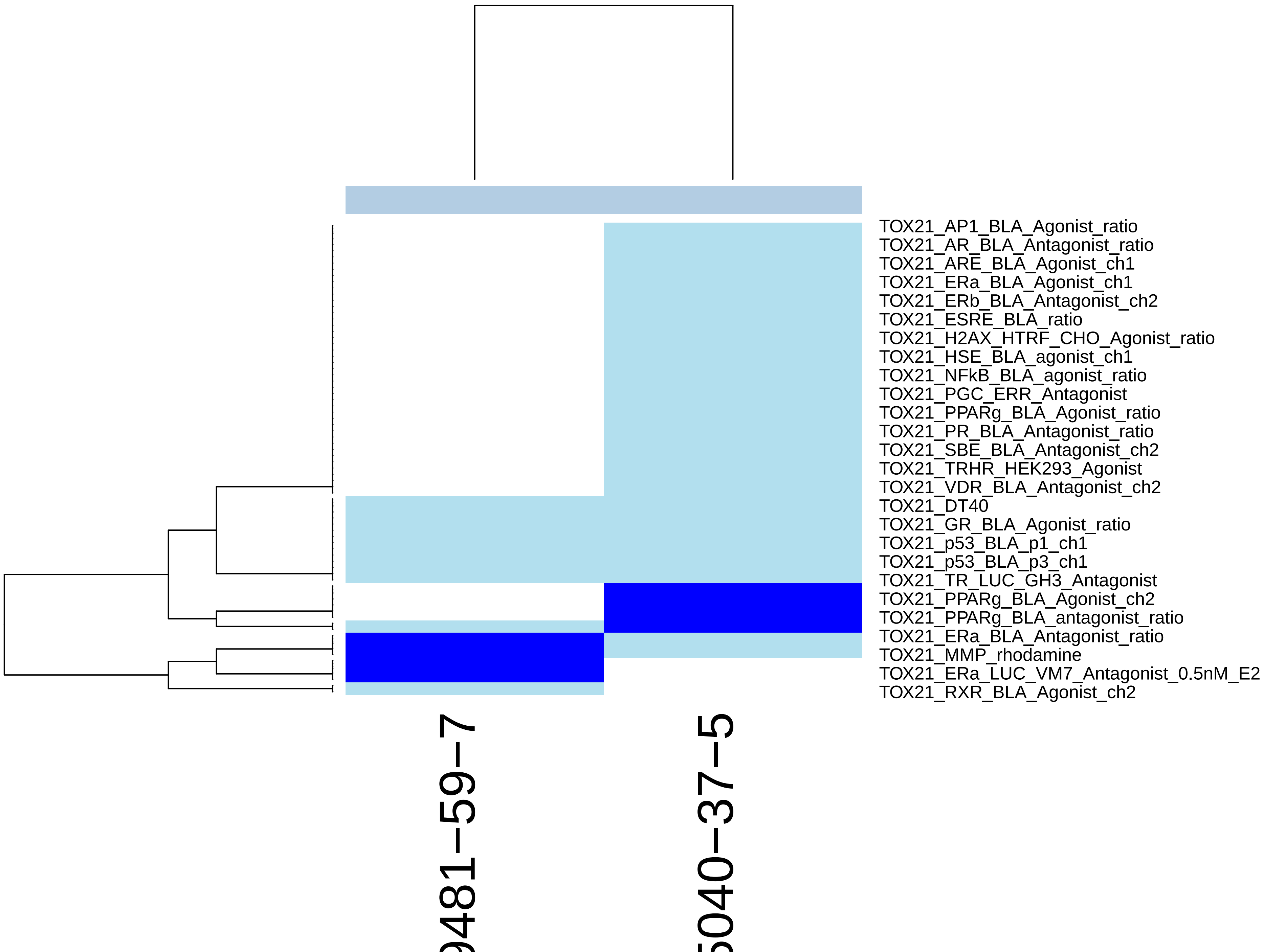

All Activity Heatmap for Cluster 1083

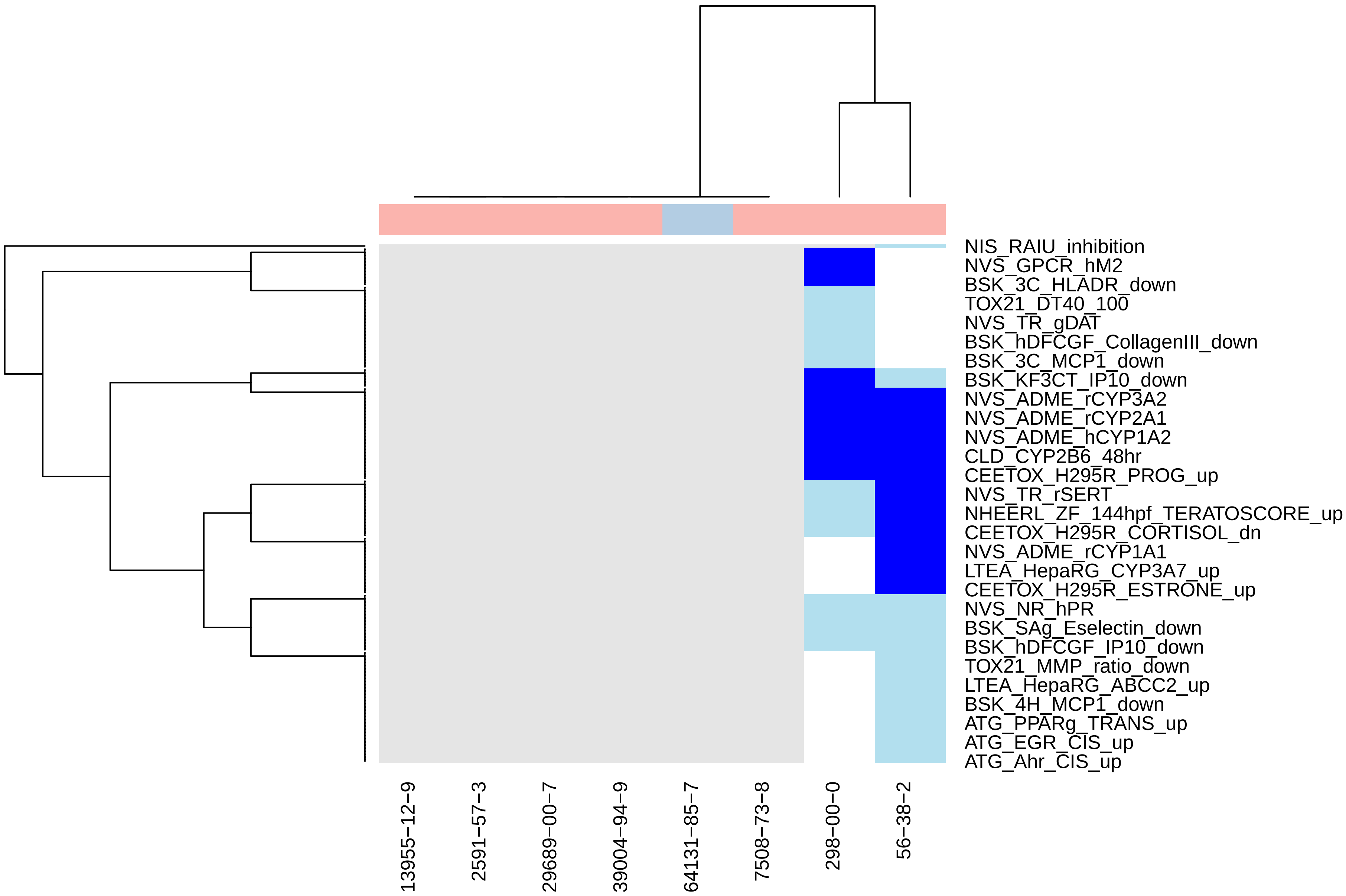

All Activity Heatmap for Cluster 1084

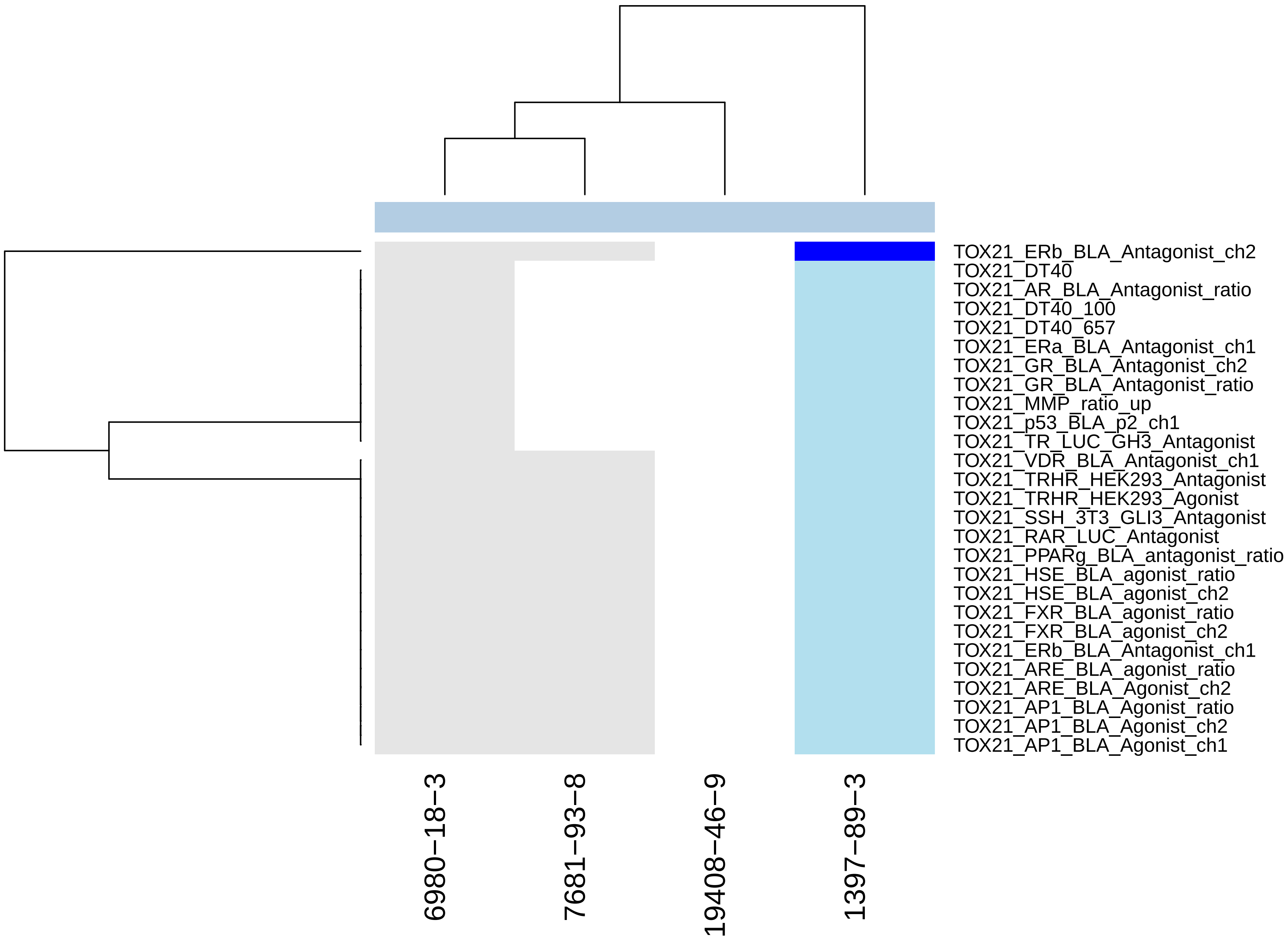

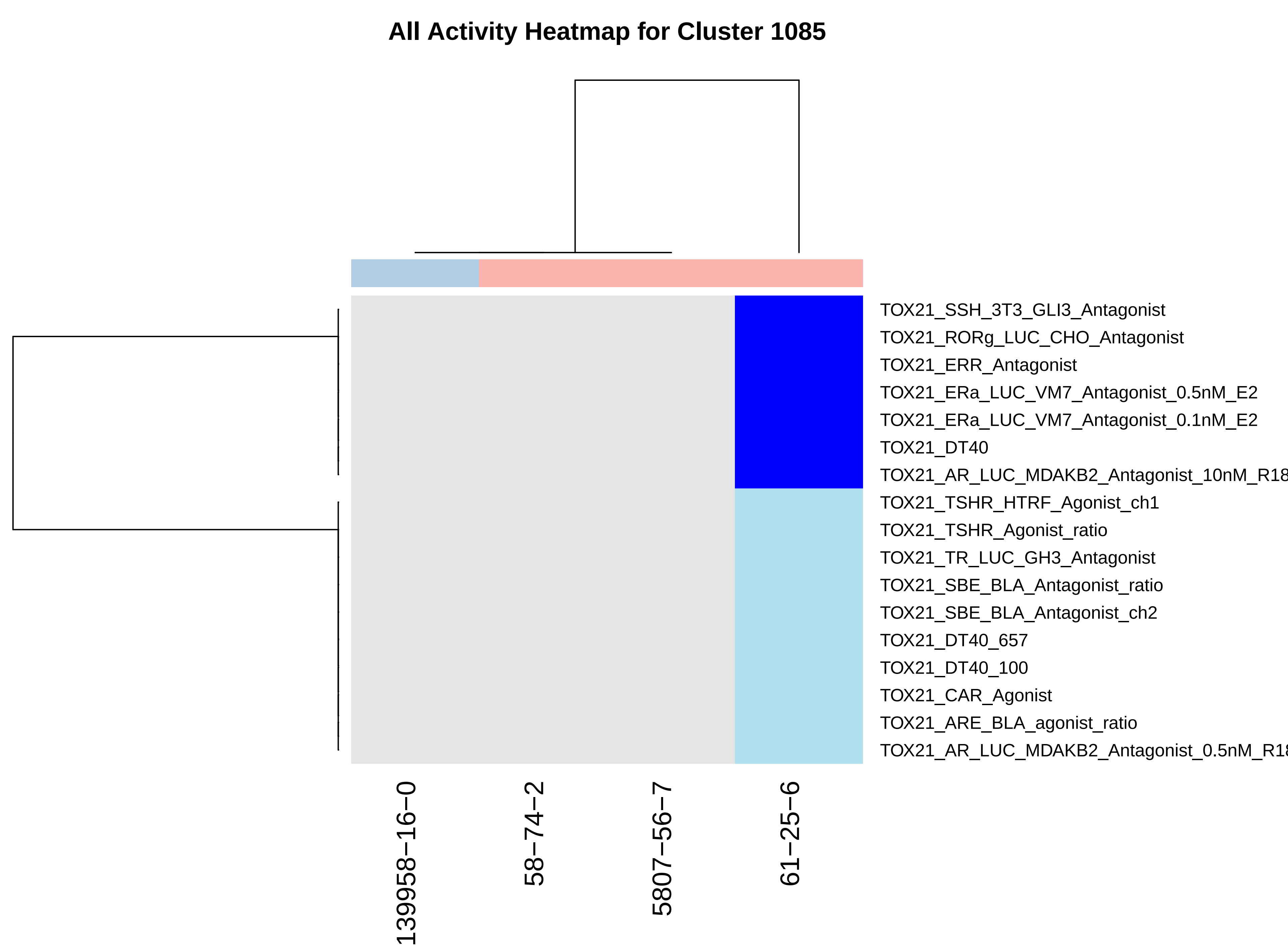
All Activity Heatmap for Cluster 1085
TOX21_SSH_3T3_GLI3_Antagonist
TOX21_RORg_LUC_CHO_Antagonist
TOX21_ERR_Antagonist
TOX21_ERa_LUC_VM7_Antagonist_0.5nM_E2
TOX21_ERa_LUC_VM7_Antagonist_0.1nM_E2
TOX21_DT40
TOX21_AR_LUC_MDAKB2_Antagonist_10nM_R18
TOX21_TSHR_HTRF_Agonist_ch1
TOX21_TSHR_Agonist_ratio
TOX21_TR_LUC_GH3_Antagonist
TOX21_SBE_BLA_Antagonist_ratio
TOX21_SBE_BLA_Antagonist_ch2
TOX21_DT40_657
TOX21_DT40_100
TOX21_CAR_Agonist
TOX21_ARE_BLA_agonist_ratio
TOX21_AR_LUC_MDAKB2_Antagonist_0.5nM_R18
139958−16−0
58−74−2
5807−56−7
61−25−6

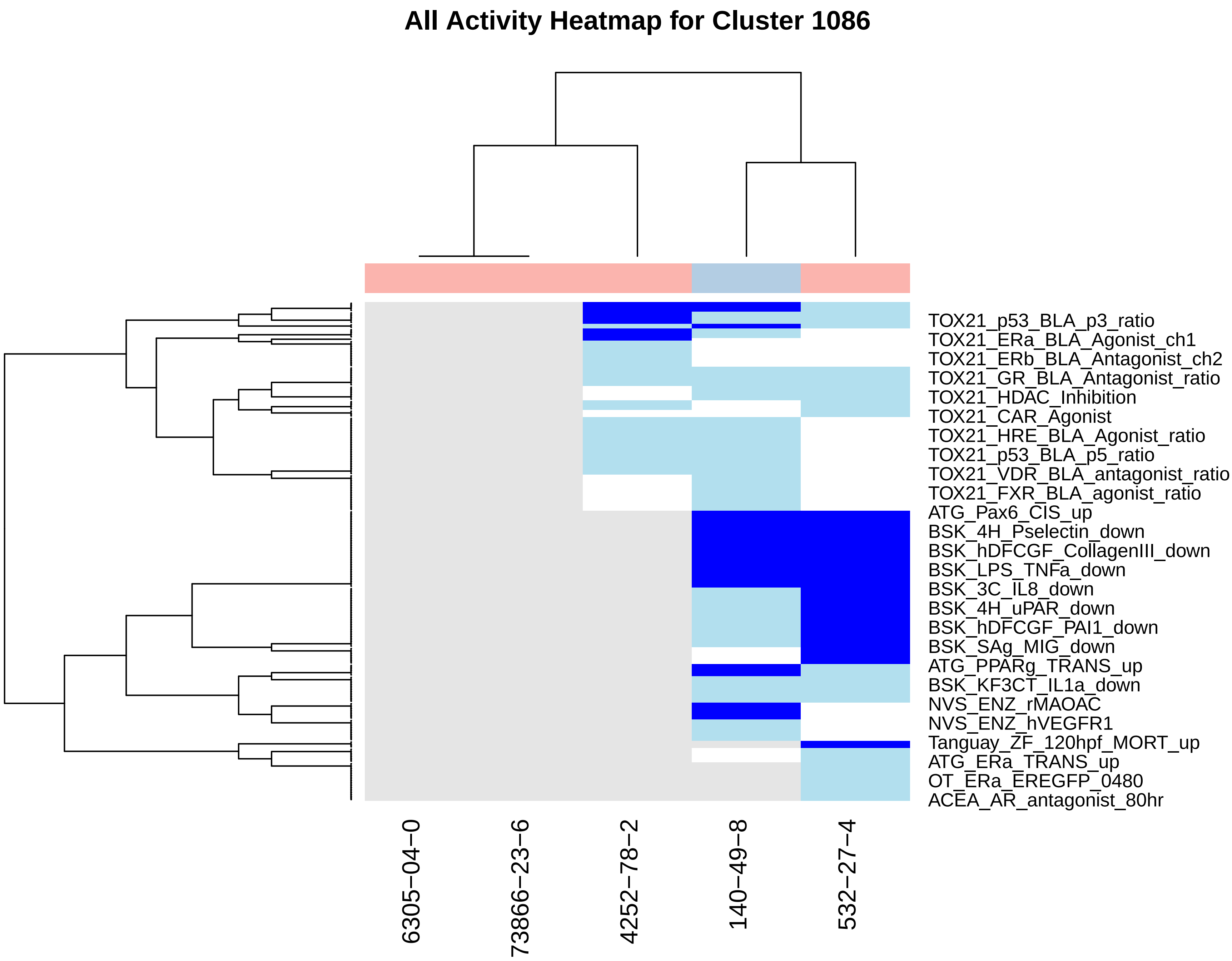
All Activity Heatmap for Cluster 1086
TOX21_p53_BLA_p3_ratio
TOX21_ERa_BLA_Agonist_ch1
TOX21_ERb_BLA_Antagonist_ch2
TOX21_GR_BLA_Antagonist_ratio
TOX21_HDAC_Inhibition
TOX21_CAR_Agonist
TOX21_HRE_BLA_Agonist_ratio
TOX21_p53_BLA_p5_ratio
TOX21_VDR_BLA_antagonist_ratio
TOX21_FXR_BLA_agonist_ratio
ATG_Pax6_CIS_up
BSK_4H_Pselectin_down
BSK_hDFCGF_CollagenIII_down
BSK_LPS_TNFa_down
BSK_3C_IL8_down
BSK_4H_uPAR_down
BSK_hDFCGF_PAI1_down
BSK_SAg_MIG_down
ATG_PPARg_TRANS_up
BSK_KF3CT_IL1a_down
NVS_ENZ_rMAOAC
NVS_ENZ_hVEGFR1
Tanguay_ZF_120hpf_MORT_up
ATG_ERa_TRANS_up
OT_ERa_EREGFP_0480
ACEA_AR_antagonist_80hr
6305−04−0
73866−23−6
4252−78−2
140−49−8
532−27−4

All Activity Heatmap for Cluster 1087

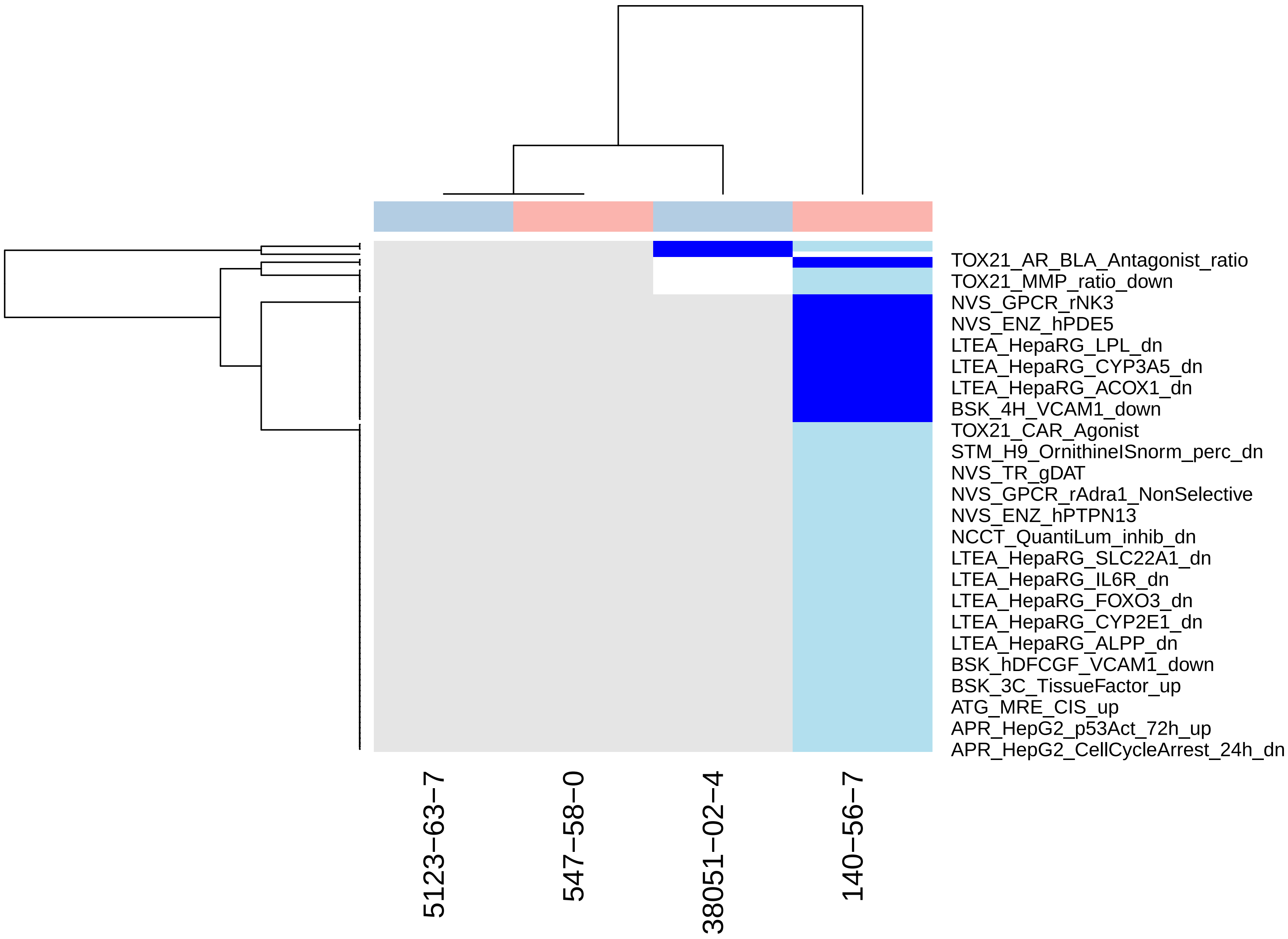

All Activity Heatmap for Cluster 1088

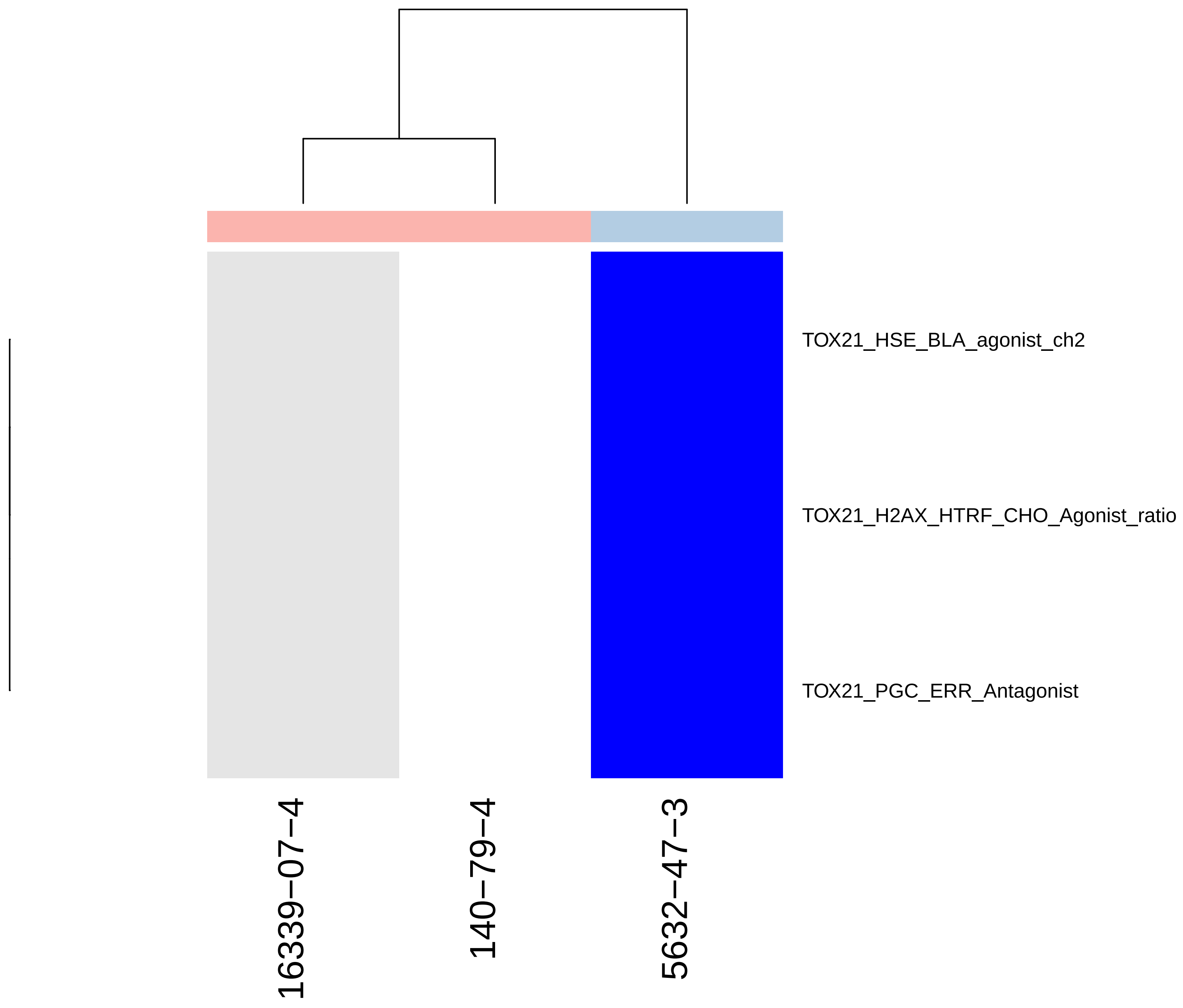

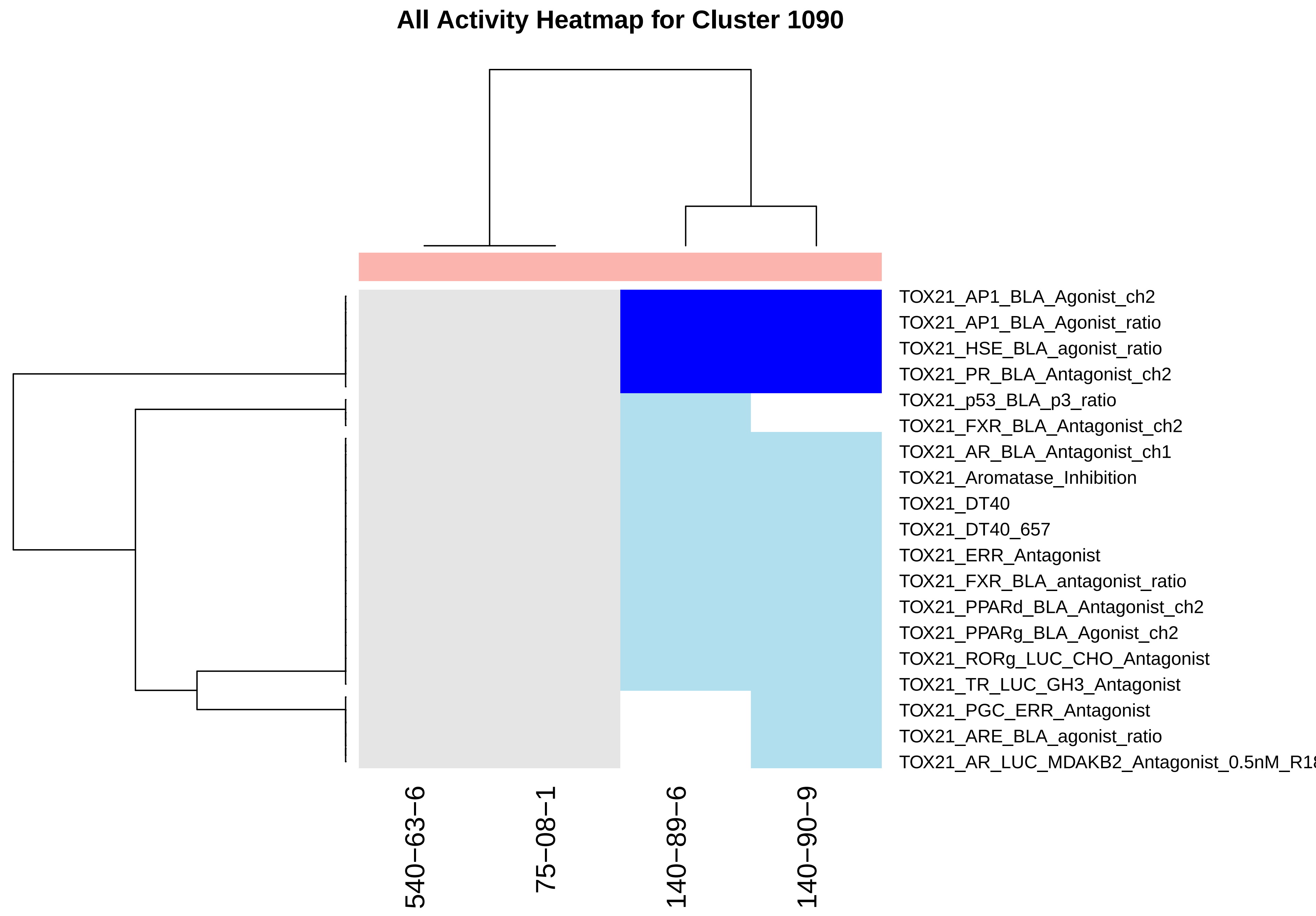
All Activity Heatmap for Cluster 1090
TOX21_AP1_BLA_Agonist_ch2
TOX21_AP1_BLA_Agonist_ratio
TOX21_HSE_BLA_agonist_ratio
TOX21_PR_BLA_Antagonist_ch2
TOX21_p53_BLA_p3_ratio
TOX21_FXR_BLA_Antagonist_ch2
TOX21_AR_BLA_Antagonist_ch1
TOX21_Aromatase_Inhibition
TOX21_DT40
TOX21_DT40_657
TOX21_ERR_Antagonist
TOX21_FXR_BLA_antagonist_ratio
TOX21_PPARd_BLA_Antagonist_ch2
TOX21_PPARg_BLA_Agonist_ch2
TOX21_RORg_LUC_CHO_Antagonist
TOX21_TR_LUC_GH3_Antagonist
TOX21_PGC_ERR_Antagonist
TOX21_ARE_BLA_agonist_ratio
540−63−6
75−08−1
140−89−6
140−90−9

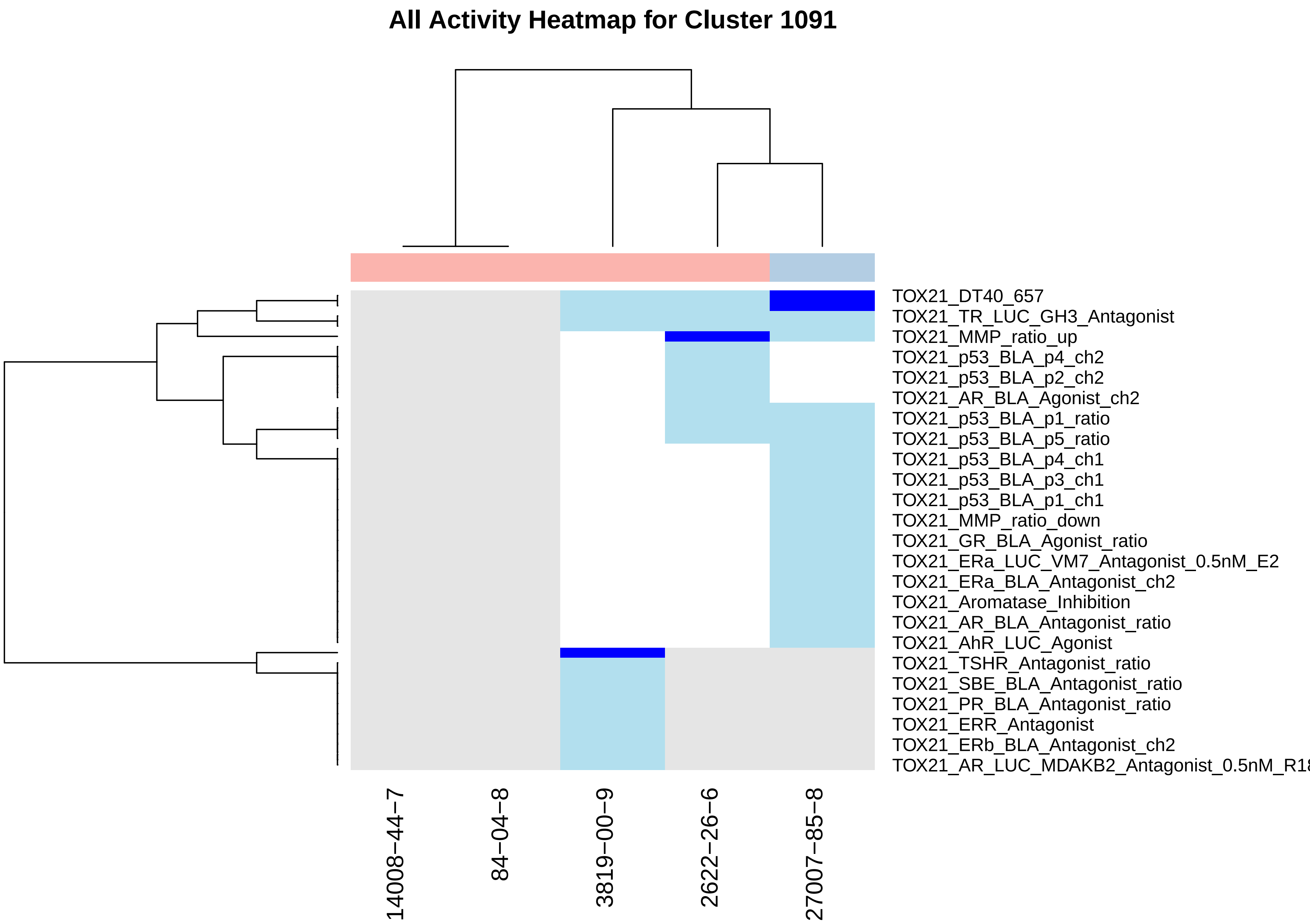
All Activity Heatmap for Cluster 1091
TOX21_DT40_657
TOX21_TR_LUC_GH3_Antagonist
TOX21_MMP_ratio_up
TOX21_p53_BLA_p4_ch2
TOX21_p53_BLA_p2_ch2
TOX21_AR_BLA_Agonist_ch2
TOX21_p53_BLA_p1_ratio
TOX21_p53_BLA_p5_ratio
TOX21_p53_BLA_p4_ch1
TOX21_p53_BLA_p3_ch1
TOX21_p53_BLA_p1_ch1
TOX21_MMP_ratio_down
TOX21_GR_BLA_Agonist_ratio
TOX21_ERa_LUC_VM7_Antagonist_0.5nM_E2
TOX21_ERa_BLA_Antagonist_ch2
TOX21_Aromatase_Inhibition
TOX21_AR_BLA_Antagonist_ratio
TOX21_AhR_LUC_Agonist
TOX21_TSHR_Antagonist_ratio
TOX21_SBE_BLA_Antagonist_ratio
TOX21_PR_BLA_Antagonist_ratio
TOX21_ERR_Antagonist
TOX21_ERb_BLA_Antagonist_ch2
TOX21_AR_LUC_MDAKB2_Antagonist_0.5nM_R18
14008−44−7
84−04−8
3819−00−9
2622−26−6
27007−85−8

All Activity Heatmap for Cluster 1092

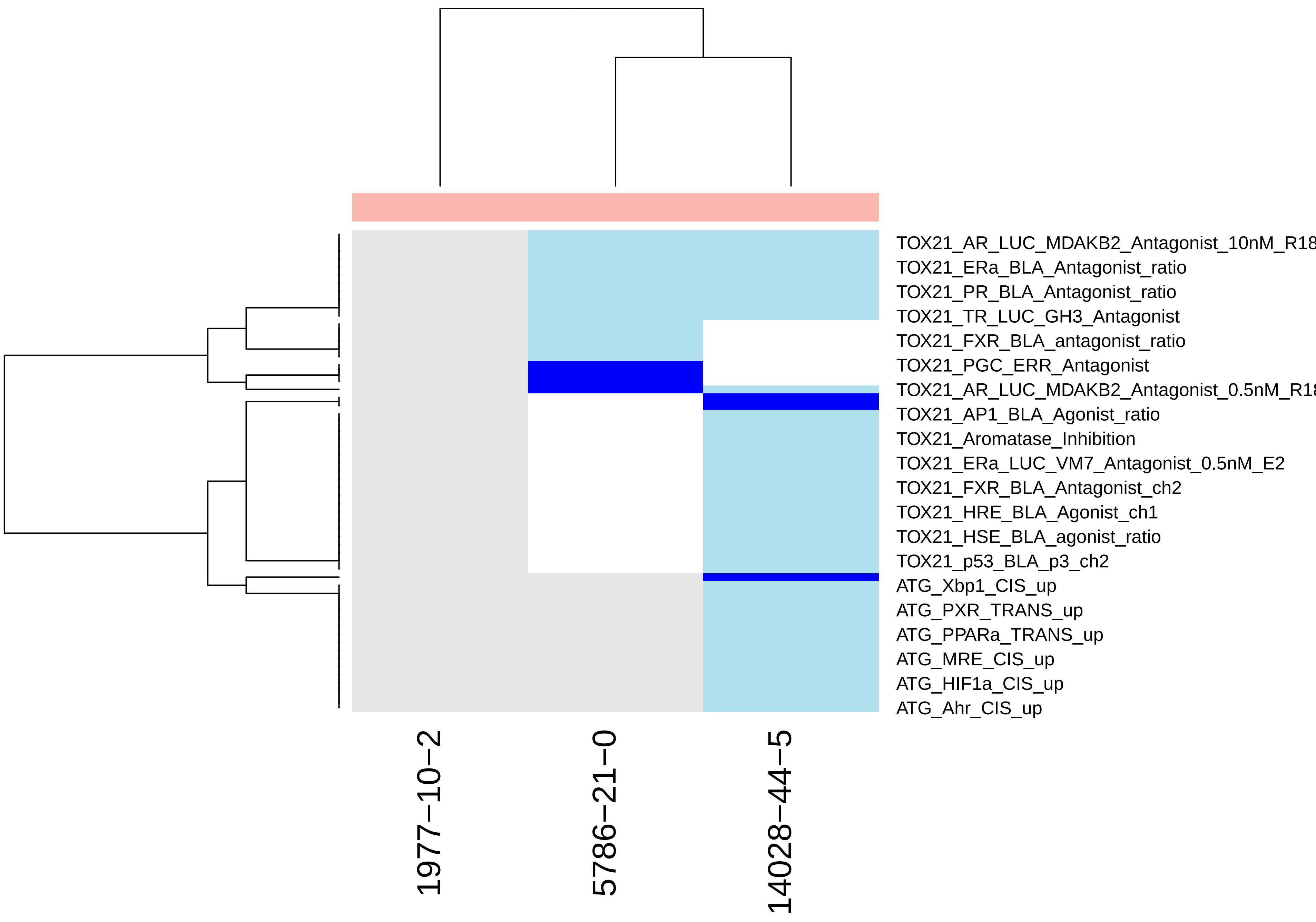

All Activity Heatmap for Cluster 1093

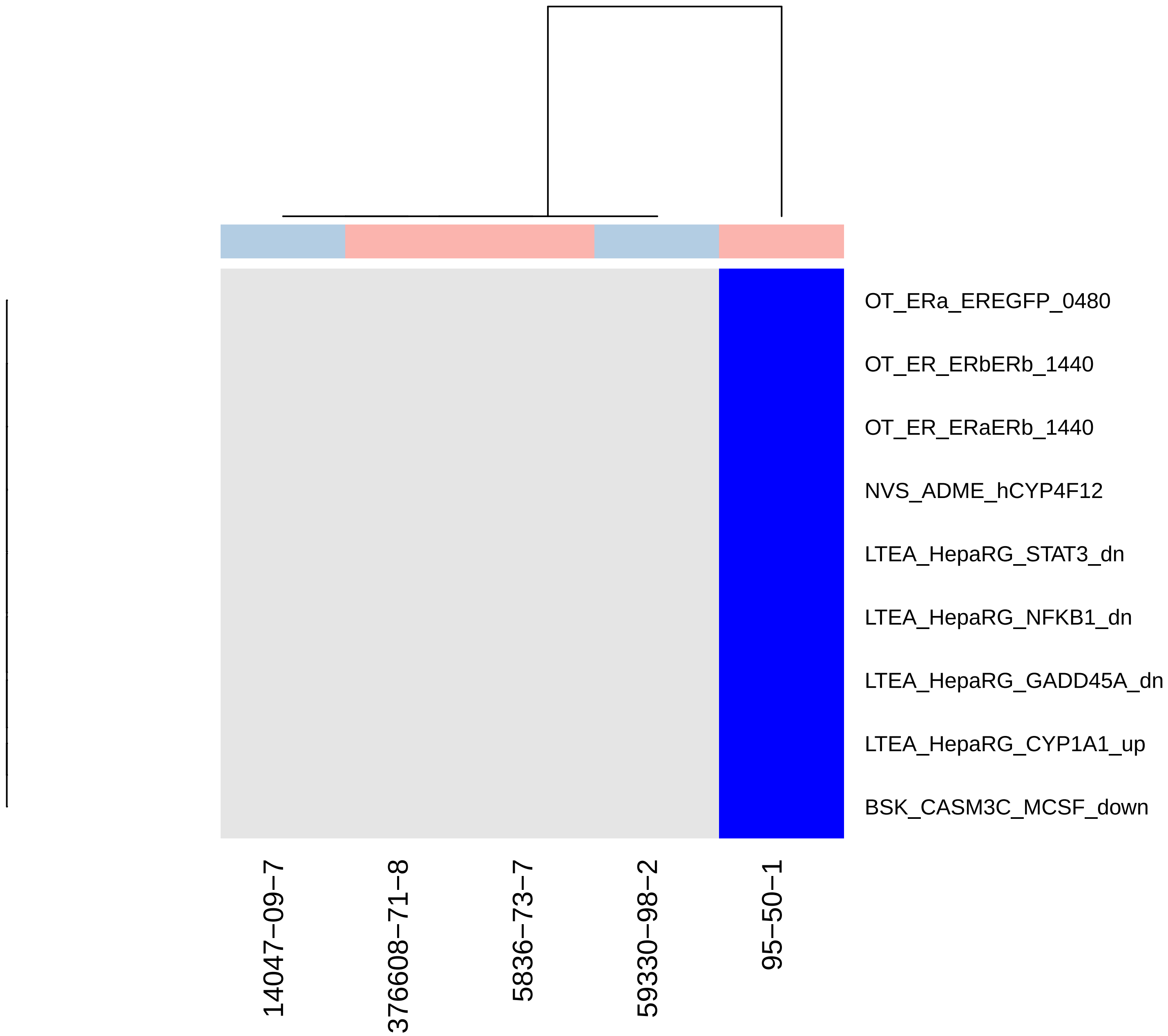

All Activity Heatmap for Cluster 1099

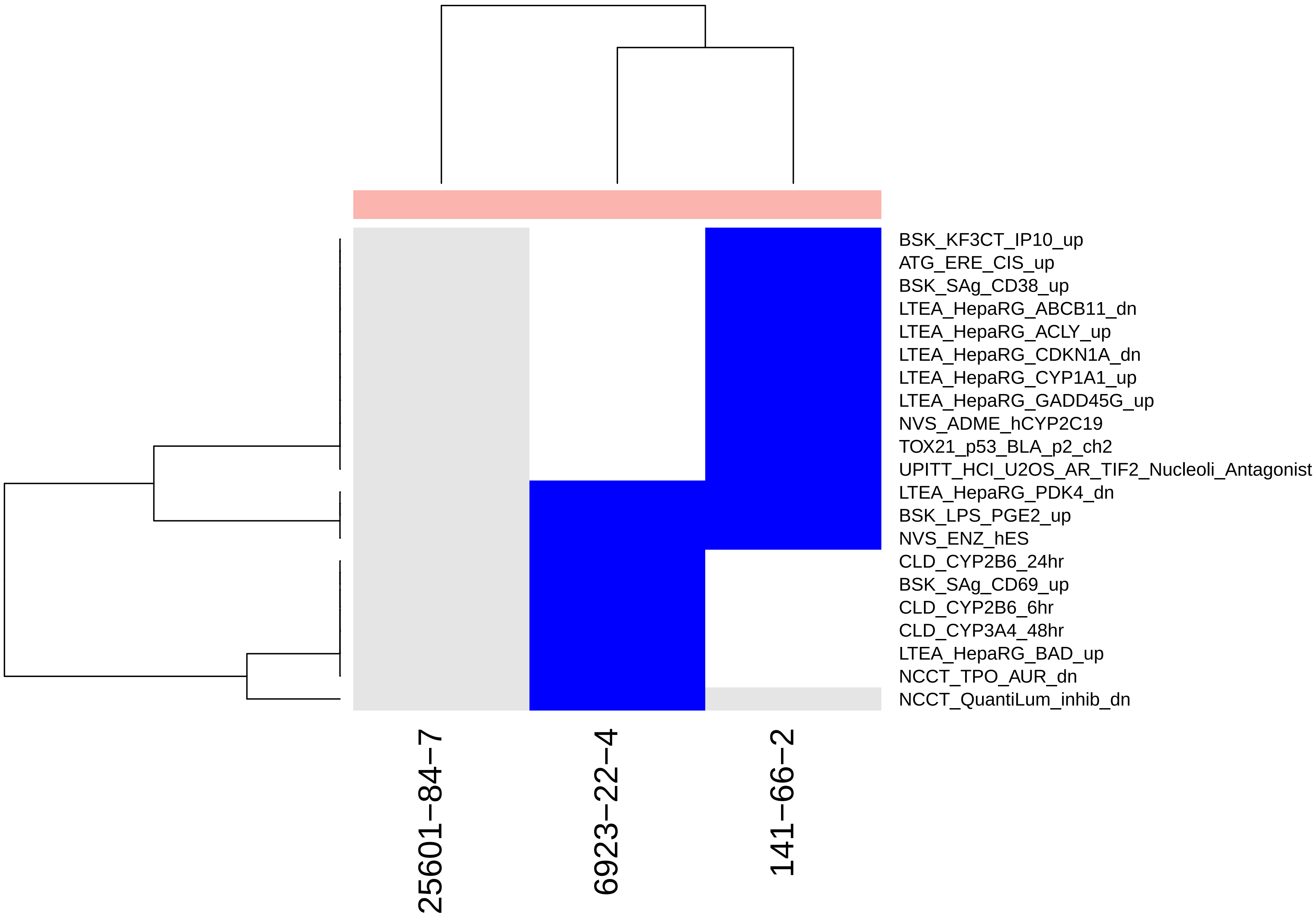

All Activity Heatmap for Cluster 1100

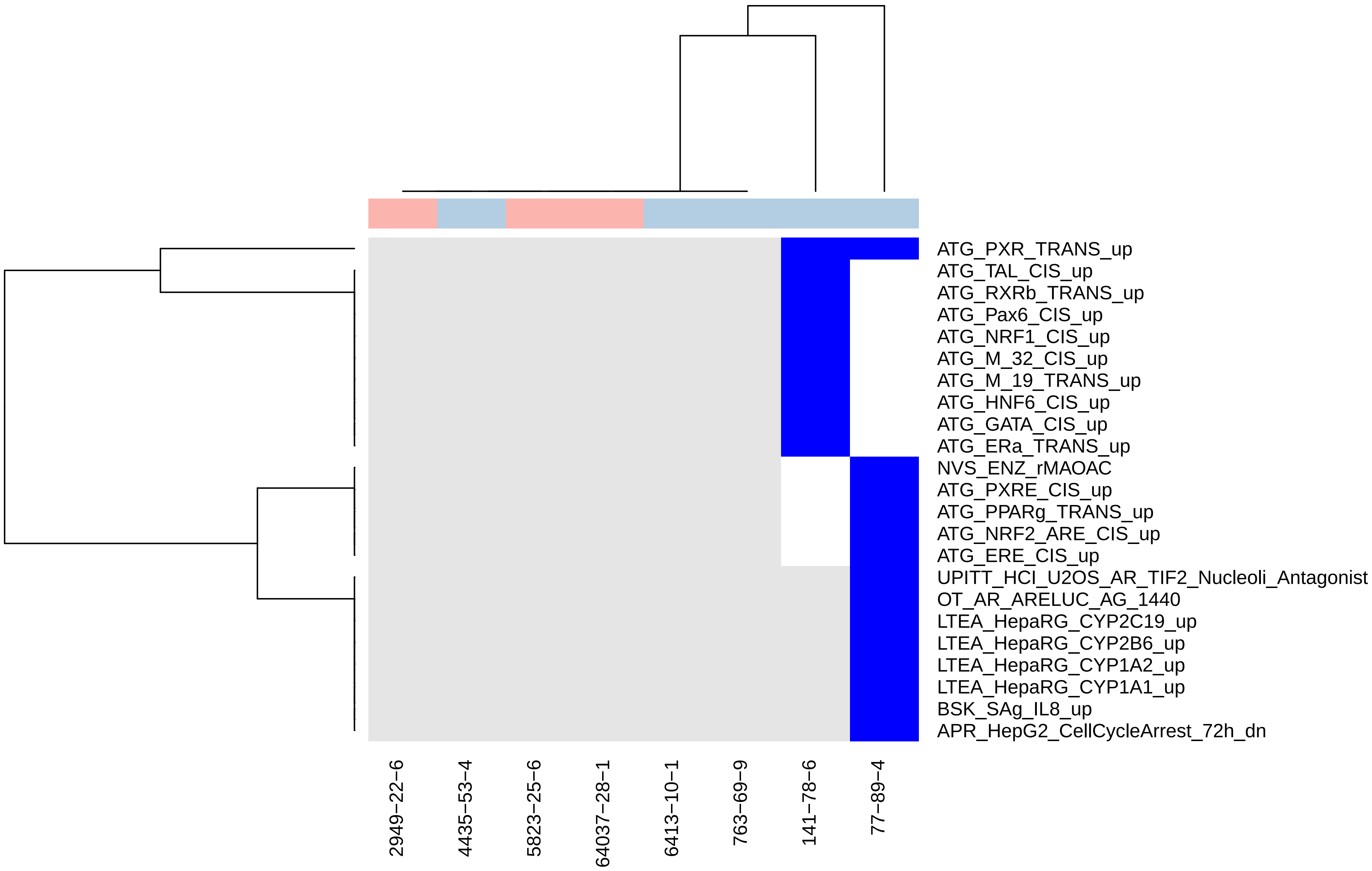

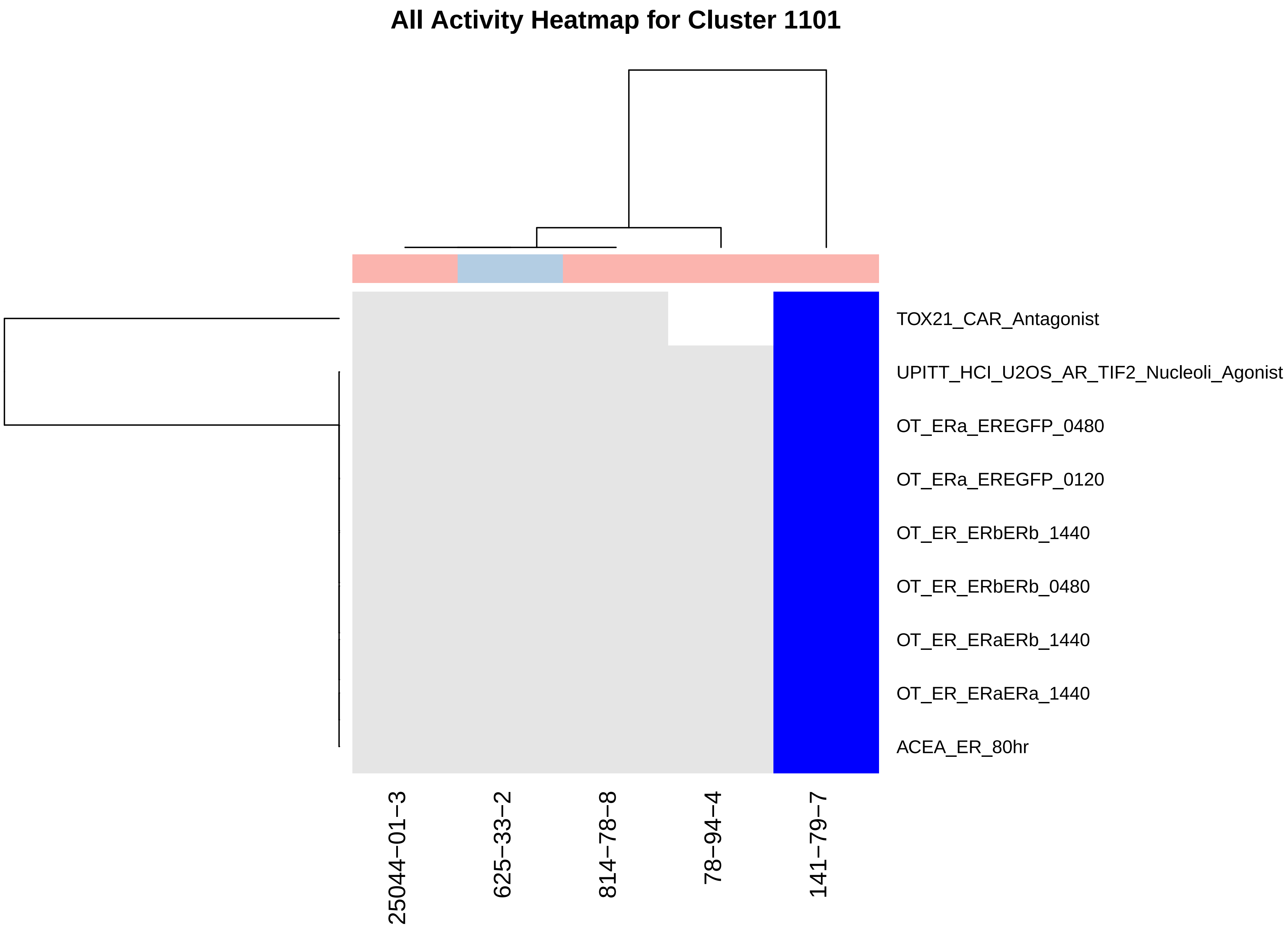
All Activity Heatmap for Cluster 1101
TOX21_CAR_Antagonist
UPITT_HCI_U2OS_AR_TIF2_Nucleoli_Agonist
OT_ERa_EREGFP_0480
OT_ERa_EREGFP_0120
OT_ER_ERbERb_1440
OT_ER_ERbERb_0480
OT_ER_ERaERb_1440
OT_ER_ERaERa_1440
ACEA_ER_80hr
25044−01−3
625−33−2
814−78−8
78−94−4
141−79−7

All Activity Heatmap for Cluster 1104

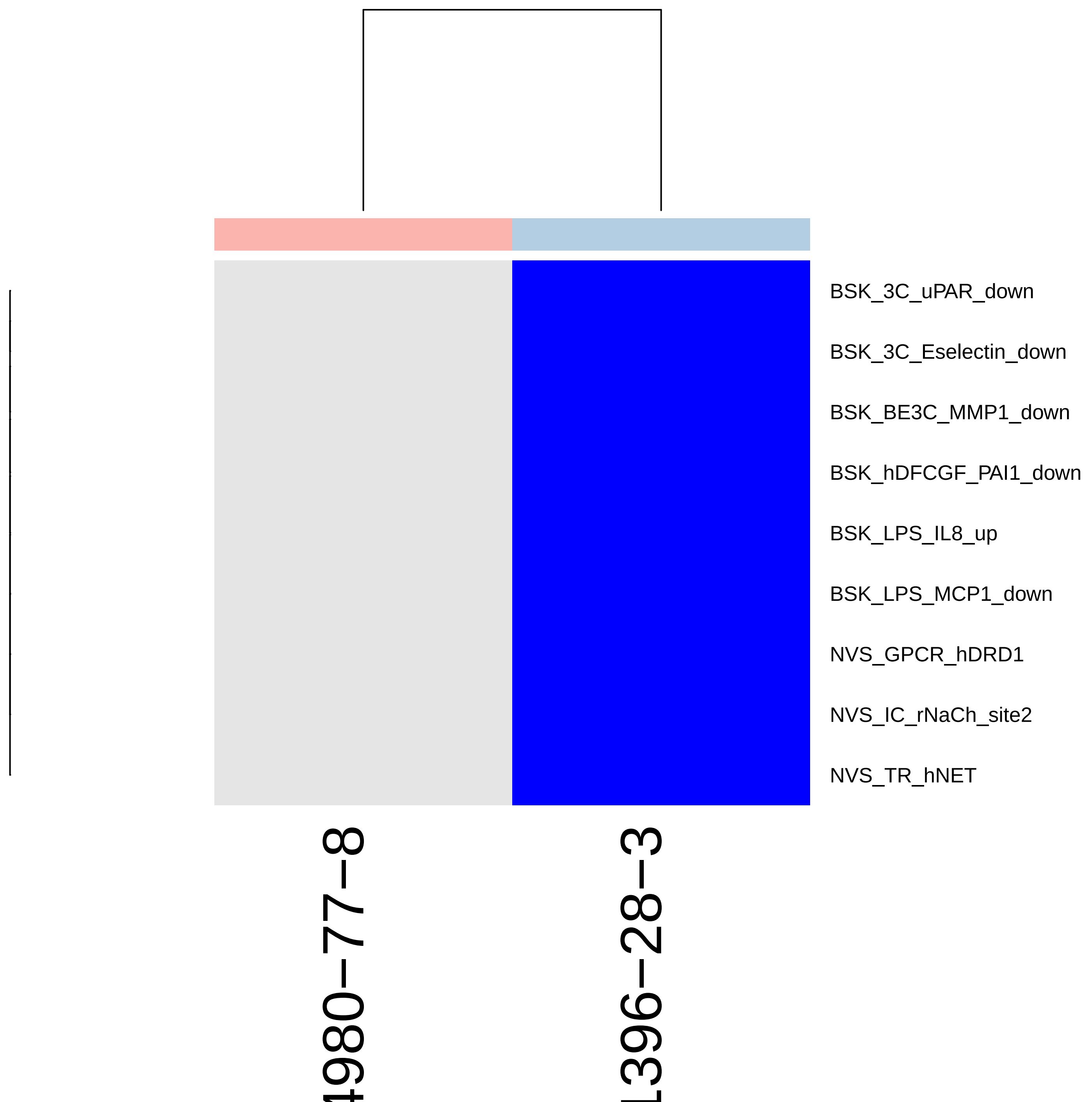

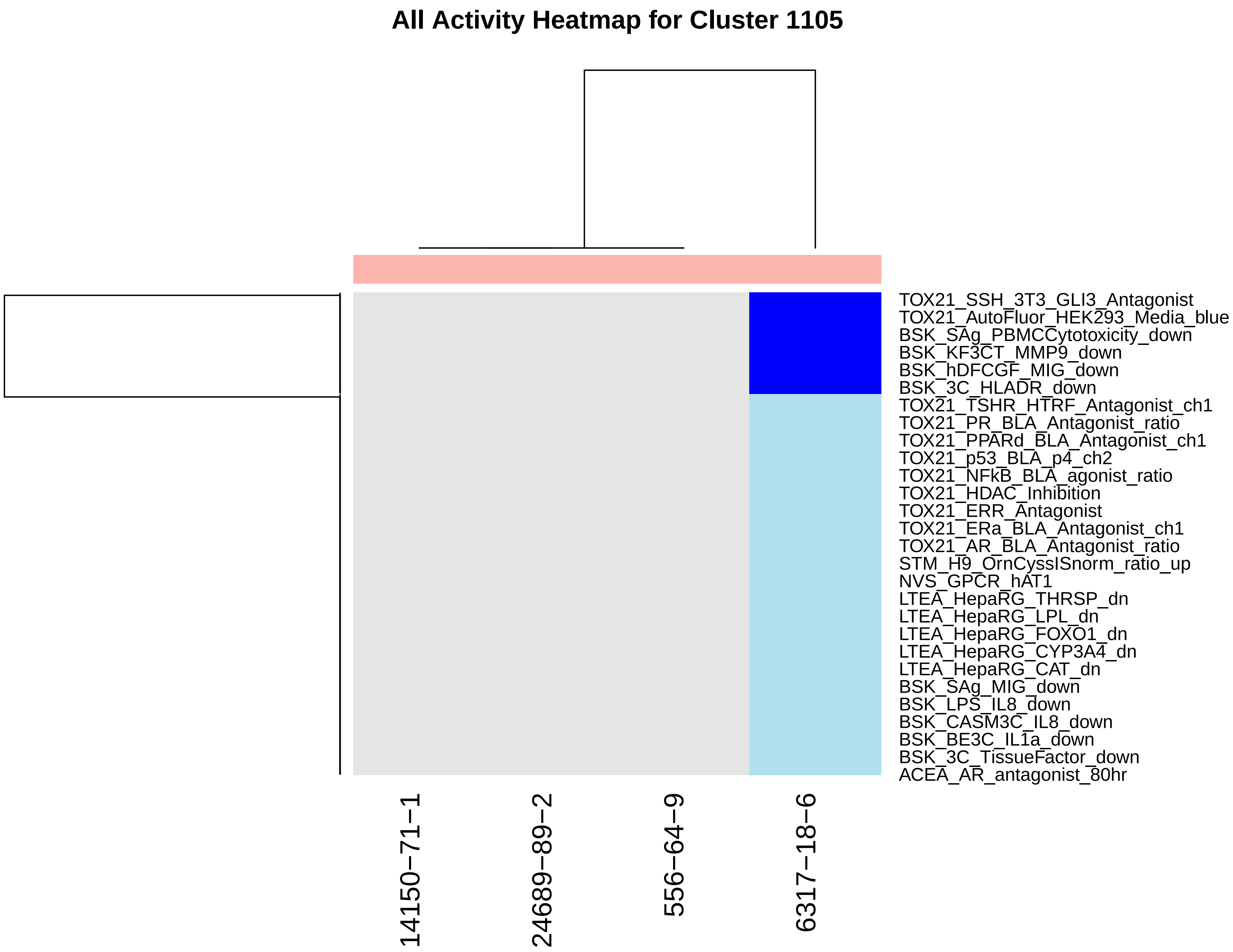
All Activity Heatmap for Cluster 1105
TOX21_SSH_3T3_GLI3_Antagonist
TOX21_AutoFluor_HEK293_Media_blue
BSK_SAg_PBMCCytotoxicity_down
BSK_KF3CT_MMP9_down
BSK_hDFCGF_MIG_down
BSK_3C_HLADR_down
TOX21_TSHR_HTRF_Antagonist_ch1
TOX21_PR_BLA_Antagonist_ratio
TOX21_PPARd_BLA_Antagonist_ch1
TOX21_p53_BLA_p4_ch2
TOX21_NFkB_BLA_agonist_ratio
TOX21_HDAC_Inhibition
TOX21_ERR_Antagonist
TOX21_ERa_BLA_Antagonist_ch1
TOX21_AR_BLA_Antagonist_ratio
STM_H9_OrnCyssISnorm_ratio_up
NVS_GPCR_hAT1
LTEA_HepaRG_THRSP_dn
LTEA_HepaRG_LPL_dn
LTEA_HepaRG_FOXO1_dn
LTEA_HepaRG_CYP3A4_dn
LTEA_HepaRG_CAT_dn
BSK_SAg_MIG_down
BSK_LPS_IL8_down
BSK_CASM3C_IL8_down
BSK_BE3C_IL1a_down
BSK_3C_TissueFactor_down
ACEA_AR_antagonist_80hr
14150−71−1
24689−89−2
556−64−9
6317−18−6

All Activity Heatmap for Cluster 1106

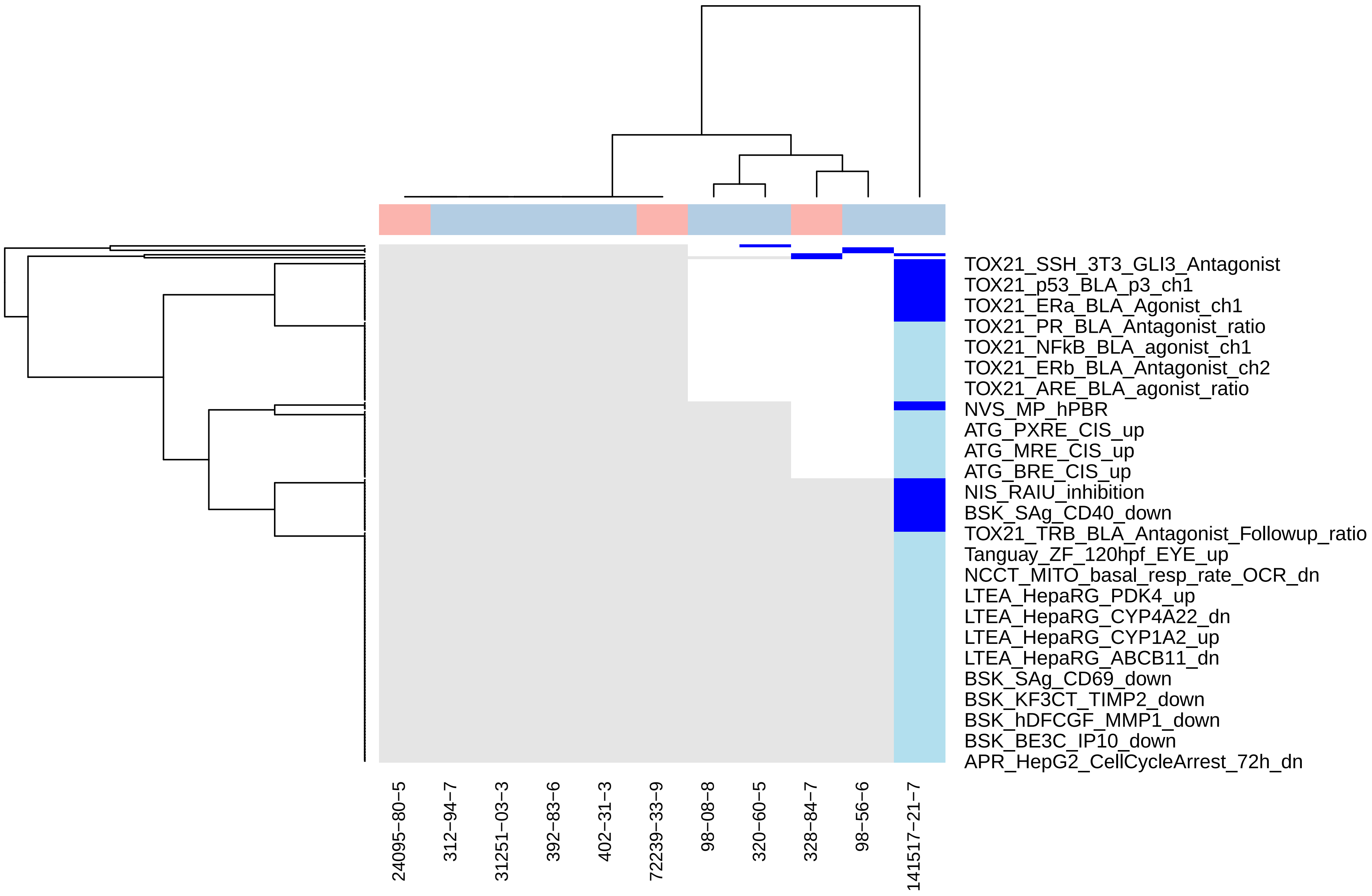

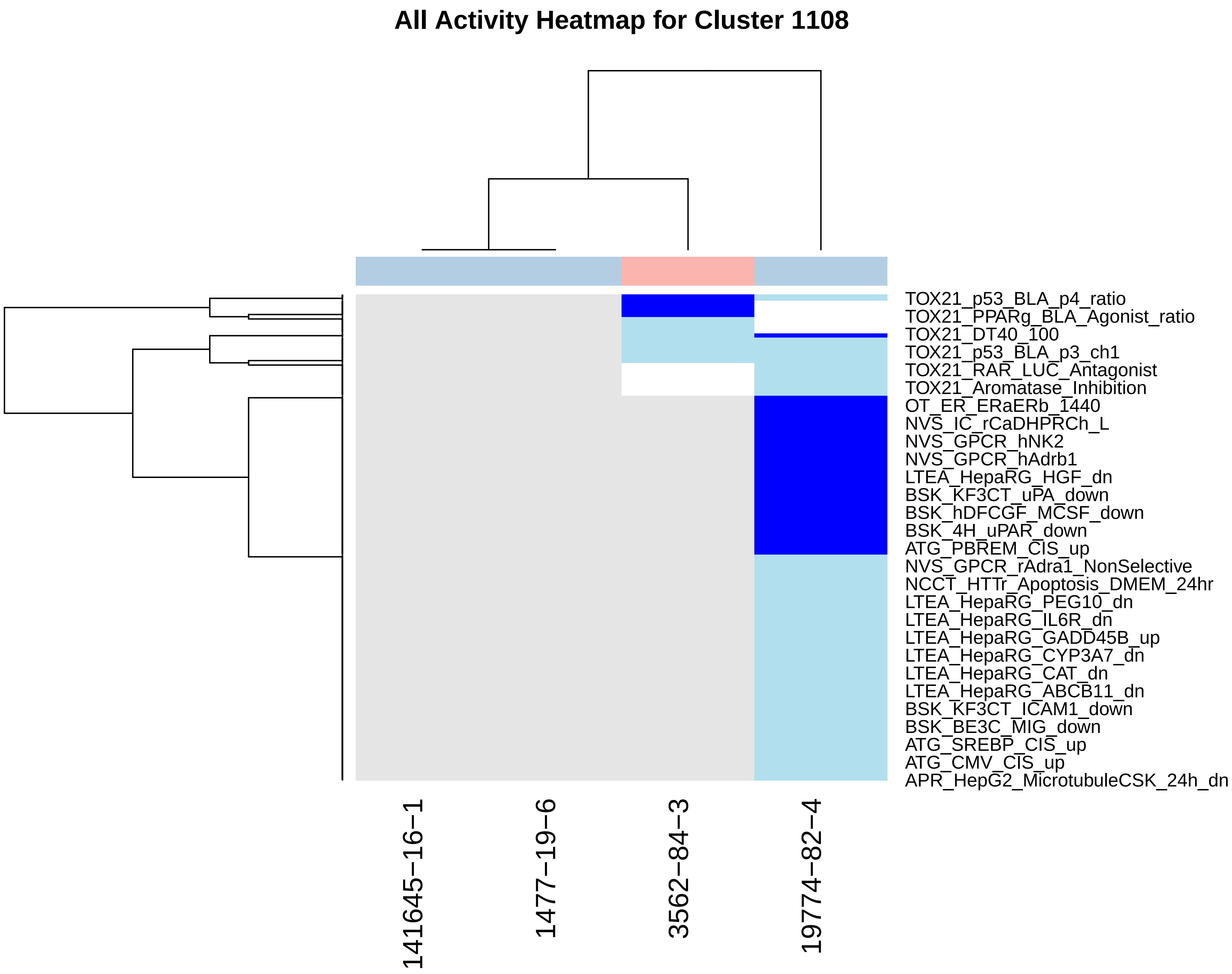
All Activity Heatmap for Cluster 1108
TOX21_p53_BLA_p4_ratio
TOX21_PPARg_BLA_Agonist_ratio
TOX21_DT40_100
TOX21_p53_BLA_p3_ch1
TOX21_RAR_LUC_Antagonist
TOX21_Aromatase_Inhibition
OT_ER_ERaERb_1440
NVS_IC_rCaDHPRCh_L
NVS_GPCR_hNK2
NVS_GPCR_hAdrb1
LTEA_HepaRG_HGF_dn
BSK_KF3CT_uPA_down
BSK_hDFCGF_MCSF_down
BSK_4H_uPAR_down
ATG_PBREM_CIS_up
NVS_GPCR_rAdra1_NonSelective
NCCT_HTTr_Apoptosis_DMEM_24hr
LTEA_HepaRG_PEG10_dn
LTEA_HepaRG_IL6R_dn
LTEA_HepaRG_GADD45B_up
LTEA_HepaRG_CYP3A7_dn
LTEA_HepaRG_CAT_dn
LTEA_HepaRG_ABCB11_dn
BSK_KF3CT_ICAM1_down
BSK_BE3C_MIG_down
ATG_SREBP_CIS_up
ATG_CMV_CIS_up
APR_HepG2_MicrotubuleCSK_24h_dn
141645−16−1
1477−19−6
3562−84−3
19774−82−4

All Activity Heatmap for Cluster 1109

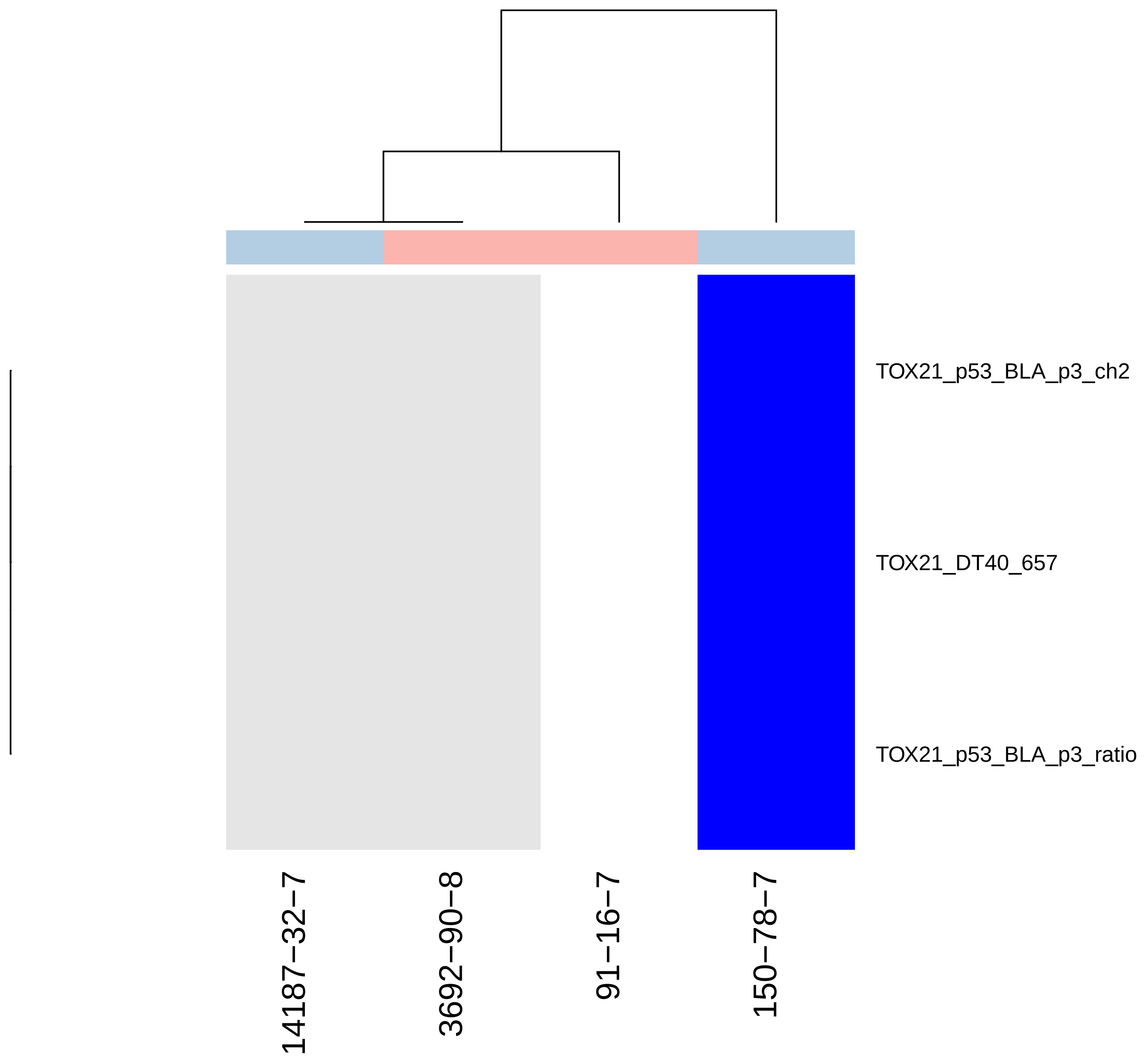

All Activity Heatmap for Cluster 1110

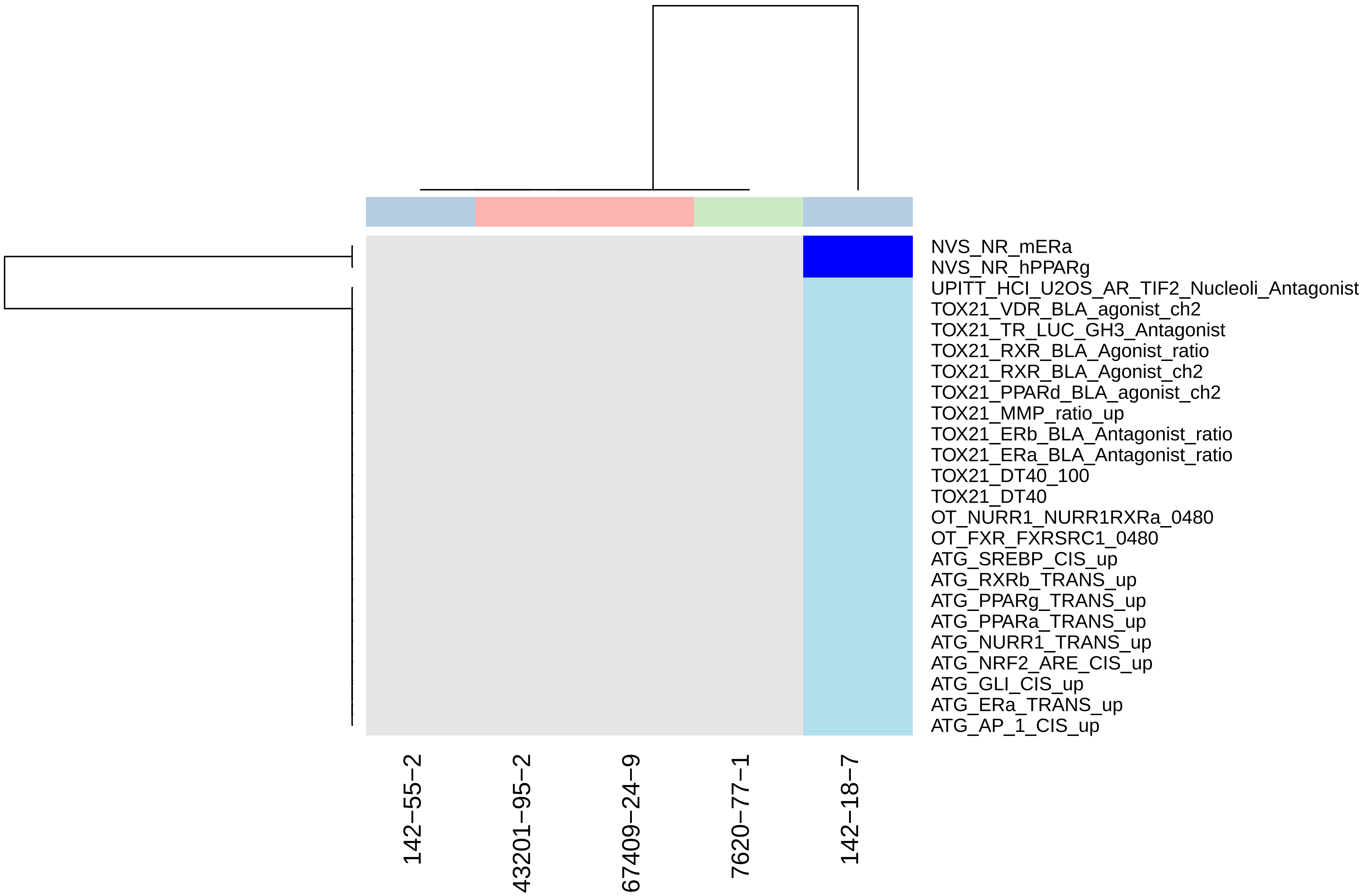

All Activity Heatmap for Cluster 1111

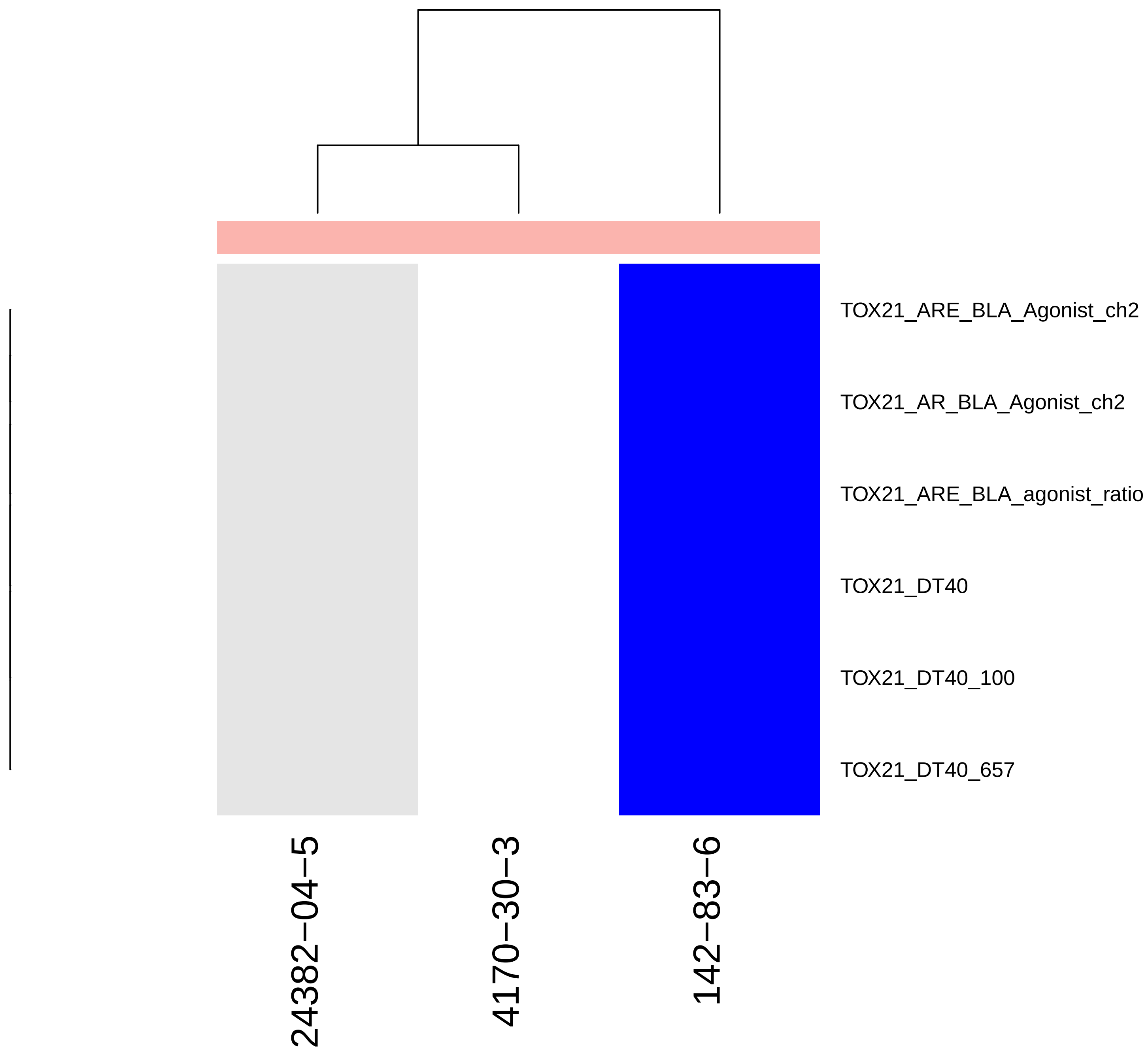

All Activity Heatmap for Cluster 1112

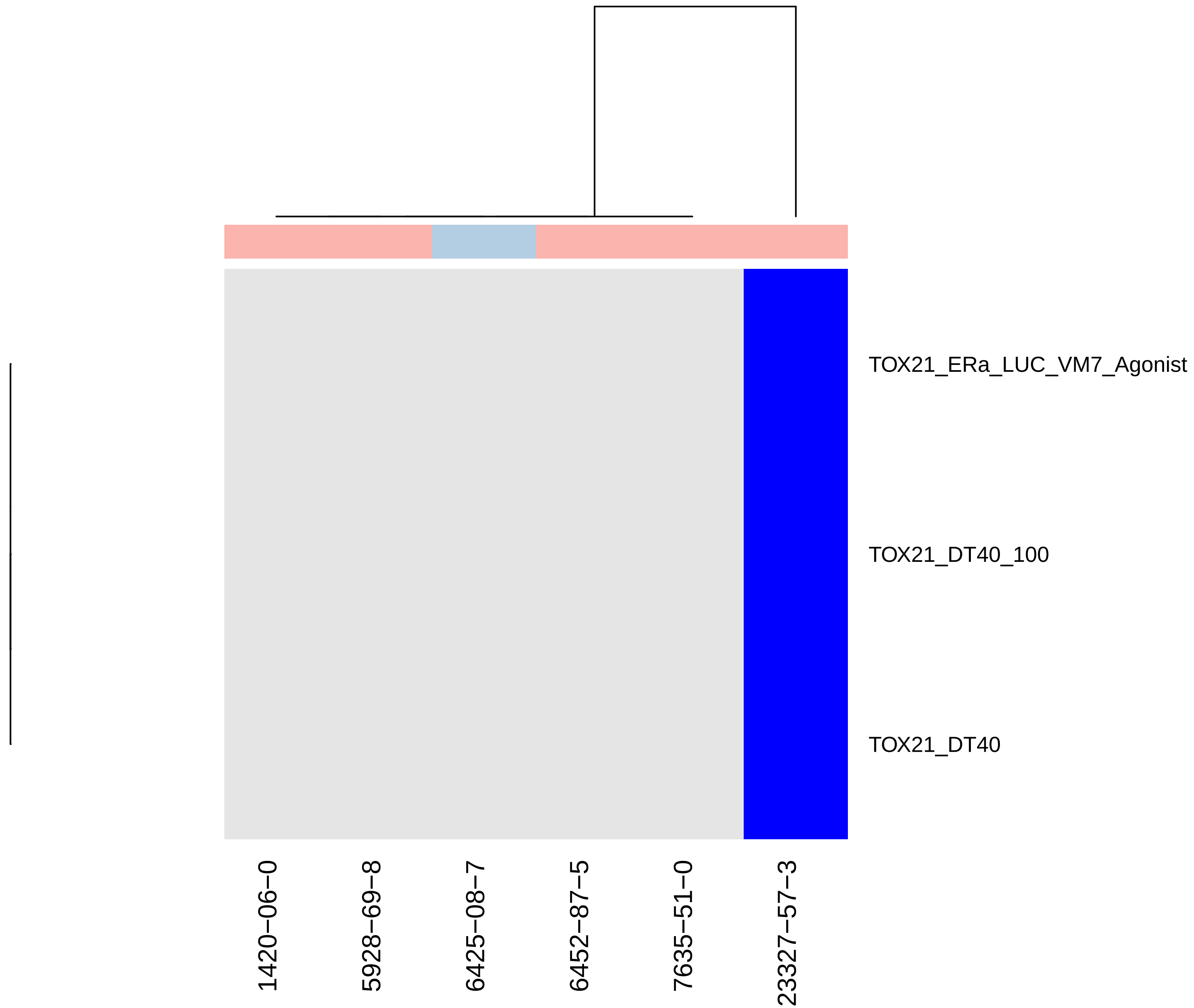

All Activity Heatmap for Cluster 1113

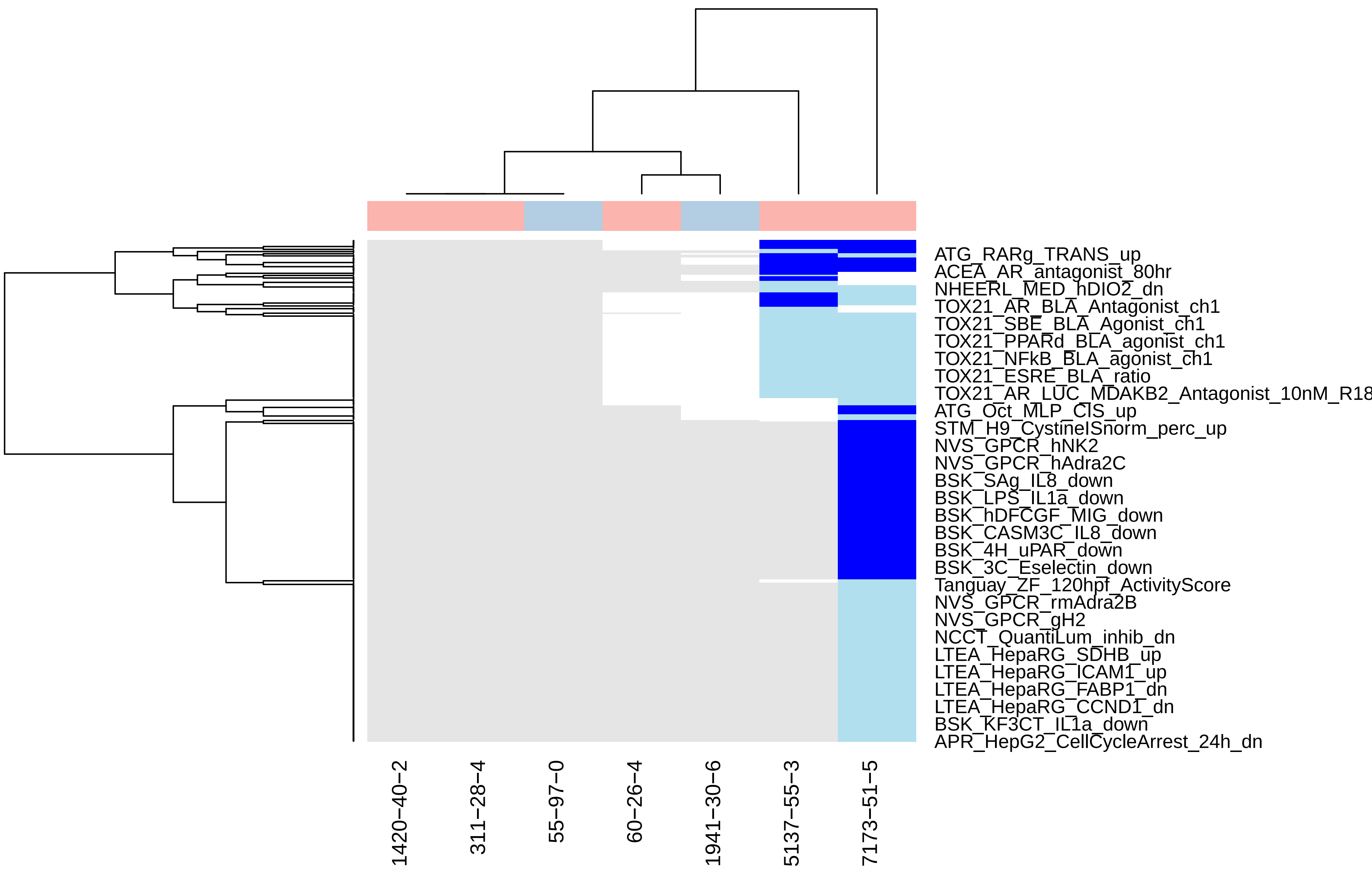

All Activity Heatmap for Cluster 1114

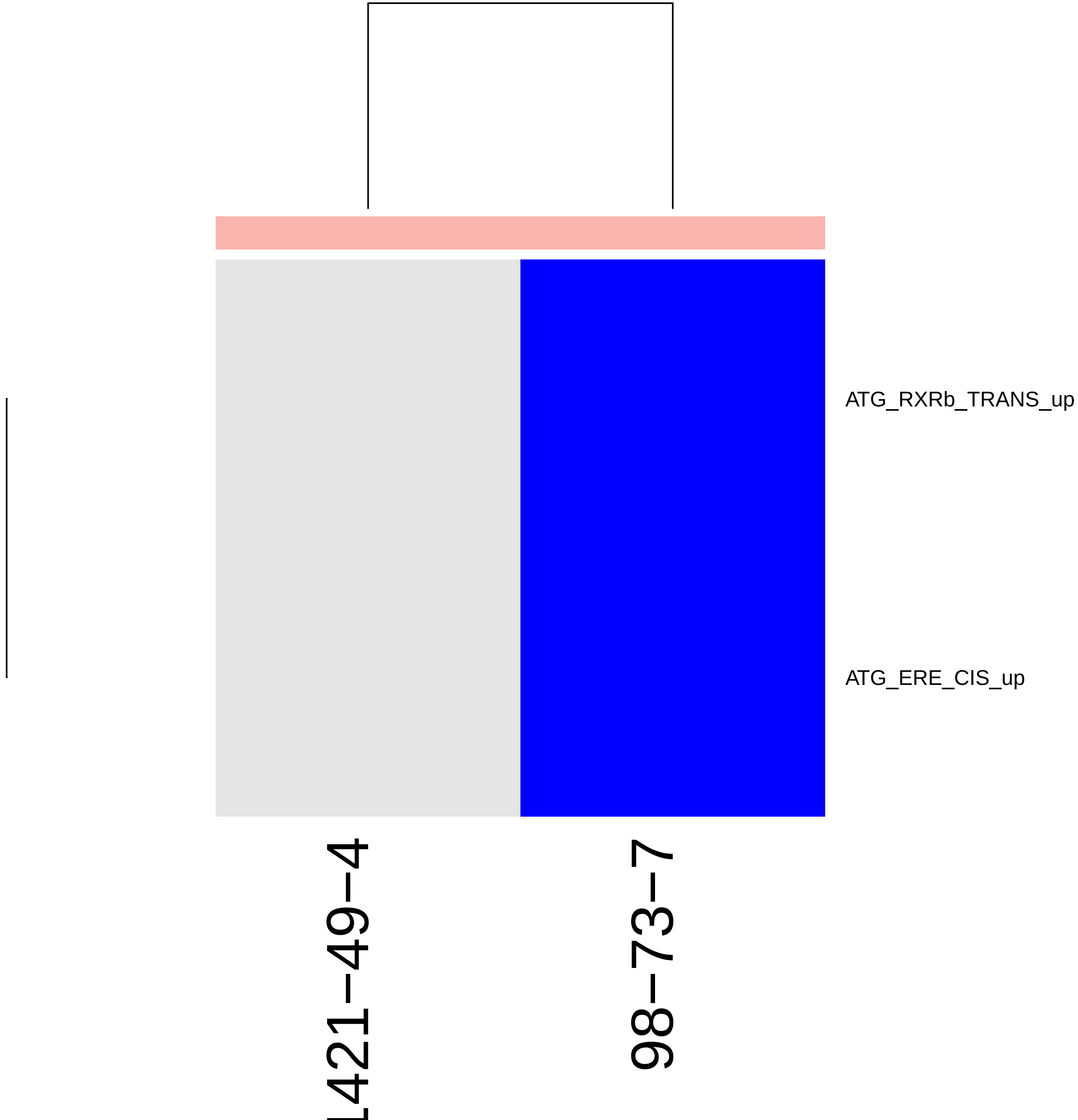

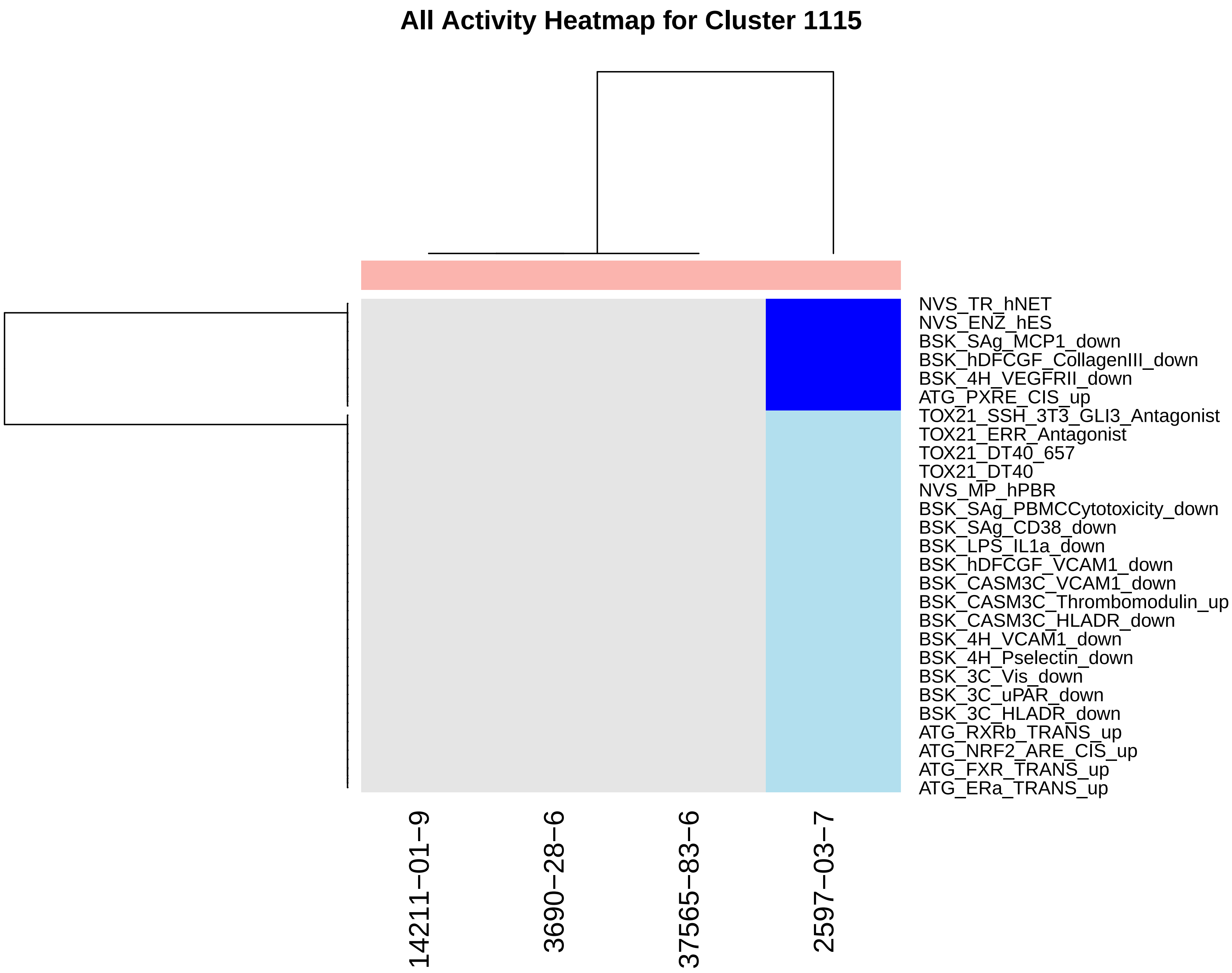
All Activity Heatmap for Cluster 1115
NVS_TR_hNET
NVS_ENZ_hES
BSK_SAg_MCP1_down
BSK_hDFCGF_CollagenIII_down
BSK_4H_VEGFRII_down
ATG_PXRE_CIS_up
TOX21_SSH_3T3_GLI3_Antagonist
TOX21_ERR_Antagonist
TOX21_DT40_657
TOX21_DT40
NVS_MP_hPBR
BSK_SAg_PBMCCytotoxicity_down
BSK_SAg_CD38_down
BSK_LPS_IL1a_down
BSK_hDFCGF_VCAM1_down
BSK_CASM3C_VCAM1_down
BSK_CASM3C_Thrombomodulin_up
BSK_CASM3C_HLADR_down
BSK_4H_VCAM1_down
BSK_4H_Pselectin_down
BSK_3C_Vis_down
BSK_3C_uPAR_down
BSK_3C_HLADR_down
ATG_RXRb_TRANS_up
ATG_NRF2_ARE_CIS_up
ATG_FXR_TRANS_up
ATG_ERa_TRANS_up
14211−01−9
3690−28−6
37565−83−6
2597−03−7

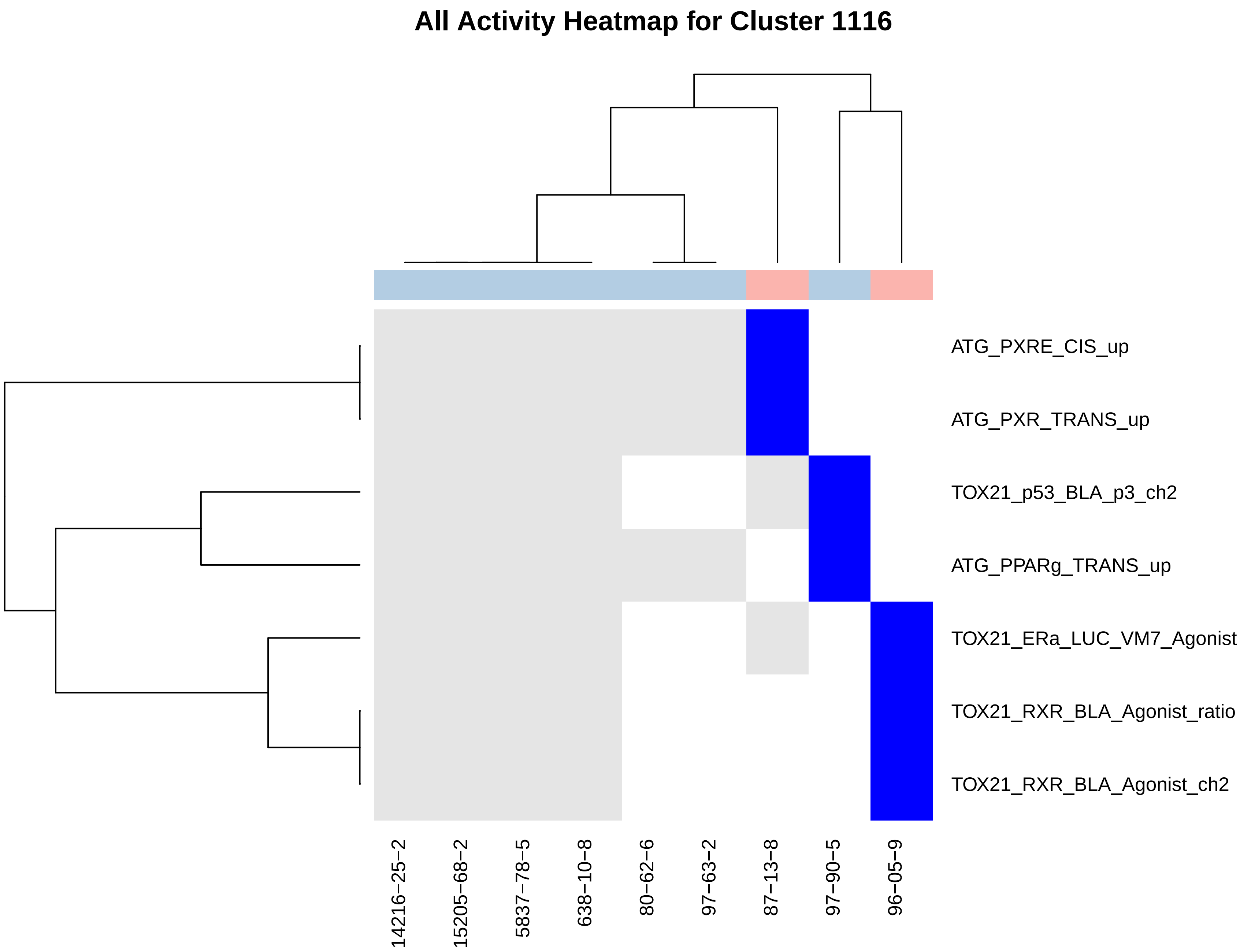

All Activity Heatmap for Cluster 1116
ATG_PXRE_CIS_up
ATG_PXR_TRANS_up
TOX21_p53_BLA_p3_ch2
ATG_PPARg_TRANS_up
TOX21_ERa_LUC_VM7_Agonist
TOX21_RXR_BLA_Agonist_ratio
TOX21_RXR_BLA_Agonist_ch2
14216−25−2
15205−68−2
5837−78−5
638−10−8
80−62−6
97−63−2
87−13−8
97−90−5
96−05−9

All Activity Heatmap for Cluster 1118

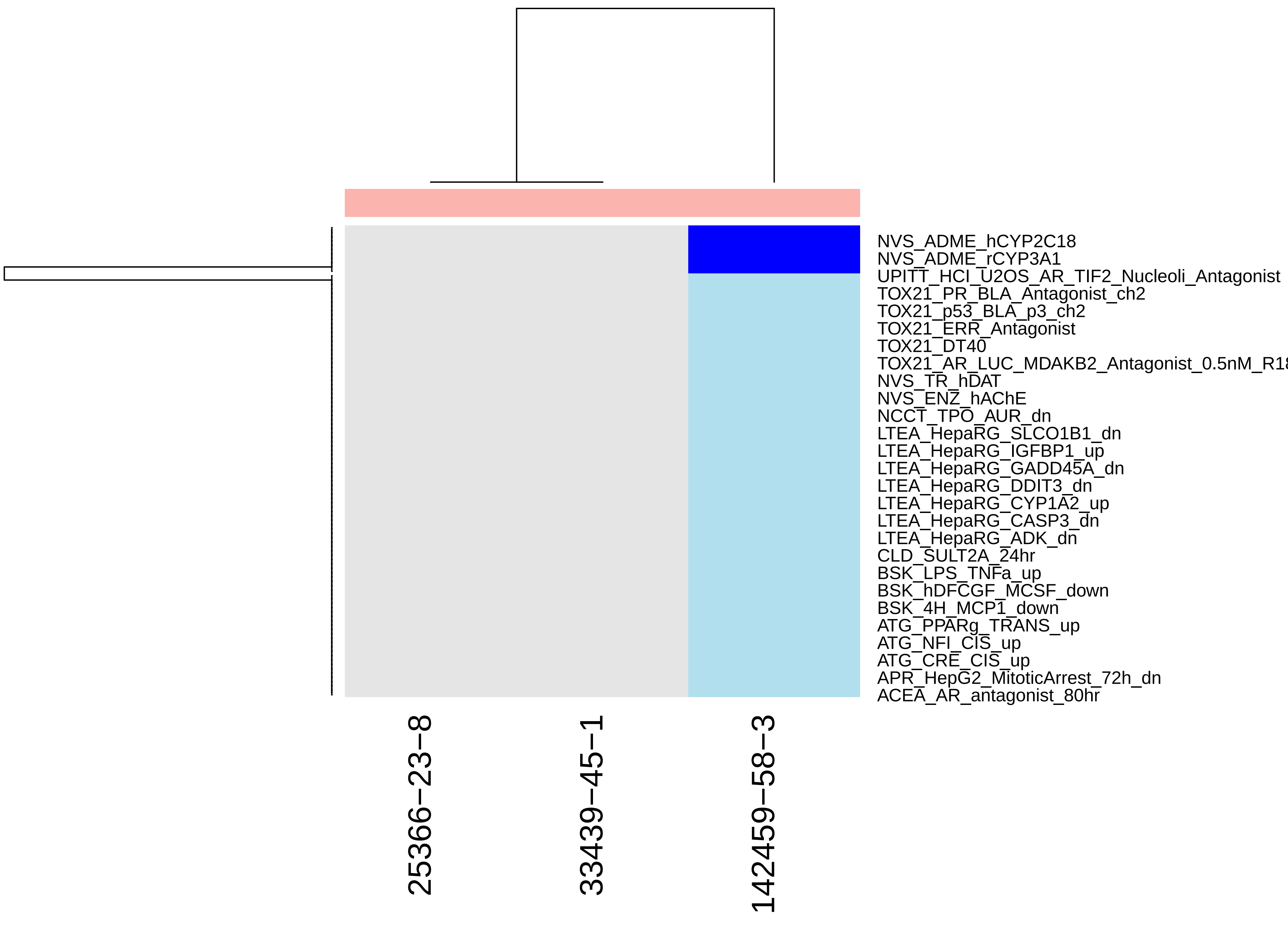

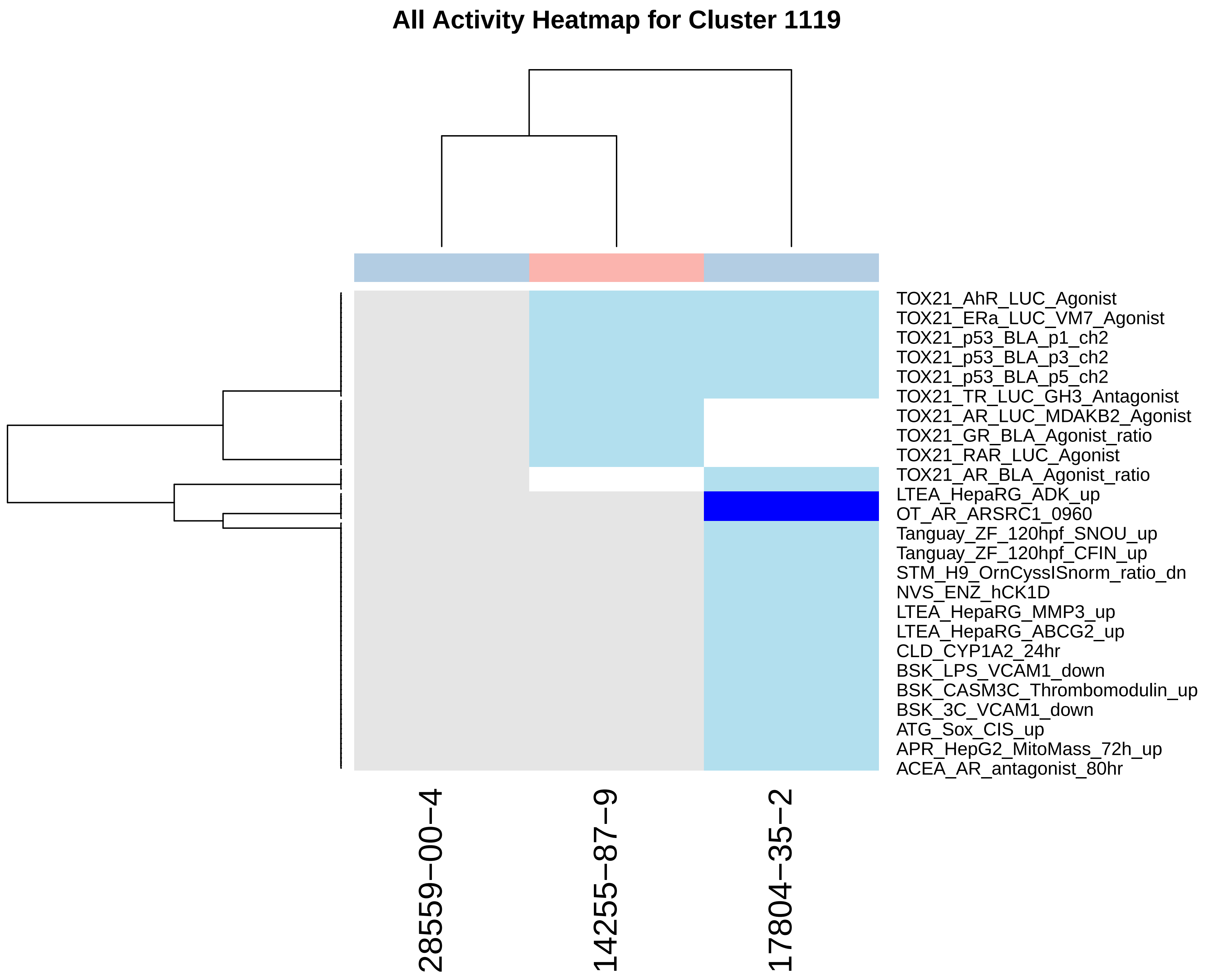
All Activity Heatmap for Cluster 1119
TOX21_AhR_LUC_Agonist
TOX21_ERa_LUC_VM7_Agonist
TOX21_p53_BLA_p1_ch2
TOX21_p53_BLA_p3_ch2
TOX21_p53_BLA_p5_ch2
TOX21_TR_LUC_GH3_Antagonist
TOX21_AR_LUC_MDAKB2_Agonist
TOX21_GR_BLA_Agonist_ratio
TOX21_RAR_LUC_Agonist
TOX21_AR_BLA_Agonist_ratio
LTEA_HepaRG_ADK_up
OT_AR_ARSRC1_0960
Tanguay_ZF_120hpf_SNOU_up
Tanguay_ZF_120hpf_CFIN_up
STM_H9_OrnCyssISnorm_ratio_dn
NVS_ENZ_hCK1D
LTEA_HepaRG_MMP3_up
LTEA_HepaRG_ABCG2_up
CLD_CYP1A2_24hr
BSK_LPS_VCAM1_down
BSK_CASM3C_Thrombomodulin_up
BSK_3C_VCAM1_down
ATG_Sox_CIS_up
APR_HepG2_MitoMass_72h_up
ACEA_AR_antagonist_80hr
28559−00−4
14255−87−9
17804−35−2

All Activity Heatmap for Cluster 1123

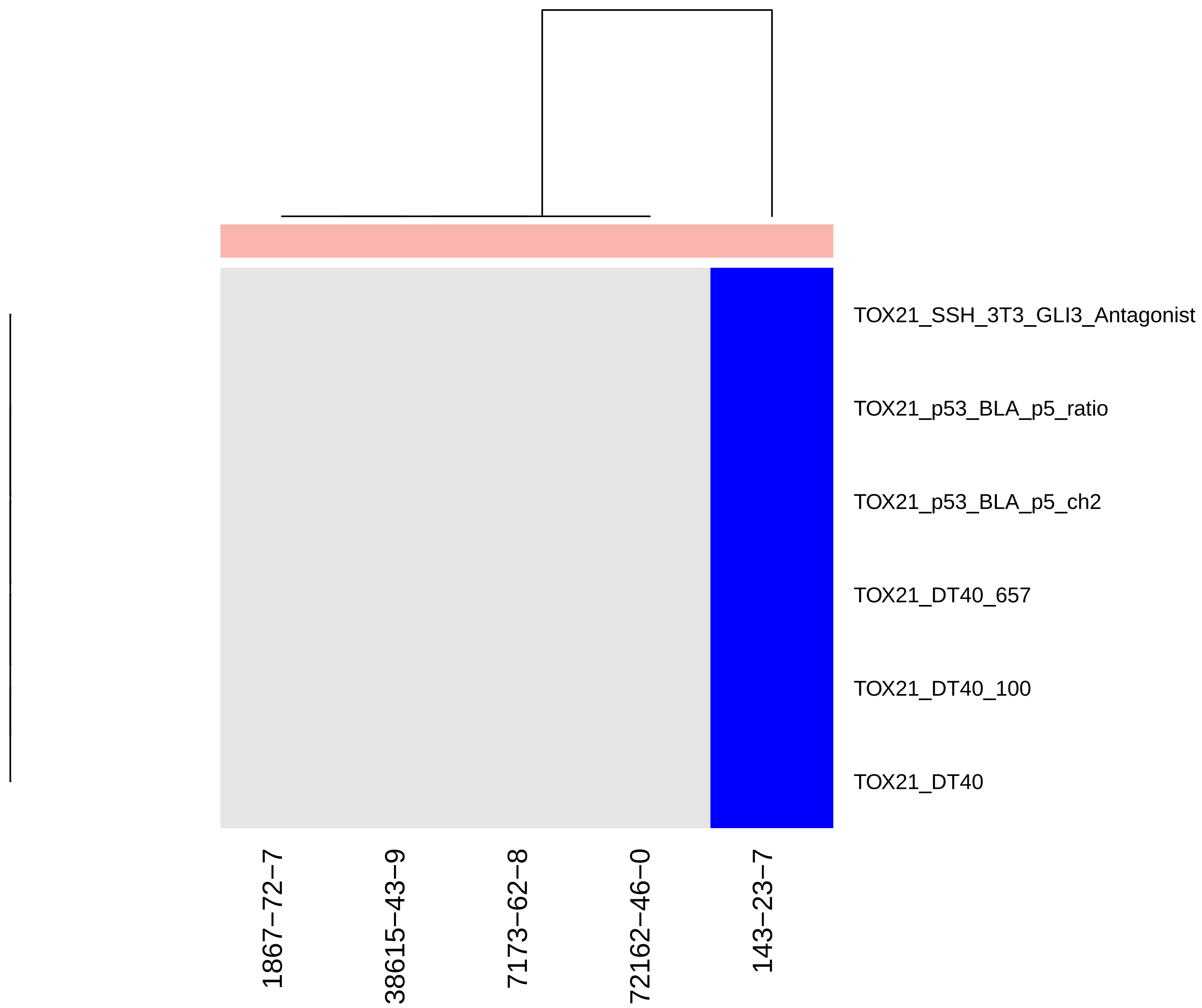

All Activity Heatmap for Cluster 1124

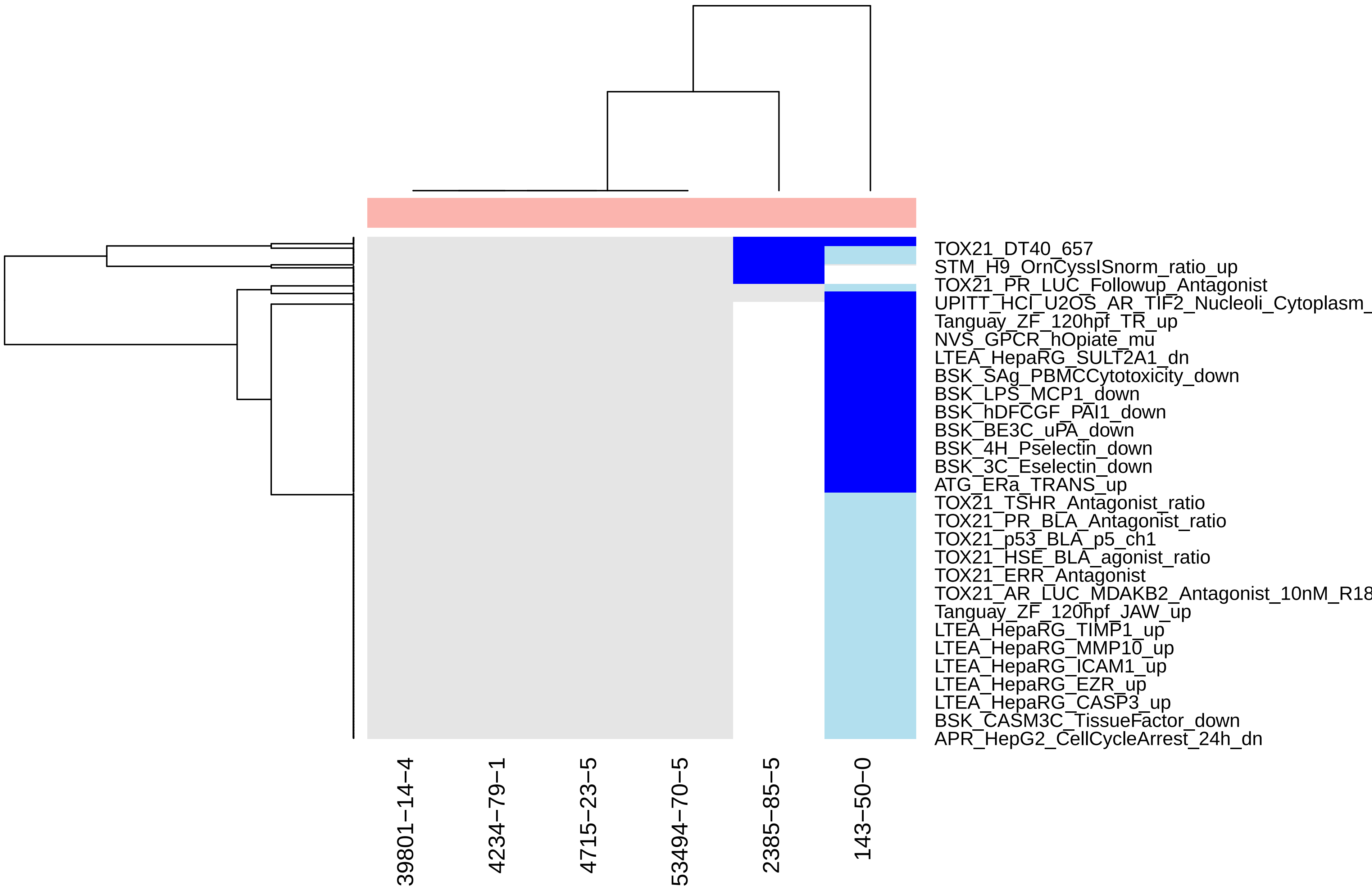

All Activity Heatmap for Cluster 1125

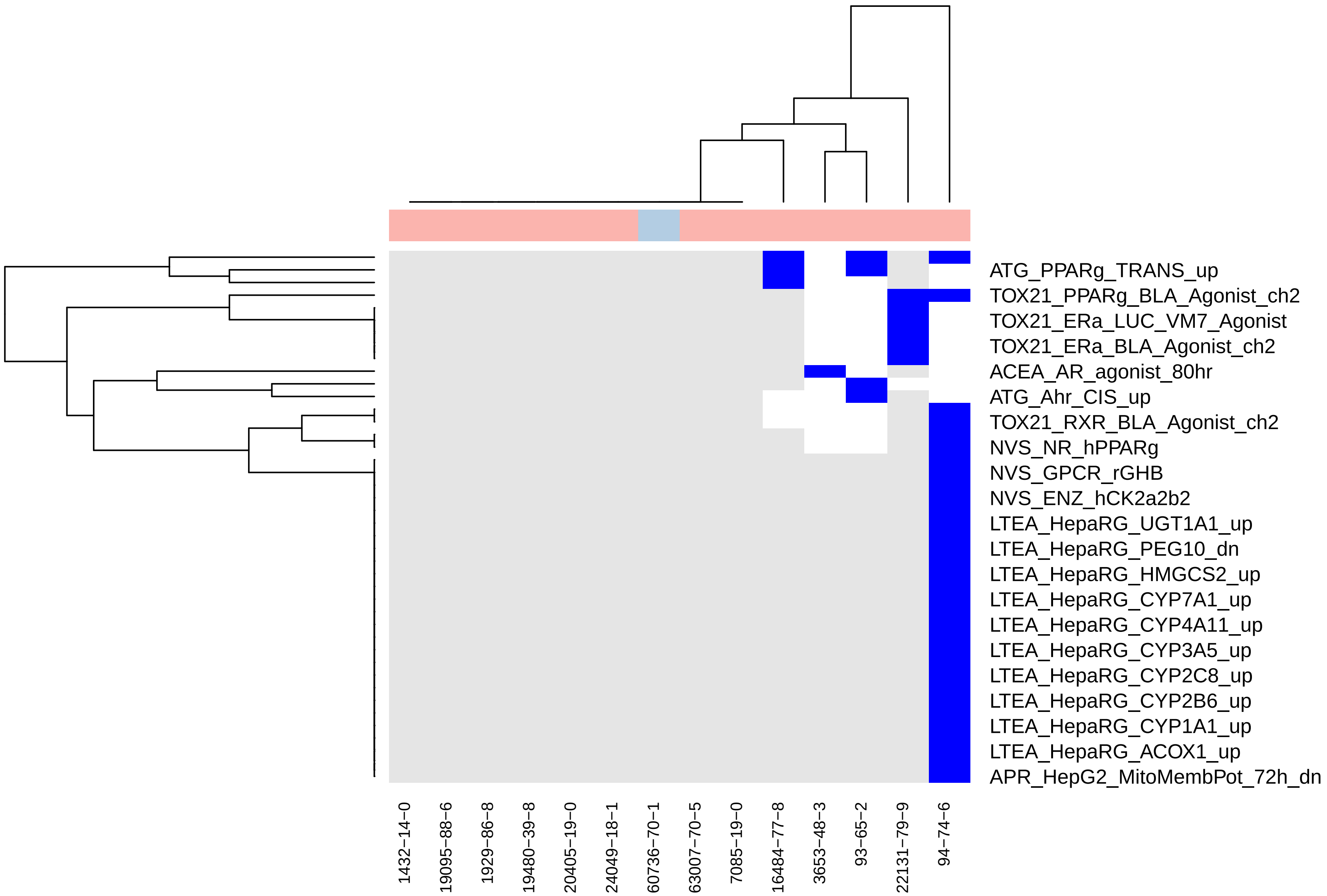

All Activity Heatmap for Cluster 1126

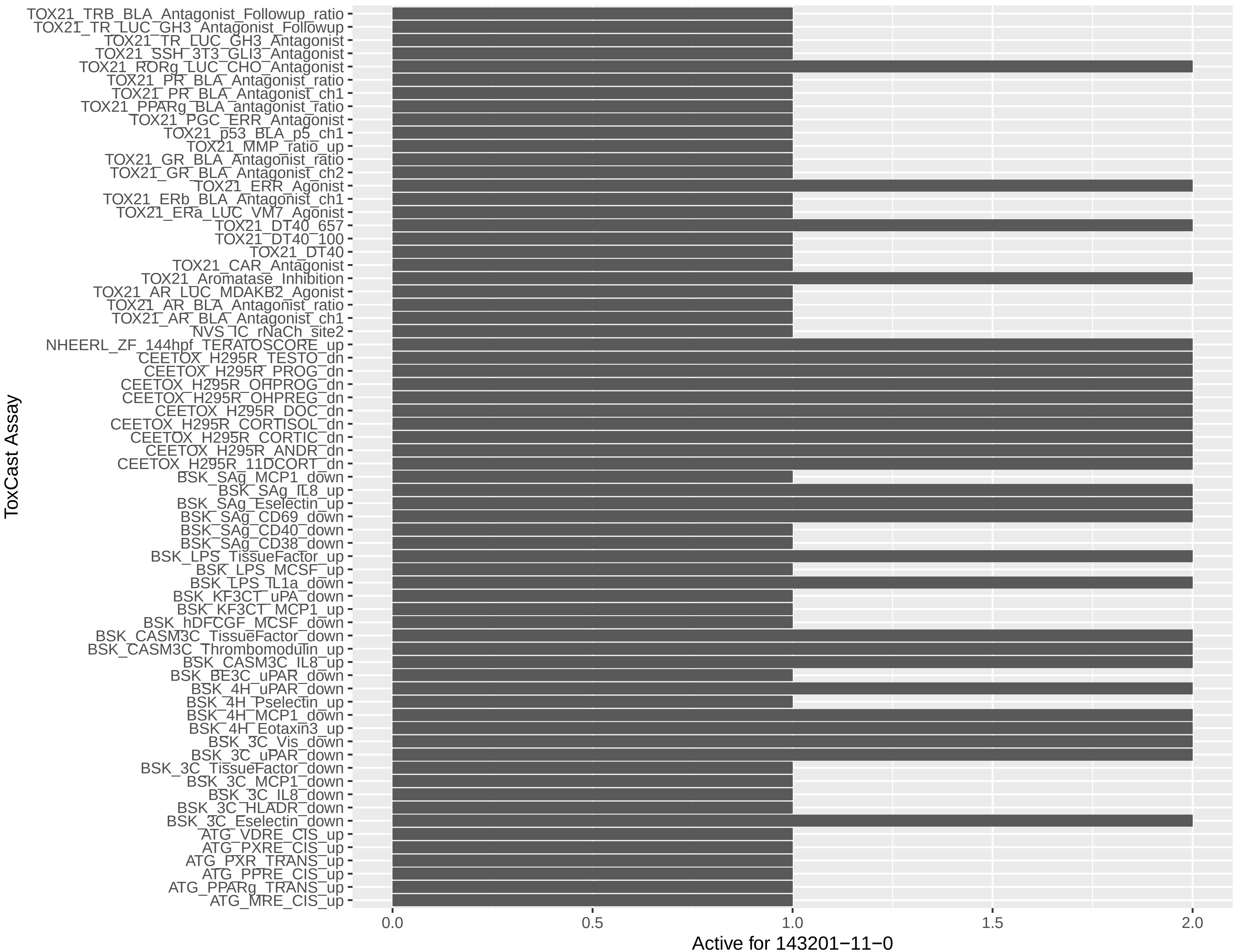

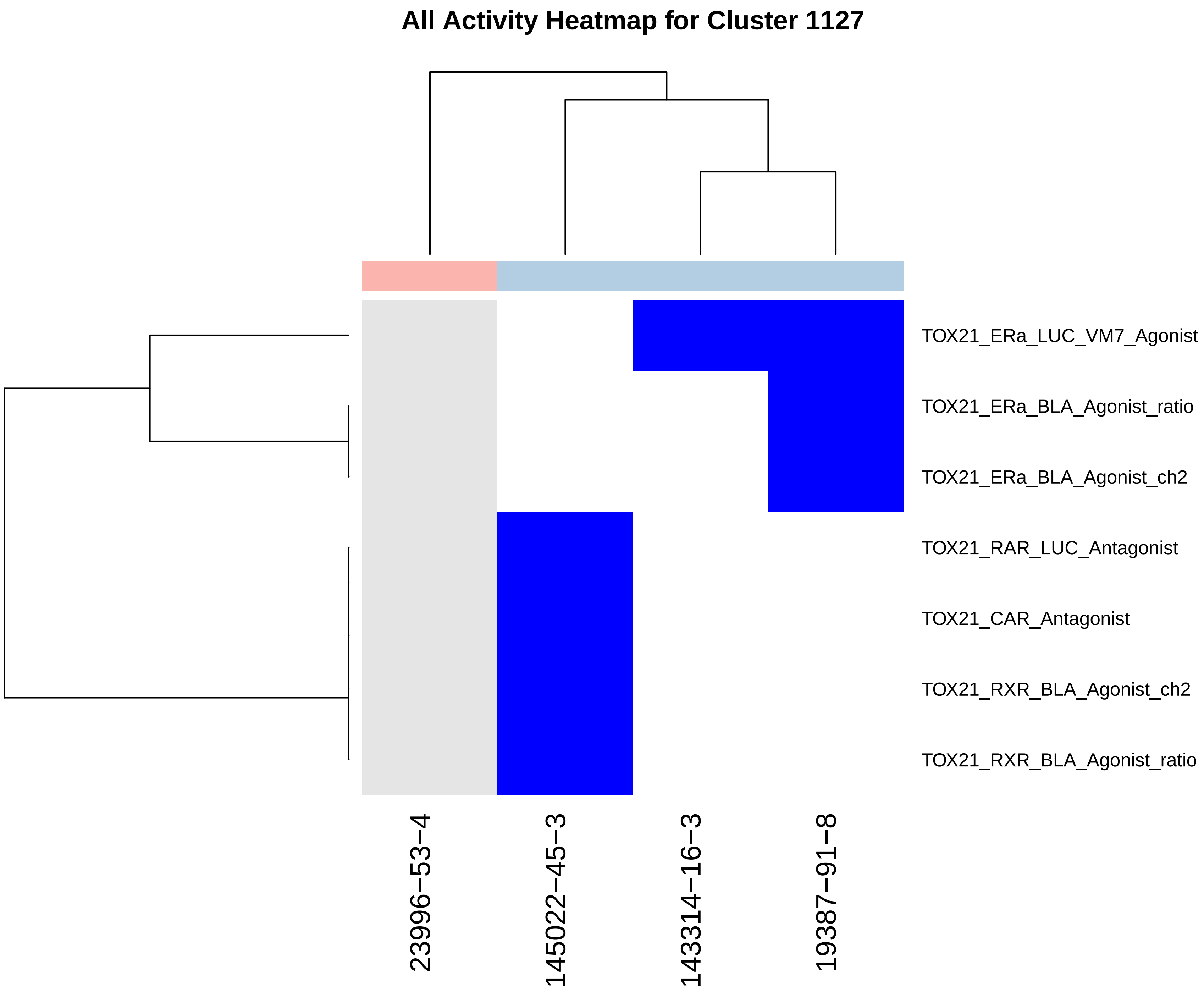

All Activity Heatmap for Cluster 1127
TOX21_ERa_LUC_VM7_Agonist
TOX21_ERa_BLA_Agonist_ratio
TOX21_ERa_BLA_Agonist_ch2
TOX21_RAR_LUC_Antagonist
TOX21_CAR_Antagonist
TOX21_RXR_BLA_Agonist_ch2
TOX21_RXR_BLA_Agonist_ratio
23996−53−4
145022−45−3
143314−16−3
19387−91−8

All Activity Heatmap for Cluster 1128

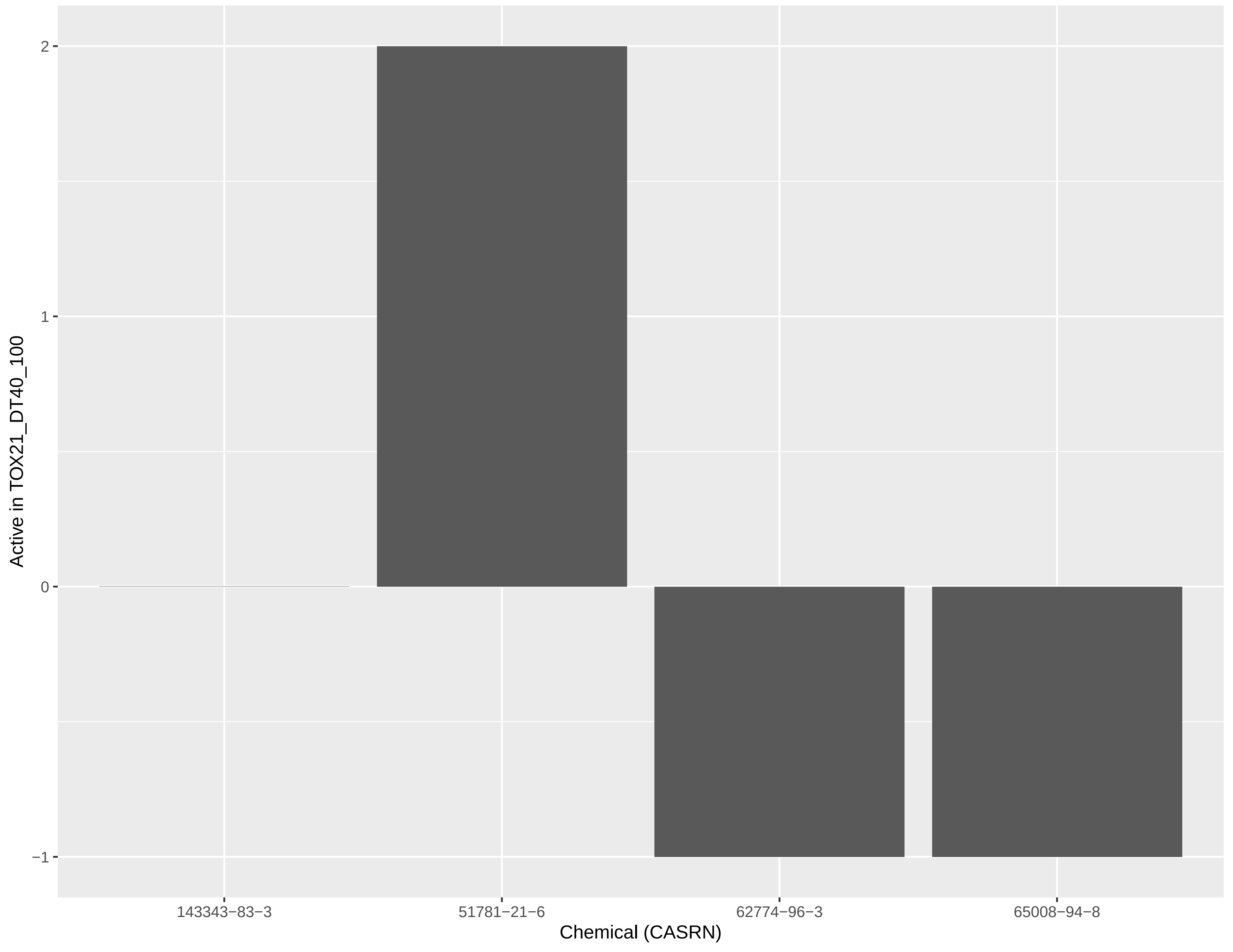

All Activity Heatmap for Cluster 1130

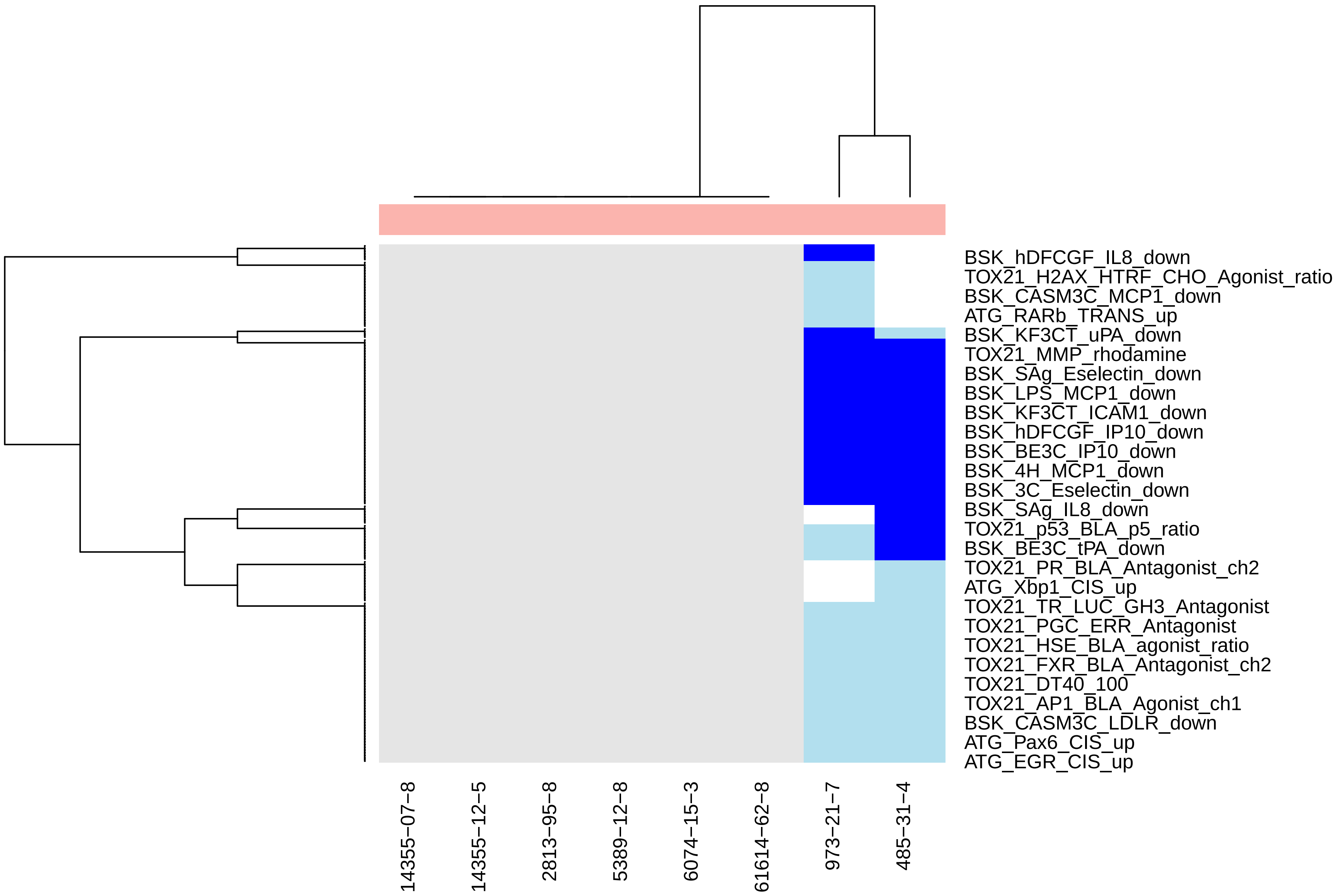

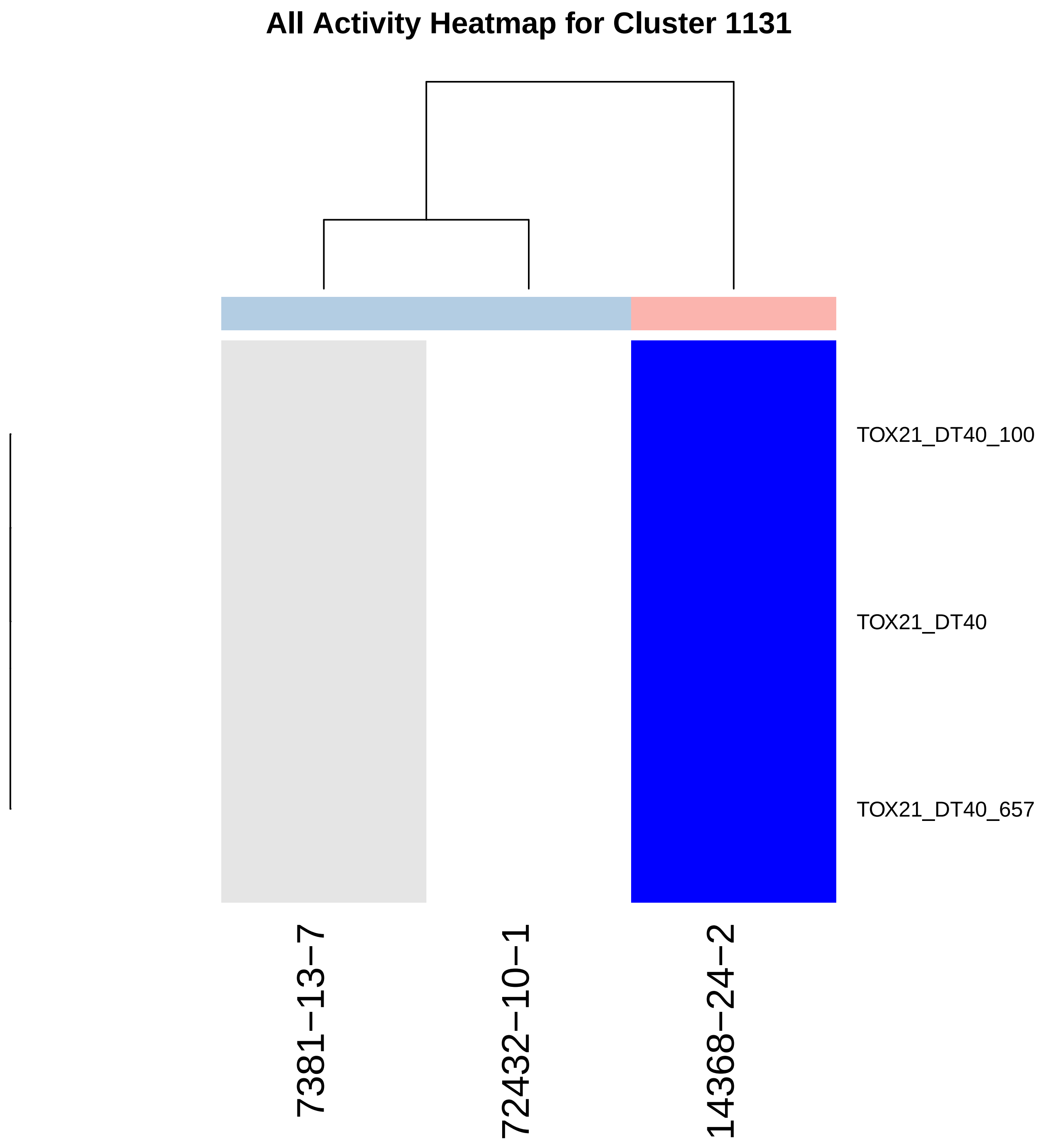
All Activity Heatmap for Cluster 1131
TOX21_DT40_100
TOX21_DT40
TOX21_DT40_657
7381−13−7
72432−10−1
14368−24−2

All Activity Heatmap for Cluster 1133

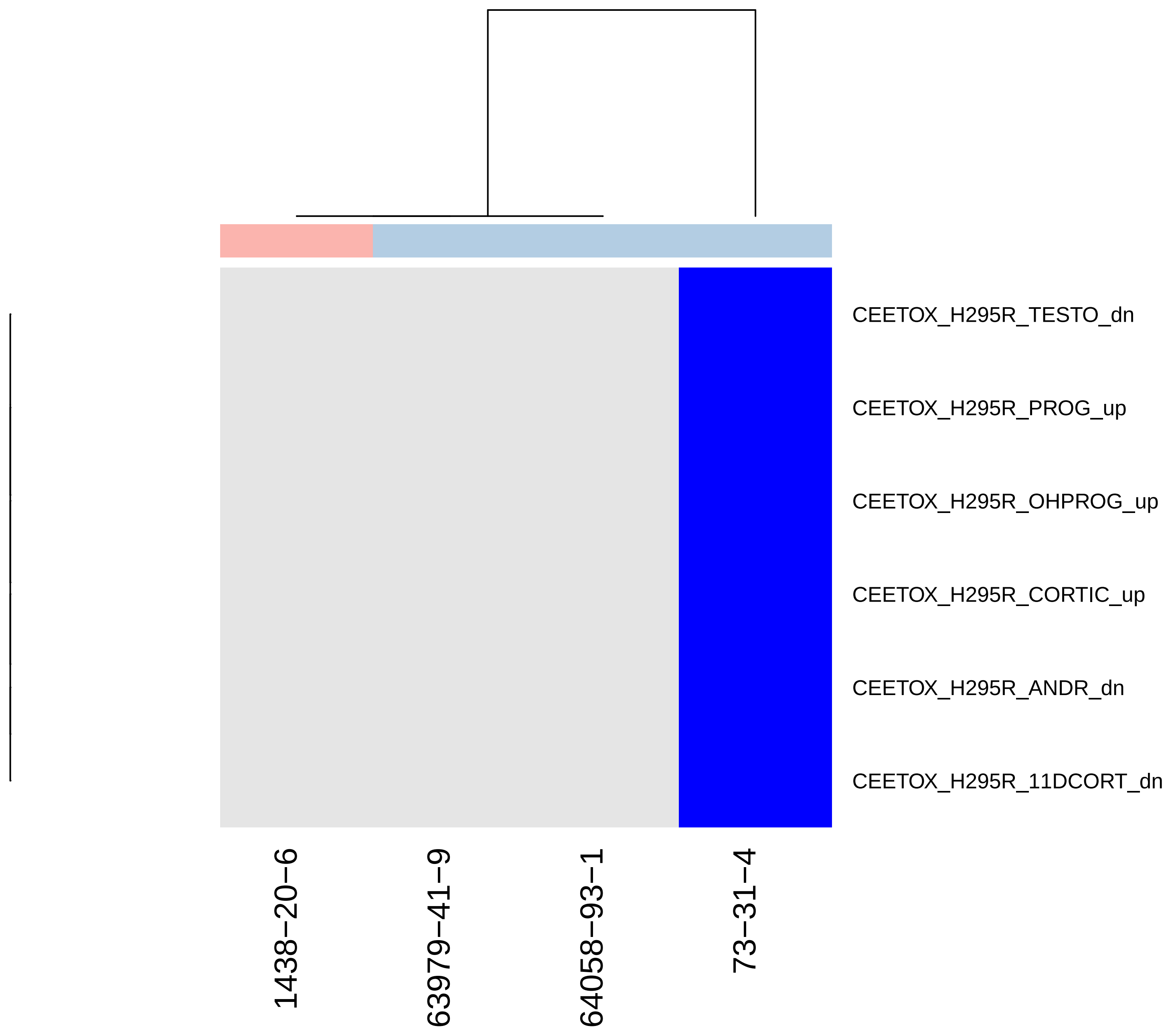

All Activity Heatmap for Cluster 1134

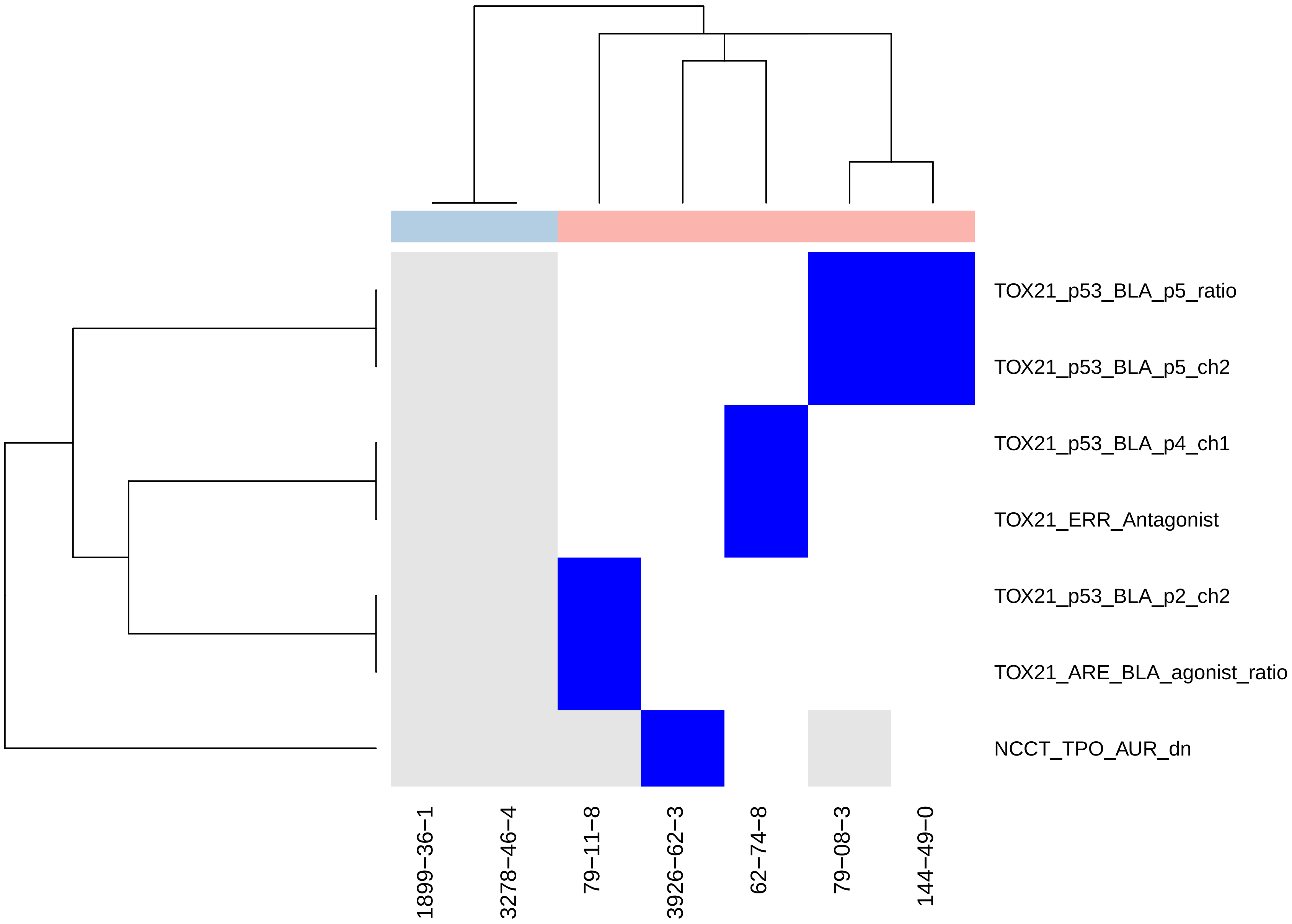

All Activity Heatmap for Cluster 1135

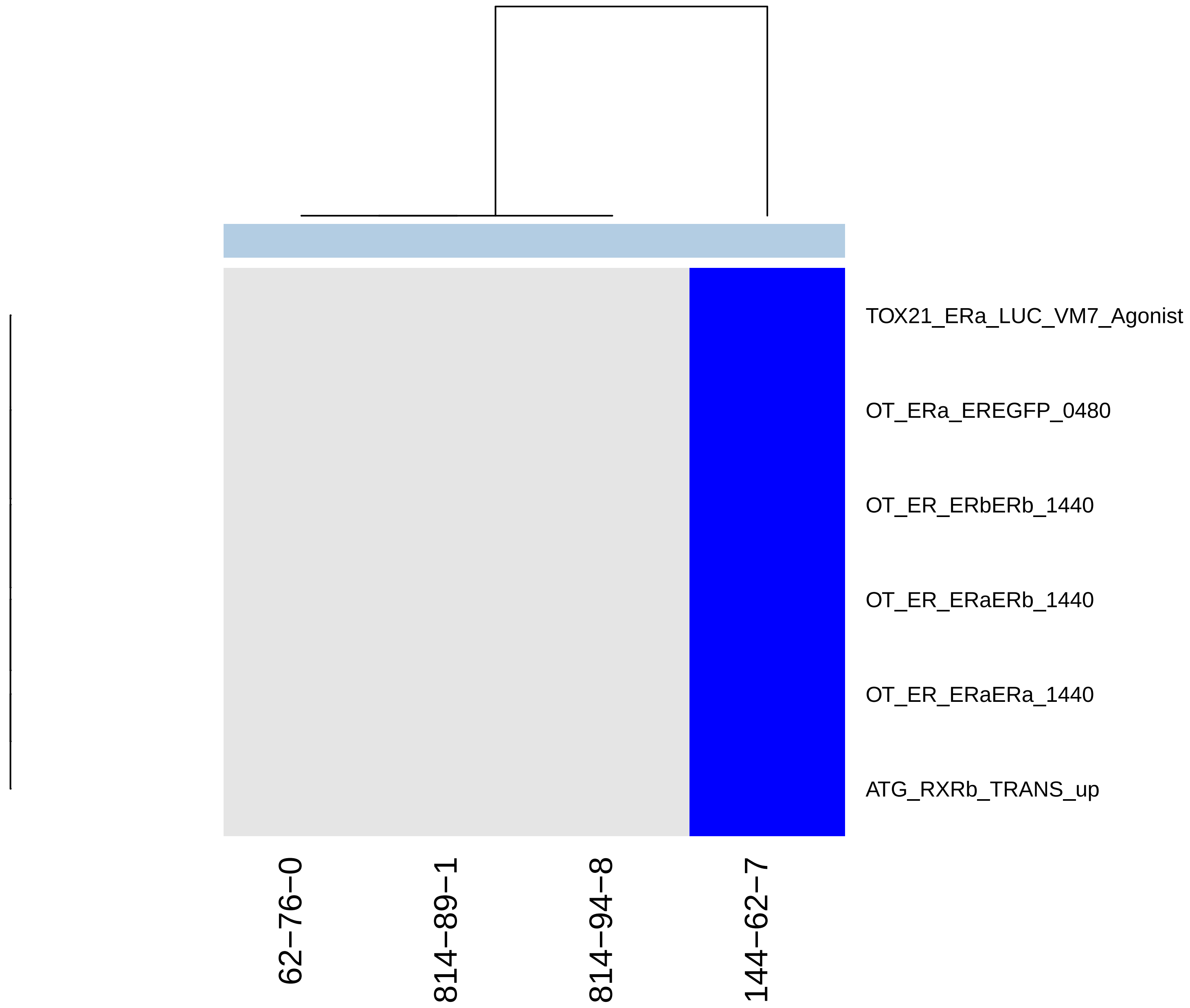

All Activity Heatmap for Cluster 1136

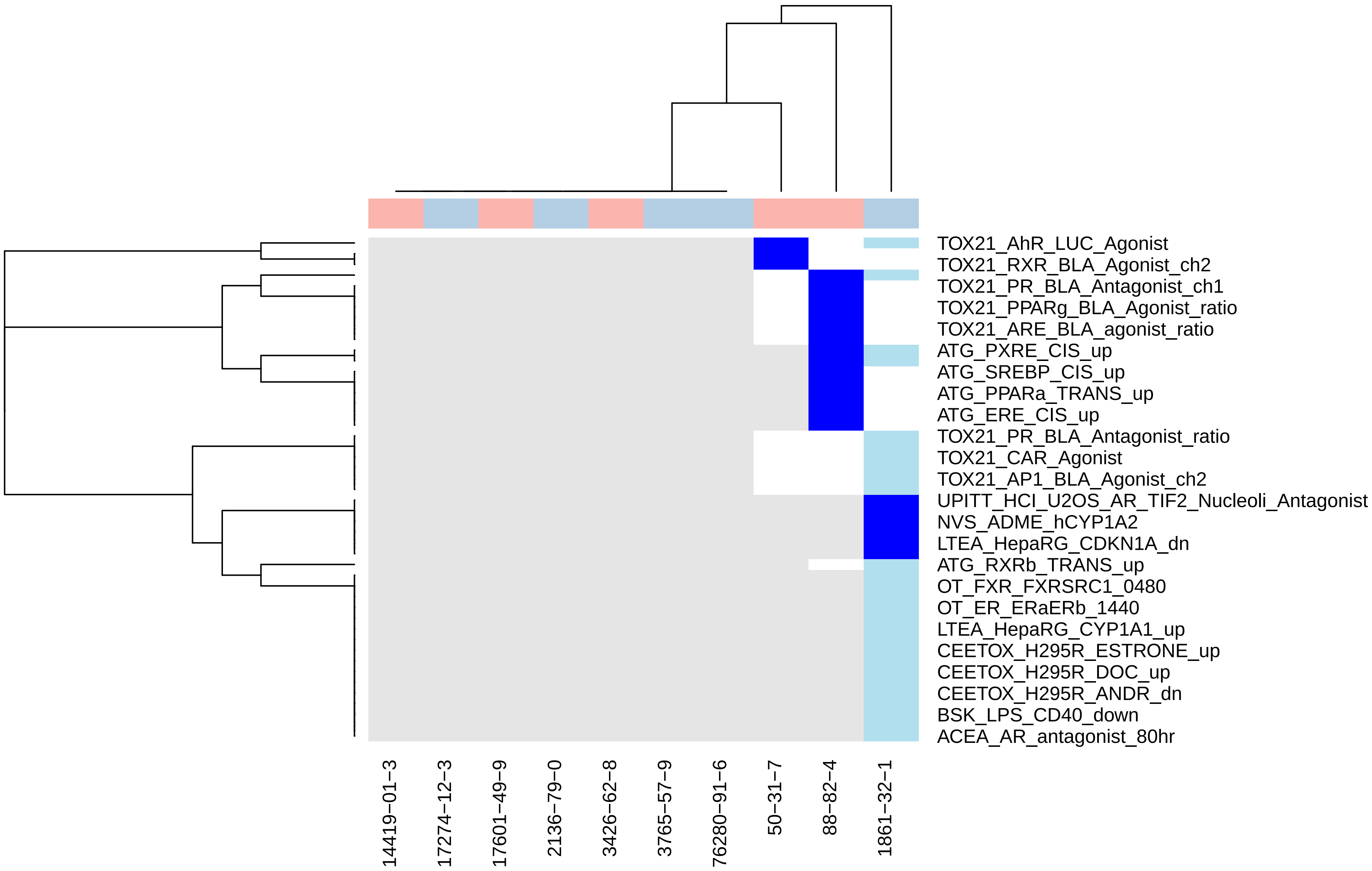

All Activity Heatmap for Cluster 1138

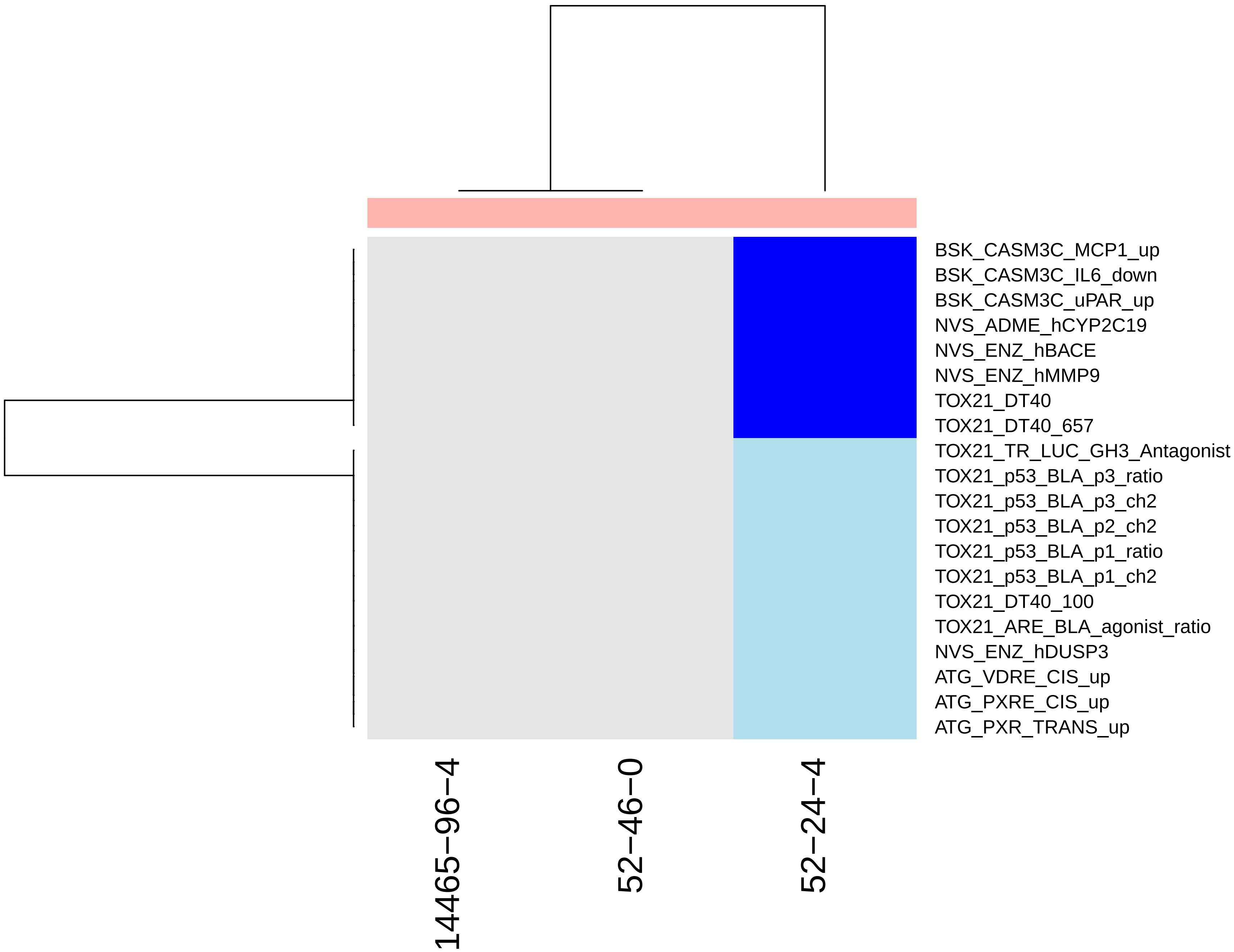

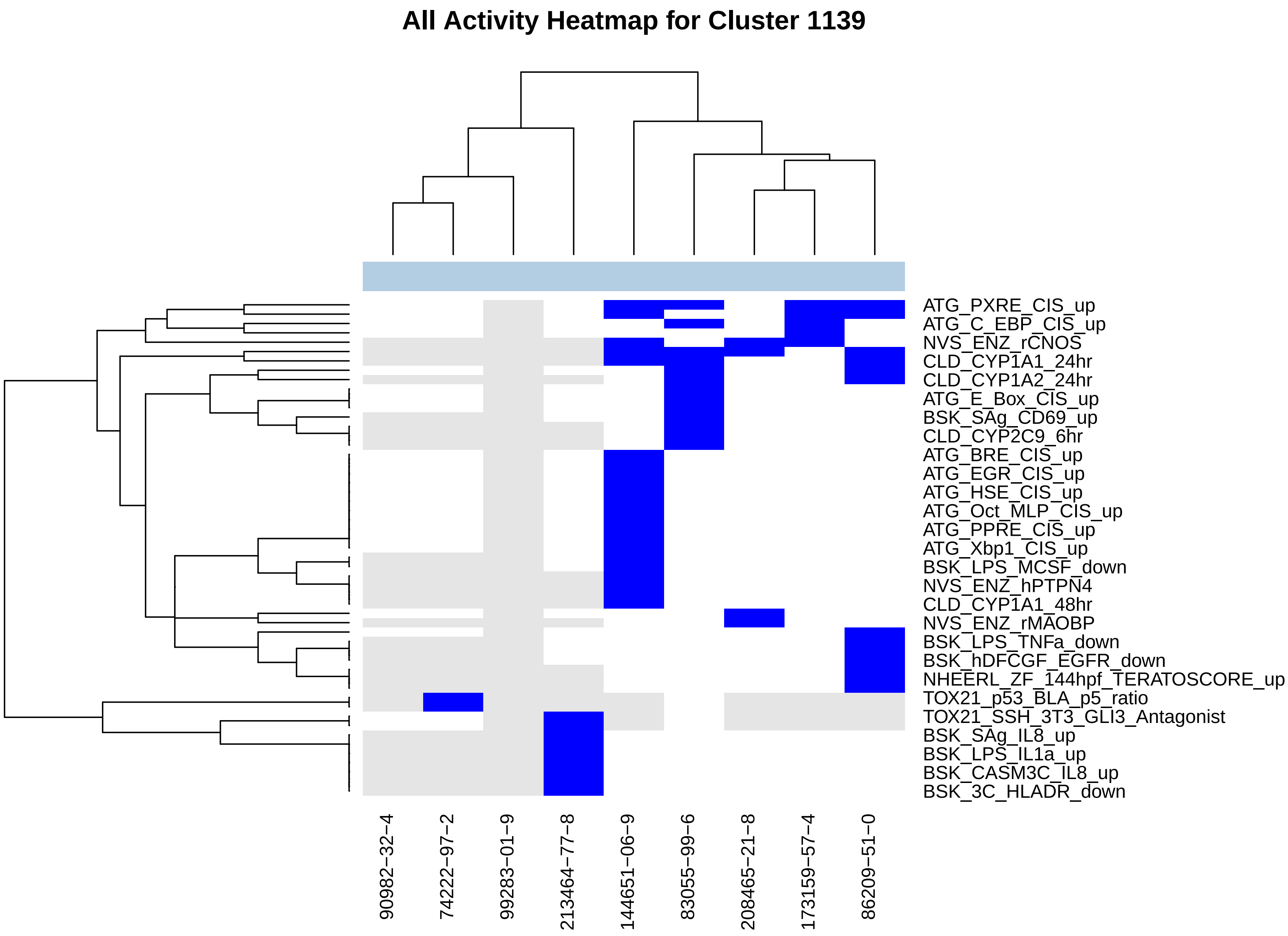
All Activity Heatmap for Cluster 1139
ATG_PXRE_CIS_up
ATG_C_EBP_CIS_up
NVS_ENZ_rCNOS
CLD_CYP1A1_24hr
CLD_CYP1A2_24hr
ATG_E_Box_CIS_up
BSK_SAg_CD69_up
CLD_CYP2C9_6hr
ATG_BRE_CIS_up
ATG_EGR_CIS_up
ATG_HSE_CIS_up
ATG_Oct_MLP_CIS_up
ATG_PPRE_CIS_up
ATG_Xbp1_CIS_up
BSK_LPS_MCSF_down
NVS_ENZ_hPTPN4
CLD_CYP1A1_48hr
NVS_ENZ_rMAOBP
BSK_LPS_TNFa_down
BSK_hDFCGF_EGFR_down
NHEERL_ZF_144hpf_TERATOSCORE_up
TOX21_p53_BLA_p5_ratio
TOX21_SSH_3T3_GLI3_Antagonist
BSK_SAg_IL8_up
BSK_LPS_IL1a_up
BSK_CASM3C_IL8_up
BSK_3C_HLADR_down
90982−32−4
74222−97−2
99283−01−9
213464−77−8
144651−06−9
83055−99−6
208465−21−8
173159−57−4
86209−51−0

All Activity Heatmap for Cluster 1142

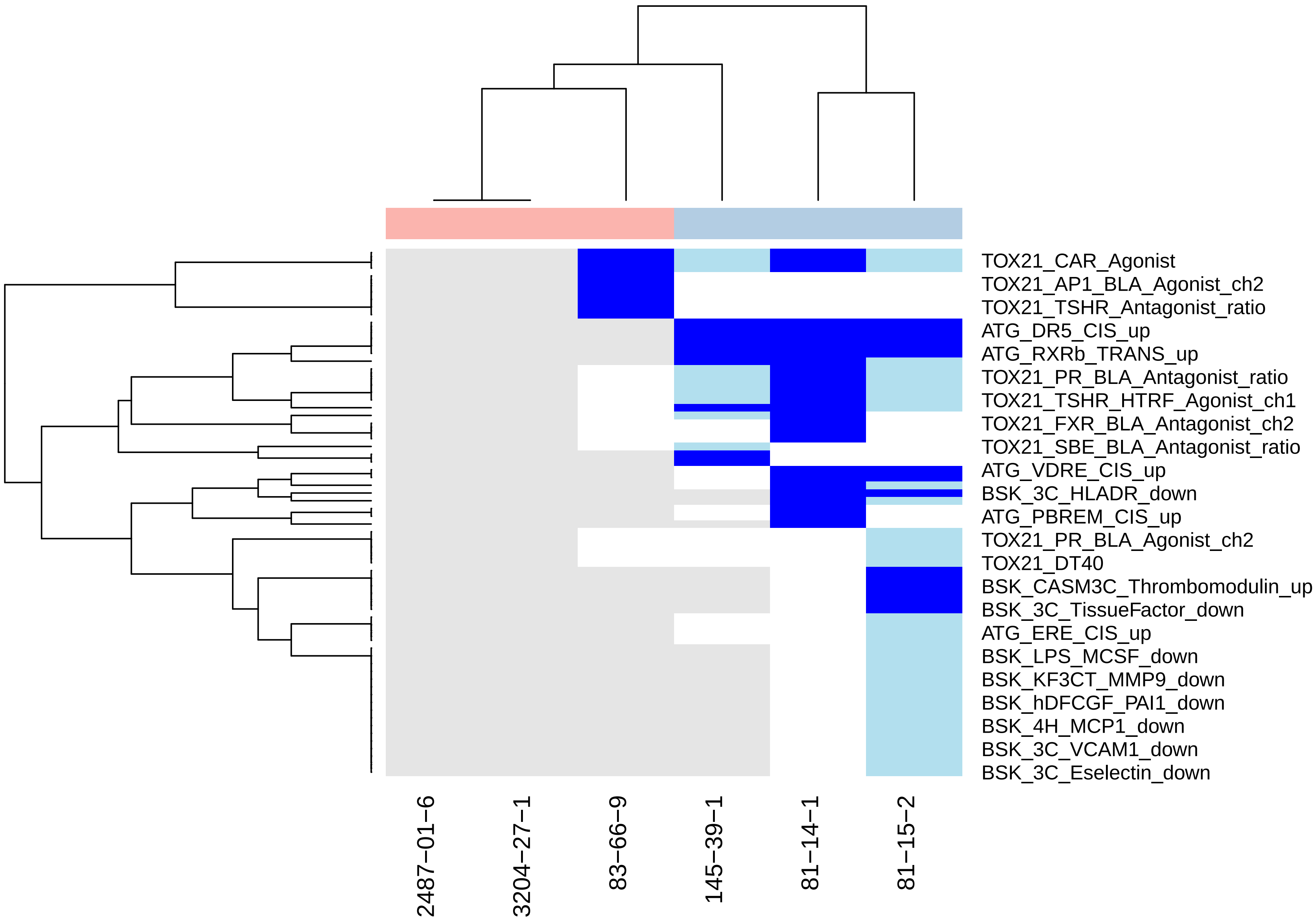

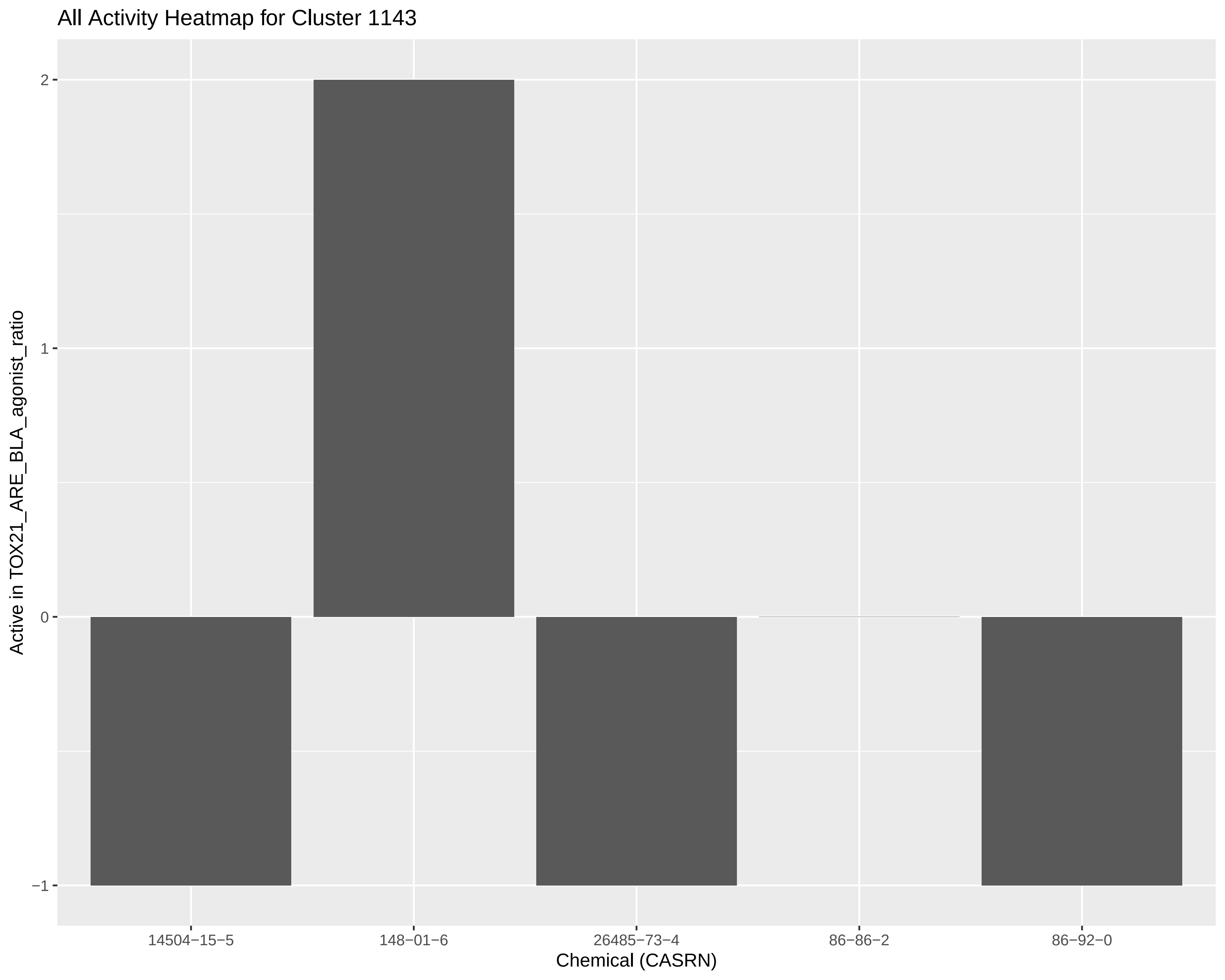
All Activity Heatmap for Cluster 1143
Active in TOX21_ARE_BLA_agonist_ratio
2
1
0
−1
14504−15−5
148−01−6
26485−73−4
86−86−2
86−92−0
Chemical (CASRN)

All Activity Heatmap for Cluster 1149

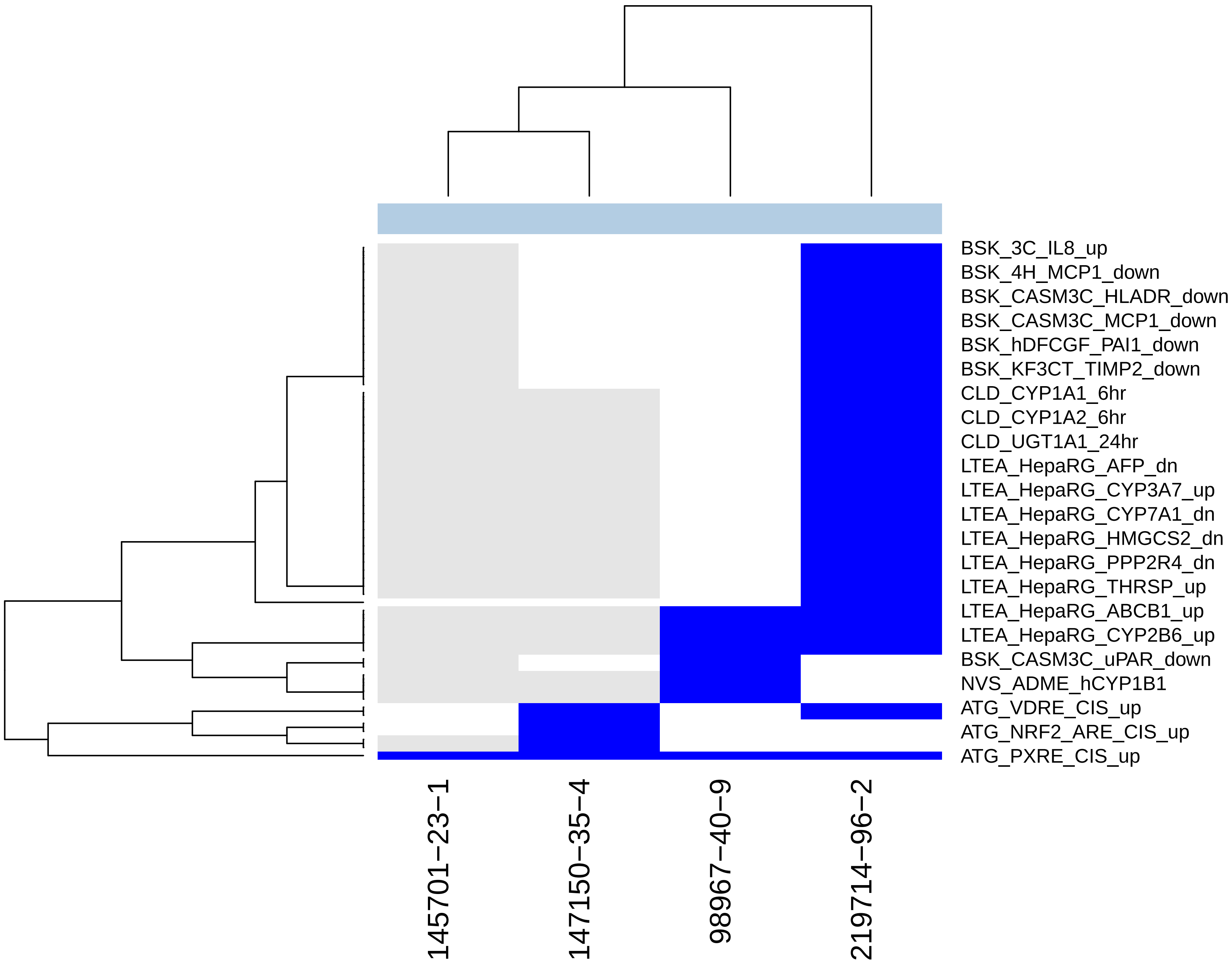

All Activity Heatmap for Cluster 1151

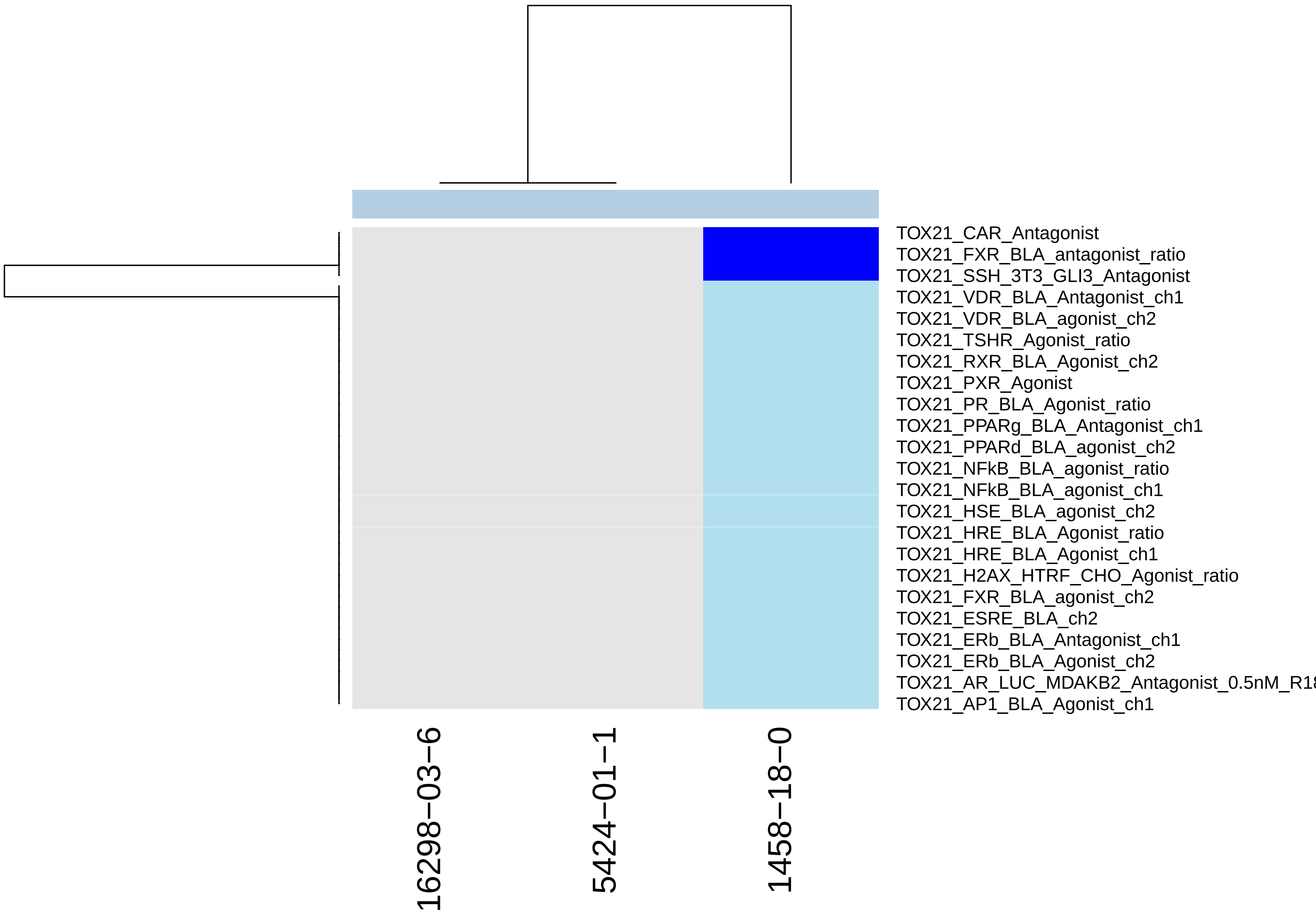

All Activity Heatmap for Cluster 1152

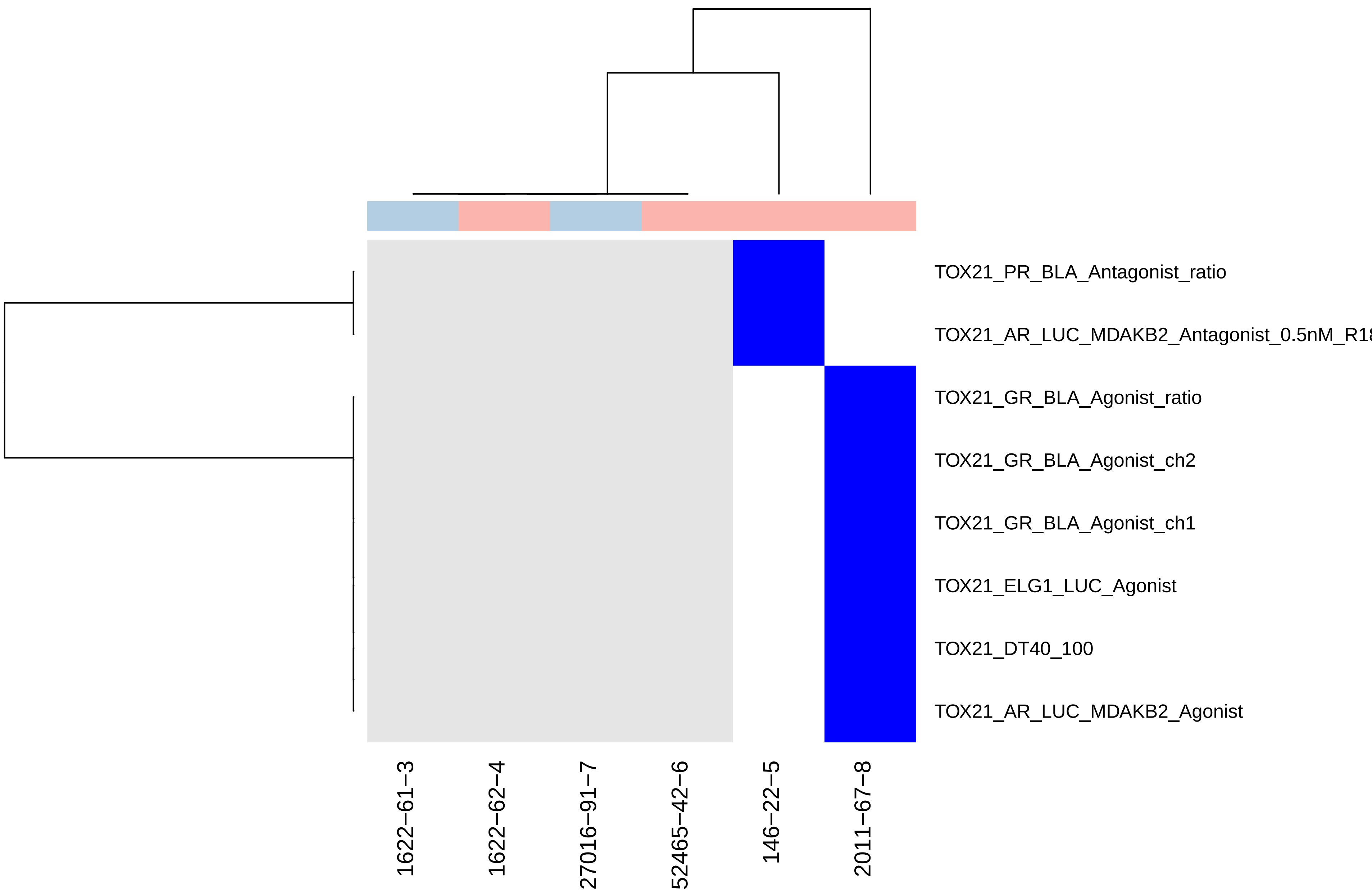

All Activity Heatmap for Cluster 1153

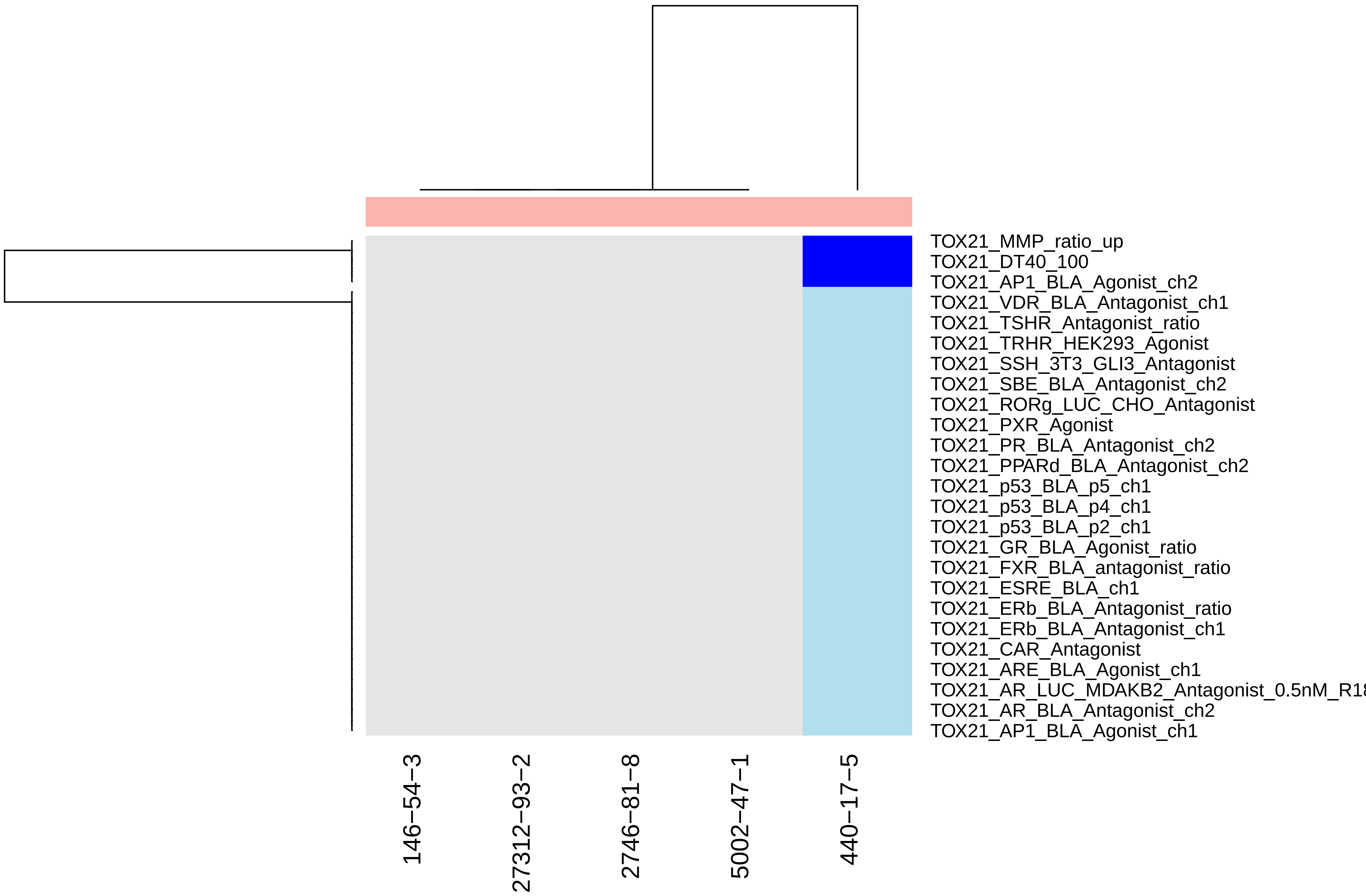

All Activity Heatmap for Cluster 1155

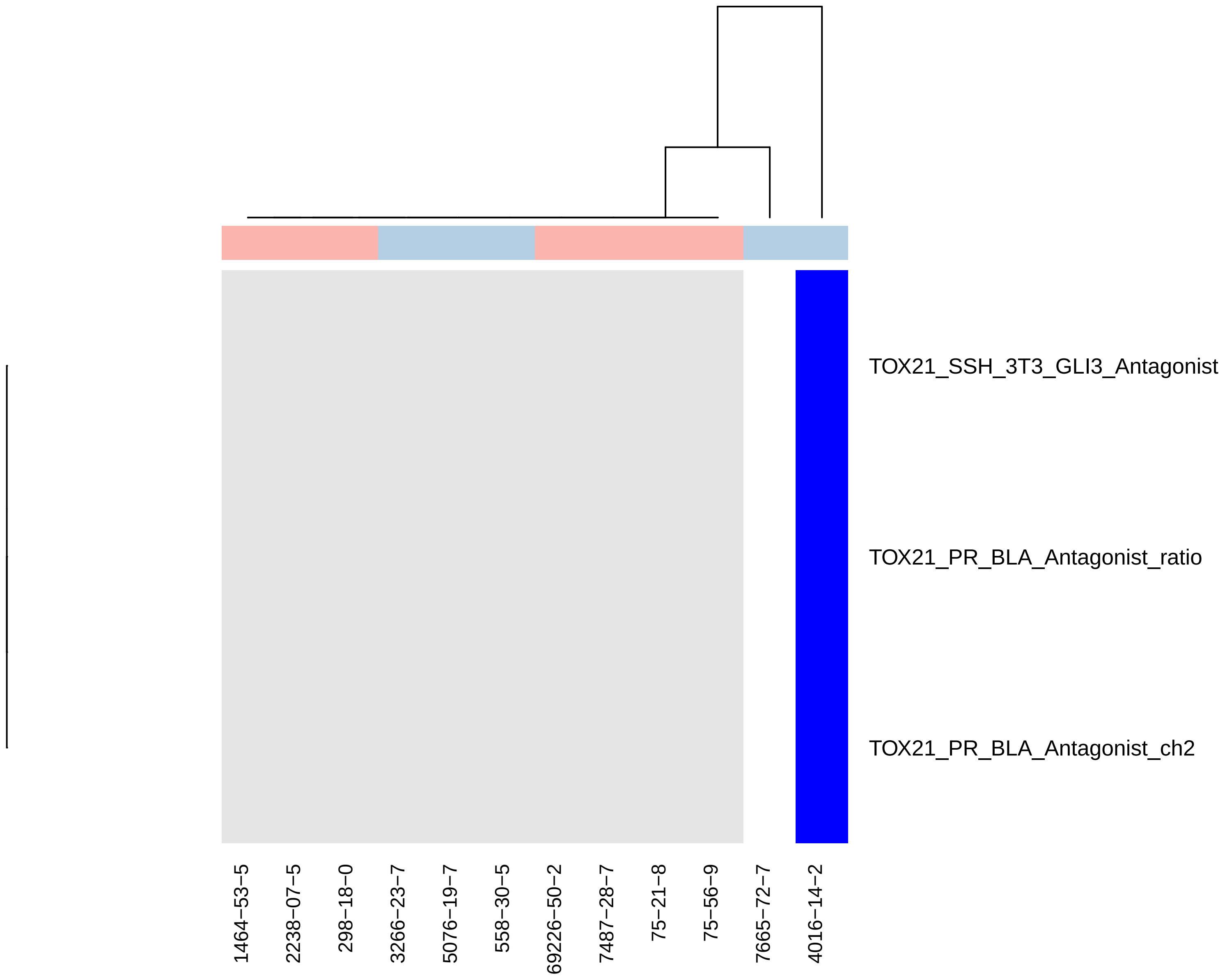

All Activity Heatmap for Cluster 1156

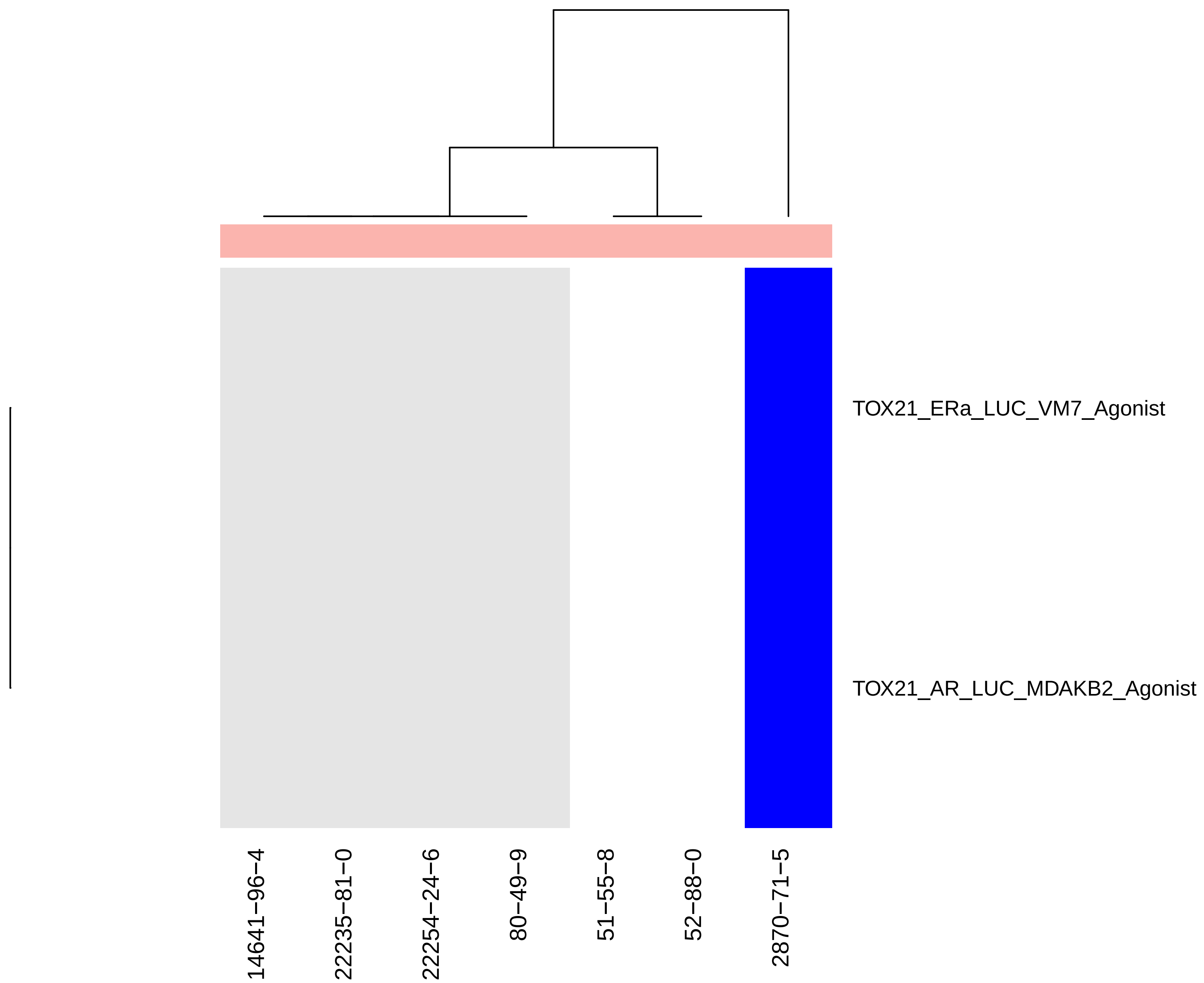

All Activity Heatmap for Cluster 1157

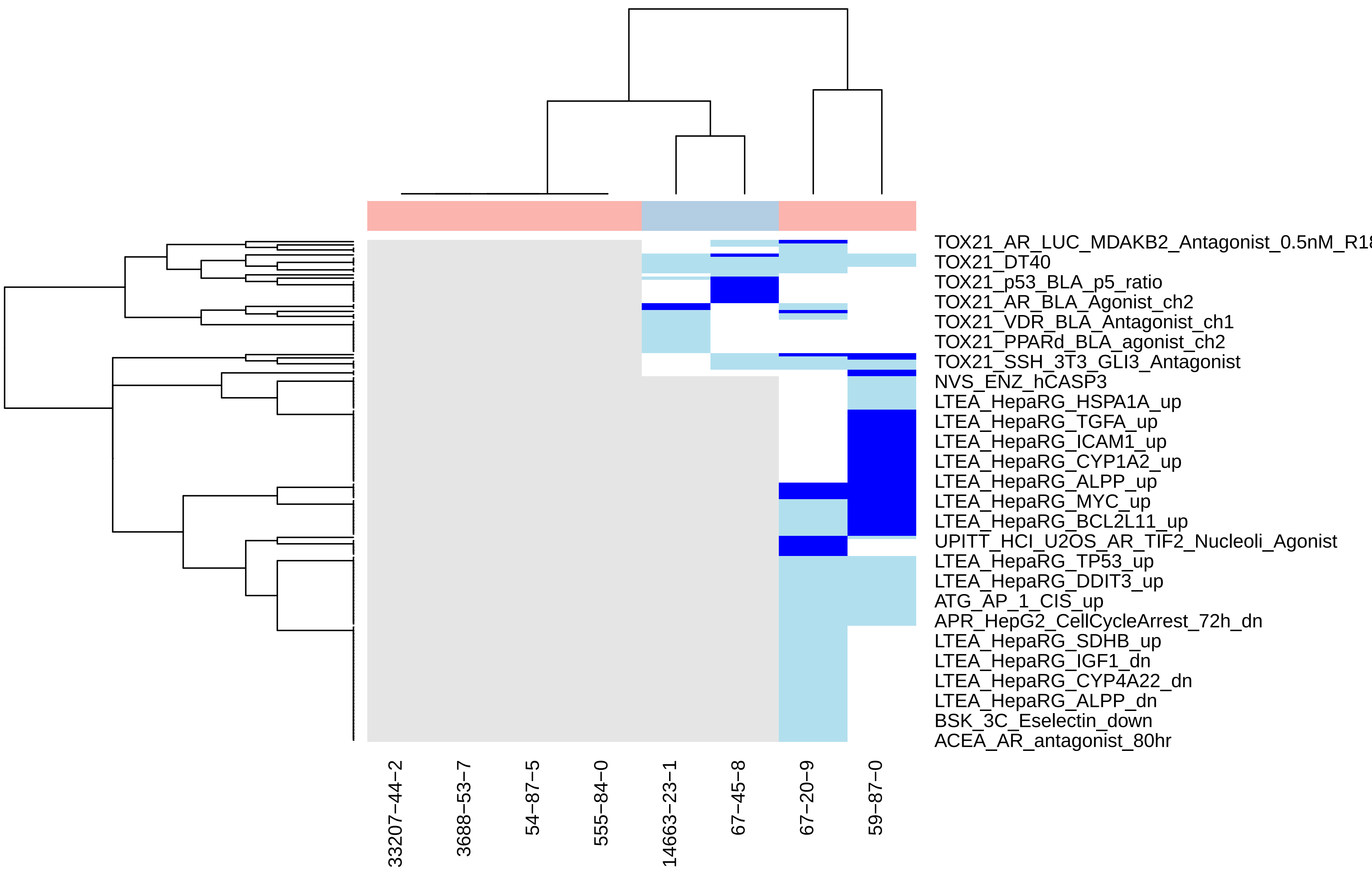

All Activity Heatmap for Cluster 1158

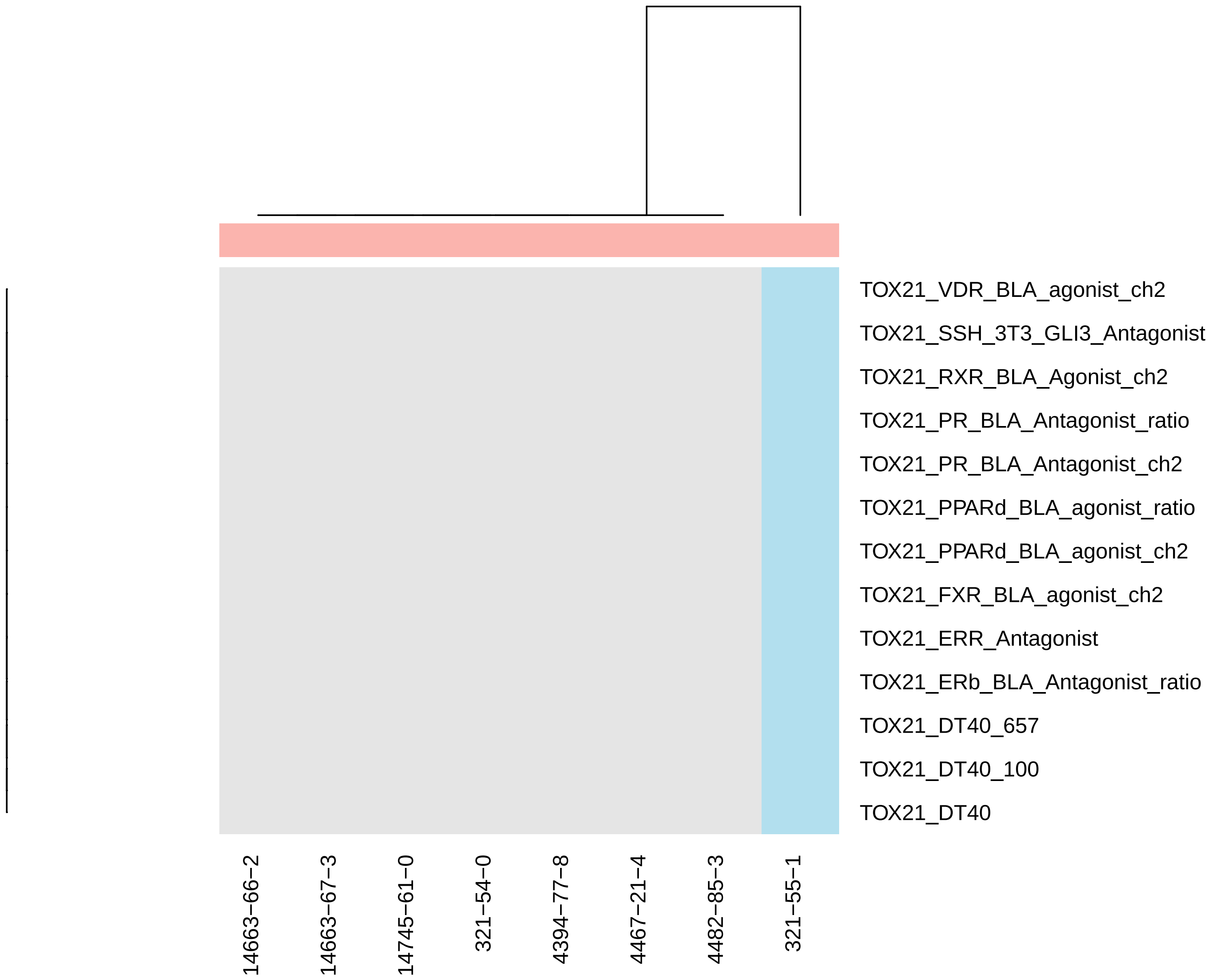

All Activity Heatmap for Cluster 1159

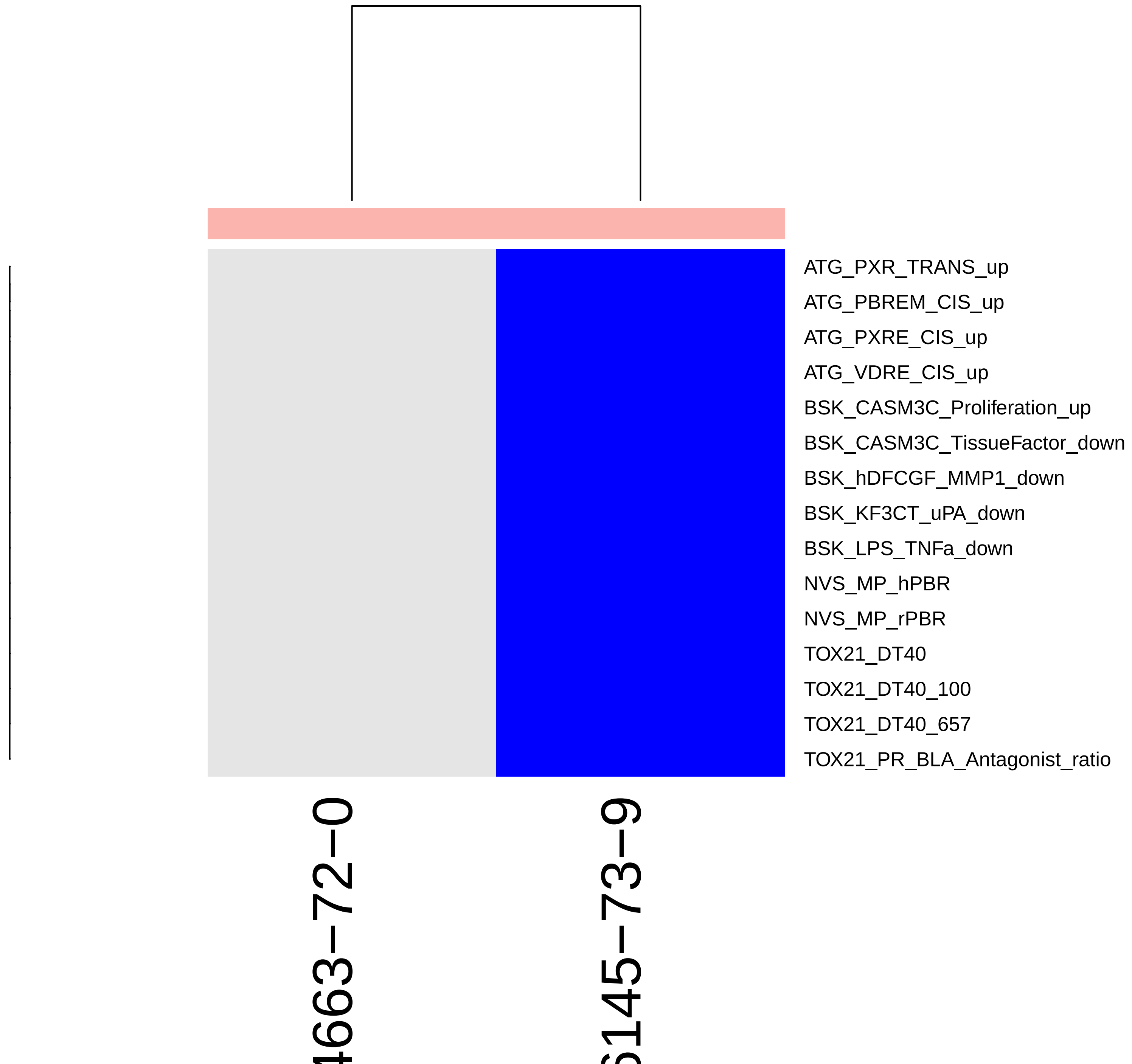

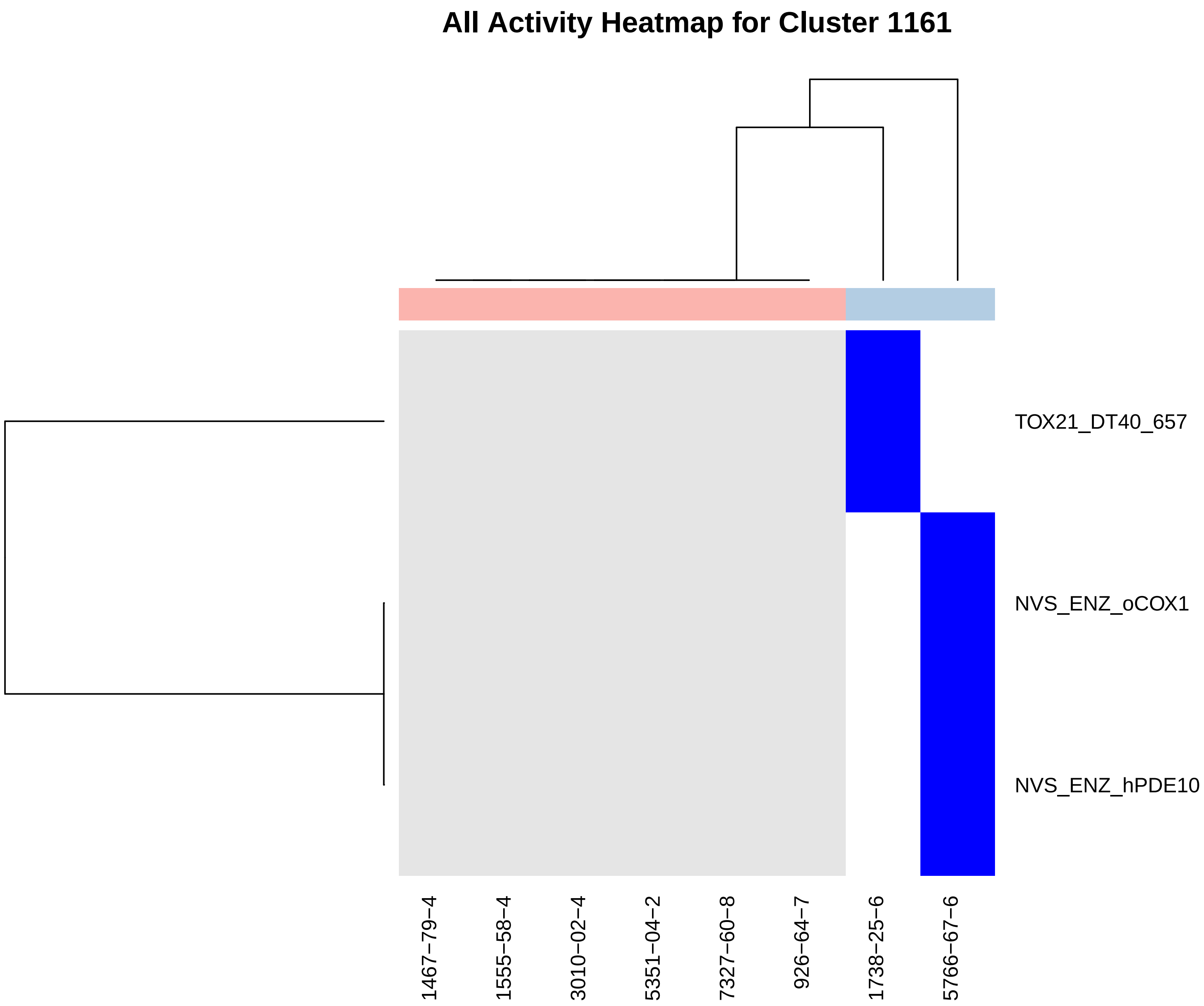
All Activity Heatmap for Cluster 1161
TOX21_DT40_657
NVS_ENZ_oCOX1
NVS_ENZ_hPDE10
1467−79−4
1555−58−4
3010−02−4
5351−04−2
7327−60−8
926−64−7
1738−25−6
5766−67−6

All Activity Heatmap for Cluster 1162

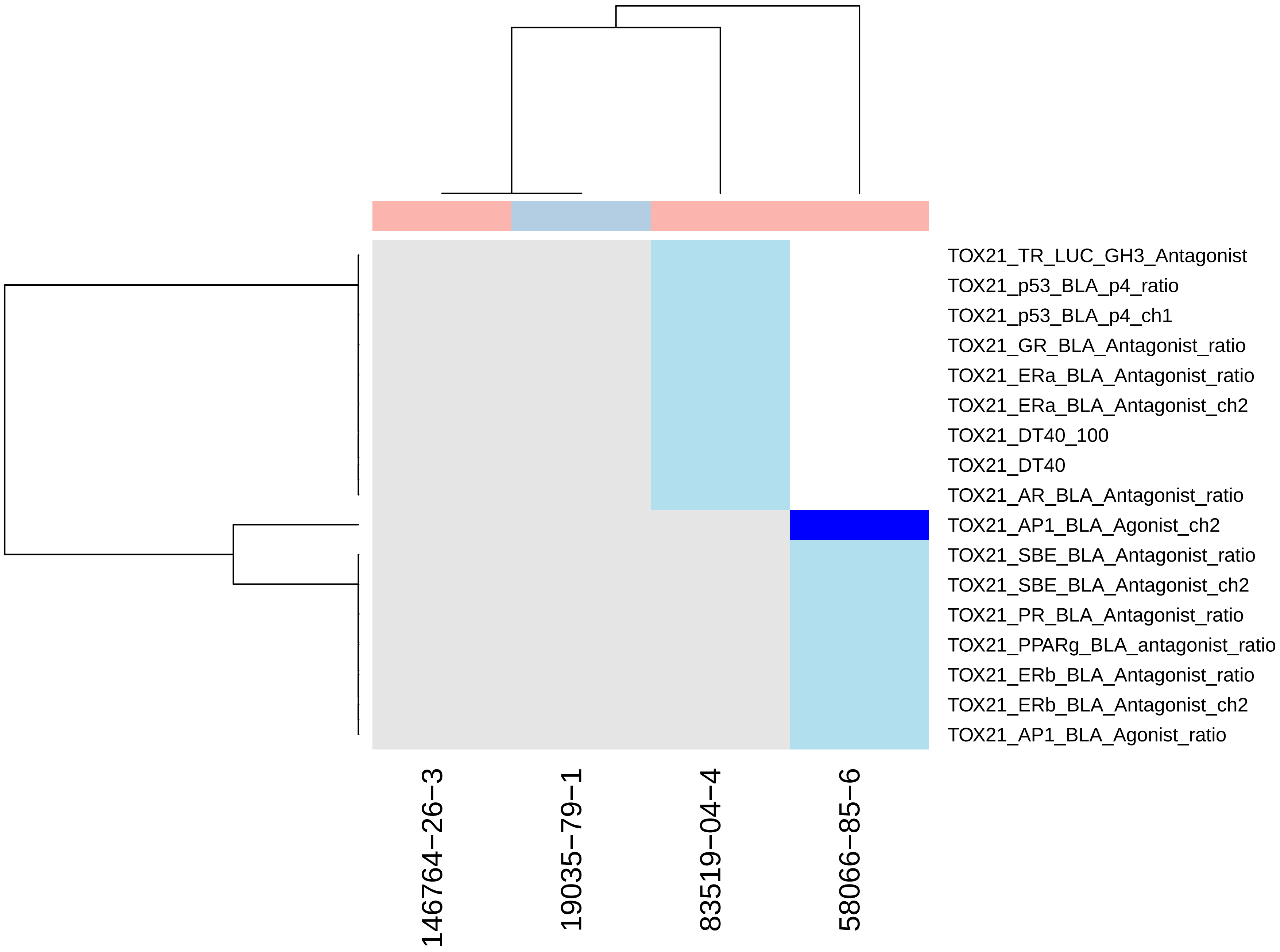

All Activity Heatmap for Cluster 1167

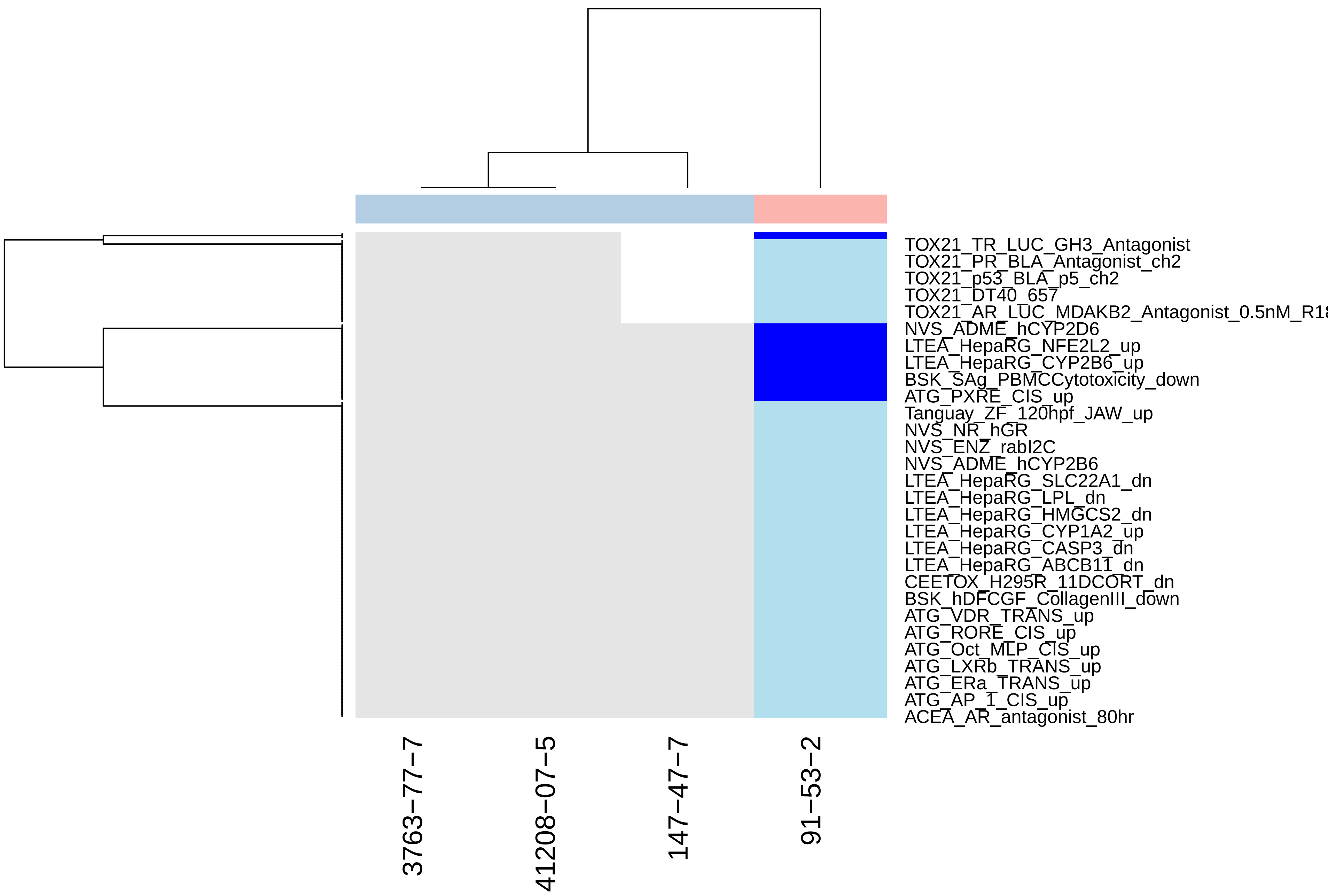

All Activity Heatmap for Cluster 1168

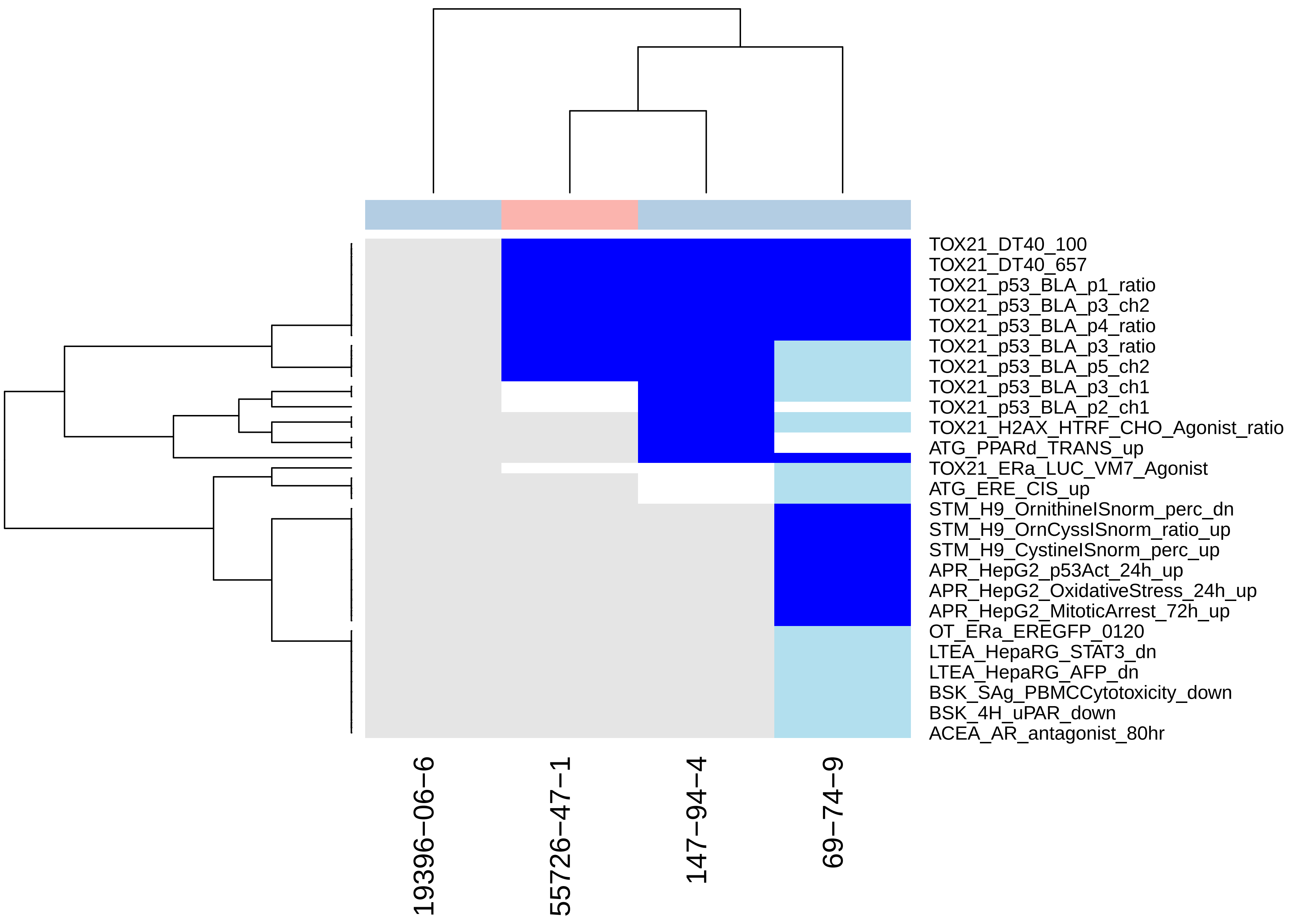

All Activity Heatmap for Cluster 1169

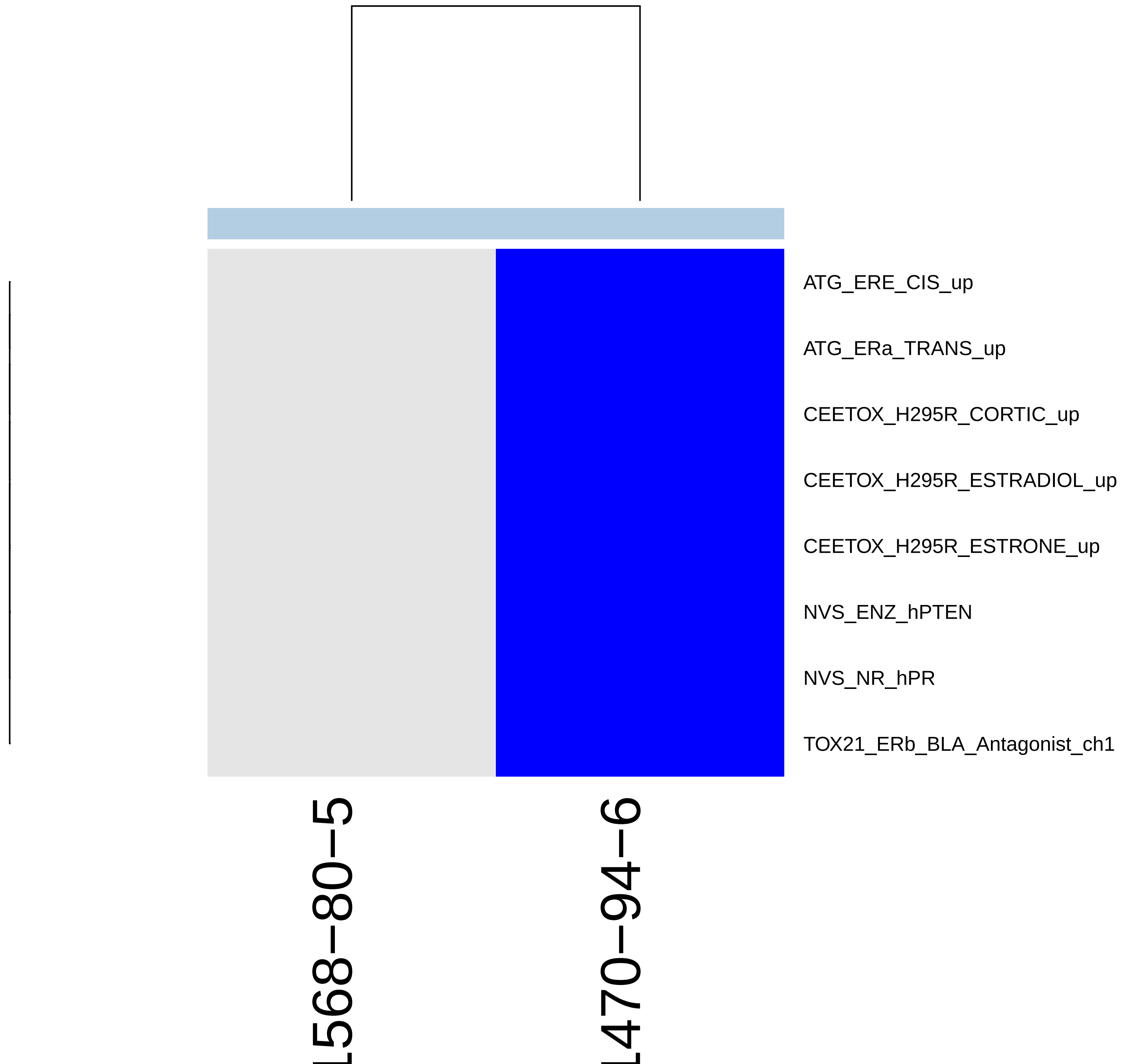

All Activity Heatmap for Cluster 1170

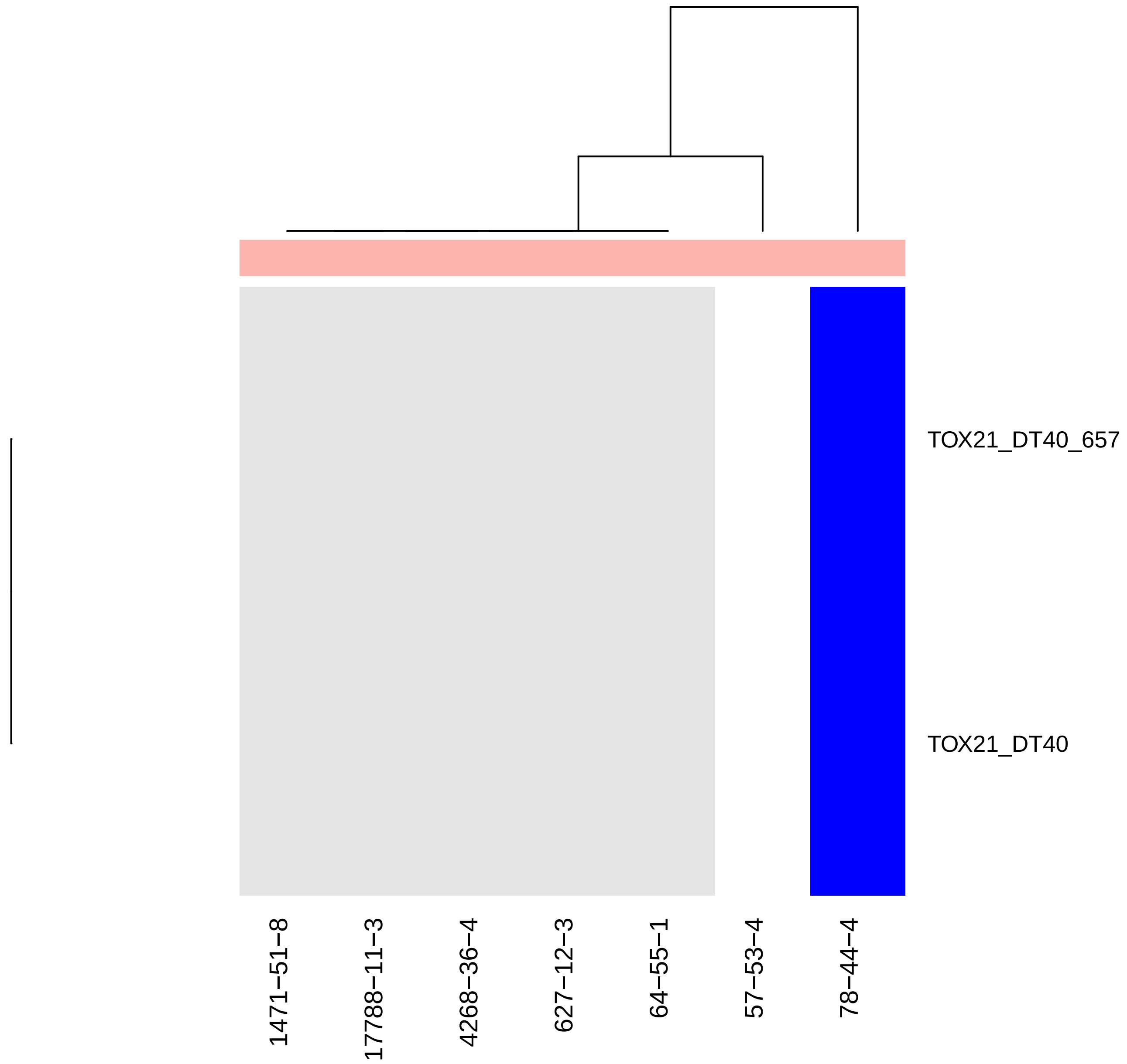

All Activity Heatmap for Cluster 1171

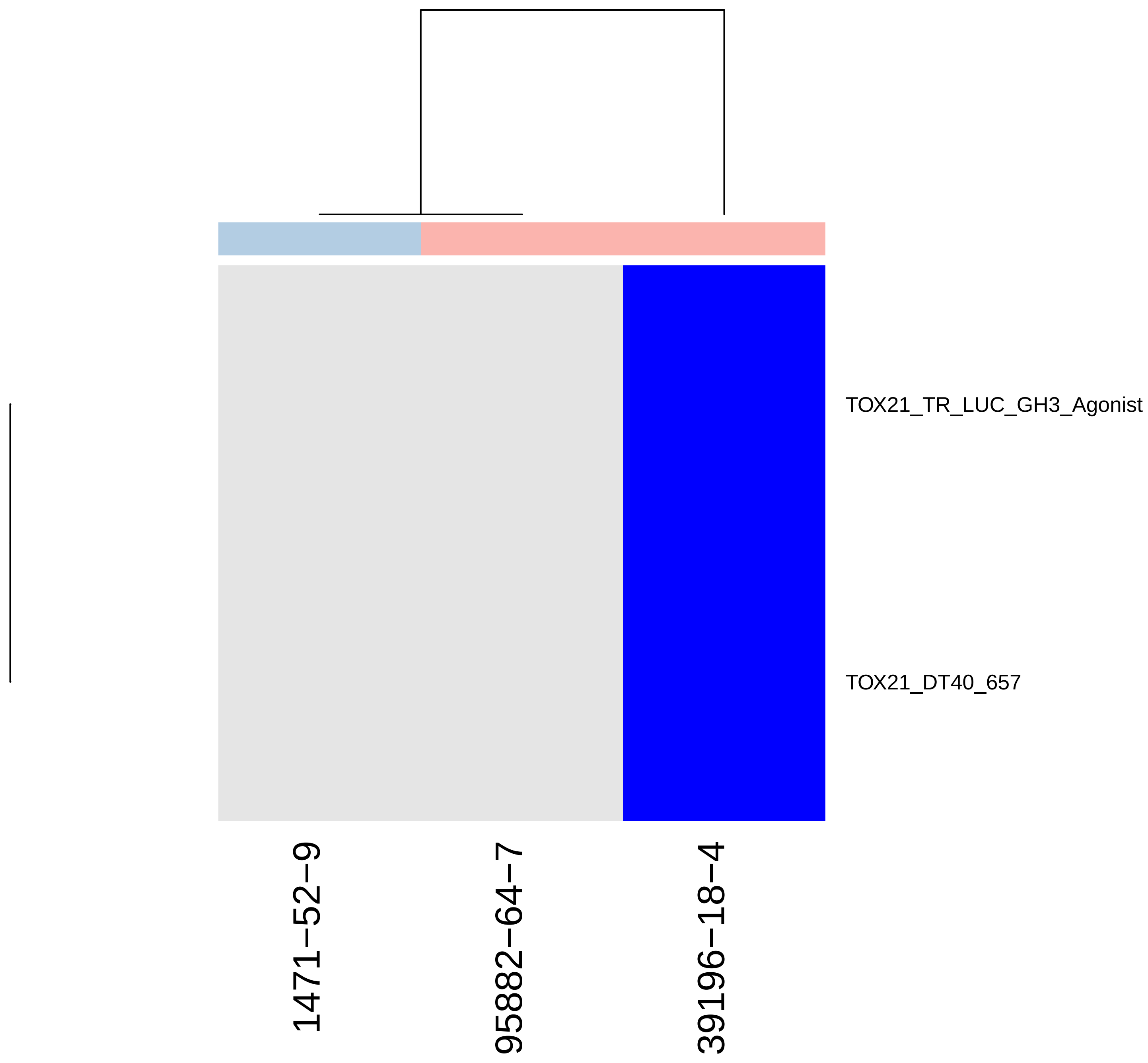

All Activity Heatmap for Cluster 1173

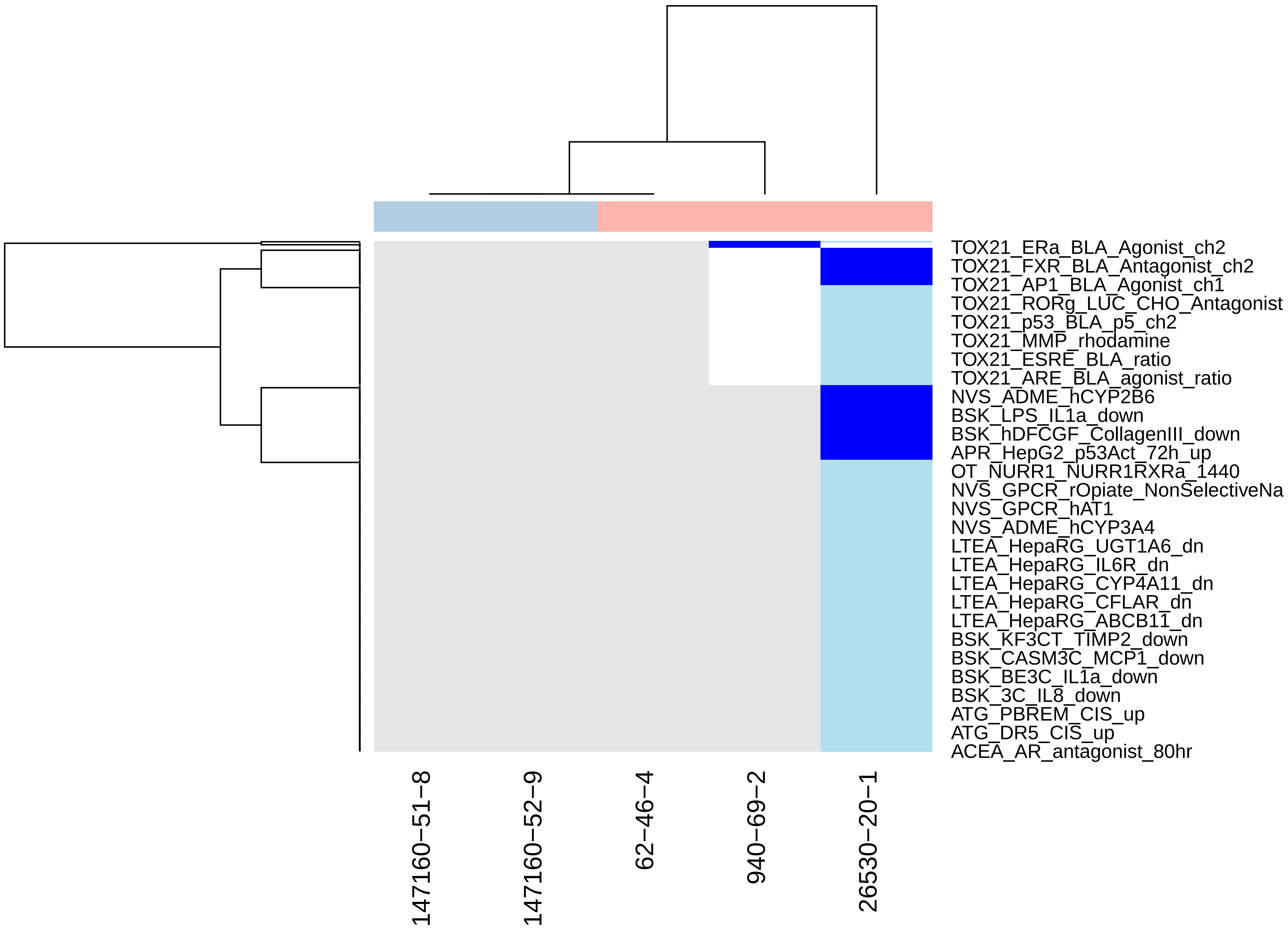

All Activity Heatmap for Cluster 1174

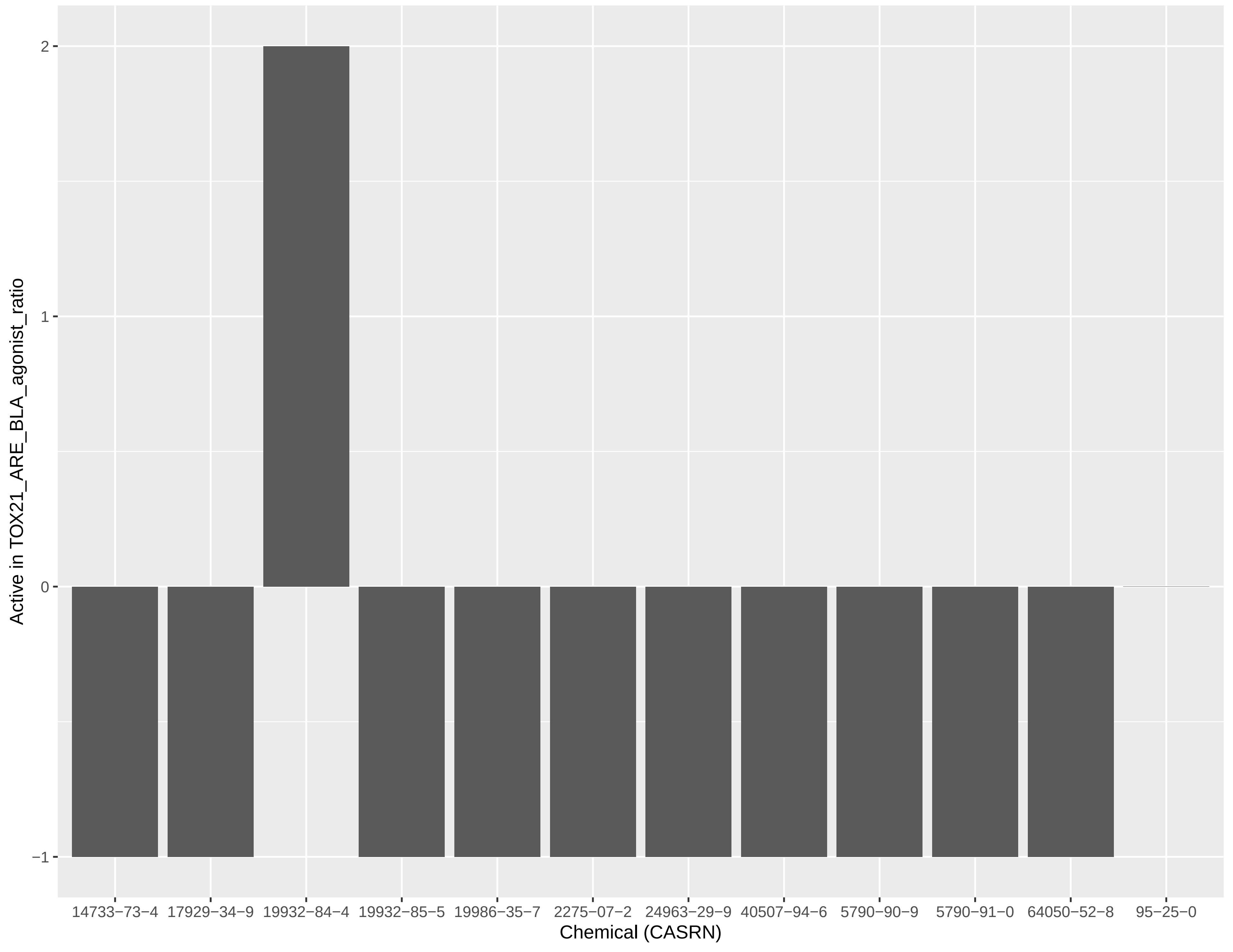

All Activity Heatmap for Cluster 1176

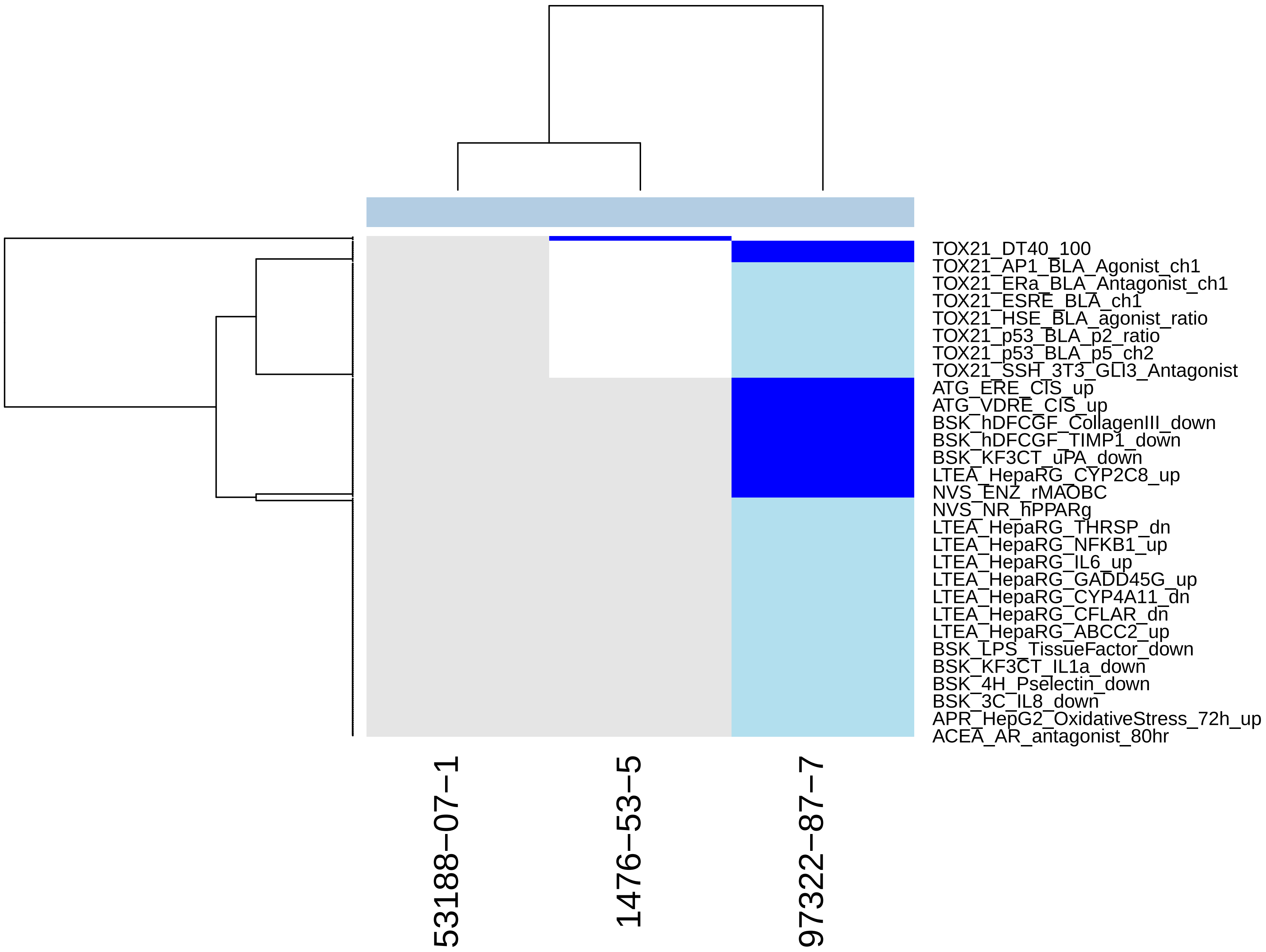

All Activity Heatmap for Cluster 1177

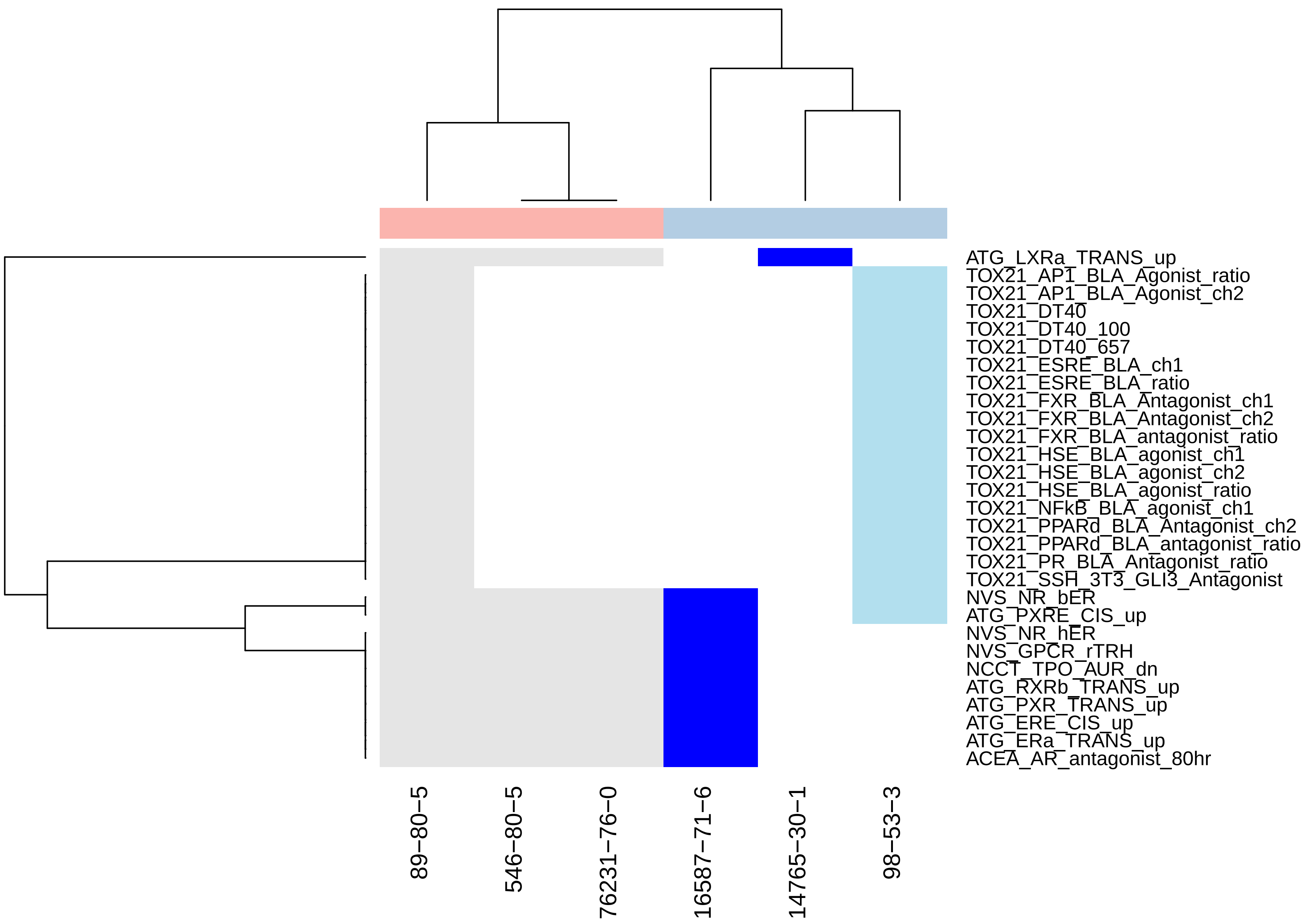

All Activity Heatmap for Cluster 1178

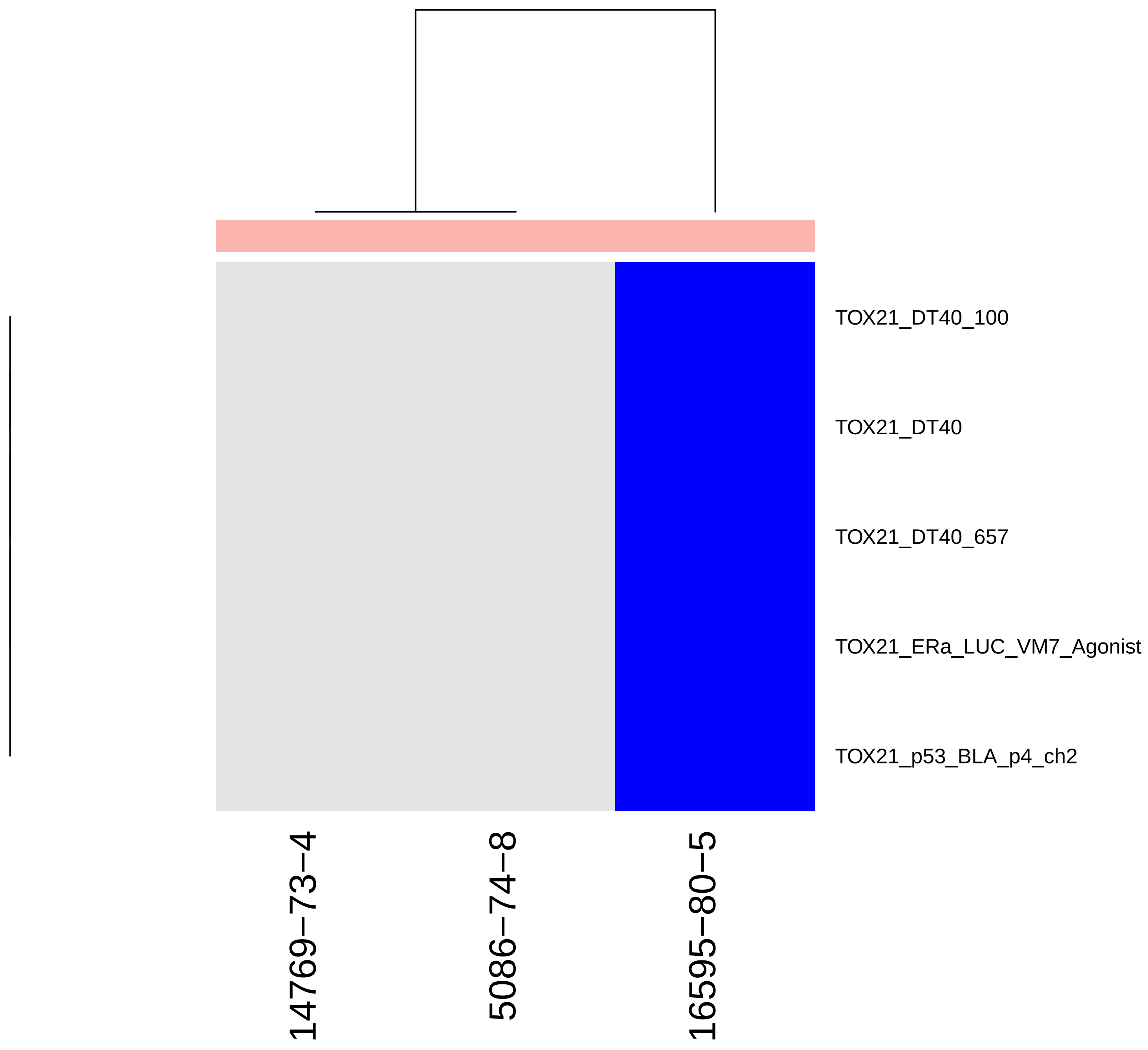

All Activity Heatmap for Cluster 1179

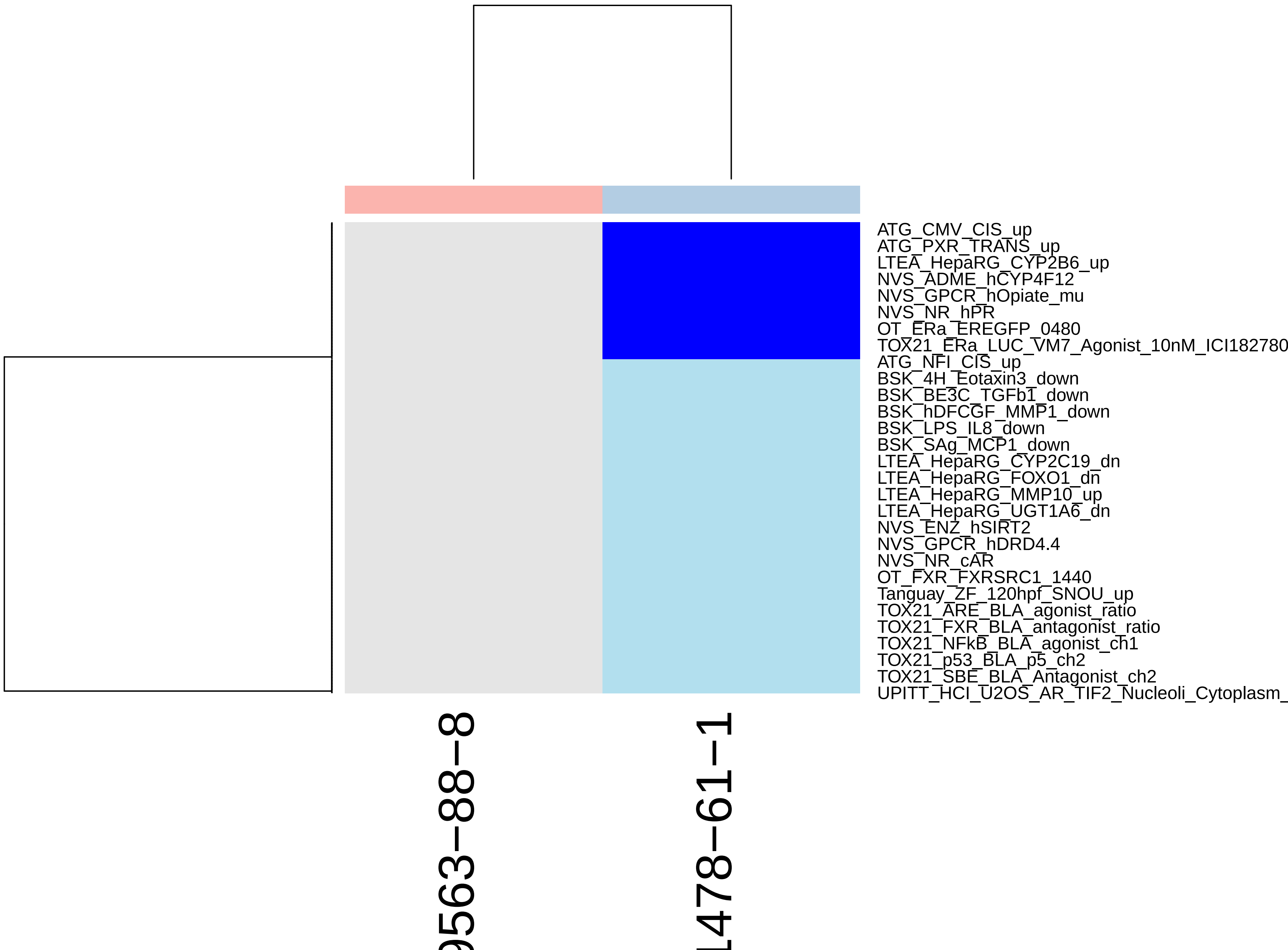

All Activity Heatmap for Cluster 1181

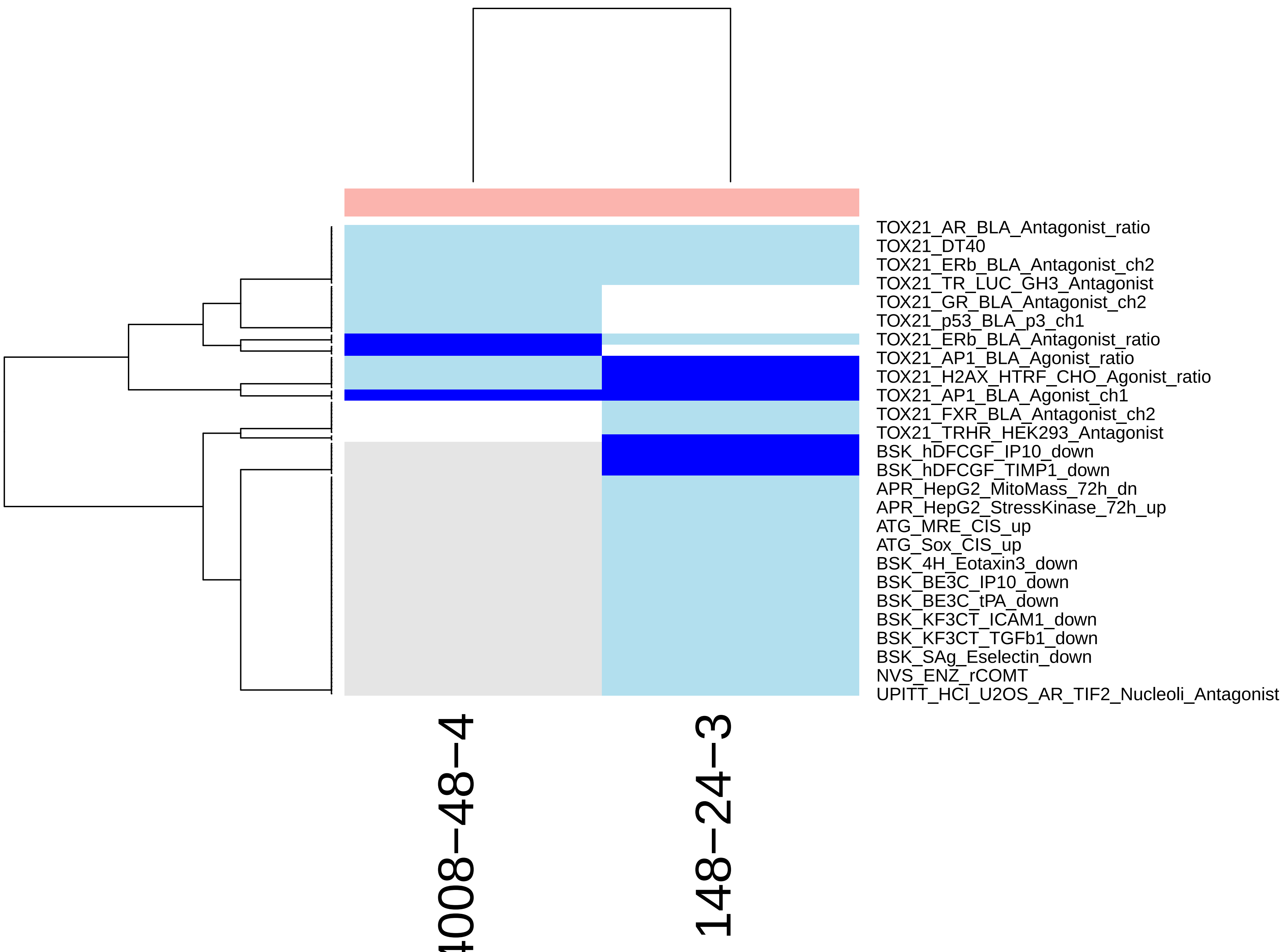

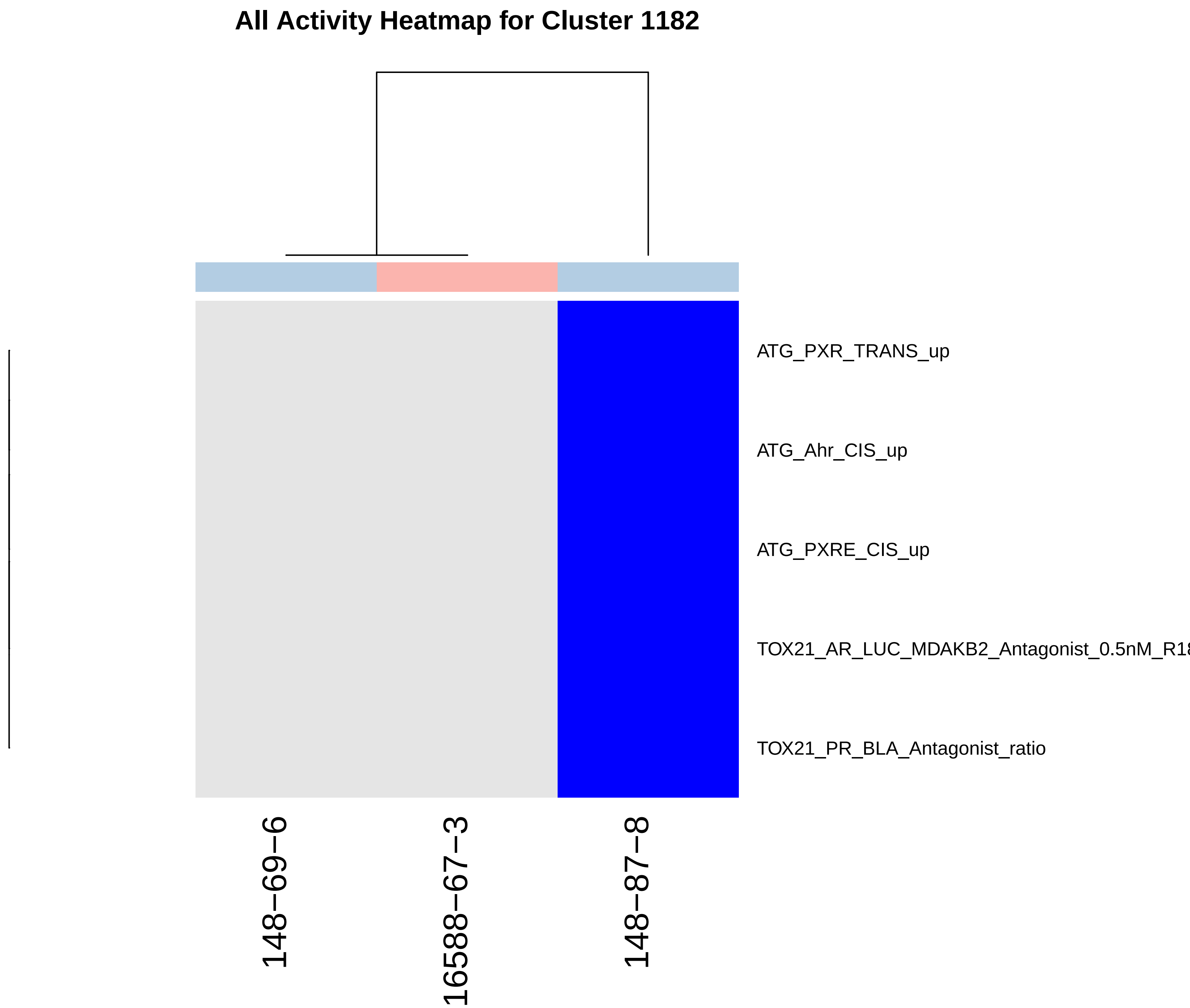
All Activity Heatmap for Cluster 1182
ATG_PXR_TRANS_up
ATG_Ahr_CIS_up
ATG_PXRE_CIS_up
TOX21_AR_LUC_MDAKB2_Antagonist_0.5nM_R1
TOX21_PR_BLA_Antagonist_ratio
148−69−6
16588−67−3
148−87−8

All Activity Heatmap for Cluster 1184

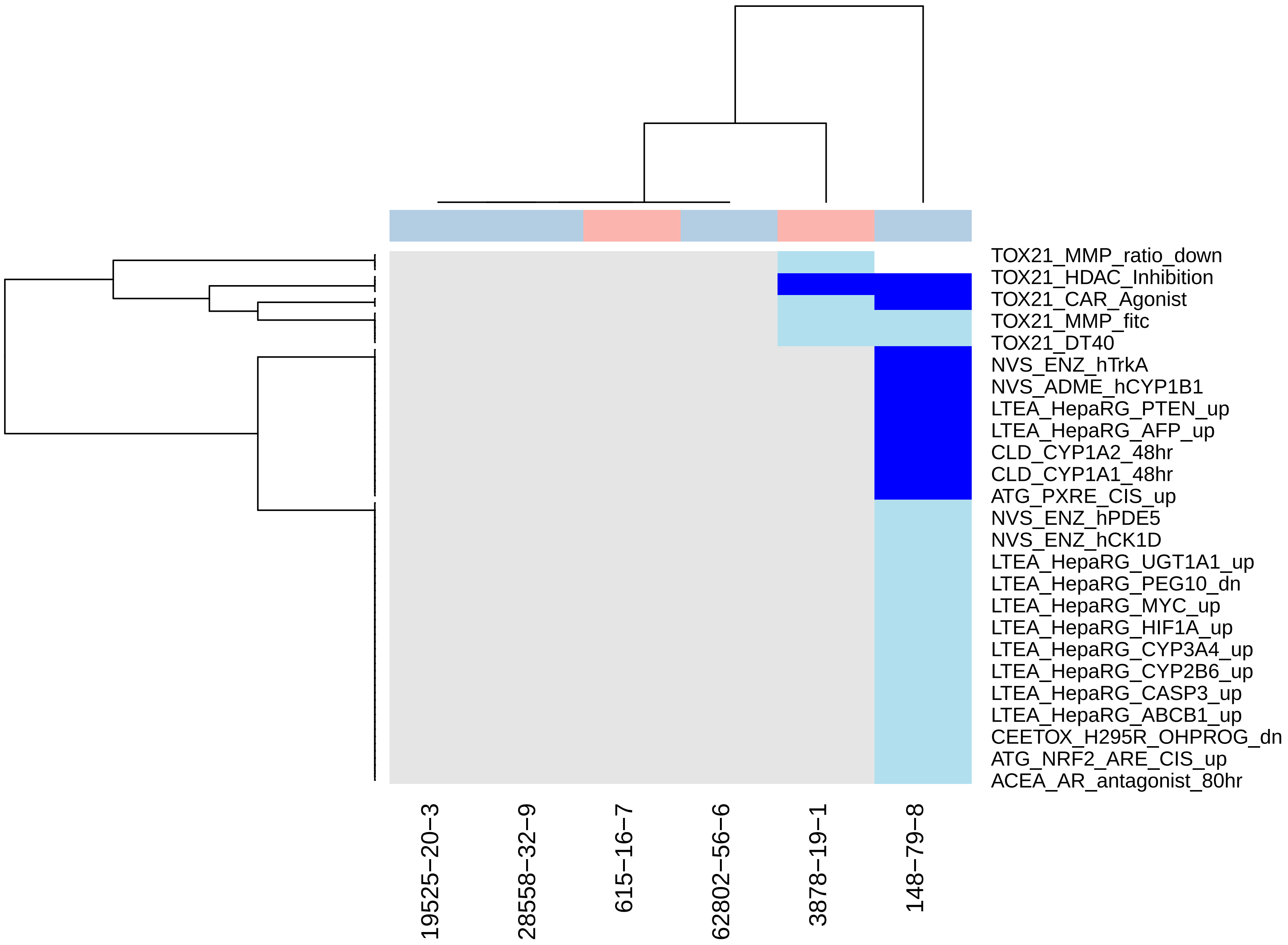

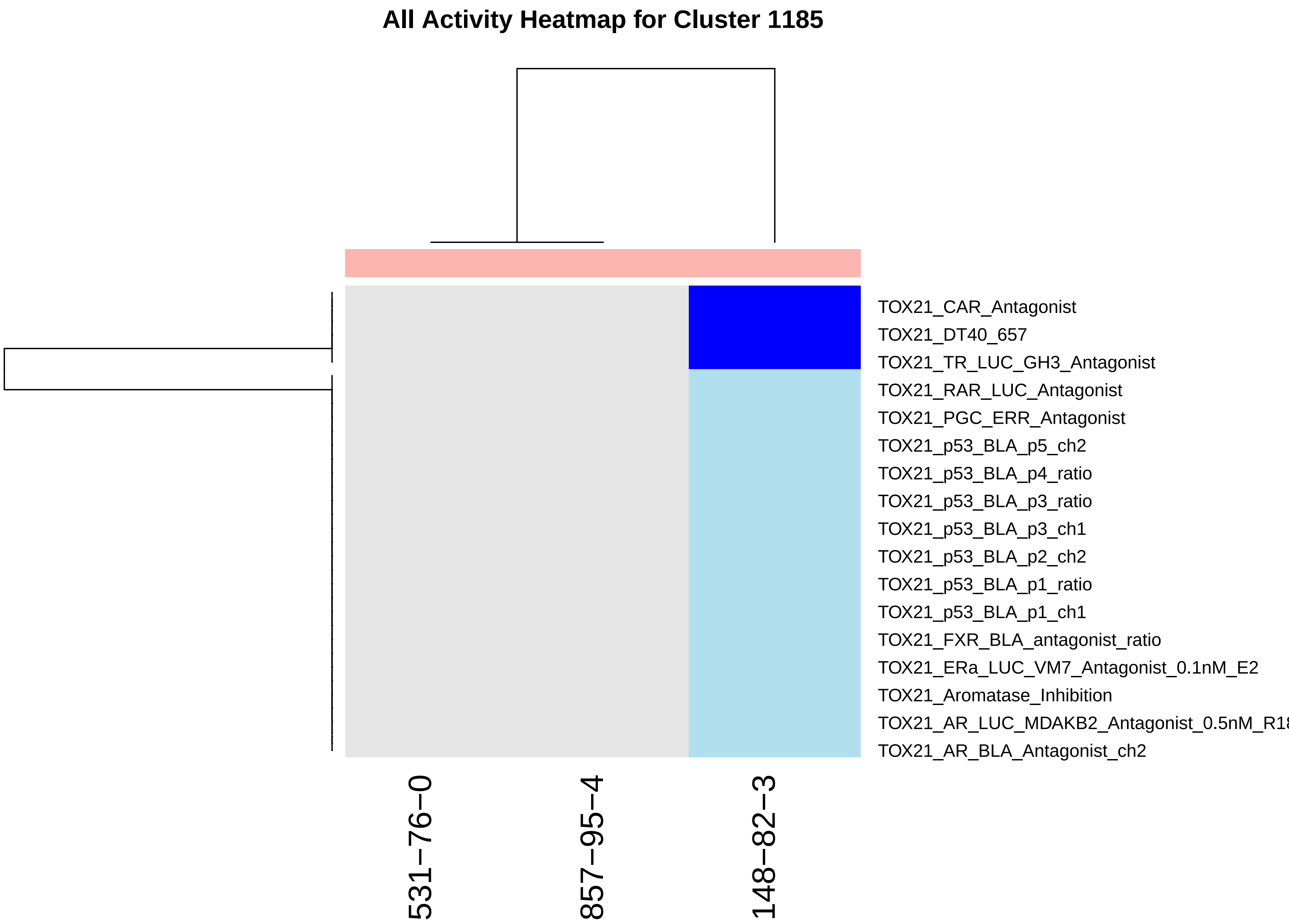

All Activity Heatmap for Cluster 1185
TOX21_CAR_Antagonist
TOX21_DT40_657
TOX21_TR_LUC_GH3_Antagonist
TOX21_RAR_LUC_Antagonist
TOX21_PGC_ERR_Antagonist
TOX21_p53_BLA_p5_ch2
TOX21_p53_BLA_p4_ratio
TOX21_p53_BLA_p3_ratio
TOX21_p53_BLA_p3_ch1
TOX21_p53_BLA_p2_ch2
TOX21_p53_BLA_p1_ratio
TOX21_p53_BLA_p1_ch1
TOX21_FXR_BLA_antagonist_ratio
TOX21_ERa_LUC_VM7_Antagonist_0.1nM_E2
TOX21_Aromatase_Inhibition
TOX21_AR_LUC_MDAKB2_Antagonist_0.5nM_R1
TOX21_AR_BLA_Antagonist_ch2
531−76−0
857−95−4
148−82−3

All Activity Heatmap for Cluster 1186

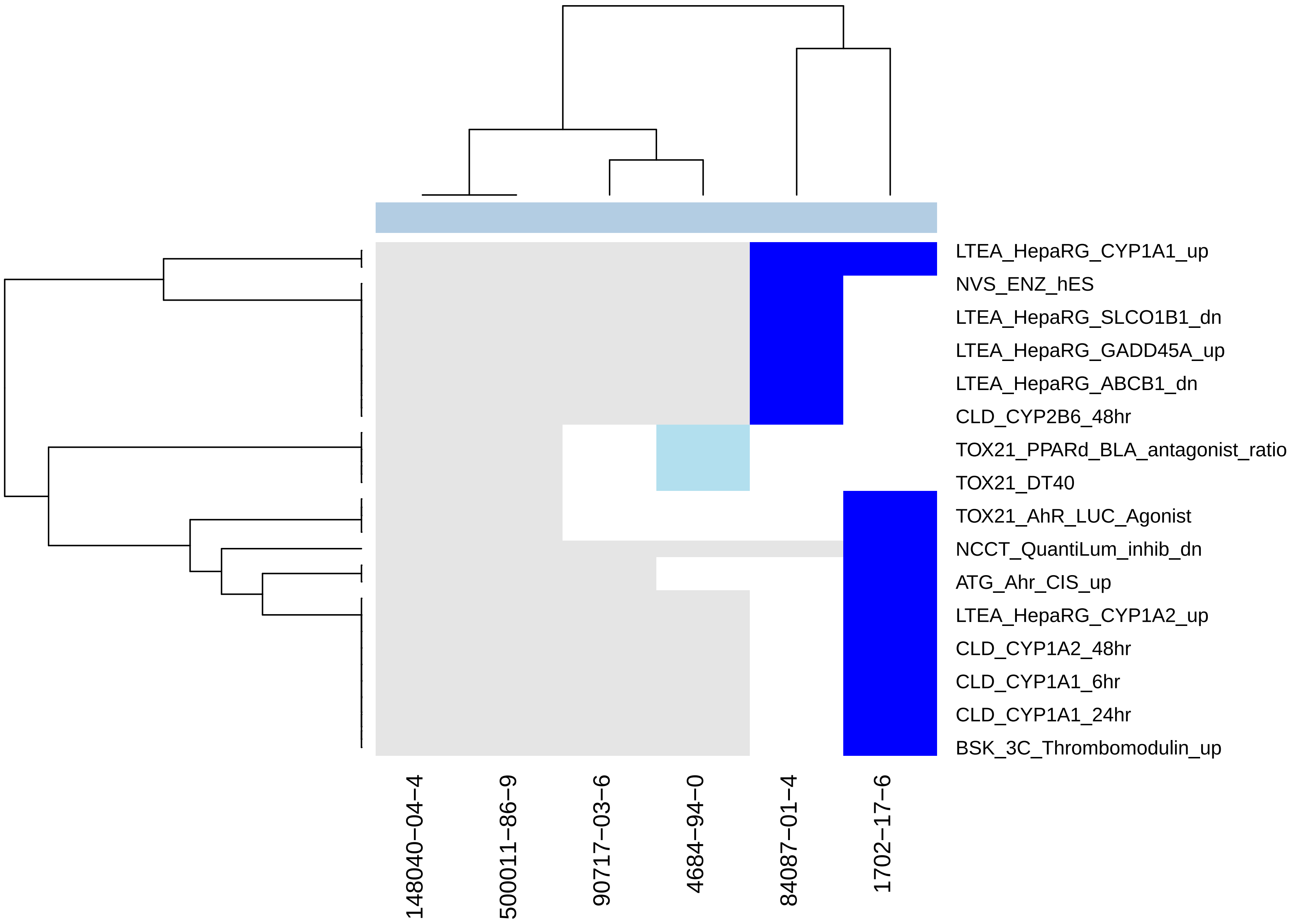

All Activity Heatmap for Cluster 1187

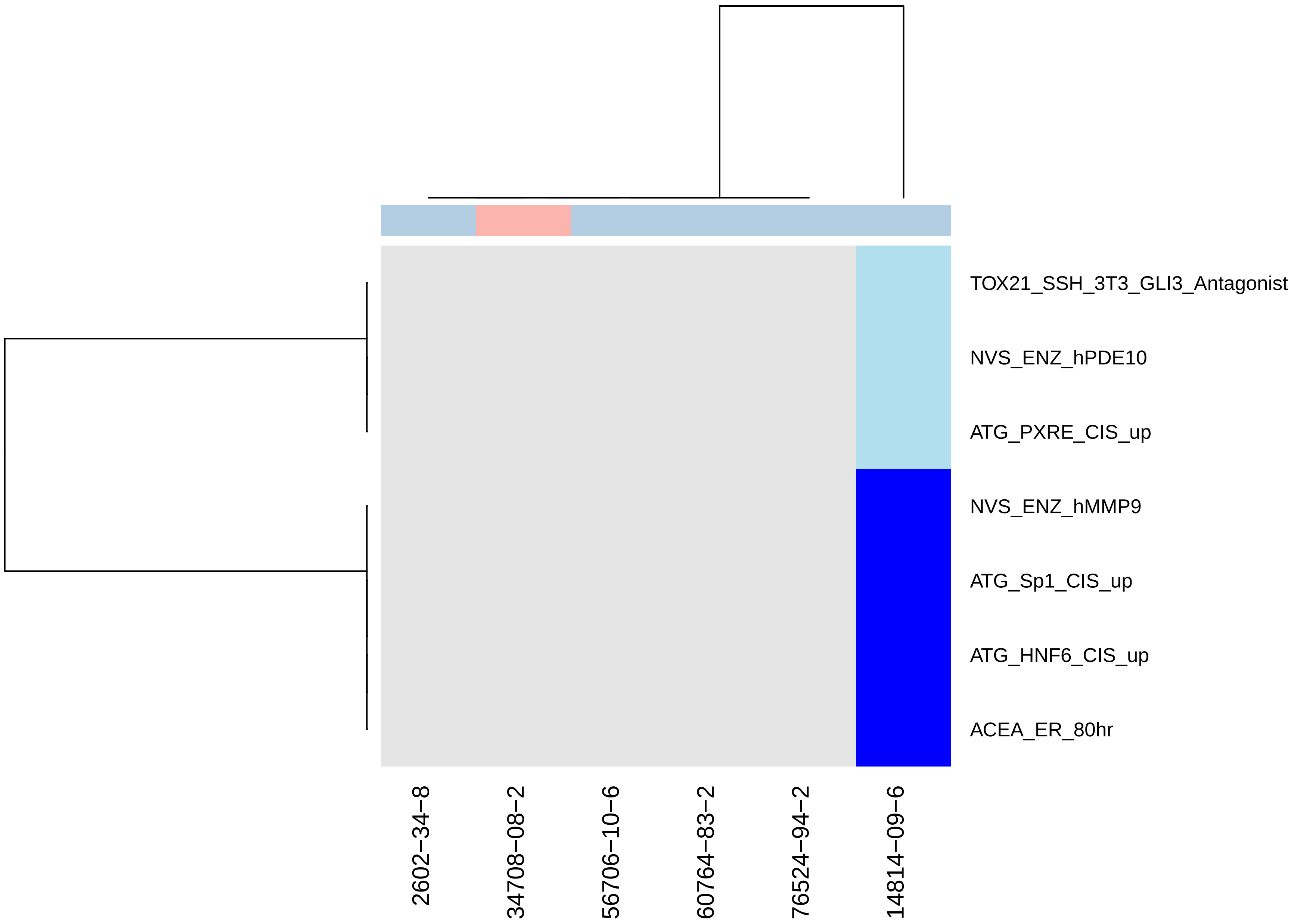

All Activity Heatmap for Cluster 1188

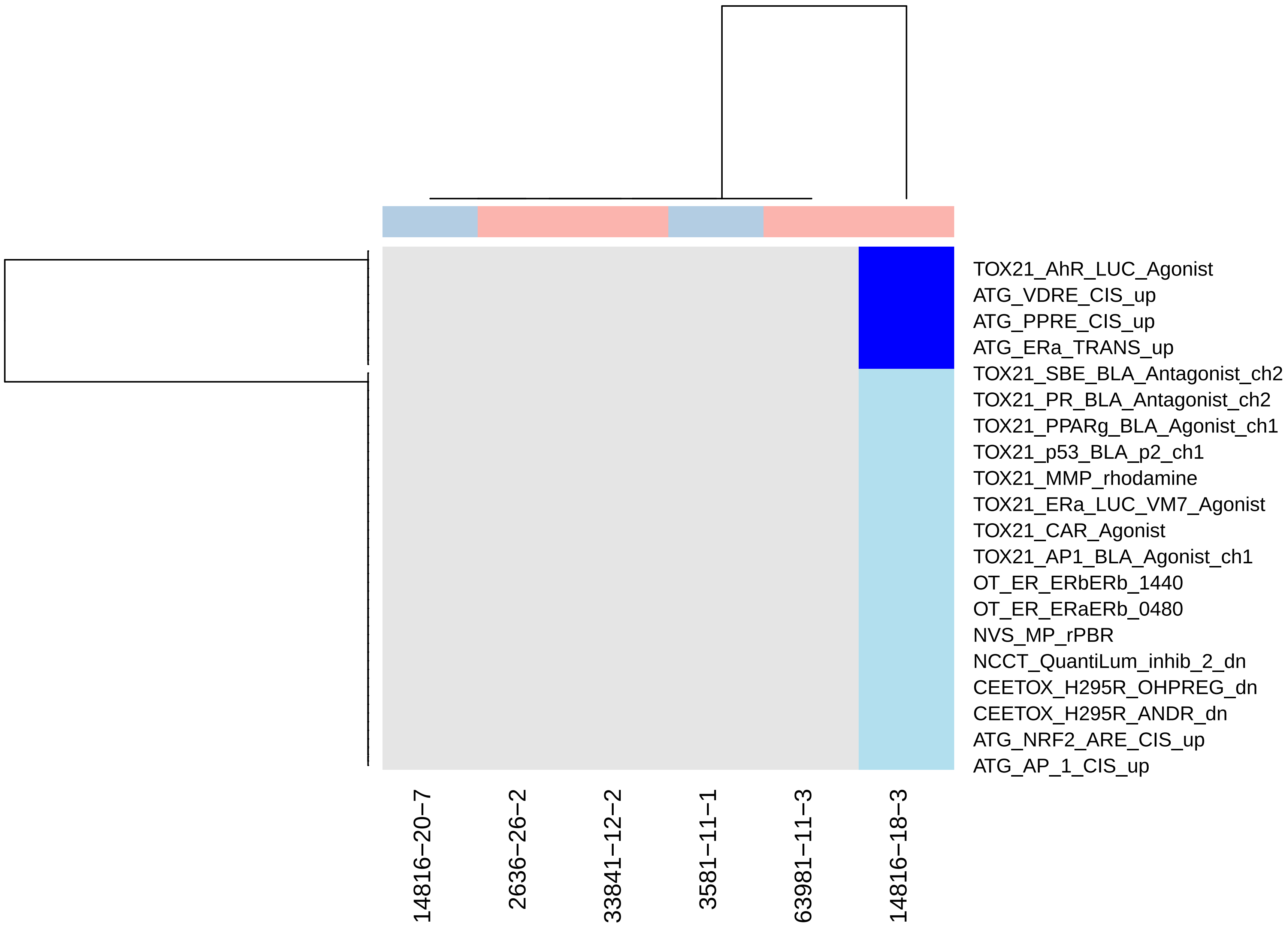

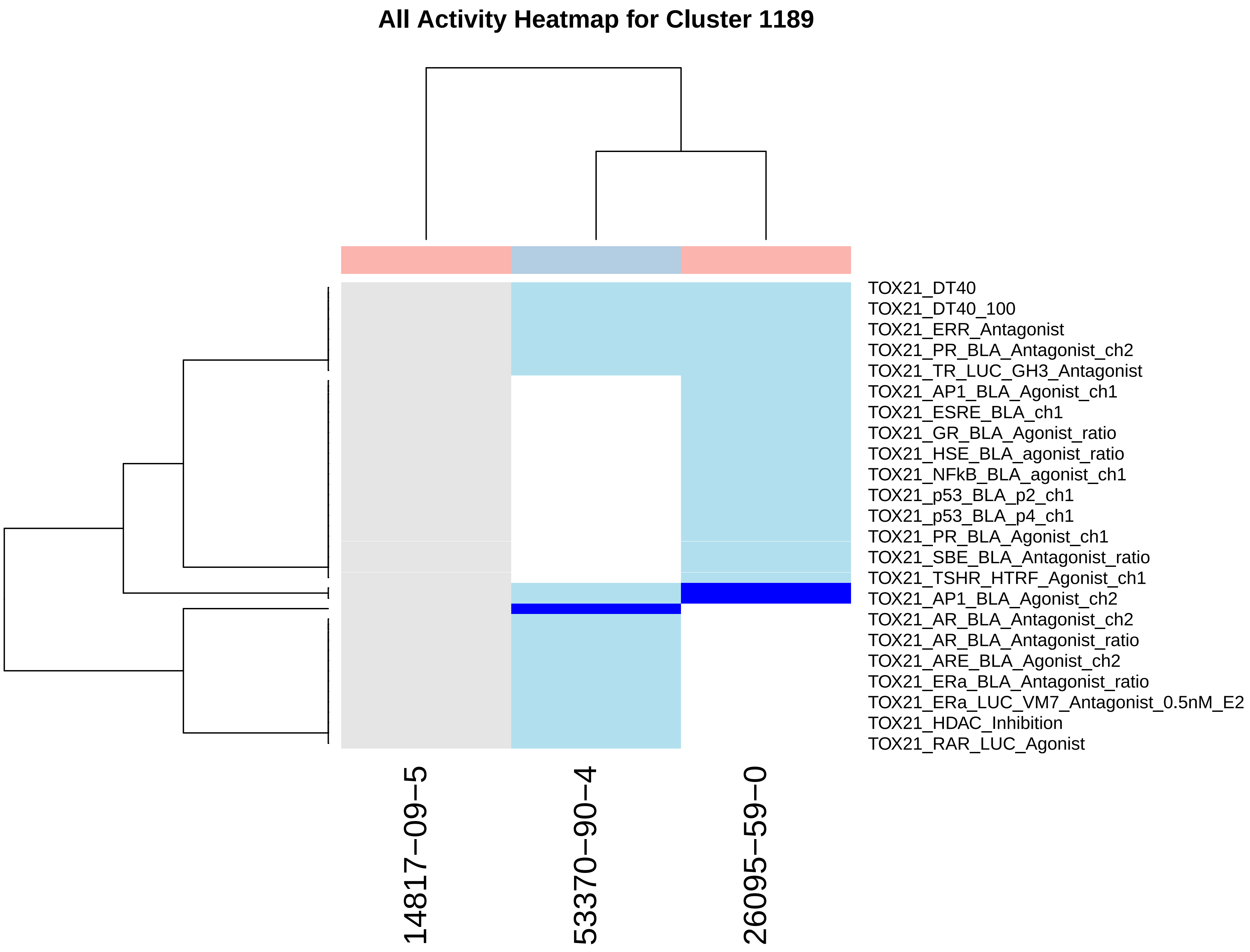
All Activity Heatmap for Cluster 1189
TOX21_DT40
TOX21_DT40_100
TOX21_ERR_Antagonist
TOX21_PR_BLA_Antagonist_ch2
TOX21_TR_LUC_GH3_Antagonist
TOX21_AP1_BLA_Agonist_ch1
TOX21_ESRE_BLA_ch1
TOX21_GR_BLA_Agonist_ratio
TOX21_HSE_BLA_agonist_ratio
TOX21_NFkB_BLA_agonist_ch1
TOX21_p53_BLA_p2_ch1
TOX21_p53_BLA_p4_ch1
TOX21_PR_BLA_Agonist_ch1
TOX21_SBE_BLA_Antagonist_ratio
TOX21_TSHR_HTRF_Agonist_ch1
TOX21_AP1_BLA_Agonist_ch2
TOX21_AR_BLA_Antagonist_ch2
TOX21_AR_BLA_Antagonist_ratio
TOX21_ARE_BLA_Agonist_ch2
TOX21_ERa_BLA_Antagonist_ratio
TOX21_ERa_LUC_VM7_Antagonist_0.5nM_E2
TOX21_HDAC_Inhibition
TOX21_RAR_LUC_Agonist
14817−09−5
53370−90−4
26095−59−0

All Activity Heatmap for Cluster 1190

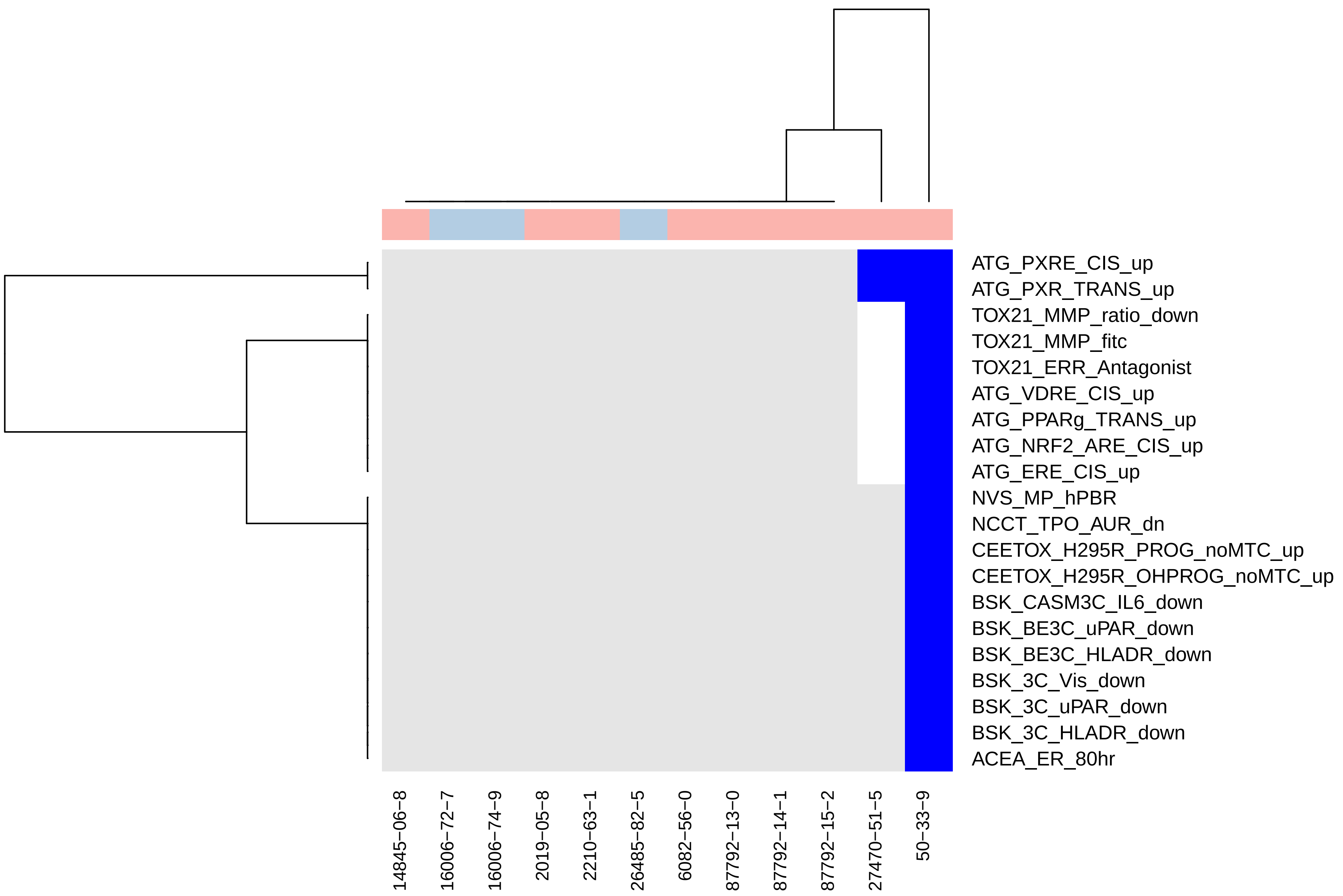

All Activity Heatmap for Cluster 1191

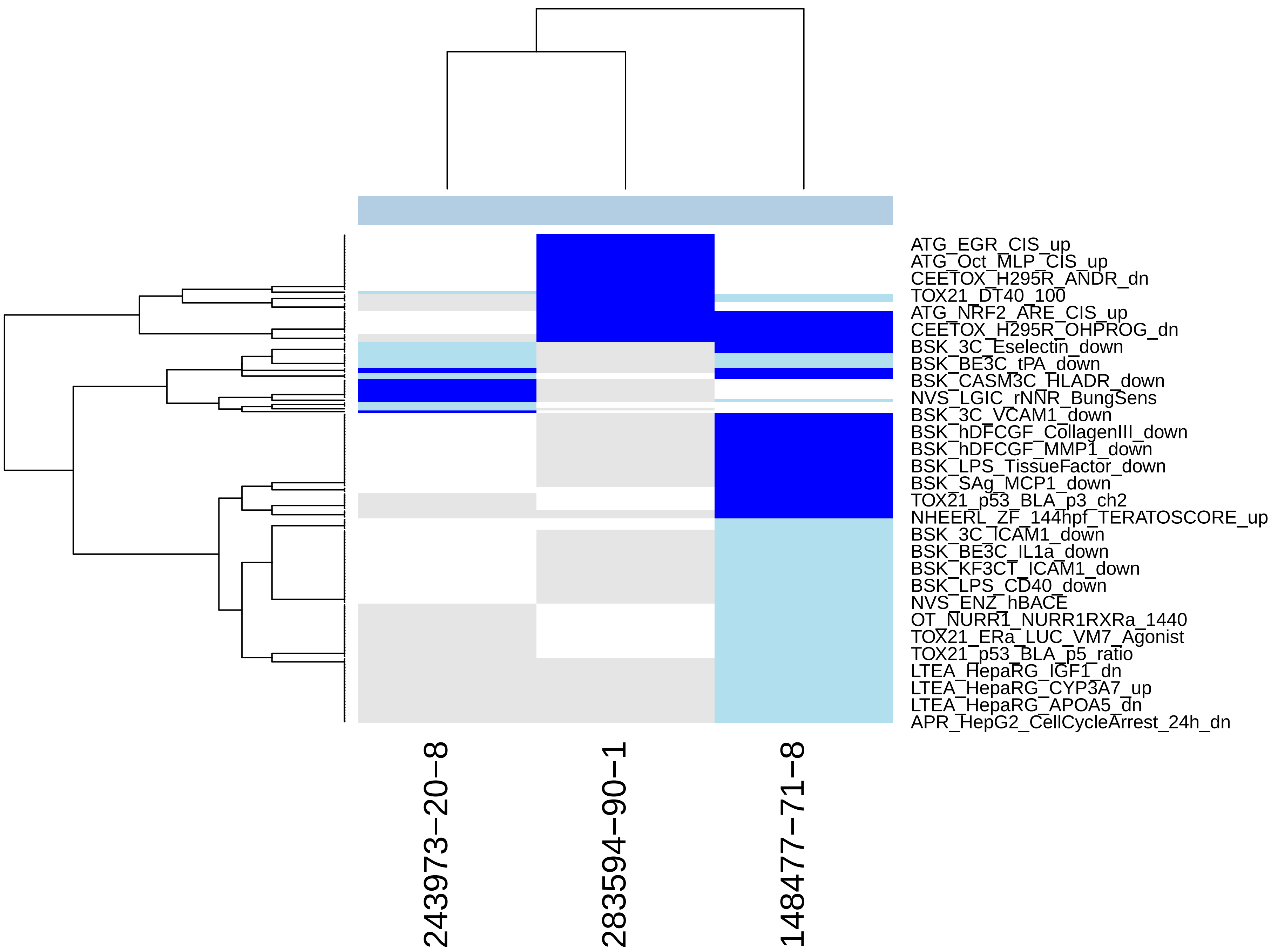

All Activity Heatmap for Cluster 1197

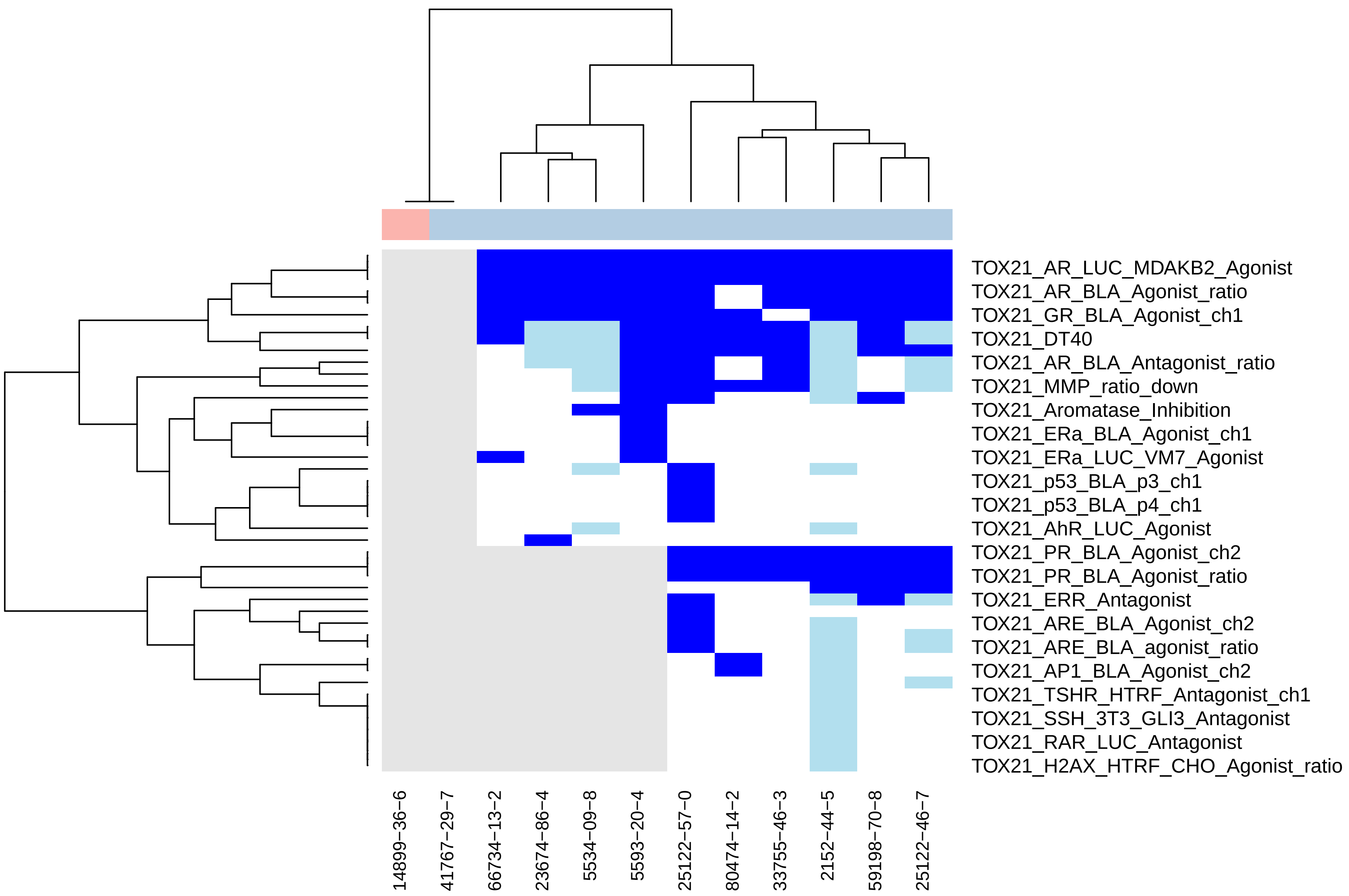

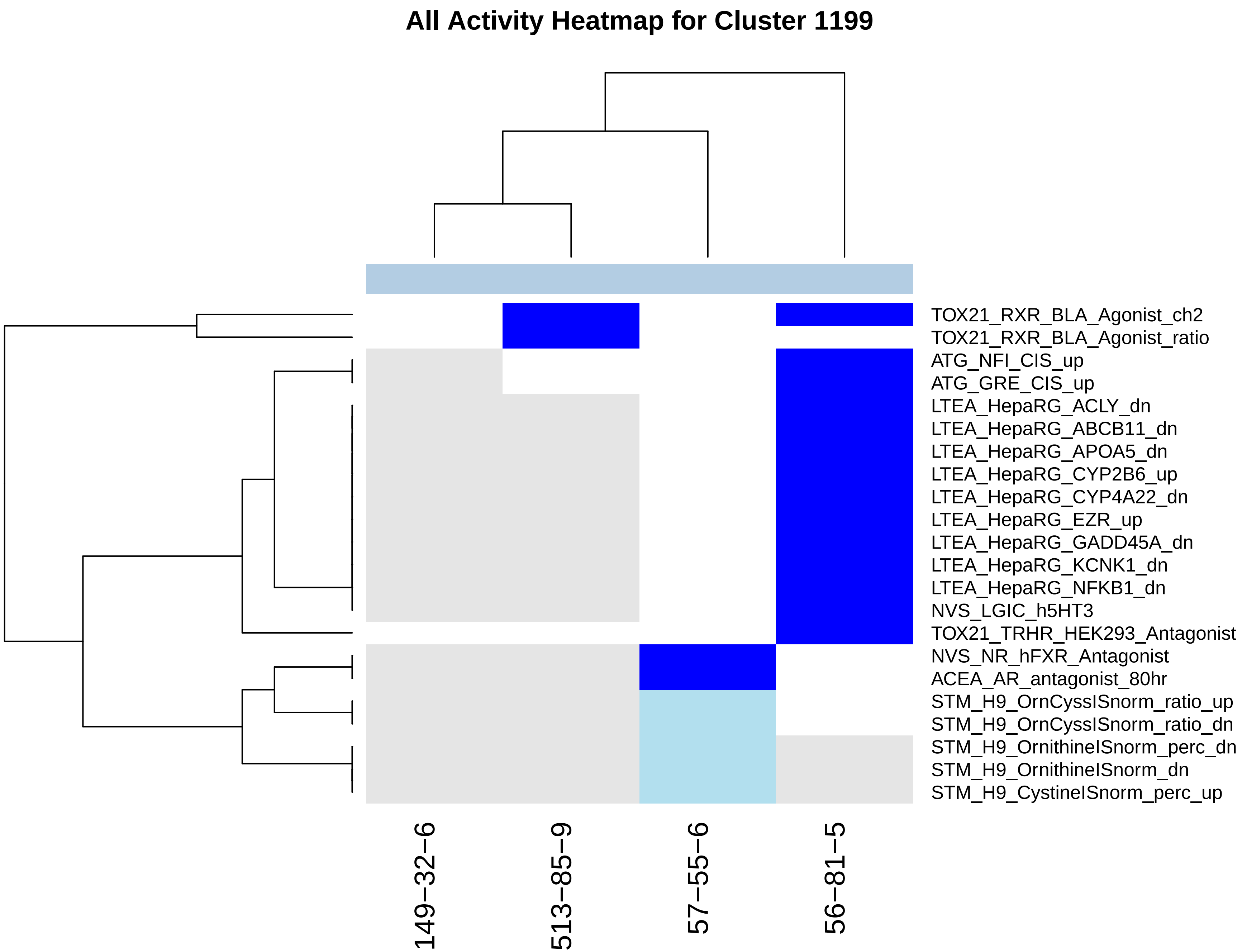
All Activity Heatmap for Cluster 1199
TOX21_RXR_BLA_Agonist_ch2
TOX21_RXR_BLA_Agonist_ratio
ATG_NFI_CIS_up
ATG_GRE_CIS_up
LTEA_HepaRG_ACLY_dn
LTEA_HepaRG_ABCB11_dn
LTEA_HepaRG_APOA5_dn
LTEA_HepaRG_CYP2B6_up
LTEA_HepaRG_CYP4A22_dn
LTEA_HepaRG_EZR_up
LTEA_HepaRG_GADD45A_dn
LTEA_HepaRG_KCNK1_dn
LTEA_HepaRG_NFKB1_dn
NVS_LGIC_h5HT3
TOX21_TRHR_HEK293_Antagonist
NVS_NR_hFXR_Antagonist
ACEA_AR_antagonist_80hr
STM_H9_OrnCyssISnorm_ratio_up
STM_H9_OrnCyssISnorm_ratio_dn
STM_H9_OrnithineISnorm_perc_dn
STM_H9_OrnithineISnorm_dn
STM_H9_CystineISnorm_perc_up
149−32−6
513−85−9
57−55−6
56−81−5

All Activity Heatmap for Cluster 1201

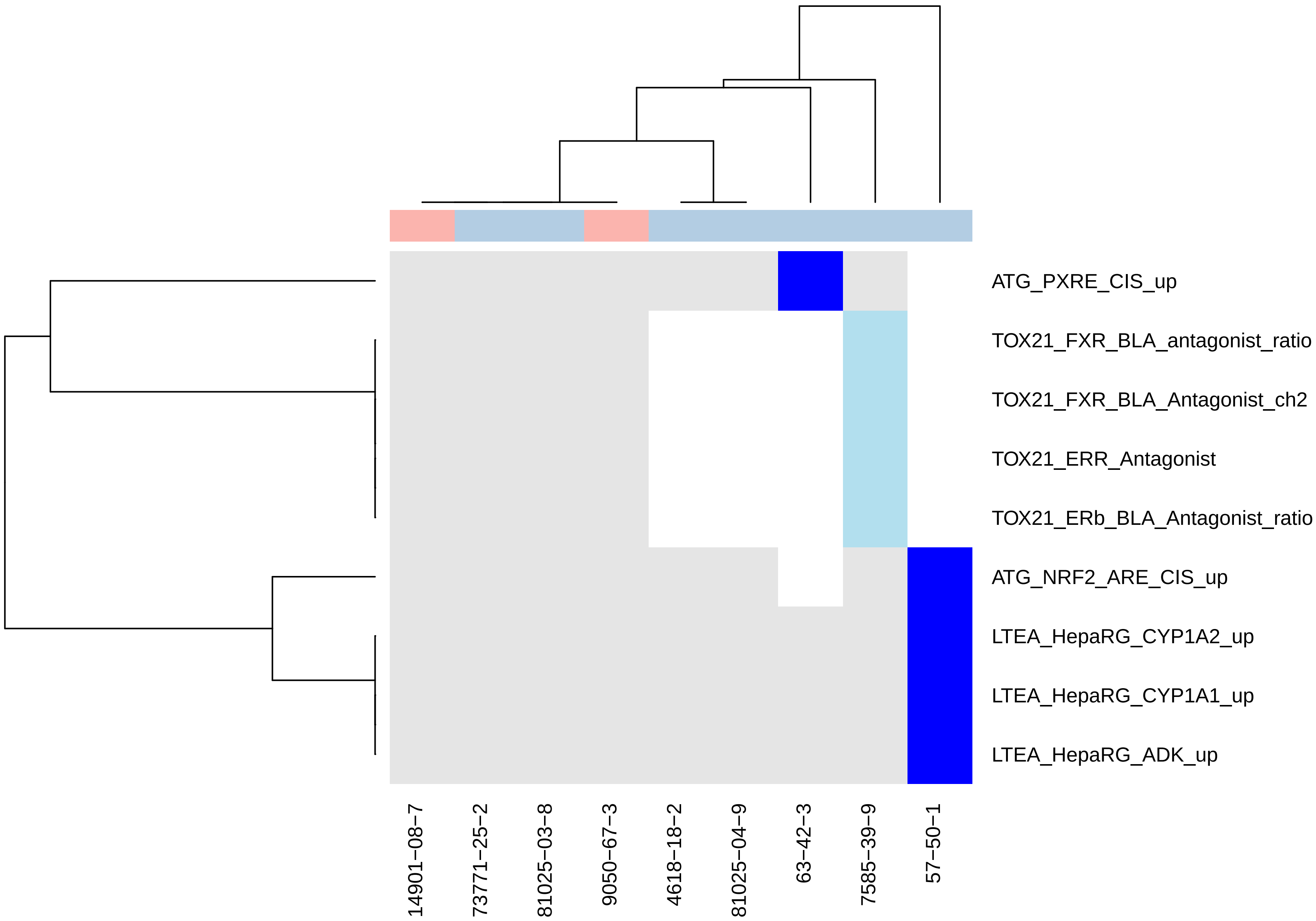

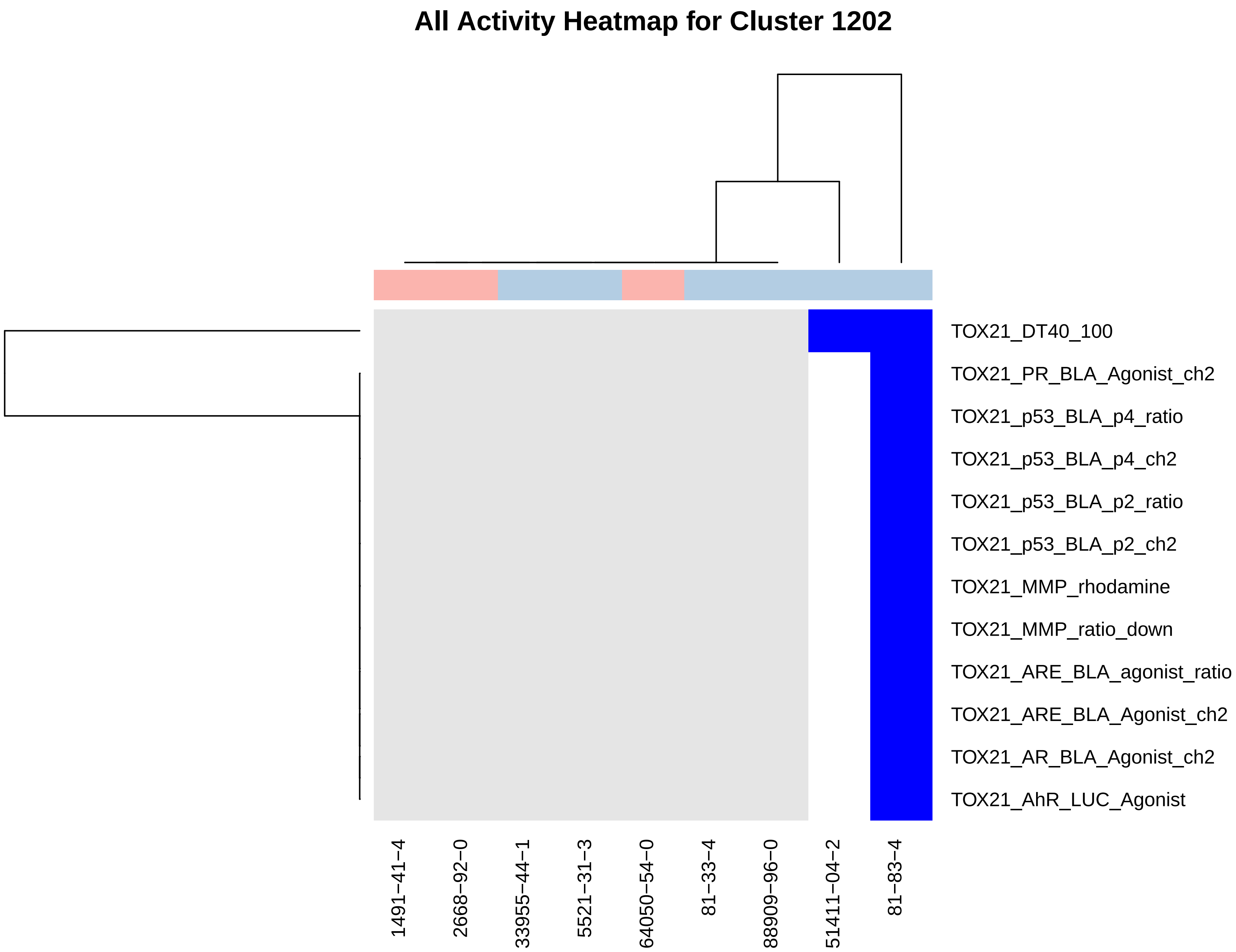
All Activity Heatmap for Cluster 1202
TOX21_DT40_100
TOX21_PR_BLA_Agonist_ch2
TOX21_p53_BLA_p4_ratio
TOX21_p53_BLA_p4_ch2
TOX21_p53_BLA_p2_ratio
TOX21_p53_BLA_p2_ch2
TOX21_MMP_rhodamine
TOX21_MMP_ratio_down
TOX21_ARE_BLA_agonist_ratio
TOX21_ARE_BLA_Agonist_ch2
TOX21_AR_BLA_Agonist_ch2
TOX21_AhR_LUC_Agonist
1491−41−4
2668−92−0
33955−44−1
5521−31−3
64050−54−0
81−33−4
88909−96−0
51411−04−2
81−83−4

All Activity Heatmap for Cluster 1203

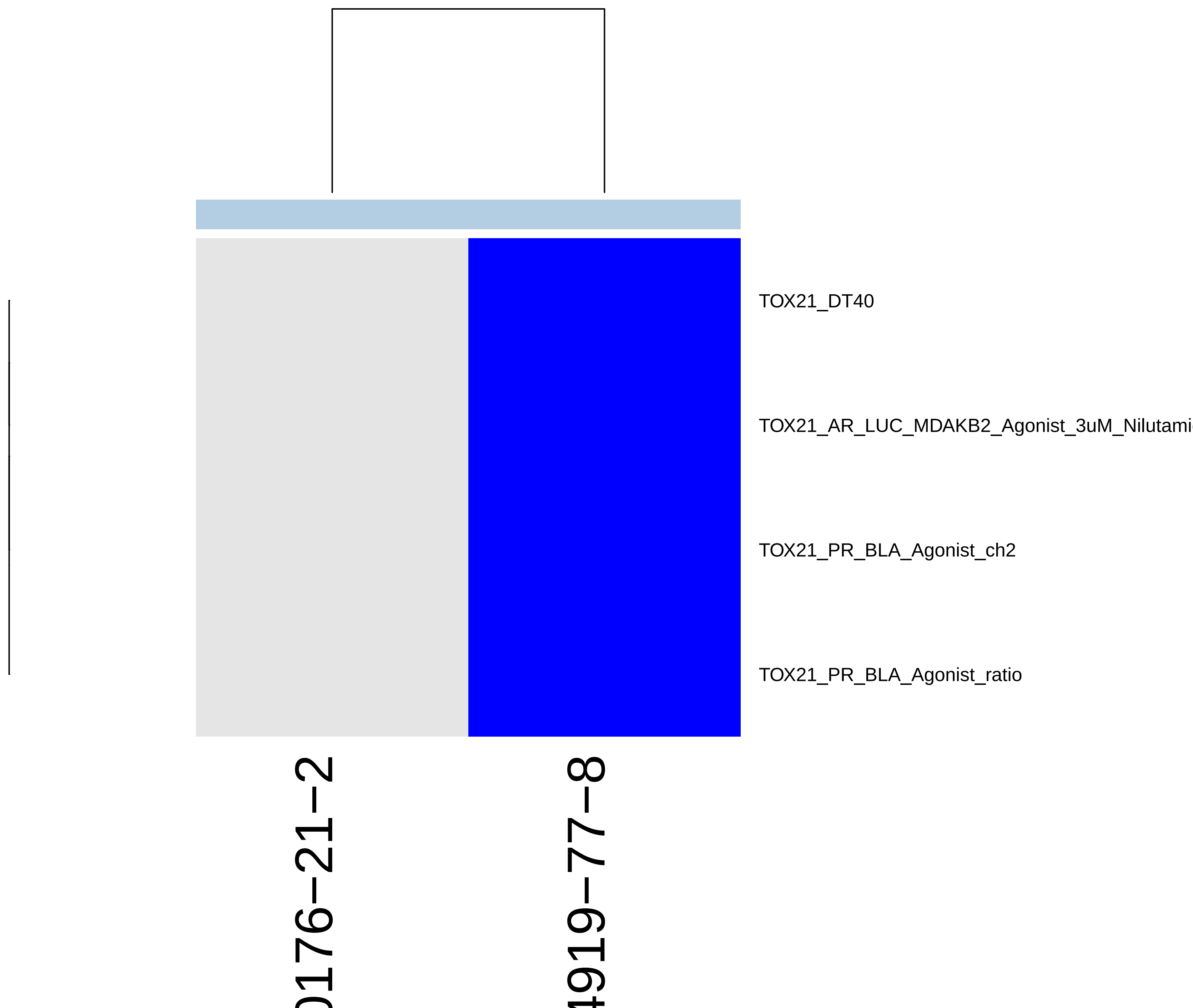

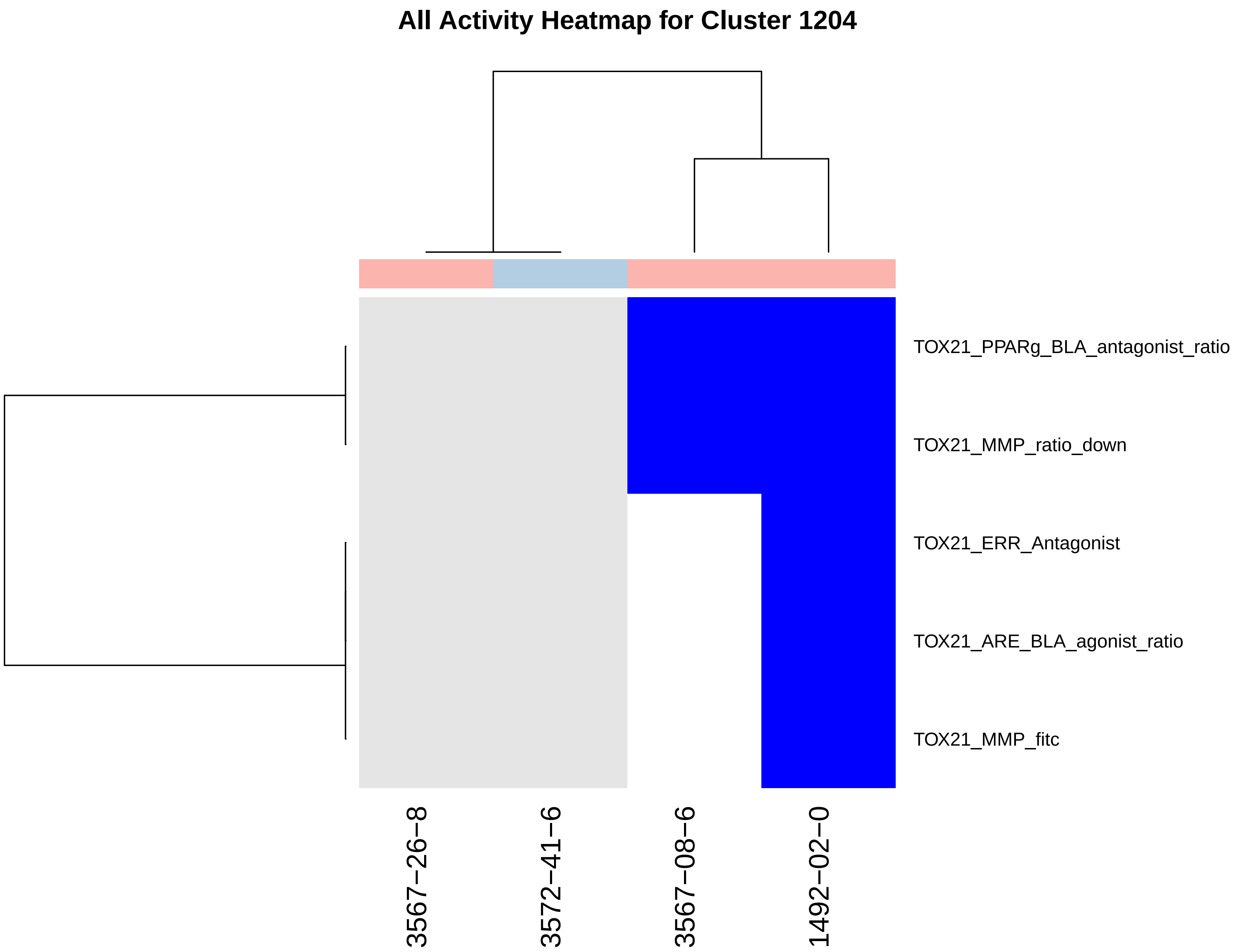
All Activity Heatmap for Cluster 1204
TOX21_PPARg_BLA_antagonist_ratio
TOX21_MMP_ratio_down
TOX21_ERR_Antagonist
TOX21_ARE_BLA_agonist_ratio
TOX21_MMP_fitc
3567−26−8
3572−41−6
3567−08−6
1492−02−0

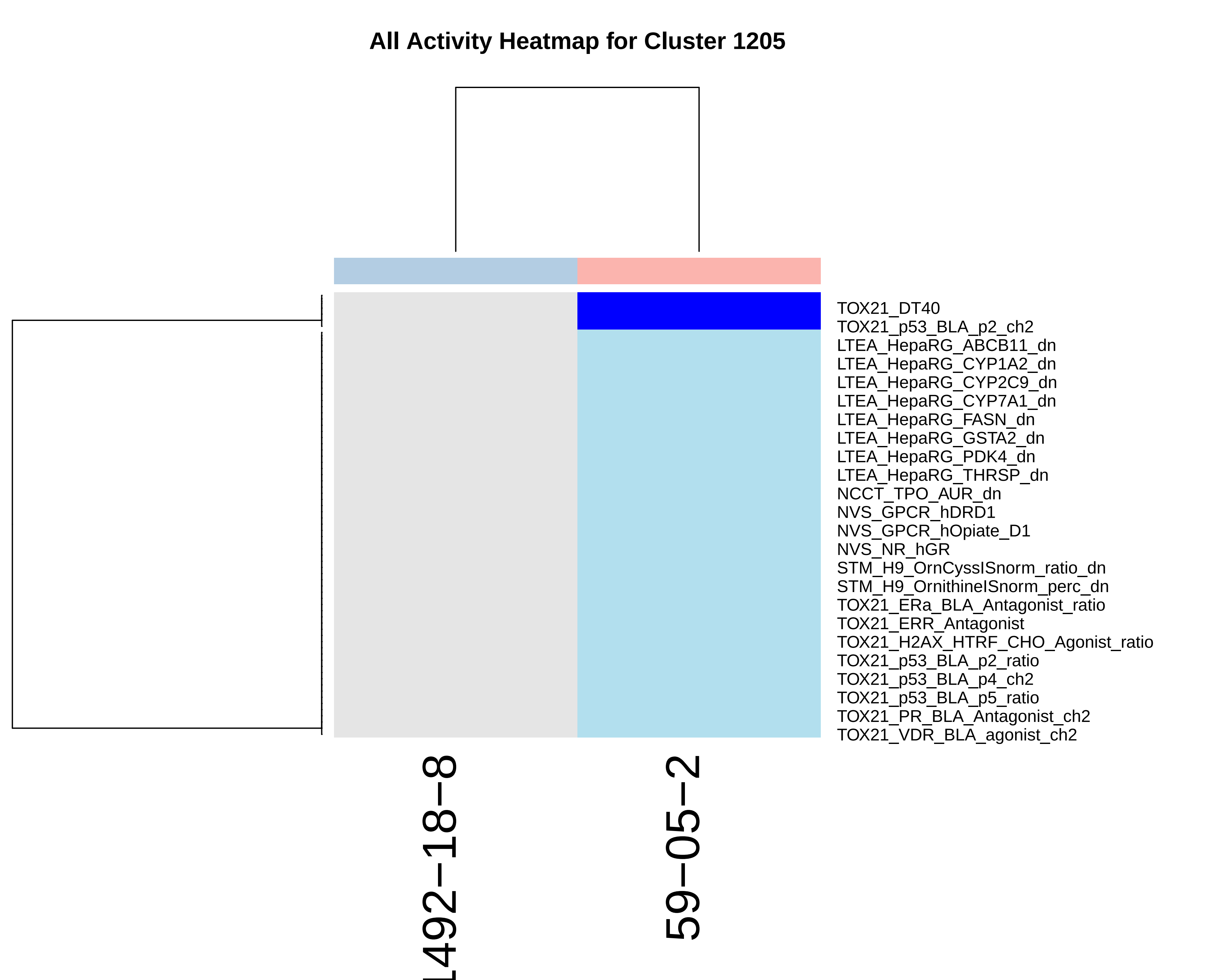
All Activity Heatmap for Cluster 1205
TOX21_DT40
TOX21_p53_BLA_p2_ch2
LTEA_HepaRG_ABCB11_dn
LTEA_HepaRG_CYP1A2_dn
LTEA_HepaRG_CYP2C9_dn
LTEA_HepaRG_CYP7A1_dn
LTEA_HepaRG_FASN_dn
LTEA_HepaRG_GSTA2_dn
LTEA_HepaRG_PDK4_dn
LTEA_HepaRG_THRSP_dn
NCCT_TPO_AUR_dn
NVS_GPCR_hDRD1
NVS_GPCR_hOpiate_D1
NVS_NR_hGR
STM_H9_OrnCyssISnorm_ratio_dn
STM_H9_OrnithineISnorm_perc_dn
TOX21_ERa_BLA_Antagonist_ratio
TOX21_ERR_Antagonist
TOX21_H2AX_HTRF_CHO_Agonist_ratio
TOX21_p53_BLA_p2_ratio
TOX21_p53_BLA_p4_ch2
TOX21_p53_BLA_p5_ratio
TOX21_PR_BLA_Antagonist_ch2
TOX21_VDR_BLA_agonist_ch2
1492−18−8
59−05−2

All Activity Heatmap for Cluster 1207

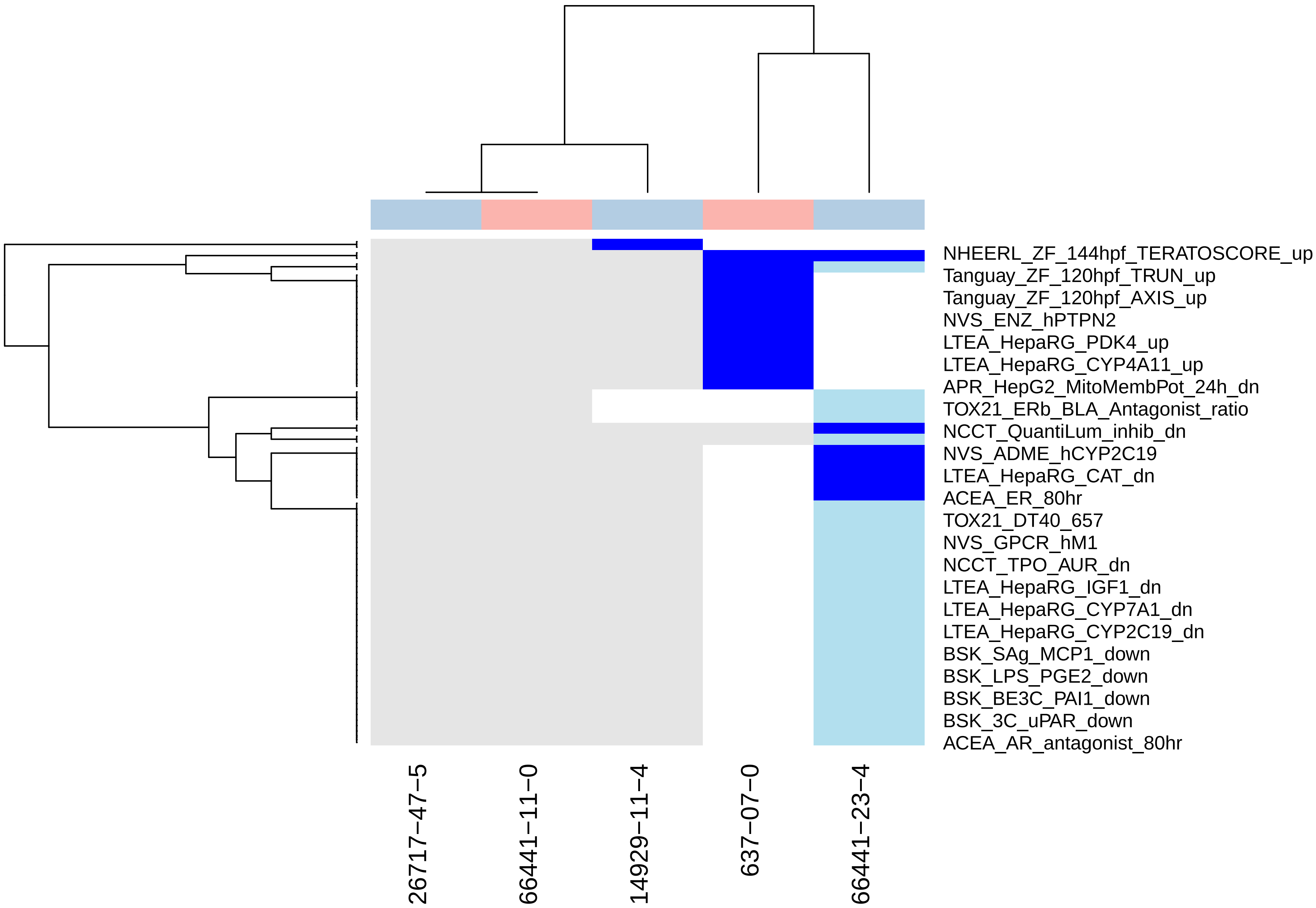

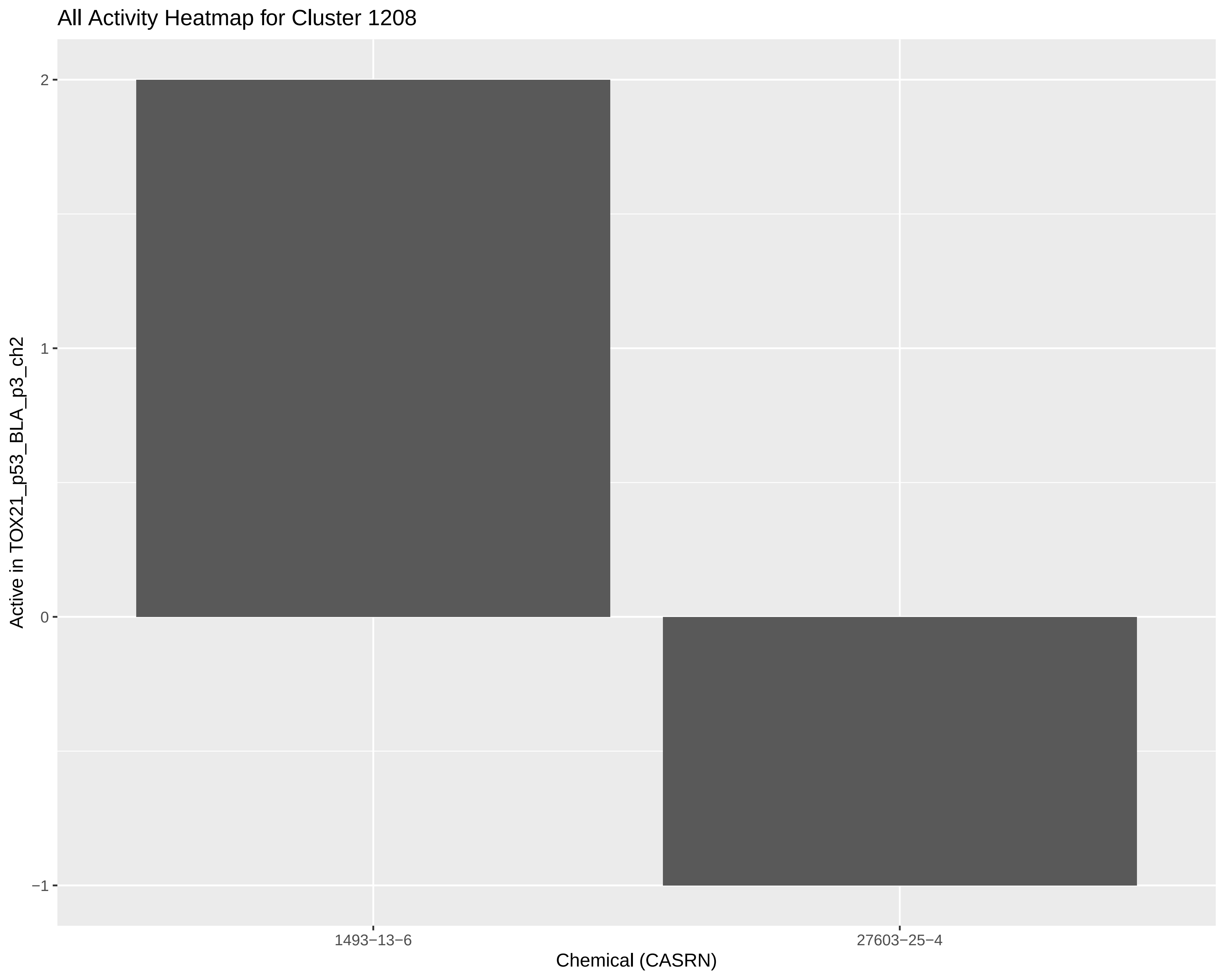
All Activity Heatmap for Cluster 1208
Active in TOX21_p53_BLA_p3_ch2
2
1
0
−1
1493−13−6
27603−25−4
Chemical (CASRN)

All Activity Heatmap for Cluster 1209

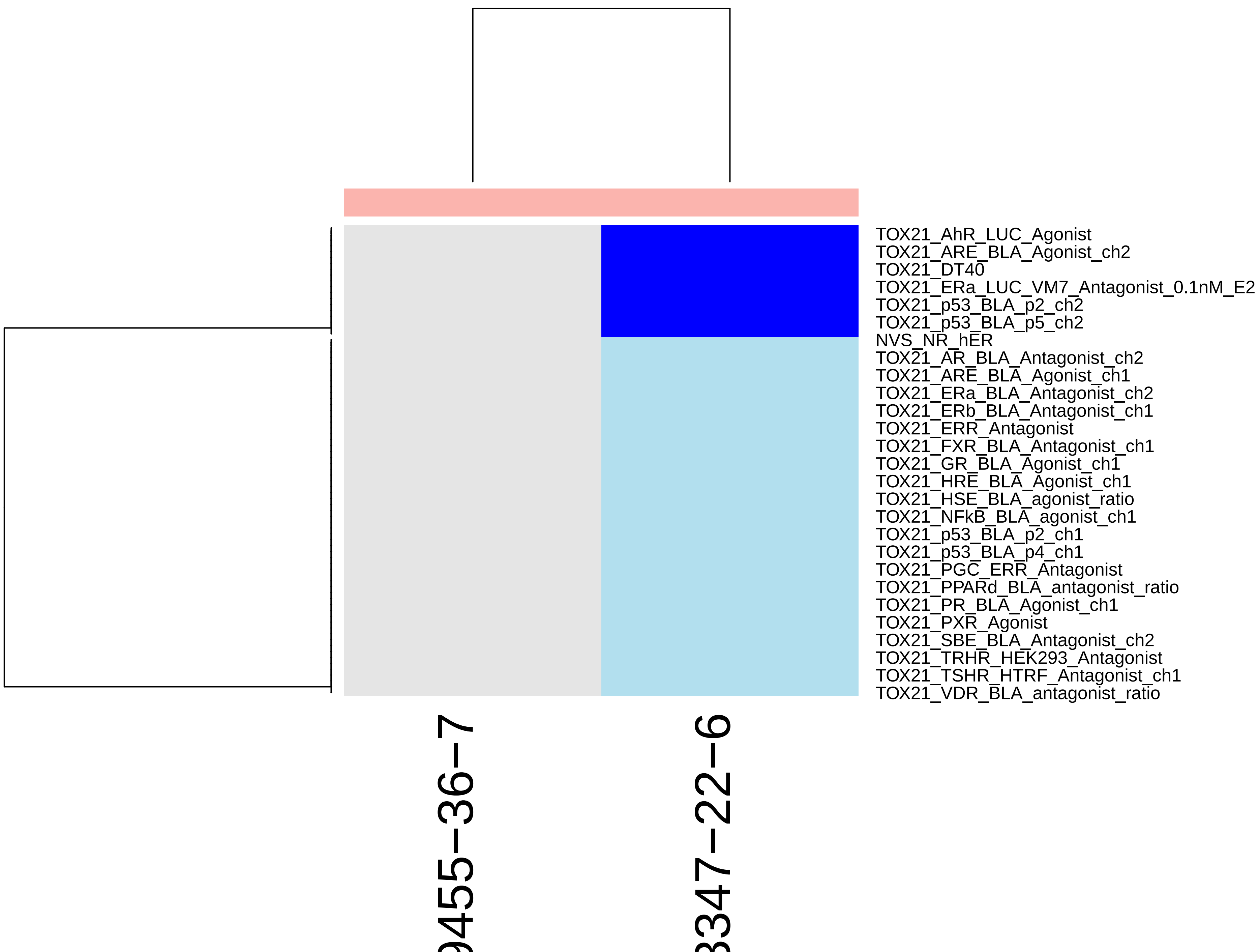

All Activity Heatmap for Cluster 1210

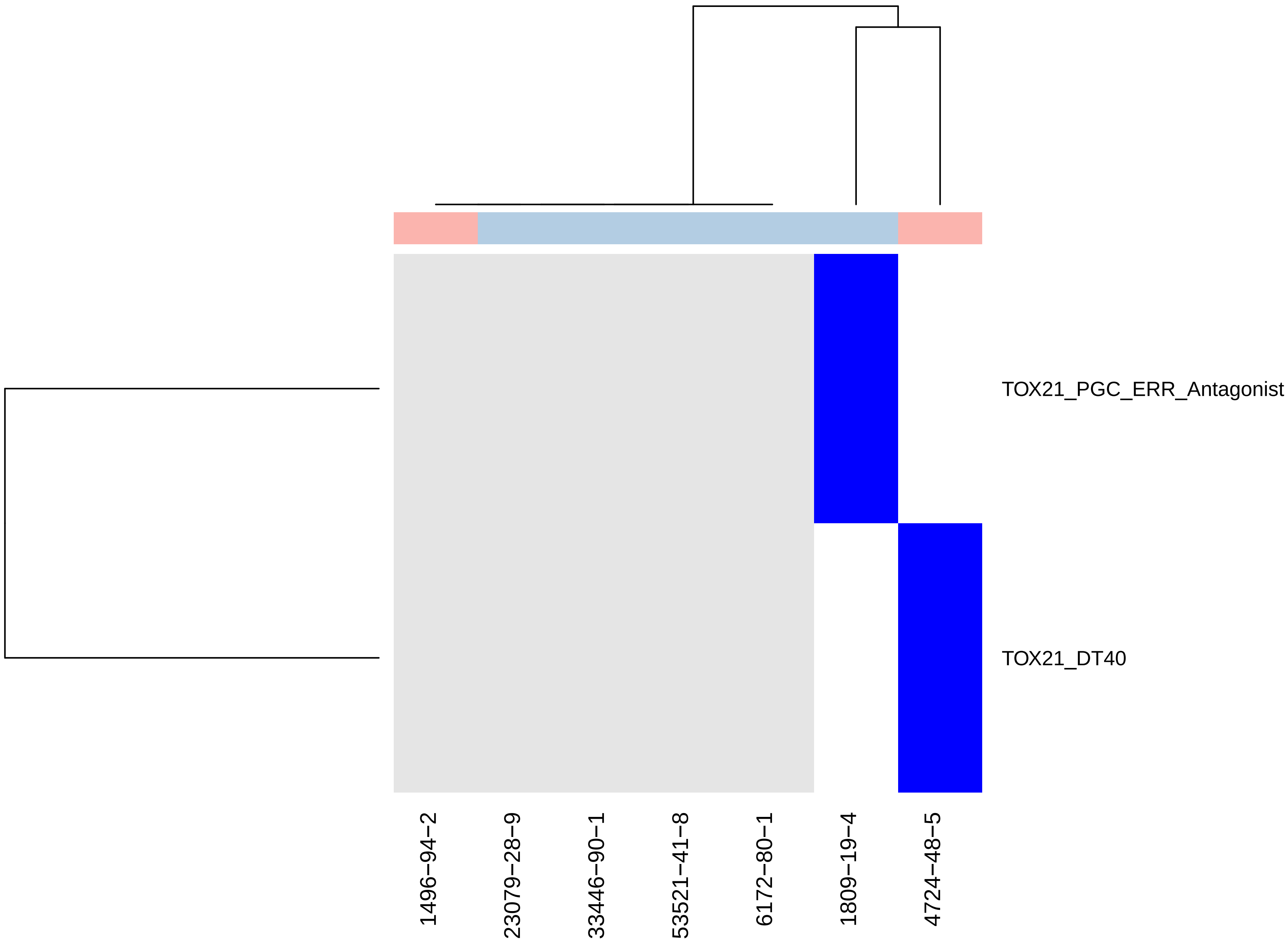

All Activity Heatmap for Cluster 1211

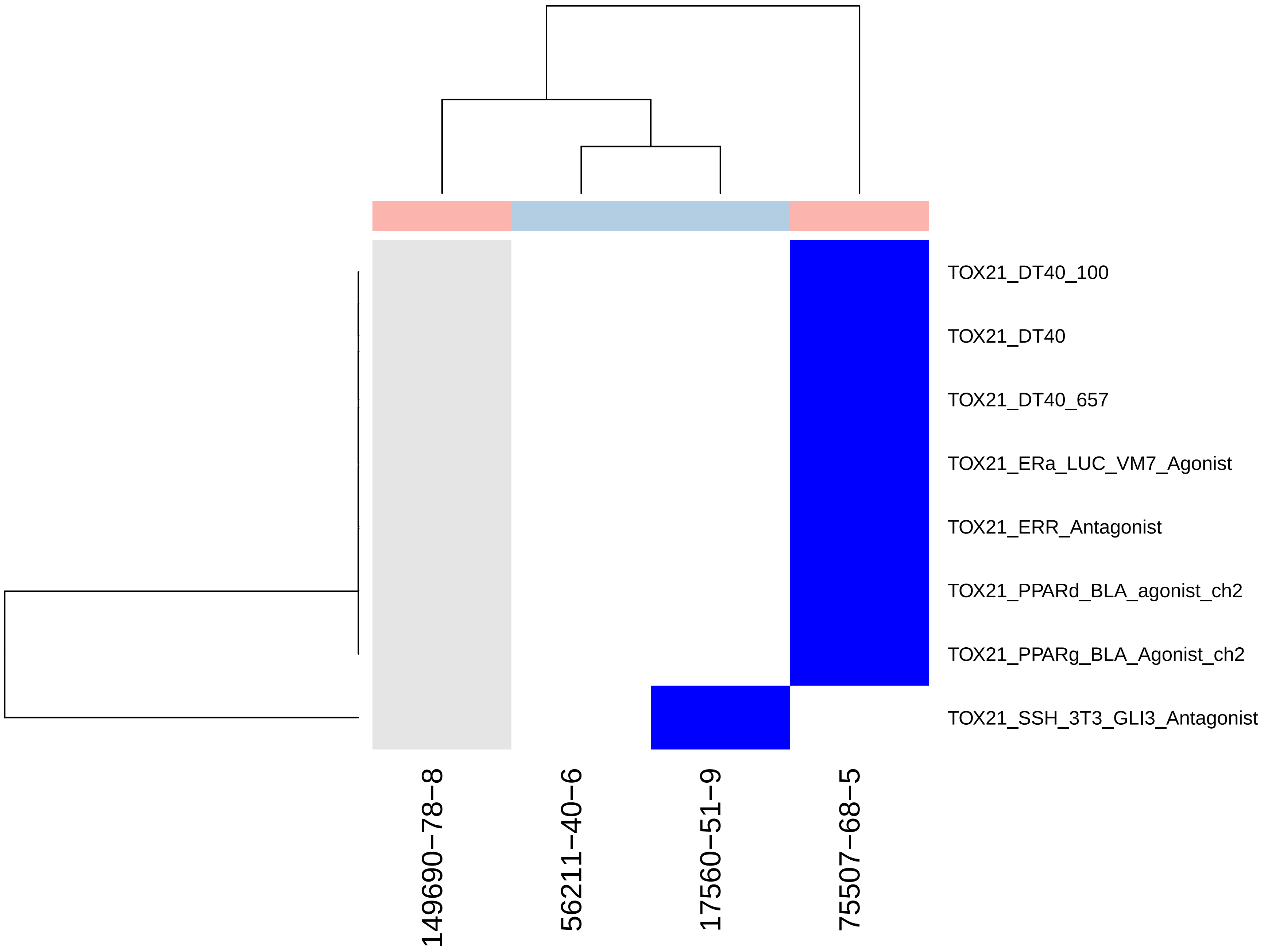

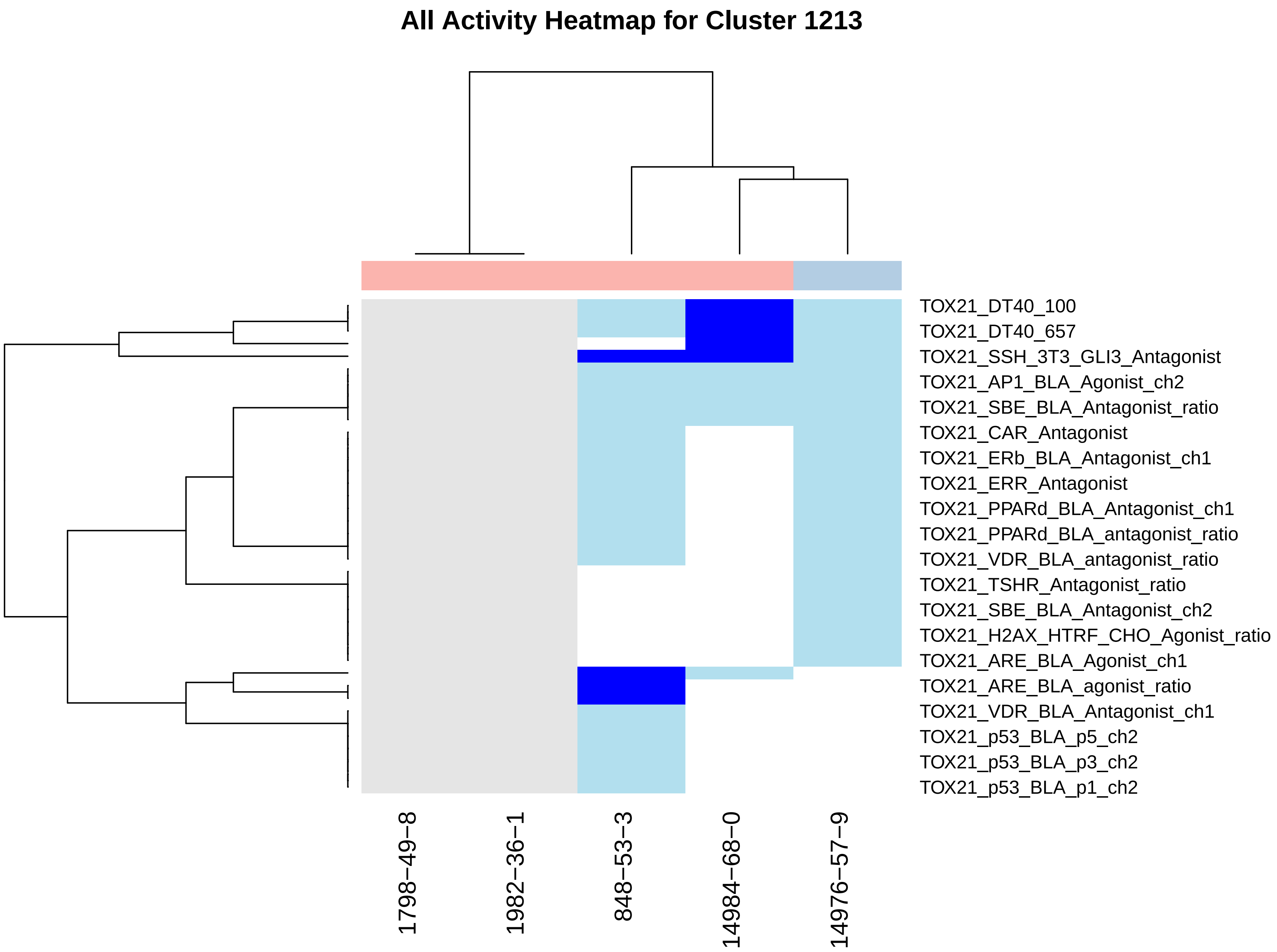

All Activity Heatmap for Cluster 1213
TOX21_DT40_100
TOX21_DT40_657
TOX21_SSH_3T3_GLI3_Antagonist
TOX21_AP1_BLA_Agonist_ch2
TOX21_SBE_BLA_Antagonist_ratio
TOX21_CAR_Antagonist
TOX21_ERb_BLA_Antagonist_ch1
TOX21_ERR_Antagonist
TOX21_PPARd_BLA_Antagonist_ch1
TOX21_PPARd_BLA_antagonist_ratio
TOX21_VDR_BLA_antagonist_ratio
TOX21_TSHR_Antagonist_ratio
TOX21_SBE_BLA_Antagonist_ch2
TOX21_H2AX_HTRF_CHO_Agonist_ratio
TOX21_ARE_BLA_Agonist_ch1
TOX21_ARE_BLA_agonist_ratio
TOX21_VDR_BLA_Antagonist_ch1
TOX21_p53_BLA_p5_ch2
TOX21_p53_BLA_p3_ch2
TOX21_p53_BLA_p1_ch2
1798−49−8
1982−36−1
848−53−3
14984−68−0
14976−57−9

All Activity Heatmap for Cluster 1215

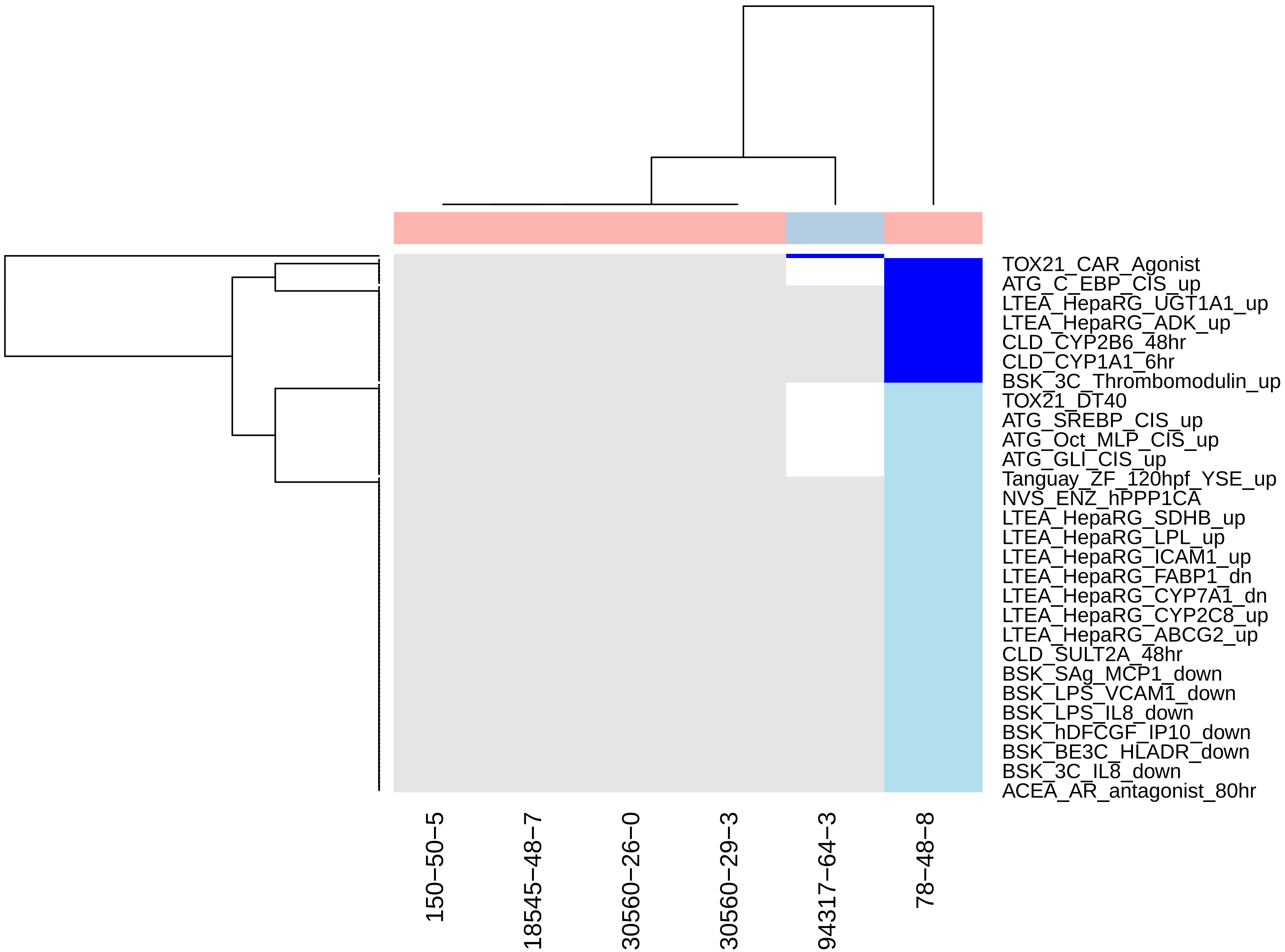

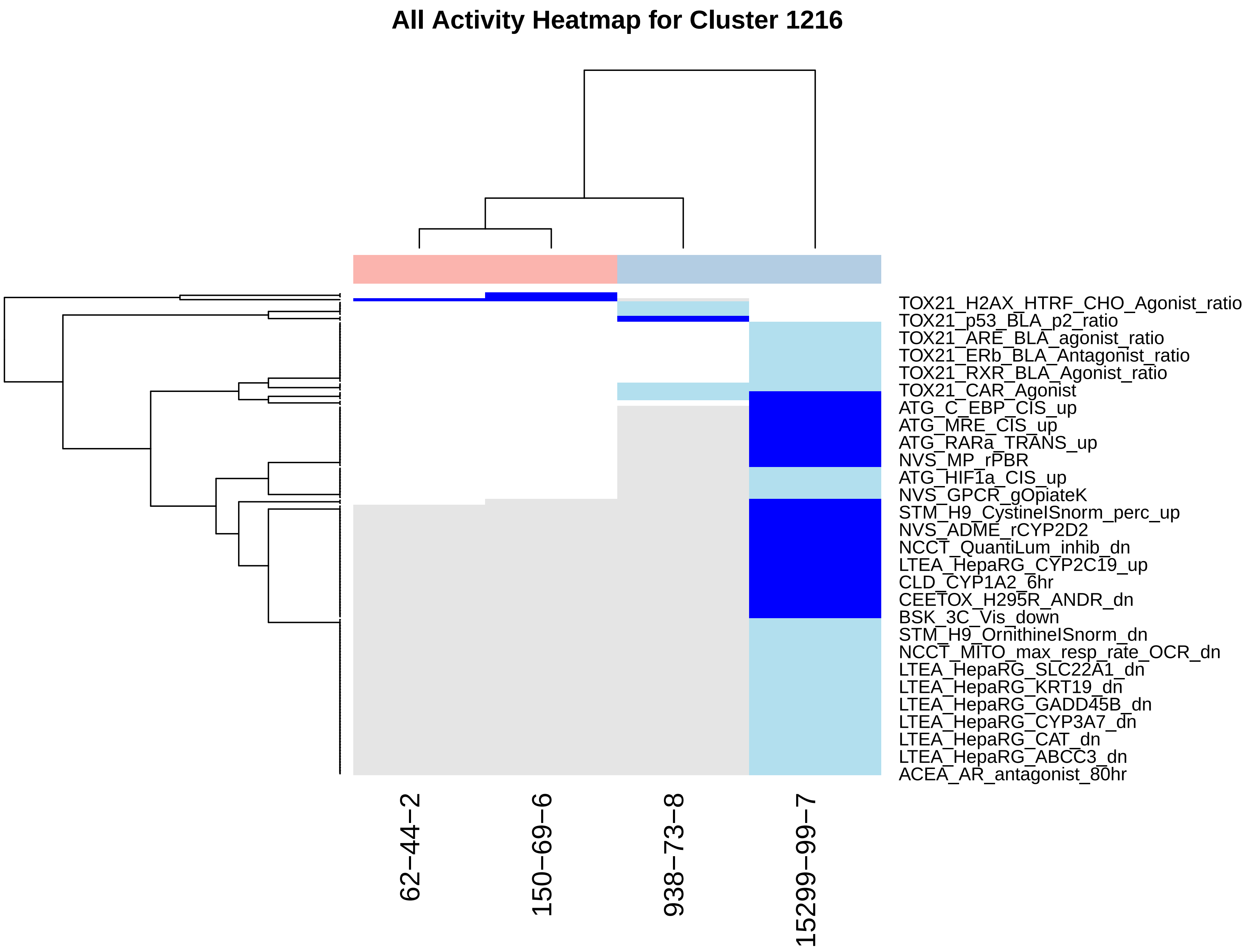
All Activity Heatmap for Cluster 1216
TOX21_H2AX_HTRF_CHO_Agonist_ratio
TOX21_p53_BLA_p2_ratio
TOX21_ARE_BLA_agonist_ratio
TOX21_ERb_BLA_Antagonist_ratio
TOX21_RXR_BLA_Agonist_ratio
TOX21_CAR_Agonist
ATG_C_EBP_CIS_up
ATG_MRE_CIS_up
ATG_RARa_TRANS_up
NVS_MP_rPBR
ATG_HIF1a_CIS_up
NVS_GPCR_gOpiateK
STM_H9_CystineISnorm_perc_up
NVS_ADME_rCYP2D2
NCCT_QuantiLum_inhib_dn
LTEA_HepaRG_CYP2C19_up
CLD_CYP1A2_6hr
CEETOX_H295R_ANDR_dn
BSK_3C_Vis_down
STM_H9_OrnithineISnorm_dn
NCCT_MITO_max_resp_rate_OCR_dn
LTEA_HepaRG_SLC22A1_dn
LTEA_HepaRG_KRT19_dn
LTEA_HepaRG_GADD45B_dn
LTEA_HepaRG_CYP3A7_dn
LTEA_HepaRG_CAT_dn
LTEA_HepaRG_ABCC3_dn
ACEA_AR_antagonist_80hr
62−44−2
150−69−6
938−73−8
15299−99−7

All Activity Heatmap for Cluster 1217

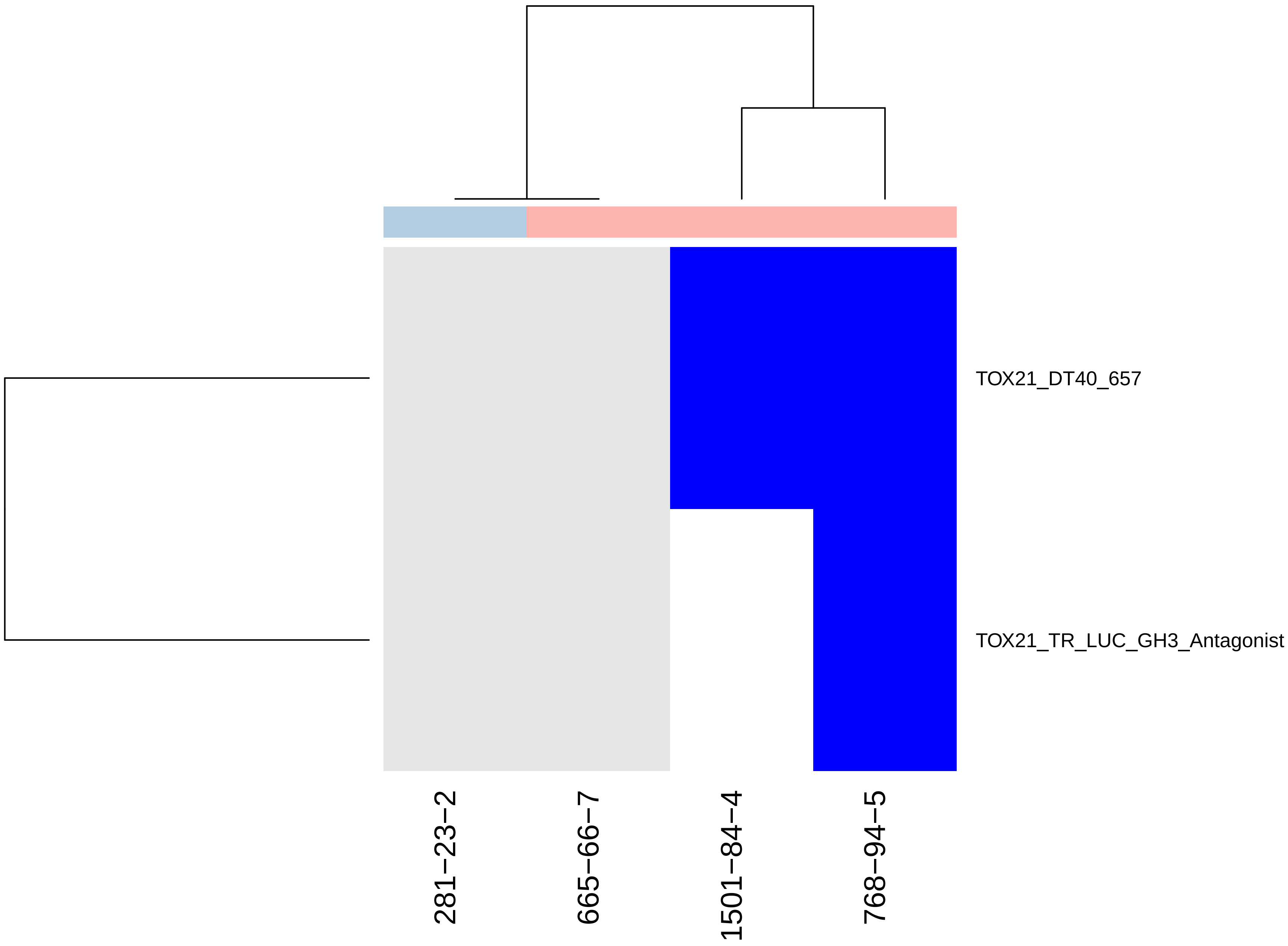

All Activity Heatmap for Cluster 1218

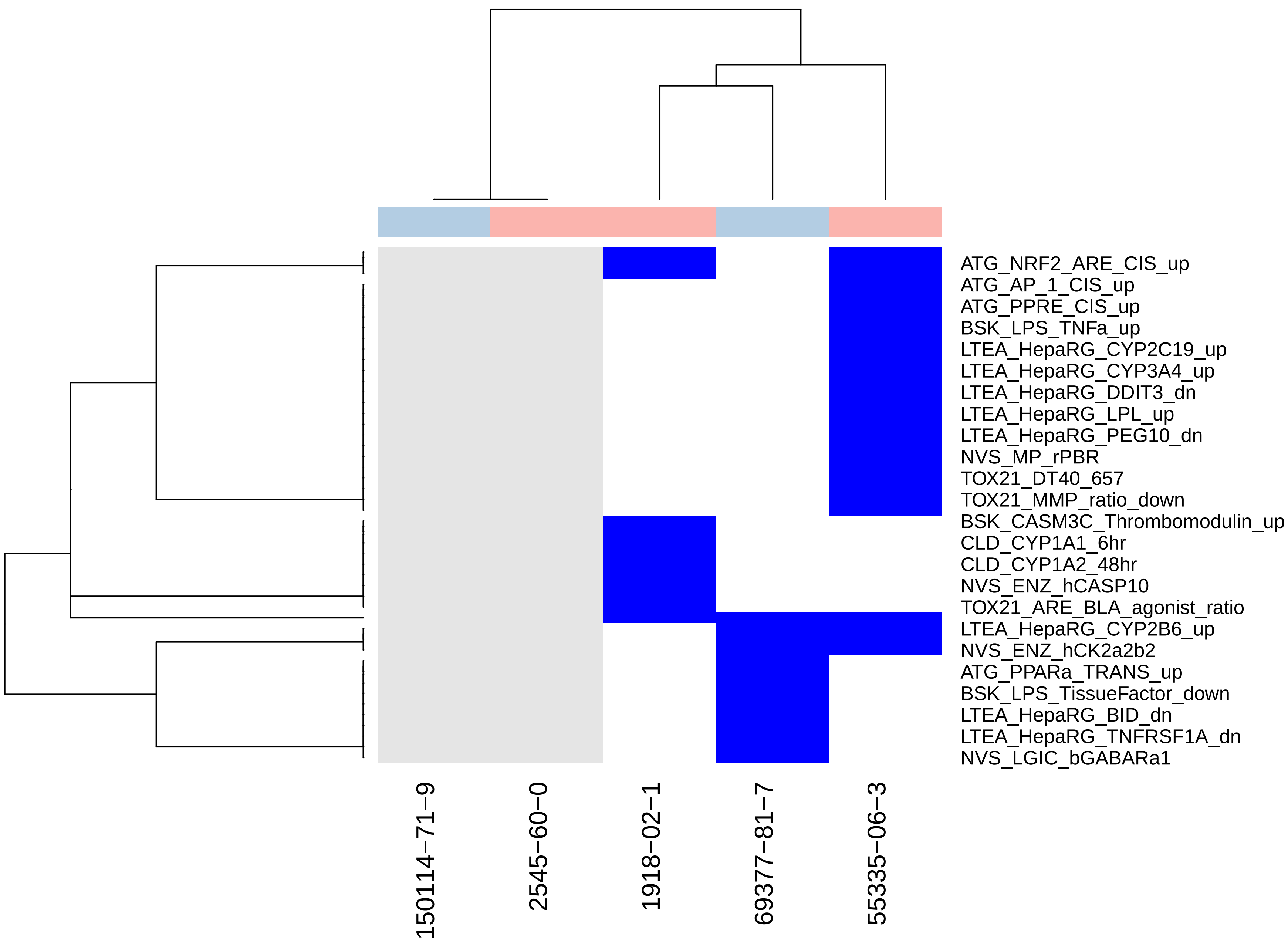

All Activity Heatmap for Cluster 1219

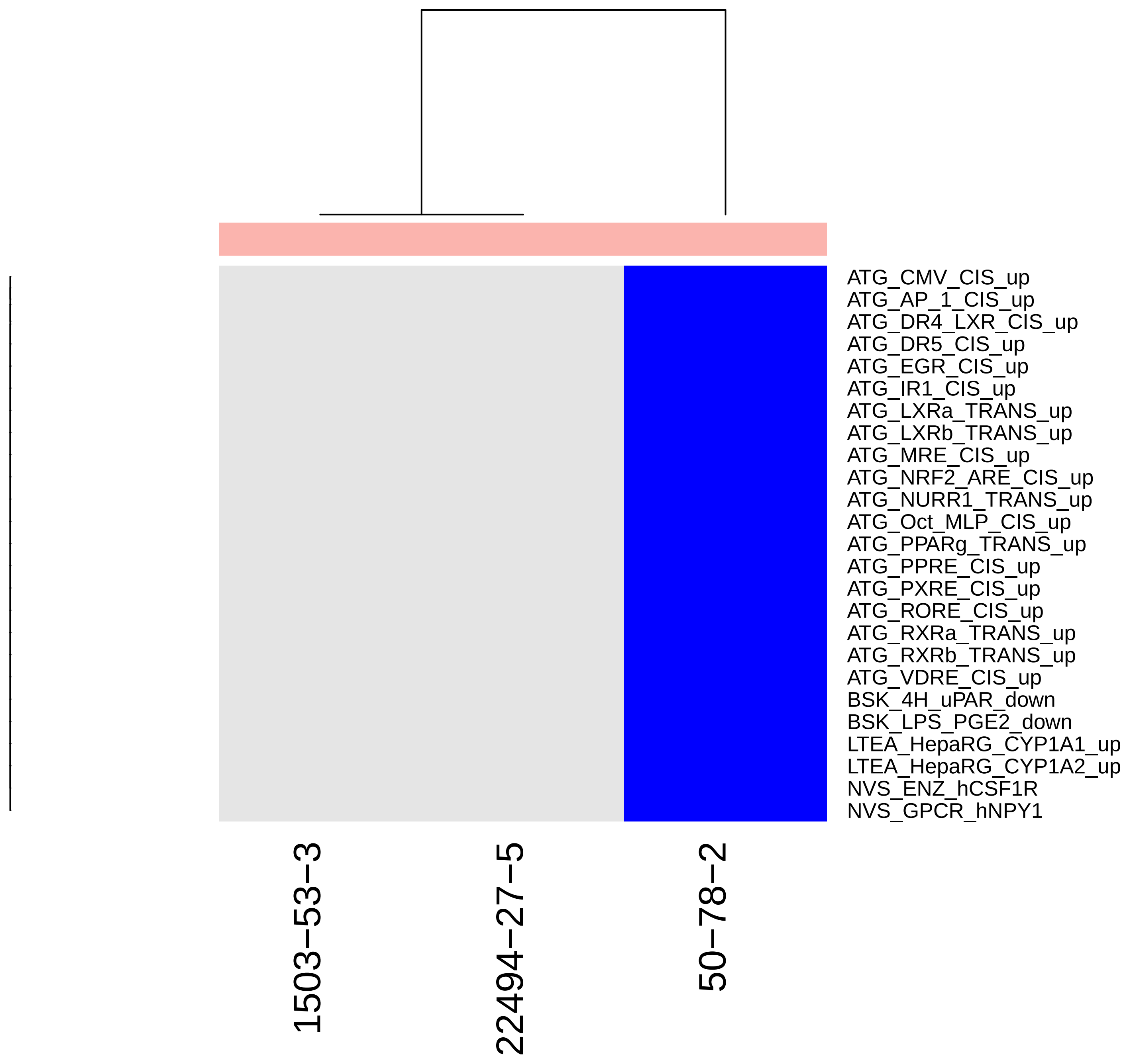

All Activity Heatmap for Cluster 1220

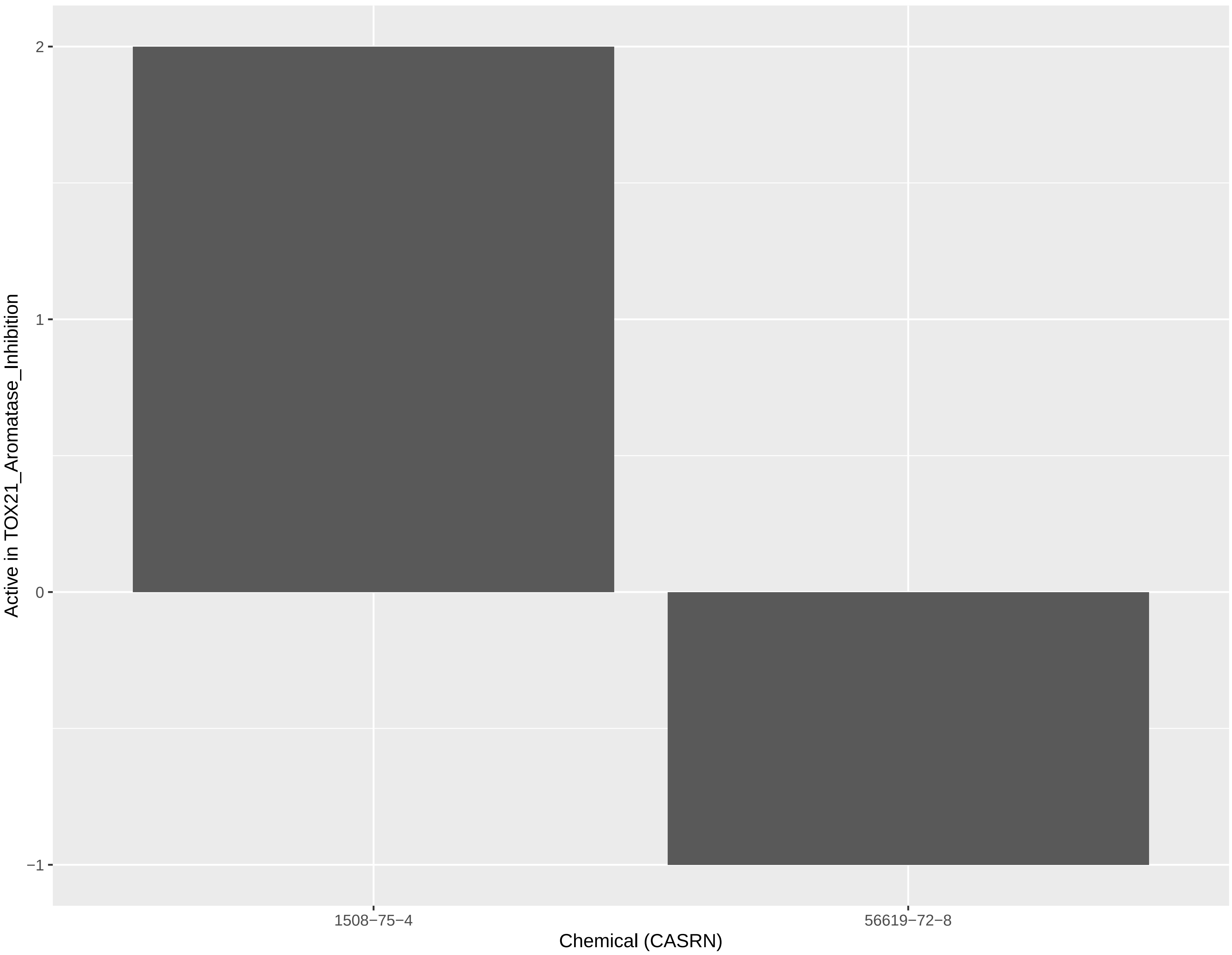

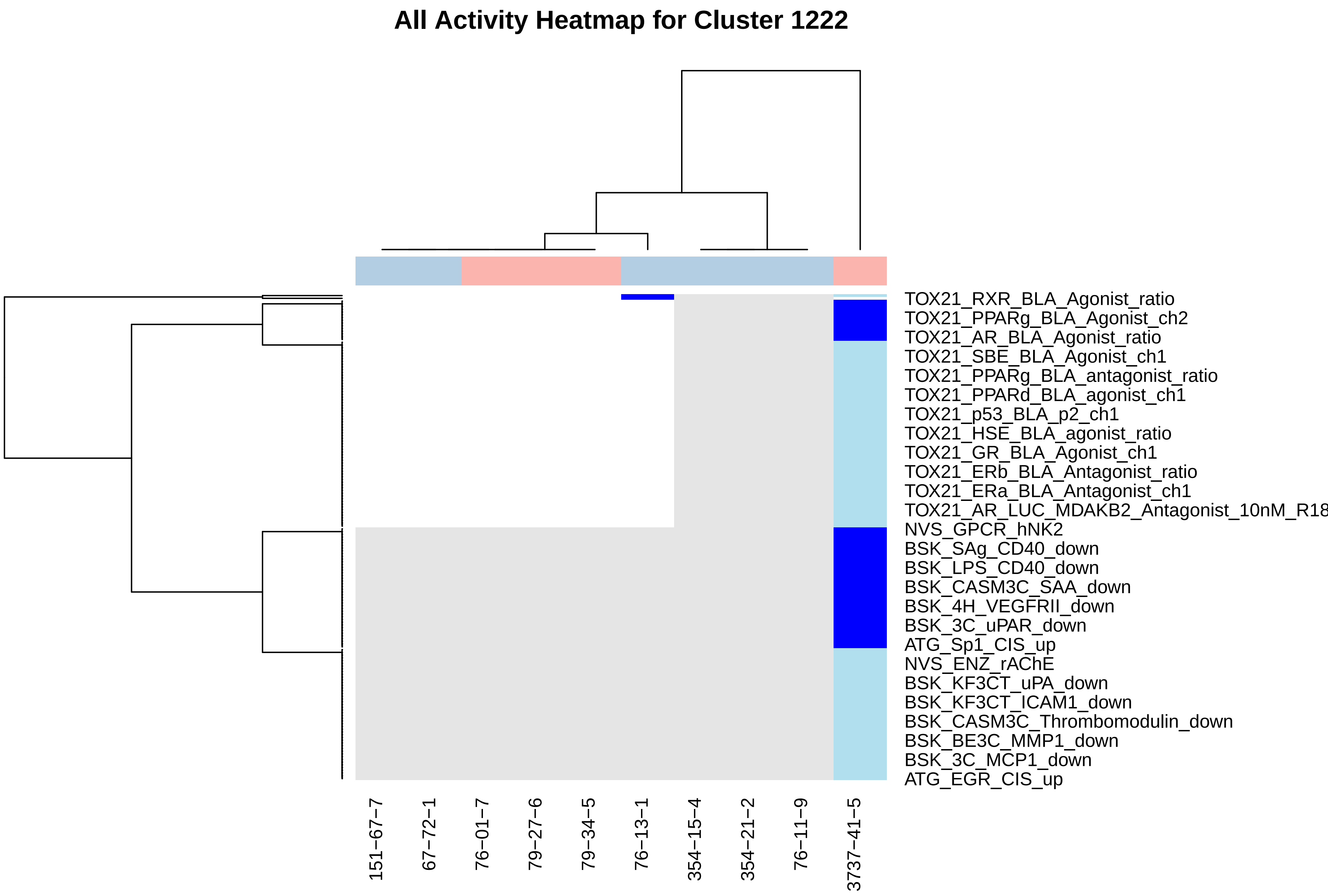
All Activity Heatmap for Cluster 1222
TOX21_RXR_BLA_Agonist_ratio
TOX21_PPARg_BLA_Agonist_ch2
TOX21_AR_BLA_Agonist_ratio
TOX21_SBE_BLA_Agonist_ch1
TOX21_PPARg_BLA_antagonist_ratio
TOX21_PPARd_BLA_agonist_ch1
TOX21_p53_BLA_p2_ch1
TOX21_HSE_BLA_agonist_ratio
TOX21_GR_BLA_Agonist_ch1
TOX21_ERb_BLA_Antagonist_ratio
TOX21_ERa_BLA_Antagonist_ch1
TOX21_AR_LUC_MDAKB2_Antagonist_10nM_R18
NVS_GPCR_hNK2
BSK_SAg_CD40_down
BSK_LPS_CD40_down
BSK_CASM3C_SAA_down
BSK_4H_VEGFRII_down
BSK_3C_uPAR_down
ATG_Sp1_CIS_up
NVS_ENZ_rAChE
BSK_KF3CT_uPA_down
BSK_KF3CT_ICAM1_down
BSK_CASM3C_Thrombomodulin_down
BSK_BE3C_MMP1_down
BSK_3C_MCP1_down
ATG_EGR_CIS_up
151−67−7
67−72−1
76−01−7
79−27−6
79−34−5
76−13−1
354−15−4
354−21−2
76−11−9
3737−41−5

All Activity Heatmap for Cluster 1223

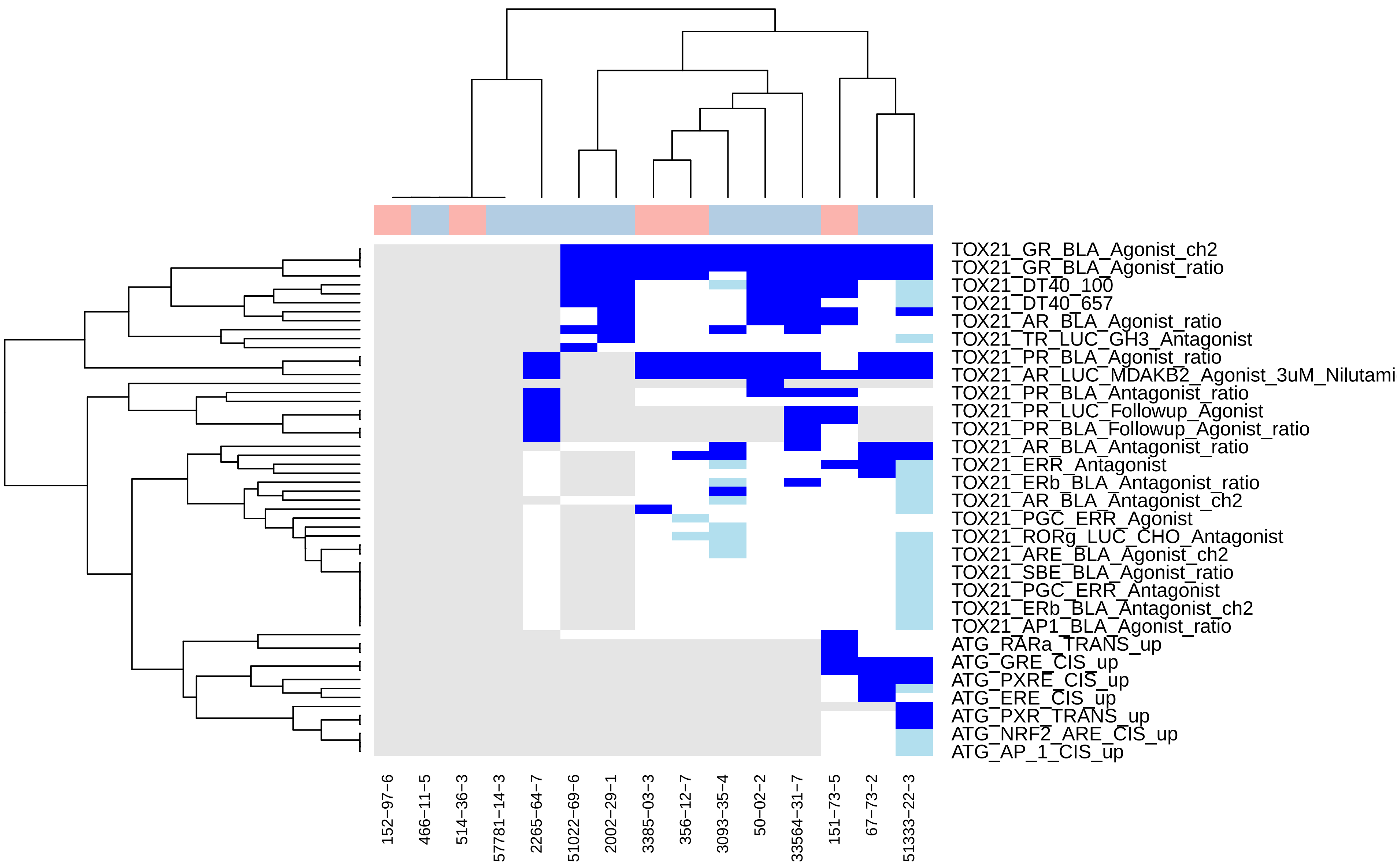

All Activity Heatmap for Cluster 1225

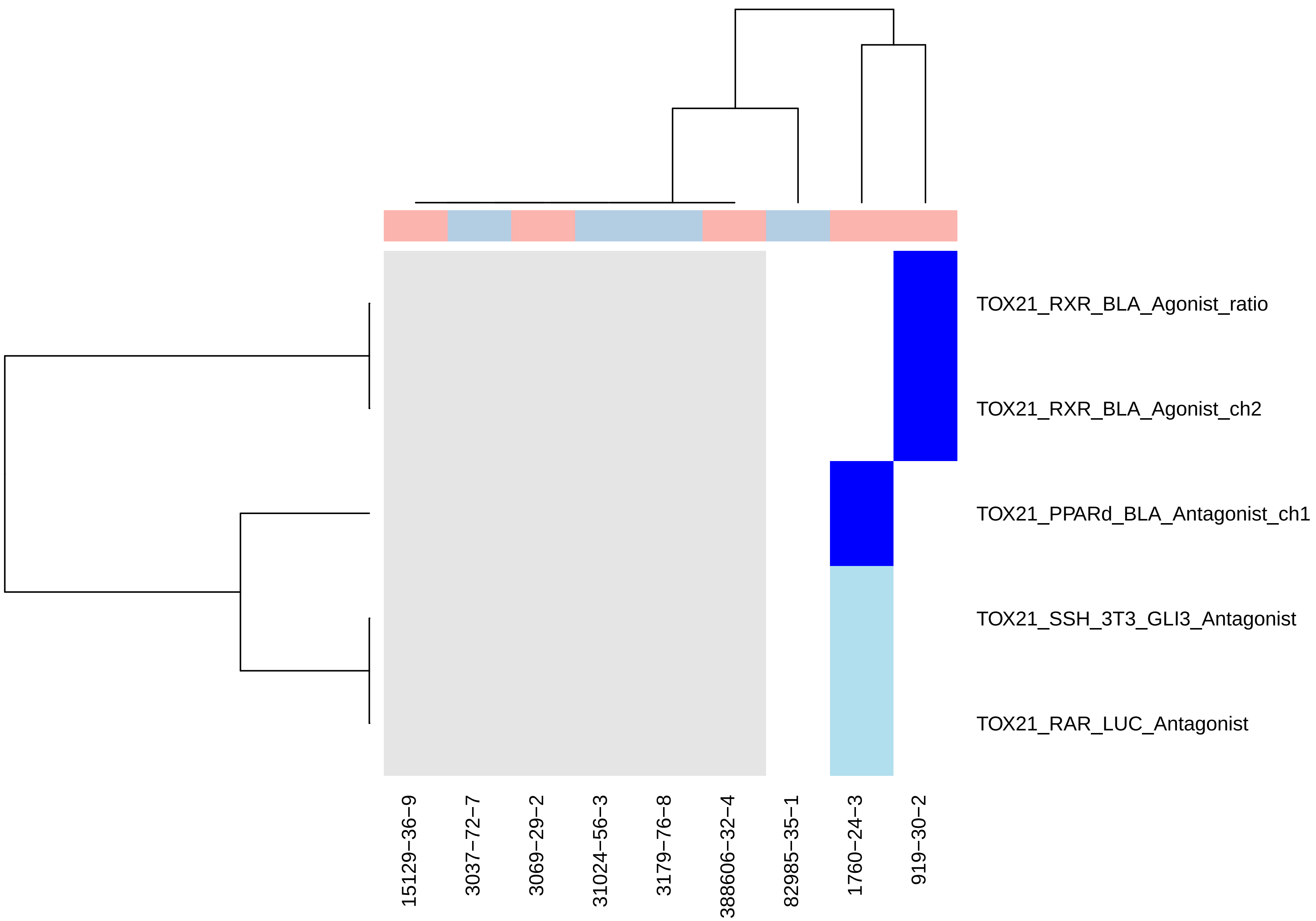

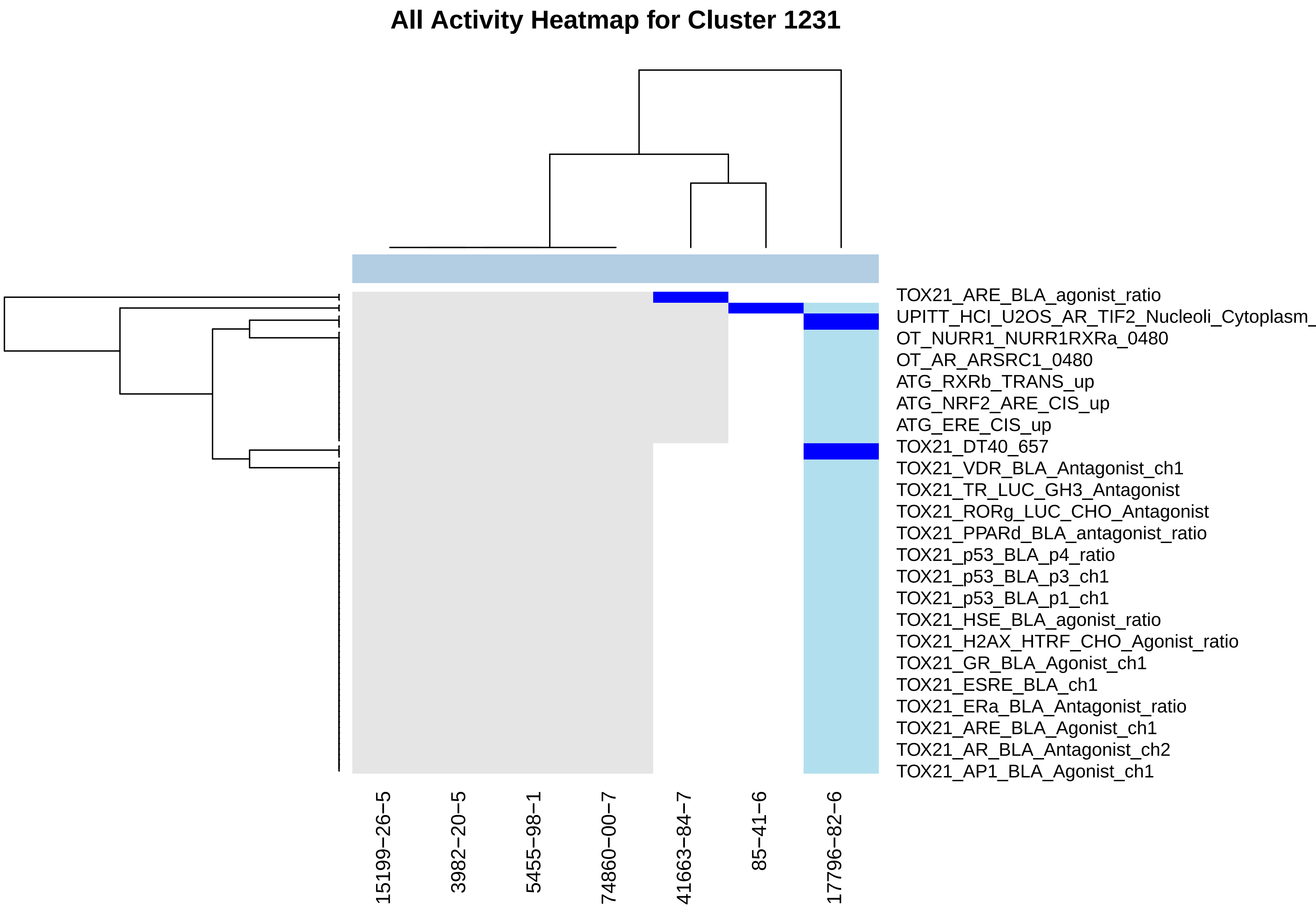
All Activity Heatmap for Cluster 1231
TOX21_ARE_BLA_agonist_ratio
UPITT_HCI_U2OS_AR_TIF2_Nucleoli_Cytoplasm_
OT_NURR1_NURR1RXRa_0480
OT_AR_ARSRC1_0480
ATG_RXRb_TRANS_up
ATG_NRF2_ARE_CIS_up
ATG_ERE_CIS_up
TOX21_DT40_657
TOX21_VDR_BLA_Antagonist_ch1
TOX21_TR_LUC_GH3_Antagonist
TOX21_RORg_LUC_CHO_Antagonist
TOX21_PPARd_BLA_antagonist_ratio
TOX21_p53_BLA_p4_ratio
TOX21_p53_BLA_p3_ch1
TOX21_p53_BLA_p1_ch1
TOX21_HSE_BLA_agonist_ratio
TOX21_H2AX_HTRF_CHO_Agonist_ratio
TOX21_GR_BLA_Agonist_ch1
TOX21_ESRE_BLA_ch1
TOX21_ERa_BLA_Antagonist_ratio
TOX21_ARE_BLA_Agonist_ch1
TOX21_AR_BLA_Antagonist_ch2
TOX21_AP1_BLA_Agonist_ch1
15199−26−5
3982−20−5
5455−98−1
74860−00−7
41663−84−7
85−41−6
17796−82−6

All Activity Heatmap for Cluster 1234

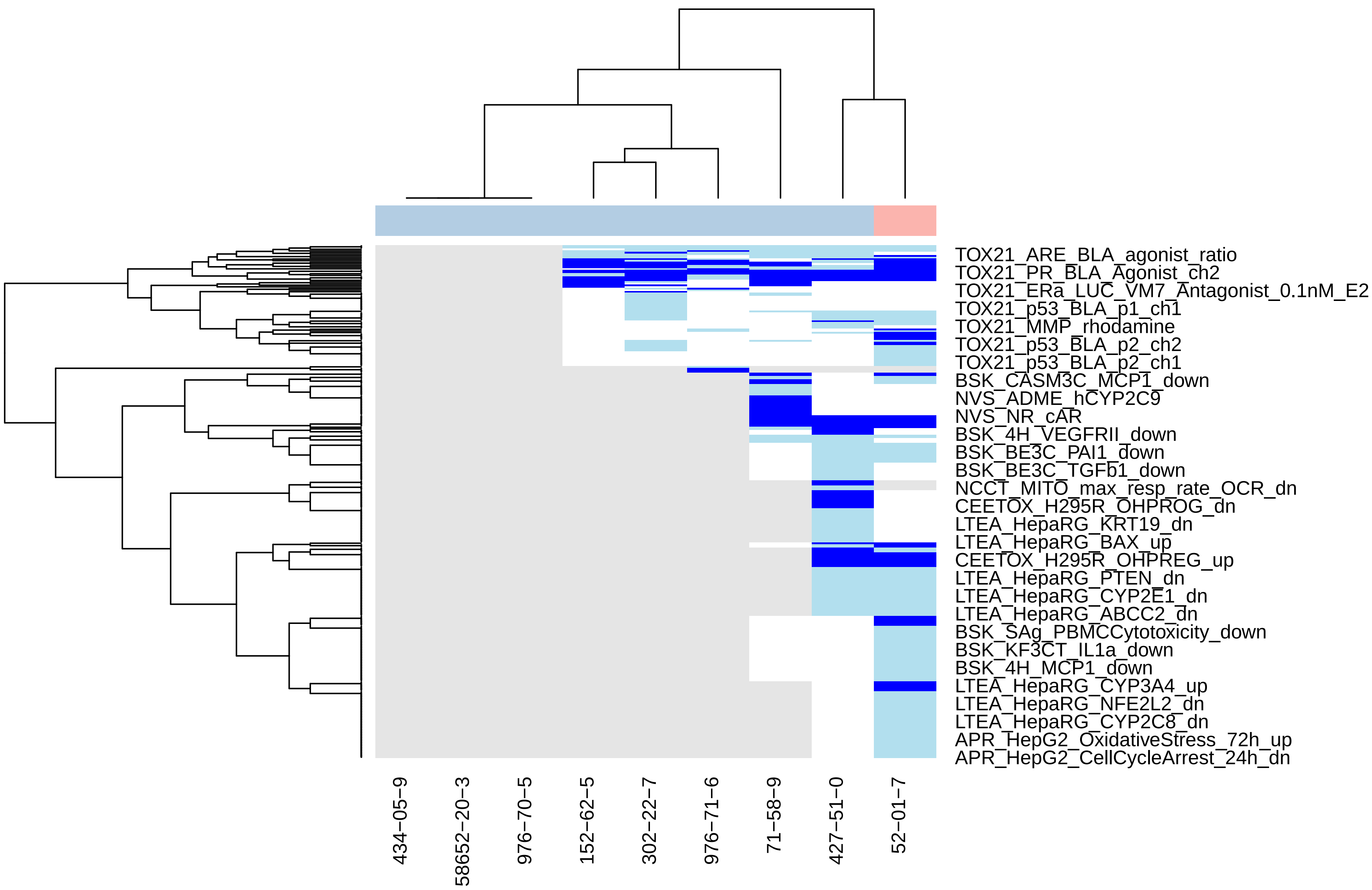

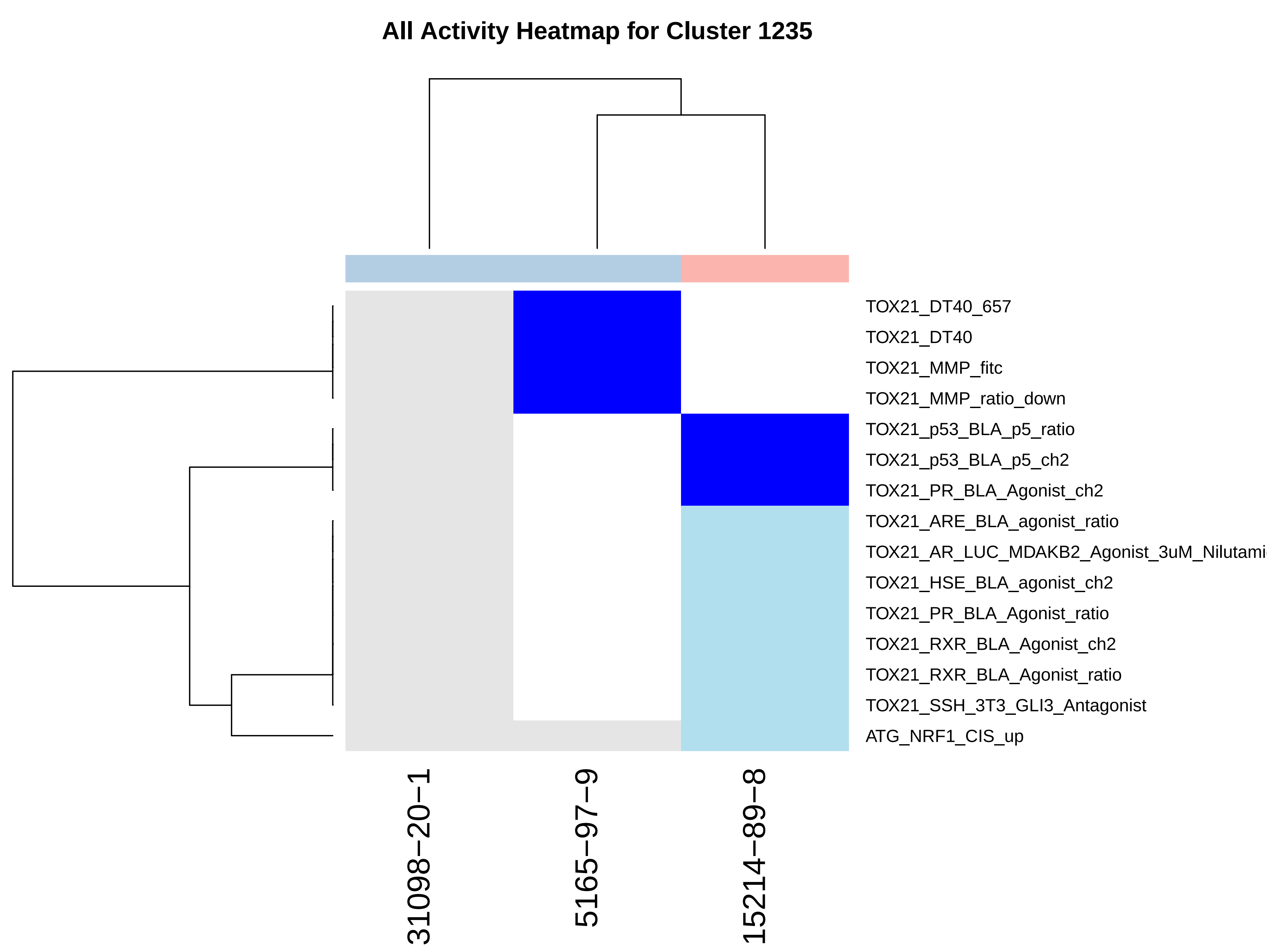
All Activity Heatmap for Cluster 1235
TOX21_DT40_657
TOX21_DT40
TOX21_MMP_fitc
TOX21_MMP_ratio_down
TOX21_p53_BLA_p5_ratio
TOX21_p53_BLA_p5_ch2
TOX21_PR_BLA_Agonist_ch2
TOX21_ARE_BLA_agonist_ratio
TOX21_AR_LUC_MDAKB2_Agonist_3uM_Nilutami
TOX21_HSE_BLA_agonist_ch2
TOX21_PR_BLA_Agonist_ratio
TOX21_RXR_BLA_Agonist_ch2
TOX21_RXR_BLA_Agonist_ratio
TOX21_SSH_3T3_GLI3_Antagonist
ATG_NRF1_CIS_up
31098−20−1
5165−97−9
15214−89−8

All Activity Heatmap for Cluster 1236

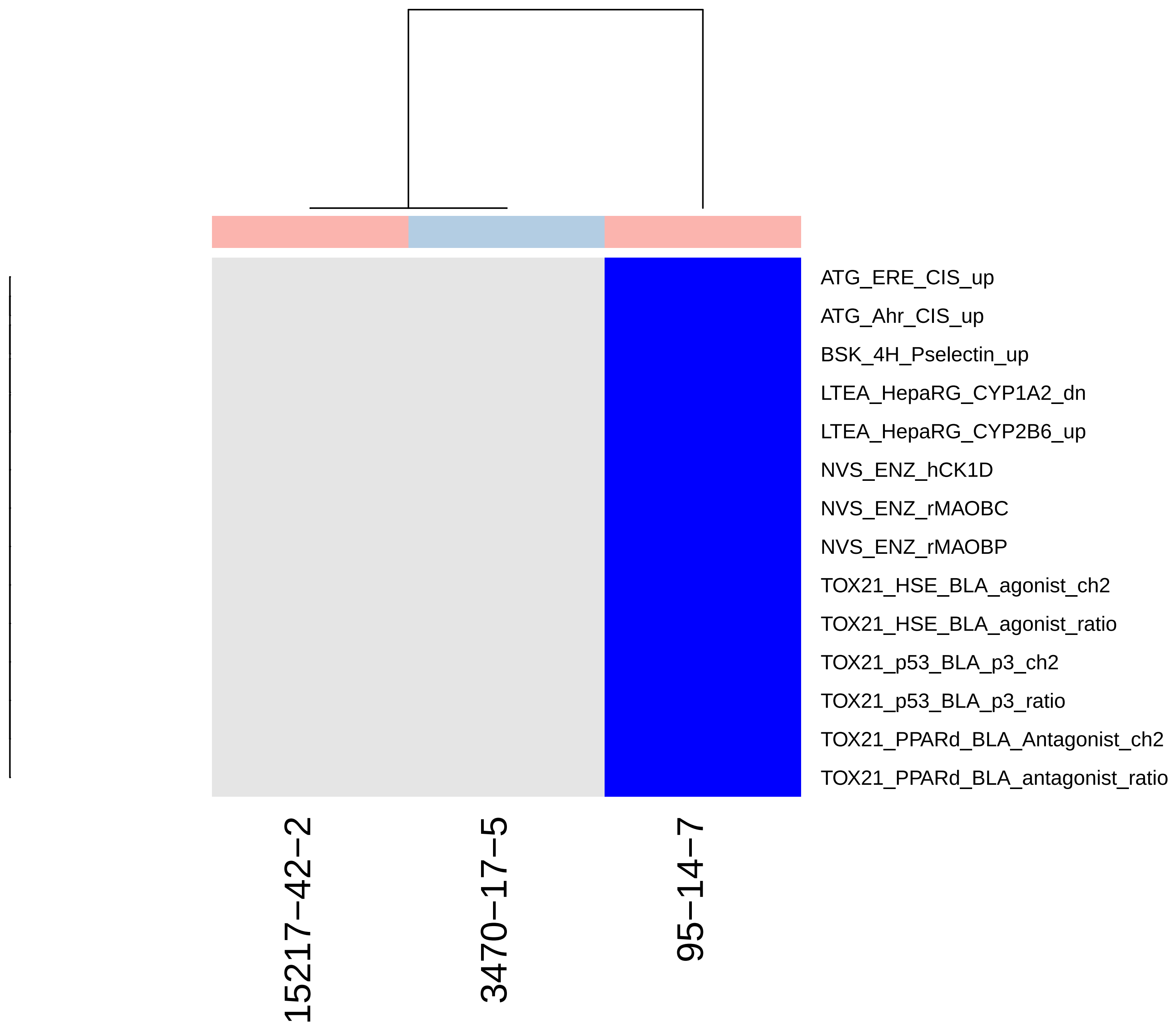

All Activity Heatmap for Cluster 1237

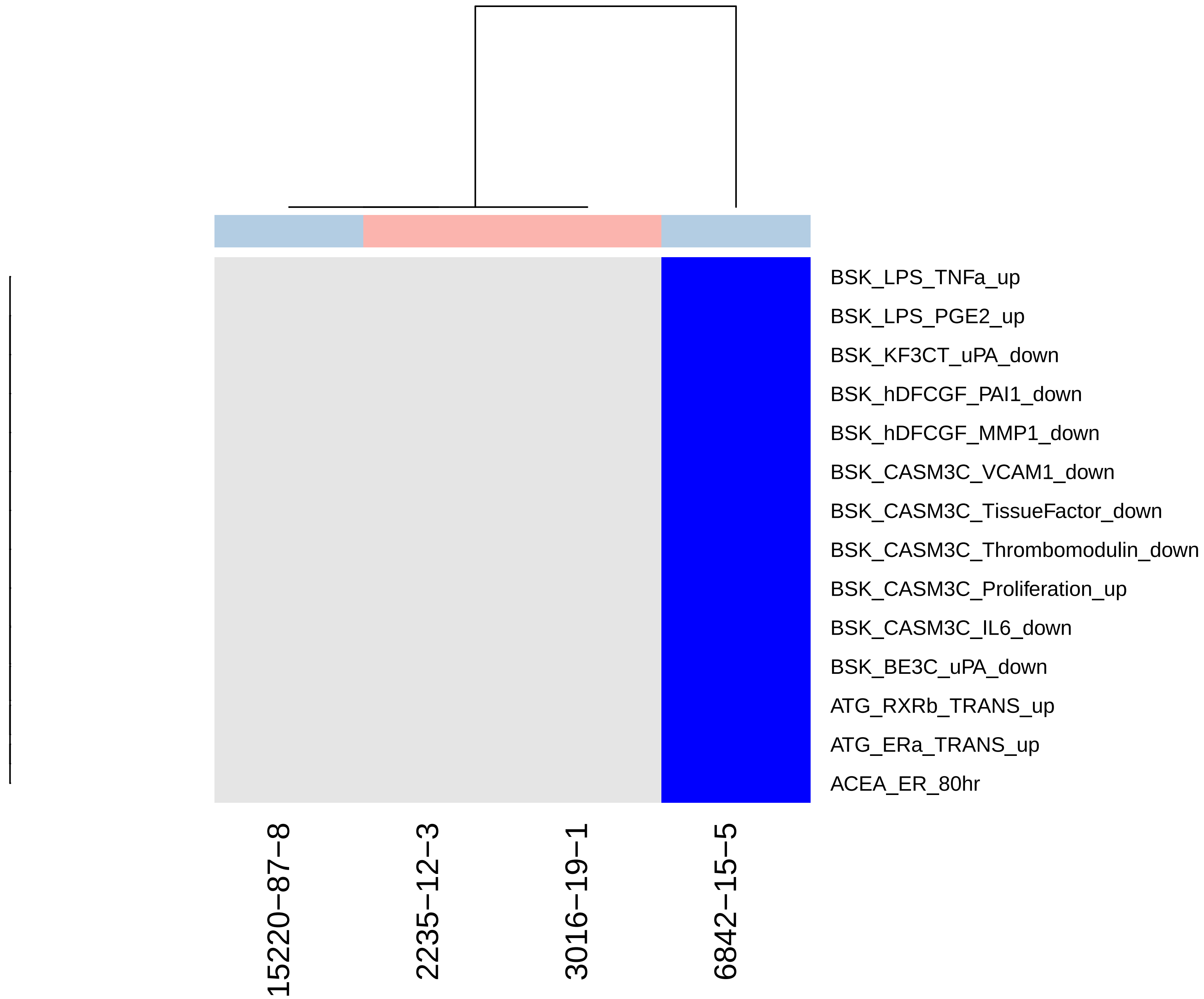

All Activity Heatmap for Cluster 1241

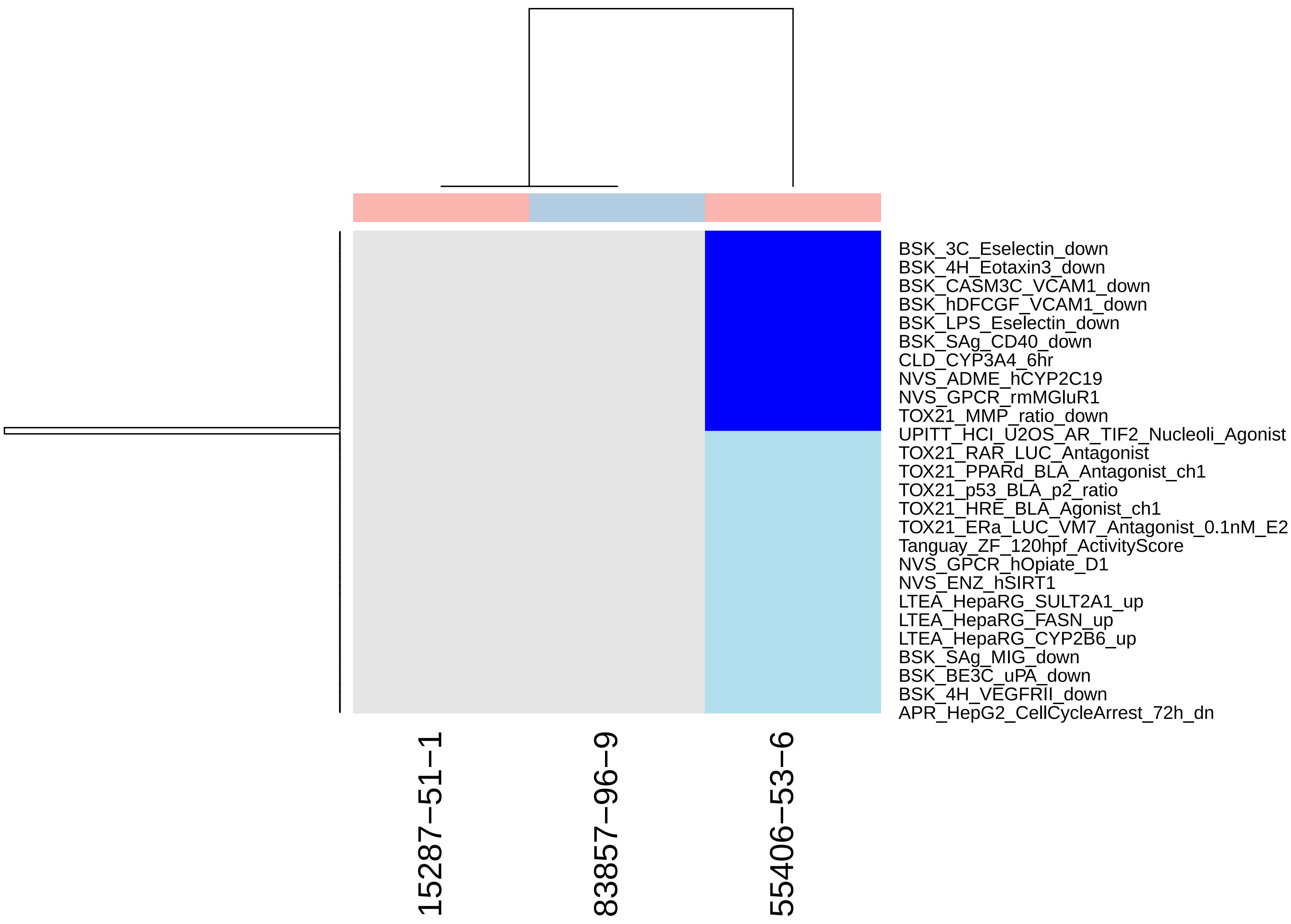

All Activity Heatmap for Cluster 1242

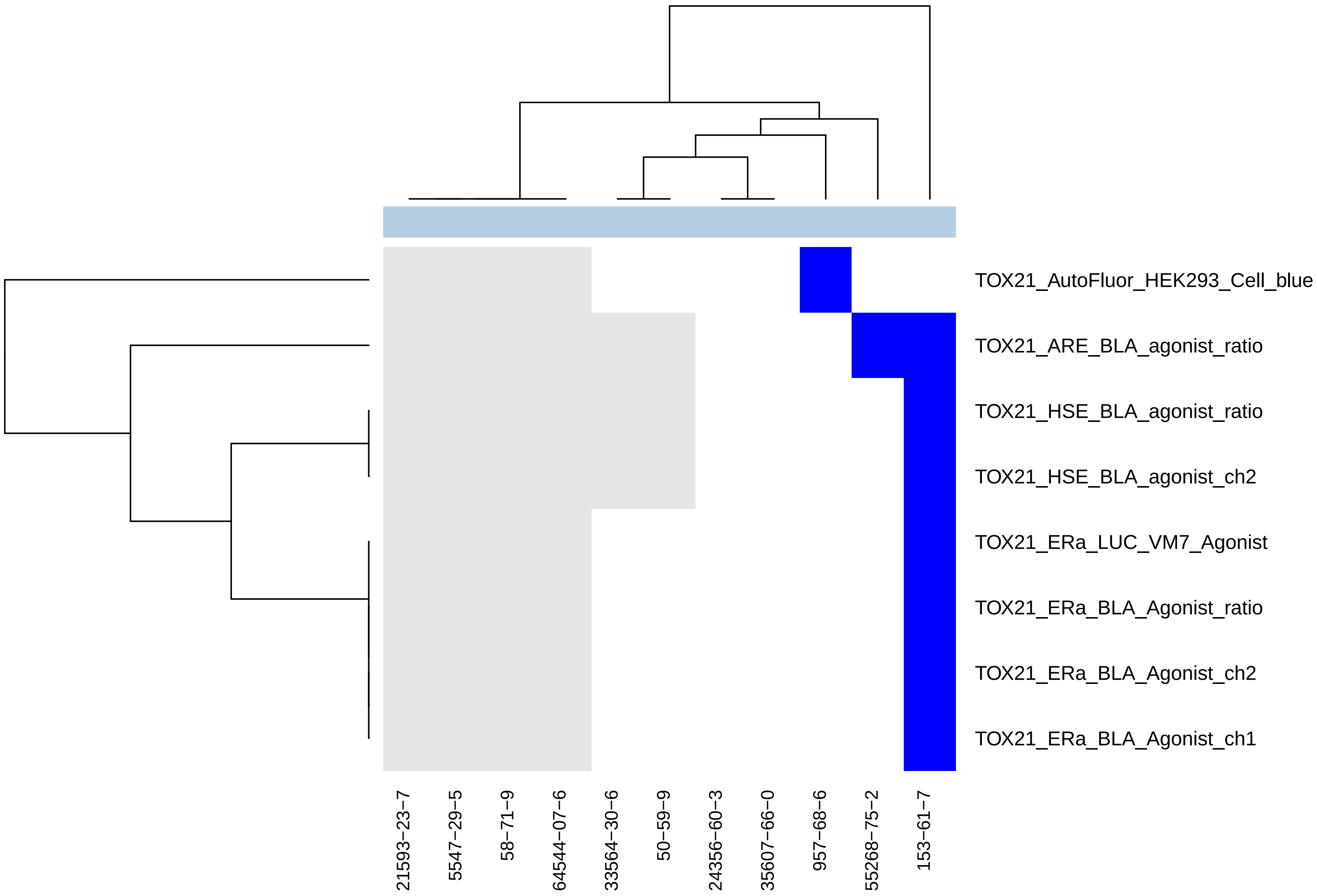

All Activity Heatmap for Cluster 1244

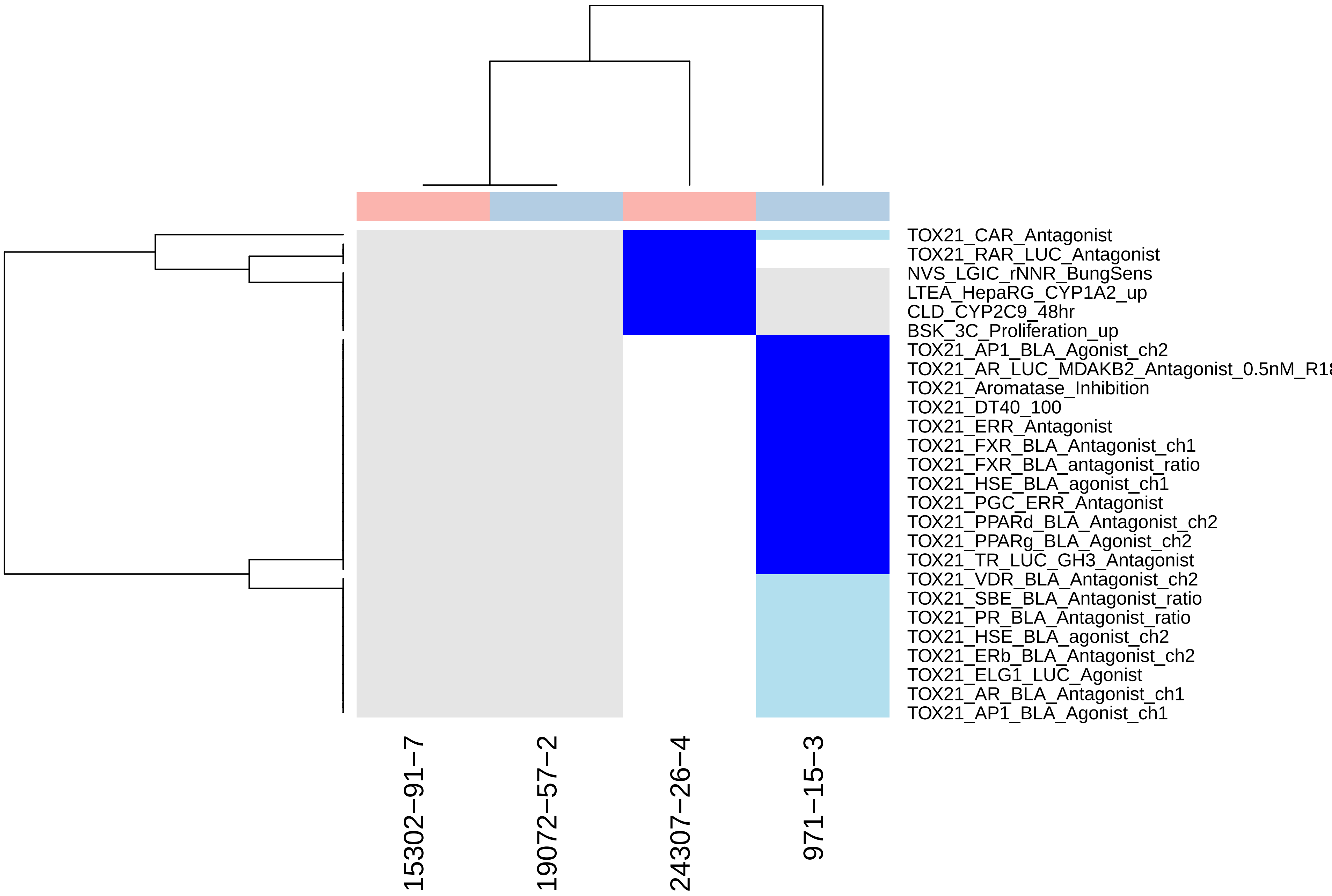

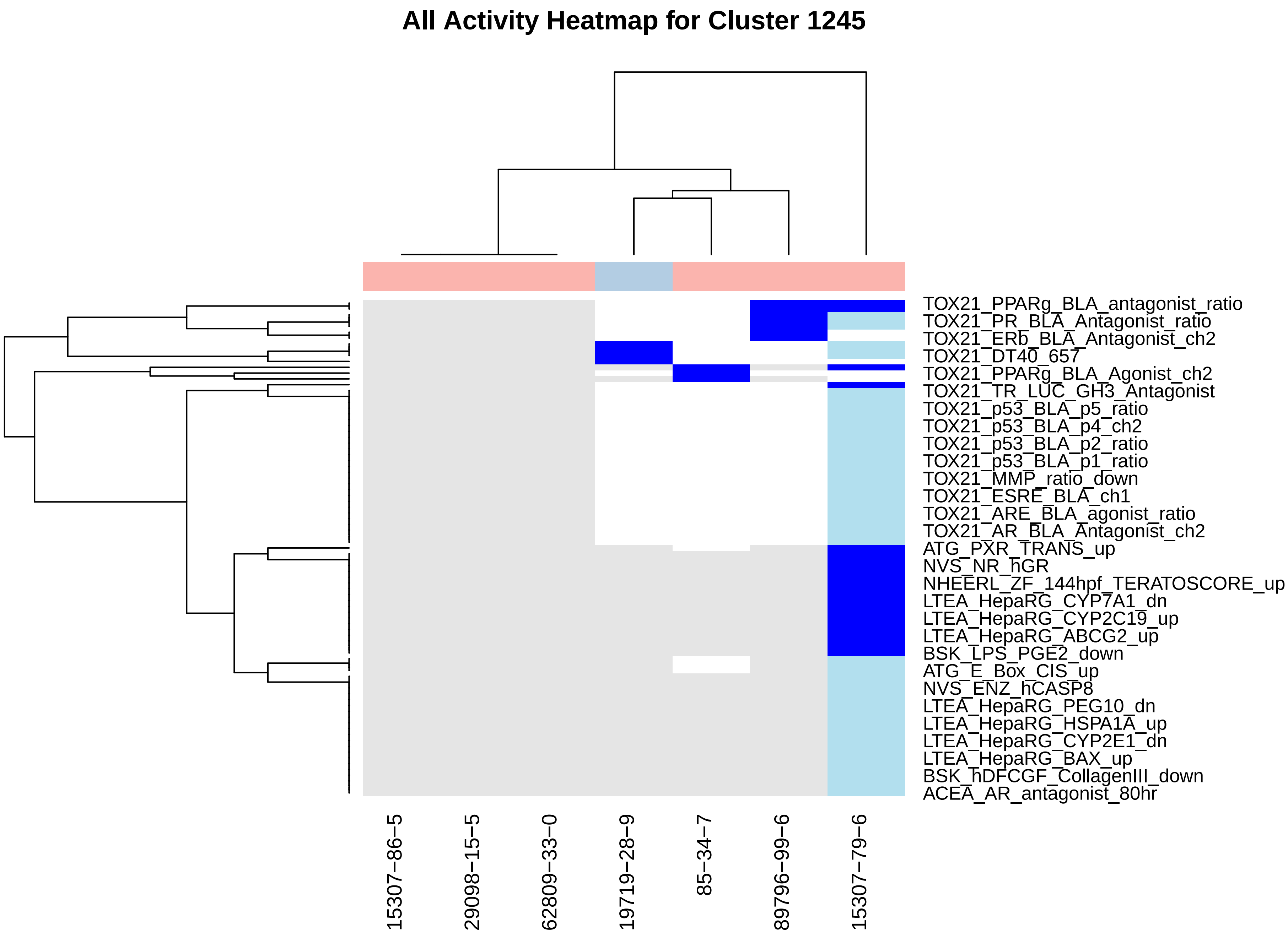
All Activity Heatmap for Cluster 1245
TOX21_PPARg_BLA_antagonist_ratio
TOX21_PR_BLA_Antagonist_ratio
TOX21_ERb_BLA_Antagonist_ch2
TOX21_DT40_657
TOX21_PPARg_BLA_Agonist_ch2
TOX21_TR_LUC_GH3_Antagonist
TOX21_p53_BLA_p5_ratio
TOX21_p53_BLA_p4_ch2
TOX21_p53_BLA_p2_ratio
TOX21_p53_BLA_p1_ratio
TOX21_MMP_ratio_down
TOX21_ESRE_BLA_ch1
TOX21_ARE_BLA_agonist_ratio
TOX21_AR_BLA_Antagonist_ch2
ATG_PXR_TRANS_up
NVS_NR_hGR
NHEERL_ZF_144hpf_TERATOSCORE_up
LTEA_HepaRG_CYP7A1_dn
LTEA_HepaRG_CYP2C19_up
LTEA_HepaRG_ABCG2_up
BSK_LPS_PGE2_down
ATG_E_Box_CIS_up
NVS_ENZ_hCASP8
LTEA_HepaRG_PEG10_dn
LTEA_HepaRG_HSPA1A_up
LTEA_HepaRG_CYP2E1_dn
LTEA_HepaRG_BAX_up
BSK_hDFCGF_CollagenIII_down
ACEA_AR_antagonist_80hr
15307−86−5
29098−15−5
62809−33−0
19719−28−9
85−34−7
89796−99−6
15307−79−6

All Activity Heatmap for Cluster 1247

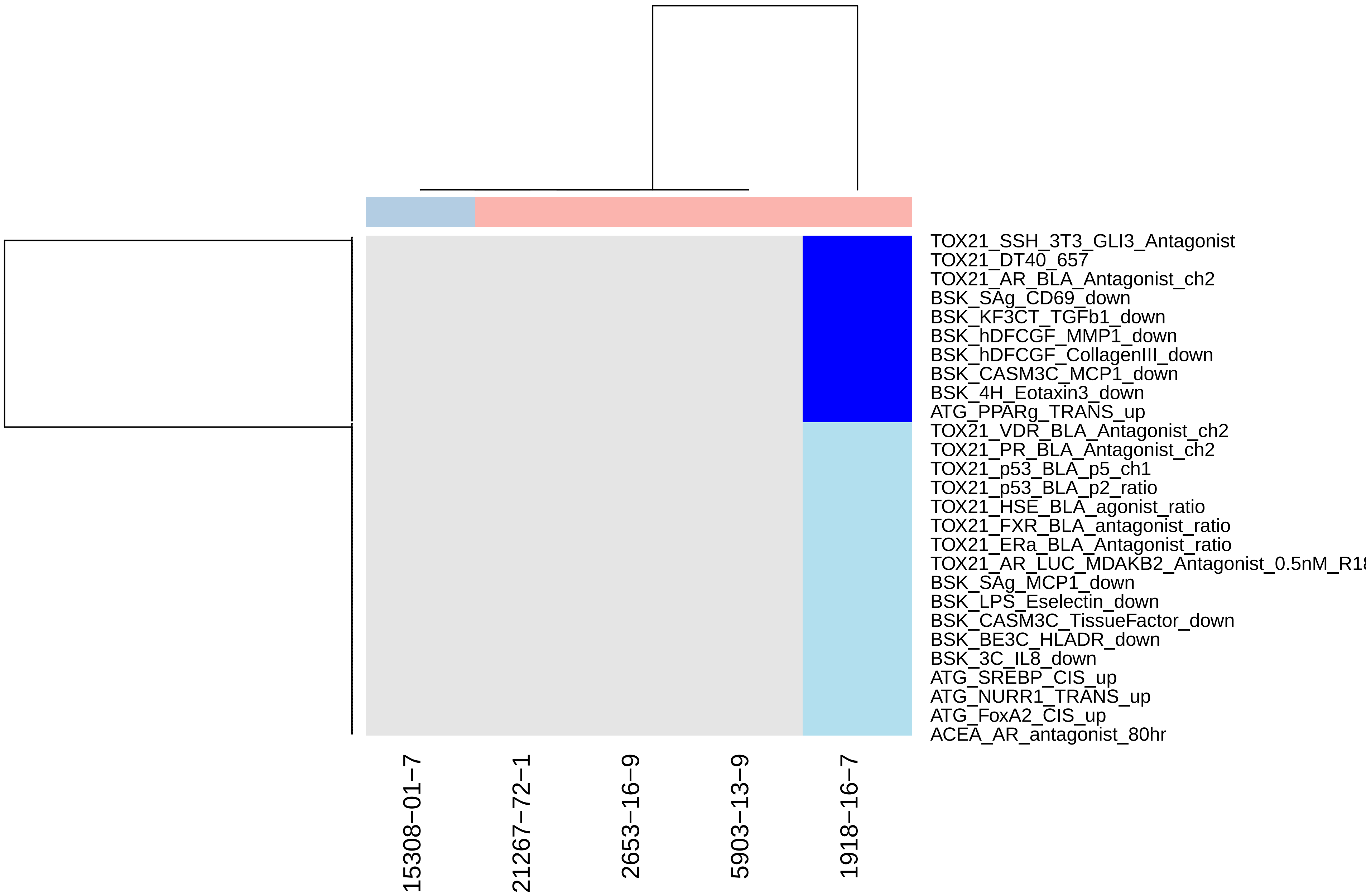

All Activity Heatmap for Cluster 1250

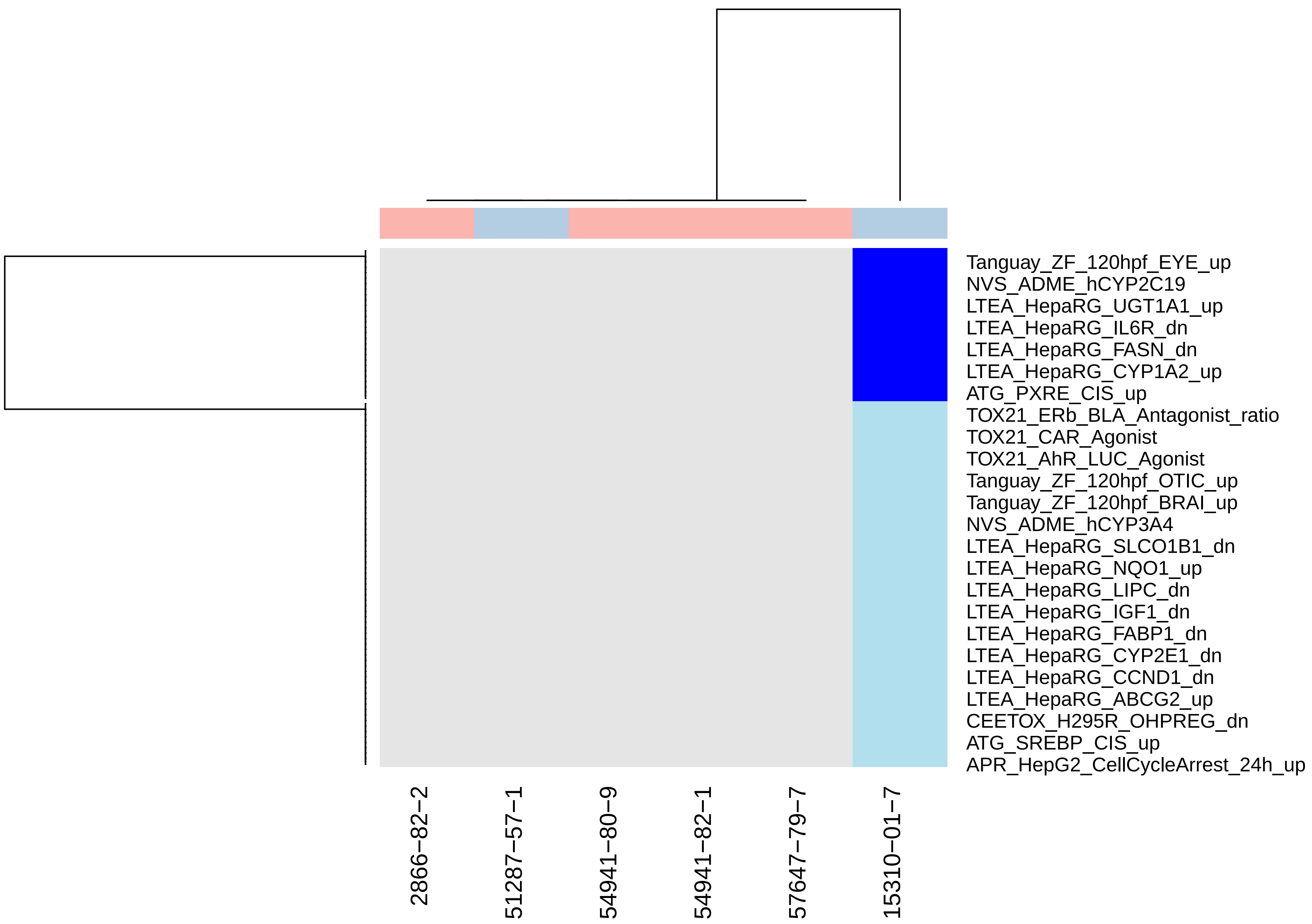

All Activity Heatmap for Cluster 1251

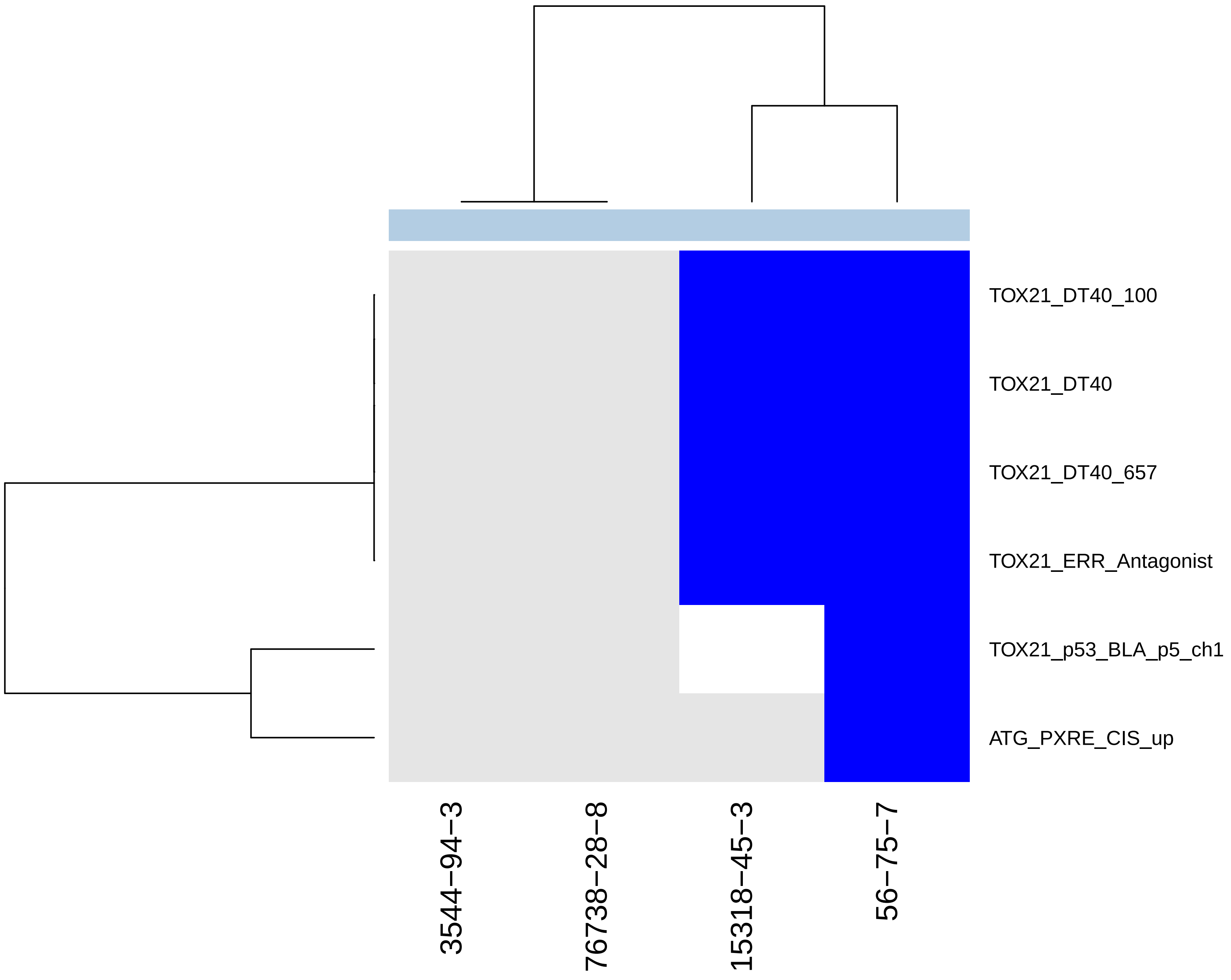

All Activity Heatmap for Cluster 1253

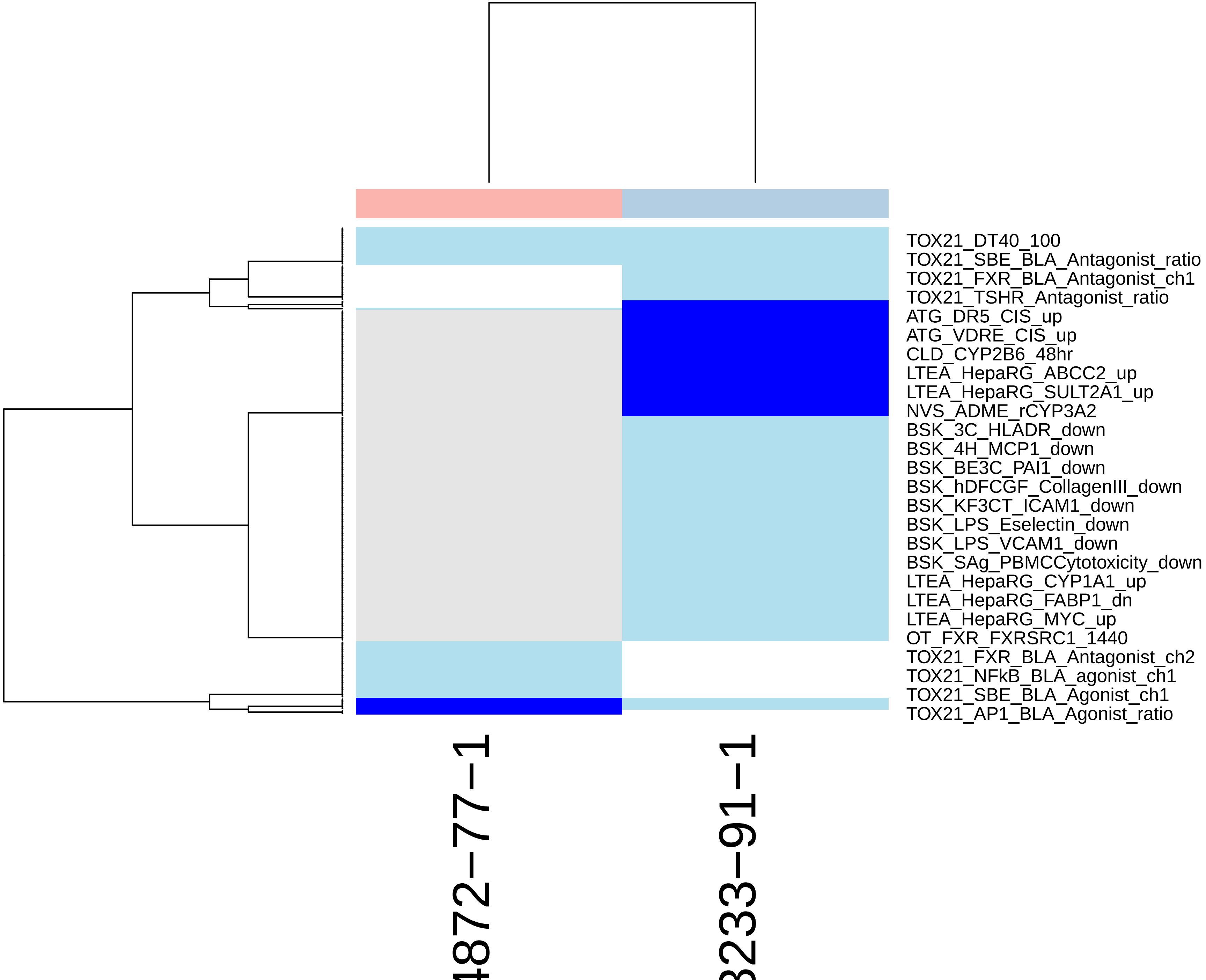

All Activity Heatmap for Cluster 1256

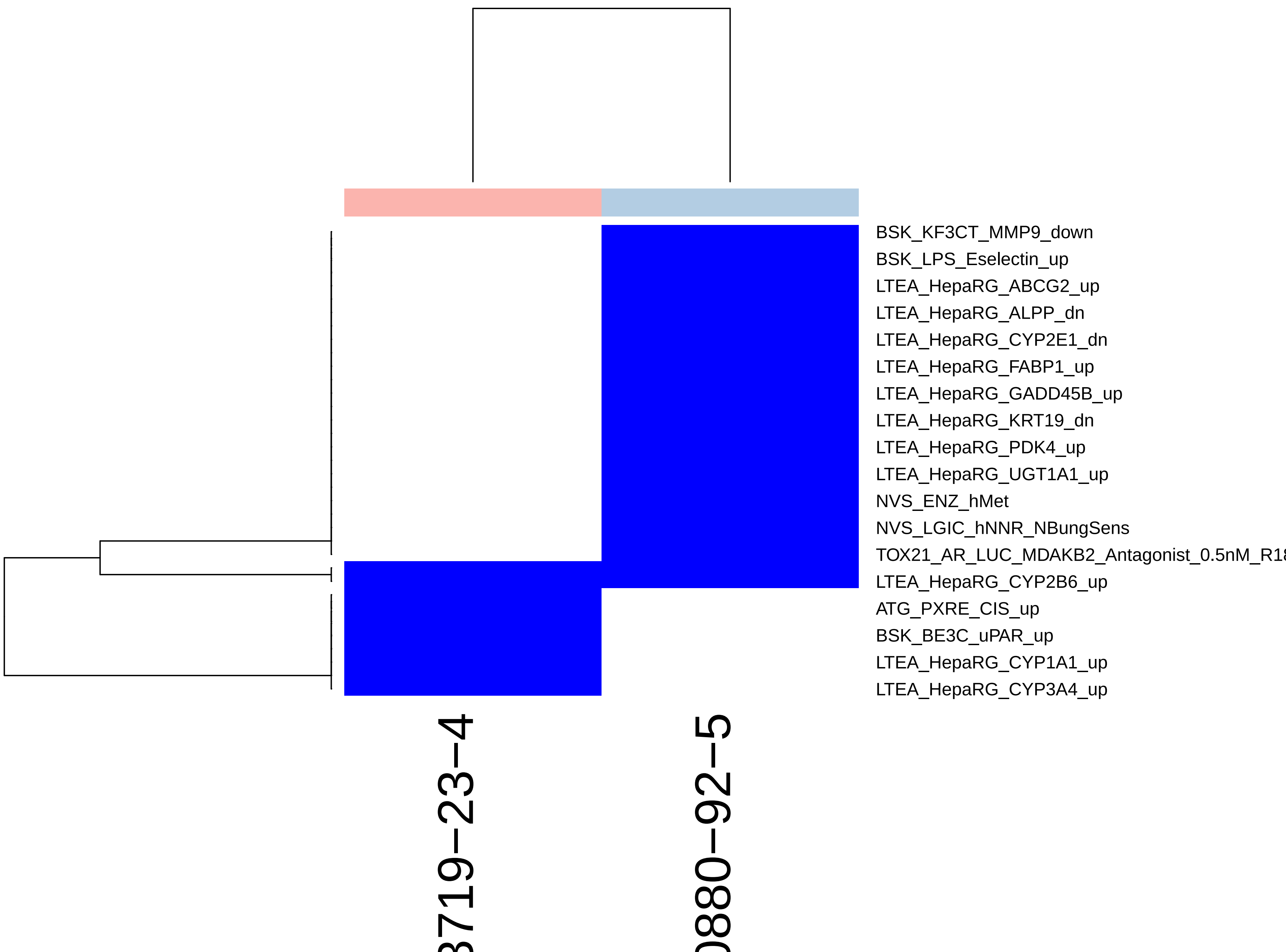

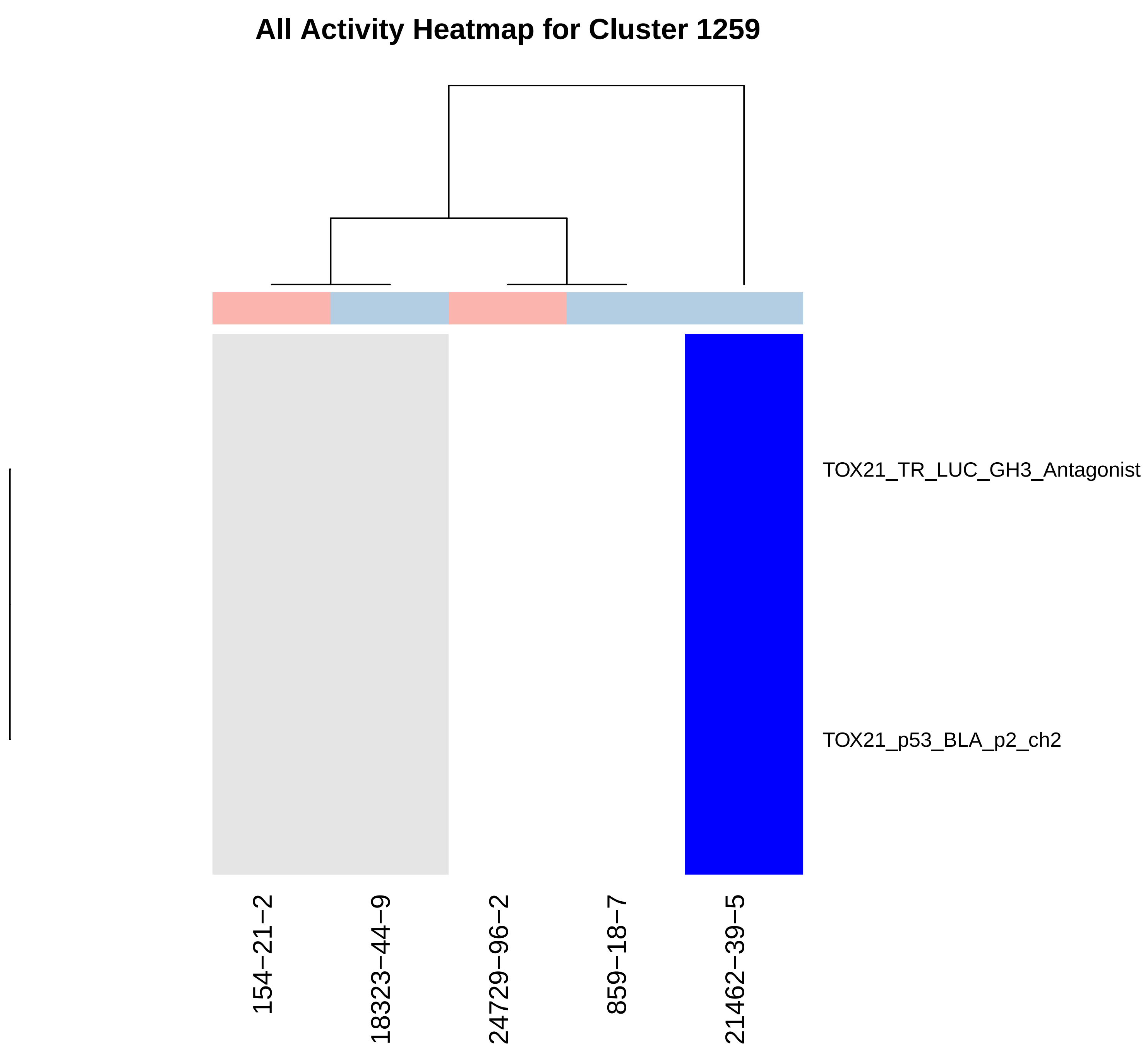
All Activity Heatmap for Cluster 1259
TOX21_TR_LUC_GH3_Antagonist
TOX21_p53_BLA_p2_ch2
154−21−2
18323−44−9
24729−96−2
859−18−7
21462−39−5

All Activity Heatmap for Cluster 1260

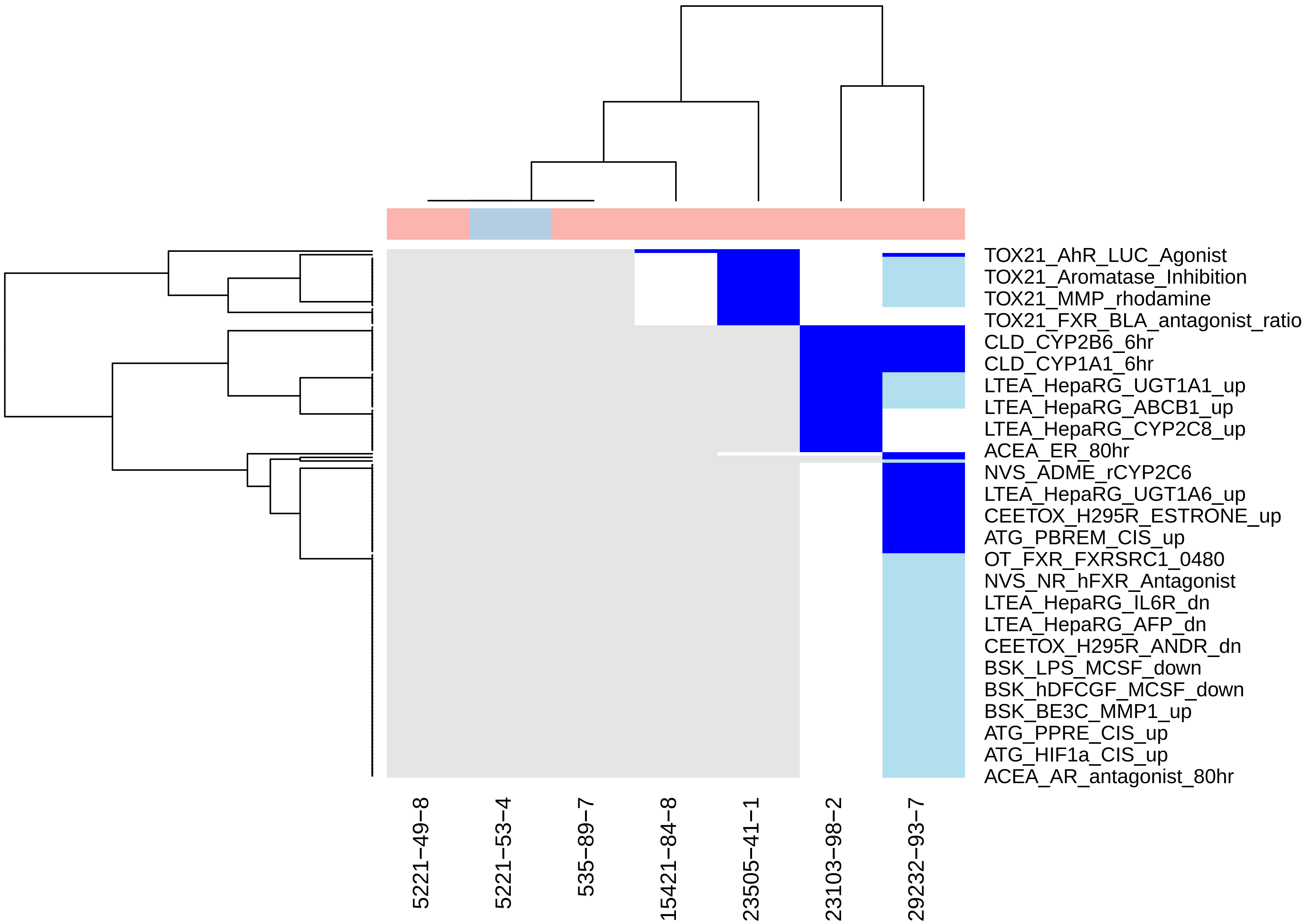

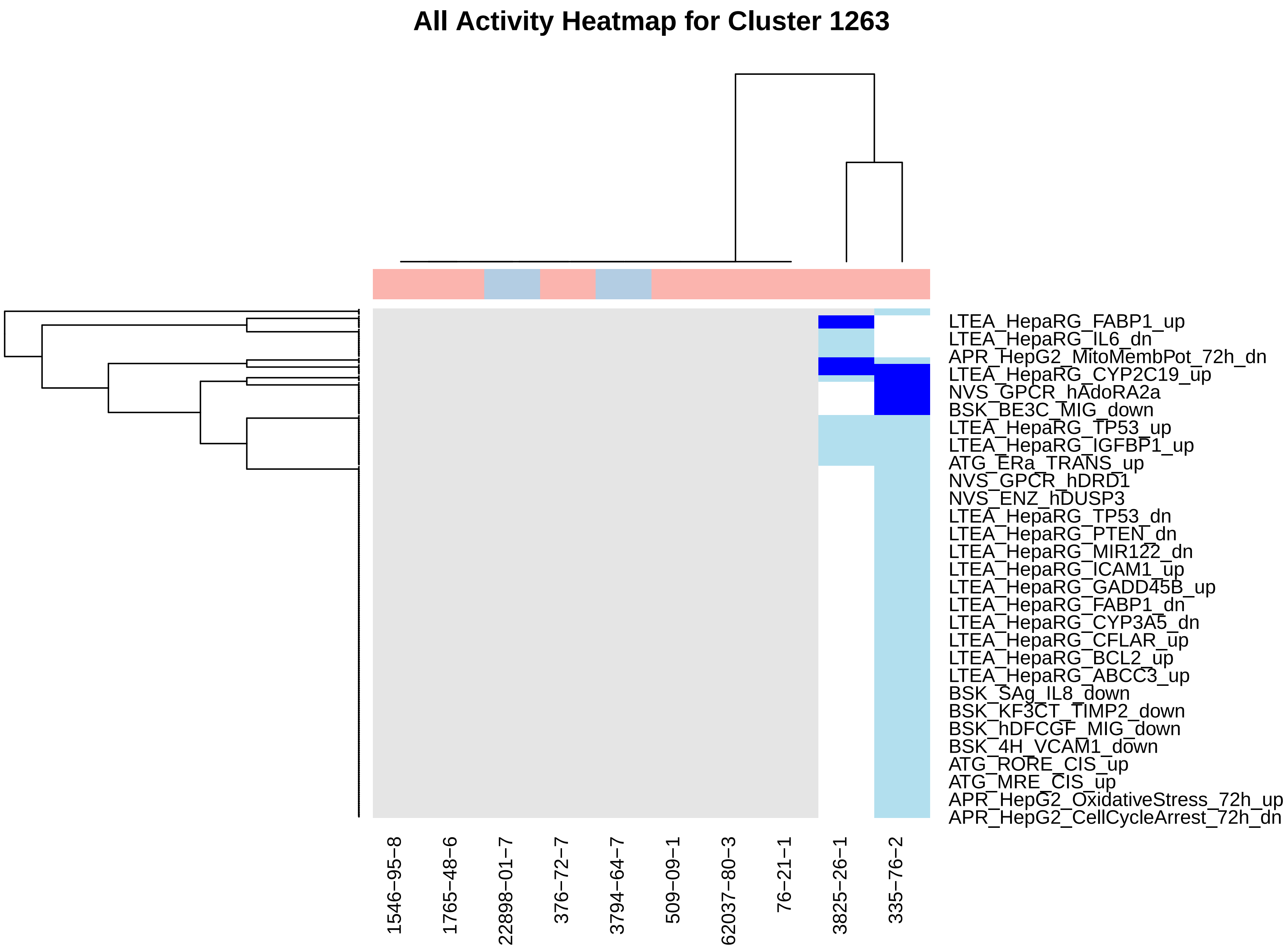
All Activity Heatmap for Cluster 1263
LTEA_HepaRG_FABP1_up
LTEA_HepaRG_IL6_dn
APR_HepG2_MitoMembPot_72h_dn
LTEA_HepaRG_CYP2C19_up
NVS_GPCR_hAdoRA2a
BSK_BE3C_MIG_down
LTEA_HepaRG_TP53_up
LTEA_HepaRG_IGFBP1_up
ATG_ERa_TRANS_up
NVS_GPCR_hDRD1
NVS_ENZ_hDUSP3
LTEA_HepaRG_TP53_dn
LTEA_HepaRG_PTEN_dn
LTEA_HepaRG_MIR122_dn
LTEA_HepaRG_ICAM1_up
LTEA_HepaRG_GADD45B_up
LTEA_HepaRG_FABP1_dn
LTEA_HepaRG_CYP3A5_dn
LTEA_HepaRG_CFLAR_up
LTEA_HepaRG_BCL2_up
LTEA_HepaRG_ABCC3_up
BSK_SAg_IL8_down
BSK_KF3CT_TIMP2_down
BSK_hDFCGF_MIG_down
BSK_4H_VCAM1_down
ATG_RORE_CIS_up
ATG_MRE_CIS_up
APR_HepG2_OxidativeStress_72h_up
APR_HepG2_CellCycleArrest_72h_dn
1546−95−8
1765−48−6
22898−01−7
376−72−7
3794−64−7
509−09−1
62037−80−3
76−21−1
3825−26−1
335−76−2

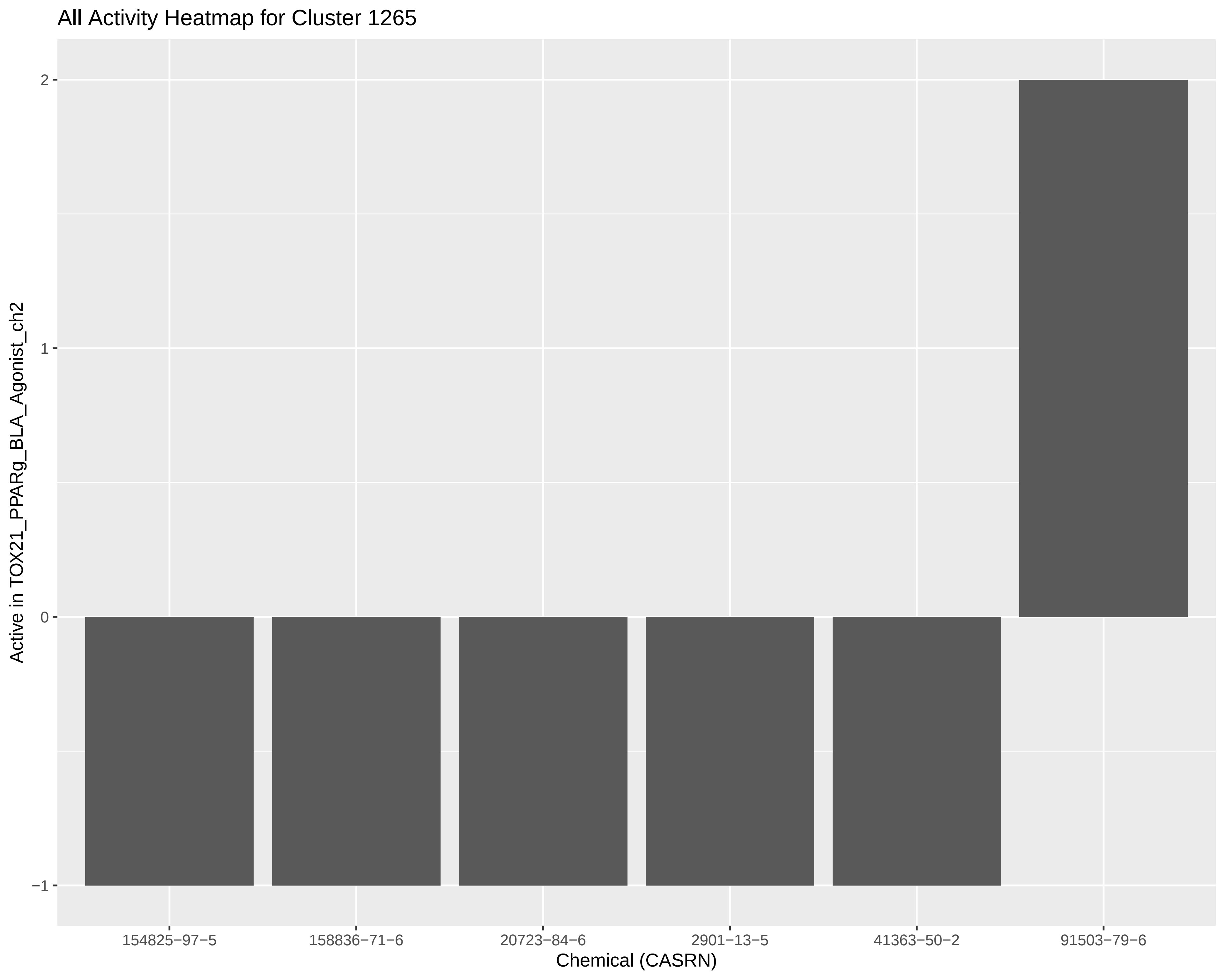
All Activity Heatmap for Cluster 1265
Active in TOX21_PPARg_BLA_Agonist_ch2
2
1
0
−1
154825−97−5
158836−71−6
20723−84−6
2901−13−5
41363−50−2
91503−79−6
Chemical (CASRN)

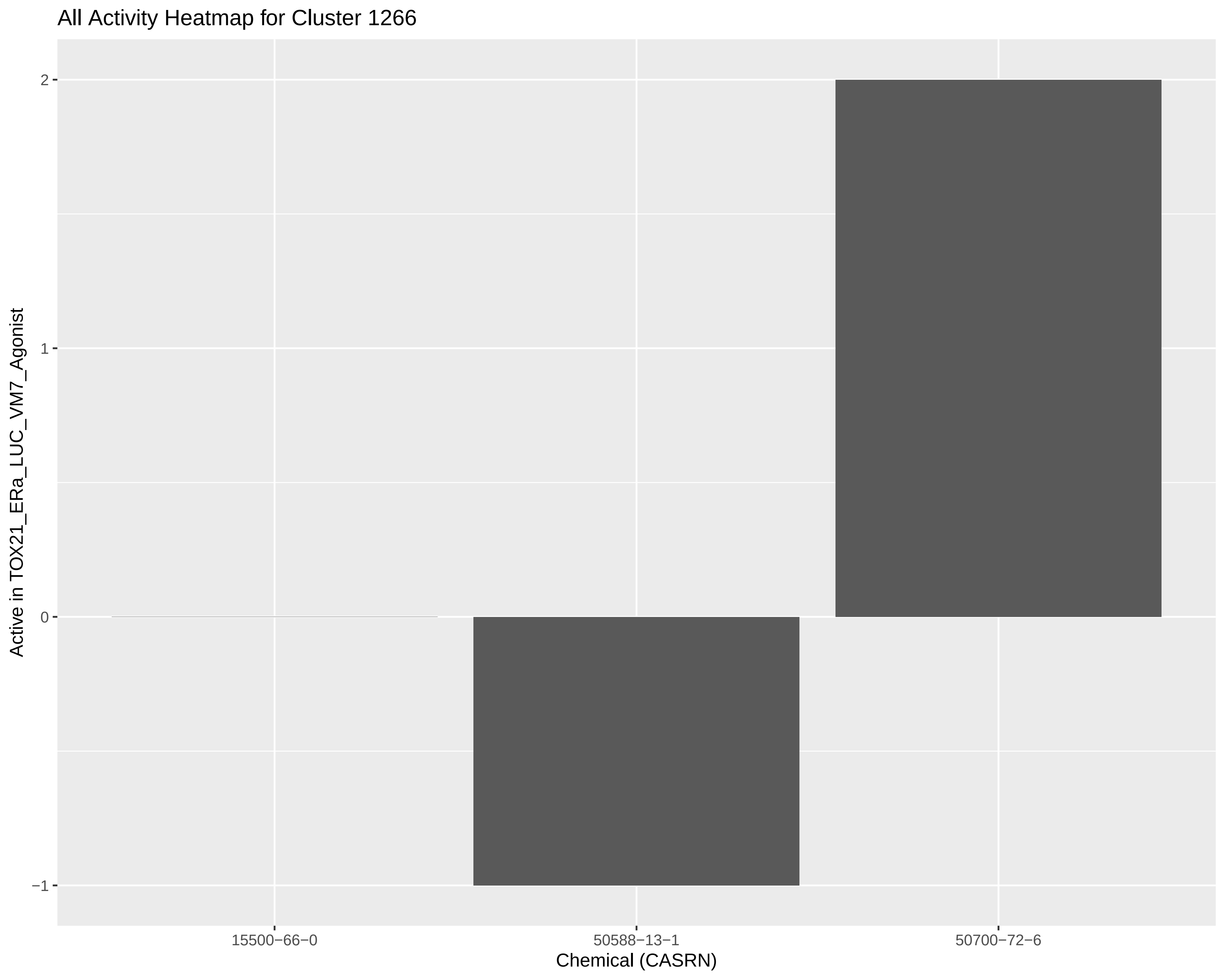

All Activity Heatmap for Cluster 1266
Active in TOX21_ERa_LUC_VM7_Agonist
2
1
0
−1
15500−66−0
50588−13−1
50700−72−6
Chemical (CASRN)

All Activity Heatmap for Cluster 1268

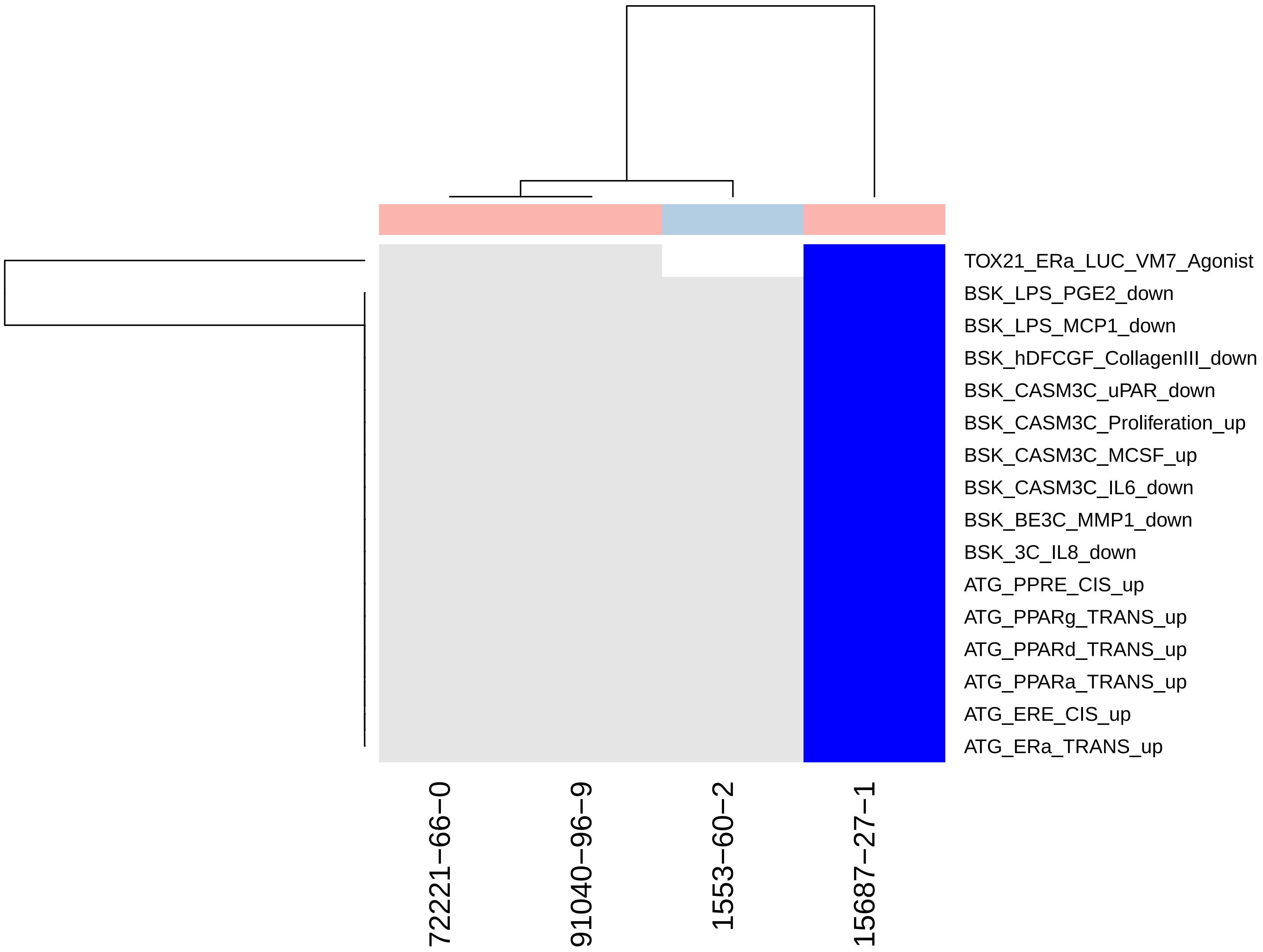

All Activity Heatmap for Cluster 1269

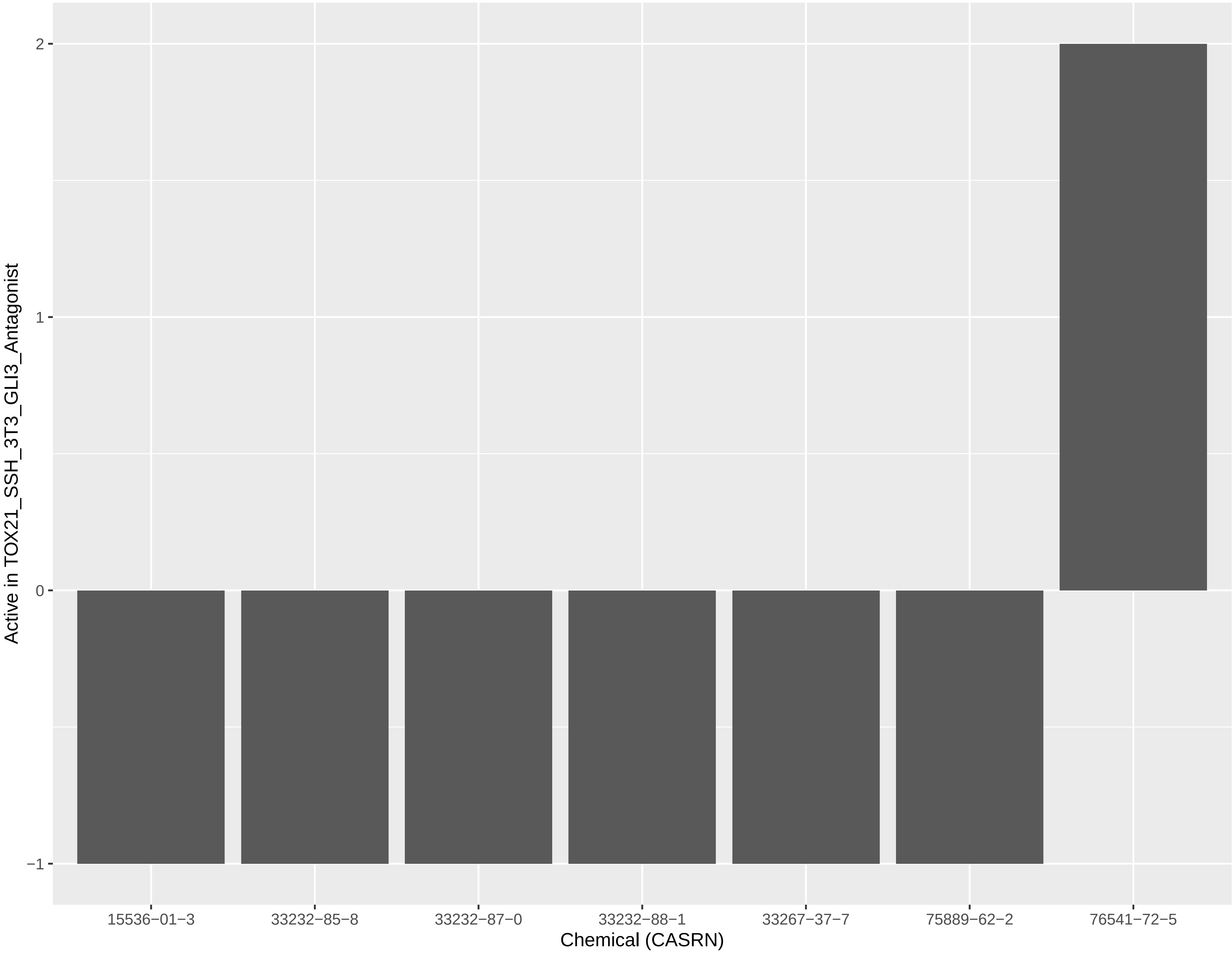

All Activity Heatmap for Cluster 1271

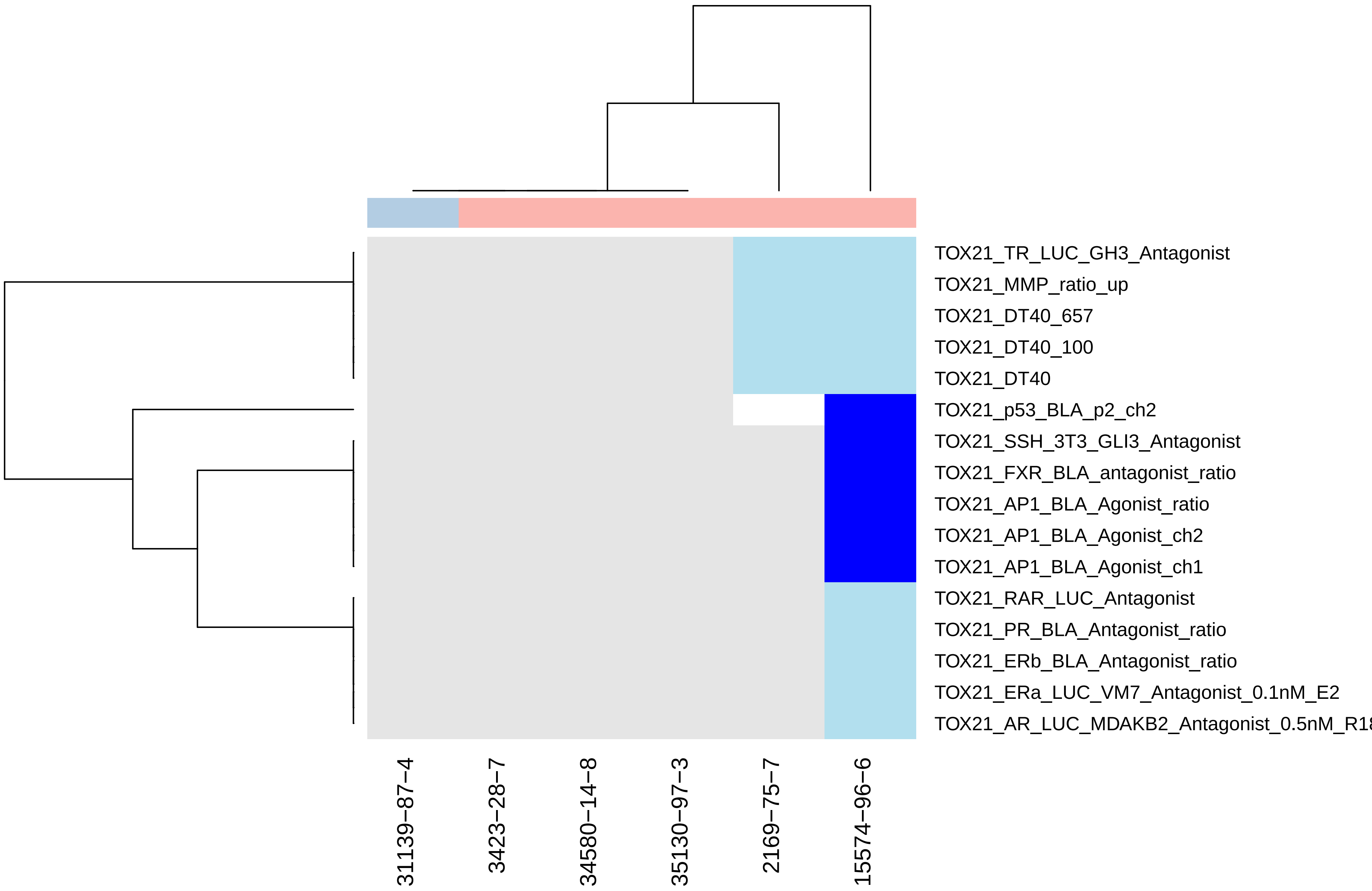

All Activity Heatmap for Cluster 1272

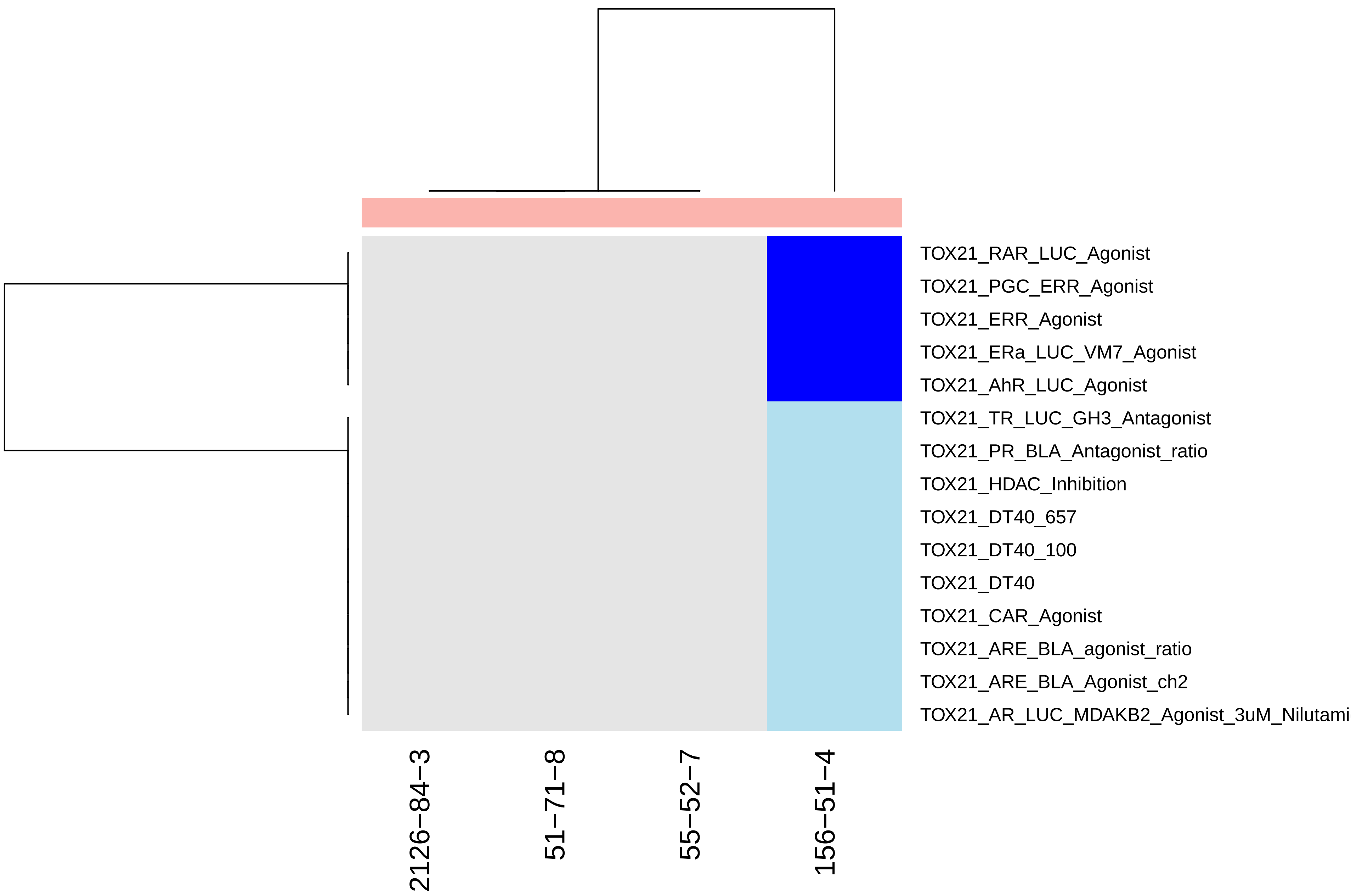

All Activity Heatmap for Cluster 1273

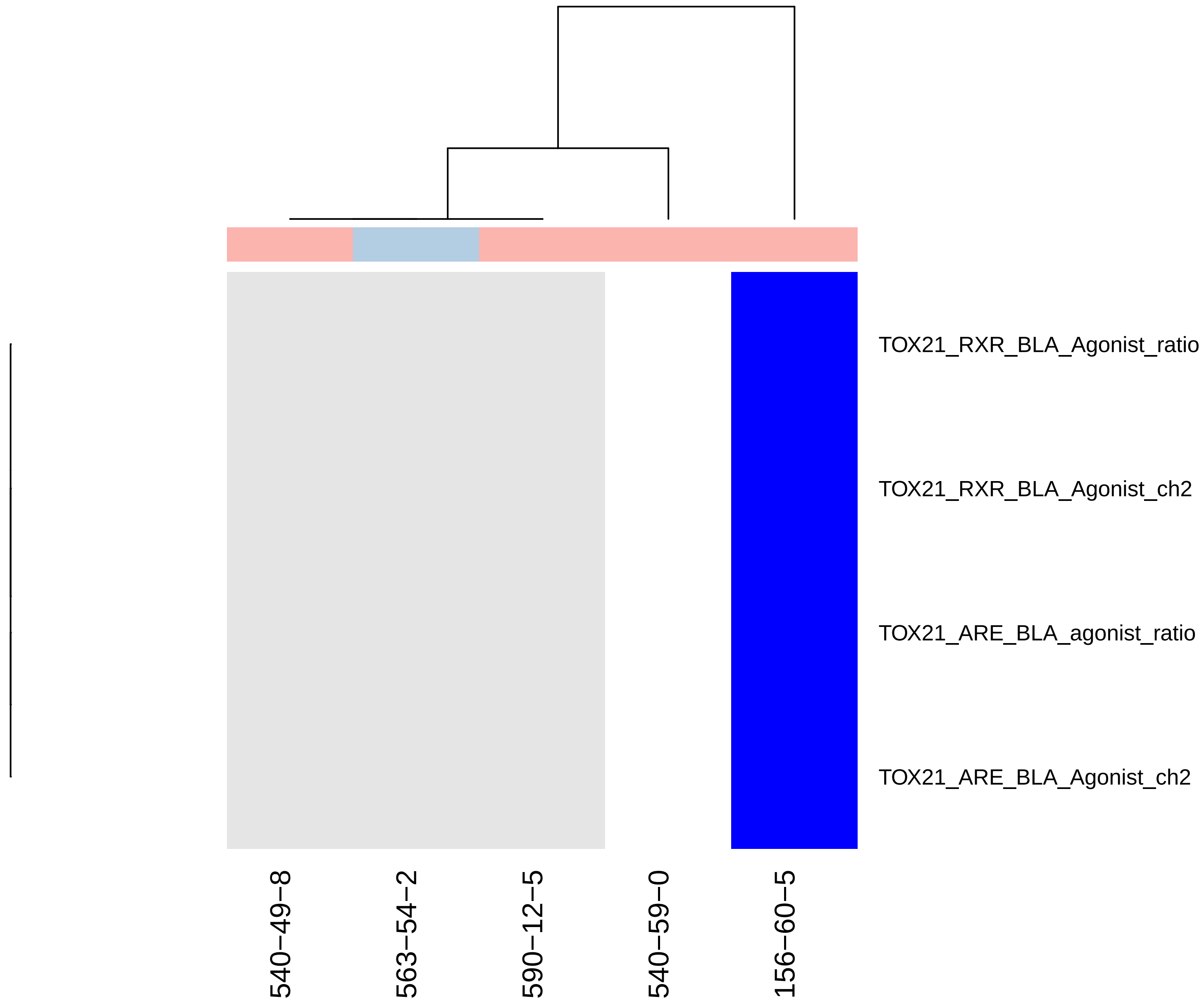

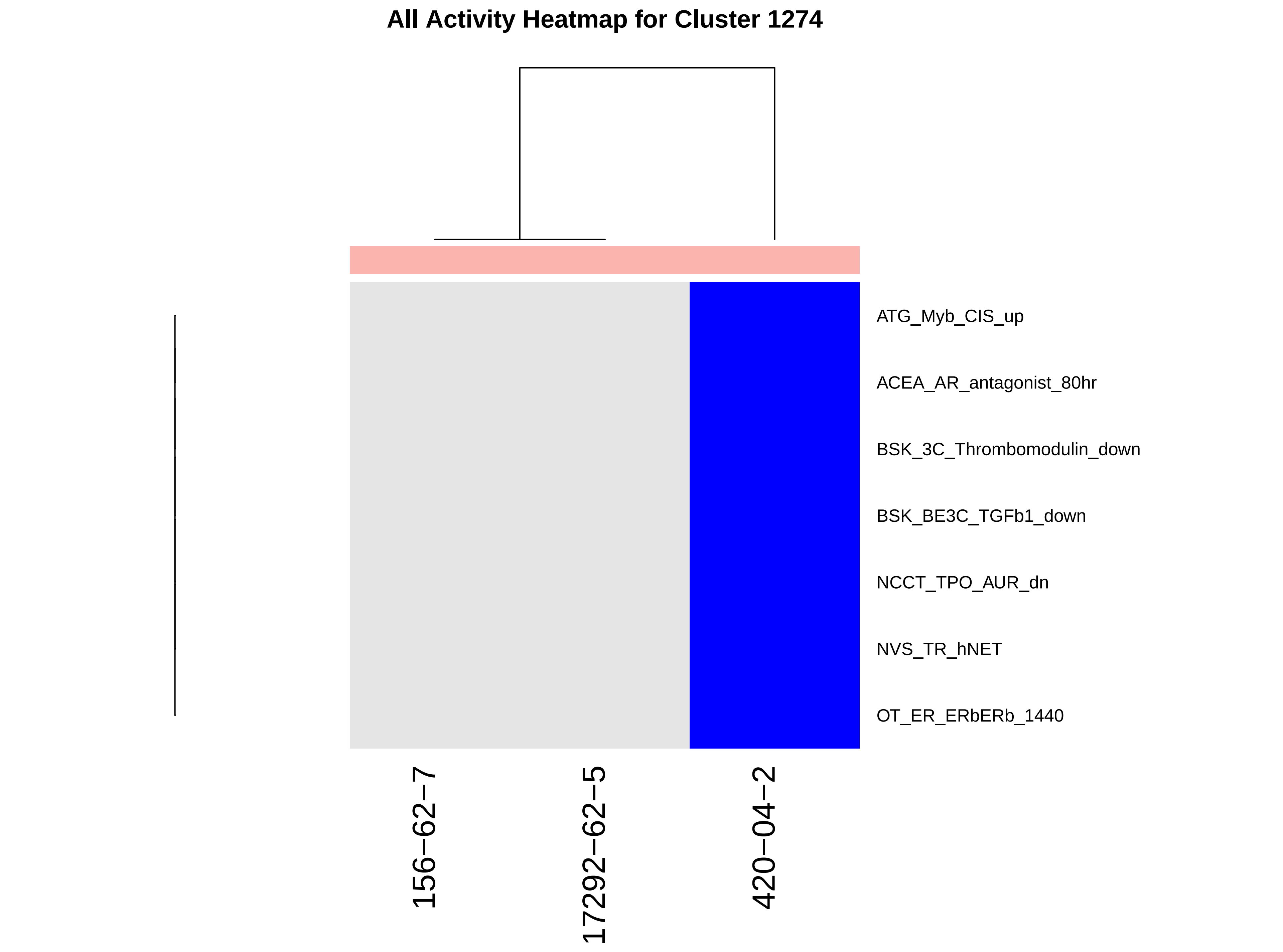
All Activity Heatmap for Cluster 1274
ATG_Myb_CIS_up
ACEA_AR_antagonist_80hr
BSK_3C_Thrombomodulin_down
BSK_BE3C_TGFb1_down
NCCT_TPO_AUR_dn
NVS_TR_hNET
OT_ER_ERbERb_1440
156−62−7
17292−62−5
420−04−2

All Activity Heatmap for Cluster 1275

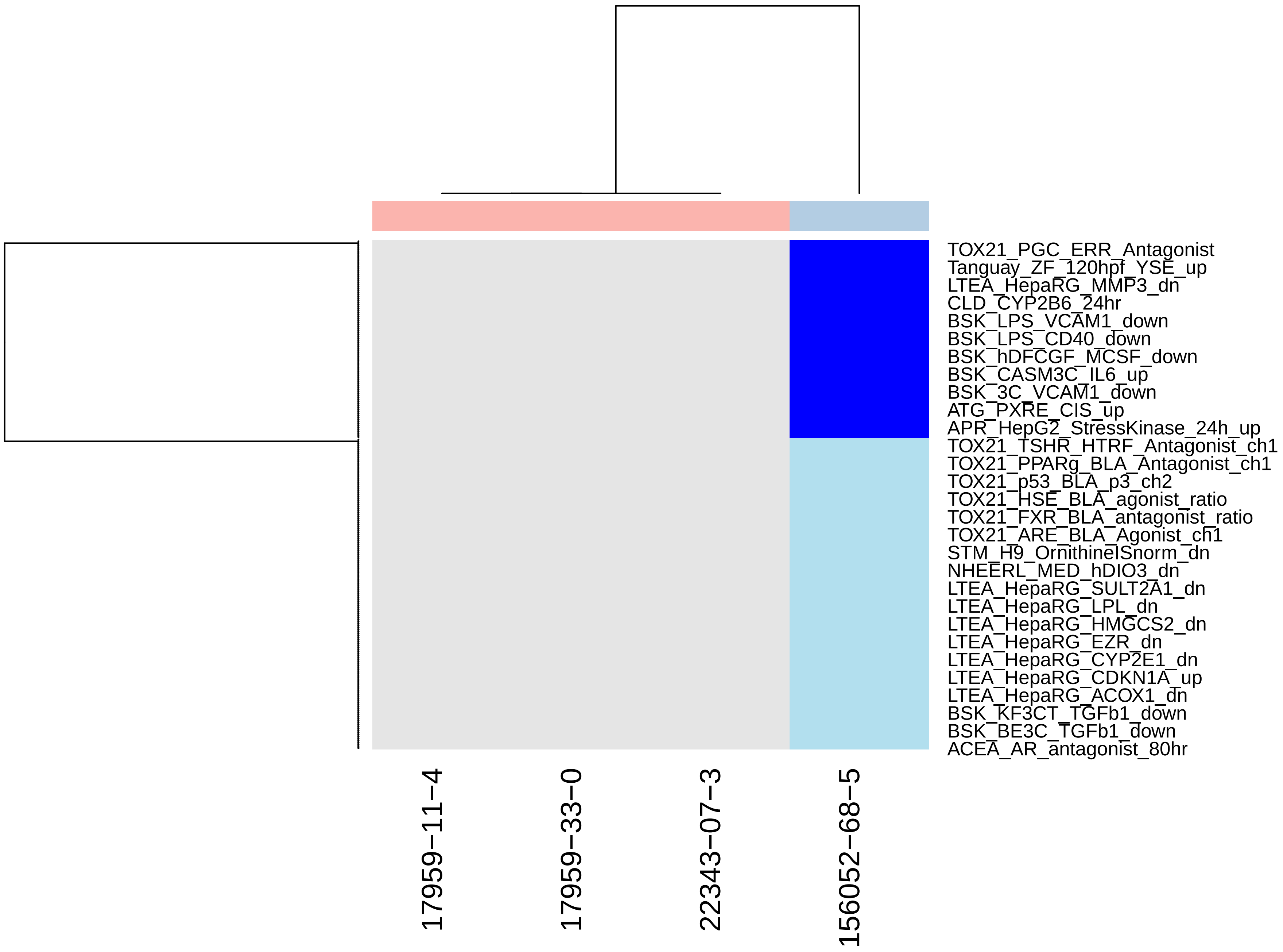

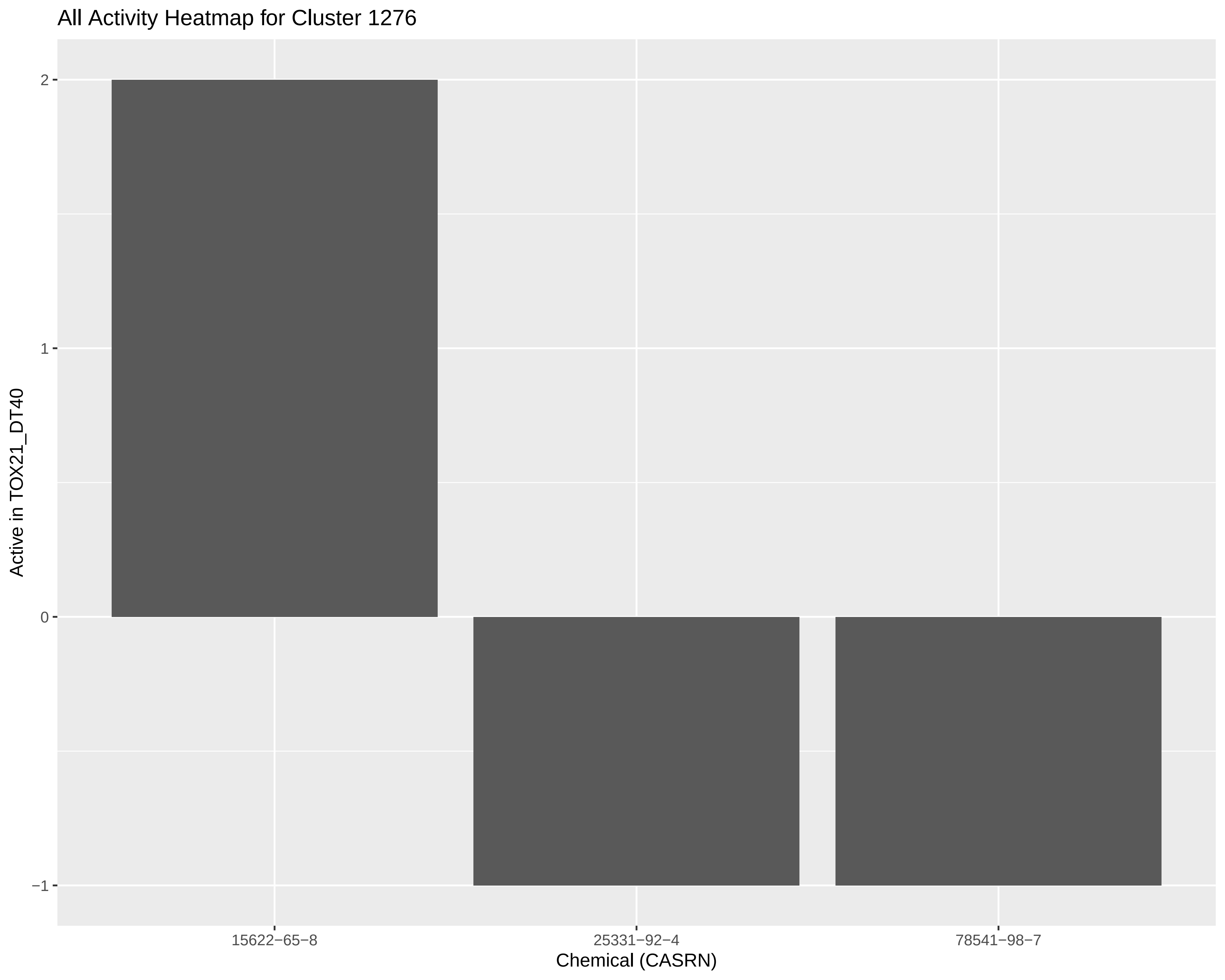
All Activity Heatmap for Cluster 1276
Active in TOX21_DT40
2
1
0
−1
15622−65−8
25331−92−4
78541−98−7
Chemical (CASRN)

All Activity Heatmap for Cluster 1277

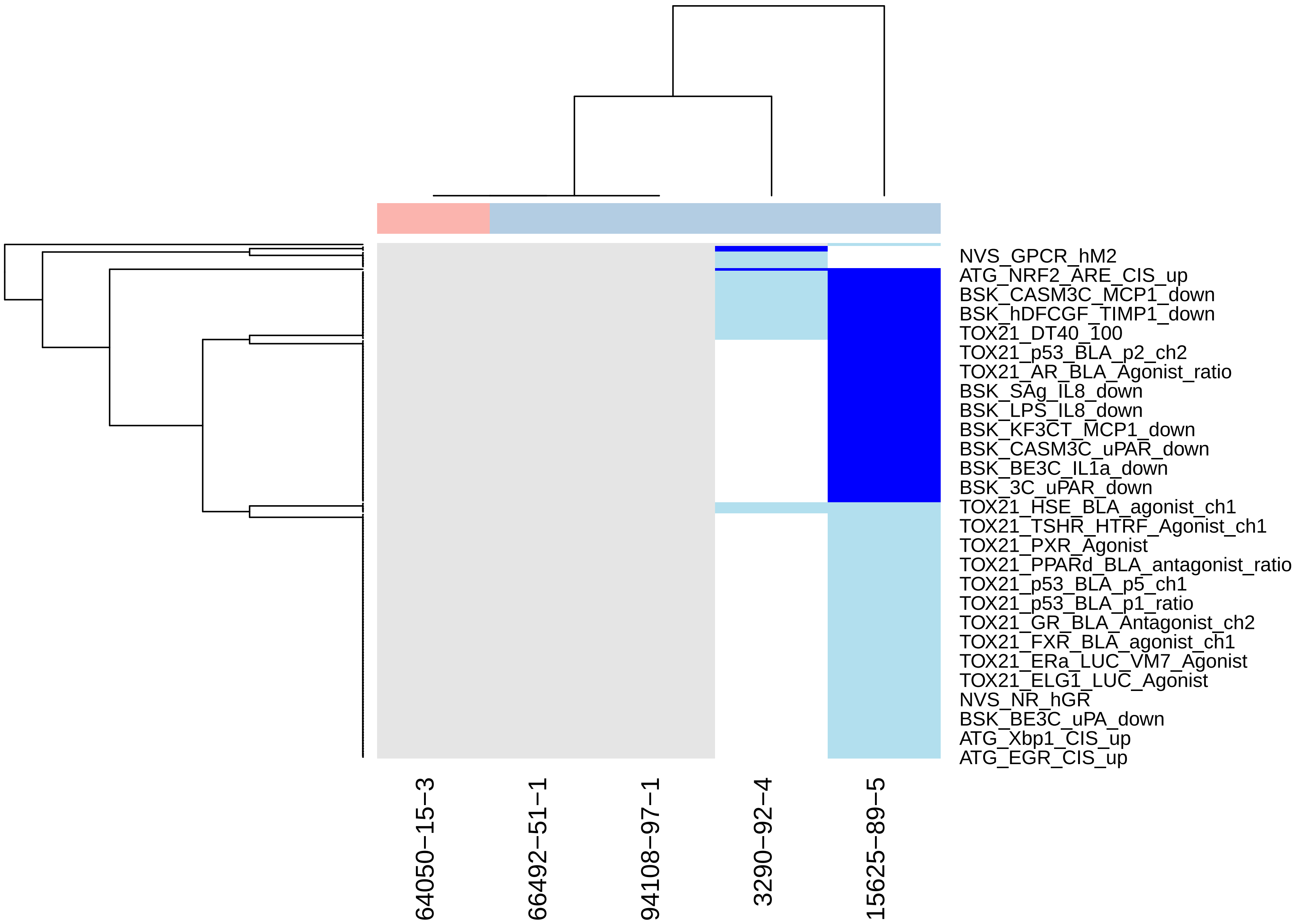

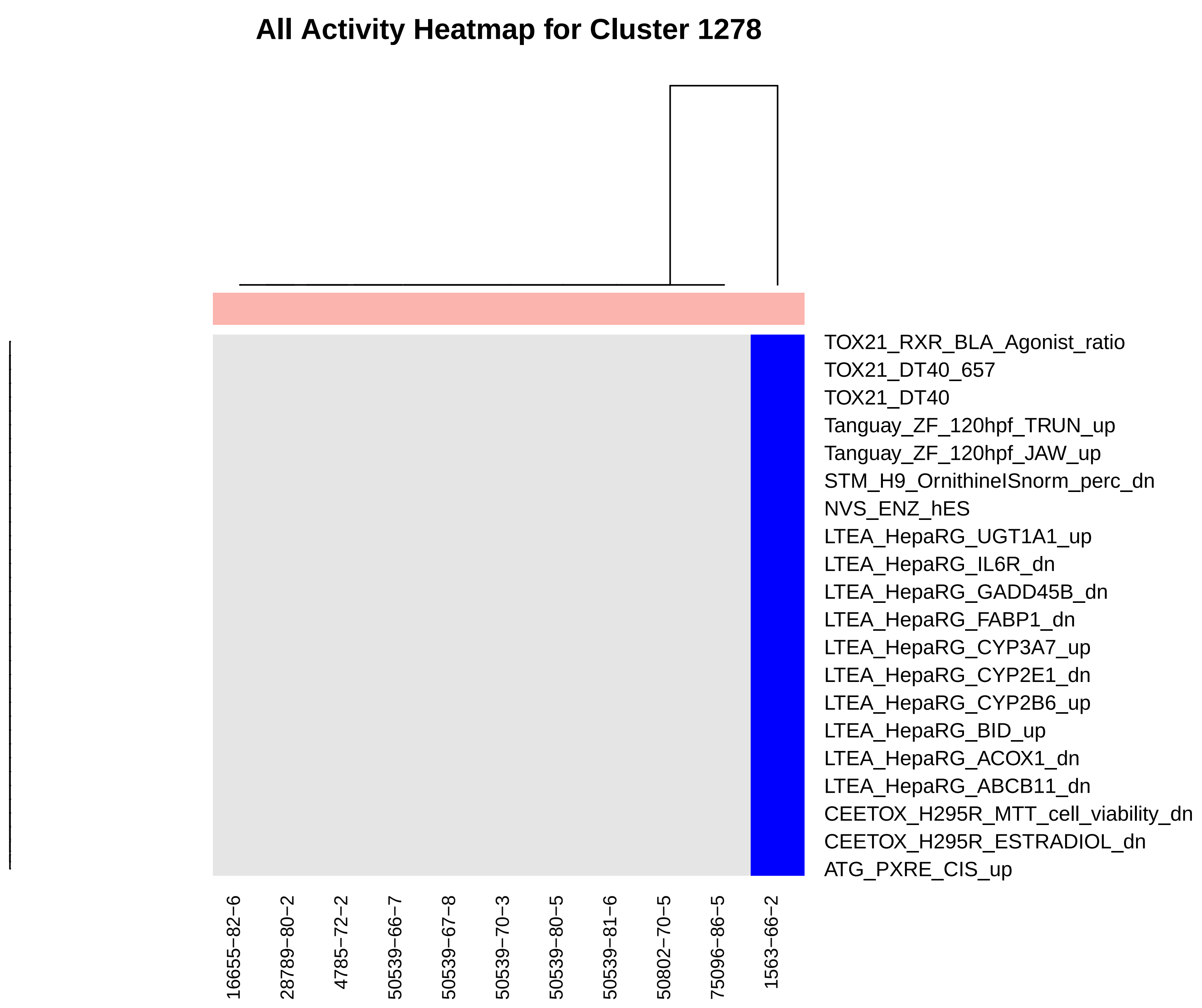
All Activity Heatmap for Cluster 1278
TOX21_RXR_BLA_Agonist_ratio
TOX21_DT40_657
TOX21_DT40
Tanguay_ZF_120hpf_TRUN_up
Tanguay_ZF_120hpf_JAW_up
STM_H9_OrnithineISnorm_perc_dn
NVS_ENZ_hES
LTEA_HepaRG_UGT1A1_up
LTEA_HepaRG_IL6R_dn
LTEA_HepaRG_GADD45B_dn
LTEA_HepaRG_FABP1_dn
LTEA_HepaRG_CYP3A7_up
LTEA_HepaRG_CYP2E1_dn
LTEA_HepaRG_CYP2B6_up
LTEA_HepaRG_BID_up
LTEA_HepaRG_ACOX1_dn
LTEA_HepaRG_ABCB11_dn
CEETOX_H295R_MTT_cell_viability_dn
CEETOX_H295R_ESTRADIOL_dn
ATG_PXRE_CIS_up
16655−82−6
28789−80−2
4785−72−2
50539−66−7
50539−67−8
50539−70−3
50539−80−5
50539−81−6
50802−70−5
75096−86−5
1563−66−2

All Activity Heatmap for Cluster 1279

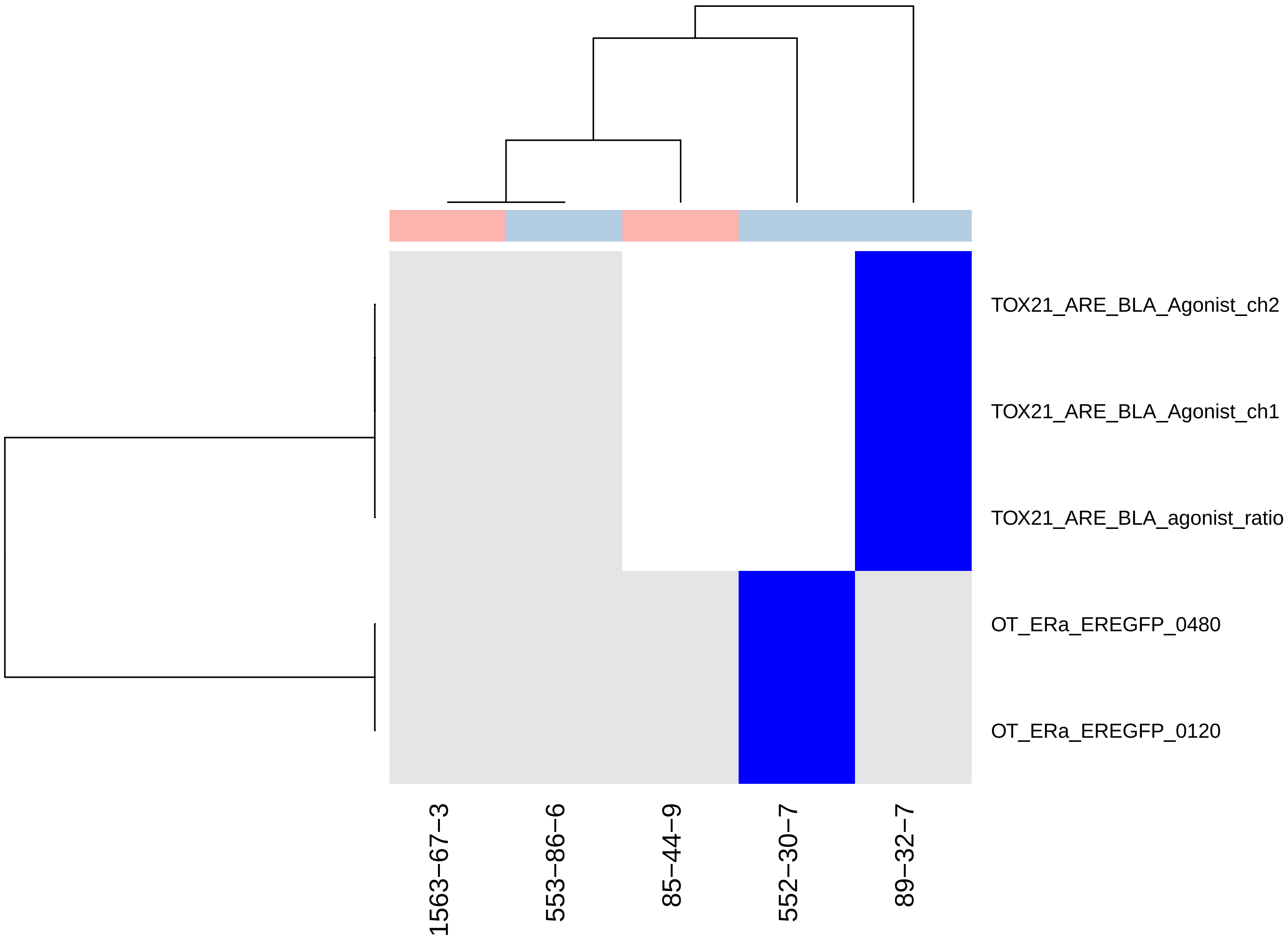

All Activity Heatmap for Cluster 1281

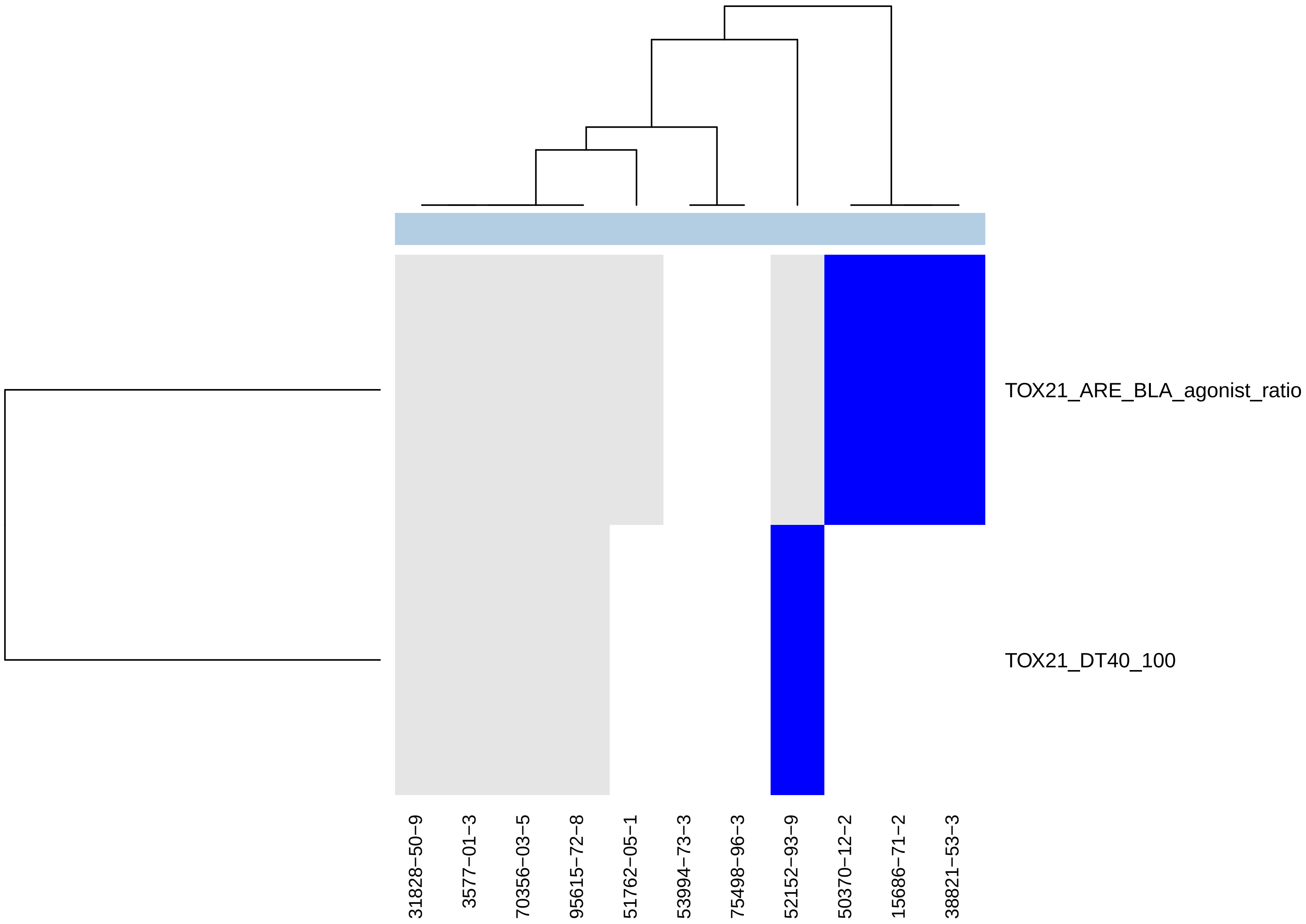

All Activity Heatmap for Cluster 1282

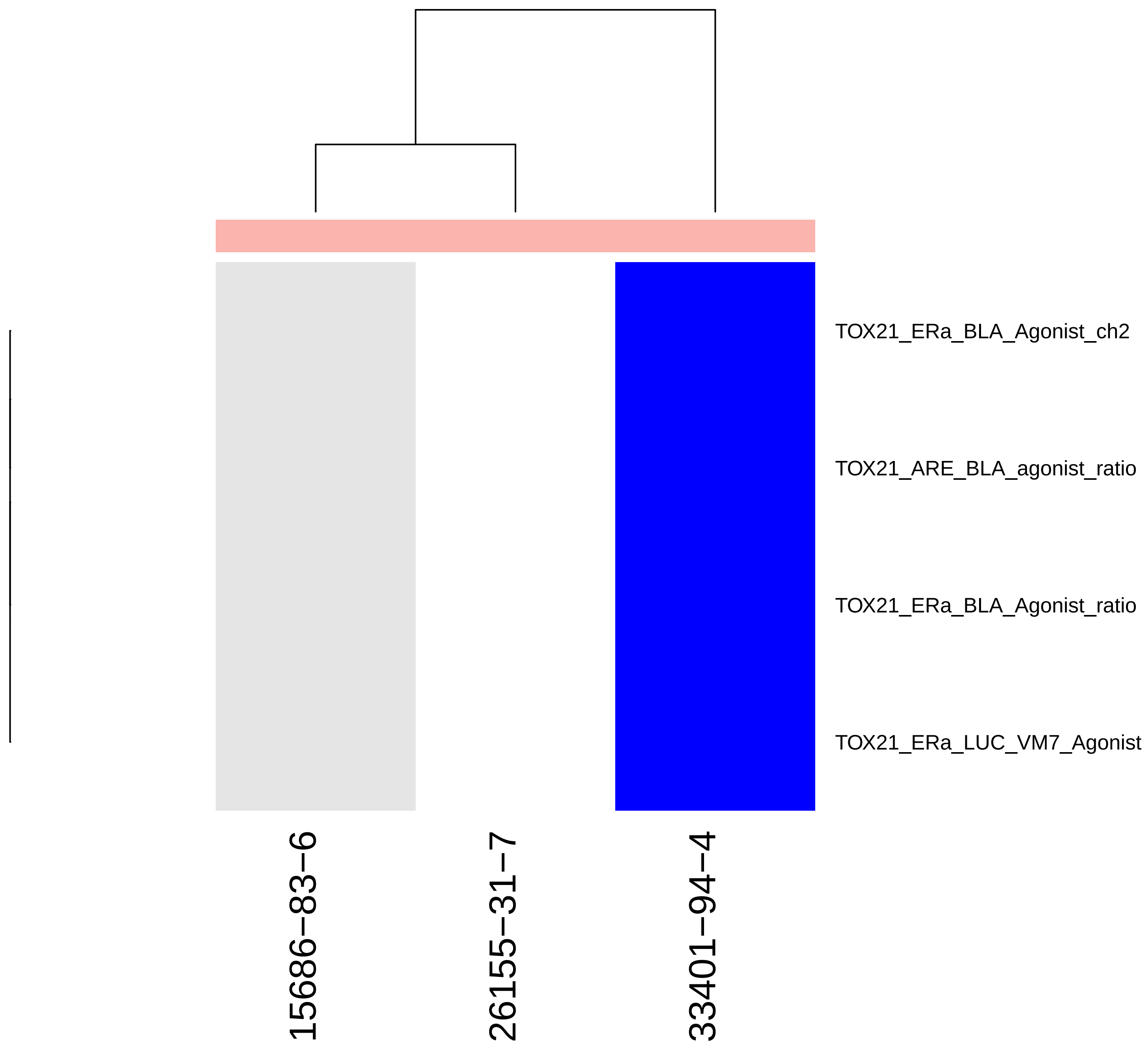

All Activity Heatmap for Cluster 1283

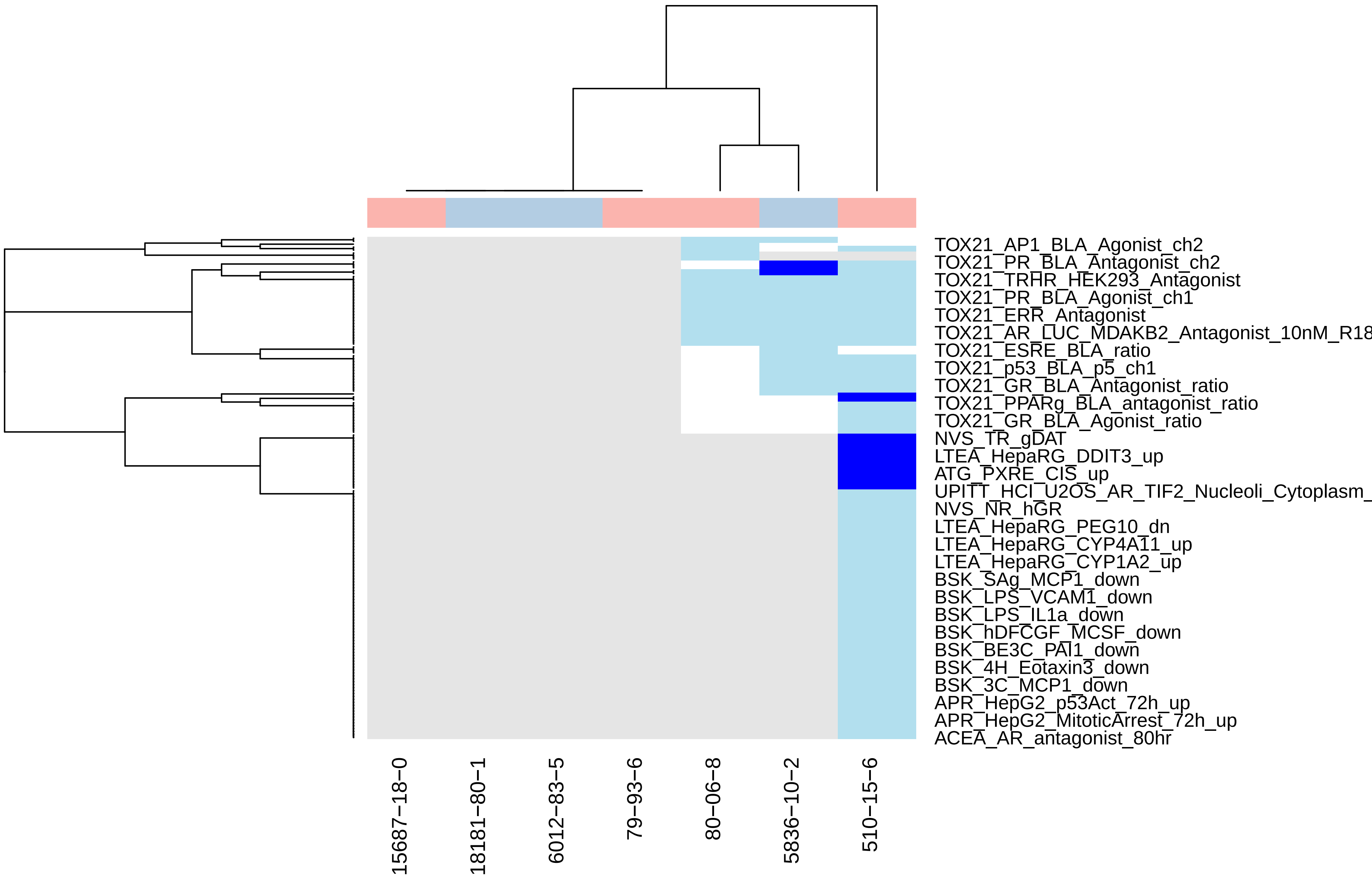

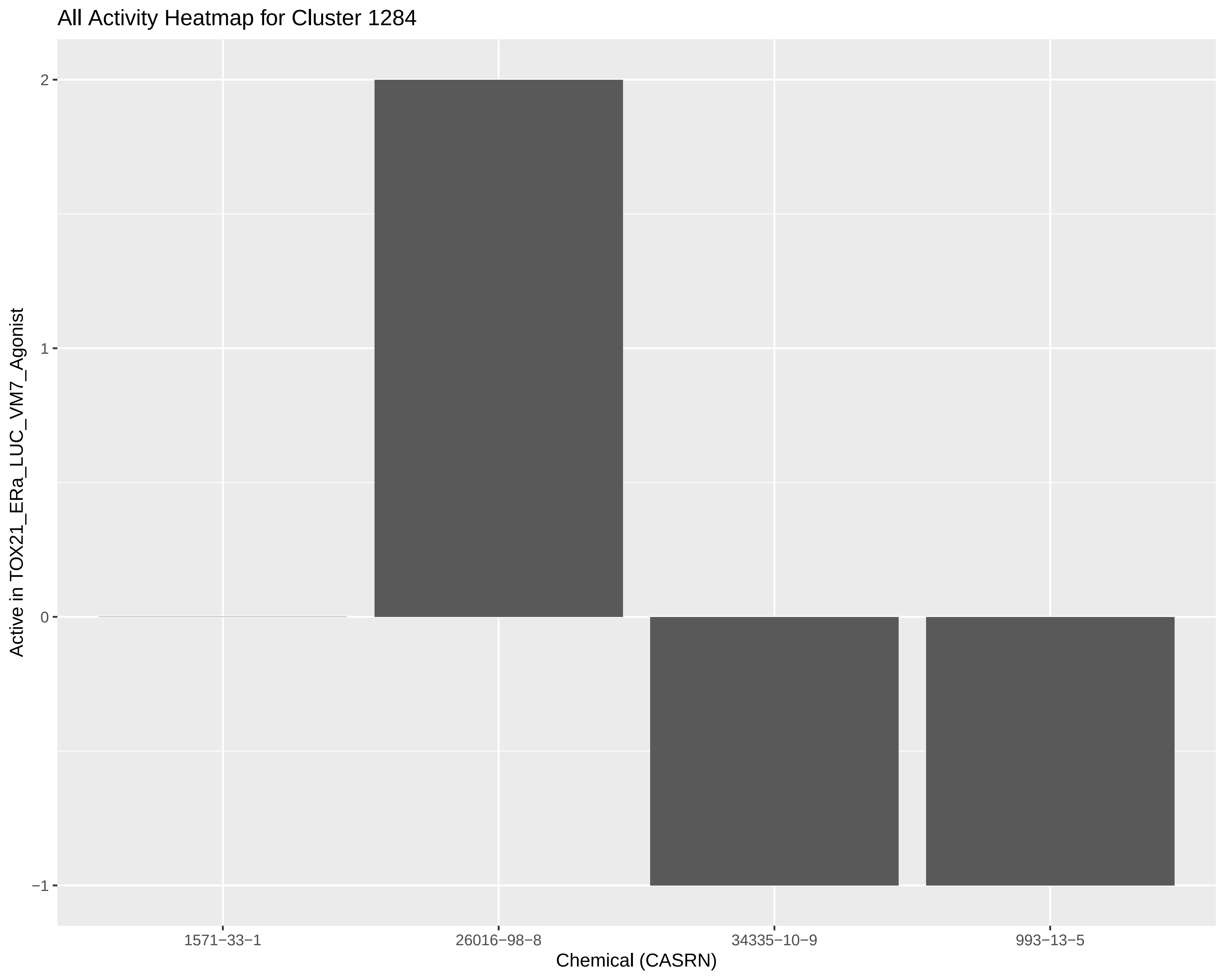
All Activity Heatmap for Cluster 1284
Active in TOX21_ERa_LUC_VM7_Agonist
2
1
0
−1
1571−33−1
26016−98−8
34335−10−9
993−13−5
Chemical (CASRN)

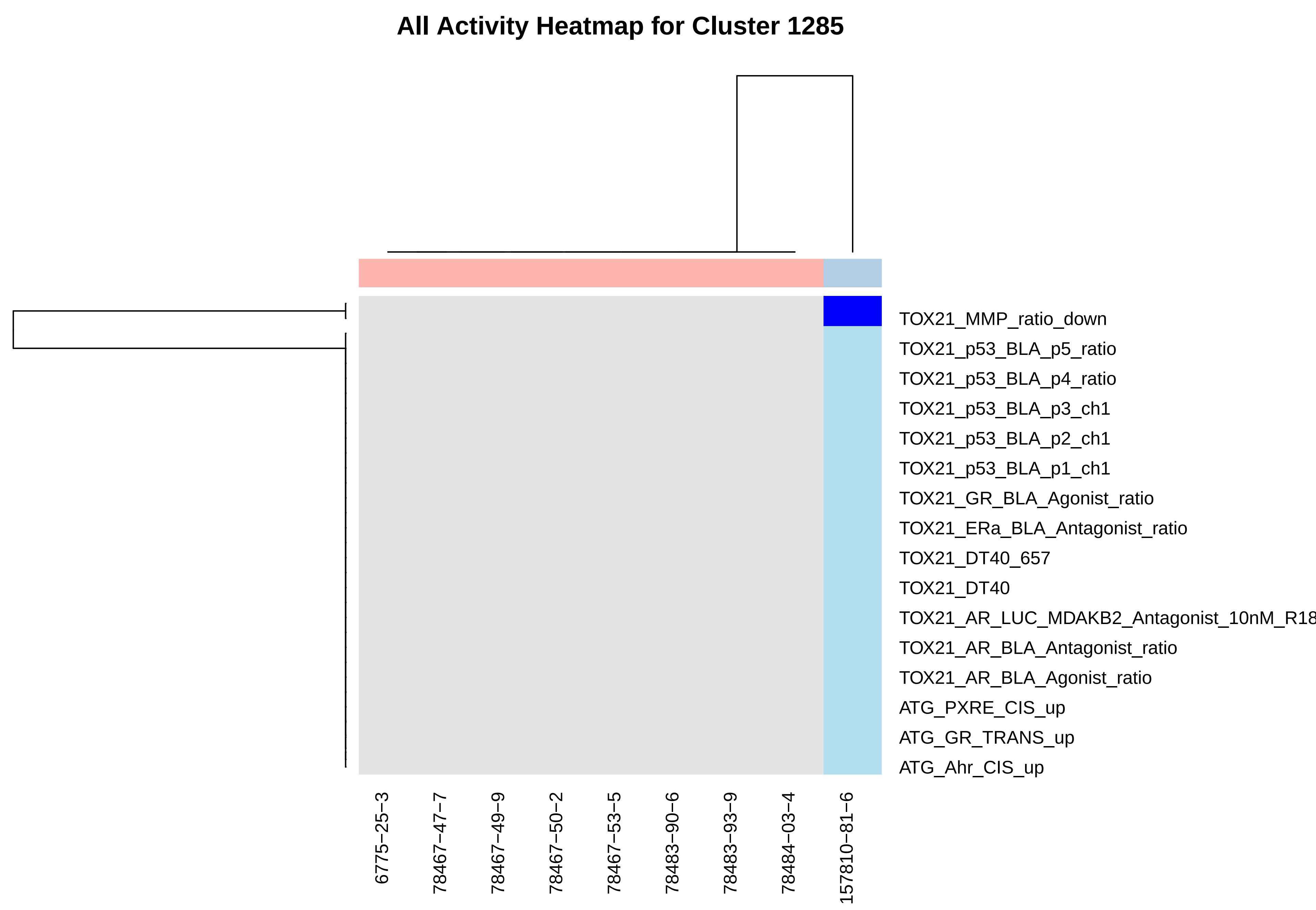
All Activity Heatmap for Cluster 1285
TOX21_MMP_ratio_down
TOX21_p53_BLA_p5_ratio
TOX21_p53_BLA_p4_ratio
TOX21_p53_BLA_p3_ch1
TOX21_p53_BLA_p2_ch1
TOX21_p53_BLA_p1_ch1
TOX21_GR_BLA_Agonist_ratio
TOX21_ERa_BLA_Antagonist_ratio
TOX21_DT40_657
TOX21_DT40
TOX21_AR_LUC_MDAKB2_Antagonist_10nM_R18
TOX21_AR_BLA_Antagonist_ratio
TOX21_AR_BLA_Agonist_ratio
ATG_PXRE_CIS_up
ATG_GR_TRANS_up
ATG_Ahr_CIS_up
6775−25−3
78467−47−7
78467−49−9
78467−50−2
78467−53−5
78483−90−6
78483−93−9
78484−03−4
157810−81−6

All Activity Heatmap for Cluster 1286

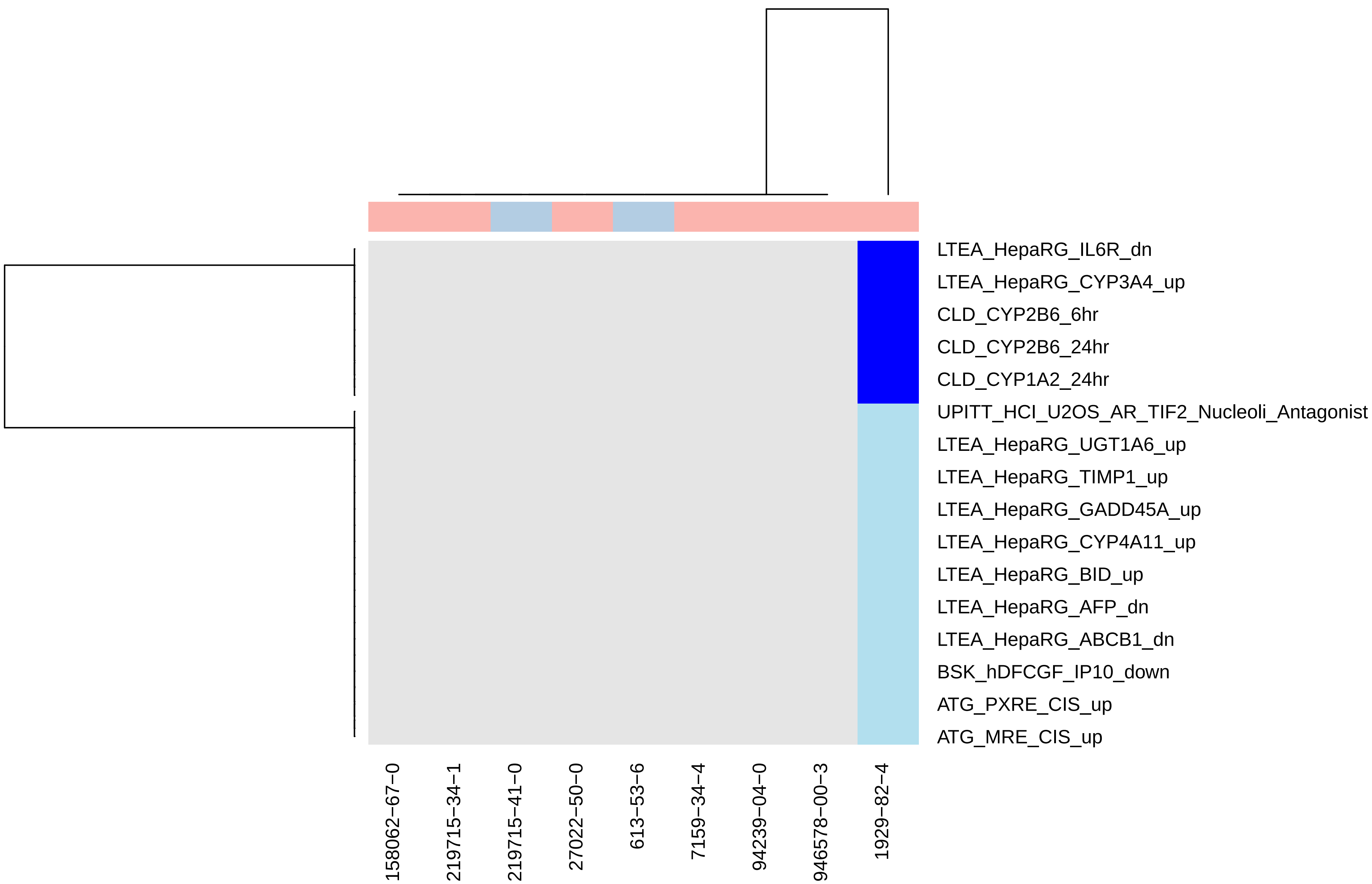

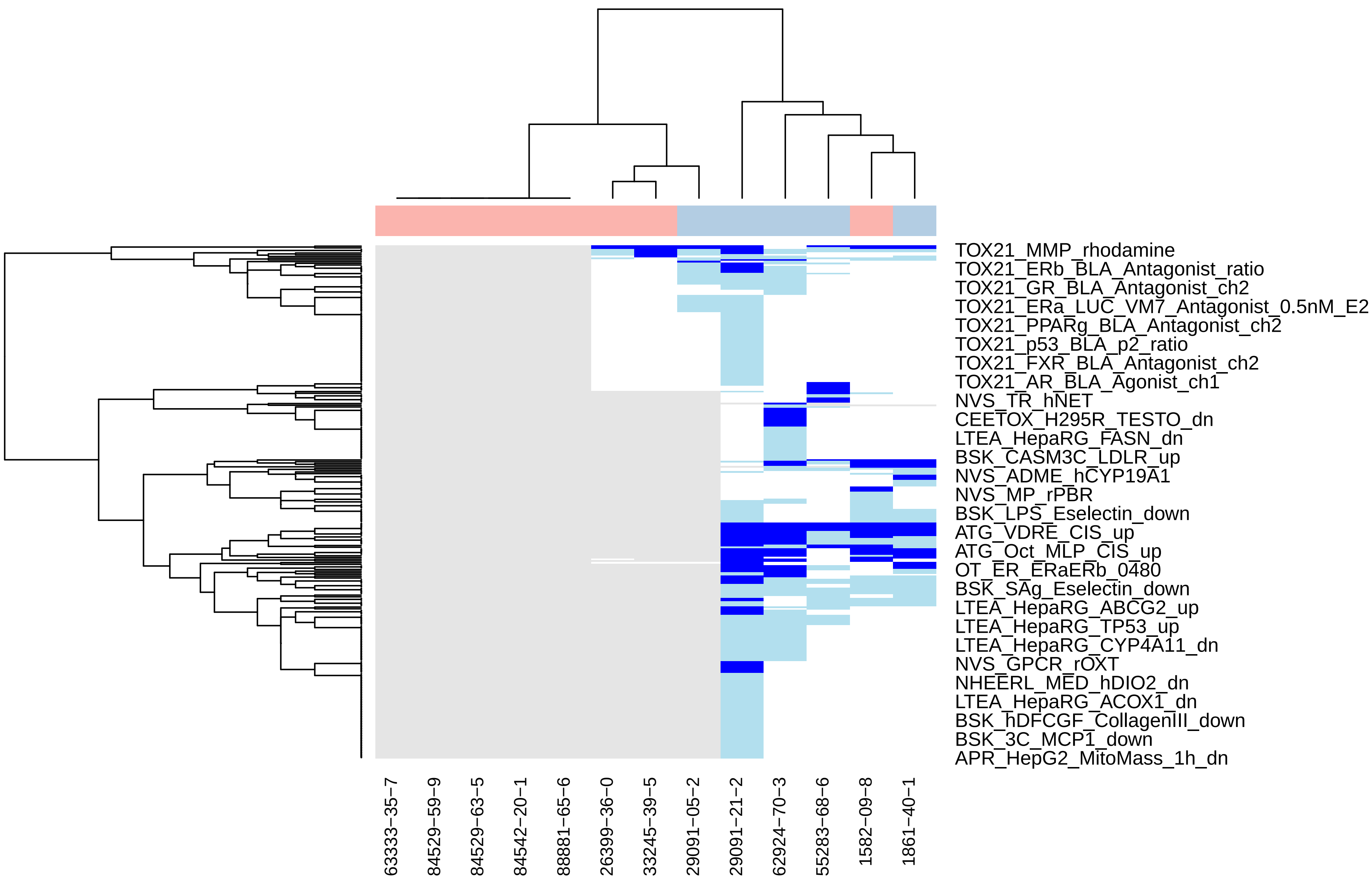

All Activity Heatmap for Cluster 1287
TOX21_MMP_rhodamine
TOX21_ERb_BLA_Antagonist_ratio
TOX21_GR_BLA_Antagonist_ch2
TOX21_ERa_LUC_VM7_Antagonist_0.5nM_E2
TOX21_PPARg_BLA_Antagonist_ch2
TOX21_p53_BLA_p2_ratio
TOX21_FXR_BLA_Antagonist_ch2
TOX21_AR_BLA_Agonist_ch1
NVS_TR_hNET
CEETOX_H295R_TESTO_dn
LTEA_HepaRG_FASN_dn
BSK_CASM3C_LDLR_up
NVS_ADME_hCYP19A1
NVS_MP_rPBR
BSK_LPS_Eselectin_down
ATG_VDRE_CIS_up
ATG_Oct_MLP_CIS_up
OT_ER_ERaERb_0480
BSK_SAg_Eselectin_down
LTEA_HepaRG_ABCG2_up
LTEA_HepaRG_TP53_up
LTEA_HepaRG_CYP4A11_dn
NVS_GPCR_rOXT
NHEERL_MED_hDIO2_dn
LTEA_HepaRG_ACOX1_dn
BSK_hDFCGF_CollagenIII_down
BSK_3C_MCP1_down
APR_HepG2_MitoMass_1h_dn
63333−35−7
84529−59−9
84529−63−5
84542−20−1
88881−65−6
26399−36−0
33245−39−5
29091−05−2
29091−21−2
62924−70−3
55283−68−6
1582−09−8
1861−40−1

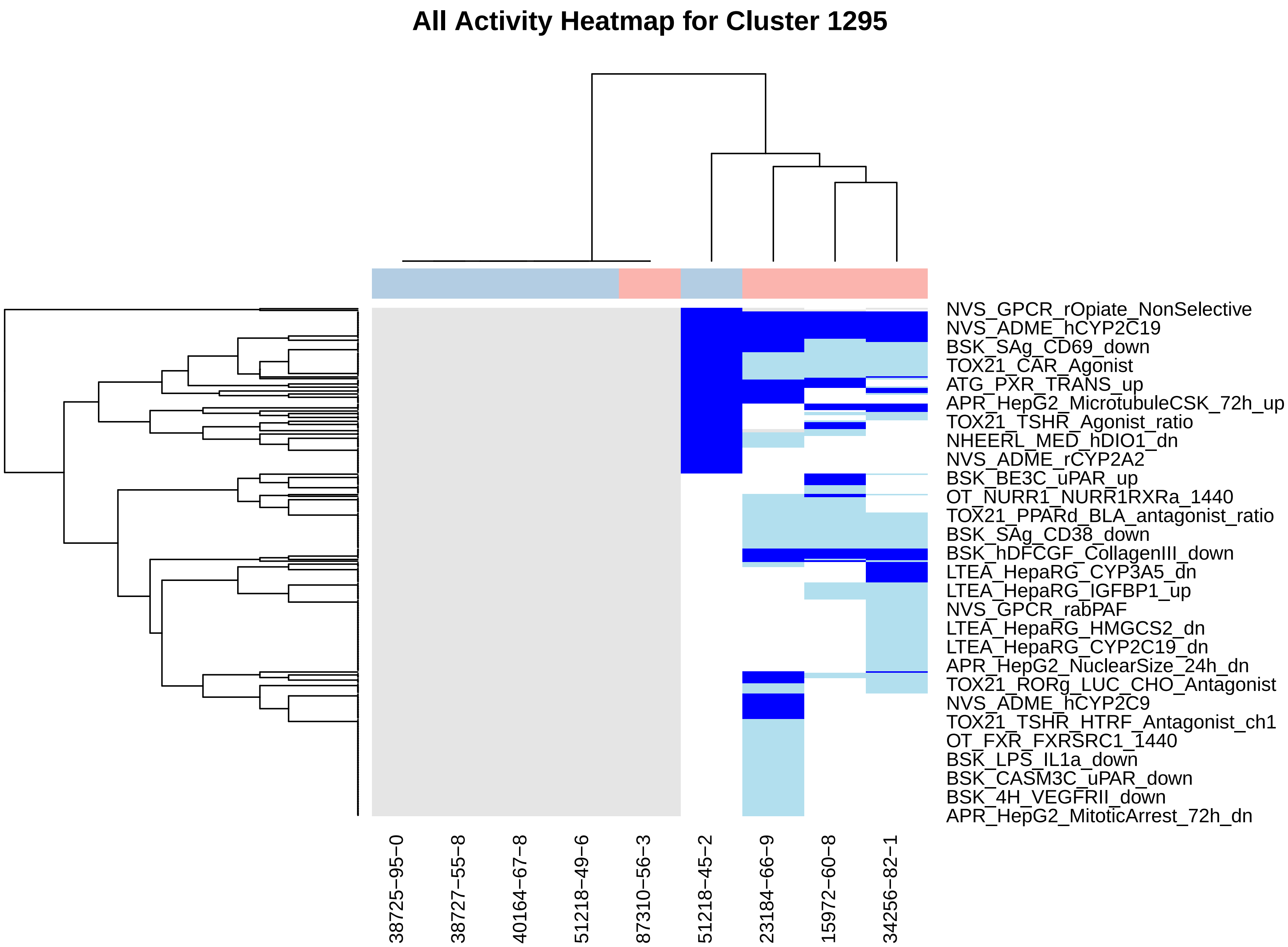

All Activity Heatmap for Cluster 1295
NVS_GPCR_rOpiate_NonSelective
NVS_ADME_hCYP2C19
BSK_SAg_CD69_down
TOX21_CAR_Agonist
ATG_PXR_TRANS_up
APR_HepG2_MicrotubuleCSK_72h_up
TOX21_TSHR_Agonist_ratio
NHEERL_MED_hDIO1_dn
NVS_ADME_rCYP2A2
BSK_BE3C_uPAR_up
OT_NURR1_NURR1RXRa_1440
TOX21_PPARd_BLA_antagonist_ratio
BSK_SAg_CD38_down
BSK_hDFCGF_CollagenIII_down
LTEA_HepaRG_CYP3A5_dn
LTEA_HepaRG_IGFBP1_up
NVS_GPCR_rabPAF
LTEA_HepaRG_HMGCS2_dn
LTEA_HepaRG_CYP2C19_dn
APR_HepG2_NuclearSize_24h_dn
TOX21_RORg_LUC_CHO_Antagonist
NVS_ADME_hCYP2C9
TOX21_TSHR_HTRF_Antagonist_ch1
OT_FXR_FXRSRC1_1440
BSK_LPS_IL1a_down
BSK_CASM3C_uPAR_down
BSK_4H_VEGFRII_down
APR_HepG2_MitoticArrest_72h_dn
38725−95−0
38727−55−8
40164−67−8
51218−49−6
87310−56−3
51218−45−2
23184−66−9
15972−60−8
34256−82−1

All Activity Heatmap for Cluster 1296

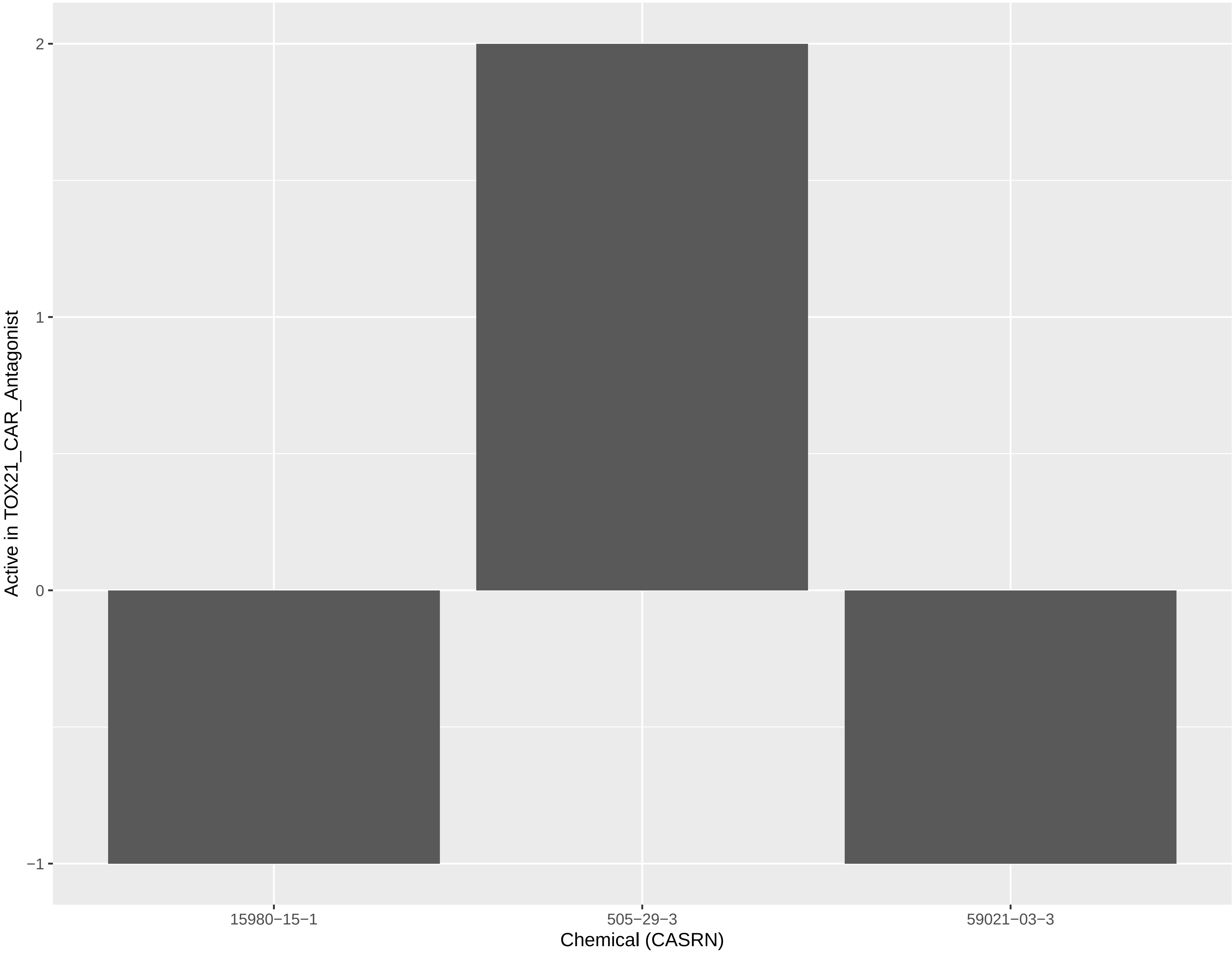

All Activity Heatmap for Cluster 1297

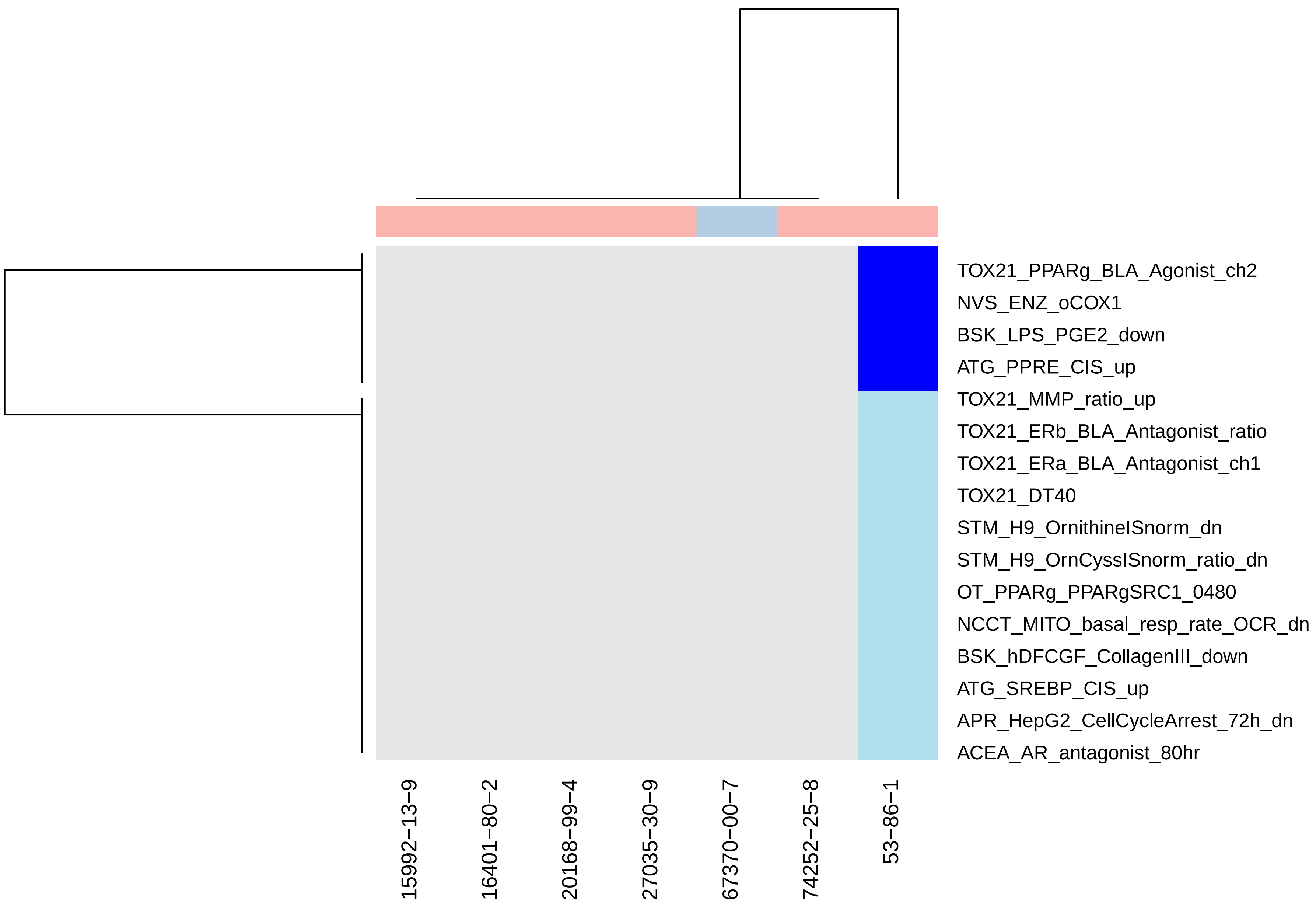

All Activity Heatmap for Cluster 1298

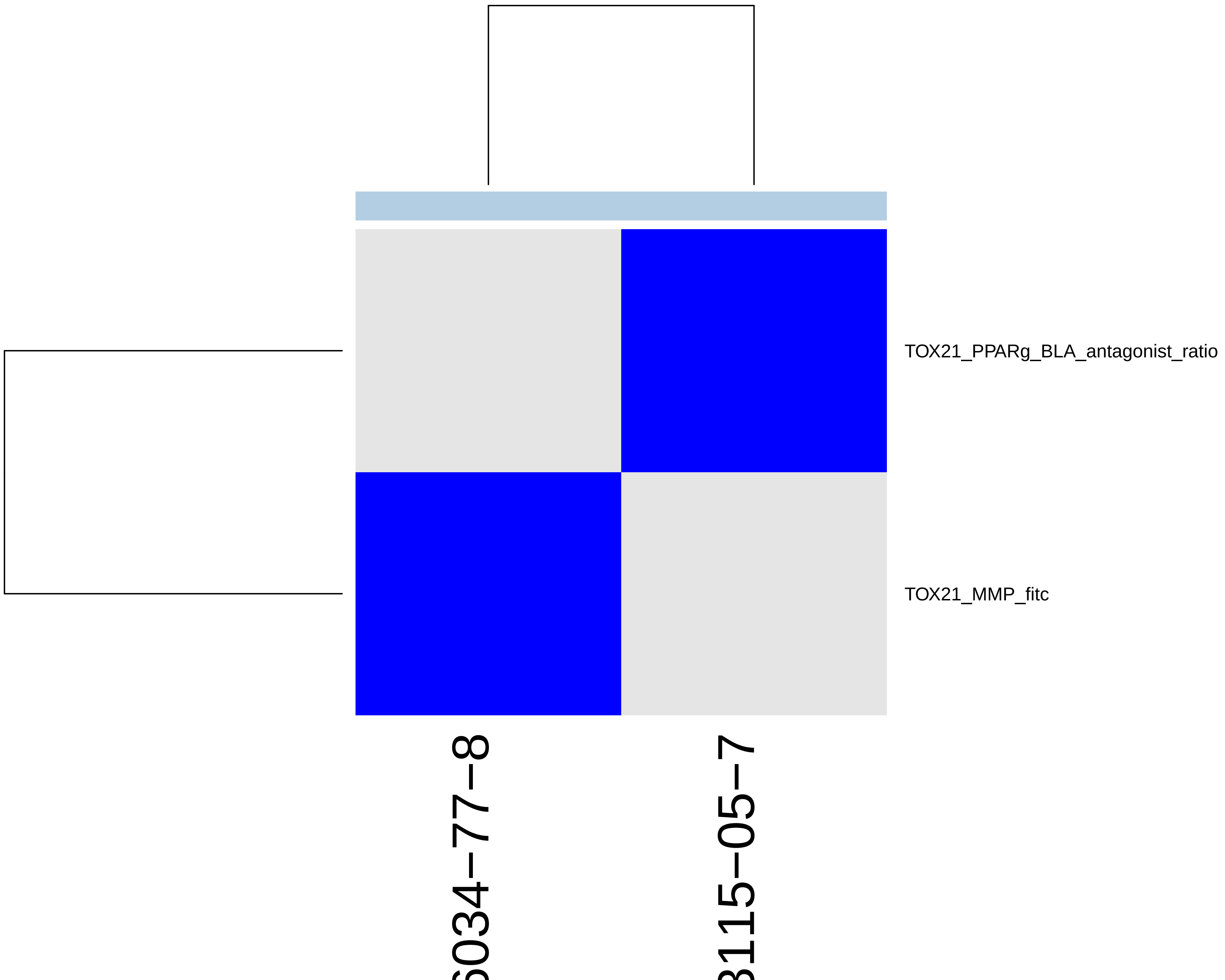

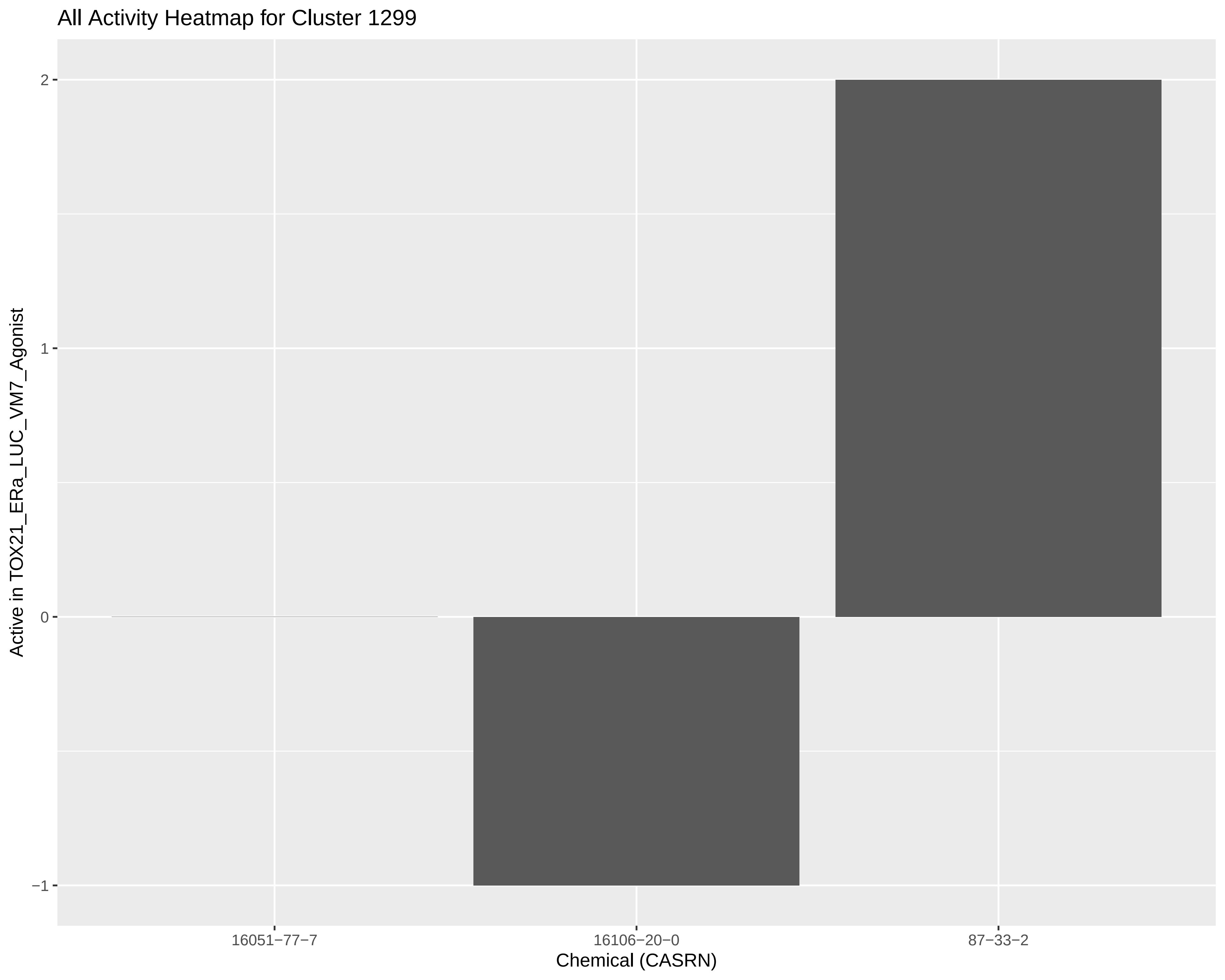

All Activity Heatmap for Cluster 1299
Active in TOX21_ERa_LUC_VM7_Agonist
2
1
0
−1
16051−77−7
16106−20−0
87−33−2
Chemical (CASRN)

All Activity Heatmap for Cluster 1301

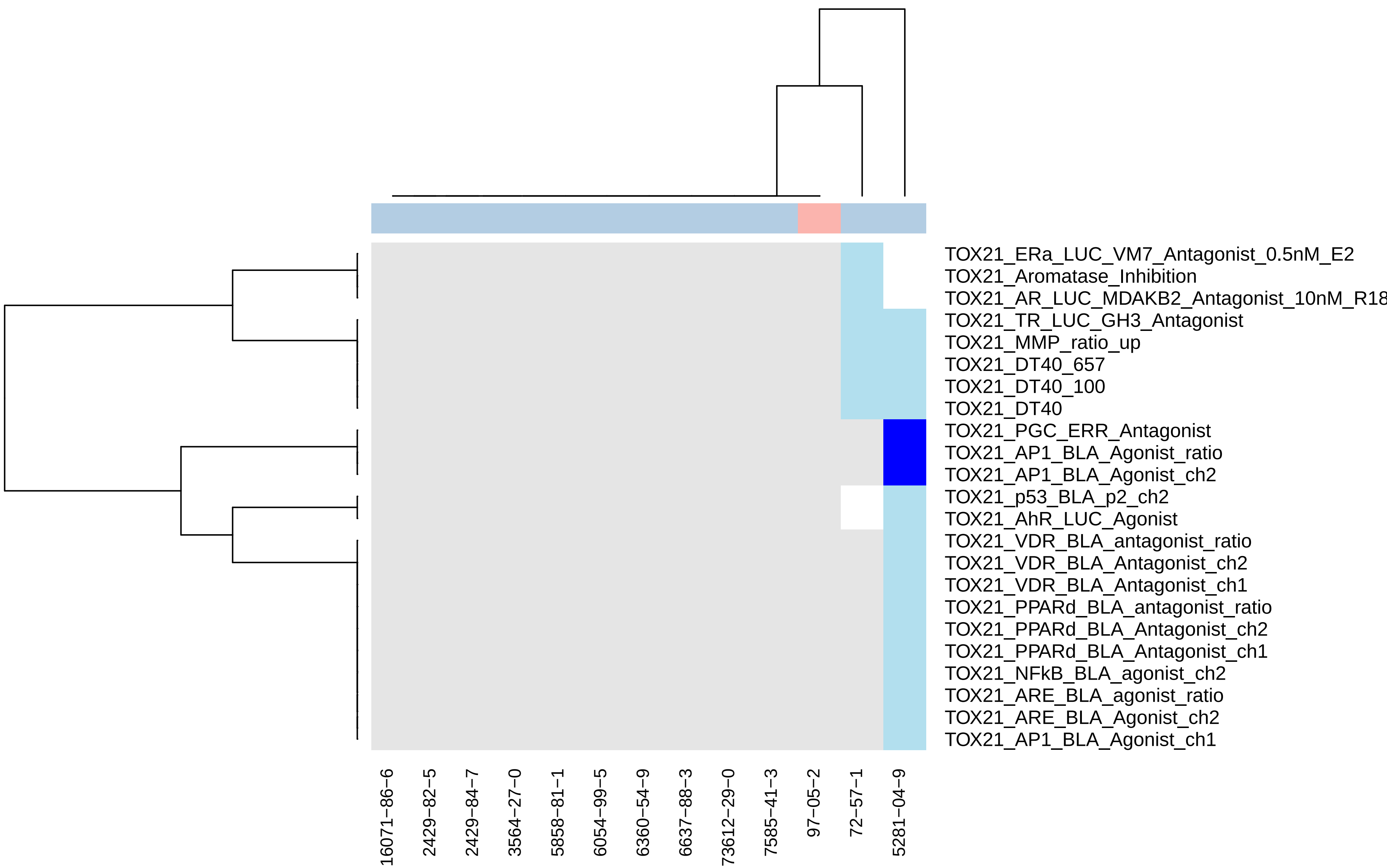

All Activity Heatmap for Cluster 1304

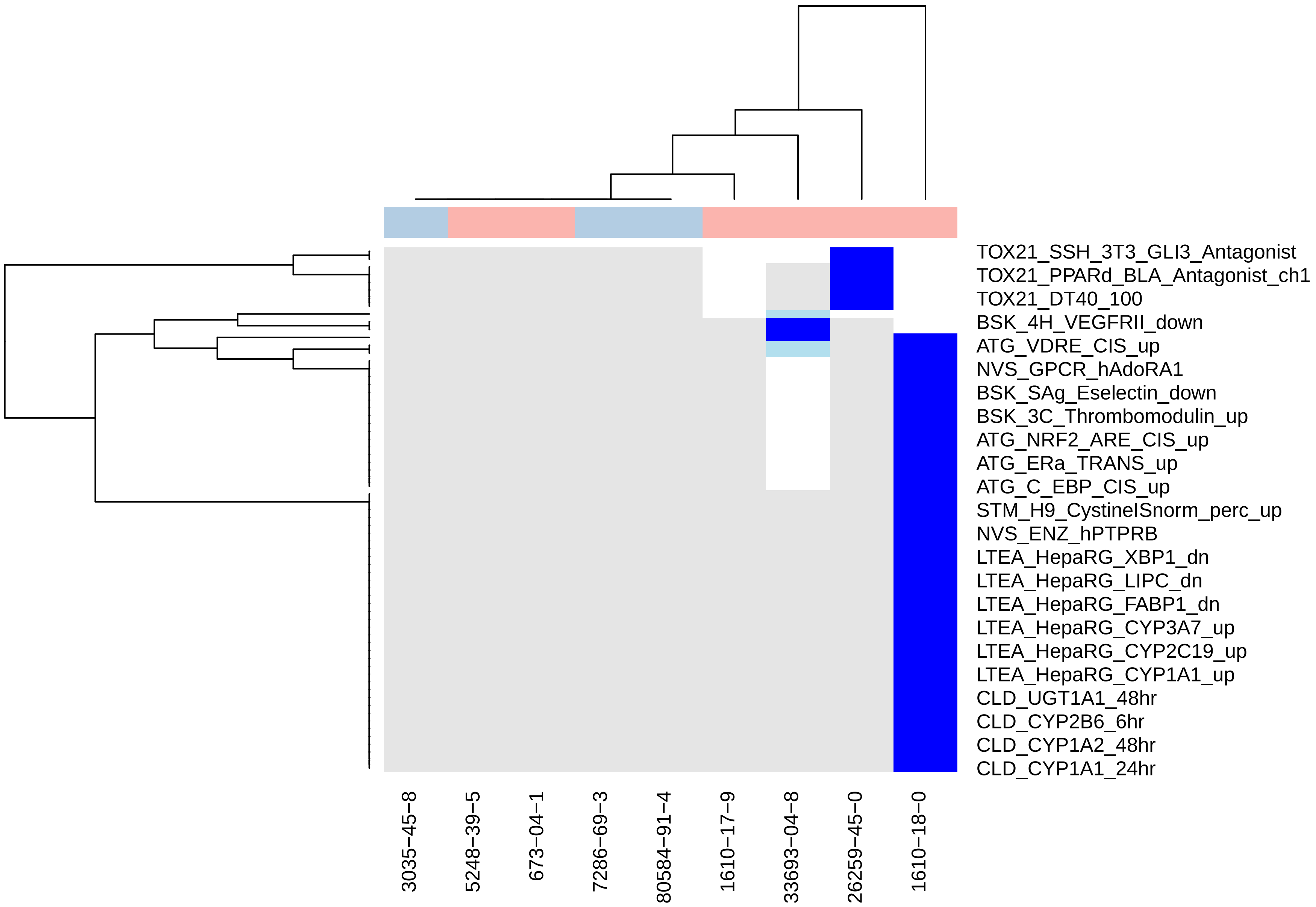

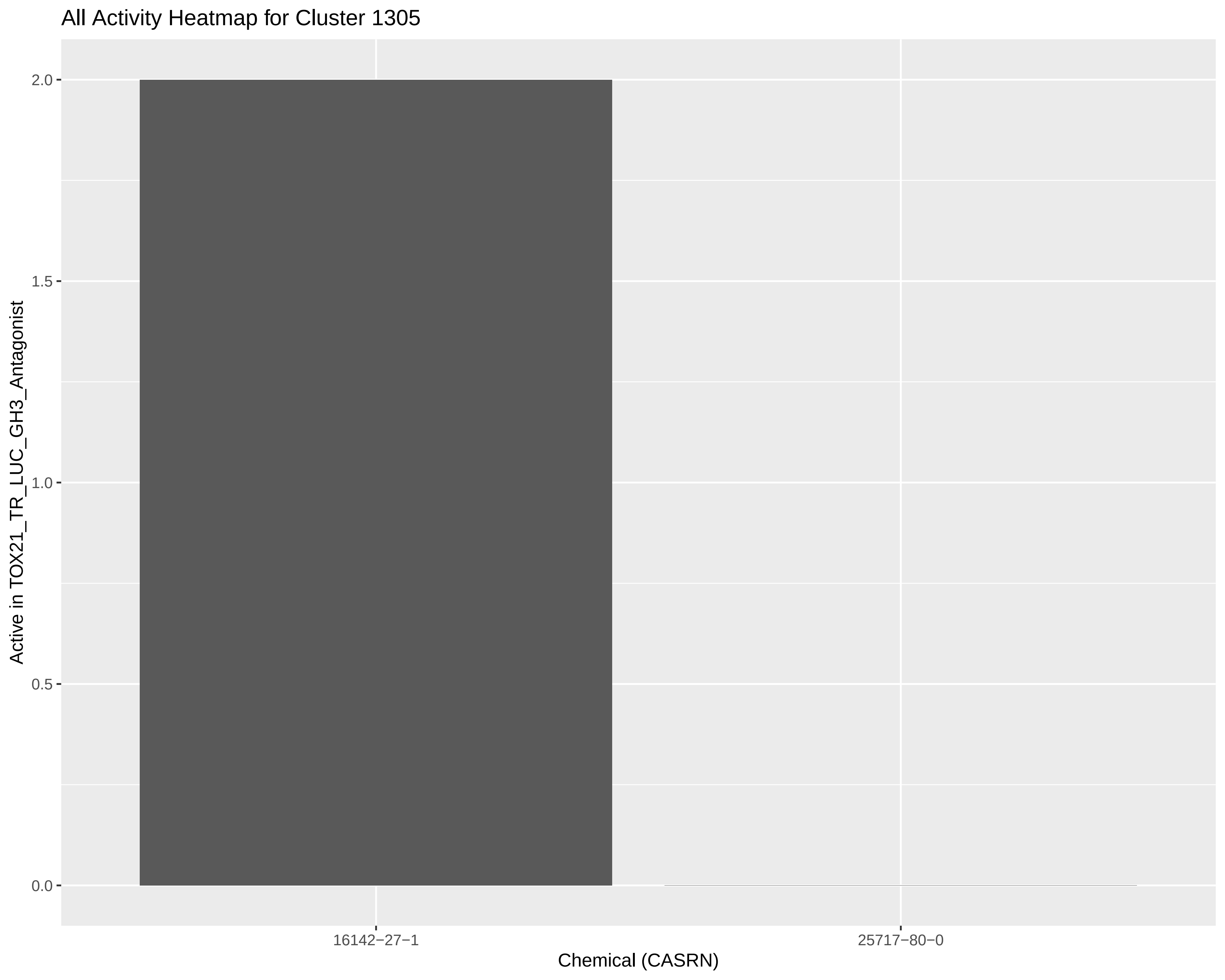
All Activity Heatmap for Cluster 1305
Active in TOX21_TR_LUC_GH3_Antagonist
2.0
1.5
1.0
0.5
0.0
16142−27−1
25717−80−0
Chemical (CASRN)

All Activity Heatmap for Cluster 1311

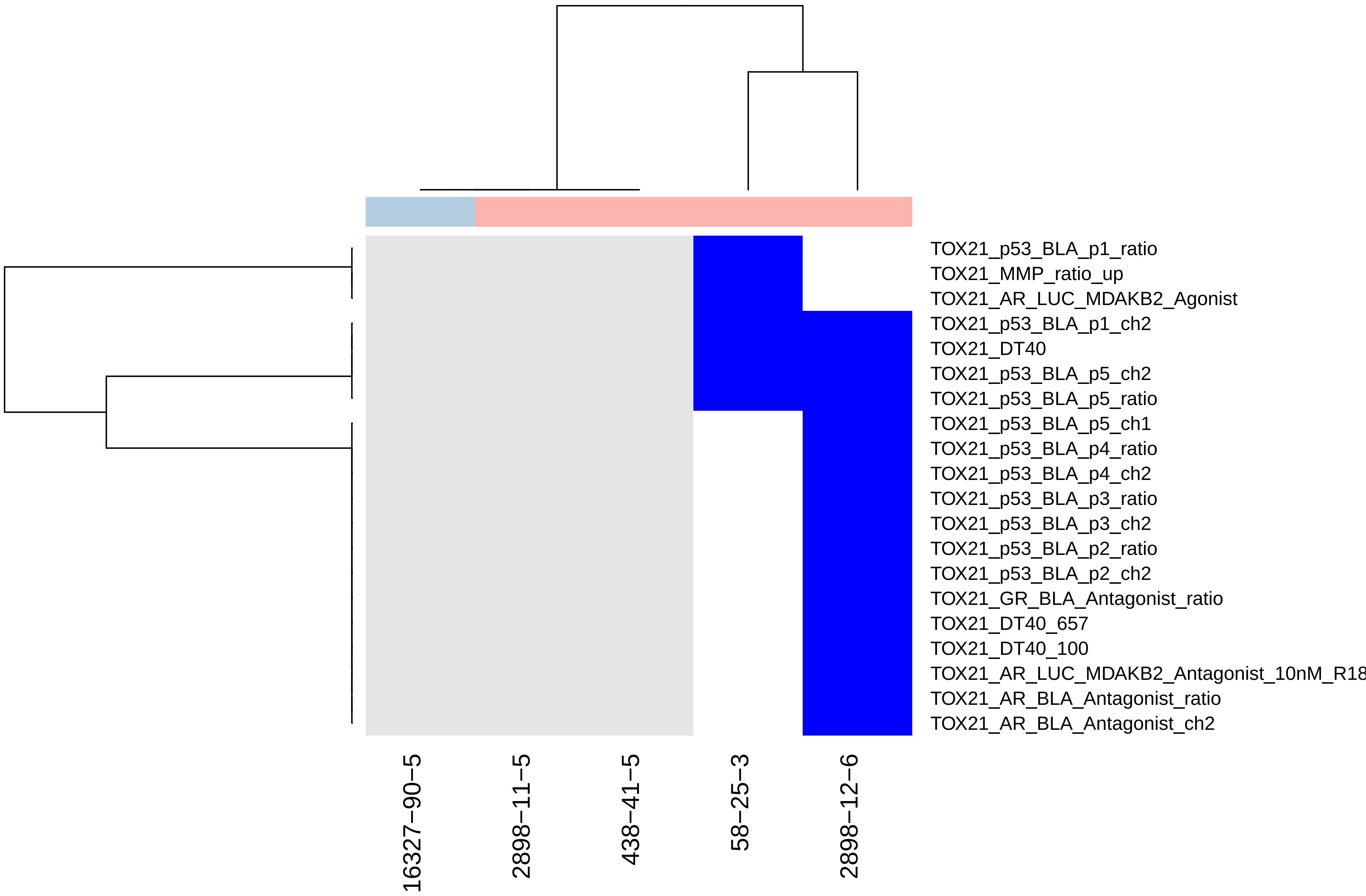

All Activity Heatmap for Cluster 1312

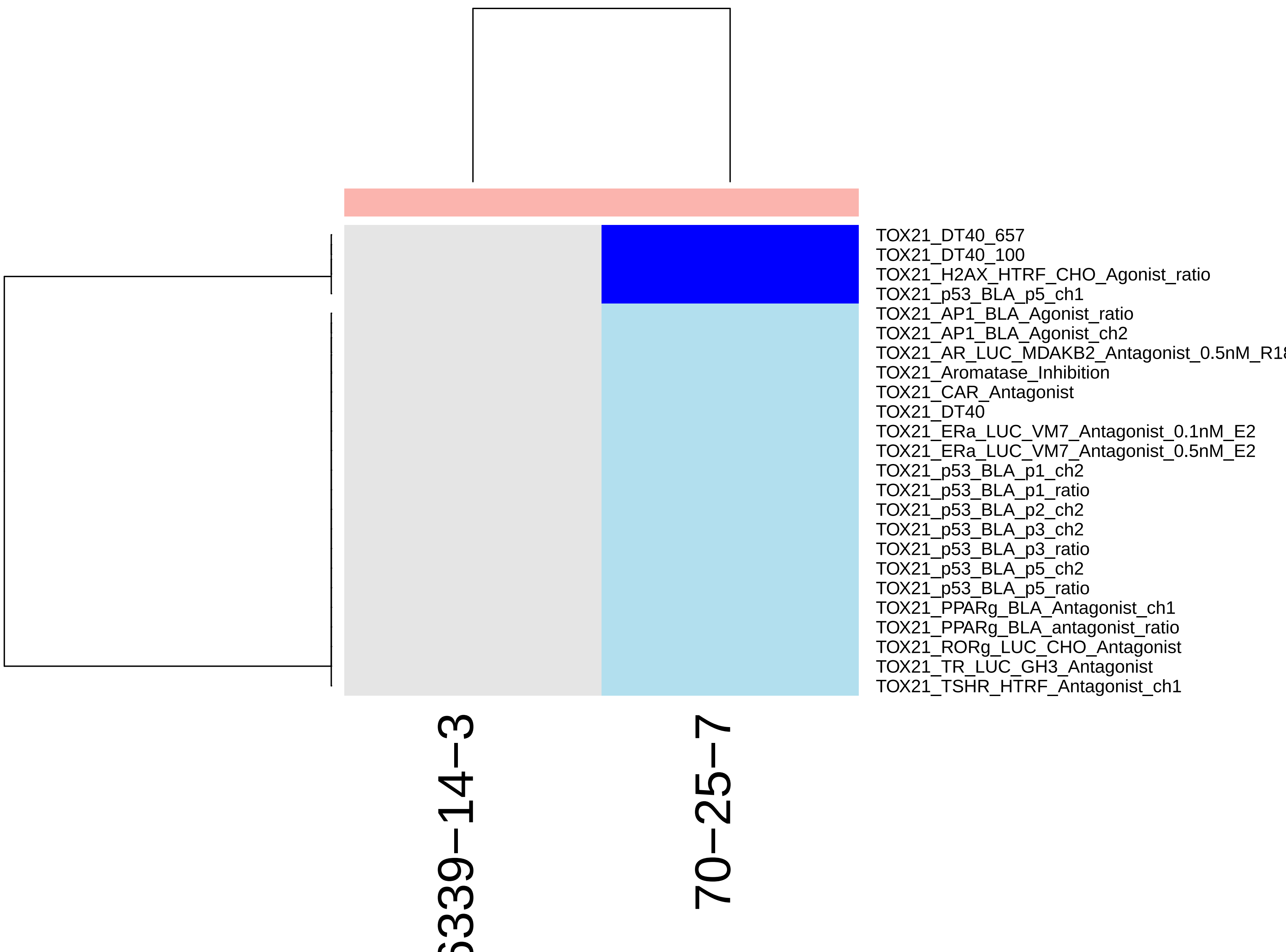

All Activity Heatmap for Cluster 1314

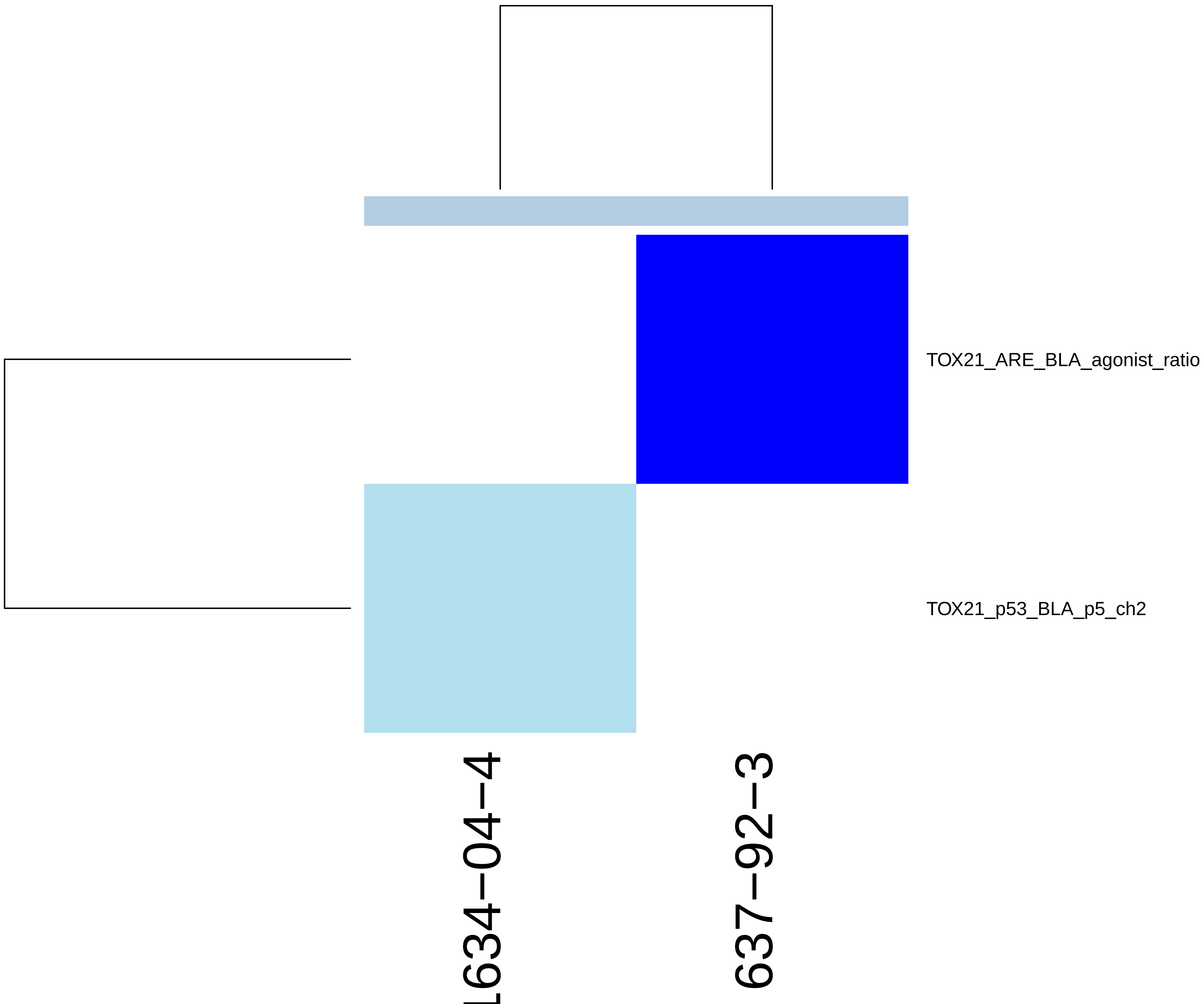

All Activity Heatmap for Cluster 1315

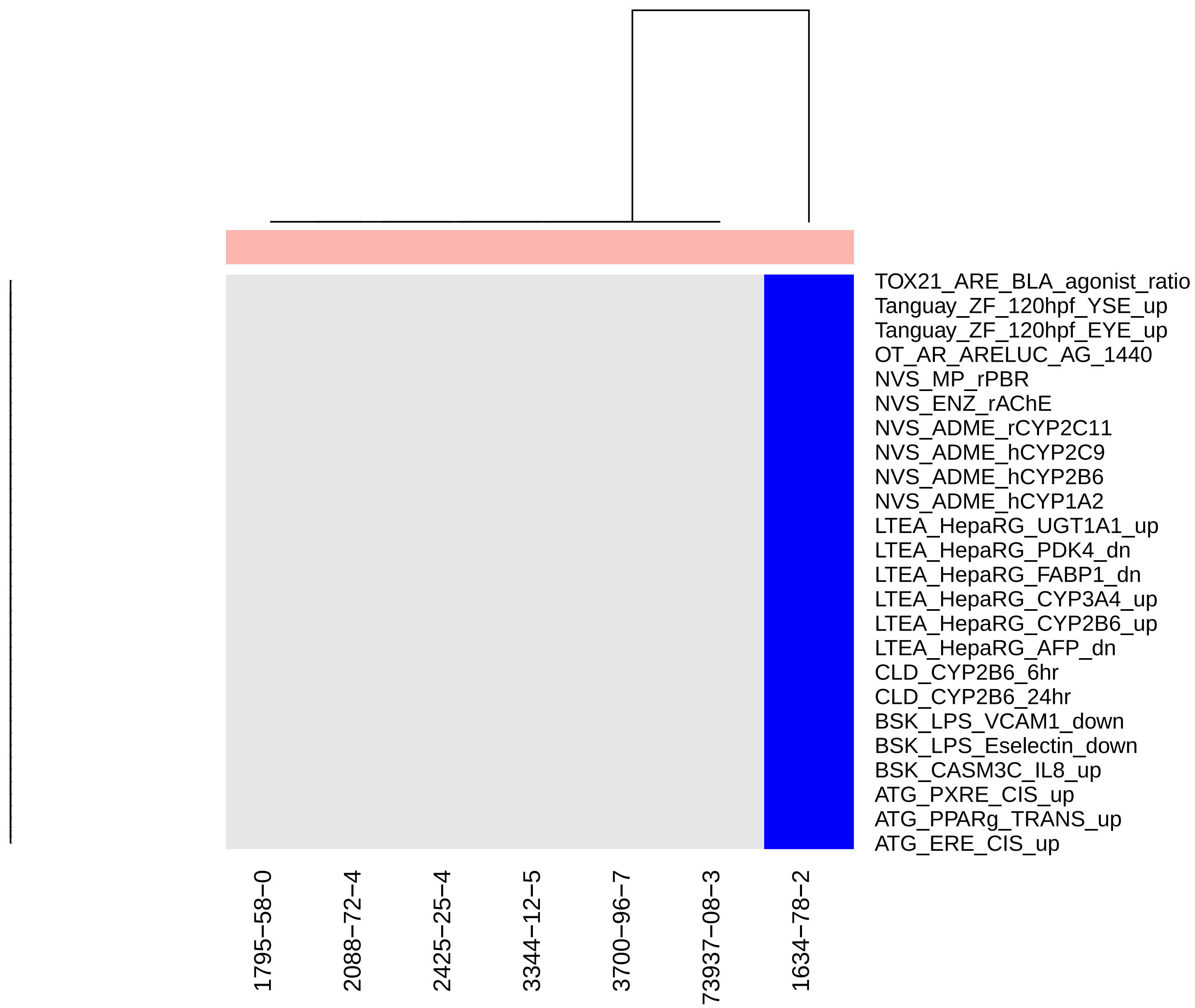

All Activity Heatmap for Cluster 1316

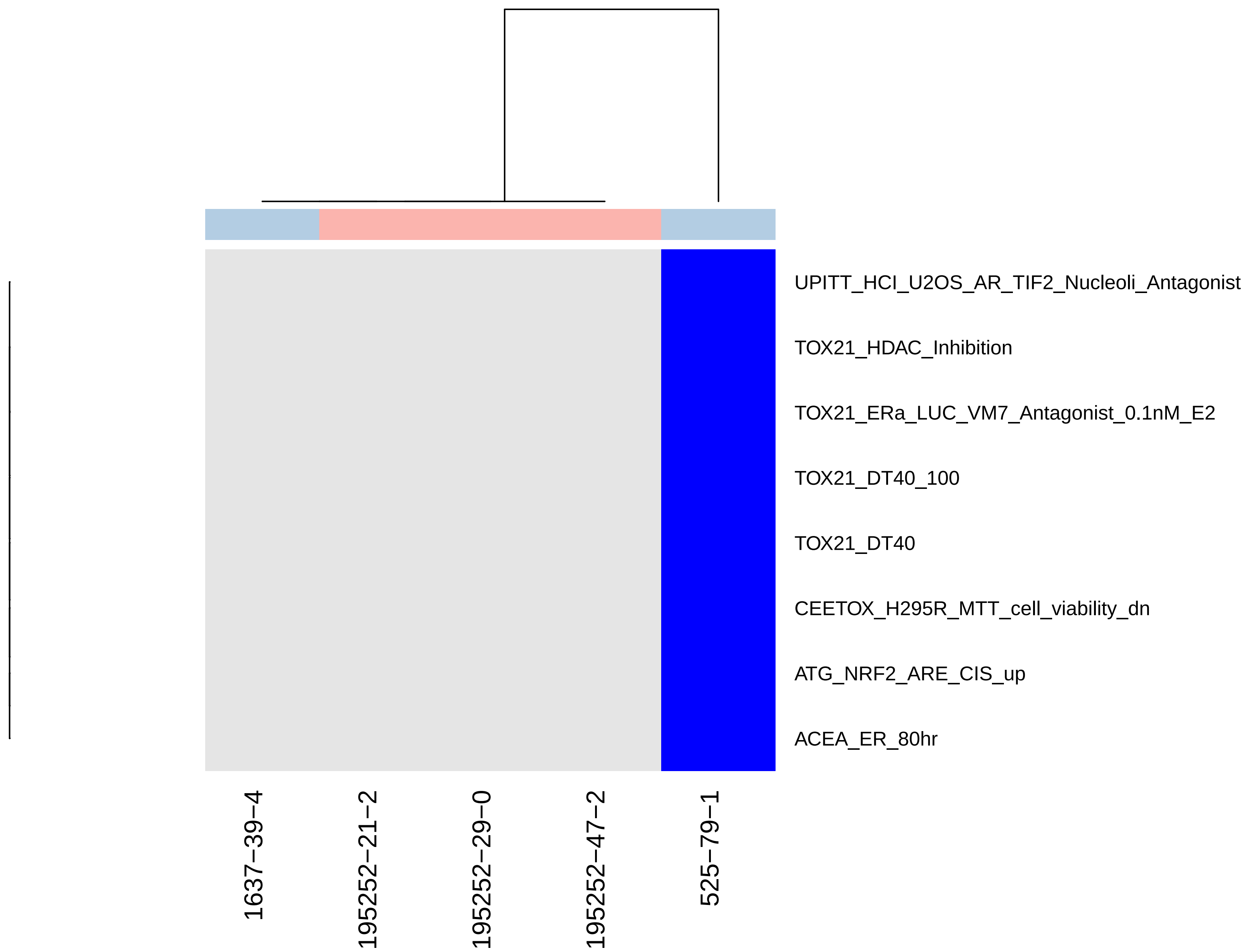

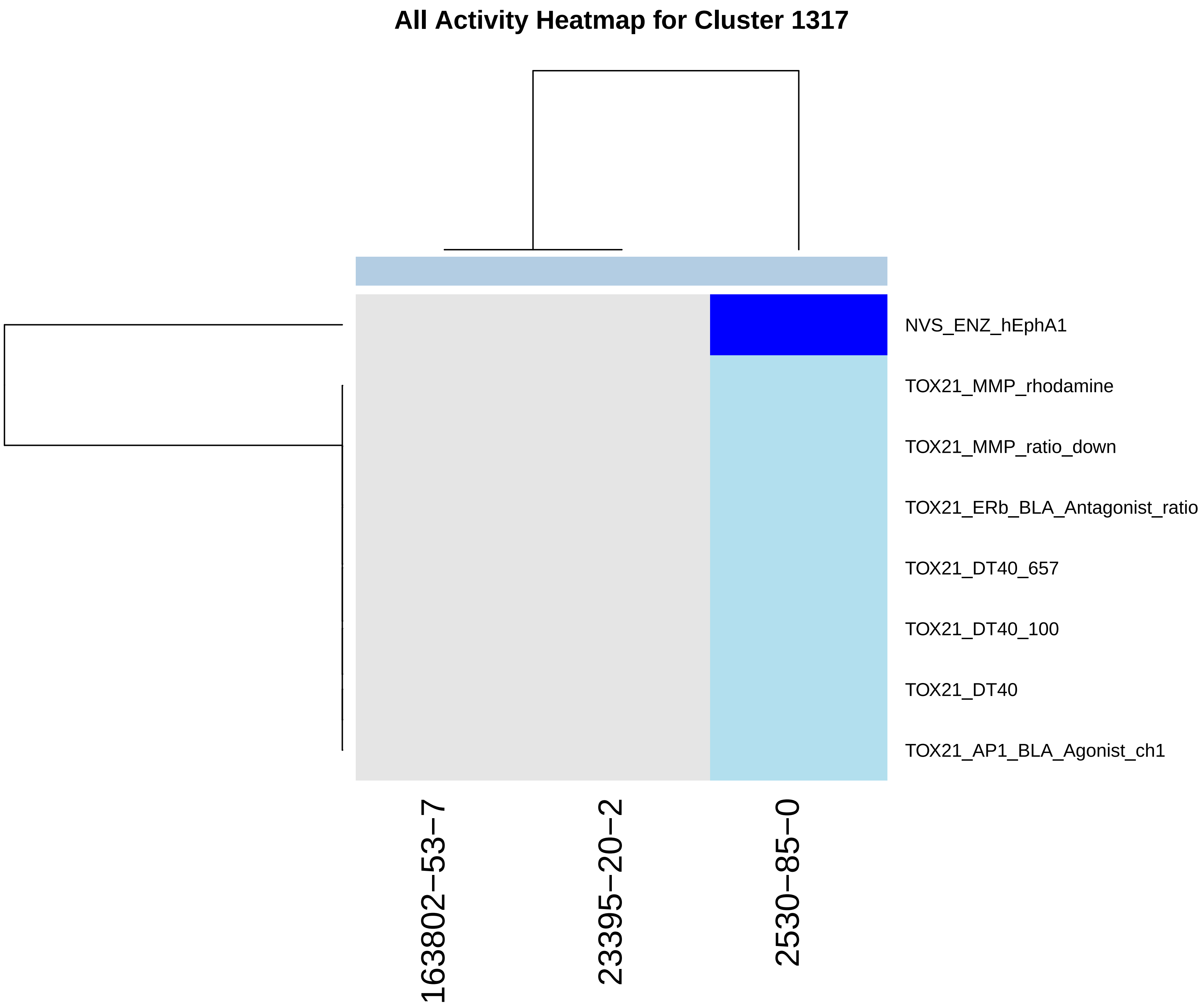
All Activity Heatmap for Cluster 1317
NVS_ENZ_hEphA1
TOX21_MMP_rhodamine
TOX21_MMP_ratio_down
TOX21_ERb_BLA_Antagonist_ratio
TOX21_DT40_657
TOX21_DT40_100
TOX21_DT40
TOX21_AP1_BLA_Agonist_ch1
163802−53−7
23395−20−2
2530−85−0

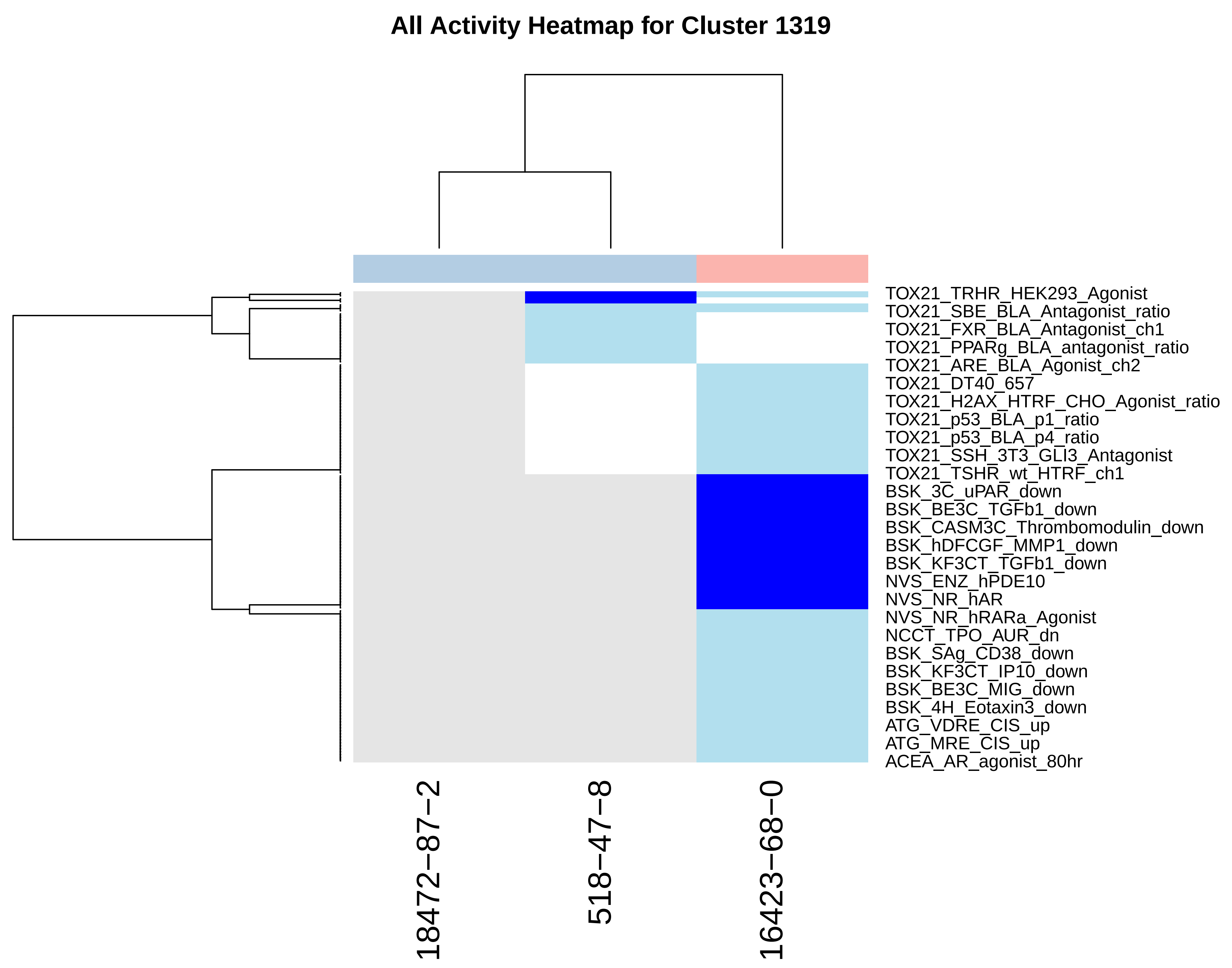

All Activity Heatmap for Cluster 1319
TOX21_TRHR_HEK293_Agonist
TOX21_SBE_BLA_Antagonist_ratio
TOX21_FXR_BLA_Antagonist_ch1
TOX21_PPARg_BLA_antagonist_ratio
TOX21_ARE_BLA_Agonist_ch2
TOX21_DT40_657
TOX21_H2AX_HTRF_CHO_Agonist_ratio
TOX21_p53_BLA_p1_ratio
TOX21_p53_BLA_p4_ratio
TOX21_SSH_3T3_GLI3_Antagonist
TOX21_TSHR_wt_HTRF_ch1
BSK_3C_uPAR_down
BSK_BE3C_TGFb1_down
BSK_CASM3C_Thrombomodulin_down
BSK_hDFCGF_MMP1_down
BSK_KF3CT_TGFb1_down
NVS_ENZ_hPDE10
NVS_NR_hAR
NVS_NR_hRARa_Agonist
NCCT_TPO_AUR_dn
BSK_SAg_CD38_down
BSK_KF3CT_IP10_down
BSK_BE3C_MIG_down
BSK_4H_Eotaxin3_down
ATG_VDRE_CIS_up
ATG_MRE_CIS_up
ACEA_AR_agonist_80hr
18472−87−2
518−47−8
16423−68−0

All Activity Heatmap for Cluster 1321

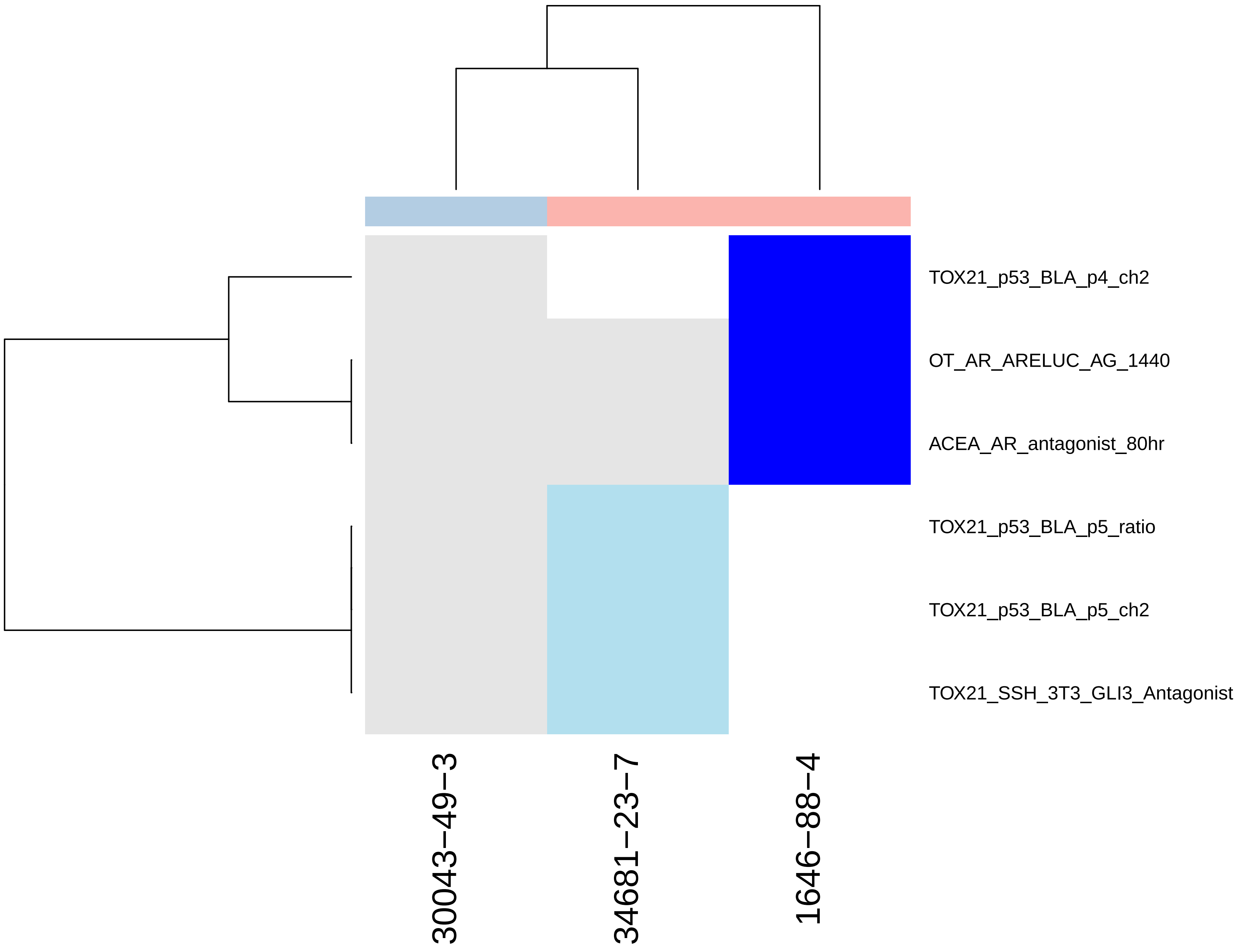

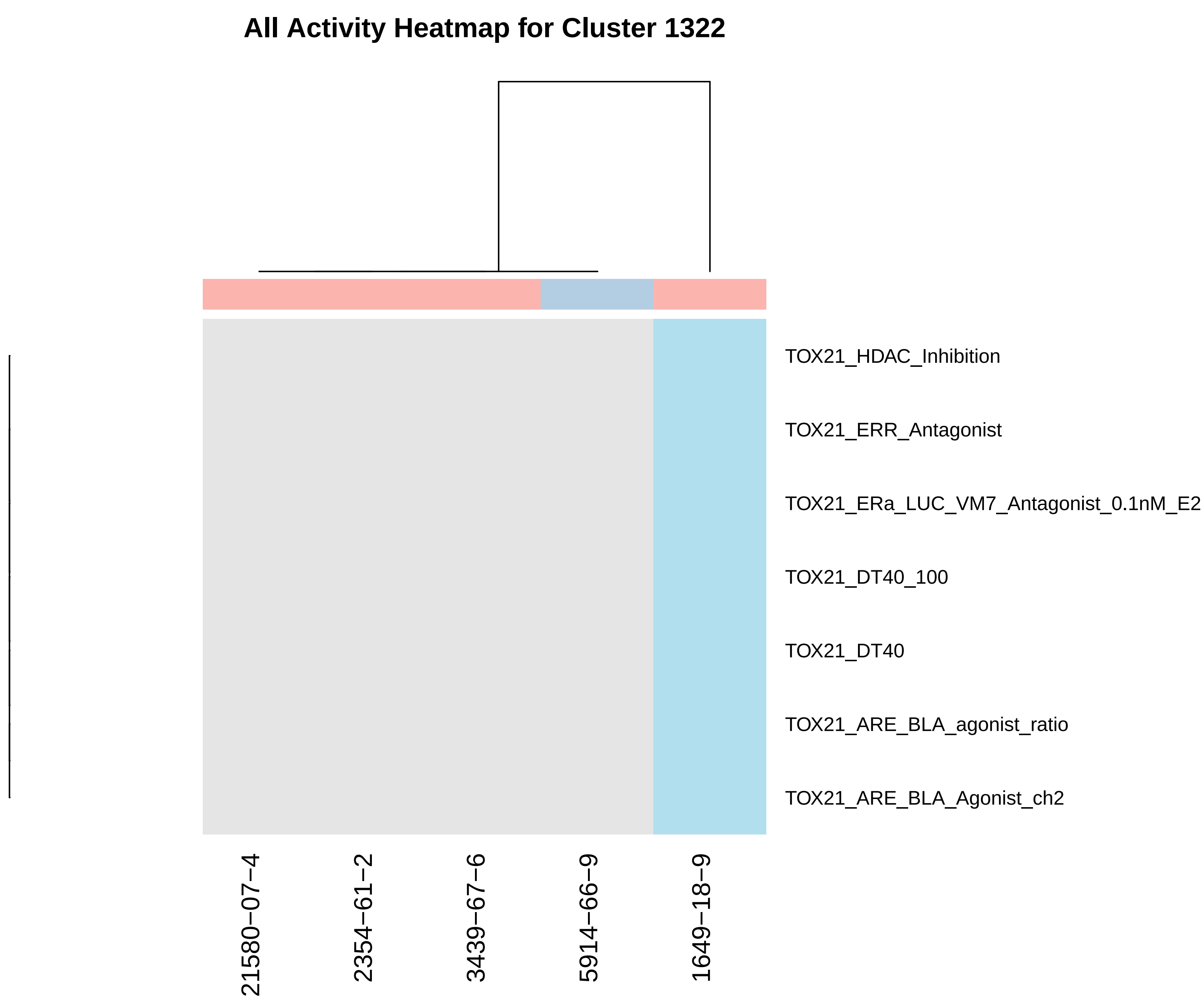

All Activity Heatmap for Cluster 1322
TOX21_HDAC_Inhibition
TOX21_ERR_Antagonist
TOX21_ERa_LUC_VM7_Antagonist_0.1nM_E2
TOX21_DT40_100
TOX21_DT40
TOX21_ARE_BLA_agonist_ratio
TOX21_ARE_BLA_Agonist_ch2
21580−07−4
2354−61−2
3439−67−6
5914−66−9
1649−18−9

All Activity Heatmap for Cluster 1323

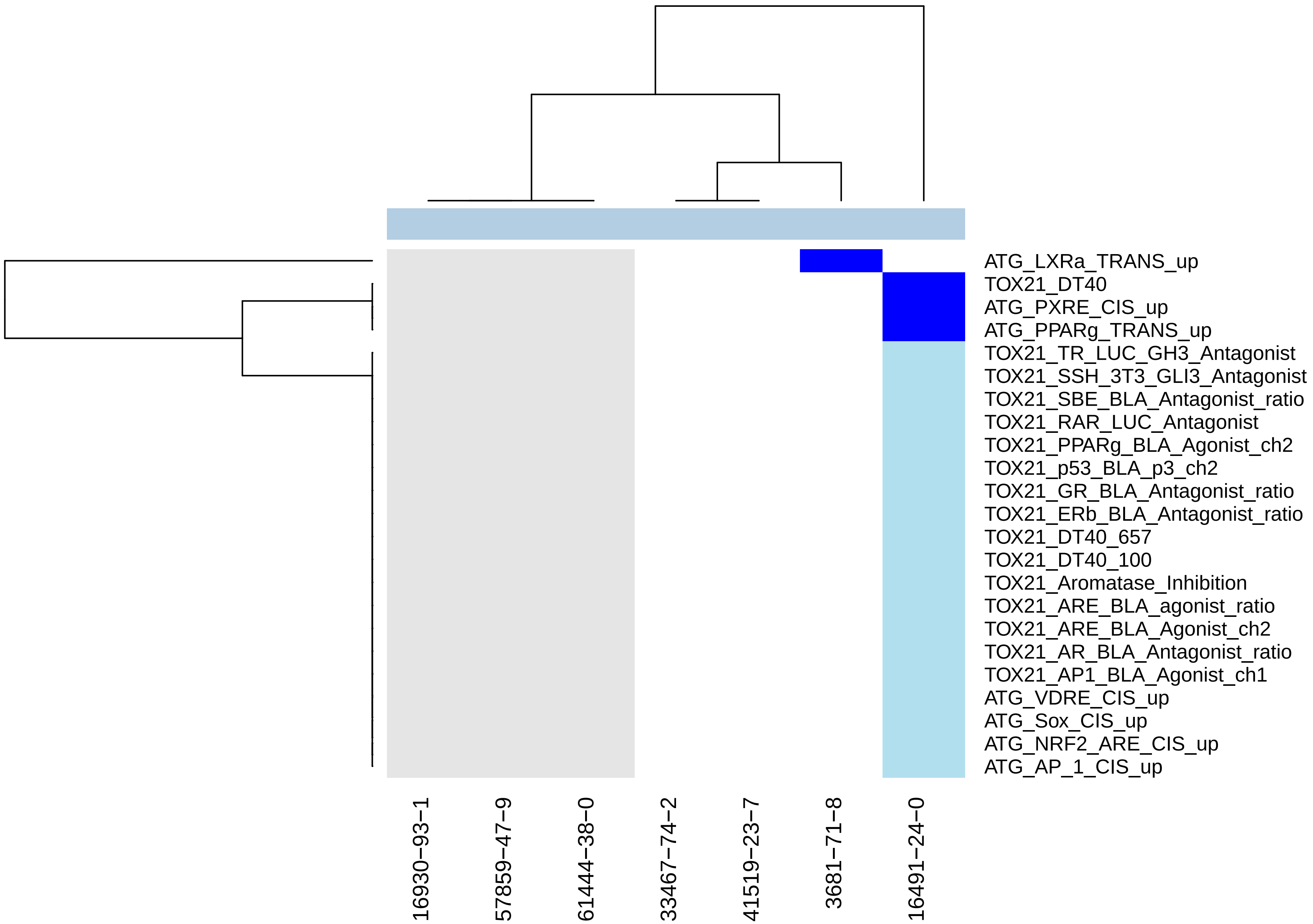

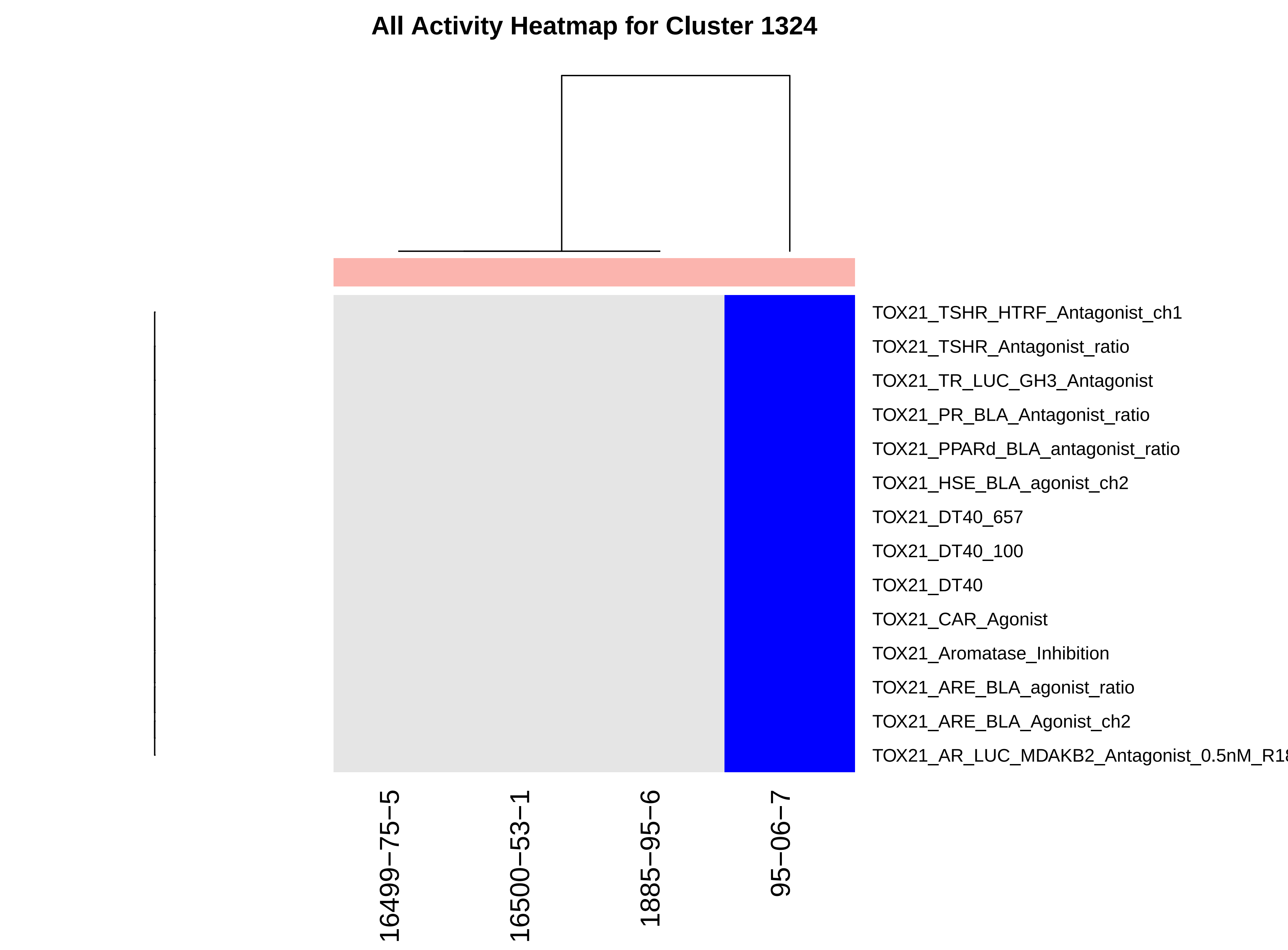
All Activity Heatmap for Cluster 1324
TOX21_TSHR_HTRF_Antagonist_ch1
TOX21_TSHR_Antagonist_ratio
TOX21_TR_LUC_GH3_Antagonist
TOX21_PR_BLA_Antagonist_ratio
TOX21_PPARd_BLA_antagonist_ratio
TOX21_HSE_BLA_agonist_ch2
TOX21_DT40_657
TOX21_DT40_100
TOX21_DT40
TOX21_CAR_Agonist
TOX21_Aromatase_Inhibition
TOX21_ARE_BLA_agonist_ratio
TOX21_ARE_BLA_Agonist_ch2
TOX21_AR_LUC_MDAKB2_Antagonist_0.5nM_R18
16499−75−5
16500−53−1
1885−95−6
95−06−7

All Activity Heatmap for Cluster 1325

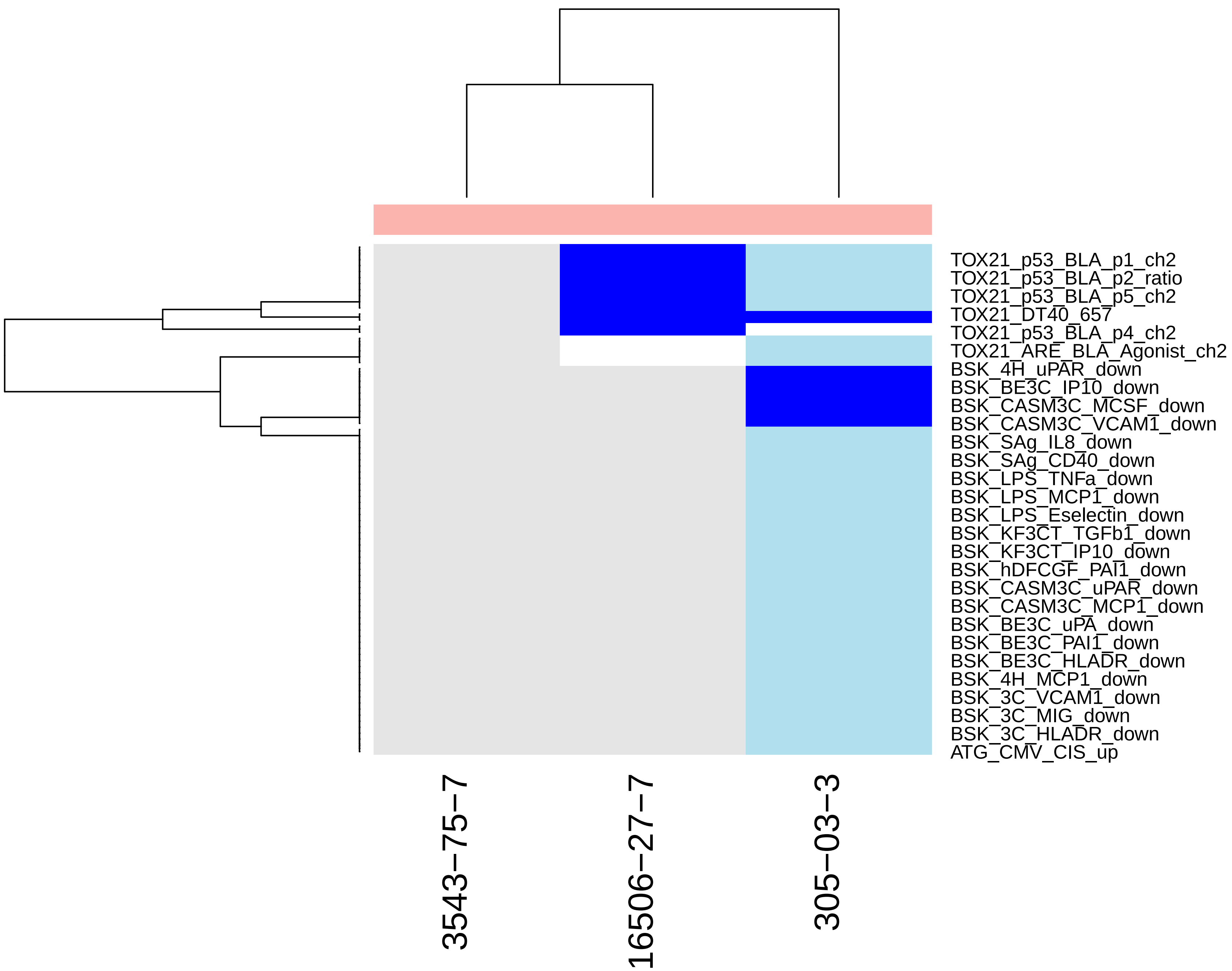

All Activity Heatmap for Cluster 1328

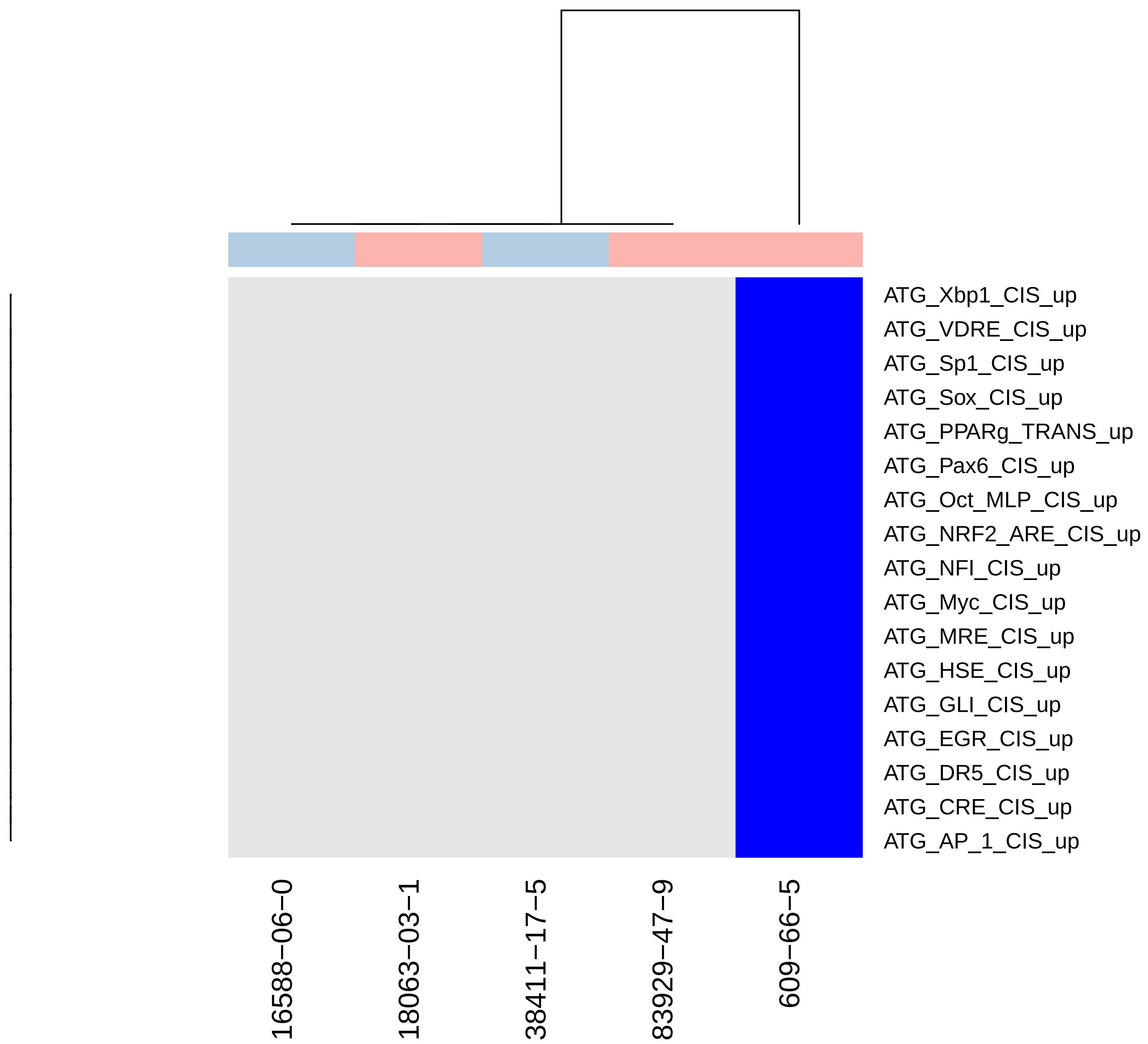

All Activity Heatmap for Cluster 1329

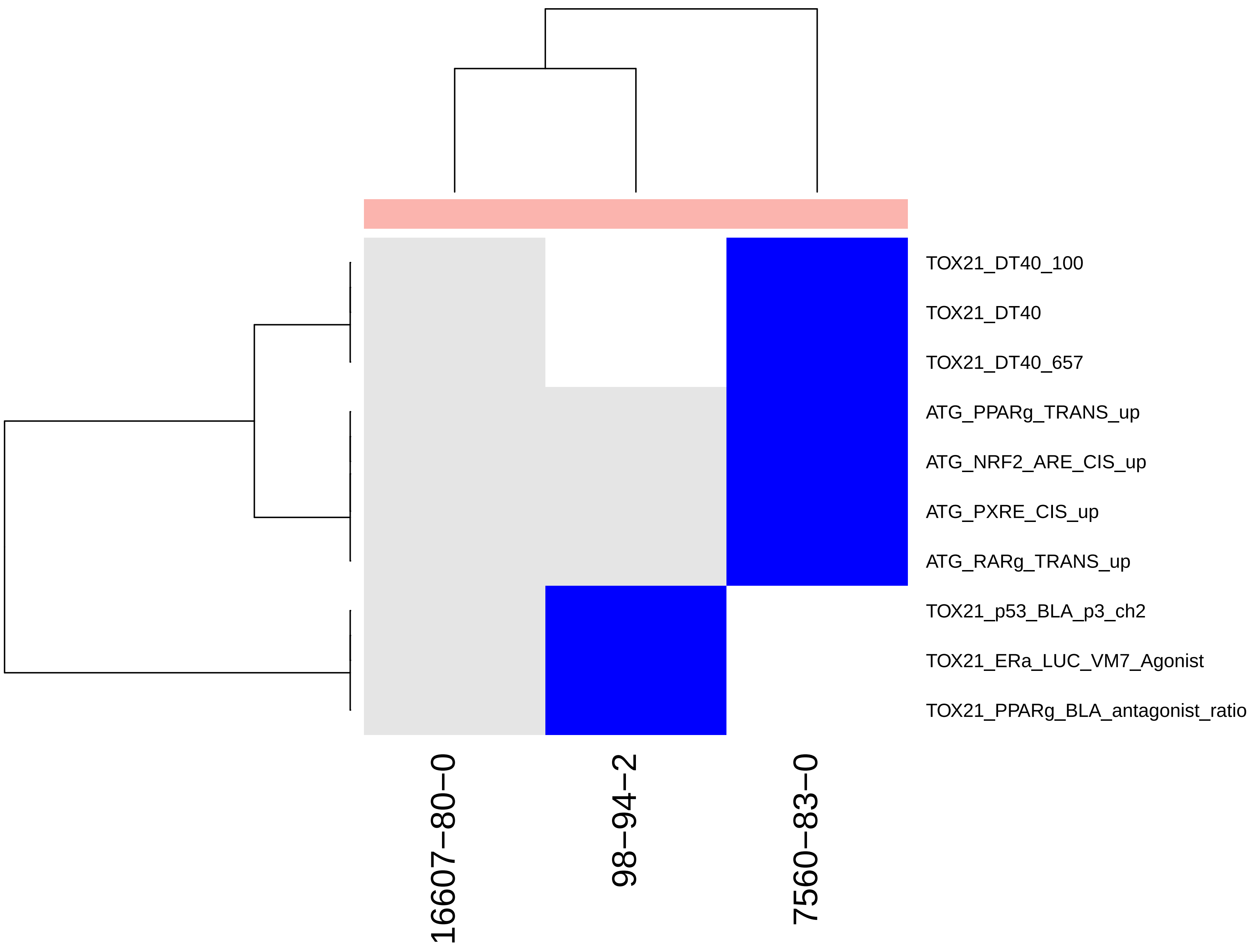

All Activity Heatmap for Cluster 1333

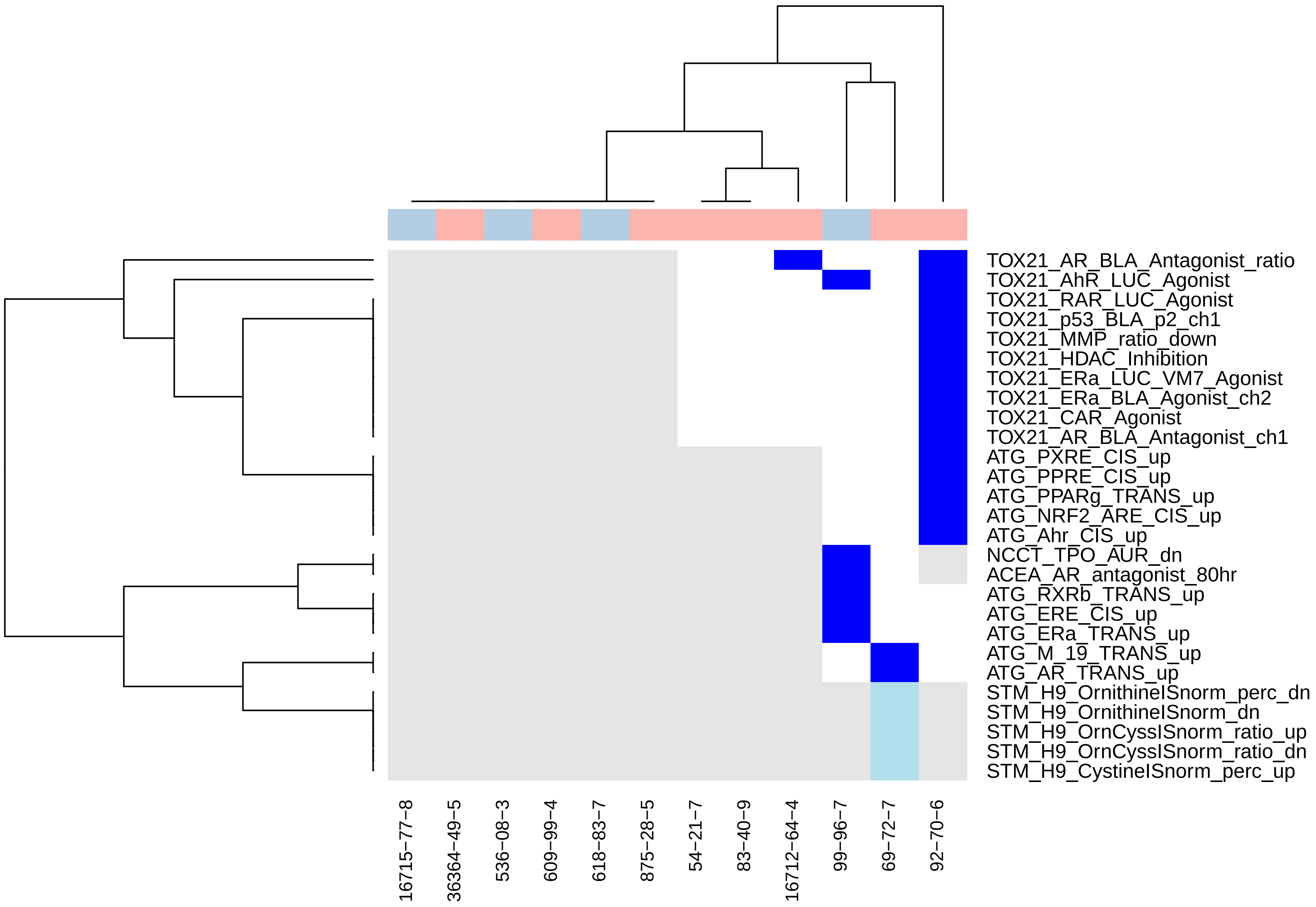

All Activity Heatmap for Cluster 1334

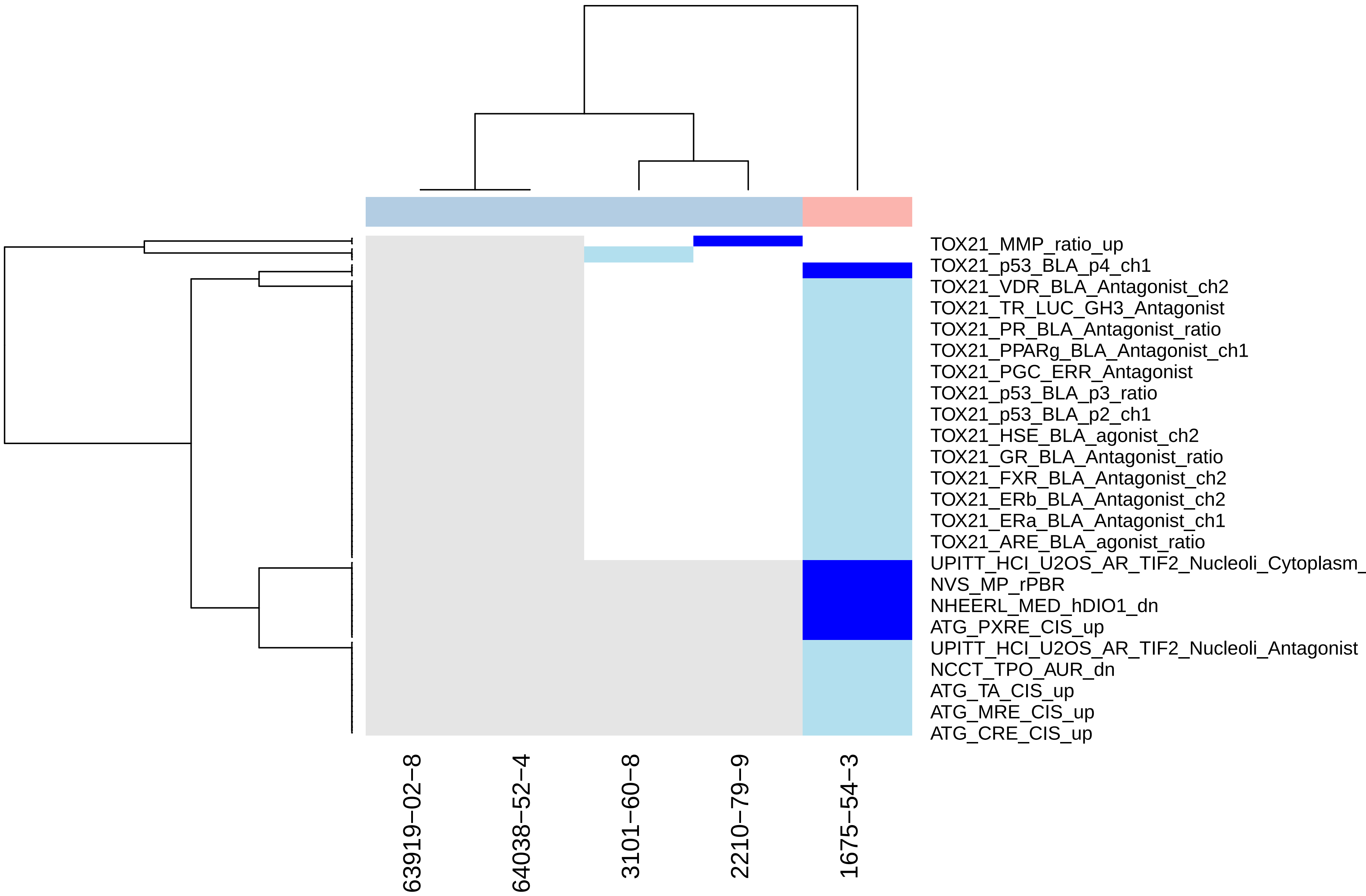

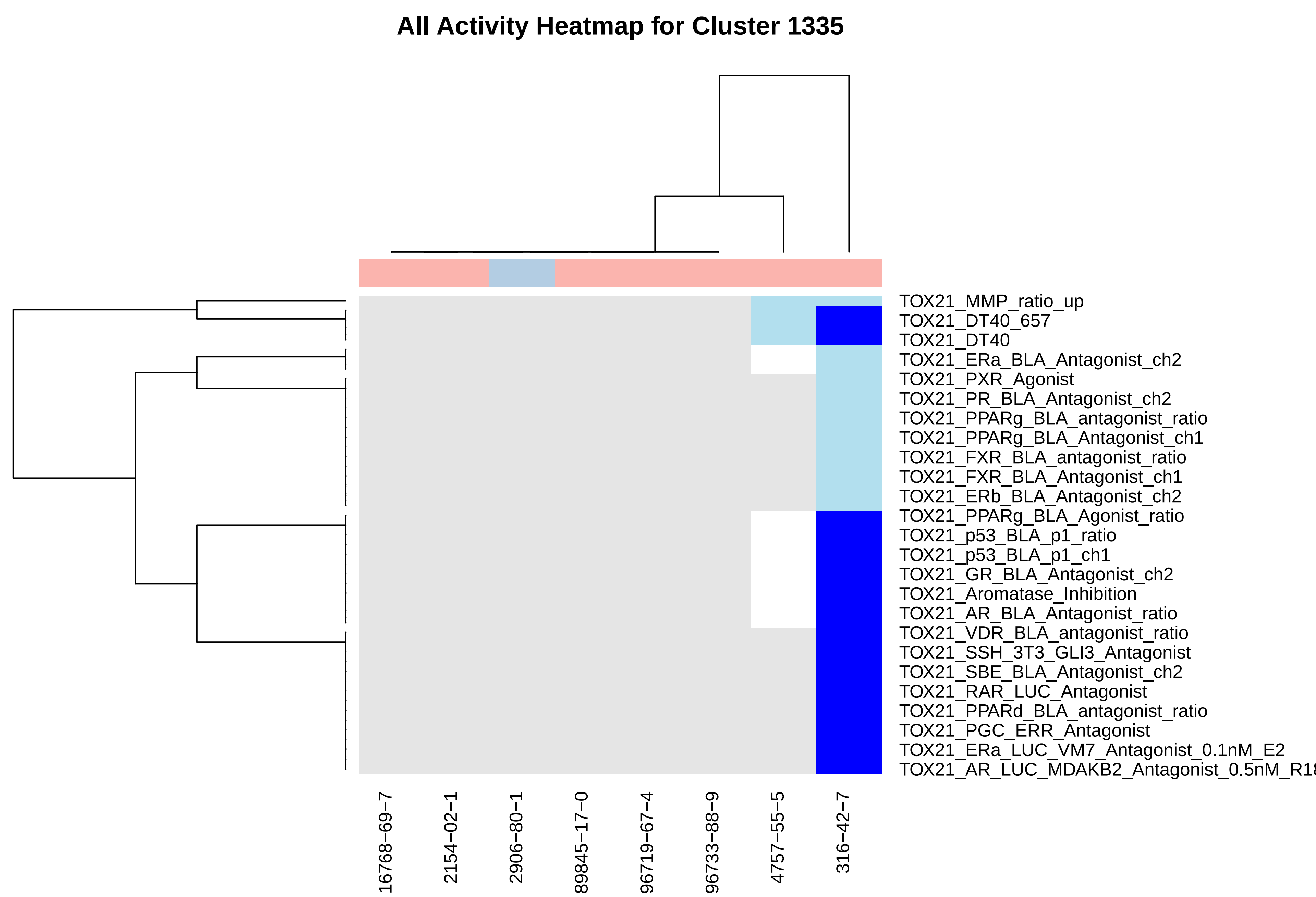

All Activity Heatmap for Cluster 1335
TOX21_MMP_ratio_up
TOX21_DT40_657
TOX21_DT40
TOX21_ERa_BLA_Antagonist_ch2
TOX21_PXR_Agonist
TOX21_PR_BLA_Antagonist_ch2
TOX21_PPARg_BLA_antagonist_ratio
TOX21_PPARg_BLA_Antagonist_ch1
TOX21_FXR_BLA_antagonist_ratio
TOX21_FXR_BLA_Antagonist_ch1
TOX21_ERb_BLA_Antagonist_ch2
TOX21_PPARg_BLA_Agonist_ratio
TOX21_p53_BLA_p1_ratio
TOX21_p53_BLA_p1_ch1
TOX21_GR_BLA_Antagonist_ch2
TOX21_Aromatase_Inhibition
TOX21_AR_BLA_Antagonist_ratio
TOX21_VDR_BLA_antagonist_ratio
TOX21_SSH_3T3_GLI3_Antagonist
TOX21_SBE_BLA_Antagonist_ch2
TOX21_RAR_LUC_Antagonist
TOX21_PPARd_BLA_antagonist_ratio
TOX21_PGC_ERR_Antagonist
TOX21_ERa_LUC_VM7_Antagonist_0.1nM_E2
TOX21_AR_LUC_MDAKB2_Antagonist_0.5nM_R18
16768−69−7
2154−02−1
2906−80−1
89845−17−0
96719−67−4
96733−88−9
4757−55−5
316−42−7

All Activity Heatmap for Cluster 1337

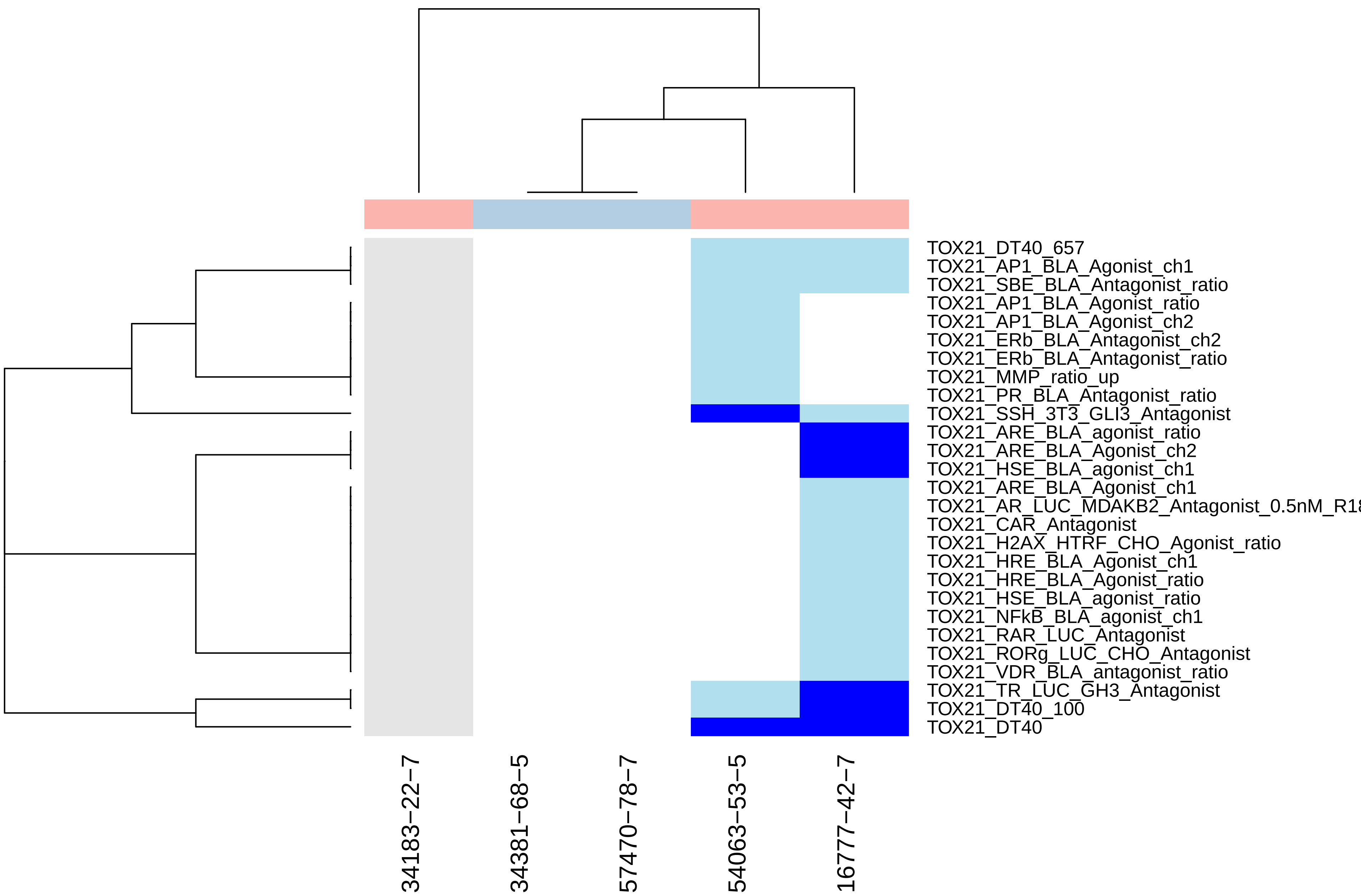

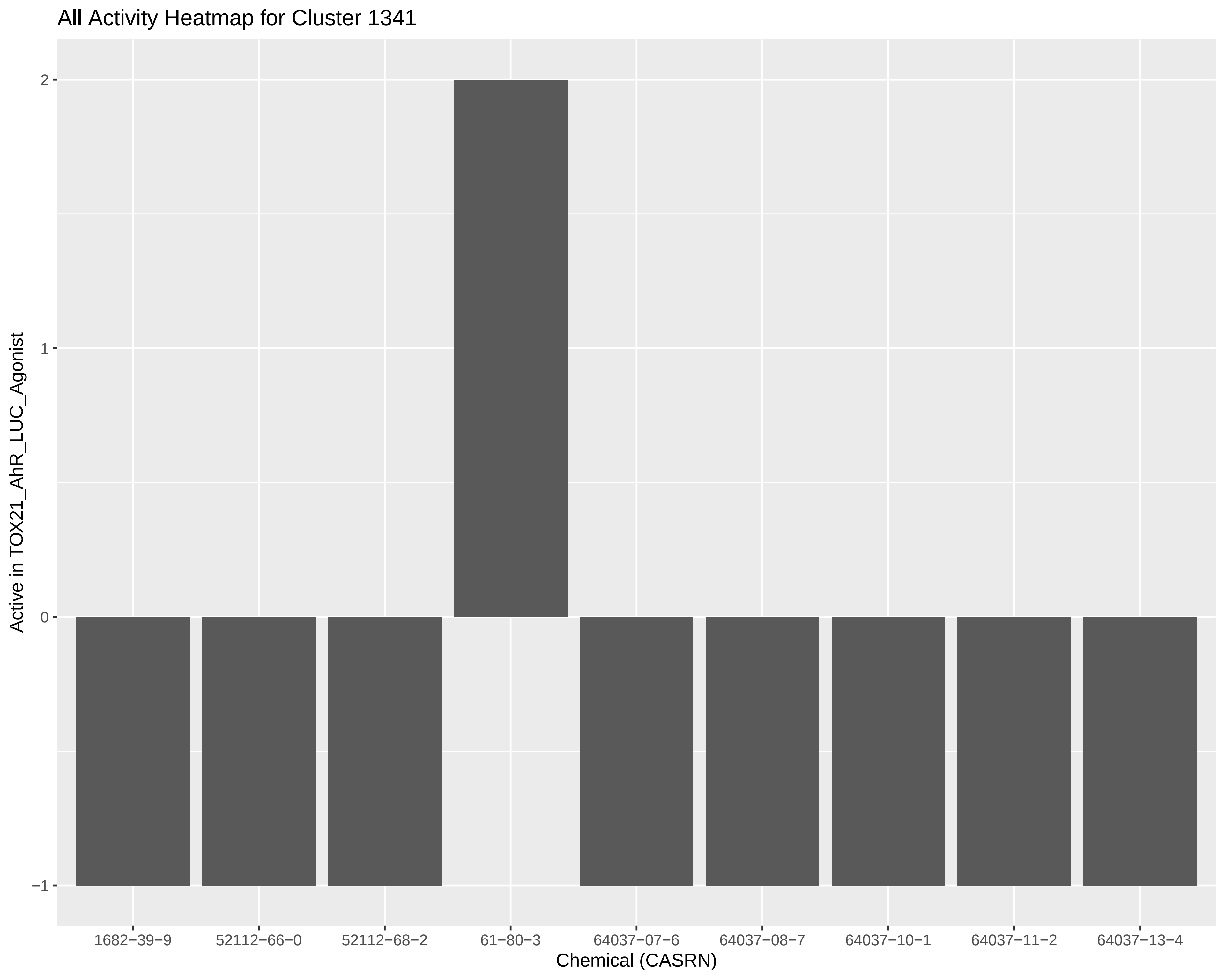
All Activity Heatmap for Cluster 1341
Active in TOX21_AhR_LUC_Agonist
2
1
0
−1
1682−39−9
52112−66−0
52112−68−2
61−80−3
64037−07−6
64037−08−7
64037−10−1
64037−11−2
64037−13−4
Chemical (CASRN)

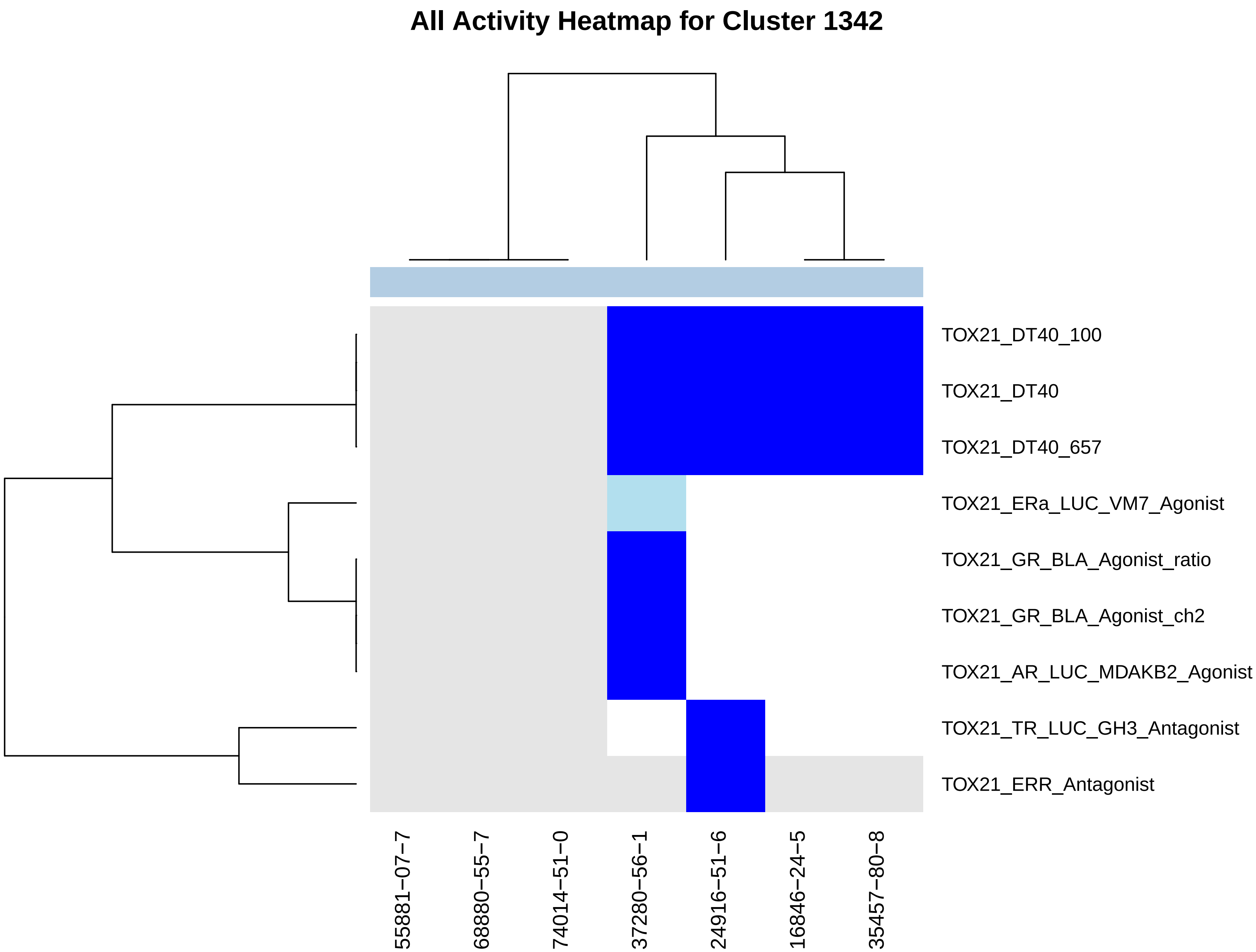
All Activity Heatmap for Cluster 1342
TOX21_DT40_100
TOX21_DT40
TOX21_DT40_657
TOX21_ERa_LUC_VM7_Agonist
TOX21_GR_BLA_Agonist_ratio
TOX21_GR_BLA_Agonist_ch2
TOX21_AR_LUC_MDAKB2_Agonist
TOX21_TR_LUC_GH3_Antagonist
TOX21_ERR_Antagonist
55881−07−7
68880−55−7
74014−51−0
37280−56−1
24916−51−6
16846−24−5
35457−80−8

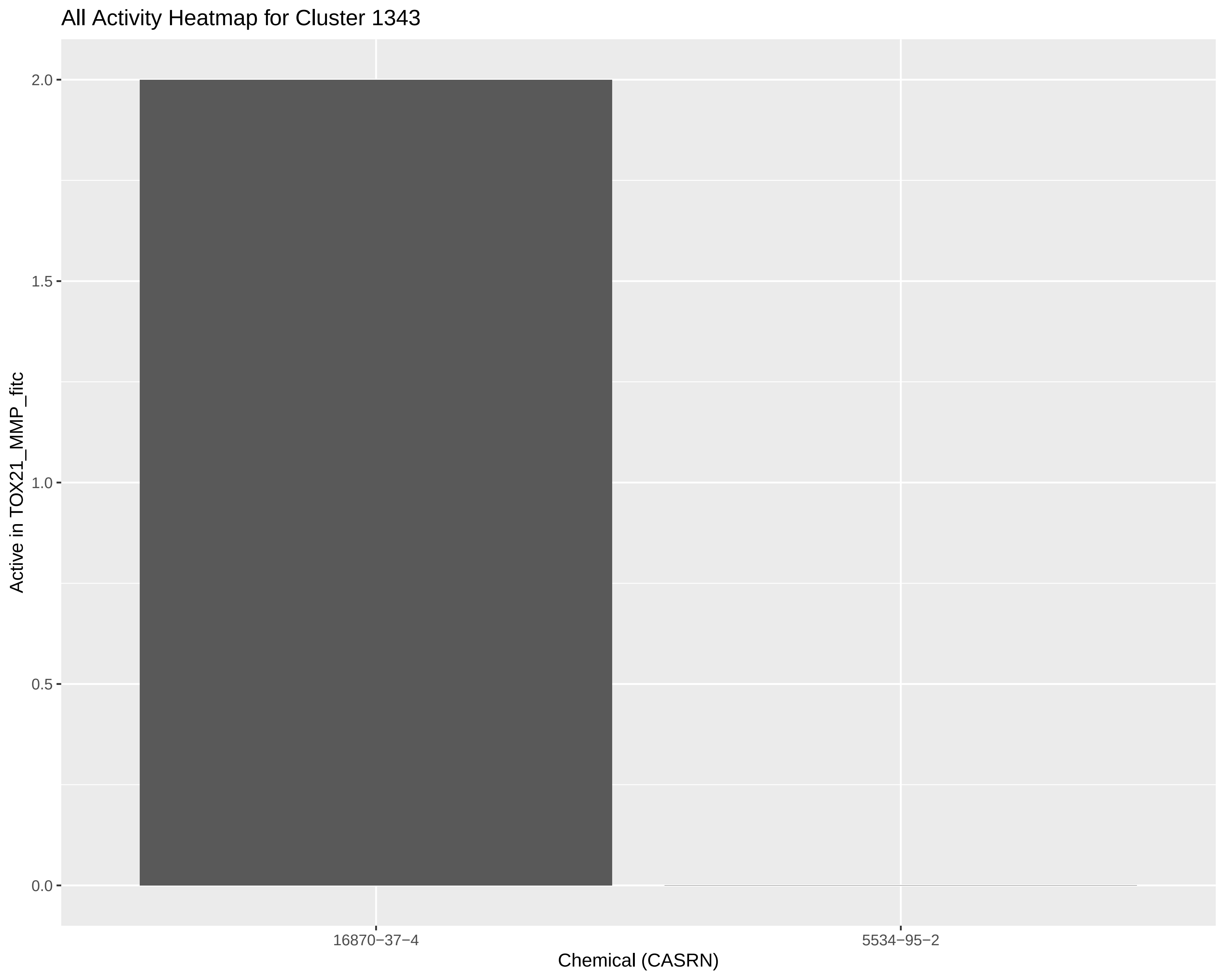

All Activity Heatmap for Cluster 1343
Active in TOX21_MMP_fitc
2.0
1.5
1.0
0.5
0.0
16870−37−4
5534−95−2
Chemical (CASRN)

All Activity Heatmap for Cluster 1344

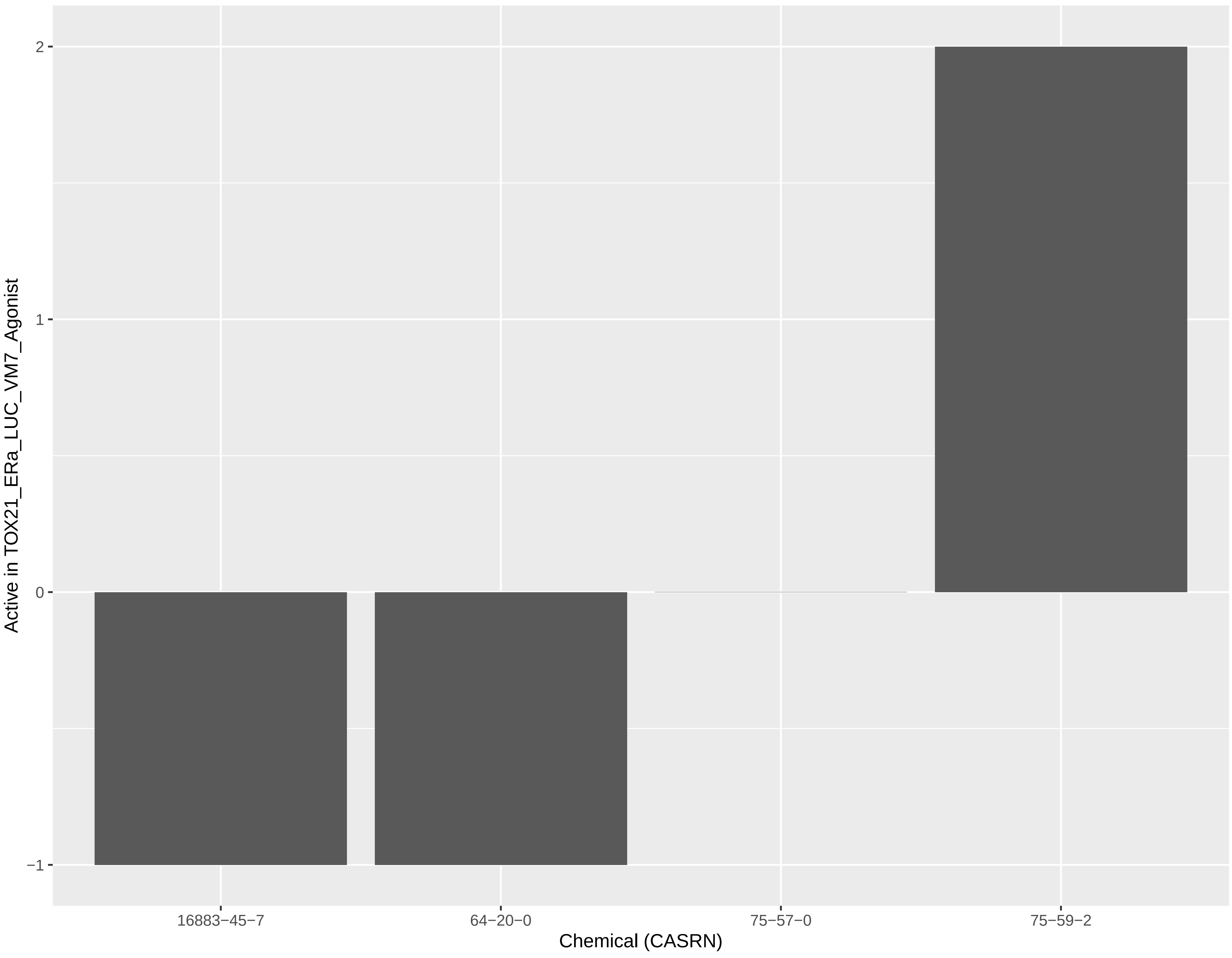

All Activity Heatmap for Cluster 1345

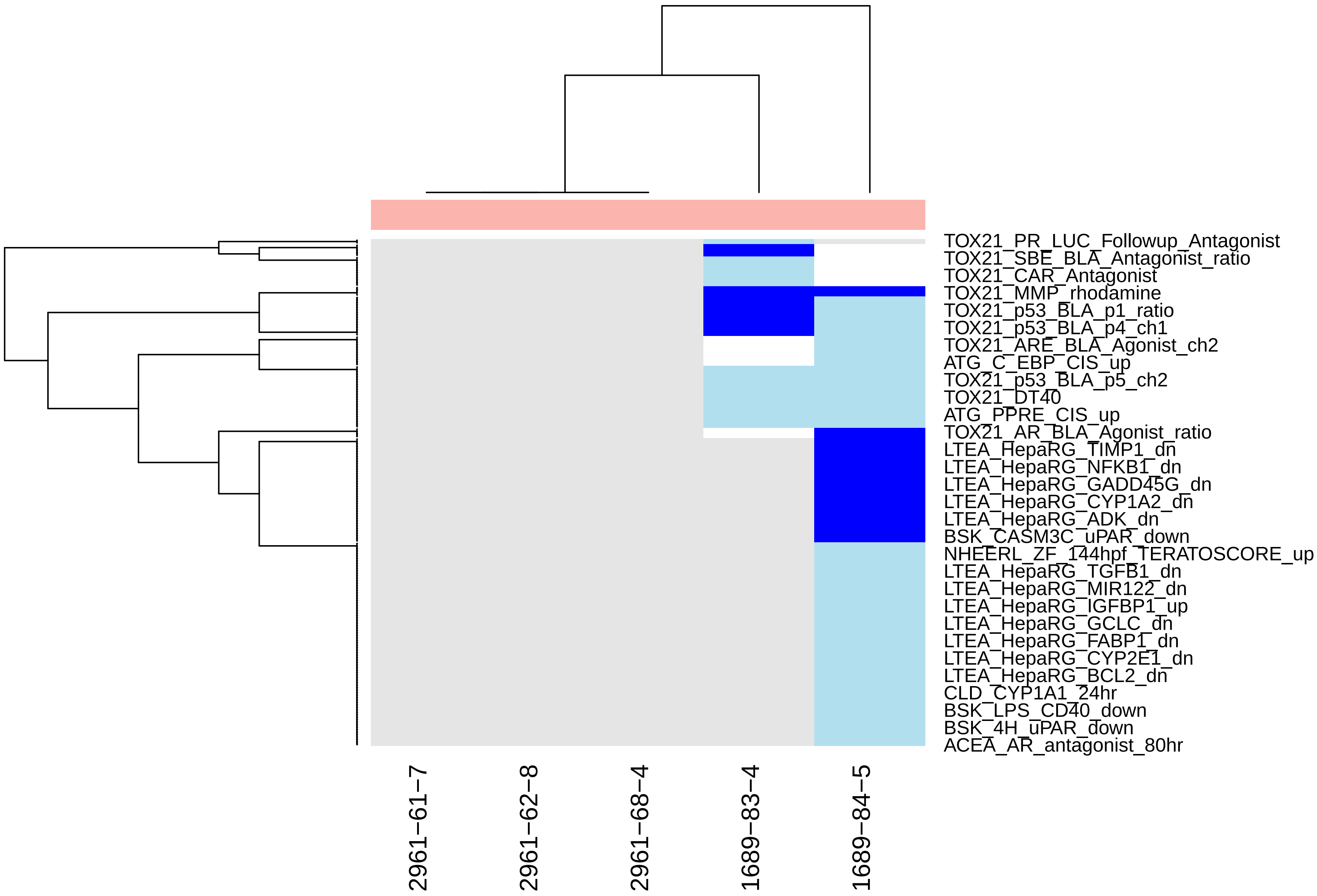

All Activity Heatmap for Cluster 1347

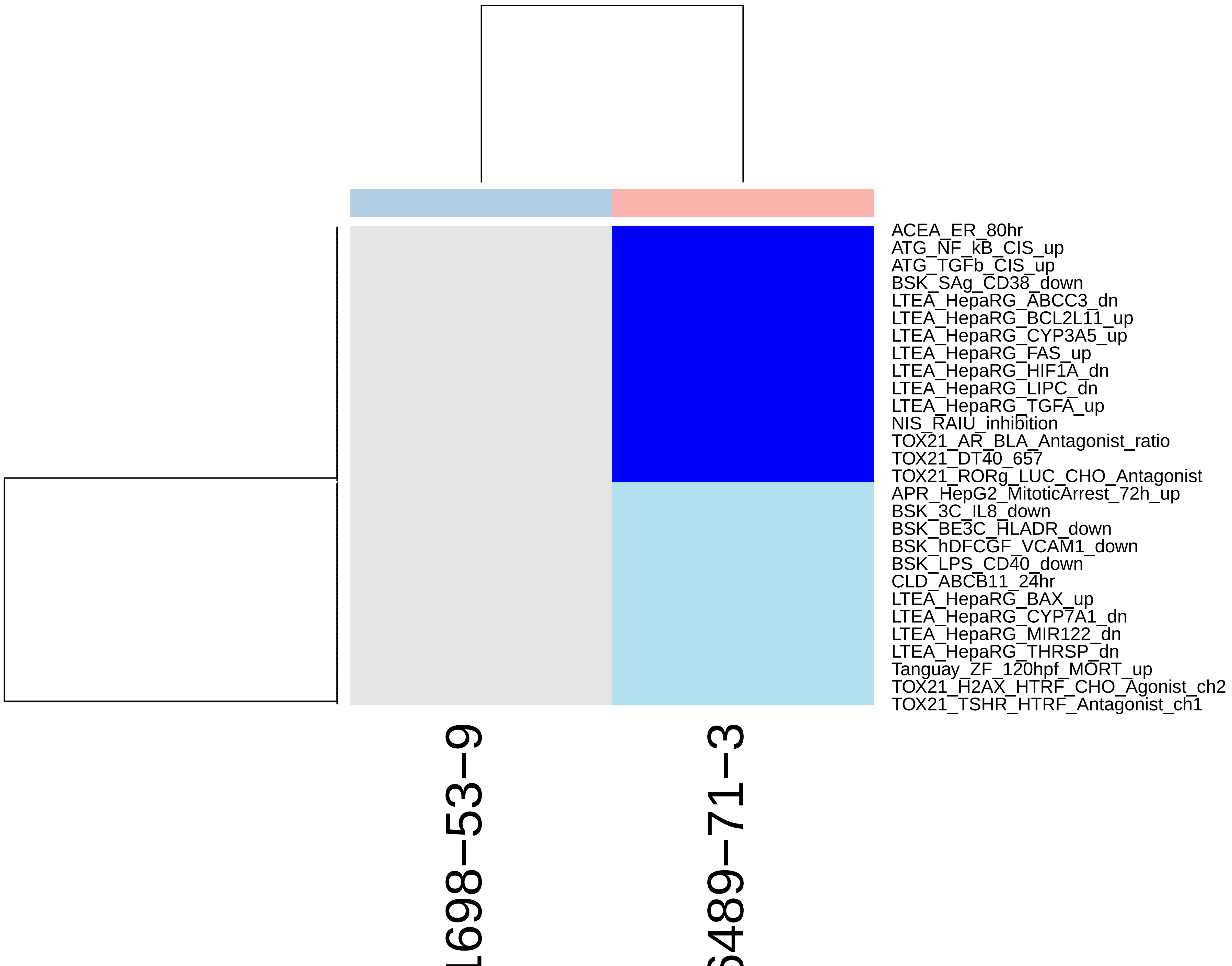

All Activity Heatmap for Cluster 1348

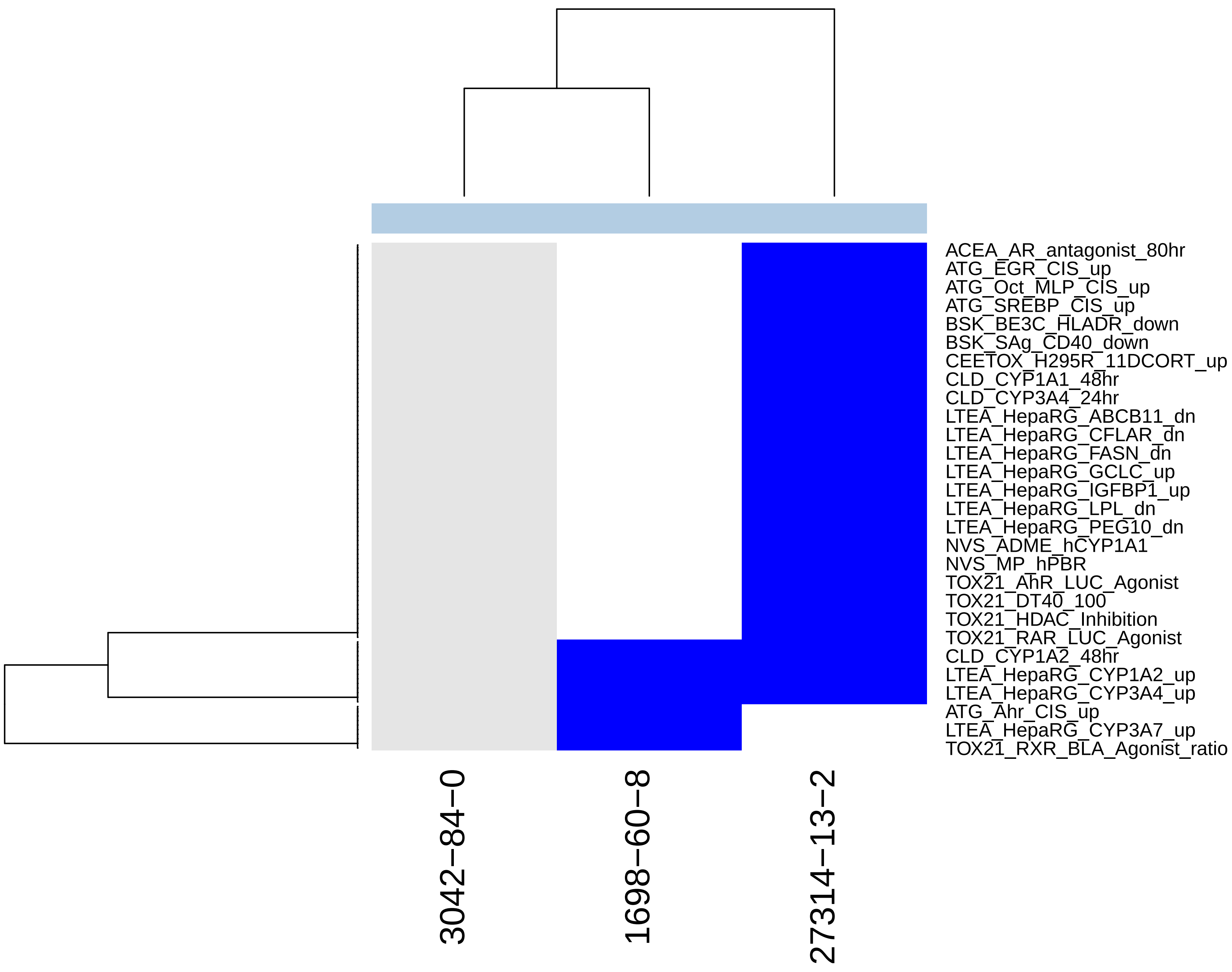

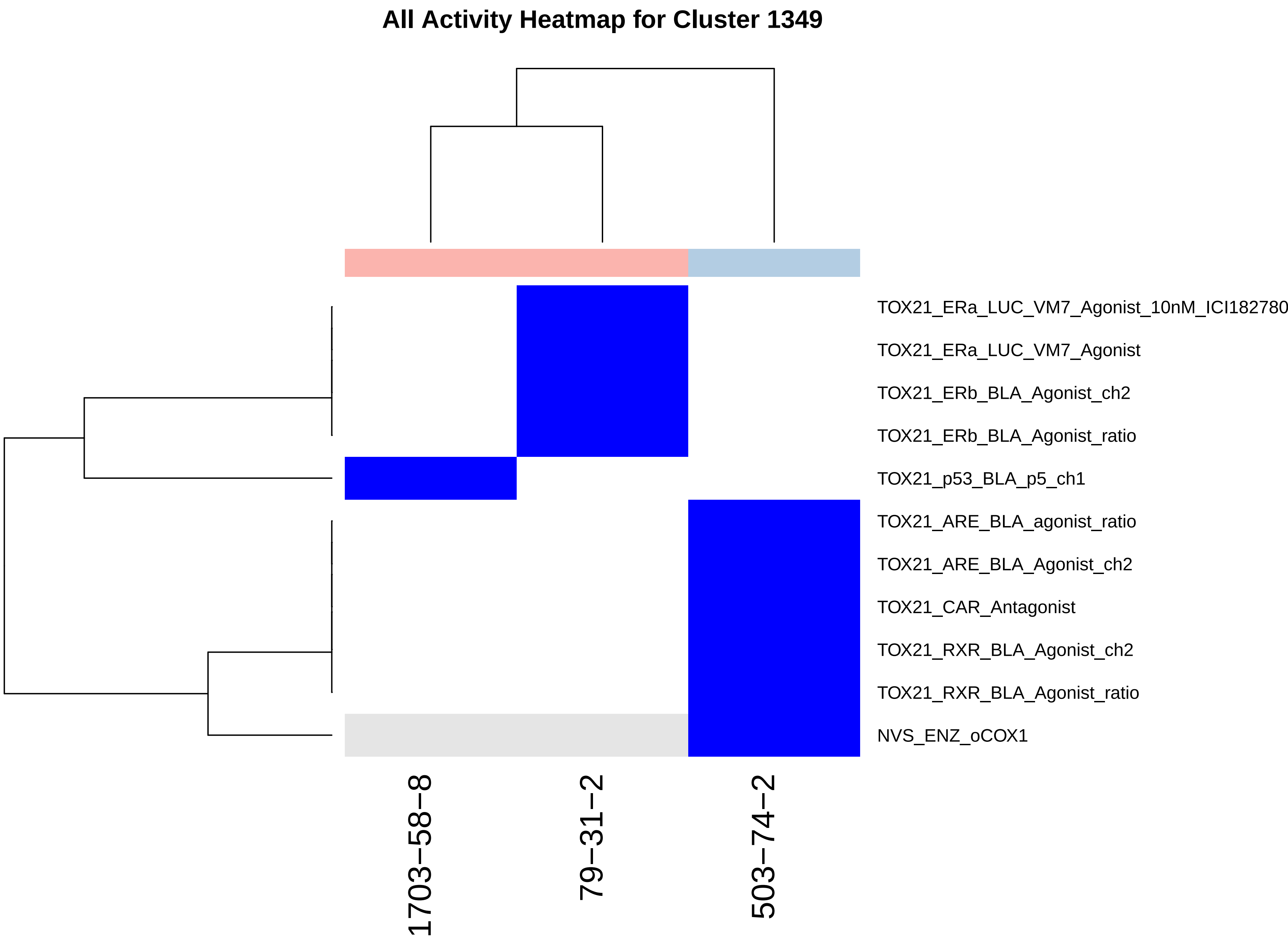
All Activity Heatmap for Cluster 1349
TOX21_ERa_LUC_VM7_Agonist_10nM_ICI182780
TOX21_ERa_LUC_VM7_Agonist
TOX21_ERb_BLA_Agonist_ch2
TOX21_ERb_BLA_Agonist_ratio
TOX21_p53_BLA_p5_ch1
TOX21_ARE_BLA_agonist_ratio
TOX21_ARE_BLA_Agonist_ch2
TOX21_CAR_Antagonist
TOX21_RXR_BLA_Agonist_ch2
TOX21_RXR_BLA_Agonist_ratio
NVS_ENZ_oCOX1
1703−58−8
79−31−2
503−74−2

All Activity Heatmap for Cluster 1350

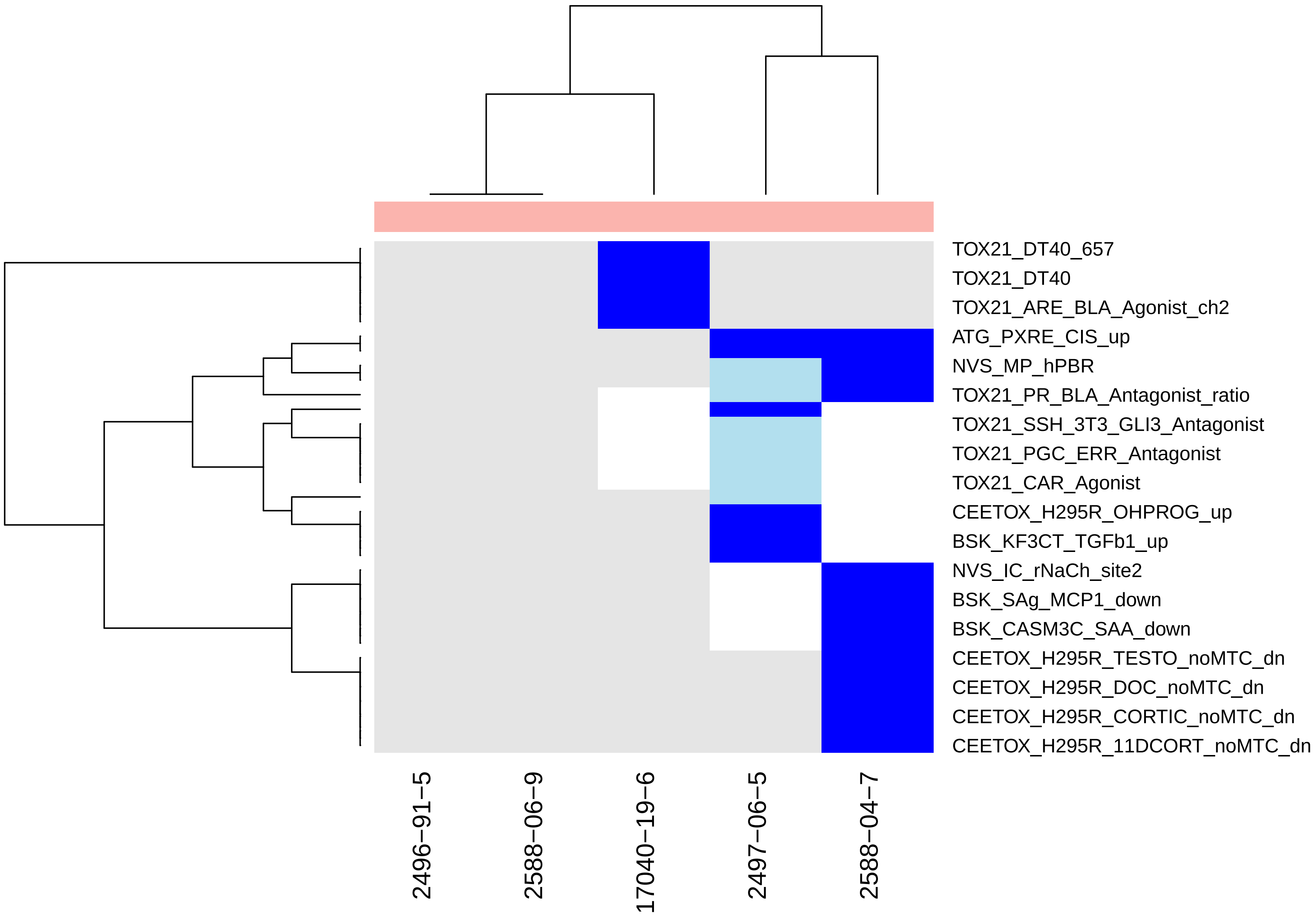

All Activity Heatmap for Cluster 1352

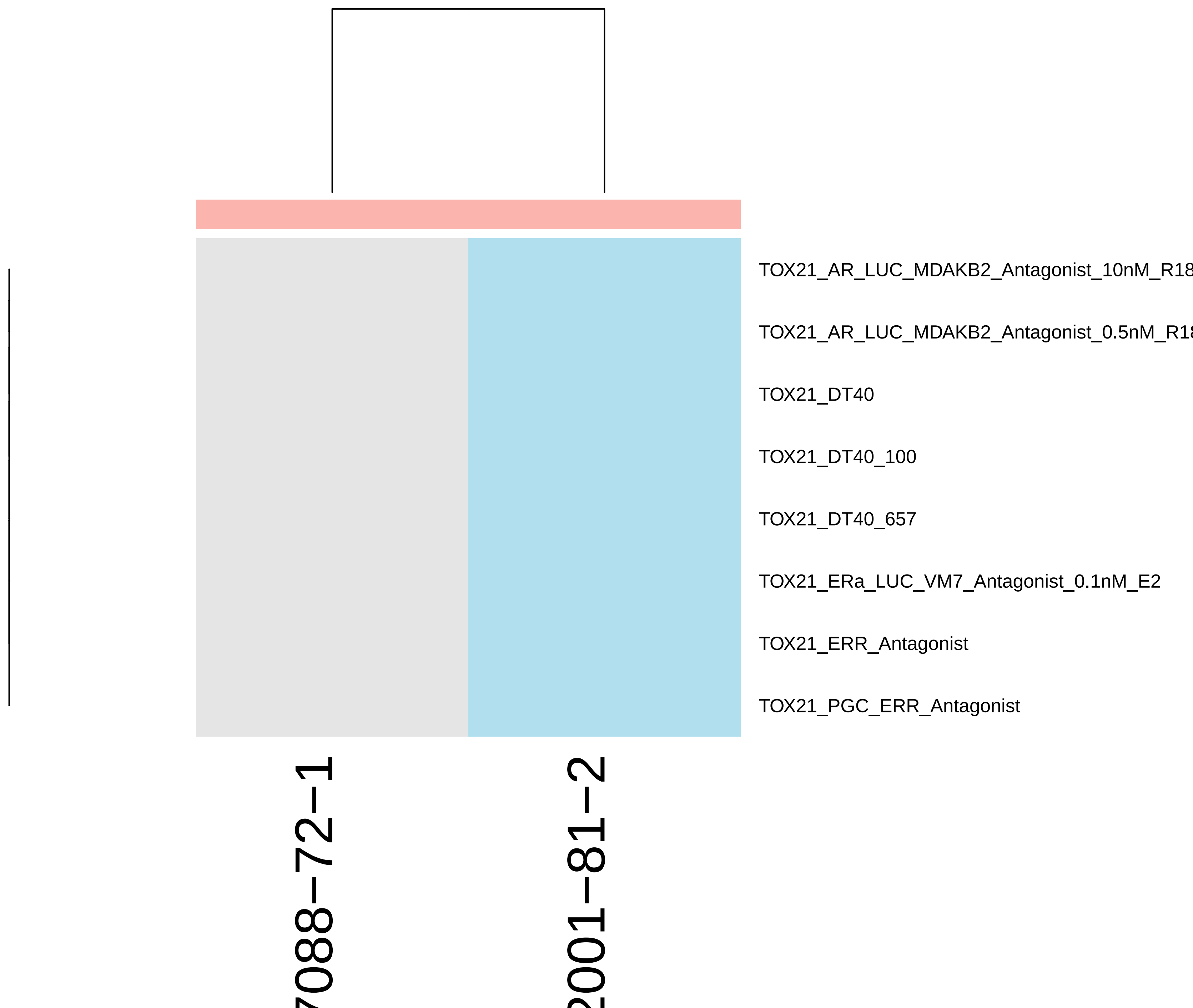

All Activity Heatmap for Cluster 1353

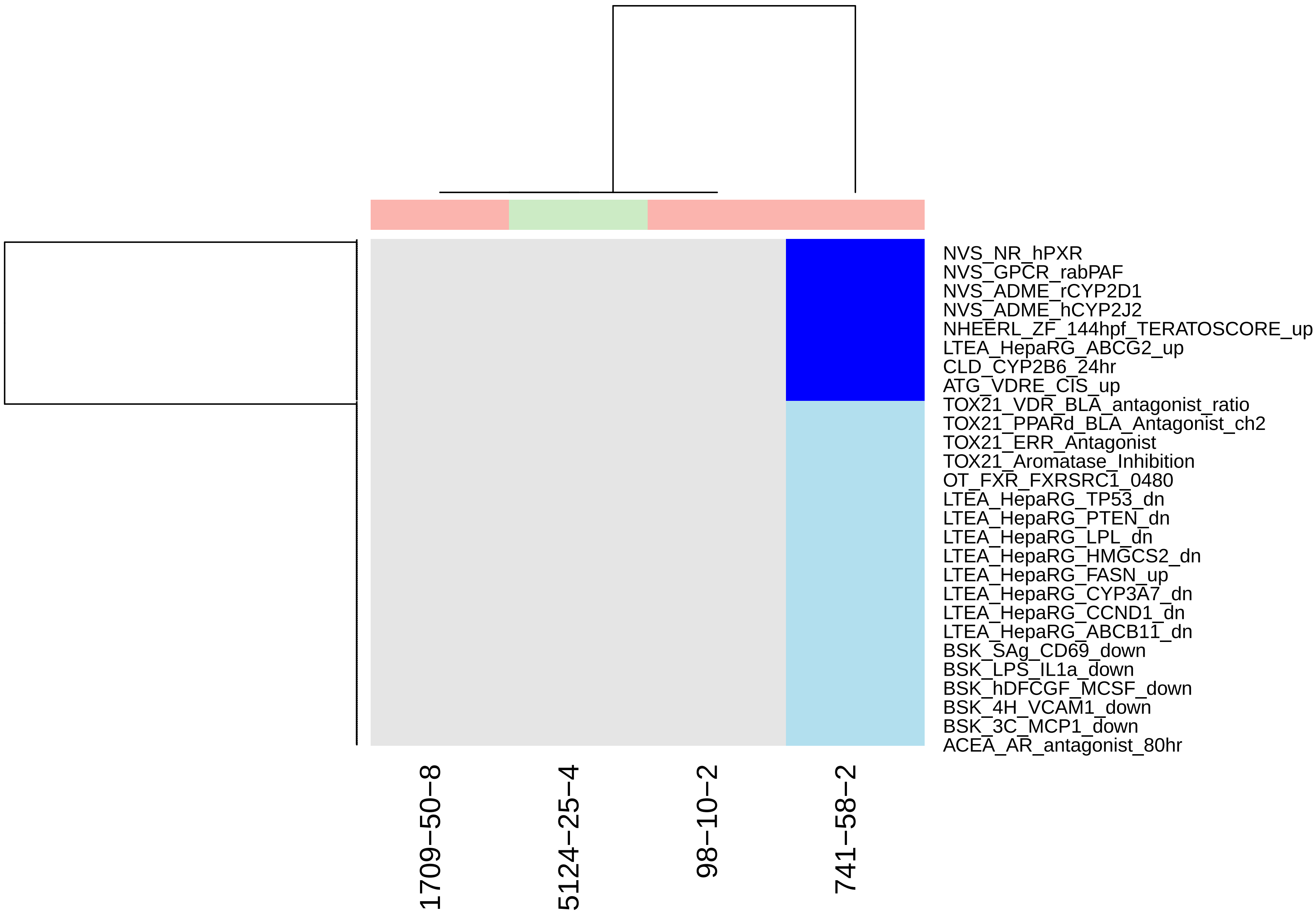

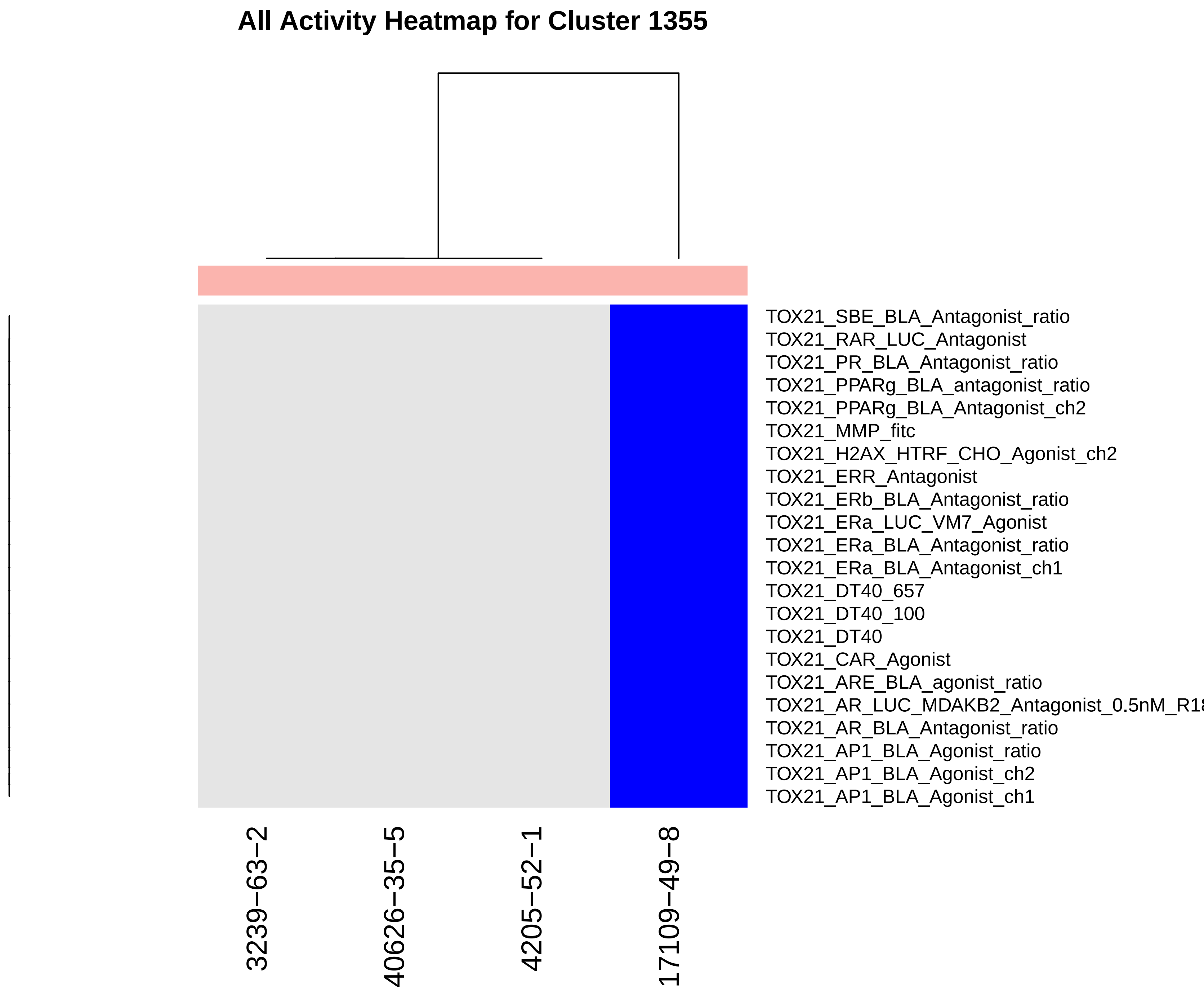

All Activity Heatmap for Cluster 1355
TOX21_SBE_BLA_Antagonist_ratio
TOX21_RAR_LUC_Antagonist
TOX21_PR_BLA_Antagonist_ratio
TOX21_PPARg_BLA_antagonist_ratio
TOX21_PPARg_BLA_Antagonist_ch2
TOX21_MMP_fitc
TOX21_H2AX_HTRF_CHO_Agonist_ch2
TOX21_ERR_Antagonist
TOX21_ERb_BLA_Antagonist_ratio
TOX21_ERa_LUC_VM7_Agonist
TOX21_ERa_BLA_Antagonist_ratio
TOX21_ERa_BLA_Antagonist_ch1
TOX21_DT40_657
TOX21_DT40_100
TOX21_DT40
TOX21_CAR_Agonist
TOX21_ARE_BLA_agonist_ratio
TOX21_AR_LUC_MDAKB2_Antagonist_0.5nM_R18
TOX21_AR_BLA_Antagonist_ratio
TOX21_AP1_BLA_Agonist_ratio
TOX21_AP1_BLA_Agonist_ch2
TOX21_AP1_BLA_Agonist_ch1
3239−63−2
40626−35−5
4205−52−1
17109−49−8

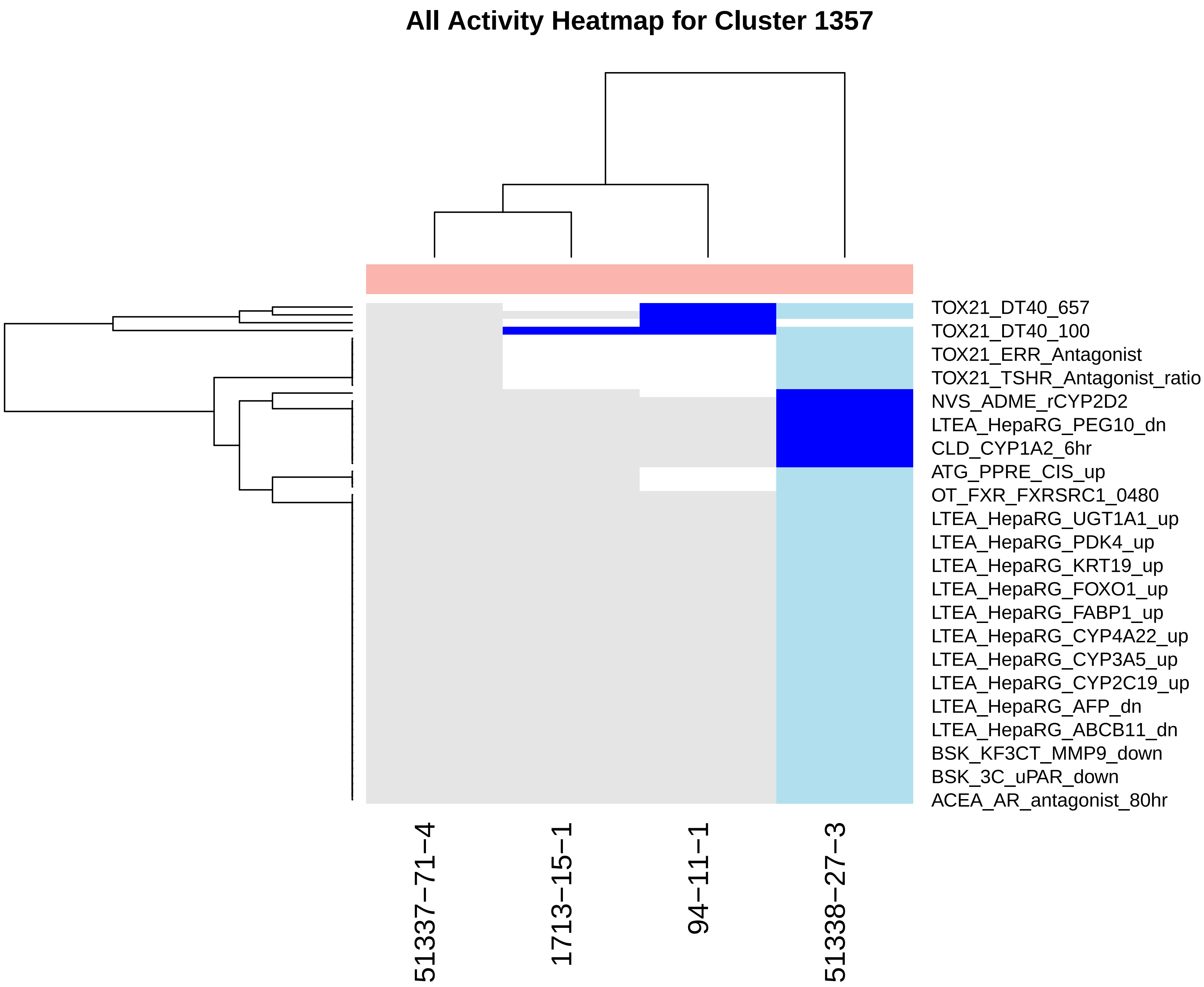
All Activity Heatmap for Cluster 1357
TOX21_DT40_657
TOX21_DT40_100
TOX21_ERR_Antagonist
TOX21_TSHR_Antagonist_ratio
NVS_ADME_rCYP2D2
LTEA_HepaRG_PEG10_dn
CLD_CYP1A2_6hr
ATG_PPRE_CIS_up
OT_FXR_FXRSRC1_0480
LTEA_HepaRG_UGT1A1_up
LTEA_HepaRG_PDK4_up
LTEA_HepaRG_KRT19_up
LTEA_HepaRG_FOXO1_up
LTEA_HepaRG_FABP1_up
LTEA_HepaRG_CYP4A22_up
LTEA_HepaRG_CYP3A5_up
LTEA_HepaRG_CYP2C19_up
LTEA_HepaRG_AFP_dn
LTEA_HepaRG_ABCB11_dn
BSK_KF3CT_MMP9_down
BSK_3C_uPAR_down
ACEA_AR_antagonist_80hr
51337−71−4
1713−15−1
94−11−1
51338−27−3

All Activity Heatmap for Cluster 1360

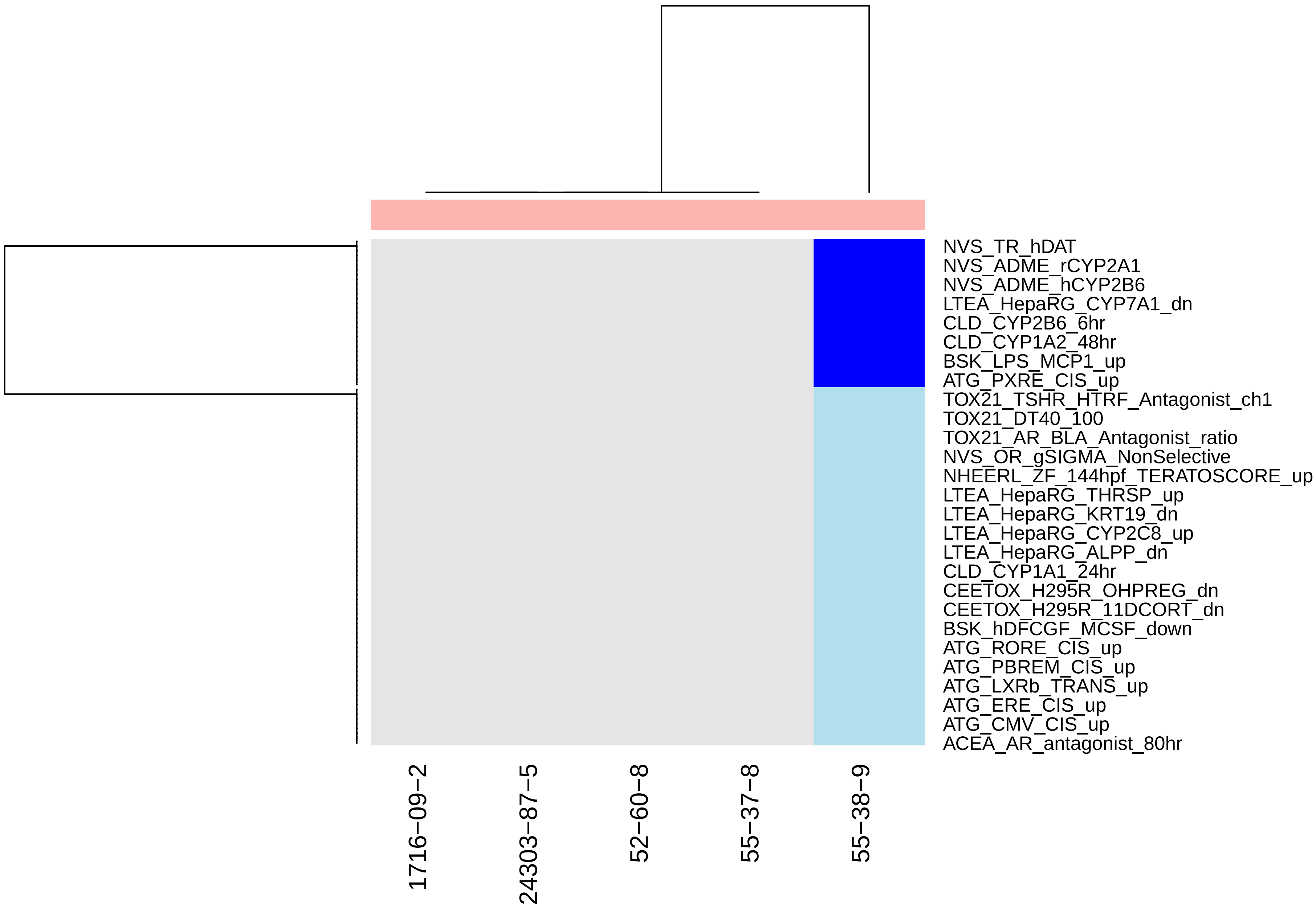

All Activity Heatmap for Cluster 1362

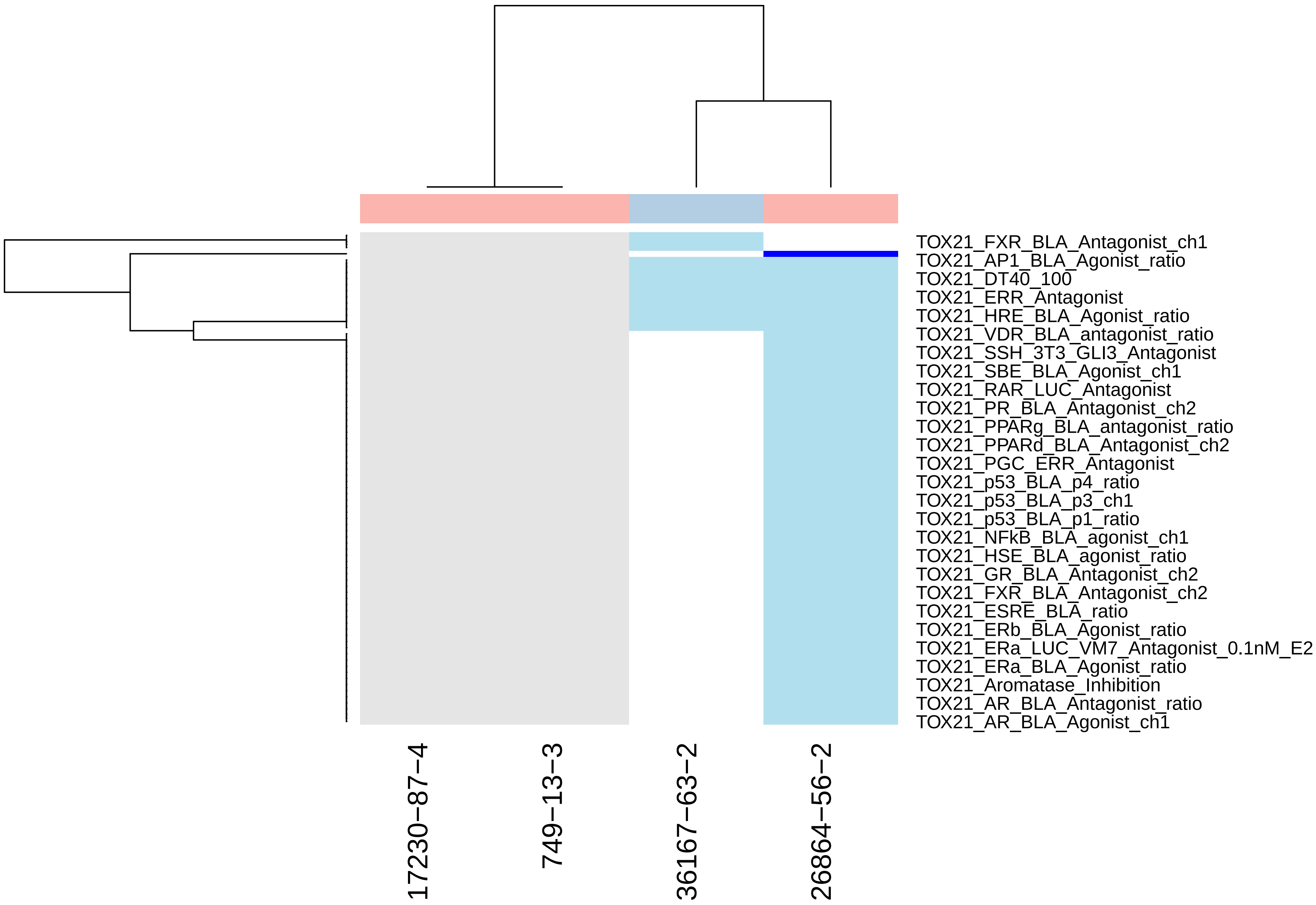

All Activity Heatmap for Cluster 1363

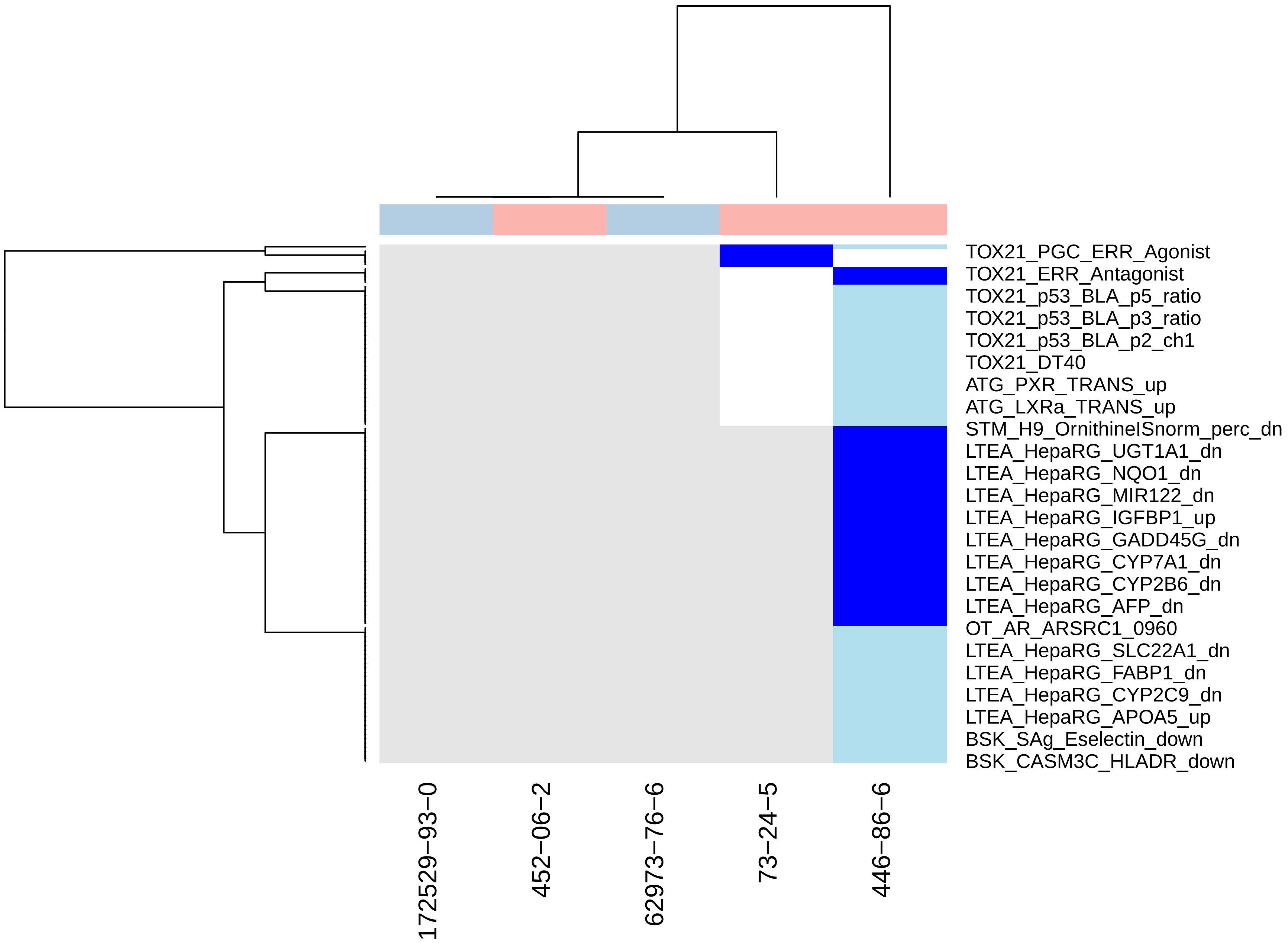

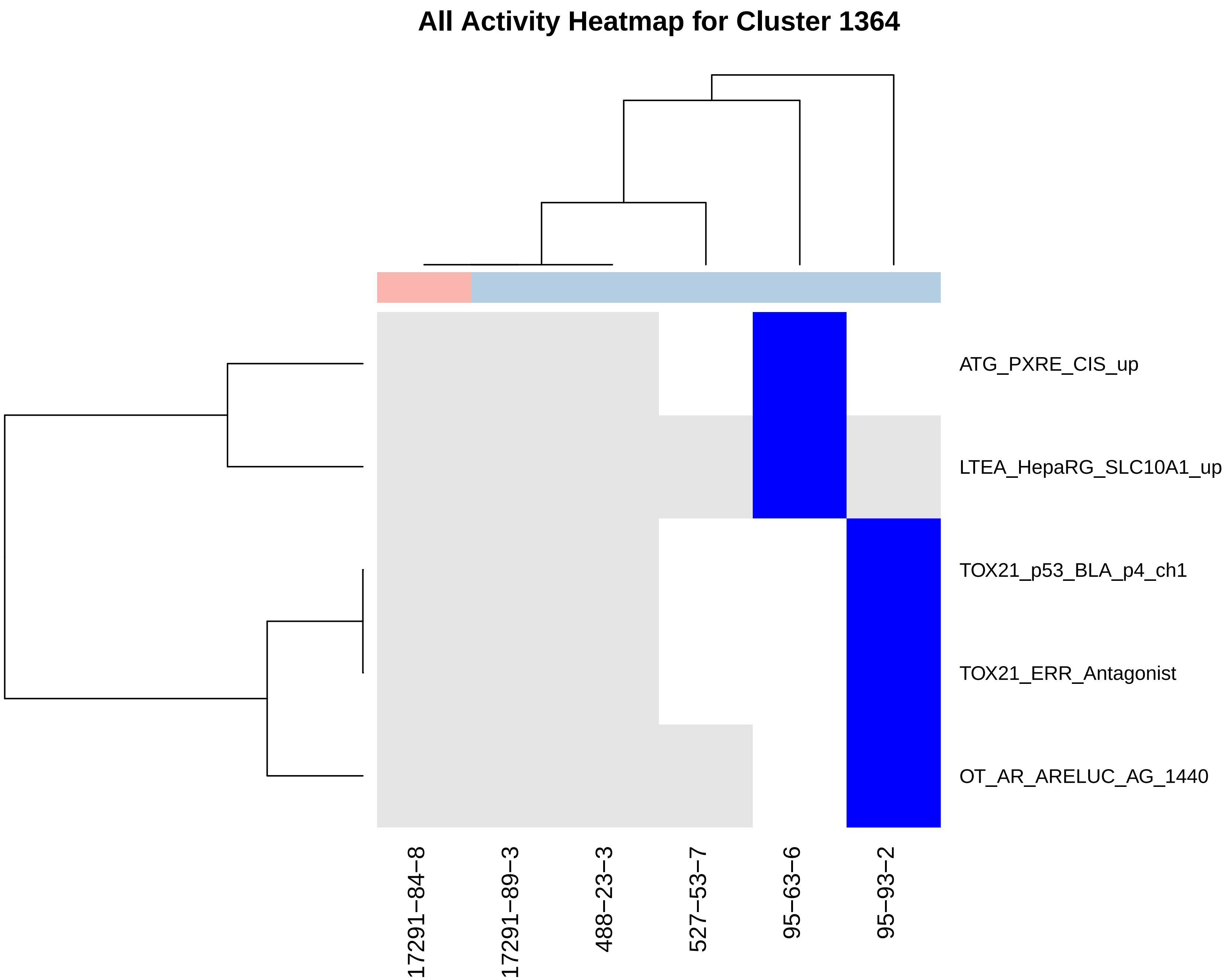
All Activity Heatmap for Cluster 1364
ATG_PXRE_CIS_up
LTEA_HepaRG_SLC10A1_up
TOX21_p53_BLA_p4_ch1
TOX21_ERR_Antagonist
OT_AR_ARELUC_AG_1440
17291−84−8
17291−89−3
488−23−3
527−53−7
95−63−6
95−93−2

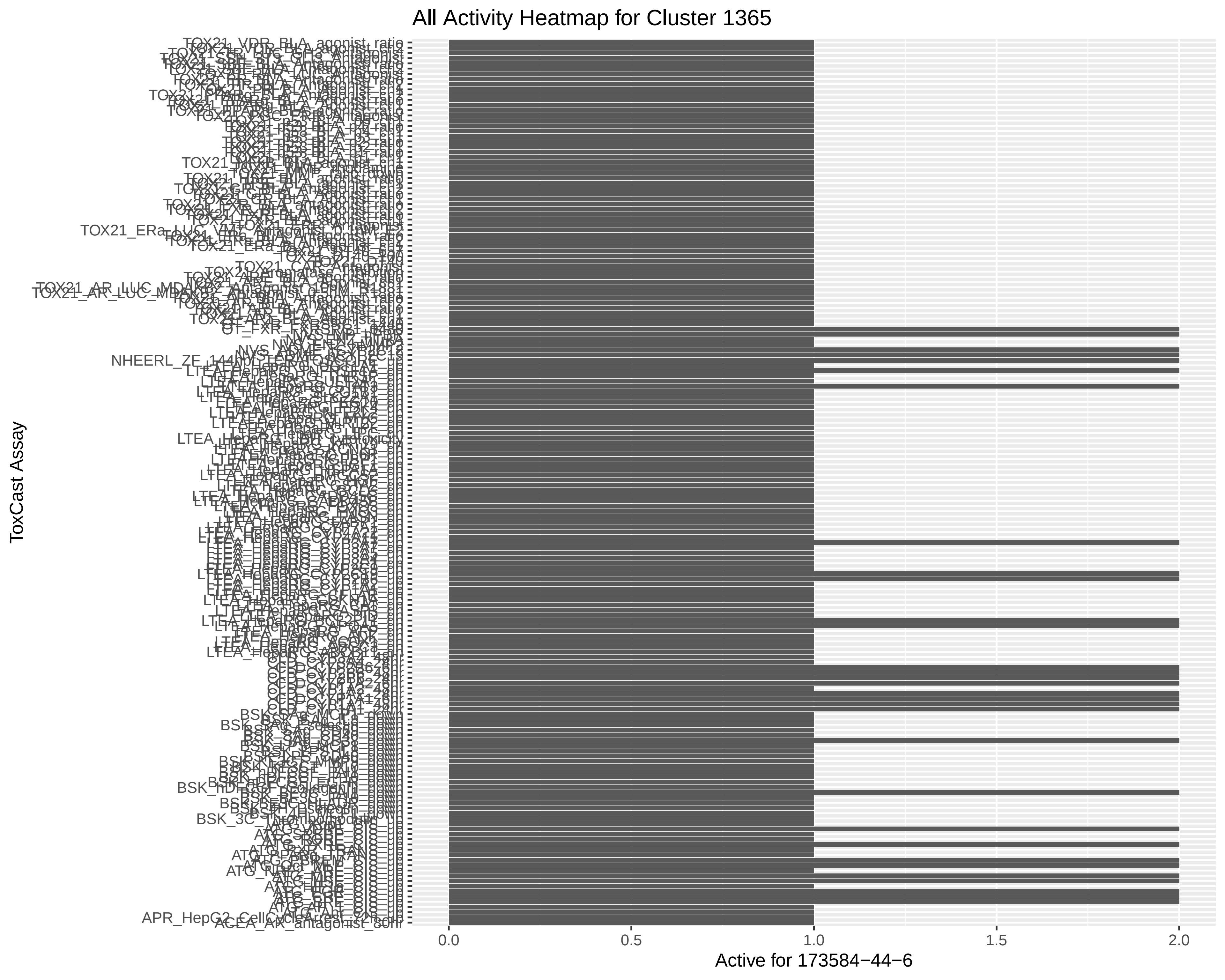
All Activity Heatmap for Cluster 1365
ToxCast Assay
Active for 173584−44−6
0.0
0.5
1.0
1.5
2.0

All Activity Heatmap for Cluster 1367

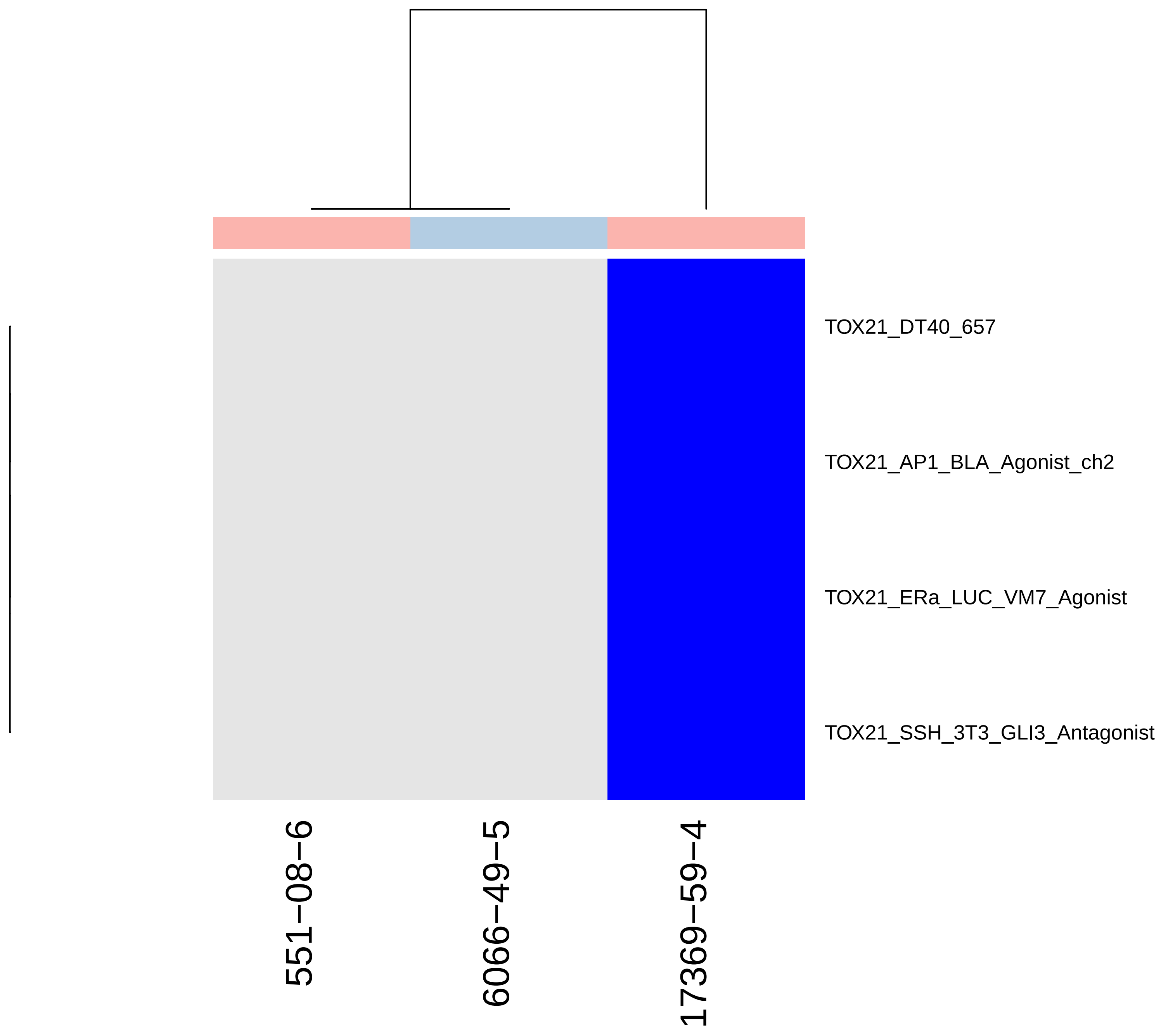

All Activity Heatmap for Cluster 1369

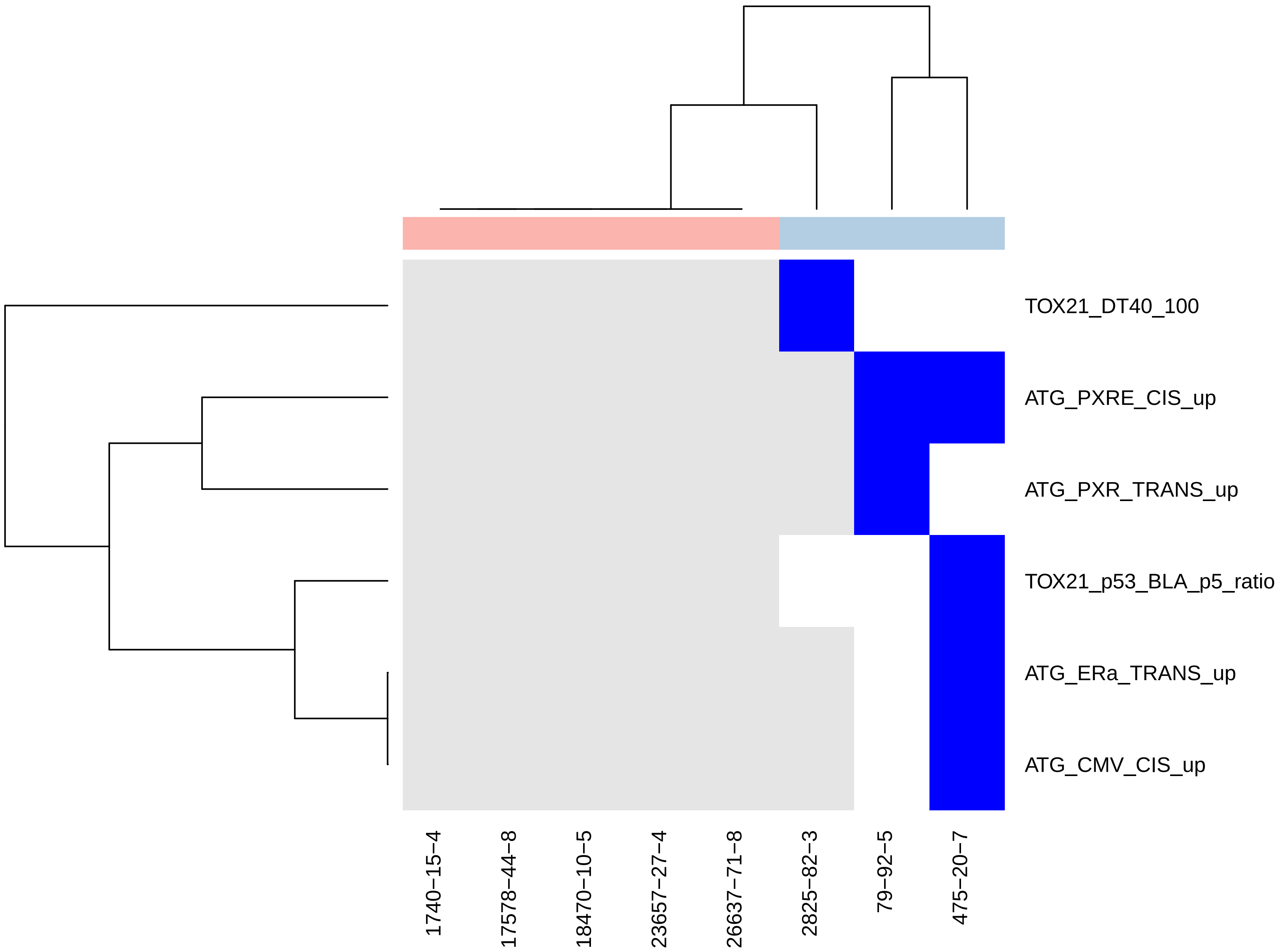

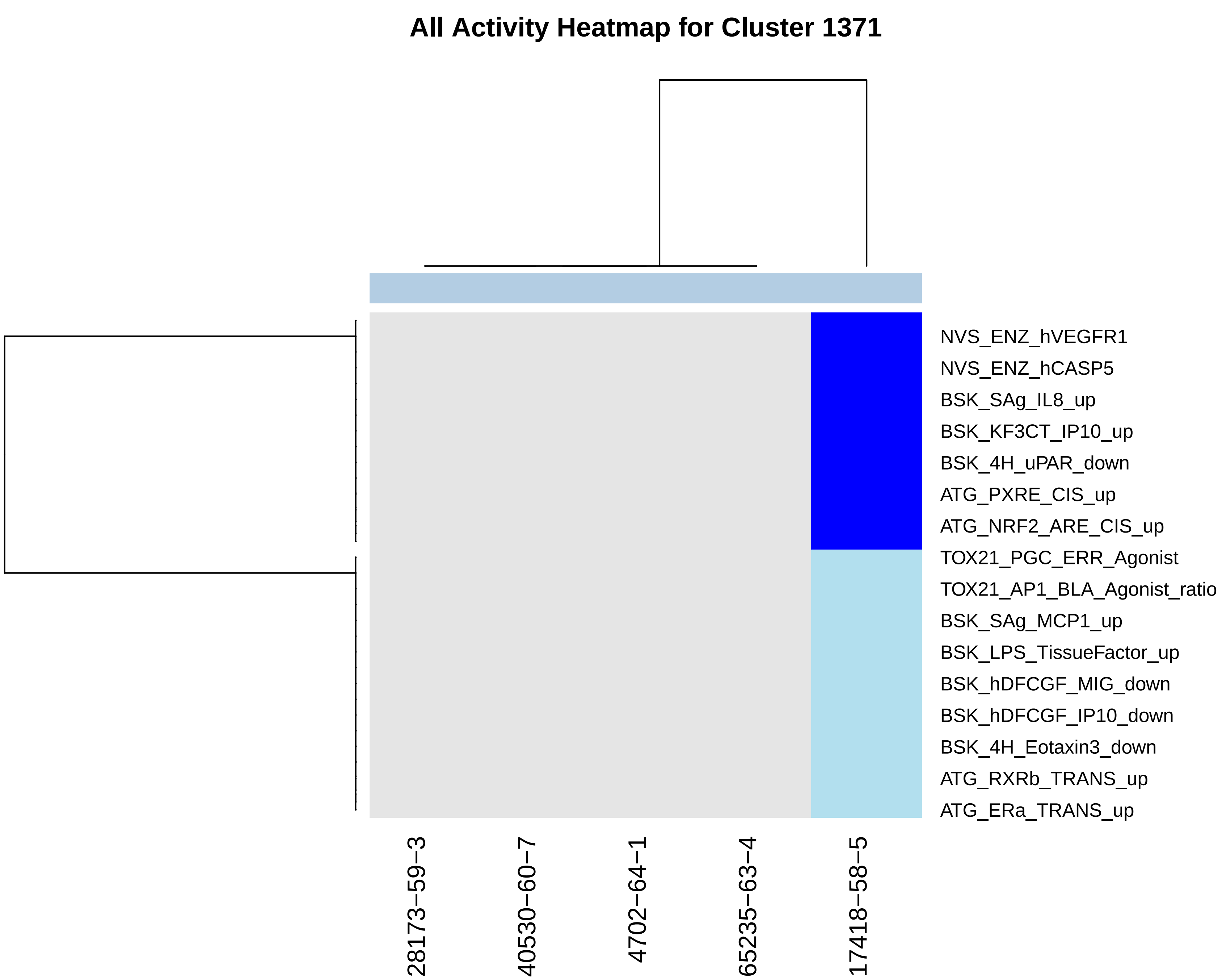
All Activity Heatmap for Cluster 1371
NVS_ENZ_hVEGFR1
NVS_ENZ_hCASP5
BSK_SAg_IL8_up
BSK_KF3CT_IP10_up
BSK_4H_uPAR_down
ATG_PXRE_CIS_up
ATG_NRF2_ARE_CIS_up
TOX21_PGC_ERR_Agonist
TOX21_AP1_BLA_Agonist_ratio
BSK_SAg_MCP1_up
BSK_LPS_TissueFactor_up
BSK_hDFCGF_MIG_down
BSK_hDFCGF_IP10_down
BSK_4H_Eotaxin3_down
ATG_RXRb_TRANS_up
ATG_ERa_TRANS_up
28173–59–3
40530–60–7
4702–64–1
65235–63–4
17418–58–5

All Activity Heatmap for Cluster 1372

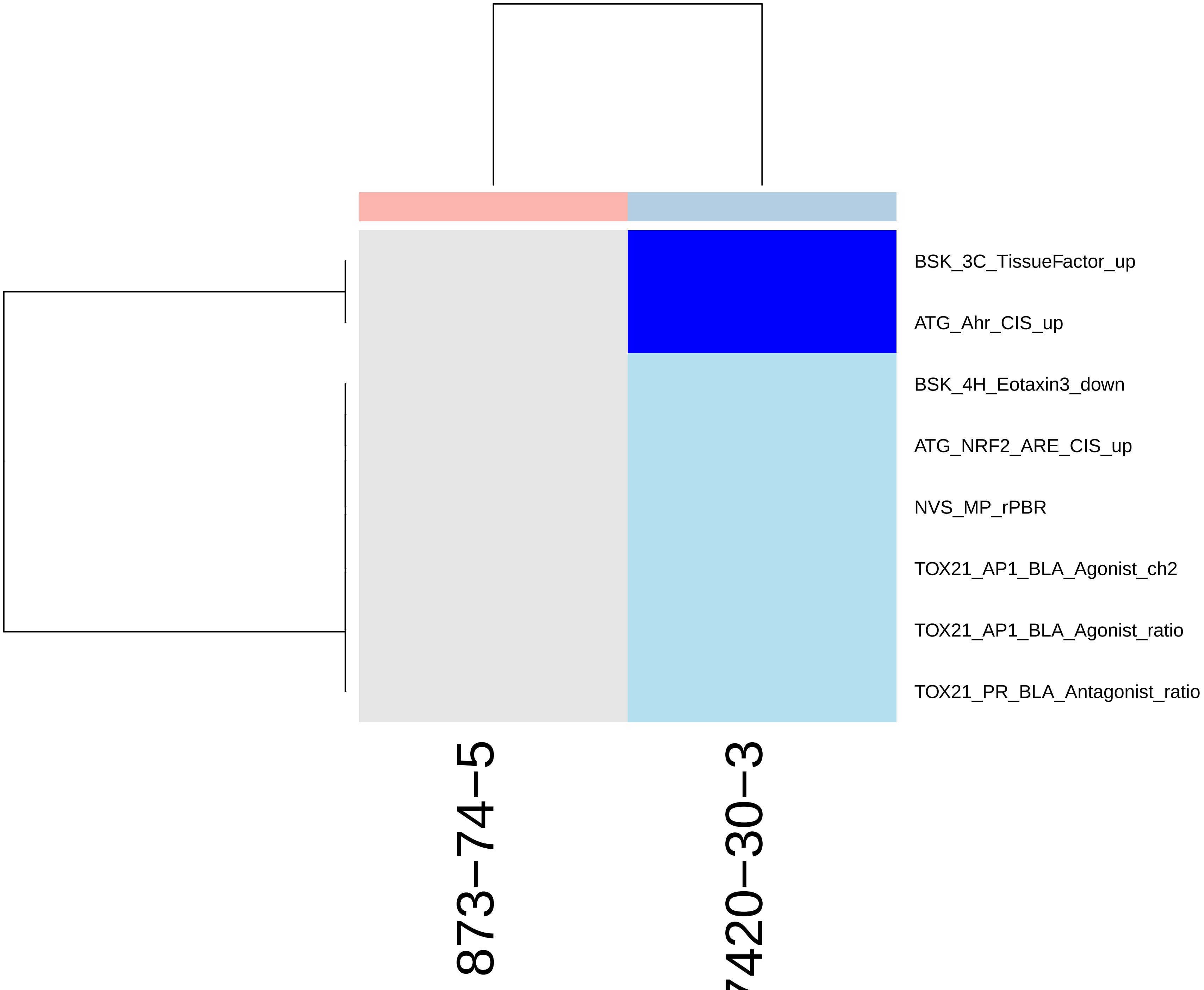

All Activity Heatmap for Cluster 1373

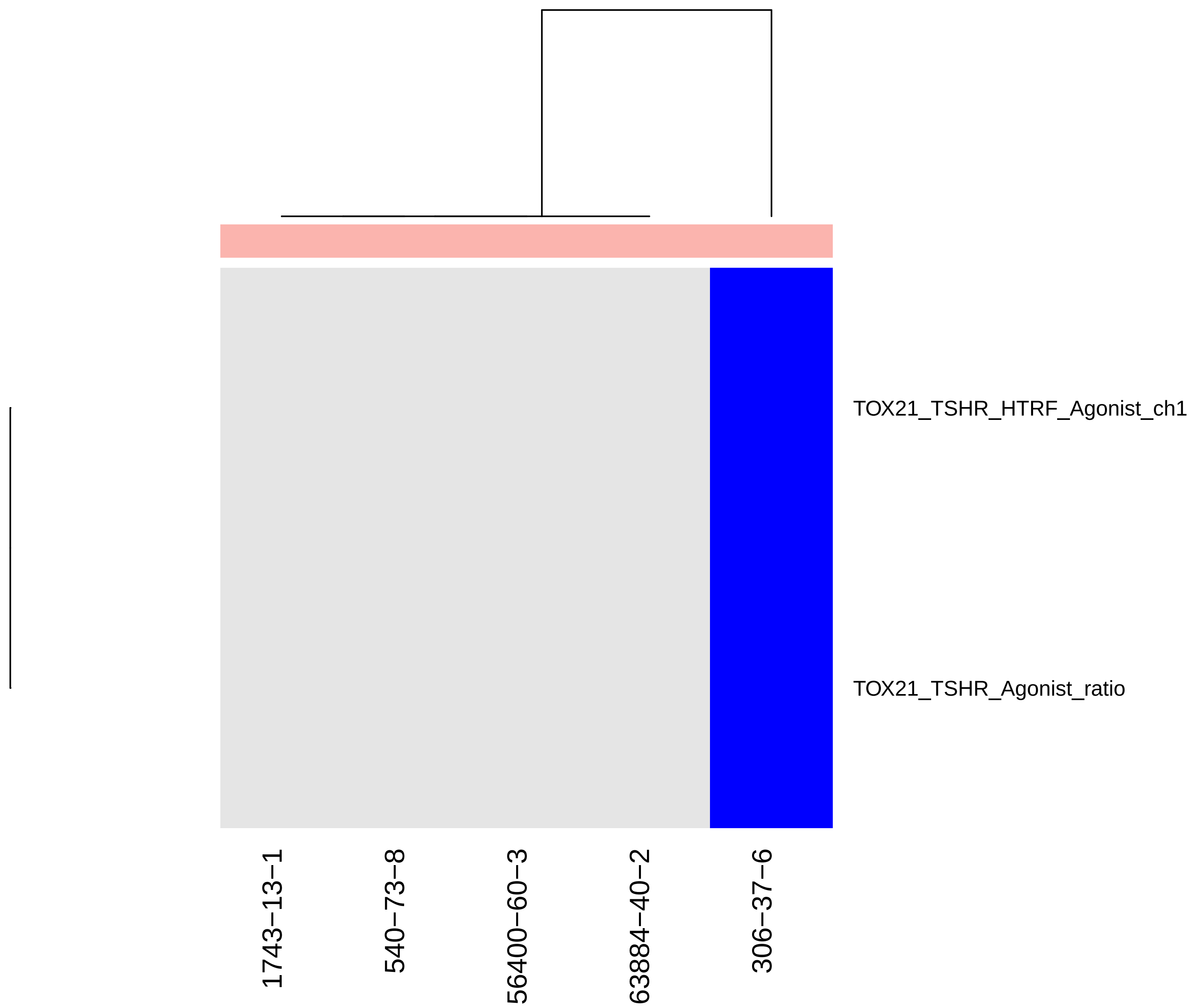

All Activity Heatmap for Cluster 1374

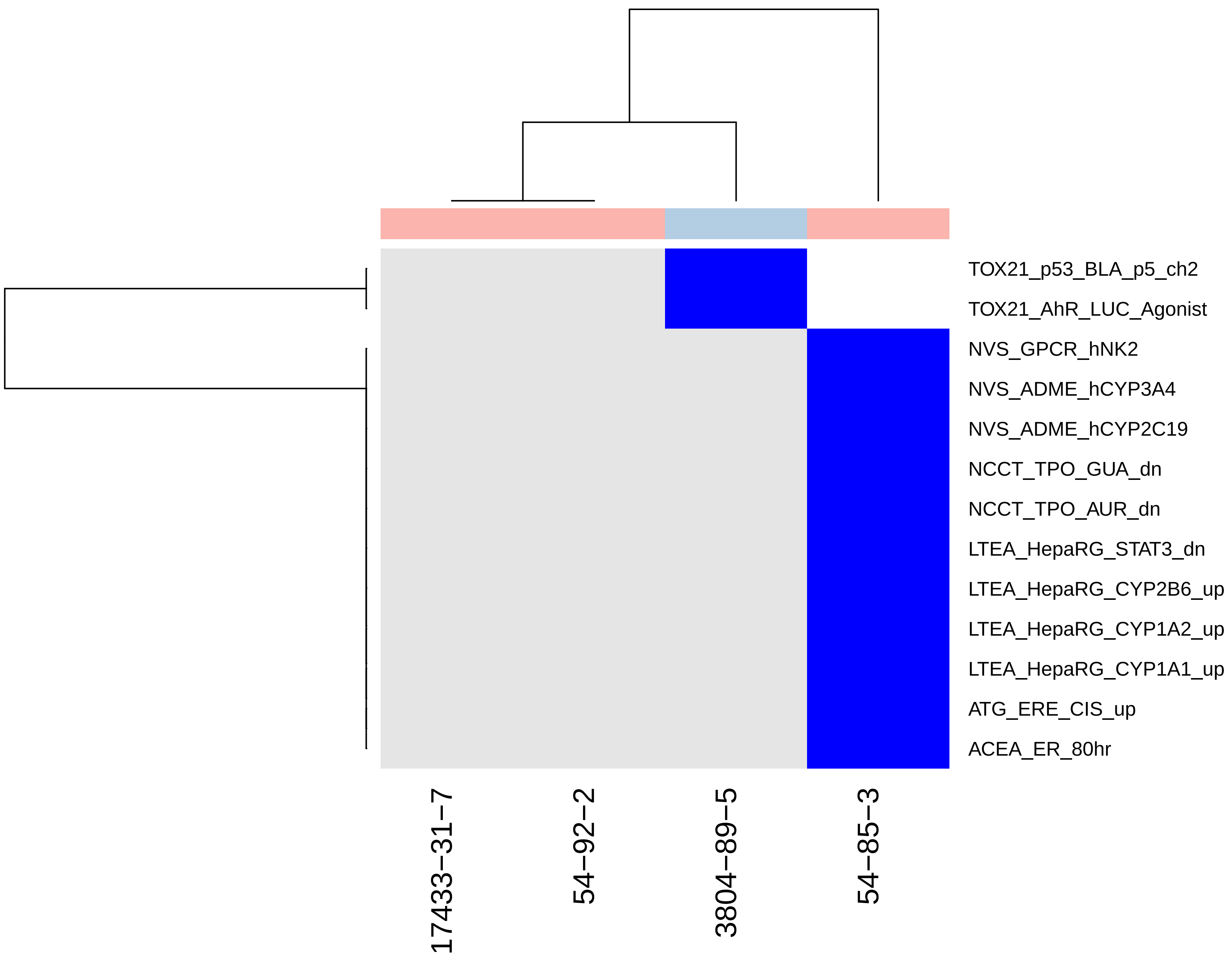

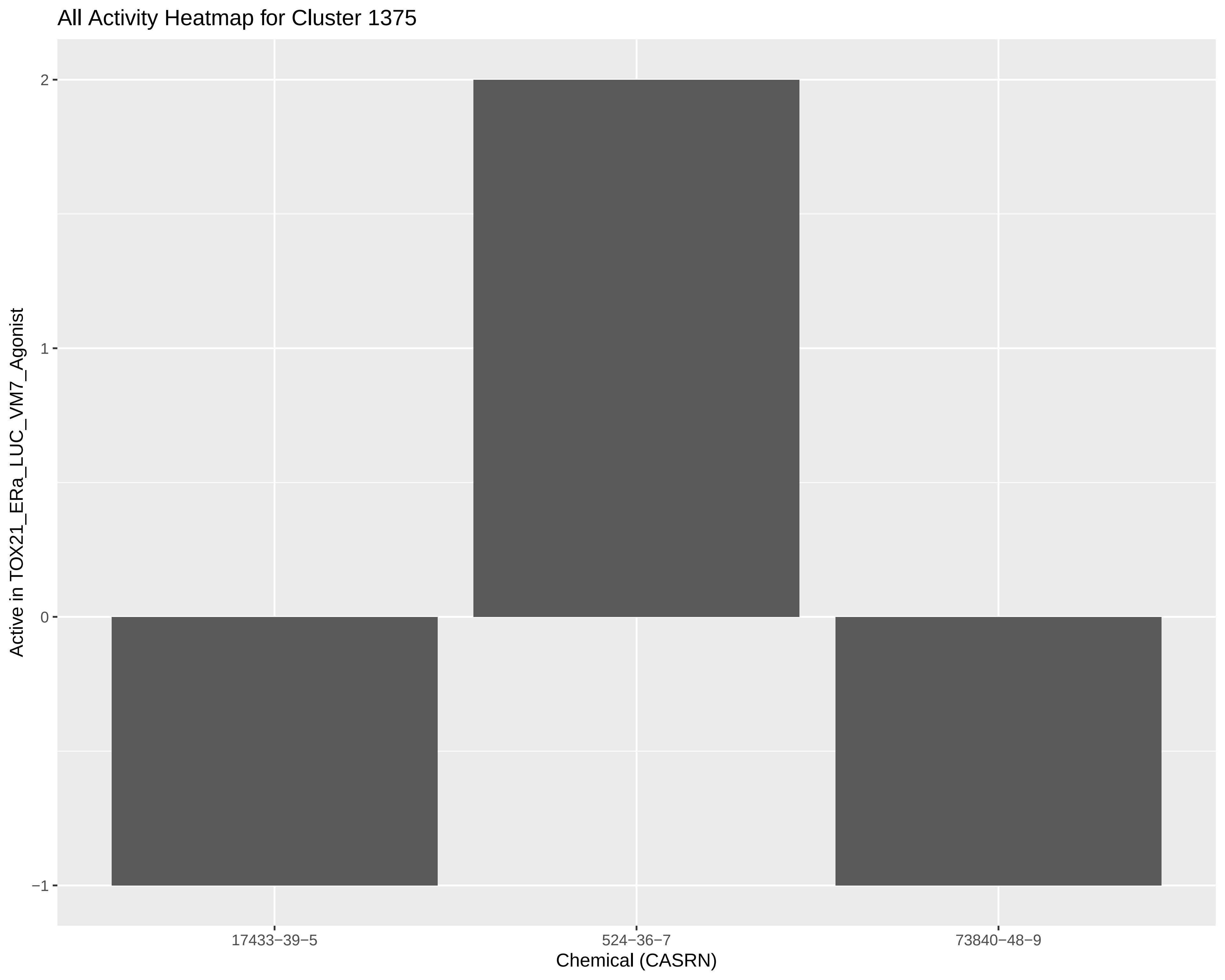
All Activity Heatmap for Cluster 1375
Active in TOX21_ERa_LUC_VM7_Agonist
2
1
0
−1
17433−39−5
524−36−7
73840−48−9
Chemical (CASRN)

All Activity Heatmap for Cluster 1377

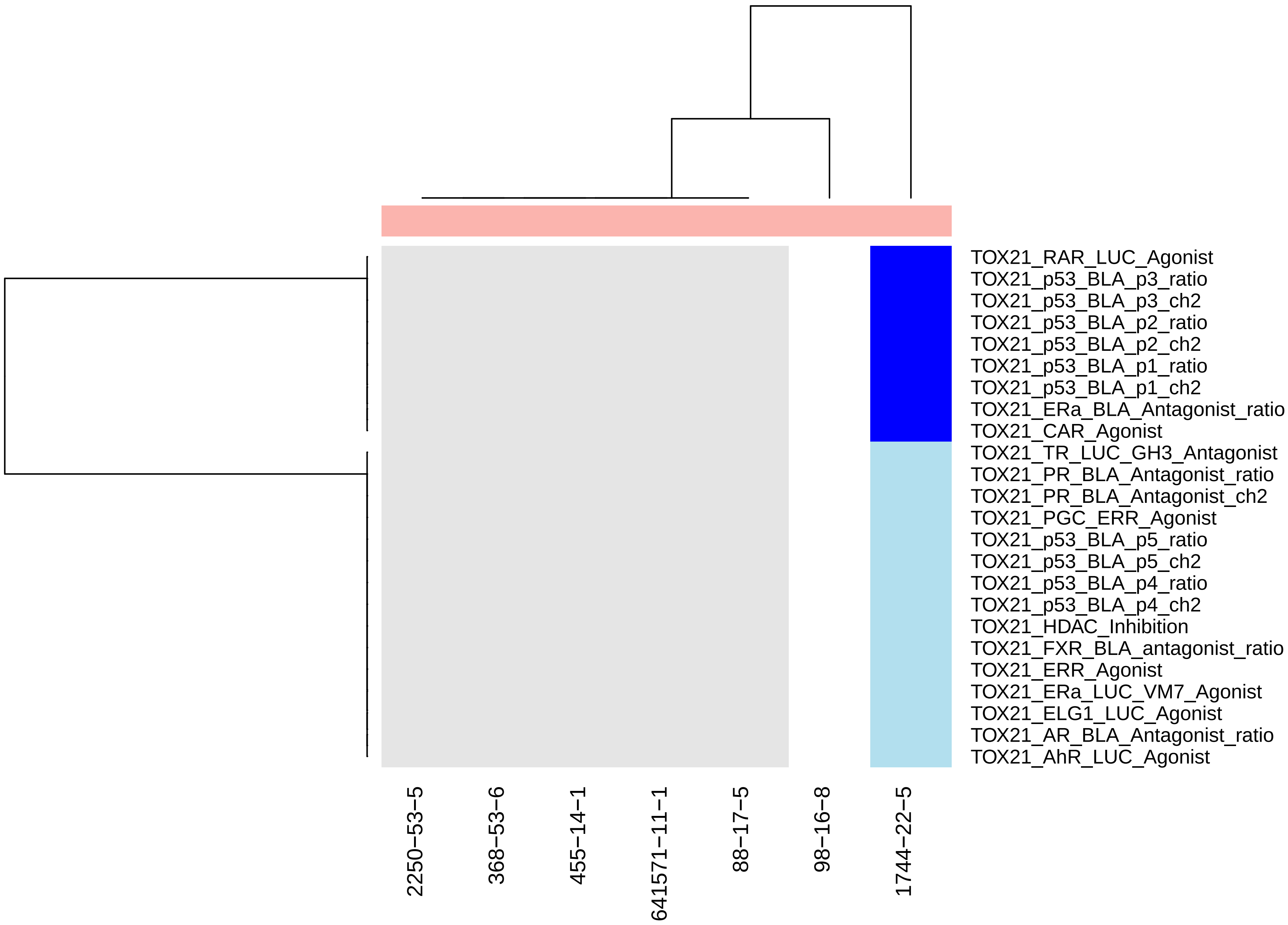

All Activity Heatmap for Cluster 1378

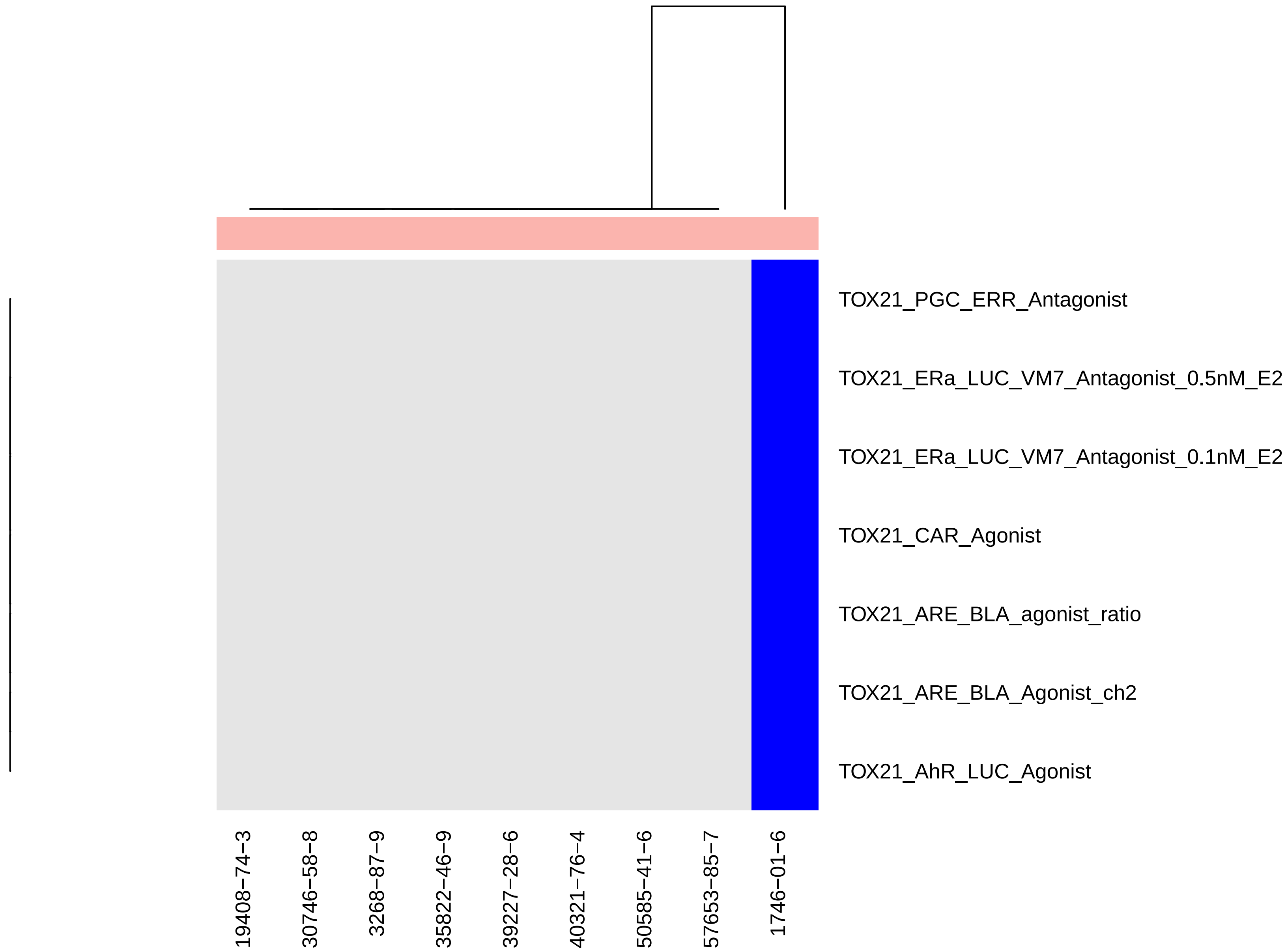

All Activity Heatmap for Cluster 1380

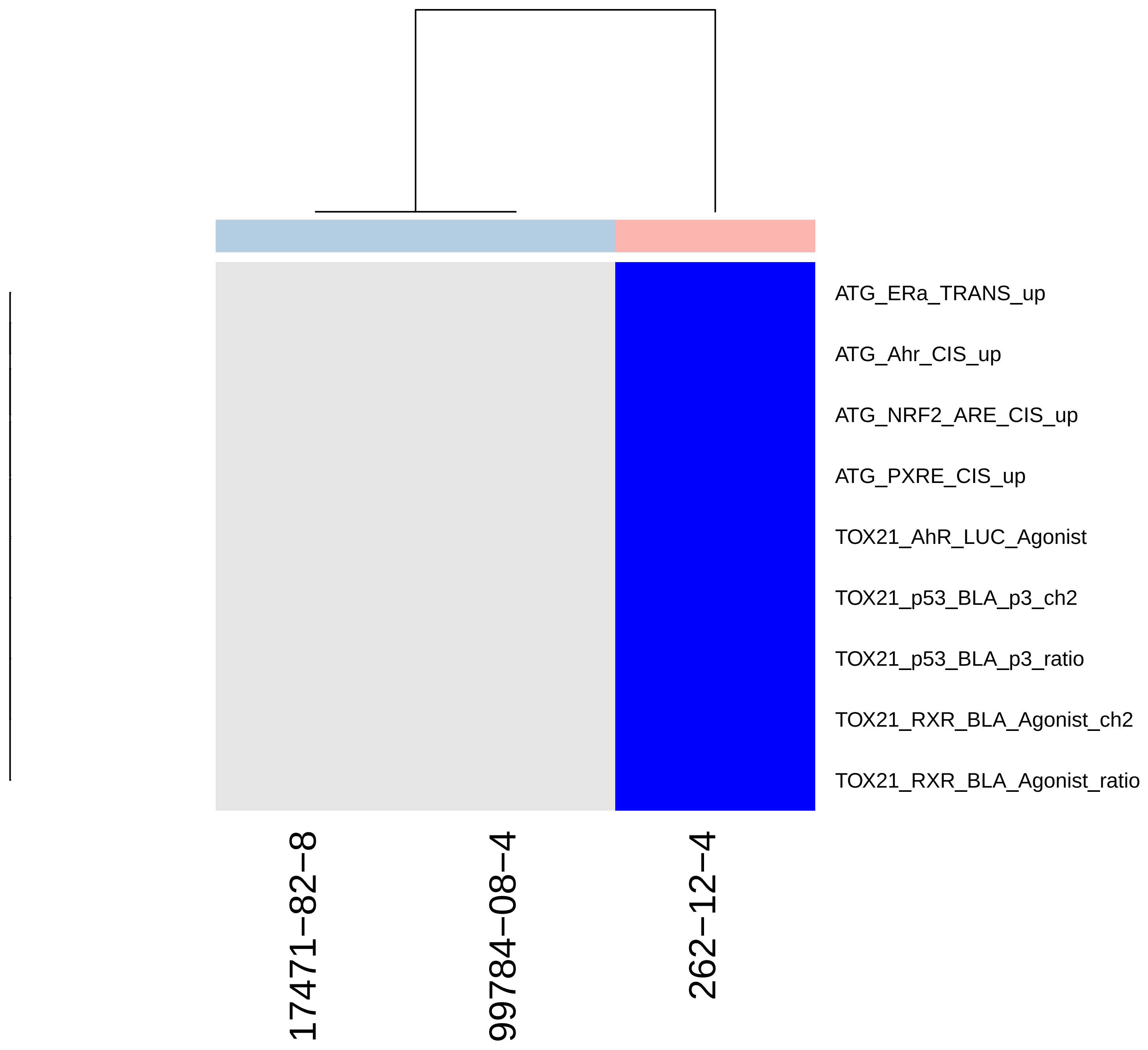

All Activity Heatmap for Cluster 1382

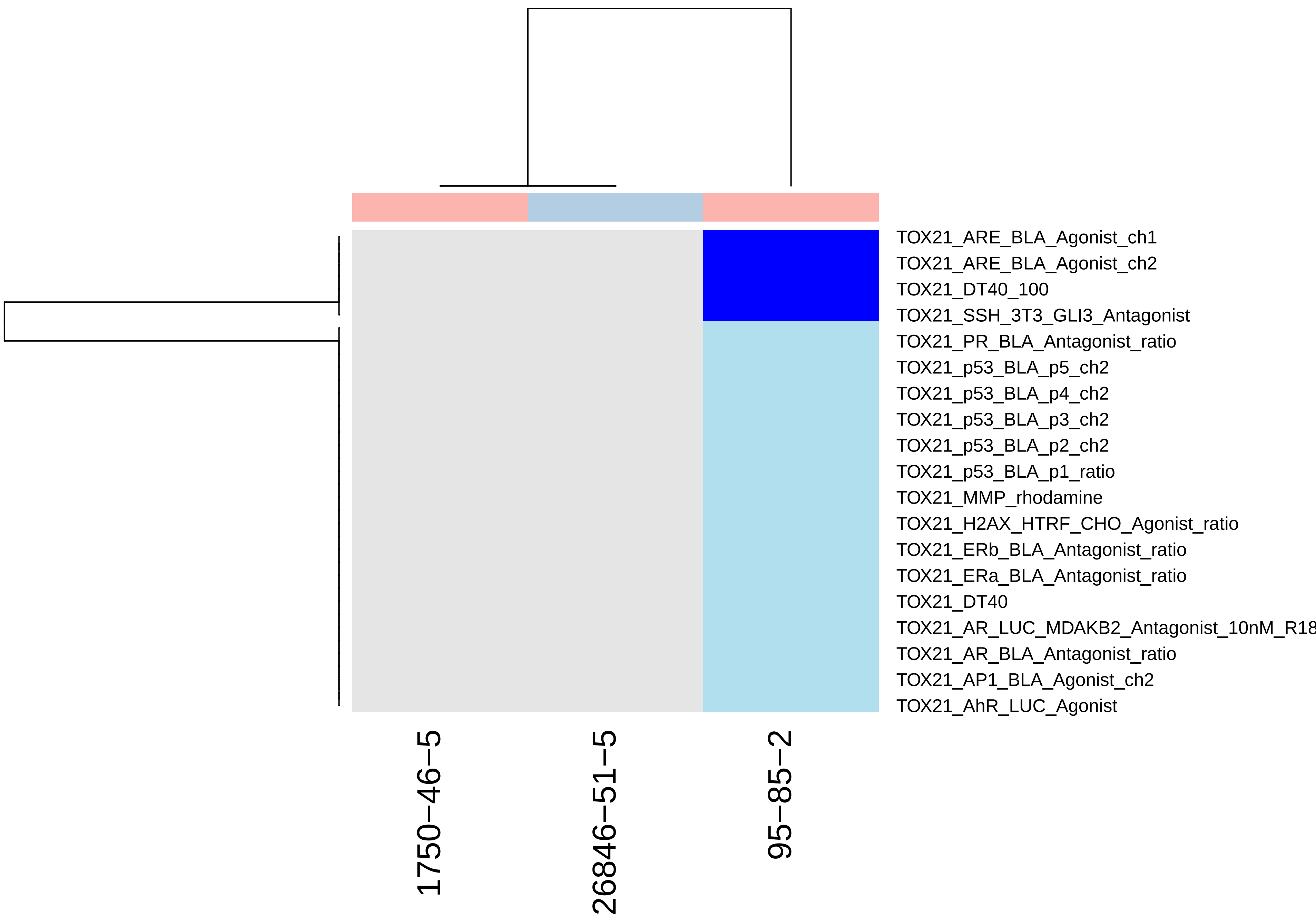

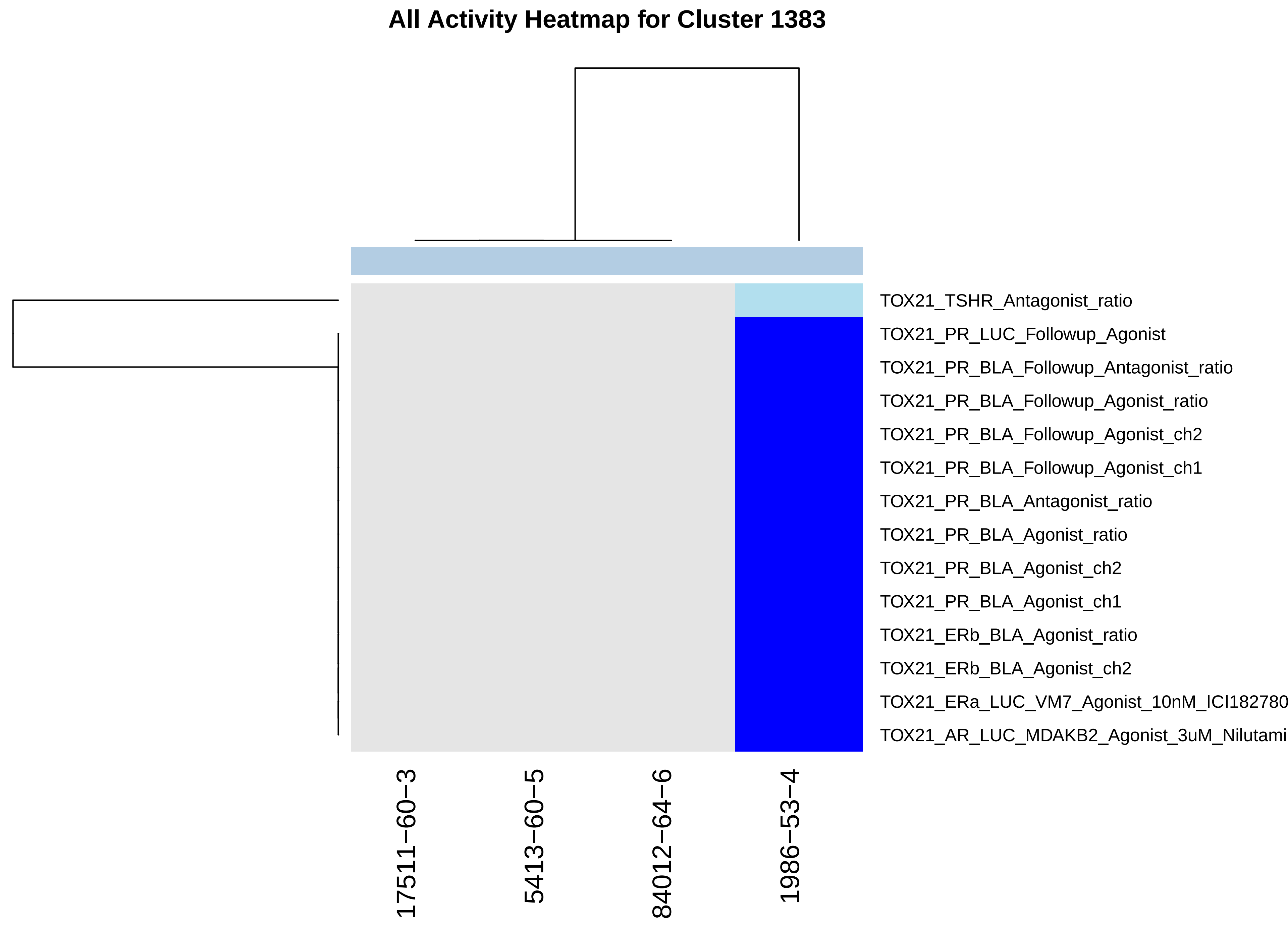
All Activity Heatmap for Cluster 1383
TOX21_TSHR_Antagonist_ratio
TOX21_PR_LUC_Followup_Agonist
TOX21_PR_BLA_Followup_Antagonist_ratio
TOX21_PR_BLA_Followup_Agonist_ratio
TOX21_PR_BLA_Followup_Agonist_ch2
TOX21_PR_BLA_Followup_Agonist_ch1
TOX21_PR_BLA_Antagonist_ratio
TOX21_PR_BLA_Agonist_ratio
TOX21_PR_BLA_Agonist_ch2
TOX21_PR_BLA_Agonist_ch1
TOX21_ERb_BLA_Agonist_ratio
TOX21_ERb_BLA_Agonist_ch2
TOX21_ERa_LUC_VM7_Agonist_10nM_ICI182780
TOX21_AR_LUC_MDAKB2_Agonist_3uM_Nilutami
17511−60−3
5413−60−5
84012−64−6
1986−53−4

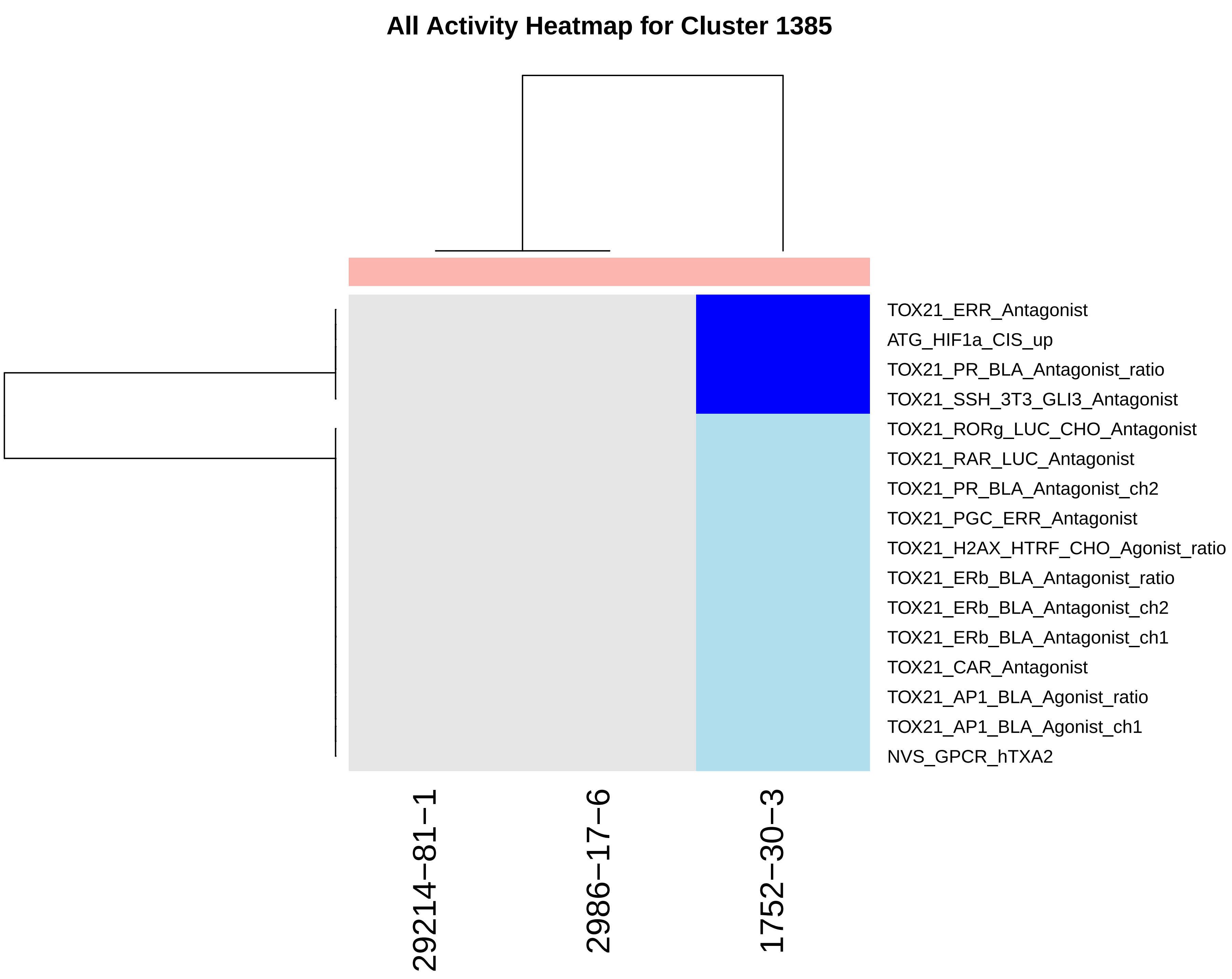

All Activity Heatmap for Cluster 1385
TOX21_ERR_Antagonist
ATG_HIF1a_CIS_up
TOX21_PR_BLA_Antagonist_ratio
TOX21_SSH_3T3_GLI3_Antagonist
TOX21_RORg_LUC_CHO_Antagonist
TOX21_RAR_LUC_Antagonist
TOX21_PR_BLA_Antagonist_ch2
TOX21_PGC_ERR_Antagonist
TOX21_H2AX_HTRF_CHO_Agonist_ratio
TOX21_ERb_BLA_Antagonist_ratio
TOX21_ERb_BLA_Antagonist_ch2
TOX21_ERb_BLA_Antagonist_ch1
TOX21_CAR_Antagonist
TOX21_AP1_BLA_Agonist_ratio
TOX21_AP1_BLA_Agonist_ch1
NVS_GPCR_hTXA2
29214−81−1
2986−17−6
1752−30−3

All Activity Heatmap for Cluster 1393

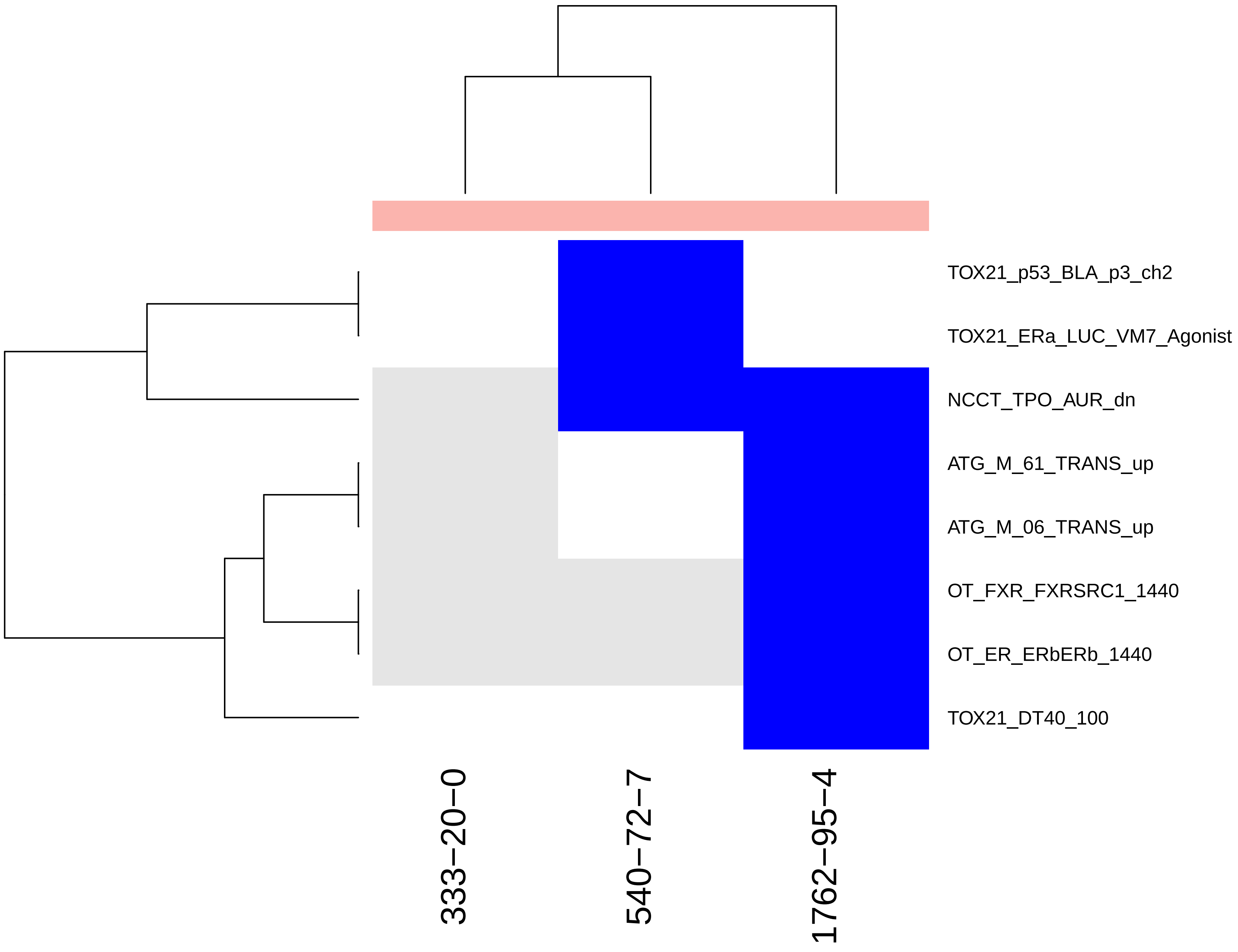

All Activity Heatmap for Cluster 1394

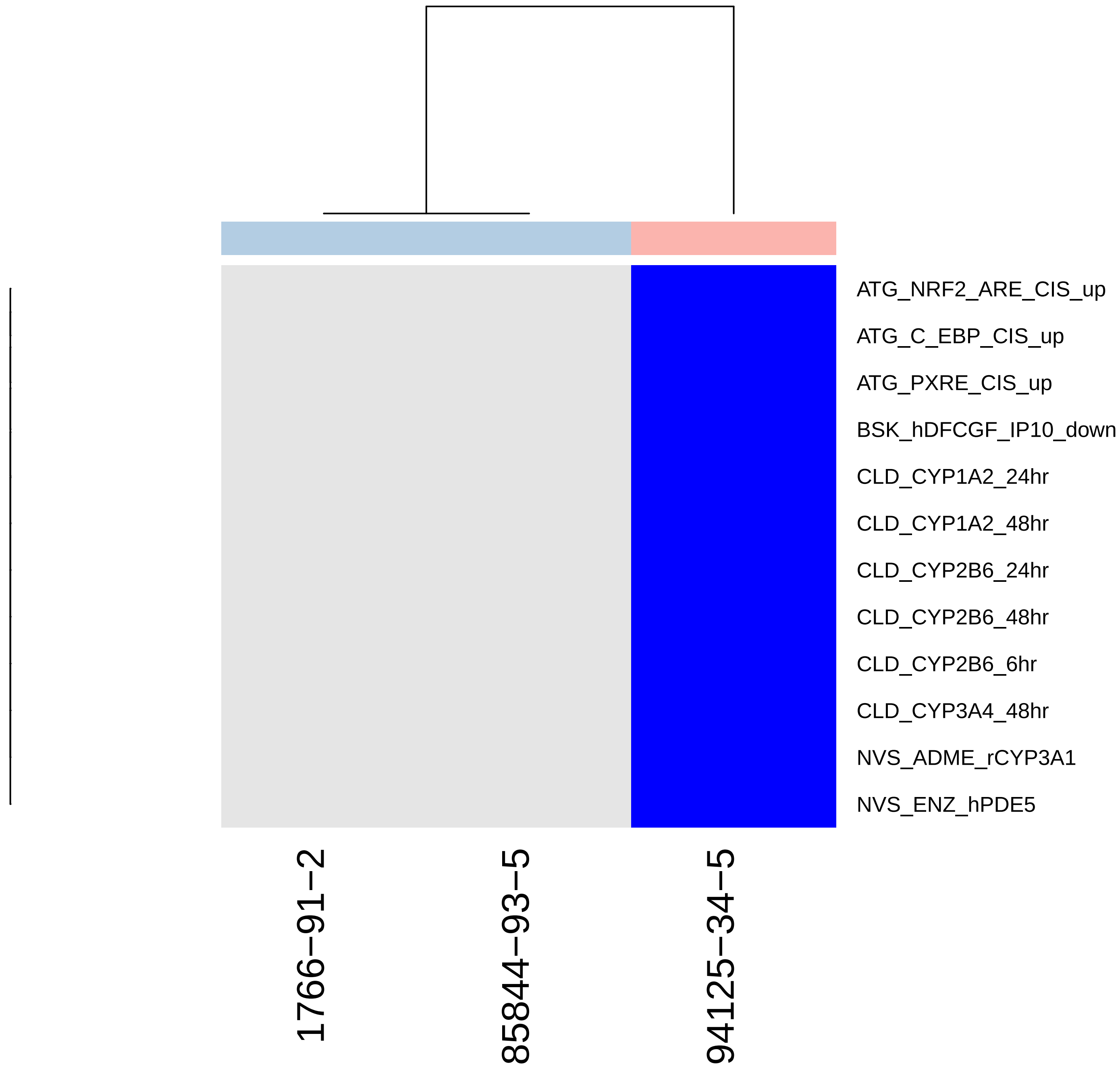

All Activity Heatmap for Cluster 1396

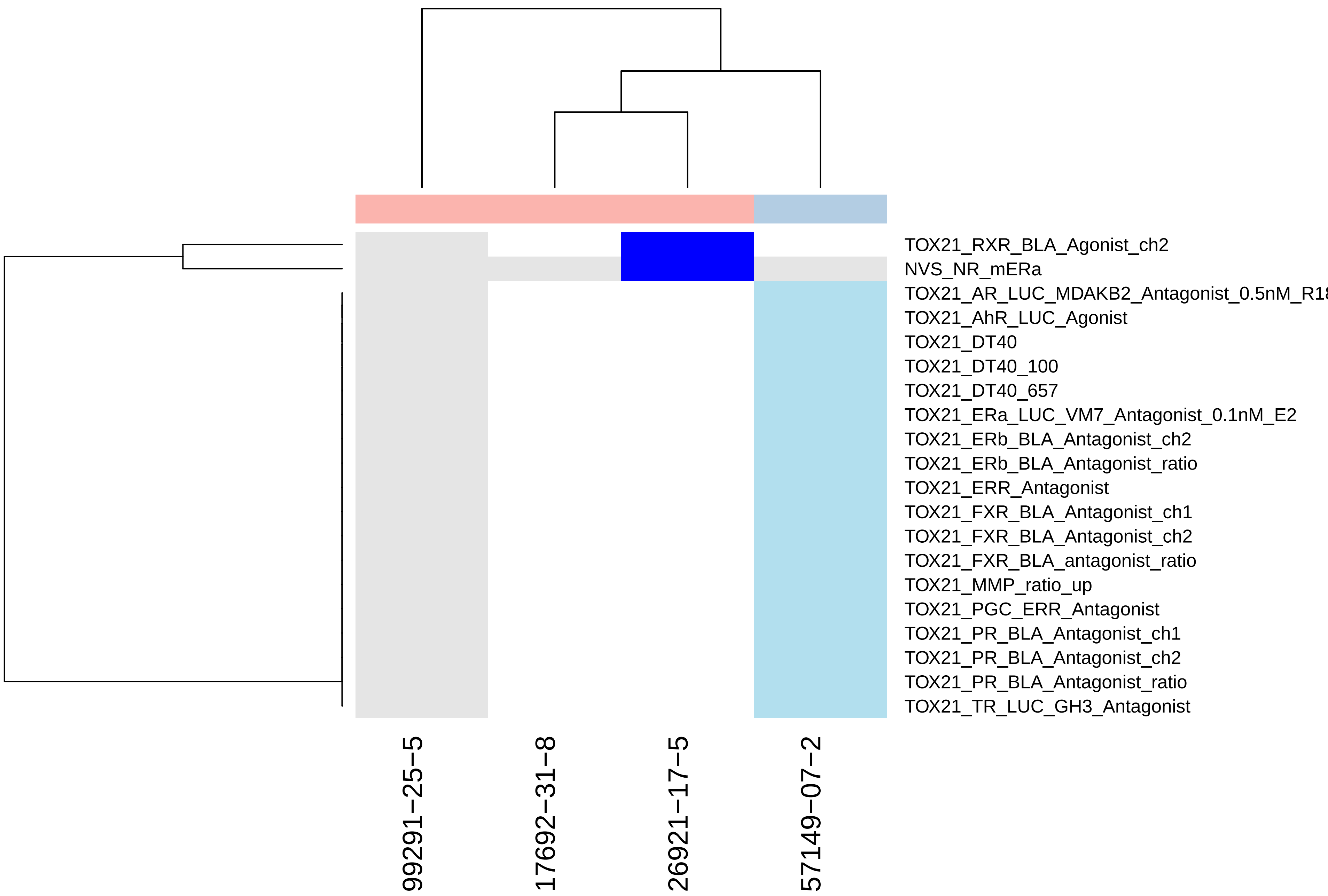

All Activity Heatmap for Cluster 1397

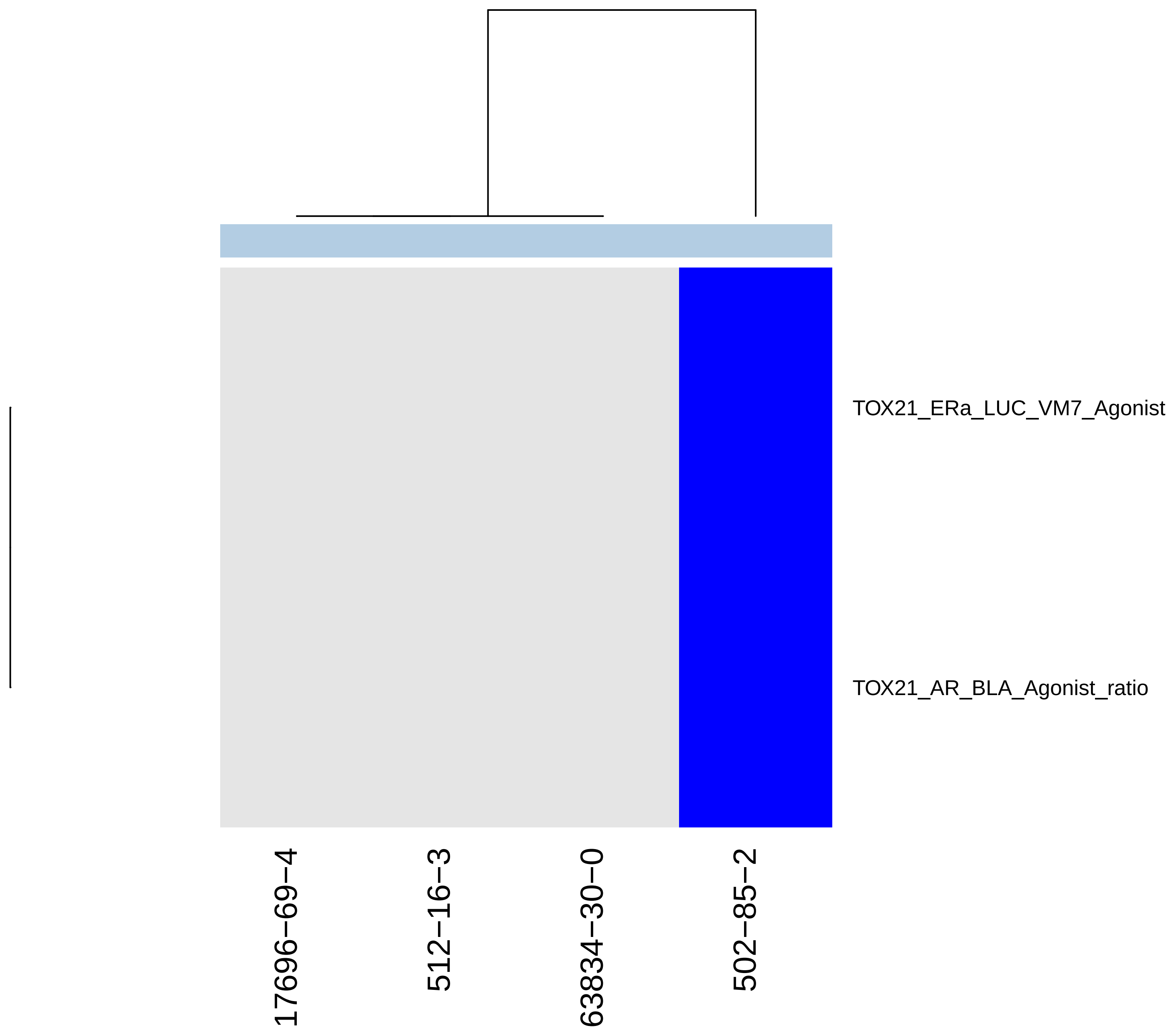

All Activity Heatmap for Cluster 1398

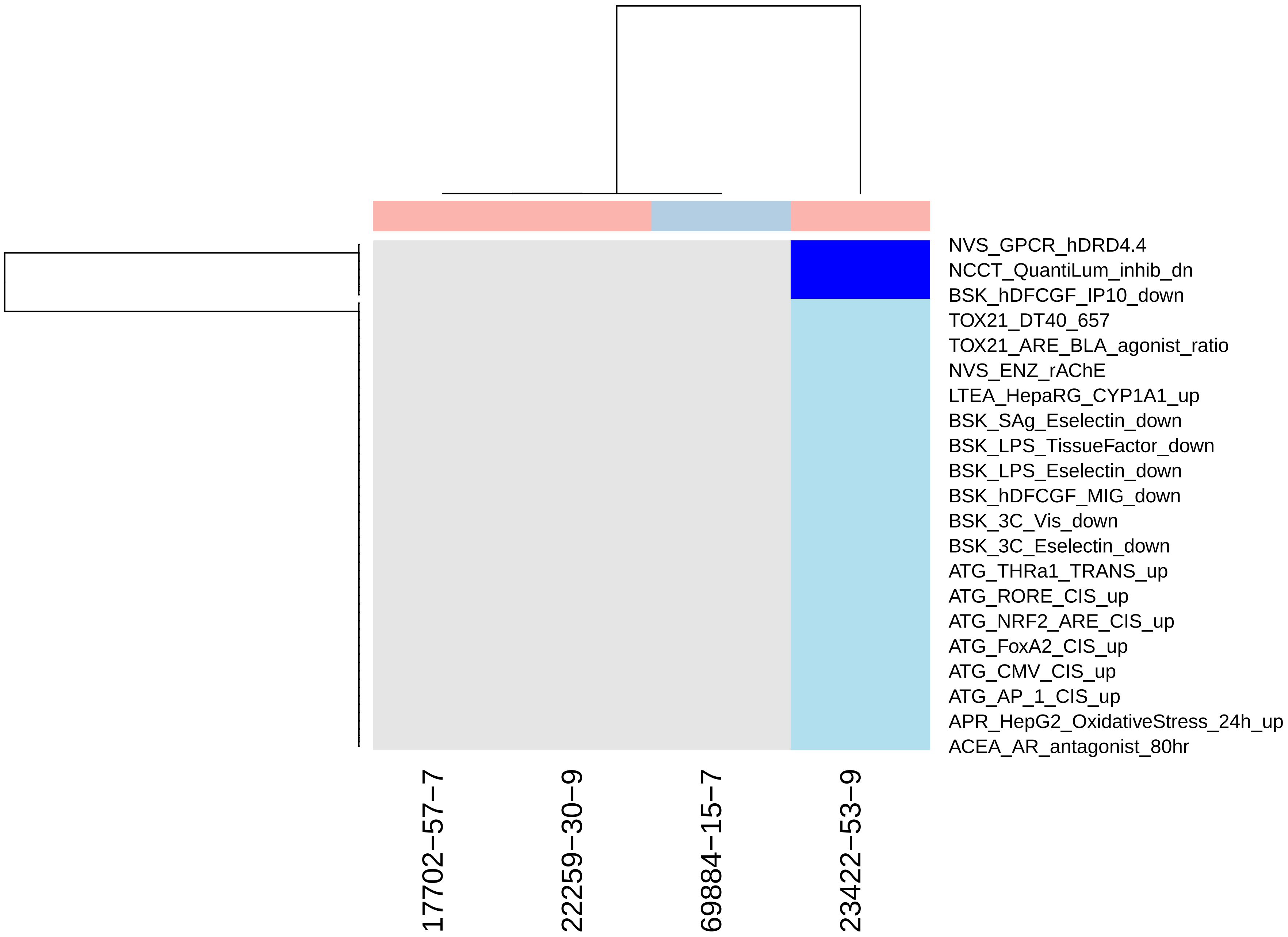

All Activity Heatmap for Cluster 1406

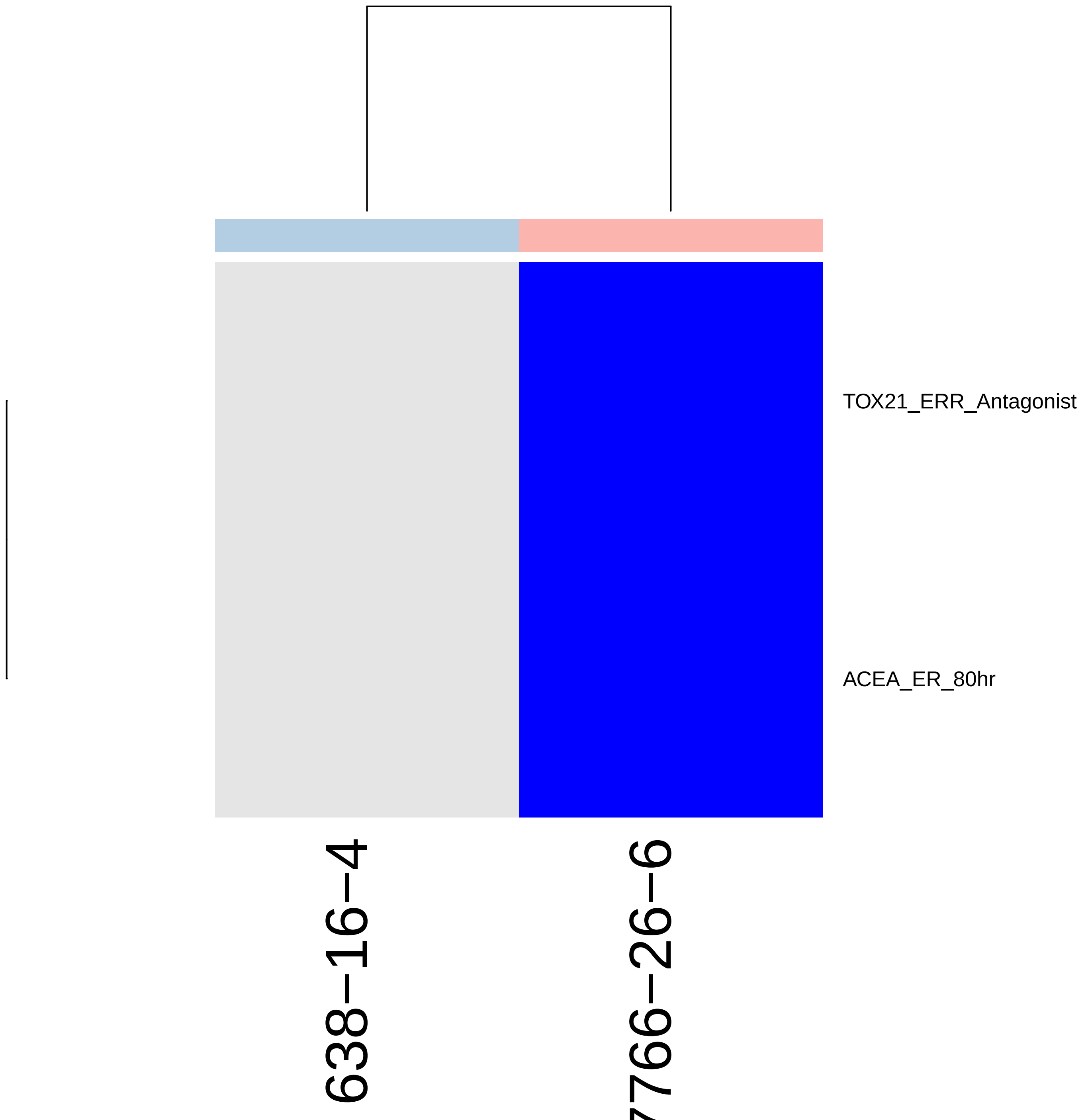

All Activity Heatmap for Cluster 1407

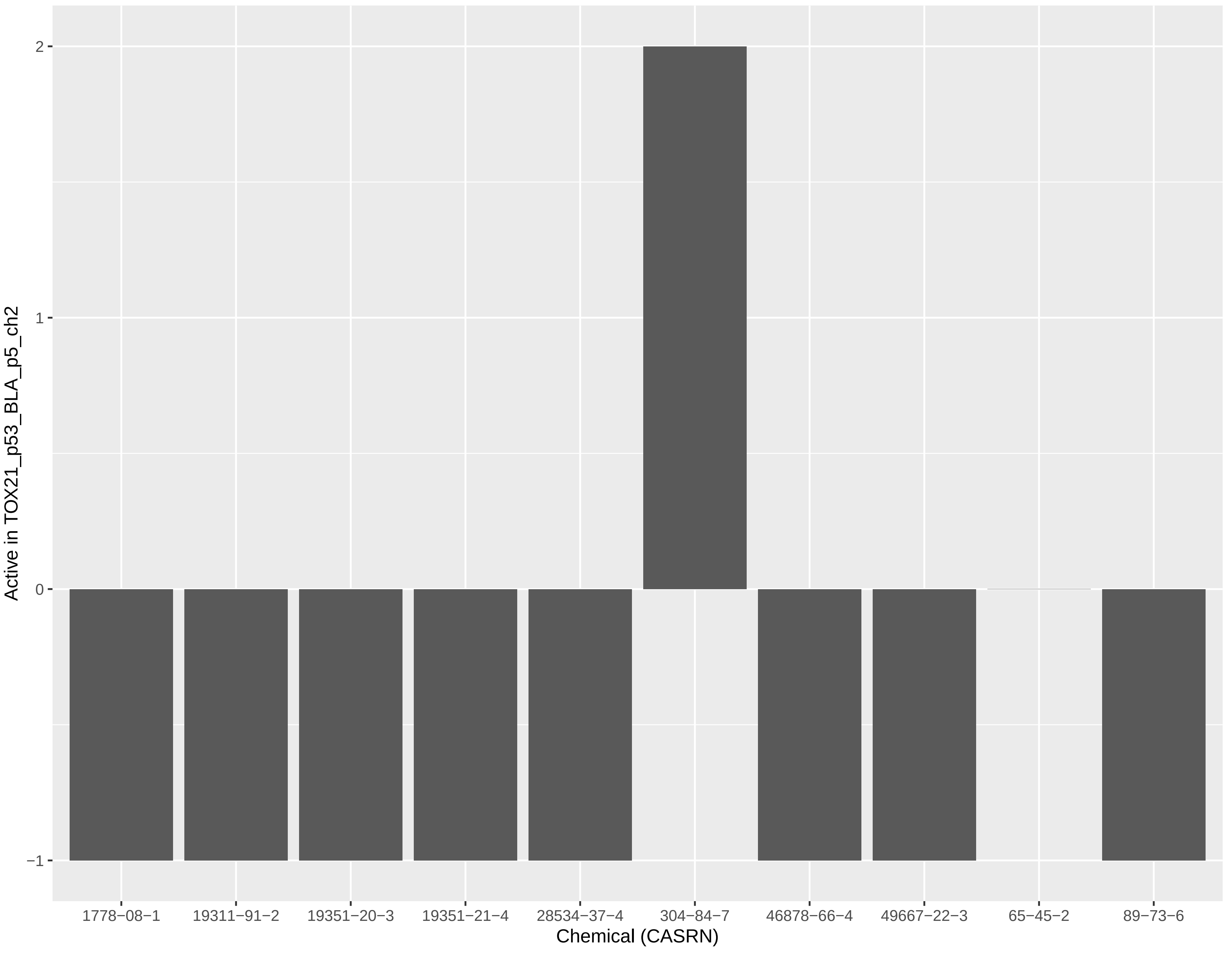

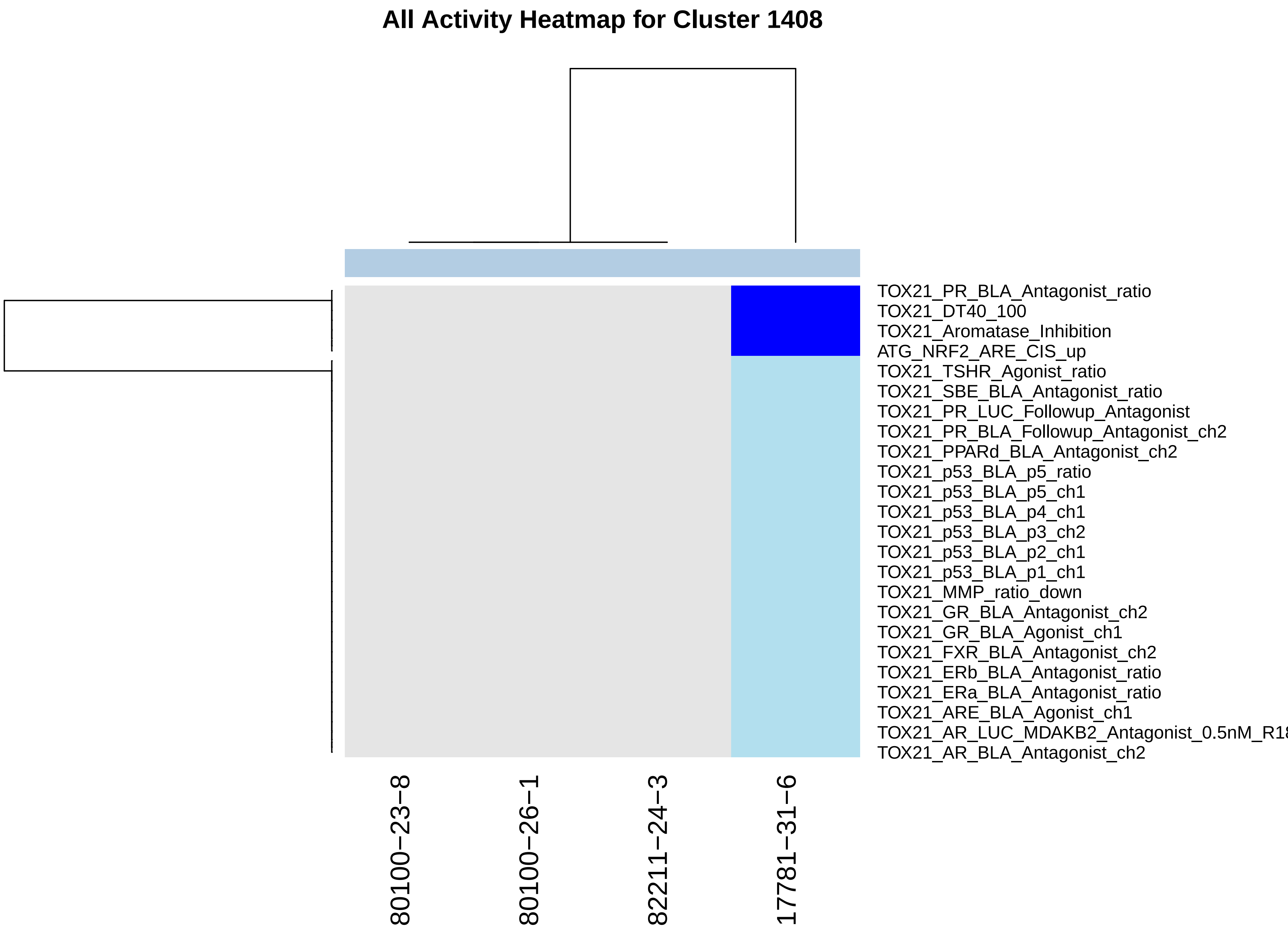

All Activity Heatmap for Cluster 1408
TOX21_PR_BLA_Antagonist_ratio
TOX21_DT40_100
TOX21_Aromatase_Inhibition
ATG_NRF2_ARE_CIS_up
TOX21_TSHR_Agonist_ratio
TOX21_SBE_BLA_Antagonist_ratio
TOX21_PR_LUC_Followup_Antagonist
TOX21_PR_BLA_Followup_Antagonist_ch2
TOX21_PPARd_BLA_Antagonist_ch2
TOX21_p53_BLA_p5_ratio
TOX21_p53_BLA_p5_ch1
TOX21_p53_BLA_p4_ch1
TOX21_p53_BLA_p3_ch2
TOX21_p53_BLA_p2_ch1
TOX21_p53_BLA_p1_ch1
TOX21_MMP_ratio_down
TOX21_GR_BLA_Antagonist_ch2
TOX21_GR_BLA_Agonist_ch1
TOX21_FXR_BLA_Antagonist_ch2
TOX21_ERb_BLA_Antagonist_ratio
TOX21_ERa_BLA_Antagonist_ratio
TOX21_ARE_BLA_Agonist_ch1
TOX21_AR_LUC_MDAKB2_Antagonist_0.5nM_R18
TOX21_AR_BLA_Antagonist_ch2
80100−23−8
80100−26−1
82211−24−3
17781−31−6

All Activity Heatmap for Cluster 1409

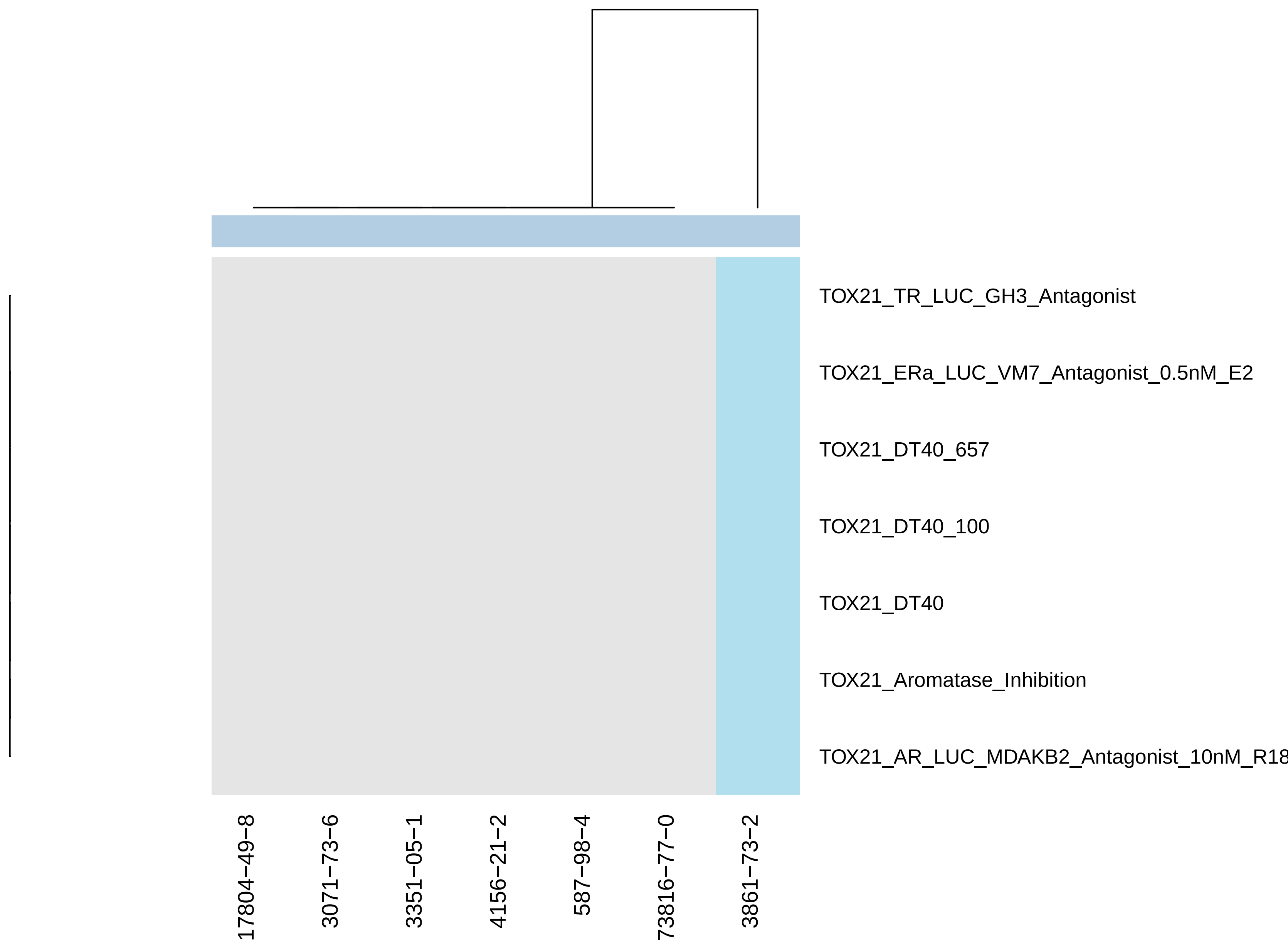

All Activity Heatmap for Cluster 1410

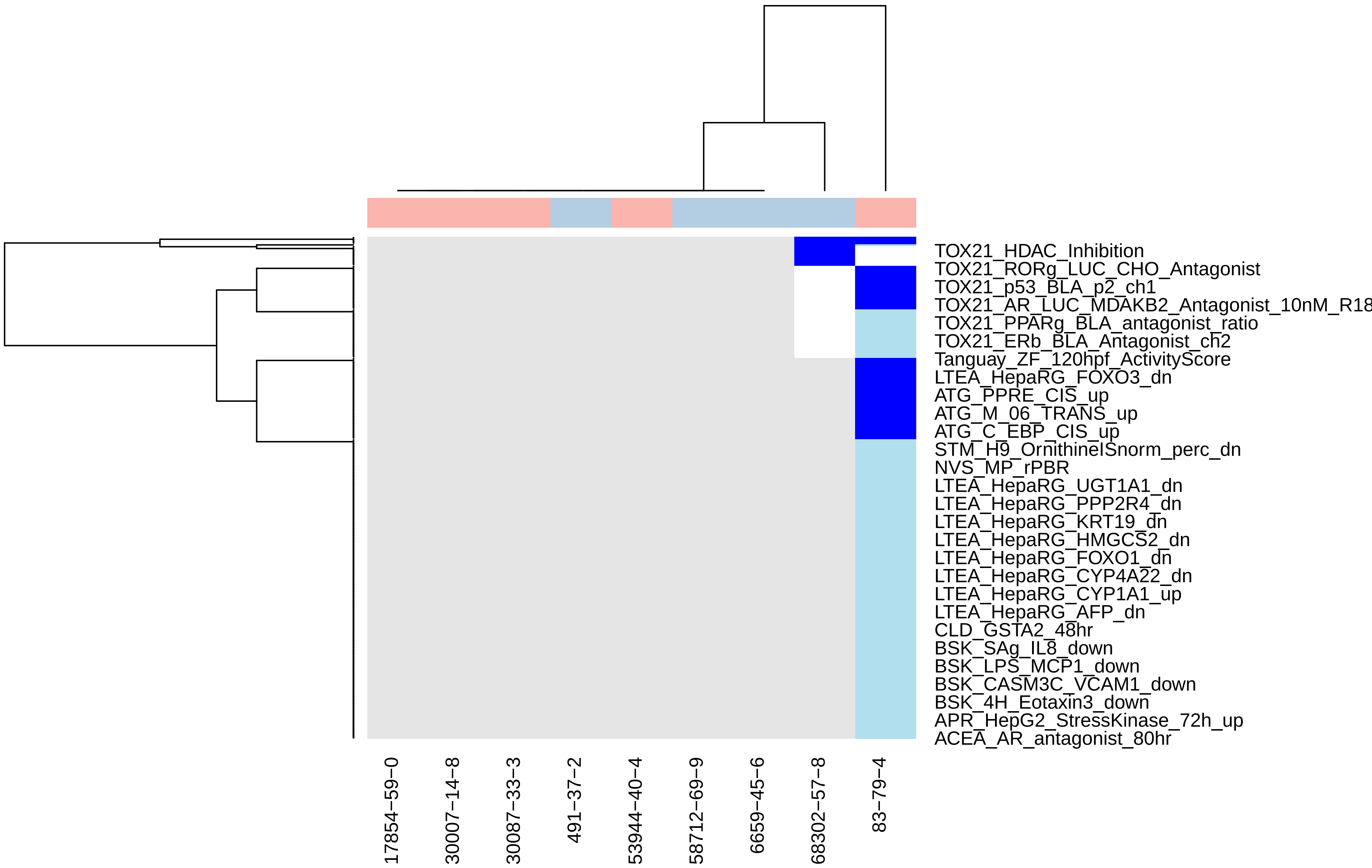

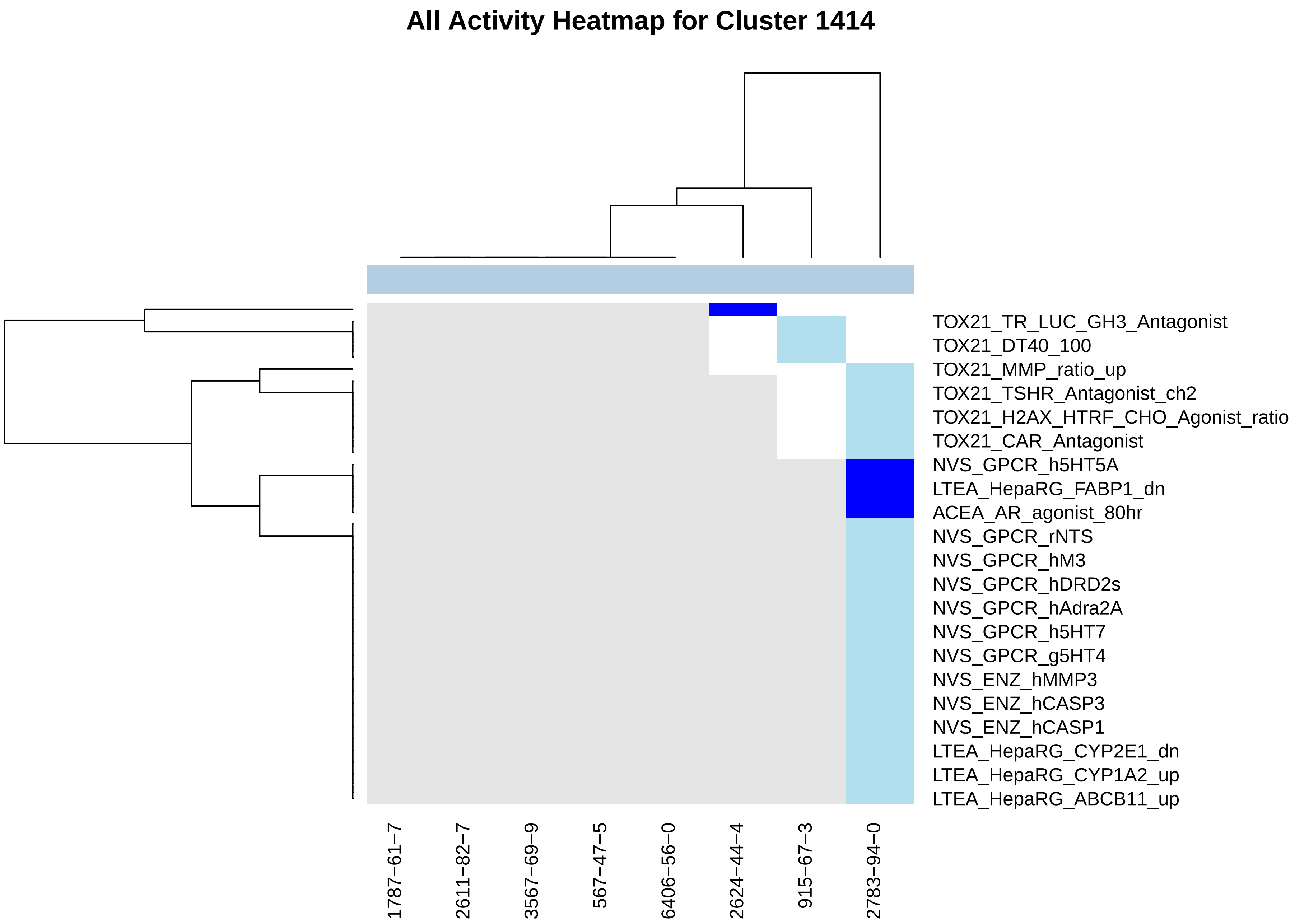
All Activity Heatmap for Cluster 1414
TOX21_TR_LUC_GH3_Antagonist
TOX21_DT40_100
TOX21_MMP_ratio_up
TOX21_TSHR_Antagonist_ch2
TOX21_H2AX_HTRF_CHO_Agonist_ratio
TOX21_CAR_Antagonist
NVS_GPCR_h5HT5A
LTEA_HepaRG_FABP1_dn
ACEA_AR_agonist_80hr
NVS_GPCR_rNTS
NVS_GPCR_hM3
NVS_GPCR_hDRD2s
NVS_GPCR_hAdra2A
NVS_GPCR_h5HT7
NVS_GPCR_g5HT4
NVS_ENZ_hMMP3
NVS_ENZ_hCASP3
NVS_ENZ_hCASP1
LTEA_HepaRG_CYP2E1_dn
LTEA_HepaRG_CYP1A2_up
LTEA_HepaRG_ABCB11_up
1787−61−7
2611−82−7
3567−69−9
567−47−5
6406−56−0
2624−44−4
915−67−3
2783−94−0

All Activity Heatmap for Cluster 1415

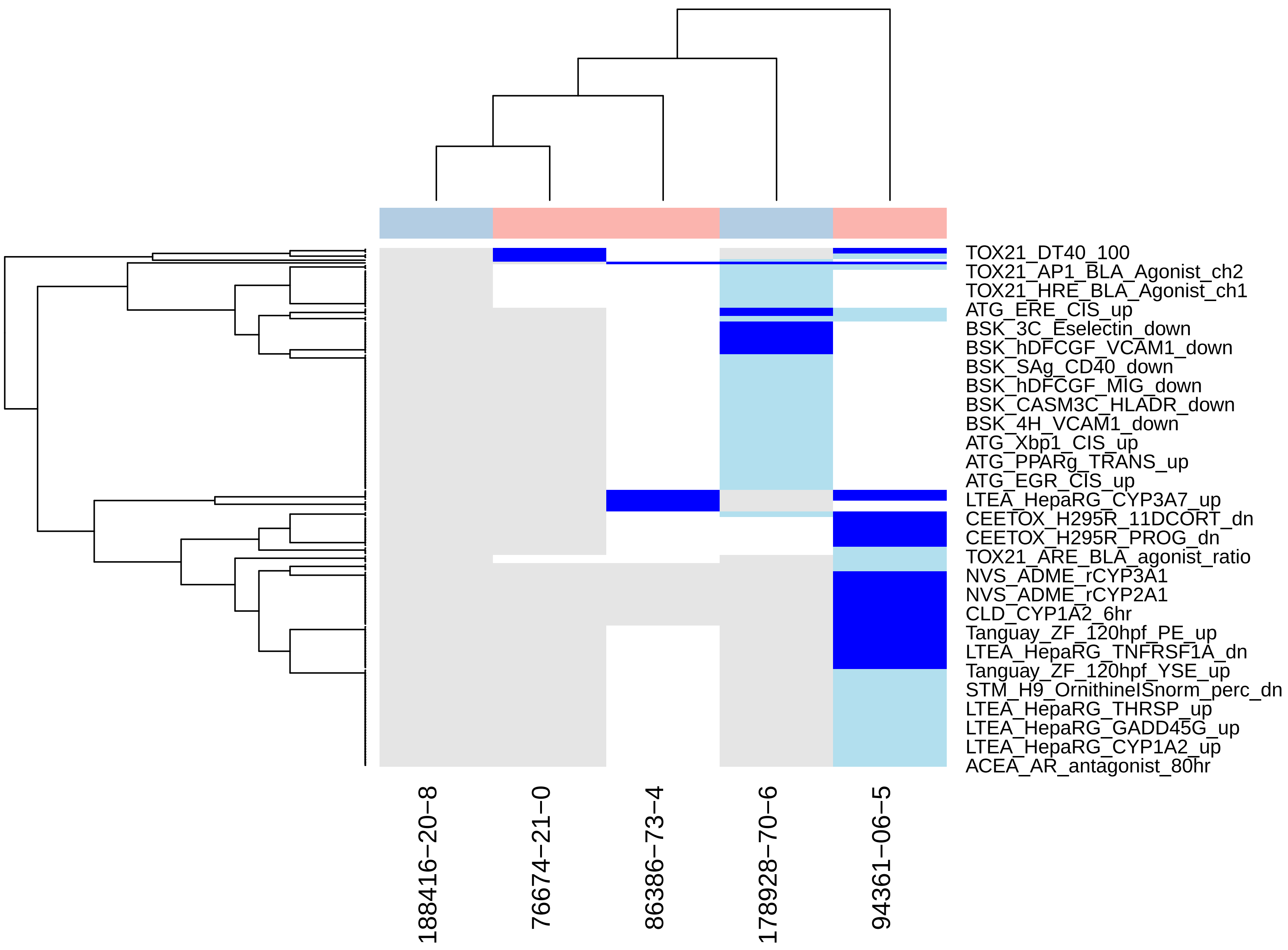

All Activity Heatmap for Cluster 1417

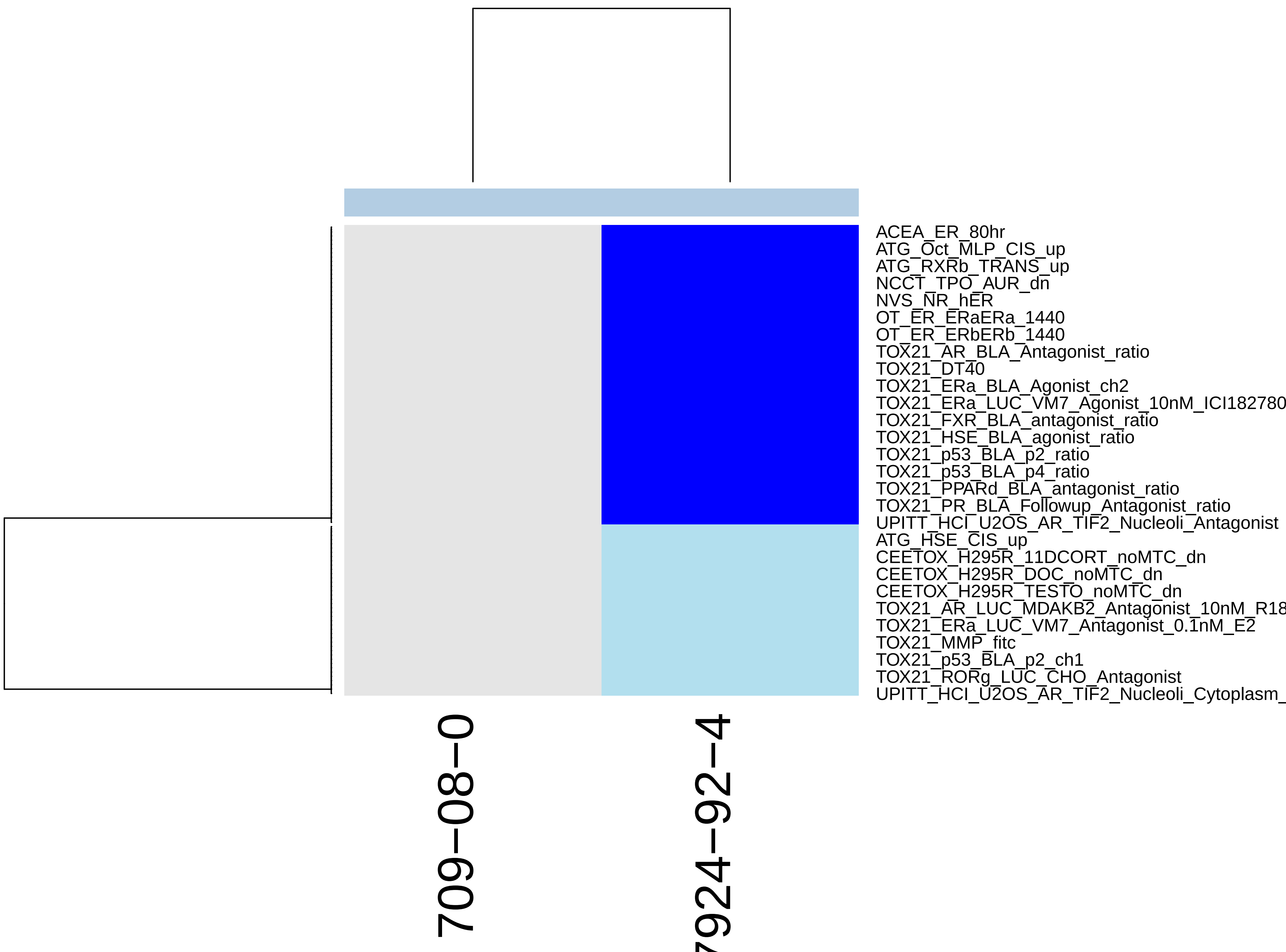

All Activity Heatmap for Cluster 1418

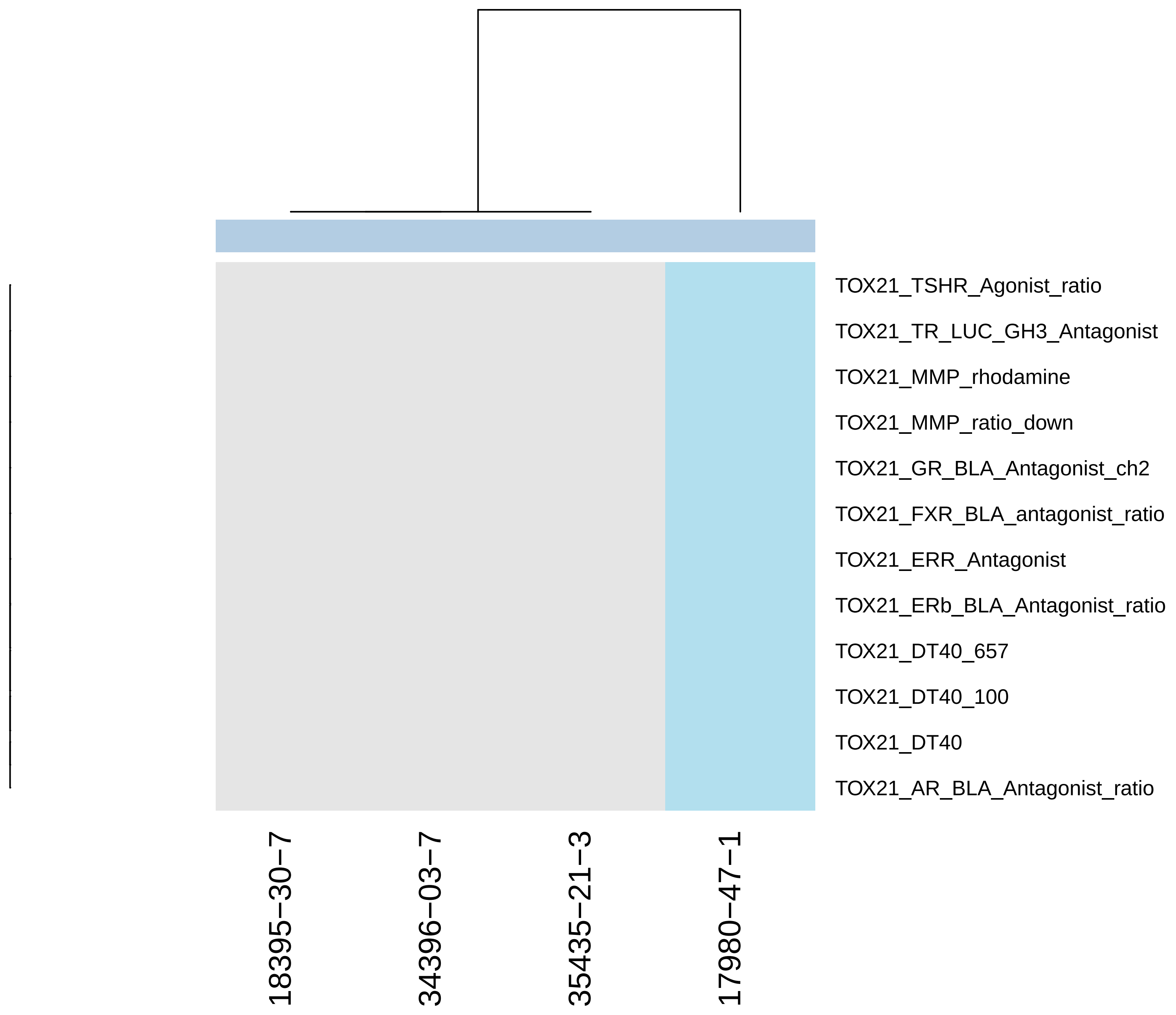

All Activity Heatmap for Cluster 1420

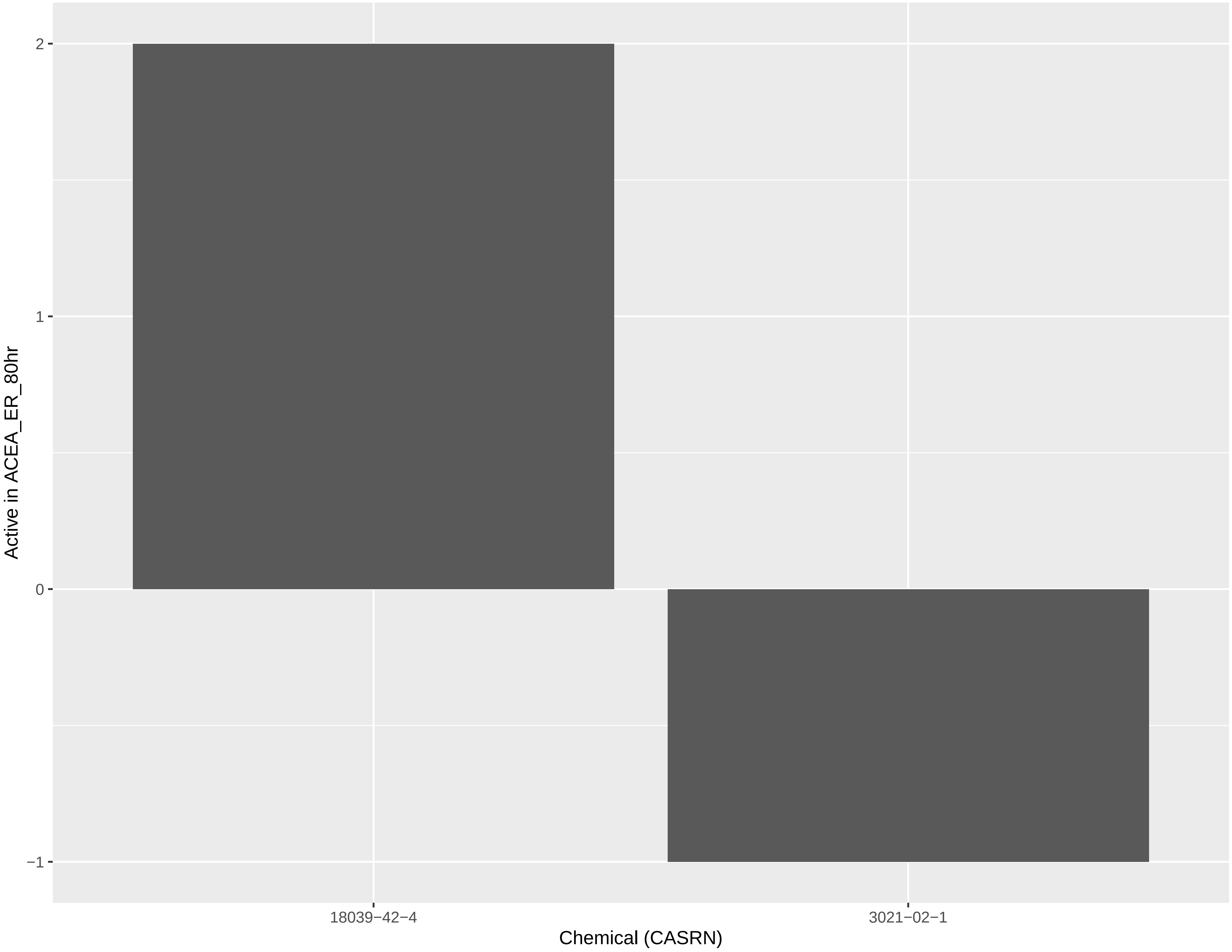

All Activity Heatmap for Cluster 1421

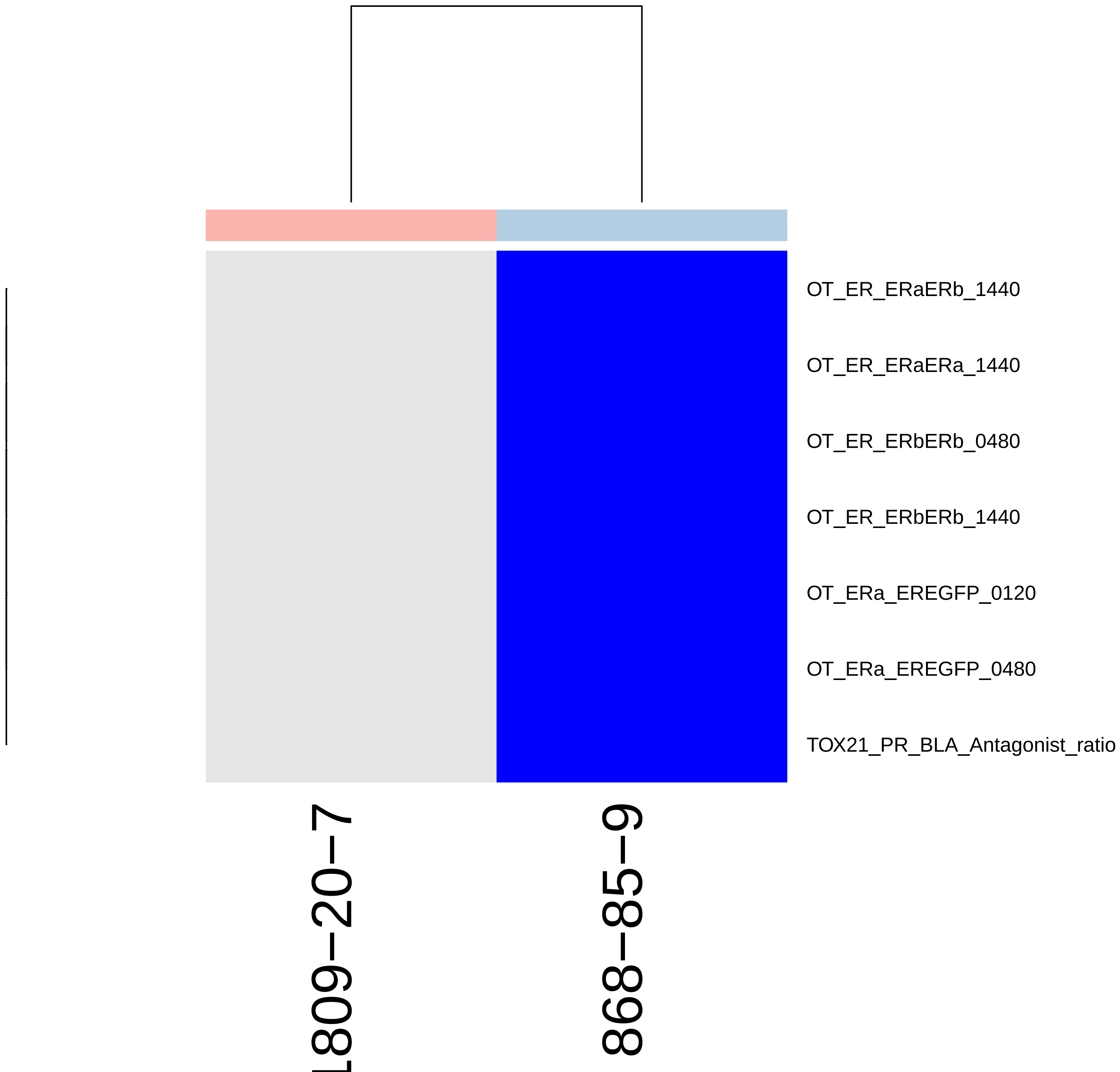

All Activity Heatmap for Cluster 1422

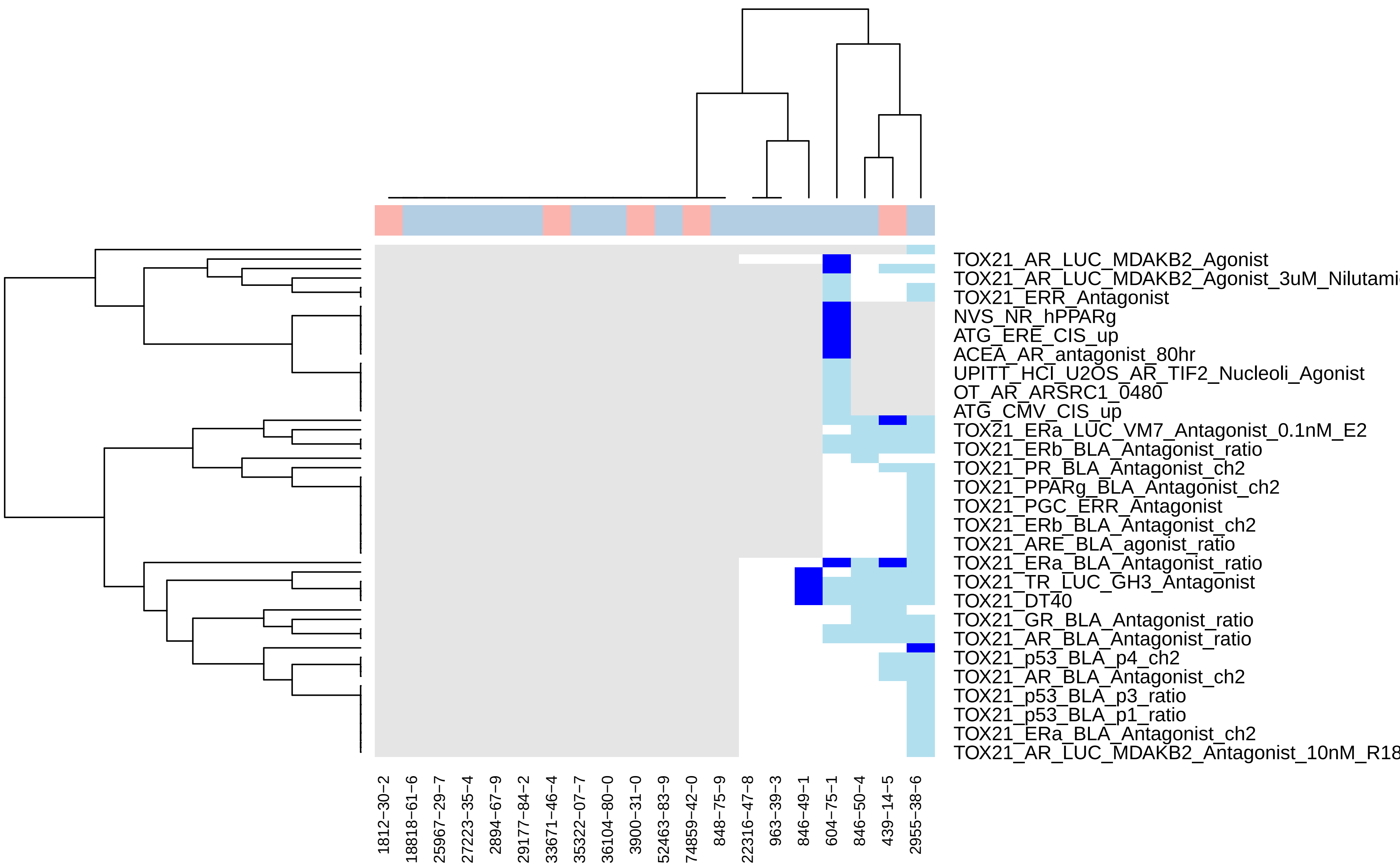

All Activity Heatmap for Cluster 1423

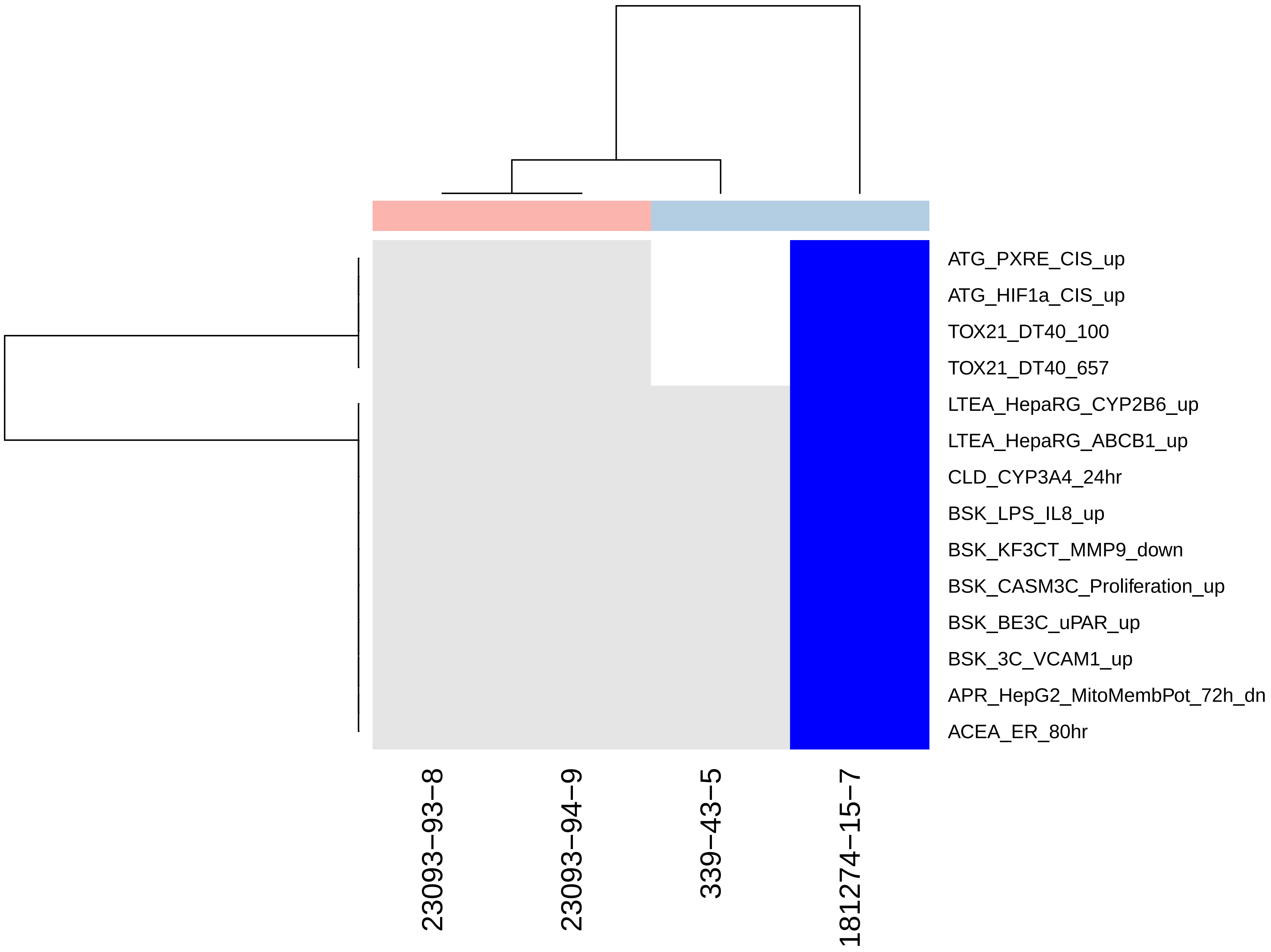

All Activity Heatmap for Cluster 1425

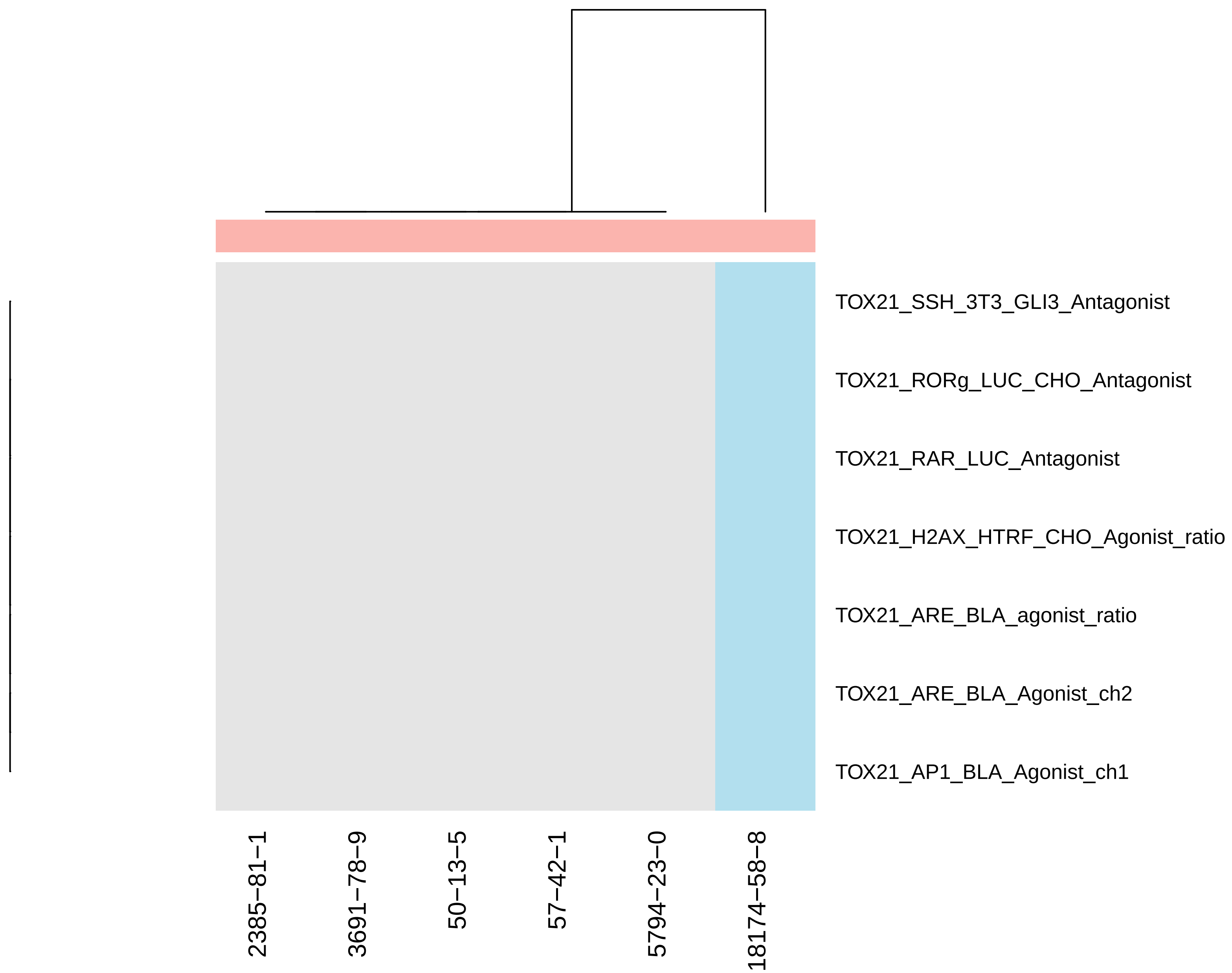

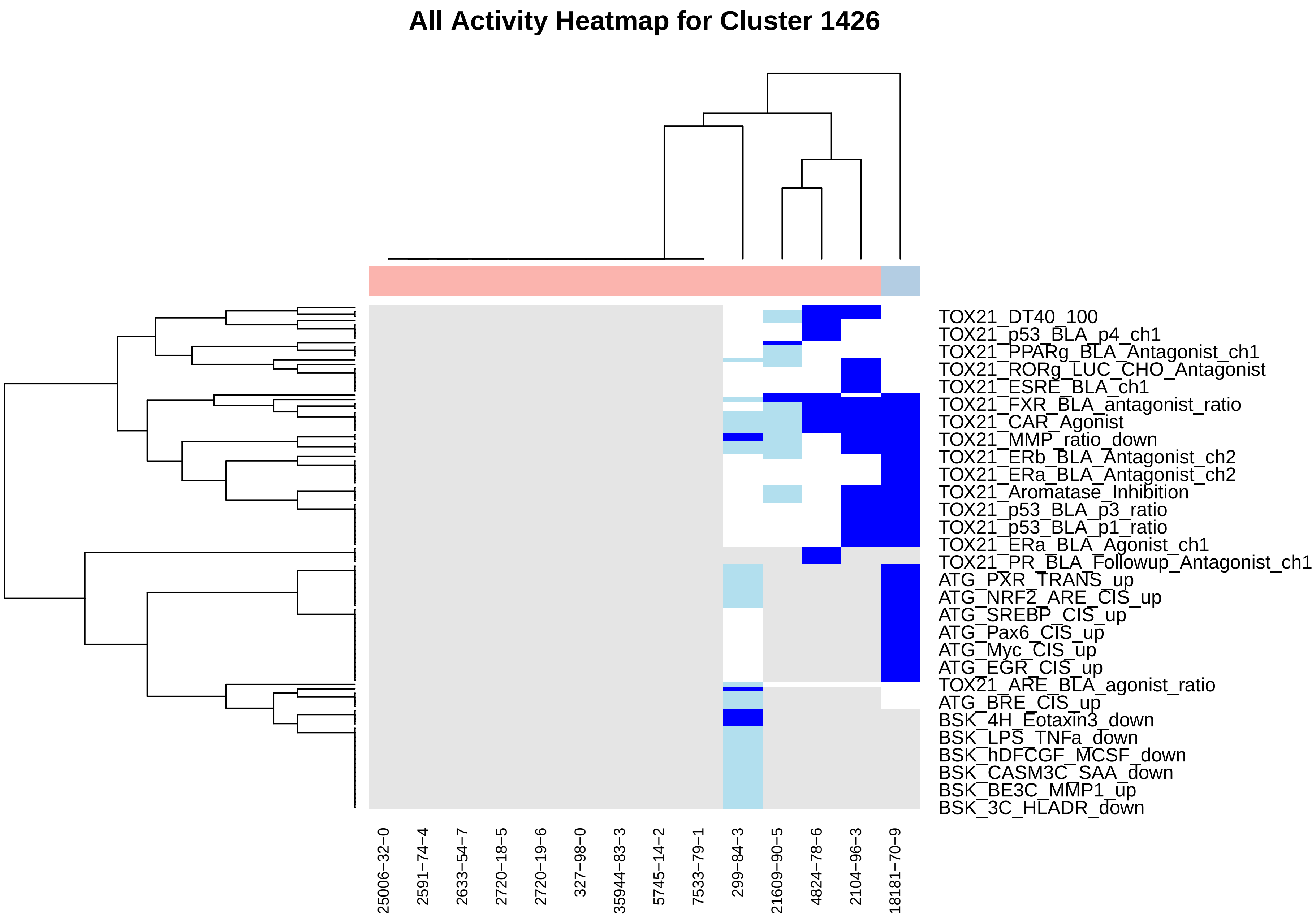
All Activity Heatmap for Cluster 1426
TOX21_DT40_100
TOX21_p53_BLA_p4_ch1
TOX21_PPARg_BLA_Antagonist_ch1
TOX21_RORg_LUC_CHO_Antagonist
TOX21_ESRE_BLA_ch1
TOX21_FXR_BLA_antagonist_ratio
TOX21_CAR_Agonist
TOX21_MMP_ratio_down
TOX21_ERb_BLA_Antagonist_ch2
TOX21_ERa_BLA_Antagonist_ch2
TOX21_Aromatase_Inhibition
TOX21_p53_BLA_p3_ratio
TOX21_p53_BLA_p1_ratio
TOX21_ERa_BLA_Agonist_ch1
TOX21_PR_BLA_Followup_Antagonist_ch1
ATG_PXR_TRANS_up
ATG_NRF2_ARE_CIS_up
ATG_SREBP_CIS_up
ATG_Pax6_CIS_up
ATG_Myc_CIS_up
ATG_EGR_CIS_up
TOX21_ARE_BLA_agonist_ratio
ATG_BRE_CIS_up
BSK_4H_Eotaxin3_down
BSK_LPS_TNFa_down
BSK_hDFCGF_MCSF_down
BSK_CASM3C_SAA_down
BSK_BE3C_MMP1_up
BSK_3C_HLADR_down
25006−32−0
2591−74−4
2633−54−7
2720−18−5
2720−19−6
327−98−0
35944−83−3
5745−14−2
7533−79−1
299−84−3
21609−90−5
4824−78−6
2104−96−3
18181−70−9

All Activity Heatmap for Cluster 1427

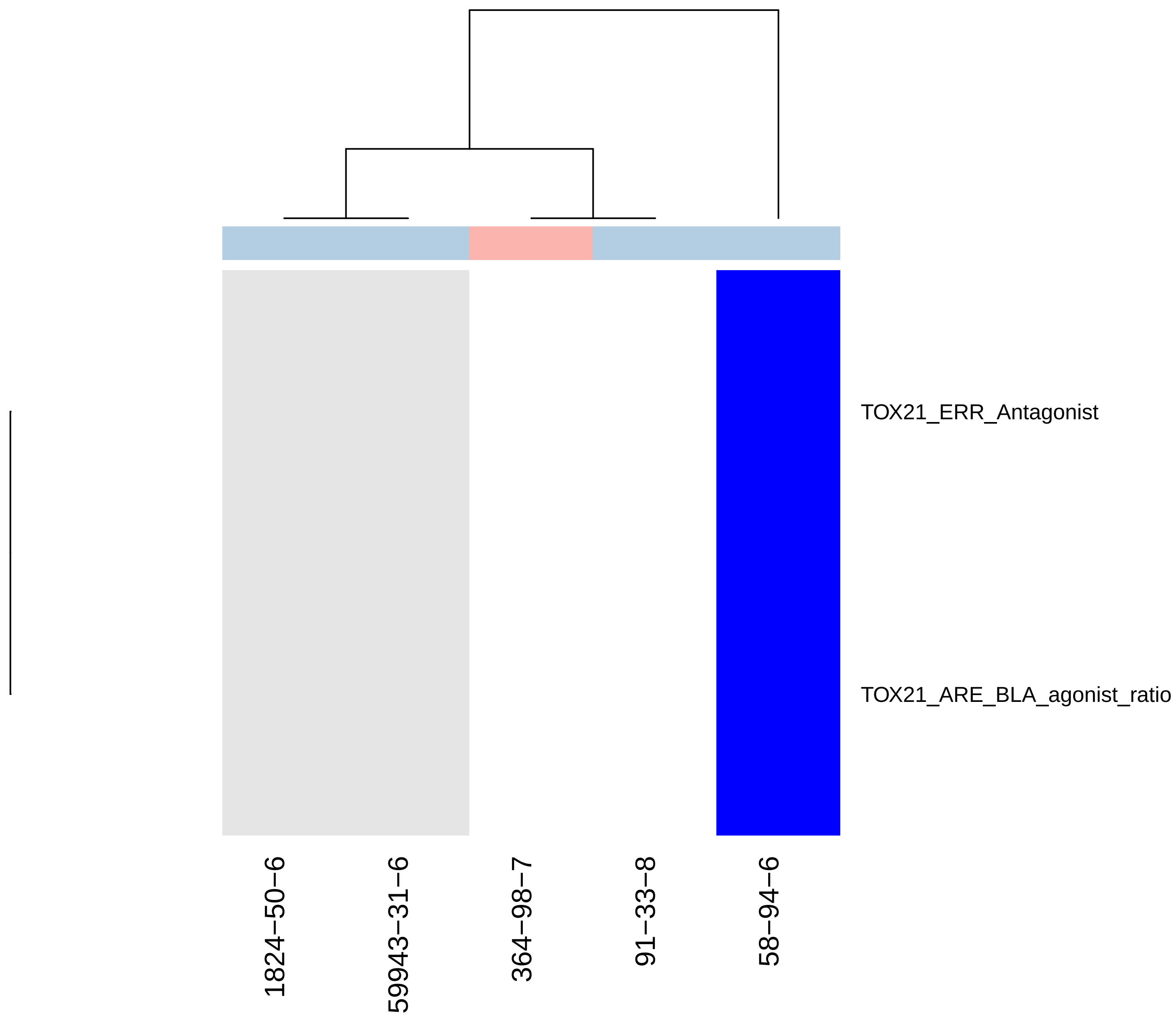

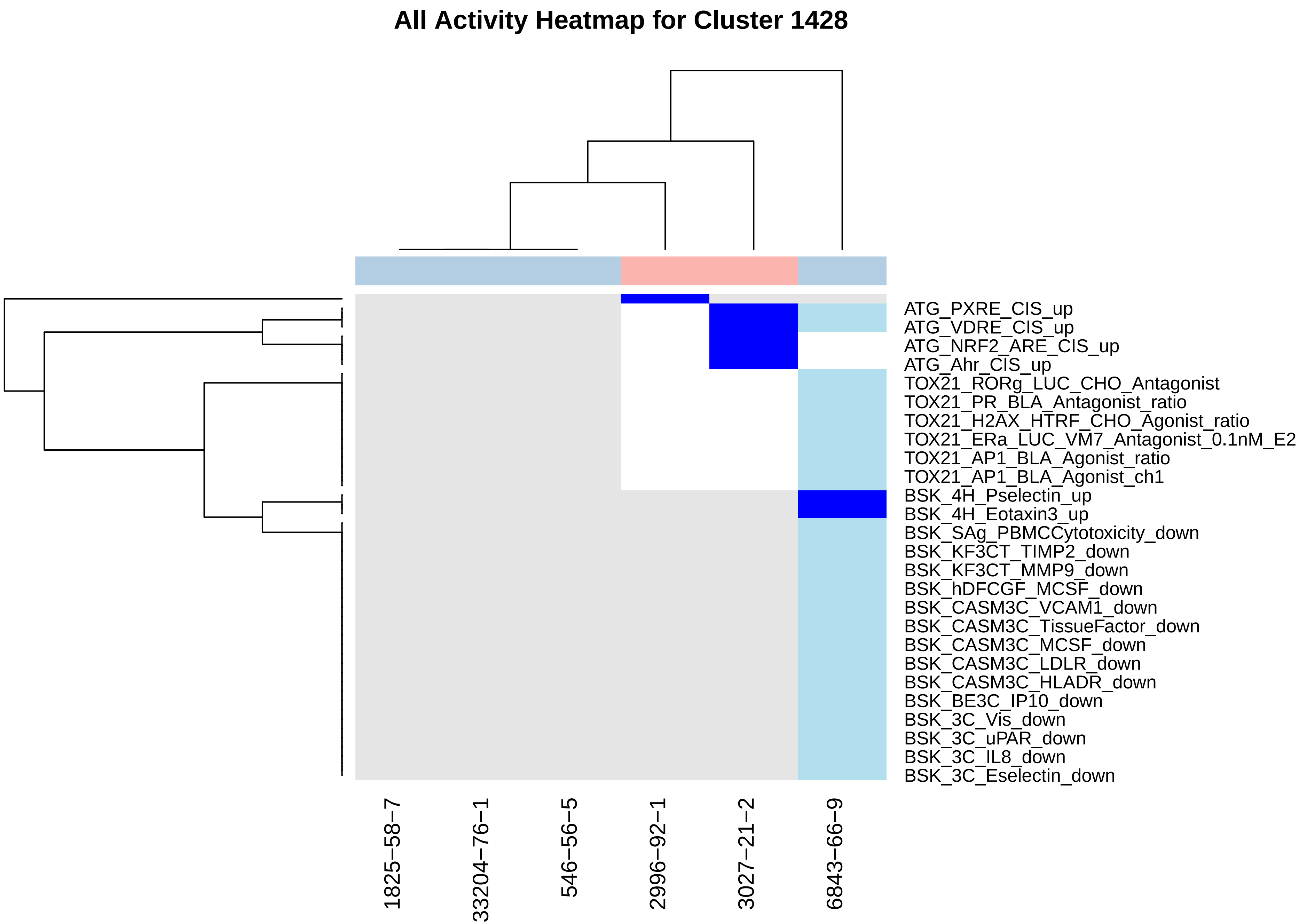
All Activity Heatmap for Cluster 1428
ATG_PXRE_CIS_up
ATG_VDRE_CIS_up
ATG_NRF2_ARE_CIS_up
ATG_Ahr_CIS_up
TOX21_RORg_LUC_CHO_Antagonist
TOX21_PR_BLA_Antagonist_ratio
TOX21_H2AX_HTRF_CHO_Agonist_ratio
TOX21_ERa_LUC_VM7_Antagonist_0.1nM_E2
TOX21_AP1_BLA_Agonist_ratio
TOX21_AP1_BLA_Agonist_ch1
BSK_4H_Pselectin_up
BSK_4H_Eotaxin3_up
BSK_SAg_PBMCCytotoxicity_down
BSK_KF3CT_TIMP2_down
BSK_KF3CT_MMP9_down
BSK_hDFCGF_MCSF_down
BSK_CASM3C_VCAM1_down
BSK_CASM3C_TissueFactor_down
BSK_CASM3C_MCSF_down
BSK_CASM3C_LDLR_down
BSK_CASM3C_HLADR_down
BSK_BE3C_IP10_down
BSK_3C_Vis_down
BSK_3C_uPAR_down
BSK_3C_IL8_down
BSK_3C_Eselectin_down
1825−58−7
33204−76−1
546−56−5
2996−92−1
3027−21−2
6843−66−9

All Activity Heatmap for Cluster 1432

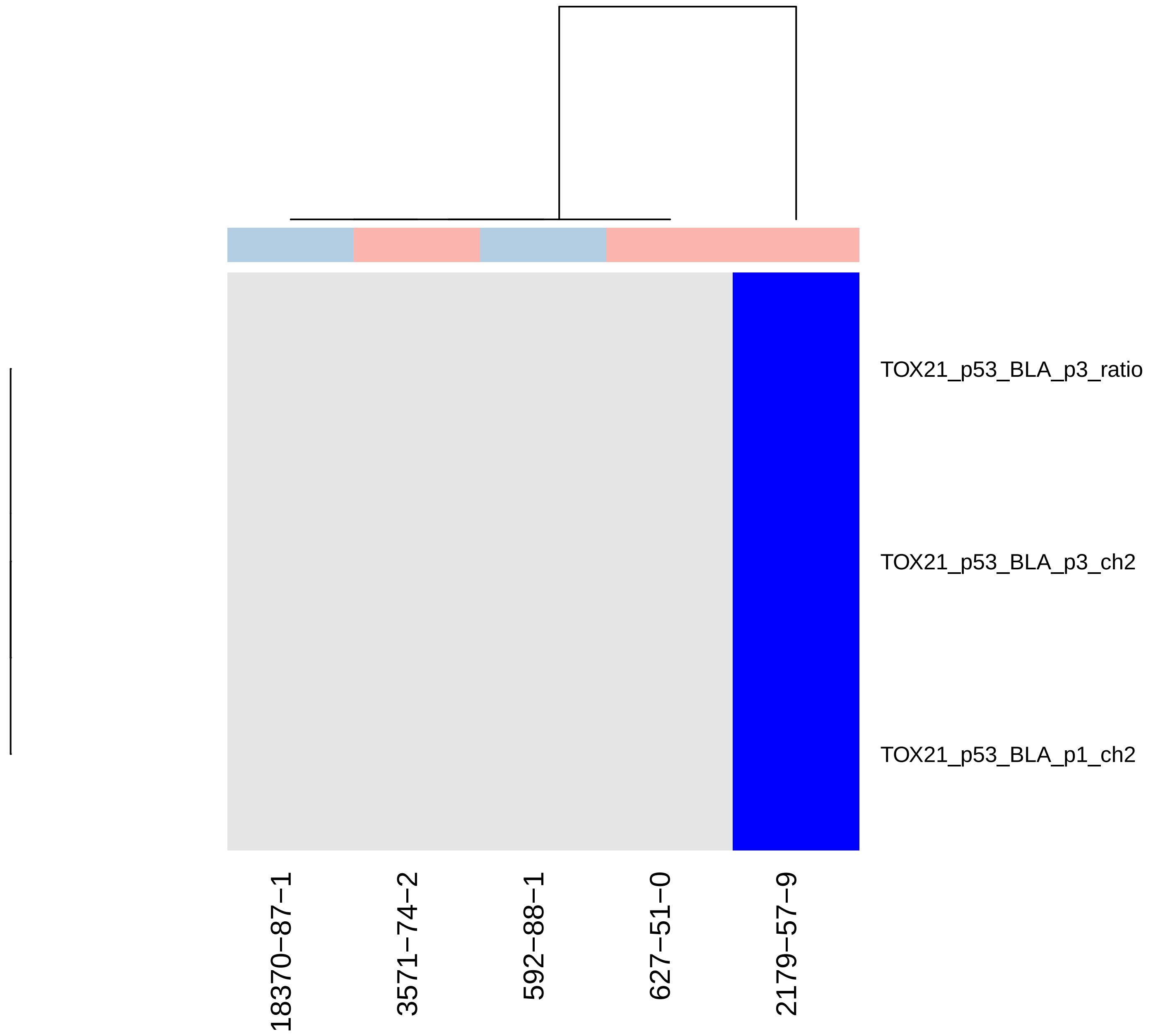

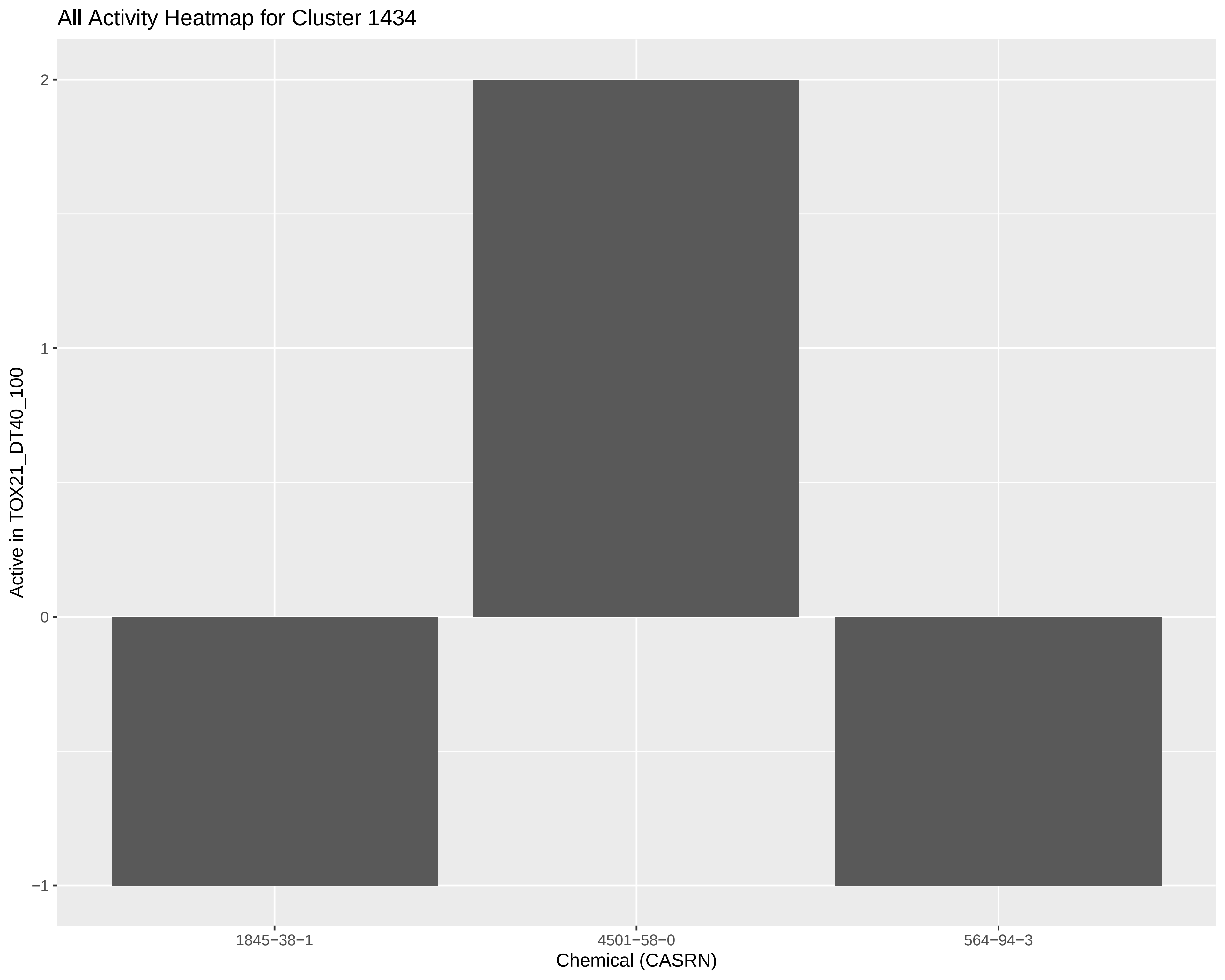
All Activity Heatmap for Cluster 1434
Active in TOX21_DT40_100
2
1
0
−1
1845−38−1
4501−58−0
564−94−3
Chemical (CASRN)

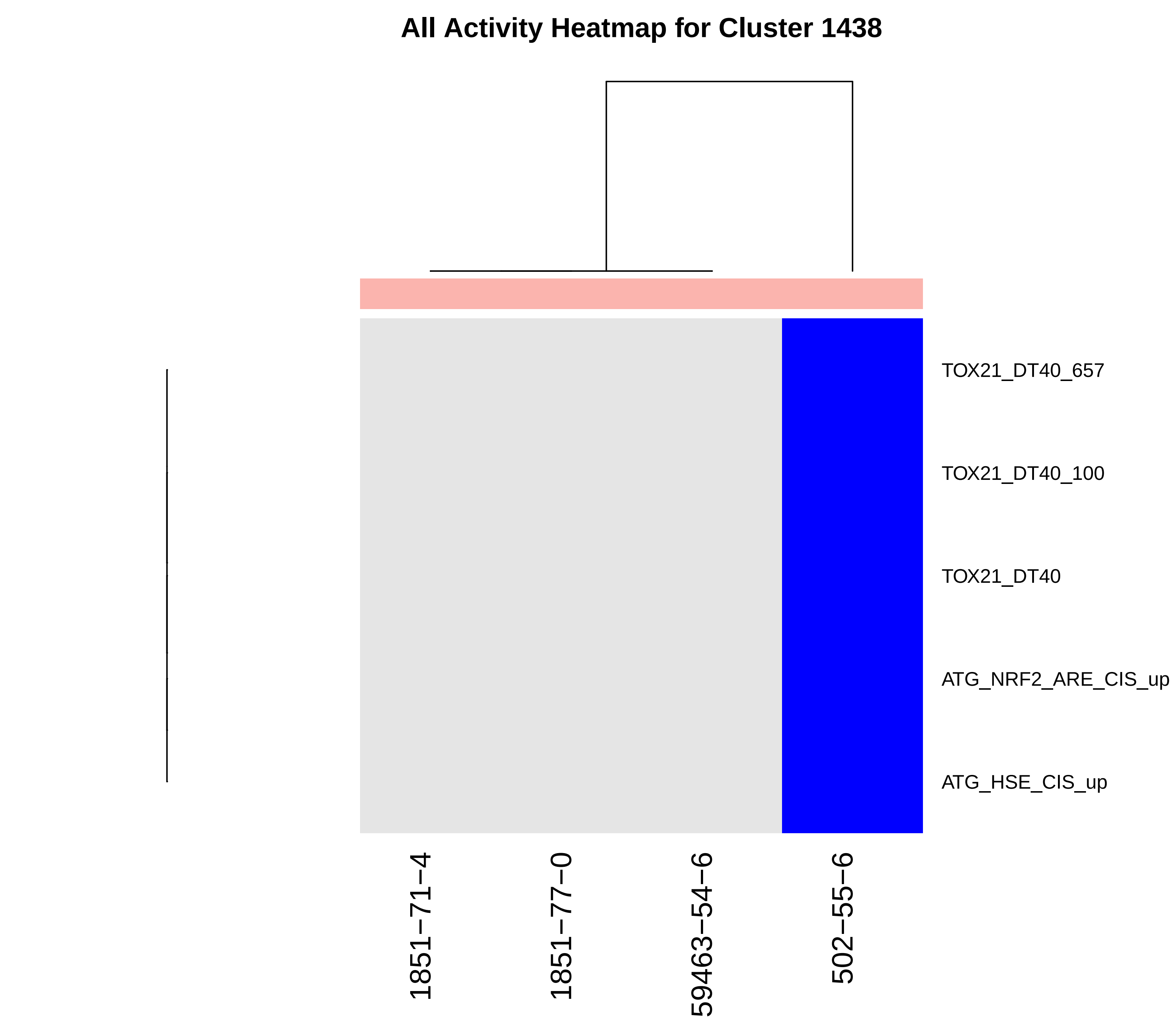

All Activity Heatmap for Cluster 1438
TOX21_DT40_657
TOX21_DT40_100
TOX21_DT40
ATG_NRF2_ARE_CIS_up
ATG_HSE_CIS_up
1851−71−4
1851−77−0
59463−54−6
502−55−6

All Activity Heatmap for Cluster 1439

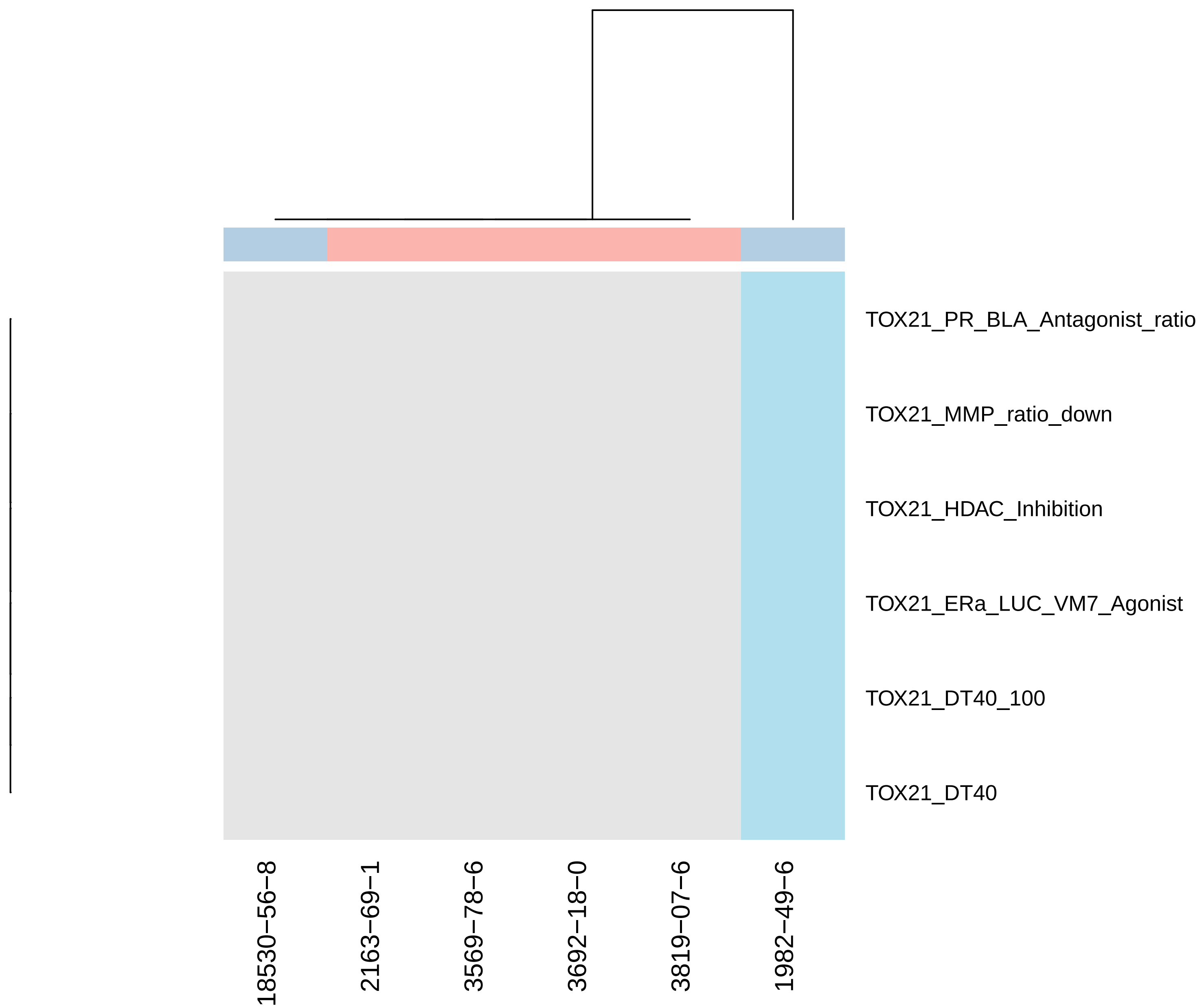

All Activity Heatmap for Cluster 1442

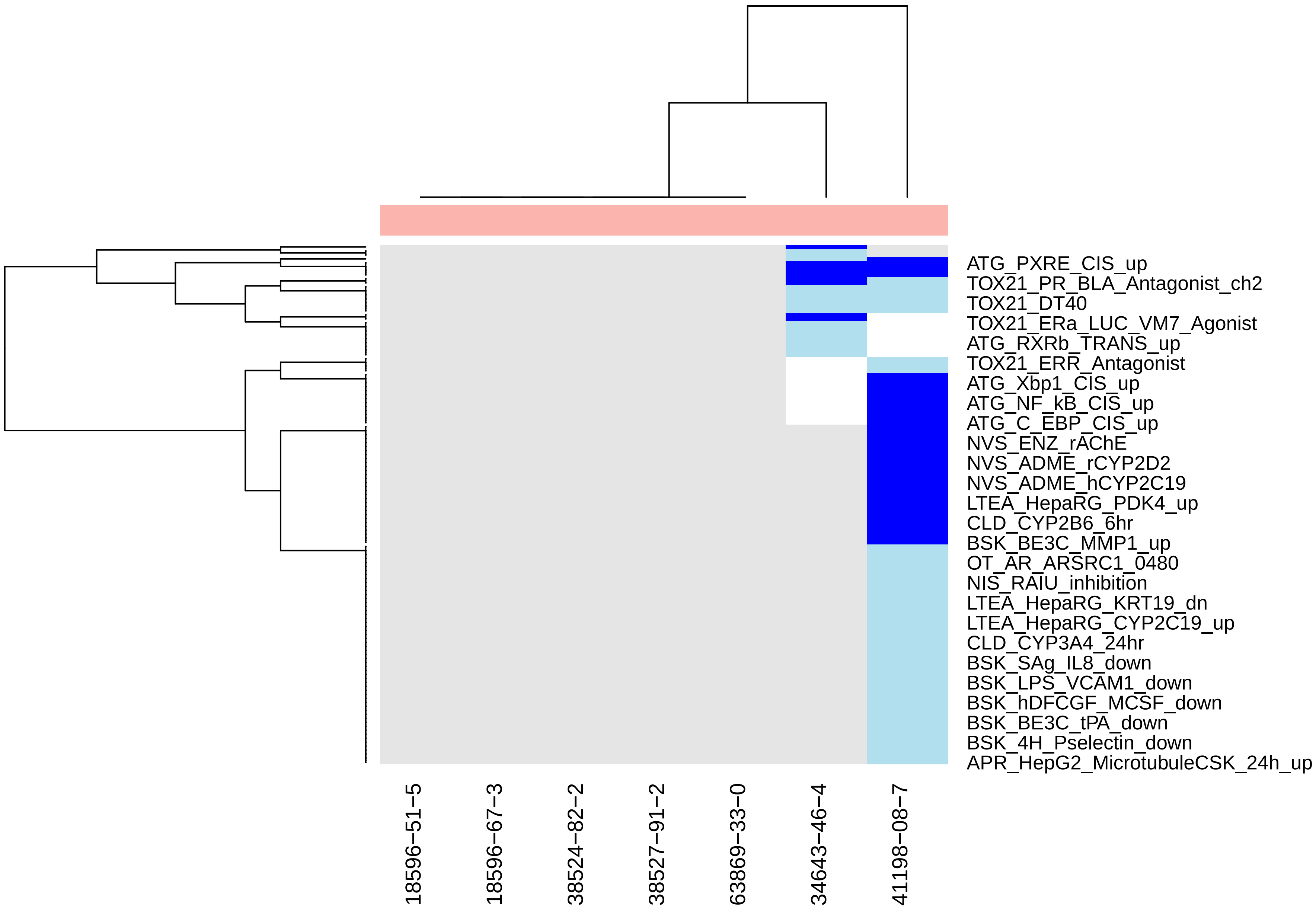

All Activity Heatmap for Cluster 1446

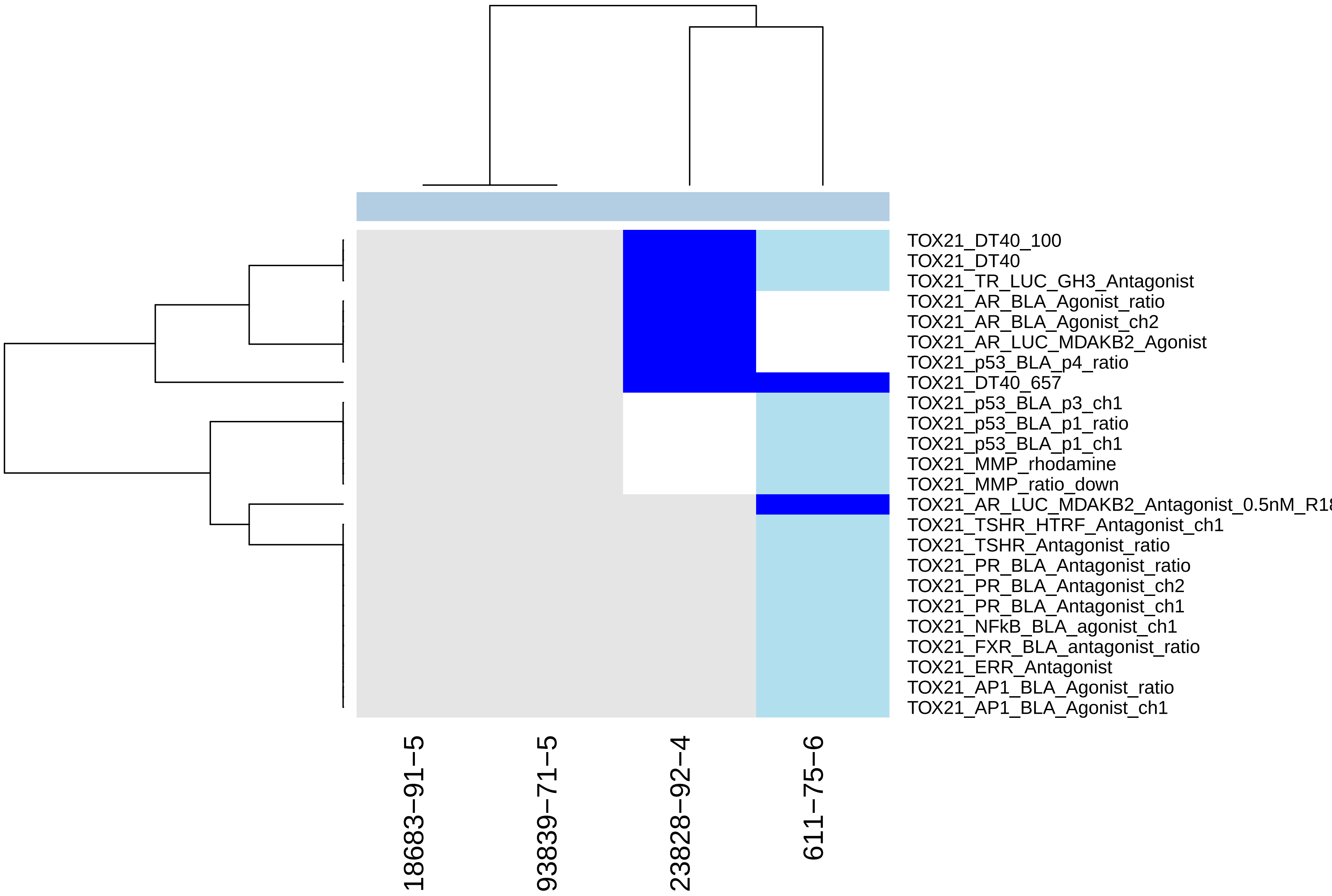

All Activity Heatmap for Cluster 1447

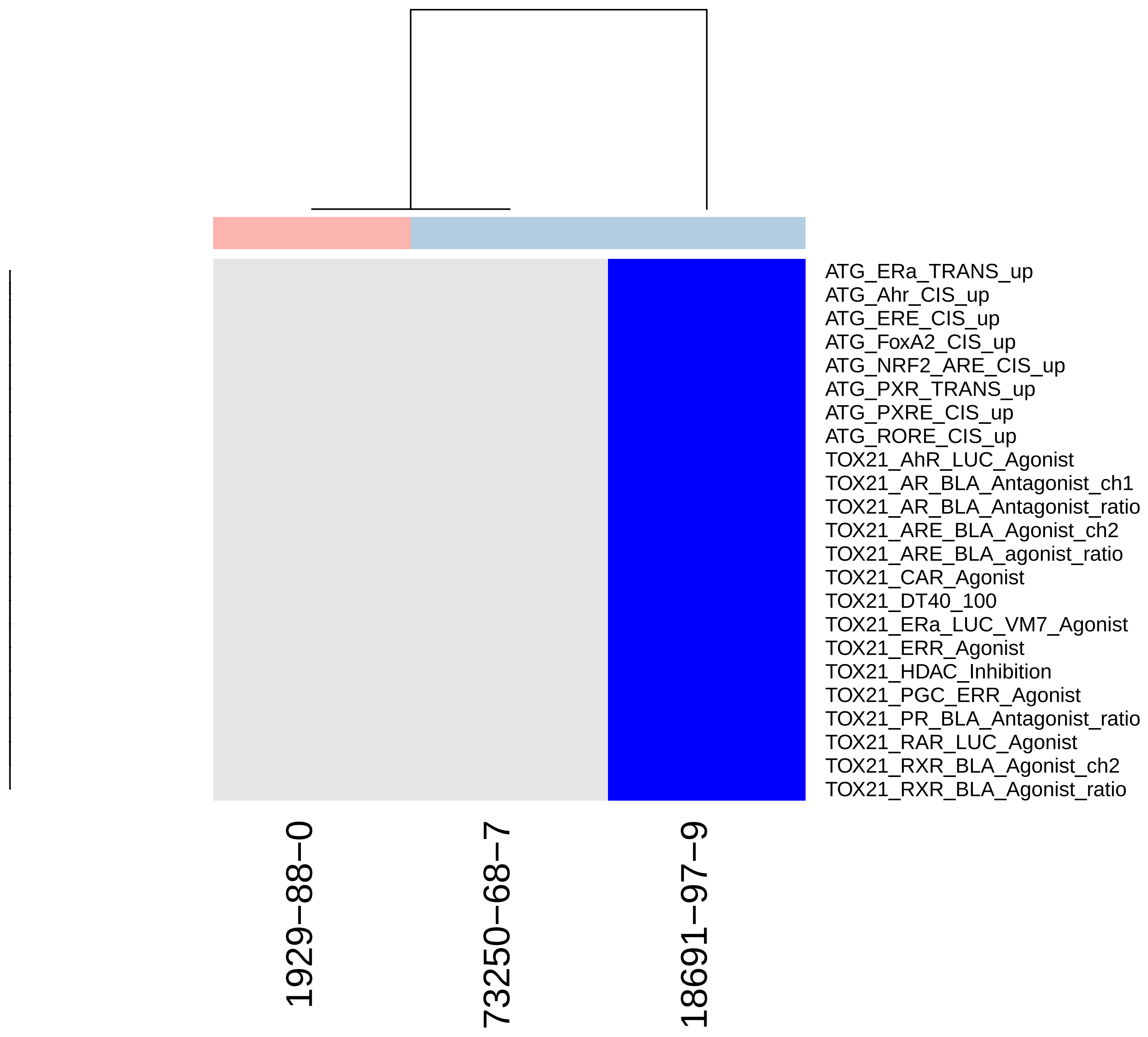

All Activity Heatmap for Cluster 1448

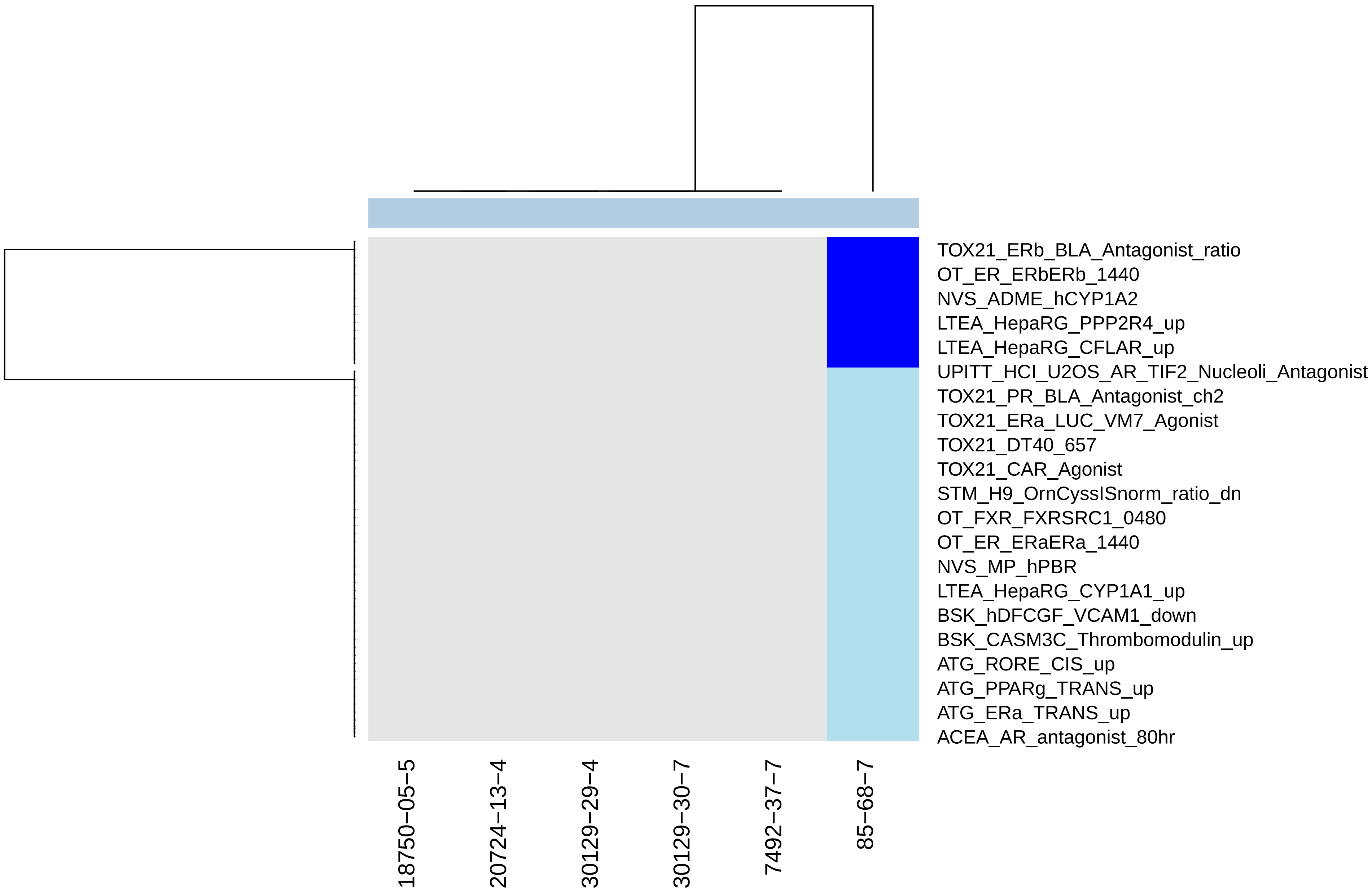

All Activity Heatmap for Cluster 1449

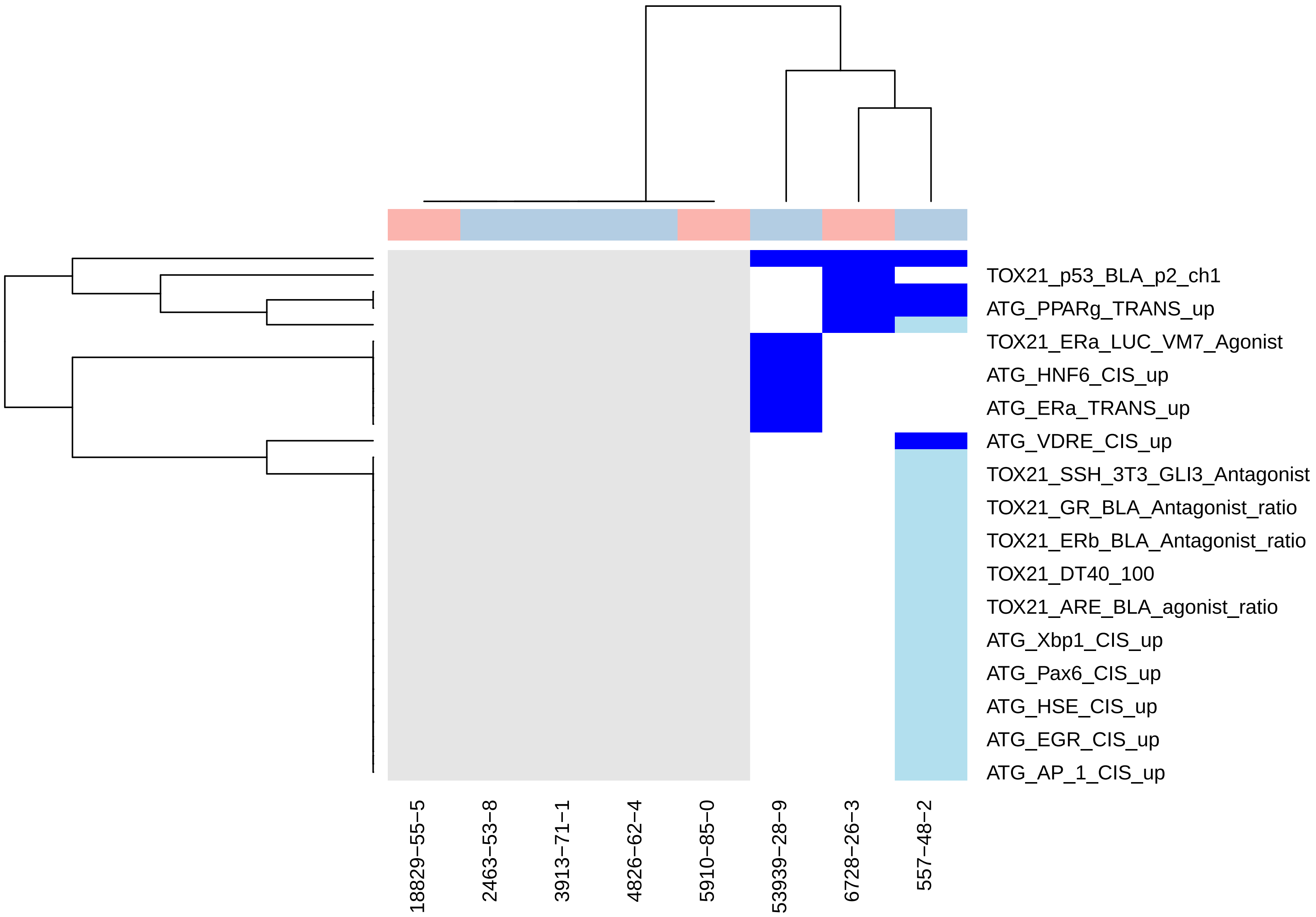

All Activity Heatmap for Cluster 1450

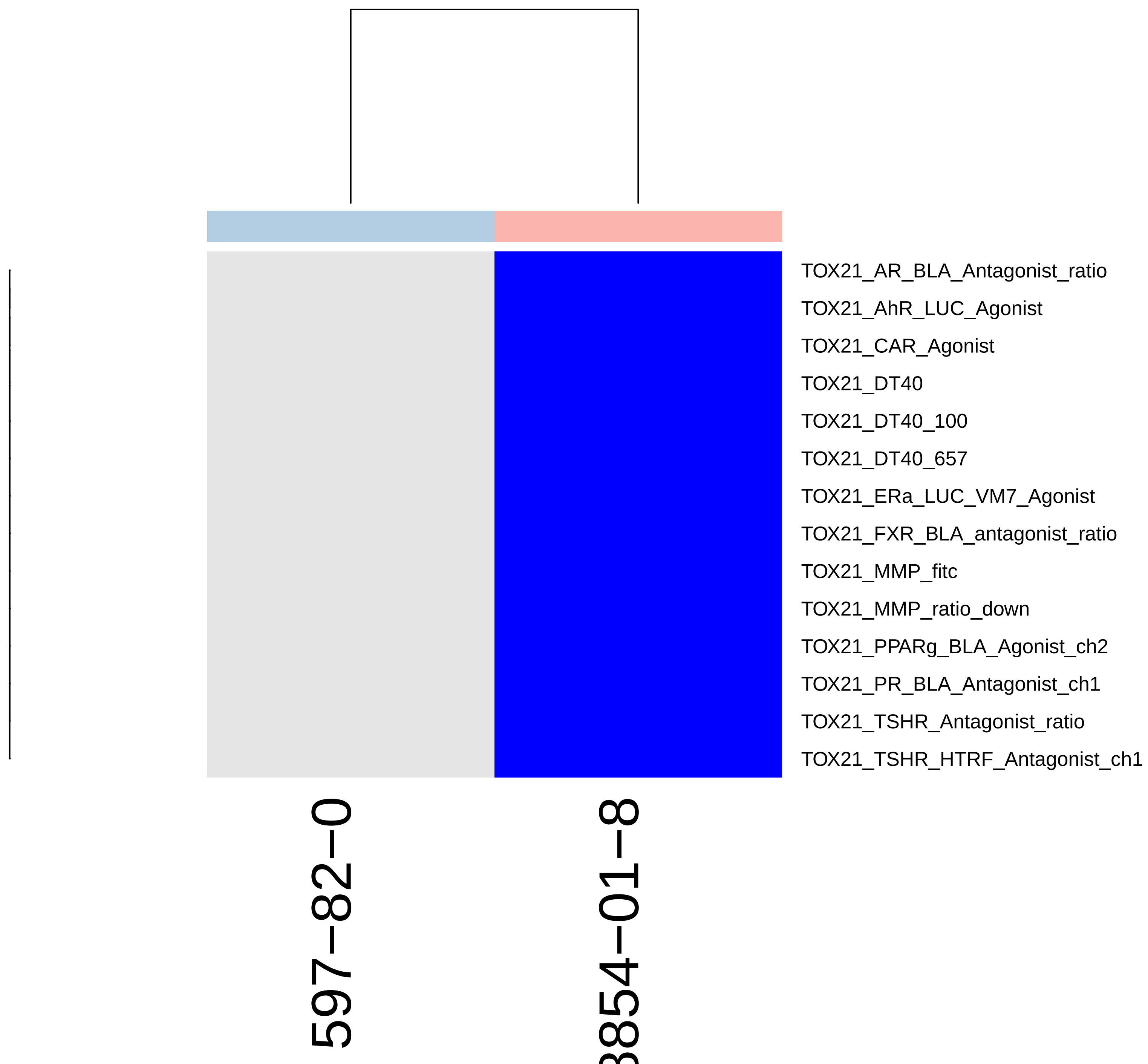

All Activity Heatmap for Cluster 1452

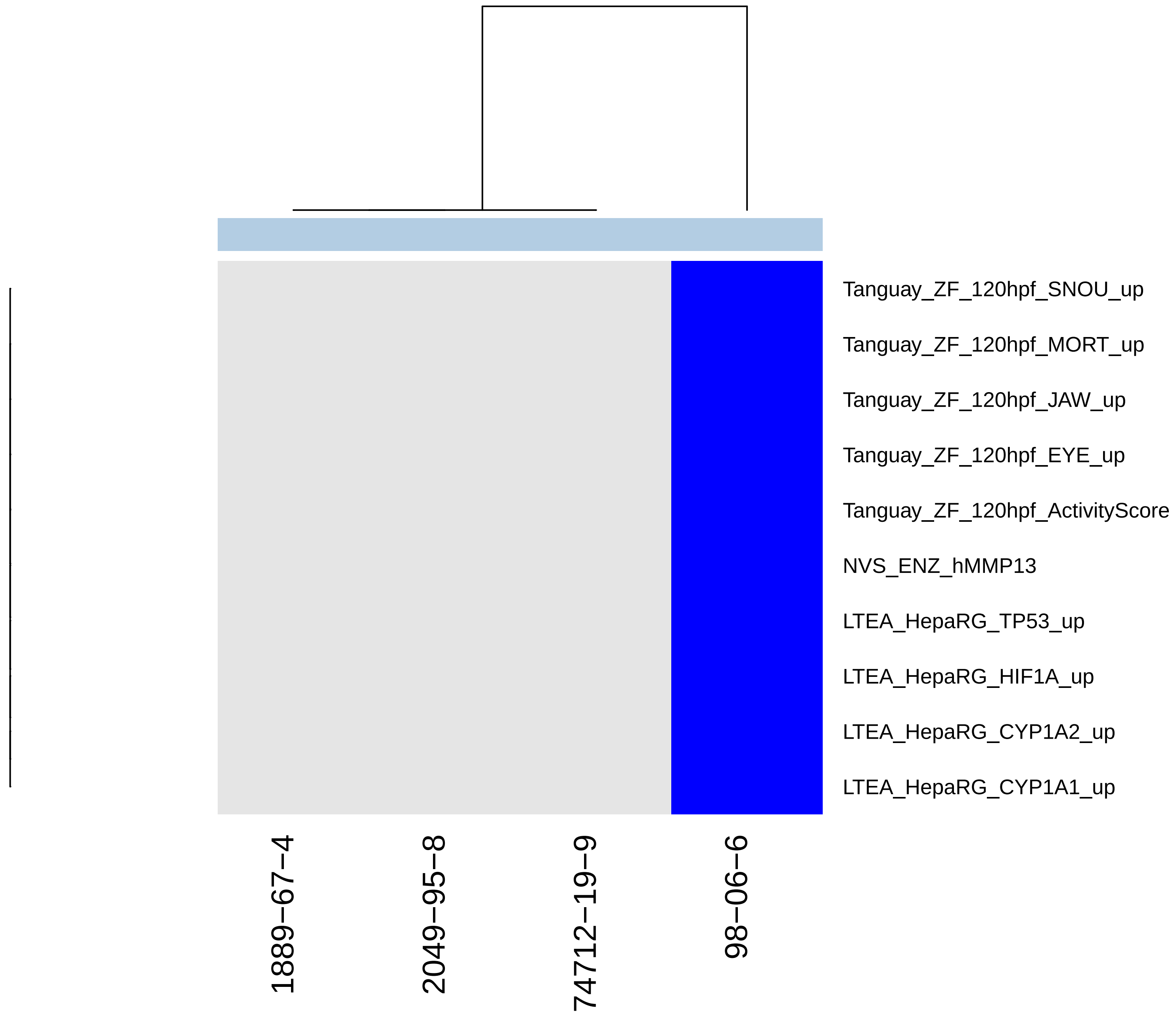

All Activity Heatmap for Cluster 1453

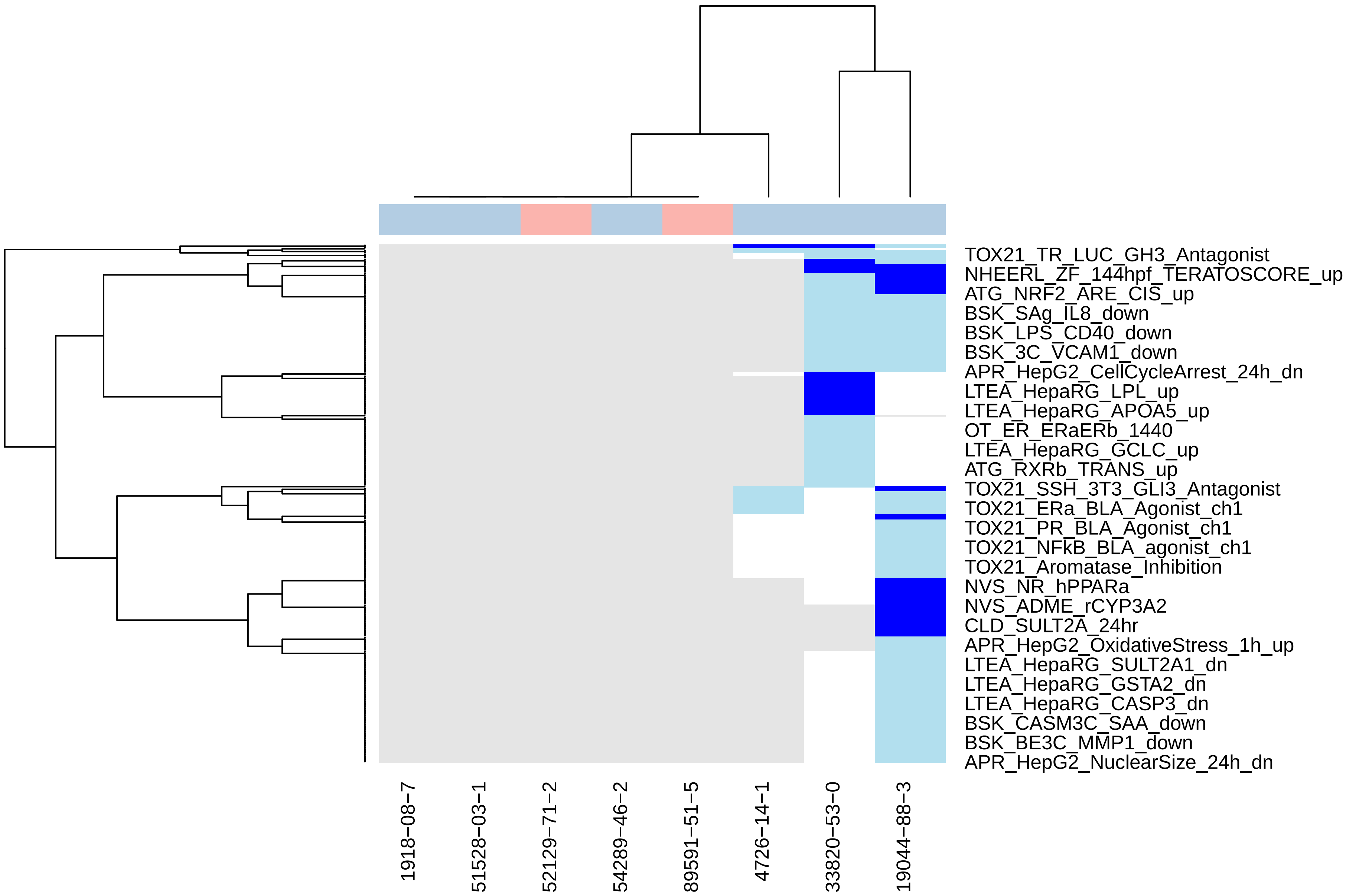

All Activity Heatmap for Cluster 1457

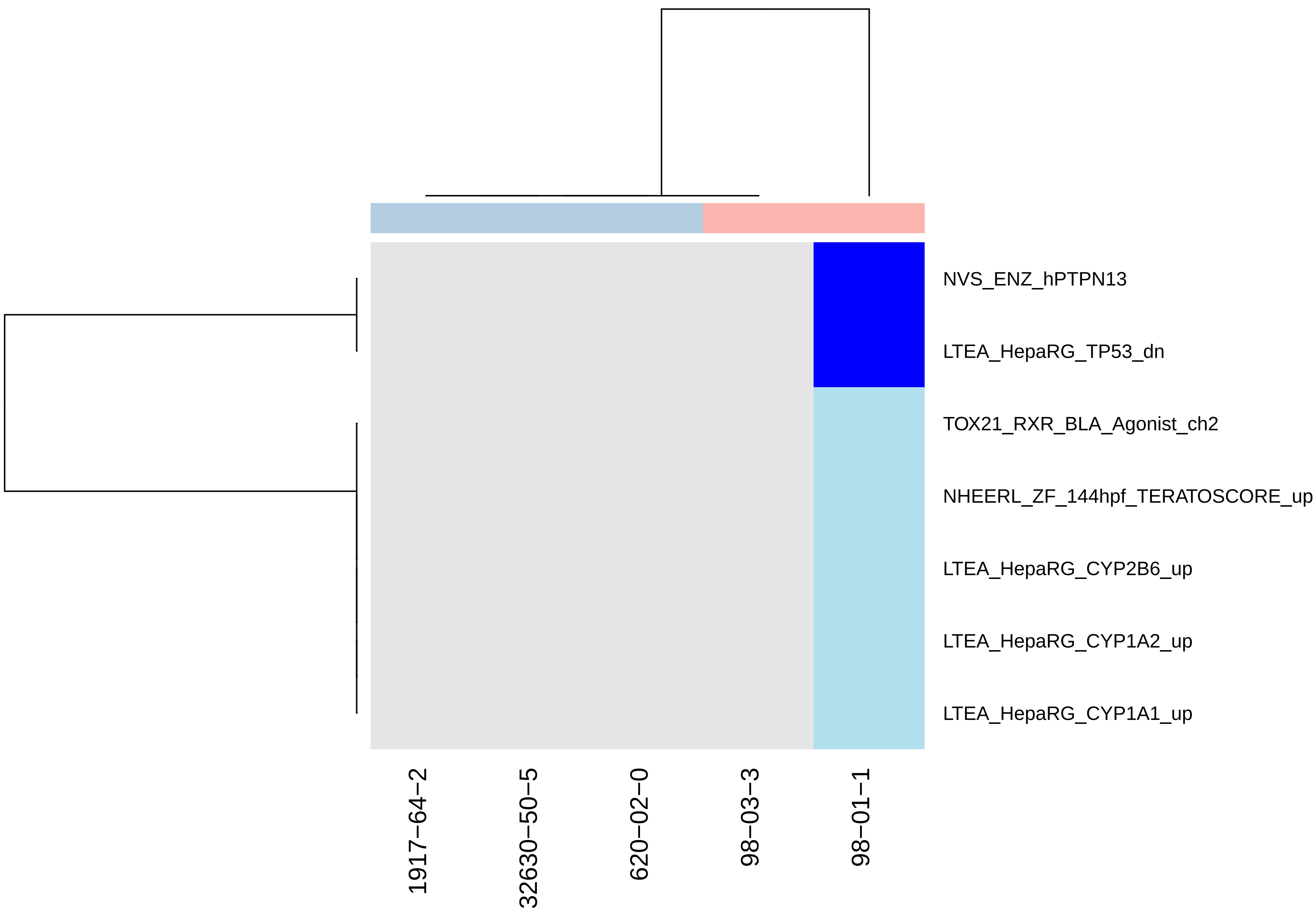

All Activity Heatmap for Cluster 1458

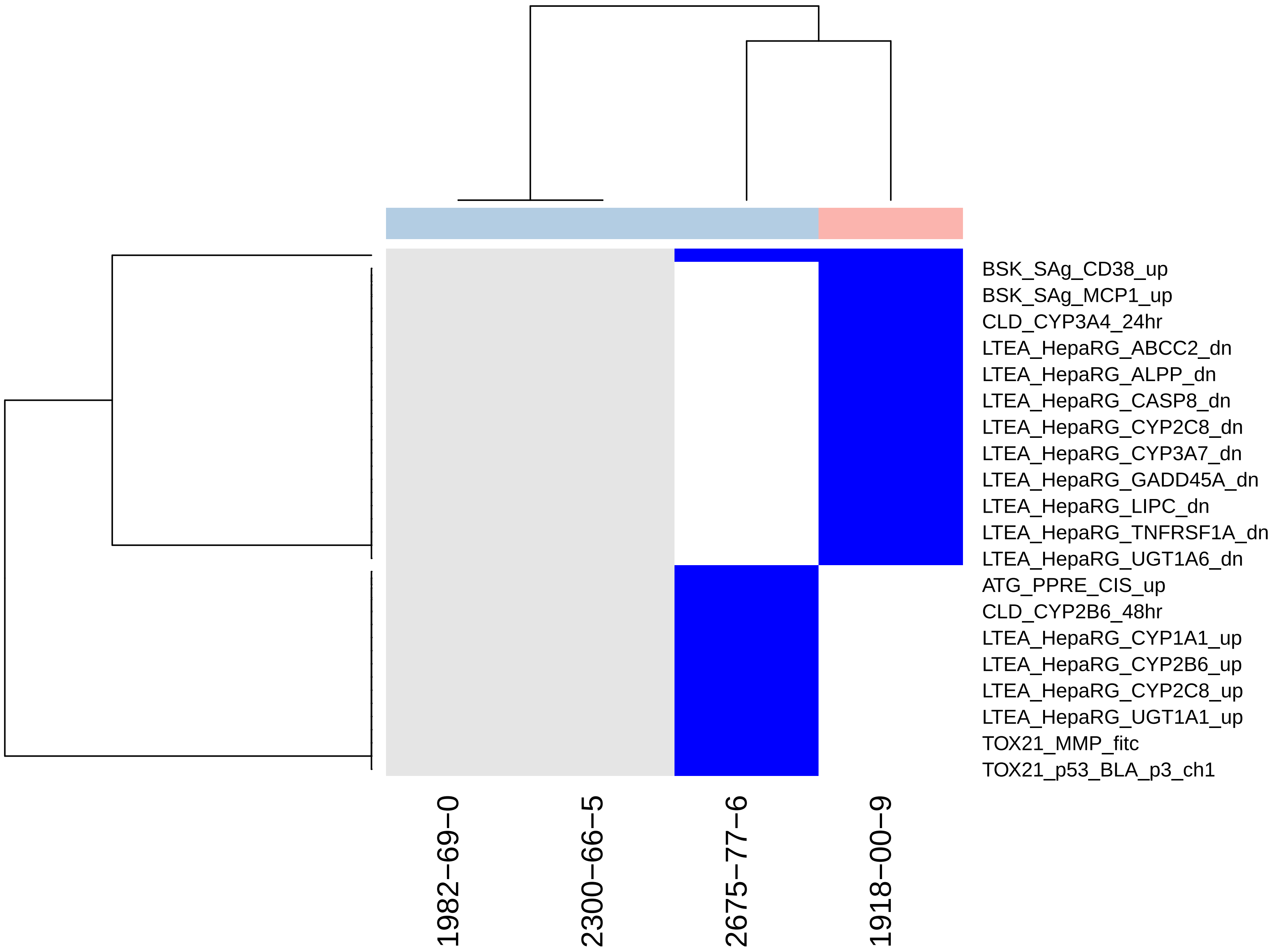

All Activity Heatmap for Cluster 1459

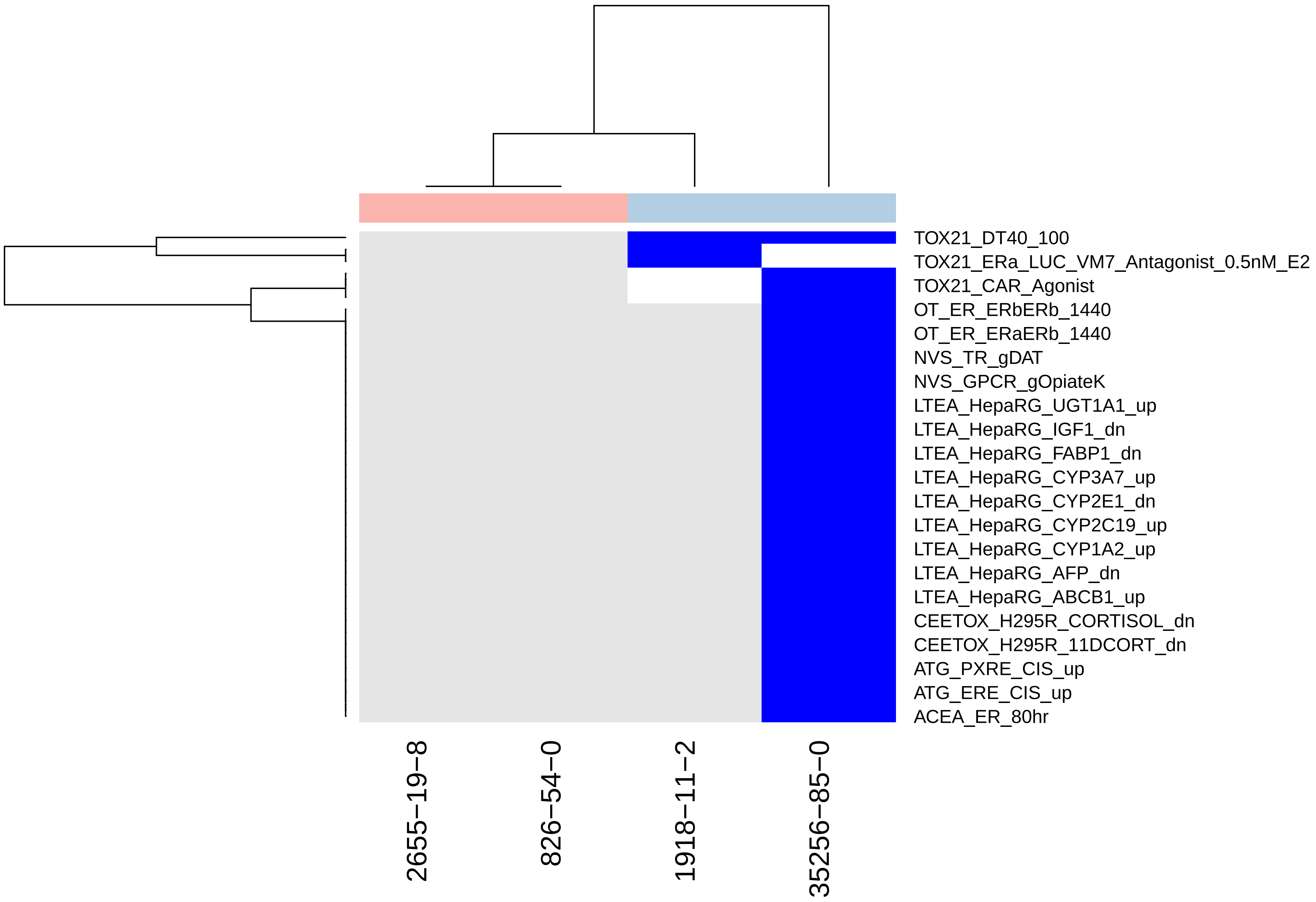

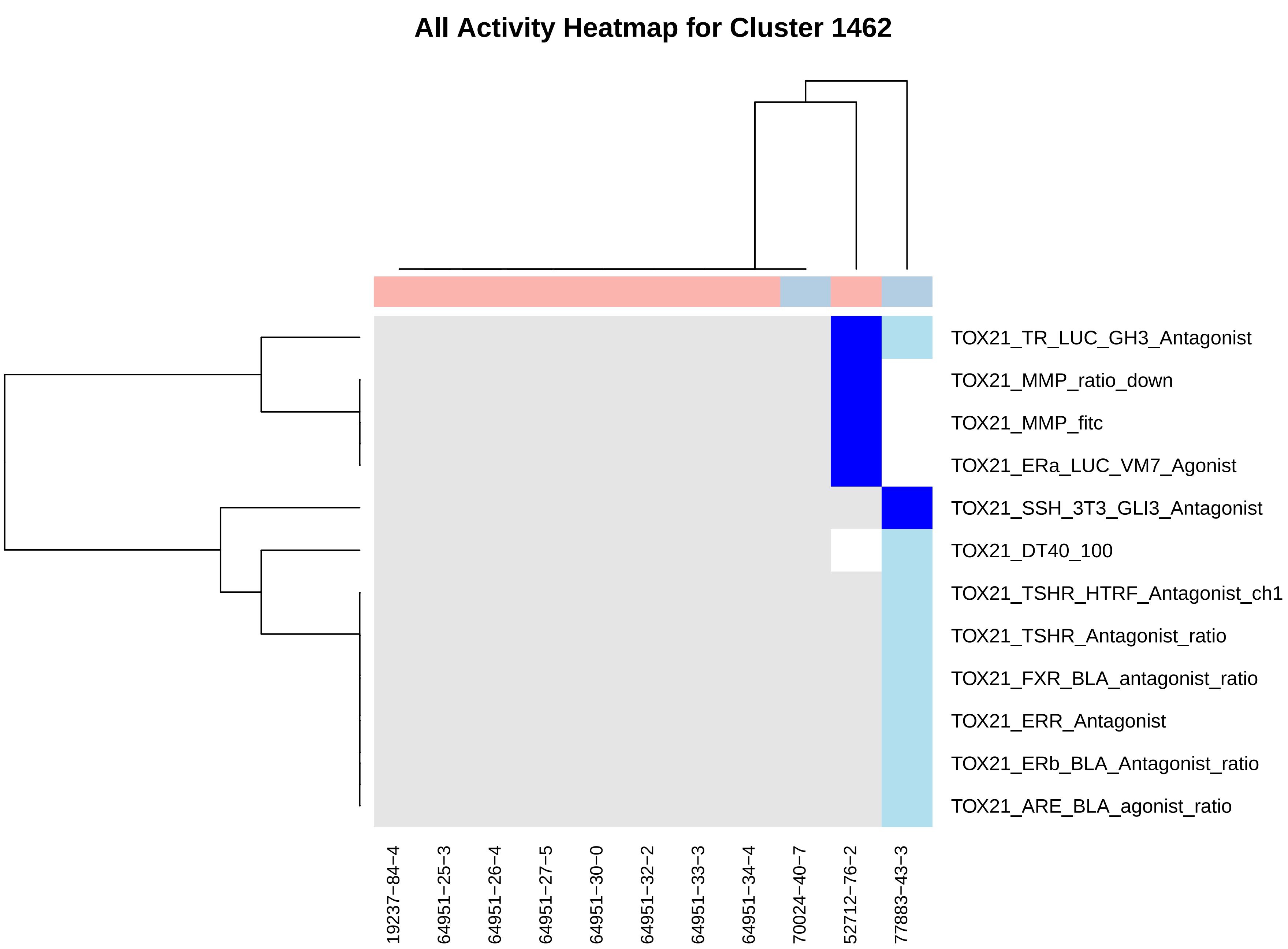
All Activity Heatmap for Cluster 1462
TOX21_TR_LUC_GH3_Antagonist
TOX21_MMP_ratio_down
TOX21_MMP_fitc
TOX21_ERa_LUC_VM7_Agonist
TOX21_SSH_3T3_GLI3_Antagonist
TOX21_DT40_100
TOX21_TSHR_HTRF_Antagonist_ch1
TOX21_TSHR_Antagonist_ratio
TOX21_FXR_BLA_antagonist_ratio
TOX21_ERR_Antagonist
TOX21_ERb_BLA_Antagonist_ratio
TOX21_ARE_BLA_agonist_ratio
19237−84−4
64951−25−3
64951−26−4
64951−27−5
64951−30−0
64951−32−2
64951−33−3
64951−34−4
70024−40−7
52712−76−2
77883−43−3

All Activity Heatmap for Cluster 1464

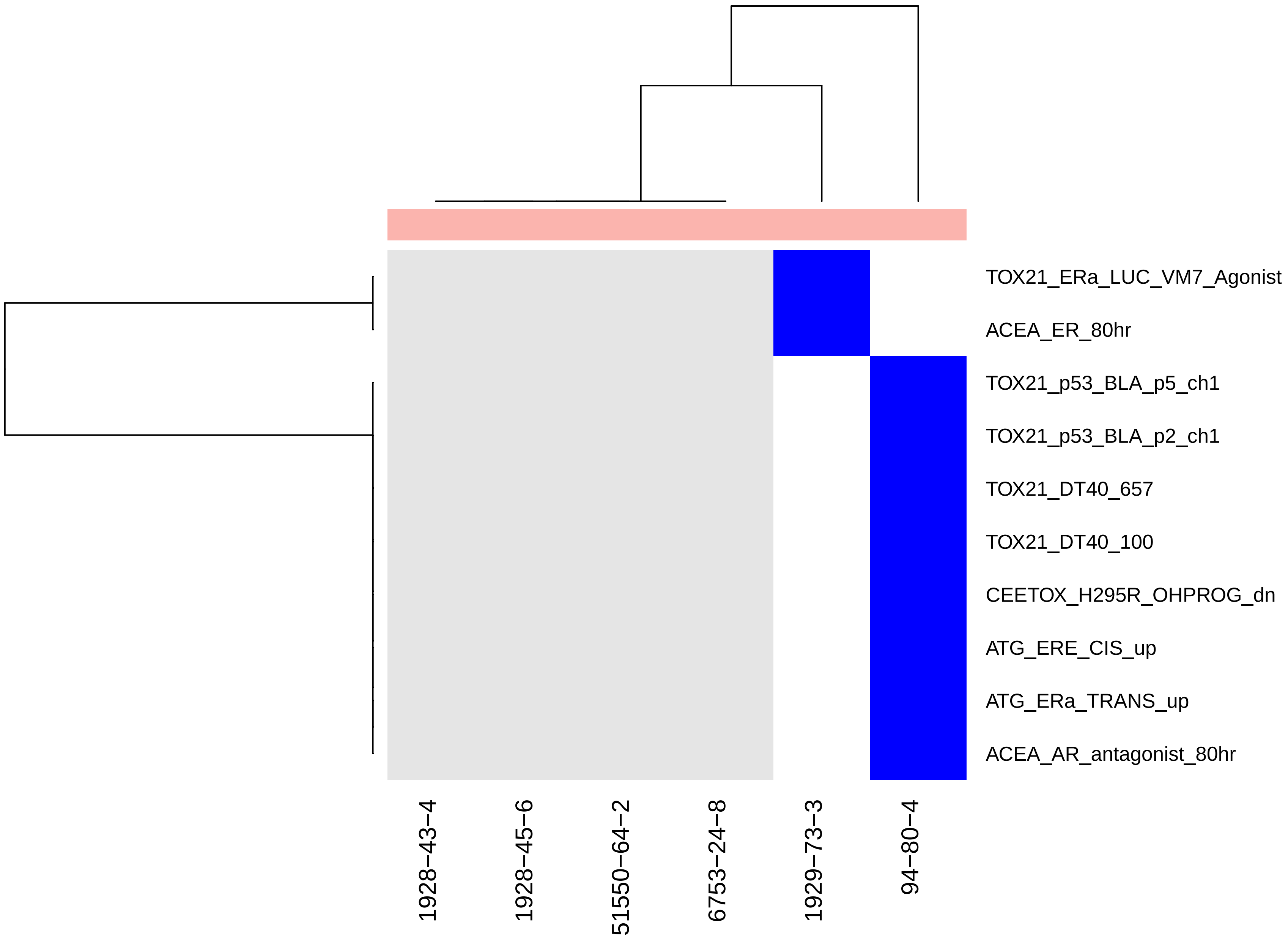

All Activity Heatmap for Cluster 1465

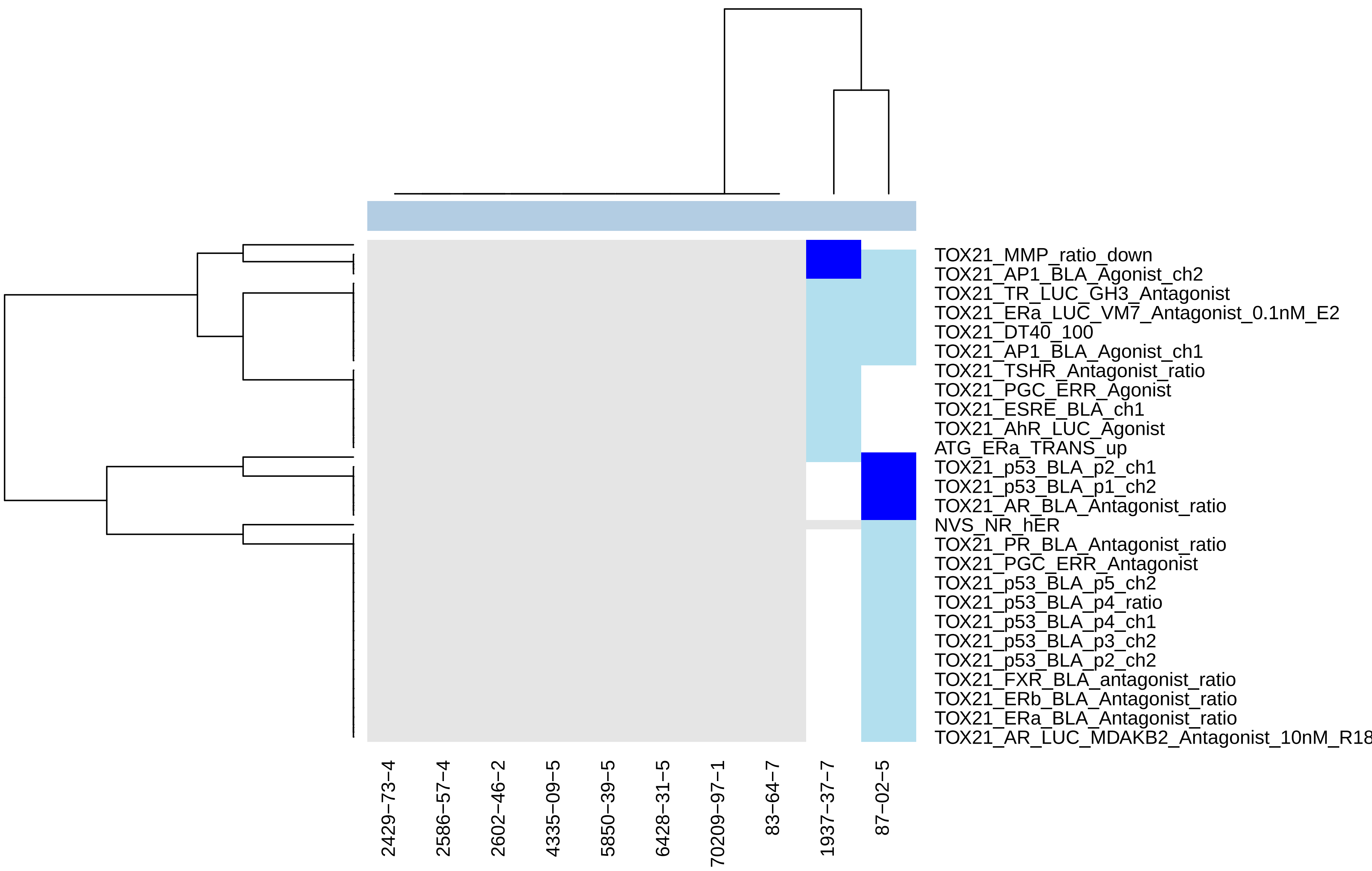

All Activity Heatmap for Cluster 1466

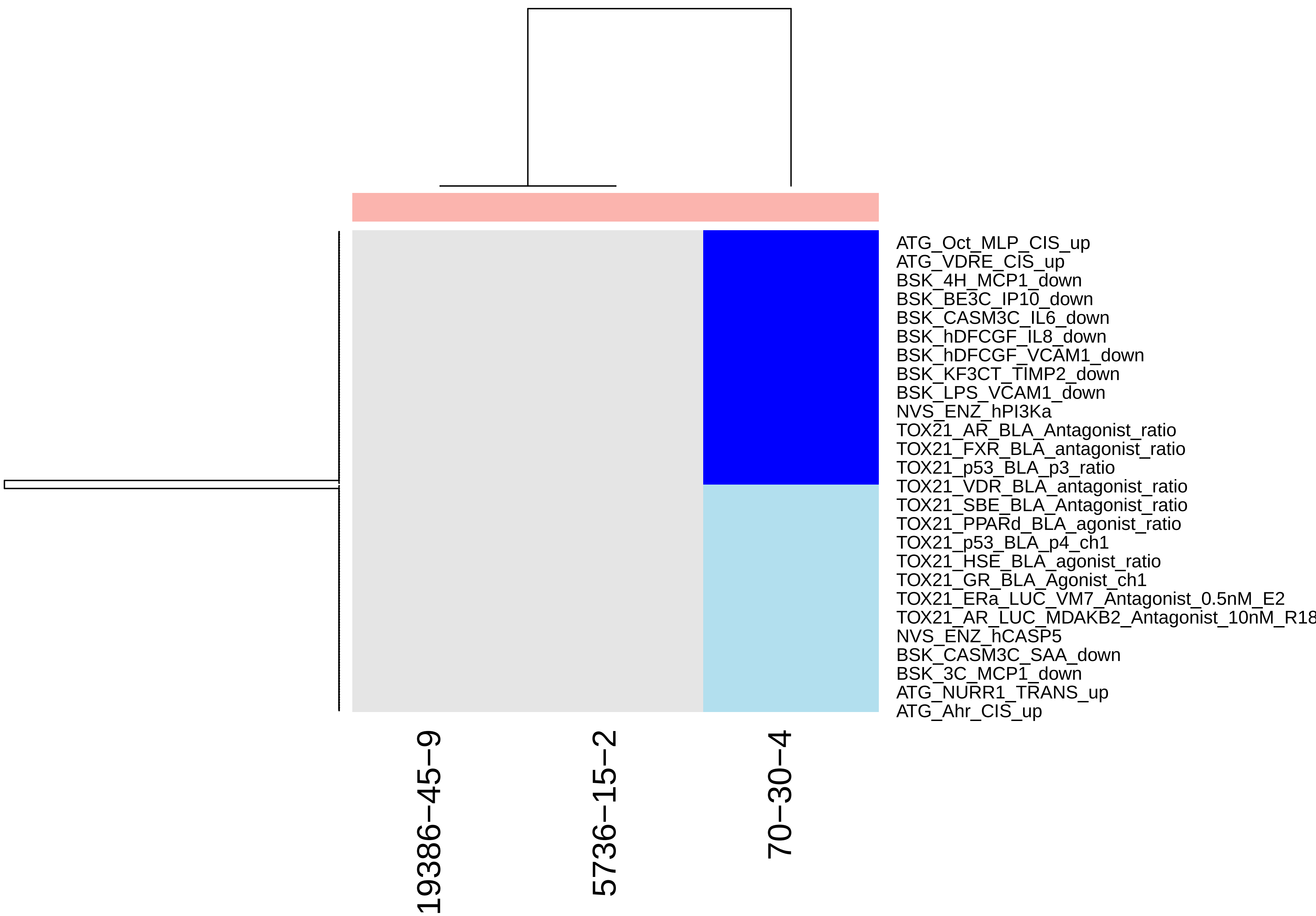

All Activity Heatmap for Cluster 1467

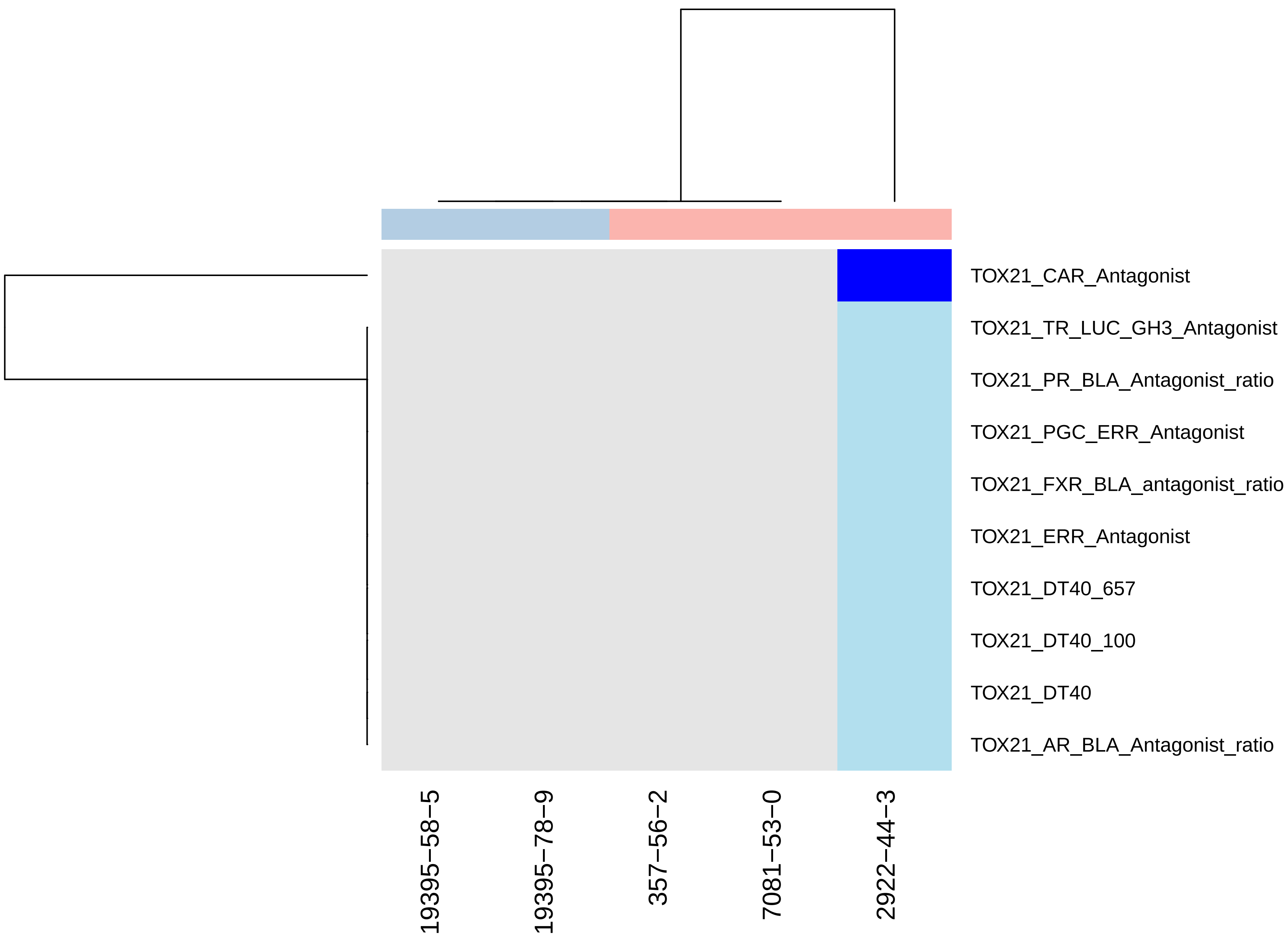

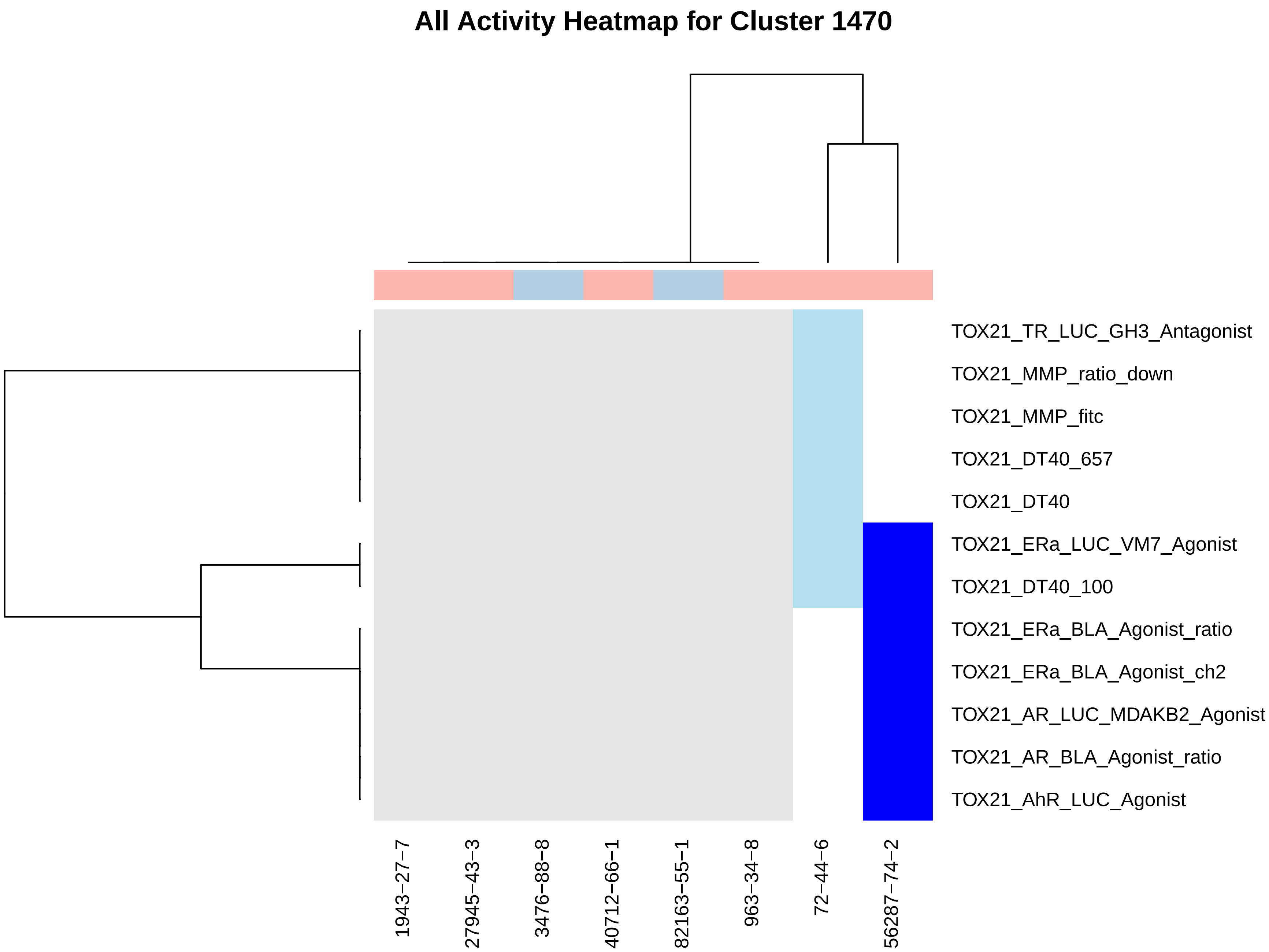
All Activity Heatmap for Cluster 1470
TOX21_TR_LUC_GH3_Antagonist
TOX21_MMP_ratio_down
TOX21_MMP_fitc
TOX21_DT40_657
TOX21_DT40
TOX21_ERa_LUC_VM7_Agonist
TOX21_DT40_100
TOX21_ERa_BLA_Agonist_ratio
TOX21_ERa_BLA_Agonist_ch2
TOX21_AR_LUC_MDAKB2_Agonist
TOX21_AR_BLA_Agonist_ratio
TOX21_AhR_LUC_Agonist
1943−27−7
27945−43−3
3476−88−8
40712−66−1
82163−55−1
963−34−8
72−44−6
56287−74−2

All Activity Heatmap for Cluster 1471

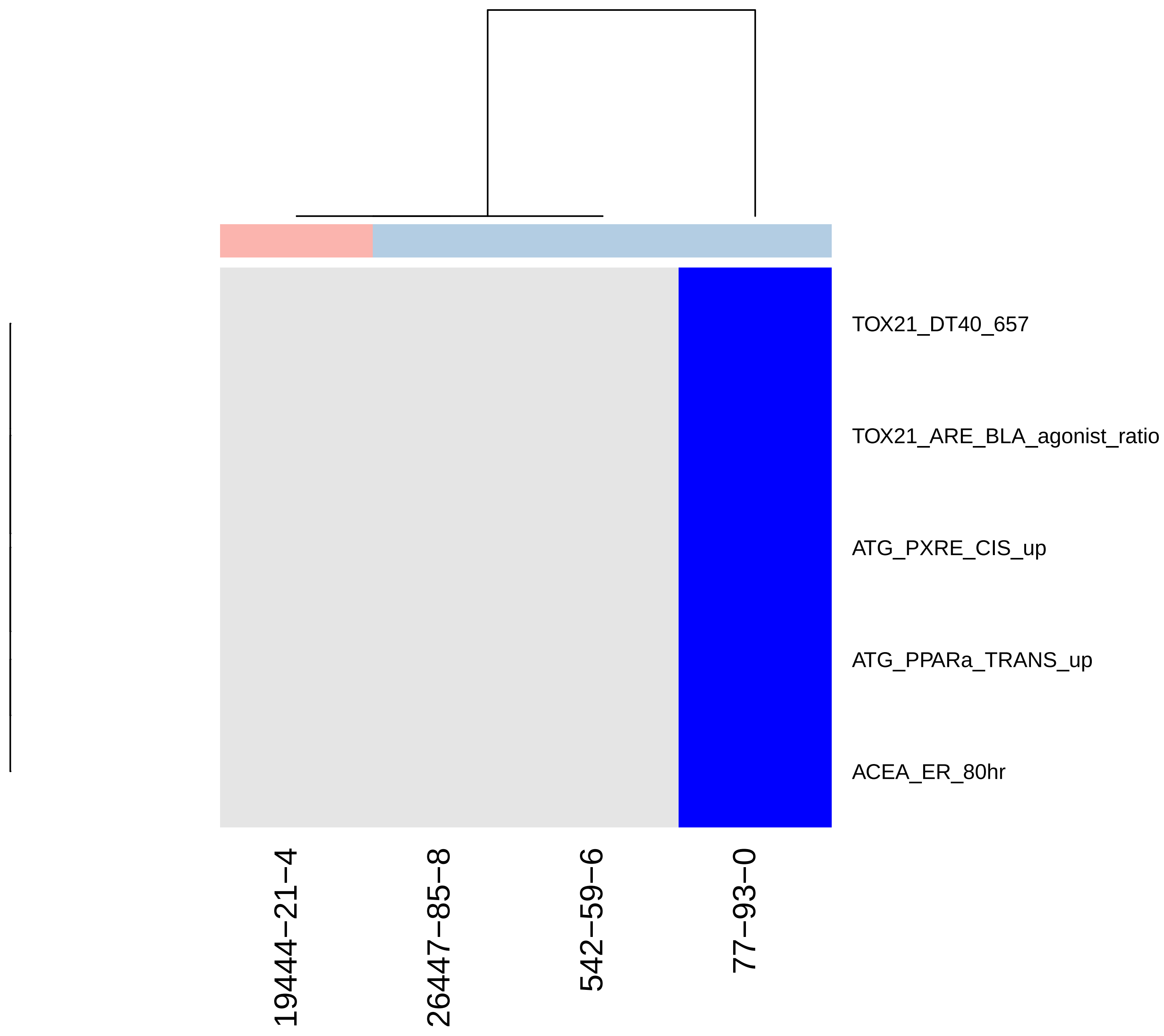

All Activity Heatmap for Cluster 1472

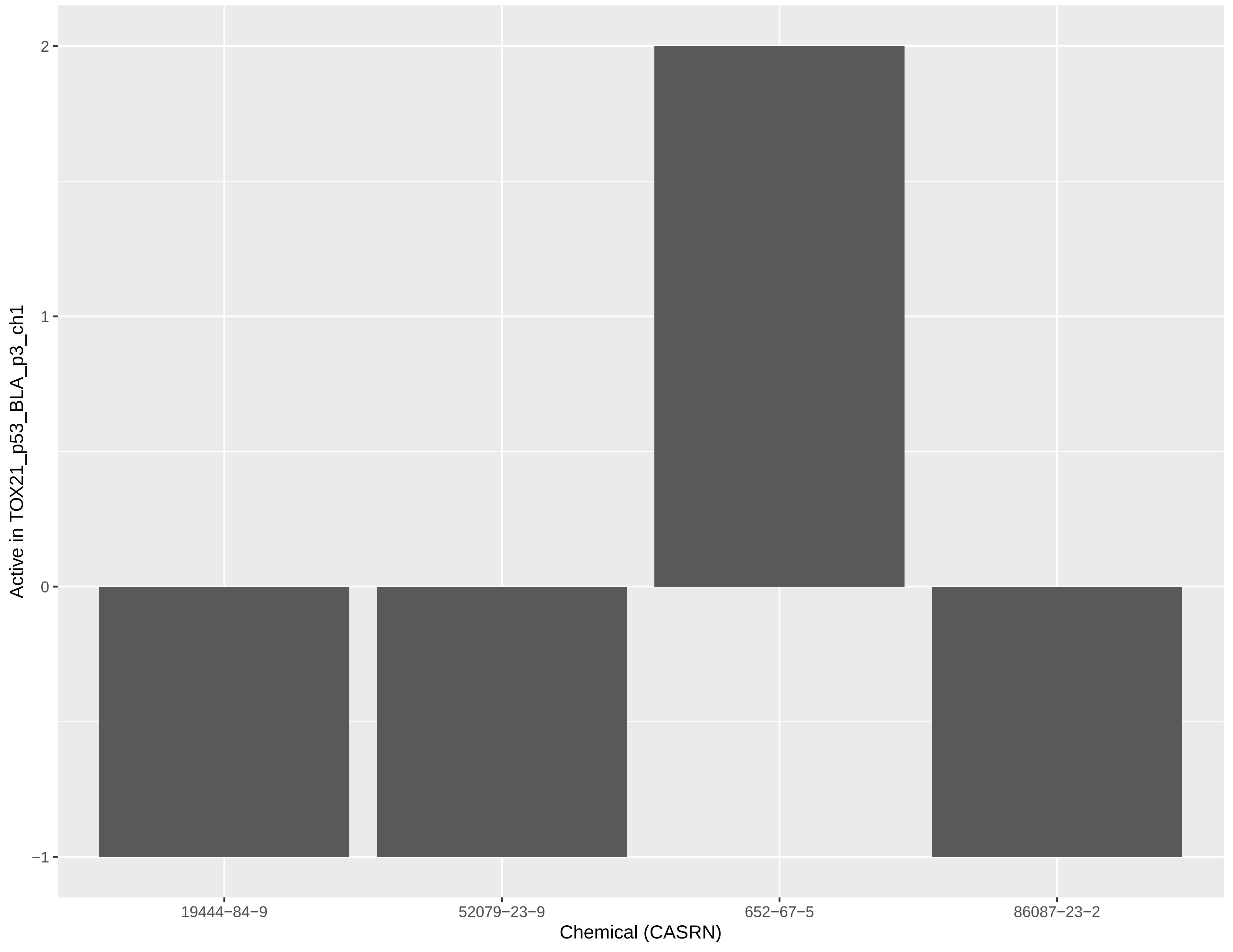

All Activity Heatmap for Cluster 1473

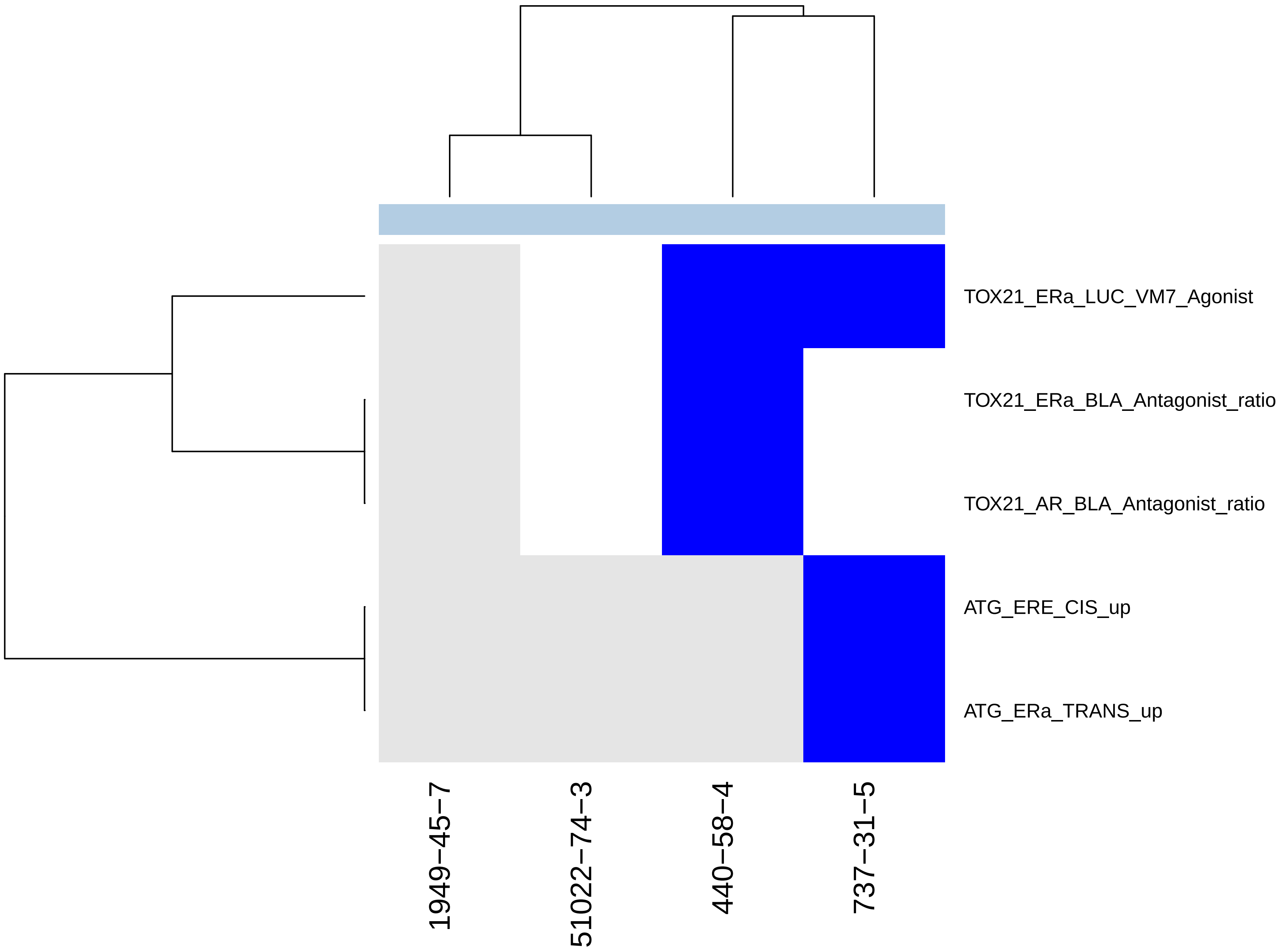

All Activity Heatmap for Cluster 1475

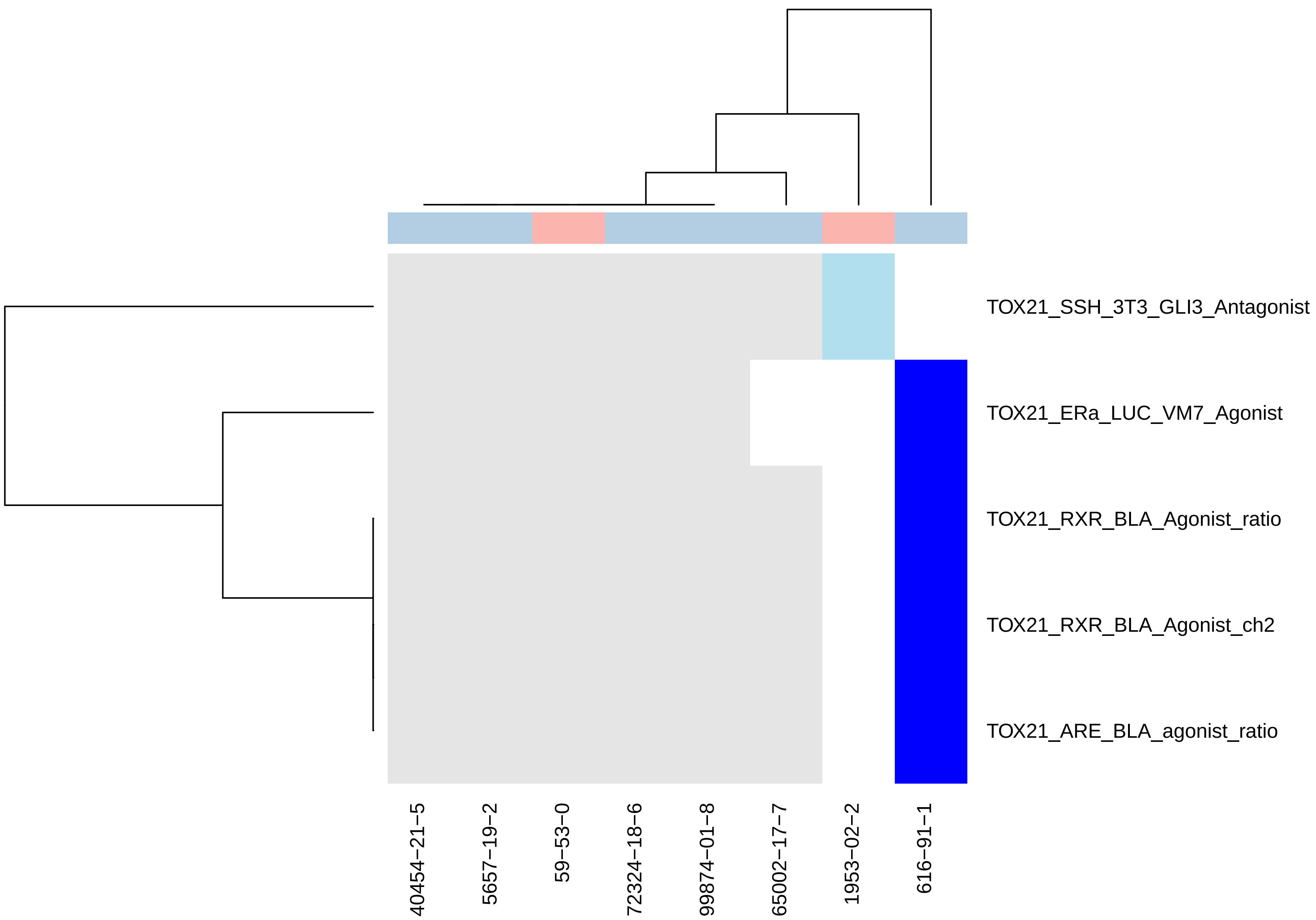

All Activity Heatmap for Cluster 1477

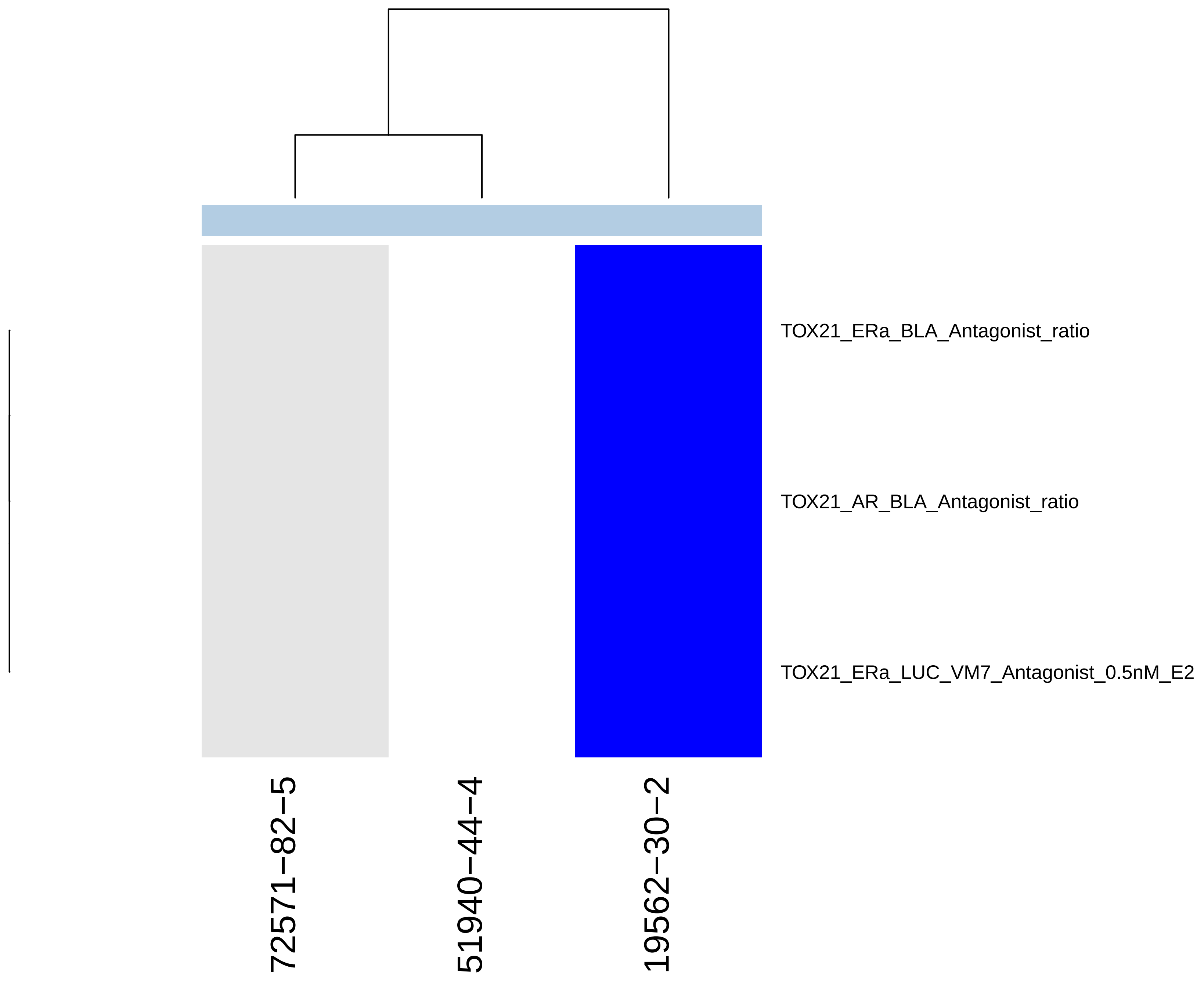

All Activity Heatmap for Cluster 1478

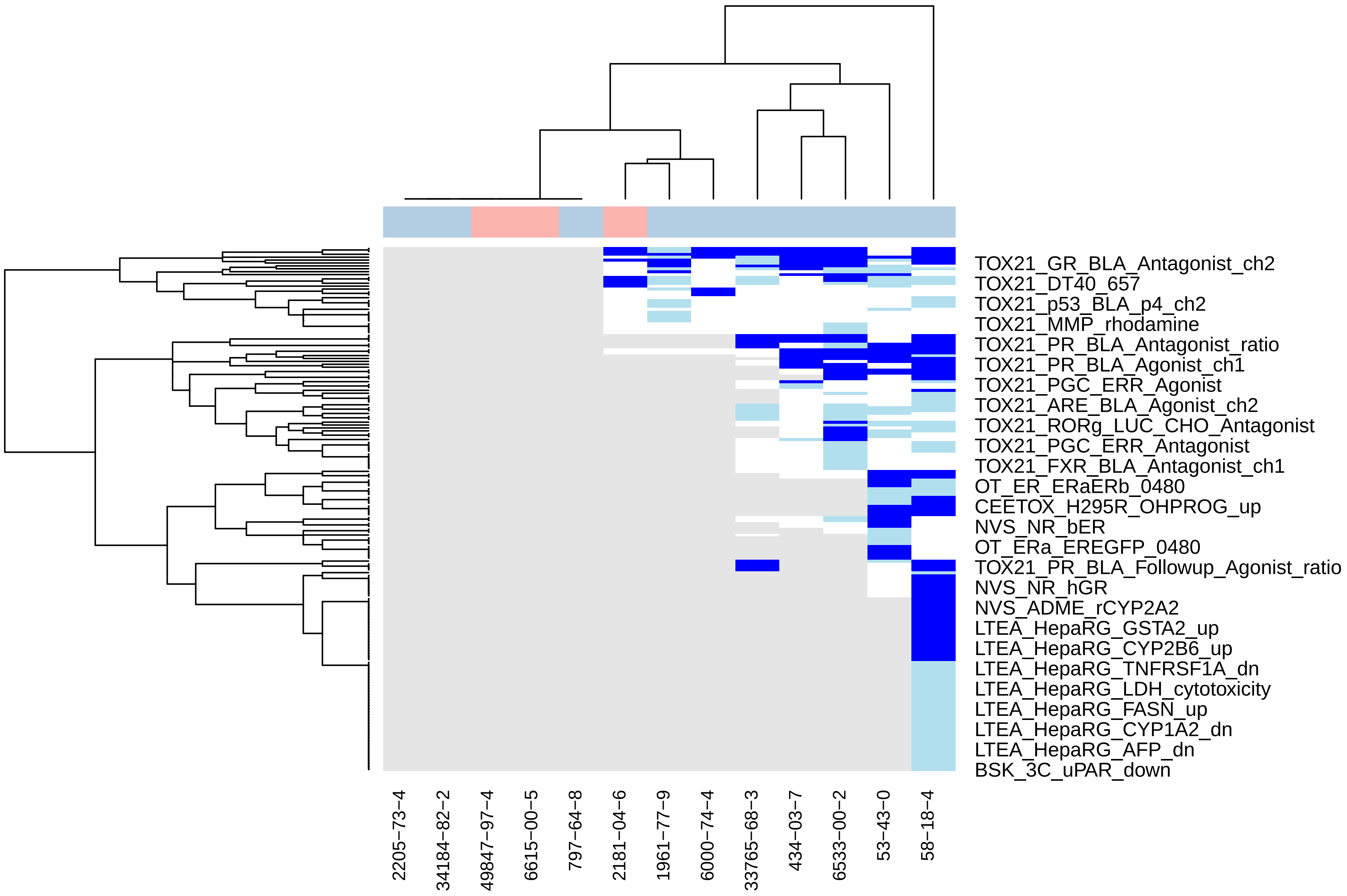

All Activity Heatmap for Cluster 1479

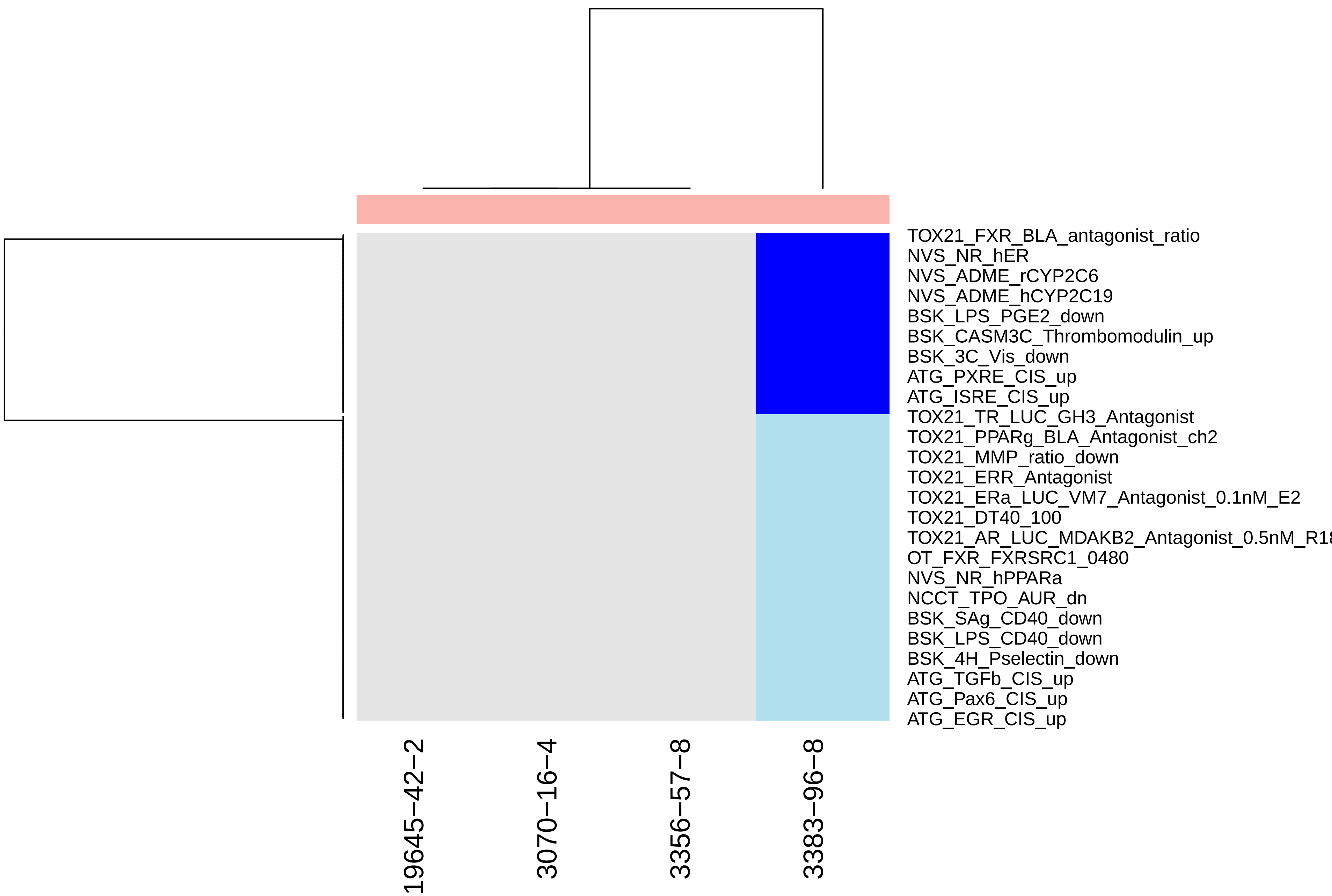

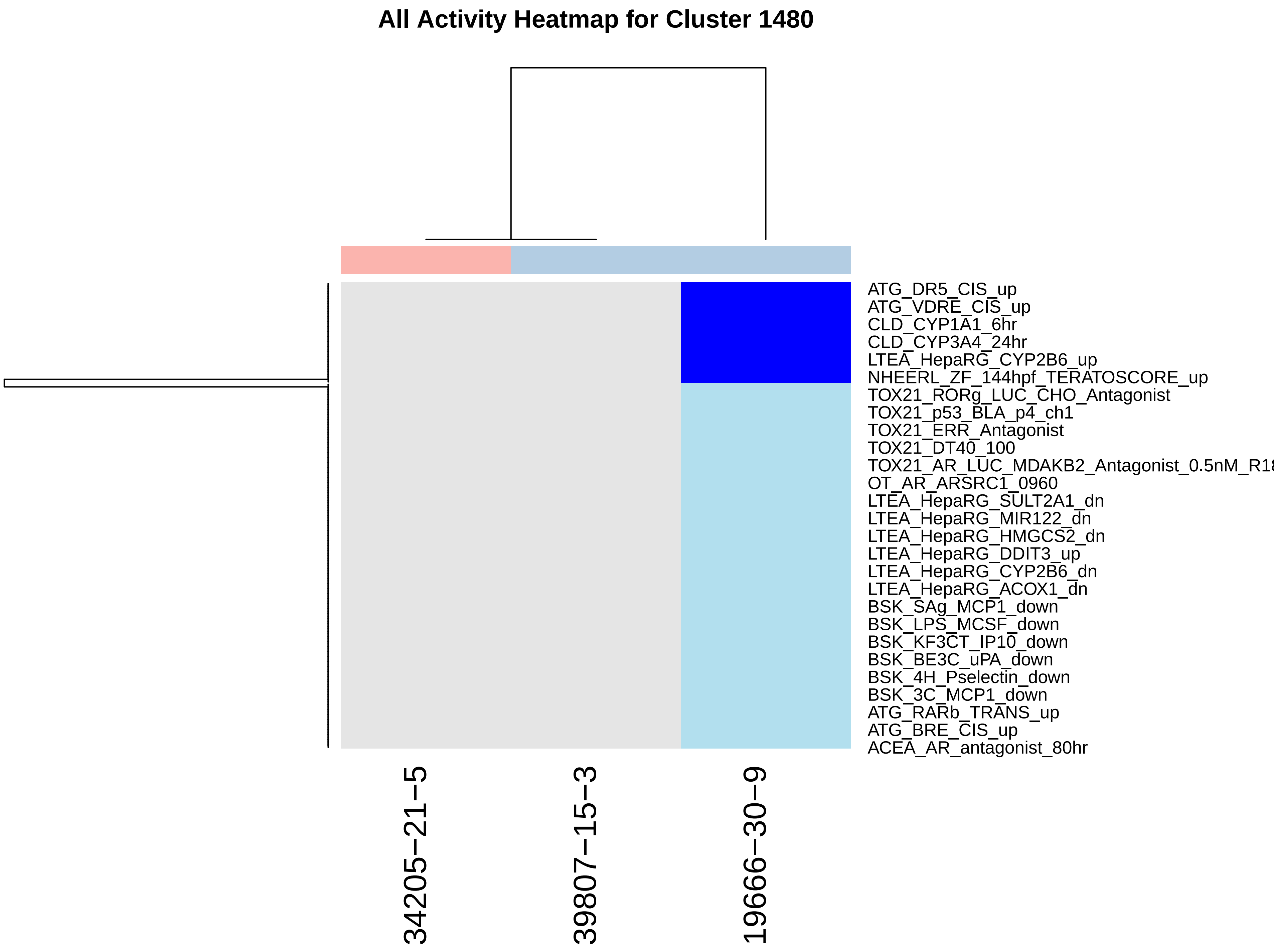
All Activity Heatmap for Cluster 1480
ATG_DR5_CIS_up
ATG_VDRE_CIS_up
CLD_CYP1A1_6hr
CLD_CYP3A4_24hr
LTEA_HepaRG_CYP2B6_up
NHEERL_ZF_144hpf_TERATOSCORE_up
TOX21_RORg_LUC_CHO_Antagonist
TOX21_p53_BLA_p4_ch1
TOX21_ERR_Antagonist
TOX21_DT40_100
TOX21_AR_LUC_MDAKB2_Antagonist_0.5nM_R18
OT_AR_ARSRC1_0960
LTEA_HepaRG_SULT2A1_dn
LTEA_HepaRG_MIR122_dn
LTEA_HepaRG_HMGCS2_dn
LTEA_HepaRG_DDIT3_up
LTEA_HepaRG_CYP2B6_dn
LTEA_HepaRG_ACOX1_dn
BSK_SAg_MCP1_down
BSK_LPS_MCSF_down
BSK_KF3CT_IP10_down
BSK_BE3C_uPA_down
BSK_4H_Pselectin_down
BSK_3C_MCP1_down
ATG_RARb_TRANS_up
ATG_BRE_CIS_up
ACEA_AR_antagonist_80hr
34205−21−5
39807−15−3
19666−30−9

All Activity Heatmap for Cluster 1482

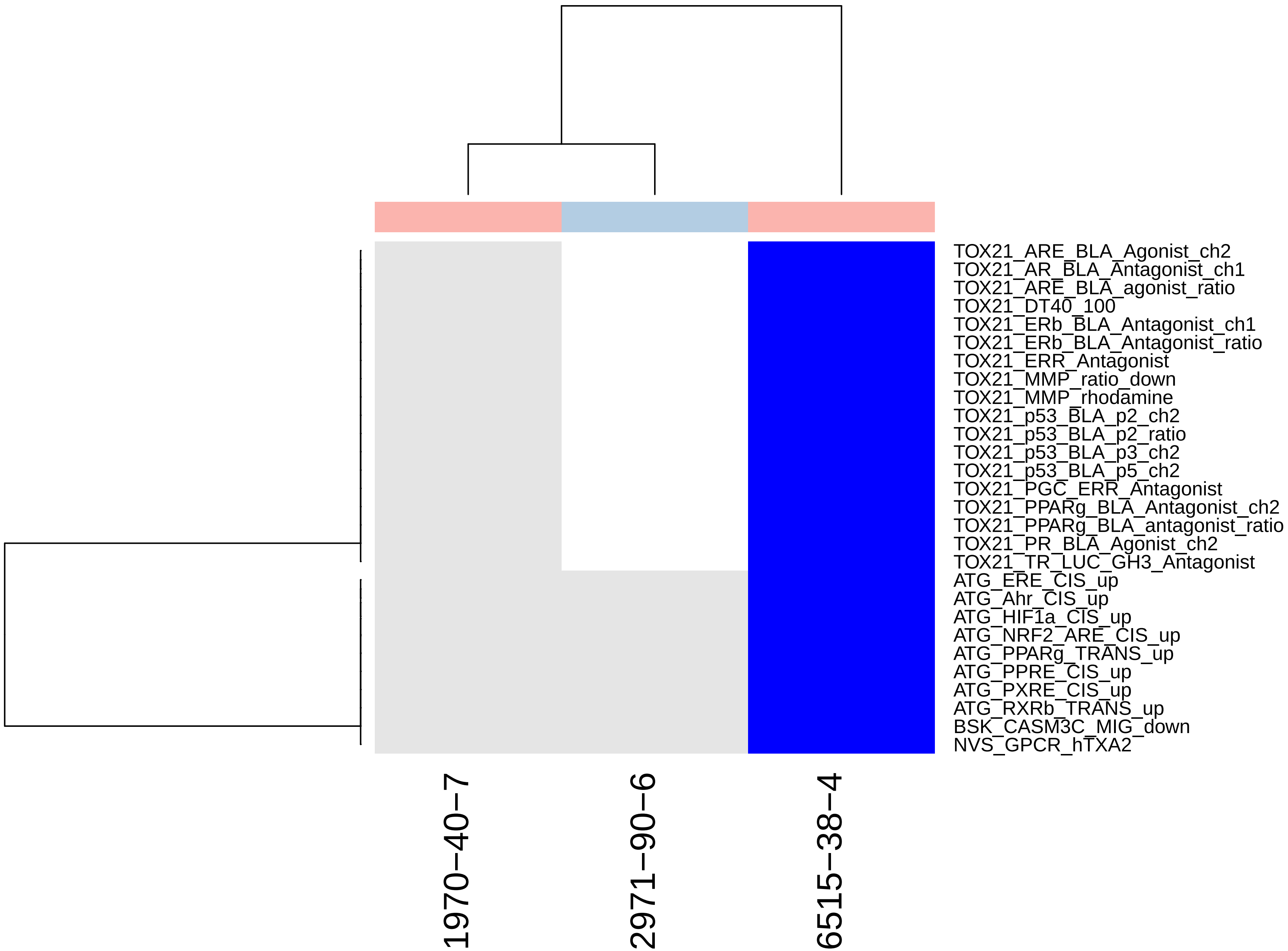

All Activity Heatmap for Cluster 1483

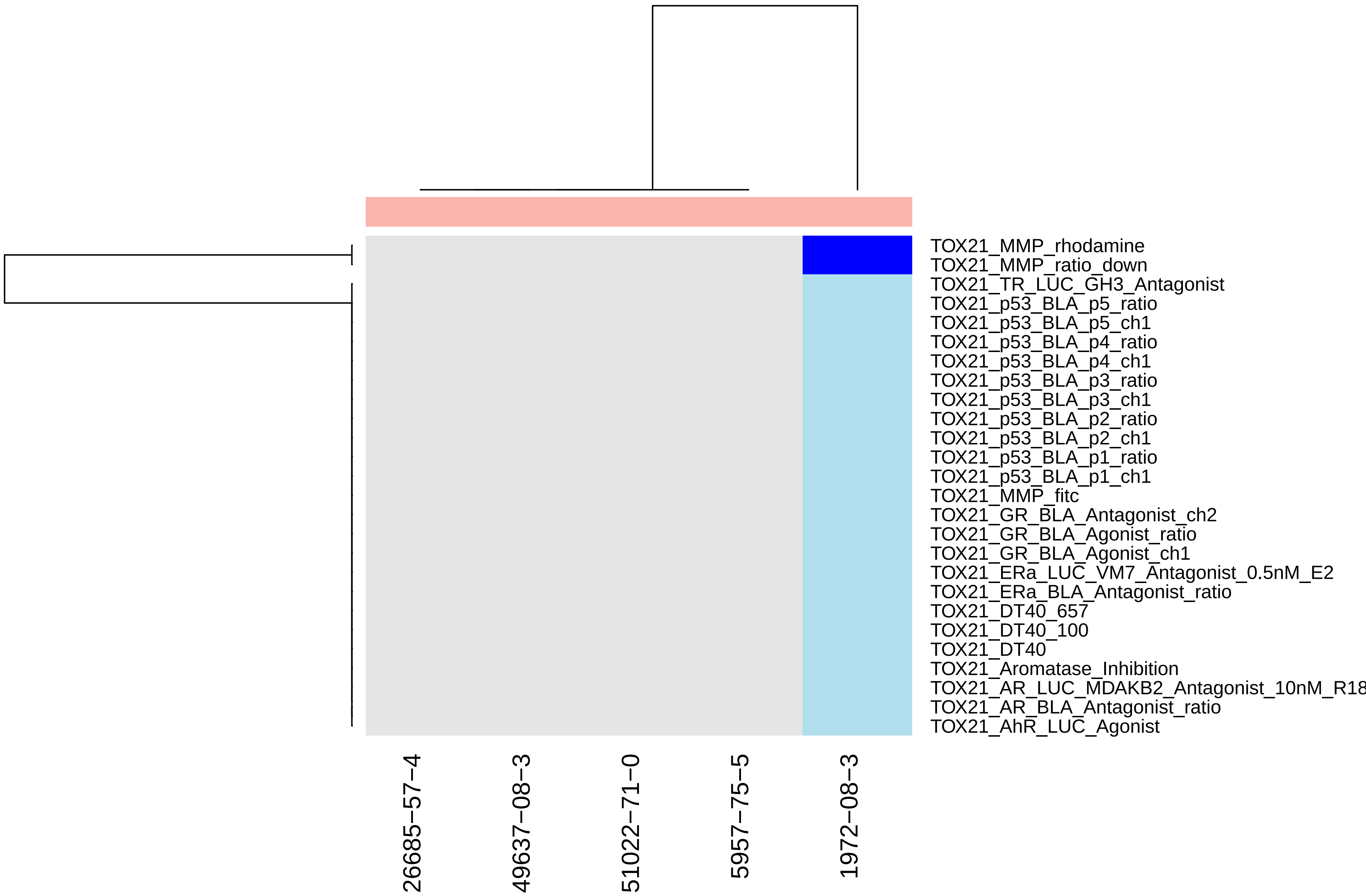

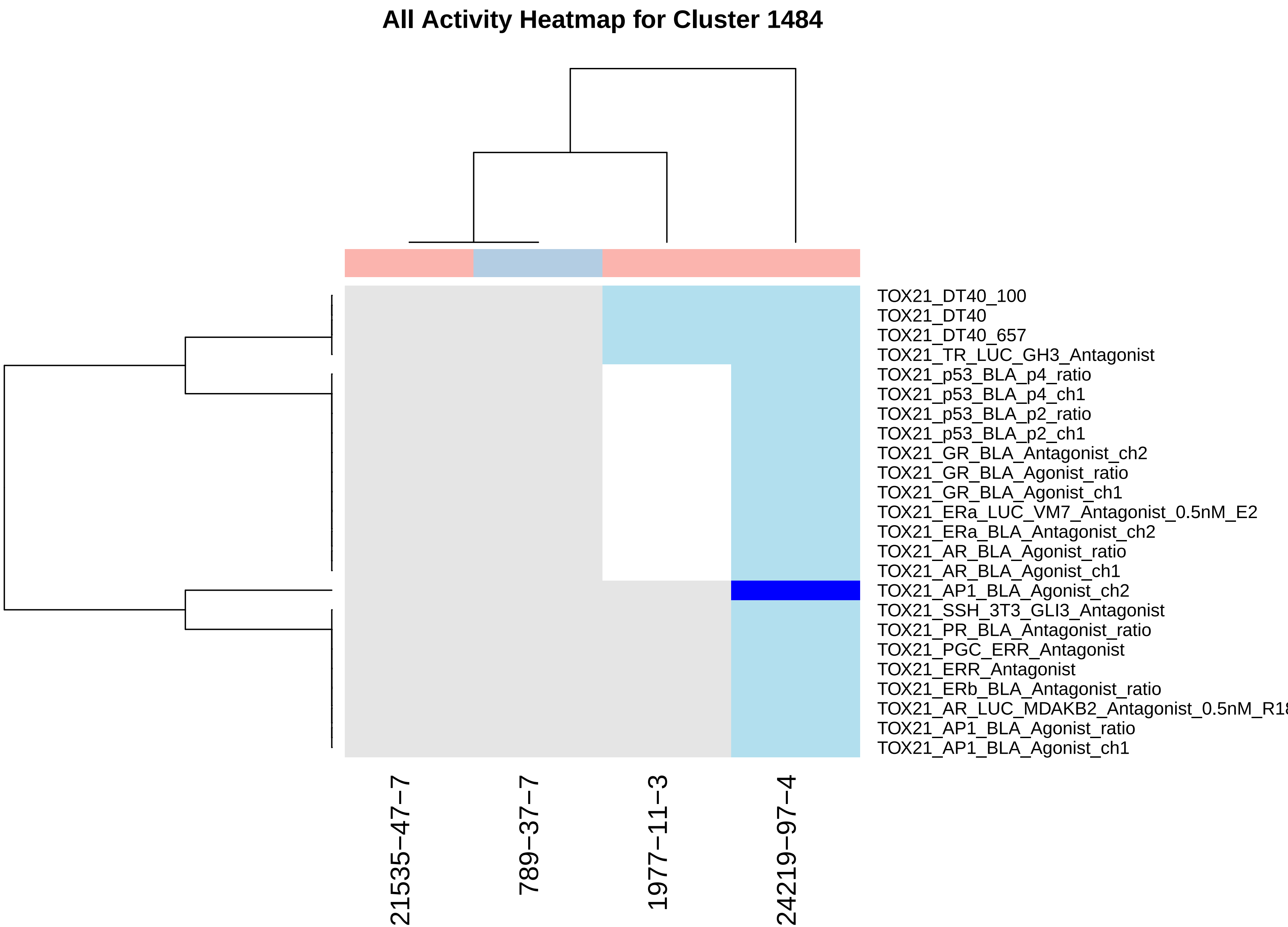
All Activity Heatmap for Cluster 1484
TOX21_DT40_100
TOX21_DT40
TOX21_DT40_657
TOX21_TR_LUC_GH3_Antagonist
TOX21_p53_BLA_p4_ratio
TOX21_p53_BLA_p4_ch1
TOX21_p53_BLA_p2_ratio
TOX21_p53_BLA_p2_ch1
TOX21_GR_BLA_Antagonist_ch2
TOX21_GR_BLA_Agonist_ratio
TOX21_GR_BLA_Agonist_ch1
TOX21_ERa_LUC_VM7_Antagonist_0.5nM_E2
TOX21_ERa_BLA_Antagonist_ch2
TOX21_AR_BLA_Agonist_ratio
TOX21_AR_BLA_Agonist_ch1
TOX21_AP1_BLA_Agonist_ch2
TOX21_SSH_3T3_GLI3_Antagonist
TOX21_PR_BLA_Antagonist_ratio
TOX21_PGC_ERR_Antagonist
TOX21_ERR_Antagonist
TOX21_ERb_BLA_Antagonist_ratio
TOX21_AR_LUC_MDAKB2_Antagonist_0.5nM_R1
TOX21_AP1_BLA_Agonist_ratio
TOX21_AP1_BLA_Agonist_ch1
21535−47−7
789−37−7
1977−11−3
24219−97−4

All Activity Heatmap for Cluster 1485

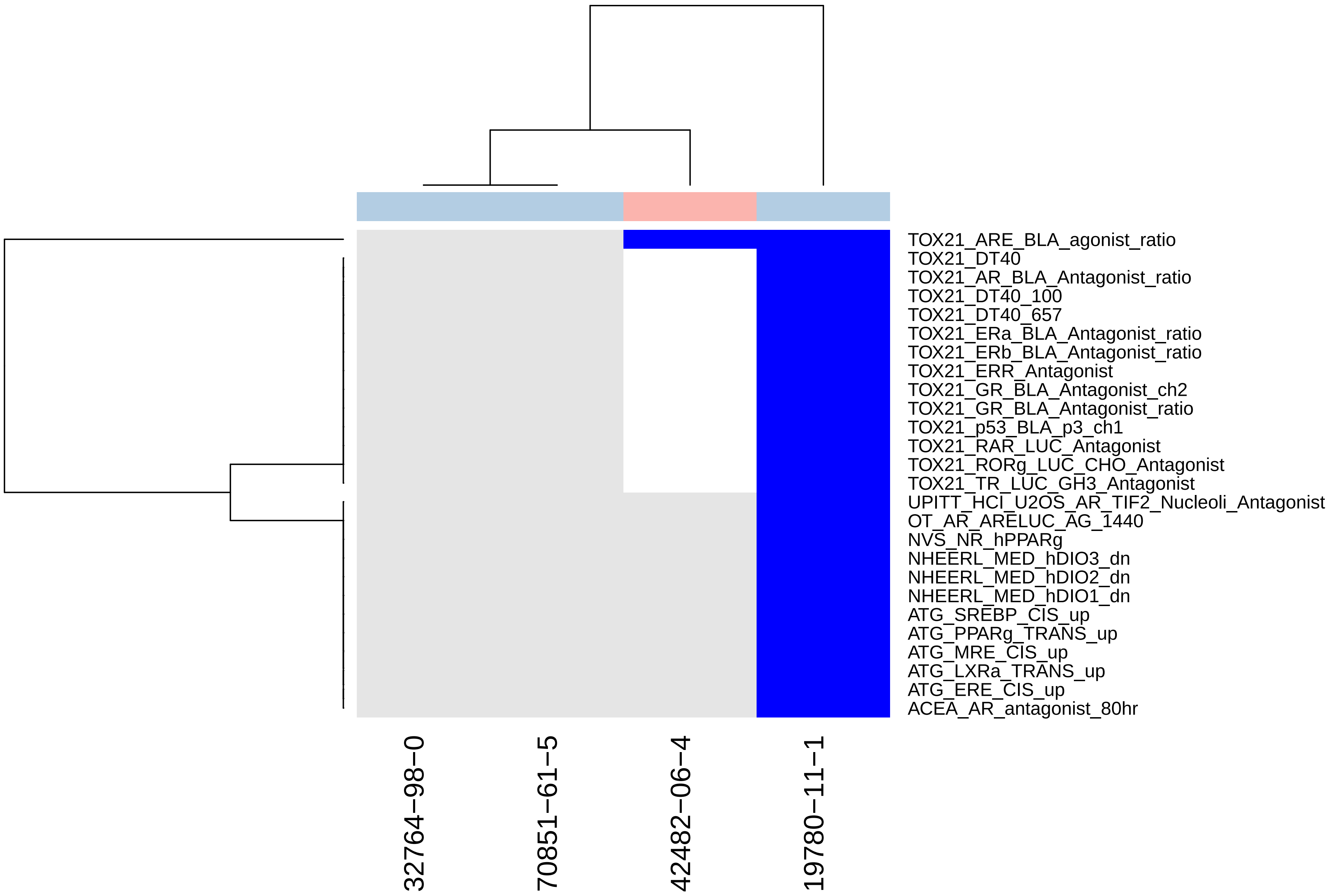

All Activity Heatmap for Cluster 1486

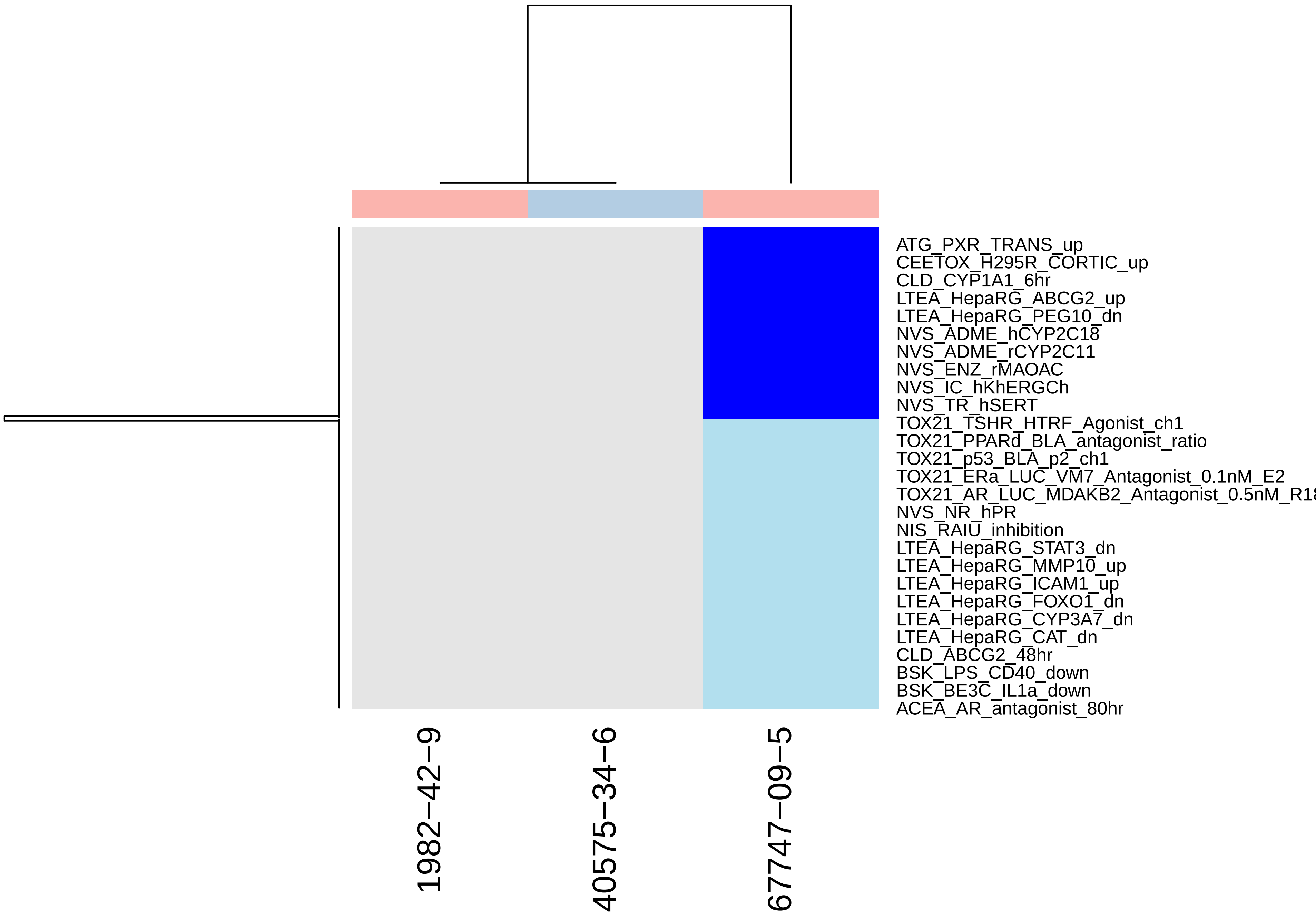

All Activity Heatmap for Cluster 1487

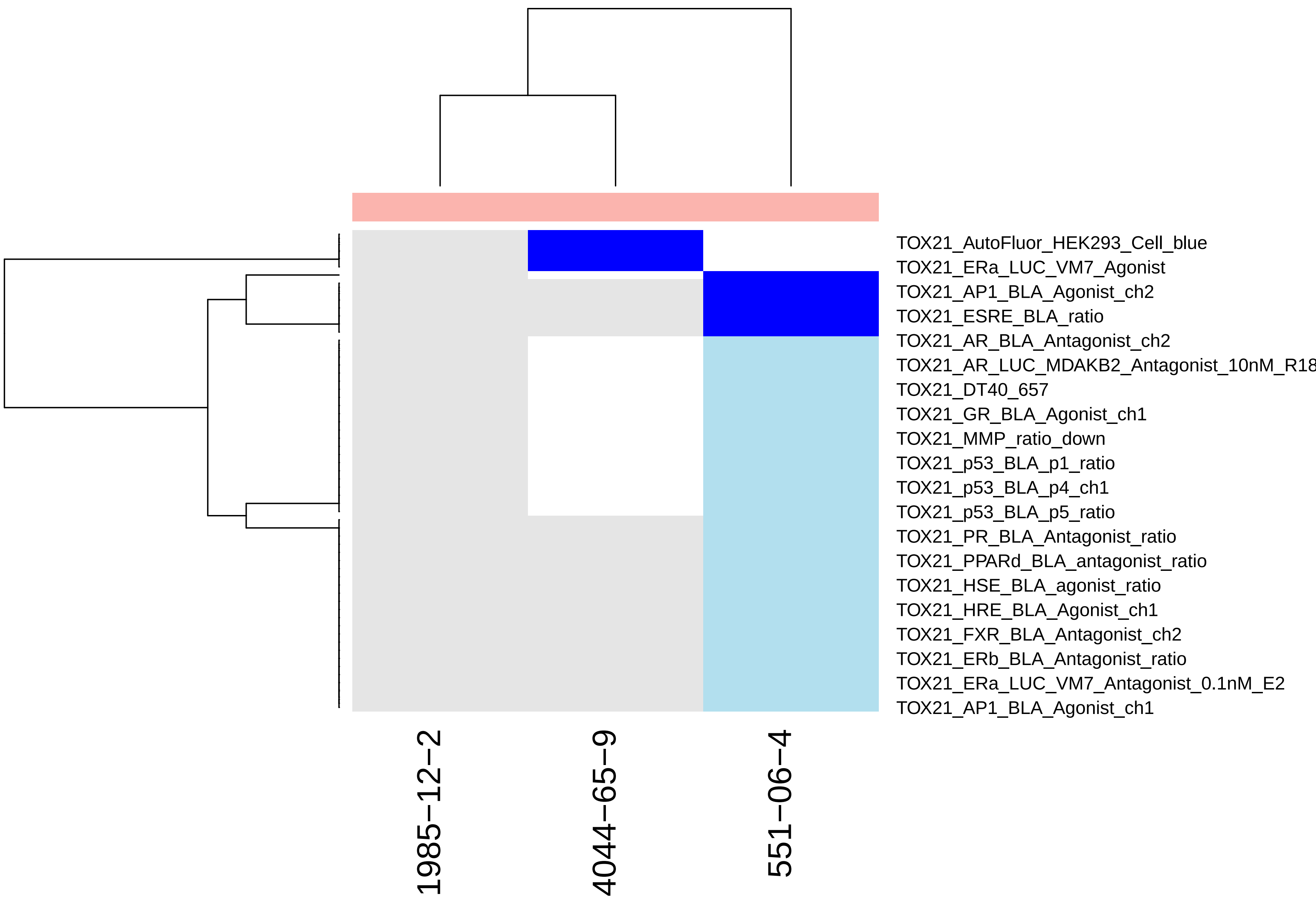

All Activity Heatmap for Cluster 1488

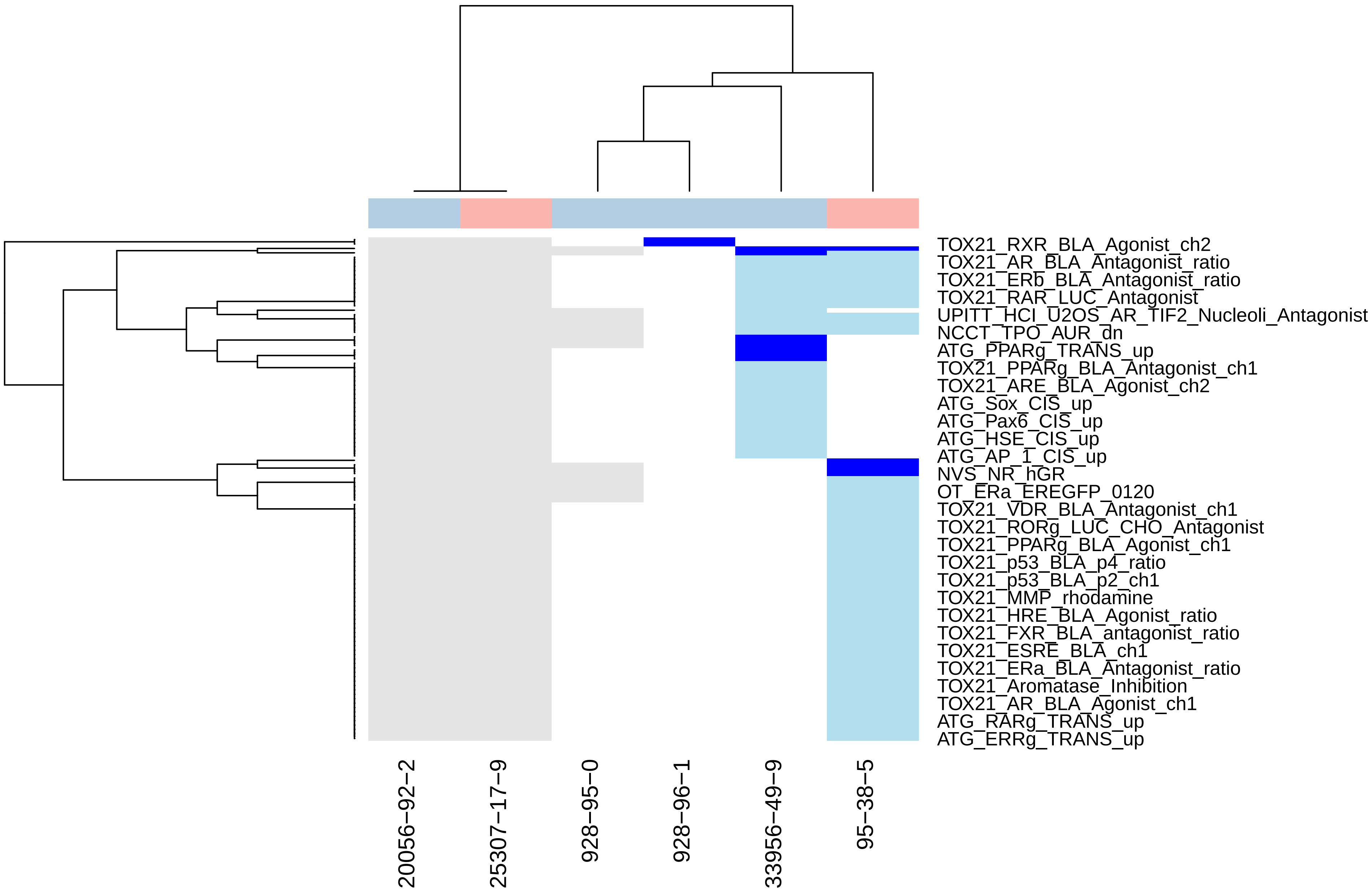

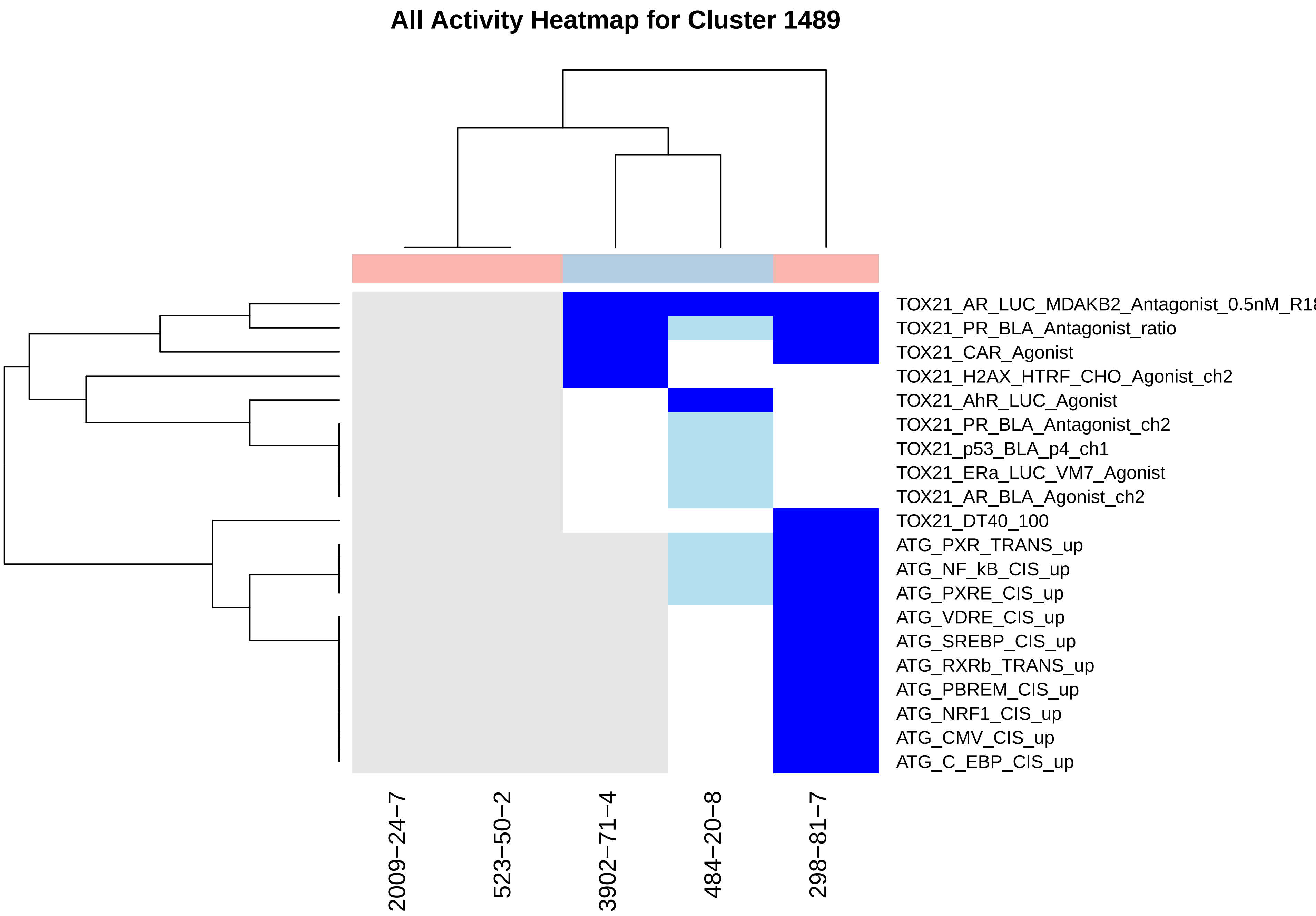

All Activity Heatmap for Cluster 1489
TOX21_AR_LUC_MDAKB2_Antagonist_0.5nM_R18
TOX21_PR_BLA_Antagonist_ratio
TOX21_CAR_Agonist
TOX21_H2AX_HTRF_CHO_Agonist_ch2
TOX21_AhR_LUC_Agonist
TOX21_PR_BLA_Antagonist_ch2
TOX21_p53_BLA_p4_ch1
TOX21_ERa_LUC_VM7_Agonist
TOX21_AR_BLA_Agonist_ch2
TOX21_DT40_100
ATG_PXR_TRANS_up
ATG_NF_kB_CIS_up
ATG_PXRE_CIS_up
ATG_VDRE_CIS_up
ATG_SREBP_CIS_up
ATG_RXRb_TRANS_up
ATG_PBREM_CIS_up
ATG_NRF1_CIS_up
ATG_CMV_CIS_up
ATG_C_EBP_CIS_up
2009−24−7
523−50−2
3902−71−4
484−20−8
298−81−7

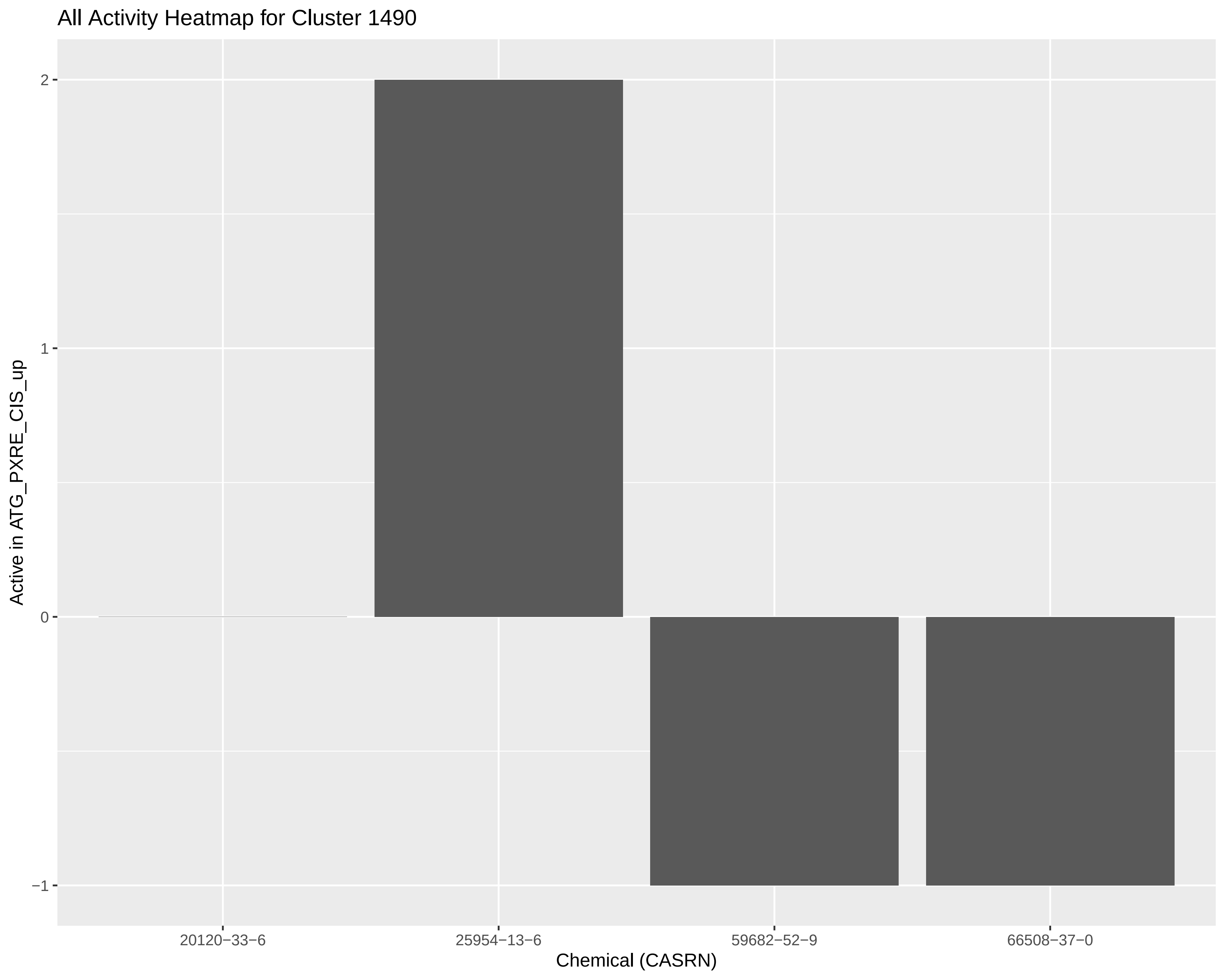
All Activity Heatmap for Cluster 1490
Active in ATG_PXRE_CIS_up
2
1
0
−1
20120−33−6
25954−13−6
59682−52−9
66508−37−0
Chemical (CASRN)

All Activity Heatmap for Cluster 1491

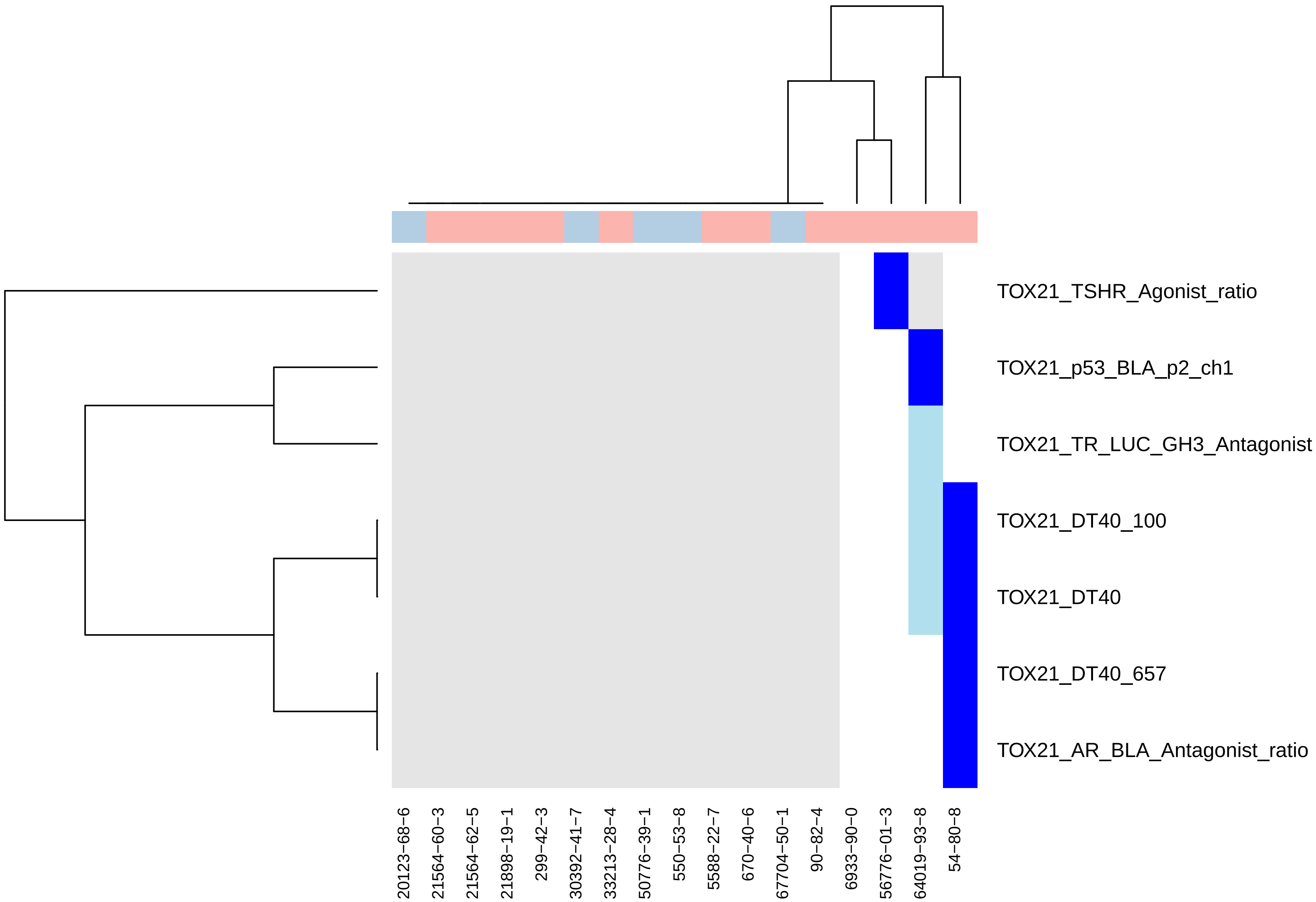

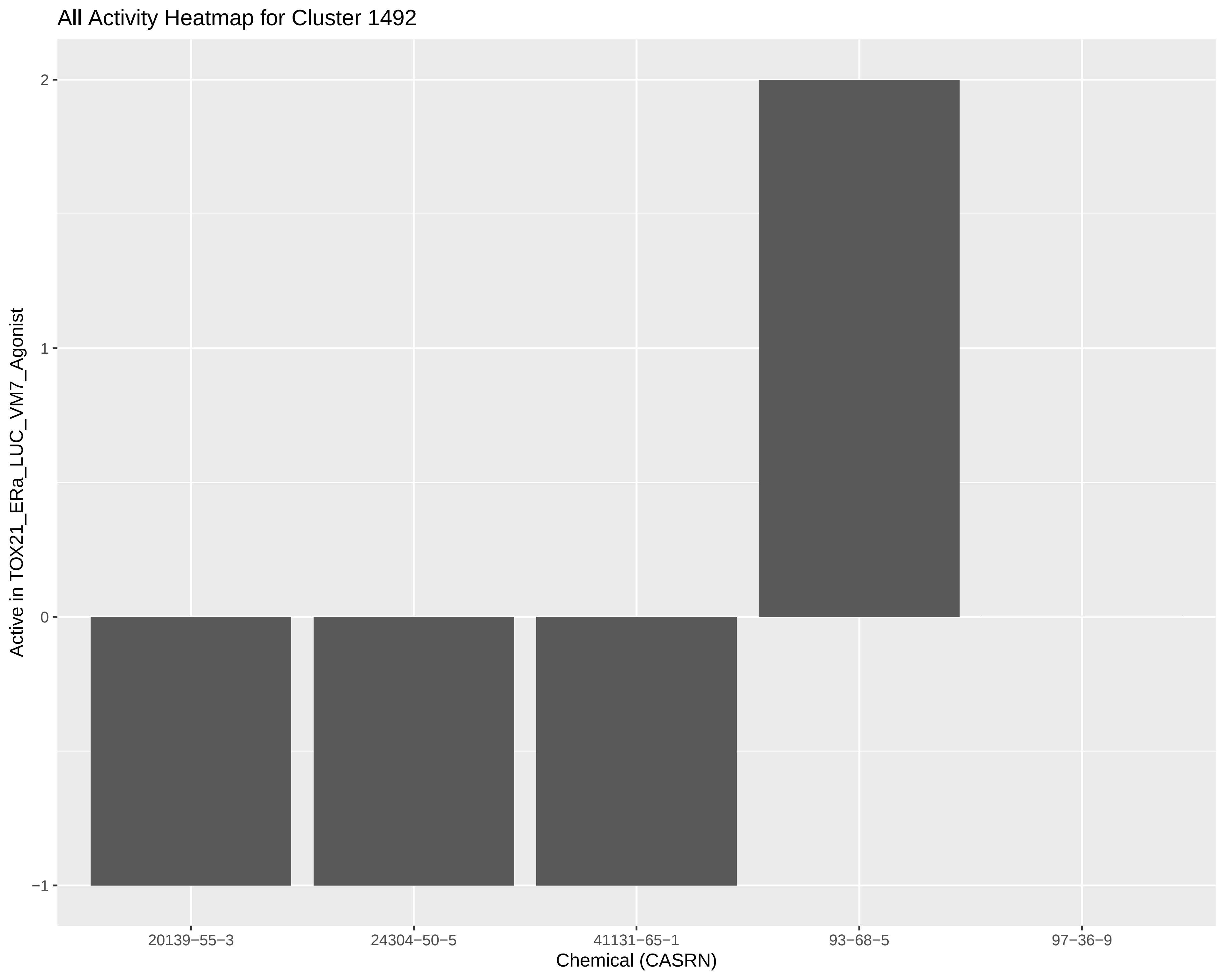
All Activity Heatmap for Cluster 1492
Active in TOX21_ERa_LUC_VM7_Agonist
2
1
0
−1
20139−55−3
24304−50−5
41131−65−1
93−68−5
97−36−9
Chemical (CASRN)

All Activity Heatmap for Cluster 1493

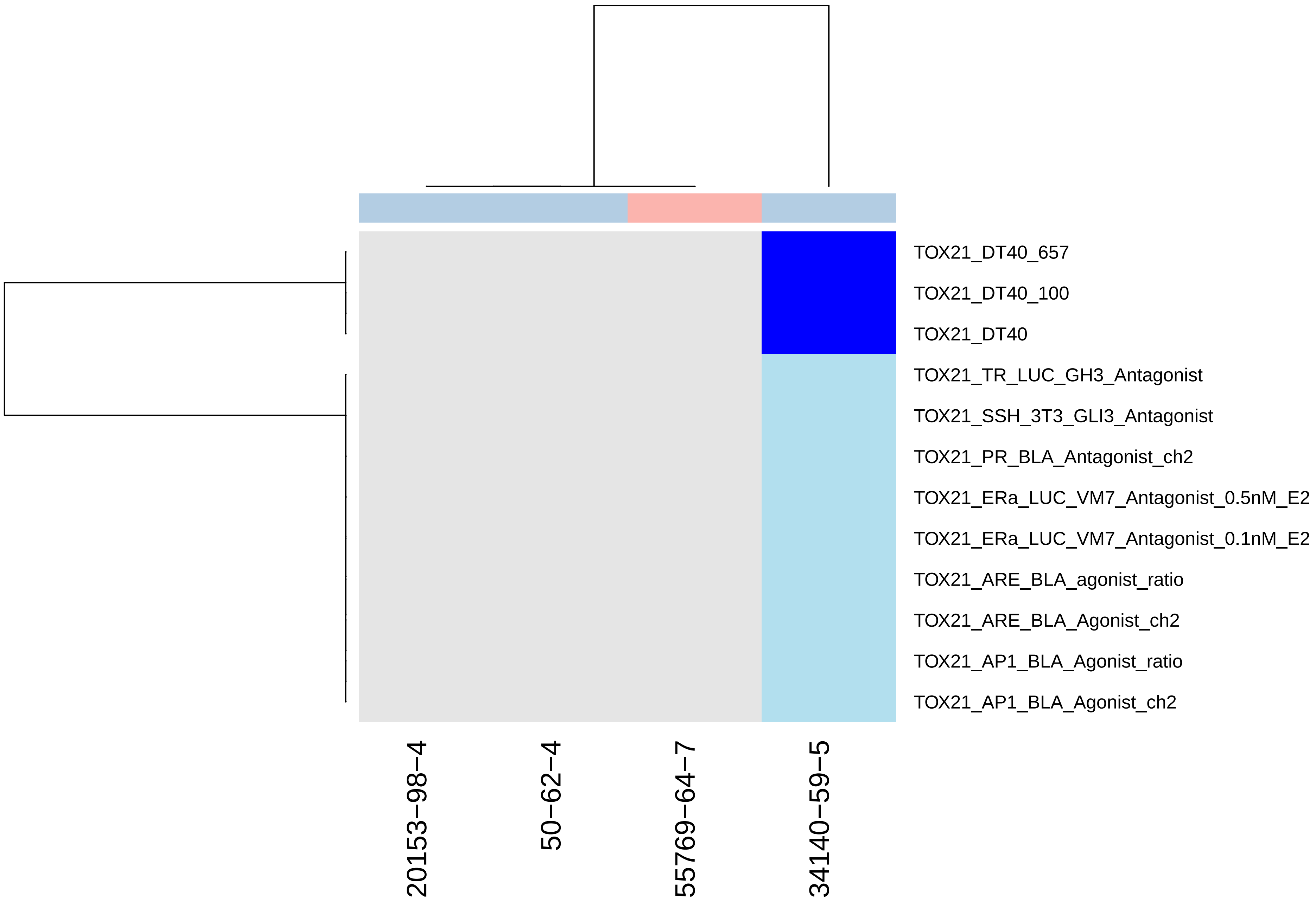

All Activity Heatmap for Cluster 1494

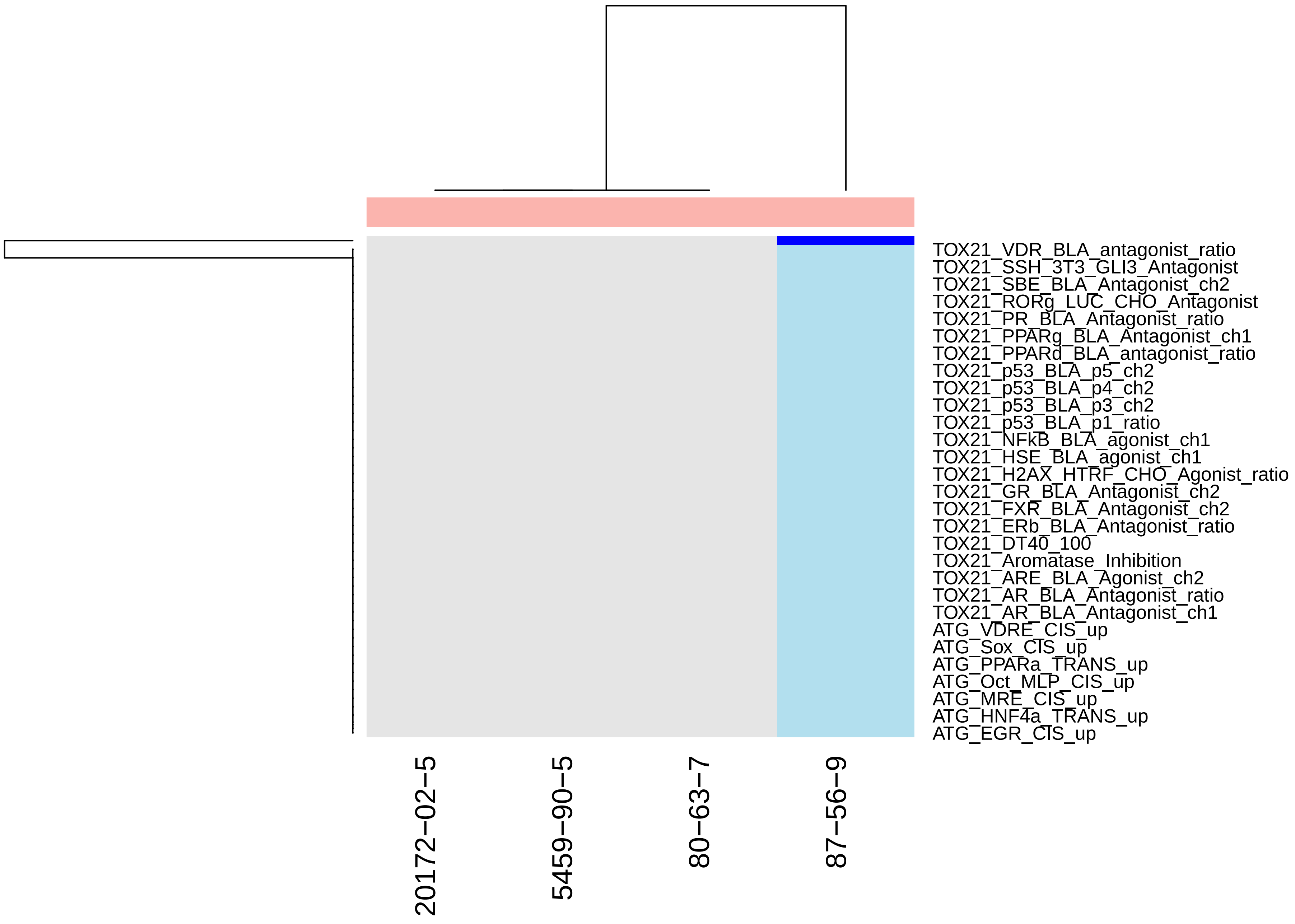

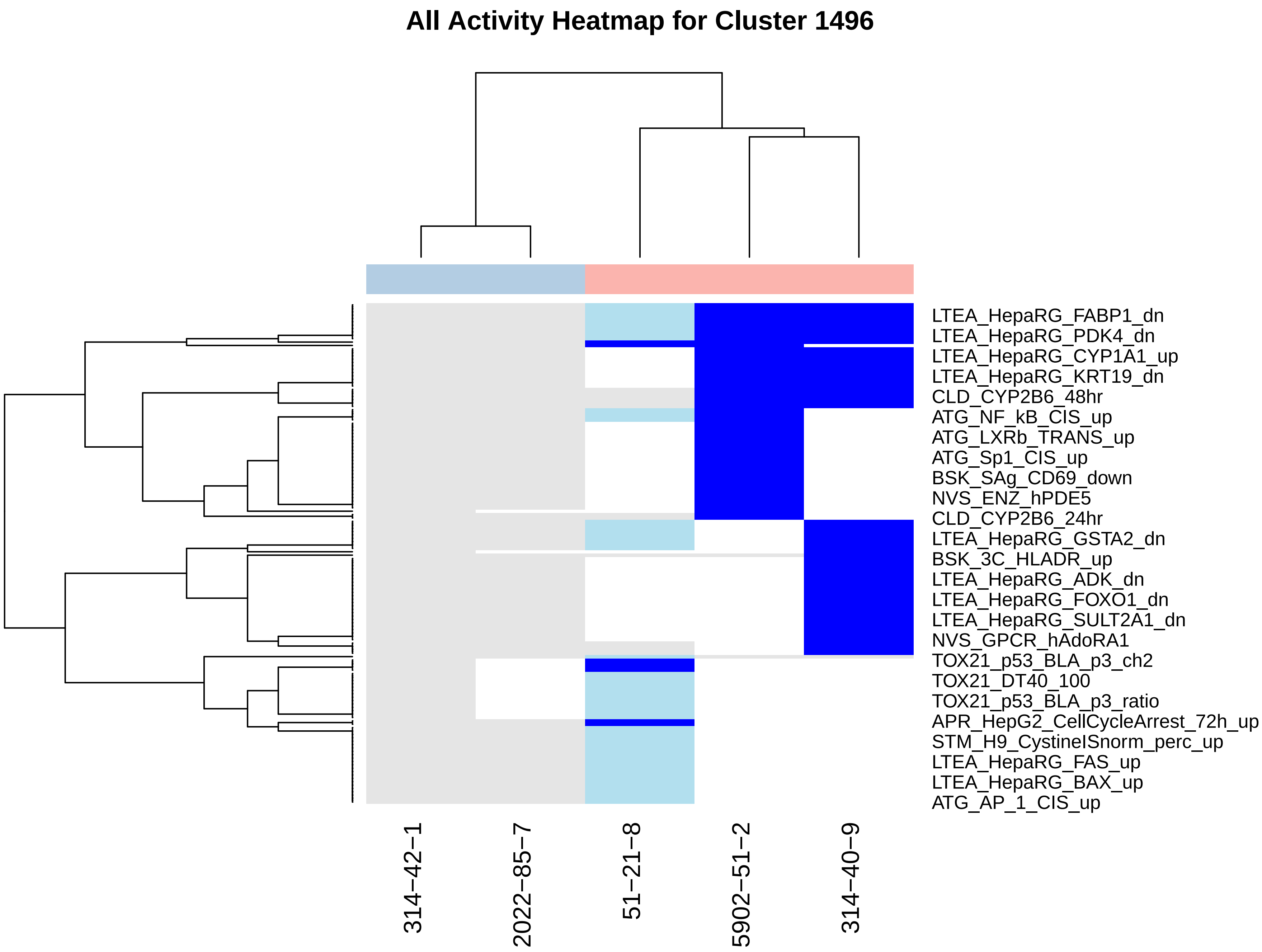
All Activity Heatmap for Cluster 1496
LTEA_HepaRG_FABP1_dn
LTEA_HepaRG_PDK4_dn
LTEA_HepaRG_CYP1A1_up
LTEA_HepaRG_KRT19_dn
CLD_CYP2B6_48hr
ATG_NF_kB_CIS_up
ATG_LXRb_TRANS_up
ATG_Sp1_CIS_up
BSK_SAg_CD69_down
NVS_ENZ_hPDE5
CLD_CYP2B6_24hr
LTEA_HepaRG_GSTA2_dn
BSK_3C_HLADR_up
LTEA_HepaRG_ADK_dn
LTEA_HepaRG_FOXO1_dn
LTEA_HepaRG_SULT2A1_dn
NVS_GPCR_hAdoRA1
TOX21_p53_BLA_p3_ch2
TOX21_DT40_100
TOX21_p53_BLA_p3_ratio
APR_HepG2_CellCycleArrest_72h_up
STM_H9_CystineISnorm_perc_up
LTEA_HepaRG_FAS_up
LTEA_HepaRG_BAX_up
ATG_AP_1_CIS_up
314−42−1
2022−85−7
51−21−8
5902−51−2
314−40−9

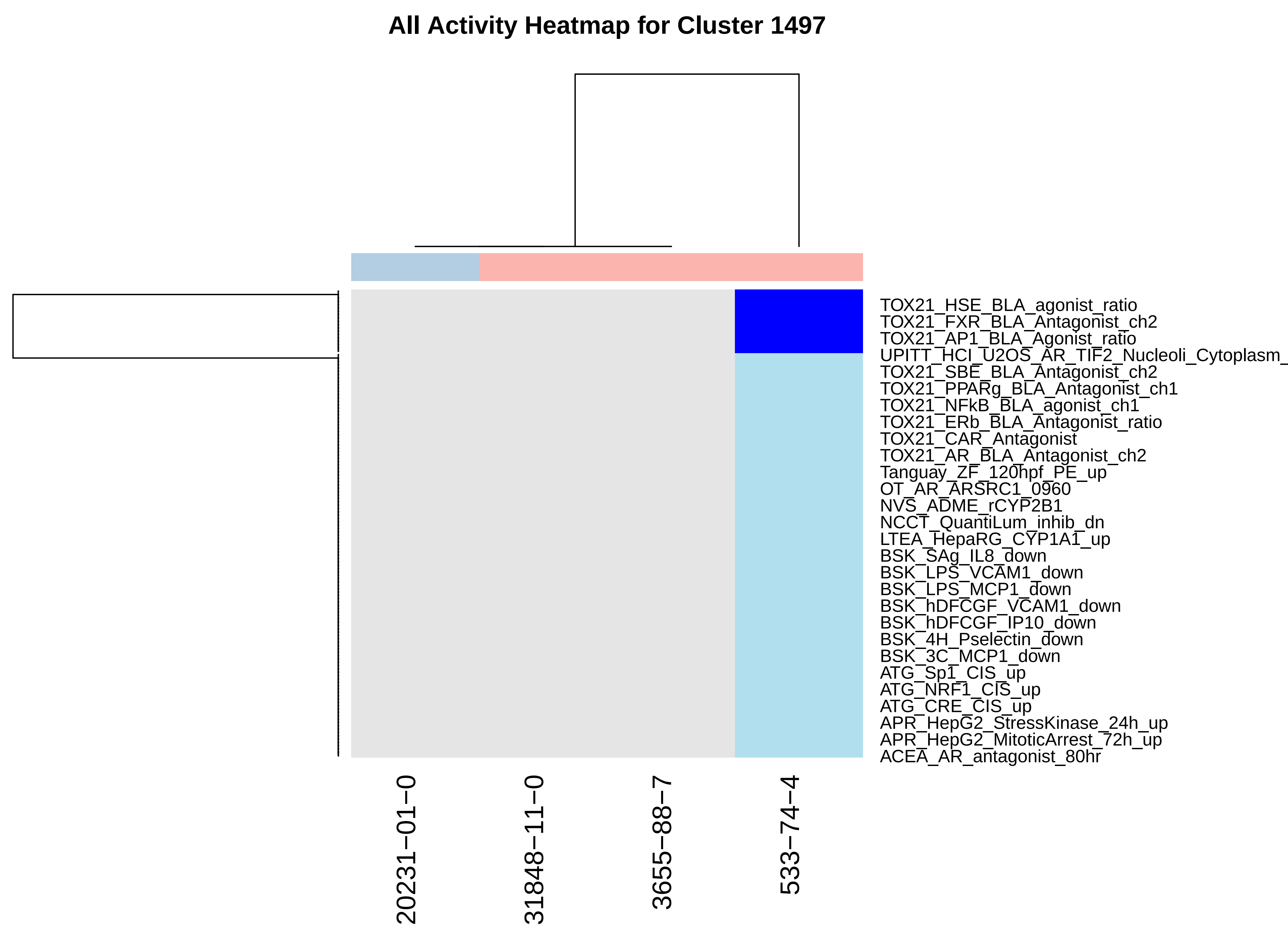

All Activity Heatmap for Cluster 1497
TOX21_HSE_BLA_agonist_ratio
TOX21_FXR_BLA_Antagonist_ch2
TOX21_AP1_BLA_Agonist_ratio
UPITT_HCI_U2OS_AR_TIF2_Nucleoli_Cytoplasm_
TOX21_SBE_BLA_Antagonist_ch2
TOX21_PPARg_BLA_Antagonist_ch1
TOX21_NFkB_BLA_agonist_ch1
TOX21_ERb_BLA_Antagonist_ratio
TOX21_CAR_Antagonist
TOX21_AR_BLA_Antagonist_ch2
Tanguay_ZF_120hpf_PE_up
OT_AR_ARSRC1_0960
NVS_ADME_rCYP2B1
NCCT_QuantiLum_inhib_dn
LTEA_HepaRG_CYP1A1_up
BSK_SAg_IL8_down
BSK_LPS_VCAM1_down
BSK_LPS_MCP1_down
BSK_hDFCGF_VCAM1_down
BSK_hDFCGF_IP10_down
BSK_4H_Pselectin_down
BSK_3C_MCP1_down
ATG_Sp1_CIS_up
ATG_NRF1_CIS_up
ATG_CRE_CIS_up
APR_HepG2_StressKinase_24h_up
APR_HepG2_MitoticArrest_72h_up
ACEA_AR_antagonist_80hr
20231−01−0
31848−11−0
3655−88−7
533−74−4

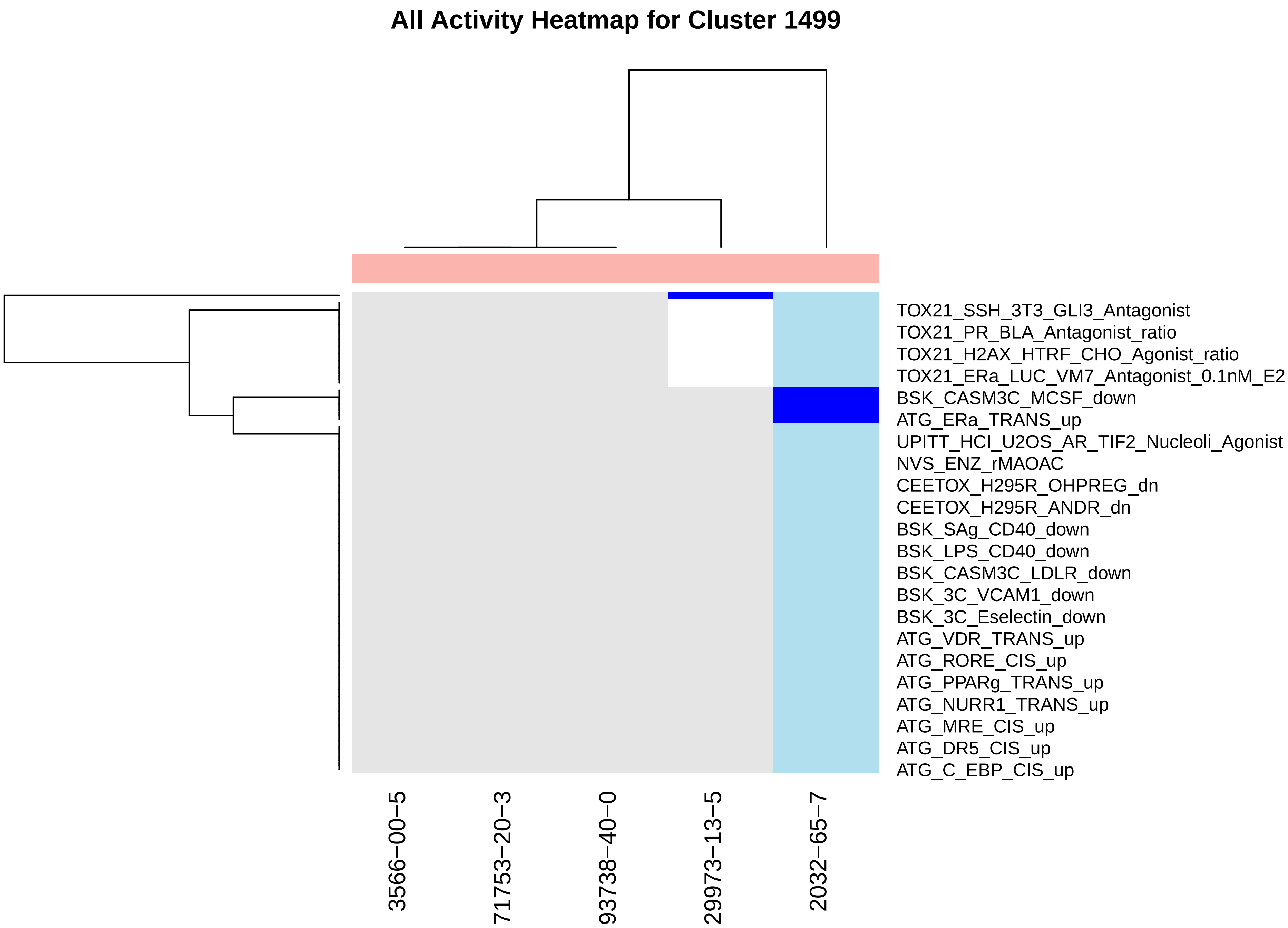
All Activity Heatmap for Cluster 1499
TOX21_SSH_3T3_GLI3_Antagonist
TOX21_PR_BLA_Antagonist_ratio
TOX21_H2AX_HTRF_CHO_Agonist_ratio
TOX21_ERa_LUC_VM7_Antagonist_0.1nM_E2
BSK_CASM3C_MCSF_down
ATG_ERa_TRANS_up
UPITT_HCI_U2OS_AR_TIF2_Nucleoli_Agonist
NVS_ENZ_rMAOAC
CEETOX_H295R_OHPREG_dn
CEETOX_H295R_ANDR_dn
BSK_SAg_CD40_down
BSK_LPS_CD40_down
BSK_CASM3C_LDLR_down
BSK_3C_VCAM1_down
BSK_3C_Eselectin_down
ATG_VDR_TRANS_up
ATG_RORE_CIS_up
ATG_PPARg_TRANS_up
ATG_NURR1_TRANS_up
ATG_MRE_CIS_up
ATG_DR5_CIS_up
ATG_C_EBP_CIS_up
3566−00−5
71753−20−3
93738−40−0
29973−13−5
2032−65−7

All Activity Heatmap for Cluster 1500

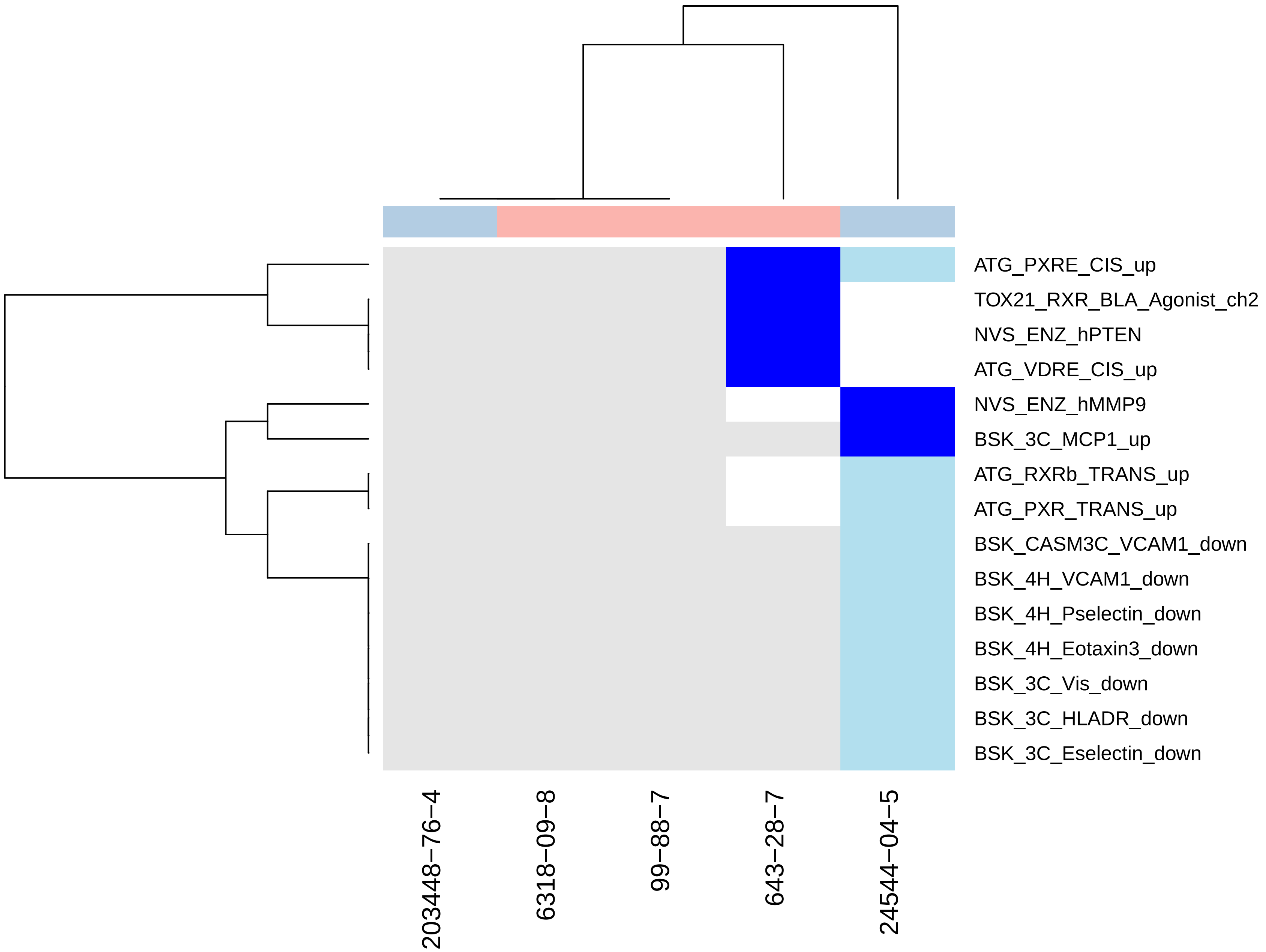

All Activity Heatmap for Cluster 1501

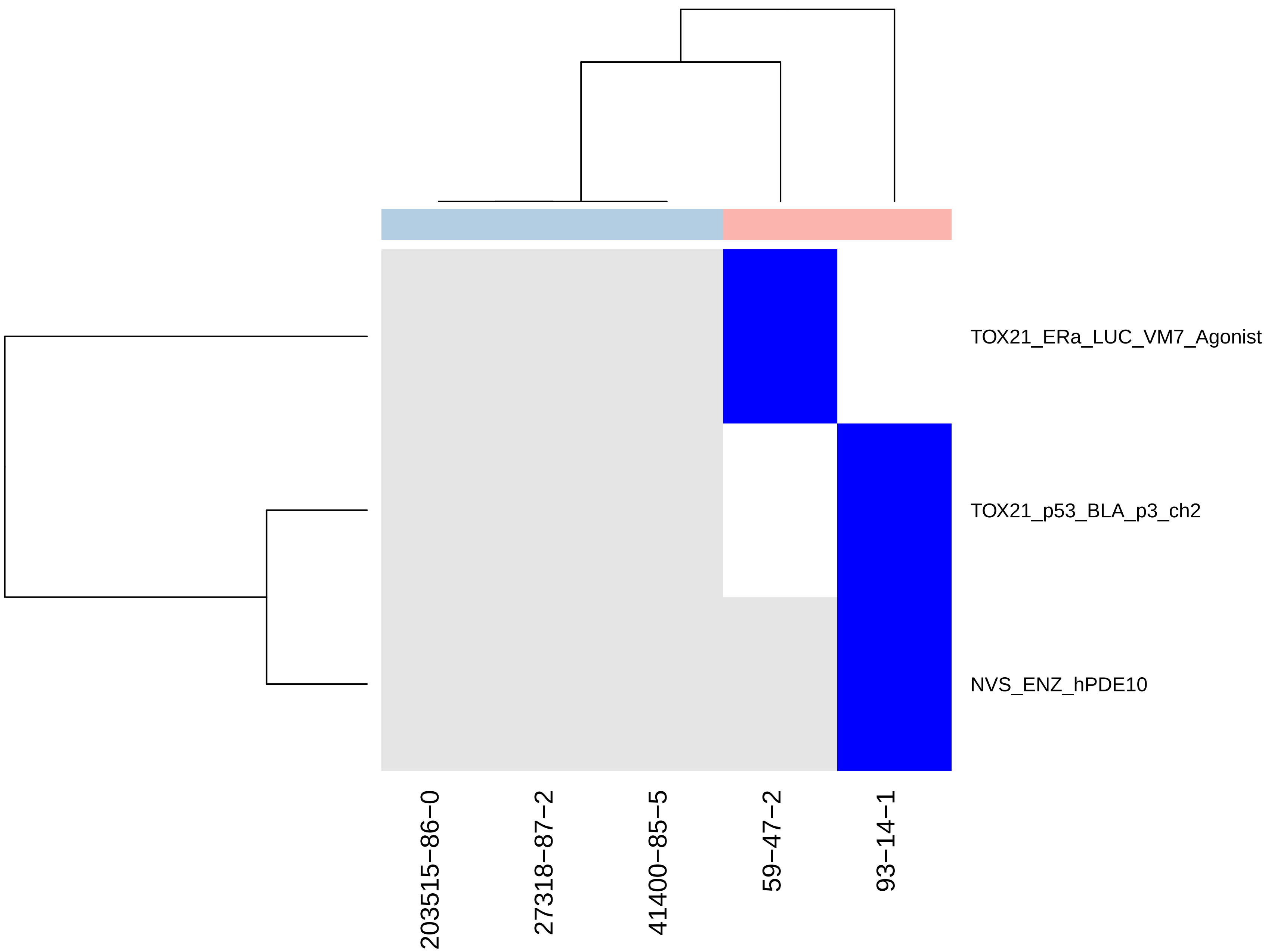

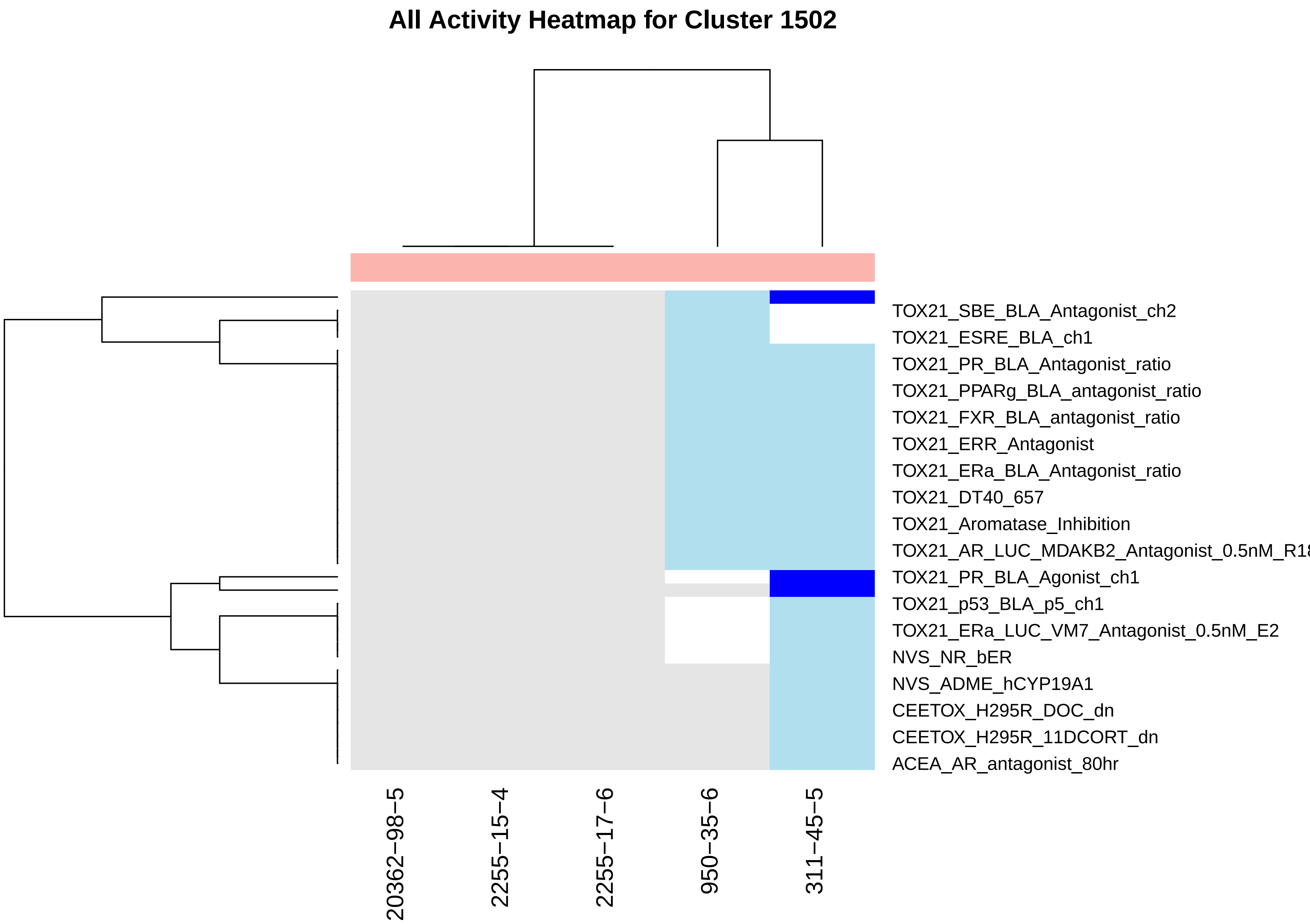
All Activity Heatmap for Cluster 1502
TOX21_SBE_BLA_Antagonist_ch2
TOX21_ESRE_BLA_ch1
TOX21_PR_BLA_Antagonist_ratio
TOX21_PPARg_BLA_antagonist_ratio
TOX21_FXR_BLA_antagonist_ratio
TOX21_ERR_Antagonist
TOX21_ERa_BLA_Antagonist_ratio
TOX21_DT40_657
TOX21_Aromatase_Inhibition
TOX21_AR_LUC_MDAKB2_Antagonist_0.5nM_R18
TOX21_PR_BLA_Agonist_ch1
TOX21_p53_BLA_p5_ch1
TOX21_ERa_LUC_VM7_Antagonist_0.5nM_E2
NVS_NR_bER
NVS_ADME_hCYP19A1
CEETOX_H295R_DOC_dn
CEETOX_H295R_11DCORT_dn
ACEA_AR_antagonist_80hr
20362−98−5
2255−15−4
2255−17−6
950−35−6
311−45−5

All Activity Heatmap for Cluster 1504

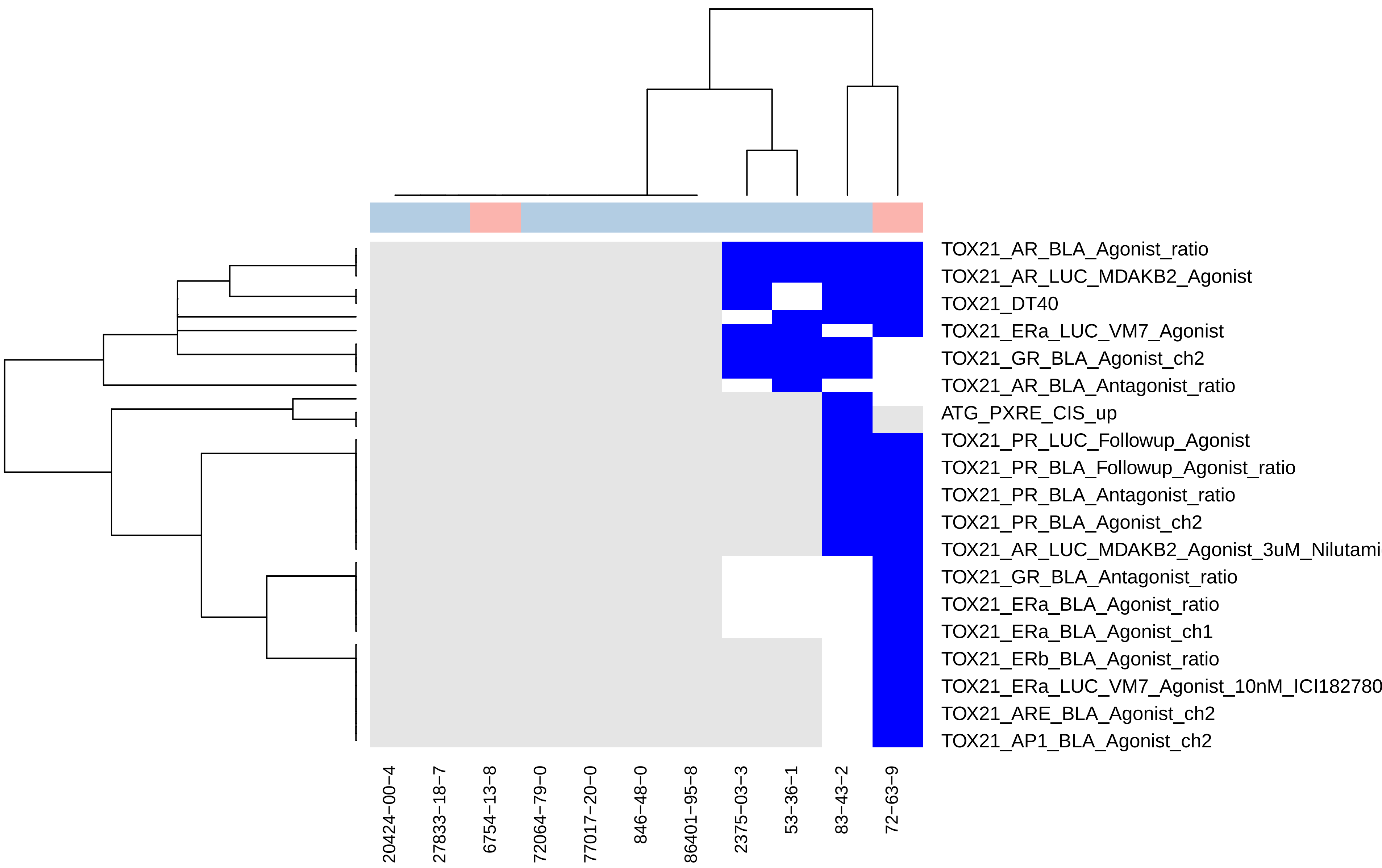

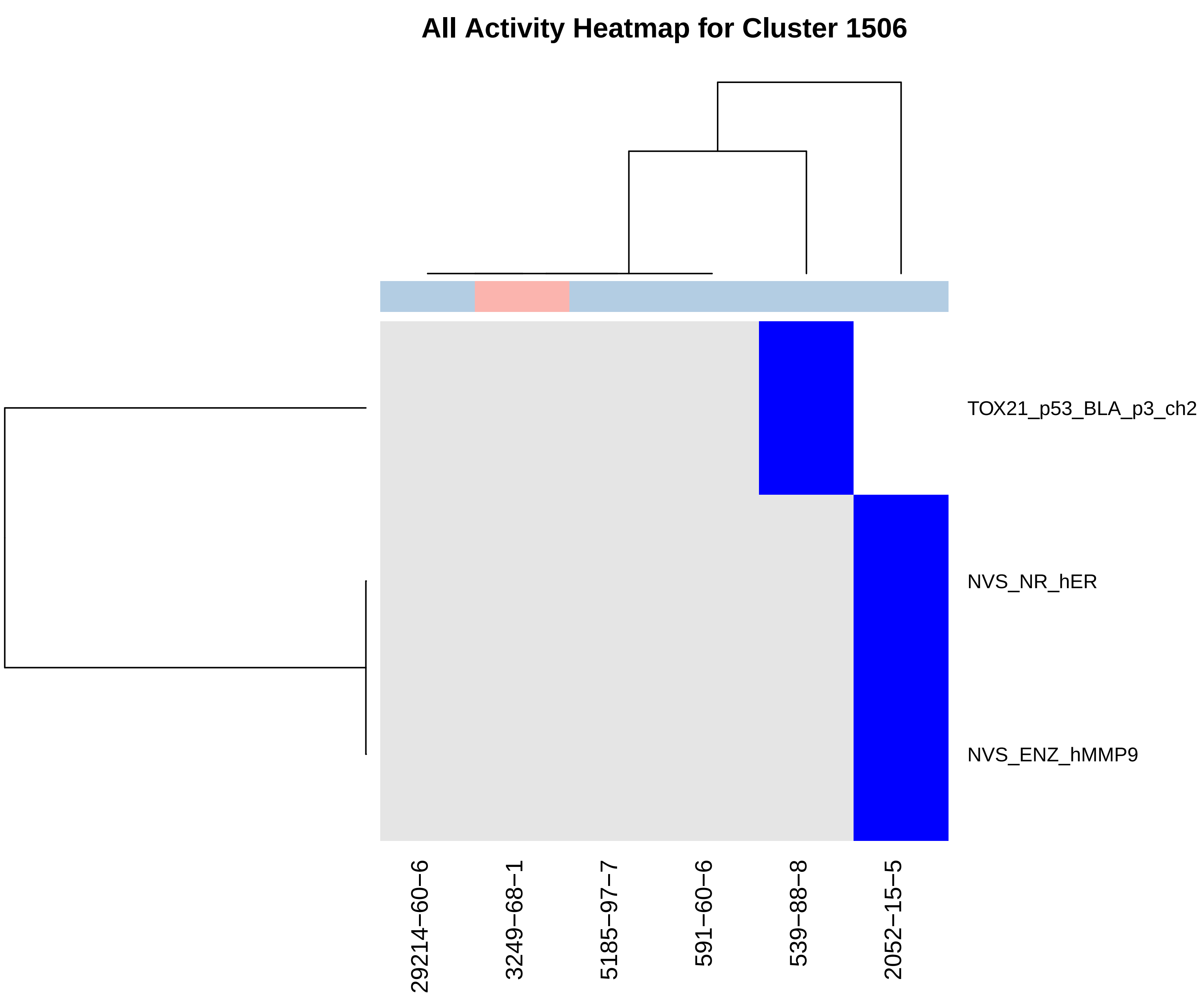
All Activity Heatmap for Cluster 1506
TOX21_p53_BLA_p3_ch2
NVS_NR_hER
NVS_ENZ_hMMP9
29214−60−6
3249−68−1
5185−97−7
591−60−6
539−88−8
2052−15−5

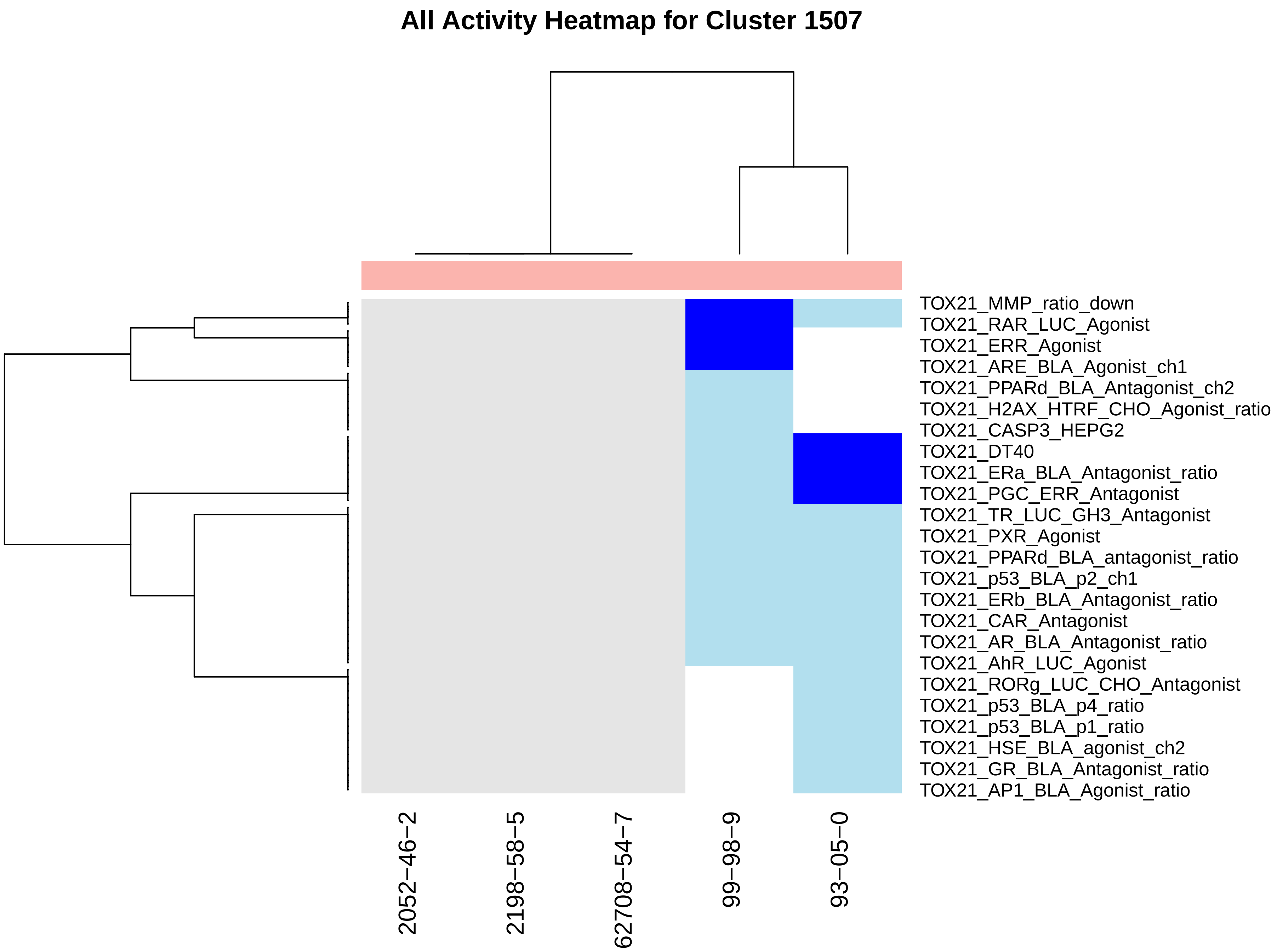
All Activity Heatmap for Cluster 1507
TOX21_MMP_ratio_down
TOX21_RAR_LUC_Agonist
TOX21_ERR_Agonist
TOX21_ARE_BLA_Agonist_ch1
TOX21_PPARd_BLA_Antagonist_ch2
TOX21_H2AX_HTRF_CHO_Agonist_ratio
TOX21_CASP3_HEPG2
TOX21_DT40
TOX21_ERa_BLA_Antagonist_ratio
TOX21_PGC_ERR_Antagonist
TOX21_TR_LUC_GH3_Antagonist
TOX21_PXR_Agonist
TOX21_PPARd_BLA_antagonist_ratio
TOX21_p53_BLA_p2_ch1
TOX21_ERb_BLA_Antagonist_ratio
TOX21_CAR_Antagonist
TOX21_AR_BLA_Antagonist_ratio
TOX21_AhR_LUC_Agonist
TOX21_RORg_LUC_CHO_Antagonist
TOX21_p53_BLA_p4_ratio
TOX21_p53_BLA_p1_ratio
TOX21_HSE_BLA_agonist_ch2
TOX21_GR_BLA_Antagonist_ratio
TOX21_AP1_BLA_Agonist_ratio
2052−46−2
2198−58−5
62708−54−7
99−98−9
93−05−0

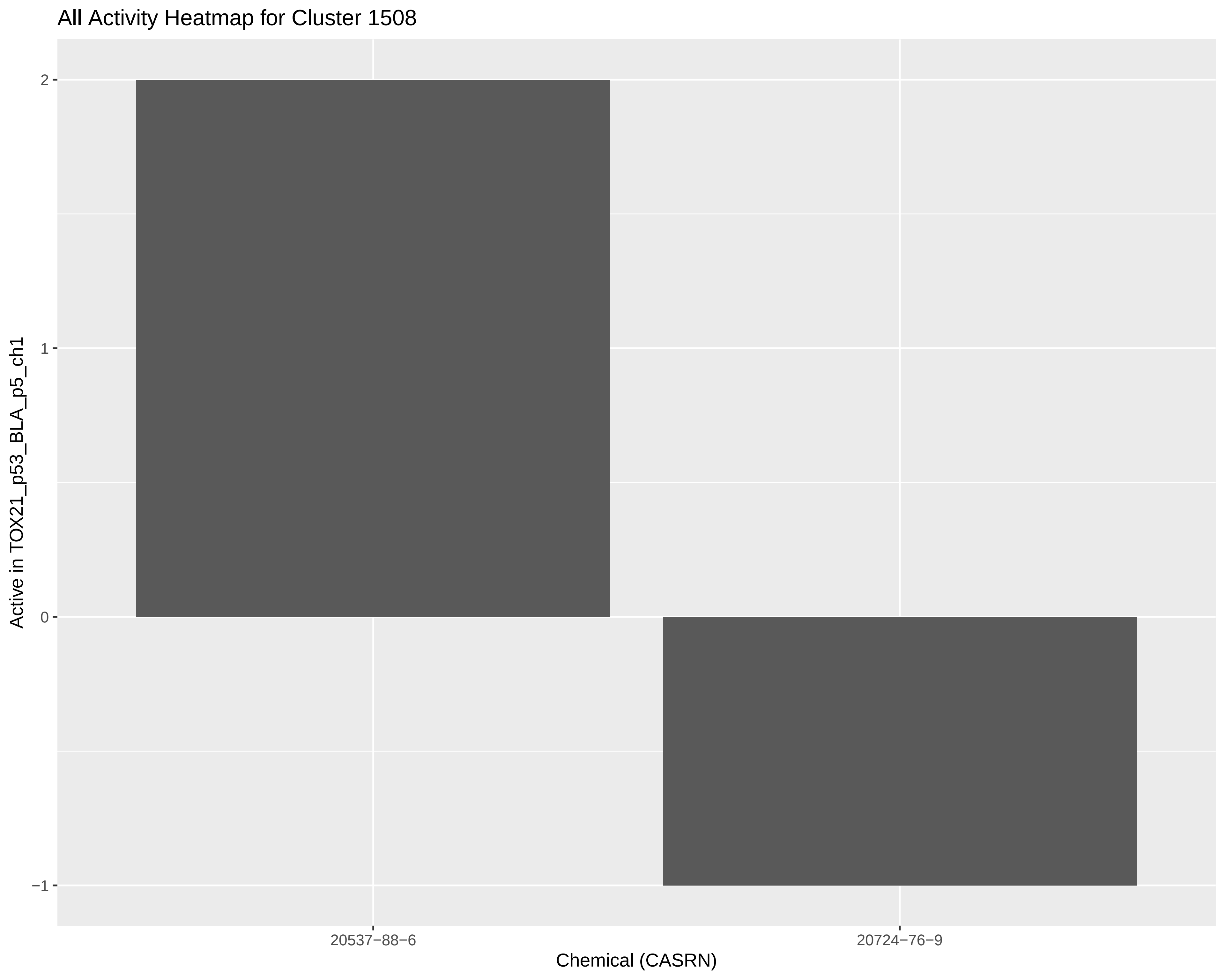
All Activity Heatmap for Cluster 1508
Active in TOX21_p53_BLA_p5_ch1
2
1
0
−1
20537−88−6
20724−76−9
Chemical (CASRN)

All Activity Heatmap for Cluster 1510

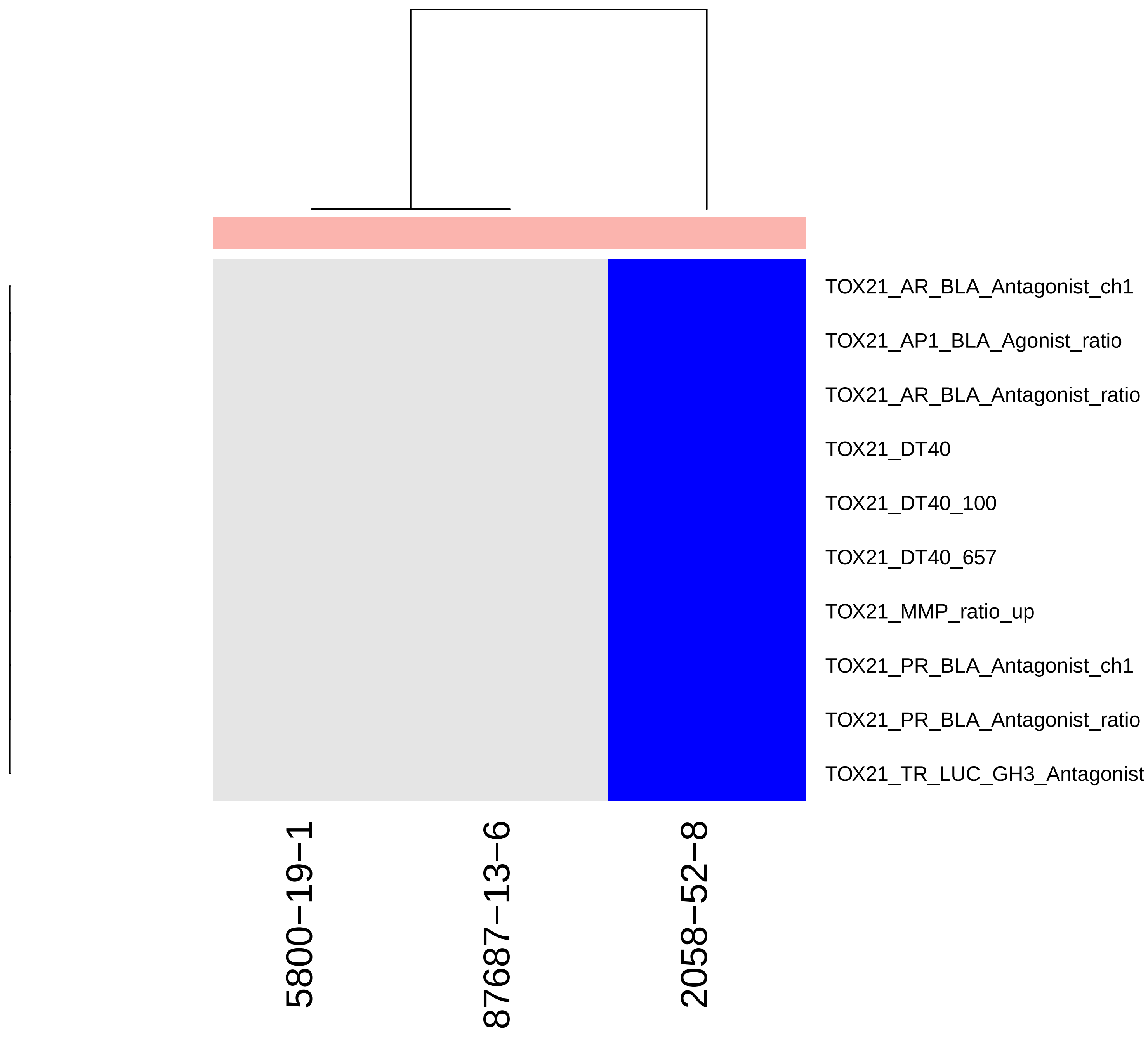

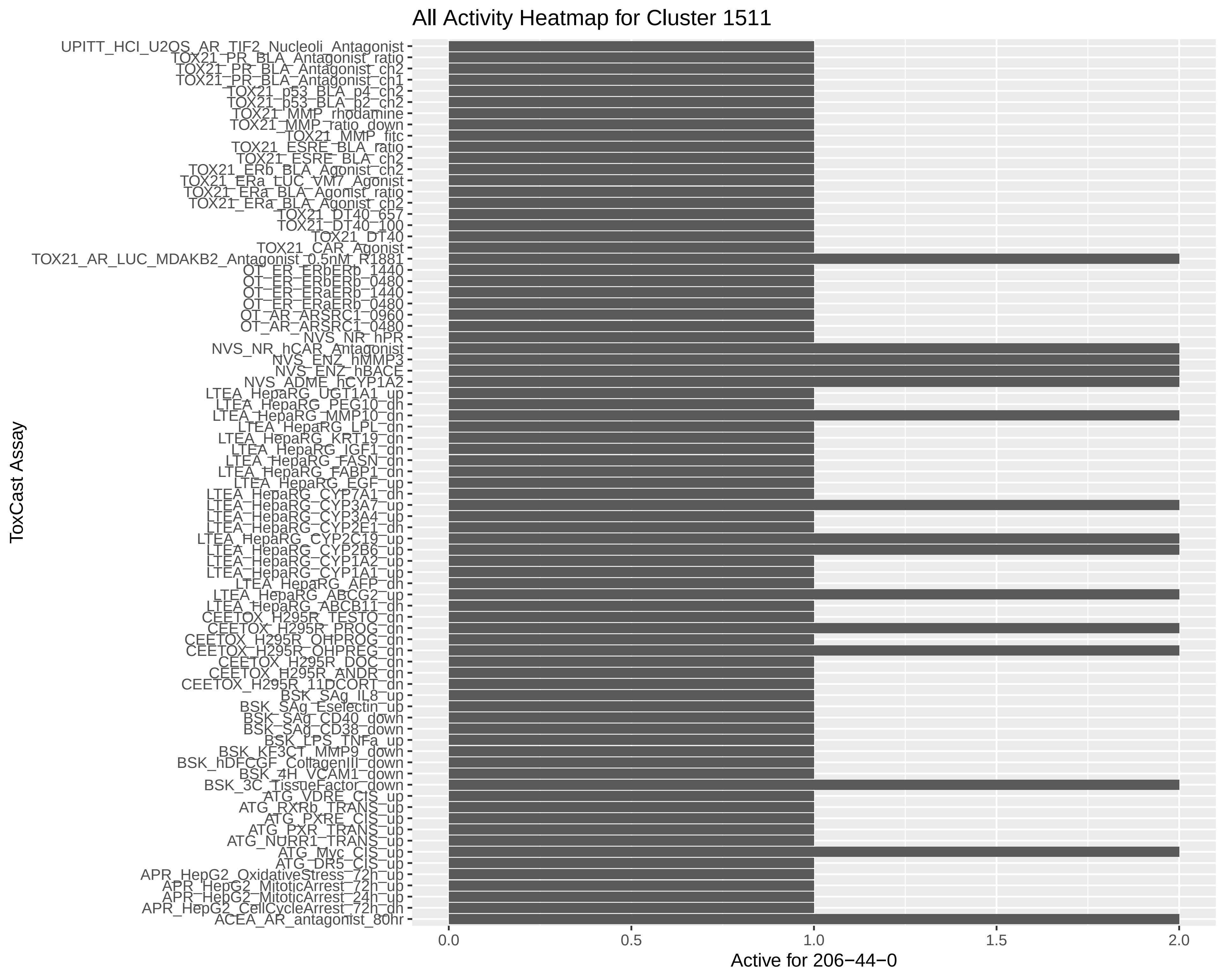
All Activity Heatmap for Cluster 1511
ToxCast Assay
Active for 206−44−0
UPITT_HCI_U2OS_AR_TIF2_Nucleoli_Antagonist
TOX21_PR_BLA_Antagonist_ratio
TOX21_PR_BLA_Antagonist_ch2
TOX21_PR_BLA_Antagonist_ch1
TOX21_p53_BLA_p4_ch2
TOX21_p53_BLA_p2_ch2
TOX21_MMP_rhodamine
TOX21_MMP_ratio_down
TOX21_MMP_fitc
TOX21_ESRE_BLA_ratio
TOX21_ESRE_BLA_ch2
TOX21_ERb_BLA_Agonist_ch2
TOX21_ERa_LUC_VM7_Agonist
TOX21_ERa_BLA_Agonist_ratio
TOX21_ERa_BLA_Agonist_ch2
TOX21_DT40_657
TOX21_DT40_100
TOX21_DT40
TOX21_CAR_Agonist
TOX21_AR_LUC_MDAKB2_Antagonist_0.5nM_R1881
OT_ER_ERbERb_1440
OT_ER_ERbERb_0480
OT_ER_ERaERb_1440
OT_ER_ERaERb_0480
OT_AR_ARSRC1_0960
OT_AR_ARSRC1_0480
NVS_NR_hPR
NVS_NR_hCAR_Antagonist
NVS_ENZ_hMMP3
NVS_ENZ_hBACE
NVS_ADME_hCYP1A2
LTEA_HepaRG_UGT1A1_up
LTEA_HepaRG_PEG10_dn
LTEA_HepaRG_MMP10_dn
LTEA_HepaRG_LPL_dn
LTEA_HepaRG_KRT19_dn
LTEA_HepaRG_IGF1_dn
LTEA_HepaRG_FASN_dn
LTEA_HepaRG_FABP1_dn
LTEA_HepaRG_EGF_up
LTEA_HepaRG_CYP7A1_dn
LTEA_HepaRG_CYP3A7_up
LTEA_HepaRG_CYP3A4_up
LTEA_HepaRG_CYP2E1_dn
LTEA_HepaRG_CYP2C19_up
LTEA_HepaRG_CYP2B6_up
LTEA_HepaRG_CYP1A2_up
LTEA_HepaRG_CYP1A1_up
LTEA_HepaRG_AFP_dn
LTEA_HepaRG_ABCG2_up
LTEA_HepaRG_ABCB11_dn
CEETOX_H295R_TESTO_dn
CEETOX_H295R_PROG_dn
CEETOX_H295R_OHPROG_dn
CEETOX_H295R_OHPREG_dn
CEETOX_H295R_DOC_dn
CEETOX_H295R_ANDR_dn
CEETOX_H295R_11DCORT_dn
BSK_SAg_IL8_up
BSK_SAg_Eselectin_up
BSK_SAg_CD40_down
BSK_SAg_CD38_down
BSK_LPS_TNFa_up
BSK_KF3CT_MMP9_down
BSK_hDFCGF_CollagenIII_down
BSK_4H_VCAM1_down
BSK_3C_TissueFactor_down
ATG_VDRE_CIS_up
ATG_RXRb_TRANS_up
ATG_PXRE_CIS_up
ATG_PXR_TRANS_up
ATG_NURR1_TRANS_up
ATG_Myc_CIS_up
ATG_DR5_CIS_up
APR_HepG2_OxidativeStress_72h_up
APR_HepG2_MitoticArrest_72h_up
APR_HepG2_MitoticArrest_24h_up
APR_HepG2_CellCycleArrest_72h_dn
ACEA_AR_antagonist_80hr
0.0
0.5
1.0
1.5
2.0

All Activity Heatmap for Cluster 1512

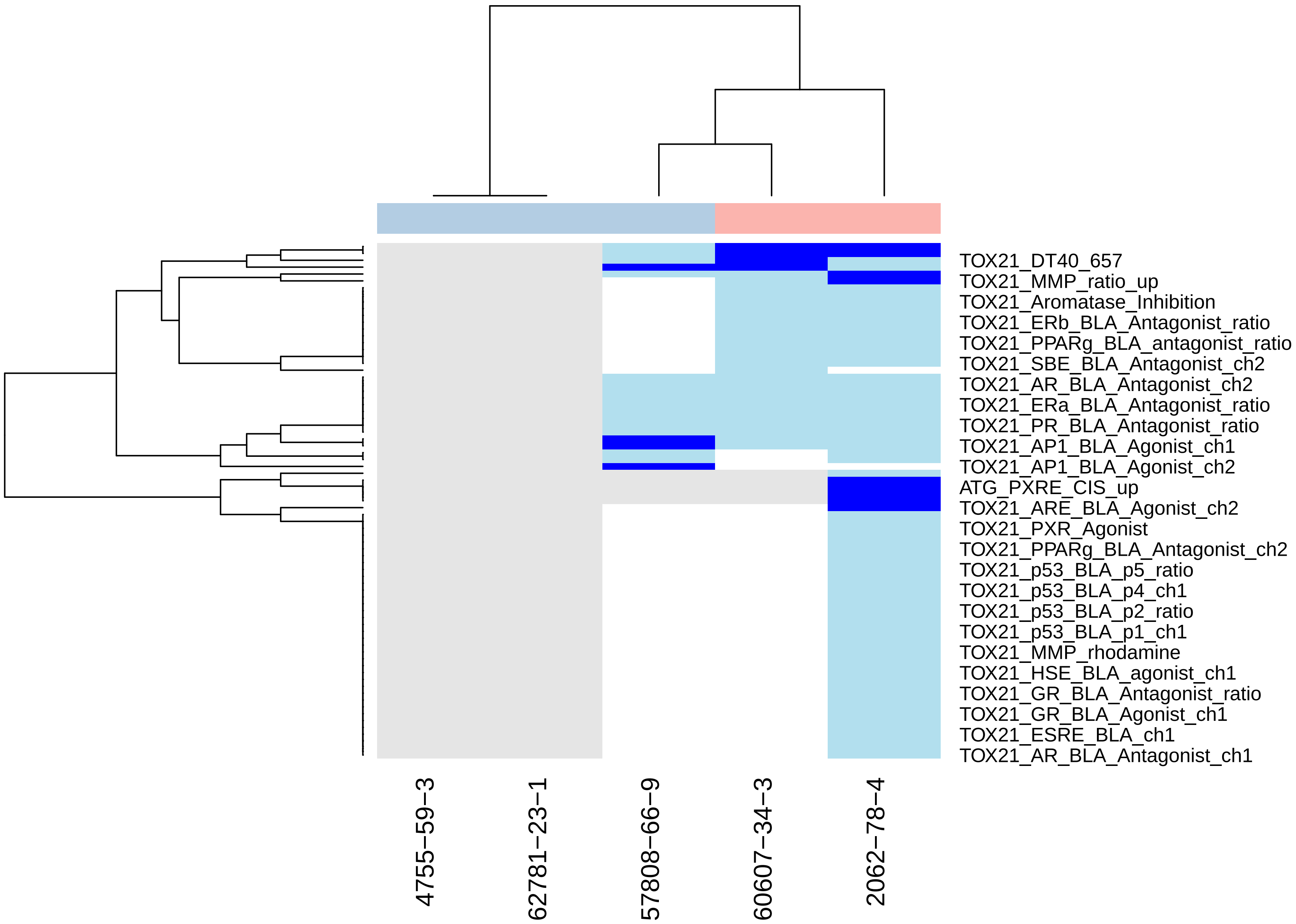

All Activity Heatmap for Cluster 1516

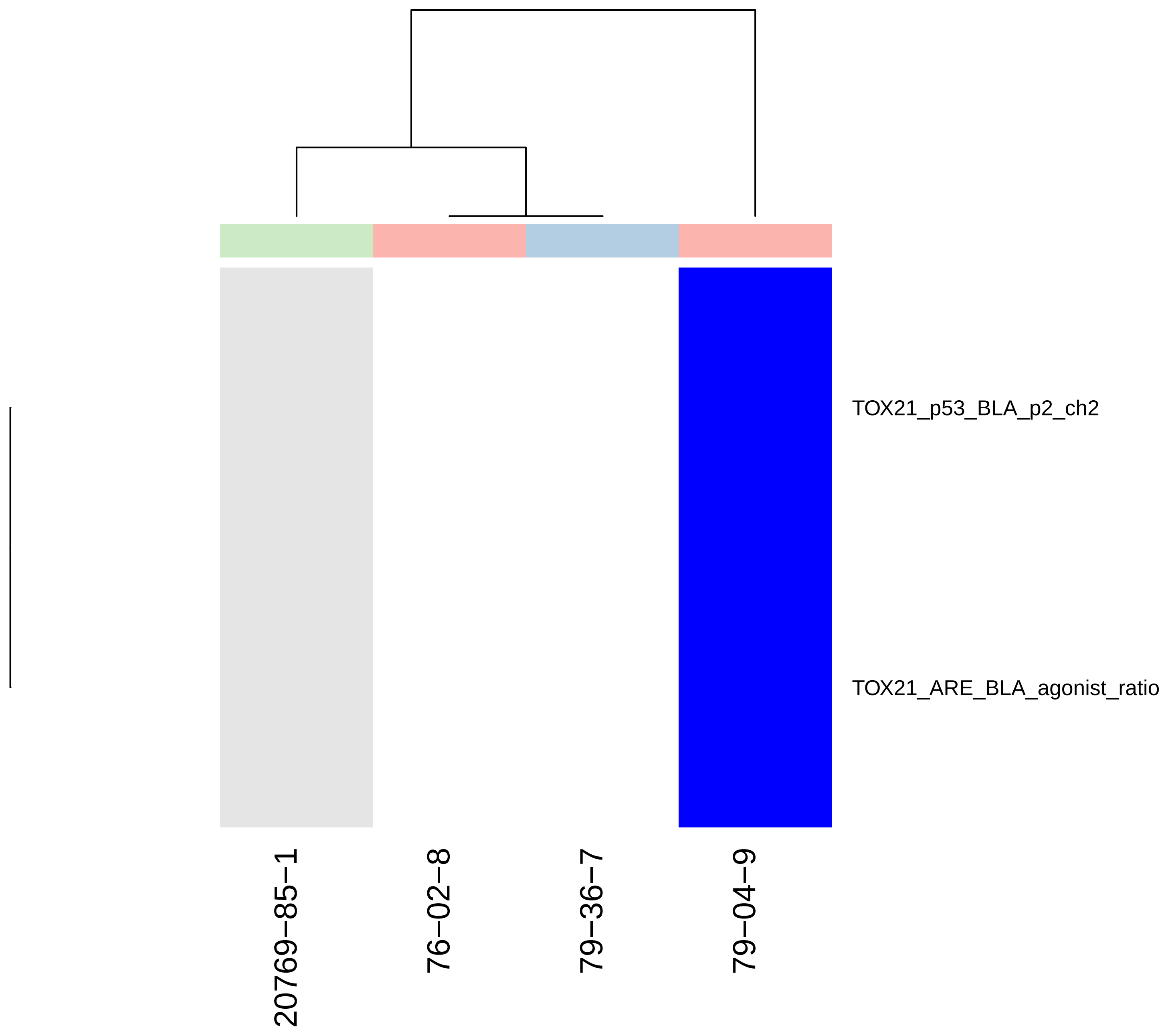

All Activity Heatmap for Cluster 1517

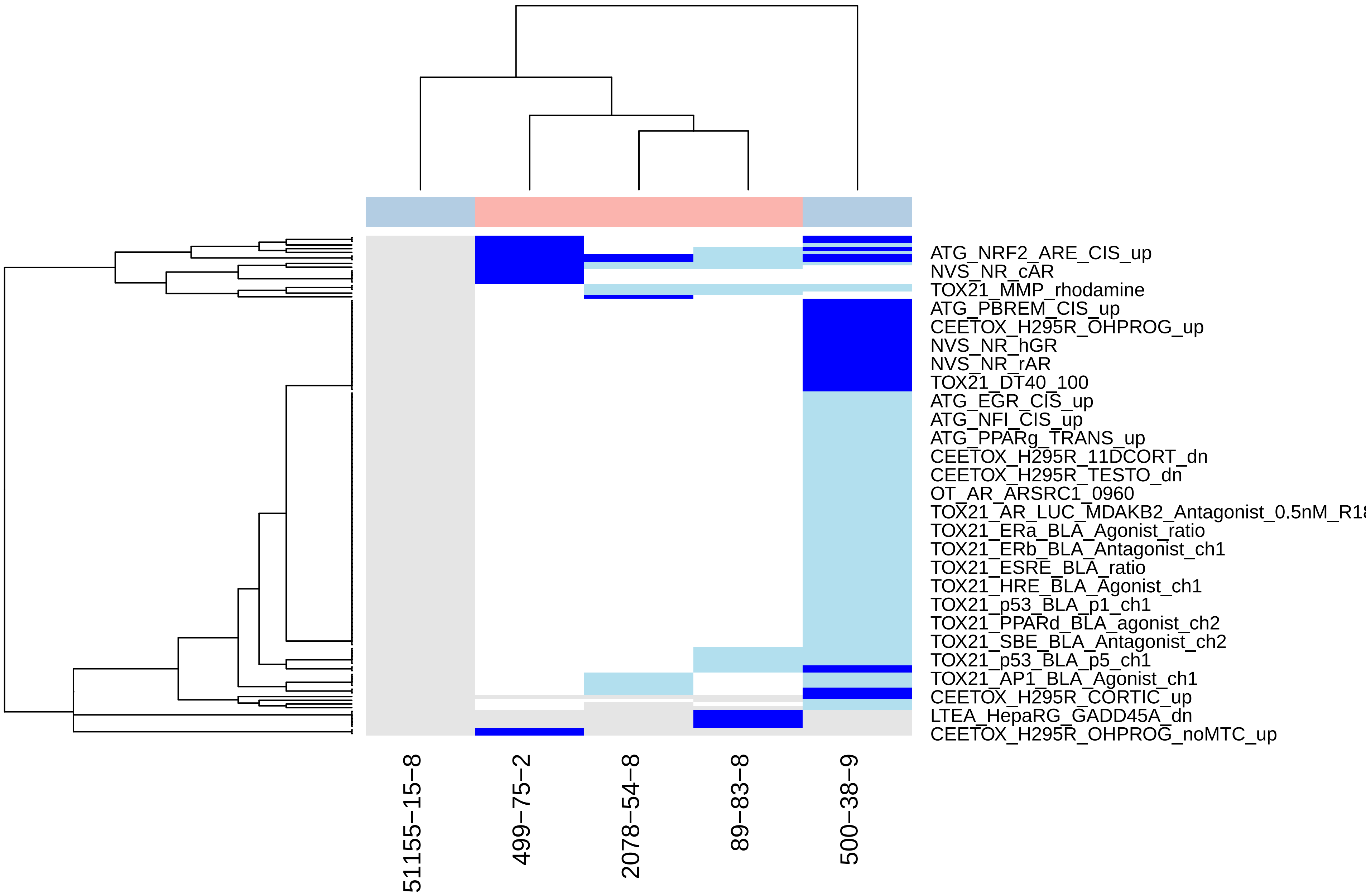

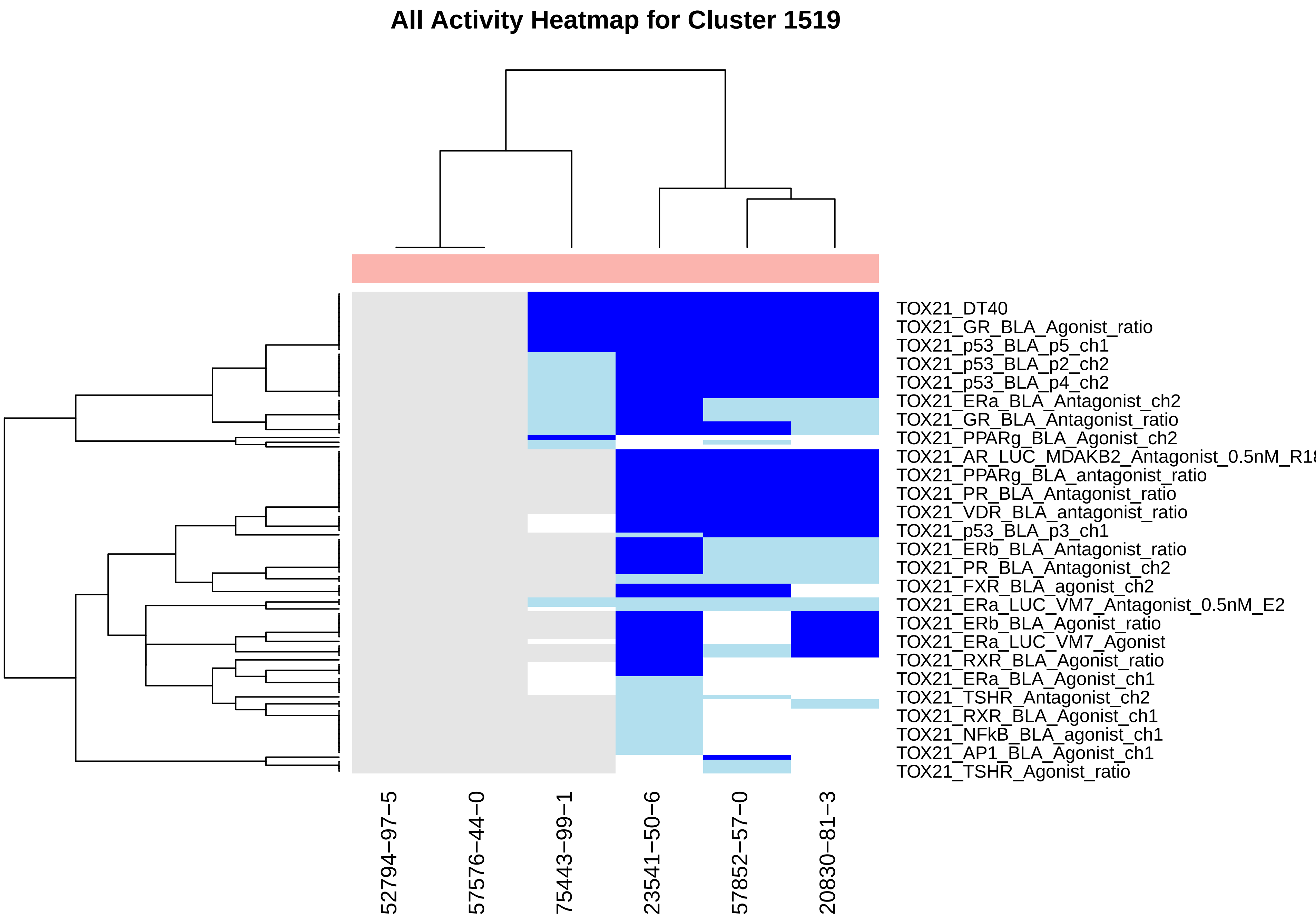

All Activity Heatmap for Cluster 1519
TOX21_DT40
TOX21_GR_BLA_Agonist_ratio
TOX21_p53_BLA_p5_ch1
TOX21_p53_BLA_p2_ch2
TOX21_p53_BLA_p4_ch2
TOX21_ERa_BLA_Antagonist_ch2
TOX21_GR_BLA_Antagonist_ratio
TOX21_PPARg_BLA_Agonist_ch2
TOX21_AR_LUC_MDAKB2_Antagonist_0.5nM_R18
TOX21_PPARg_BLA_antagonist_ratio
TOX21_PR_BLA_Antagonist_ratio
TOX21_VDR_BLA_antagonist_ratio
TOX21_p53_BLA_p3_ch1
TOX21_ERb_BLA_Antagonist_ratio
TOX21_PR_BLA_Antagonist_ch2
TOX21_FXR_BLA_agonist_ch2
TOX21_ERa_LUC_VM7_Antagonist_0.5nM_E2
TOX21_ERb_BLA_Agonist_ratio
TOX21_ERa_LUC_VM7_Agonist
TOX21_RXR_BLA_Agonist_ratio
TOX21_ERa_BLA_Agonist_ch1
TOX21_TSHR_Antagonist_ch2
TOX21_RXR_BLA_Agonist_ch1
TOX21_NFkB_BLA_agonist_ch1
TOX21_AP1_BLA_Agonist_ch1
TOX21_TSHR_Agonist_ratio
52794-97-5
57576-44-0
75443-99-1
23541-50-6
57852-57-0
20830-81-3

All Activity Heatmap for Cluster 1521

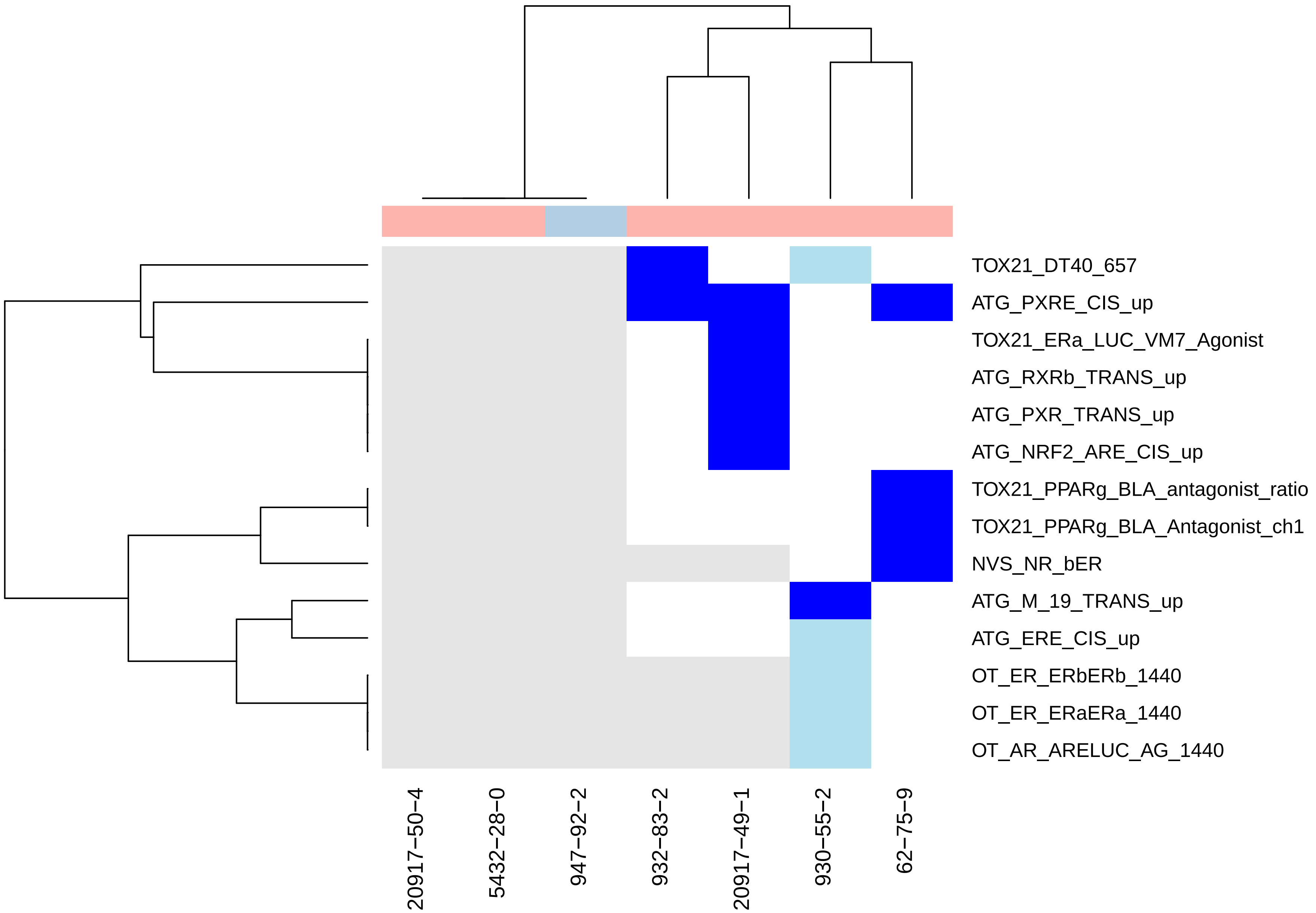

All Activity Heatmap for Cluster 1524

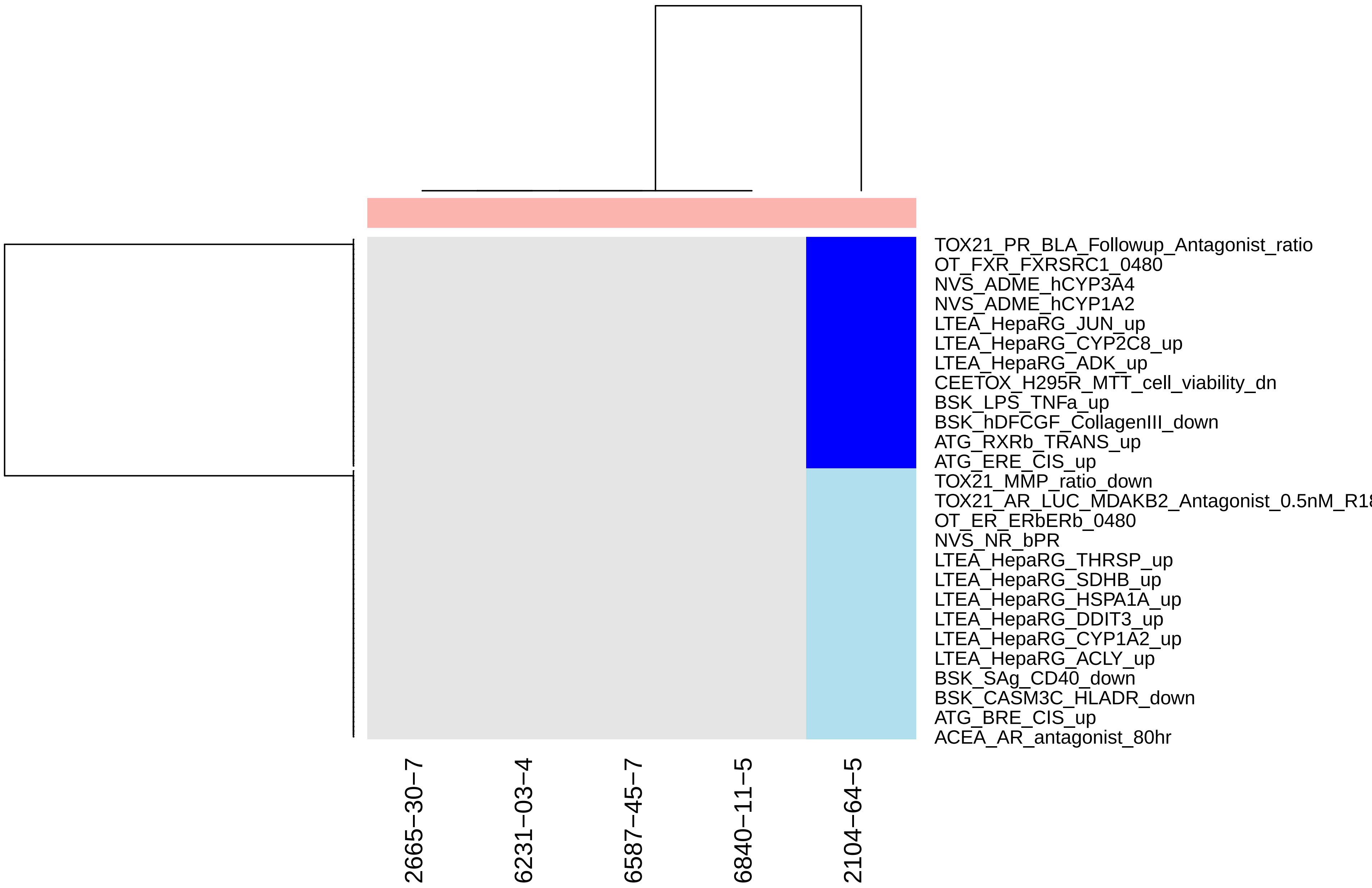

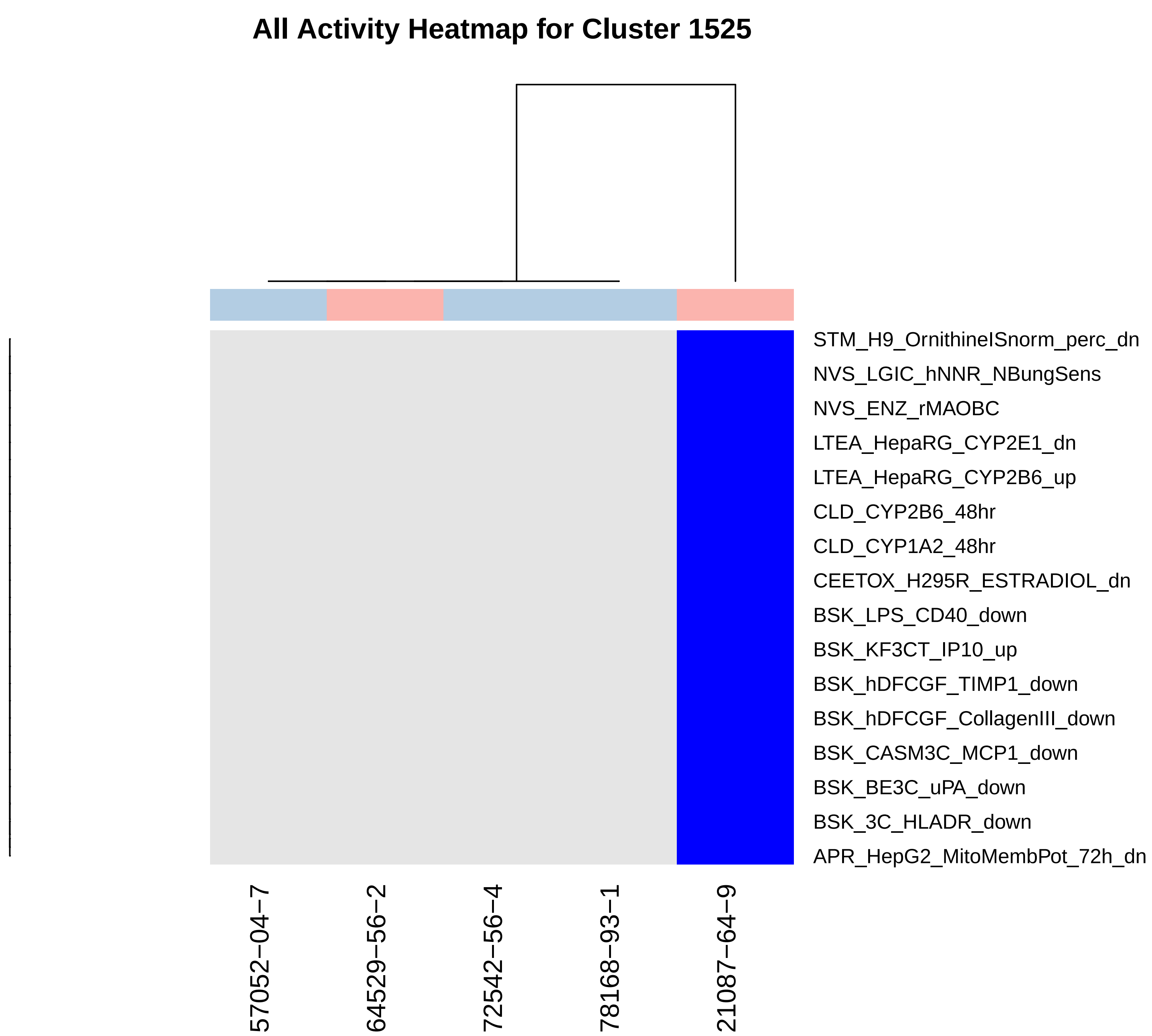
All Activity Heatmap for Cluster 1525
STM_H9_OrnithineISnorm_perc_dn
NVS_LGIC_hNNR_NBungSens
NVS_ENZ_rMAOBC
LTEA_HepaRG_CYP2E1_dn
LTEA_HepaRG_CYP2B6_up
CLD_CYP2B6_48hr
CLD_CYP1A2_48hr
CEETOX_H295R_ESTRADIOL_dn
BSK_LPS_CD40_down
BSK_KF3CT_IP10_up
BSK_hDFCGF_TIMP1_down
BSK_hDFCGF_CollagenIII_down
BSK_CASM3C_MCP1_down
BSK_BE3C_uPA_down
BSK_3C_HLADR_down
APR_HepG2_MitoMembPot_72h_dn
57052−04−7
64529−56−2
72542−56−4
78168−93−1
21087−64−9

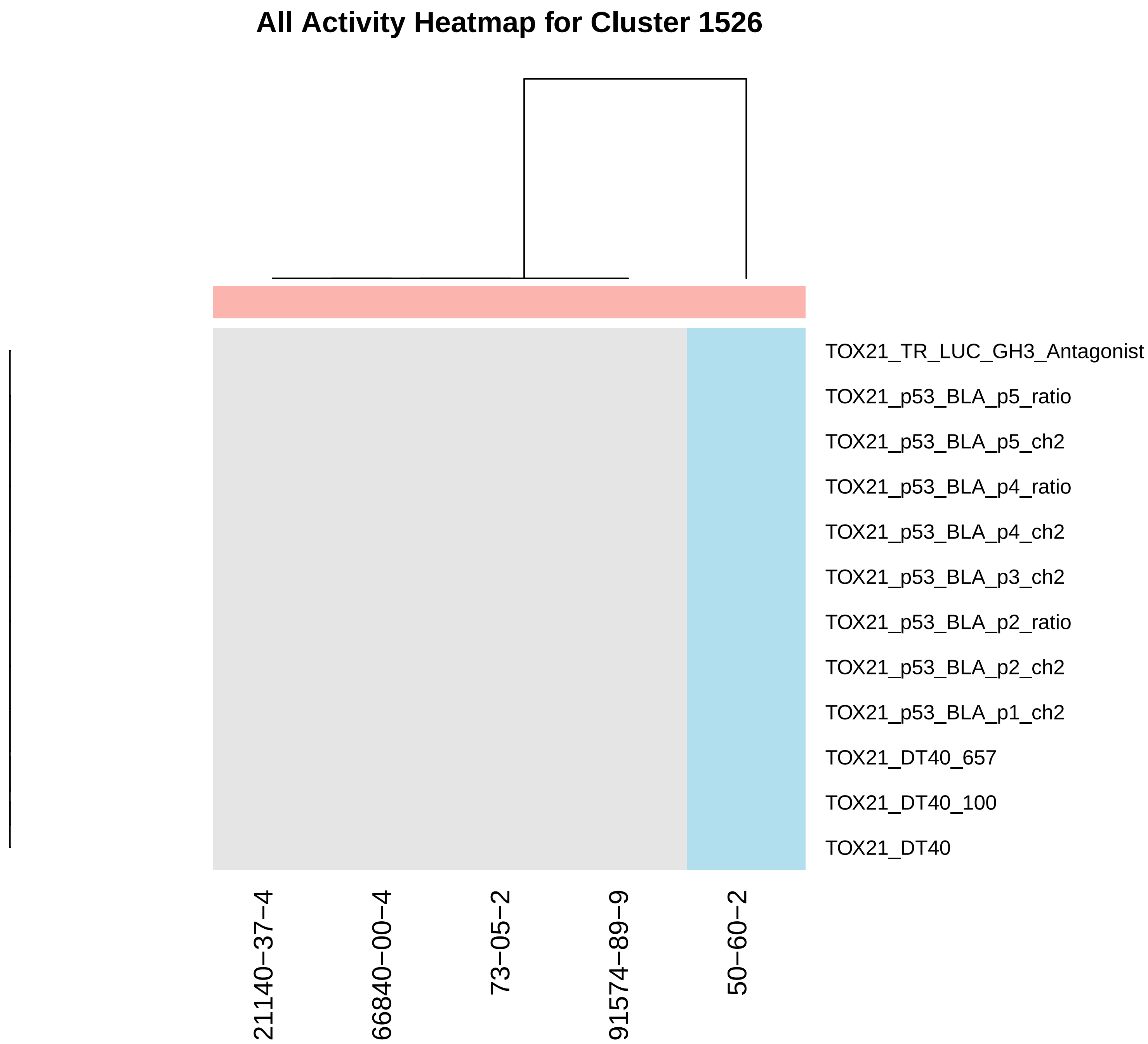
All Activity Heatmap for Cluster 1526
TOX21_TR_LUC_GH3_Antagonist
TOX21_p53_BLA_p5_ratio
TOX21_p53_BLA_p5_ch2
TOX21_p53_BLA_p4_ratio
TOX21_p53_BLA_p4_ch2
TOX21_p53_BLA_p3_ch2
TOX21_p53_BLA_p2_ratio
TOX21_p53_BLA_p2_ch2
TOX21_p53_BLA_p1_ch2
TOX21_DT40_657
TOX21_DT40_100
TOX21_DT40
21140−37−4
66840−00−4
73−05−2
91574−89−9
50−60−2

All Activity Heatmap for Cluster 1527

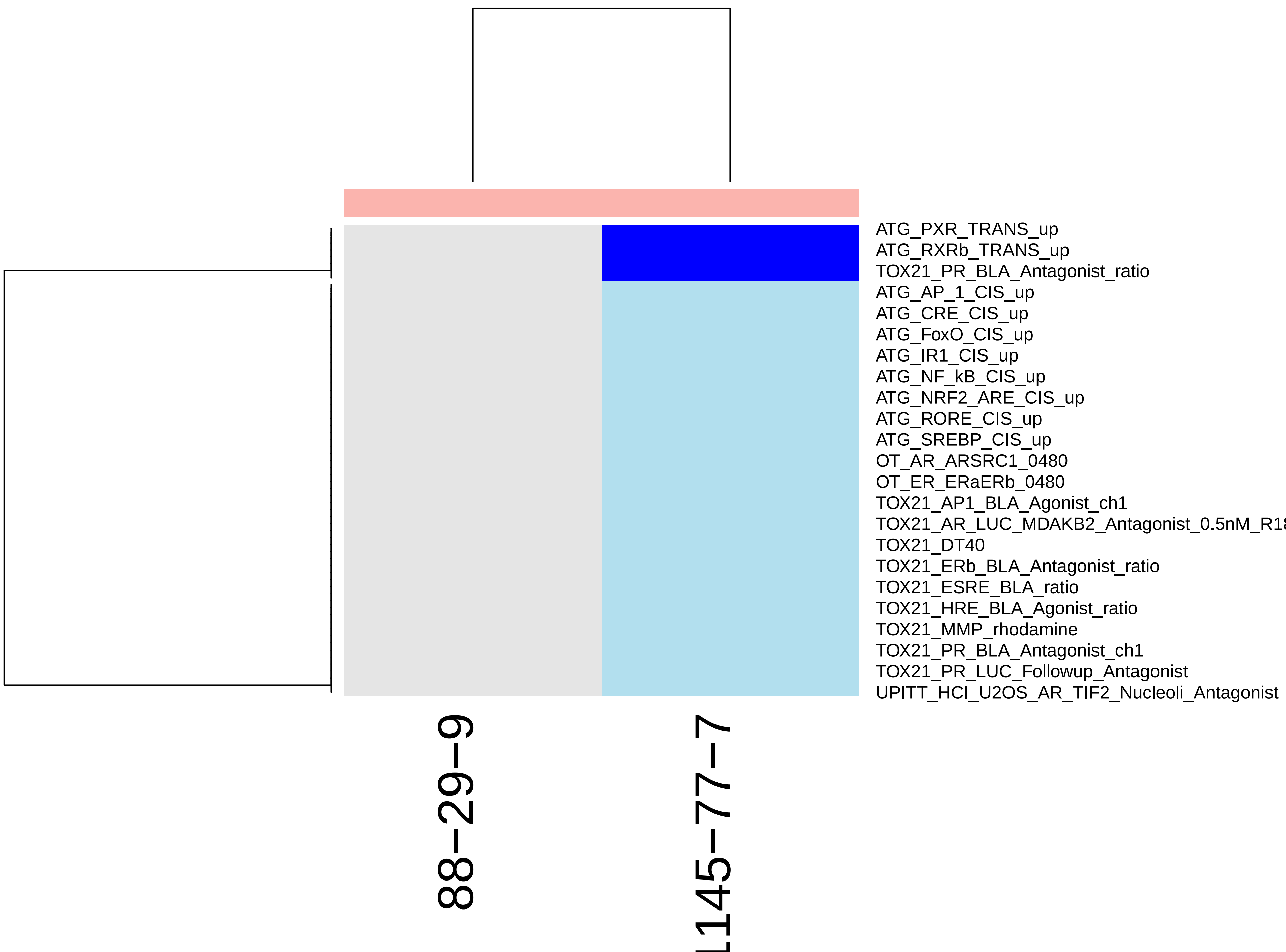

All Activity Heatmap for Cluster 1528

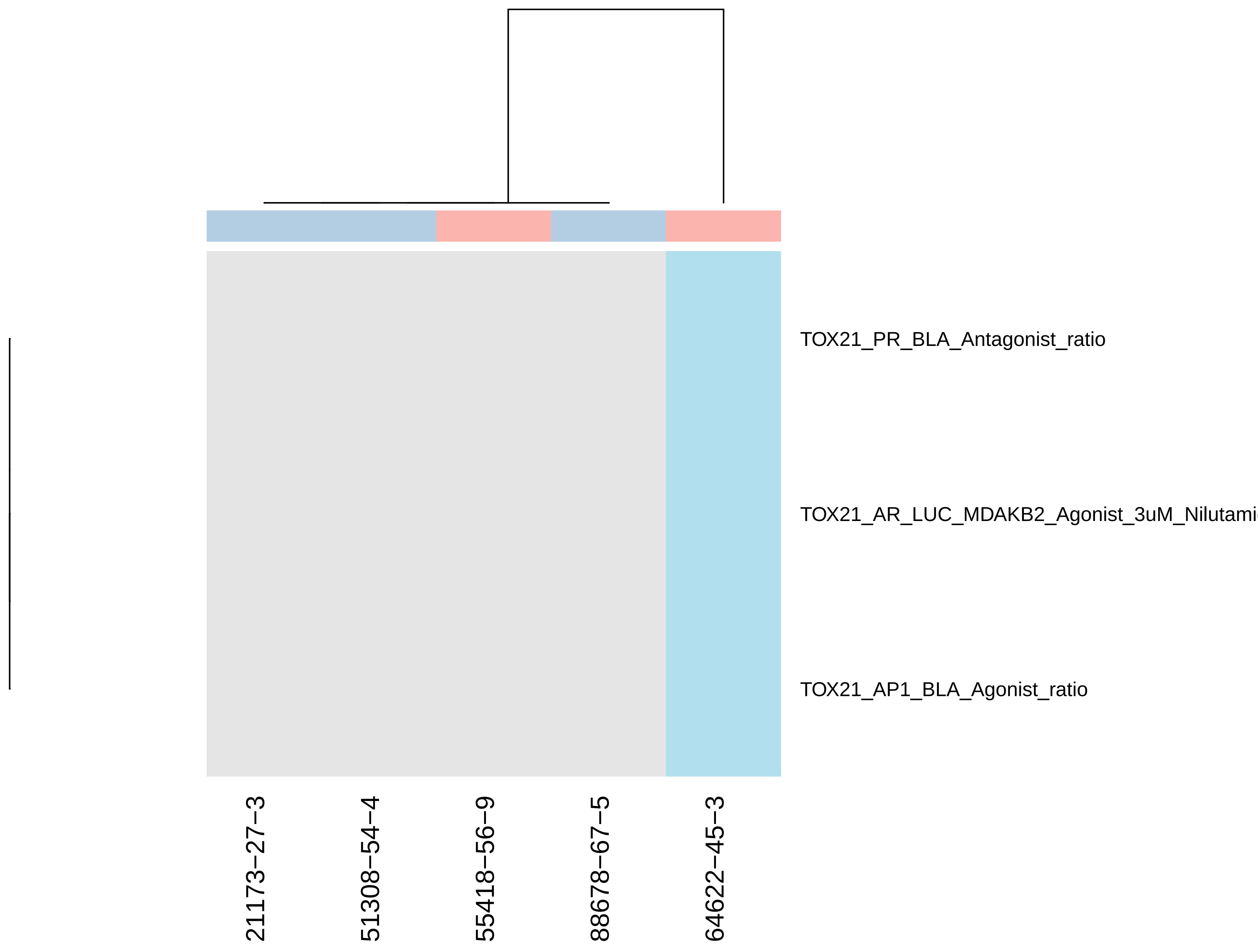

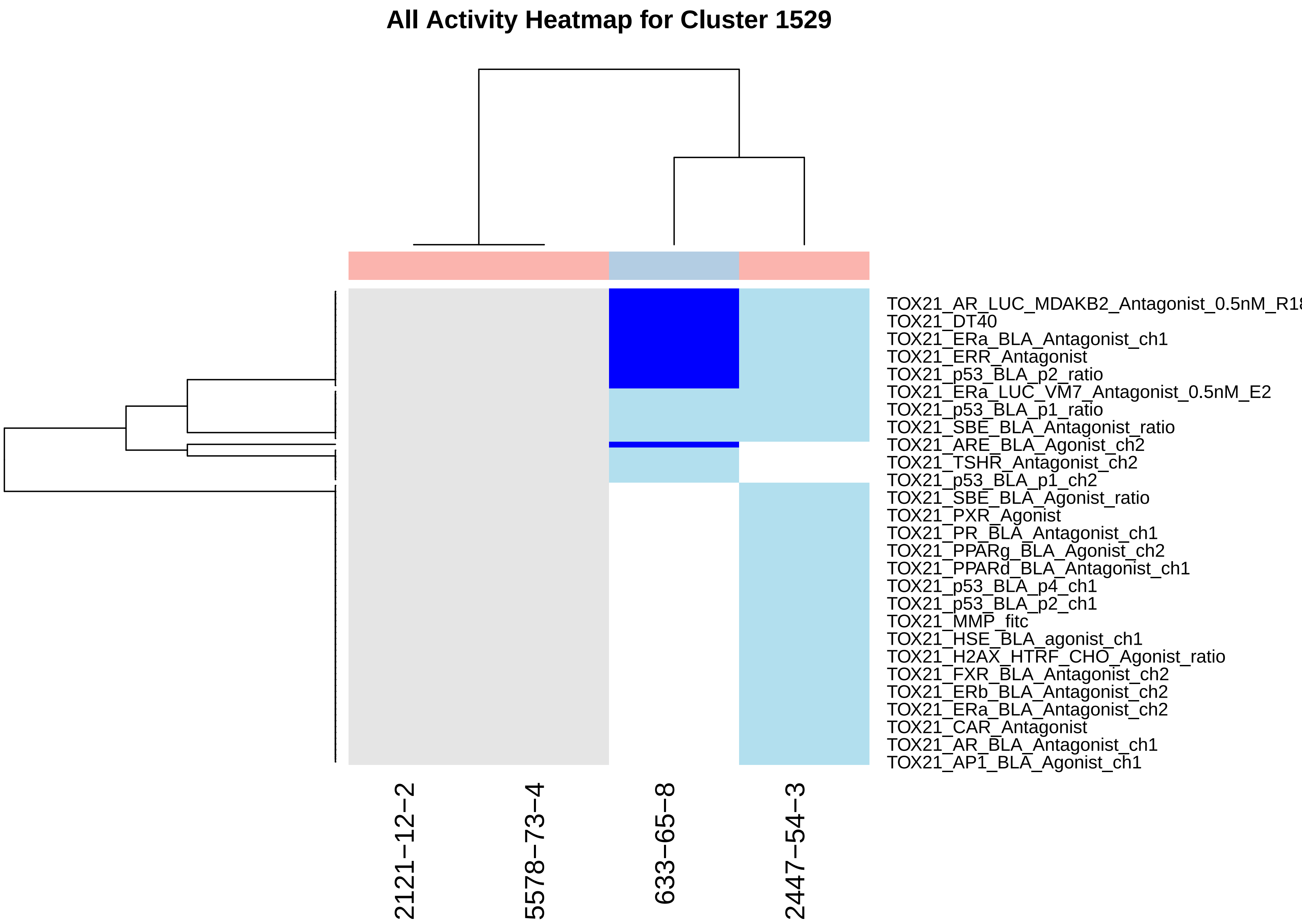

All Activity Heatmap for Cluster 1529
TOX21_AR_LUC_MDAKB2_Antagonist_0.5nM_R18
TOX21_DT40
TOX21_ERa_BLA_Antagonist_ch1
TOX21_ERR_Antagonist
TOX21_p53_BLA_p2_ratio
TOX21_ERa_LUC_VM7_Antagonist_0.5nM_E2
TOX21_p53_BLA_p1_ratio
TOX21_SBE_BLA_Antagonist_ratio
TOX21_ARE_BLA_Agonist_ch2
TOX21_TSHR_Antagonist_ch2
TOX21_p53_BLA_p1_ch2
TOX21_SBE_BLA_Agonist_ratio
TOX21_PXR_Agonist
TOX21_PR_BLA_Antagonist_ch1
TOX21_PPARg_BLA_Agonist_ch2
TOX21_PPARd_BLA_Antagonist_ch1
TOX21_p53_BLA_p4_ch1
TOX21_p53_BLA_p2_ch1
TOX21_MMP_fitc
TOX21_HSE_BLA_agonist_ch1
TOX21_H2AX_HTRF_CHO_Agonist_ratio
TOX21_FXR_BLA_Antagonist_ch2
TOX21_ERb_BLA_Antagonist_ch2
TOX21_ERa_BLA_Antagonist_ch2
TOX21_CAR_Antagonist
TOX21_AR_BLA_Antagonist_ch1
TOX21_AP1_BLA_Agonist_ch1
2121−12−2
5578−73−4
633−65−8
2447−54−3

All Activity Heatmap for Cluster 1530

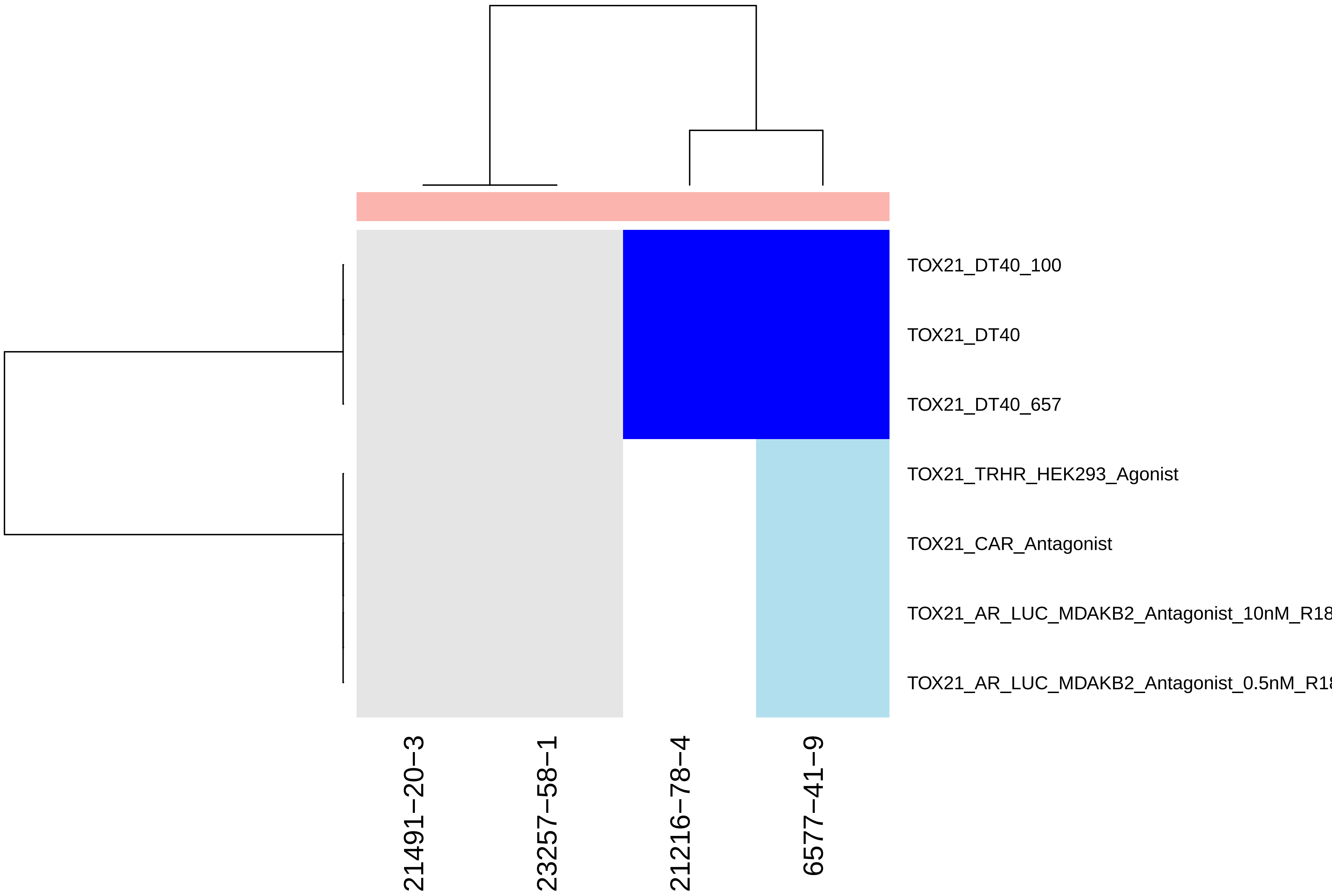

All Activity Heatmap for Cluster 1531

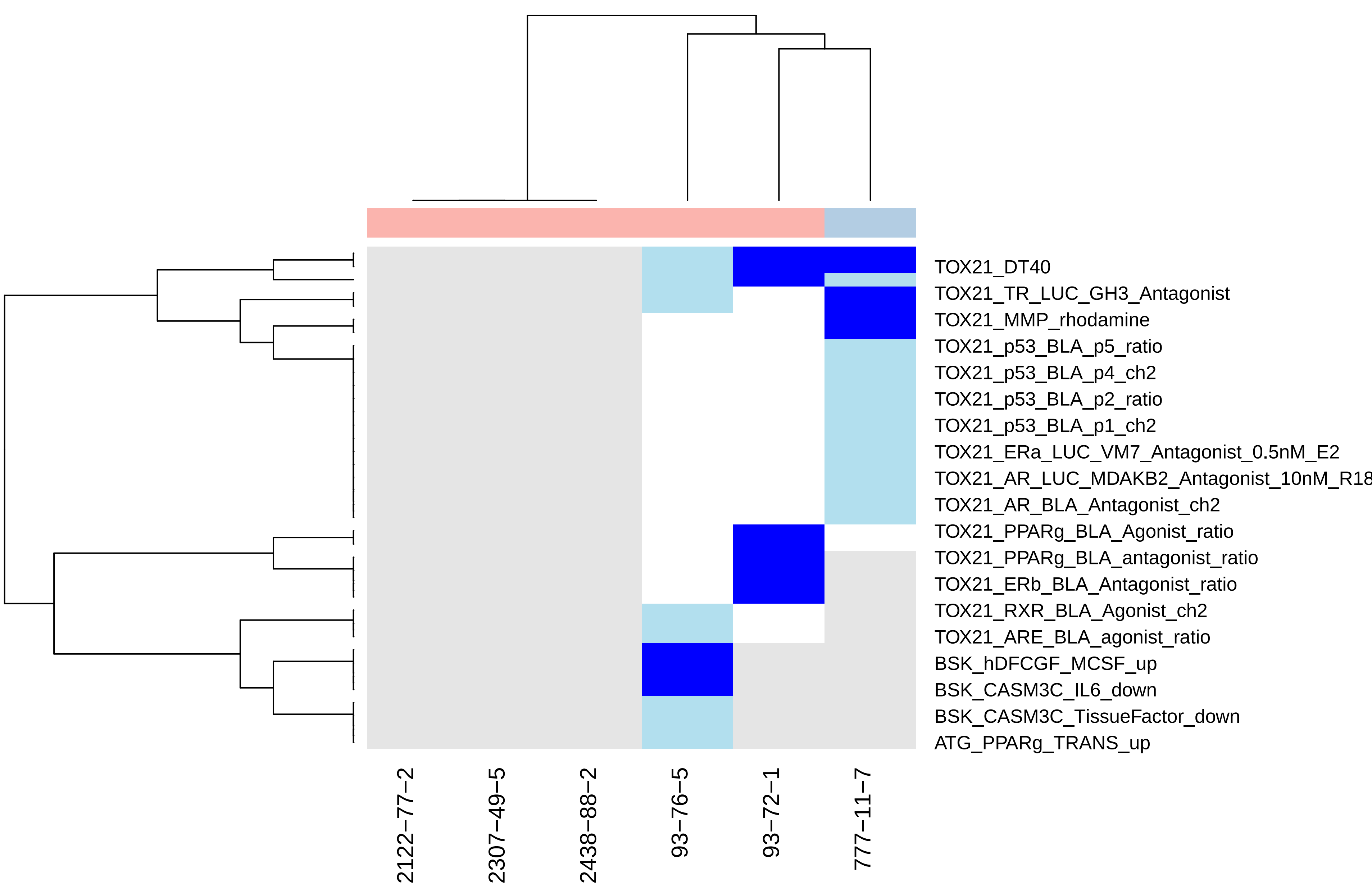

All Activity Heatmap for Cluster 1532

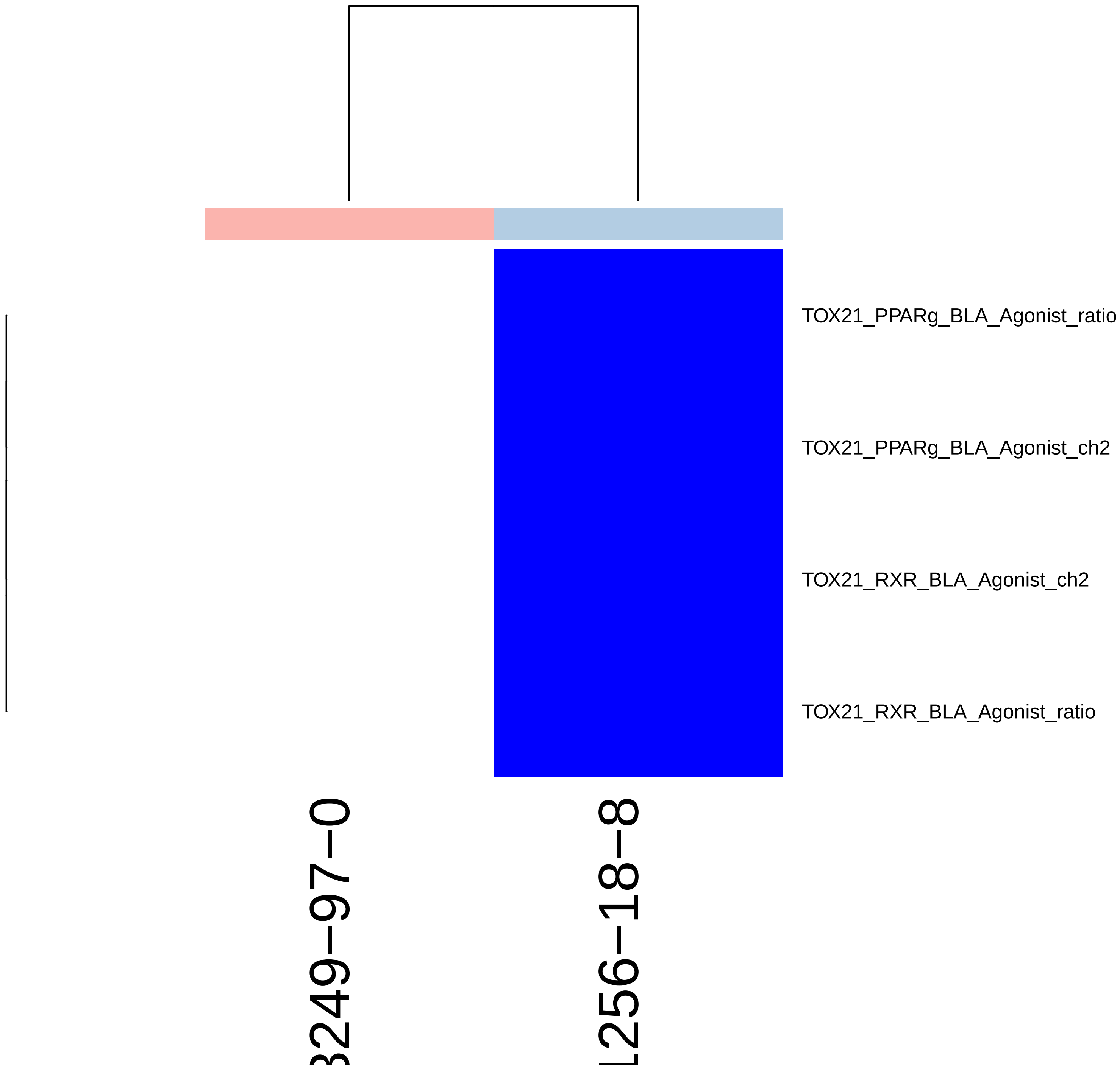

All Activity Heatmap for Cluster 1534

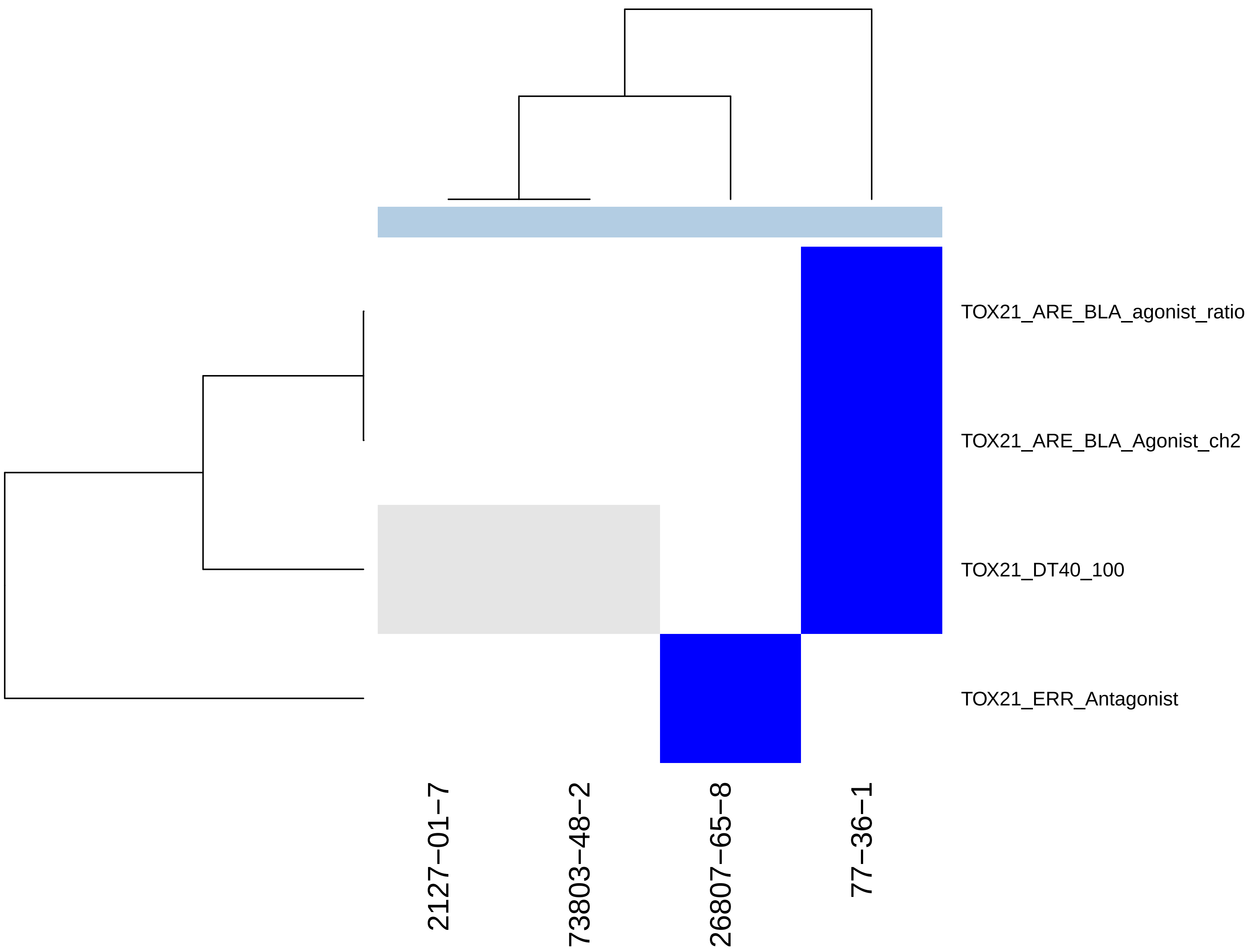

All Activity Heatmap for Cluster 1538

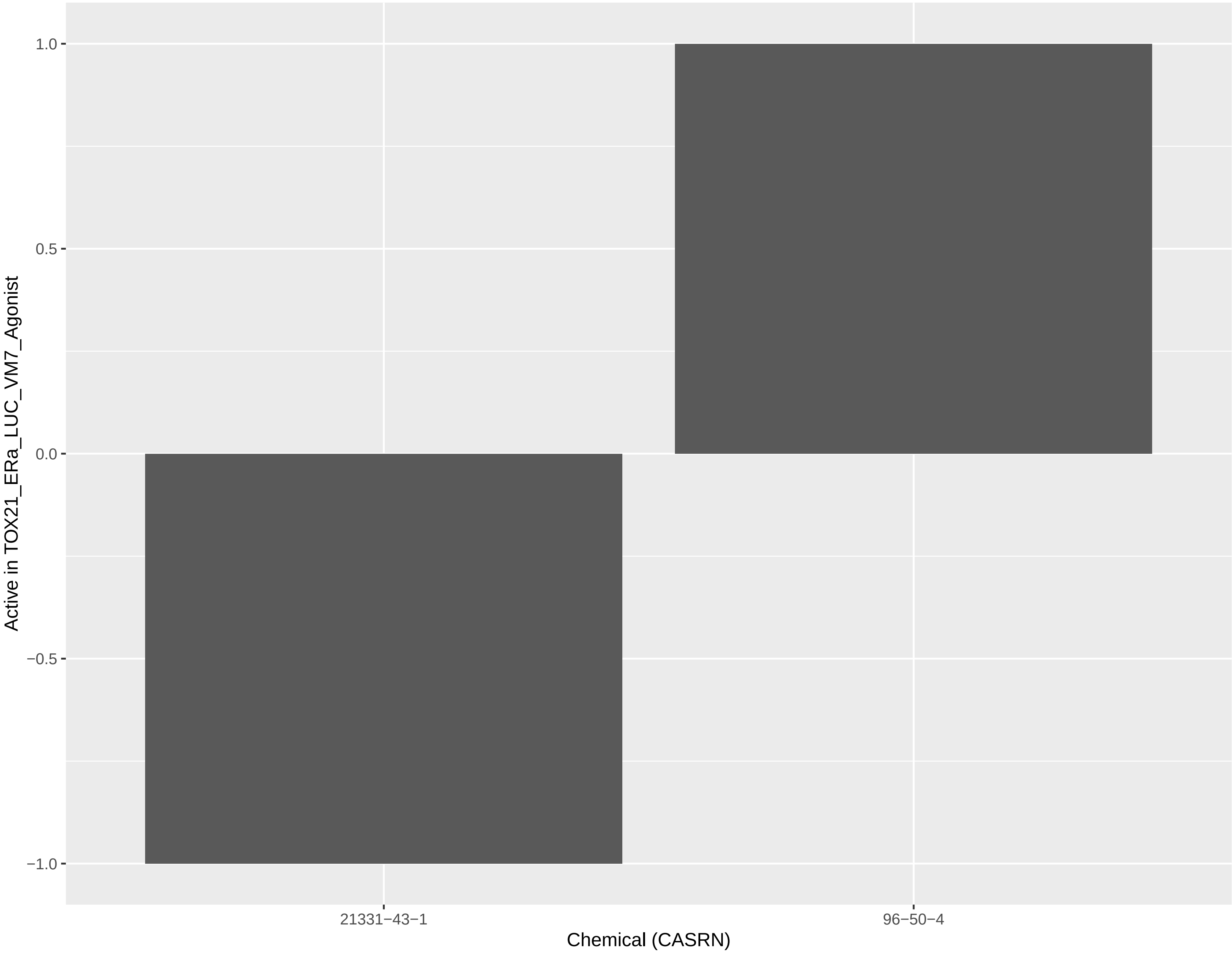

All Activity Heatmap for Cluster 1540

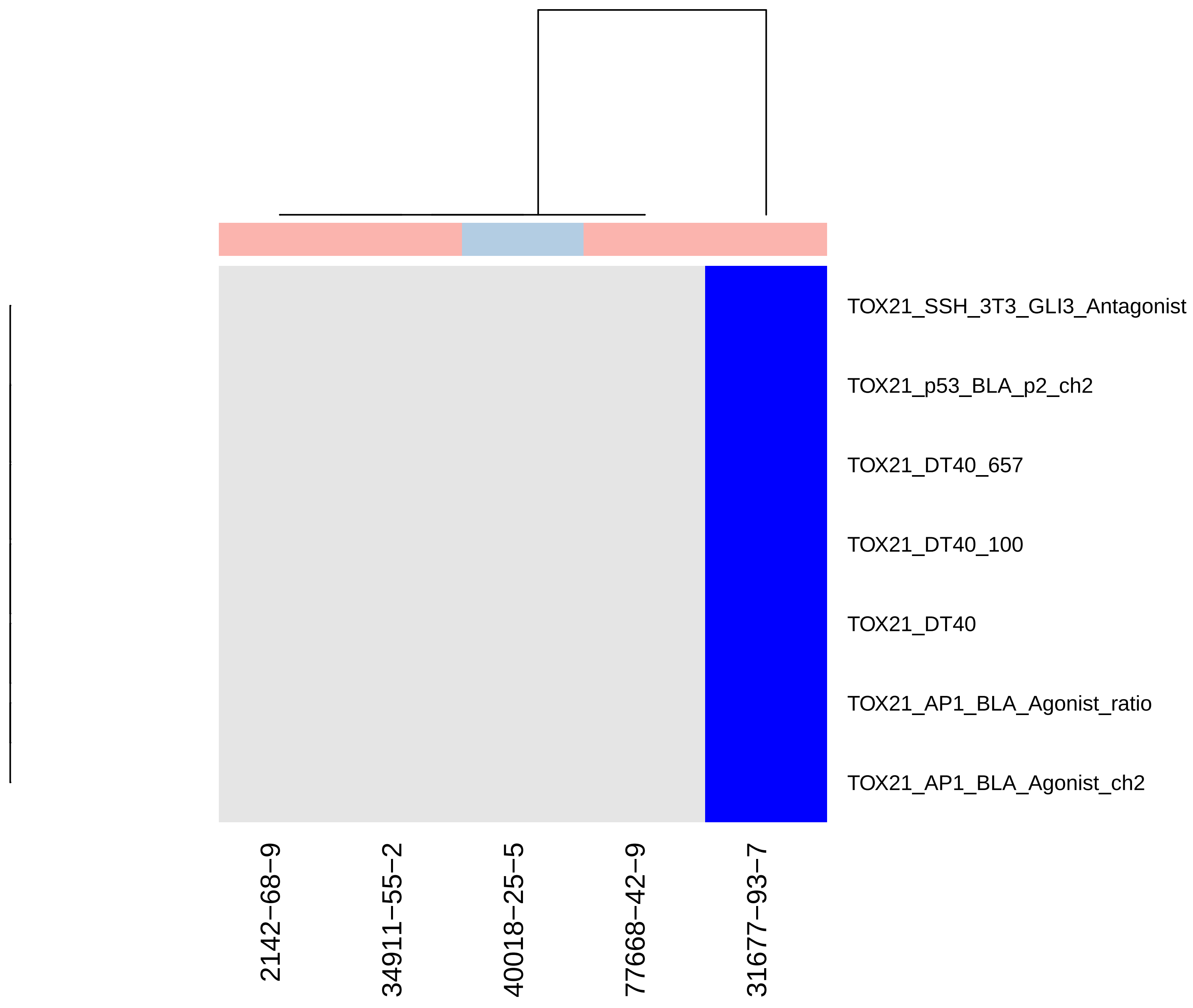

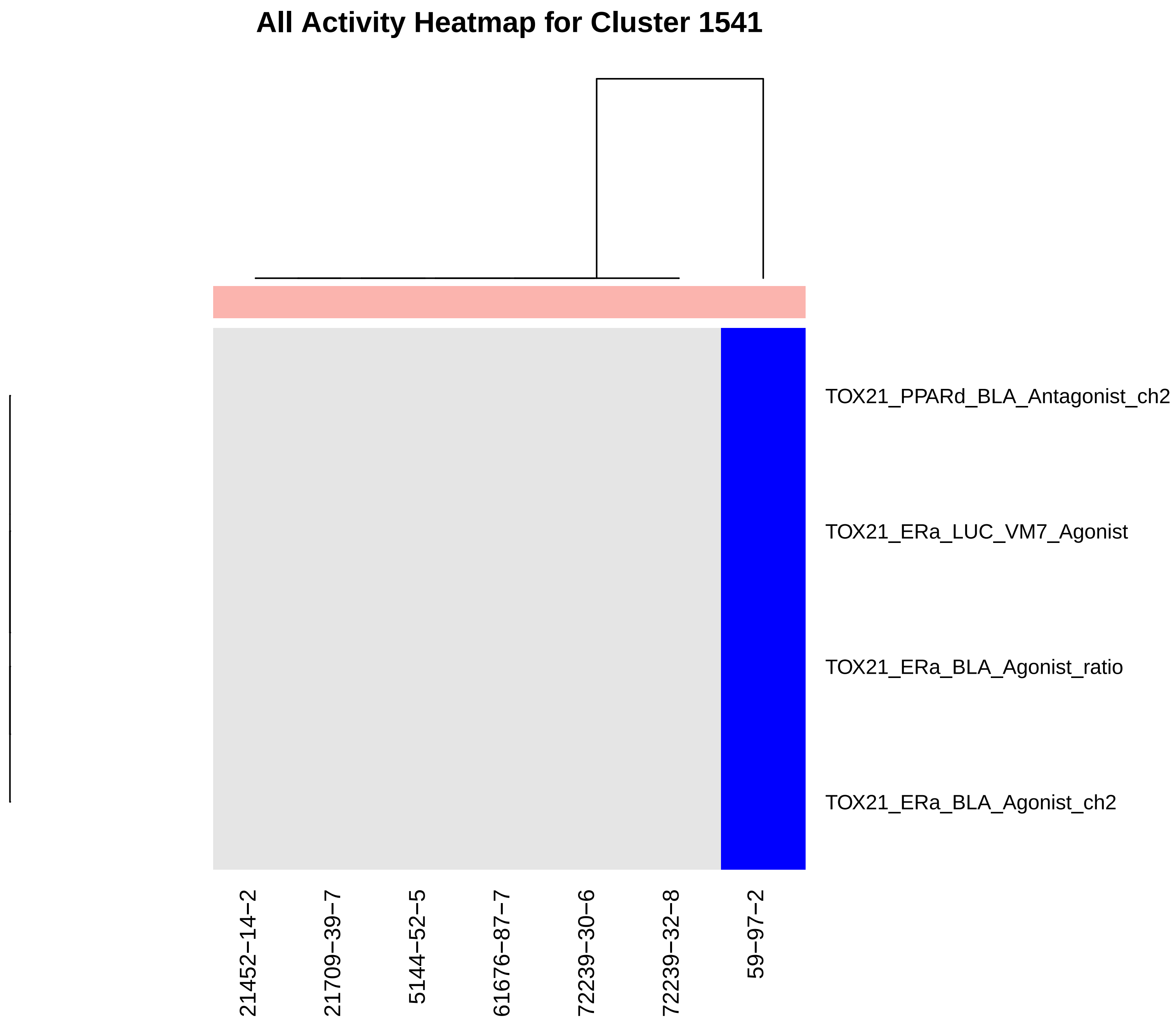
All Activity Heatmap for Cluster 1541
TOX21_PPARd_BLA_Antagonist_ch2
TOX21_ERa_LUC_VM7_Agonist
TOX21_ERa_BLA_Agonist_ratio
TOX21_ERa_BLA_Agonist_ch2
21452−14−2
21709−39−7
5144−52−5
61676−87−7
72239−30−6
72239−32−8
59−97−2

All Activity Heatmap for Cluster 1544

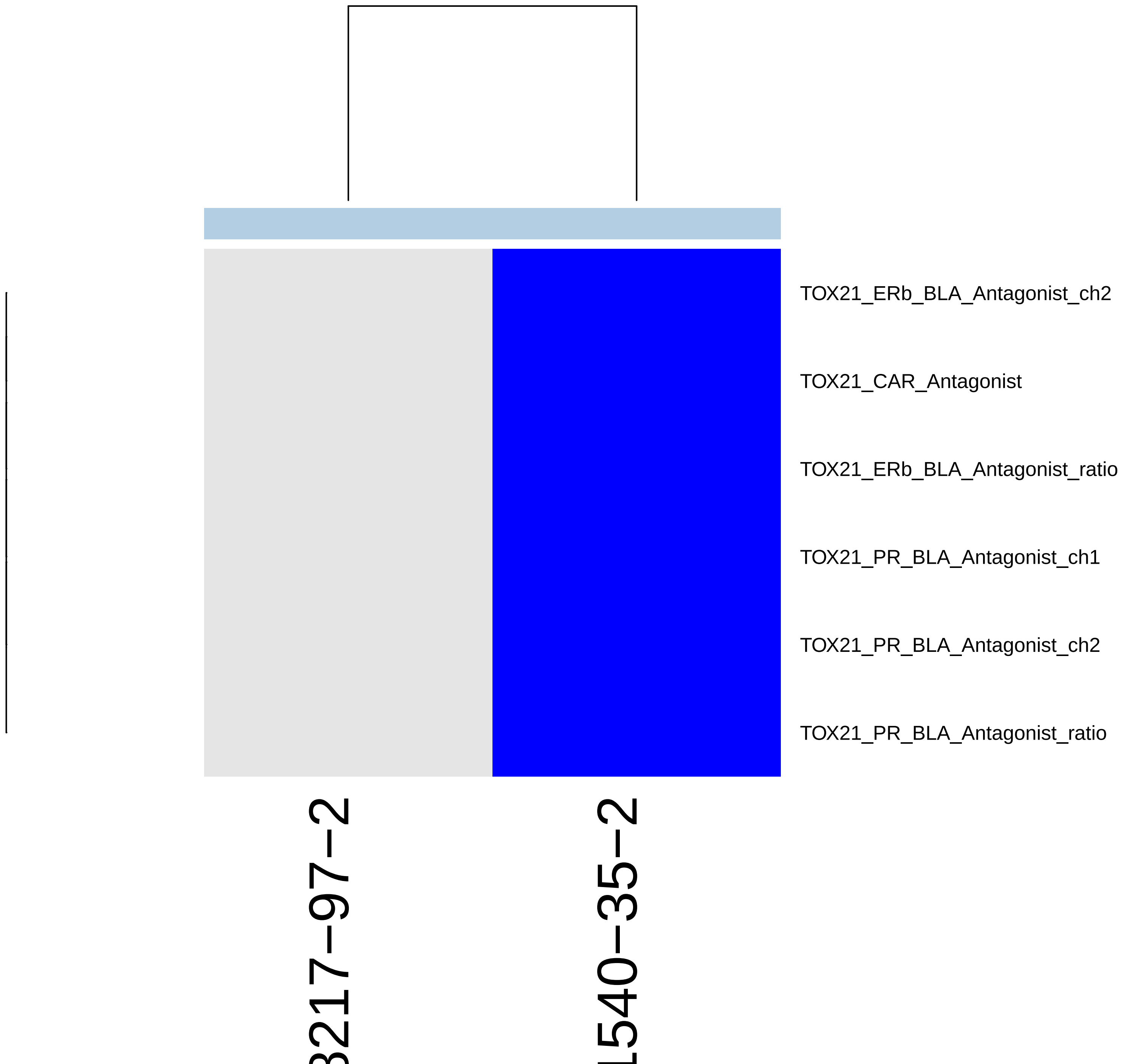

All Activity Heatmap for Cluster 1546

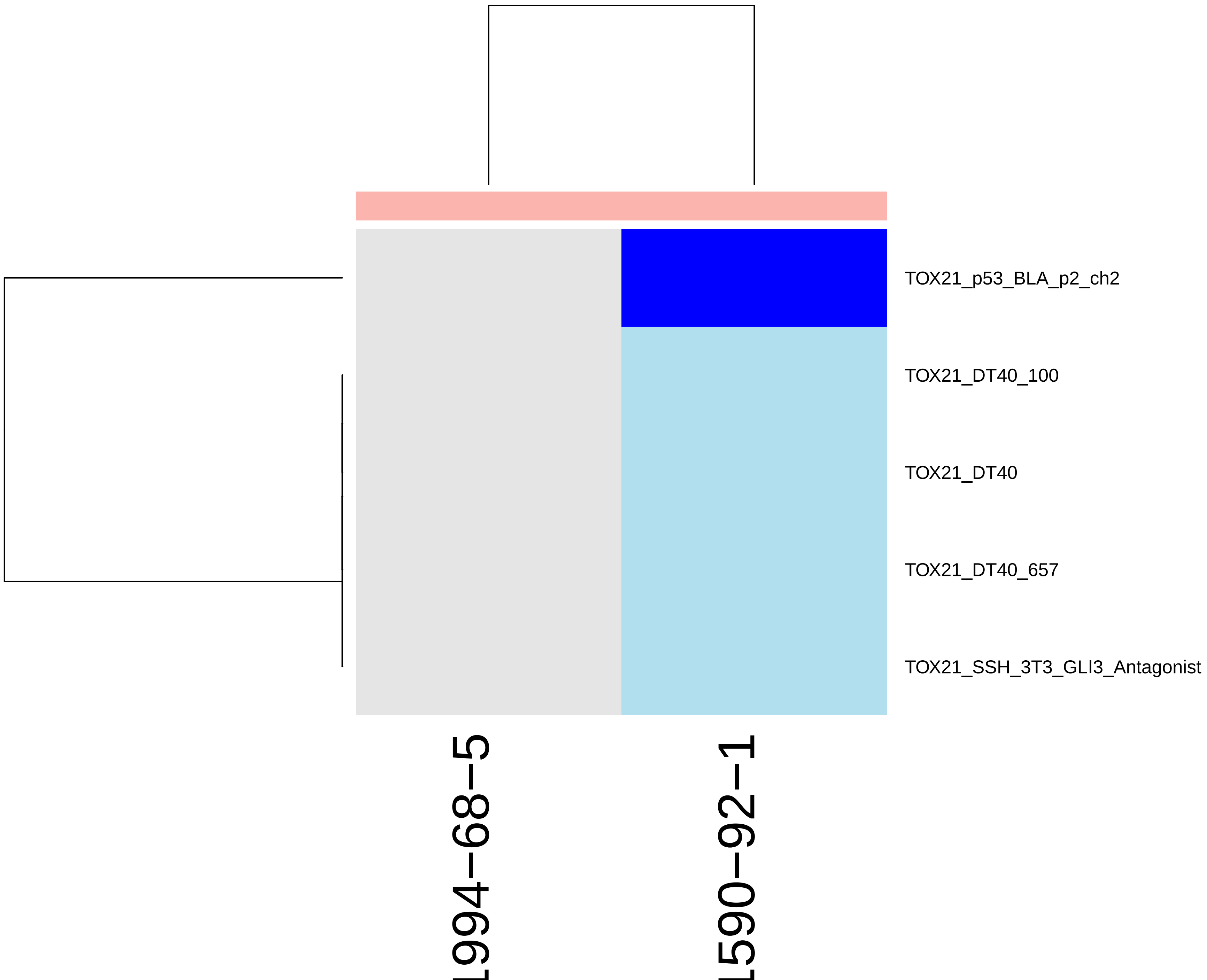

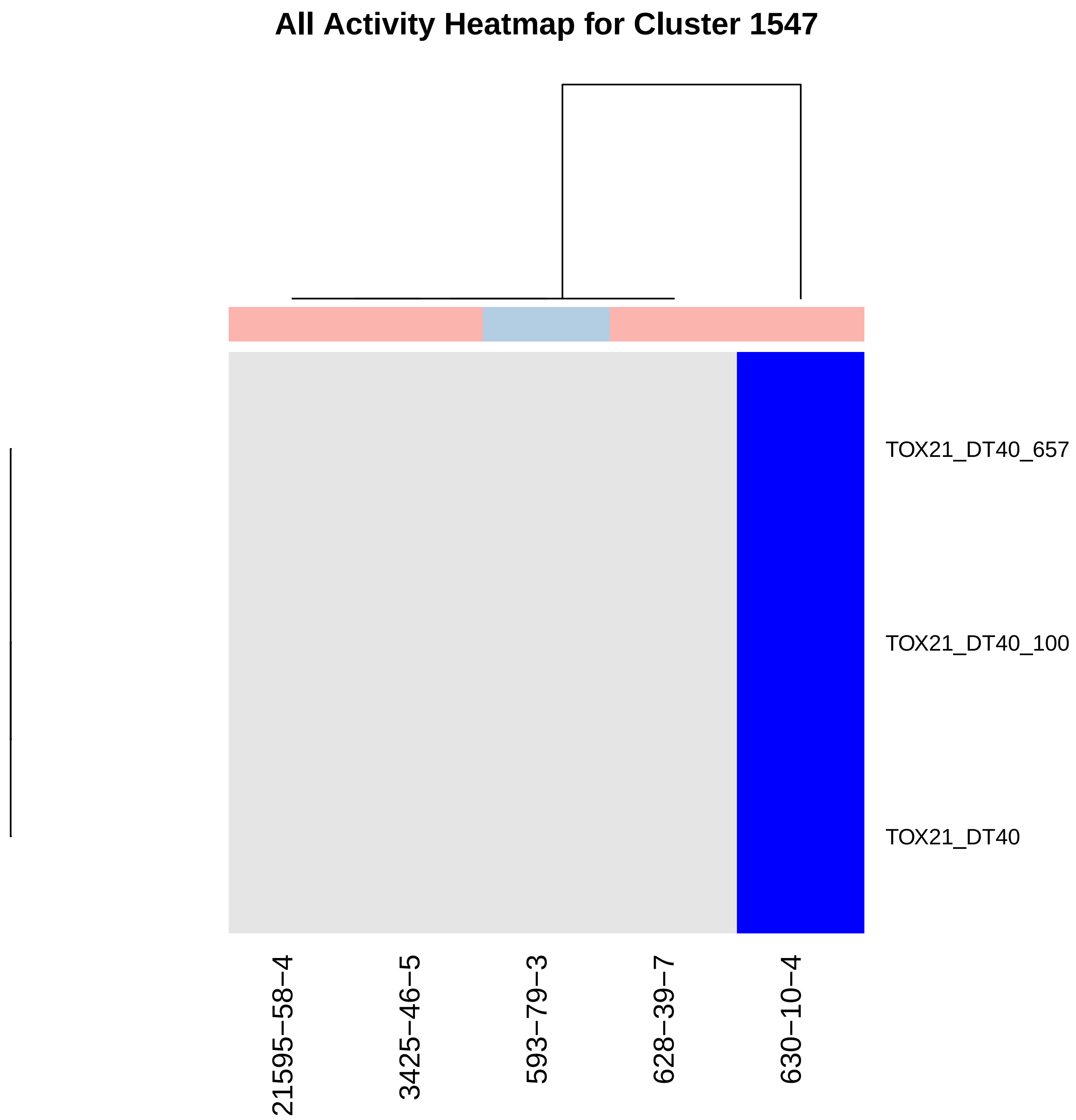
All Activity Heatmap for Cluster 1547
TOX21_DT40_657
TOX21_DT40_100
TOX21_DT40
21595−58−4
3425−46−5
593−79−3
628−39−7
630−10−4

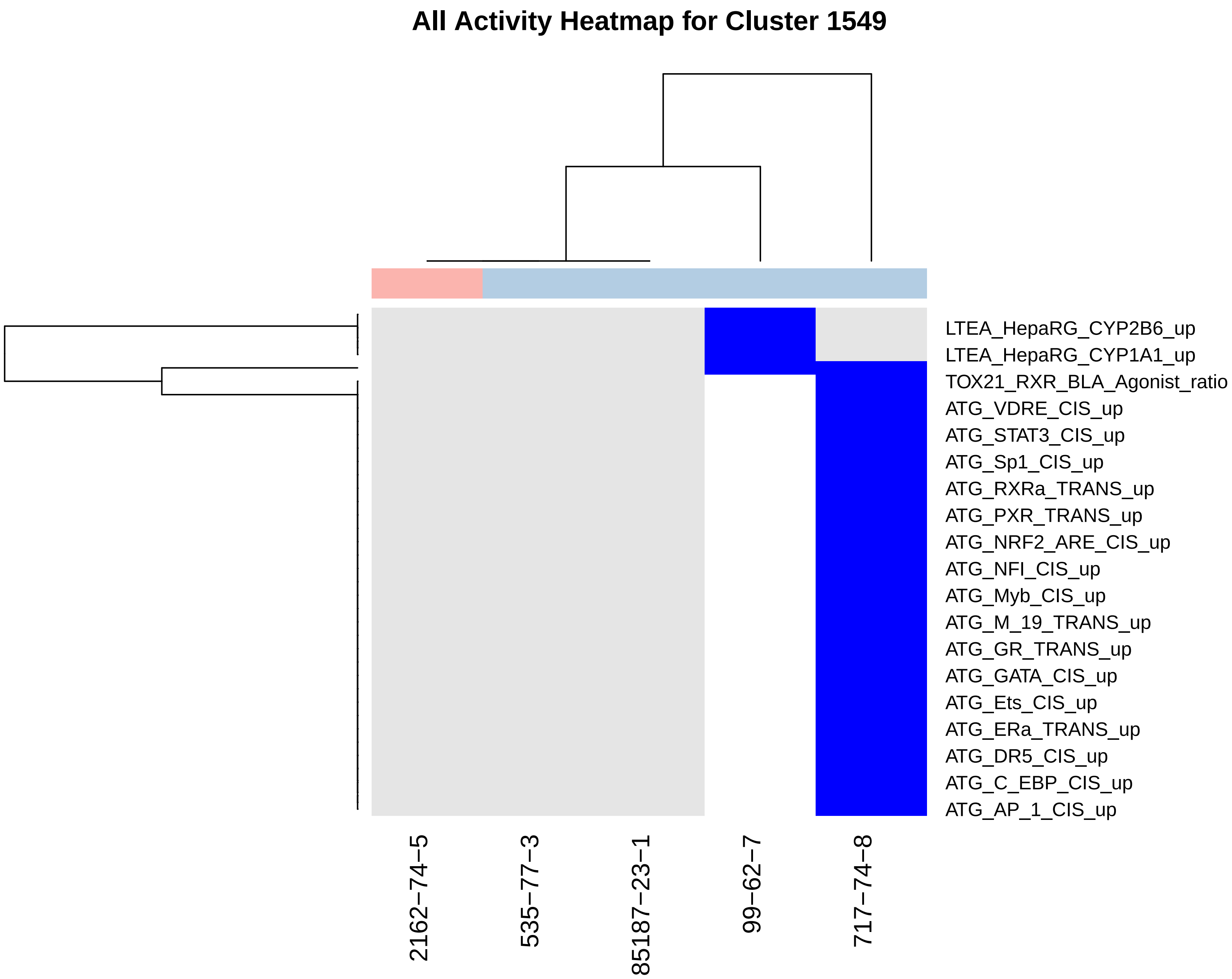

All Activity Heatmap for Cluster 1549
LTEA_HepaRG_CYP2B6_up
LTEA_HepaRG_CYP1A1_up
TOX21_RXR_BLA_Agonist_ratio
ATG_VDRE_CIS_up
ATG_STAT3_CIS_up
ATG_Sp1_CIS_up
ATG_RXRa_TRANS_up
ATG_PXR_TRANS_up
ATG_NRF2_ARE_CIS_up
ATG_NFI_CIS_up
ATG_Myb_CIS_up
ATG_M_19_TRANS_up
ATG_GR_TRANS_up
ATG_GATA_CIS_up
ATG_Ets_CIS_up
ATG_ERa_TRANS_up
ATG_DR5_CIS_up
ATG_C_EBP_CIS_up
ATG_AP_1_CIS_up
2162−74−5
535−77−3
85187−23−1
99−62−7
717−74−8

All Activity Heatmap for Cluster 1551

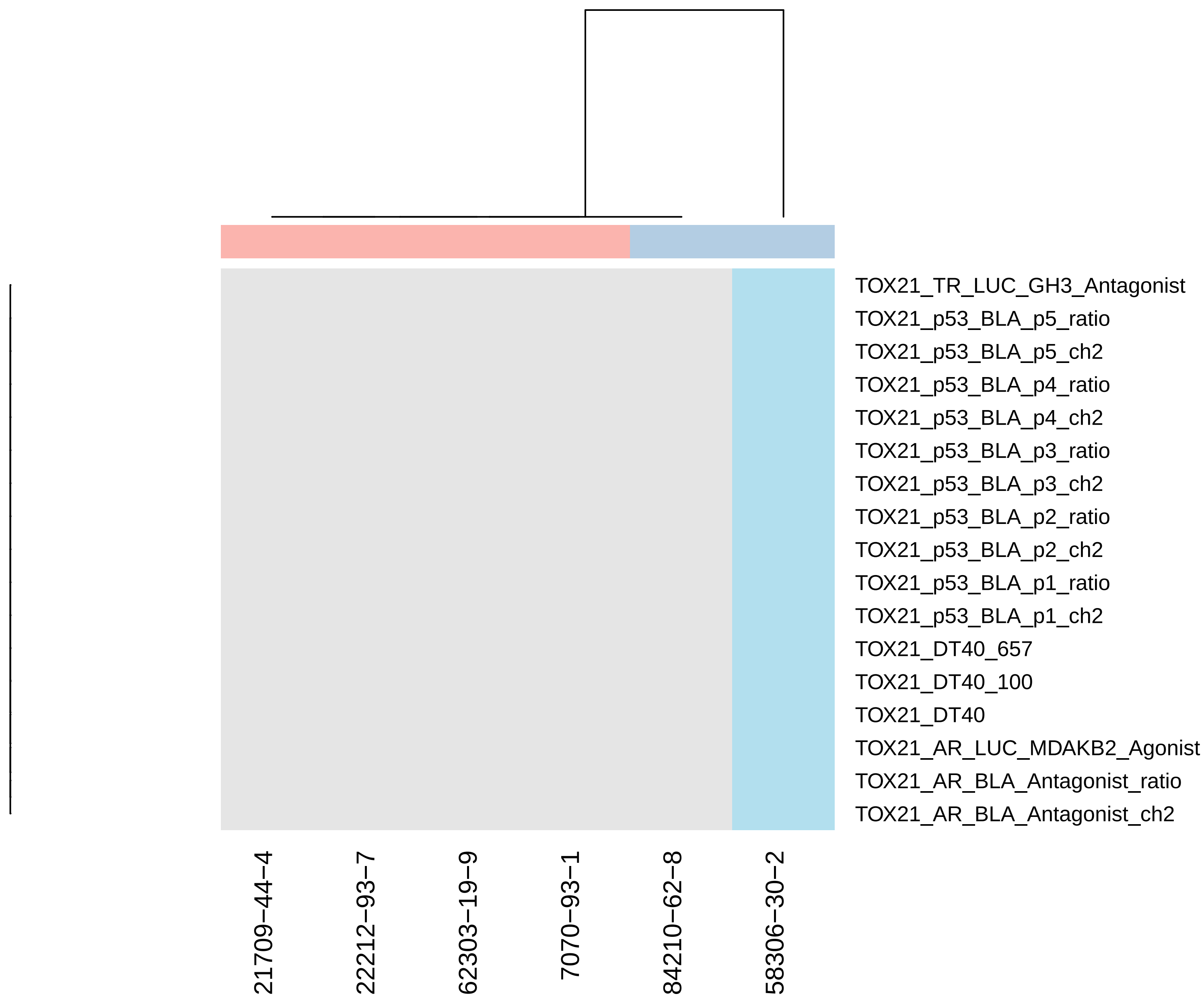

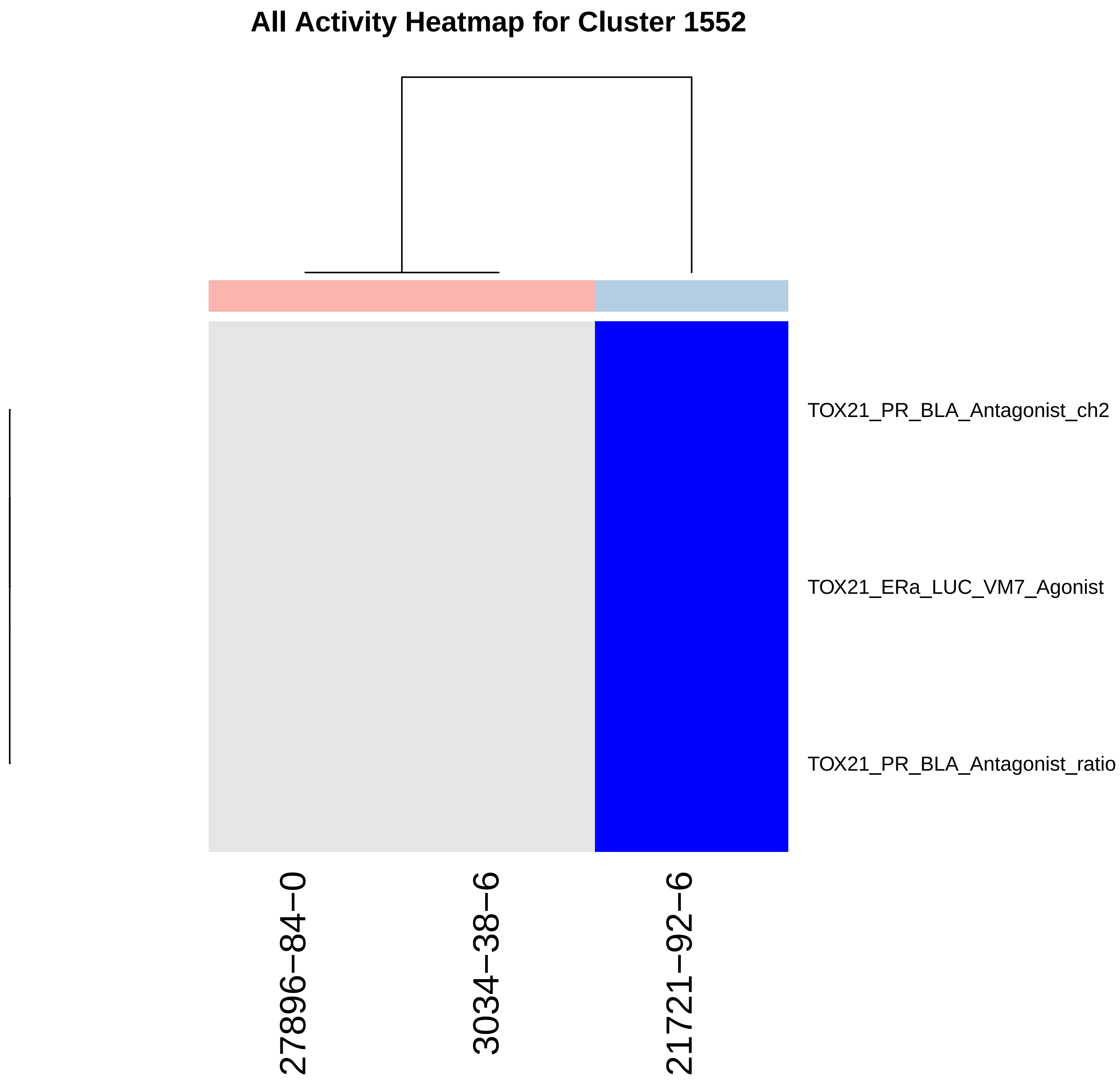
All Activity Heatmap for Cluster 1552
TOX21_PR_BLA_Antagonist_ch2
TOX21_ERa_LUC_VM7_Agonist
TOX21_PR_BLA_Antagonist_ratio
27896−84−0
3034−38−6
21721−92−6

All Activity Heatmap for Cluster 1553

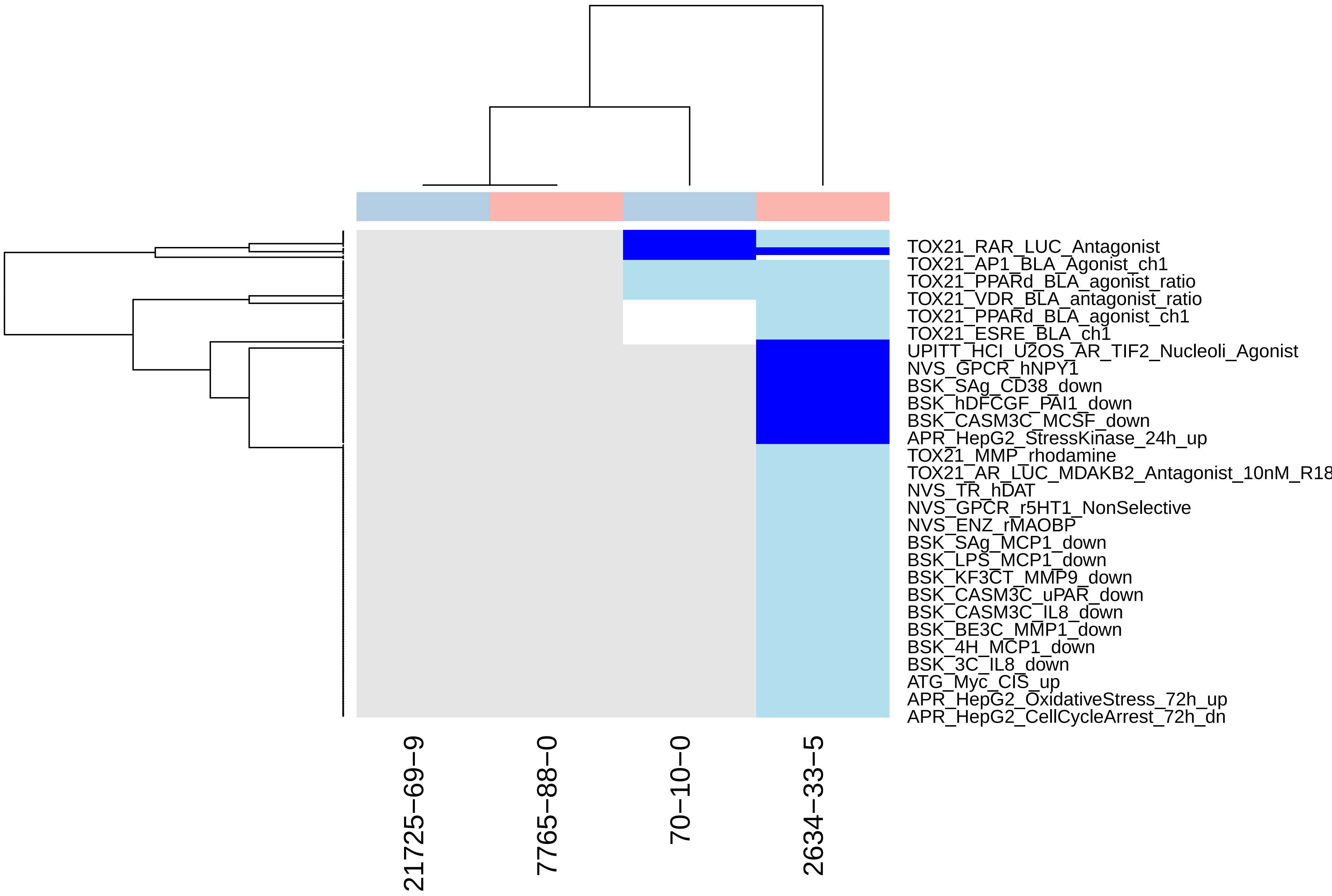

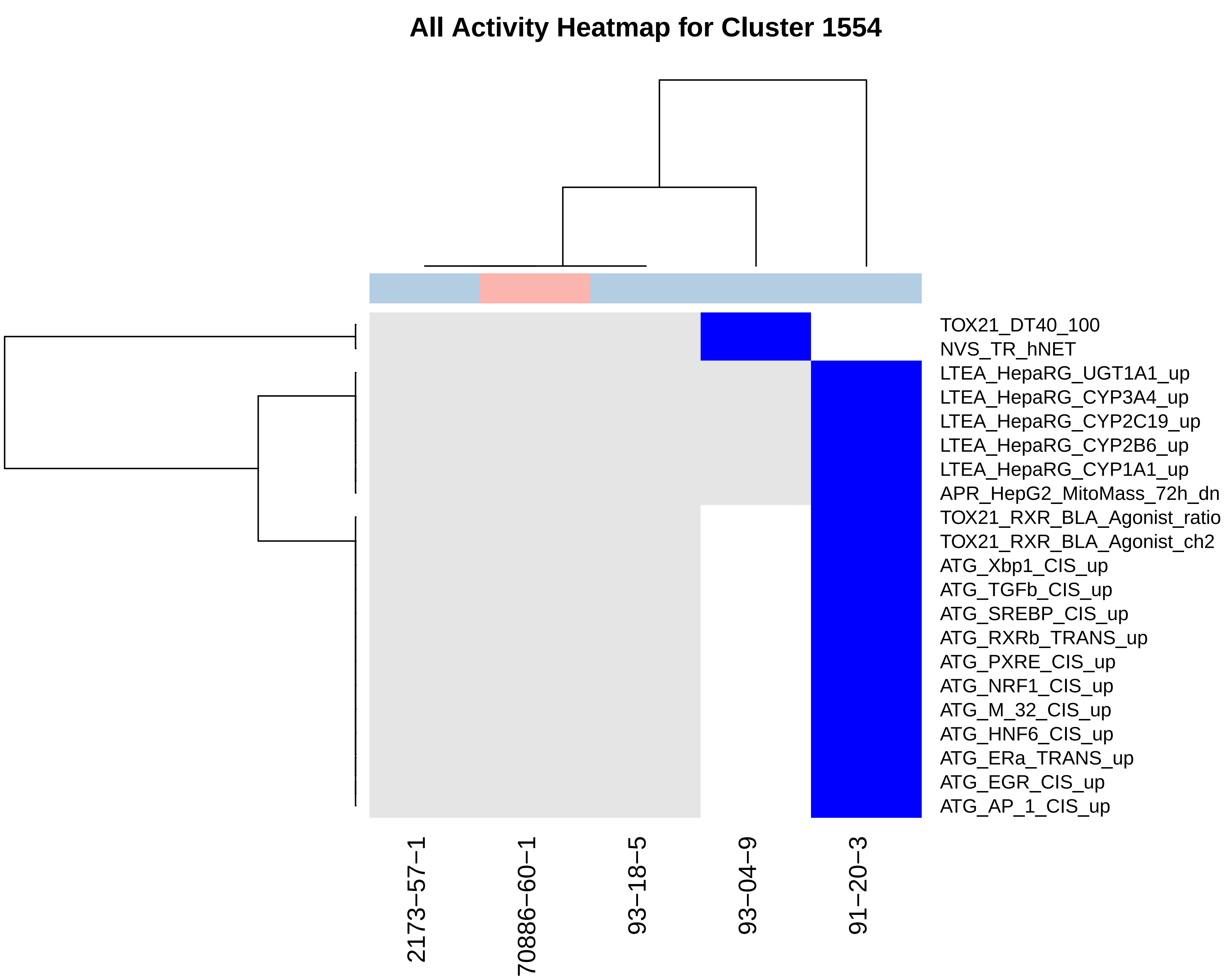
All Activity Heatmap for Cluster 1554
TOX21_DT40_100
NVS_TR_hNET
LTEA_HepaRG_UGT1A1_up
LTEA_HepaRG_CYP3A4_up
LTEA_HepaRG_CYP2C19_up
LTEA_HepaRG_CYP2B6_up
LTEA_HepaRG_CYP1A1_up
APR_HepG2_MitoMass_72h_dn
TOX21_RXR_BLA_Agonist_ratio
TOX21_RXR_BLA_Agonist_ch2
ATG_Xbp1_CIS_up
ATG_TGFb_CIS_up
ATG_SREBP_CIS_up
ATG_RXRb_TRANS_up
ATG_PXRE_CIS_up
ATG_NRF1_CIS_up
ATG_M_32_CIS_up
ATG_HNF6_CIS_up
ATG_ERa_TRANS_up
ATG_EGR_CIS_up
ATG_AP_1_CIS_up
2173−57−1
70886−60−1
93−18−5
93−04−9
91−20−3

All Activity Heatmap for Cluster 1556

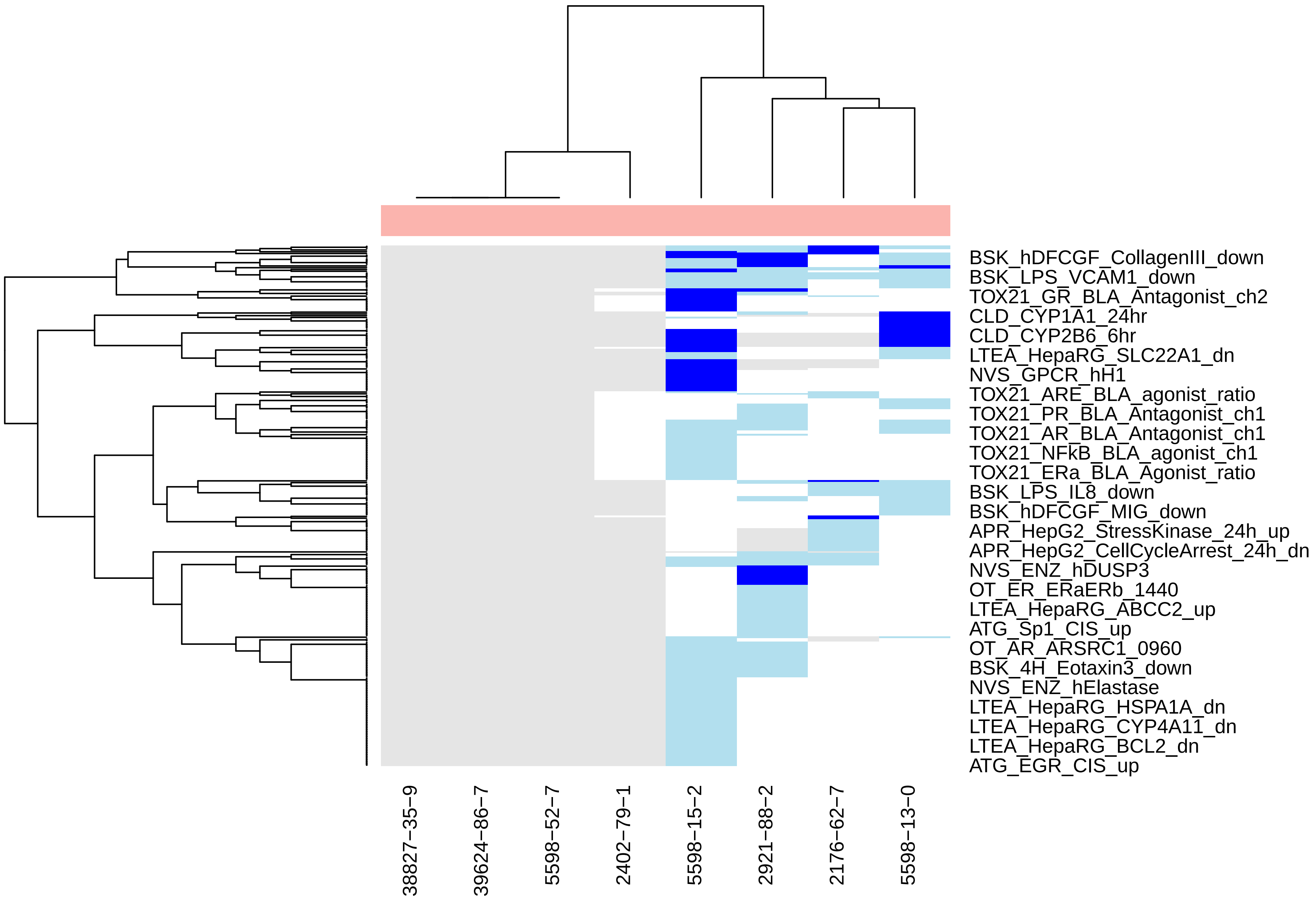

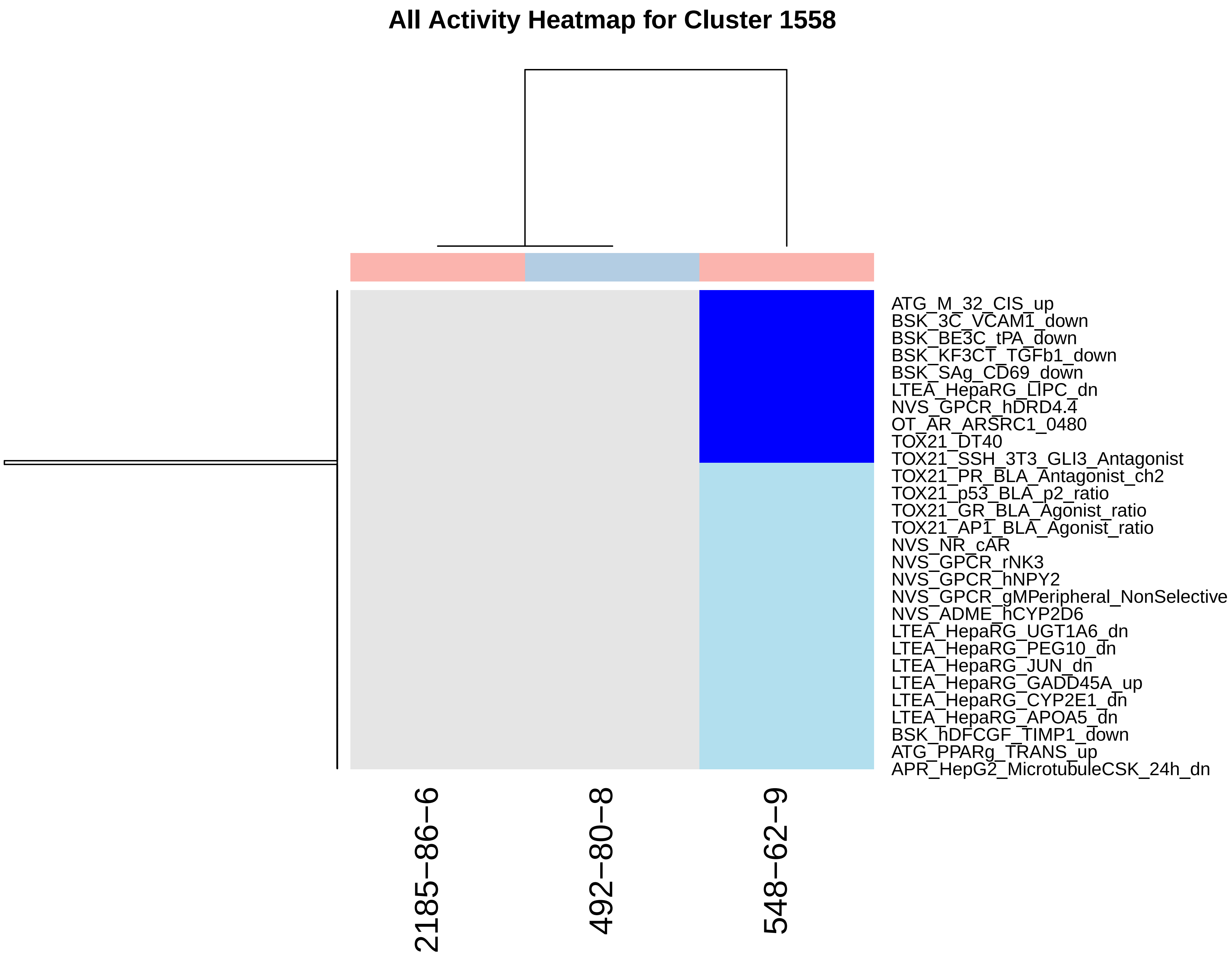
All Activity Heatmap for Cluster 1558
ATG_M_32_CIS_up
BSK_3C_VCAM1_down
BSK_BE3C_tPA_down
BSK_KF3CT_TGFb1_down
BSK_SAg_CD69_down
LTEA_HepaRG_LIPC_dn
NVS_GPCR_hDRD4.4
OT_AR_ARSRC1_0480
TOX21_DT40
TOX21_SSH_3T3_GLI3_Antagonist
TOX21_PR_BLA_Antagonist_ch2
TOX21_p53_BLA_p2_ratio
TOX21_GR_BLA_Agonist_ratio
TOX21_AP1_BLA_Agonist_ratio
NVS_NR_cAR
NVS_GPCR_rNK3
NVS_GPCR_hNPY2
NVS_GPCR_gMPeripheral_NonSelective
NVS_ADME_hCYP2D6
LTEA_HepaRG_UGT1A6_dn
LTEA_HepaRG_PEG10_dn
LTEA_HepaRG_JUN_dn
LTEA_HepaRG_GADD45A_up
LTEA_HepaRG_CYP2E1_dn
LTEA_HepaRG_APOA5_dn
BSK_hDFCGF_TIMP1_down
ATG_PPARg_TRANS_up
APR_HepG2_MicrotubuleCSK_24h_dn
2185−86−6
492−80−8
548−62−9

All Activity Heatmap for Cluster 1559

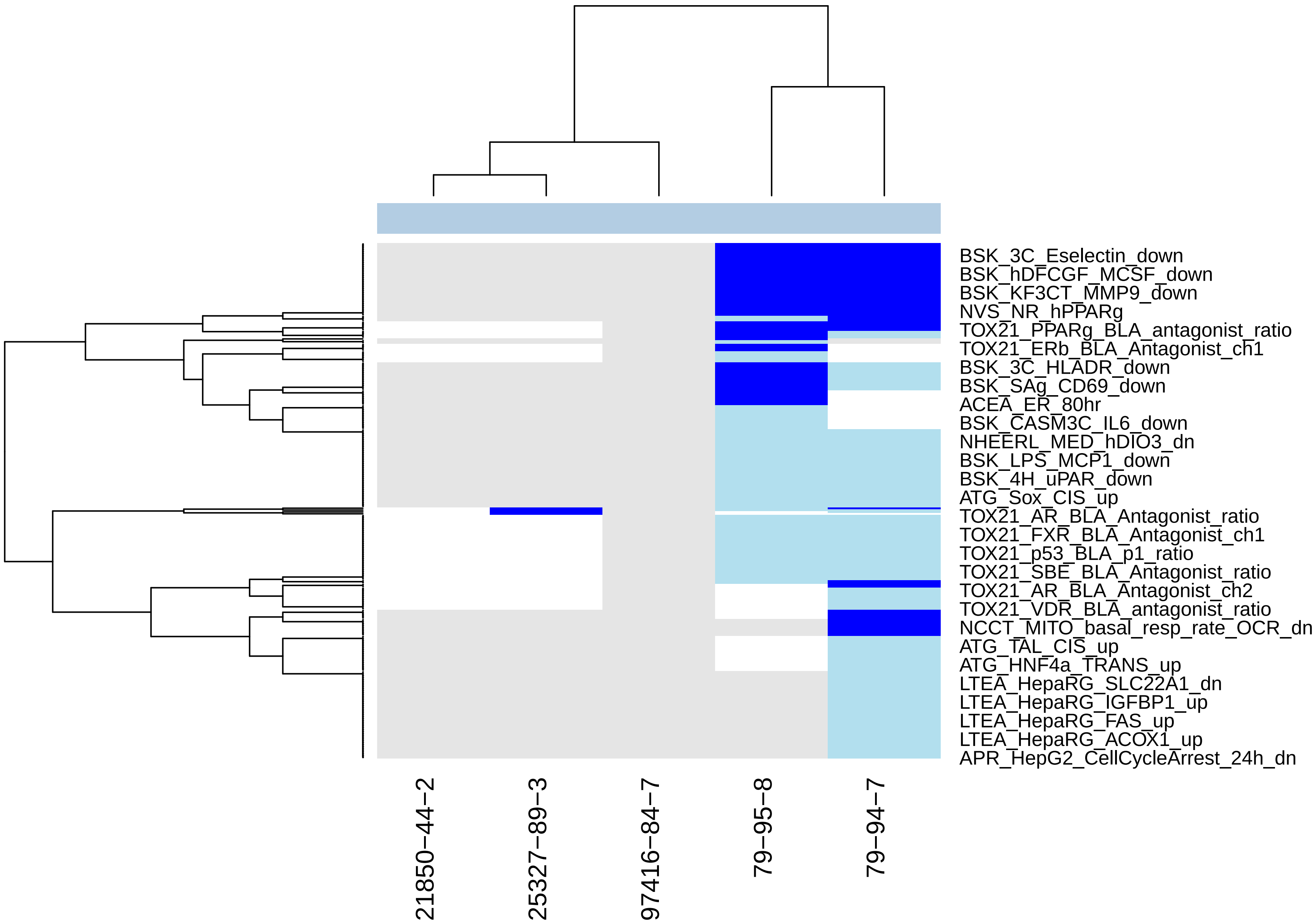

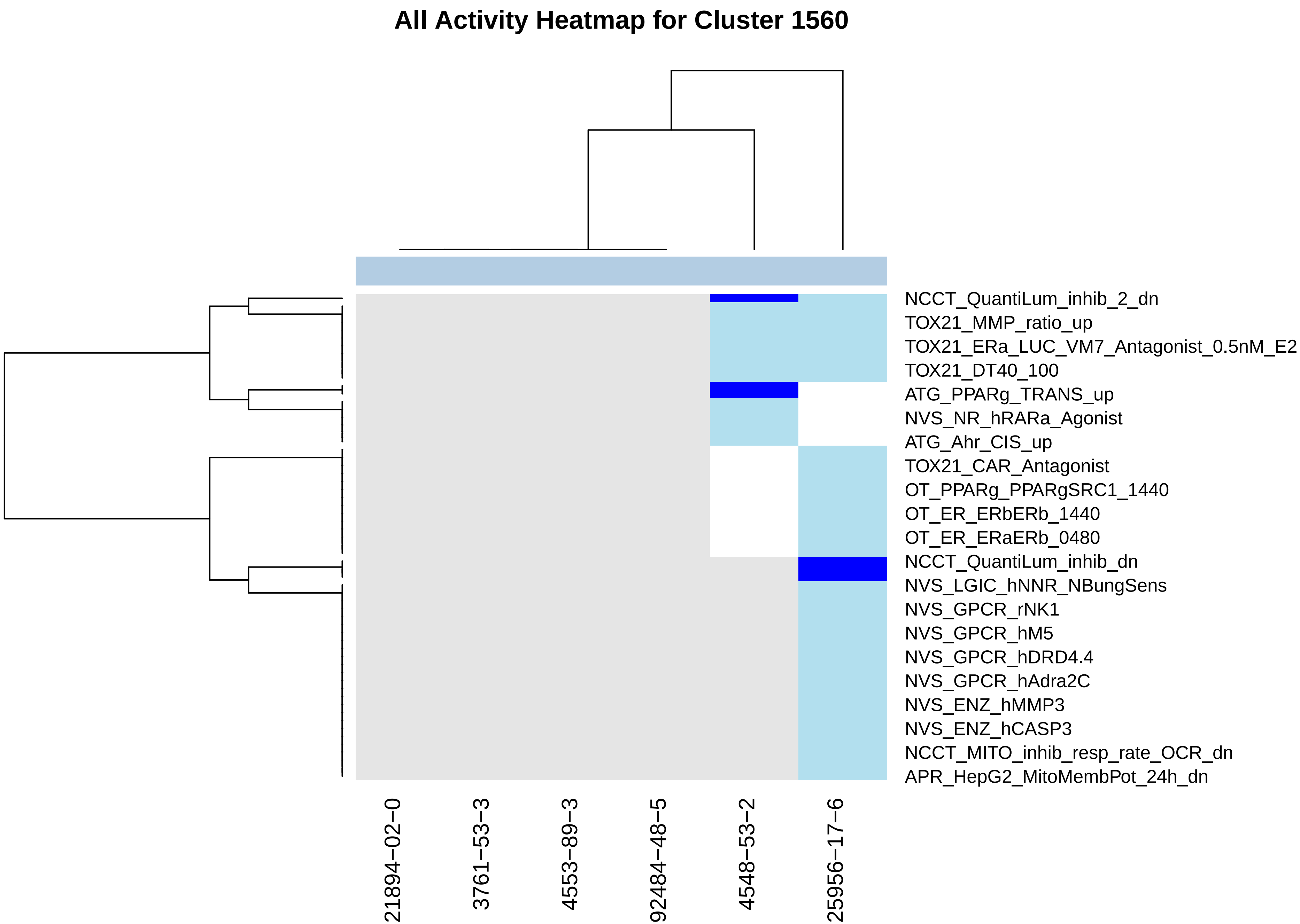
All Activity Heatmap for Cluster 1560
NCCT_QuantiLum_inhib_2_dn
TOX21_MMP_ratio_up
TOX21_ERa_LUC_VM7_Antagonist_0.5nM_E2
TOX21_DT40_100
ATG_PPARg_TRANS_up
NVS_NR_hRARa_Agonist
ATG_Ahr_CIS_up
TOX21_CAR_Antagonist
OT_PPARg_PPARgSRC1_1440
OT_ER_ERbERb_1440
OT_ER_ERaERb_0480
NCCT_QuantiLum_inhib_dn
NVS_LGIC_hNNR_NBungSens
NVS_GPCR_rNK1
NVS_GPCR_hM5
NVS_GPCR_hDRD4.4
NVS_GPCR_hAdra2C
NVS_ENZ_hMMP3
NVS_ENZ_hCASP3
NCCT_MITO_inhib_resp_rate_OCR_dn
APR_HepG2_MitoMembPot_24h_dn
21894−02−0
3761−53−3
4553−89−3
92484−48−5
4548−53−2
25956−17−6

All Activity Heatmap for Cluster 1563

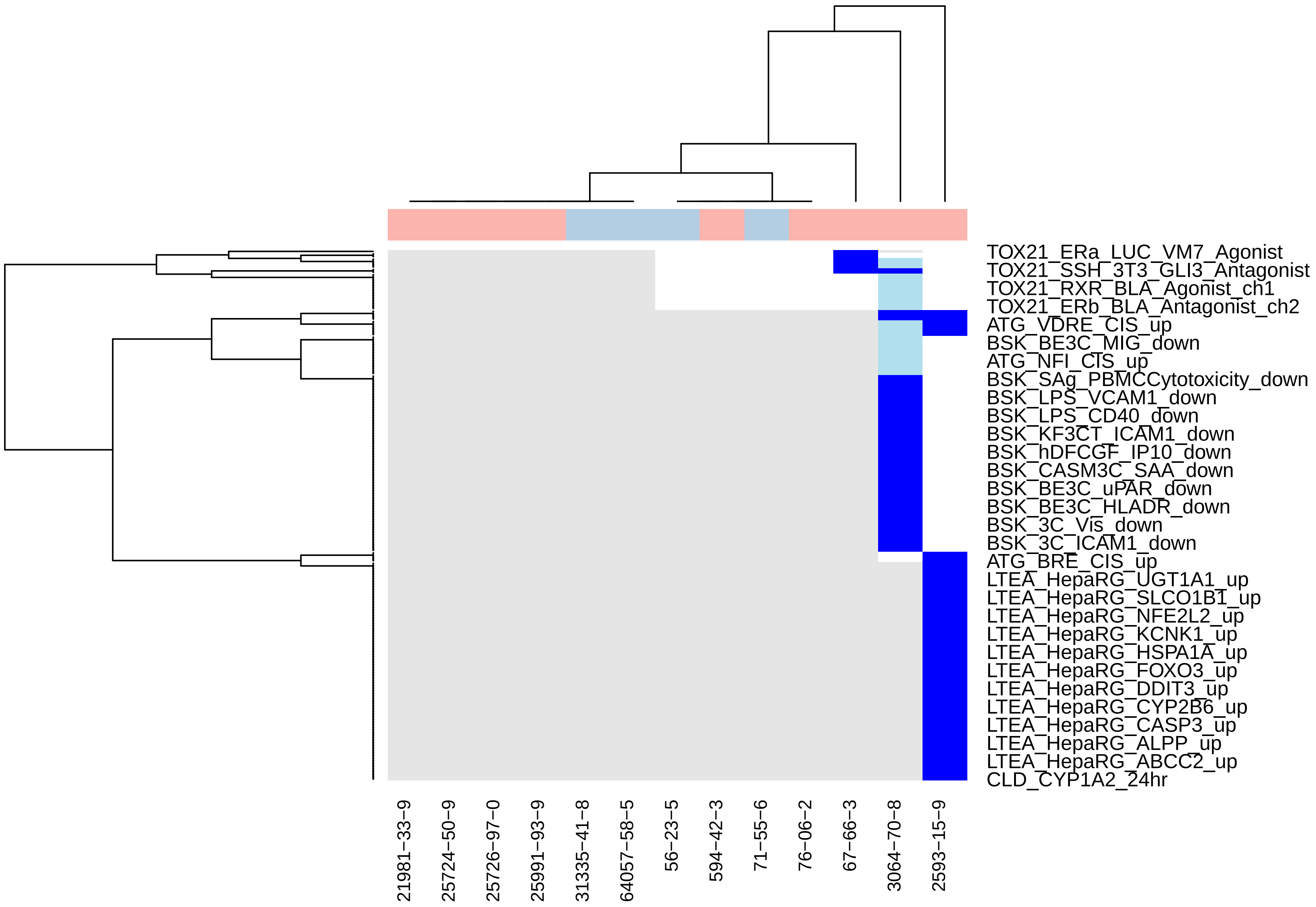

All Activity Heatmap for Cluster 1566

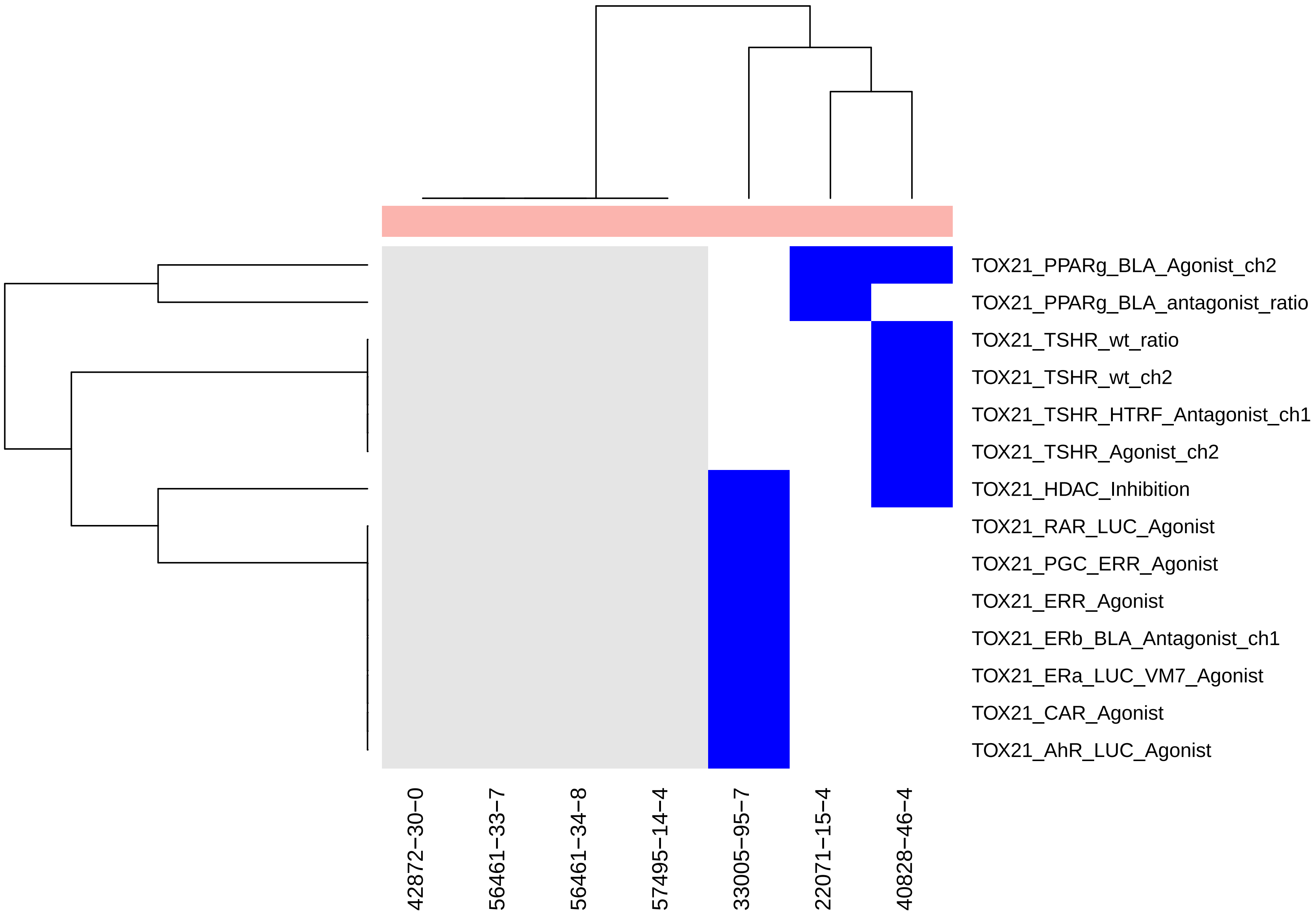

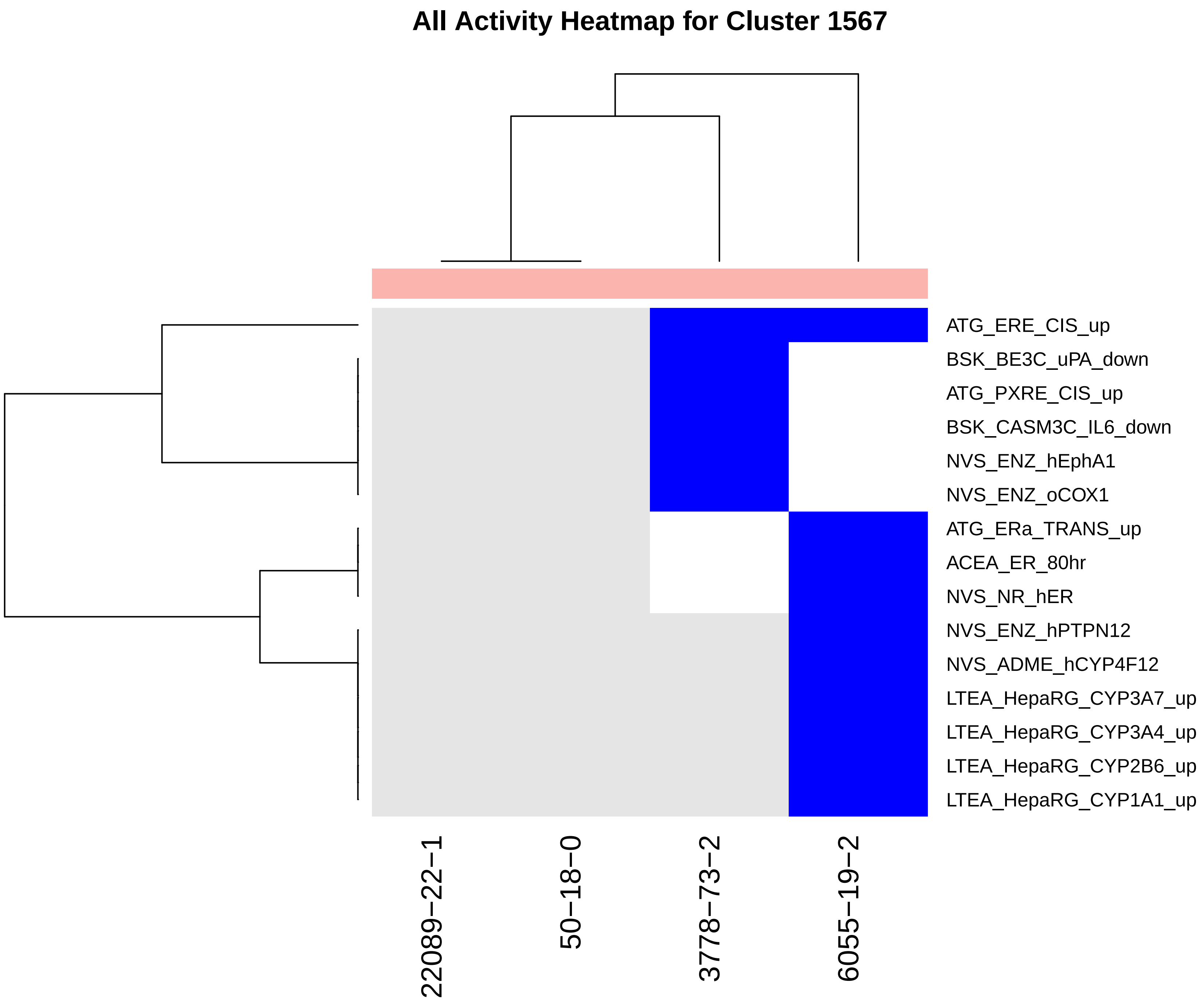
All Activity Heatmap for Cluster 1567
ATG_ERE_CIS_up
BSK_BE3C_uPA_down
ATG_PXRE_CIS_up
BSK_CASM3C_IL6_down
NVS_ENZ_hEphA1
NVS_ENZ_oCOX1
ATG_ERa_TRANS_up
ACEA_ER_80hr
NVS_NR_hER
NVS_ENZ_hPTPN12
NVS_ADME_hCYP4F12
LTEA_HepaRG_CYP3A7_up
LTEA_HepaRG_CYP3A4_up
LTEA_HepaRG_CYP2B6_up
LTEA_HepaRG_CYP1A1_up
22089−22−1
50−18−0
3778−73−2
6055−19−2

All Activity Heatmap for Cluster 1568

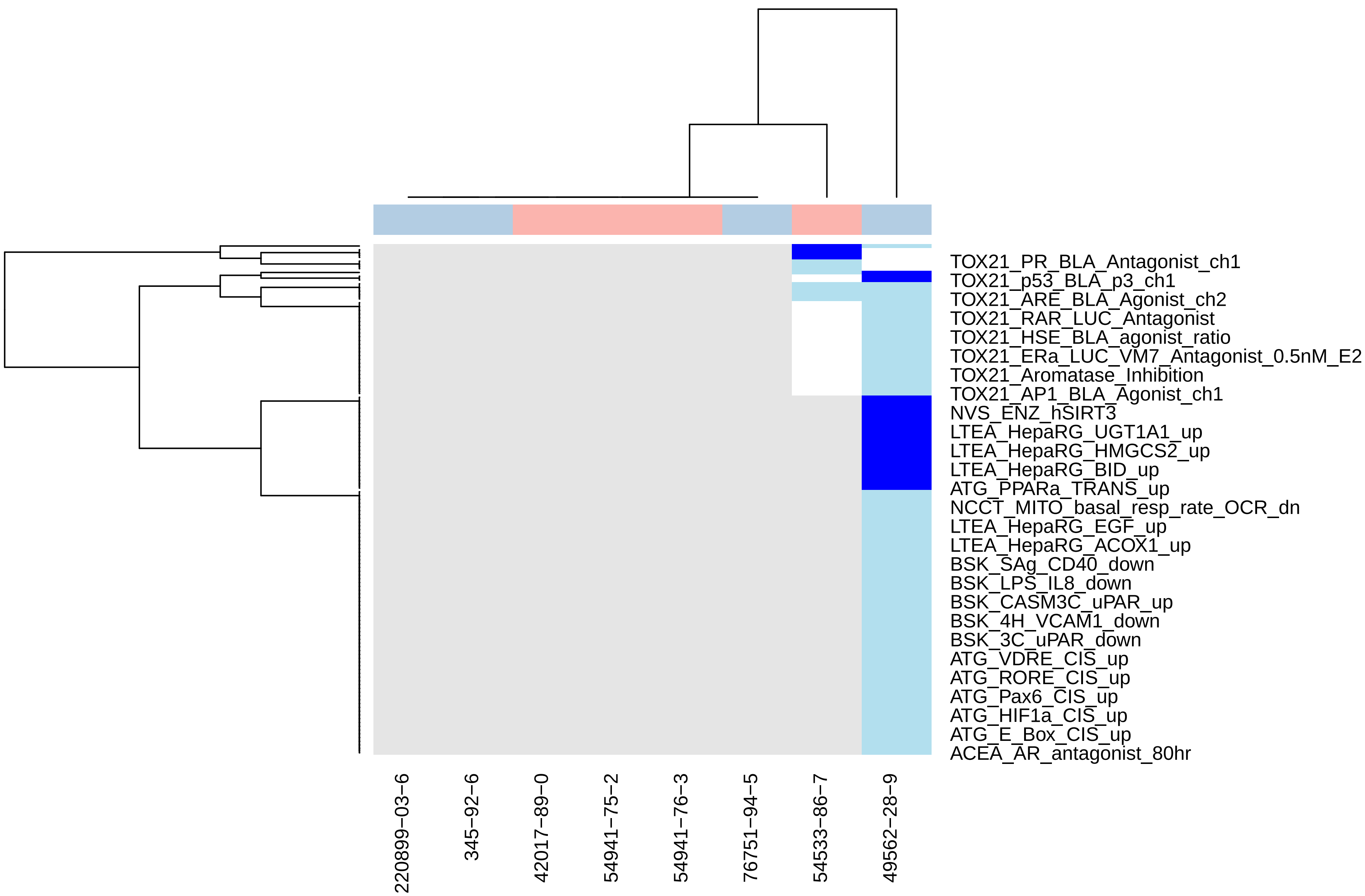

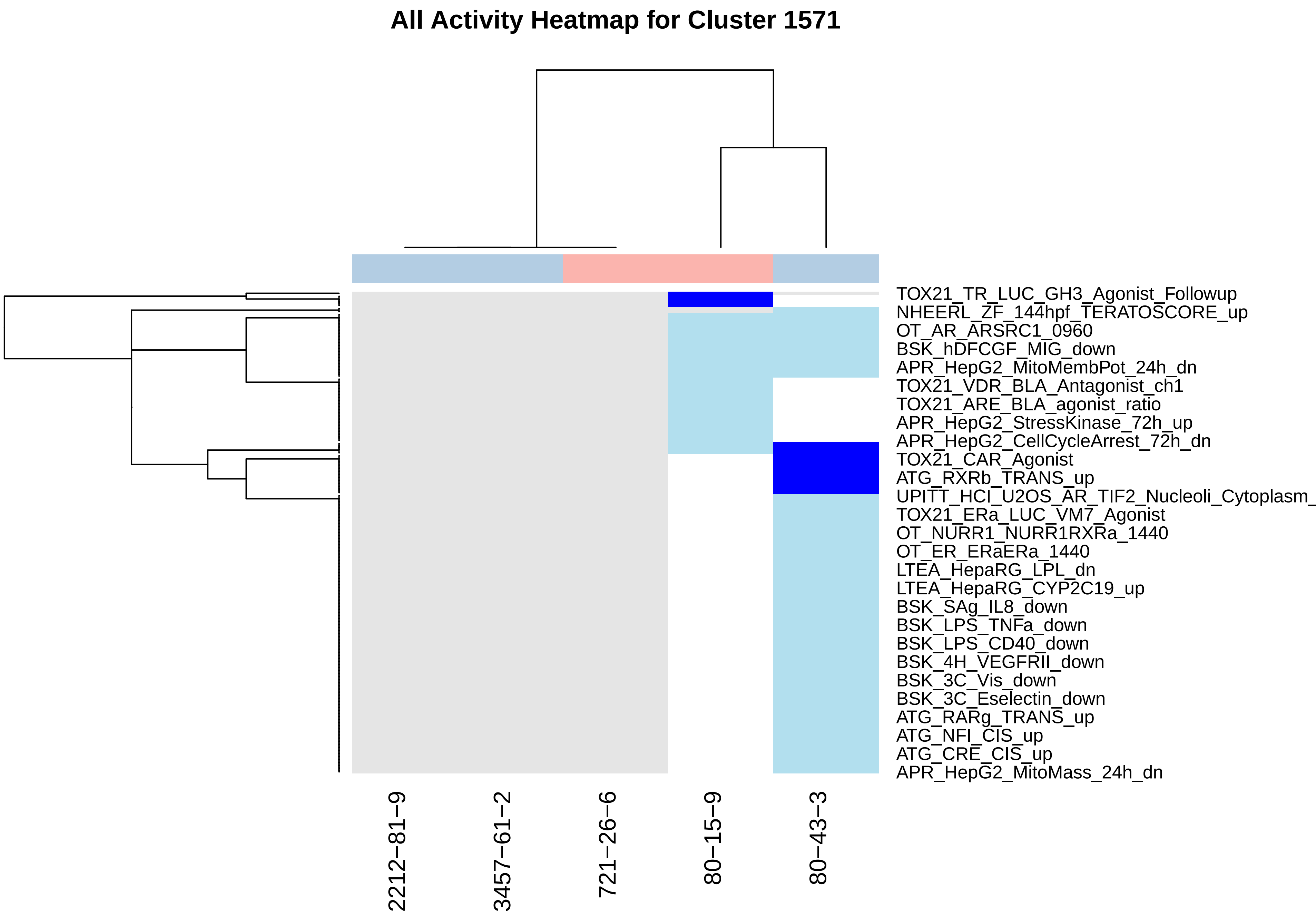

All Activity Heatmap for Cluster 1571
TOX21_TR_LUC_GH3_Agonist_Followup
NHEERL_ZF_144hpf_TERATOSCORE_up
OT_AR_ARSRC1_0960
BSK_hDFCGF_MIG_down
APR_HepG2_MitoMembPot_24h_dn
TOX21_VDR_BLA_Antagonist_ch1
TOX21_ARE_BLA_agonist_ratio
APR_HepG2_StressKinase_72h_up
APR_HepG2_CellCycleArrest_72h_dn
TOX21_CAR_Agonist
ATG_RXRb_TRANS_up
UPITT_HCI_U2OS_AR_TIF2_Nucleoli_Cytoplasm_
TOX21_ERa_LUC_VM7_Agonist
OT_NURR1_NURR1RXRa_1440
OT_ER_ERaERa_1440
LTEA_HepaRG_LPL_dn
LTEA_HepaRG_CYP2C19_up
BSK_SAg_IL8_down
BSK_LPS_TNFa_down
BSK_LPS_CD40_down
BSK_4H_VEGFRII_down
BSK_3C_Vis_down
BSK_3C_Eselectin_down
ATG_RARg_TRANS_up
ATG_NFI_CIS_up
ATG_CRE_CIS_up
APR_HepG2_MitoMass_24h_dn
2212−81−9
3457−61−2
721−26−6
80−15−9
80−43−3

All Activity Heatmap for Cluster 1573

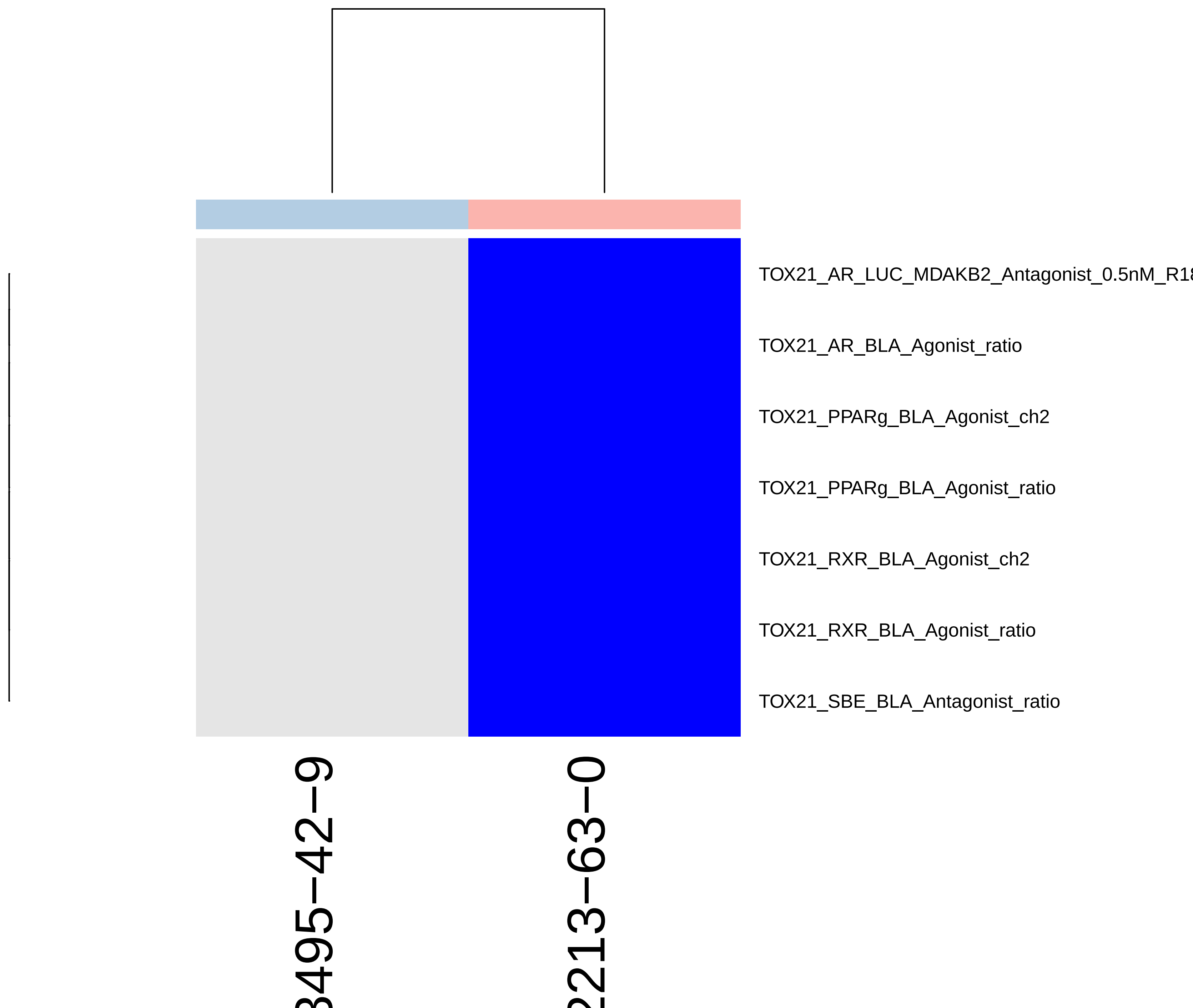

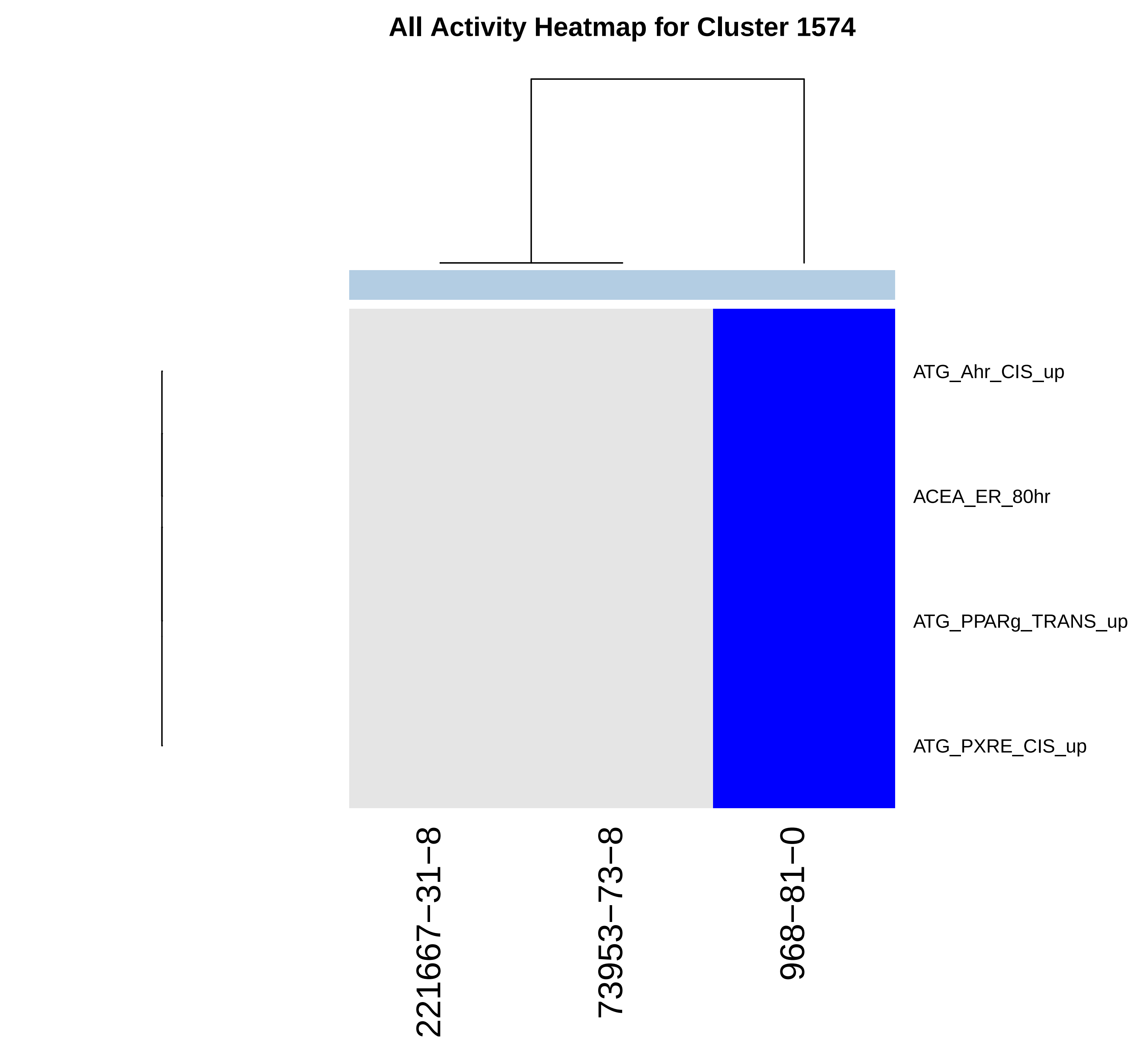
All Activity Heatmap for Cluster 1574
ATG_Ahr_CIS_up
ACEA_ER_80hr
ATG_PPARg_TRANS_up
ATG_PXRE_CIS_up
221667−31−8
73953−73−8
968−81−0

All Activity Heatmap for Cluster 1575

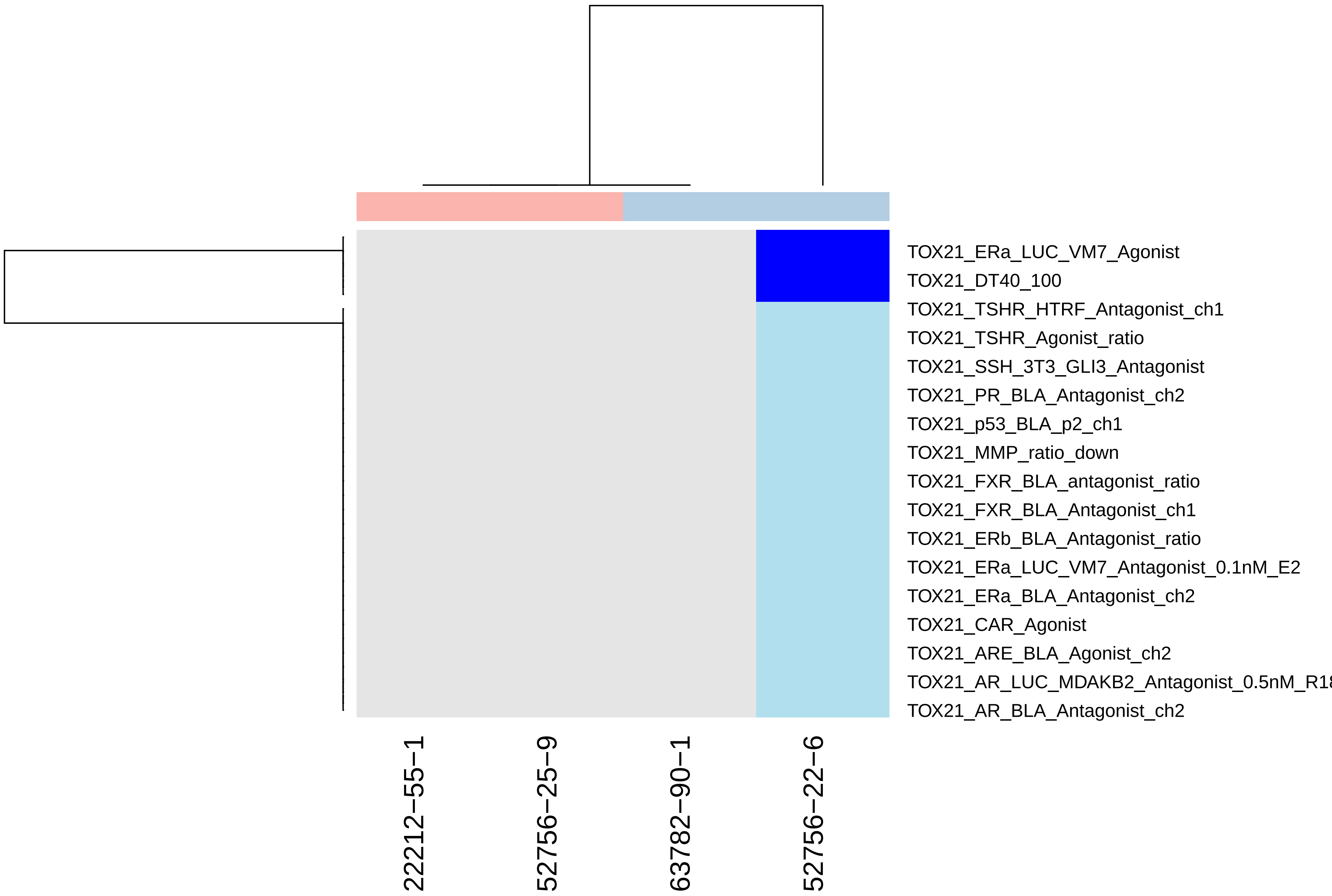

All Activity Heatmap for Cluster 1576

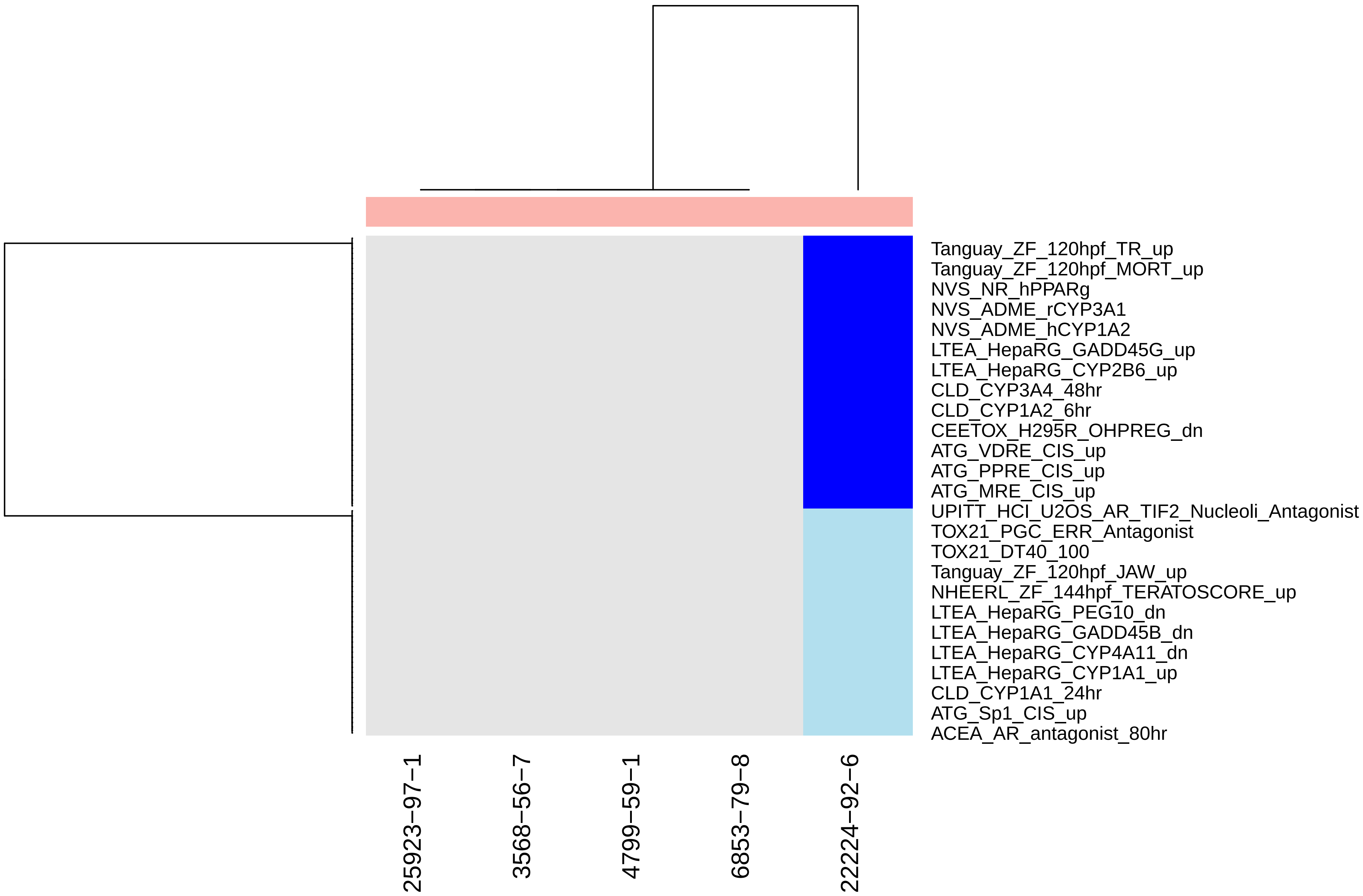

All Activity Heatmap for Cluster 1577

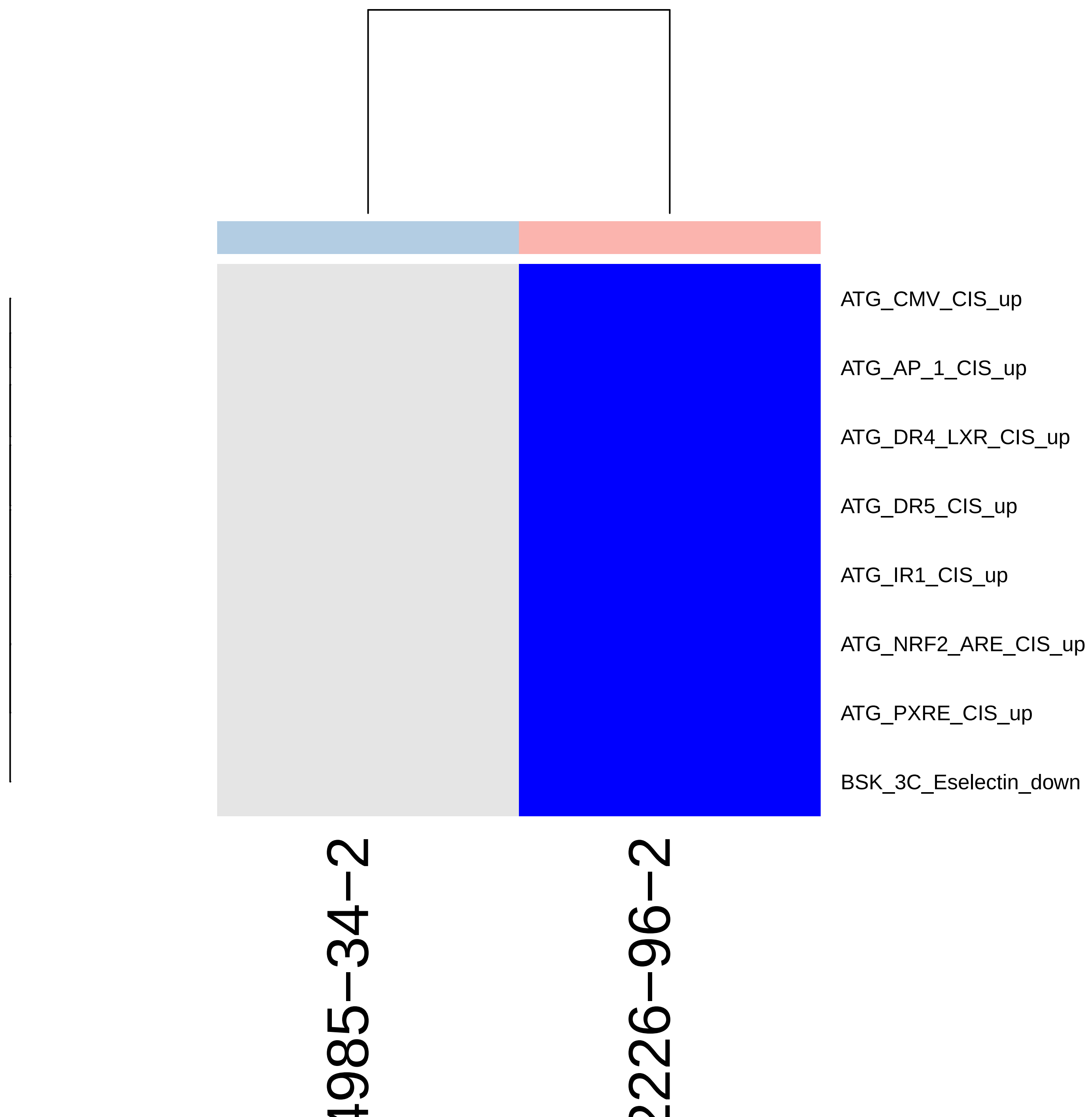

All Activity Heatmap for Cluster 1578

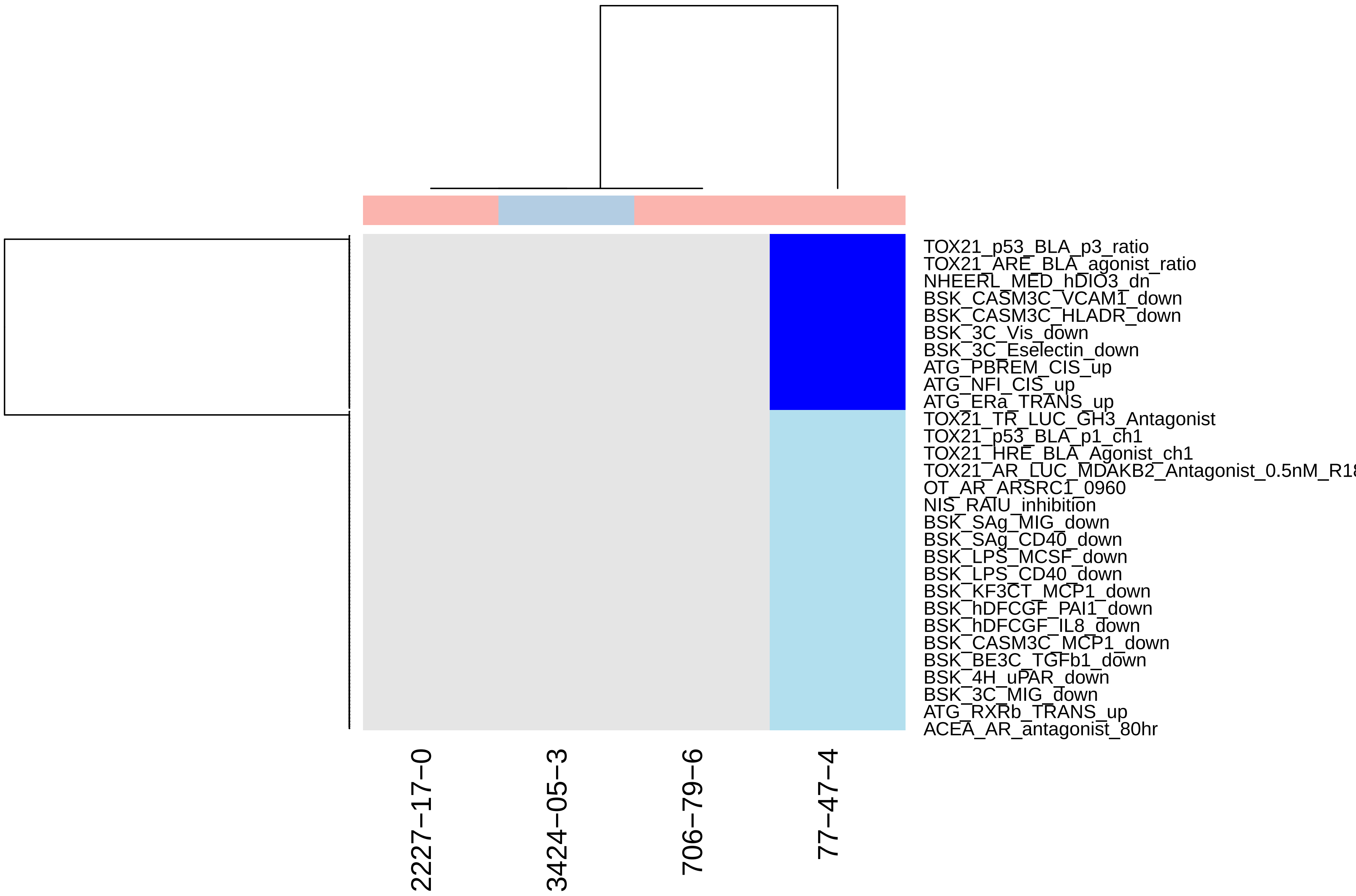

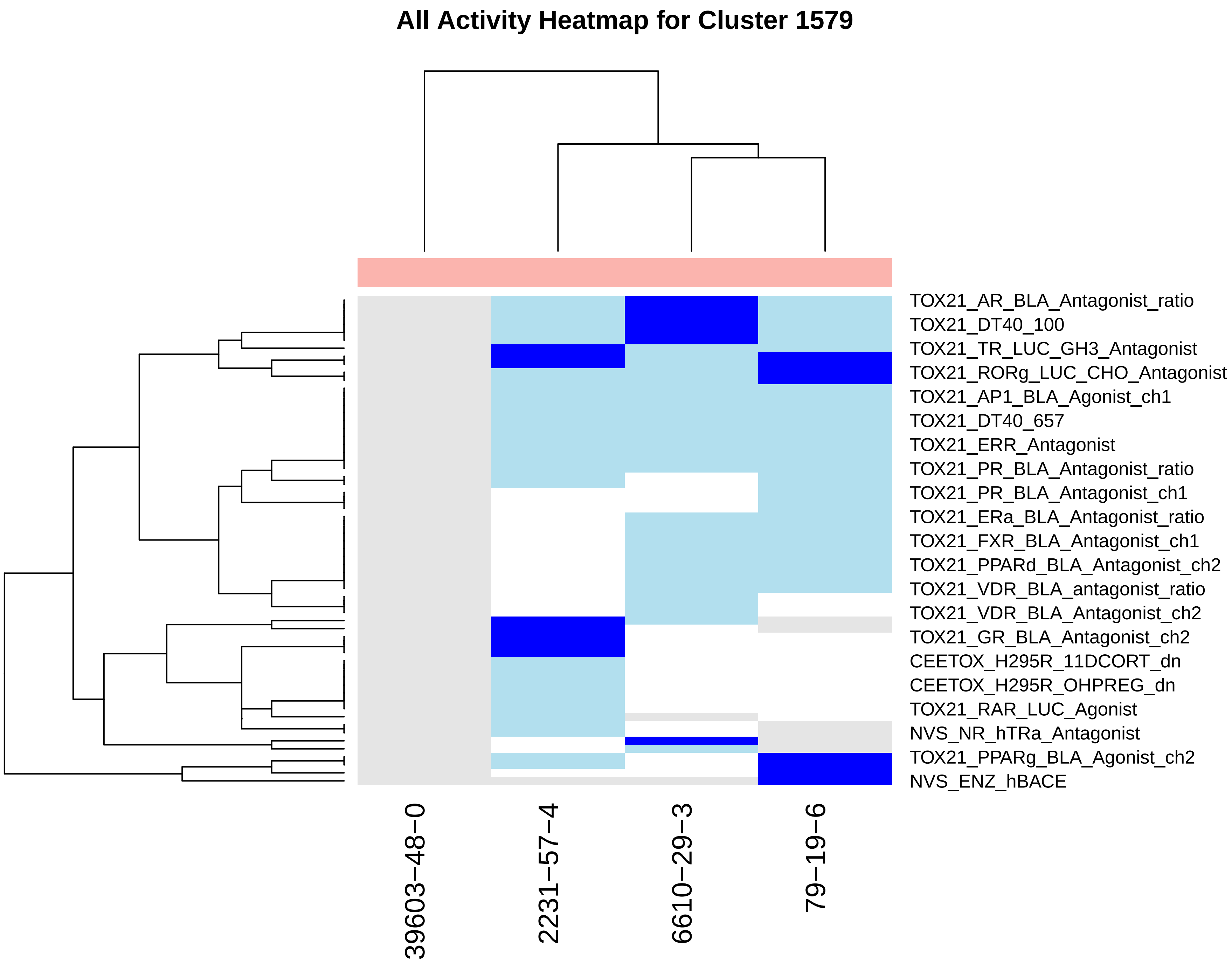
All Activity Heatmap for Cluster 1579
TOX21_AR_BLA_Antagonist_ratio
TOX21_DT40_100
TOX21_TR_LUC_GH3_Antagonist
TOX21_RORg_LUC_CHO_Antagonist
TOX21_AP1_BLA_Agonist_ch1
TOX21_DT40_657
TOX21_ERR_Antagonist
TOX21_PR_BLA_Antagonist_ratio
TOX21_PR_BLA_Antagonist_ch1
TOX21_ERa_BLA_Antagonist_ratio
TOX21_FXR_BLA_Antagonist_ch1
TOX21_PPARd_BLA_Antagonist_ch2
TOX21_VDR_BLA_antagonist_ratio
TOX21_VDR_BLA_Antagonist_ch2
TOX21_GR_BLA_Antagonist_ch2
CEETOX_H295R_11DCORT_dn
CEETOX_H295R_OHPREG_dn
TOX21_RAR_LUC_Agonist
NVS_NR_hTRa_Antagonist
TOX21_PPARg_BLA_Agonist_ch2
NVS_ENZ_hBACE
39603−48−0
2231−57−4
6610−29−3
79−19−6

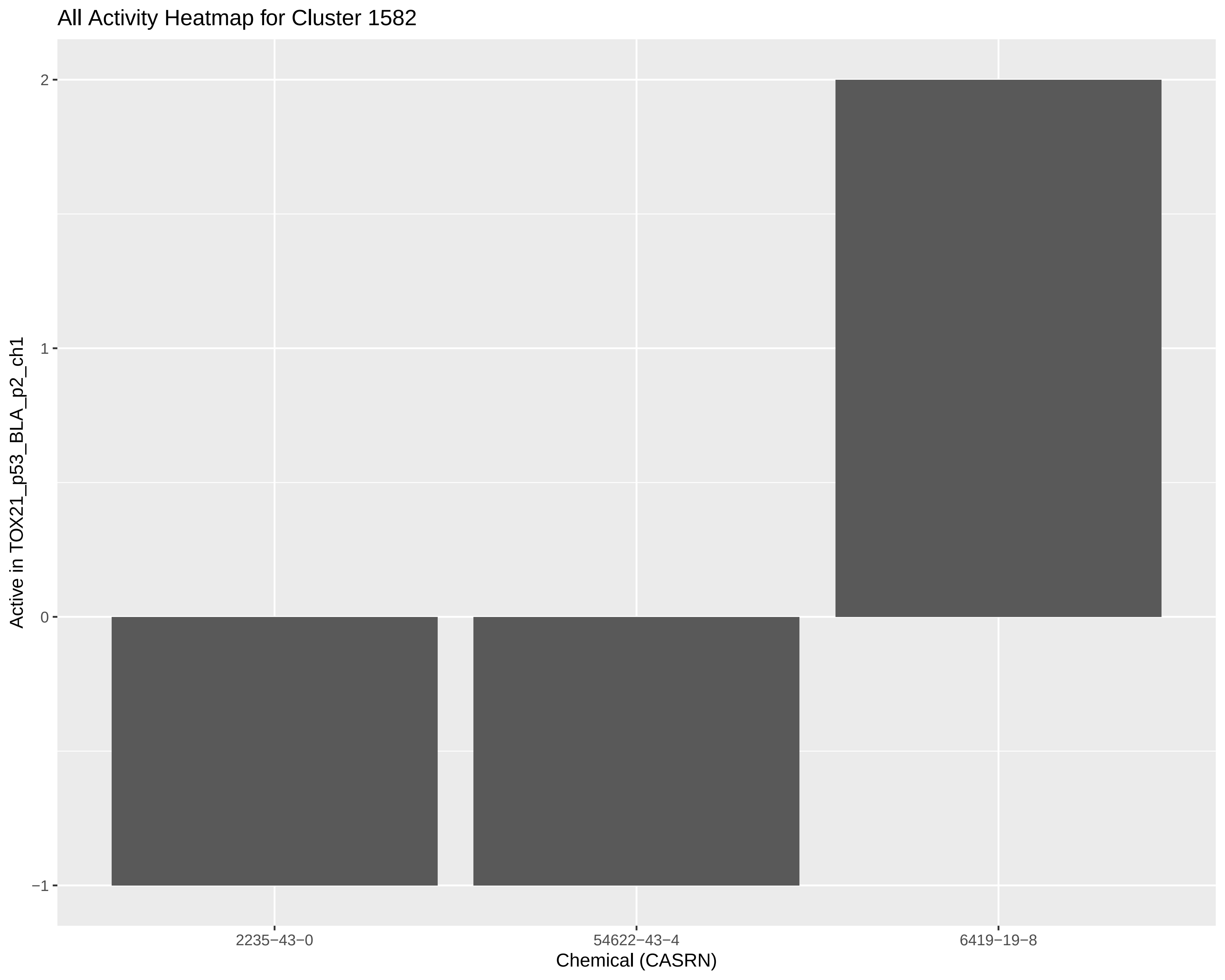
All Activity Heatmap for Cluster 1582
Active in TOX21_p53_BLA_p2_ch1
2
1
0
−1
2235−43−0
54622−43−4
6419−19−8
Chemical (CASRN)

All Activity Heatmap for Cluster 1584

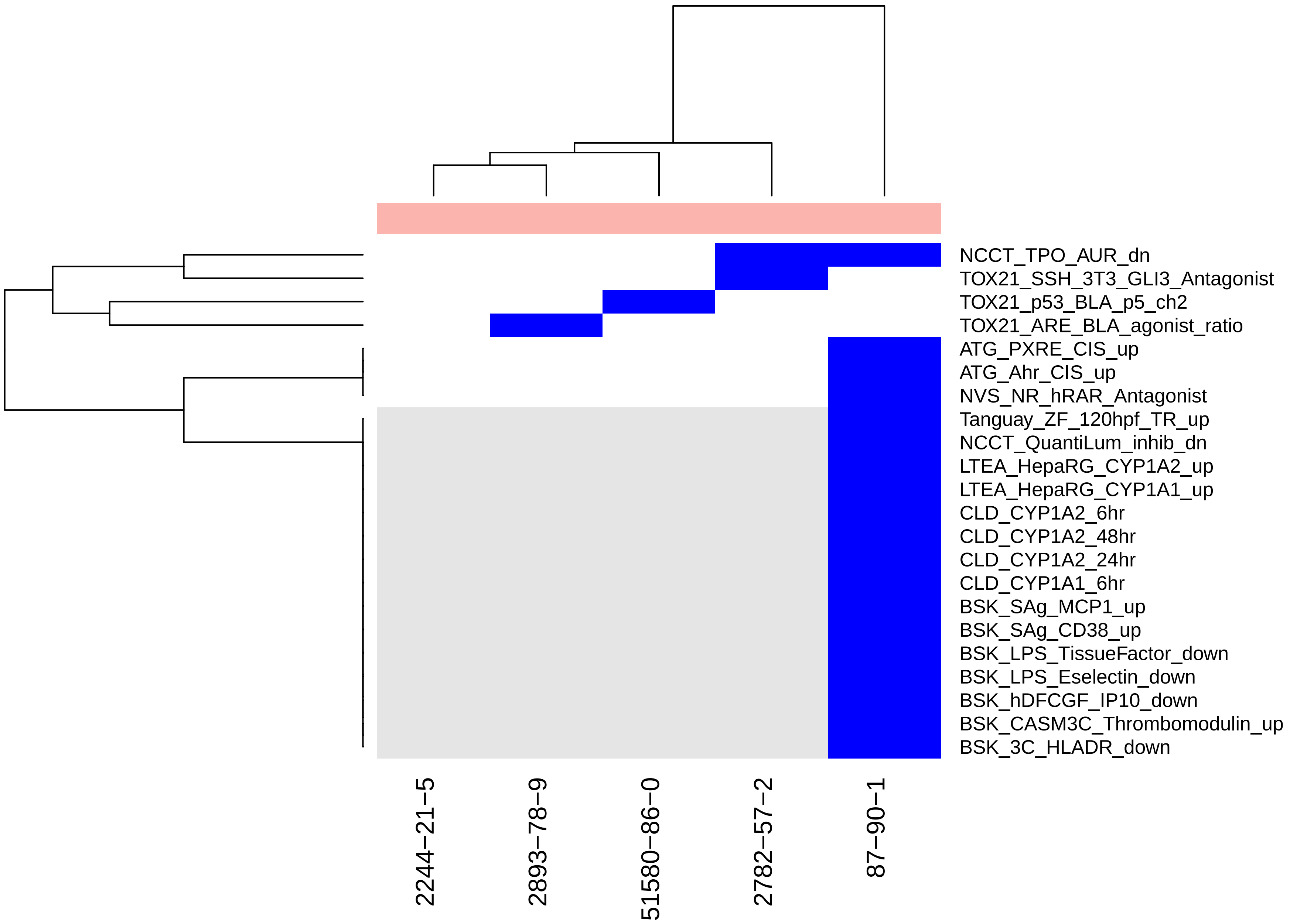

All Activity Heatmap for Cluster 1585

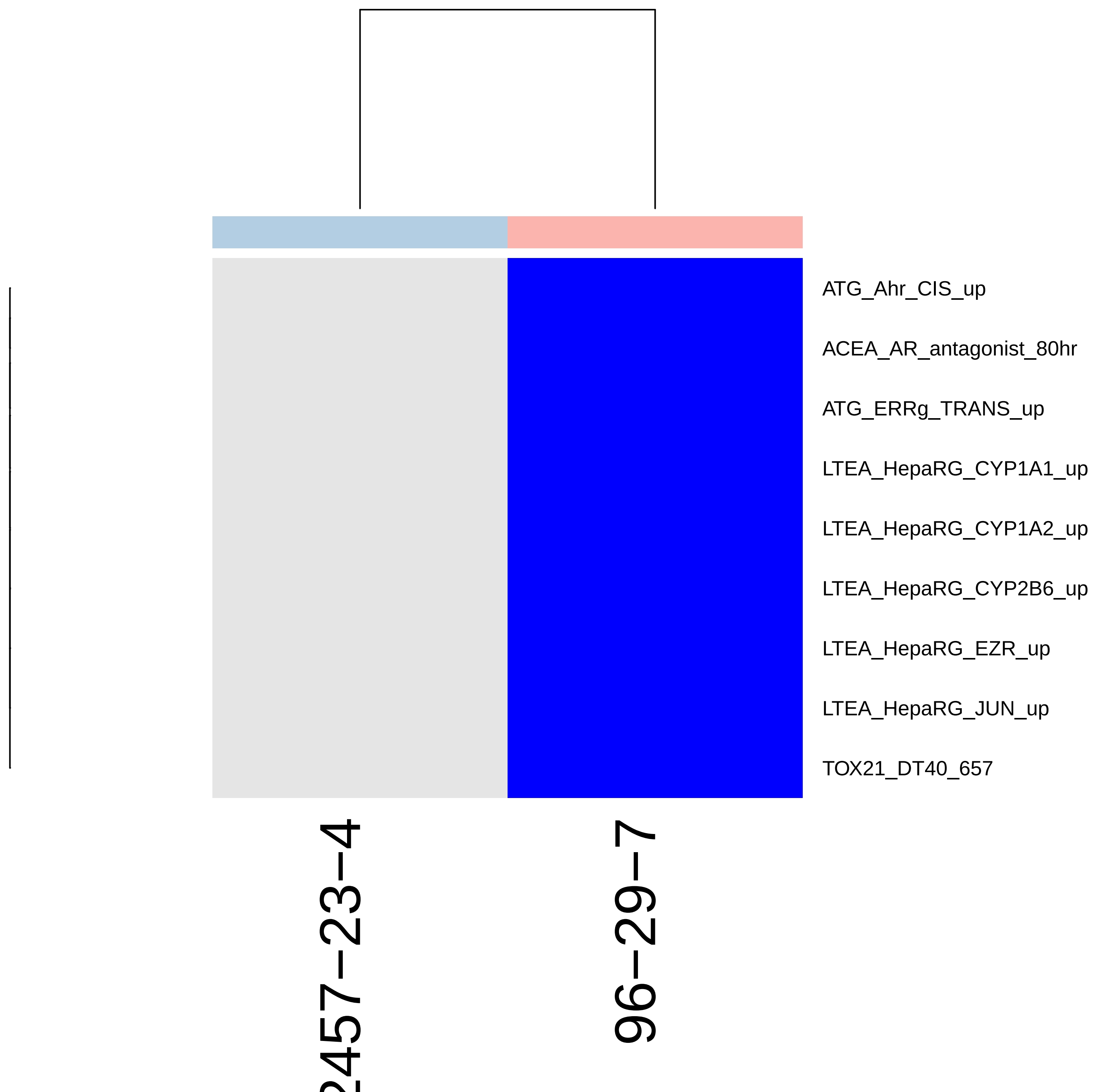

All Activity Heatmap for Cluster 1586

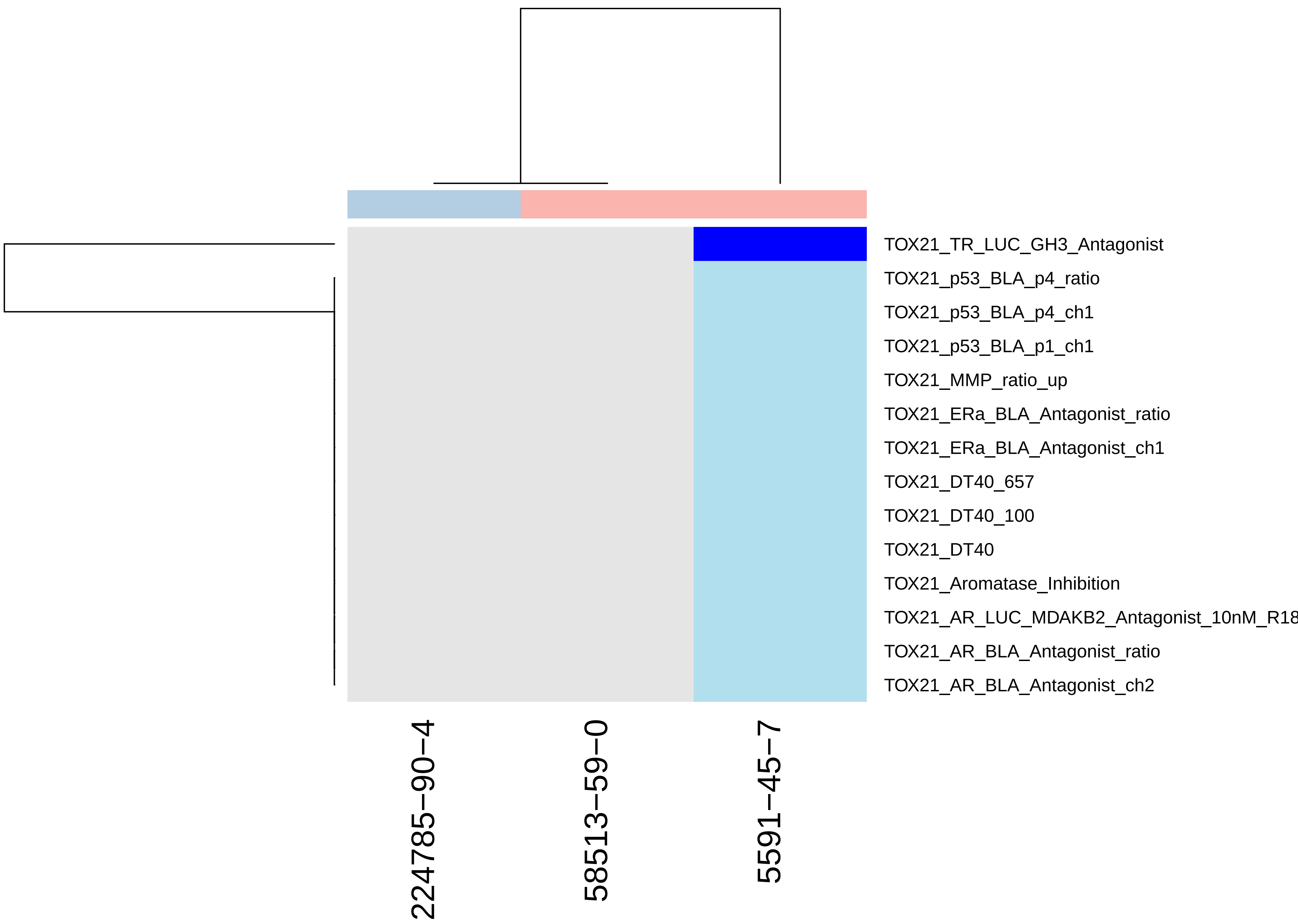

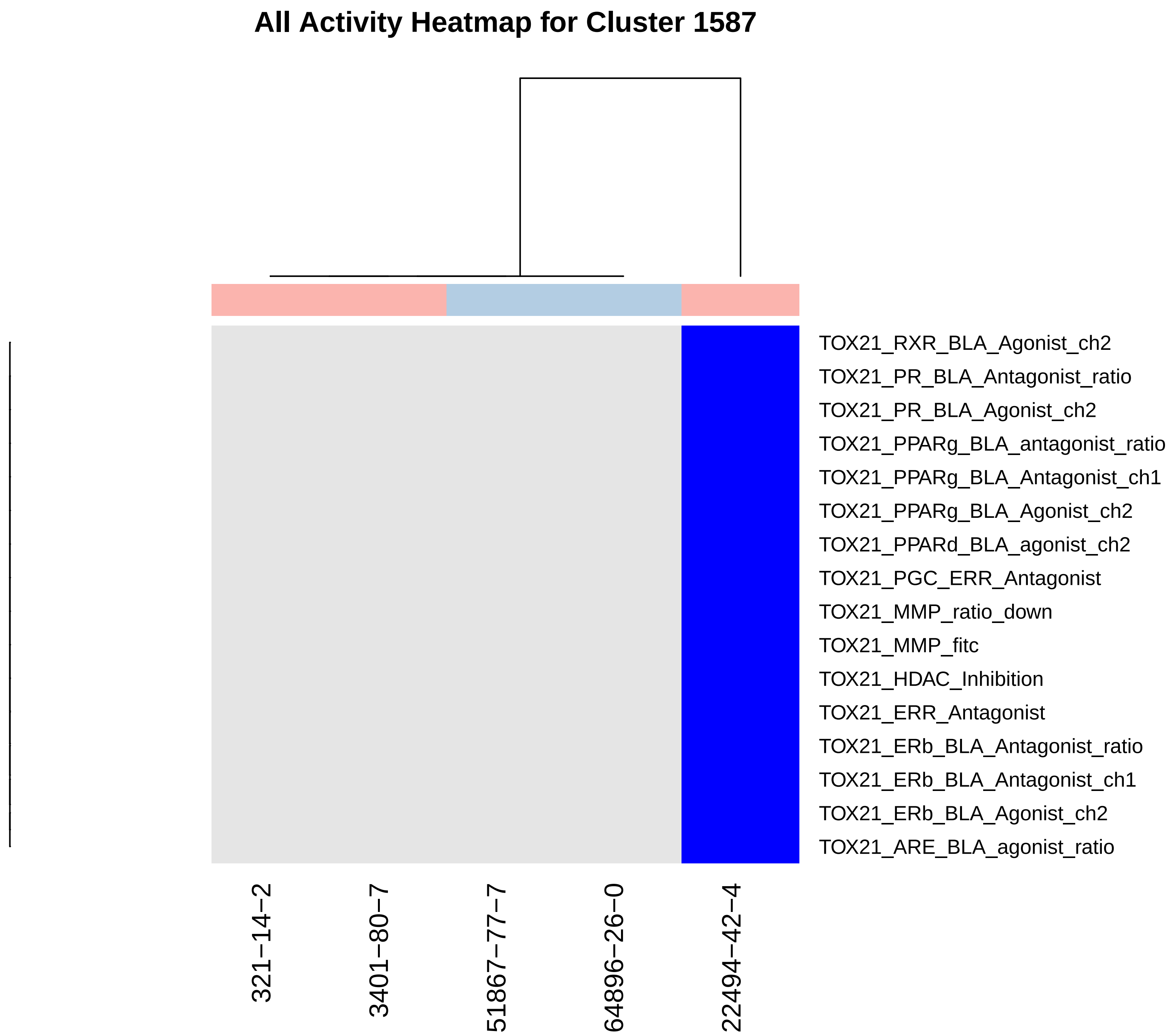
All Activity Heatmap for Cluster 1587
TOX21_RXR_BLA_Agonist_ch2
TOX21_PR_BLA_Antagonist_ratio
TOX21_PR_BLA_Agonist_ch2
TOX21_PPARg_BLA_antagonist_ratio
TOX21_PPARg_BLA_Antagonist_ch1
TOX21_PPARg_BLA_Agonist_ch2
TOX21_PPARd_BLA_agonist_ch2
TOX21_PGC_ERR_Antagonist
TOX21_MMP_ratio_down
TOX21_MMP_fitc
TOX21_HDAC_Inhibition
TOX21_ERR_Antagonist
TOX21_ERb_BLA_Antagonist_ratio
TOX21_ERb_BLA_Antagonist_ch1
TOX21_ERb_BLA_Agonist_ch2
TOX21_ARE_BLA_agonist_ratio
321−14−2
3401−80−7
51867−77−7
64896−26−0
22494−42−4

All Activity Heatmap for Cluster 1589

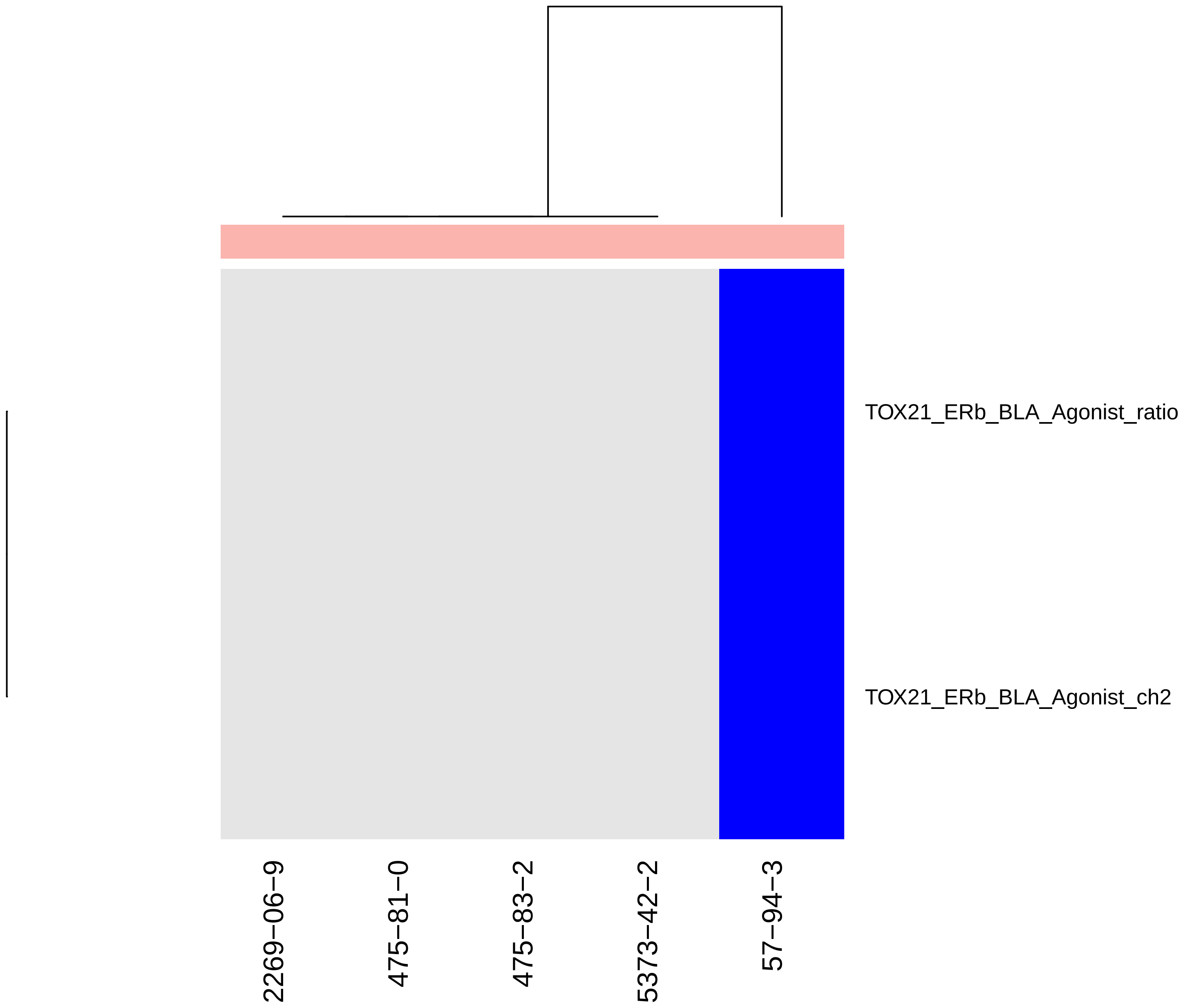

All Activity Heatmap for Cluster 1591

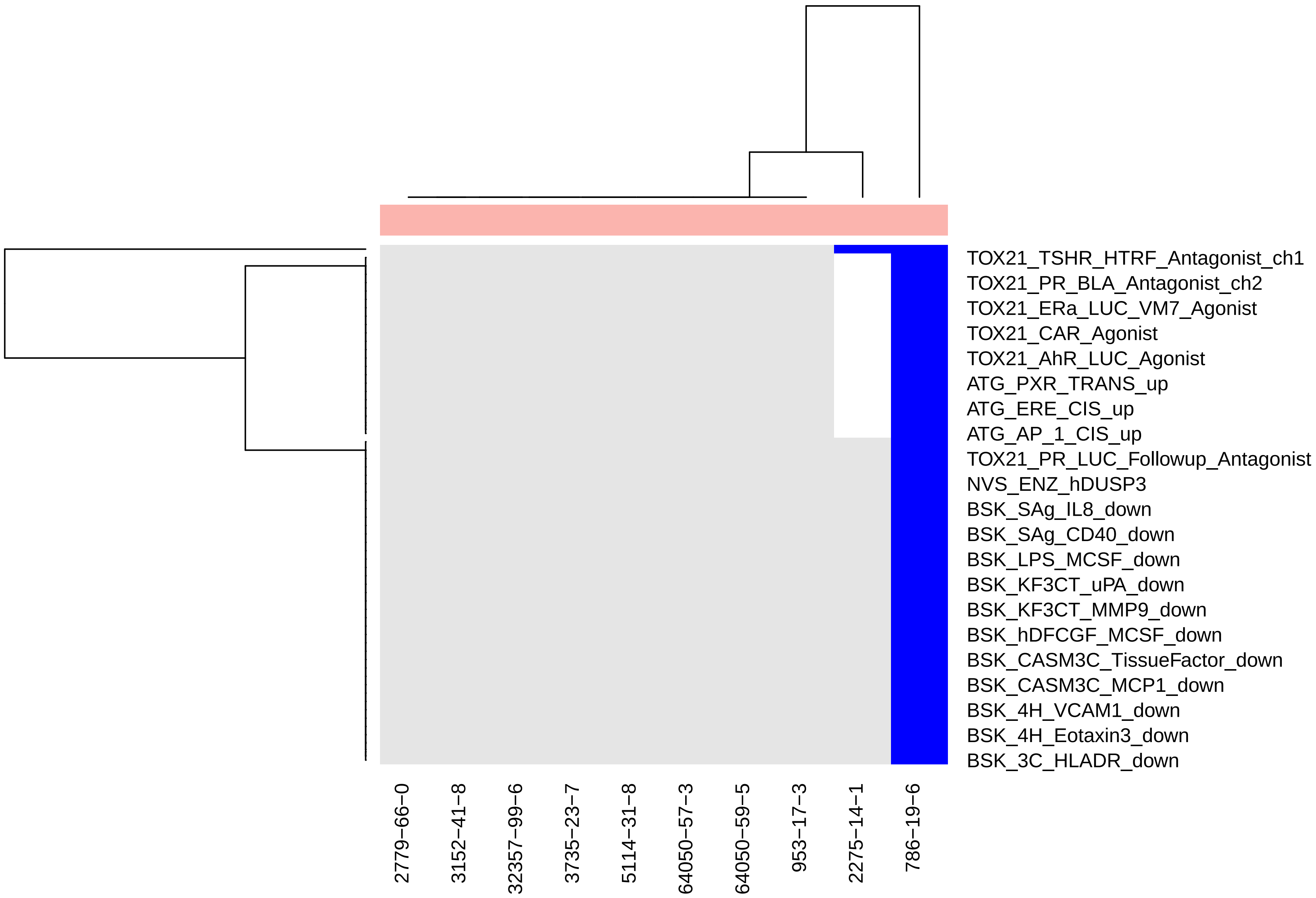

All Activity Heatmap for Cluster 1593

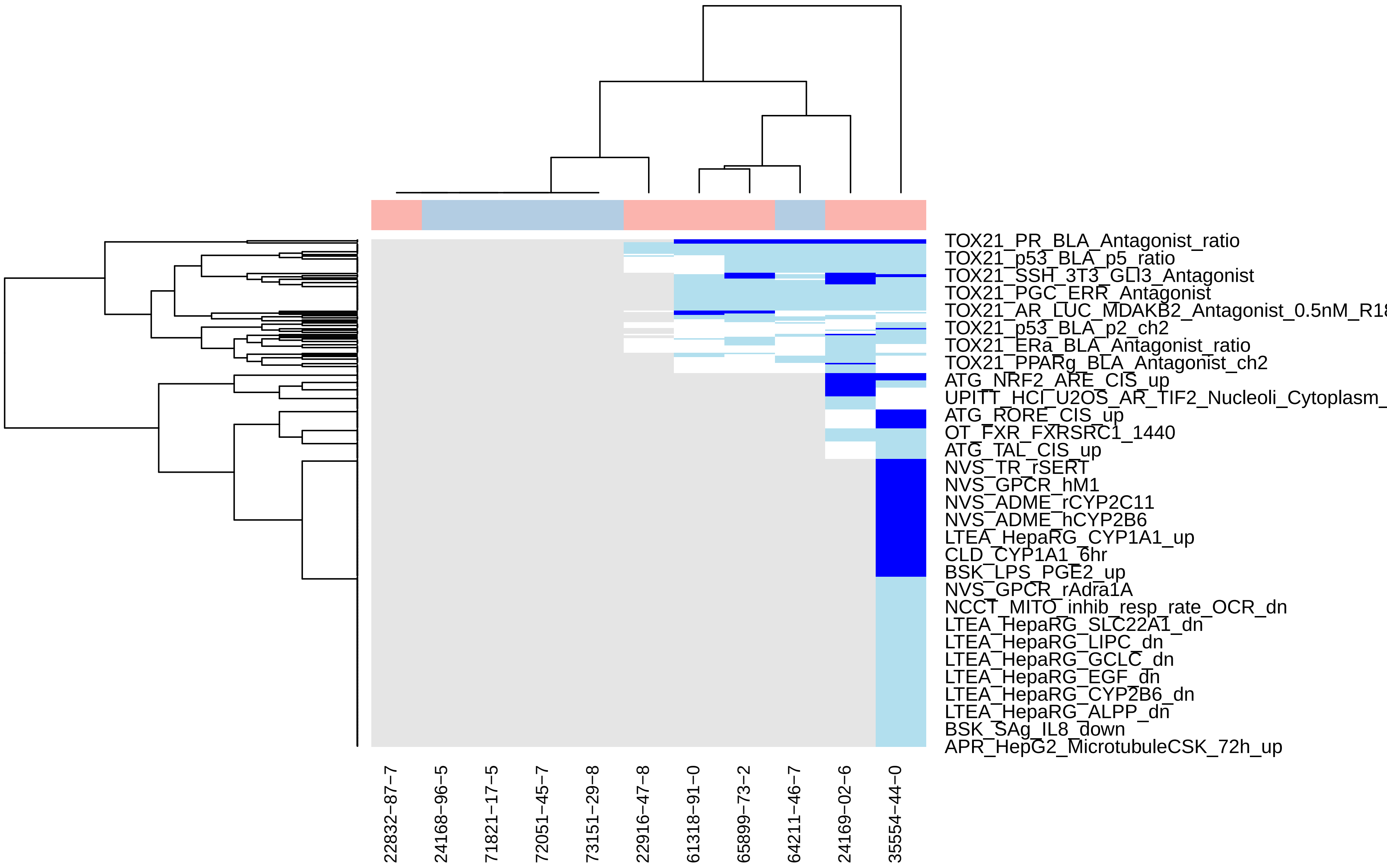

All Activity Heatmap for Cluster 1594

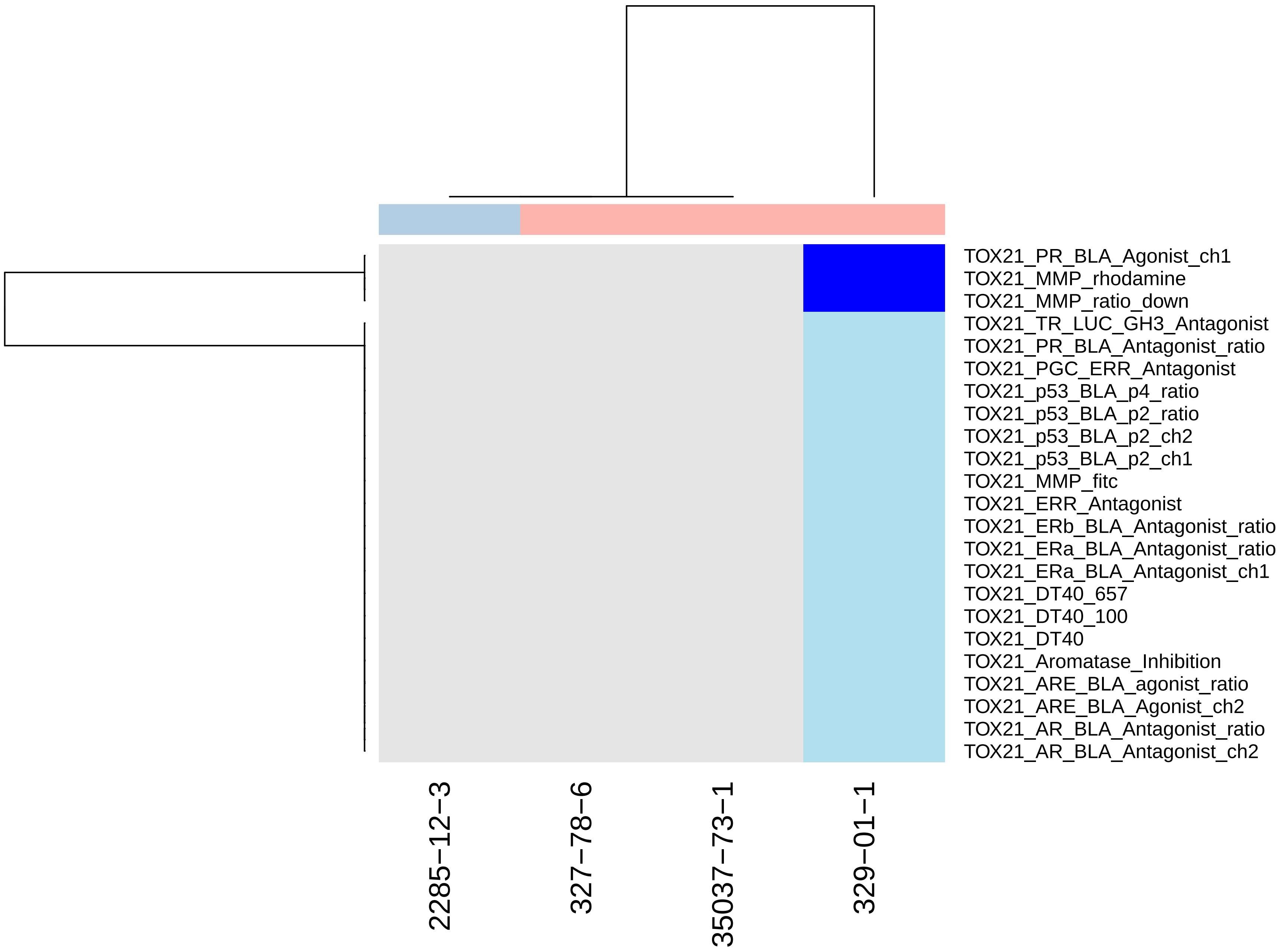

All Activity Heatmap for Cluster 1596

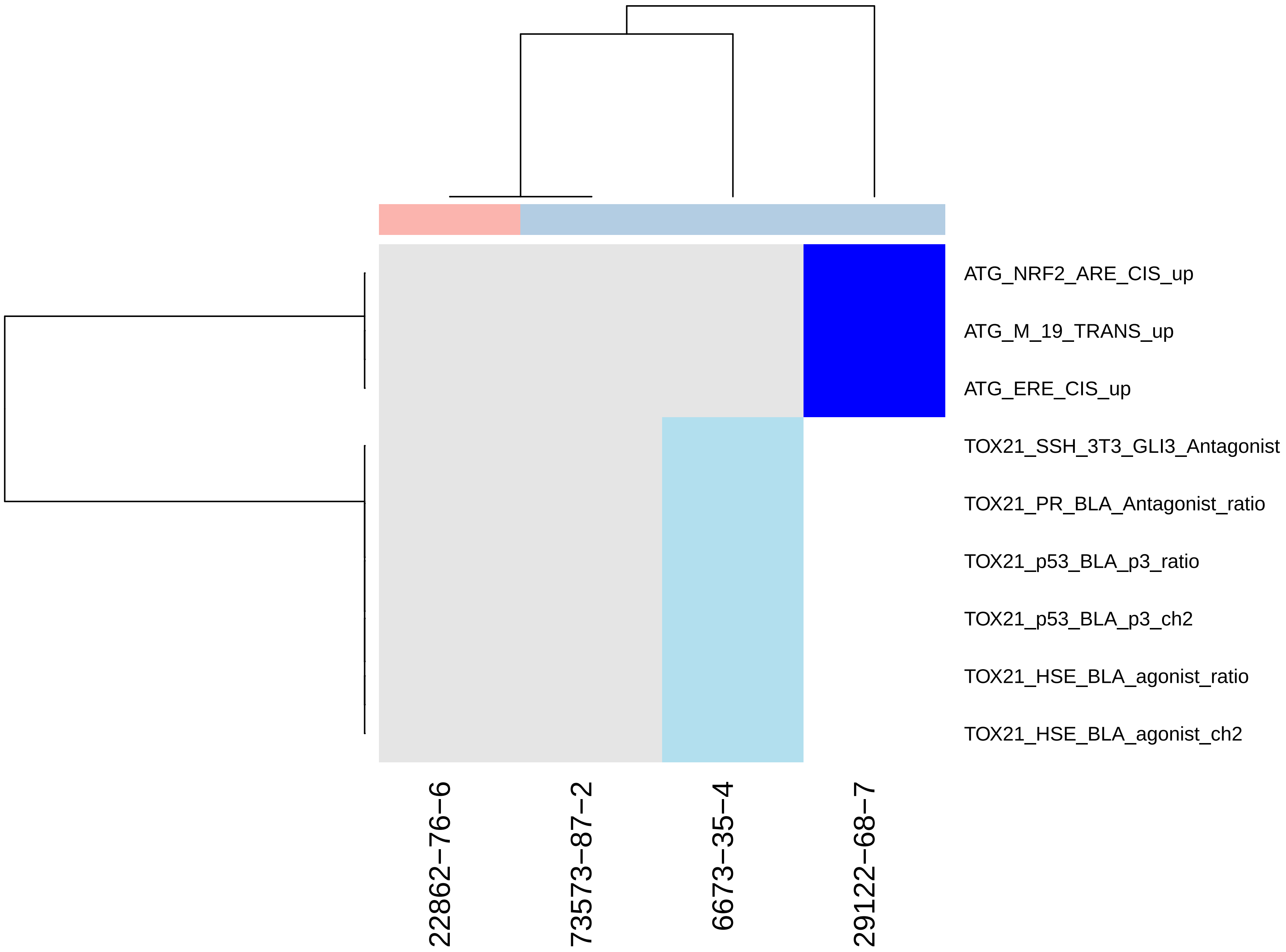

All Activity Heatmap for Cluster 1597

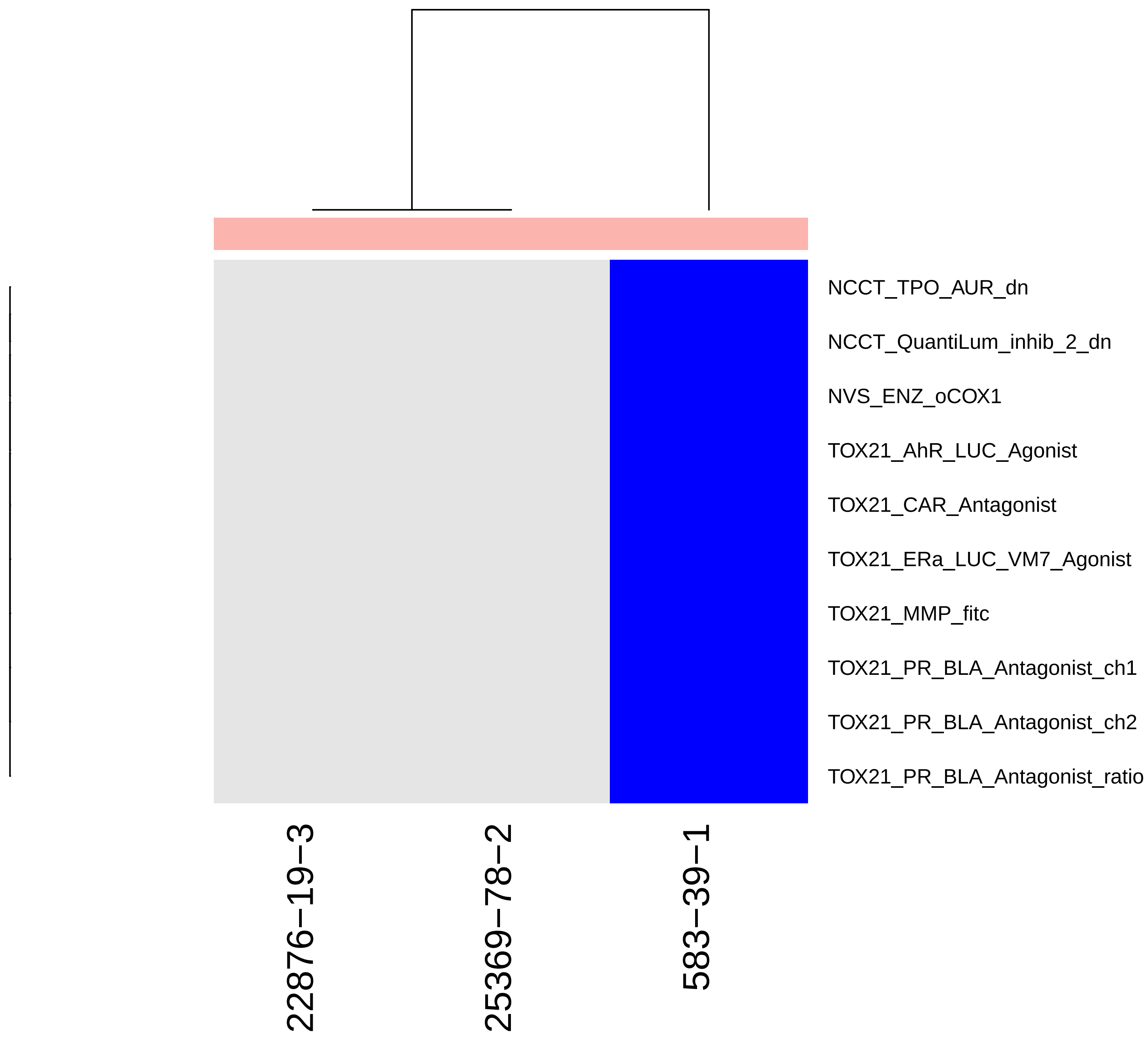

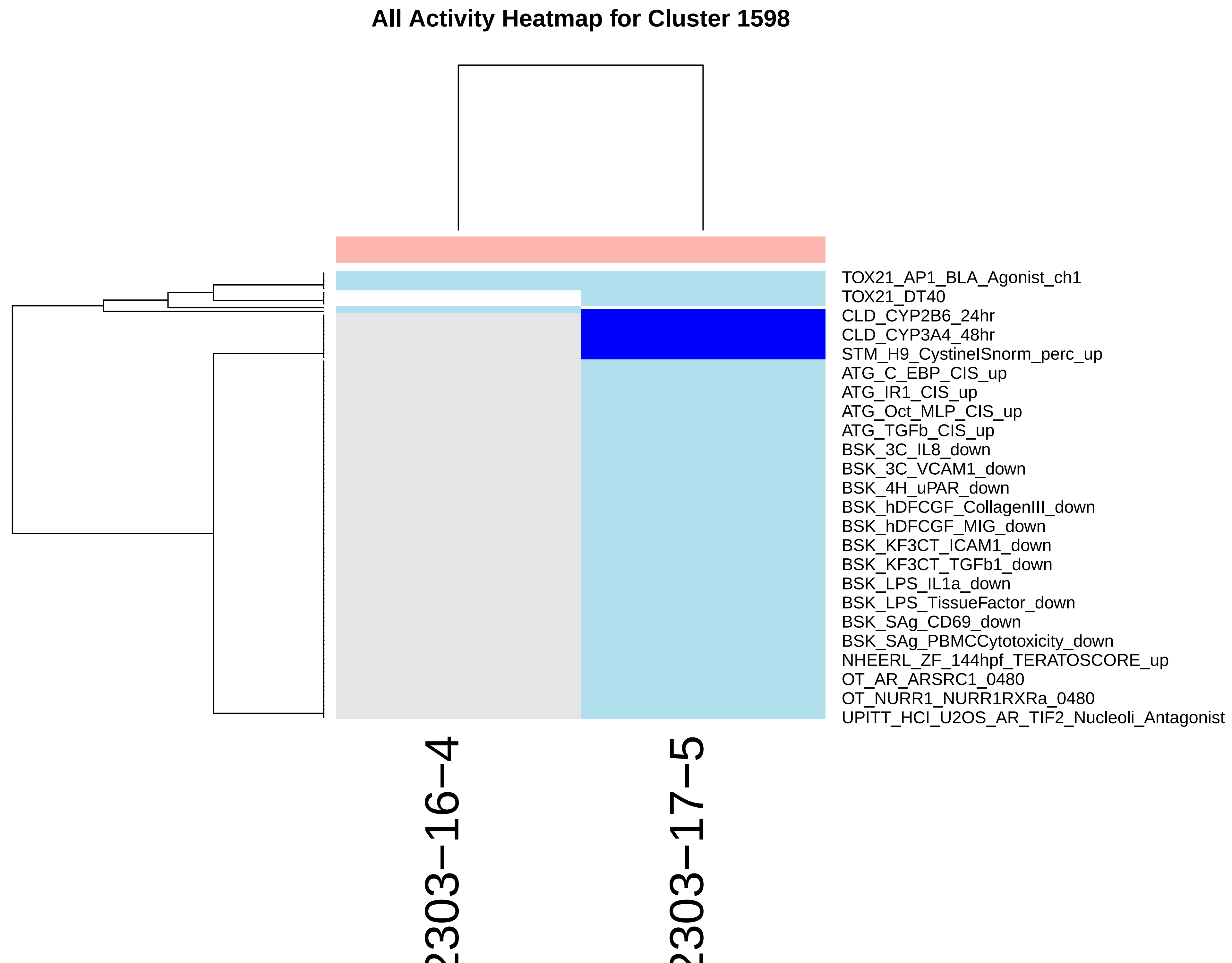
All Activity Heatmap for Cluster 1598
TOX21_AP1_BLA_Agonist_ch1
TOX21_DT40
CLD_CYP2B6_24hr
CLD_CYP3A4_48hr
STM_H9_CystineISnorm_perc_up
ATG_C_EBP_CIS_up
ATG_IR1_CIS_up
ATG_Oct_MLP_CIS_up
ATG_TGFb_CIS_up
BSK_3C_IL8_down
BSK_3C_VCAM1_down
BSK_4H_uPAR_down
BSK_hDFCGF_CollagenIII_down
BSK_hDFCGF_MIG_down
BSK_KF3CT_ICAM1_down
BSK_KF3CT_TGFb1_down
BSK_LPS_IL1a_down
BSK_LPS_TissueFactor_down
BSK_SAg_CD69_down
BSK_SAg_PBMCCytotoxicity_down
NHEERL_ZF_144hpf_TERATOSCORE_up
OT_AR_ARSRC1_0480
OT_NURR1_NURR1RXRa_0480
UPITT_HCI_U2OS_AR_TIF2_Nucleoli_Antagonist
2303−16−4
2303−17−5

All Activity Heatmap for Cluster 1599

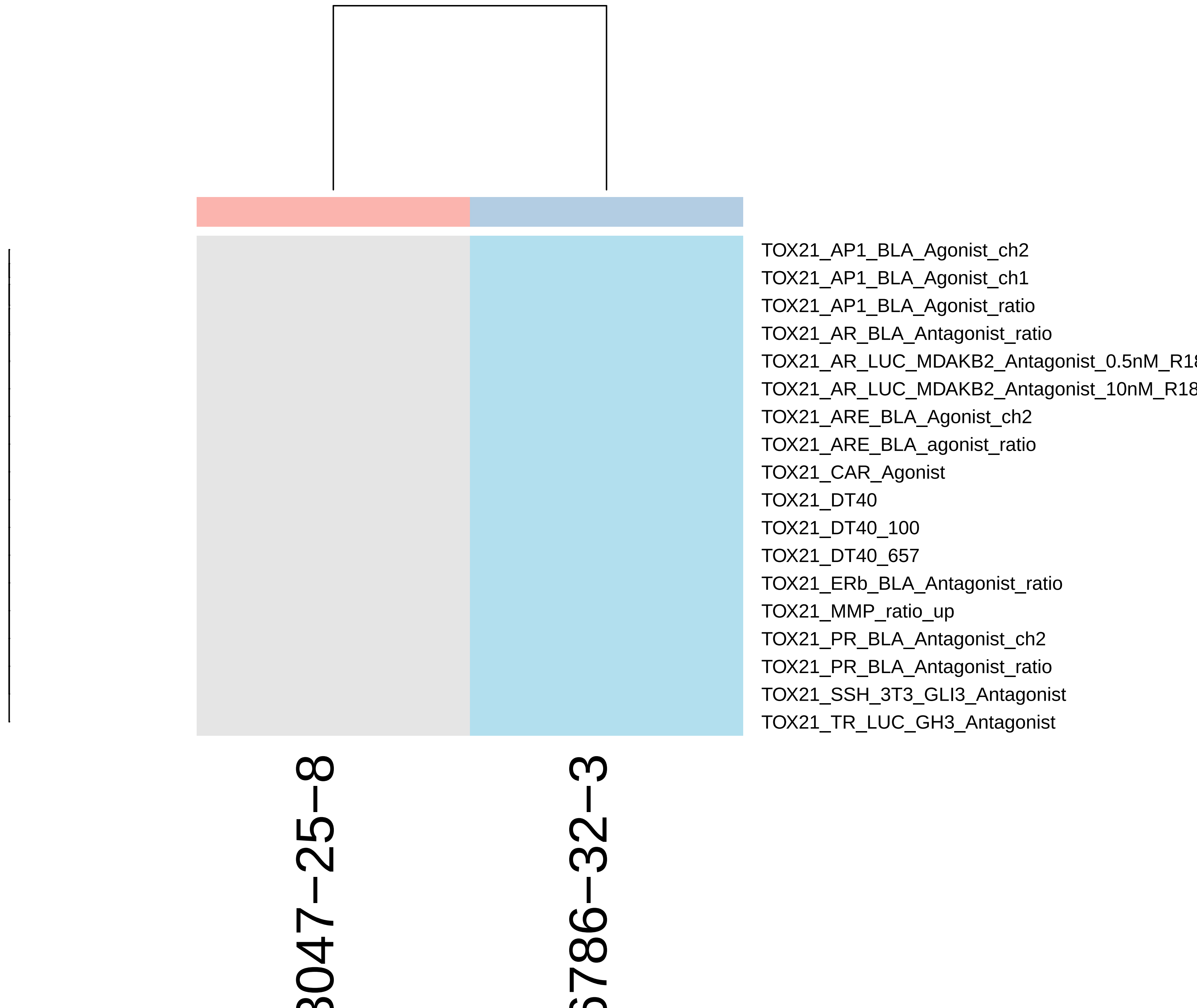

All Activity Heatmap for Cluster 1600

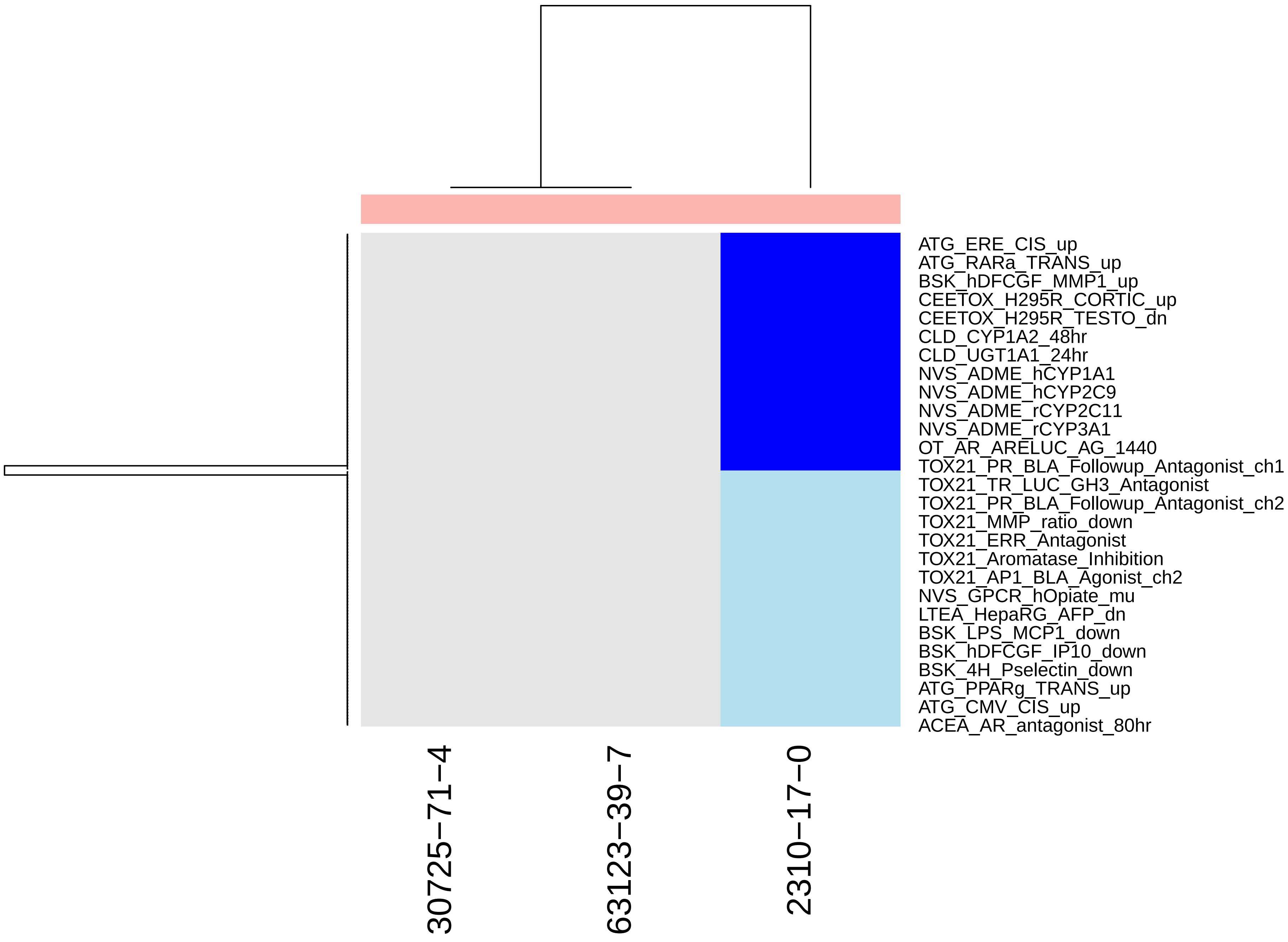

All Activity Heatmap for Cluster 1601

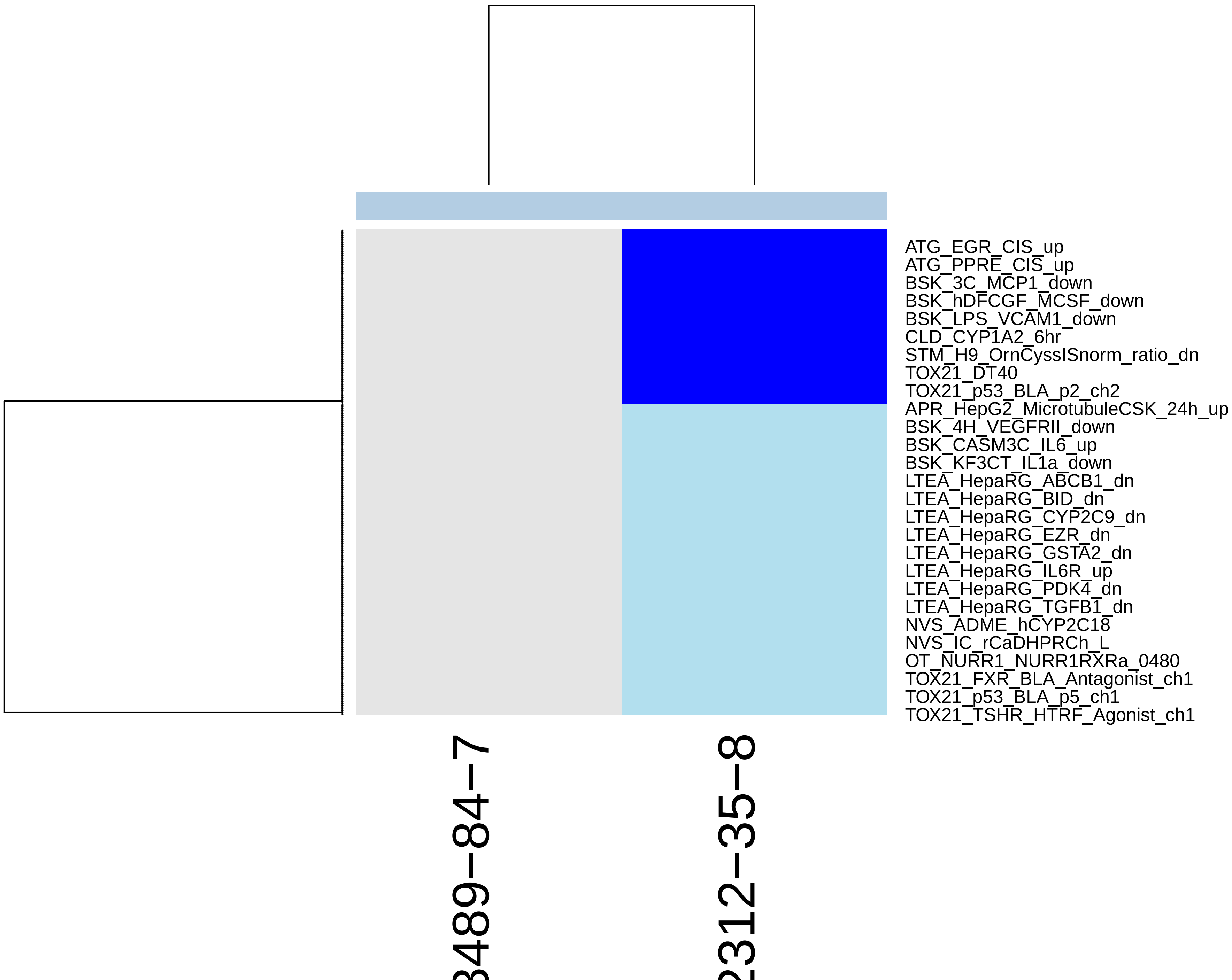

All Activity Heatmap for Cluster 1602

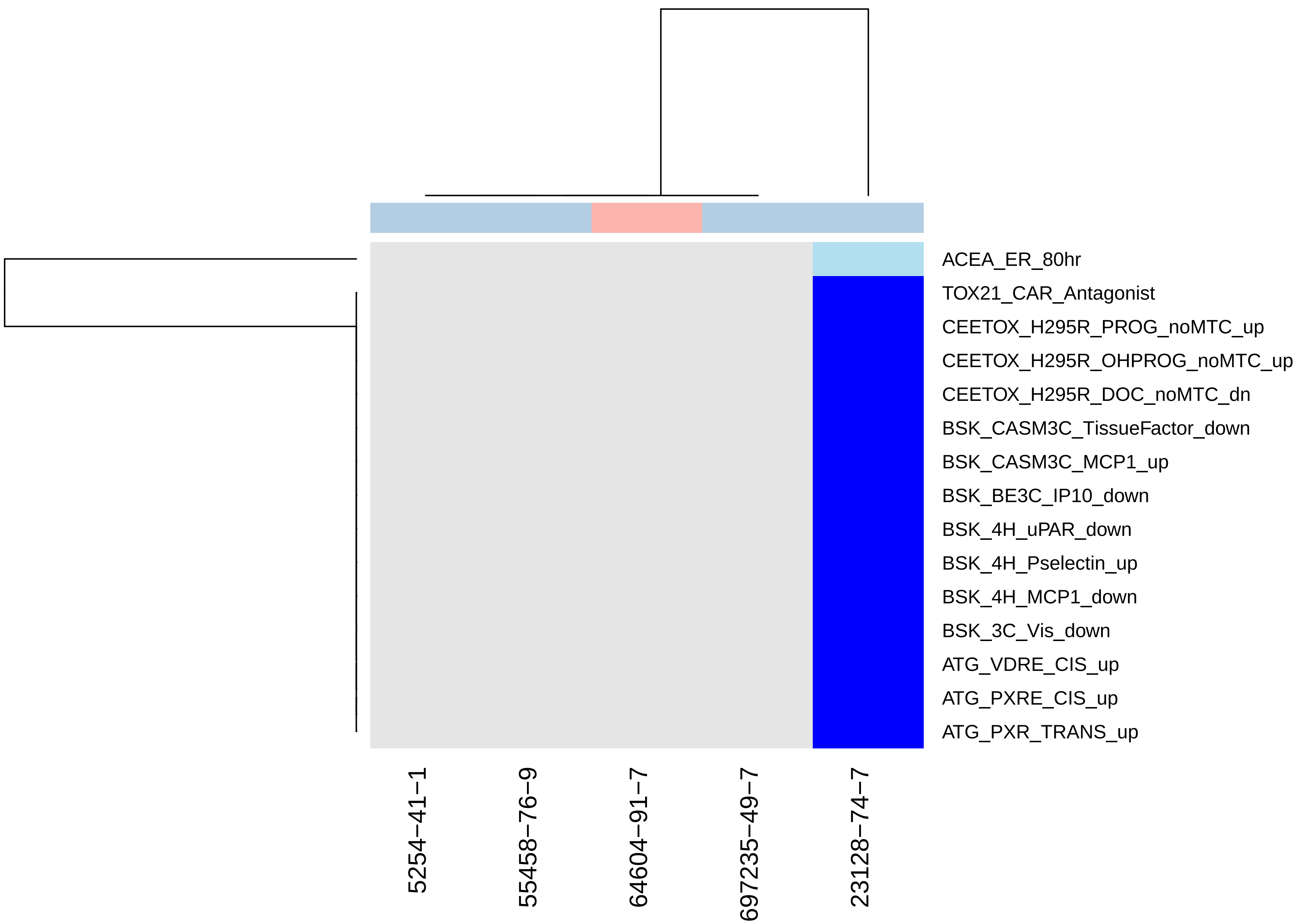

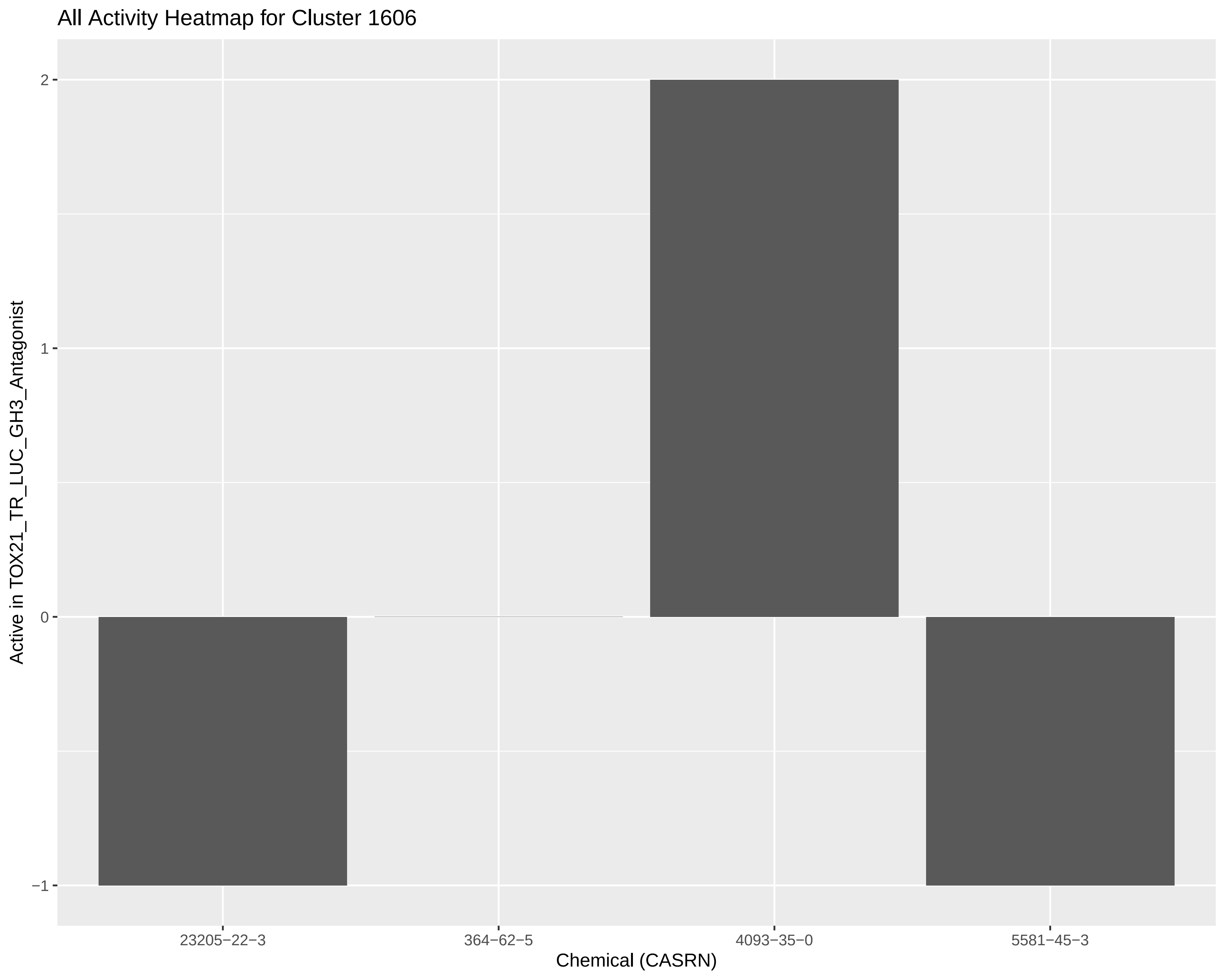

All Activity Heatmap for Cluster 1606
Active in TOX21_TR_LUC_GH3_Antagonist
2
1
0
−1
23205−22−3
364−62−5
4093−35−0
5581−45−3
Chemical (CASRN)

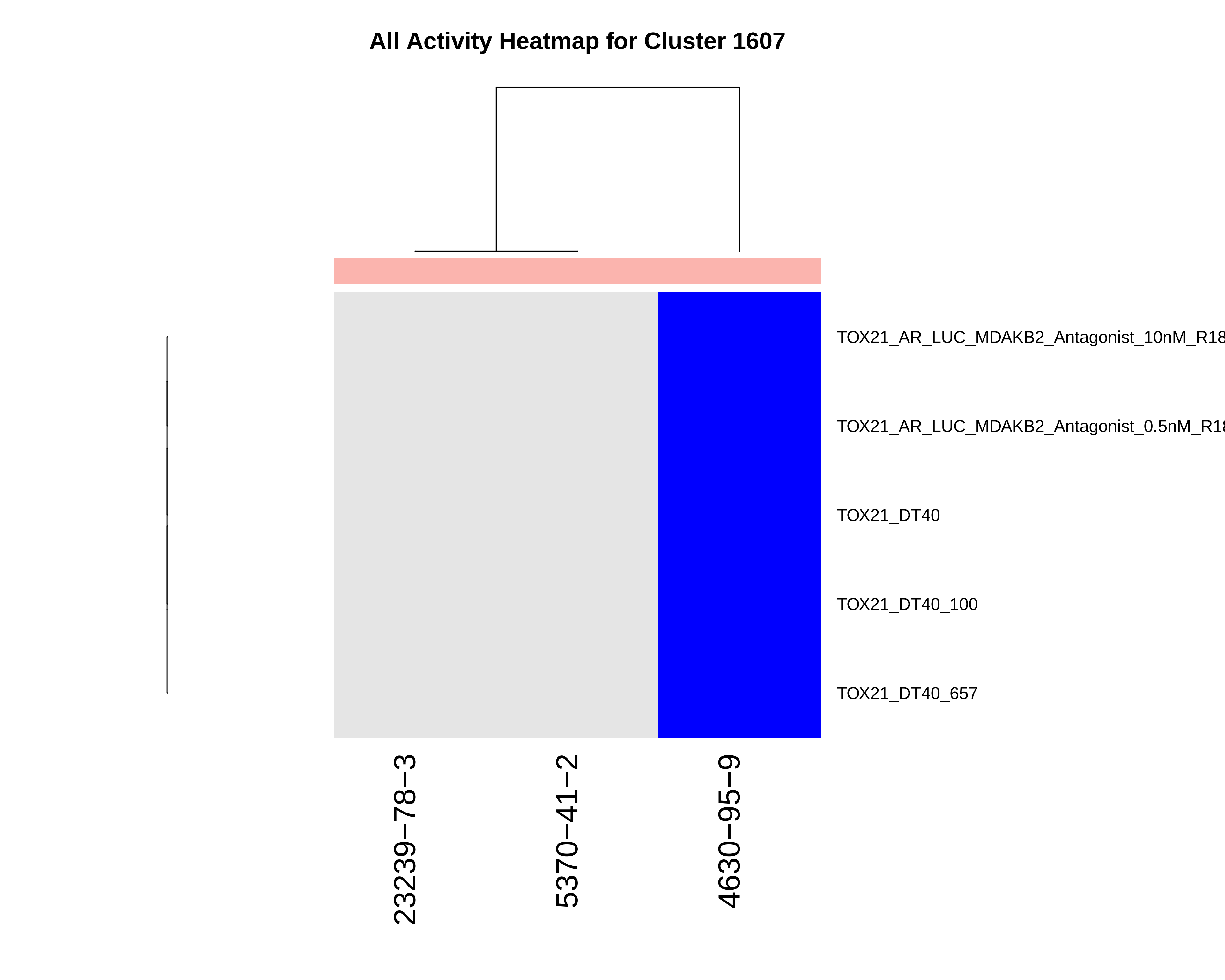

All Activity Heatmap for Cluster 1607
TOX21_AR_LUC_MDAKB2_Antagonist_10nM_R18
TOX21_AR_LUC_MDAKB2_Antagonist_0.5nM_R18
TOX21_DT40
TOX21_DT40_100
TOX21_DT40_657
23239−78−3
5370−41−2
4630−95−9

All Activity Heatmap for Cluster 1609

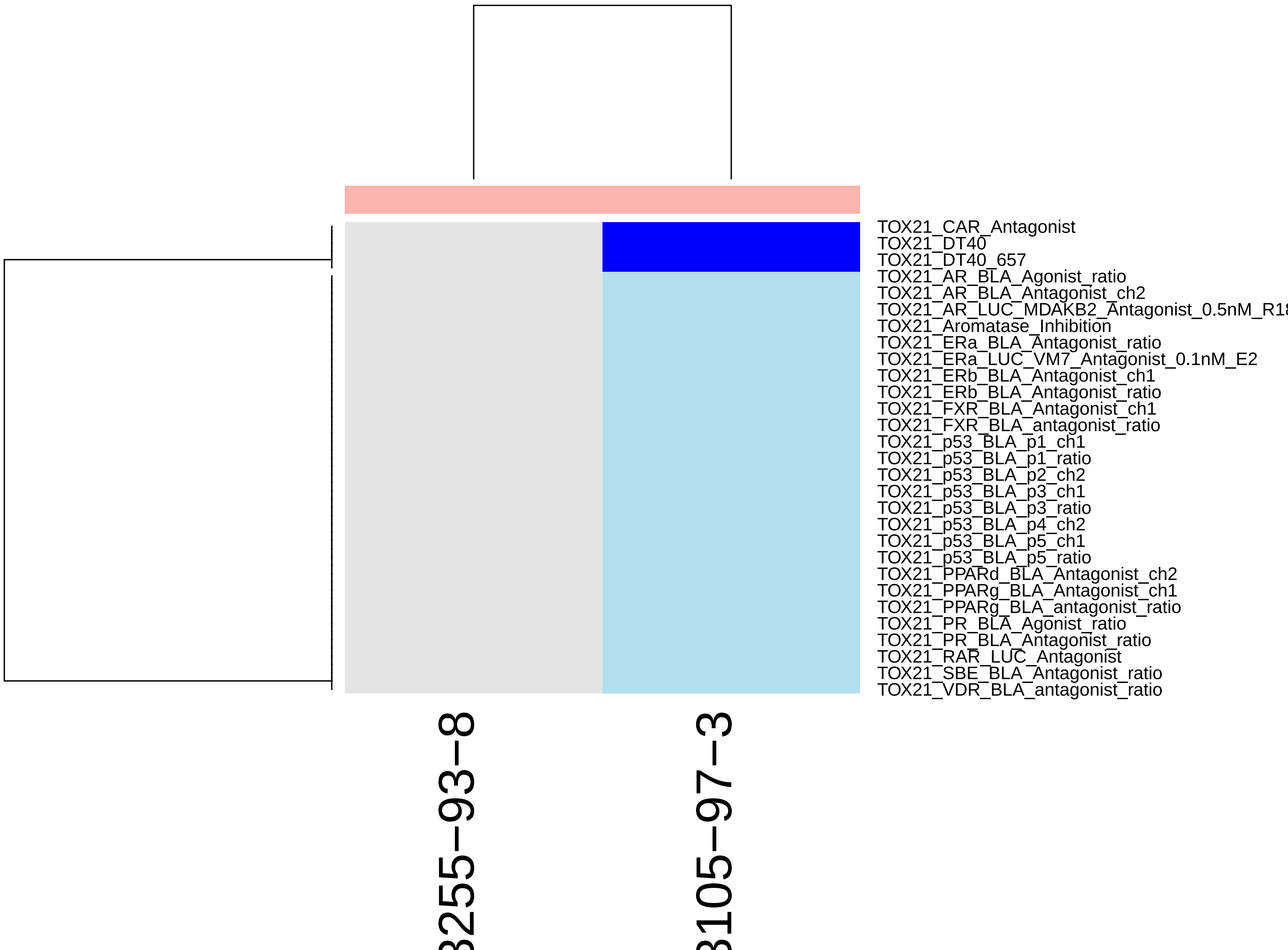

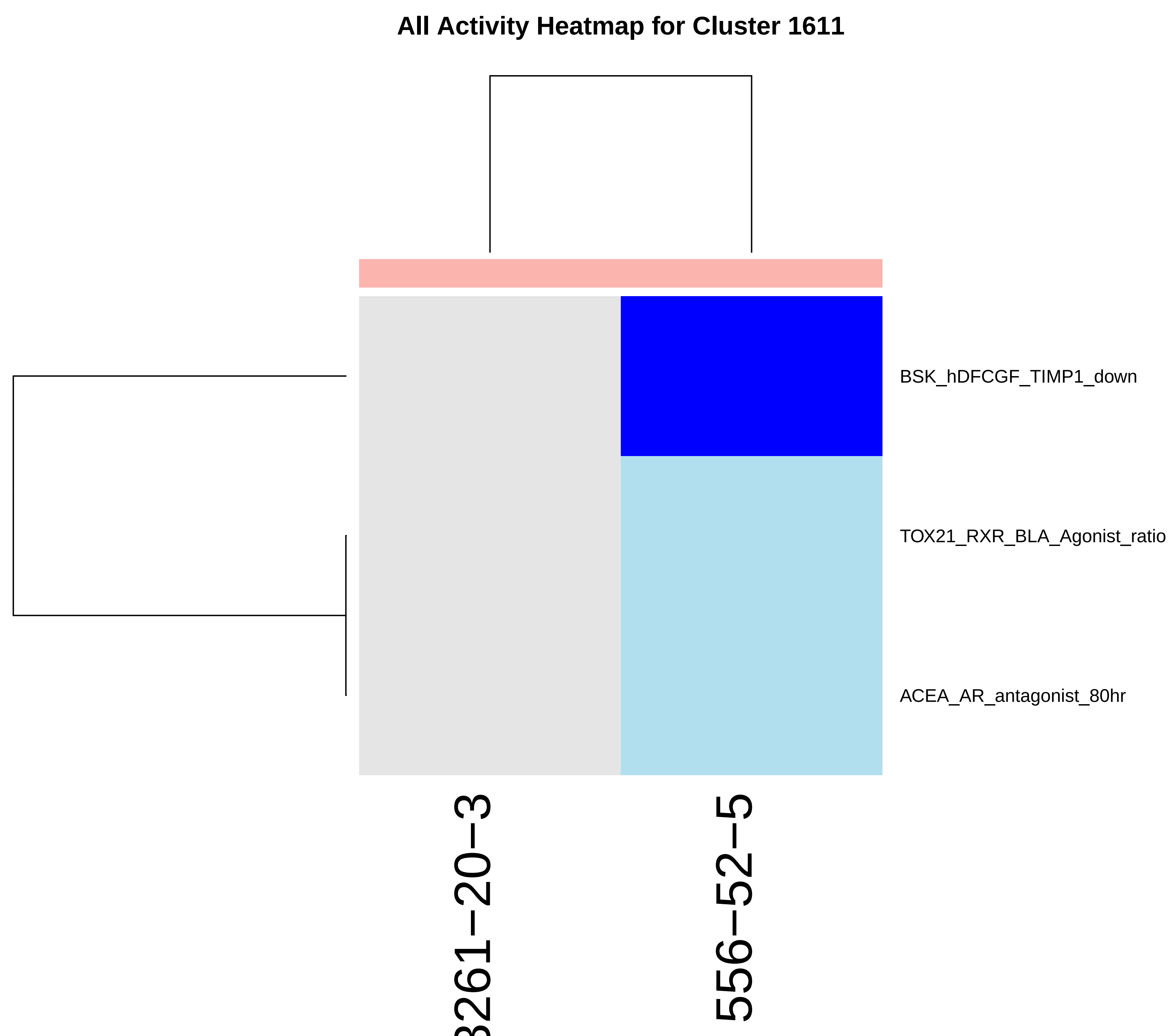
All Activity Heatmap for Cluster 1611
BSK_hDFCGF_TIMP1_down
TOX21_RXR_BLA_Agonist_ratio
ACEA_AR_antagonist_80hr
3261−20−3
556−52−5

All Activity Heatmap for Cluster 1616

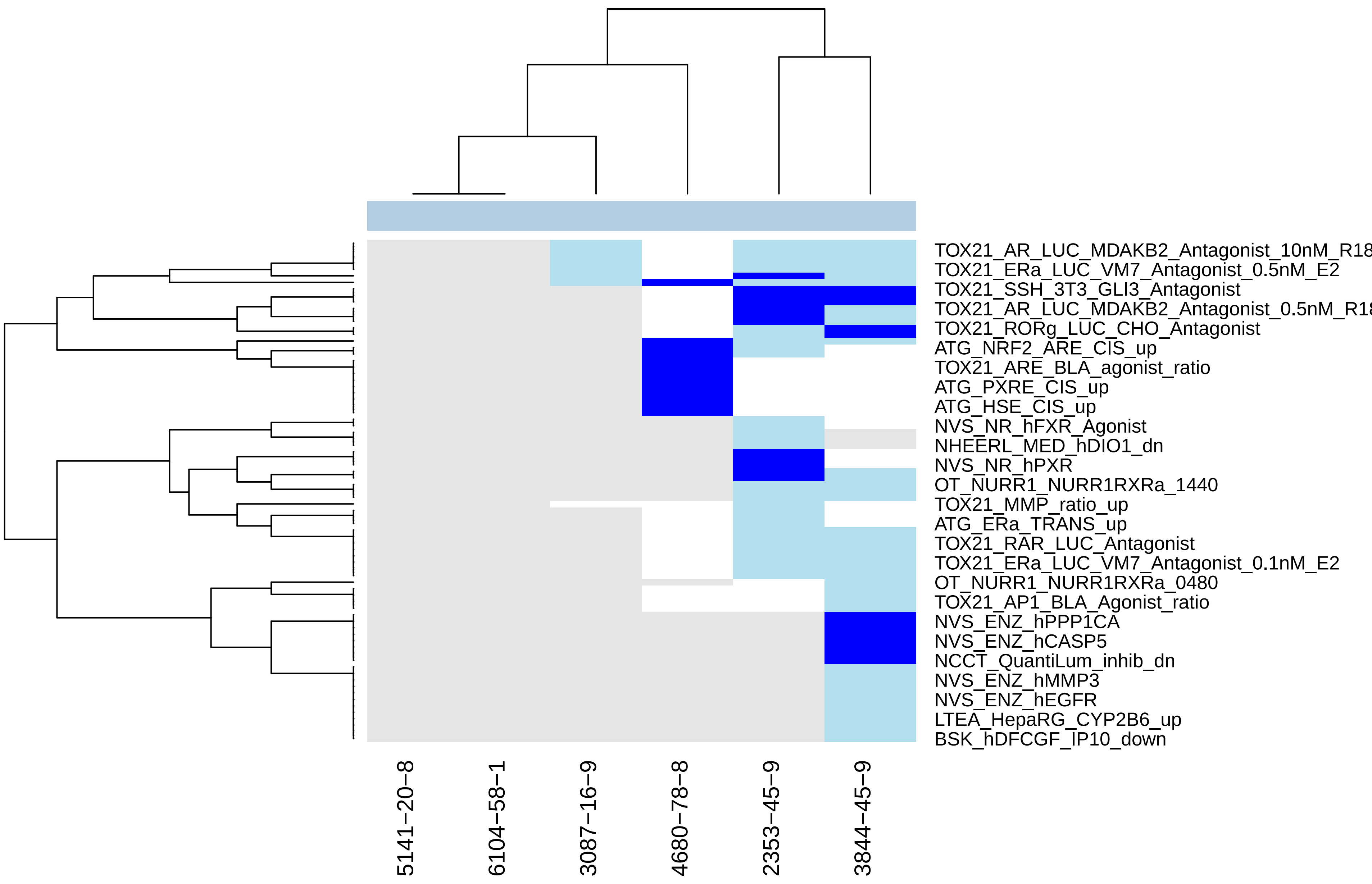

All Activity Heatmap for Cluster 1618

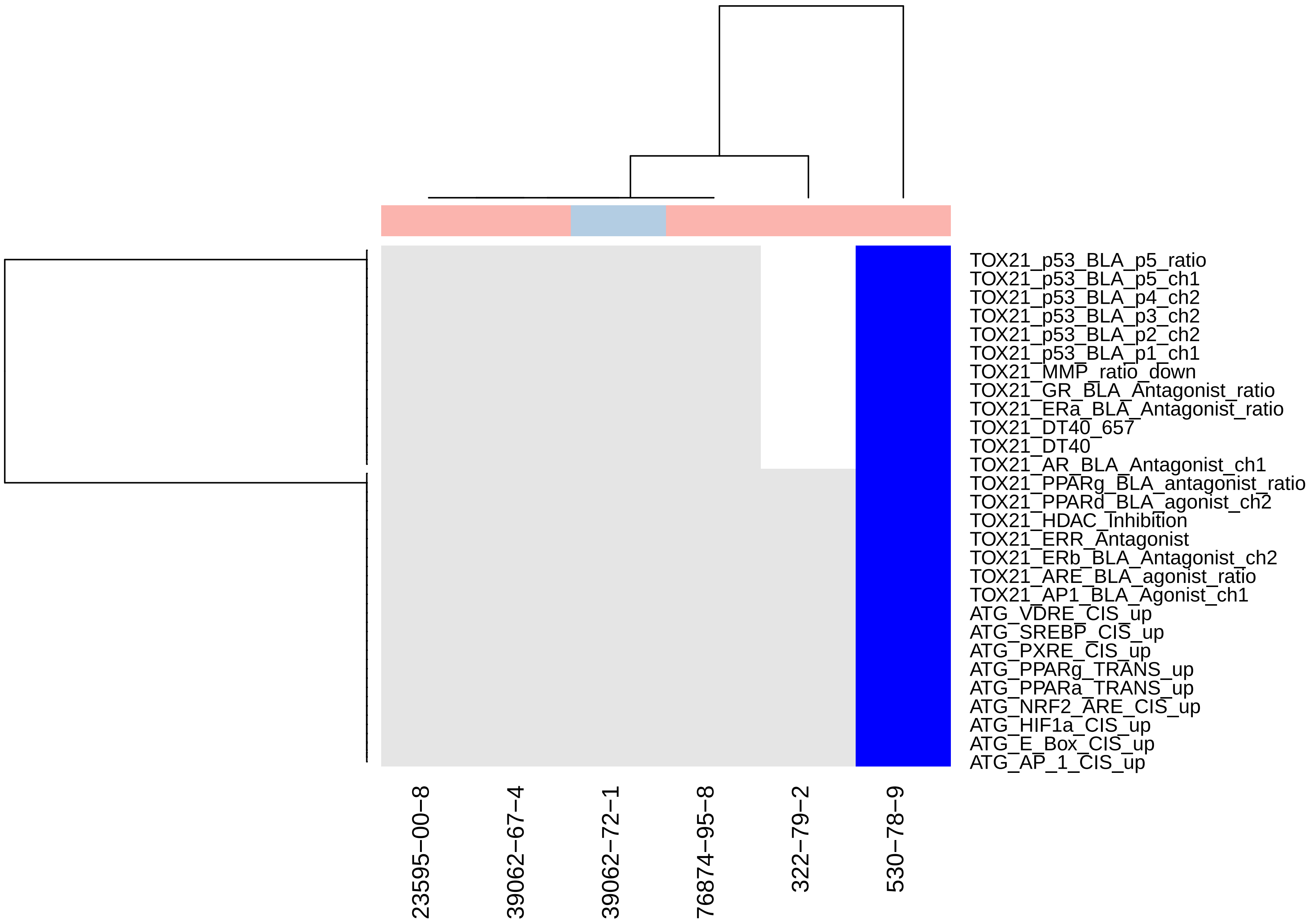

All Activity Heatmap for Cluster 1619

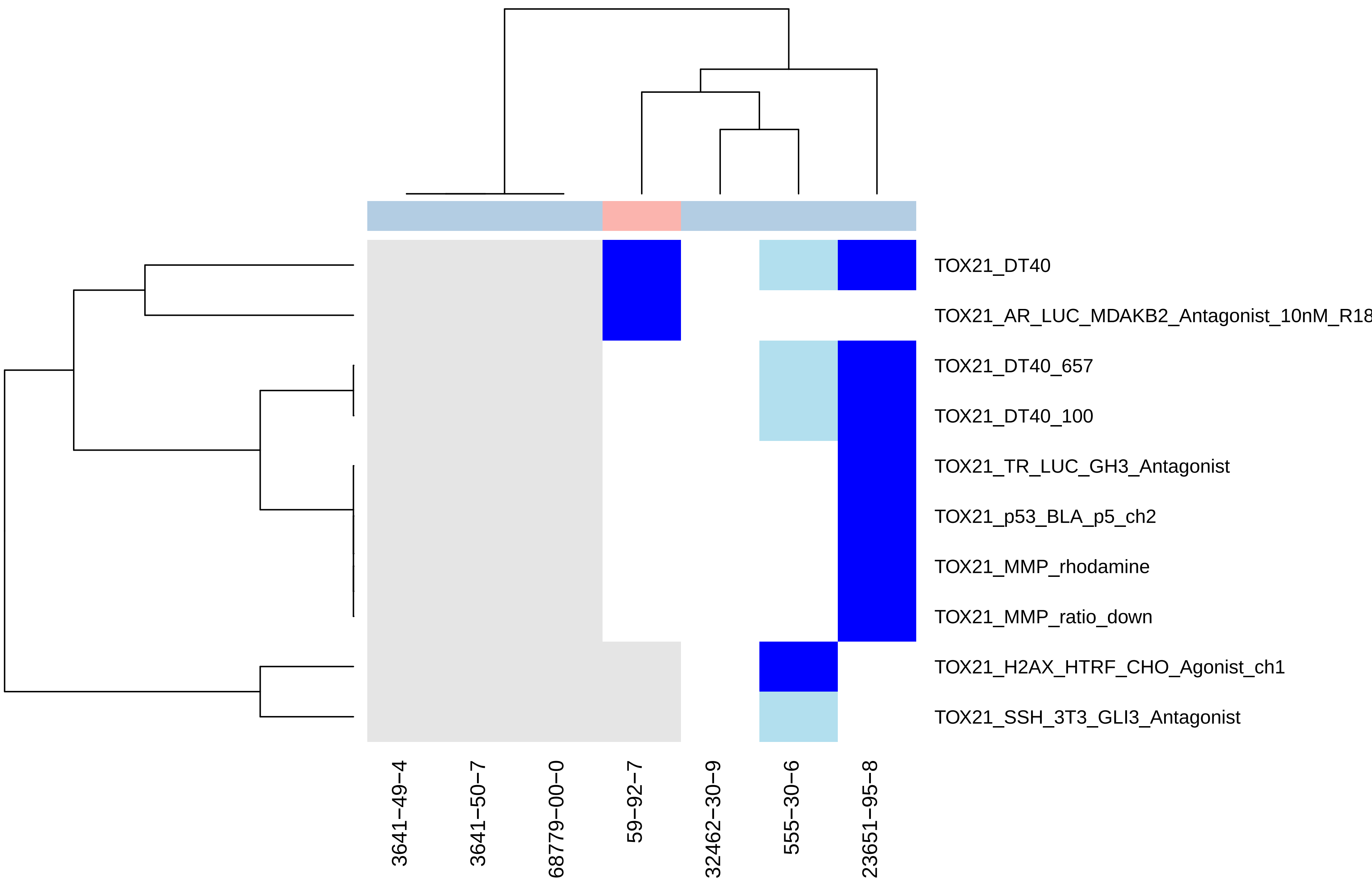

All Activity Heatmap for Cluster 1622

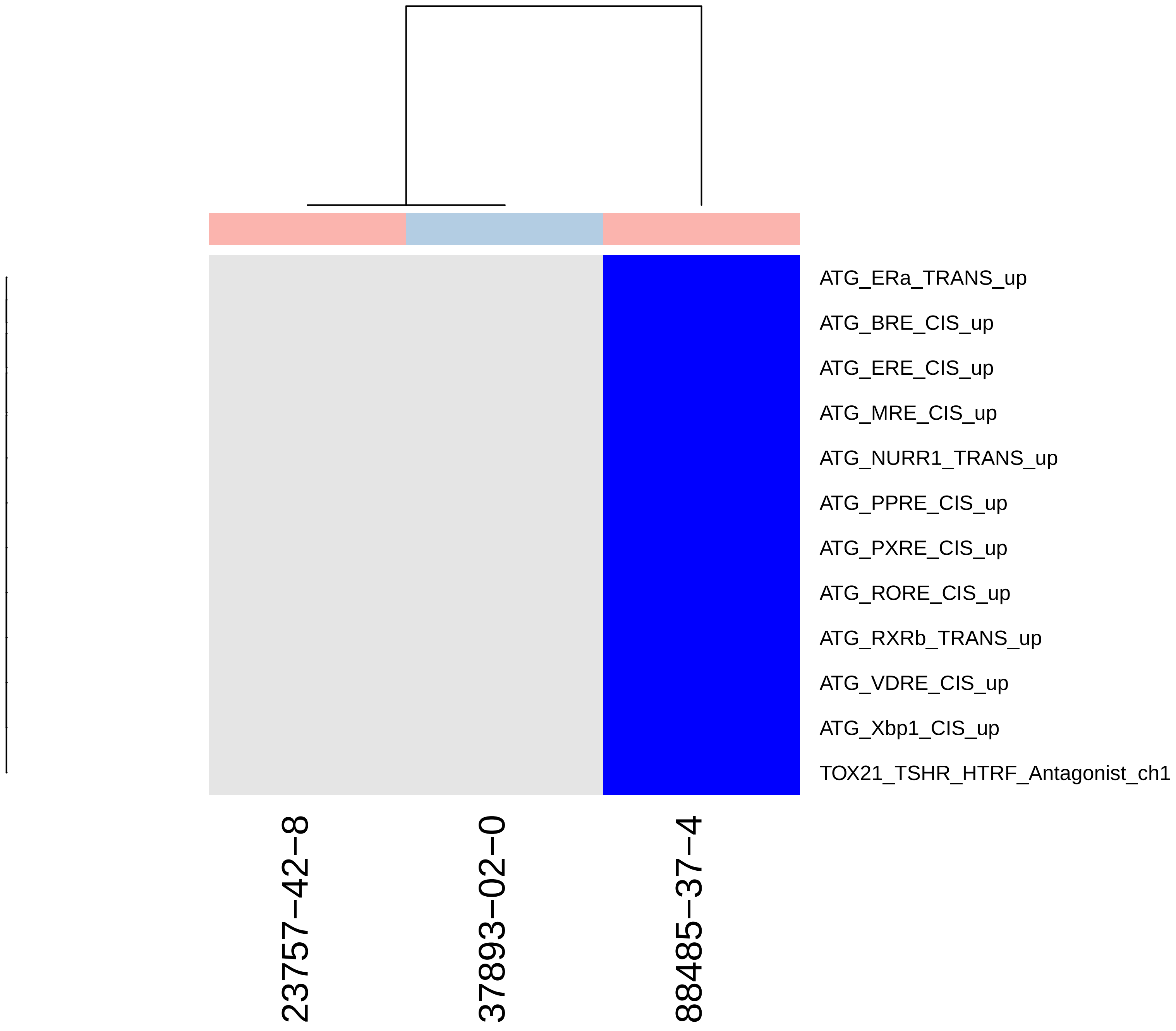

All Activity Heatmap for Cluster 1623

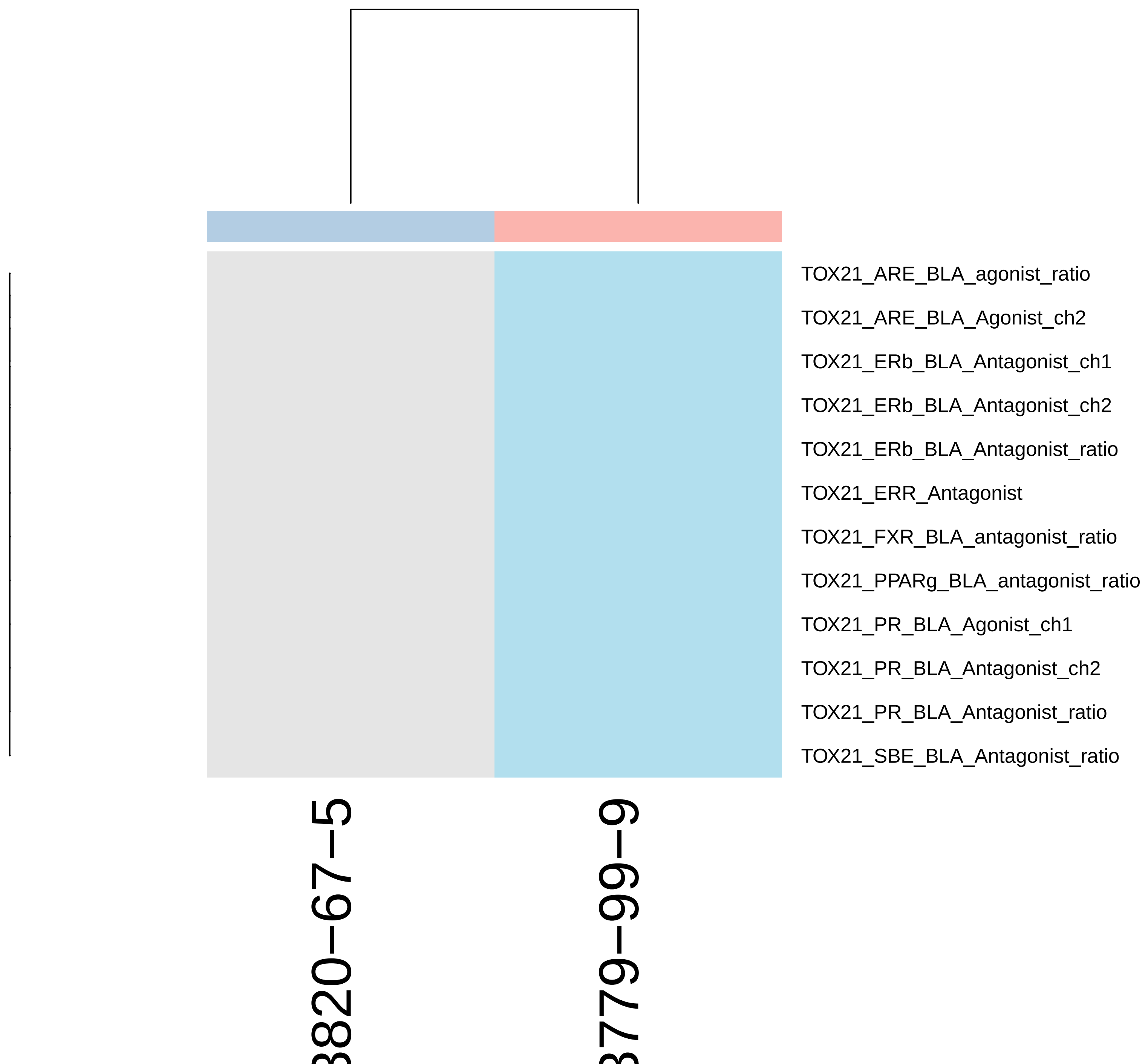

All Activity Heatmap for Cluster 1624

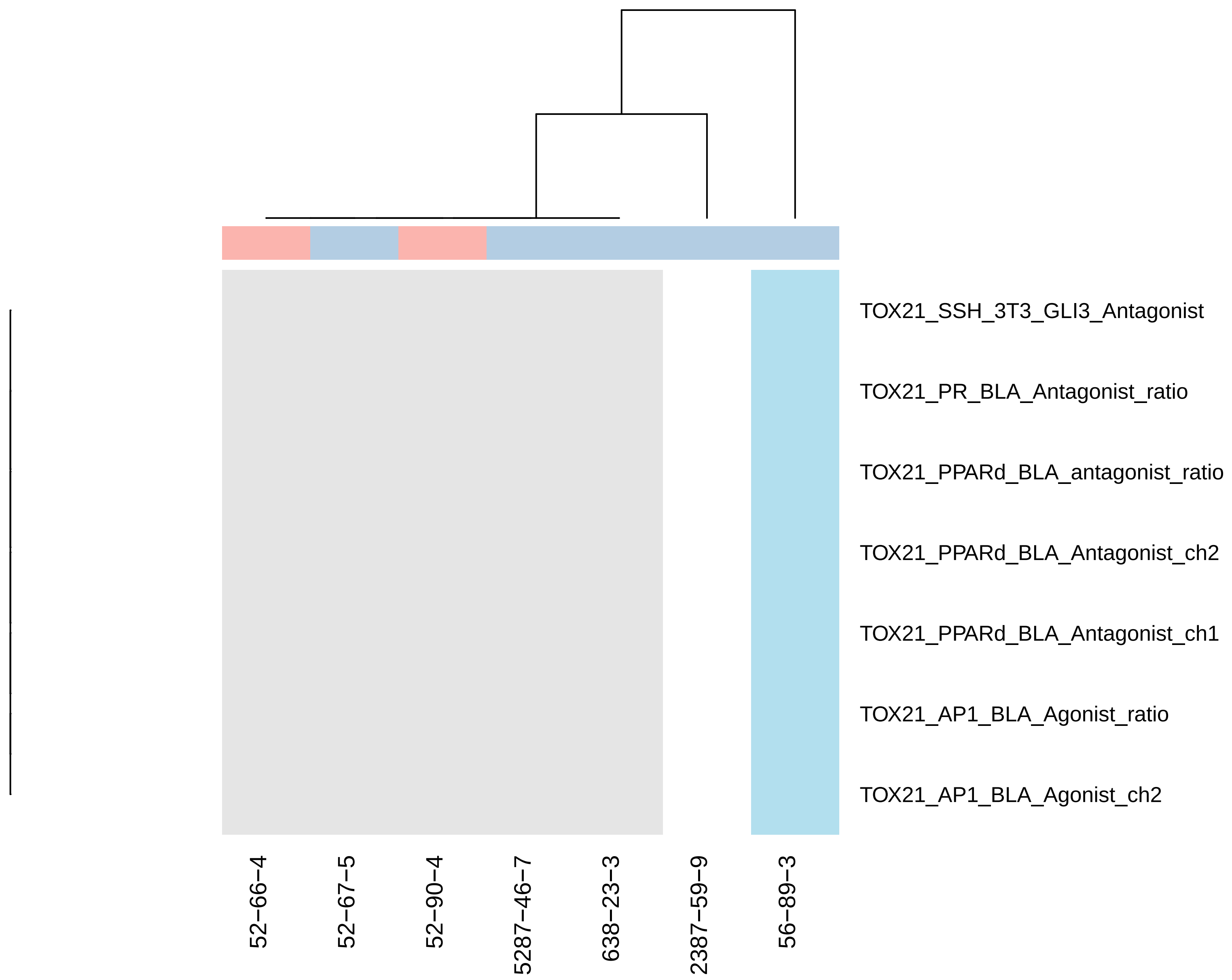

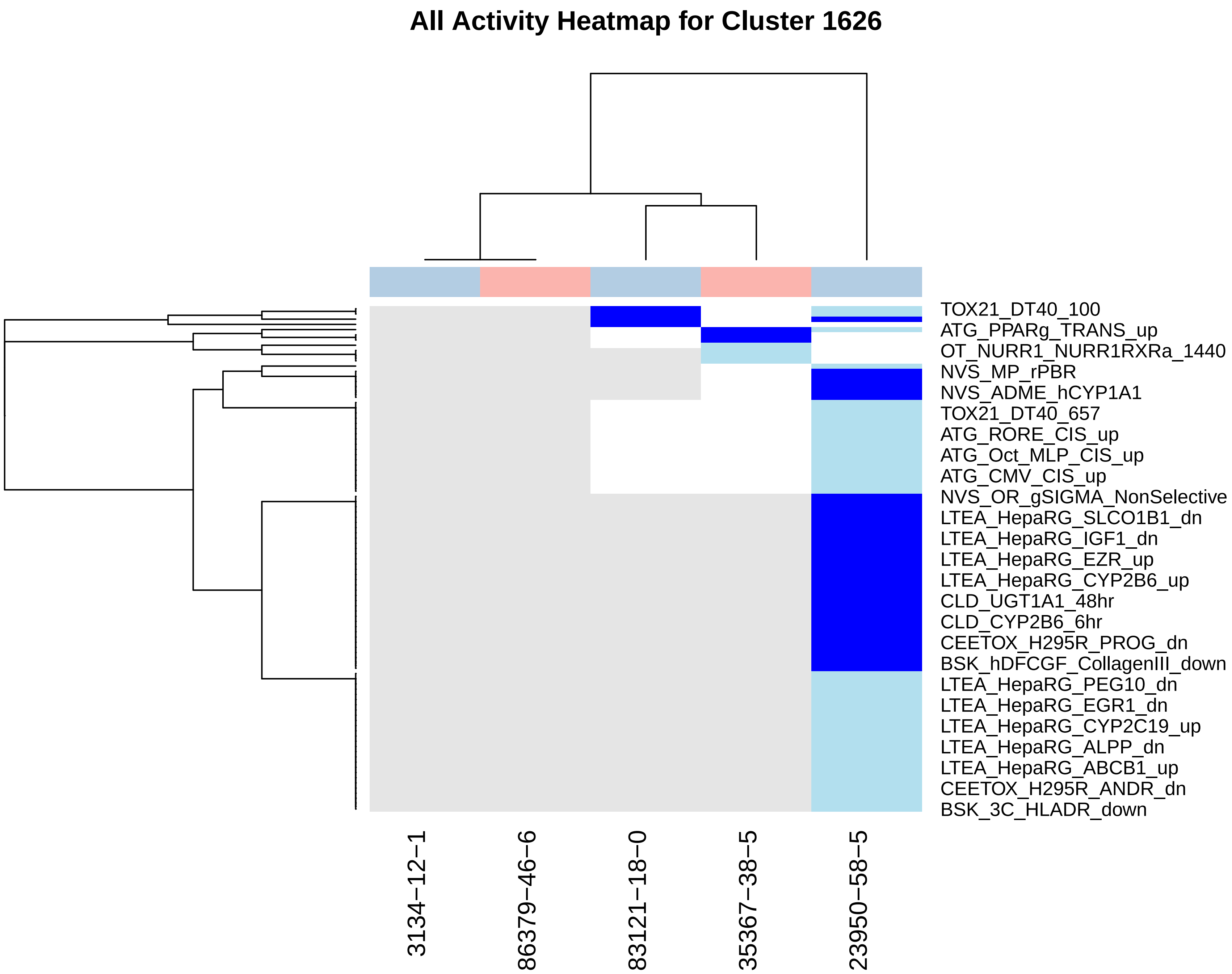

All Activity Heatmap for Cluster 1626
TOX21_DT40_100
ATG_PPARg_TRANS_up
OT_NURR1_NURR1RXRa_1440
NVS_MP_rPBR
NVS_ADME_hCYP1A1
TOX21_DT40_657
ATG_RORE_CIS_up
ATG_Oct_MLP_CIS_up
ATG_CMV_CIS_up
NVS_OR_gSIGMA_NonSelective
LTEA_HepaRG_SLCO1B1_dn
LTEA_HepaRG_IGF1_dn
LTEA_HepaRG_EZR_up
LTEA_HepaRG_CYP2B6_up
CLD_UGT1A1_48hr
CLD_CYP2B6_6hr
CEETOX_H295R_PROG_dn
BSK_hDFCGF_CollagenIII_down
LTEA_HepaRG_PEG10_dn
LTEA_HepaRG_EGR1_dn
LTEA_HepaRG_CYP2C19_up
LTEA_HepaRG_ALPP_dn
LTEA_HepaRG_ABCB1_up
CEETOX_H295R_ANDR_dn
BSK_3C_HLADR_down
3134−12−1
86379−46−6
83121−18−0
35367−38−5
23950−58−5

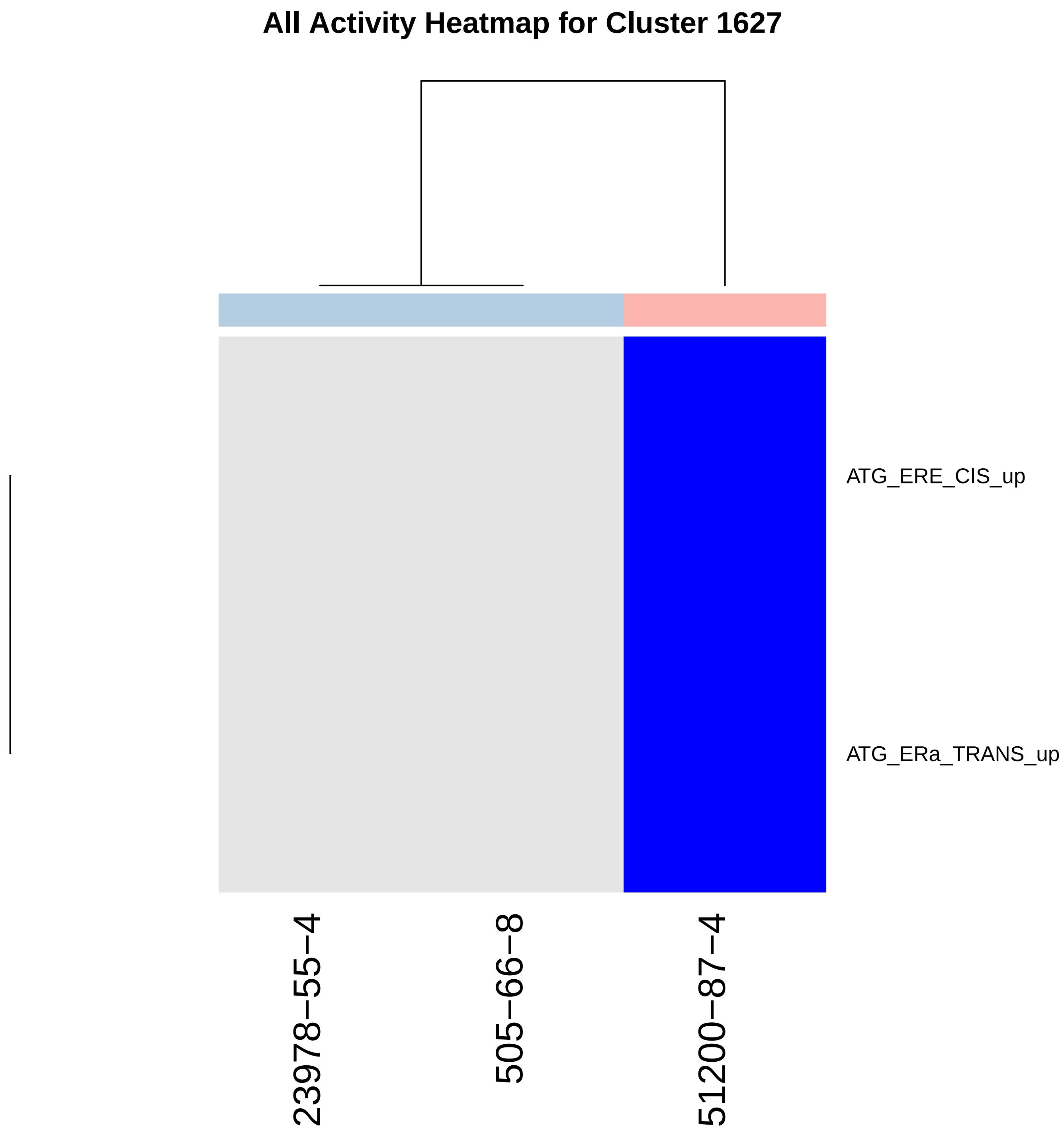
All Activity Heatmap for Cluster 1627
ATG_ERE_CIS_up
ATG_ERa_TRANS_up
23978−55−4
505−66−8
51200−87−4

All Activity Heatmap for Cluster 1628

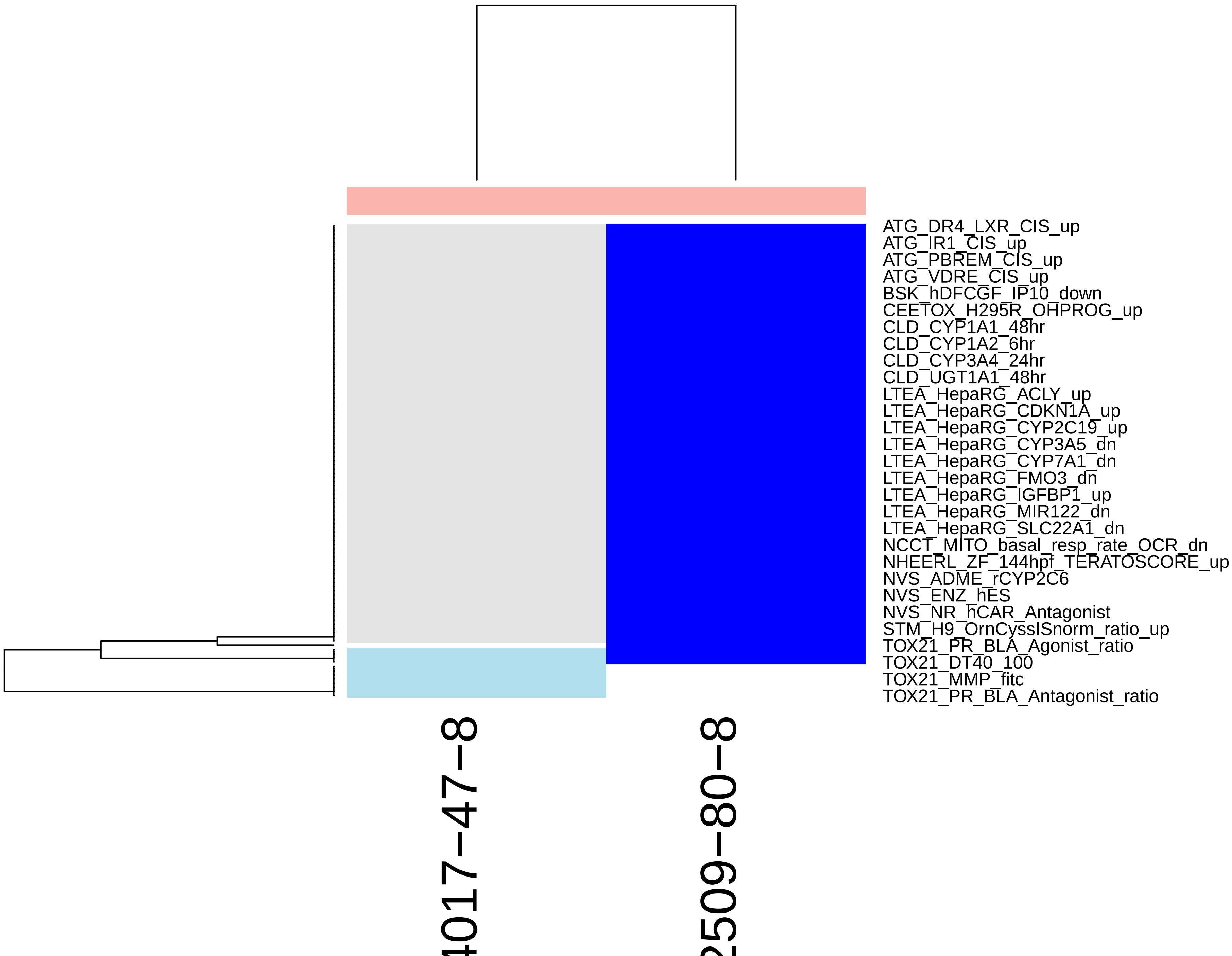

All Activity Heatmap for Cluster 1629

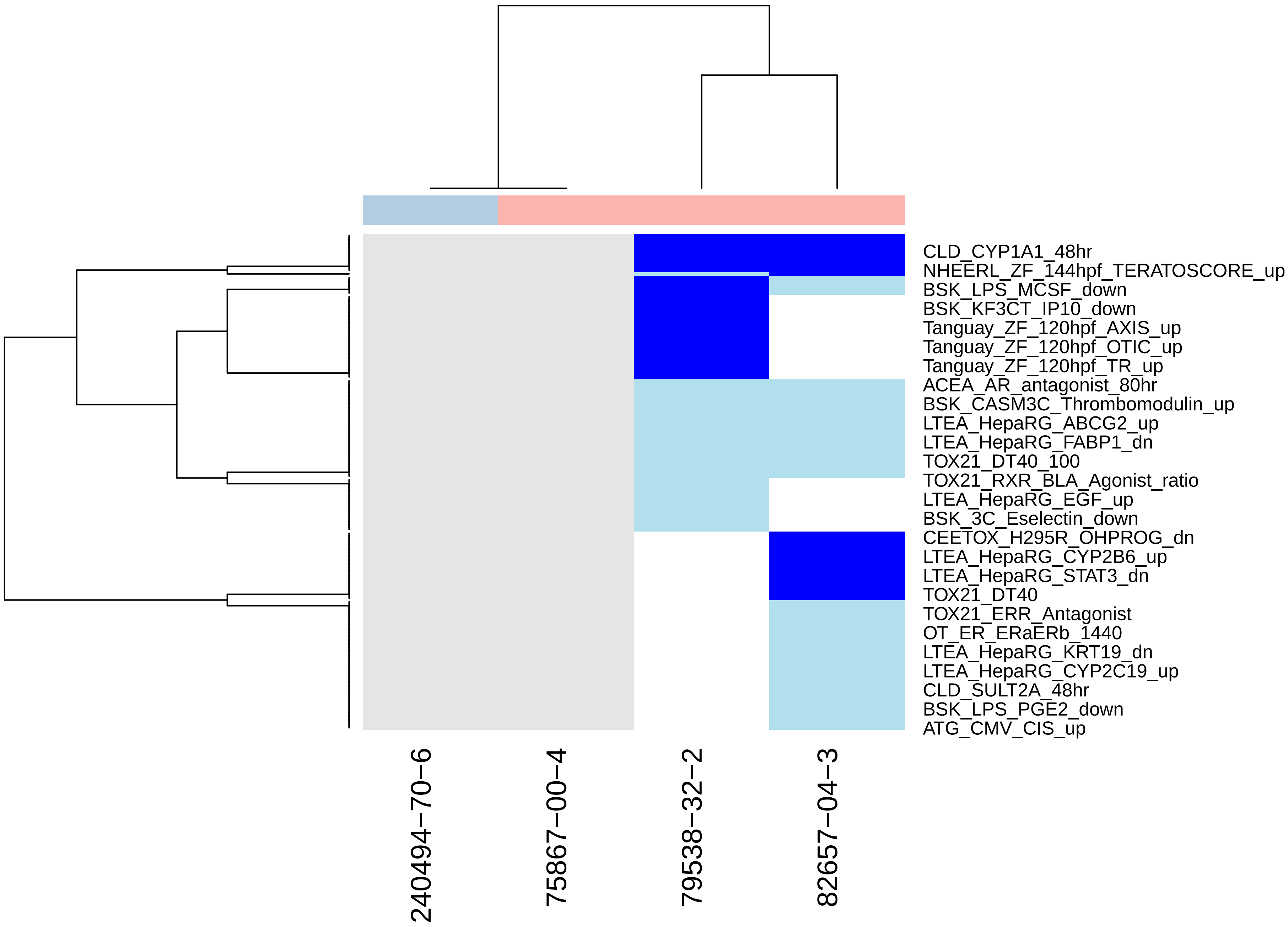

All Activity Heatmap for Cluster 1630

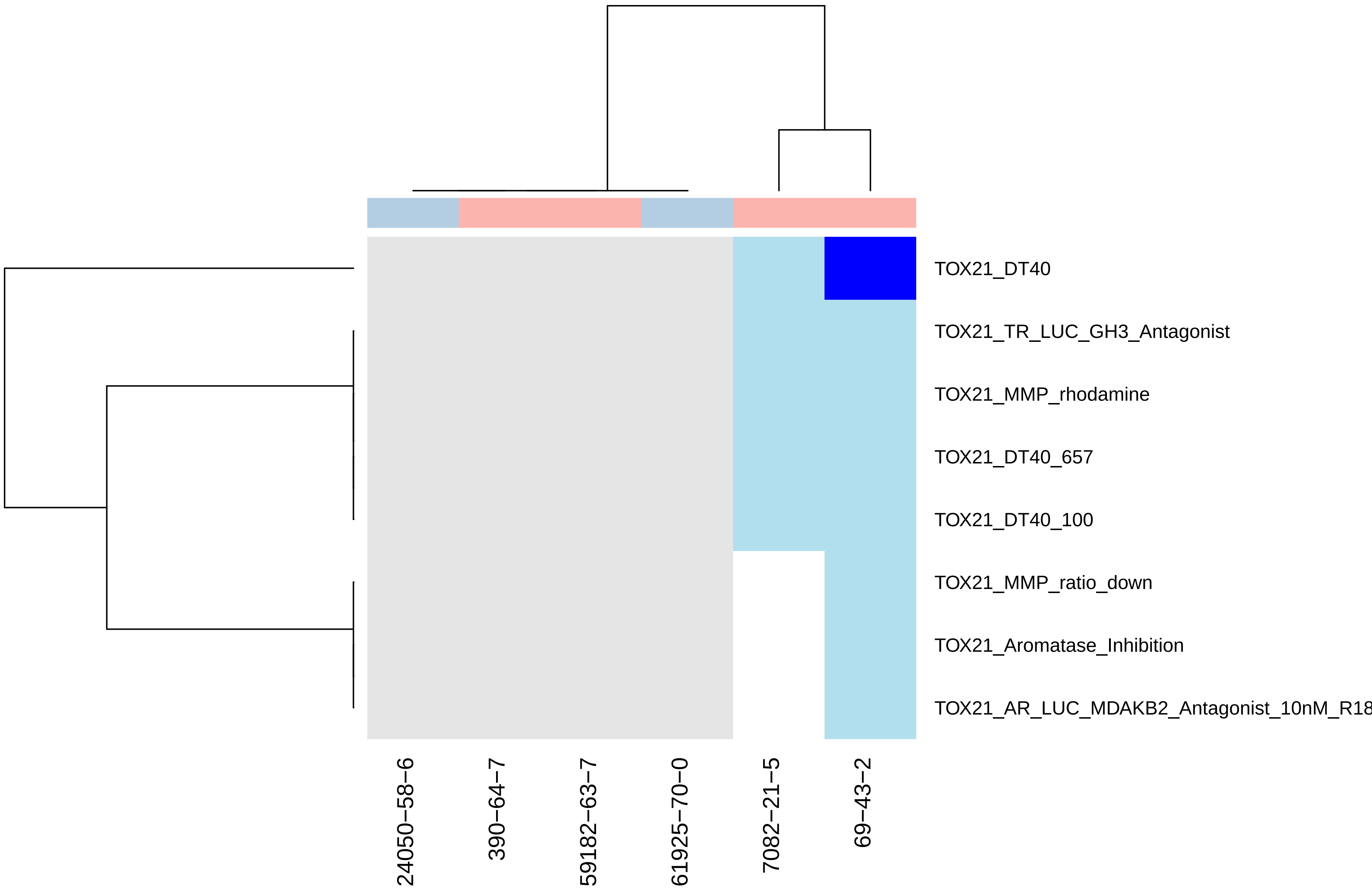

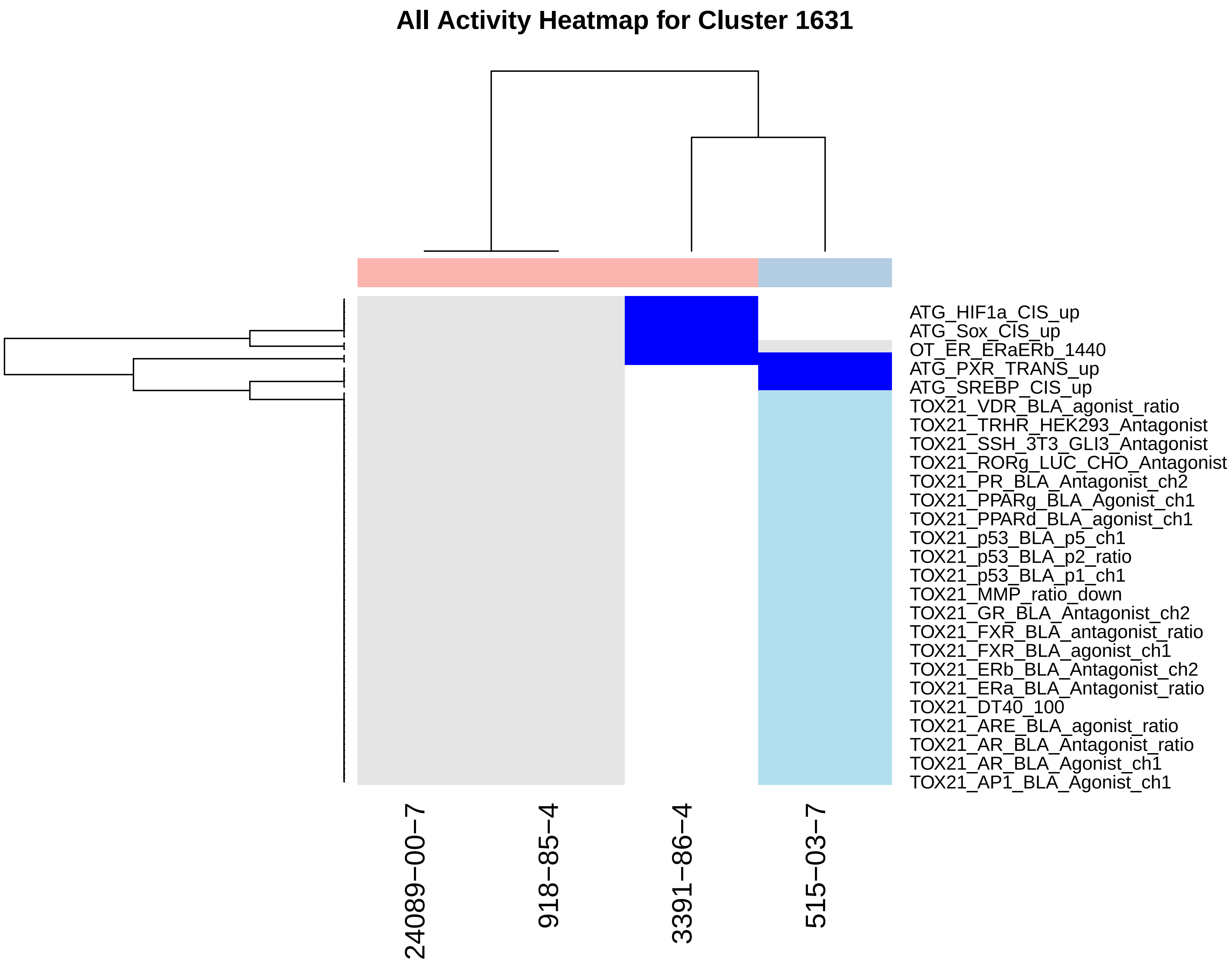

All Activity Heatmap for Cluster 1631
ATG_HIF1a_CIS_up
ATG_Sox_CIS_up
OT_ER_ERaERb_1440
ATG_PXR_TRANS_up
ATG_SREBP_CIS_up
TOX21_VDR_BLA_agonist_ratio
TOX21_TRHR_HEK293_Antagonist
TOX21_SSH_3T3_GLI3_Antagonist
TOX21_RORg_LUC_CHO_Antagonist
TOX21_PR_BLA_Antagonist_ch2
TOX21_PPARg_BLA_Agonist_ch1
TOX21_PPARd_BLA_agonist_ch1
TOX21_p53_BLA_p5_ch1
TOX21_p53_BLA_p2_ratio
TOX21_p53_BLA_p1_ch1
TOX21_MMP_ratio_down
TOX21_GR_BLA_Antagonist_ch2
TOX21_FXR_BLA_antagonist_ratio
TOX21_FXR_BLA_agonist_ch1
TOX21_ERb_BLA_Antagonist_ch2
TOX21_ERa_BLA_Antagonist_ratio
TOX21_DT40_100
TOX21_ARE_BLA_agonist_ratio
TOX21_AR_BLA_Antagonist_ratio
TOX21_AR_BLA_Agonist_ch1
TOX21_AP1_BLA_Agonist_ch1
24089−00−7
918−85−4
3391−86−4
515−03−7

All Activity Heatmap for Cluster 1634

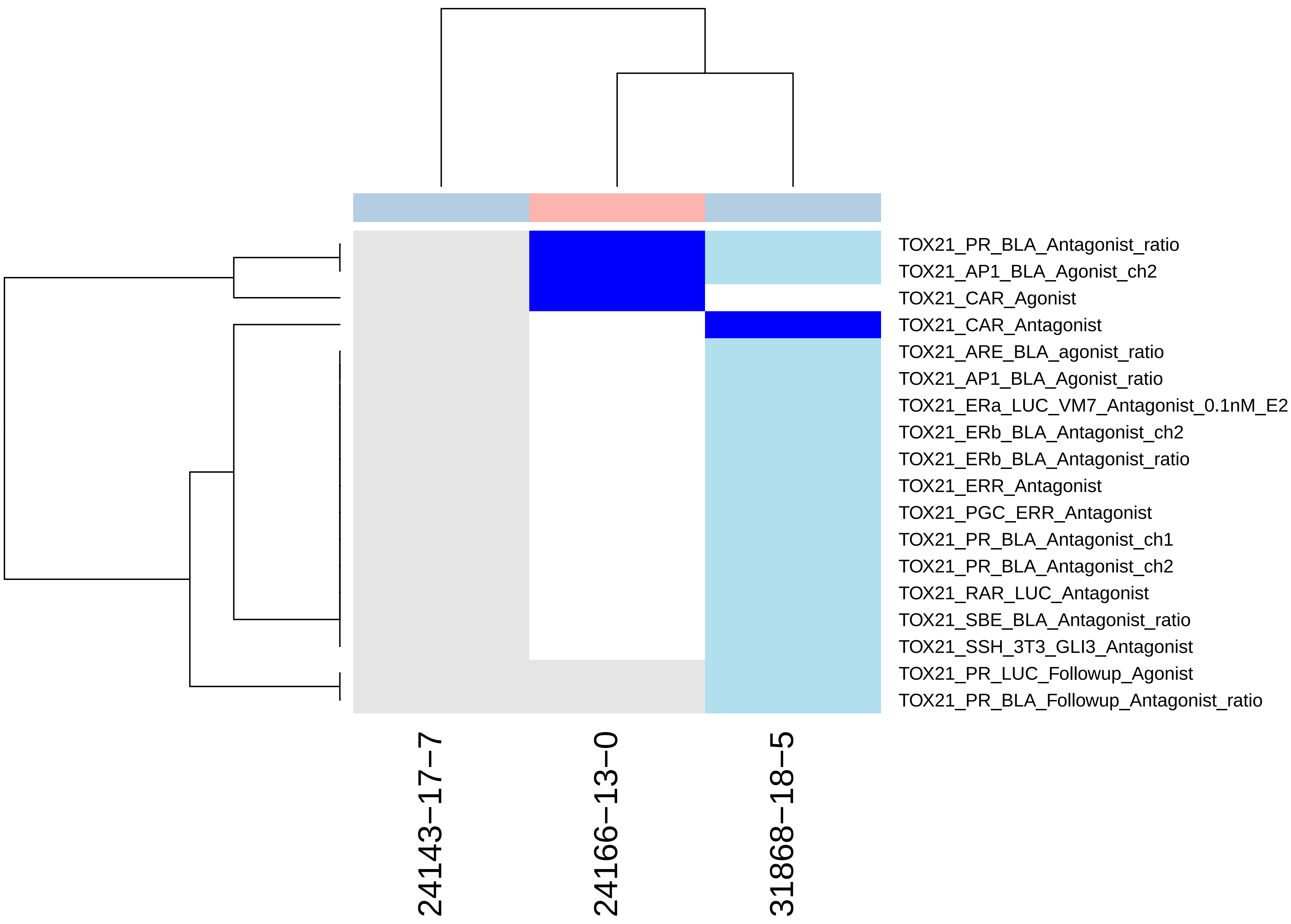

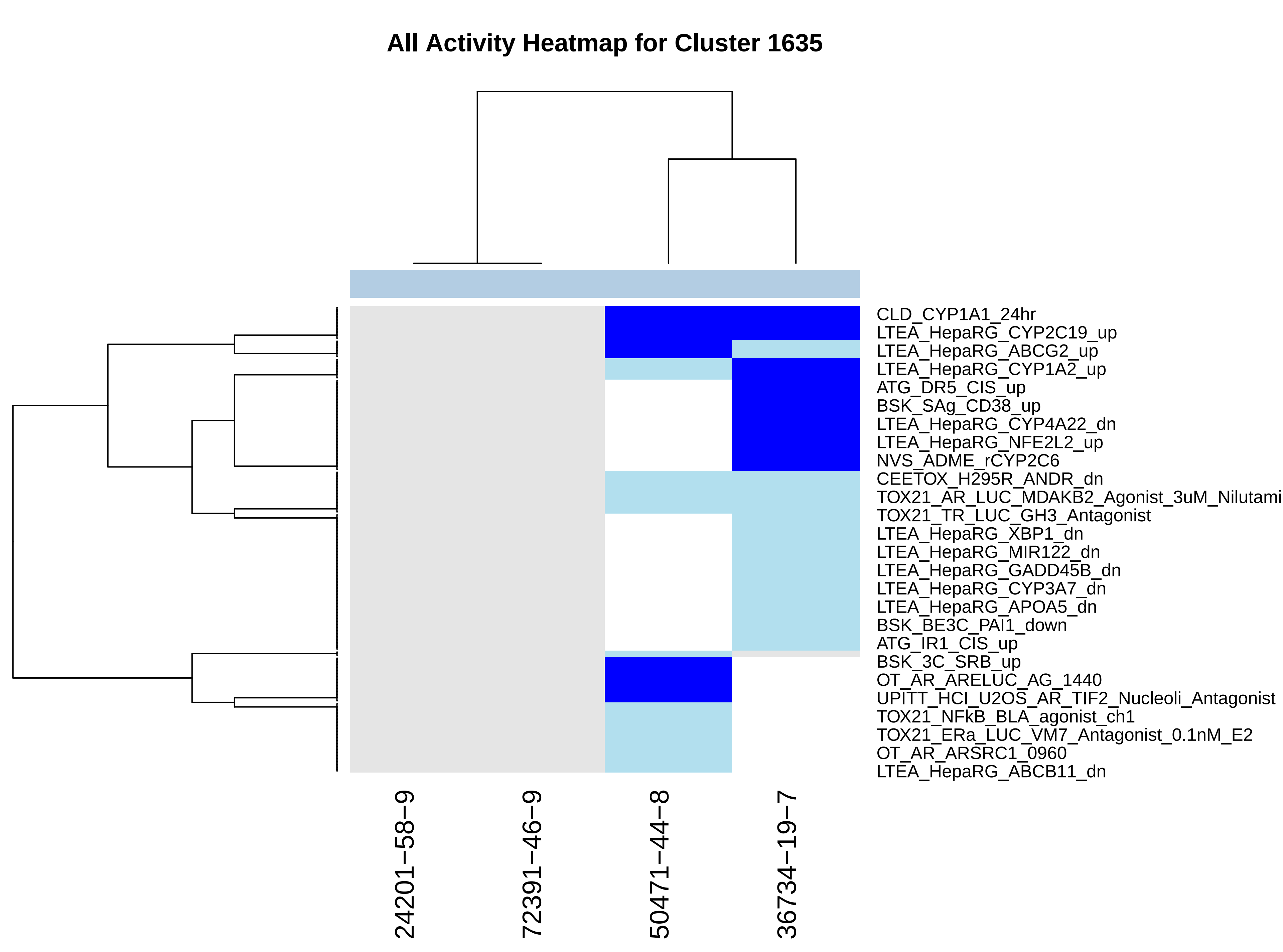

All Activity Heatmap for Cluster 1635
CLD_CYP1A1_24hr
LTEA_HepaRG_CYP2C19_up
LTEA_HepaRG_ABCG2_up
LTEA_HepaRG_CYP1A2_up
ATG_DR5_CIS_up
BSK_SAg_CD38_up
LTEA_HepaRG_CYP4A22_dn
LTEA_HepaRG_NFE2L2_up
NVS_ADME_rCYP2C6
CEETOX_H295R_ANDR_dn
TOX21_AR_LUC_MDAKB2_Agonist_3uM_Nilutami
TOX21_TR_LUC_GH3_Antagonist
LTEA_HepaRG_XBP1_dn
LTEA_HepaRG_MIR122_dn
LTEA_HepaRG_GADD45B_dn
LTEA_HepaRG_CYP3A7_dn
LTEA_HepaRG_APOA5_dn
BSK_BE3C_PAI1_down
ATG_IR1_CIS_up
BSK_3C_SRB_up
OT_AR_ARELUC_AG_1440
UPITT_HCI_U2OS_AR_TIF2_Nucleoli_Antagonist
TOX21_NFkB_BLA_agonist_ch1
TOX21_ERa_LUC_VM7_Antagonist_0.1nM_E2
OT_AR_ARSRC1_0960
LTEA_HepaRG_ABCB11_dn
24201−58−9
72391−46−9
50471−44−8
36734−19−7

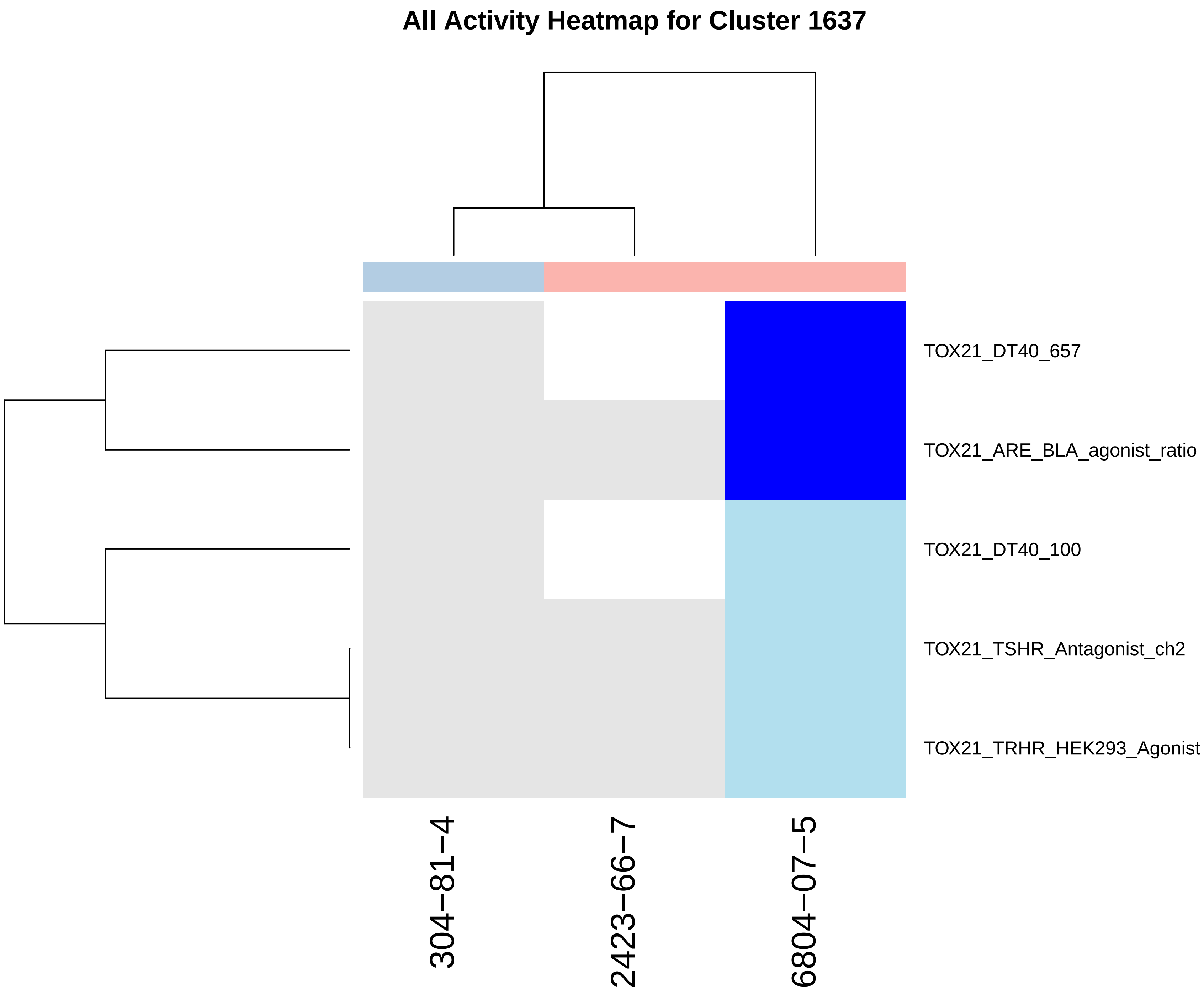
All Activity Heatmap for Cluster 1637
TOX21_DT40_657
TOX21_ARE_BLA_agonist_ratio
TOX21_DT40_100
TOX21_TSHR_Antagonist_ch2
TOX21_TRHR_HEK293_Agonist
304−81−4
2423−66−7
6804−07−5

All Activity Heatmap for Cluster 1639

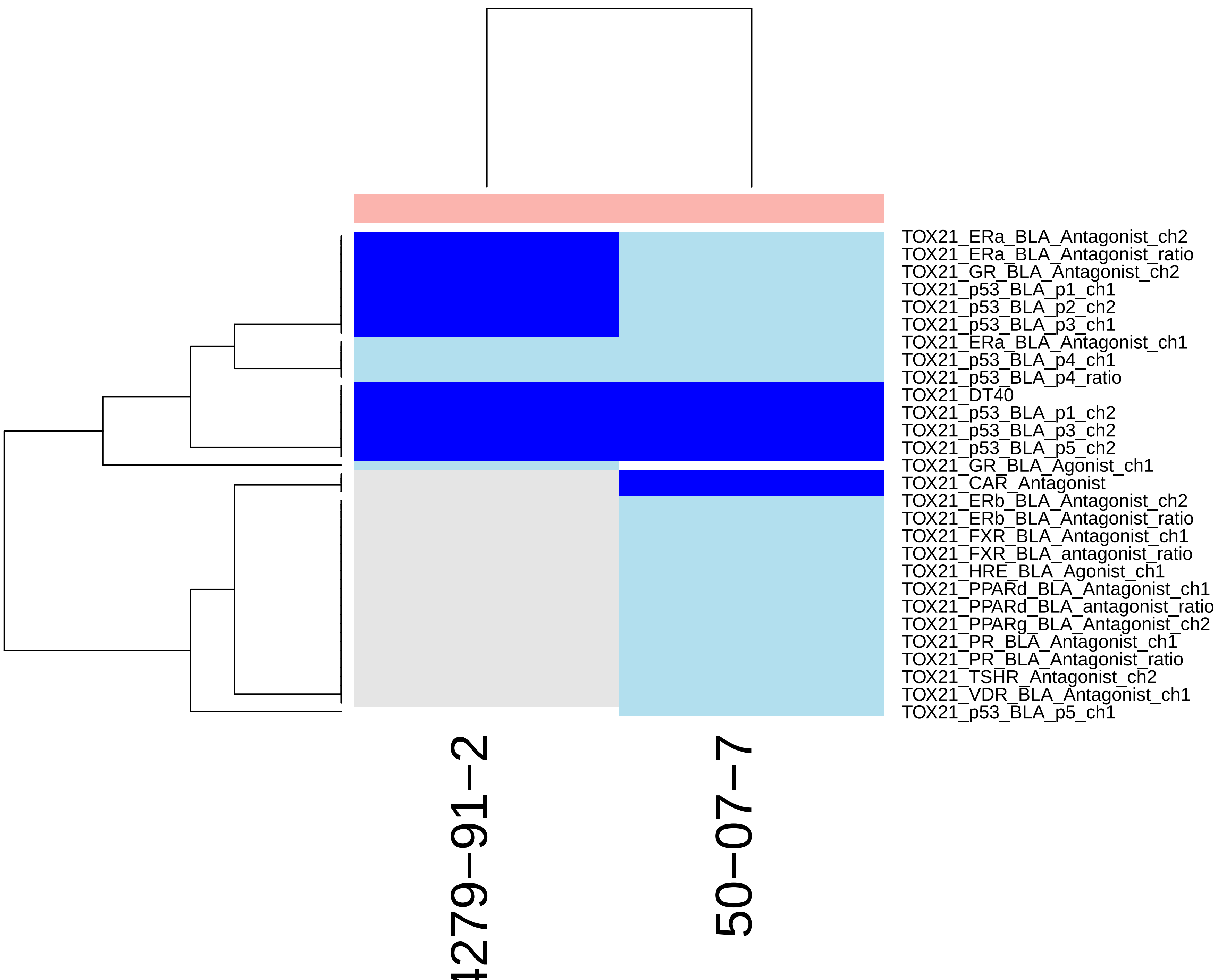

All Activity Heatmap for Cluster 1640

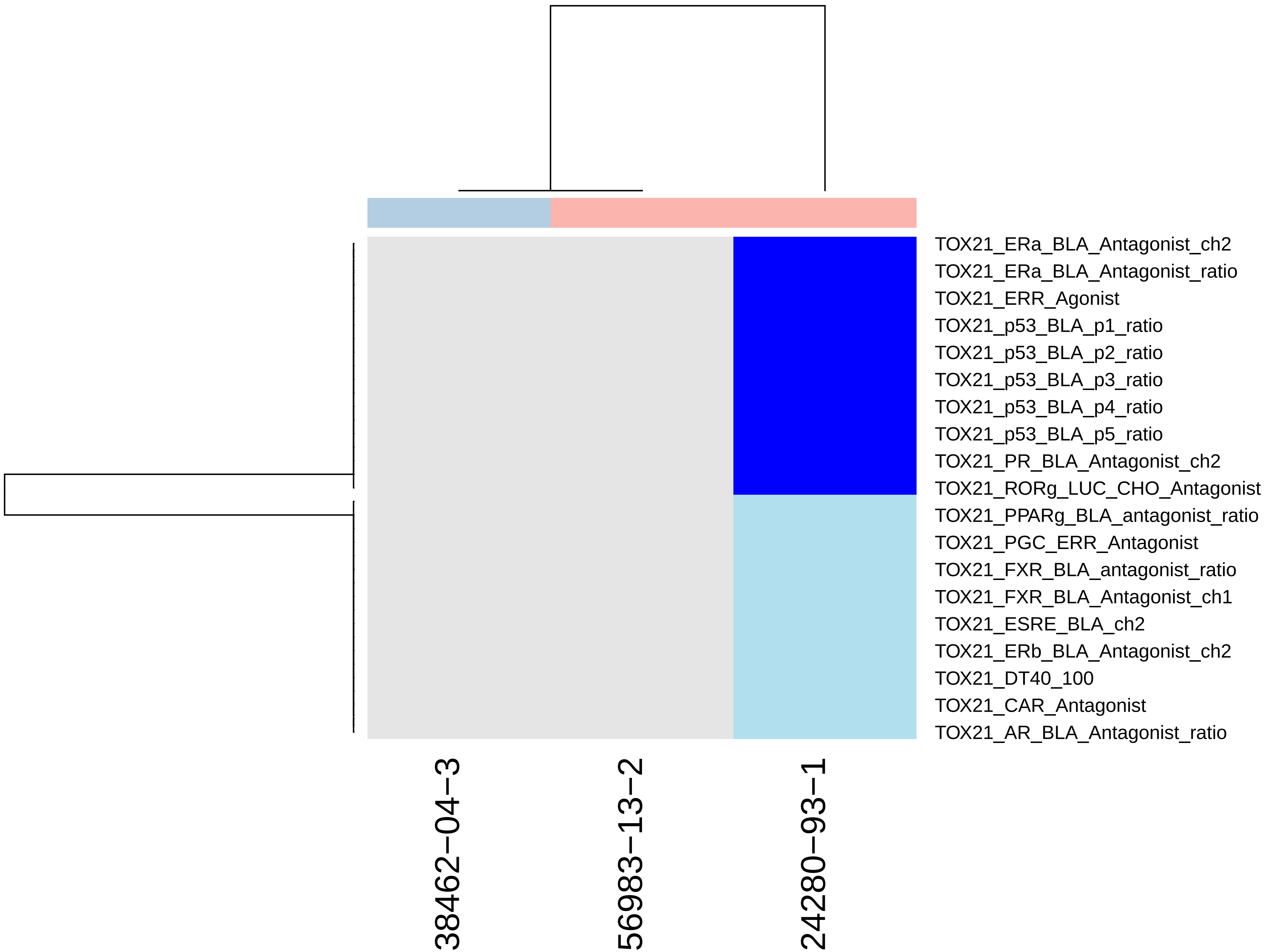

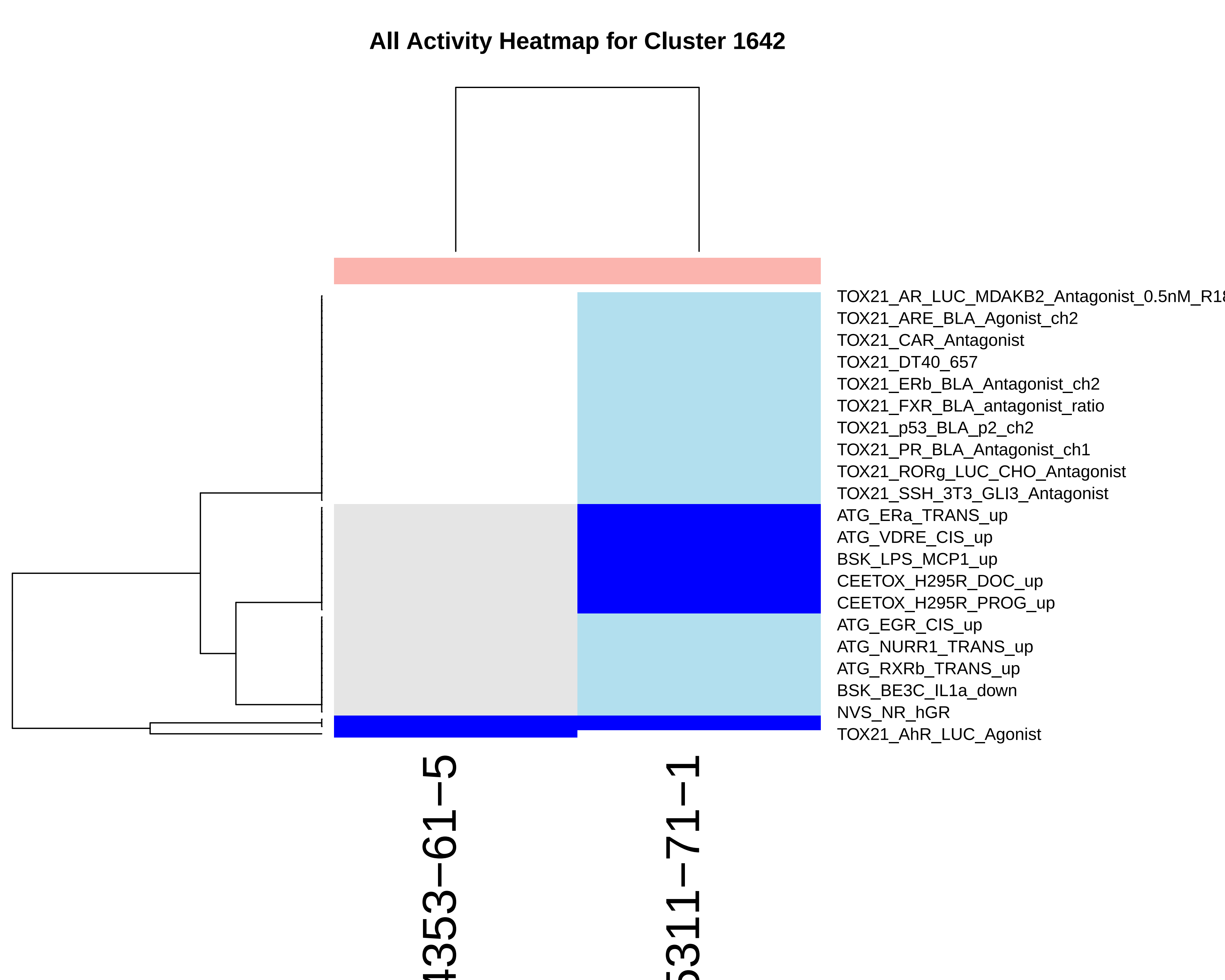

All Activity Heatmap for Cluster 1642
TOX21_AR_LUC_MDAKB2_Antagonist_0.5nM_R18
TOX21_ARE_BLA_Agonist_ch2
TOX21_CAR_Antagonist
TOX21_DT40_657
TOX21_ERb_BLA_Antagonist_ch2
TOX21_FXR_BLA_antagonist_ratio
TOX21_p53_BLA_p2_ch2
TOX21_PR_BLA_Antagonist_ch1
TOX21_RORg_LUC_CHO_Antagonist
TOX21_SSH_3T3_GLI3_Antagonist
ATG_ERa_TRANS_up
ATG_VDRE_CIS_up
BSK_LPS_MCP1_up
CEETOX_H295R_DOC_up
CEETOX_H295R_PROG_up
ATG_EGR_CIS_up
ATG_NURR1_TRANS_up
ATG_RXRb_TRANS_up
BSK_BE3C_IL1a_down
NVS_NR_hGR
TOX21_AhR_LUC_Agonist
4353−61−5
5311−71−1

All Activity Heatmap for Cluster 1643

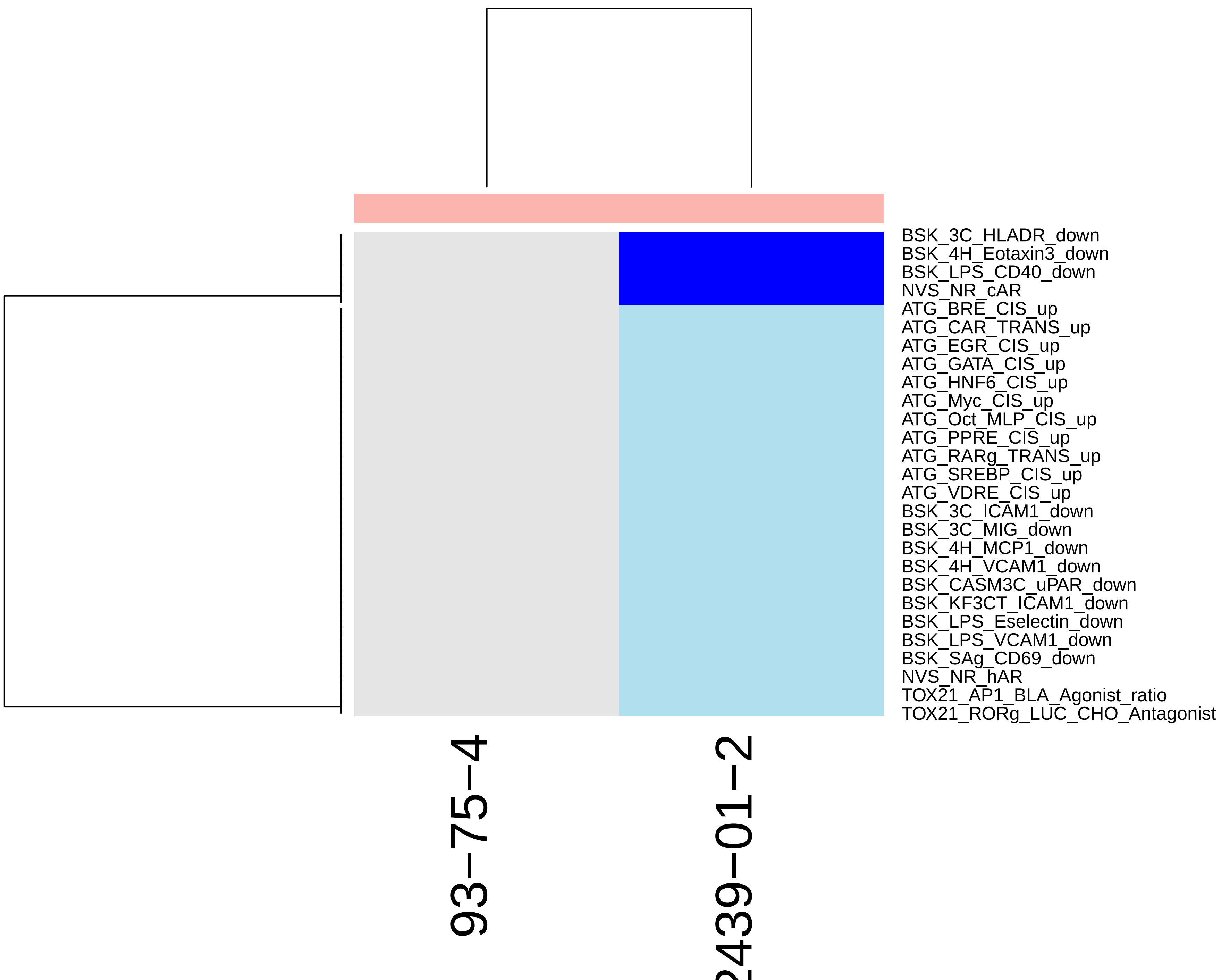

All Activity Heatmap for Cluster 1644

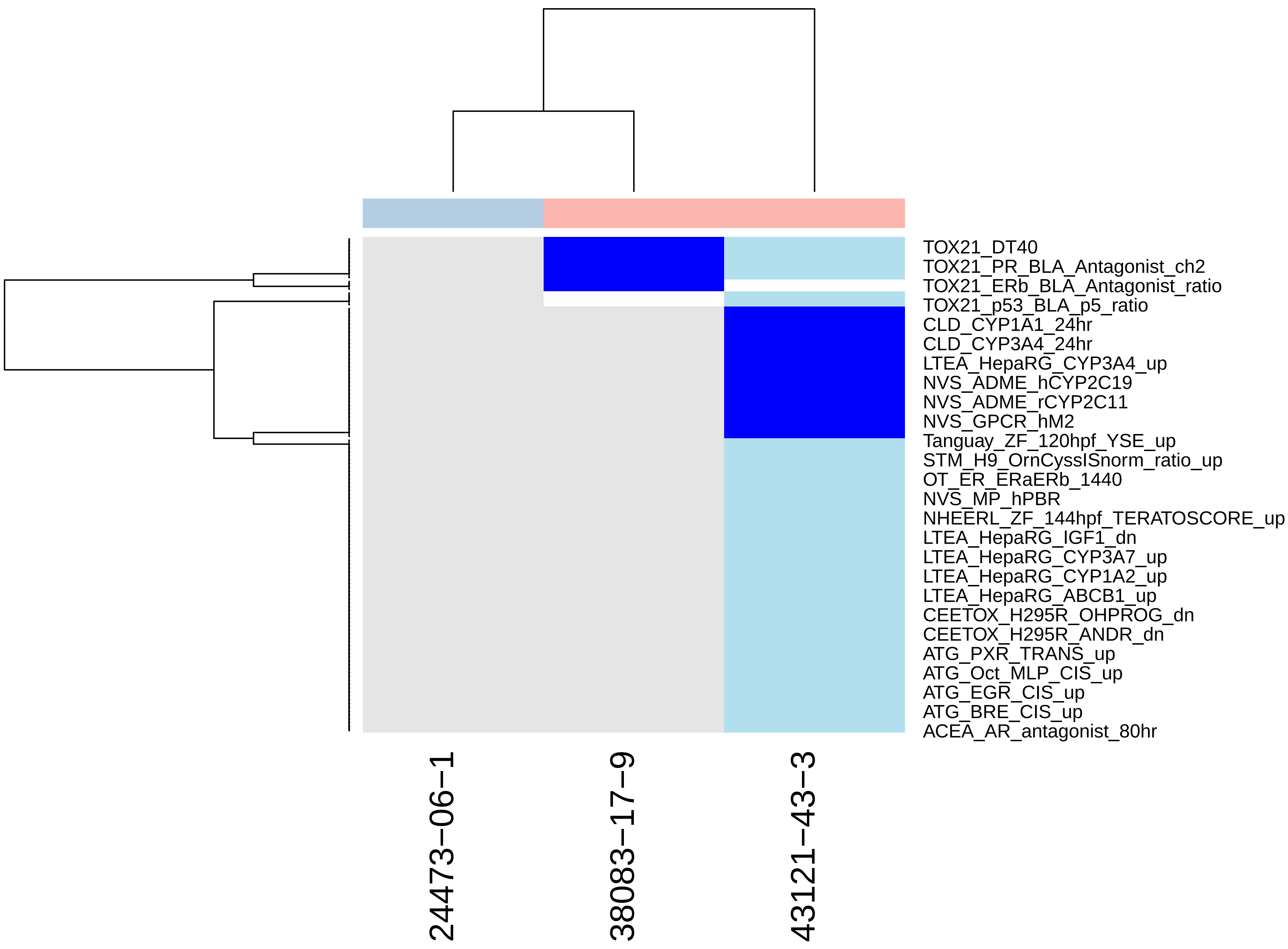

All Activity Heatmap for Cluster 1646

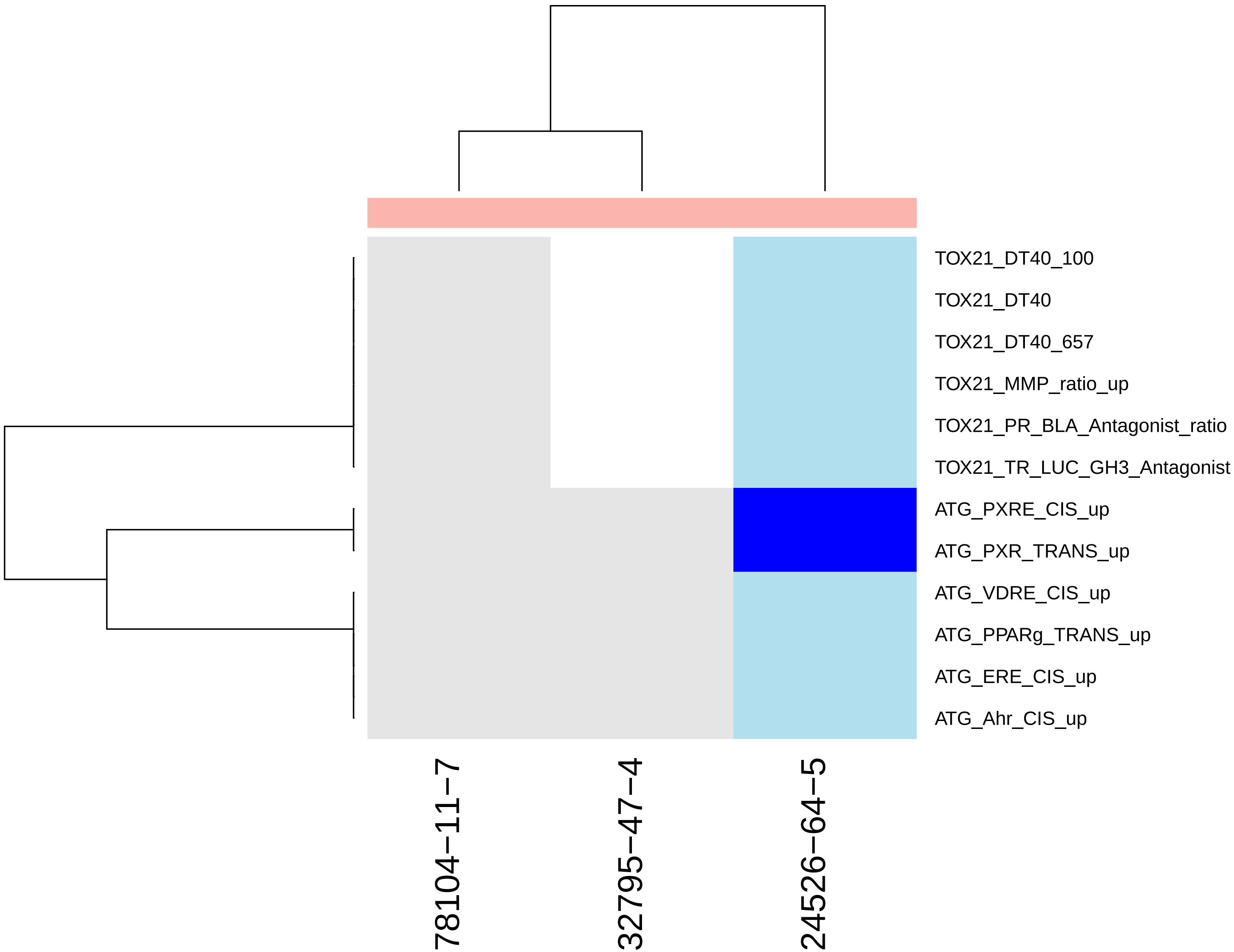

All Activity Heatmap for Cluster 1647

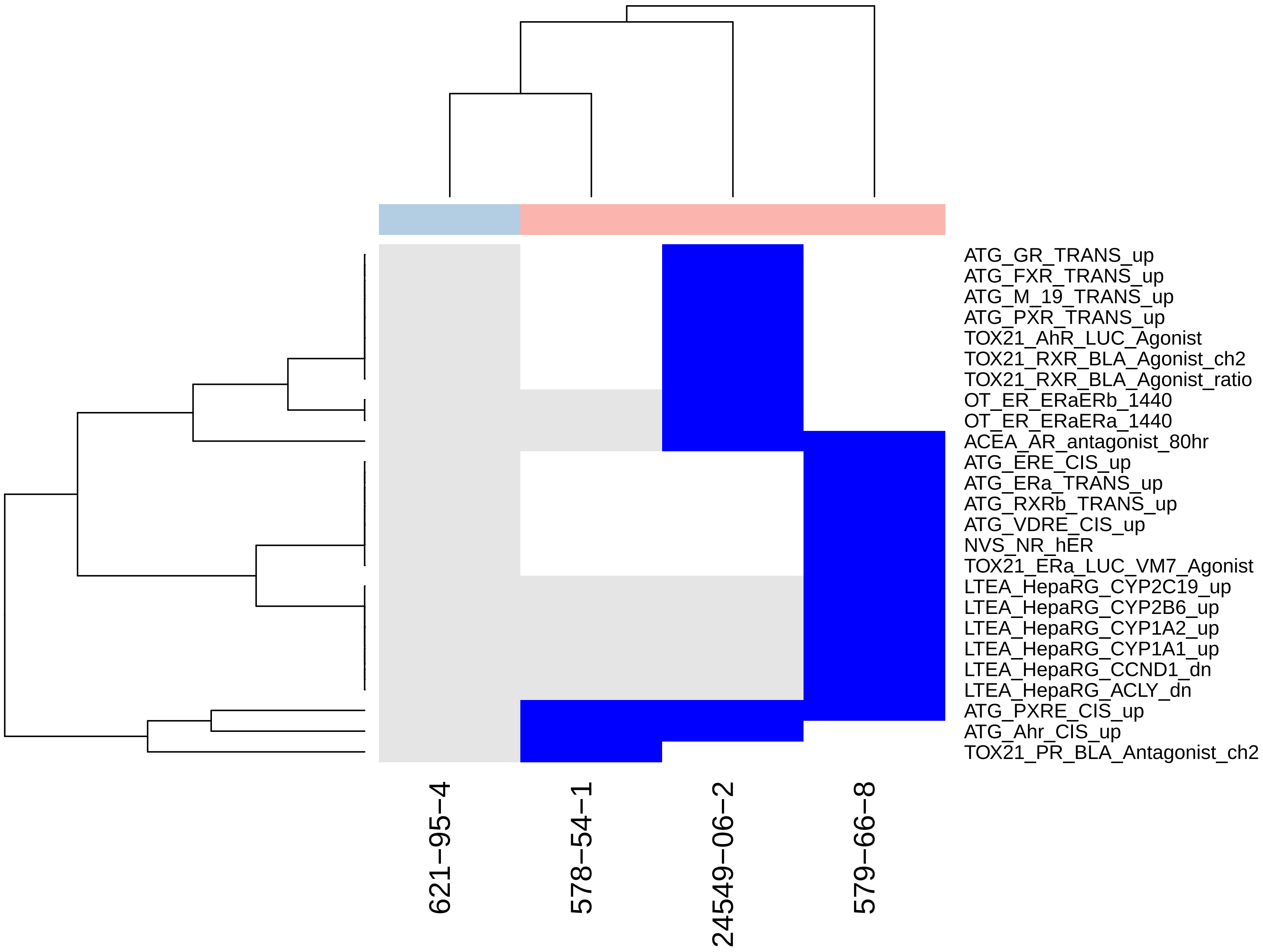

All Activity Heatmap for Cluster 1649

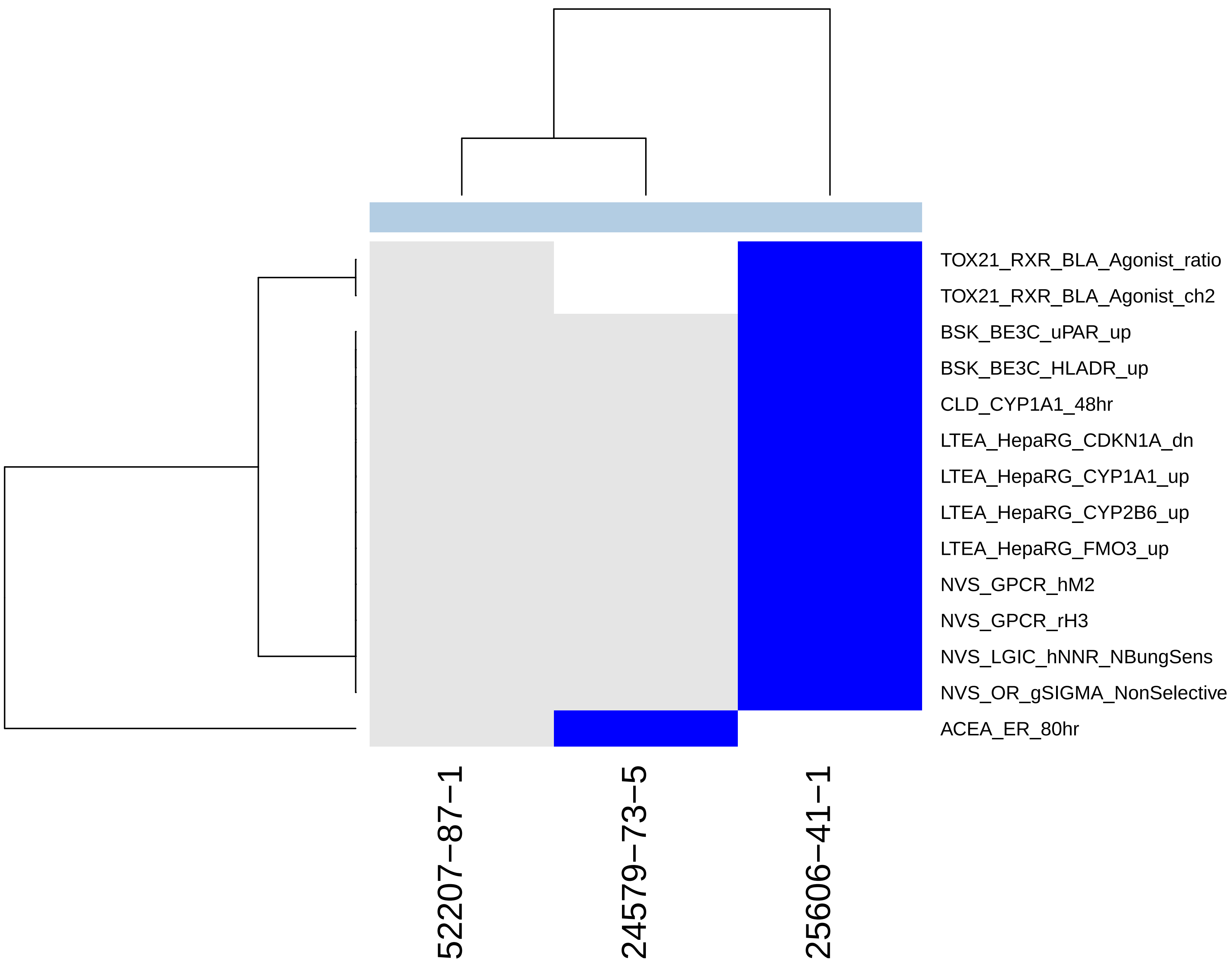

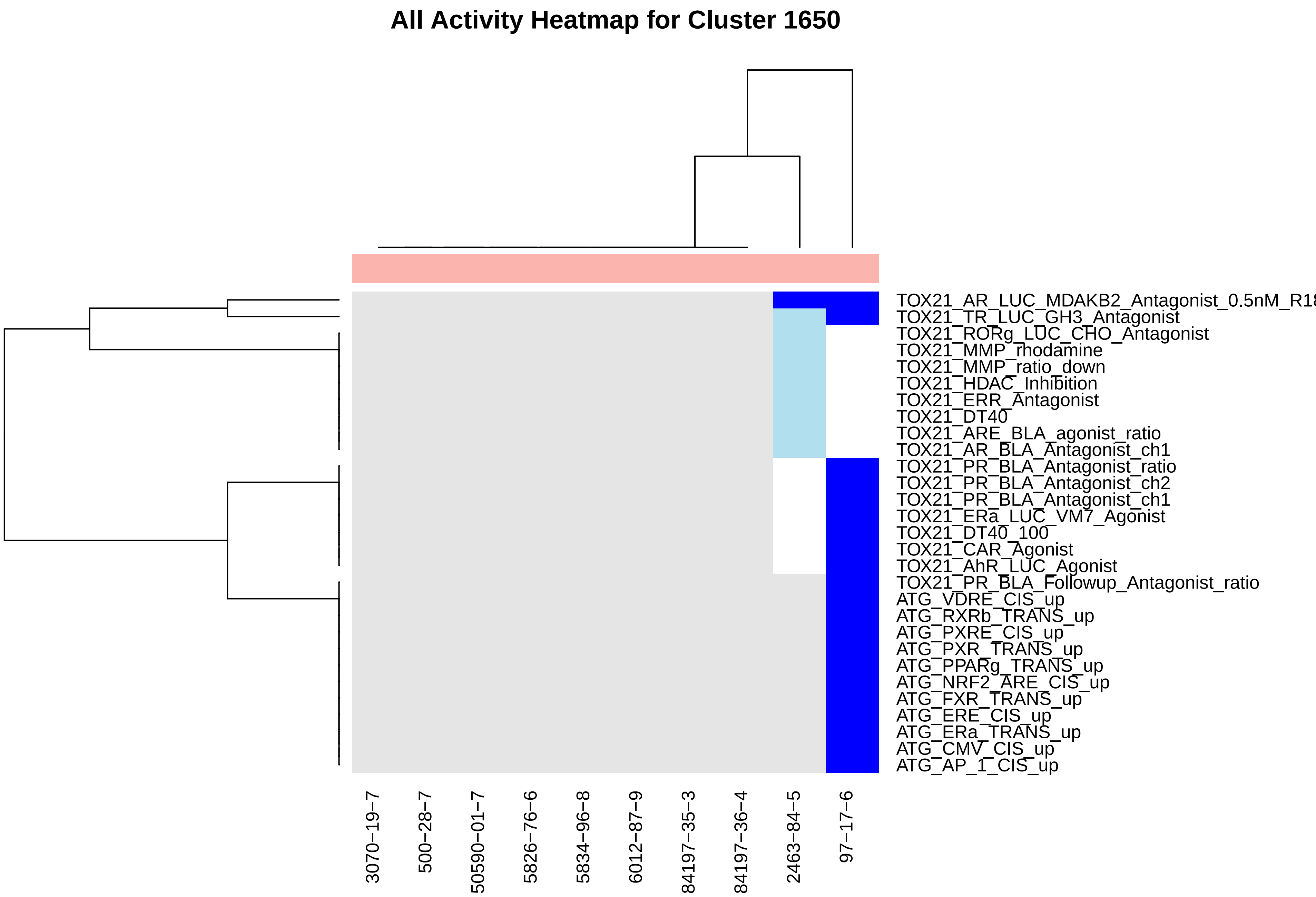
All Activity Heatmap for Cluster 1650
TOX21_AR_LUC_MDAKB2_Antagonist_0.5nM_R18
TOX21_TR_LUC_GH3_Antagonist
TOX21_RORg_LUC_CHO_Antagonist
TOX21_MMP_rhodamine
TOX21_MMP_ratio_down
TOX21_HDAC_Inhibition
TOX21_ERR_Antagonist
TOX21_DT40
TOX21_ARE_BLA_agonist_ratio
TOX21_AR_BLA_Antagonist_ch1
TOX21_PR_BLA_Antagonist_ratio
TOX21_PR_BLA_Antagonist_ch2
TOX21_PR_BLA_Antagonist_ch1
TOX21_ERa_LUC_VM7_Agonist
TOX21_DT40_100
TOX21_CAR_Agonist
TOX21_AhR_LUC_Agonist
TOX21_PR_BLA_Followup_Antagonist_ratio
ATG_VDRE_CIS_up
ATG_RXRb_TRANS_up
ATG_PXRE_CIS_up
ATG_PXR_TRANS_up
ATG_PPARg_TRANS_up
ATG_NRF2_ARE_CIS_up
ATG_FXR_TRANS_up
ATG_ERE_CIS_up
ATG_ERa_TRANS_up
ATG_CMV_CIS_up
ATG_AP_1_CIS_up
3070−19−7
500−28−7
50590−01−7
5826−76−6
5834−96−8
6012−87−9
84197−35−3
84197−36−4
2463−84−5
97−17−6

All Activity Heatmap for Cluster 1652

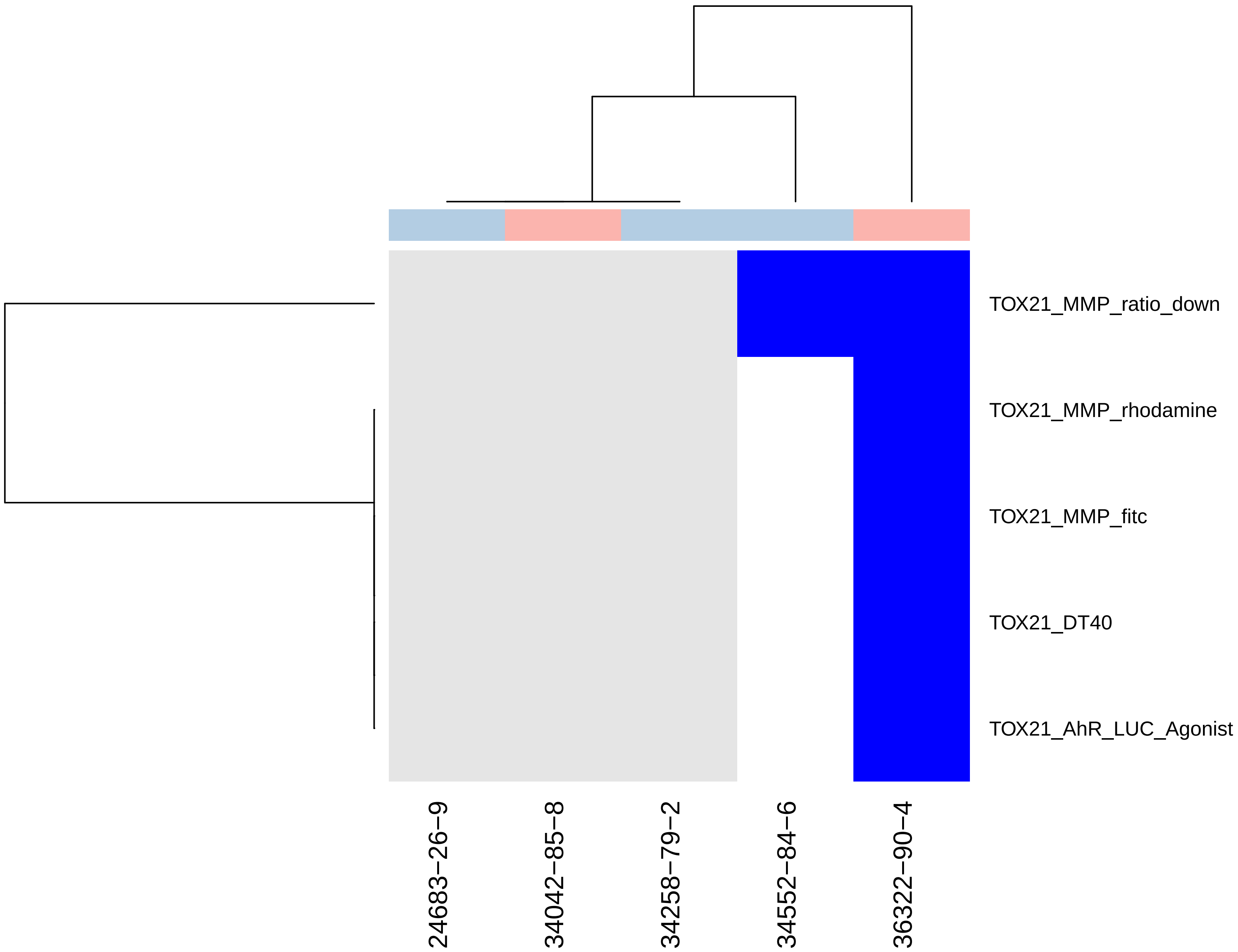

All Activity Heatmap for Cluster 1653

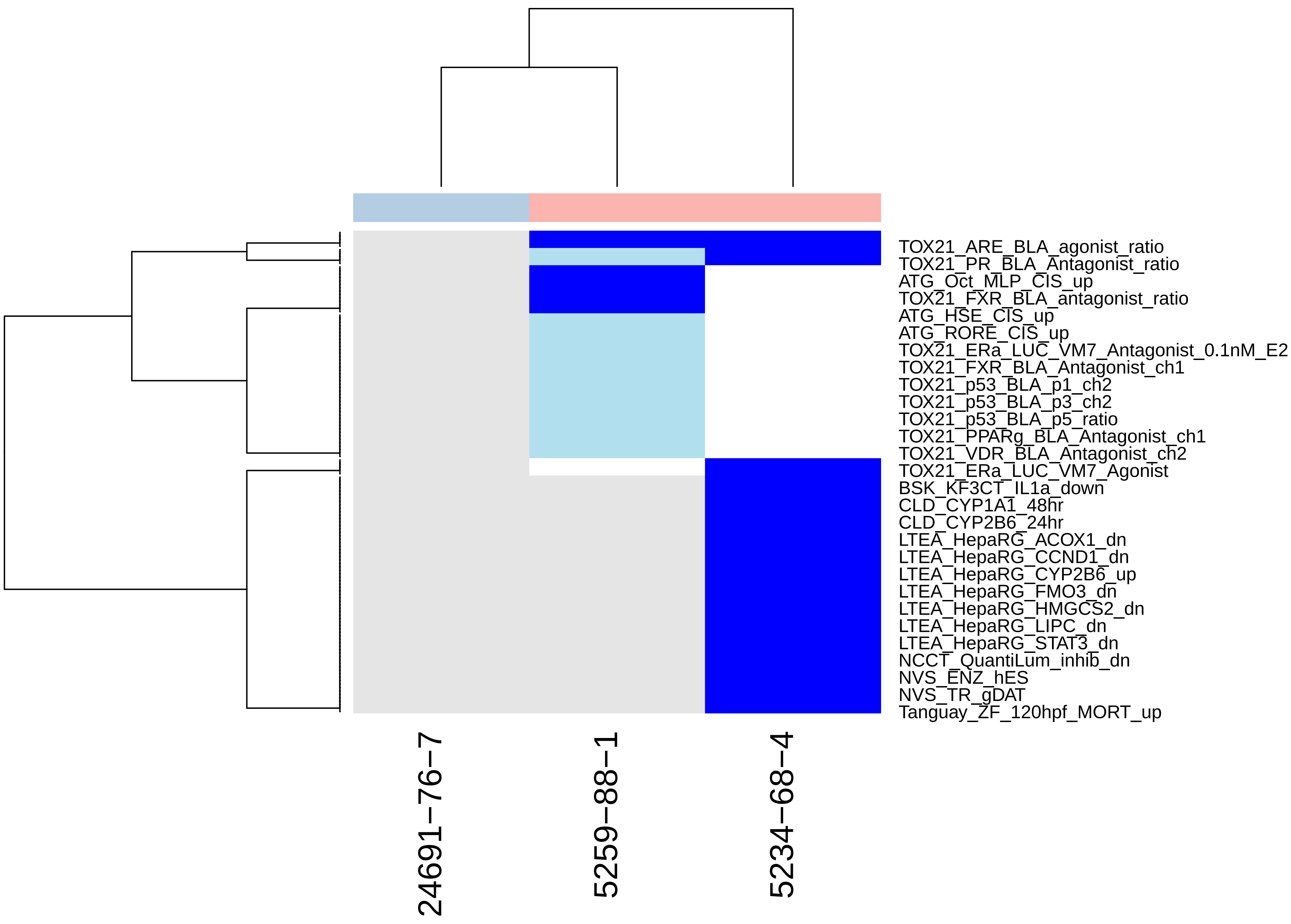

All Activity Heatmap for Cluster 1654

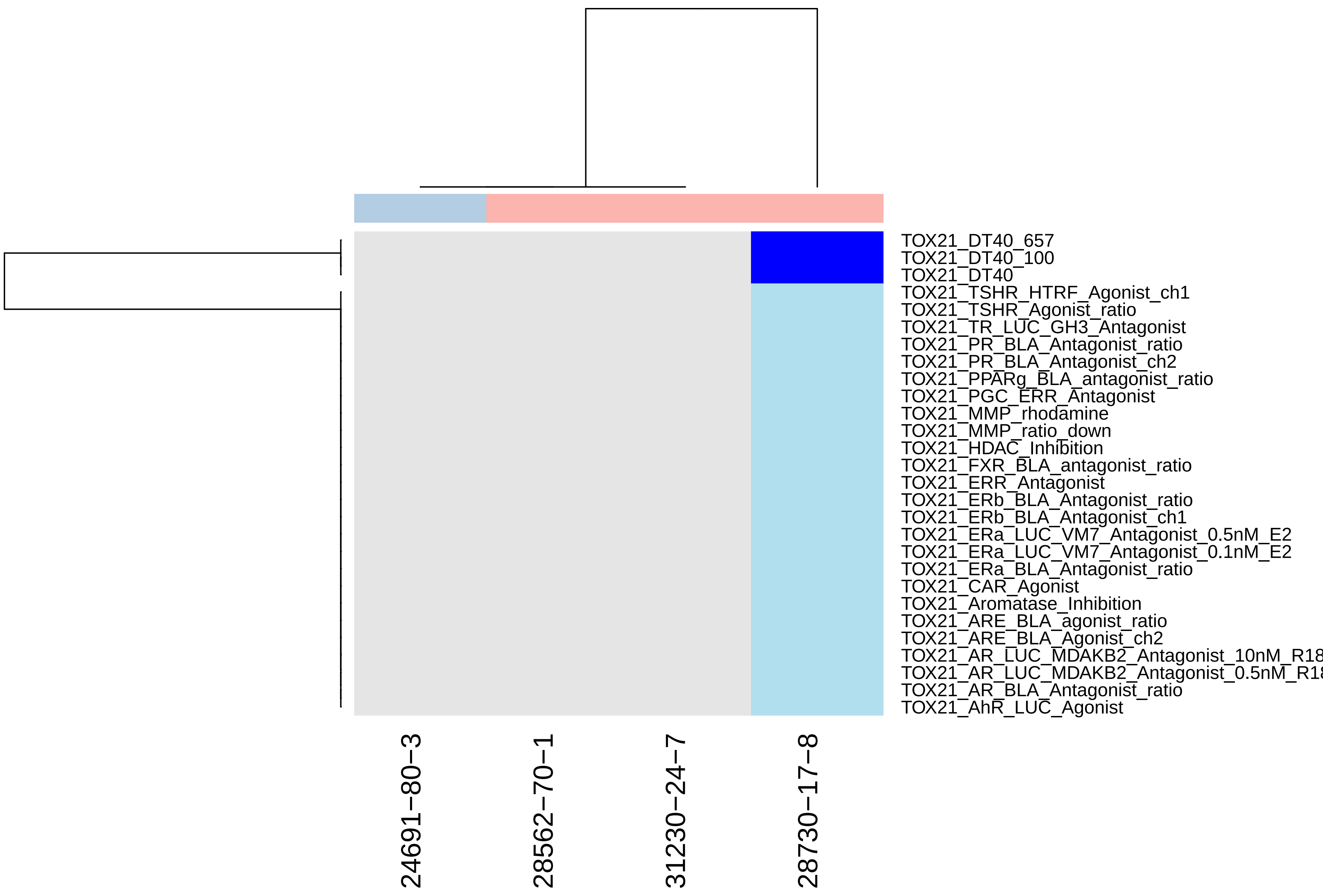

All Activity Heatmap for Cluster 1658

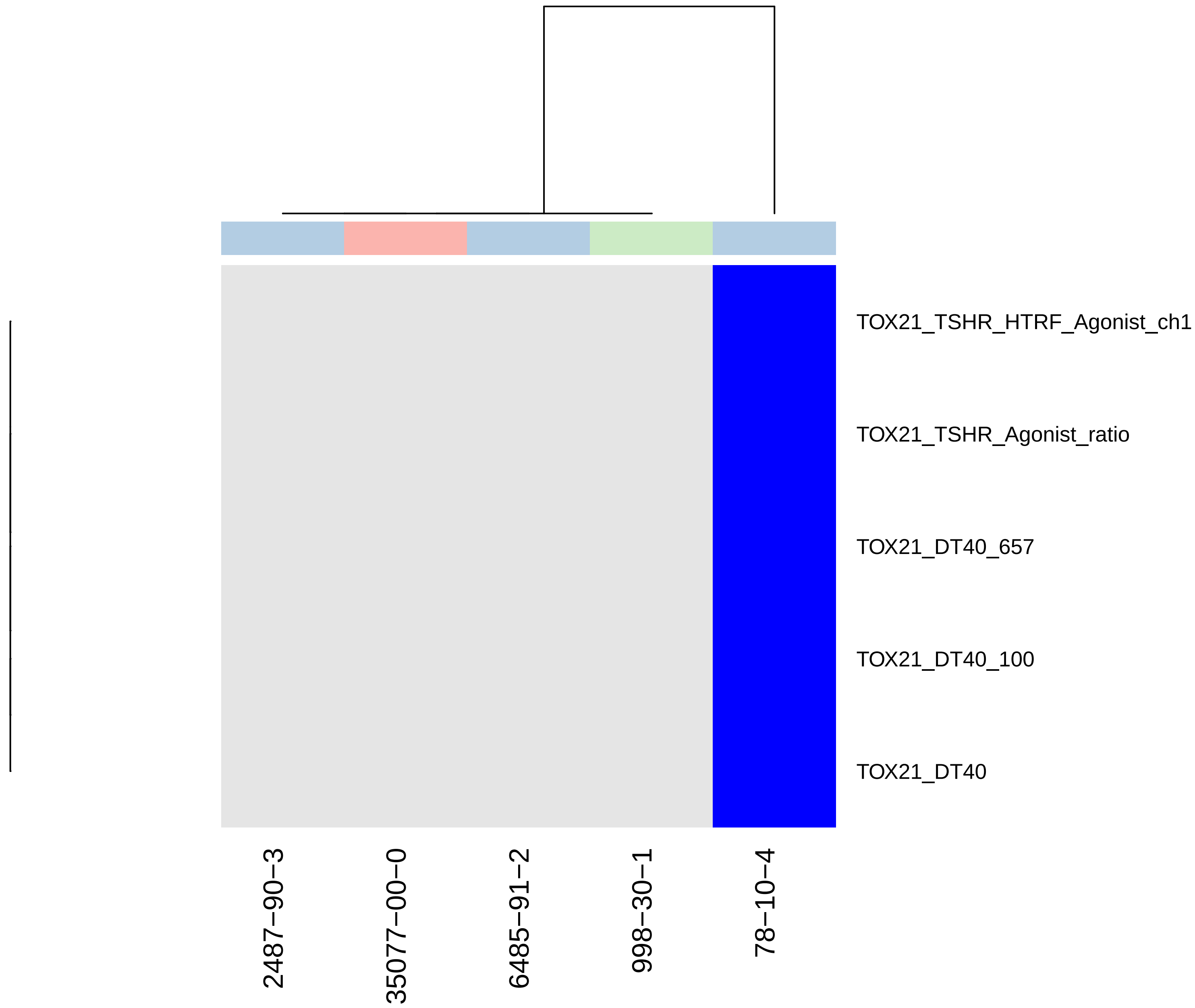

All Activity Heatmap for Cluster 1659

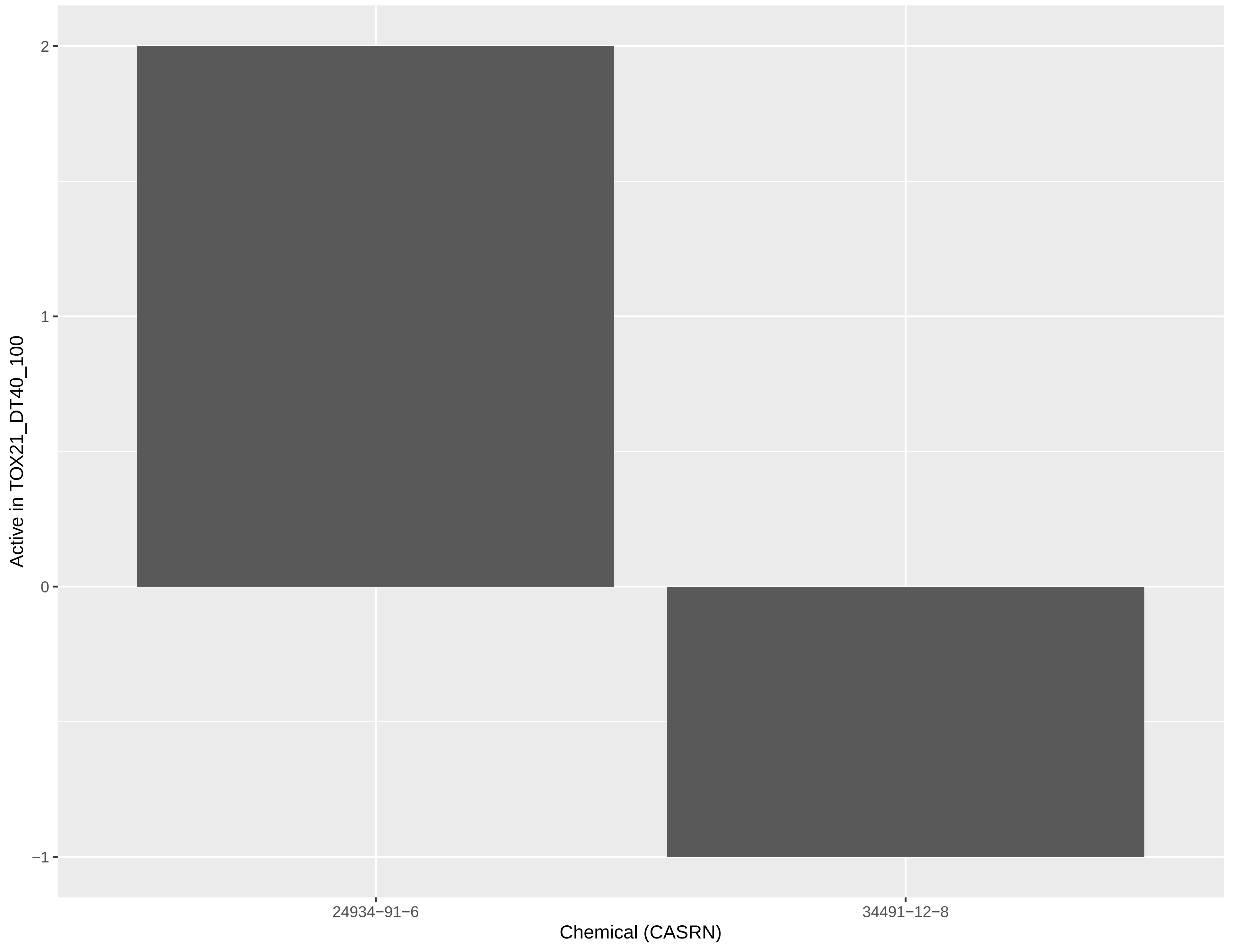

All Activity Heatmap for Cluster 1660

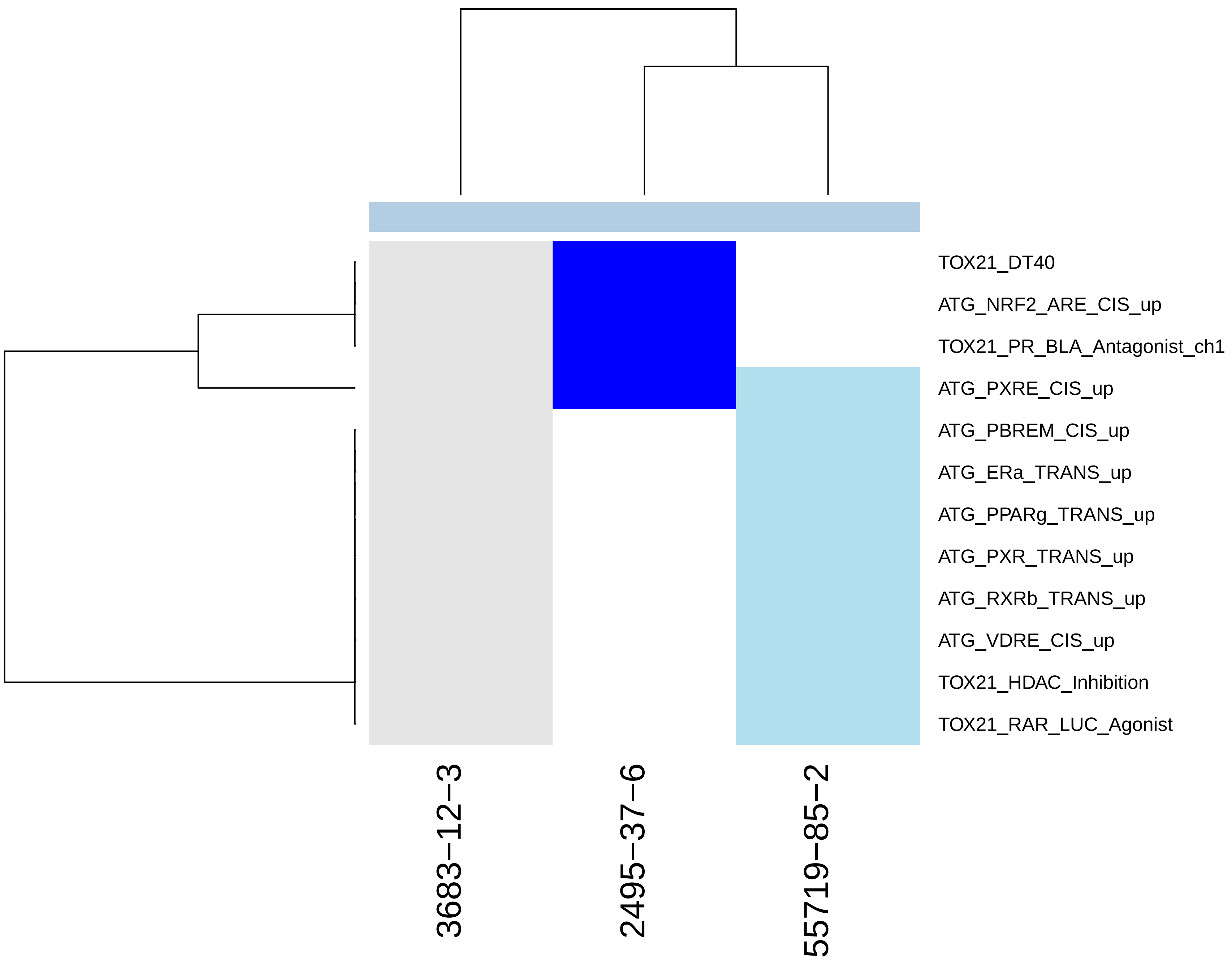

All Activity Heatmap for Cluster 1661

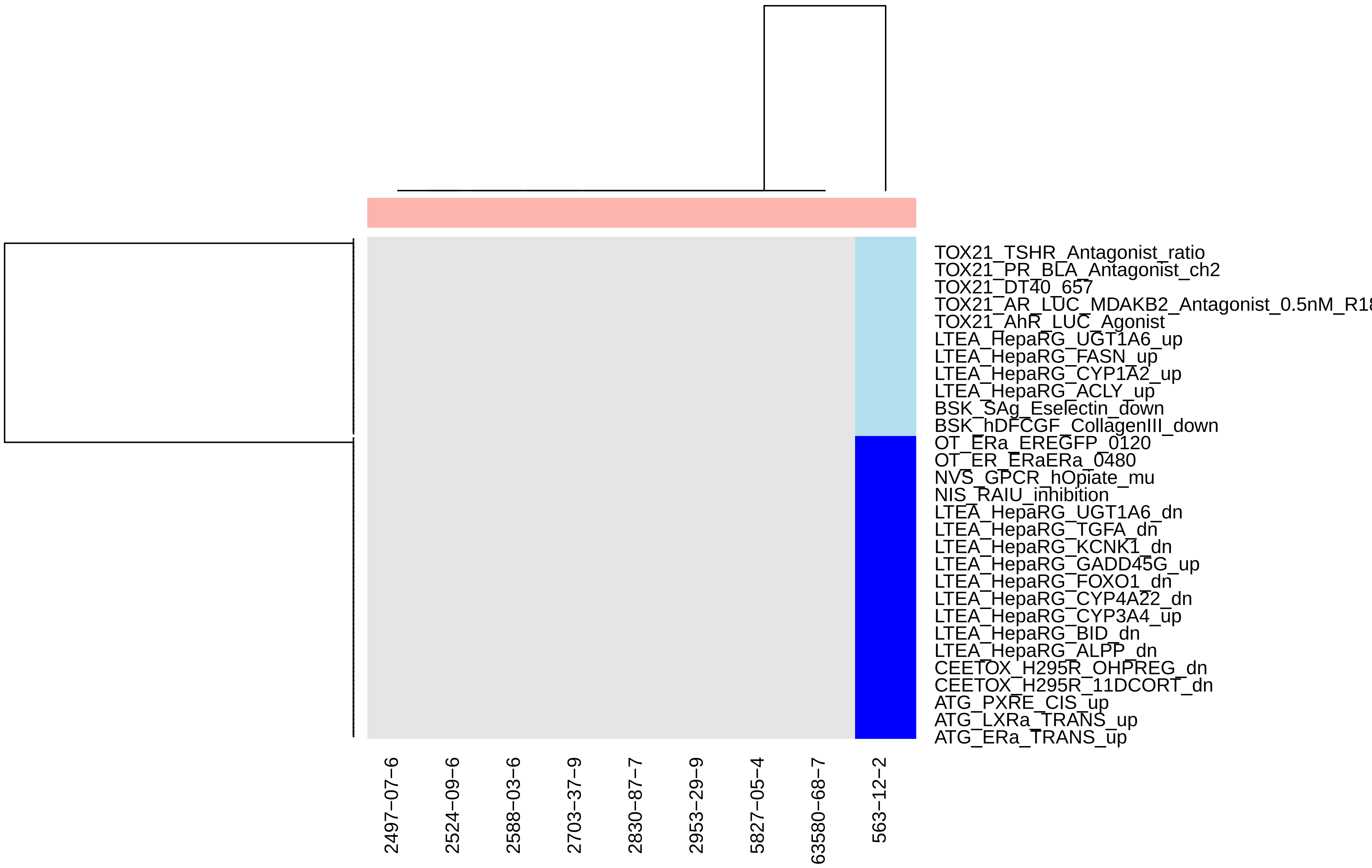

All Activity Heatmap for Cluster 1662

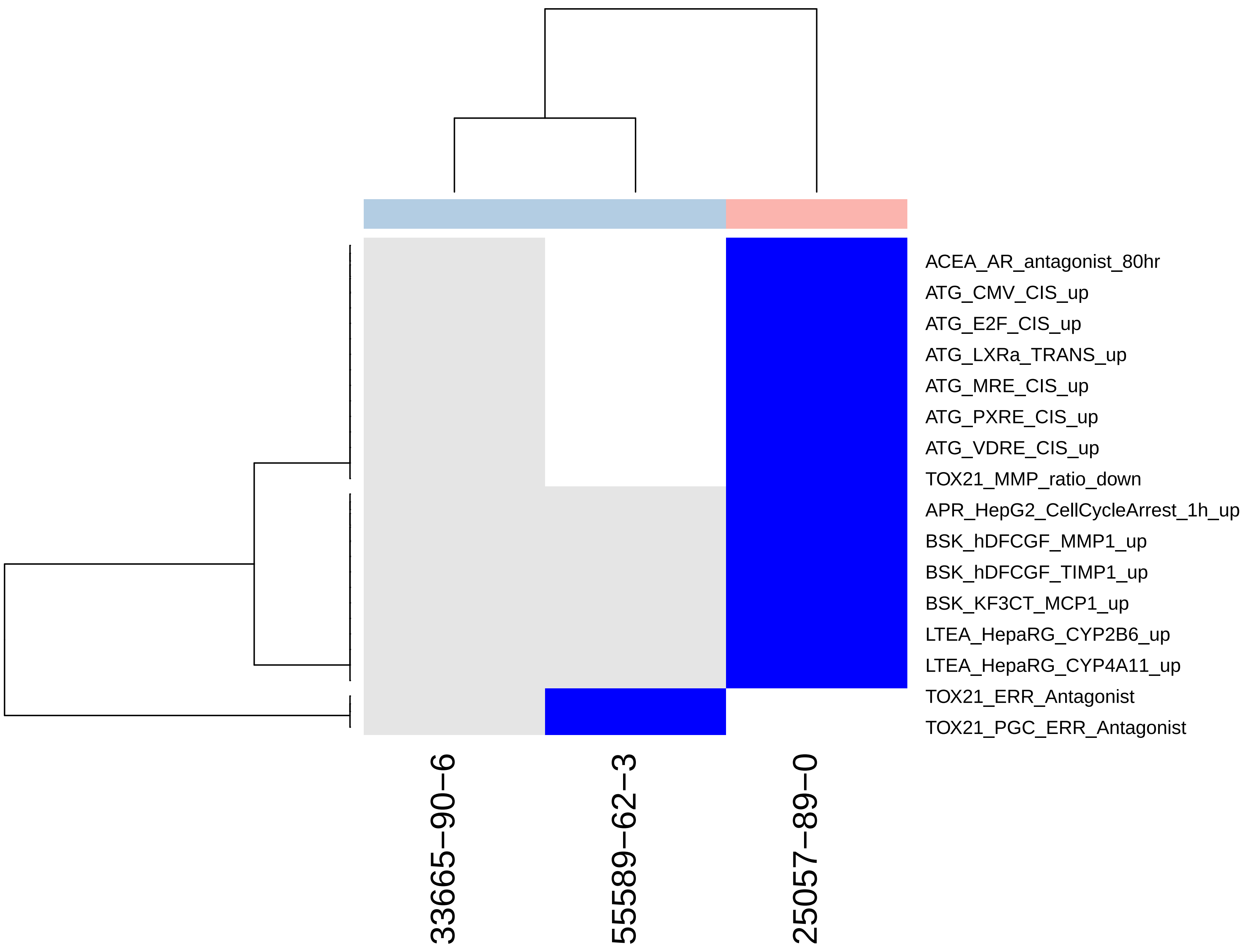

All Activity Heatmap for Cluster 1663

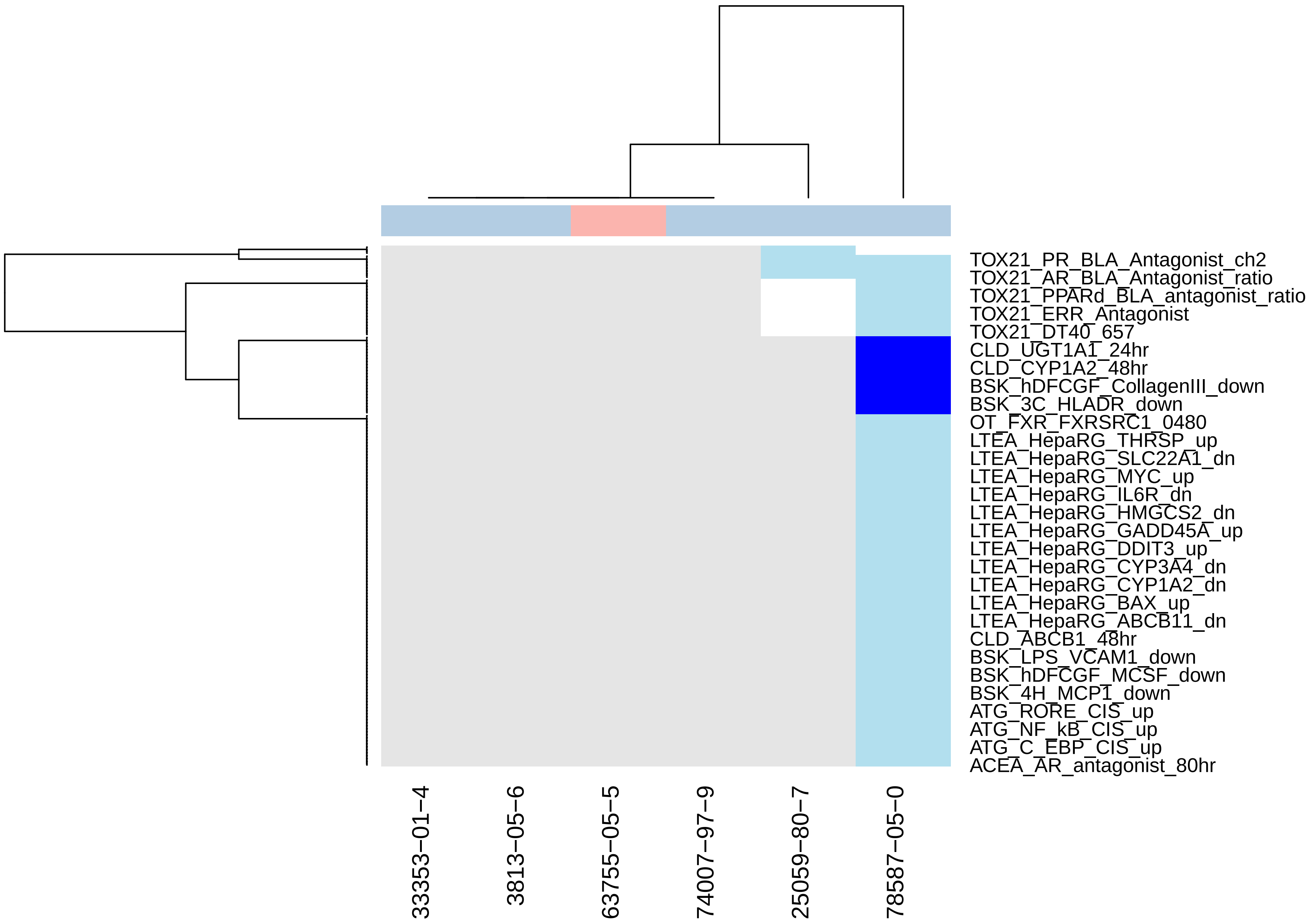

All Activity Heatmap for Cluster 1665

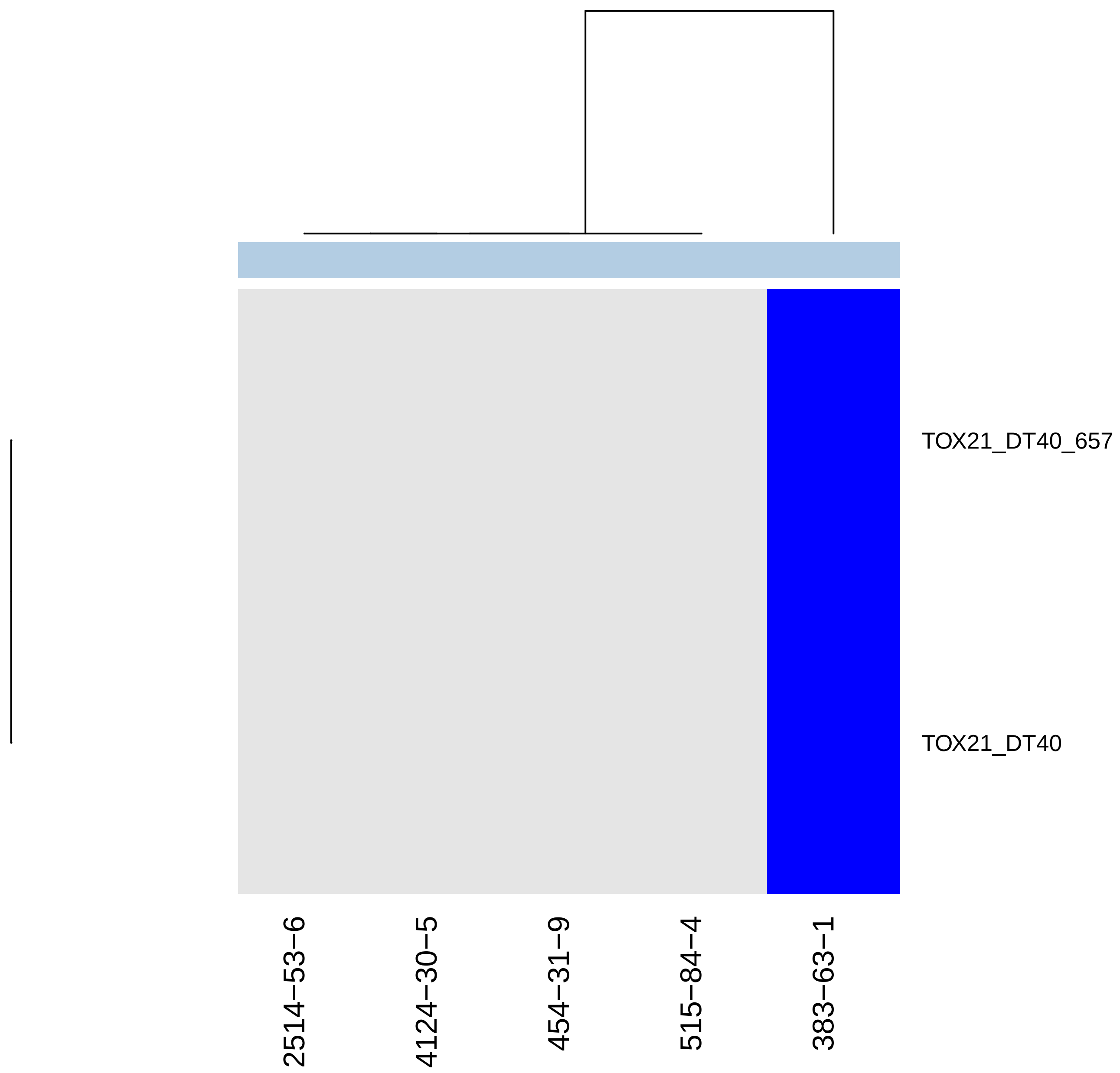

All Activity Heatmap for Cluster 1666

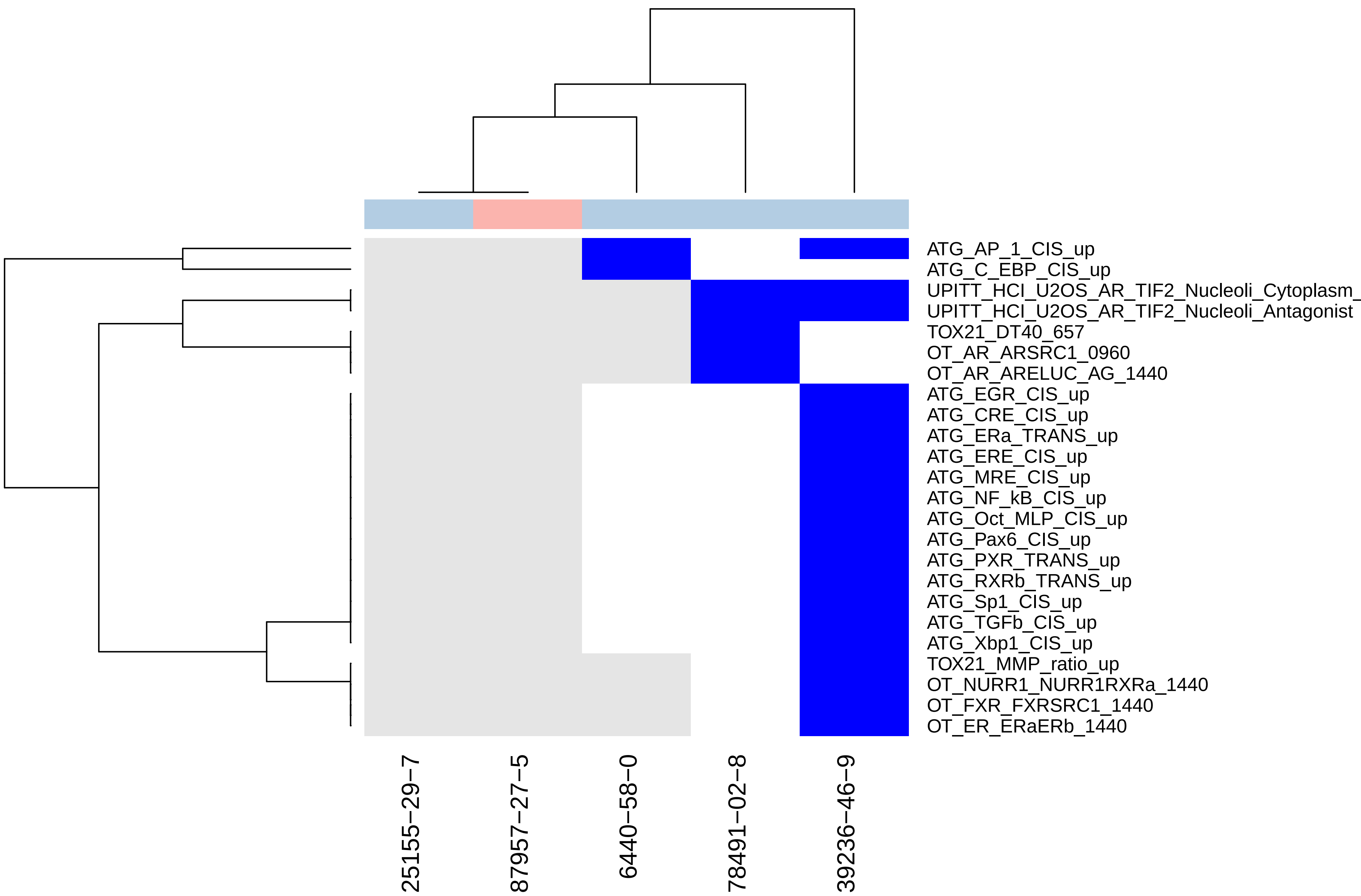

All Activity Heatmap for Cluster 1667

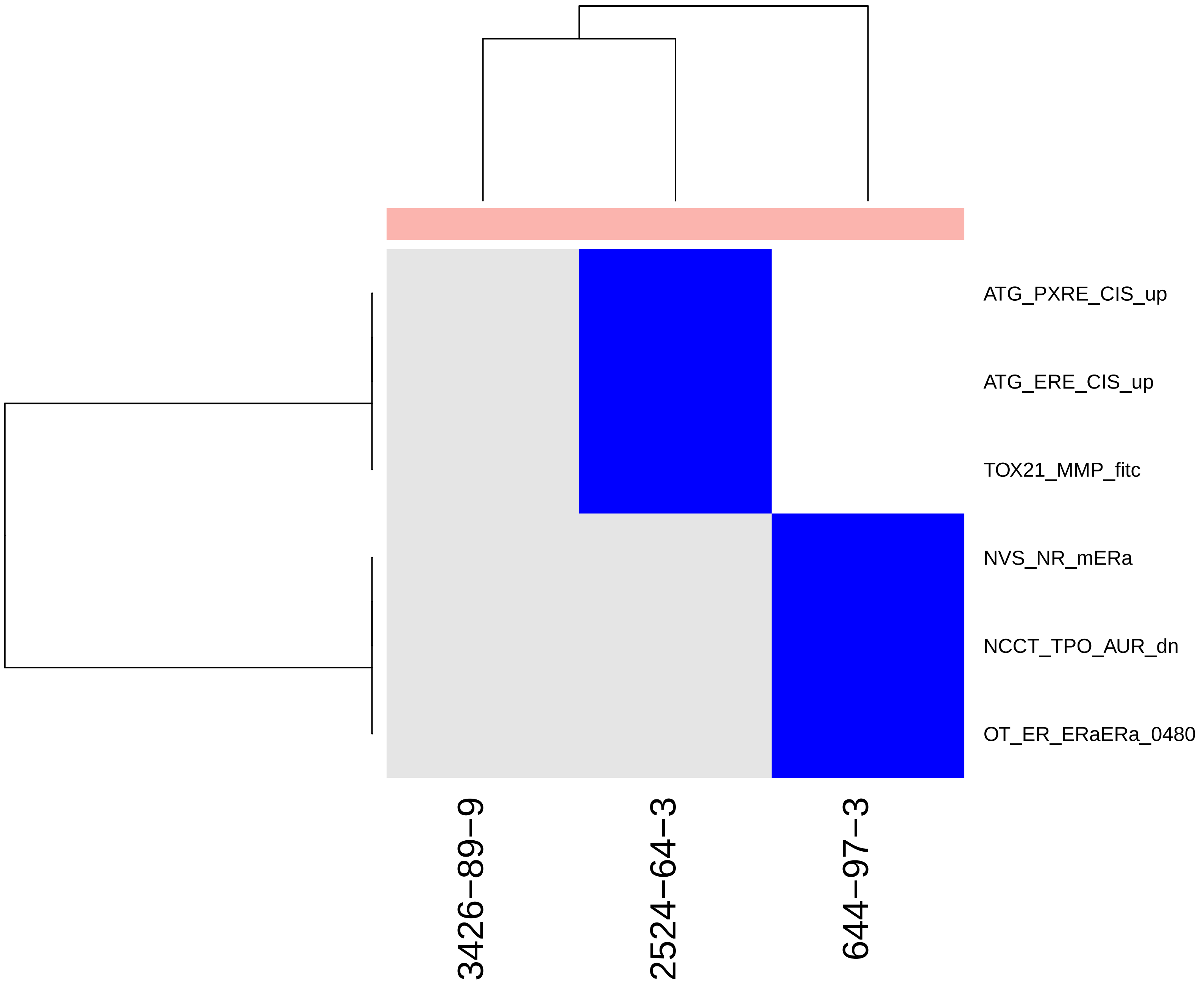

All Activity Heatmap for Cluster 1670

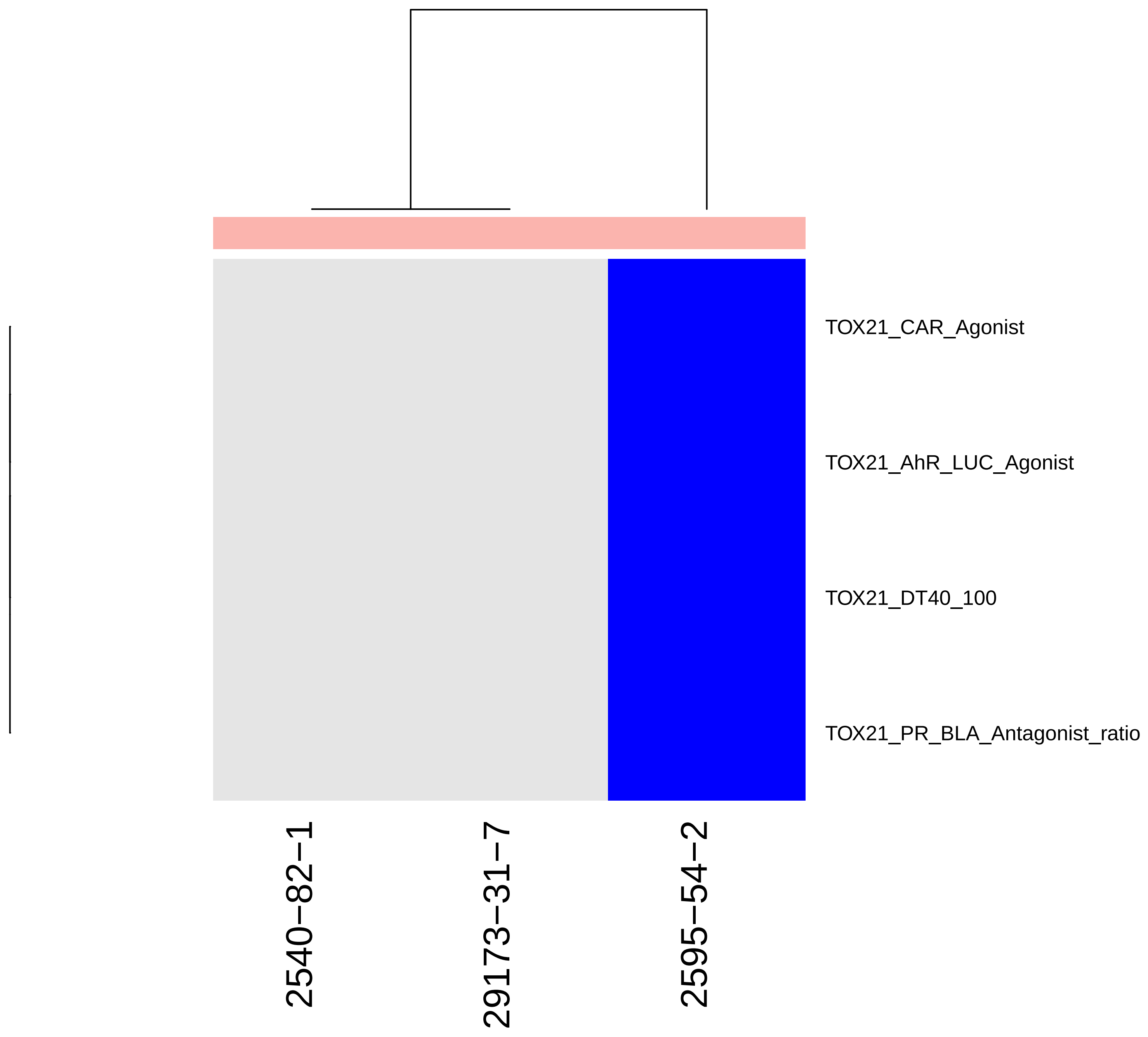

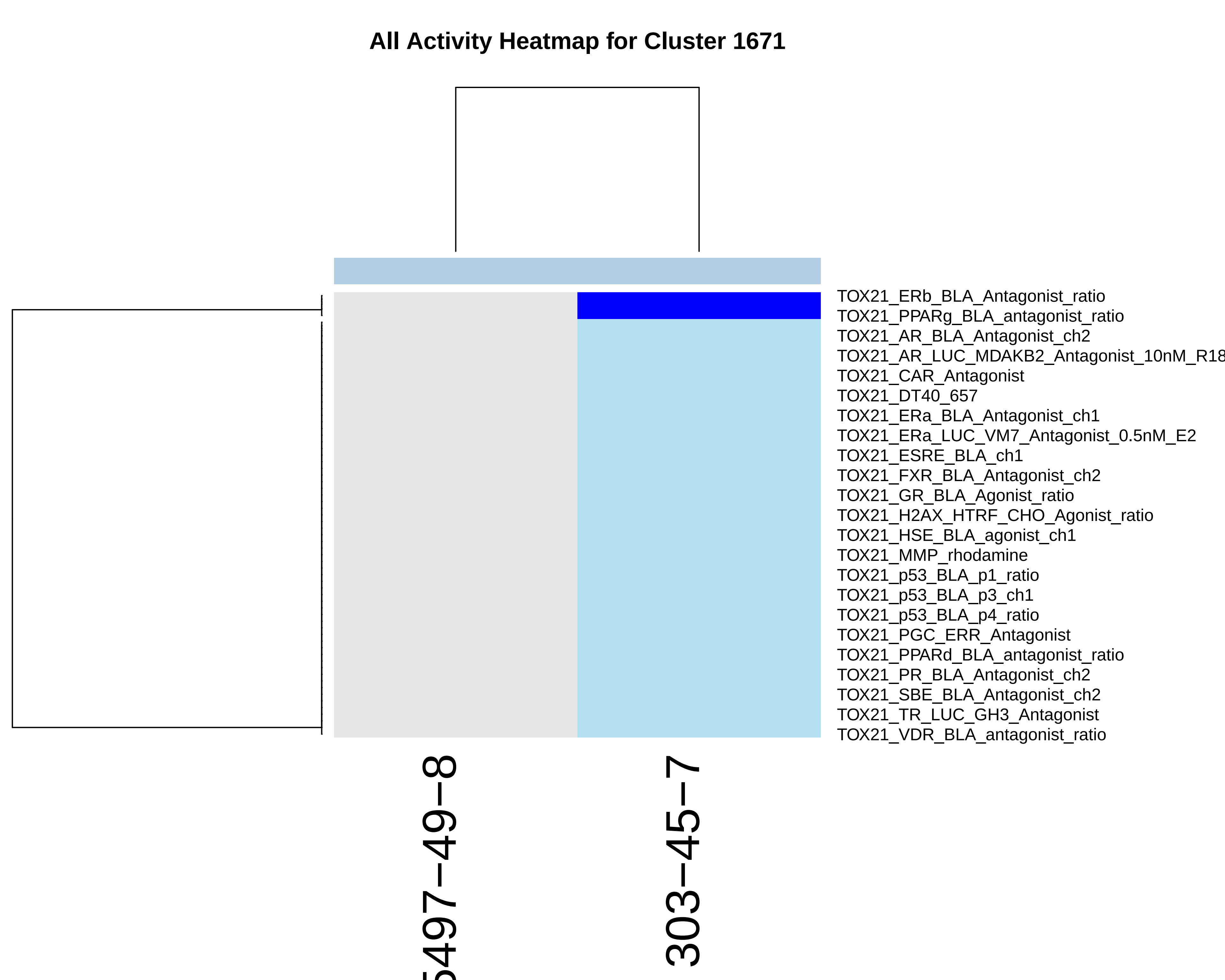
All Activity Heatmap for Cluster 1671
TOX21_ERb_BLA_Antagonist_ratio
TOX21_PPARg_BLA_antagonist_ratio
TOX21_AR_BLA_Antagonist_ch2
TOX21_AR_LUC_MDAKB2_Antagonist_10nM_R18
TOX21_CAR_Antagonist
TOX21_DT40_657
TOX21_ERa_BLA_Antagonist_ch1
TOX21_ERa_LUC_VM7_Antagonist_0.5nM_E2
TOX21_ESRE_BLA_ch1
TOX21_FXR_BLA_Antagonist_ch2
TOX21_GR_BLA_Agonist_ratio
TOX21_H2AX_HTRF_CHO_Agonist_ratio
TOX21_HSE_BLA_agonist_ch1
TOX21_MMP_rhodamine
TOX21_p53_BLA_p1_ratio
TOX21_p53_BLA_p3_ch1
TOX21_p53_BLA_p4_ratio
TOX21_PGC_ERR_Antagonist
TOX21_PPARd_BLA_antagonist_ratio
TOX21_PR_BLA_Antagonist_ch2
TOX21_SBE_BLA_Antagonist_ch2
TOX21_TR_LUC_GH3_Antagonist
TOX21_VDR_BLA_antagonist_ratio
5497−49−8
303−45−7

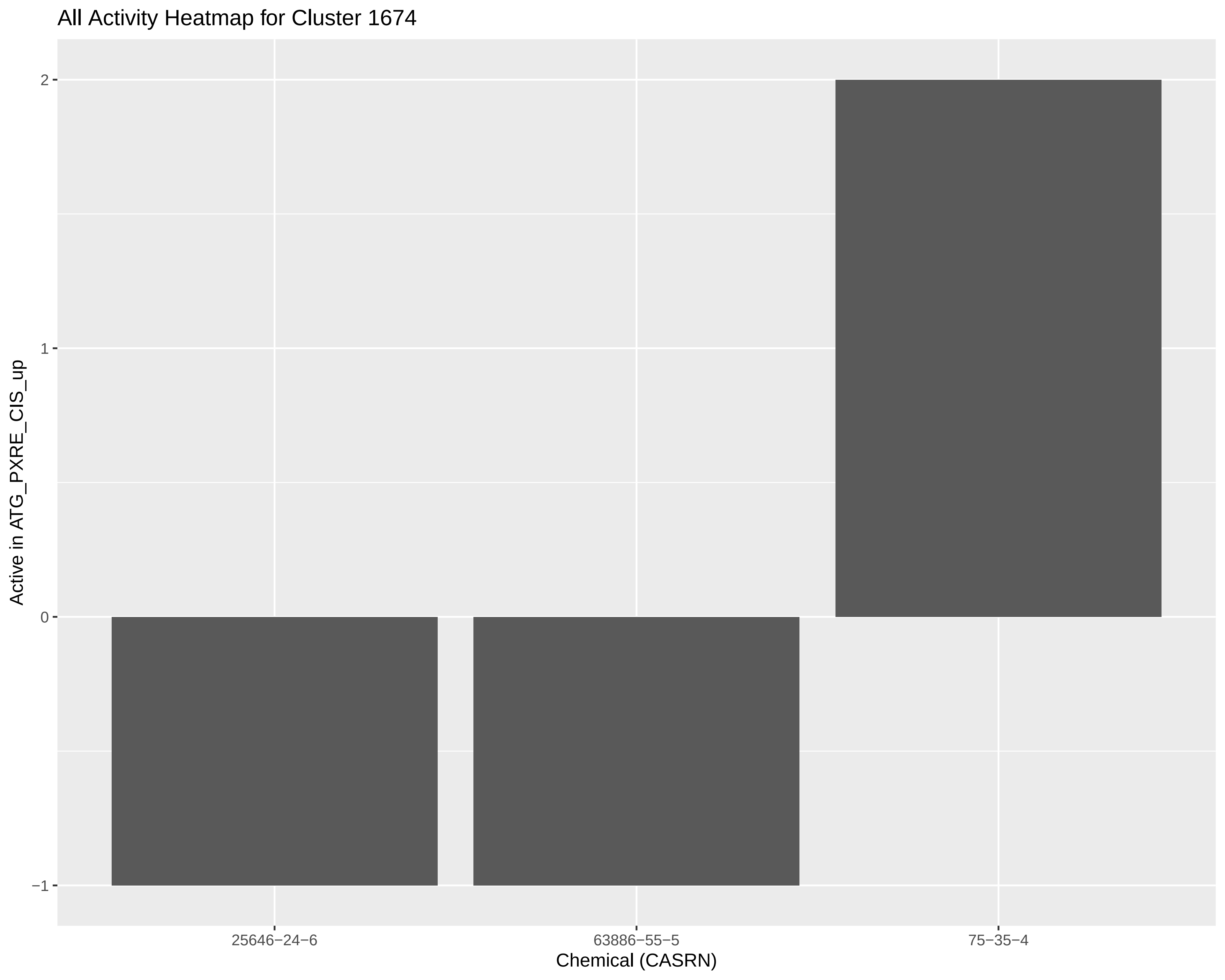
All Activity Heatmap for Cluster 1674
Active in ATG_PXRE_CIS_up
2
1
0
−1
25646−24−6
63886−55−5
75−35−4
Chemical (CASRN)

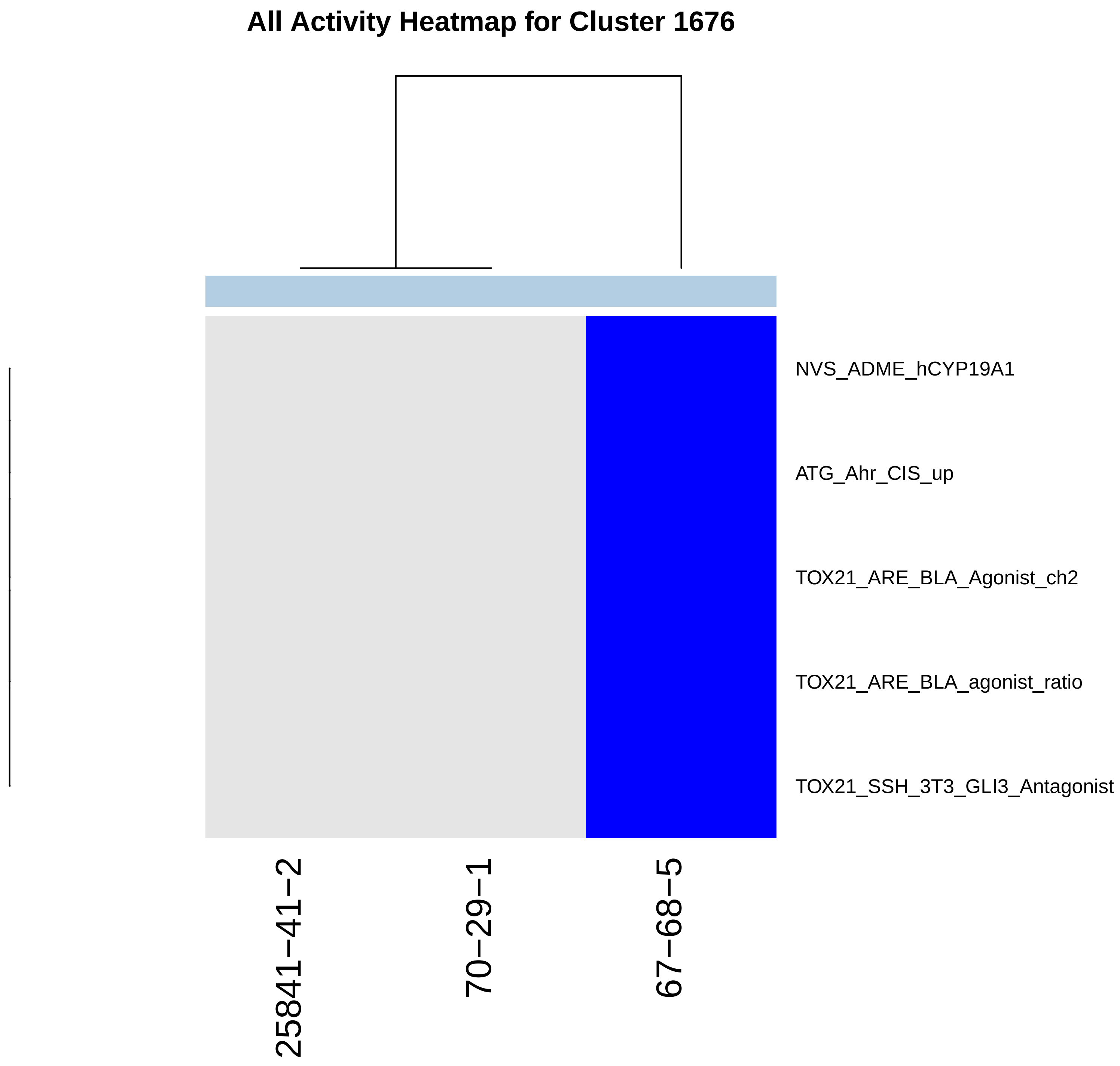
All Activity Heatmap for Cluster 1676
NVS_ADME_hCYP19A1
ATG_Ahr_CIS_up
TOX21_ARE_BLA_Agonist_ch2
TOX21_ARE_BLA_agonist_ratio
TOX21_SSH_3T3_GLI3_Antagonist
25841−41−2
70−29−1
67−68−5

All Activity Heatmap for Cluster 1677

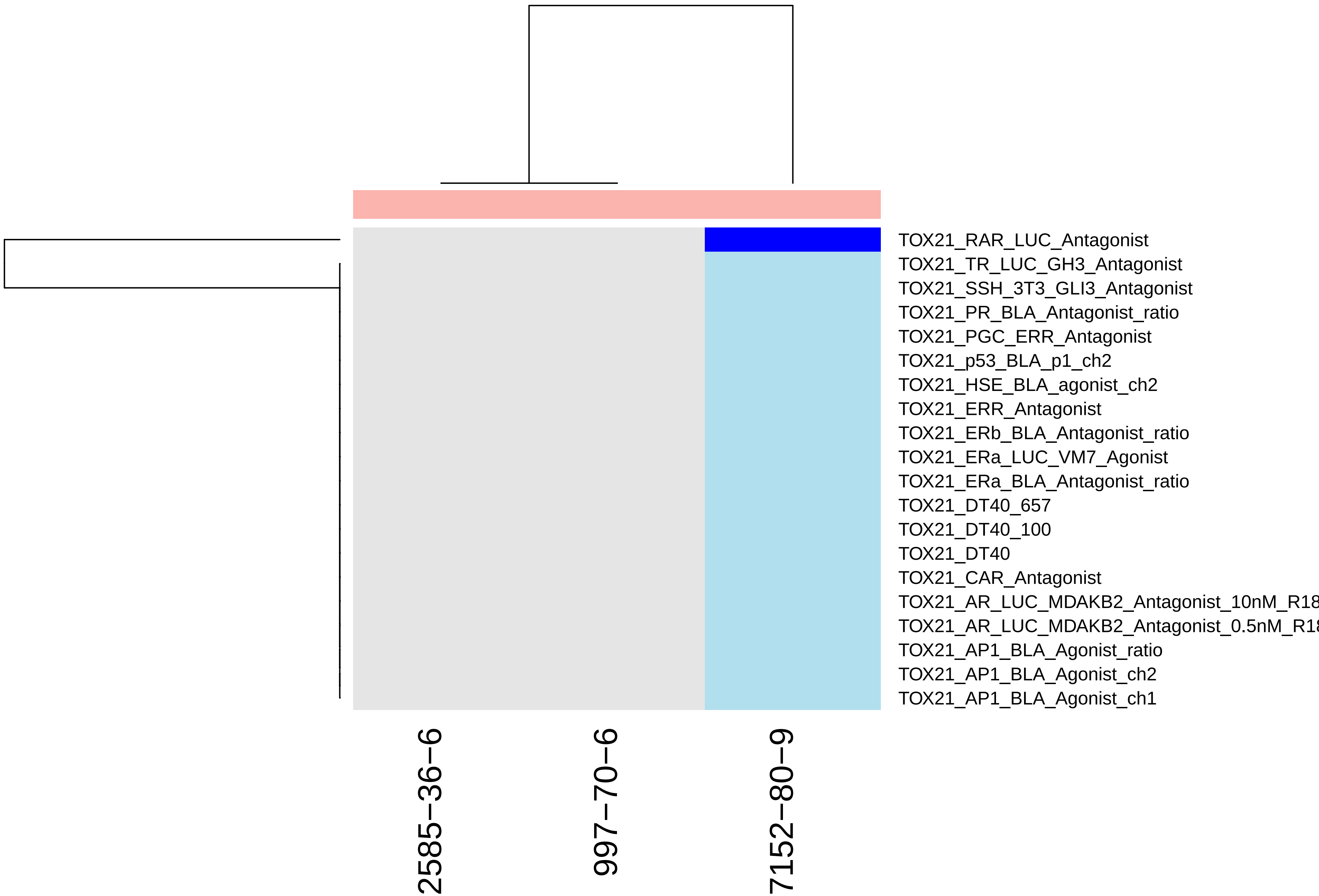

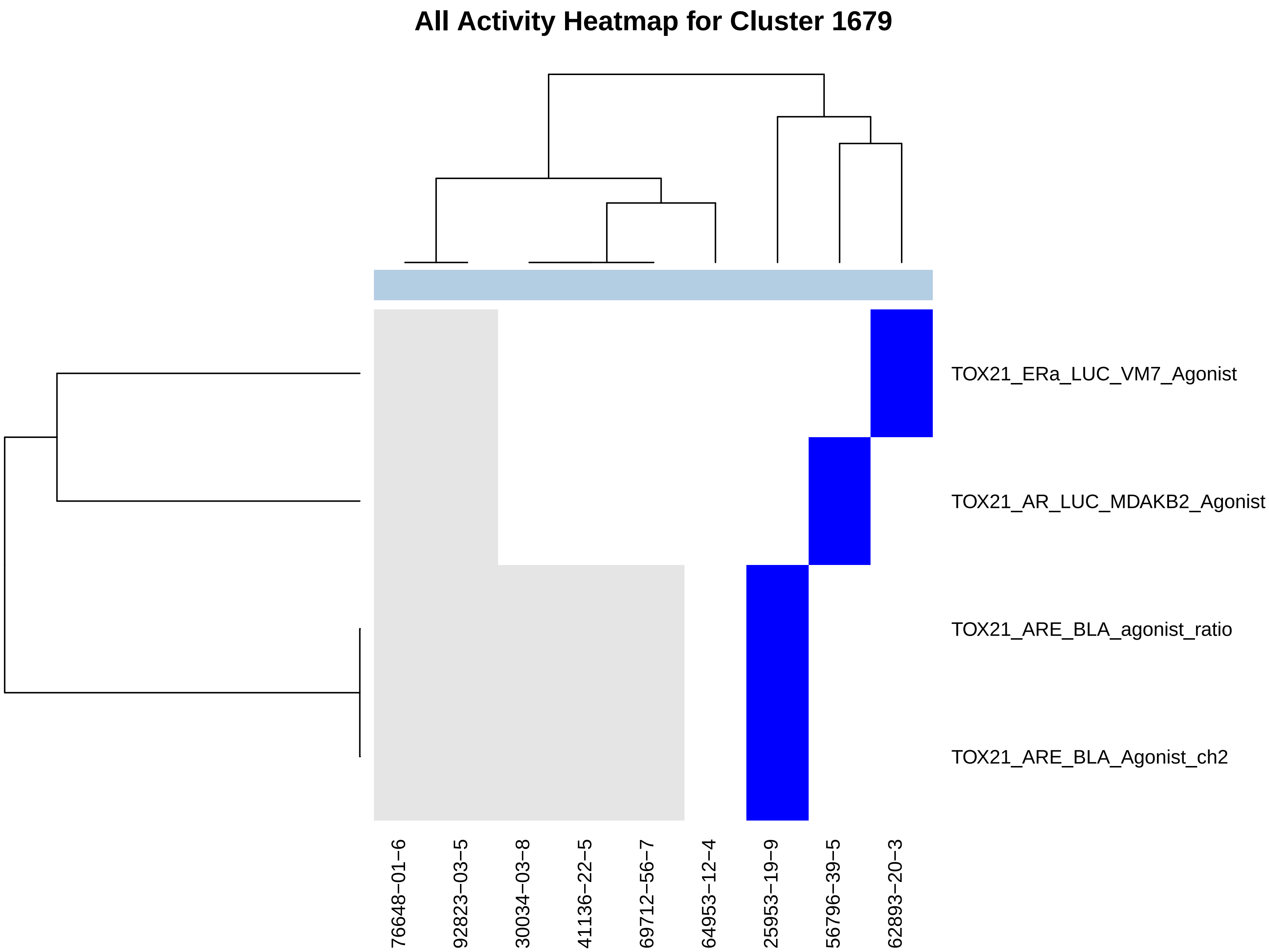
All Activity Heatmap for Cluster 1679
TOX21_ERa_LUC_VM7_Agonist
TOX21_AR_LUC_MDAKB2_Agonist
TOX21_ARE_BLA_agonist_ratio
TOX21_ARE_BLA_Agonist_ch2
76648−01−6
92823−03−5
30034−03−8
41136−22−5
69712−56−7
64953−12−4
25953−19−9
56796−39−5
62893−20−3

All Activity Heatmap for Cluster 1680

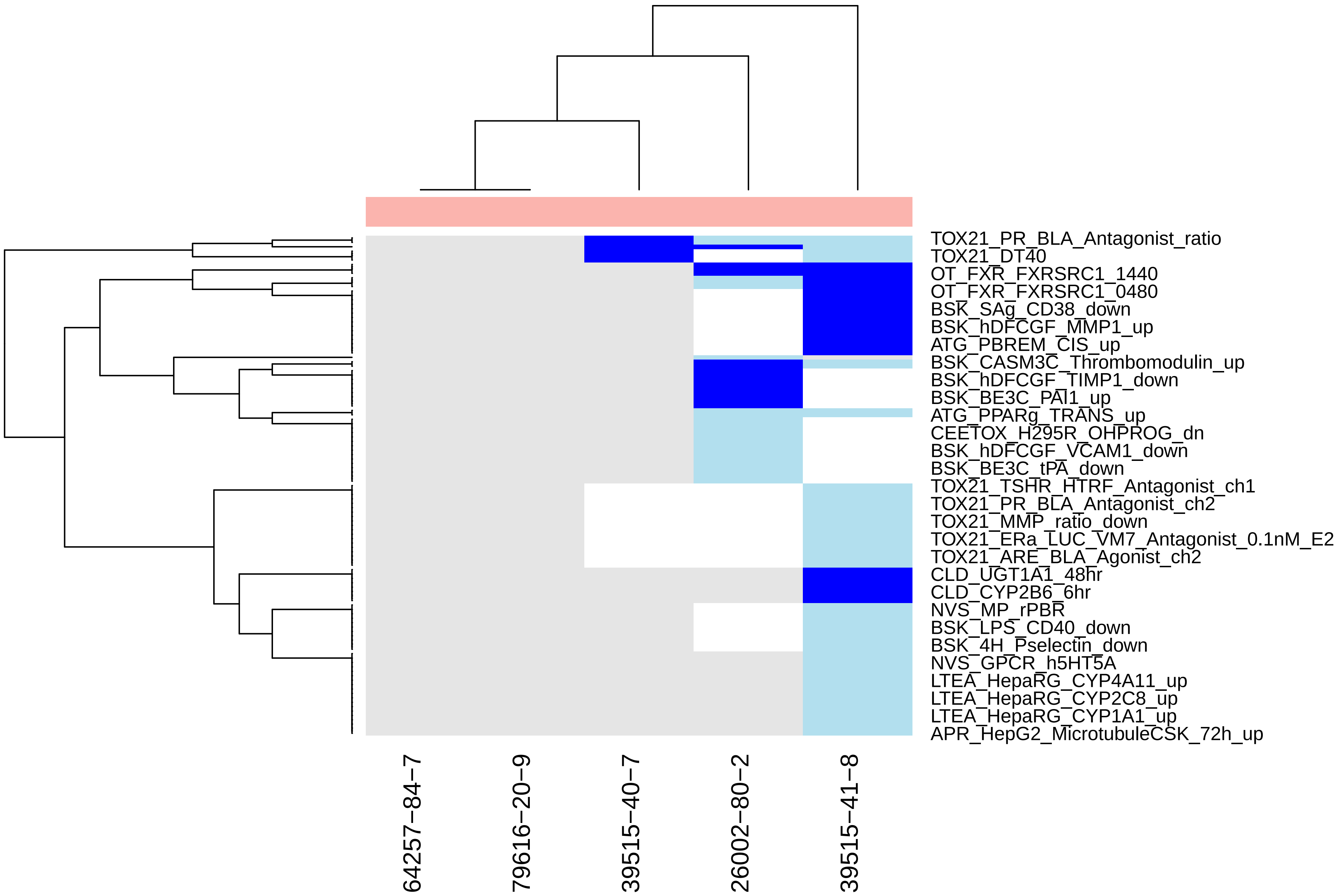

All Activity Heatmap for Cluster 1682

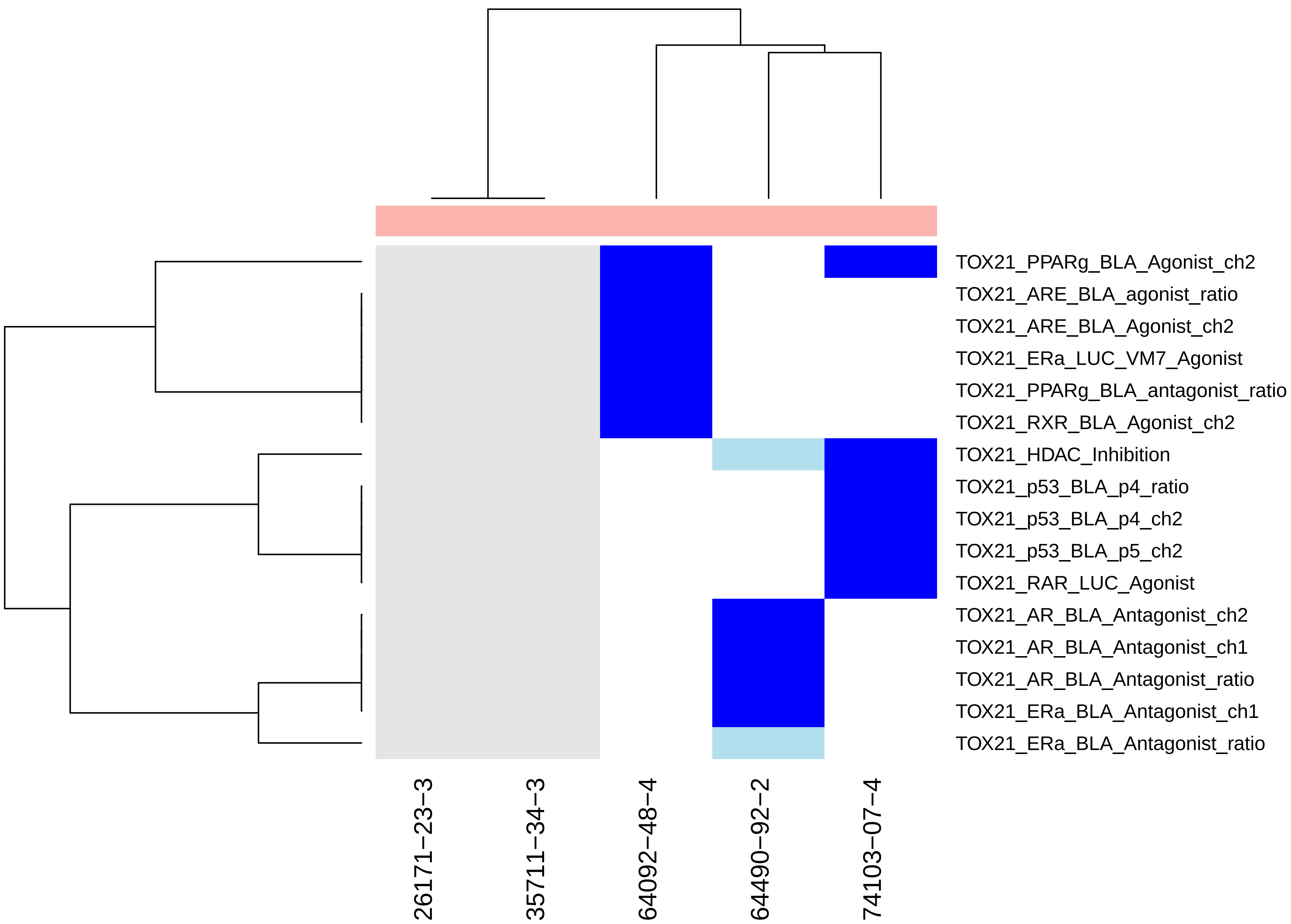

All Activity Heatmap for Cluster 1683

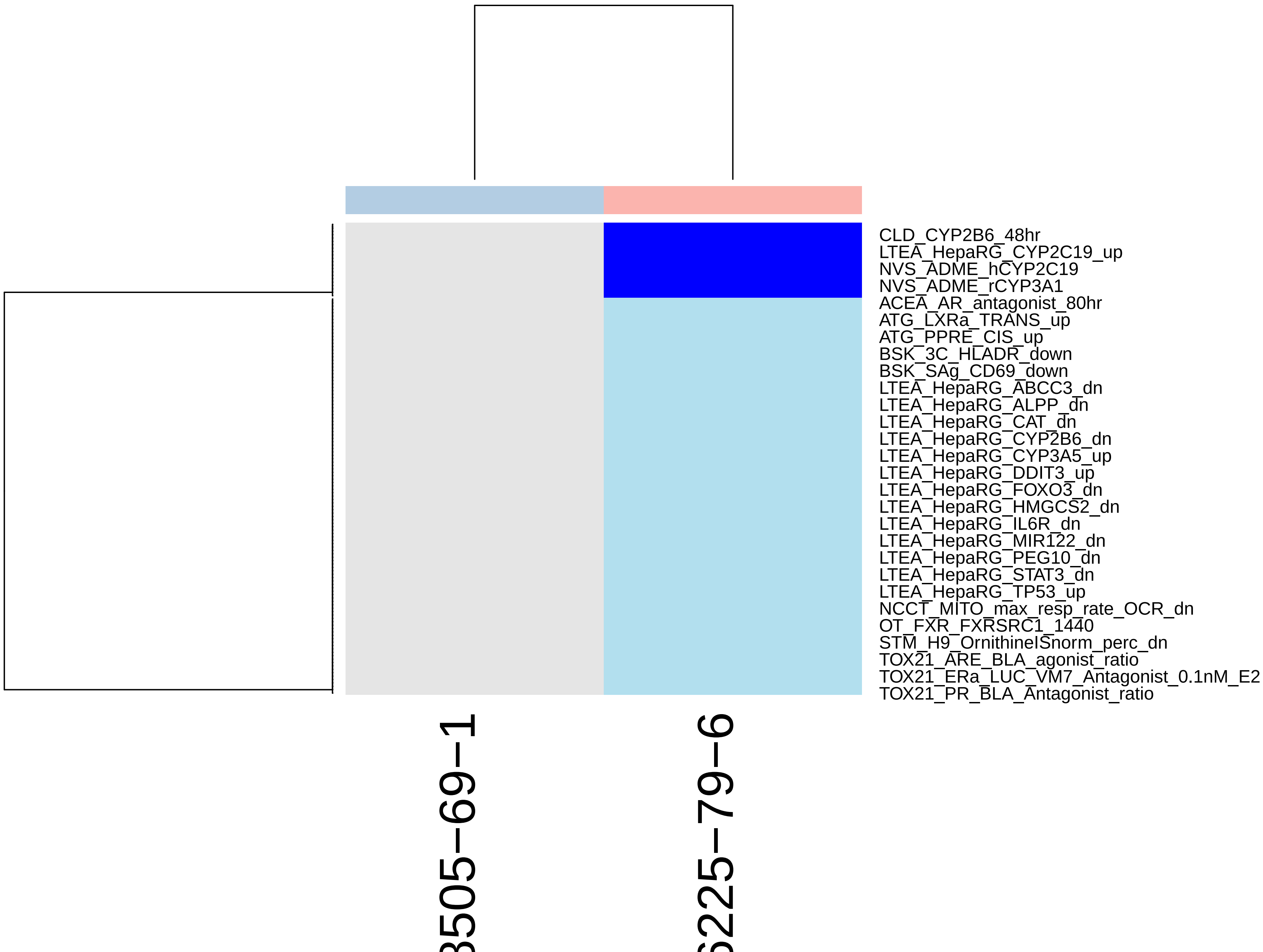

All Activity Heatmap for Cluster 1684

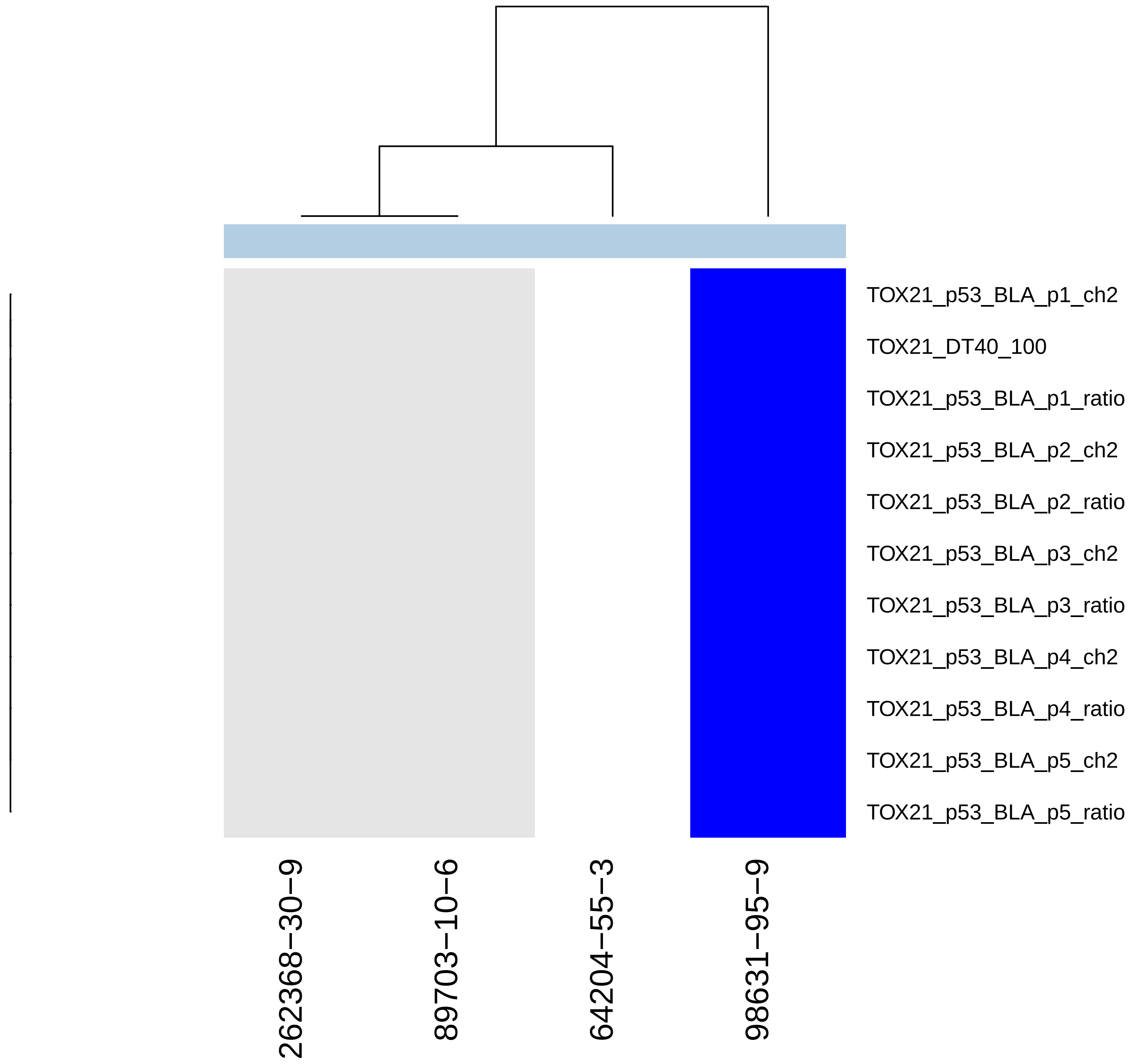

All Activity Heatmap for Cluster 1685

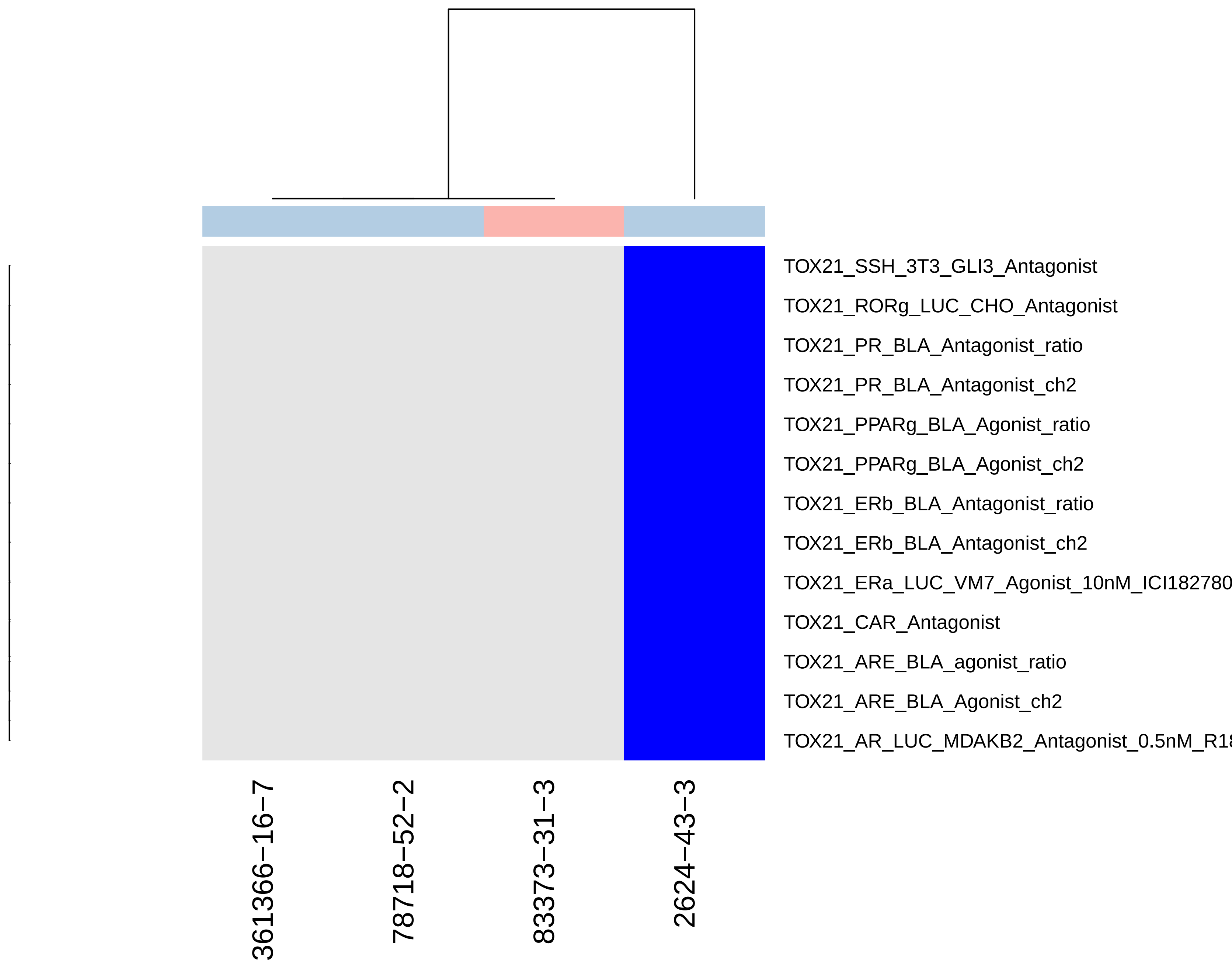

All Activity Heatmap for Cluster 1686

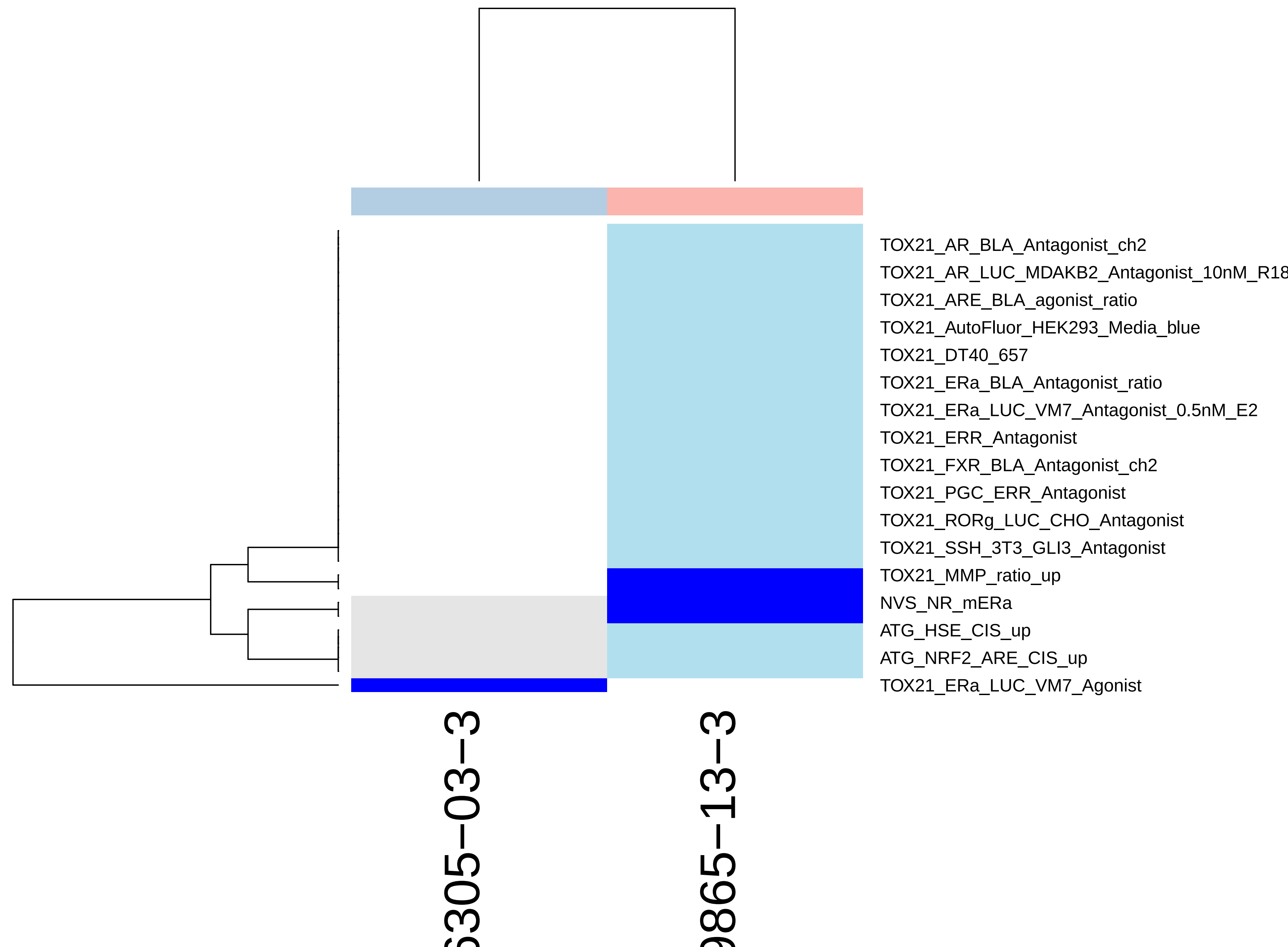

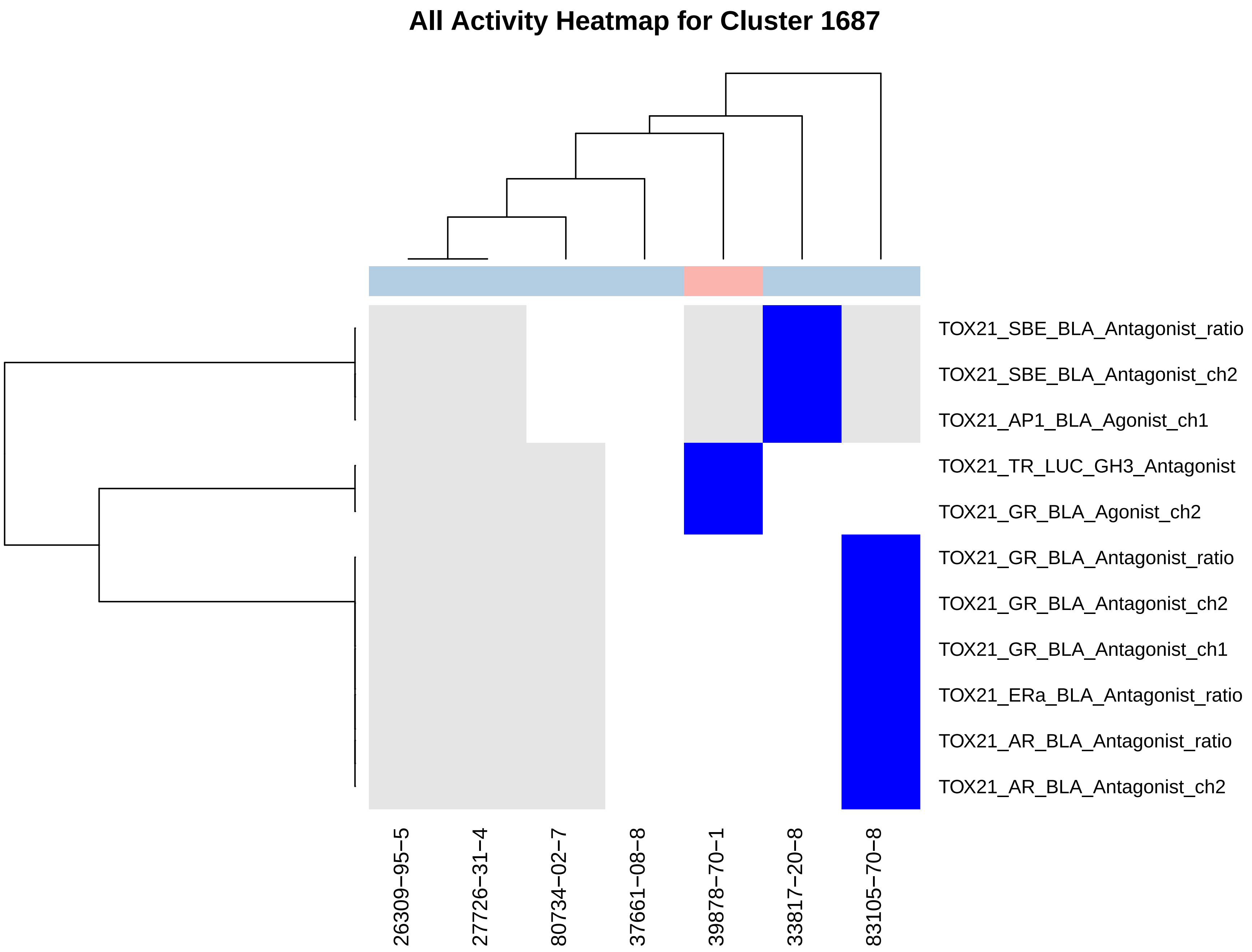

All Activity Heatmap for Cluster 1687
TOX21_SBE_BLA_Antagonist_ratio
TOX21_SBE_BLA_Antagonist_ch2
TOX21_AP1_BLA_Agonist_ch1
TOX21_TR_LUC_GH3_Antagonist
TOX21_GR_BLA_Agonist_ch2
TOX21_GR_BLA_Antagonist_ratio
TOX21_GR_BLA_Antagonist_ch2
TOX21_GR_BLA_Antagonist_ch1
TOX21_ERa_BLA_Antagonist_ratio
TOX21_AR_BLA_Antagonist_ratio
TOX21_AR_BLA_Antagonist_ch2
26309−95−5
27726−31−4
80734−02−7
37661−08−8
39878−70−1
33817−20−8
83105−70−8

All Activity Heatmap for Cluster 1688

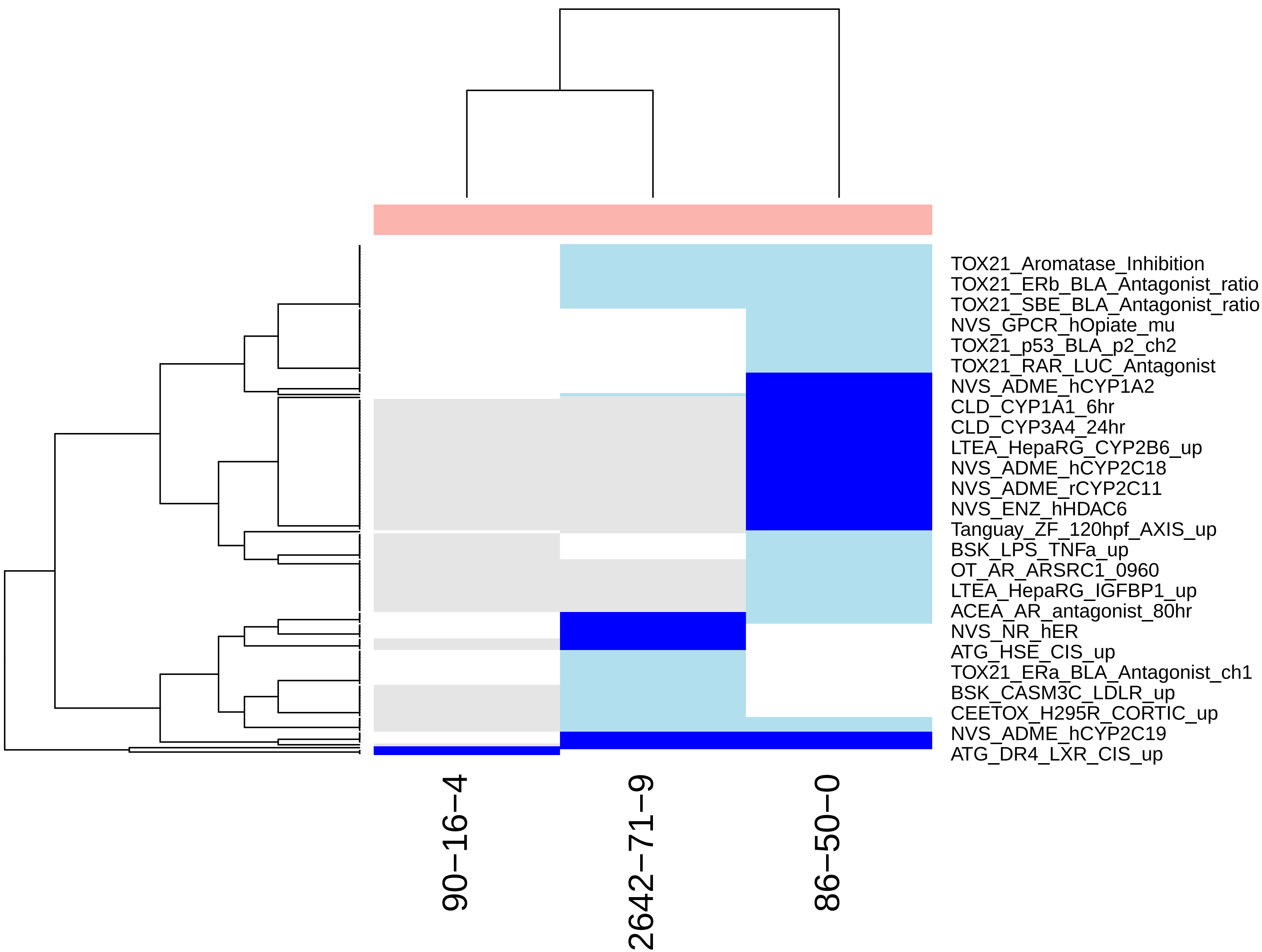

All Activity Heatmap for Cluster 1689

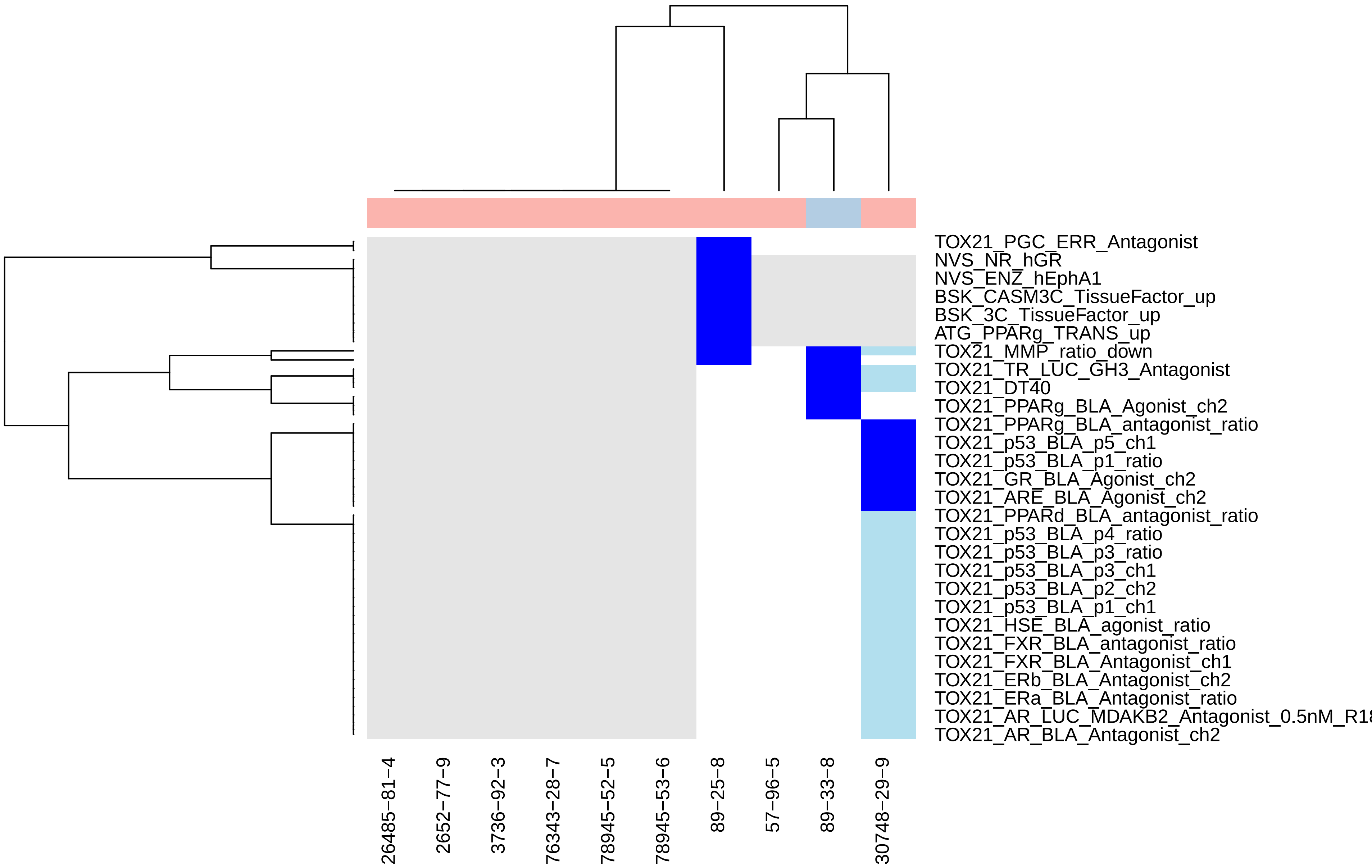

All Activity Heatmap for Cluster 1691

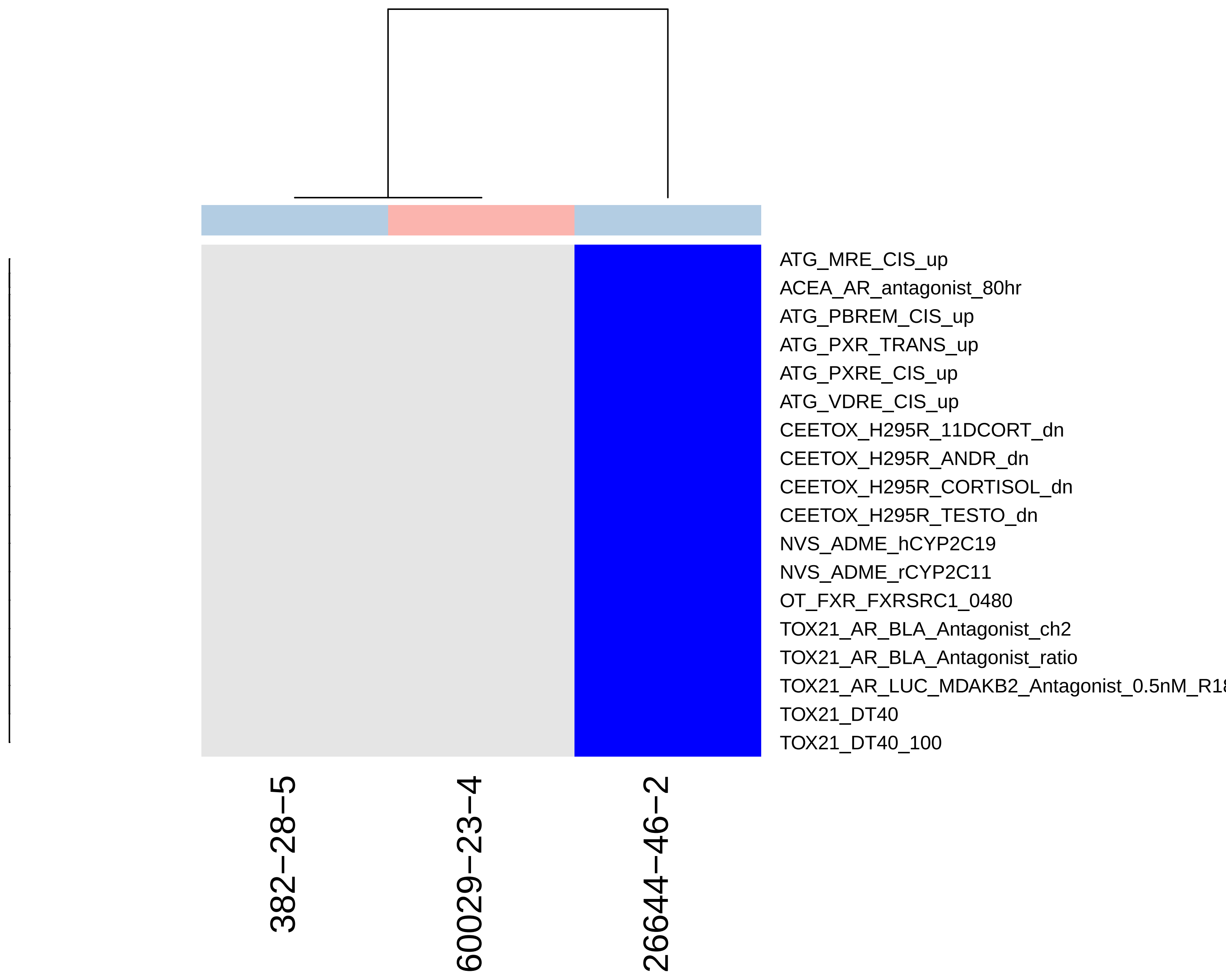

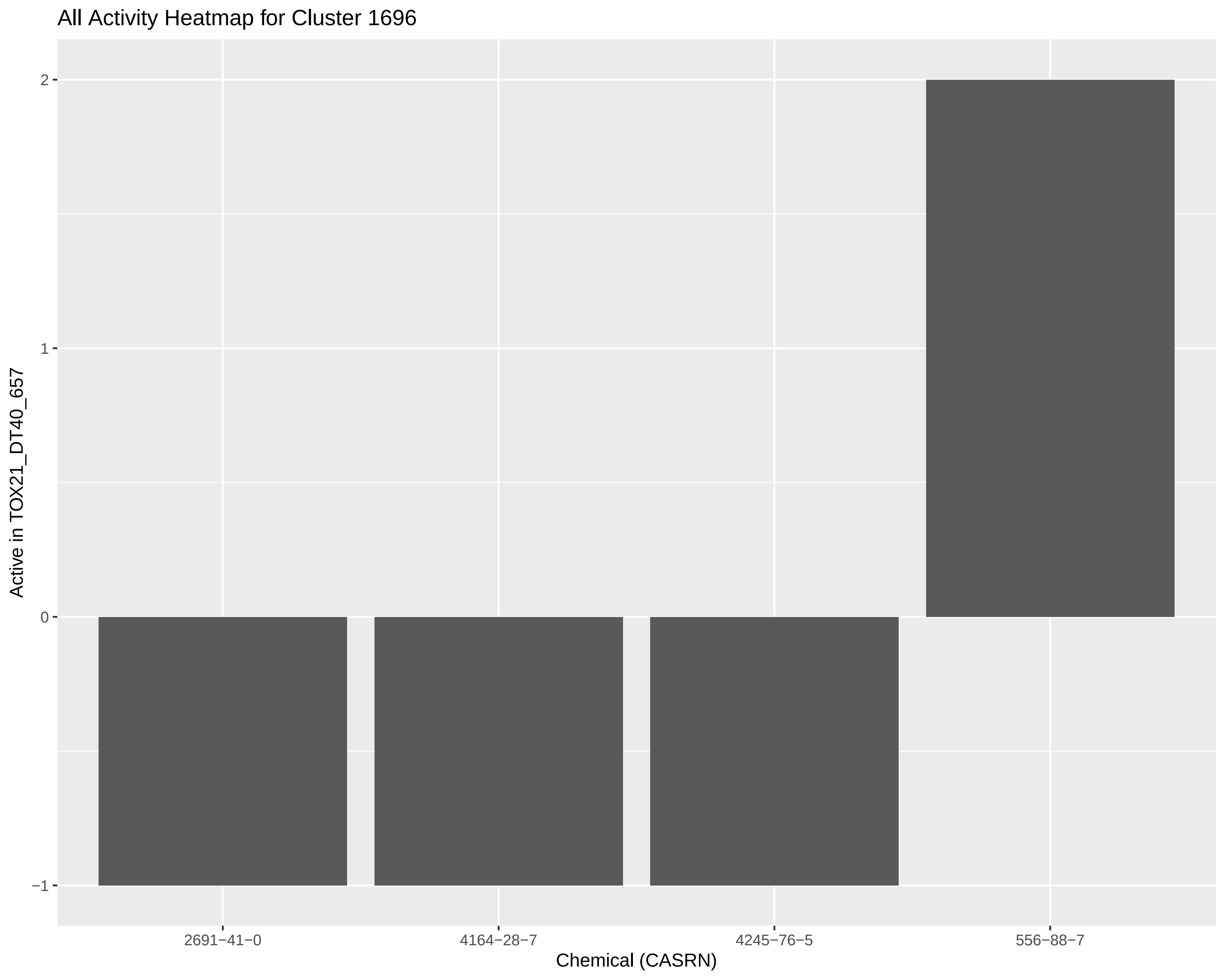

All Activity Heatmap for Cluster 1696
Active in TOX21_DT40_657
2
1
0
−1
2691−41−0
4164−28−7
4245−76−5
556−88−7
Chemical (CASRN)

All Activity Heatmap for Cluster 1697

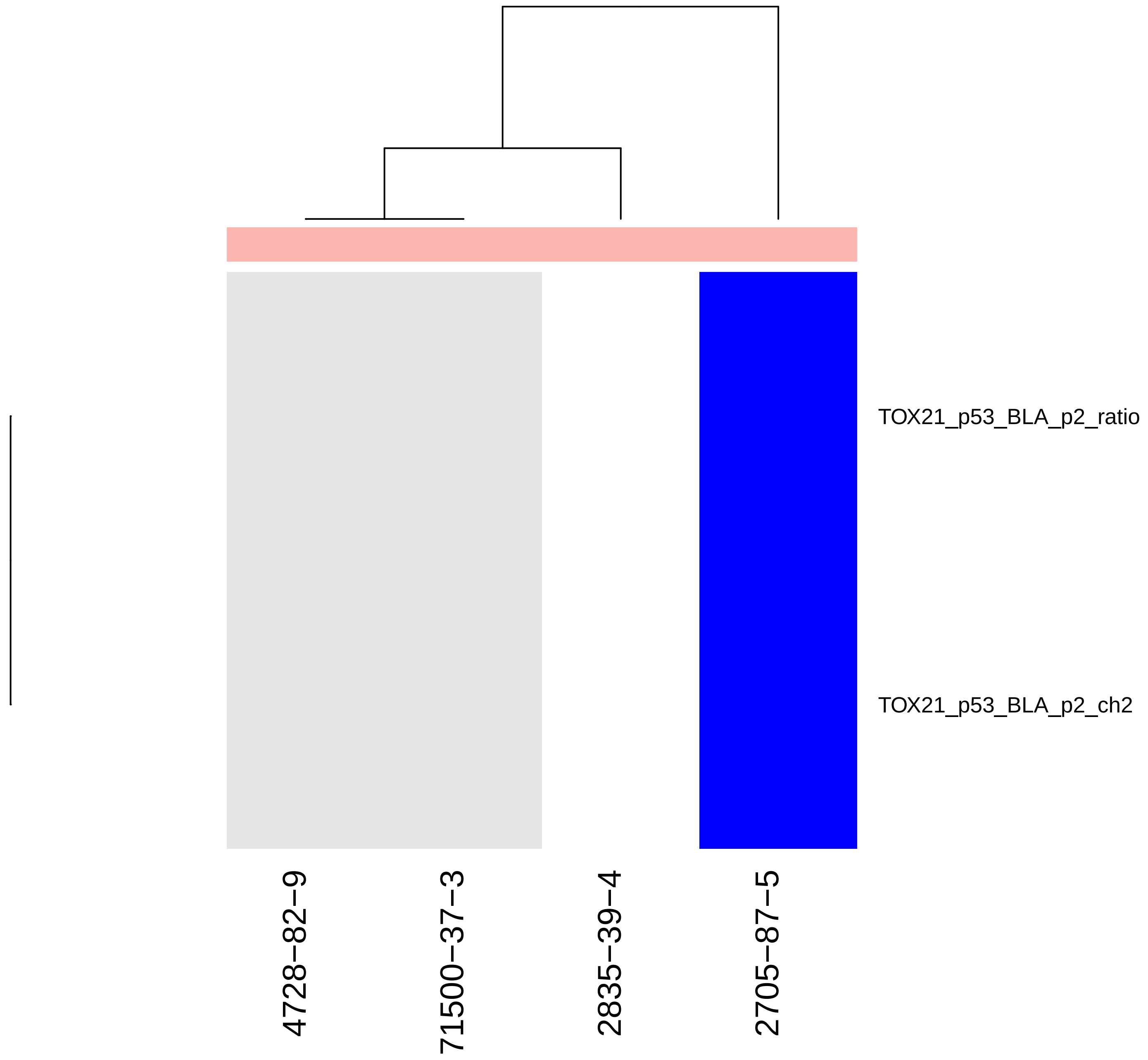

All Activity Heatmap for Cluster 1698

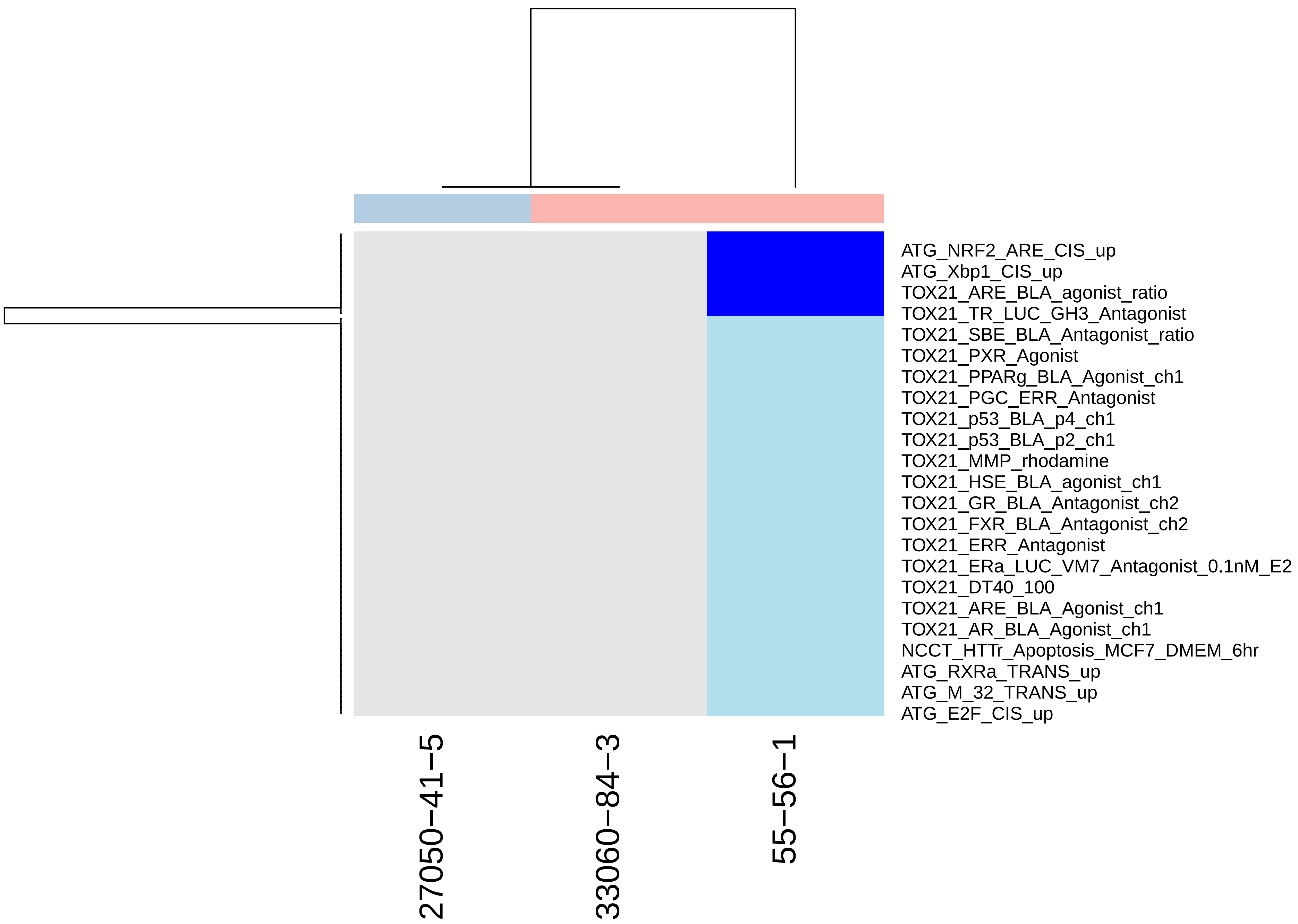

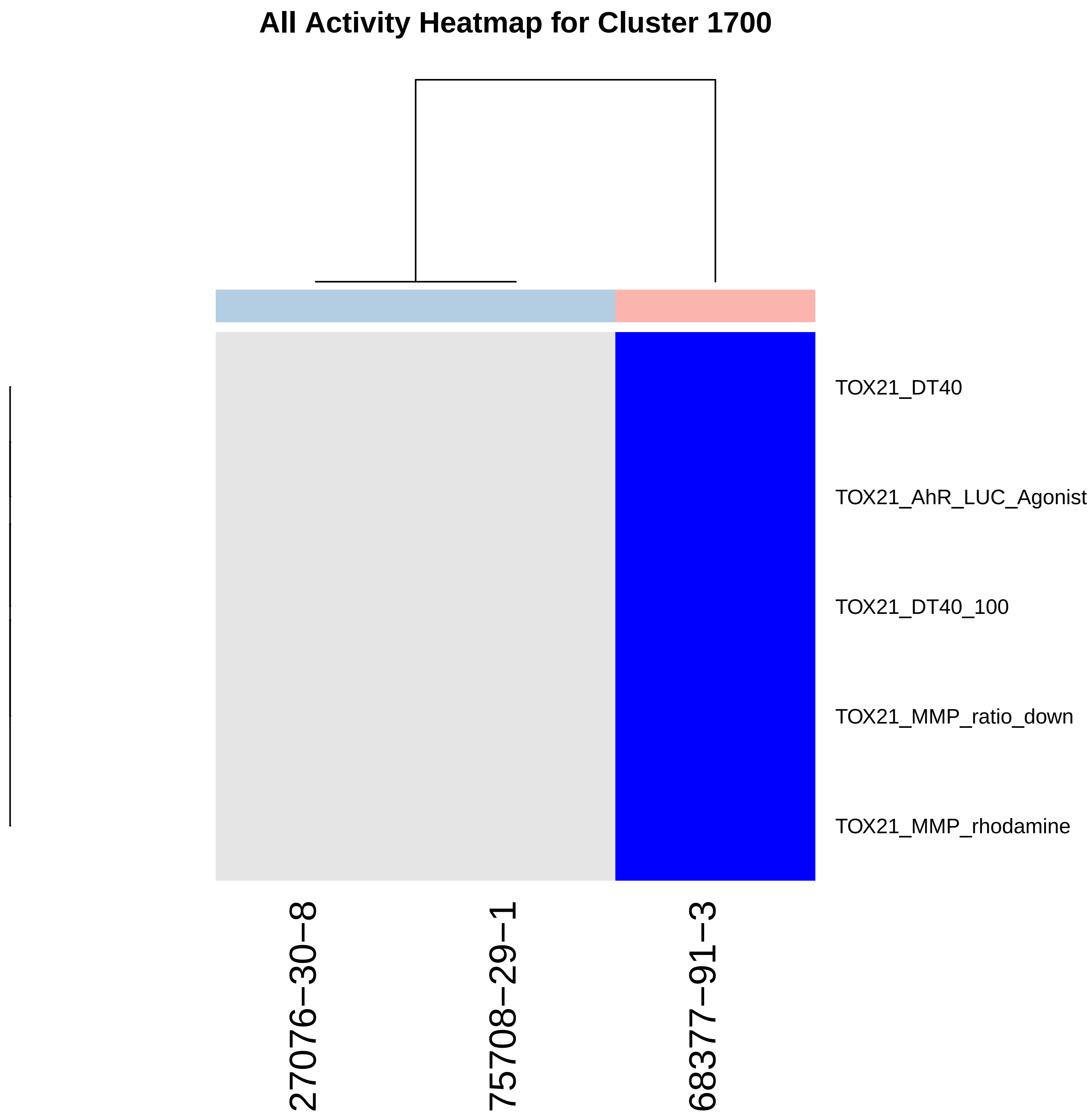
All Activity Heatmap for Cluster 1700
TOX21_DT40
TOX21_AhR_LUC_Agonist
TOX21_DT40_100
TOX21_MMP_ratio_down
TOX21_MMP_rhodamine
27076−30−8
75708−29−1
68377−91−3

All Activity Heatmap for Cluster 1702

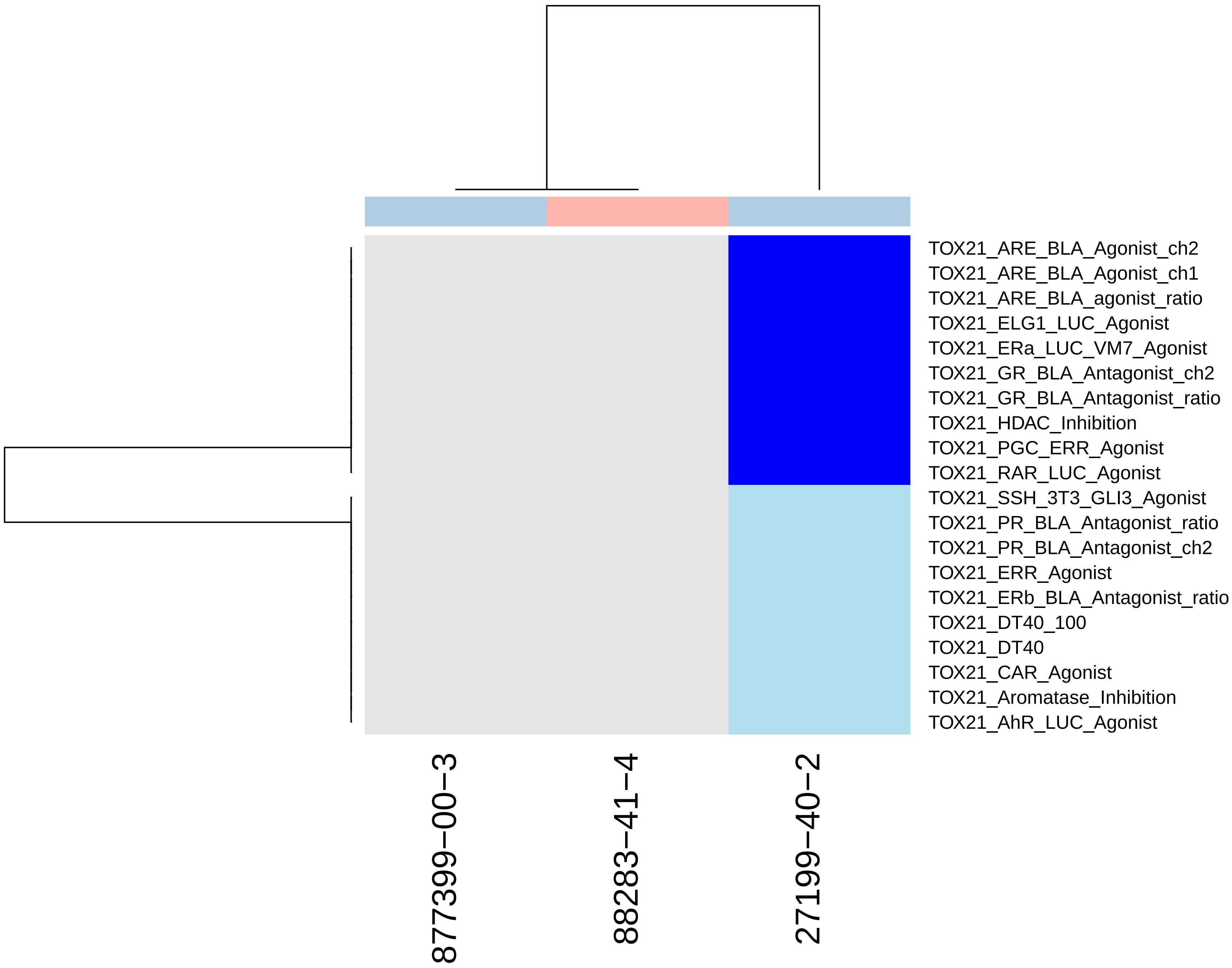

All Activity Heatmap for Cluster 1703

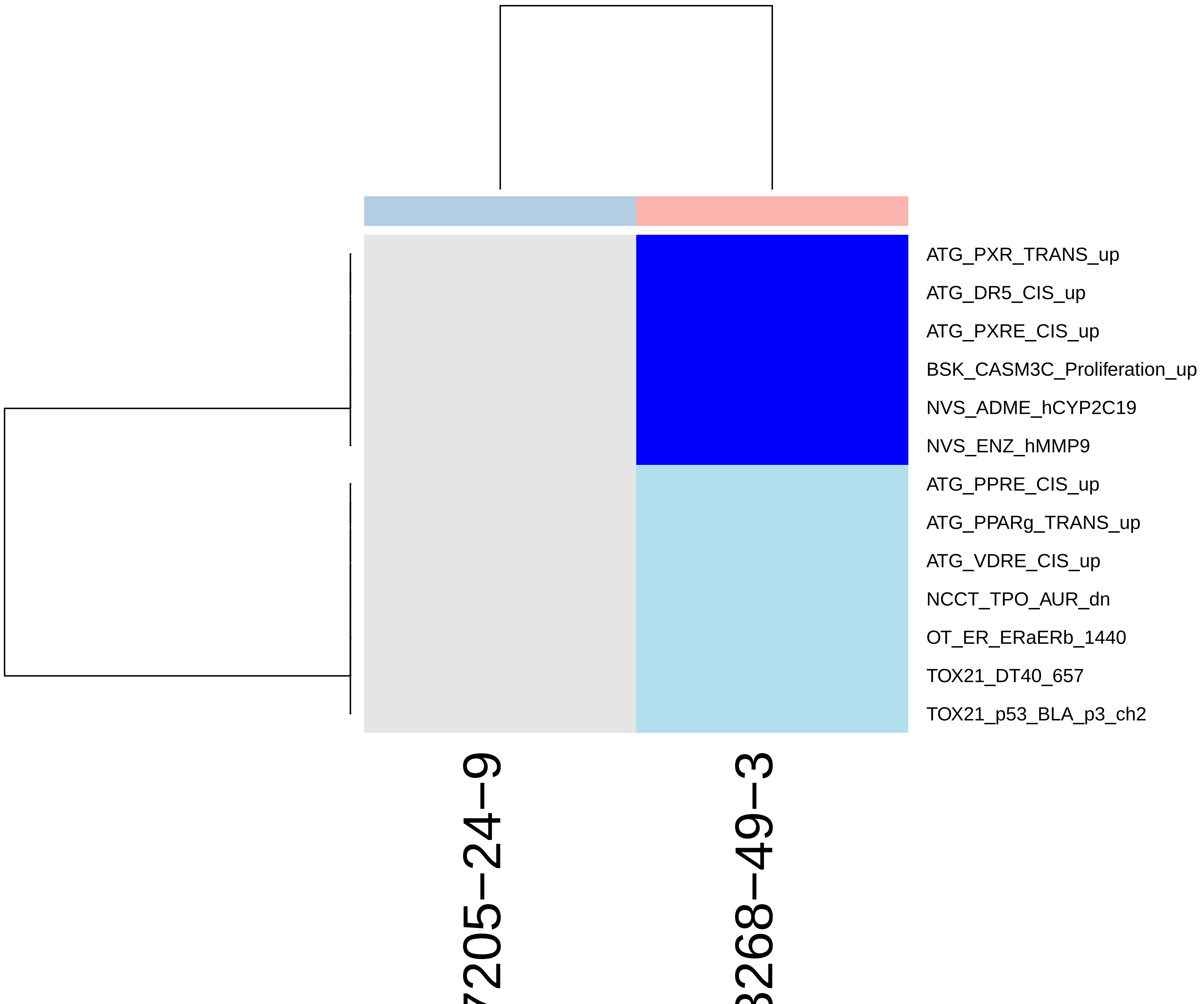

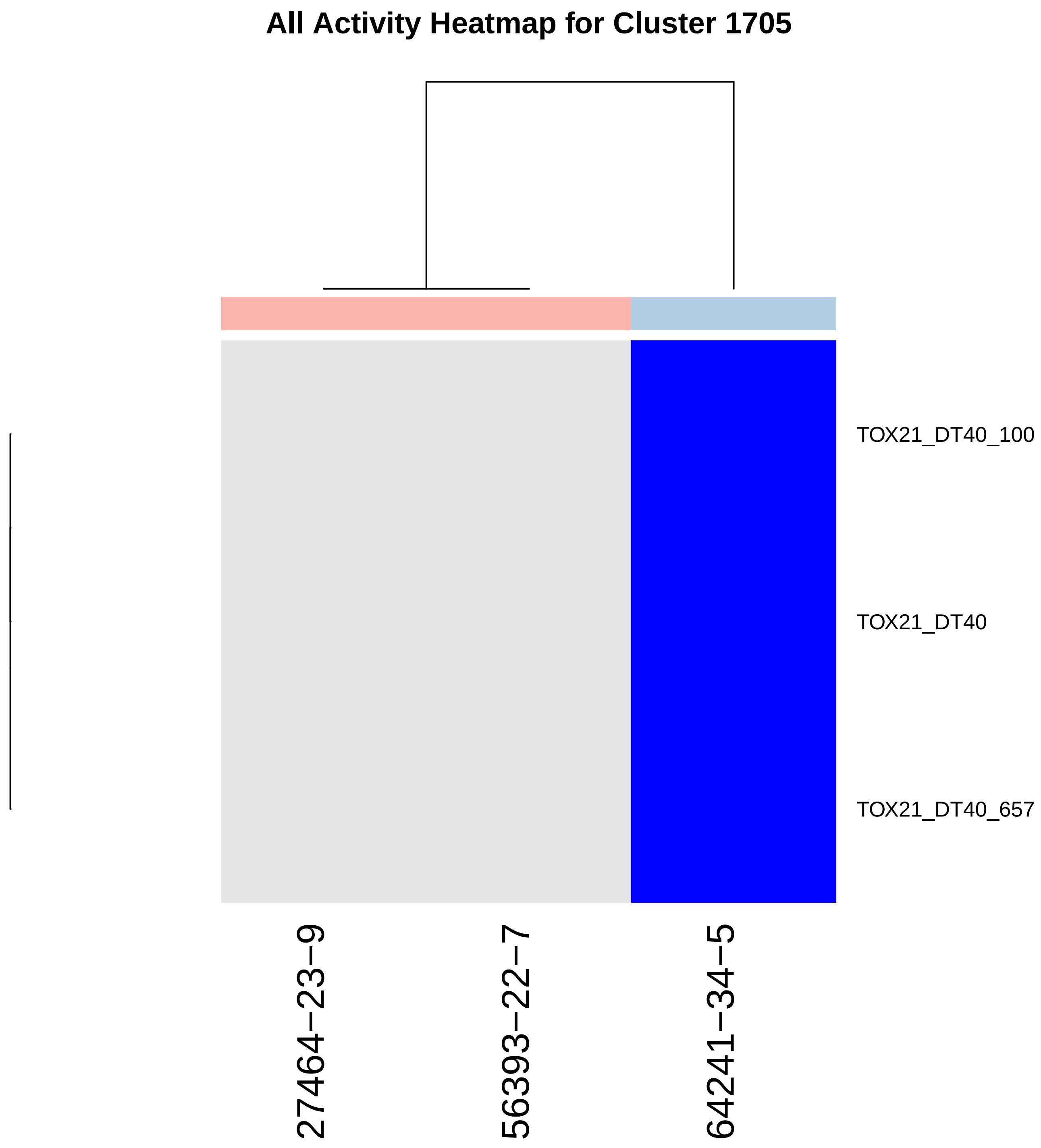
All Activity Heatmap for Cluster 1705
TOX21_DT40_100
TOX21_DT40
TOX21_DT40_657
27464−23−9
56393−22−7
64241−34−5

All Activity Heatmap for Cluster 1706

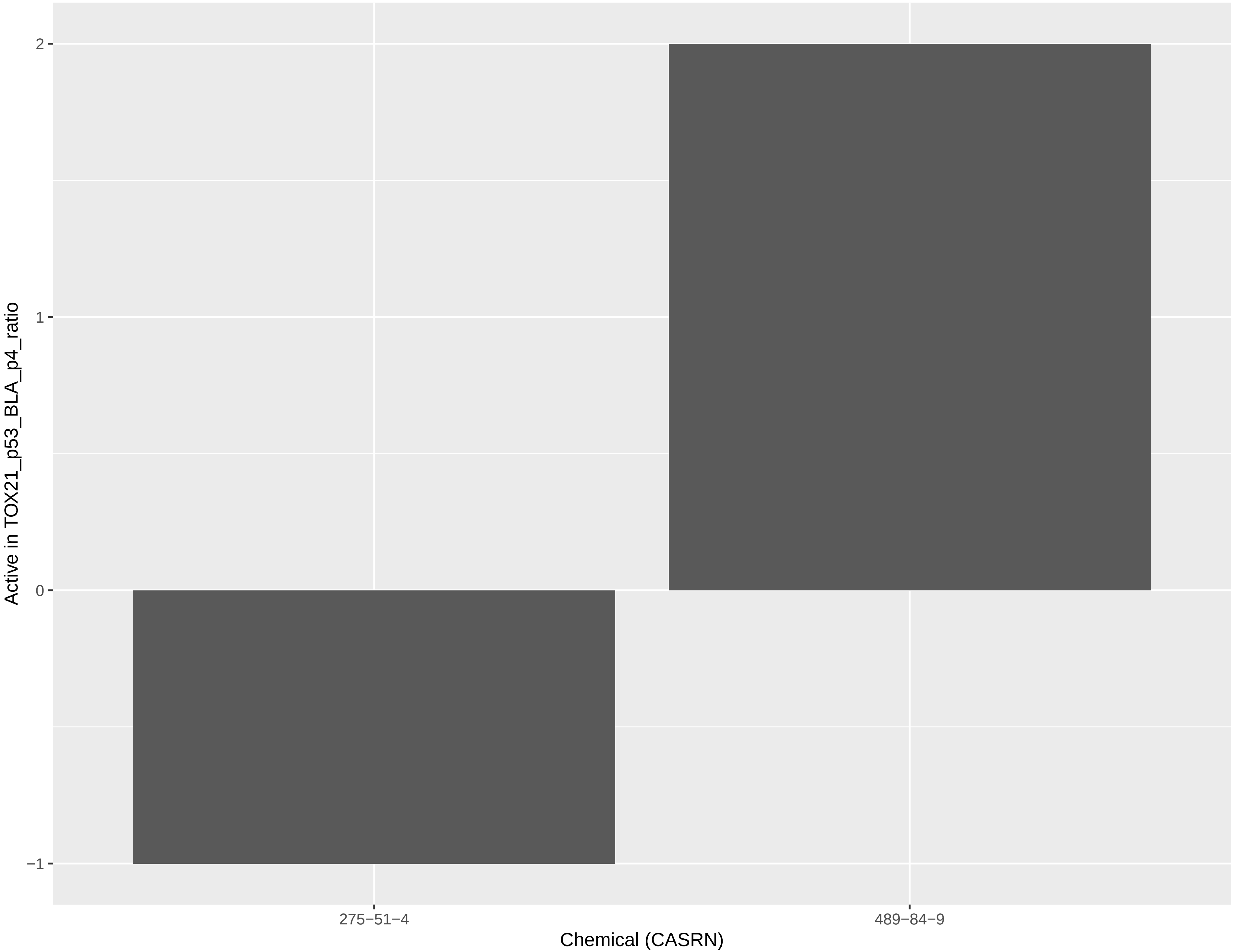

All Activity Heatmap for Cluster 1707

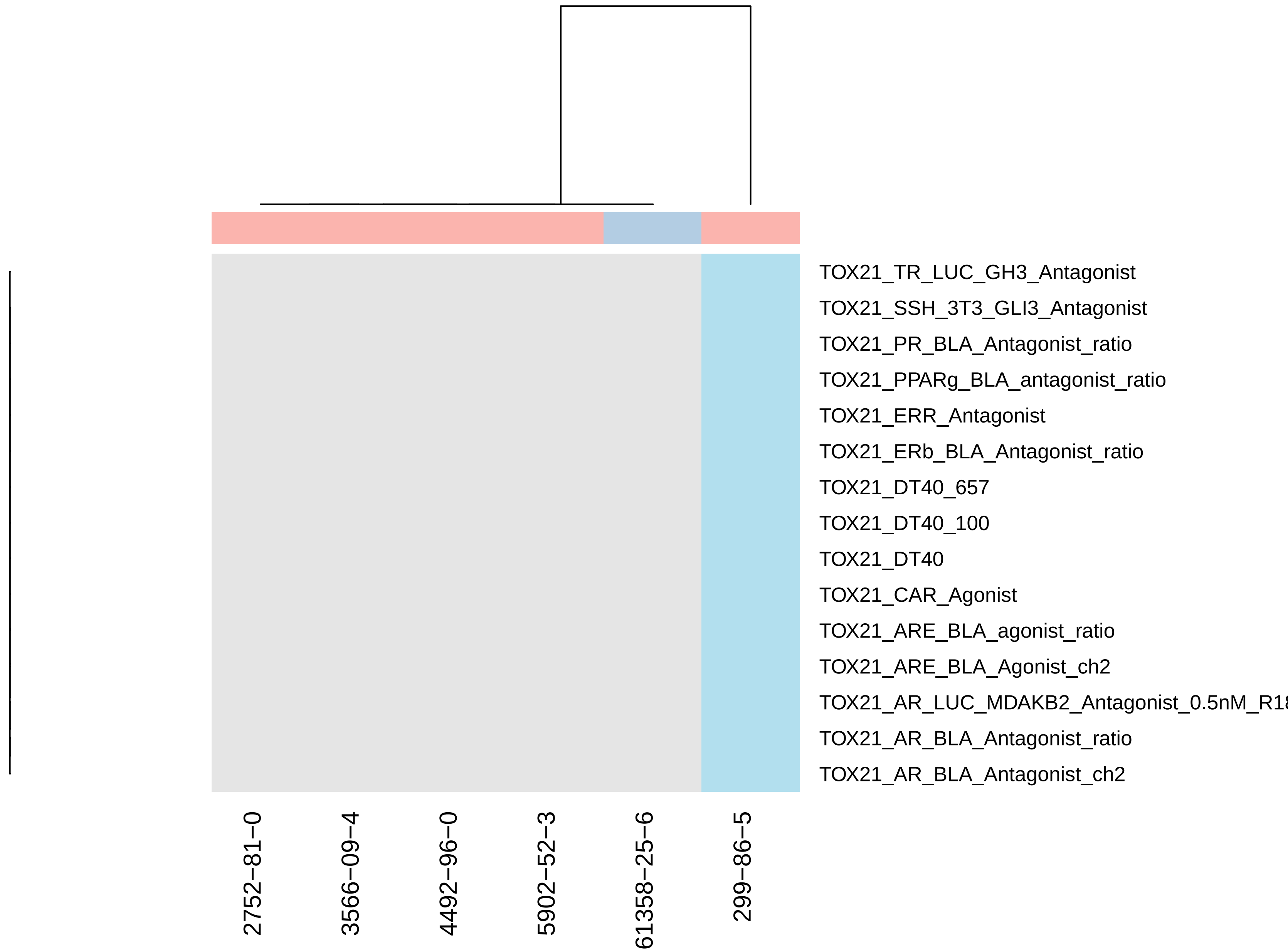

All Activity Heatmap for Cluster 1709

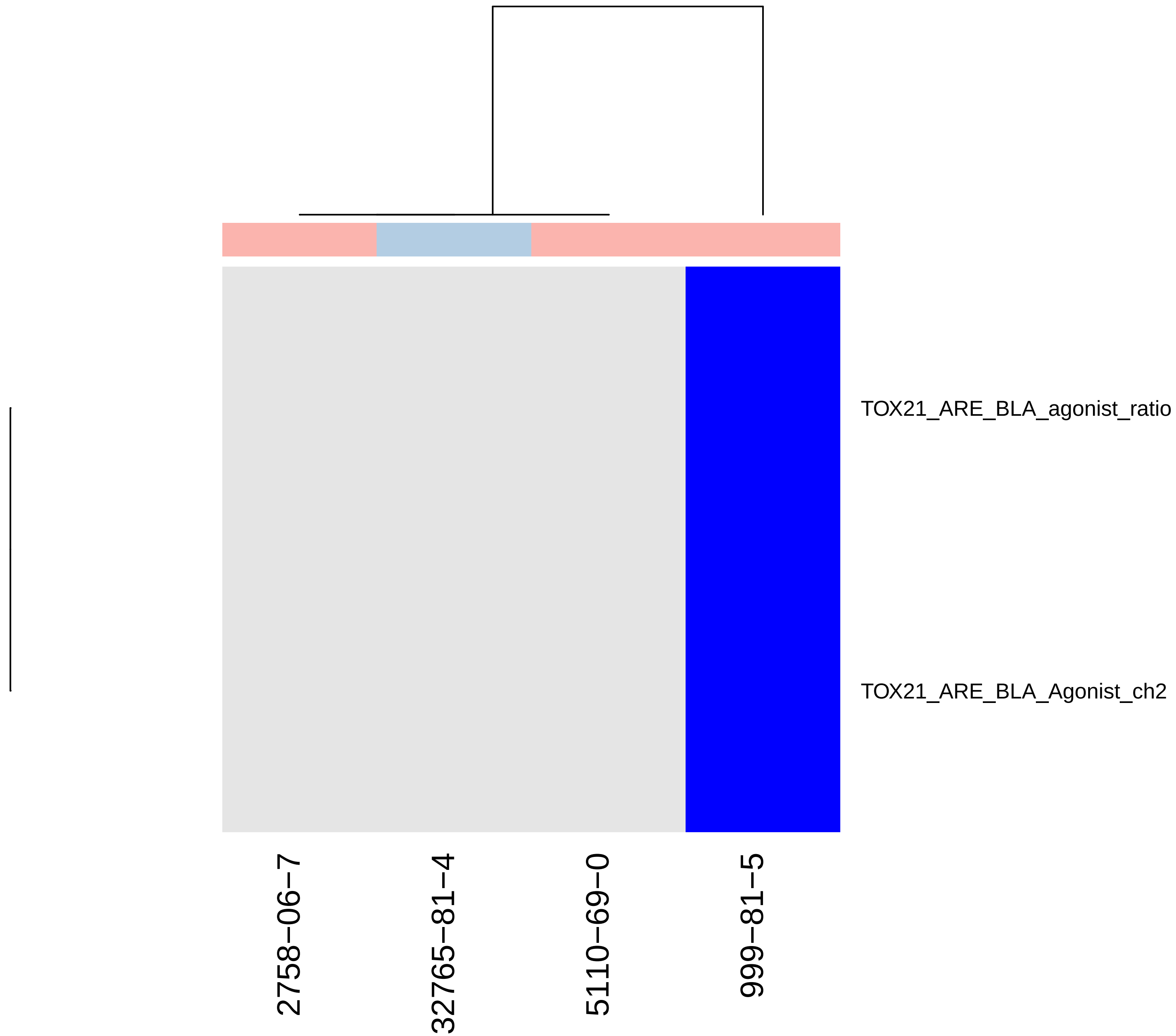

All Activity Heatmap for Cluster 1710

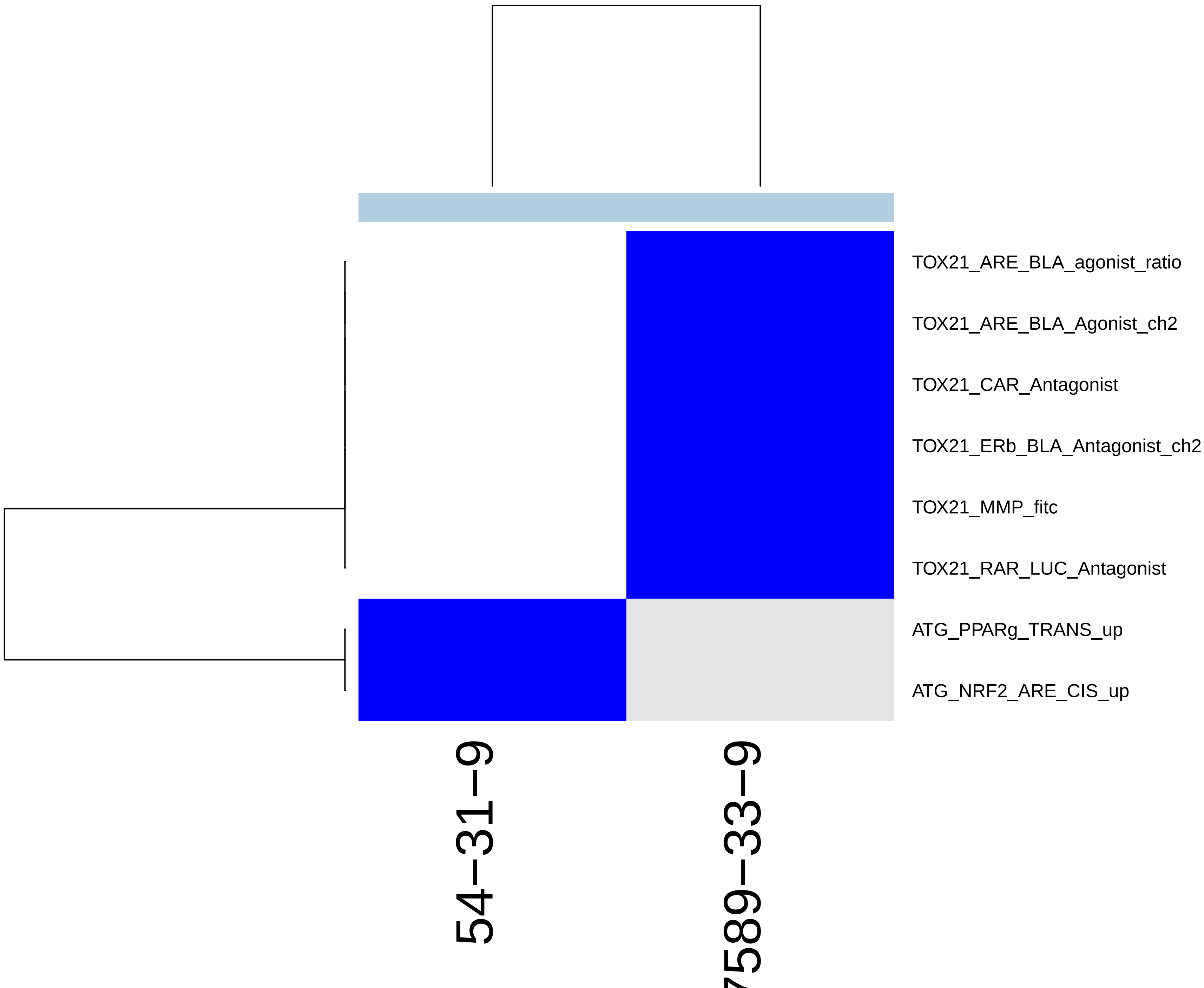

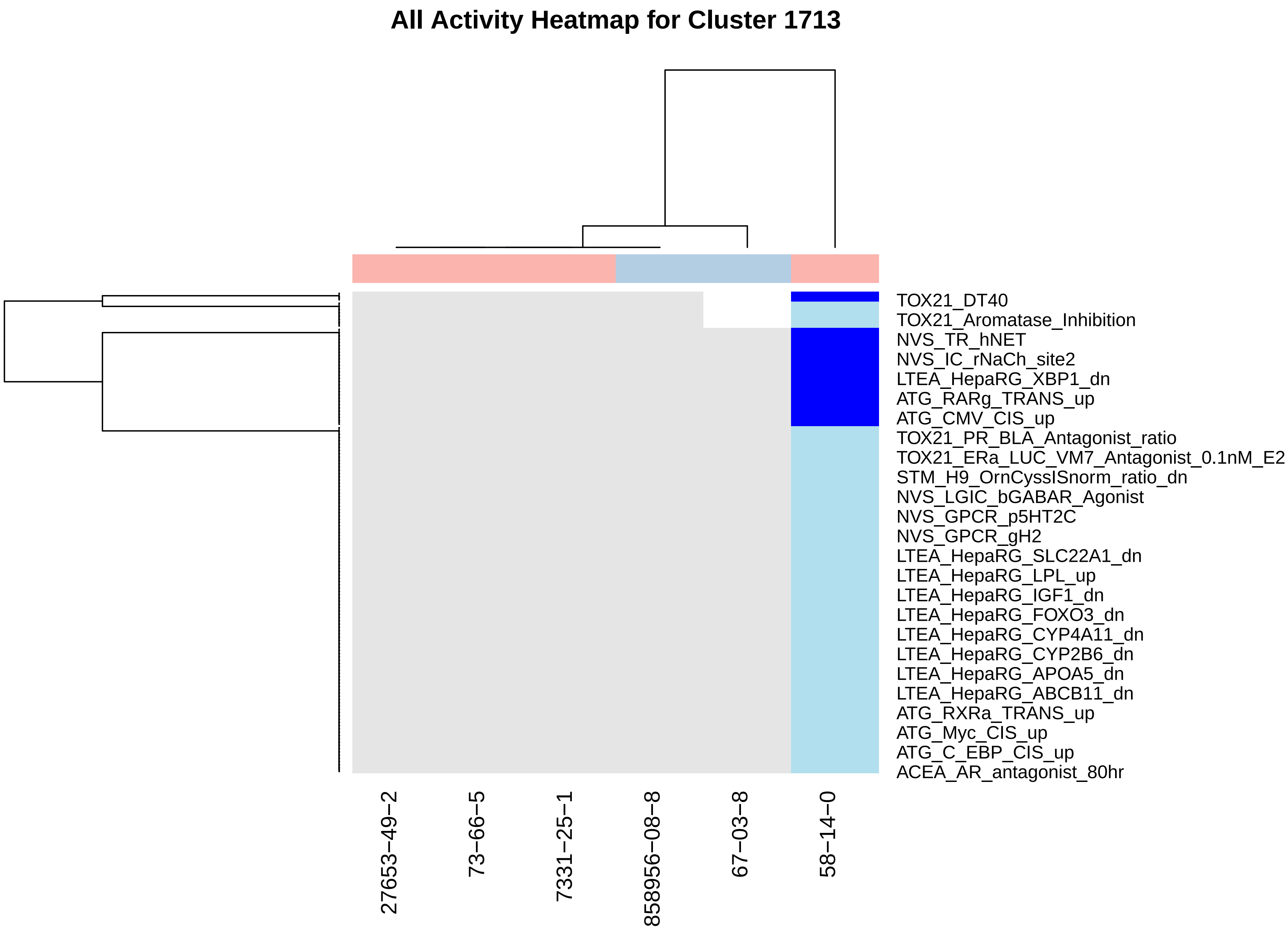
All Activity Heatmap for Cluster 1713
TOX21_DT40
TOX21_Aromatase_Inhibition
NVS_TR_hNET
NVS_IC_rNaCh_site2
LTEA_HepaRG_XBP1_dn
ATG_RARg_TRANS_up
ATG_CMV_CIS_up
TOX21_PR_BLA_Antagonist_ratio
TOX21_ERa_LUC_VM7_Antagonist_0.1nM_E2
STM_H9_OrnCyssISnorm_ratio_dn
NVS_LGIC_bGABAR_Agonist
NVS_GPCR_p5HT2C
NVS_GPCR_gH2
LTEA_HepaRG_SLC22A1_dn
LTEA_HepaRG_LPL_up
LTEA_HepaRG_IGF1_dn
LTEA_HepaRG_FOXO3_dn
LTEA_HepaRG_CYP4A11_dn
LTEA_HepaRG_CYP2B6_dn
LTEA_HepaRG_APOA5_dn
LTEA_HepaRG_ABCB11_dn
ATG_RXRa_TRANS_up
ATG_Myc_CIS_up
ATG_C_EBP_CIS_up
ACEA_AR_antagonist_80hr
27653−49−2
73−66−5
7331−25−1
858956−08−8
67−03−8
58−14−0

All Activity Heatmap for Cluster 1714

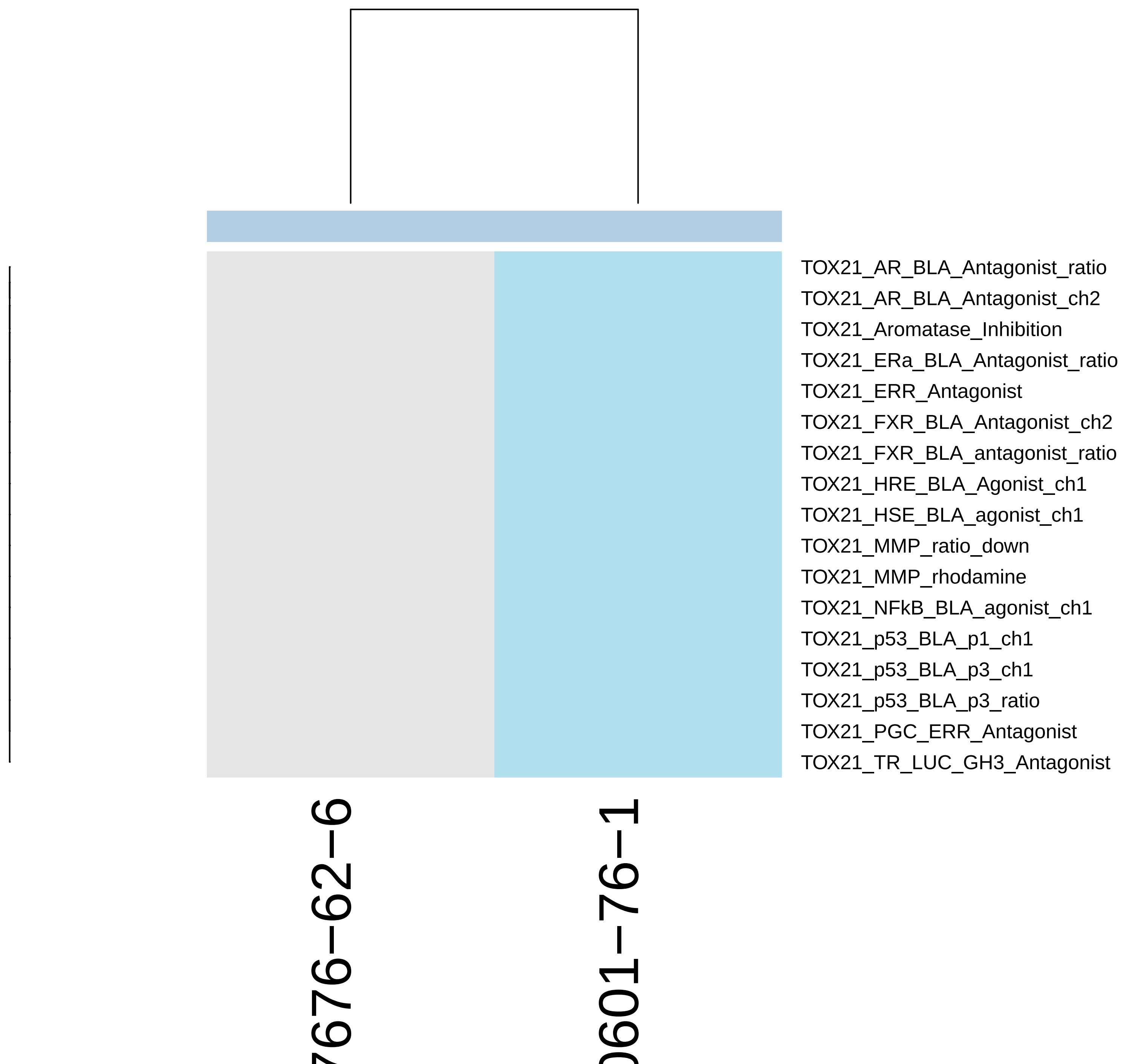

All Activity Heatmap for Cluster 1716

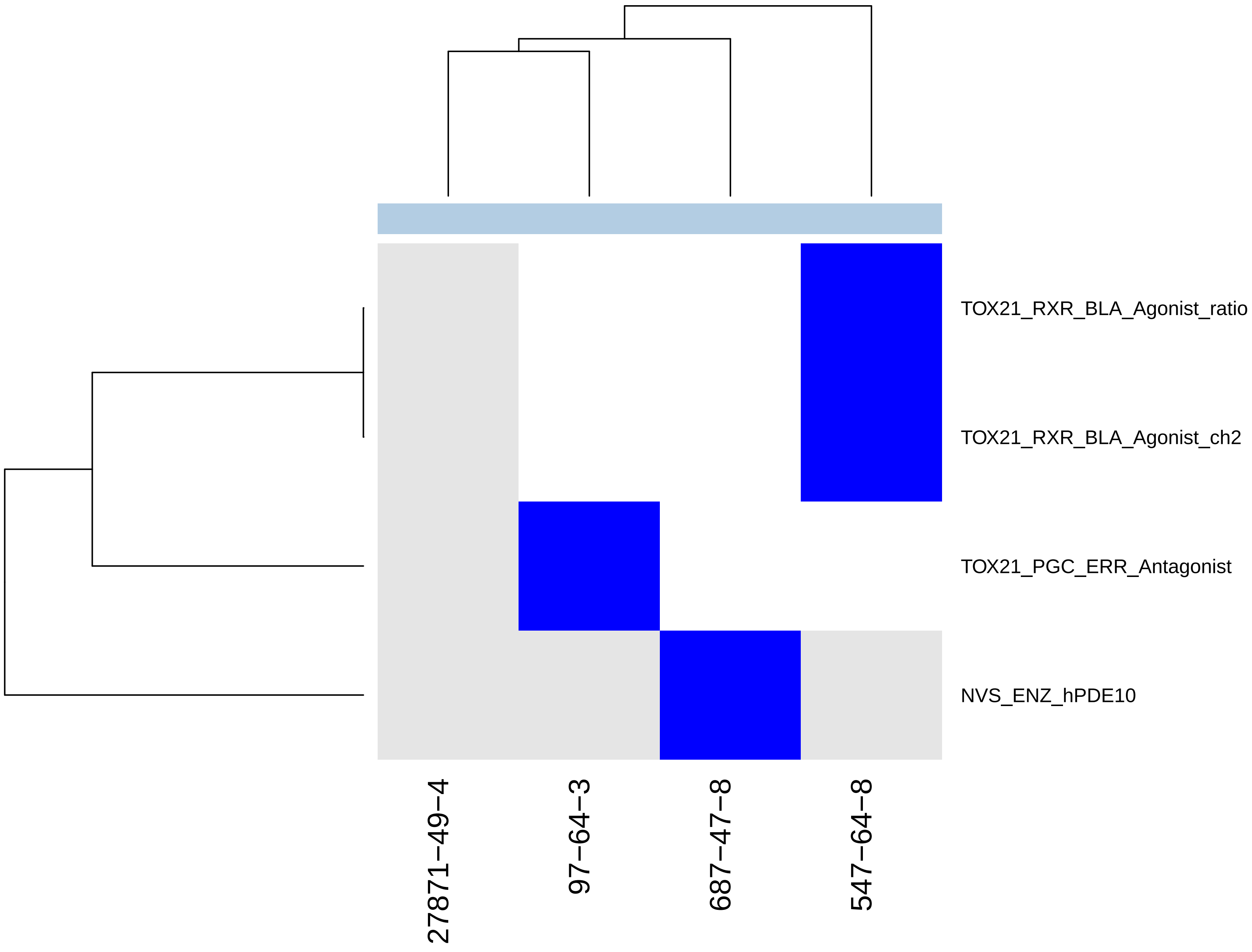

All Activity Heatmap for Cluster 1719

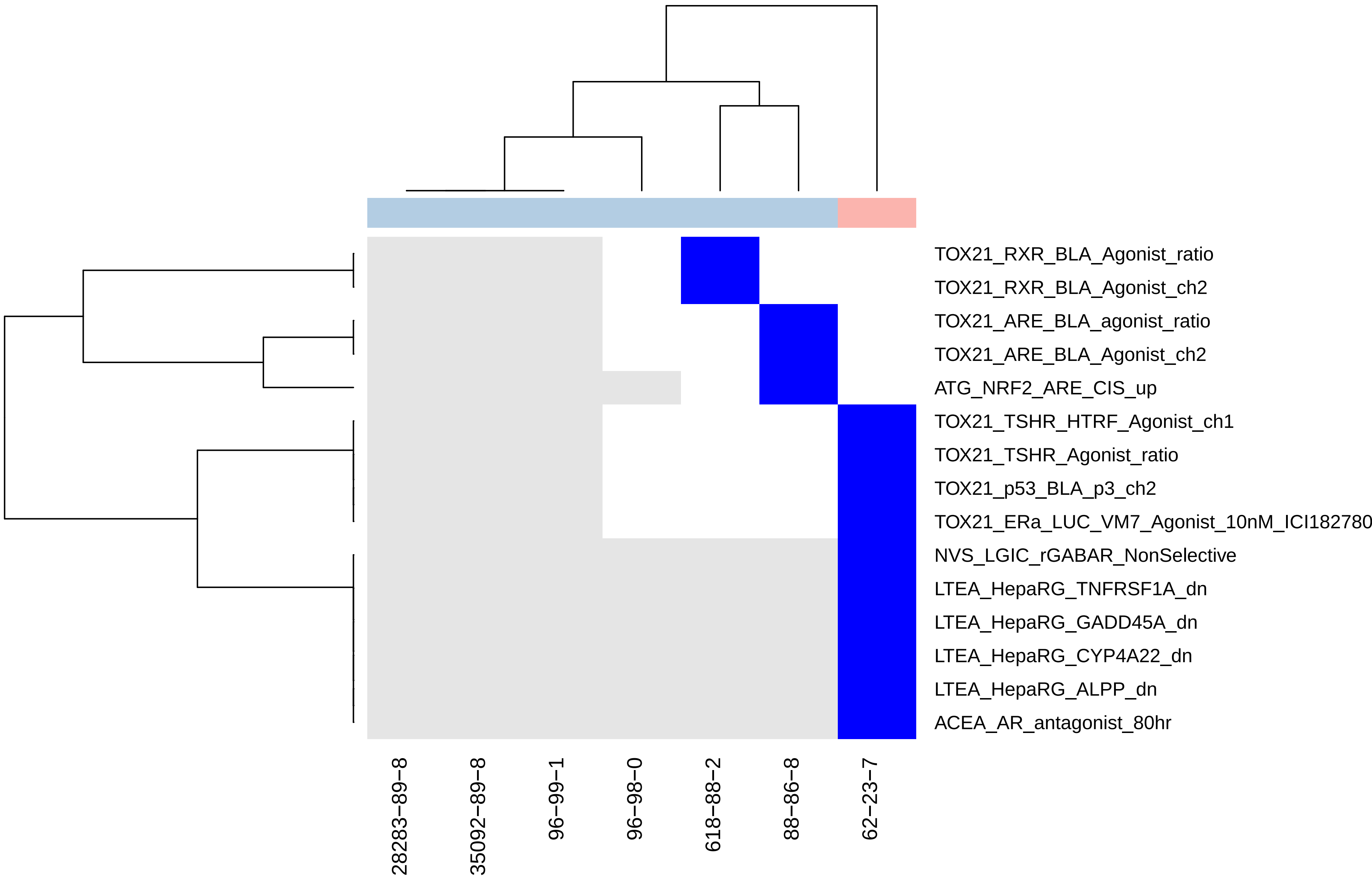

All Activity Heatmap for Cluster 1720

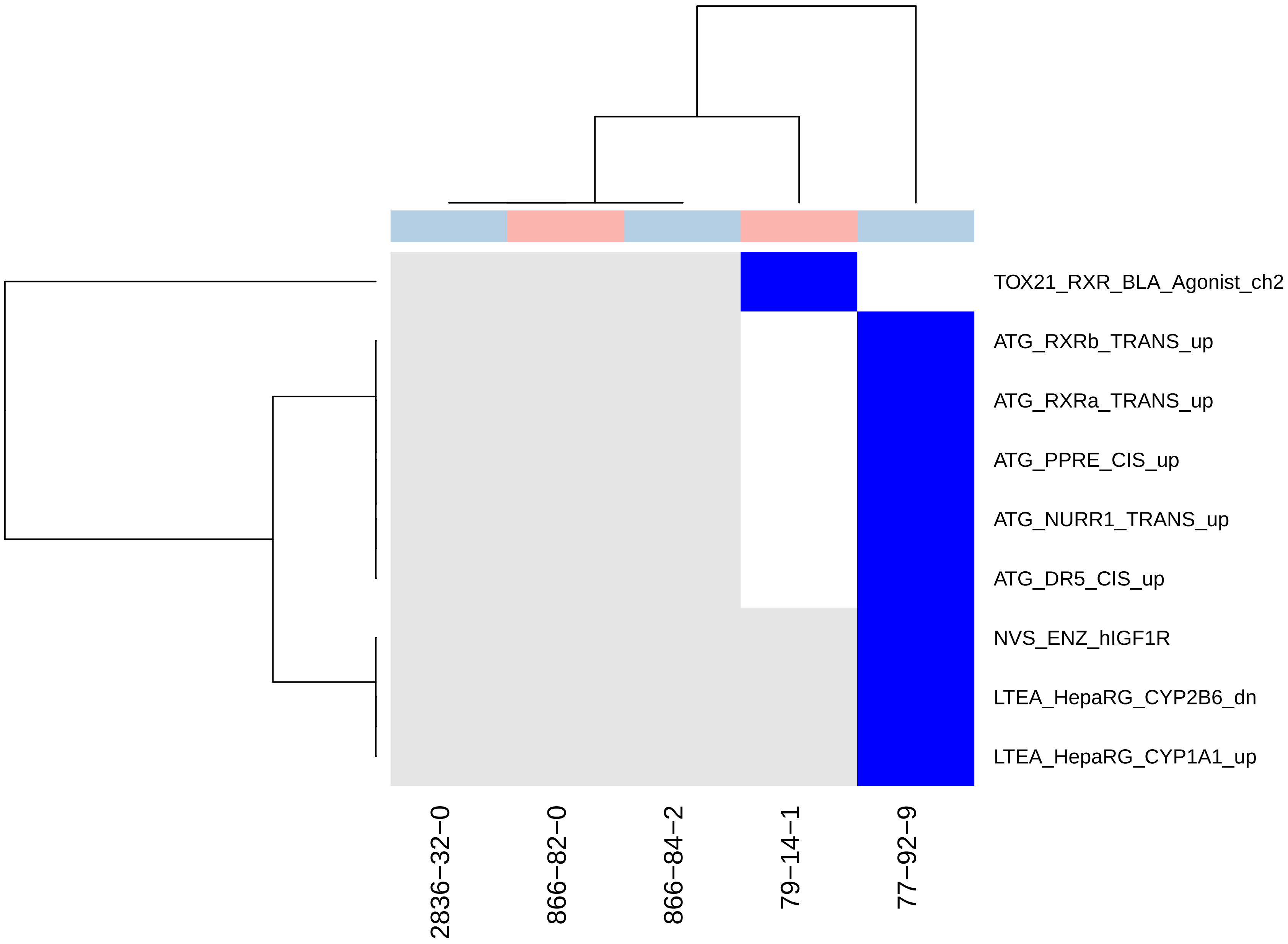

All Activity Heatmap for Cluster 1721

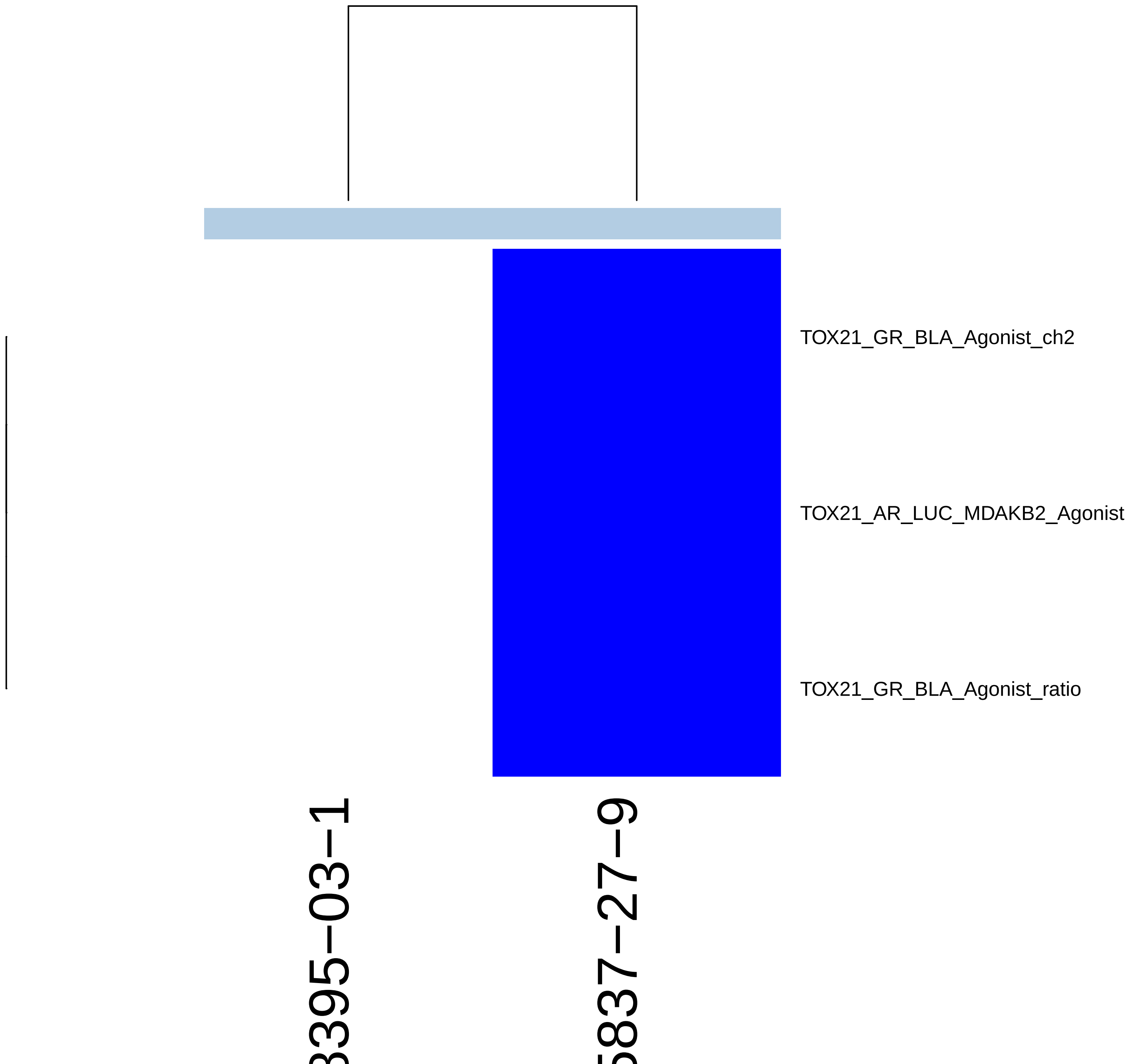

All Activity Heatmap for Cluster 1722

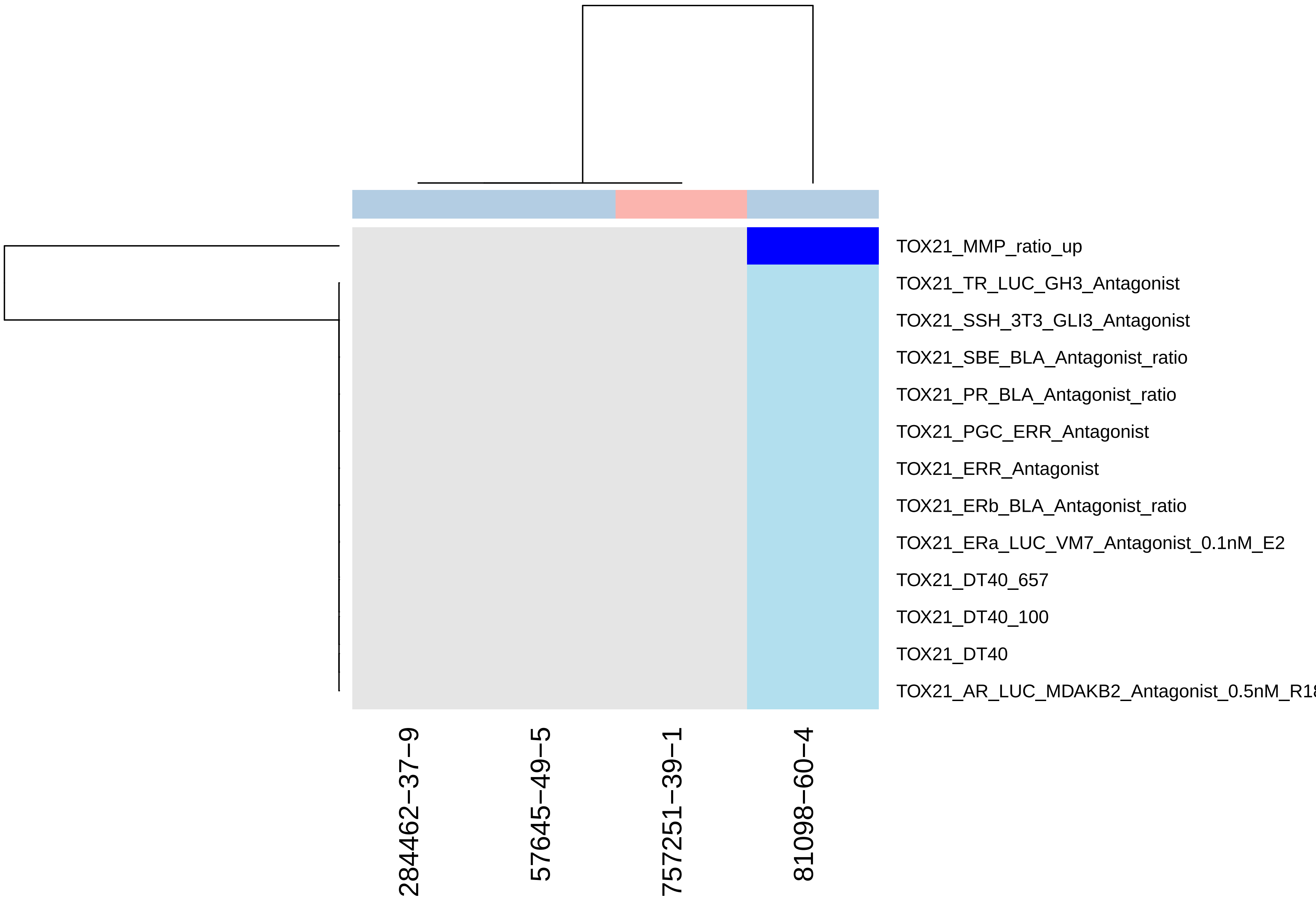

All Activity Heatmap for Cluster 1724

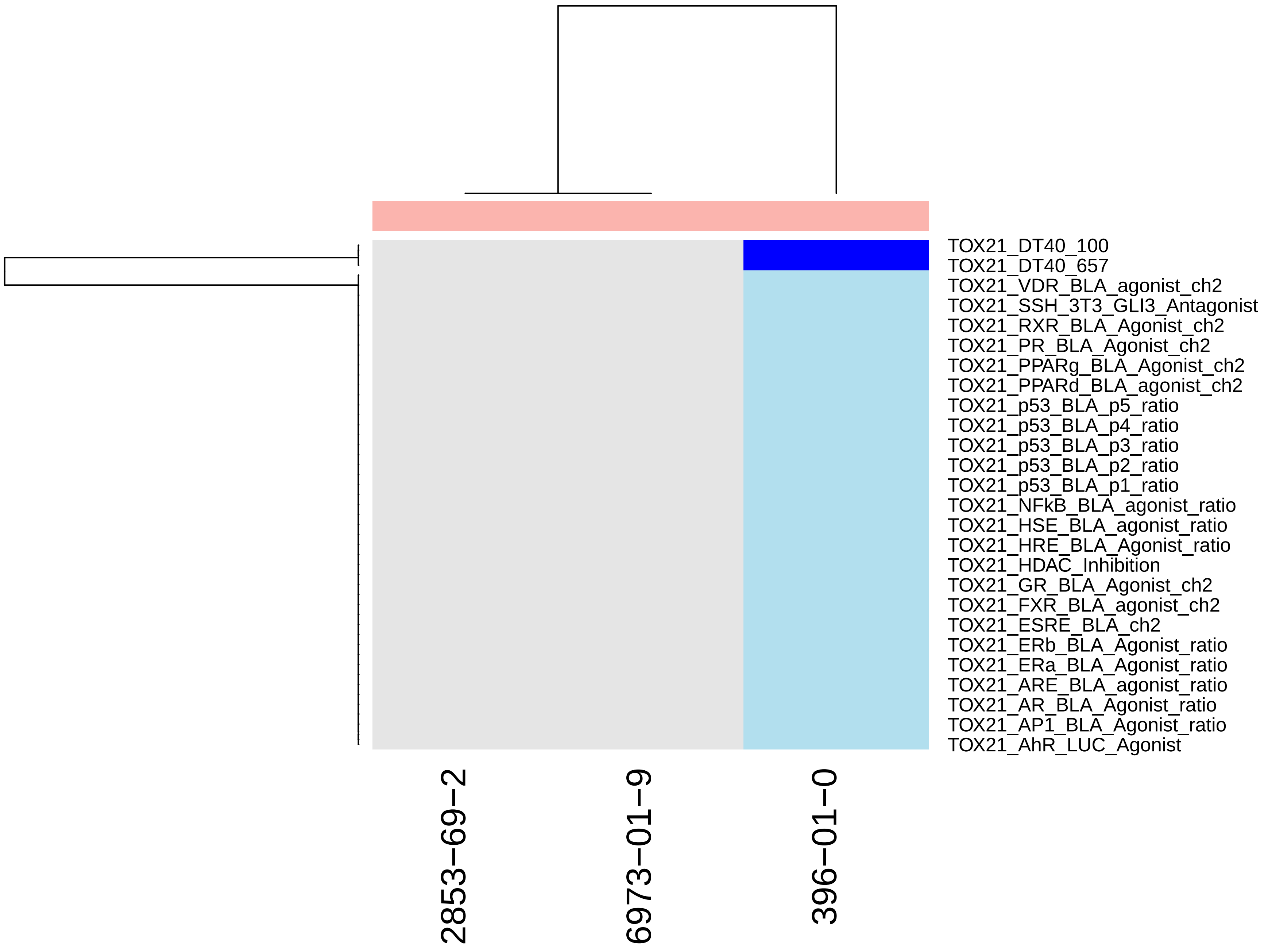

All Activity Heatmap for Cluster 1727

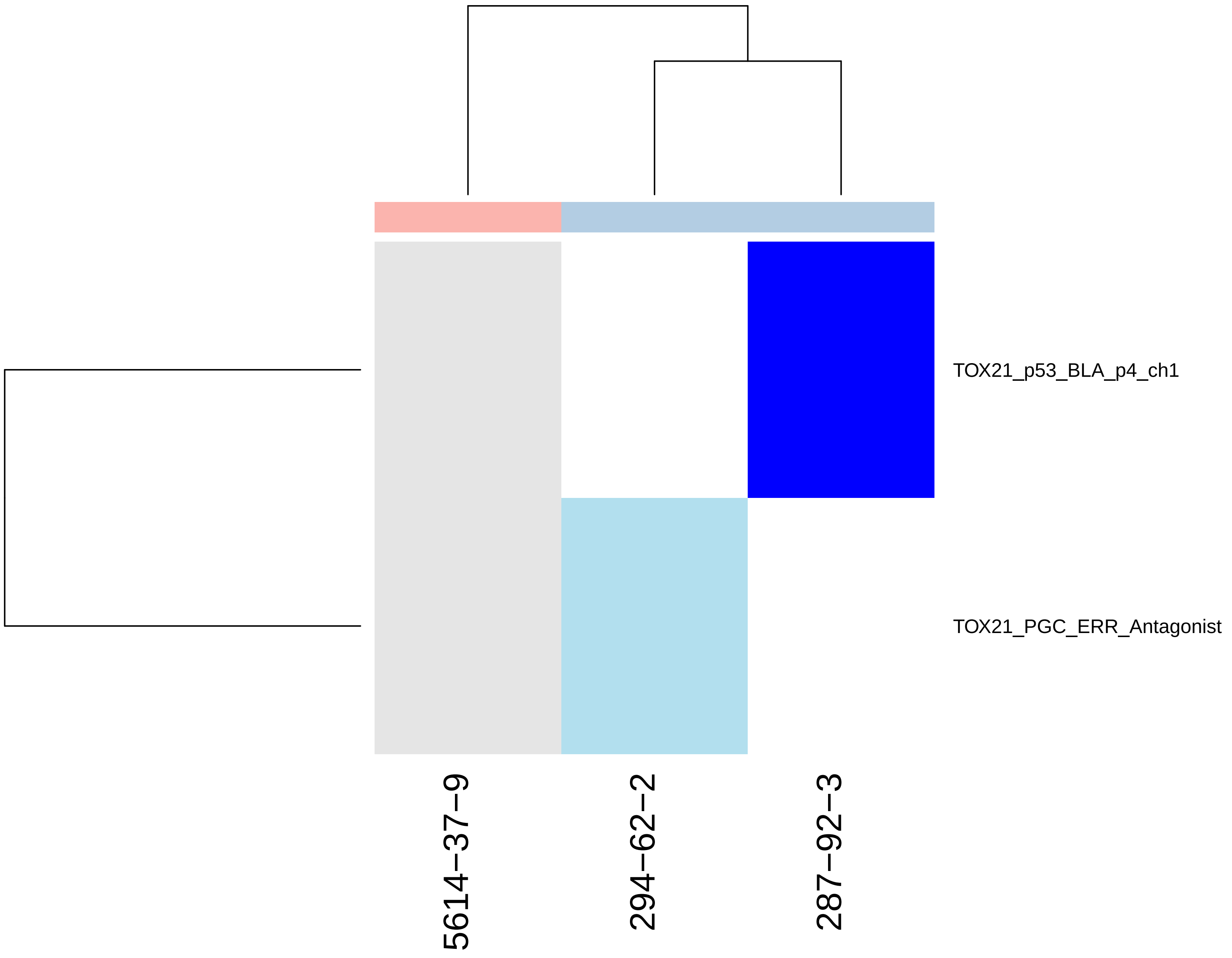

All Activity Heatmap for Cluster 1728

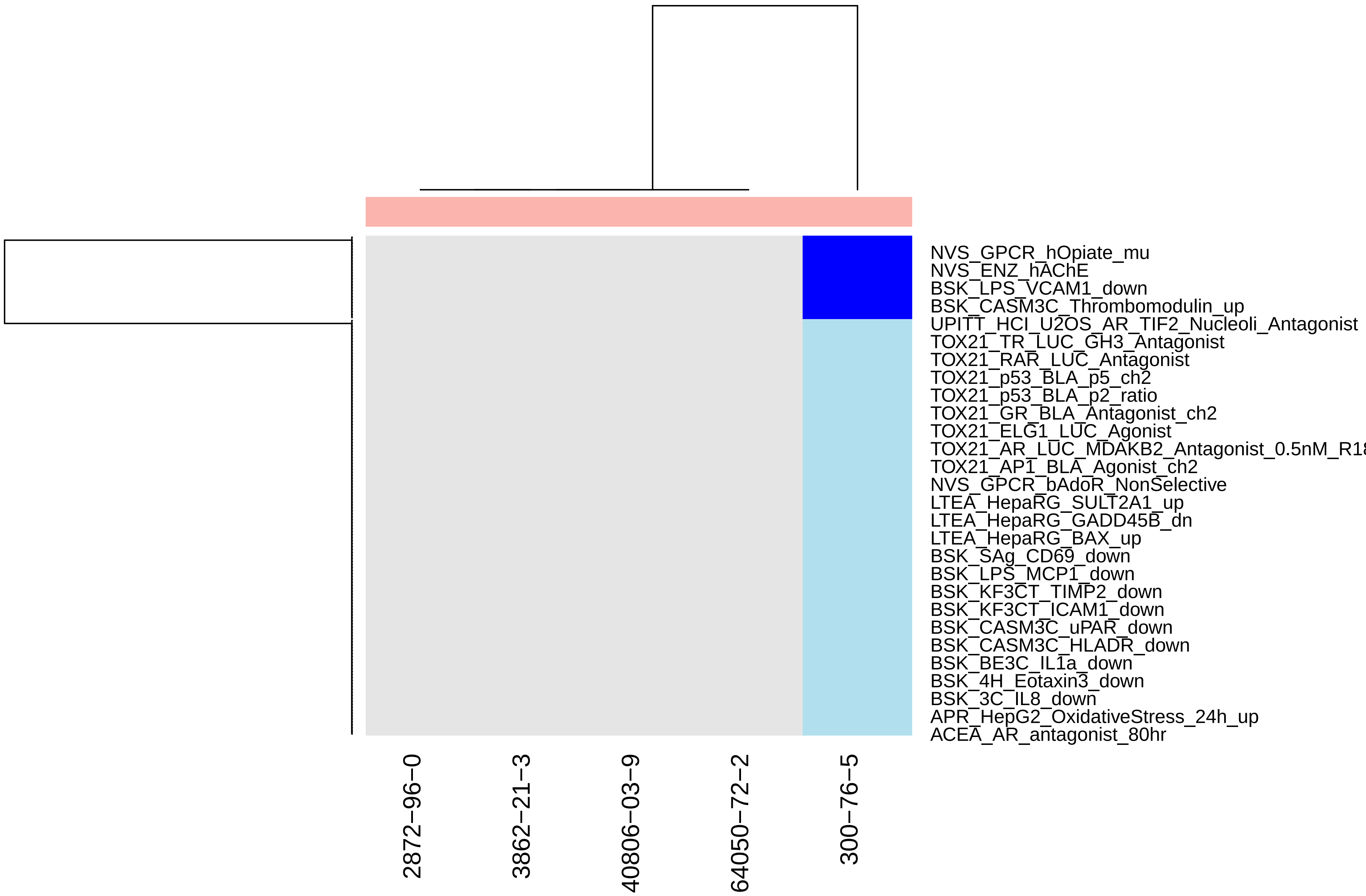

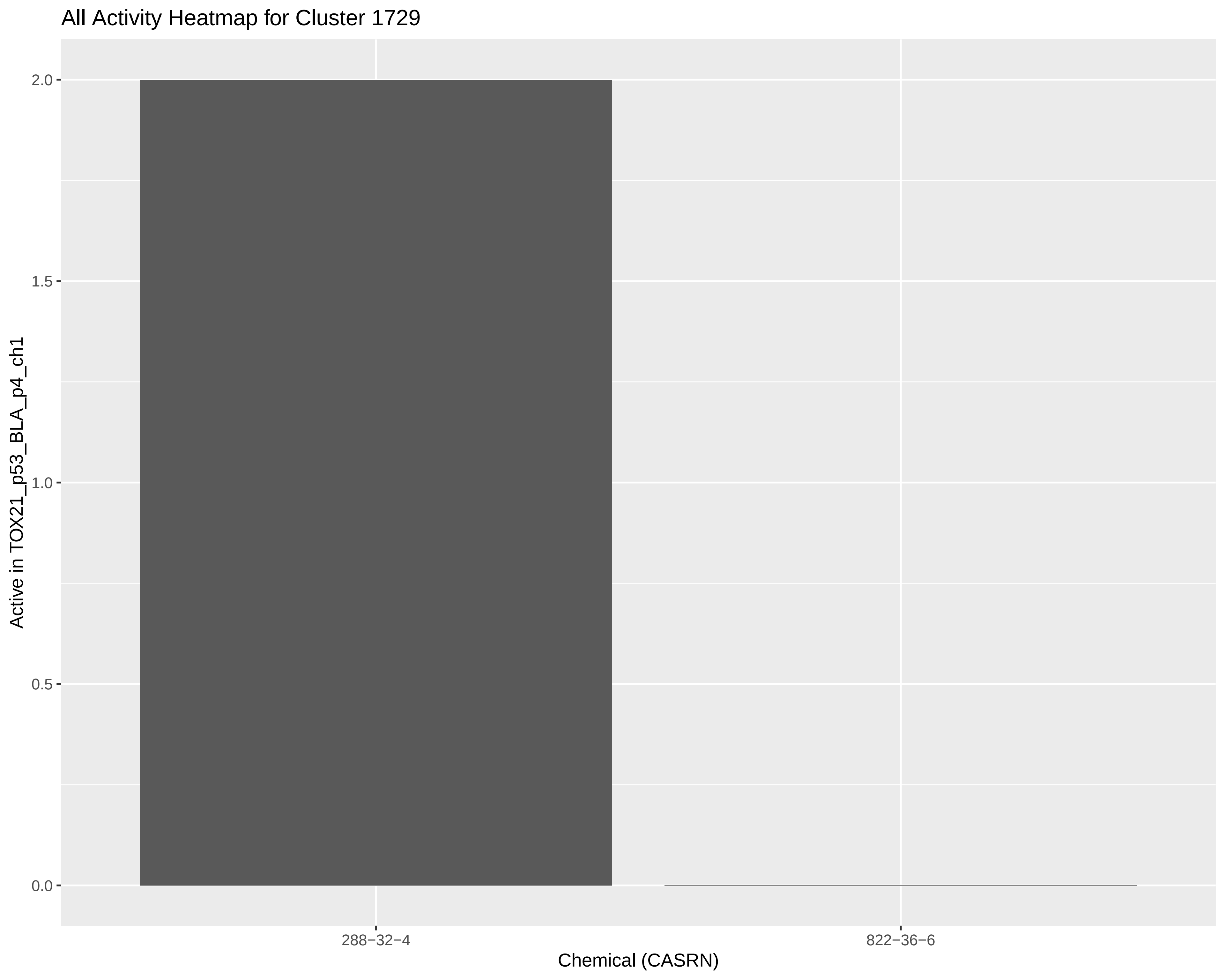

All Activity Heatmap for Cluster 1729
Active in TOX21_p53_BLA_p4_ch1
2.0
1.5
1.0
0.5
0.0
288−32−4
822−36−6
Chemical (CASRN)

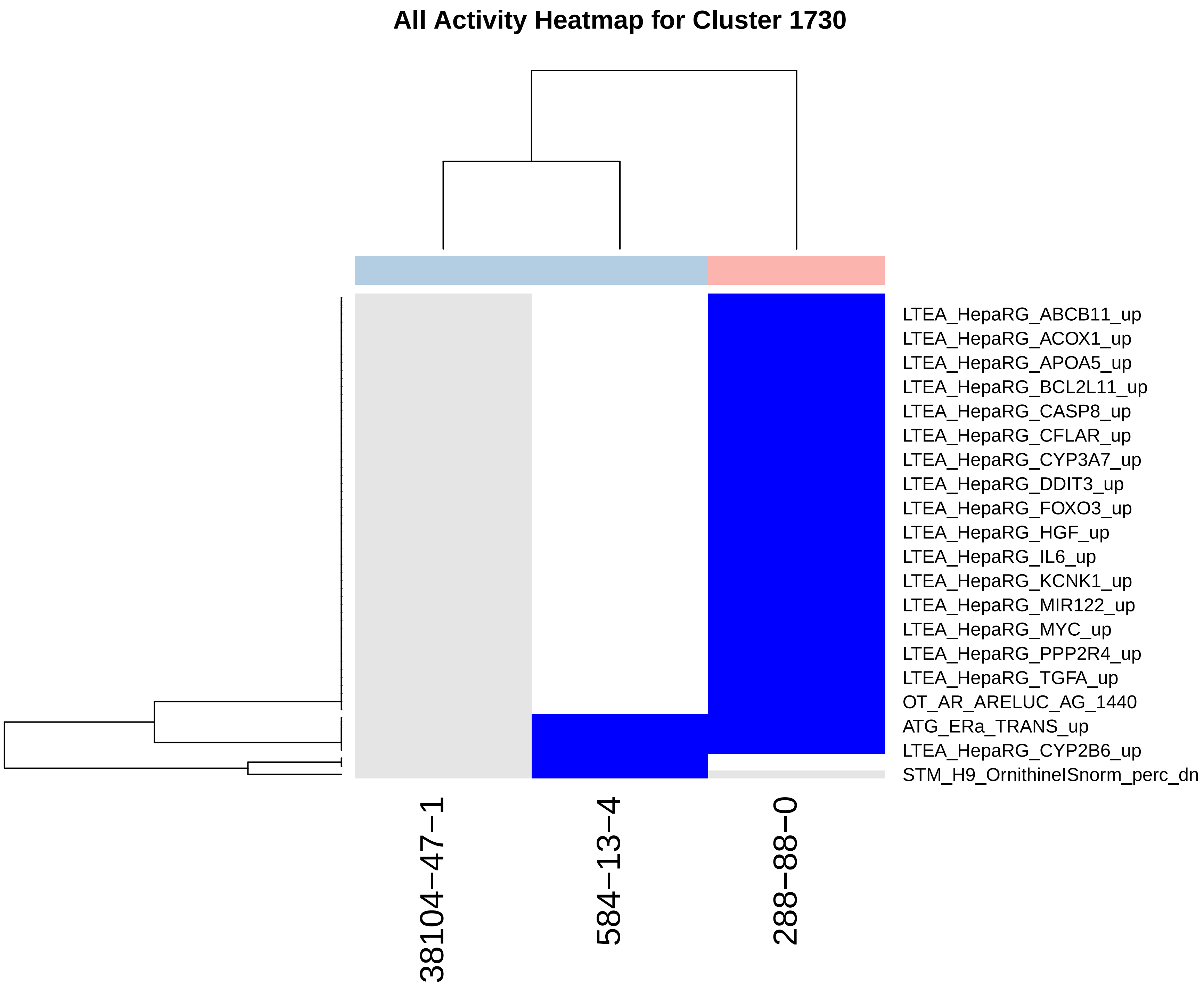
All Activity Heatmap for Cluster 1730
LTEA_HepaRG_ABCB11_up
LTEA_HepaRG_ACOX1_up
LTEA_HepaRG_APOA5_up
LTEA_HepaRG_BCL2L11_up
LTEA_HepaRG_CASP8_up
LTEA_HepaRG_CFLAR_up
LTEA_HepaRG_CYP3A7_up
LTEA_HepaRG_DDIT3_up
LTEA_HepaRG_FOXO3_up
LTEA_HepaRG_HGF_up
LTEA_HepaRG_IL6_up
LTEA_HepaRG_KCNK1_up
LTEA_HepaRG_MIR122_up
LTEA_HepaRG_MYC_up
LTEA_HepaRG_PPP2R4_up
LTEA_HepaRG_TGFA_up
OT_AR_ARELUC_AG_1440
ATG_ERa_TRANS_up
LTEA_HepaRG_CYP2B6_up
STM_H9_OrnithineISnorm_perc_dn
38104−47−1
584−13−4
288−88−0

All Activity Heatmap for Cluster 1732

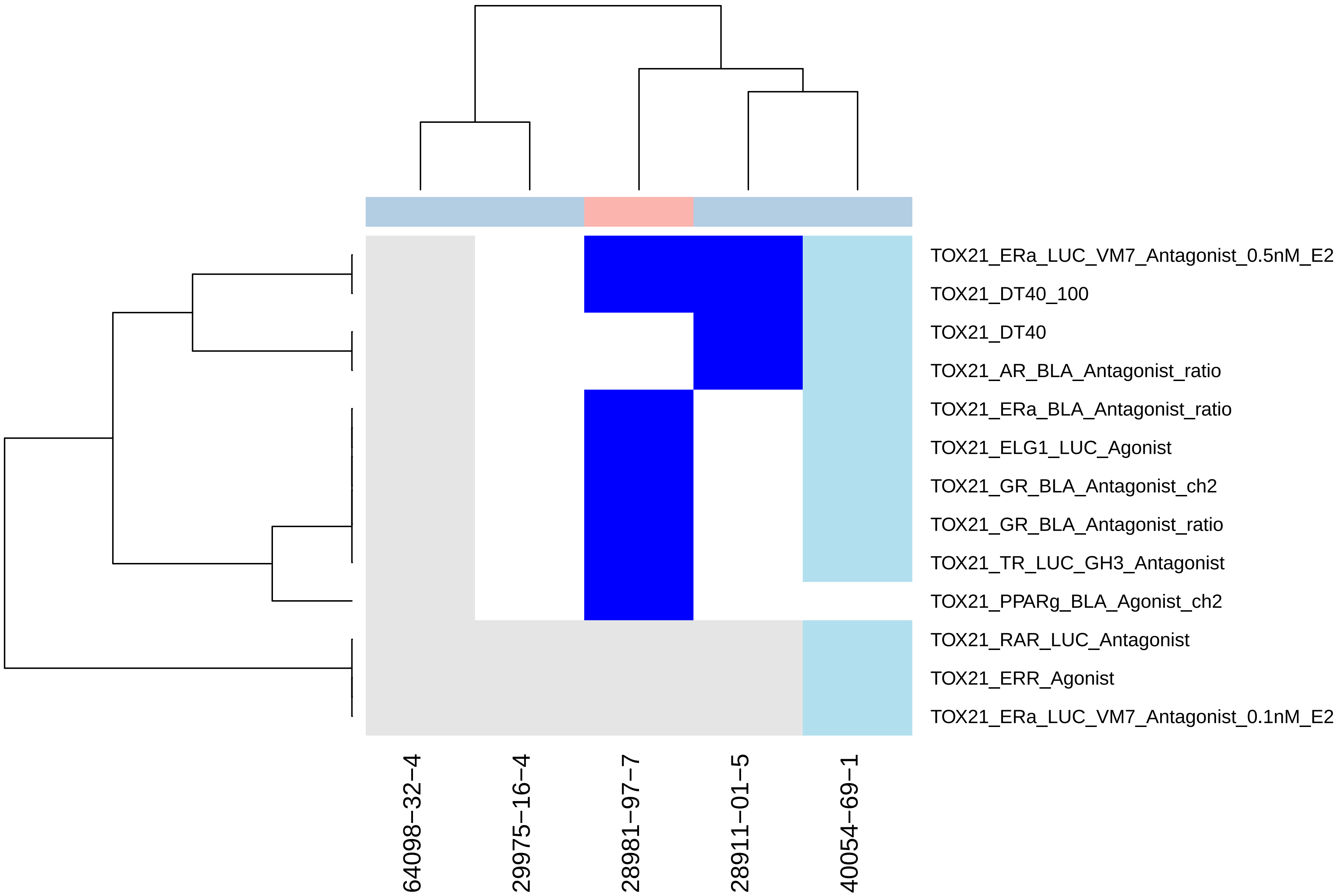

All Activity Heatmap for Cluster 1733

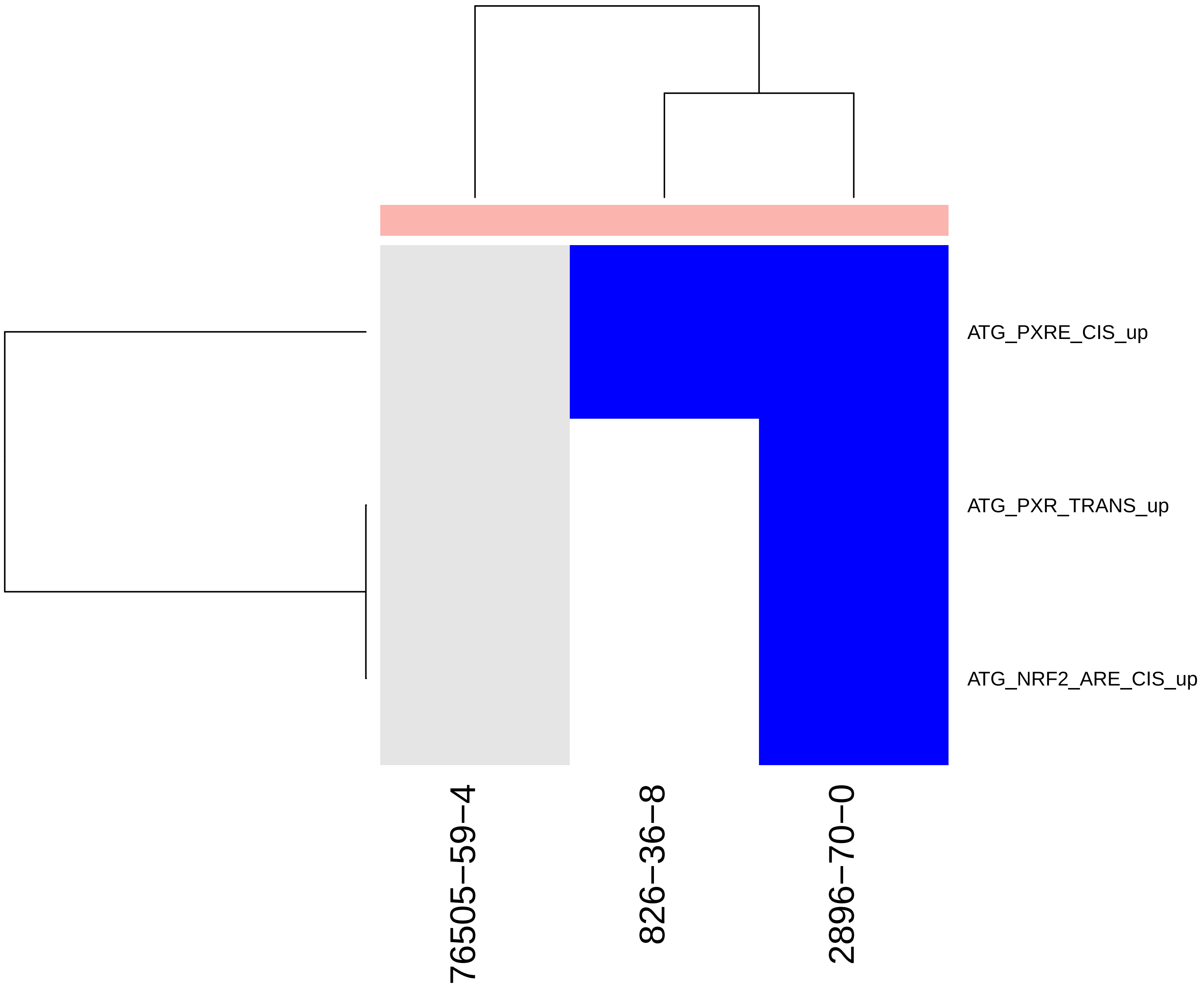

All Activity Heatmap for Cluster 1737

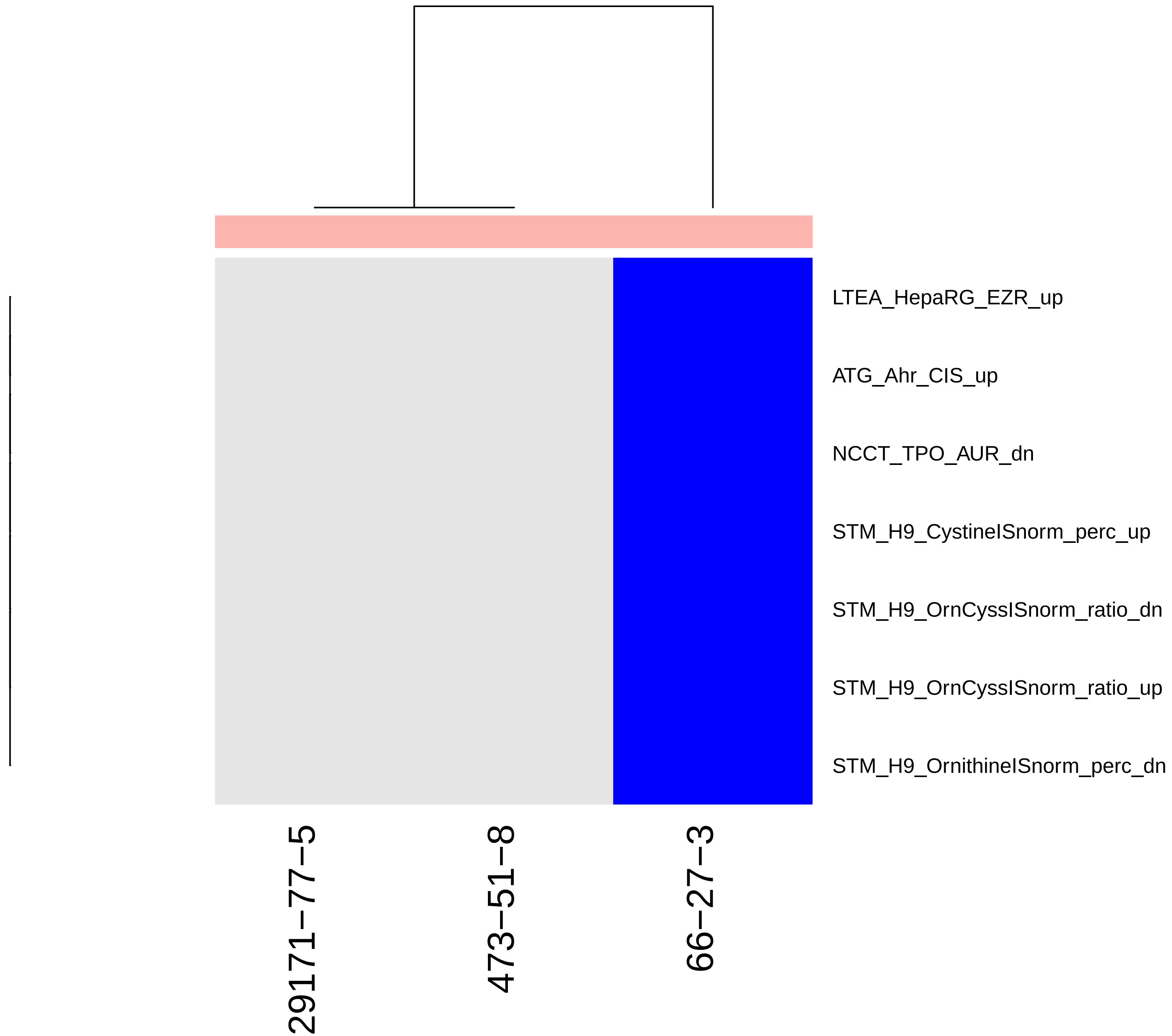

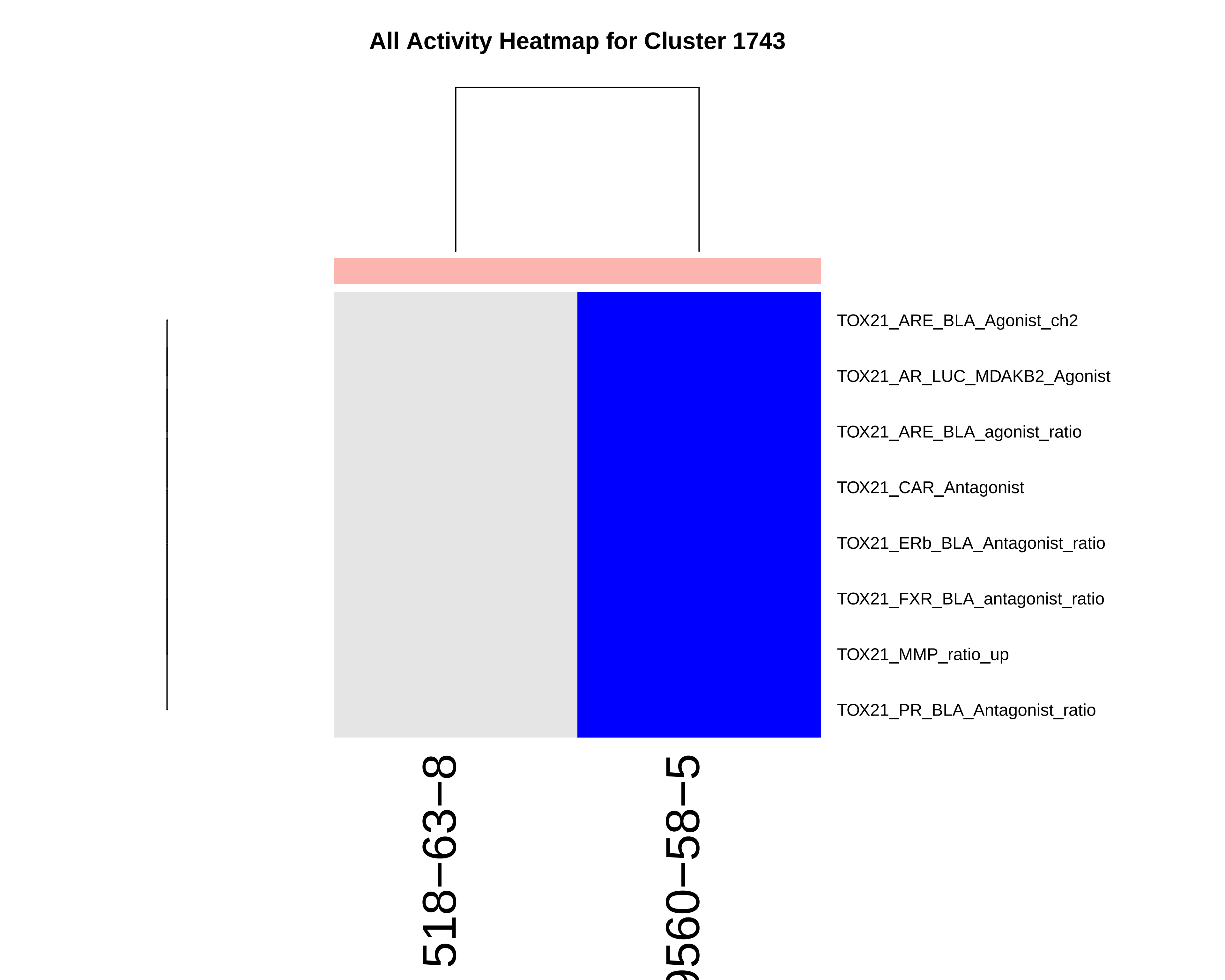
All Activity Heatmap for Cluster 1743
TOX21_ARE_BLA_Agonist_ch2
TOX21_AR_LUC_MDAKB2_Agonist
TOX21_ARE_BLA_agonist_ratio
TOX21_CAR_Antagonist
TOX21_ERb_BLA_Antagonist_ratio
TOX21_FXR_BLA_antagonist_ratio
TOX21_MMP_ratio_up
TOX21_PR_BLA_Antagonist_ratio
518−63−8
9560−58−5

All Activity Heatmap for Cluster 1745

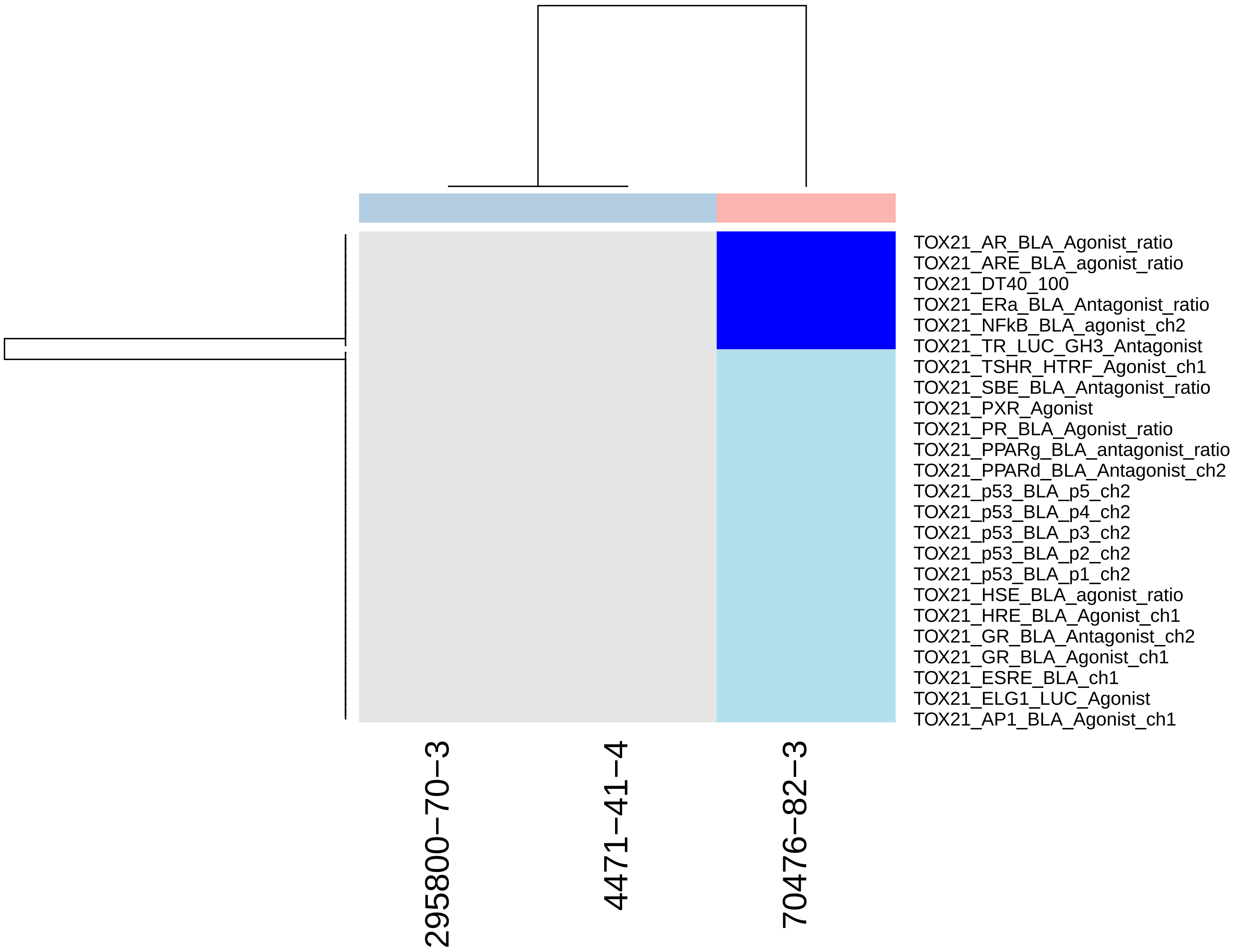

All Activity Heatmap for Cluster 1747

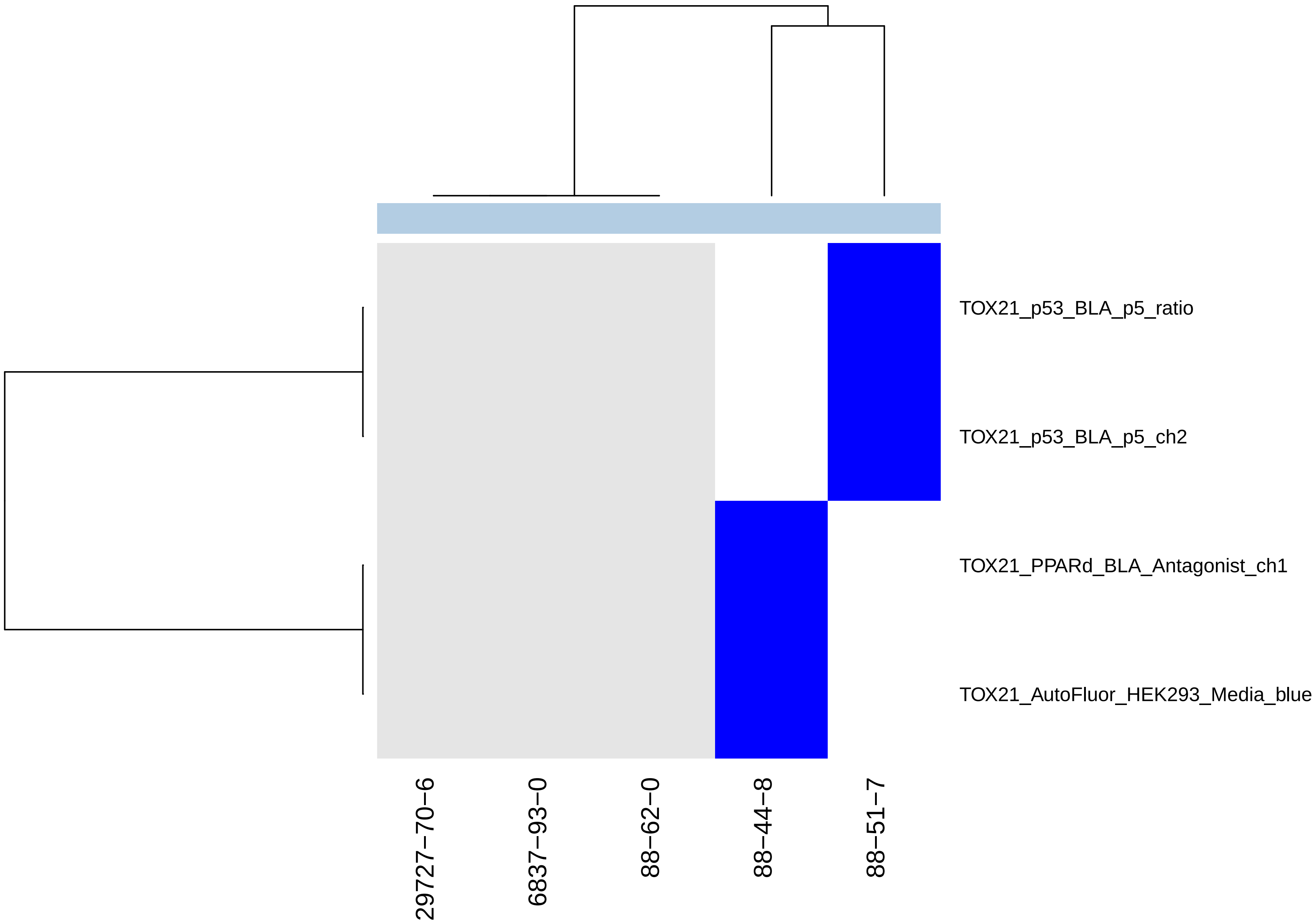

All Activity Heatmap for Cluster 1749

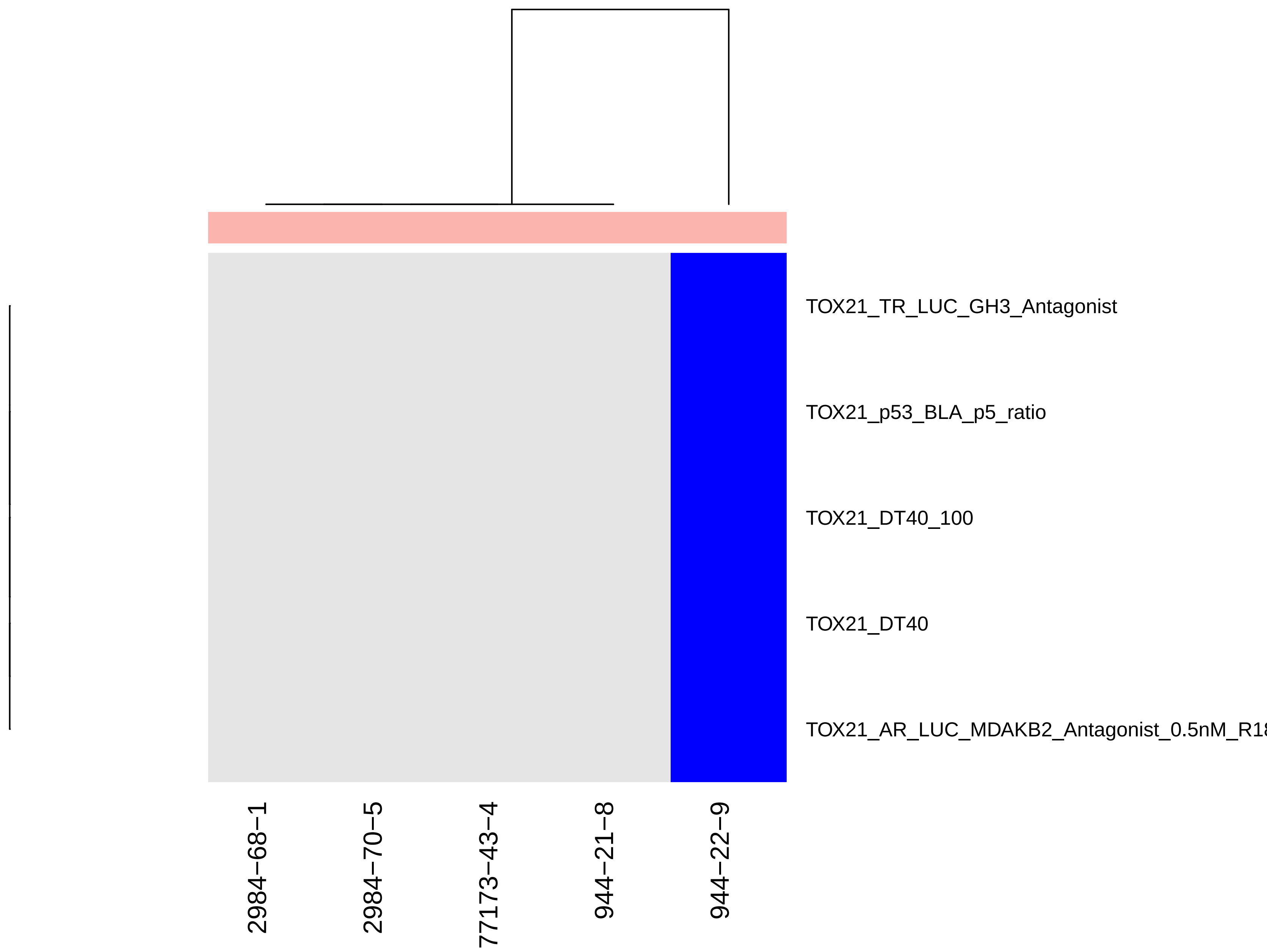

All Activity Heatmap for Cluster 1750

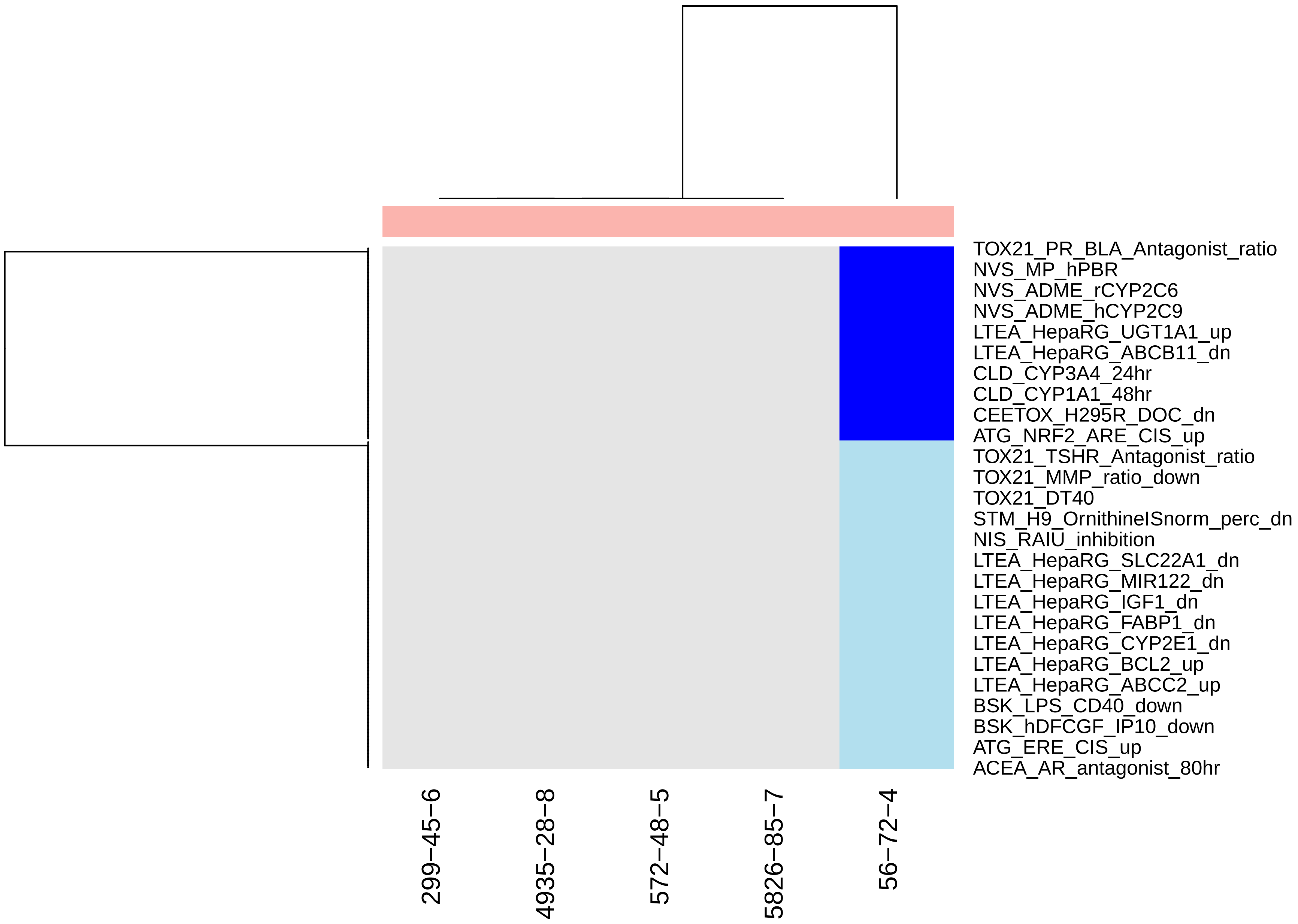

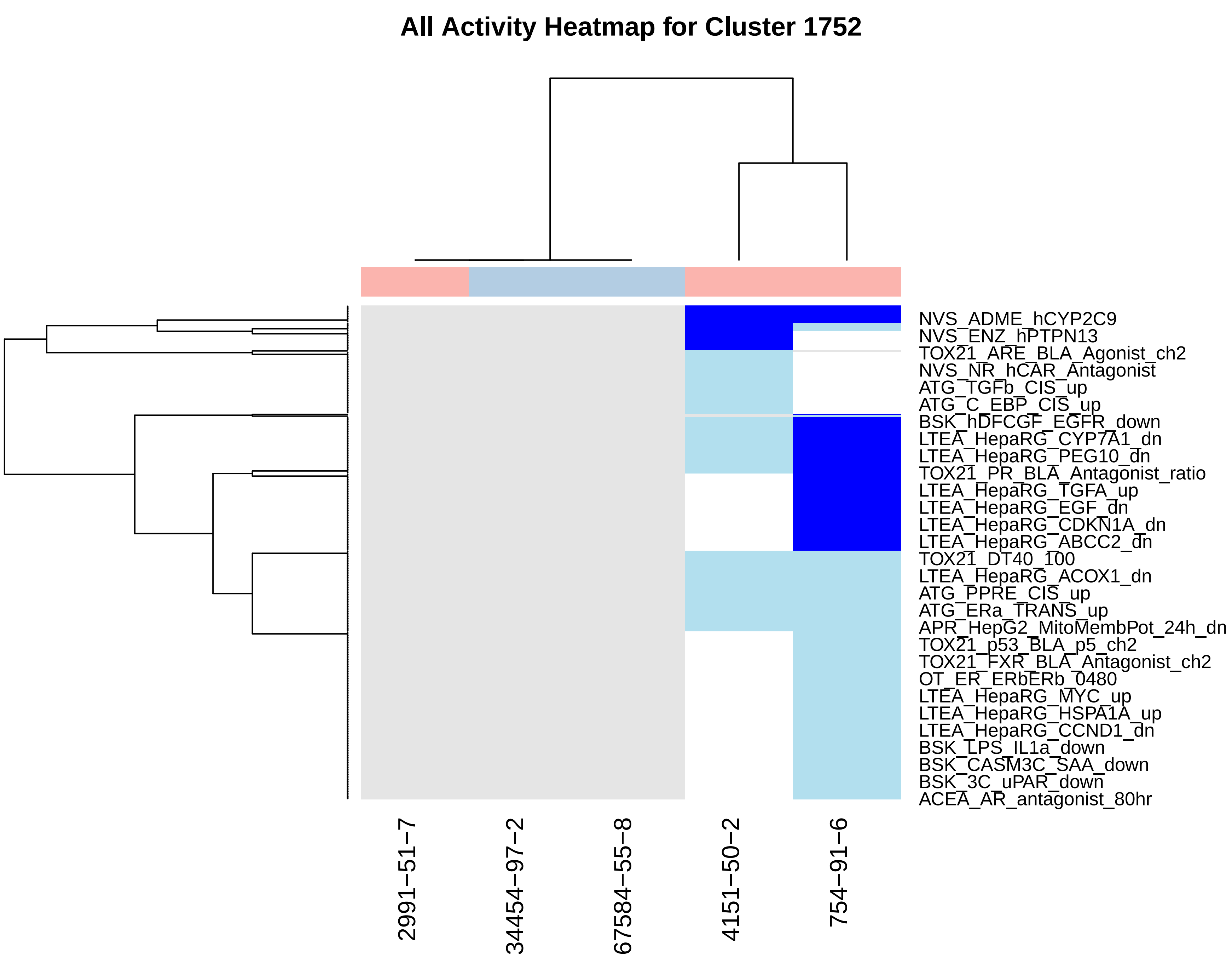

All Activity Heatmap for Cluster 1752
NVS_ADME_hCYP2C9
NVS_ENZ_hPTPN13
TOX21_ARE_BLA_Agonist_ch2
NVS_NR_hCAR_Antagonist
ATG_TGFb_CIS_up
ATG_C_EBP_CIS_up
BSK_hDFCGF_EGFR_down
LTEA_HepaRG_CYP7A1_dn
LTEA_HepaRG_PEG10_dn
TOX21_PR_BLA_Antagonist_ratio
LTEA_HepaRG_TGFA_up
LTEA_HepaRG_EGF_dn
LTEA_HepaRG_CDKN1A_dn
LTEA_HepaRG_ABCC2_dn
TOX21_DT40_100
LTEA_HepaRG_ACOX1_dn
ATG_PPRE_CIS_up
ATG_ERa_TRANS_up
APR_HepG2_MitoMembPot_24h_dn
TOX21_p53_BLA_p5_ch2
TOX21_FXR_BLA_Antagonist_ch2
OT_ER_ERbERb_0480
LTEA_HepaRG_MYC_up
LTEA_HepaRG_HSPA1A_up
LTEA_HepaRG_CCND1_dn
BSK_LPS_IL1a_down
BSK_CASM3C_SAA_down
BSK_3C_uPAR_down
ACEA_AR_antagonist_80hr
2991−51−7
34454−97−2
67584−55−8
4151−50−2
754−91−6

All Activity Heatmap for Cluster 1753

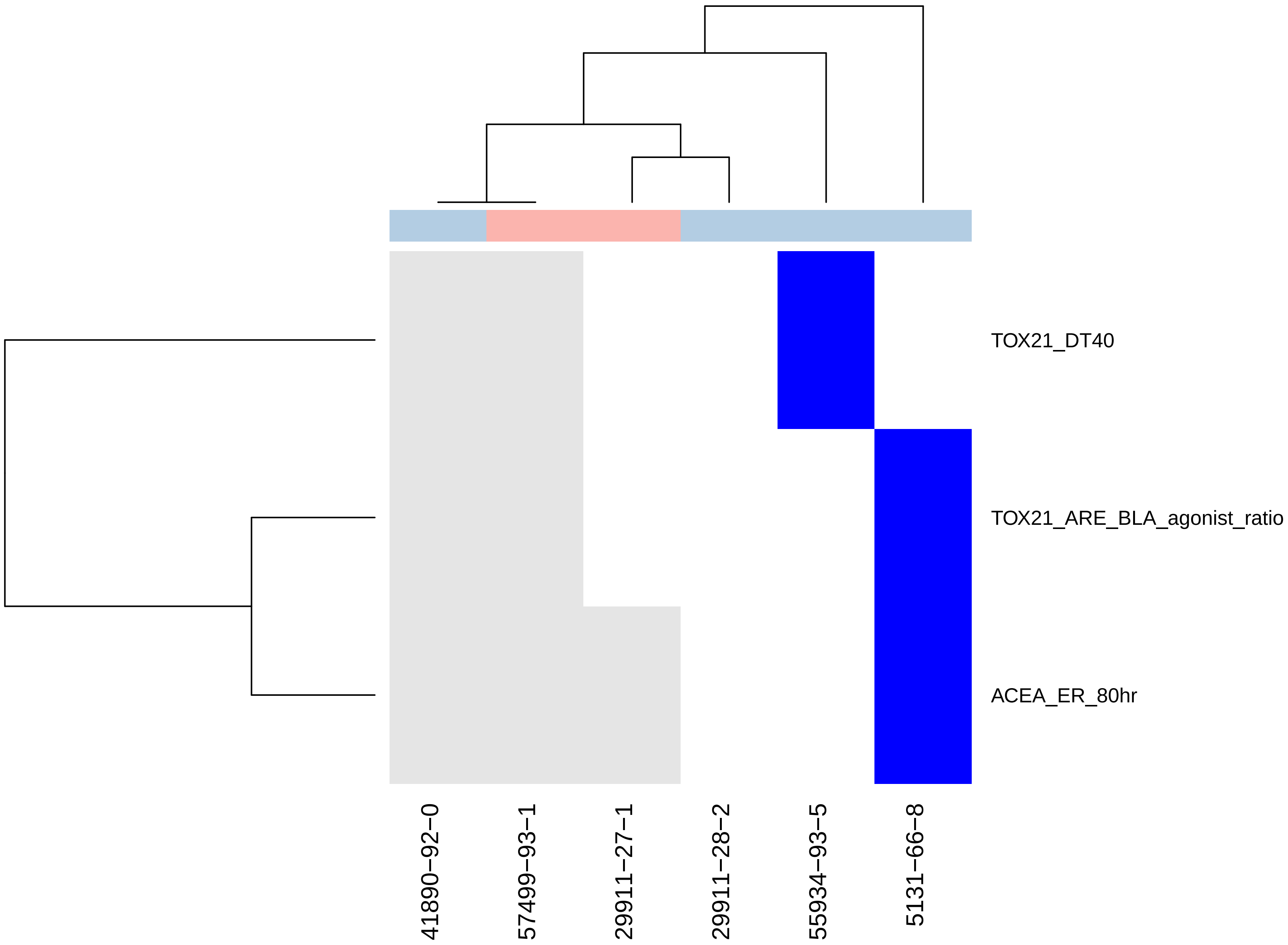

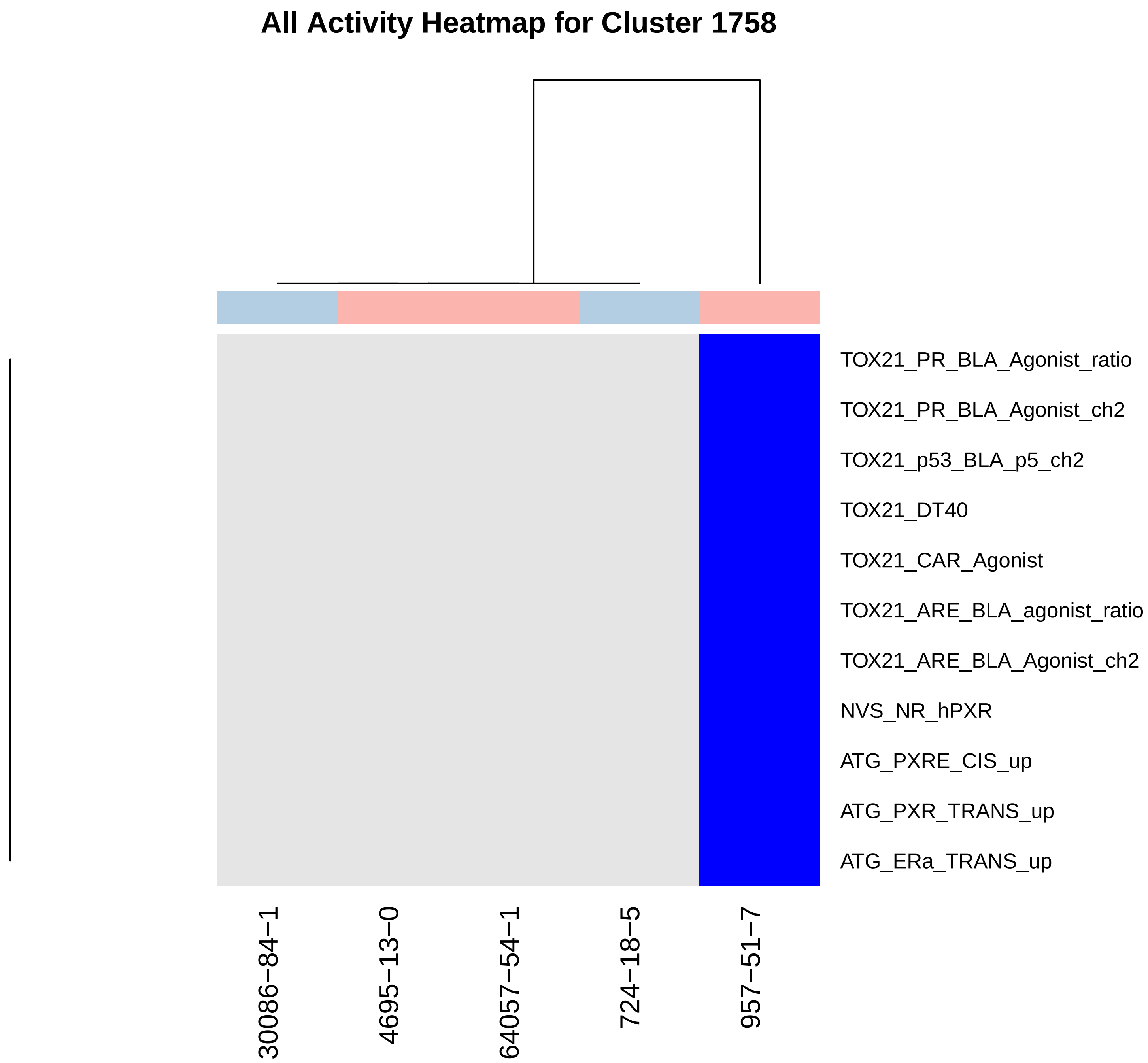
All Activity Heatmap for Cluster 1758
TOX21_PR_BLA_Agonist_ratio
TOX21_PR_BLA_Agonist_ch2
TOX21_p53_BLA_p5_ch2
TOX21_DT40
TOX21_CAR_Agonist
TOX21_ARE_BLA_agonist_ratio
TOX21_ARE_BLA_Agonist_ch2
NVS_NR_hPXR
ATG_PXRE_CIS_up
ATG_PXR_TRANS_up
ATG_ERa_TRANS_up
30086−84−1
4695−13−0
64057−54−1
724−18−5
957−51−7

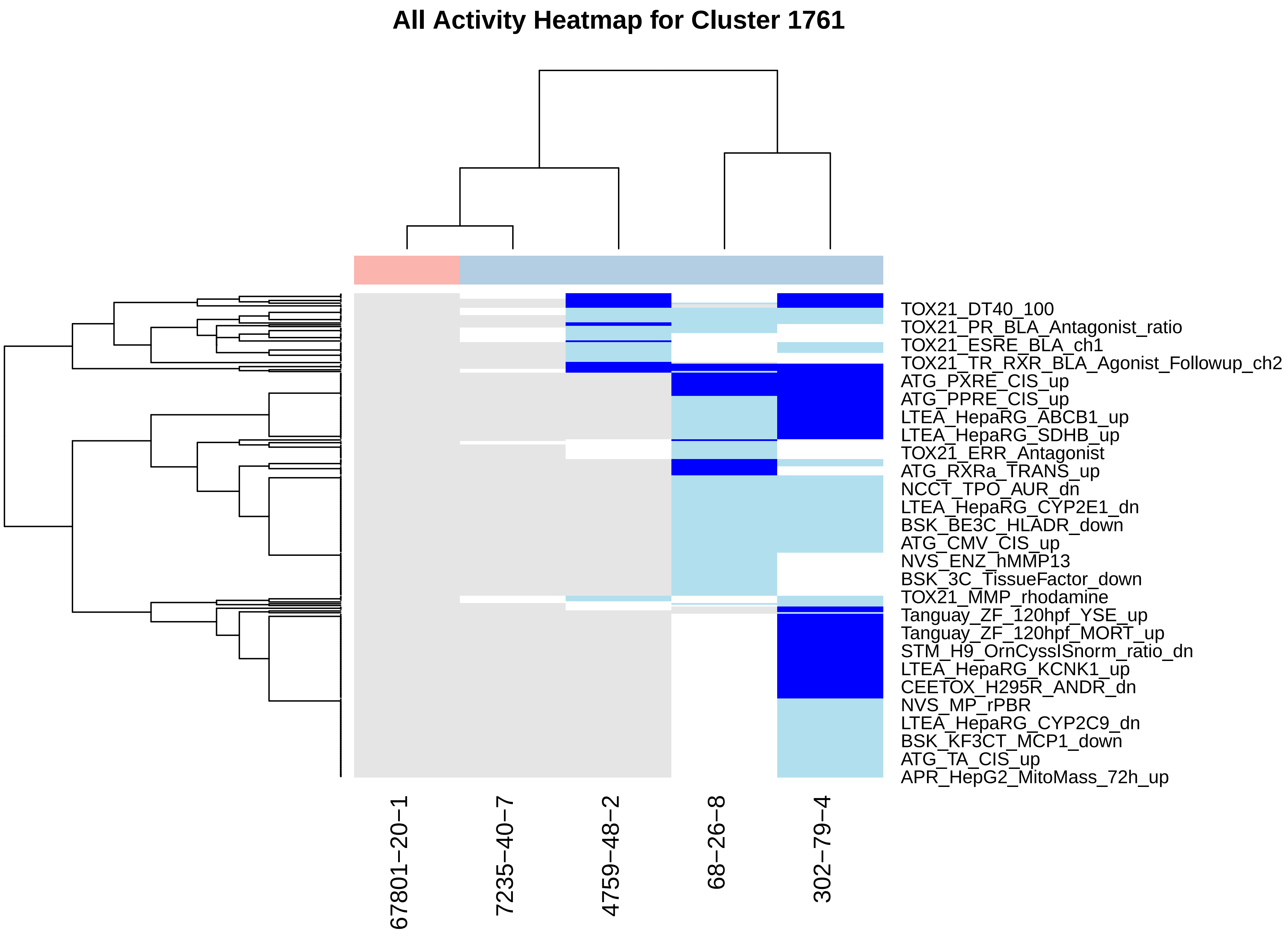
All Activity Heatmap for Cluster 1761
TOX21_DT40_100
TOX21_PR_BLA_Antagonist_ratio
TOX21_ESRE_BLA_ch1
TOX21_TR_RXR_BLA_Agonist_Followup_ch2
ATG_PXRE_CIS_up
ATG_PPRE_CIS_up
LTEA_HepaRG_ABCB1_up
LTEA_HepaRG_SDHB_up
TOX21_ERR_Antagonist
ATG_RXRa_TRANS_up
NCCT_TPO_AUR_dn
LTEA_HepaRG_CYP2E1_dn
BSK_BE3C_HLADR_down
ATG_CMV_CIS_up
NVS_ENZ_hMMP13
BSK_3C_TissueFactor_down
TOX21_MMP_rhodamine
Tanguay_ZF_120hpf_YSE_up
Tanguay_ZF_120hpf_MORT_up
STM_H9_OrnCyssISnorm_ratio_dn
LTEA_HepaRG_KCNK1_up
CEETOX_H295R_ANDR_dn
NVS_MP_rPBR
LTEA_HepaRG_CYP2C9_dn
BSK_KF3CT_MCP1_down
ATG_TA_CIS_up
APR_HepG2_MitoMass_72h_up
67801−20−1
7235−40−7
4759−48−2
68−26−8
302−79−4

All Activity Heatmap for Cluster 1762

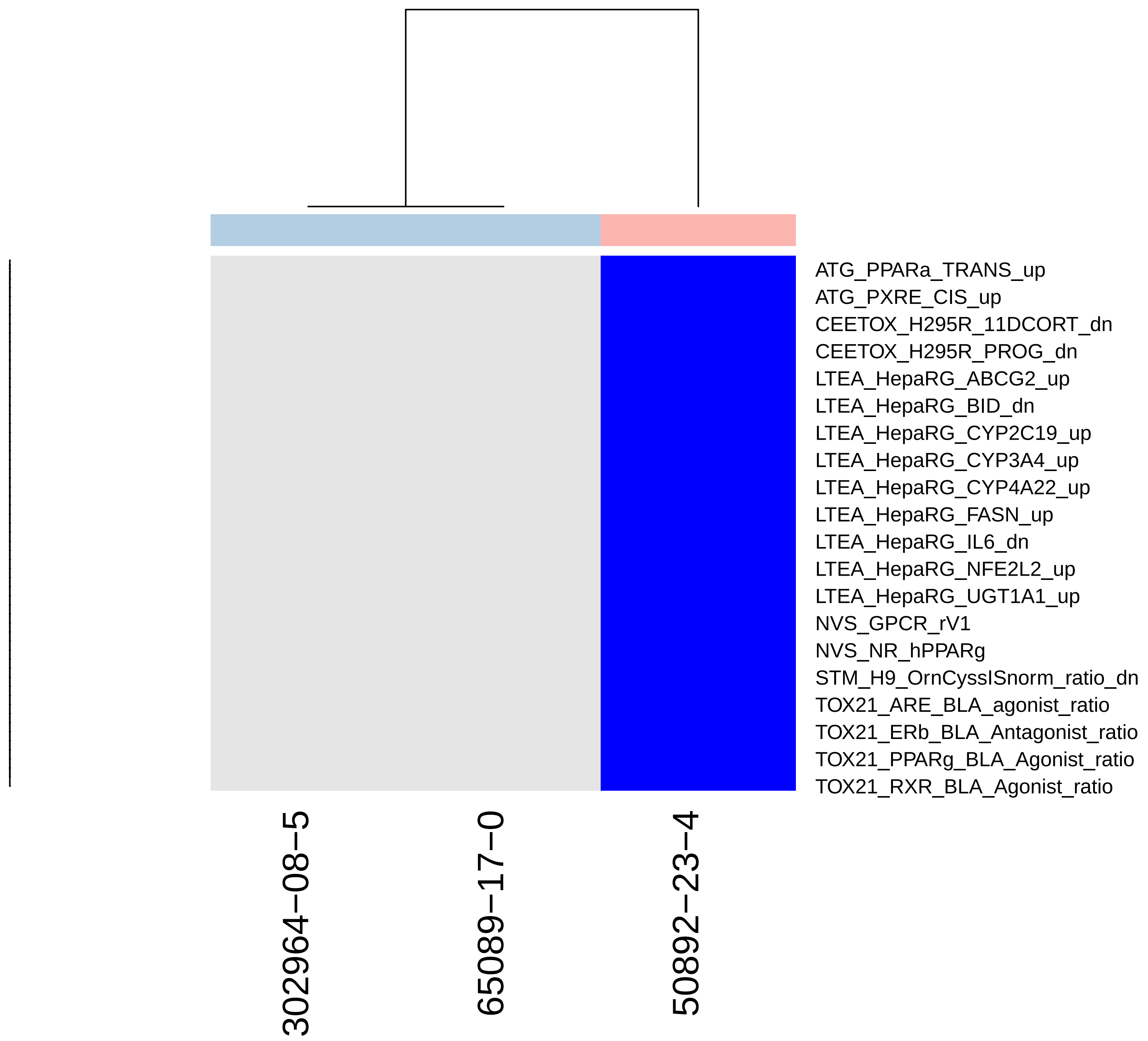

All Activity Heatmap for Cluster 1764

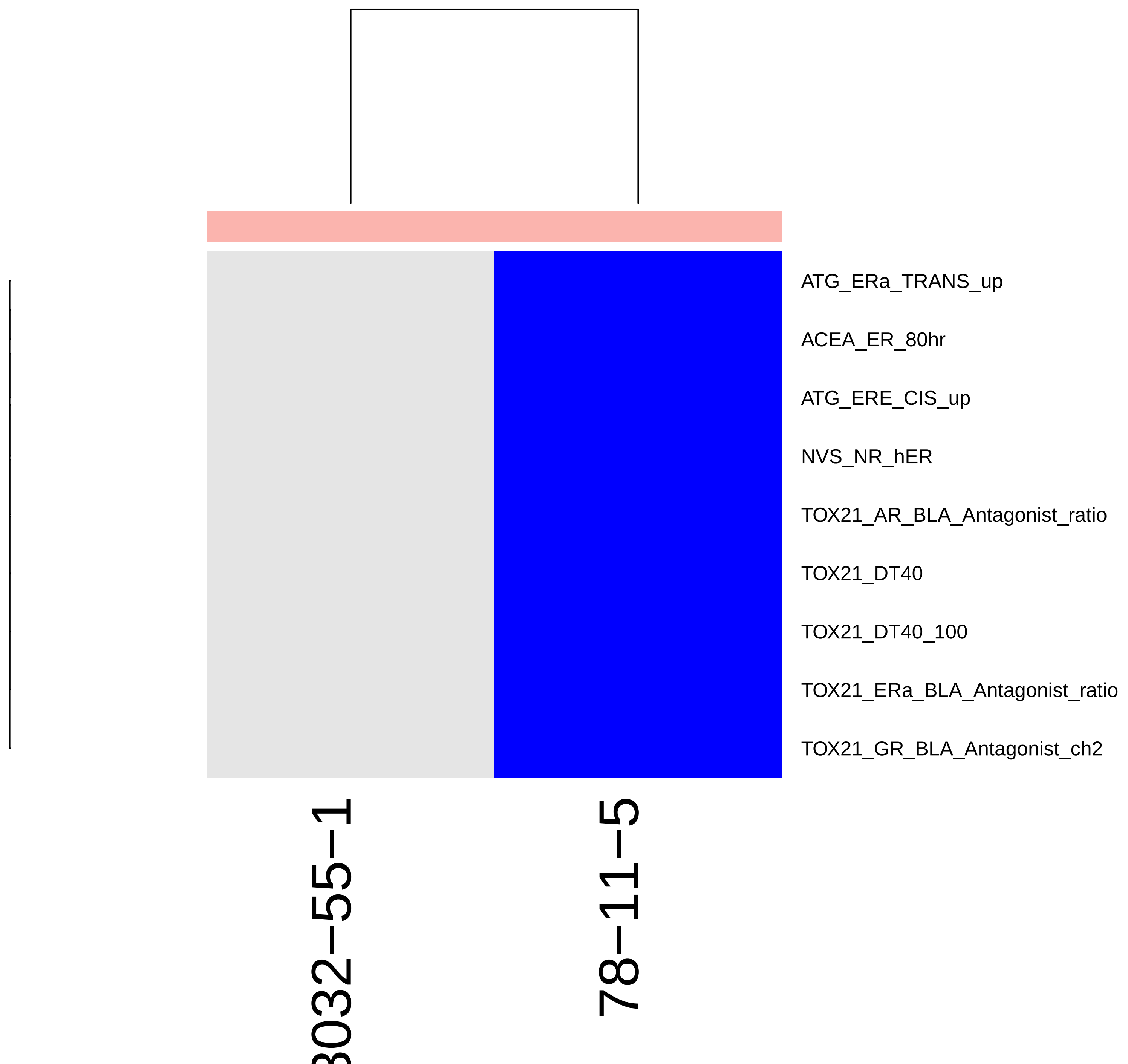

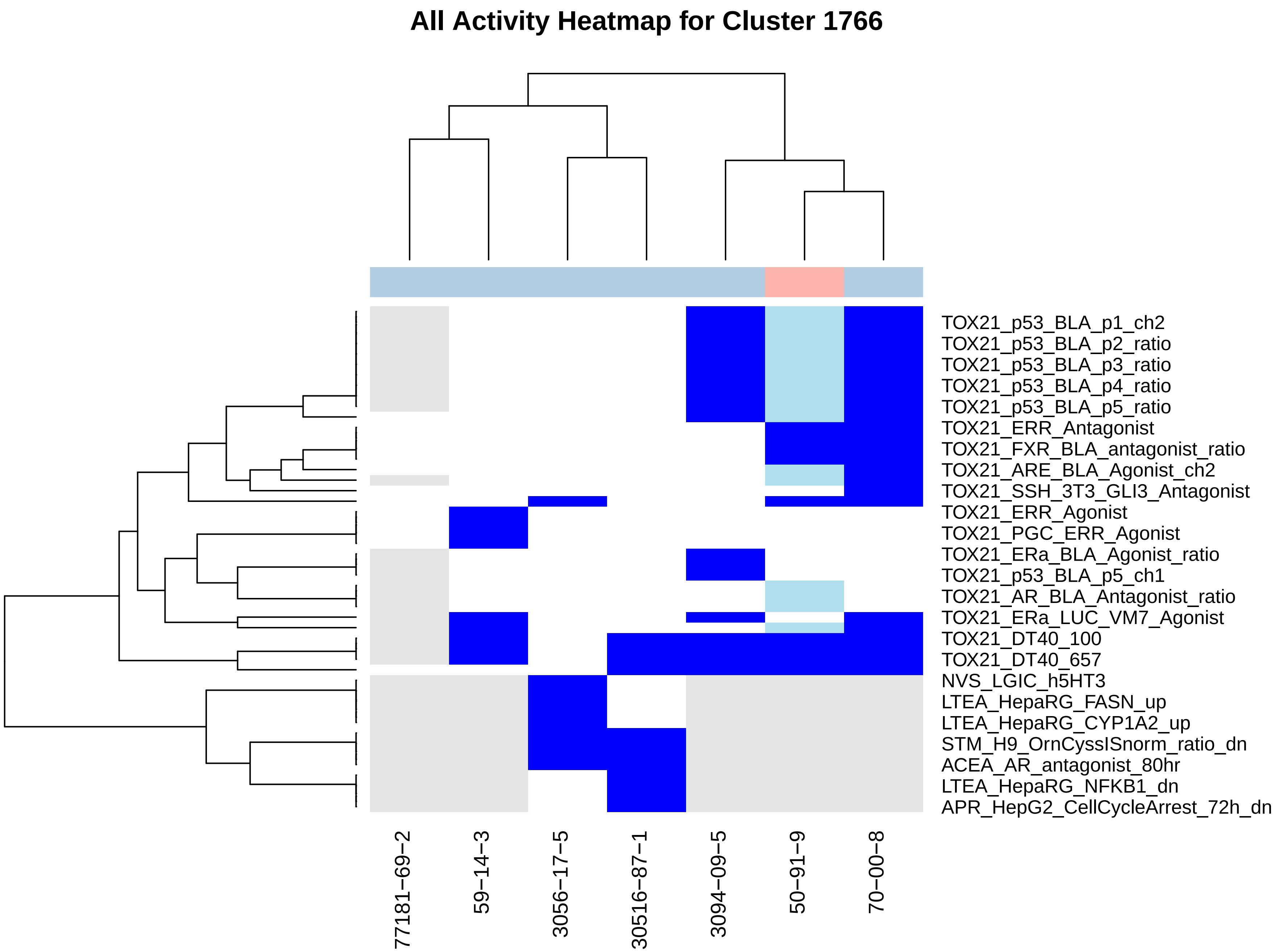

All Activity Heatmap for Cluster 1766
TOX21_p53_BLA_p1_ch2
TOX21_p53_BLA_p2_ratio
TOX21_p53_BLA_p3_ratio
TOX21_p53_BLA_p4_ratio
TOX21_p53_BLA_p5_ratio
TOX21_ERR_Antagonist
TOX21_FXR_BLA_antagonist_ratio
TOX21_ARE_BLA_Agonist_ch2
TOX21_SSH_3T3_GLI3_Antagonist
TOX21_ERR_Agonist
TOX21_PGC_ERR_Agonist
TOX21_ERa_BLA_Agonist_ratio
TOX21_p53_BLA_p5_ch1
TOX21_AR_BLA_Antagonist_ratio
TOX21_ERa_LUC_VM7_Agonist
TOX21_DT40_100
TOX21_DT40_657
NVS_LGIC_h5HT3
LTEA_HepaRG_FASN_up
LTEA_HepaRG_CYP1A2_up
STM_H9_OrnCyssISnorm_ratio_dn
ACEA_AR_antagonist_80hr
LTEA_HepaRG_NFKB1_dn
APR_HepG2_CellCycleArrest_72h_dn
77181−69−2
59−14−3
3056−17−5
30516−87−1
3094−09−5
50−91−9
70−00−8

All Activity Heatmap for Cluster 1767

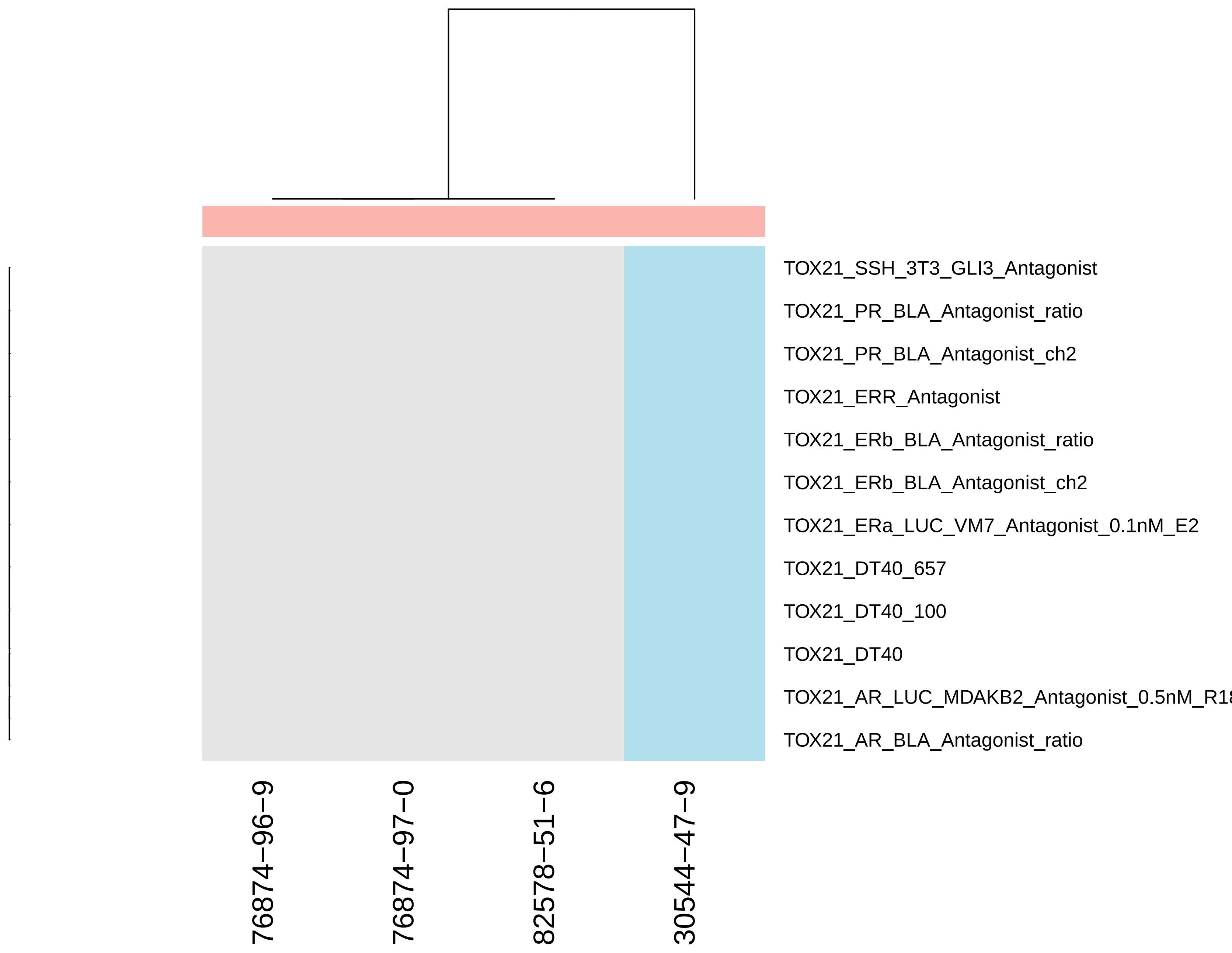

All Activity Heatmap for Cluster 1774

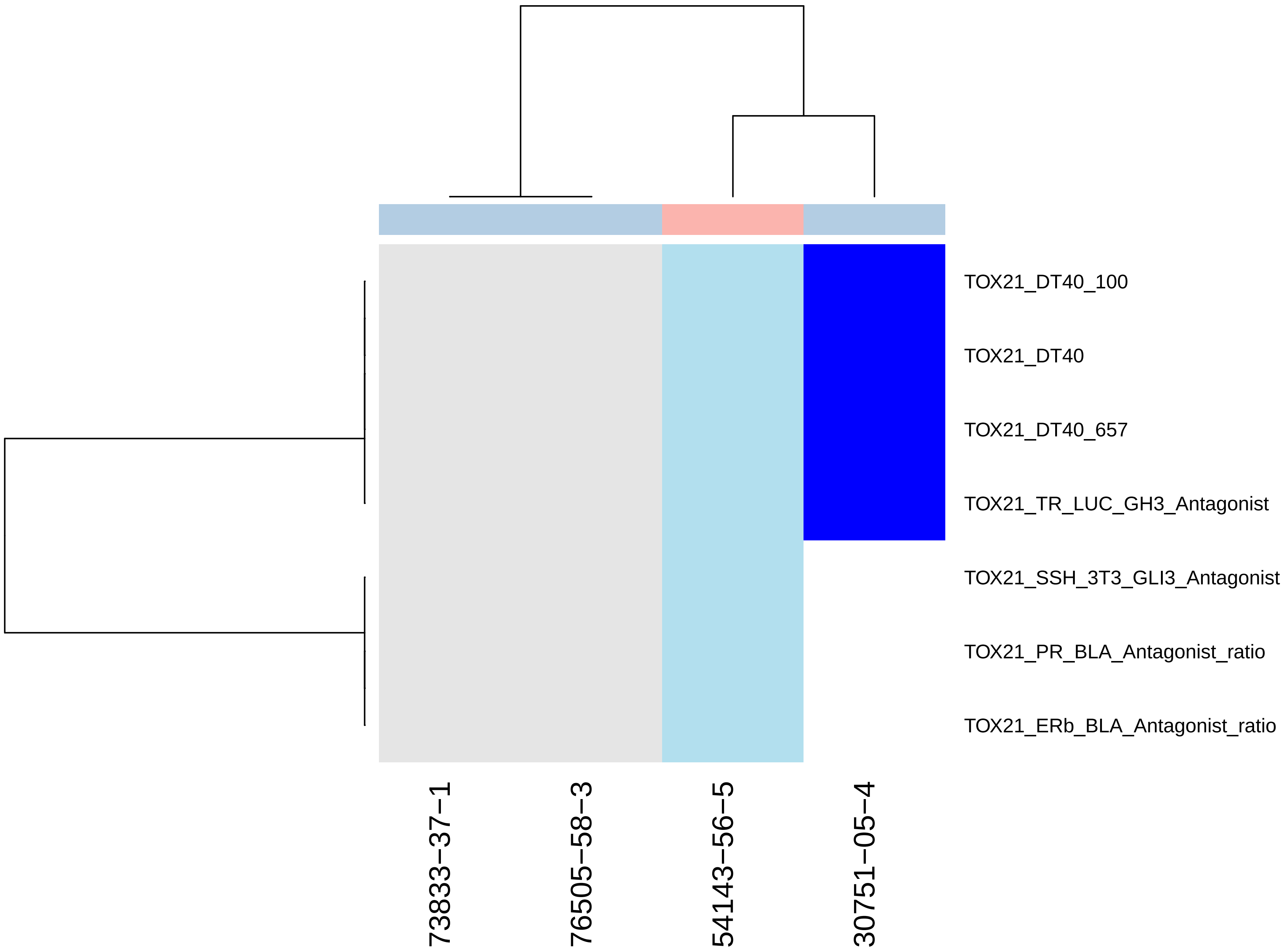

All Activity Heatmap for Cluster 1779

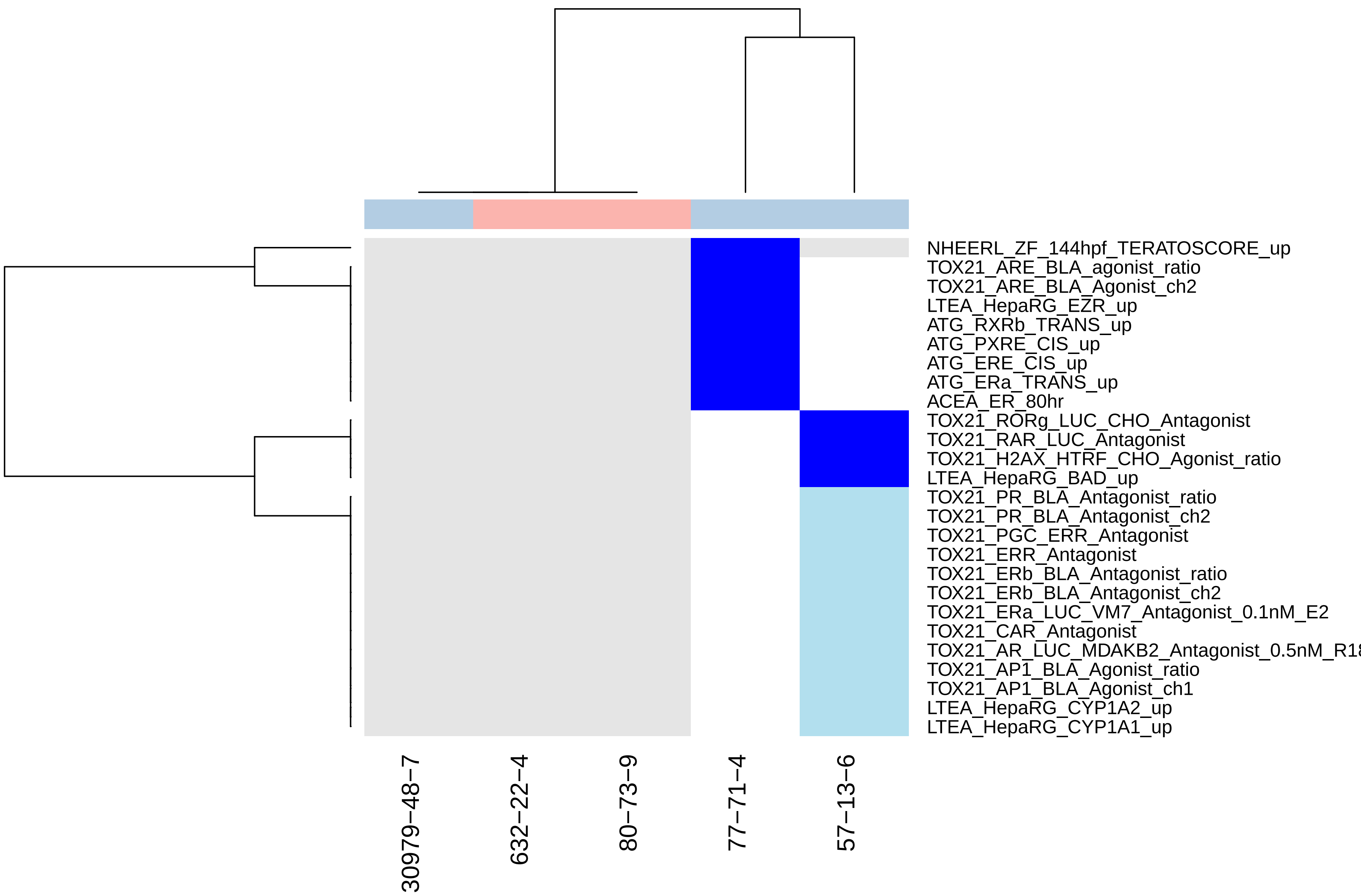

All Activity Heatmap for Cluster 1780

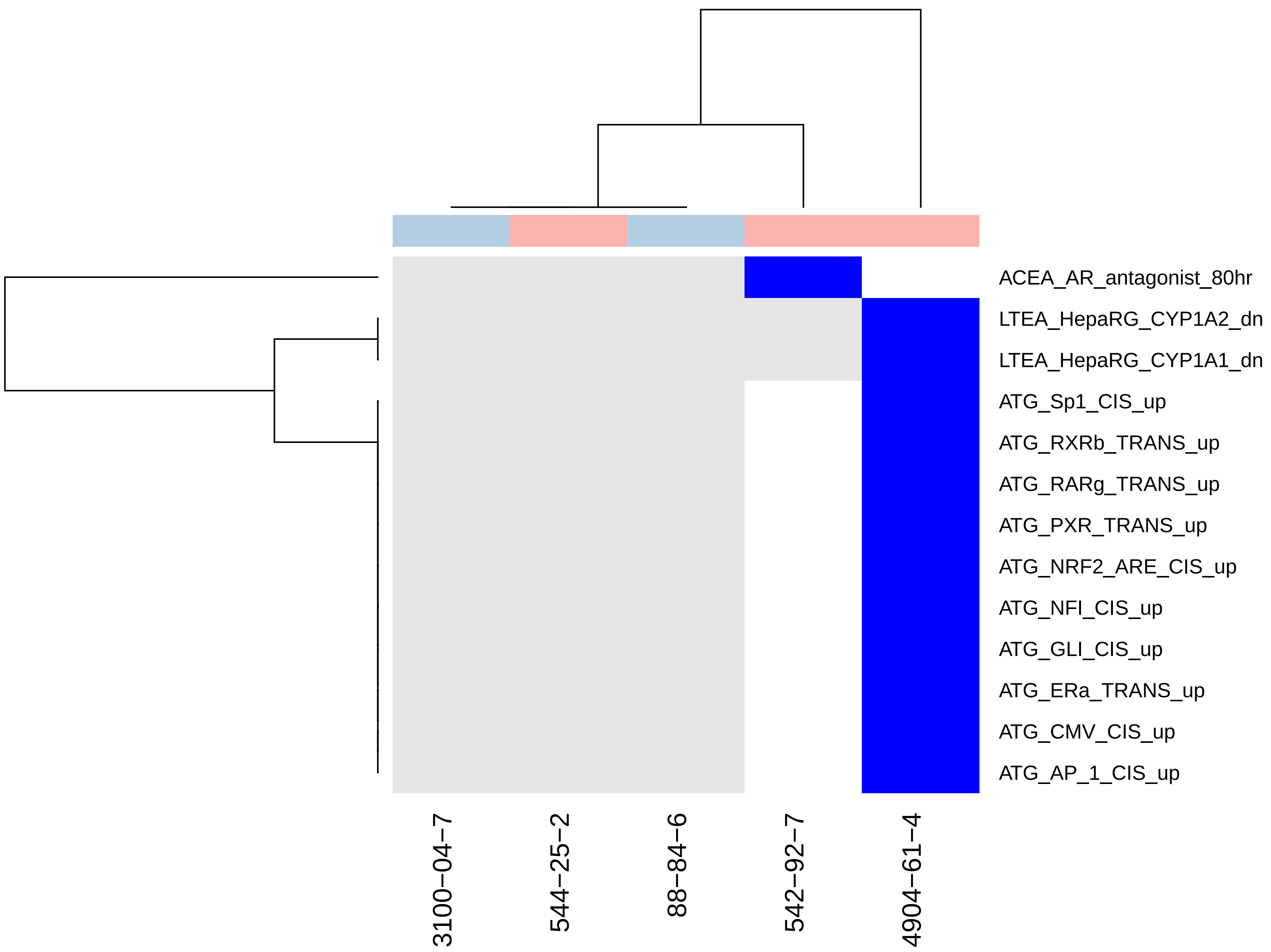

All Activity Heatmap for Cluster 1782

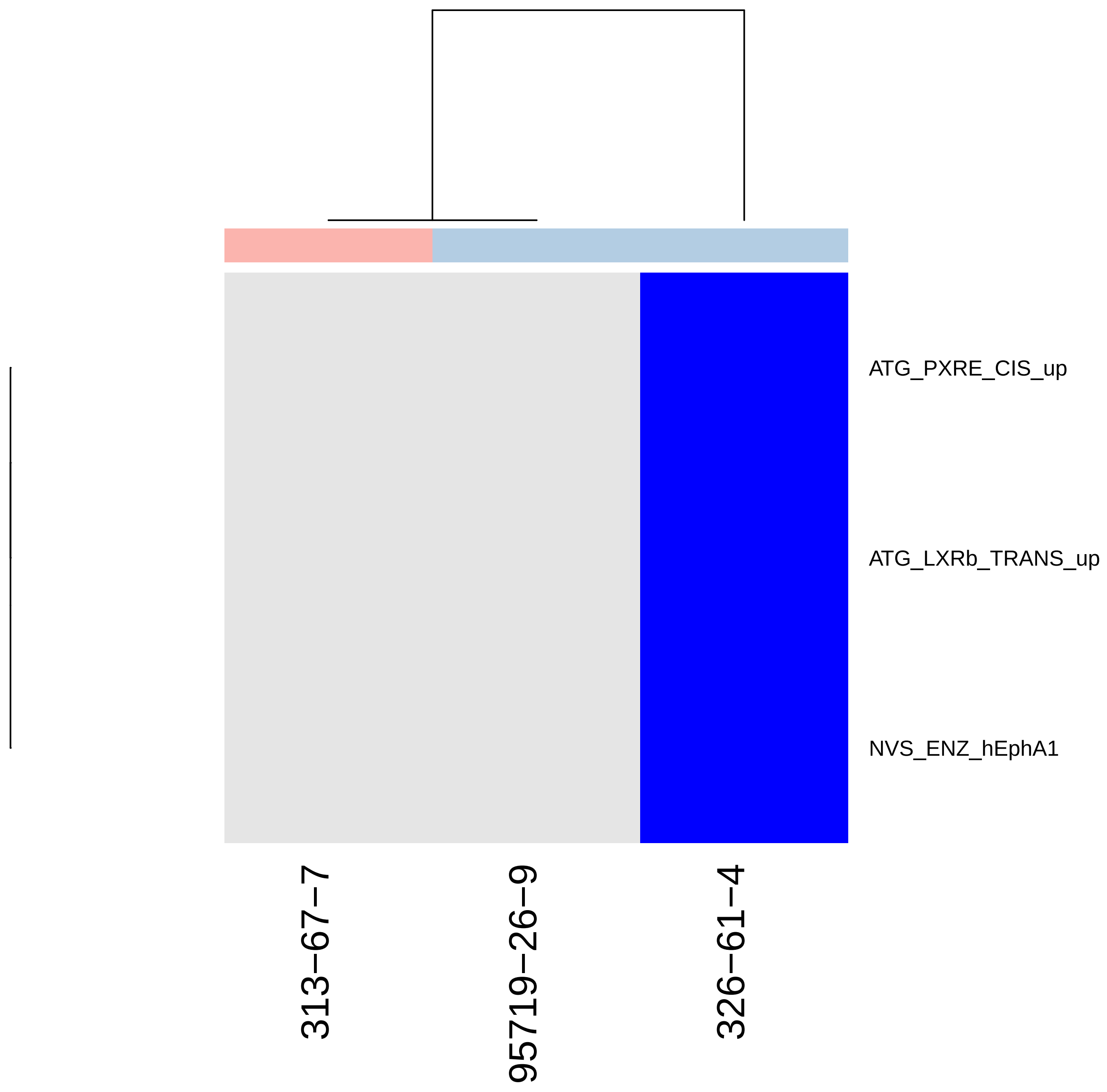

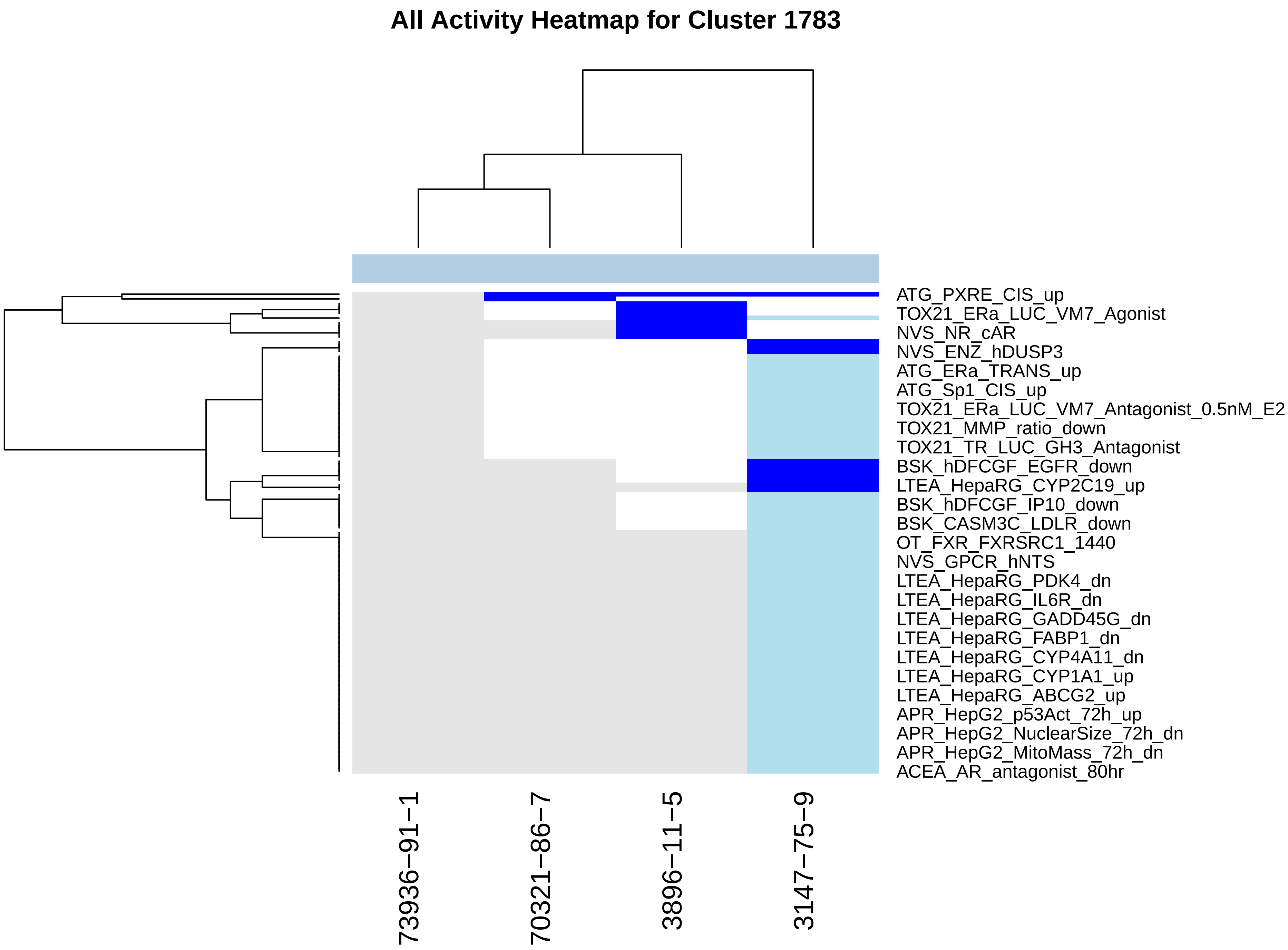
All Activity Heatmap for Cluster 1783
ATG_PXRE_CIS_up
TOX21_ERa_LUC_VM7_Agonist
NVS_NR_cAR
NVS_ENZ_hDUSP3
ATG_ERa_TRANS_up
ATG_Sp1_CIS_up
TOX21_ERa_LUC_VM7_Antagonist_0.5nM_E2
TOX21_MMP_ratio_down
TOX21_TR_LUC_GH3_Antagonist
BSK_hDFCGF_EGFR_down
LTEA_HepaRG_CYP2C19_up
BSK_hDFCGF_IP10_down
BSK_CASM3C_LDLR_down
OT_FXR_FXRSRC1_1440
NVS_GPCR_hNTS
LTEA_HepaRG_PDK4_dn
LTEA_HepaRG_IL6R_dn
LTEA_HepaRG_GADD45G_dn
LTEA_HepaRG_FABP1_dn
LTEA_HepaRG_CYP4A11_dn
LTEA_HepaRG_CYP1A1_up
LTEA_HepaRG_ABCG2_up
APR_HepG2_p53Act_72h_up
APR_HepG2_NuclearSize_72h_dn
APR_HepG2_MitoMass_72h_dn
ACEA_AR_antagonist_80hr
73936−91−1
70321−86−7
3896−11−5
3147−75−9

All Activity Heatmap for Cluster 1785

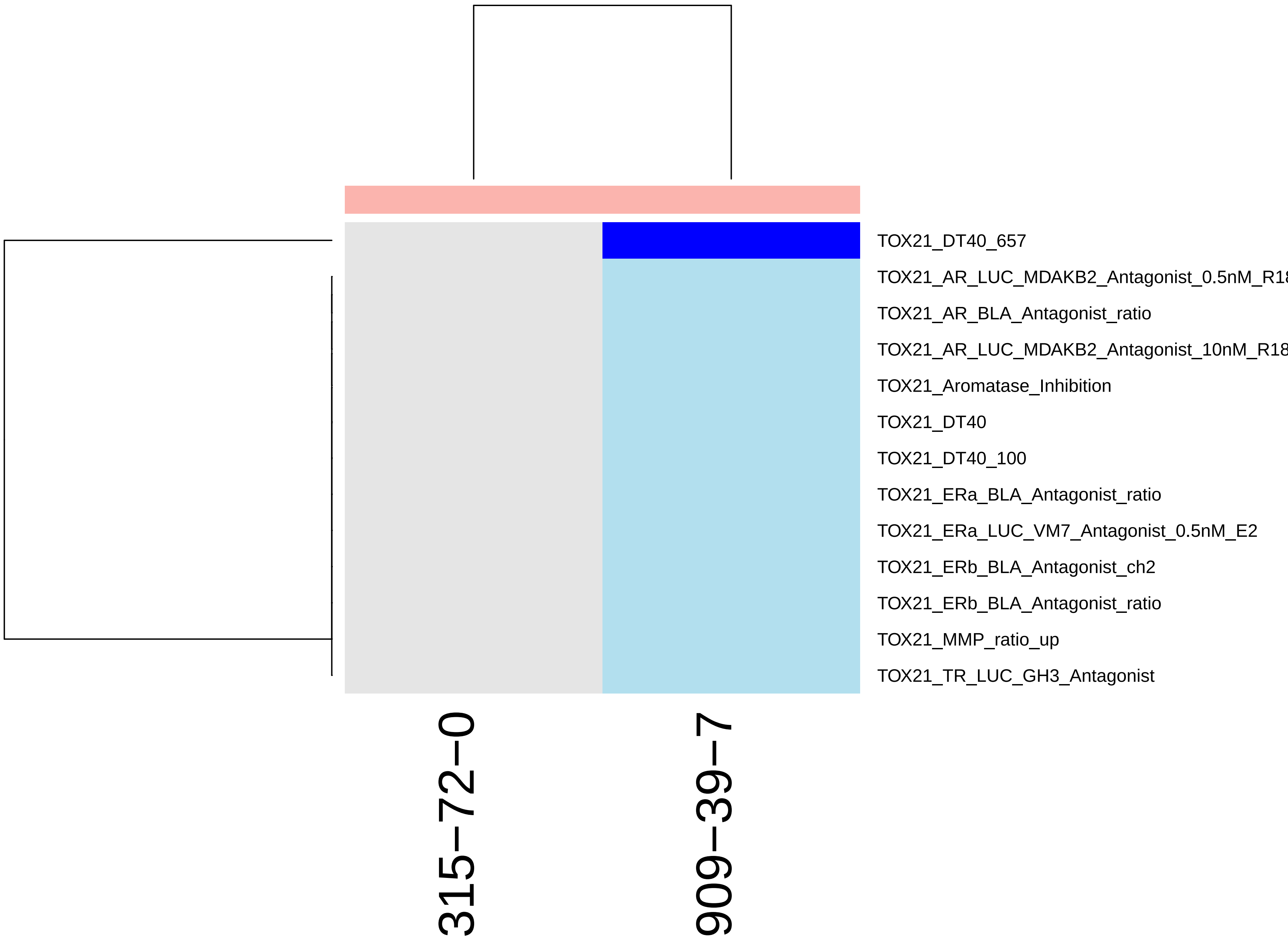

All Activity Heatmap for Cluster 1789

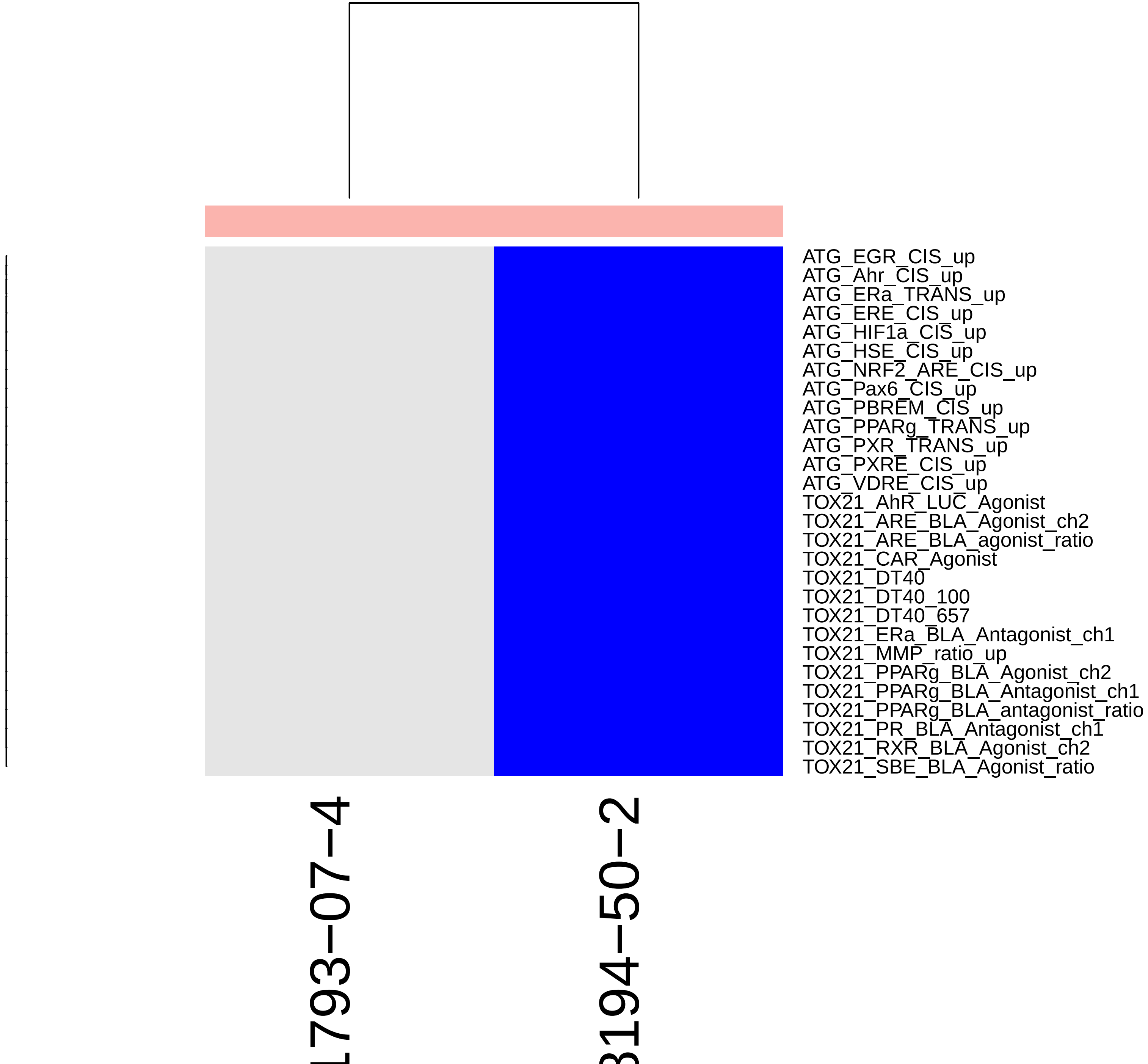

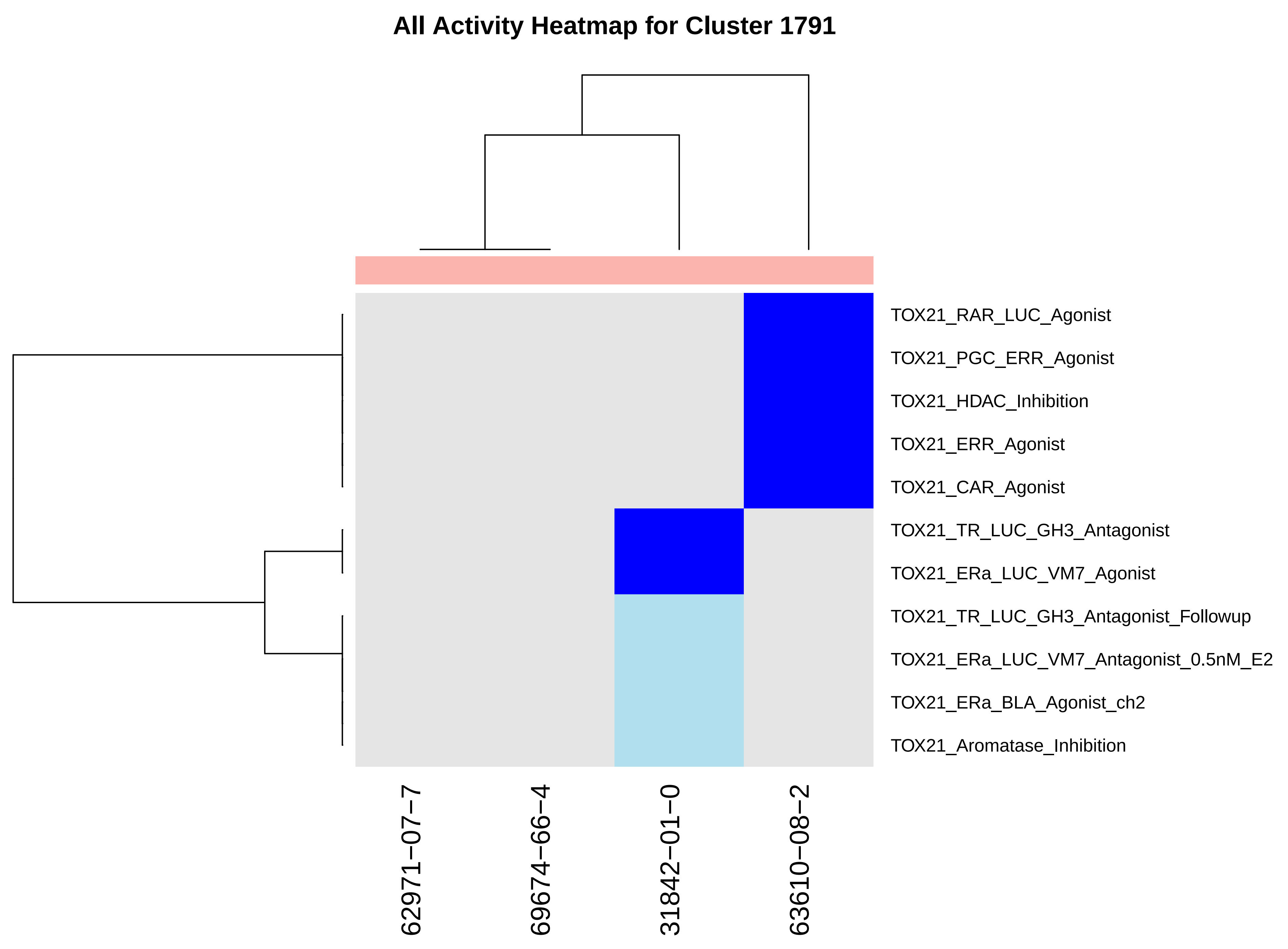
All Activity Heatmap for Cluster 1791
TOX21_RAR_LUC_Agonist
TOX21_PGC_ERR_Agonist
TOX21_HDAC_Inhibition
TOX21_ERR_Agonist
TOX21_CAR_Agonist
TOX21_TR_LUC_GH3_Antagonist
TOX21_ERa_LUC_VM7_Agonist
TOX21_TR_LUC_GH3_Antagonist_Followup
TOX21_ERa_LUC_VM7_Antagonist_0.5nM_E2
TOX21_ERa_BLA_Agonist_ch2
TOX21_Aromatase_Inhibition
62971−07−7
69674−66−4
31842−01−0
63610−08−2

All Activity Heatmap for Cluster 1792

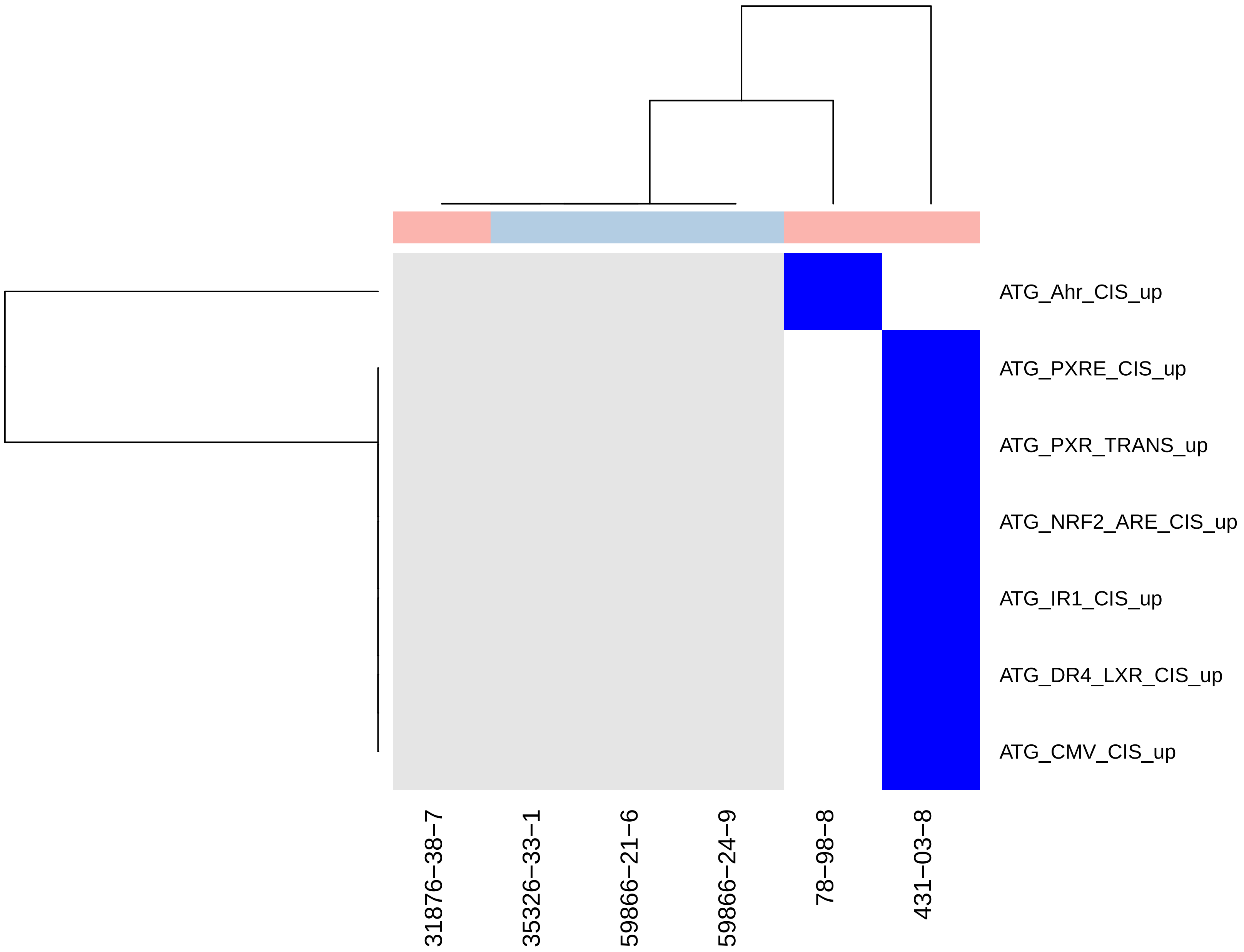

All Activity Heatmap for Cluster 1794

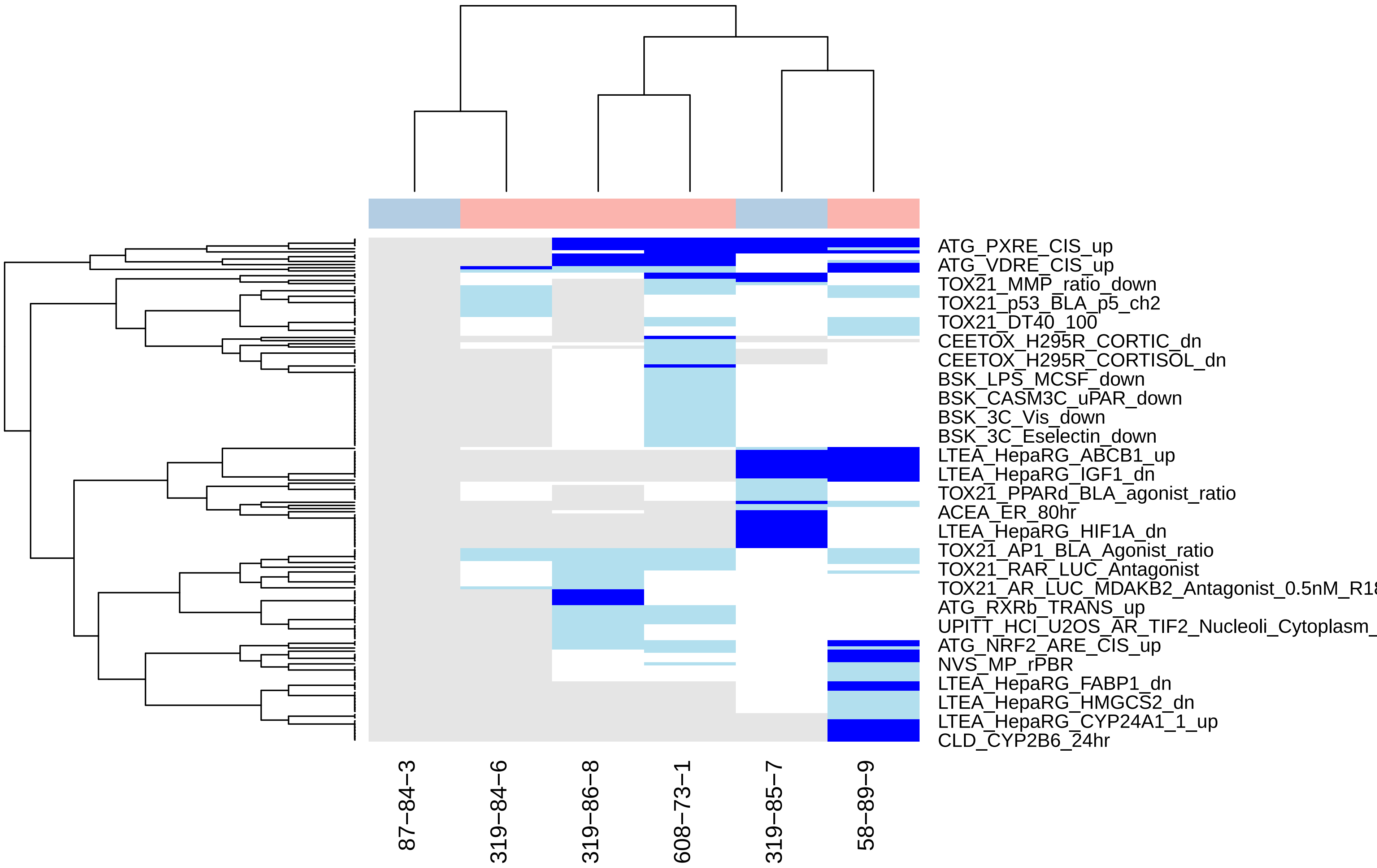

All Activity Heatmap for Cluster 1795

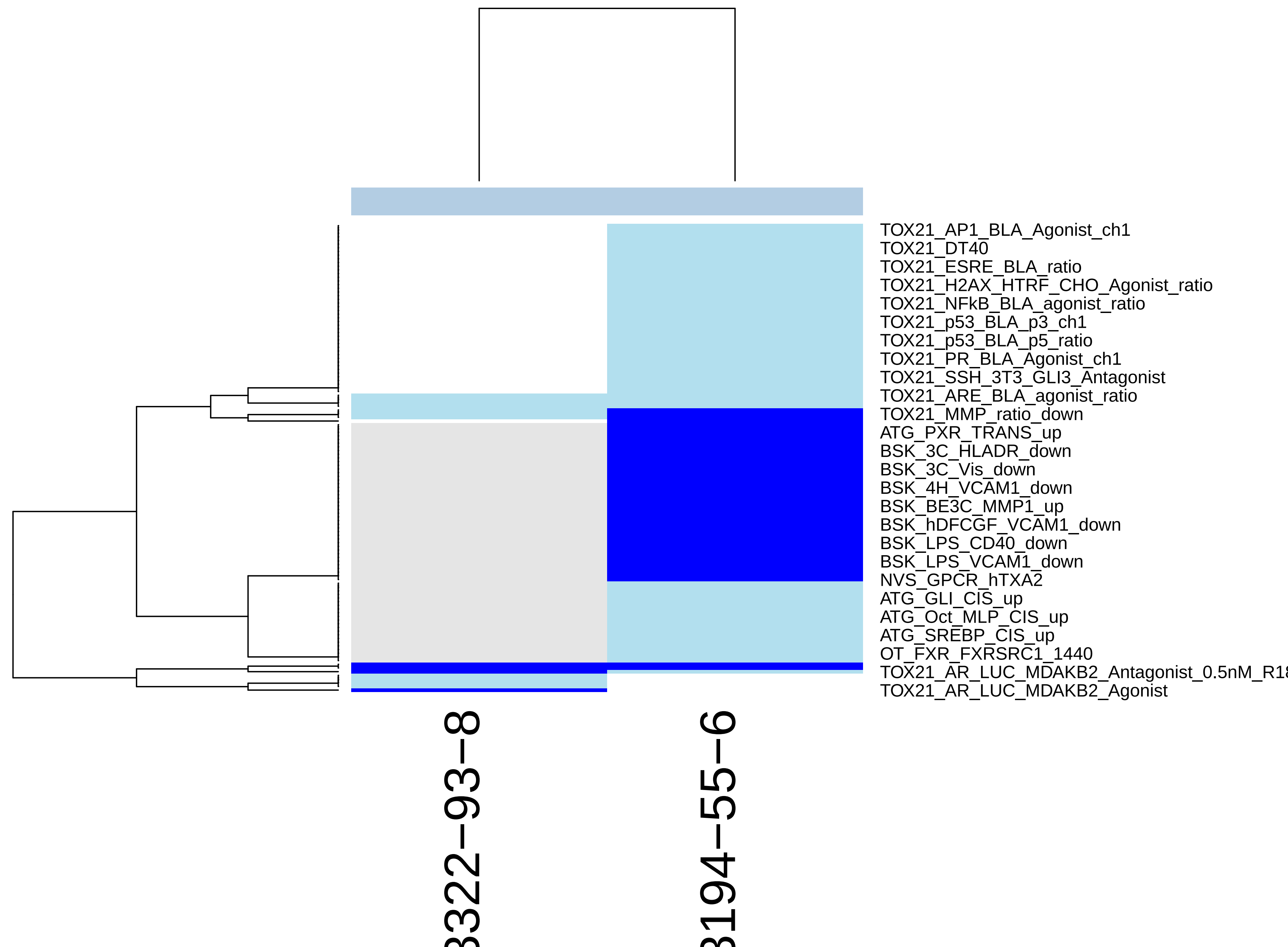

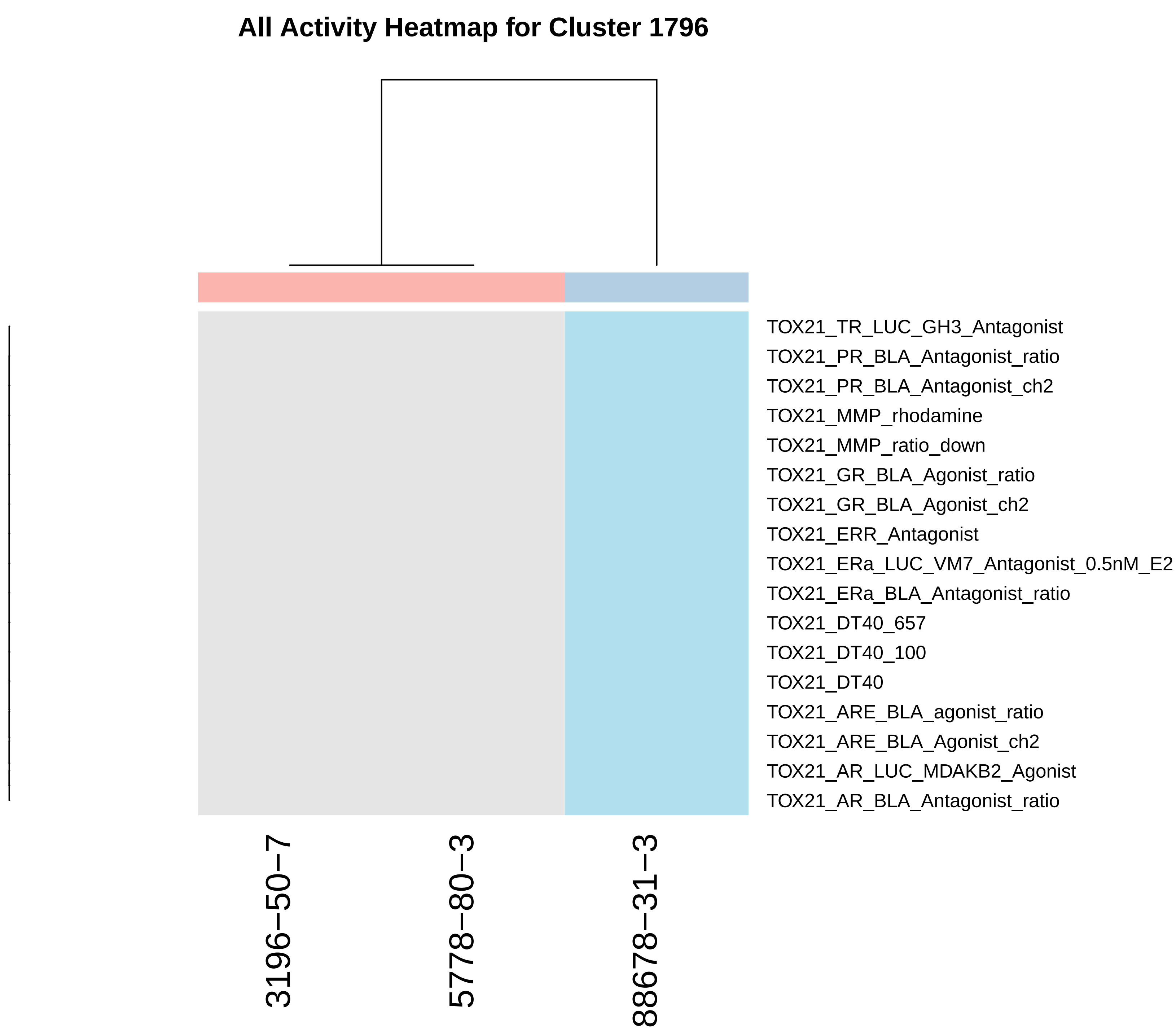
All Activity Heatmap for Cluster 1796
TOX21_TR_LUC_GH3_Antagonist
TOX21_PR_BLA_Antagonist_ratio
TOX21_PR_BLA_Antagonist_ch2
TOX21_MMP_rhodamine
TOX21_MMP_ratio_down
TOX21_GR_BLA_Agonist_ratio
TOX21_GR_BLA_Agonist_ch2
TOX21_ERR_Antagonist
TOX21_ERa_LUC_VM7_Antagonist_0.5nM_E2
TOX21_ERa_BLA_Antagonist_ratio
TOX21_DT40_657
TOX21_DT40_100
TOX21_DT40
TOX21_ARE_BLA_agonist_ratio
TOX21_ARE_BLA_Agonist_ch2
TOX21_AR_LUC_MDAKB2_Agonist
TOX21_AR_BLA_Antagonist_ratio
3196−50−7
5778−80−3
88678−31−3

All Activity Heatmap for Cluster 1798

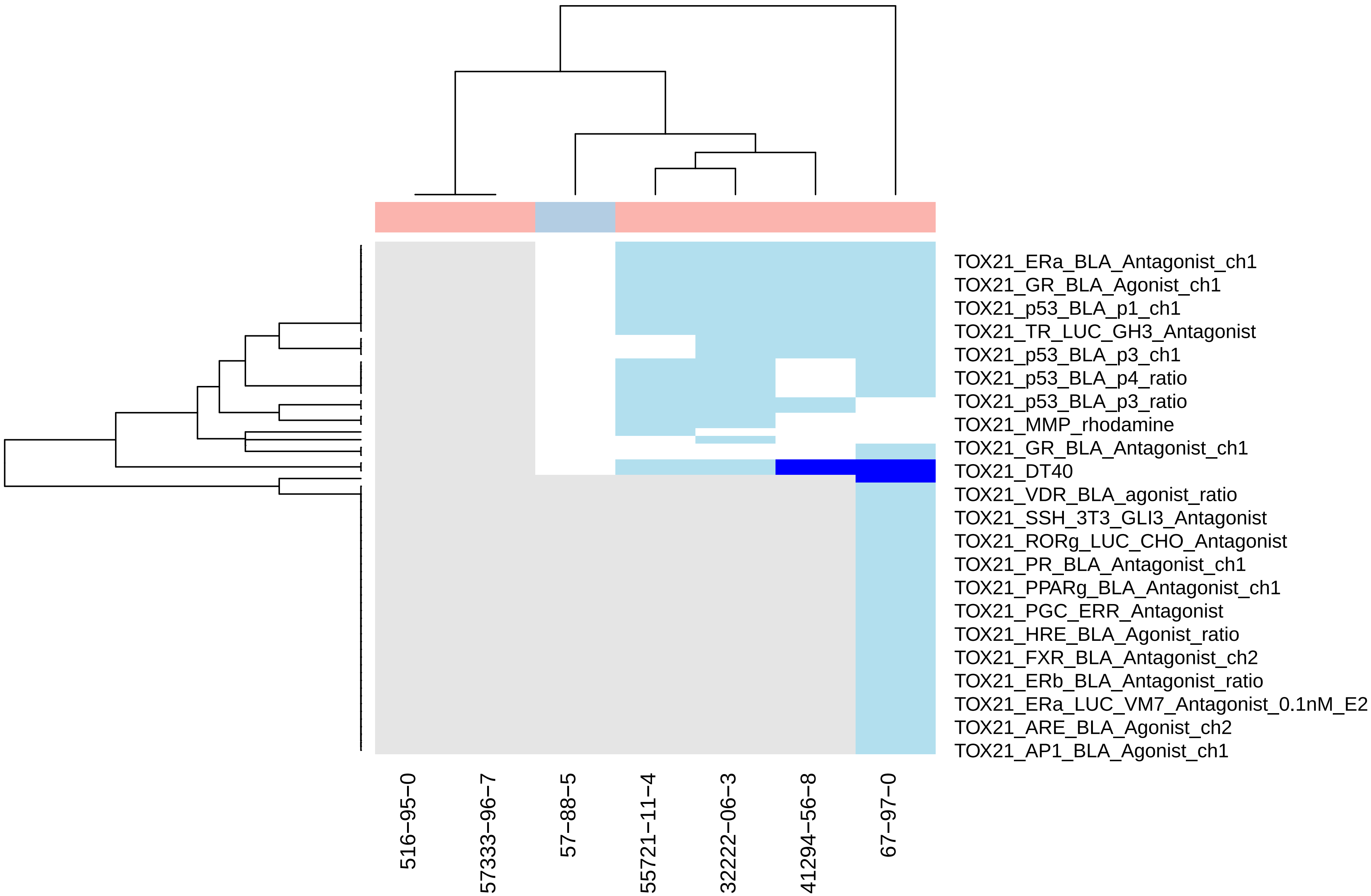

All Activity Heatmap for Cluster 1799

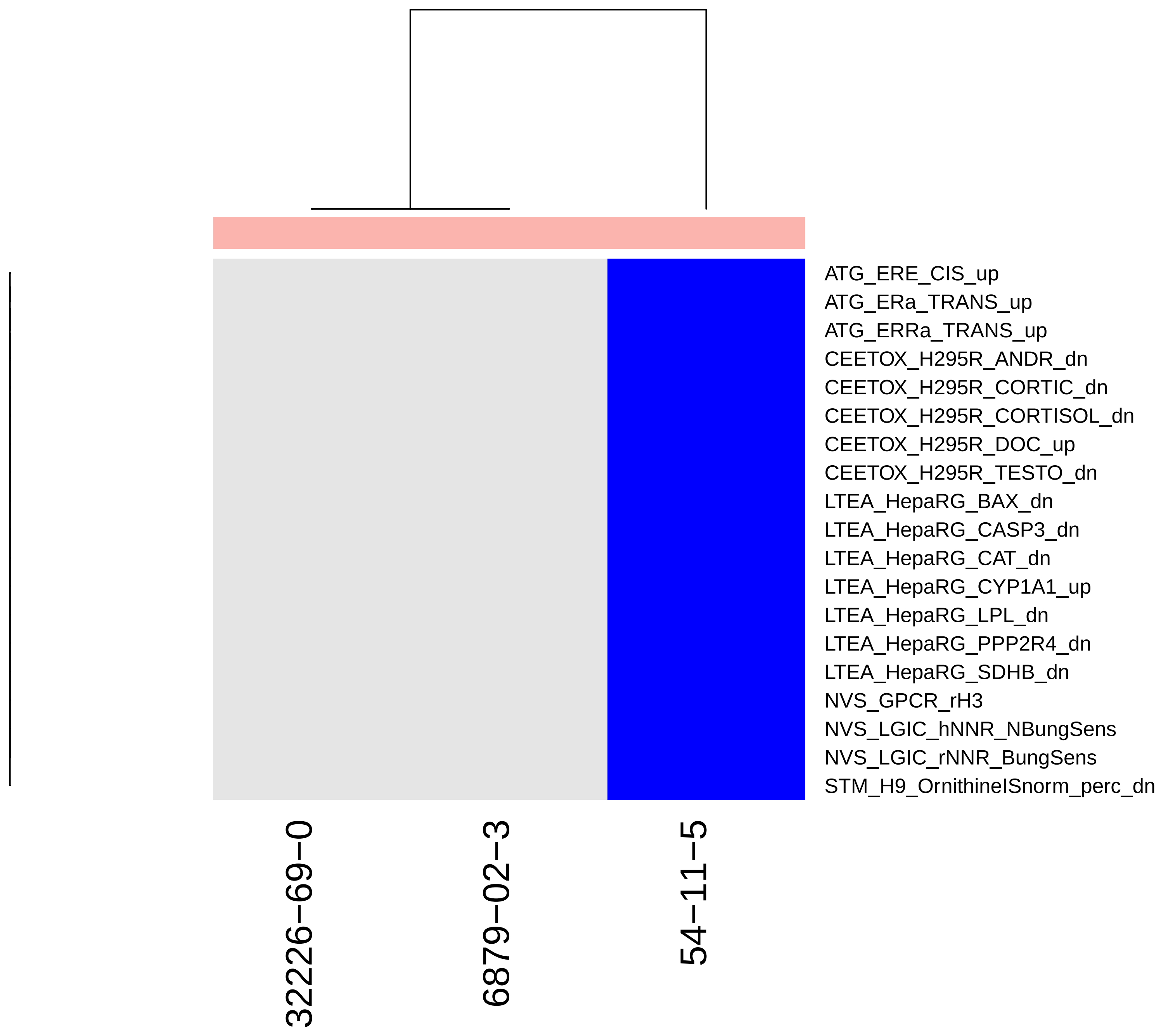

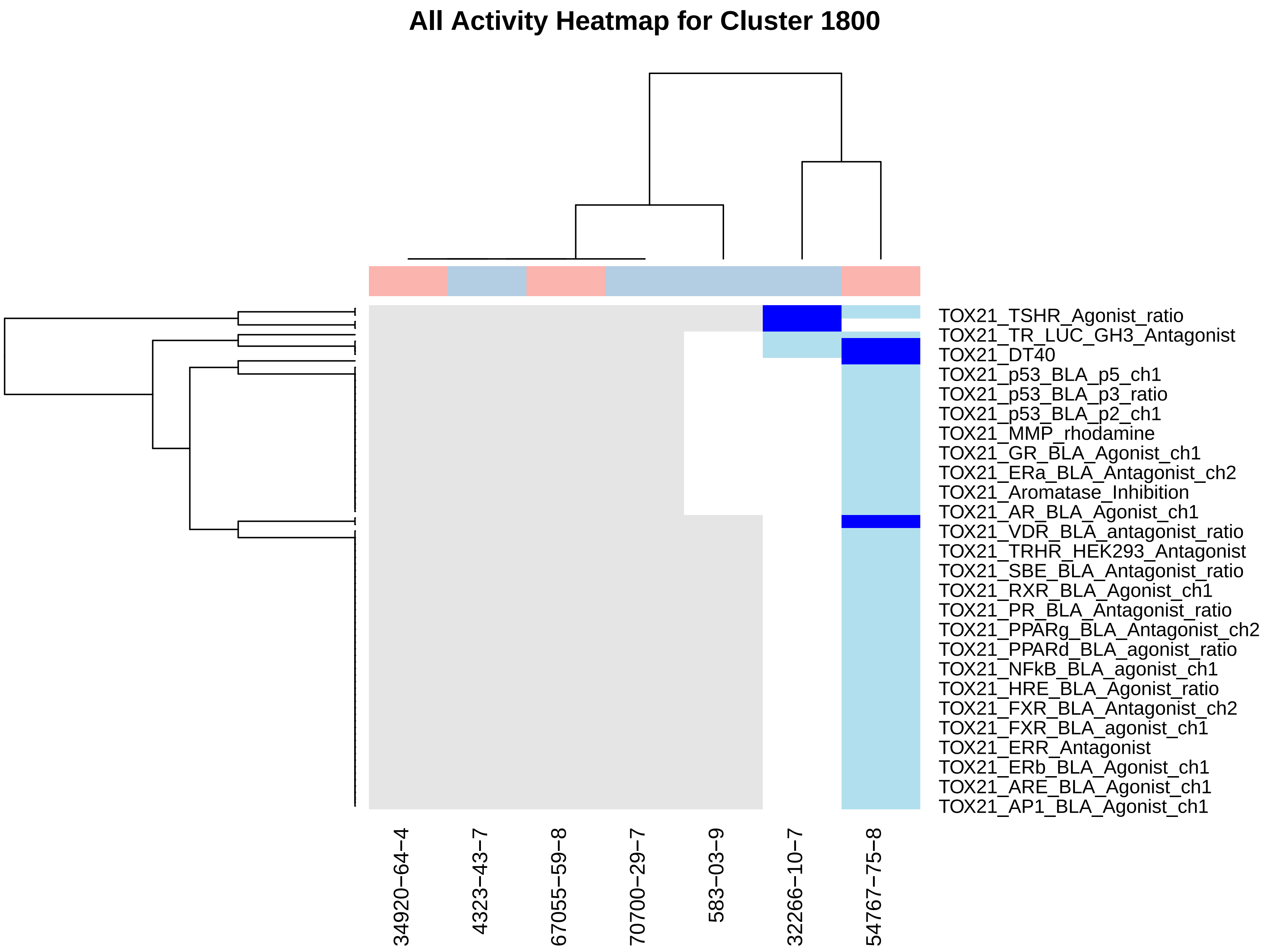

All Activity Heatmap for Cluster 1800
TOX21_TSHR_Agonist_ratio
TOX21_TR_LUC_GH3_Antagonist
TOX21_DT40
TOX21_p53_BLA_p5_ch1
TOX21_p53_BLA_p3_ratio
TOX21_p53_BLA_p2_ch1
TOX21_MMP_rhodamine
TOX21_GR_BLA_Agonist_ch1
TOX21_ERa_BLA_Antagonist_ch2
TOX21_Aromatase_Inhibition
TOX21_AR_BLA_Agonist_ch1
TOX21_VDR_BLA_antagonist_ratio
TOX21_TRHR_HEK293_Antagonist
TOX21_SBE_BLA_Antagonist_ratio
TOX21_RXR_BLA_Agonist_ch1
TOX21_PR_BLA_Antagonist_ratio
TOX21_PPARg_BLA_Antagonist_ch2
TOX21_PPARd_BLA_agonist_ratio
TOX21_NFkB_BLA_agonist_ch1
TOX21_HRE_BLA_Agonist_ratio
TOX21_FXR_BLA_Antagonist_ch2
TOX21_FXR_BLA_agonist_ch1
TOX21_ERR_Antagonist
TOX21_ERb_BLA_Agonist_ch1
TOX21_ARE_BLA_Agonist_ch1
TOX21_AP1_BLA_Agonist_ch1
34920−64−4
4323−43−7
67055−59−8
70700−29−7
583−03−9
32266−10−7
54767−75−8

All Activity Heatmap for Cluster 1803

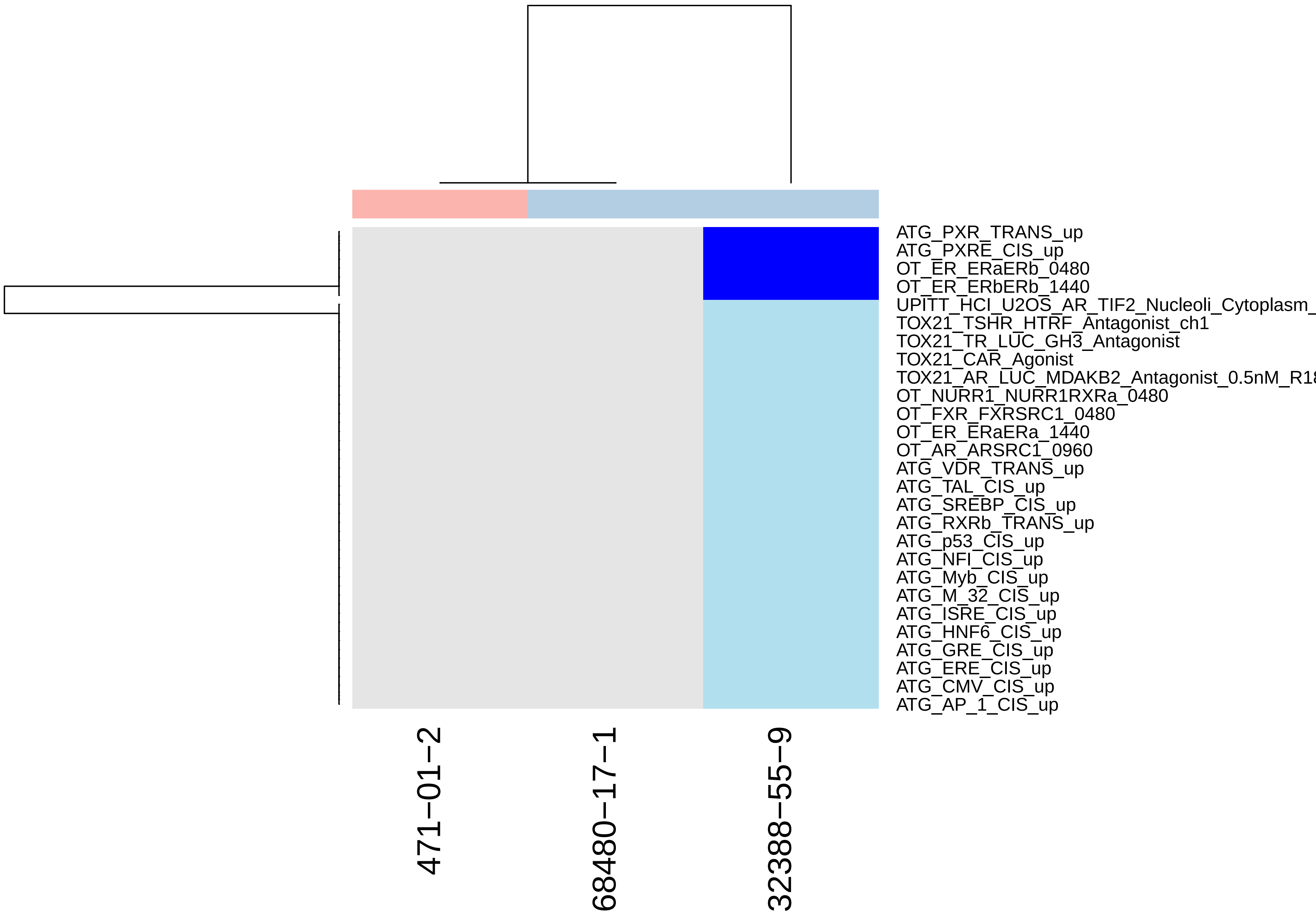

All Activity Heatmap for Cluster 1804

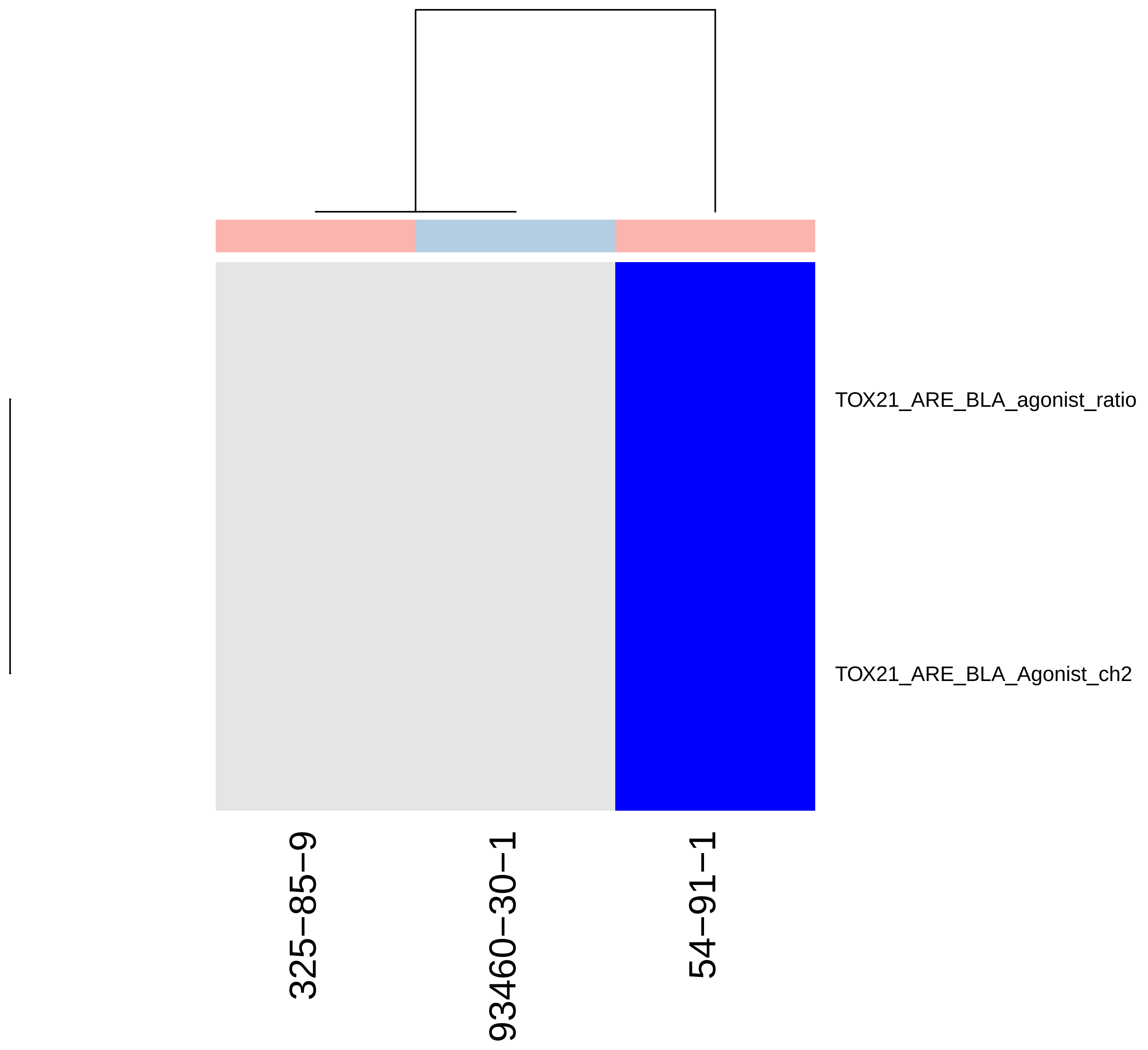

All Activity Heatmap for Cluster 1809

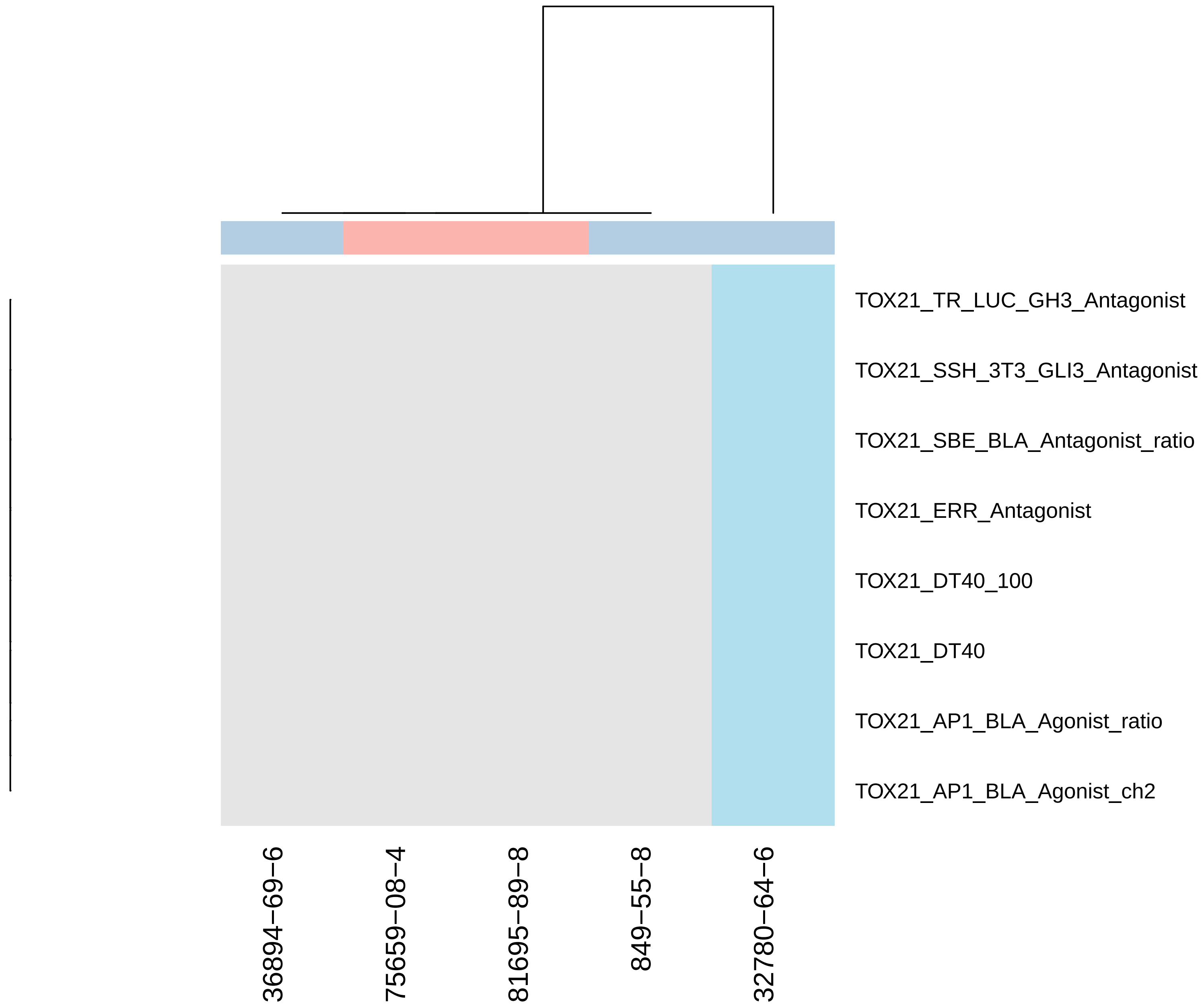

All Activity Heatmap for Cluster 1811

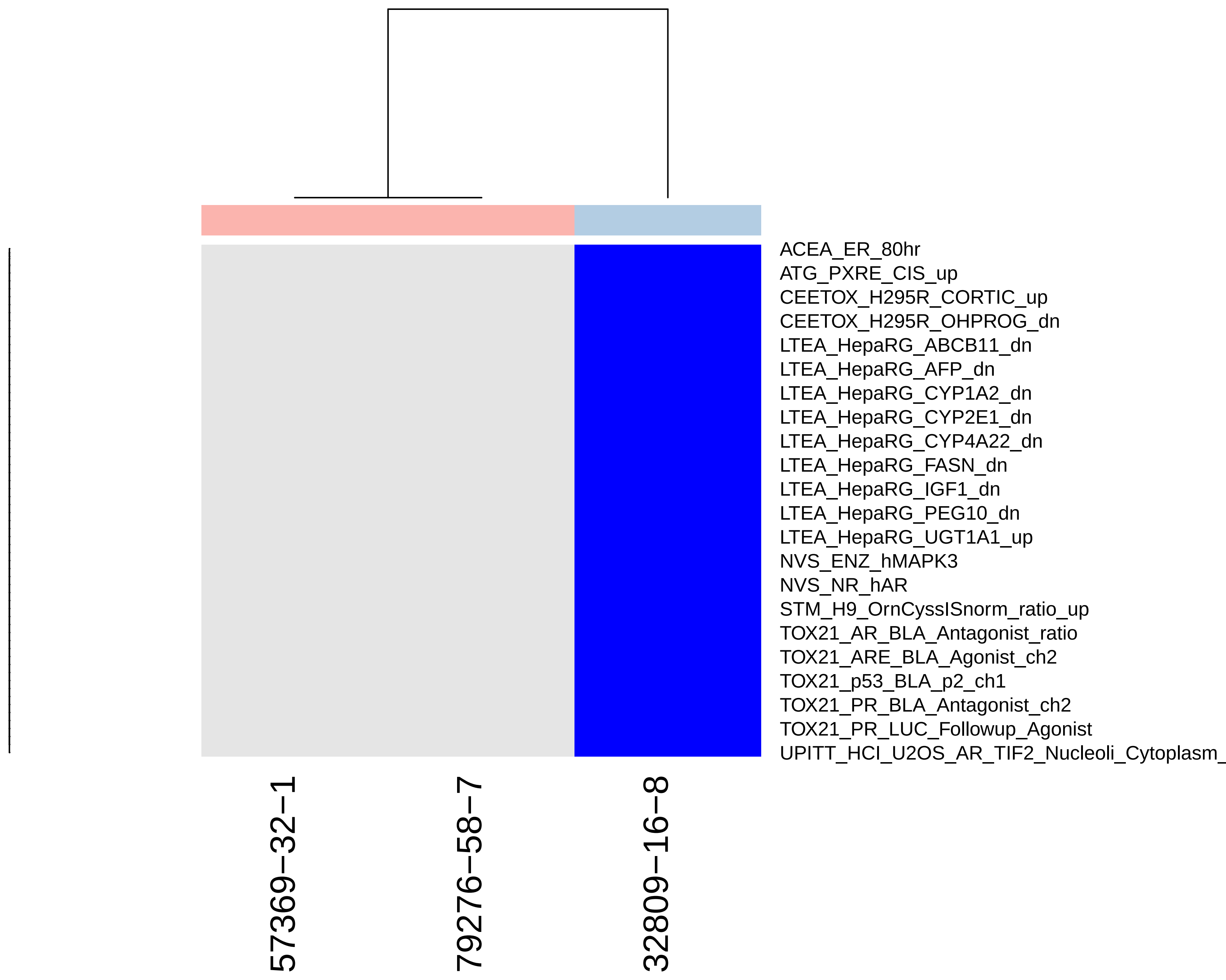

All Activity Heatmap for Cluster 1812

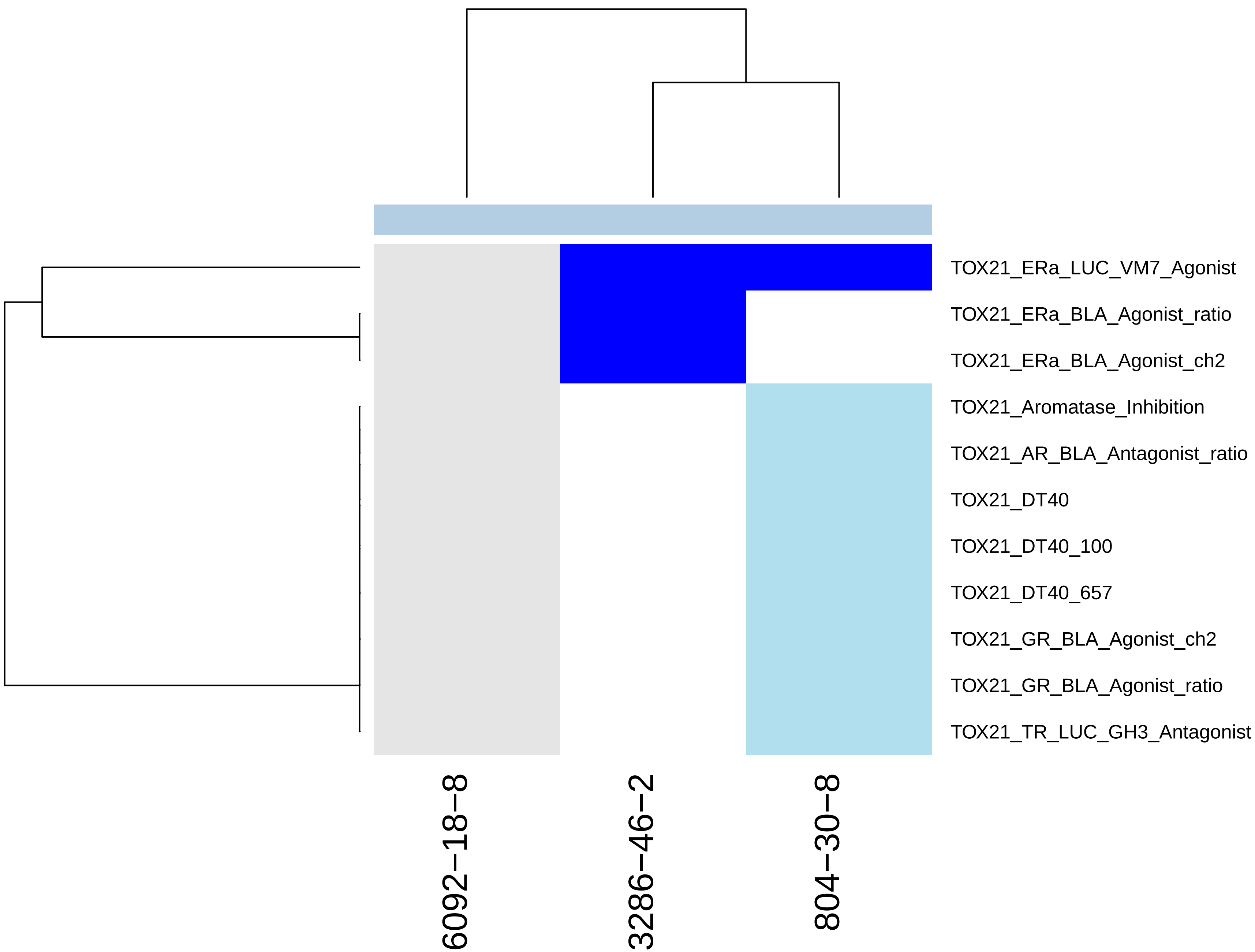

All Activity Heatmap for Cluster 1813

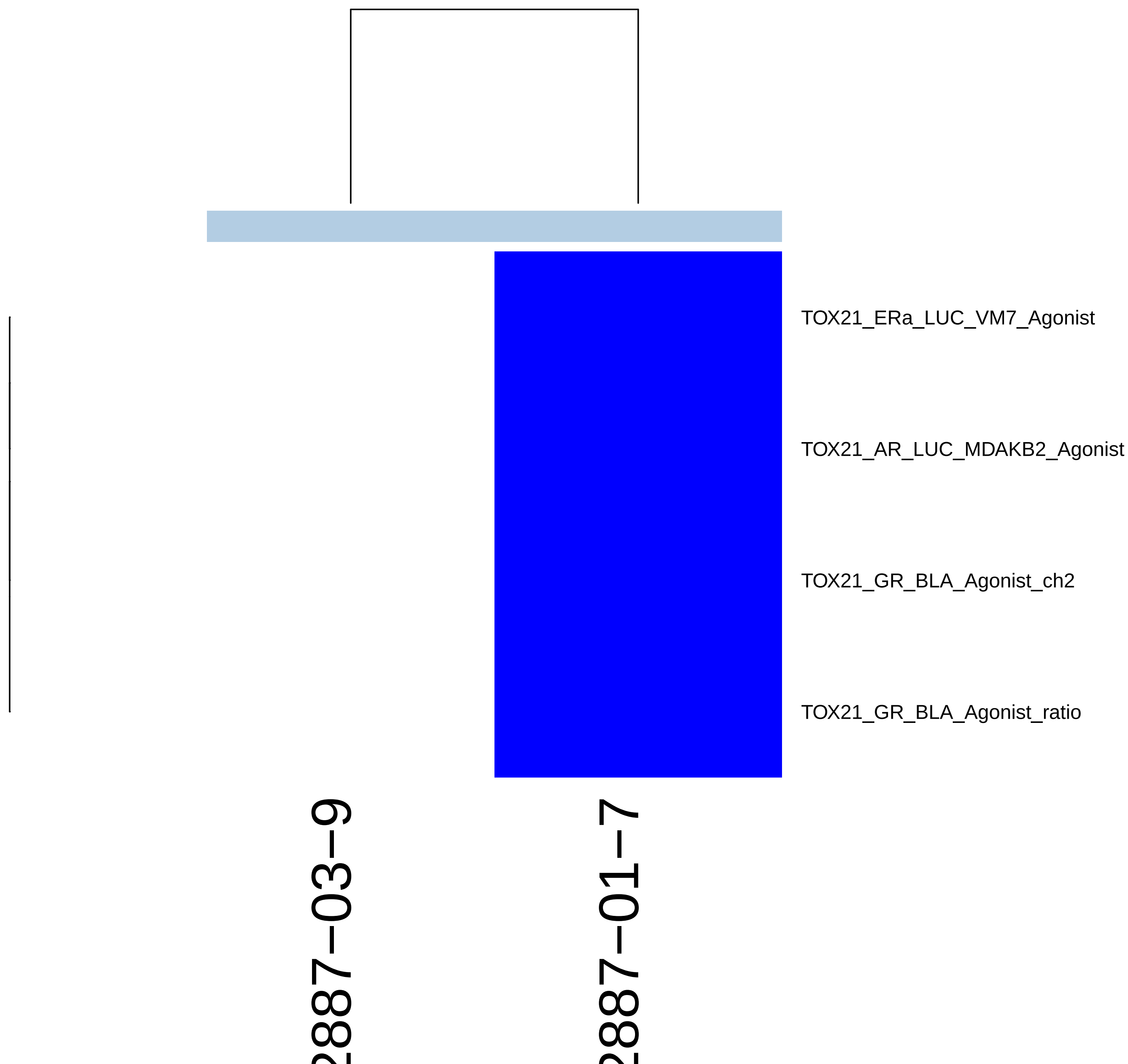

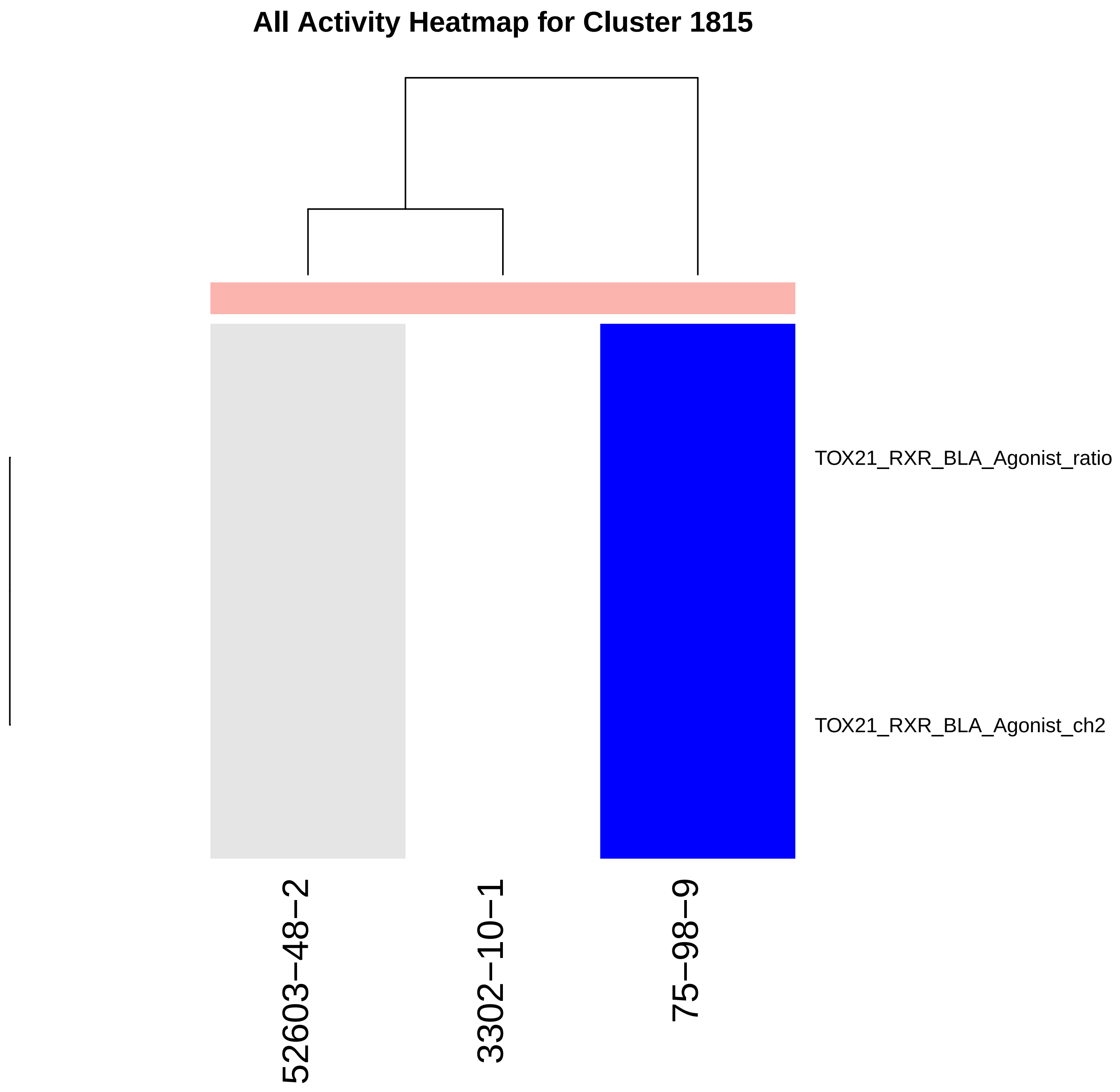
All Activity Heatmap for Cluster 1815
TOX21_RXR_BLA_Agonist_ratio
TOX21_RXR_BLA_Agonist_ch2
52603−48−2
3302−10−1
75−98−9

All Activity Heatmap for Cluster 1816

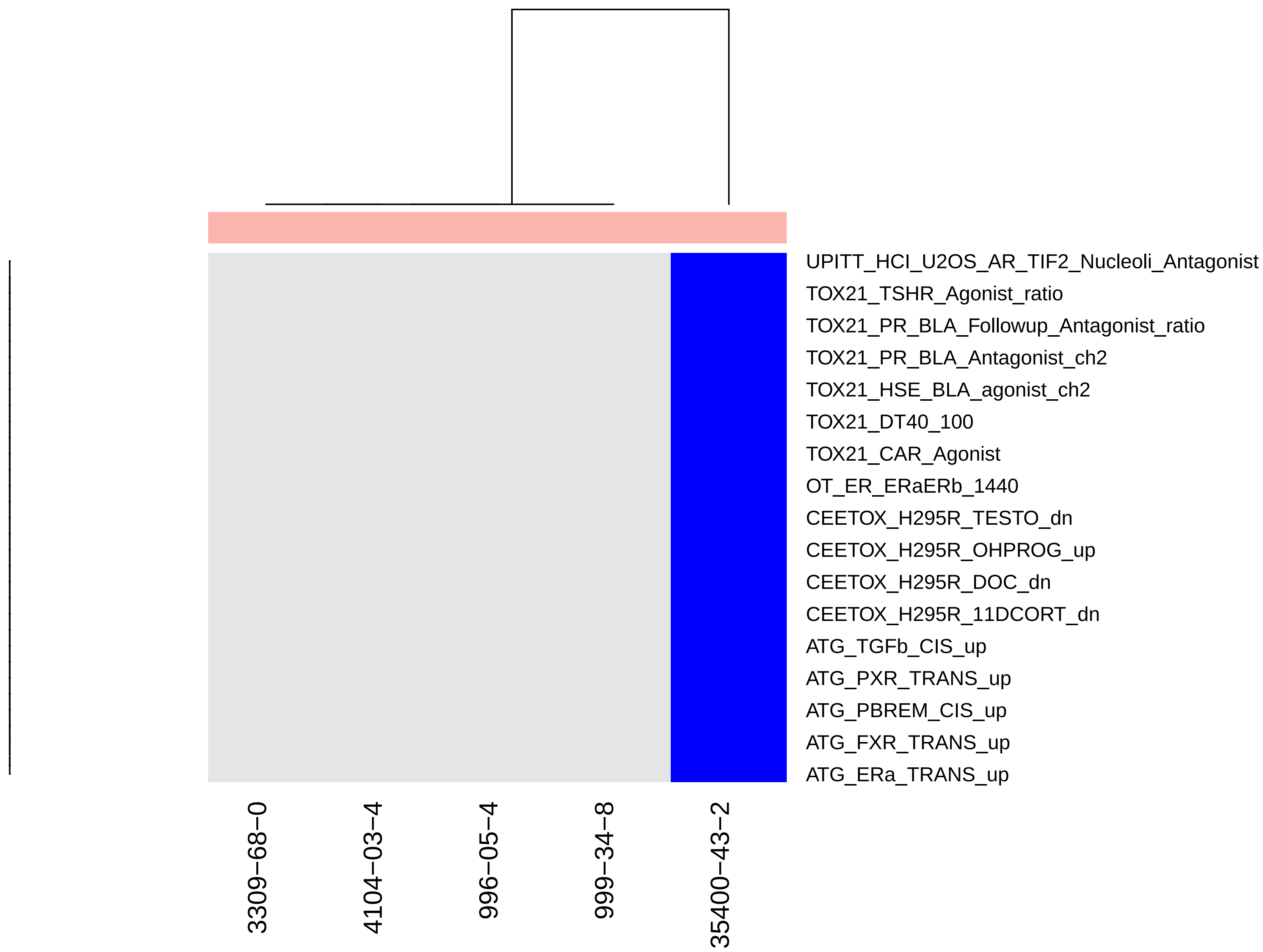

All Activity Heatmap for Cluster 1817

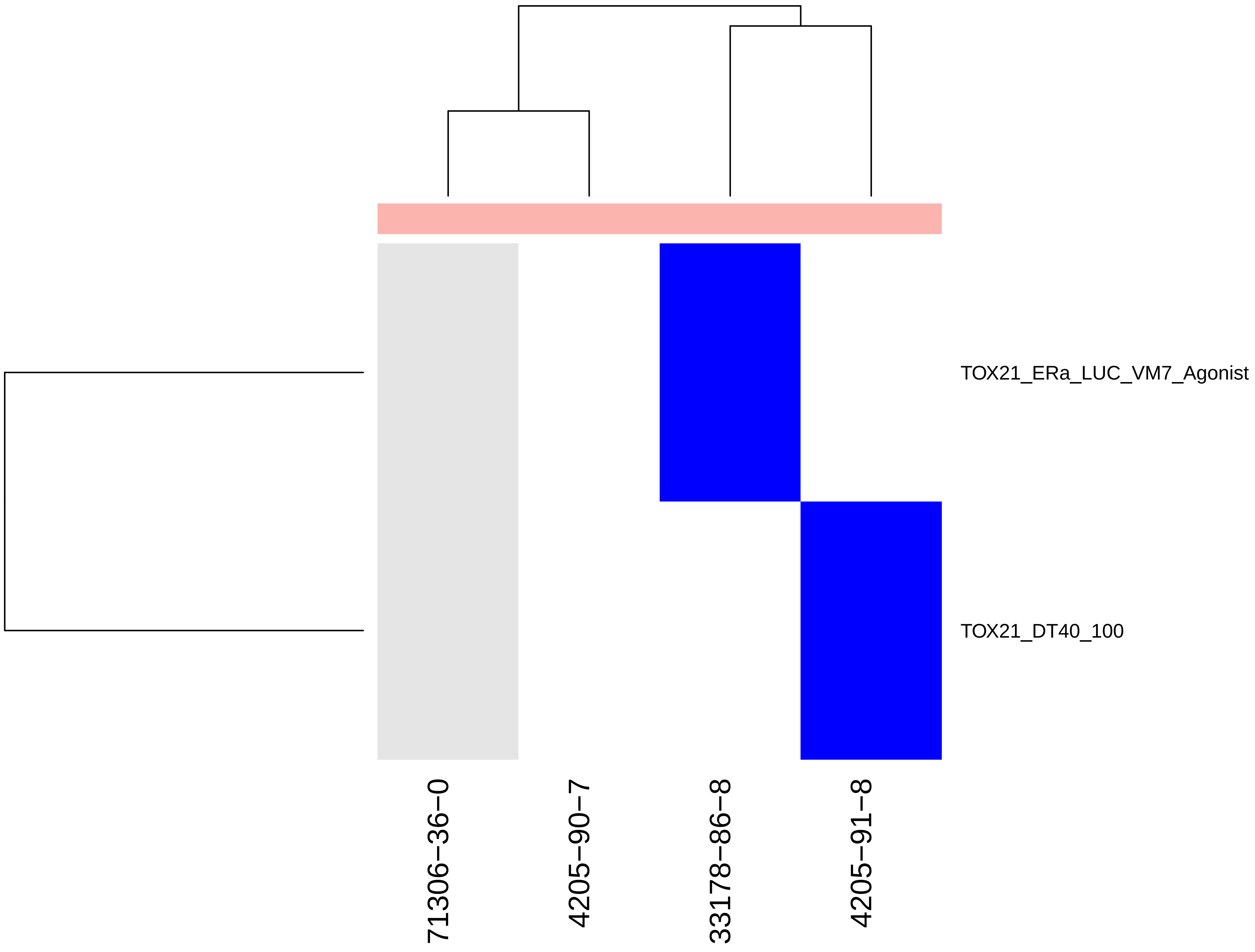

All Activity Heatmap for Cluster 1821

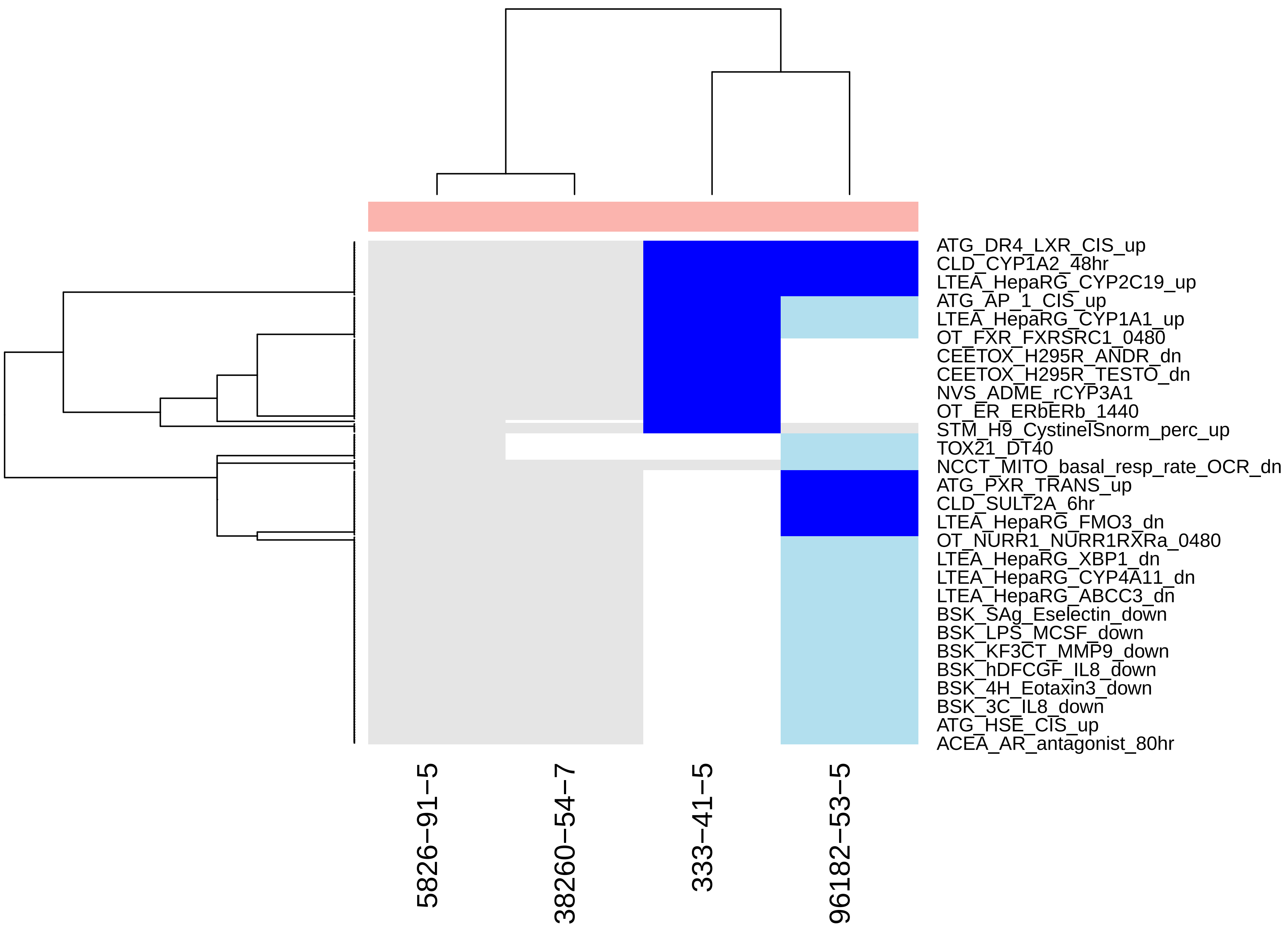

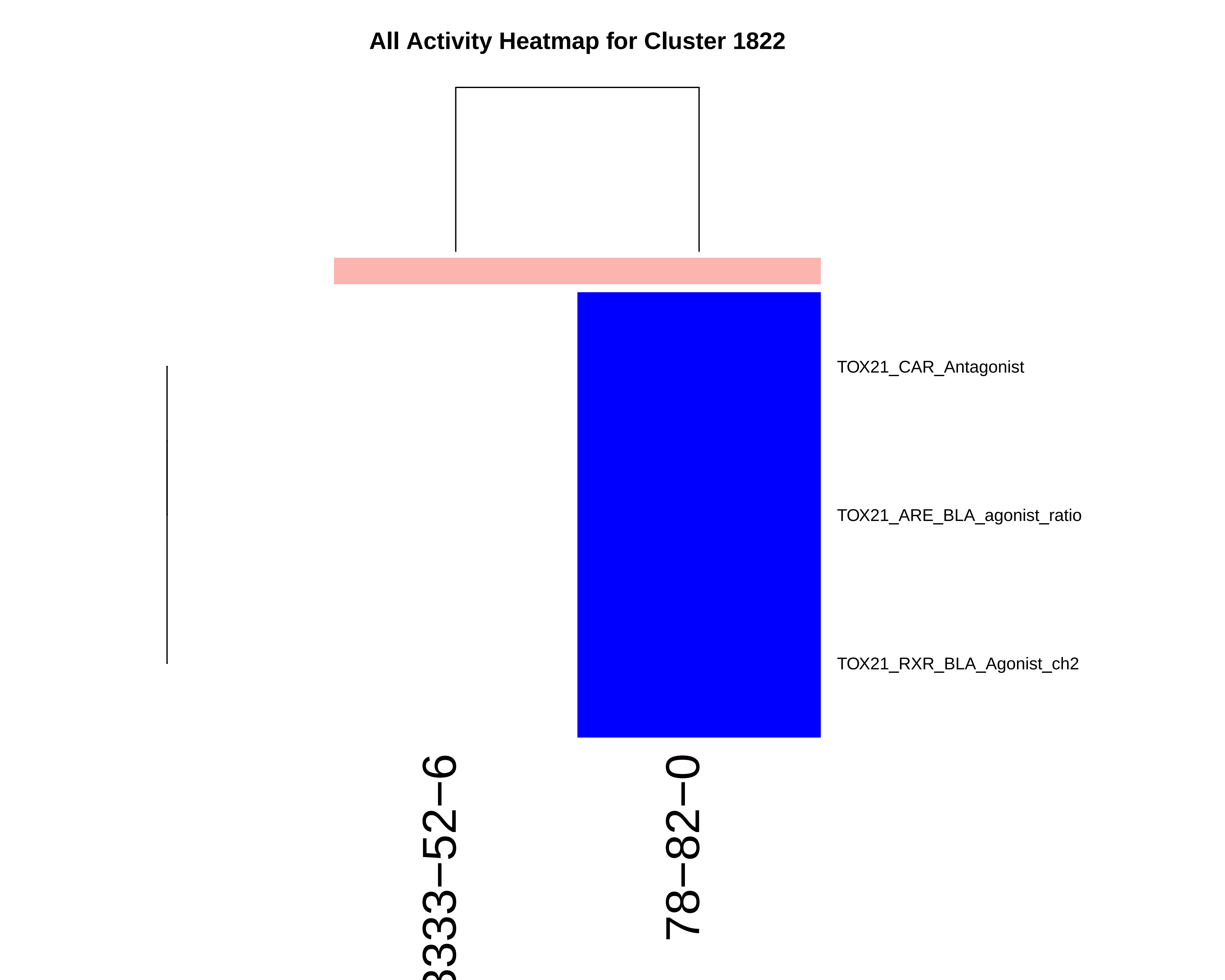

All Activity Heatmap for Cluster 1822
TOX21_CAR_Antagonist
TOX21_ARE_BLA_agonist_ratio
TOX21_RXR_BLA_Agonist_ch2
333−52−6
78−82−0

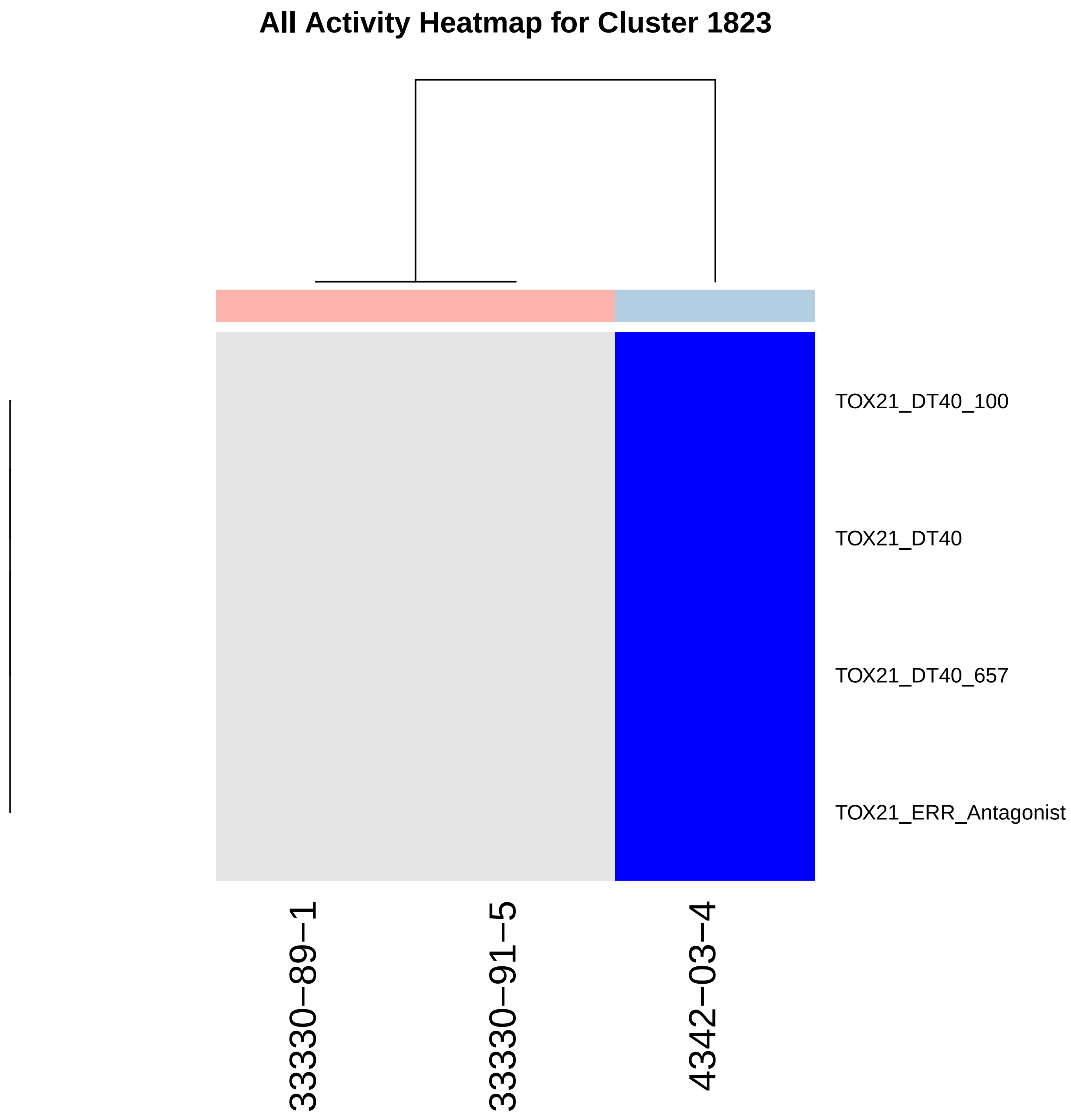
All Activity Heatmap for Cluster 1823
TOX21_DT40_100
TOX21_DT40
TOX21_DT40_657
TOX21_ERR_Antagonist
33330−89−1
33330−91−5
4342−03−4

All Activity Heatmap for Cluster 1826

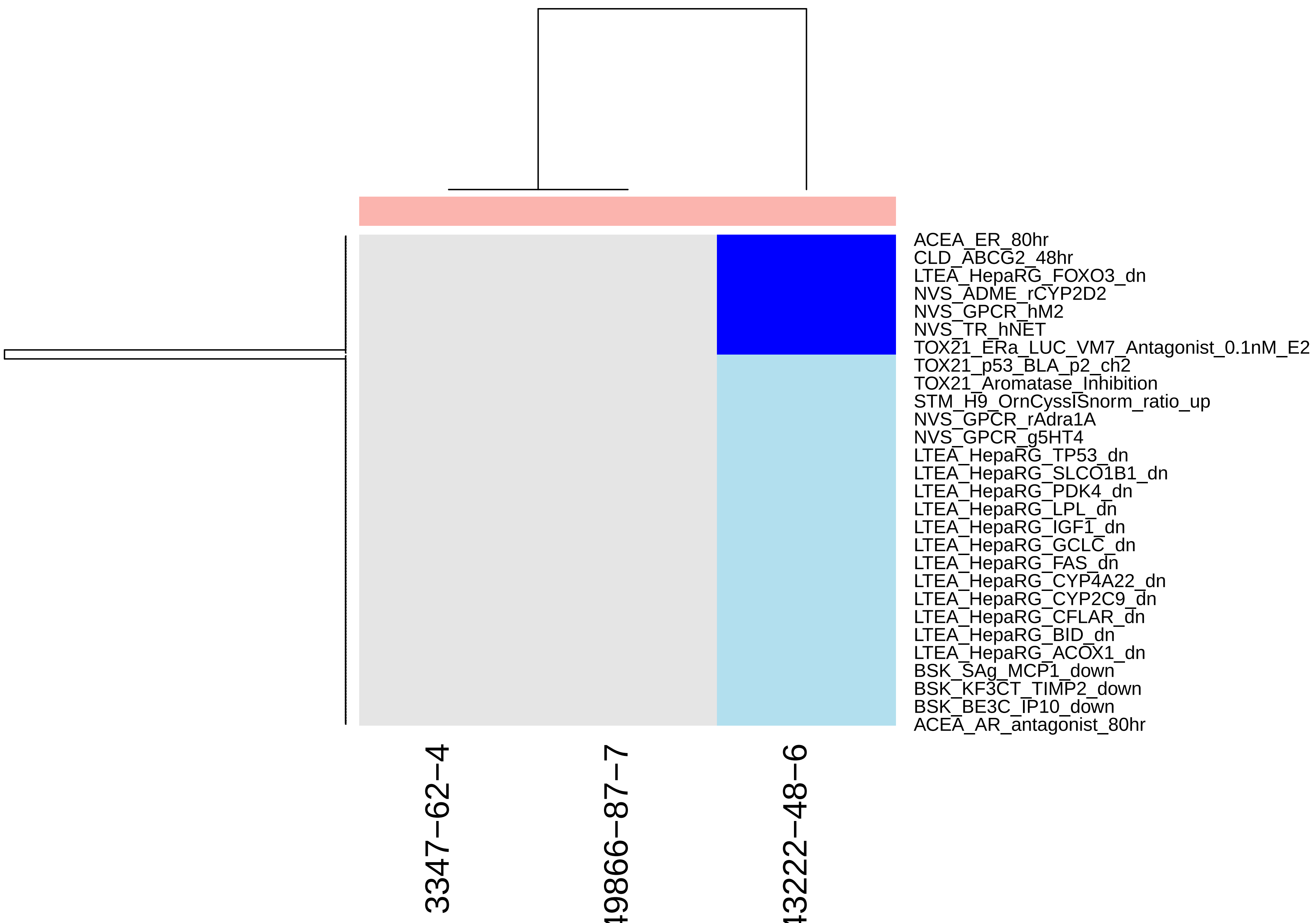

All Activity Heatmap for Cluster 1827

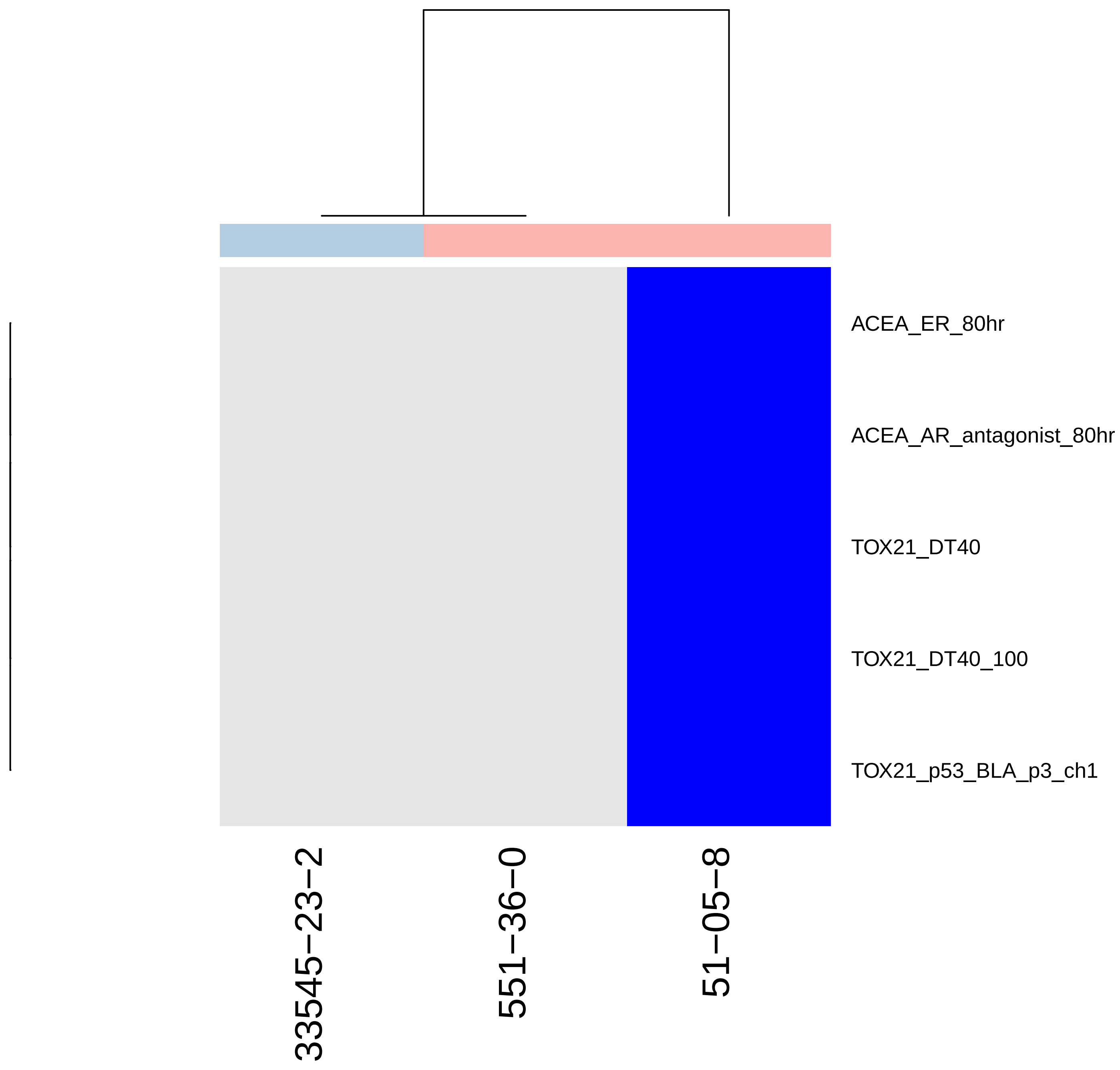

All Activity Heatmap for Cluster 1832

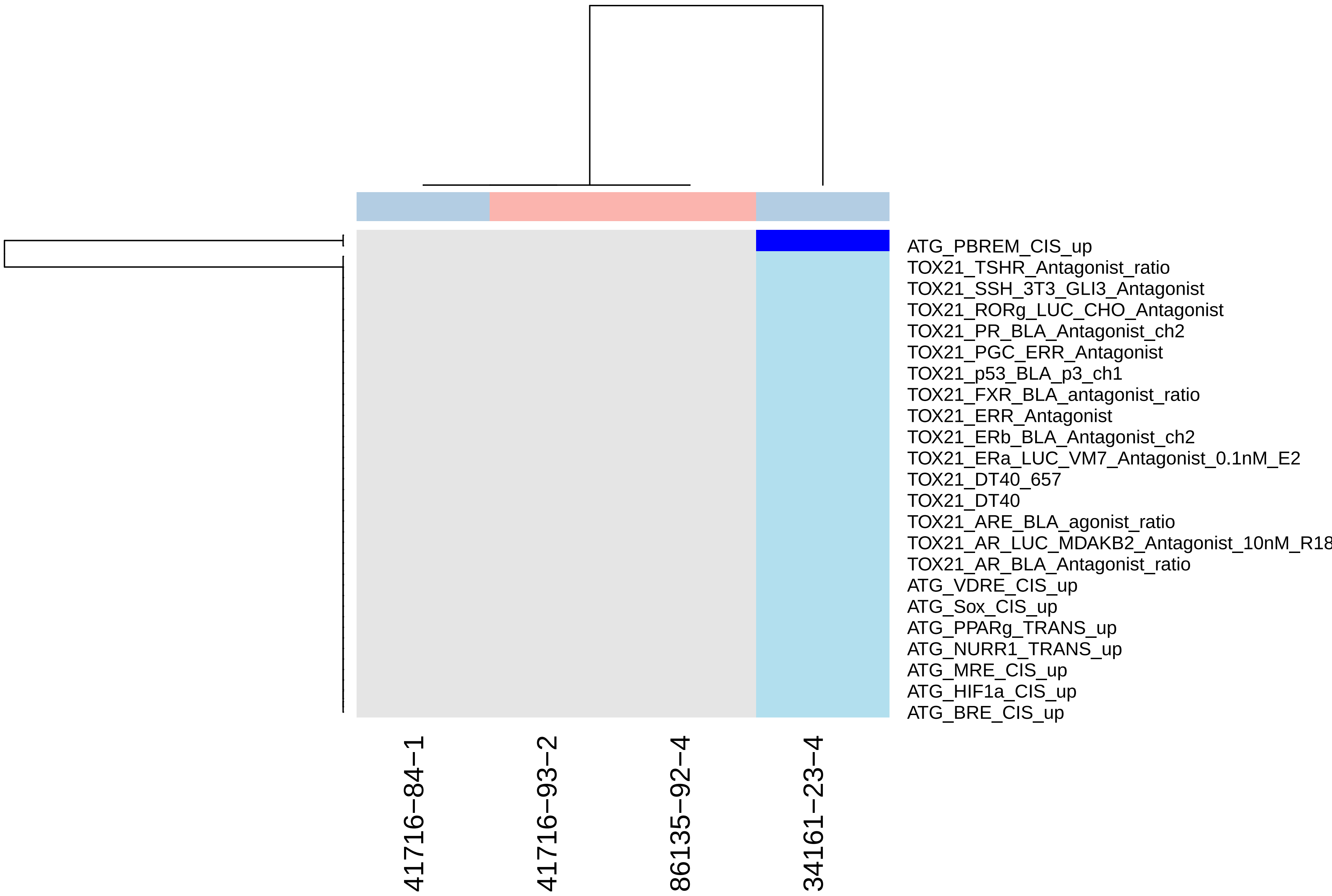

All Activity Heatmap for Cluster 1833

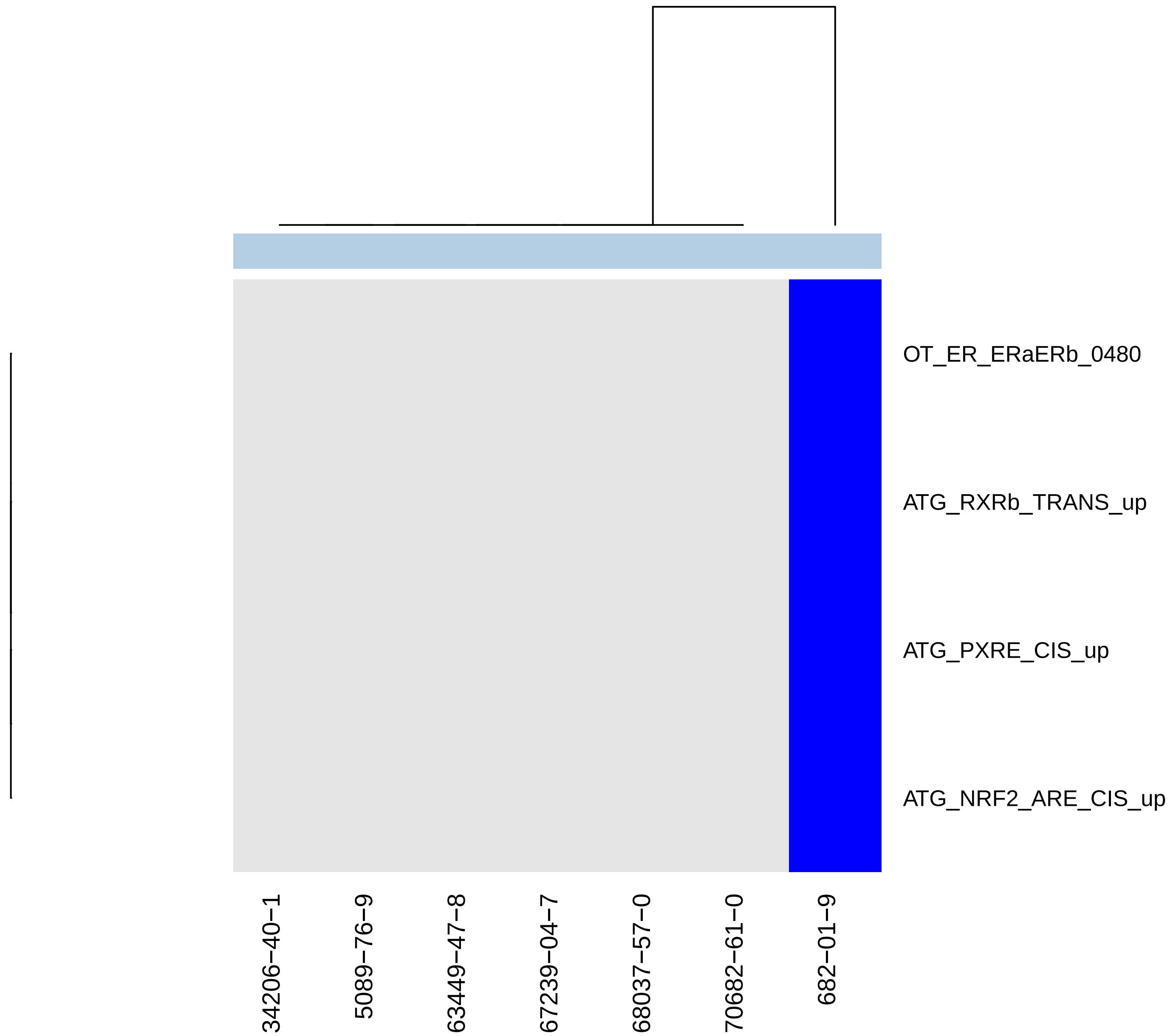

All Activity Heatmap for Cluster 1834

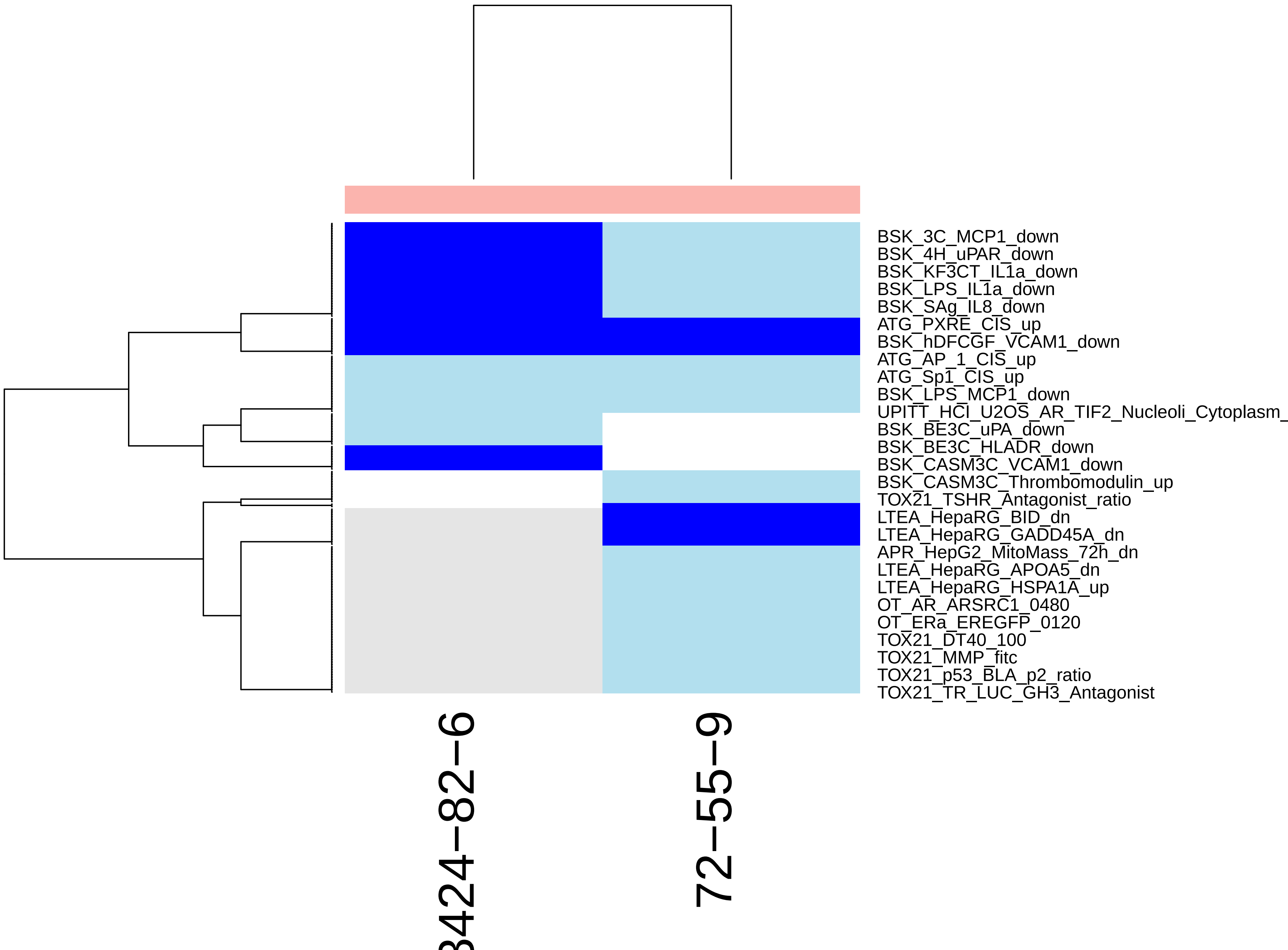

All Activity Heatmap for Cluster 1836

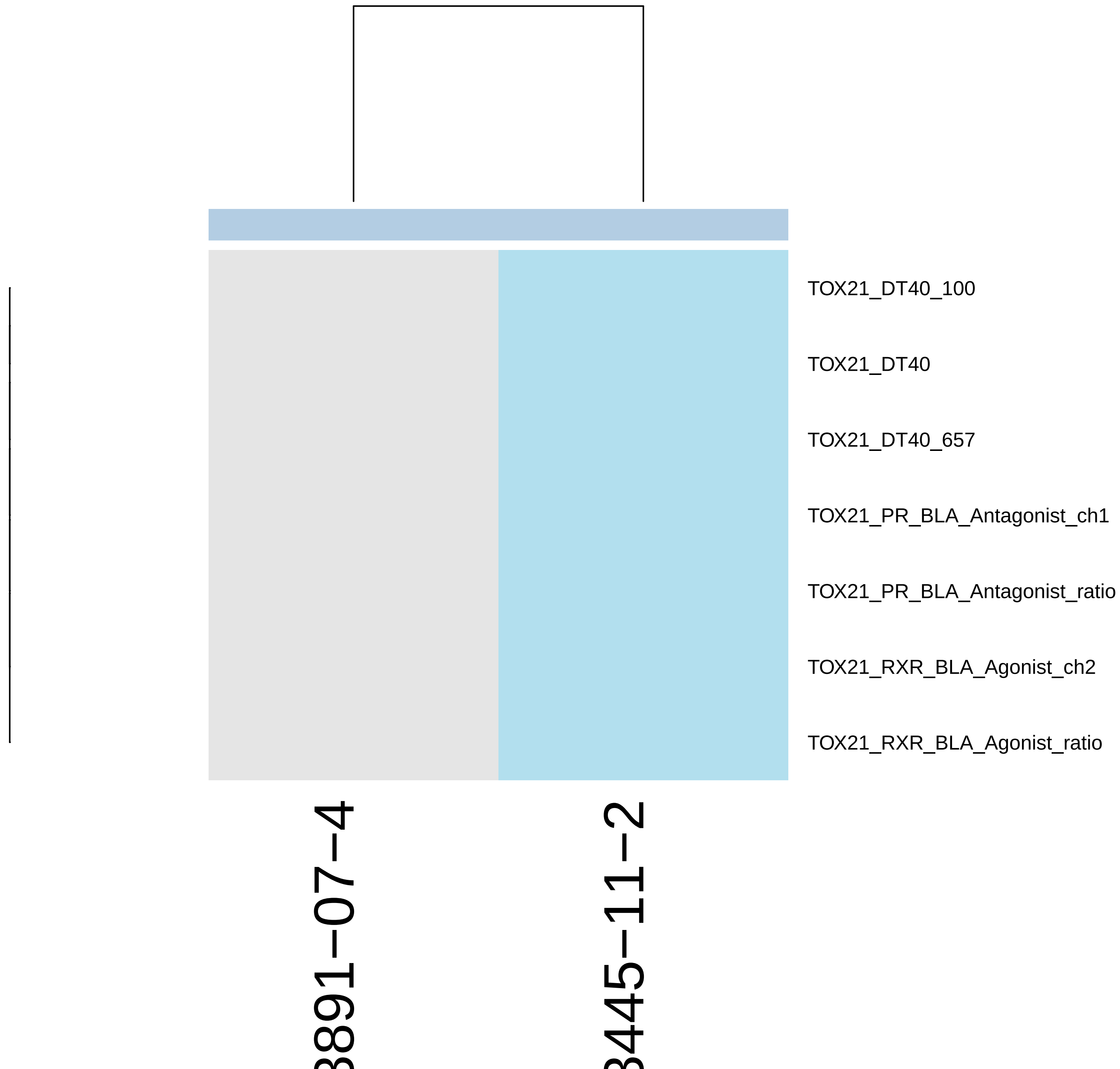

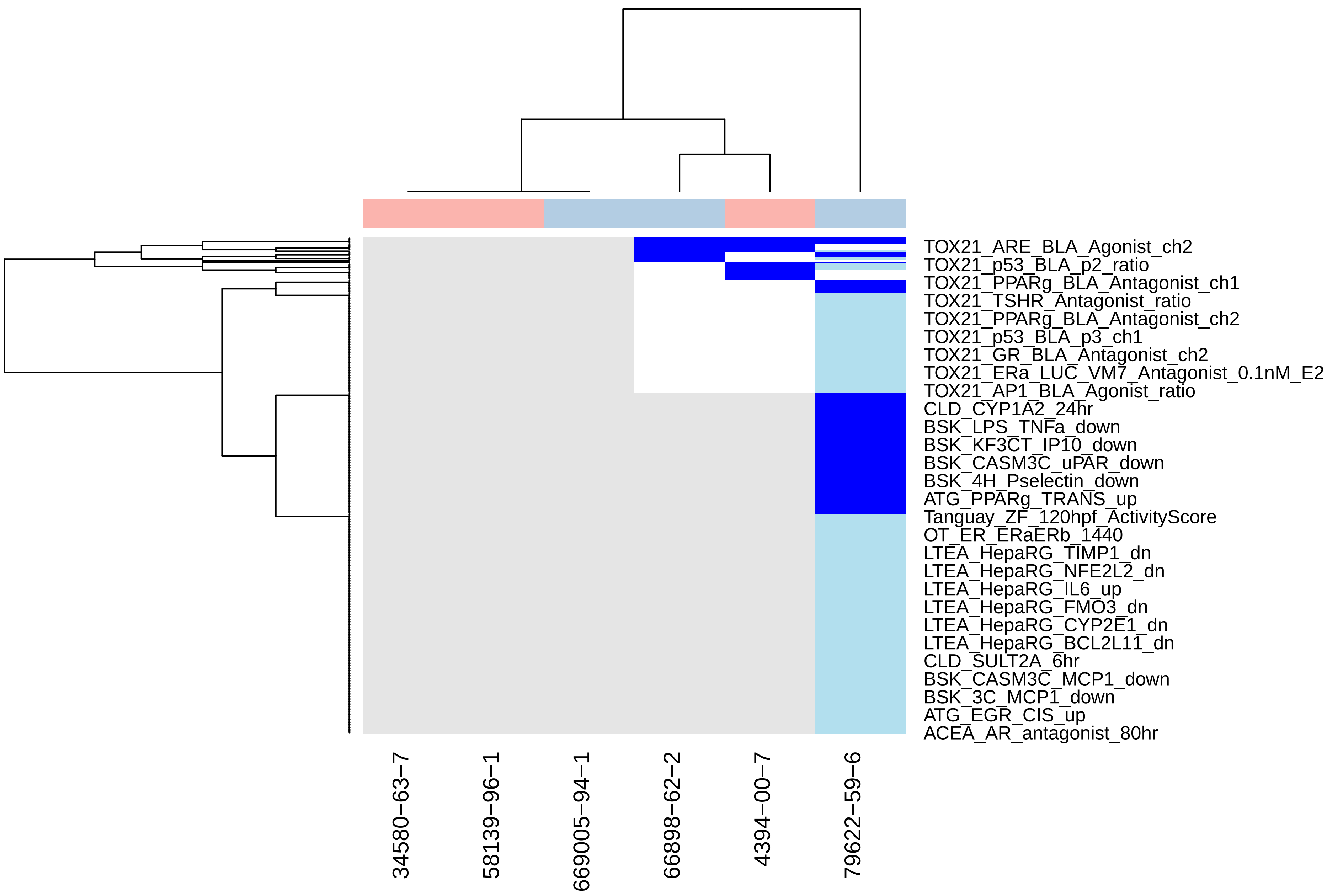
All Activity Heatmap for Cluster 1837
TOX21_ARE_BLA_Agonist_ch2
TOX21_p53_BLA_p2_ratio
TOX21_PPARg_BLA_Antagonist_ch1
TOX21_TSHR_Antagonist_ratio
TOX21_PPARg_BLA_Antagonist_ch2
TOX21_p53_BLA_p3_ch1
TOX21_GR_BLA_Antagonist_ch2
TOX21_ERa_LUC_VM7_Antagonist_0.1nM_E2
TOX21_AP1_BLA_Agonist_ratio
CLD_CYP1A2_24hr
BSK_LPS_TNFa_down
BSK_KF3CT_IP10_down
BSK_CASM3C_uPAR_down
BSK_4H_Pselectin_down
ATG_PPARg_TRANS_up
Tanguay_ZF_120hpf_ActivityScore
OT_ER_ERaERb_1440
LTEA_HepaRG_TIMP1_dn
LTEA_HepaRG_NFE2L2_dn
LTEA_HepaRG_IL6_up
LTEA_HepaRG_FMO3_dn
LTEA_HepaRG_CYP2E1_dn
LTEA_HepaRG_BCL2L11_dn
CLD_SULT2A_6hr
BSK_CASM3C_MCP1_down
BSK_3C_MCP1_down
ATG_EGR_CIS_up
ACEA_AR_antagonist_80hr
34580−63−7
58139−96−1
669005−94−1
66898−62−2
4394−00−7
79622−59−6

All Activity Heatmap for Cluster 1840

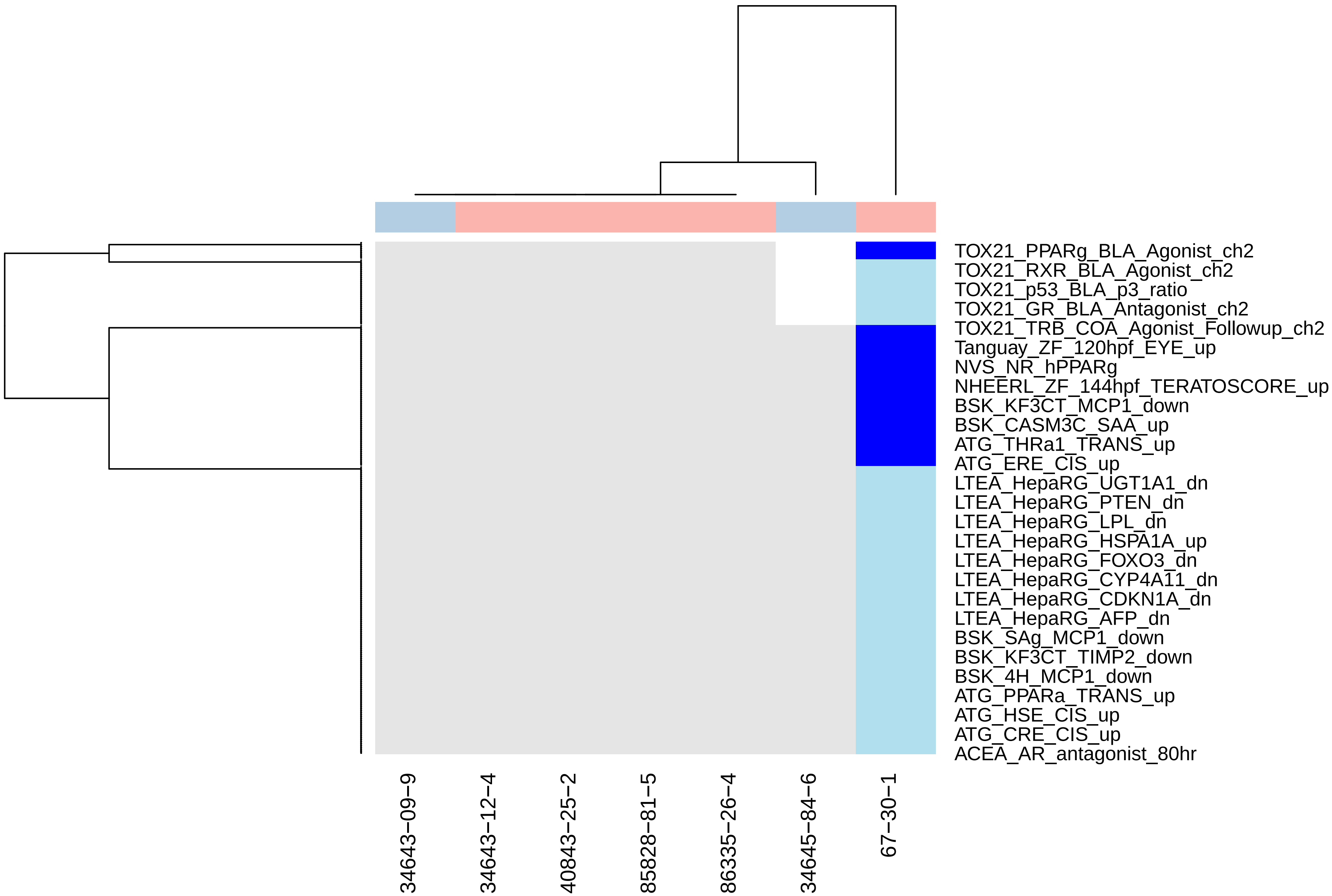

All Activity Heatmap for Cluster 1841

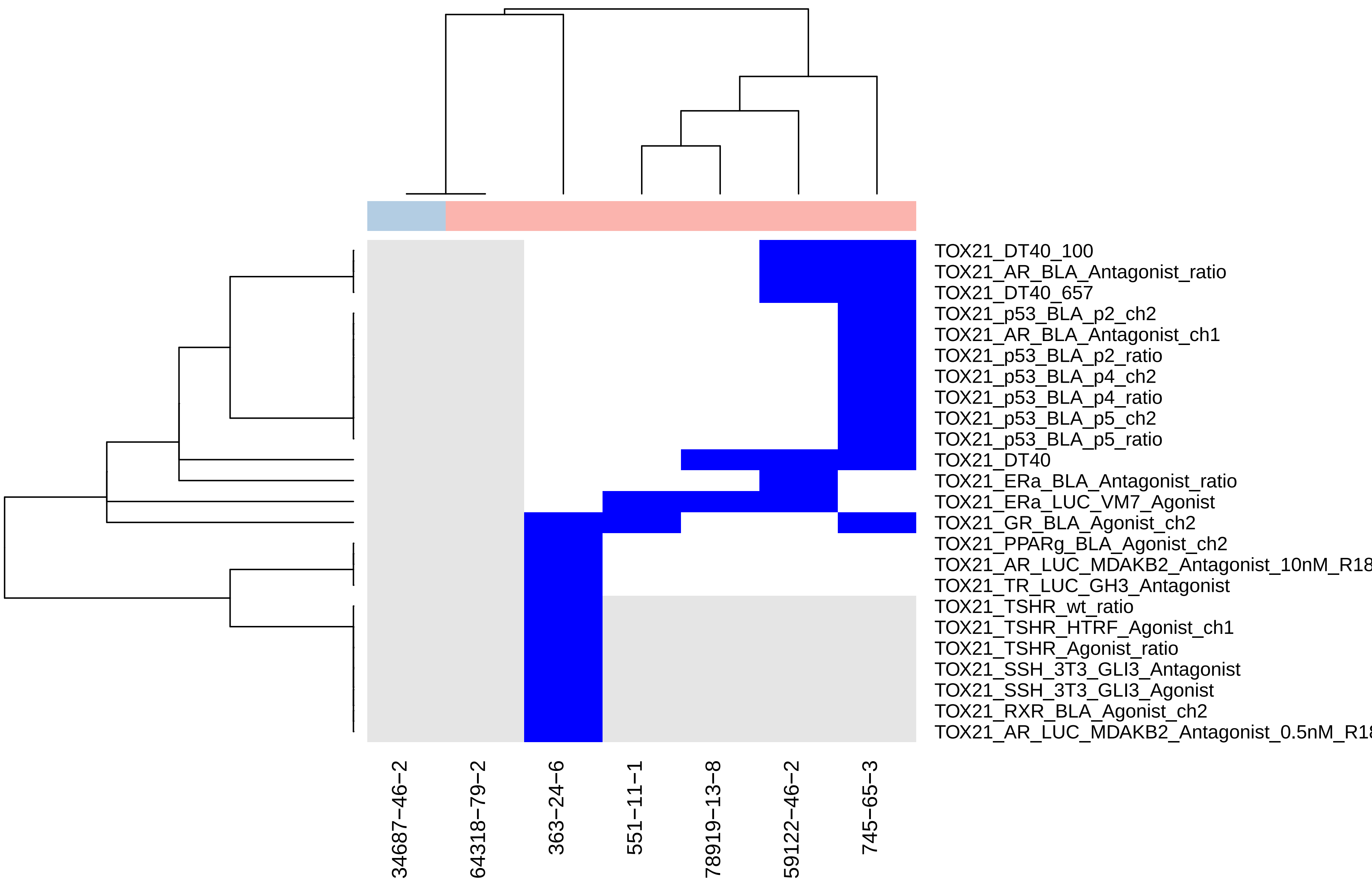

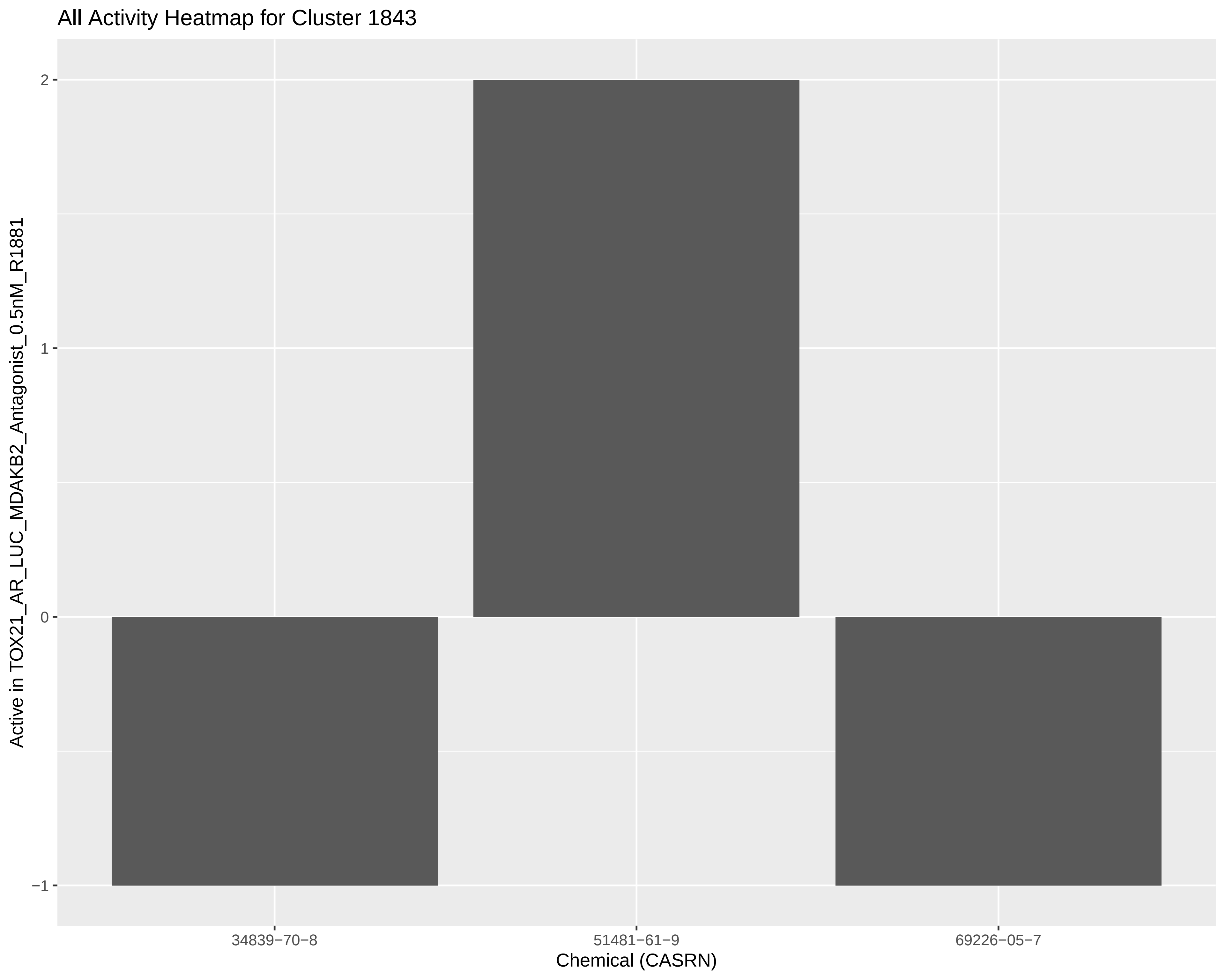
All Activity Heatmap for Cluster 1843
Active in TOX21_AR_LUC_MDAKB2_Antagonist_0.5nM_R1881
2
1
0
−1
34839−70−8
51481−61−9
69226−05−7
Chemical (CASRN)

All Activity Heatmap for Cluster 1848

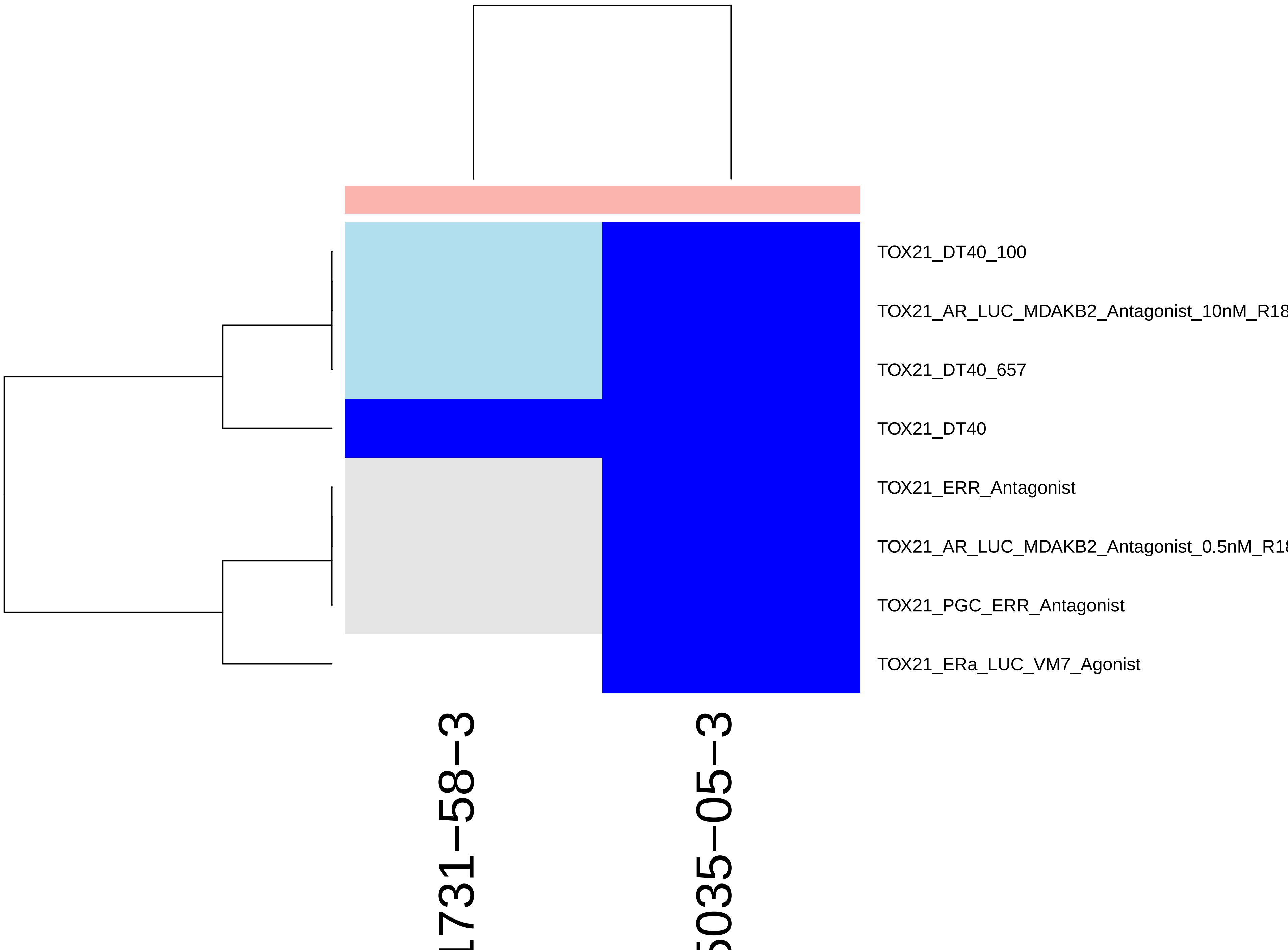

All Activity Heatmap for Cluster 1849

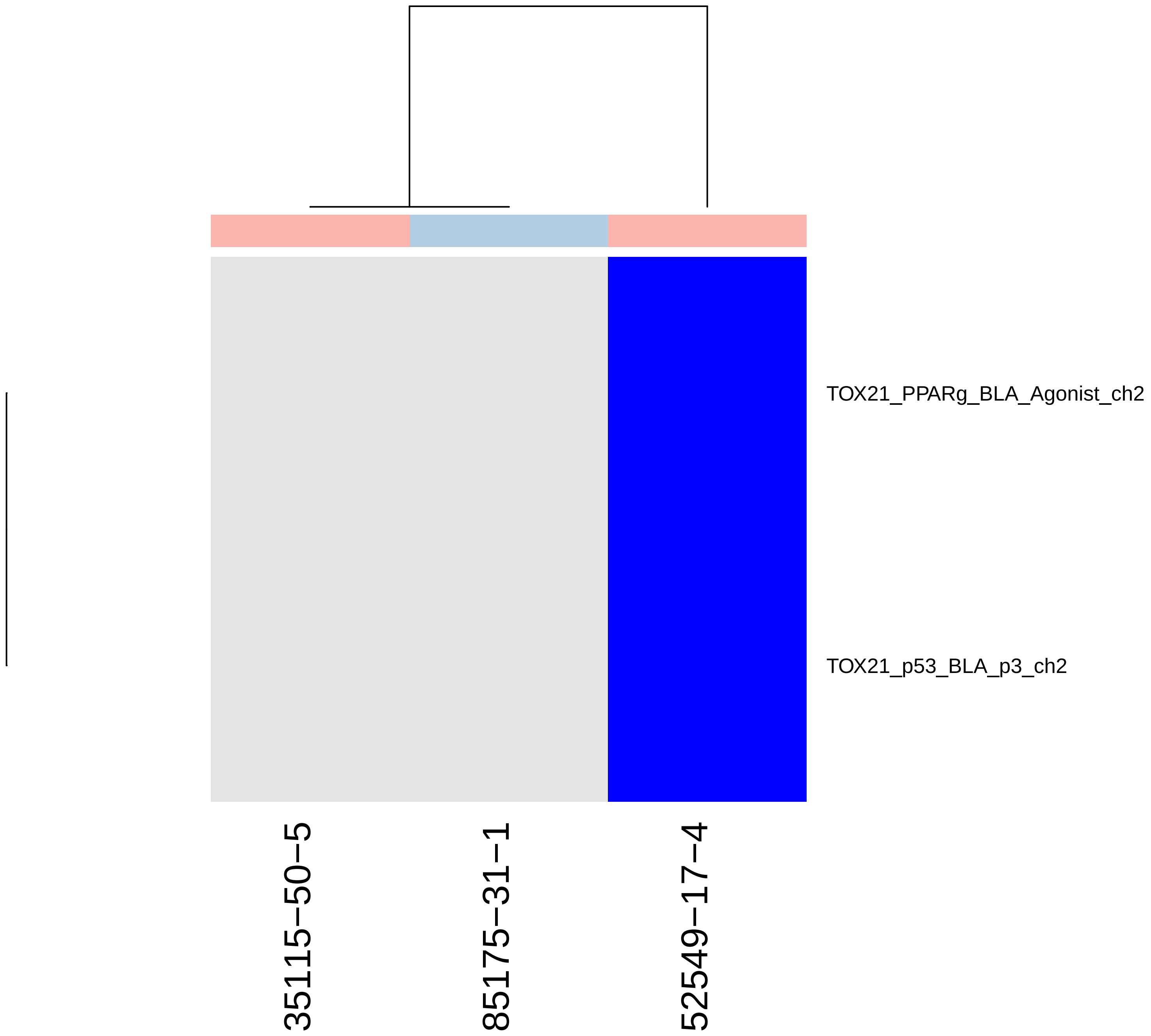

All Activity Heatmap for Cluster 1853

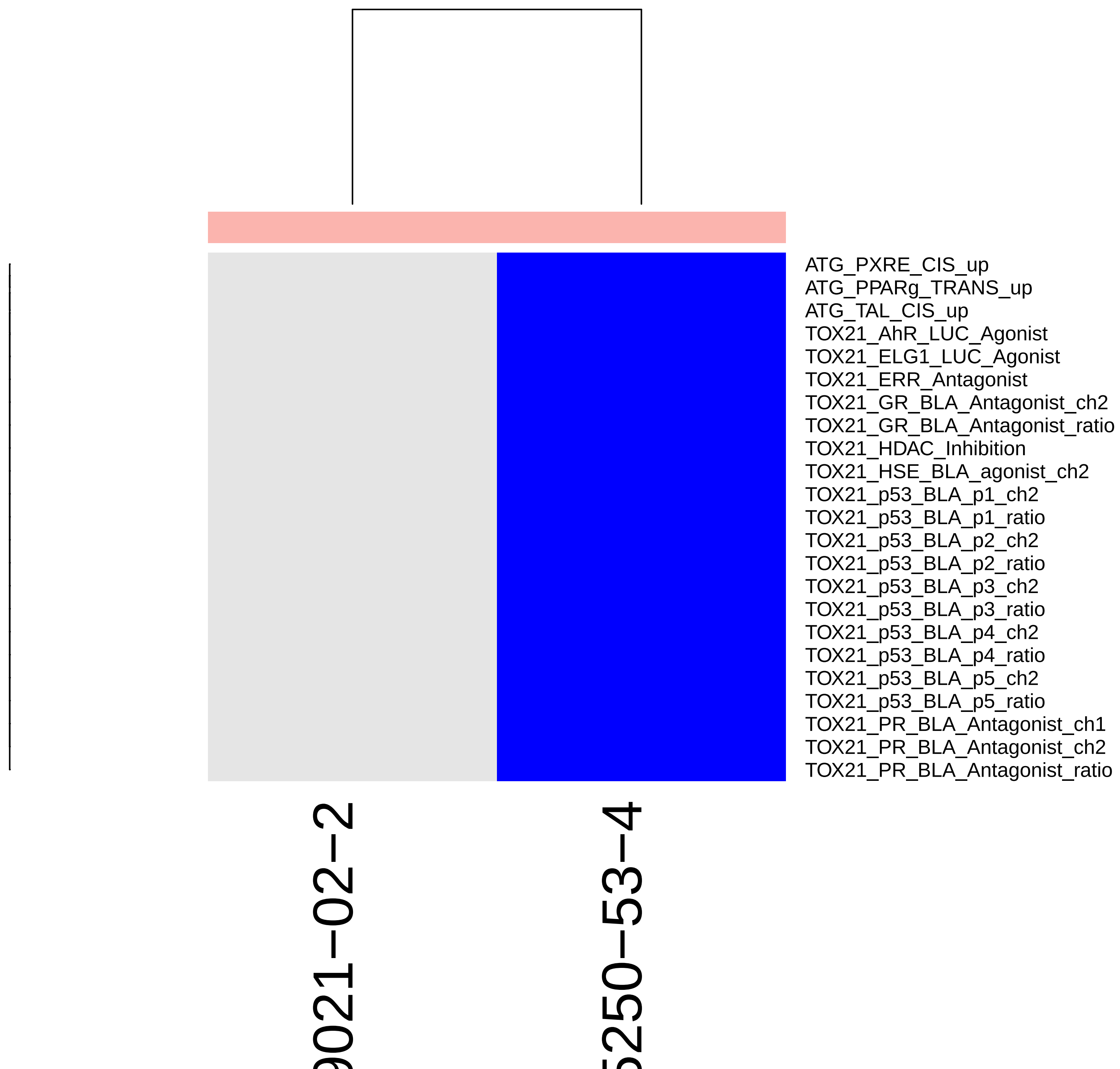

All Activity Heatmap for Cluster 1854

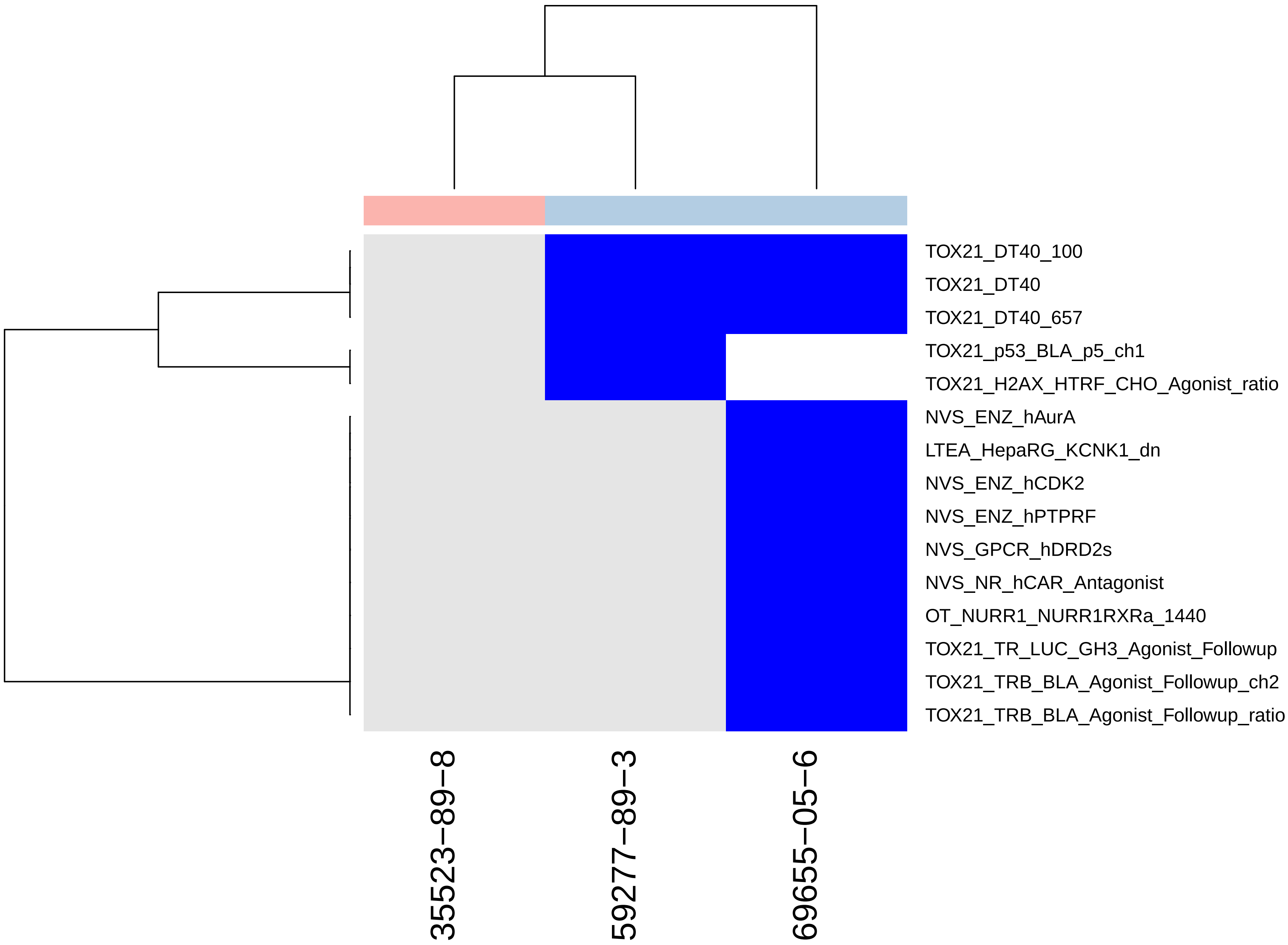

All Activity Heatmap for Cluster 1855

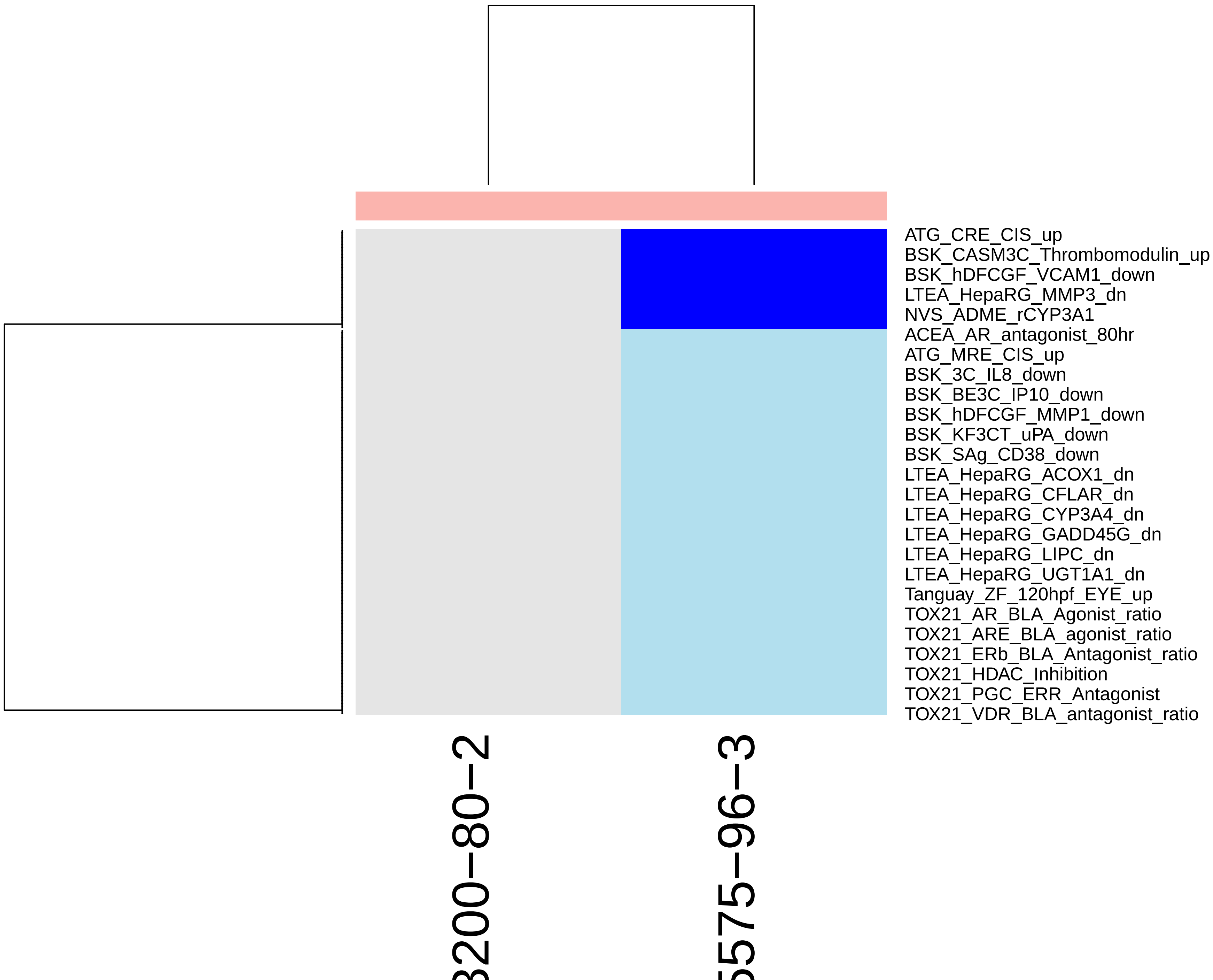

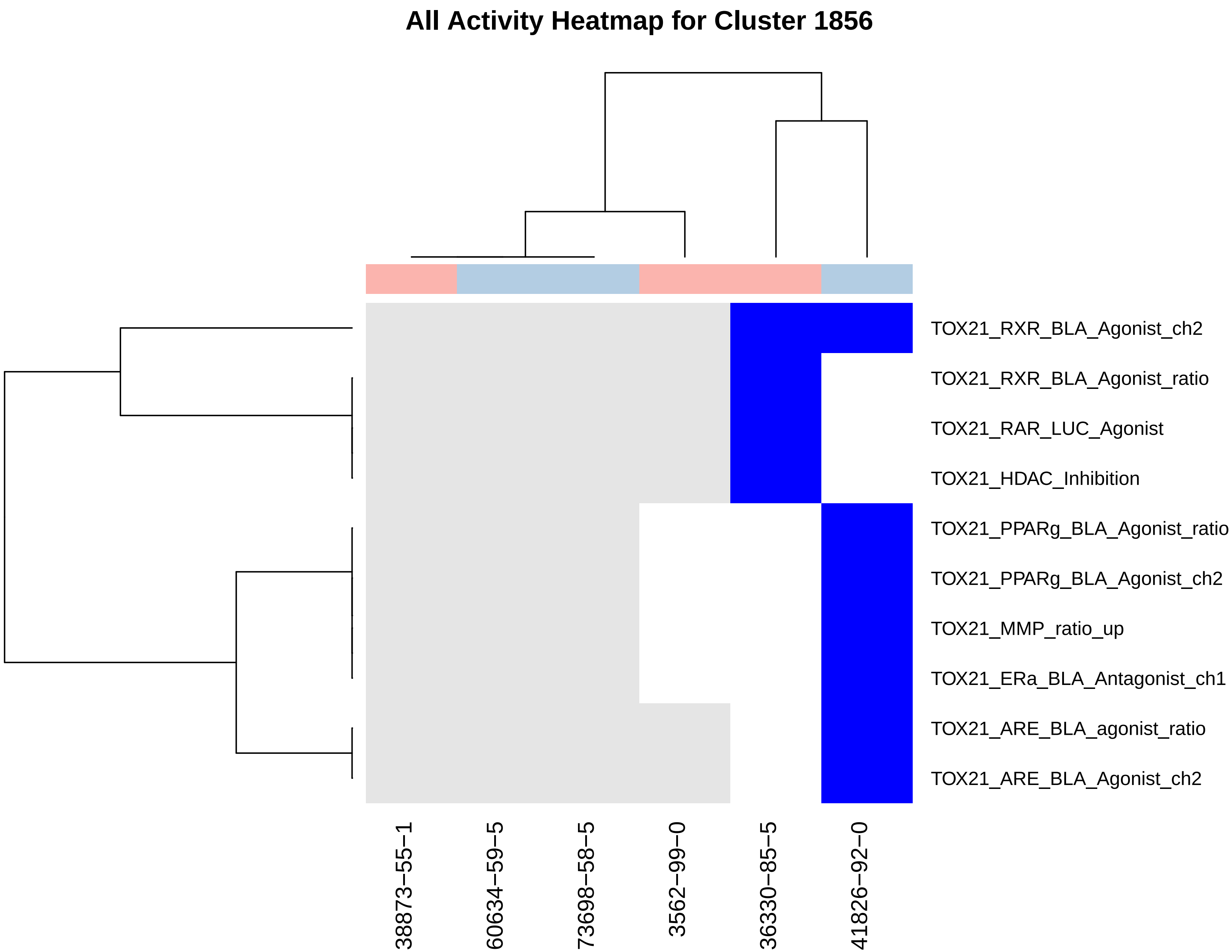
All Activity Heatmap for Cluster 1856
TOX21_RXR_BLA_Agonist_ch2
TOX21_RXR_BLA_Agonist_ratio
TOX21_RAR_LUC_Agonist
TOX21_HDAC_Inhibition
TOX21_PPARg_BLA_Agonist_ratio
TOX21_PPARg_BLA_Agonist_ch2
TOX21_MMP_ratio_up
TOX21_ERa_BLA_Antagonist_ch1
TOX21_ARE_BLA_agonist_ratio
TOX21_ARE_BLA_Agonist_ch2
38873−55−1
60634−59−5
73698−58−5
3562−99−0
36330−85−5
41826−92−0

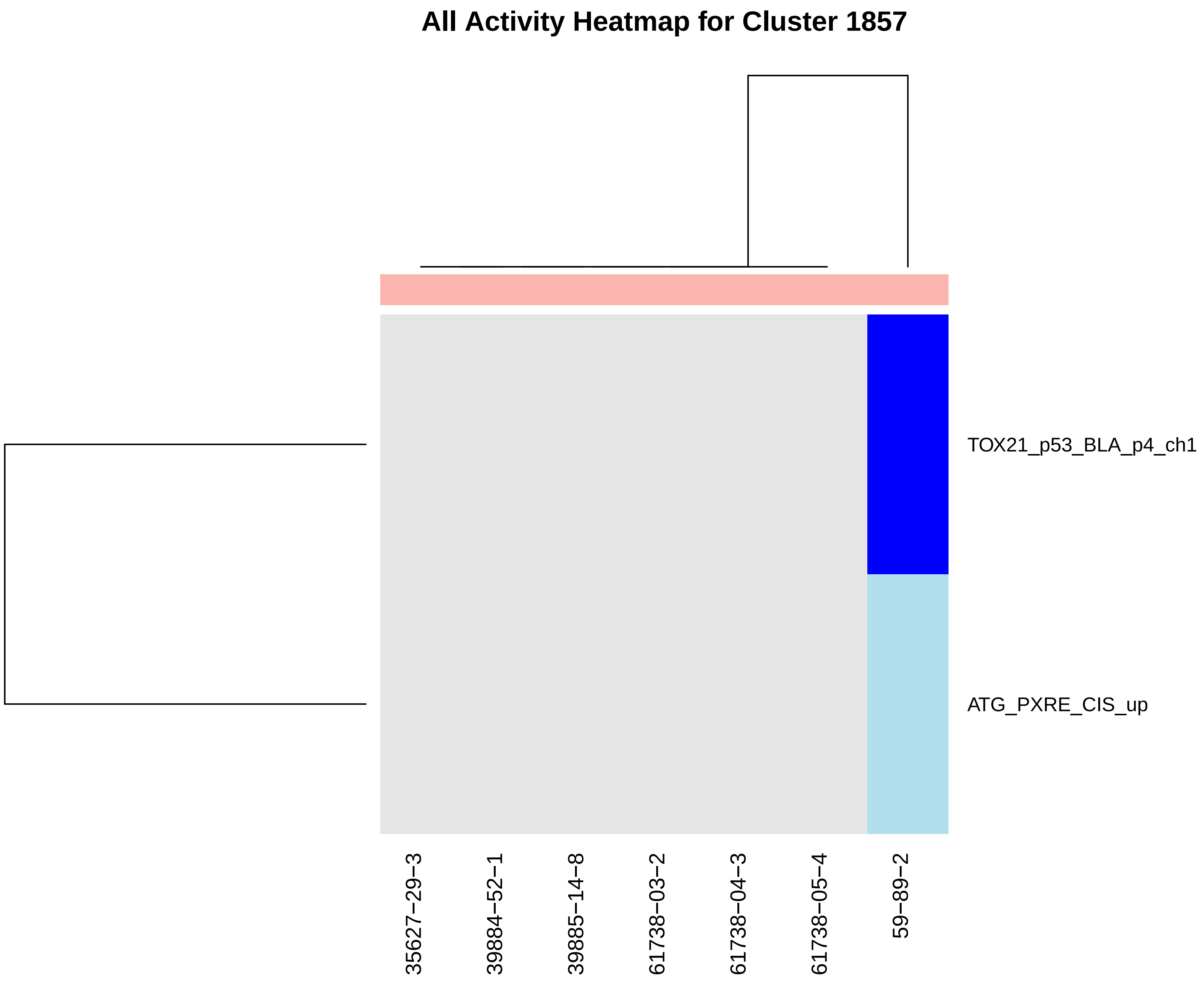
All Activity Heatmap for Cluster 1857
TOX21_p53_BLA_p4_ch1
ATG_PXRE_CIS_up
35627−29−3
39884−52−1
39885−14−8
61738−03−2
61738−04−3
61738−05−4
59−89−2

All Activity Heatmap for Cluster 1860

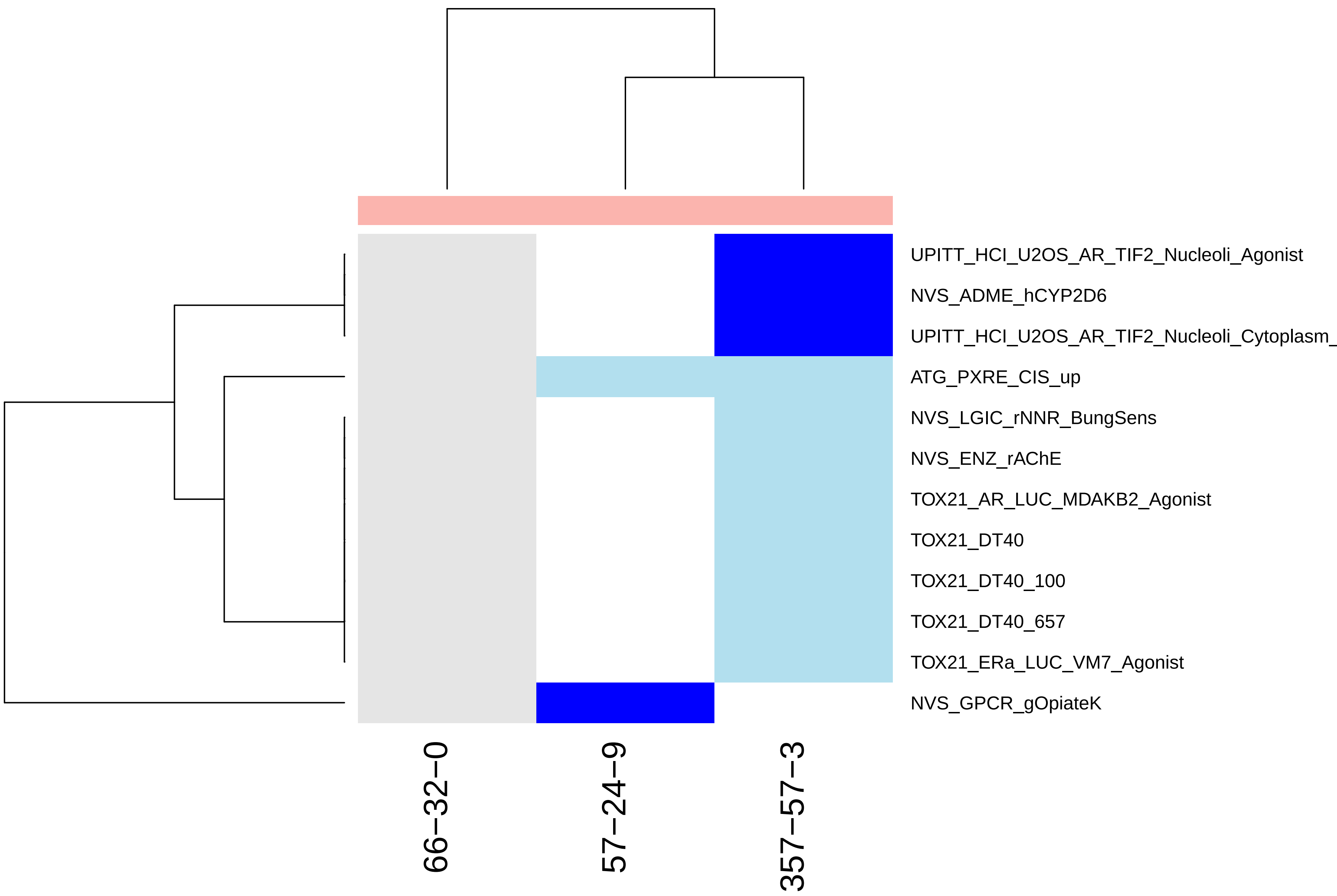

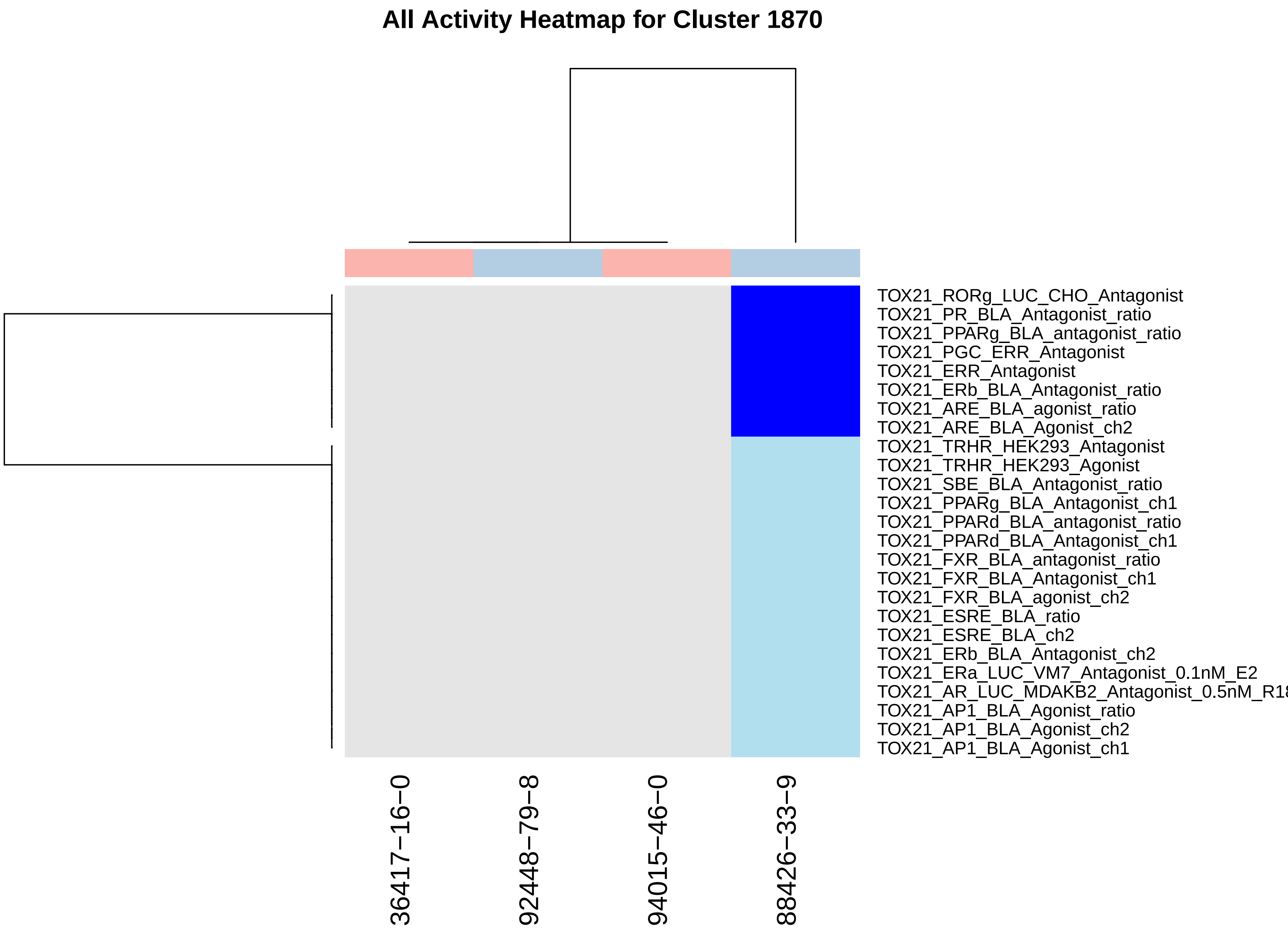

All Activity Heatmap for Cluster 1870
TOX21_RORg_LUC_CHO_Antagonist
TOX21_PR_BLA_Antagonist_ratio
TOX21_PPARg_BLA_antagonist_ratio
TOX21_PGC_ERR_Antagonist
TOX21_ERR_Antagonist
TOX21_ERb_BLA_Antagonist_ratio
TOX21_ARE_BLA_agonist_ratio
TOX21_ARE_BLA_Agonist_ch2
TOX21_TRHR_HEK293_Antagonist
TOX21_TRHR_HEK293_Agonist
TOX21_SBE_BLA_Antagonist_ratio
TOX21_PPARg_BLA_Antagonist_ch1
TOX21_PPARd_BLA_antagonist_ratio
TOX21_PPARd_BLA_Antagonist_ch1
TOX21_FXR_BLA_antagonist_ratio
TOX21_FXR_BLA_Antagonist_ch1
TOX21_FXR_BLA_agonist_ch2
TOX21_ESRE_BLA_ratio
TOX21_ESRE_BLA_ch2
TOX21_ERb_BLA_Antagonist_ch2
TOX21_ERa_LUC_VM7_Antagonist_0.1nM_E2
TOX21_AR_LUC_MDAKB2_Antagonist_0.5nM_R18
TOX21_AP1_BLA_Agonist_ratio
TOX21_AP1_BLA_Agonist_ch2
TOX21_AP1_BLA_Agonist_ch1
36417−16−0
92448−79−8
94015−46−0
88426−33−9

All Activity Heatmap for Cluster 1875

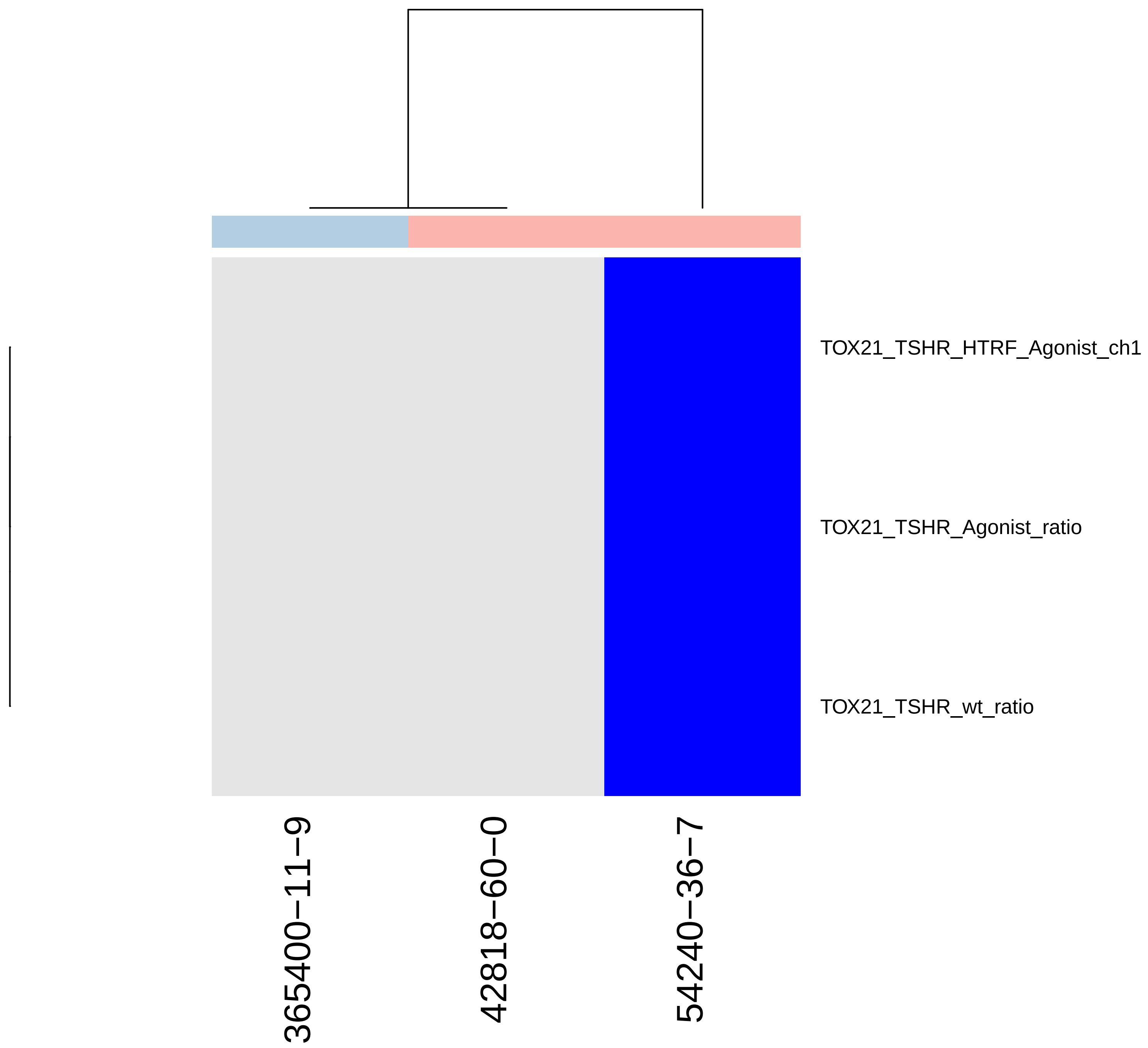

All Activity Heatmap for Cluster 1879

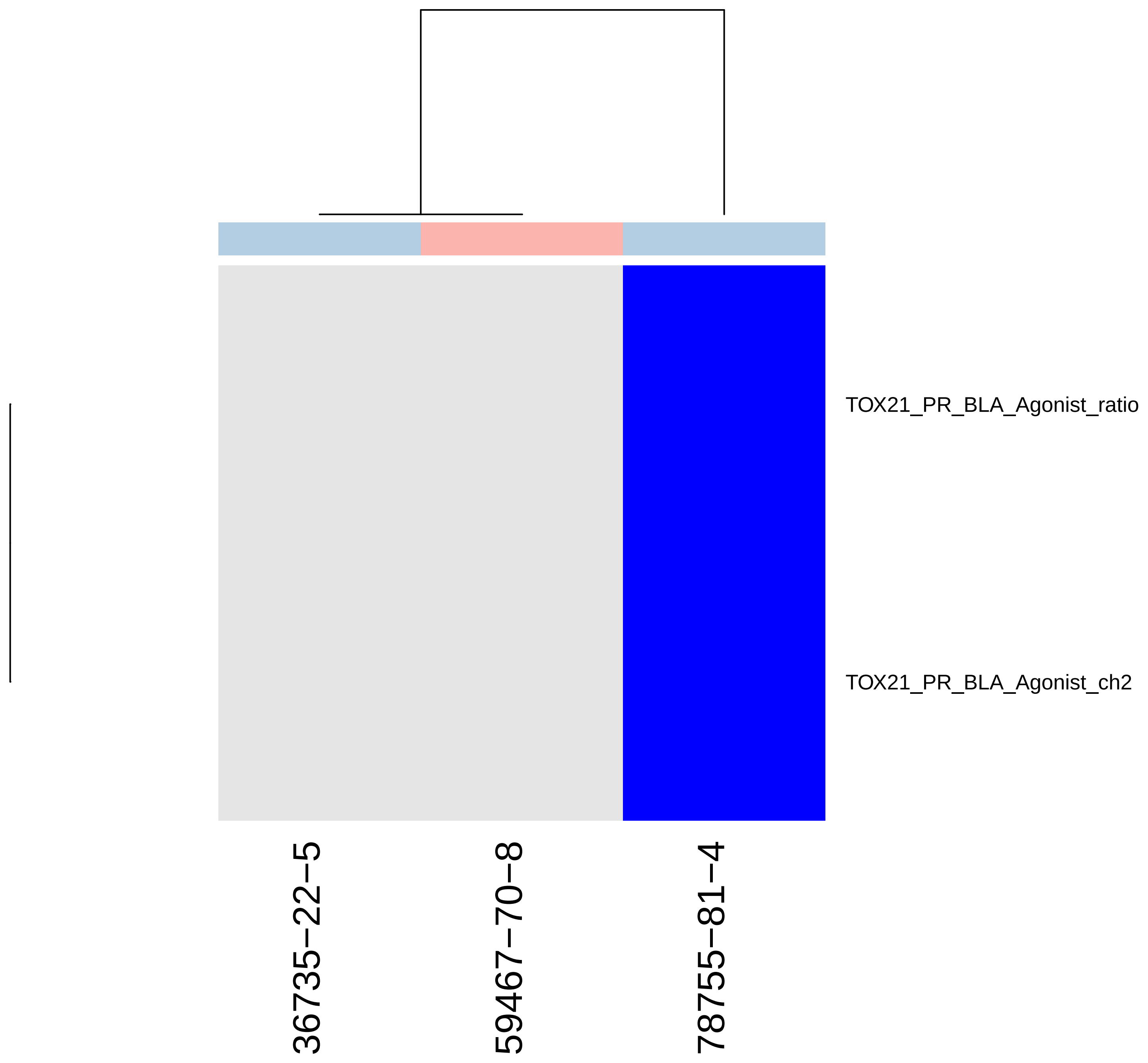

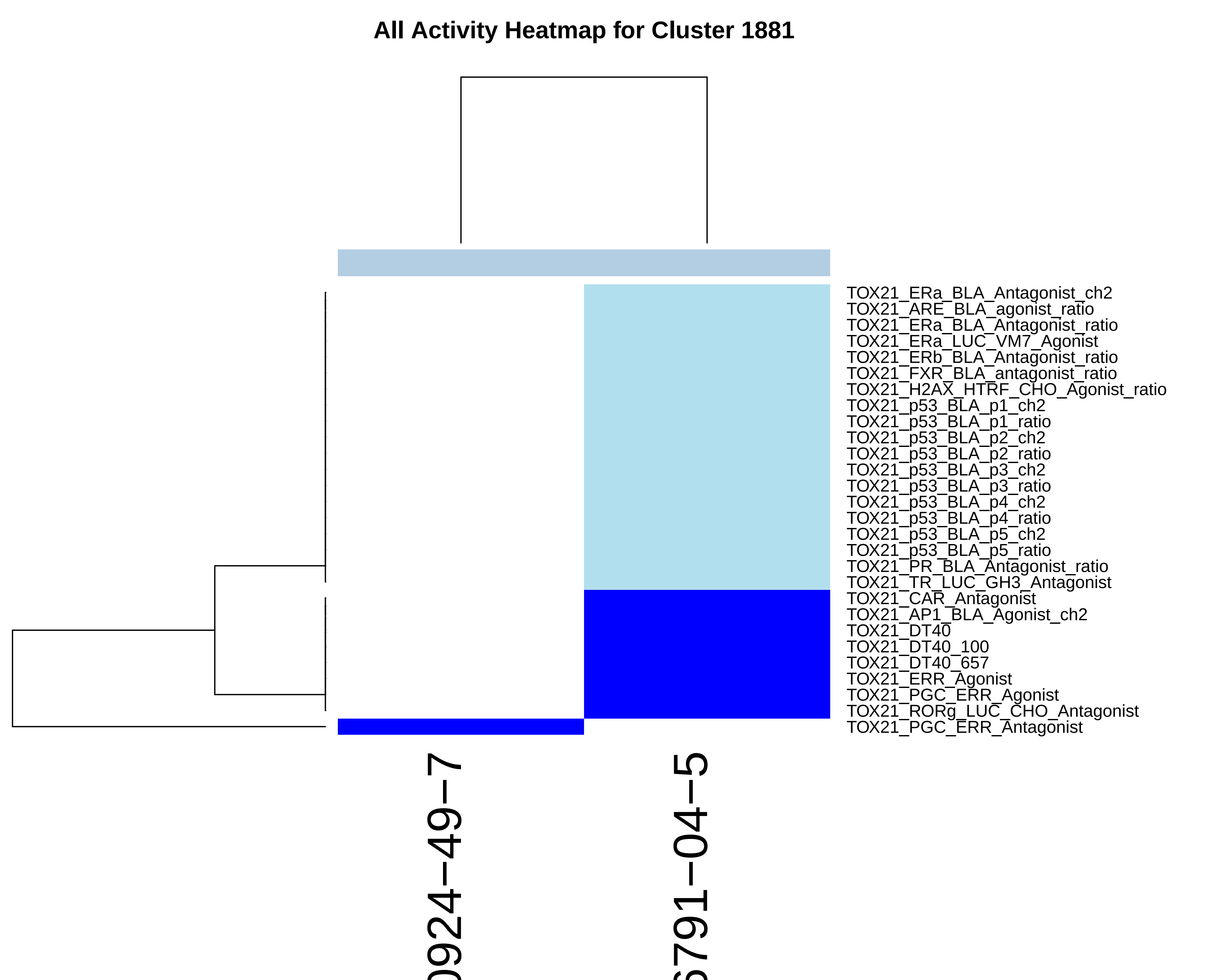
All Activity Heatmap for Cluster 1881
TOX21_ERa_BLA_Antagonist_ch2
TOX21_ARE_BLA_agonist_ratio
TOX21_ERa_BLA_Antagonist_ratio
TOX21_ERa_LUC_VM7_Agonist
TOX21_ERb_BLA_Antagonist_ratio
TOX21_FXR_BLA_antagonist_ratio
TOX21_H2AX_HTRF_CHO_Agonist_ratio
TOX21_p53_BLA_p1_ch2
TOX21_p53_BLA_p1_ratio
TOX21_p53_BLA_p2_ch2
TOX21_p53_BLA_p2_ratio
TOX21_p53_BLA_p3_ch2
TOX21_p53_BLA_p3_ratio
TOX21_p53_BLA_p4_ch2
TOX21_p53_BLA_p4_ratio
TOX21_p53_BLA_p5_ch2
TOX21_p53_BLA_p5_ratio
TOX21_PR_BLA_Antagonist_ratio
TOX21_TR_LUC_GH3_Antagonist
TOX21_CAR_Antagonist
TOX21_AP1_BLA_Agonist_ch2
TOX21_DT40
TOX21_DT40_100
TOX21_DT40_657
TOX21_ERR_Agonist
TOX21_PGC_ERR_Agonist
TOX21_RORg_LUC_CHO_Antagonist
TOX21_PGC_ERR_Antagonist
0924−49−7
6791−04−5

All Activity Heatmap for Cluster 1882

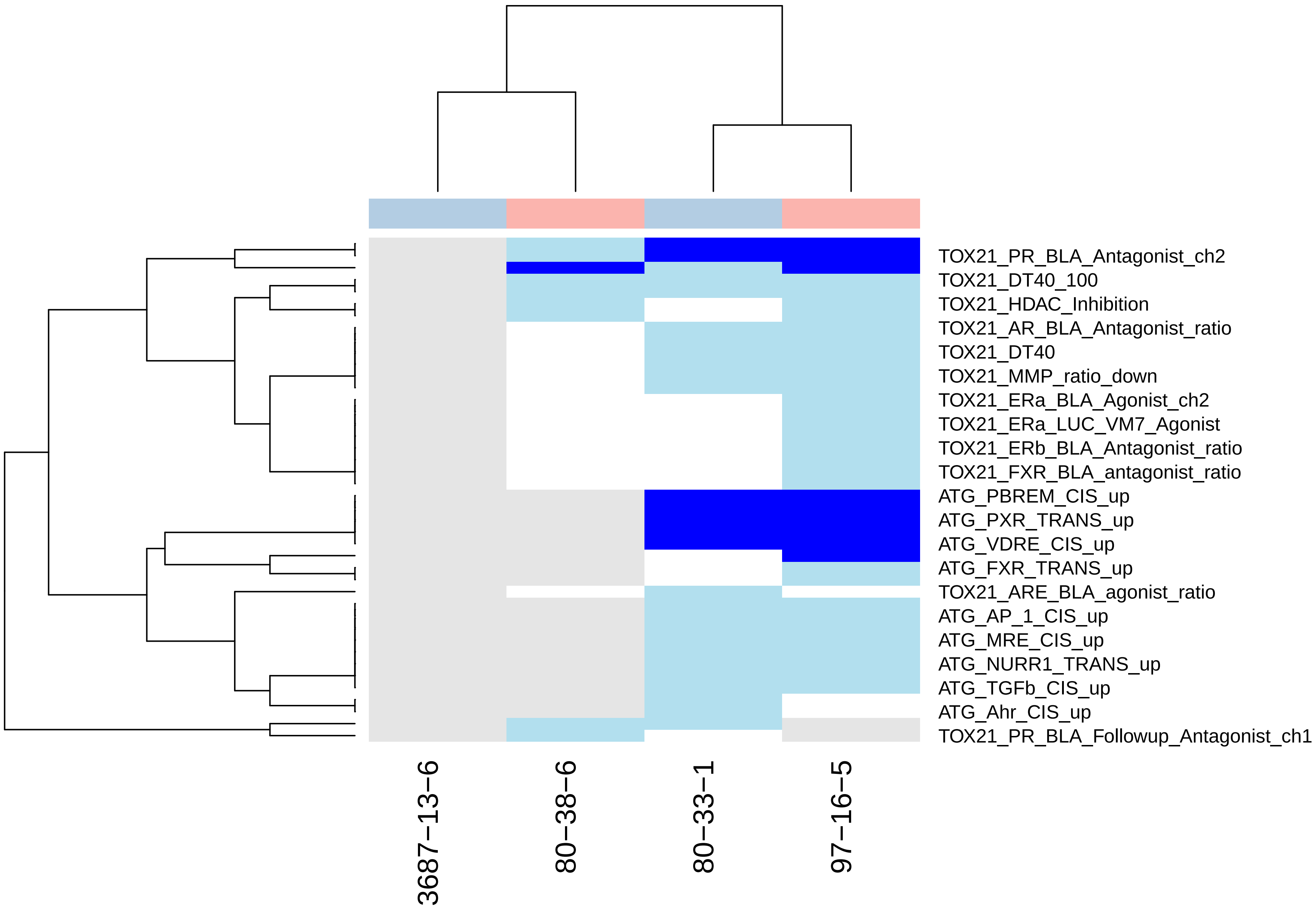

All Activity Heatmap for Cluster 1883

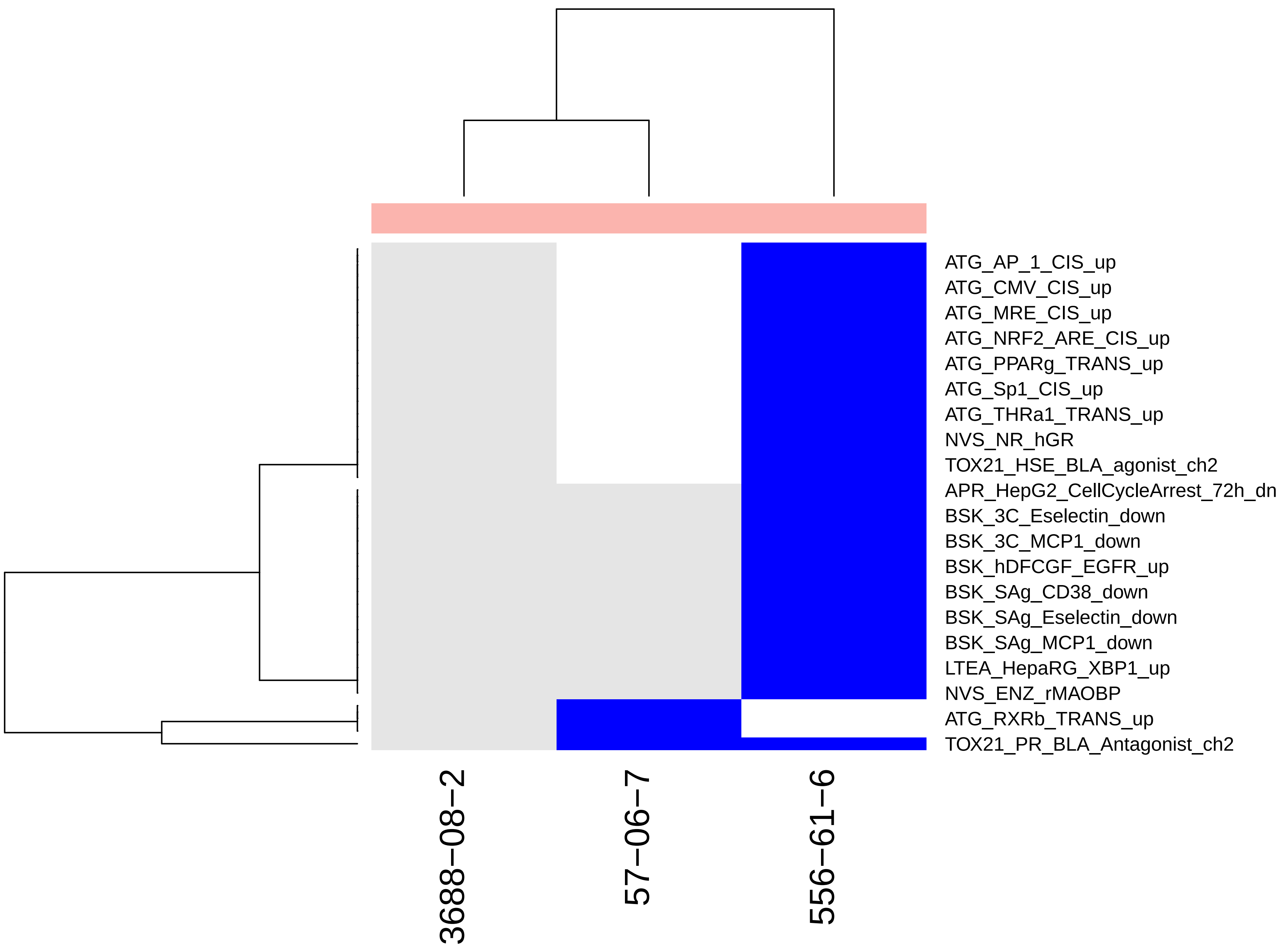

All Activity Heatmap for Cluster 1885

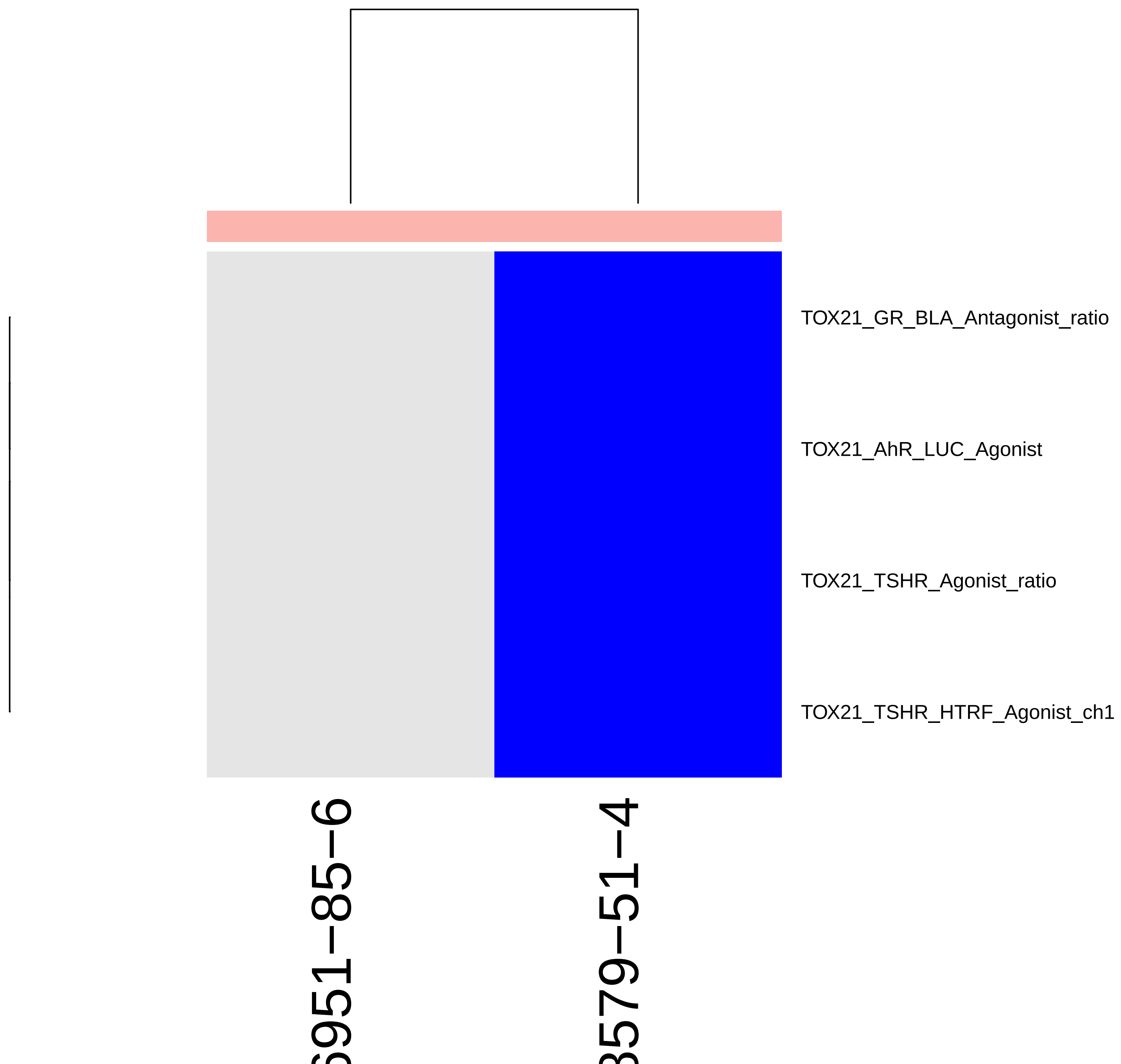

All Activity Heatmap for Cluster 1890

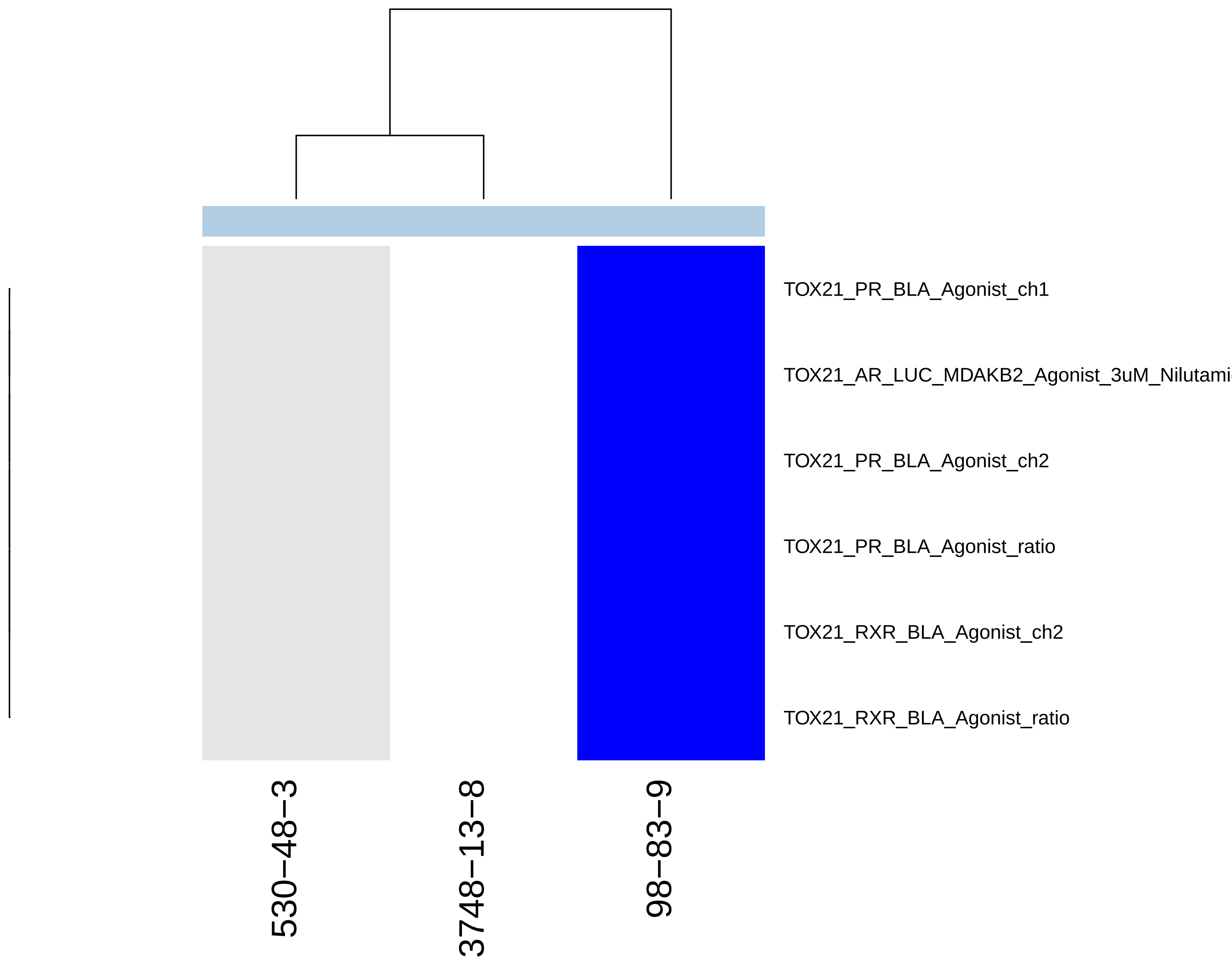

All Activity Heatmap for Cluster 1891

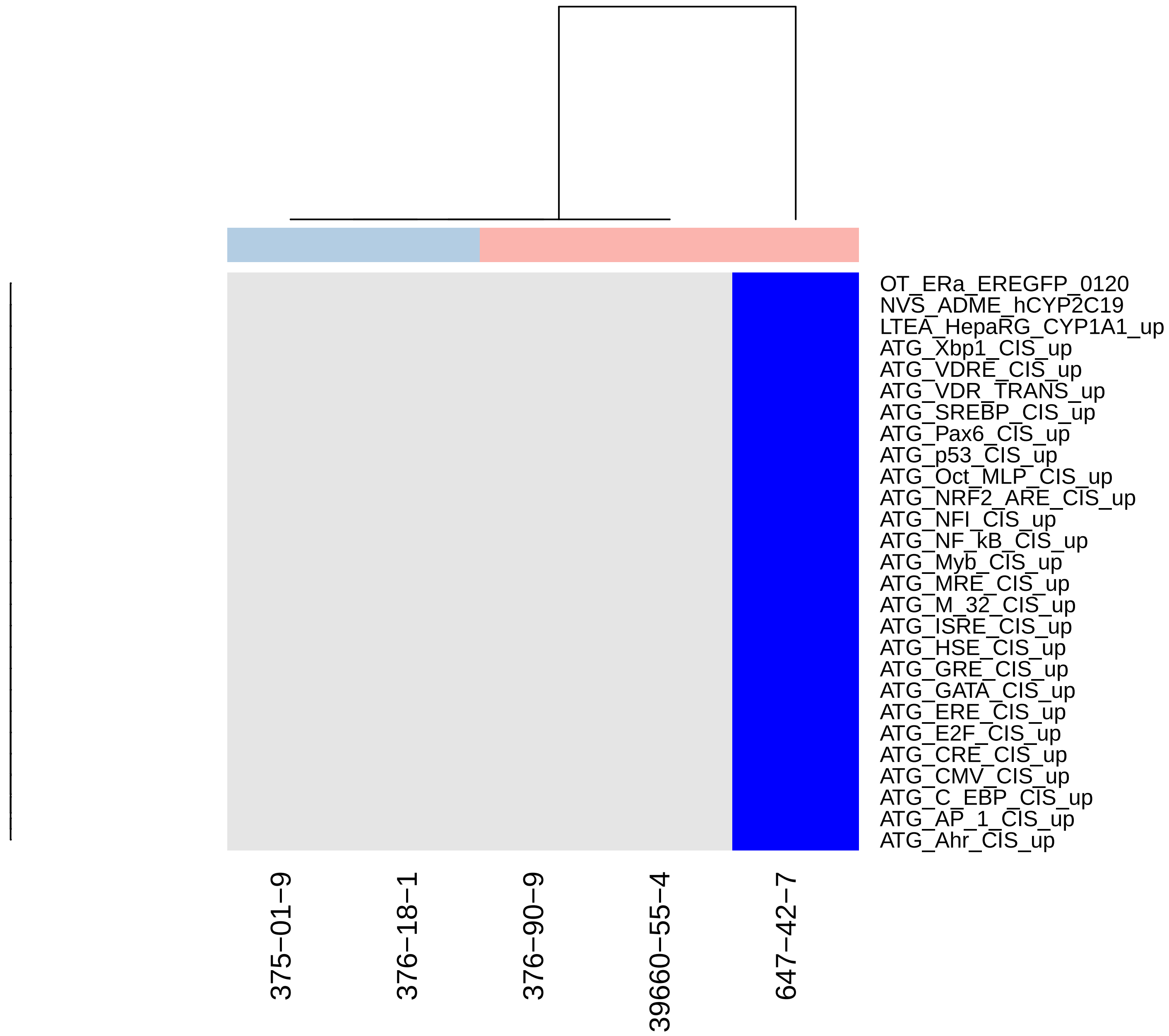

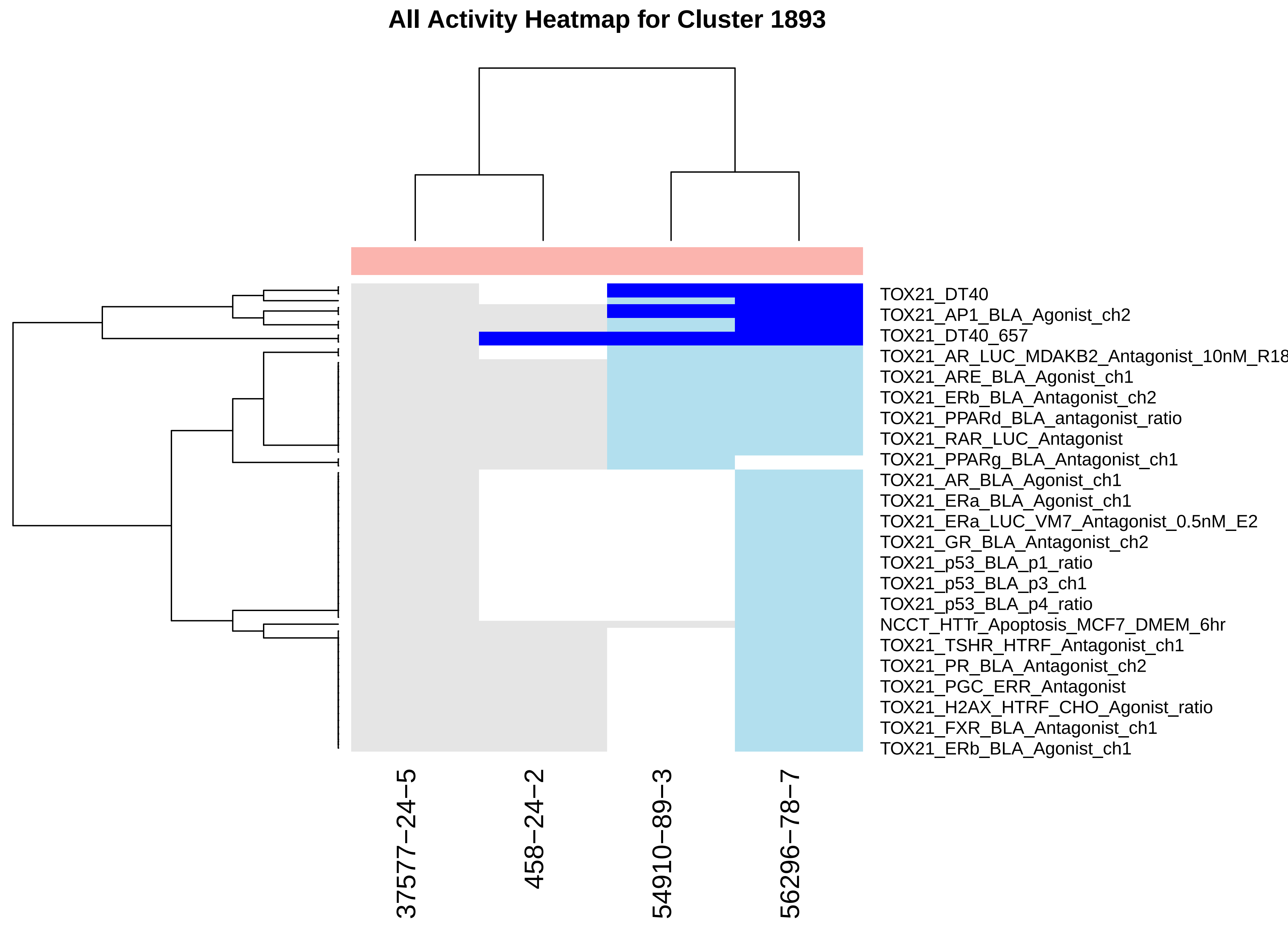

All Activity Heatmap for Cluster 1893
TOX21_DT40
TOX21_AP1_BLA_Agonist_ch2
TOX21_DT40_657
TOX21_AR_LUC_MDAKB2_Antagonist_10nM_R18
TOX21_ARE_BLA_Agonist_ch1
TOX21_ERb_BLA_Antagonist_ch2
TOX21_PPARd_BLA_antagonist_ratio
TOX21_RAR_LUC_Antagonist
TOX21_PPARg_BLA_Antagonist_ch1
TOX21_AR_BLA_Agonist_ch1
TOX21_ERa_BLA_Agonist_ch1
TOX21_ERa_LUC_VM7_Antagonist_0.5nM_E2
TOX21_GR_BLA_Antagonist_ch2
TOX21_p53_BLA_p1_ratio
TOX21_p53_BLA_p3_ch1
TOX21_p53_BLA_p4_ratio
NCCT_HTTr_Apoptosis_MCF7_DMEM_6hr
TOX21_TSHR_HTRF_Antagonist_ch1
TOX21_PR_BLA_Antagonist_ch2
TOX21_PGC_ERR_Antagonist
TOX21_H2AX_HTRF_CHO_Agonist_ratio
TOX21_FXR_BLA_Antagonist_ch1
TOX21_ERb_BLA_Agonist_ch1
37577−24−5
458−24−2
54910−89−3
56296−78−7

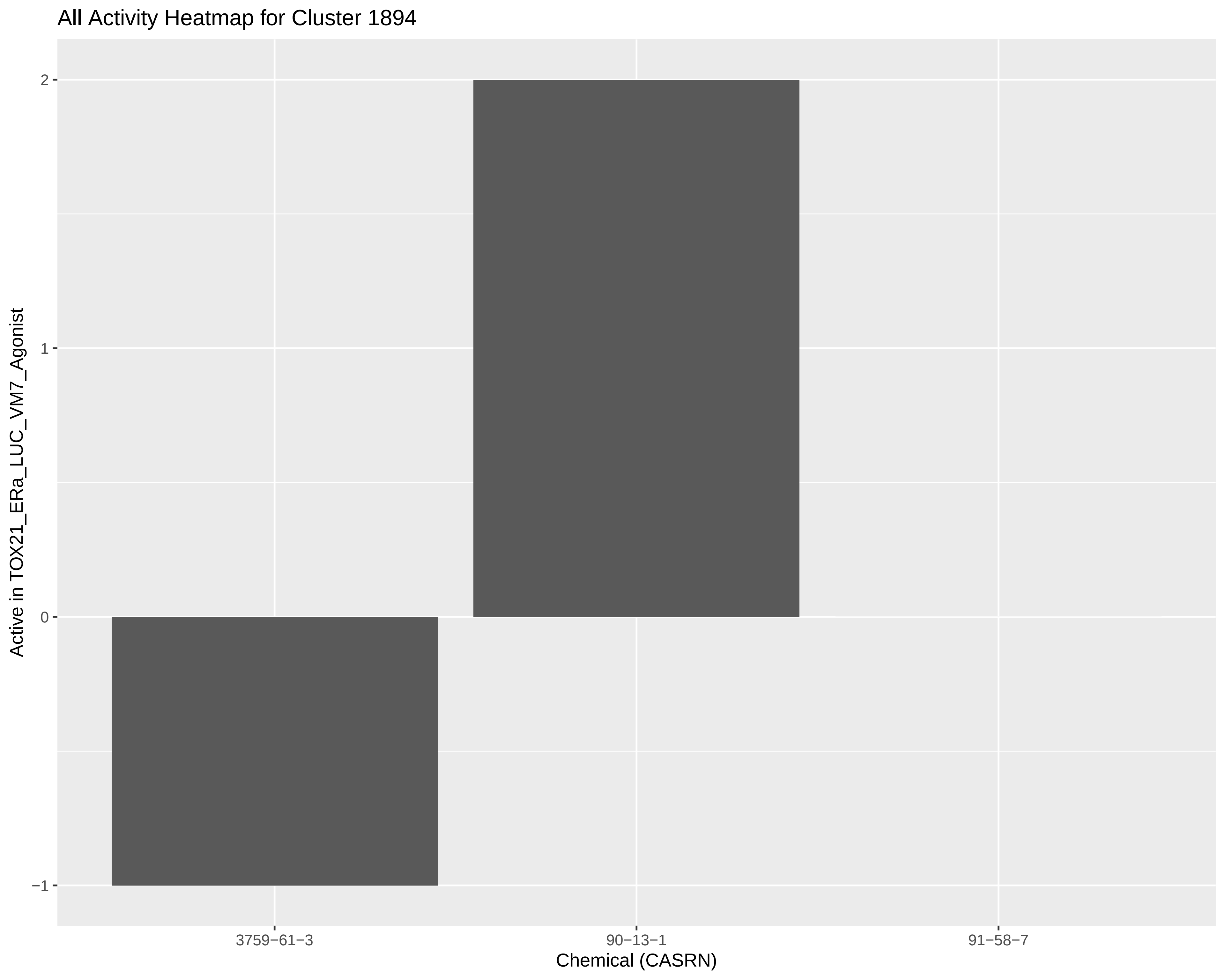
All Activity Heatmap for Cluster 1894
Active in TOX21_ERa_LUC_VM7_Agonist
2
1
0
−1
3759−61−3
90−13−1
91−58−7
Chemical (CASRN)

All Activity Heatmap for Cluster 1902

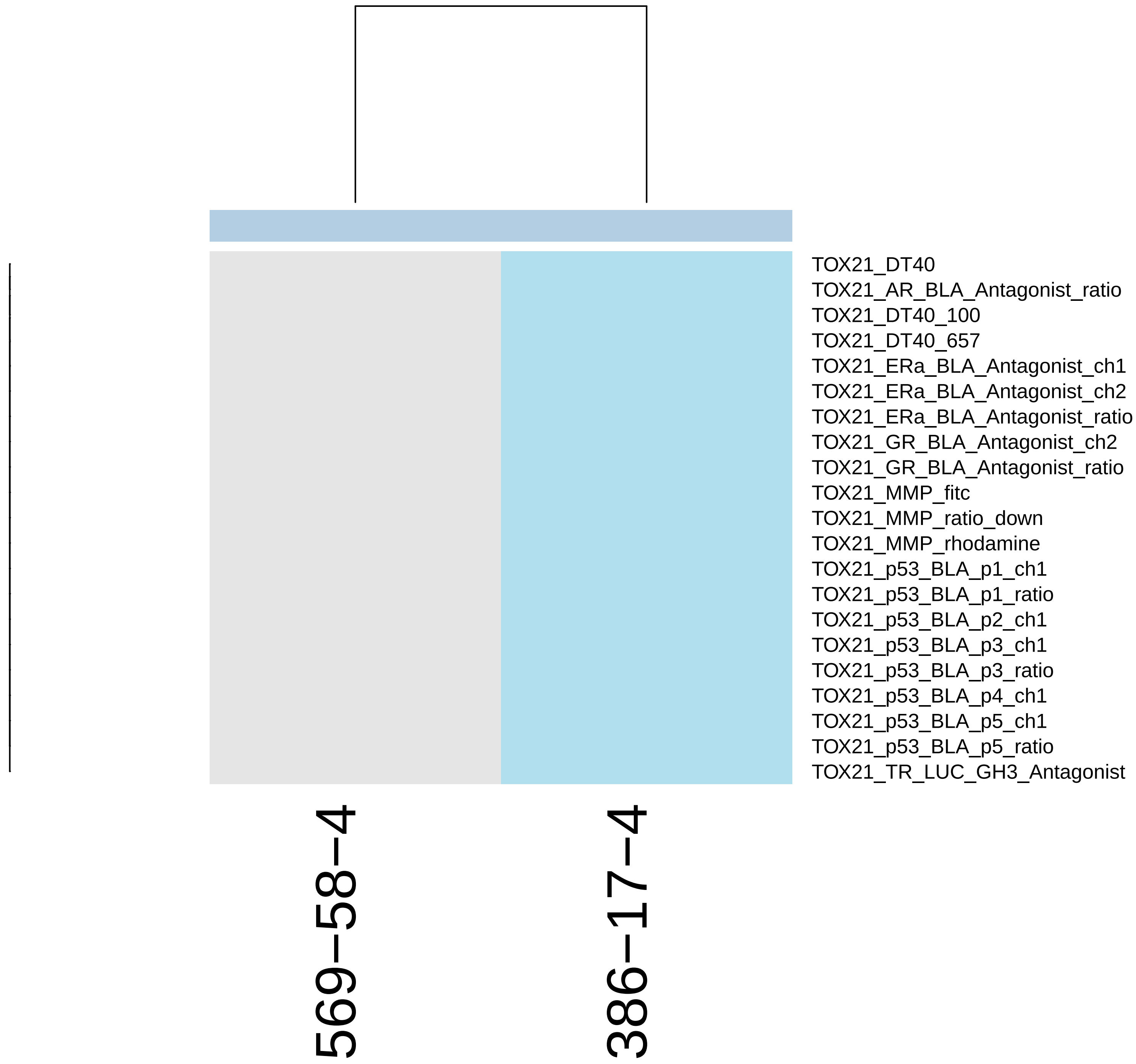

All Activity Heatmap for Cluster 1903

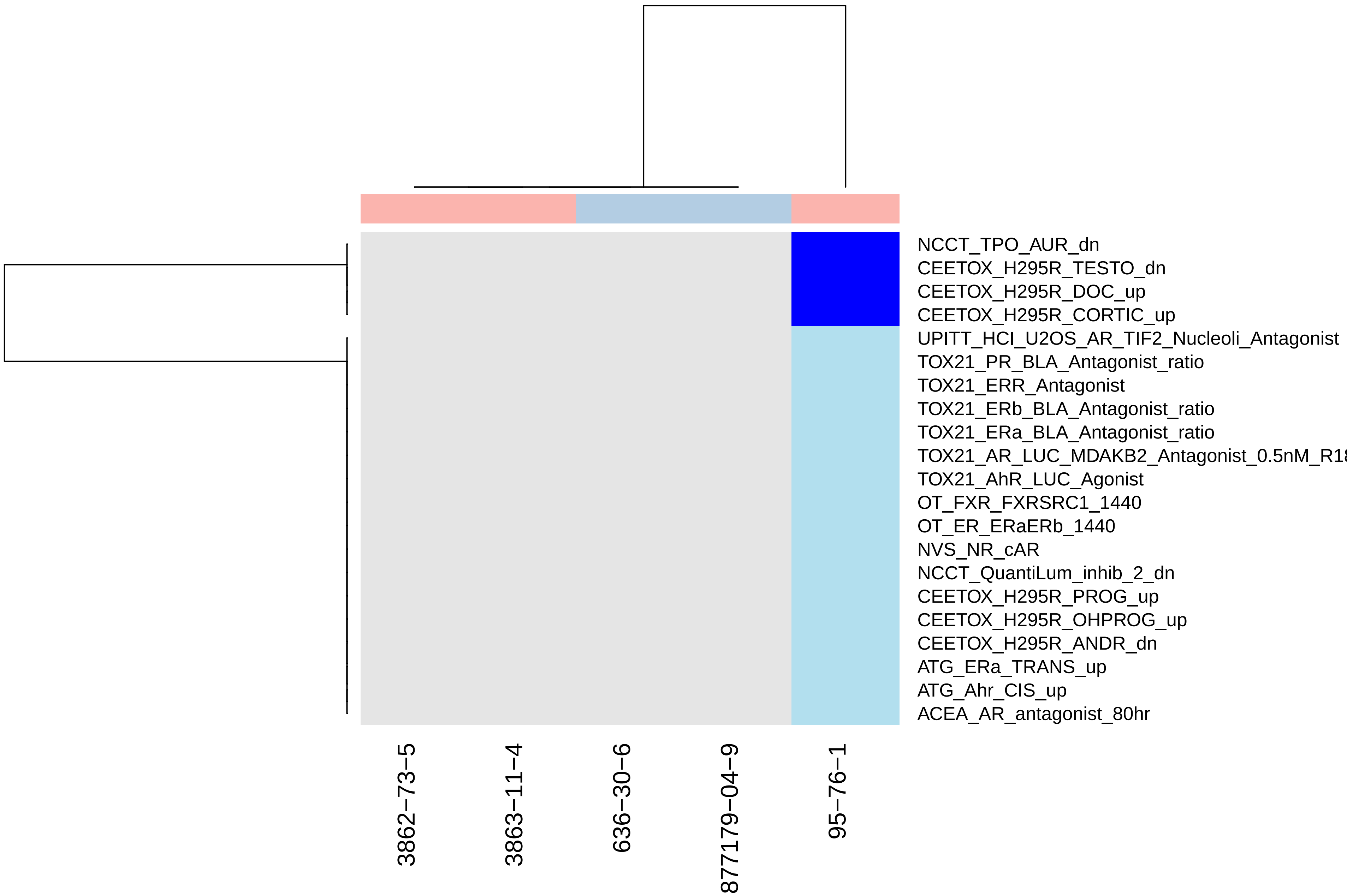

All Activity Heatmap for Cluster 1904

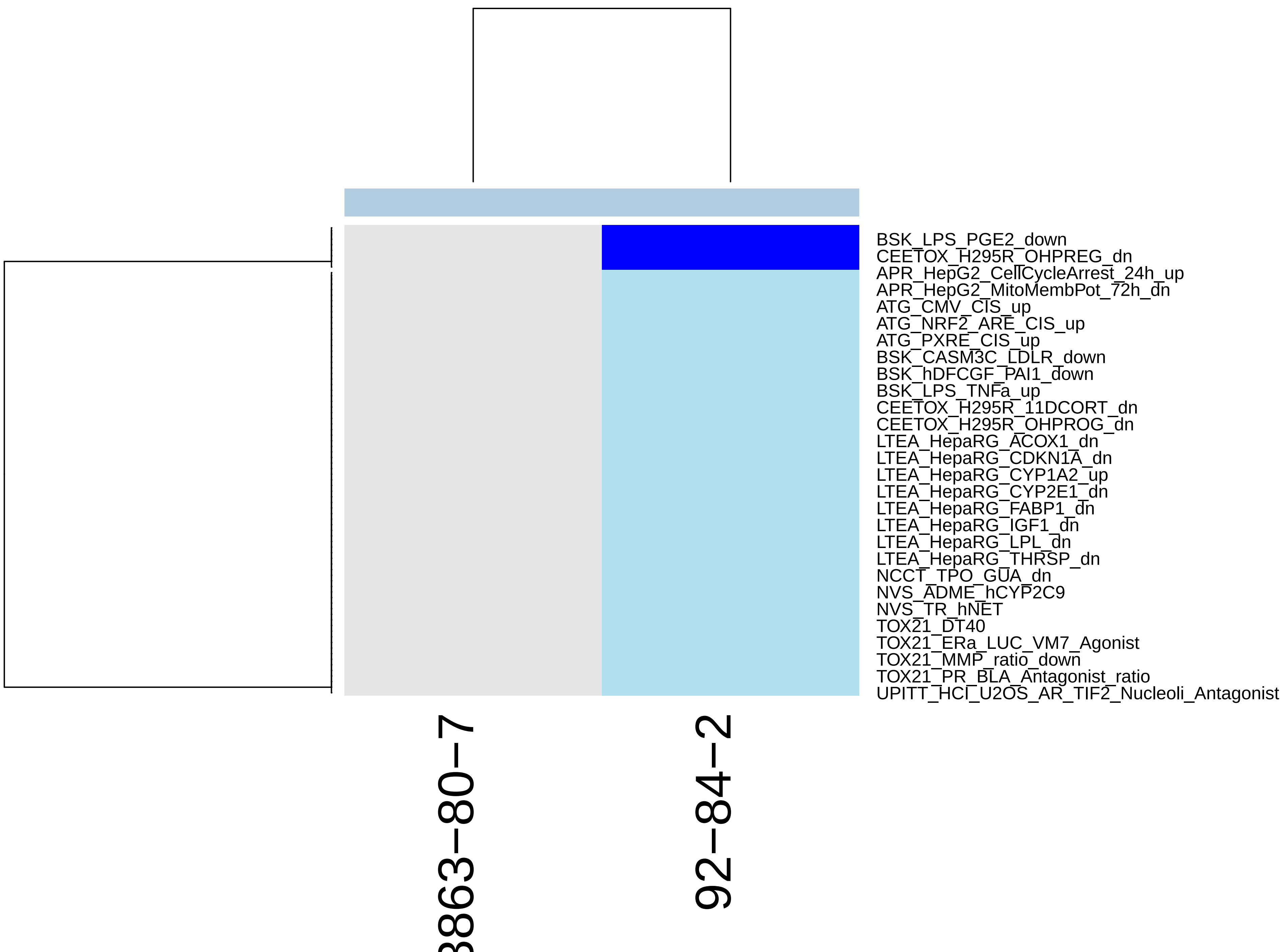

All Activity Heatmap for Cluster 1907

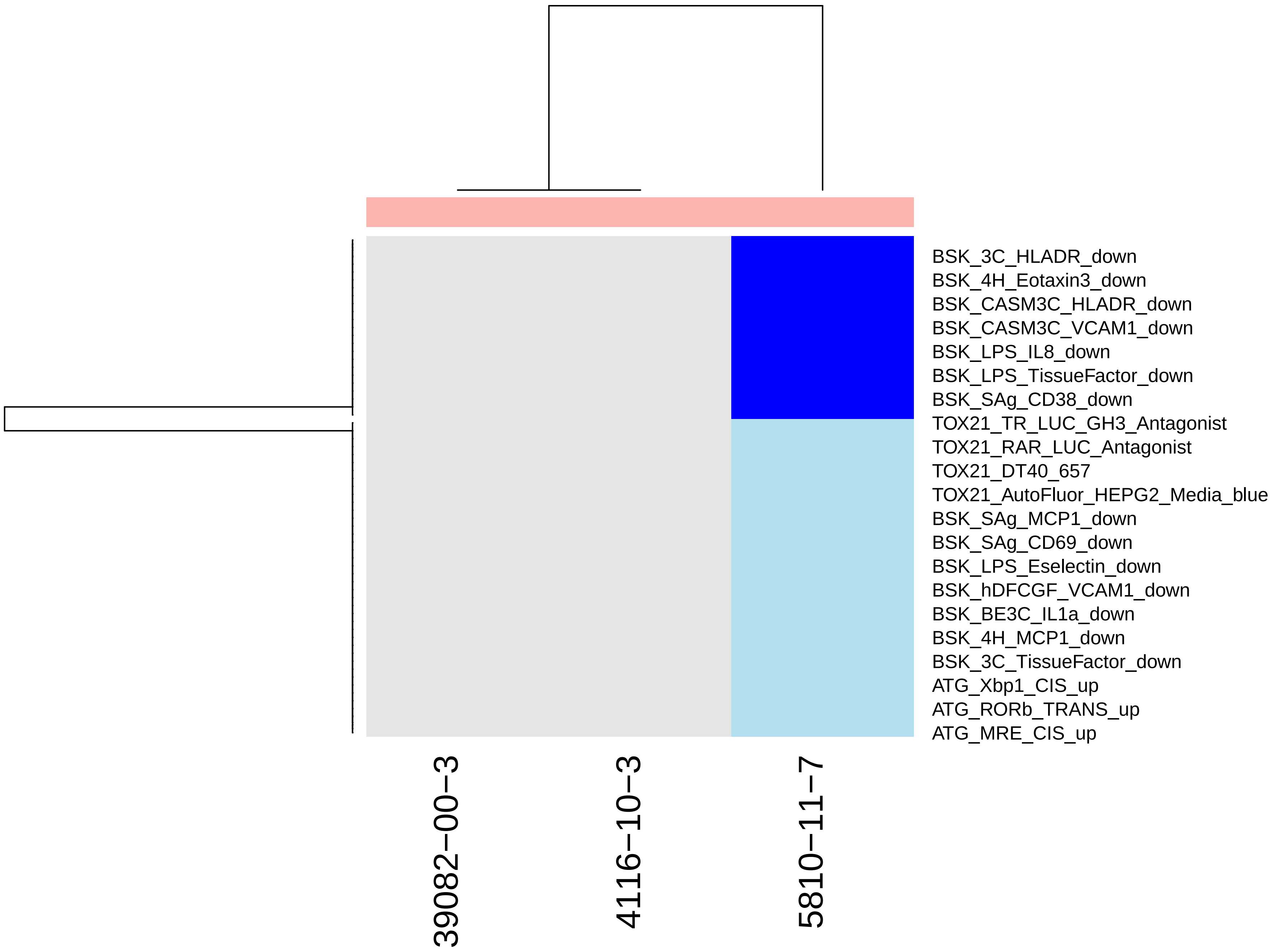

All Activity Heatmap for Cluster 1908

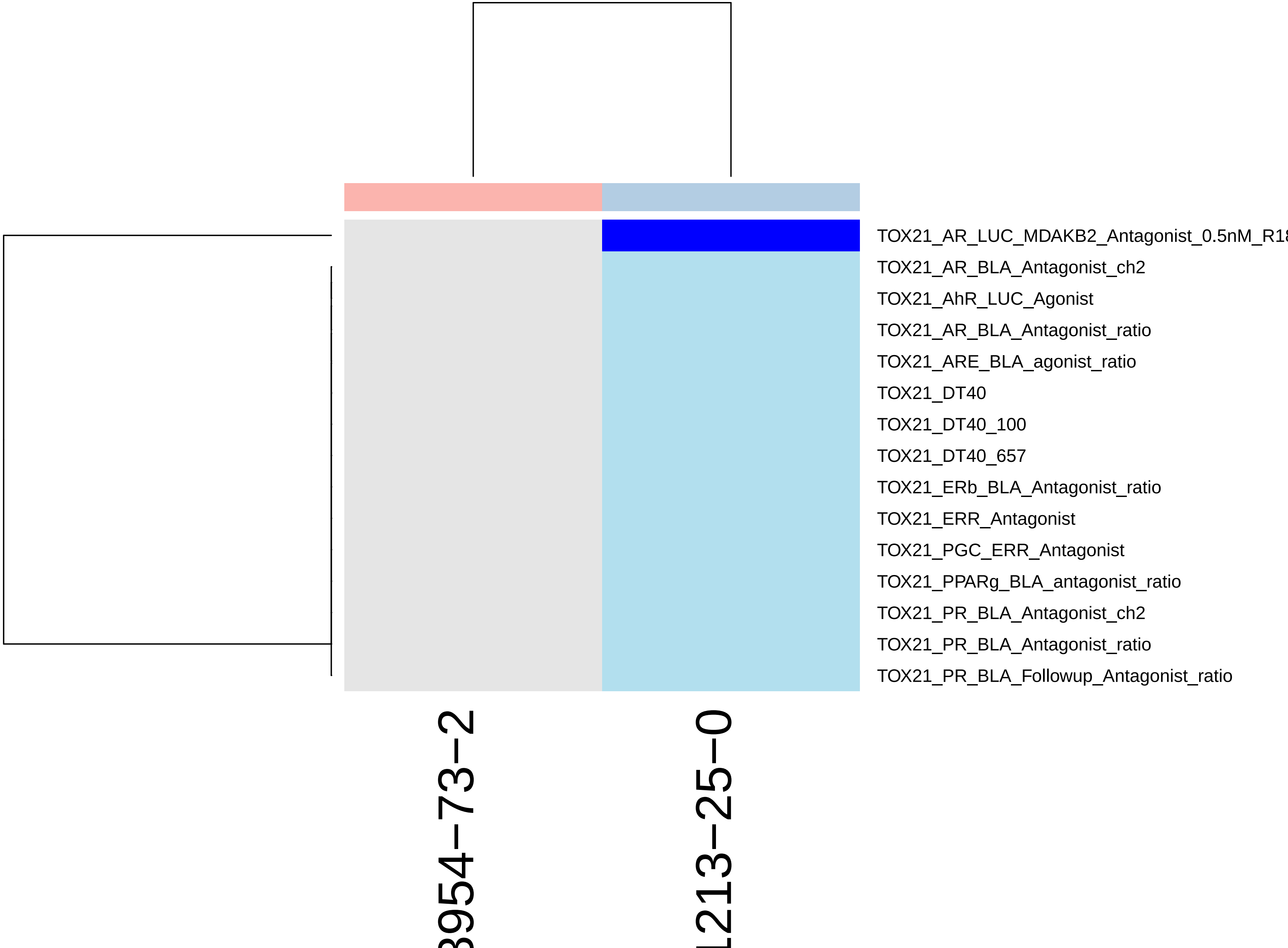

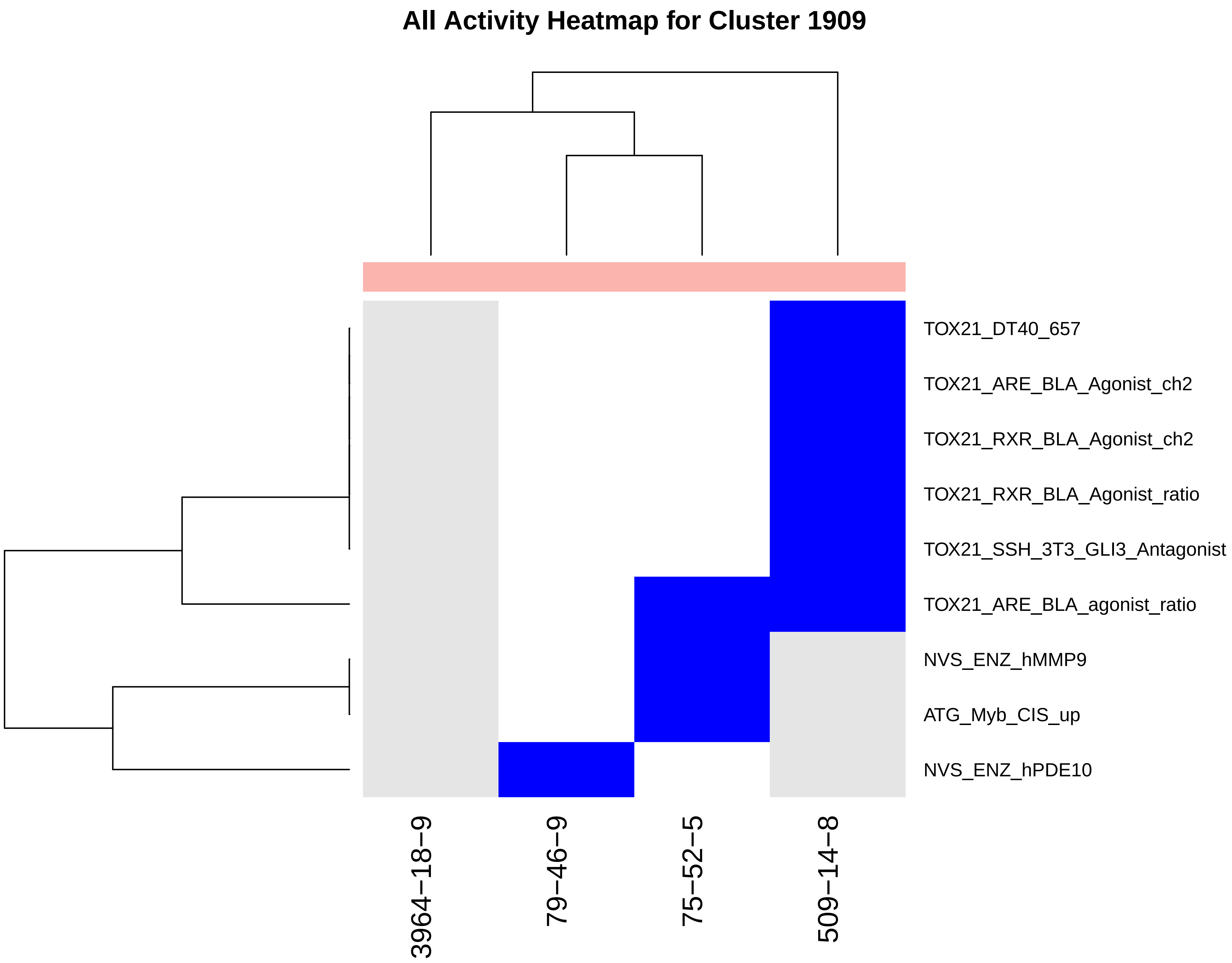

All Activity Heatmap for Cluster 1909
TOX21_DT40_657
TOX21_ARE_BLA_Agonist_ch2
TOX21_RXR_BLA_Agonist_ch2
TOX21_RXR_BLA_Agonist_ratio
TOX21_SSH_3T3_GLI3_Antagonist
TOX21_ARE_BLA_agonist_ratio
NVS_ENZ_hMMP9
ATG_Myb_CIS_up
NVS_ENZ_hPDE10
3964−18−9
79−46−9
75−52−5
509−14−8

All Activity Heatmap for Cluster 1911

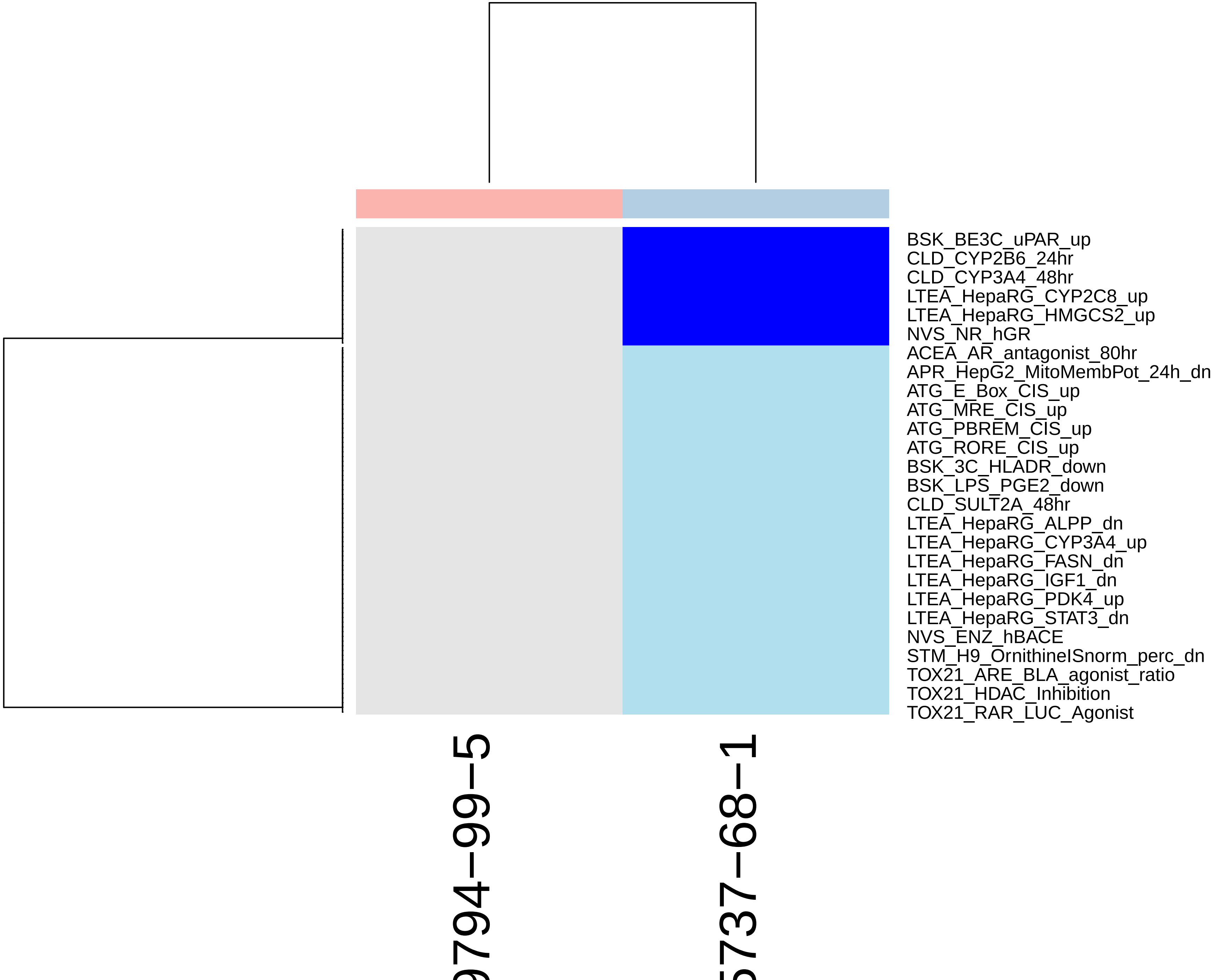

All Activity Heatmap for Cluster 1913

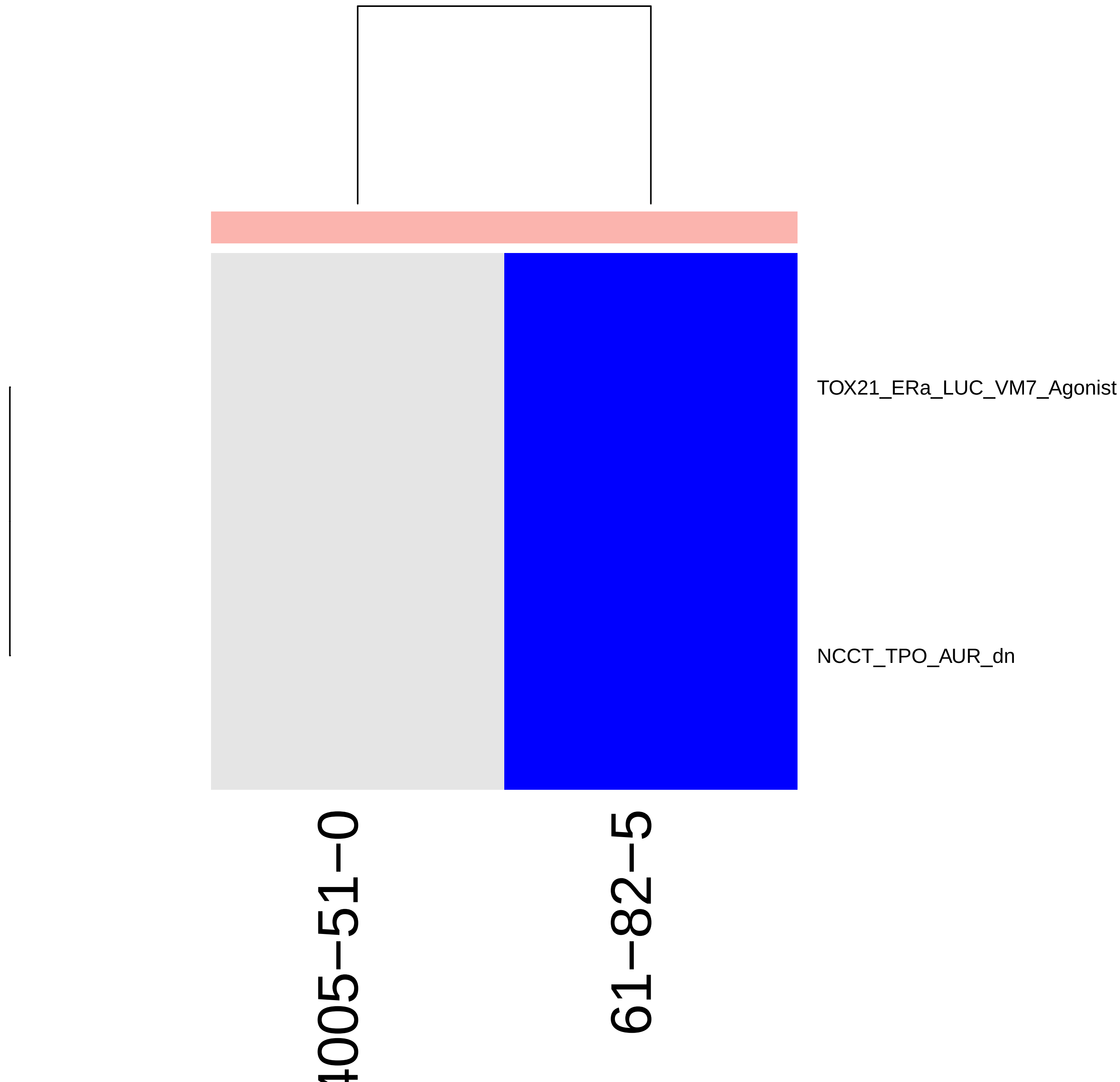

All Activity Heatmap for Cluster 1915

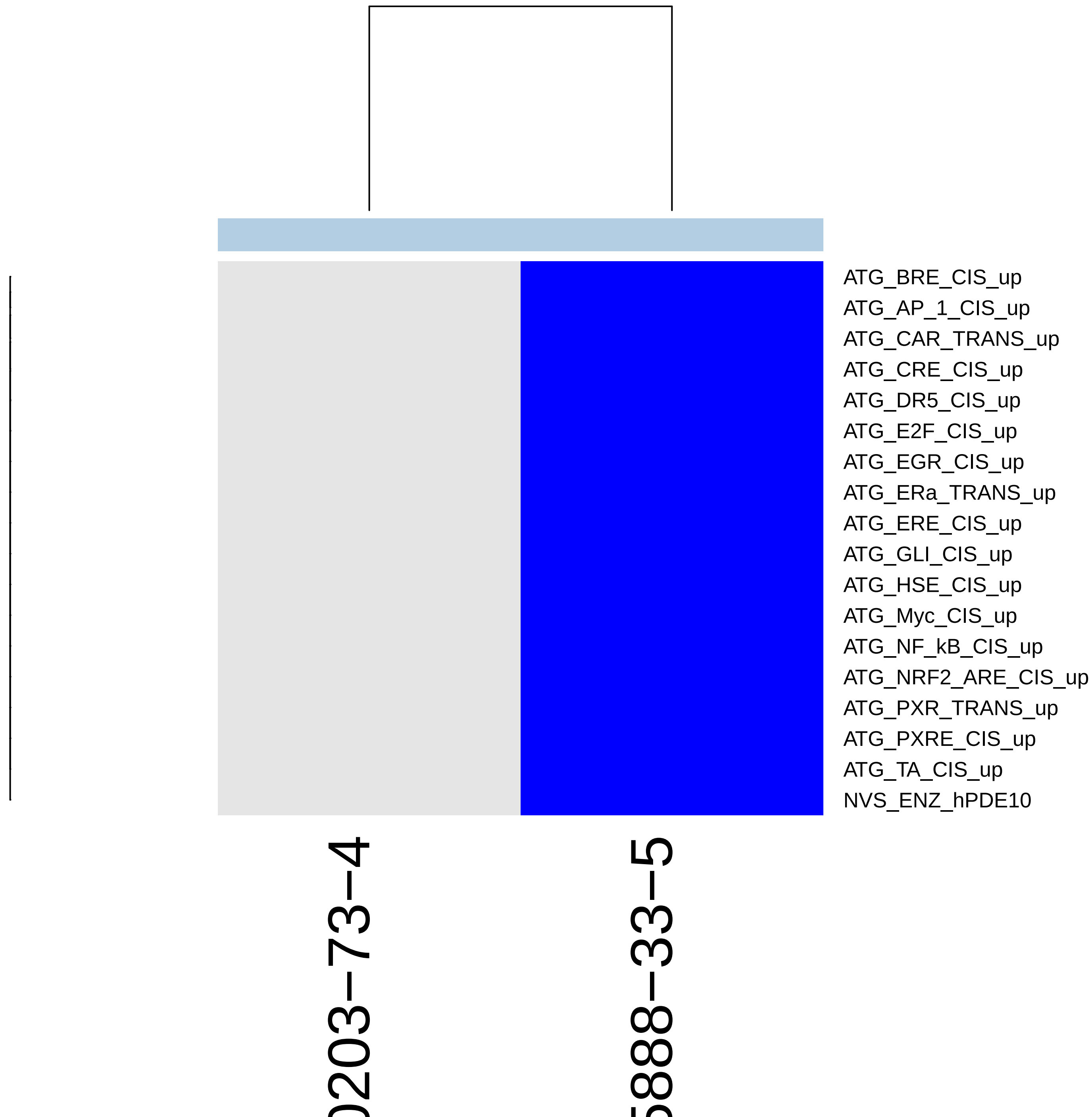

All Activity Heatmap for Cluster 1916

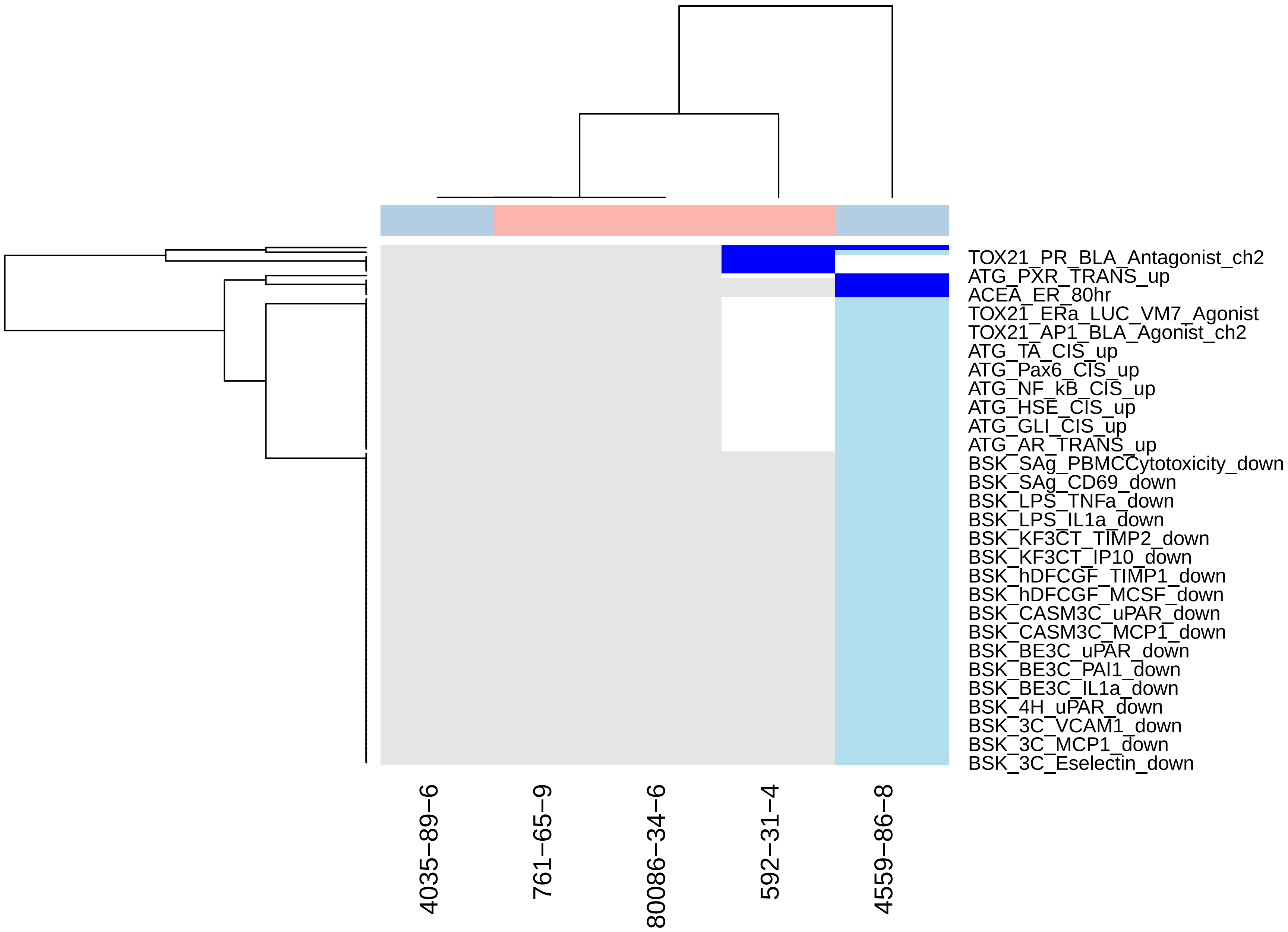

All Activity Heatmap for Cluster 1918

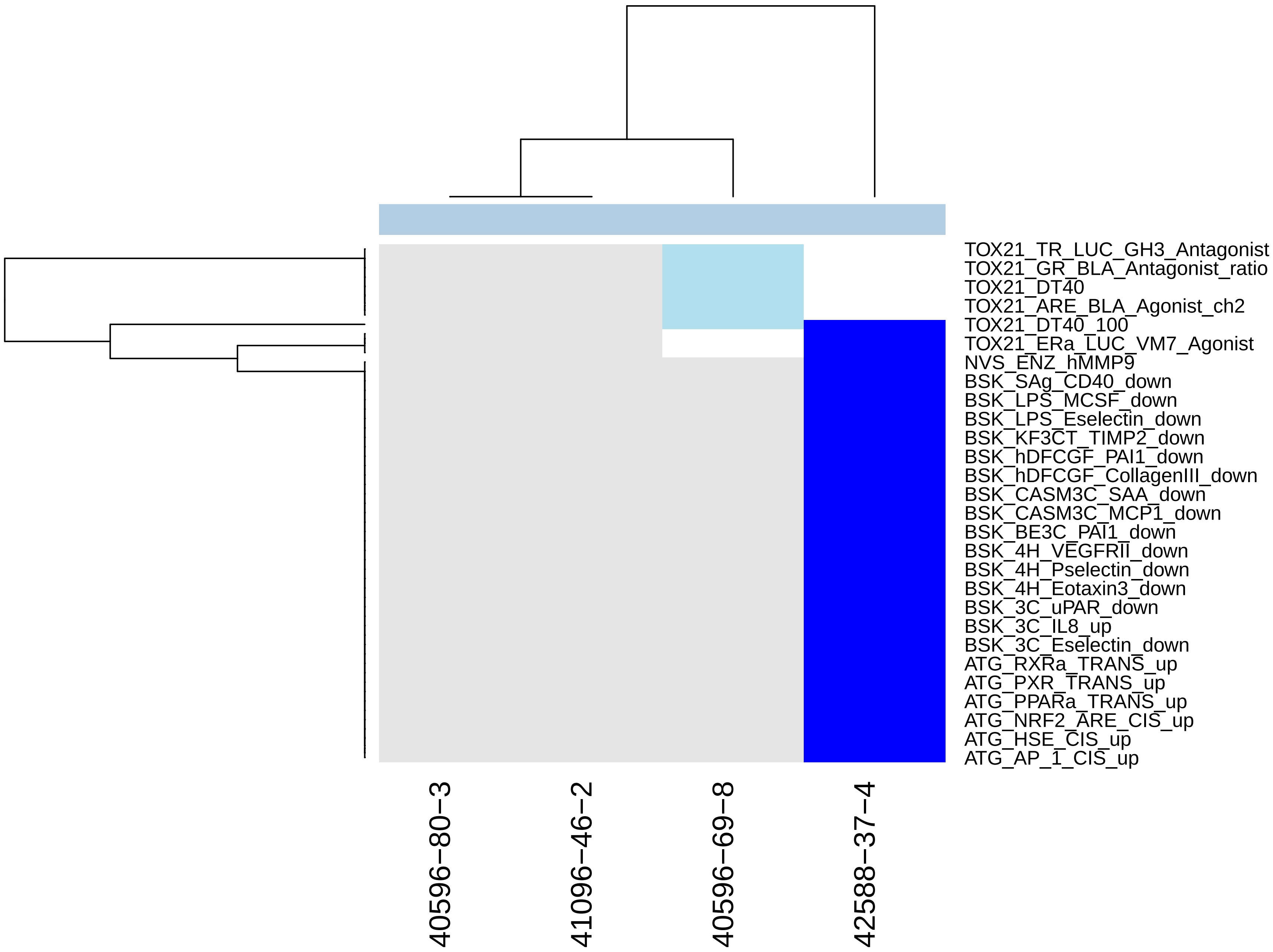

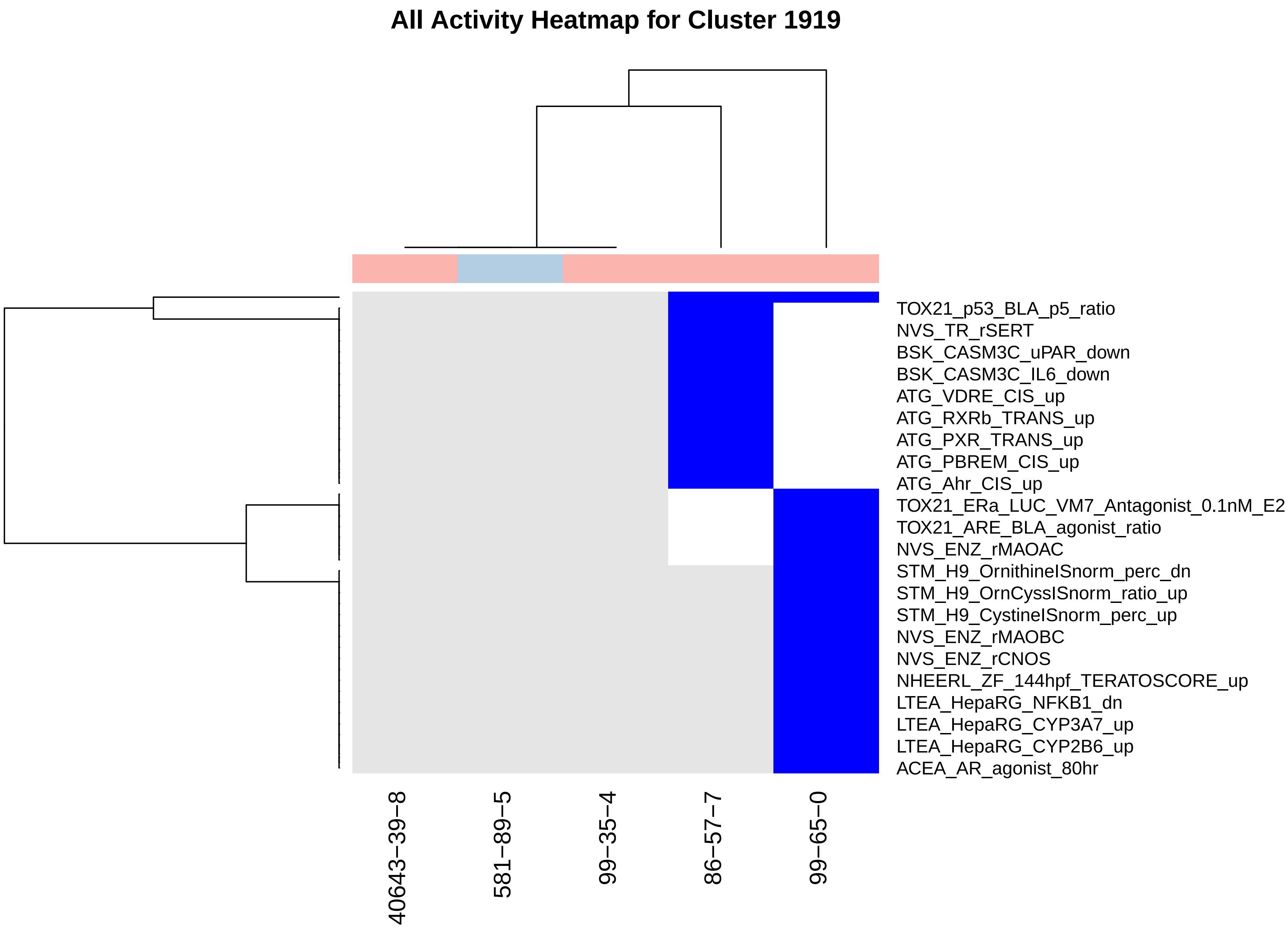
All Activity Heatmap for Cluster 1919
TOX21_p53_BLA_p5_ratio
NVS_TR_rSERT
BSK_CASM3C_uPAR_down
BSK_CASM3C_IL6_down
ATG_VDRE_CIS_up
ATG_RXRb_TRANS_up
ATG_PXR_TRANS_up
ATG_PBREM_CIS_up
ATG_Ahr_CIS_up
TOX21_ERa_LUC_VM7_Antagonist_0.1nM_E2
TOX21_ARE_BLA_agonist_ratio
NVS_ENZ_rMAOAC
STM_H9_OrnithineISnorm_perc_dn
STM_H9_OrnCyssISnorm_ratio_up
STM_H9_CystineISnorm_perc_up
NVS_ENZ_rMAOBC
NVS_ENZ_rCNOS
NHEERL_ZF_144hpf_TERATOSCORE_up
LTEA_HepaRG_NFKB1_dn
LTEA_HepaRG_CYP3A7_up
LTEA_HepaRG_CYP2B6_up
ACEA_AR_agonist_80hr
40643−39−8
581−89−5
99−35−4
86−57−7
99−65−0

All Activity Heatmap for Cluster 1923

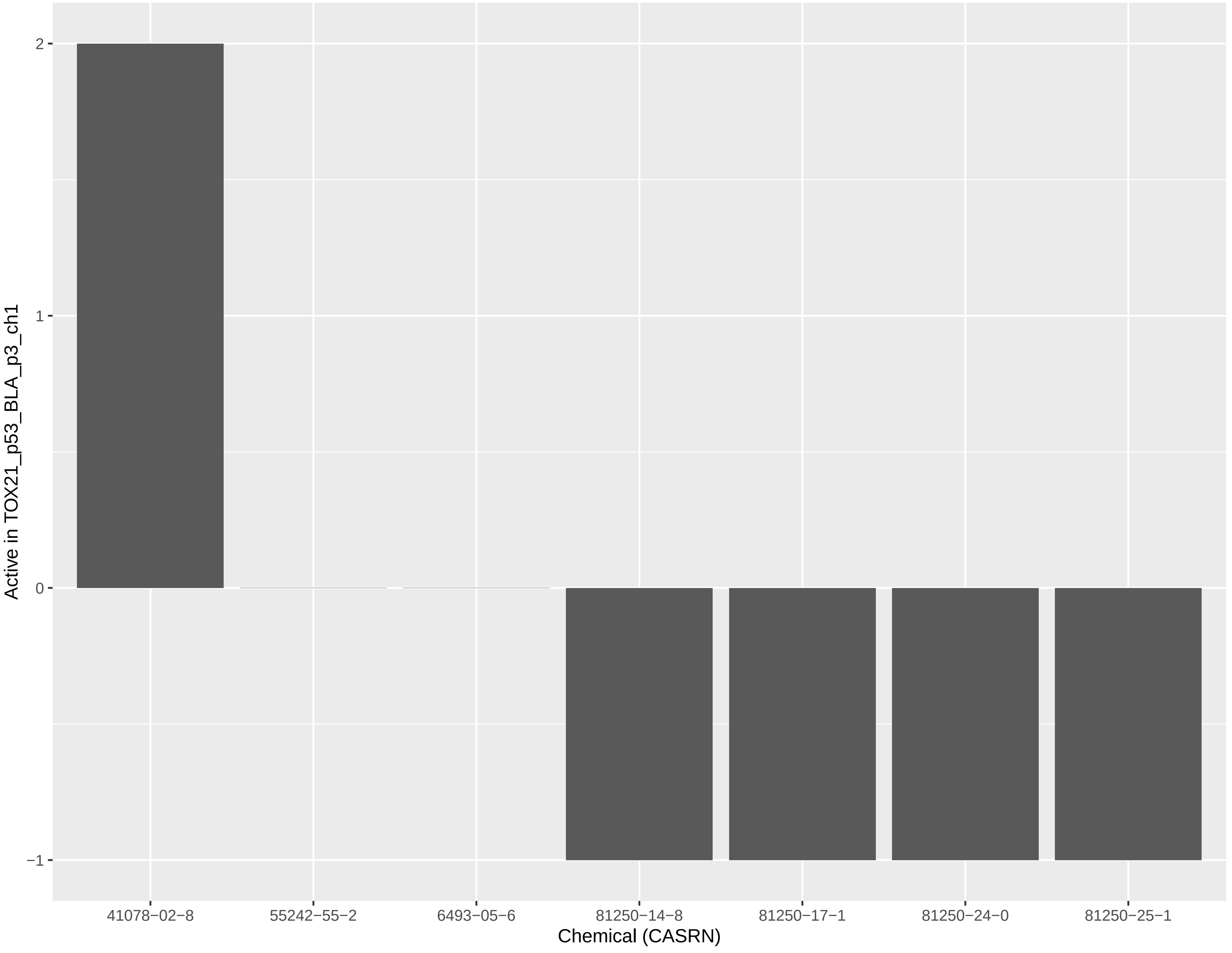

All Activity Heatmap for Cluster 1927

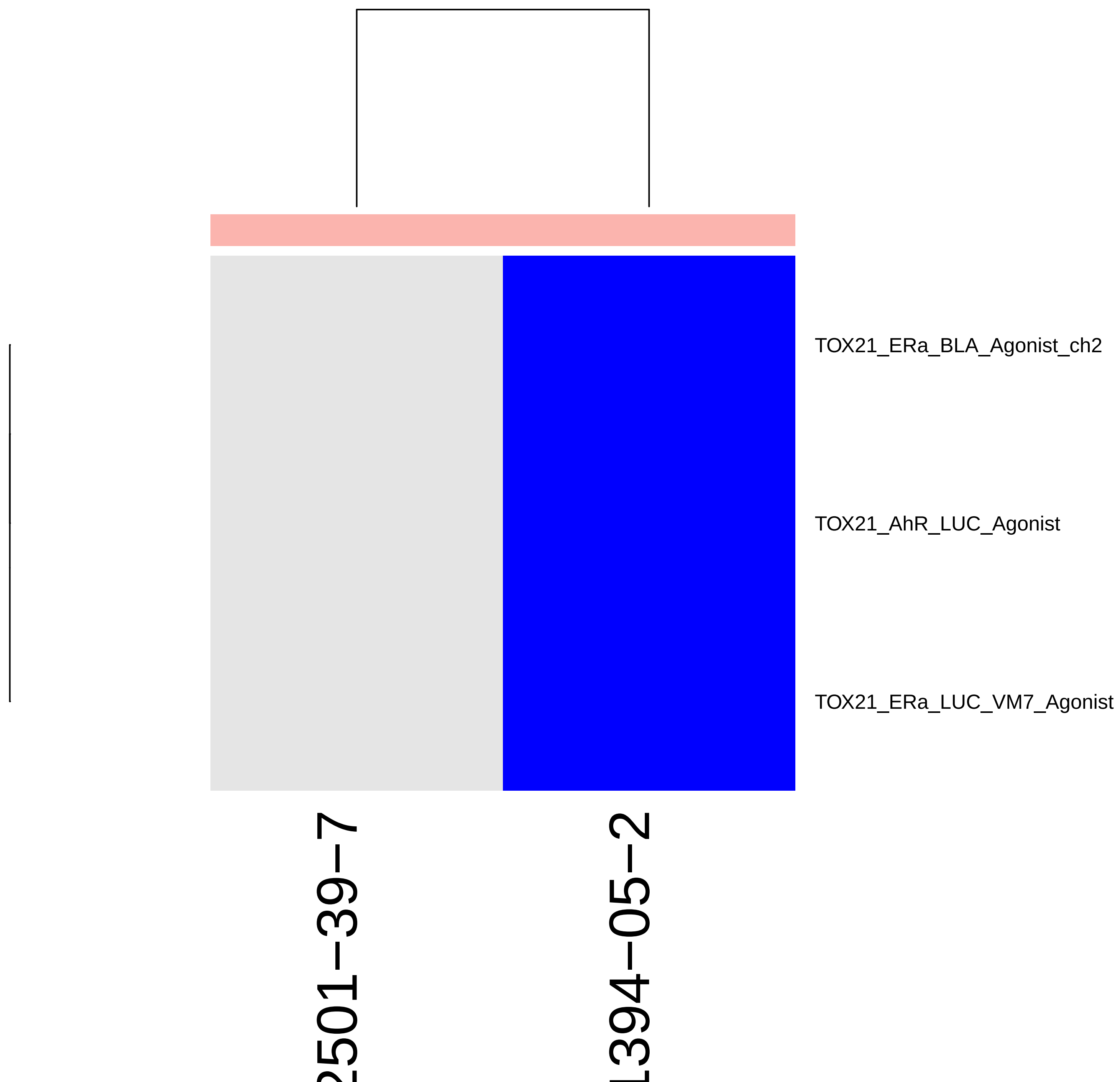

All Activity Heatmap for Cluster 1930

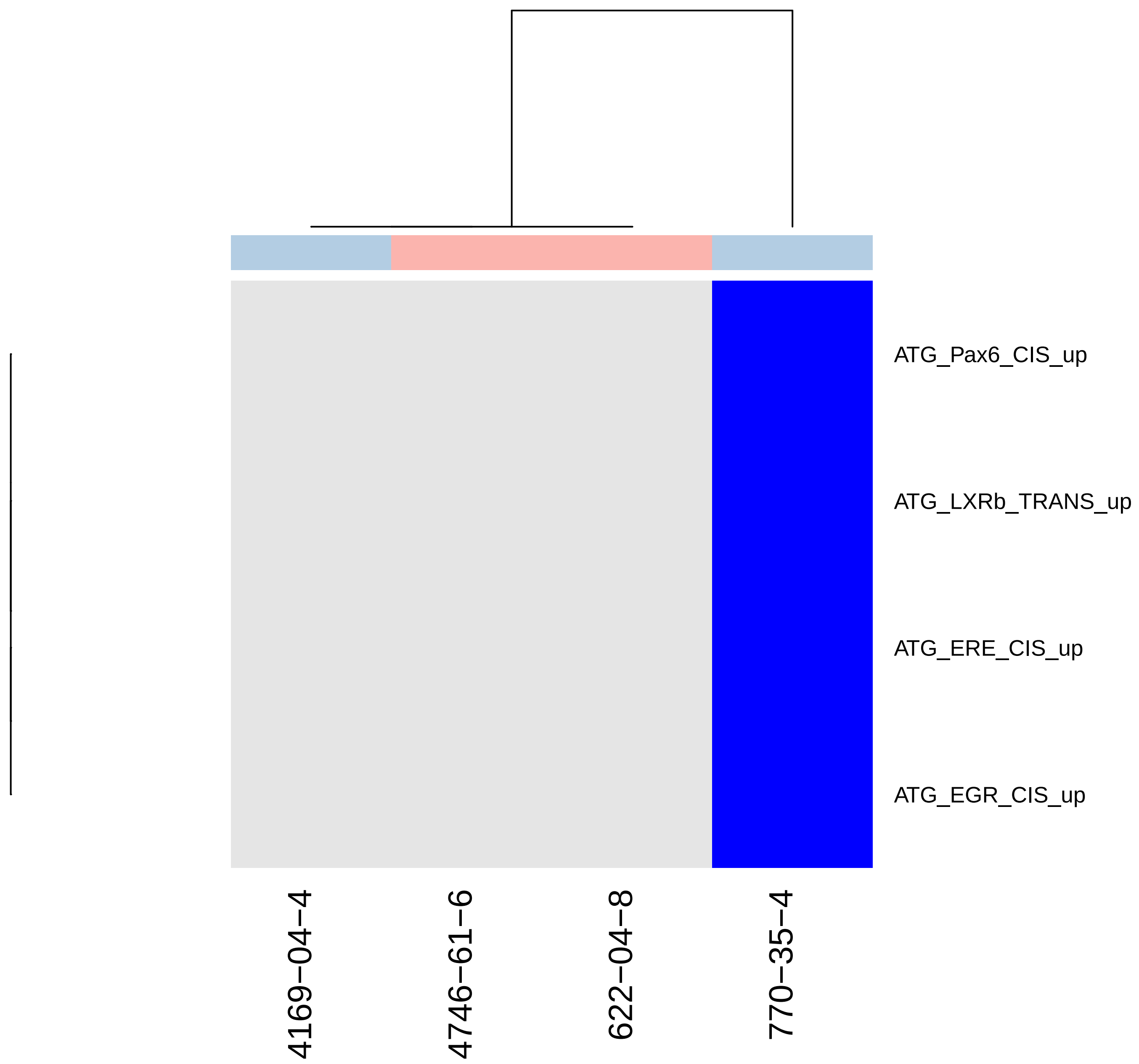

All Activity Heatmap for Cluster 1935

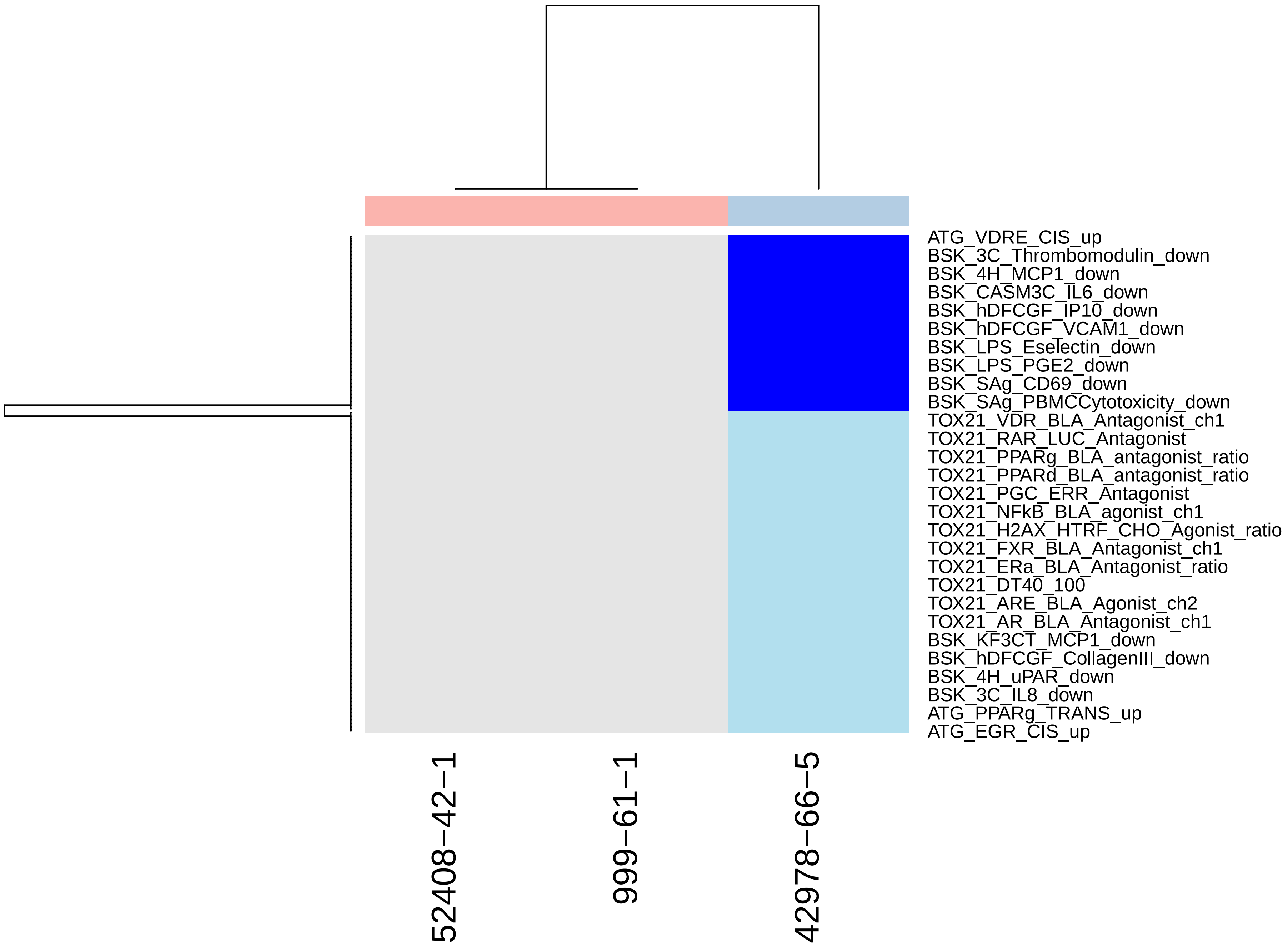

All Activity Heatmap for Cluster 1939

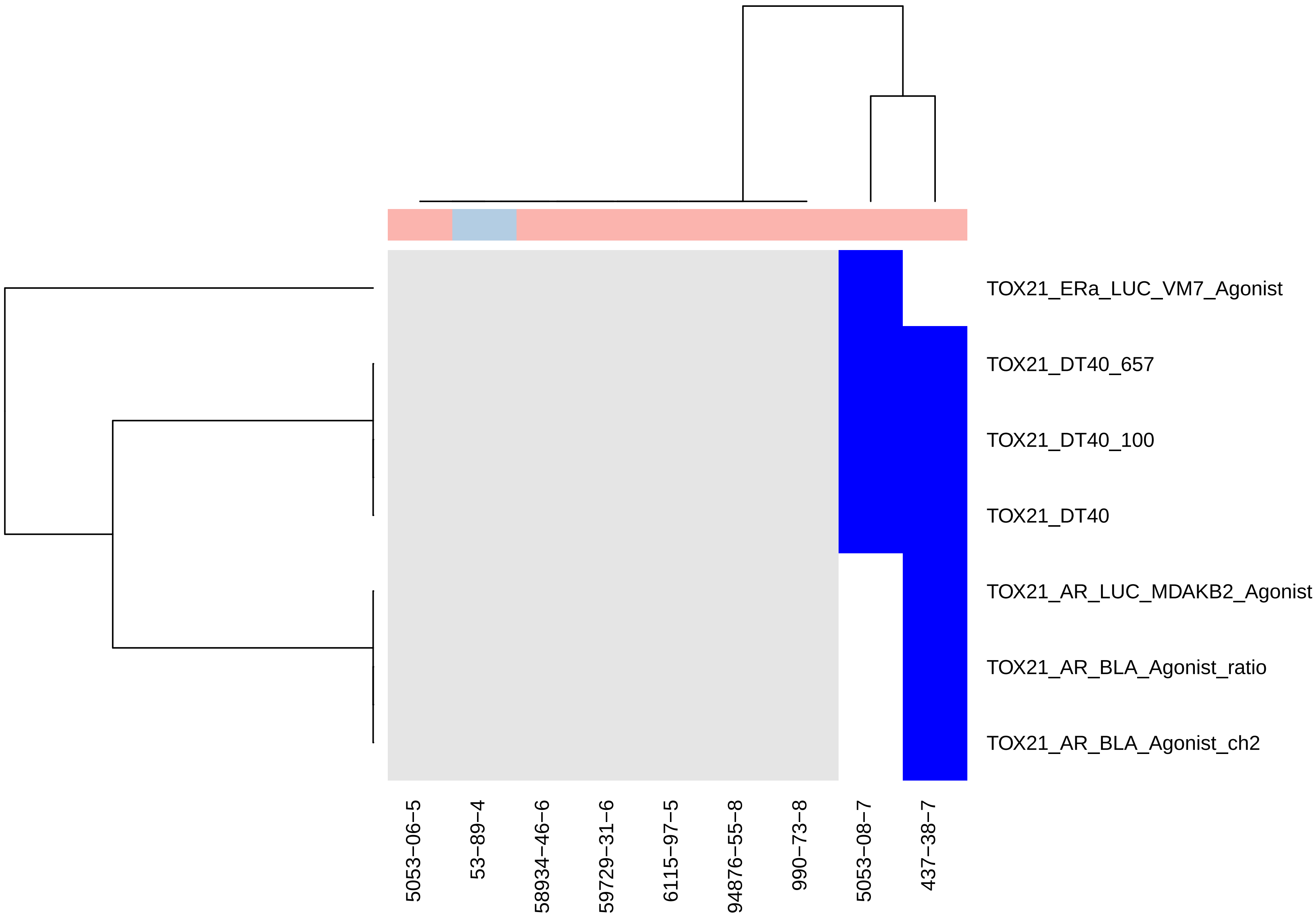

All Activity Heatmap for Cluster 1941

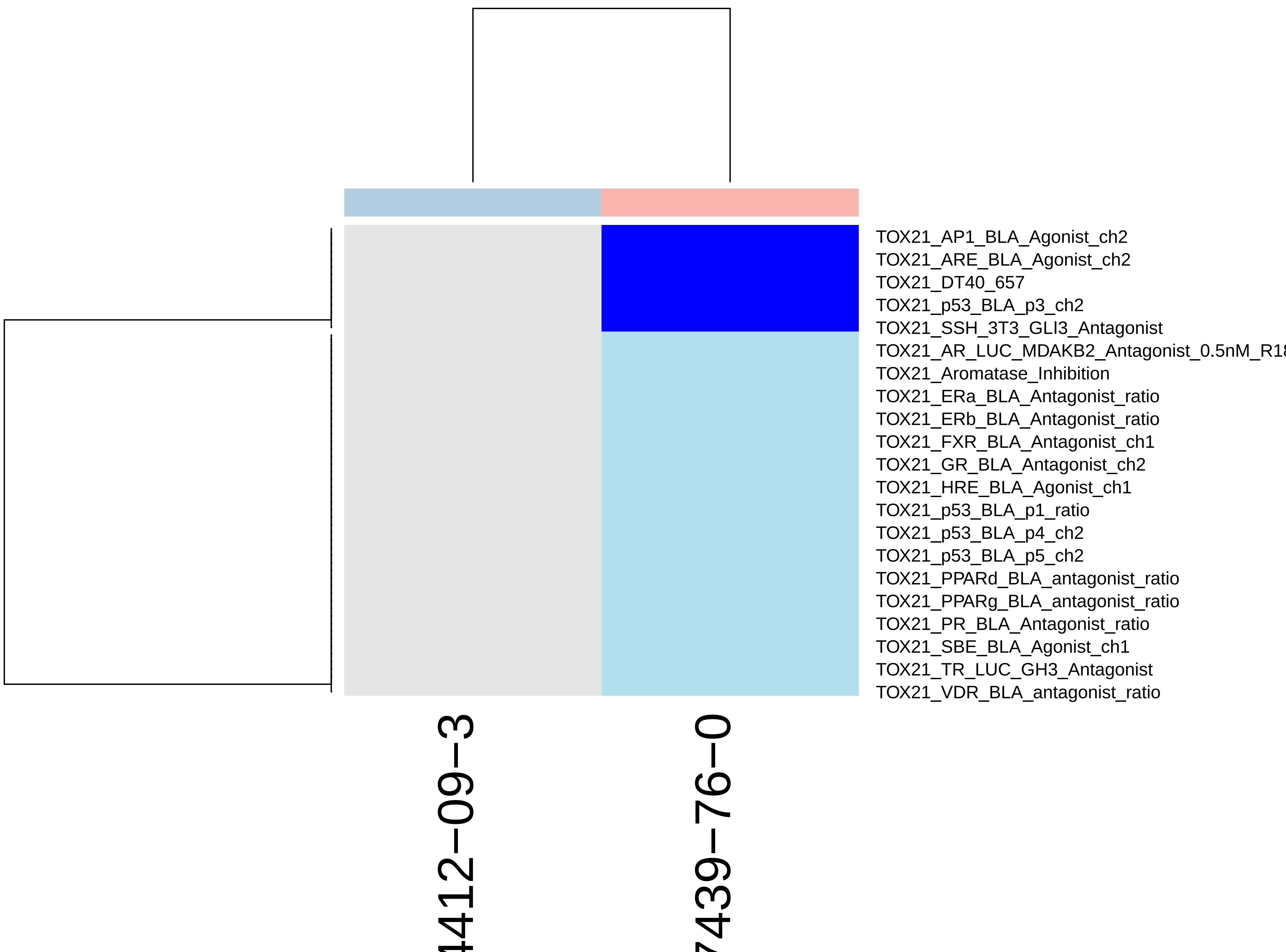

All Activity Heatmap for Cluster 1942

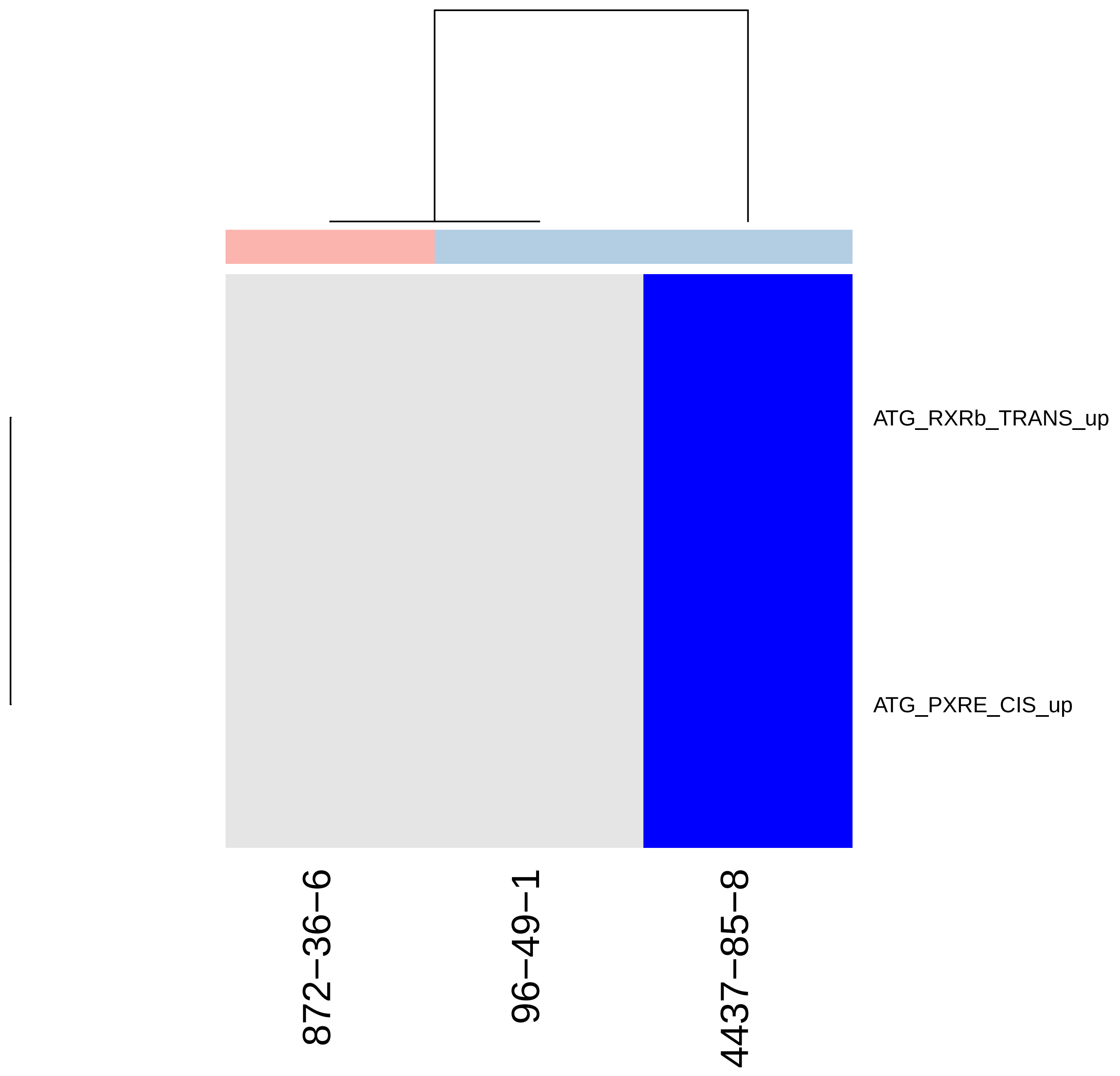

All Activity Heatmap for Cluster 1943

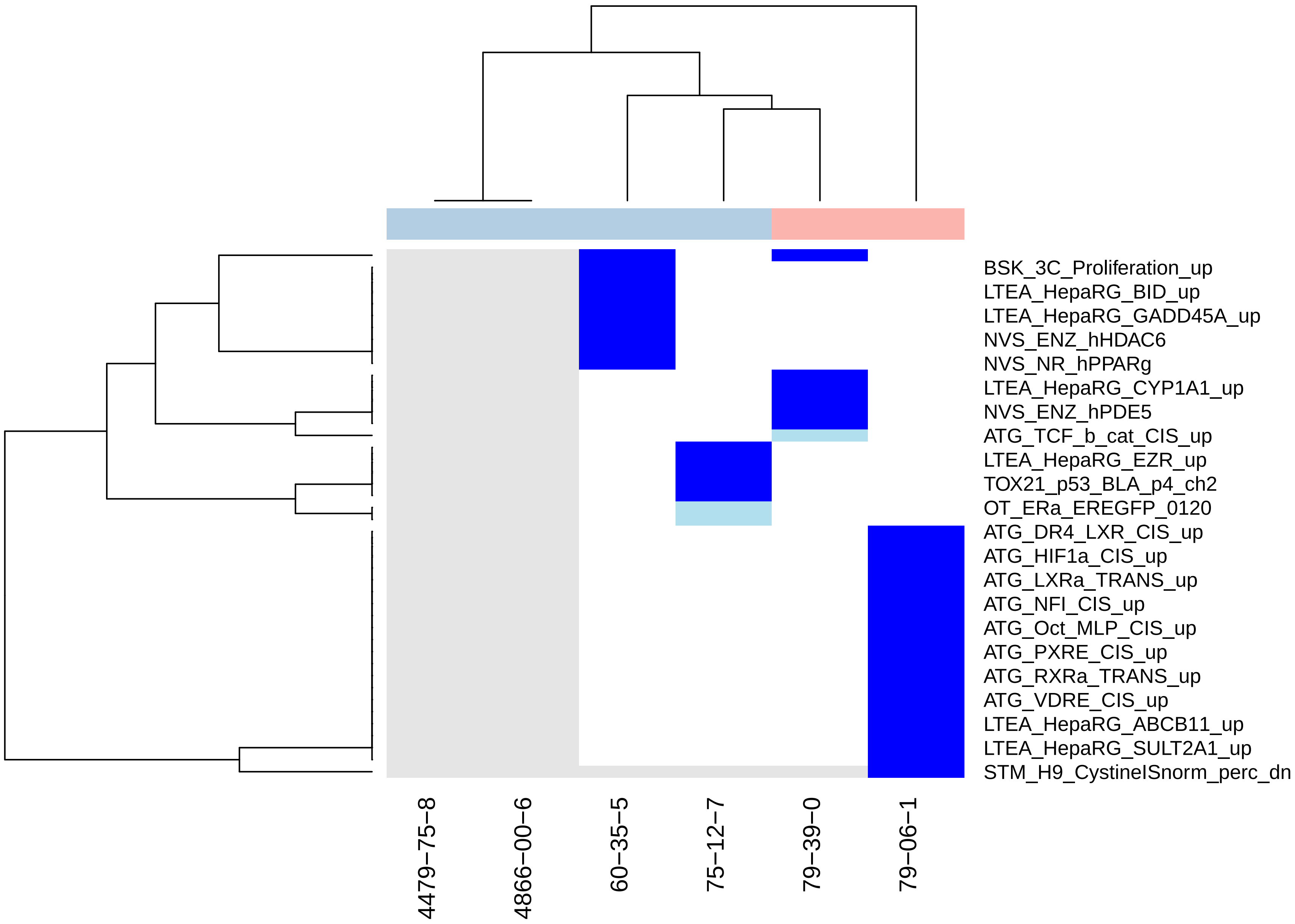

All Activity Heatmap for Cluster 1944

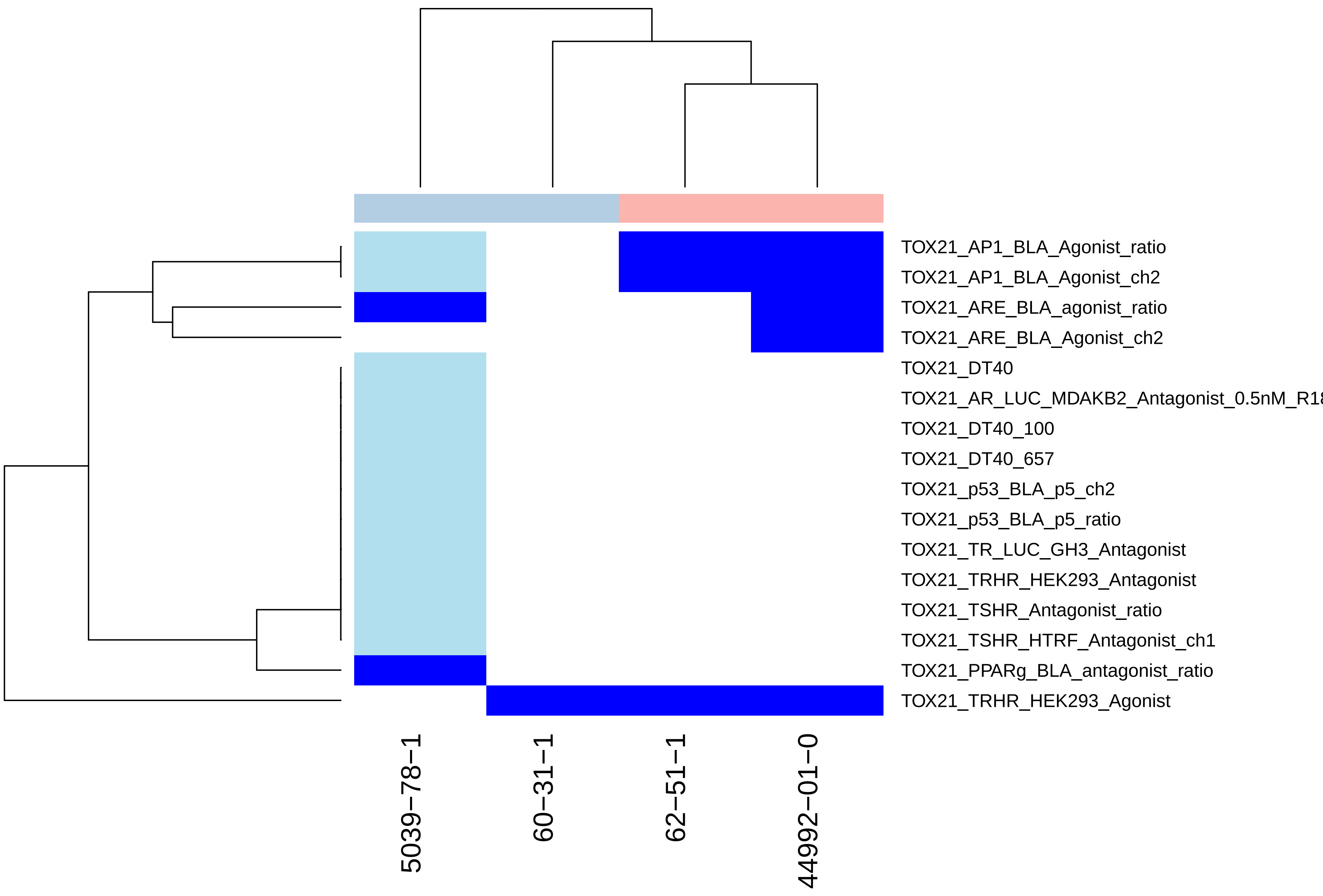

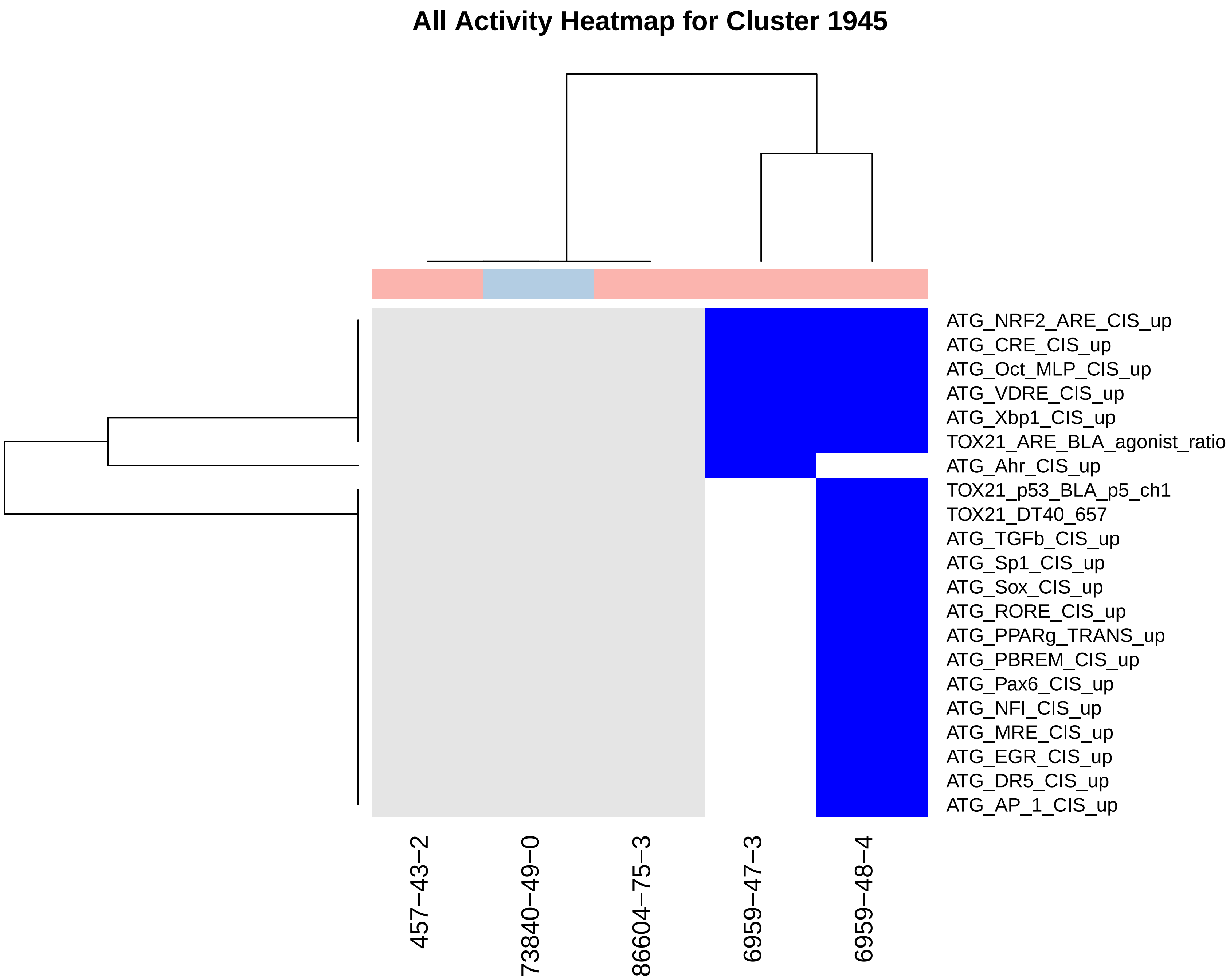
All Activity Heatmap for Cluster 1945
ATG_NRF2_ARE_CIS_up
ATG_CRE_CIS_up
ATG_Oct_MLP_CIS_up
ATG_VDRE_CIS_up
ATG_Xbp1_CIS_up
TOX21_ARE_BLA_agonist_ratio
ATG_Ahr_CIS_up
TOX21_p53_BLA_p5_ch1
TOX21_DT40_657
ATG_TGFb_CIS_up
ATG_Sp1_CIS_up
ATG_Sox_CIS_up
ATG_RORE_CIS_up
ATG_PPARg_TRANS_up
ATG_PBREM_CIS_up
ATG_Pax6_CIS_up
ATG_NFI_CIS_up
ATG_MRE_CIS_up
ATG_EGR_CIS_up
ATG_DR5_CIS_up
ATG_AP_1_CIS_up
457−43−2
73840−49−0
86604−75−3
6959−47−3
6959−48−4

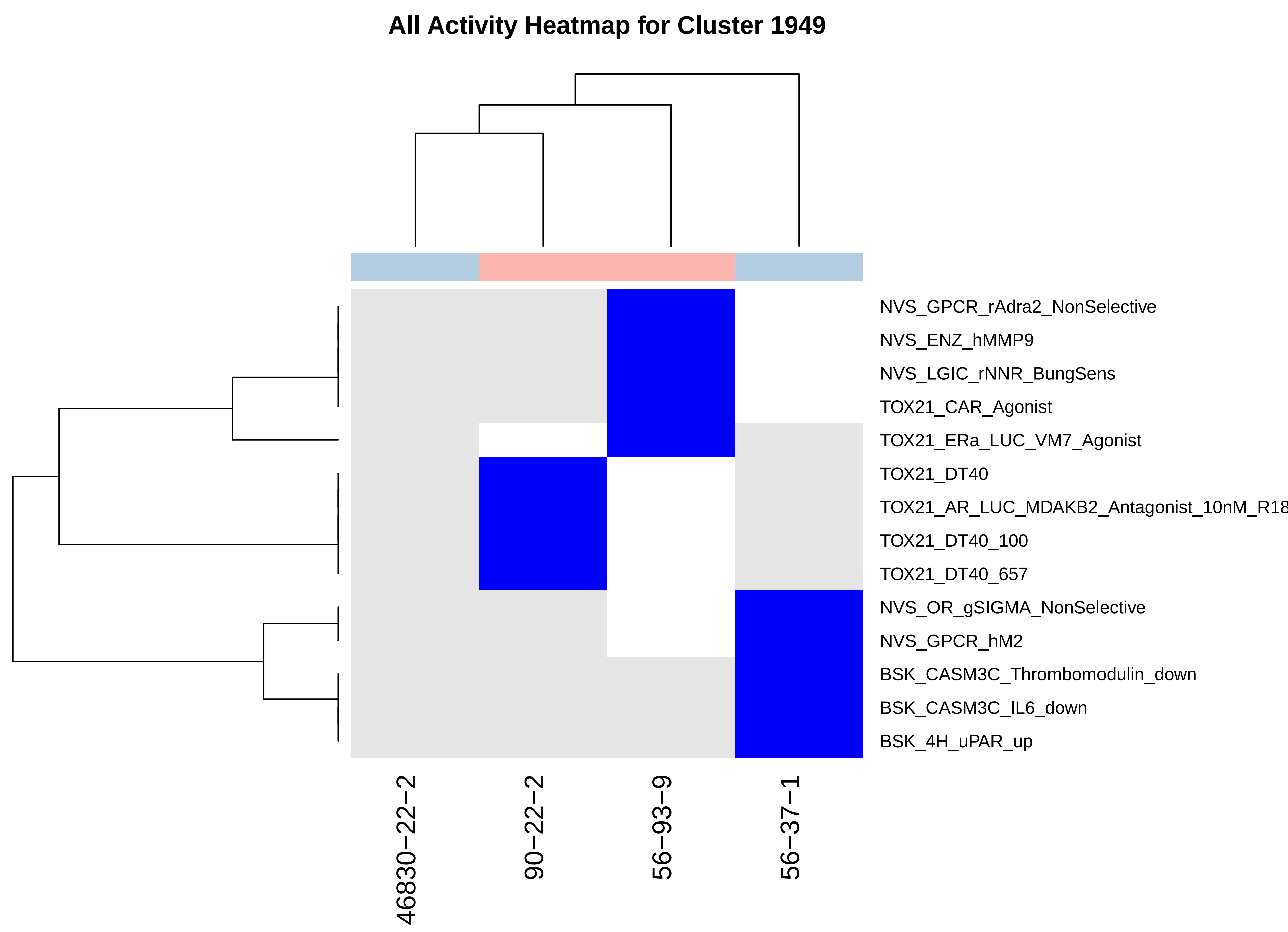

All Activity Heatmap for Cluster 1949
NVS_GPCR_rAdra2_NonSelective
NVS_ENZ_hMMP9
NVS_LGIC_rNNR_BungSens
TOX21_CAR_Agonist
TOX21_ERa_LUC_VM7_Agonist
TOX21_DT40
TOX21_AR_LUC_MDAKB2_Antagonist_10nM_R18
TOX21_DT40_100
TOX21_DT40_657
NVS_OR_gSIGMA_NonSelective
NVS_GPCR_hM2
BSK_CASM3C_Thrombomodulin_down
BSK_CASM3C_IL6_down
BSK_4H_uPAR_up
46830−22−2
90−22−2
56−93−9
56−37−1

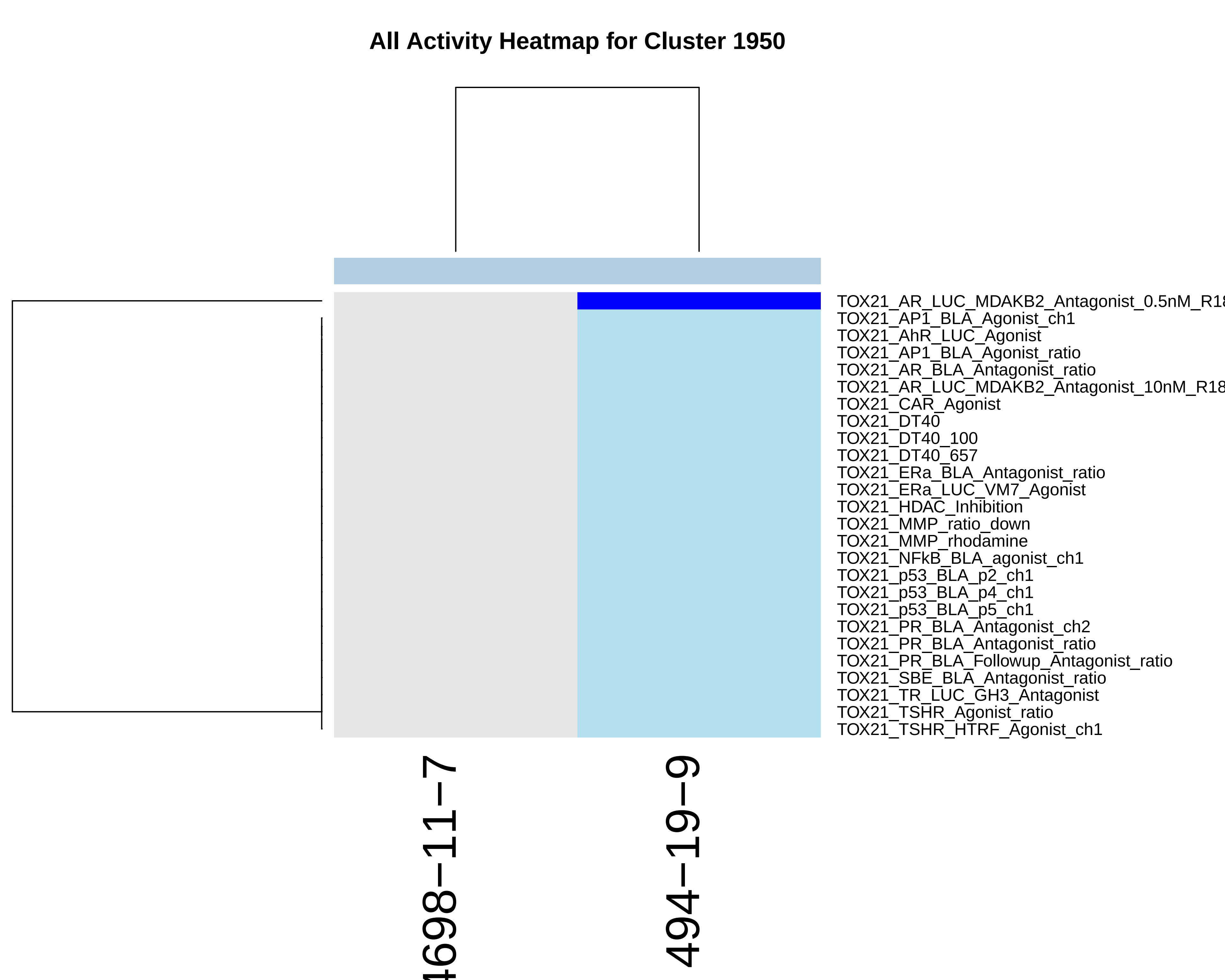

All Activity Heatmap for Cluster 1950
TOX21_AR_LUC_MDAKB2_Antagonist_0.5nM_R18
TOX21_AP1_BLA_Agonist_ch1
TOX21_AhR_LUC_Agonist
TOX21_AP1_BLA_Agonist_ratio
TOX21_AR_BLA_Antagonist_ratio
TOX21_AR_LUC_MDAKB2_Antagonist_10nM_R18
TOX21_CAR_Agonist
TOX21_DT40
TOX21_DT40_100
TOX21_DT40_657
TOX21_ERa_BLA_Antagonist_ratio
TOX21_ERa_LUC_VM7_Agonist
TOX21_HDAC_Inhibition
TOX21_MMP_ratio_down
TOX21_MMP_rhodamine
TOX21_NFkB_BLA_agonist_ch1
TOX21_p53_BLA_p2_ch1
TOX21_p53_BLA_p4_ch1
TOX21_p53_BLA_p5_ch1
TOX21_PR_BLA_Antagonist_ch2
TOX21_PR_BLA_Antagonist_ratio
TOX21_PR_BLA_Followup_Antagonist_ratio
TOX21_SBE_BLA_Antagonist_ratio
TOX21_TR_LUC_GH3_Antagonist
TOX21_TSHR_Agonist_ratio
TOX21_TSHR_HTRF_Agonist_ch1
4698−11−7
494−19−9

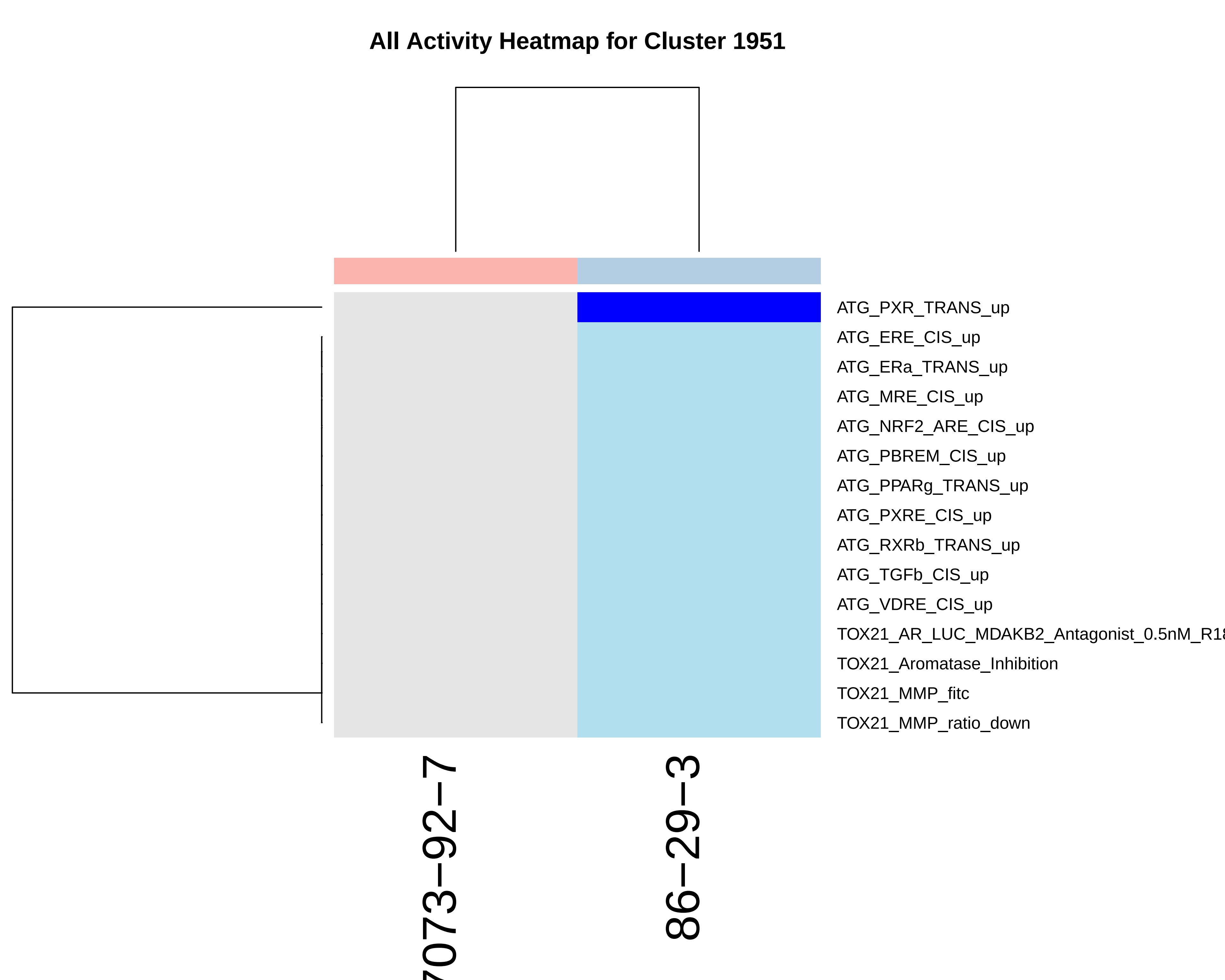
All Activity Heatmap for Cluster 1951
ATG_PXR_TRANS_up
ATG_ERE_CIS_up
ATG_ERa_TRANS_up
ATG_MRE_CIS_up
ATG_NRF2_ARE_CIS_up
ATG_PBREM_CIS_up
ATG_PPARg_TRANS_up
ATG_PXRE_CIS_up
ATG_RXRb_TRANS_up
ATG_TGFb_CIS_up
ATG_VDRE_CIS_up
TOX21_AR_LUC_MDAKB2_Antagonist_0.5nM_R1
TOX21_Aromatase_Inhibition
TOX21_MMP_fitc
TOX21_MMP_ratio_down
7073−92−7
86−29−3

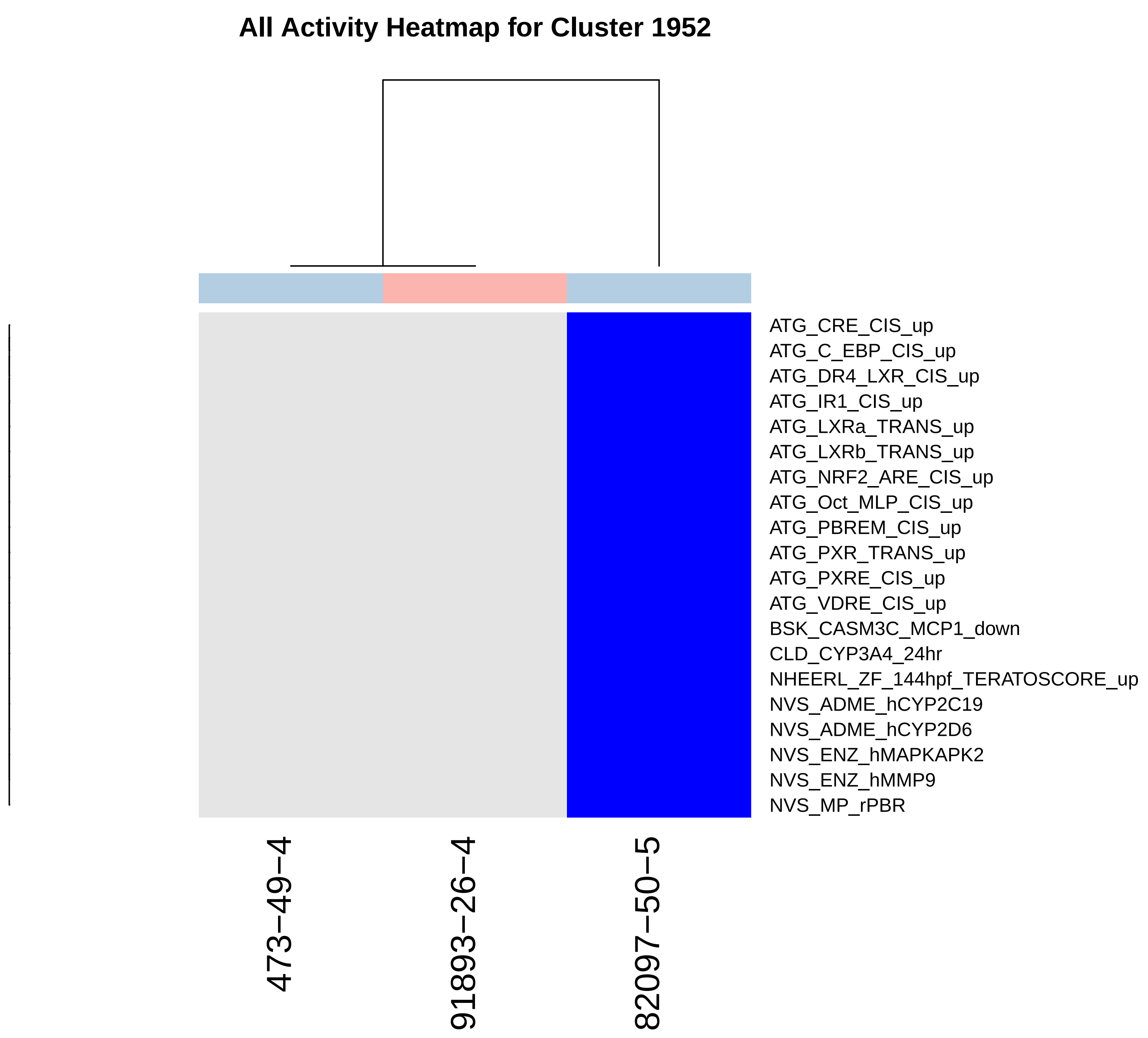
All Activity Heatmap for Cluster 1952
ATG_CRE_CIS_up
ATG_C_EBP_CIS_up
ATG_DR4_LXR_CIS_up
ATG_IR1_CIS_up
ATG_LXRa_TRANS_up
ATG_LXRb_TRANS_up
ATG_NRF2_ARE_CIS_up
ATG_Oct_MLP_CIS_up
ATG_PBREM_CIS_up
ATG_PXR_TRANS_up
ATG_PXRE_CIS_up
ATG_VDRE_CIS_up
BSK_CASM3C_MCP1_down
CLD_CYP3A4_24hr
NHEERL_ZF_144hpf_TERATOSCORE_up
NVS_ADME_hCYP2C19
NVS_ADME_hCYP2D6
NVS_ENZ_hMAPKAPK2
NVS_ENZ_hMMP9
NVS_MP_rPBR
473−49−4
91893−26−4
82097−50−5

All Activity Heatmap for Cluster 1955

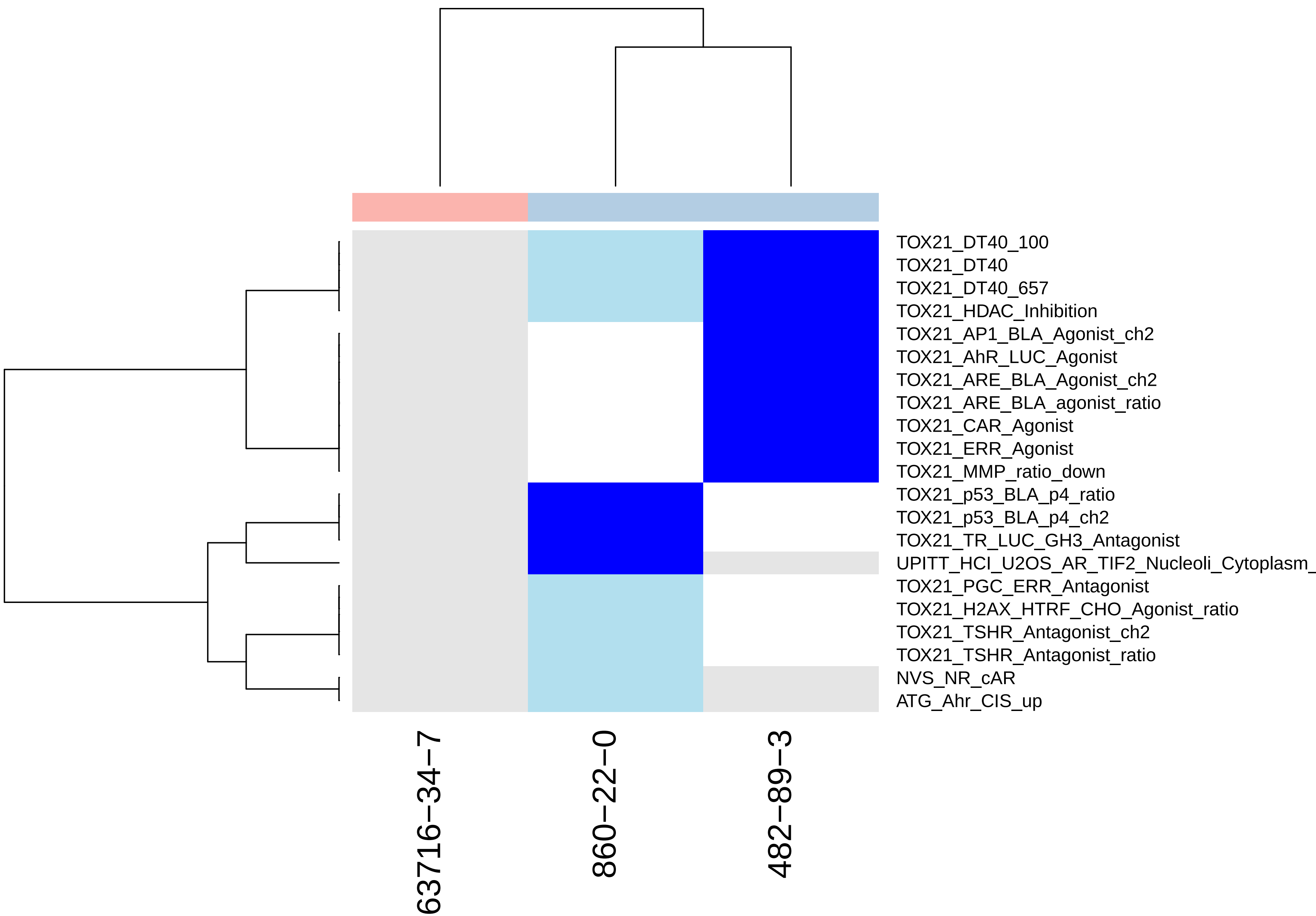

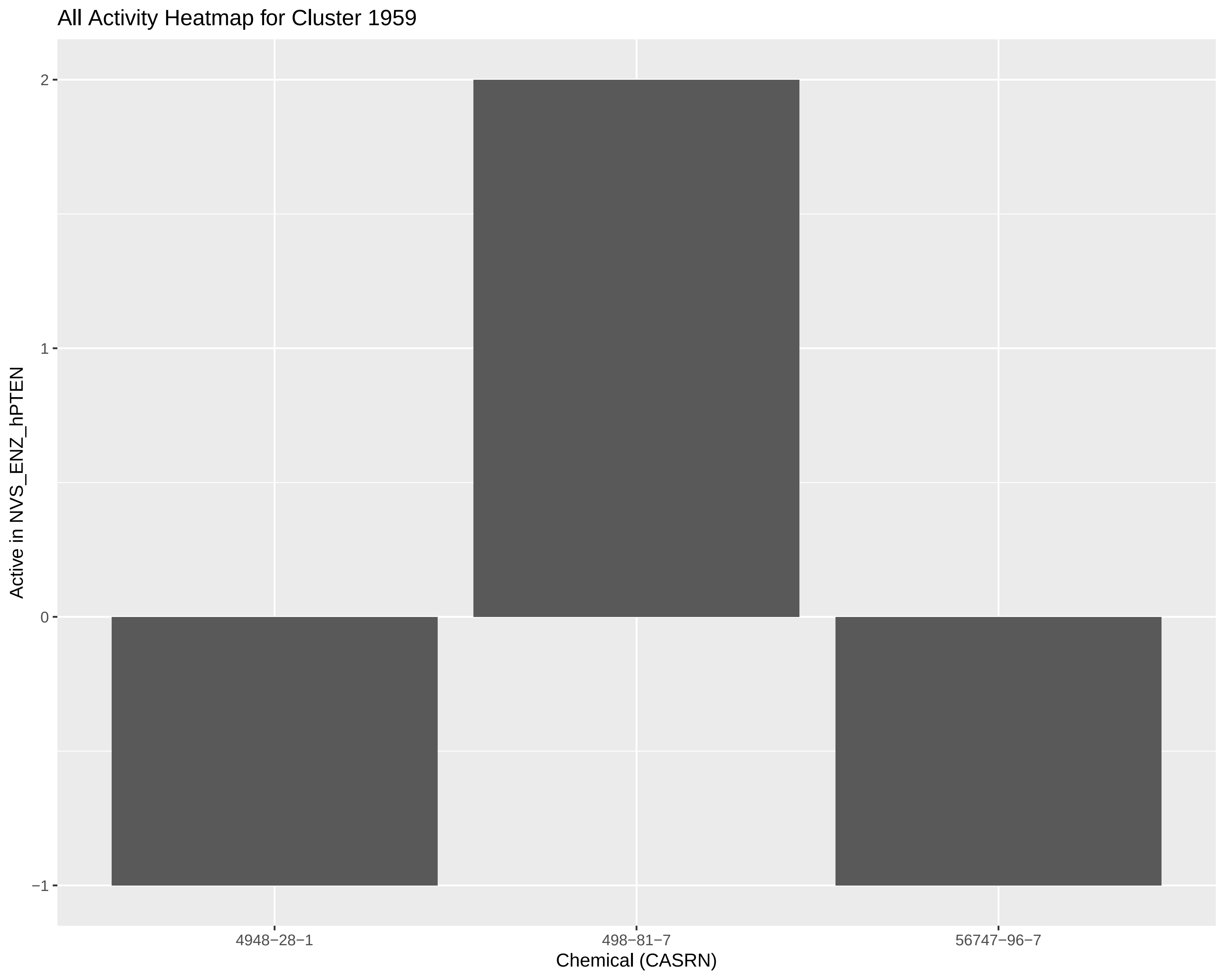
All Activity Heatmap for Cluster 1959
Active in NVS_ENZ_hPTEN
2
1
0
−1
4948−28−1
498−81−7
56747−96−7
Chemical (CASRN)

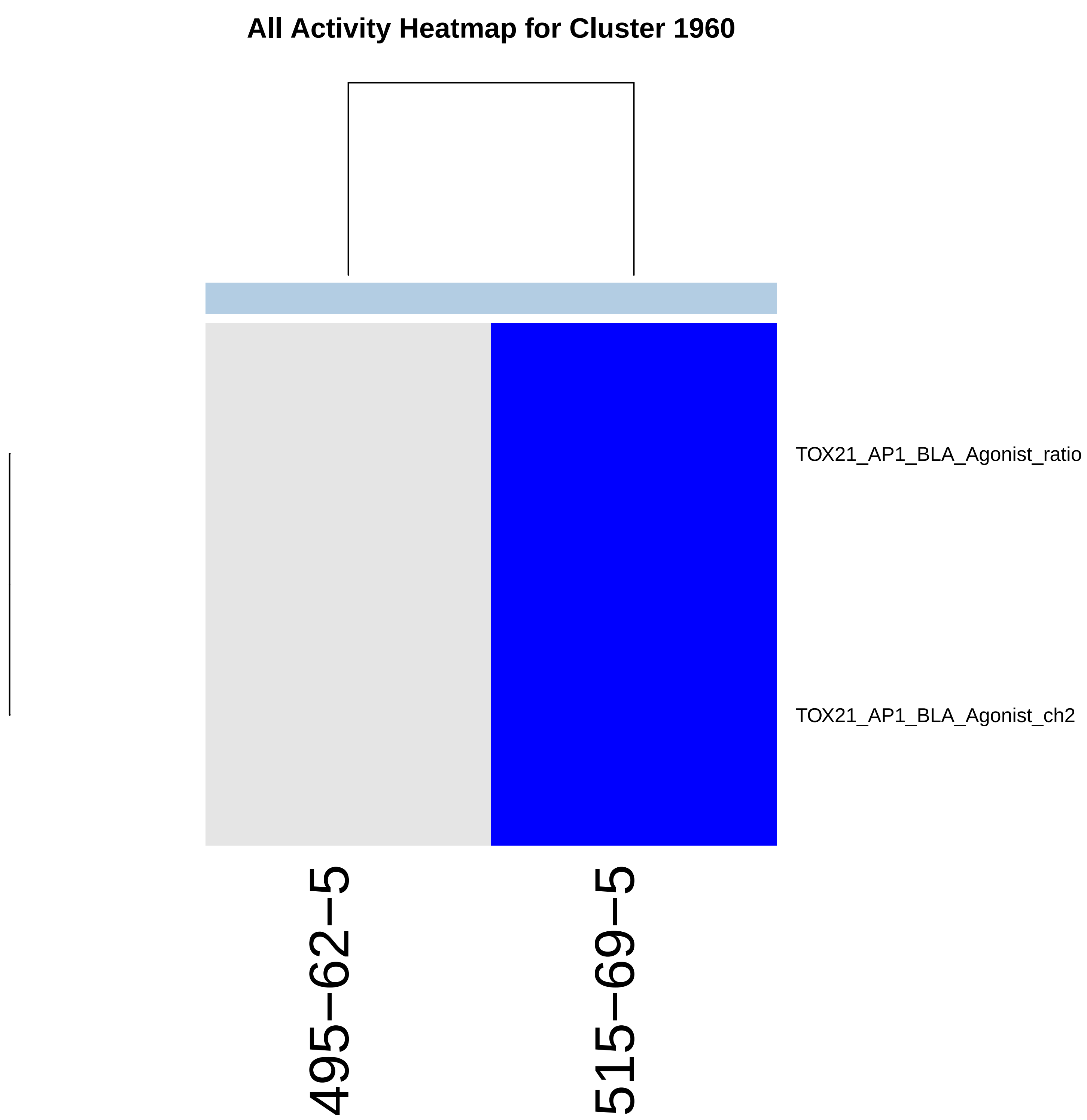
All Activity Heatmap for Cluster 1960
TOX21_AP1_BLA_Agonist_ratio
TOX21_AP1_BLA_Agonist_ch2
495−62−5
515−69−5

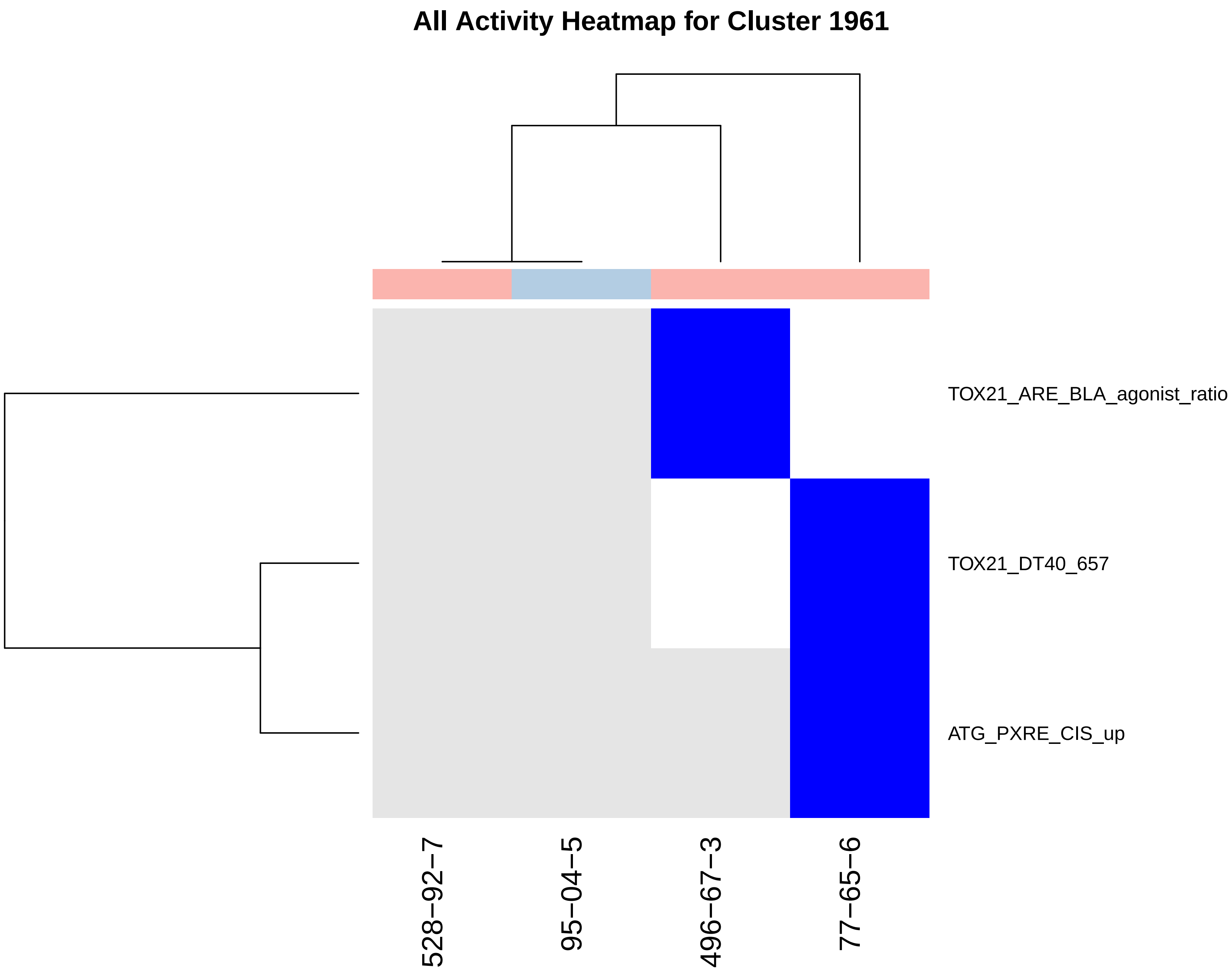

All Activity Heatmap for Cluster 1961
TOX21_ARE_BLA_agonist_ratio
TOX21_DT40_657
ATG_PXRE_CIS_up
528−92−7
95−04−5
496−67−3
77−65−6

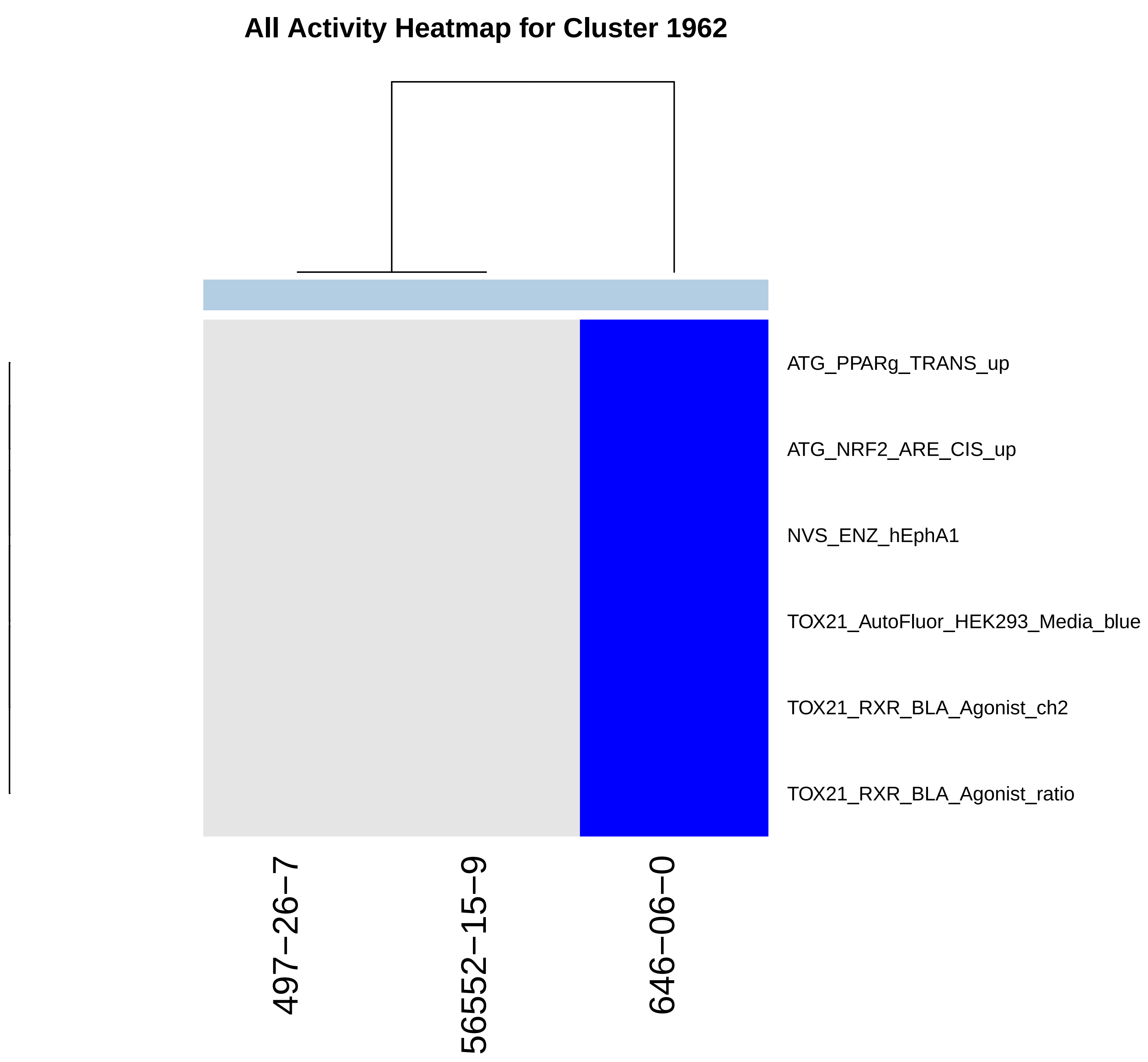
All Activity Heatmap for Cluster 1962
ATG_PPARg_TRANS_up
ATG_NRF2_ARE_CIS_up
NVS_ENZ_hEphA1
TOX21_AutoFluor_HEK293_Media_blue
TOX21_RXR_BLA_Agonist_ch2
TOX21_RXR_BLA_Agonist_ratio
497−26−7
56552−15−9
646−06−0

All Activity Heatmap for Cluster 1964

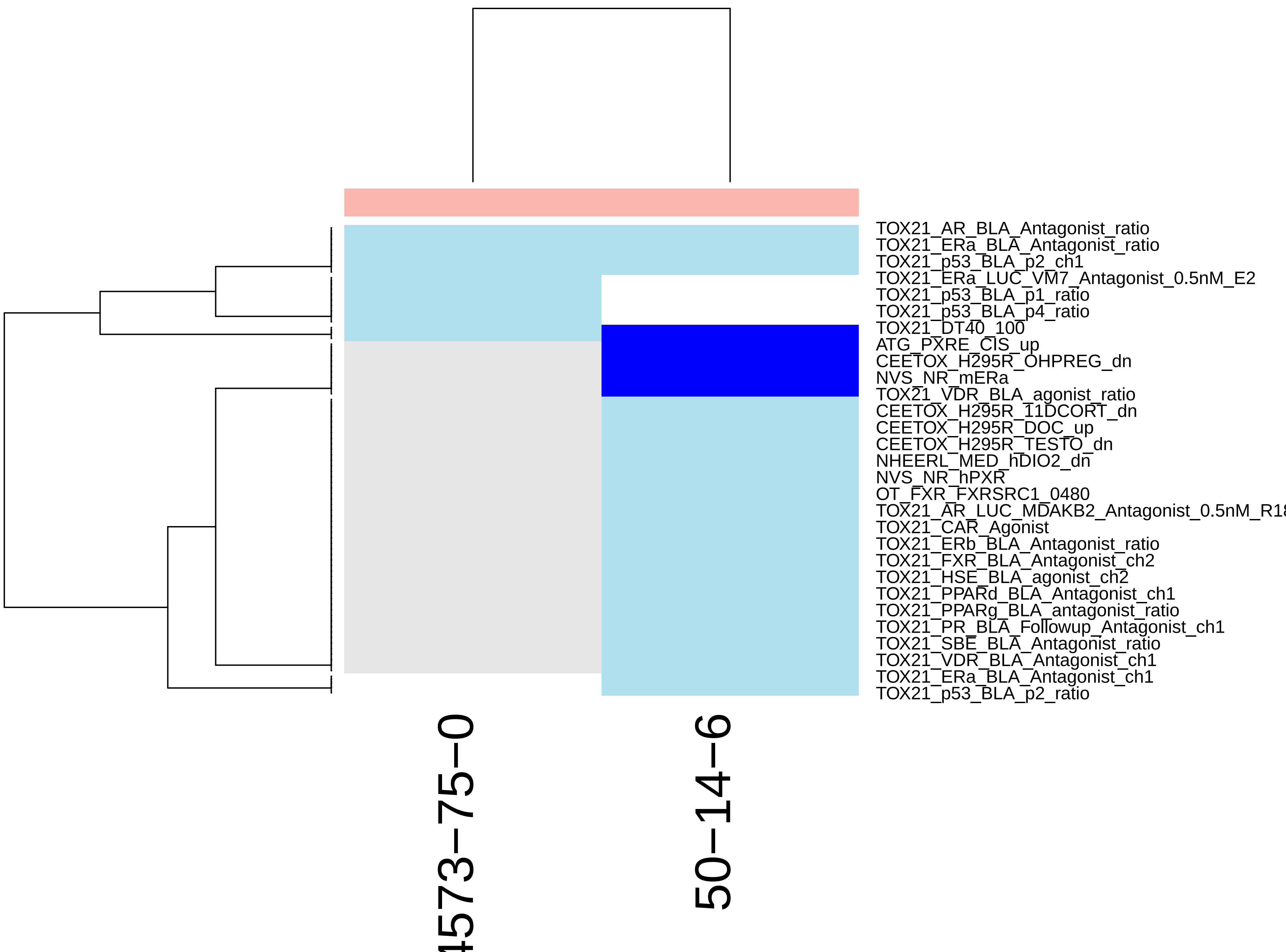

All Activity Heatmap for Cluster 1965

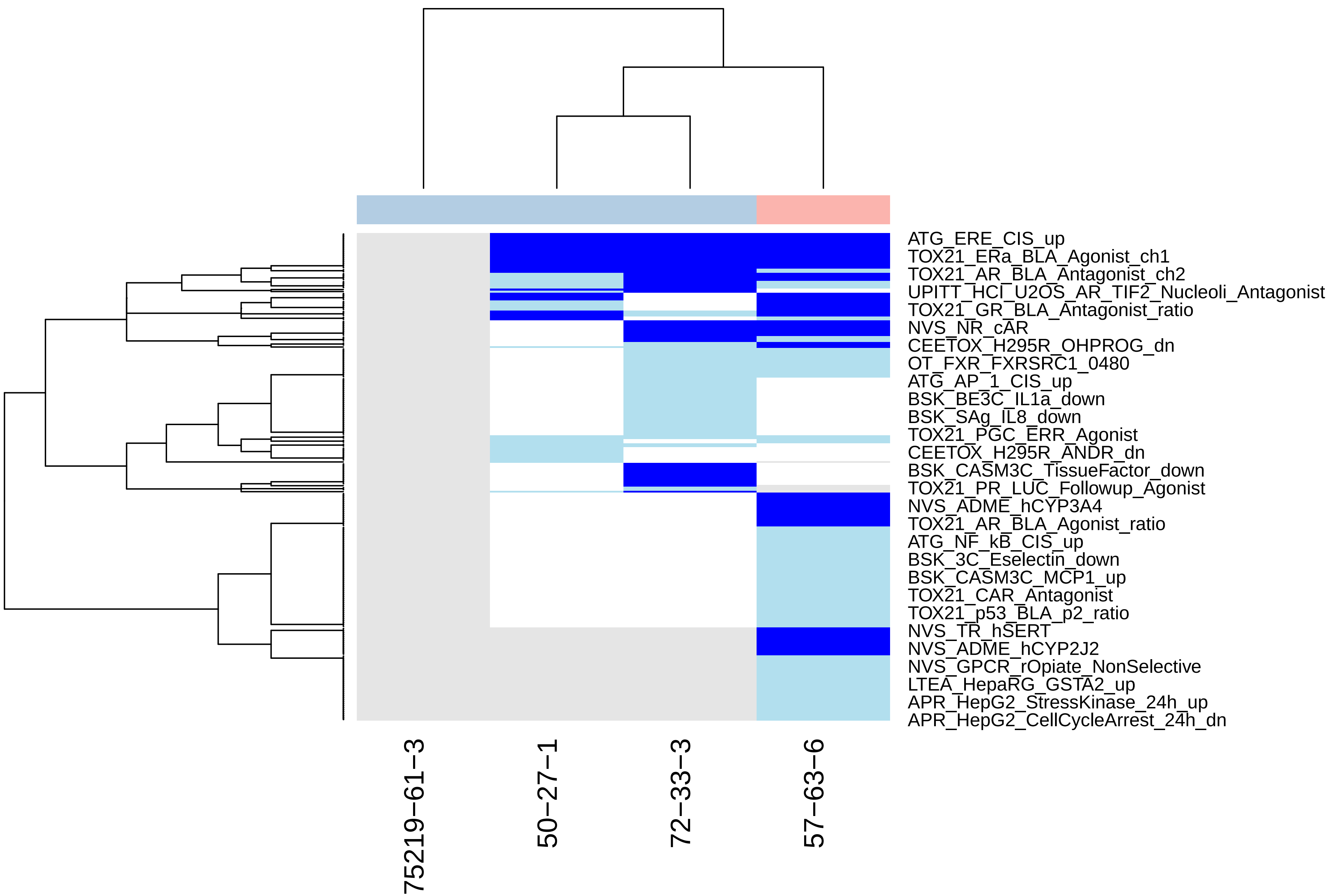

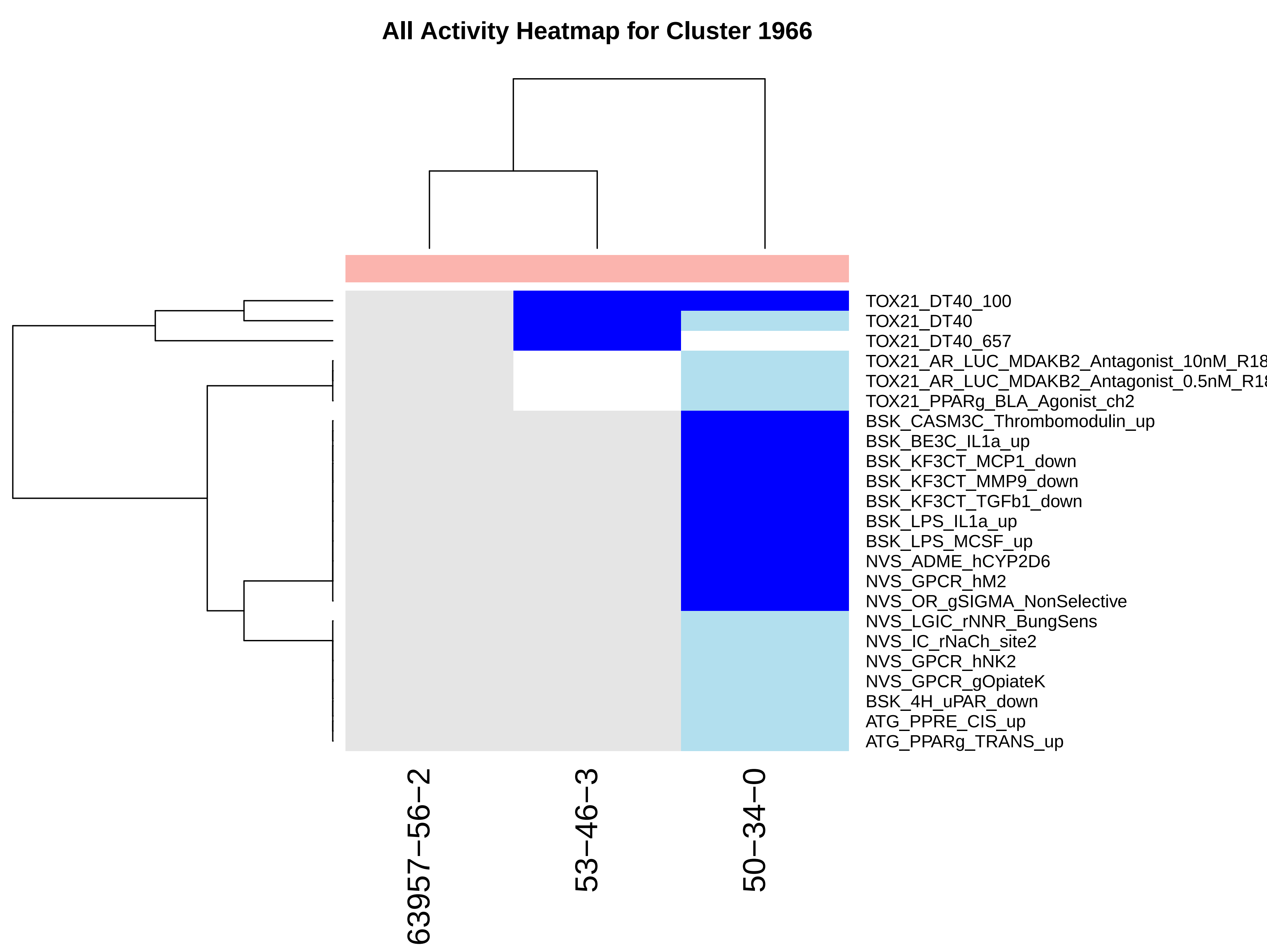
All Activity Heatmap for Cluster 1966
TOX21_DT40_100
TOX21_DT40
TOX21_DT40_657
TOX21_AR_LUC_MDAKB2_Antagonist_10nM_R18
TOX21_AR_LUC_MDAKB2_Antagonist_0.5nM_R18
TOX21_PPARg_BLA_Agonist_ch2
BSK_CASM3C_Thrombomodulin_up
BSK_BE3C_IL1a_up
BSK_KF3CT_MCP1_down
BSK_KF3CT_MMP9_down
BSK_KF3CT_TGFb1_down
BSK_LPS_IL1a_up
BSK_LPS_MCSF_up
NVS_ADME_hCYP2D6
NVS_GPCR_hM2
NVS_OR_gSIGMA_NonSelective
NVS_LGIC_rNNR_BungSens
NVS_IC_rNaCh_site2
NVS_GPCR_hNK2
NVS_GPCR_gOpiateK
BSK_4H_uPAR_down
ATG_PPRE_CIS_up
ATG_PPARg_TRANS_up
63957−56−2
53−46−3
50−34−0

All Activity Heatmap for Cluster 1967

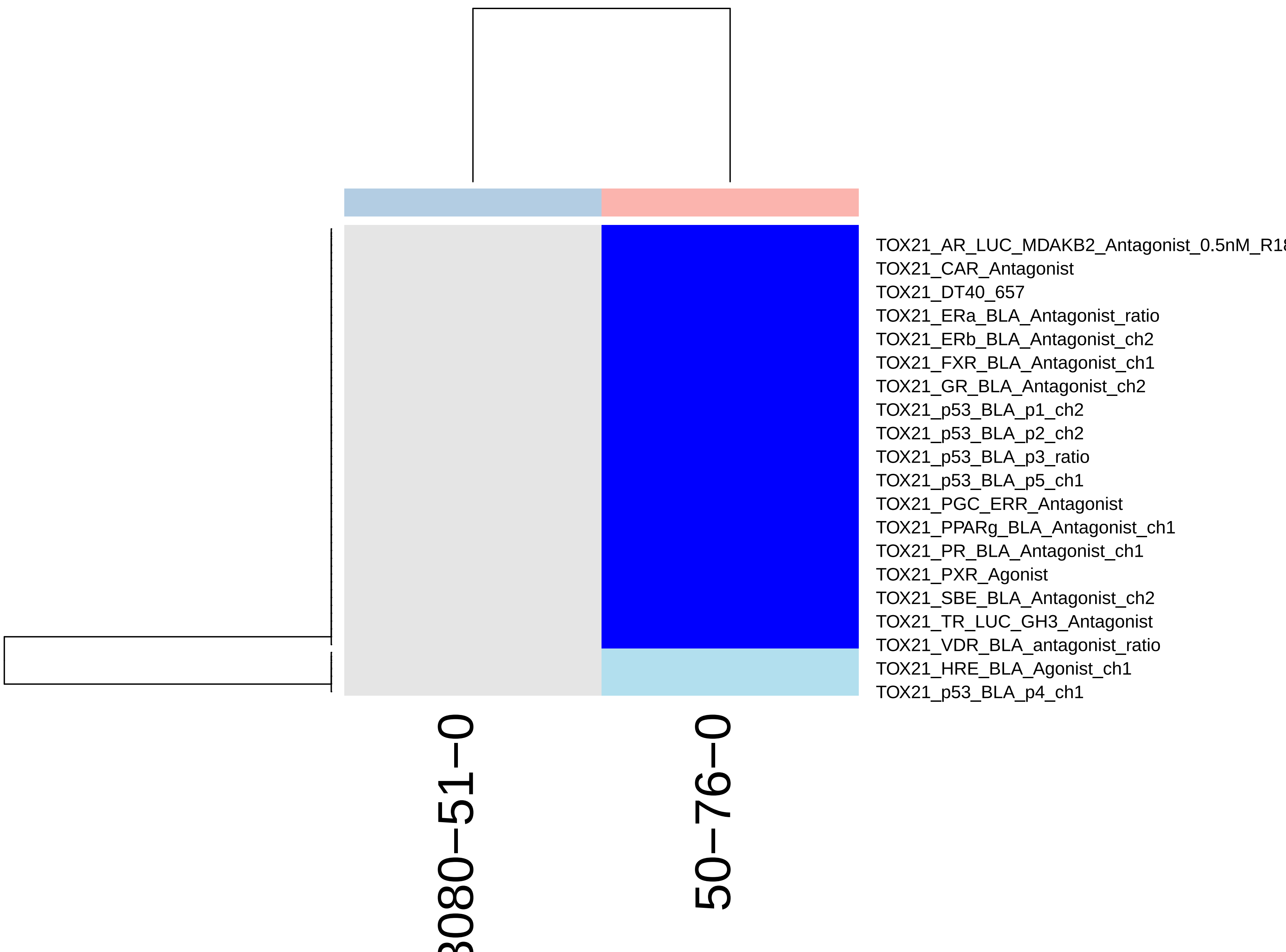

All Activity Heatmap for Cluster 1968

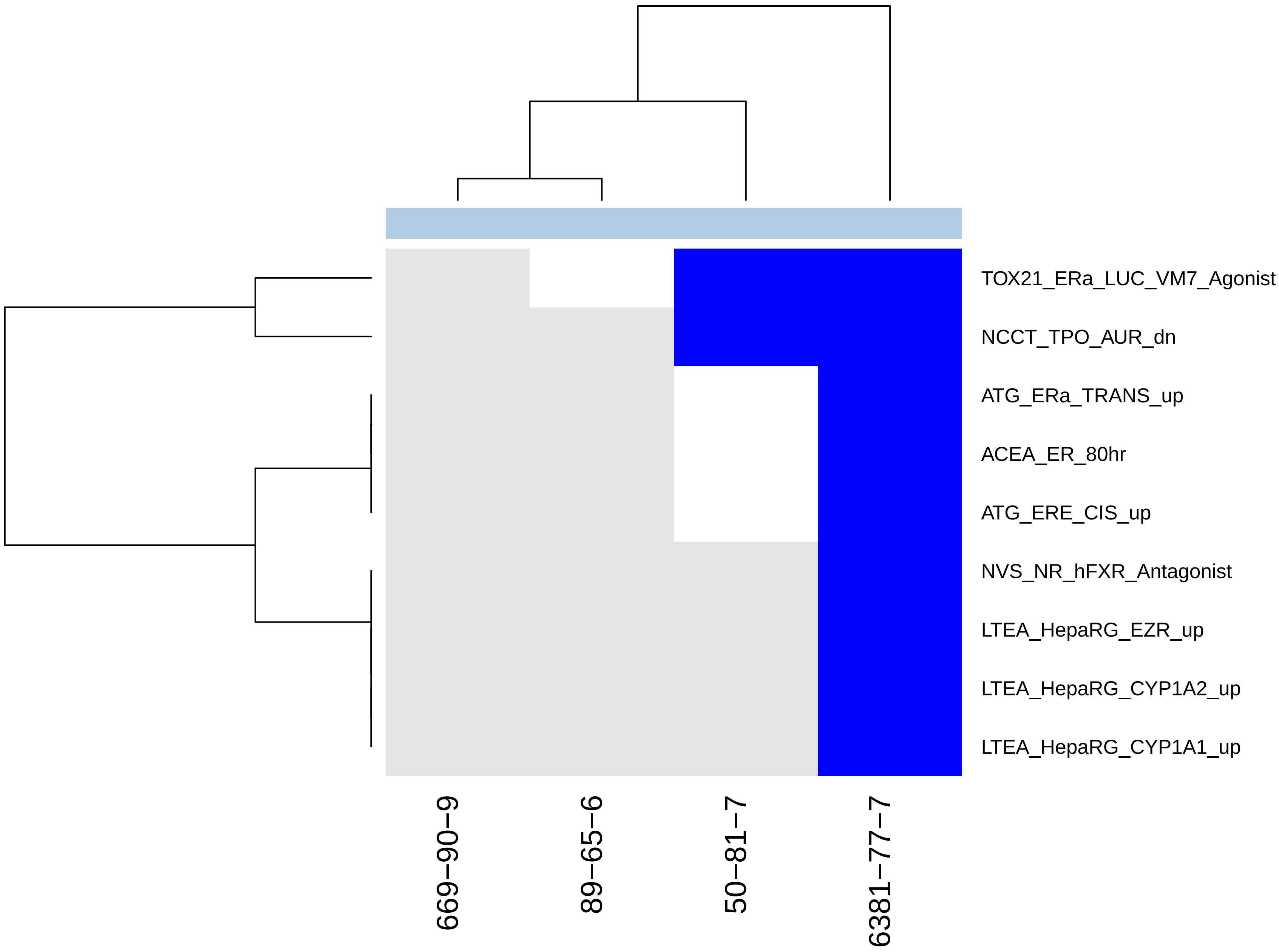

All Activity Heatmap for Cluster 1969

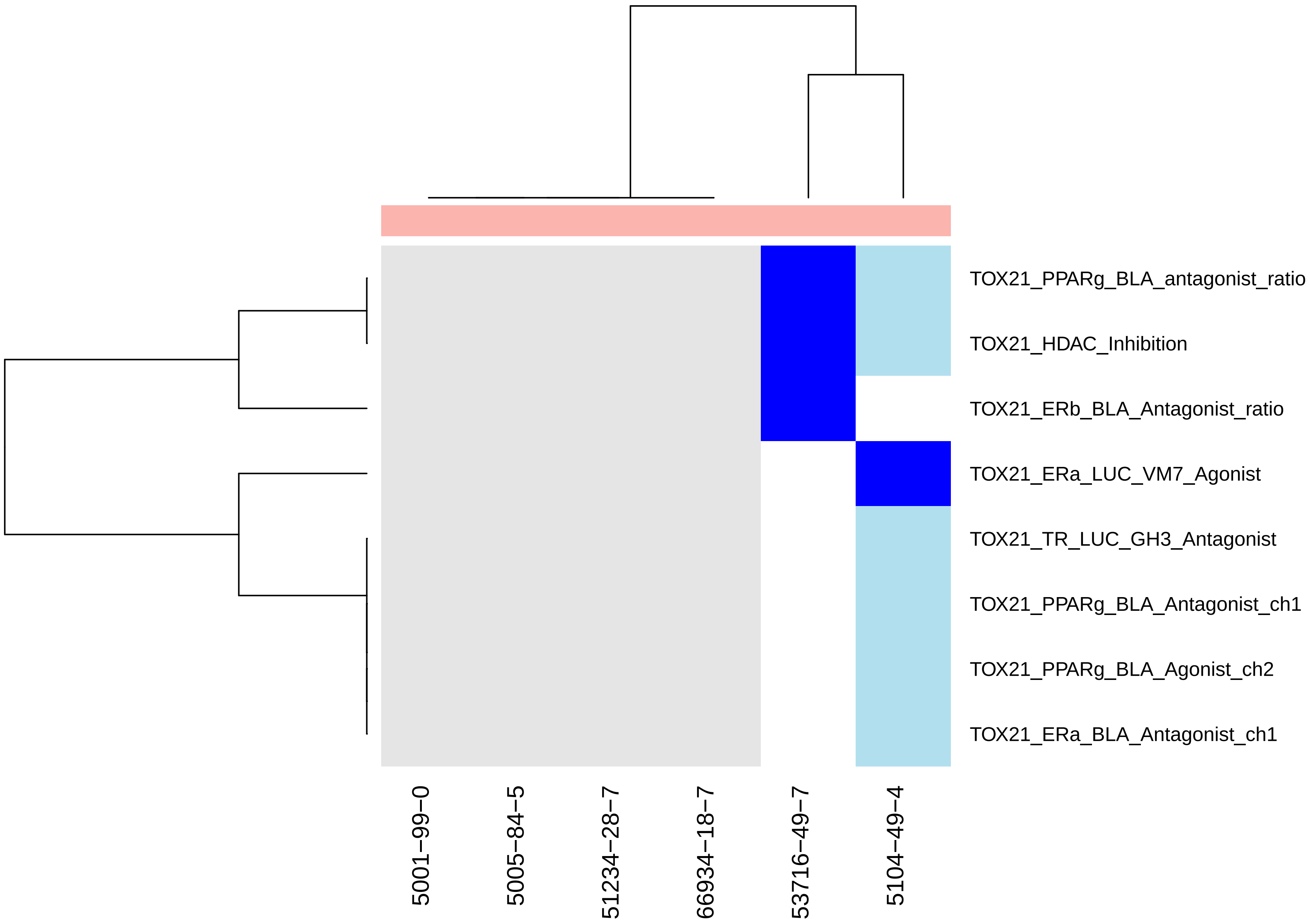

All Activity Heatmap for Cluster 1970

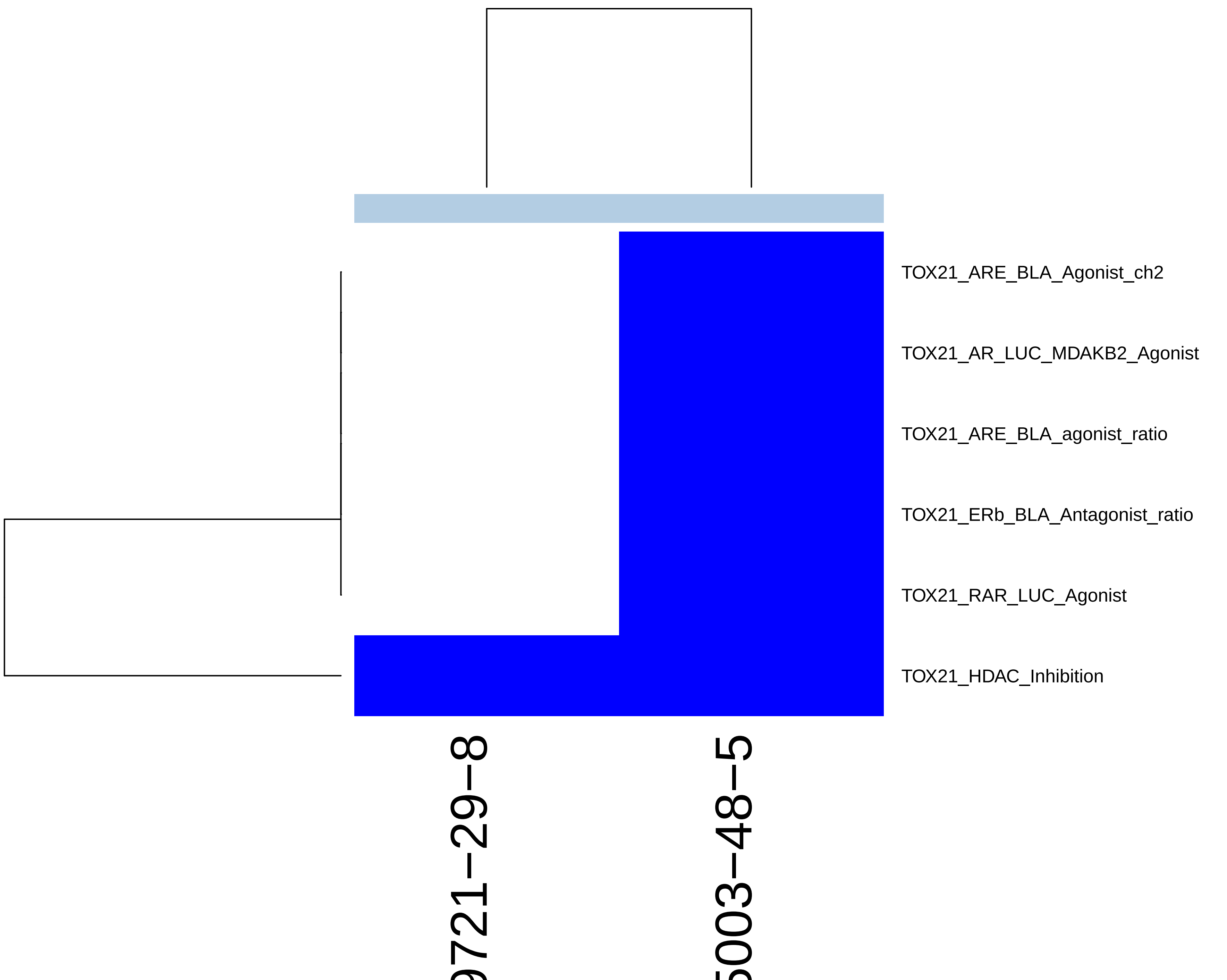

All Activity Heatmap for Cluster 1971

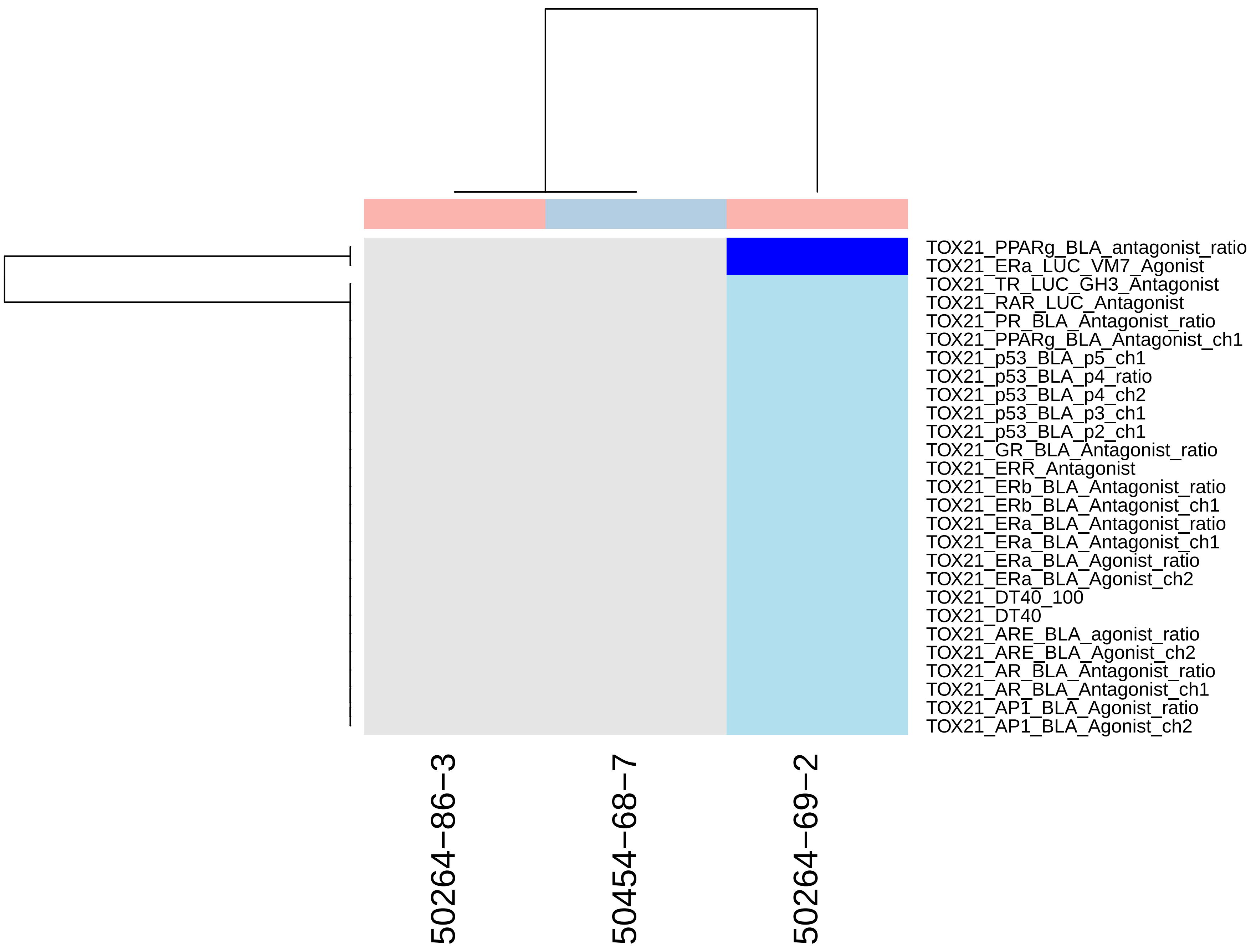

All Activity Heatmap for Cluster 1973

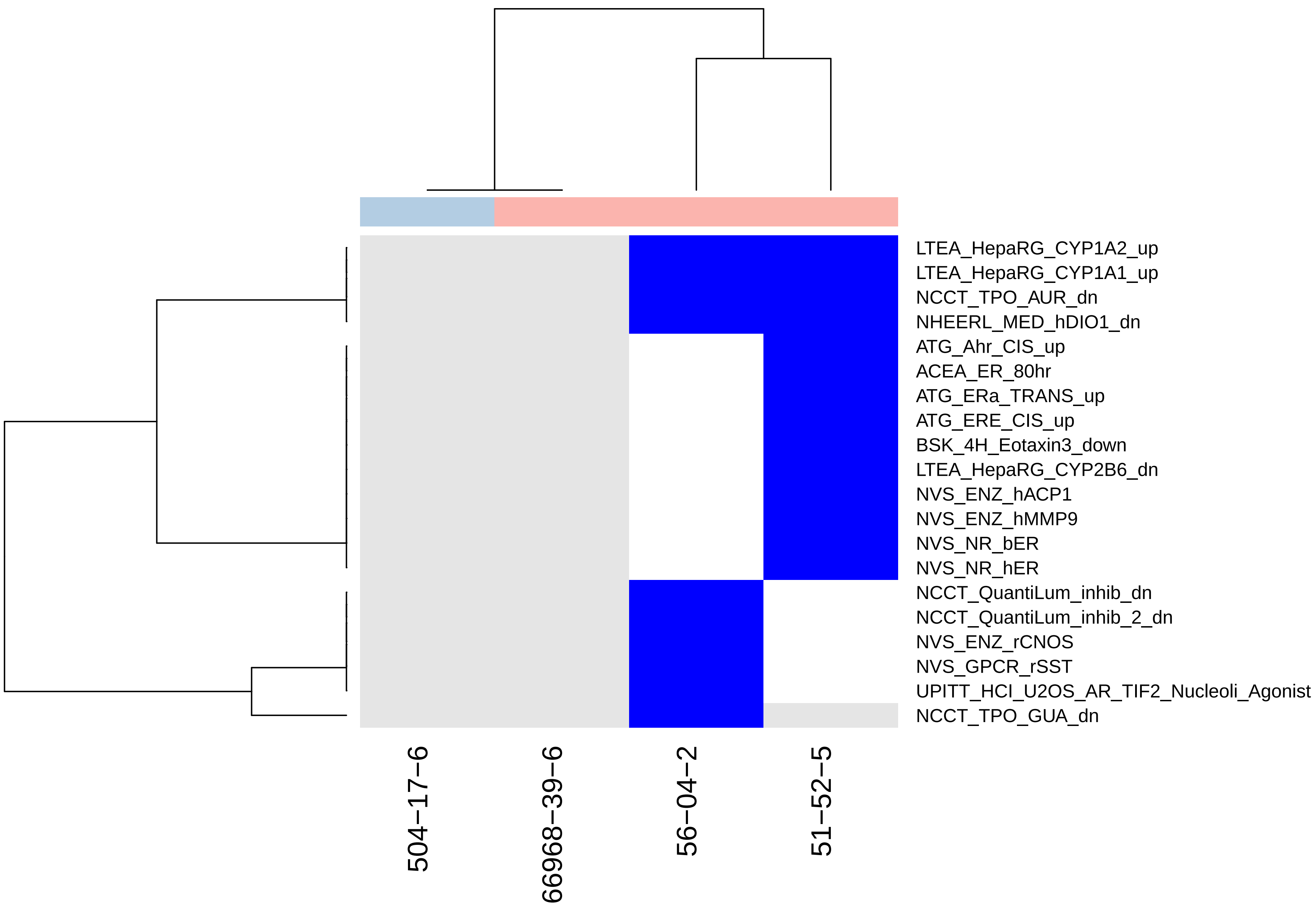

All Activity Heatmap for Cluster 1974

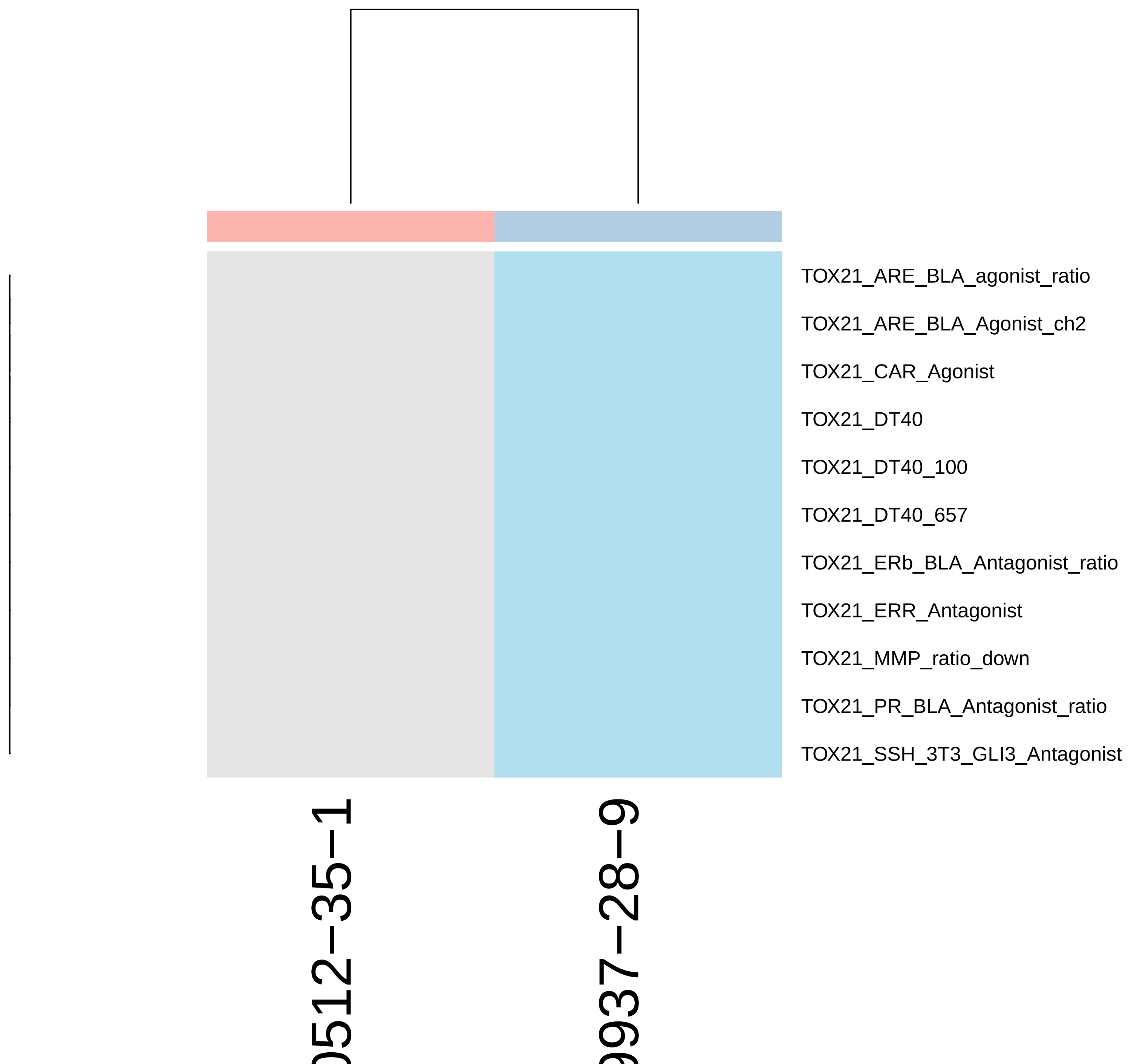

All Activity Heatmap for Cluster 1975

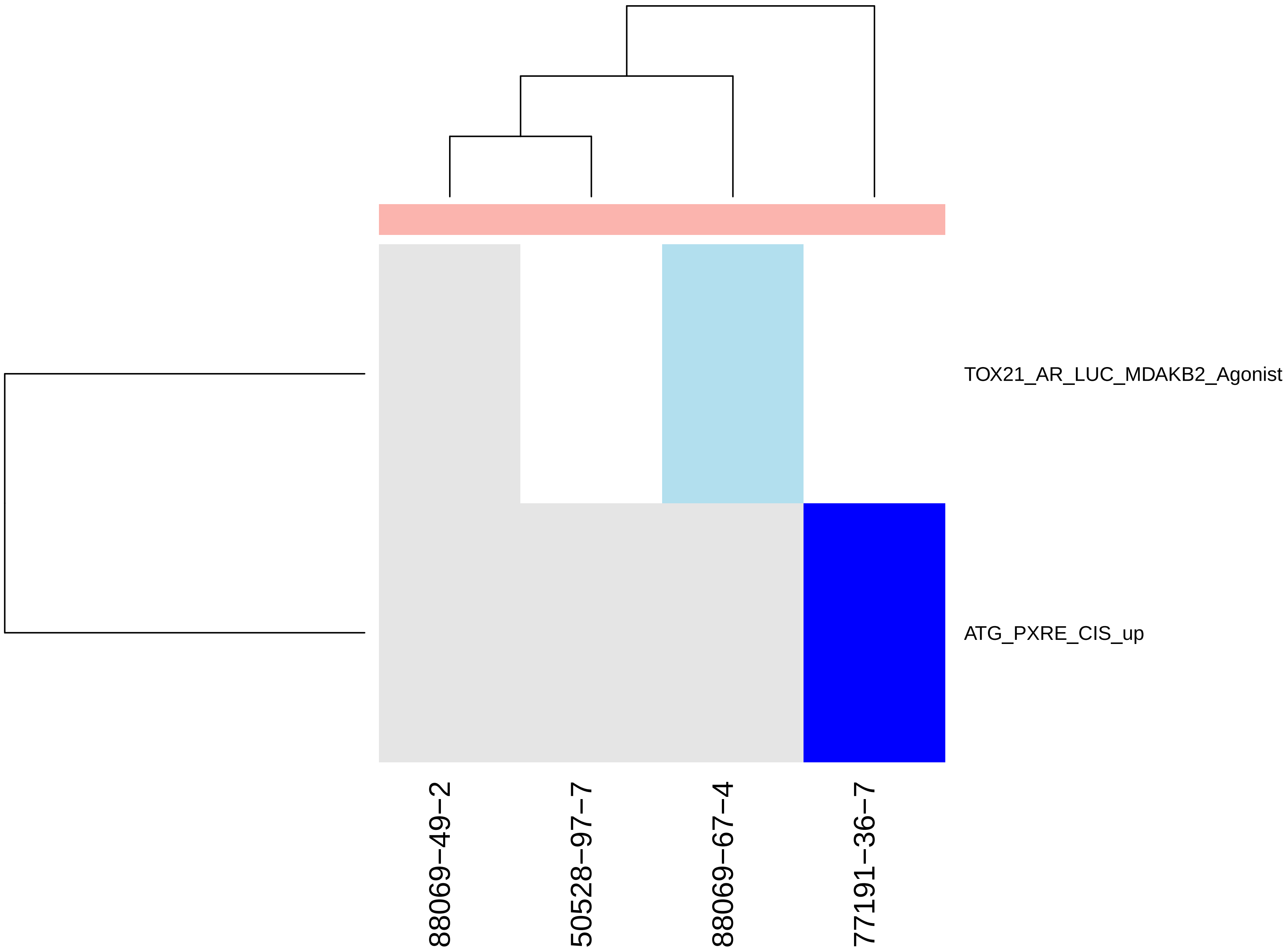

All Activity Heatmap for Cluster 1976

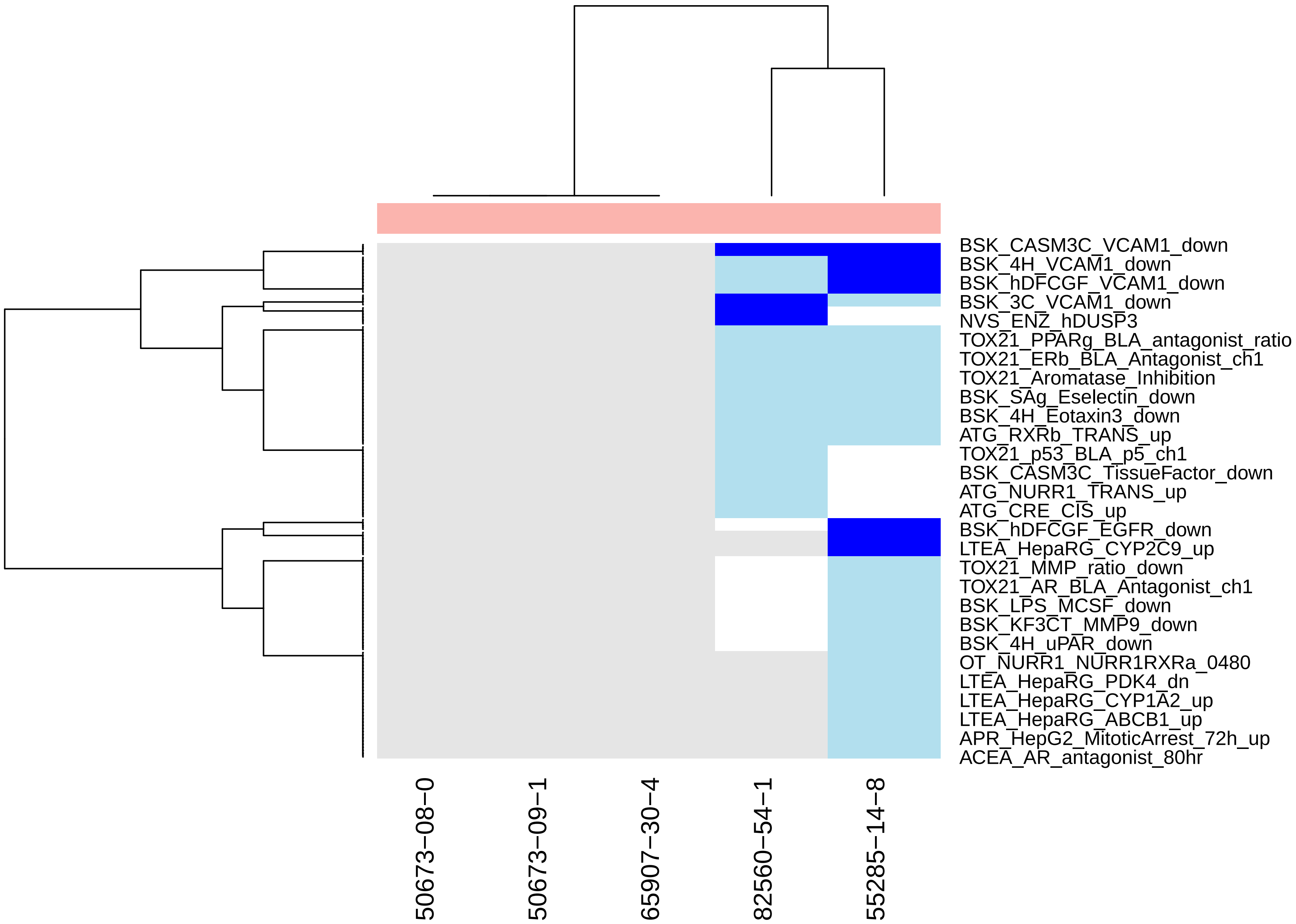

All Activity Heatmap for Cluster 1978

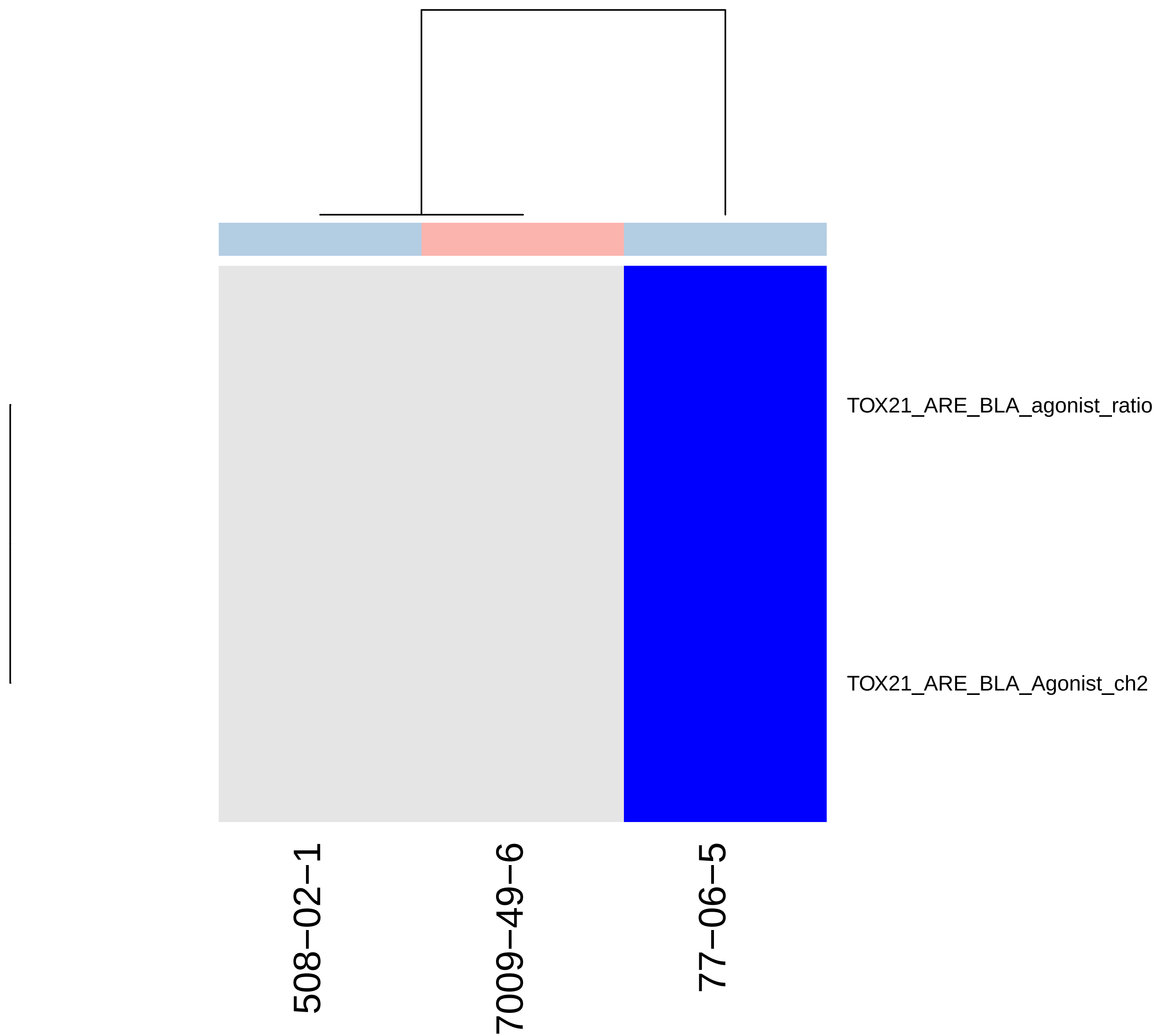

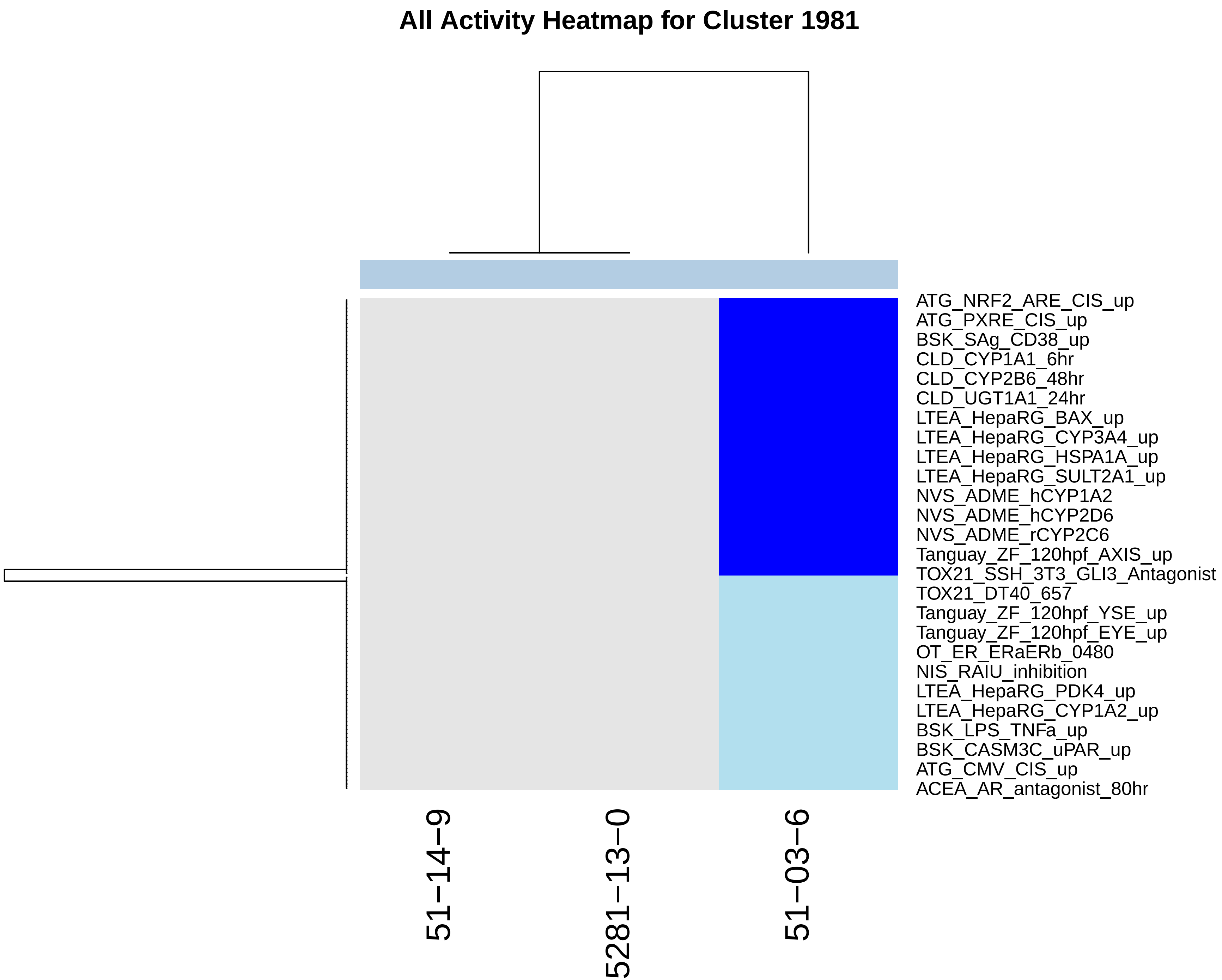
All Activity Heatmap for Cluster 1981
ATG_NRF2_ARE_CIS_up
ATG_PXRE_CIS_up
BSK_SAg_CD38_up
CLD_CYP1A1_6hr
CLD_CYP2B6_48hr
CLD_UGT1A1_24hr
LTEA_HepaRG_BAX_up
LTEA_HepaRG_CYP3A4_up
LTEA_HepaRG_HSPA1A_up
LTEA_HepaRG_SULT2A1_up
NVS_ADME_hCYP1A2
NVS_ADME_hCYP2D6
NVS_ADME_rCYP2C6
Tanguay_ZF_120hpf_AXIS_up
TOX21_SSH_3T3_GLI3_Antagonist
TOX21_DT40_657
Tanguay_ZF_120hpf_YSE_up
Tanguay_ZF_120hpf_EYE_up
OT_ER_ERaERb_0480
NIS_RAIU_inhibition
LTEA_HepaRG_PDK4_up
LTEA_HepaRG_CYP1A2_up
BSK_LPS_TNFa_up
BSK_CASM3C_uPAR_up
ATG_CMV_CIS_up
ACEA_AR_antagonist_80hr
51−14−9
5281−13−0
51−03−6

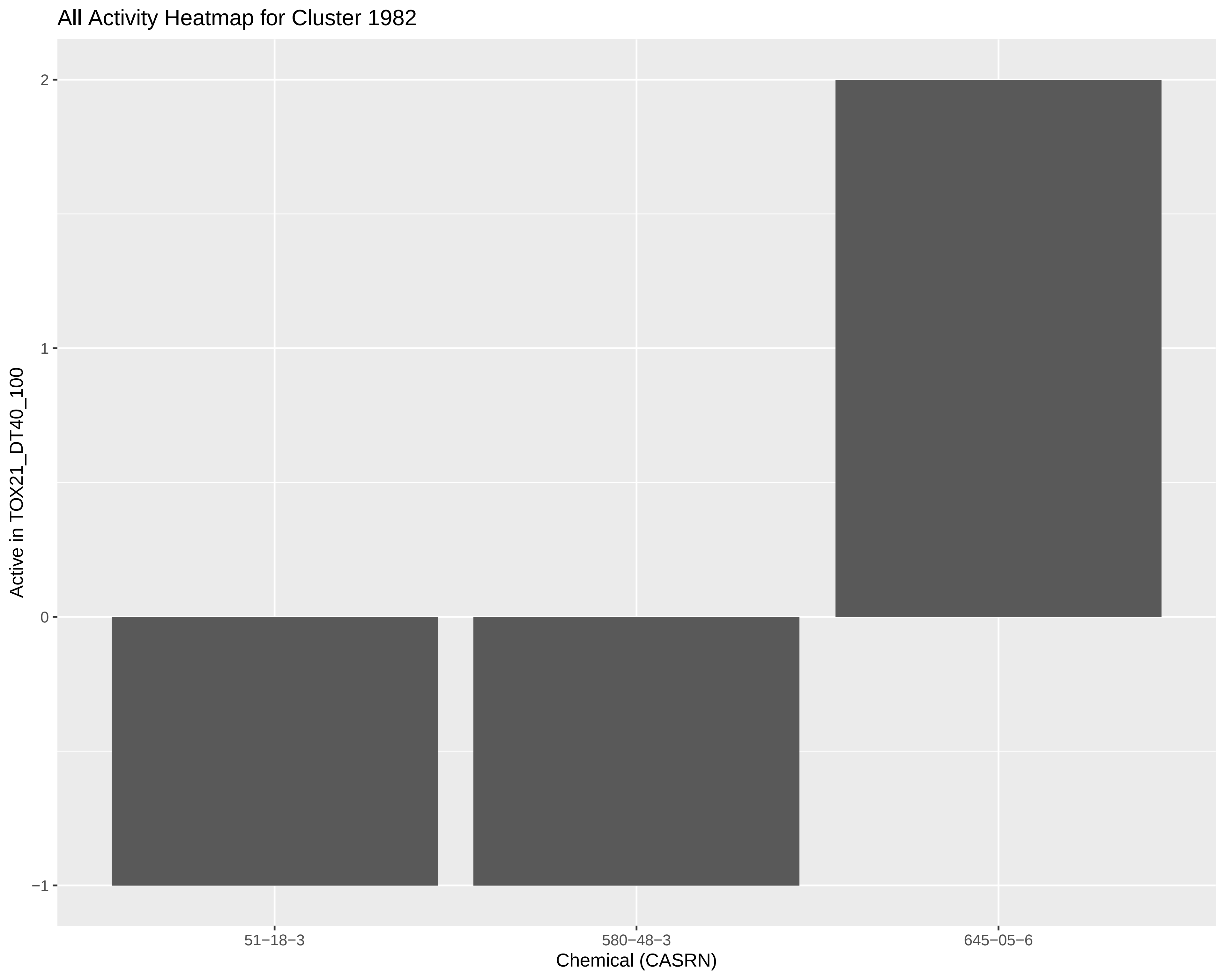
All Activity Heatmap for Cluster 1982
Active in TOX21_DT40_100
2
1
0
−1
51−18−3
580−48−3
645−05−6
Chemical (CASRN)

All Activity Heatmap for Cluster 1983

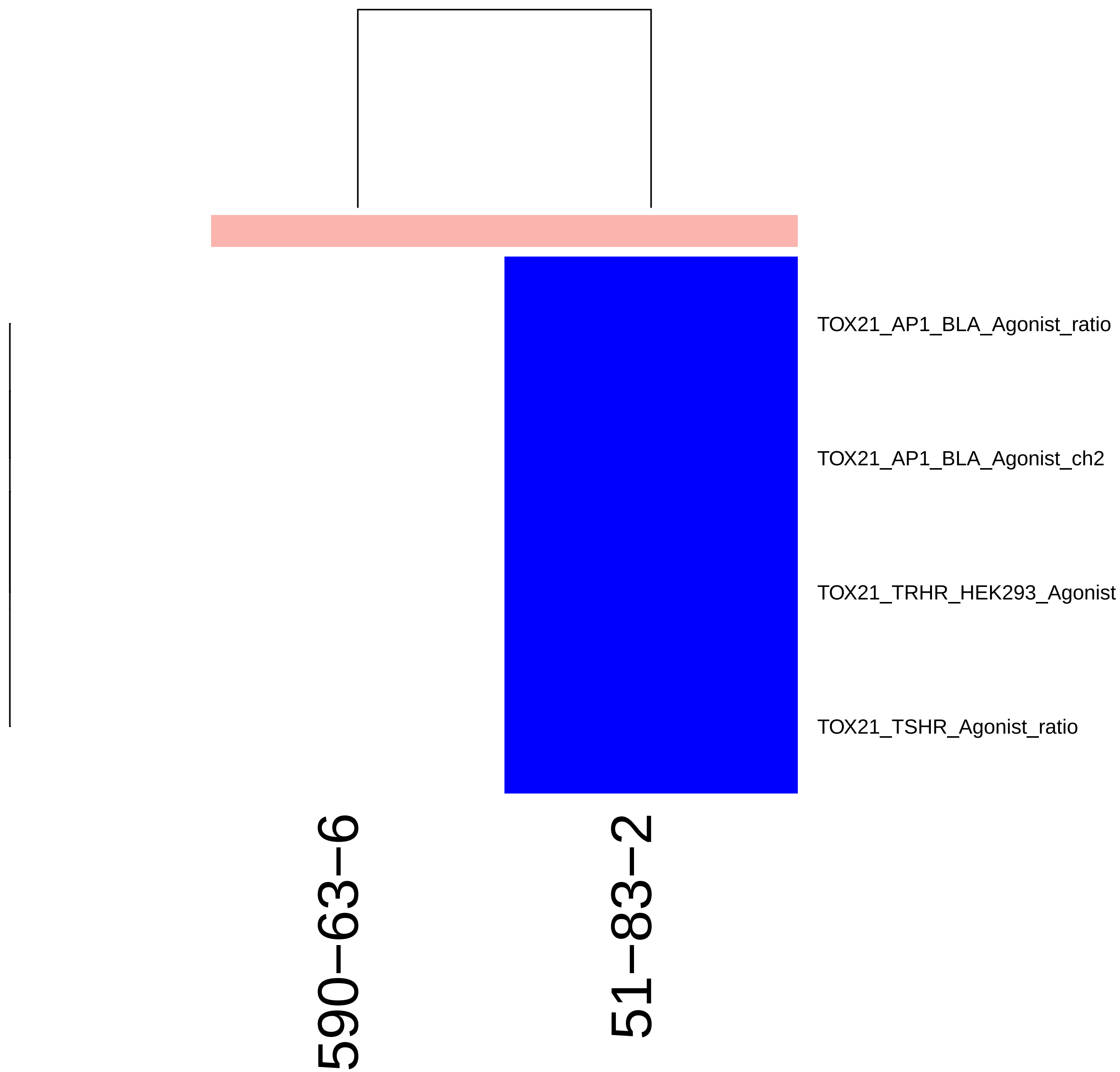

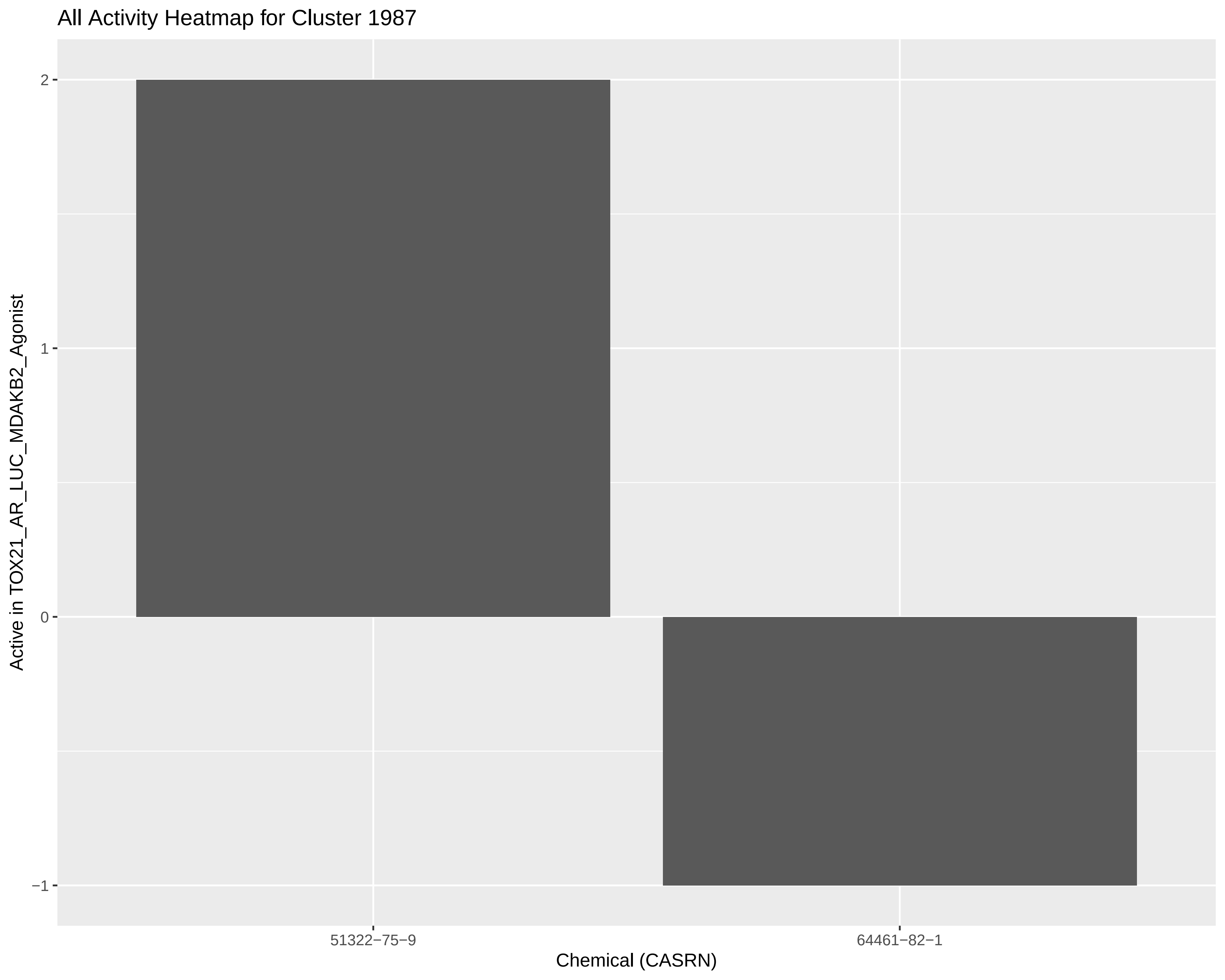
All Activity Heatmap for Cluster 1987
Active in TOX21_AR_LUC_MDAKB2_Agonist
2
1
0
−1
51322−75−9
64461−82−1
Chemical (CASRN)

All Activity Heatmap for Cluster 1988

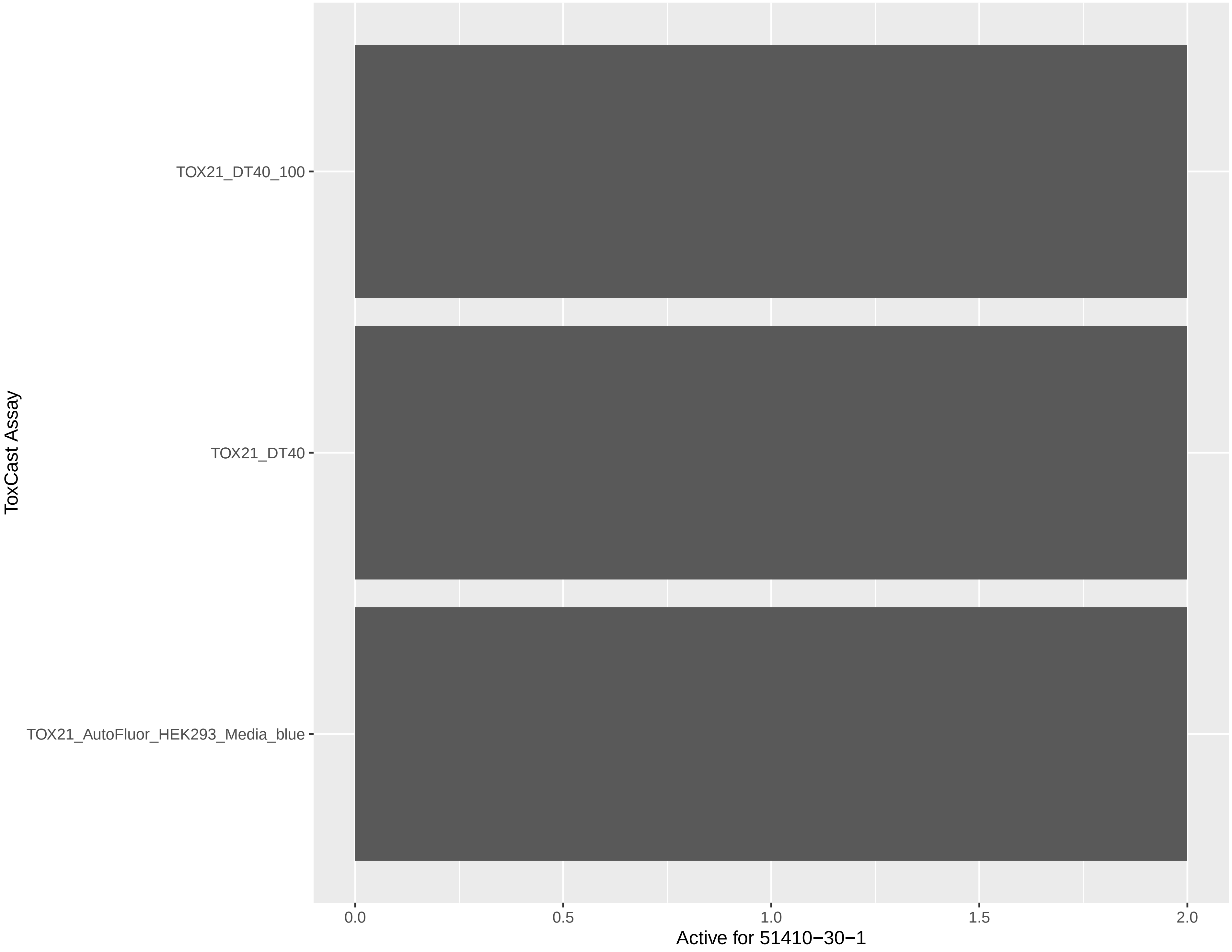

All Activity Heatmap for Cluster 1992

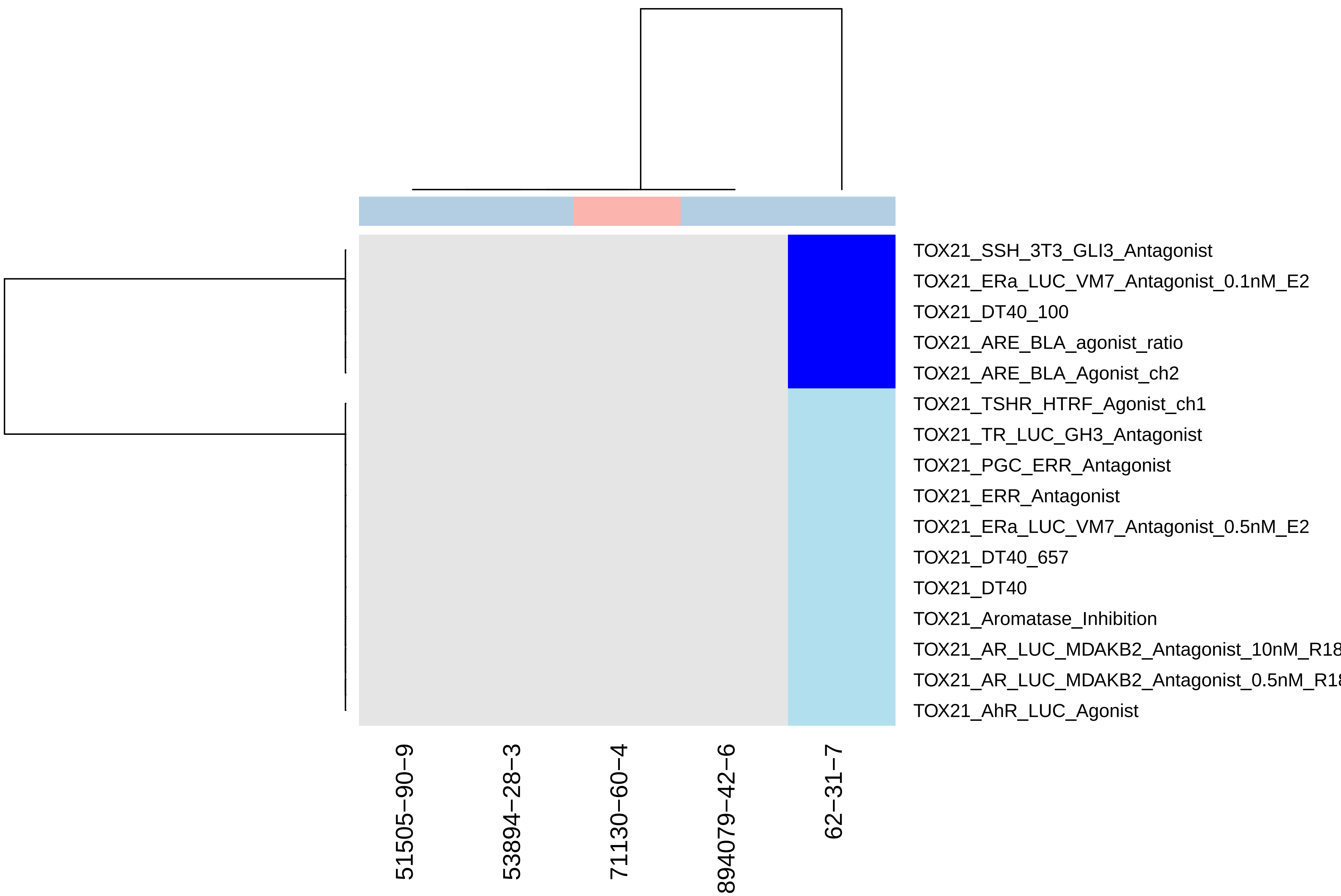

All Activity Heatmap for Cluster 1994

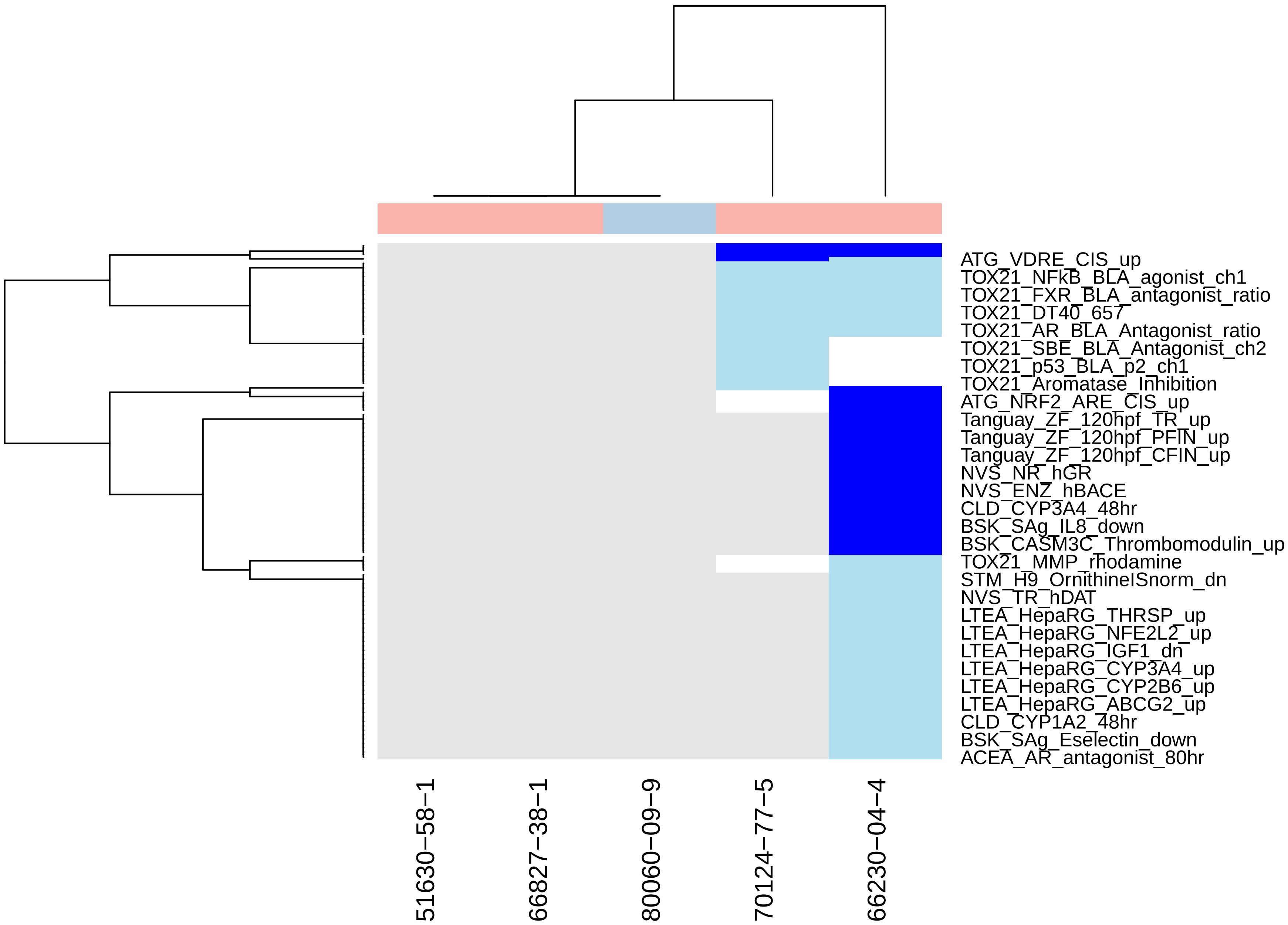

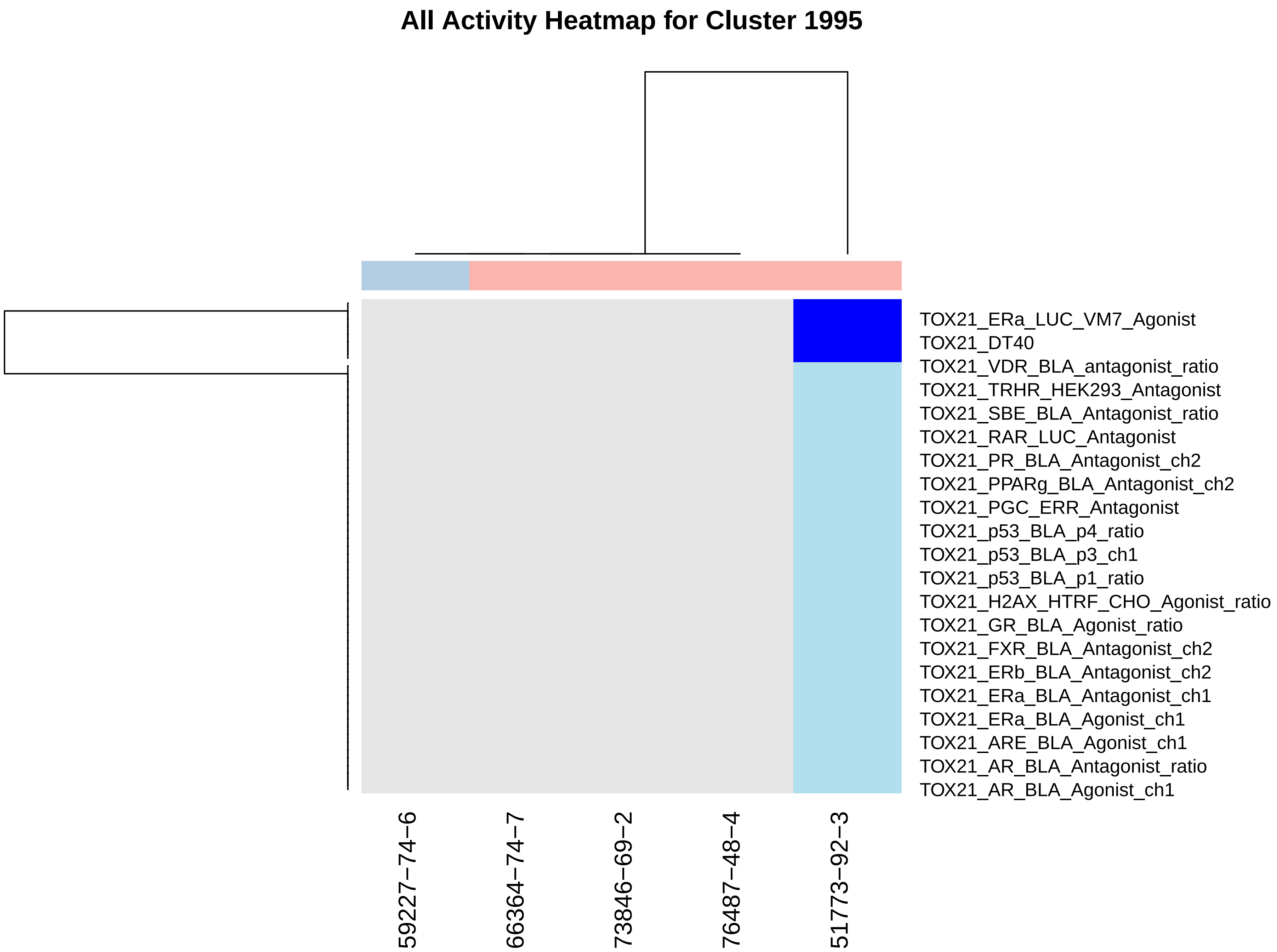
All Activity Heatmap for Cluster 1995
TOX21_ERa_LUC_VM7_Agonist
TOX21_DT40
TOX21_VDR_BLA_antagonist_ratio
TOX21_TRHR_HEK293_Antagonist
TOX21_SBE_BLA_Antagonist_ratio
TOX21_RAR_LUC_Antagonist
TOX21_PR_BLA_Antagonist_ch2
TOX21_PPARg_BLA_Antagonist_ch2
TOX21_PGC_ERR_Antagonist
TOX21_p53_BLA_p4_ratio
TOX21_p53_BLA_p3_ch1
TOX21_p53_BLA_p1_ratio
TOX21_H2AX_HTRF_CHO_Agonist_ratio
TOX21_GR_BLA_Agonist_ratio
TOX21_FXR_BLA_Antagonist_ch2
TOX21_ERb_BLA_Antagonist_ch2
TOX21_ERa_BLA_Antagonist_ch1
TOX21_ERa_BLA_Agonist_ch1
TOX21_ARE_BLA_Agonist_ch1
TOX21_AR_BLA_Antagonist_ratio
TOX21_AR_BLA_Agonist_ch1
59227−74−6
66364−74−7
73846−69−2
76487−48−4
51773−92−3

All Activity Heatmap for Cluster 1996

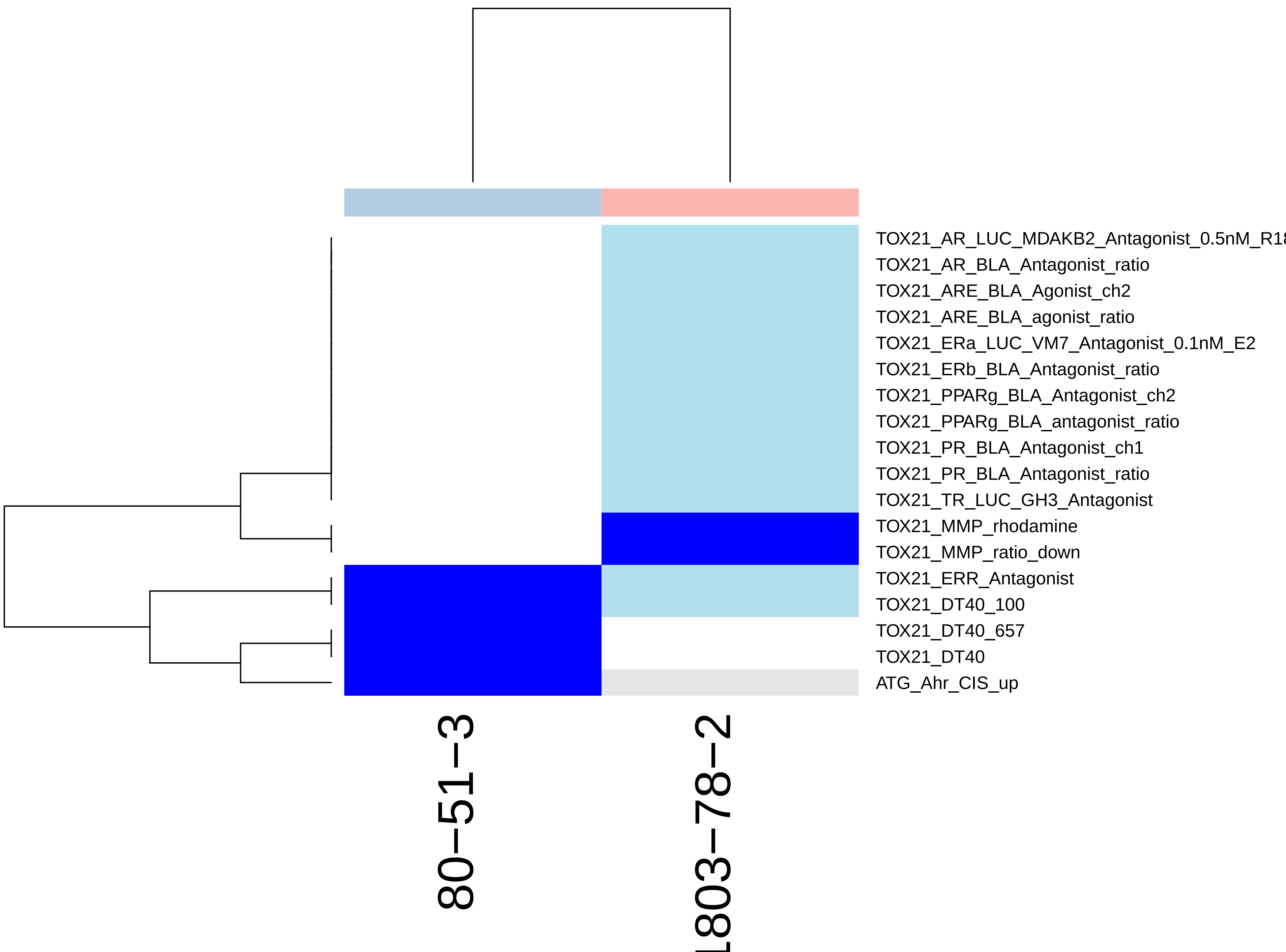

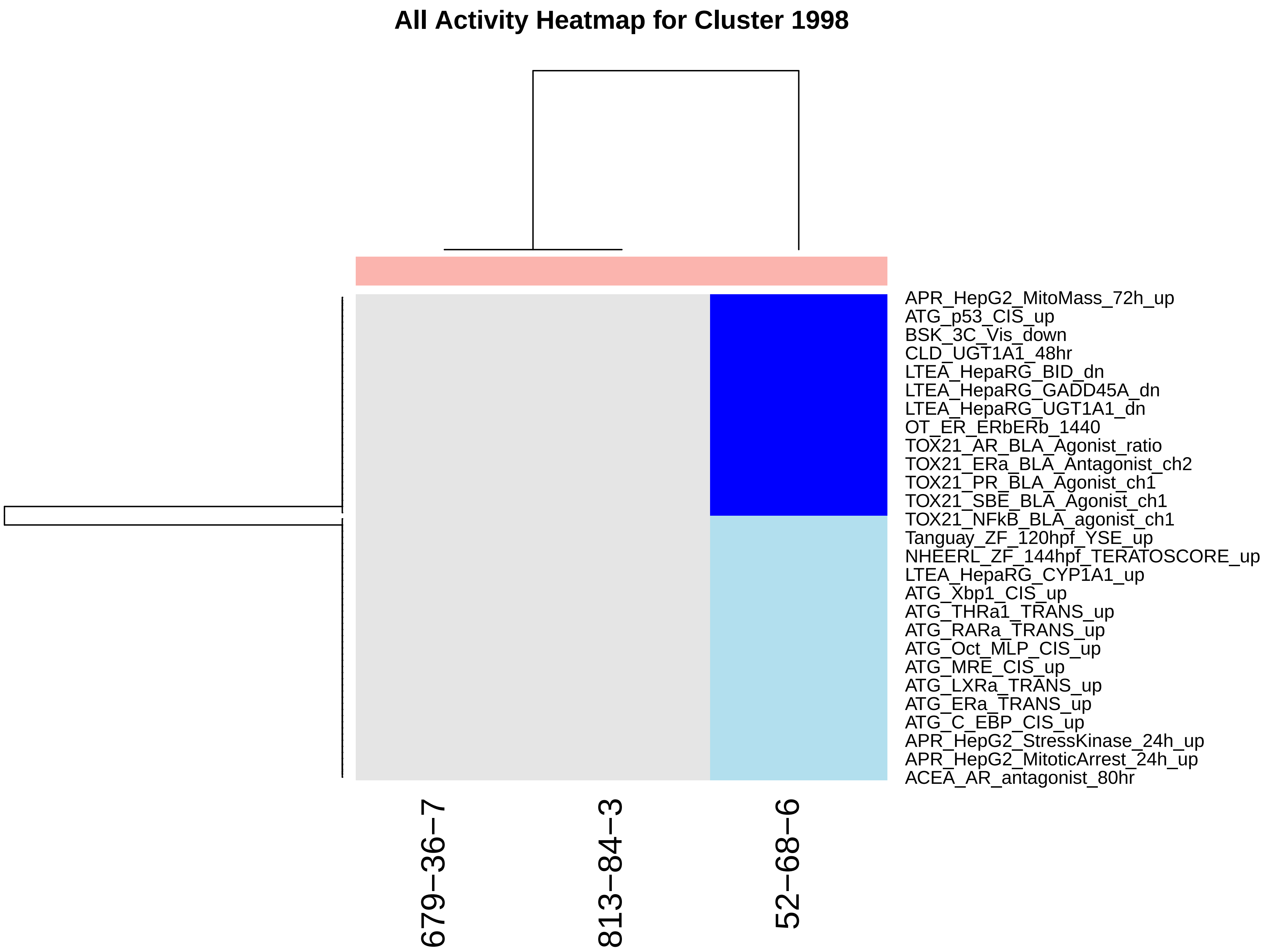
All Activity Heatmap for Cluster 1998
APR_HepG2_MitoMass_72h_up
ATG_p53_CIS_up
BSK_3C_Vis_down
CLD_UGT1A1_48hr
LTEA_HepaRG_BID_dn
LTEA_HepaRG_GADD45A_dn
LTEA_HepaRG_UGT1A1_dn
OT_ER_ERbERb_1440
TOX21_AR_BLA_Agonist_ratio
TOX21_ERa_BLA_Antagonist_ch2
TOX21_PR_BLA_Agonist_ch1
TOX21_SBE_BLA_Agonist_ch1
TOX21_NFkB_BLA_agonist_ch1
Tanguay_ZF_120hpf_YSE_up
NHEERL_ZF_144hpf_TERATOSCORE_up
LTEA_HepaRG_CYP1A1_up
ATG_Xbp1_CIS_up
ATG_THRa1_TRANS_up
ATG_RARa_TRANS_up
ATG_Oct_MLP_CIS_up
ATG_MRE_CIS_up
ATG_LXRa_TRANS_up
ATG_ERa_TRANS_up
ATG_C_EBP_CIS_up
APR_HepG2_StressKinase_24h_up
APR_HepG2_MitoticArrest_24h_up
ACEA_AR_antagonist_80hr
679−36−7
813−84−3
52−68−6

All Activity Heatmap for Cluster 1999

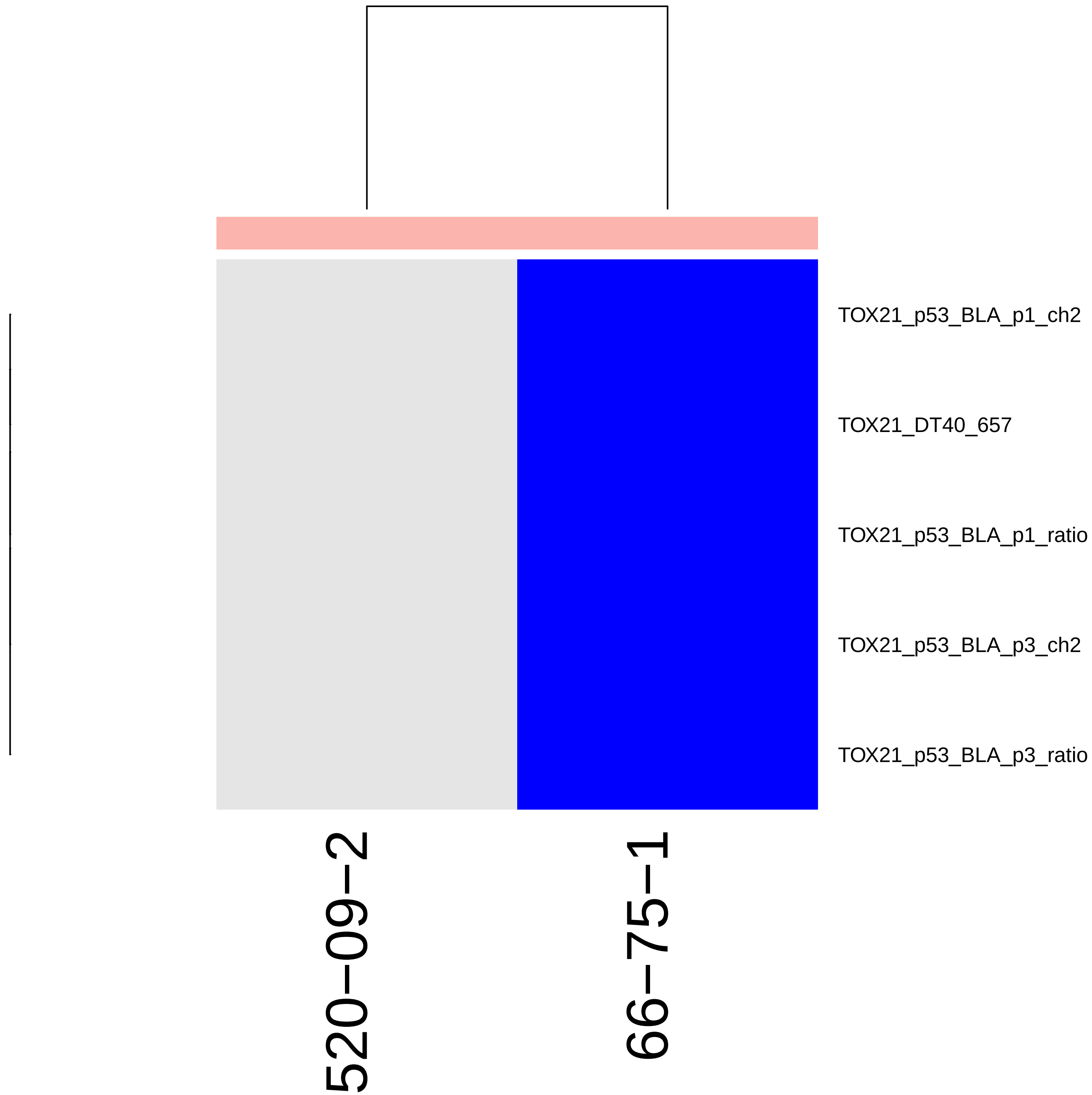

All Activity Heatmap for Cluster 2001

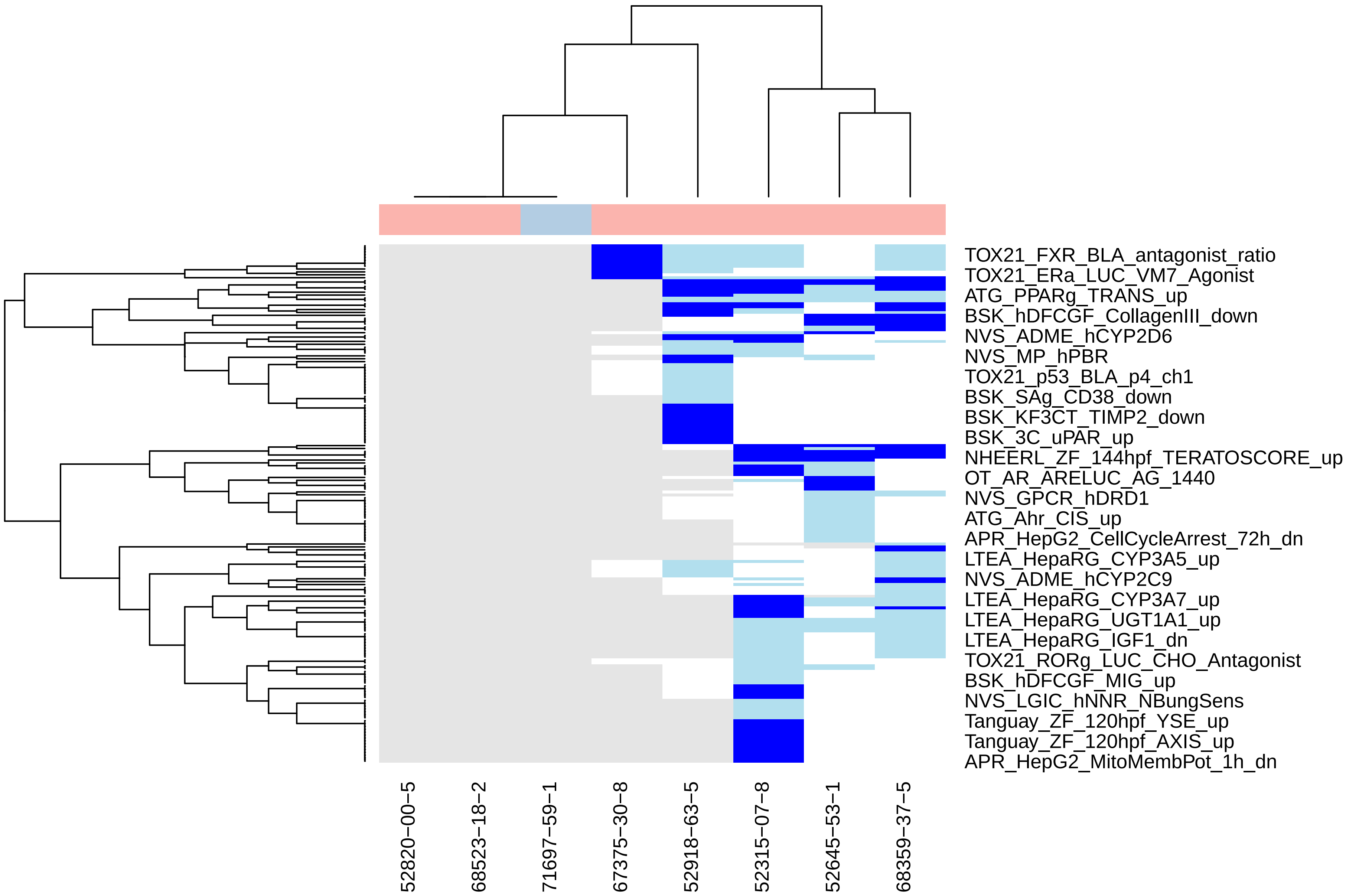

All Activity Heatmap for Cluster 2005

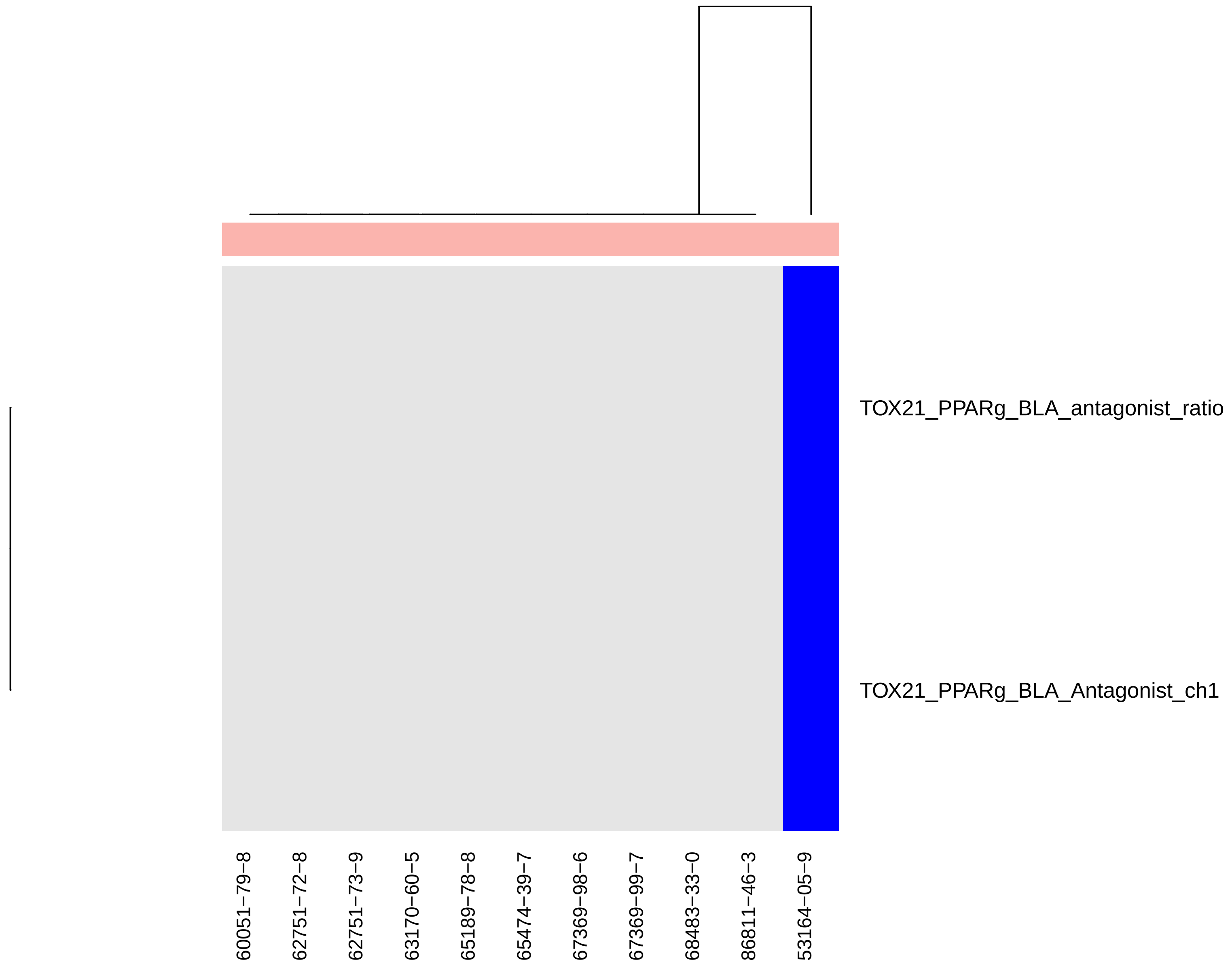

All Activity Heatmap for Cluster 2008

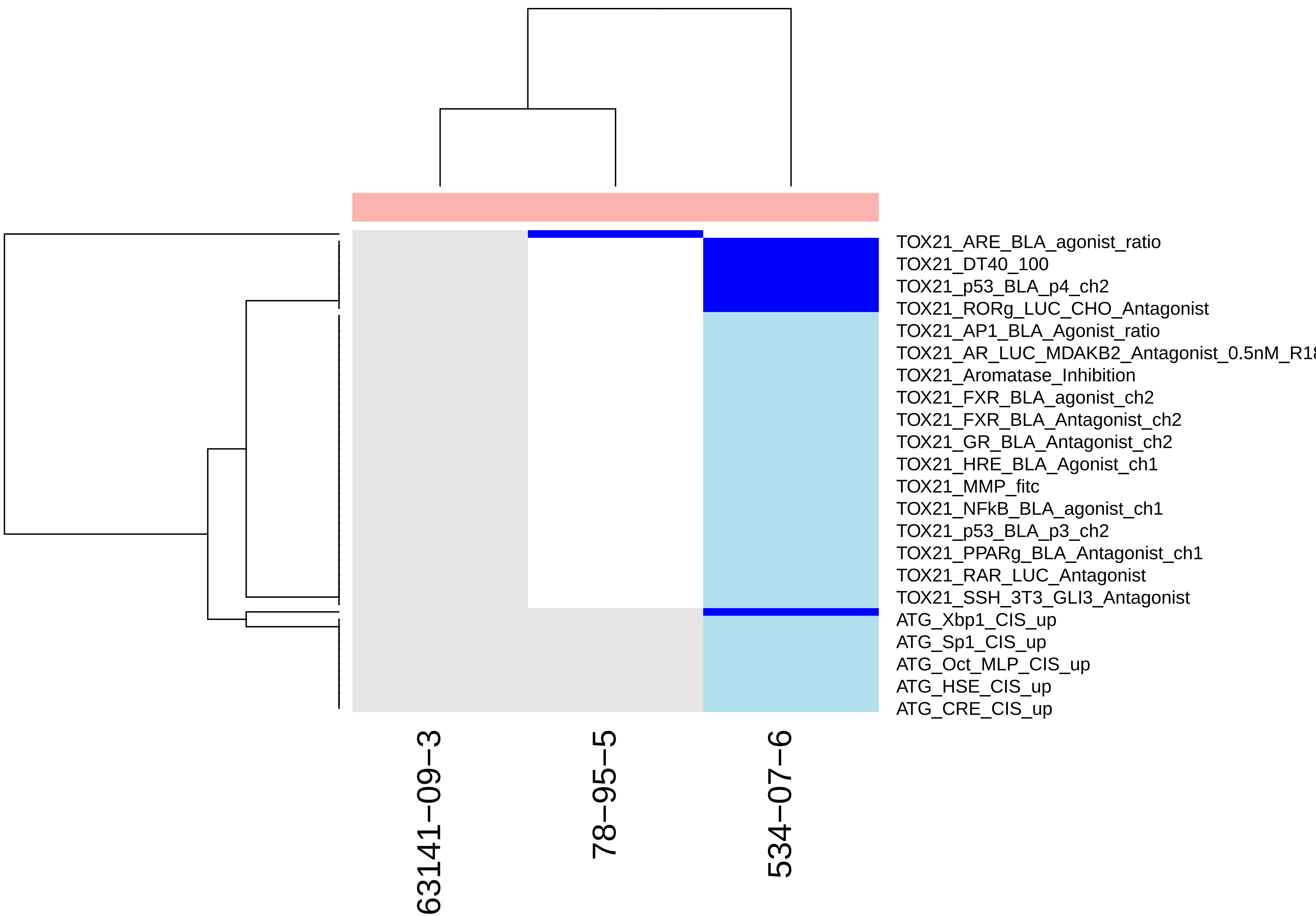

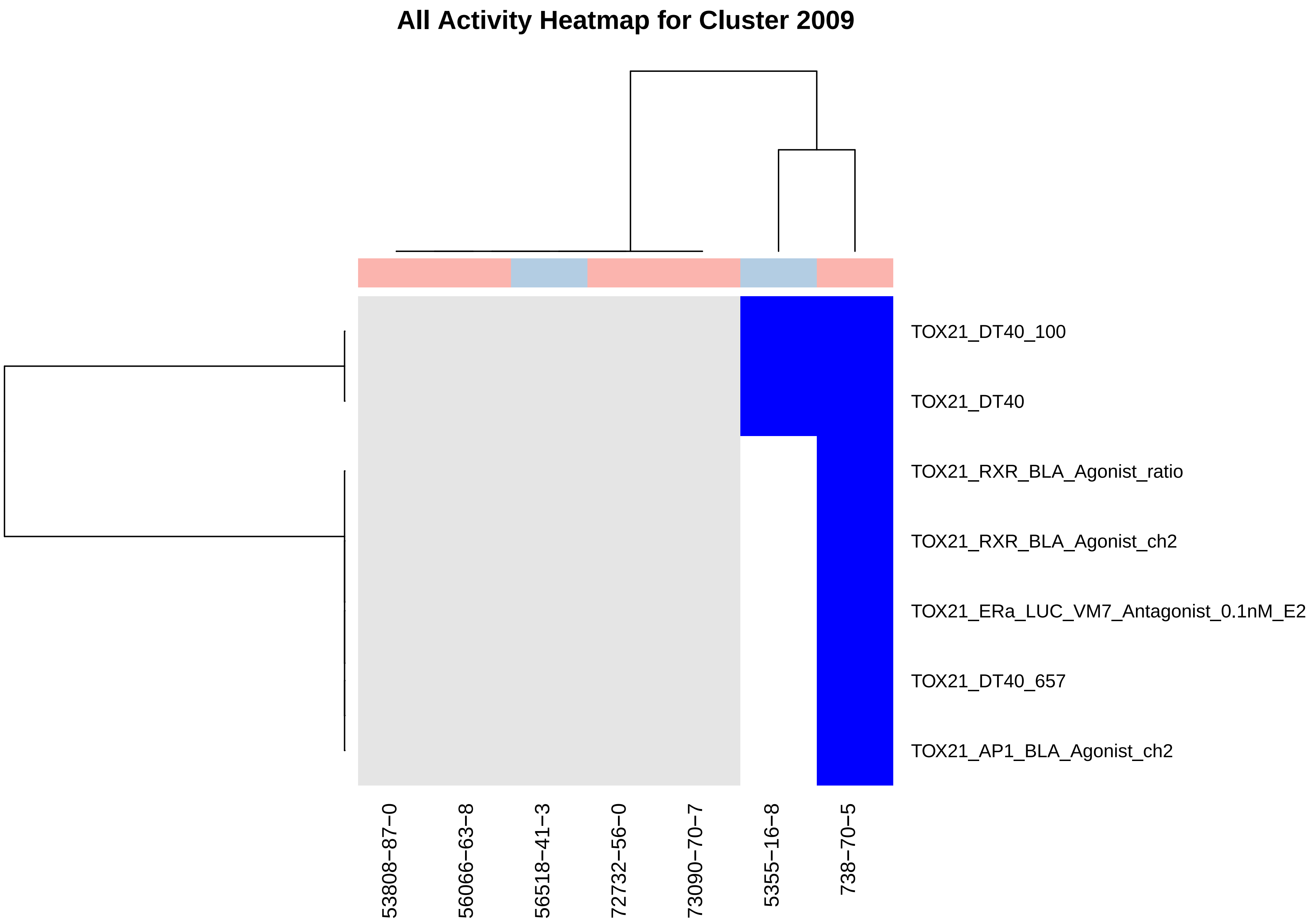

All Activity Heatmap for Cluster 2009
TOX21_DT40_100
TOX21_DT40
TOX21_RXR_BLA_Agonist_ratio
TOX21_RXR_BLA_Agonist_ch2
TOX21_ERa_LUC_VM7_Antagonist_0.1nM_E2
TOX21_DT40_657
TOX21_AP1_BLA_Agonist_ch2
53808−87−0
56066−63−8
56518−41−3
72732−56−0
73090−70−7
5355−16−8
738−70−5

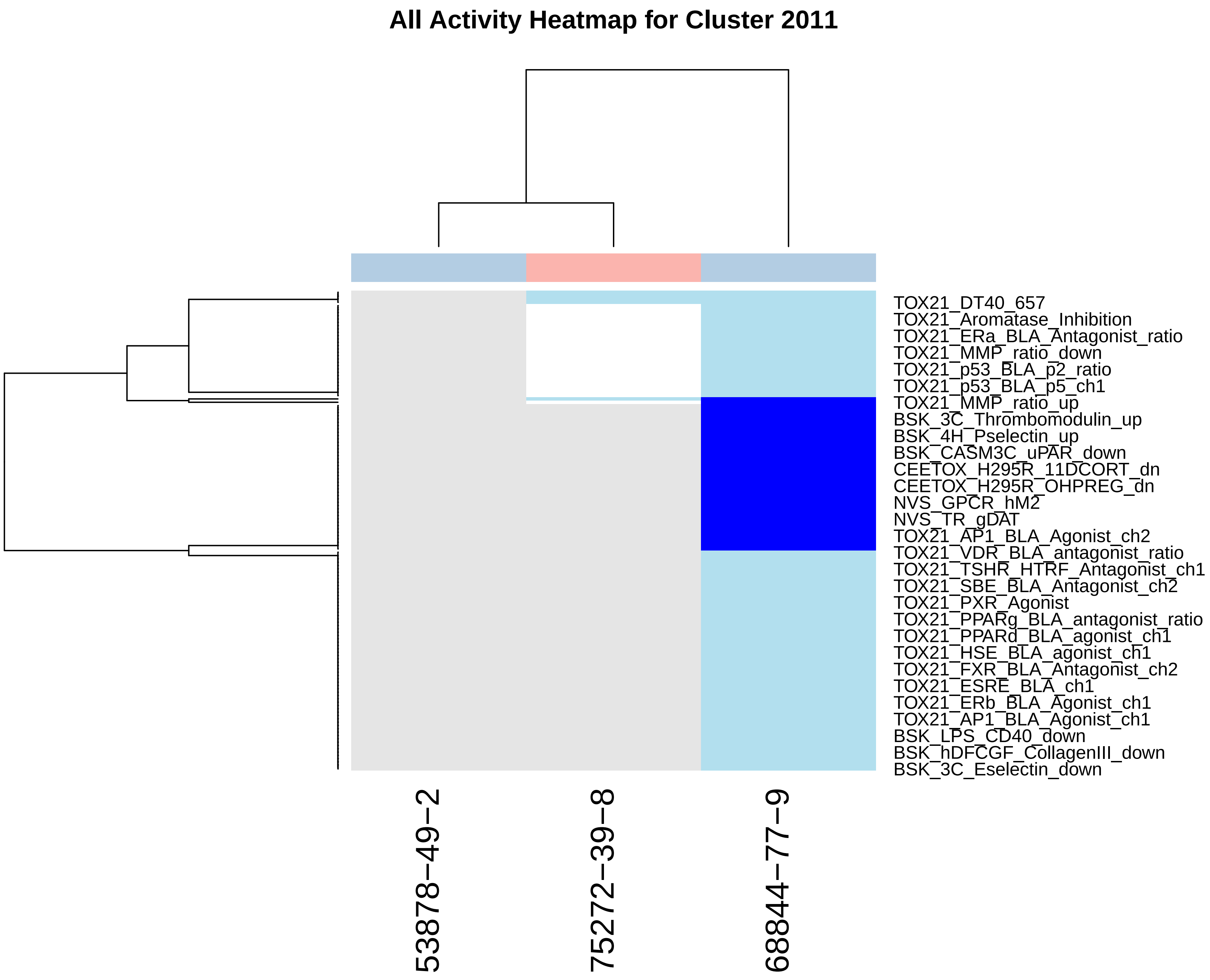
All Activity Heatmap for Cluster 2011
TOX21_DT40_657
TOX21_Aromatase_Inhibition
TOX21_ERa_BLA_Antagonist_ratio
TOX21_MMP_ratio_down
TOX21_p53_BLA_p2_ratio
TOX21_p53_BLA_p5_ch1
TOX21_MMP_ratio_up
BSK_3C_Thrombomodulin_up
BSK_4H_Pselectin_up
BSK_CASM3C_uPAR_down
CEETOX_H295R_11DCORT_dn
CEETOX_H295R_OHPREG_dn
NVS_GPCR_hM2
NVS_TR_gDAT
TOX21_AP1_BLA_Agonist_ch2
TOX21_VDR_BLA_antagonist_ratio
TOX21_TSHR_HTRF_Antagonist_ch1
TOX21_SBE_BLA_Antagonist_ch2
TOX21_PXR_Agonist
TOX21_PPARg_BLA_antagonist_ratio
TOX21_PPARd_BLA_agonist_ch1
TOX21_HSE_BLA_agonist_ch1
TOX21_FXR_BLA_Antagonist_ch2
TOX21_ESRE_BLA_ch1
TOX21_ERb_BLA_Agonist_ch1
TOX21_AP1_BLA_Agonist_ch1
BSK_LPS_CD40_down
BSK_hDFCGF_CollagenIII_down
BSK_3C_Eselectin_down
53878−49−2
75272−39−8
68844−77−9

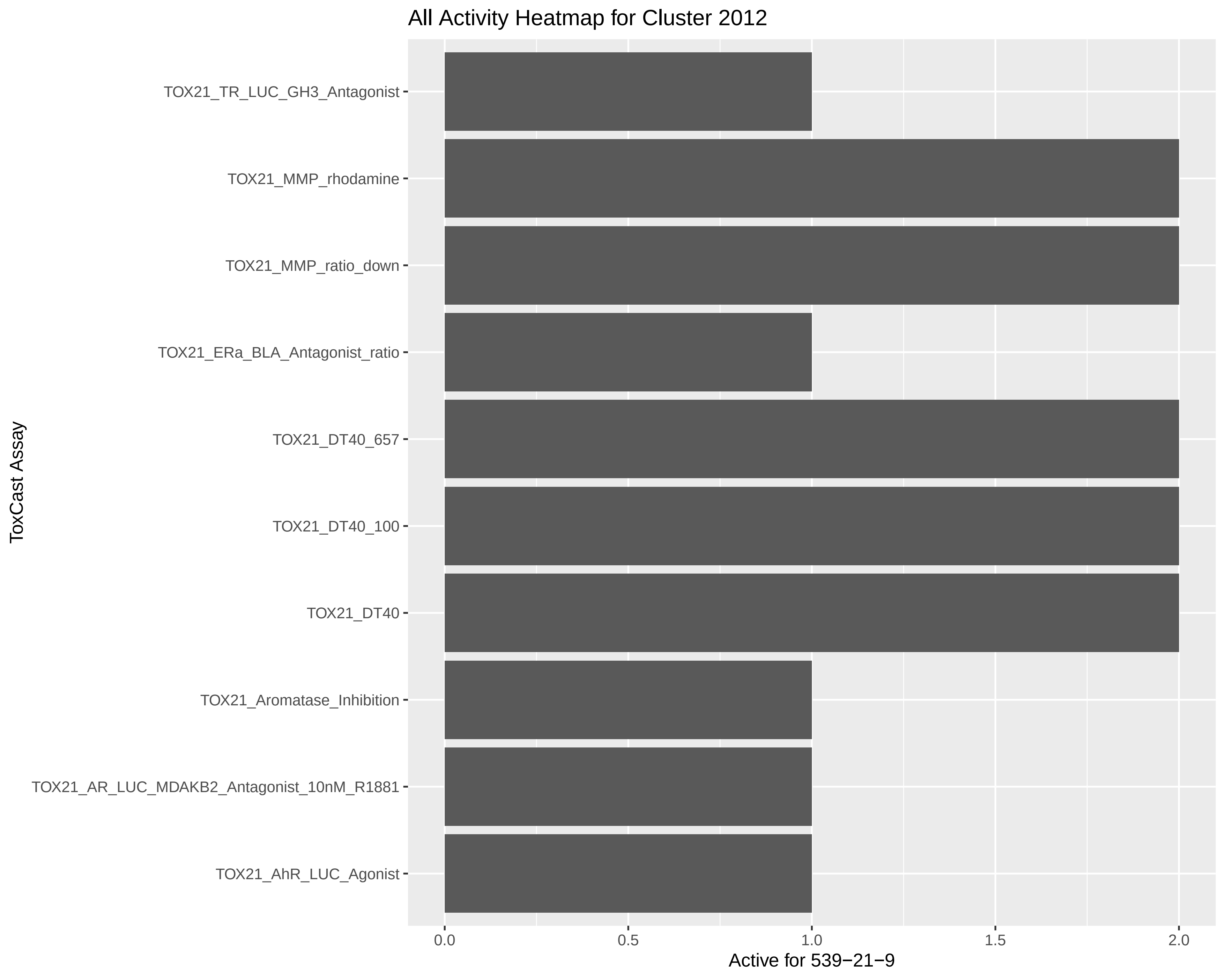
All Activity Heatmap for Cluster 2012
TOX21_TR_LUC_GH3_Antagonist
TOX21_MMP_rhodamine
TOX21_MMP_ratio_down
TOX21_ERa_BLA_Antagonist_ratio
TOX21_DT40_657
TOX21_DT40_100
TOX21_DT40
TOX21_Aromatase_Inhibition
TOX21_AR_LUC_MDAKB2_Antagonist_10nM_R1881
TOX21_AhR_LUC_Agonist
ToxCast Assay
0.0
0.5
1.0
1.5
2.0
Active for 539−21−9

All Activity Heatmap for Cluster 2014

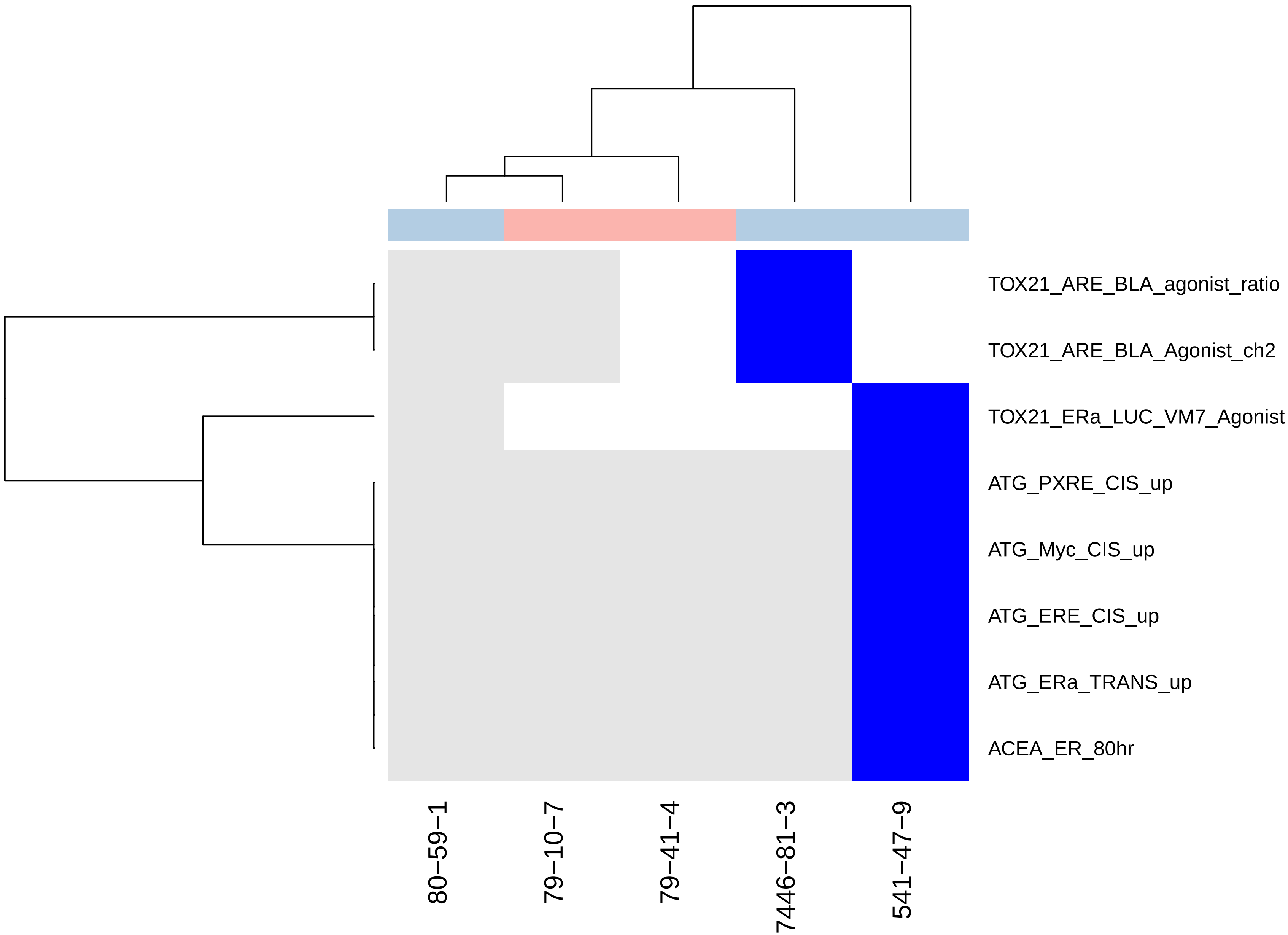

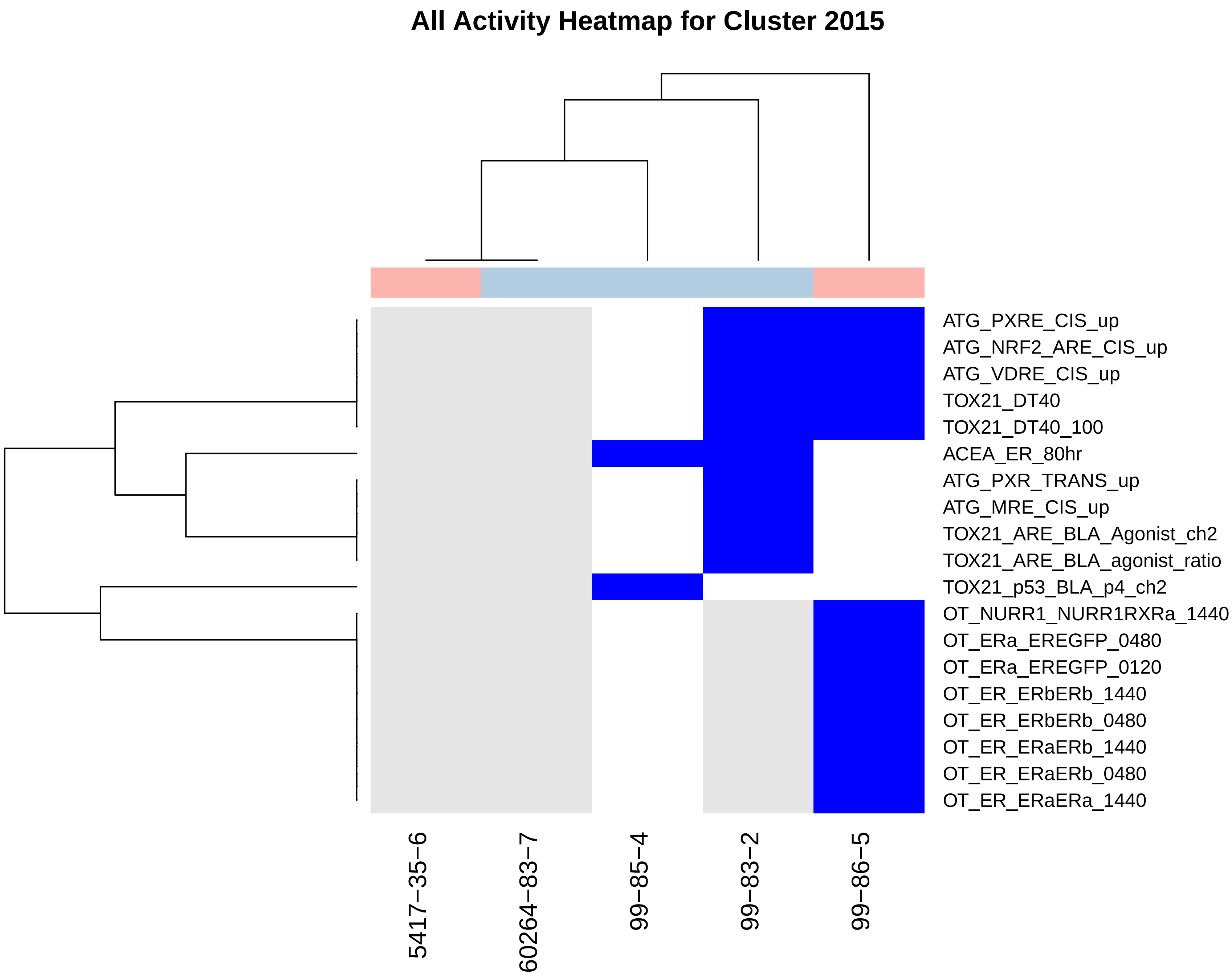
All Activity Heatmap for Cluster 2015
ATG_PXRE_CIS_up
ATG_NRF2_ARE_CIS_up
ATG_VDRE_CIS_up
TOX21_DT40
TOX21_DT40_100
ACEA_ER_80hr
ATG_PXR_TRANS_up
ATG_MRE_CIS_up
TOX21_ARE_BLA_Agonist_ch2
TOX21_ARE_BLA_agonist_ratio
TOX21_p53_BLA_p4_ch2
OT_NURR1_NURR1RXRa_1440
OT_ERa_EREGFP_0480
OT_ERa_EREGFP_0120
OT_ER_ERbERb_1440
OT_ER_ERbERb_0480
OT_ER_ERaERb_1440
OT_ER_ERaERb_0480
OT_ER_ERaERa_1440
5417−35−6
60264−83−7
99−85−4
99−83−2
99−86−5

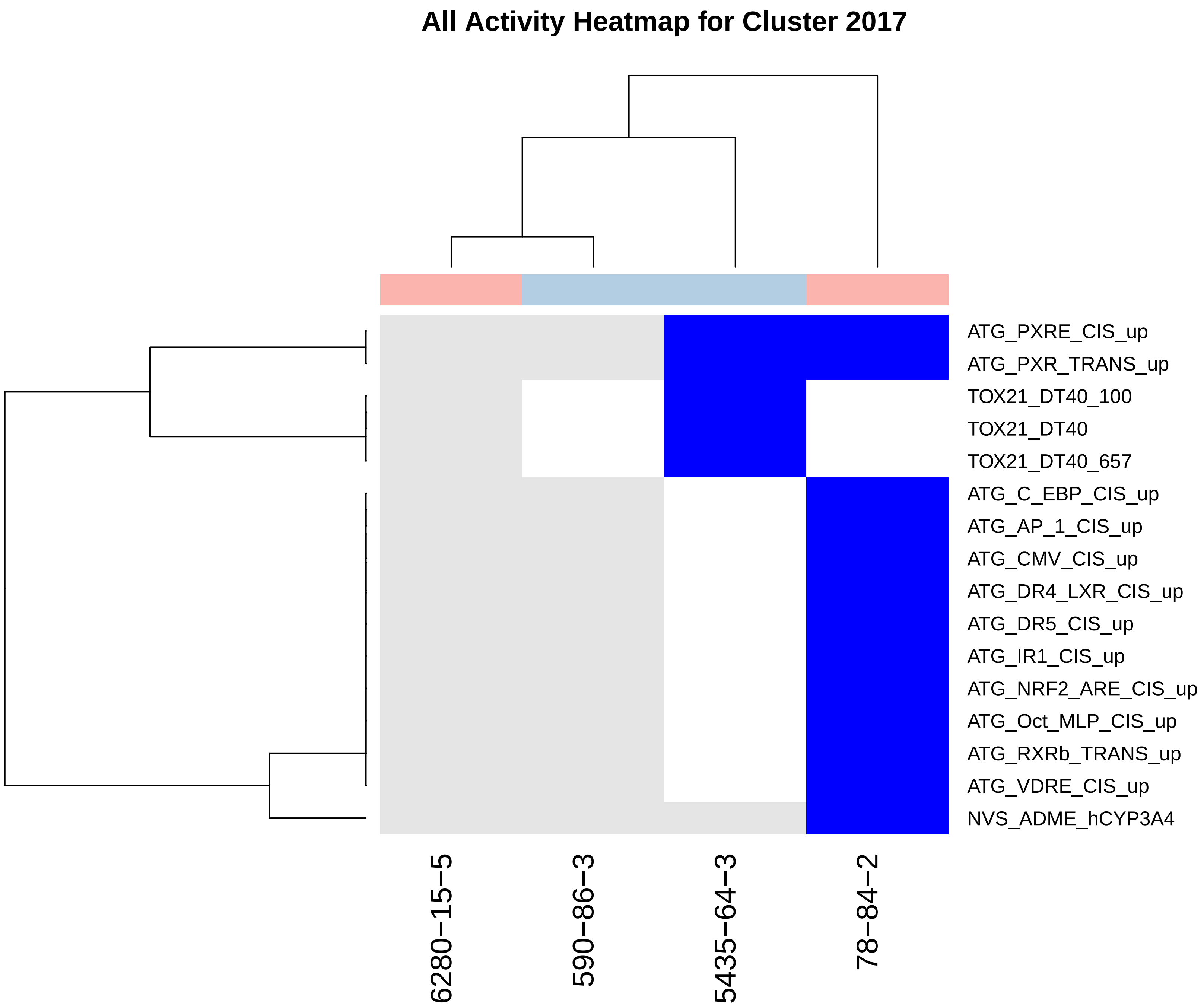

All Activity Heatmap for Cluster 2017
ATG_PXRE_CIS_up
ATG_PXR_TRANS_up
TOX21_DT40_100
TOX21_DT40
TOX21_DT40_657
ATG_C_EBP_CIS_up
ATG_AP_1_CIS_up
ATG_CMV_CIS_up
ATG_DR4_LXR_CIS_up
ATG_DR5_CIS_up
ATG_IR1_CIS_up
ATG_NRF2_ARE_CIS_up
ATG_Oct_MLP_CIS_up
ATG_RXRb_TRANS_up
ATG_VDRE_CIS_up
NVS_ADME_hCYP3A4
6280−15−5
590−86−3
5435−64−3
78−84−2

All Activity Heatmap for Cluster 2018

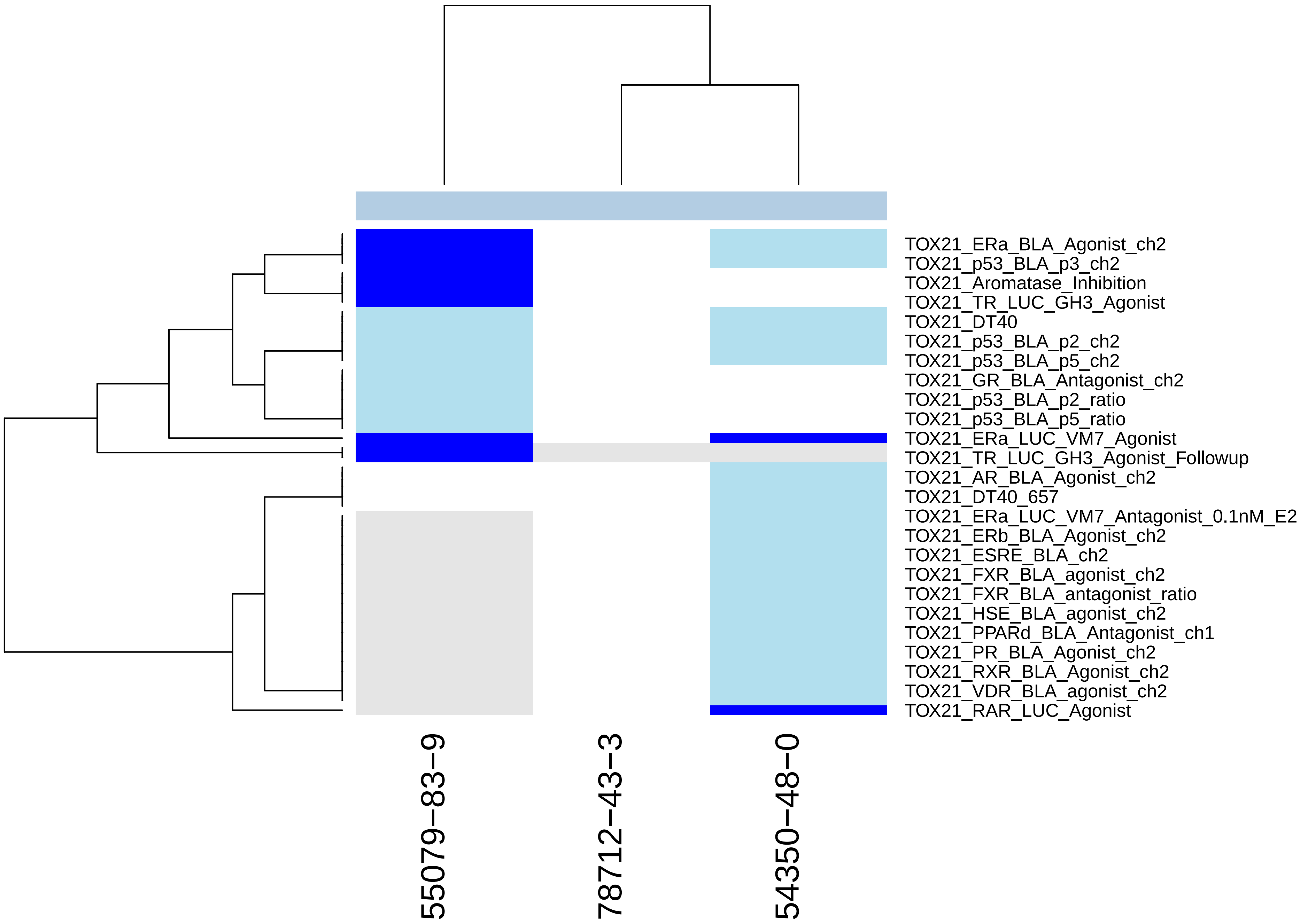

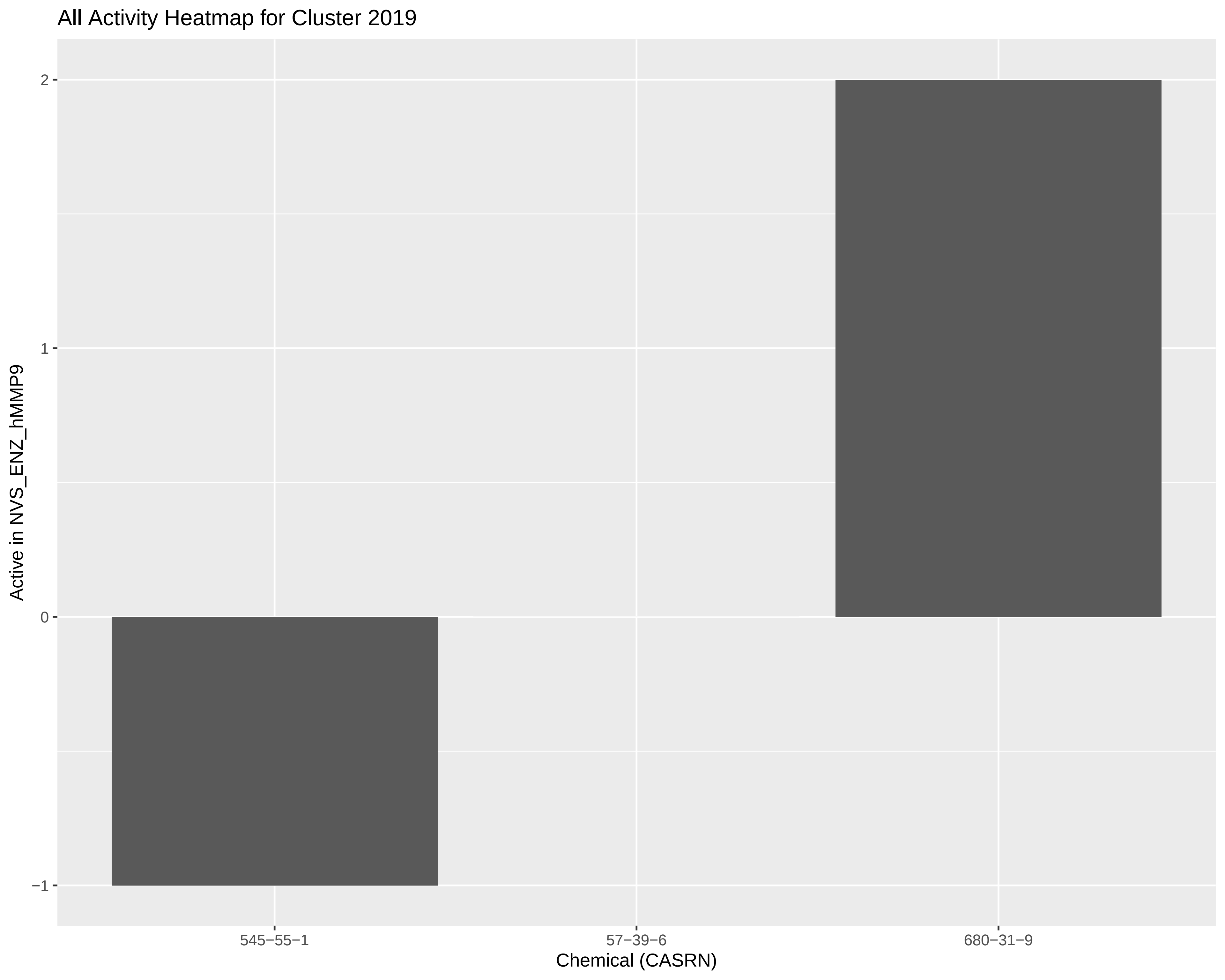
All Activity Heatmap for Cluster 2019
Active in NVS_ENZ_hMMP9
2
1
0
−1
545−55−1
57−39−6
680−31−9
Chemical (CASRN)

All Activity Heatmap for Cluster 2025

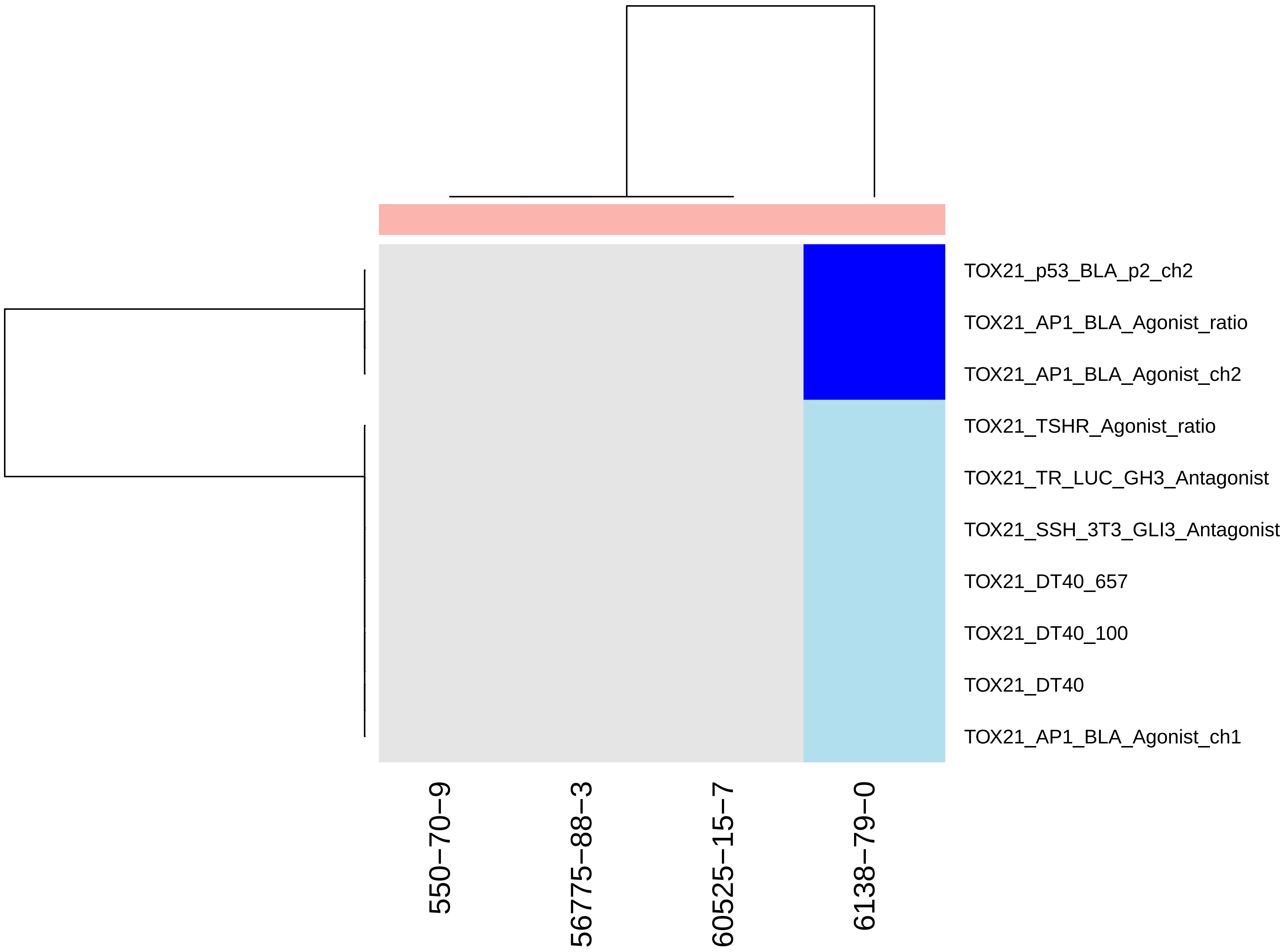

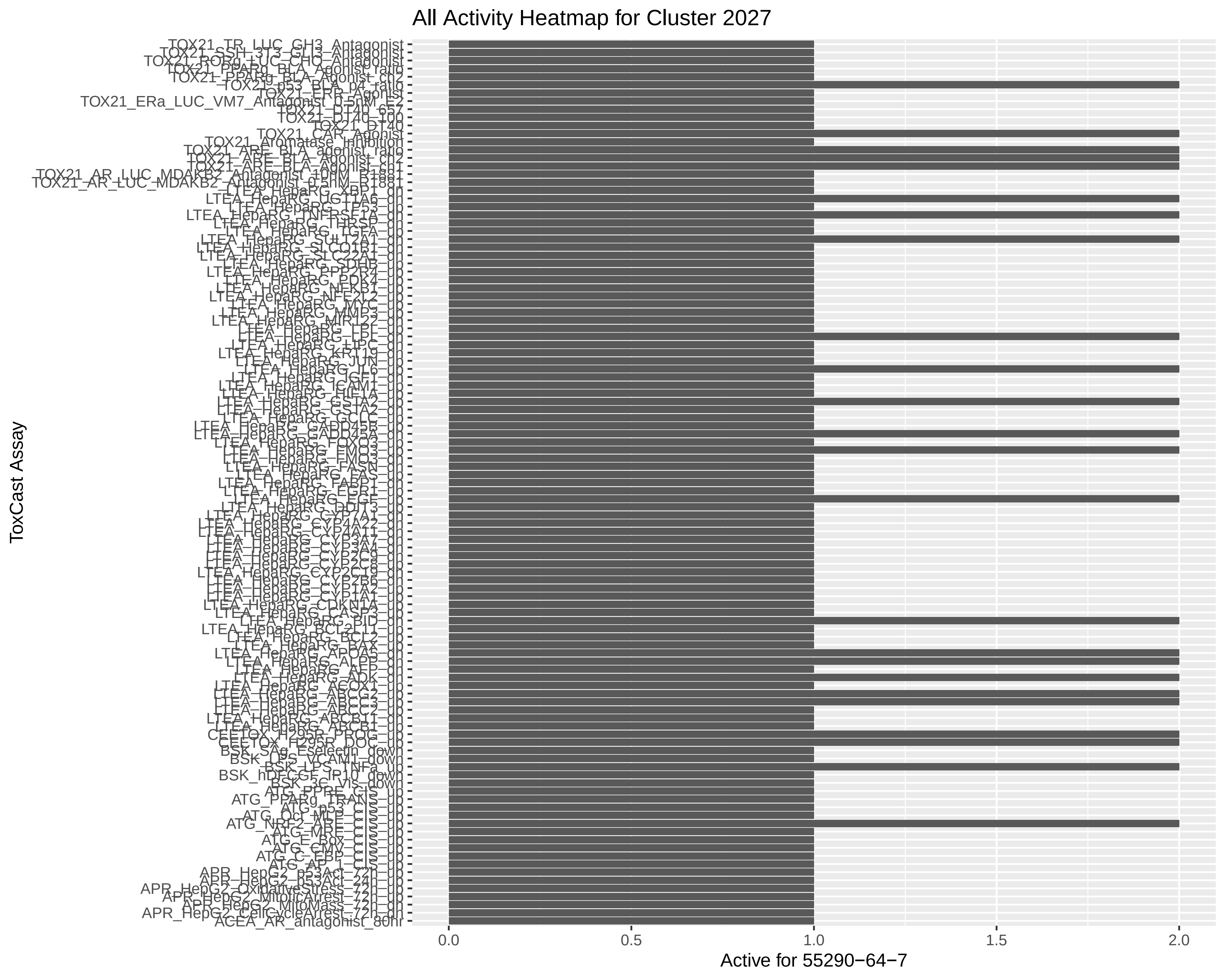
All Activity Heatmap for Cluster 2027
ToxCast Assay
Active for 55290−64−7
0.0
0.5
1.0
1.5
2.0
TOX21_TR_LUC_GH3_Antagonist
TOX21_RORg_LUC_CHO_Antagonist
TOX21_PPARg_BLA_Agonist_ch2
TOX21_ERa_LUC_VM7_Antagonist_0.5nM_E2
TOX21_CAR_Agonist
TOX21_Aromatase_Inhibition
TOX21_AR_LUC_MDAKB2_Antagonist_10nM_R1881
LTEA_HepaRG_TNFRSF1B_dn
LTEA_HepaRG_MIR122_dn
LTEA_HepaRG_LIPC_dn
LTEA_HepaRG_KRT19_dn
LTEA_HepaRG_ICAM1_up
LTEA_HepaRG_GADD45B_up
LTEA_HepaRG_CYP2C19_dn
LTEA_HepaRG_CDKN1A_up
LTEA_HepaRG_BCL2L11_up
LTEA_HepaRG_APOA5_dn
LTEA_HepaRG_ABCB11_dn
CEETOX_H295R_PROG_up
BSK_SAg_Eselectin_down
BSK_LPS_VCAM1_down
BSK_LPS_TNFa_up
BSK_hDFCGF_IP10_down
BSK_3C_Vis_down
ATG_PPARg_TRANS_up
ATG_NRF2_ARE_CIS_up
ATG_E_Box_CIS_up
ATG_C_EBP_CIS_up
ATG_AP_1_CIS_up
APR_HepG2_p53Act_72h_up
APR_HepG2_OxidativeStress_72h_up
APR_HepG2_MitoMass_72h_dn
APR_HepG2_CellCycleArrest_72h_dn
ACEA_AR_antagonist_80hr

All Activity Heatmap for Cluster 2028

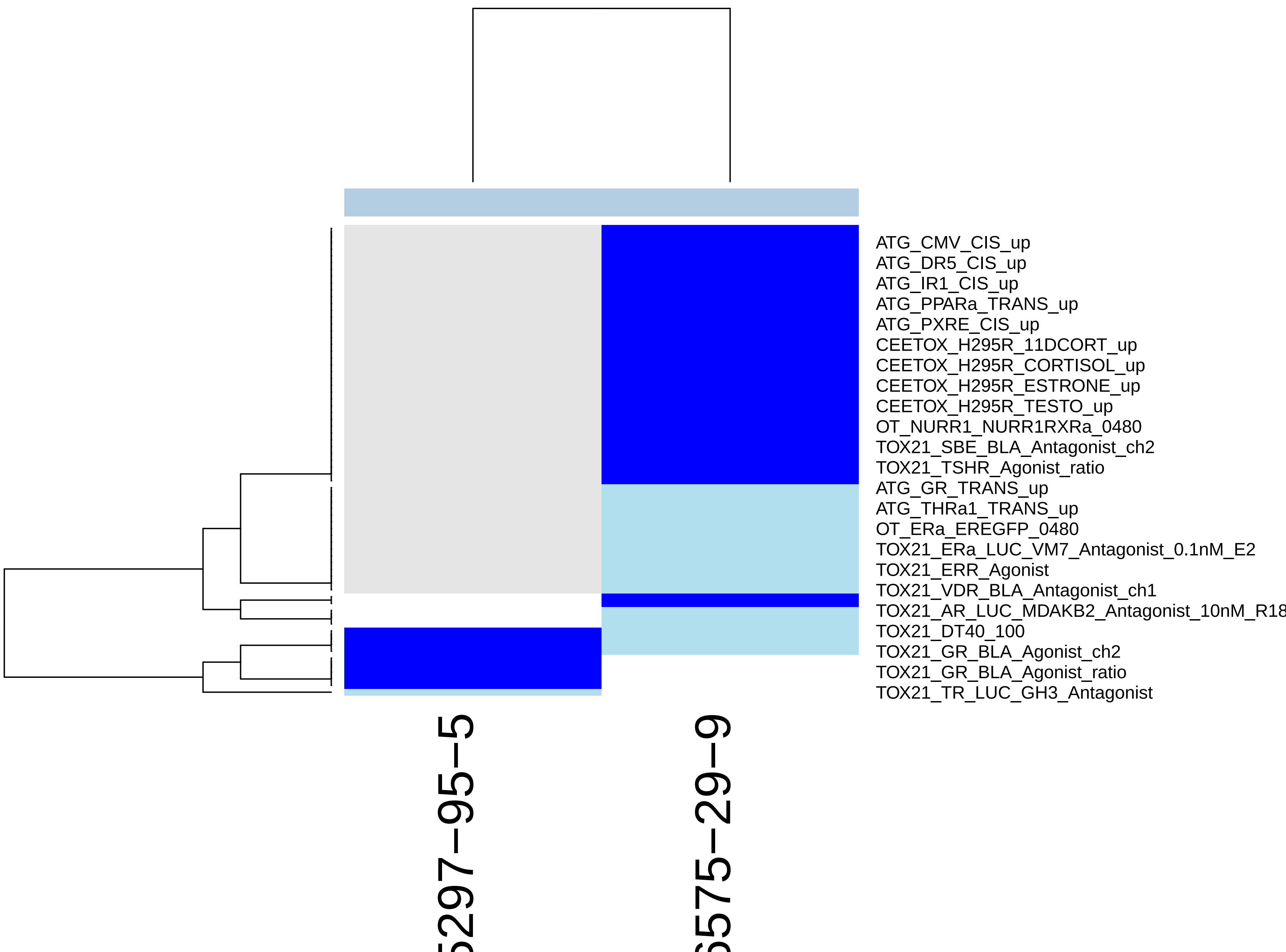

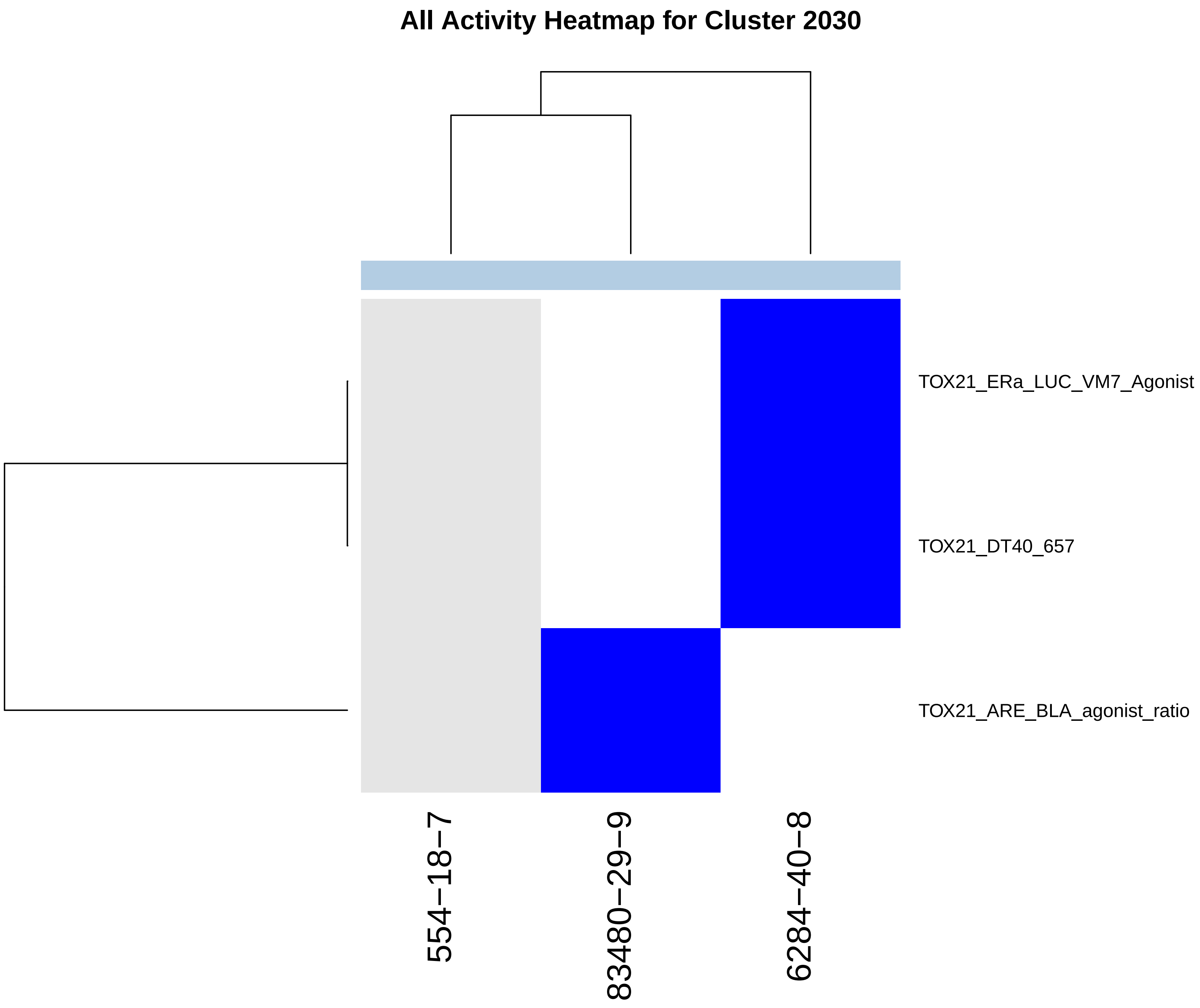
All Activity Heatmap for Cluster 2030
TOX21_ERa_LUC_VM7_Agonist
TOX21_DT40_657
TOX21_ARE_BLA_agonist_ratio
554−18−7
83480−29−9
6284−40−8

All Activity Heatmap for Cluster 2031

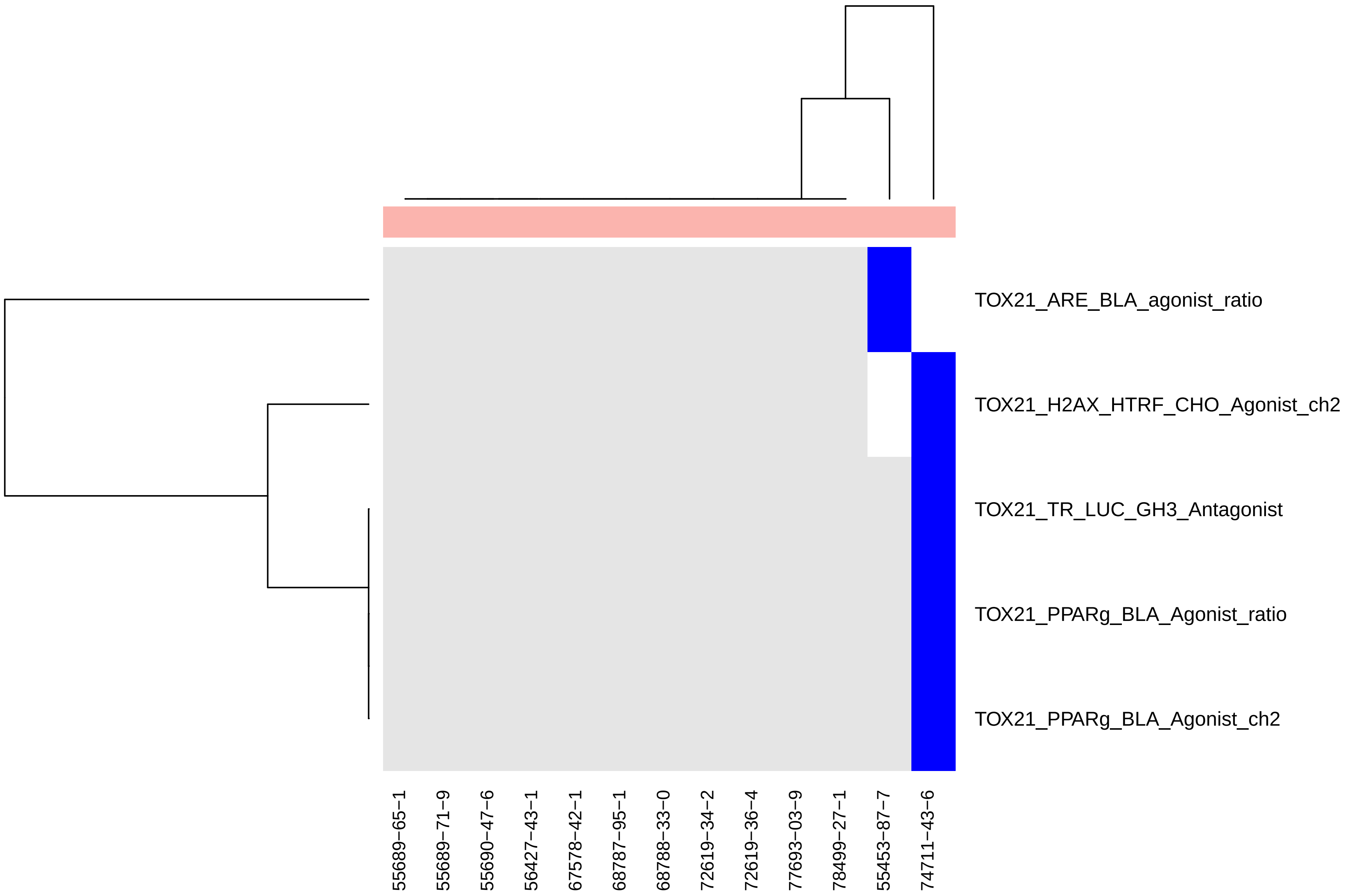

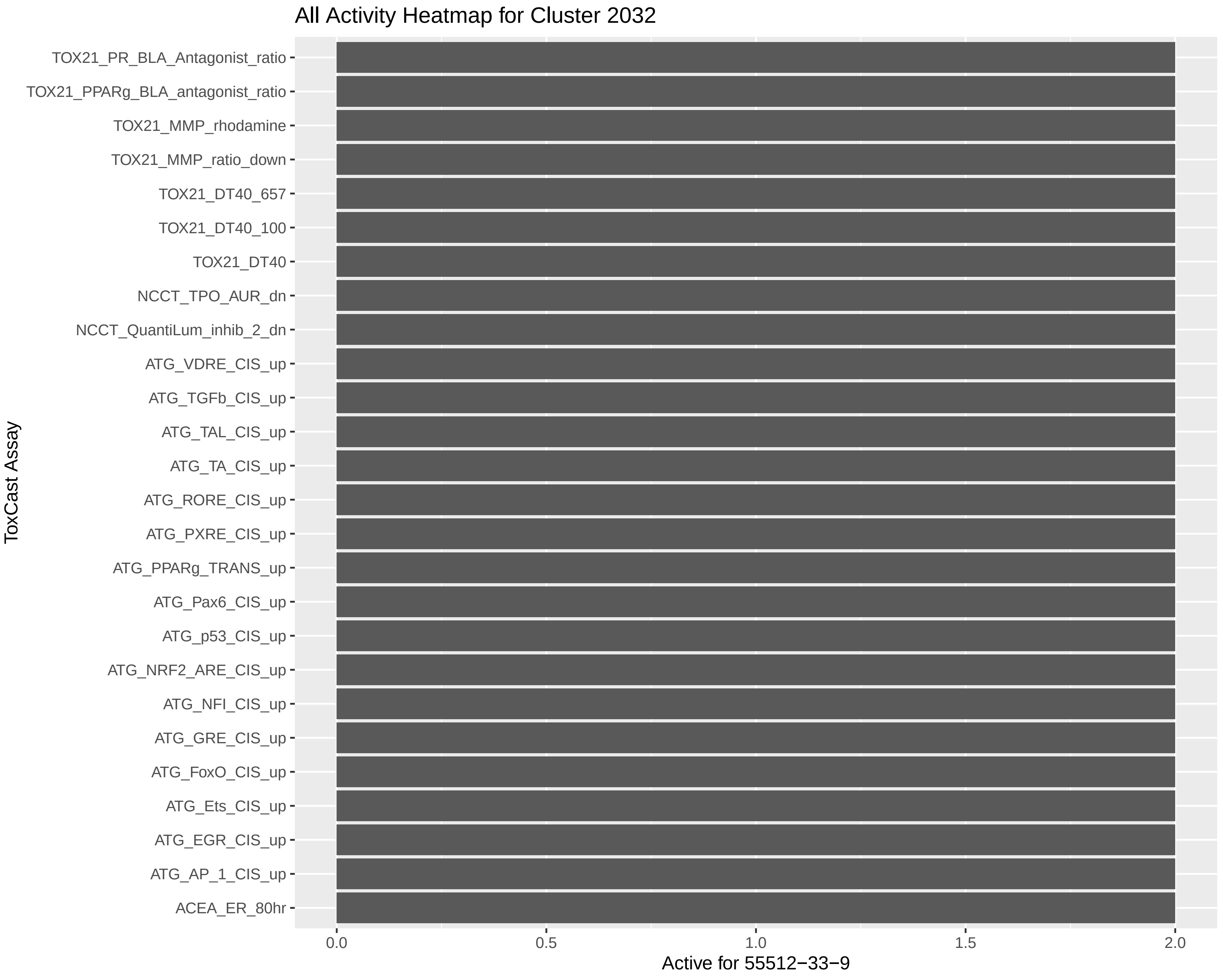

All Activity Heatmap for Cluster 2032
ToxCast Assay
TOX21_PR_BLA_Antagonist_ratio
TOX21_PPARg_BLA_antagonist_ratio
TOX21_MMP_rhodamine
TOX21_MMP_ratio_down
TOX21_DT40_657
TOX21_DT40_100
TOX21_DT40
NCCT_TPO_AUR_dn
NCCT_QuantiLum_inhib_2_dn
ATG_VDRE_CIS_up
ATG_TGFb_CIS_up
ATG_TAL_CIS_up
ATG_TA_CIS_up
ATG_RORE_CIS_up
ATG_PXRE_CIS_up
ATG_PPARg_TRANS_up
ATG_Pax6_CIS_up
ATG_p53_CIS_up
ATG_NRF2_ARE_CIS_up
ATG_NFI_CIS_up
ATG_GRE_CIS_up
ATG_FoxO_CIS_up
ATG_Ets_CIS_up
ATG_EGR_CIS_up
ATG_AP_1_CIS_up
ACEA_ER_80hr
0.0
0.5
1.0
1.5
2.0
Active for 55512−33−9

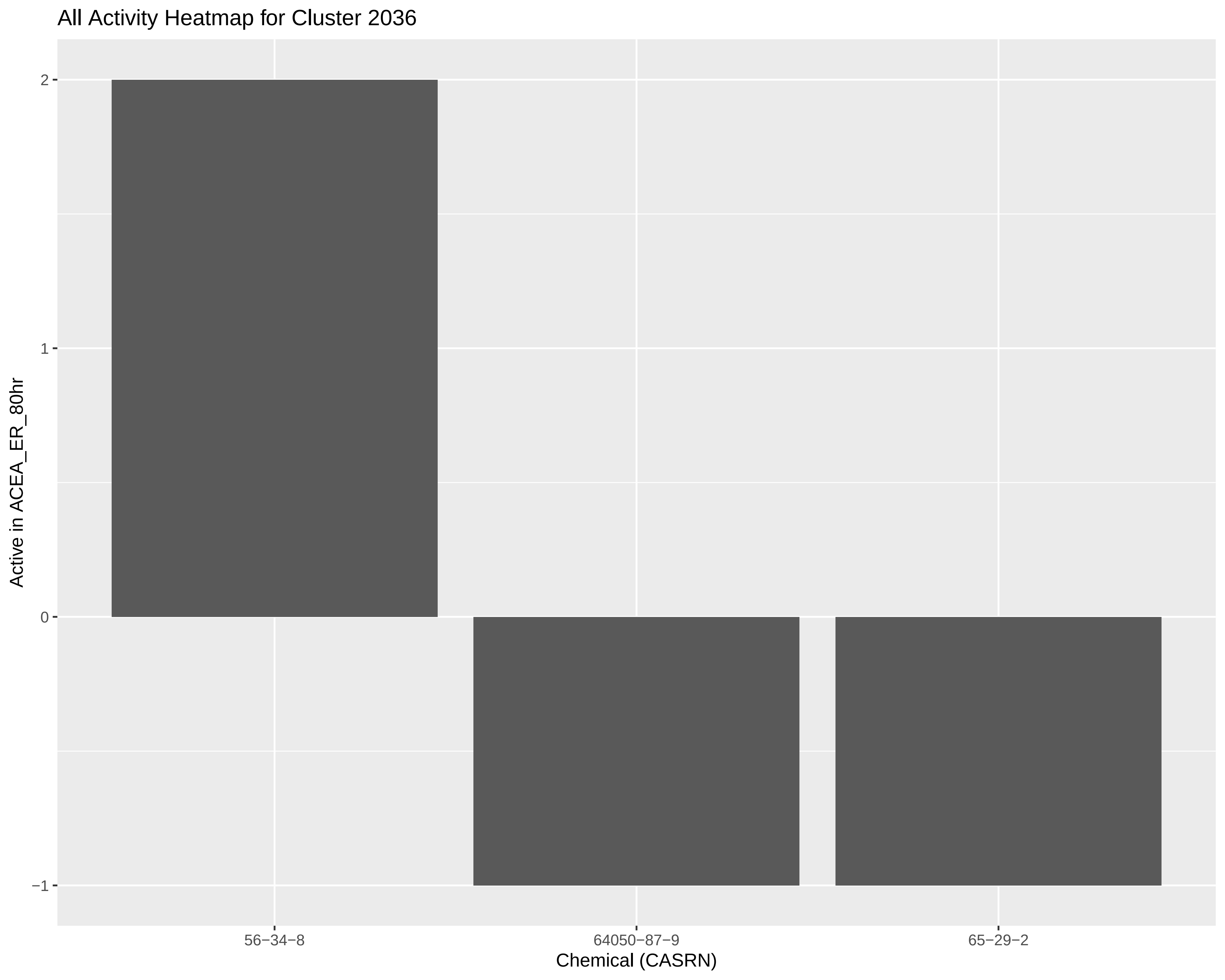
All Activity Heatmap for Cluster 2036
Active in ACEA_ER_80hr
2
1
0
−1
56−34−8
64050−87−9
65−29−2
Chemical (CASRN)

All Activity Heatmap for Cluster 2041

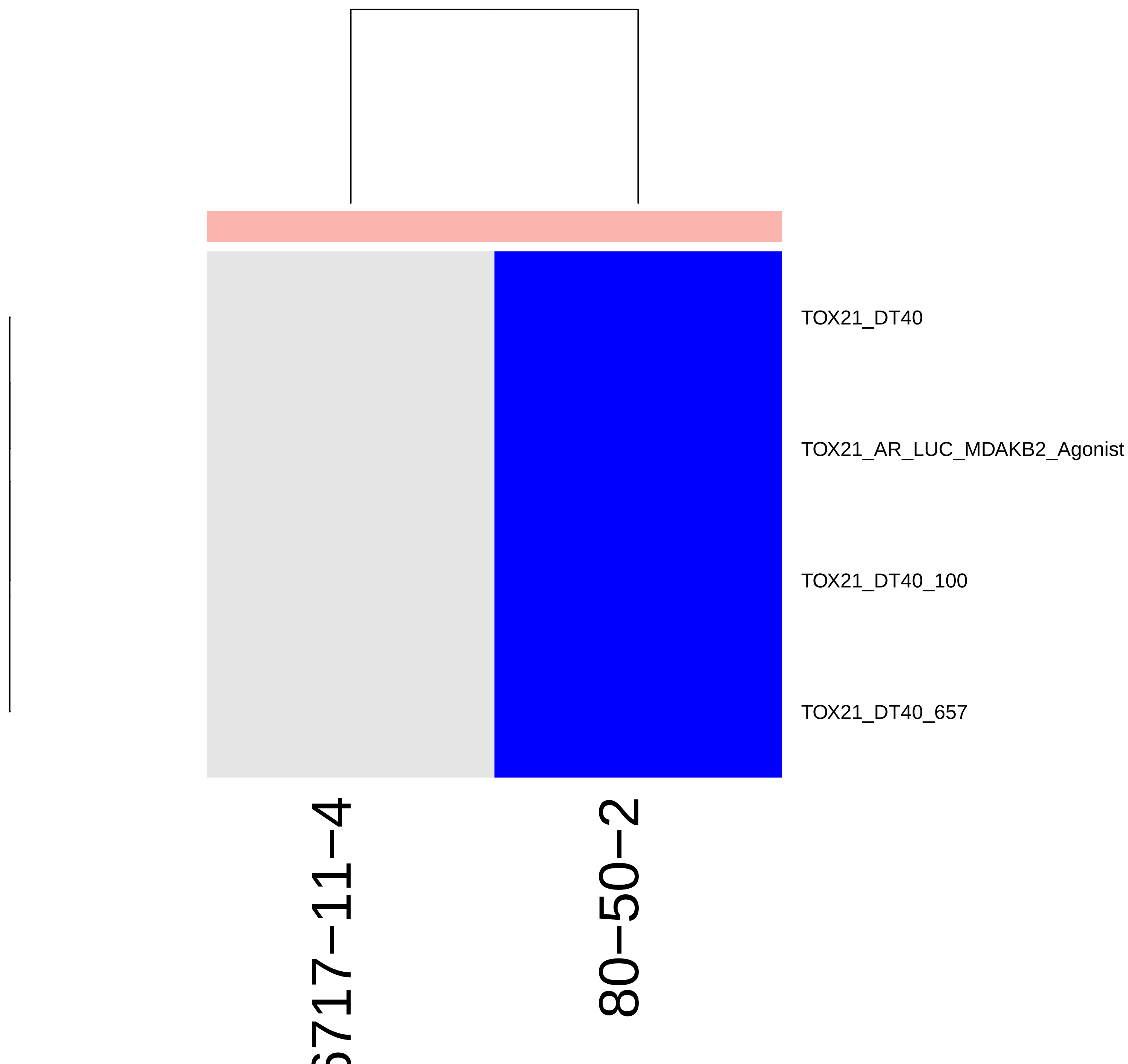

All Activity Heatmap for Cluster 2043

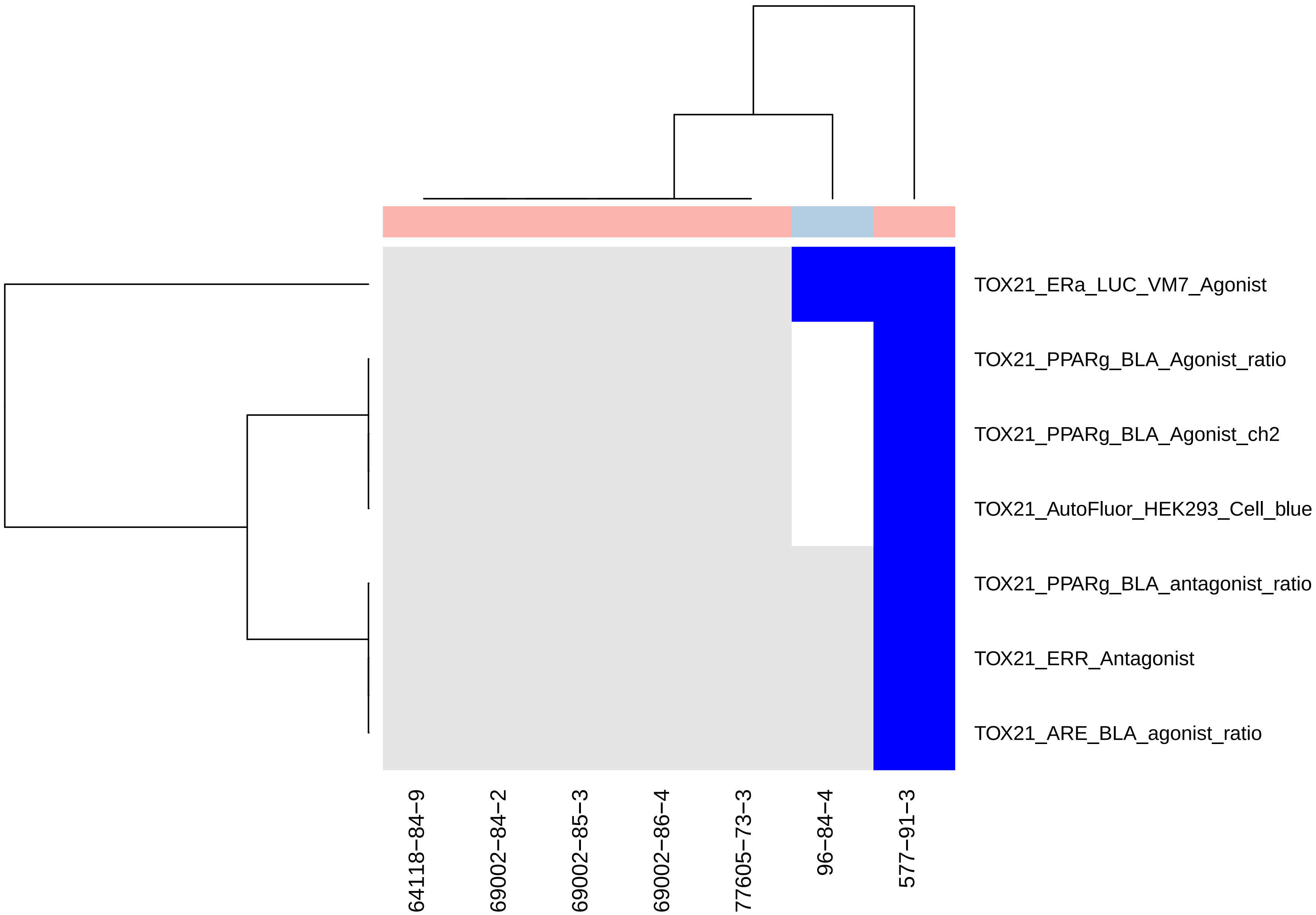

All Activity Heatmap for Cluster 2046

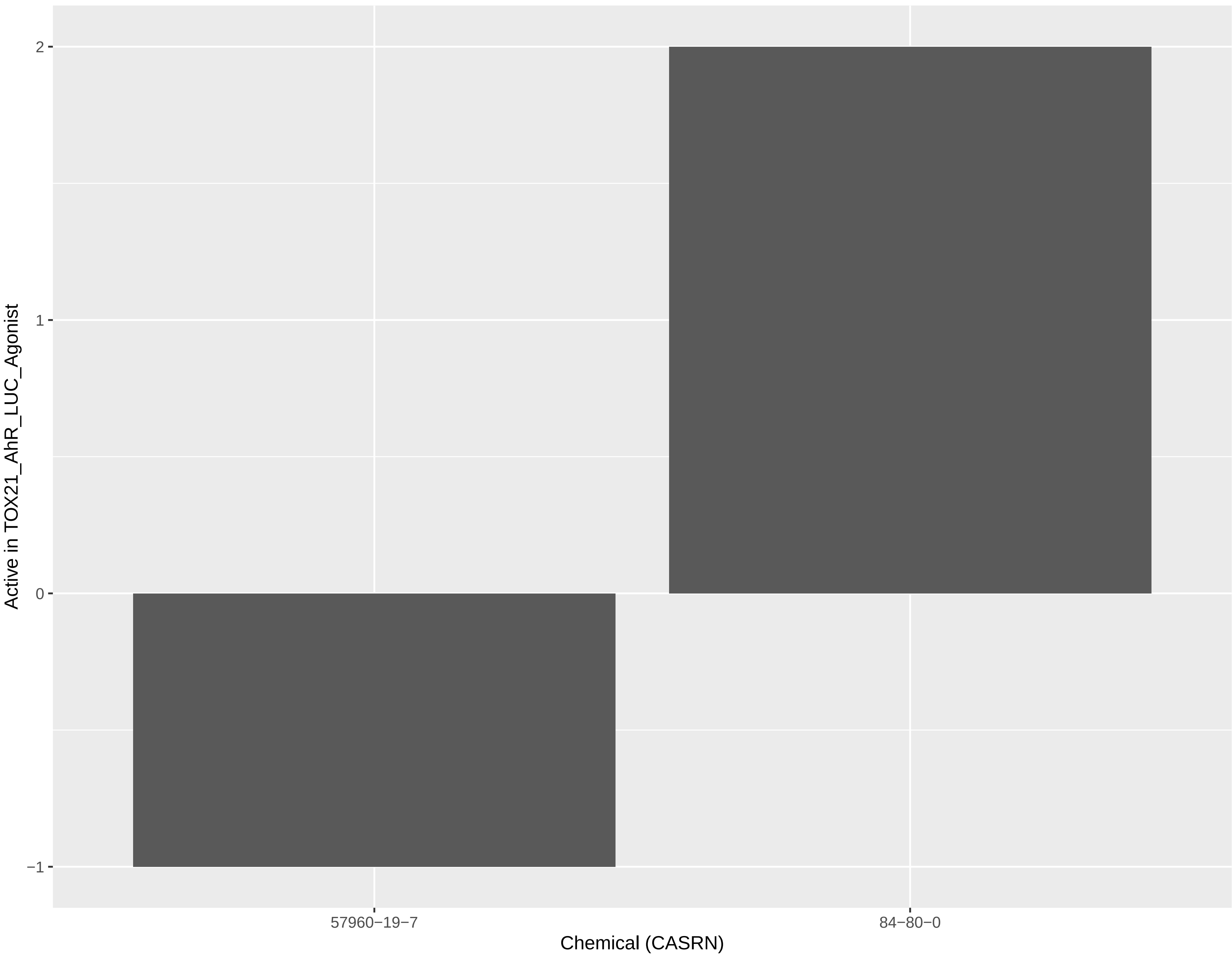

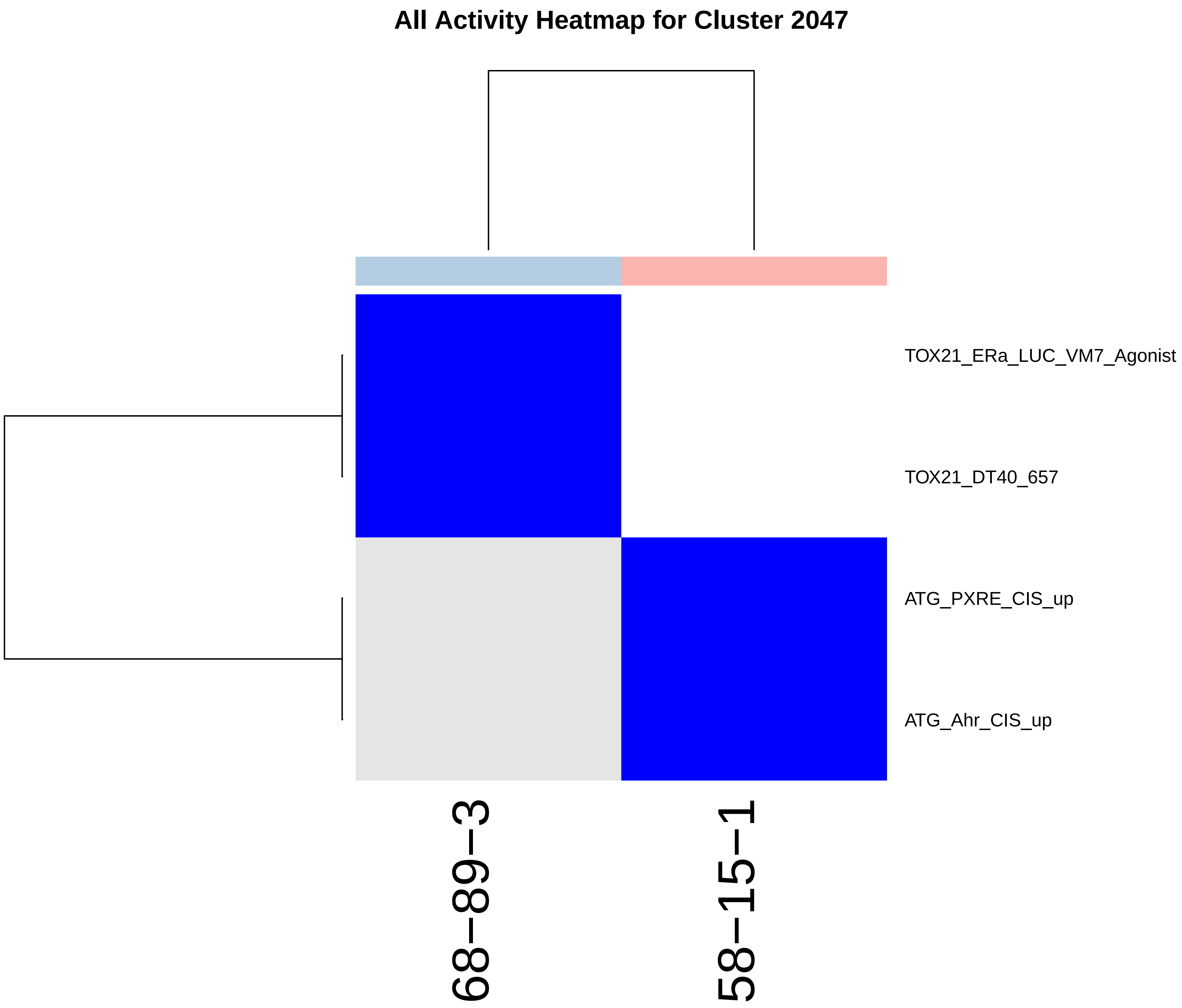
All Activity Heatmap for Cluster 2047
TOX21_ERa_LUC_VM7_Agonist
TOX21_DT40_657
ATG_PXRE_CIS_up
ATG_Ahr_CIS_up
68−89−3
58−15−1

All Activity Heatmap for Cluster 2052

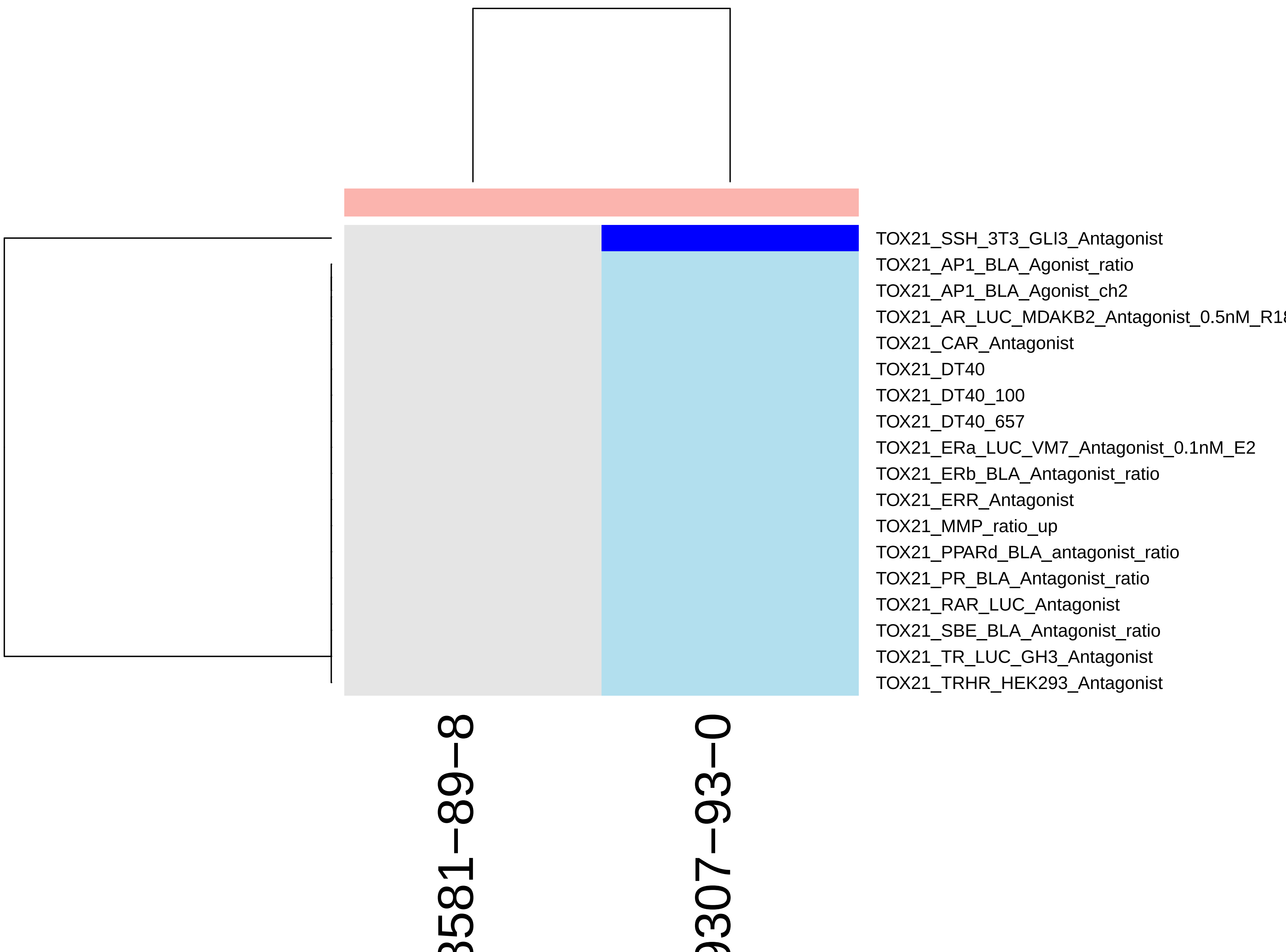

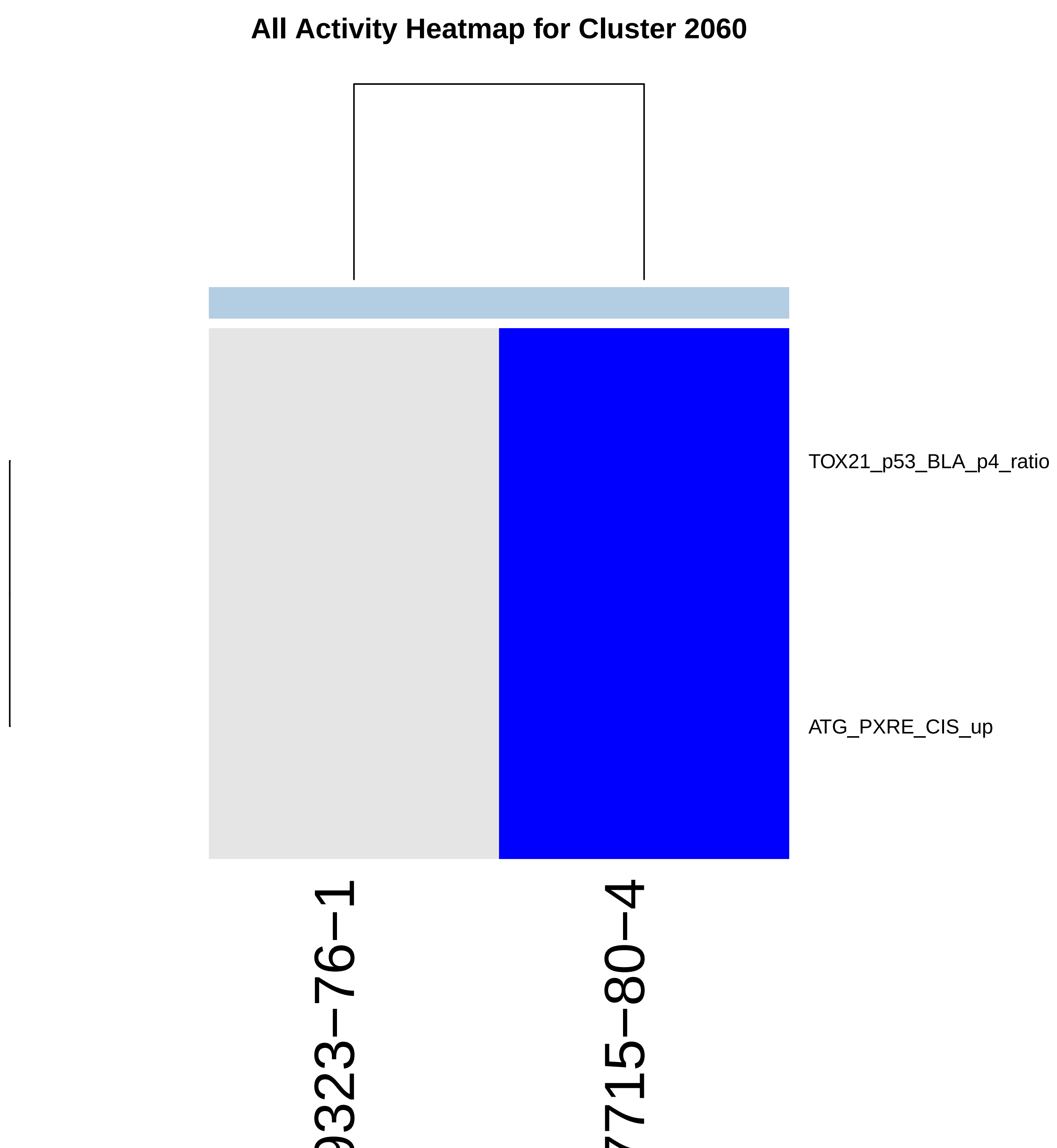
All Activity Heatmap for Cluster 2060
TOX21_p53_BLA_p4_ratio
ATG_PXRE_CIS_up
923−76−1
7715−80−4

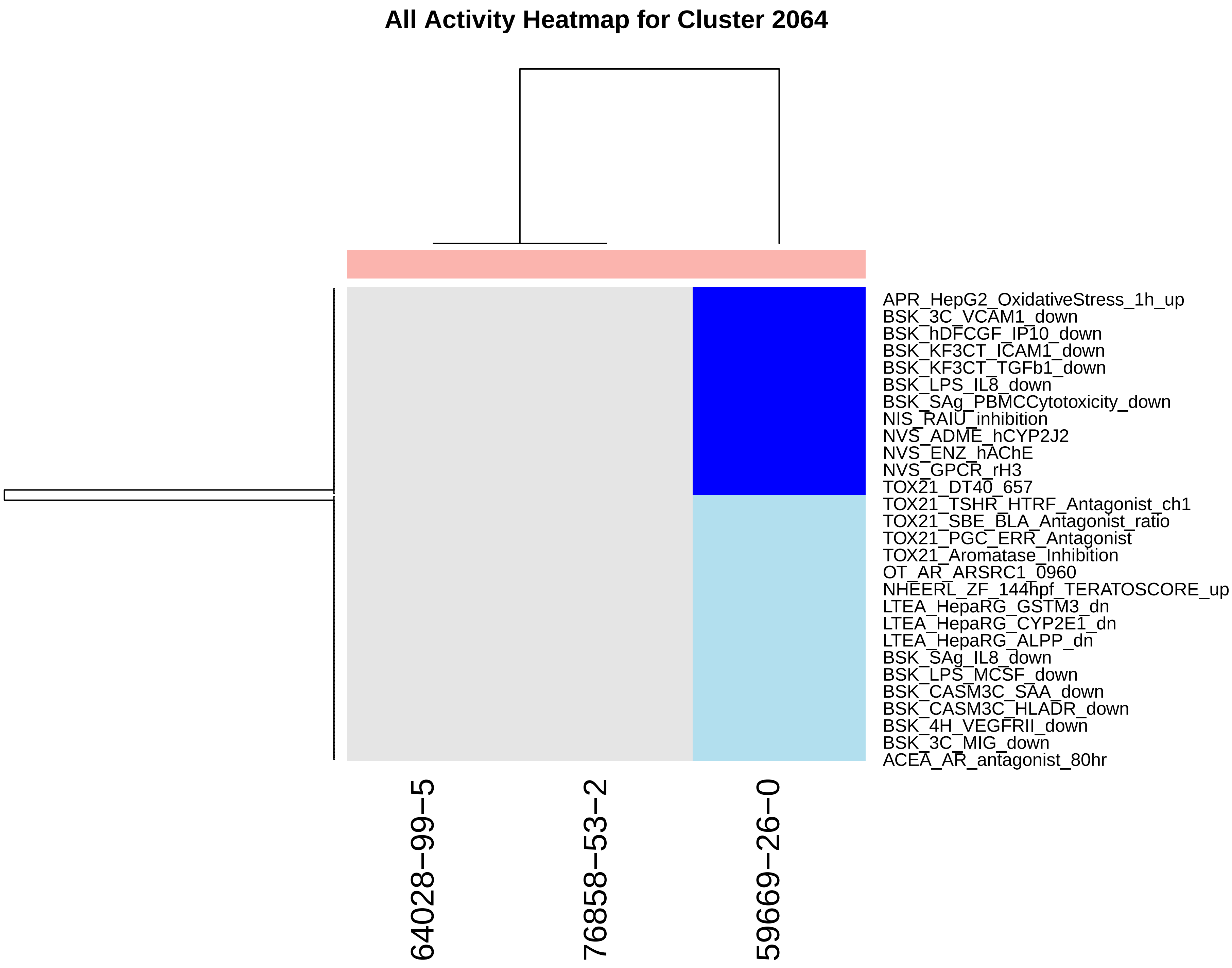
All Activity Heatmap for Cluster 2064
APR_HepG2_OxidativeStress_1h_up
BSK_3C_VCAM1_down
BSK_hDFCGF_IP10_down
BSK_KF3CT_ICAM1_down
BSK_KF3CT_TGFb1_down
BSK_LPS_IL8_down
BSK_SAg_PBMCCytotoxicity_down
NIS_RAIU_inhibition
NVS_ADME_hCYP2J2
NVS_ENZ_hAChE
NVS_GPCR_rH3
TOX21_DT40_657
TOX21_TSHR_HTRF_Antagonist_ch1
TOX21_SBE_BLA_Antagonist_ratio
TOX21_PGC_ERR_Antagonist
TOX21_Aromatase_Inhibition
OT_AR_ARSRC1_0960
NHEERL_ZF_144hpf_TERATOSCORE_up
LTEA_HepaRG_GSTM3_dn
LTEA_HepaRG_CYP2E1_dn
LTEA_HepaRG_ALPP_dn
BSK_SAg_IL8_down
BSK_LPS_MCSF_down
BSK_CASM3C_SAA_down
BSK_CASM3C_HLADR_down
BSK_4H_VEGFRII_down
BSK_3C_MIG_down
ACEA_AR_antagonist_80hr
64028−99−5
76858−53−2
59669−26−0

All Activity Heatmap for Cluster 2065

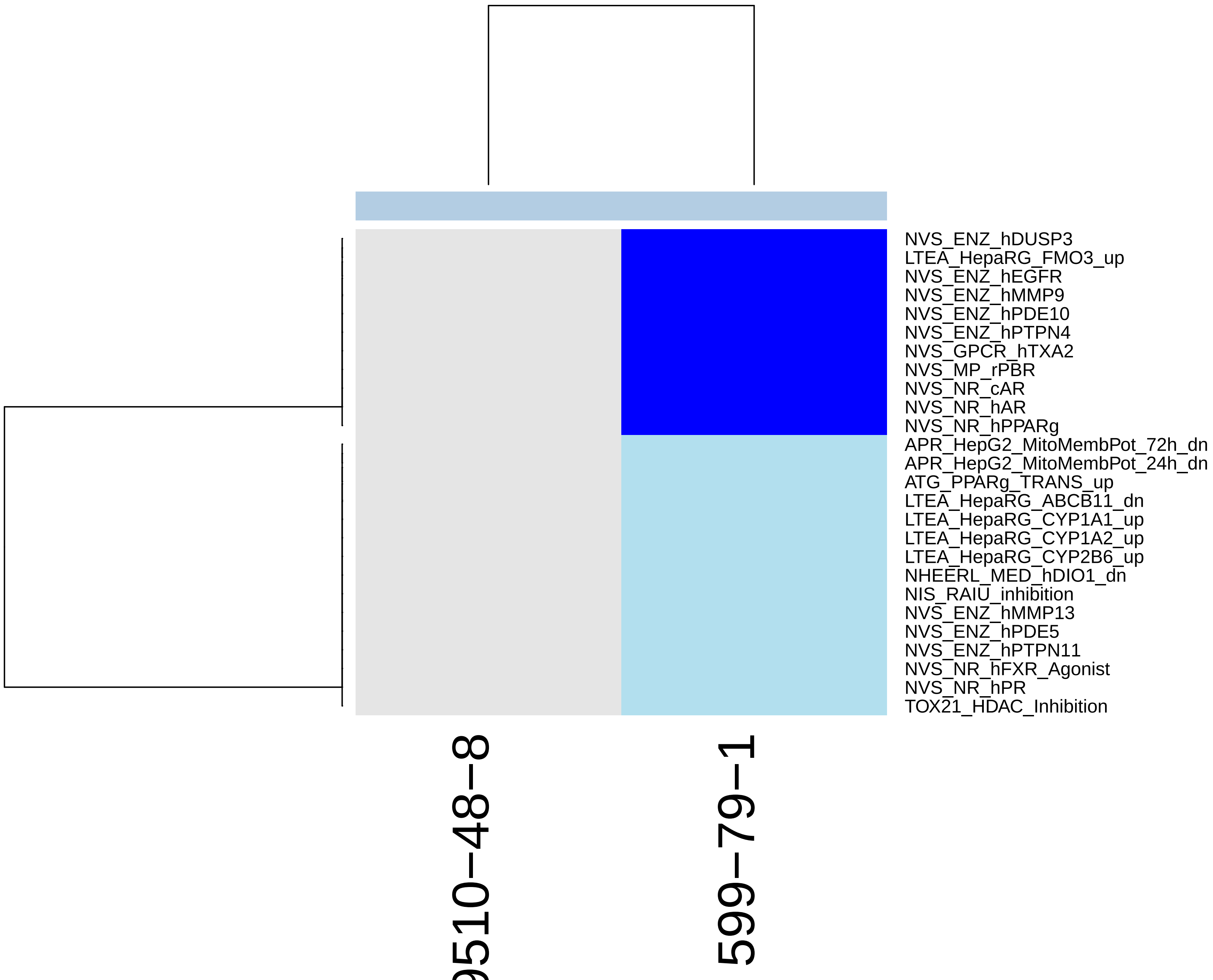

All Activity Heatmap for Cluster 2066

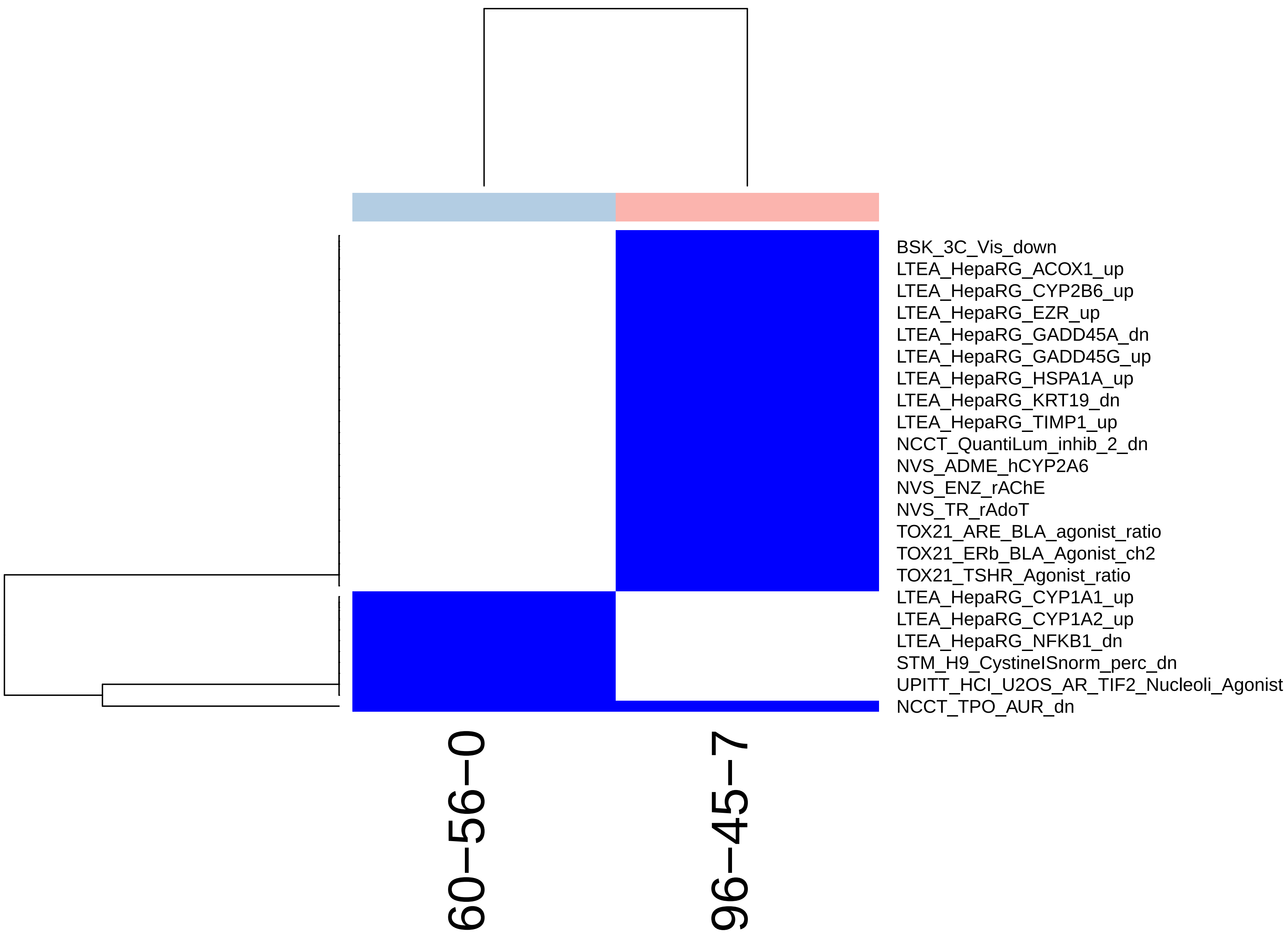

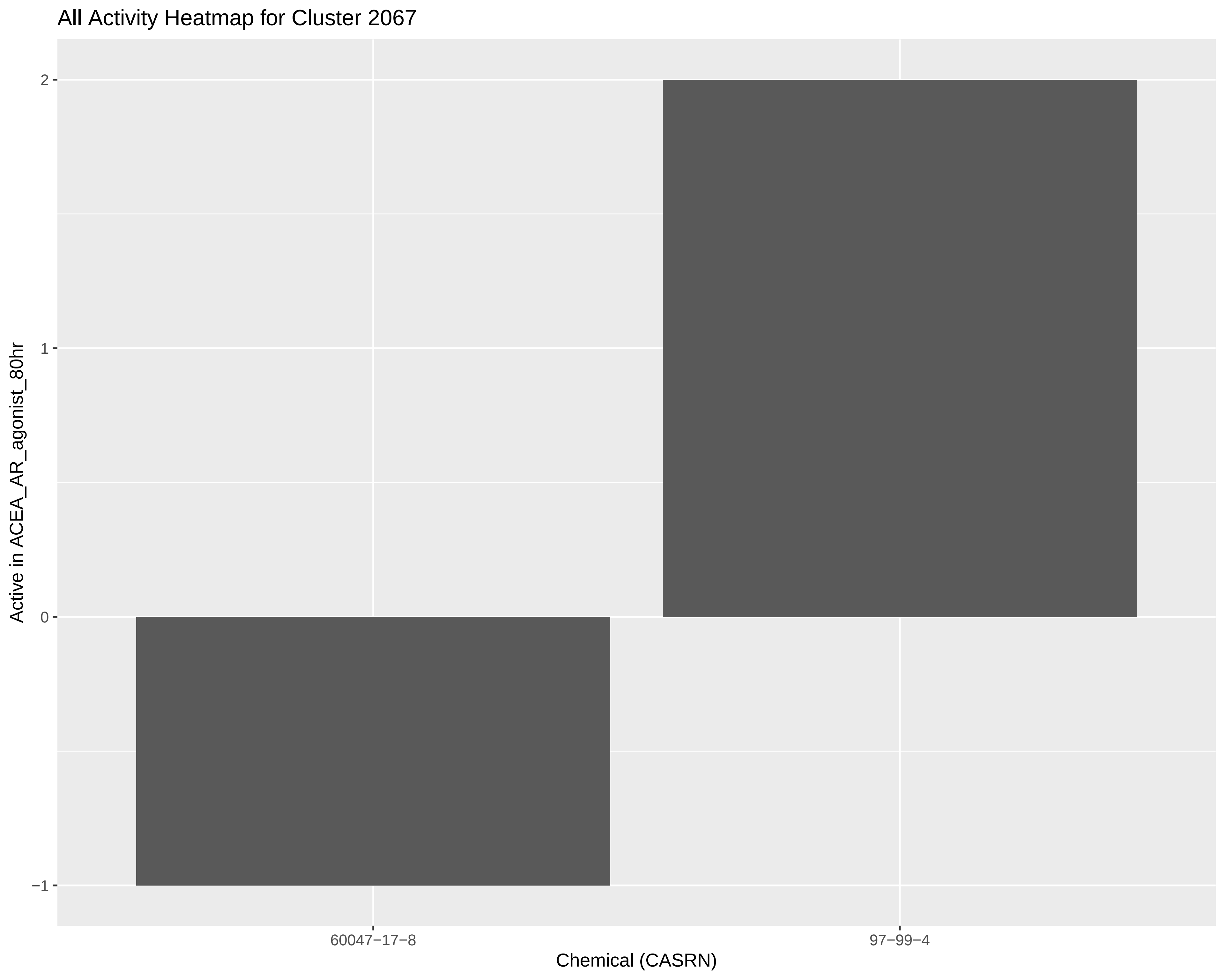
All Activity Heatmap for Cluster 2067
Active in ACEA_AR_agonist_80hr
2
1
0
−1
60047−17−8
97−99−4
Chemical (CASRN)

All Activity Heatmap for Cluster 2069

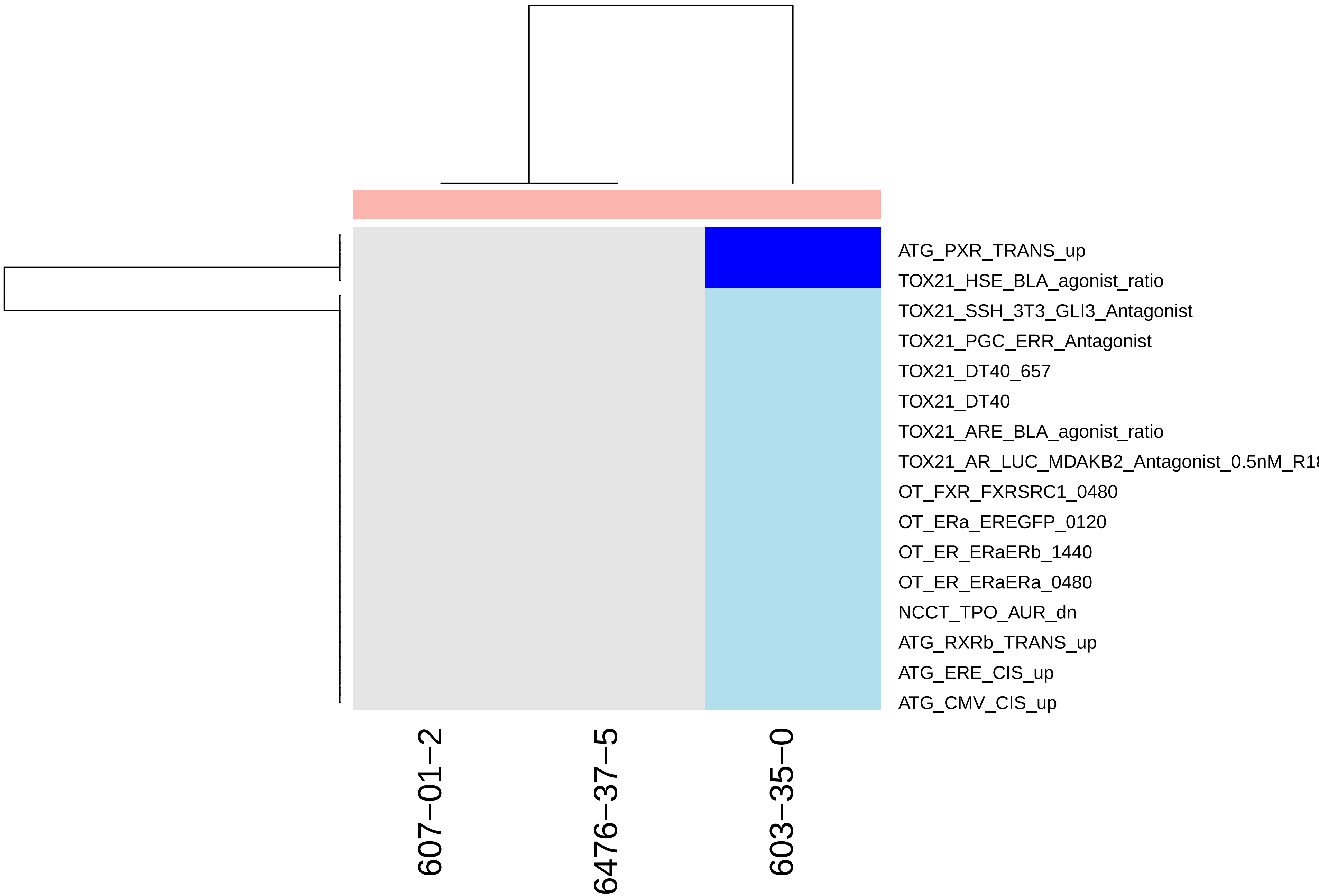

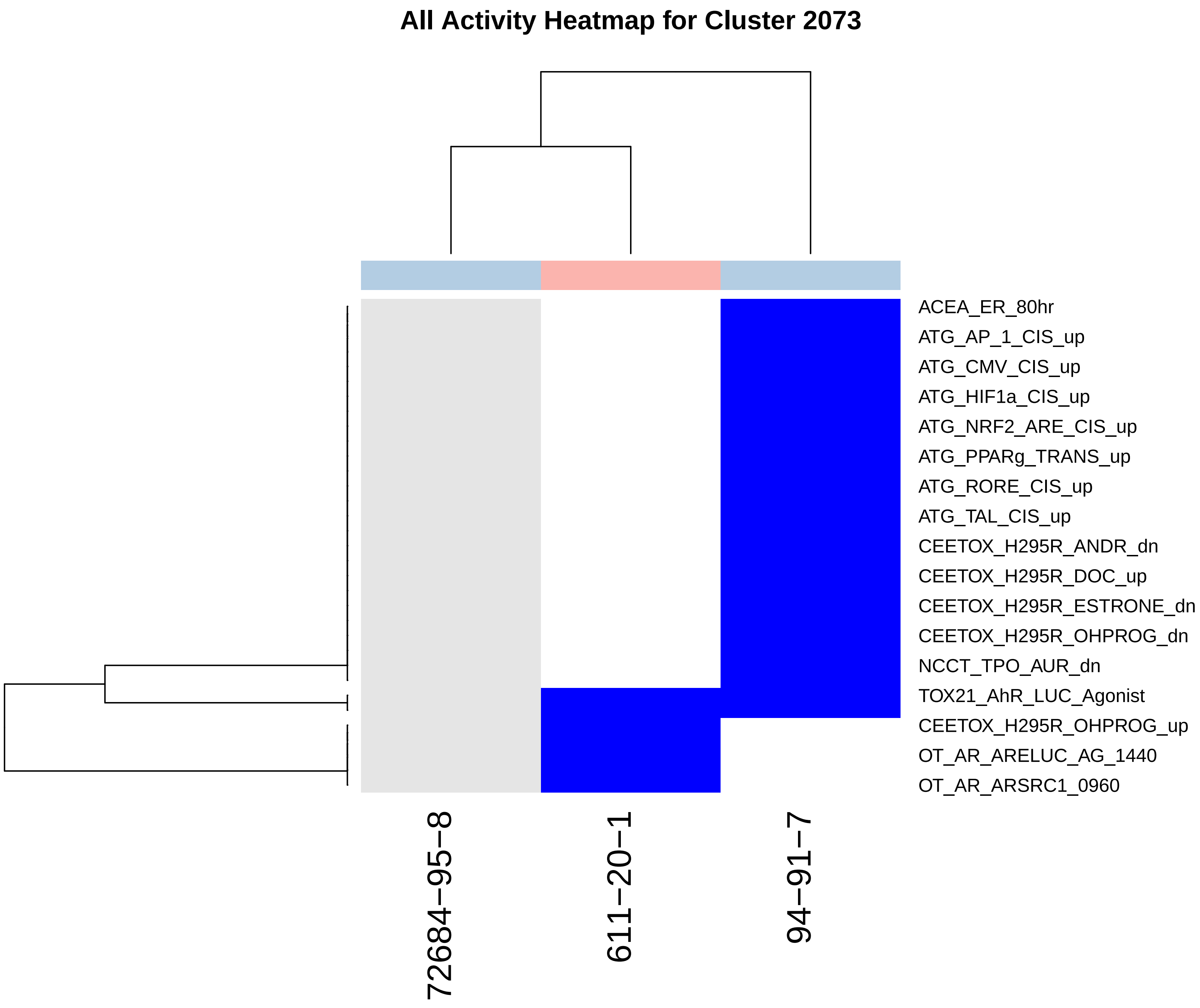
All Activity Heatmap for Cluster 2073
ACEA_ER_80hr
ATG_AP_1_CIS_up
ATG_CMV_CIS_up
ATG_HIF1a_CIS_up
ATG_NRF2_ARE_CIS_up
ATG_PPARg_TRANS_up
ATG_RORE_CIS_up
ATG_TAL_CIS_up
CEETOX_H295R_ANDR_dn
CEETOX_H295R_DOC_up
CEETOX_H295R_ESTRONE_dn
CEETOX_H295R_OHPROG_dn
NCCT_TPO_AUR_dn
TOX21_AhR_LUC_Agonist
CEETOX_H295R_OHPROG_up
OT_AR_ARELUC_AG_1440
OT_AR_ARSRC1_0960
72684−95−8
611−20−1
94−91−7

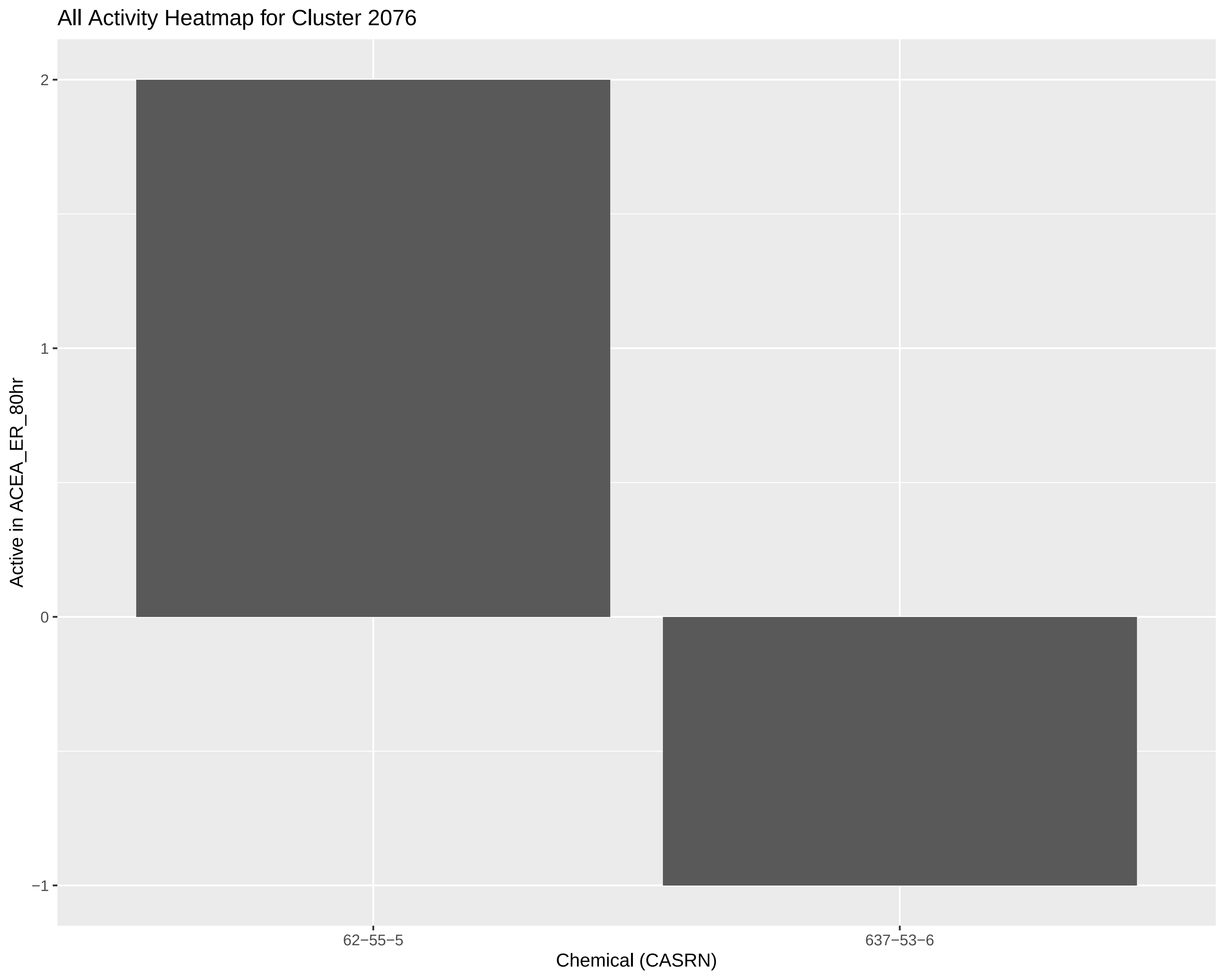

All Activity Heatmap for Cluster 2076
Active in ACEA_ER_80hr
2
1
0
−1
62−55−5
637−53−6
Chemical (CASRN)

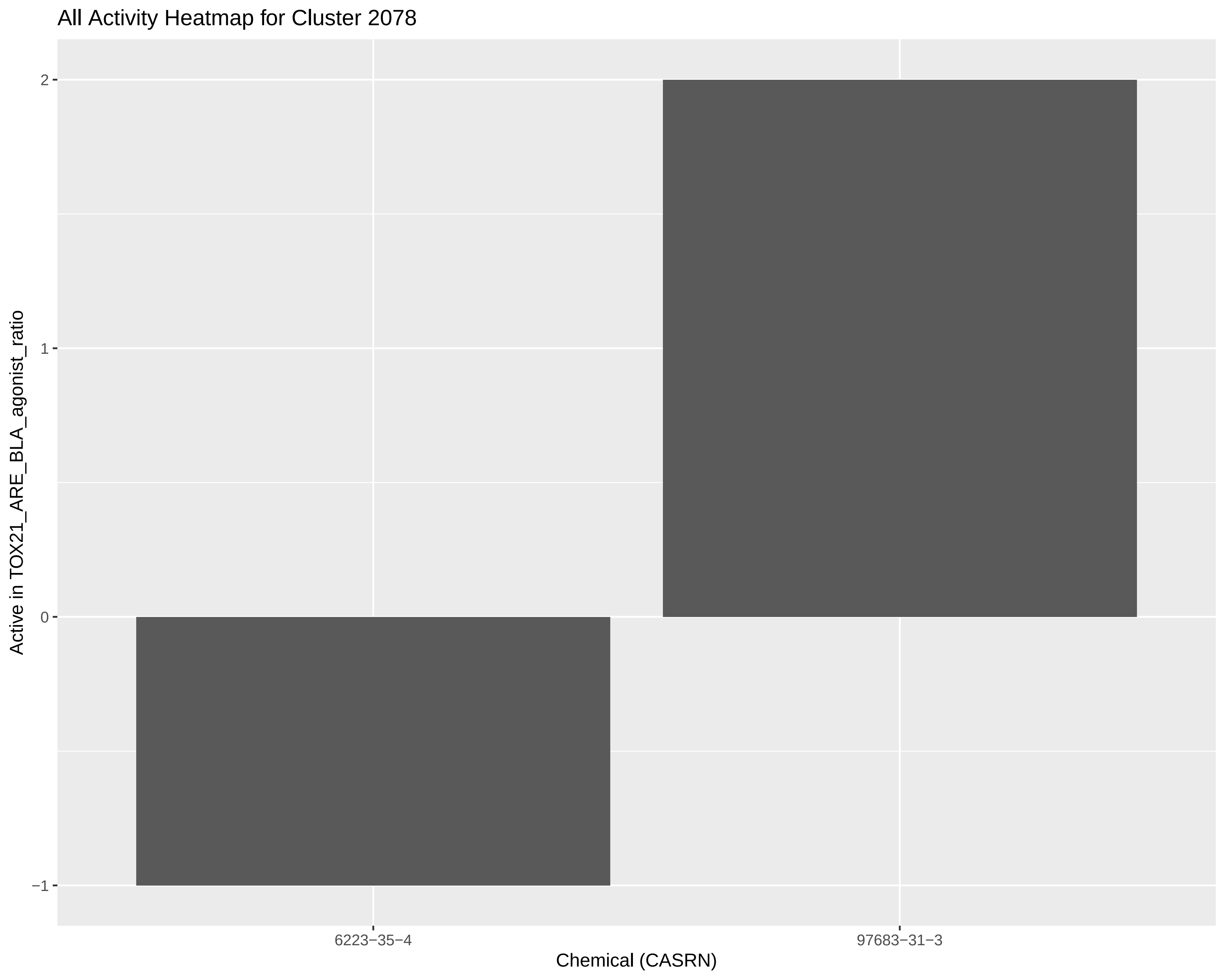
All Activity Heatmap for Cluster 2078
Active in TOX21_ARE_BLA_agonist_ratio
2
1
0
−1
6223−35−4
97683−31−3
Chemical (CASRN)

All Activity Heatmap for Cluster 2079

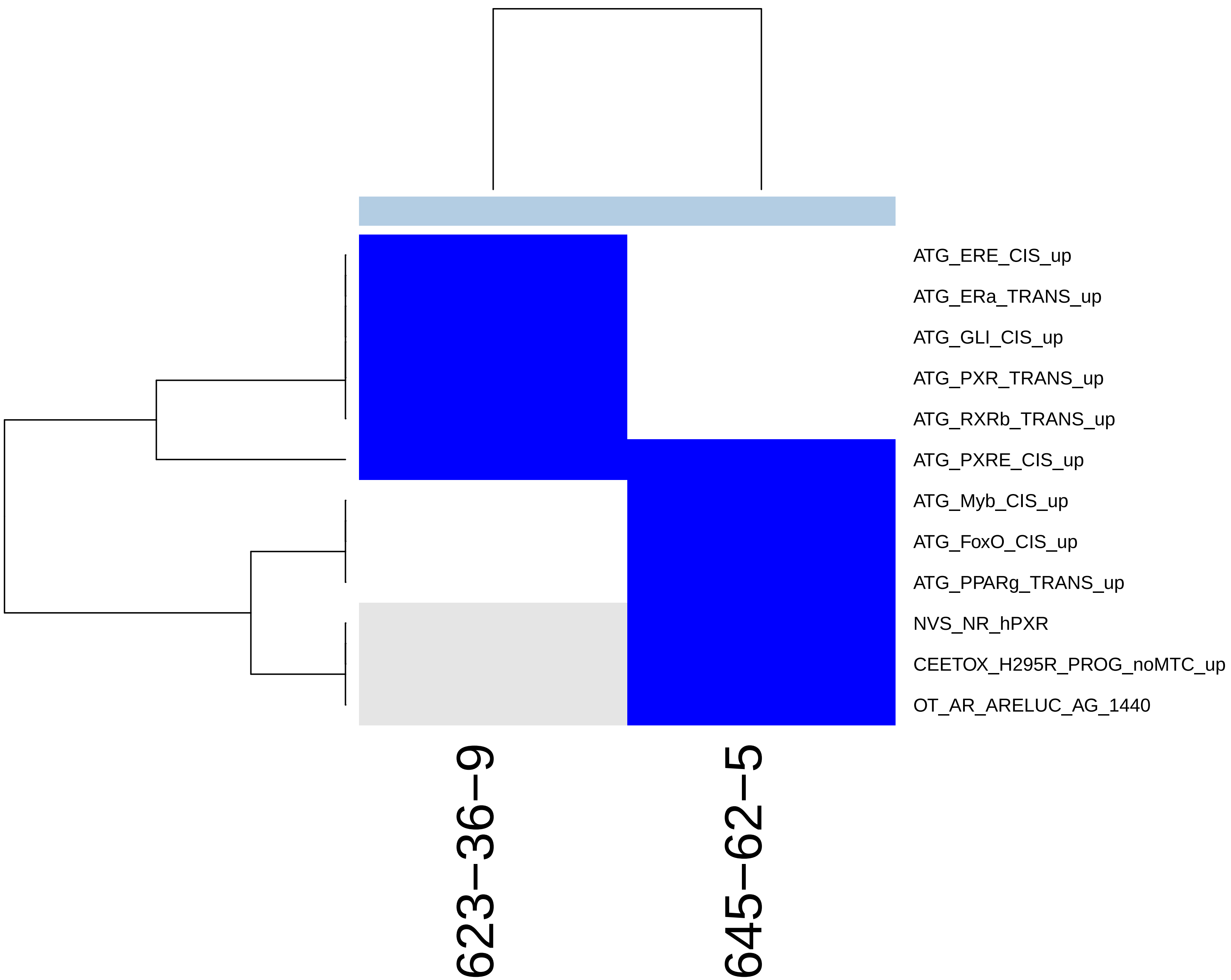

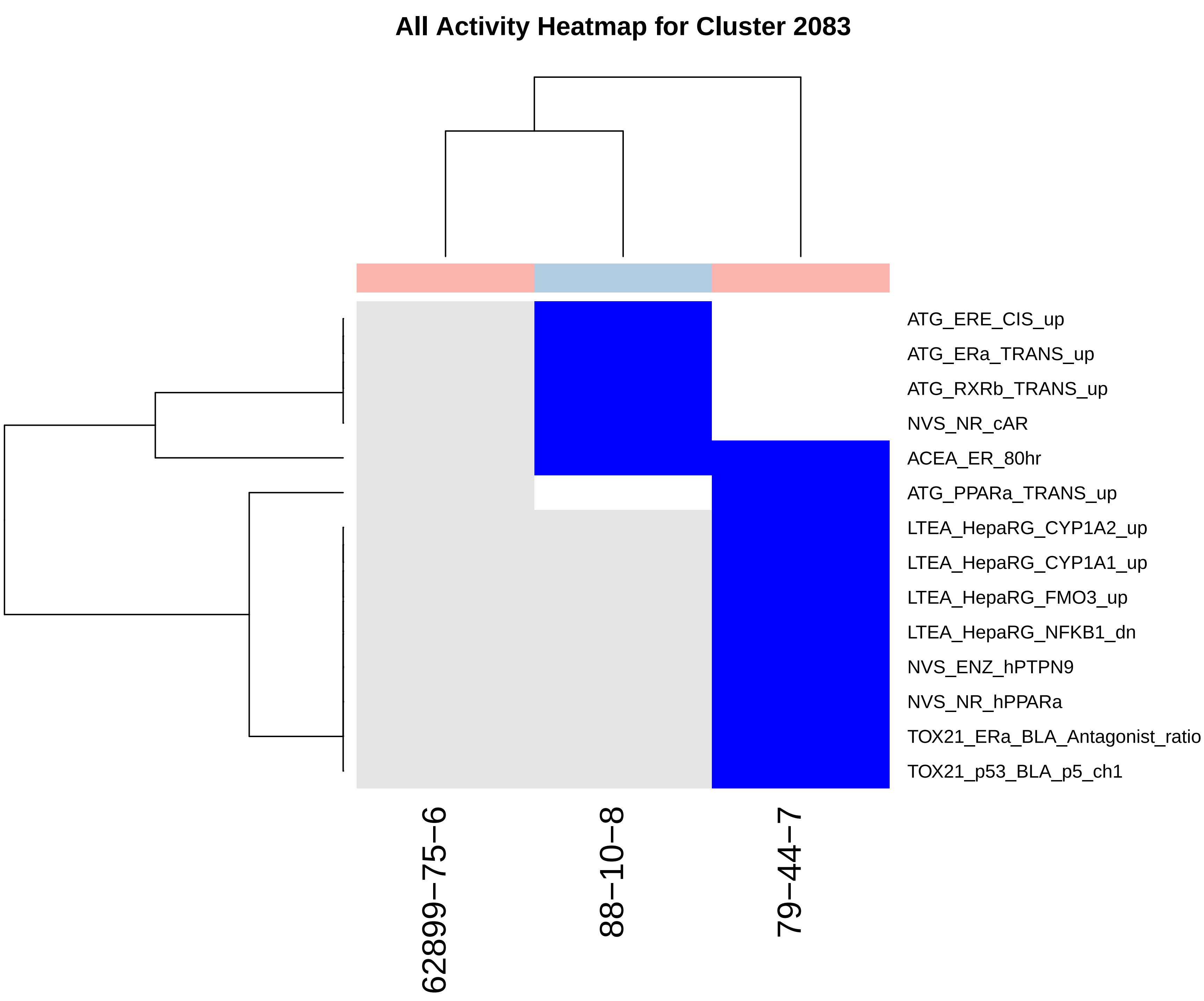
All Activity Heatmap for Cluster 2083
ATG_ERE_CIS_up
ATG_ERa_TRANS_up
ATG_RXRb_TRANS_up
NVS_NR_cAR
ACEA_ER_80hr
ATG_PPARa_TRANS_up
LTEA_HepaRG_CYP1A2_up
LTEA_HepaRG_CYP1A1_up
LTEA_HepaRG_FMO3_up
LTEA_HepaRG_NFKB1_dn
NVS_ENZ_hPTPN9
NVS_NR_hPPARa
TOX21_ERa_BLA_Antagonist_ratio
TOX21_p53_BLA_p5_ch1
62899−75−6
88−10−8
79−44−7

All Activity Heatmap for Cluster 2098

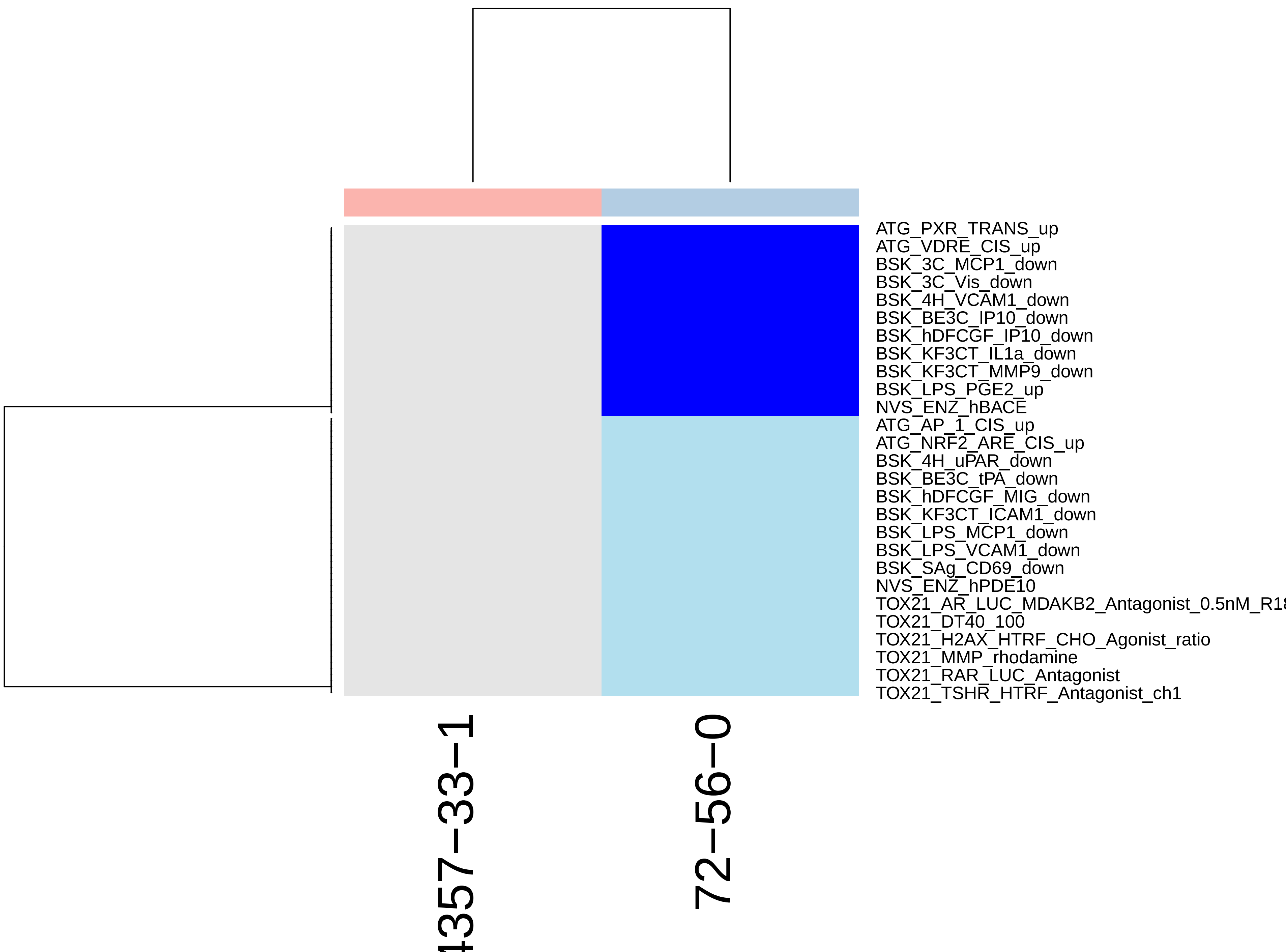

All Activity Heatmap for Cluster 2101

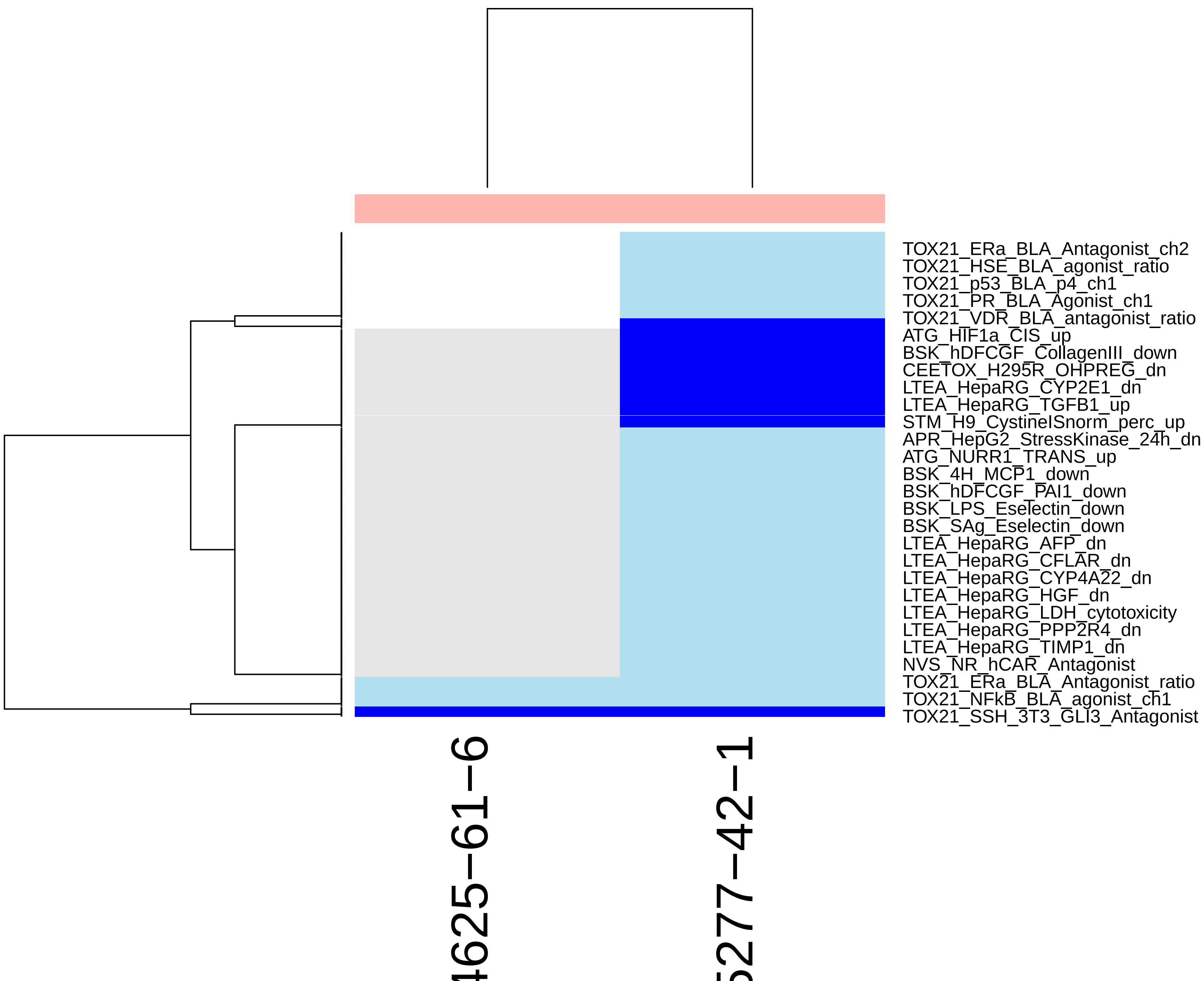

All Activity Heatmap for Cluster 2102

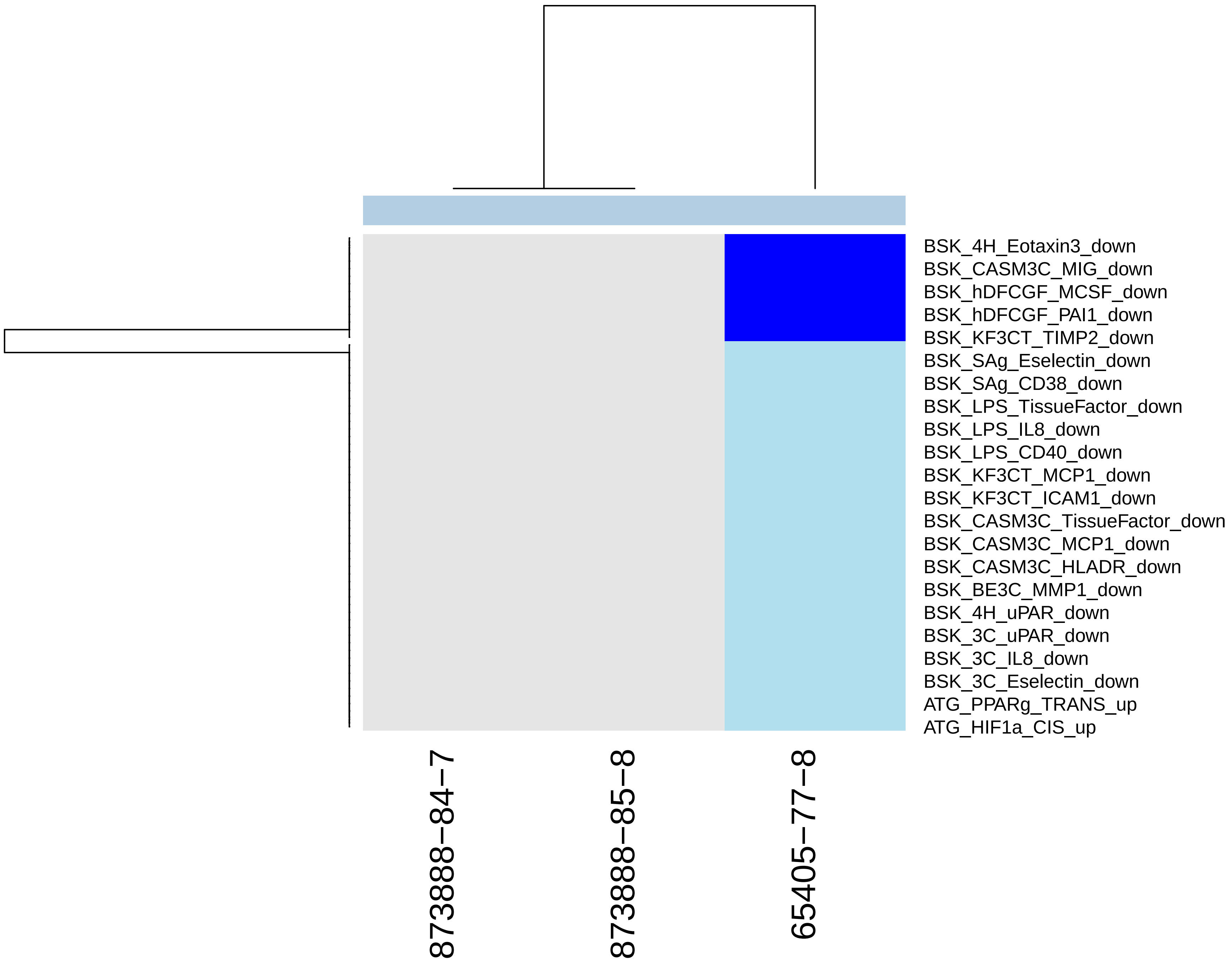

All Activity Heatmap for Cluster 2104

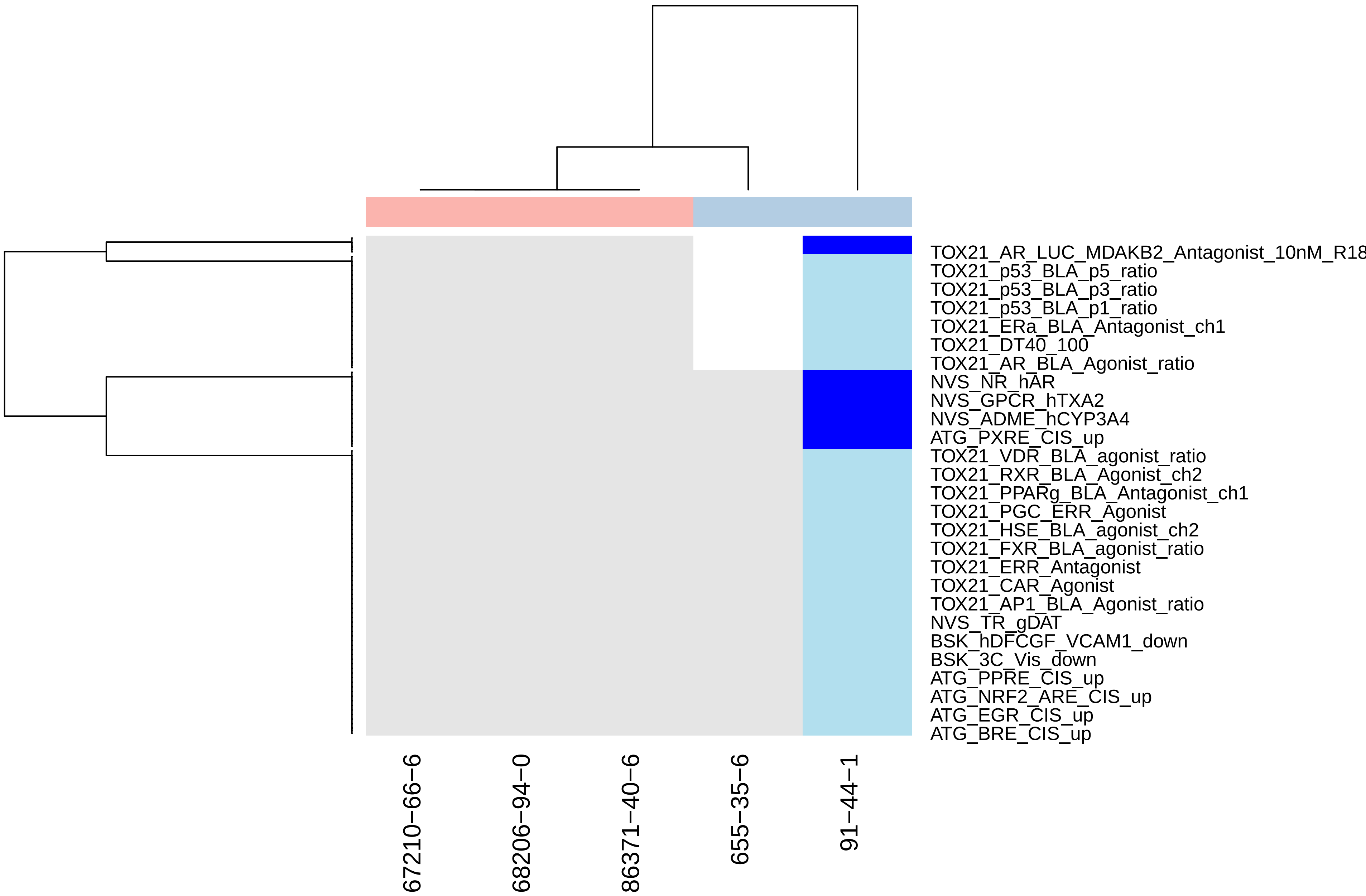

All Activity Heatmap for Cluster 2106

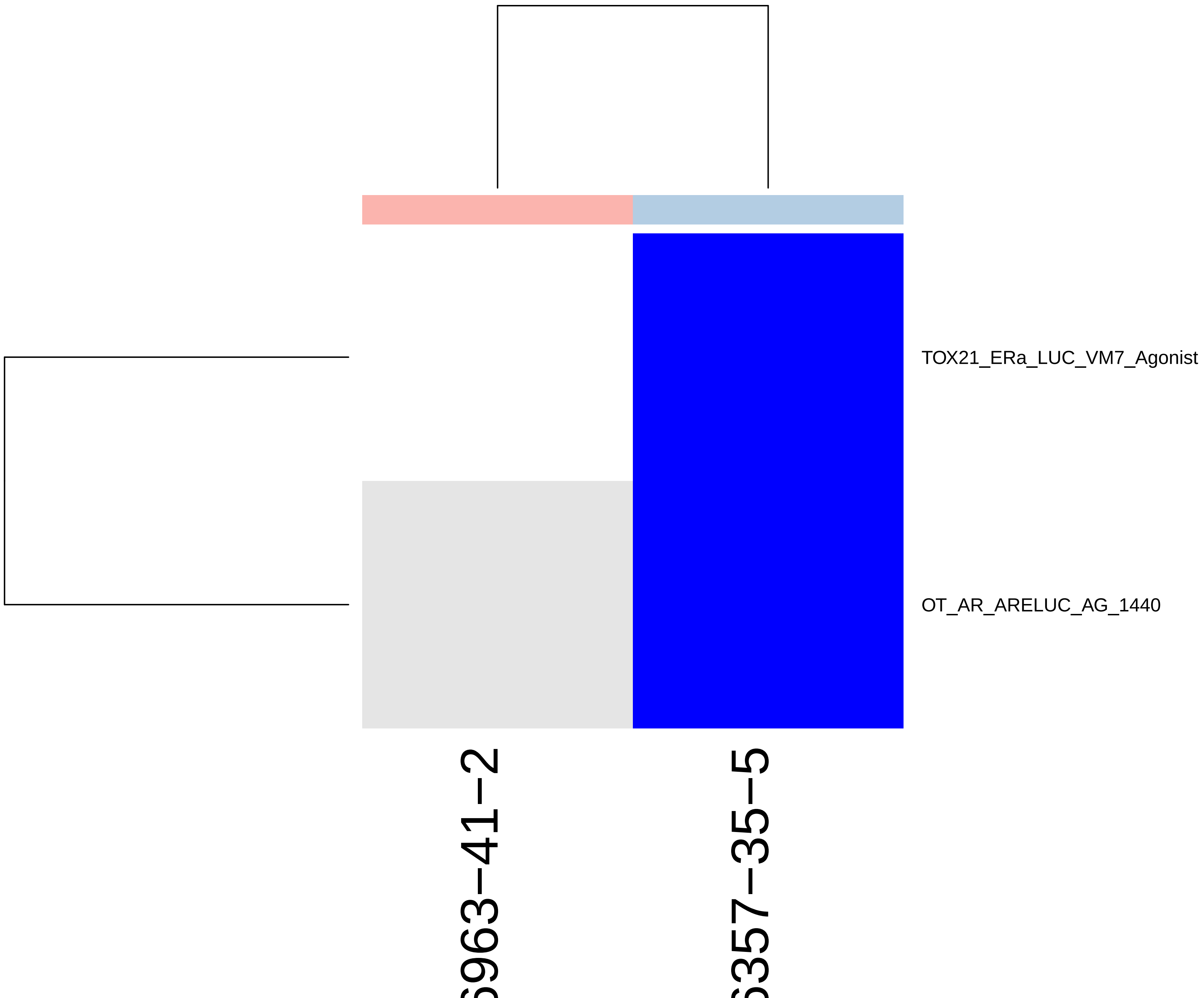

All Activity Heatmap for Cluster 2108

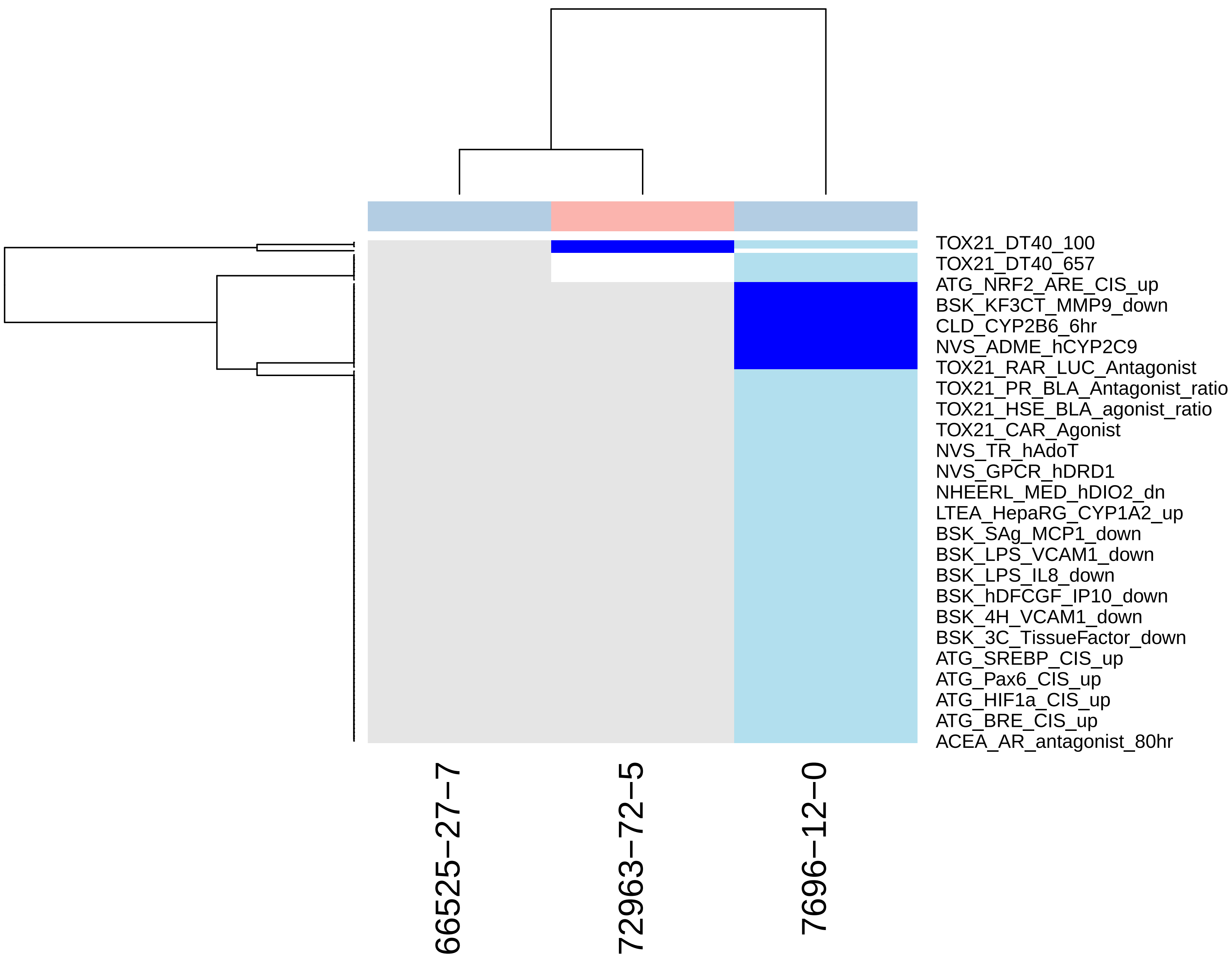

All Activity Heatmap for Cluster 2111

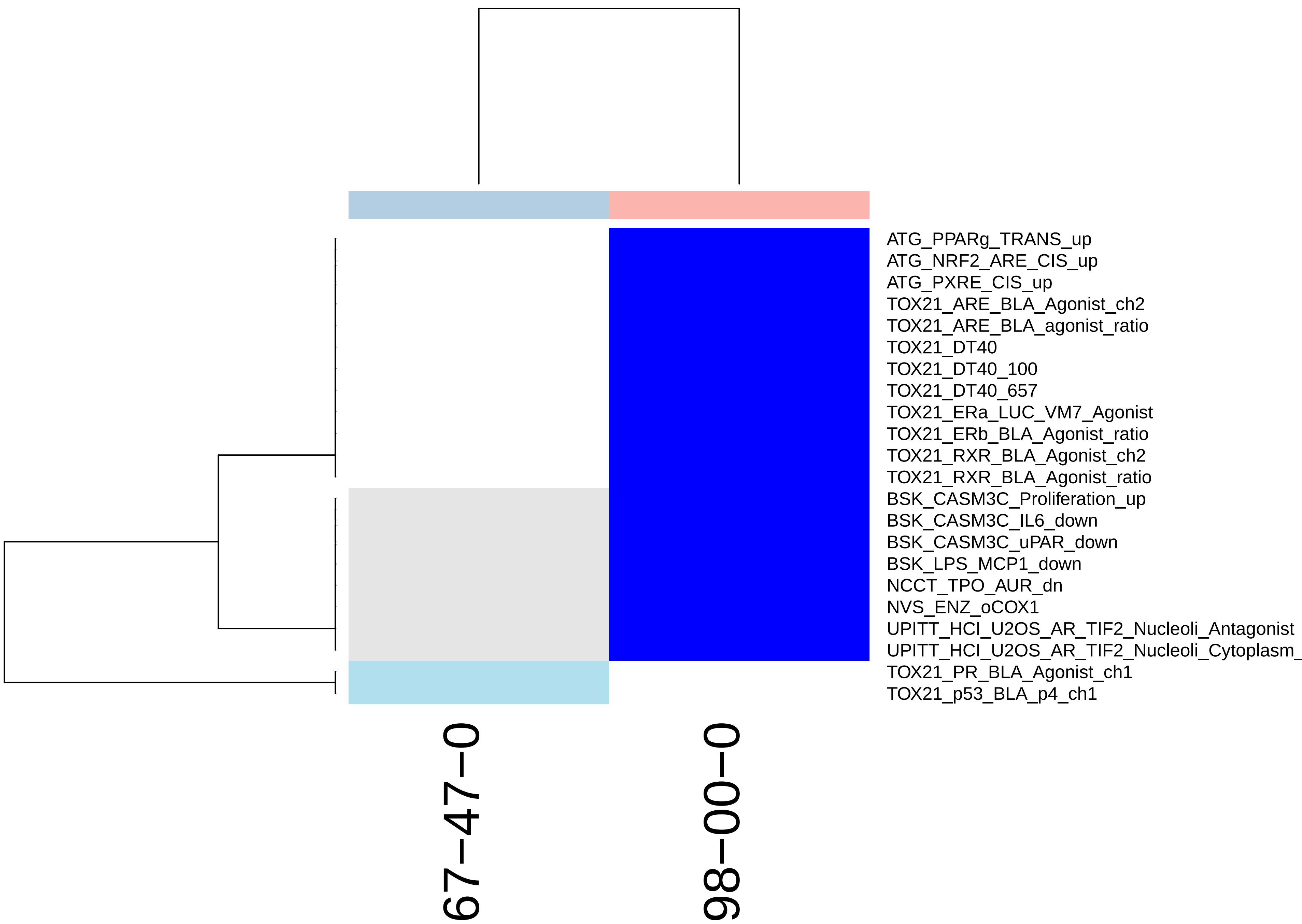

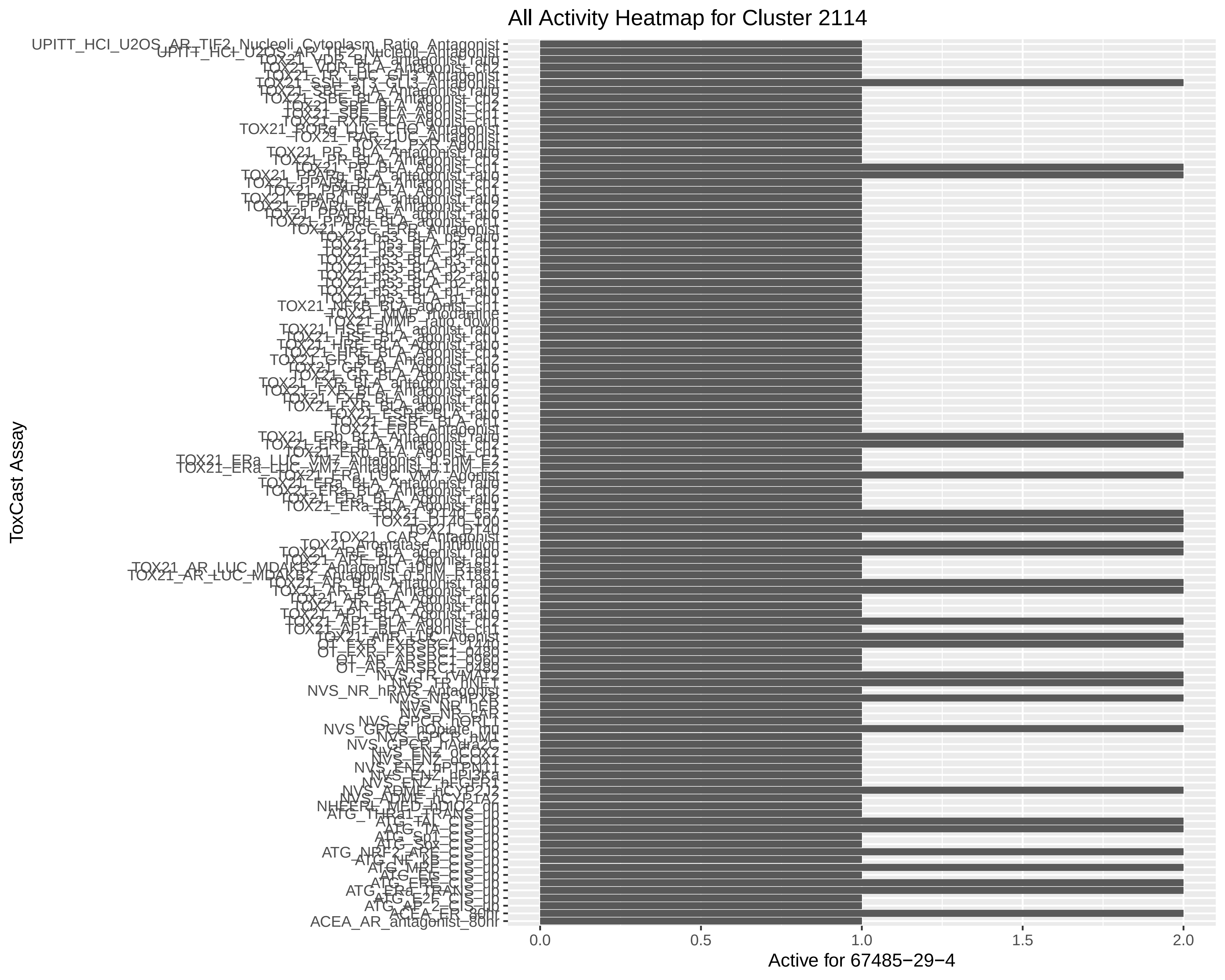
All Activity Heatmap for Cluster 2114
ToxCast Assay
Active for 67485−29−4
0.0
0.5
1.0
1.5
2.0
UPITT_HCI_U2OS_AR_TIF2_Nucleoli_Cytoplasm_Ratio_Antagonist
TOX21_RORg_LUC_CHO_Antagonist
TOX21_NFkB_BLA_agonist_ch1
TOX21_MMP_ratio_down
TOX21_HSE_BLA_agonist_ratio
TOX21_CAR_Antagonist
TOX21_AR_LUC_MDAKB2_Antagonist_10nM_R1881
NVS_NR_hRAR_Antagonist
NVS_GPCR_hOpiate_mu
NVS_GPCR_hAdra2C
NVS_ENZ_hPTPN11
NHEERL_MED_hDIO2_dn
ATG_THRa1_TRANS_up
ATG_NRF2_ARE_CIS_up
ATG_ERa_TRANS_up
ACEA_AR_antagonist_80hr

All Activity Heatmap for Cluster 2117

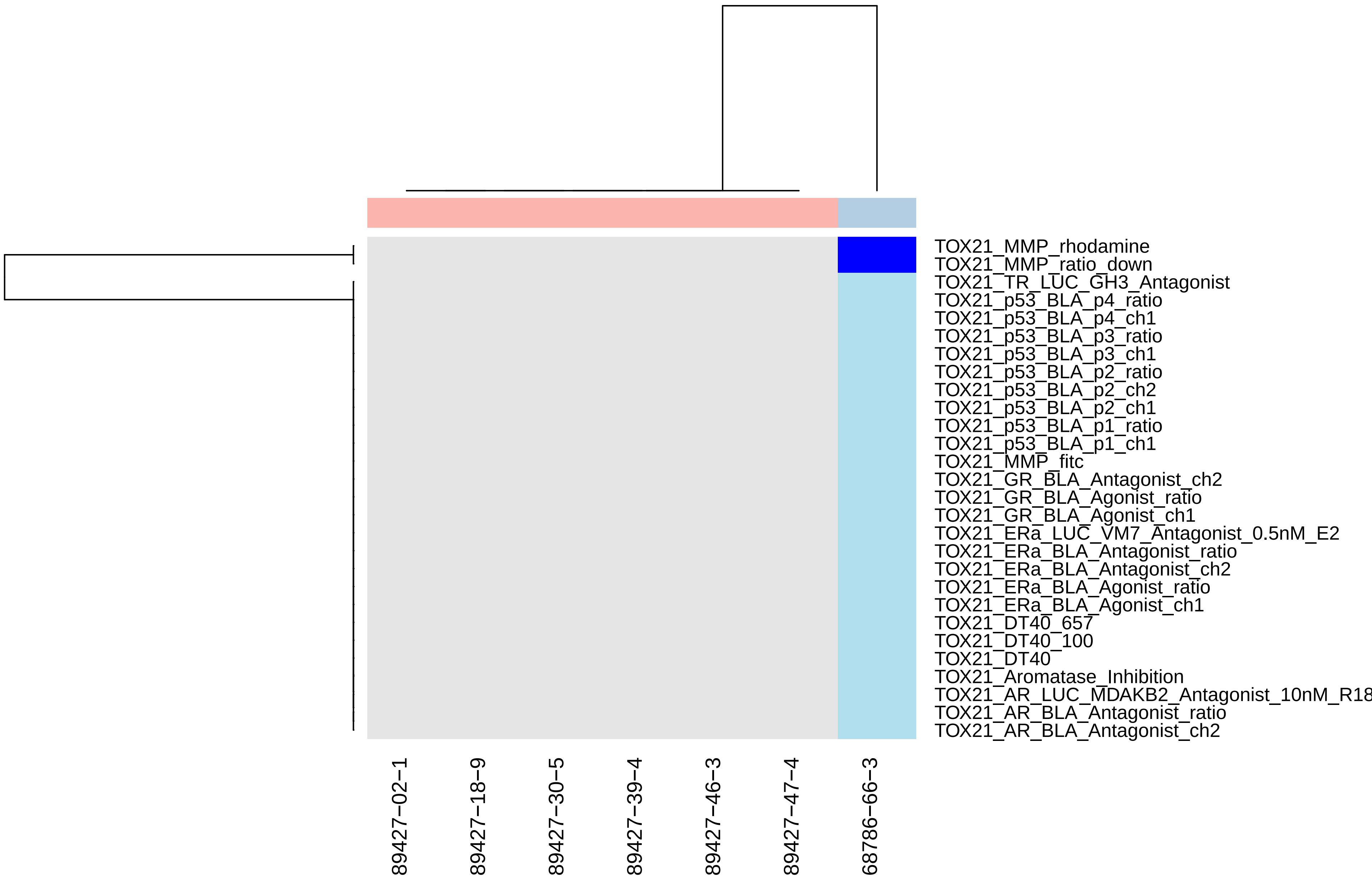

All Activity Heatmap for Cluster 2121

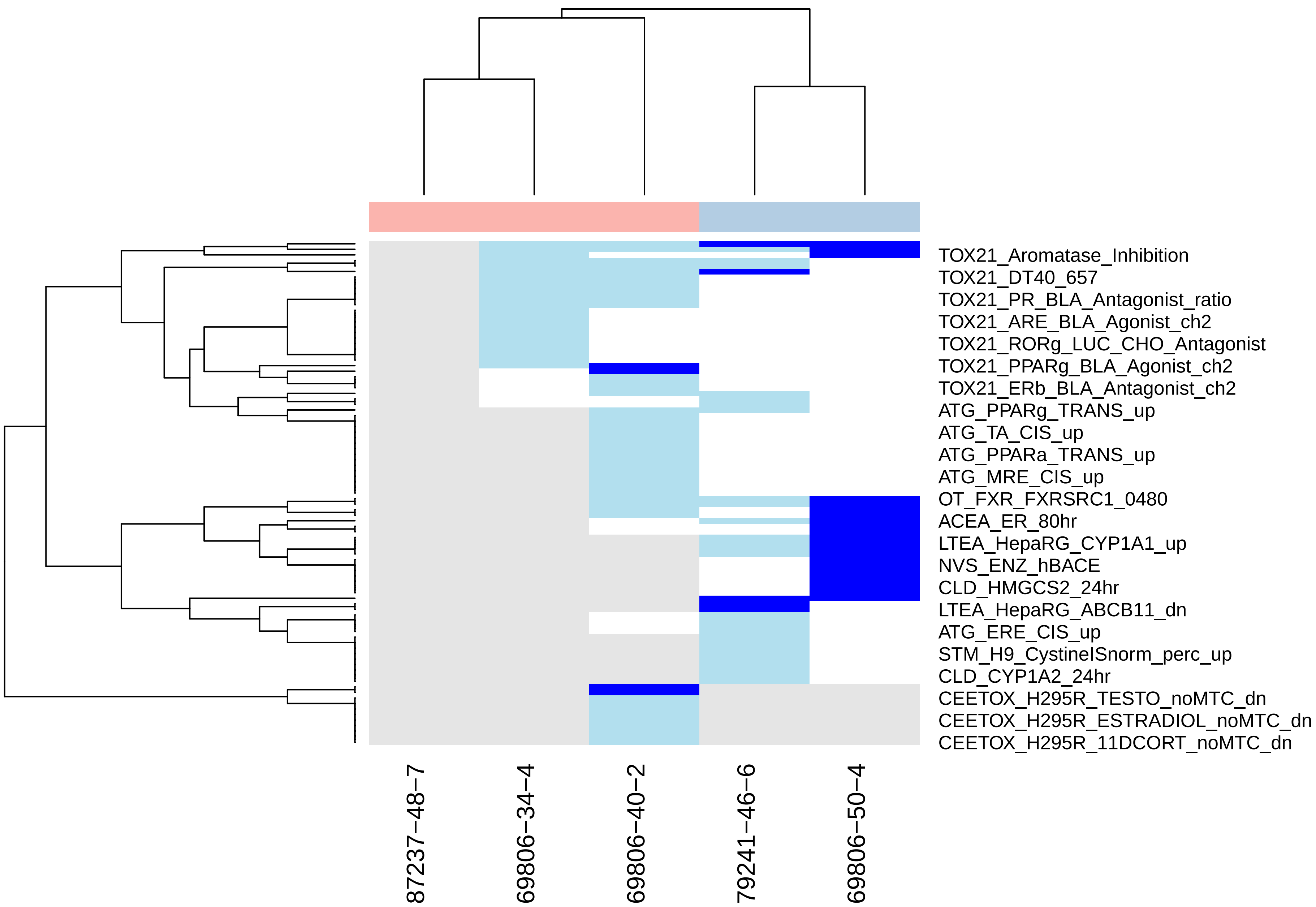

All Activity Heatmap for Cluster 2142

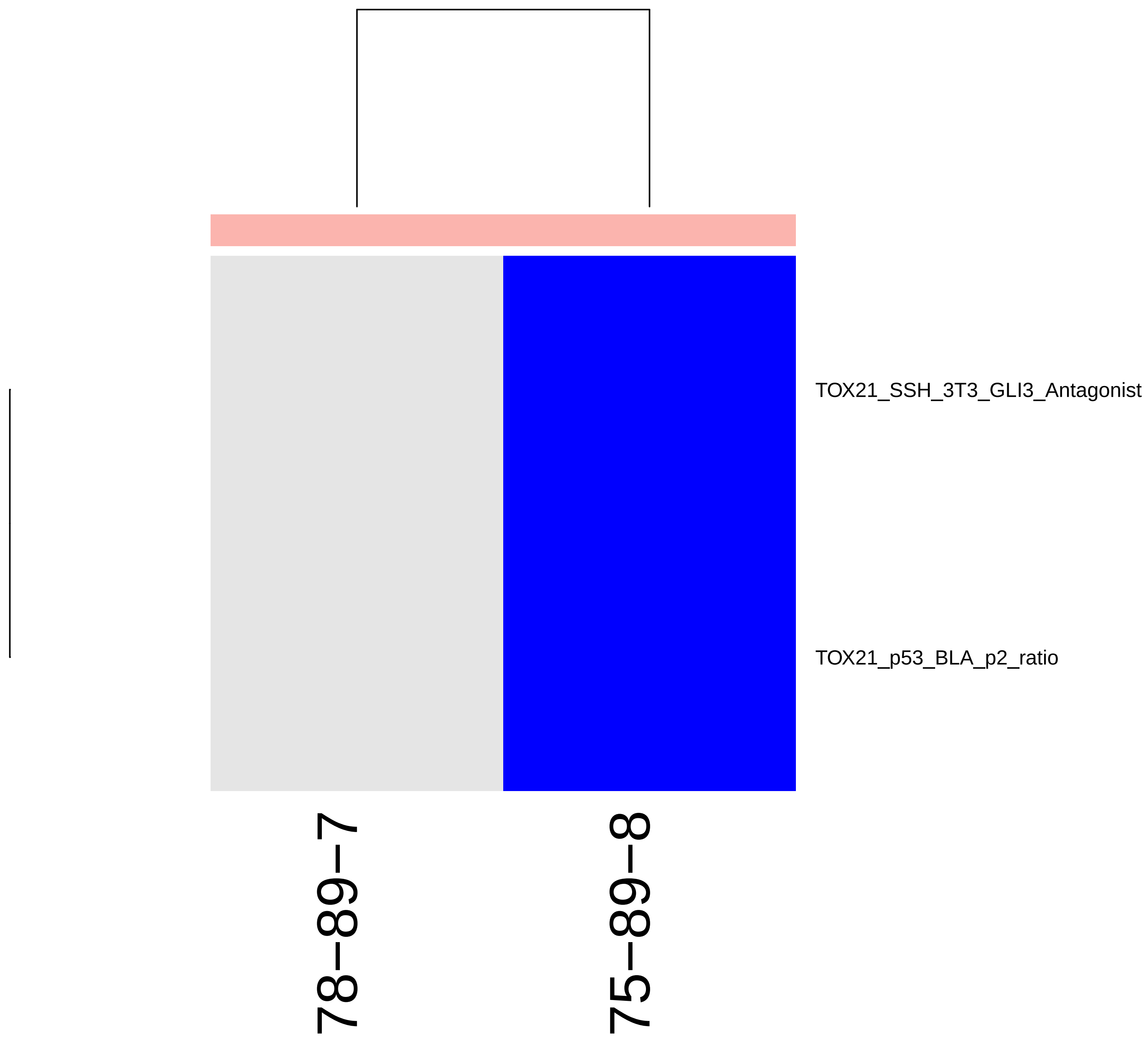

All Activity Heatmap for Cluster 2143

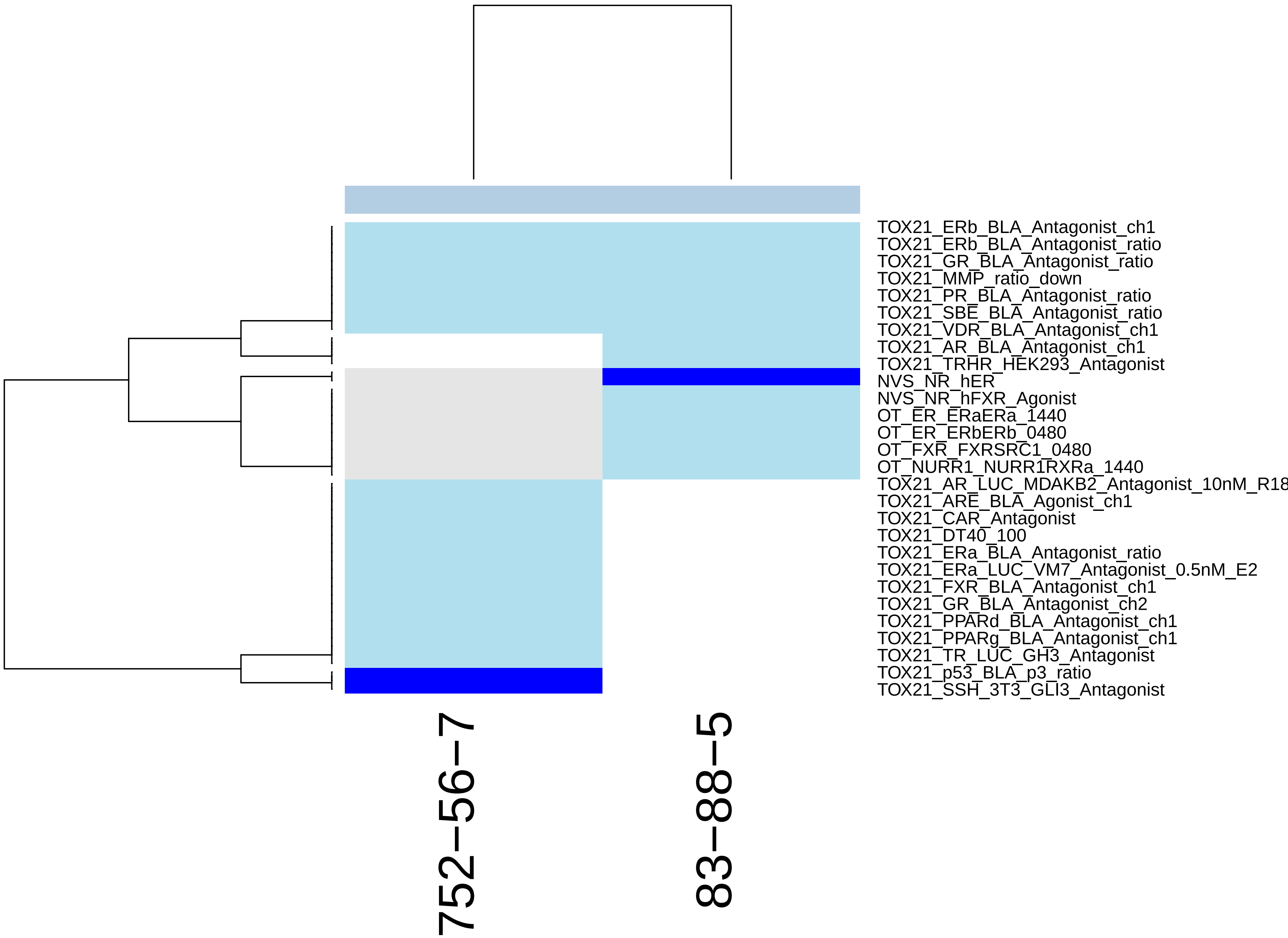

All Activity Heatmap for Cluster 2145

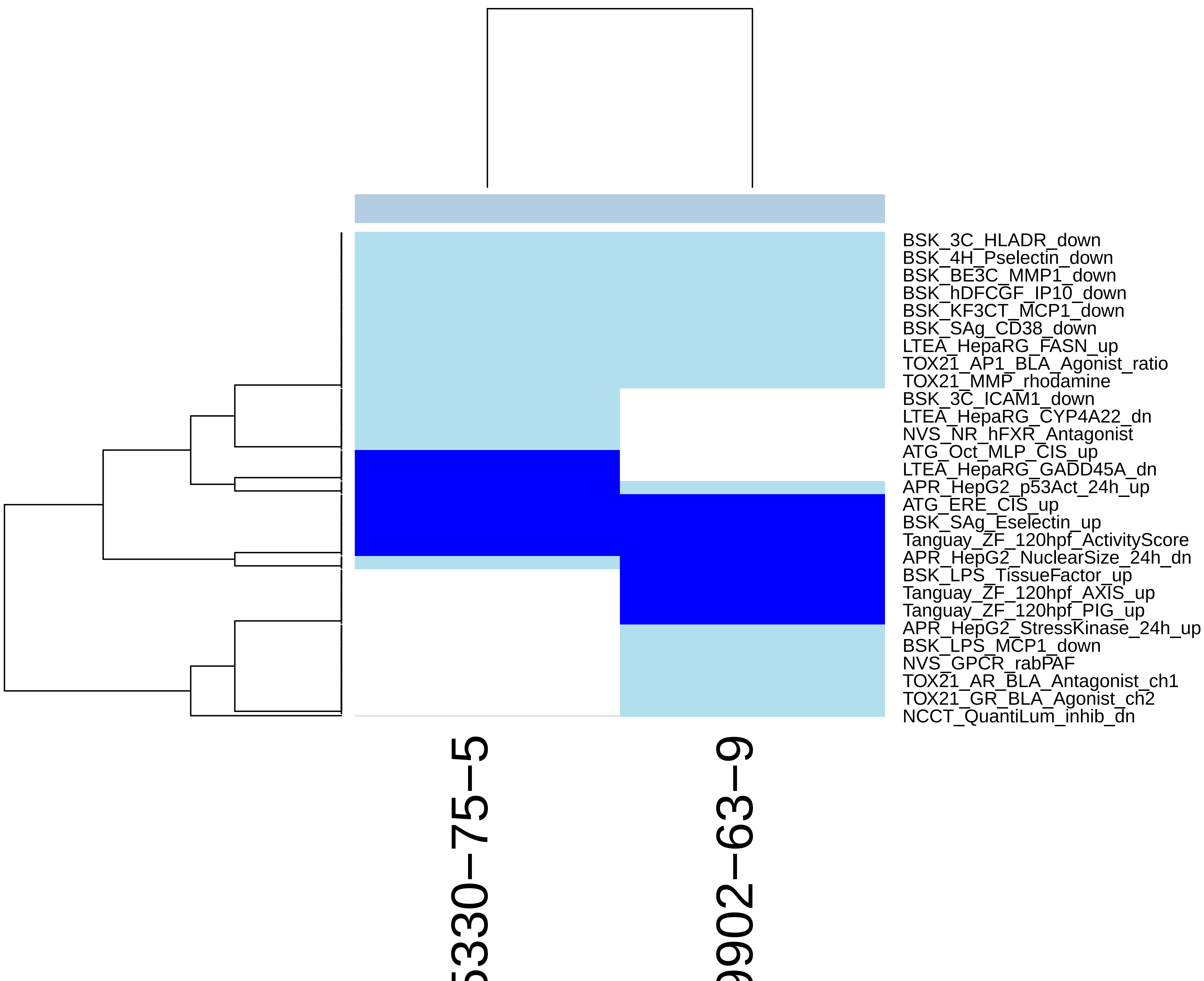

All Activity Heatmap for Cluster 2146

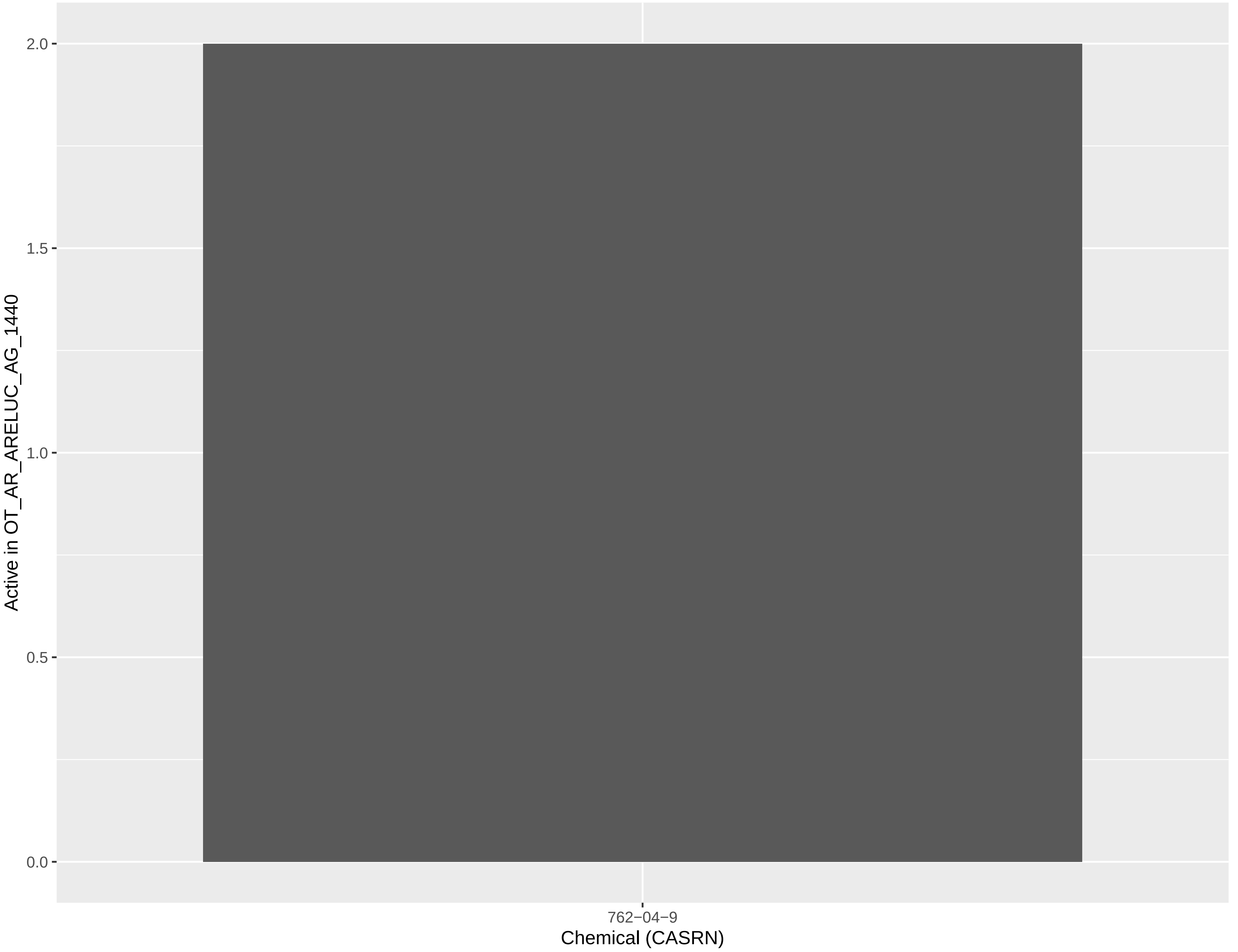

All Activity Heatmap for Cluster 2148

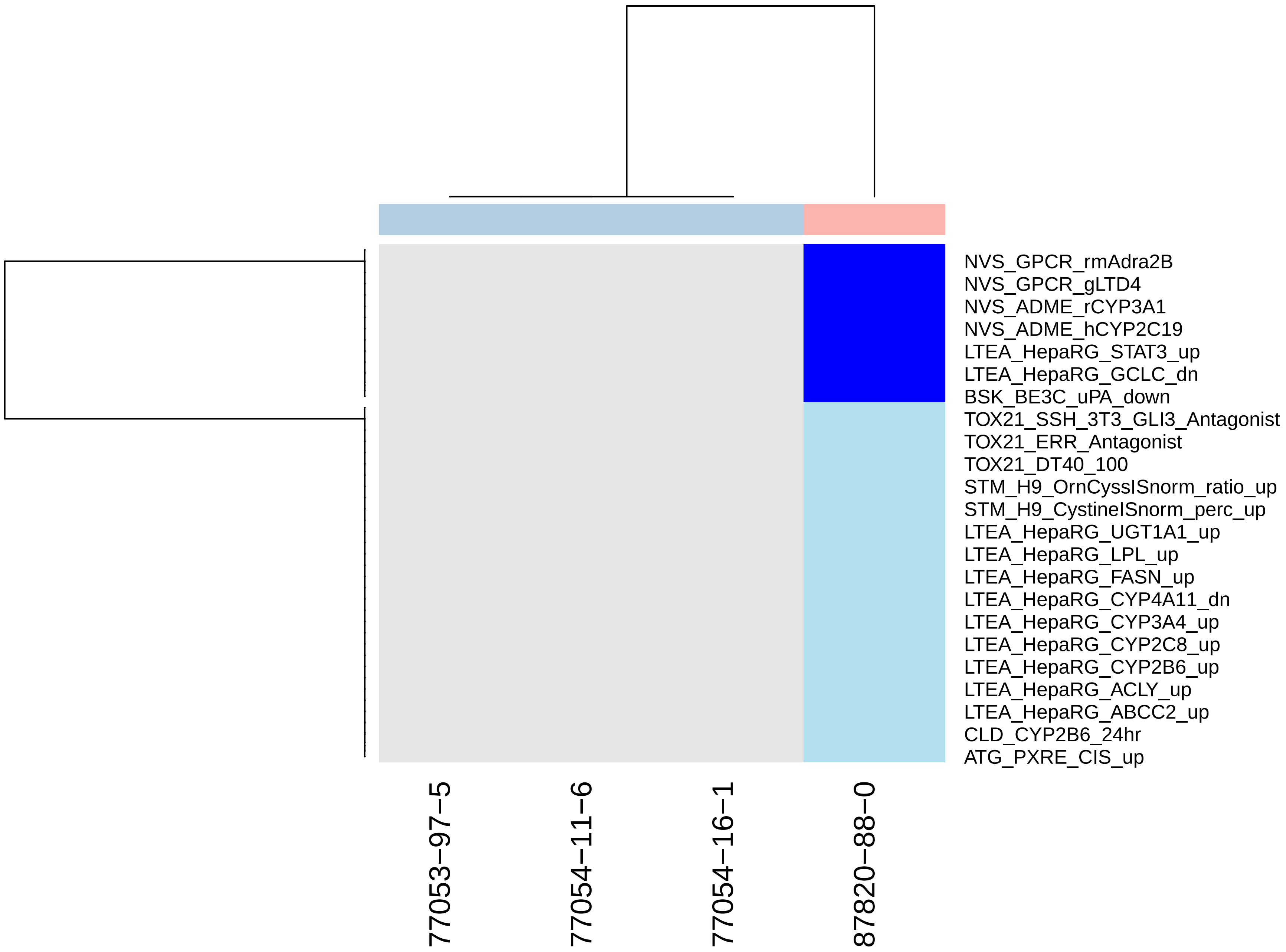

All Activity Heatmap for Cluster 2149

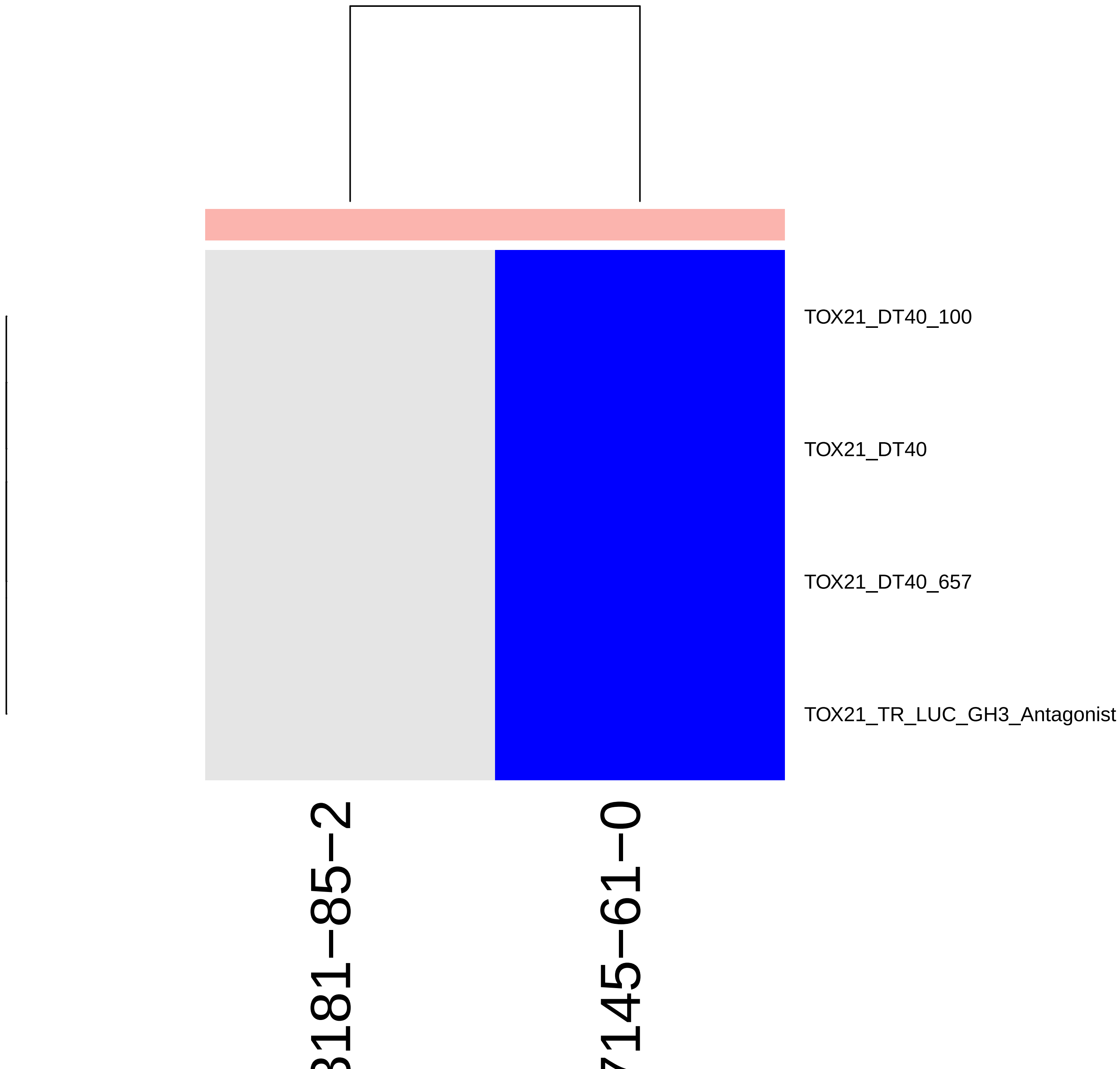

All Activity Heatmap for Cluster 2151

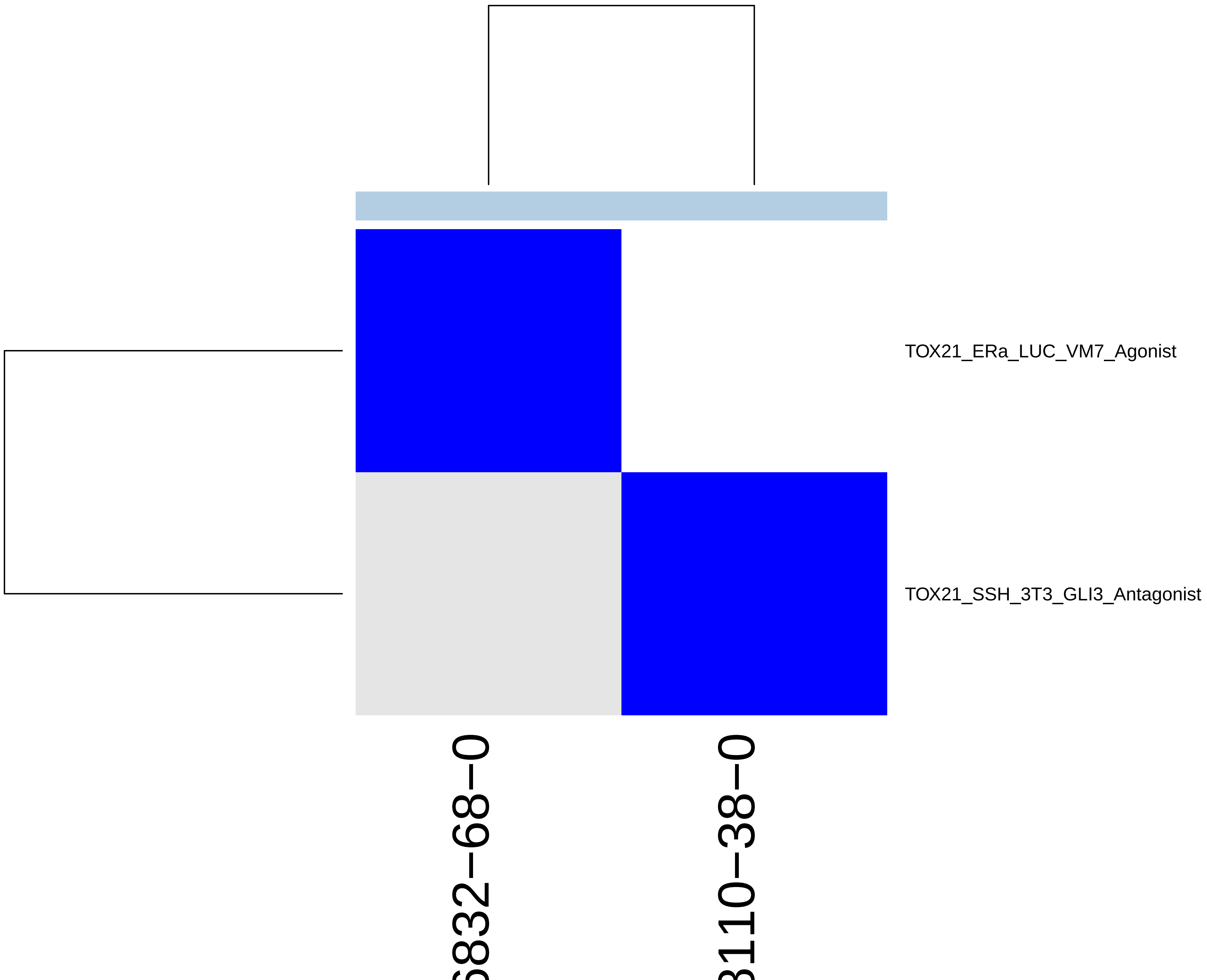

All Activity Heatmap for Cluster 2153

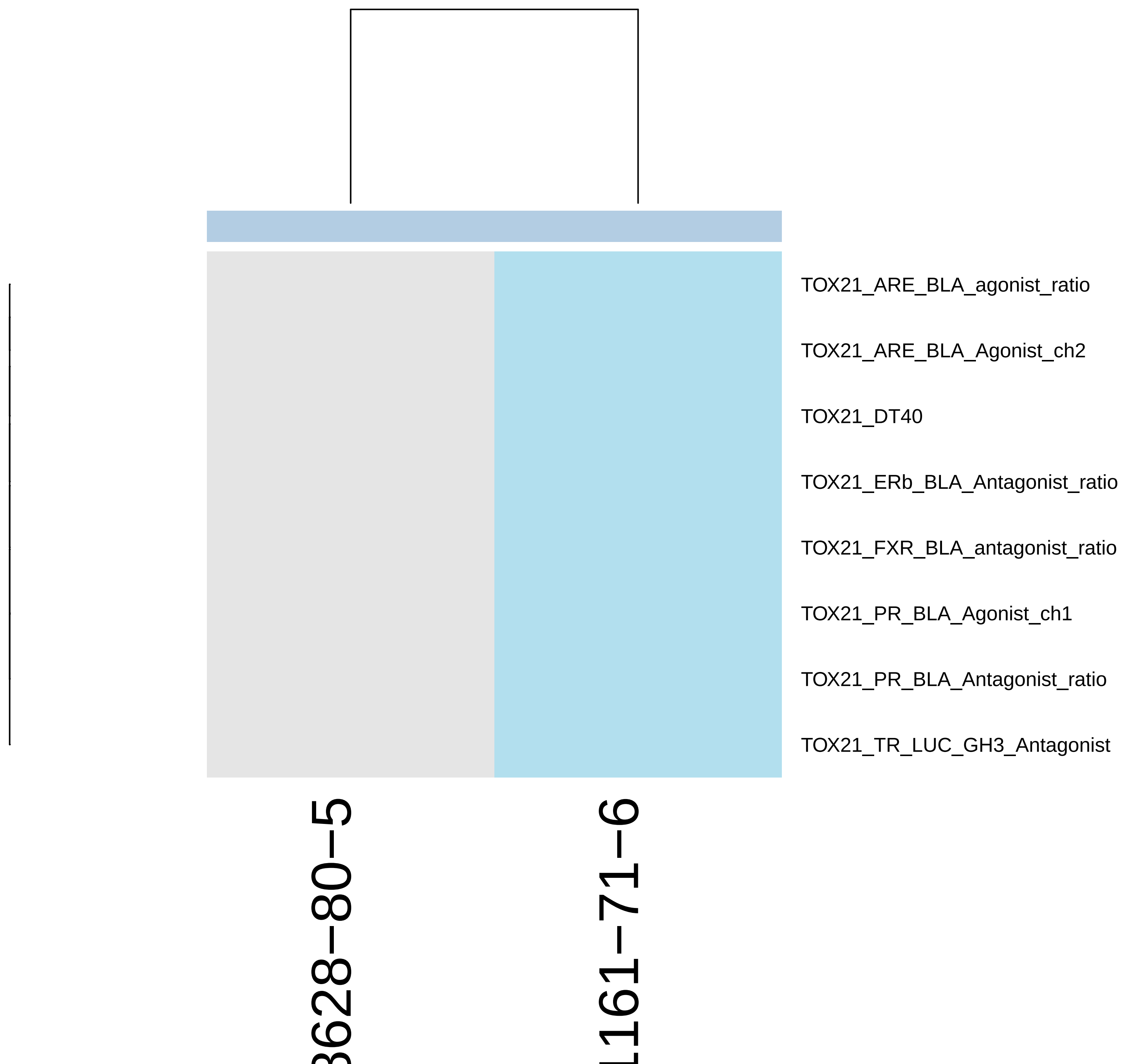

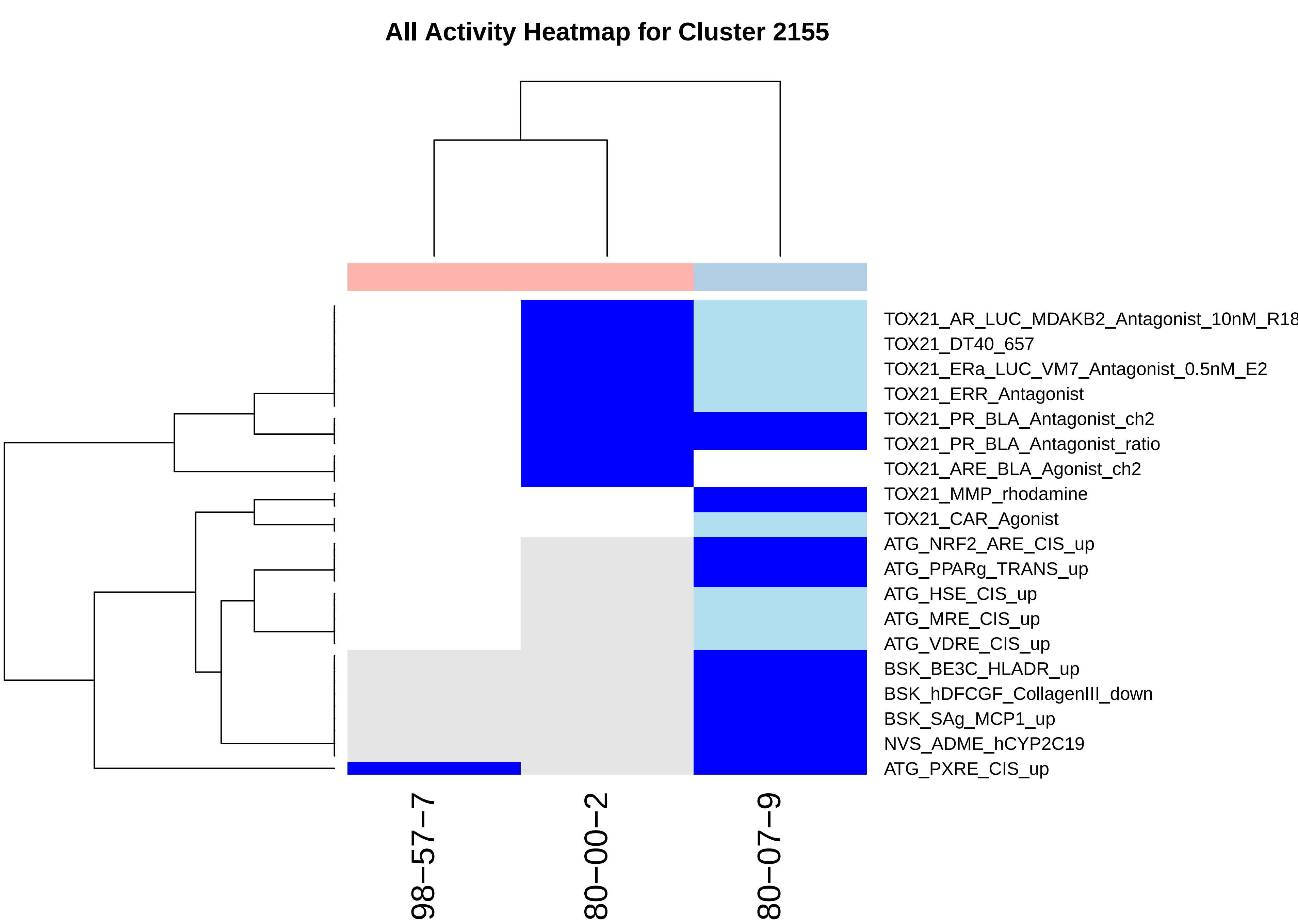
All Activity Heatmap for Cluster 2155
TOX21_AR_LUC_MDAKB2_Antagonist_10nM_R18
TOX21_DT40_657
TOX21_ERa_LUC_VM7_Antagonist_0.5nM_E2
TOX21_ERR_Antagonist
TOX21_PR_BLA_Antagonist_ch2
TOX21_PR_BLA_Antagonist_ratio
TOX21_ARE_BLA_Agonist_ch2
TOX21_MMP_rhodamine
TOX21_CAR_Agonist
ATG_NRF2_ARE_CIS_up
ATG_PPARg_TRANS_up
ATG_HSE_CIS_up
ATG_MRE_CIS_up
ATG_VDRE_CIS_up
BSK_BE3C_HLADR_up
BSK_hDFCGF_CollagenIII_down
BSK_SAg_MCP1_up
NVS_ADME_hCYP2C19
ATG_PXRE_CIS_up
98−57−7
80−00−2
80−07−9

All Activity Heatmap for Cluster 2160

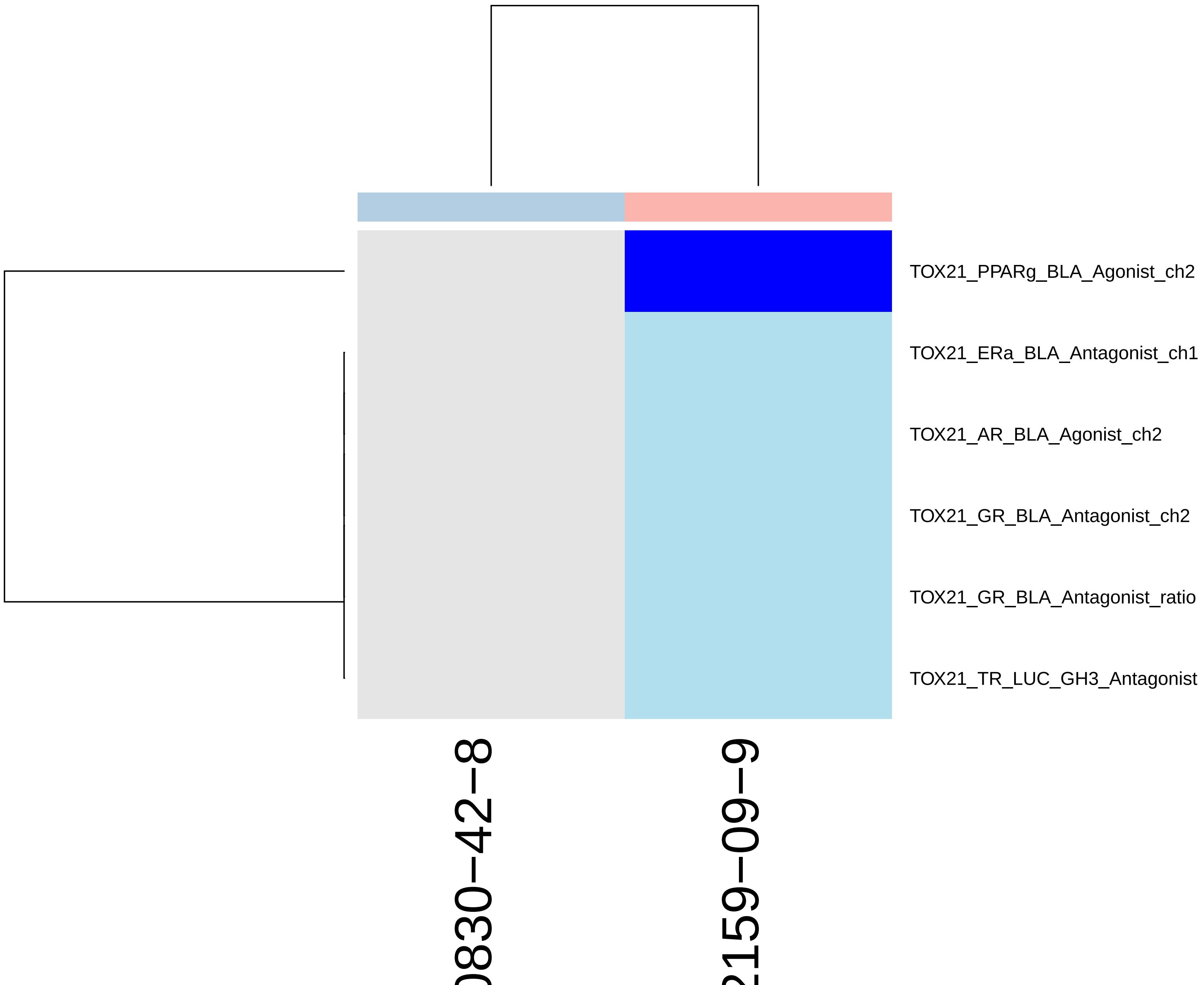

All Activity Heatmap for Cluster 2162

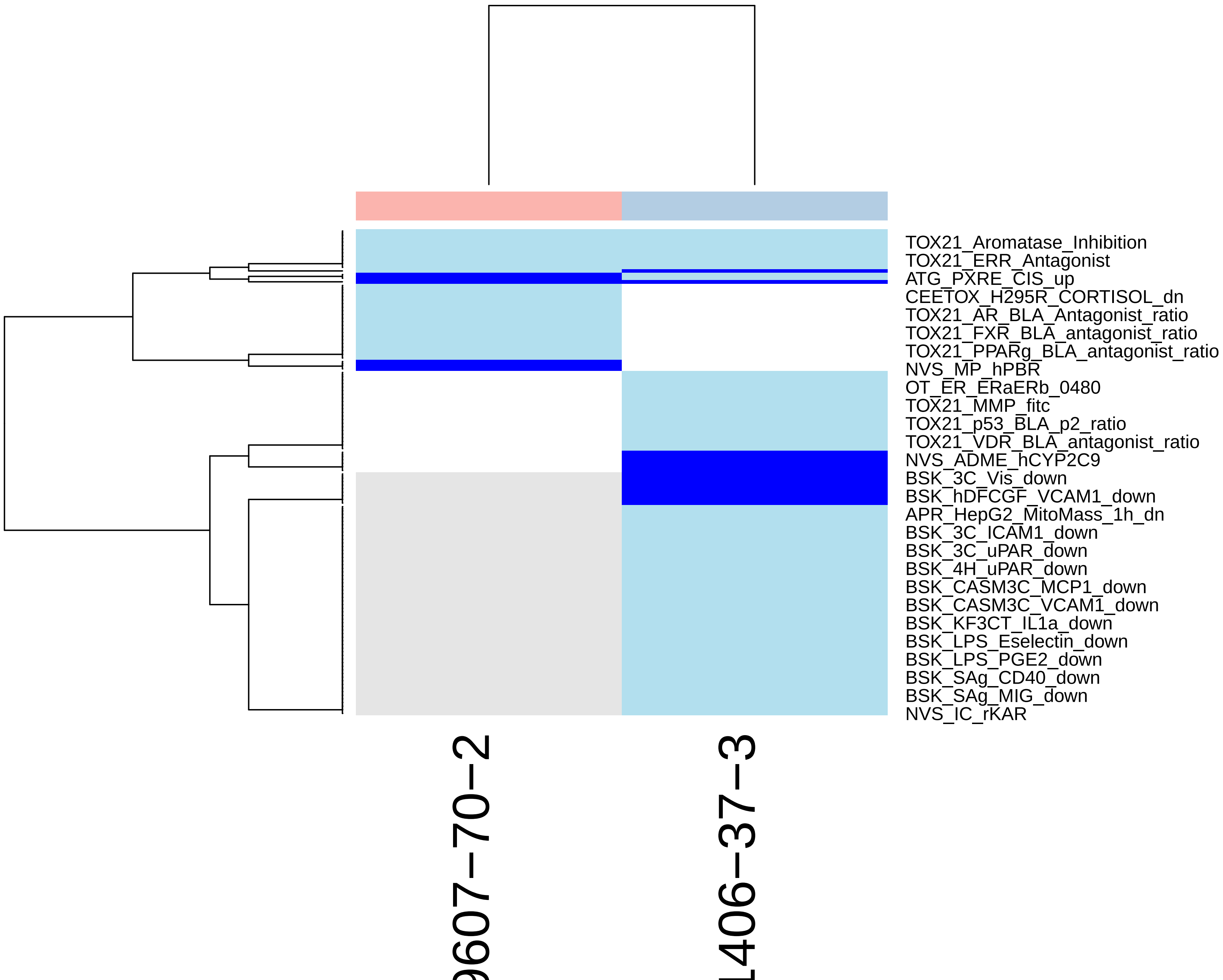

All Activity Heatmap for Cluster 2163

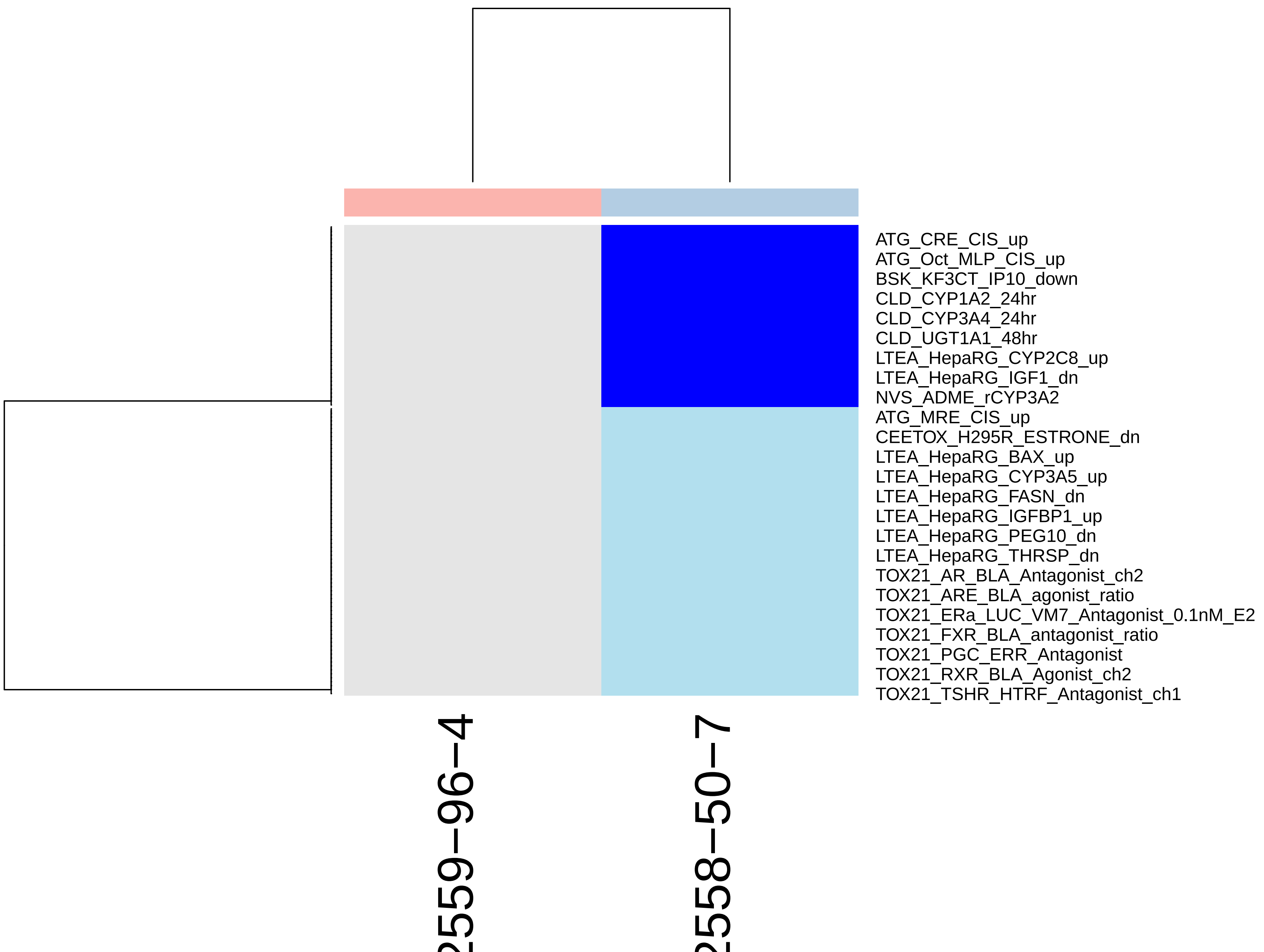

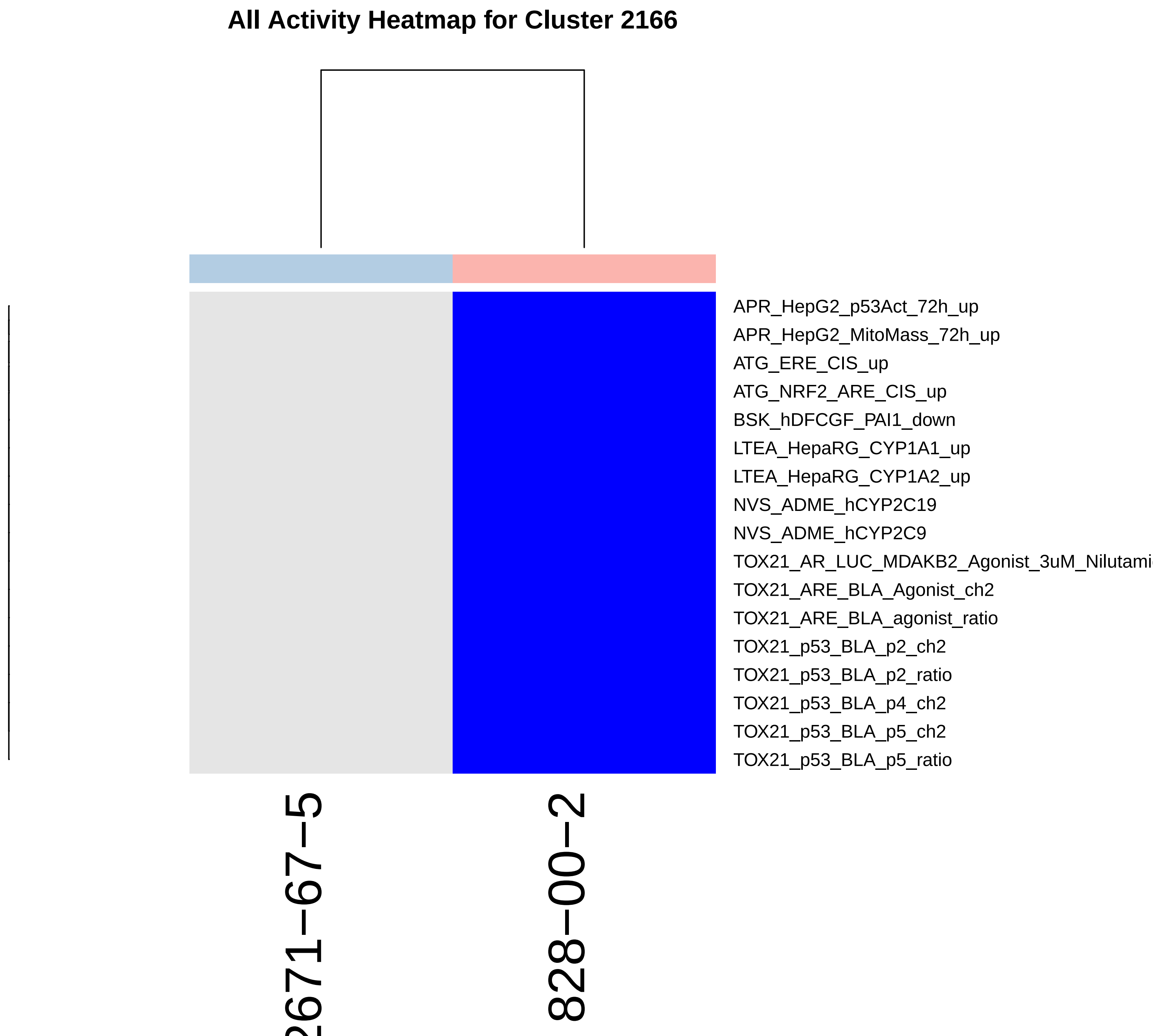
All Activity Heatmap for Cluster 2166
APR_HepG2_p53Act_72h_up
APR_HepG2_MitoMass_72h_up
ATG_ERE_CIS_up
ATG_NRF2_ARE_CIS_up
BSK_hDFCGF_PAI1_down
LTEA_HepaRG_CYP1A1_up
LTEA_HepaRG_CYP1A2_up
NVS_ADME_hCYP2C19
NVS_ADME_hCYP2C9
TOX21_AR_LUC_MDAKB2_Agonist_3uM_Nilutami
TOX21_ARE_BLA_Agonist_ch2
TOX21_ARE_BLA_agonist_ratio
TOX21_p53_BLA_p2_ch2
TOX21_p53_BLA_p2_ratio
TOX21_p53_BLA_p4_ch2
TOX21_p53_BLA_p5_ch2
TOX21_p53_BLA_p5_ratio
2671−67−5
828−00−2

All Activity Heatmap for Cluster 2169

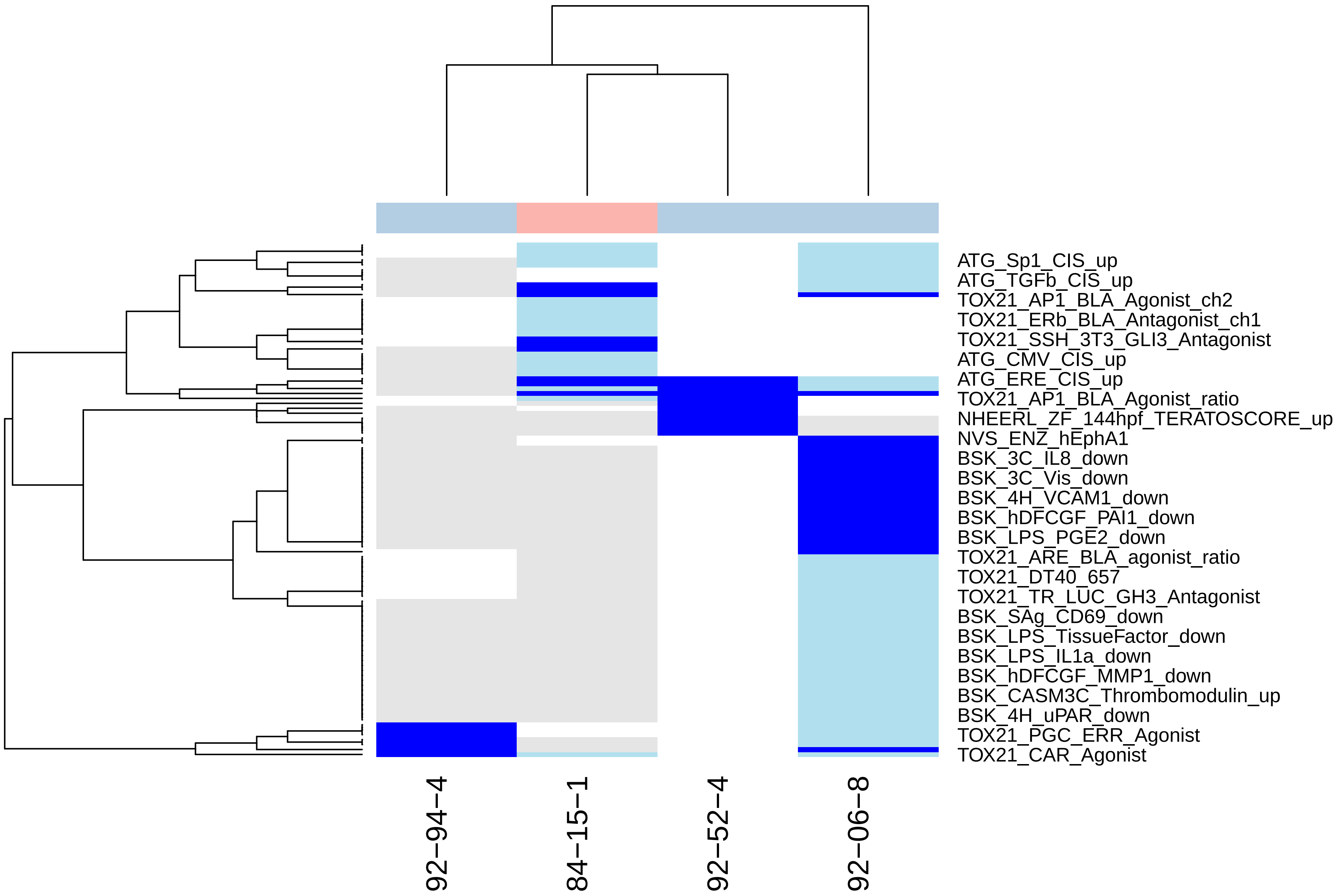

All Activity Heatmap for Cluster 2170

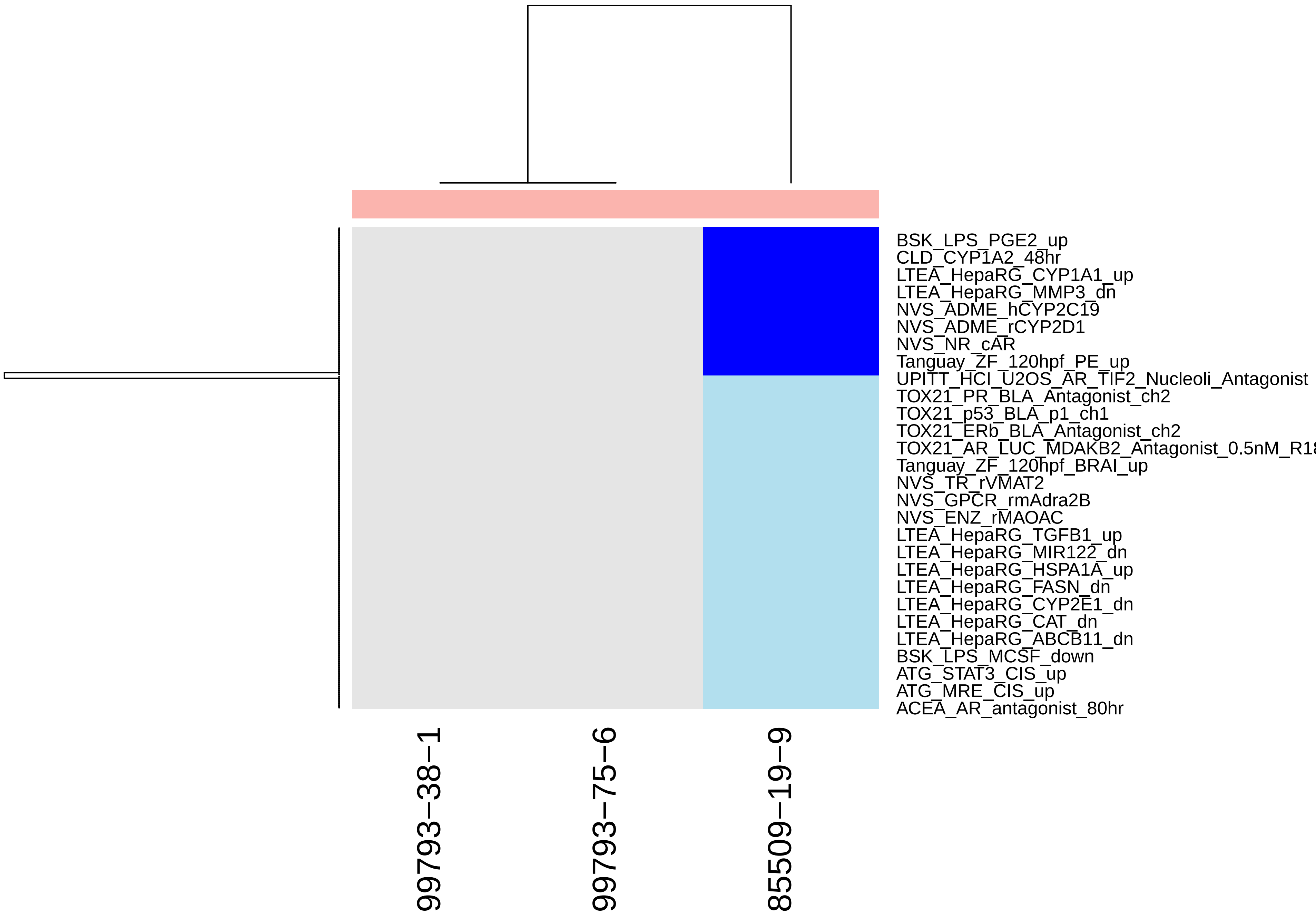

All Activity Heatmap for Cluster 2173

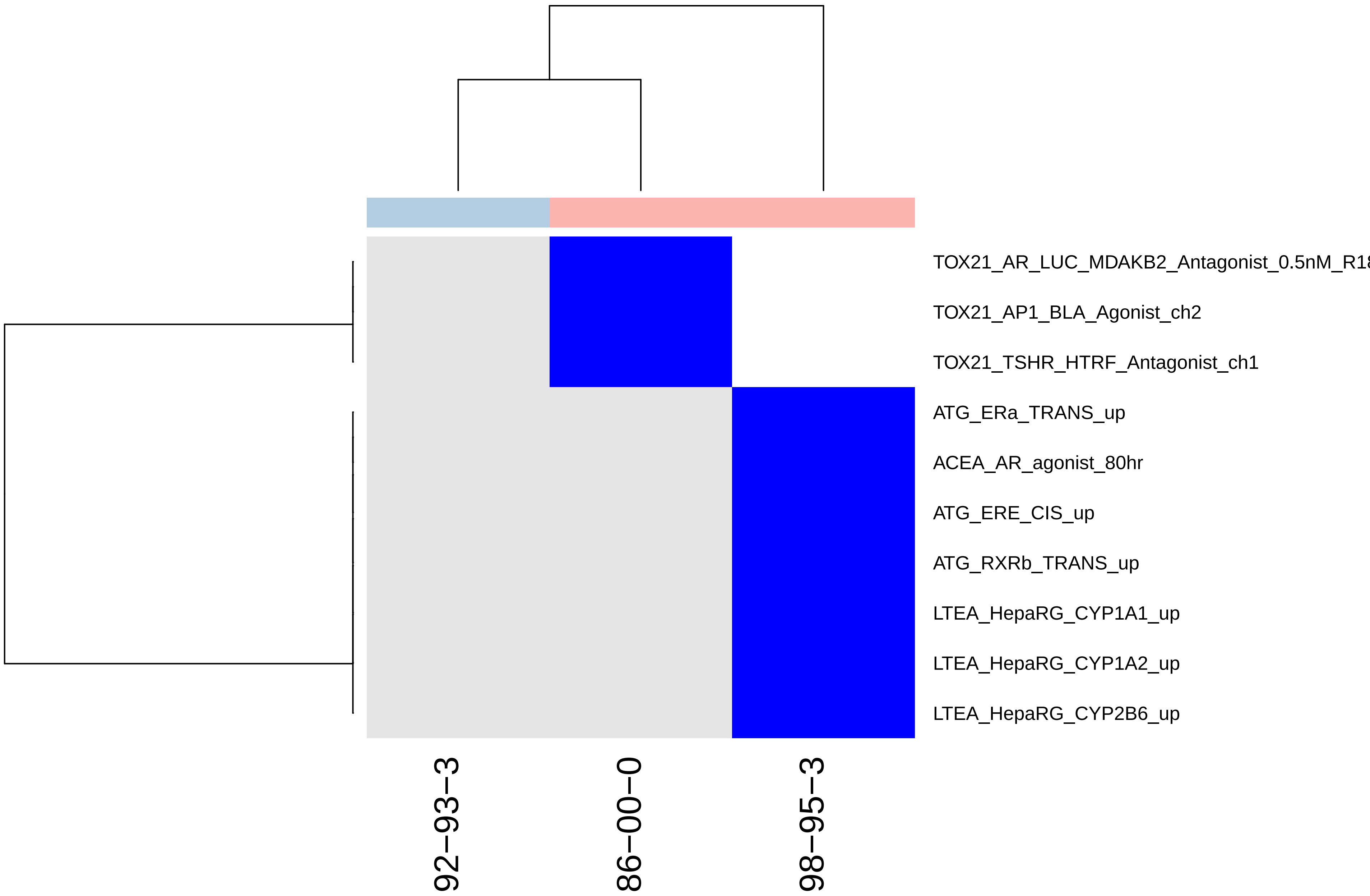

All Activity Heatmap for Cluster 2179

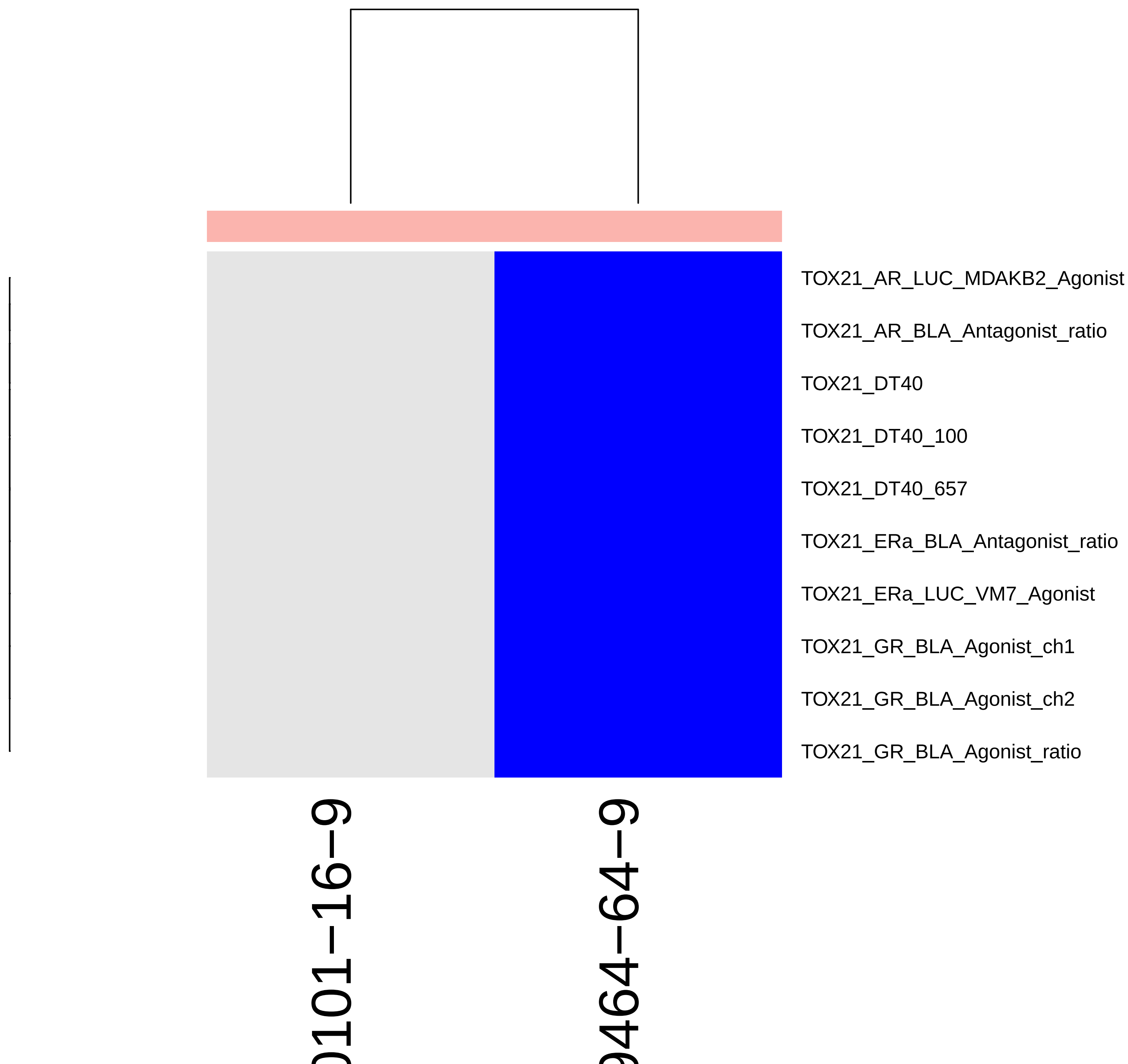

Supplement: Supplementary file 1 [file DataSheet2.PDF]
