# Supplementary material for: Mapping Mechanistic Pathways of Acute Oral Systemic Toxicity Using Chemical Structure and Bioactivity Measurements
Source: Front Toxicol. 2022 Mar 7;4:824094. doi: 10.3389/ftox.2022.824094 (PMC8915918; doi:10.3389/ftox.2022.824094)
Supplement: Supplementary file 2 [file DataSheet1.PDF]

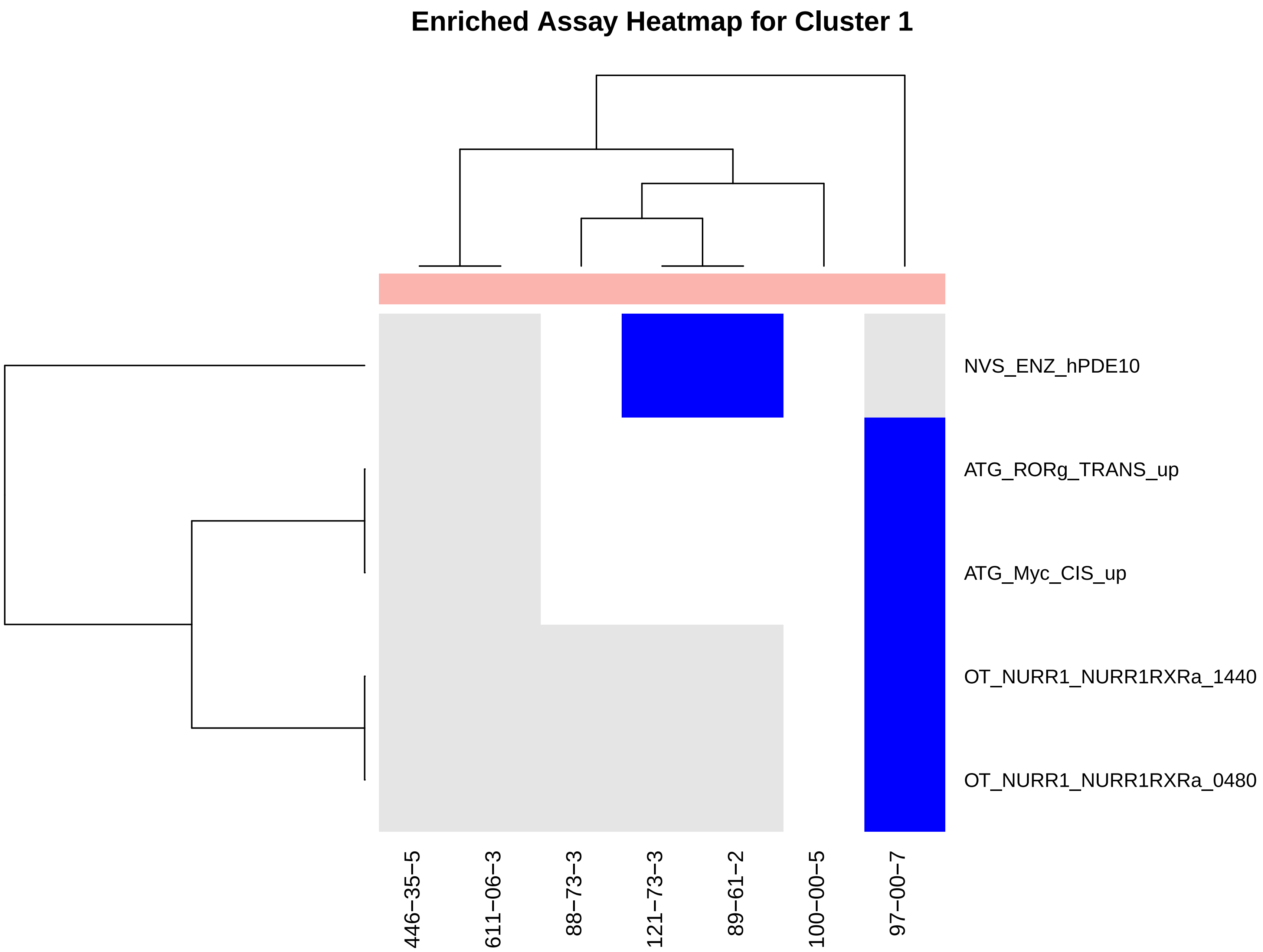

Enriched Assay Heatmap for Cluster 1
NVS_ENZ_hPDE10
ATG_RORg_TRANS_up
ATG_Myc_CIS_up
OT_NURR1_NURR1RXRa_1440
OT_NURR1_NURR1RXRa_0480
446−35−5
611−06−3
88−73−3
121−73−3
89−61−2
100−00−5
97−00−7

Enriched Assay Heatmap for Cluster 2

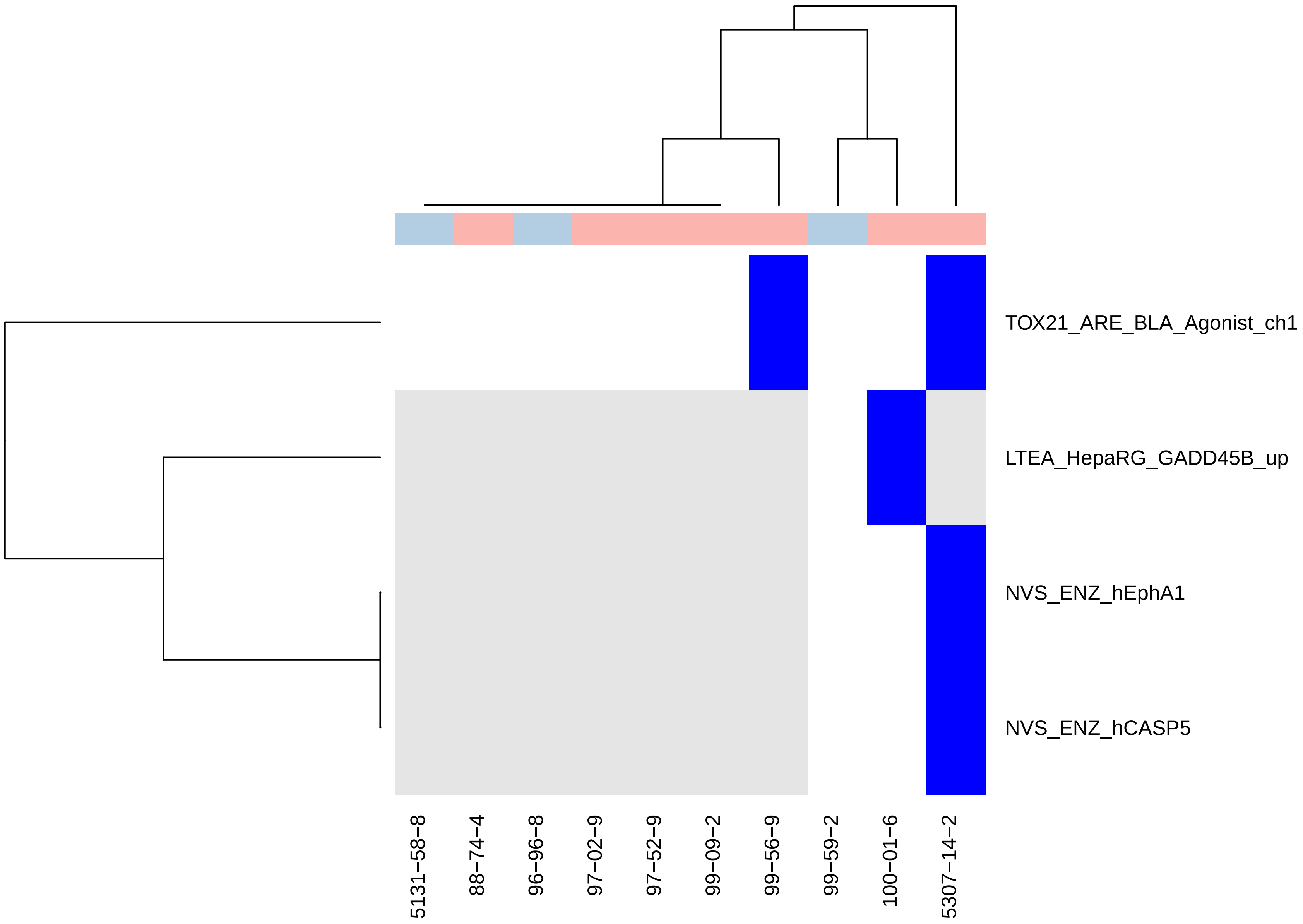

Enriched Assay Heatmap for Cluster 3

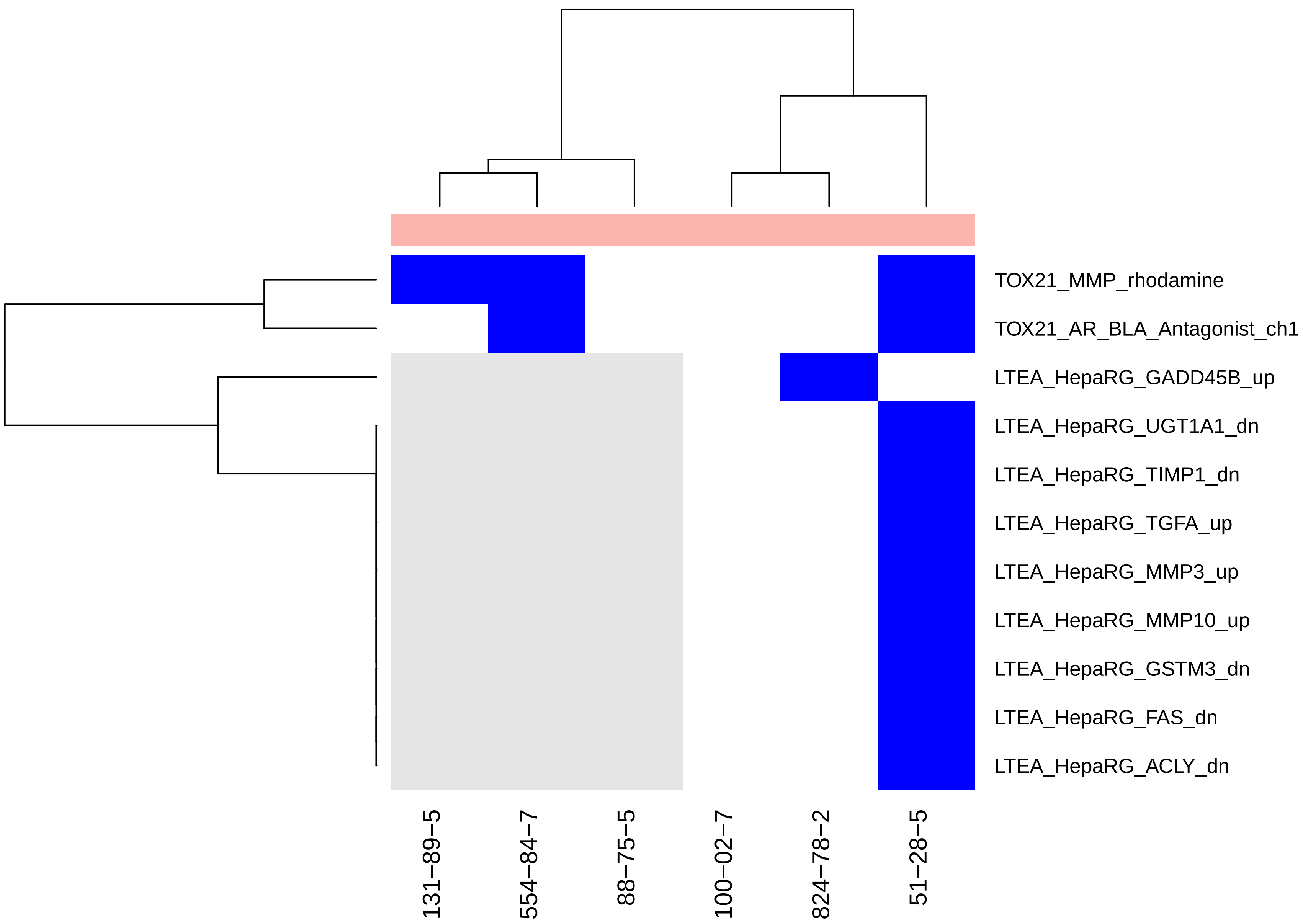

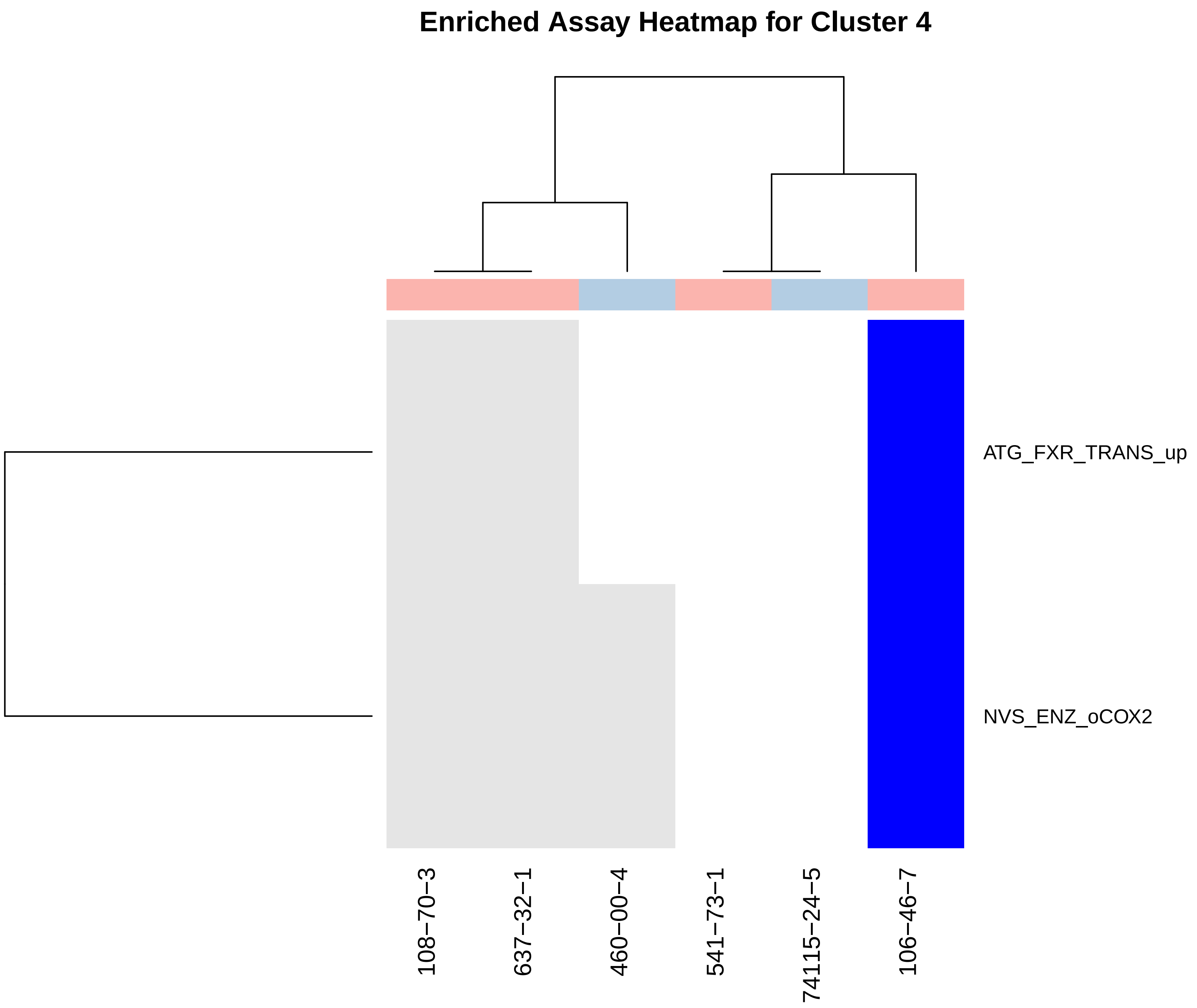

Enriched Assay Heatmap for Cluster 4
ATG_FXR_TRANS_up
NVS_ENZ_oCOX2
108−70−3
637−32−1
460−00−4
541−73−1
74115−24−5
106−46−7

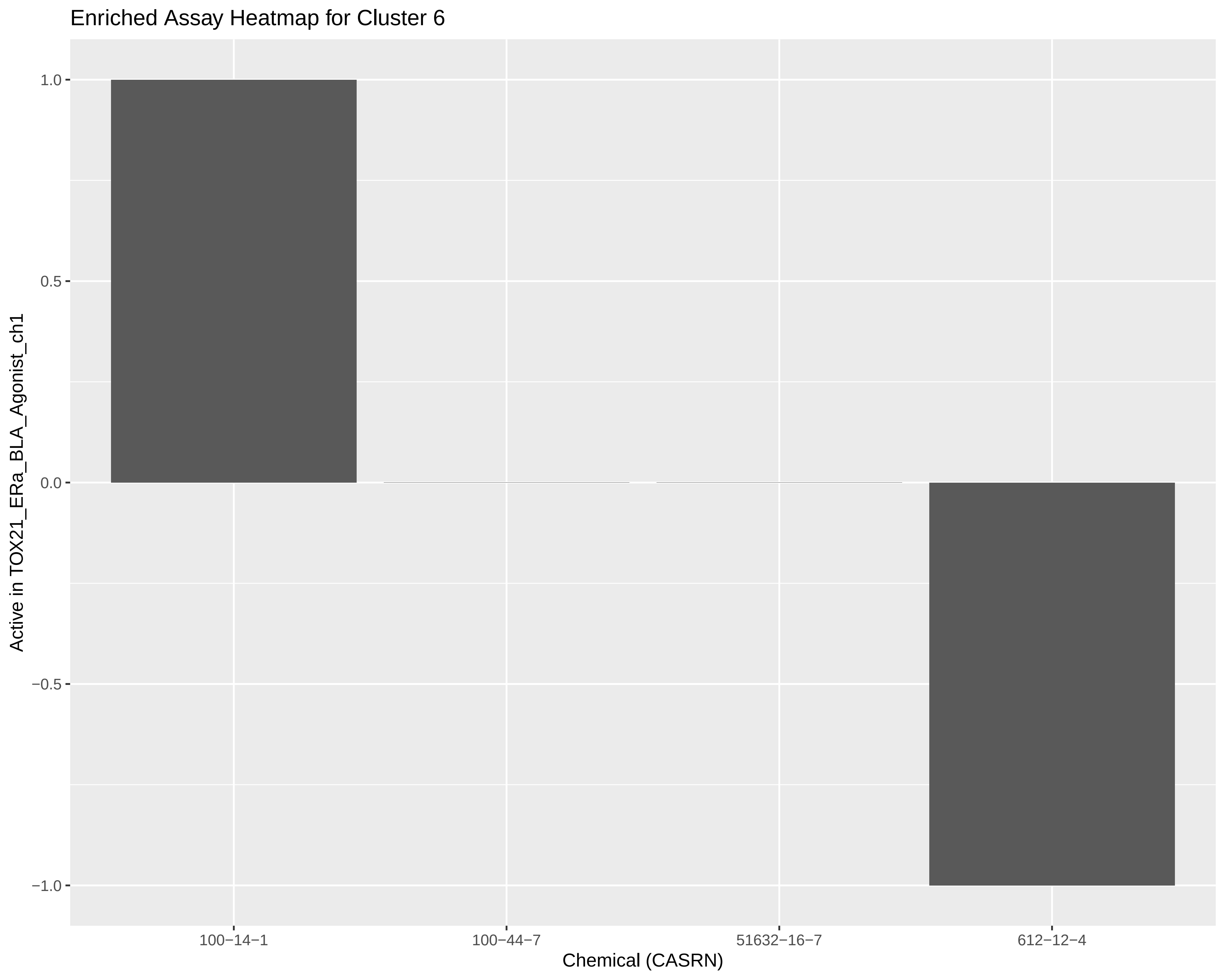

Enriched Assay Heatmap for Cluster 6
1.0
0.5
0.0
−0.5
−1.0
Active in TOX21_ERa_BLA_Agonist_ch1
100−14−1
100−44−7
51632−16−7
612−12−4
Chemical (CASRN)

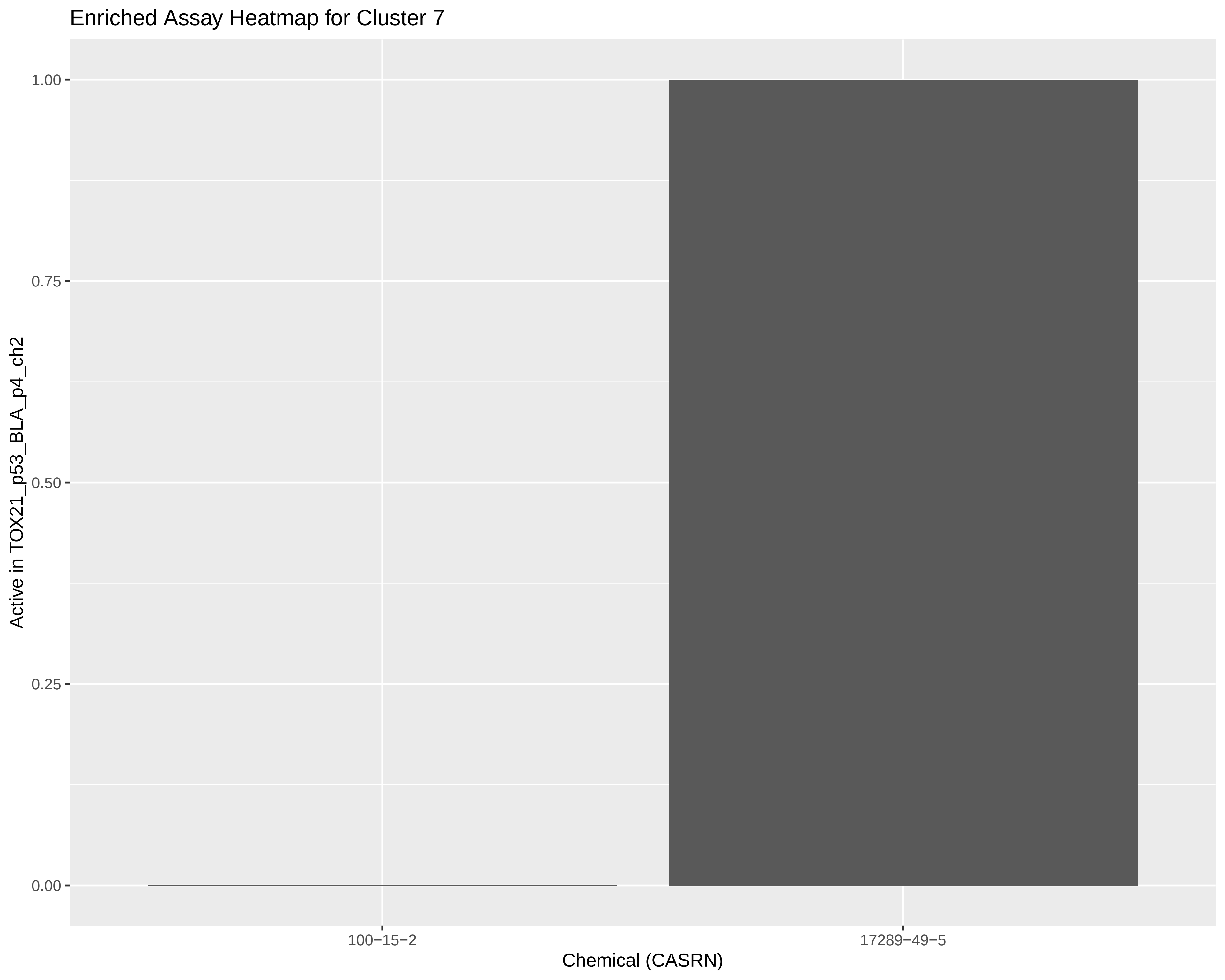
Enriched Assay Heatmap for Cluster 7
Active in TOX21_p53_BLA_p4_ch2
1.00
0.75
0.50
0.25
0.00
100−15−2
17289−49−5
Chemical (CASRN)

Enriched Assay Heatmap for Cluster 11

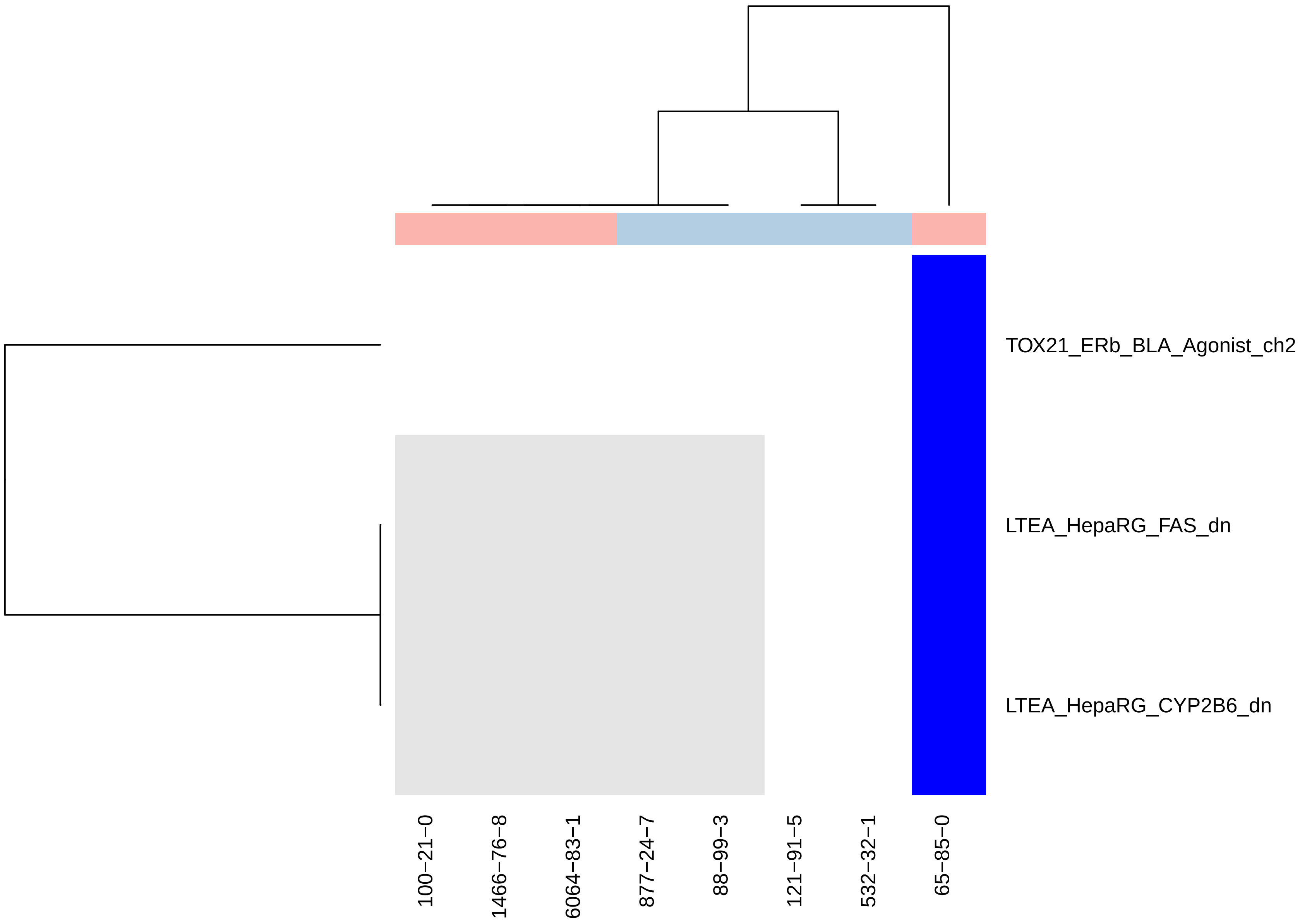

Enriched Assay Heatmap for Cluster 13

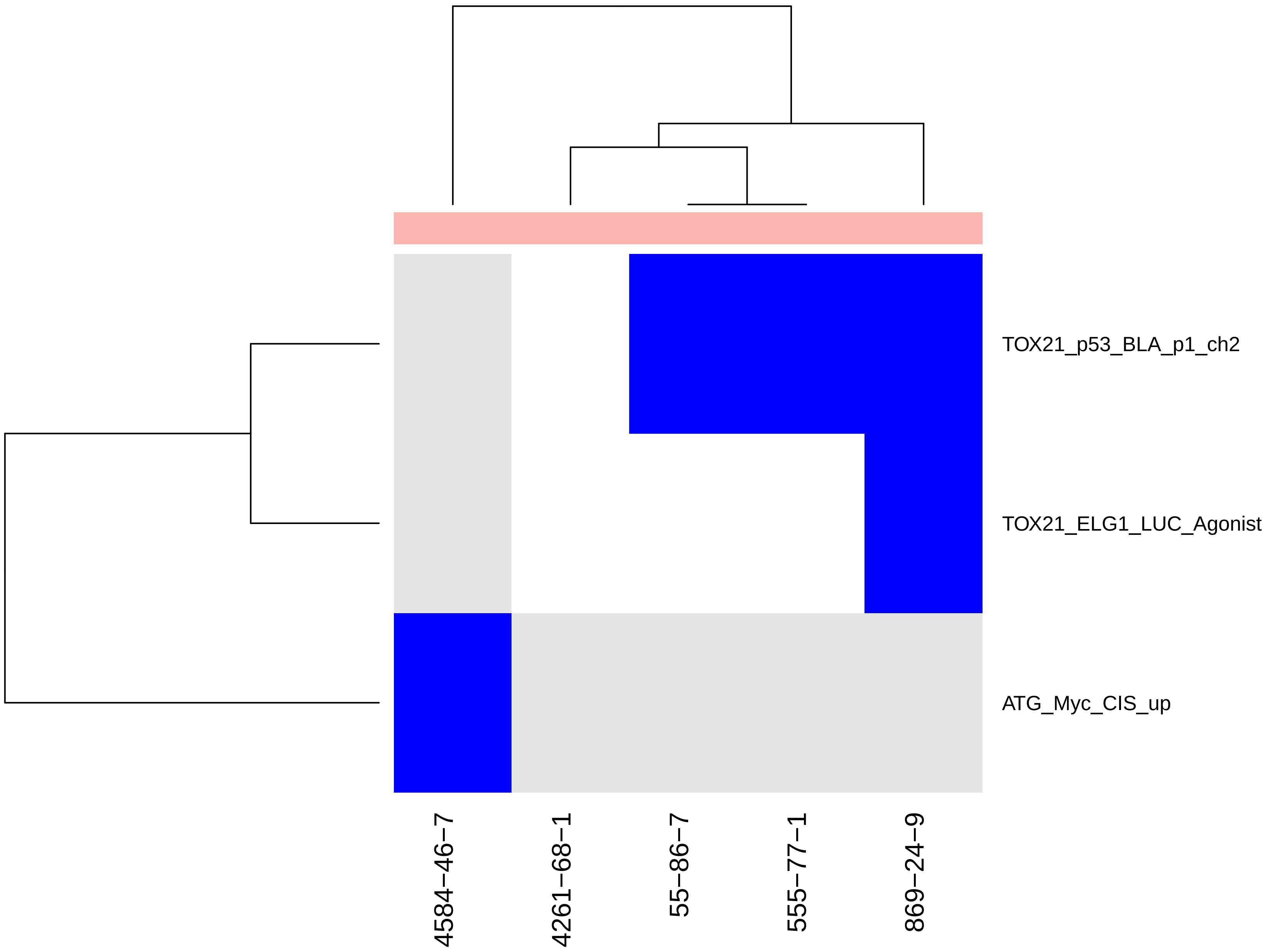

Enriched Assay Heatmap for Cluster 15

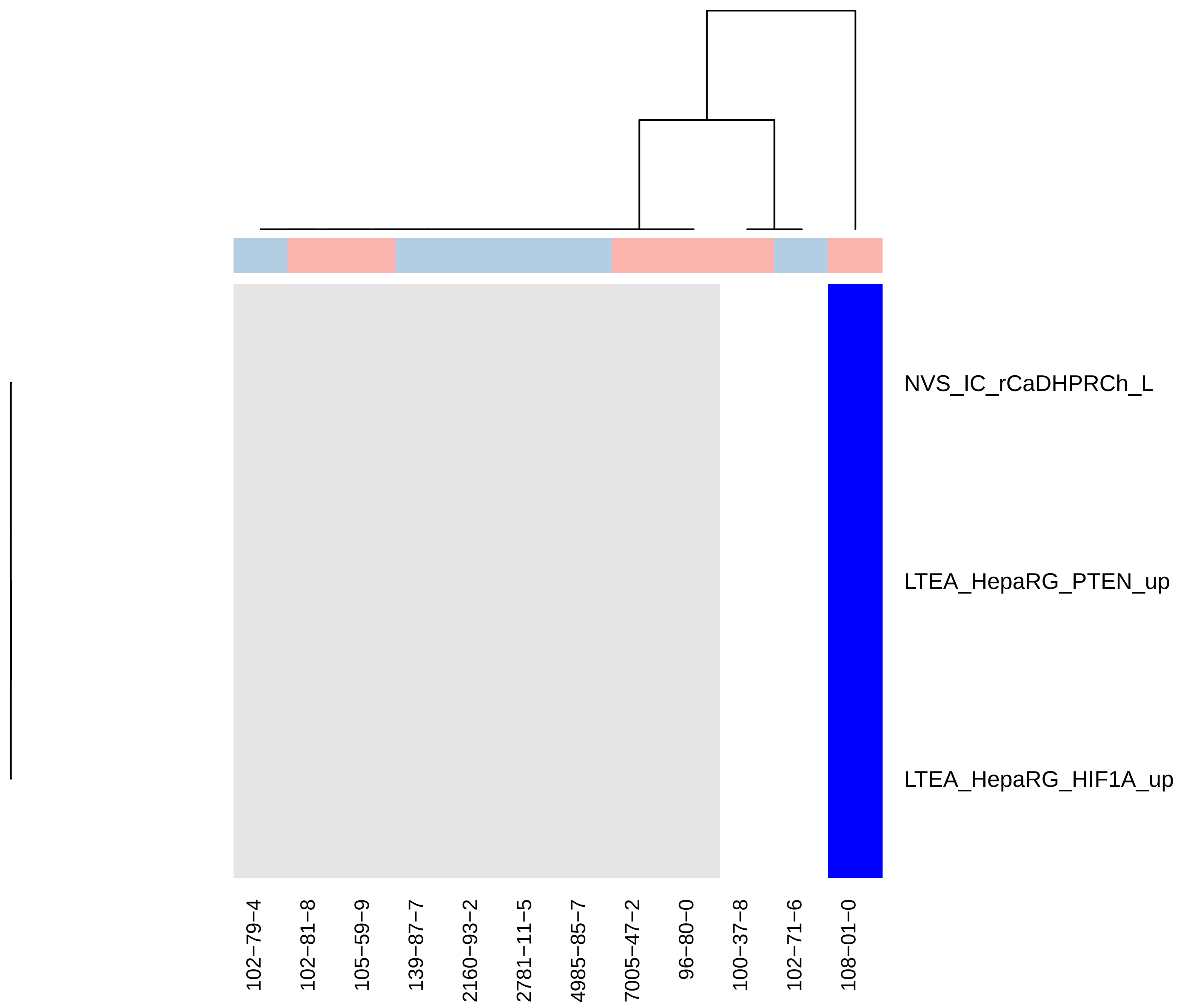

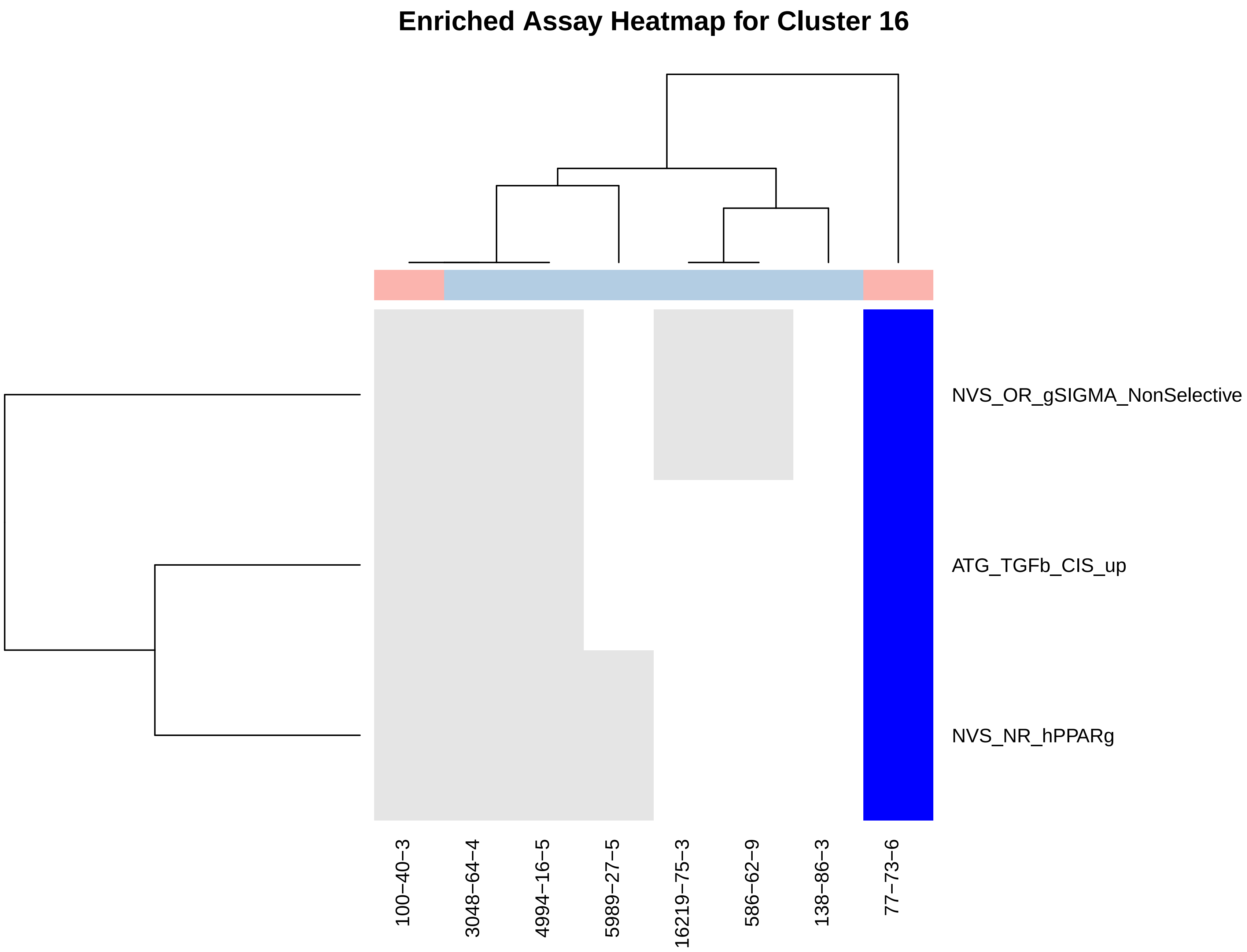
Enriched Assay Heatmap for Cluster 16
NVS_OR_gSIGMA_NonSelective
ATG_TGFb_CIS_up
NVS_NR_hPPARg
100−40−3
3048−64−4
4994−16−5
5989−27−5
16219−75−3
586−62−9
138−86−3
77−73−6

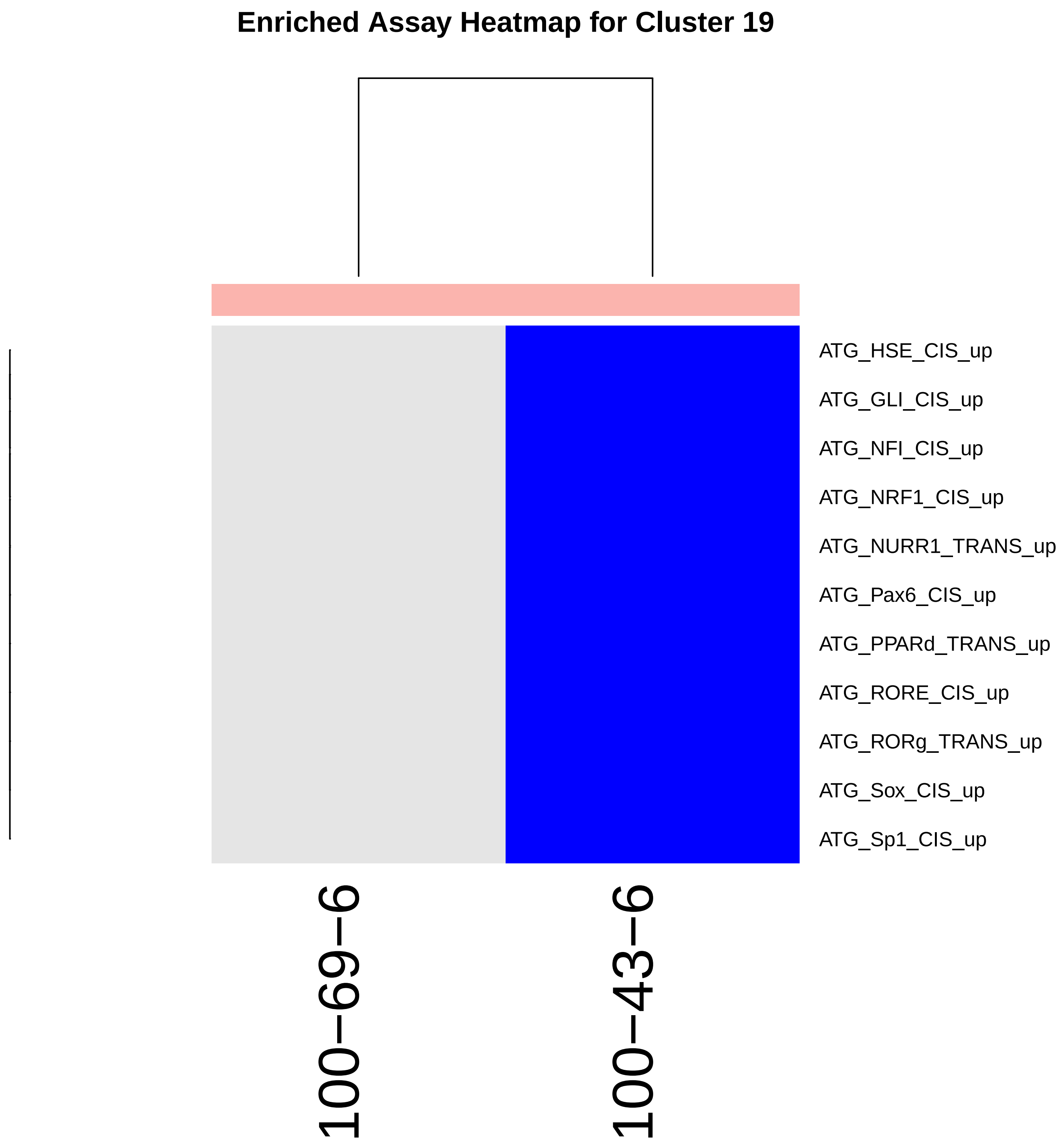
Enriched Assay Heatmap for Cluster 19
ATG_HSE_CIS_up
ATG_GLI_CIS_up
ATG_NFI_CIS_up
ATG_NRF1_CIS_up
ATG_NURR1_TRANS_up
ATG_Pax6_CIS_up
ATG_PPARd_TRANS_up
ATG_RORE_CIS_up
ATG_RORg_TRANS_up
ATG_Sox_CIS_up
ATG_Sp1_CIS_up
100−69−6
100−43−6

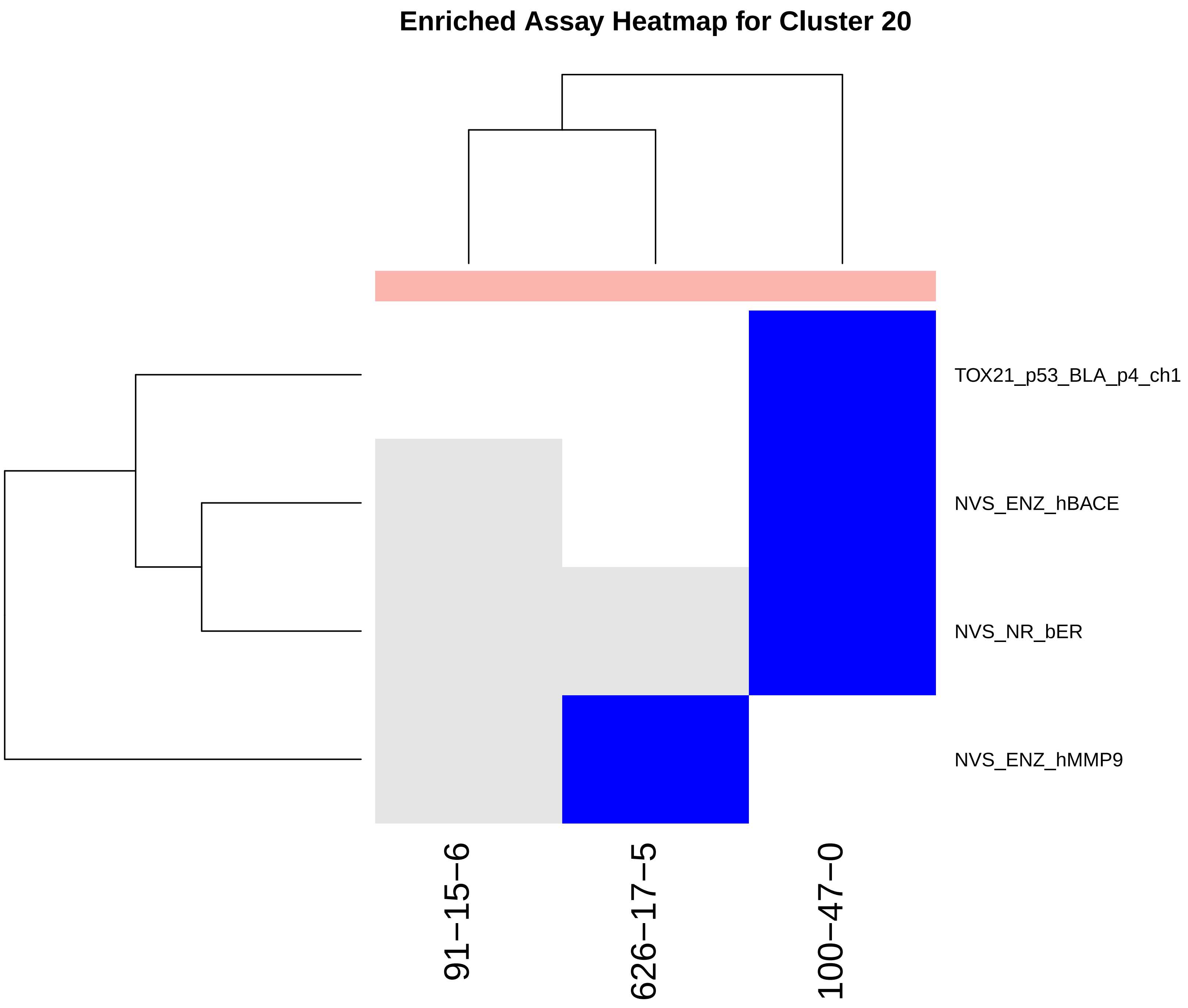
Enriched Assay Heatmap for Cluster 20
TOX21_p53_BLA_p4_ch1
NVS_ENZ_hBACE
NVS_NR_bER
NVS_ENZ_hMMP9
91−15−6
626−17−5
100−47−0

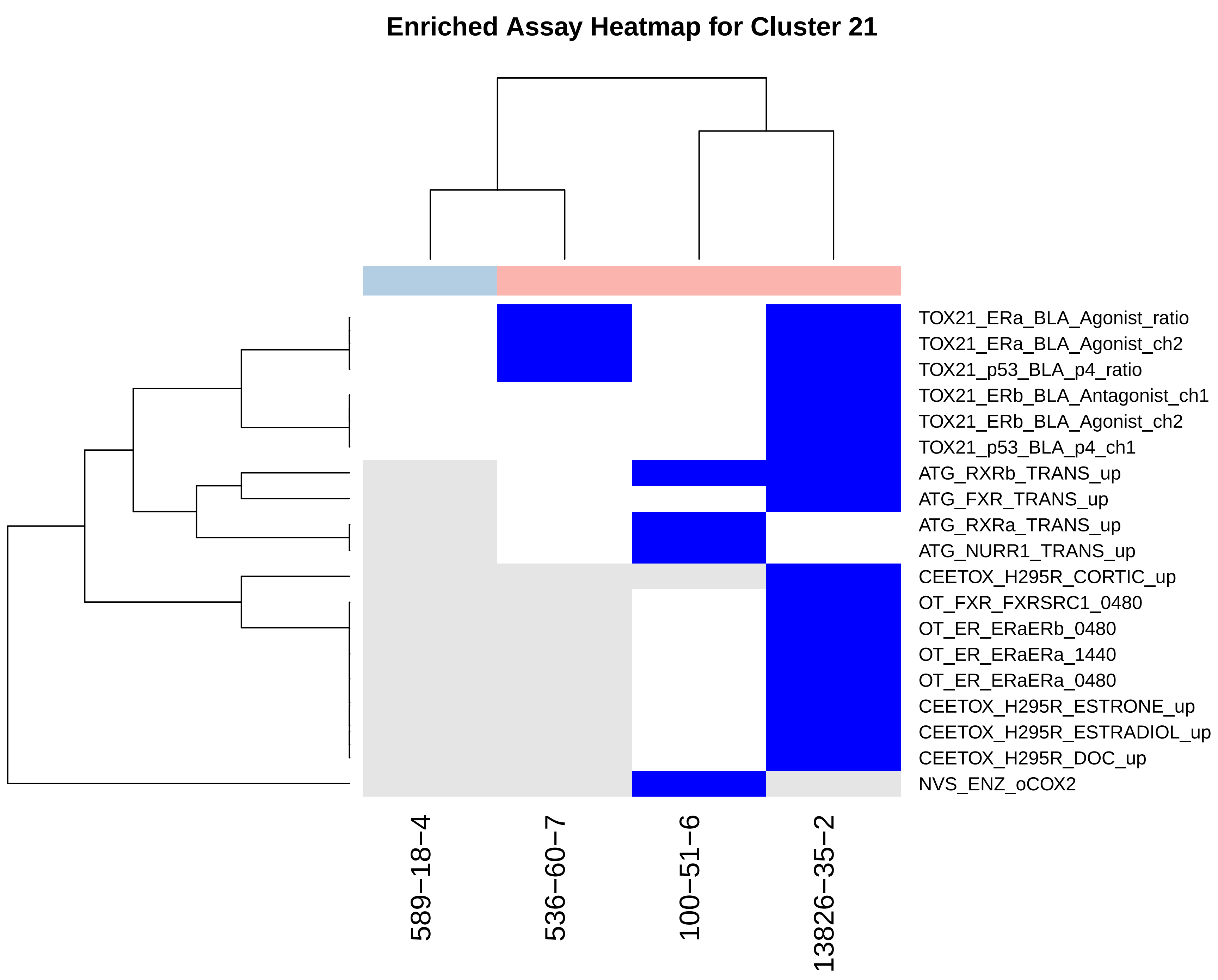
Enriched Assay Heatmap for Cluster 21
TOX21_ERa_BLA_Agonist_ratio
TOX21_ERa_BLA_Agonist_ch2
TOX21_p53_BLA_p4_ratio
TOX21_ERb_BLA_Antagonist_ch1
TOX21_ERb_BLA_Agonist_ch2
TOX21_p53_BLA_p4_ch1
ATG_RXRb_TRANS_up
ATG_FXR_TRANS_up
ATG_RXRa_TRANS_up
ATG_NURR1_TRANS_up
CEETOX_H295R_CORTIC_up
OT_FXR_FXRSRC1_0480
OT_ER_ERaERb_0480
OT_ER_ERaERa_1440
OT_ER_ERaERa_0480
CEETOX_H295R_ESTRONE_up
CEETOX_H295R_ESTRADIOL_up
CEETOX_H295R_DOC_up
NVS_ENZ_oCOX2
589−18−4
536−60−7
100−51−6
13826−35−2

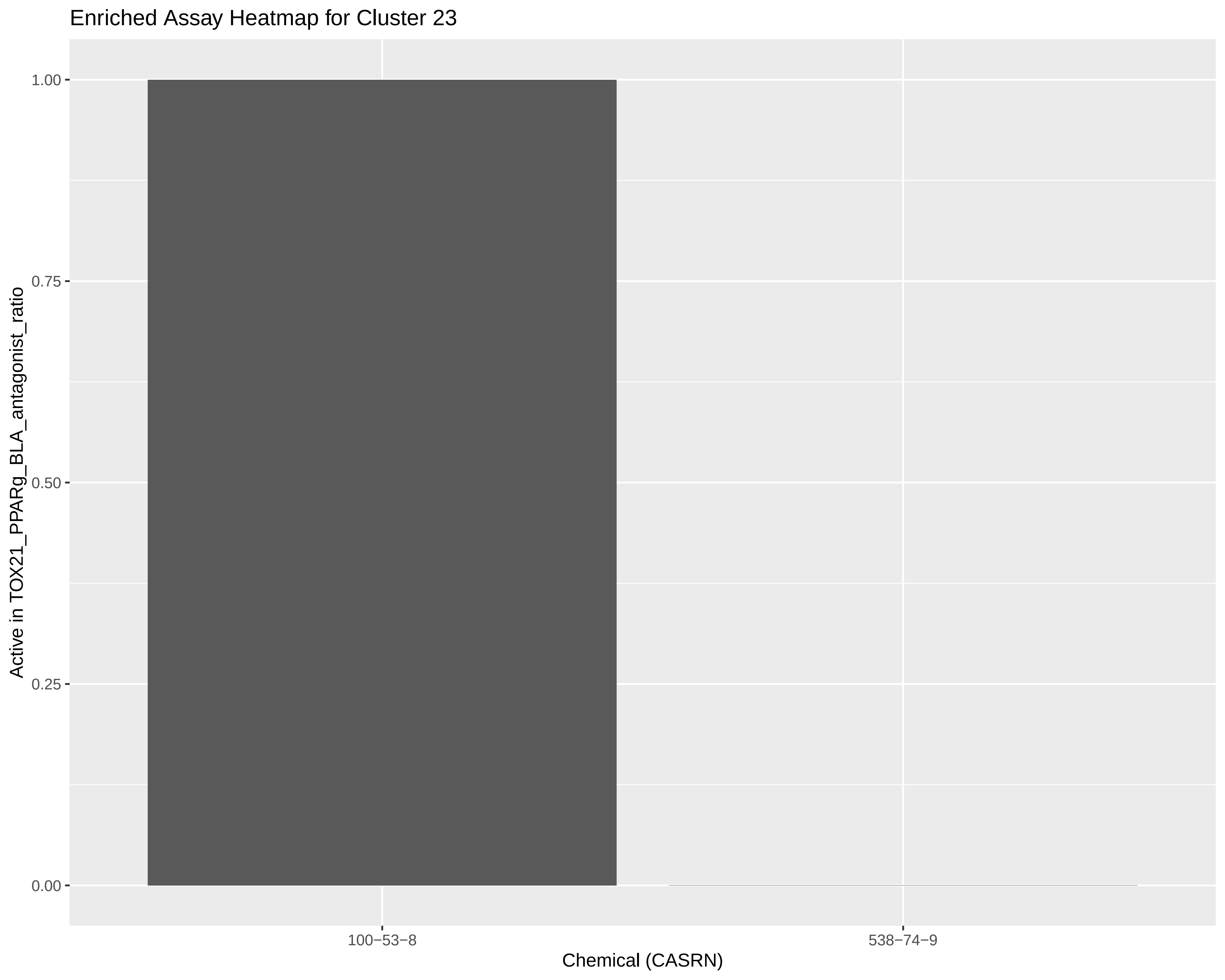
Enriched Assay Heatmap for Cluster 23
Active in TOX21_PPARg_BLA_antagonist_ratio
1.00
0.75
0.50
0.25
0.00
100−53−8
538−74−9
Chemical (CASRN)

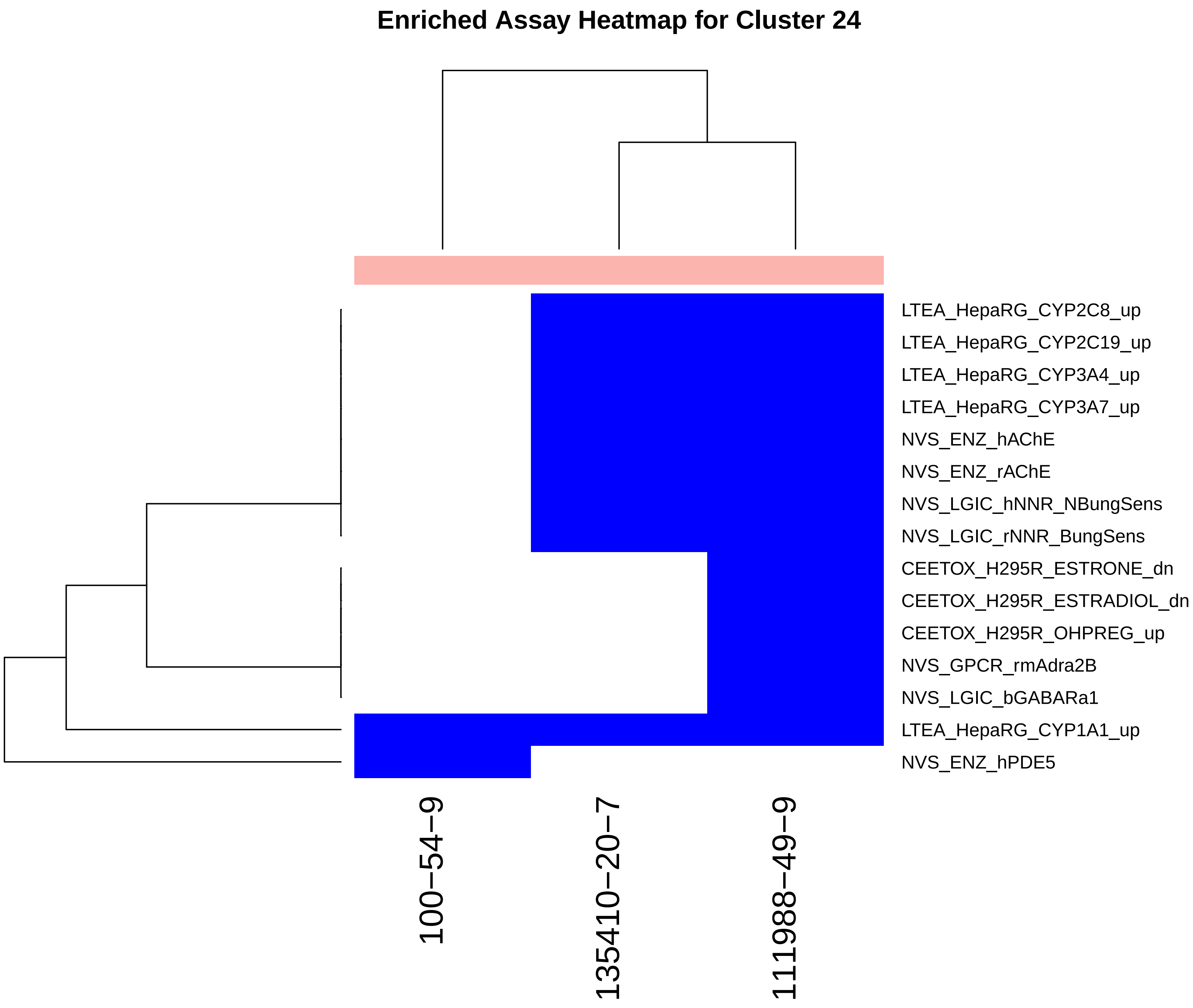
Enriched Assay Heatmap for Cluster 24
LTEA_HepaRG_CYP2C8_up
LTEA_HepaRG_CYP2C19_up
LTEA_HepaRG_CYP3A4_up
LTEA_HepaRG_CYP3A7_up
NVS_ENZ_hAChE
NVS_ENZ_rAChE
NVS_LGIC_hNNR_NBungSens
NVS_LGIC_rNNR_BungSens
CEETOX_H295R_ESTRONE_dn
CEETOX_H295R_ESTRADIOL_dn
CEETOX_H295R_OHPREG_up
NVS_GPCR_rmAdra2B
NVS_LGIC_bGABARa1
LTEA_HepaRG_CYP1A1_up
NVS_ENZ_hPDE5
100−54−9
135410−20−7
111988−49−9

Enriched Assay Heatmap for Cluster 25

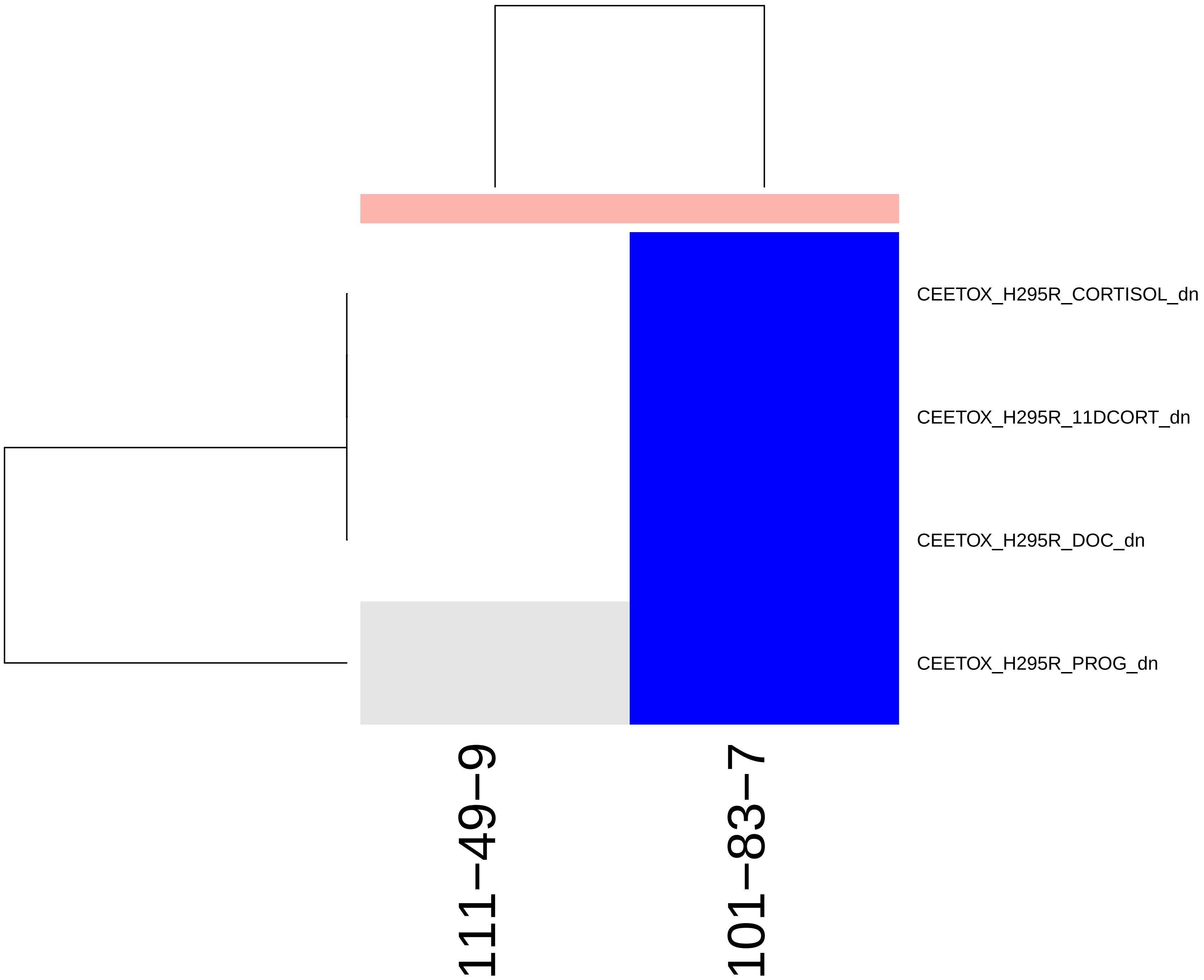

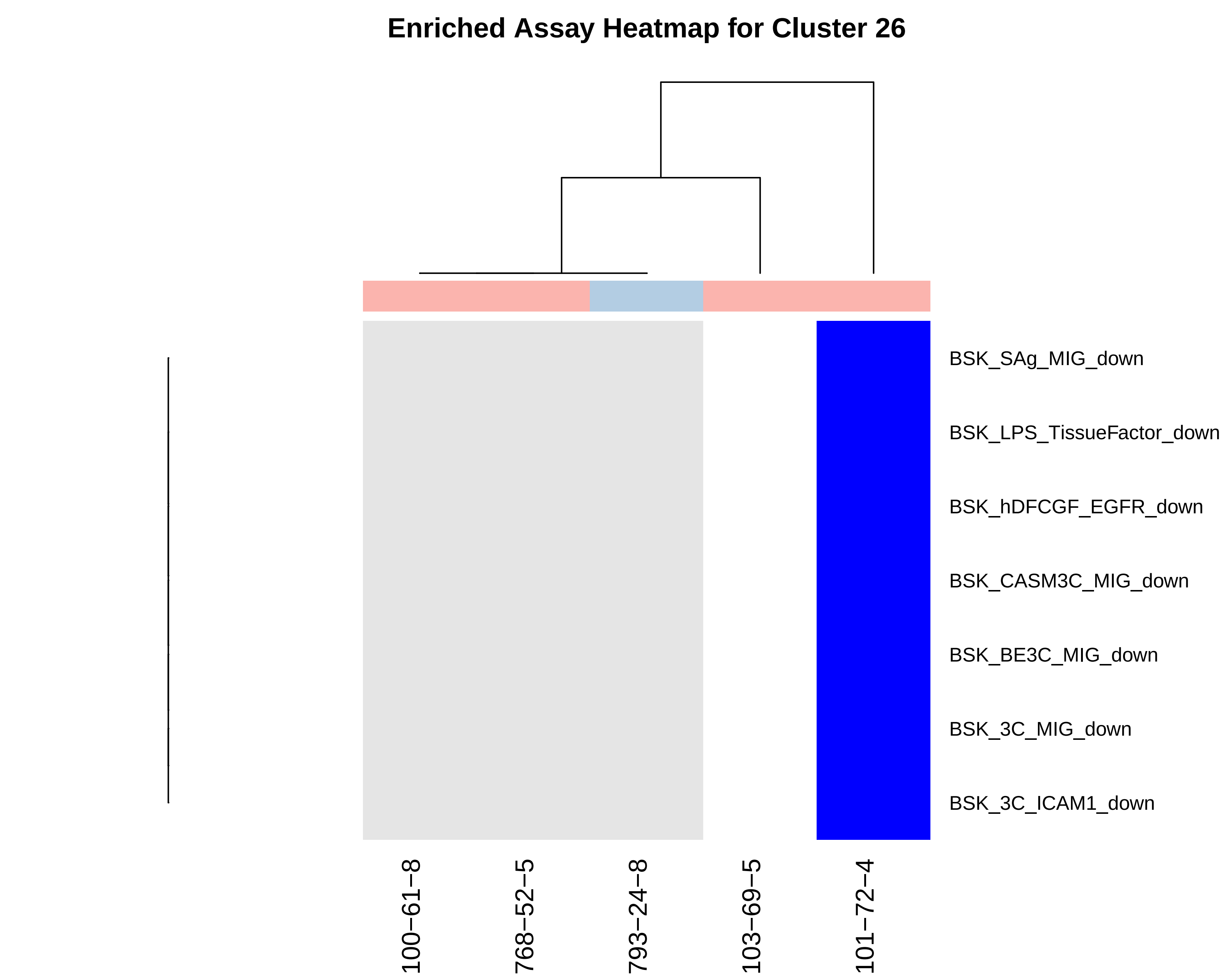
Enriched Assay Heatmap for Cluster 26
BSK_SAg_MIG_down
BSK_LPS_TissueFactor_down
BSK_hDFCGF_EGFR_down
BSK_CASM3C_MIG_down
BSK_BE3C_MIG_down
BSK_3C_MIG_down
BSK_3C_ICAM1_down
100−61−8
768−52−5
793−24−8
103−69−5
101−72−4

Enriched Assay Heatmap for Cluster 27

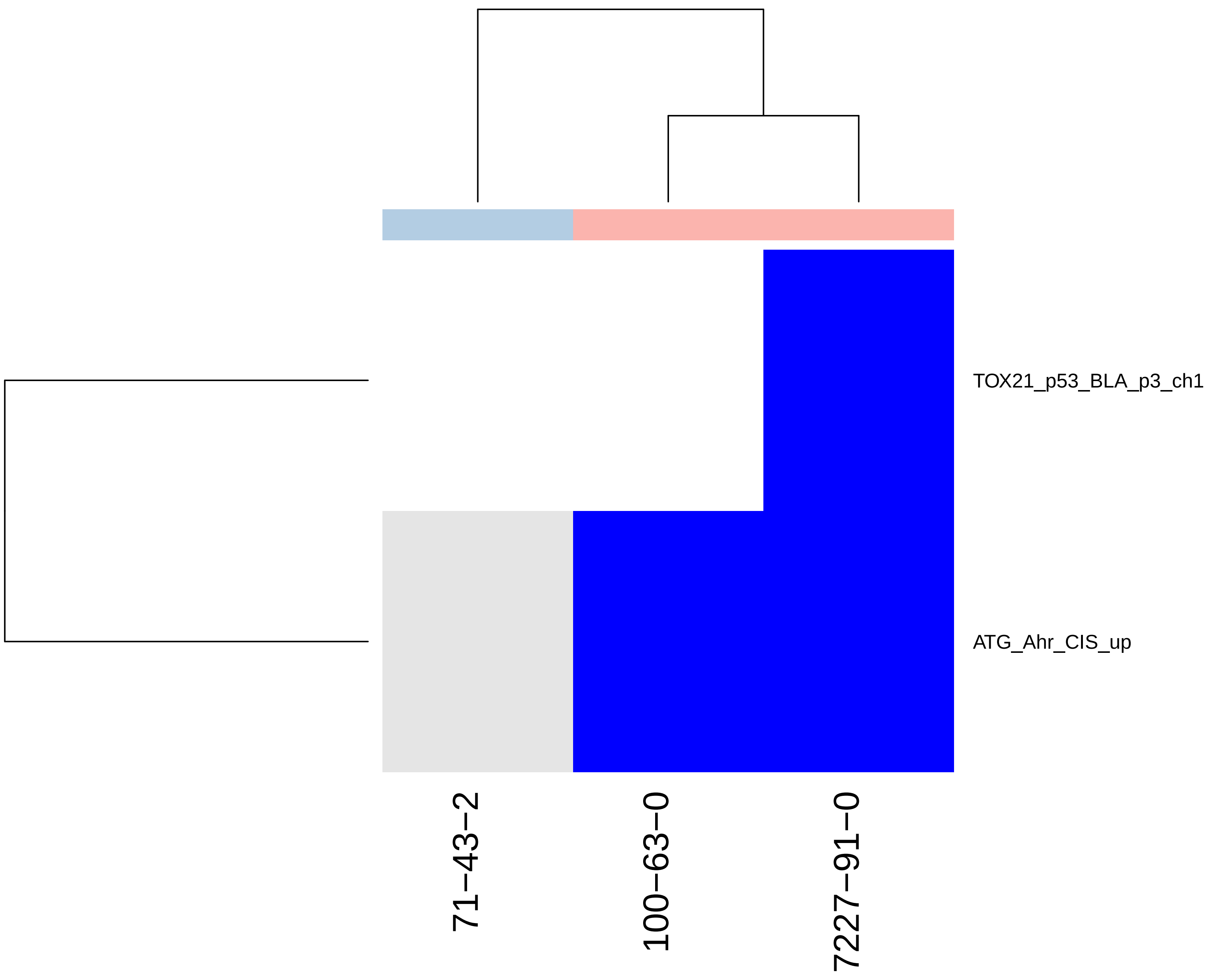

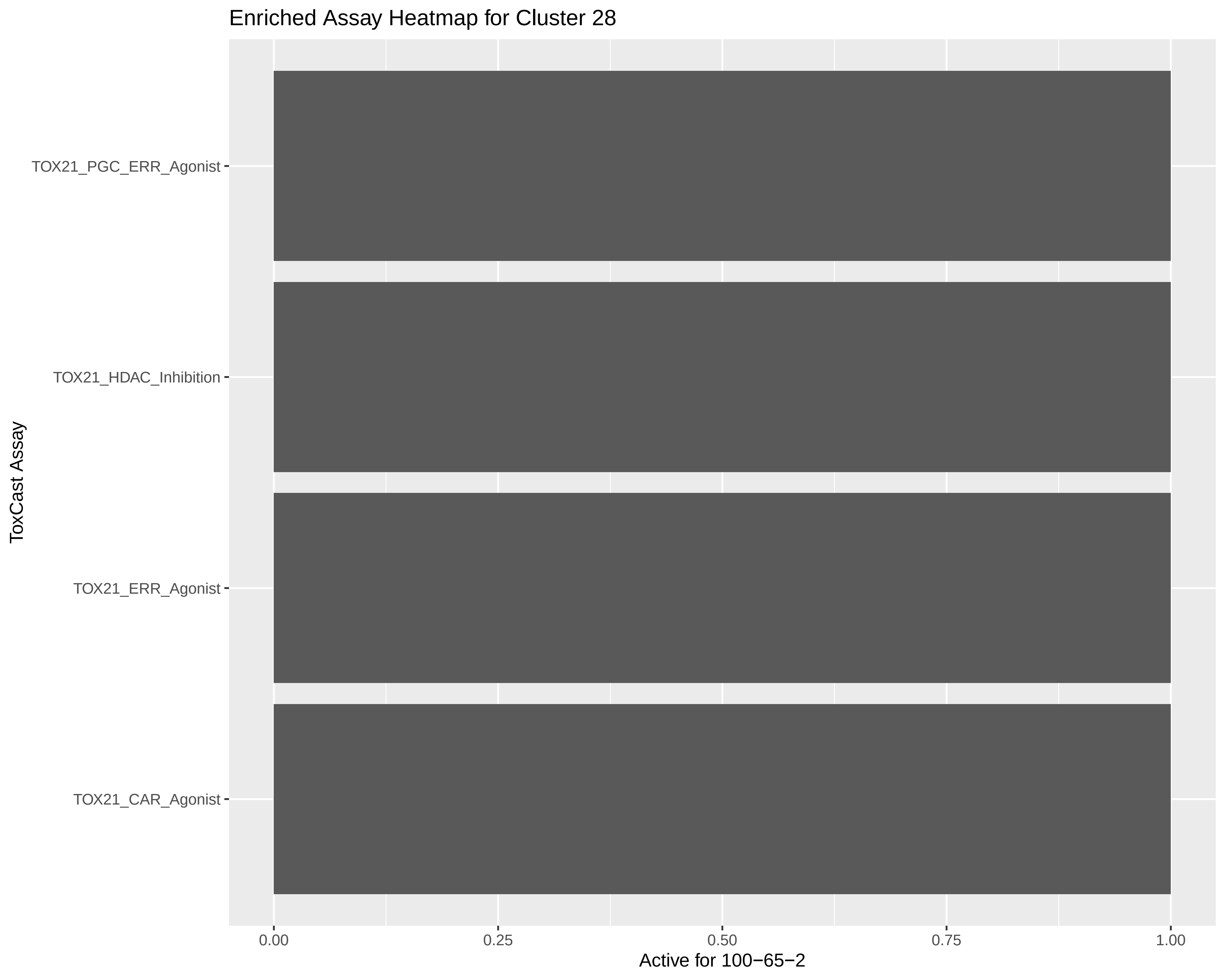

Enriched Assay Heatmap for Cluster 28
TOX21_PGC_ERR_Agonist
TOX21_HDAC_Inhibition
TOX21_ERR_Agonist
TOX21_CAR_Agonist
ToxCast Assay
0.00
0.25
0.50
0.75
1.00
Active for 100−65−2

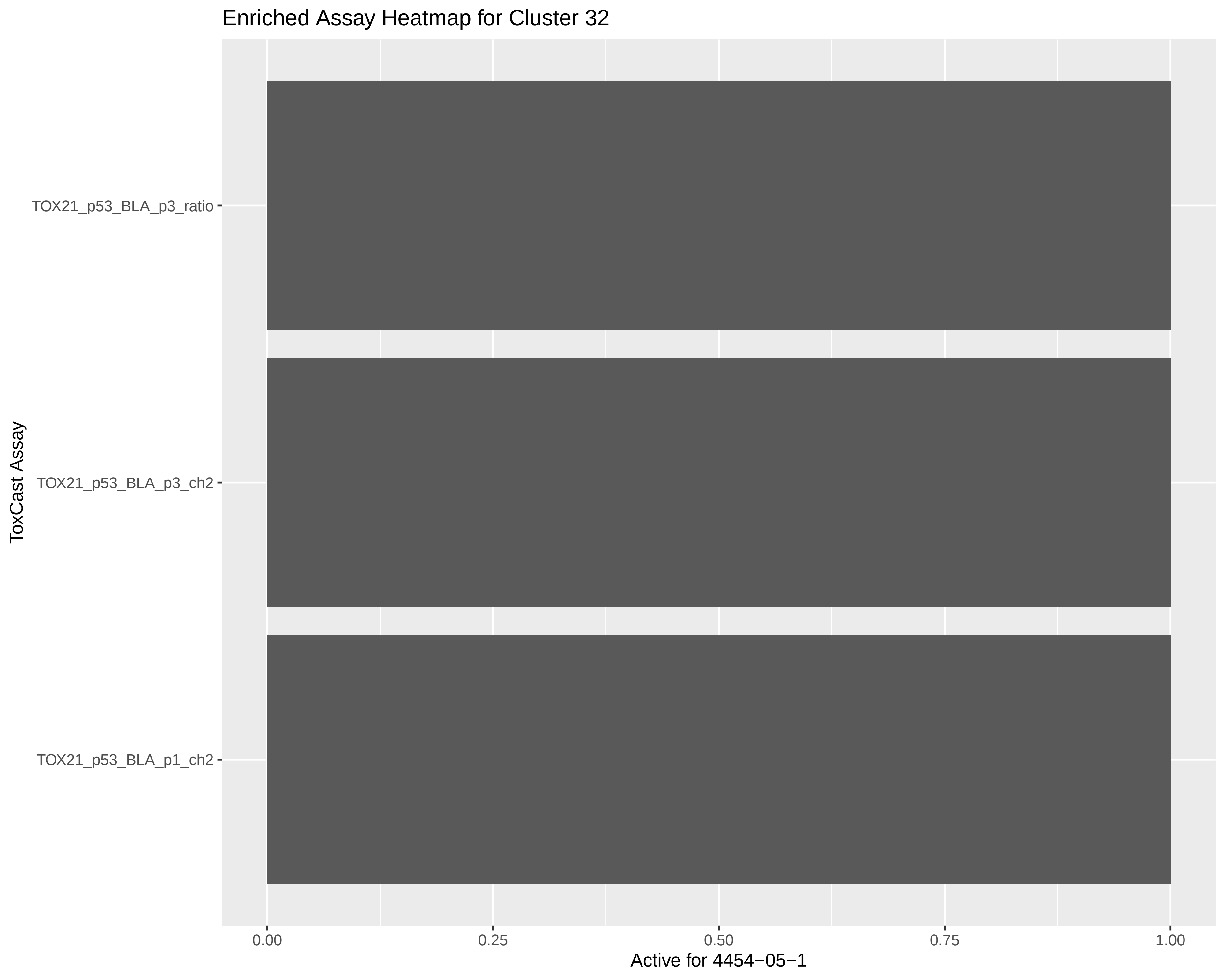
Enriched Assay Heatmap for Cluster 32
ToxCast Assay
TOX21_p53_BLA_p3_ratio
TOX21_p53_BLA_p3_ch2
TOX21_p53_BLA_p1_ch2
0.00
0.25
0.50
0.75
1.00
Active for 4454−05−1

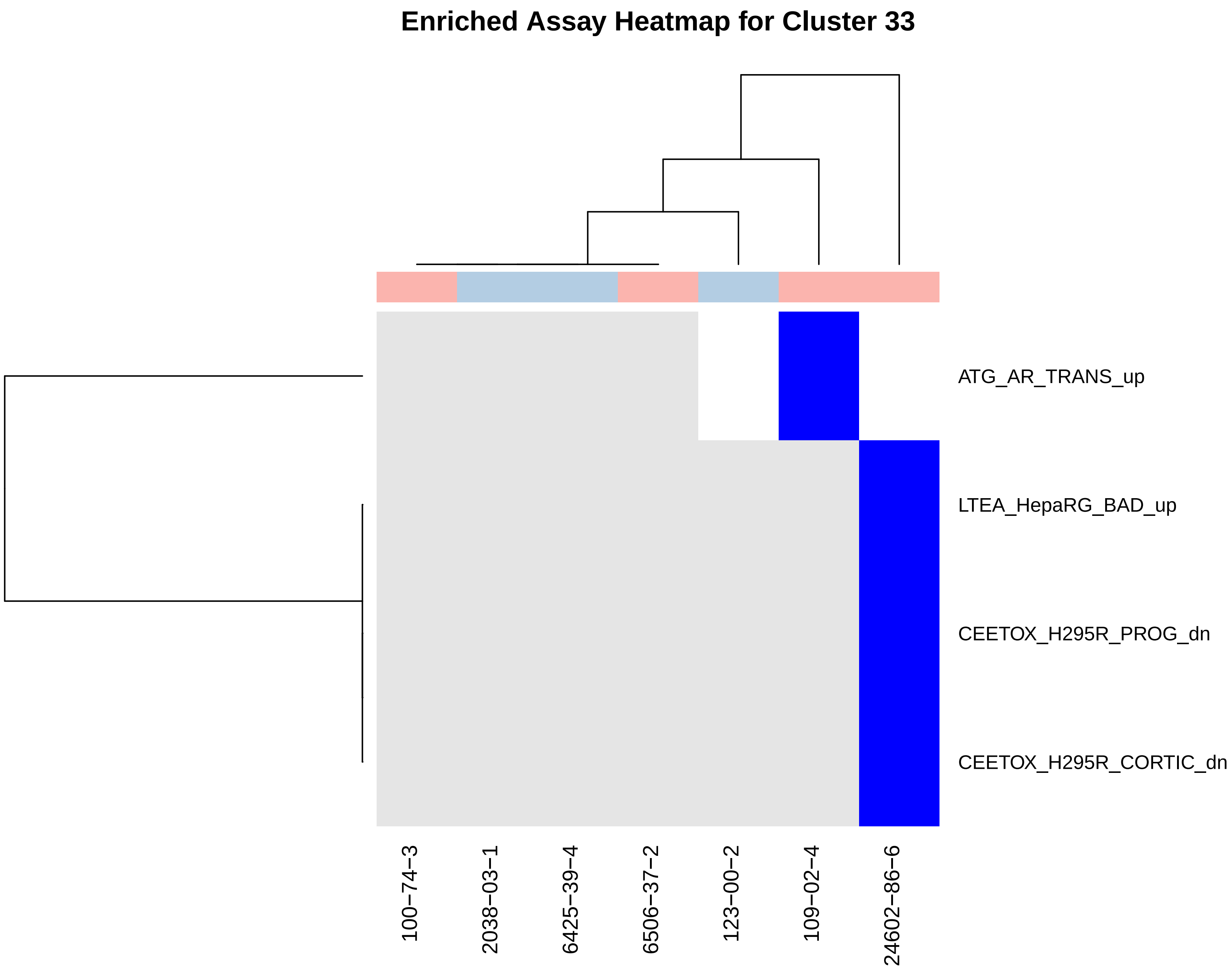
Enriched Assay Heatmap for Cluster 33
ATG_AR_TRANS_up
LTEA_HepaRG_BAD_up
CEETOX_H295R_PROG_dn
CEETOX_H295R_CORTIC_dn
100−74−3
2038−03−1
6425−39−4
6506−37−2
123−00−2
109−02−4
24602−86−6

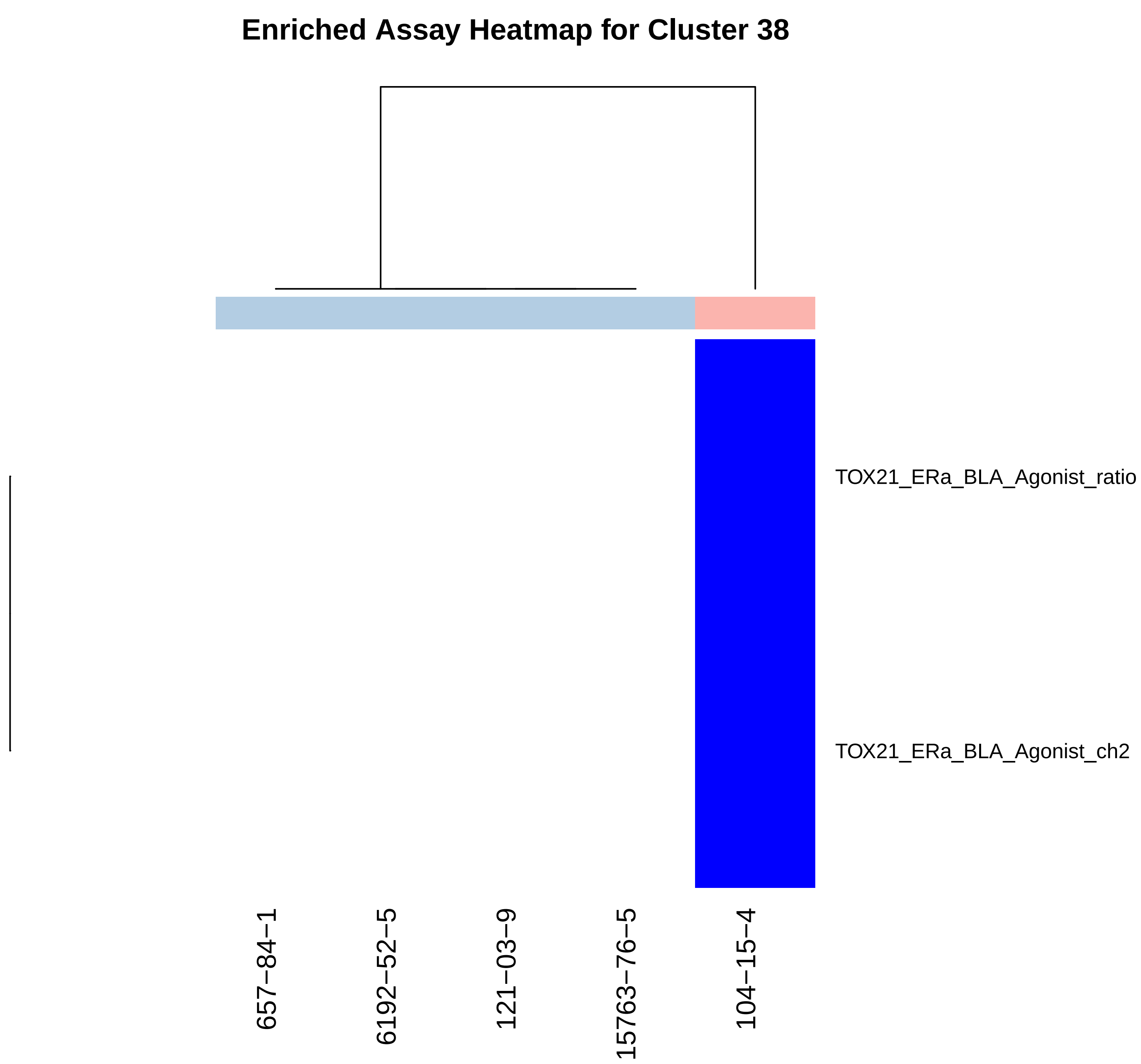
Enriched Assay Heatmap for Cluster 38
TOX21_ERa_BLA_Agonist_ratio
TOX21_ERa_BLA_Agonist_ch2
657−84−1
6192−52−5
121−03−9
15763−76−5
104−15−4

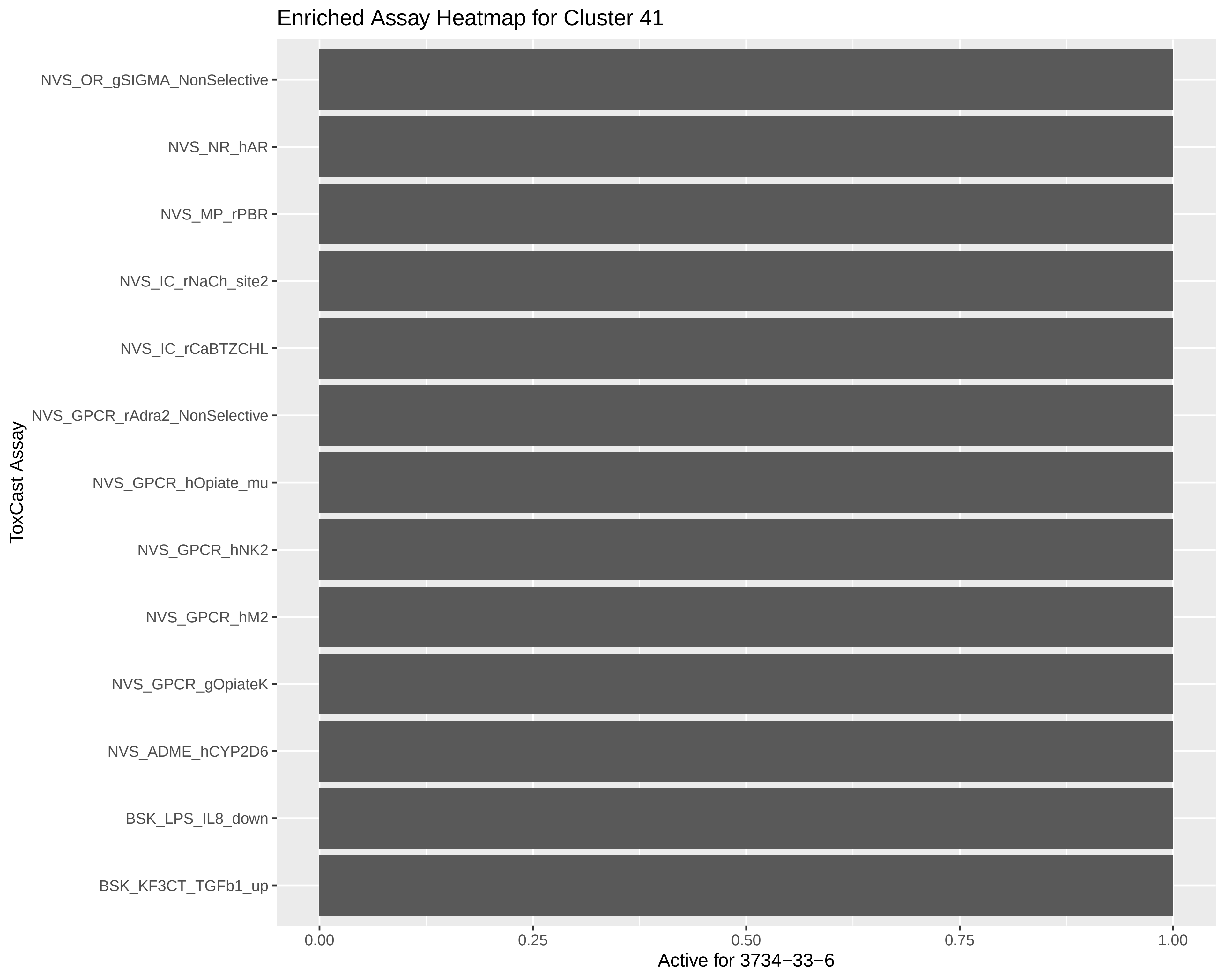

Enriched Assay Heatmap for Cluster 41
ToxCast Assay
NVS_OR_gSIGMA_NonSelective
NVS_NR_hAR
NVS_MP_rPBR
NVS_IC_rNaCh_site2
NVS_IC_rCaBTZCHL
NVS_GPCR_rAdra2_NonSelective
NVS_GPCR_hOpiate_mu
NVS_GPCR_hNK2
NVS_GPCR_hM2
NVS_GPCR_gOpiateK
NVS_ADME_hCYP2D6
BSK_LPS_IL8_down
BSK_KF3CT_TGFb1_up
0.00
0.25
0.50
0.75
1.00
Active for 3734−33−6

Enriched Assay Heatmap for Cluster 42

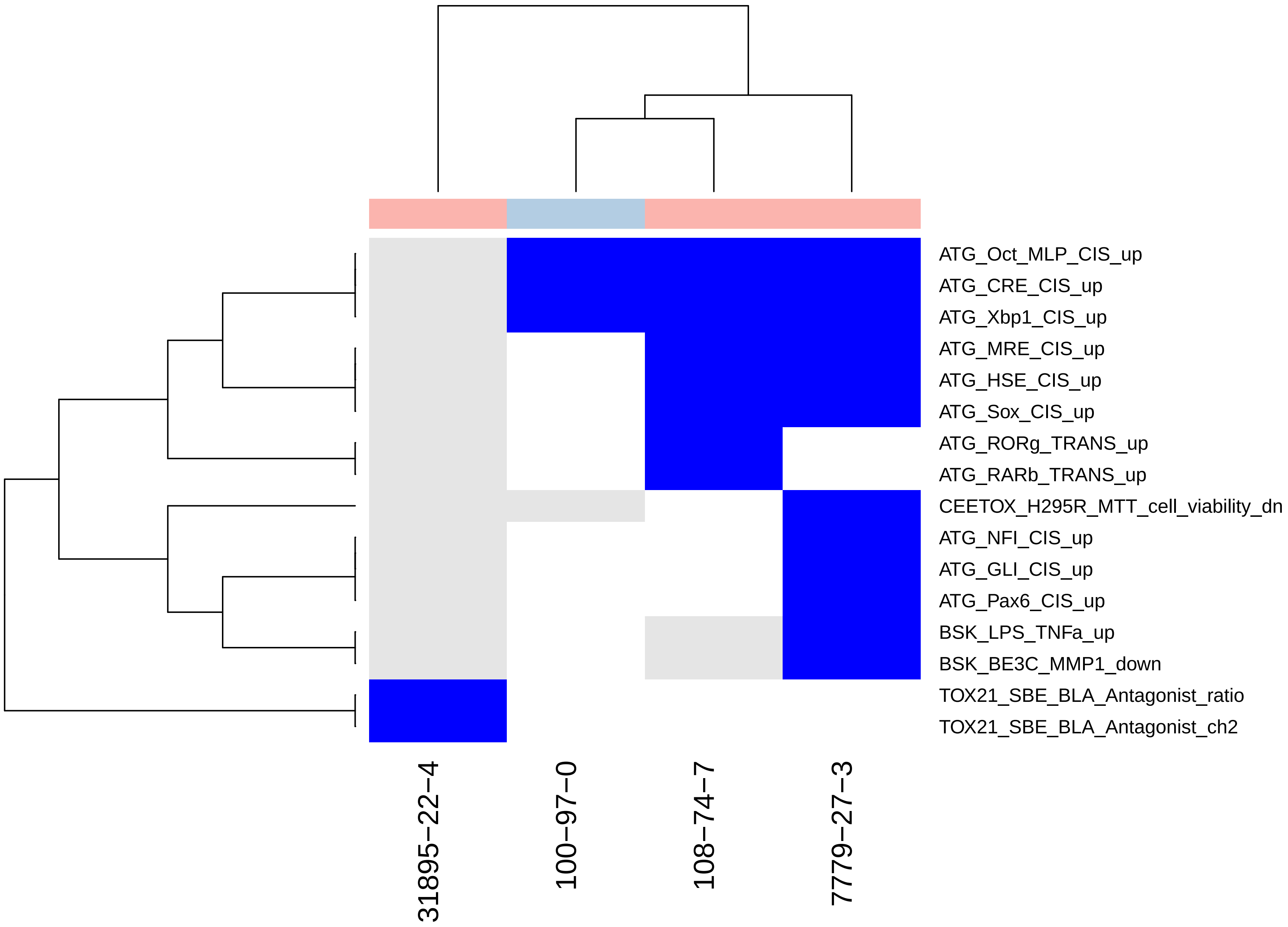

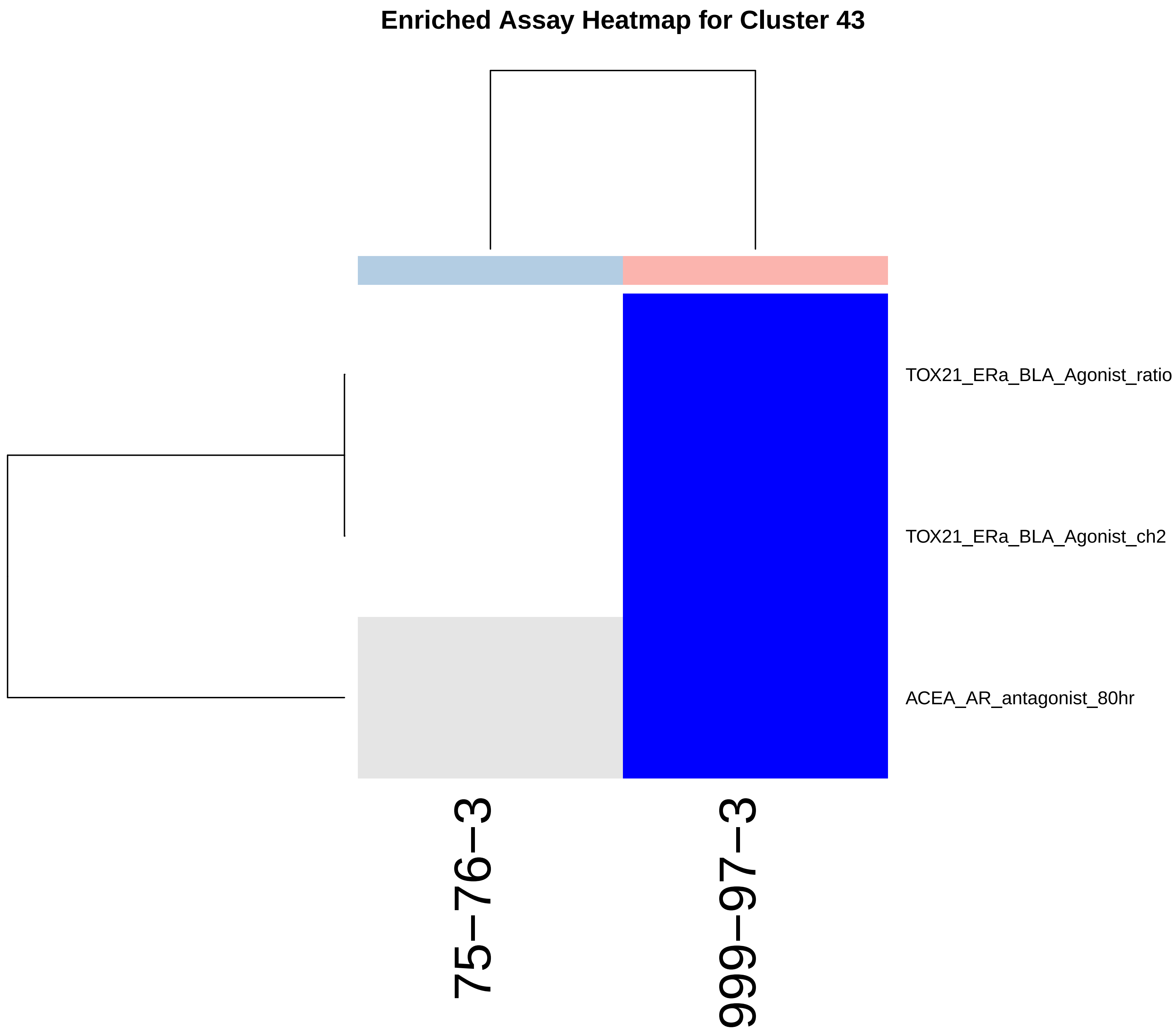
Enriched Assay Heatmap for Cluster 43
TOX21_ERa_BLA_Agonist_ratio
TOX21_ERa_BLA_Agonist_ch2
ACEA_AR_antagonist_80hr
75−76−3
999−97−3

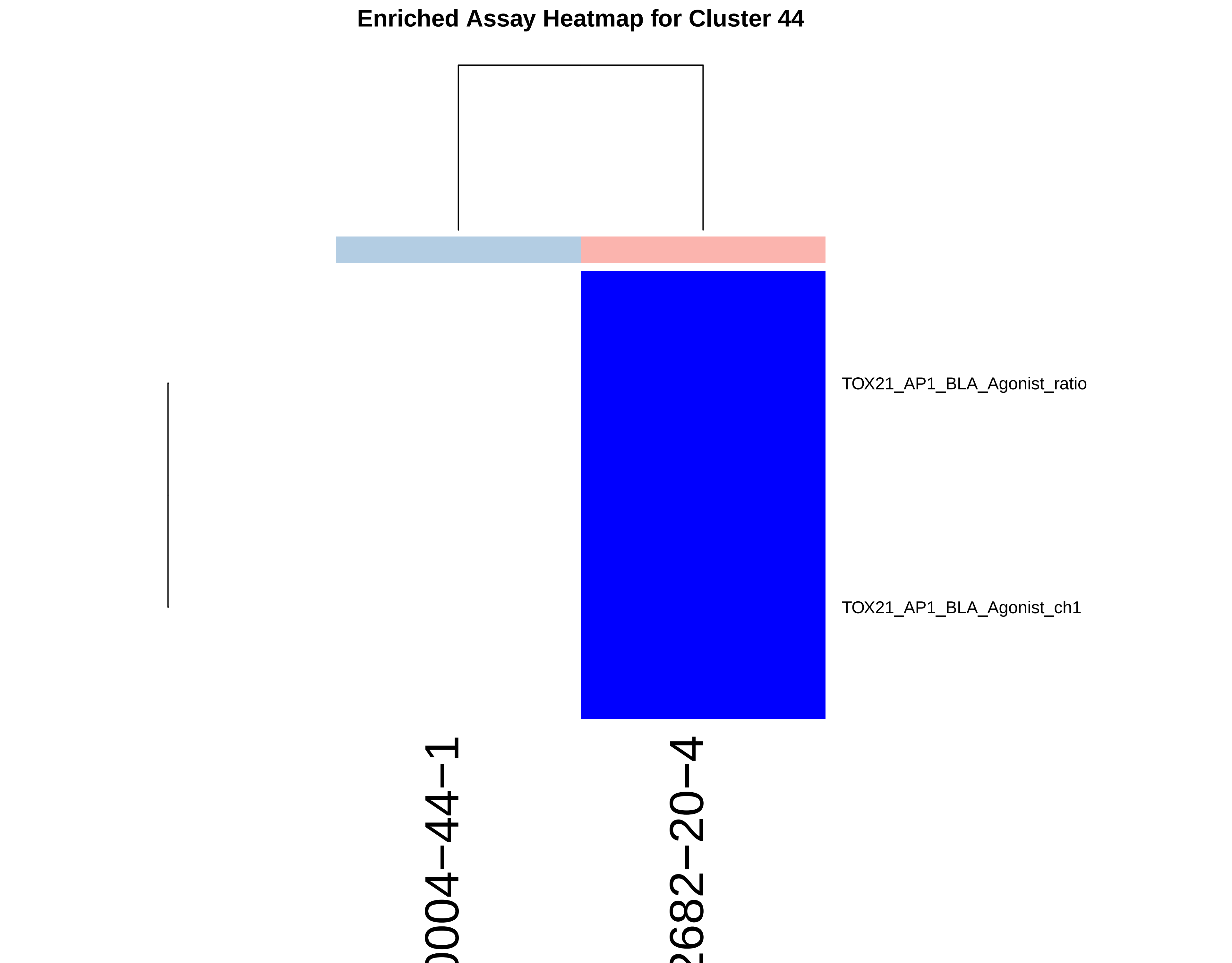

Enriched Assay Heatmap for Cluster 44
TOX21_AP1_BLA_Agonist_ratio
TOX21_AP1_BLA_Agonist_ch1
004−44−1
2682−20−4

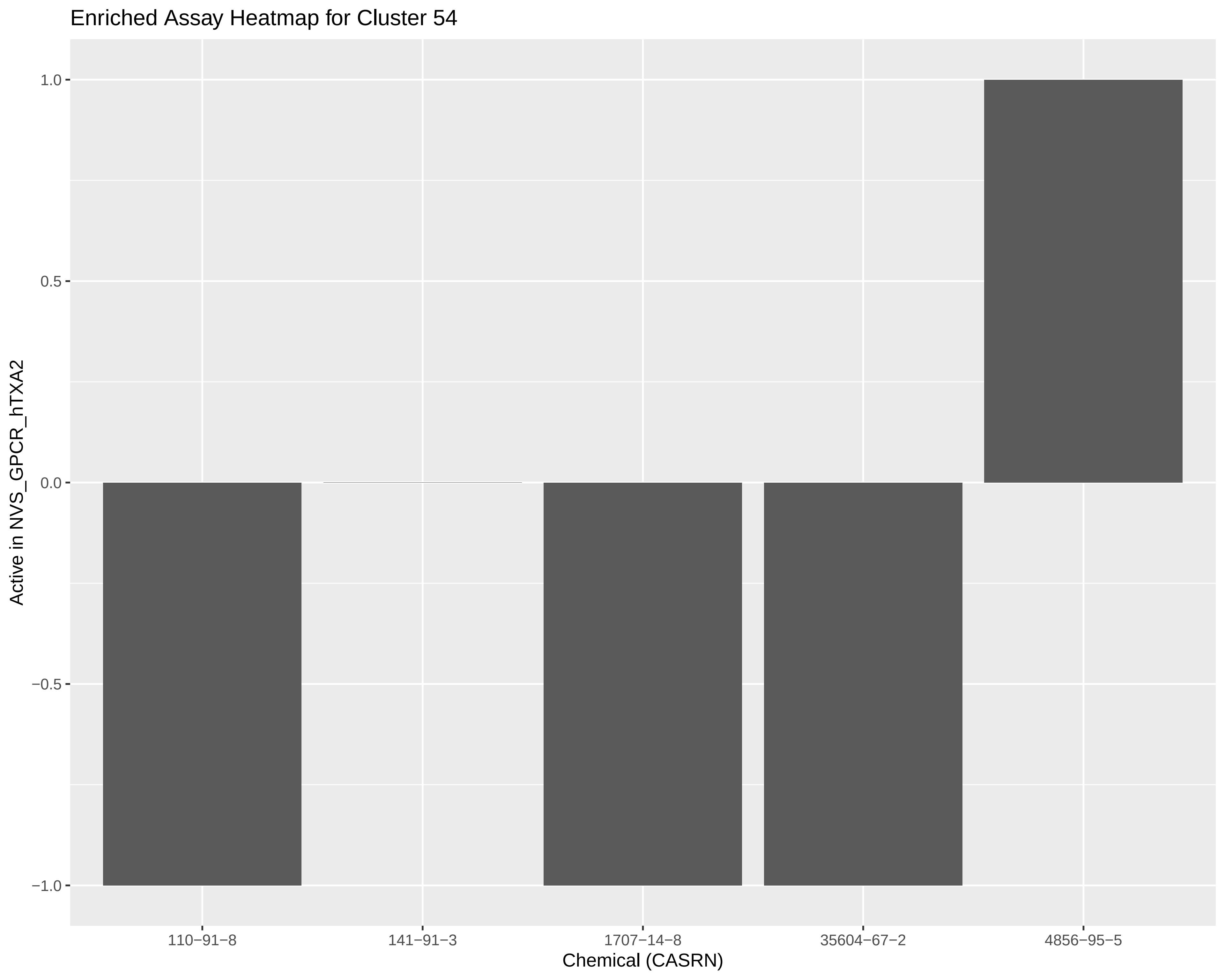

Enriched Assay Heatmap for Cluster 54
Active in NVS_GPCR_hTXA2
1.0
0.5
0.0
−0.5
−1.0
110−91−8
141−91−3
1707−14−8
35604−67−2
4856−95−5
Chemical (CASRN)

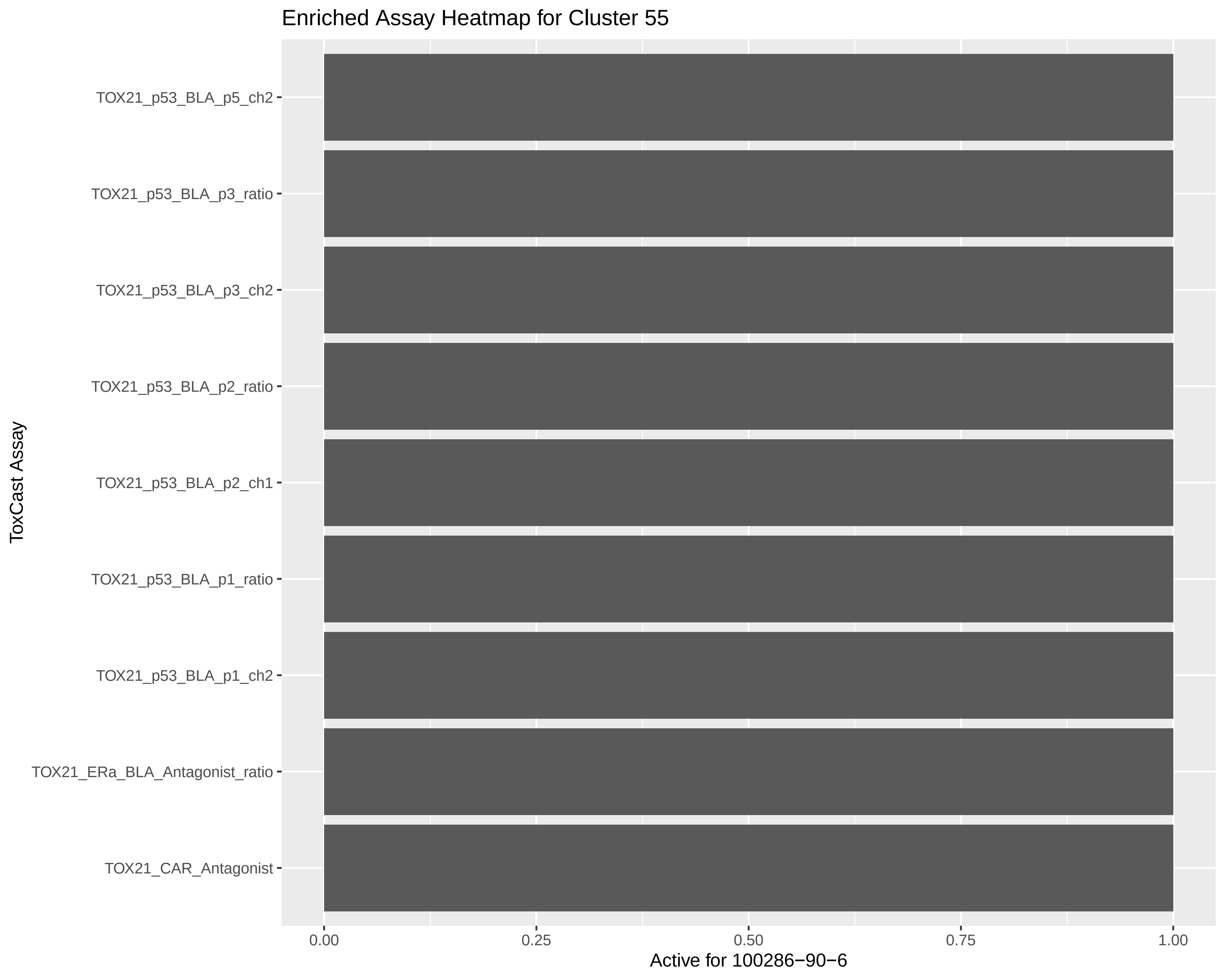
Enriched Assay Heatmap for Cluster 55
ToxCast Assay
TOX21_p53_BLA_p5_ch2
TOX21_p53_BLA_p3_ratio
TOX21_p53_BLA_p3_ch2
TOX21_p53_BLA_p2_ratio
TOX21_p53_BLA_p2_ch1
TOX21_p53_BLA_p1_ratio
TOX21_p53_BLA_p1_ch2
TOX21_ERa_BLA_Antagonist_ratio
TOX21_CAR_Antagonist
0.00
0.25
0.50
0.75
1.00
Active for 100286−90−6

Enriched Assay Heatmap for Cluster 56

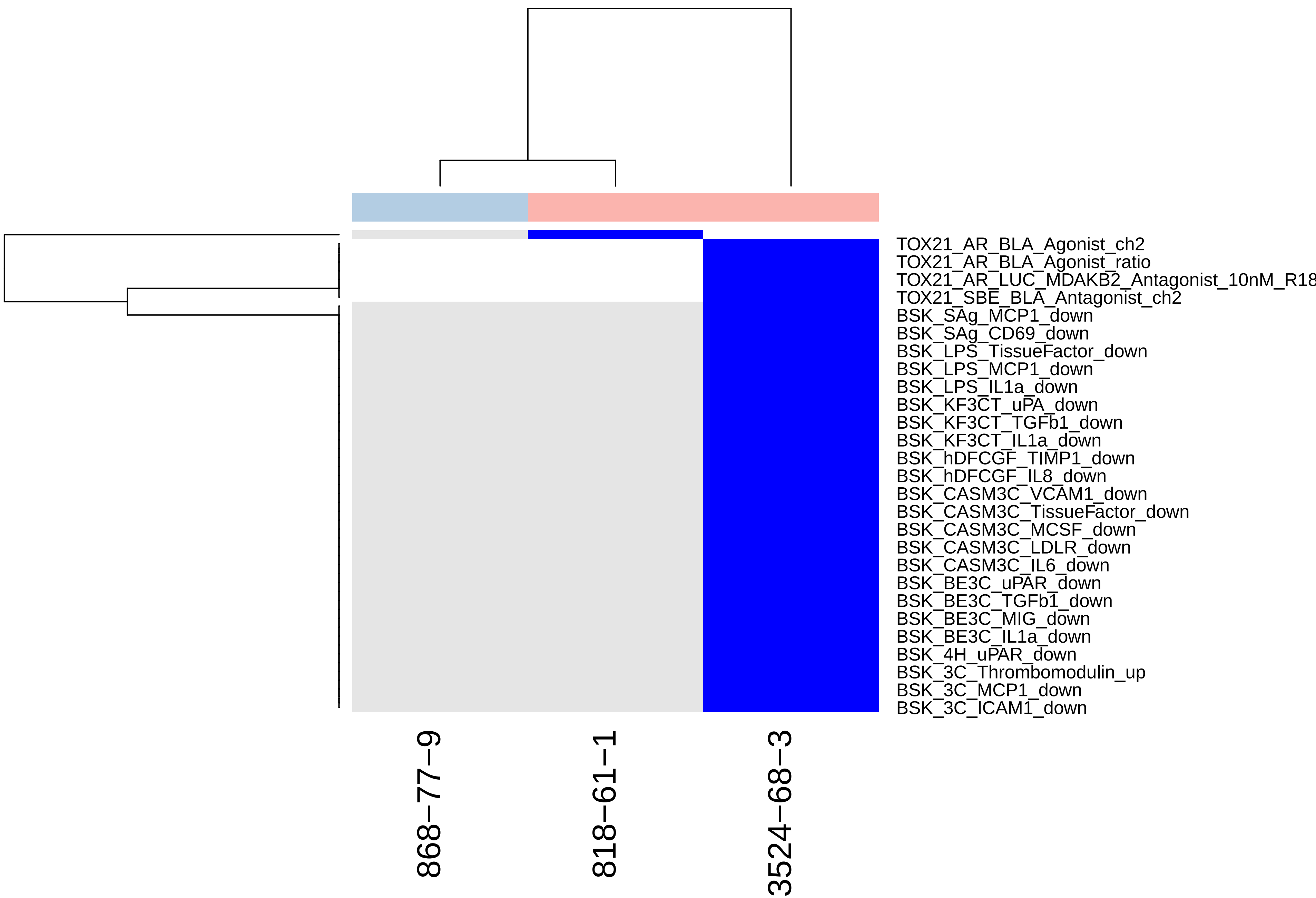

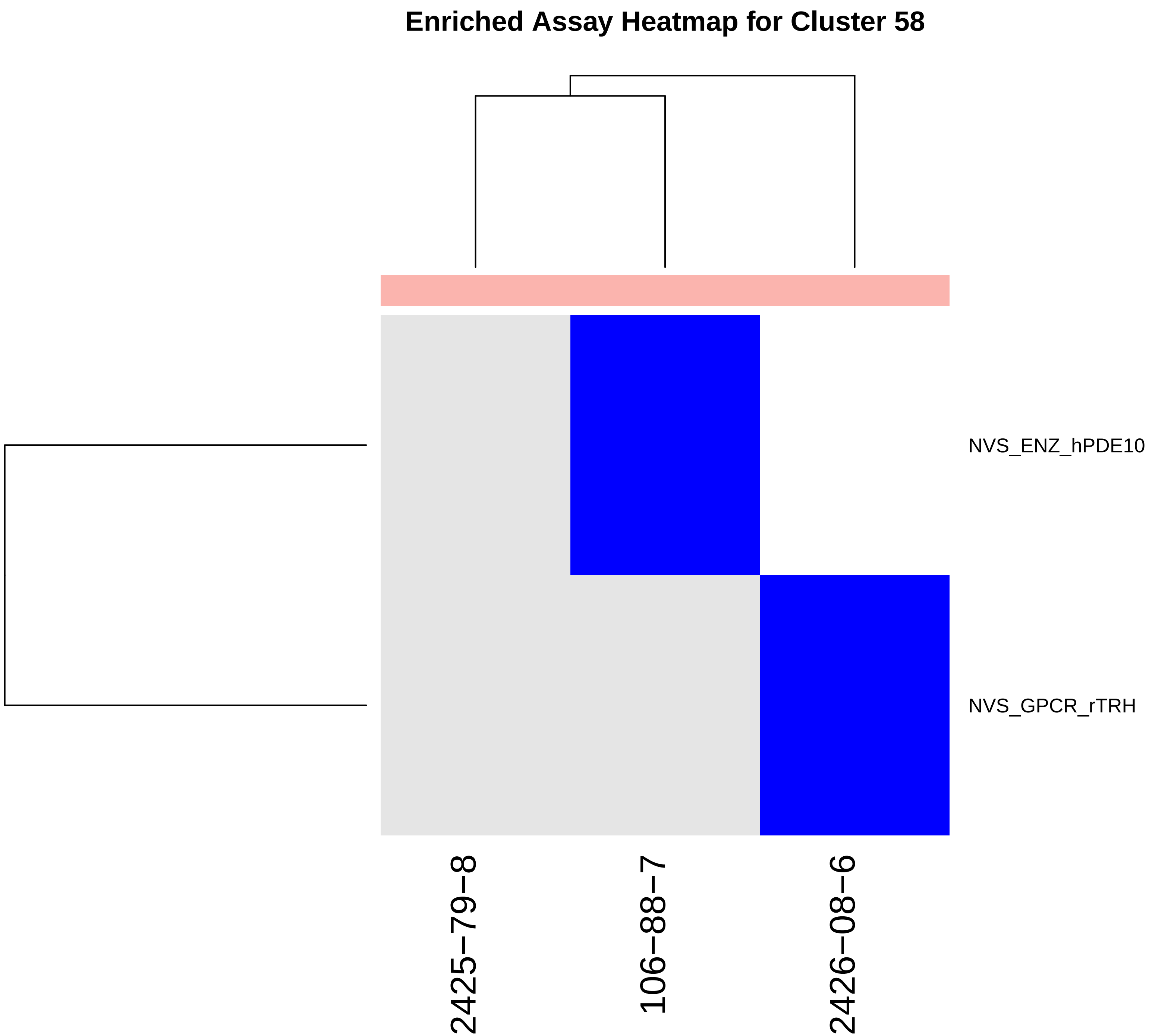

Enriched Assay Heatmap for Cluster 58
NVS_ENZ_hPDE10
NVS_GPCR_rTRH
2425−79−8
106−88−7
2426−08−6

Enriched Assay Heatmap for Cluster 60

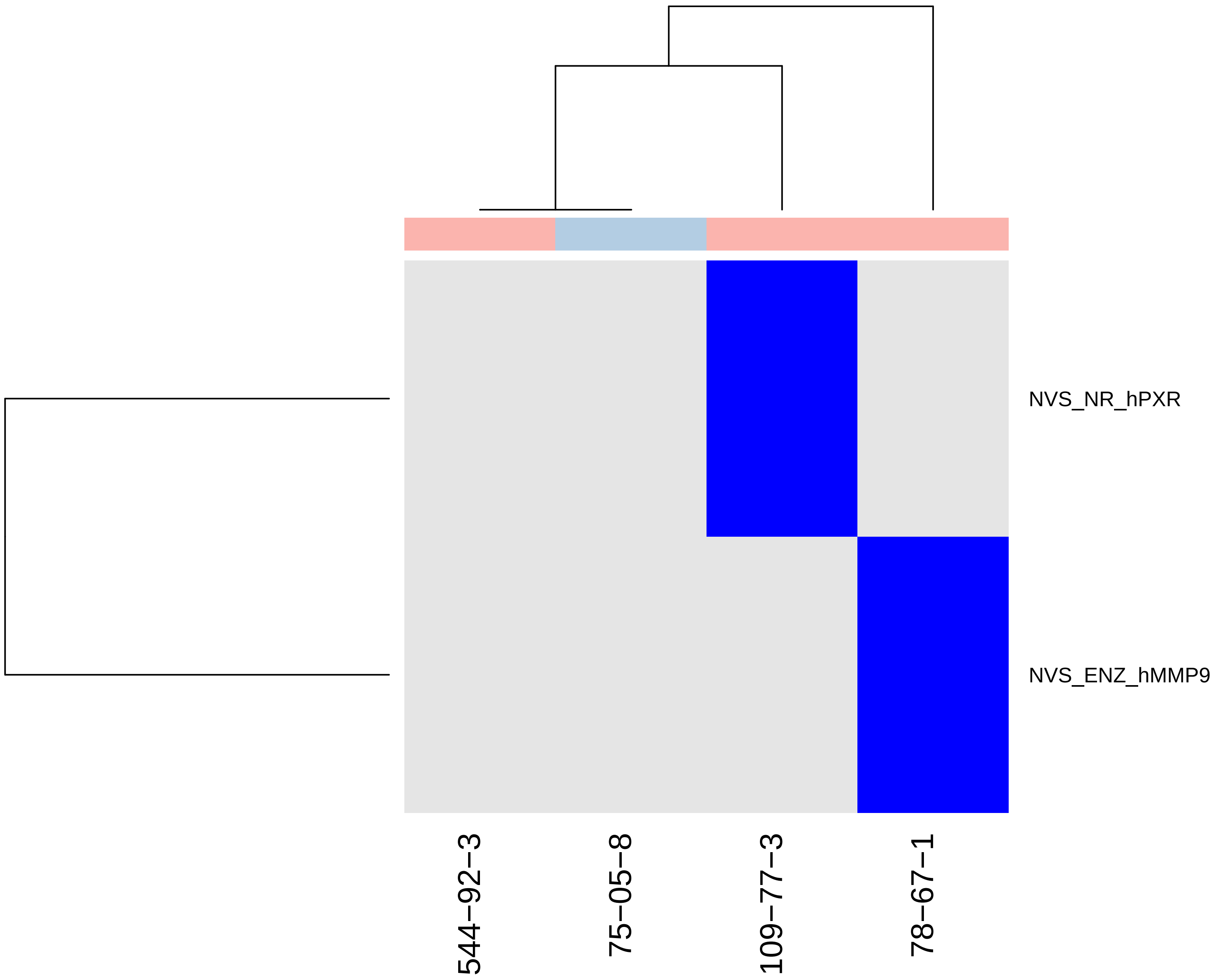

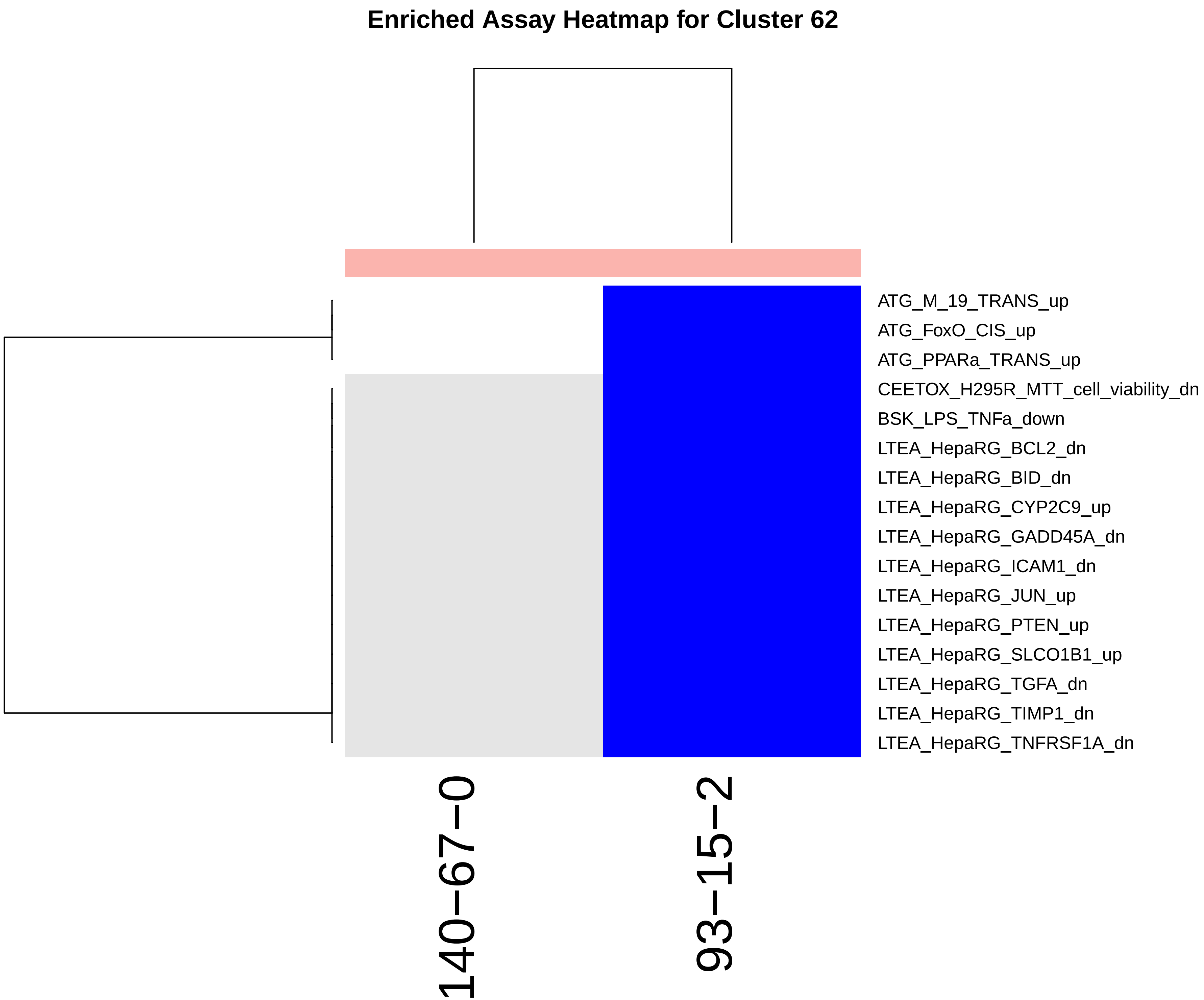
Enriched Assay Heatmap for Cluster 62
ATG_M_19_TRANS_up
ATG_FoxO_CIS_up
ATG_PPARa_TRANS_up
CEETOX_H295R_MTT_cell_viability_dn
BSK_LPS_TNFa_down
LTEA_HepaRG_BCL2_dn
LTEA_HepaRG_BID_dn
LTEA_HepaRG_CYP2C9_up
LTEA_HepaRG_GADD45A_dn
LTEA_HepaRG_ICAM1_dn
LTEA_HepaRG_JUN_up
LTEA_HepaRG_PTEN_up
LTEA_HepaRG_SLCO1B1_up
LTEA_HepaRG_TGFA_dn
LTEA_HepaRG_TIMP1_dn
LTEA_HepaRG_TNFRSF1A_dn
140−67−0
93−15−2

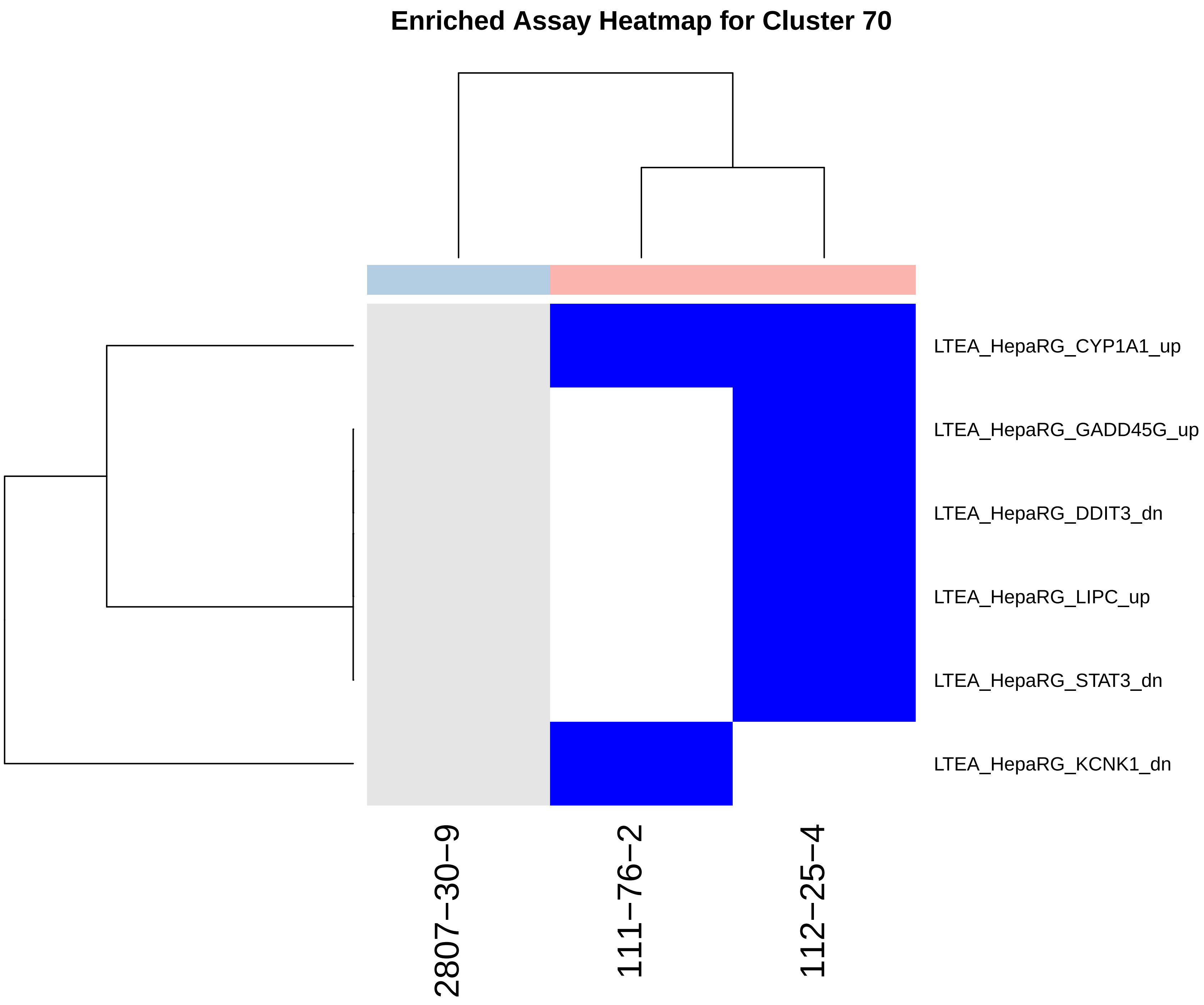
Enriched Assay Heatmap for Cluster 70
LTEA_HepaRG_CYP1A1_up
LTEA_HepaRG_GADD45G_up
LTEA_HepaRG_DDIT3_dn
LTEA_HepaRG_LIPC_up
LTEA_HepaRG_STAT3_dn
LTEA_HepaRG_KCNK1_dn
2807−30−9
111−76−2
112−25−4

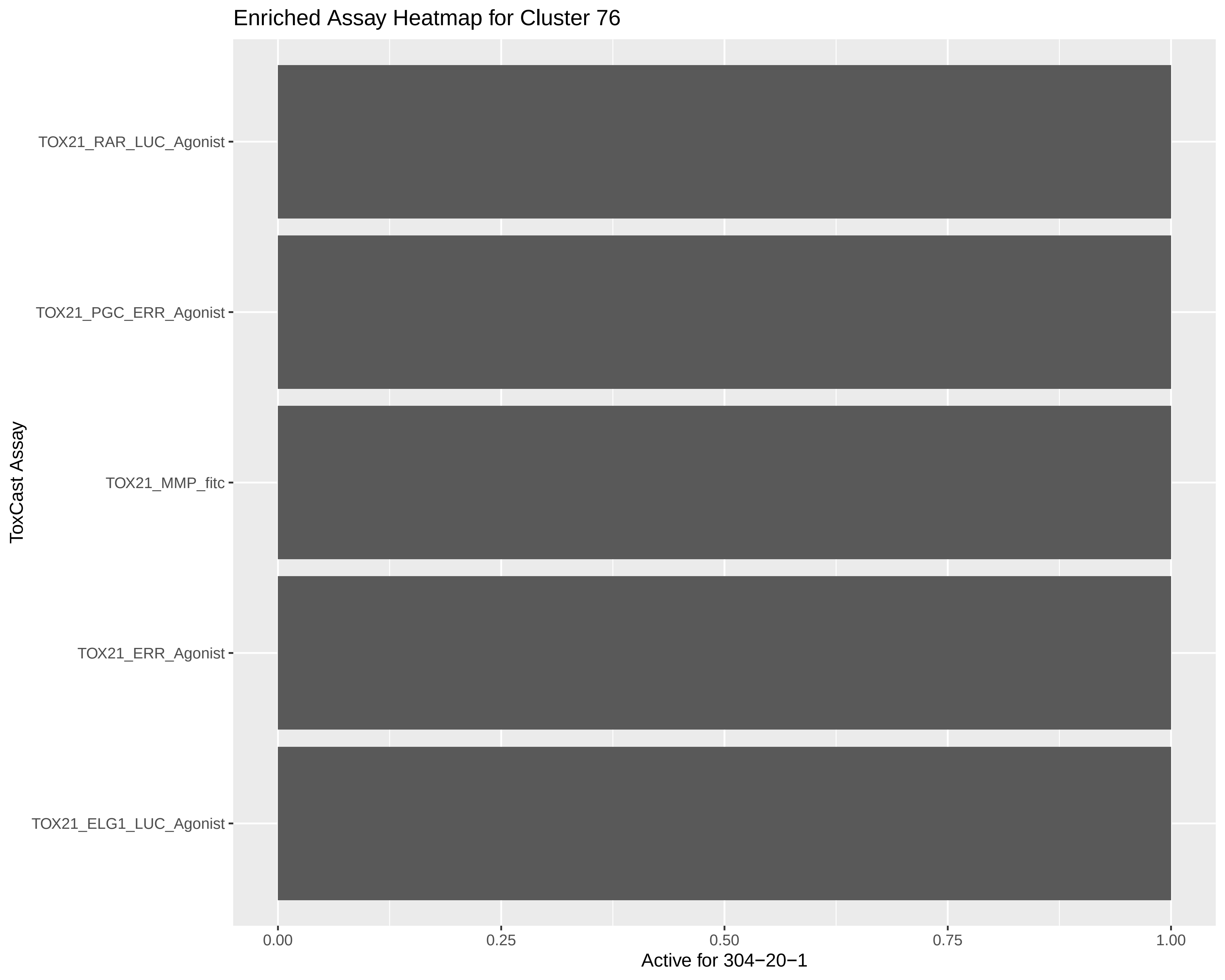
Enriched Assay Heatmap for Cluster 76
ToxCast Assay
TOX21_RAR_LUC_Agonist
TOX21_PGC_ERR_Agonist
TOX21_MMP_fitc
TOX21_ERR_Agonist
TOX21_ELG1_LUC_Agonist
0.00
0.25
0.50
0.75
1.00
Active for 304−20−1

Enriched Assay Heatmap for Cluster 78

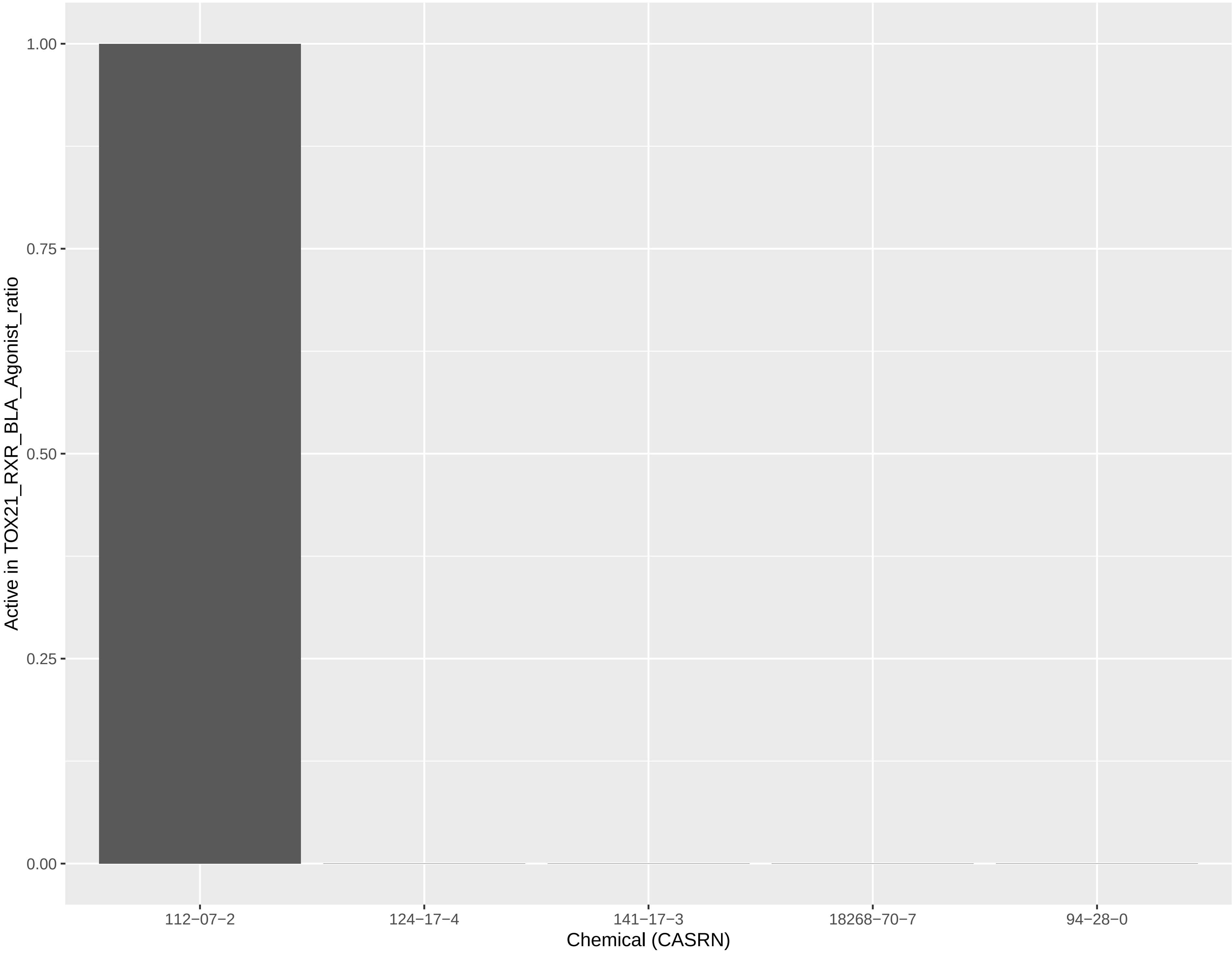

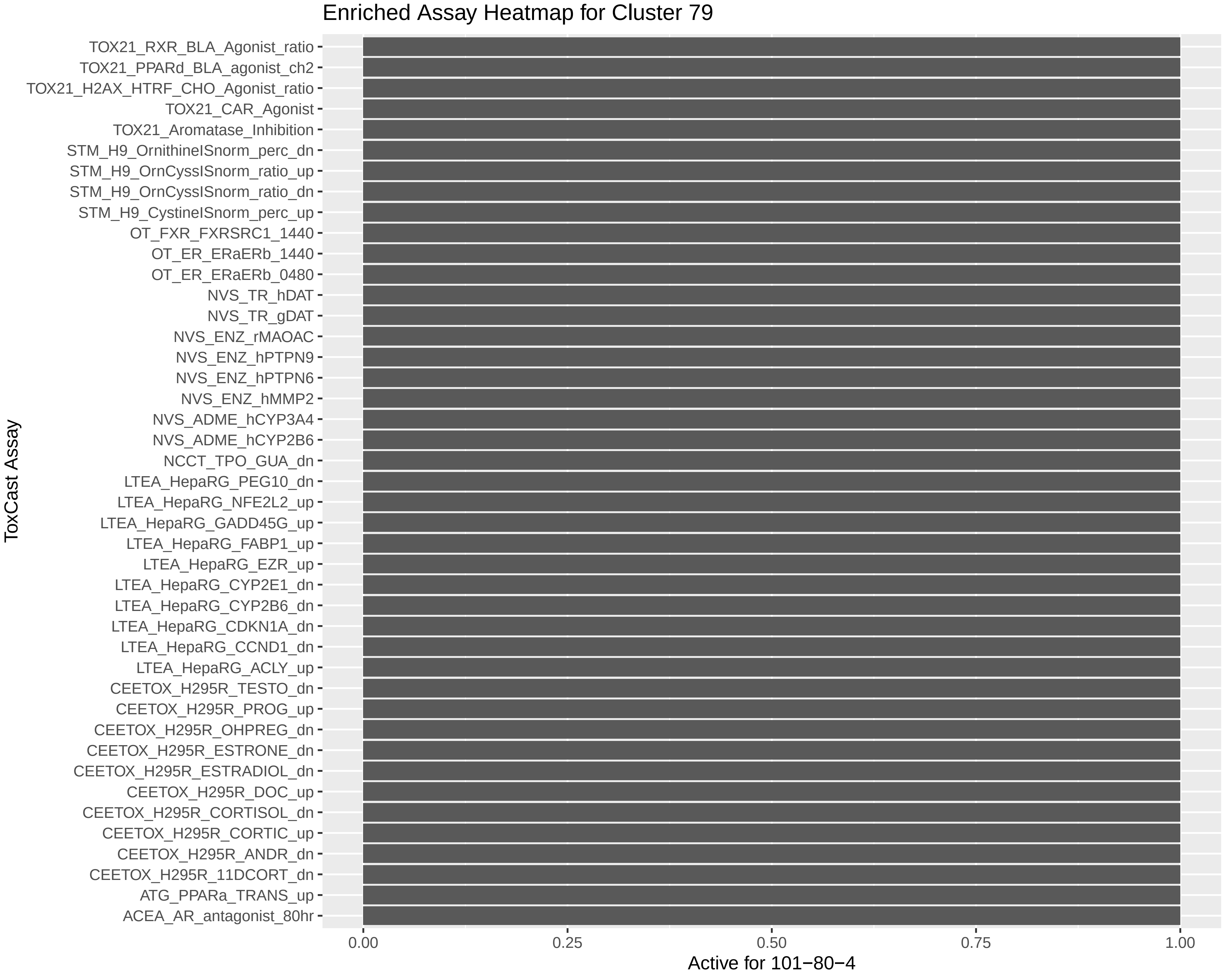
Enriched Assay Heatmap for Cluster 79
ToxCast Assay
TOX21_RXR_BLA_Agonist_ratio
TOX21_PPARd_BLA_agonist_ch2
TOX21_H2AX_HTRF_CHO_Agonist_ratio
TOX21_CAR_Agonist
TOX21_Aromatase_Inhibition
STM_H9_OrnithineISnorm_perc_dn
STM_H9_OrnCyssISnorm_ratio_up
STM_H9_OrnCyssISnorm_ratio_dn
STM_H9_CystineISnorm_perc_up
OT_FXR_FXRSRC1_1440
OT_ER_ERaERb_1440
OT_ER_ERaERb_0480
NVS_TR_hDAT
NVS_TR_gDAT
NVS_ENZ_rMAOAC
NVS_ENZ_hPTPN9
NVS_ENZ_hPTPN6
NVS_ENZ_hMMP2
NVS_ADME_hCYP3A4
NVS_ADME_hCYP2B6
NCCT_TPO_GUA_dn
LTEA_HepaRG_PEG10_dn
LTEA_HepaRG_NFE2L2_up
LTEA_HepaRG_GADD45G_up
LTEA_HepaRG_FABP1_up
LTEA_HepaRG_EZR_up
LTEA_HepaRG_CYP2E1_dn
LTEA_HepaRG_CYP2B6_dn
LTEA_HepaRG_CDKN1A_dn
LTEA_HepaRG_CCND1_dn
LTEA_HepaRG_ACLY_up
CEETOX_H295R_TESTO_dn
CEETOX_H295R_PROG_up
CEETOX_H295R_OHPREG_dn
CEETOX_H295R_ESTRONE_dn
CEETOX_H295R_ESTRADIOL_dn
CEETOX_H295R_DOC_up
CEETOX_H295R_CORTISOL_dn
CEETOX_H295R_CORTIC_up
CEETOX_H295R_ANDR_dn
CEETOX_H295R_11DCORT_dn
ATG_PPARa_TRANS_up
ACEA_AR_antagonist_80hr
0.00
0.25
0.50
0.75
1.00
Active for 101−80−4

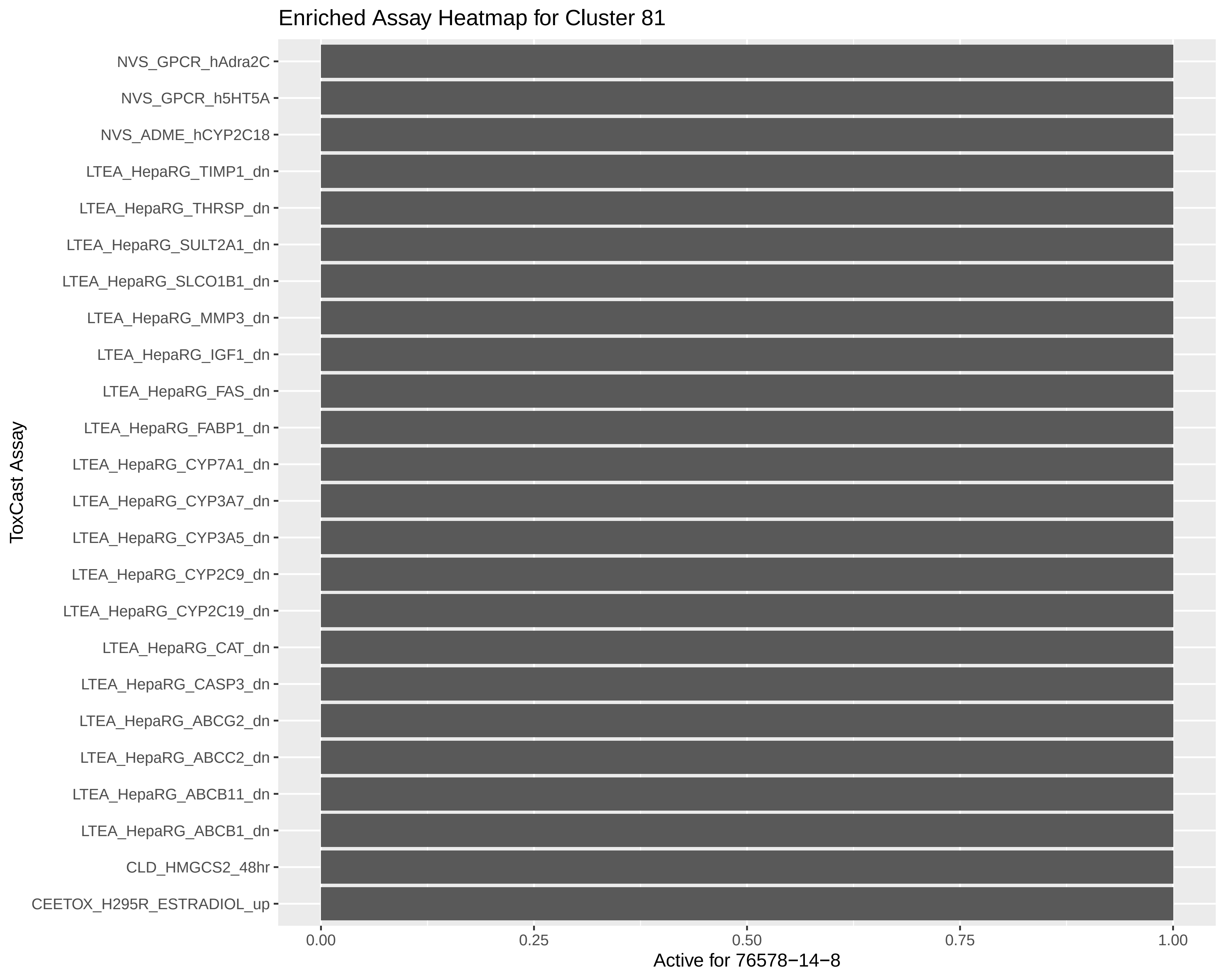

Enriched Assay Heatmap for Cluster 81
ToxCast Assay
NVS_GPCR_hAdra2C
NVS_GPCR_h5HT5A
NVS_ADME_hCYP2C18
LTEA_HepaRG_TIMP1_dn
LTEA_HepaRG_THRSP_dn
LTEA_HepaRG_SULT2A1_dn
LTEA_HepaRG_SLCO1B1_dn
LTEA_HepaRG_MMP3_dn
LTEA_HepaRG_IGF1_dn
LTEA_HepaRG_FAS_dn
LTEA_HepaRG_FABP1_dn
LTEA_HepaRG_CYP7A1_dn
LTEA_HepaRG_CYP3A7_dn
LTEA_HepaRG_CYP3A5_dn
LTEA_HepaRG_CYP2C9_dn
LTEA_HepaRG_CYP2C19_dn
LTEA_HepaRG_CAT_dn
LTEA_HepaRG_CASP3_dn
LTEA_HepaRG_ABCG2_dn
LTEA_HepaRG_ABCC2_dn
LTEA_HepaRG_ABCB11_dn
LTEA_HepaRG_ABCB1_dn
CLD_HMGCS2_48hr
CEETOX_H295R_ESTRADIOL_up
0.00
0.25
0.50
0.75
1.00
Active for 76578−14−8

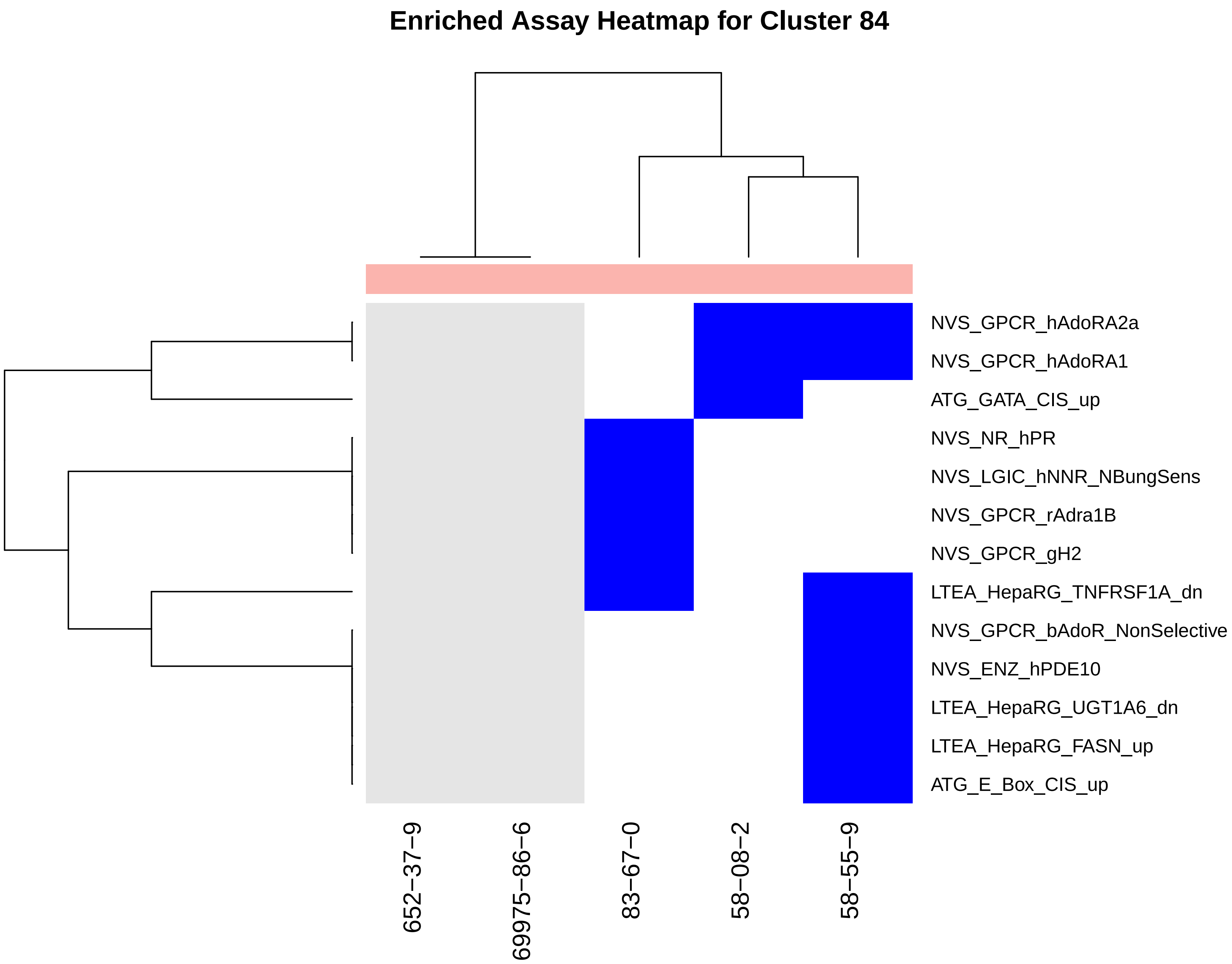
Enriched Assay Heatmap for Cluster 84
NVS_GPCR_hAdoRA2a
NVS_GPCR_hAdoRA1
ATG_GATA_CIS_up
NVS_NR_hPR
NVS_LGIC_hNNR_NBungSens
NVS_GPCR_rAdra1B
NVS_GPCR_gH2
LTEA_HepaRG_TNFRSF1A_dn
NVS_GPCR_bAdoR_NonSelective
NVS_ENZ_hPDE10
LTEA_HepaRG_UGT1A6_dn
LTEA_HepaRG_FASN_up
ATG_E_Box_CIS_up
652–37–9
69975–86–6
83–67–0
58–08–2
58–55–9

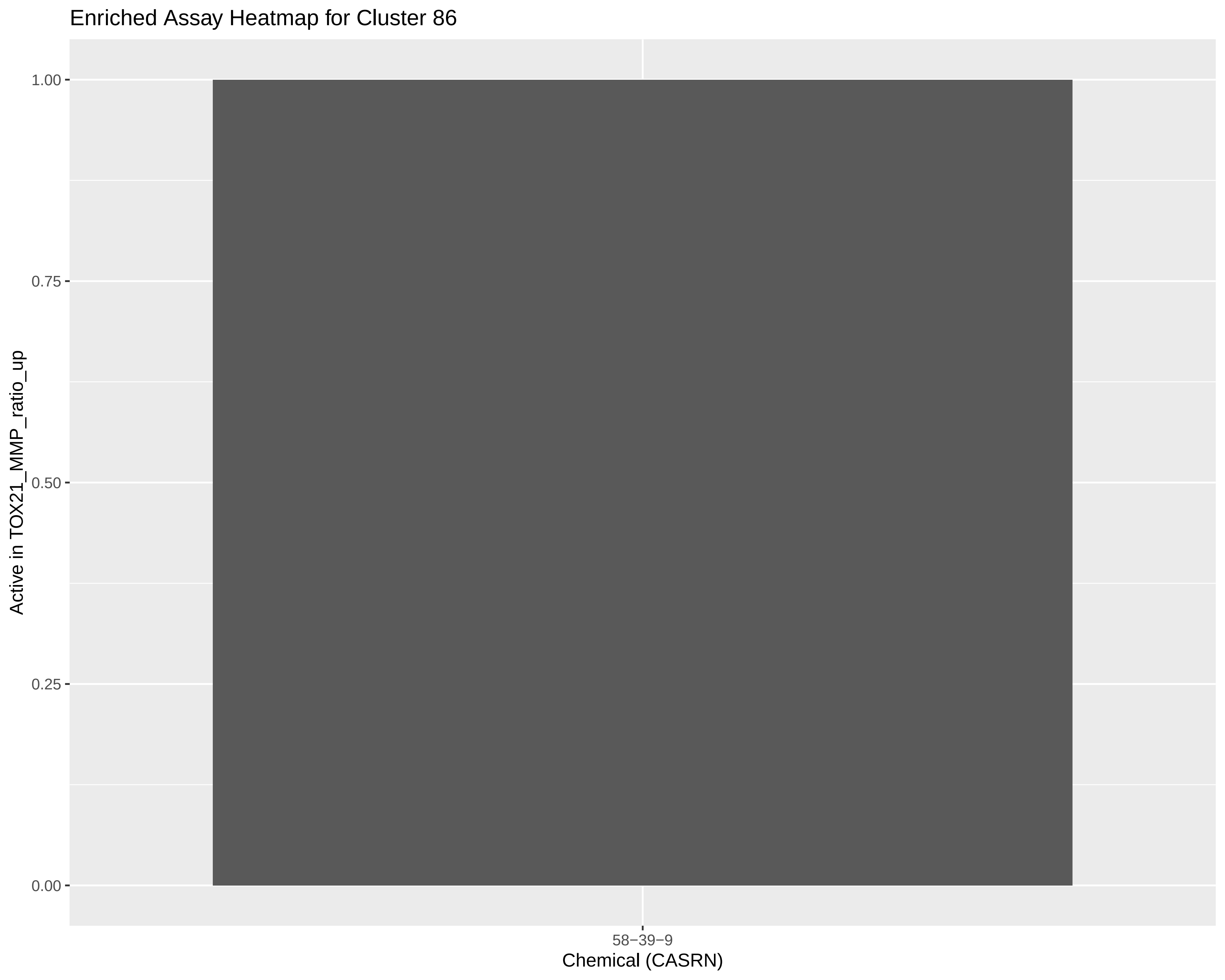
Enriched Assay Heatmap for Cluster 86
Active in TOX21_MMP_ratio_up
1.00
0.75
0.50
0.25
0.00
58−39−9
Chemical (CASRN)

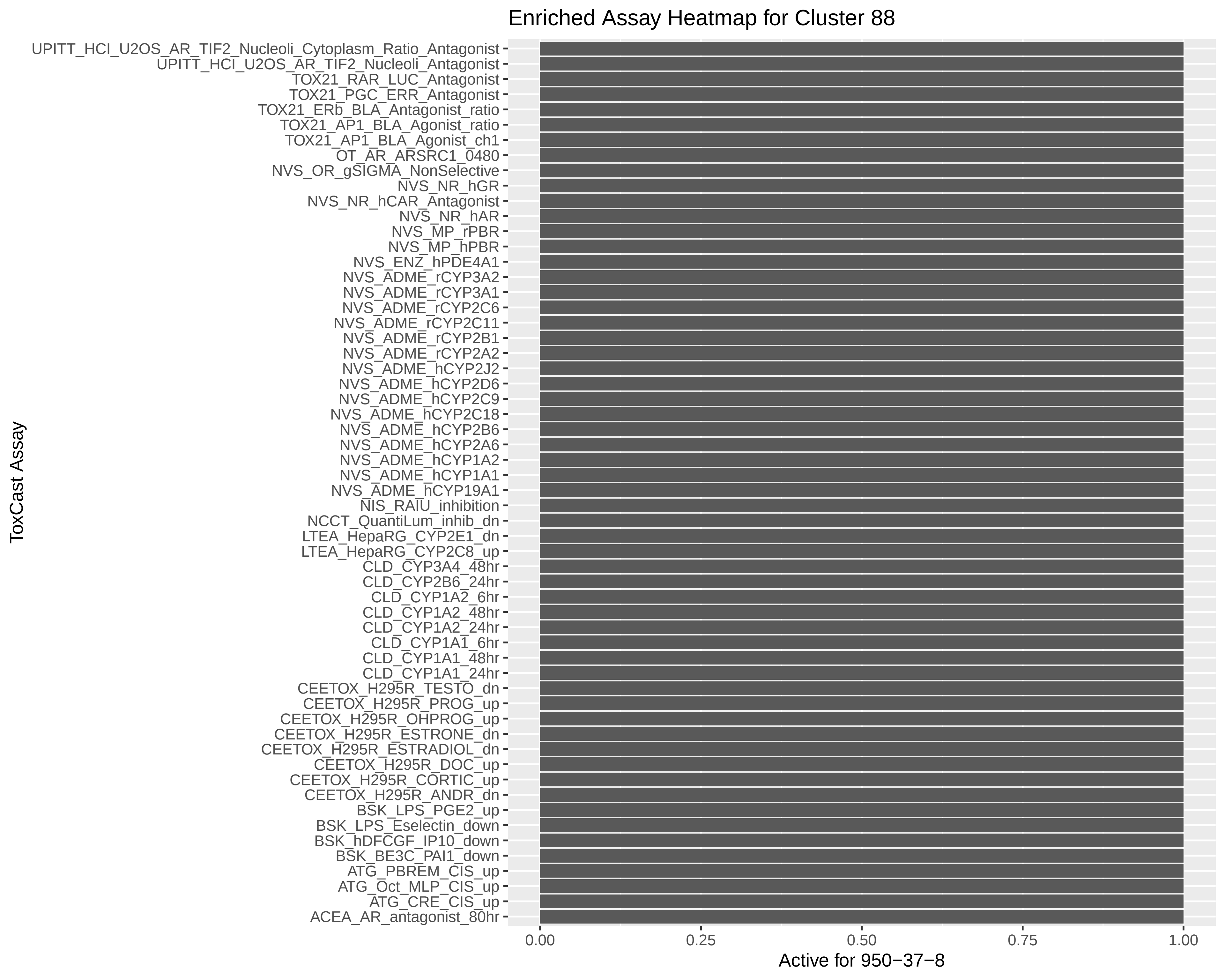
Enriched Assay Heatmap for Cluster 88
UPITT_HCI_U2OS_AR_TIF2_Nucleoli_Cytoplasm_Ratio_Antagonist
UPITT_HCI_U2OS_AR_TIF2_Nucleoli_Antagonist
TOX21_RAR_LUC_Antagonist
TOX21_PGC_ERR_Antagonist
TOX21_ERb_BLA_Antagonist_ratio
TOX21_AP1_BLA_Agonist_ratio
TOX21_AP1_BLA_Agonist_ch1
OT_AR_ARSRC1_0480
NVS_OR_gSIGMA_NonSelective
NVS_NR_hGR
NVS_NR_hCAR_Antagonist
NVS_NR_hAR
NVS_MP_rPBR
NVS_MP_hPBR
NVS_ENZ_hPDE4A1
NVS_ADME_rCYP3A2
NVS_ADME_rCYP3A1
NVS_ADME_rCYP2C6
NVS_ADME_rCYP2C11
NVS_ADME_rCYP2B1
NVS_ADME_rCYP2A2
NVS_ADME_hCYP2J2
NVS_ADME_hCYP2D6
NVS_ADME_hCYP2C9
NVS_ADME_hCYP2C18
NVS_ADME_hCYP2B6
NVS_ADME_hCYP2A6
NVS_ADME_hCYP1A2
NVS_ADME_hCYP1A1
NVS_ADME_hCYP19A1
NIS_RAIU_inhibition
NCCT_QuantiLum_inhib_dn
LTEA_HepaRG_CYP2E1_dn
LTEA_HepaRG_CYP2C8_up
CLD_CYP3A4_48hr
CLD_CYP2B6_24hr
CLD_CYP1A2_6hr
CLD_CYP1A2_48hr
CLD_CYP1A2_24hr
CLD_CYP1A1_6hr
CLD_CYP1A1_48hr
CLD_CYP1A1_24hr
CEETOX_H295R_TESTO_dn
CEETOX_H295R_PROG_up
CEETOX_H295R_OHPROG_up
CEETOX_H295R_ESTRONE_dn
CEETOX_H295R_ESTRADIOL_dn
CEETOX_H295R_DOC_up
CEETOX_H295R_CORTIC_up
CEETOX_H295R_ANDR_dn
BSK_LPS_PGE2_up
BSK_LPS_Eselectin_down
BSK_hDFCGF_IP10_down
BSK_BE3C_PAI1_down
ATG_PBREM_CIS_up
ATG_Oct_MLP_CIS_up
ATG_CRE_CIS_up
ACEA_AR_antagonist_80hr
ToxCast Assay
0.00
0.25
0.50
0.75
1.00
Active for 950−37−8

Enriched Assay Heatmap for Cluster 90

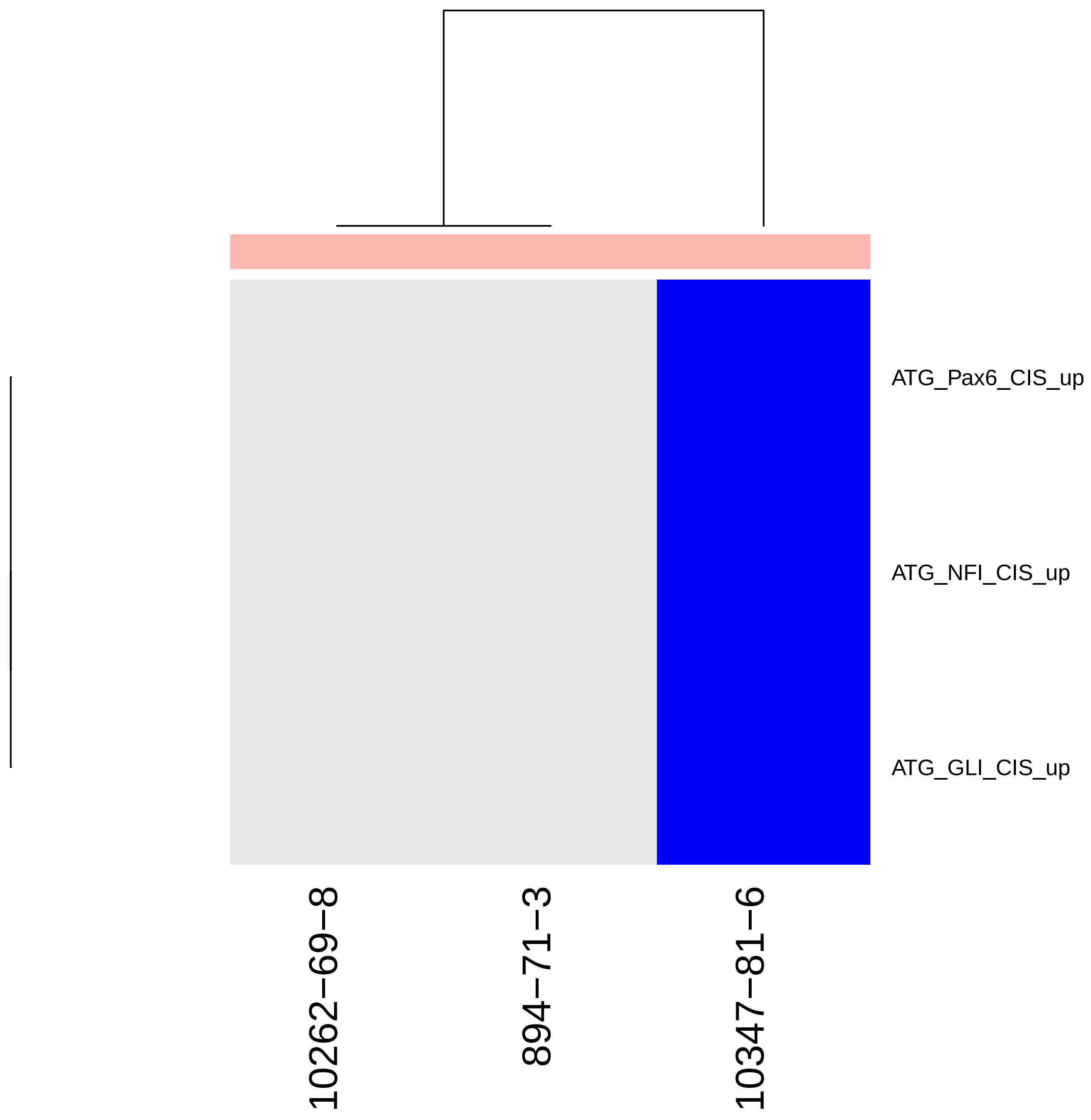

Enriched Assay Heatmap for Cluster 93

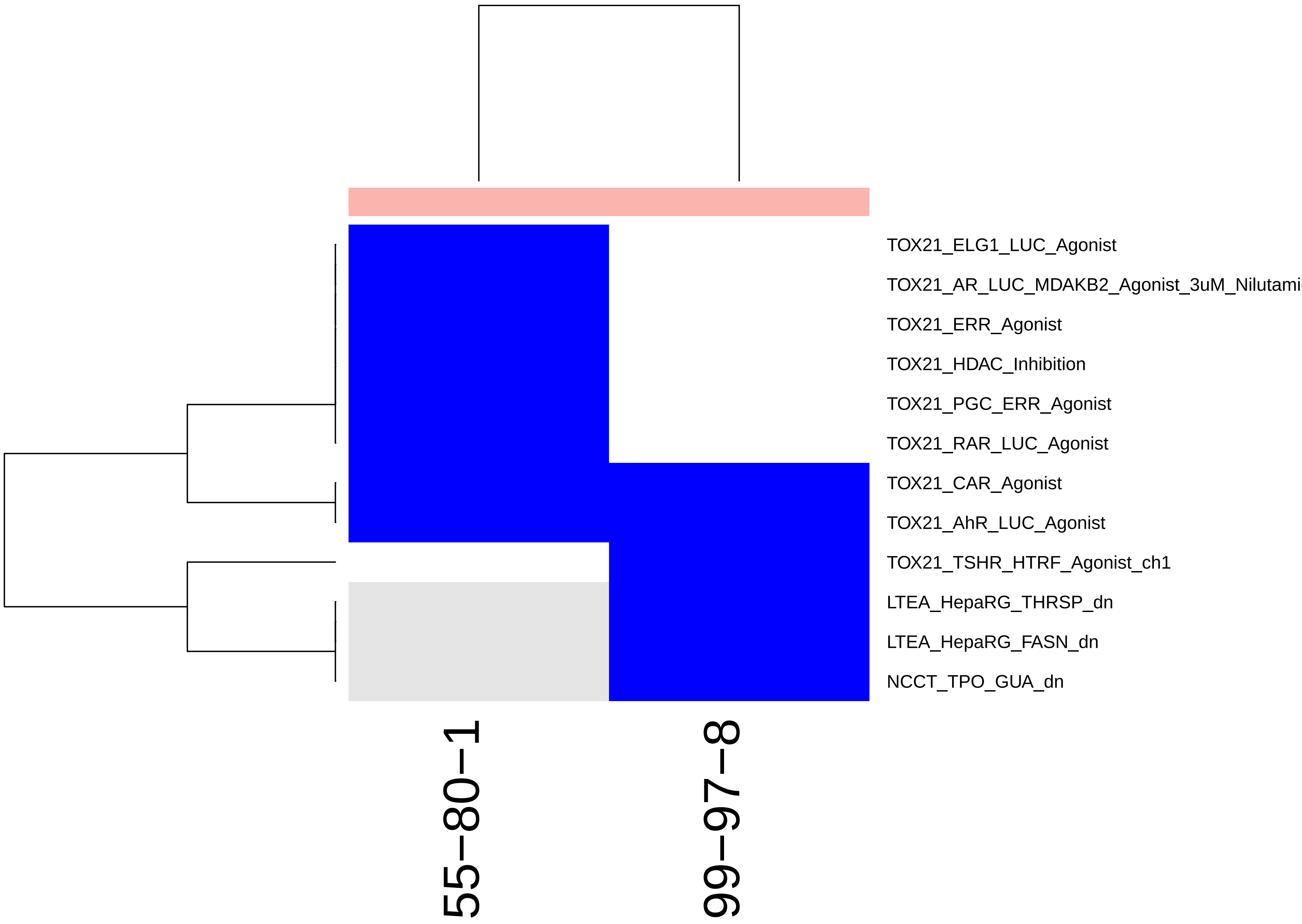

Enriched Assay Heatmap for Cluster 98

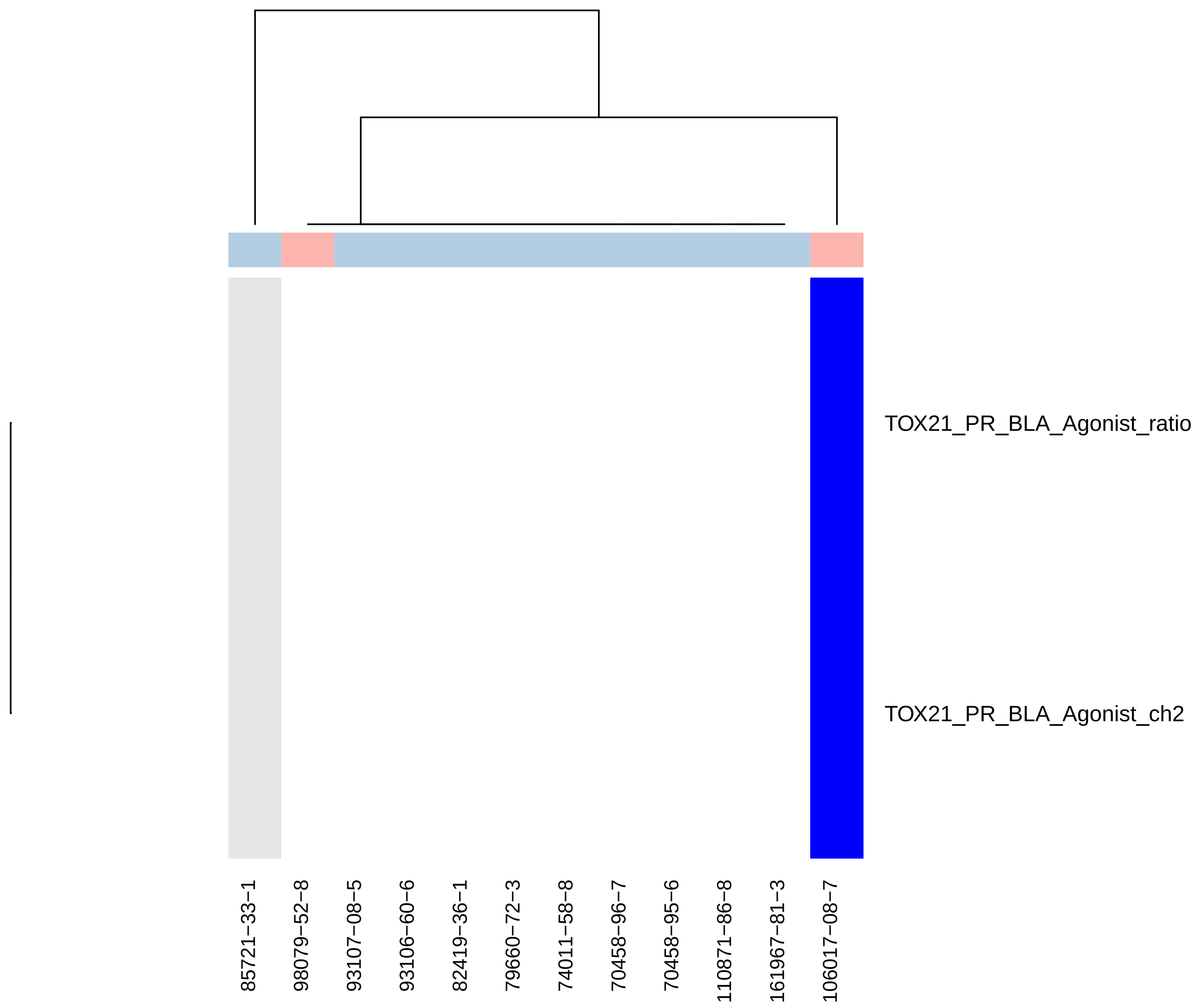

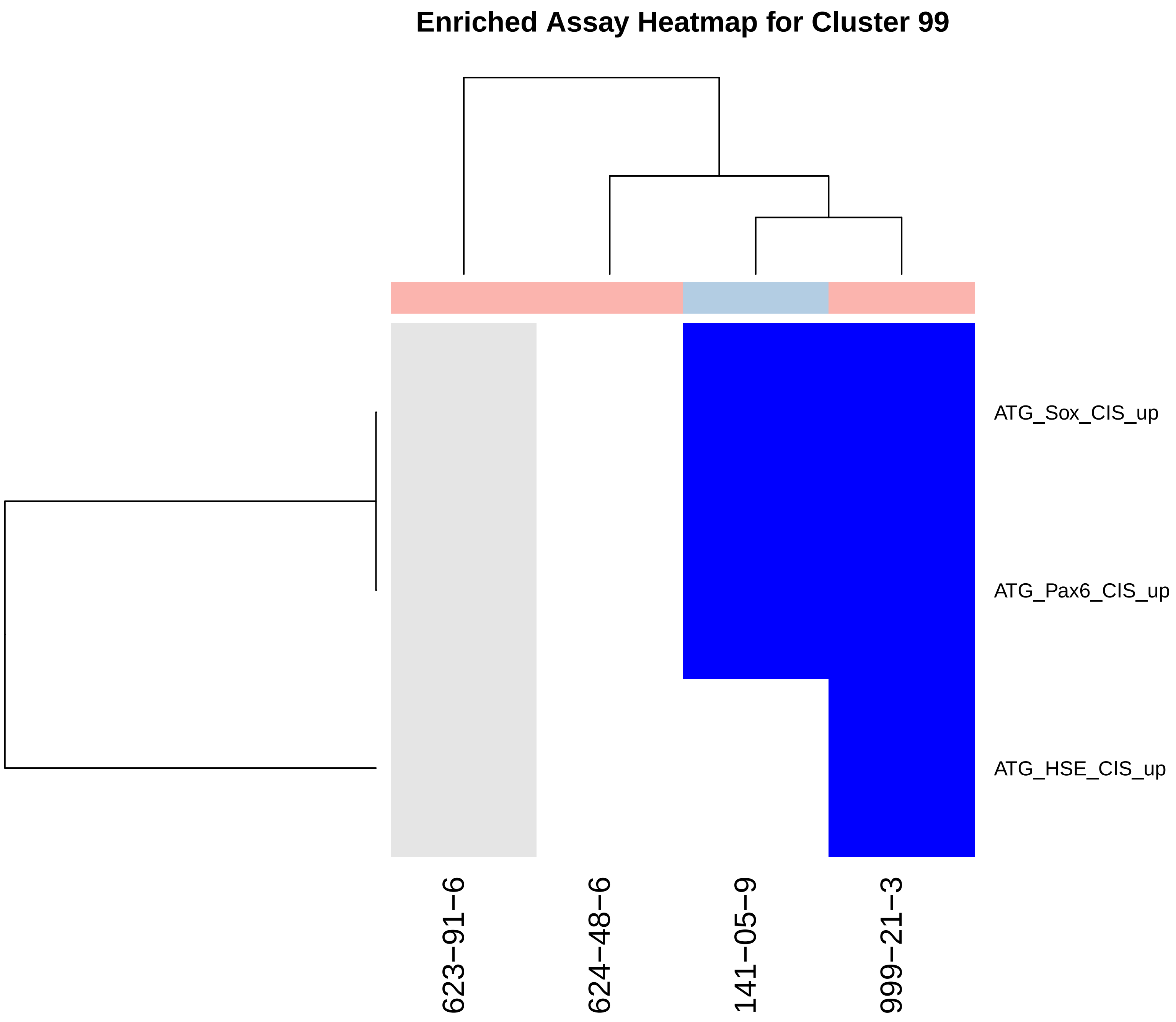
Enriched Assay Heatmap for Cluster 99
ATG_Sox_CIS_up
ATG_Pax6_CIS_up
ATG_HSE_CIS_up
623−91−6
624−48−6
141−05−9
999−21−3

Enriched Assay Heatmap for Cluster 100

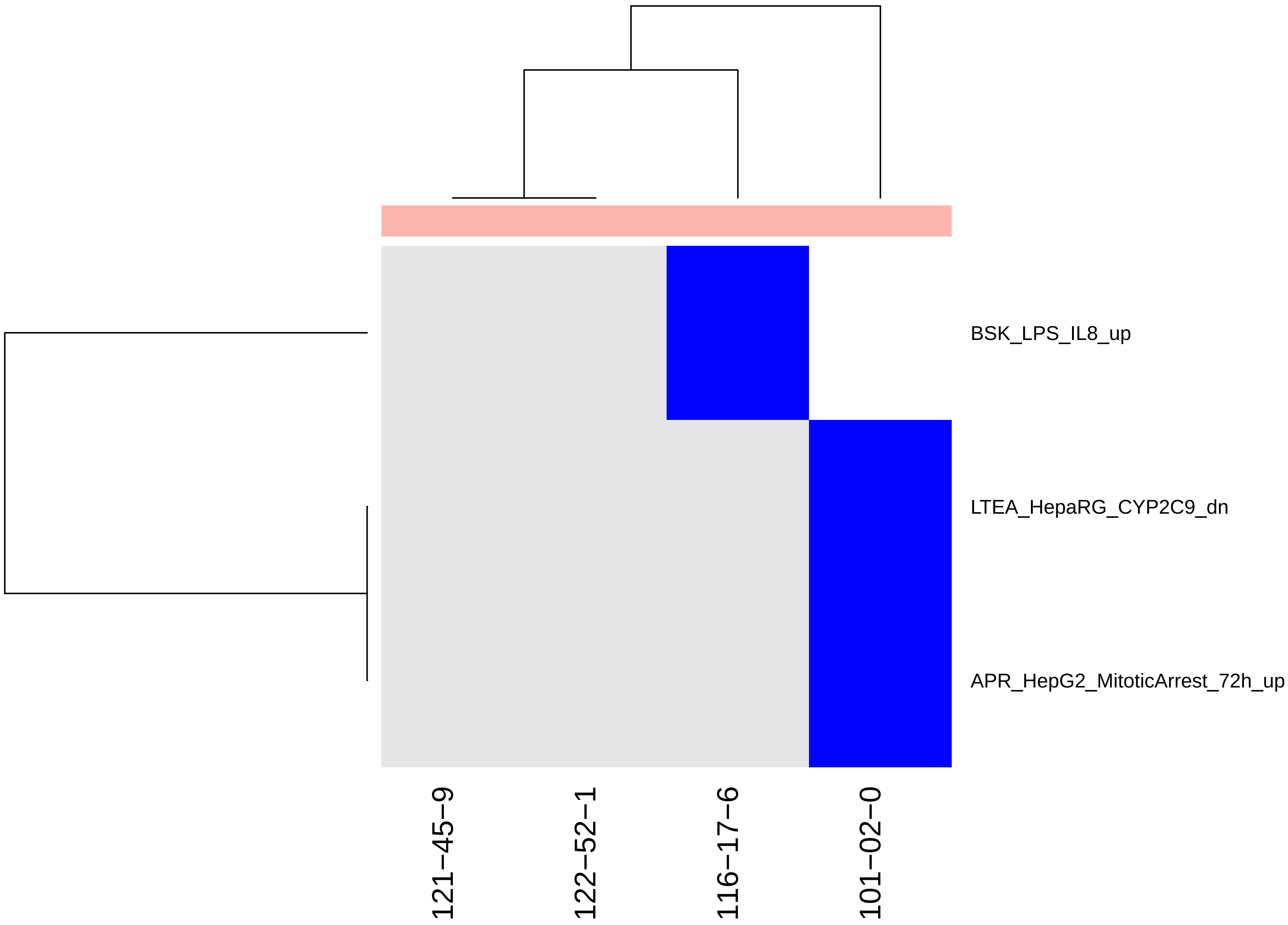

Enriched Assay Heatmap for Cluster 102

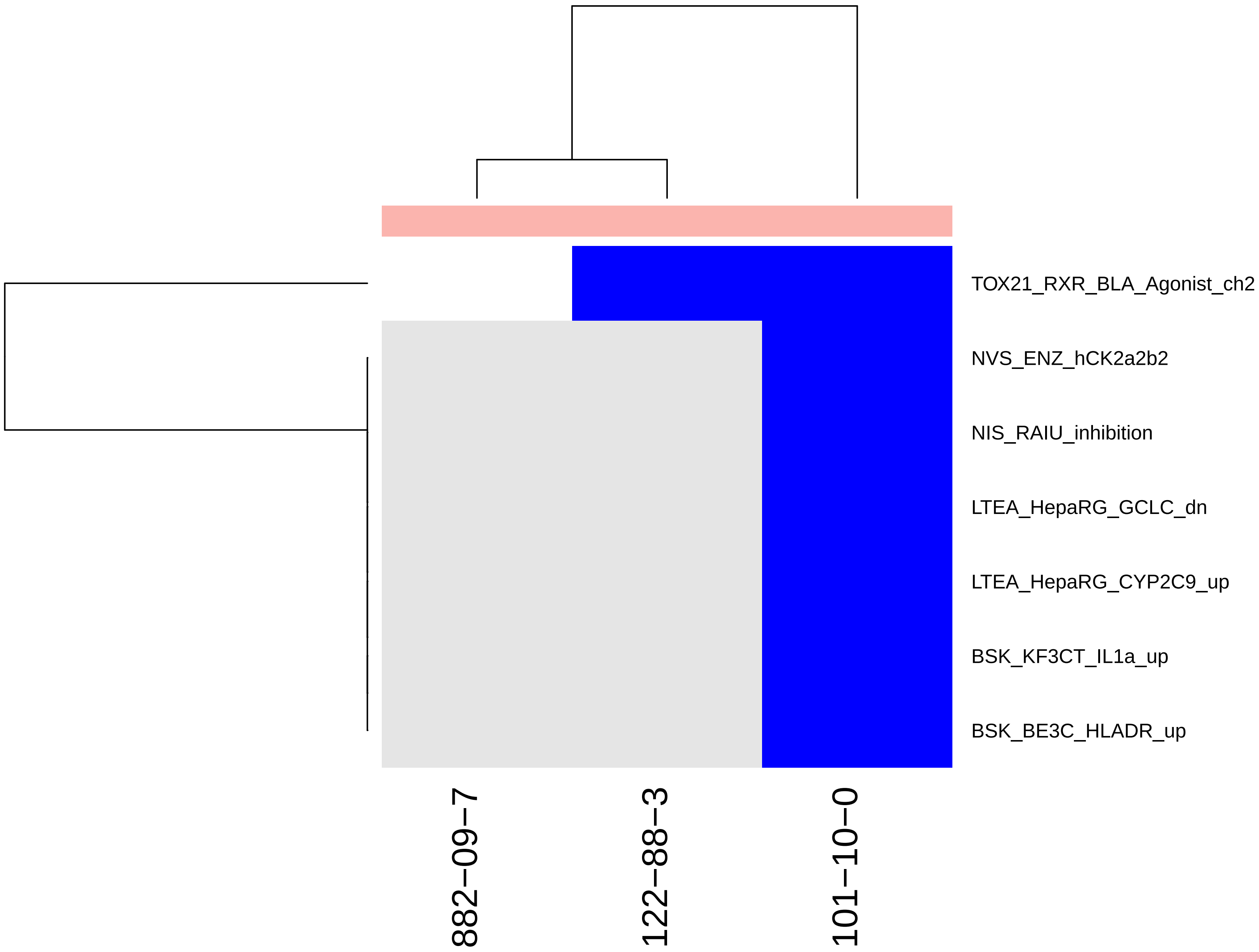

Enriched Assay Heatmap for Cluster 103

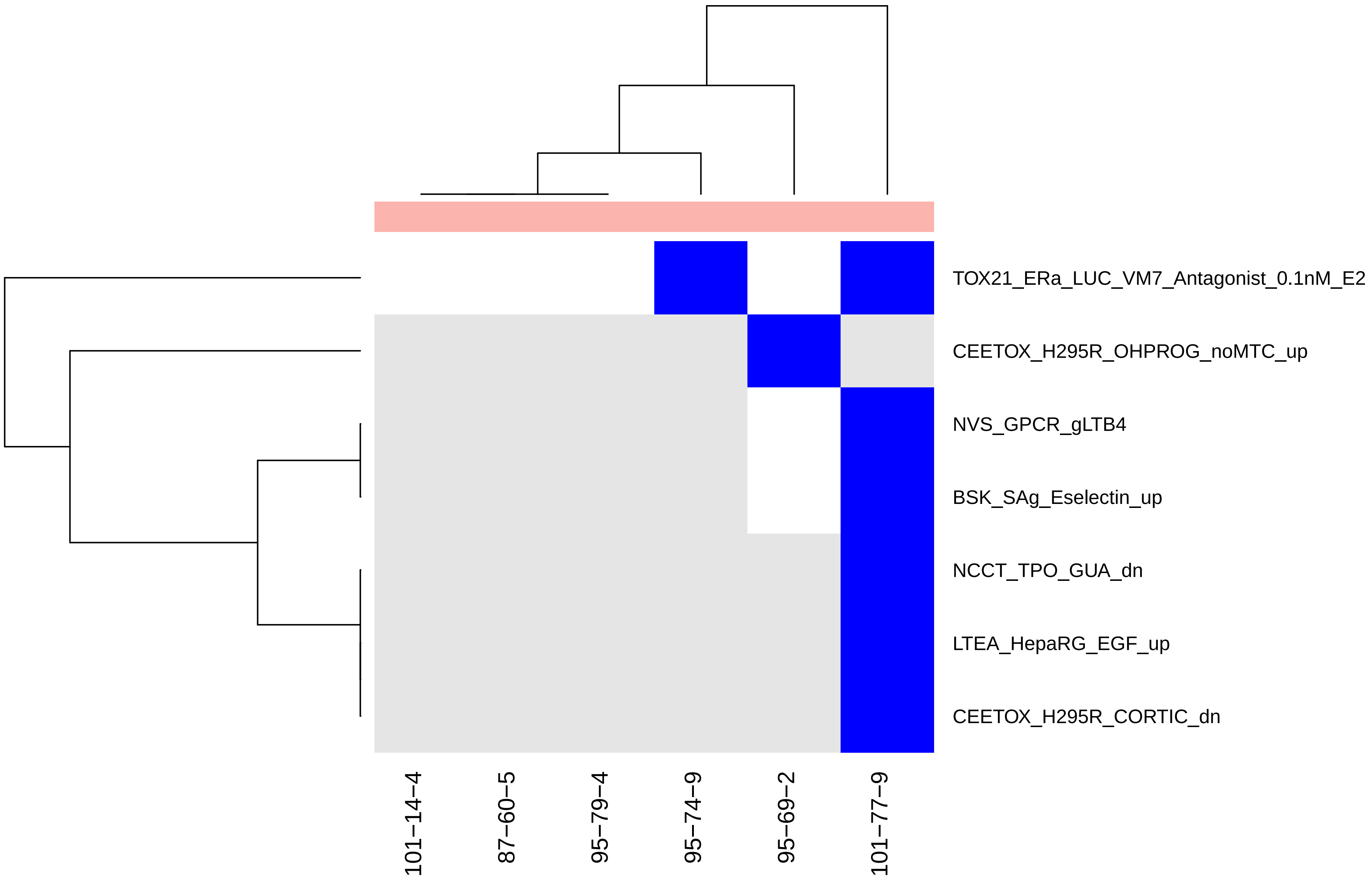

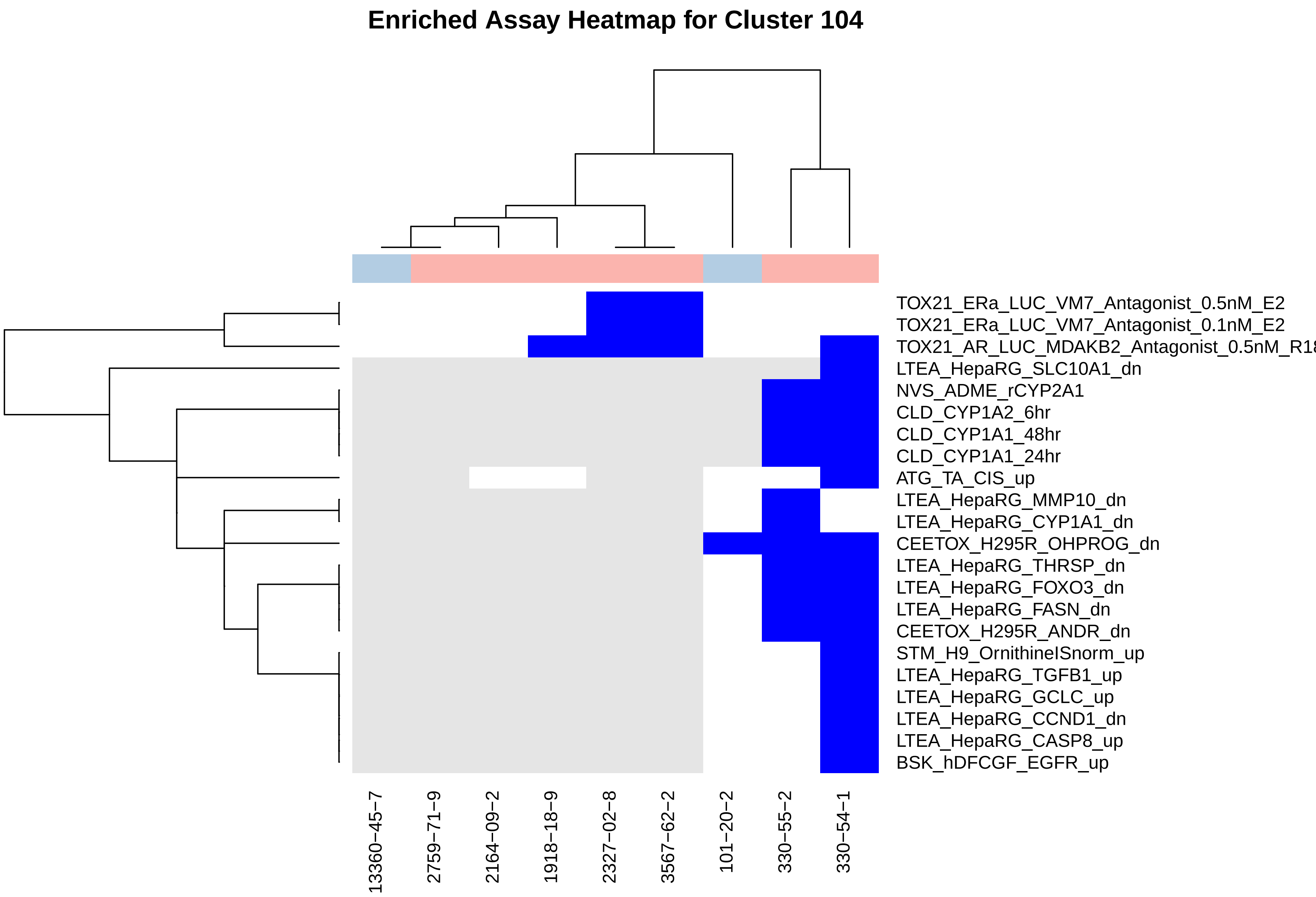

Enriched Assay Heatmap for Cluster 104
TOX21_ERa_LUC_VM7_Antagonist_0.5nM_E2
TOX21_ERa_LUC_VM7_Antagonist_0.1nM_E2
TOX21_AR_LUC_MDAKB2_Antagonist_0.5nM_R18
LTEA_HepaRG_SLC10A1_dn
NVS_ADME_rCYP2A1
CLD_CYP1A2_6hr
CLD_CYP1A1_48hr
CLD_CYP1A1_24hr
ATG_TA_CIS_up
LTEA_HepaRG_MMP10_dn
LTEA_HepaRG_CYP1A1_dn
CEETOX_H295R_OHPROG_dn
LTEA_HepaRG_THRSP_dn
LTEA_HepaRG_FOXO3_dn
LTEA_HepaRG_FASN_dn
CEETOX_H295R_ANDR_dn
STM_H9_OrnithineISnorm_up
LTEA_HepaRG_TGFB1_up
LTEA_HepaRG_GCLC_up
LTEA_HepaRG_CCND1_dn
LTEA_HepaRG_CASP8_up
BSK_hDFCGF_EGFR_up
13360−45−7
2759−71−9
2164−09−2
1918−18−9
2327−02−8
3567−62−2
101−20−2
330−55−2
330−54−1

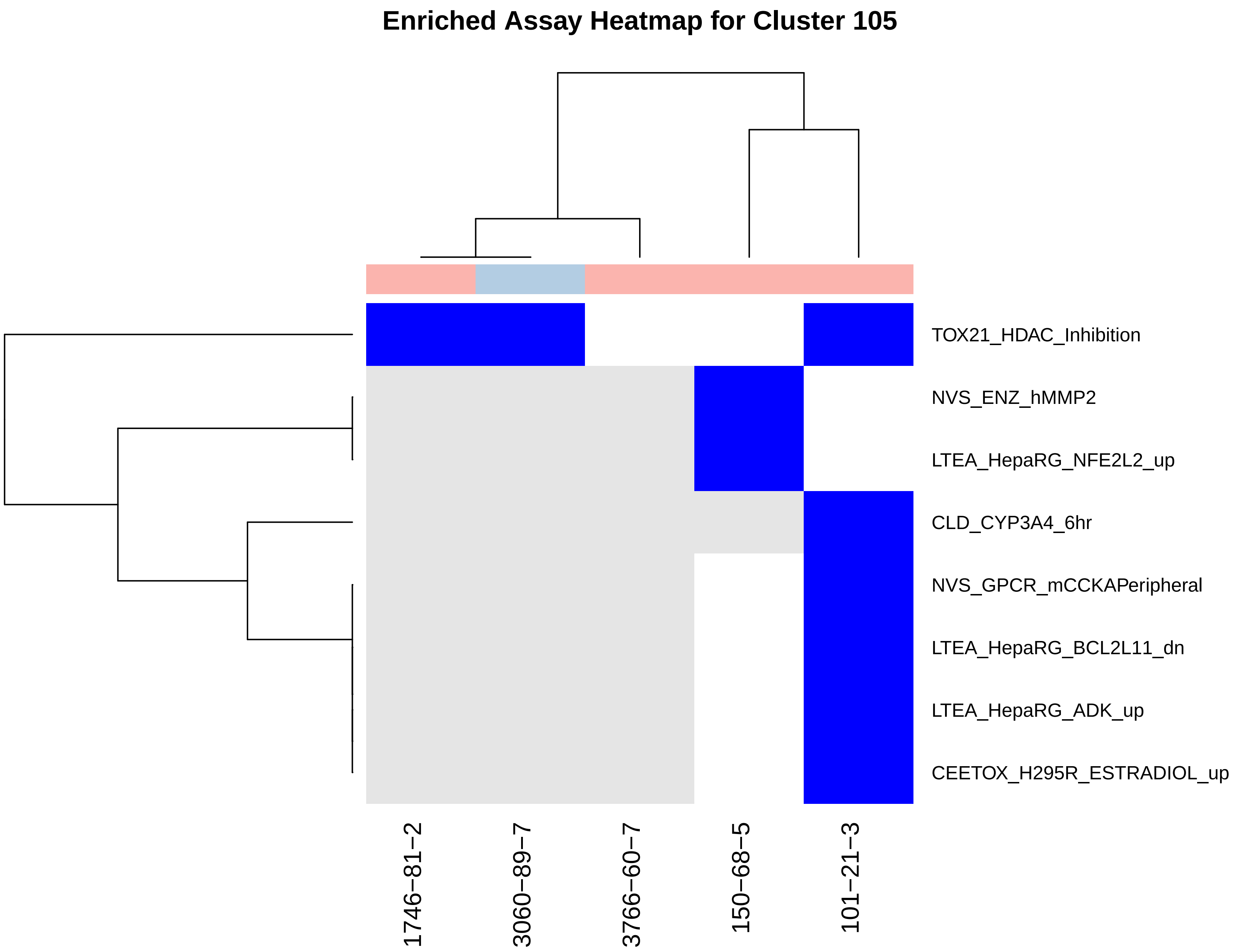

Enriched Assay Heatmap for Cluster 105
TOX21_HDAC_Inhibition
NVS_ENZ_hMMP2
LTEA_HepaRG_NFE2L2_up
CLD_CYP3A4_6hr
NVS_GPCR_mCCKAPeripheral
LTEA_HepaRG_BCL2L11_dn
LTEA_HepaRG_ADK_up
CEETOX_H295R_ESTRADIOL_up
1746−81−2
3060−89−7
3766−60−7
150−68−5
101−21−3

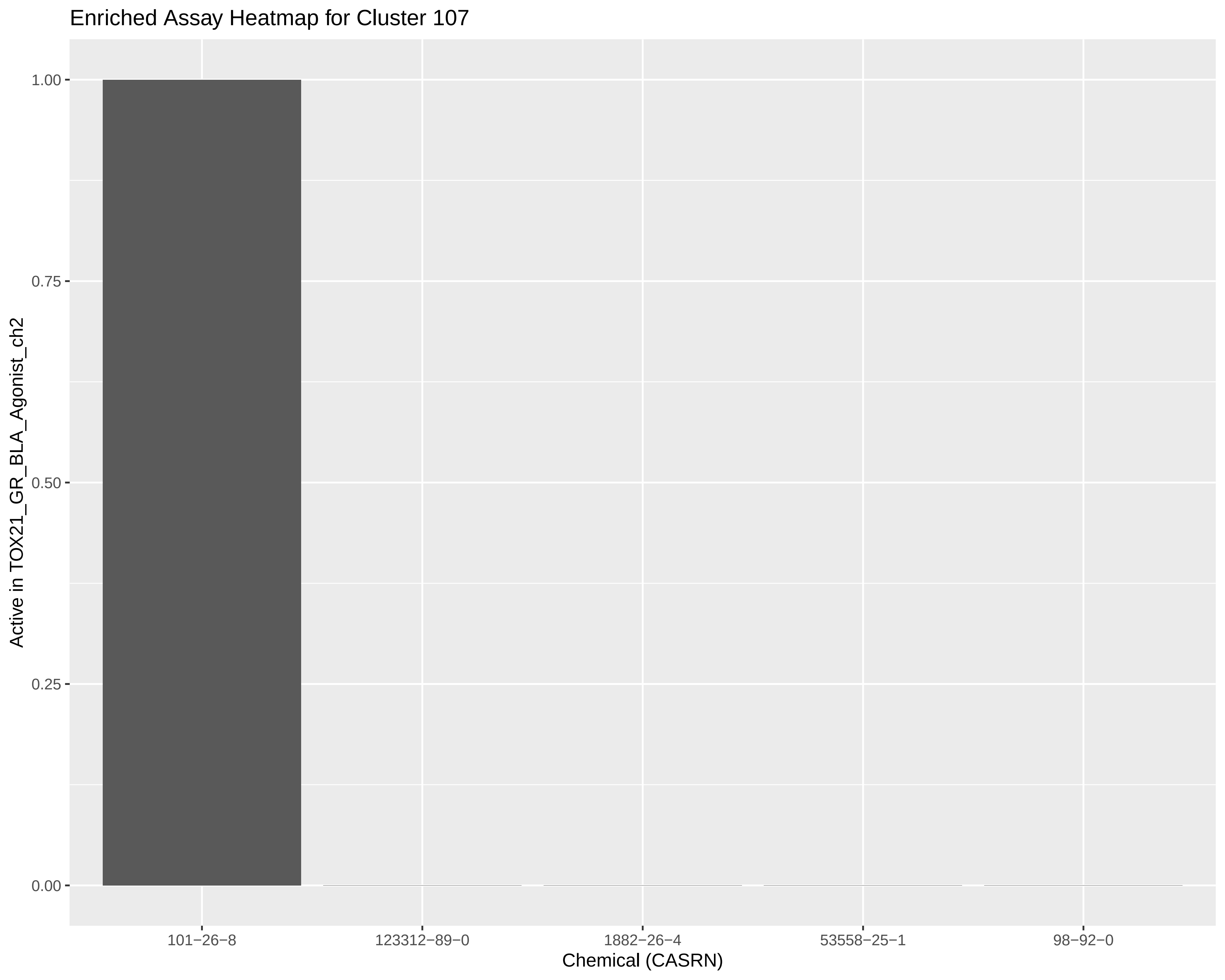

Enriched Assay Heatmap for Cluster 107
Active in TOX21_GR_BLA_Agonist_ch2
1.00
0.75
0.50
0.25
0.00
101−26−8
123312−89−0
1882−26−4
53558−25−1
98−92−0
Chemical (CASRN)

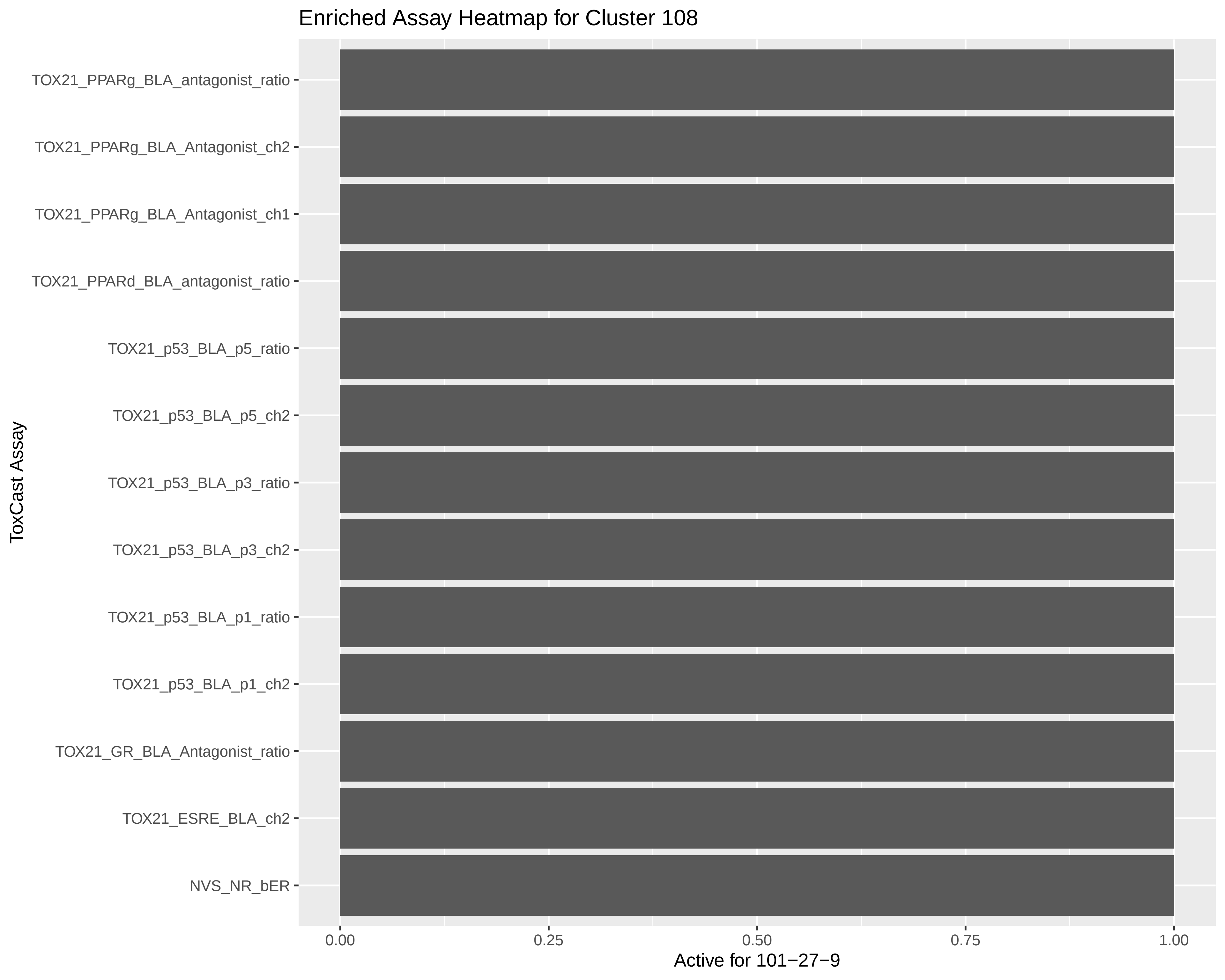

Enriched Assay Heatmap for Cluster 108
ToxCast Assay
TOX21_PPARg_BLA_antagonist_ratio
TOX21_PPARg_BLA_Antagonist_ch2
TOX21_PPARg_BLA_Antagonist_ch1
TOX21_PPARd_BLA_antagonist_ratio
TOX21_p53_BLA_p5_ratio
TOX21_p53_BLA_p5_ch2
TOX21_p53_BLA_p3_ratio
TOX21_p53_BLA_p3_ch2
TOX21_p53_BLA_p1_ratio
TOX21_p53_BLA_p1_ch2
TOX21_GR_BLA_Antagonist_ratio
TOX21_ESRE_BLA_ch2
NVS_NR_bER
0.00
0.25
0.50
0.75
1.00
Active for 101−27−9

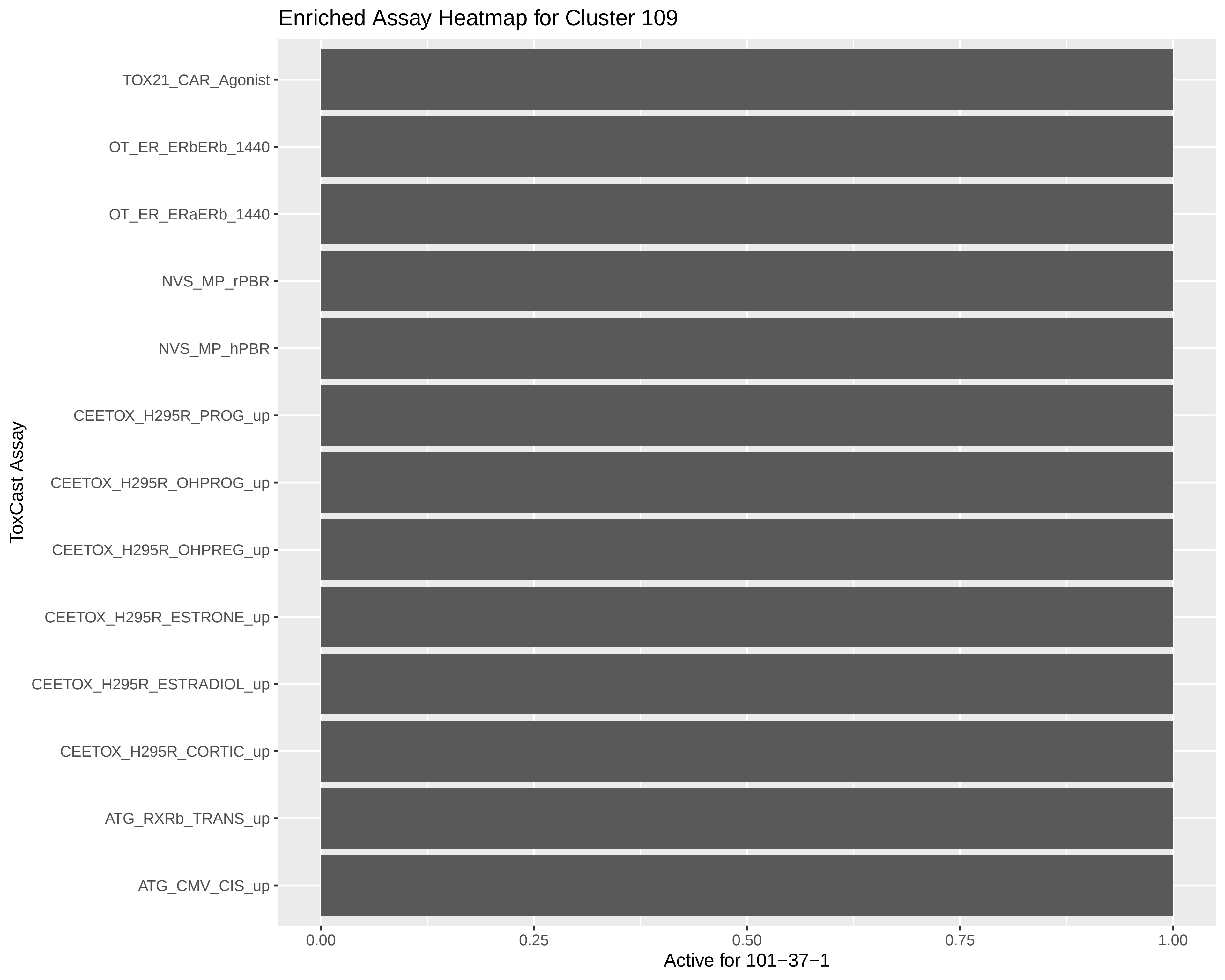
Enriched Assay Heatmap for Cluster 109
ToxCast Assay
TOX21_CAR_Agonist
OT_ER_ERbERb_1440
OT_ER_ERaERb_1440
NVS_MP_rPBR
NVS_MP_hPBR
CEETOX_H295R_PROG_up
CEETOX_H295R_OHPROG_up
CEETOX_H295R_OHPREG_up
CEETOX_H295R_ESTRONE_up
CEETOX_H295R_ESTRADIOL_up
CEETOX_H295R_CORTIC_up
ATG_RXRb_TRANS_up
ATG_CMV_CIS_up
0.00
0.25
0.50
0.75
1.00
Active for 101−37−1

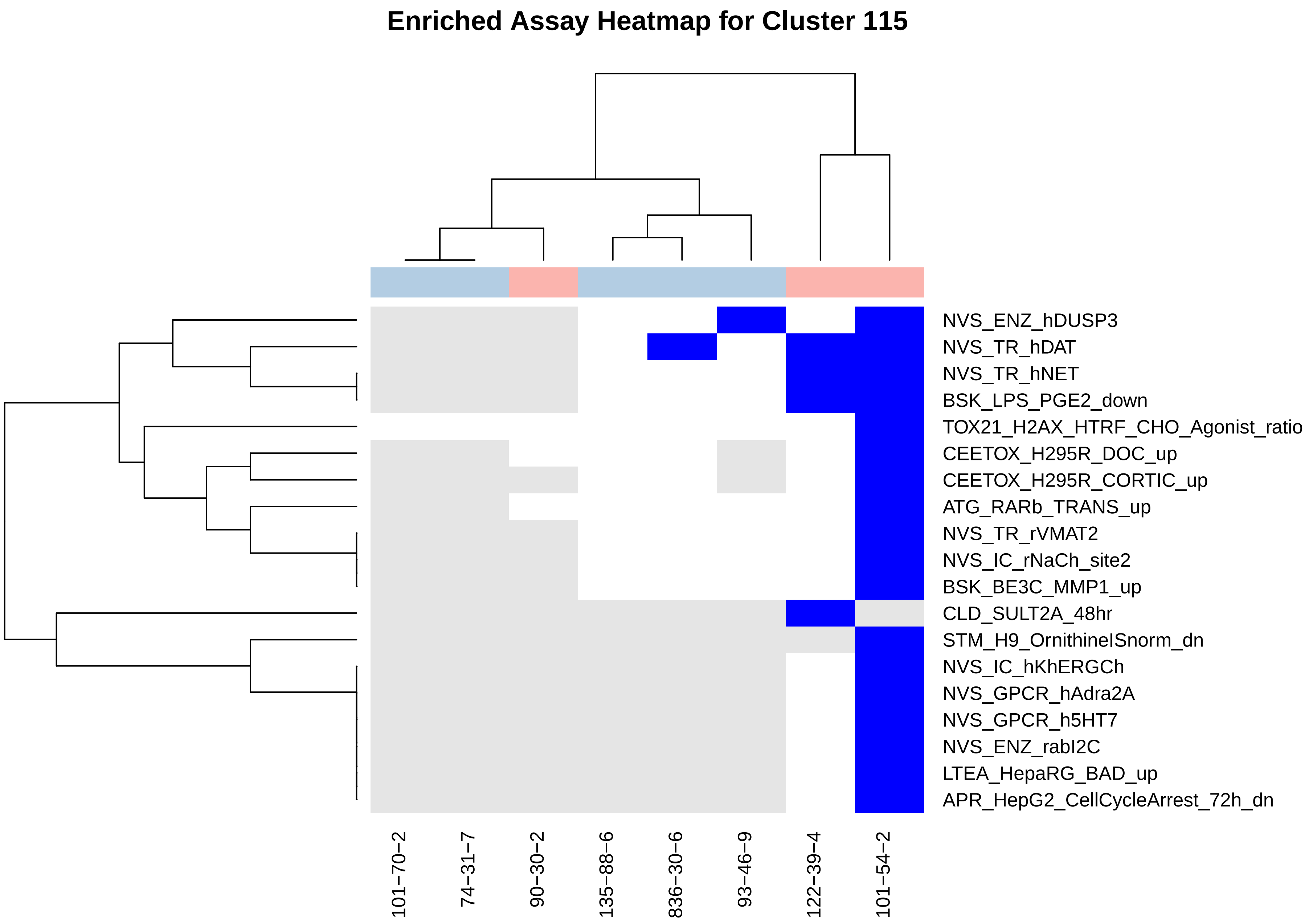

Enriched Assay Heatmap for Cluster 115
NVS_ENZ_hDUSP3
NVS_TR_hDAT
NVS_TR_hNET
BSK_LPS_PGE2_down
TOX21_H2AX_HTRF_CHO_Agonist_ratio
CEETOX_H295R_DOC_up
CEETOX_H295R_CORTIC_up
ATG_RARb_TRANS_up
NVS_TR_rVMAT2
NVS_IC_rNaCh_site2
BSK_BE3C_MMP1_up
CLD_SULT2A_48hr
STM_H9_OrnithineISnorm_dn
NVS_IC_hKhERGCh
NVS_GPCR_hAdra2A
NVS_GPCR_h5HT7
NVS_ENZ_rabI2C
LTEA_HepaRG_BAD_up
APR_HepG2_CellCycleArrest_72h_dn
101−70−2
74−31−7
90−30−2
135−88−6
836−30−6
93−46−9
122−39−4
101−54−2

Enriched Assay Heatmap for Cluster 122

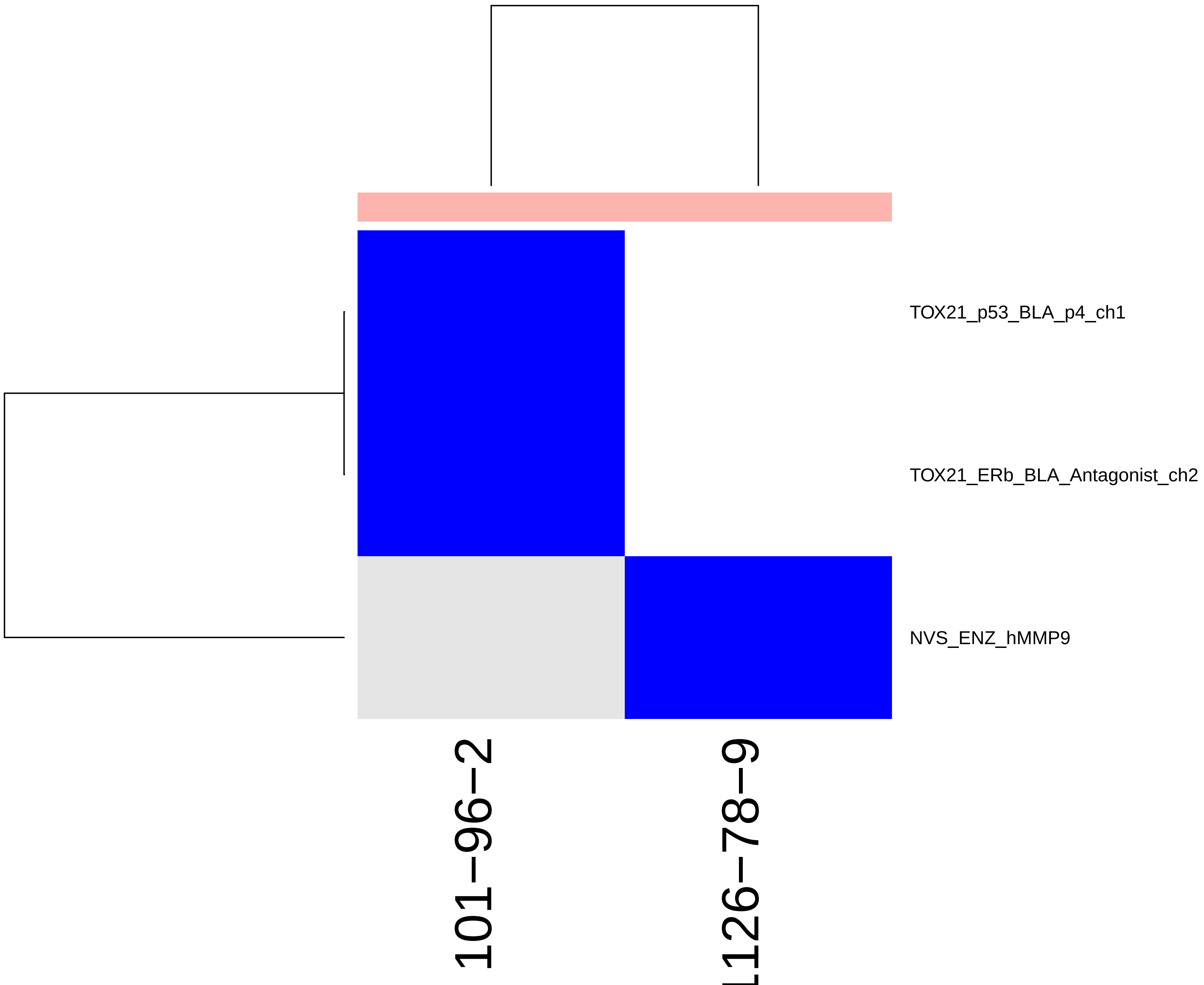

Enriched Assay Heatmap for Cluster 123

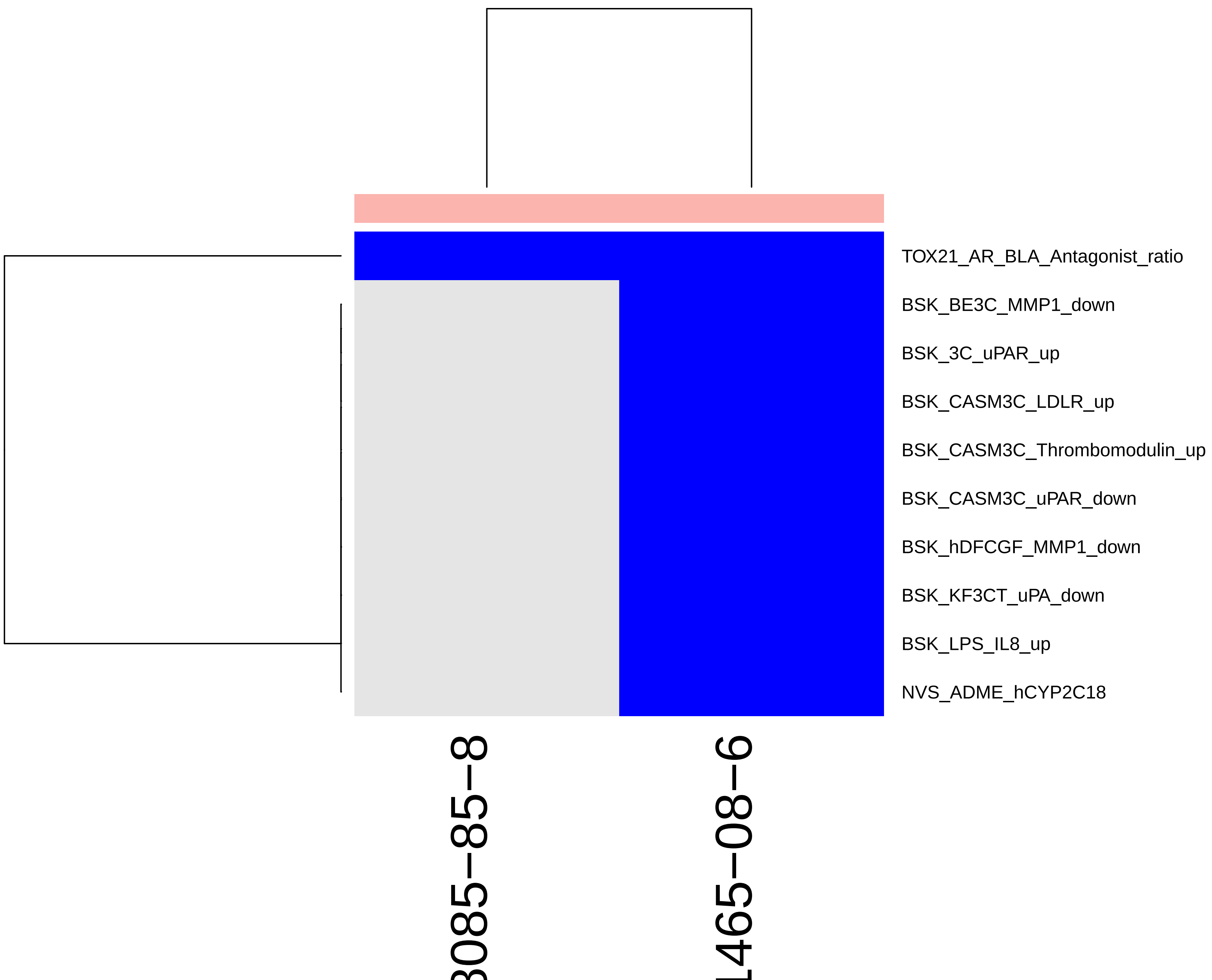

Enriched Assay Heatmap for Cluster 124

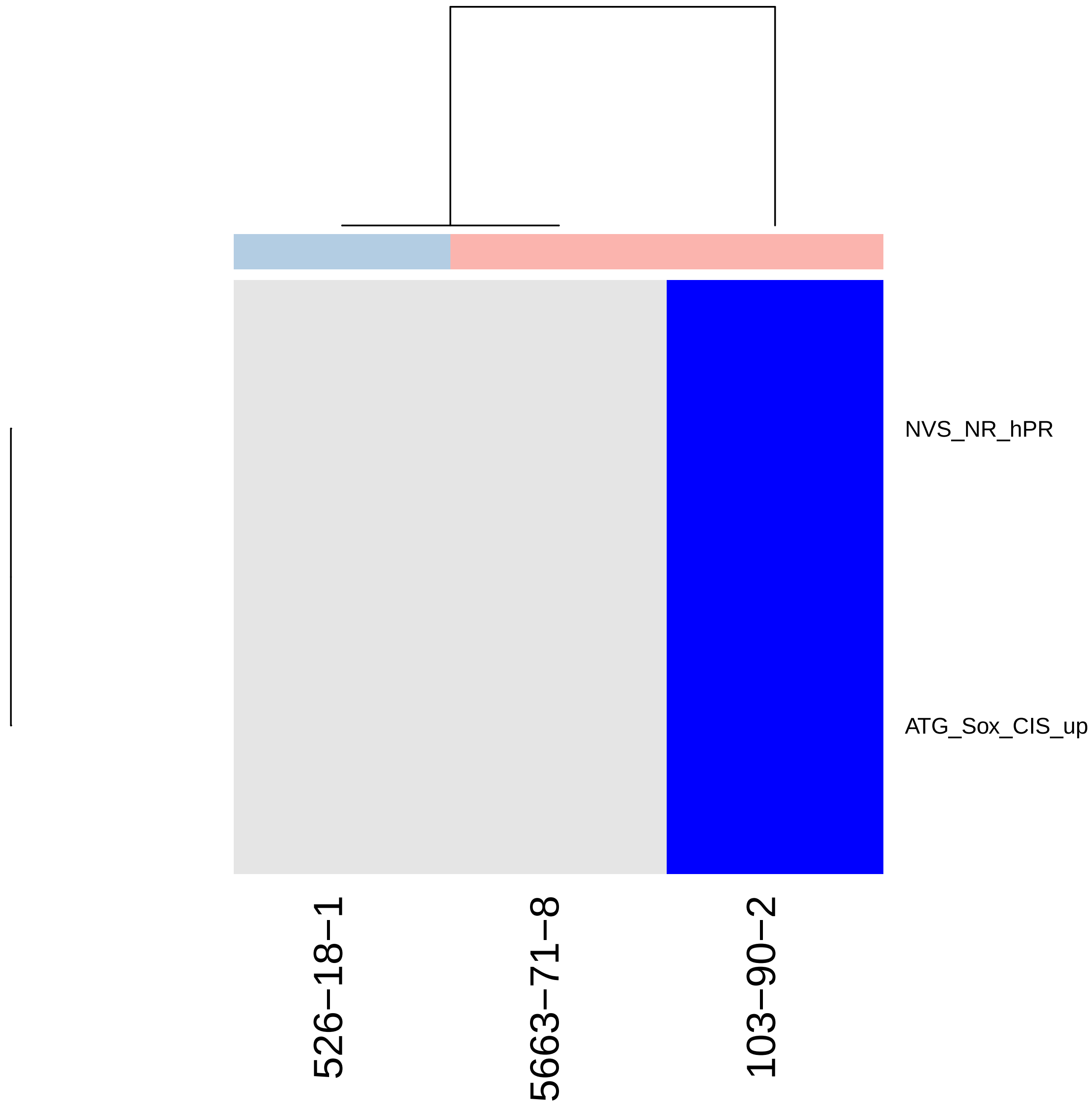

Enriched Assay Heatmap for Cluster 127

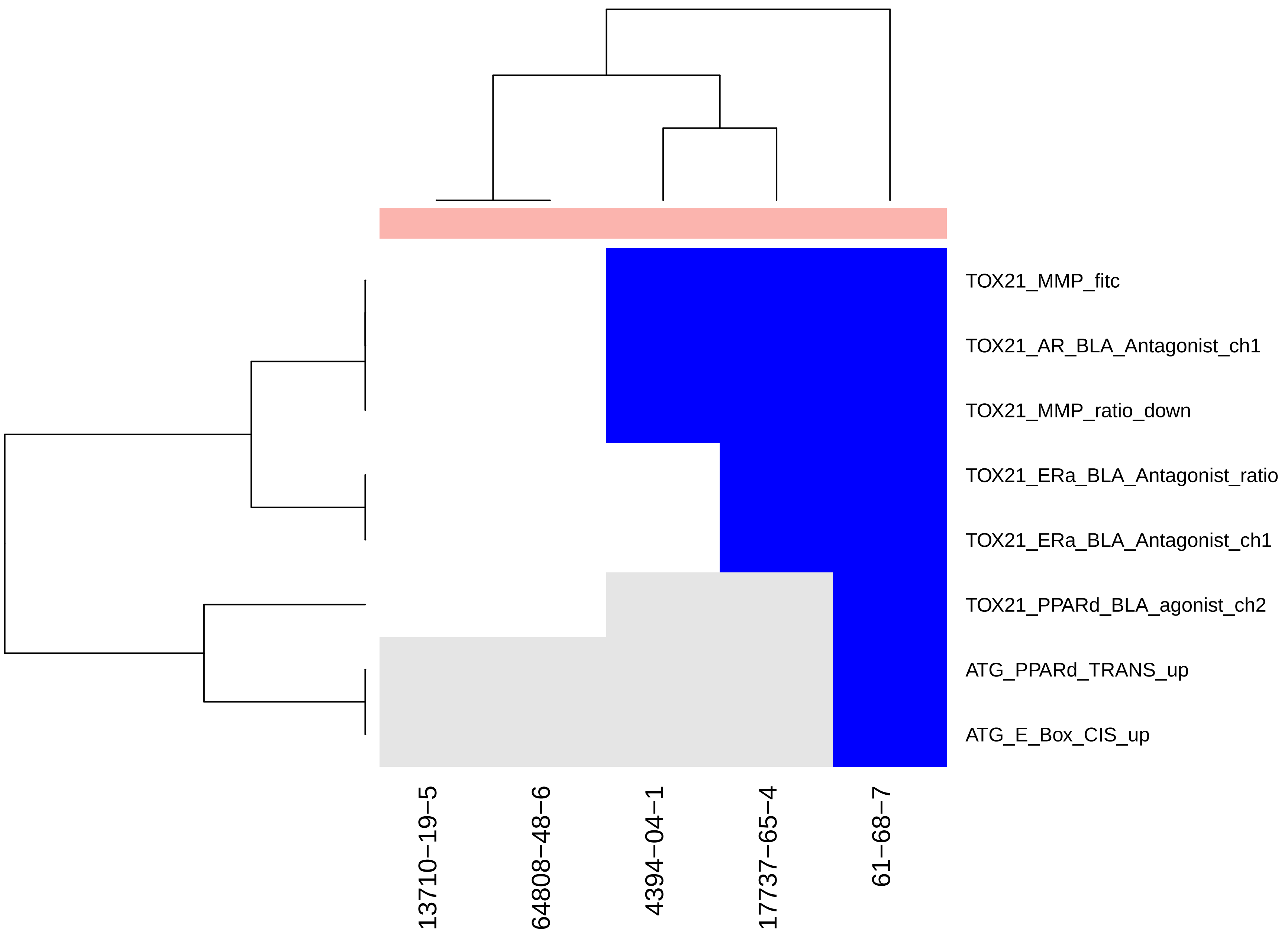

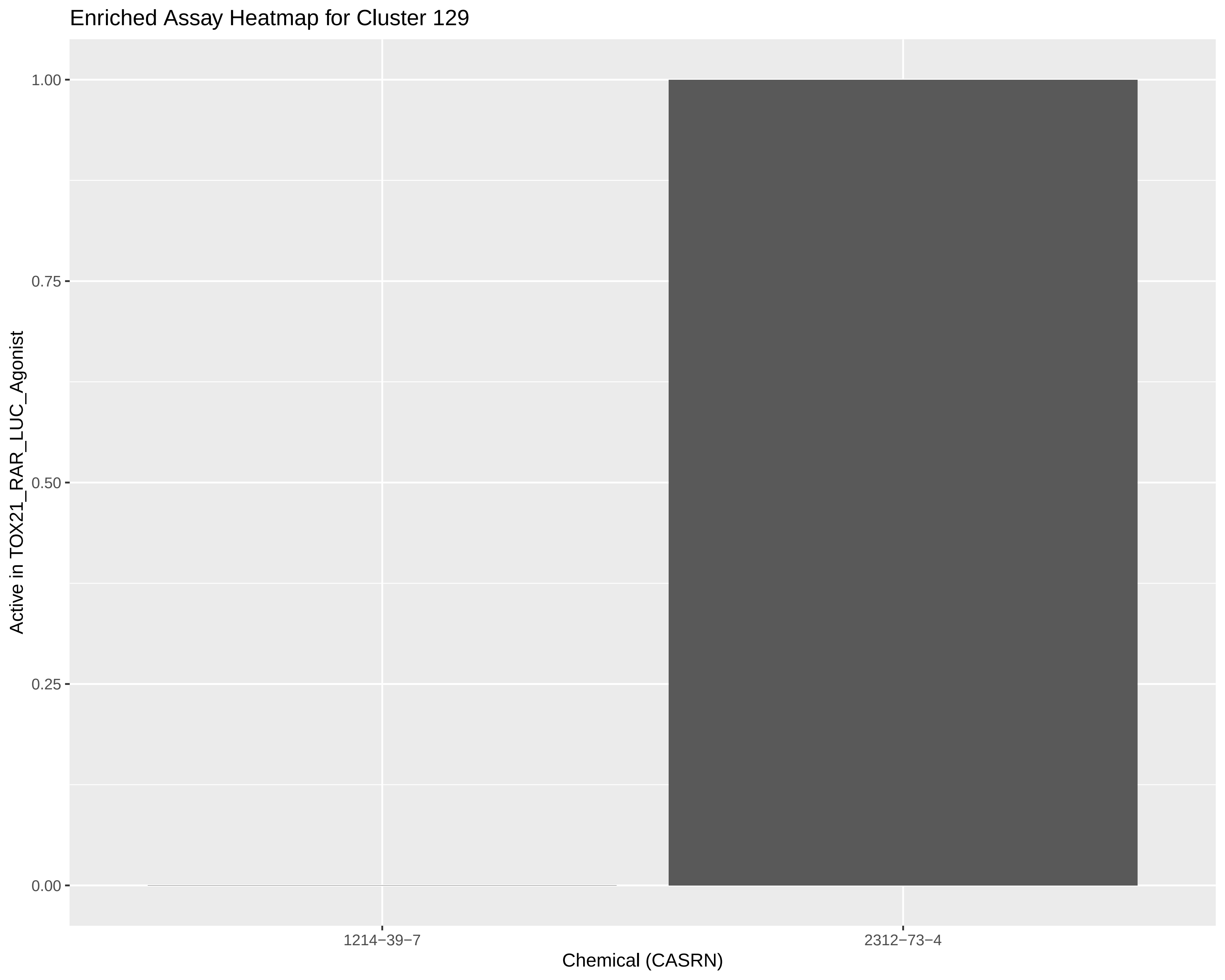

Enriched Assay Heatmap for Cluster 129
Active in TOX21_RAR_LUC_Agonist
1.00
0.75
0.50
0.25
0.00
1214−39−7
2312−73−4
Chemical (CASRN)

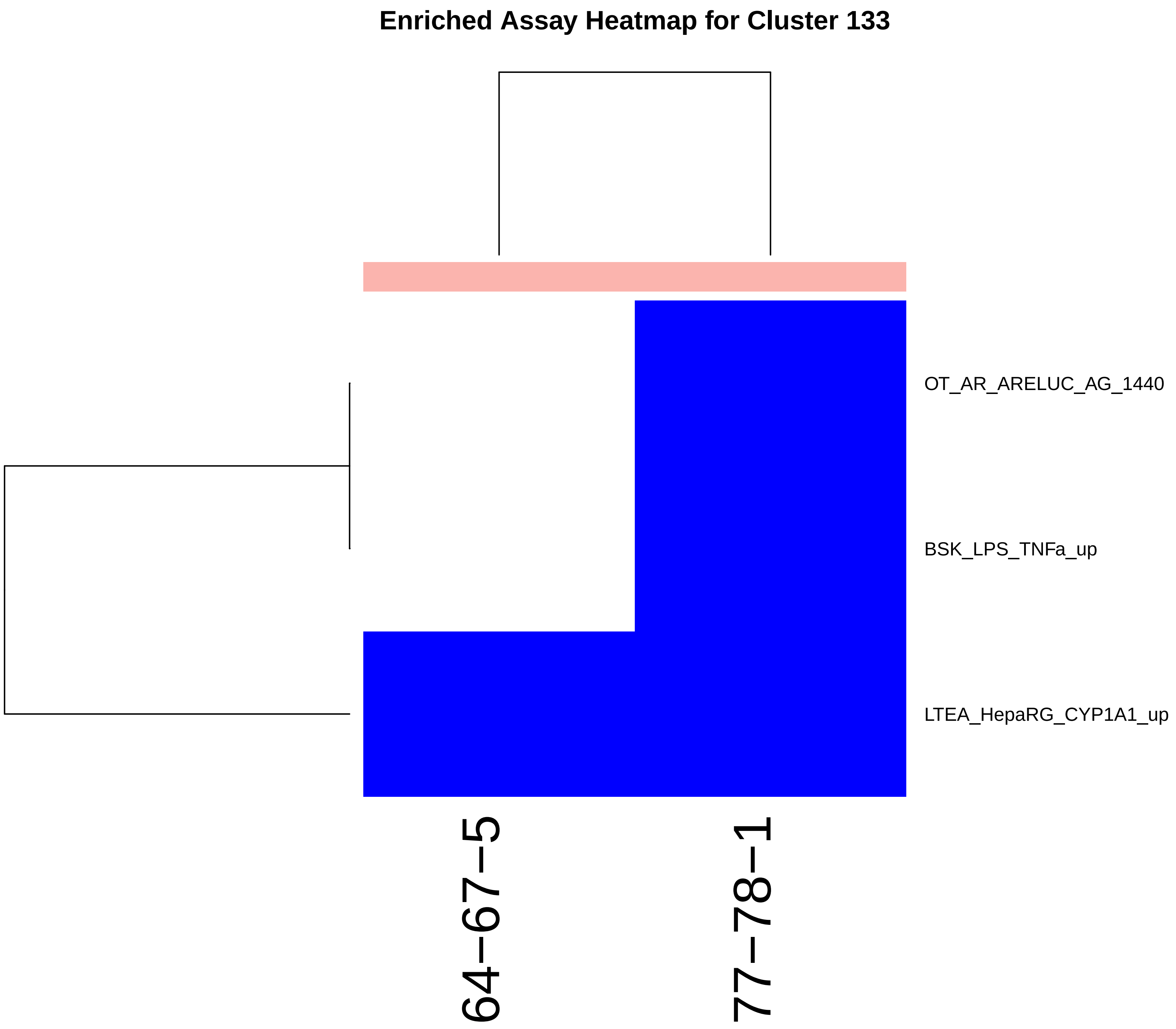

Enriched Assay Heatmap for Cluster 133
OT_AR_ARELUC_AG_1440
BSK_LPS_TNFa_up
LTEA_HepaRG_CYP1A1_up
64−67−5
77−78−1

Enriched Assay Heatmap for Cluster 134

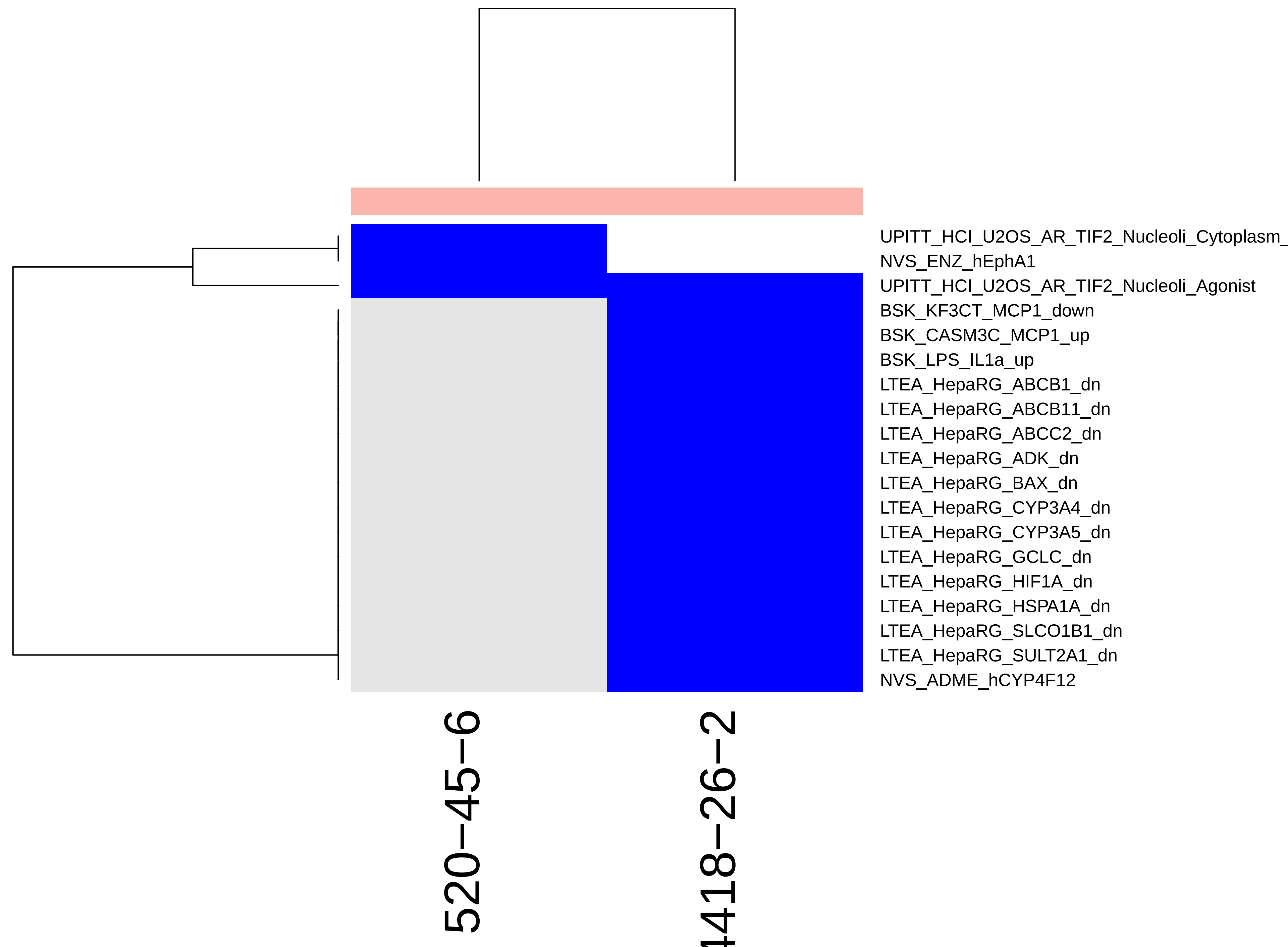

Enriched Assay Heatmap for Cluster 138

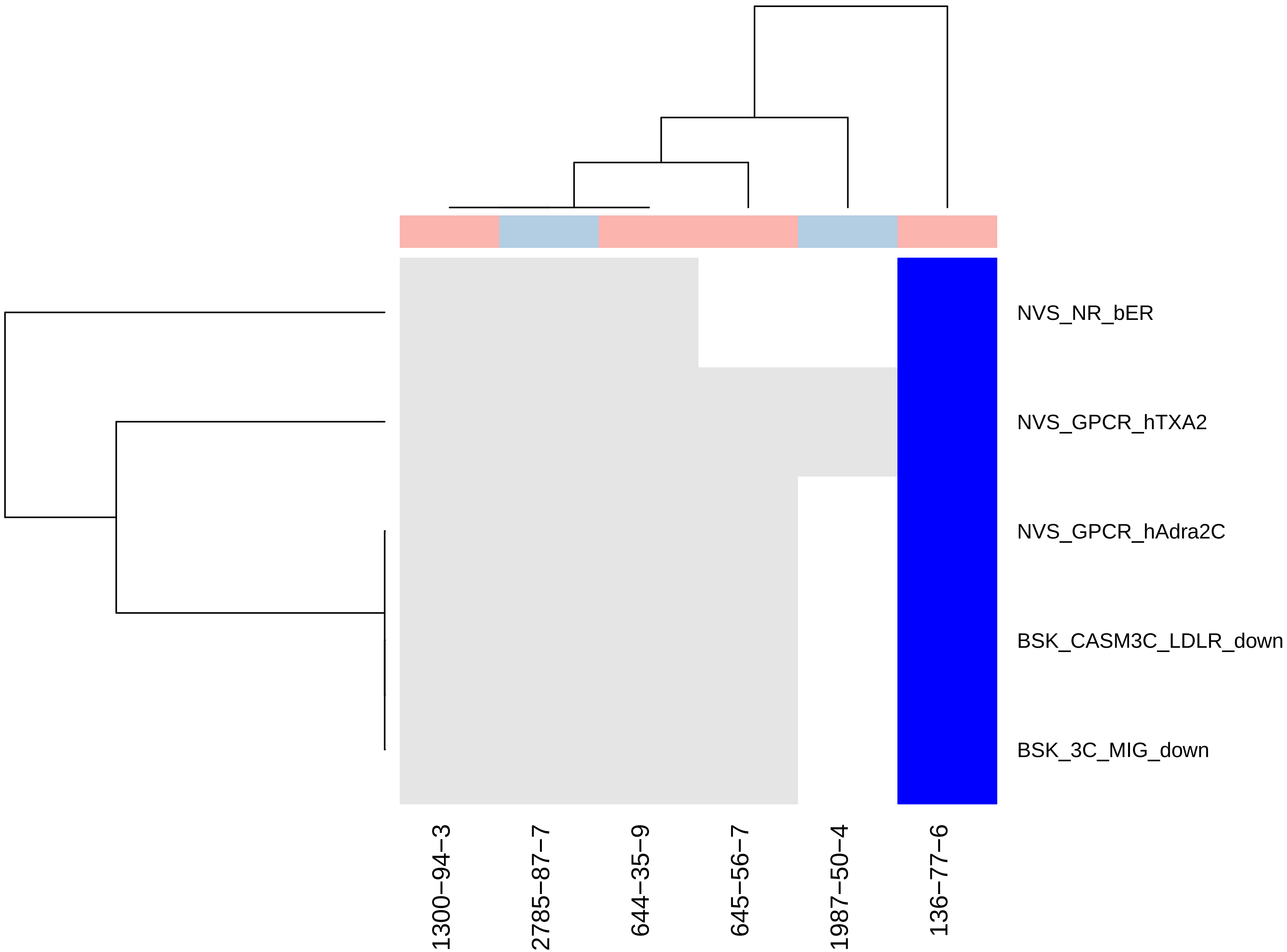

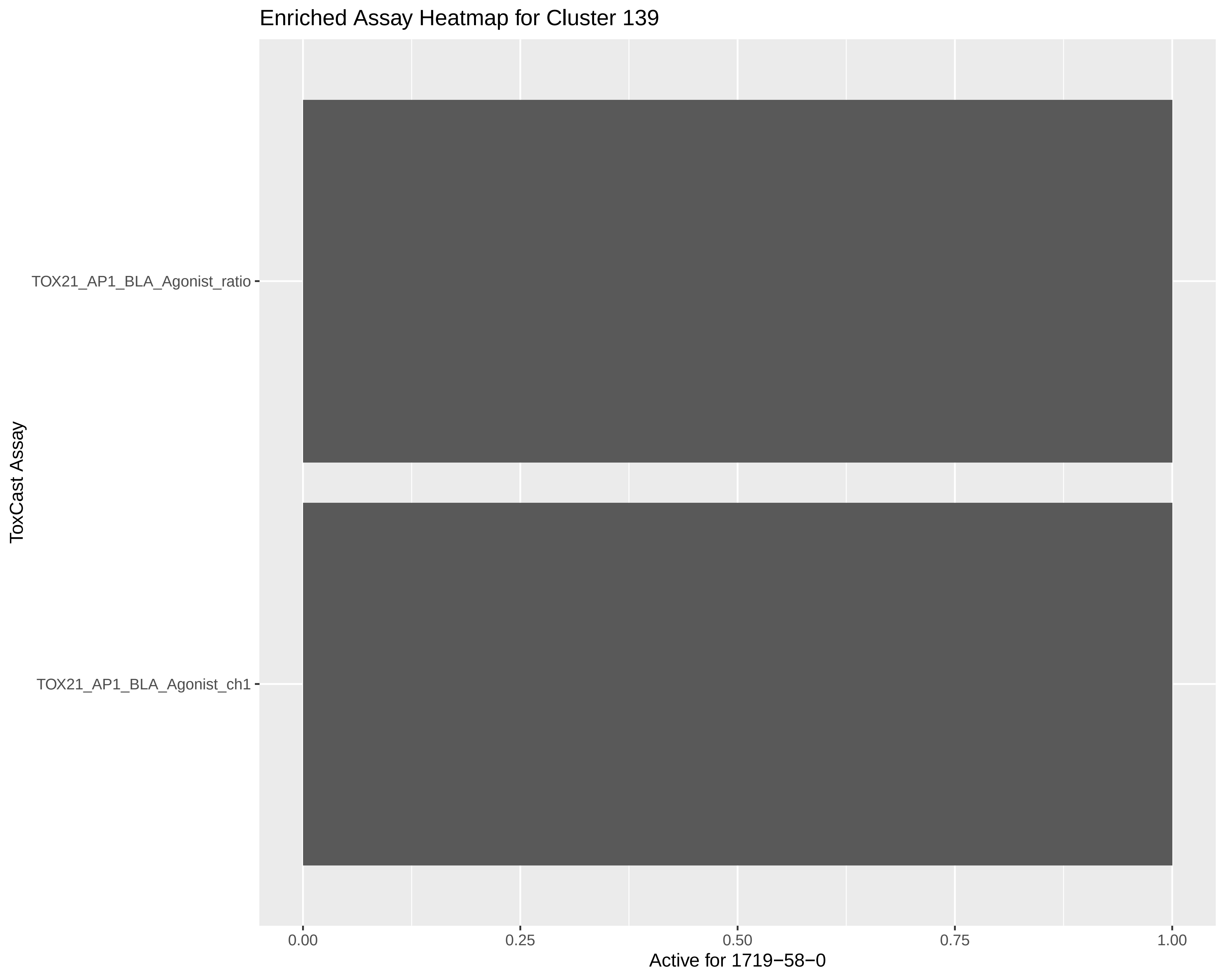
Enriched Assay Heatmap for Cluster 139
ToxCast Assay
TOX21_AP1_BLA_Agonist_ratio
TOX21_AP1_BLA_Agonist_ch1
0.00
0.25
0.50
0.75
1.00
Active for 1719−58−0

Enriched Assay Heatmap for Cluster 144

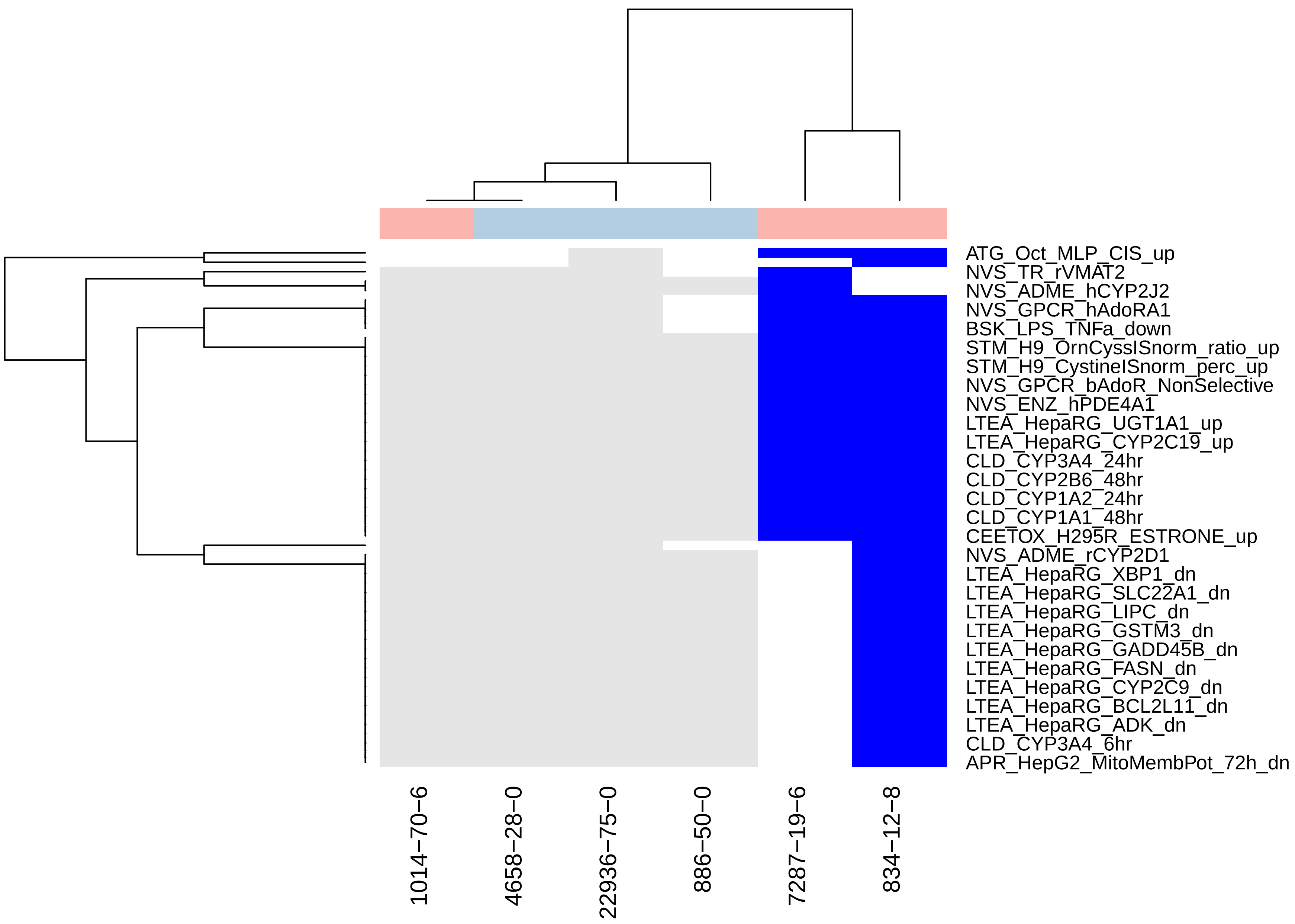

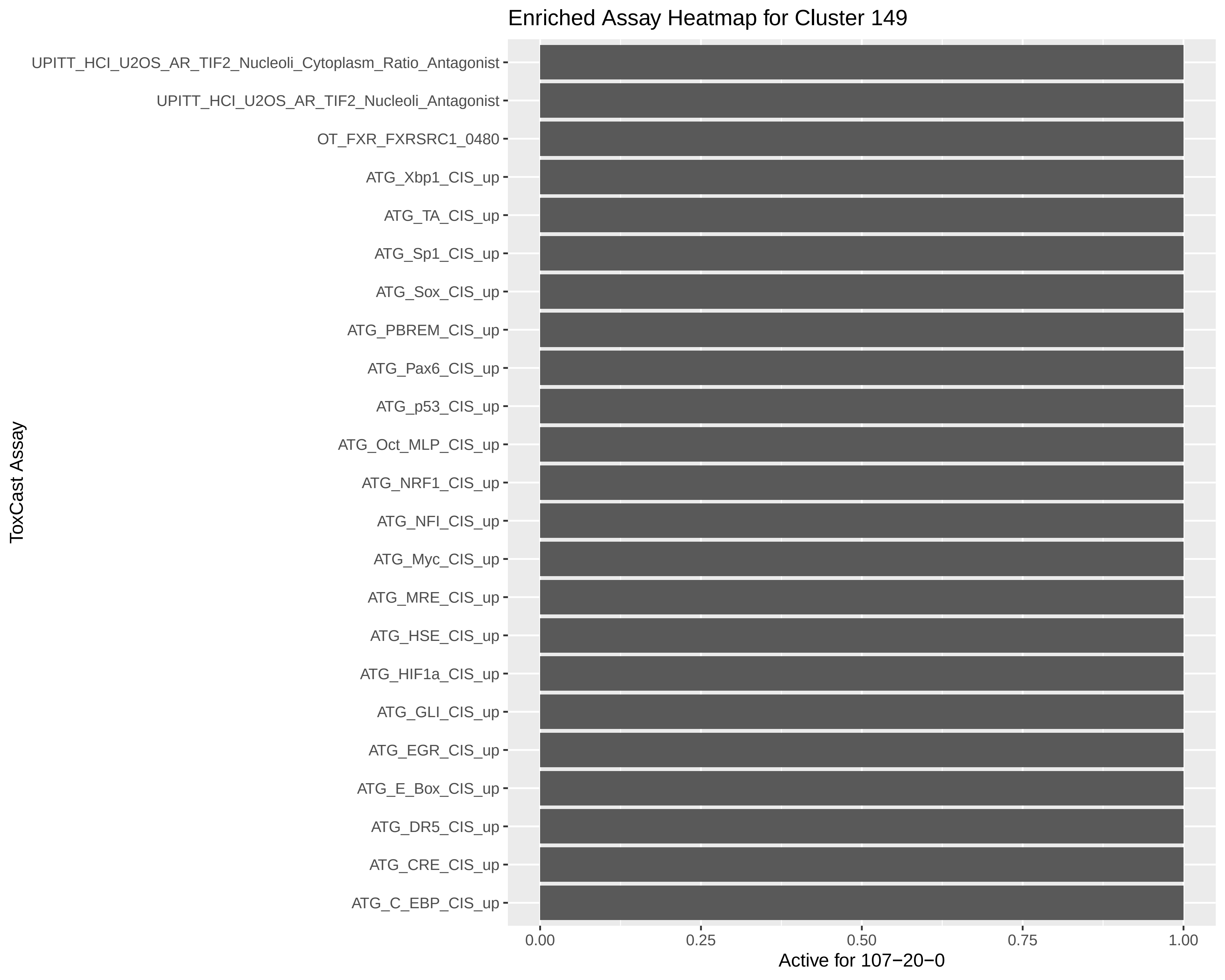
Enriched Assay Heatmap for Cluster 149
UPITT_HCI_U2OS_AR_TIF2_Nucleoli_Cytoplasm_Ratio_Antagonist
UPITT_HCI_U2OS_AR_TIF2_Nucleoli_Antagonist
OT_FXR_FXRSRC1_0480
ATG_Xbp1_CIS_up
ATG_TA_CIS_up
ATG_Sp1_CIS_up
ATG_Sox_CIS_up
ATG_PBREM_CIS_up
ATG_Pax6_CIS_up
ATG_p53_CIS_up
ATG_Oct_MLP_CIS_up
ATG_NRF1_CIS_up
ATG_NFI_CIS_up
ATG_Myc_CIS_up
ATG_MRE_CIS_up
ATG_HSE_CIS_up
ATG_HIF1a_CIS_up
ATG_GLI_CIS_up
ATG_EGR_CIS_up
ATG_E_Box_CIS_up
ATG_DR5_CIS_up
ATG_CRE_CIS_up
ATG_C_EBP_CIS_up
ToxCast Assay
0.00
0.25
0.50
0.75
1.00
Active for 107−20−0

Enriched Assay Heatmap for Cluster 157

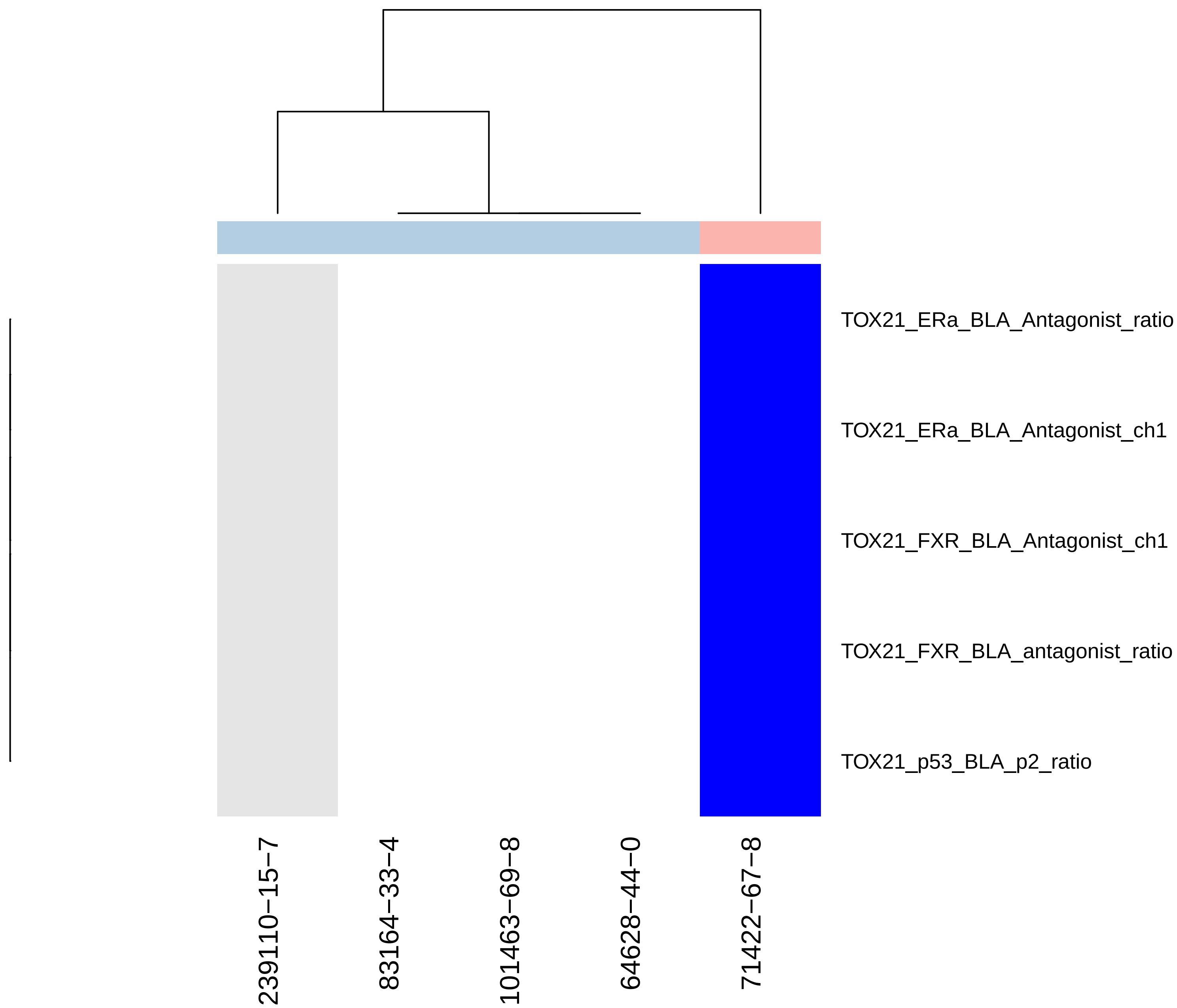

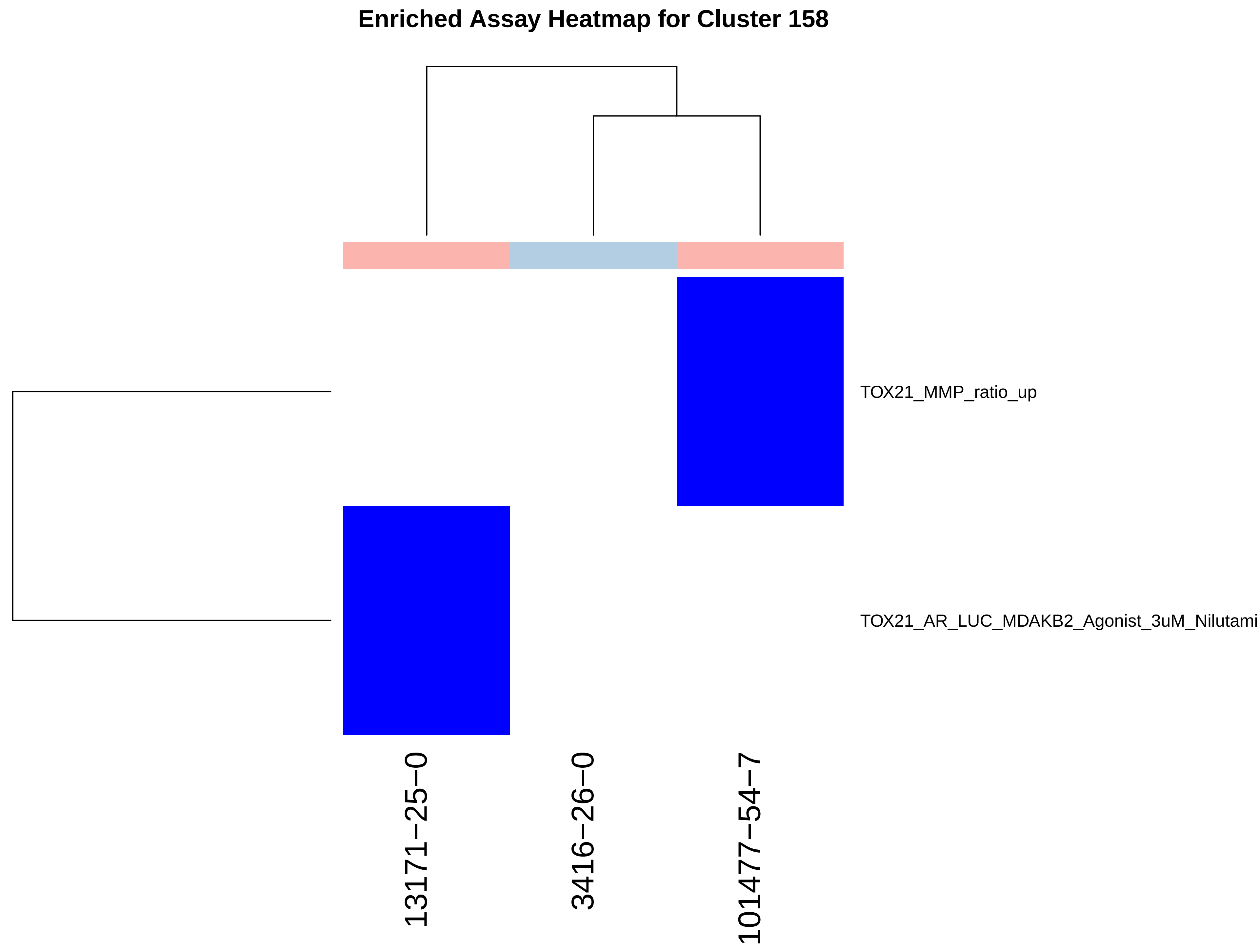

Enriched Assay Heatmap for Cluster 158
TOX21_MMP_ratio_up
TOX21_AR_LUC_MDAKB2_Agonist_3uM_Nilutami
13171−25−0
3416−26−0
101477−54−7

Enriched Assay Heatmap for Cluster 160

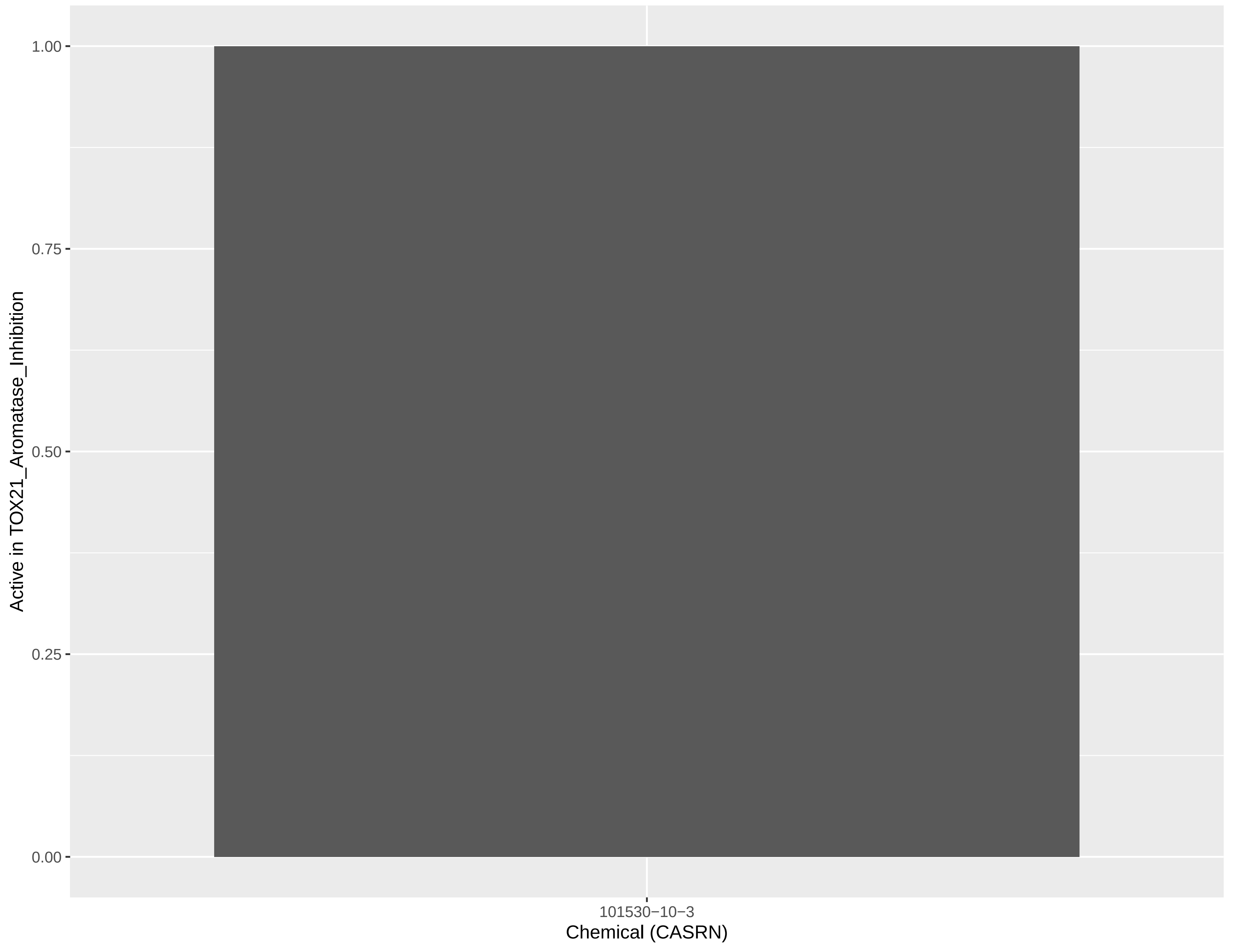

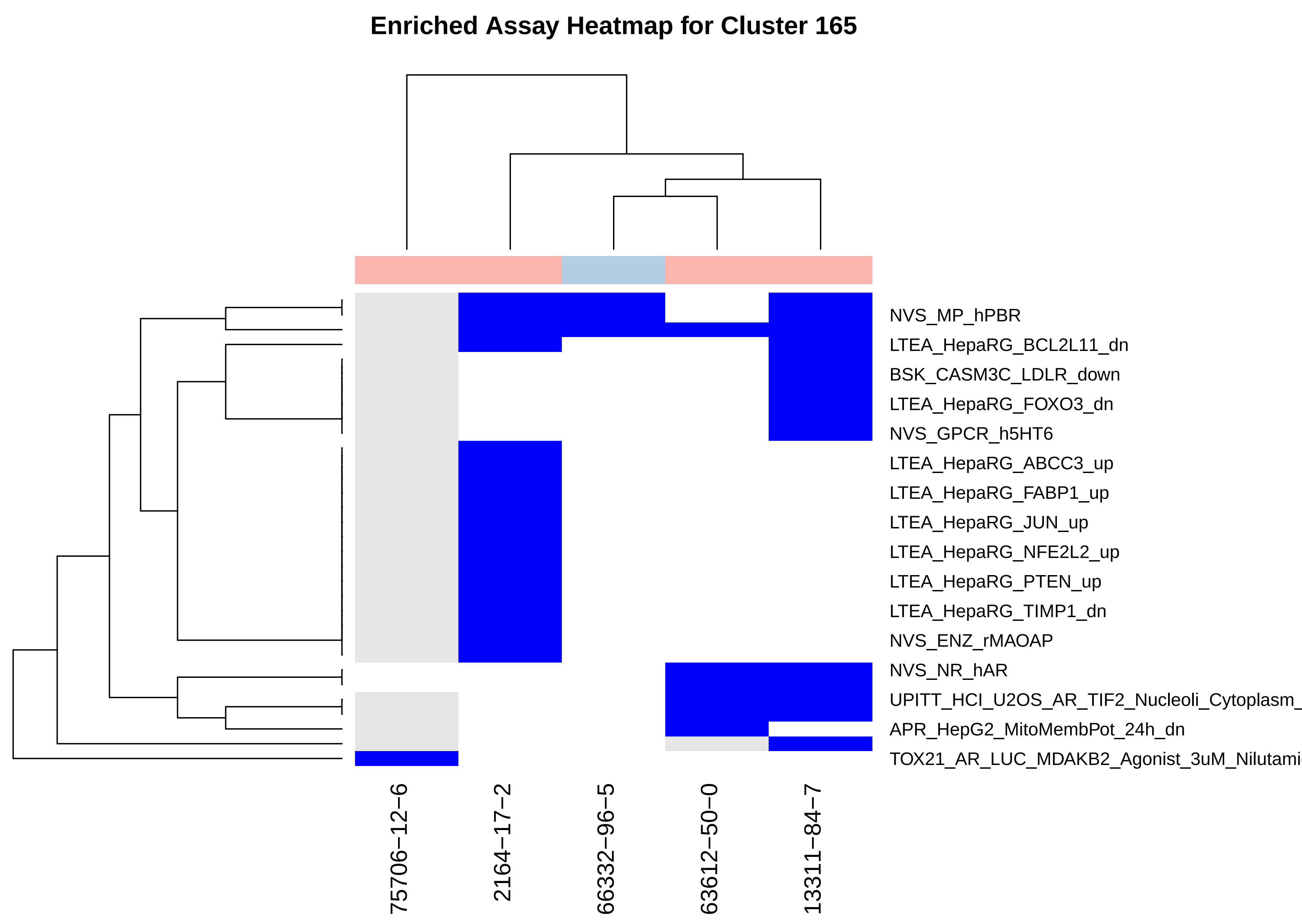

Enriched Assay Heatmap for Cluster 165
NVS_MP_hPBR
LTEA_HepaRG_BCL2L11_dn
BSK_CASM3C_LDLR_down
LTEA_HepaRG_FOXO3_dn
NVS_GPCR_h5HT6
LTEA_HepaRG_ABCC3_up
LTEA_HepaRG_FABP1_up
LTEA_HepaRG_JUN_up
LTEA_HepaRG_NFE2L2_up
LTEA_HepaRG_PTEN_up
LTEA_HepaRG_TIMP1_dn
NVS_ENZ_rMAOAP
NVS_NR_hAR
UPITT_HCI_U2OS_AR_TIF2_Nucleoli_Cytoplasm_
APR_HepG2_MitoMembPot_24h_dn
TOX21_AR_LUC_MDAKB2_Agonist_3uM_Nilutami
75706−12−6
2164−17−2
66332−96−5
63612−50−0
13311−84−7

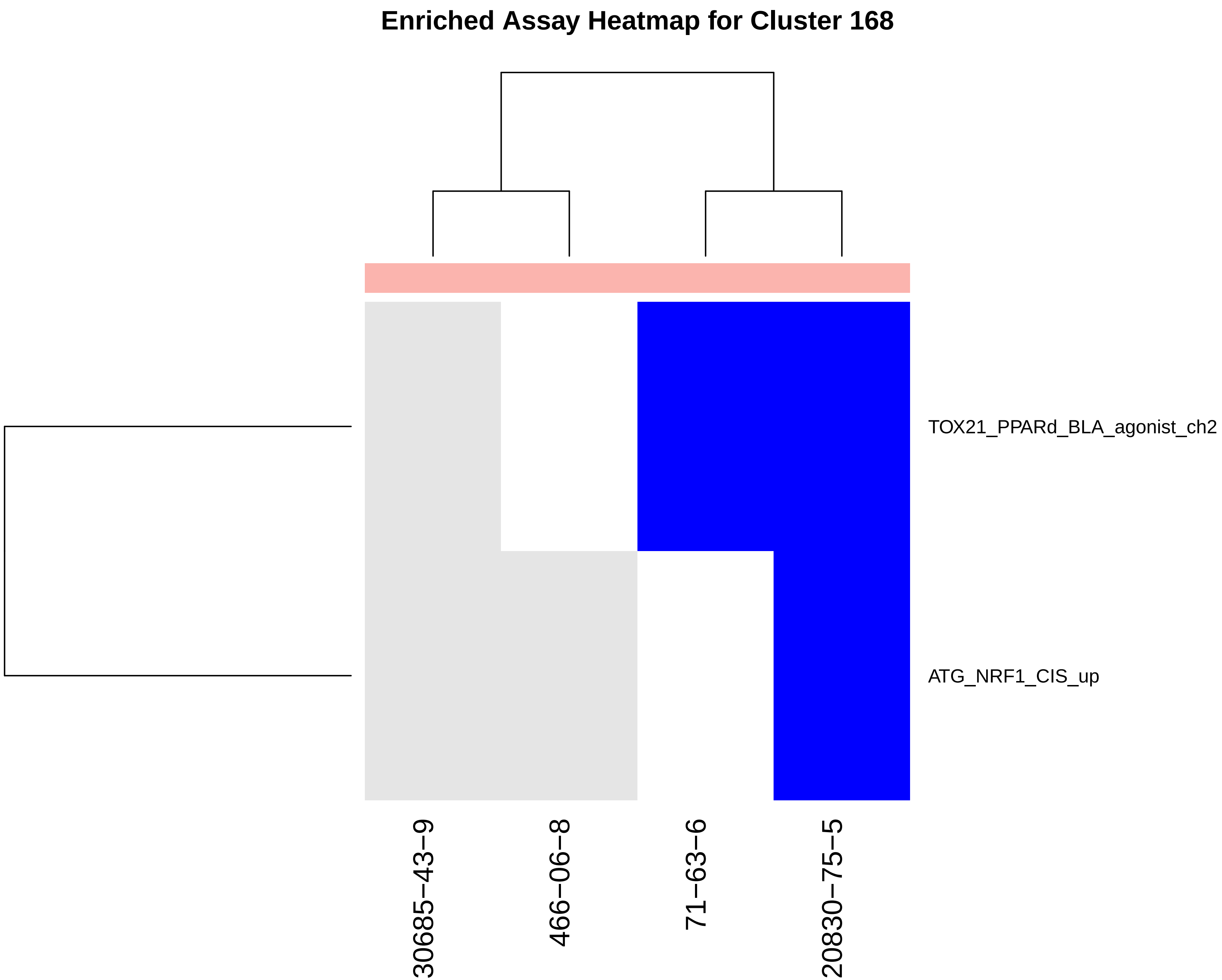

Enriched Assay Heatmap for Cluster 168
TOX21_PPARd_BLA_agonist_ch2
ATG_NRF1_CIS_up
30685−43−9
466−06−8
71−63−6
20830−75−5

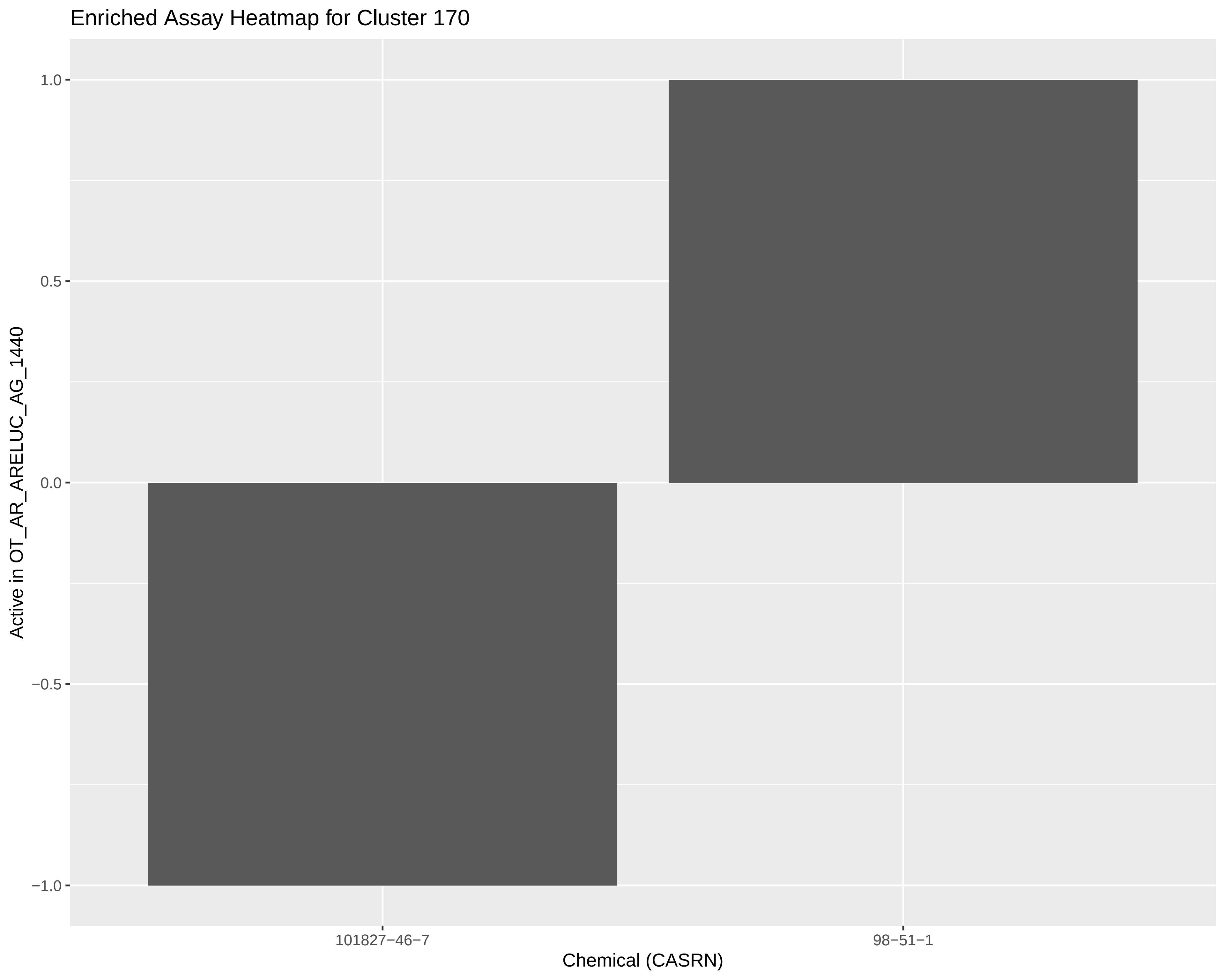

Enriched Assay Heatmap for Cluster 170
Active in OT_AR_ARELUC_AG_1440
1.0
0.5
0.0
−0.5
−1.0
101827−46−7
98−51−1
Chemical (CASRN)

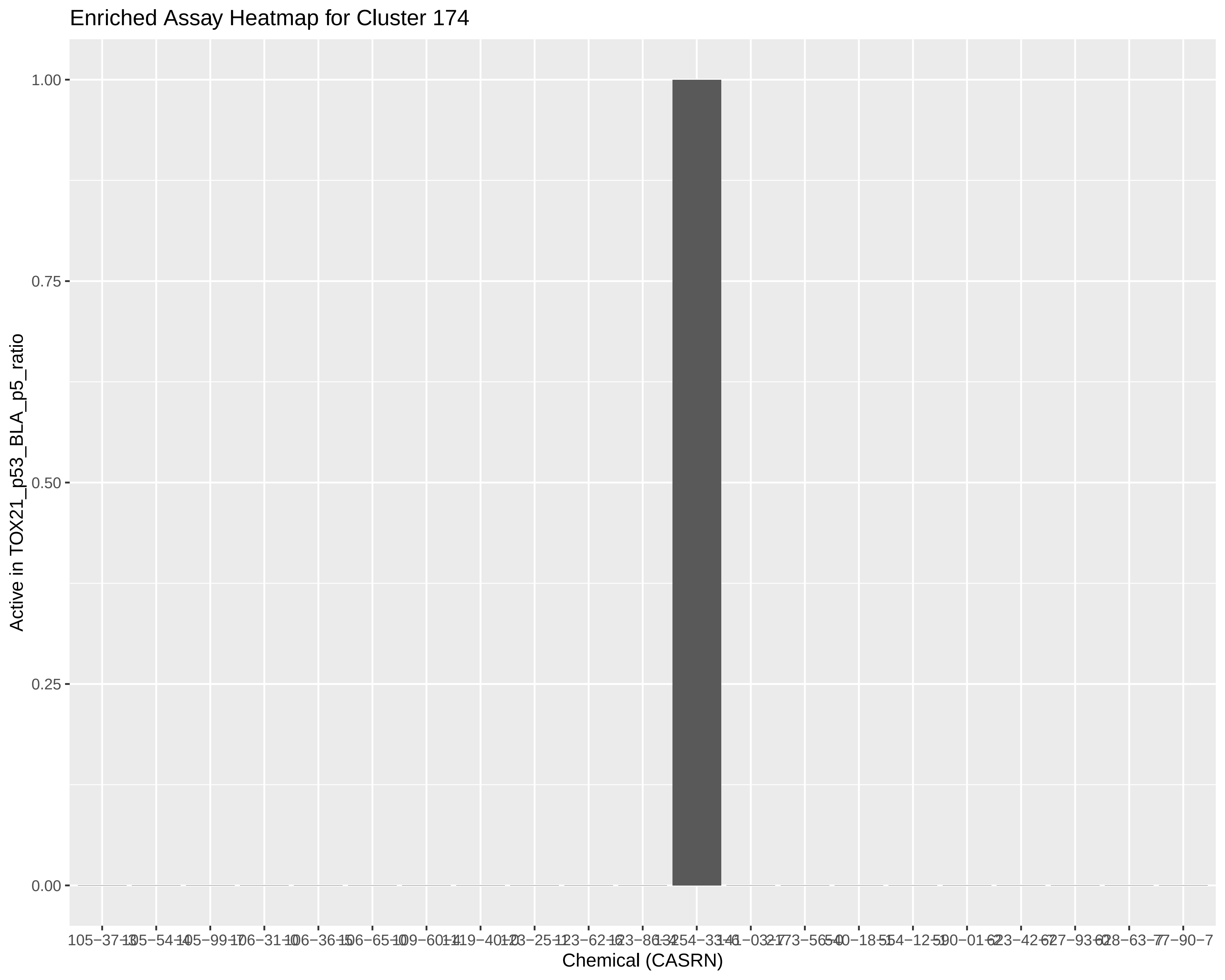
Enriched Assay Heatmap for Cluster 174
Active in TOX21_p53_BLA_p5_ratio
1.00
0.75
0.50
0.25
0.00
Chemical (CASRN)

Enriched Assay Heatmap for Cluster 176

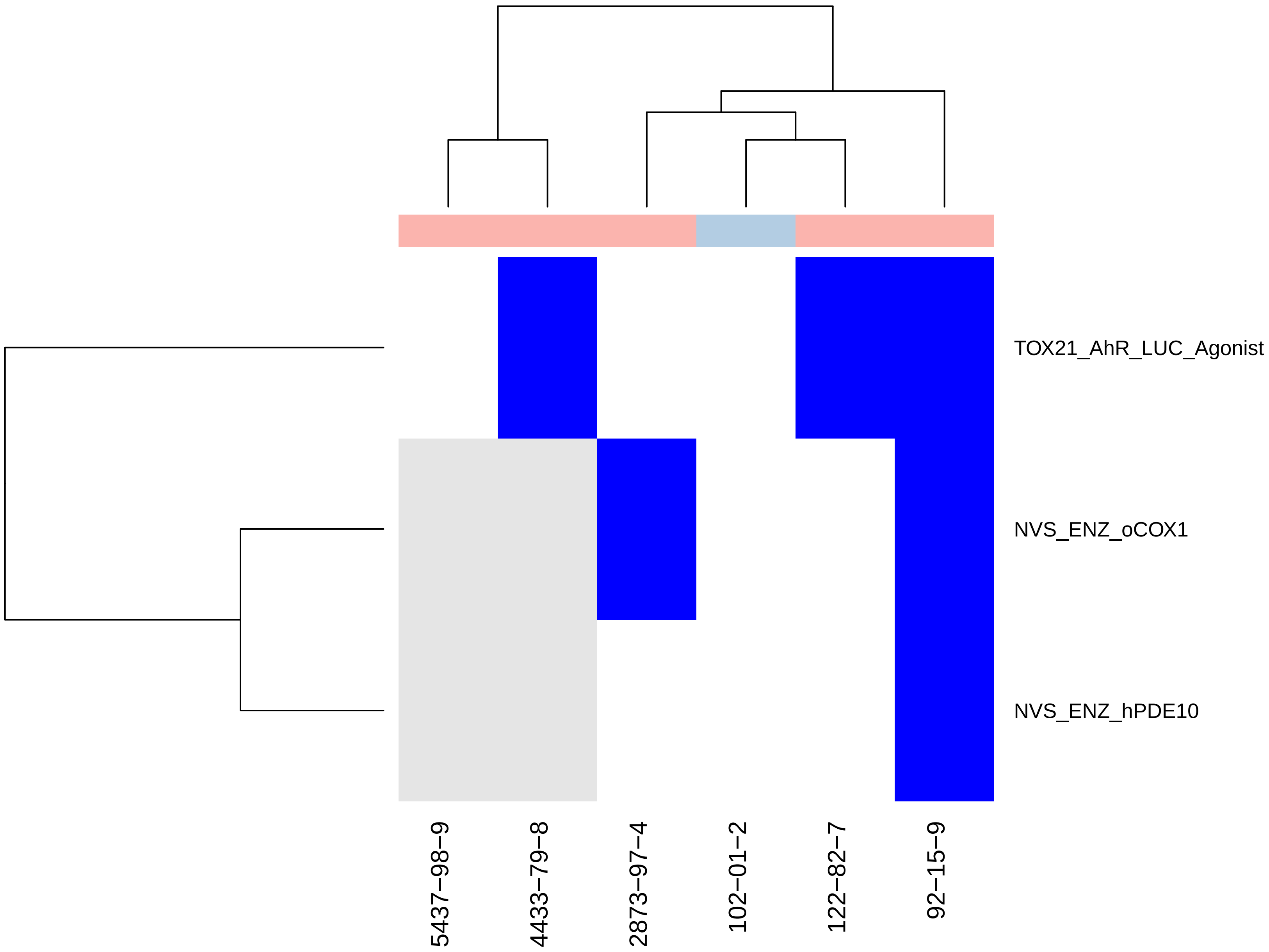

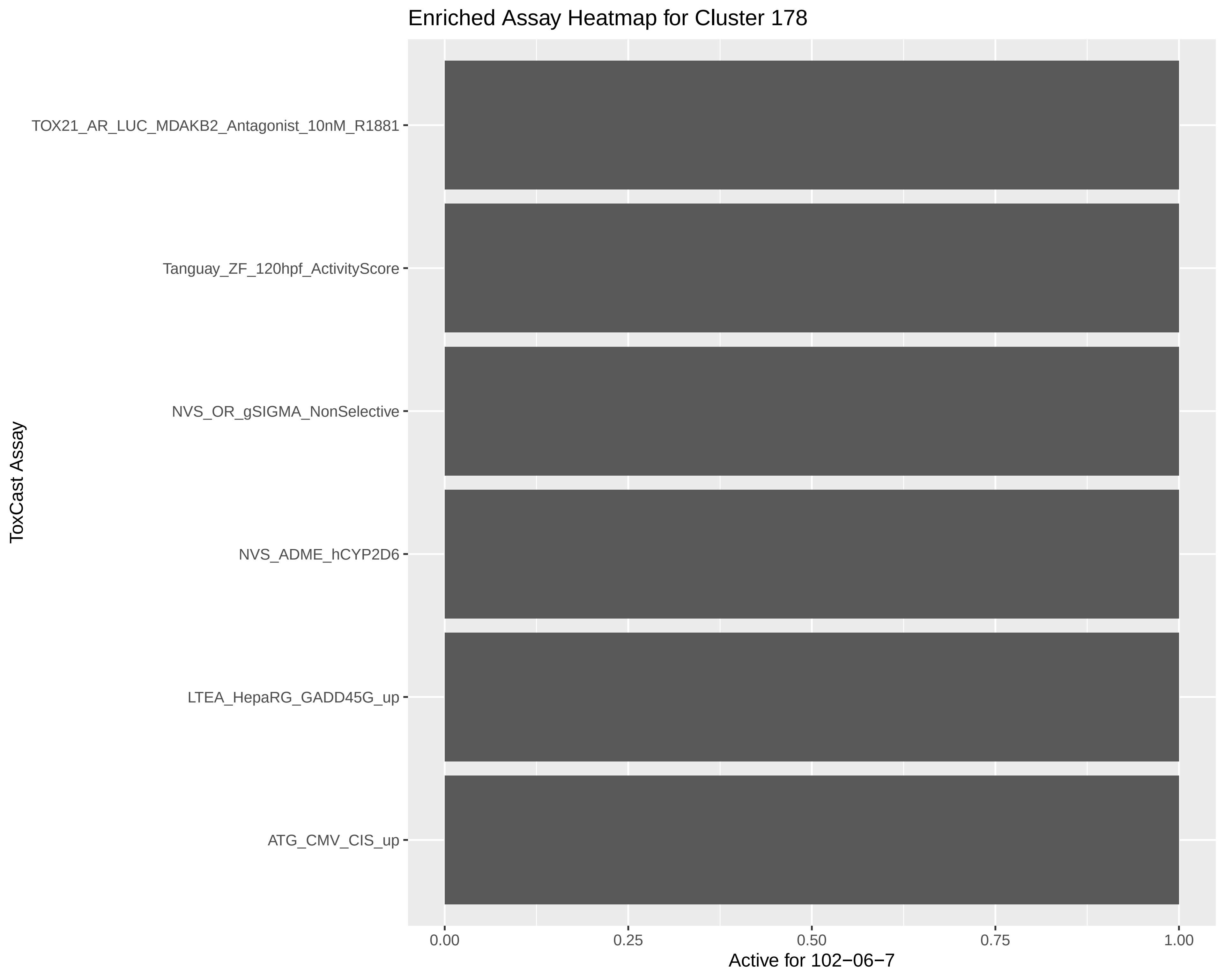

Enriched Assay Heatmap for Cluster 178
TOX21_AR_LUC_MDAKB2_Antagonist_10nM_R1881
Tanguay_ZF_120hpf_ActivityScore
NVS_OR_gSIGMA_NonSelective
NVS_ADME_hCYP2D6
LTEA_HepaRG_GADD45G_up
ATG_CMV_CIS_up
ToxCast Assay
0.00
0.25
0.50
0.75
1.00
Active for 102−06−7

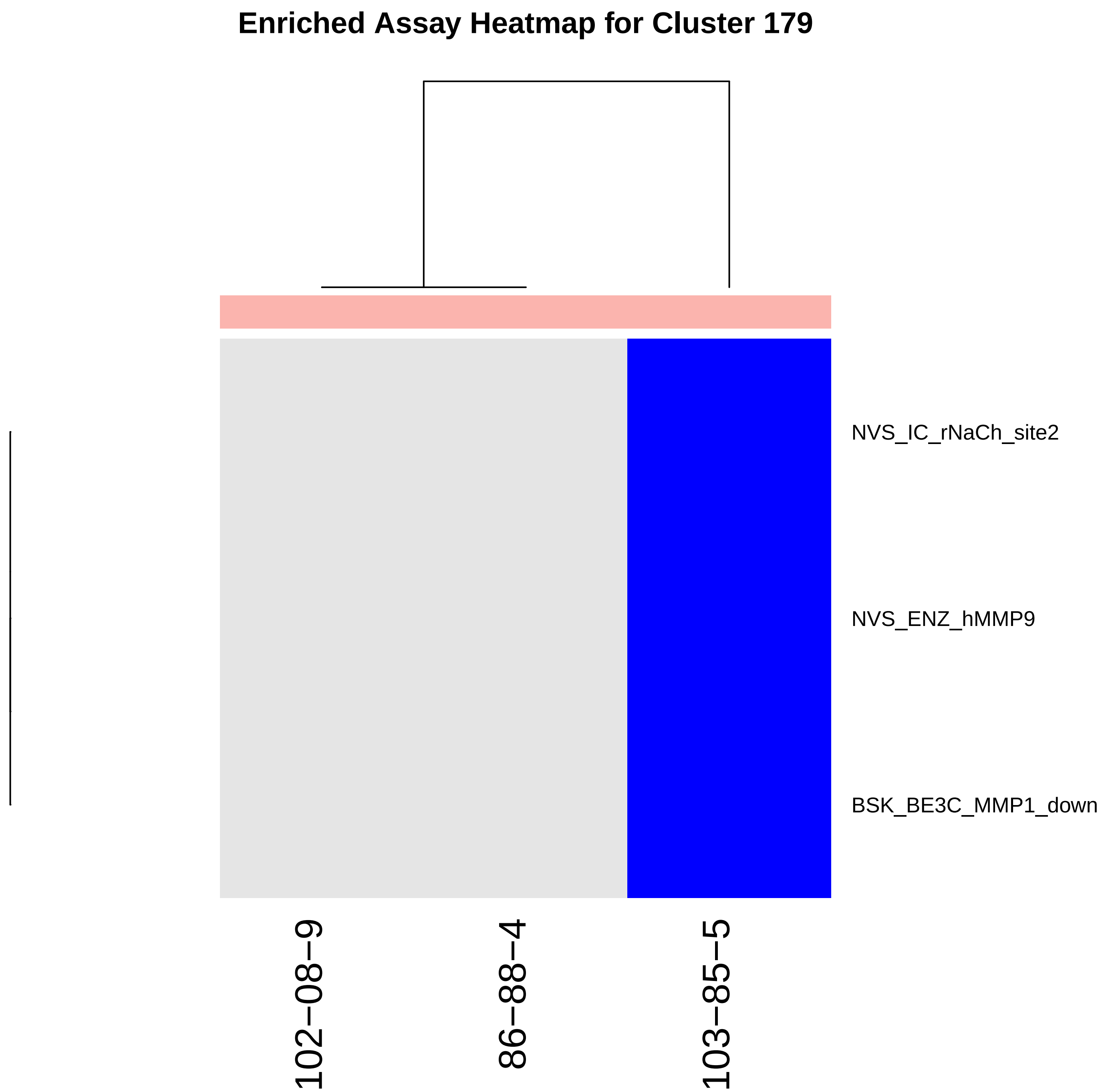
Enriched Assay Heatmap for Cluster 179
NVS_IC_rNaCh_site2
NVS_ENZ_hMMP9
BSK_BE3C_MMP1_down
102−08−9
86−88−4
103−85−5

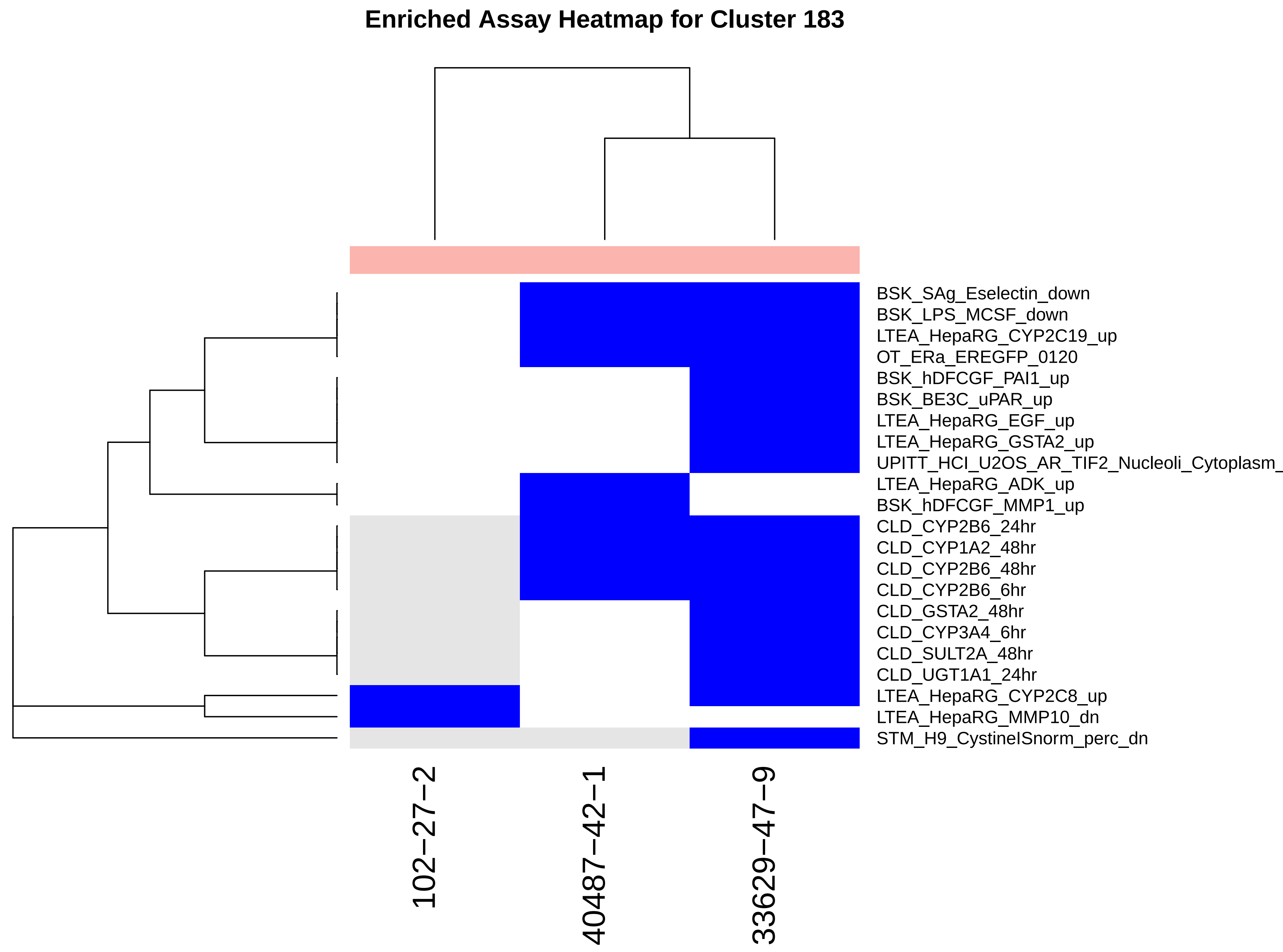

Enriched Assay Heatmap for Cluster 183
BSK_SAg_Eselectin_down
BSK_LPS_MCSF_down
LTEA_HepaRG_CYP2C19_up
OT_ERa_EREGFP_0120
BSK_hDFCGF_PAI1_up
BSK_BE3C_uPAR_up
LTEA_HepaRG_EGF_up
LTEA_HepaRG_GSTA2_up
UPITT_HCI_U2OS_AR_TIF2_Nucleoli_Cytoplasm_
LTEA_HepaRG_ADK_up
BSK_hDFCGF_MMP1_up
CLD_CYP2B6_24hr
CLD_CYP1A2_48hr
CLD_CYP2B6_48hr
CLD_CYP2B6_6hr
CLD_GSTA2_48hr
CLD_CYP3A4_6hr
CLD_SULT2A_48hr
CLD_UGT1A1_24hr
LTEA_HepaRG_CYP2C8_up
LTEA_HepaRG_MMP10_dn
STM_H9_CystineISnorm_perc_dn
102−27−2
40487−42−1
33629−47−9

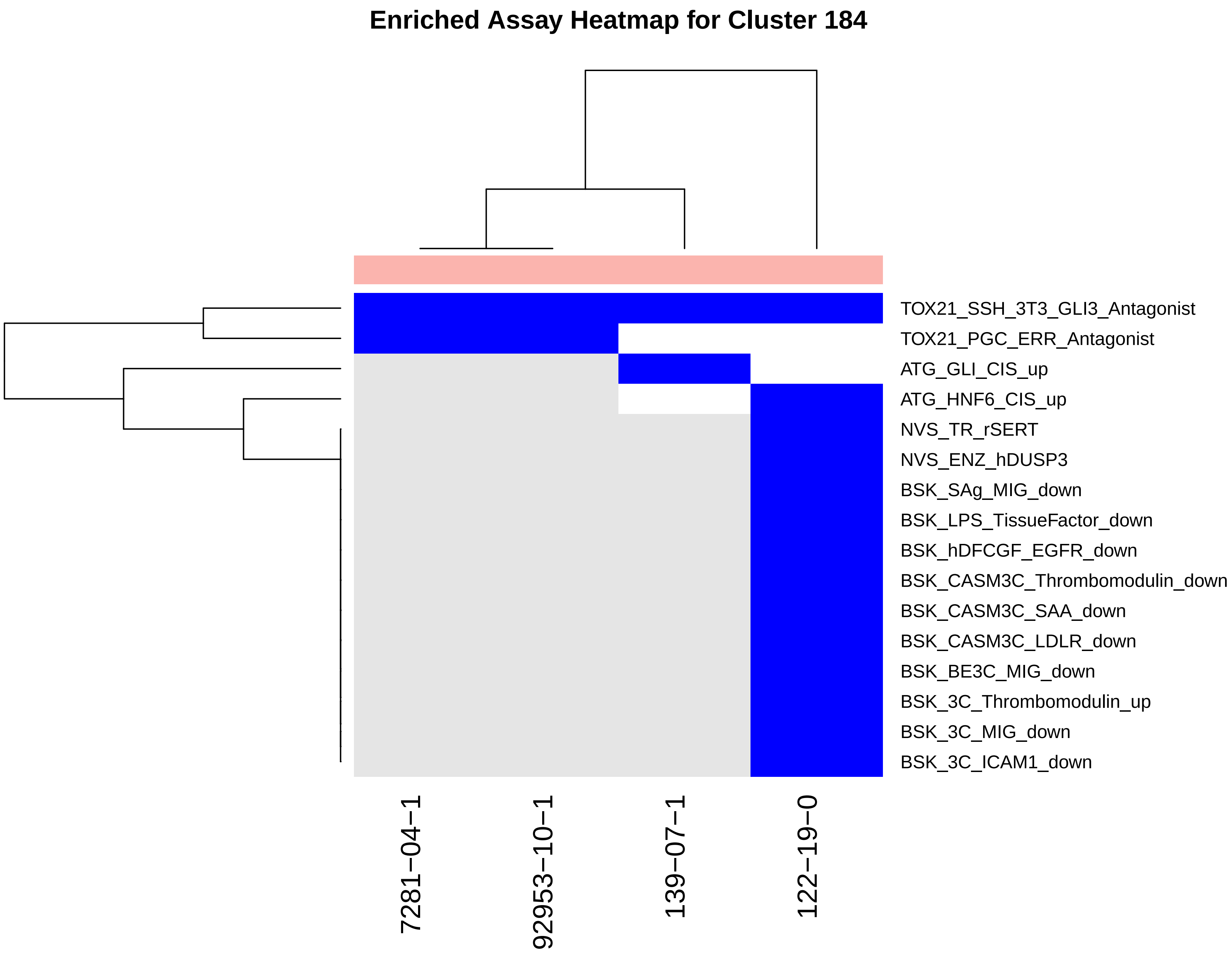
Enriched Assay Heatmap for Cluster 184
TOX21_SSH_3T3_GLI3_Antagonist
TOX21_PGC_ERR_Antagonist
ATG_GLI_CIS_up
ATG_HNF6_CIS_up
NVS_TR_rSERT
NVS_ENZ_hDUSP3
BSK_SAg_MIG_down
BSK_LPS_TissueFactor_down
BSK_hDFCGF_EGFR_down
BSK_CASM3C_Thrombomodulin_down
BSK_CASM3C_SAA_down
BSK_CASM3C_LDLR_down
BSK_BE3C_MIG_down
BSK_3C_Thrombomodulin_up
BSK_3C_MIG_down
BSK_3C_ICAM1_down
7281−04−1
92953−10−1
139−07−1
122−19−0

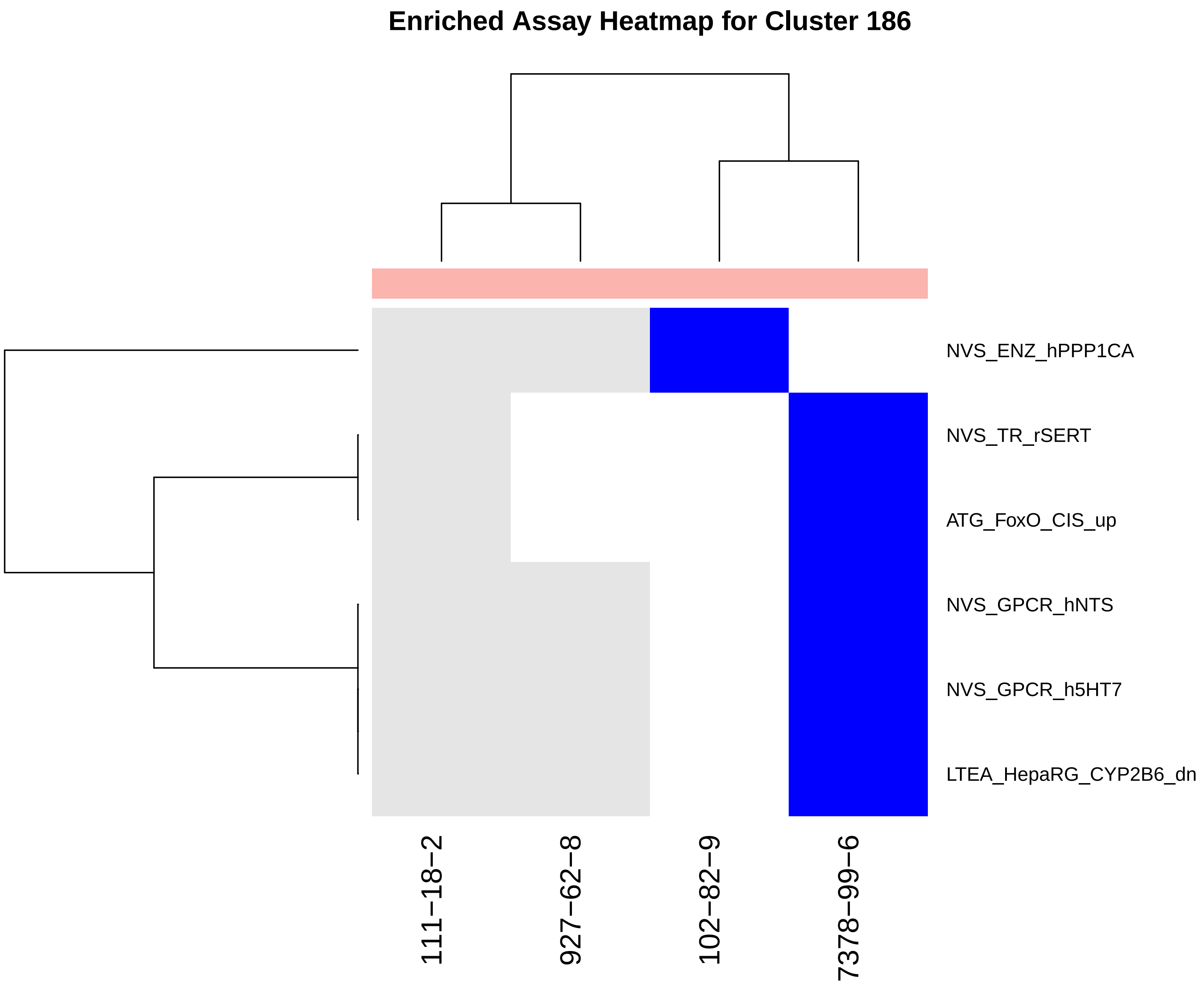
Enriched Assay Heatmap for Cluster 186
NVS_ENZ_hPPP1CA
NVS_TR_rSERT
ATG_FoxO_CIS_up
NVS_GPCR_hNTS
NVS_GPCR_h5HT7
LTEA_HepaRG_CYP2B6_dn
111−18−2
927−62−8
102−82−9
7378−99−6

Enriched Assay Heatmap for Cluster 194

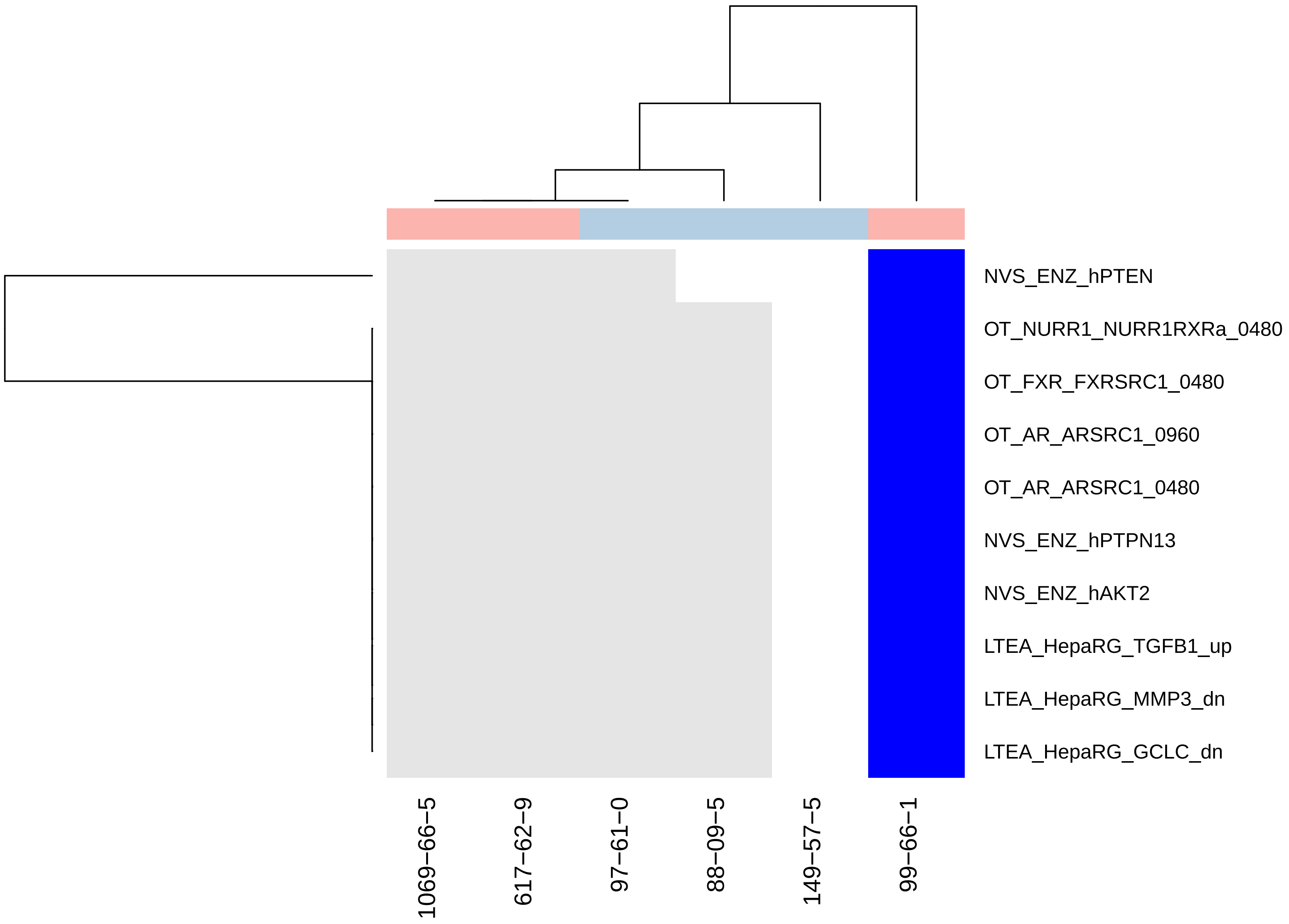

Enriched Assay Heatmap for Cluster 195

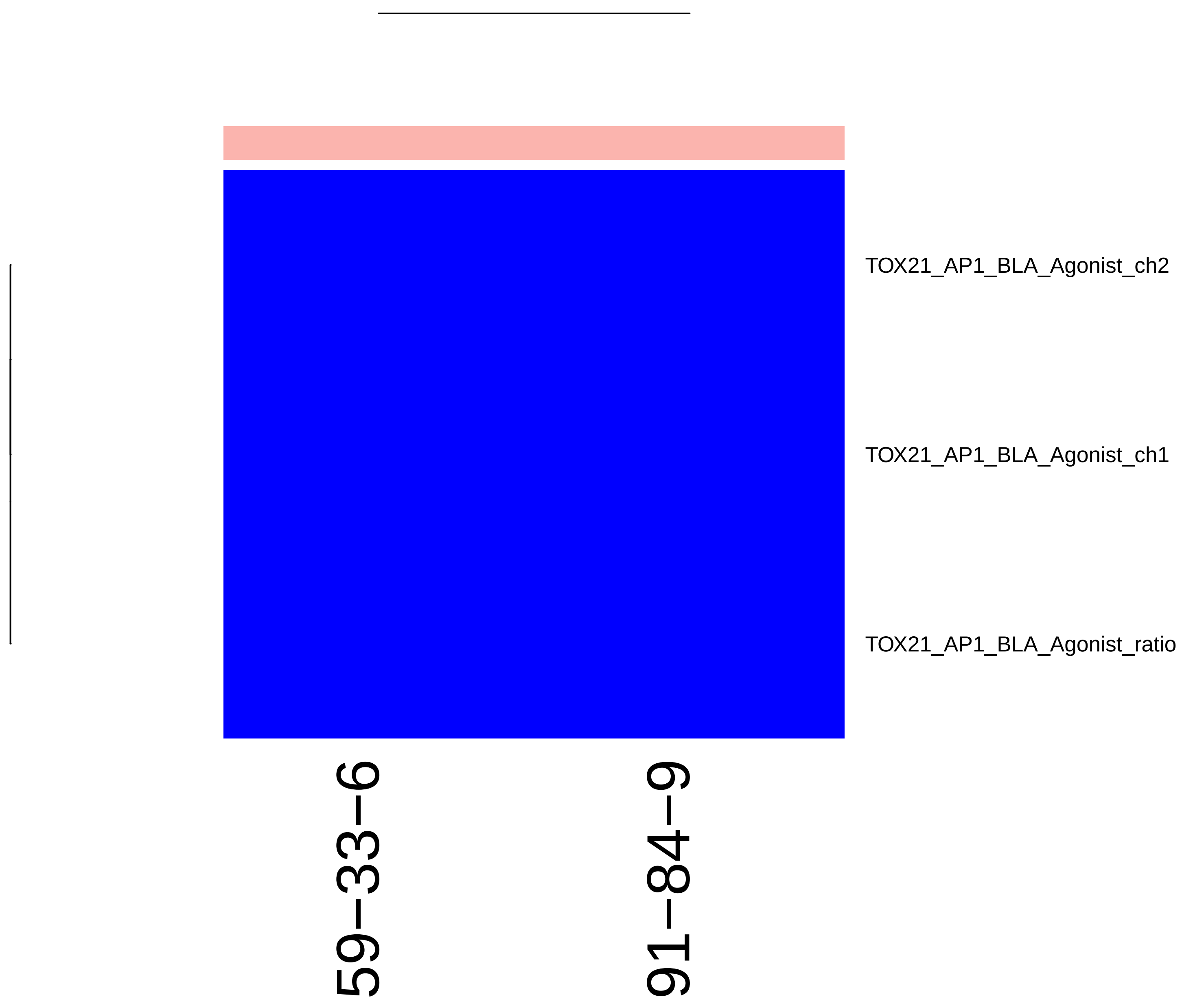

Enriched Assay Heatmap for Cluster 197

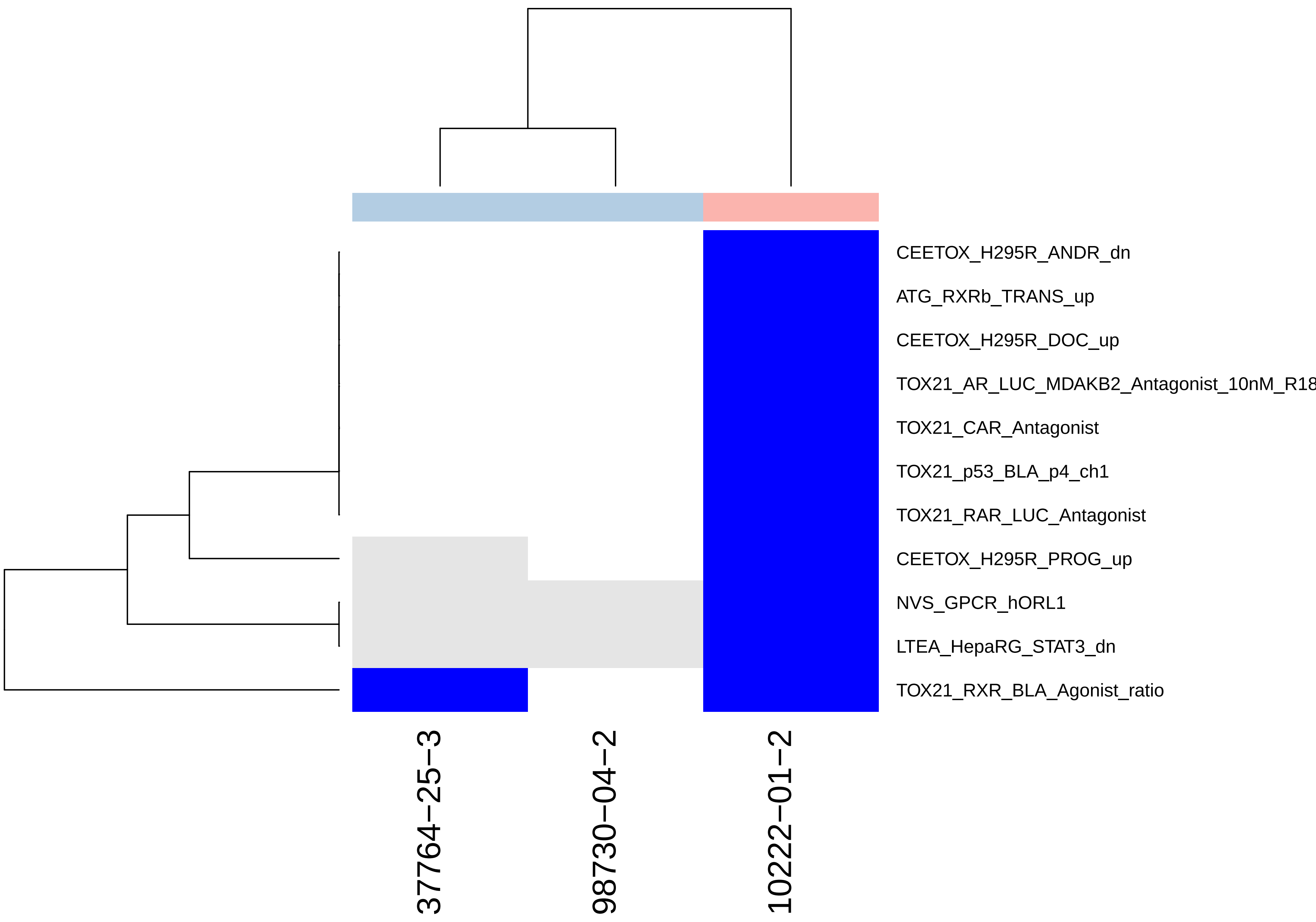

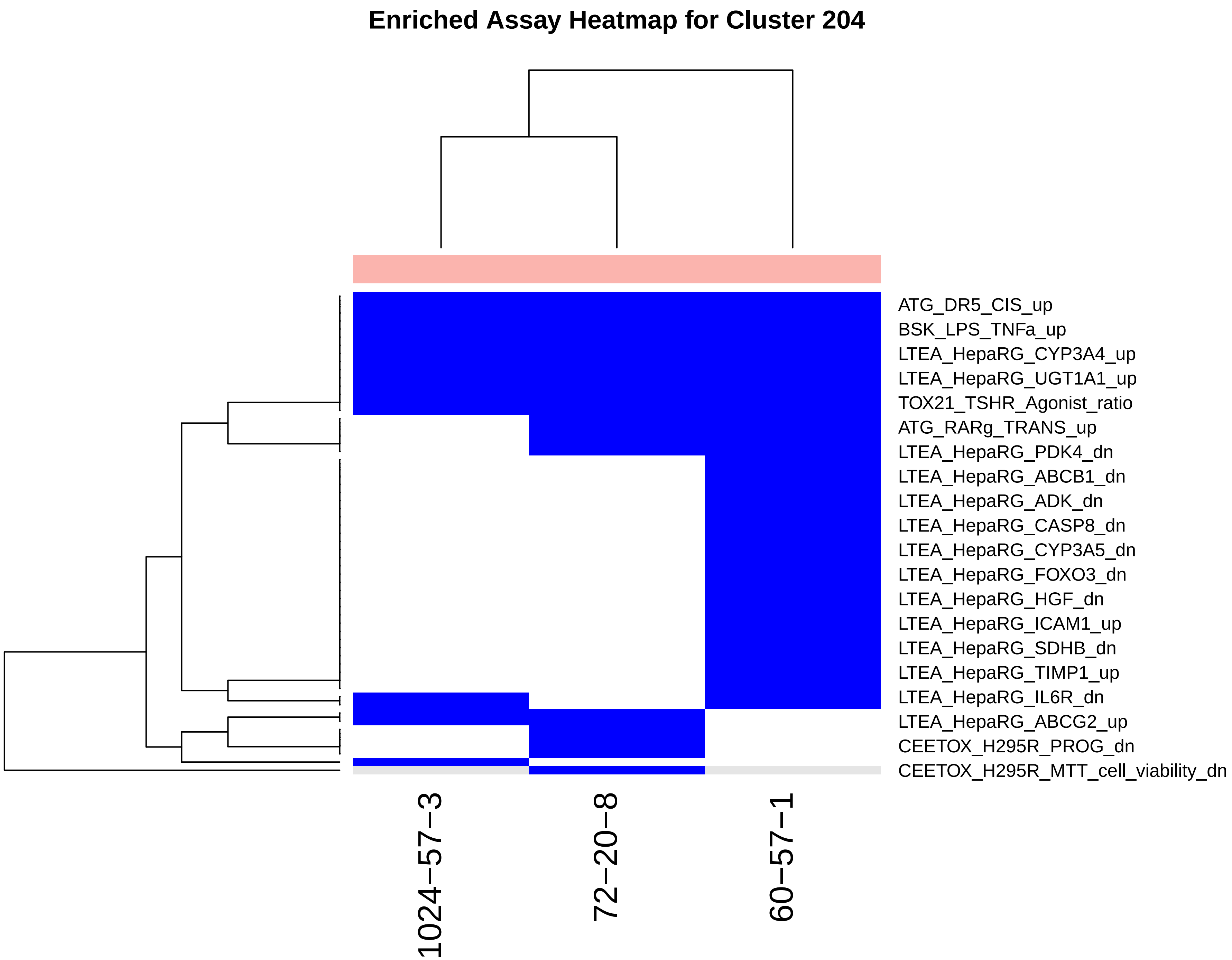

Enriched Assay Heatmap for Cluster 204
ATG_DR5_CIS_up
BSK_LPS_TNFa_up
LTEA_HepaRG_CYP3A4_up
LTEA_HepaRG_UGT1A1_up
TOX21_TSHR_Agonist_ratio
ATG_RARg_TRANS_up
LTEA_HepaRG_PDK4_dn
LTEA_HepaRG_ABCB1_dn
LTEA_HepaRG_ADK_dn
LTEA_HepaRG_CASP8_dn
LTEA_HepaRG_CYP3A5_dn
LTEA_HepaRG_FOXO3_dn
LTEA_HepaRG_HGF_dn
LTEA_HepaRG_ICAM1_up
LTEA_HepaRG_SDHB_dn
LTEA_HepaRG_TIMP1_up
LTEA_HepaRG_IL6R_dn
LTEA_HepaRG_ABCG2_up
CEETOX_H295R_PROG_dn
CEETOX_H295R_MTT_cell_viability_dn
1024−57−3
72−20−8
60−57−1

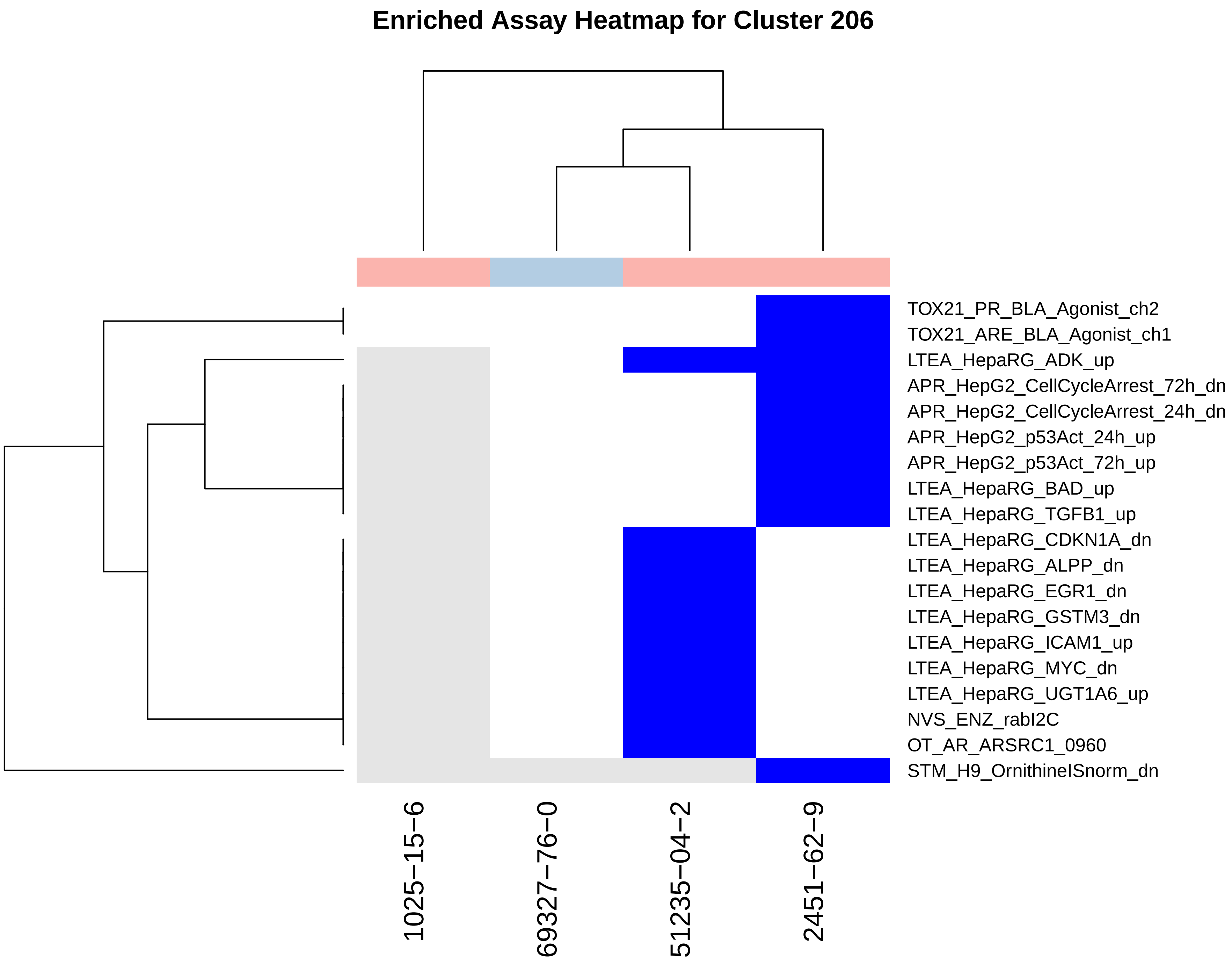
Enriched Assay Heatmap for Cluster 206
TOX21_PR_BLA_Agonist_ch2
TOX21_ARE_BLA_Agonist_ch1
LTEA_HepaRG_ADK_up
APR_HepG2_CellCycleArrest_72h_dn
APR_HepG2_CellCycleArrest_24h_dn
APR_HepG2_p53Act_24h_up
APR_HepG2_p53Act_72h_up
LTEA_HepaRG_BAD_up
LTEA_HepaRG_TGFB1_up
LTEA_HepaRG_CDKN1A_dn
LTEA_HepaRG_ALPP_dn
LTEA_HepaRG_EGR1_dn
LTEA_HepaRG_GSTM3_dn
LTEA_HepaRG_ICAM1_up
LTEA_HepaRG_MYC_dn
LTEA_HepaRG_UGT1A6_up
NVS_ENZ_rabI2C
OT_AR_ARSRC1_0960
STM_H9_OrnithineISnorm_dn
1025−15−6
69327−76−0
51235−04−2
2451−62−9

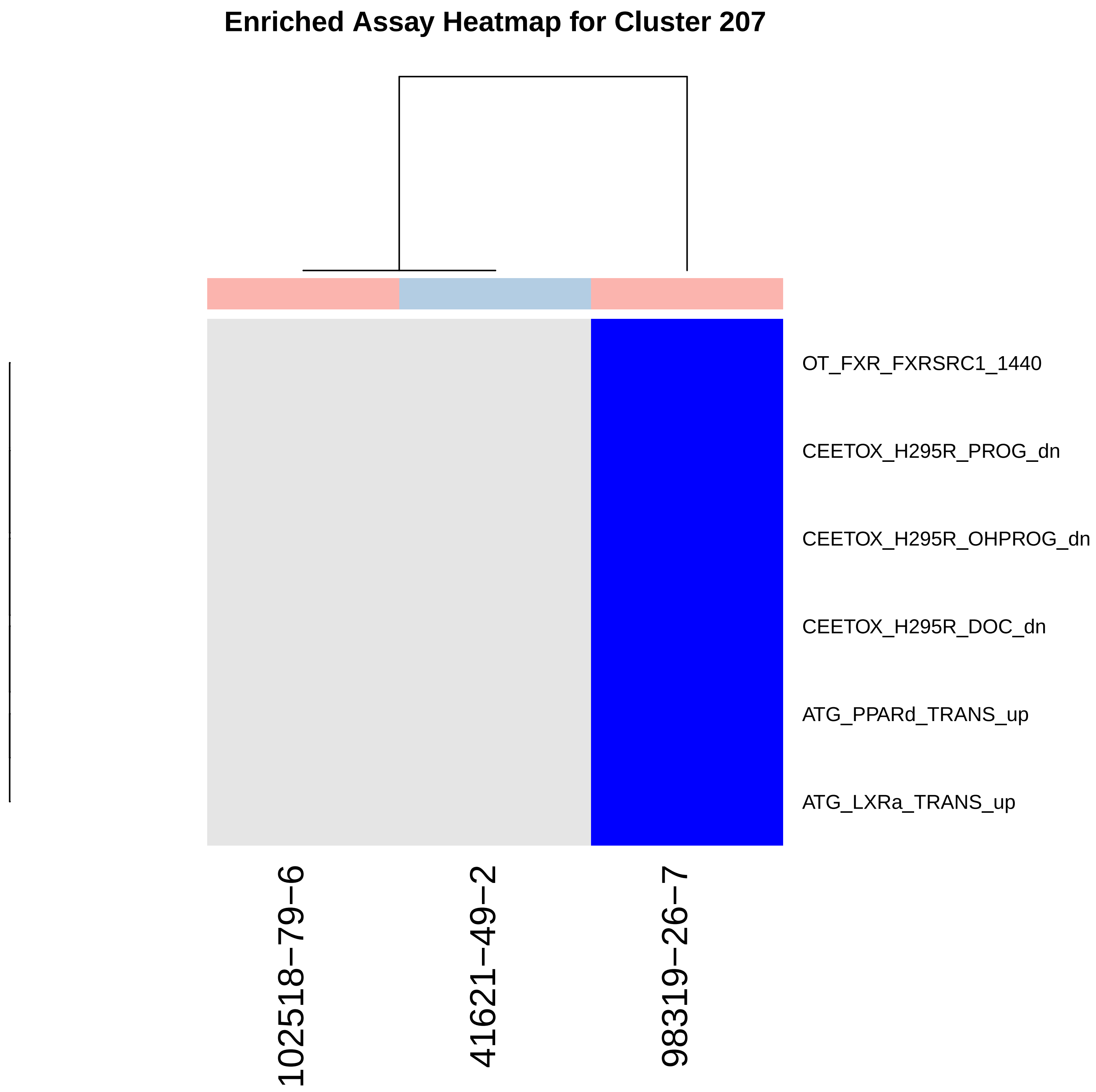

Enriched Assay Heatmap for Cluster 207
OT_FXR_FXRSRC1_1440
CEETOX_H295R_PROG_dn
CEETOX_H295R_OHPROG_dn
CEETOX_H295R_DOC_dn
ATG_PPARd_TRANS_up
ATG_LXRa_TRANS_up
102518−79−6
41621−49−2
98319−26−7

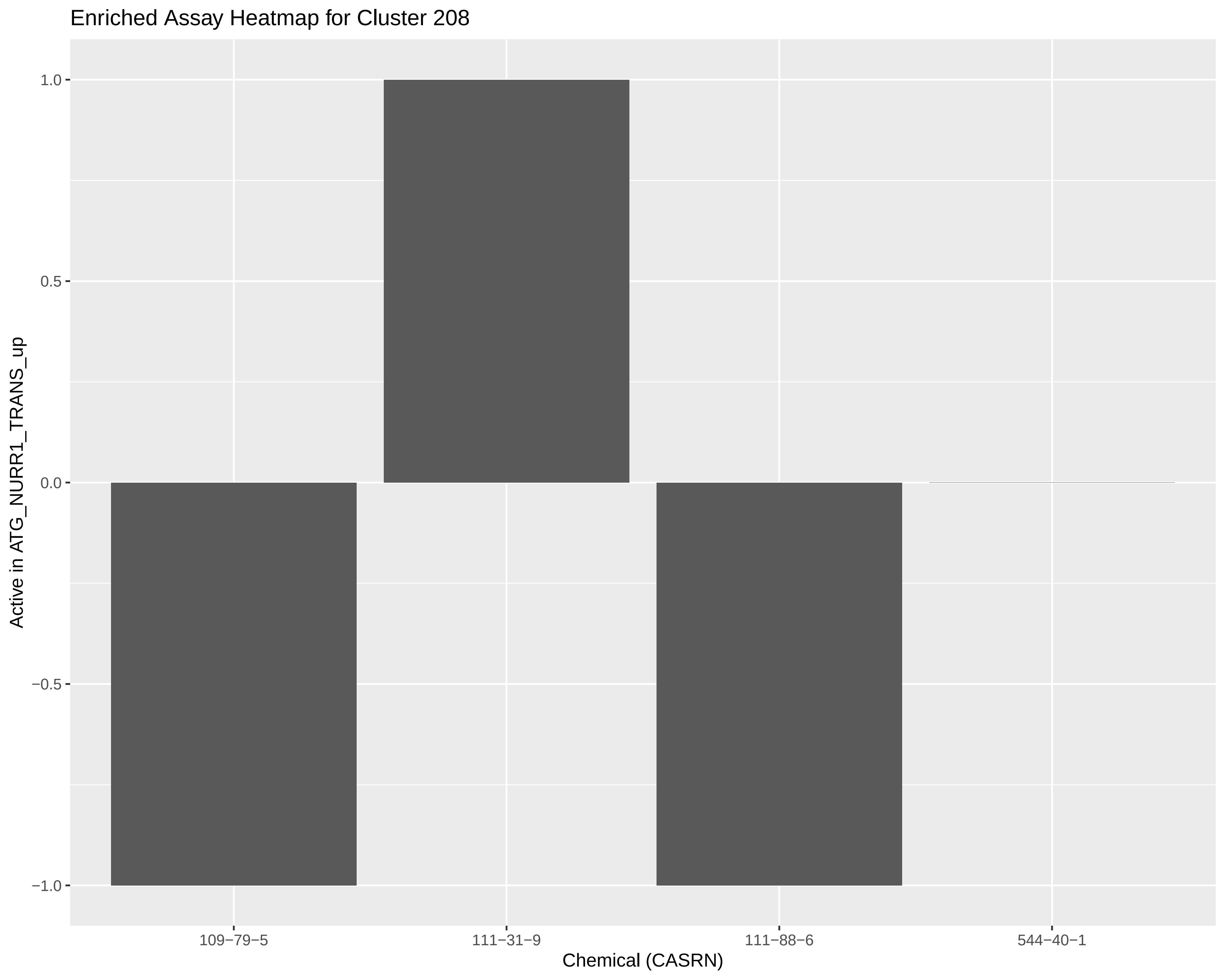
Enriched Assay Heatmap for Cluster 208
Active in ATG_NURR1_TRANS_up
1.0
0.5
0.0
−0.5
−1.0
109−79−5
111−31−9
111−88−6
544−40−1
Chemical (CASRN)

Enriched Assay Heatmap for Cluster 212

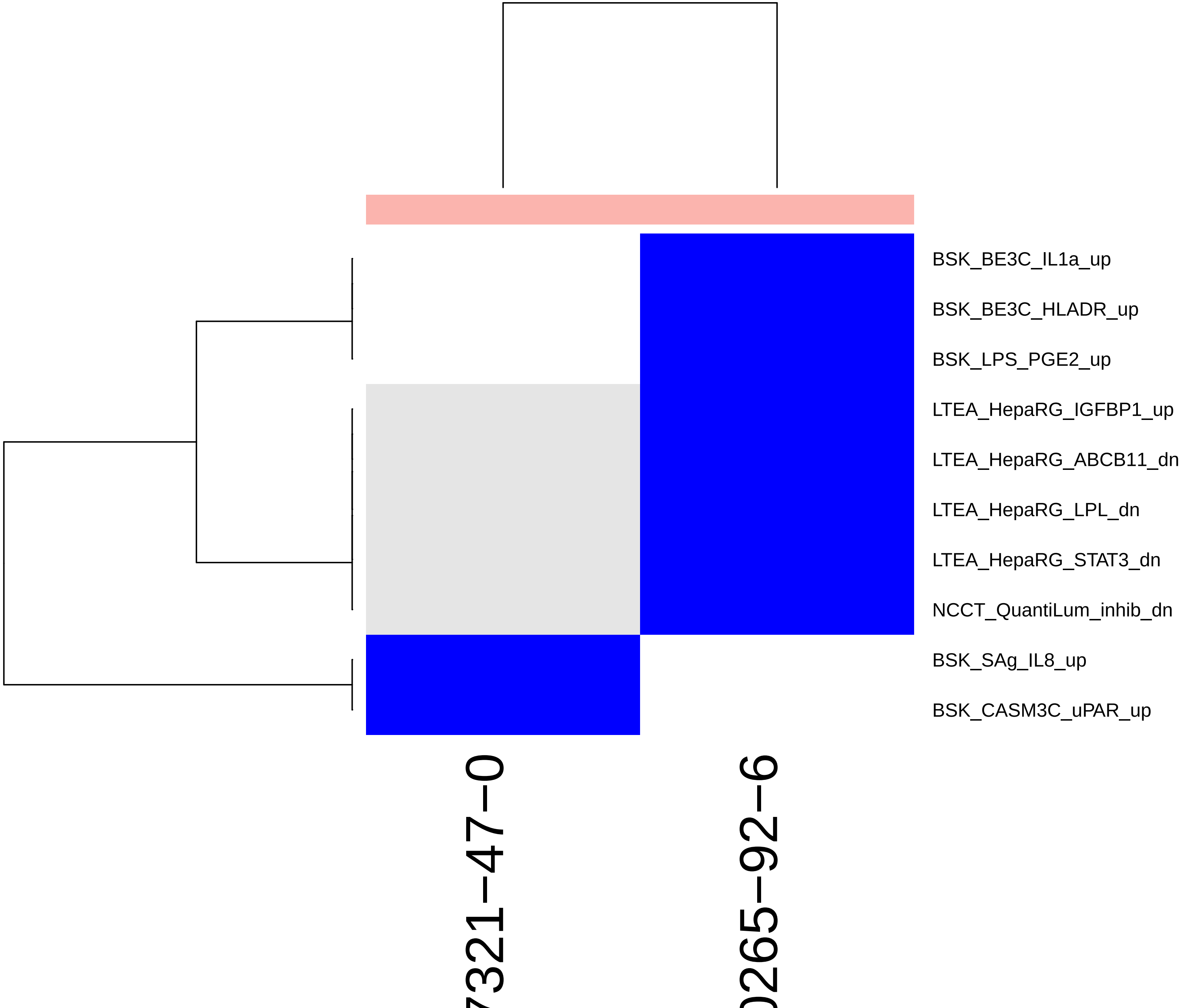

Enriched Assay Heatmap for Cluster 213

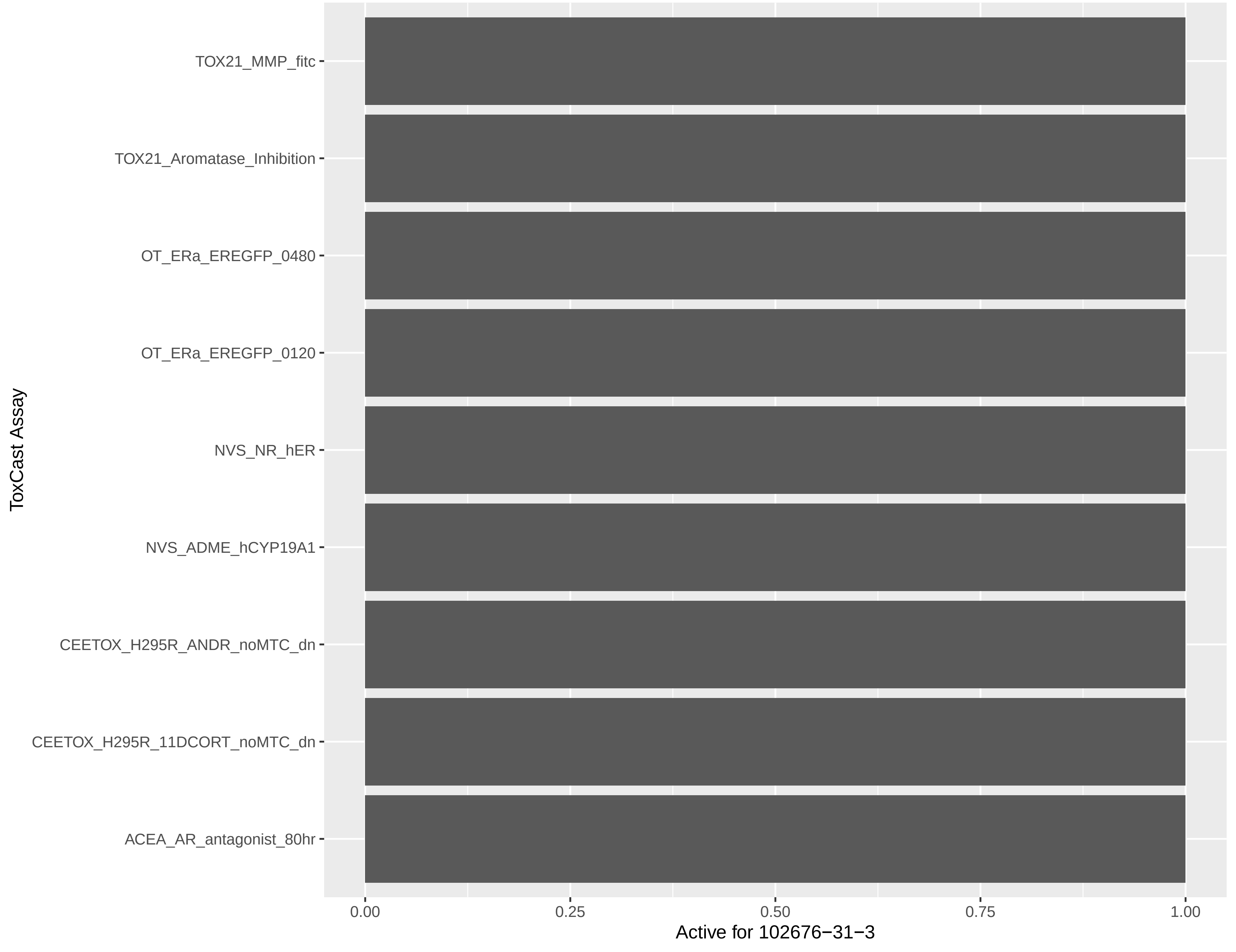

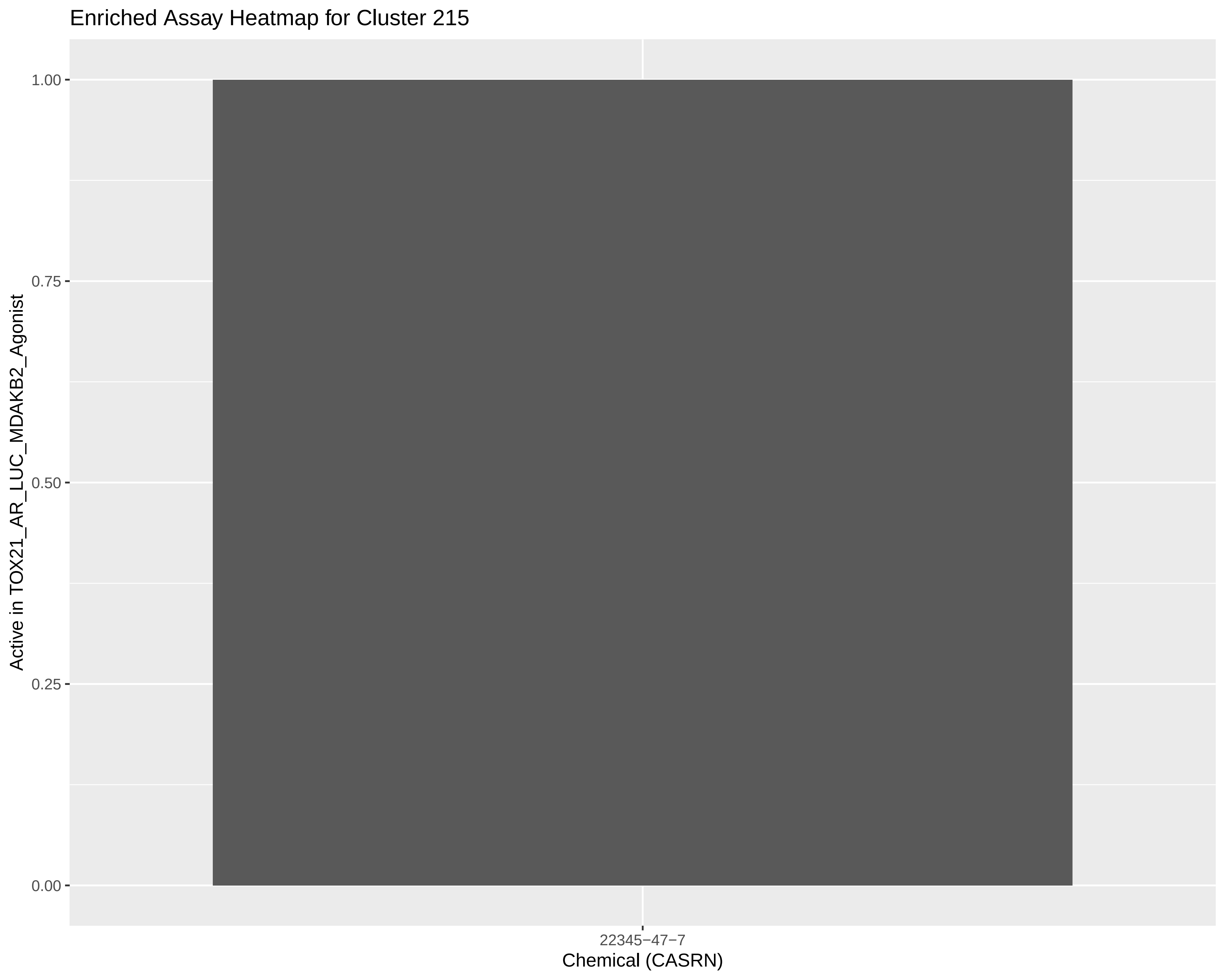

Enriched Assay Heatmap for Cluster 215
Active in TOX21_AR_LUC_MDAKB2_Agonist
1.00
0.75
0.50
0.25
0.00
22345−47−7
Chemical (CASRN)

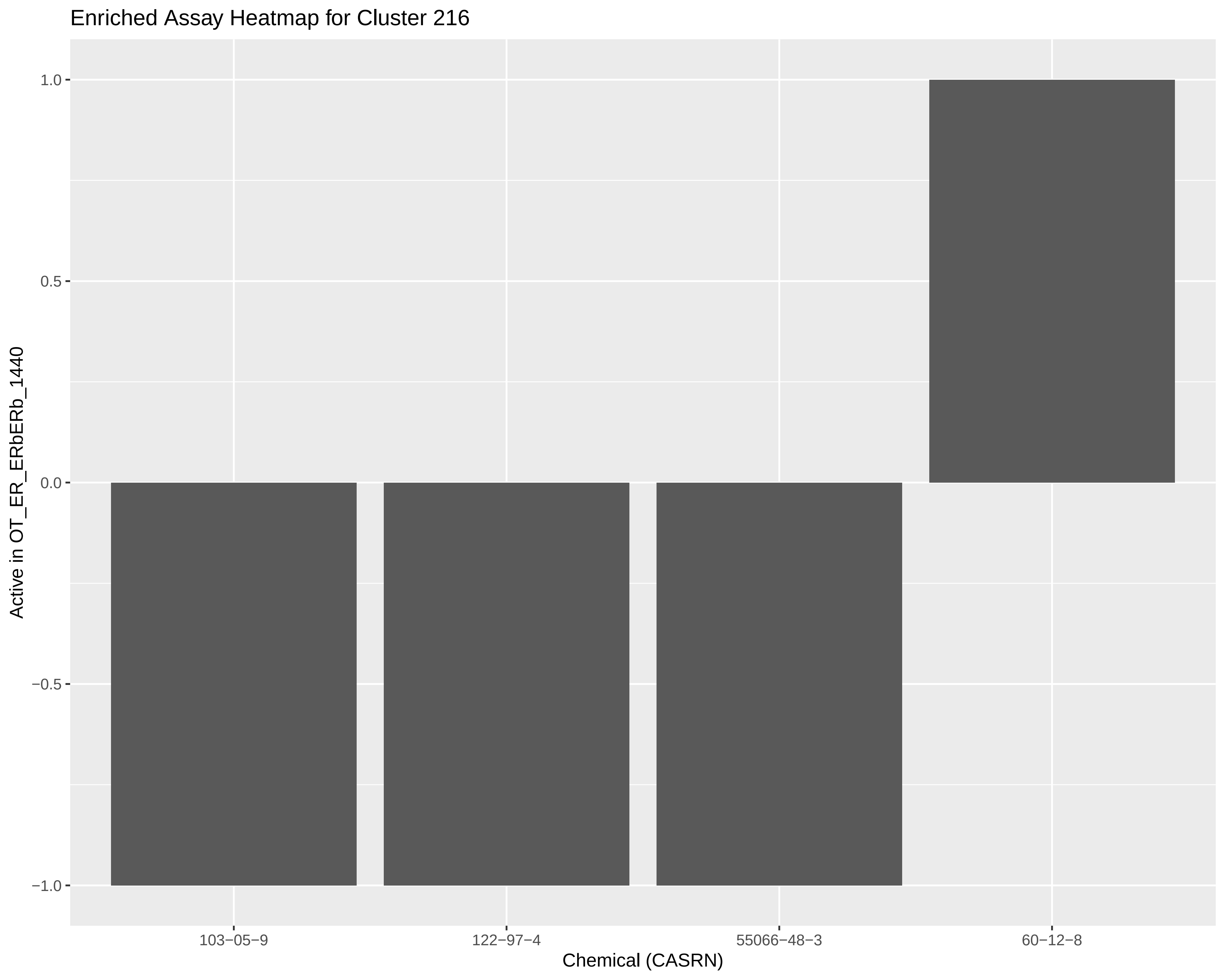
Enriched Assay Heatmap for Cluster 216
Active in OT_ER_ERbERb_1440
1.0
0.5
0.0
−0.5
−1.0
103−05−9
122−97−4
55066−48−3
60−12−8
Chemical (CASRN)

Enriched Assay Heatmap for Cluster 217

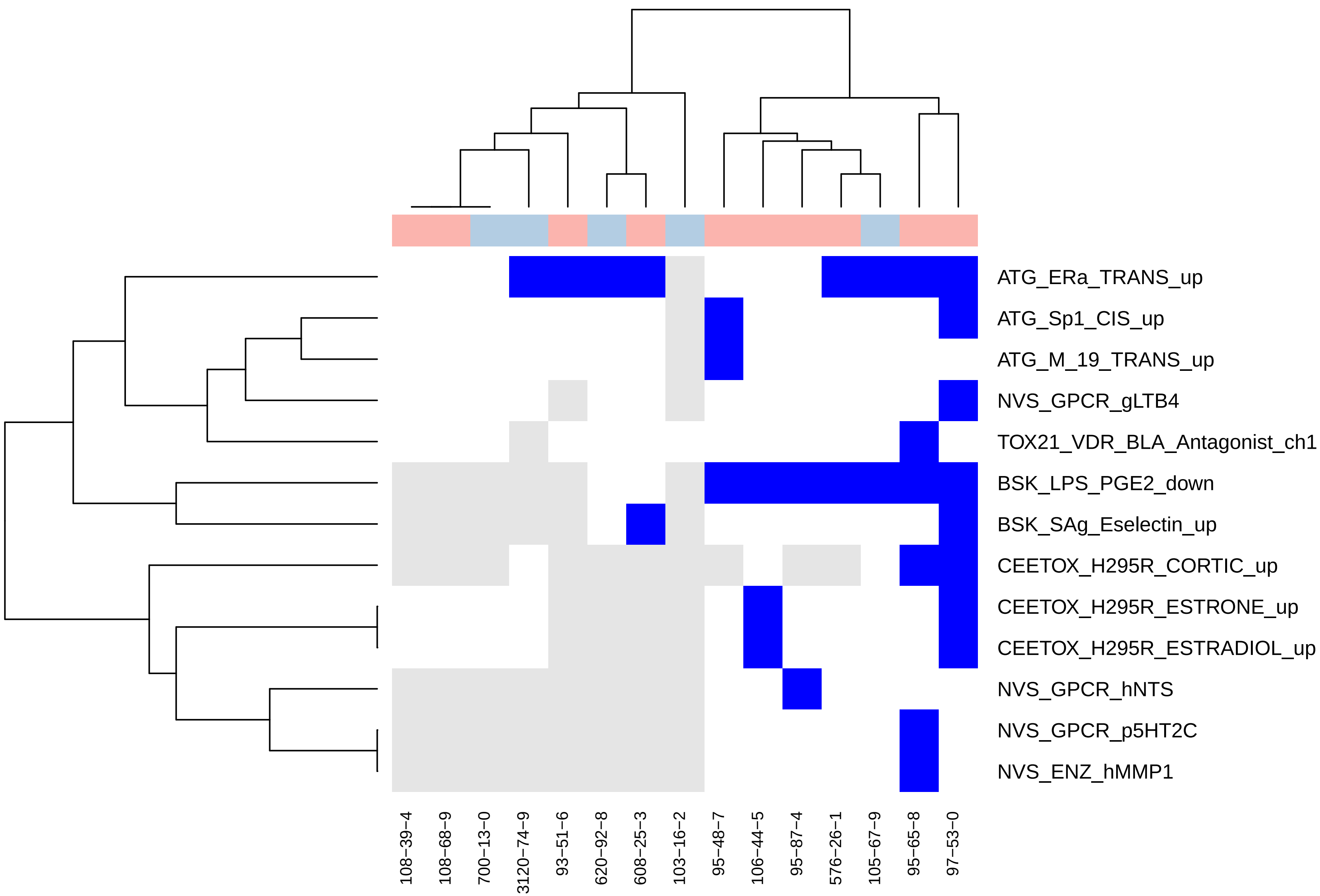

Enriched Assay Heatmap for Cluster 218

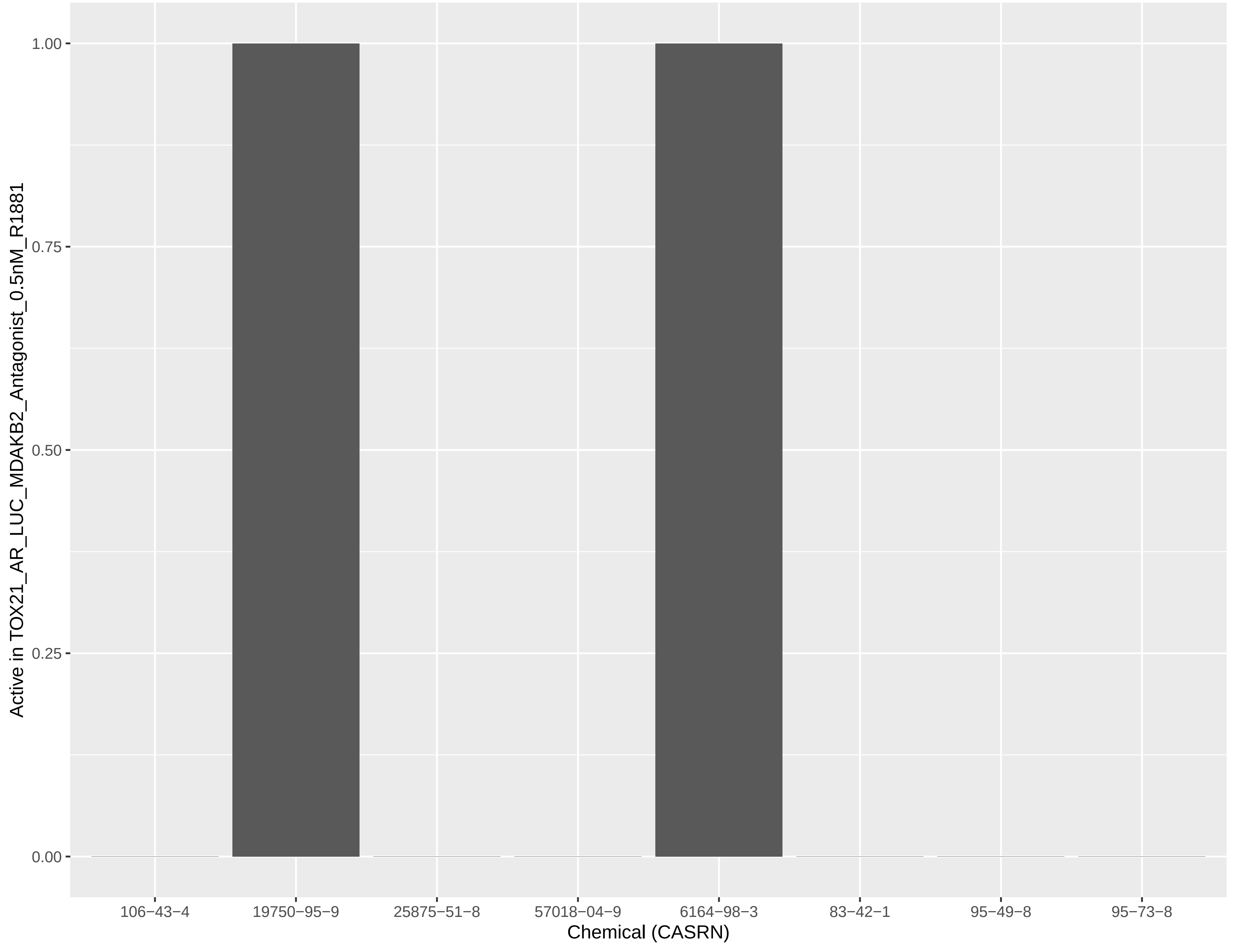

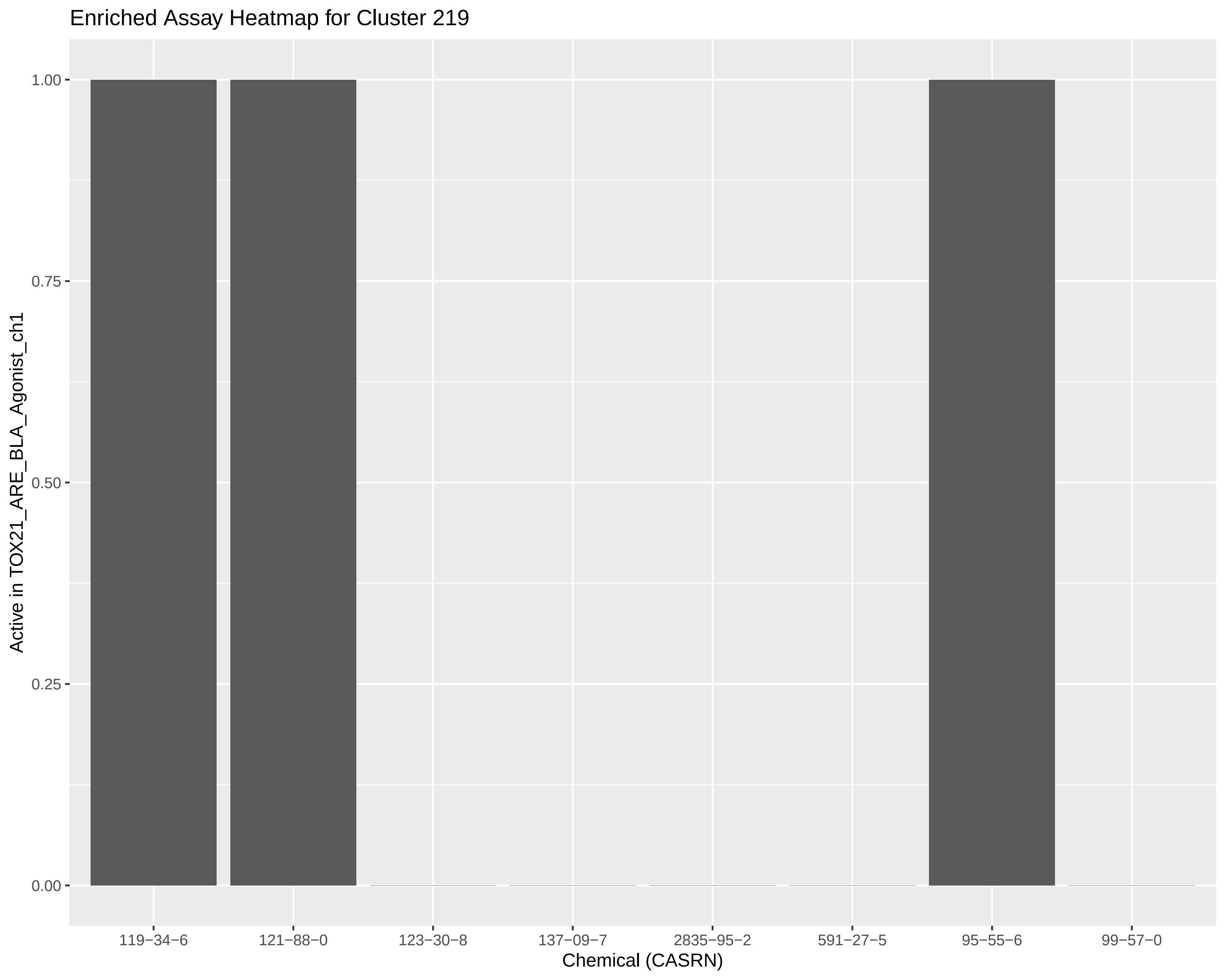

Enriched Assay Heatmap for Cluster 219
1.00
0.75
0.50
0.25
0.00
Active in TOX21_ARE_BLA_Agonist_ch1
119−34−6
121−88−0
123−30−8
137−09−7
2835−95−2
591−27−5
95−55−6
99−57−0
Chemical (CASRN)

Enriched Assay Heatmap for Cluster 221

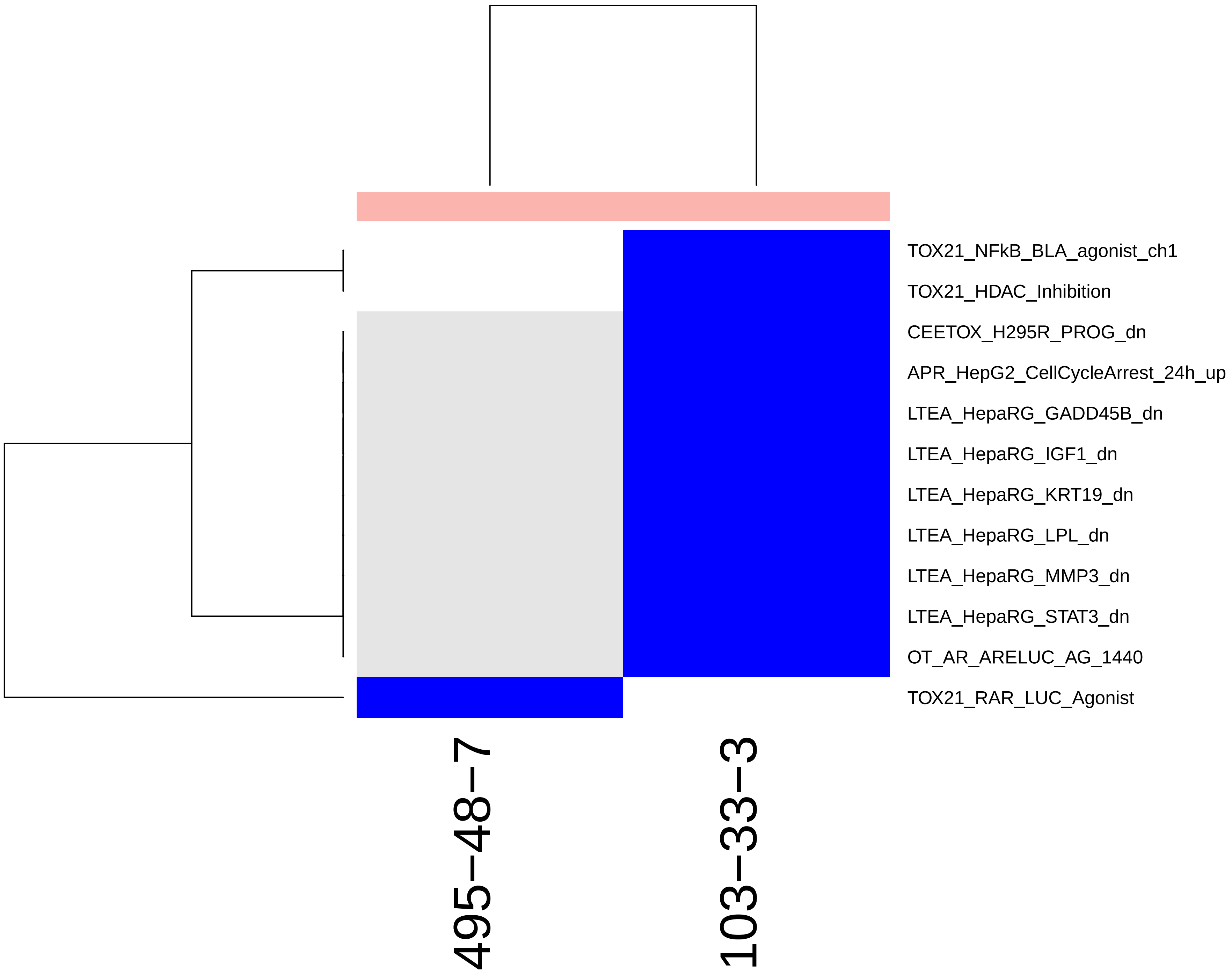

Enriched Assay Heatmap for Cluster 226

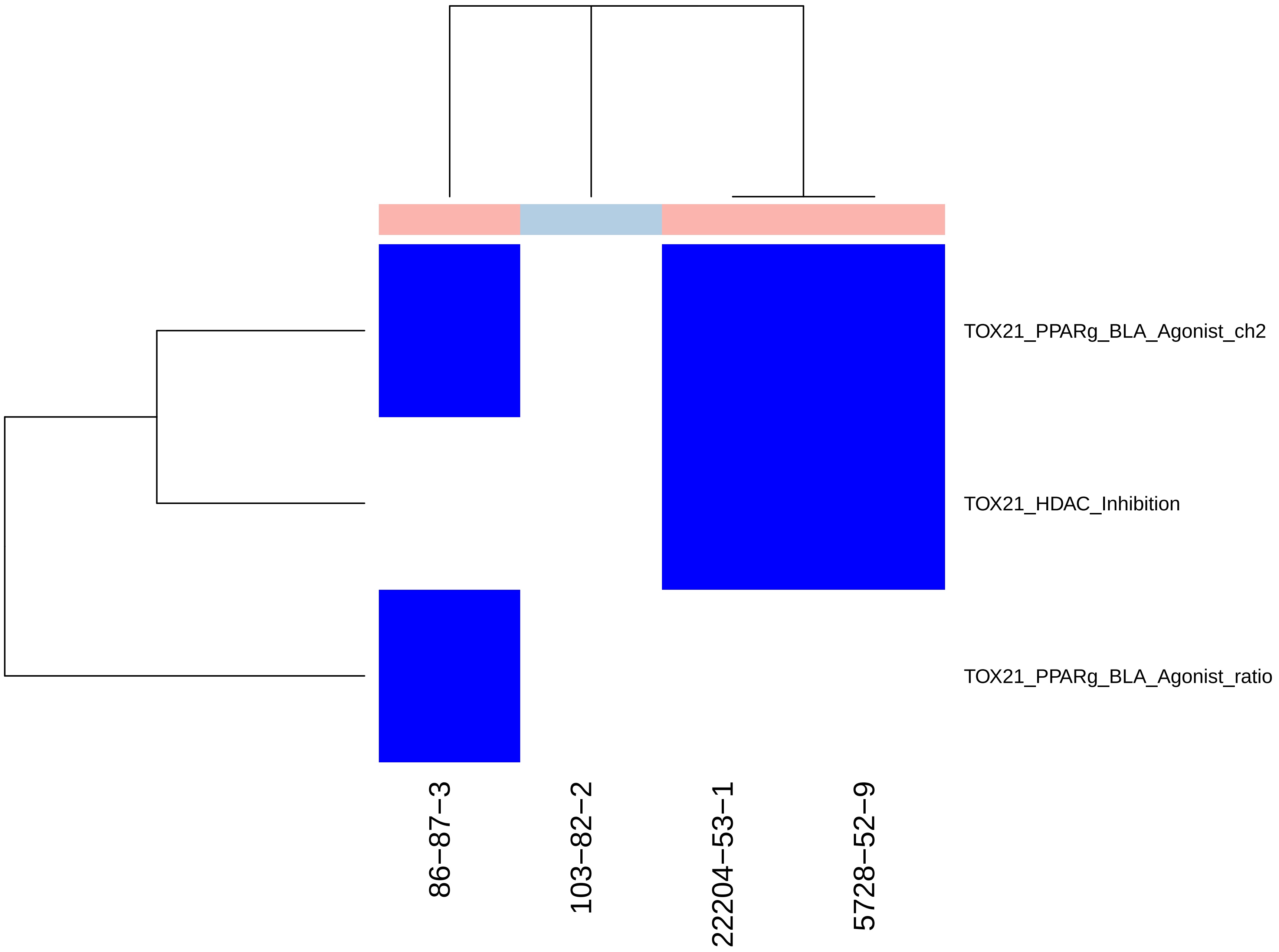

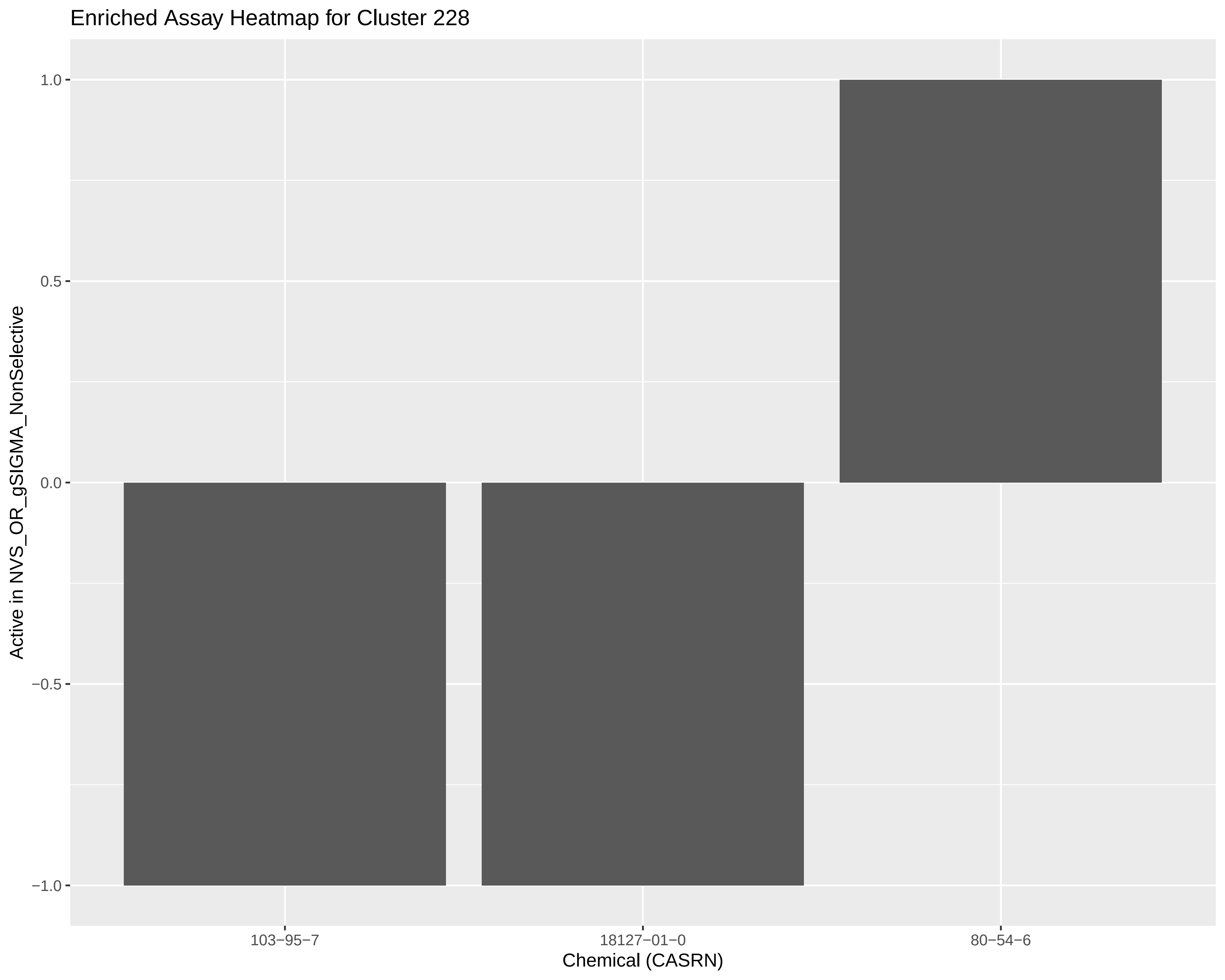
Enriched Assay Heatmap for Cluster 228
Active in NVS_OR_gSIGMA_NonSelective
1.0
0.5
0.0
−0.5
−1.0
103−95−7
18127−01−0
80−54−6
Chemical (CASRN)

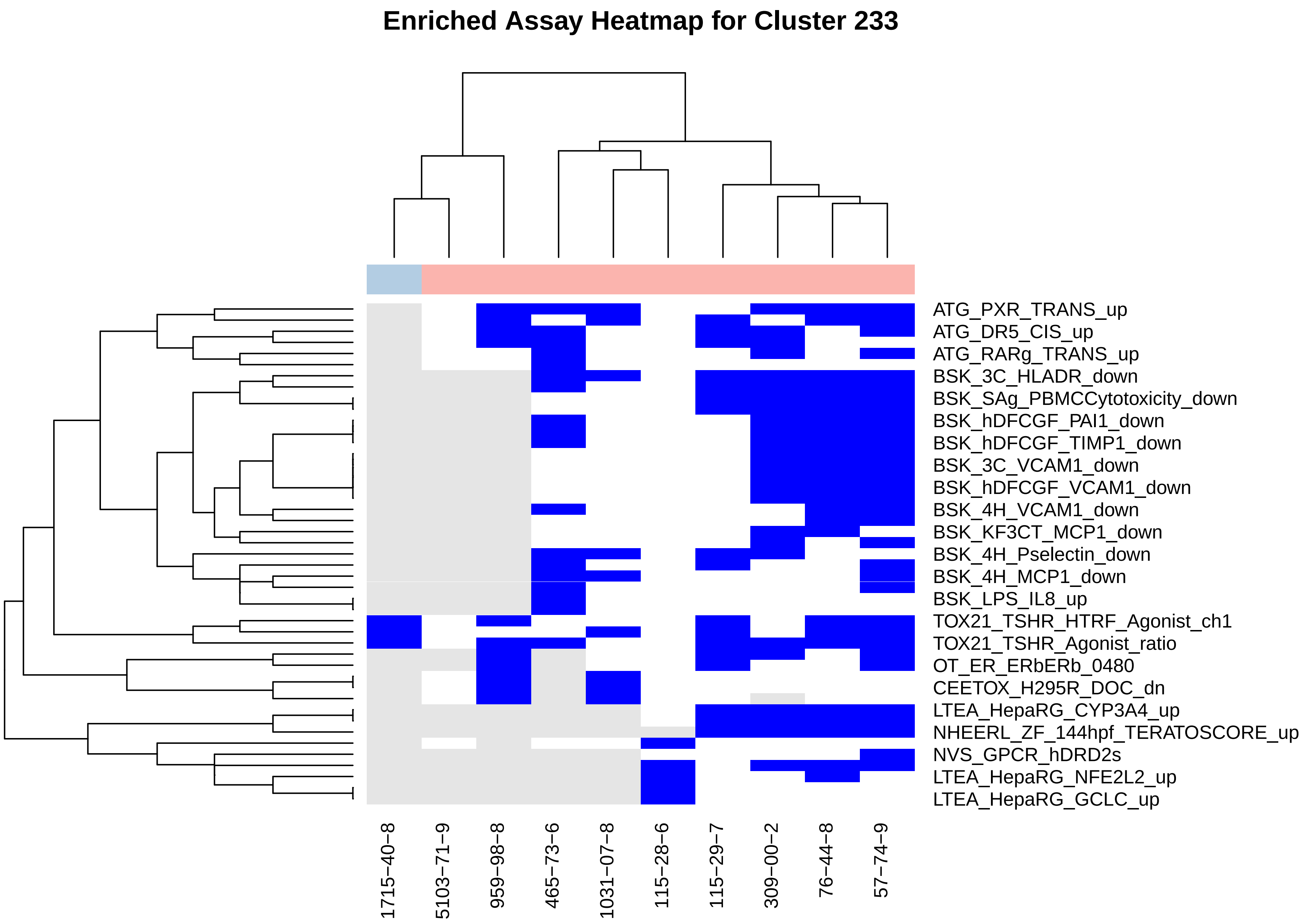
Enriched Assay Heatmap for Cluster 233
ATG_PXR_TRANS_up
ATG_DR5_CIS_up
ATG_RARg_TRANS_up
BSK_3C_HLADR_down
BSK_SAg_PBMCCytotoxicity_down
BSK_hDFCGF_PAI1_down
BSK_hDFCGF_TIMP1_down
BSK_3C_VCAM1_down
BSK_hDFCGF_VCAM1_down
BSK_4H_VCAM1_down
BSK_KF3CT_MCP1_down
BSK_4H_Pselectin_down
BSK_4H_MCP1_down
BSK_LPS_IL8_up
TOX21_TSHR_HTRF_Agonist_ch1
TOX21_TSHR_Agonist_ratio
OT_ER_ERbERb_0480
CEETOX_H295R_DOC_dn
LTEA_HepaRG_CYP3A4_up
NHEERL_ZF_144hpf_TERATOSCORE_up
NVS_GPCR_hDRD2s
LTEA_HepaRG_NFE2L2_up
LTEA_HepaRG_GCLC_up
1715−40−8
5103−71−9
959−98−8
465−73−6
1031−07−8
115−28−6
115−29−7
309−00−2
76−44−8
57−74−9

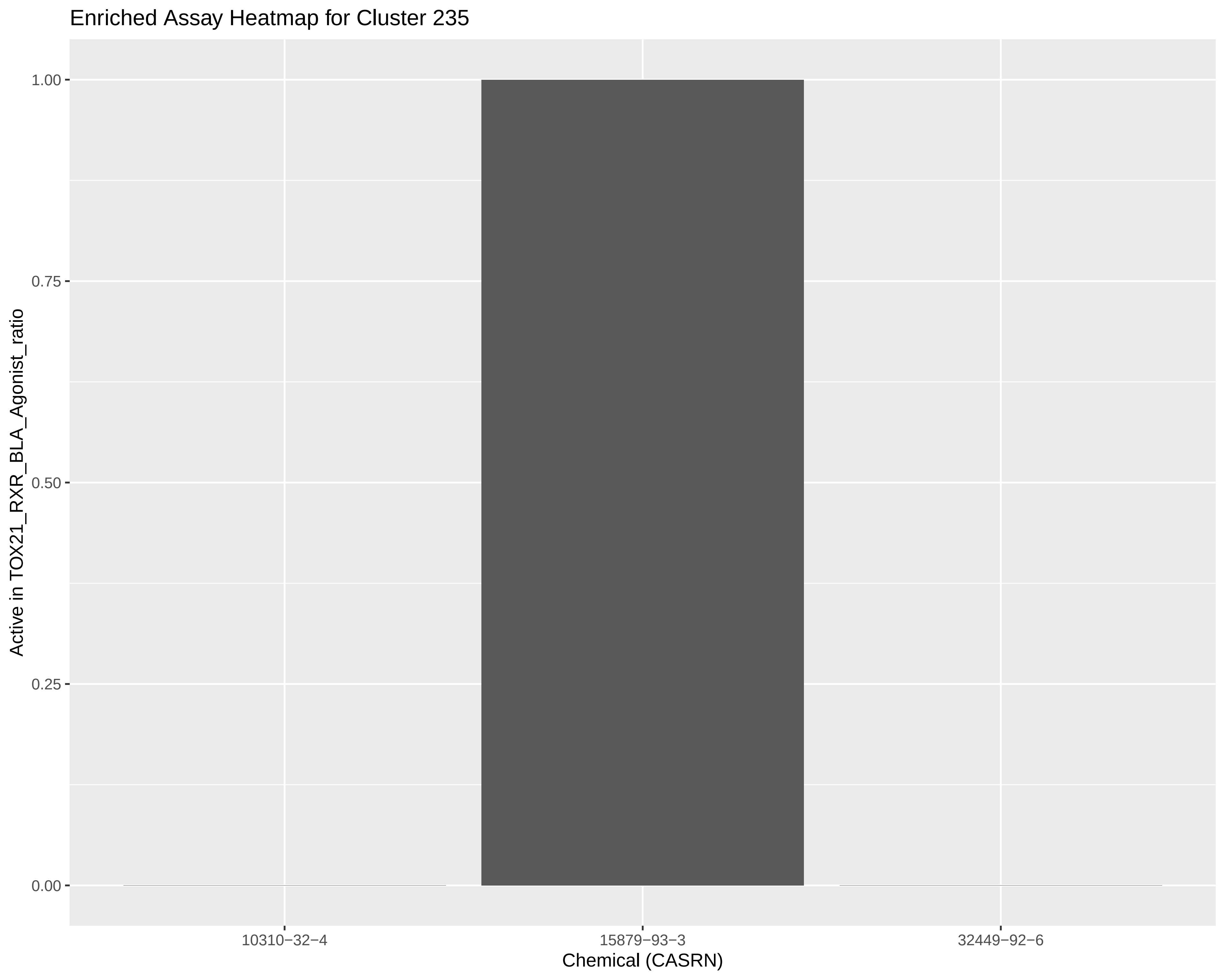
Enriched Assay Heatmap for Cluster 235
Active in TOX21_RXR_BLA_Agonist_ratio
1.00
0.75
0.50
0.25
0.00
10310−32−4
15879−93−3
32449−92−6
Chemical (CASRN)

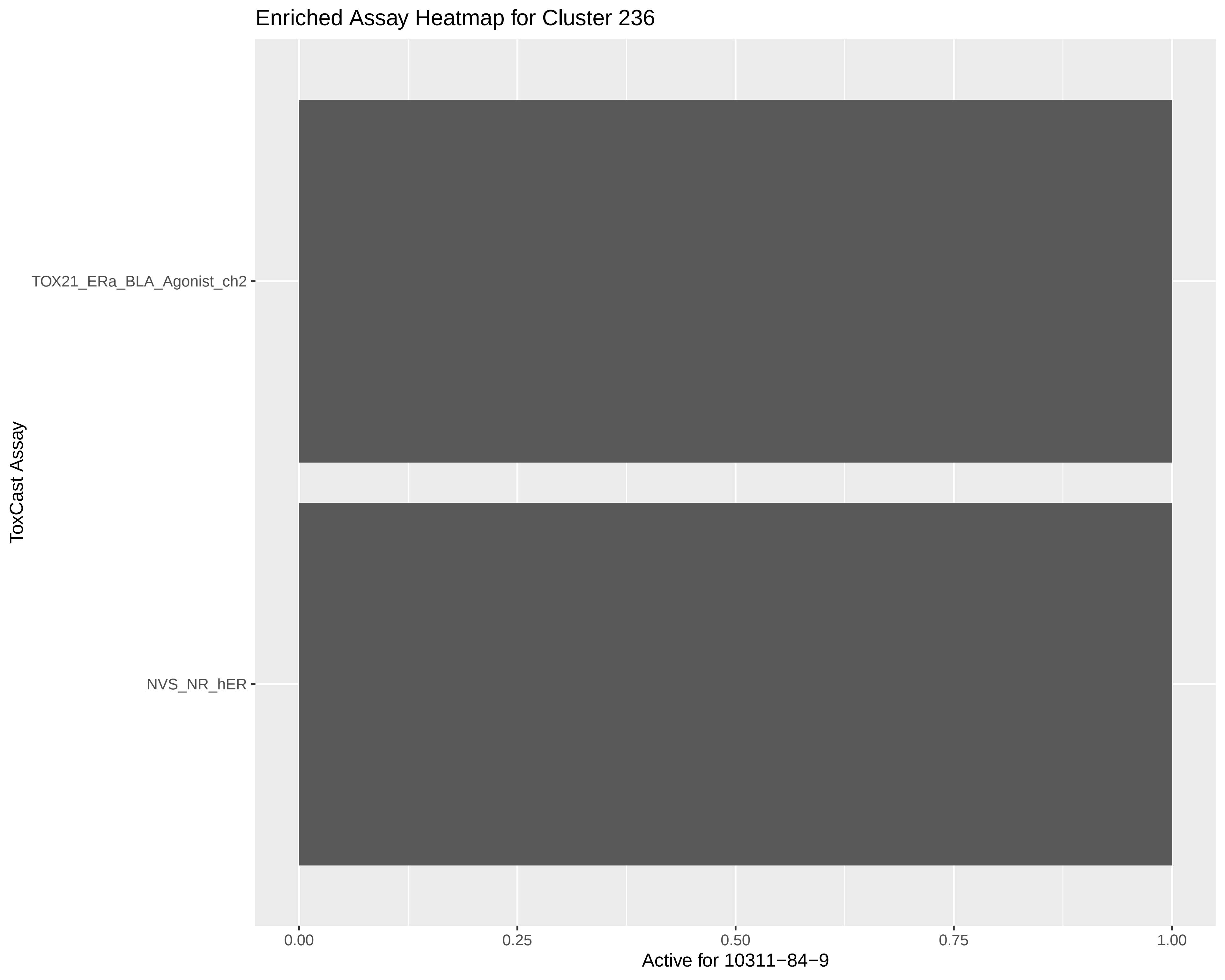
Enriched Assay Heatmap for Cluster 236
TOX21_ERa_BLA_Agonist_ch2
NVS_NR_hER
ToxCast Assay
0.00
0.25
0.50
0.75
1.00
Active for 10311−84−9

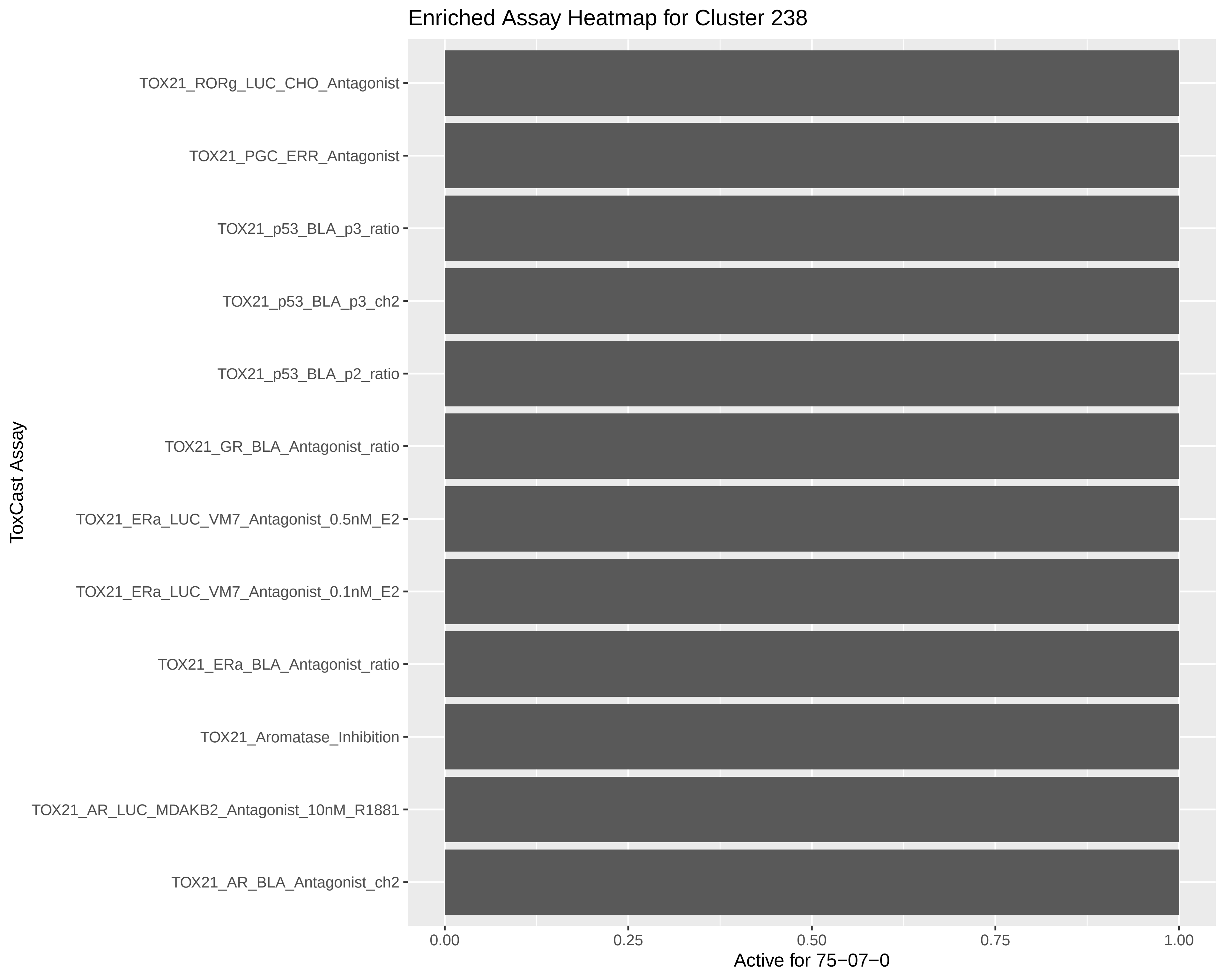

Enriched Assay Heatmap for Cluster 238
ToxCast Assay
TOX21_RORg_LUC_CHO_Antagonist
TOX21_PGC_ERR_Antagonist
TOX21_p53_BLA_p3_ratio
TOX21_p53_BLA_p3_ch2
TOX21_p53_BLA_p2_ratio
TOX21_GR_BLA_Antagonist_ratio
TOX21_ERa_LUC_VM7_Antagonist_0.5nM_E2
TOX21_ERa_LUC_VM7_Antagonist_0.1nM_E2
TOX21_ERa_BLA_Antagonist_ratio
TOX21_Aromatase_Inhibition
TOX21_AR_LUC_MDAKB2_Antagonist_10nM_R1881
TOX21_AR_BLA_Antagonist_ch2
0.00
0.25
0.50
0.75
1.00
Active for 75−07−0

Enriched Assay Heatmap for Cluster 240

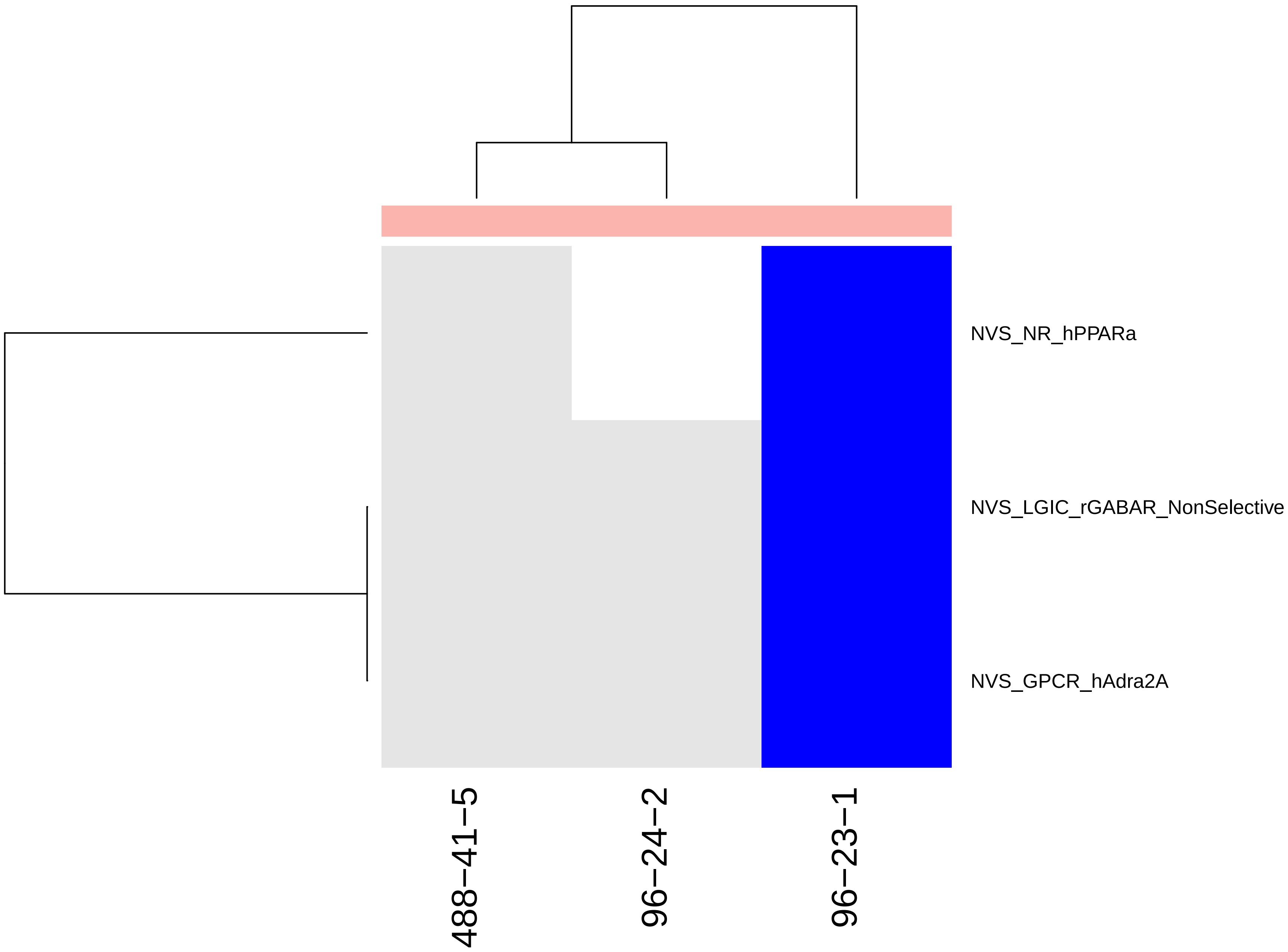

Enriched Assay Heatmap for Cluster 242

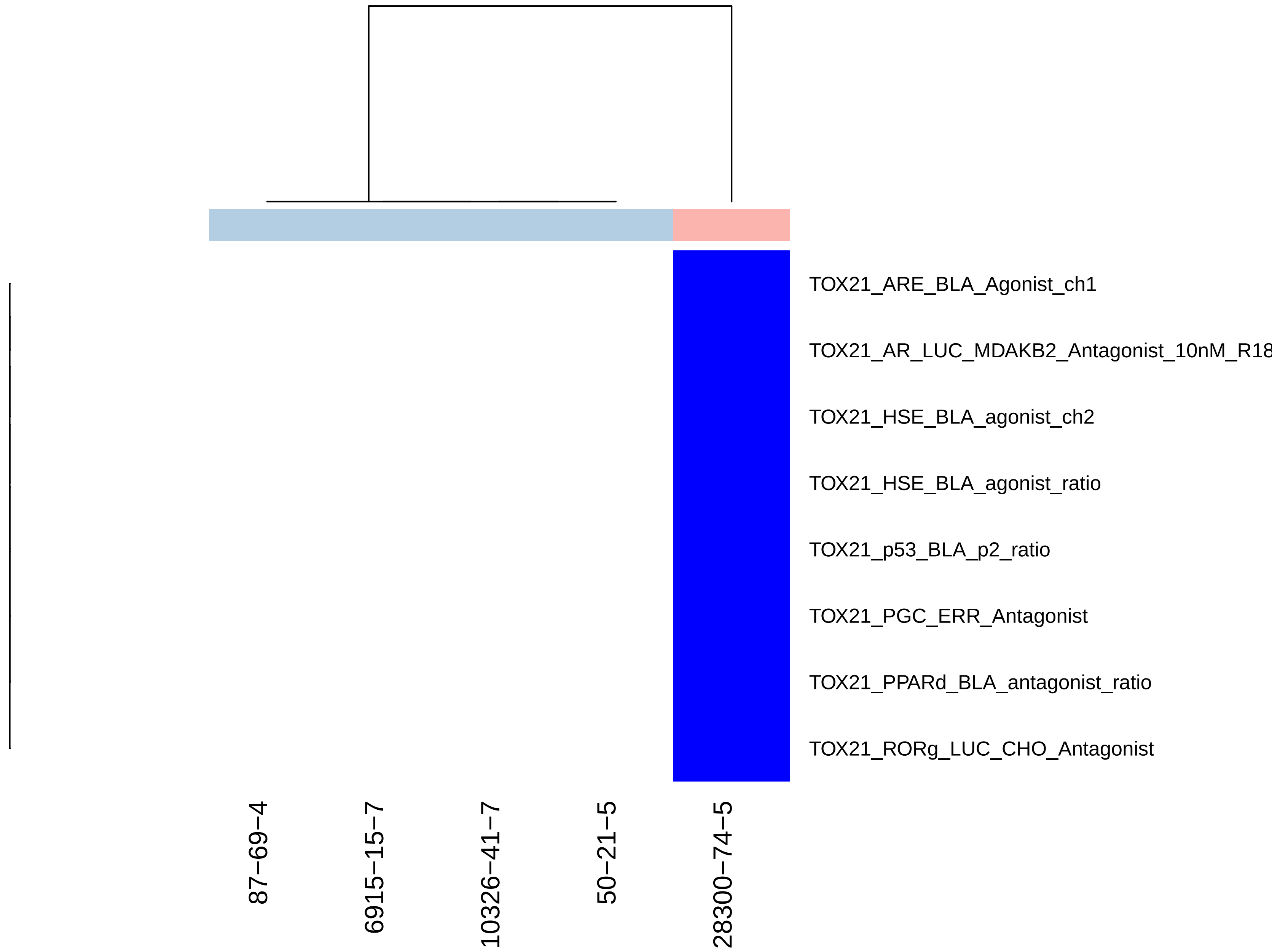

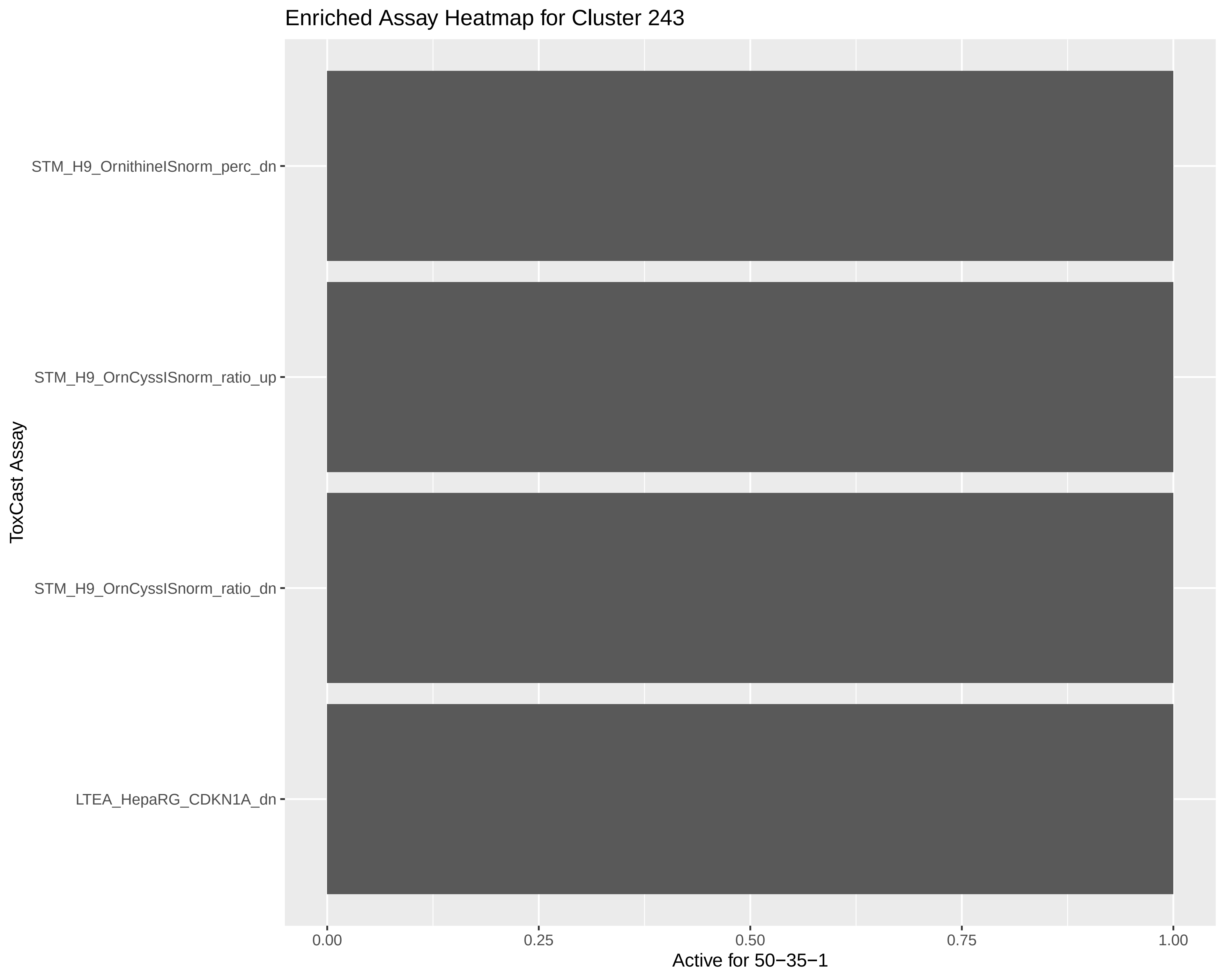

Enriched Assay Heatmap for Cluster 243
STM_H9_OrnithineISnorm_perc_dn
STM_H9_OrnCyssISnorm_ratio_up
STM_H9_OrnCyssISnorm_ratio_dn
LTEA_HepaRG_CDKN1A_dn
ToxCast Assay
0.00
0.25
0.50
0.75
1.00
Active for 50−35−1

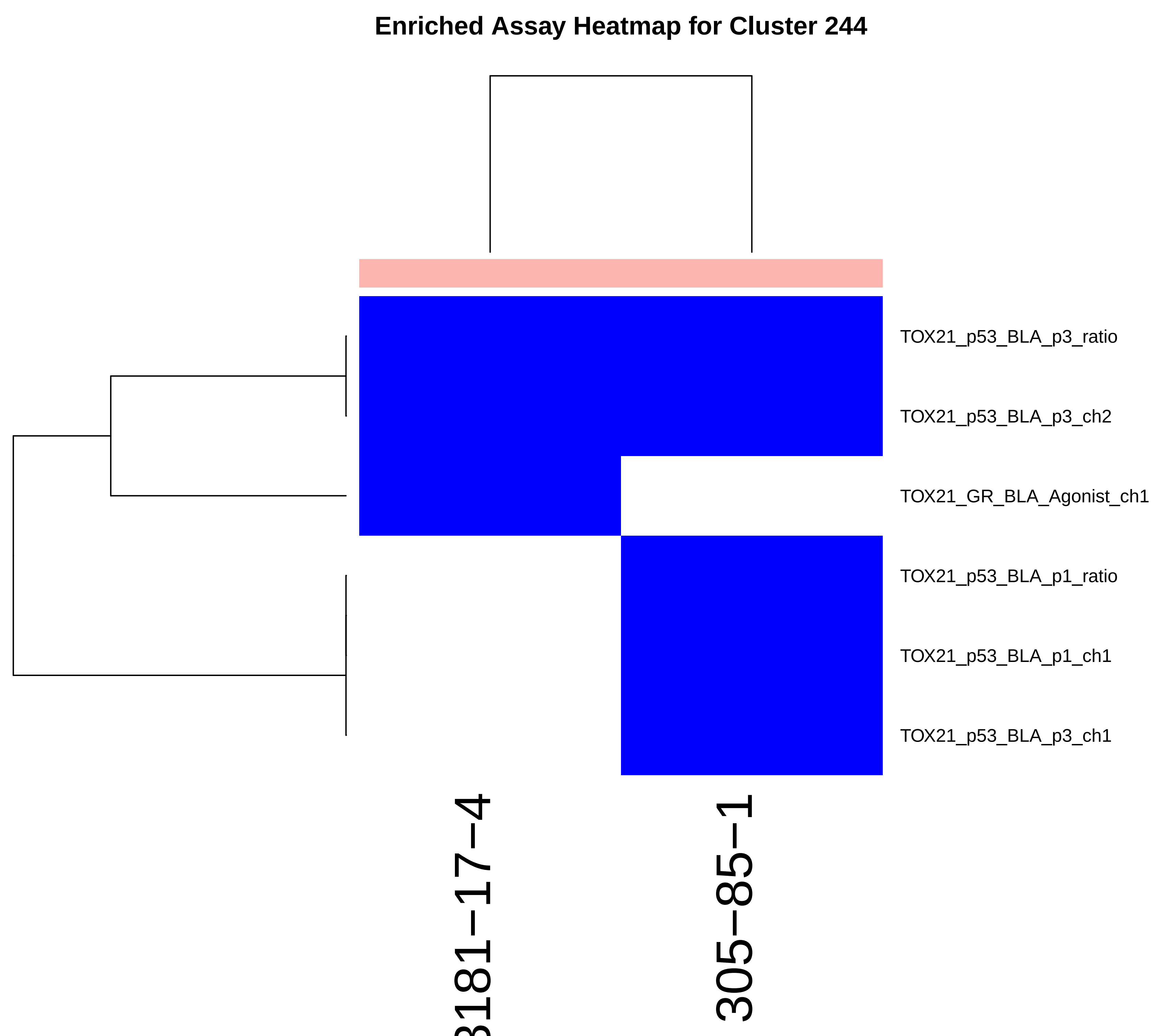
Enriched Assay Heatmap for Cluster 244
TOX21_p53_BLA_p3_ratio
TOX21_p53_BLA_p3_ch2
TOX21_GR_BLA_Agonist_ch1
TOX21_p53_BLA_p1_ratio
TOX21_p53_BLA_p1_ch1
TOX21_p53_BLA_p3_ch1
3181−17−4
305−85−1

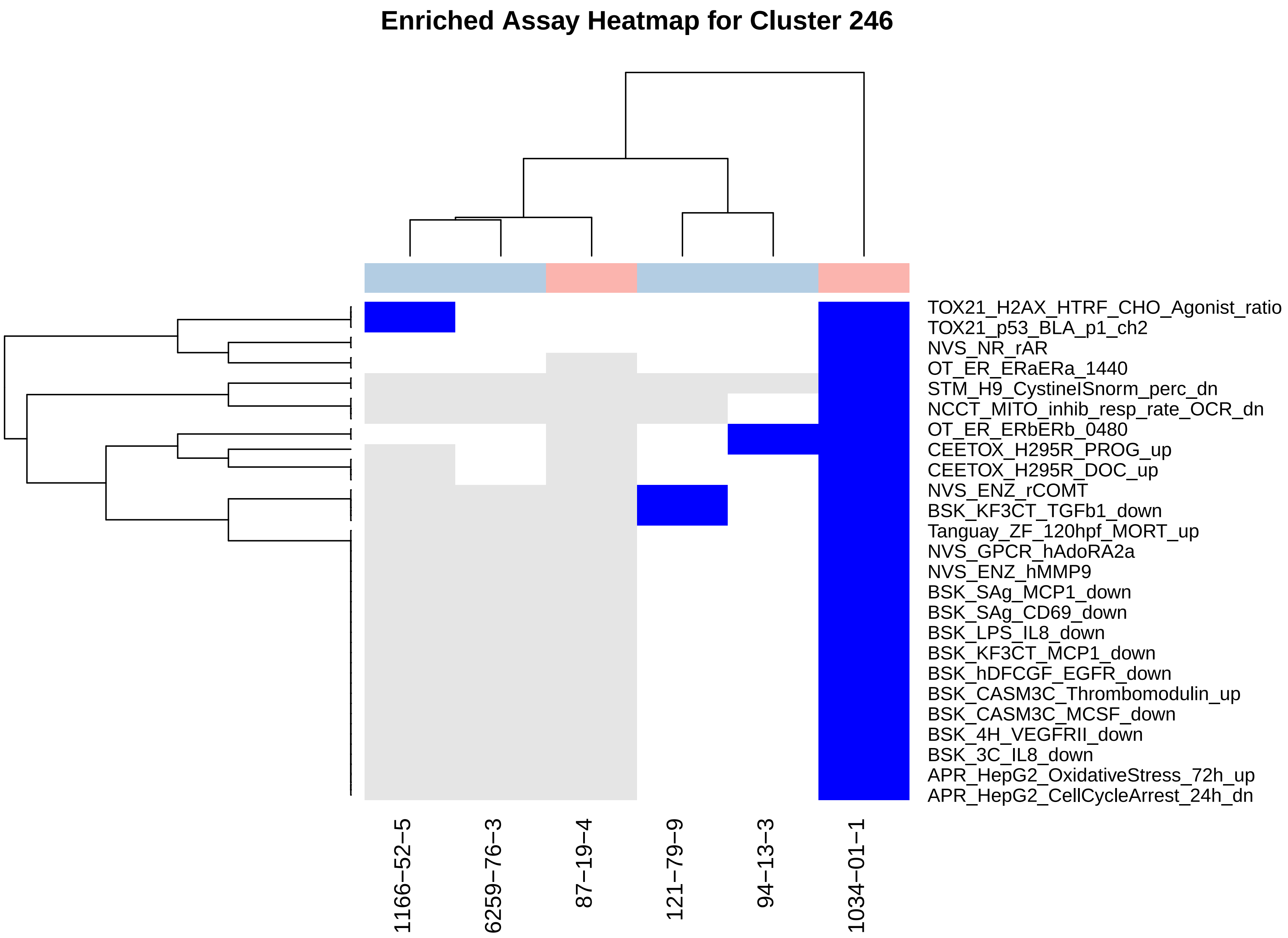
Enriched Assay Heatmap for Cluster 246
TOX21_H2AX_HTRF_CHO_Agonist_ratio
TOX21_p53_BLA_p1_ch2
NVS_NR_rAR
OT_ER_ERaERa_1440
STM_H9_CystineISnorm_perc_dn
NCCT_MITO_inhib_resp_rate_OCR_dn
OT_ER_ERbERb_0480
CEETOX_H295R_PROG_up
CEETOX_H295R_DOC_up
NVS_ENZ_rCOMT
BSK_KF3CT_TGFb1_down
Tanguay_ZF_120hpf_MORT_up
NVS_GPCR_hAdoRA2a
NVS_ENZ_hMMP9
BSK_SAg_MCP1_down
BSK_SAg_CD69_down
BSK_LPS_IL8_down
BSK_KF3CT_MCP1_down
BSK_hDFCGF_EGFR_down
BSK_CASM3C_Thrombomodulin_up
BSK_CASM3C_MCSF_down
BSK_4H_VEGFRII_down
BSK_3C_IL8_down
APR_HepG2_OxidativeStress_72h_up
APR_HepG2_CellCycleArrest_24h_dn
1166−52−5
6259−76−3
87−19−4
121−79−9
94−13−3
1034−01−1

Enriched Assay Heatmap for Cluster 248

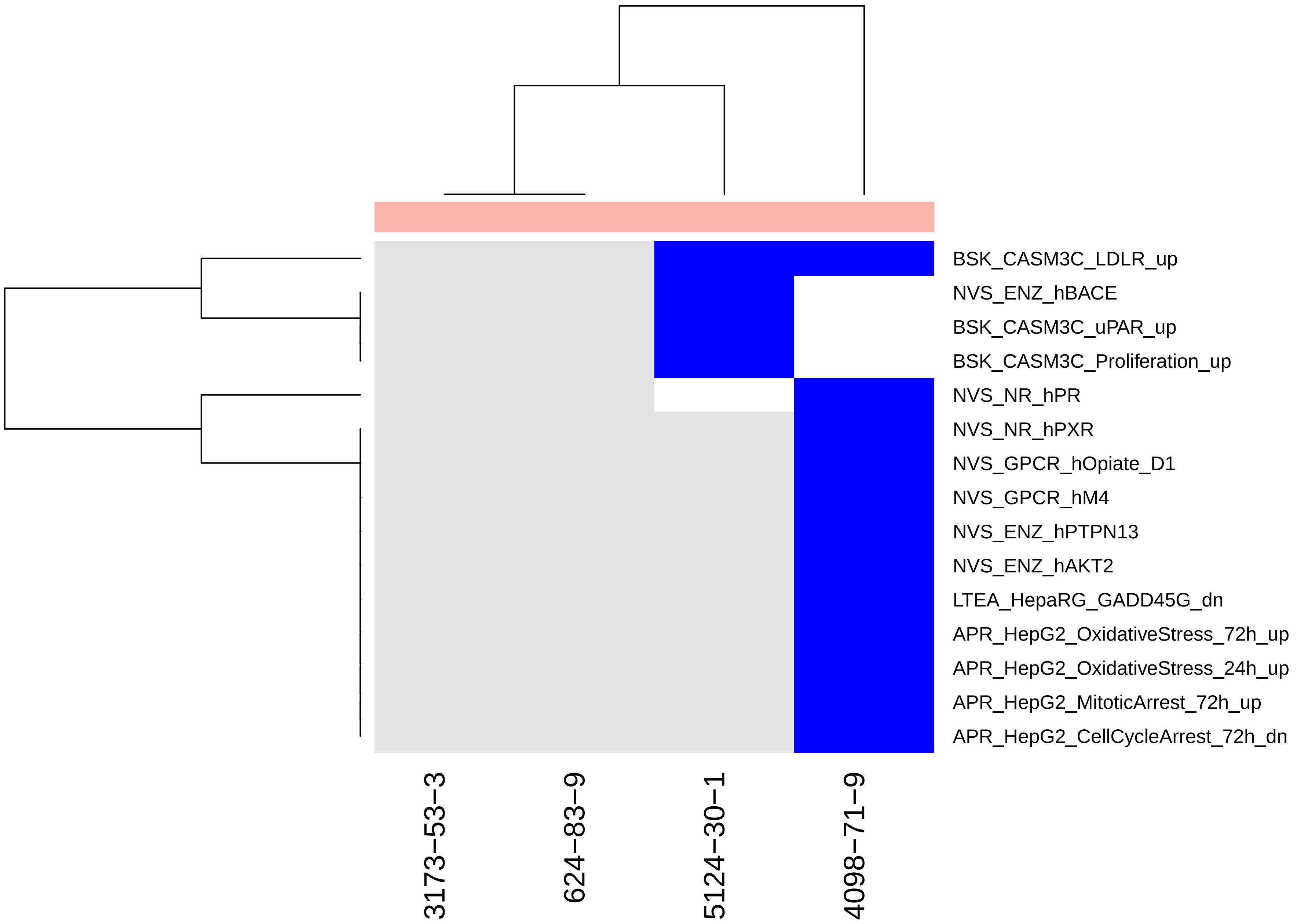

Enriched Assay Heatmap for Cluster 251

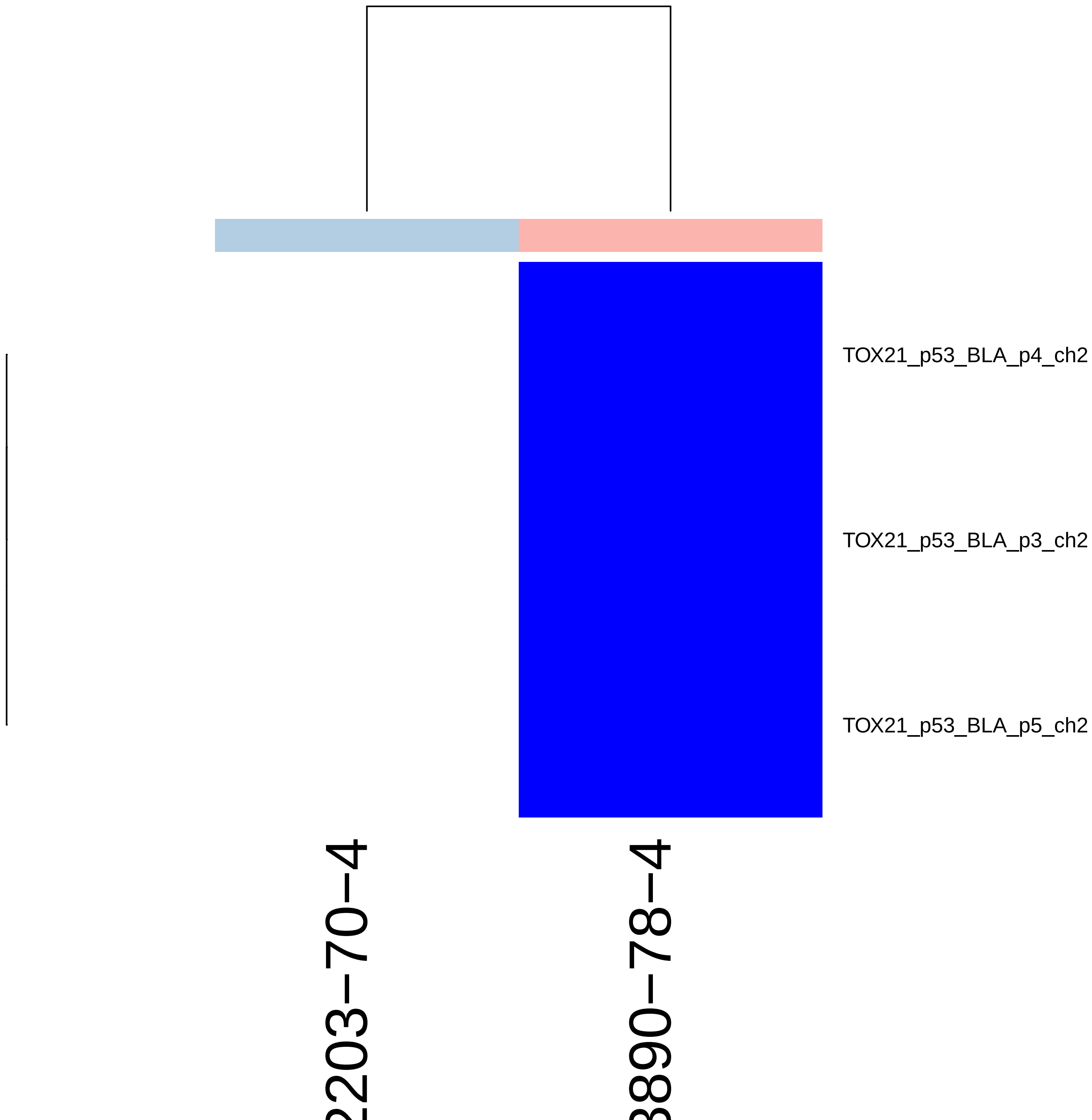

Enriched Assay Heatmap for Cluster 252

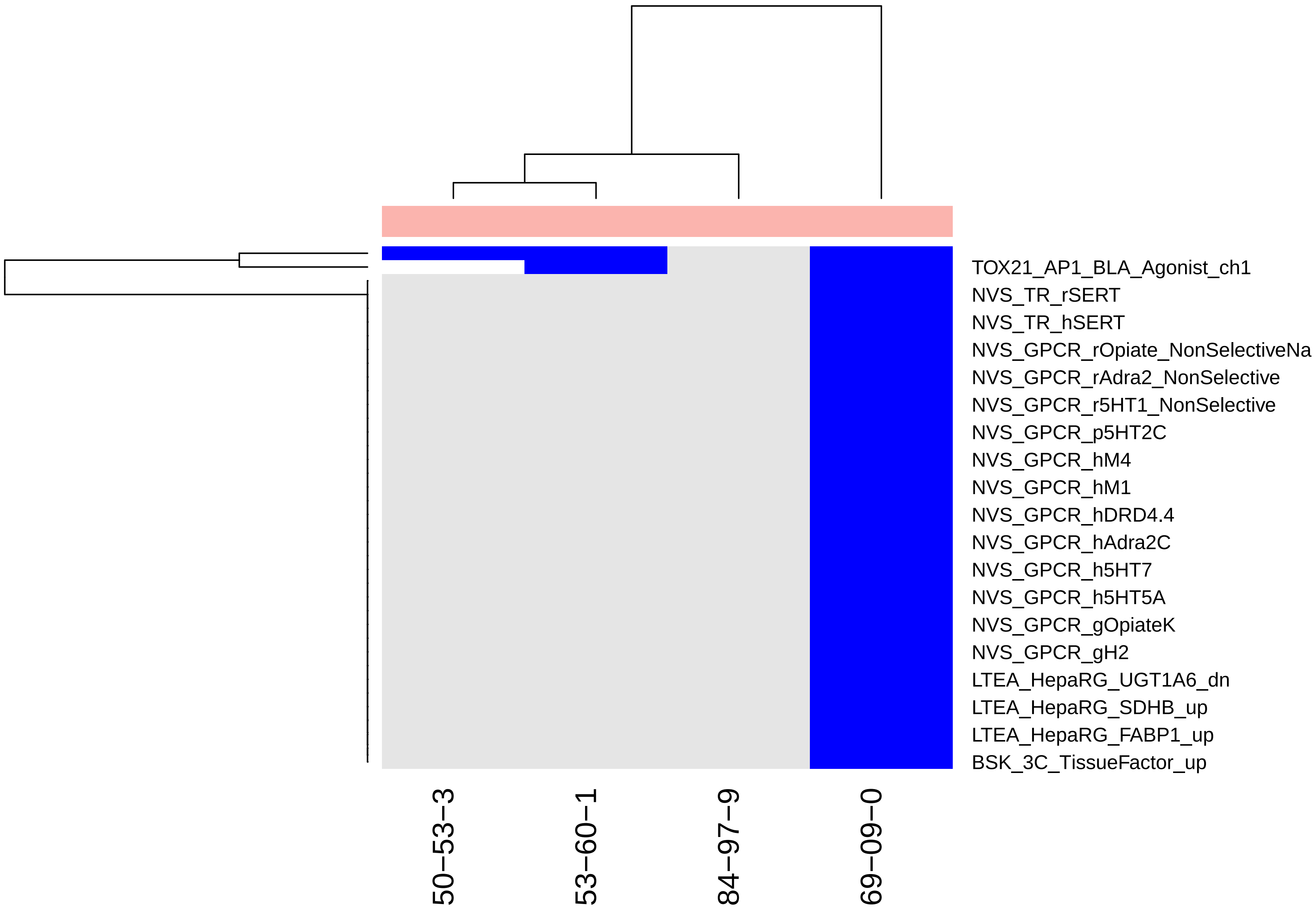

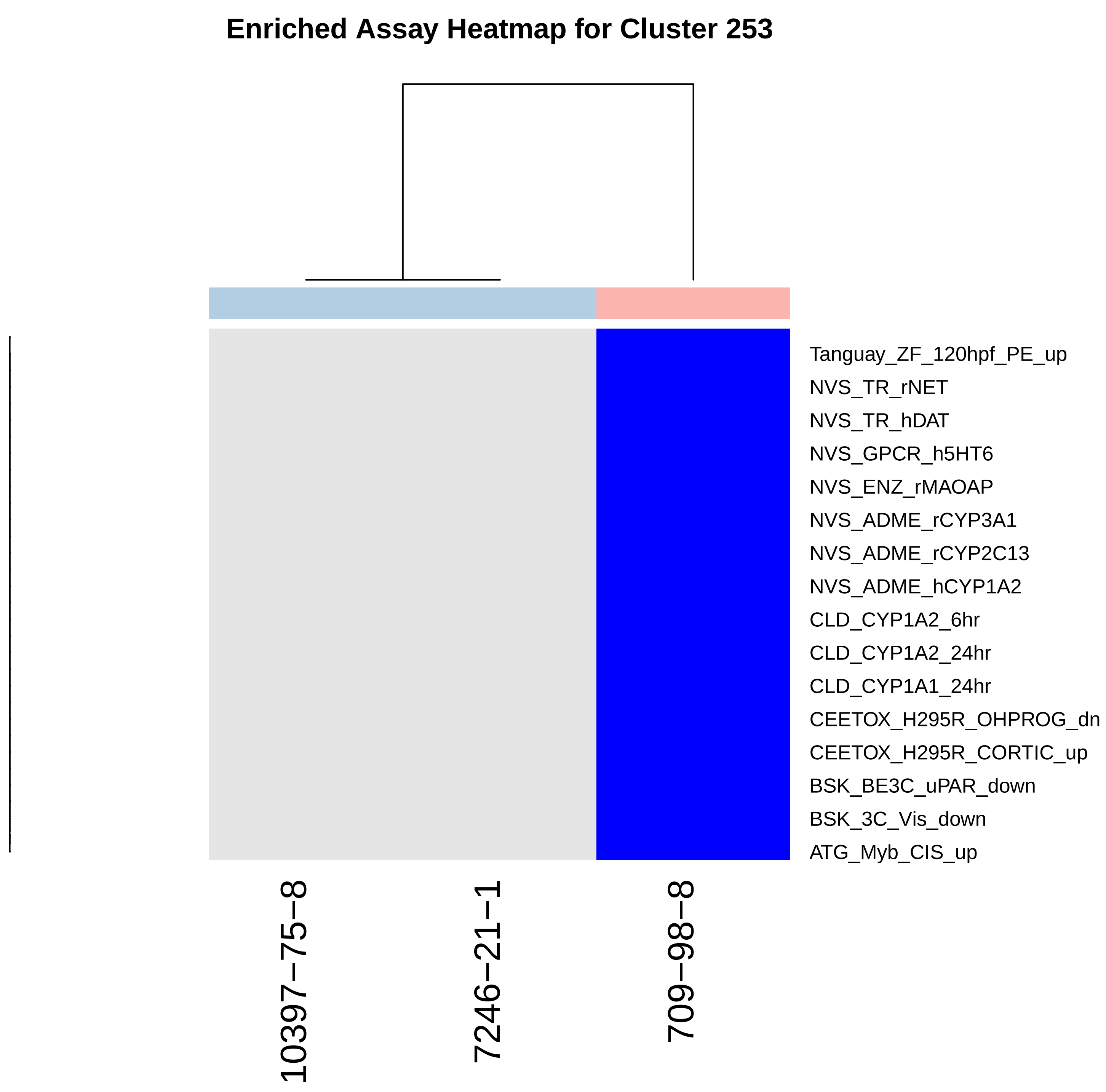
Enriched Assay Heatmap for Cluster 253
Tanguay_ZF_120hpf_PE_up
NVS_TR_rNET
NVS_TR_hDAT
NVS_GPCR_h5HT6
NVS_ENZ_rMAOAP
NVS_ADME_rCYP3A1
NVS_ADME_rCYP2C13
NVS_ADME_hCYP1A2
CLD_CYP1A2_6hr
CLD_CYP1A2_24hr
CLD_CYP1A1_24hr
CEETOX_H295R_OHPROG_dn
CEETOX_H295R_CORTIC_up
BSK_BE3C_uPAR_down
BSK_3C_Vis_down
ATG_Myb_CIS_up
10397−75−8
7246−21−1
709−98−8

Enriched Assay Heatmap for Cluster 257

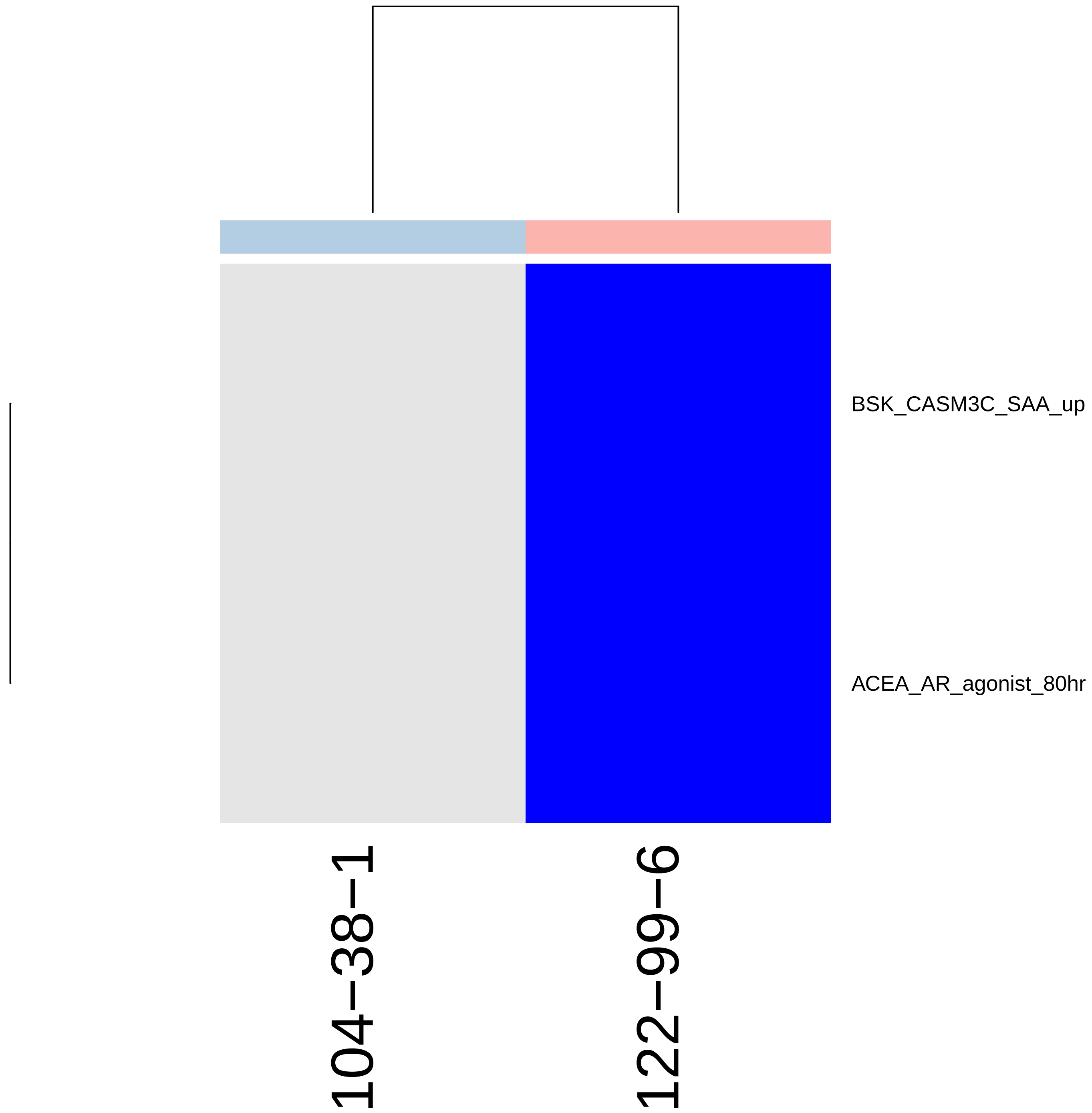

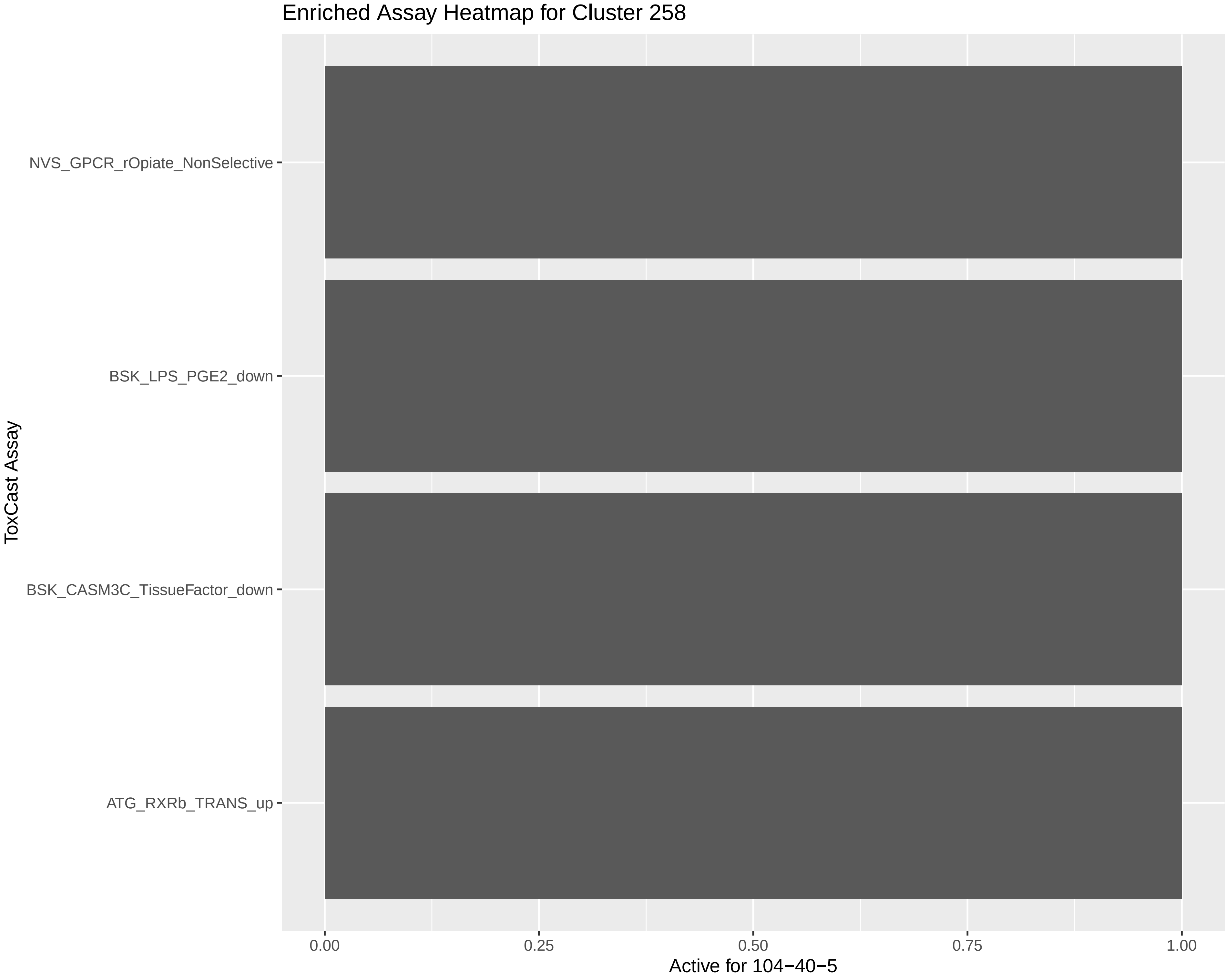

Enriched Assay Heatmap for Cluster 258
NVS_GPCR_rOpiate_NonSelective
BSK_LPS_PGE2_down
BSK_CASM3C_TissueFactor_down
ATG_RXRb_TRANS_up
ToxCast Assay
0.00
0.25
0.50
0.75
1.00
Active for 104−40−5

Enriched Assay Heatmap for Cluster 259

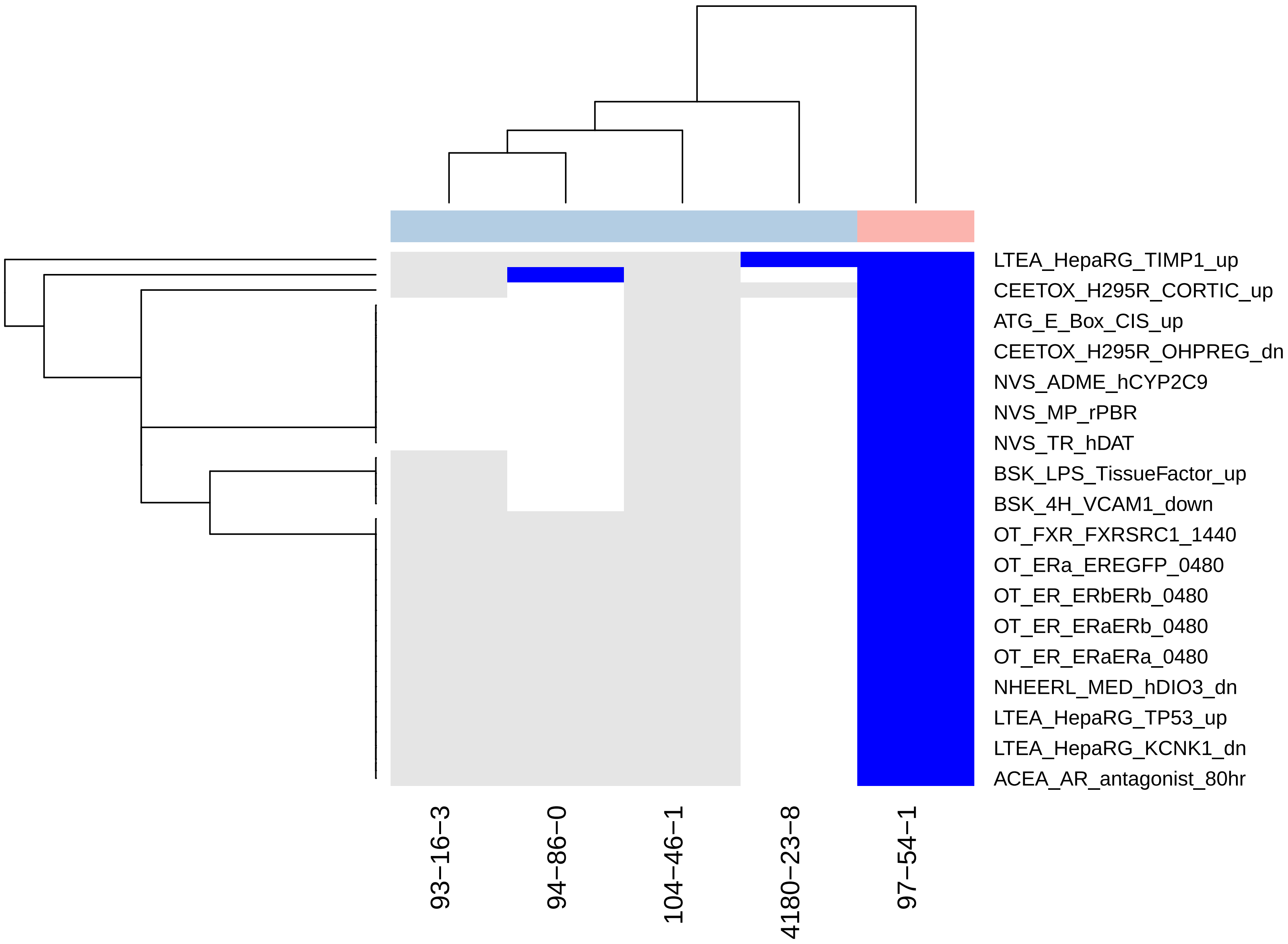

Enriched Assay Heatmap for Cluster 261

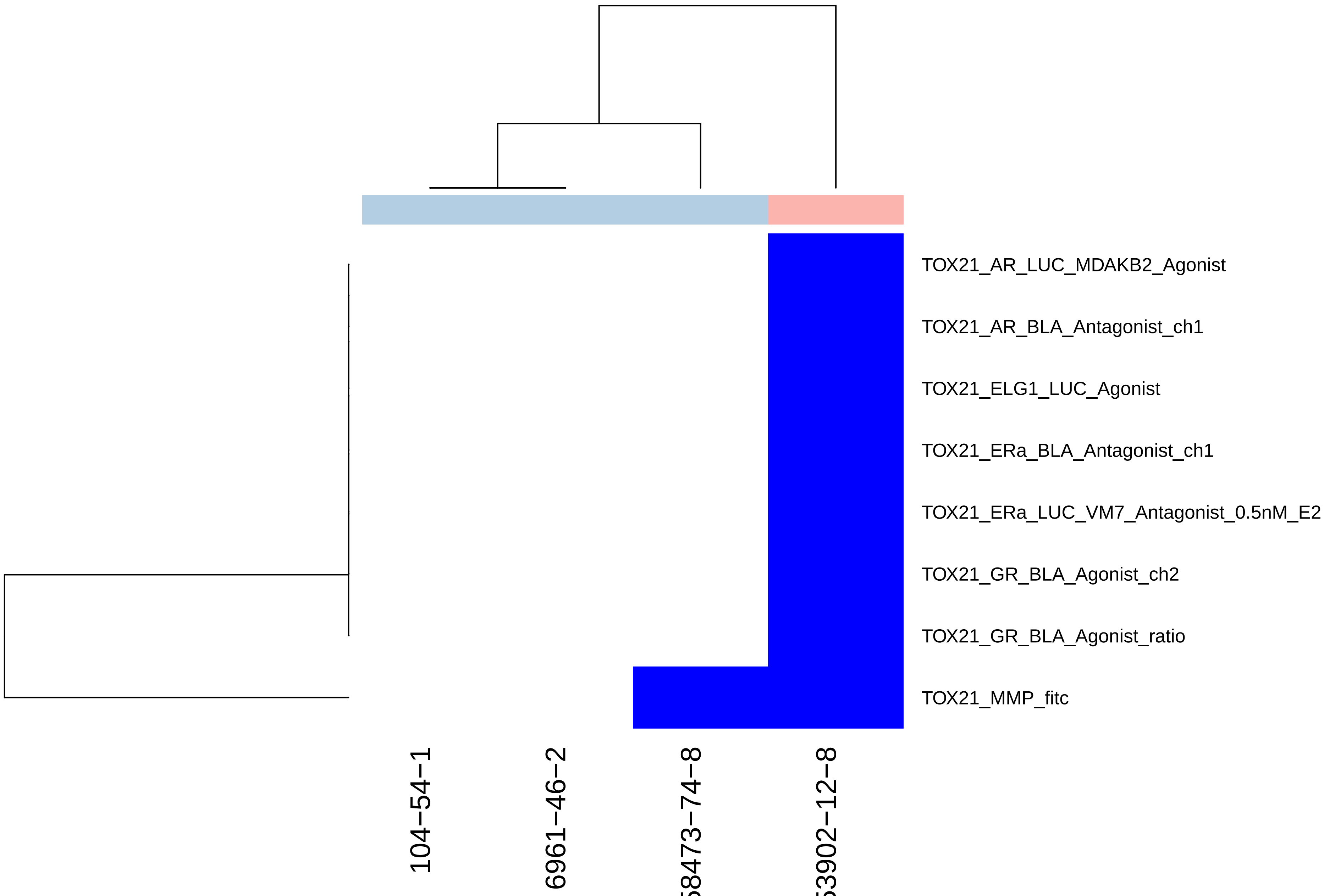

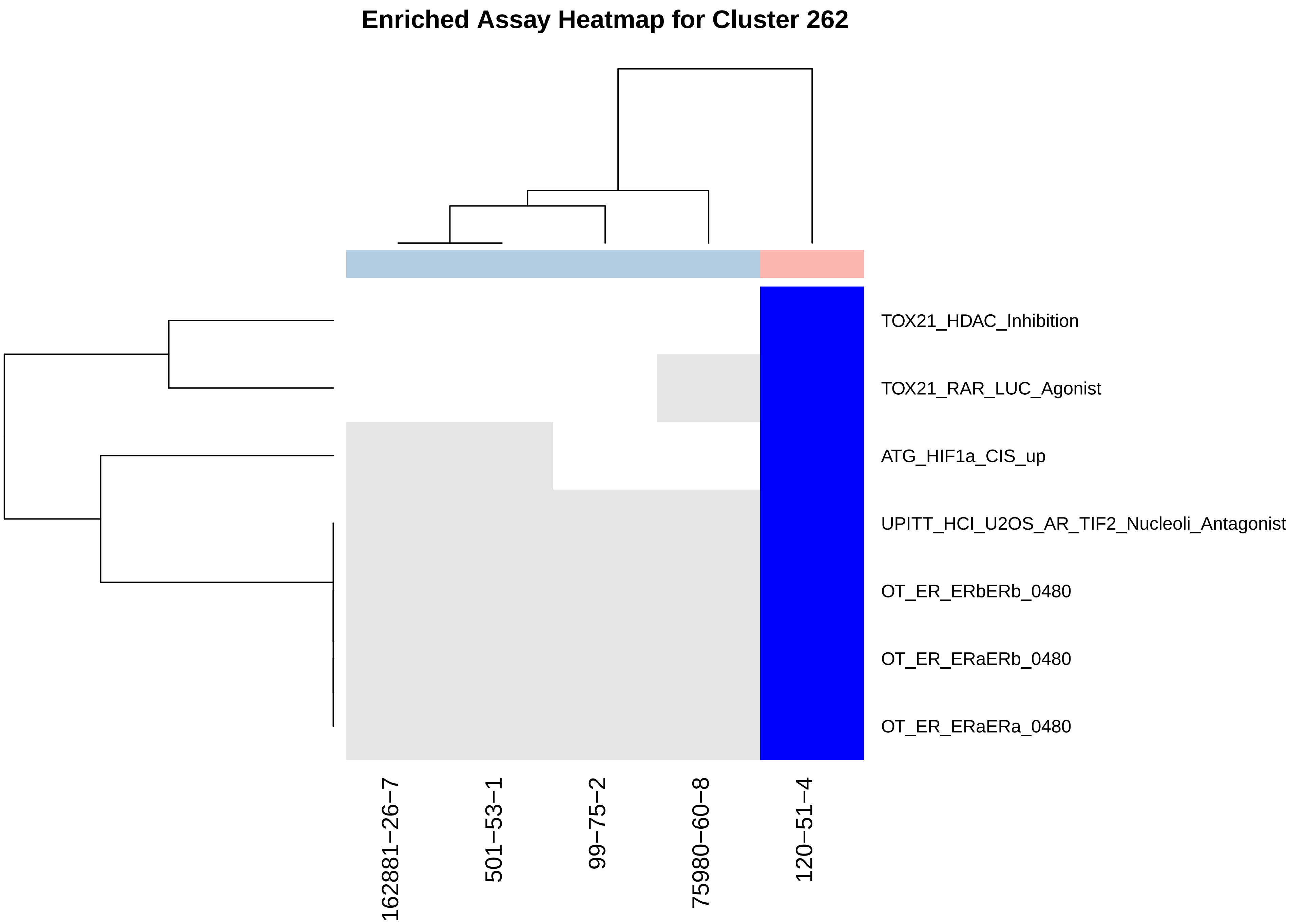
Enriched Assay Heatmap for Cluster 262
TOX21_HDAC_Inhibition
TOX21_RAR_LUC_Agonist
ATG_HIF1a_CIS_up
UPITT_HCI_U2OS_AR_TIF2_Nucleoli_Antagonist
OT_ER_ERbERb_0480
OT_ER_ERaERb_0480
OT_ER_ERaERa_0480
162881−26−7
501−53−1
99−75−2
75980−60−8
120−51−4

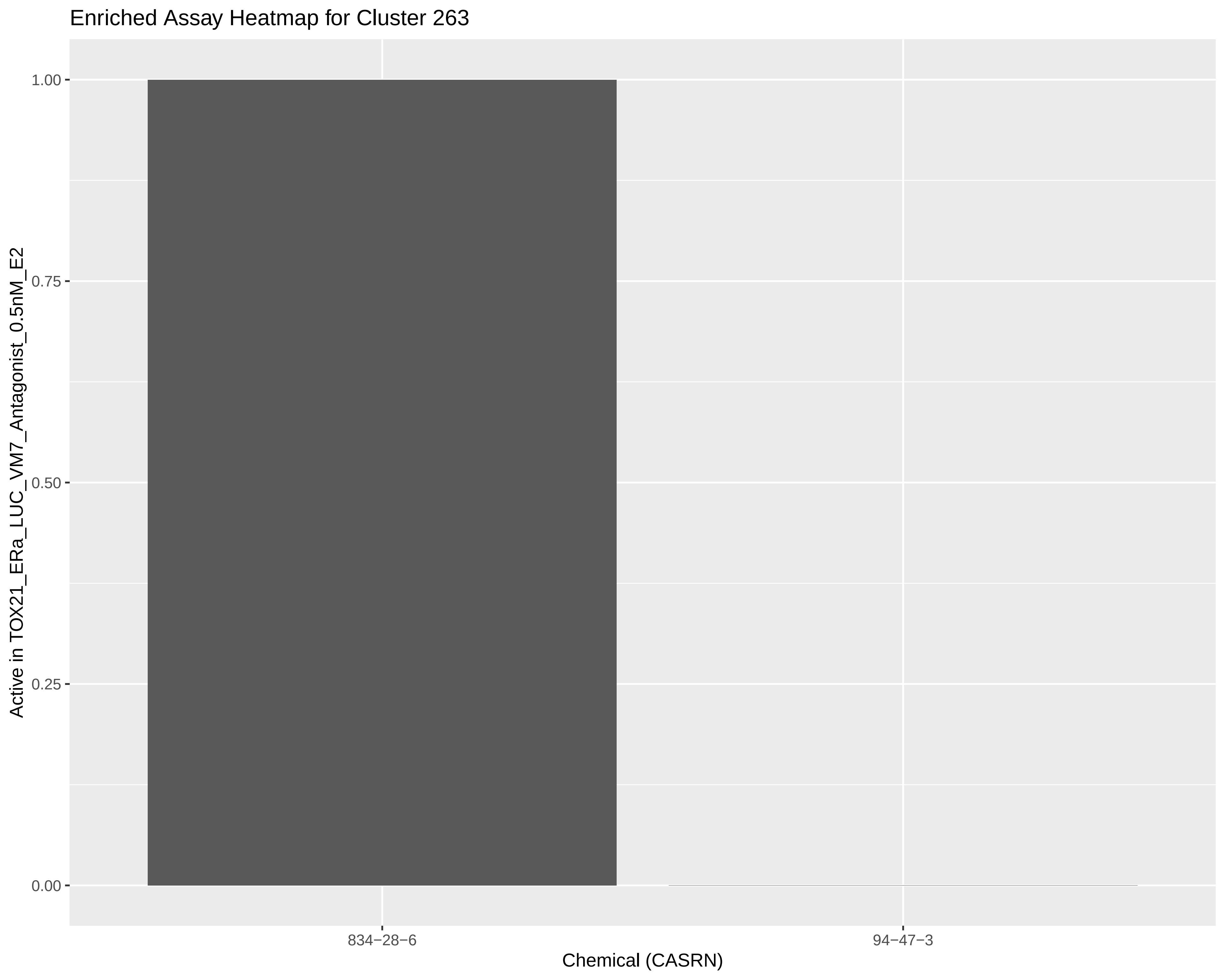
Enriched Assay Heatmap for Cluster 263
Active in TOX21_ERa_LUC_VM7_Antagonist_0.5nM_E2
1.00
0.75
0.50
0.25
0.00
834−28−6
94−47−3
Chemical (CASRN)

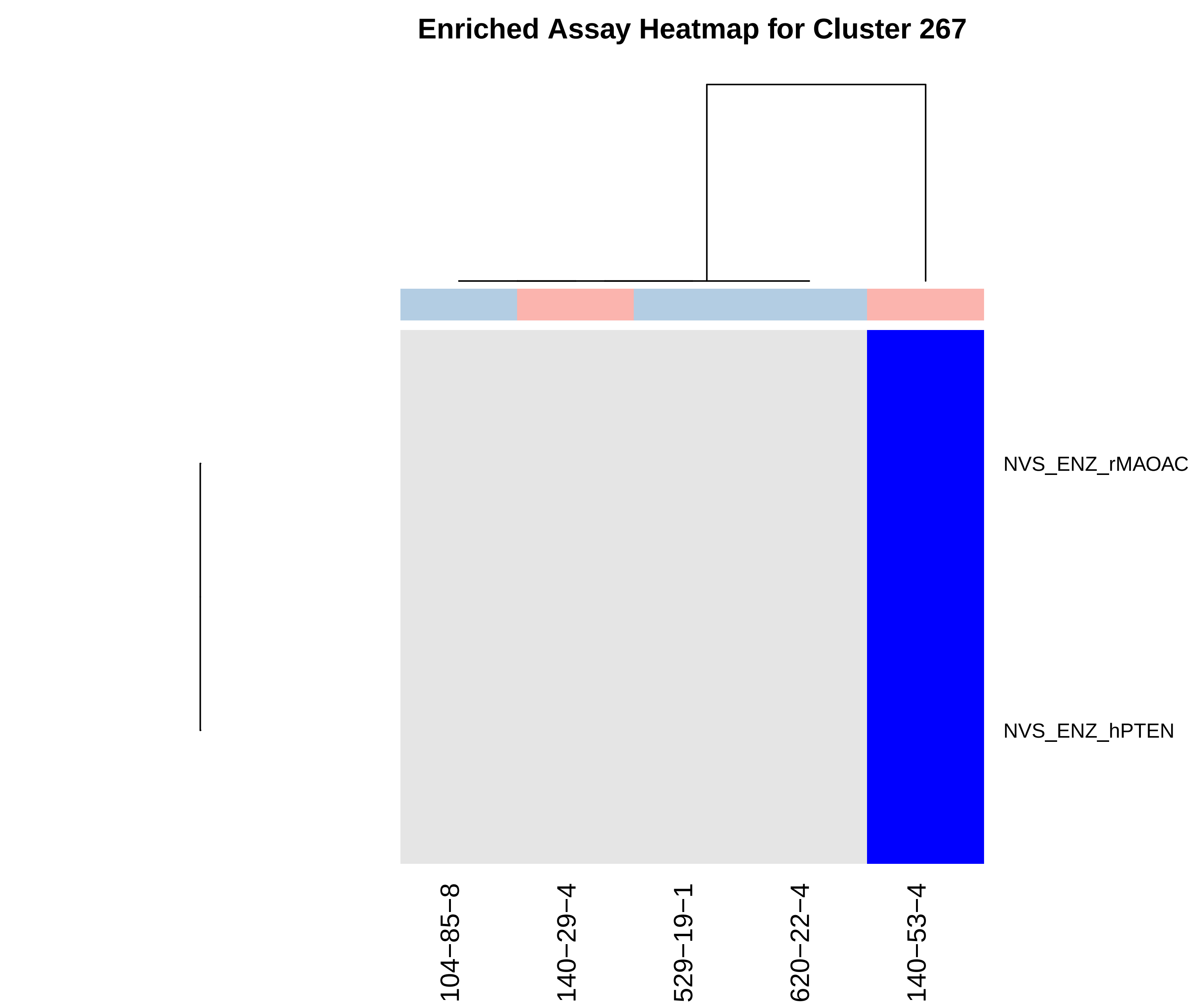
Enriched Assay Heatmap for Cluster 267
NVS_ENZ_rMAOAC
NVS_ENZ_hPTEN
104−85−8
140−29−4
529−19−1
620−22−4
140−53−4

Enriched Assay Heatmap for Cluster 270

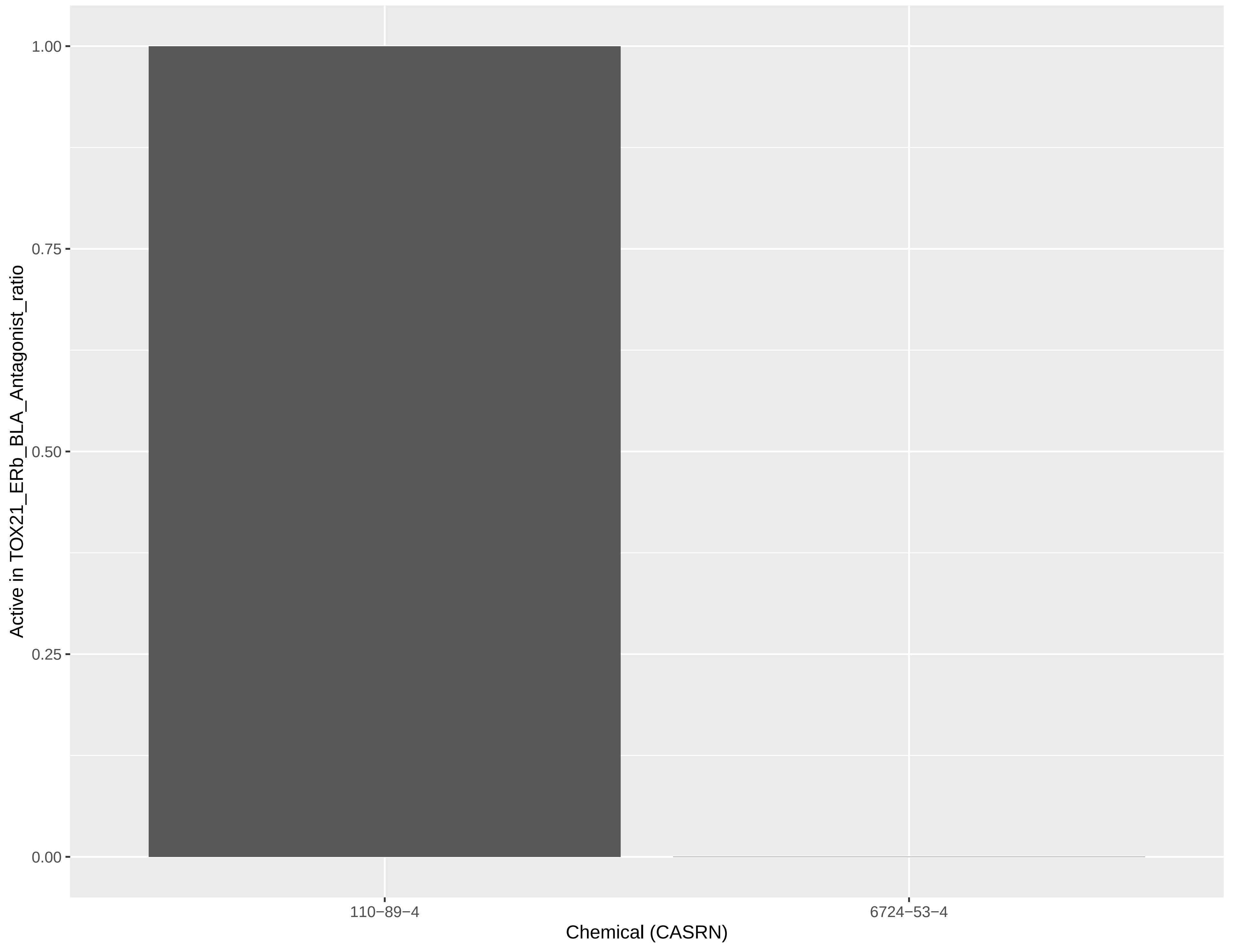

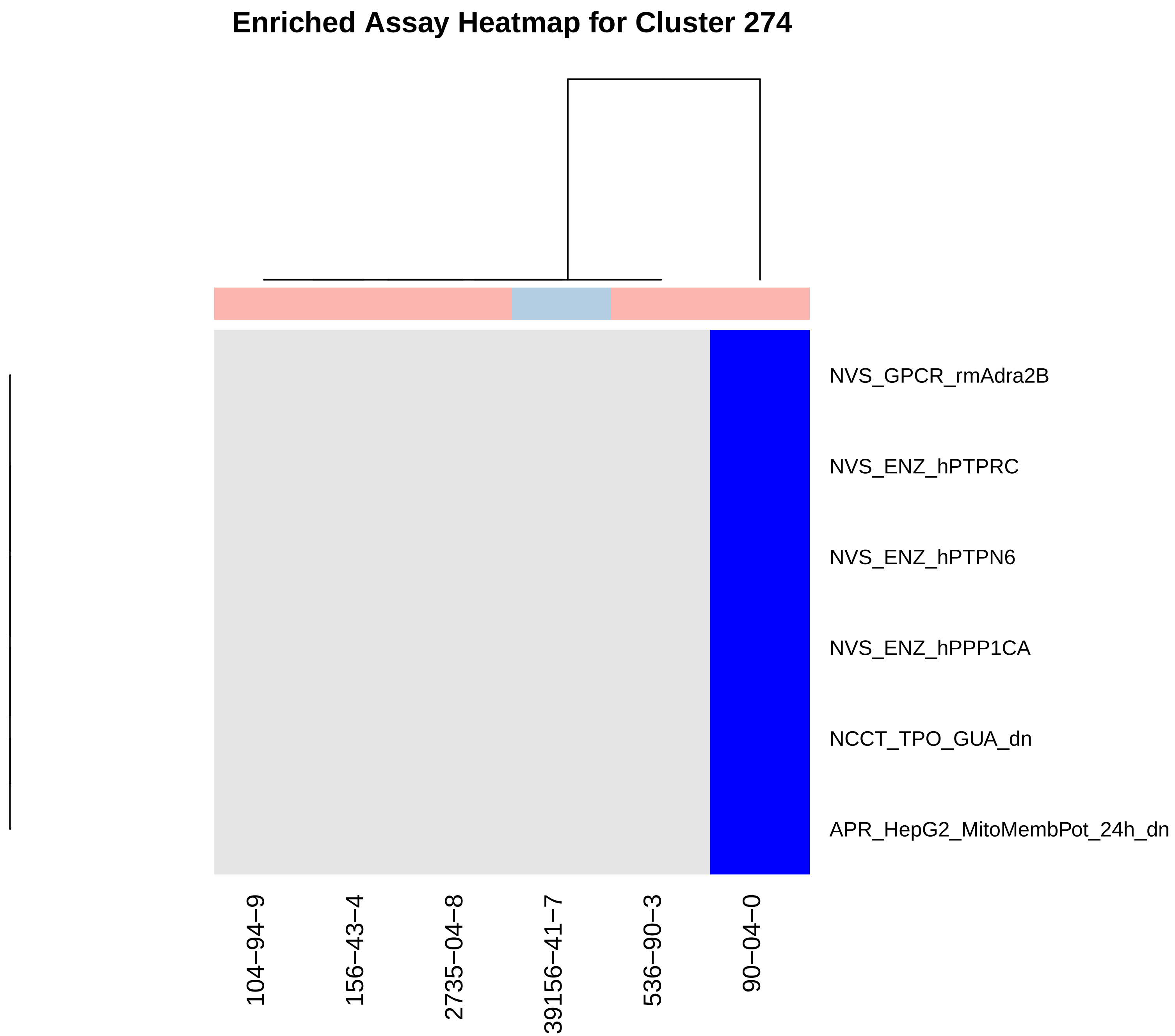
Enriched Assay Heatmap for Cluster 274
NVS_GPCR_rmAdra2B
NVS_ENZ_hPTPRC
NVS_ENZ_hPTPN6
NVS_ENZ_hPPP1CA
NCCT_TPO_GUA_dn
APR_HepG2_MitoMembPot_24h_dn
104−94−9
156−43−4
2735−04−8
39156−41−7
536−90−3
90−04−0

Enriched Assay Heatmap for Cluster 277

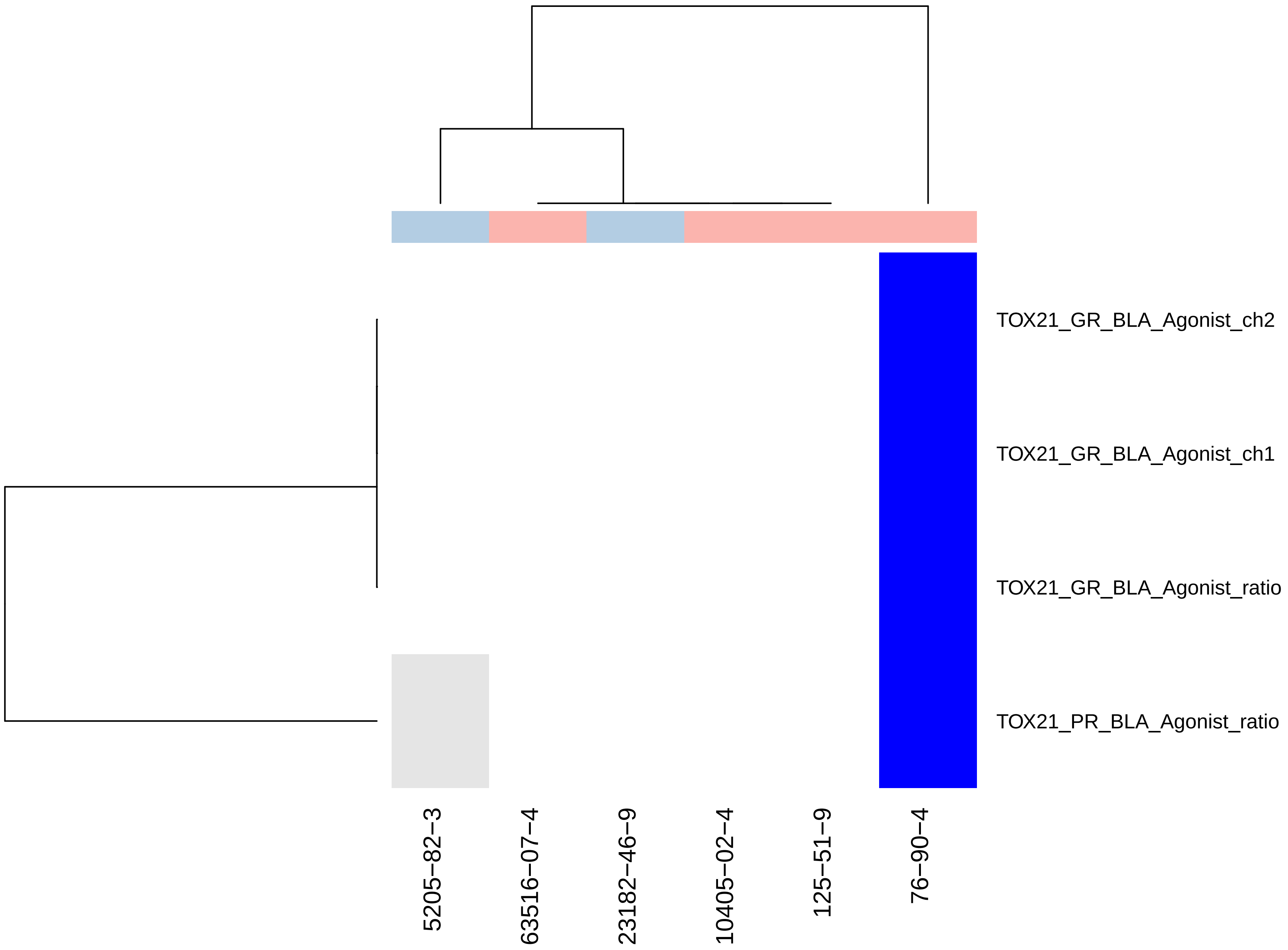

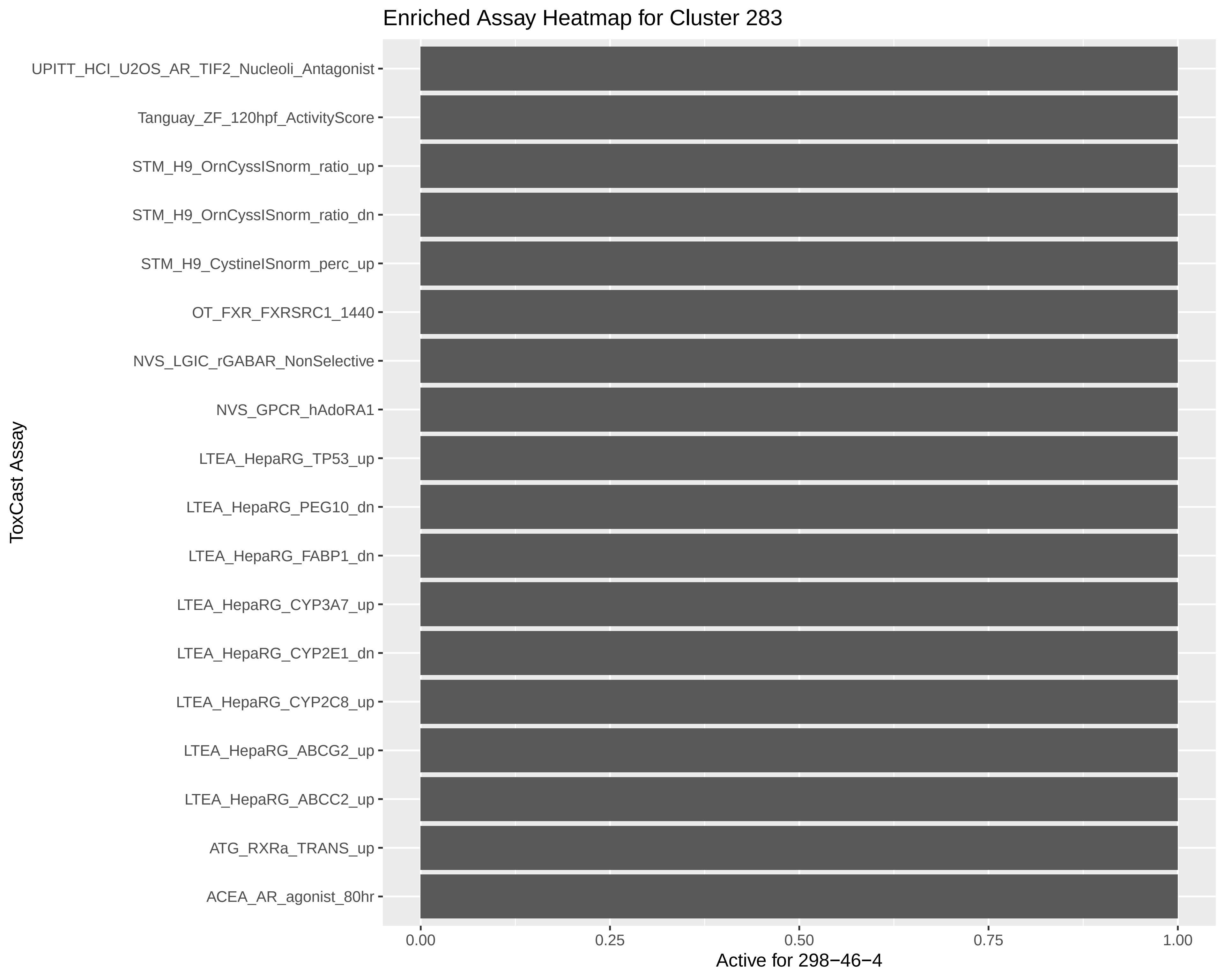
Enriched Assay Heatmap for Cluster 283
UPITT_HCI_U2OS_AR_TIF2_Nucleoli_Antagonist
Tanguay_ZF_120hpf_ActivityScore
STM_H9_OrnCyssISnorm_ratio_up
STM_H9_OrnCyssISnorm_ratio_dn
STM_H9_CystineISnorm_perc_up
OT_FXR_FXRSRC1_1440
NVS_LGIC_rGABAR_NonSelective
NVS_GPCR_hAdoRA1
LTEA_HepaRG_TP53_up
LTEA_HepaRG_PEG10_dn
LTEA_HepaRG_FABP1_dn
LTEA_HepaRG_CYP3A7_up
LTEA_HepaRG_CYP2E1_dn
LTEA_HepaRG_CYP2C8_up
LTEA_HepaRG_ABCG2_up
LTEA_HepaRG_ABCC2_up
ATG_RXRa_TRANS_up
ACEA_AR_agonist_80hr
ToxCast Assay
0.00
0.25
0.50
0.75
1.00
Active for 298−46−4

Enriched Assay Heatmap for Cluster 284

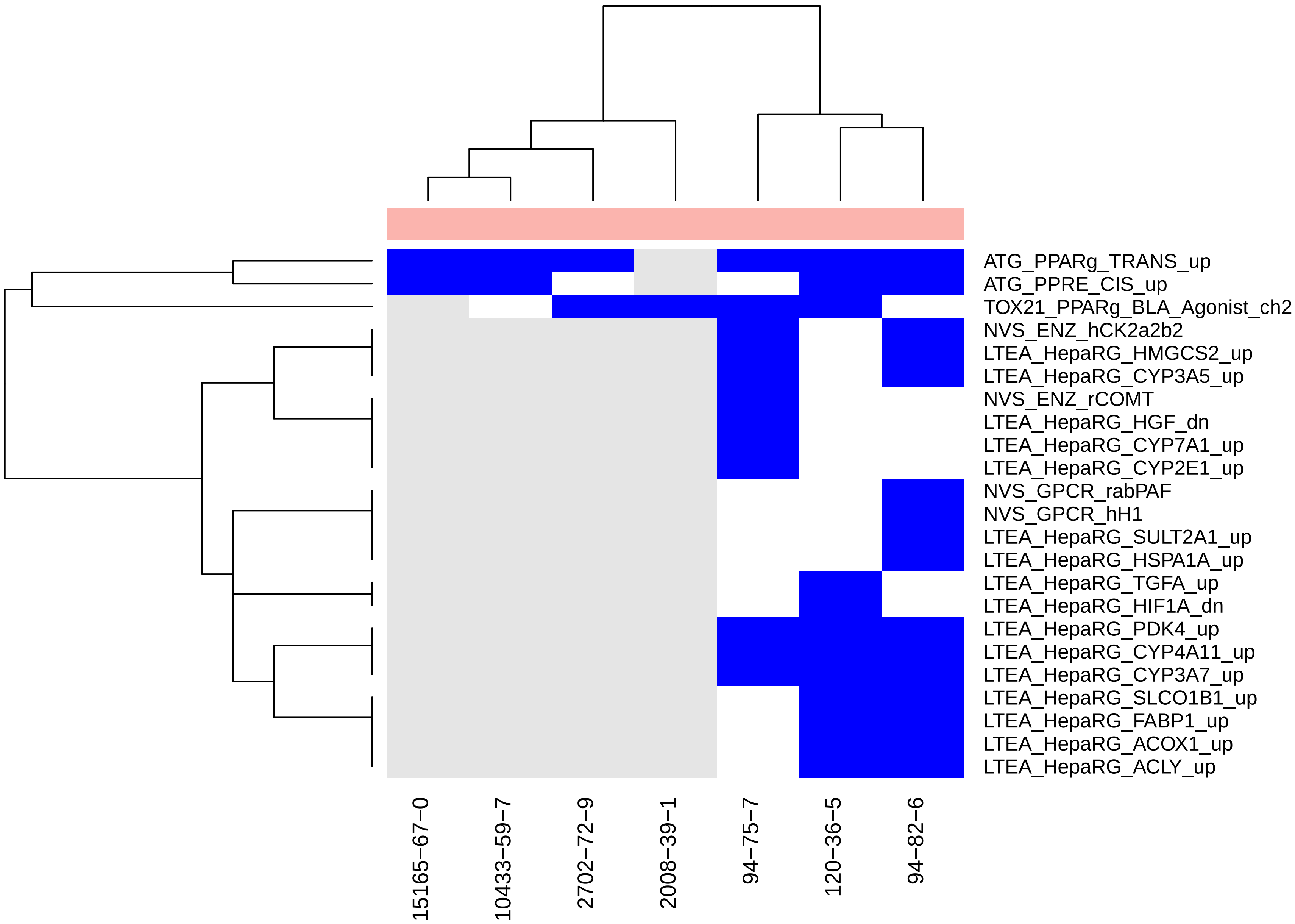

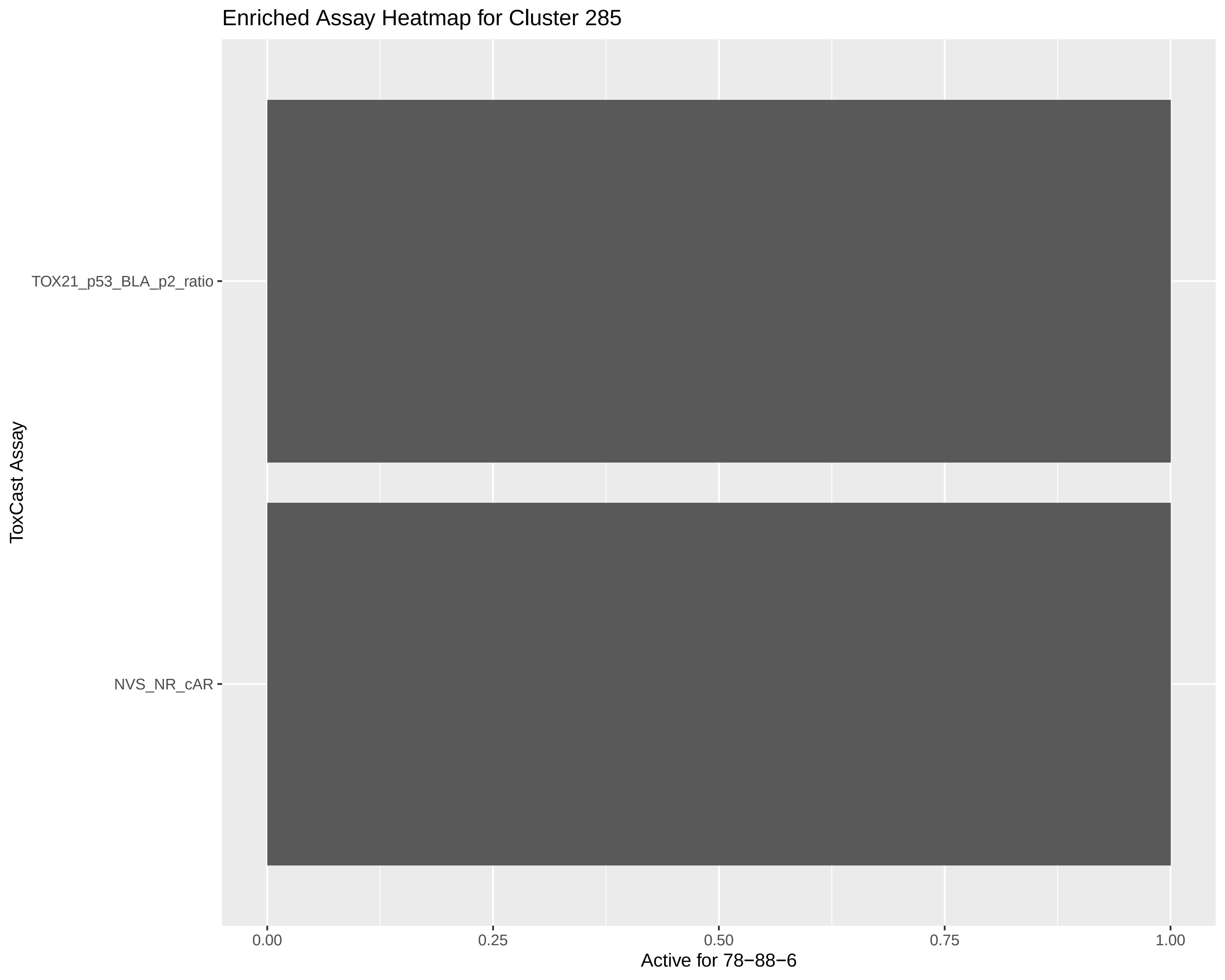

Enriched Assay Heatmap for Cluster 285
TOX21_p53_BLA_p2_ratio
NVS_NR_cAR
ToxCast Assay
0.00
0.25
0.50
0.75
1.00
Active for 78−88−6

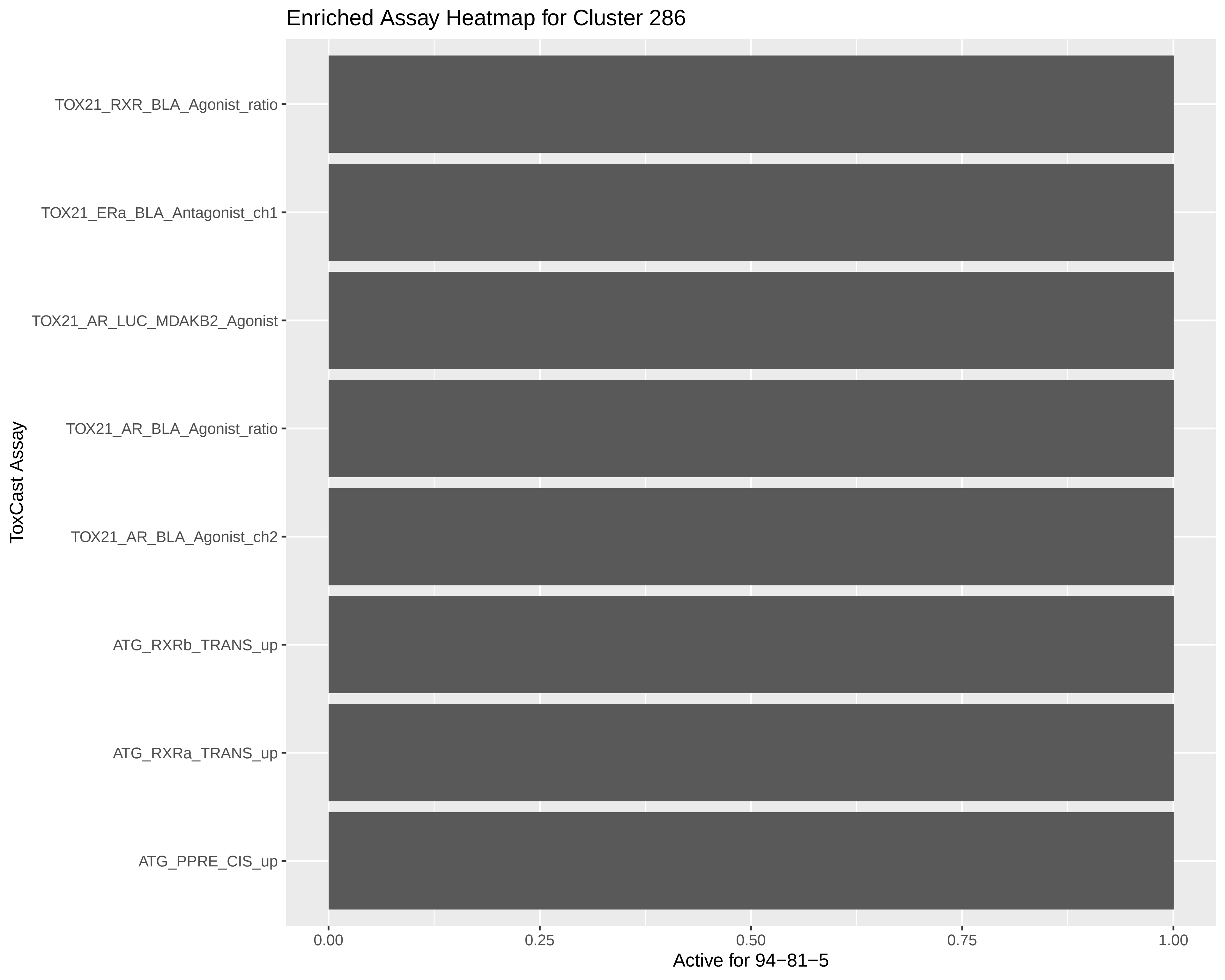
Enriched Assay Heatmap for Cluster 286
TOX21_RXR_BLA_Agonist_ratio
TOX21_ERa_BLA_Antagonist_ch1
TOX21_AR_LUC_MDAKB2_Agonist
TOX21_AR_BLA_Agonist_ratio
TOX21_AR_BLA_Agonist_ch2
ATG_RXRb_TRANS_up
ATG_RXRa_TRANS_up
ATG_PPRE_CIS_up
ToxCast Assay
0.00
0.25
0.50
0.75
1.00
Active for 94−81−5

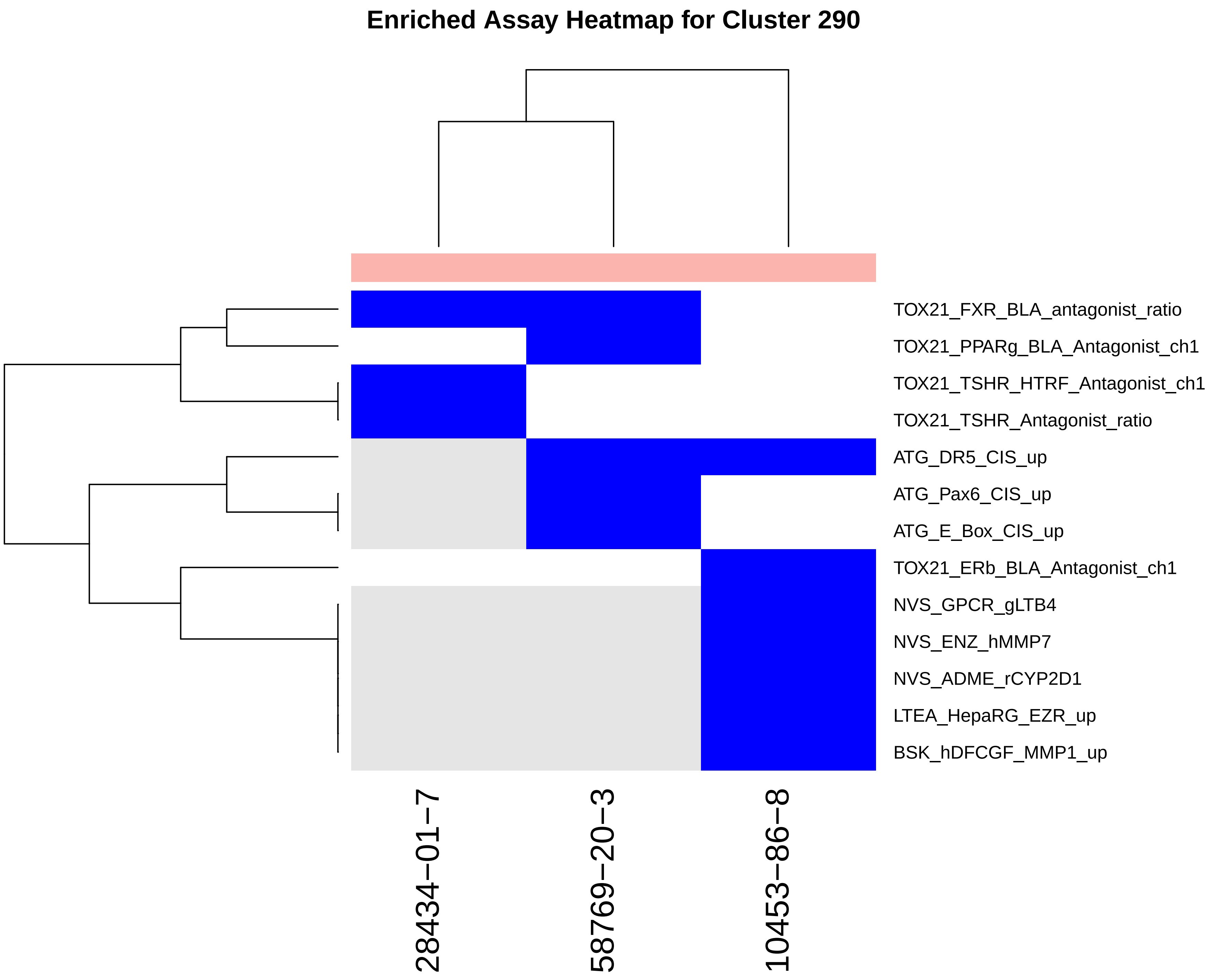
Enriched Assay Heatmap for Cluster 290
TOX21_FXR_BLA_antagonist_ratio
TOX21_PPARg_BLA_Antagonist_ch1
TOX21_TSHR_HTRF_Antagonist_ch1
TOX21_TSHR_Antagonist_ratio
ATG_DR5_CIS_up
ATG_Pax6_CIS_up
ATG_E_Box_CIS_up
TOX21_ERb_BLA_Antagonist_ch1
NVS_GPCR_gLTB4
NVS_ENZ_hMMP7
NVS_ADME_rCYP2D1
LTEA_HepaRG_EZR_up
BSK_hDFCGF_MMP1_up
28434−01−7
58769−20−3
10453−86−8

Enriched Assay Heatmap for Cluster 292

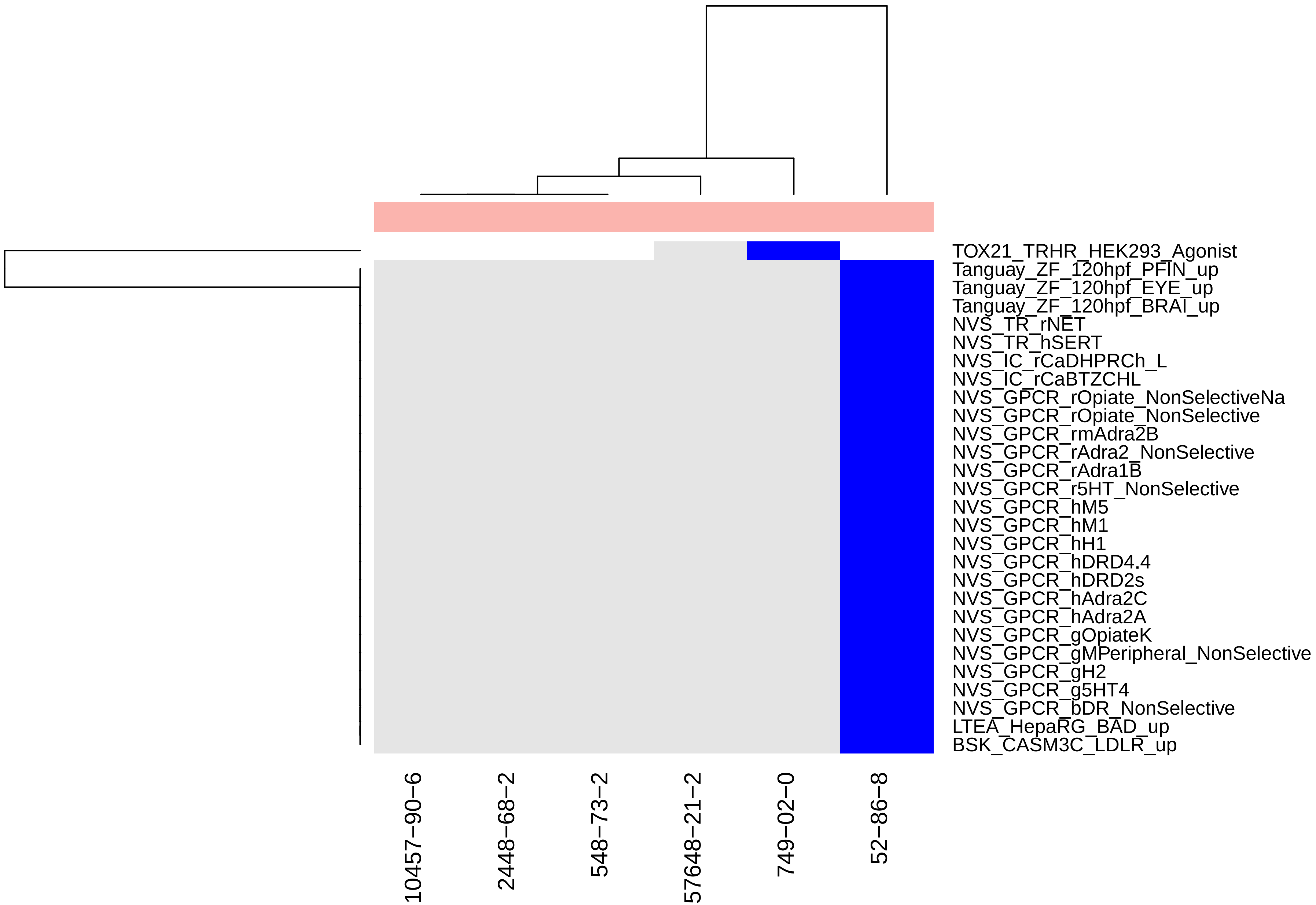

Enriched Assay Heatmap for Cluster 296

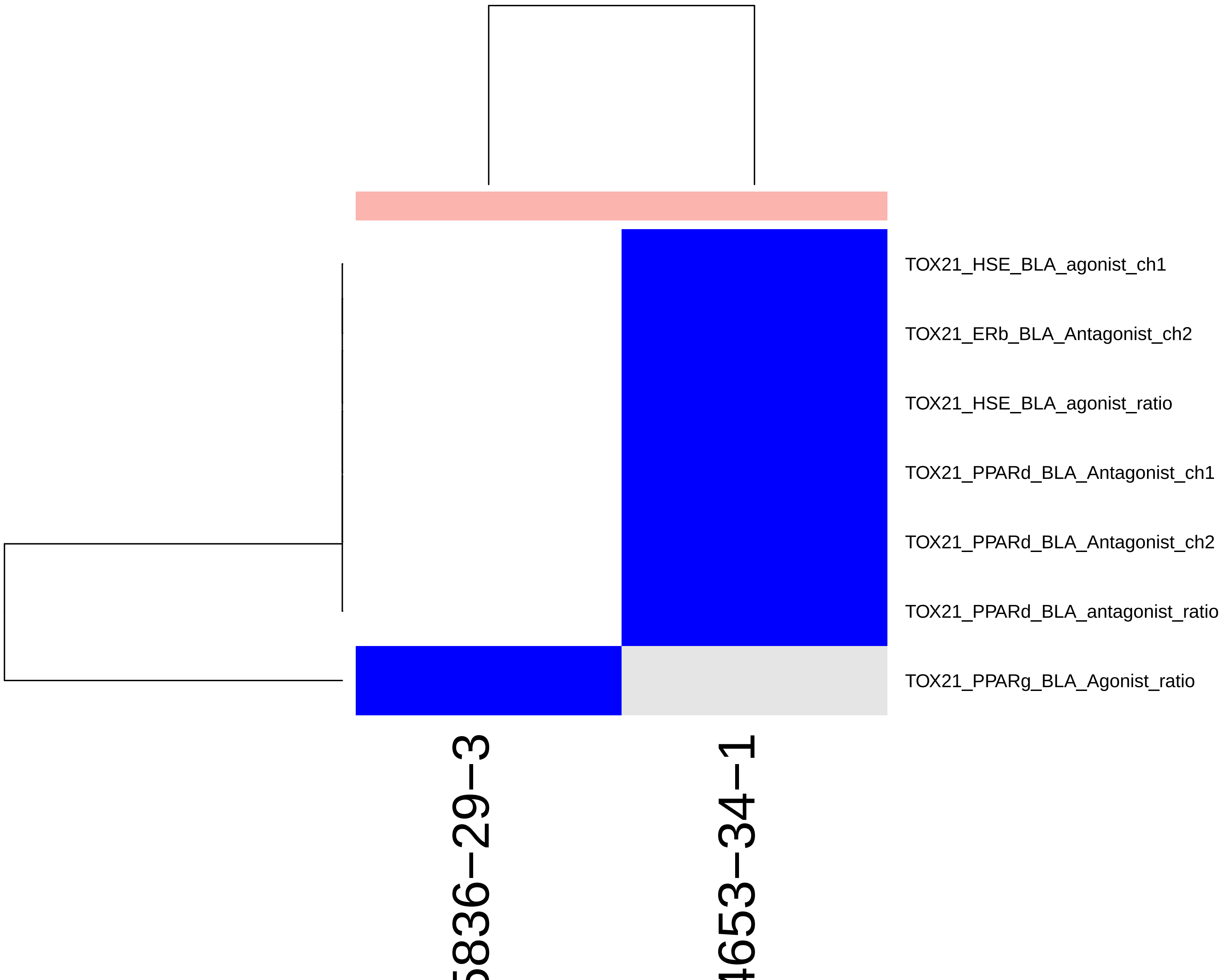

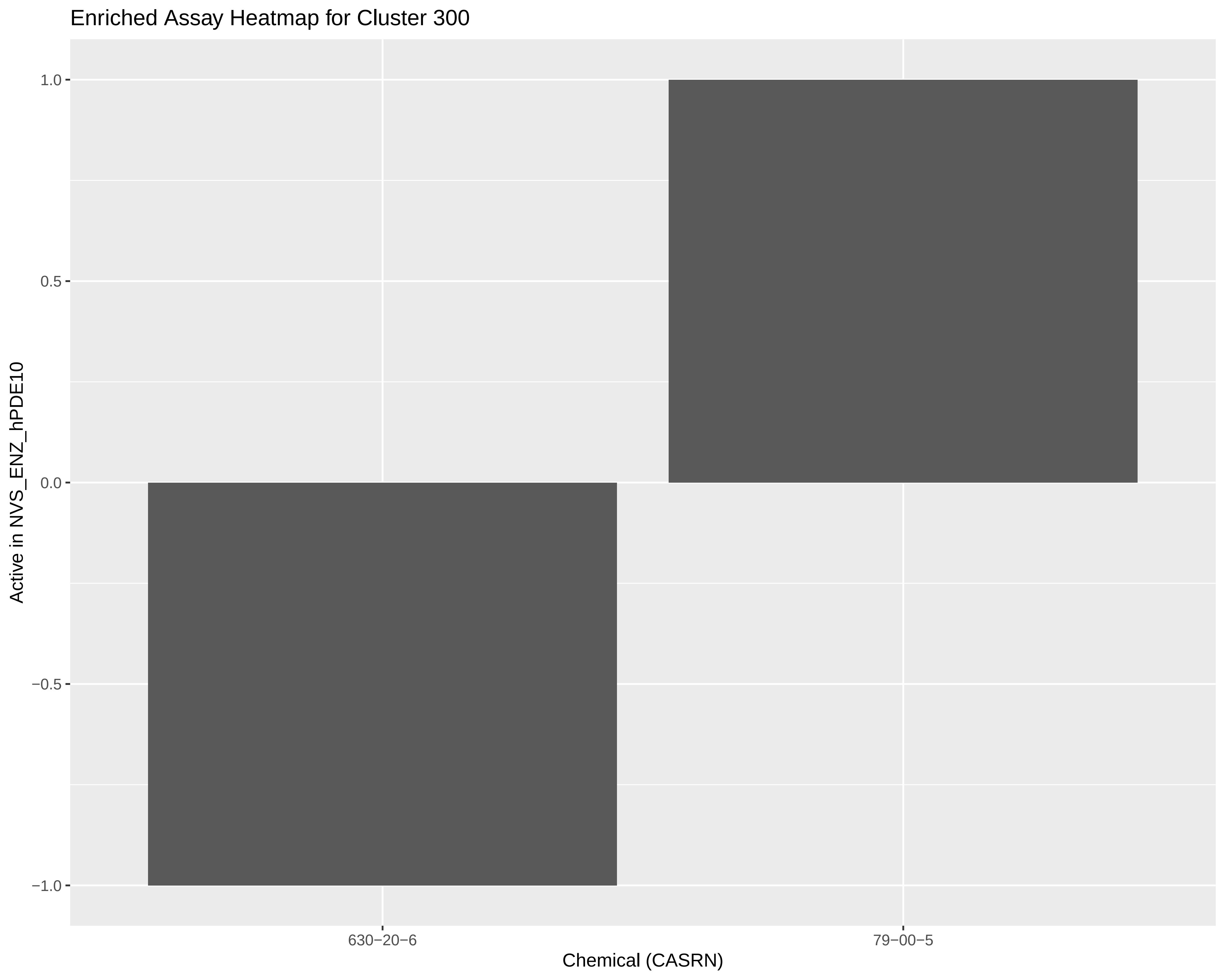

Enriched Assay Heatmap for Cluster 300
Active in NVS_ENZ_hPDE10
1.0
0.5
0.0
−0.5
−1.0
630−20−6
79−00−5
Chemical (CASRN)

Enriched Assay Heatmap for Cluster 301

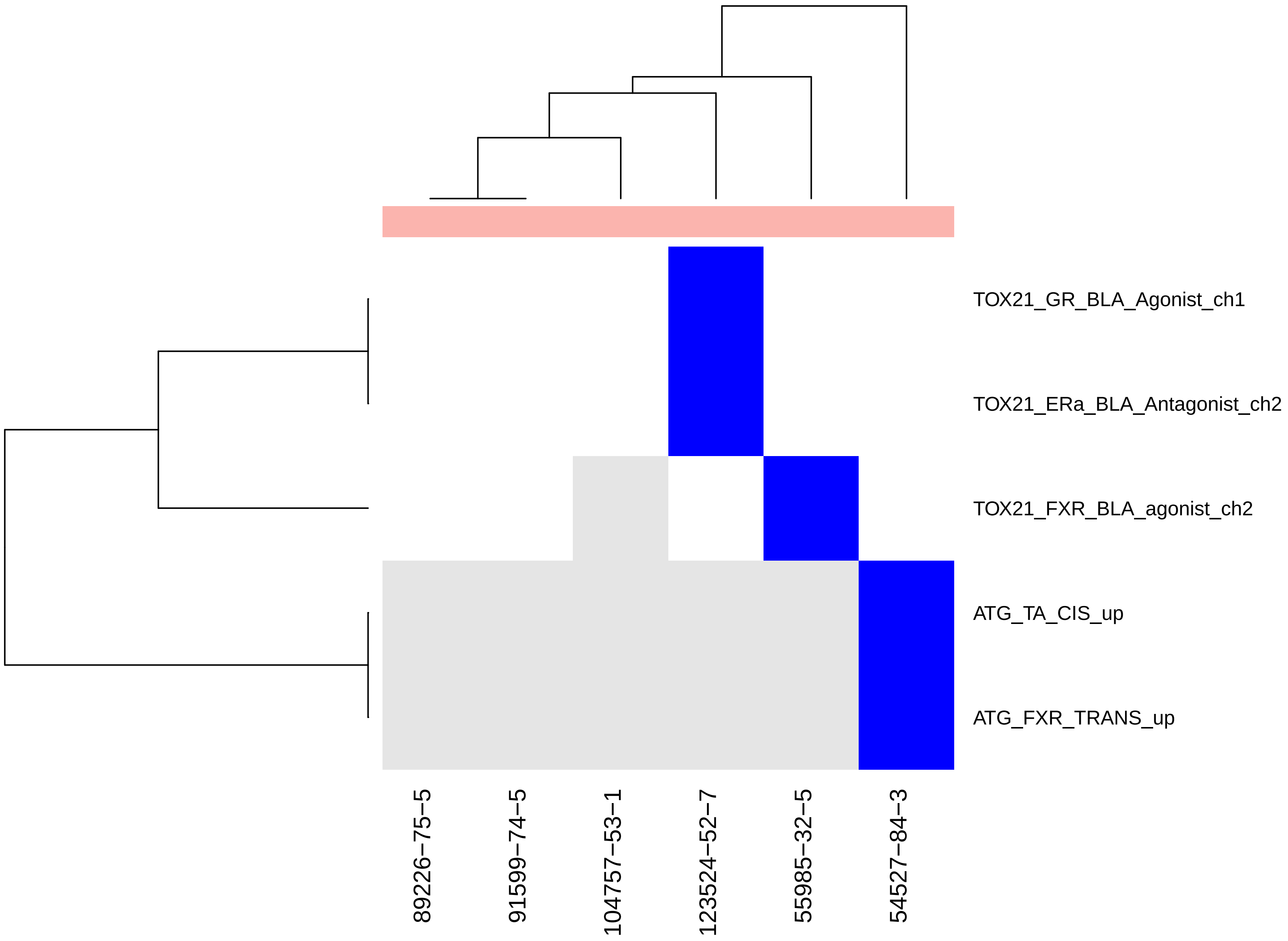

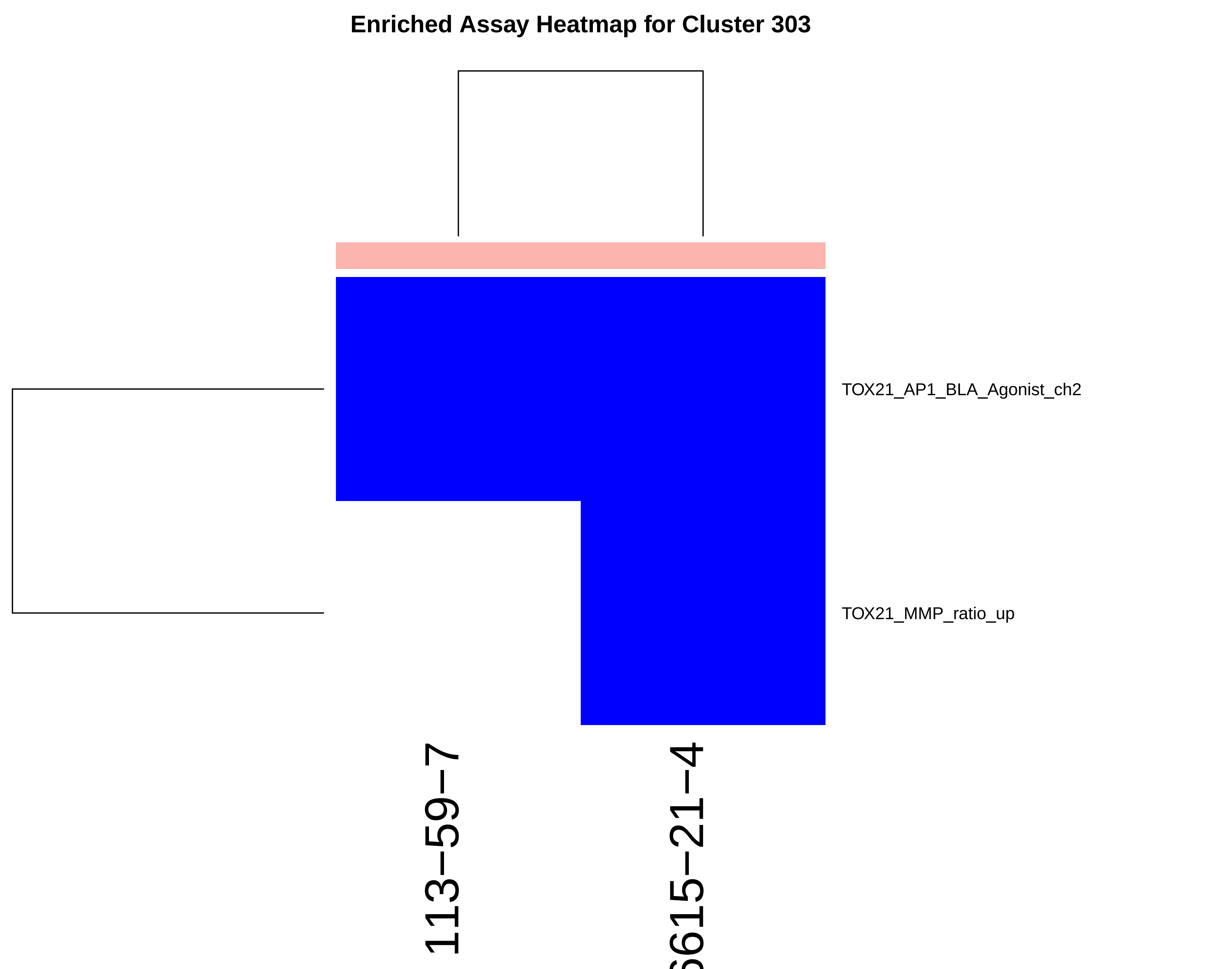

Enriched Assay Heatmap for Cluster 303
TOX21_AP1_BLA_Agonist_ch2
TOX21_MMP_ratio_up
113−59−7
6615−21−4

Enriched Assay Heatmap for Cluster 307

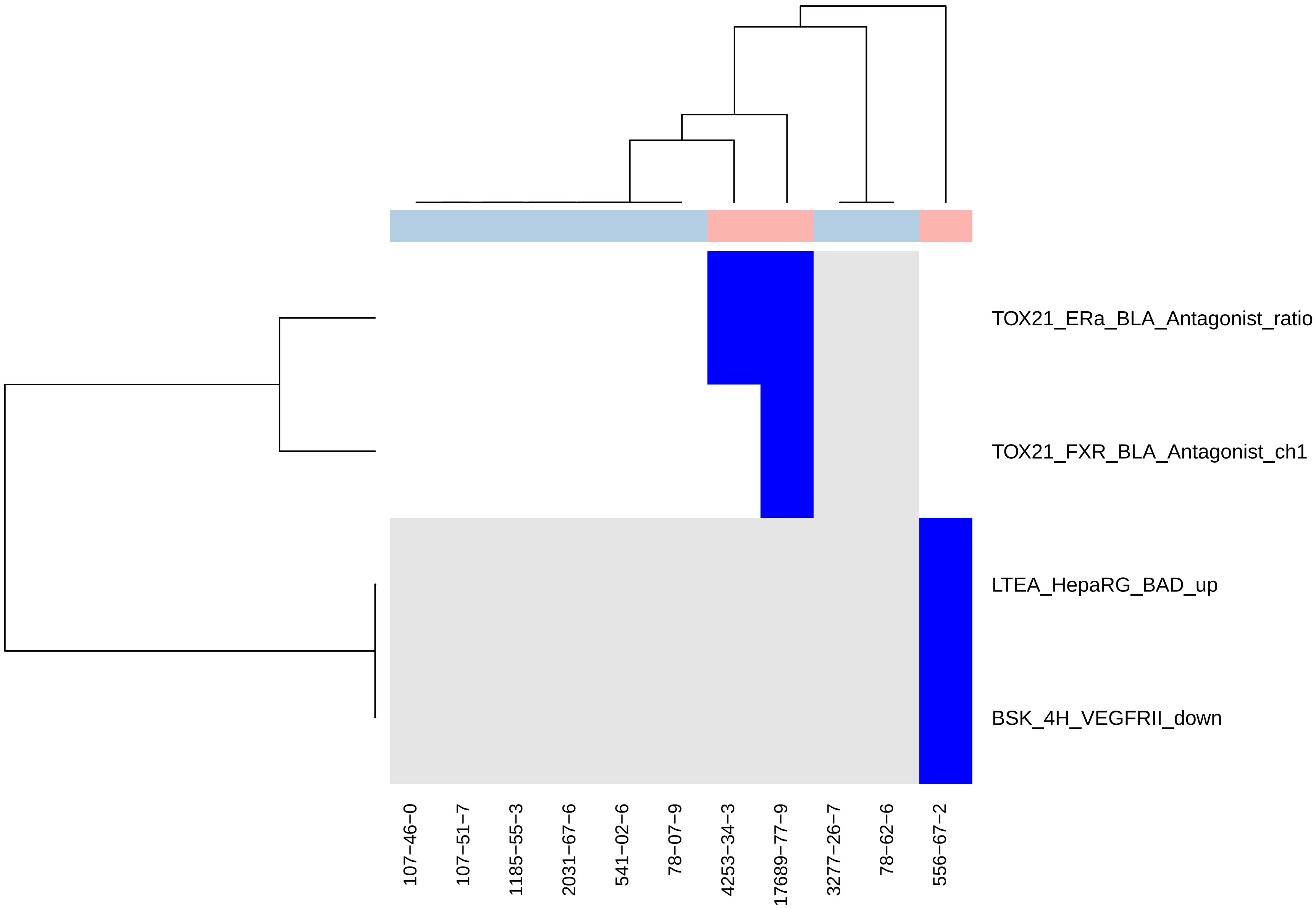

Enriched Assay Heatmap for Cluster 308

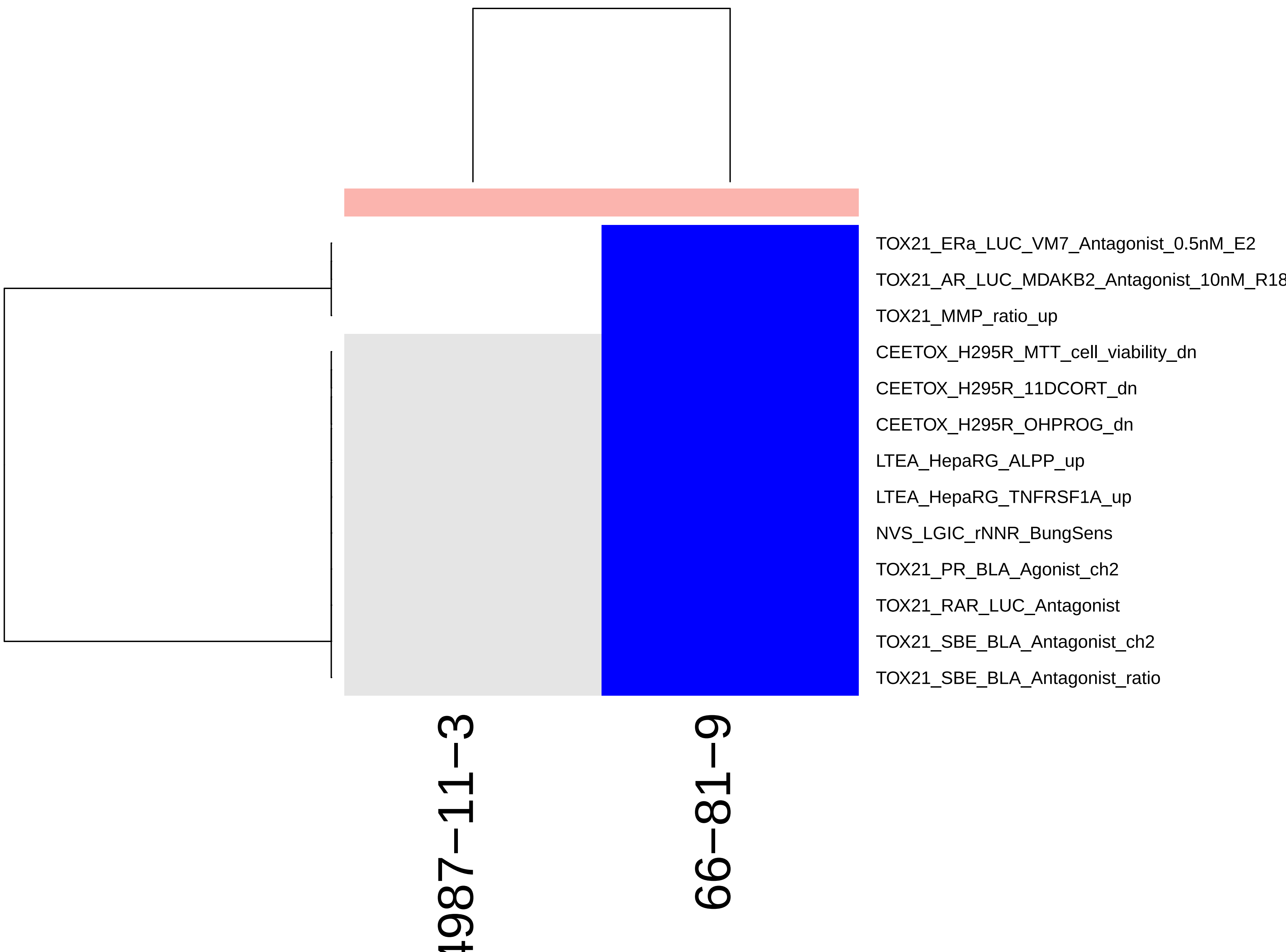

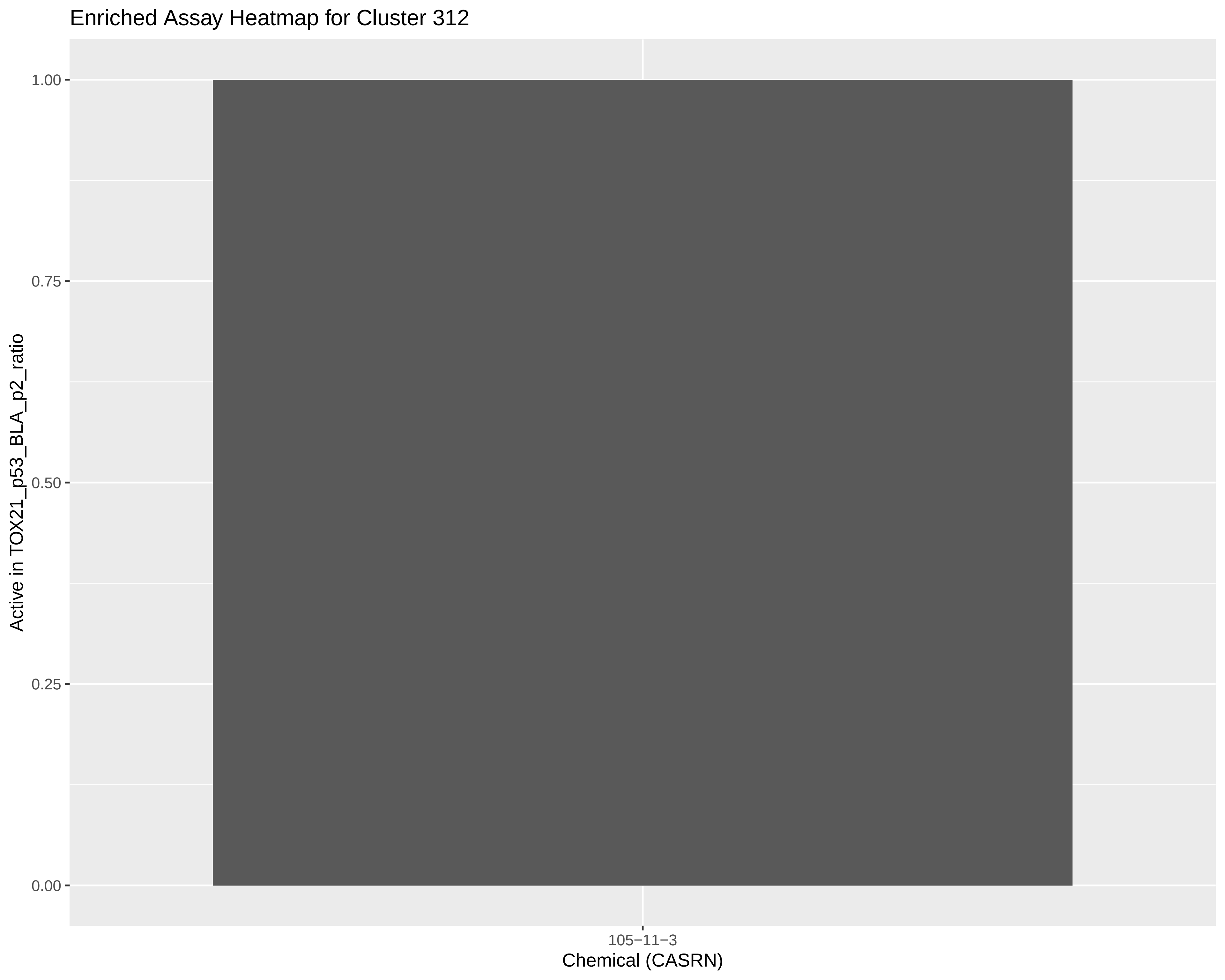

Enriched Assay Heatmap for Cluster 312
Active in TOX21_p53_BLA_p2_ratio
1.00
0.75
0.50
0.25
0.00
105−11−3
Chemical (CASRN)

Enriched Assay Heatmap for Cluster 314

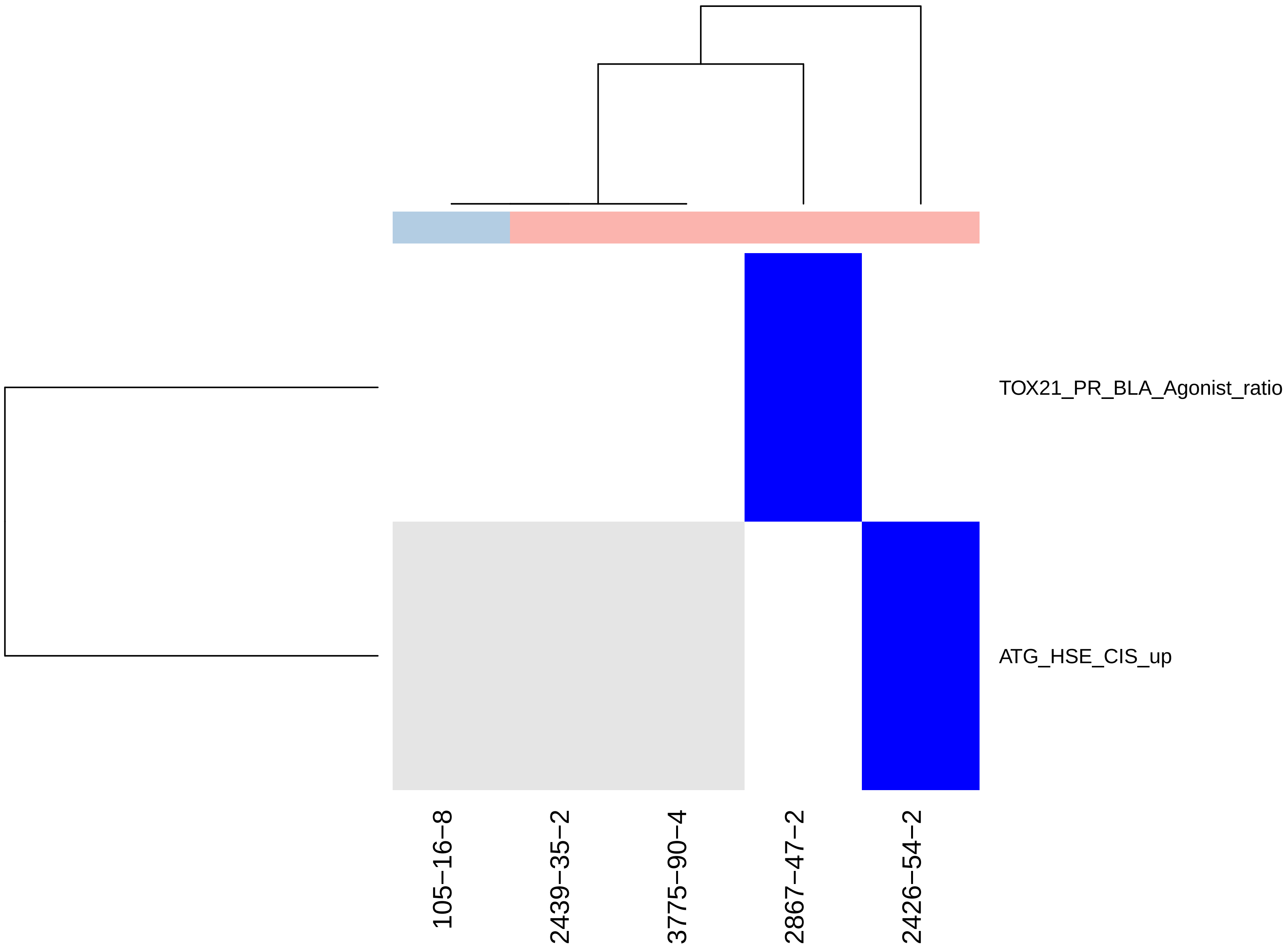

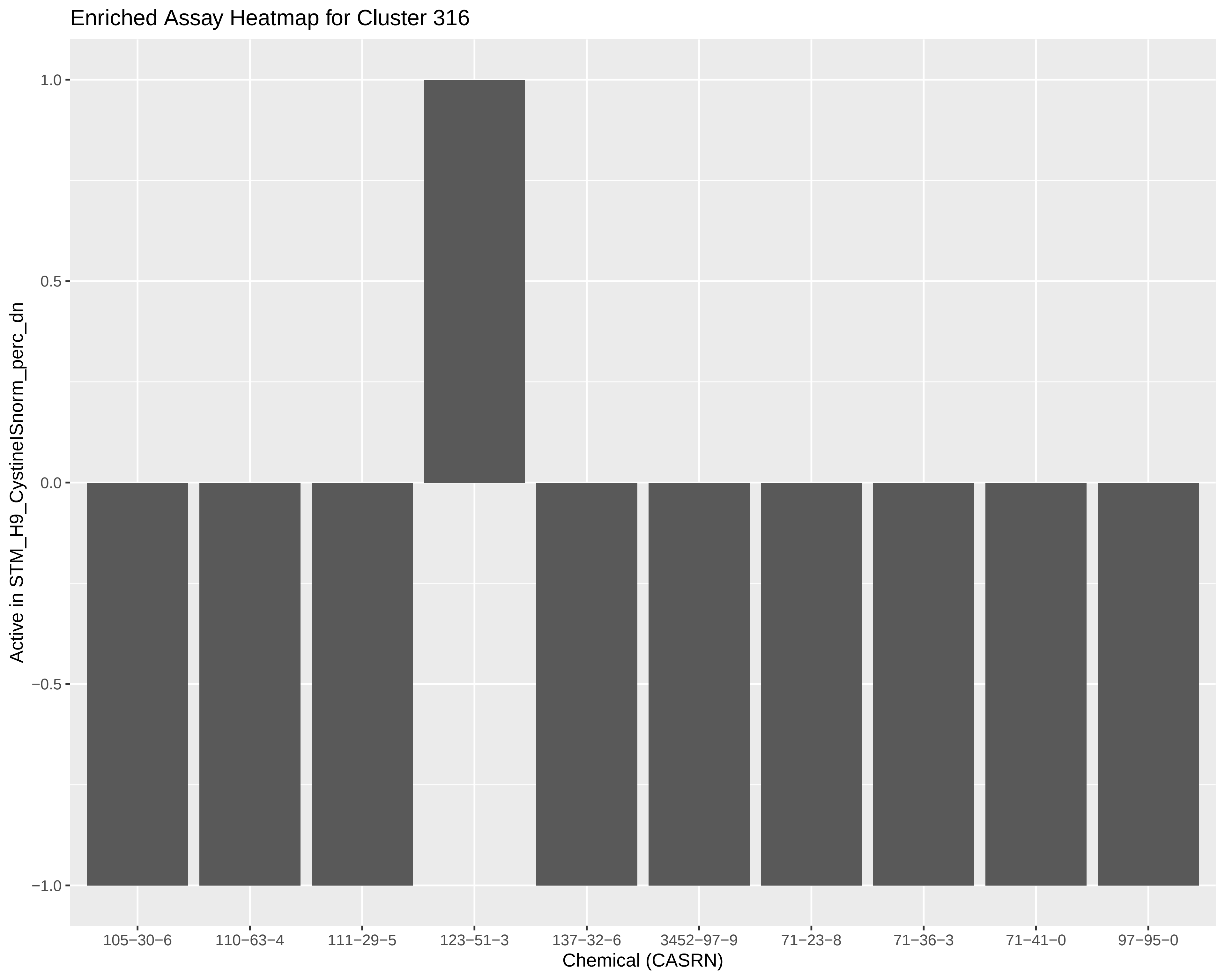
Enriched Assay Heatmap for Cluster 316
Active in STM_H9_CystineISnorm_perc_dn
1.0
0.5
0.0
−0.5
−1.0
105−30−6
110−63−4
111−29−5
123−51−3
137−32−6
3452−97−9
71−23−8
71−36−3
71−41−0
97−95−0
Chemical (CASRN)

Enriched Assay Heatmap for Cluster 317

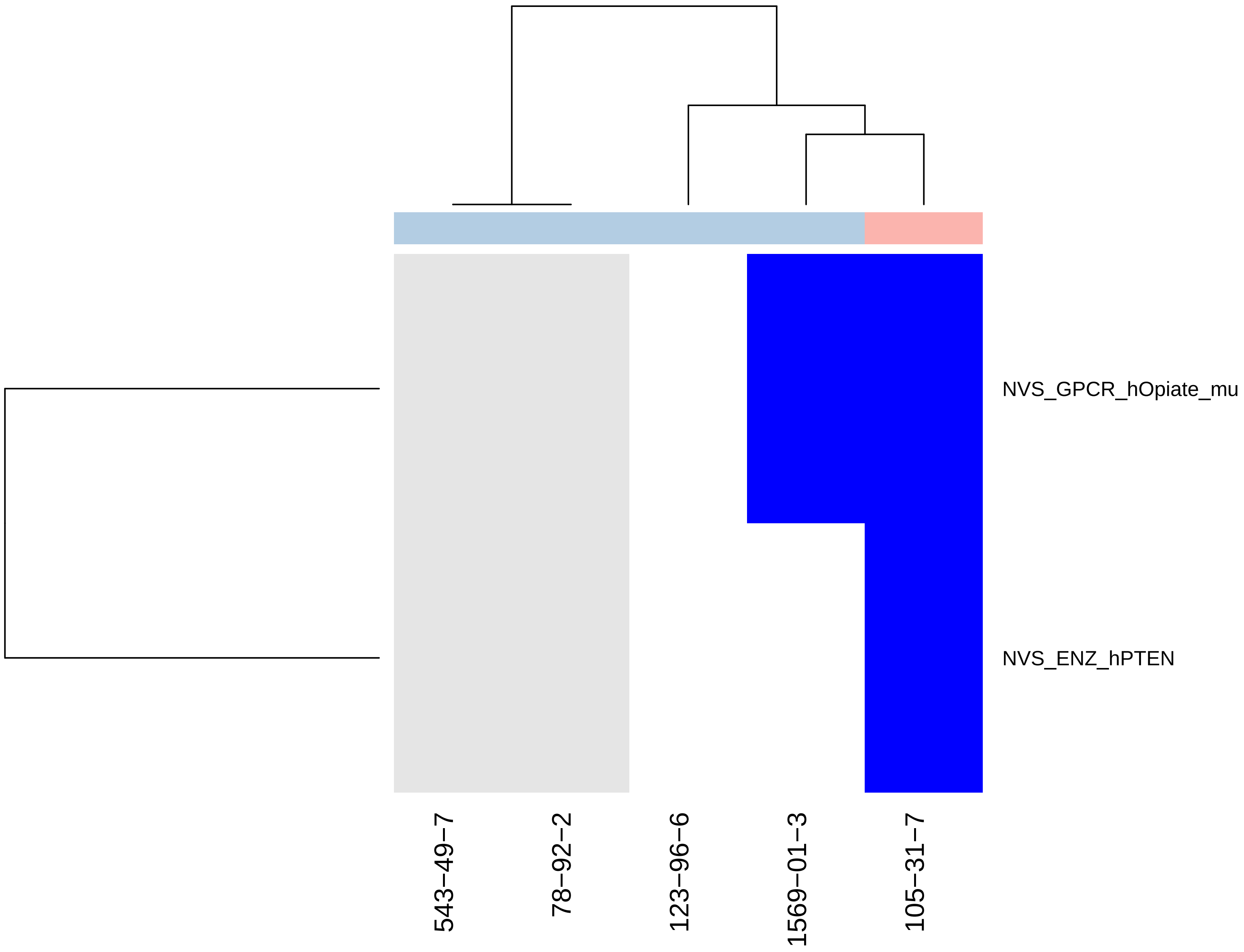

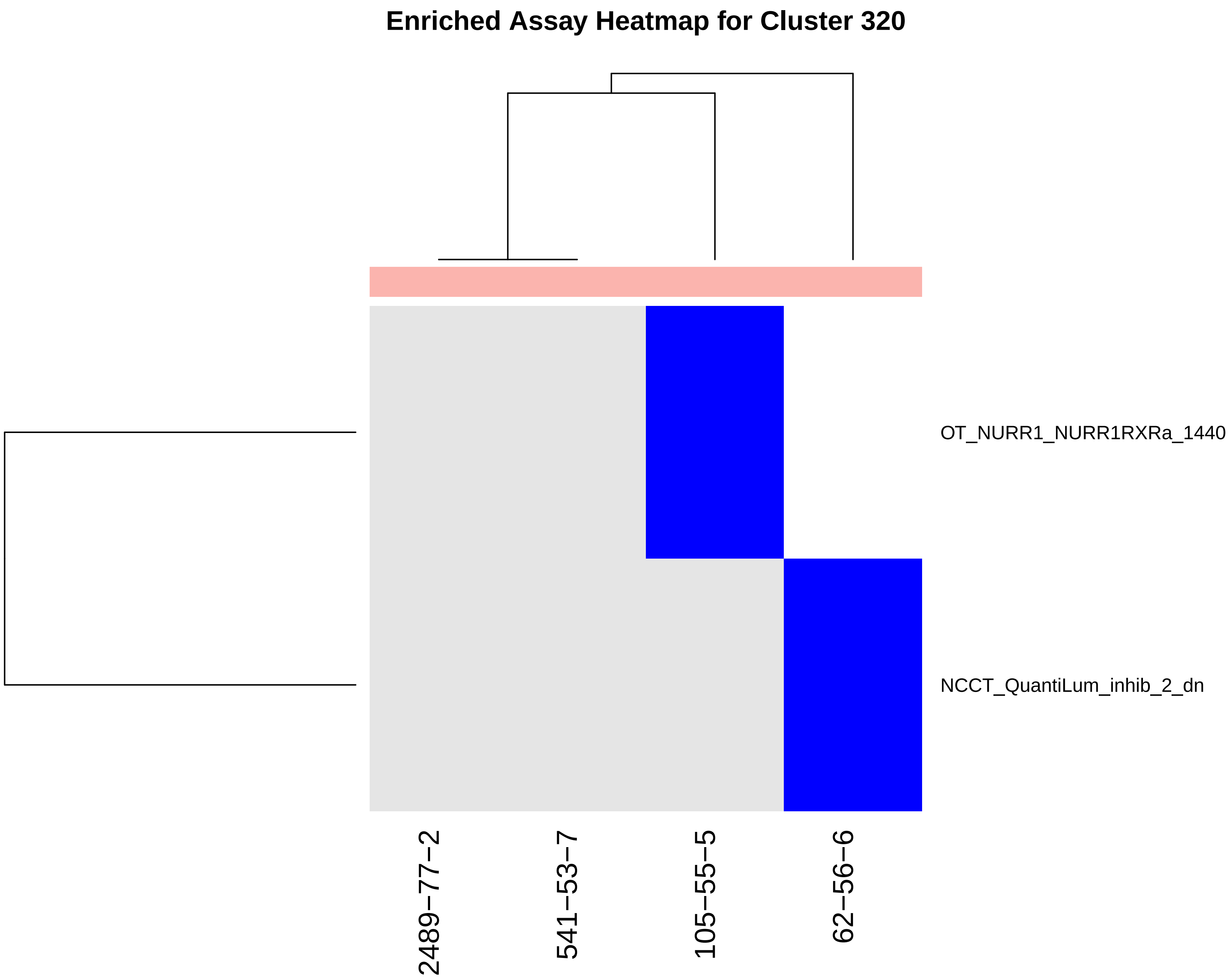

Enriched Assay Heatmap for Cluster 320
OT_NURR1_NURR1RXRa_1440
NCCT_QuantiLum_inhib_2_dn
2489−77−2
541−53−7
105−55−5
62−56−6

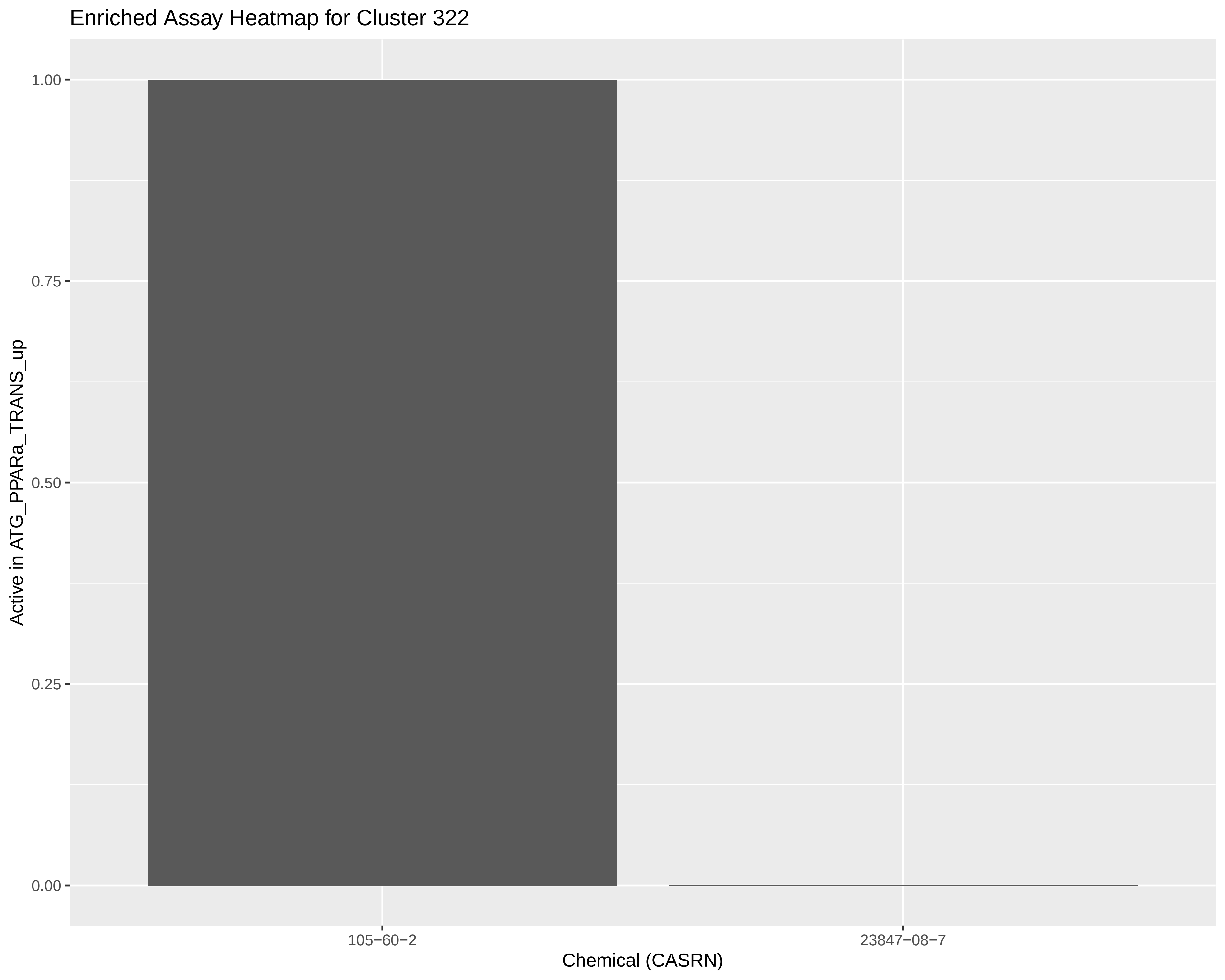

Enriched Assay Heatmap for Cluster 322
1.00
0.75
0.50
0.25
0.00
Active in ATG_PPARa_TRANS_up
105−60−2
23847−08−7
Chemical (CASRN)

Enriched Assay Heatmap for Cluster 324

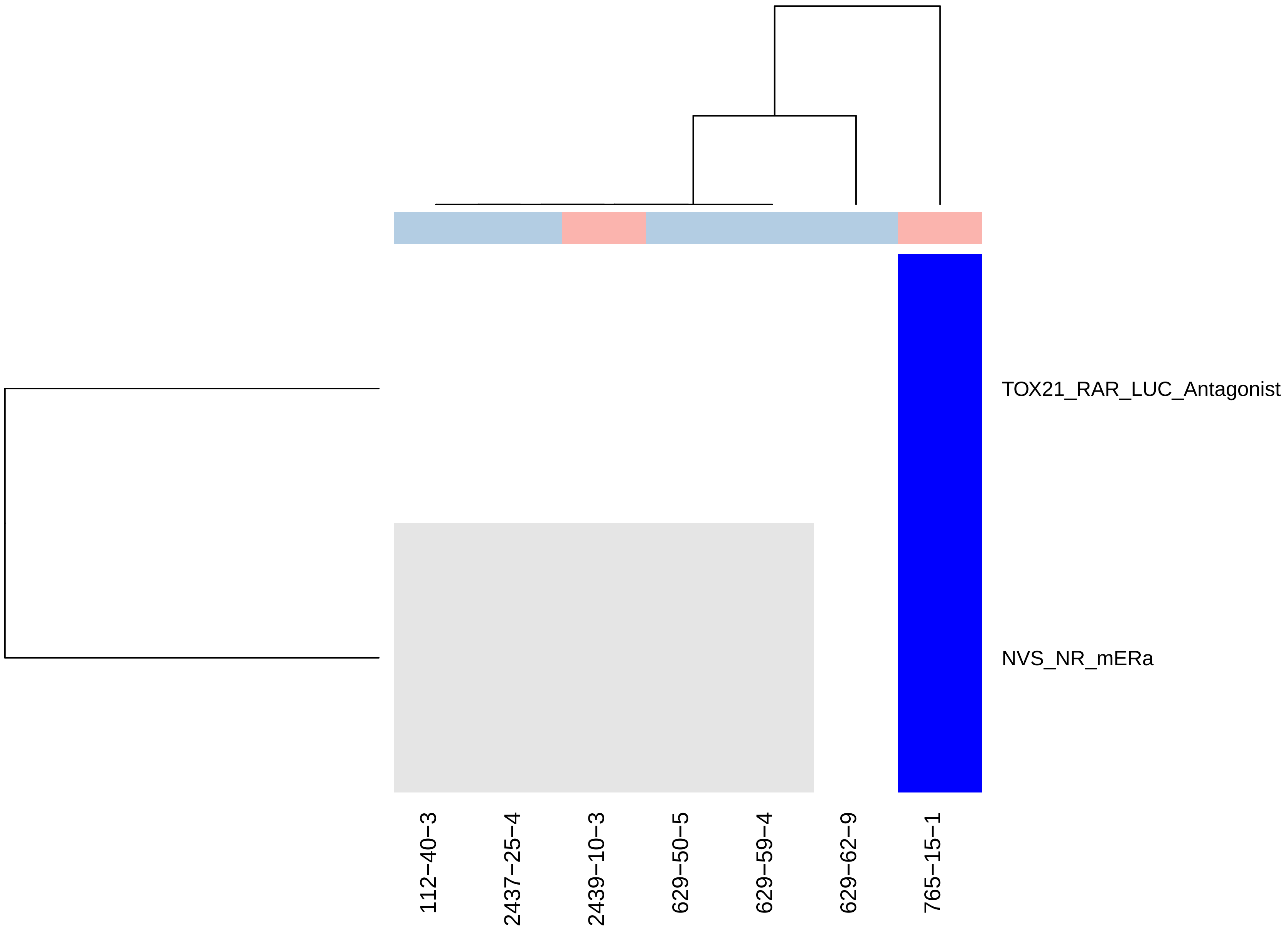

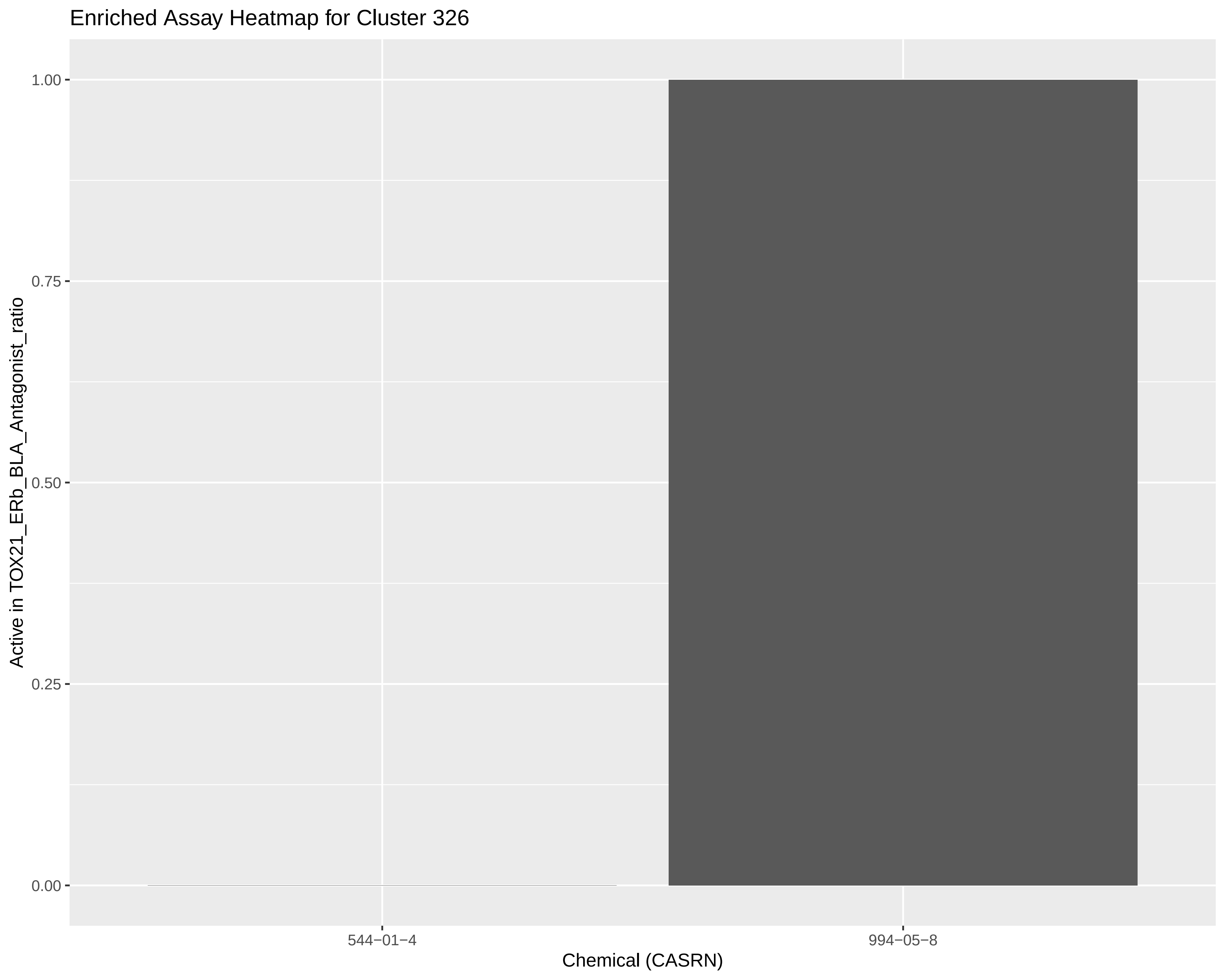

Enriched Assay Heatmap for Cluster 326
Active in TOX21_ERb_BLA_Antagonist_ratio
1.00
0.75
0.50
0.25
0.00
544−01−4
994−05−8
Chemical (CASRN)

Enriched Assay Heatmap for Cluster 331

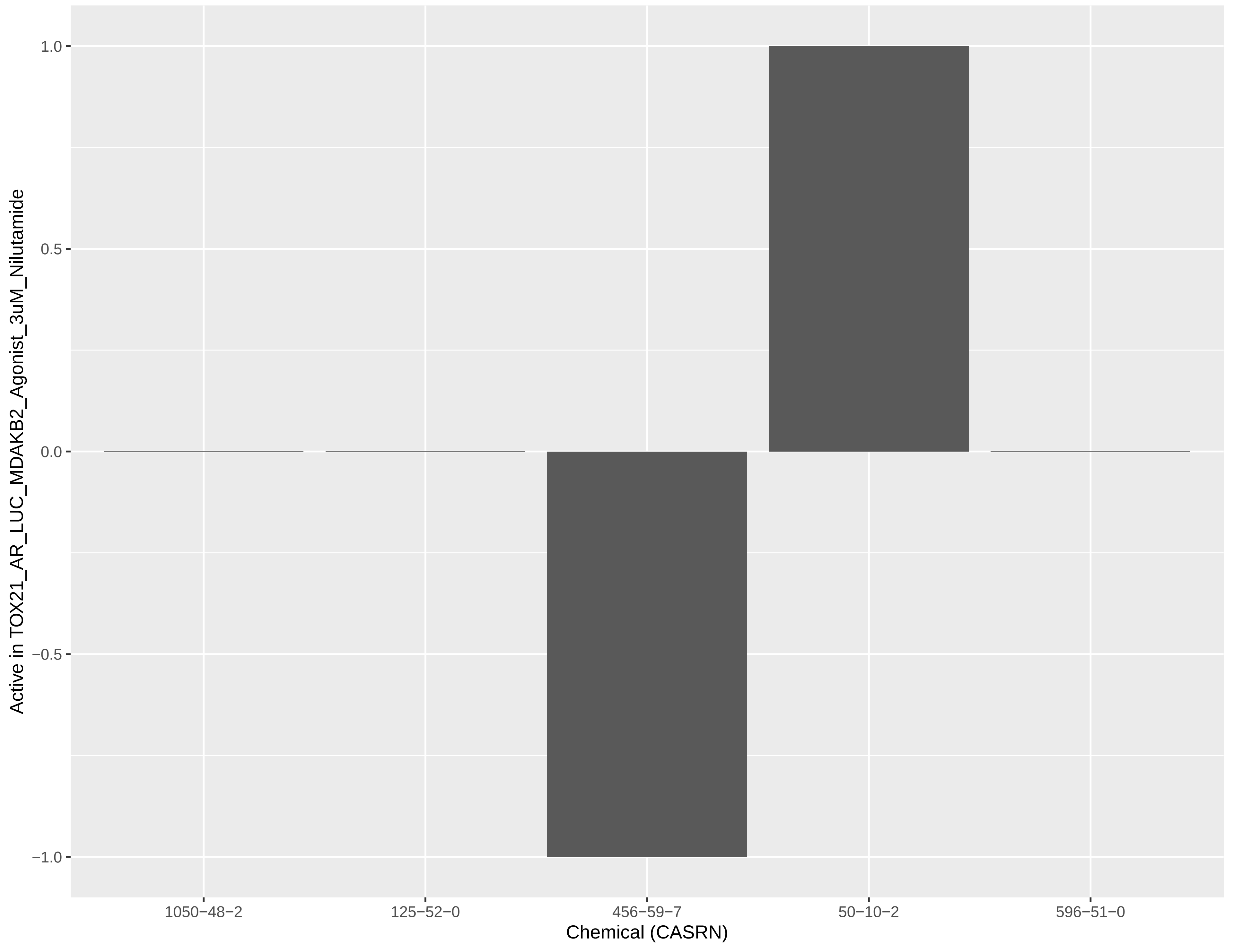

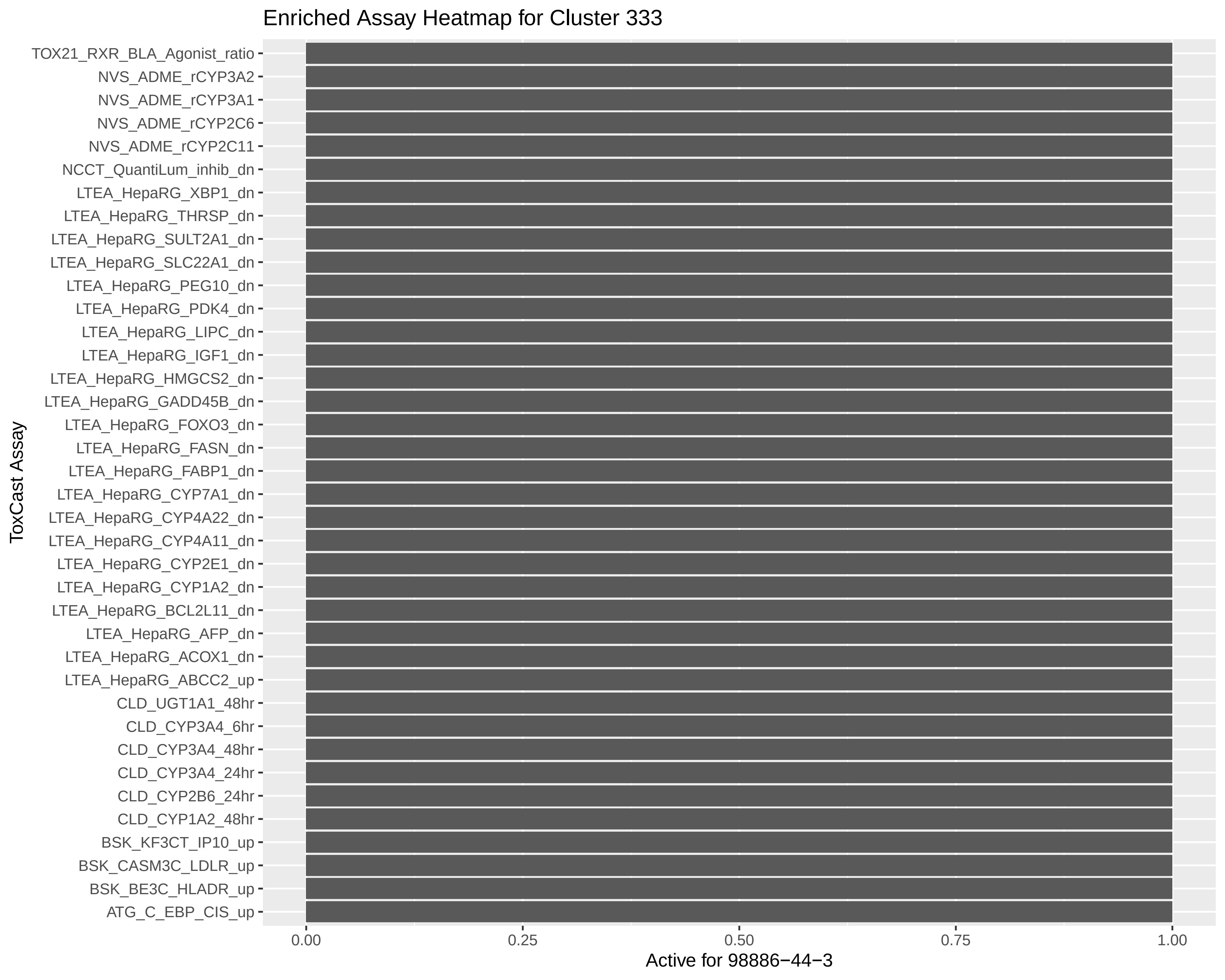
Enriched Assay Heatmap for Cluster 333
TOX21_RXR_BLA_Agonist_ratio
NVS_ADME_rCYP3A2
NVS_ADME_rCYP3A1
NVS_ADME_rCYP2C6
NVS_ADME_rCYP2C11
NCCT_QuantiLum_inhib_dn
LTEA_HepaRG_XBP1_dn
LTEA_HepaRG_THRSP_dn
LTEA_HepaRG_SULT2A1_dn
LTEA_HepaRG_SLC22A1_dn
LTEA_HepaRG_PEG10_dn
LTEA_HepaRG_PDK4_dn
LTEA_HepaRG_LIPC_dn
LTEA_HepaRG_IGF1_dn
LTEA_HepaRG_HMGCS2_dn
LTEA_HepaRG_GADD45B_dn
LTEA_HepaRG_FOXO3_dn
LTEA_HepaRG_FASN_dn
LTEA_HepaRG_FABP1_dn
LTEA_HepaRG_CYP7A1_dn
LTEA_HepaRG_CYP4A22_dn
LTEA_HepaRG_CYP4A11_dn
LTEA_HepaRG_CYP2E1_dn
LTEA_HepaRG_CYP1A2_dn
LTEA_HepaRG_BCL2L11_dn
LTEA_HepaRG_AFP_dn
LTEA_HepaRG_ACOX1_dn
LTEA_HepaRG_ABCC2_up
CLD_UGT1A1_48hr
CLD_CYP3A4_6hr
CLD_CYP3A4_48hr
CLD_CYP3A4_24hr
CLD_CYP2B6_24hr
CLD_CYP1A2_48hr
BSK_KF3CT_IP10_up
BSK_CASM3C_LDLR_up
BSK_BE3C_HLADR_up
ATG_C_EBP_CIS_up
ToxCast Assay
0.00
0.25
0.50
0.75
1.00
Active for 98886−44−3

Enriched Assay Heatmap for Cluster 337

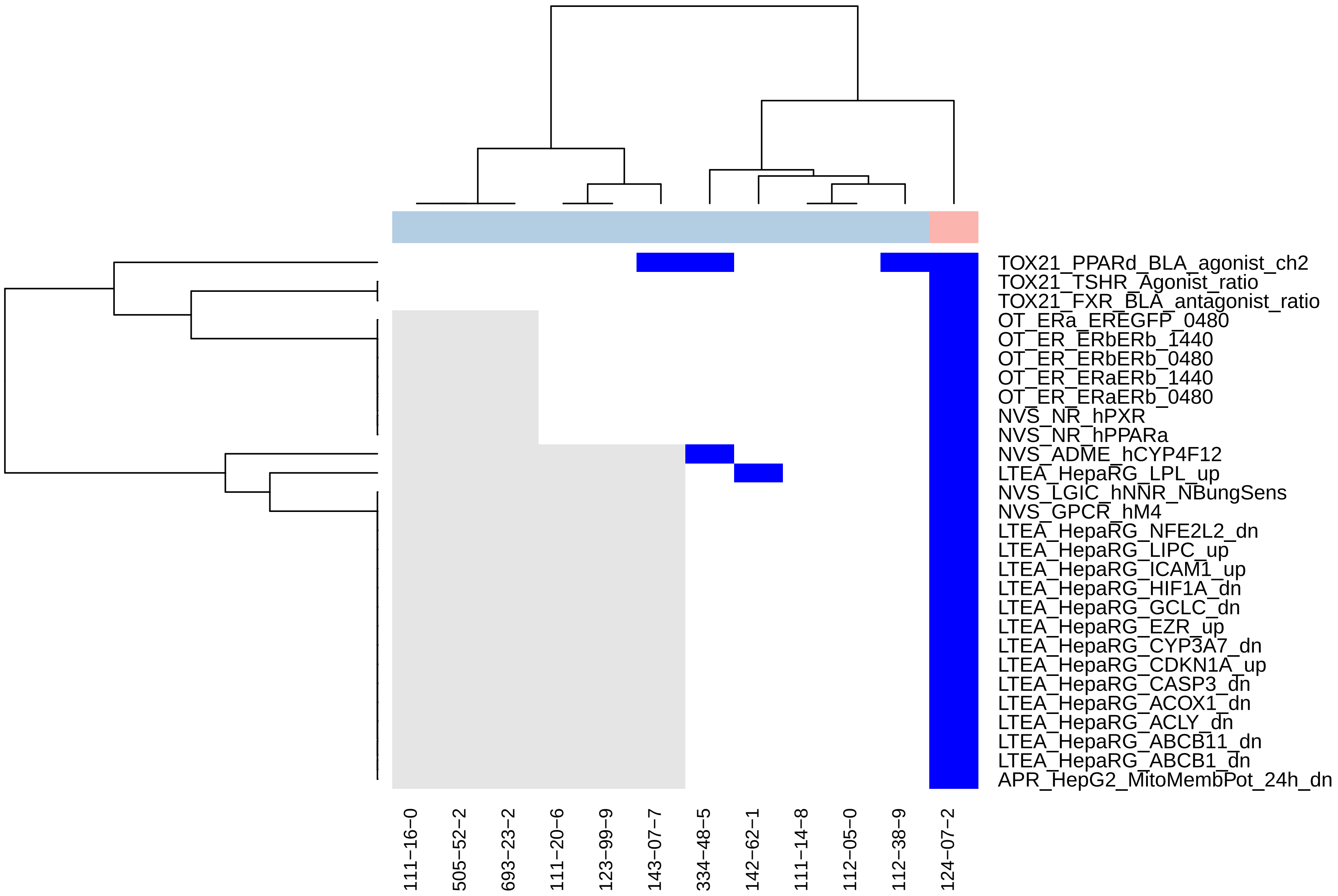

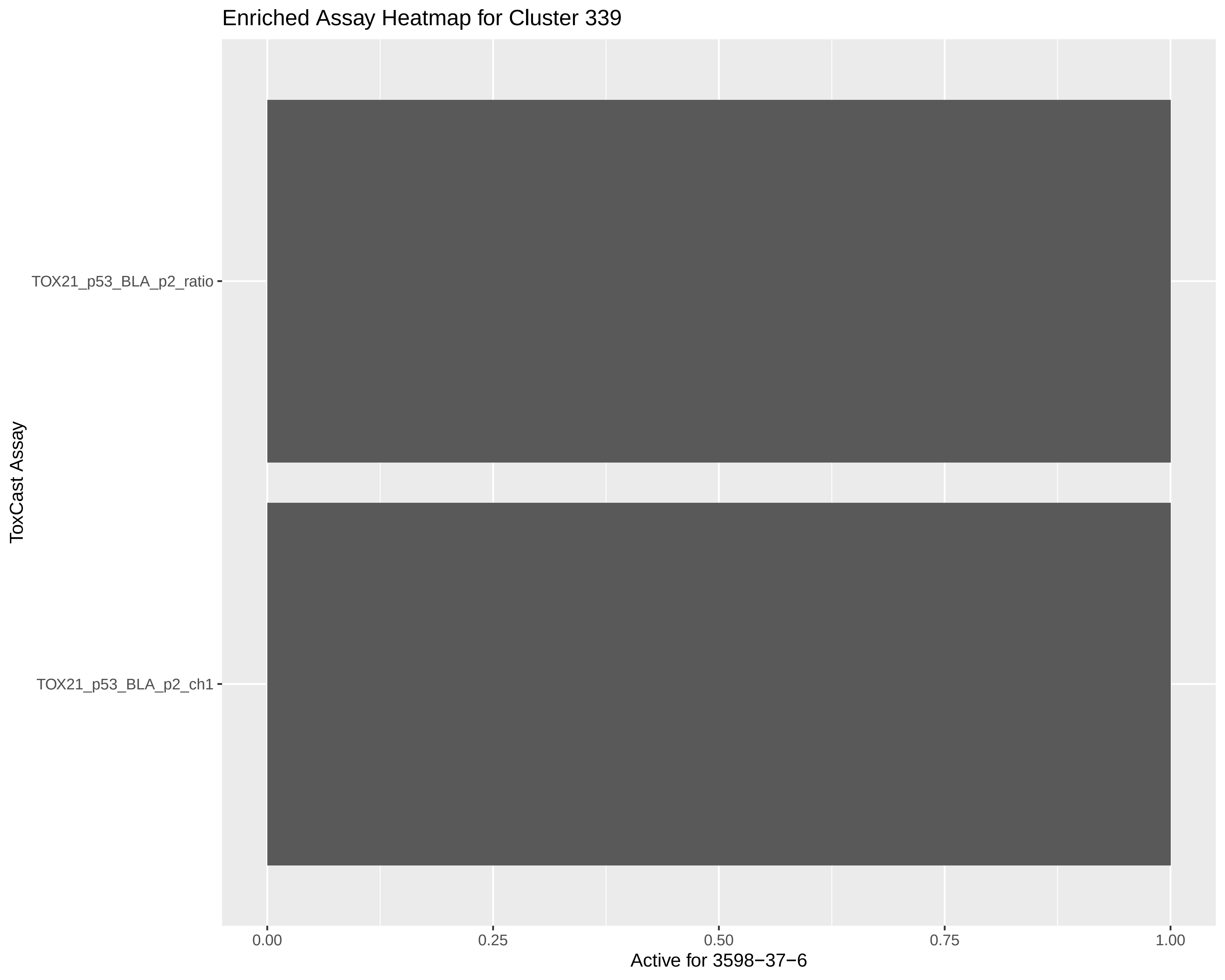

Enriched Assay Heatmap for Cluster 339
ToxCast Assay
TOX21_p53_BLA_p2_ratio
TOX21_p53_BLA_p2_ch1
0.00
0.25
0.50
0.75
1.00
Active for 3598−37−6

Enriched Assay Heatmap for Cluster 341

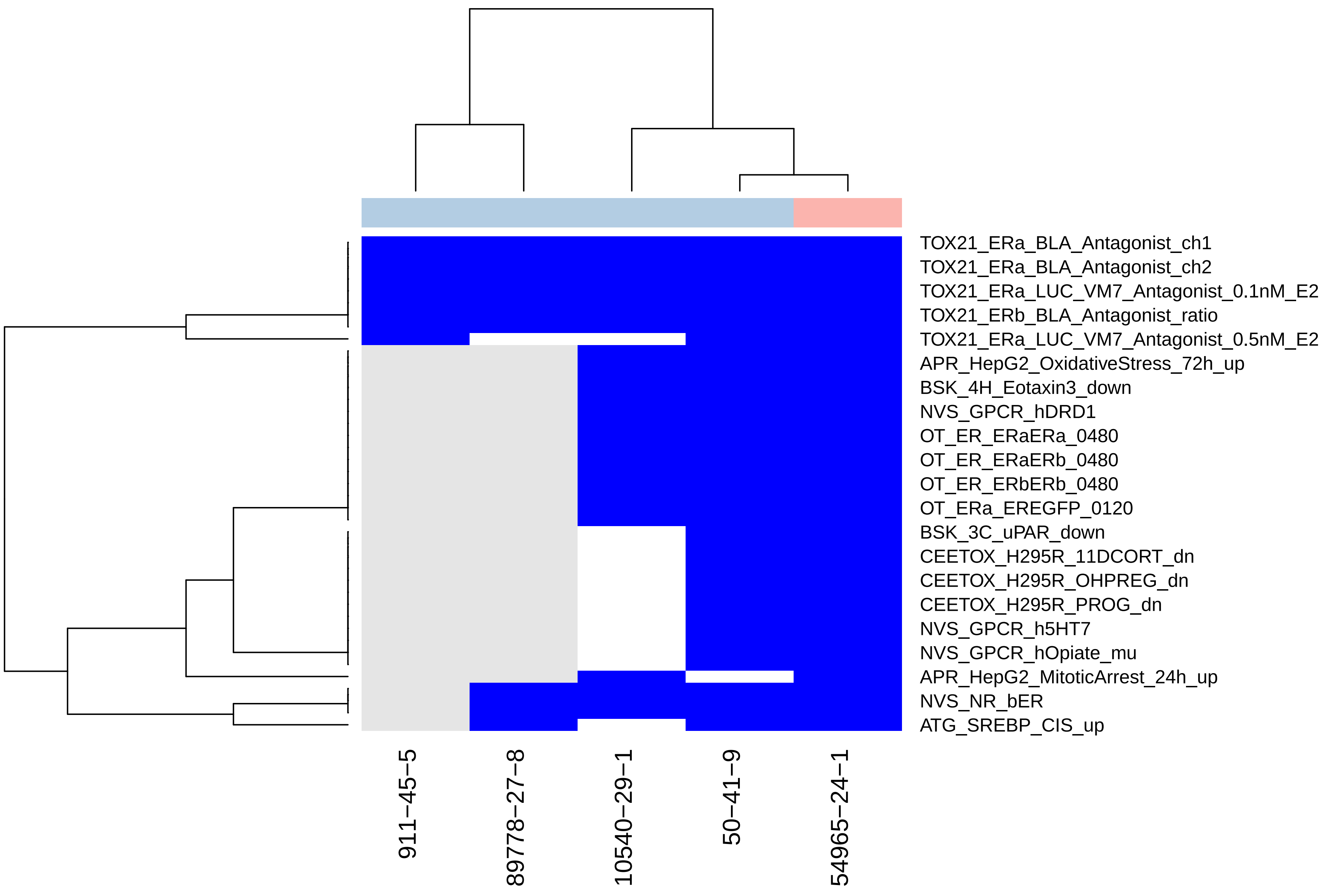

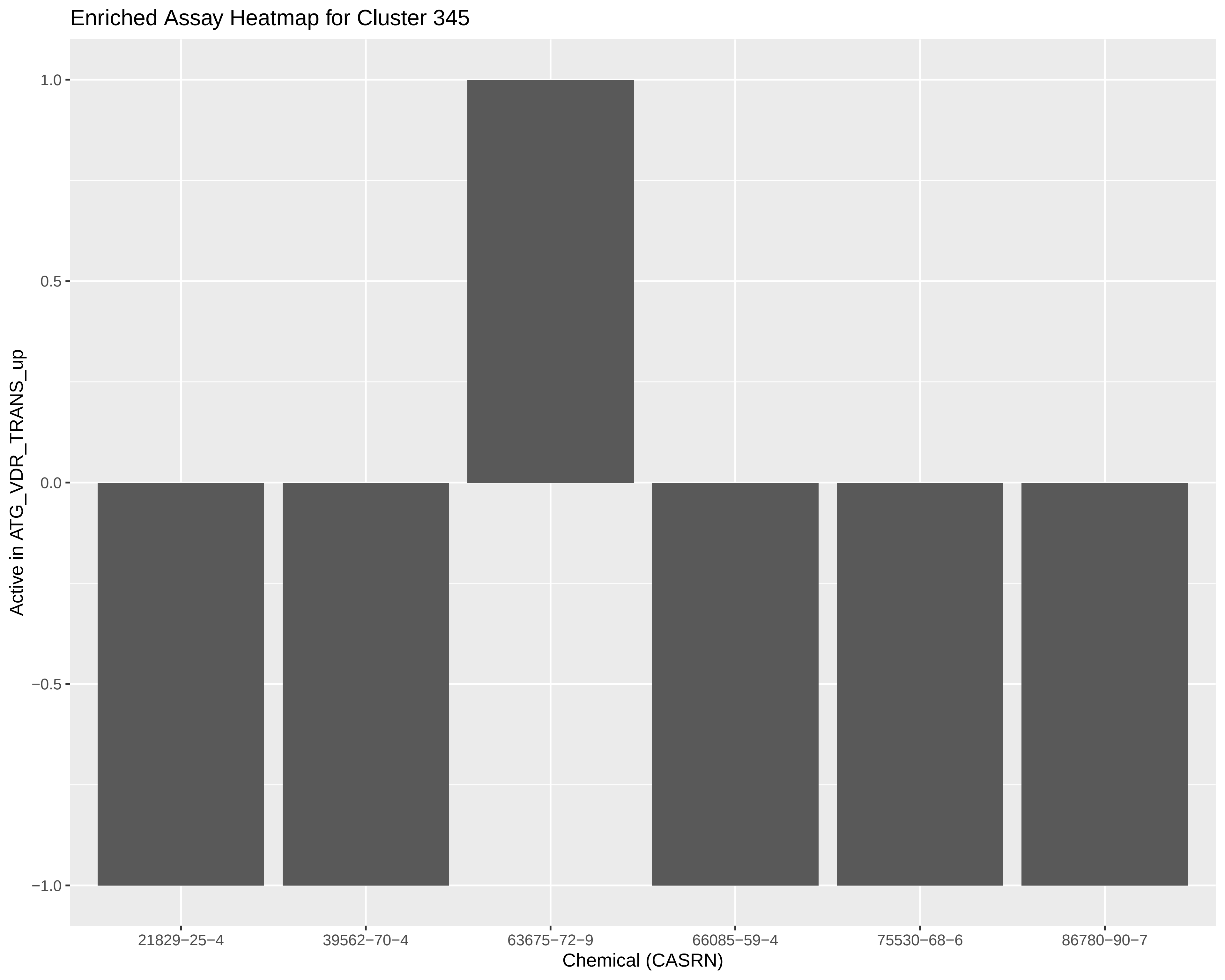

Enriched Assay Heatmap for Cluster 345
1.0
0.5
0.0
−0.5
−1.0
Active in ATG_VDR_TRANS_up
21829−25−4
39562−70−4
63675−72−9
66085−59−4
75530−68−6
86780−90−7
Chemical (CASRN)

Enriched Assay Heatmap for Cluster 352

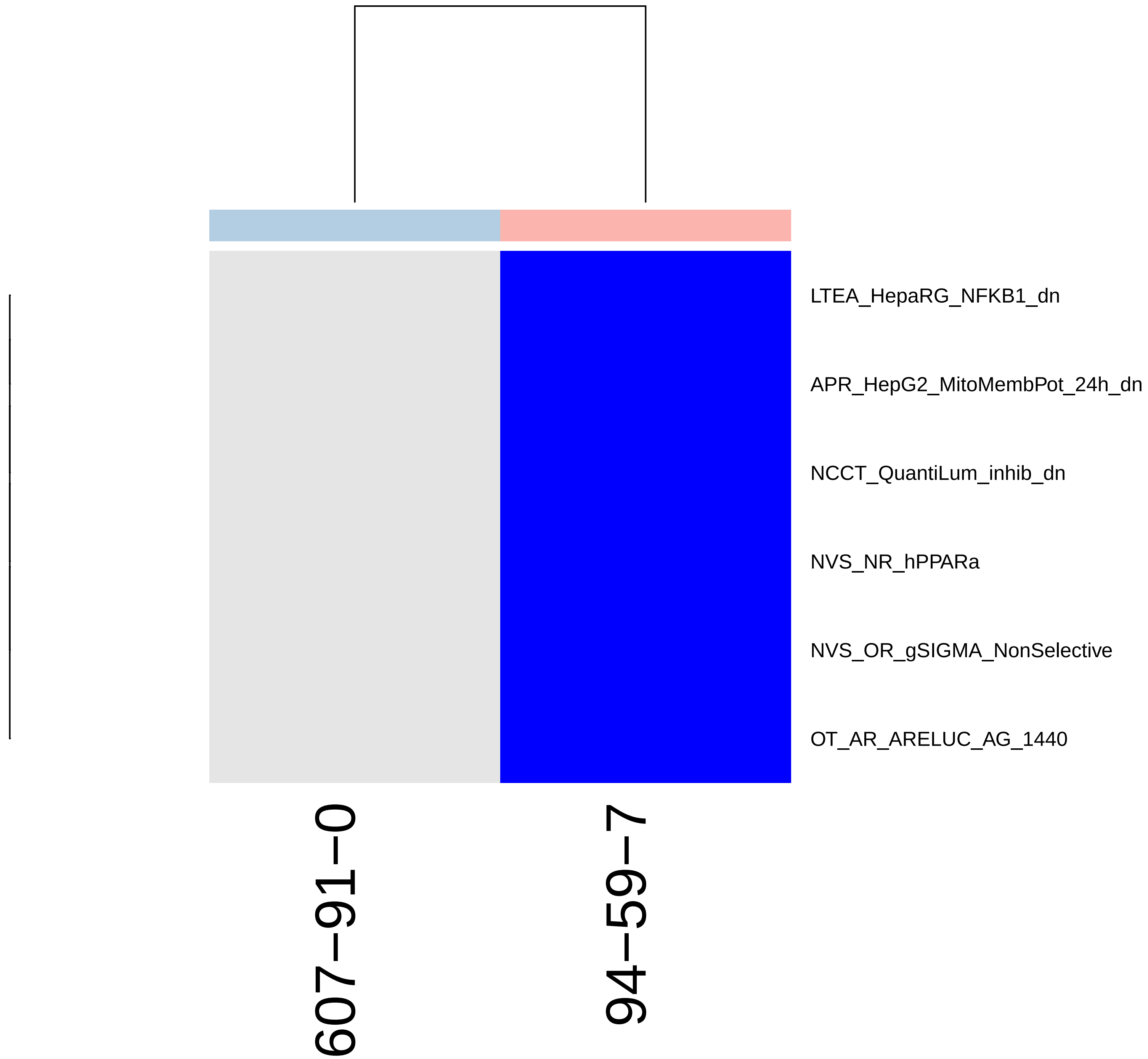

Enriched Assay Heatmap for Cluster 356

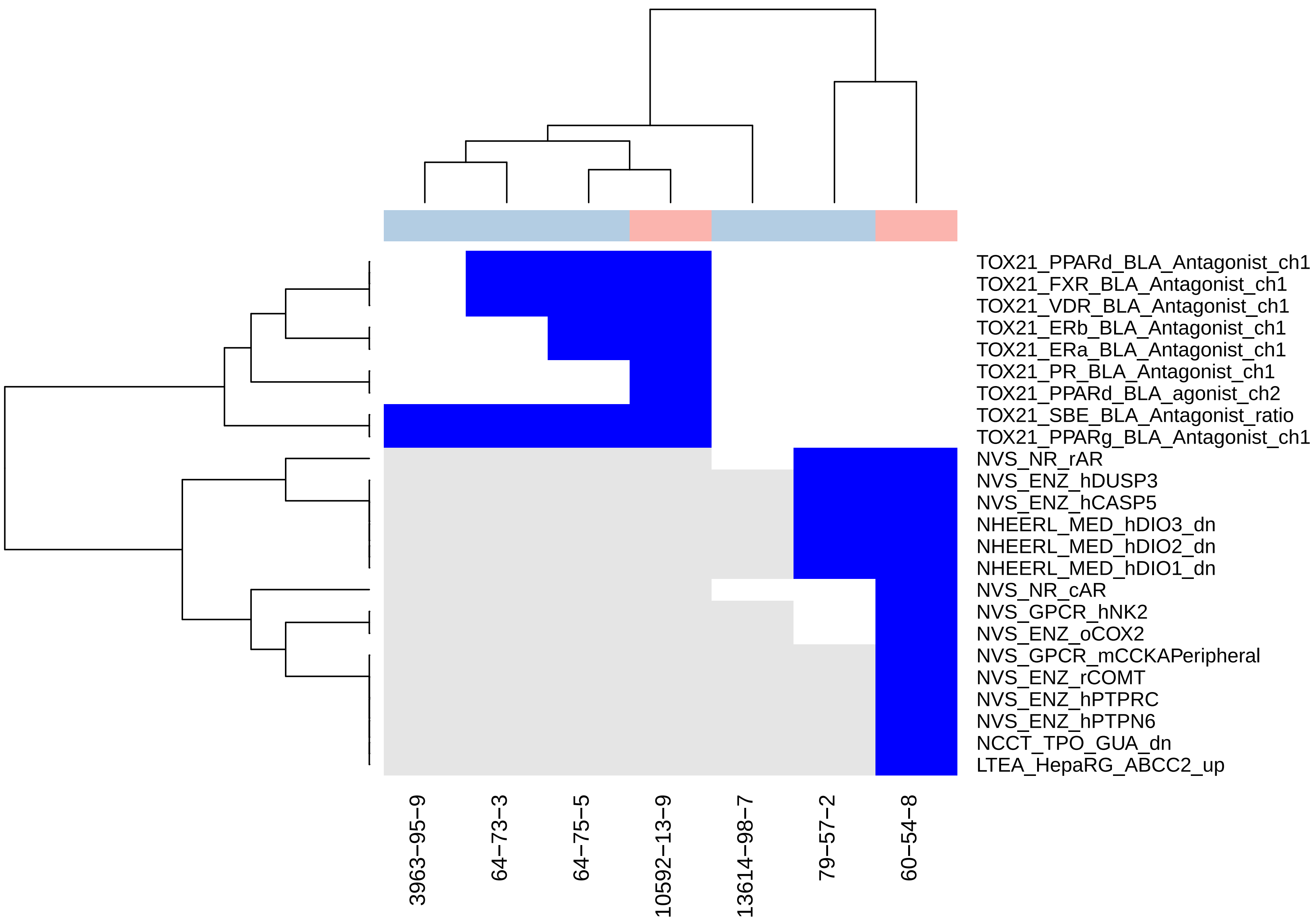

Enriched Assay Heatmap for Cluster 357

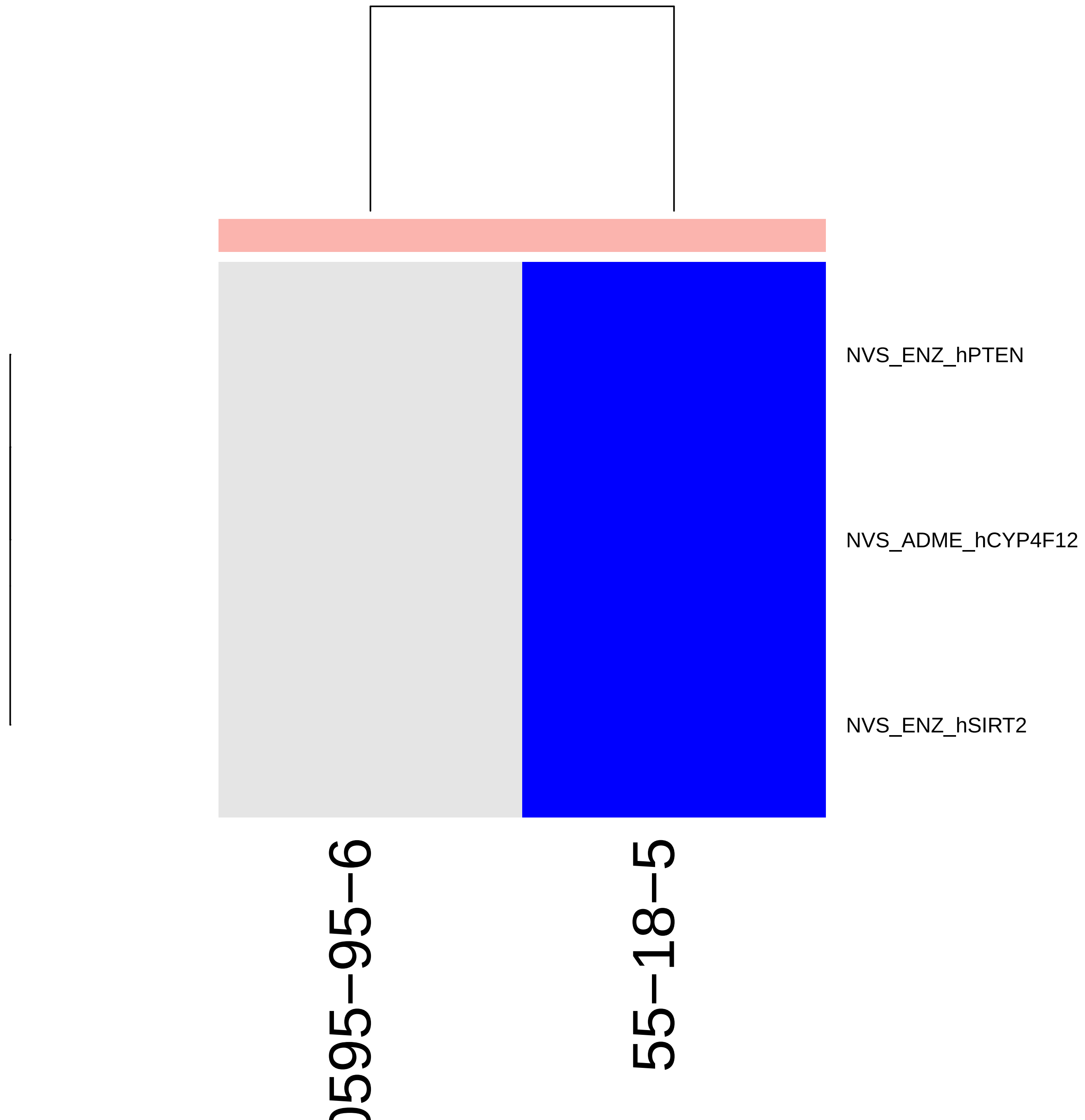

Enriched Assay Heatmap for Cluster 362

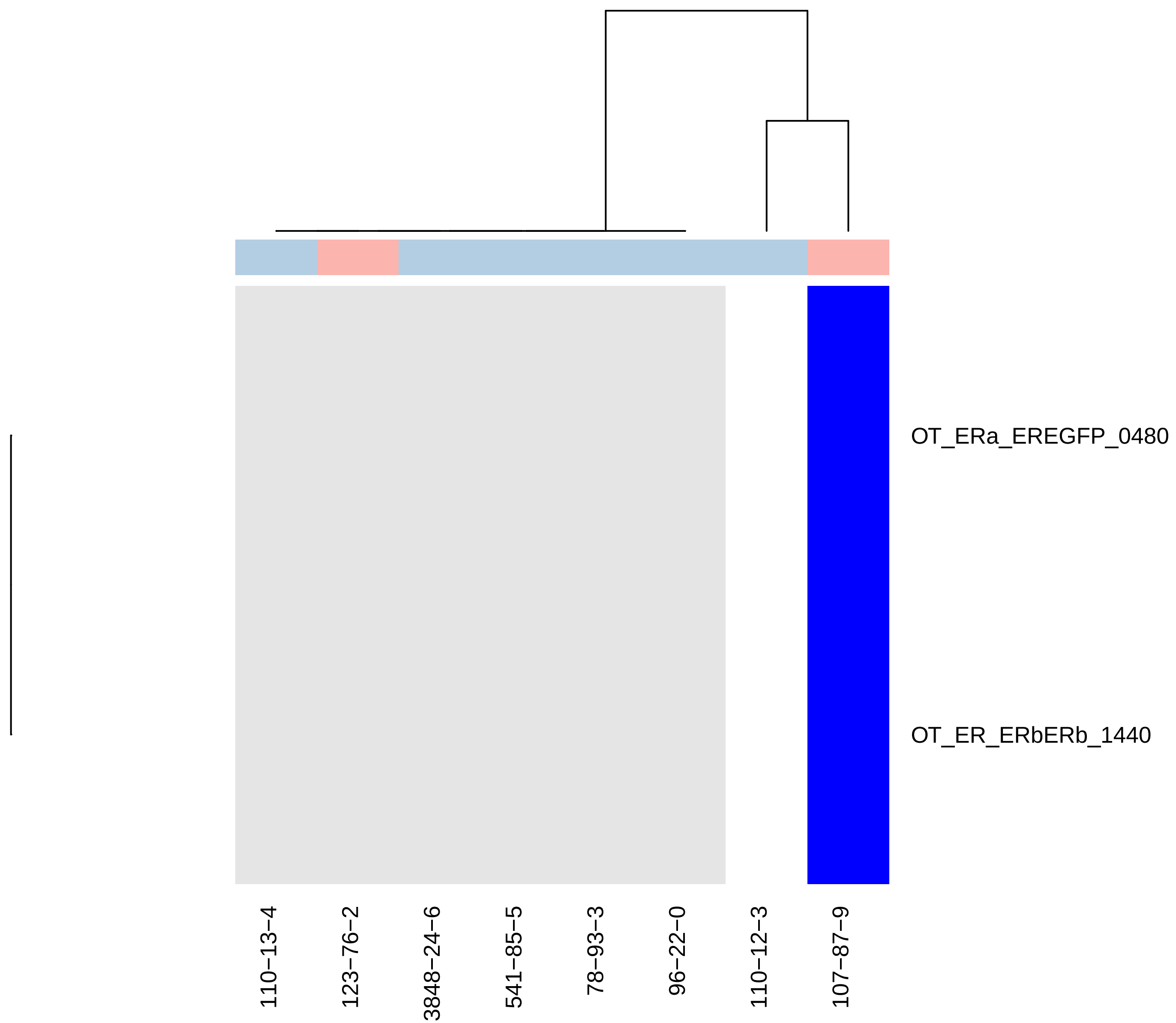

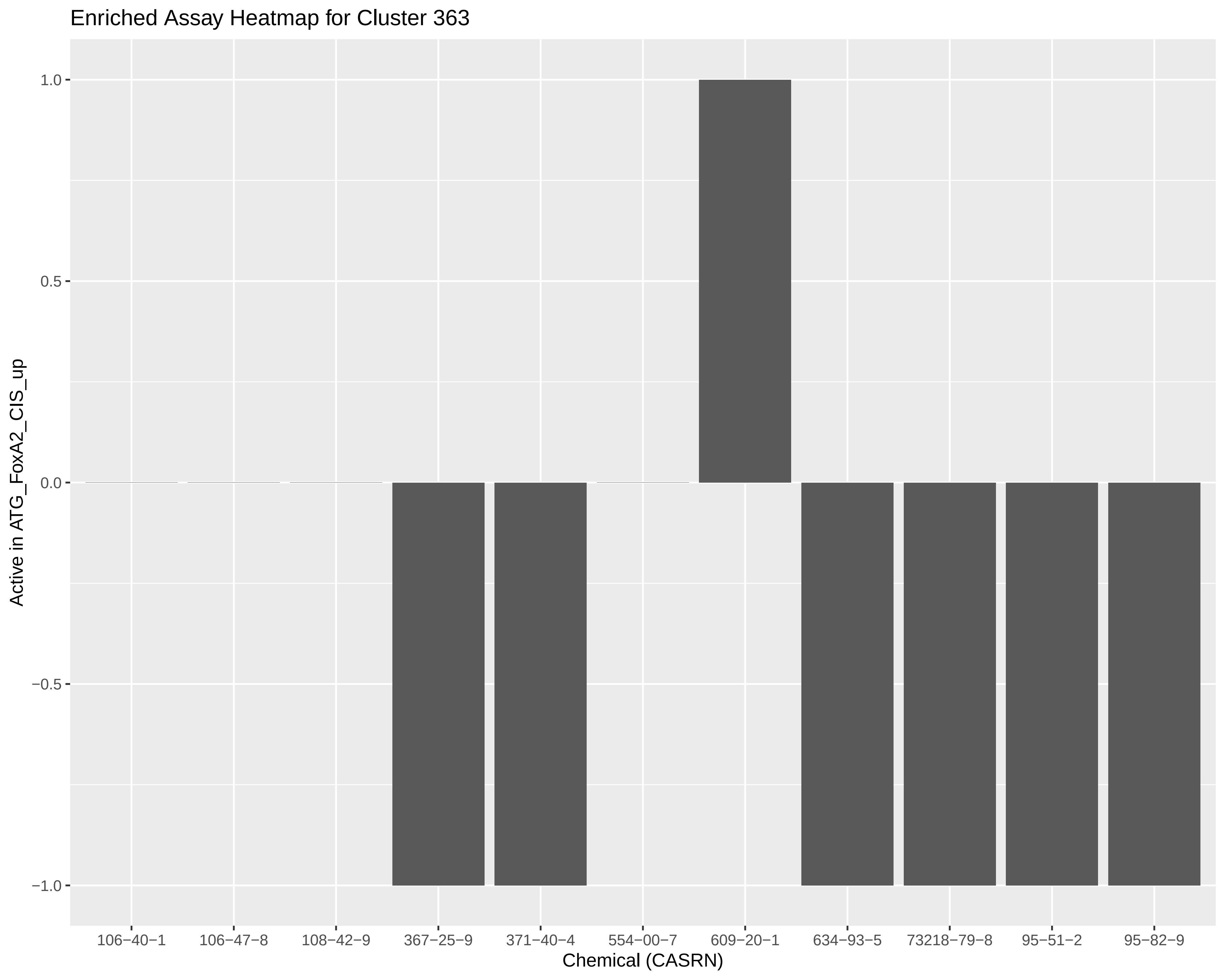

Enriched Assay Heatmap for Cluster 363
Active in ATG_FoxA2_CIS_up
1.0
0.5
0.0
−0.5
−1.0
106−40−1
106−47−8
108−42−9
367−25−9
371−40−4
554−00−7
609−20−1
634−93−5
73218−79−8
95−51−2
95−82−9
Chemical (CASRN)

Enriched Assay Heatmap for Cluster 364

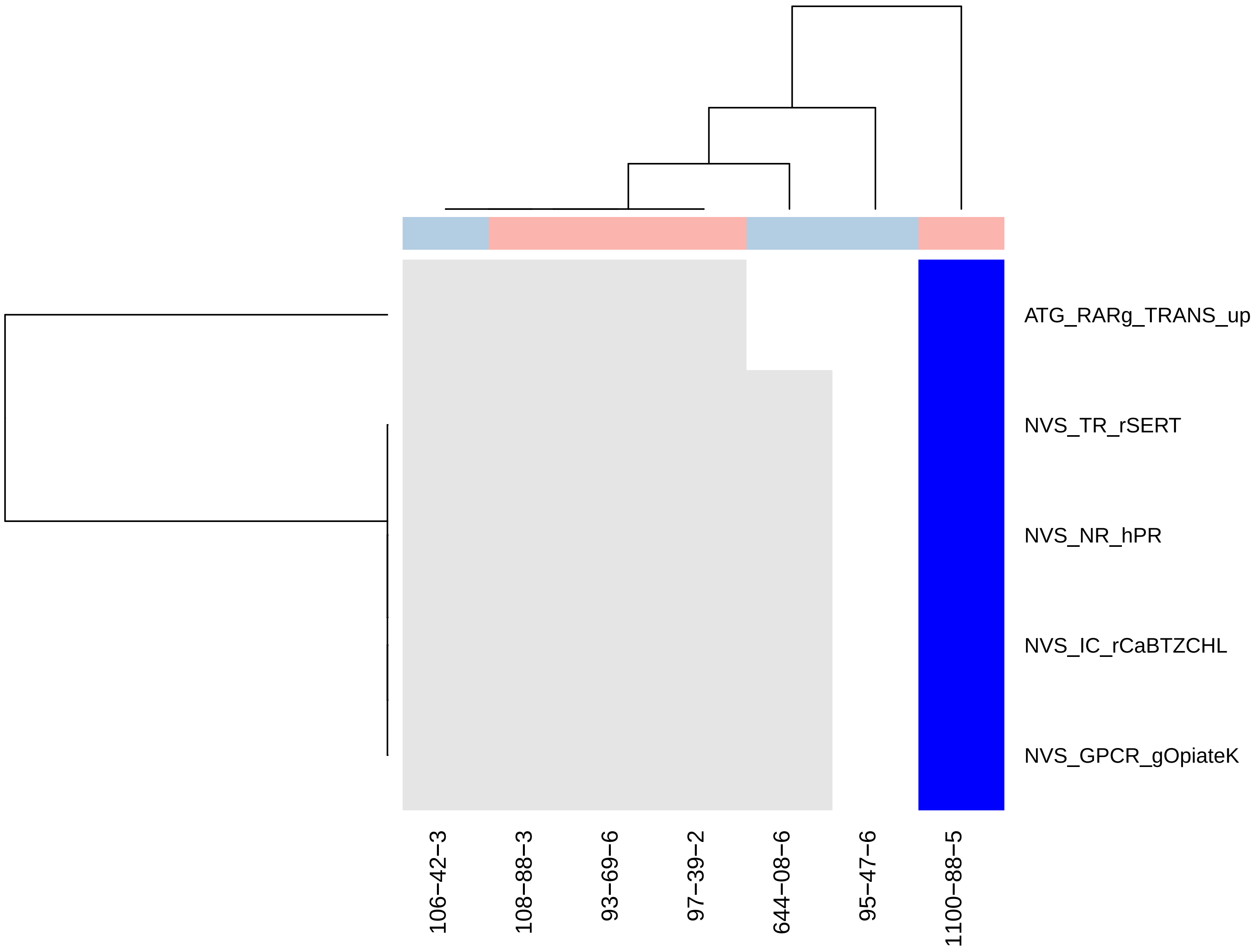

Enriched Assay Heatmap for Cluster 365

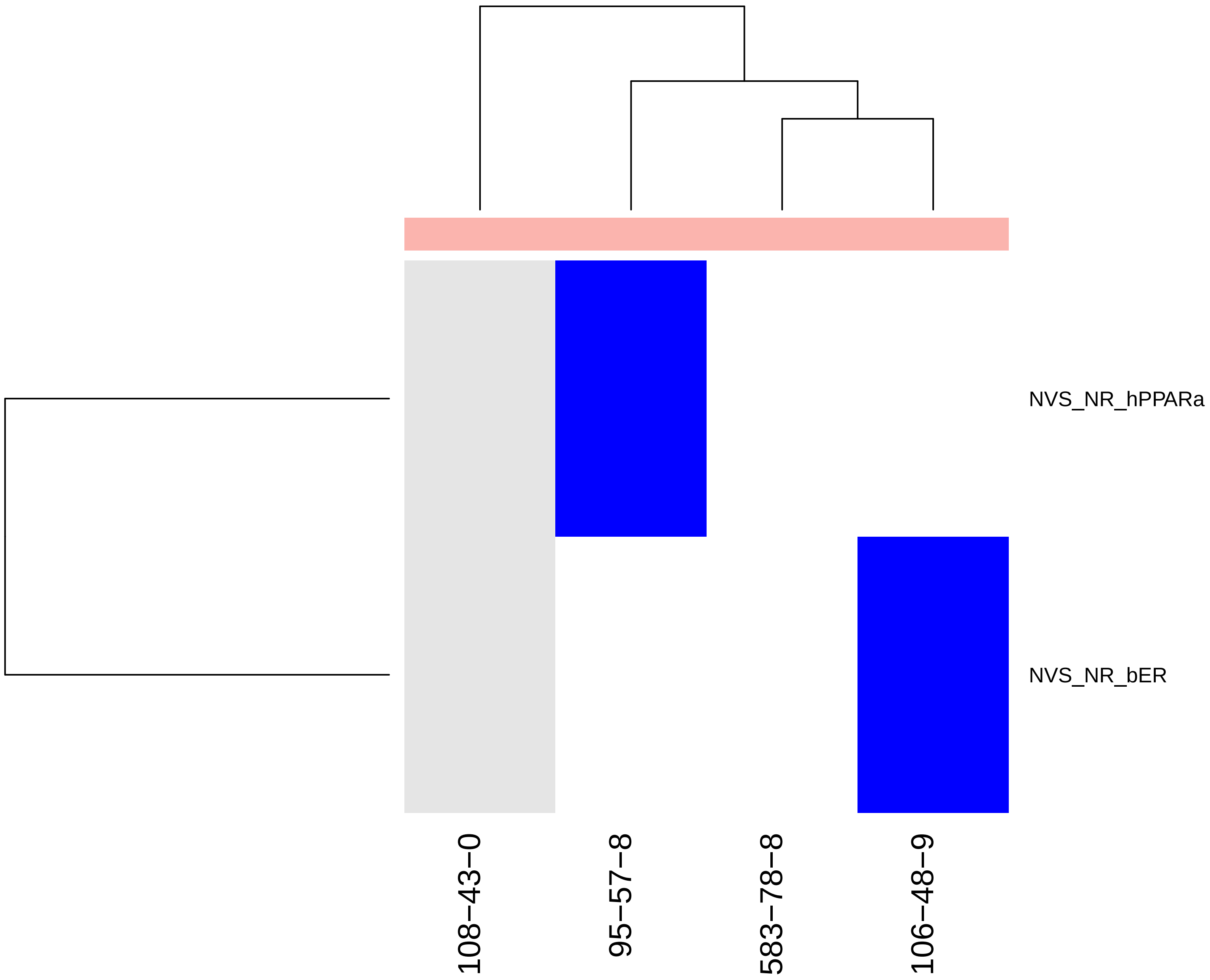

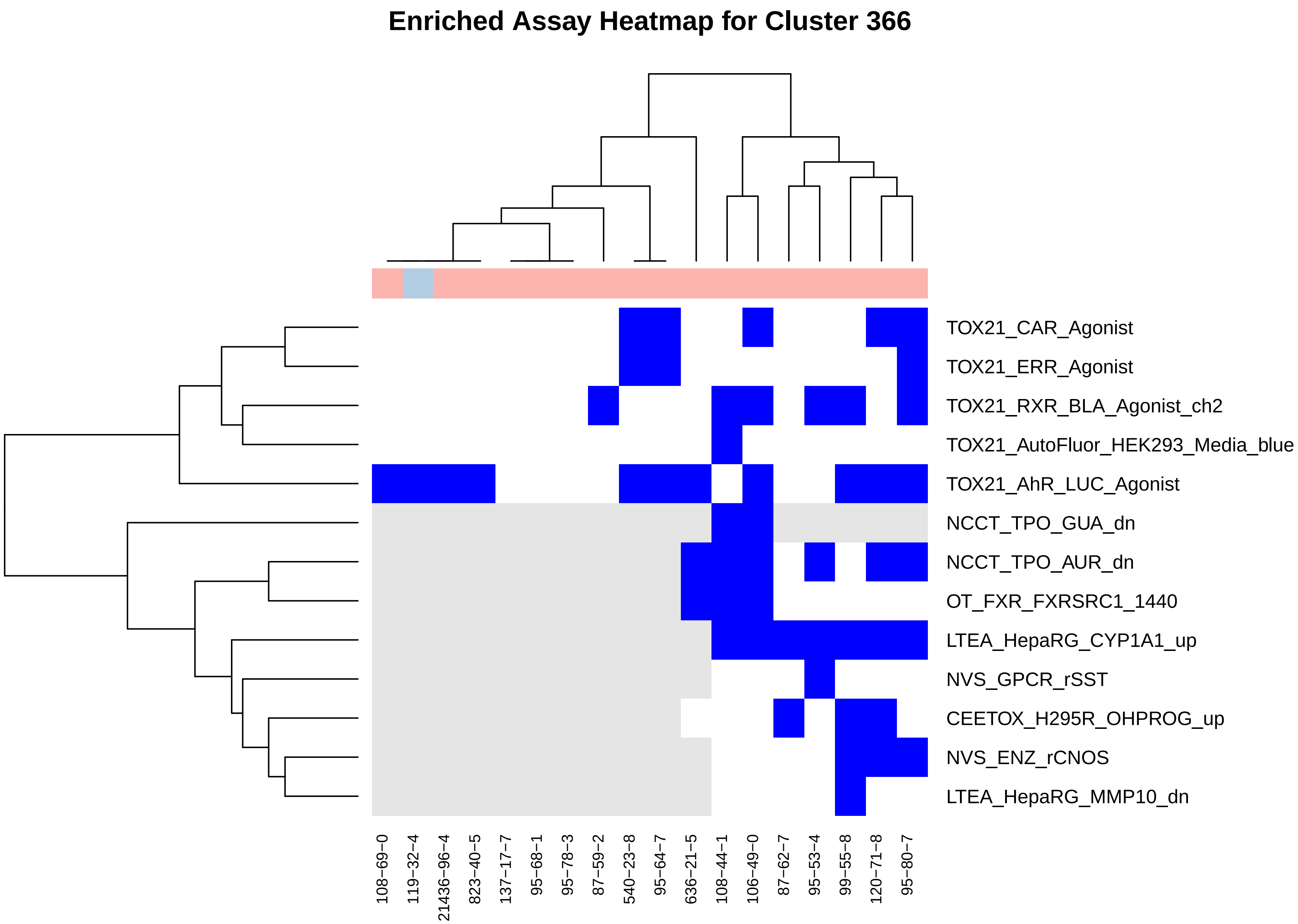
Enriched Assay Heatmap for Cluster 366
TOX21_CAR_Agonist
TOX21_ERR_Agonist
TOX21_RXR_BLA_Agonist_ch2
TOX21_AutoFluor_HEK293_Media_blue
TOX21_AhR_LUC_Agonist
NCCT_TPO_GUA_dn
NCCT_TPO_AUR_dn
OT_FXR_FXRSRC1_1440
LTEA_HepaRG_CYP1A1_up
NVS_GPCR_rSST
CEETOX_H295R_OHPROG_up
NVS_ENZ_rCNOS
LTEA_HepaRG_MMP10_dn
108−69−0
119−32−4
21436−96−4
823−40−5
137−17−7
95−68−1
95−78−3
87−59−2
540−23−8
95−64−7
636−21−5
108−44−1
106−49−0
87−62−7
95−53−4
99−55−8
120−71−8
95−80−7

Enriched Assay Heatmap for Cluster 367

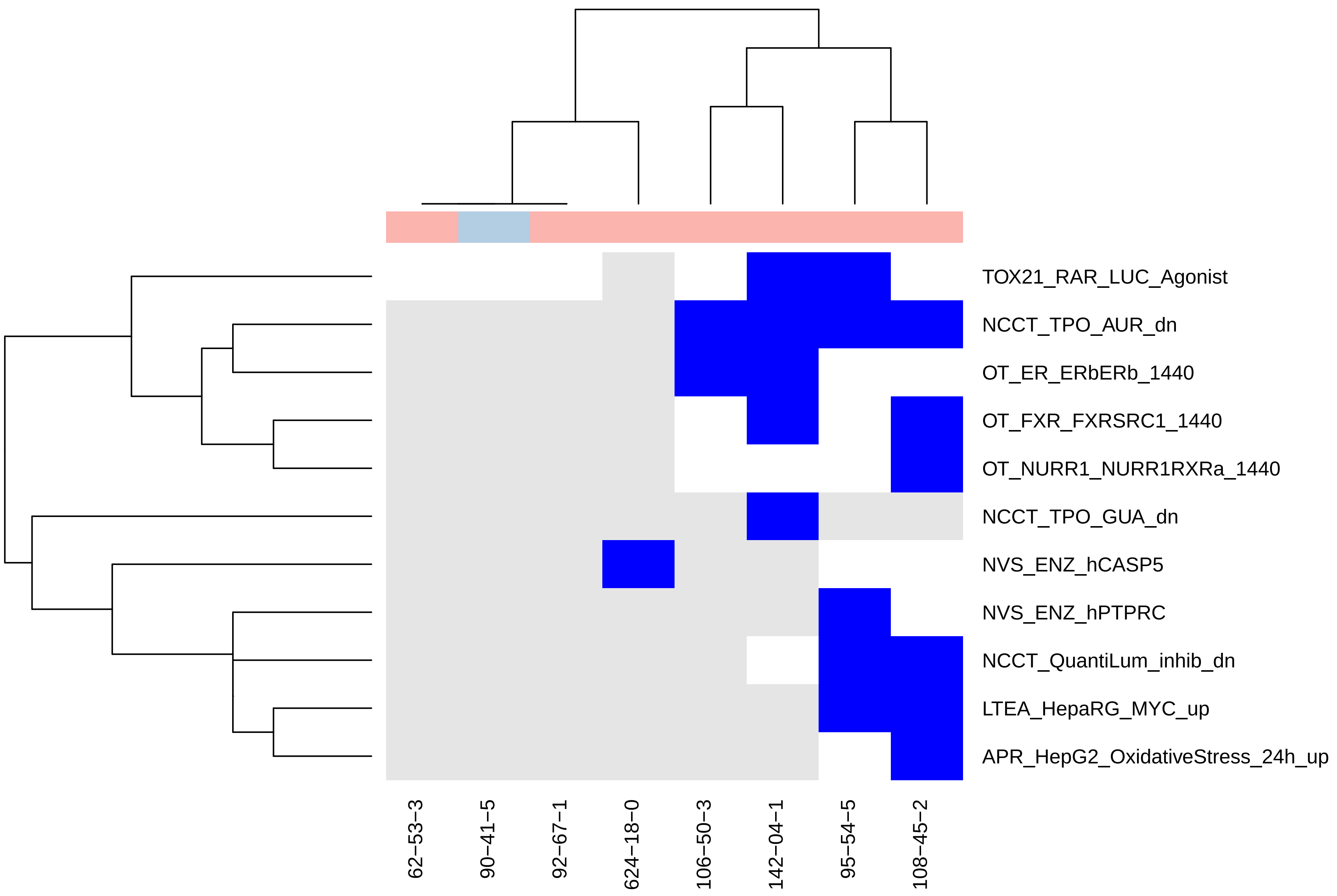

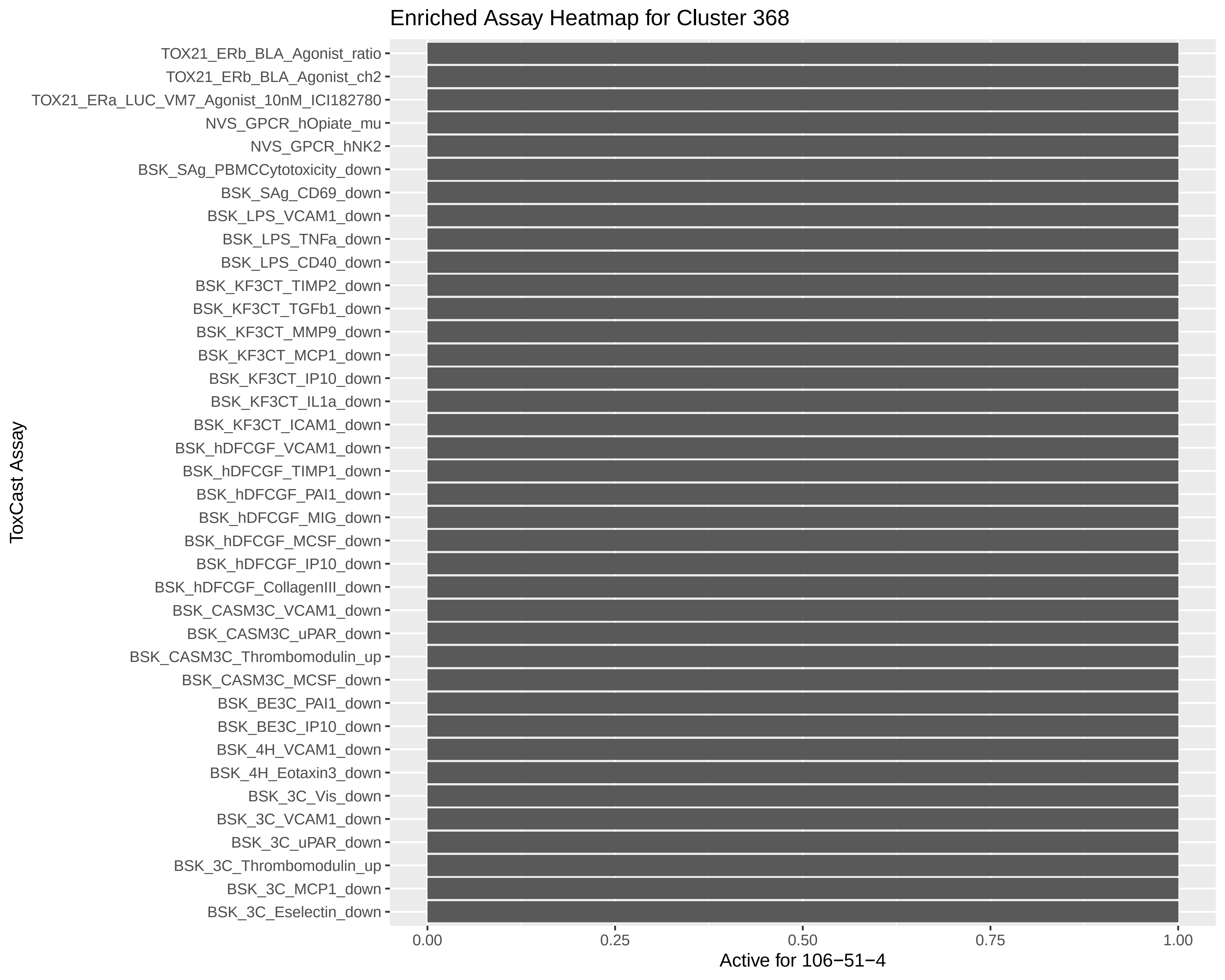
Enriched Assay Heatmap for Cluster 368
TOX21_ERb_BLA_Agonist_ratio
TOX21_ERb_BLA_Agonist_ch2
TOX21_ERa_LUC_VM7_Agonist_10nM_ICI182780
NVS_GPCR_hOpiate_mu
NVS_GPCR_hNK2
BSK_SAg_PBMCCytotoxicity_down
BSK_SAg_CD69_down
BSK_LPS_VCAM1_down
BSK_LPS_TNFa_down
BSK_LPS_CD40_down
BSK_KF3CT_TIMP2_down
BSK_KF3CT_TGFb1_down
BSK_KF3CT_MMP9_down
BSK_KF3CT_MCP1_down
BSK_KF3CT_IP10_down
BSK_KF3CT_IL1a_down
BSK_KF3CT_ICAM1_down
BSK_hDFCGF_VCAM1_down
BSK_hDFCGF_TIMP1_down
BSK_hDFCGF_PAI1_down
BSK_hDFCGF_MIG_down
BSK_hDFCGF_MCSF_down
BSK_hDFCGF_IP10_down
BSK_hDFCGF_CollagenIII_down
BSK_CASM3C_VCAM1_down
BSK_CASM3C_uPAR_down
BSK_CASM3C_Thrombomodulin_up
BSK_CASM3C_MCSF_down
BSK_BE3C_PAI1_down
BSK_BE3C_IP10_down
BSK_4H_VCAM1_down
BSK_4H_Eotaxin3_down
BSK_3C_Vis_down
BSK_3C_VCAM1_down
BSK_3C_uPAR_down
BSK_3C_Thrombomodulin_up
BSK_3C_MCP1_down
BSK_3C_Eselectin_down
ToxCast Assay
0.00
0.25
0.50
0.75
1.00
Active for 106−51−4

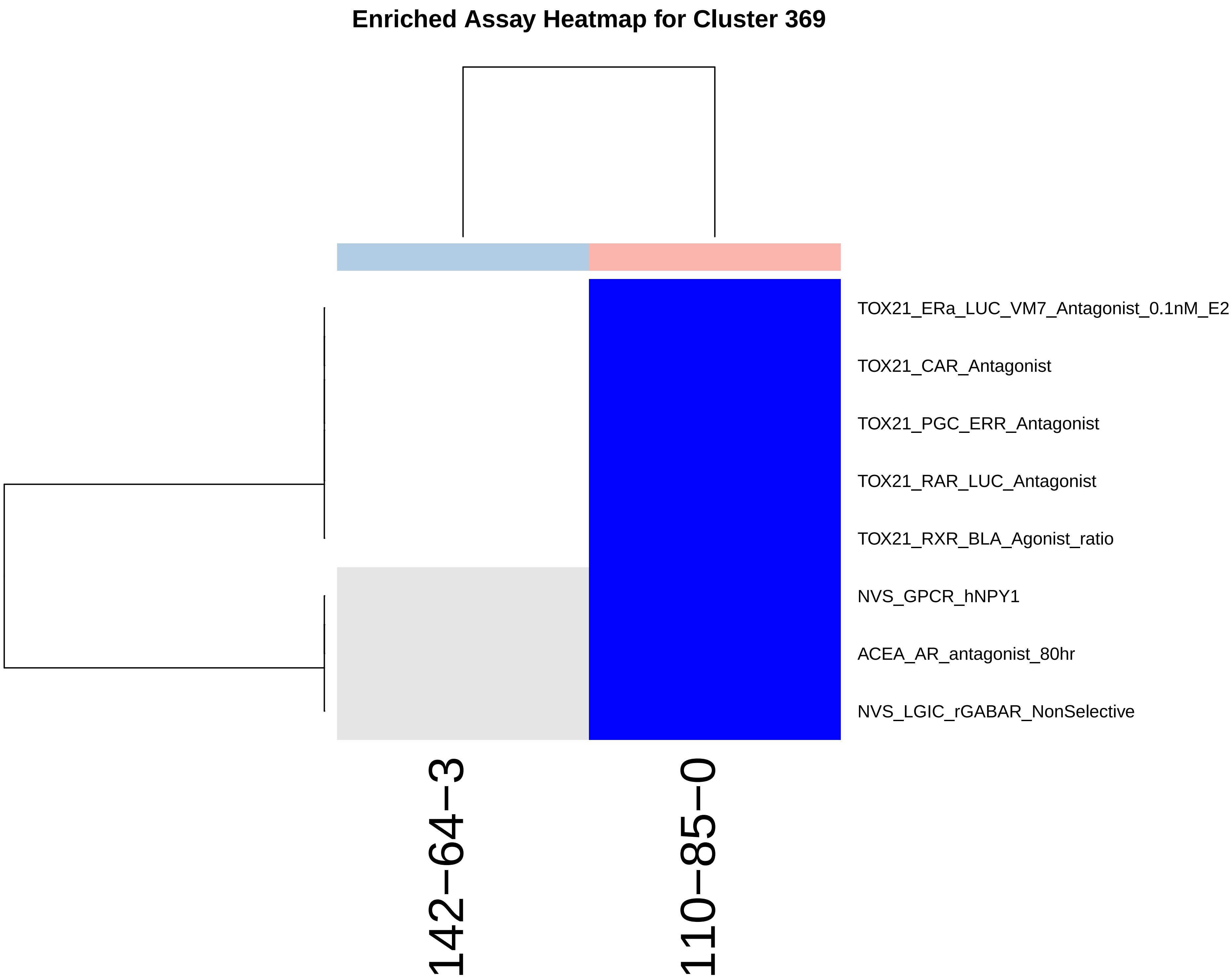
Enriched Assay Heatmap for Cluster 369
TOX21_ERa_LUC_VM7_Antagonist_0.1nM_E2
TOX21_CAR_Antagonist
TOX21_PGC_ERR_Antagonist
TOX21_RAR_LUC_Antagonist
TOX21_RXR_BLA_Agonist_ratio
NVS_GPCR_hNPY1
ACEA_AR_antagonist_80hr
NVS_LGIC_rGABAR_NonSelective
142−64−3
110−85−0

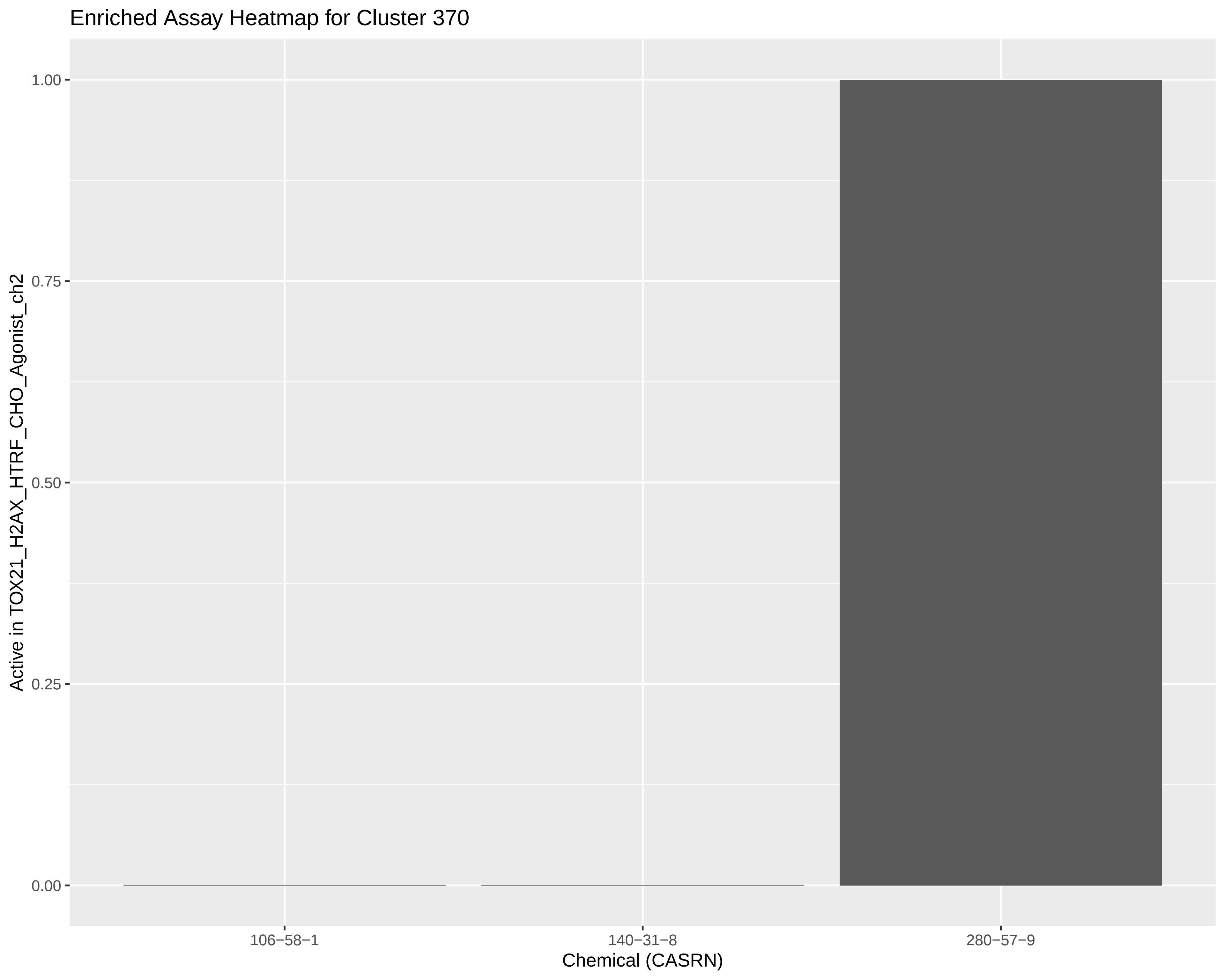

Enriched Assay Heatmap for Cluster 370
Active in TOX21_H2AX_HTRF_CHO_Agonist_ch2
1.00
0.75
0.50
0.25
0.00
106−58−1
140−31−8
280−57−9
Chemical (CASRN)

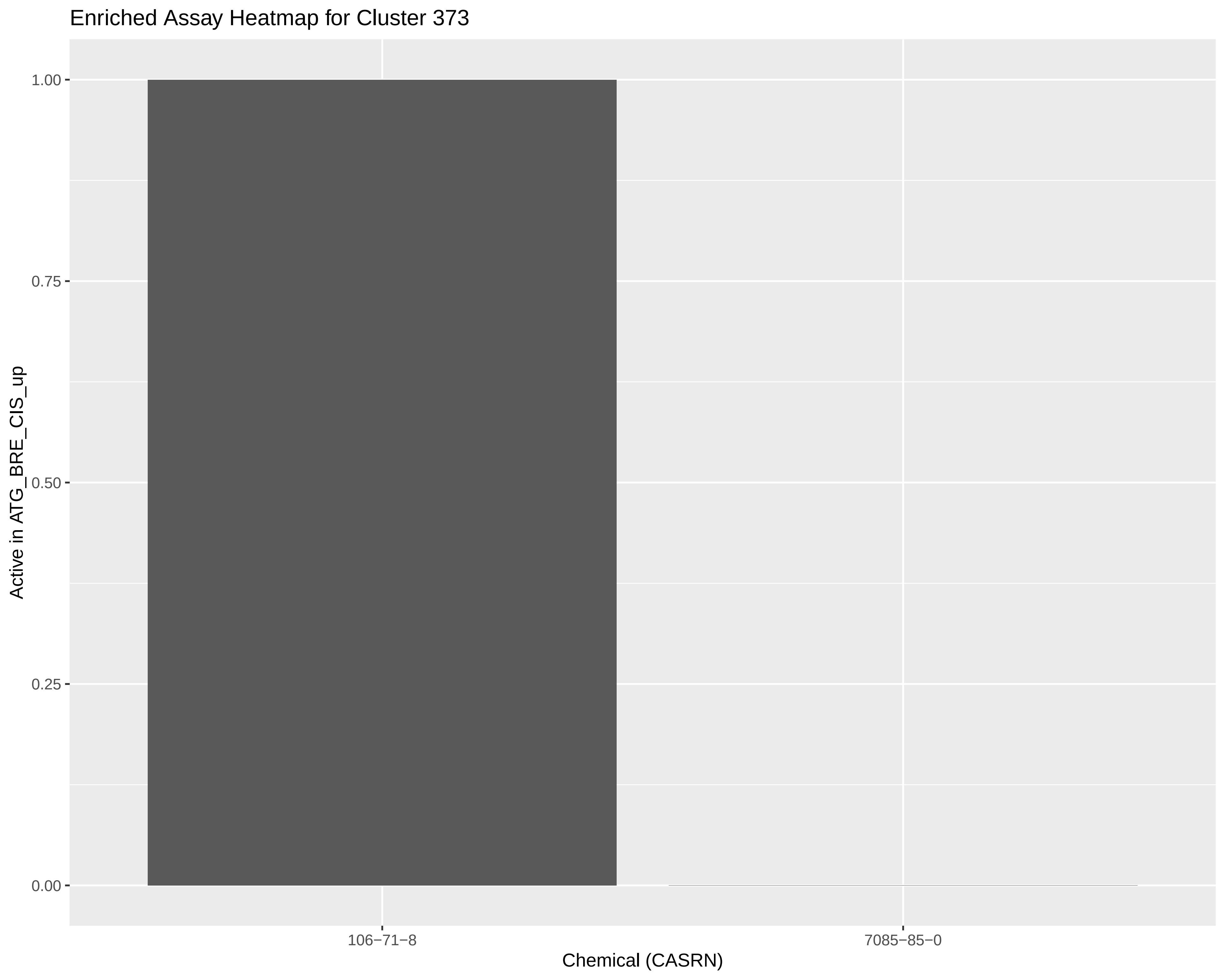

Enriched Assay Heatmap for Cluster 373
Active in ATG_BRE_CIS_up
1.00
0.75
0.50
0.25
0.00
106−71−8
7085−85−0
Chemical (CASRN)

Enriched Assay Heatmap for Cluster 374

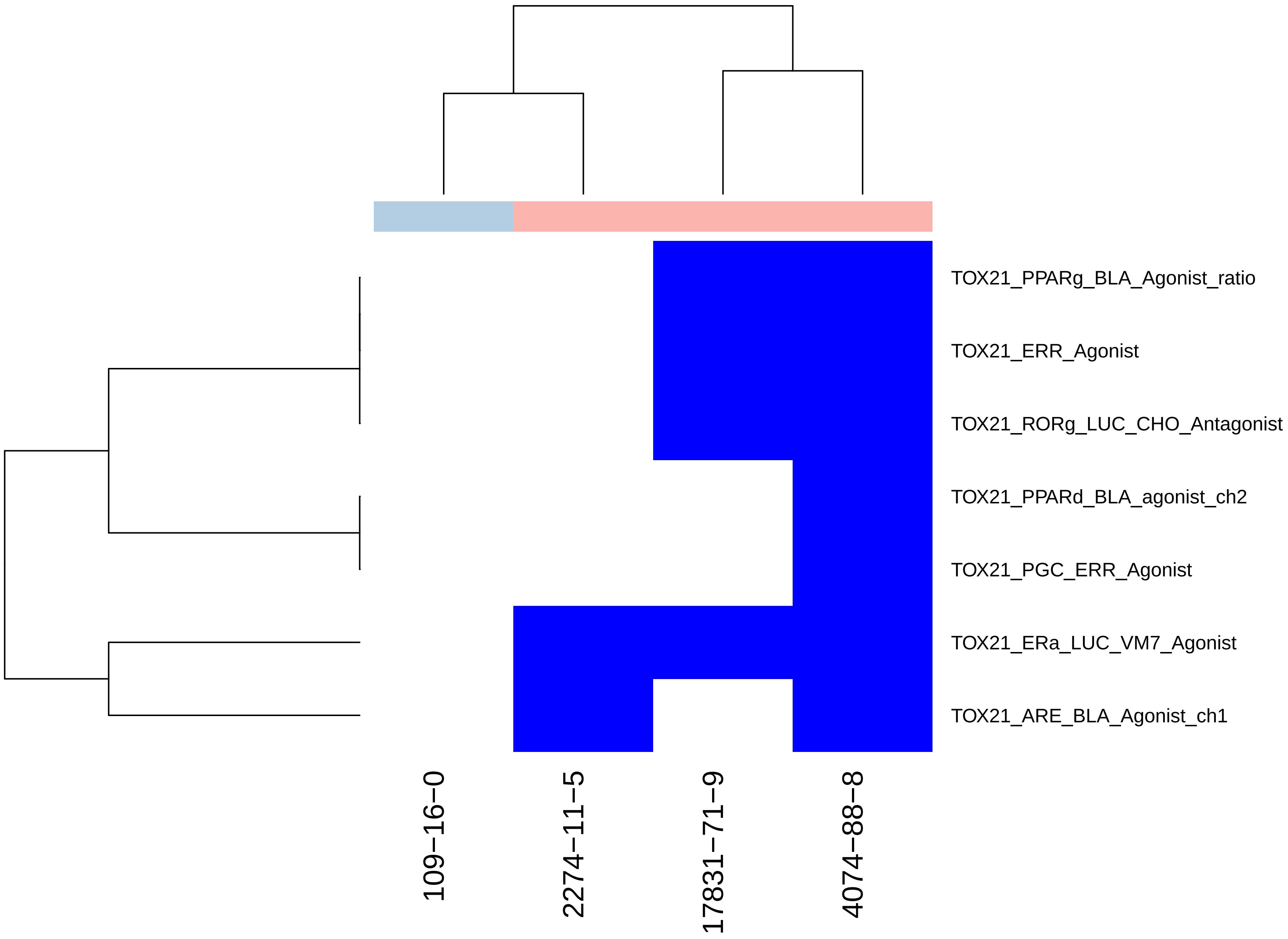

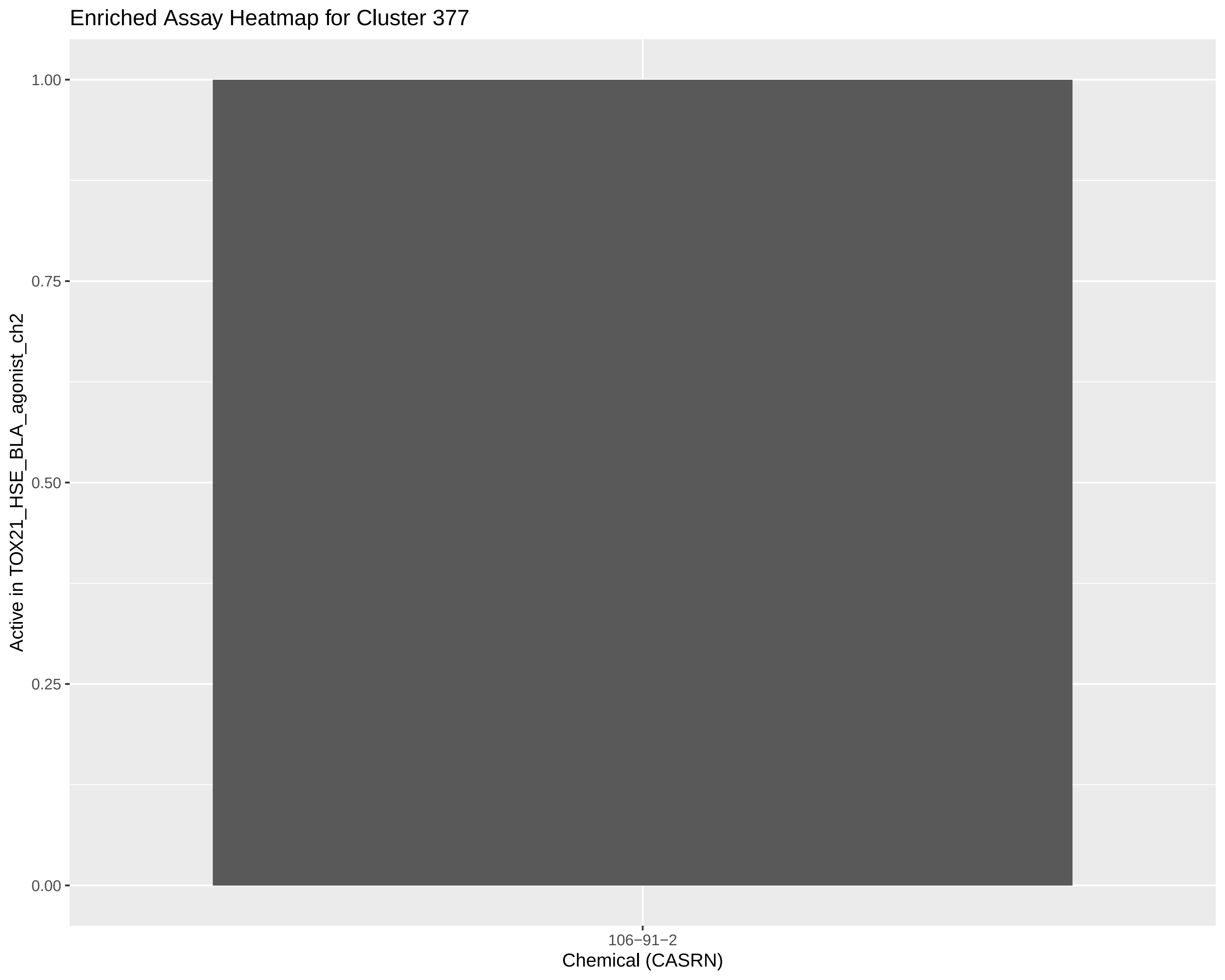

Enriched Assay Heatmap for Cluster 377
1.00
0.75
0.50
0.25
0.00
Active in TOX21_HSE_BLA_agonist_ch2
106−91−2
Chemical (CASRN)

Enriched Assay Heatmap for Cluster 379

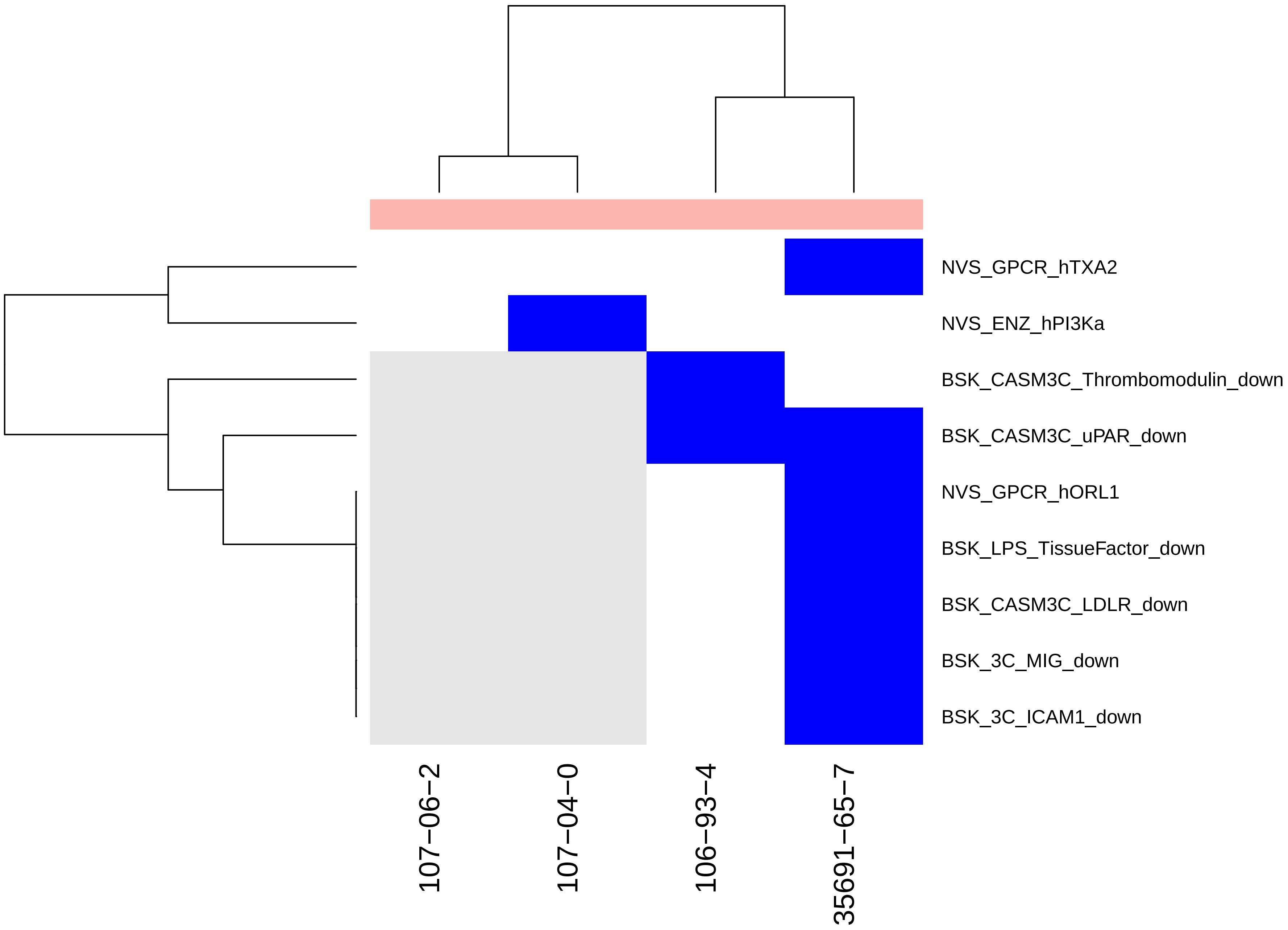

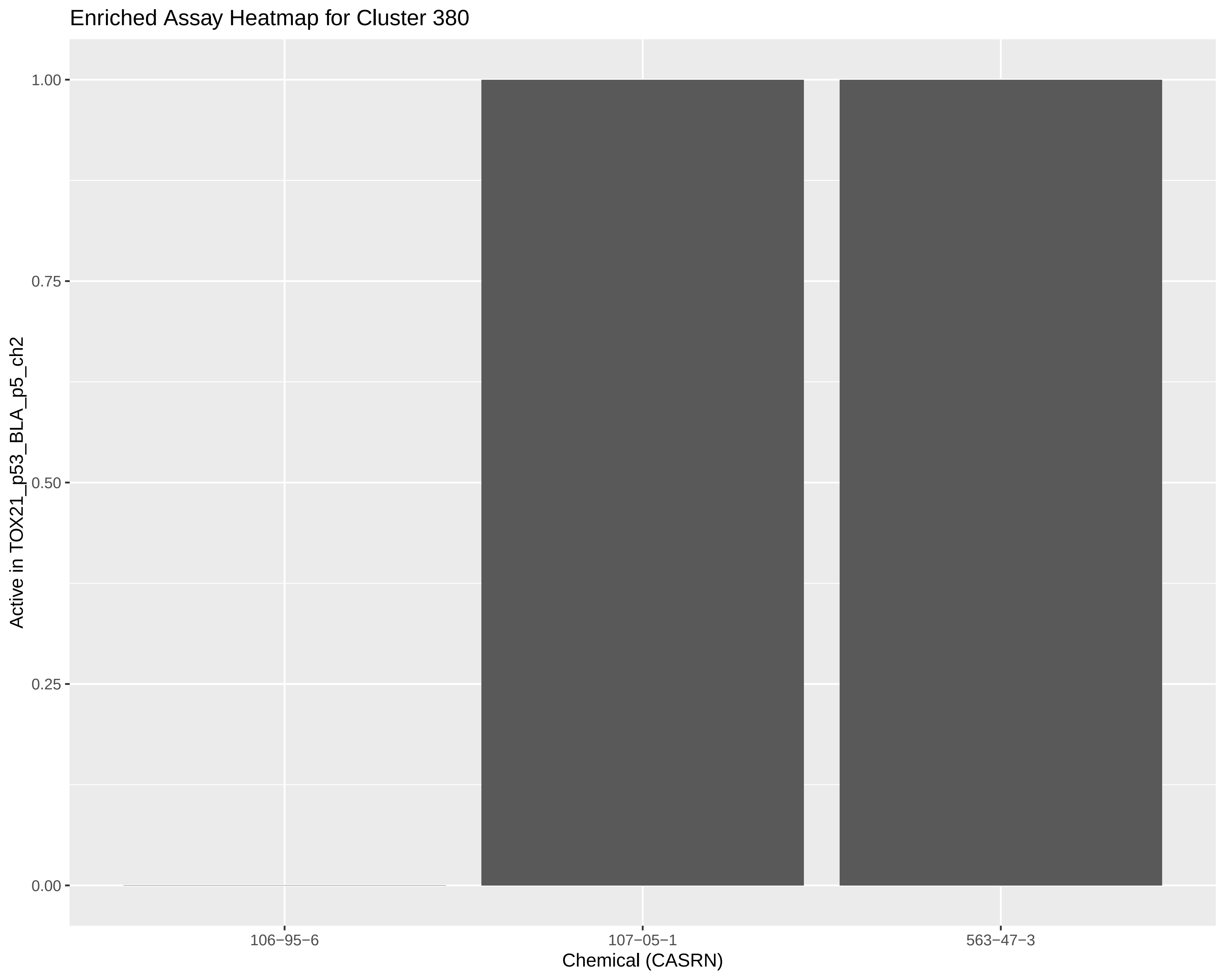

Enriched Assay Heatmap for Cluster 380
Active in TOX21_p53_BLA_p5_ch2
1.00
0.75
0.50
0.25
0.00
106−95−6
107−05−1
563−47−3
Chemical (CASRN)

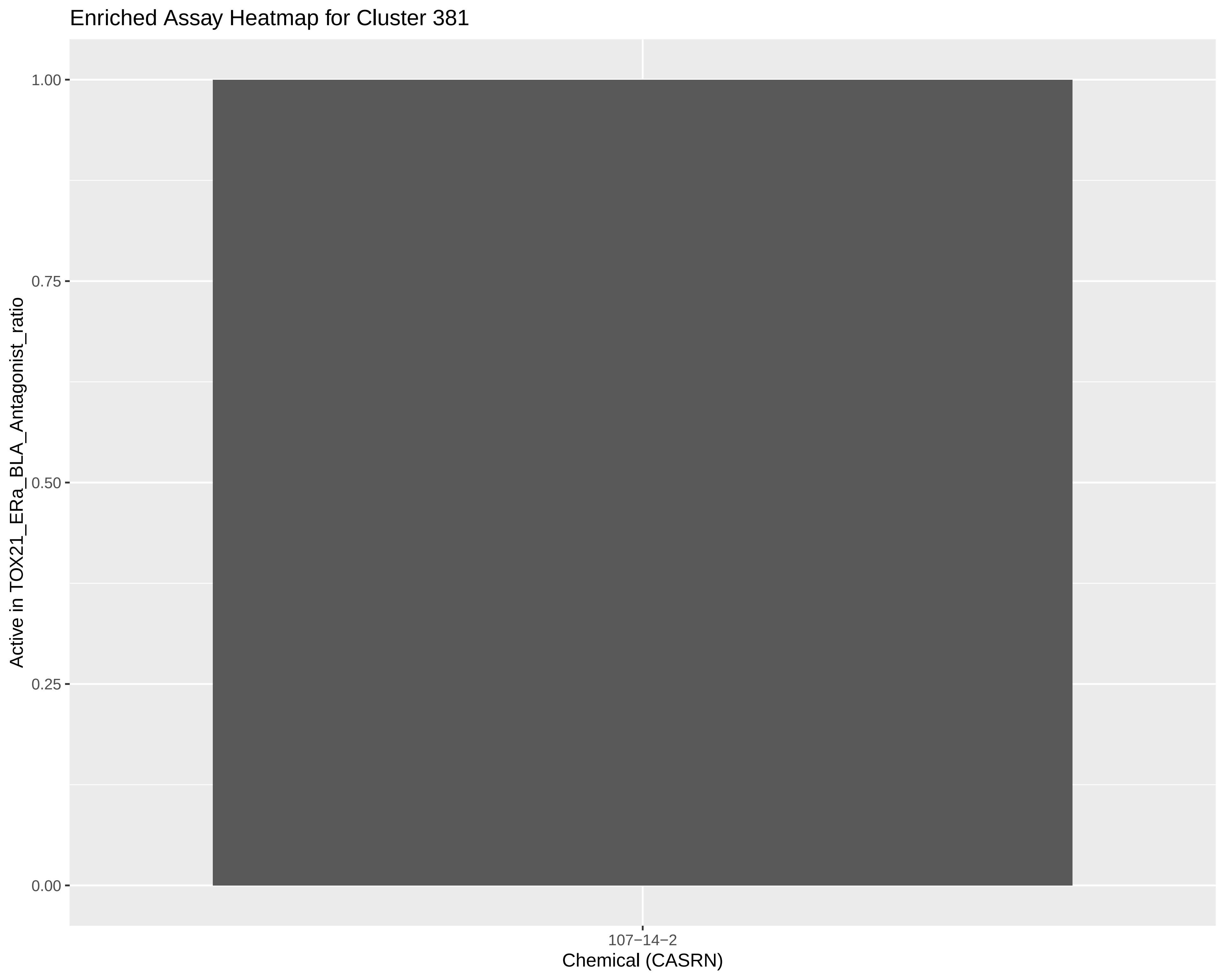

Enriched Assay Heatmap for Cluster 381
Active in TOX21_ERa_BLA_Antagonist_ratio
1.00
0.75
0.50
0.25
0.00
107−14−2
Chemical (CASRN)

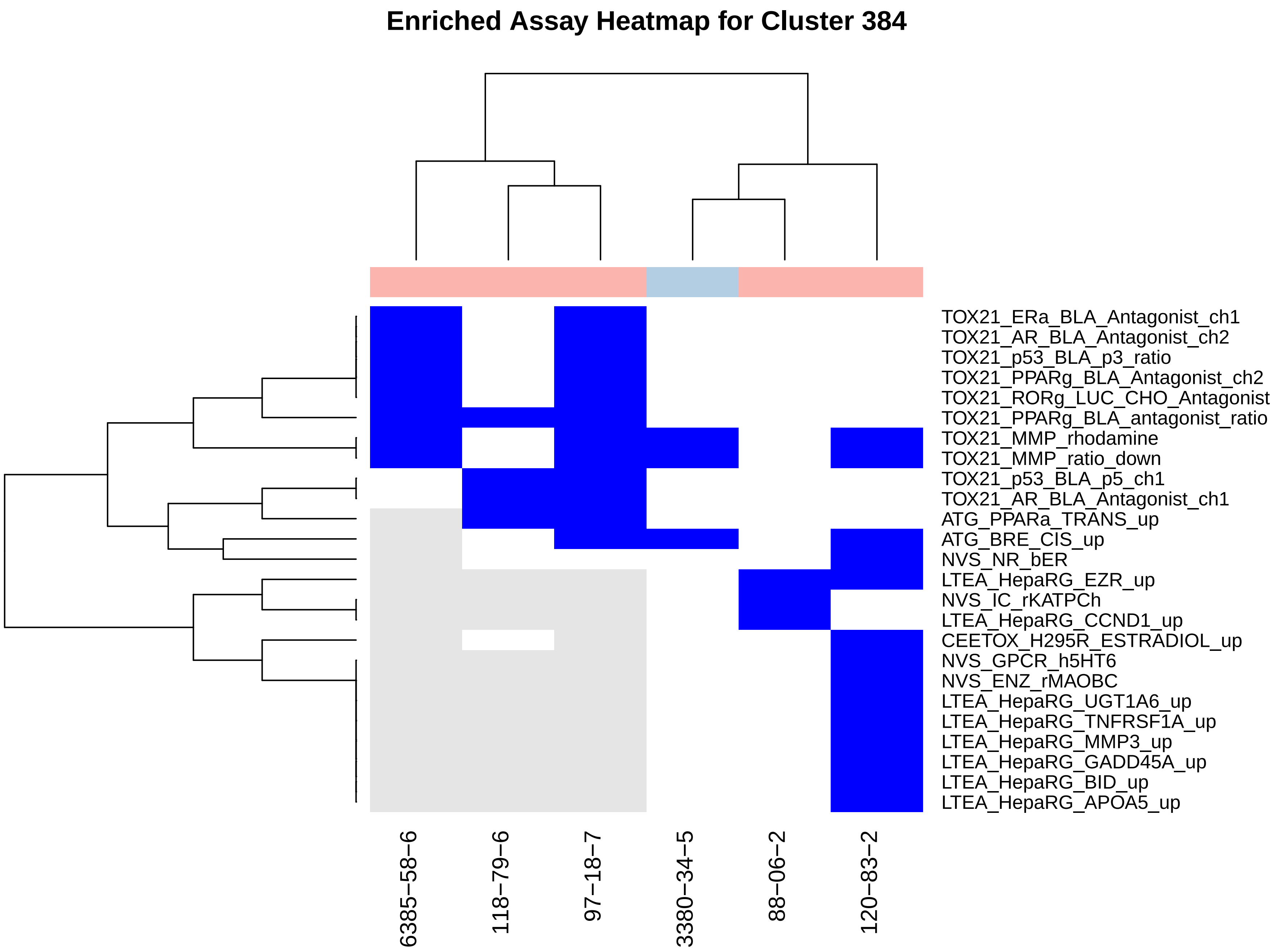
Enriched Assay Heatmap for Cluster 384
TOX21_ERa_BLA_Antagonist_ch1
TOX21_AR_BLA_Antagonist_ch2
TOX21_p53_BLA_p3_ratio
TOX21_PPARg_BLA_Antagonist_ch2
TOX21_RORg_LUC_CHO_Antagonist
TOX21_PPARg_BLA_antagonist_ratio
TOX21_MMP_rhodamine
TOX21_MMP_ratio_down
TOX21_p53_BLA_p5_ch1
TOX21_AR_BLA_Antagonist_ch1
ATG_PPARa_TRANS_up
ATG_BRE_CIS_up
NVS_NR_bER
LTEA_HepaRG_EZR_up
NVS_IC_rKATPCh
LTEA_HepaRG_CCND1_up
CEETOX_H295R_ESTRADIOL_up
NVS_GPCR_h5HT6
NVS_ENZ_rMAOBC
LTEA_HepaRG_UGT1A6_up
LTEA_HepaRG_TNFRSF1A_up
LTEA_HepaRG_MMP3_up
LTEA_HepaRG_GADD45A_up
LTEA_HepaRG_BID_up
LTEA_HepaRG_APOA5_up
6385–58–6
118–79–6
97–18–7
3380–34–5
88–06–2
120–83–2

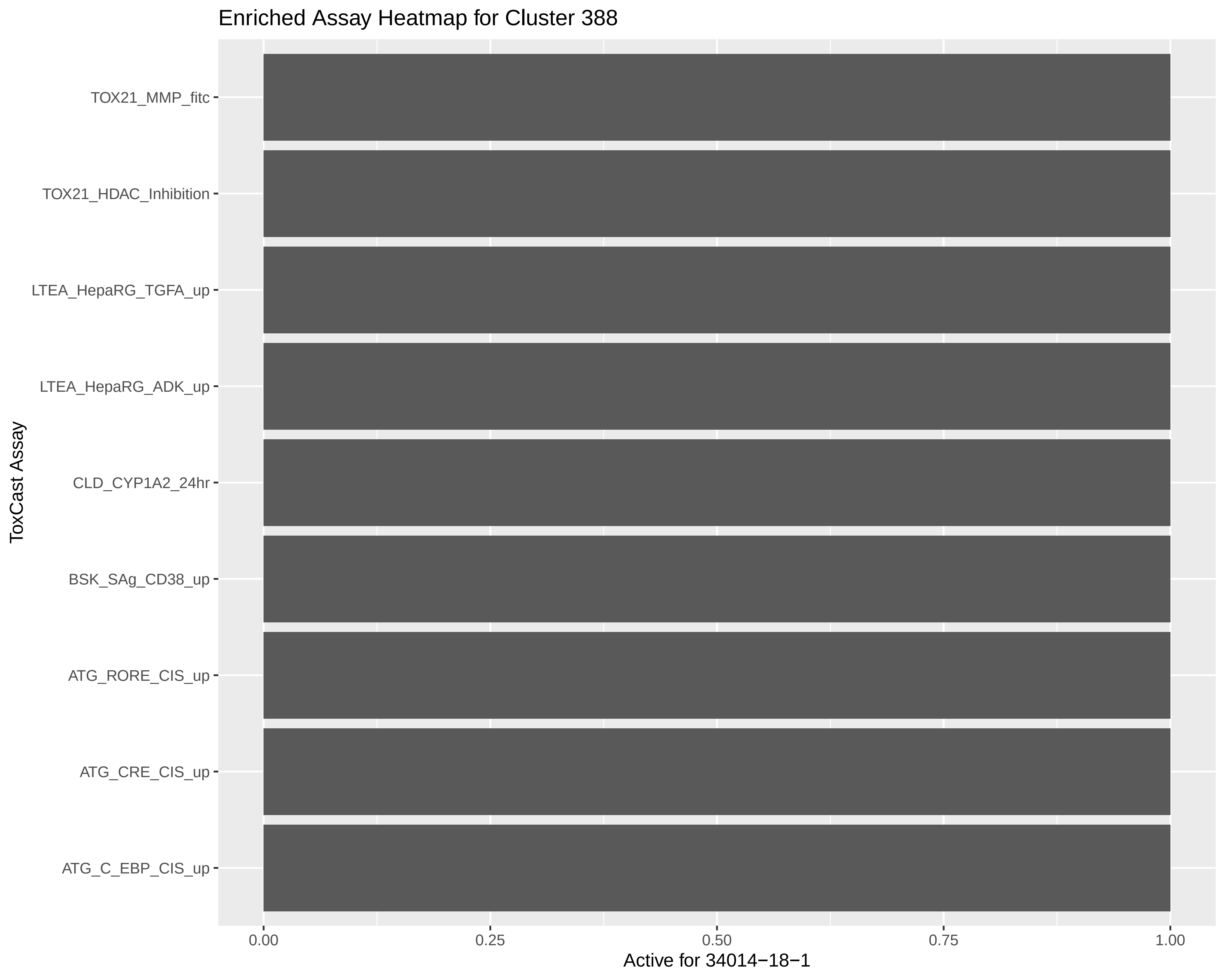
Enriched Assay Heatmap for Cluster 388
ToxCast Assay
TOX21_MMP_fitc
TOX21_HDAC_Inhibition
LTEA_HepaRG_TGFA_up
LTEA_HepaRG_ADK_up
CLD_CYP1A2_24hr
BSK_SAg_CD38_up
ATG_RORE_CIS_up
ATG_CRE_CIS_up
ATG_C_EBP_CIS_up
0.00
0.25
0.50
0.75
1.00
Active for 34014−18−1

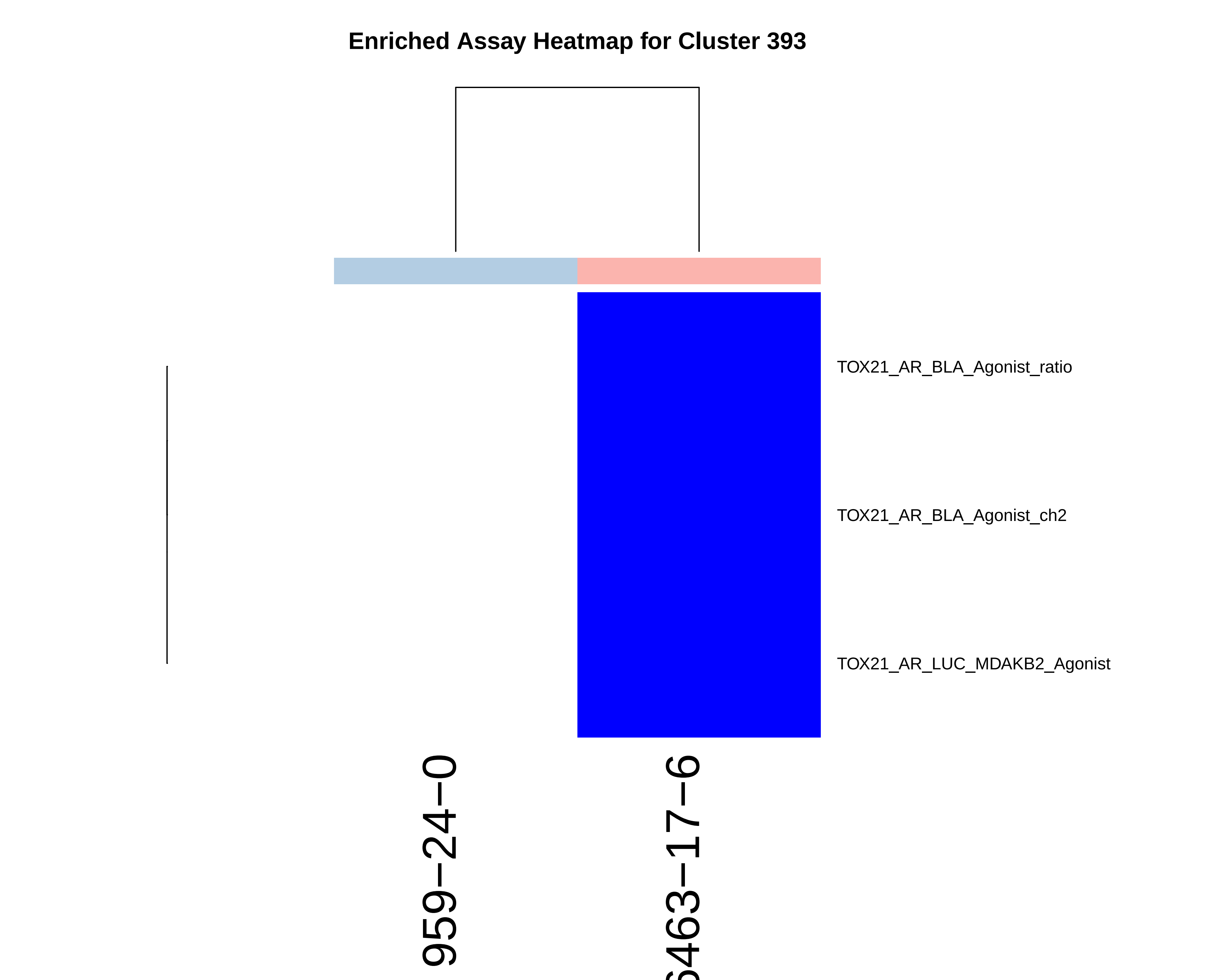

Enriched Assay Heatmap for Cluster 393
TOX21_AR_BLA_Agonist_ratio
TOX21_AR_BLA_Agonist_ch2
TOX21_AR_LUC_MDAKB2_Agonist
959−24−0

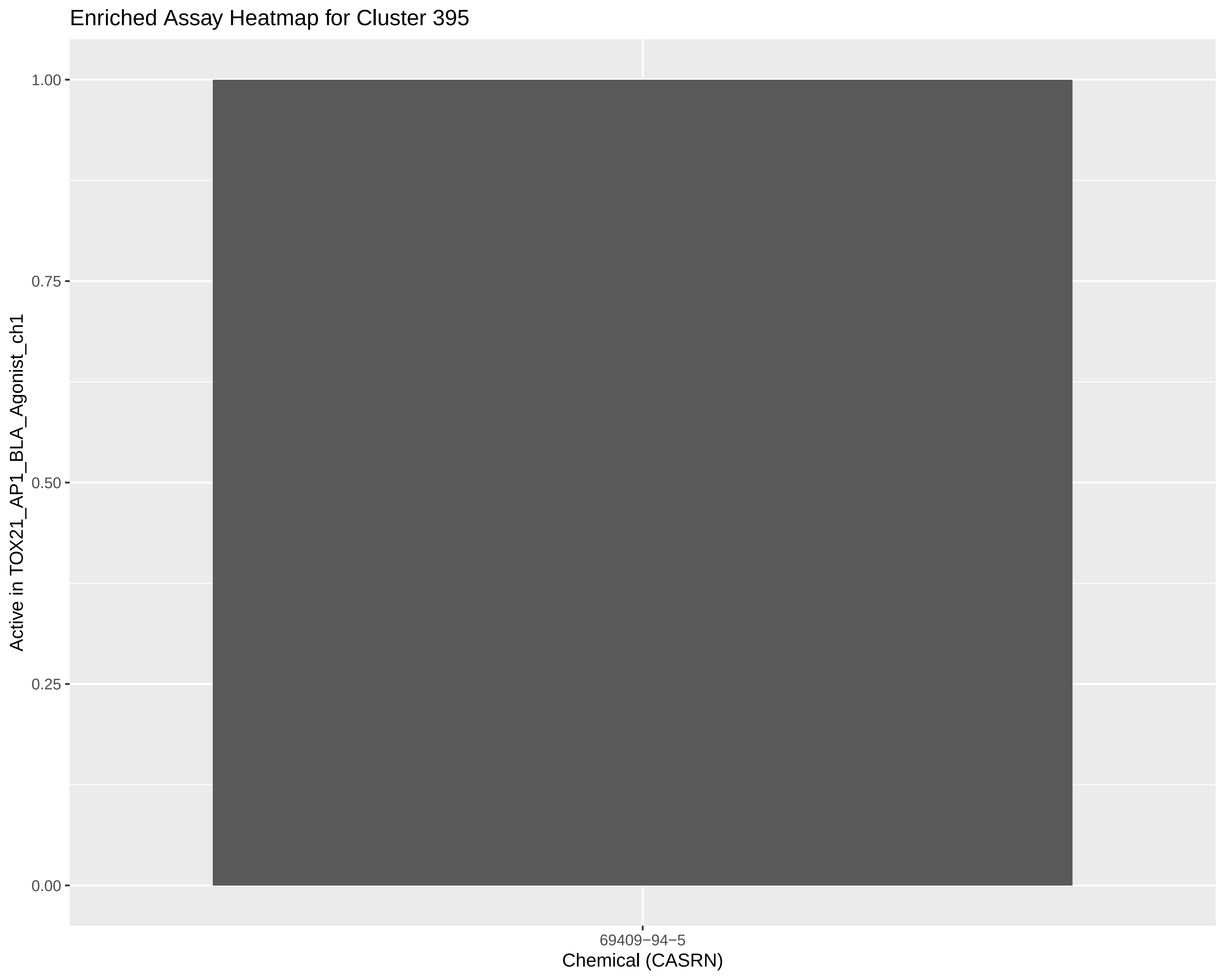
Enriched Assay Heatmap for Cluster 395
1.00
0.75
0.50
0.25
0.00
Active in TOX21_AP1_BLA_Agonist_ch1
69409−94−5
Chemical (CASRN)

Enriched Assay Heatmap for Cluster 399

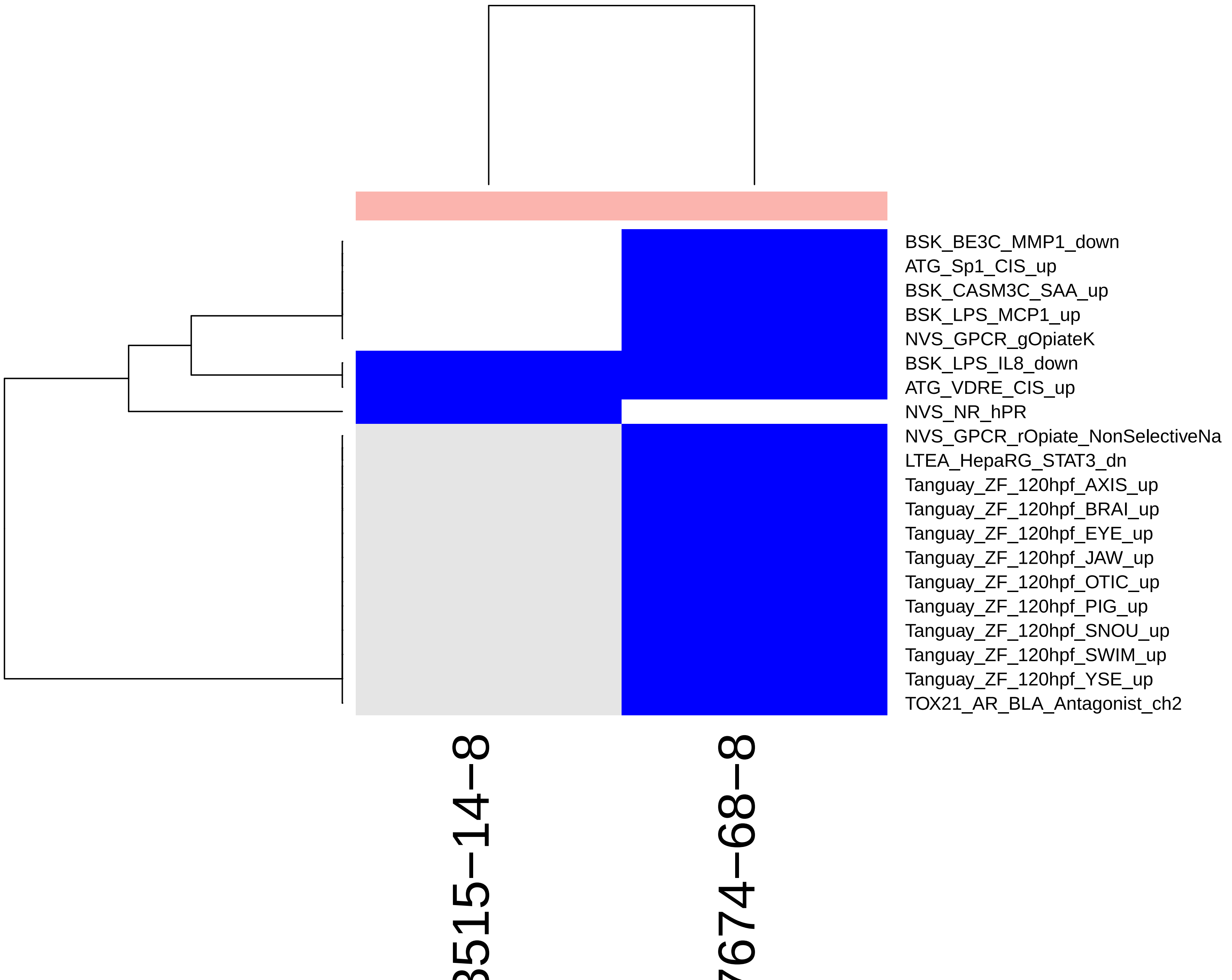

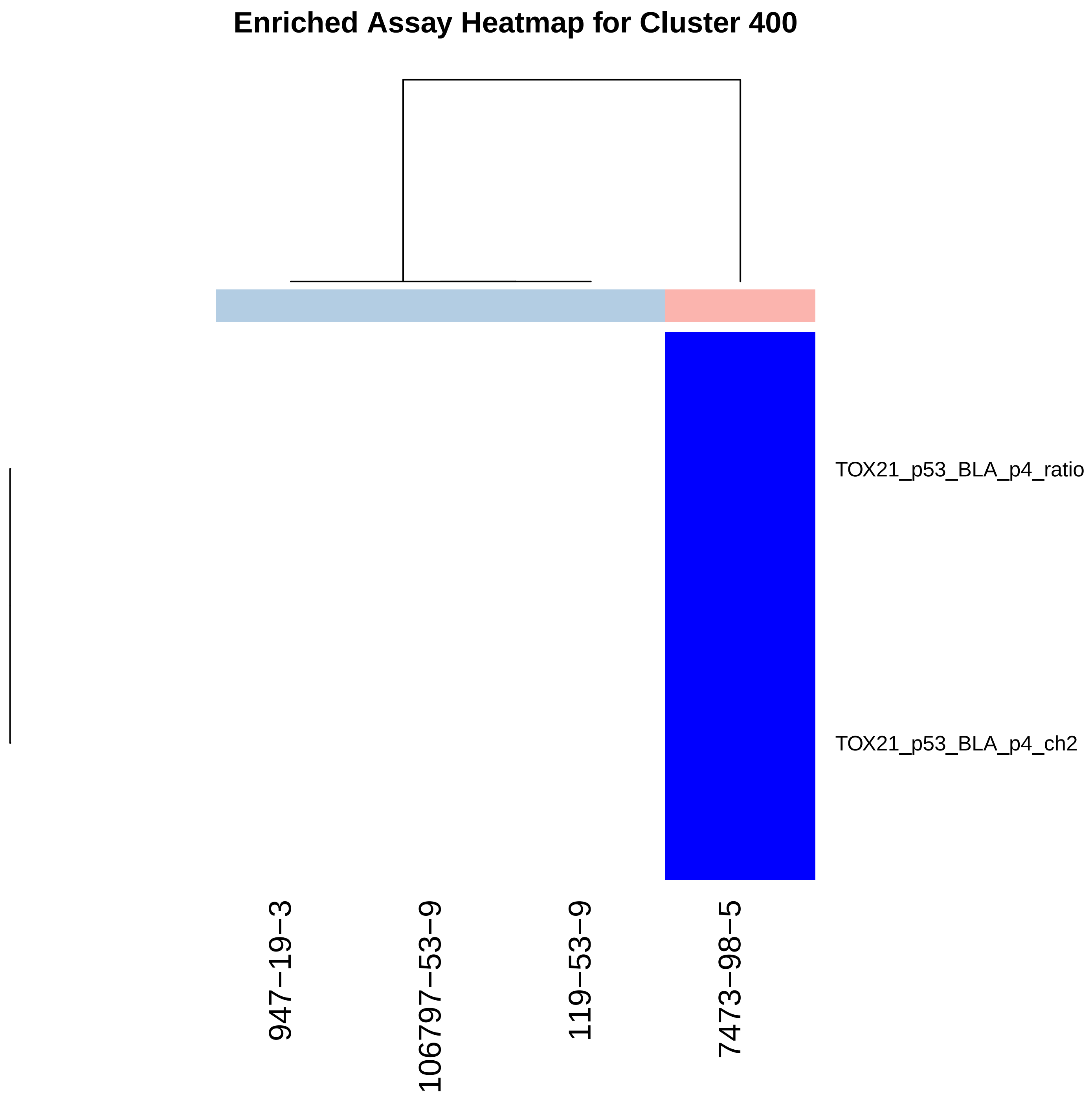
Enriched Assay Heatmap for Cluster 400
TOX21_p53_BLA_p4_ratio
TOX21_p53_BLA_p4_ch2
947−19−3
106797−53−9
119−53−9
7473−98−5

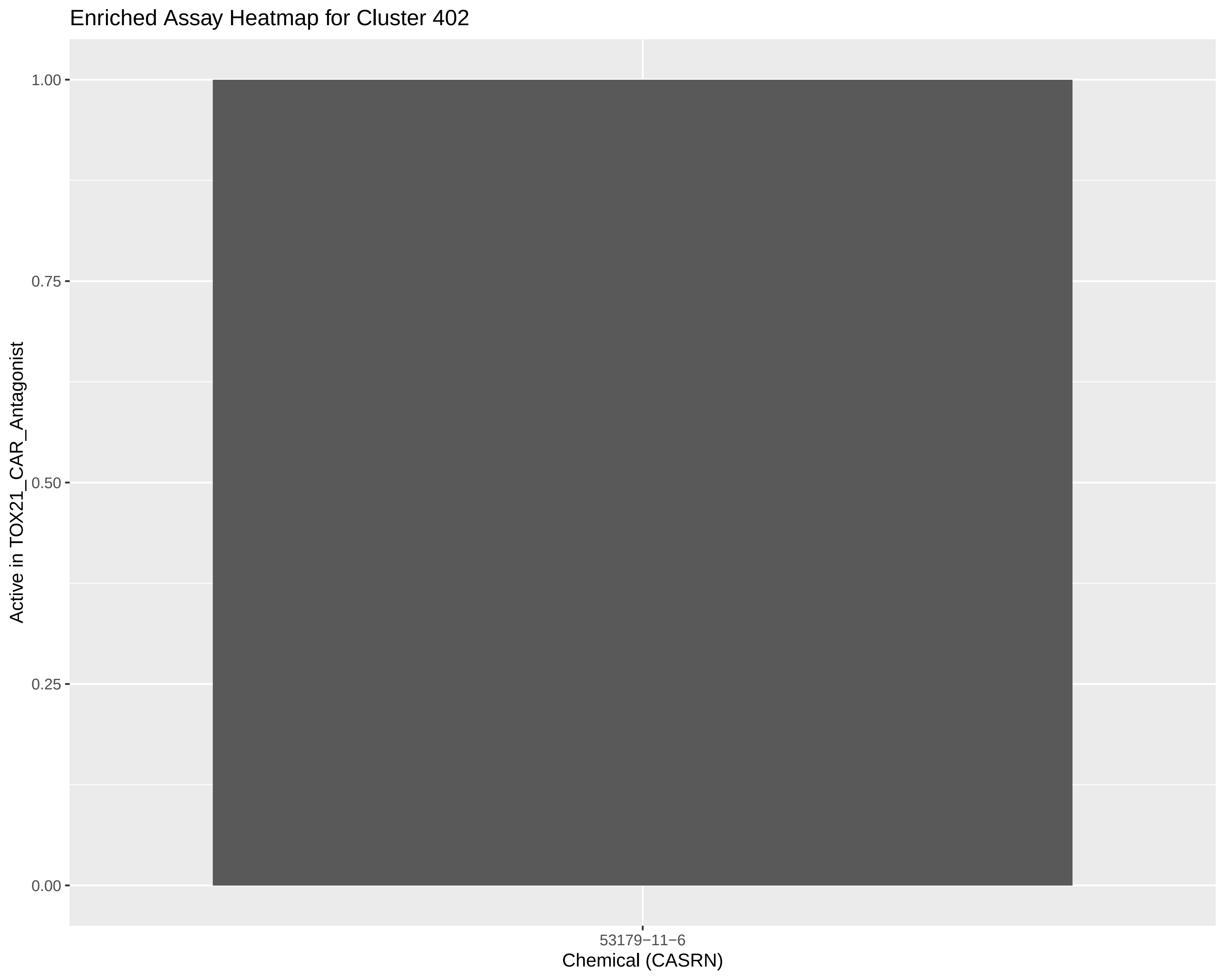

Enriched Assay Heatmap for Cluster 402
1.00
0.75
0.50
0.25
0.00
Active in TOX21_CAR_Antagonist
53179−11−6
Chemical (CASRN)

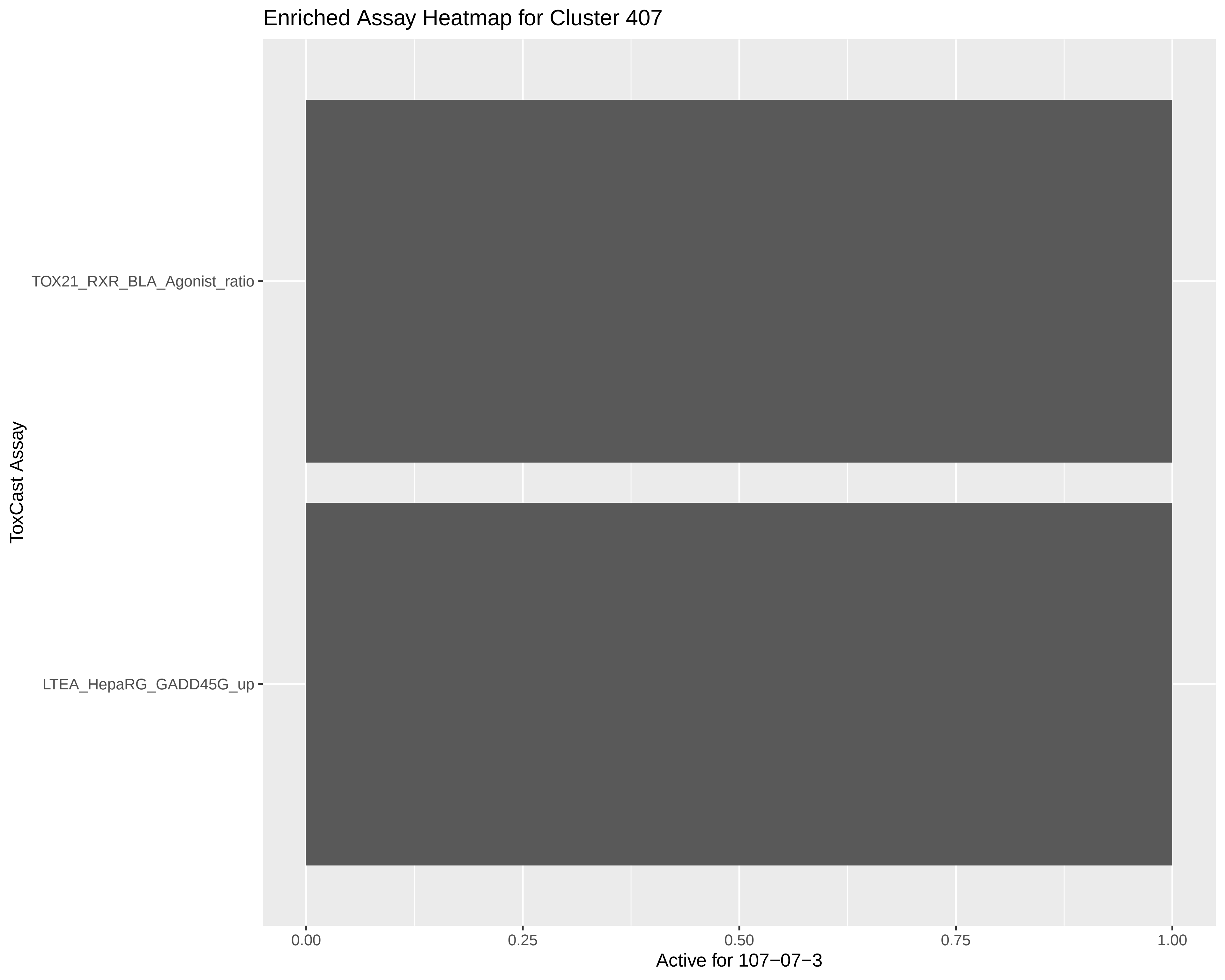
Enriched Assay Heatmap for Cluster 407
ToxCast Assay
TOX21_RXR_BLA_Agonist_ratio
LTEA_HepaRG_GADD45G_up
0.00
0.25
0.50
0.75
1.00
Active for 107−07−3

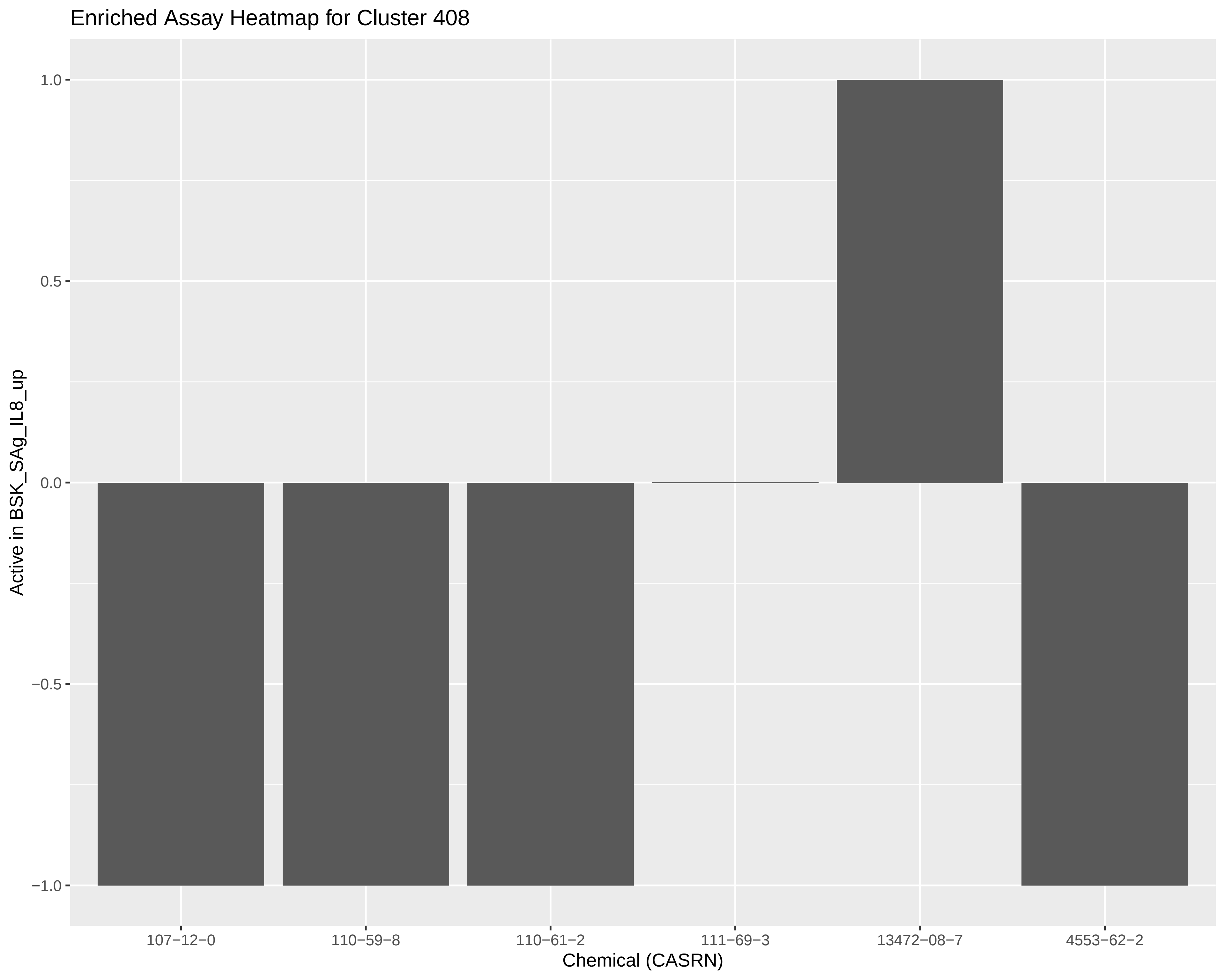
Enriched Assay Heatmap for Cluster 408
Active in BSK_SAg_IL8_up
1.0
0.5
0.0
−0.5
−1.0
107−12−0
110−59−8
110−61−2
111−69−3
13472−08−7
4553−62−2
Chemical (CASRN)

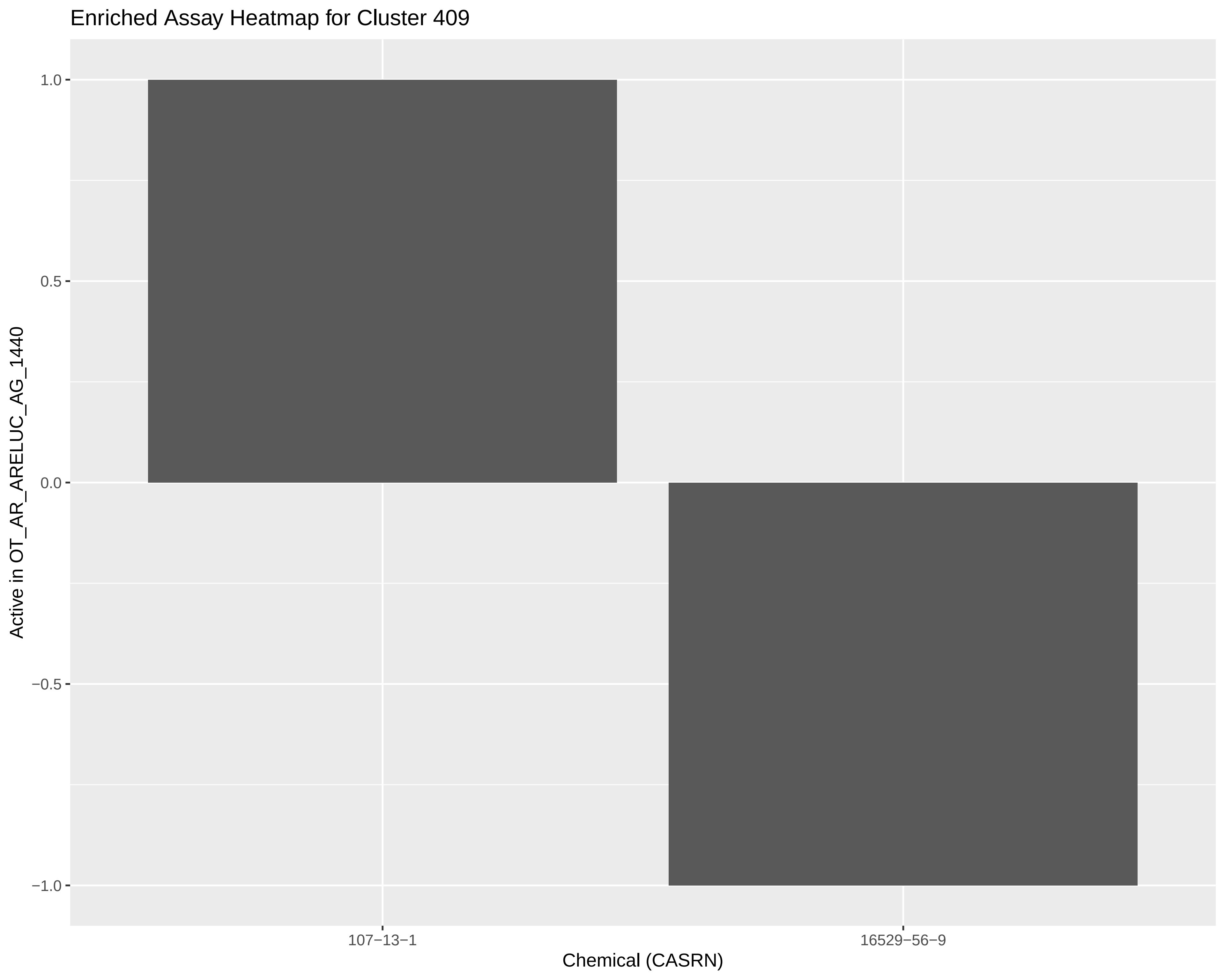

Enriched Assay Heatmap for Cluster 409
Active in OT_AR_ARELUC_AG_1440
1.0
0.5
0.0
−0.5
−1.0
107−13−1
16529−56−9
Chemical (CASRN)

Enriched Assay Heatmap for Cluster 410

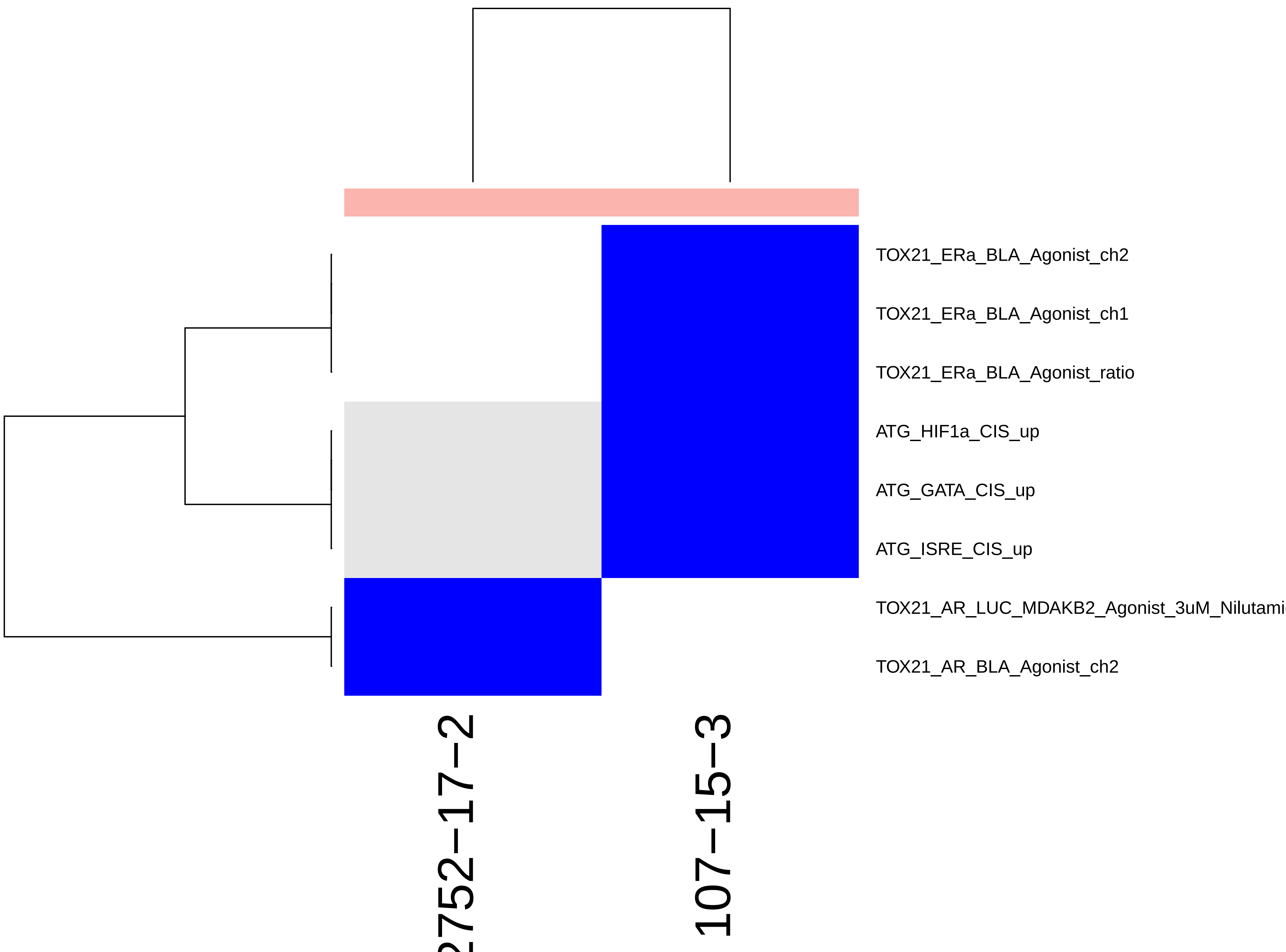

Enriched Assay Heatmap for Cluster 412

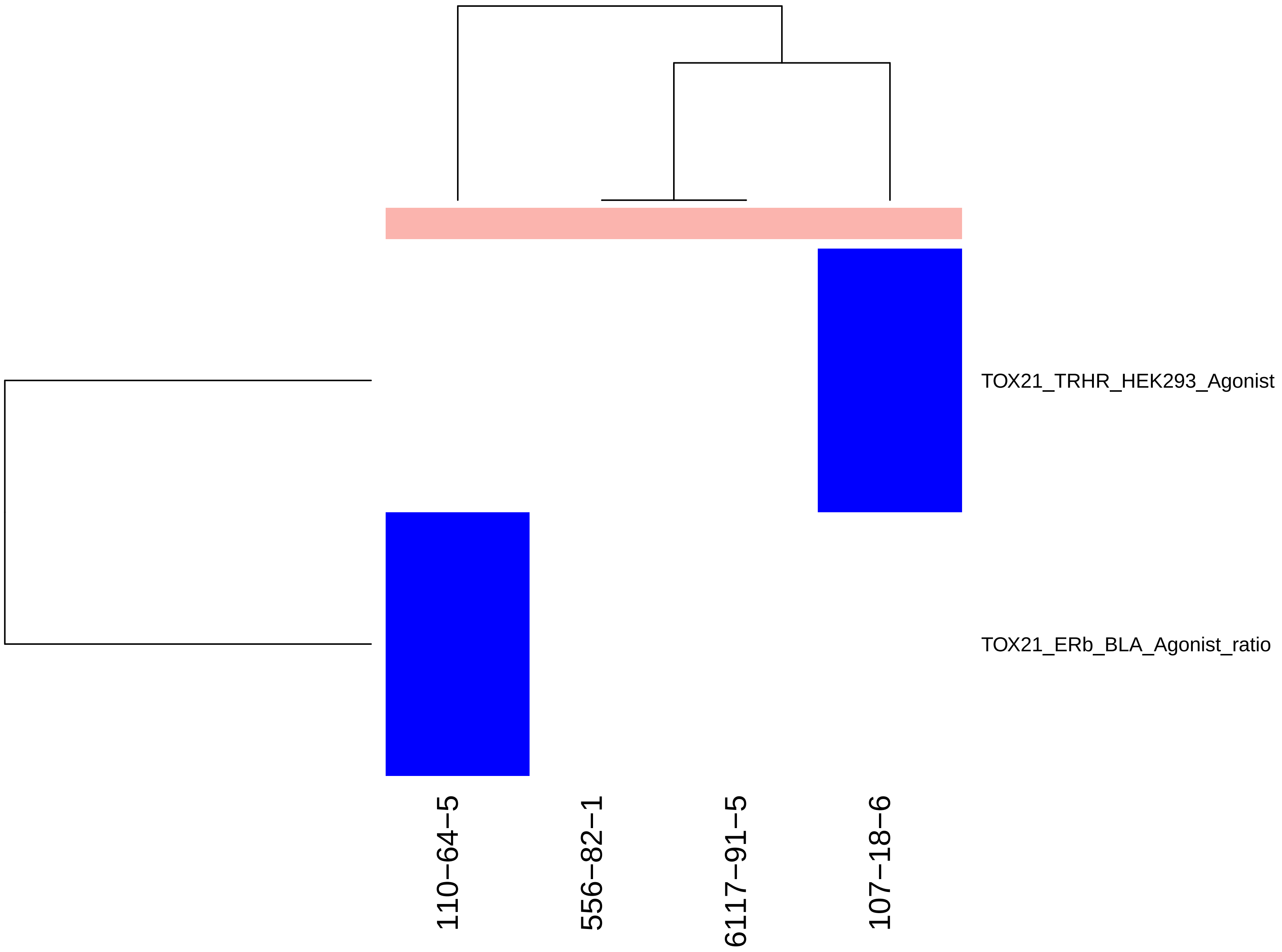

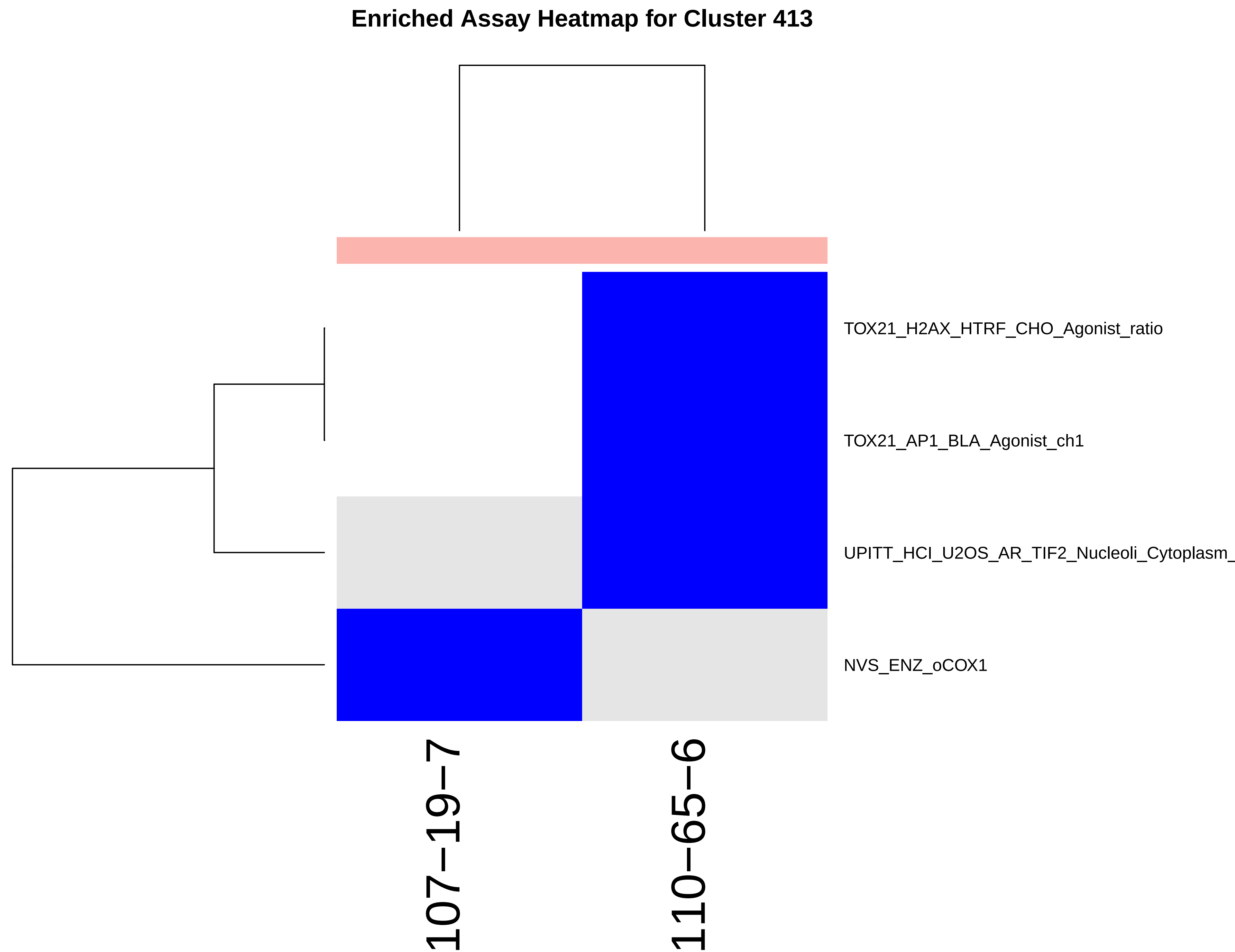
Enriched Assay Heatmap for Cluster 413
TOX21_H2AX_HTRF_CHO_Agonist_ratio
TOX21_AP1_BLA_Agonist_ch1
UPITT_HCI_U2OS_AR_TIF2_Nucleoli_Cytoplasm_
NVS_ENZ_oCOX1
107−19−7
110−65−6

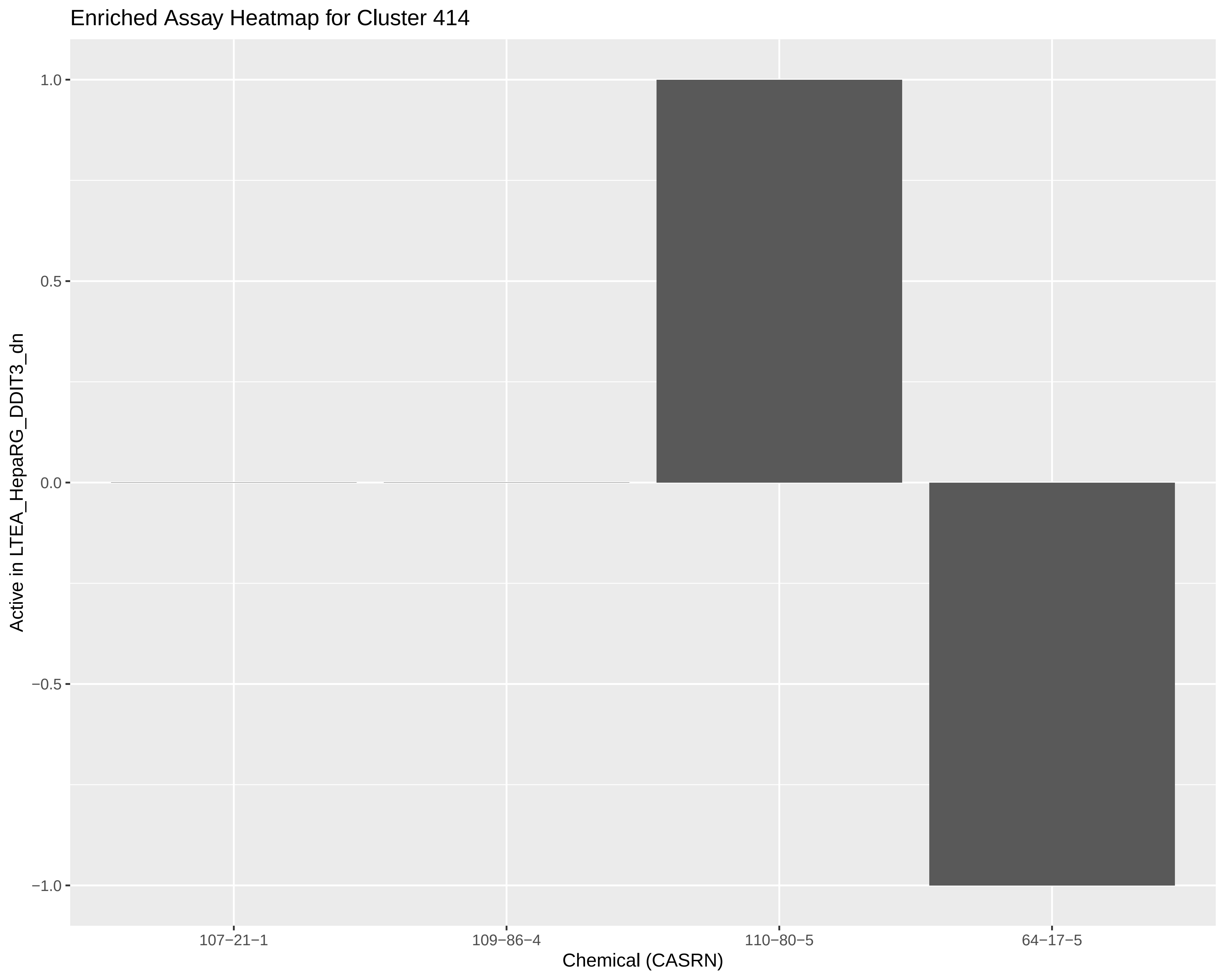

Enriched Assay Heatmap for Cluster 414
Active in LTEA_HepaRG_DDIT3_dn
1.0
0.5
0.0
−0.5
−1.0
107−21−1
109−86−4
110−80−5
64−17−5
Chemical (CASRN)

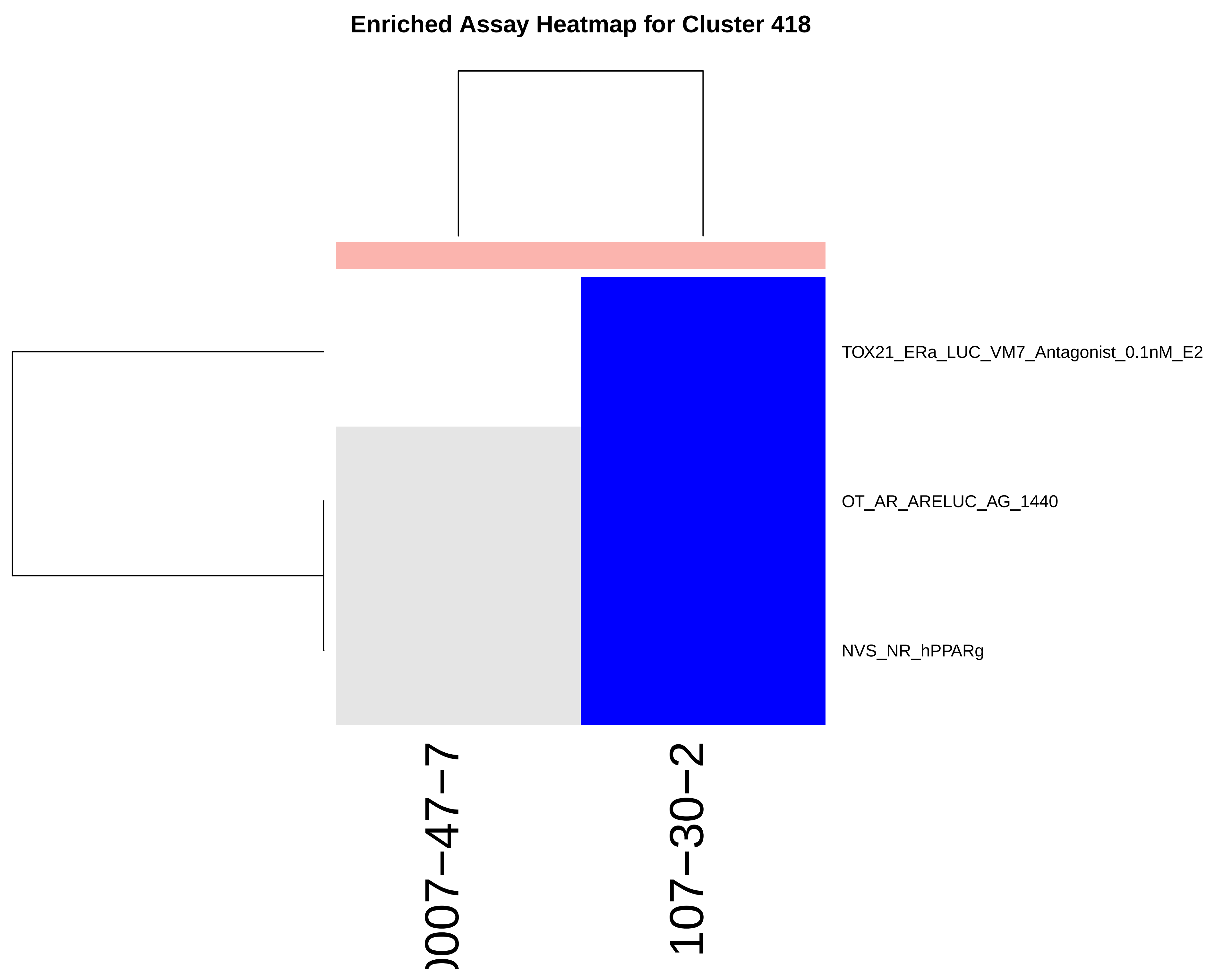
Enriched Assay Heatmap for Cluster 418
TOX21_ERa_LUC_VM7_Antagonist_0.1nM_E2
OT_AR_ARELUC_AG_1440
NVS_NR_hPPARg
0007−47−7
107−30−2

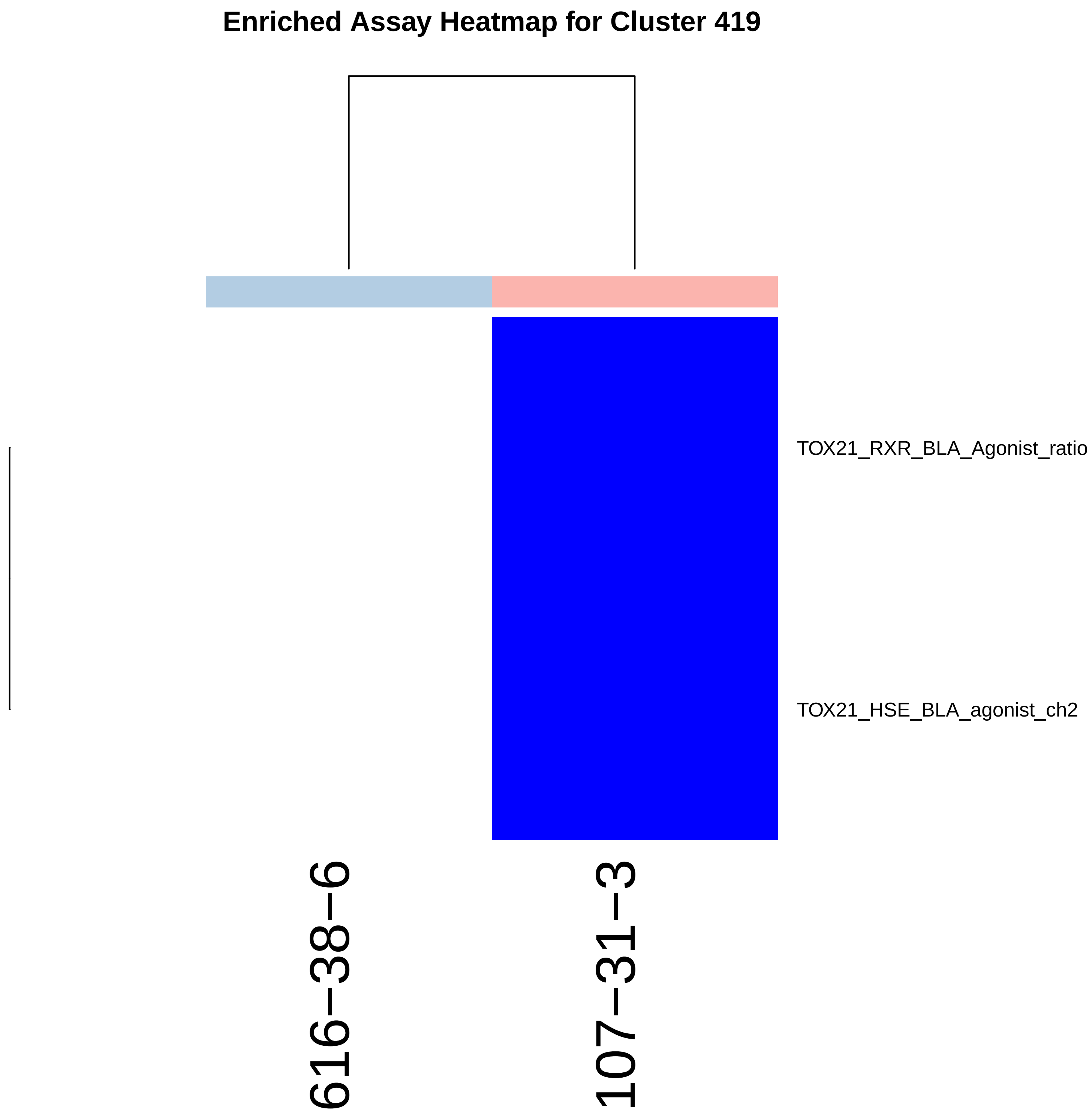

Enriched Assay Heatmap for Cluster 419
TOX21_RXR_BLA_Agonist_ratio
TOX21_HSE_BLA_agonist_ch2
616−38−6
107−31−3

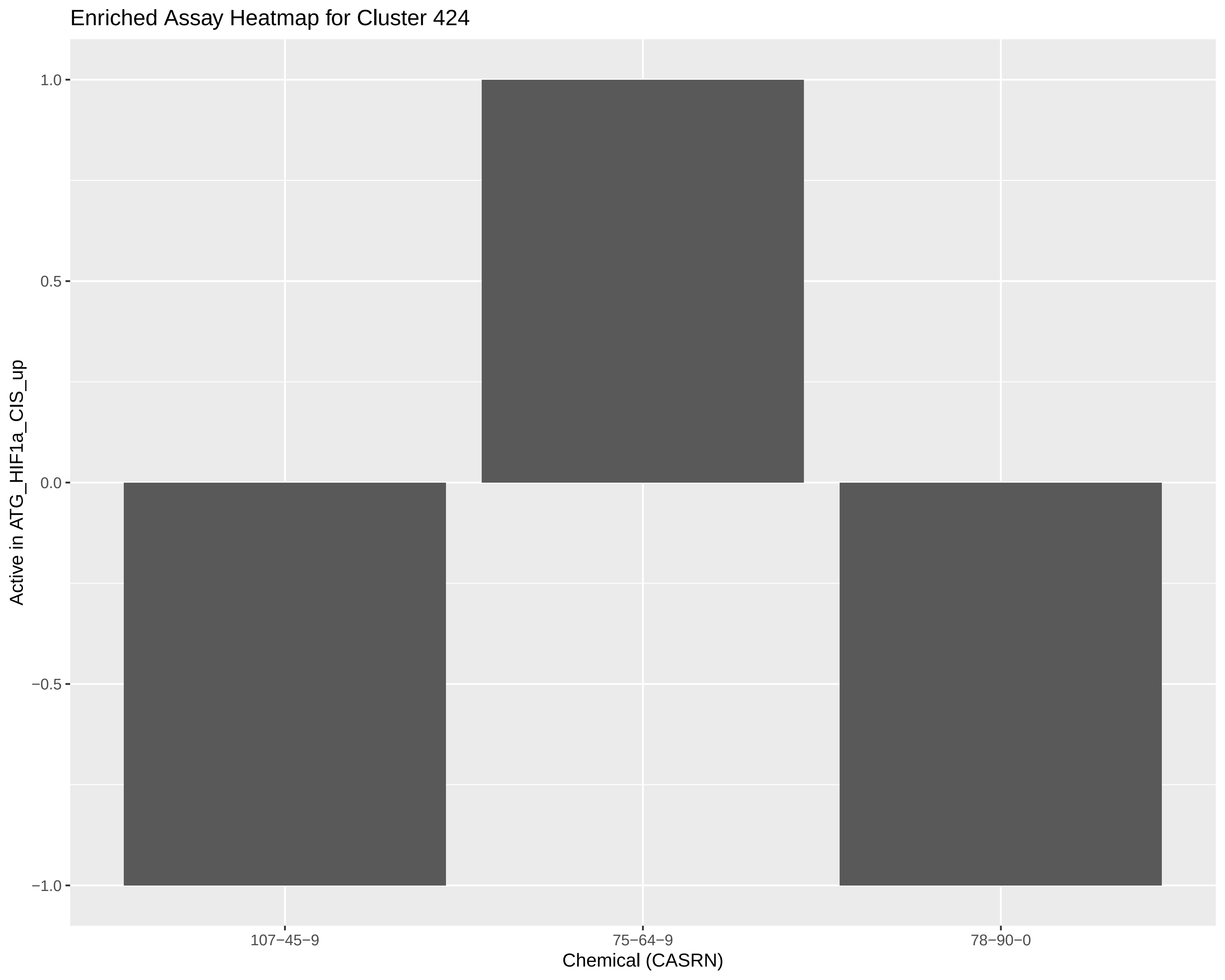
Enriched Assay Heatmap for Cluster 424
1.0
0.5
0.0
−0.5
−1.0
Active in ATG_HIF1a_CIS_up
107−45−9
75−64−9
78−90−0
Chemical (CASRN)

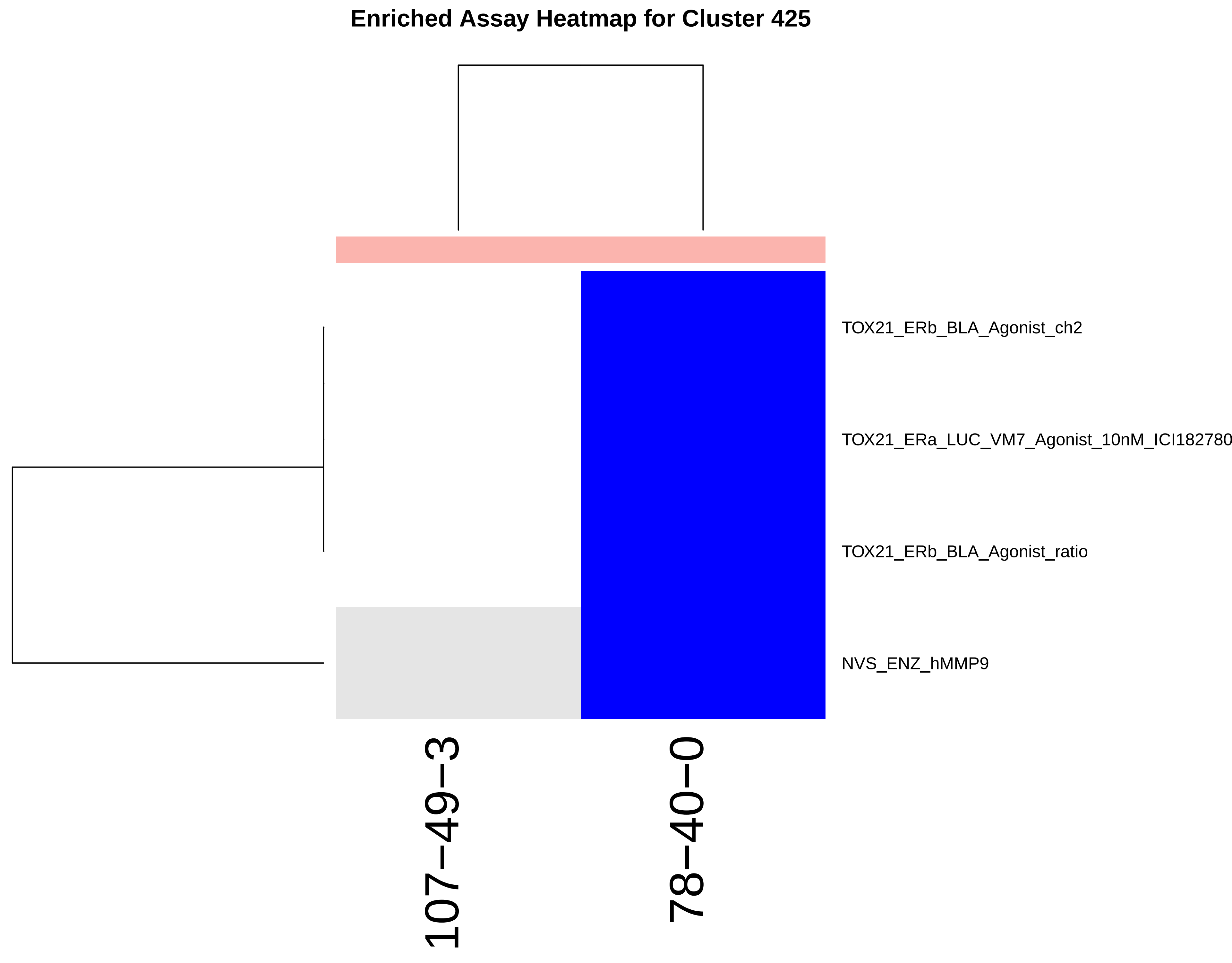

Enriched Assay Heatmap for Cluster 425
TOX21_ERb_BLA_Agonist_ch2
TOX21_ERa_LUC_VM7_Agonist_10nM_ICI182780
TOX21_ERb_BLA_Agonist_ratio
NVS_ENZ_hMMP9
107−49−3
78−40−0

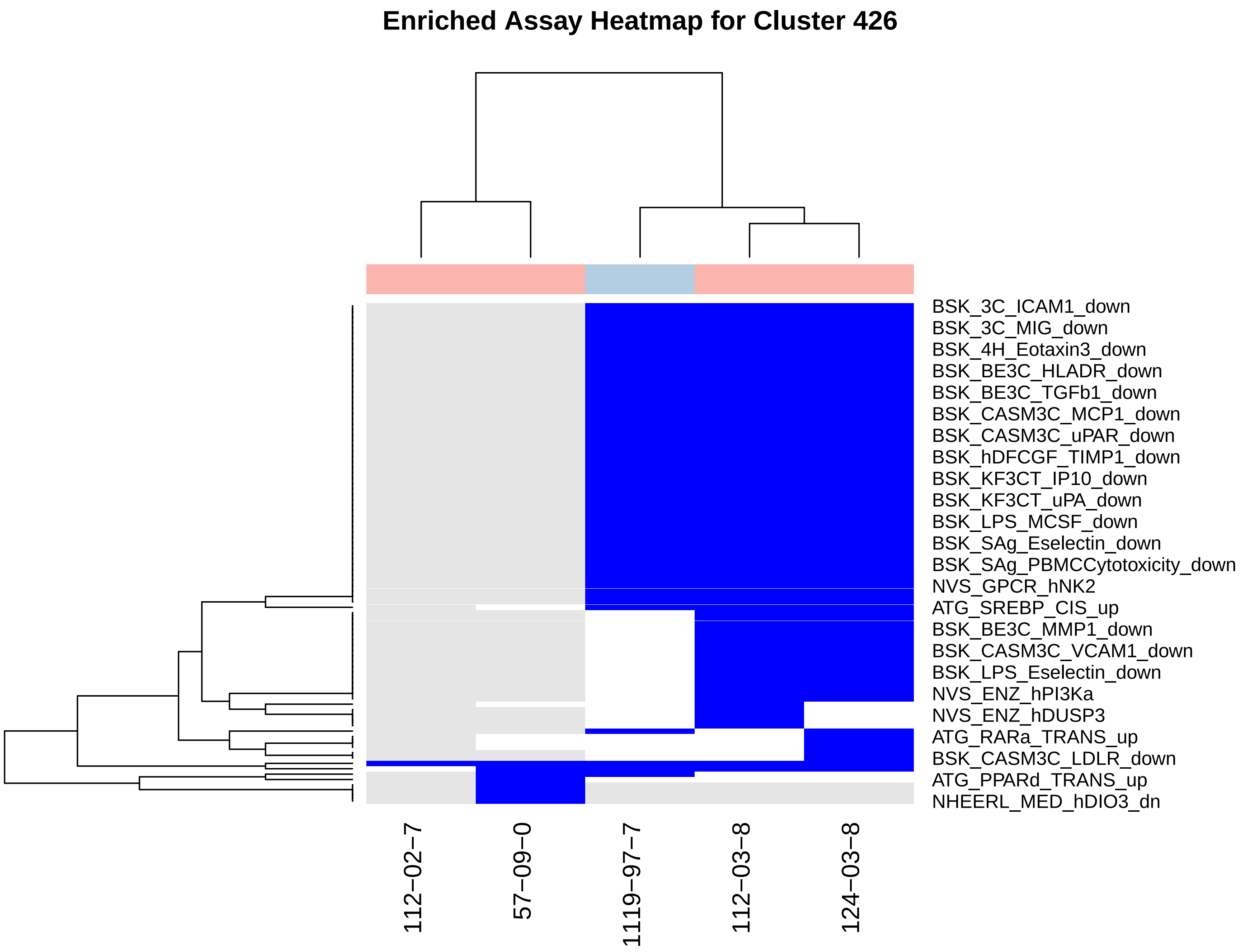
Enriched Assay Heatmap for Cluster 426
BSK_3C_ICAM1_down
BSK_3C_MIG_down
BSK_4H_Eotaxin3_down
BSK_BE3C_HLADR_down
BSK_BE3C_TGFb1_down
BSK_CASM3C_MCP1_down
BSK_CASM3C_uPAR_down
BSK_hDFCGF_TIMP1_down
BSK_KF3CT_IP10_down
BSK_KF3CT_uPA_down
BSK_LPS_MCSF_down
BSK_SAg_Eselectin_down
BSK_SAg_PBMCCytotoxicity_down
NVS_GPCR_hNK2
ATG_SREBP_CIS_up
BSK_BE3C_MMP1_down
BSK_CASM3C_VCAM1_down
BSK_LPS_Eselectin_down
NVS_ENZ_hPI3Ka
NVS_ENZ_hDUSP3
ATG_RARa_TRANS_up
BSK_CASM3C_LDLR_down
ATG_PPARd_TRANS_up
NHEERL_MED_hDIO3_dn
112−02−7
57−09−0
1119−97−7
112−03−8
124−03−8

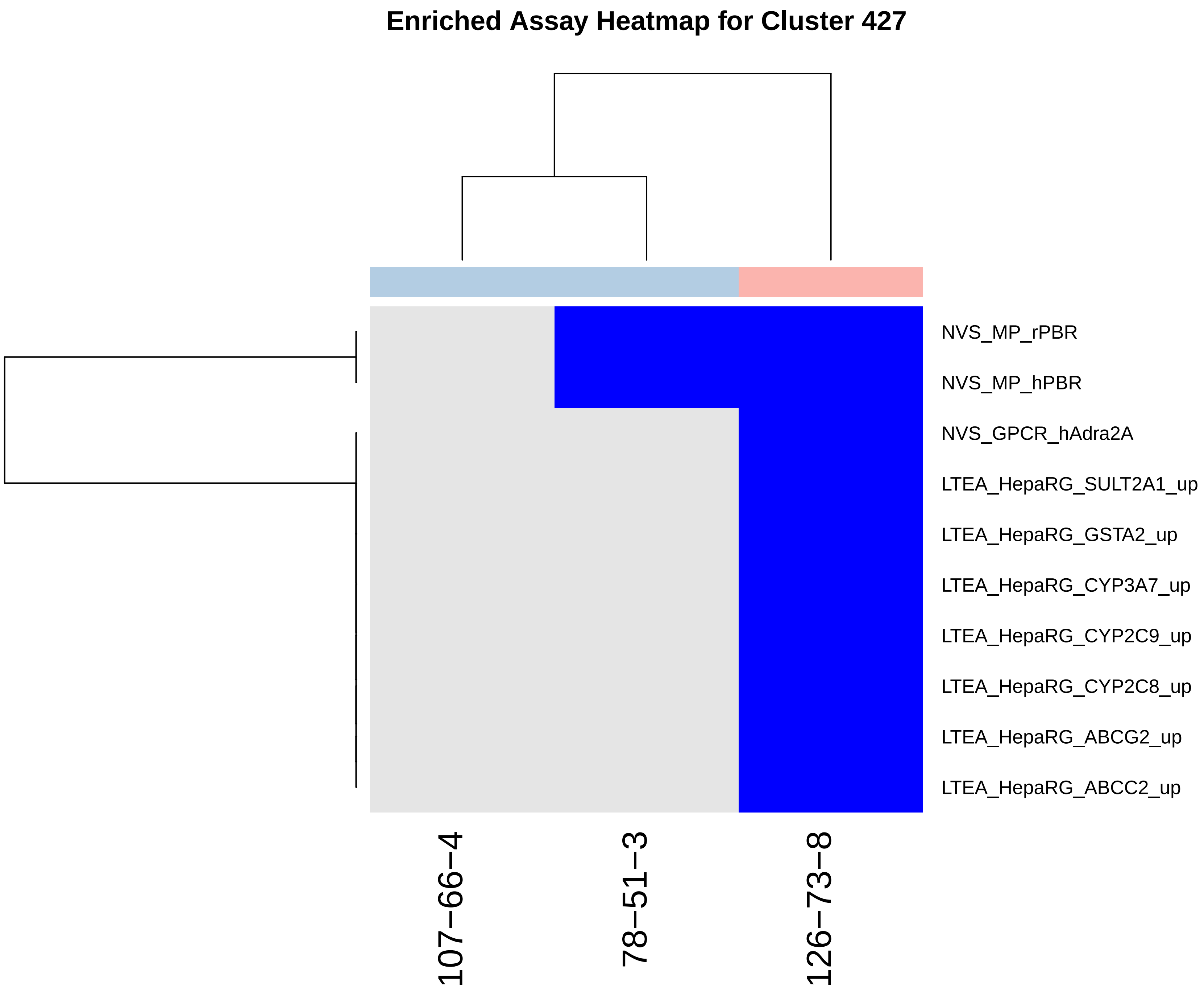
Enriched Assay Heatmap for Cluster 427
NVS_MP_rPBR
NVS_MP_hPBR
NVS_GPCR_hAdra2A
LTEA_HepaRG_SULT2A1_up
LTEA_HepaRG_GSTA2_up
LTEA_HepaRG_CYP3A7_up
LTEA_HepaRG_CYP2C9_up
LTEA_HepaRG_CYP2C8_up
LTEA_HepaRG_ABCG2_up
LTEA_HepaRG_ABCC2_up
107−66−4
78−51−3
126−73−8

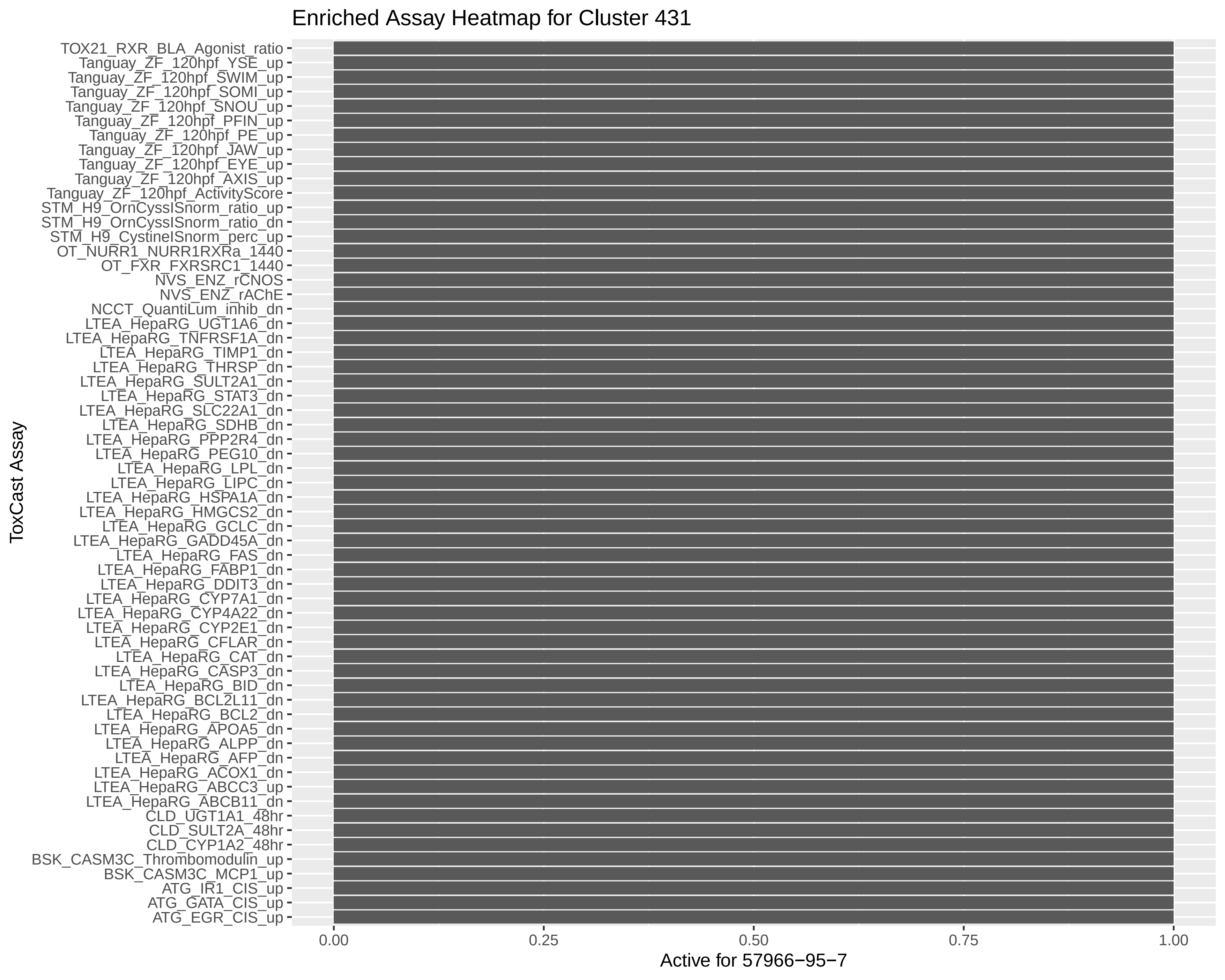

Enriched Assay Heatmap for Cluster 431
ToxCast Assay
TOX21_RXR_BLA_Agonist_ratio
Tanguay_ZF_120hpf_YSE_up
Tanguay_ZF_120hpf_SWIM_up
Tanguay_ZF_120hpf_SOMI_up
Tanguay_ZF_120hpf_SNOU_up
Tanguay_ZF_120hpf_PFIN_up
Tanguay_ZF_120hpf_PE_up
Tanguay_ZF_120hpf_JAW_up
Tanguay_ZF_120hpf_EYE_up
Tanguay_ZF_120hpf_AXIS_up
Tanguay_ZF_120hpf_ActivityScore
STM_H9_OrnCyssISnorm_ratio_up
STM_H9_OrnCyssISnorm_ratio_dn
STM_H9_CystineISnorm_perc_up
OT_NURR1_NURR1RXRa_1440
OT_FXR_FXRSRC1_1440
NVS_ENZ_rCNOS
NVS_ENZ_rAChE
NCCT_QuantiLum_inhib_dn
LTEA_HepaRG_UGT1A6_dn
LTEA_HepaRG_TNFRSF1A_dn
LTEA_HepaRG_TIMP1_dn
LTEA_HepaRG_THRSP_dn
LTEA_HepaRG_SULT2A1_dn
LTEA_HepaRG_STAT3_dn
LTEA_HepaRG_SLC22A1_dn
LTEA_HepaRG_SDHB_dn
LTEA_HepaRG_PPP2R4_dn
LTEA_HepaRG_PEG10_dn
LTEA_HepaRG_LPL_dn
LTEA_HepaRG_LIPC_dn
LTEA_HepaRG_HSPA1A_dn
LTEA_HepaRG_HMGCS2_dn
LTEA_HepaRG_GCLC_dn
LTEA_HepaRG_GADD45A_dn
LTEA_HepaRG_FAS_dn
LTEA_HepaRG_FABP1_dn
LTEA_HepaRG_DDIT3_dn
LTEA_HepaRG_CYP7A1_dn
LTEA_HepaRG_CYP4A22_dn
LTEA_HepaRG_CYP2E1_dn
LTEA_HepaRG_CFLAR_dn
LTEA_HepaRG_CAT_dn
LTEA_HepaRG_CASP3_dn
LTEA_HepaRG_BID_dn
LTEA_HepaRG_BCL2L11_dn
LTEA_HepaRG_BCL2_dn
LTEA_HepaRG_APOA5_dn
LTEA_HepaRG_ALPP_dn
LTEA_HepaRG_AFP_dn
LTEA_HepaRG_ACOX1_dn
LTEA_HepaRG_ABCC3_up
LTEA_HepaRG_ABCB11_dn
CLD_UGT1A1_48hr
CLD_SULT2A_48hr
CLD_CYP1A2_48hr
BSK_CASM3C_Thrombomodulin_up
BSK_CASM3C_MCP1_up
ATG_IR1_CIS_up
ATG_GATA_CIS_up
ATG_EGR_CIS_up
0.00
0.25
0.50
0.75
1.00
Active for 57966−95−7

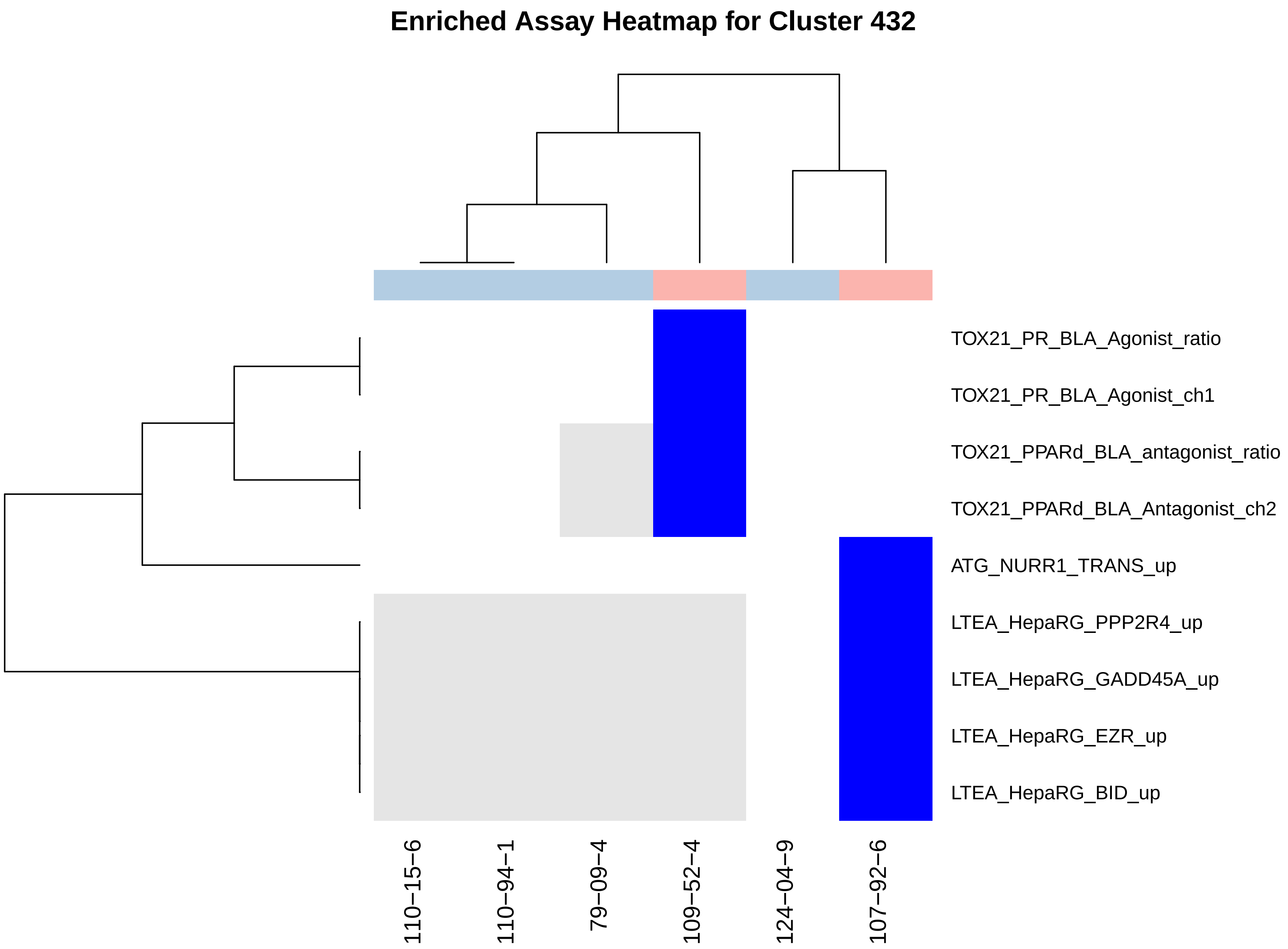
Enriched Assay Heatmap for Cluster 432
TOX21_PR_BLA_Agonist_ratio
TOX21_PR_BLA_Agonist_ch1
TOX21_PPARd_BLA_antagonist_ratio
TOX21_PPARd_BLA_Antagonist_ch2
ATG_NURR1_TRANS_up
LTEA_HepaRG_PPP2R4_up
LTEA_HepaRG_GADD45A_up
LTEA_HepaRG_EZR_up
LTEA_HepaRG_BID_up
110−15−6
110−94−1
79−09−4
109−52−4
124−04−9
107−92−6

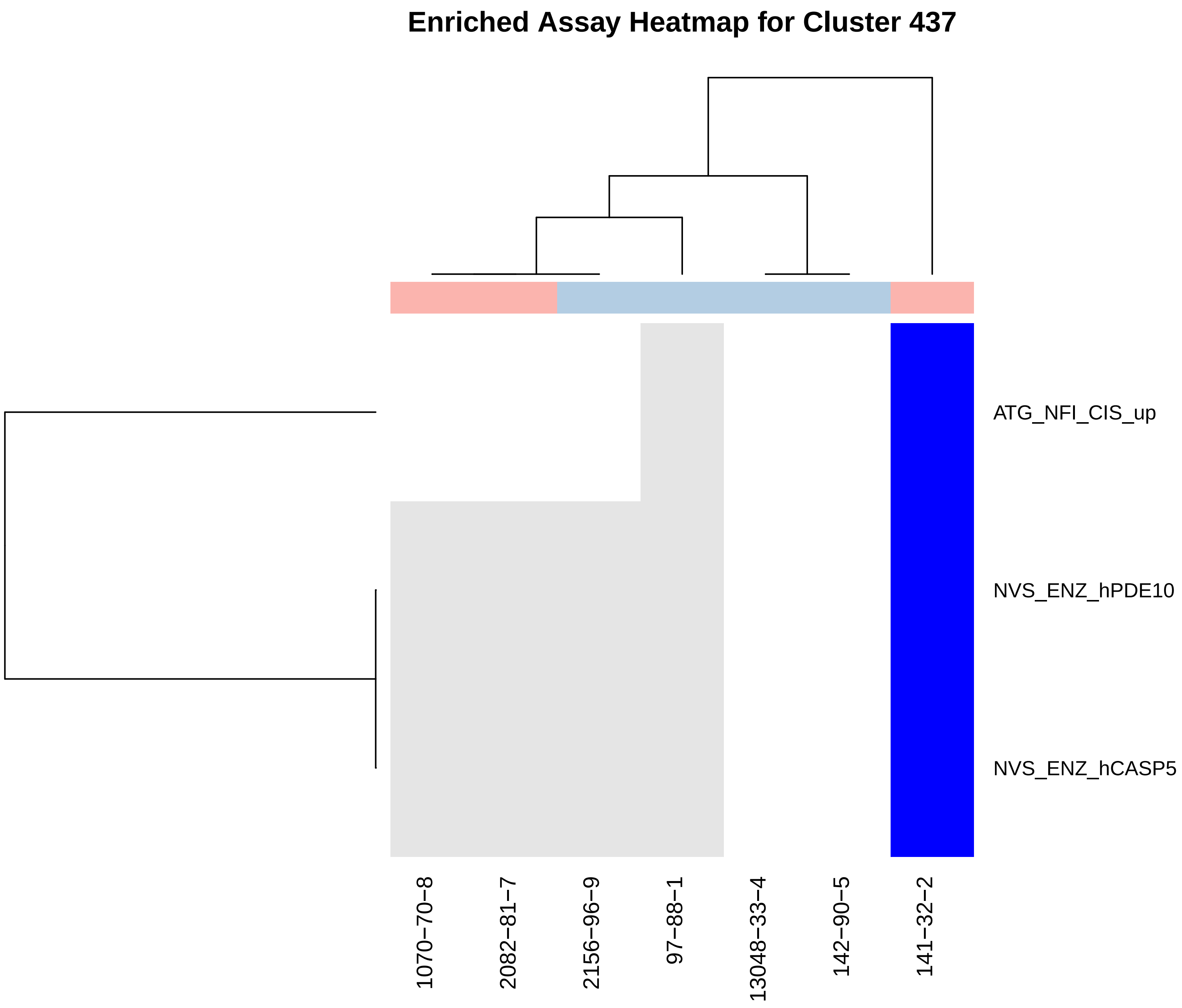

Enriched Assay Heatmap for Cluster 437
ATG_NFI_CIS_up
NVS_ENZ_hPDE10
NVS_ENZ_hCASP5
1070−70−8
2082−81−7
2156−96−9
97−88−1
13048−33−4
142−90−5
141−32−2

Enriched Assay Heatmap for Cluster 443

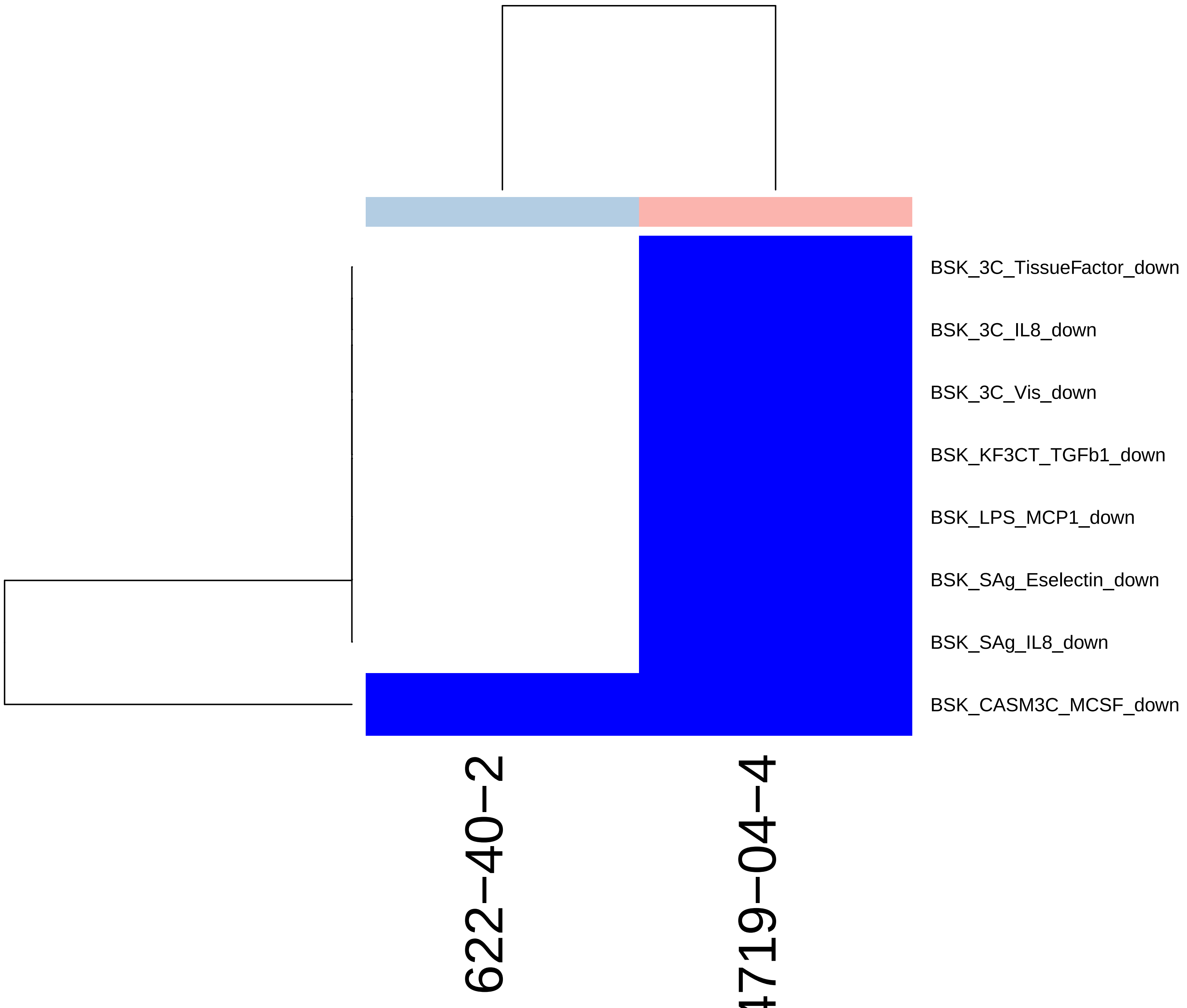

Enriched Assay Heatmap for Cluster 446

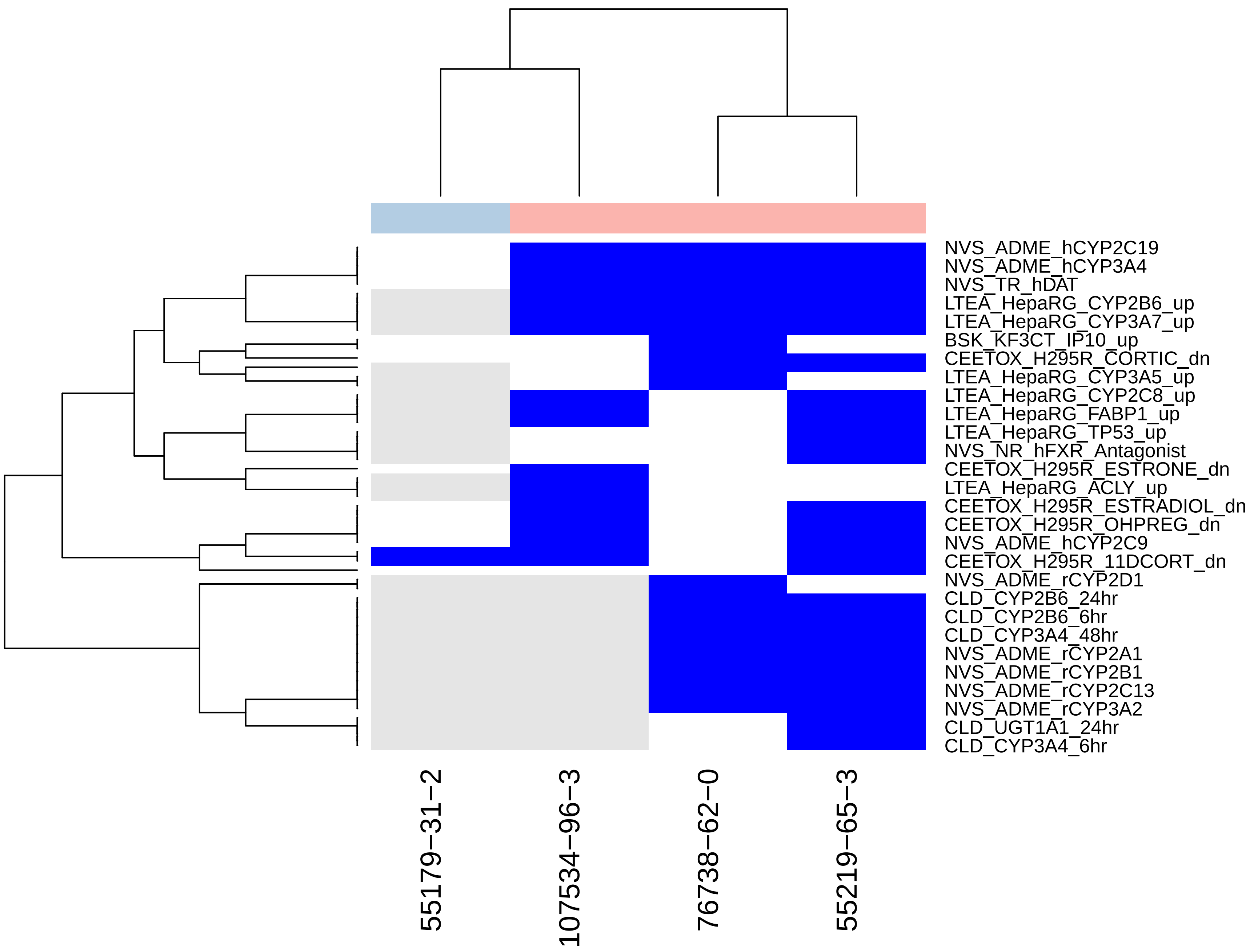

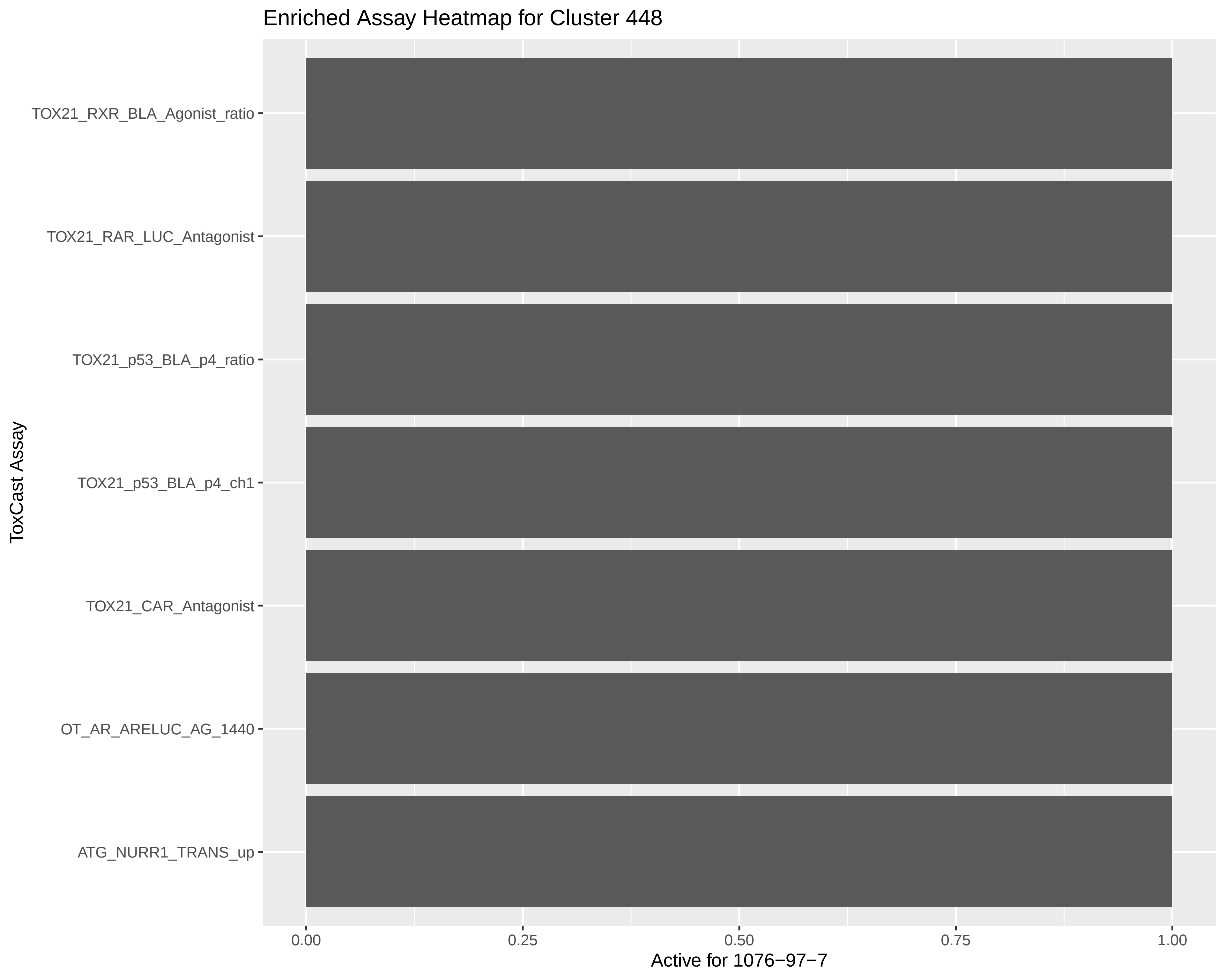

Enriched Assay Heatmap for Cluster 448
ToxCast Assay
TOX21_RXR_BLA_Agonist_ratio
TOX21_RAR_LUC_Antagonist
TOX21_p53_BLA_p4_ratio
TOX21_p53_BLA_p4_ch1
TOX21_CAR_Antagonist
OT_AR_ARELUC_AG_1440
ATG_NURR1_TRANS_up
0.00
0.25
0.50
0.75
1.00
Active for 1076−97−7

Enriched Assay Heatmap for Cluster 455

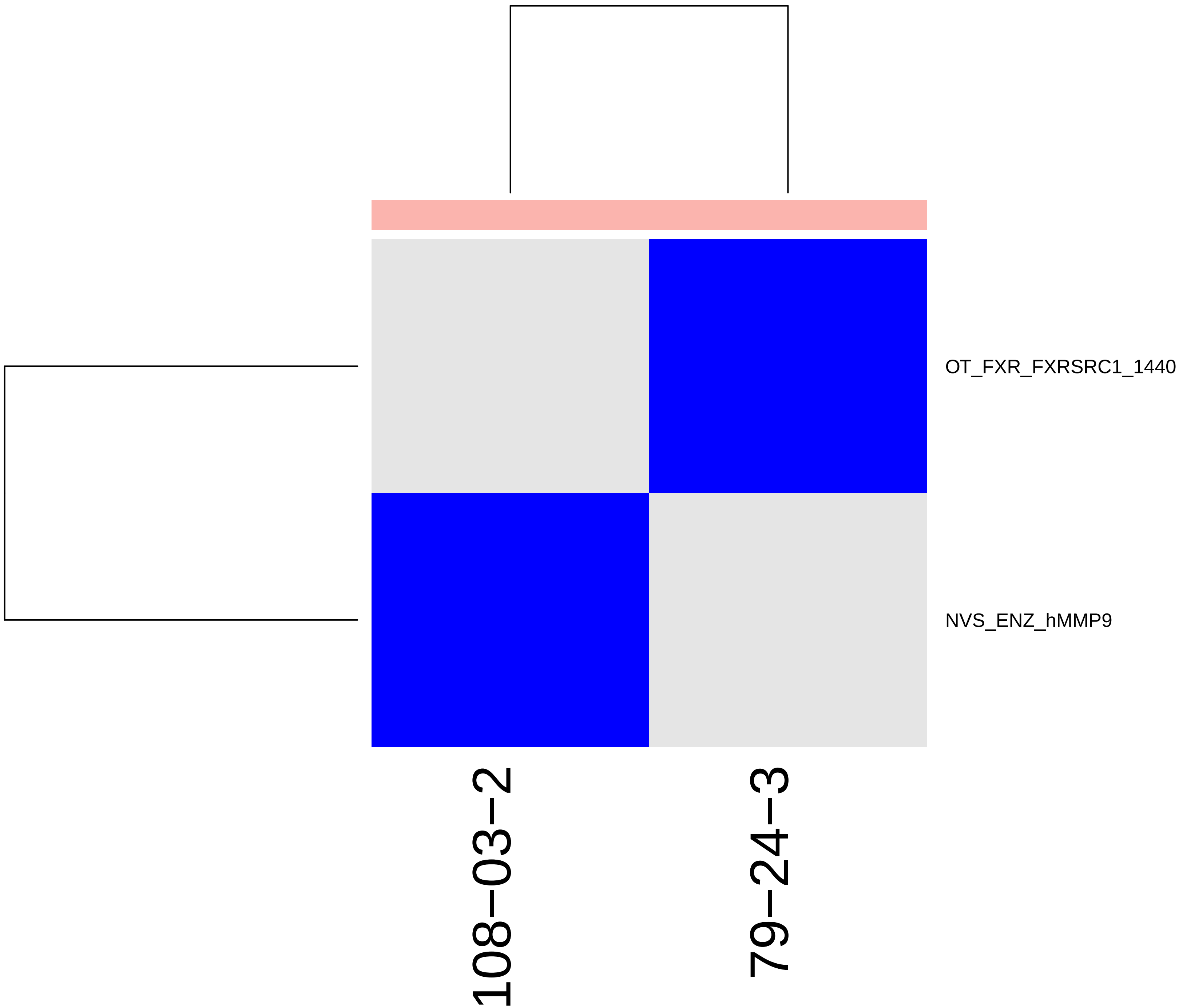

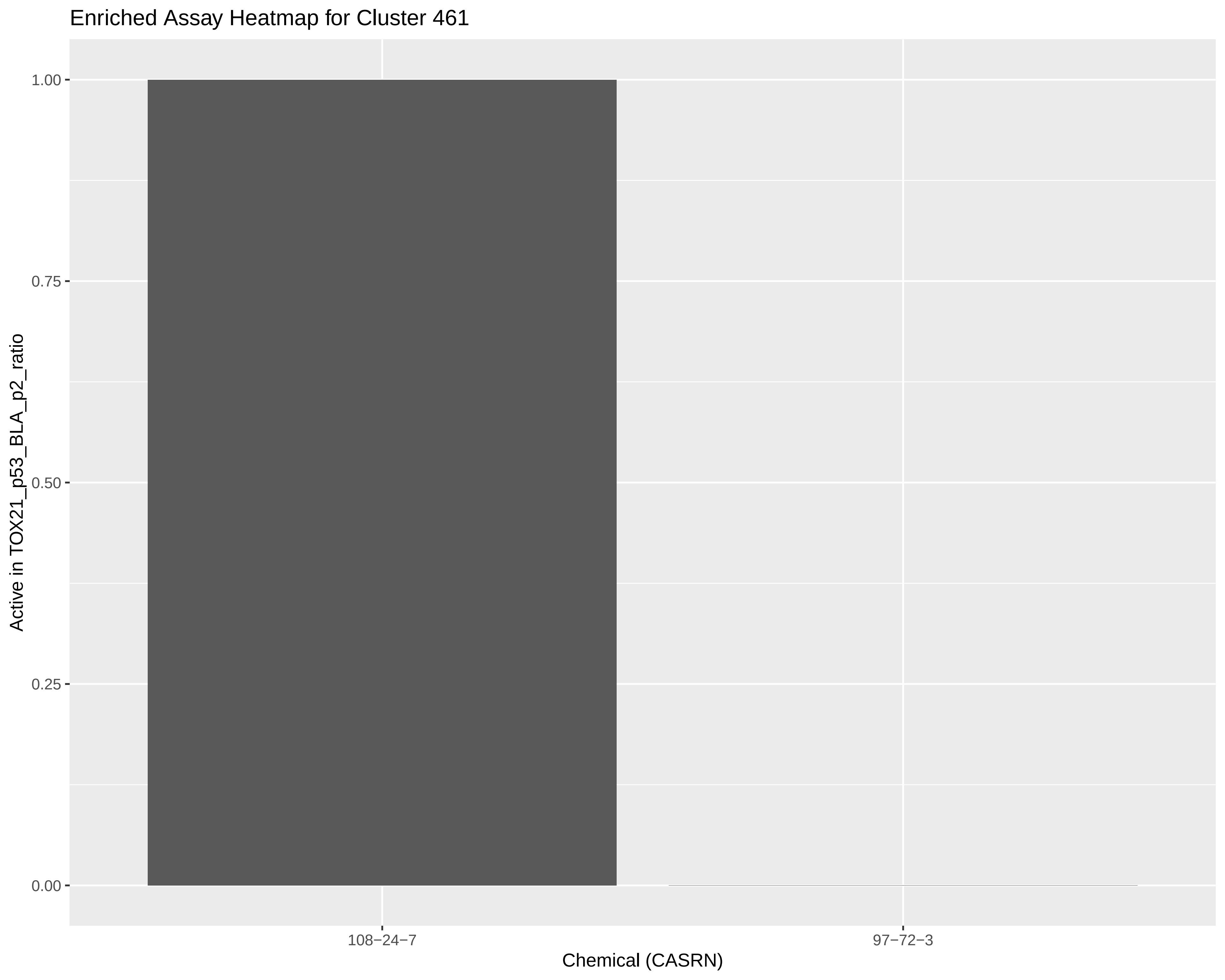
Enriched Assay Heatmap for Cluster 461
Active in TOX21_p53_BLA_p2_ratio
1.00
0.75
0.50
0.25
0.00
108−24−7
97−72−3
Chemical (CASRN)

Enriched Assay Heatmap for Cluster 462

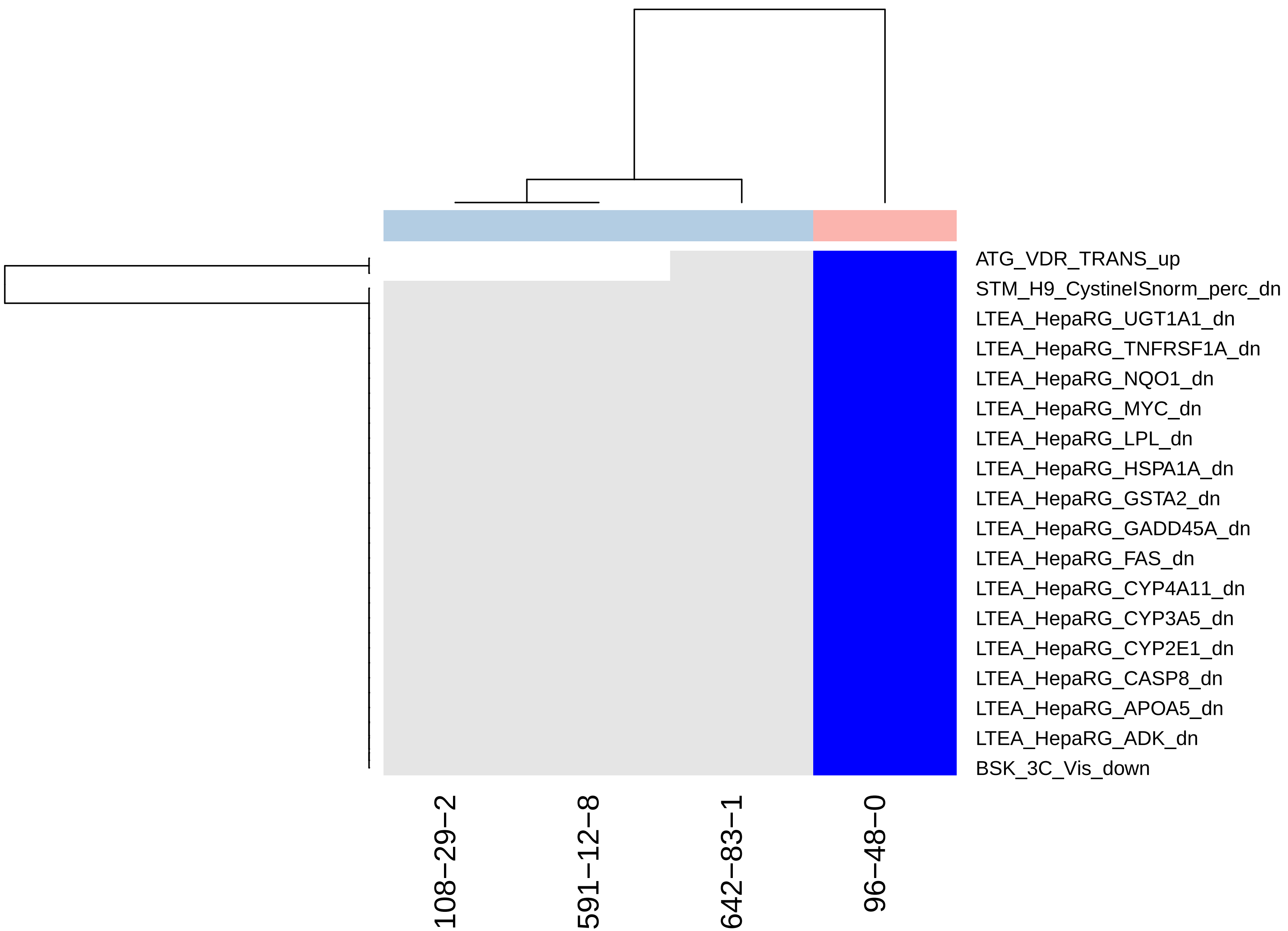

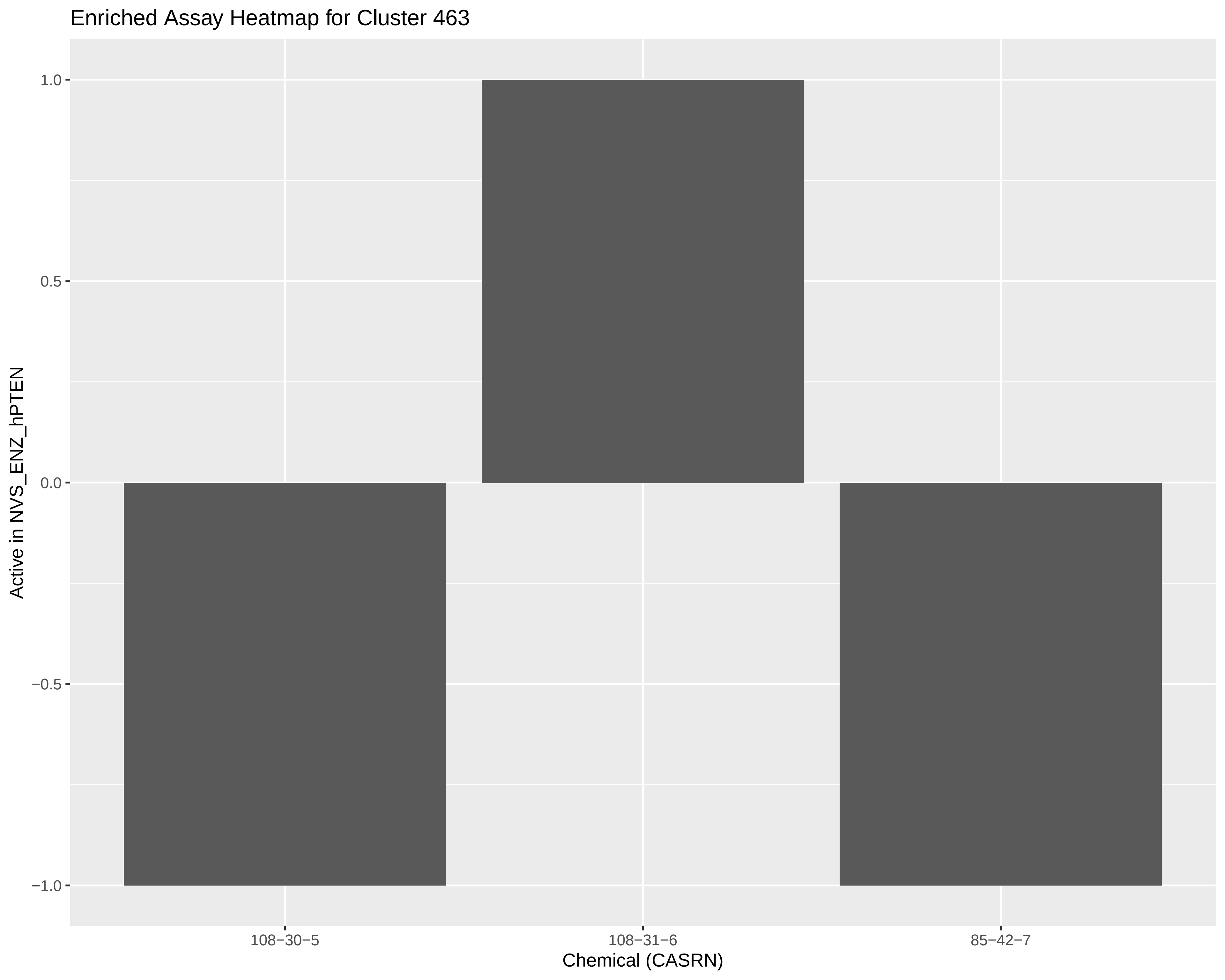
Enriched Assay Heatmap for Cluster 463
Active in NVS_ENZ_hPTEN
1.0
0.5
0.0
−0.5
−1.0
108−30−5
108−31−6
85−42−7
Chemical (CASRN)

Enriched Assay Heatmap for Cluster 465

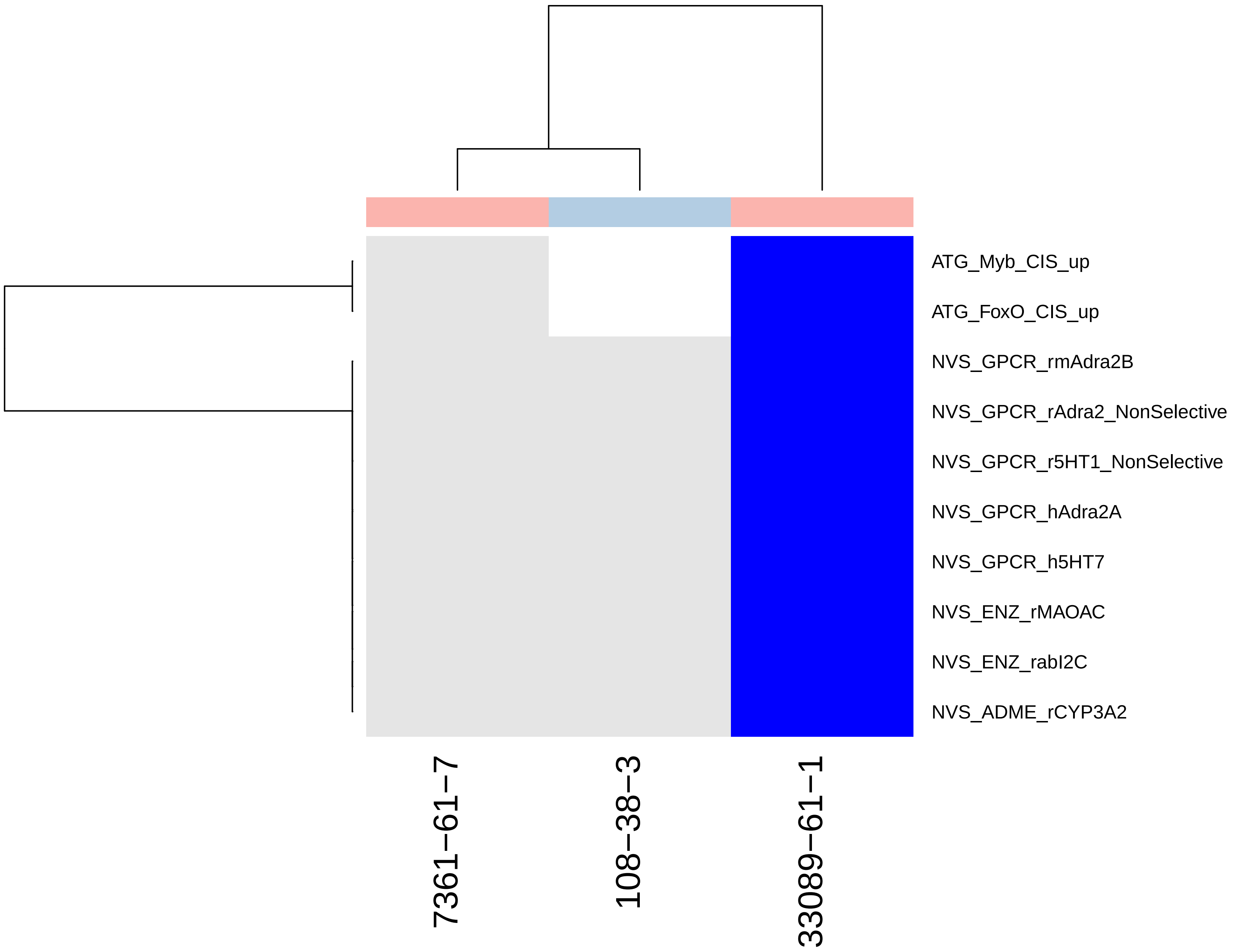

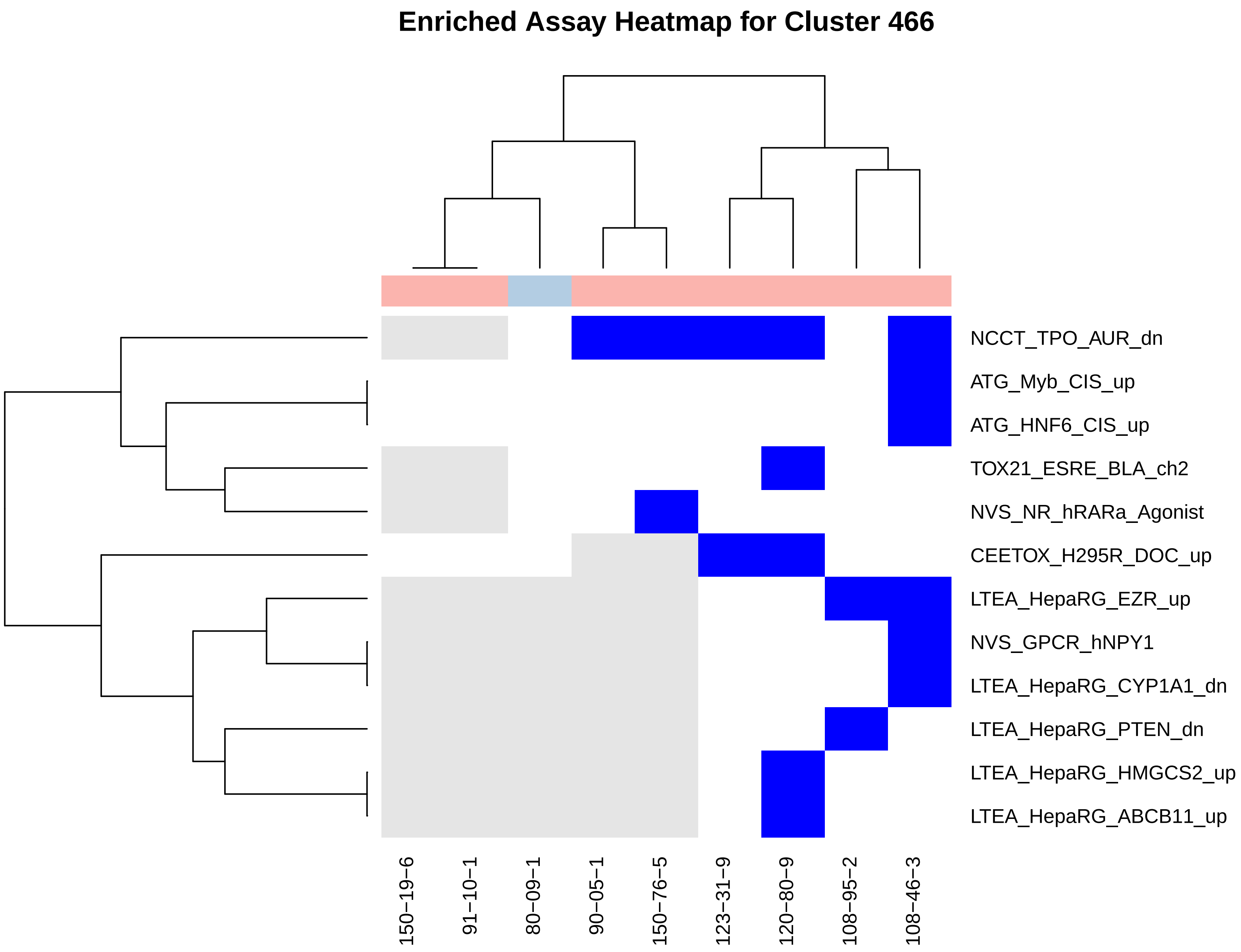

Enriched Assay Heatmap for Cluster 466
NCCT_TPO_AUR_dn
ATG_Myb_CIS_up
ATG_HNF6_CIS_up
TOX21_ESRE_BLA_ch2
NVS_NR_hRARa_Agonist
CEETOX_H295R_DOC_up
LTEA_HepaRG_EZR_up
NVS_GPCR_hNPY1
LTEA_HepaRG_CYP1A1_dn
LTEA_HepaRG_PTEN_dn
LTEA_HepaRG_HMGCS2_up
LTEA_HepaRG_ABCB11_up
150−19−6
91−10−1
80−09−1
90−05−1
150−76−5
123−31−9
120−80−9
108−95−2
108−46−3

Enriched Assay Heatmap for Cluster 467

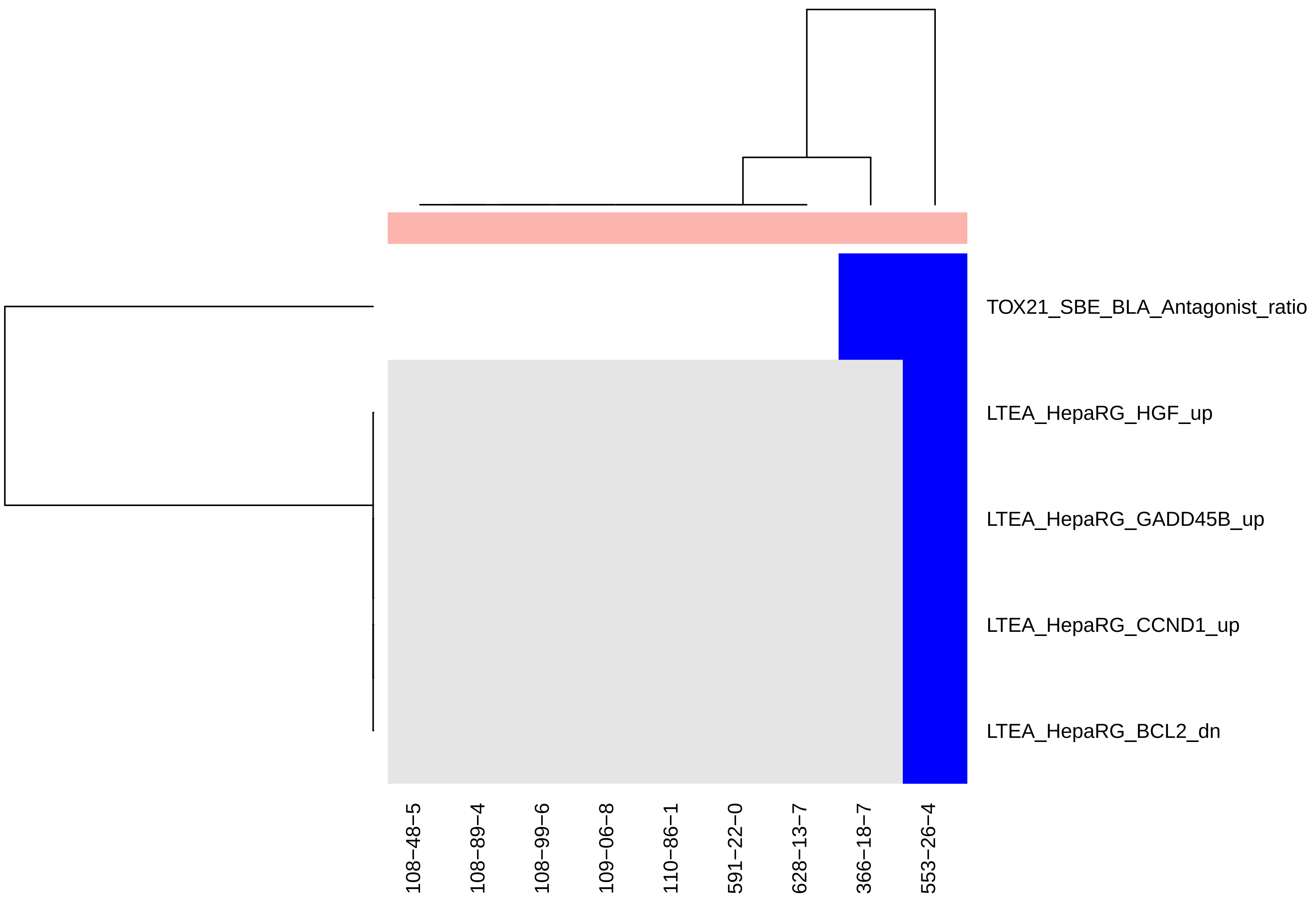

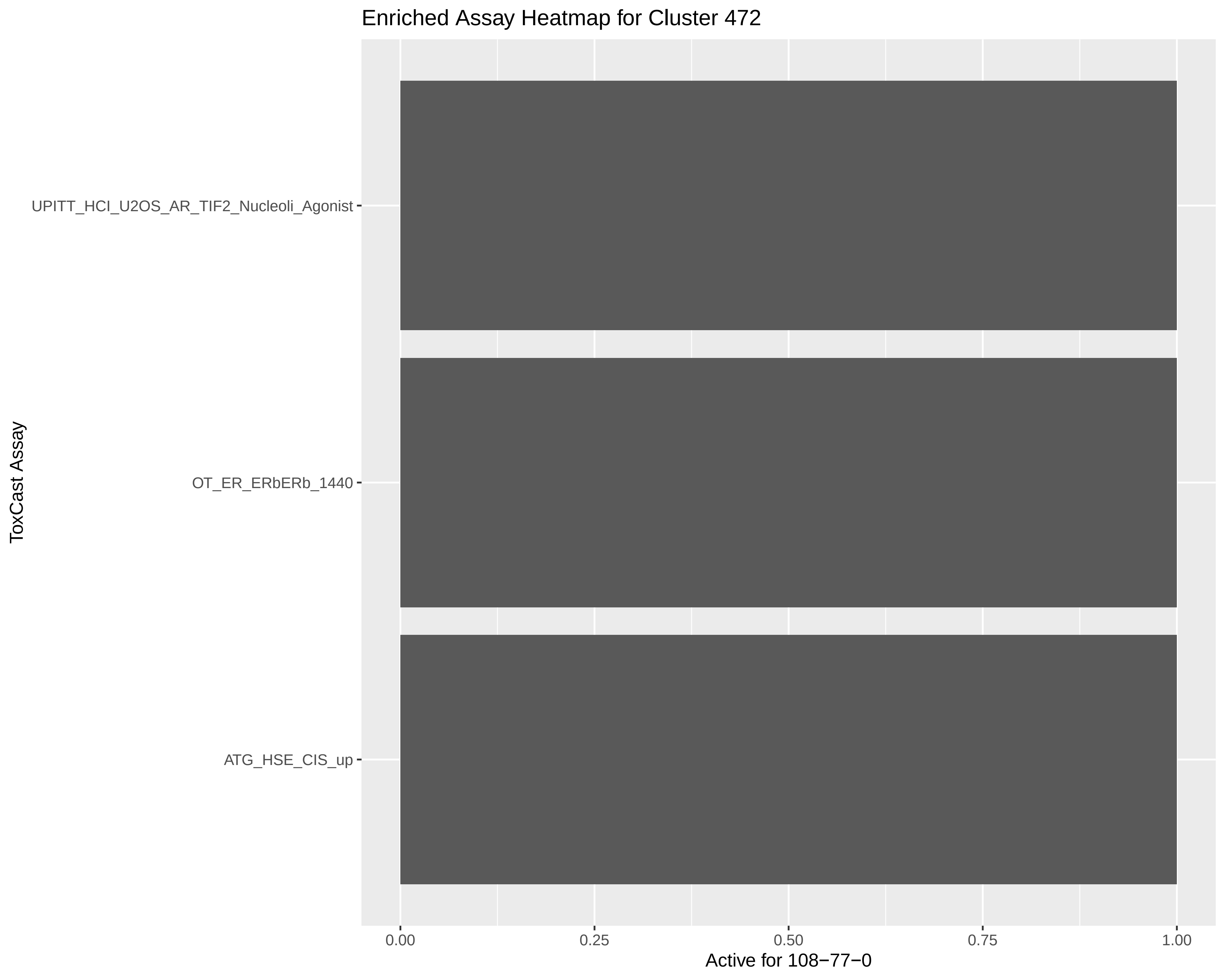
Enriched Assay Heatmap for Cluster 472
ToxCast Assay
UPITT_HCI_U2OS_AR_TIF2_Nucleoli_Agonist
OT_ER_ERbERb_1440
ATG_HSE_CIS_up
0.00
0.25
0.50
0.75
1.00
Active for 108−77−0

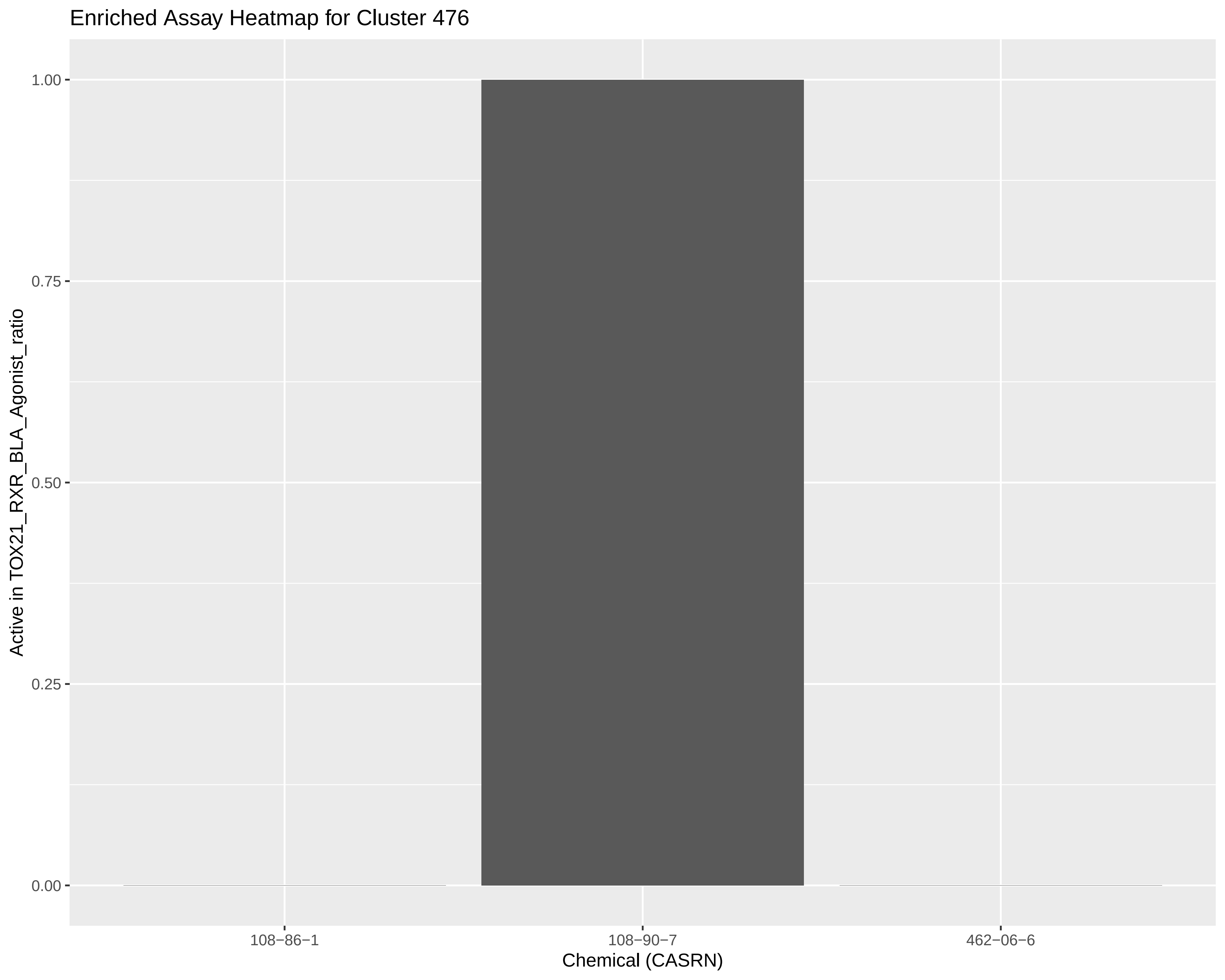

Enriched Assay Heatmap for Cluster 476
Active in TOX21_RXR_BLA_Agonist_ratio
1.00
0.75
0.50
0.25
0.00
108−86−1
108−90−7
462−06−6
Chemical (CASRN)

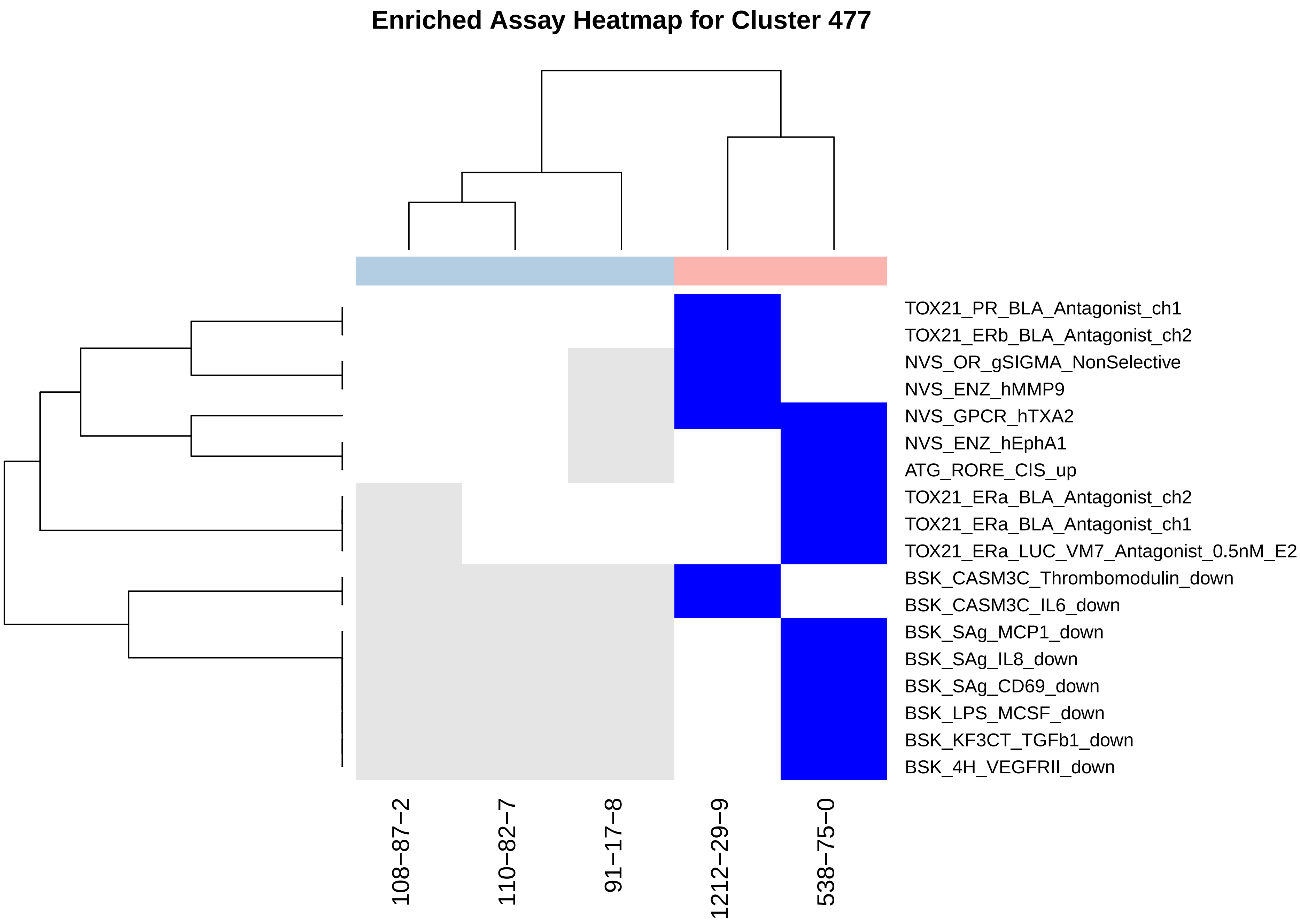
Enriched Assay Heatmap for Cluster 477
TOX21_PR_BLA_Antagonist_ch1
TOX21_ERb_BLA_Antagonist_ch2
NVS_OR_gSIGMA_NonSelective
NVS_ENZ_hMMP9
NVS_GPCR_hTXA2
NVS_ENZ_hEphA1
ATG_RORE_CIS_up
TOX21_ERa_BLA_Antagonist_ch2
TOX21_ERa_BLA_Antagonist_ch1
TOX21_ERa_LUC_VM7_Antagonist_0.5nM_E2
BSK_CASM3C_Thrombomodulin_down
BSK_CASM3C_IL6_down
BSK_SAg_MCP1_down
BSK_SAg_IL8_down
BSK_SAg_CD69_down
BSK_LPS_MCSF_down
BSK_KF3CT_TGFb1_down
BSK_4H_VEGFRII_down
108−87−2
110−82−7
91−17−8
1212−29−9
538−75−0

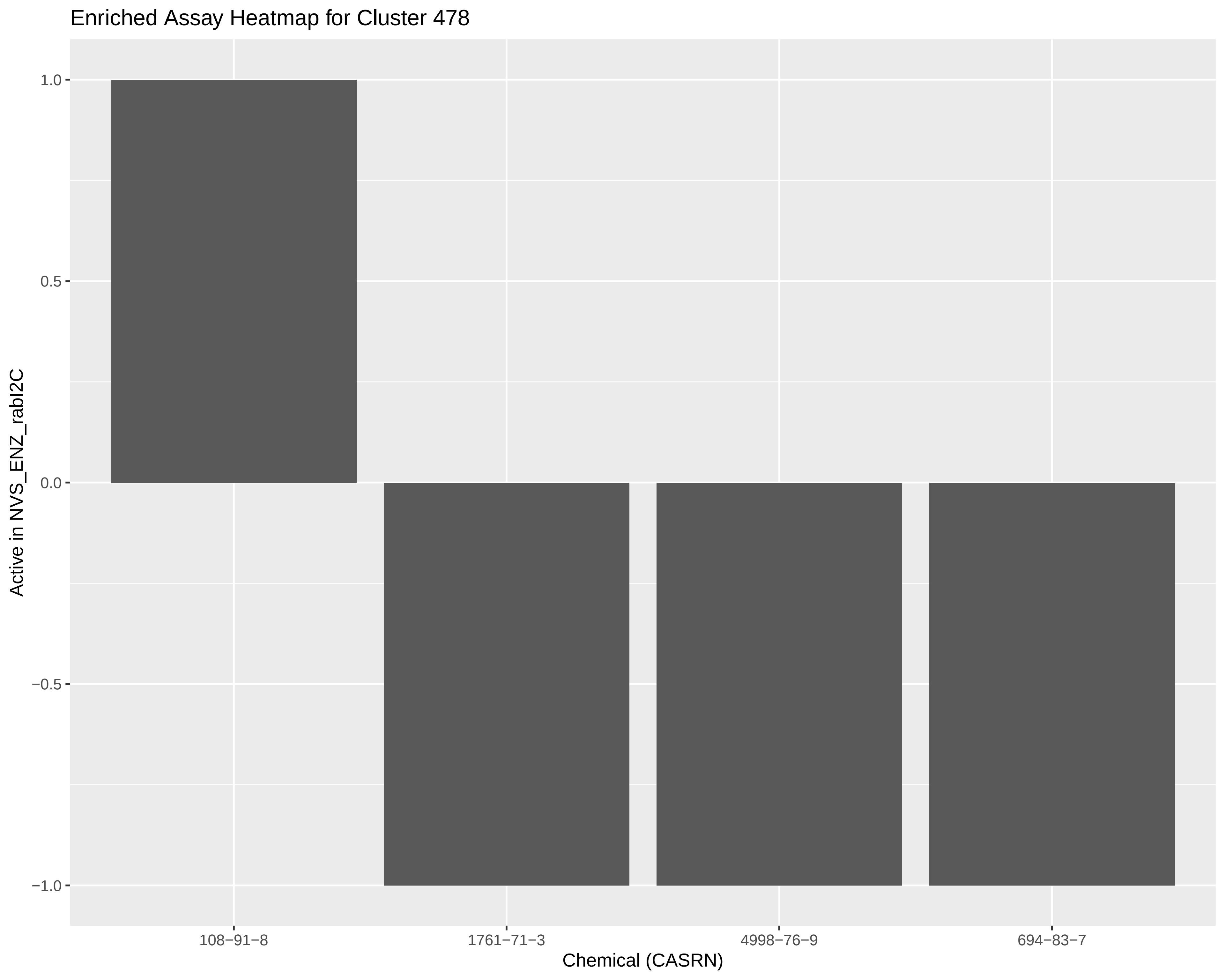
Enriched Assay Heatmap for Cluster 478
Active in NVS_ENZ_rabI2C
1.0
0.5
0.0
−0.5
−1.0
108−91−8
1761−71−3
4998−76−9
694−83−7
Chemical (CASRN)

Enriched Assay Heatmap for Cluster 479

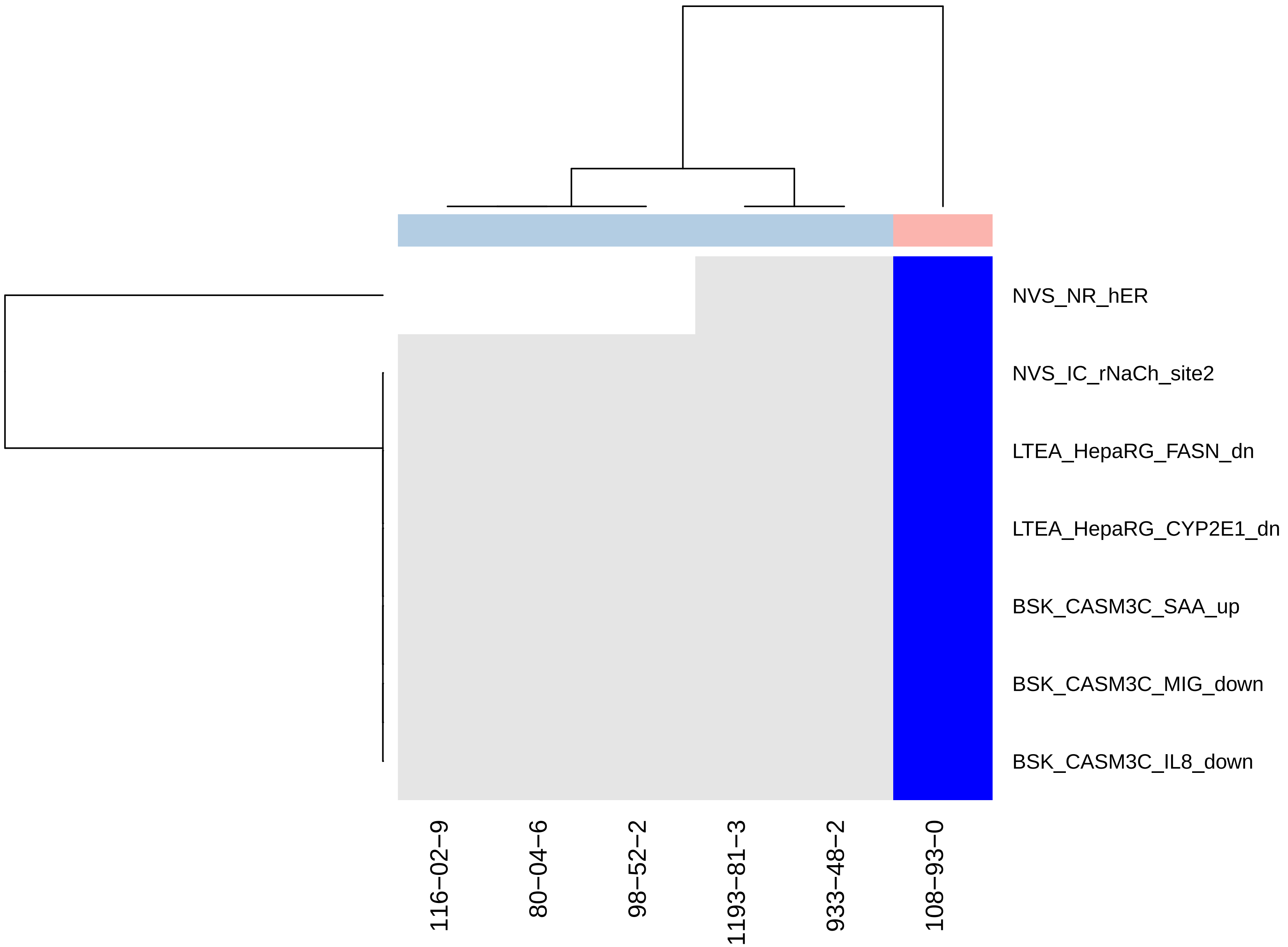

Enriched Assay Heatmap for Cluster 480

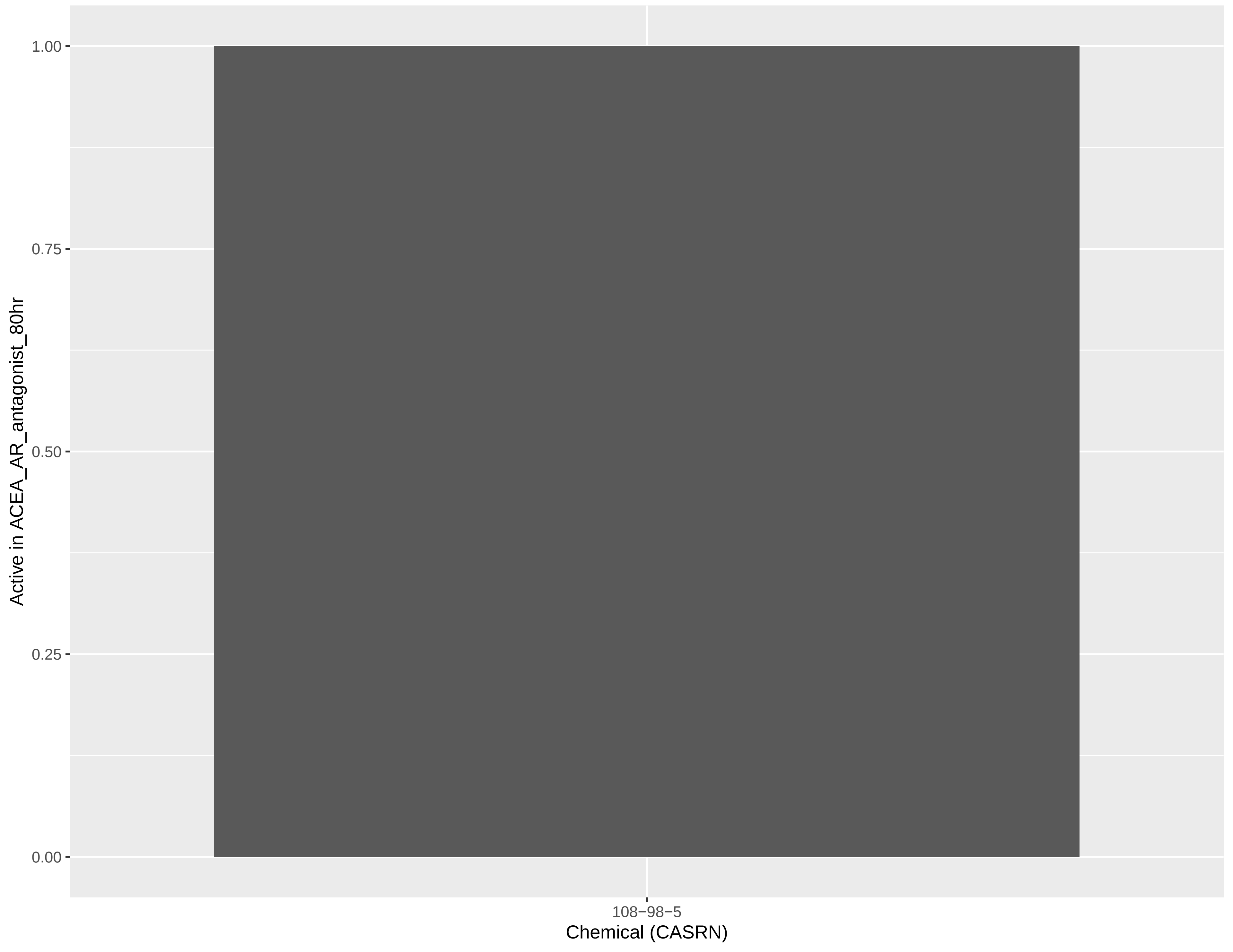

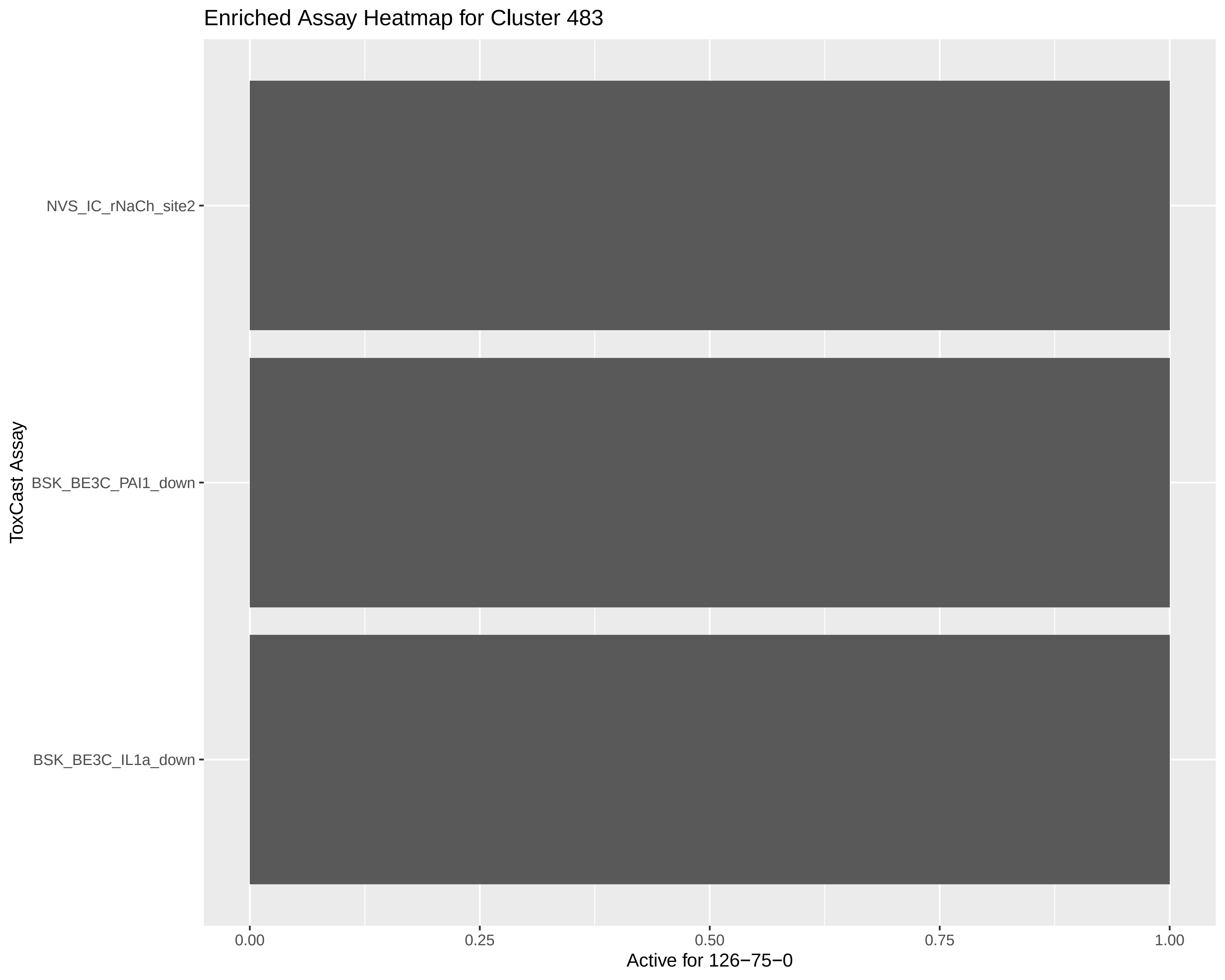

Enriched Assay Heatmap for Cluster 483
ToxCast Assay
NVS_IC_rNaCh_site2
BSK_BE3C_PAI1_down
BSK_BE3C_IL1a_down
0.00
0.25
0.50
0.75
1.00
Active for 126−75−0

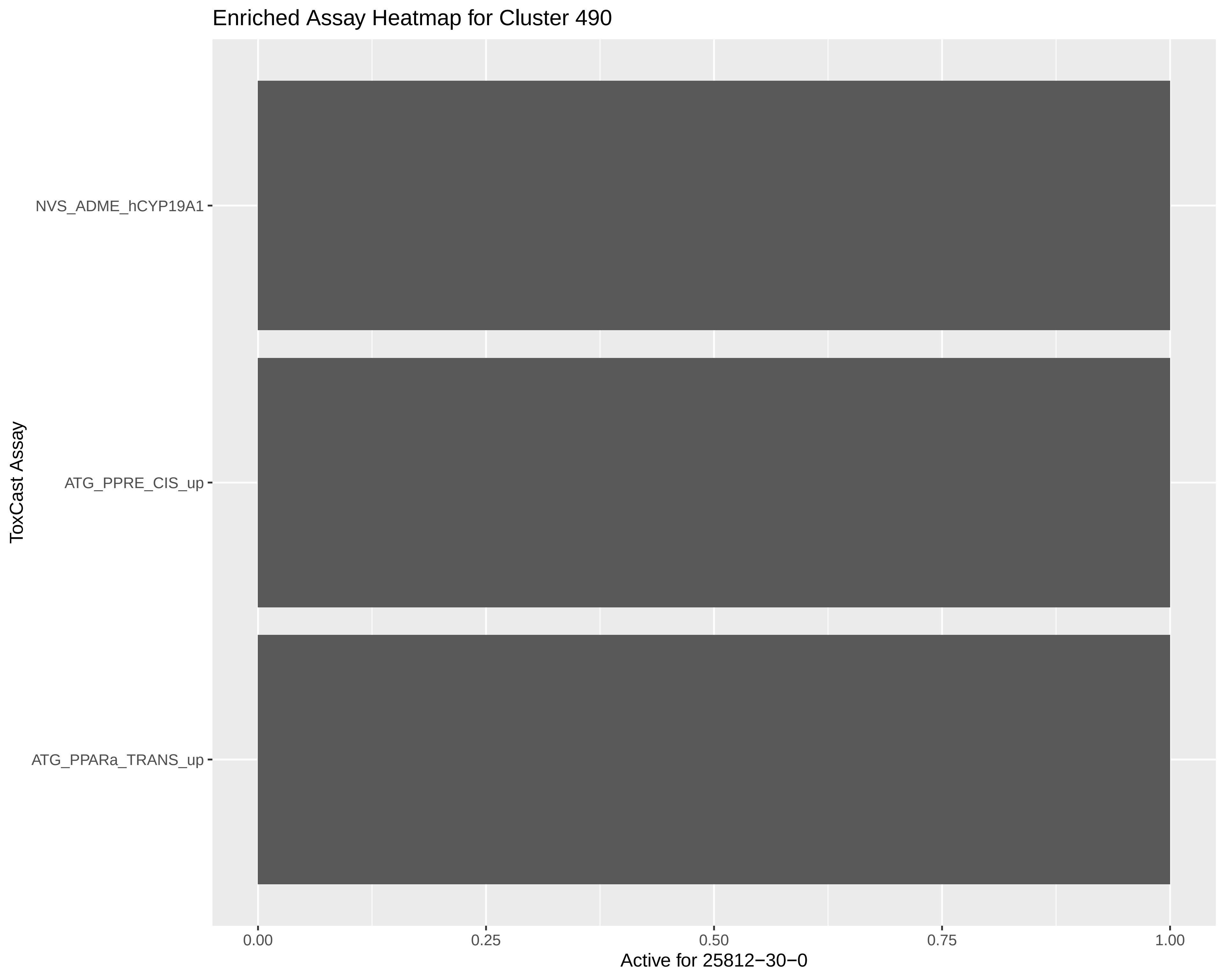

Enriched Assay Heatmap for Cluster 490
NVS_ADME_hCYP19A1
ATG_PPRE_CIS_up
ATG_PPARa_TRANS_up
ToxCast Assay
0.00
0.25
0.50
0.75
1.00
Active for 25812−30−0

Enriched Assay Heatmap for Cluster 491

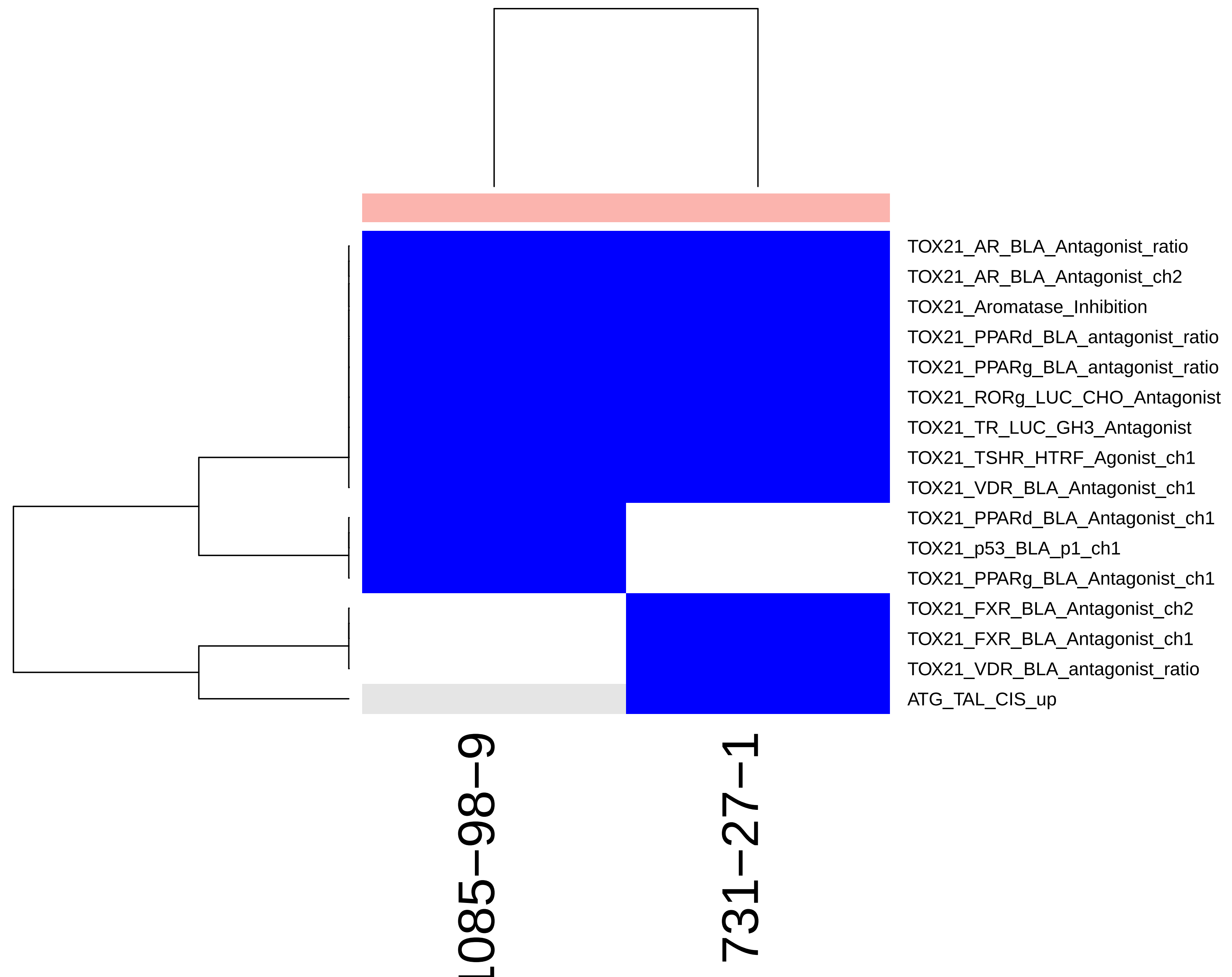

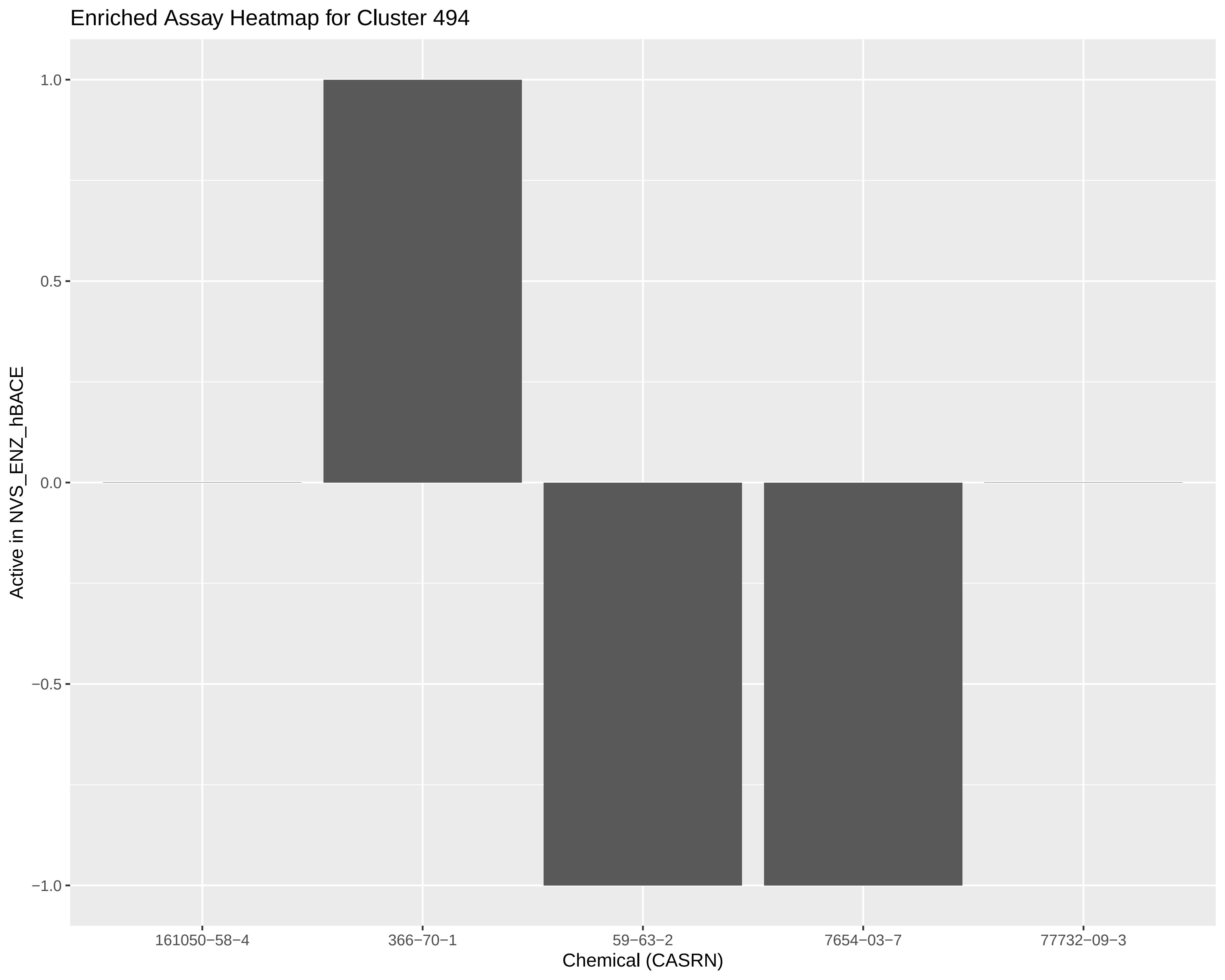
Enriched Assay Heatmap for Cluster 494
Active in NVS_ENZ_hBACE
1.0
0.5
0.0
−0.5
−1.0
161050−58−4
366−70−1
59−63−2
7654−03−7
77732−09−3
Chemical (CASRN)

Enriched Assay Heatmap for Cluster 495

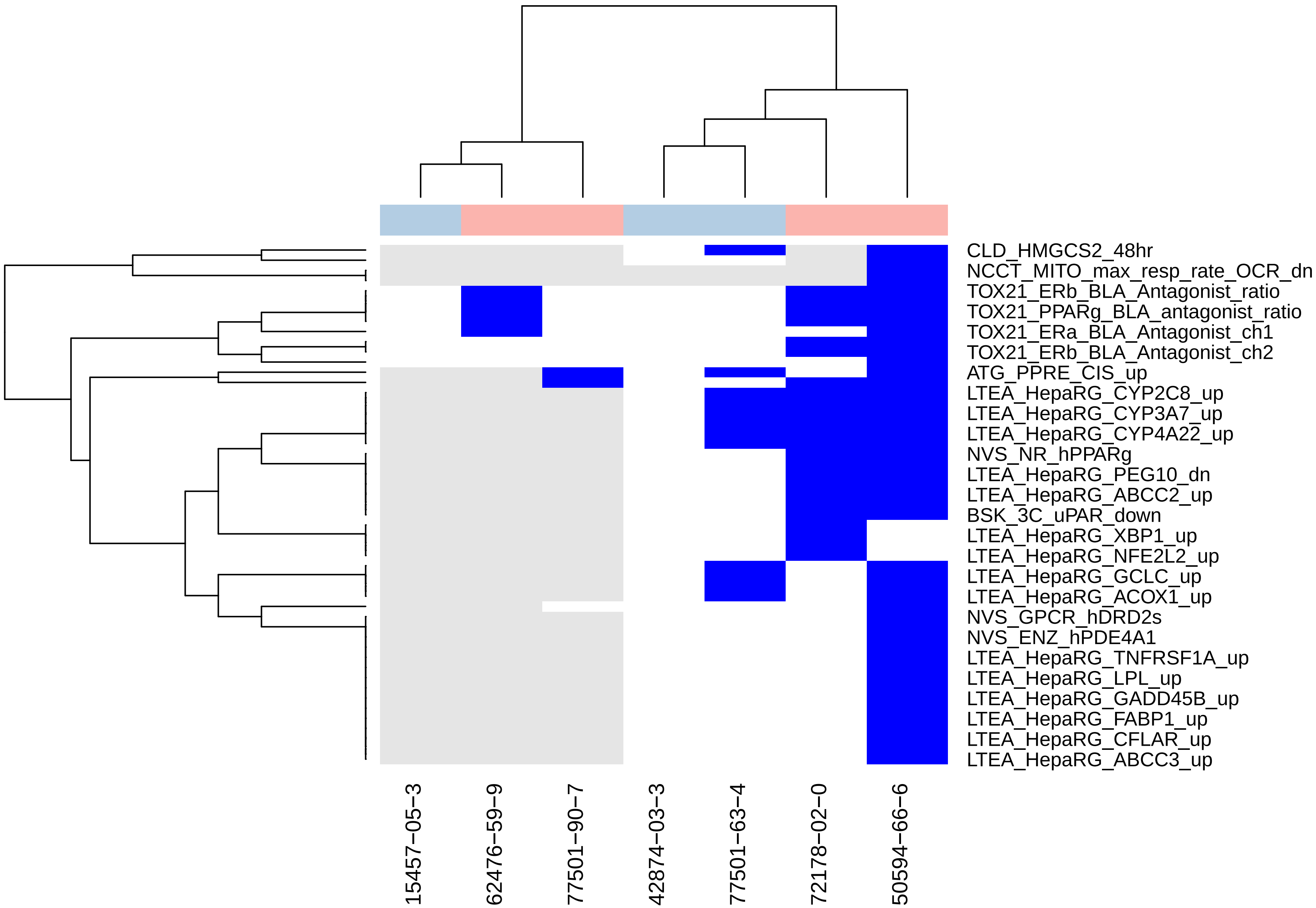

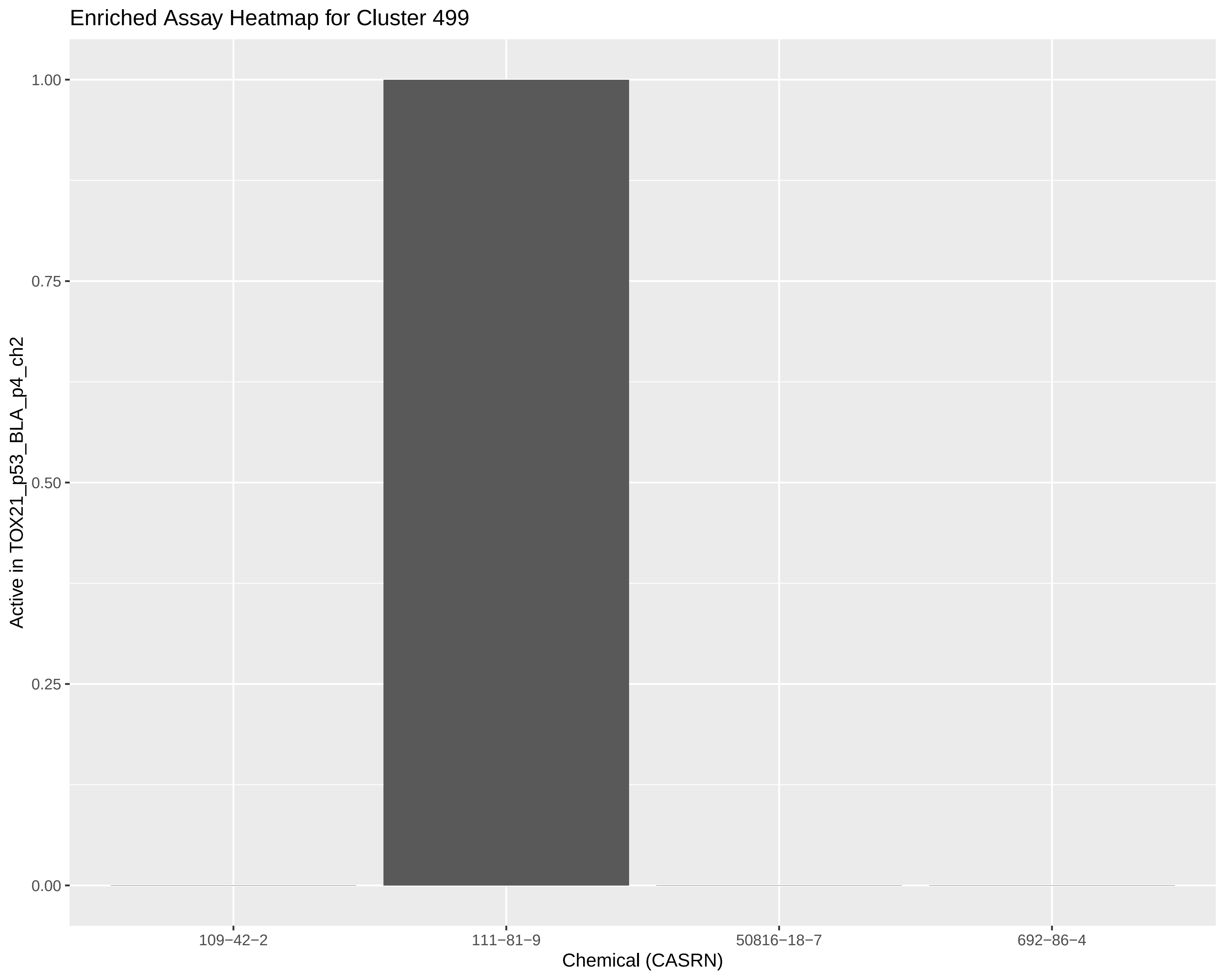

Enriched Assay Heatmap for Cluster 499
Active in TOX21_p53_BLA_p4_ch2
1.00
0.75
0.50
0.25
0.00
109−42−2
111−81−9
50816−18−7
692−86−4
Chemical (CASRN)

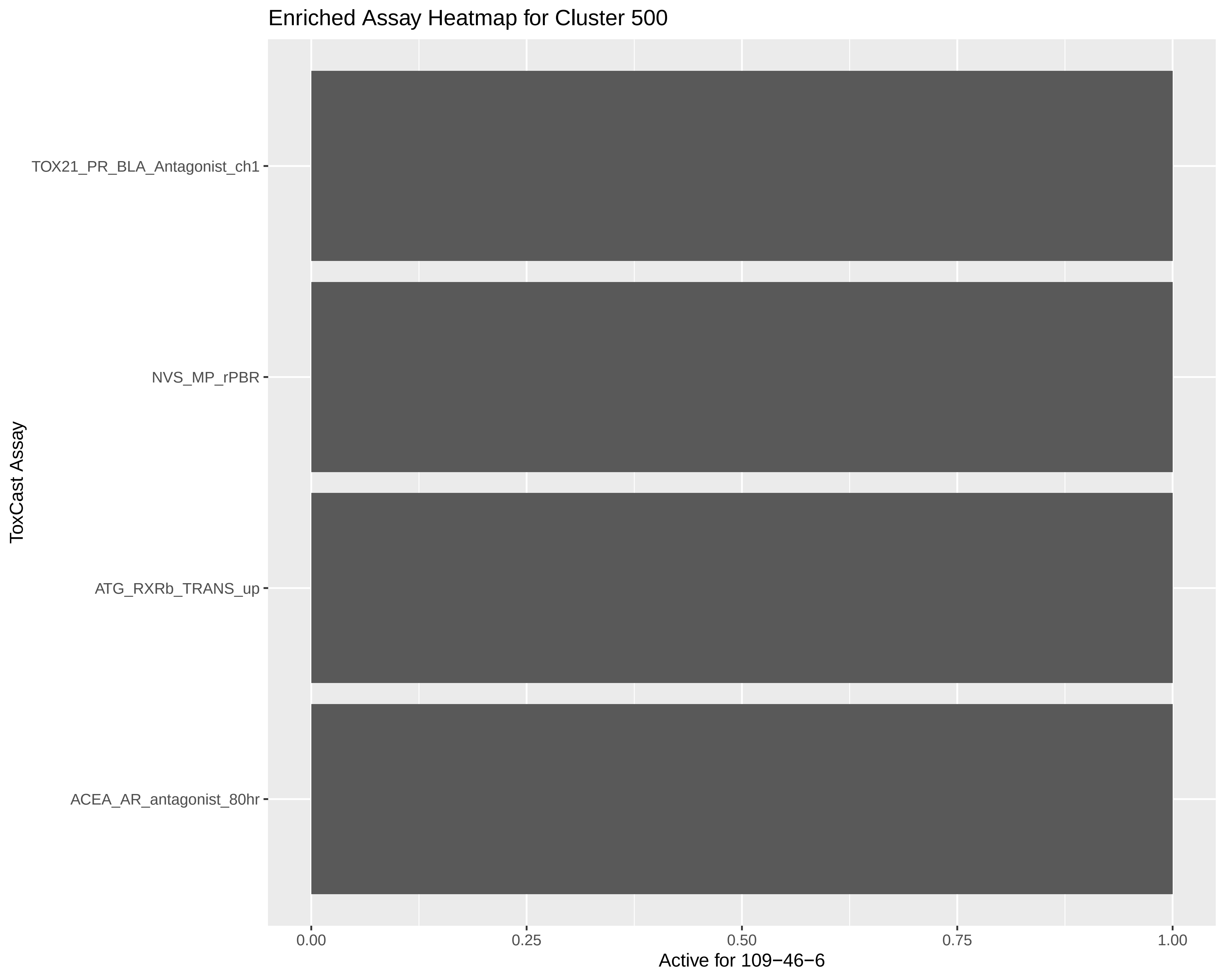
Enriched Assay Heatmap for Cluster 500
ToxCast Assay
TOX21_PR_BLA_Antagonist_ch1
NVS_MP_rPBR
ATG_RXRb_TRANS_up
ACEA_AR_antagonist_80hr
0.00
0.25
0.50
0.75
1.00
Active for 109−46−6

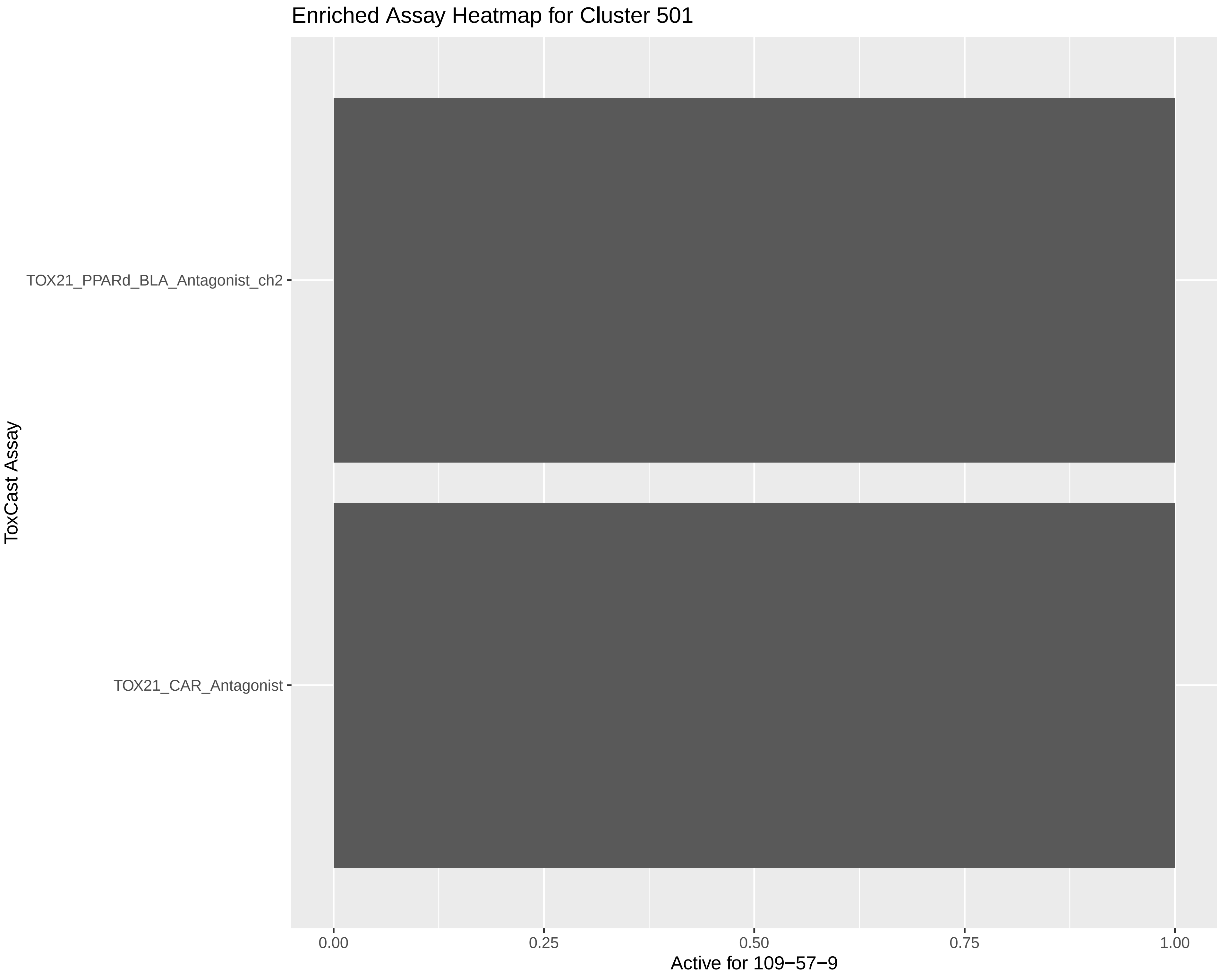

Enriched Assay Heatmap for Cluster 501
TOX21_PPARd_BLA_Antagonist_ch2
TOX21_CAR_Antagonist
ToxCast Assay
0.00
0.25
0.50
0.75
1.00
Active for 109−57−9

Enriched Assay Heatmap for Cluster 505

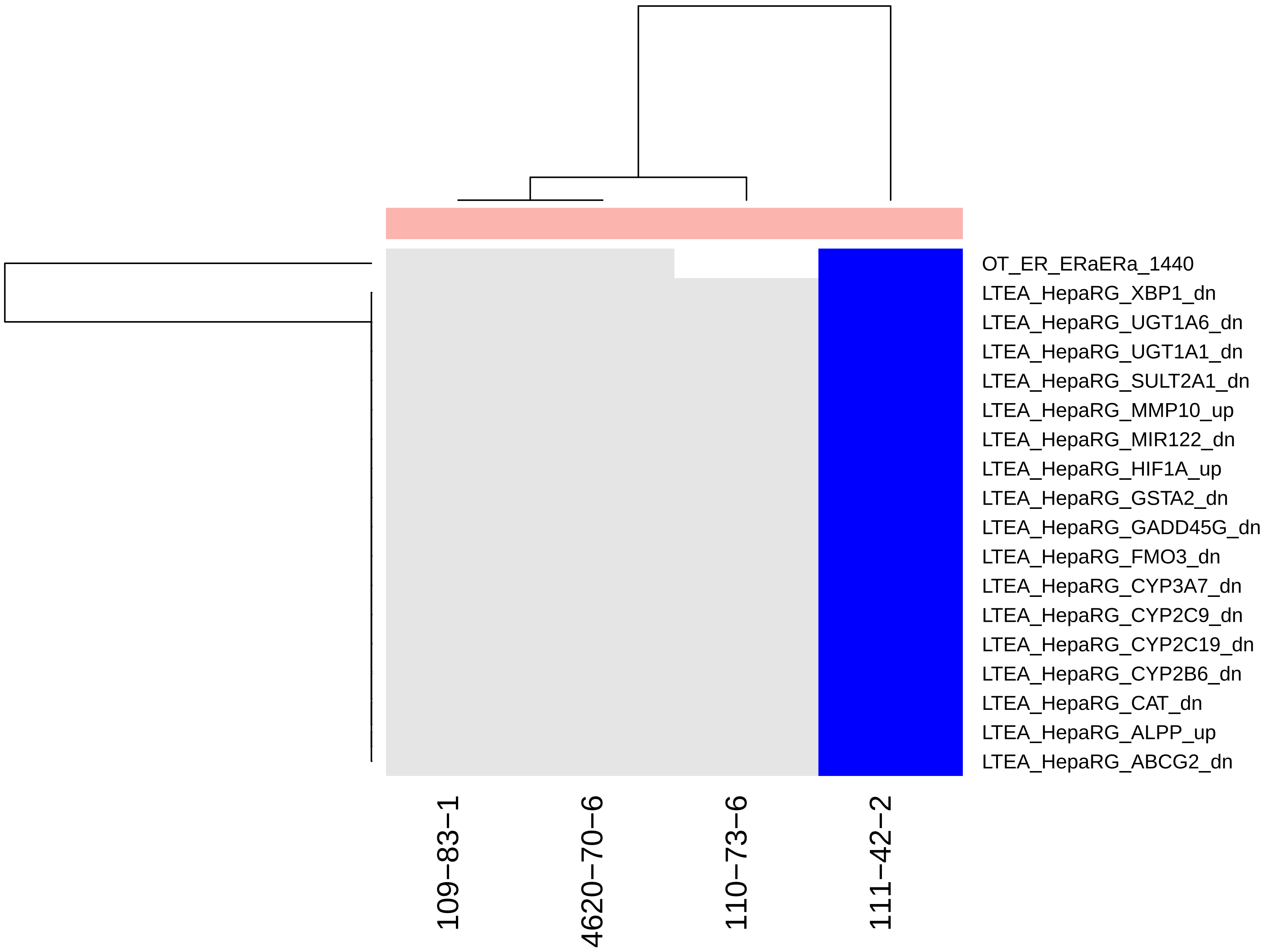

Enriched Assay Heatmap for Cluster 511

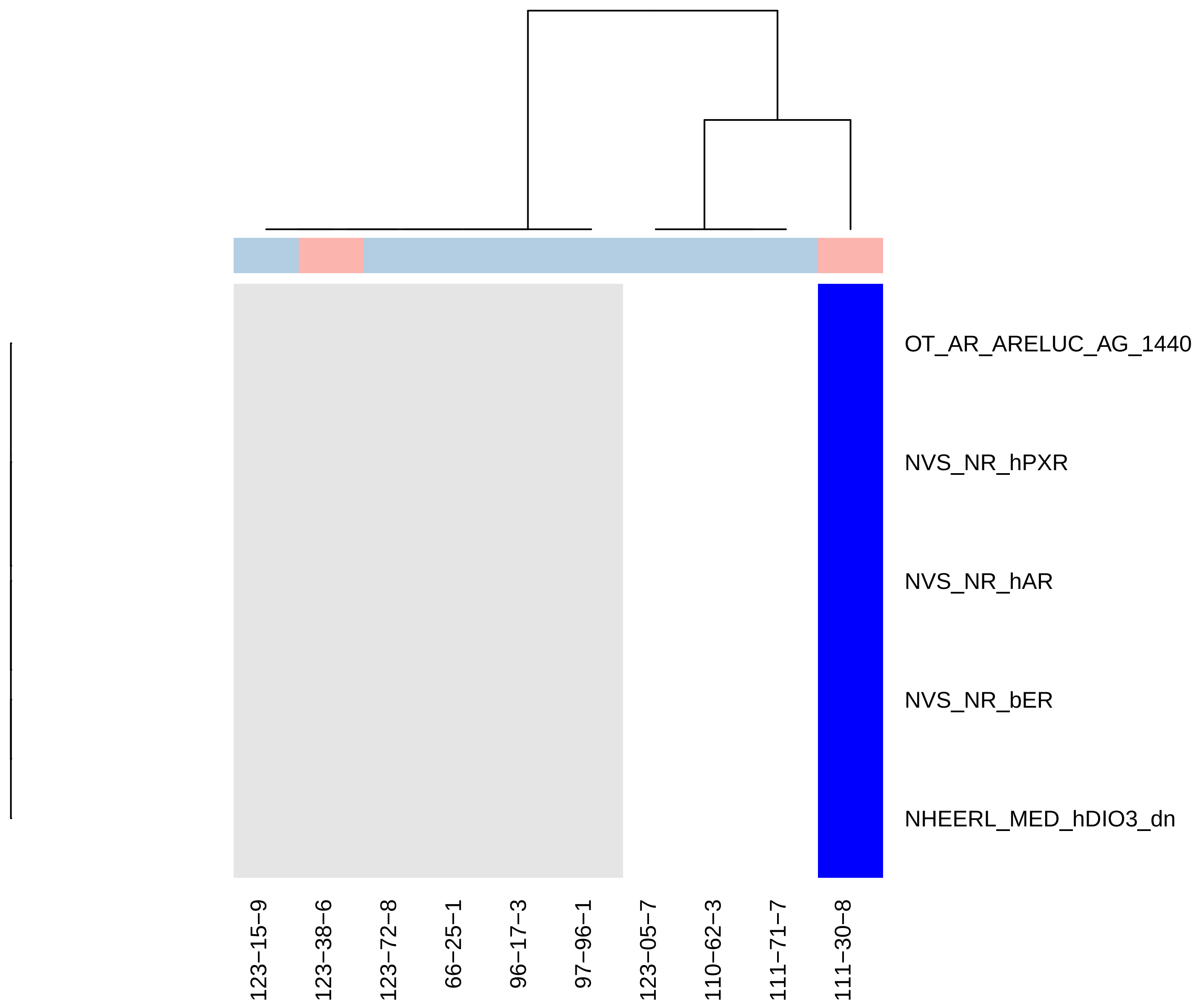

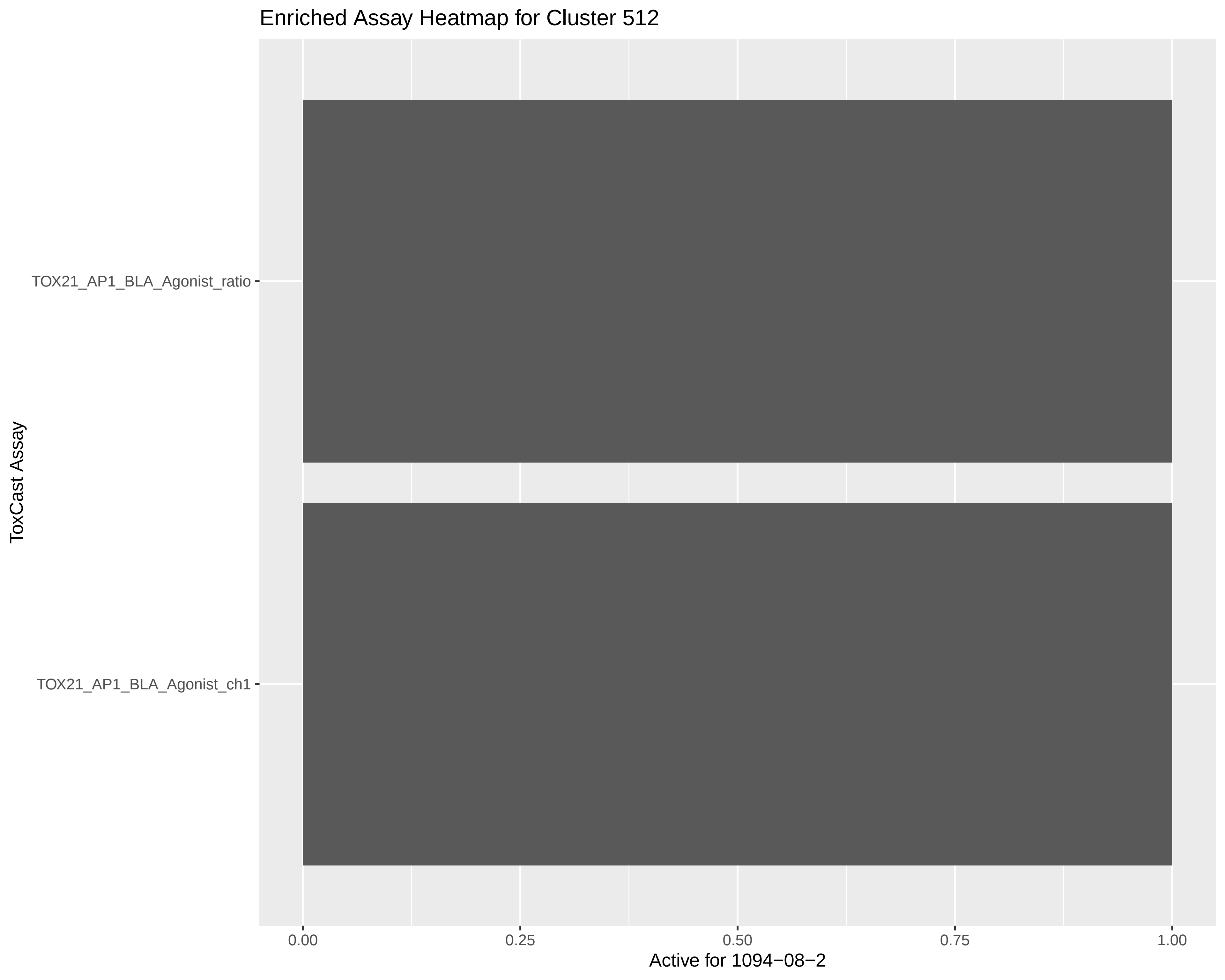

Enriched Assay Heatmap for Cluster 512
ToxCast Assay
TOX21_AP1_BLA_Agonist_ratio
TOX21_AP1_BLA_Agonist_ch1
0.00
0.25
0.50
0.75
1.00
Active for 1094−08−2

Enriched Assay Heatmap for Cluster 513

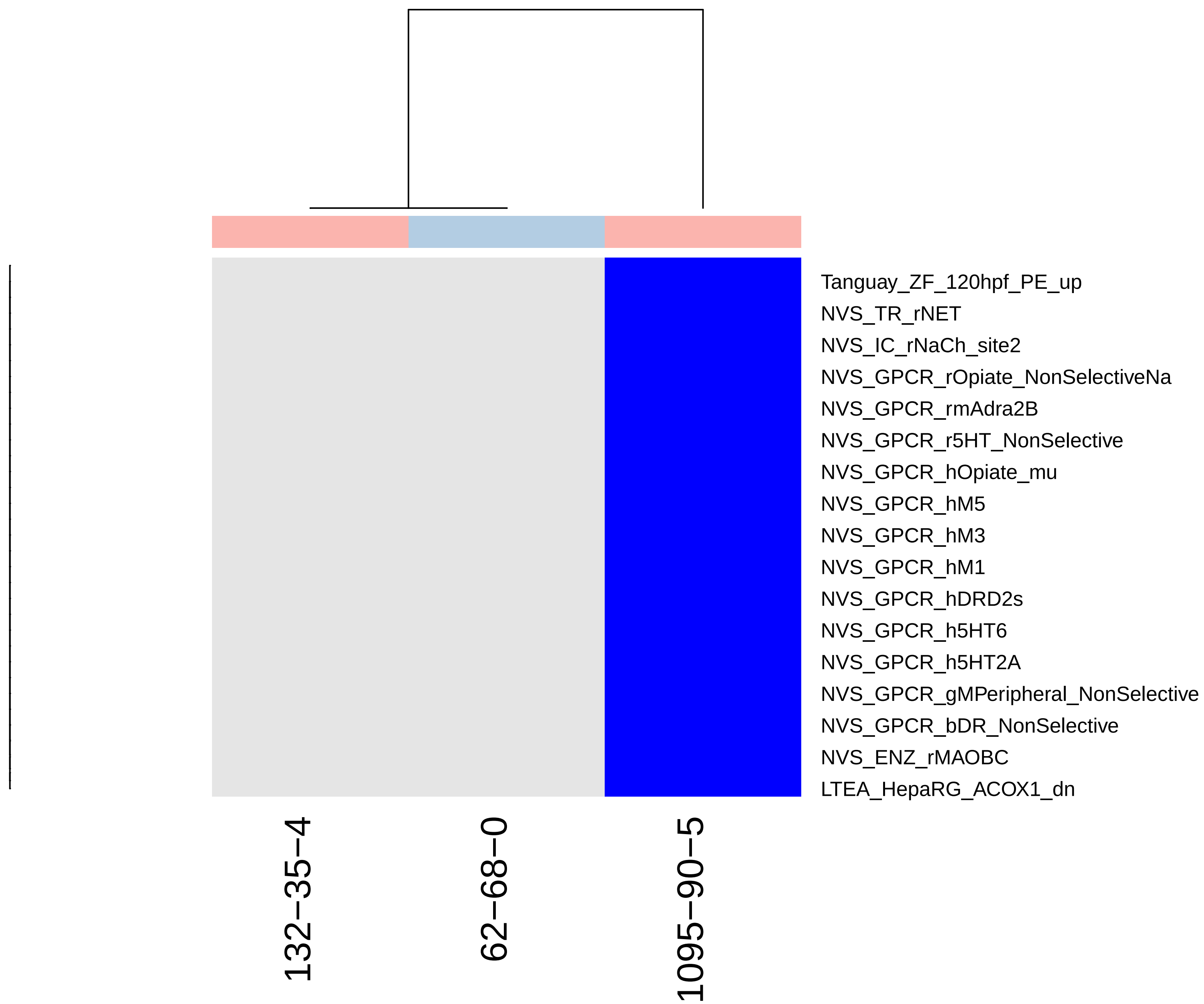

Enriched Assay Heatmap for Cluster 515

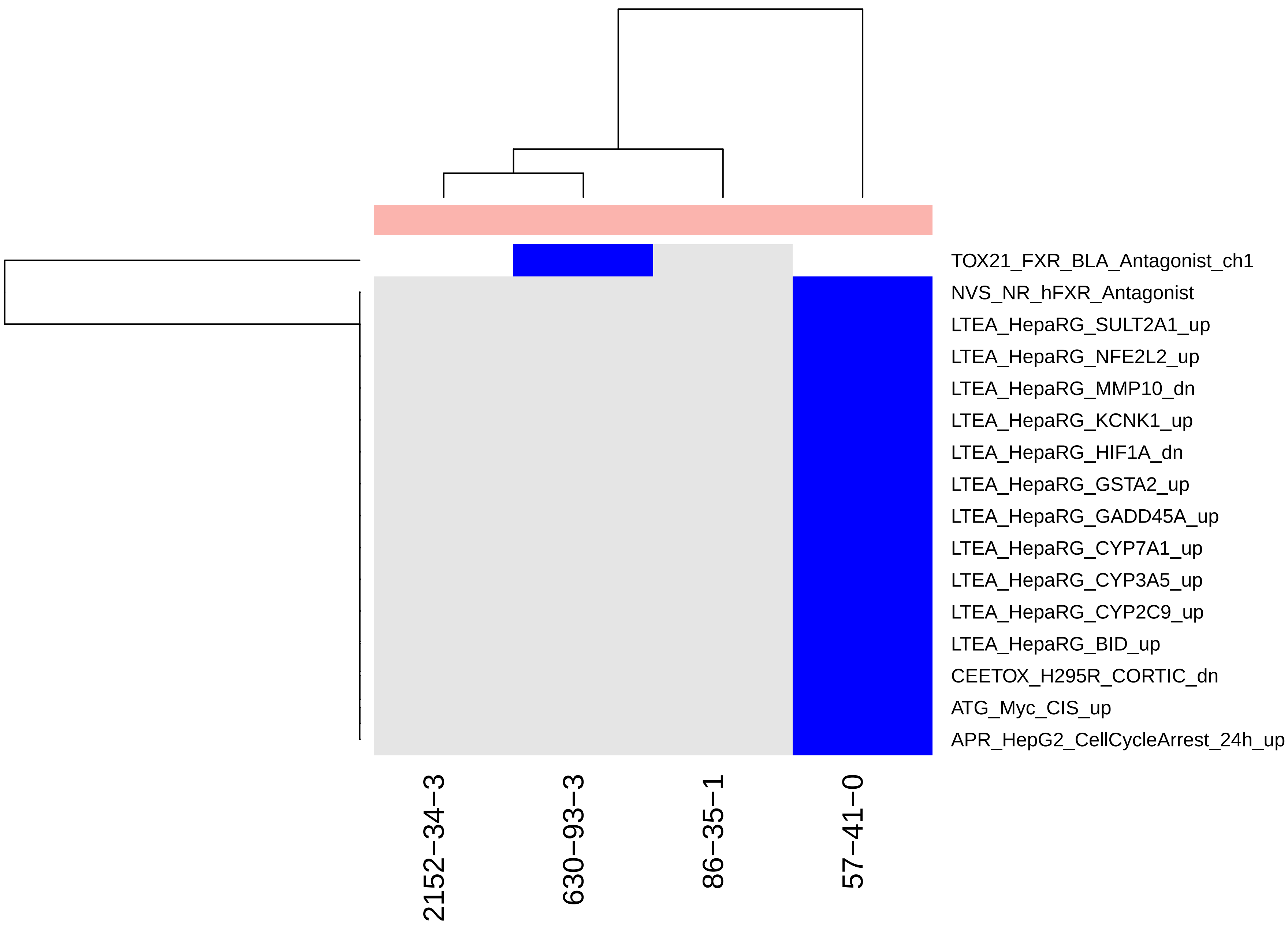

Enriched Assay Heatmap for Cluster 522

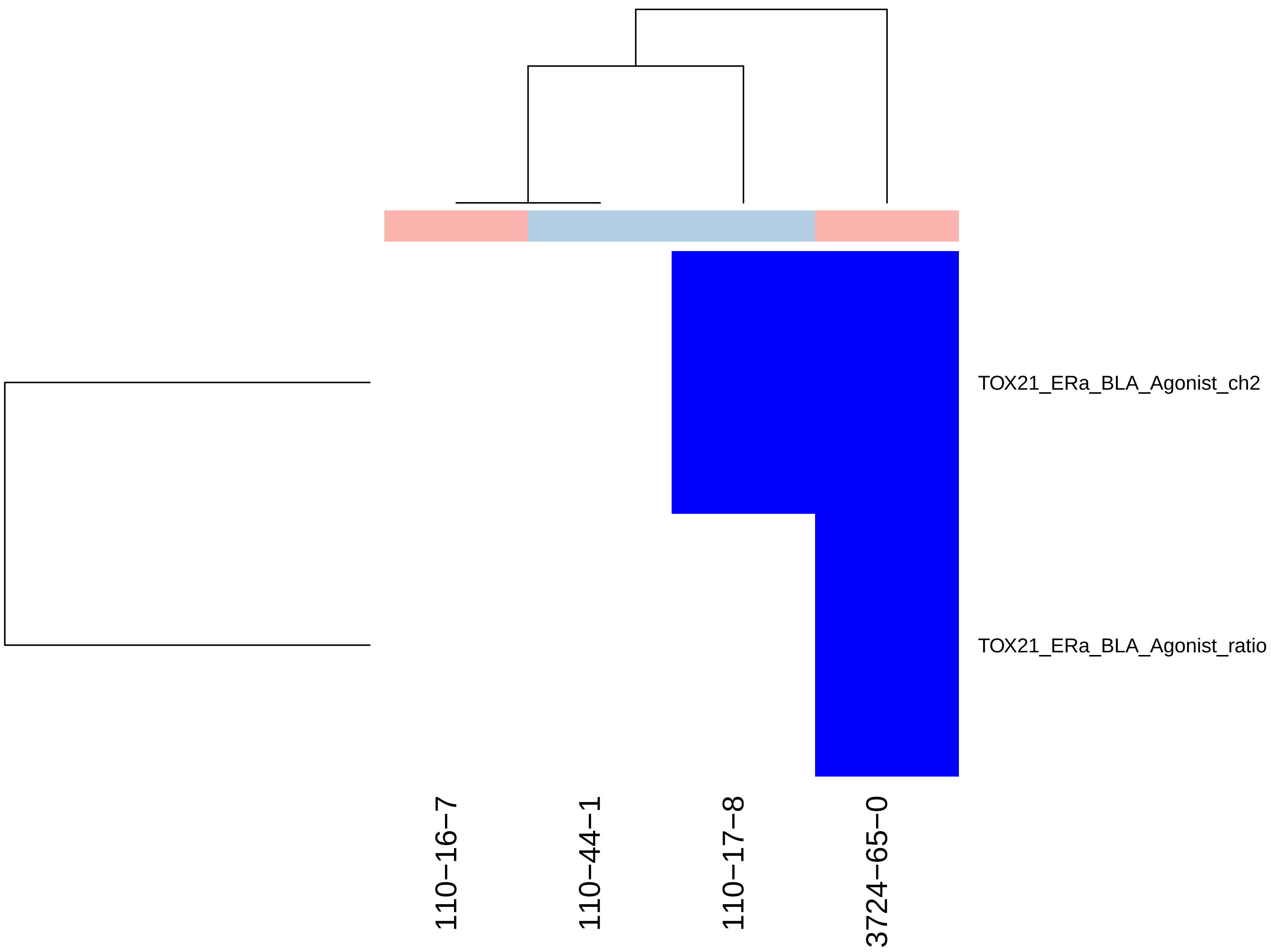

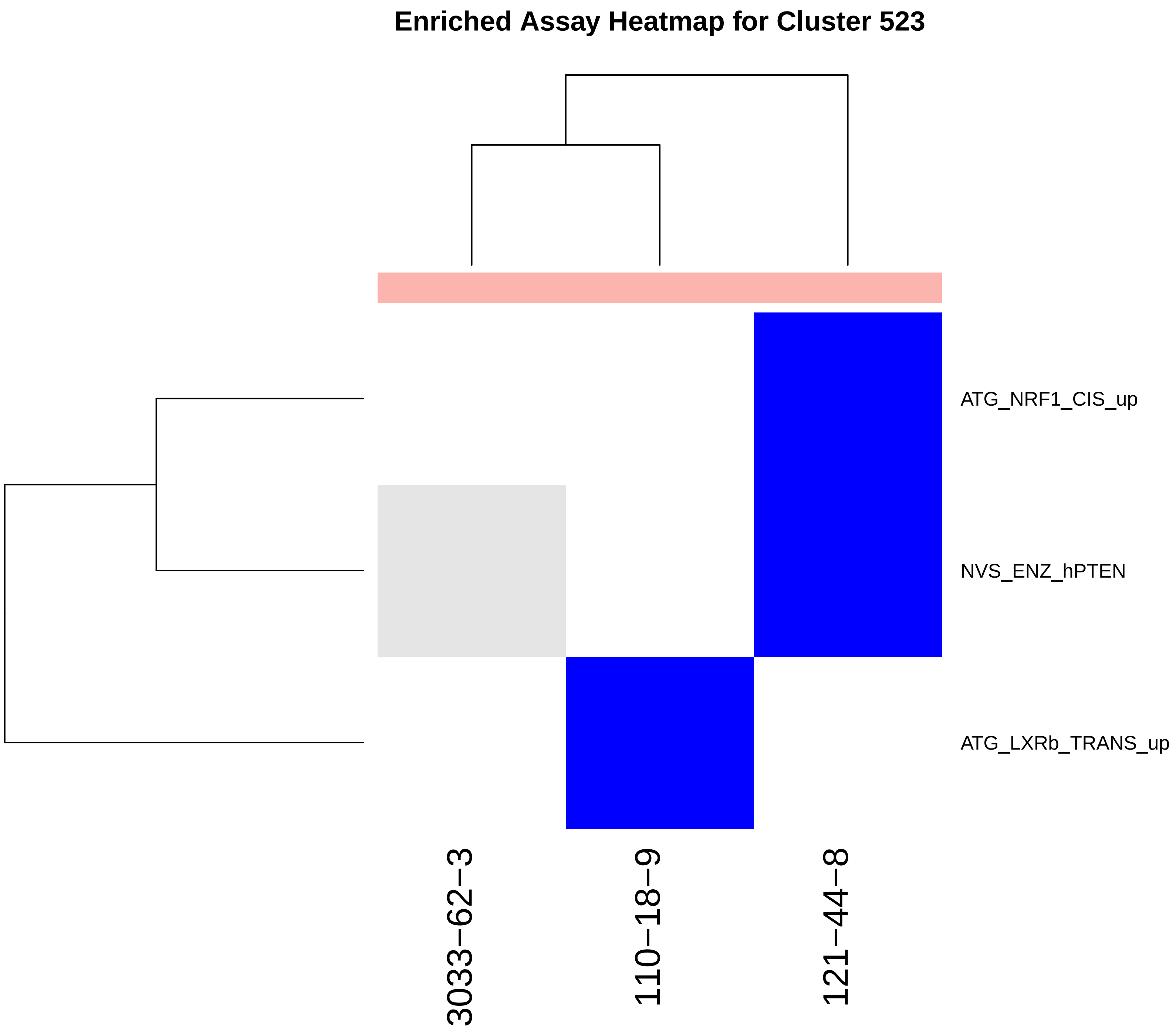

Enriched Assay Heatmap for Cluster 523
ATG_NRF1_CIS_up
NVS_ENZ_hPTEN
ATG_LXRb_TRANS_up
3033−62−3
110−18−9
121−44−8

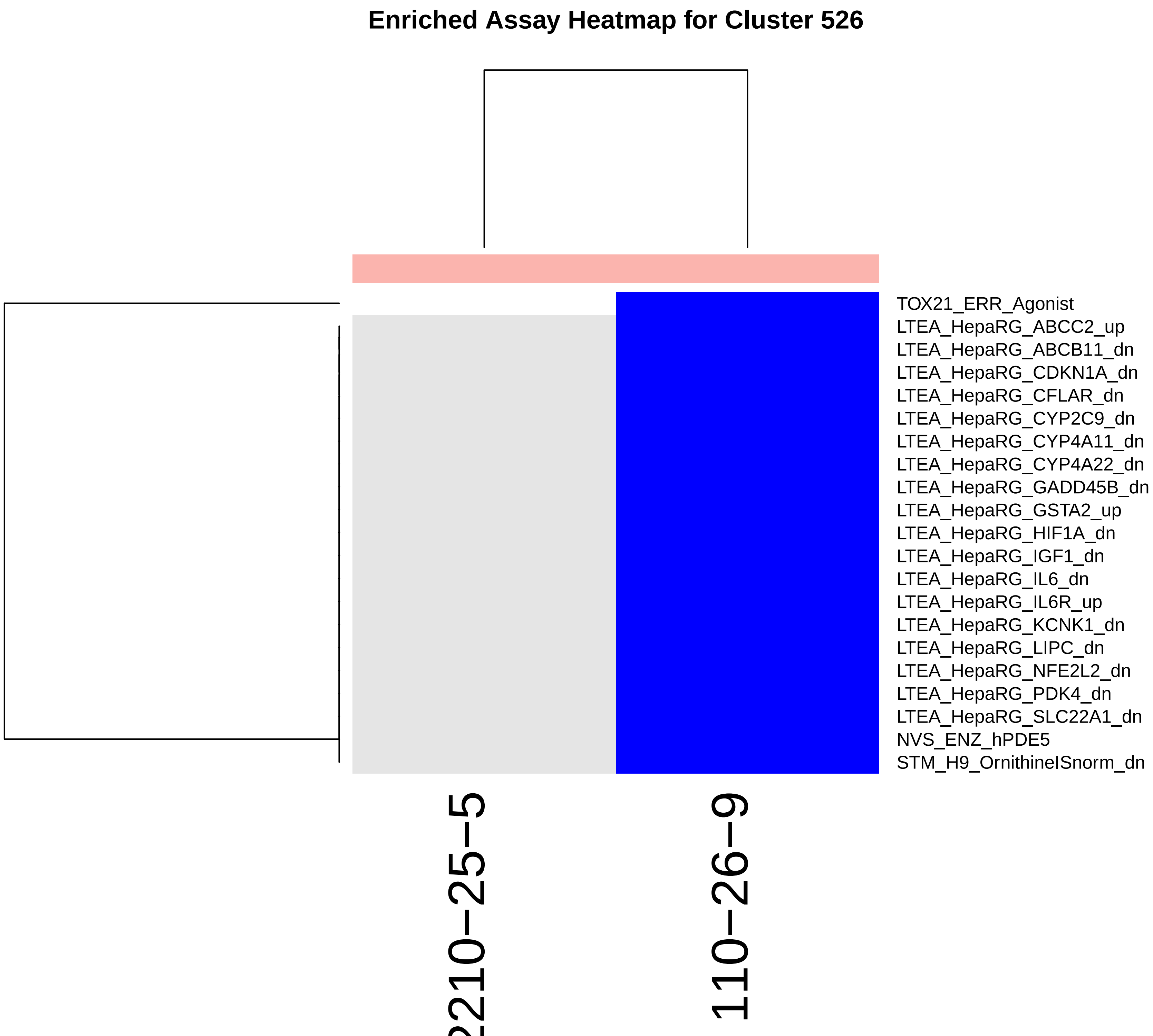

Enriched Assay Heatmap for Cluster 526
TOX21_ERR_Agonist
LTEA_HepaRG_ABCC2_up
LTEA_HepaRG_ABCB11_dn
LTEA_HepaRG_CDKN1A_dn
LTEA_HepaRG_CFLAR_dn
LTEA_HepaRG_CYP2C9_dn
LTEA_HepaRG_CYP4A11_dn
LTEA_HepaRG_CYP4A22_dn
LTEA_HepaRG_GADD45B_dn
LTEA_HepaRG_GSTA2_up
LTEA_HepaRG_HIF1A_dn
LTEA_HepaRG_IGF1_dn
LTEA_HepaRG_IL6_dn
LTEA_HepaRG_IL6R_up
LTEA_HepaRG_KCNK1_dn
LTEA_HepaRG_LIPC_dn
LTEA_HepaRG_NFE2L2_dn
LTEA_HepaRG_PDK4_dn
LTEA_HepaRG_SLC22A1_dn
NVS_ENZ_hPDE5
STM_H9_OrnithineISnorm_dn
2210−25−5
110−26−9

Enriched Assay Heatmap for Cluster 531

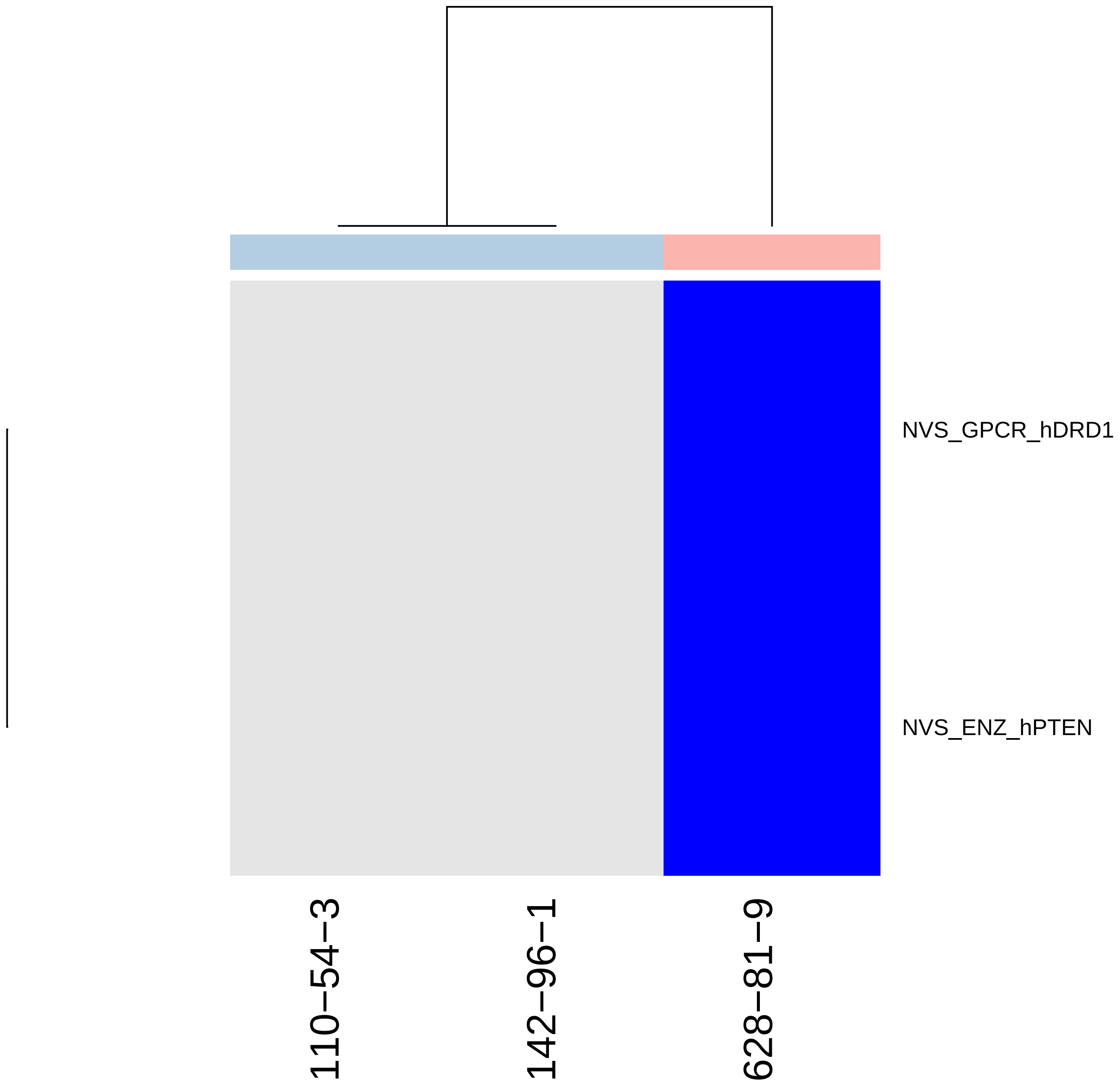

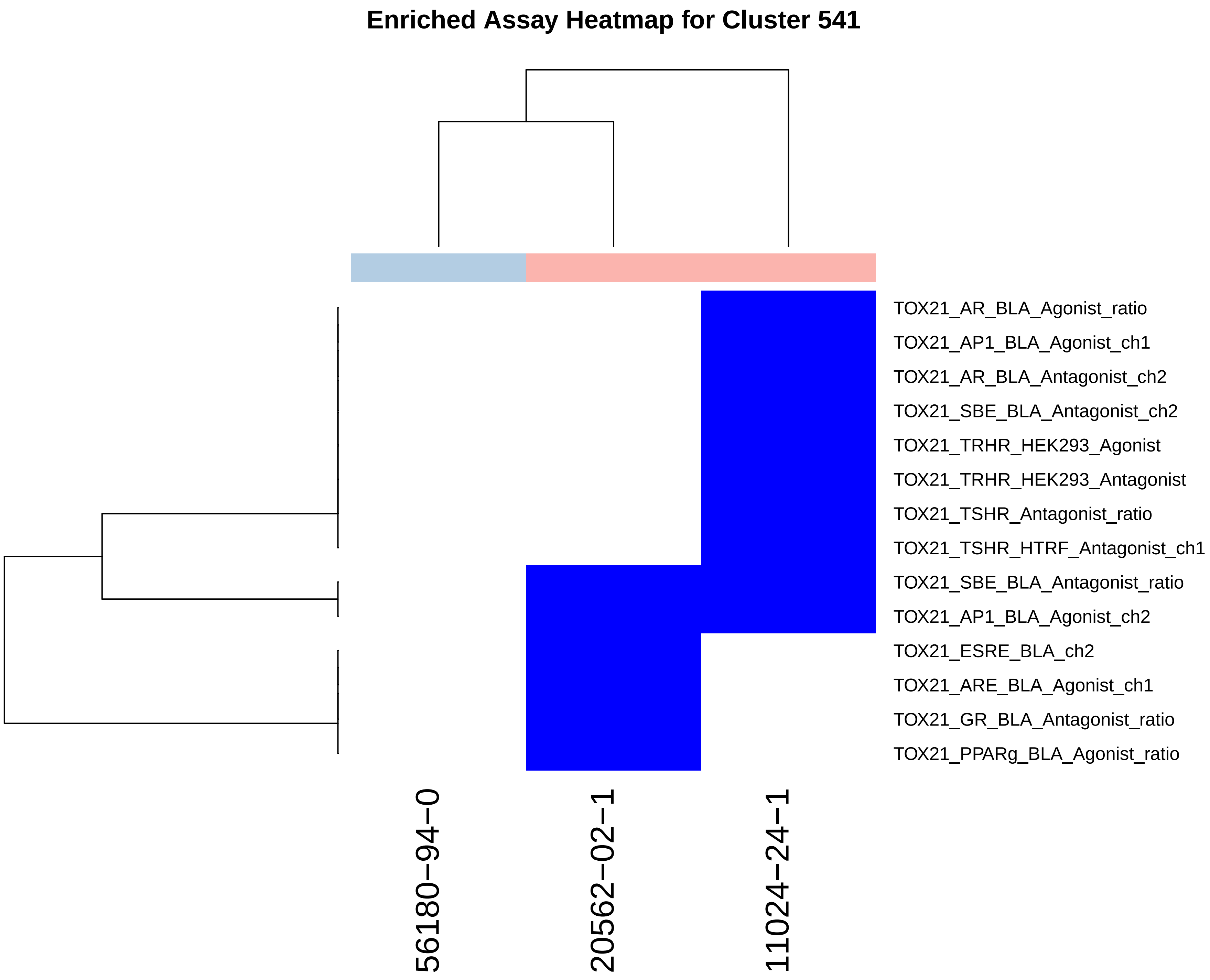
Enriched Assay Heatmap for Cluster 541
TOX21_AR_BLA_Agonist_ratio
TOX21_AP1_BLA_Agonist_ch1
TOX21_AR_BLA_Antagonist_ch2
TOX21_SBE_BLA_Antagonist_ch2
TOX21_TRHR_HEK293_Agonist
TOX21_TRHR_HEK293_Antagonist
TOX21_TSHR_Antagonist_ratio
TOX21_TSHR_HTRF_Antagonist_ch1
TOX21_SBE_BLA_Antagonist_ratio
TOX21_AP1_BLA_Agonist_ch2
TOX21_ESRE_BLA_ch2
TOX21_ARE_BLA_Agonist_ch1
TOX21_GR_BLA_Antagonist_ratio
TOX21_PPARg_BLA_Agonist_ratio
56180−94−0
20562−02−1
11024−24−1

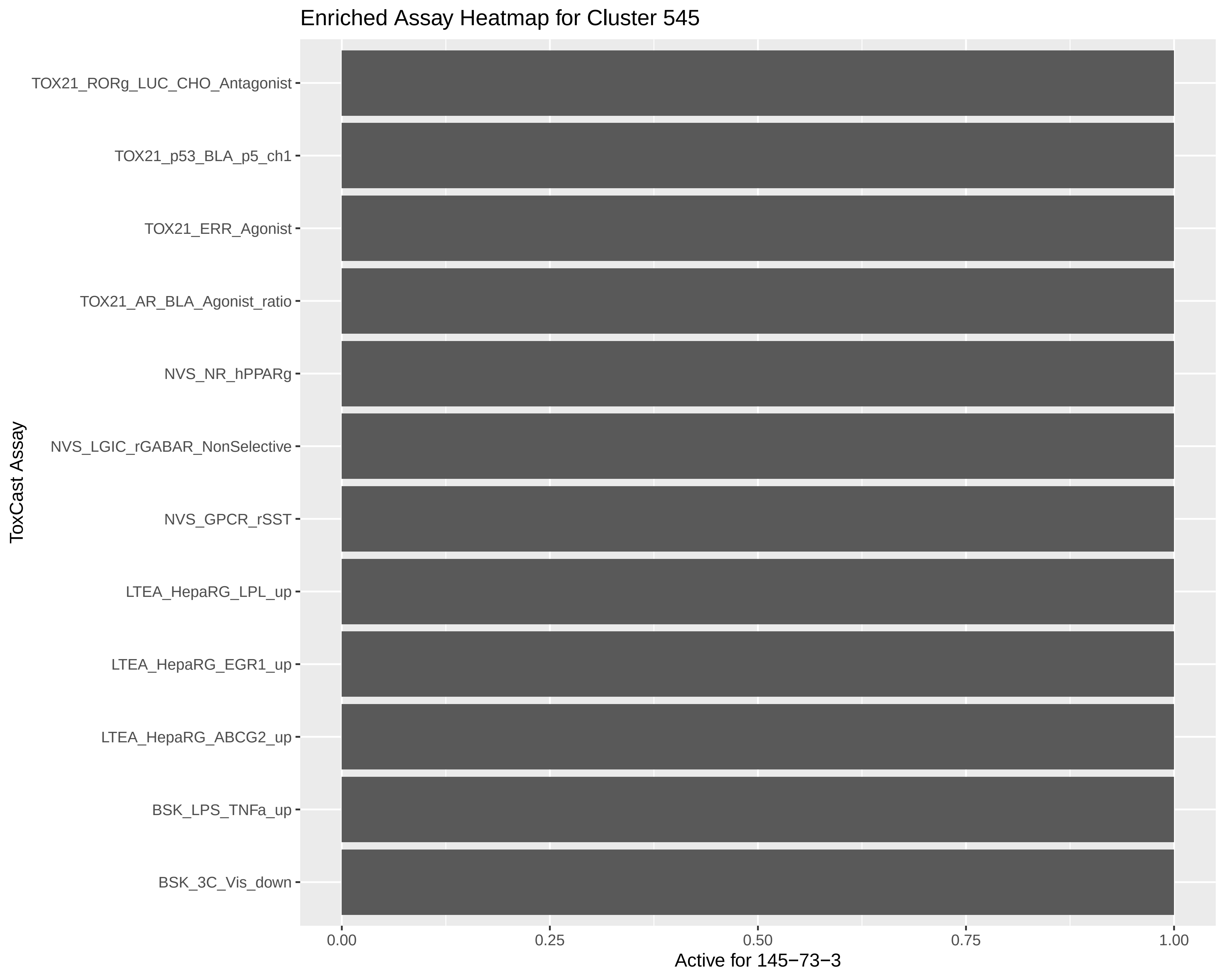
Enriched Assay Heatmap for Cluster 545
ToxCast Assay
TOX21_RORg_LUC_CHO_Antagonist
TOX21_p53_BLA_p5_ch1
TOX21_ERR_Agonist
TOX21_AR_BLA_Agonist_ratio
NVS_NR_hPPARg
NVS_LGIC_rGABAR_NonSelective
NVS_GPCR_rSST
LTEA_HepaRG_LPL_up
LTEA_HepaRG_EGR1_up
LTEA_HepaRG_ABCG2_up
BSK_LPS_TNFa_up
BSK_3C_Vis_down
0.00
0.25
0.50
0.75
1.00
Active for 145−73−3

Enriched Assay Heatmap for Cluster 546

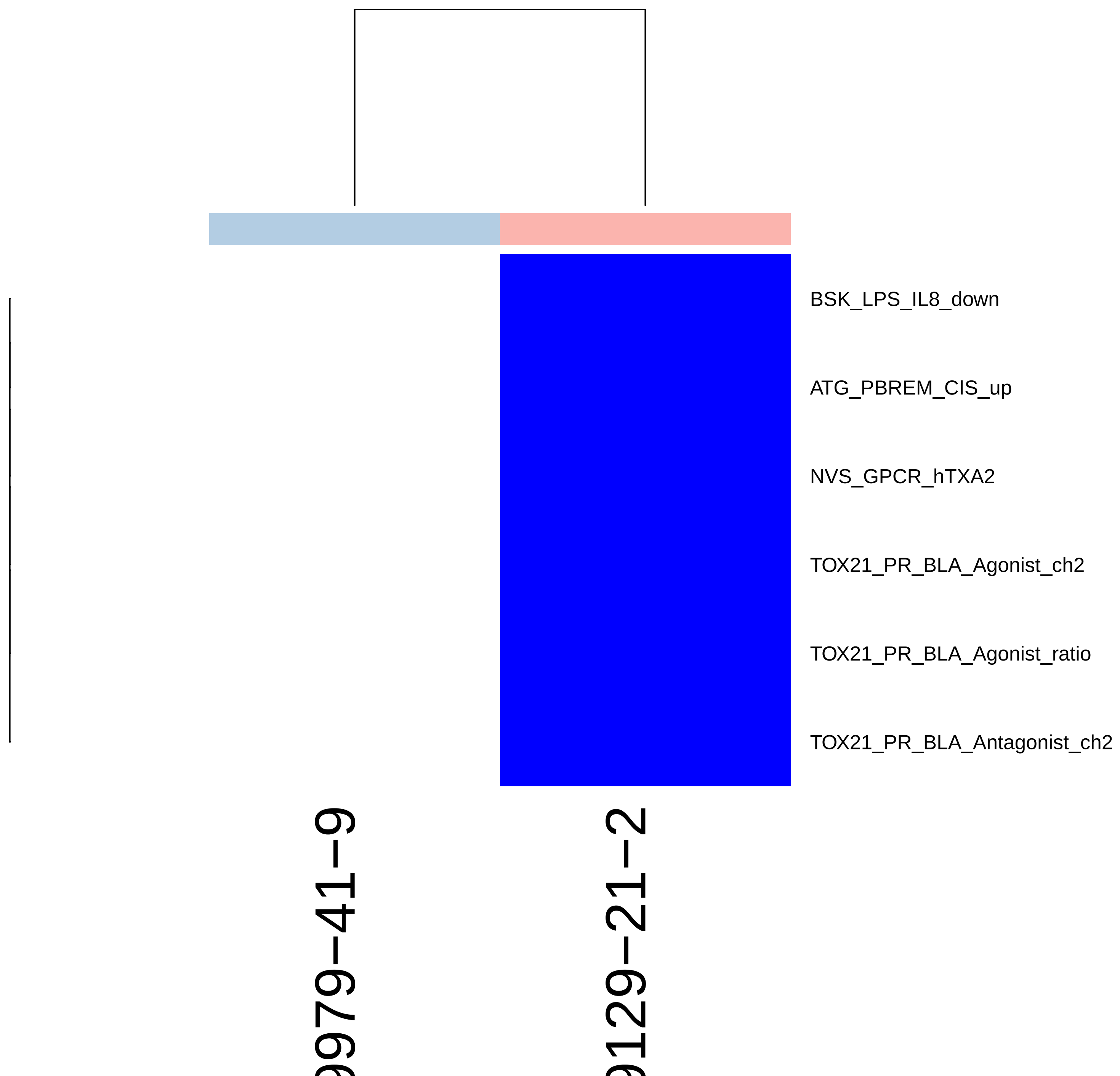

Enriched Assay Heatmap for Cluster 552

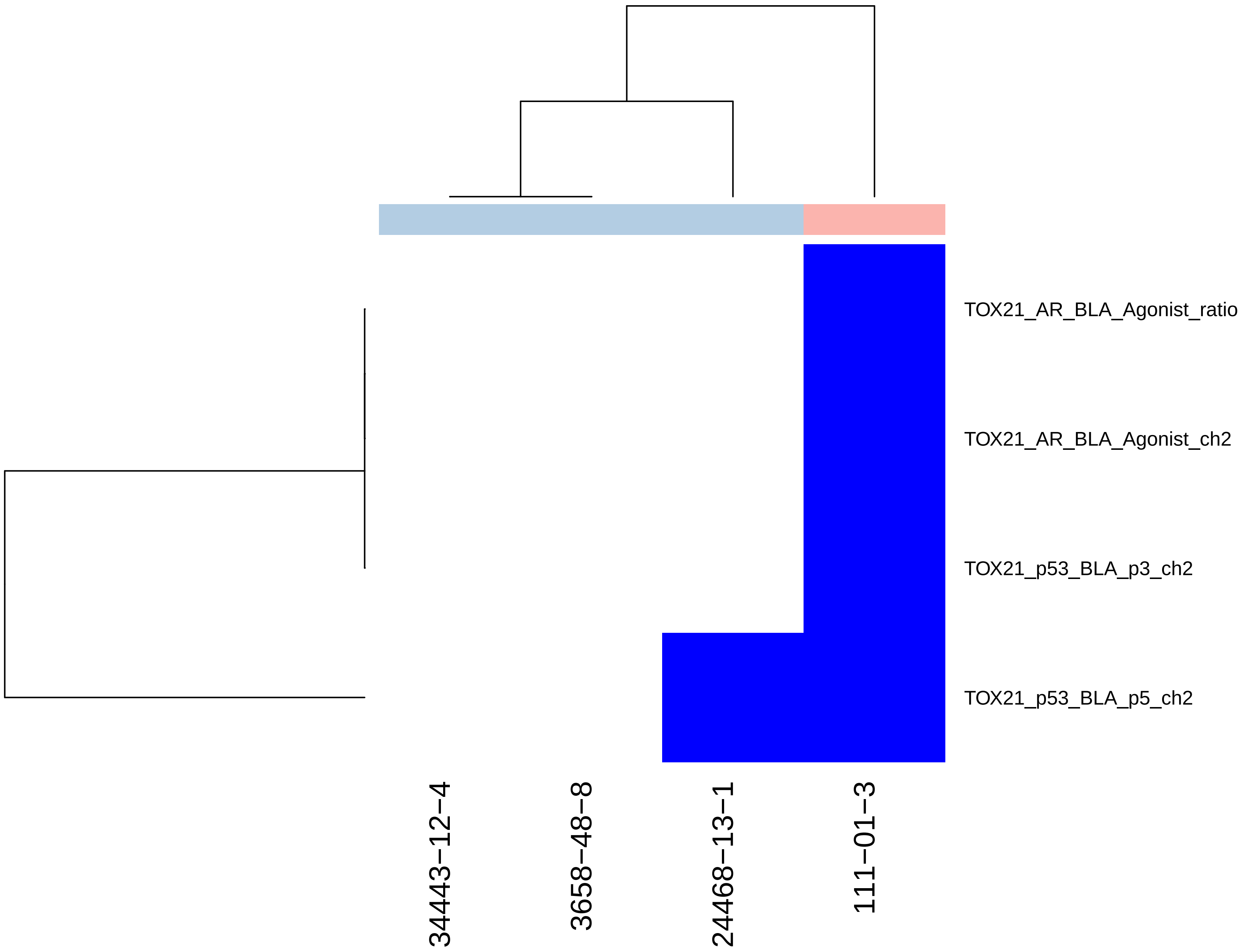

Enriched Assay Heatmap for Cluster 554

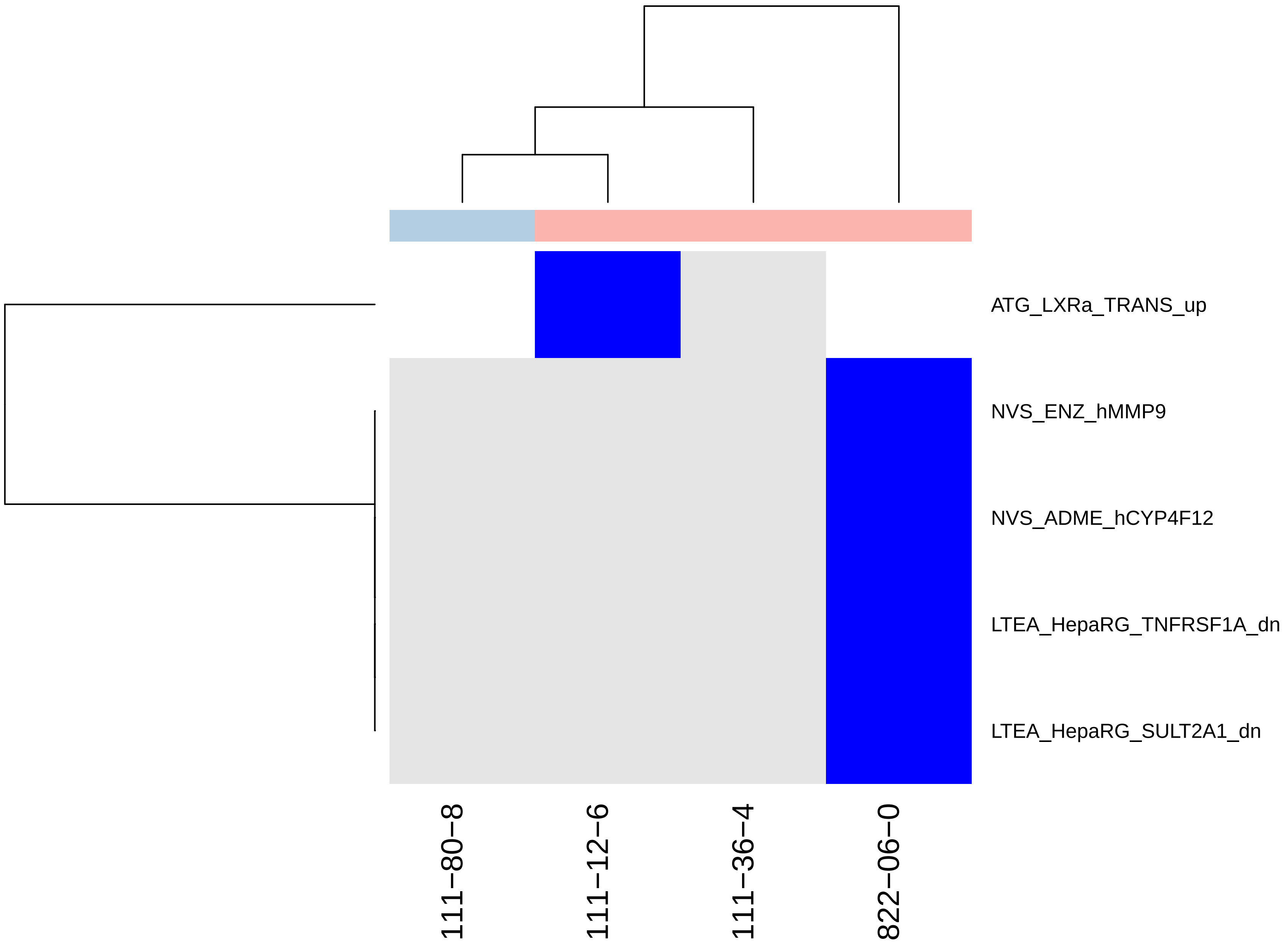

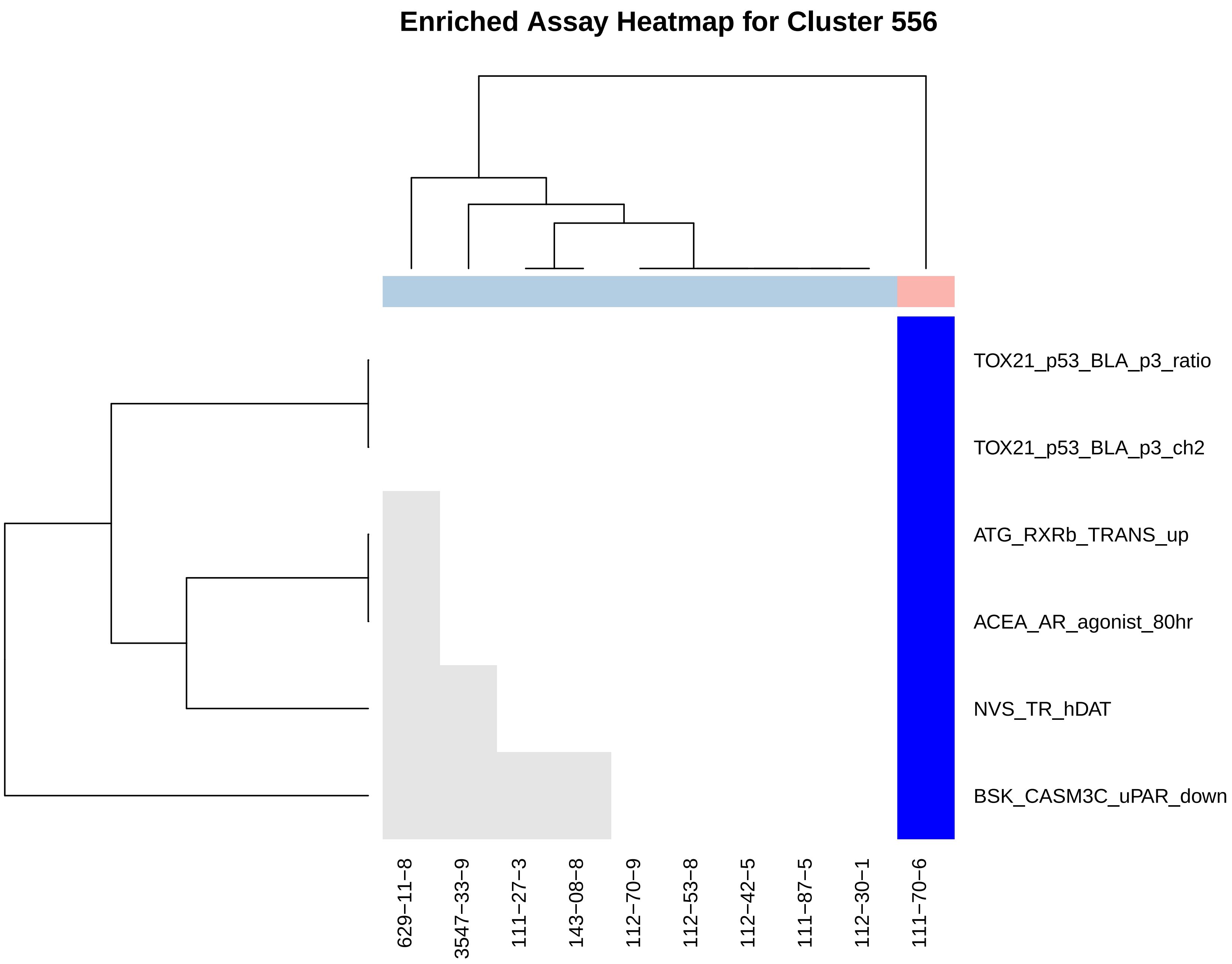
Enriched Assay Heatmap for Cluster 556
TOX21_p53_BLA_p3_ratio
TOX21_p53_BLA_p3_ch2
ATG_RXRb_TRANS_up
ACEA_AR_agonist_80hr
NVS_TR_hDAT
BSK_CASM3C_uPAR_down
629−11−8
3547−33−9
111−27−3
143−08−8
112−70−9
112−53−8
112−42−5
111−87−5
112−30−1
111−70−6

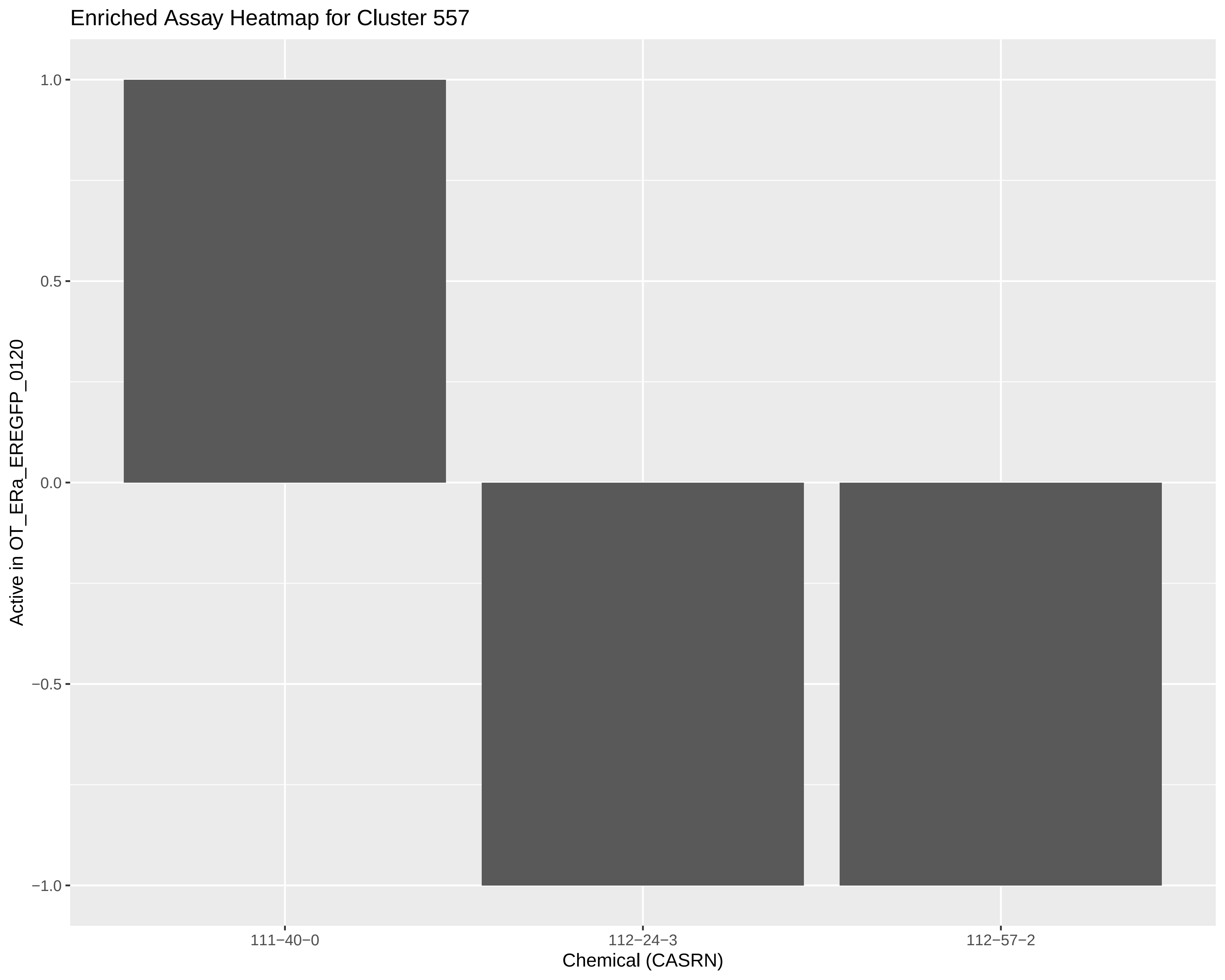
Enriched Assay Heatmap for Cluster 557
1.0
0.5
0.0
−0.5
−1.0
Active in OT_ERa_EREGFP_0120
111−40−0
112−24−3
112−57−2
Chemical (CASRN)

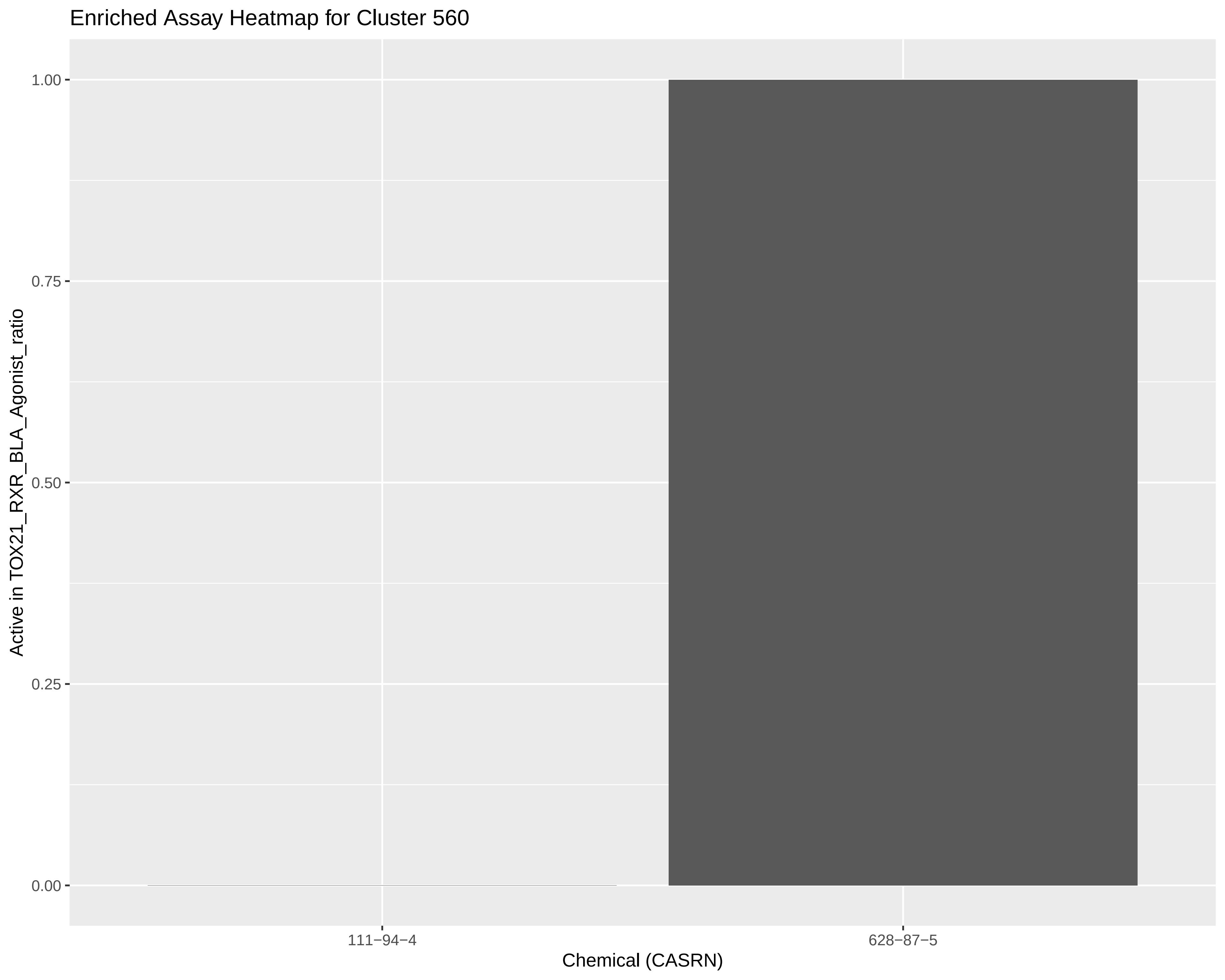
Enriched Assay Heatmap for Cluster 560
Active in TOX21_RXR_BLA_Agonist_ratio
1.00
0.75
0.50
0.25
0.00
111−94−4
628−87−5
Chemical (CASRN)

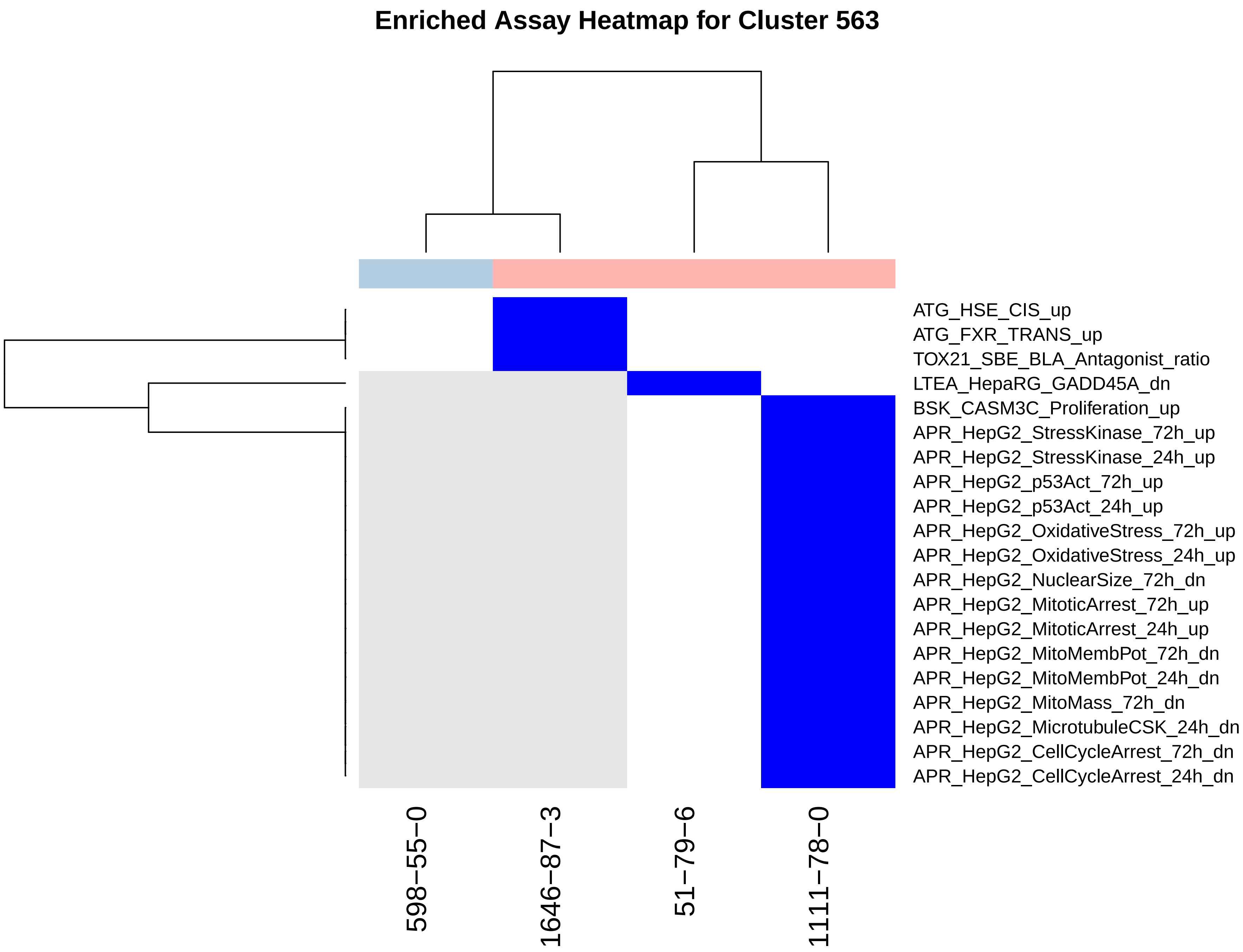

Enriched Assay Heatmap for Cluster 563
ATG_HSE_CIS_up
ATG_FXR_TRANS_up
TOX21_SBE_BLA_Antagonist_ratio
LTEA_HepaRG_GADD45A_dn
BSK_CASM3C_Proliferation_up
APR_HepG2_StressKinase_72h_up
APR_HepG2_StressKinase_24h_up
APR_HepG2_p53Act_72h_up
APR_HepG2_p53Act_24h_up
APR_HepG2_OxidativeStress_72h_up
APR_HepG2_OxidativeStress_24h_up
APR_HepG2_NuclearSize_72h_dn
APR_HepG2_MitoticArrest_72h_up
APR_HepG2_MitoticArrest_24h_up
APR_HepG2_MitoMembPot_72h_dn
APR_HepG2_MitoMembPot_24h_dn
APR_HepG2_MitoMass_72h_dn
APR_HepG2_MicrotubuleCSK_24h_dn
APR_HepG2_CellCycleArrest_72h_dn
APR_HepG2_CellCycleArrest_24h_dn
598−55−0
1646−87−3
51−79−6
1111−78−0

Enriched Assay Heatmap for Cluster 566

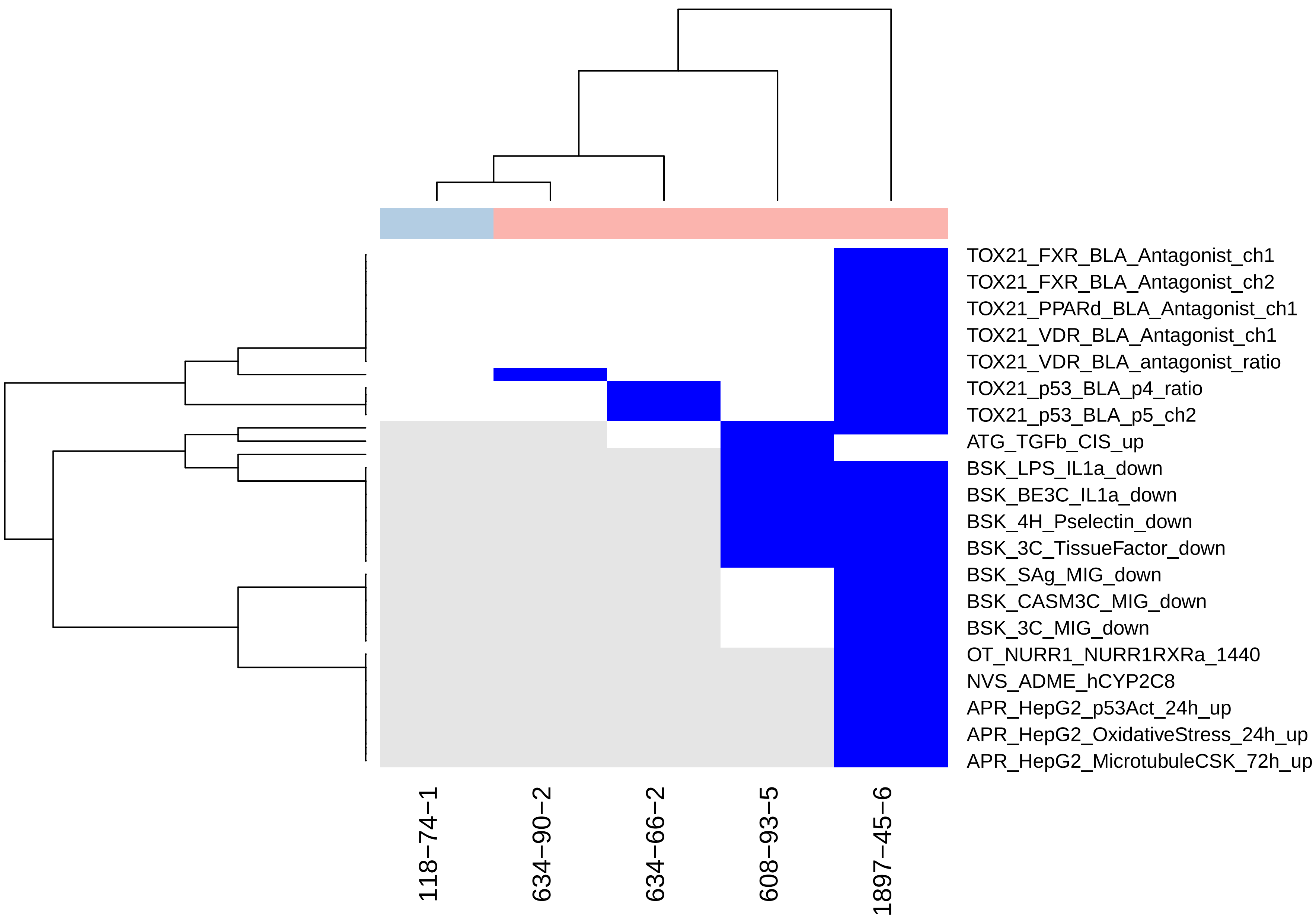

Enriched Assay Heatmap for Cluster 569

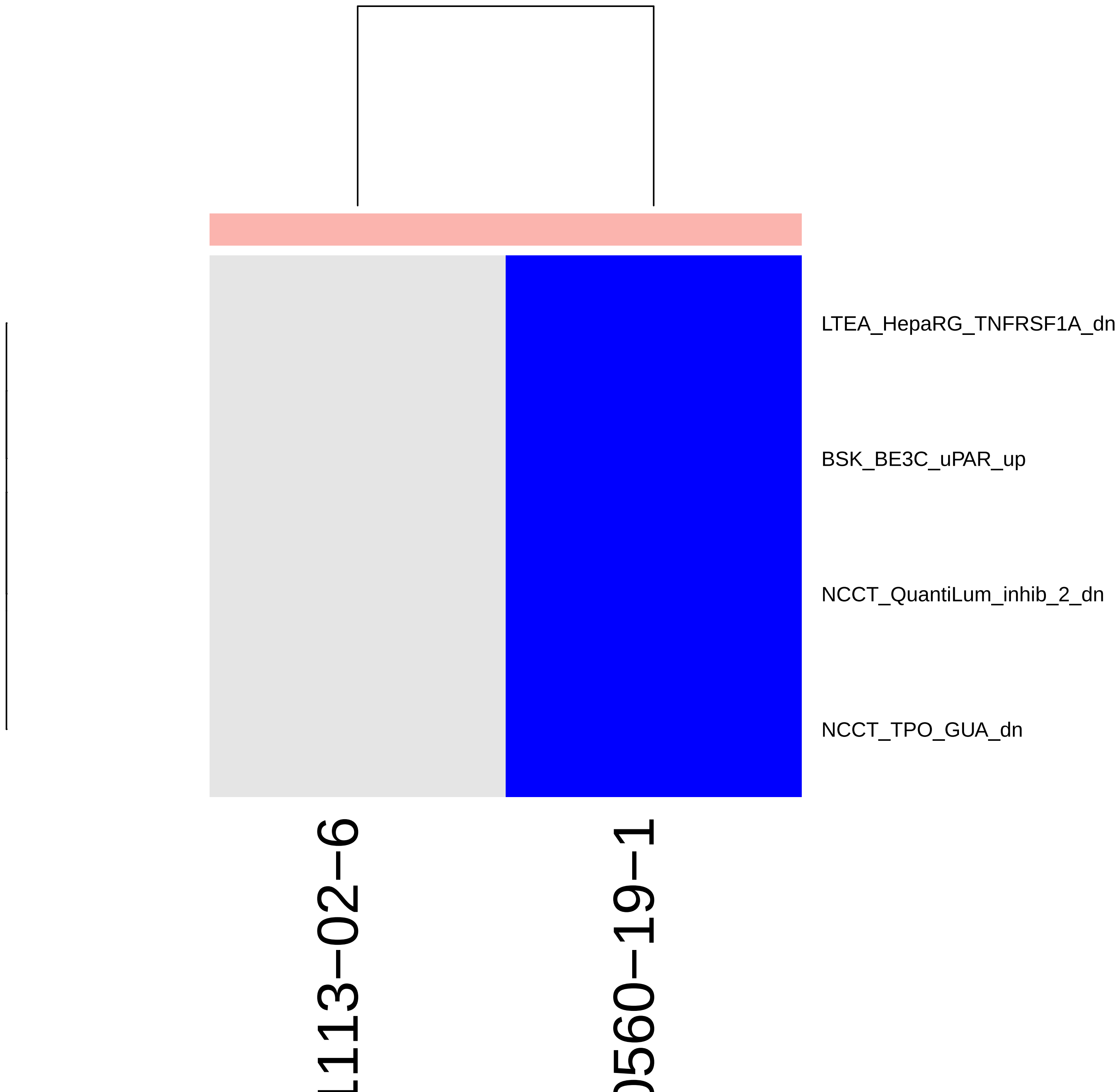

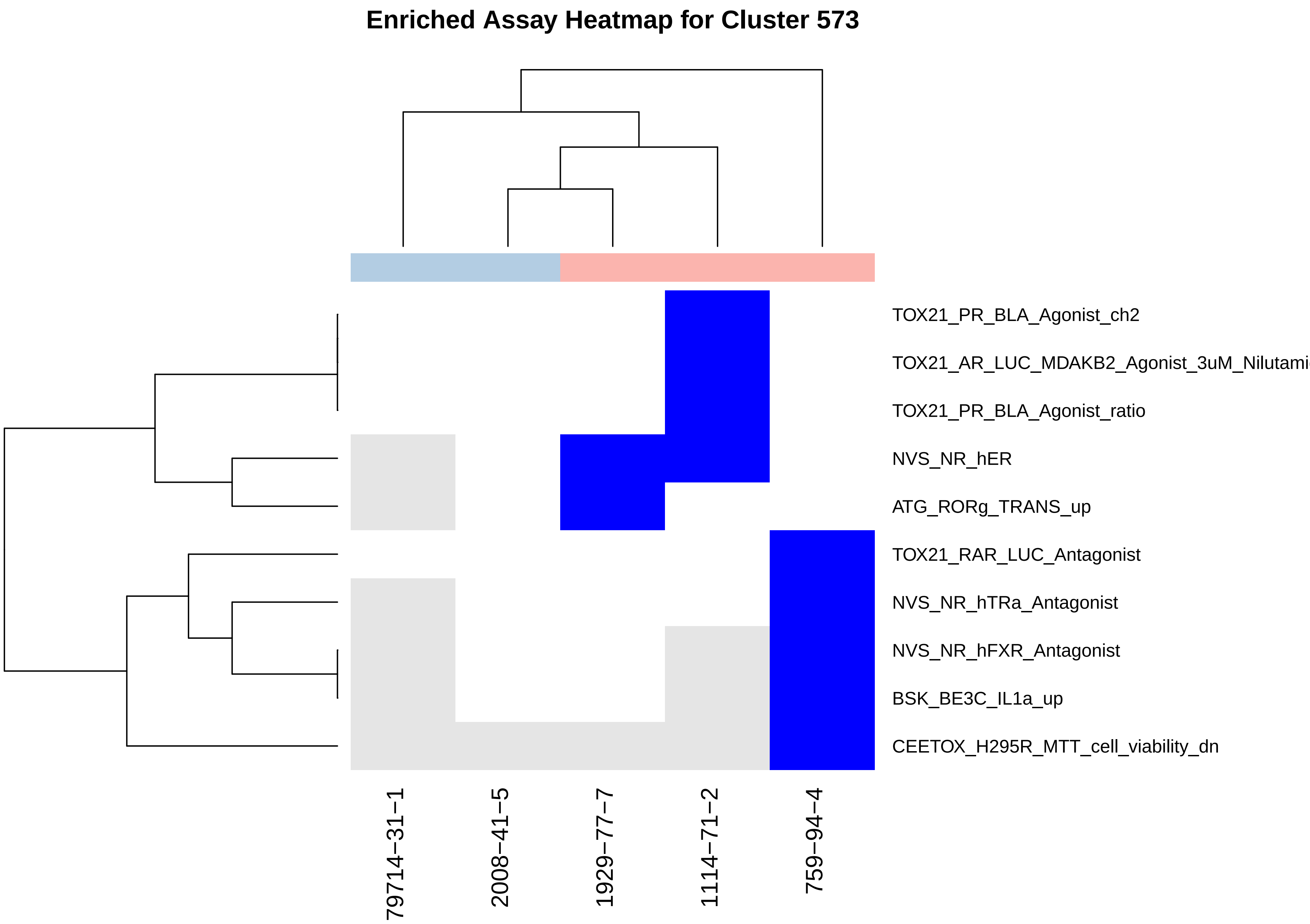
Enriched Assay Heatmap for Cluster 573
TOX21_PR_BLA_Agonist_ch2
TOX21_AR_LUC_MDAKB2_Agonist_3uM_Nilutami
TOX21_PR_BLA_Agonist_ratio
NVS_NR_hER
ATG_RORg_TRANS_up
TOX21_RAR_LUC_Antagonist
NVS_NR_hTRa_Antagonist
NVS_NR_hFXR_Antagonist
BSK_BE3C_IL1a_up
CEETOX_H295R_MTT_cell_viability_dn
79714−31−1
2008−41−5
1929−77−7
1114−71−2
759−94−4

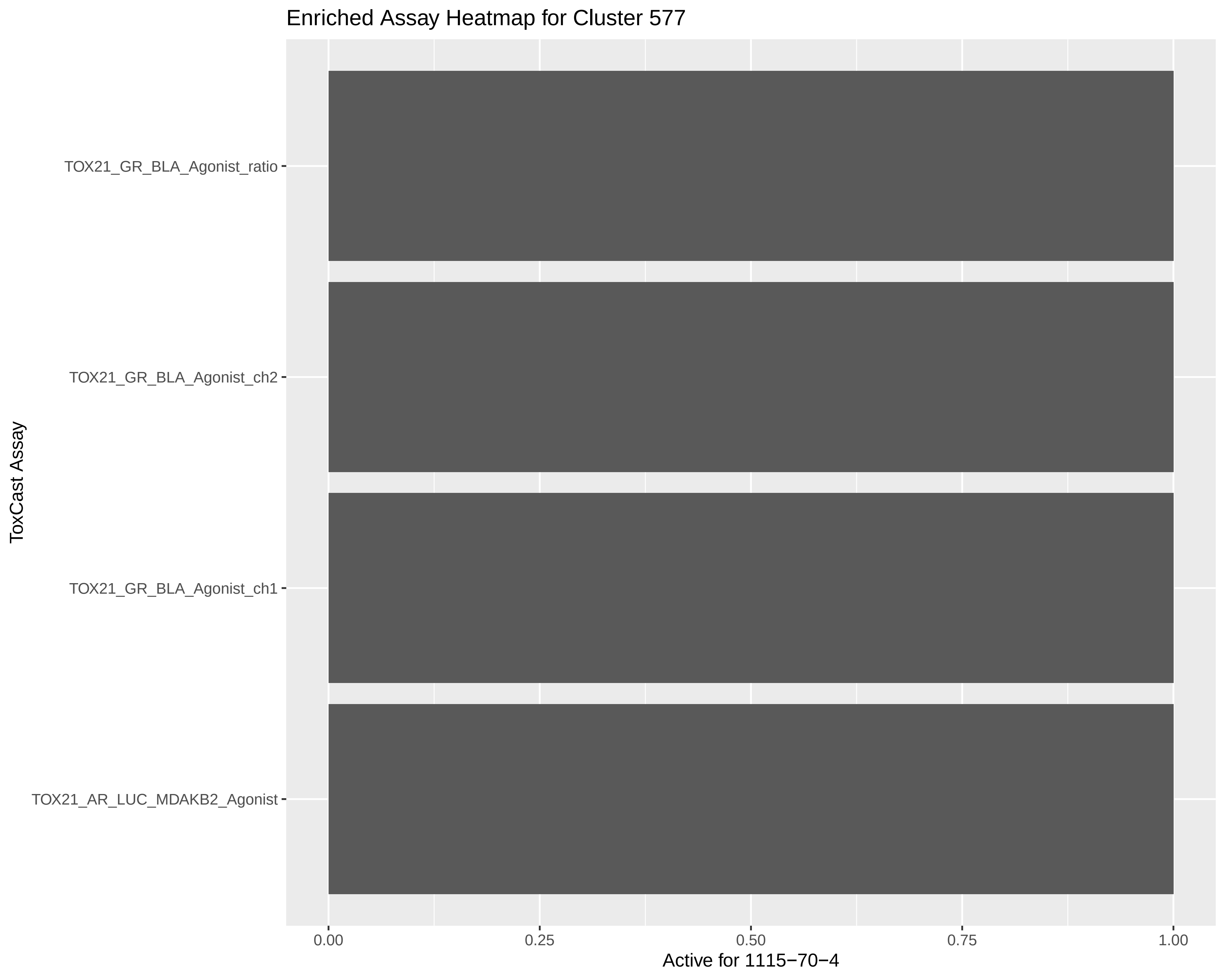

Enriched Assay Heatmap for Cluster 577
TOX21_GR_BLA_Agonist_ratio
TOX21_GR_BLA_Agonist_ch2
TOX21_GR_BLA_Agonist_ch1
TOX21_AR_LUC_MDAKB2_Agonist
ToxCast Assay
0.00
0.25
0.50
0.75
1.00
Active for 1115−70−4

Enriched Assay Heatmap for Cluster 580

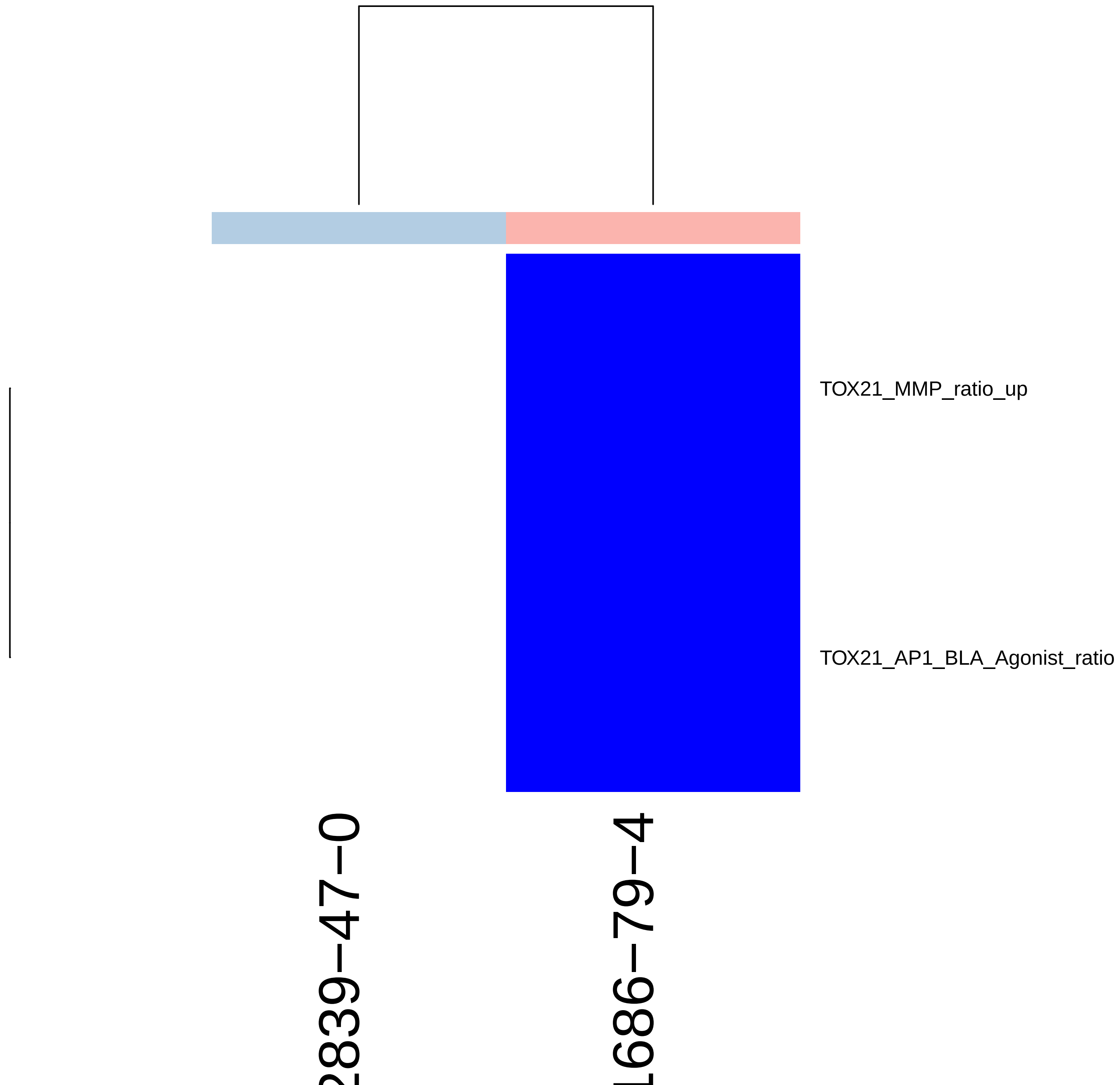

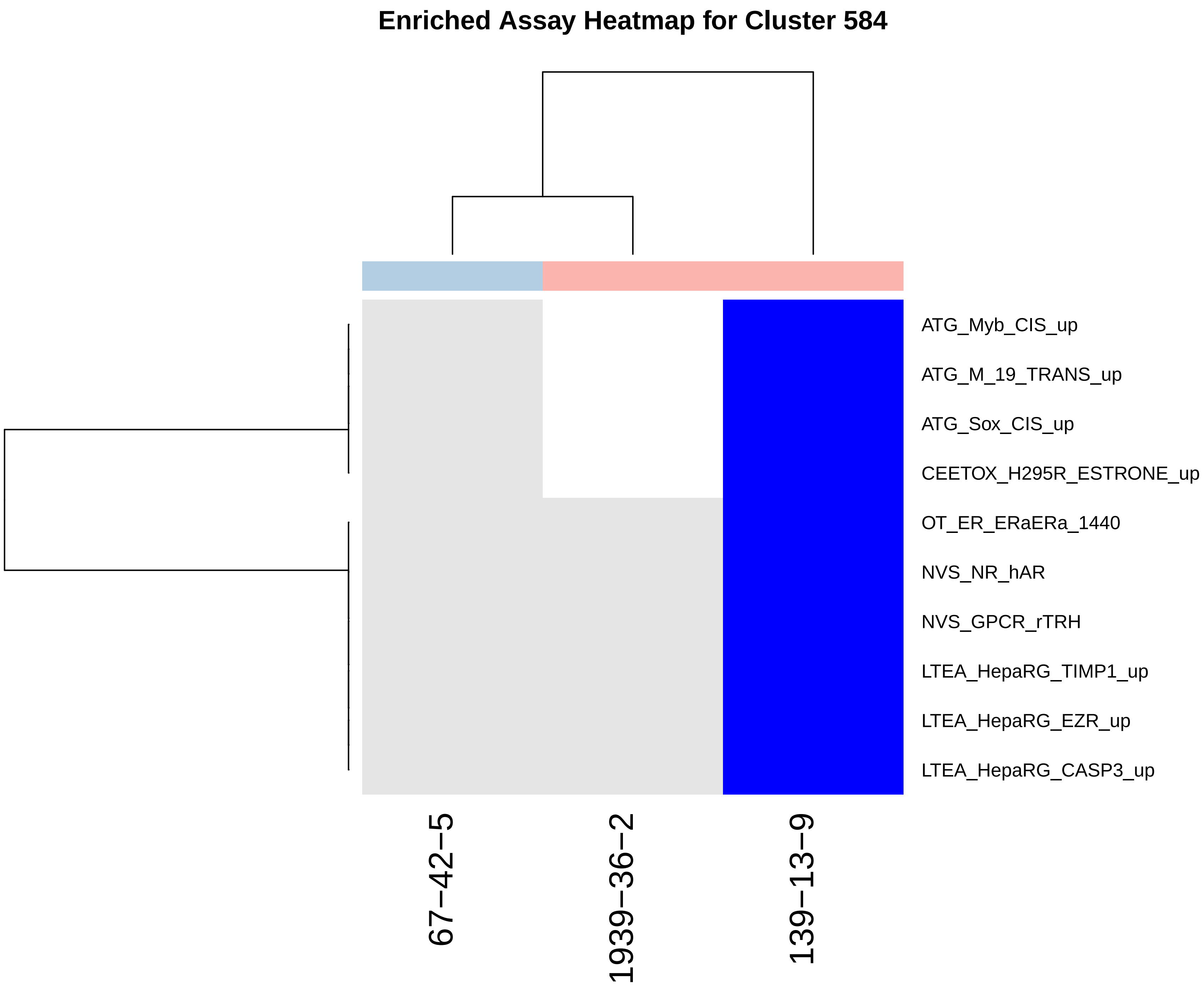

Enriched Assay Heatmap for Cluster 584
ATG_Myb_CIS_up
ATG_M_19_TRANS_up
ATG_Sox_CIS_up
CEETOX_H295R_ESTRONE_up
OT_ER_ERaERa_1440
NVS_NR_hAR
NVS_GPCR_rTRH
LTEA_HepaRG_TIMP1_up
LTEA_HepaRG_EZR_up
LTEA_HepaRG_CASP3_up
67−42−5
1939−36−2
139−13−9

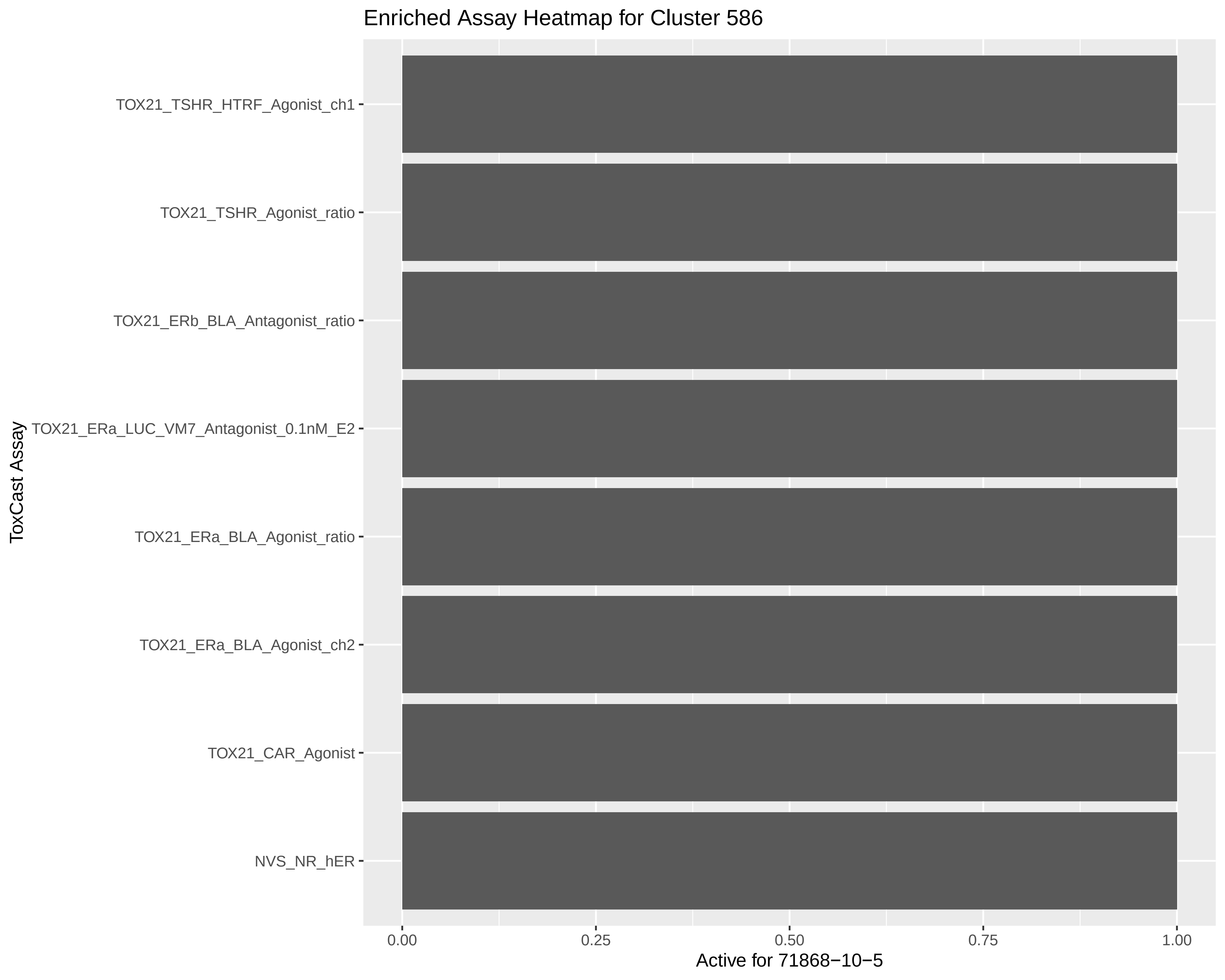
Enriched Assay Heatmap for Cluster 586
TOX21_TSHR_HTRF_Agonist_ch1
TOX21_TSHR_Agonist_ratio
TOX21_ERb_BLA_Antagonist_ratio
TOX21_ERa_LUC_VM7_Antagonist_0.1nM_E2
TOX21_ERa_BLA_Agonist_ratio
TOX21_ERa_BLA_Agonist_ch2
TOX21_CAR_Agonist
NVS_NR_hER
ToxCast Assay
0.00
0.25
0.50
0.75
1.00
Active for 71868−10−5

Enriched Assay Heatmap for Cluster 587

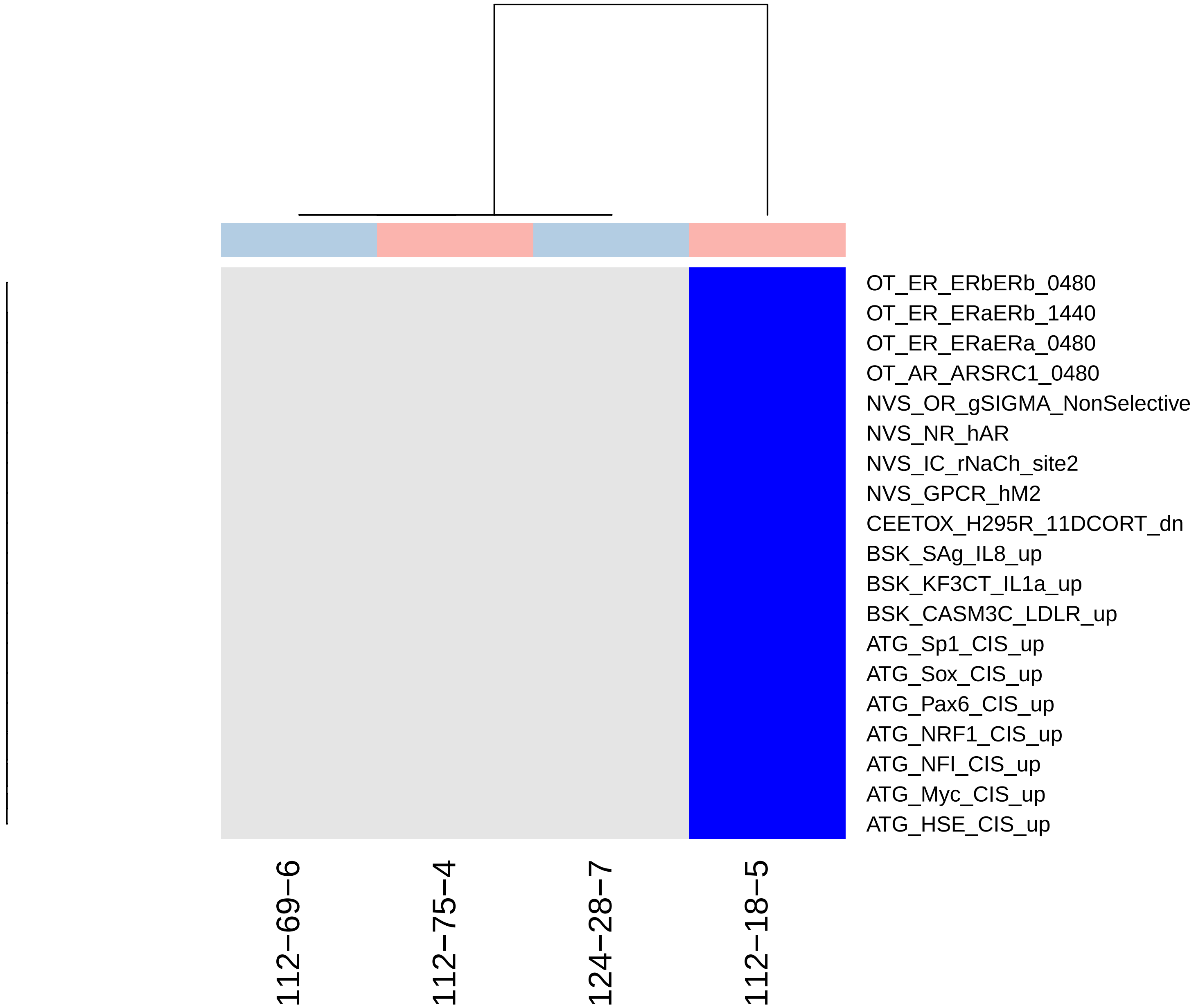

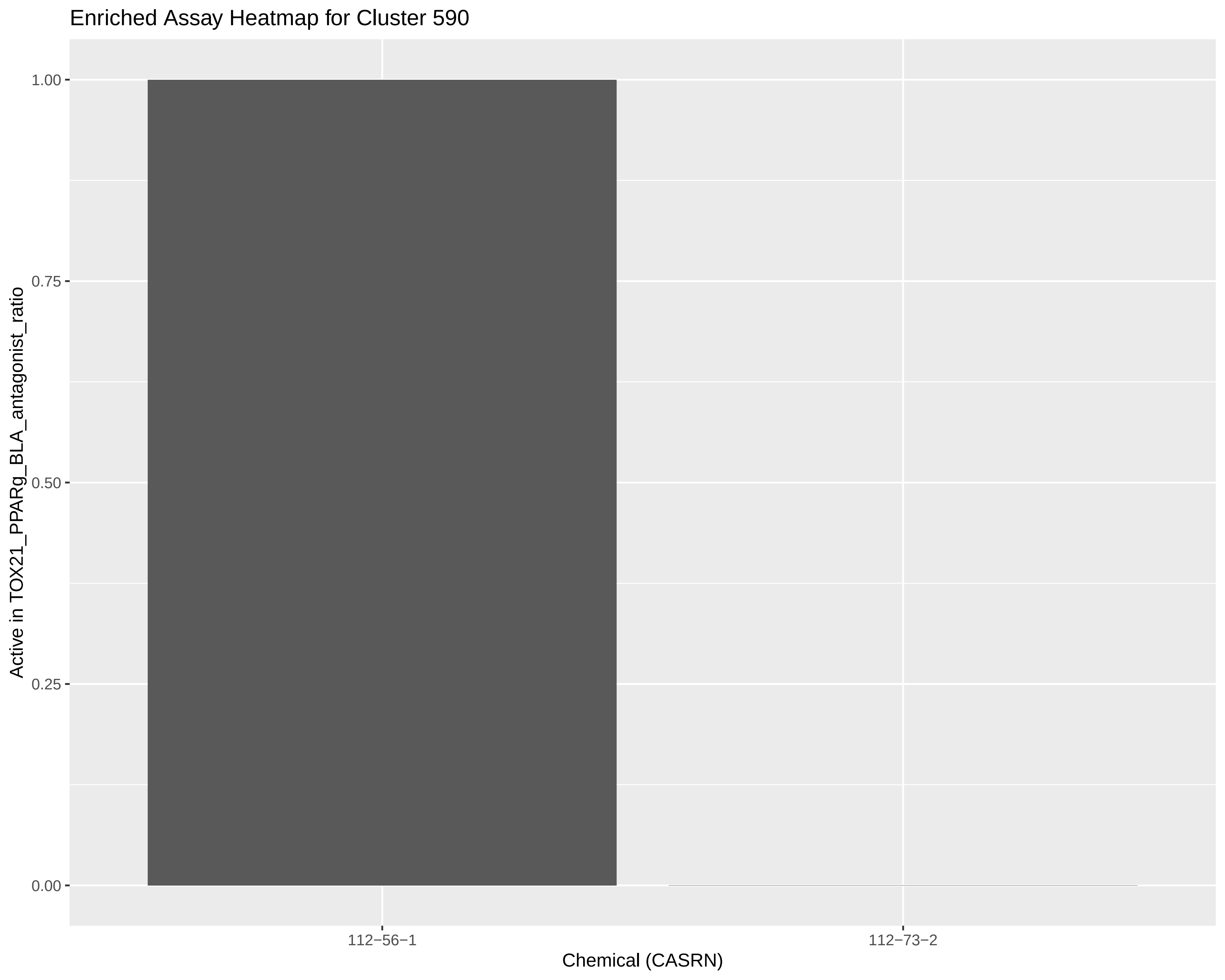
Enriched Assay Heatmap for Cluster 590
Active in TOX21_PPARg_BLA_antagonist_ratio
1.00
0.75
0.50
0.25
0.00
112−56−1
112−73−2
Chemical (CASRN)

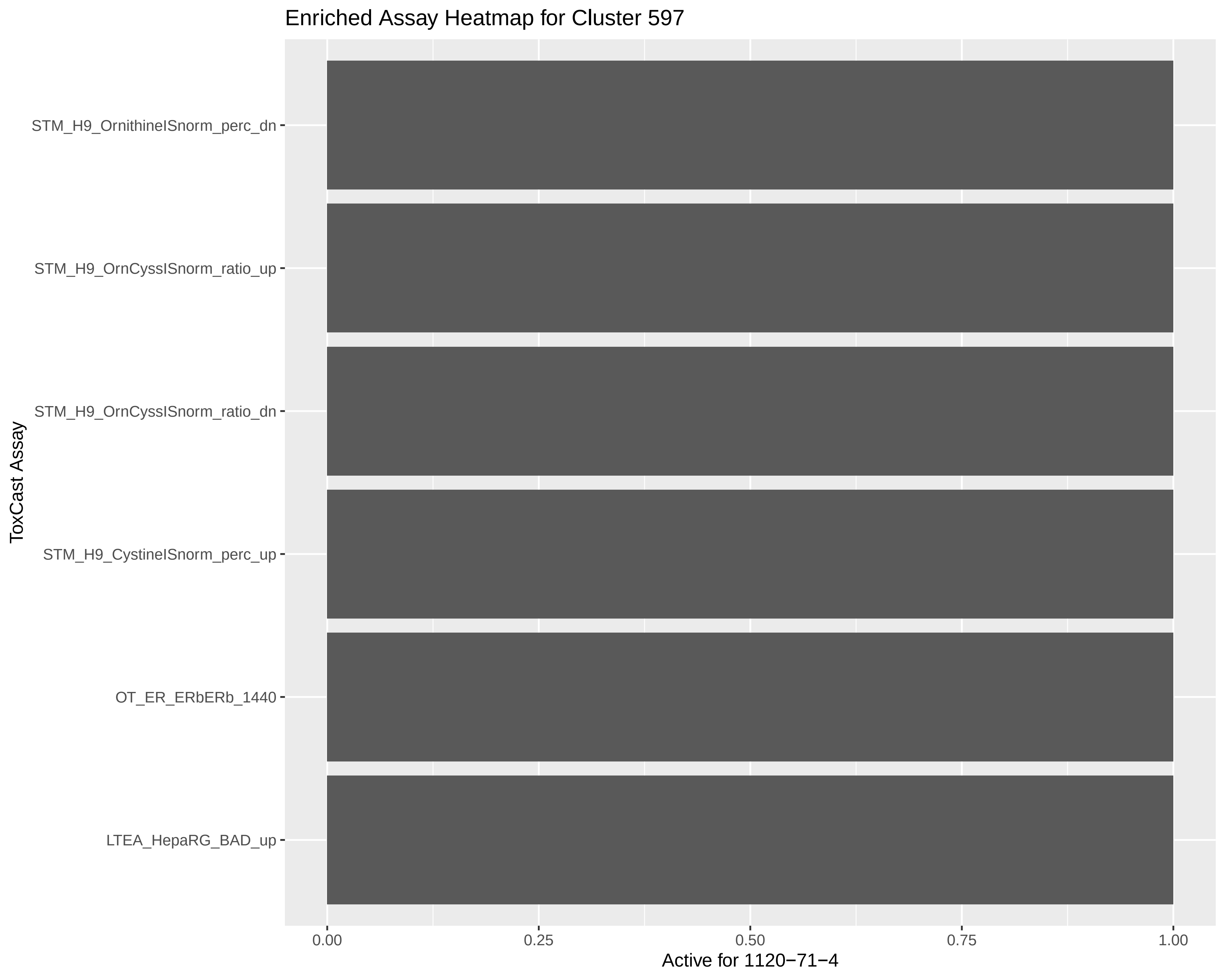
Enriched Assay Heatmap for Cluster 597
ToxCast Assay
STM_H9_OrnithineISnorm_perc_dn
STM_H9_OrnCyssISnorm_ratio_up
STM_H9_OrnCyssISnorm_ratio_dn
STM_H9_CystineISnorm_perc_up
OT_ER_ERbERb_1440
LTEA_HepaRG_BAD_up
0.00
0.25
0.50
0.75
1.00
Active for 1120−71−4

Enriched Assay Heatmap for Cluster 598

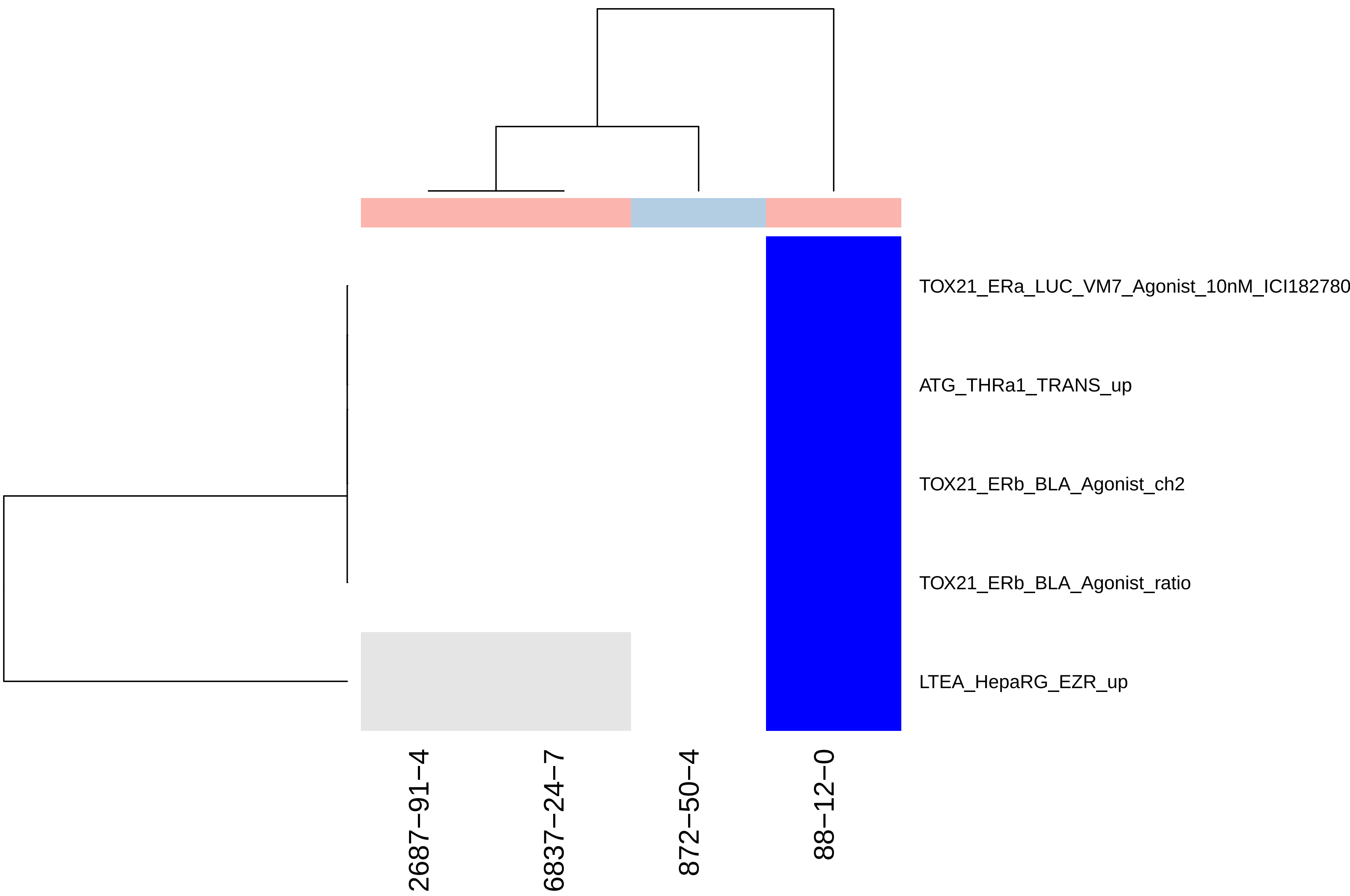

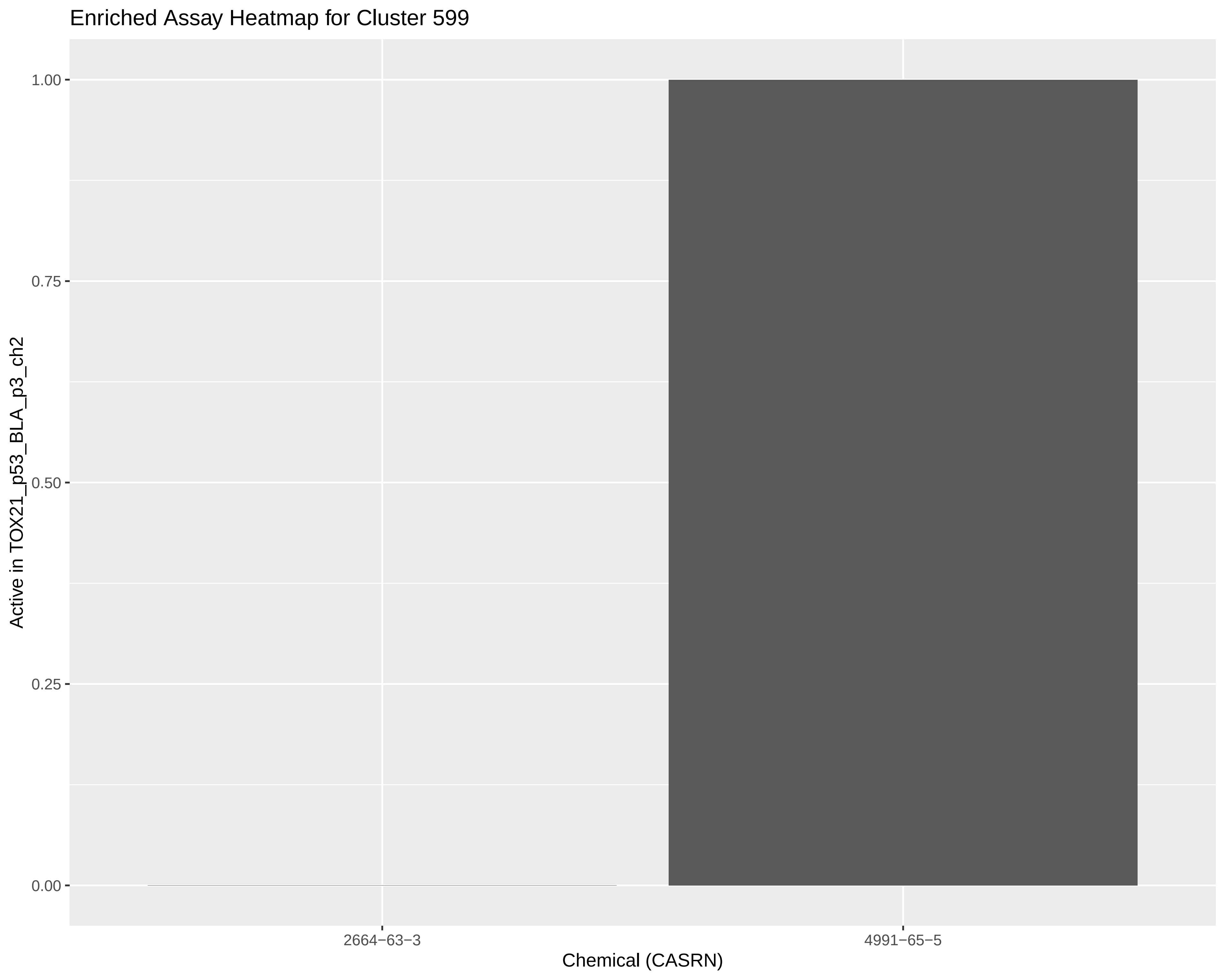

Enriched Assay Heatmap for Cluster 599
Active in TOX21_p53_BLA_p3_ch2
1.00
0.75
0.50
0.25
0.00
2664−63−3
4991−65−5
Chemical (CASRN)

Enriched Assay Heatmap for Cluster 603

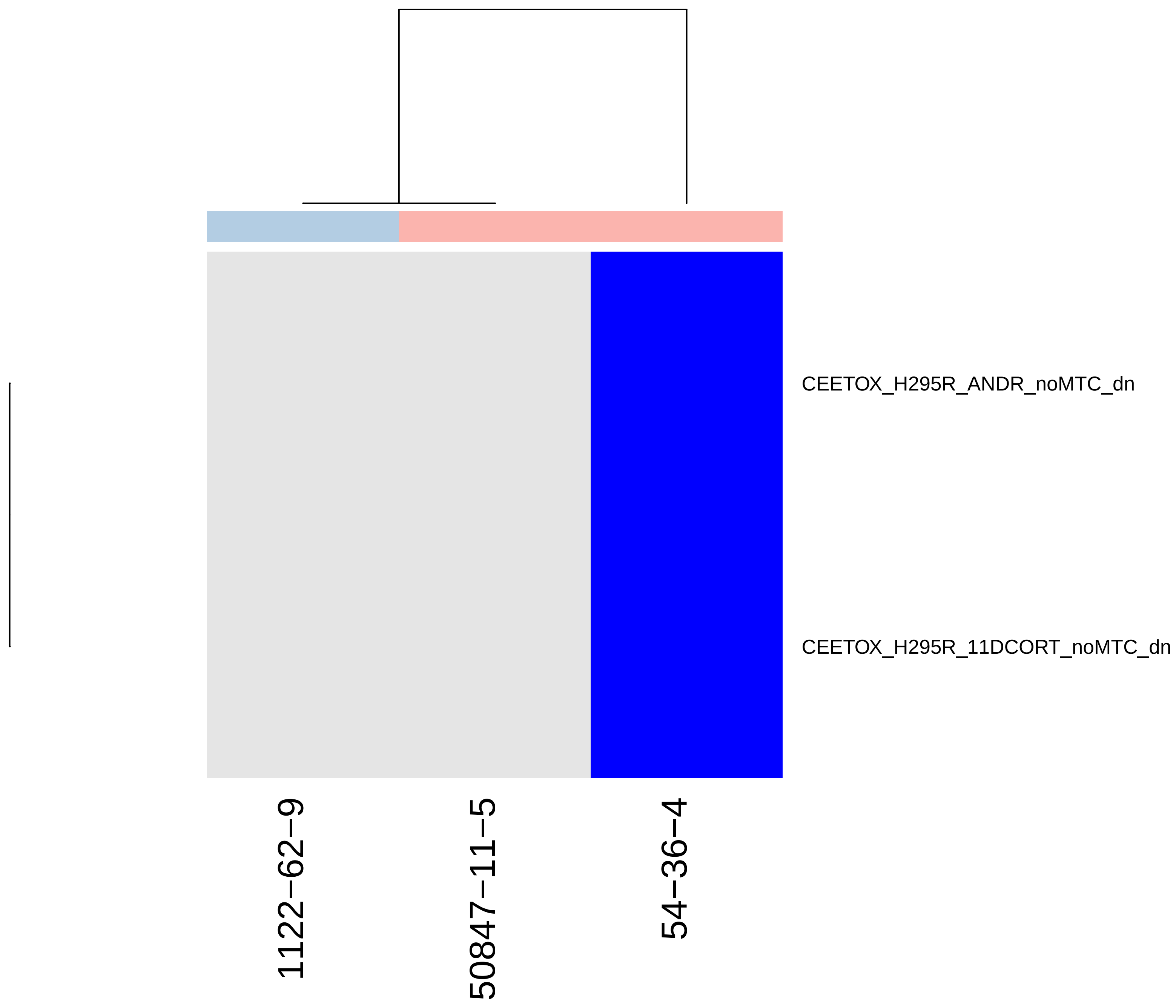

Enriched Assay Heatmap for Cluster 605

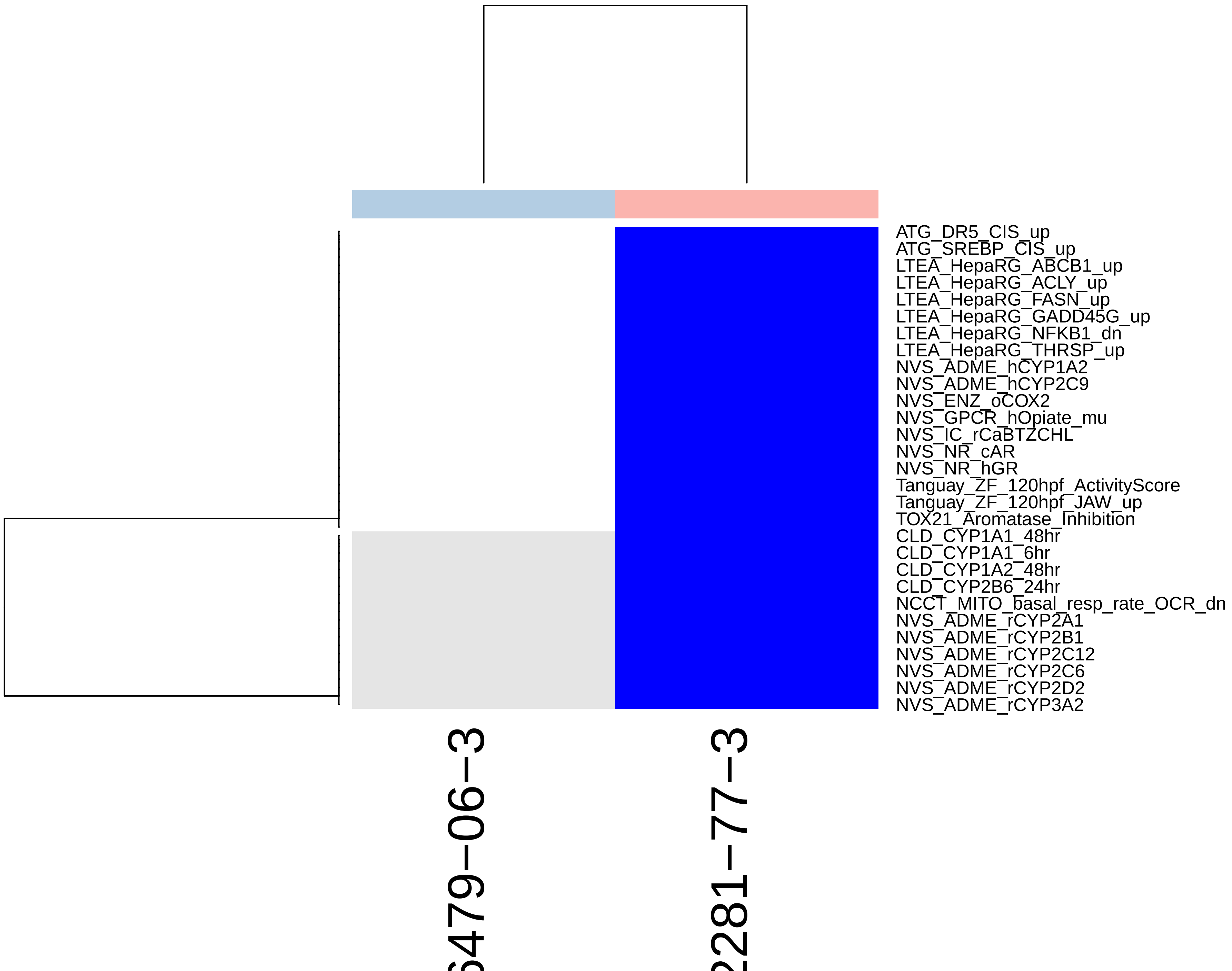

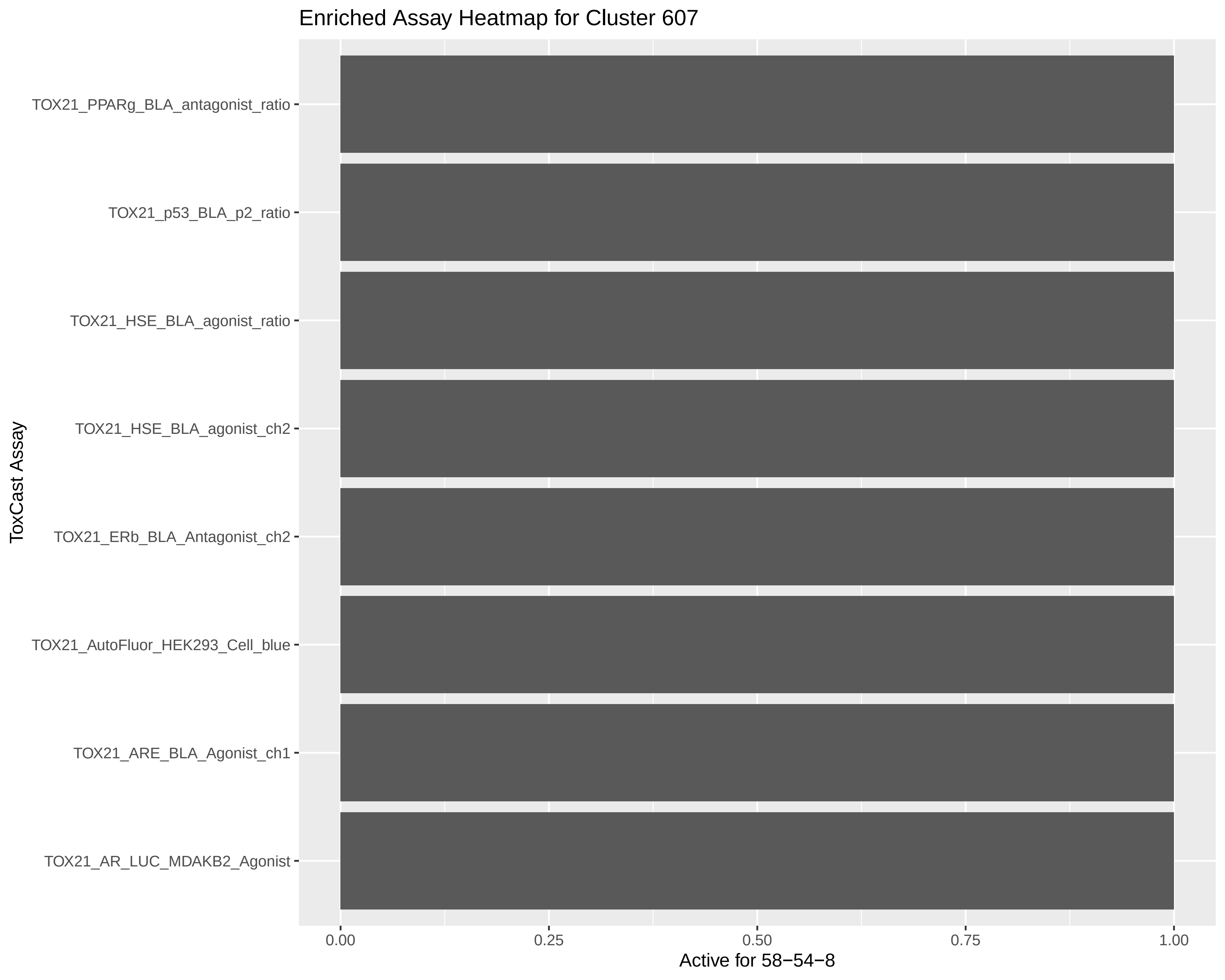

Enriched Assay Heatmap for Cluster 607
ToxCast Assay
TOX21_PPARg_BLA_antagonist_ratio
TOX21_p53_BLA_p2_ratio
TOX21_HSE_BLA_agonist_ratio
TOX21_HSE_BLA_agonist_ch2
TOX21_ERb_BLA_Antagonist_ch2
TOX21_AutoFluor_HEK293_Cell_blue
TOX21_ARE_BLA_Agonist_ch1
TOX21_AR_LUC_MDAKB2_Agonist
0.00
0.25
0.50
0.75
1.00
Active for 58−54−8

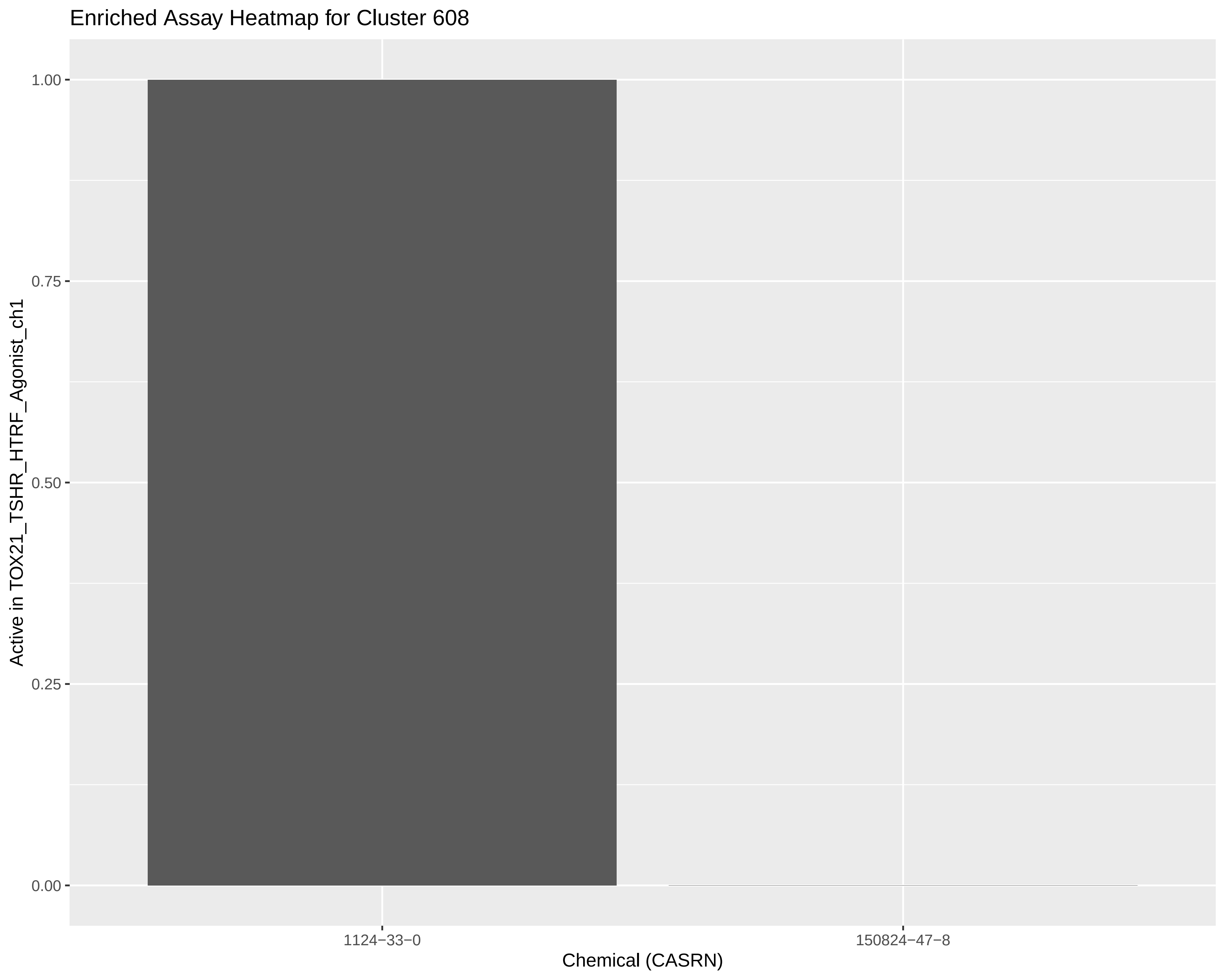

Enriched Assay Heatmap for Cluster 608
Active in TOX21_TSHR_HTRF_Agonist_ch1
1.00
0.75
0.50
0.25
0.00
1124−33−0
150824−47−8
Chemical (CASRN)

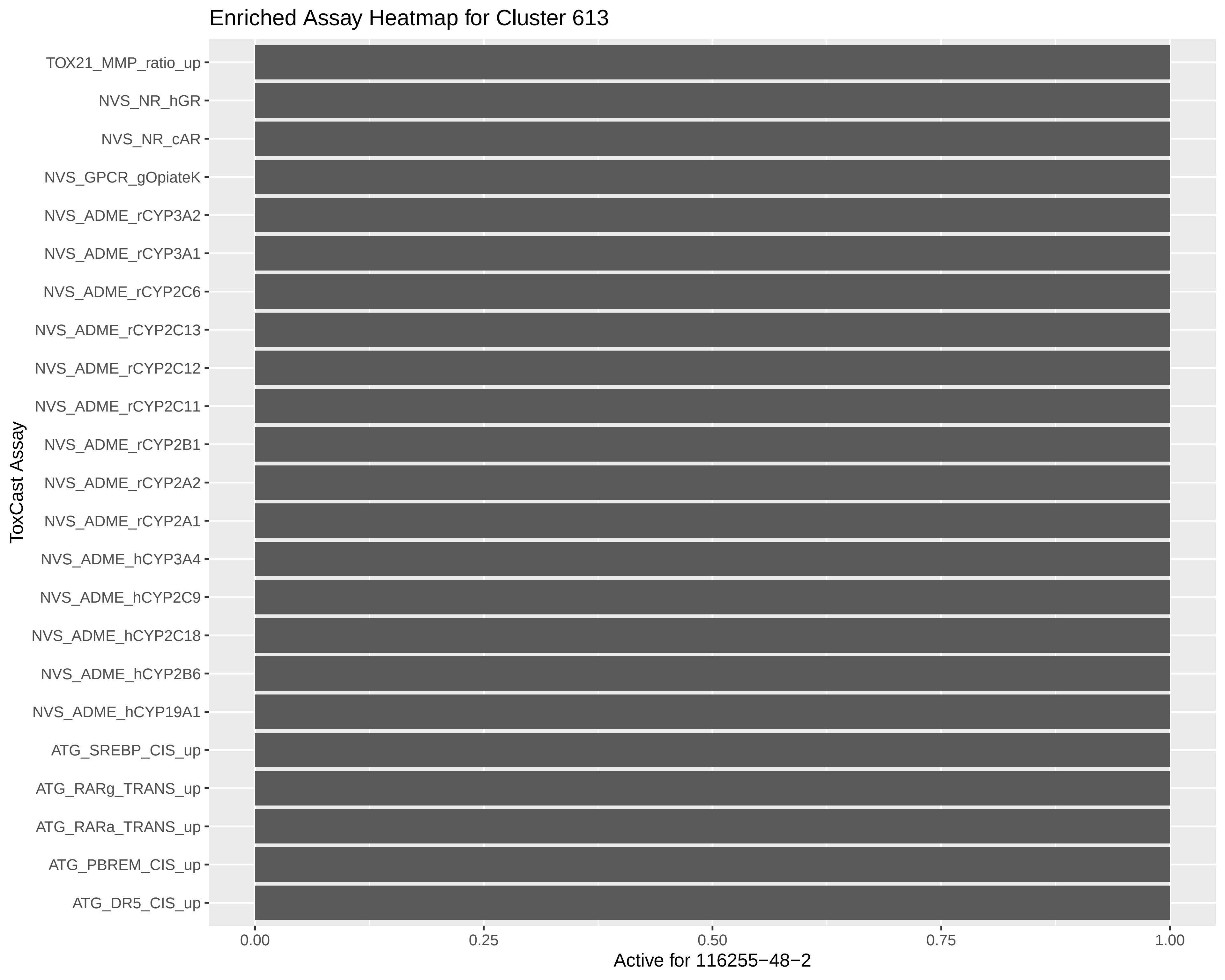

Enriched Assay Heatmap for Cluster 613
ToxCast Assay
TOX21_MMP_ratio_up
NVS_NR_hGR
NVS_NR_cAR
NVS_GPCR_gOpiateK
NVS_ADME_rCYP3A2
NVS_ADME_rCYP3A1
NVS_ADME_rCYP2C6
NVS_ADME_rCYP2C13
NVS_ADME_rCYP2C12
NVS_ADME_rCYP2C11
NVS_ADME_rCYP2B1
NVS_ADME_rCYP2A2
NVS_ADME_rCYP2A1
NVS_ADME_hCYP3A4
NVS_ADME_hCYP2C9
NVS_ADME_hCYP2C18
NVS_ADME_hCYP2B6
NVS_ADME_hCYP19A1
ATG_SREBP_CIS_up
ATG_RARg_TRANS_up
ATG_RARa_TRANS_up
ATG_PBREM_CIS_up
ATG_DR5_CIS_up
0.00
0.25
0.50
0.75
1.00
Active for 116255−48−2

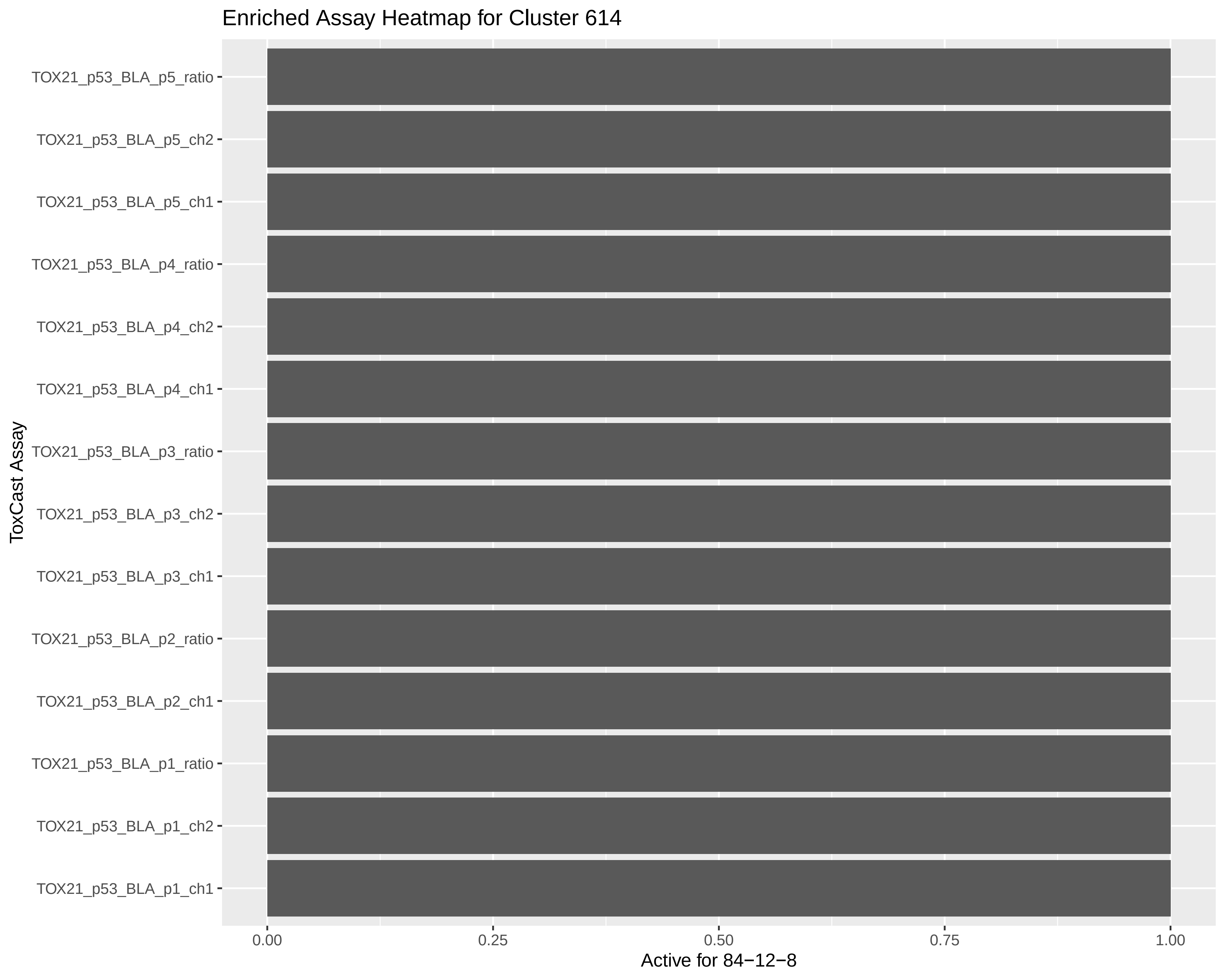

Enriched Assay Heatmap for Cluster 614
ToxCast Assay
TOX21_p53_BLA_p5_ratio
TOX21_p53_BLA_p5_ch2
TOX21_p53_BLA_p5_ch1
TOX21_p53_BLA_p4_ratio
TOX21_p53_BLA_p4_ch2
TOX21_p53_BLA_p4_ch1
TOX21_p53_BLA_p3_ratio
TOX21_p53_BLA_p3_ch2
TOX21_p53_BLA_p3_ch1
TOX21_p53_BLA_p2_ratio
TOX21_p53_BLA_p2_ch1
TOX21_p53_BLA_p1_ratio
TOX21_p53_BLA_p1_ch2
TOX21_p53_BLA_p1_ch1
0.00
0.25
0.50
0.75
1.00
Active for 84−12−8

Enriched Assay Heatmap for Cluster 615

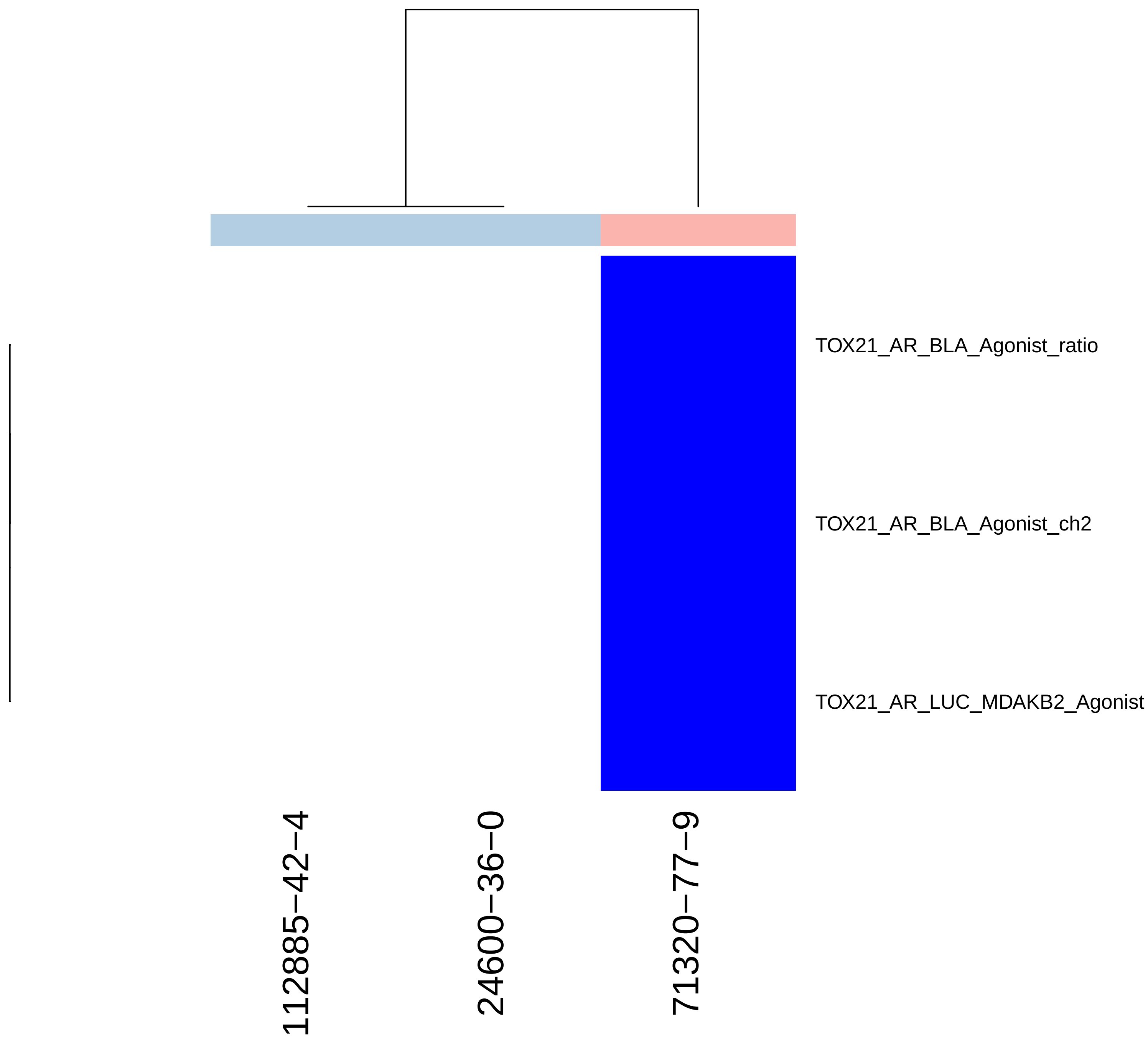

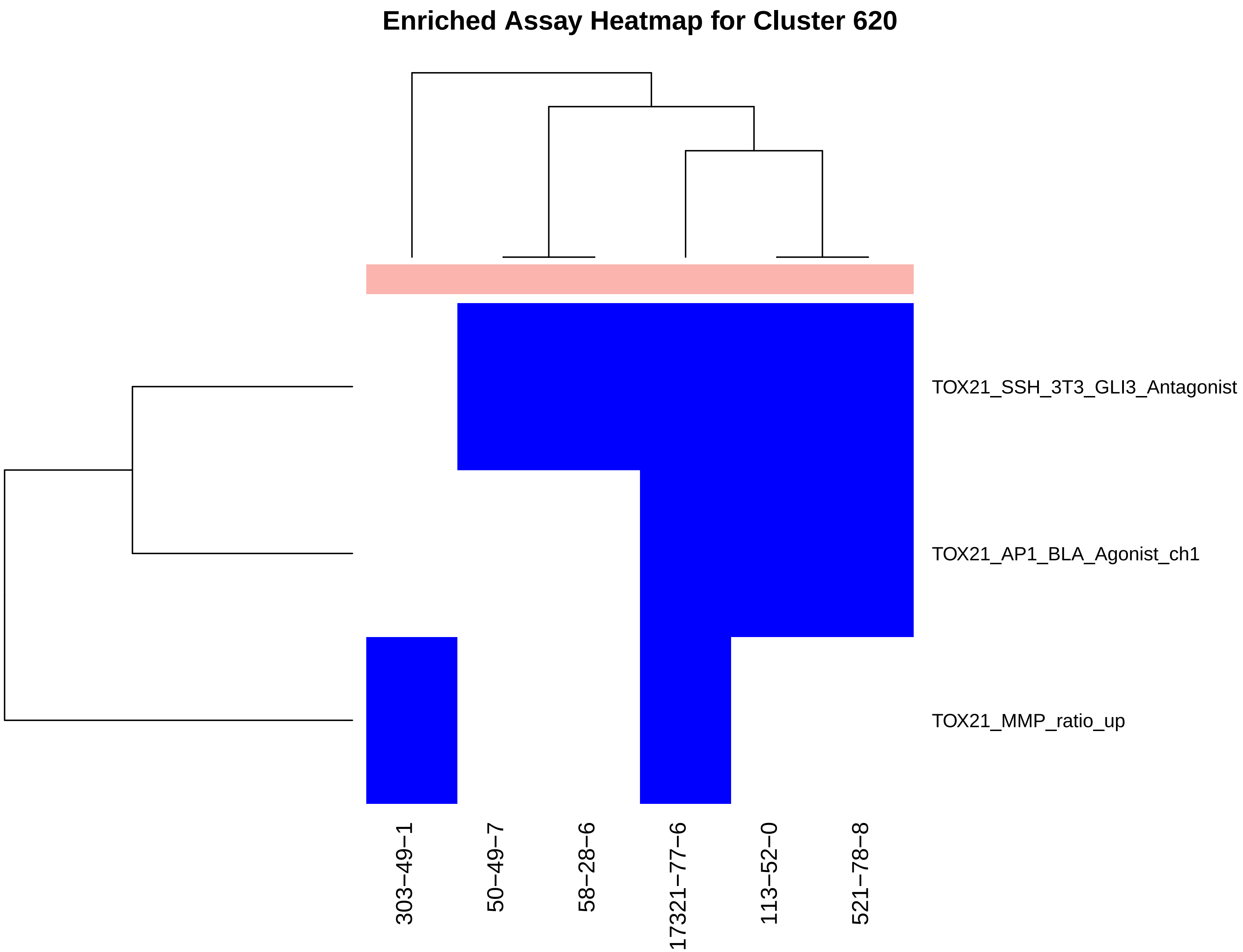
Enriched Assay Heatmap for Cluster 620
TOX21_SSH_3T3_GLI3_Antagonist
TOX21_AP1_BLA_Agonist_ch1
TOX21_MMP_ratio_up
303−49−1
50−49−7
58−28−6
17321−77−6
113−52−0
521−78−8

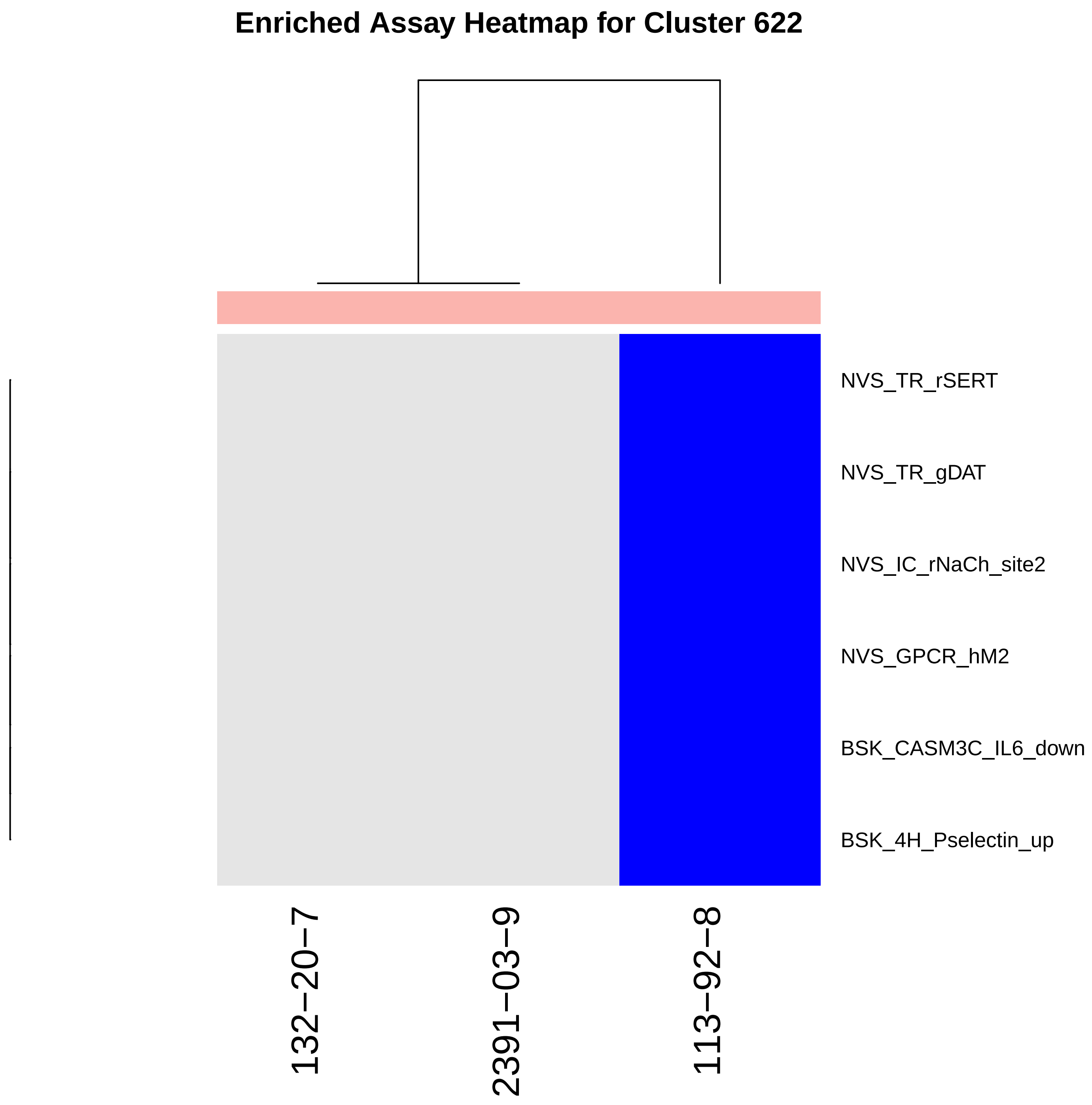
Enriched Assay Heatmap for Cluster 622
NVS_TR_rSERT
NVS_TR_gDAT
NVS_IC_rNaCh_site2
NVS_GPCR_hM2
BSK_CASM3C_IL6_down
BSK_4H_Pselectin_up
132−20−7
2391−03−9
113−92−8

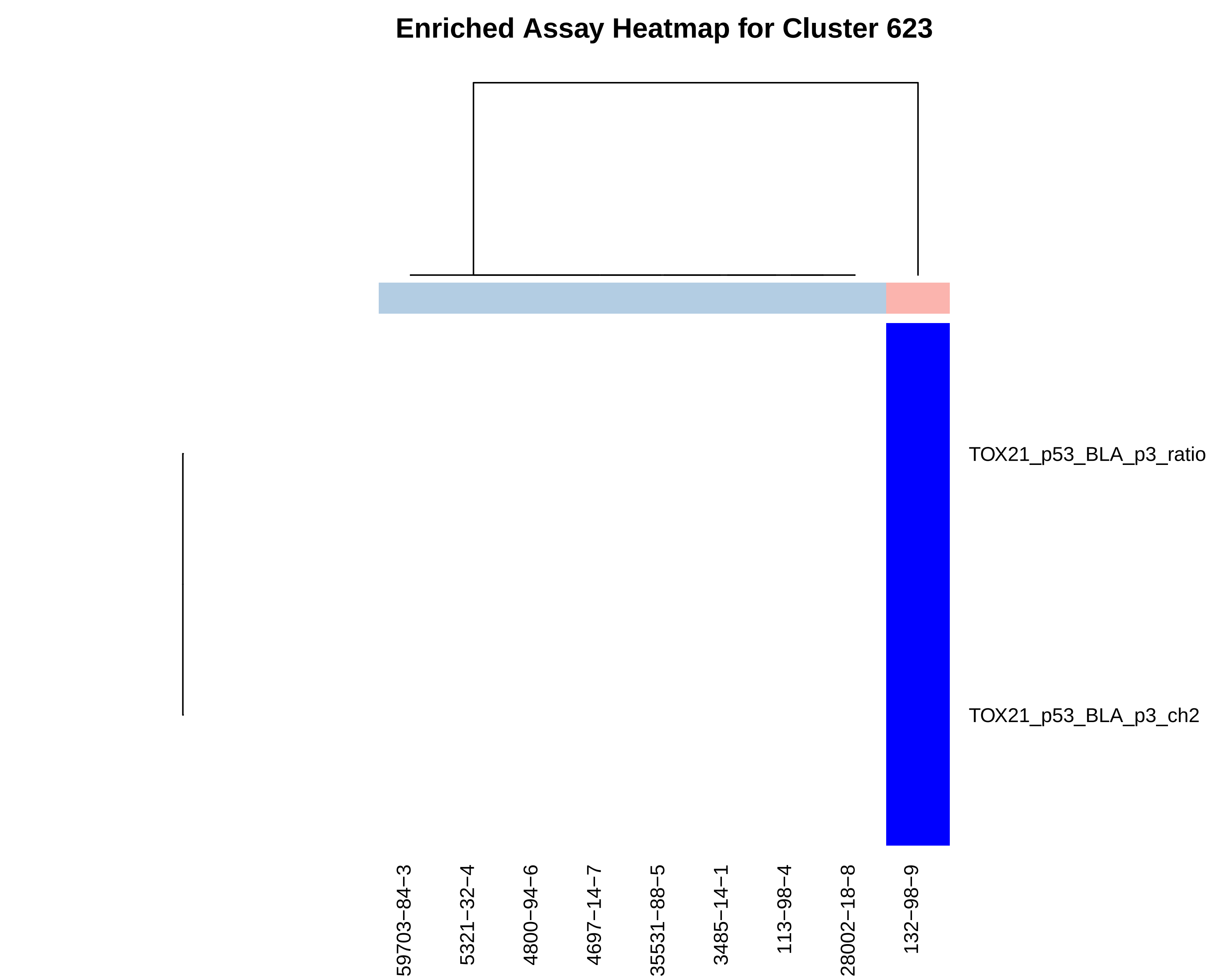
Enriched Assay Heatmap for Cluster 623
TOX21_p53_BLA_p3_ratio
TOX21_p53_BLA_p3_ch2
59703−84−3
5321−32−4
4800−94−6
4697−14−7
35531−88−5
3485−14−1
113−98−4
28002−18−8
132−98−9

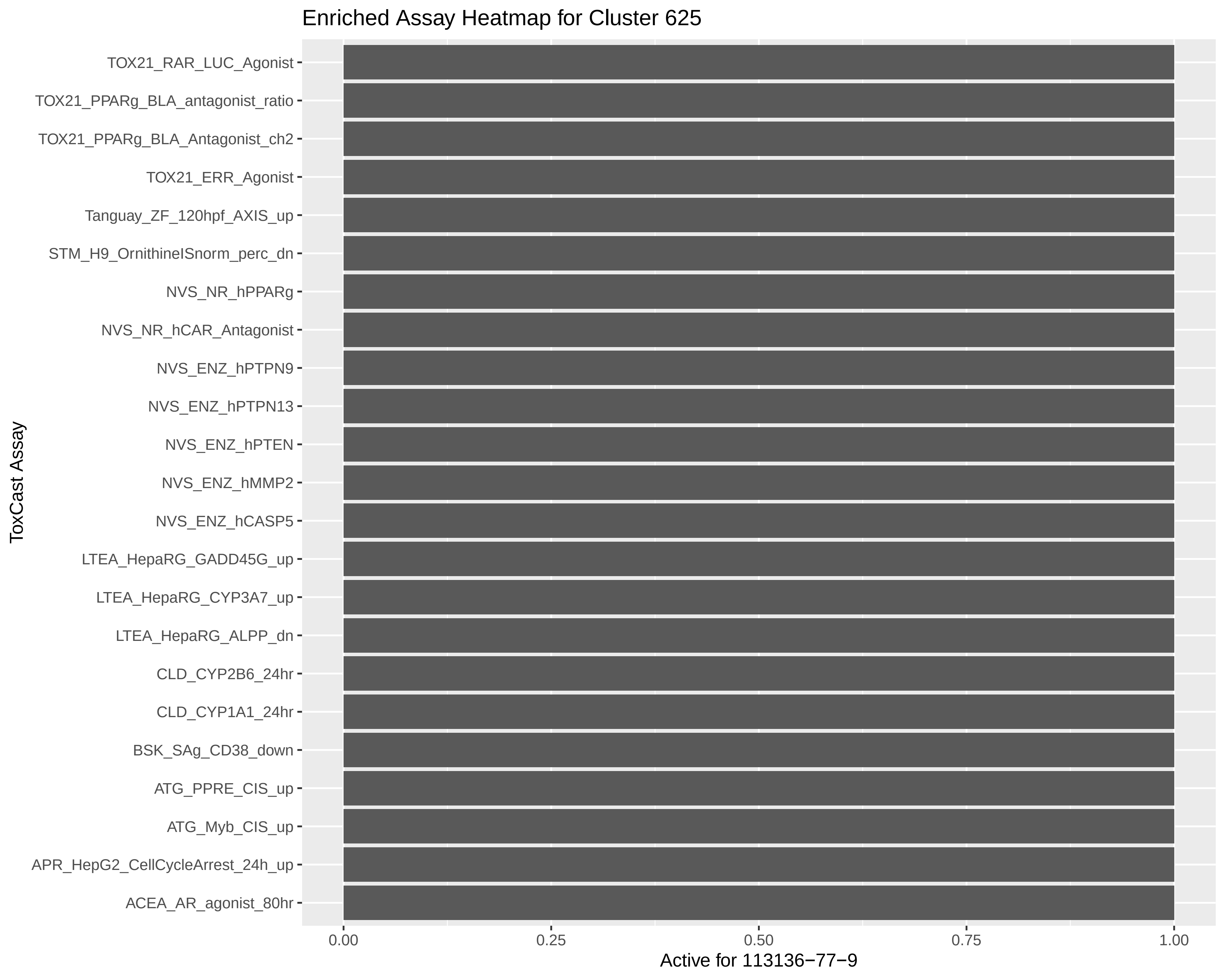

Enriched Assay Heatmap for Cluster 625
TOX21_RAR_LUC_Agonist
TOX21_PPARg_BLA_antagonist_ratio
TOX21_PPARg_BLA_Antagonist_ch2
TOX21_ERR_Agonist
Tanguay_ZF_120hpf_AXIS_up
STM_H9_OrnithineISnorm_perc_dn
NVS_NR_hPPARg
NVS_NR_hCAR_Antagonist
NVS_ENZ_hPTPN9
NVS_ENZ_hPTPN13
NVS_ENZ_hPTEN
NVS_ENZ_hMMP2
NVS_ENZ_hCASP5
LTEA_HepaRG_GADD45G_up
LTEA_HepaRG_CYP3A7_up
LTEA_HepaRG_ALPP_dn
CLD_CYP2B6_24hr
CLD_CYP1A1_24hr
BSK_SAg_CD38_down
ATG_PPRE_CIS_up
ATG_Myb_CIS_up
APR_HepG2_CellCycleArrest_24h_up
ACEA_AR_agonist_80hr
ToxCast Assay
0.00
0.25
0.50
0.75
1.00
Active for 113136−77−9

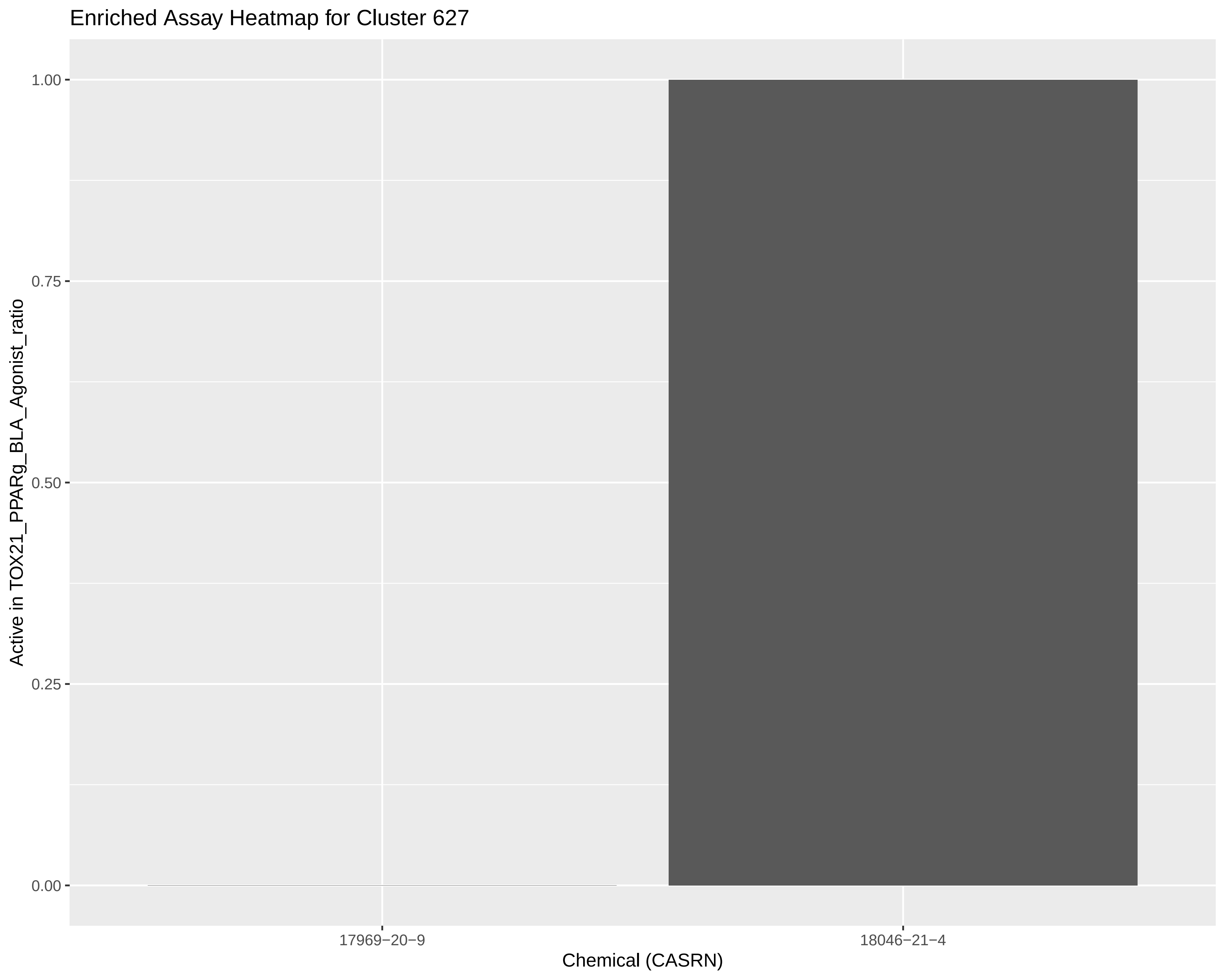
Enriched Assay Heatmap for Cluster 627
Active in TOX21_PPARg_BLA_Agonist_ratio
1.00
0.75
0.50
0.25
0.00
17969−20−9
18046−21−4
Chemical (CASRN)

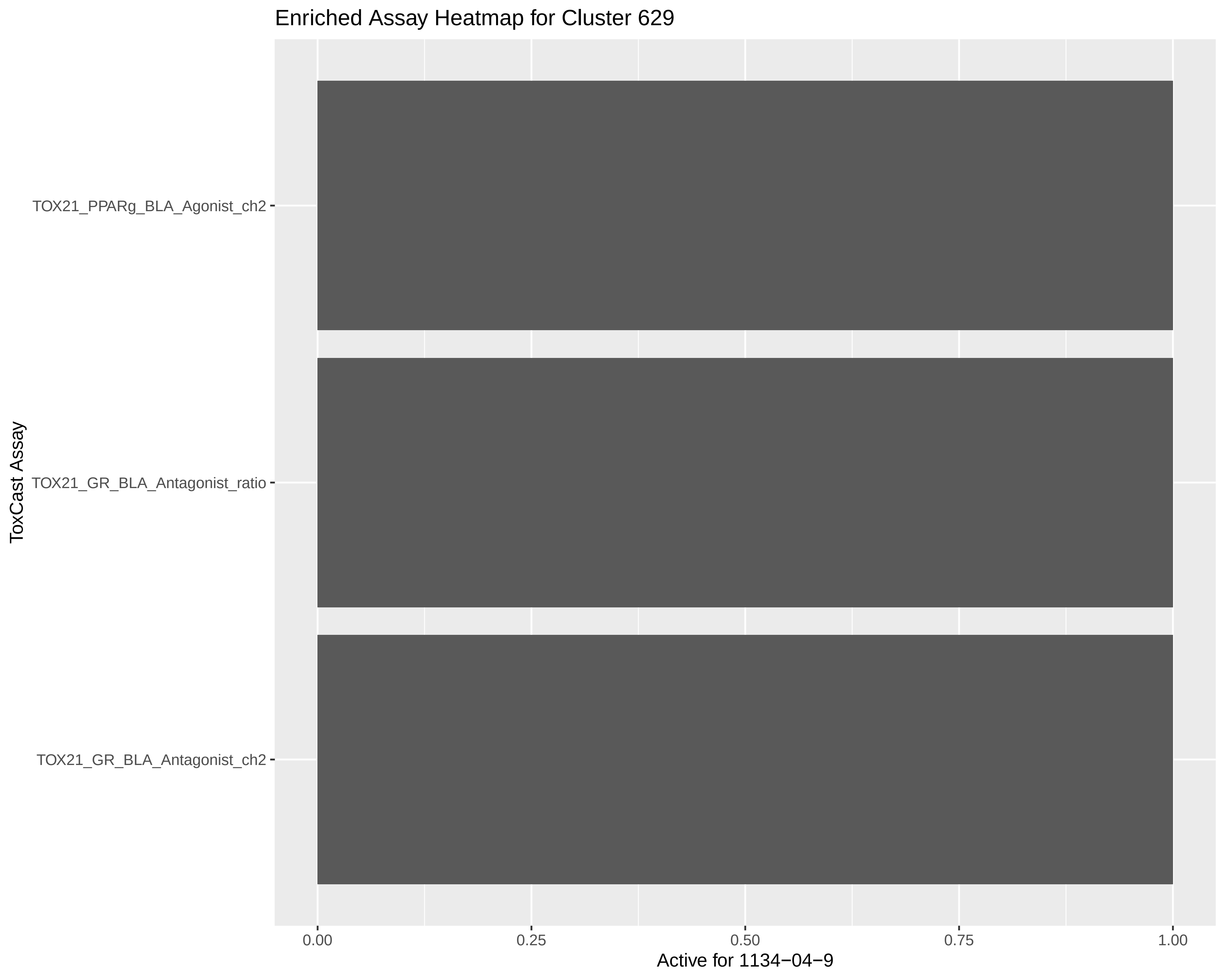

Enriched Assay Heatmap for Cluster 629
ToxCast Assay
TOX21_PPARg_BLA_Agonist_ch2
TOX21_GR_BLA_Antagonist_ratio
TOX21_GR_BLA_Antagonist_ch2
0.00
0.25
0.50
0.75
1.00
Active for 1134−04−9

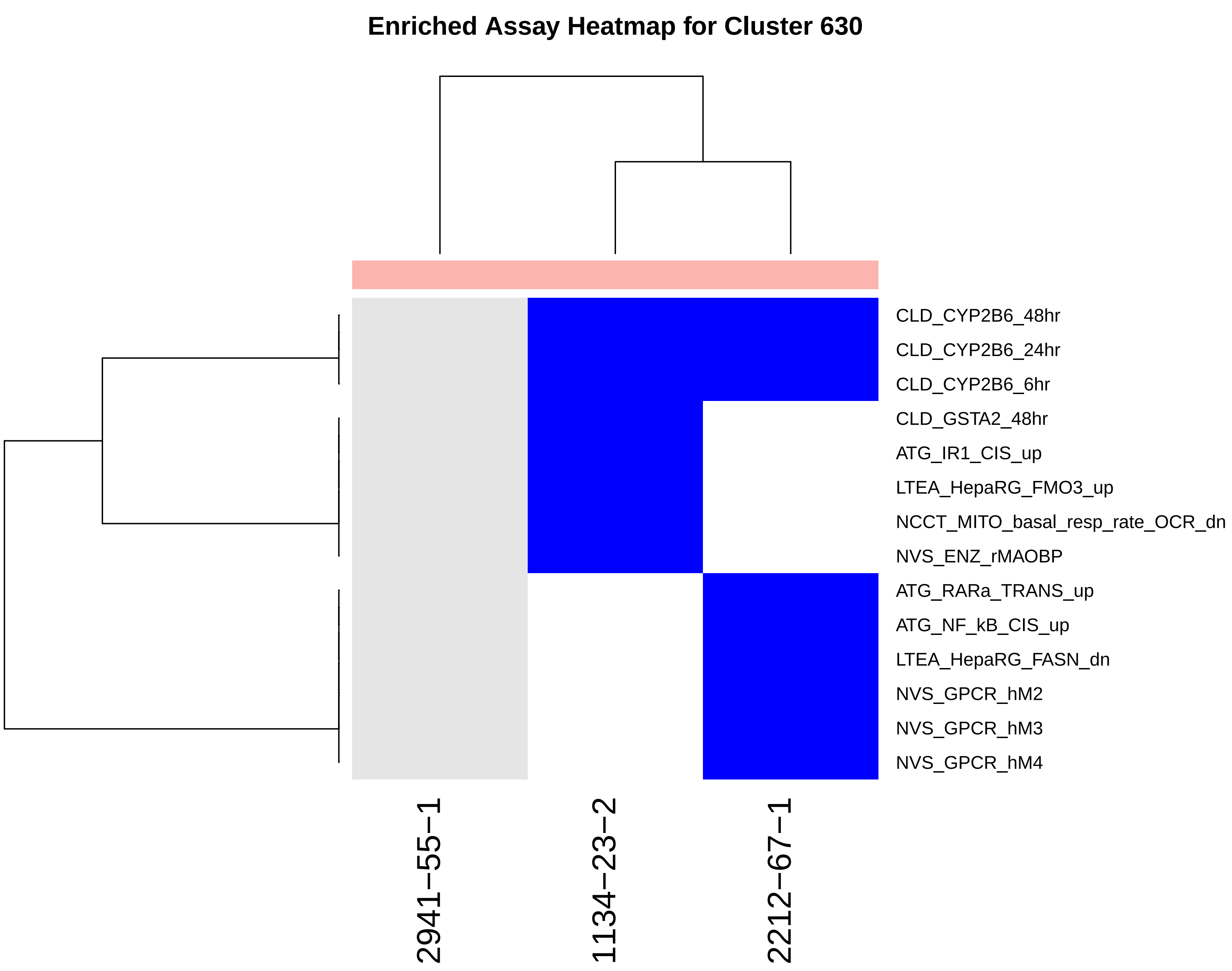
Enriched Assay Heatmap for Cluster 630
CLD_CYP2B6_48hr
CLD_CYP2B6_24hr
CLD_CYP2B6_6hr
CLD_GSTA2_48hr
ATG_IR1_CIS_up
LTEA_HepaRG_FMO3_up
NCCT_MITO_basal_resp_rate_OCR_dn
NVS_ENZ_rMAOBP
ATG_RARa_TRANS_up
ATG_NF_kB_CIS_up
LTEA_HepaRG_FASN_dn
NVS_GPCR_hM2
NVS_GPCR_hM3
NVS_GPCR_hM4
2941−55−1
1134−23−2
2212−67−1

Enriched Assay Heatmap for Cluster 631

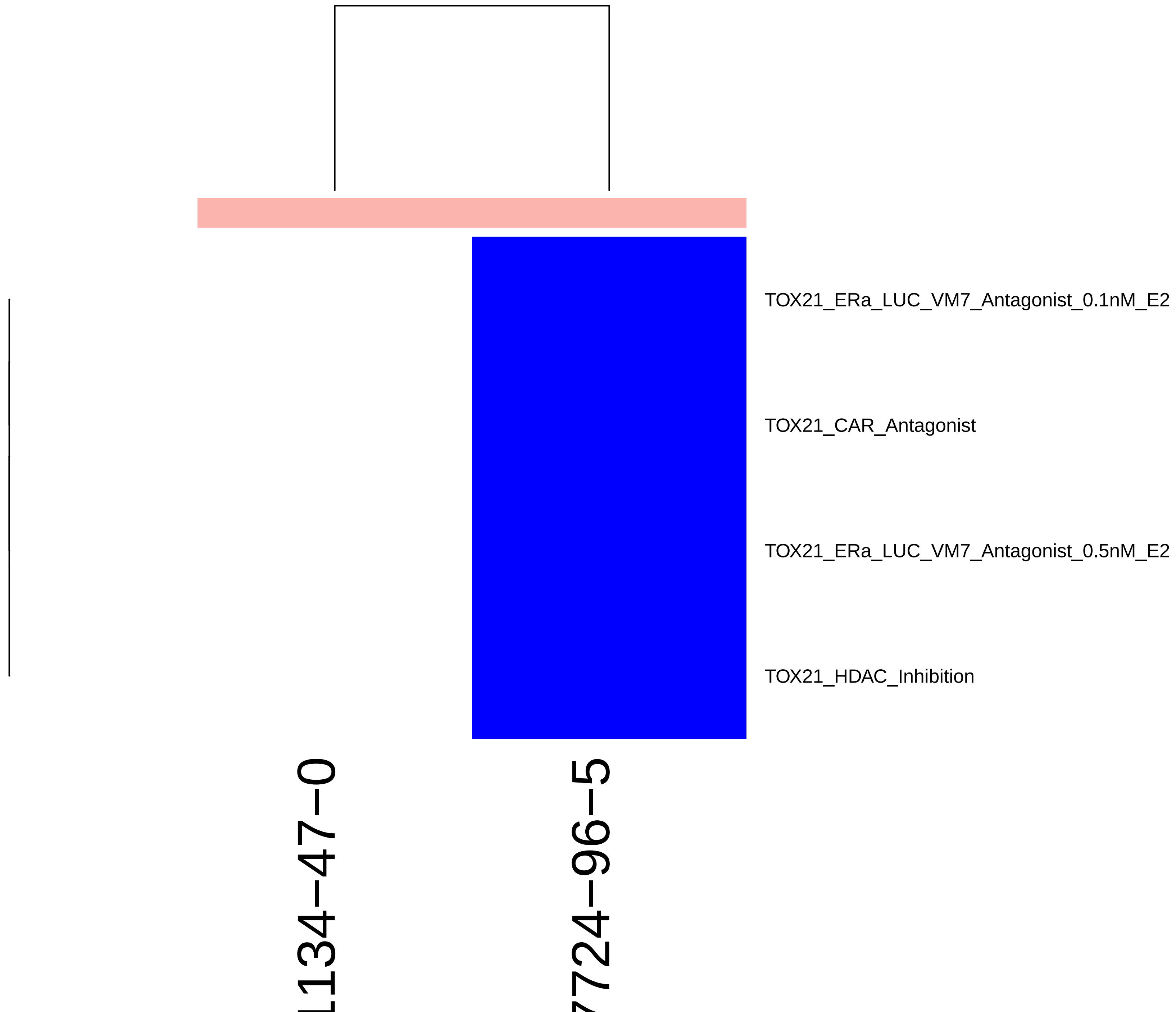

Enriched Assay Heatmap for Cluster 632

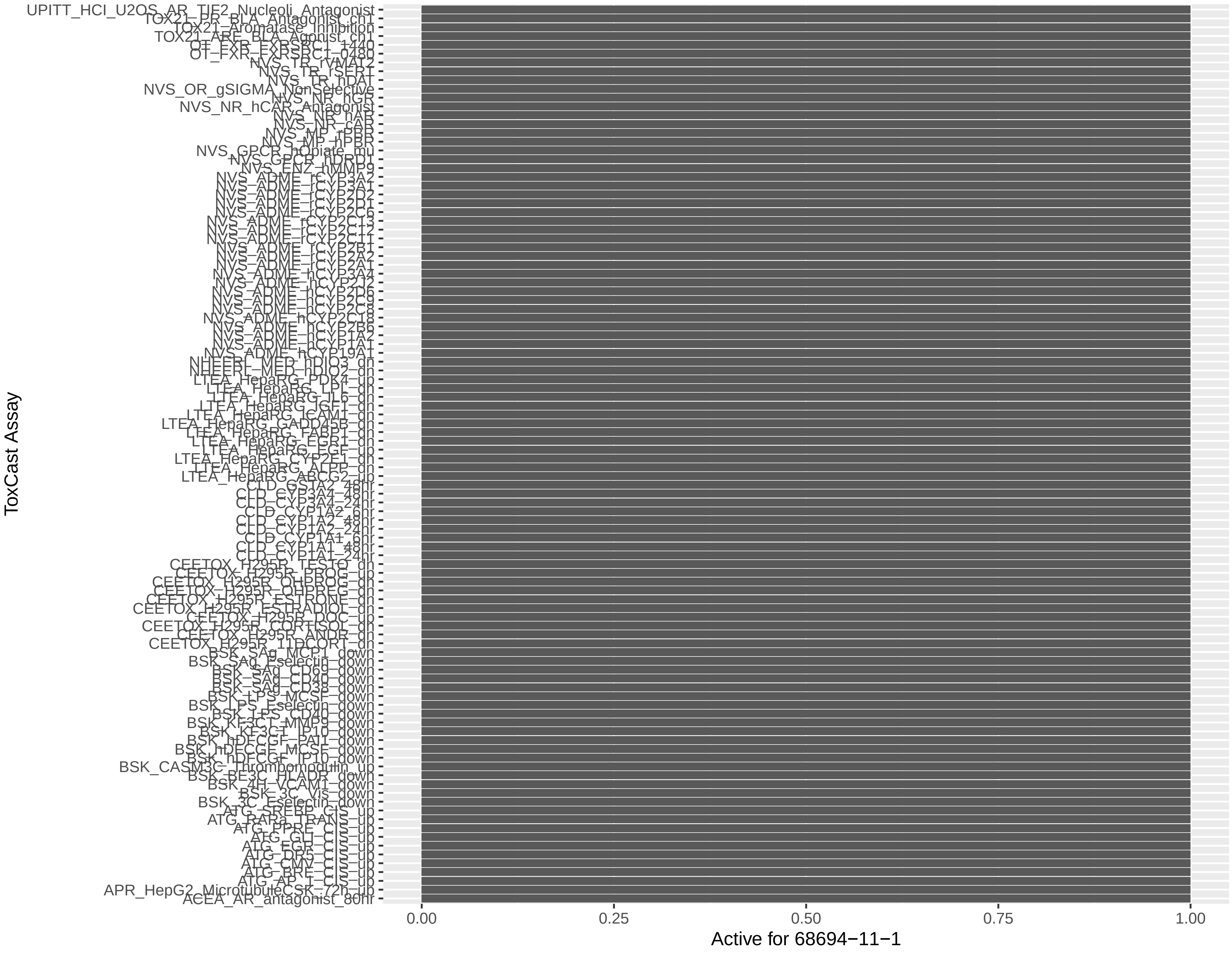

Enriched Assay Heatmap for Cluster 633

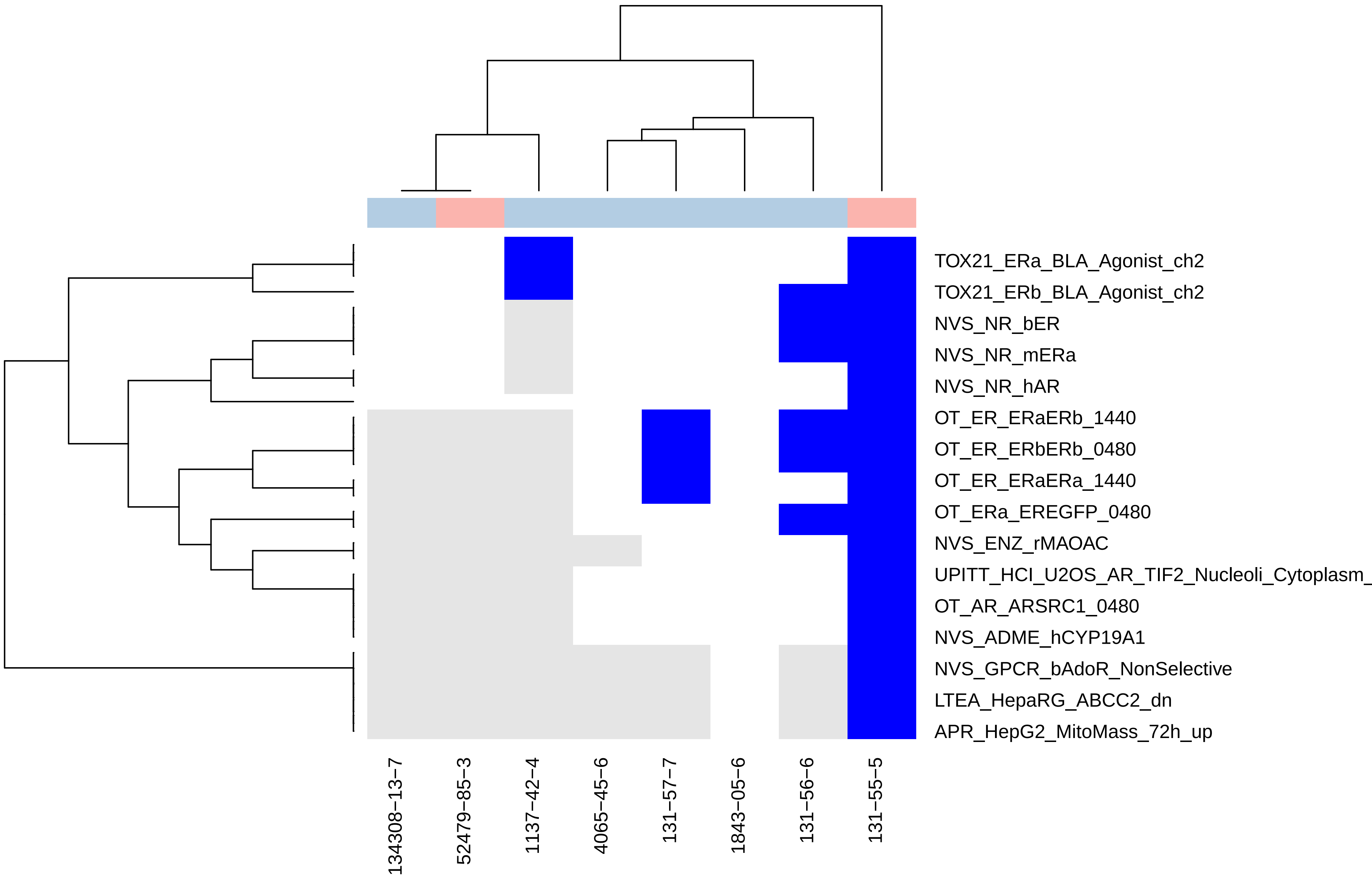

Enriched Assay Heatmap for Cluster 634

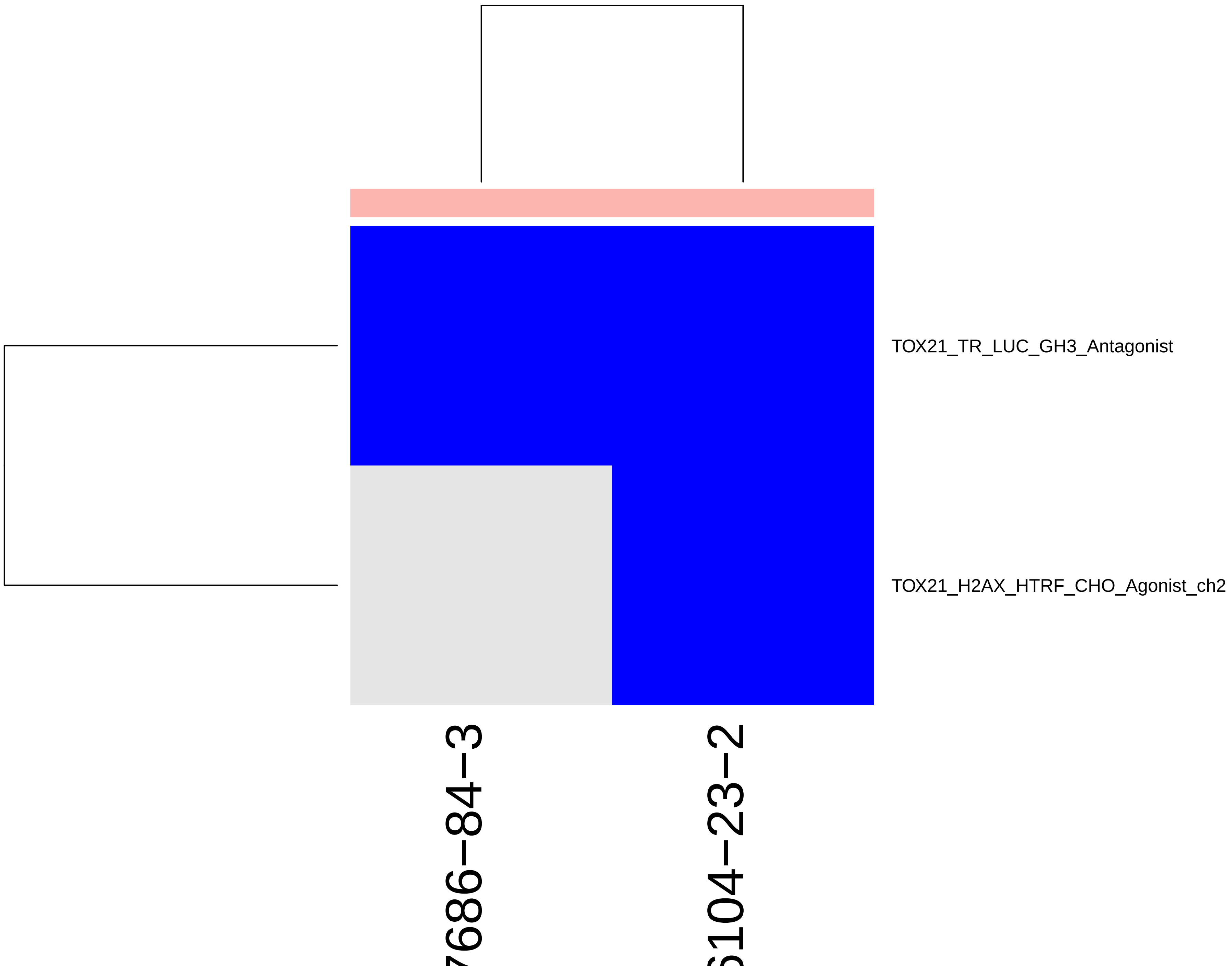

Enriched Assay Heatmap for Cluster 639

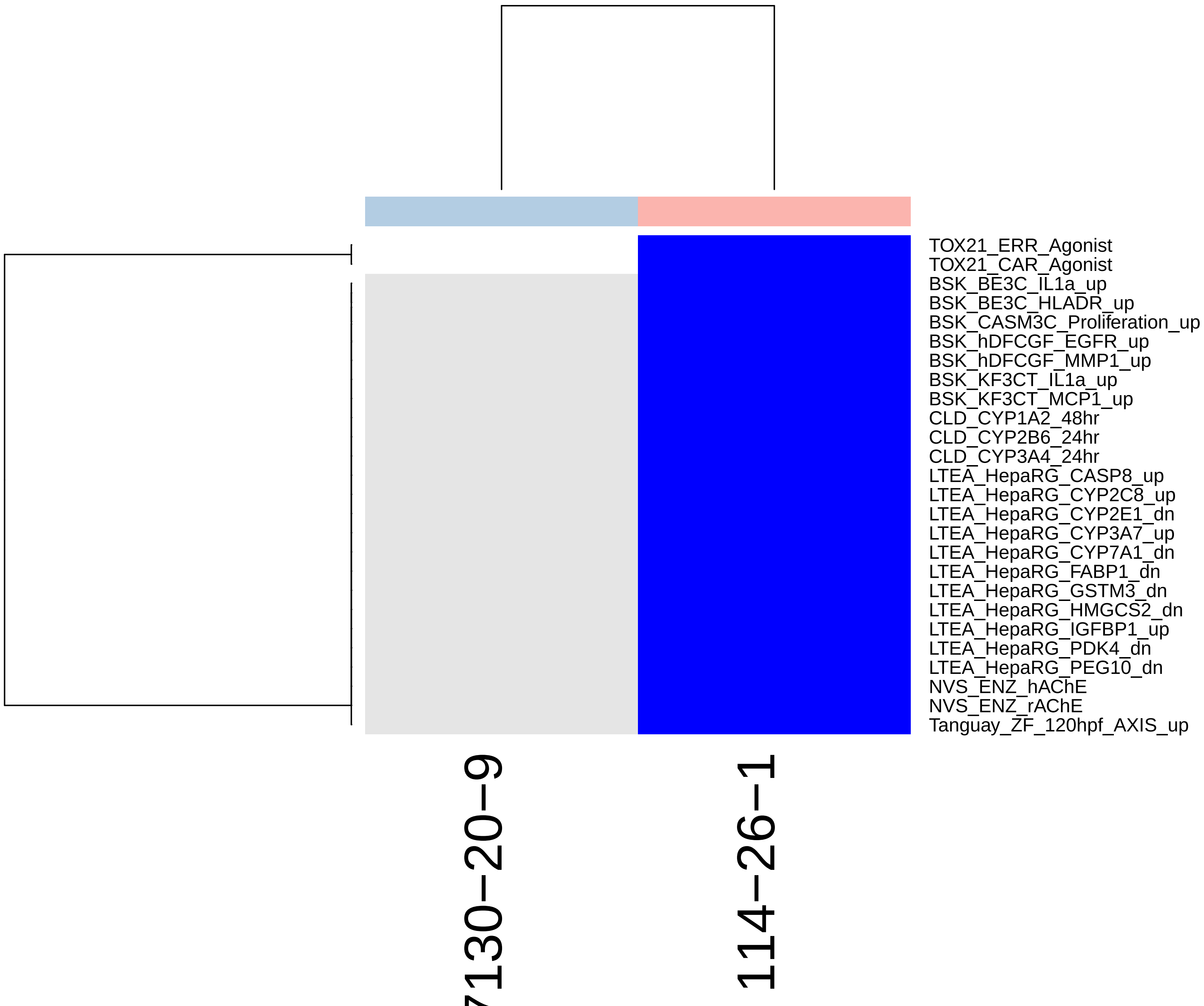

Enriched Assay Heatmap for Cluster 641

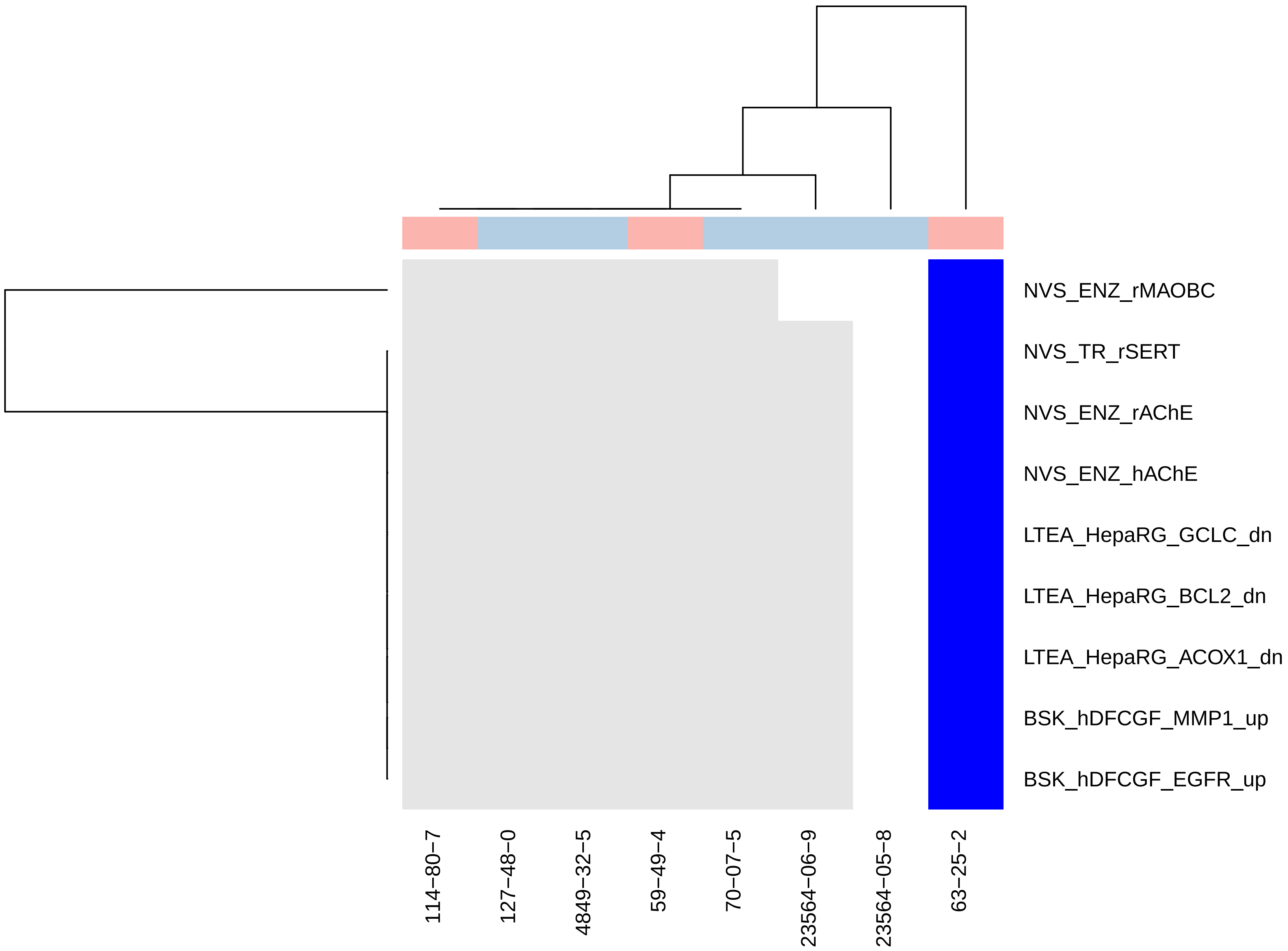

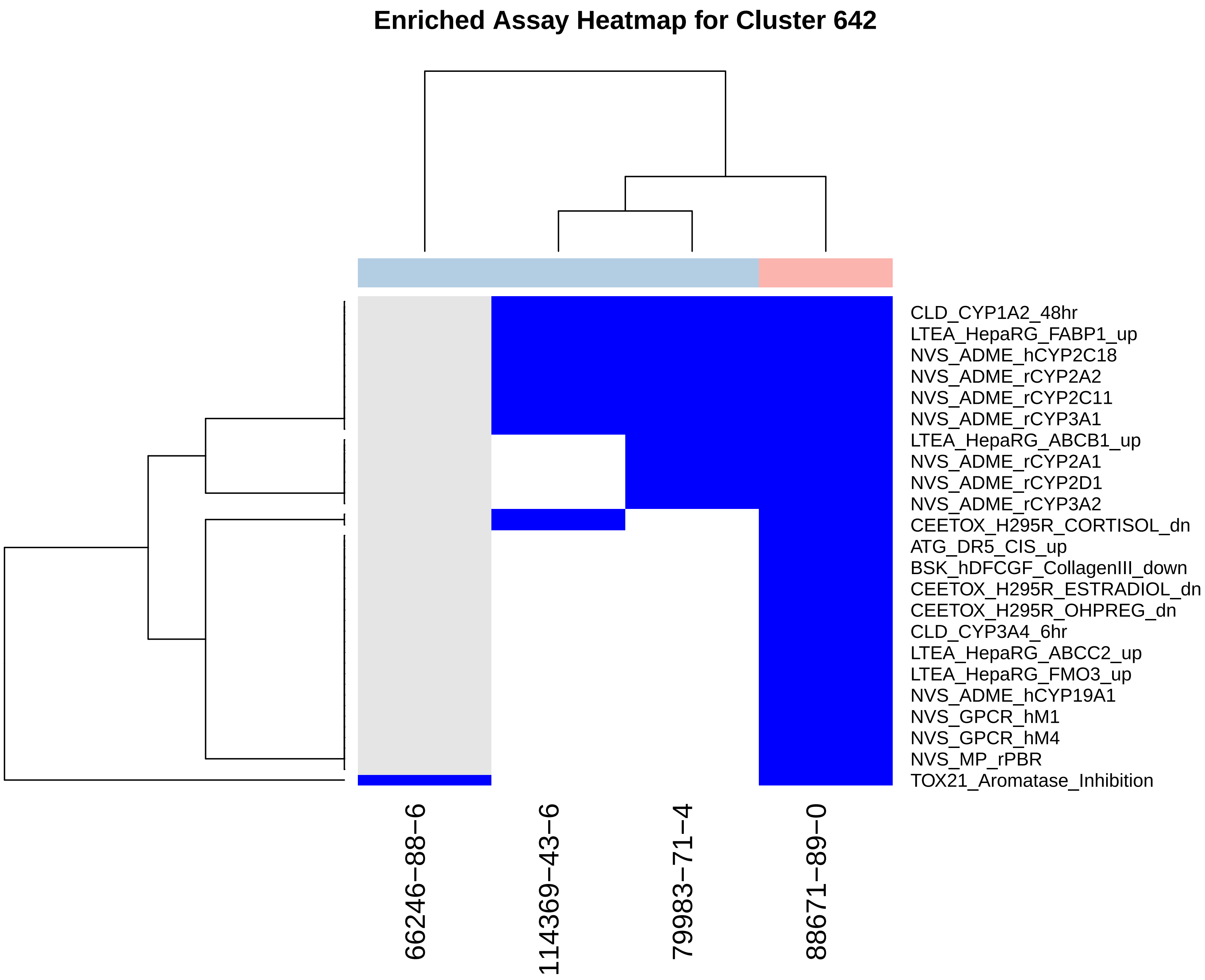
Enriched Assay Heatmap for Cluster 642
CLD_CYP1A2_48hr
LTEA_HepaRG_FABP1_up
NVS_ADME_hCYP2C18
NVS_ADME_rCYP2A2
NVS_ADME_rCYP2C11
NVS_ADME_rCYP3A1
LTEA_HepaRG_ABCB1_up
NVS_ADME_rCYP2A1
NVS_ADME_rCYP2D1
NVS_ADME_rCYP3A2
CEETOX_H295R_CORTISOL_dn
ATG_DR5_CIS_up
BSK_hDFCGF_CollagenIII_down
CEETOX_H295R_ESTRADIOL_dn
CEETOX_H295R_OHPREG_dn
CLD_CYP3A4_6hr
LTEA_HepaRG_ABCC2_up
LTEA_HepaRG_FMO3_up
NVS_ADME_hCYP19A1
NVS_GPCR_hM1
NVS_GPCR_hM4
NVS_MP_rPBR
TOX21_Aromatase_Inhibition
66246−88−6
114369−43−6
79983−71−4
88671−89−0

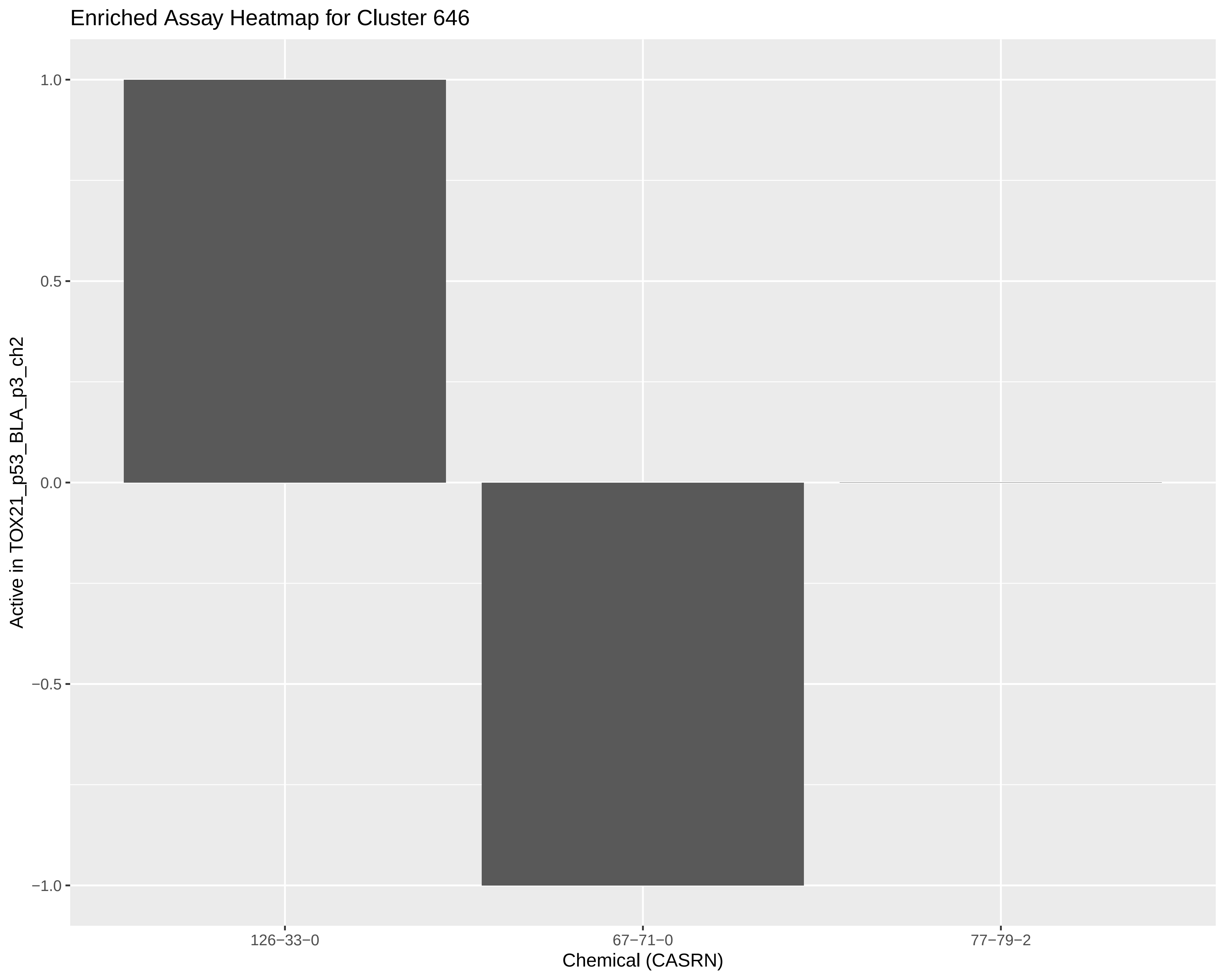

Enriched Assay Heatmap for Cluster 646
1.0
0.5
0.0
−0.5
−1.0
Active in TOX21_p53_BLA_p3_ch2
126−33−0
67−71−0
77−79−2
Chemical (CASRN)

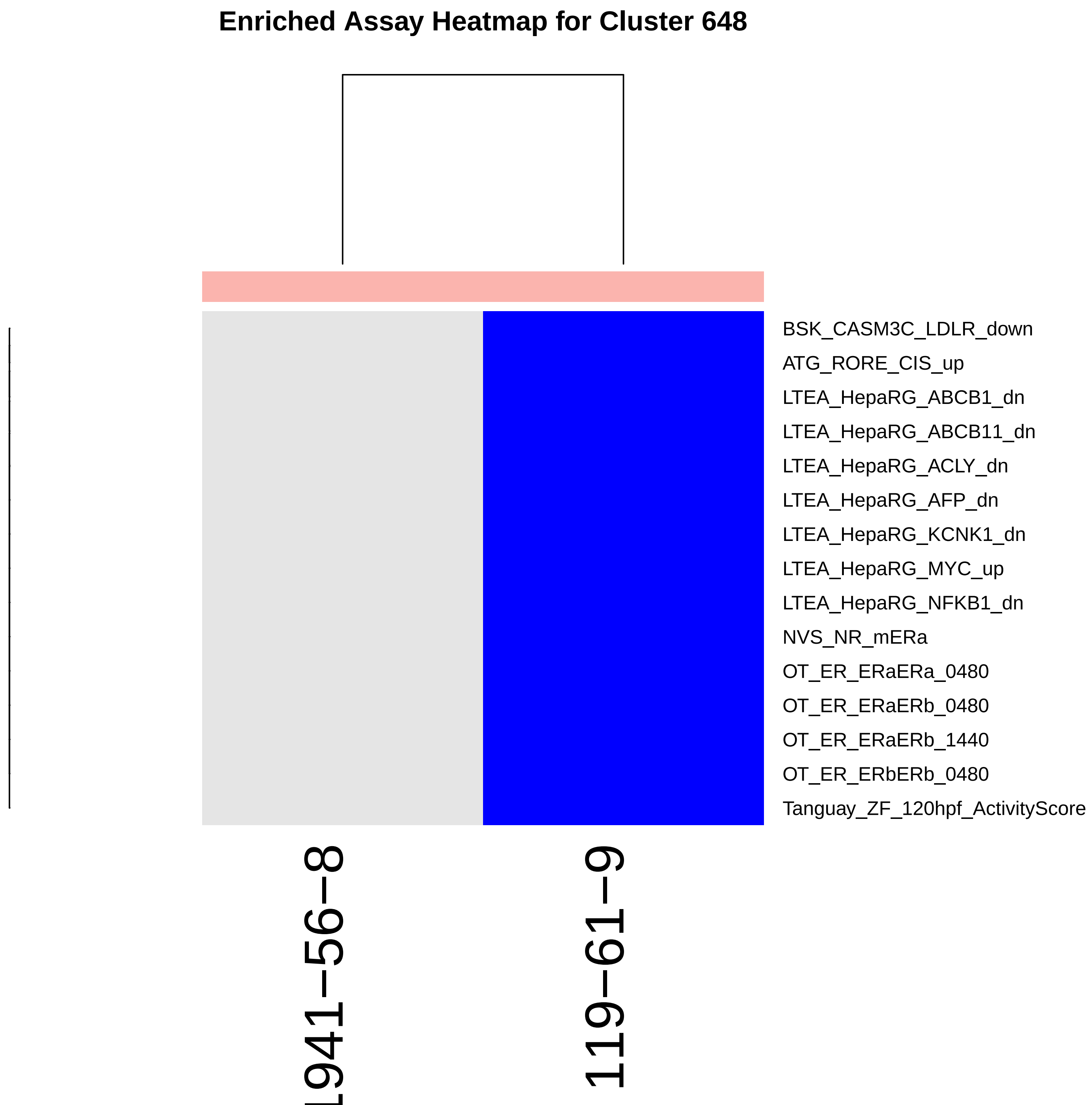
Enriched Assay Heatmap for Cluster 648
BSK_CASM3C_LDLR_down
ATG_RORE_CIS_up
LTEA_HepaRG_ABCB1_dn
LTEA_HepaRG_ABCB11_dn
LTEA_HepaRG_ACLY_dn
LTEA_HepaRG_AFP_dn
LTEA_HepaRG_KCNK1_dn
LTEA_HepaRG_MYC_up
LTEA_HepaRG_NFKB1_dn
NVS_NR_mERa
OT_ER_ERaERa_0480
OT_ER_ERaERb_0480
OT_ER_ERaERb_1440
OT_ER_ERbERb_0480
Tanguay_ZF_120hpf_ActivityScore
941−56−8
119−61−9

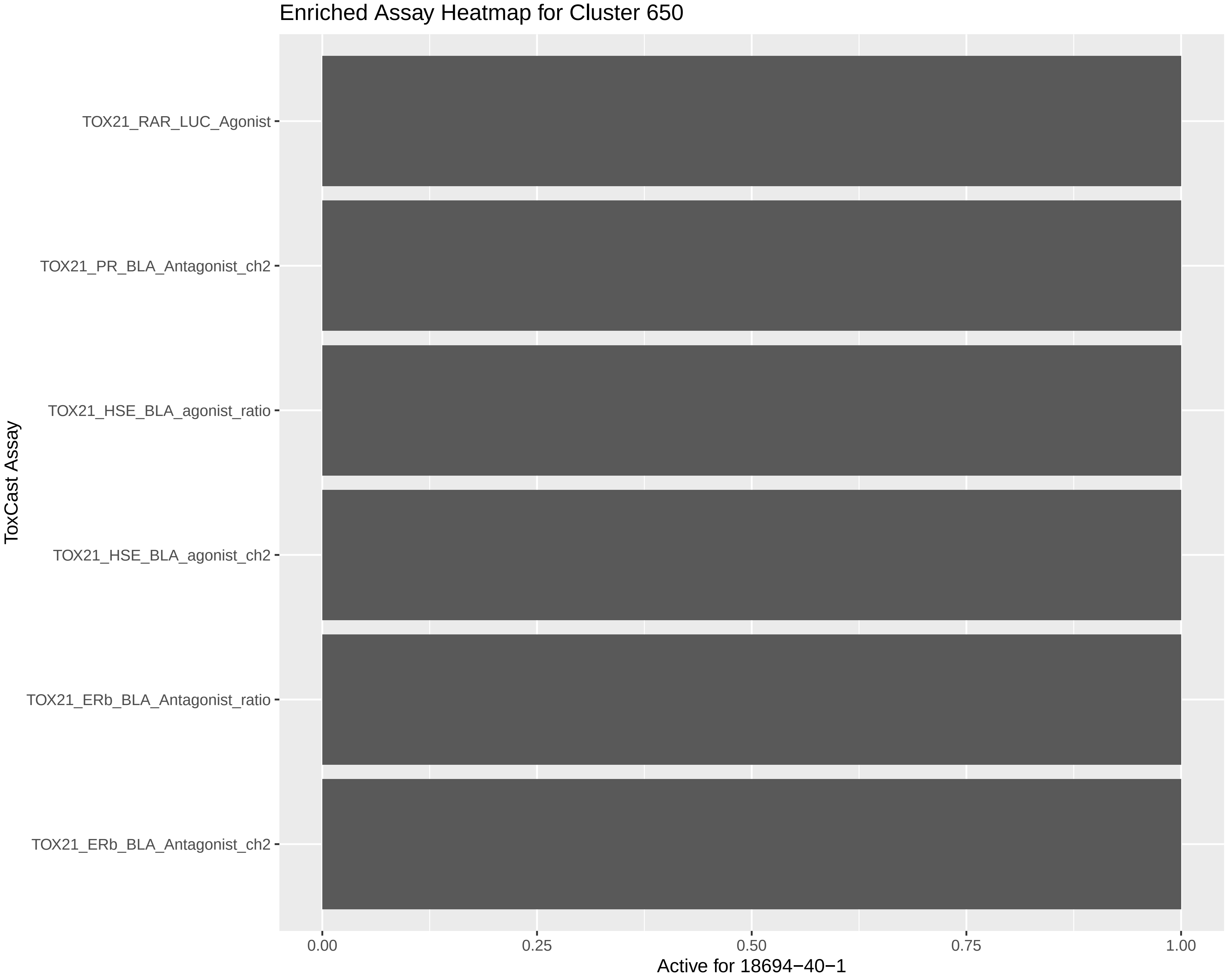

Enriched Assay Heatmap for Cluster 650
ToxCast Assay
TOX21_RAR_LUC_Agonist
TOX21_PR_BLA_Antagonist_ch2
TOX21_HSE_BLA_agonist_ratio
TOX21_HSE_BLA_agonist_ch2
TOX21_ERb_BLA_Antagonist_ratio
TOX21_ERb_BLA_Antagonist_ch2
0.00
0.25
0.50
0.75
1.00
Active for 18694−40−1

Enriched Assay Heatmap for Cluster 653

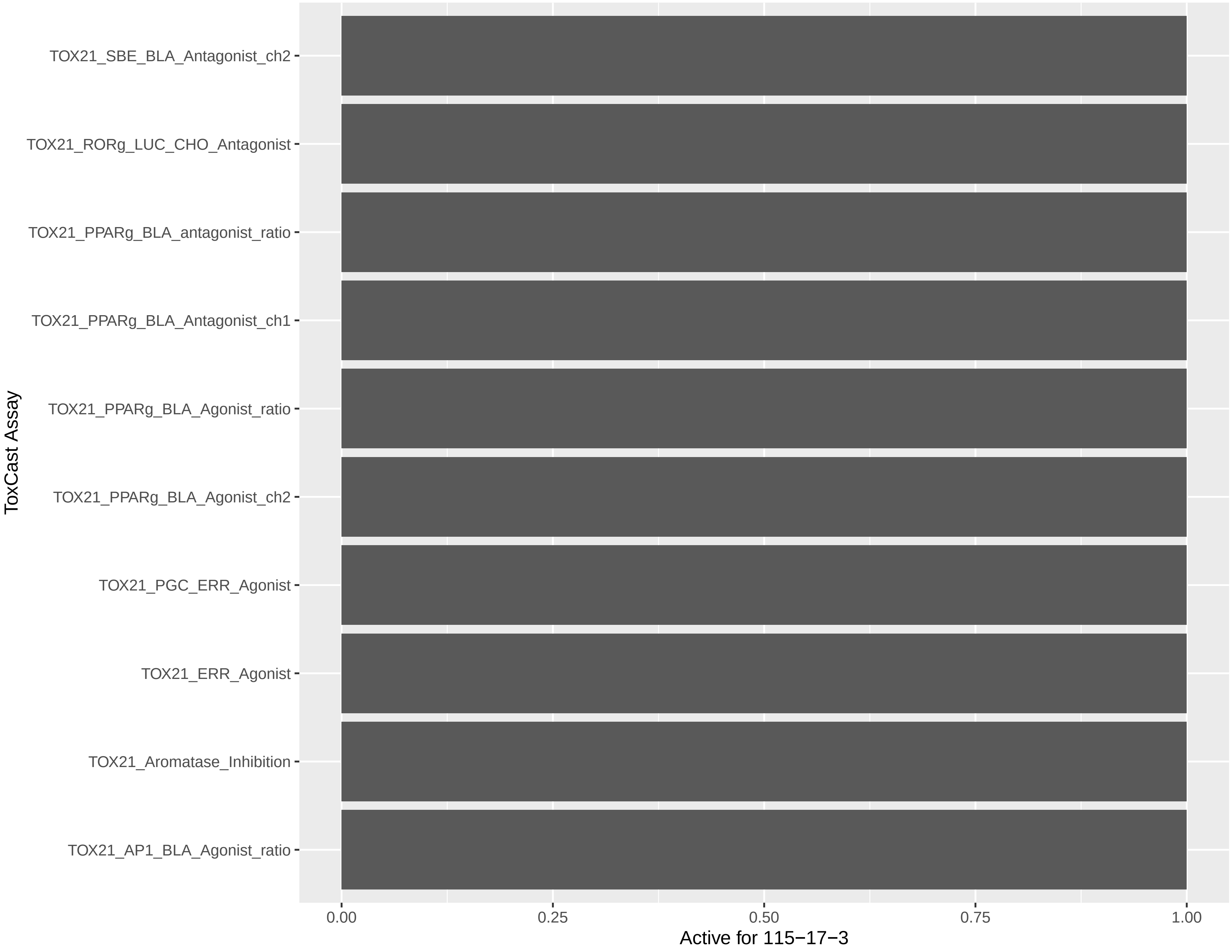

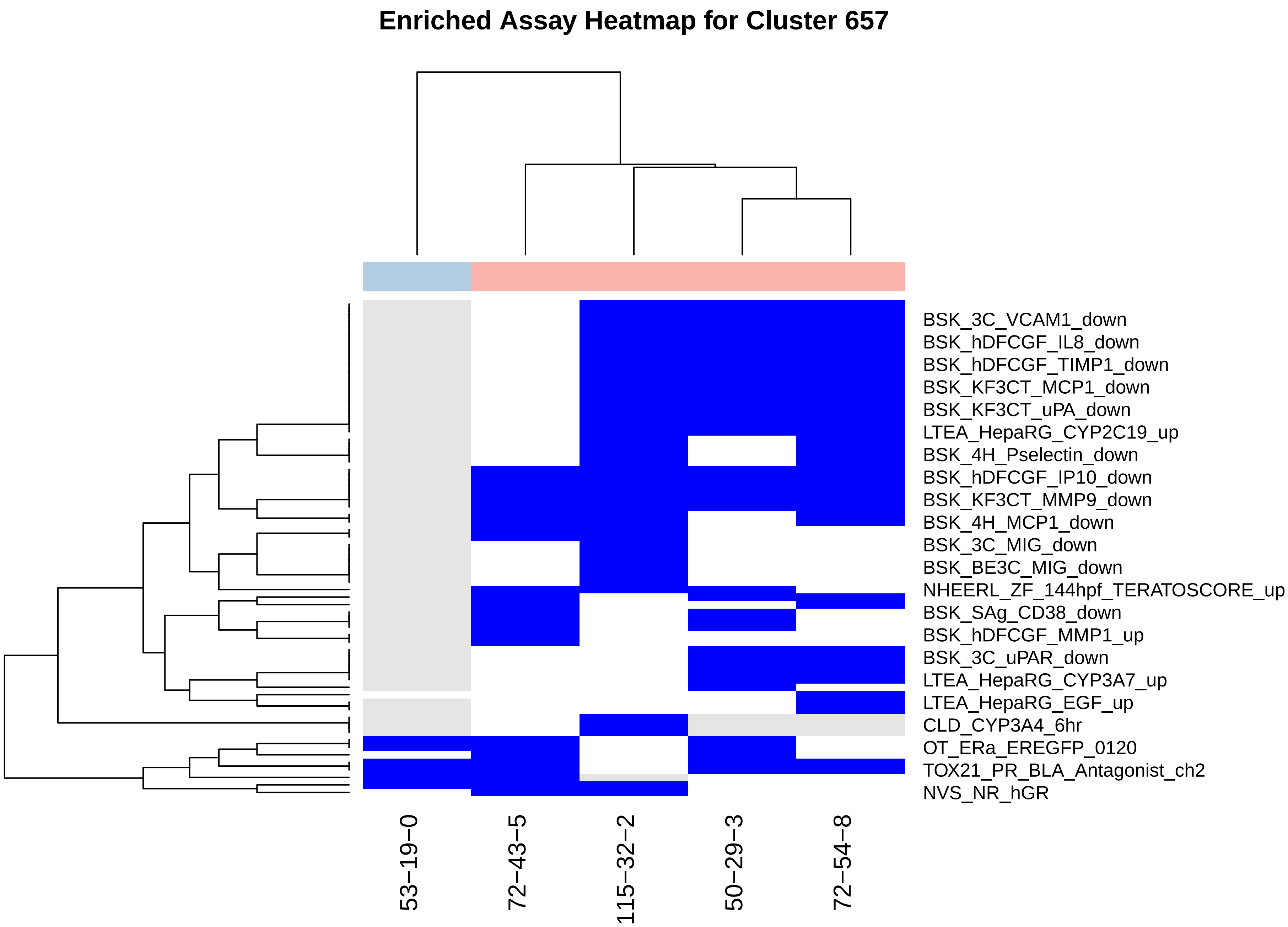

Enriched Assay Heatmap for Cluster 657
BSK_3C_VCAM1_down
BSK_hDFCGF_IL8_down
BSK_hDFCGF_TIMP1_down
BSK_KF3CT_MCP1_down
BSK_KF3CT_uPA_down
LTEA_HepaRG_CYP2C19_up
BSK_4H_Pselectin_down
BSK_hDFCGF_IP10_down
BSK_KF3CT_MMP9_down
BSK_4H_MCP1_down
BSK_3C_MIG_down
BSK_BE3C_MIG_down
NHEERL_ZF_144hpf_TERATOSCORE_up
BSK_SAg_CD38_down
BSK_hDFCGF_MMP1_up
BSK_3C_uPAR_down
LTEA_HepaRG_CYP3A7_up
LTEA_HepaRG_EGF_up
CLD_CYP3A4_6hr
OT_ERa_EREGFP_0120
TOX21_PR_BLA_Antagonist_ch2
NVS_NR_hGR
53−19−0
72−43−5
115−32−2
50−29−3
72−54−8

Enriched Assay Heatmap for Cluster 658

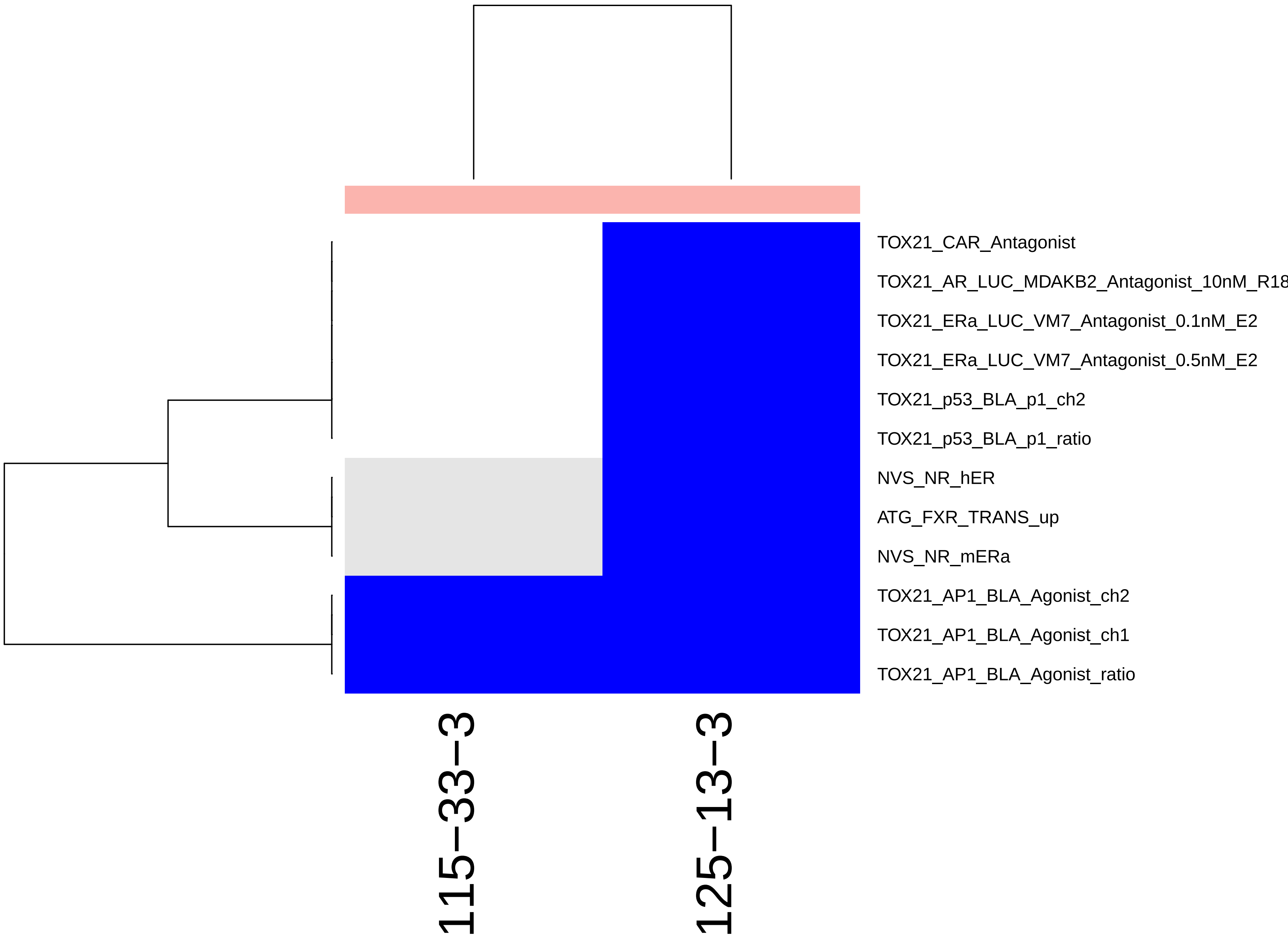

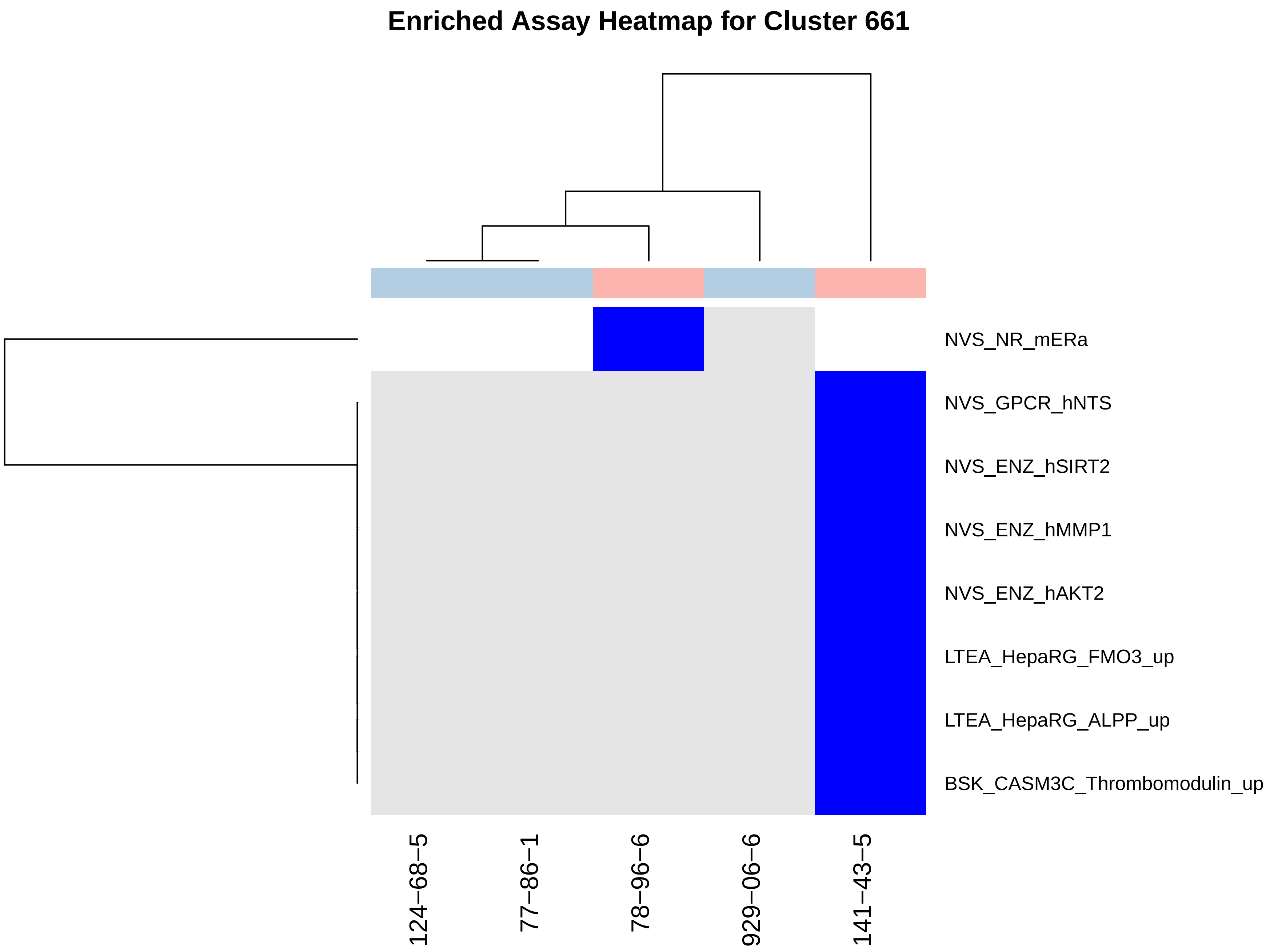

Enriched Assay Heatmap for Cluster 661
NVS_NR_mERa
NVS_GPCR_hNTS
NVS_ENZ_hSIRT2
NVS_ENZ_hMMP1
NVS_ENZ_hAKT2
LTEA_HepaRG_FMO3_up
LTEA_HepaRG_ALPP_up
BSK_CASM3C_Thrombomodulin_up
124−68−5
77−86−1
78−96−6
929−06−6
141−43−5

Enriched Assay Heatmap for Cluster 665

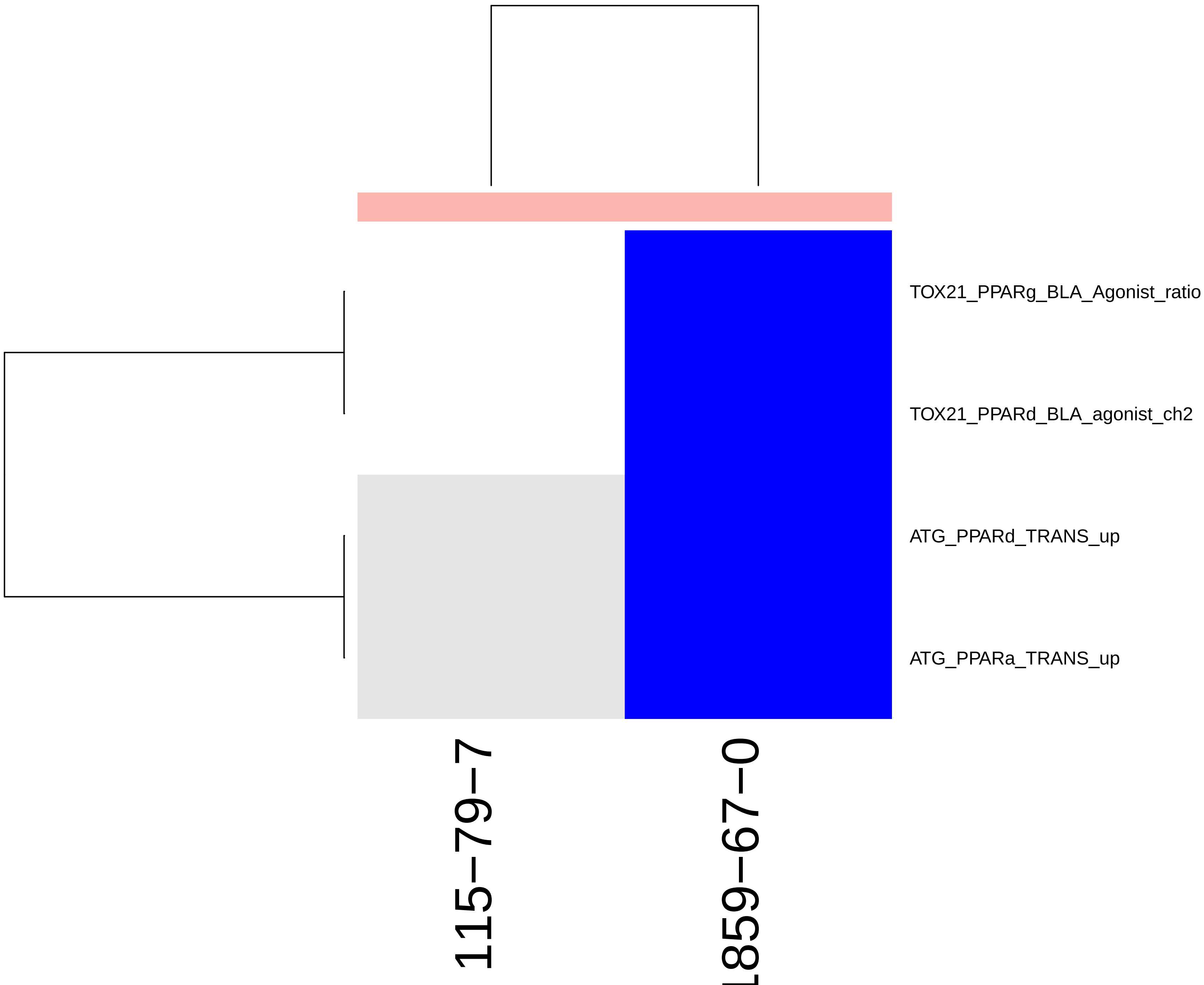

Enriched Assay Heatmap for Cluster 667

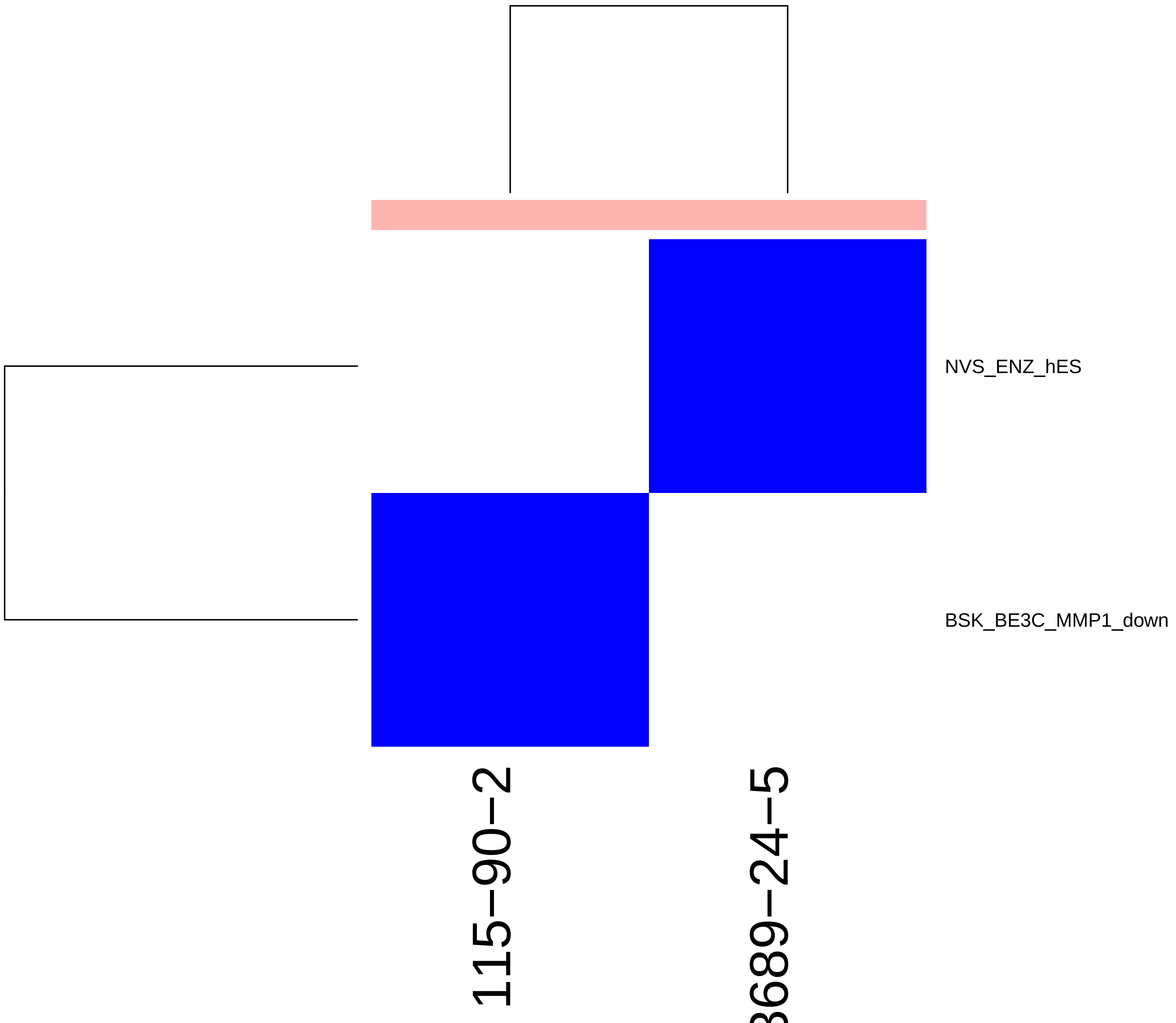

Enriched Assay Heatmap for Cluster 668

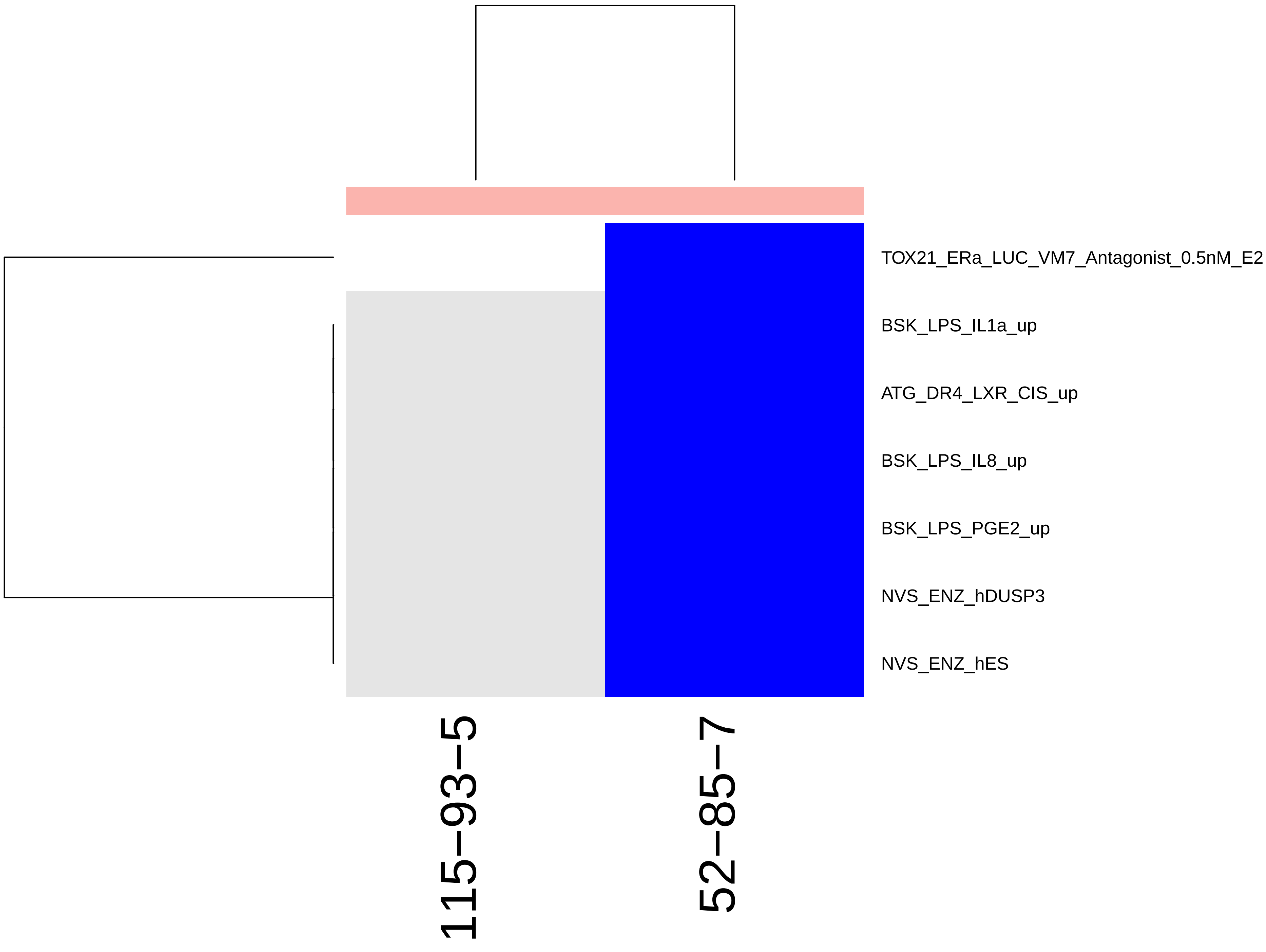

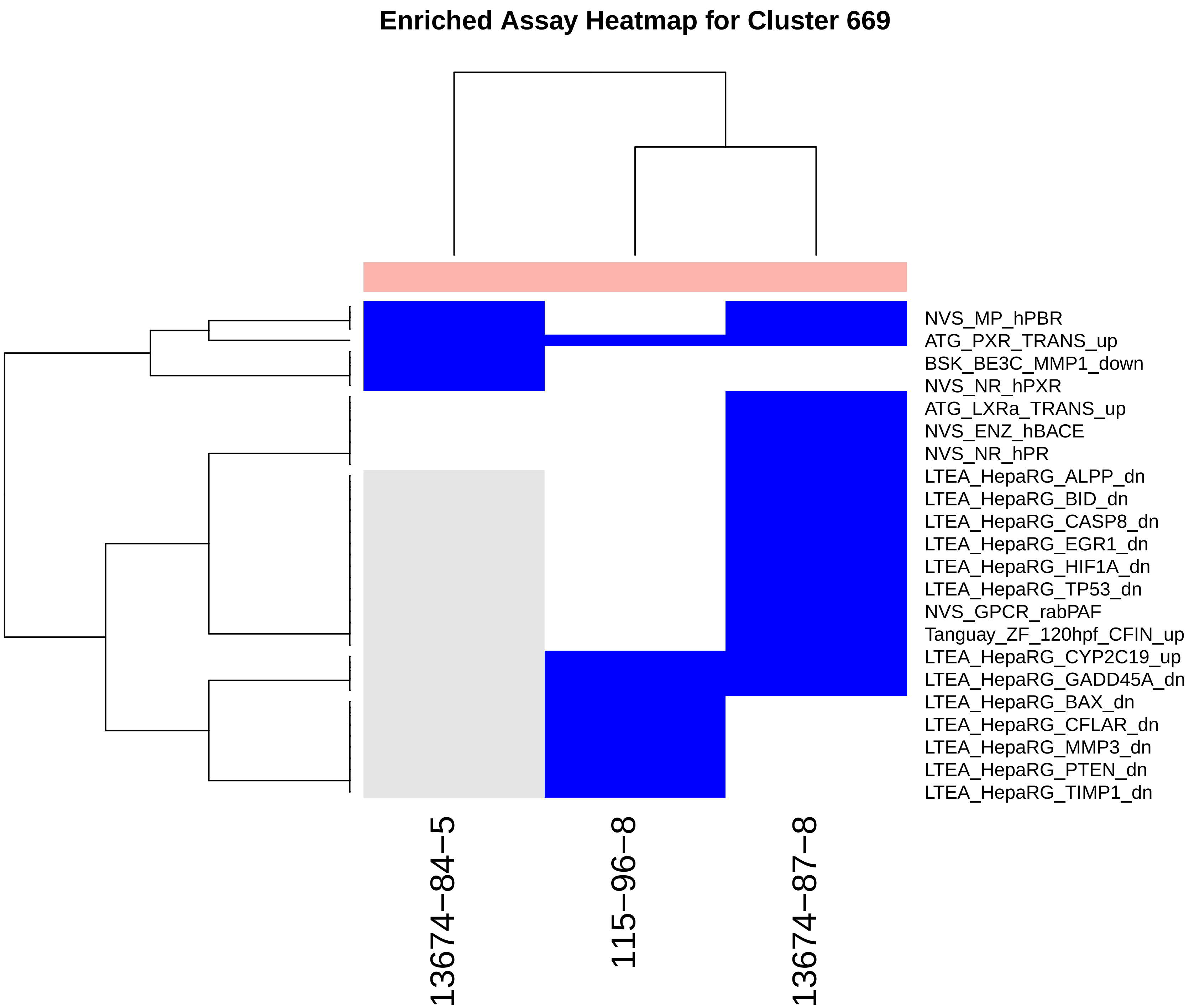
Enriched Assay Heatmap for Cluster 669
NVS_MP_hPBR
ATG_PXR_TRANS_up
BSK_BE3C_MMP1_down
NVS_NR_hPXR
ATG_LXRa_TRANS_up
NVS_ENZ_hBACE
NVS_NR_hPR
LTEA_HepaRG_ALPP_dn
LTEA_HepaRG_BID_dn
LTEA_HepaRG_CASP8_dn
LTEA_HepaRG_EGR1_dn
LTEA_HepaRG_HIF1A_dn
LTEA_HepaRG_TP53_dn
NVS_GPCR_rabPAF
Tanguay_ZF_120hpf_CFIN_up
LTEA_HepaRG_CYP2C19_up
LTEA_HepaRG_GADD45A_dn
LTEA_HepaRG_BAX_dn
LTEA_HepaRG_CFLAR_dn
LTEA_HepaRG_MMP3_dn
LTEA_HepaRG_PTEN_dn
LTEA_HepaRG_TIMP1_dn
13674−84−5
115−96−8
13674−87−8

Enriched Assay Heatmap for Cluster 671

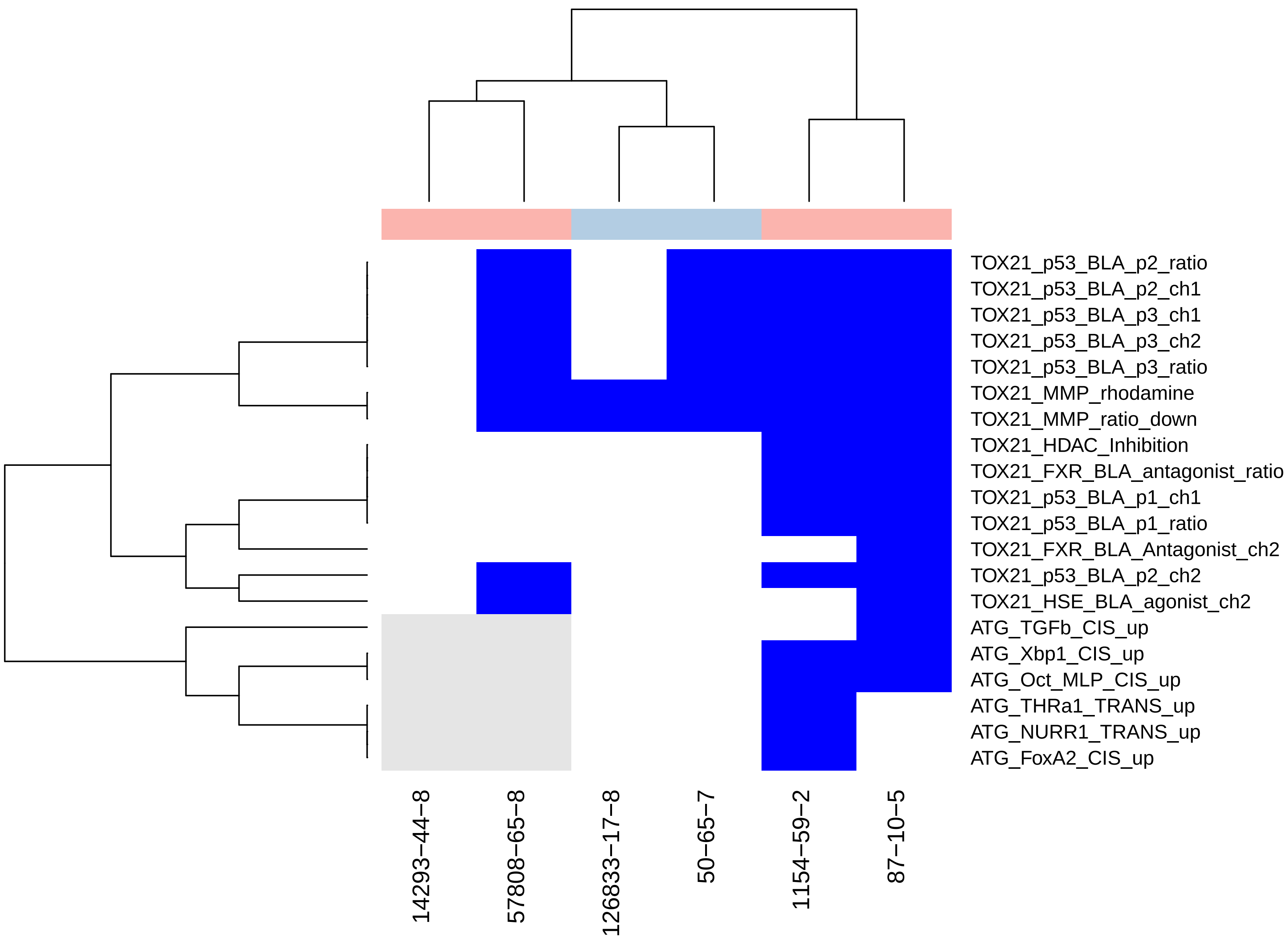

Enriched Assay Heatmap for Cluster 674

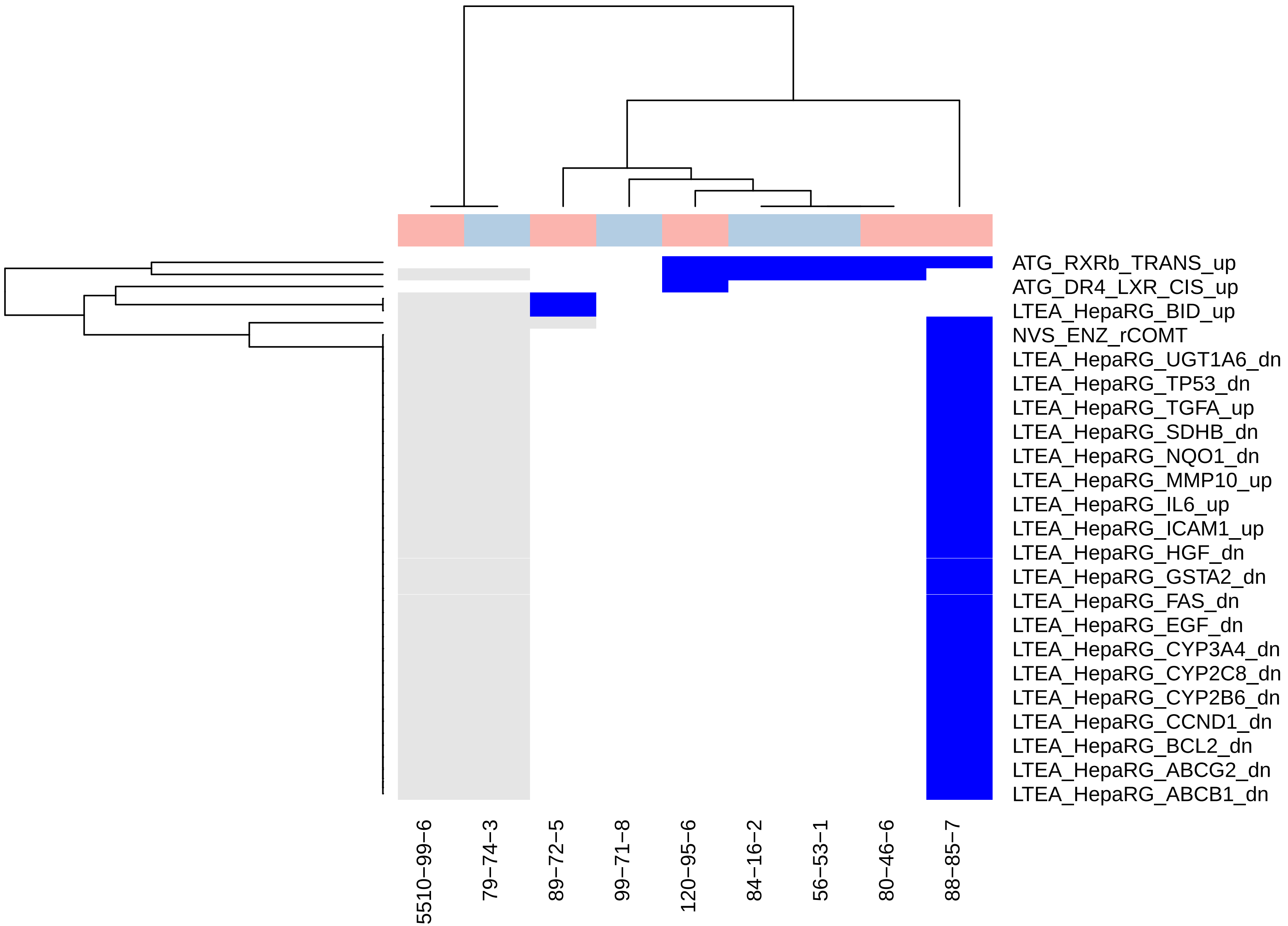

Enriched Assay Heatmap for Cluster 675

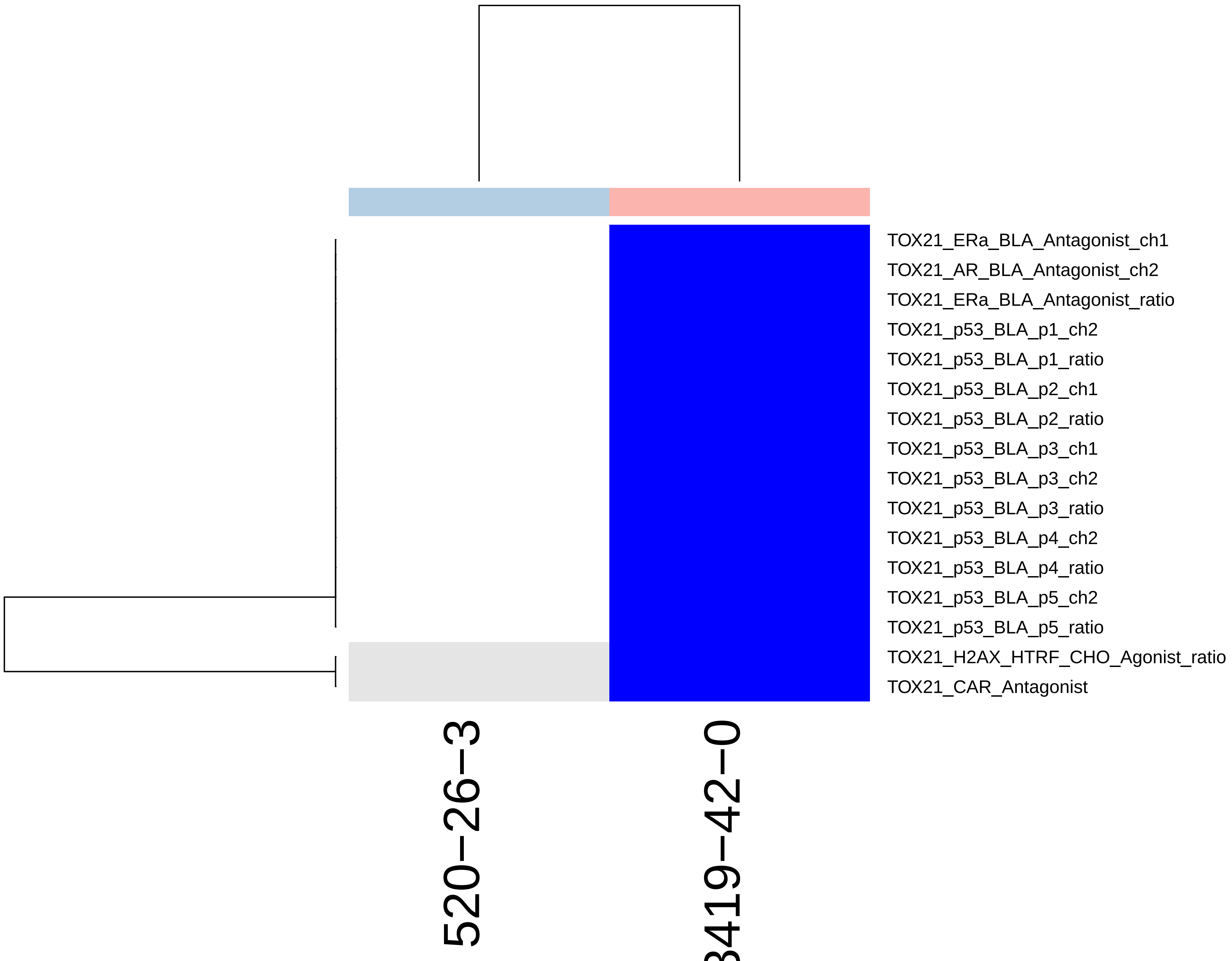

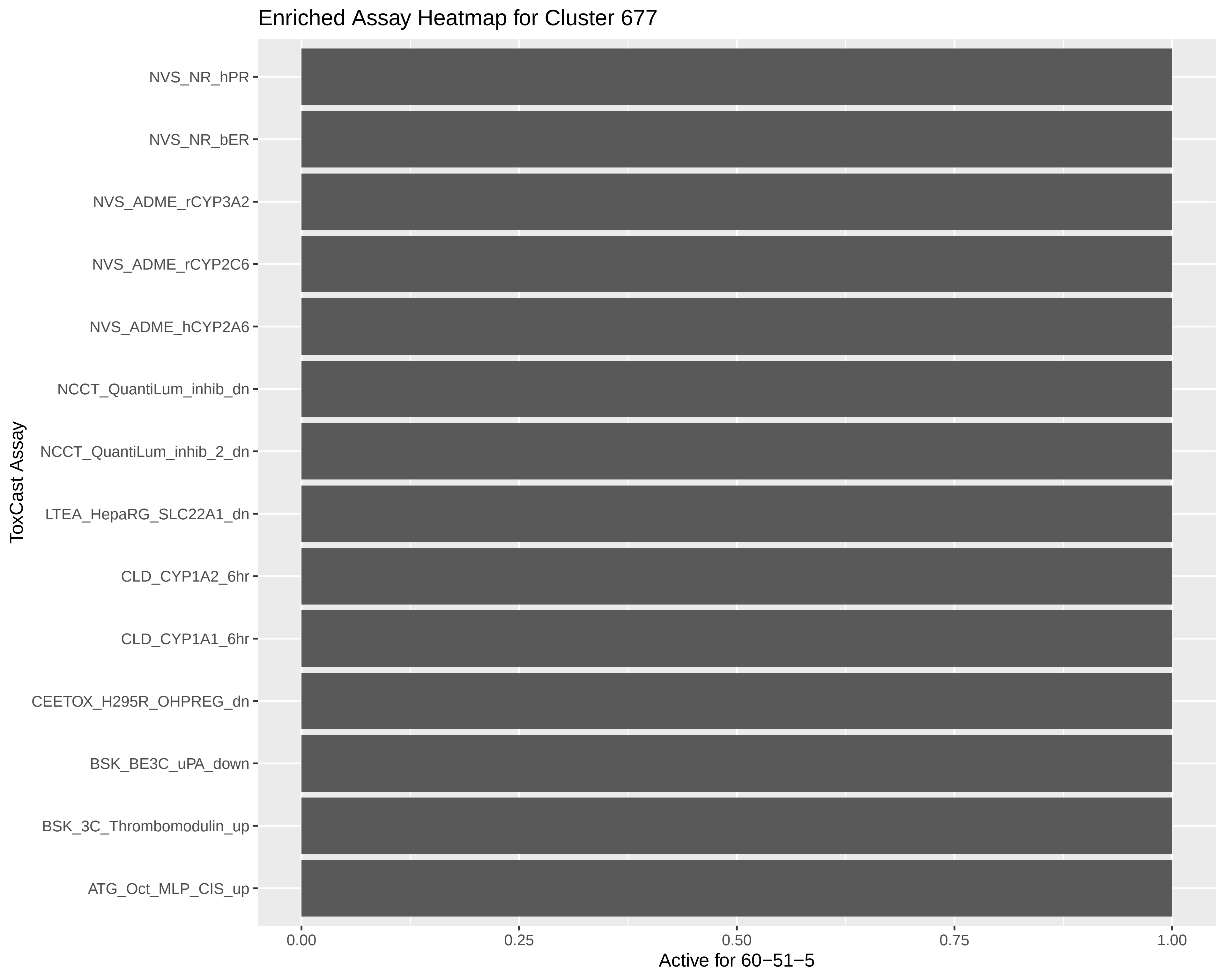
Enriched Assay Heatmap for Cluster 677
ToxCast Assay
NVS_NR_hPR
NVS_NR_bER
NVS_ADME_rCYP3A2
NVS_ADME_rCYP2C6
NVS_ADME_hCYP2A6
NCCT_QuantiLum_inhib_dn
NCCT_QuantiLum_inhib_2_dn
LTEA_HepaRG_SLC22A1_dn
CLD_CYP1A2_6hr
CLD_CYP1A1_6hr
CEETOX_H295R_OHPREG_dn
BSK_BE3C_uPA_down
BSK_3C_Thrombomodulin_up
ATG_Oct_MLP_CIS_up
0.00
0.25
0.50
0.75
1.00
Active for 60−51−5

Enriched Assay Heatmap for Cluster 678

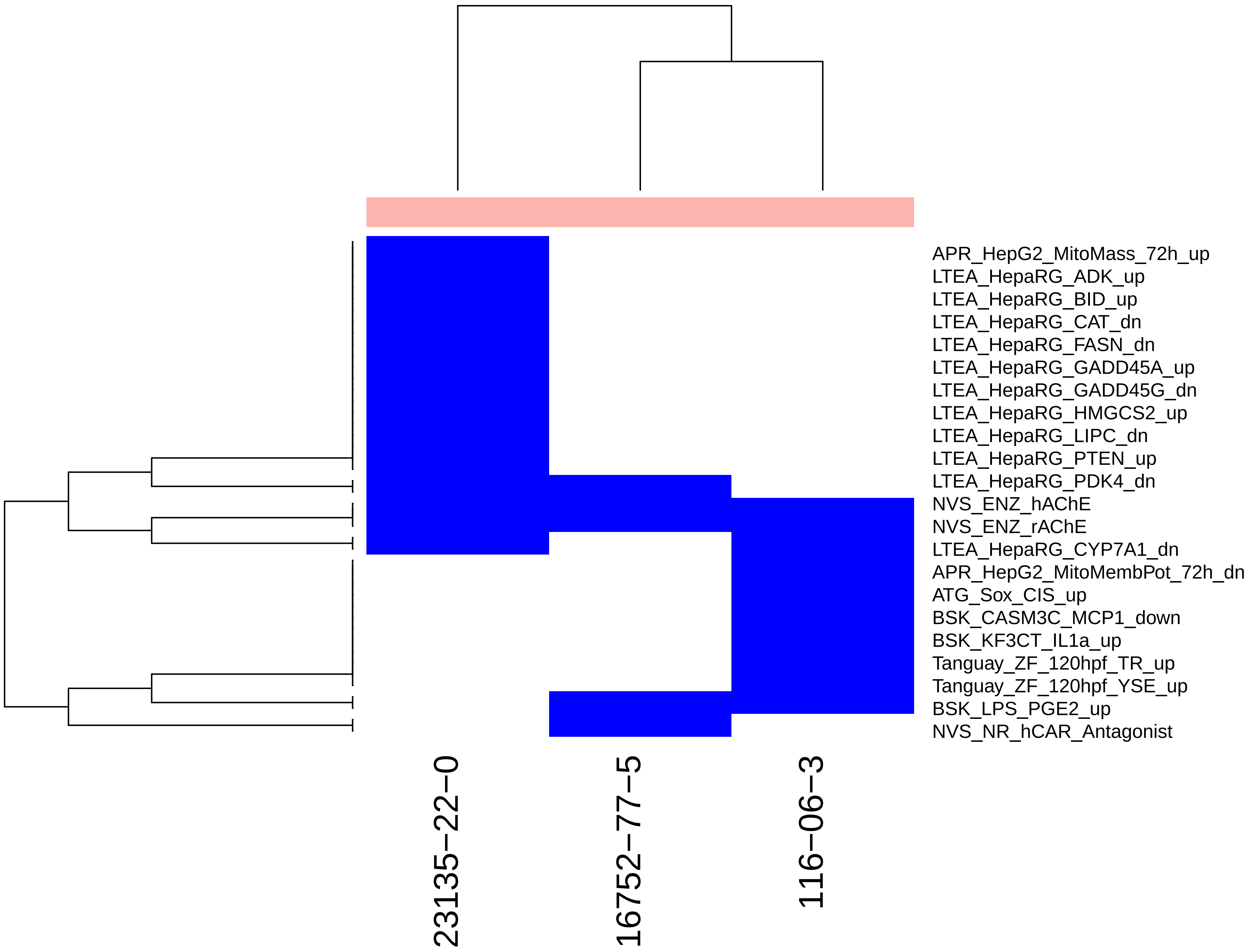

Enriched Assay Heatmap for Cluster 680

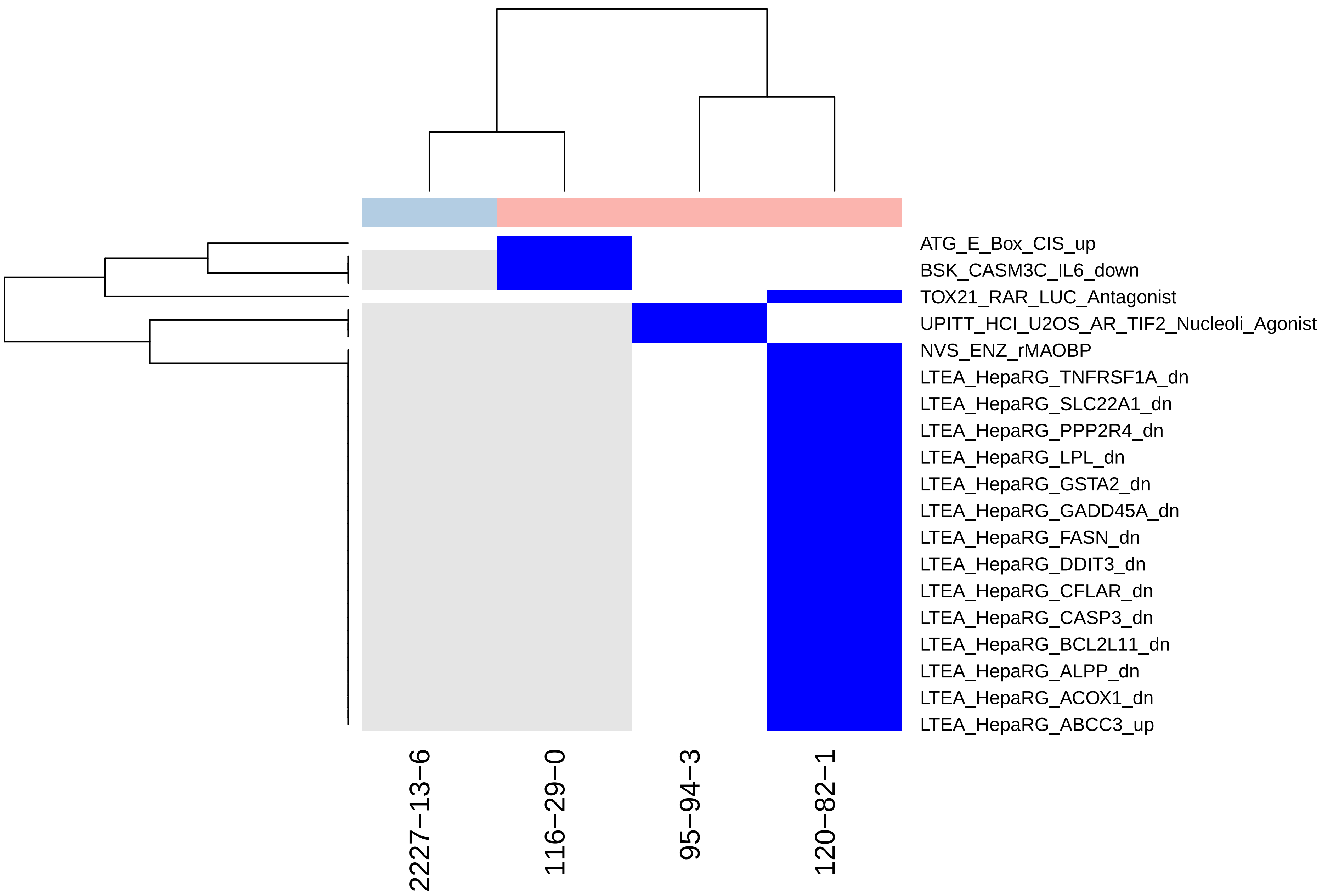

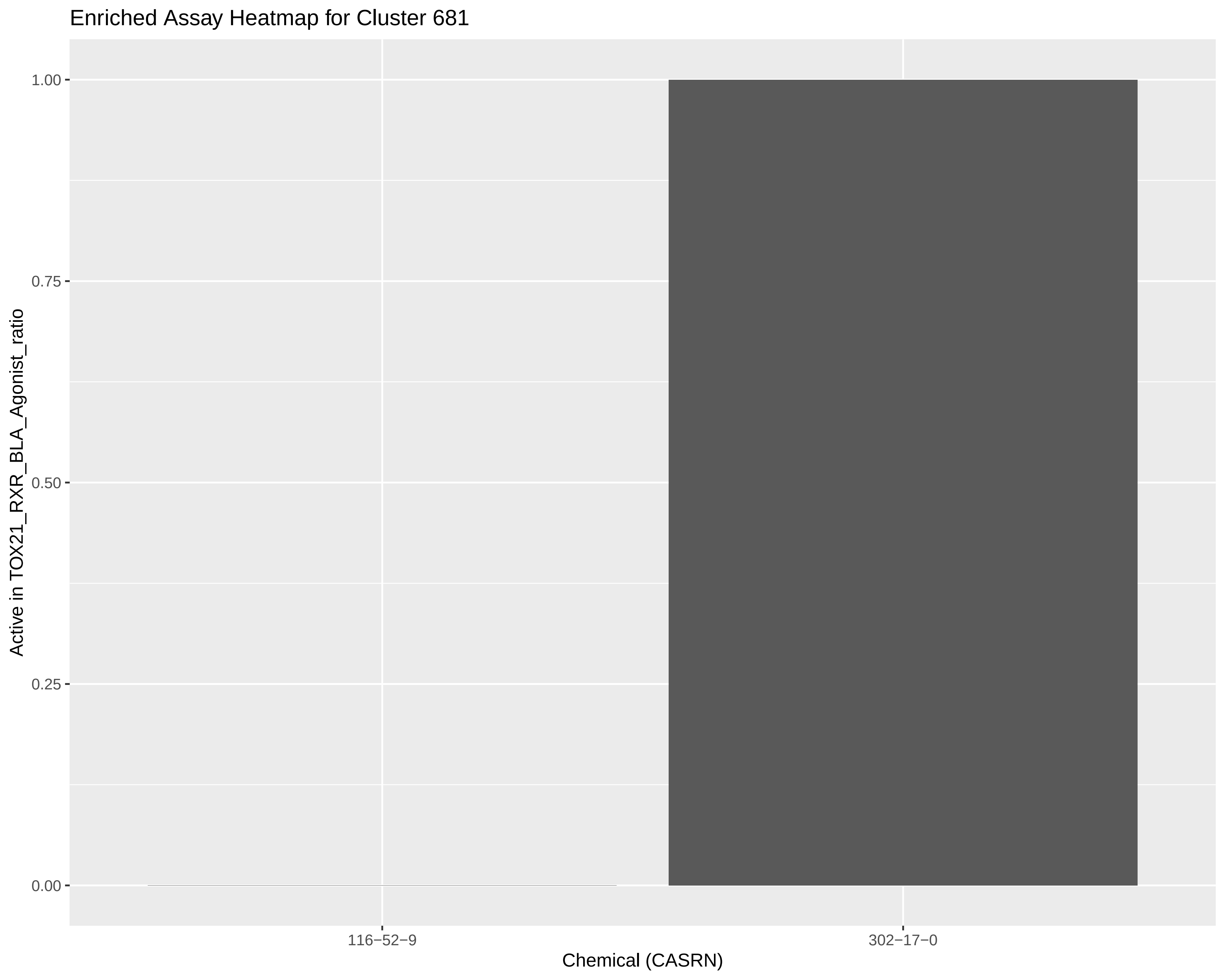
Enriched Assay Heatmap for Cluster 681
Active in TOX21_RXR_BLA_Agonist_ratio
1.00
0.75
0.50
0.25
0.00
116−52−9
302−17−0
Chemical (CASRN)

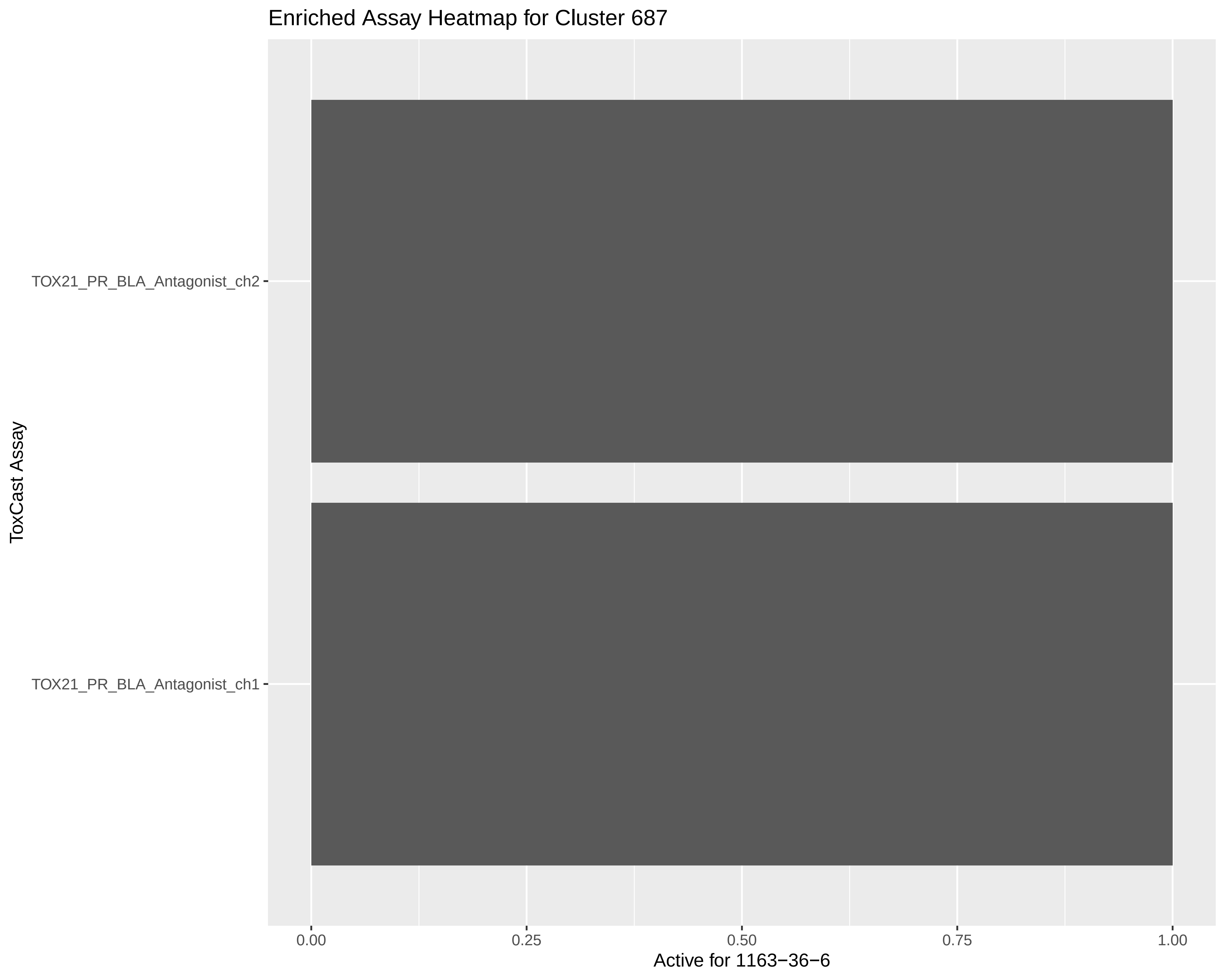
Enriched Assay Heatmap for Cluster 687
ToxCast Assay
TOX21_PR_BLA_Antagonist_ch2
TOX21_PR_BLA_Antagonist_ch1
0.00
0.25
0.50
0.75
1.00
Active for 1163−36−6

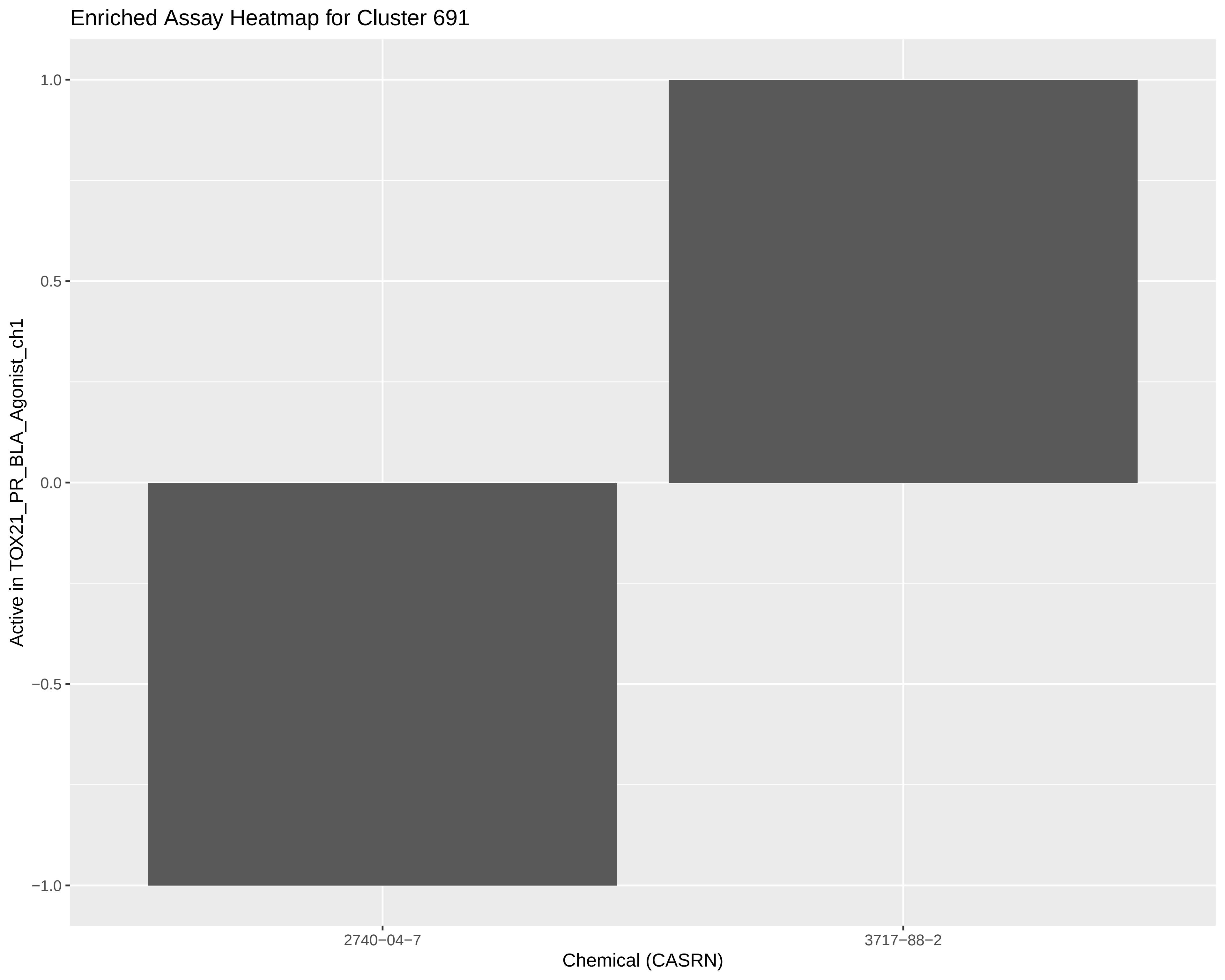

Enriched Assay Heatmap for Cluster 691
1.0
0.5
0.0
−0.5
−1.0
Active in TOX21_PR_BLA_Agonist_ch1
2740−04−7
3717−88−2
Chemical (CASRN)

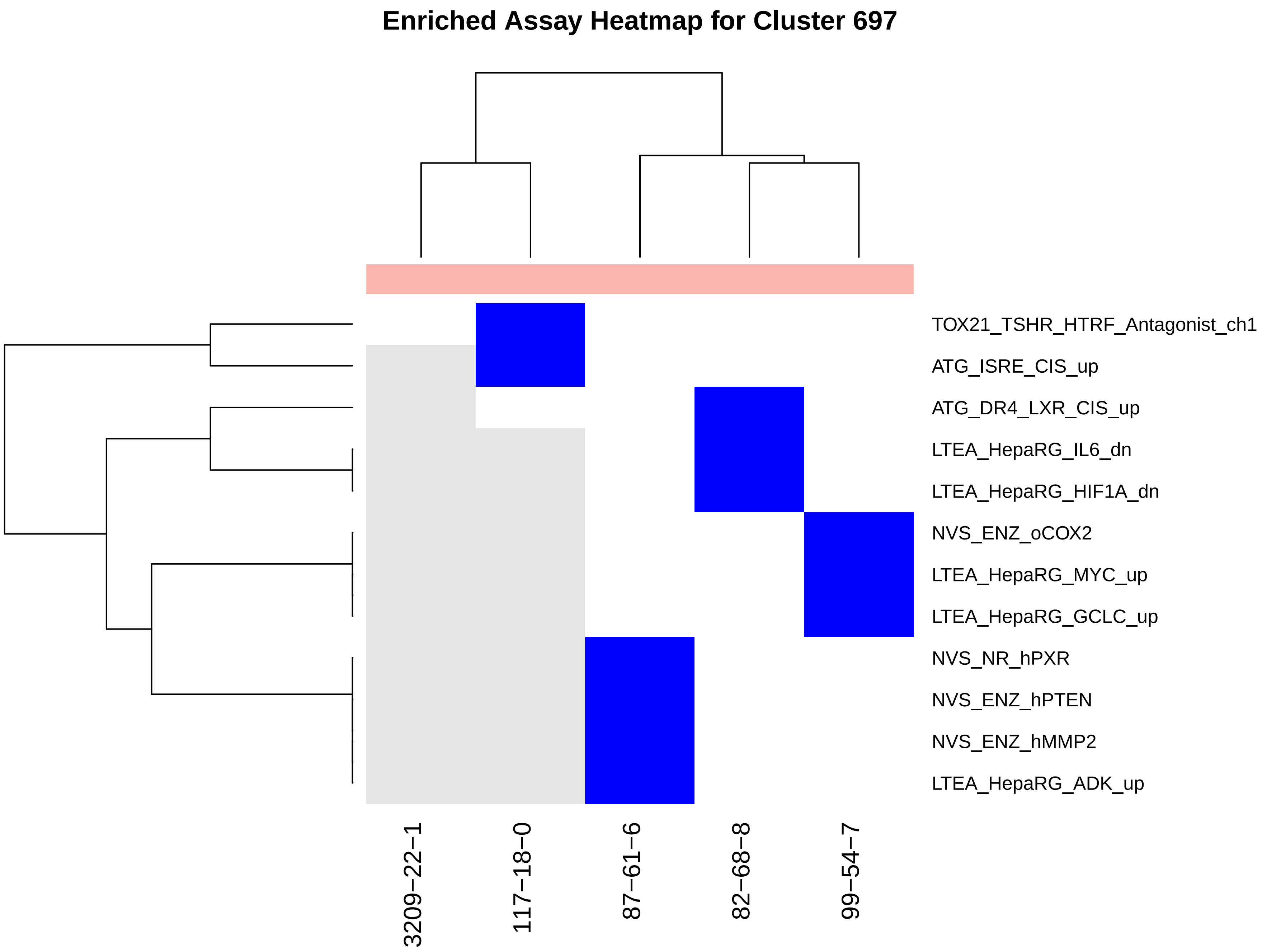

Enriched Assay Heatmap for Cluster 697
TOX21_TSHR_HTRF_Antagonist_ch1
ATG_ISRE_CIS_up
ATG_DR4_LXR_CIS_up
LTEA_HepaRG_IL6_dn
LTEA_HepaRG_HIF1A_dn
NVS_ENZ_oCOX2
LTEA_HepaRG_MYC_up
LTEA_HepaRG_GCLC_up
NVS_NR_hPXR
NVS_ENZ_hPTEN
NVS_ENZ_hMMP2
LTEA_HepaRG_ADK_up
3209−22−1
117−18−0
87−61−6
82−68−8
99−54−7

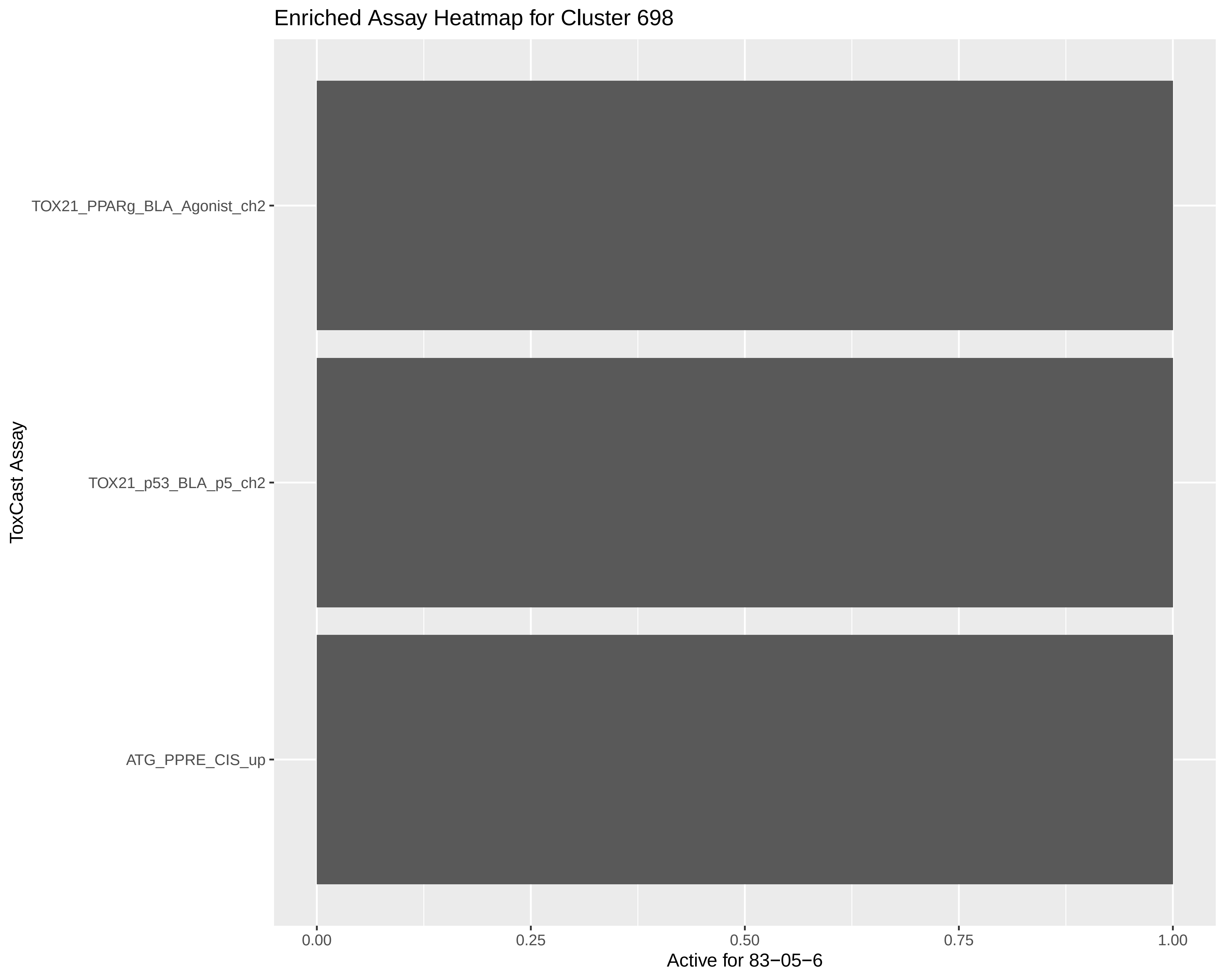
Enriched Assay Heatmap for Cluster 698
TOX21_PPARg_BLA_Agonist_ch2
TOX21_p53_BLA_p5_ch2
ATG_PPRE_CIS_up
ToxCast Assay
0.00
0.25
0.50
0.75
1.00
Active for 83−05−6

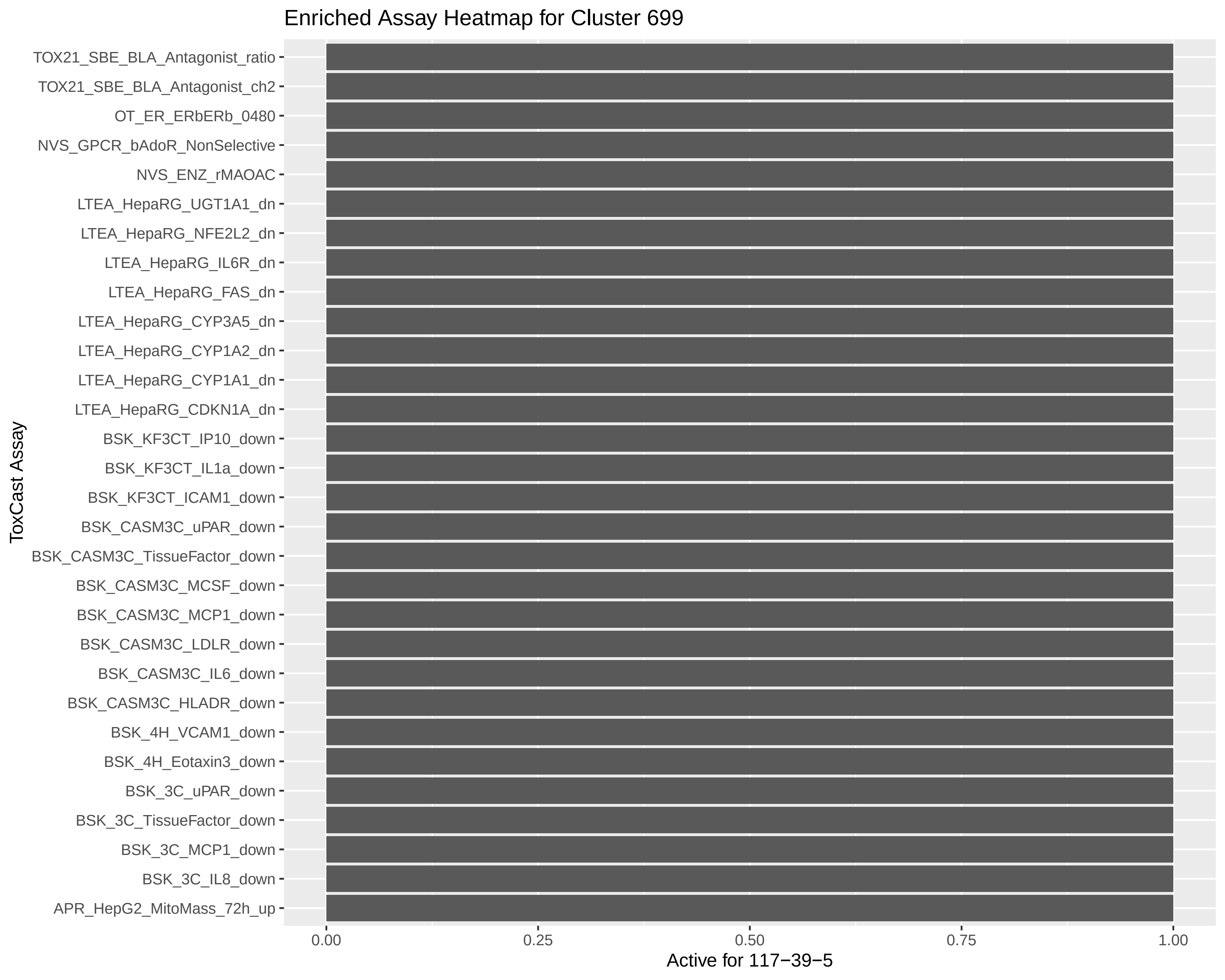

Enriched Assay Heatmap for Cluster 699
ToxCast Assay
TOX21_SBE_BLA_Antagonist_ratio
TOX21_SBE_BLA_Antagonist_ch2
OT_ER_ERbERb_0480
NVS_GPCR_bAdoR_NonSelective
NVS_ENZ_rMAOAC
LTEA_HepaRG_UGT1A1_dn
LTEA_HepaRG_NFE2L2_dn
LTEA_HepaRG_IL6R_dn
LTEA_HepaRG_FAS_dn
LTEA_HepaRG_CYP3A5_dn
LTEA_HepaRG_CYP1A2_dn
LTEA_HepaRG_CYP1A1_dn
LTEA_HepaRG_CDKN1A_dn
BSK_KF3CT_IP10_down
BSK_KF3CT_IL1a_down
BSK_KF3CT_ICAM1_down
BSK_CASM3C_uPAR_down
BSK_CASM3C_TissueFactor_down
BSK_CASM3C_MCSF_down
BSK_CASM3C_MCP1_down
BSK_CASM3C_LDLR_down
BSK_CASM3C_IL6_down
BSK_CASM3C_HLADR_down
BSK_4H_VCAM1_down
BSK_4H_Eotaxin3_down
BSK_3C_uPAR_down
BSK_3C_TissueFactor_down
BSK_3C_MCP1_down
BSK_3C_IL8_down
APR_HepG2_MitoMass_72h_up
0.00
0.25
0.50
0.75
1.00
Active for 117−39−5

Enriched Assay Heatmap for Cluster 700

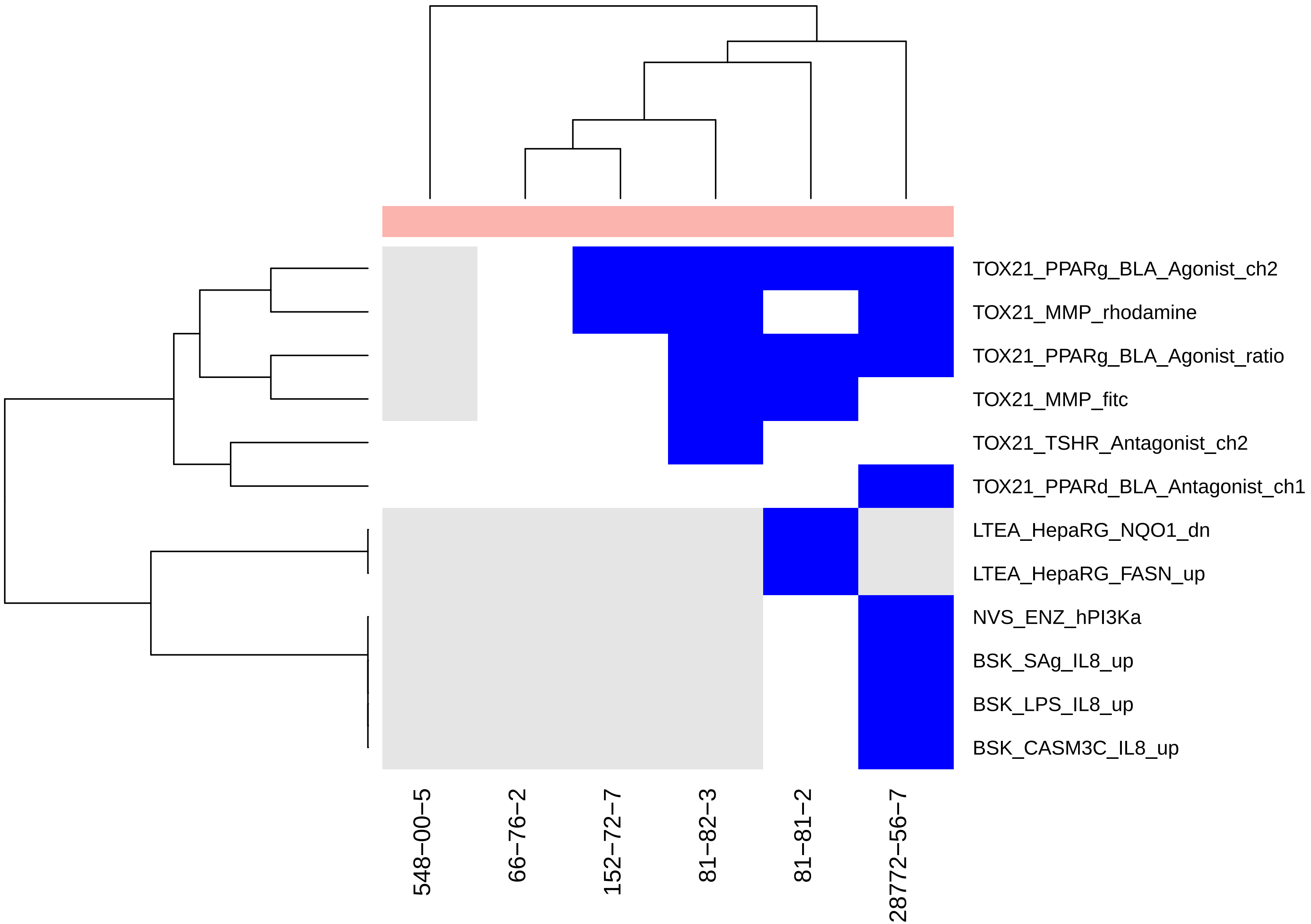

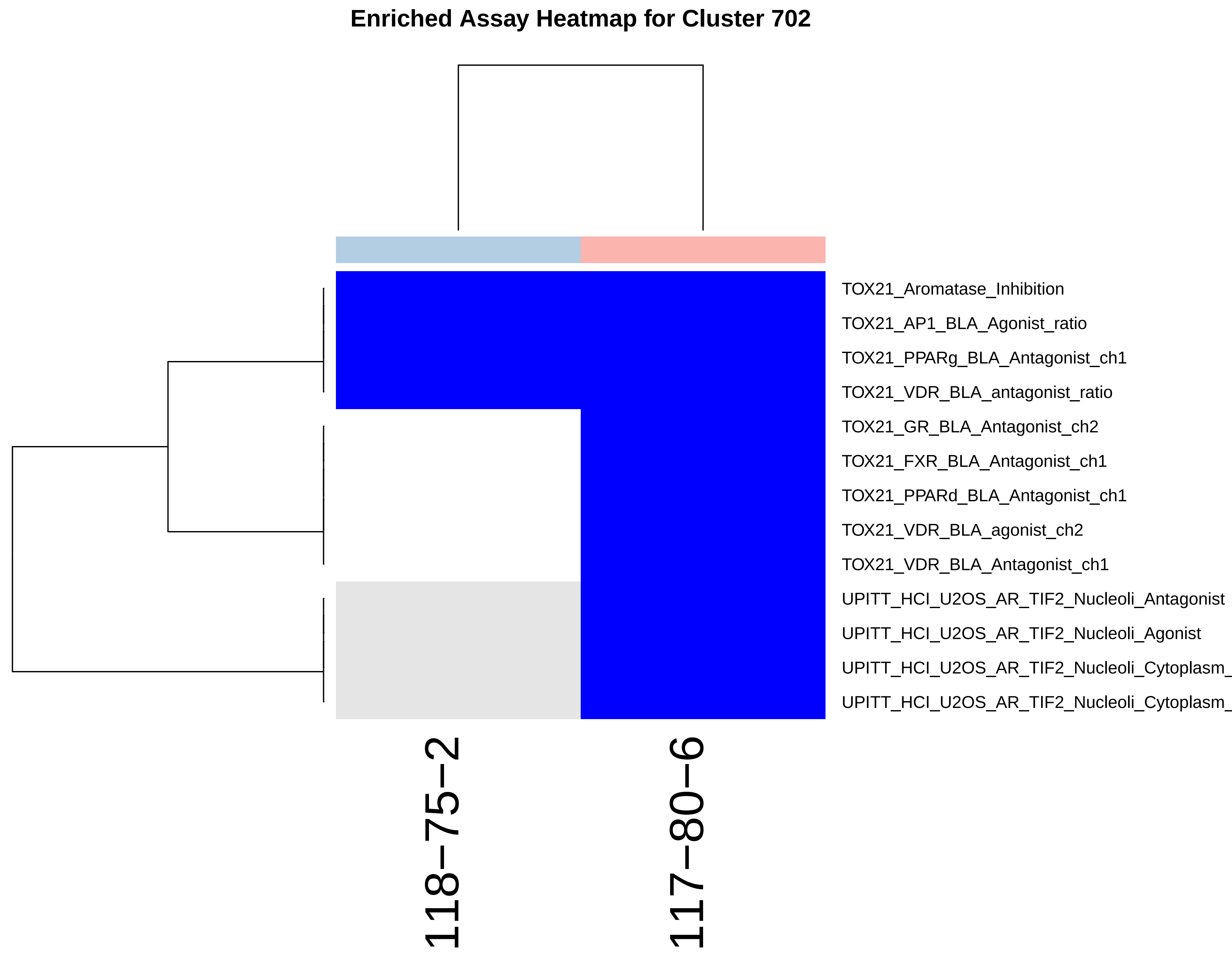

Enriched Assay Heatmap for Cluster 702
TOX21_Aromatase_Inhibition
TOX21_AP1_BLA_Agonist_ratio
TOX21_PPARg_BLA_Antagonist_ch1
TOX21_VDR_BLA_antagonist_ratio
TOX21_GR_BLA_Antagonist_ch2
TOX21_FXR_BLA_Antagonist_ch1
TOX21_PPARd_BLA_Antagonist_ch1
TOX21_VDR_BLA_agonist_ch2
TOX21_VDR_BLA_Antagonist_ch1
UPITT_HCI_U2OS_AR_TIF2_Nucleoli_Antagonist
UPITT_HCI_U2OS_AR_TIF2_Nucleoli_Agonist
UPITT_HCI_U2OS_AR_TIF2_Nucleoli_Cytoplasm_
UPITT_HCI_U2OS_AR_TIF2_Nucleoli_Cytoplasm_
118−75−2
117−80−6

Enriched Assay Heatmap for Cluster 703

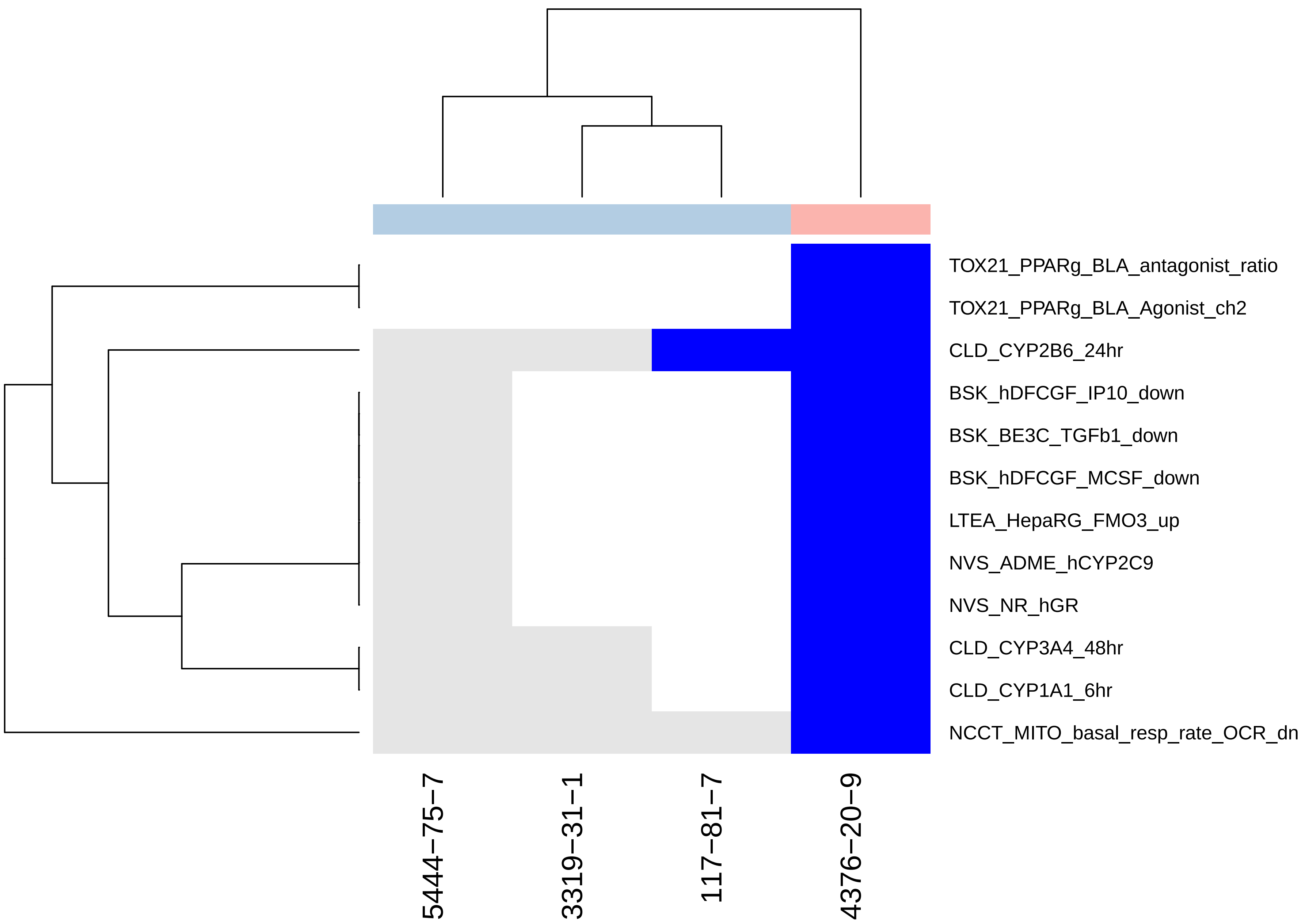

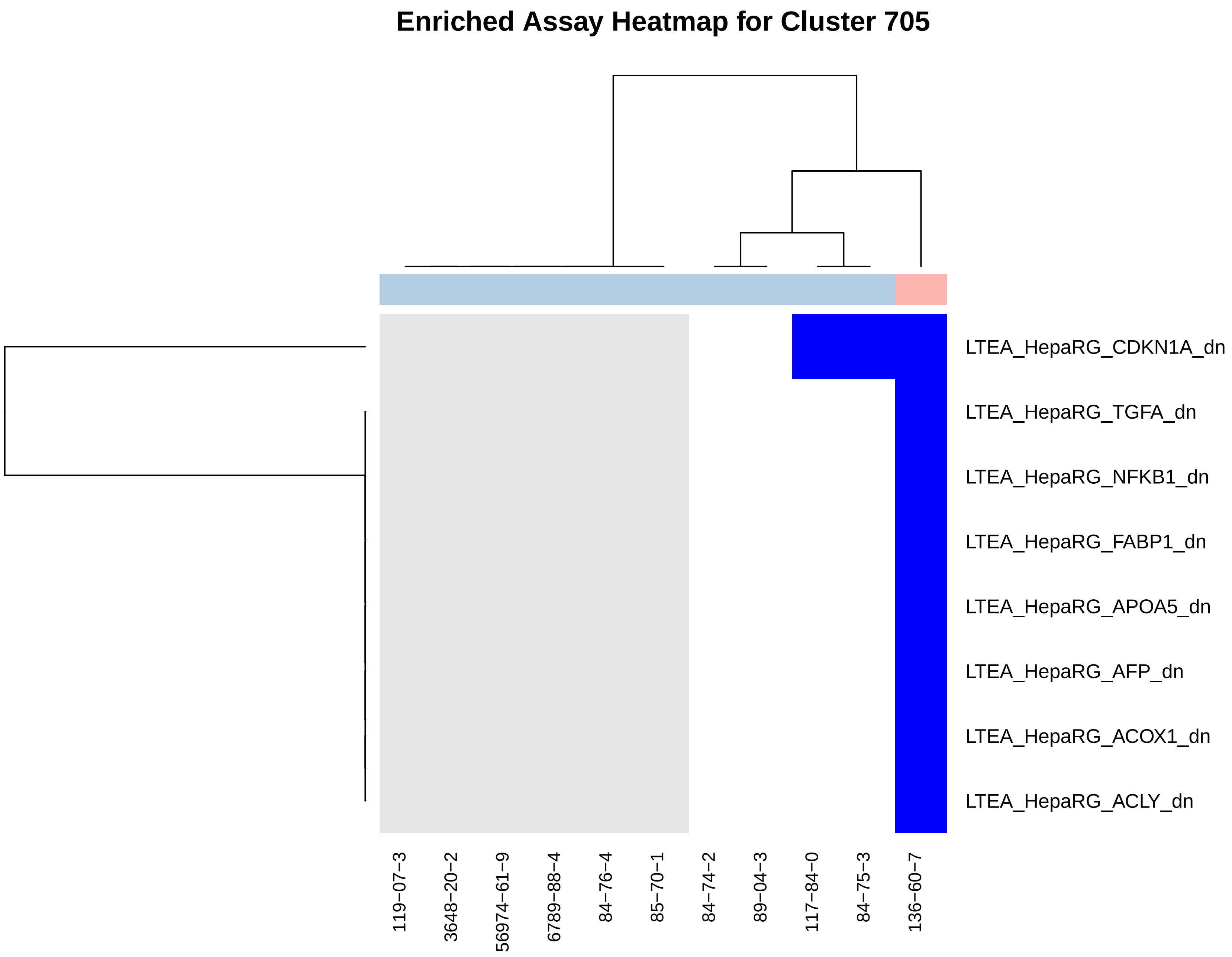
Enriched Assay Heatmap for Cluster 705
LTEA_HepaRG_CDKN1A_dn
LTEA_HepaRG_TGFA_dn
LTEA_HepaRG_NFKB1_dn
LTEA_HepaRG_FABP1_dn
LTEA_HepaRG_APOA5_dn
LTEA_HepaRG_AFP_dn
LTEA_HepaRG_ACOX1_dn
LTEA_HepaRG_ACLY_dn
119−07−3
3648−20−2
56974−61−9
6789−88−4
84−76−4
85−70−1
84−74−2
89−04−3
117−84−0
84−75−3
136−60−7

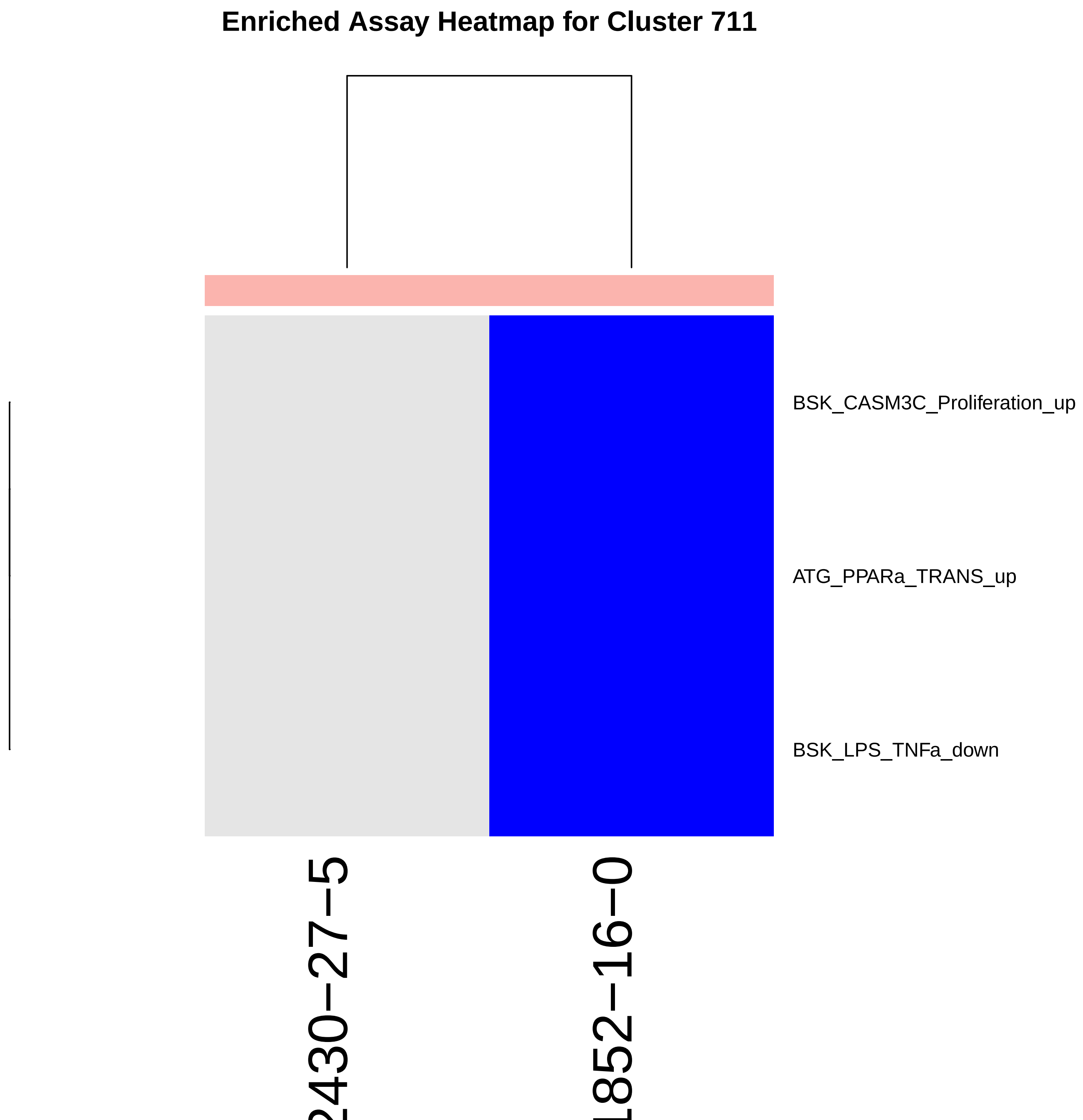
Enriched Assay Heatmap for Cluster 711
BSK_CASM3C_Proliferation_up
ATG_PPARa_TRANS_up
BSK_LPS_TNFa_down
2430−27−5
852−16−0

Enriched Assay Heatmap for Cluster 712

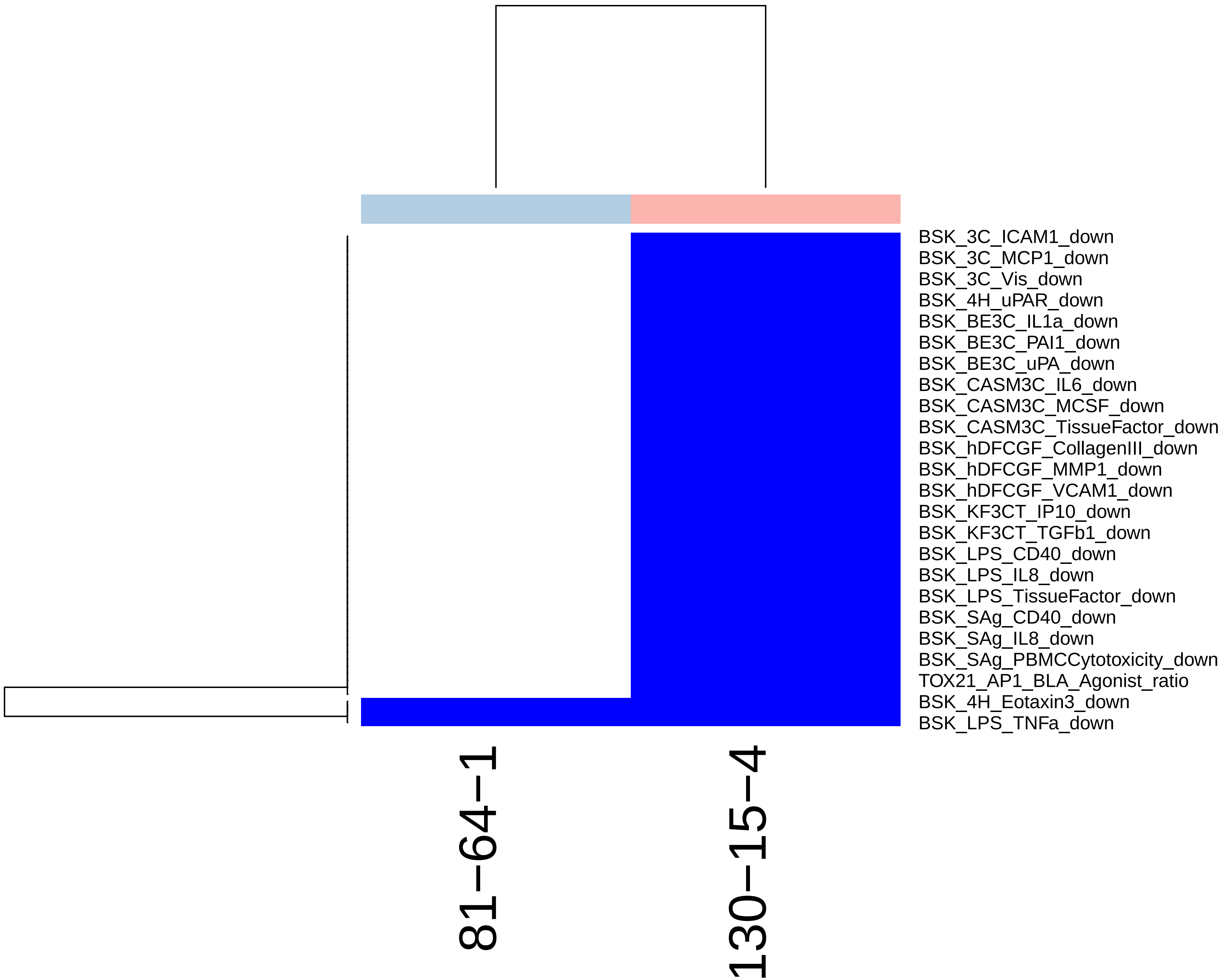

Enriched Assay Heatmap for Cluster 713

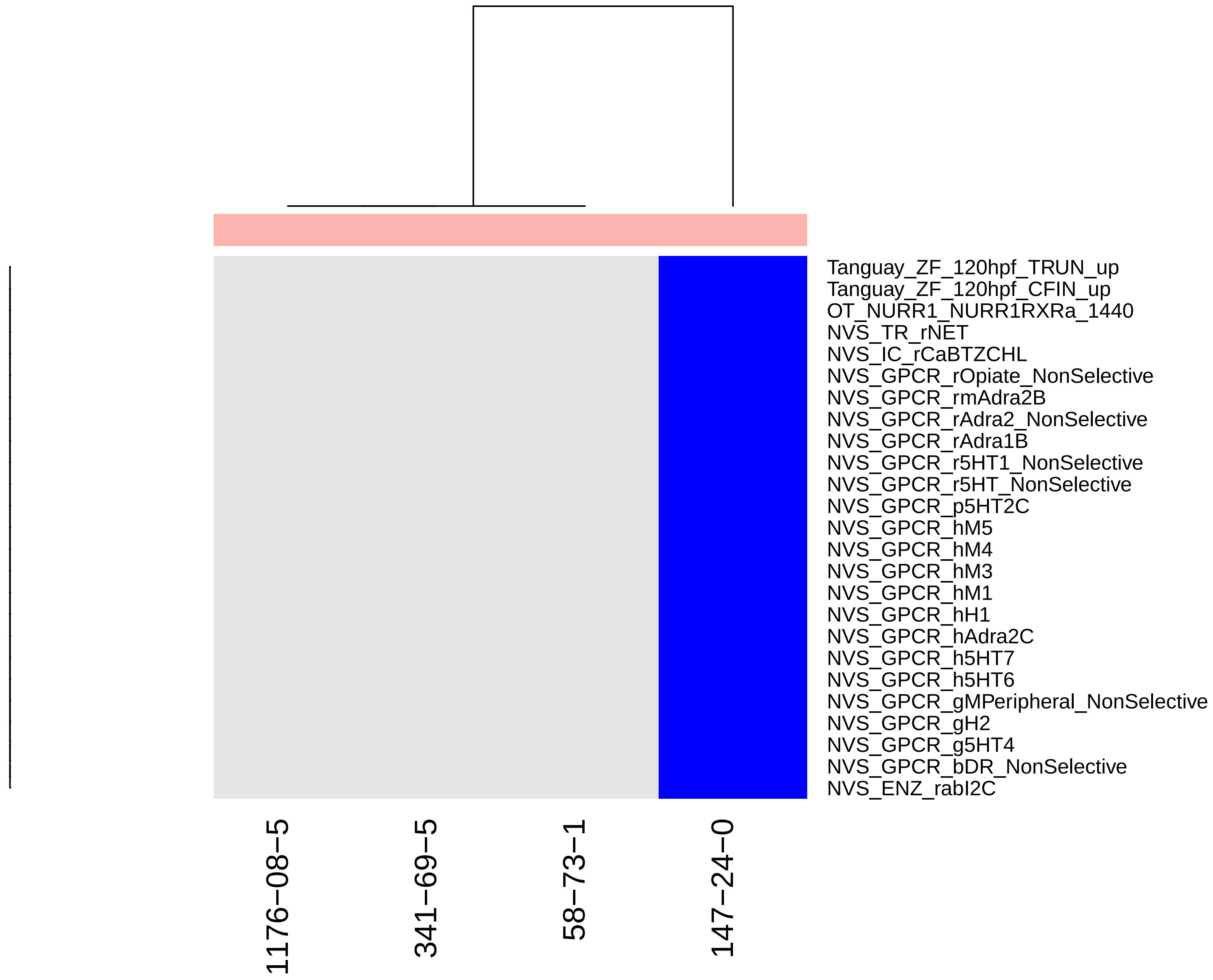

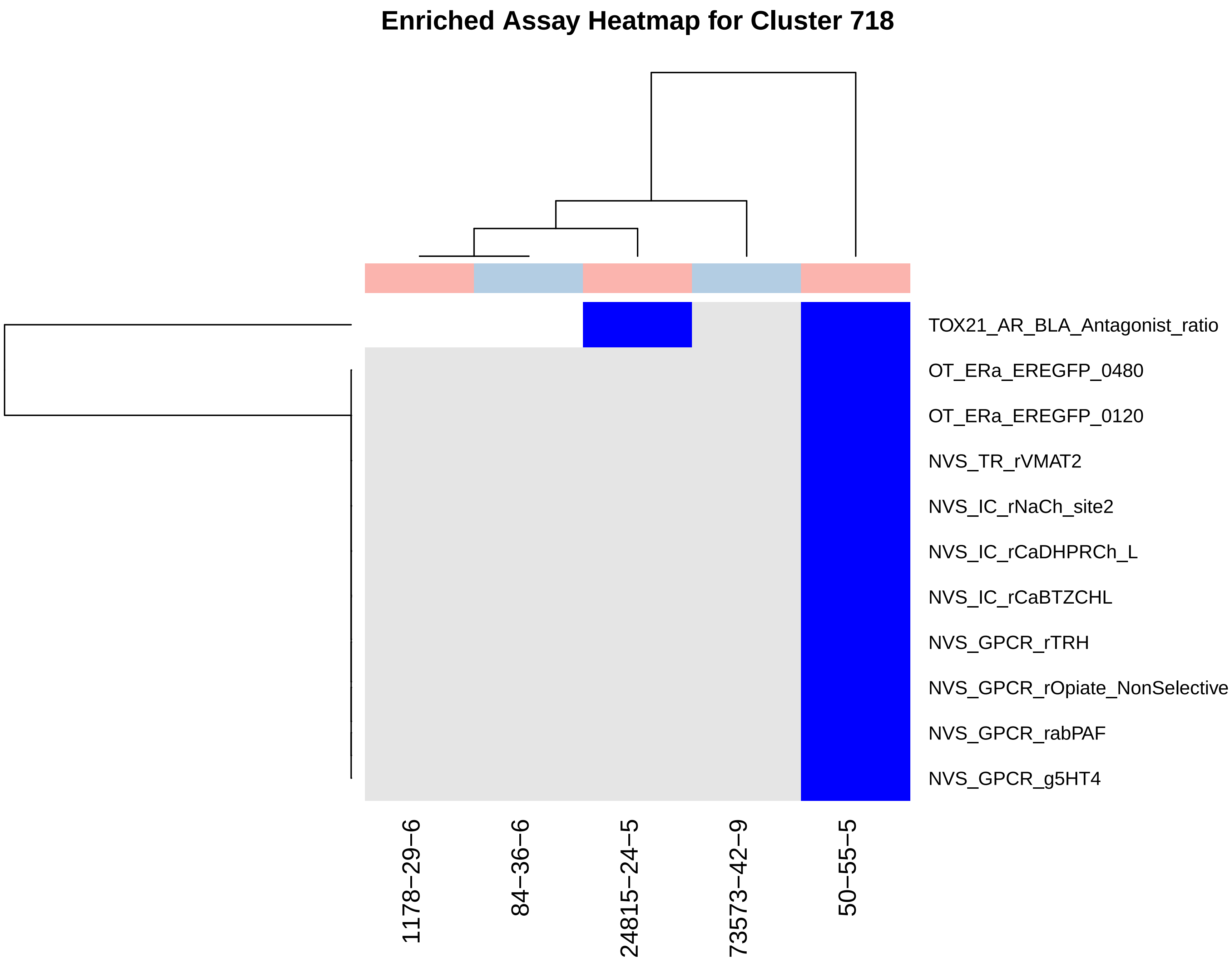
Enriched Assay Heatmap for Cluster 718
TOX21_AR_BLA_Antagonist_ratio
OT_ERa_EREGFP_0480
OT_ERa_EREGFP_0120
NVS_TR_rVMAT2
NVS_IC_rNaCh_site2
NVS_IC_rCaDHPRCh_L
NVS_IC_rCaBTZCHL
NVS_GPCR_rTRH
NVS_GPCR_rOpiate_NonSelective
NVS_GPCR_rabPAF
NVS_GPCR_g5HT4
1178−29−6
84−36−6
24815−24−5
73573−42−9
50−55−5

Enriched Assay Heatmap for Cluster 721

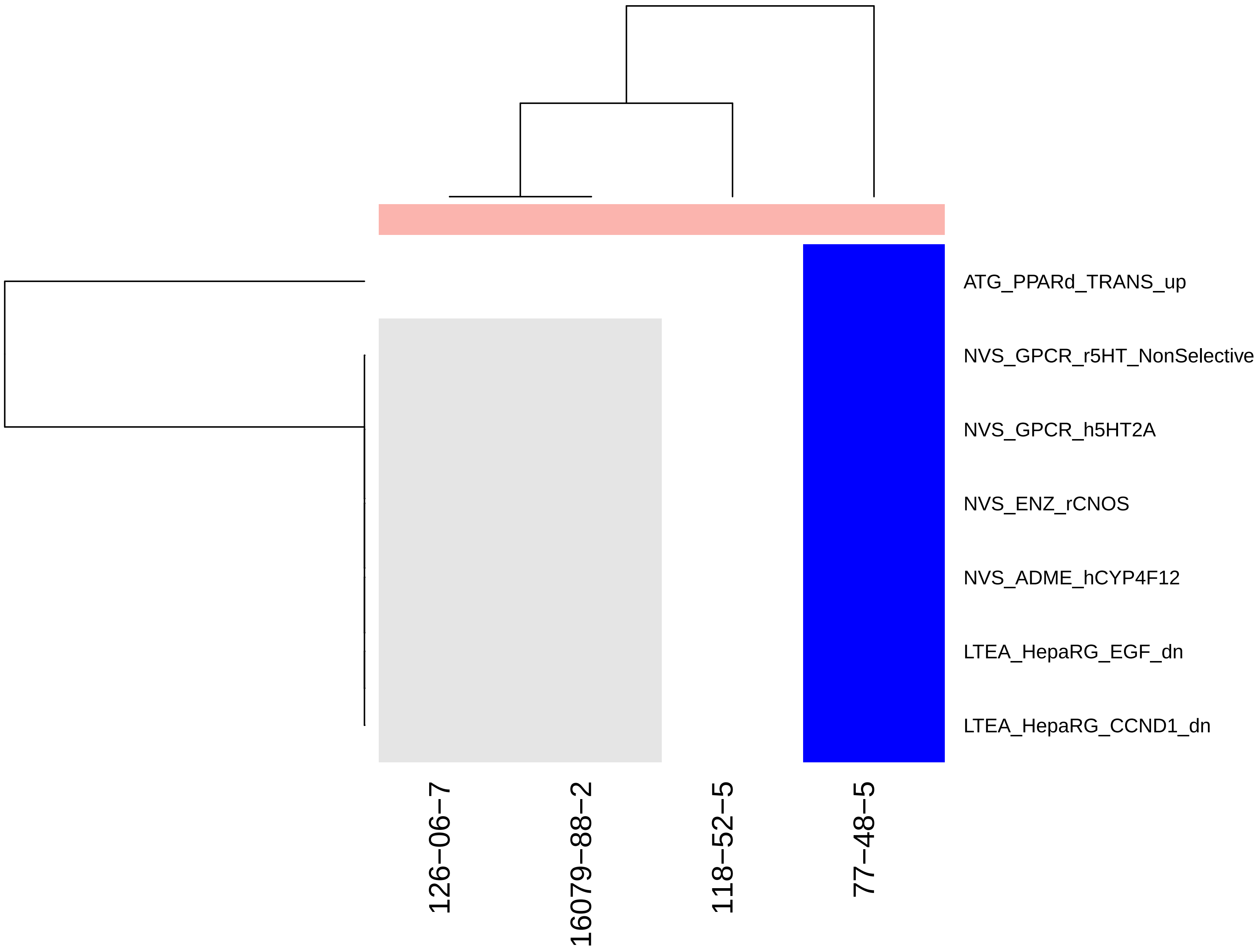

Enriched Assay Heatmap for Cluster 722

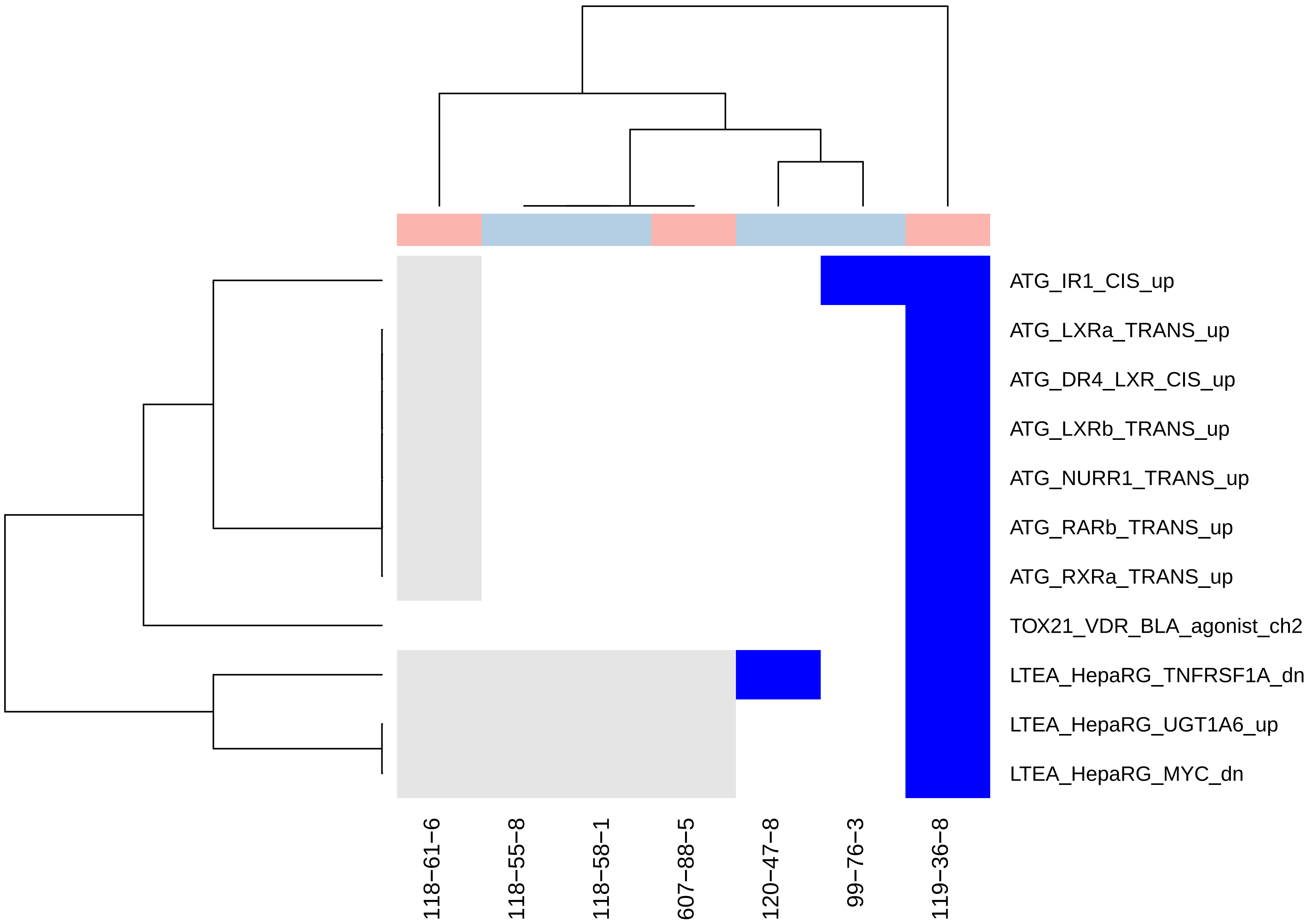

Enriched Assay Heatmap for Cluster 726

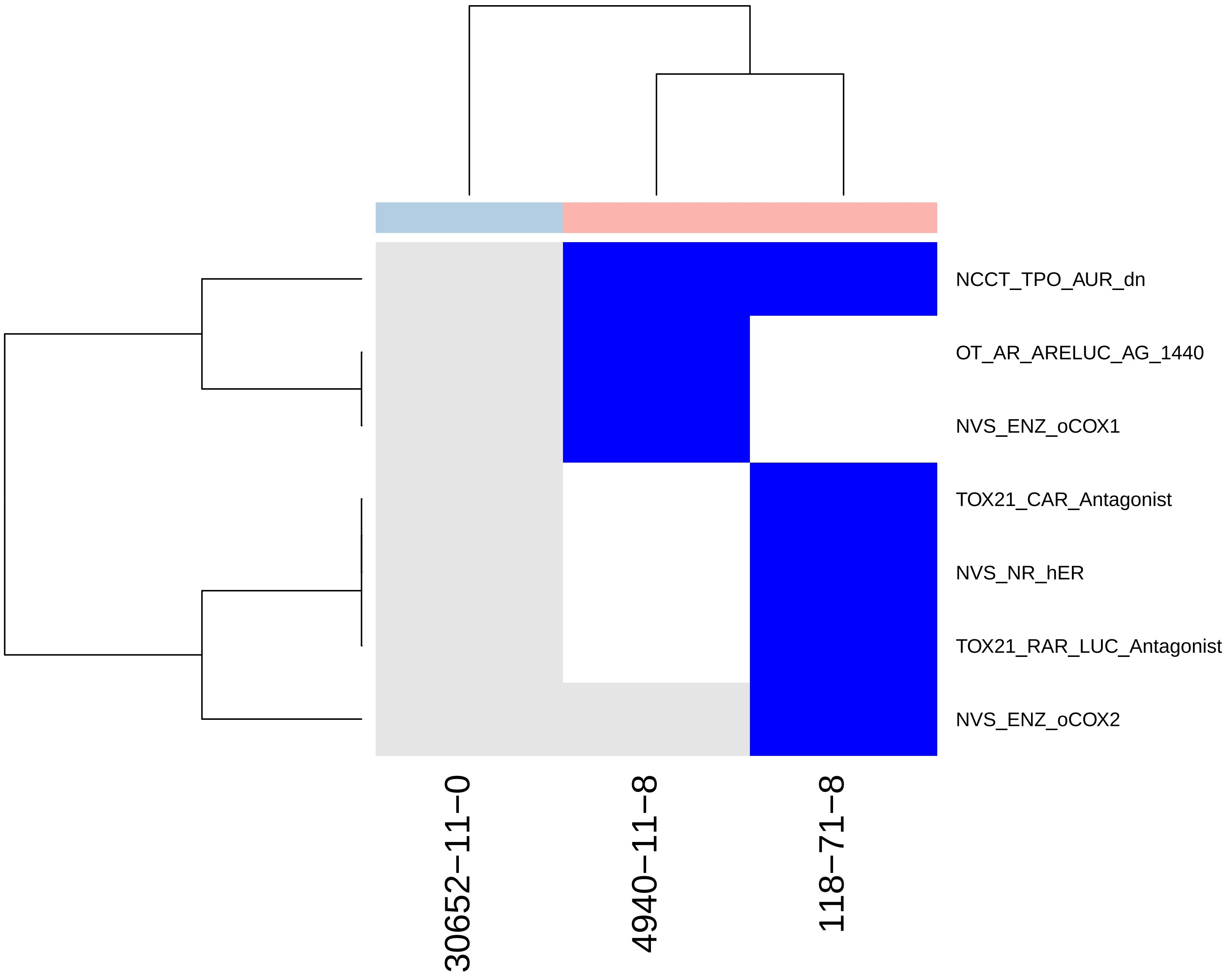

Enriched Assay Heatmap for Cluster 727

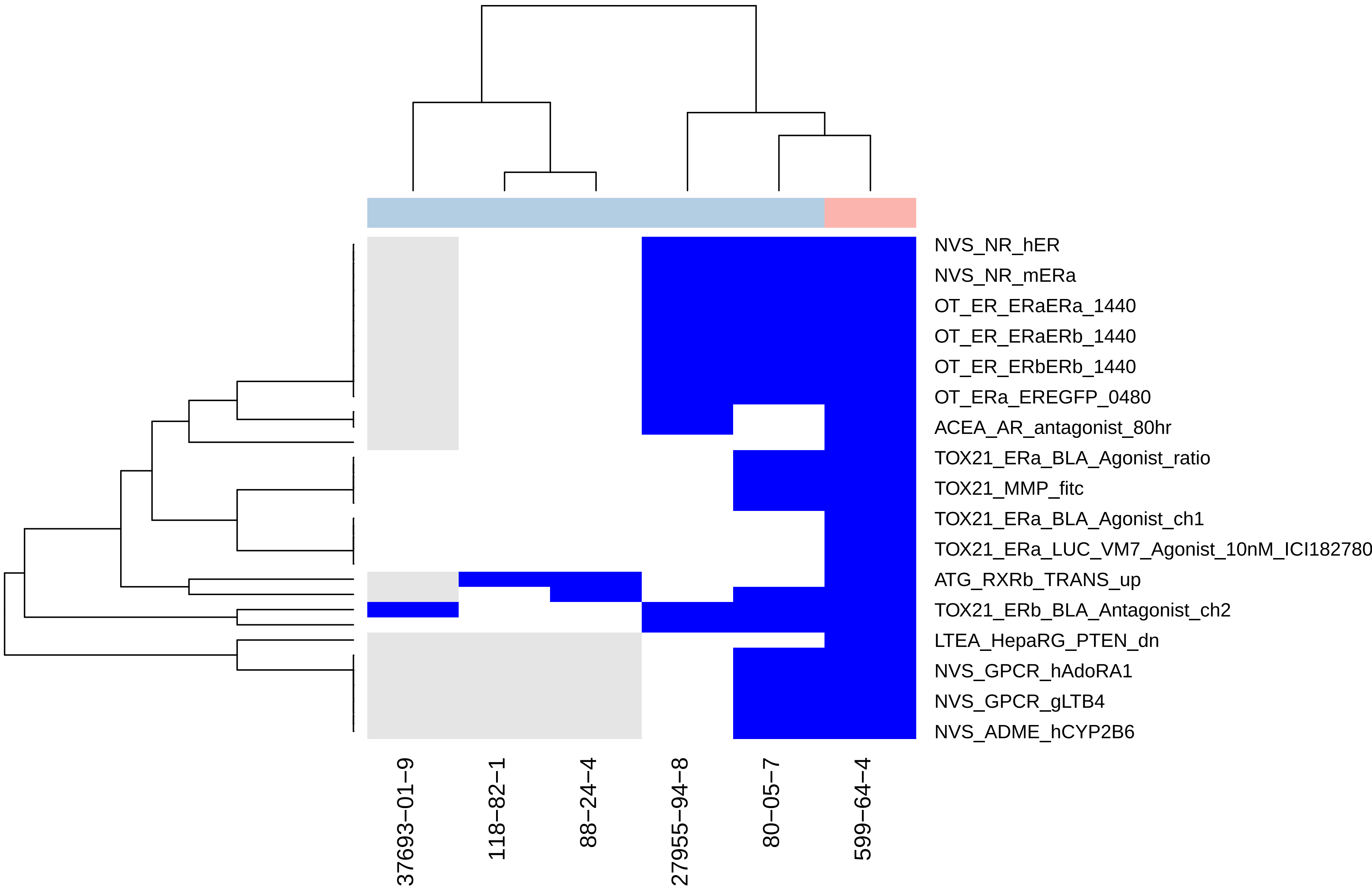

Enriched Assay Heatmap for Cluster 728

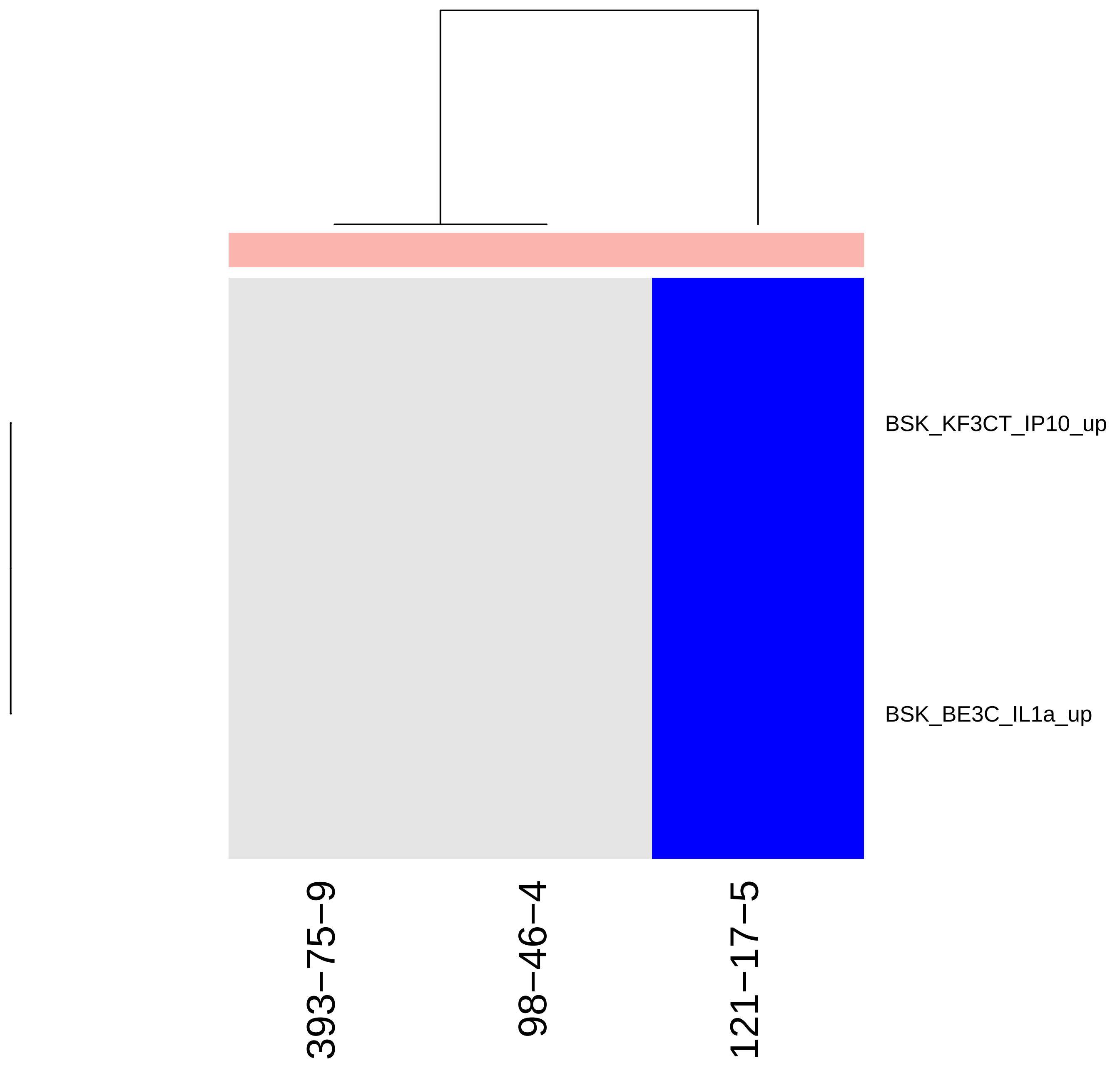

Enriched Assay Heatmap for Cluster 730

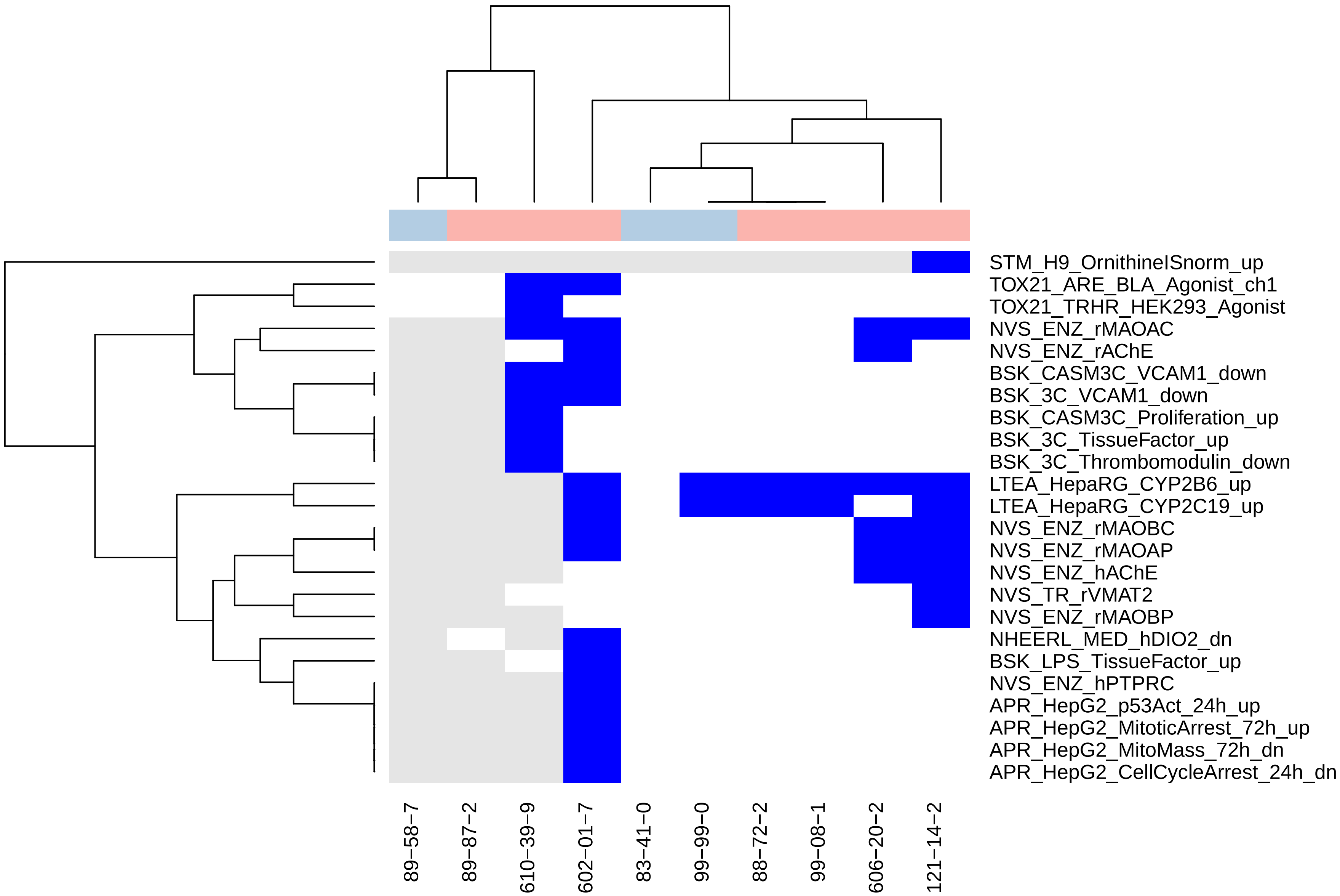

Enriched Assay Heatmap for Cluster 731

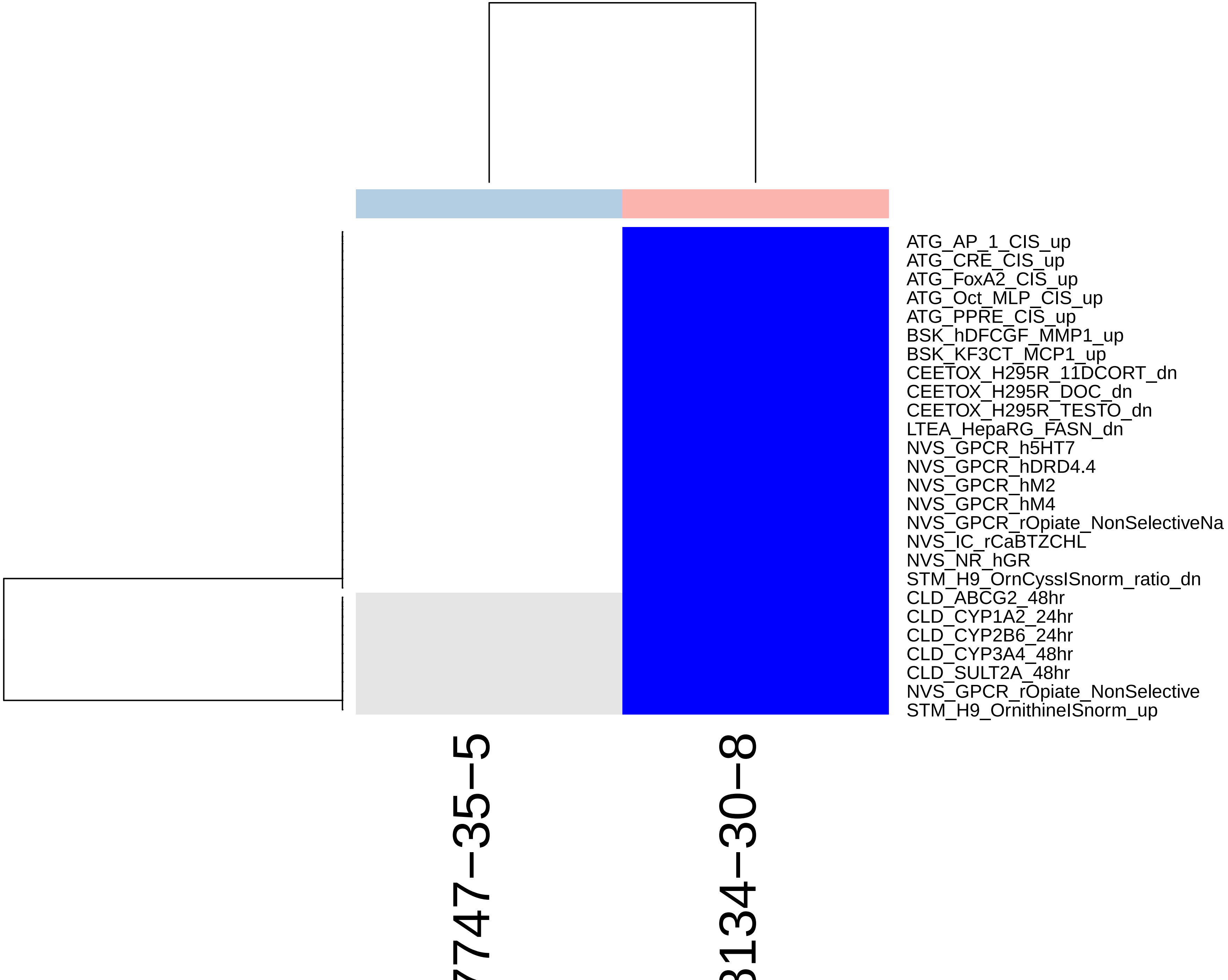

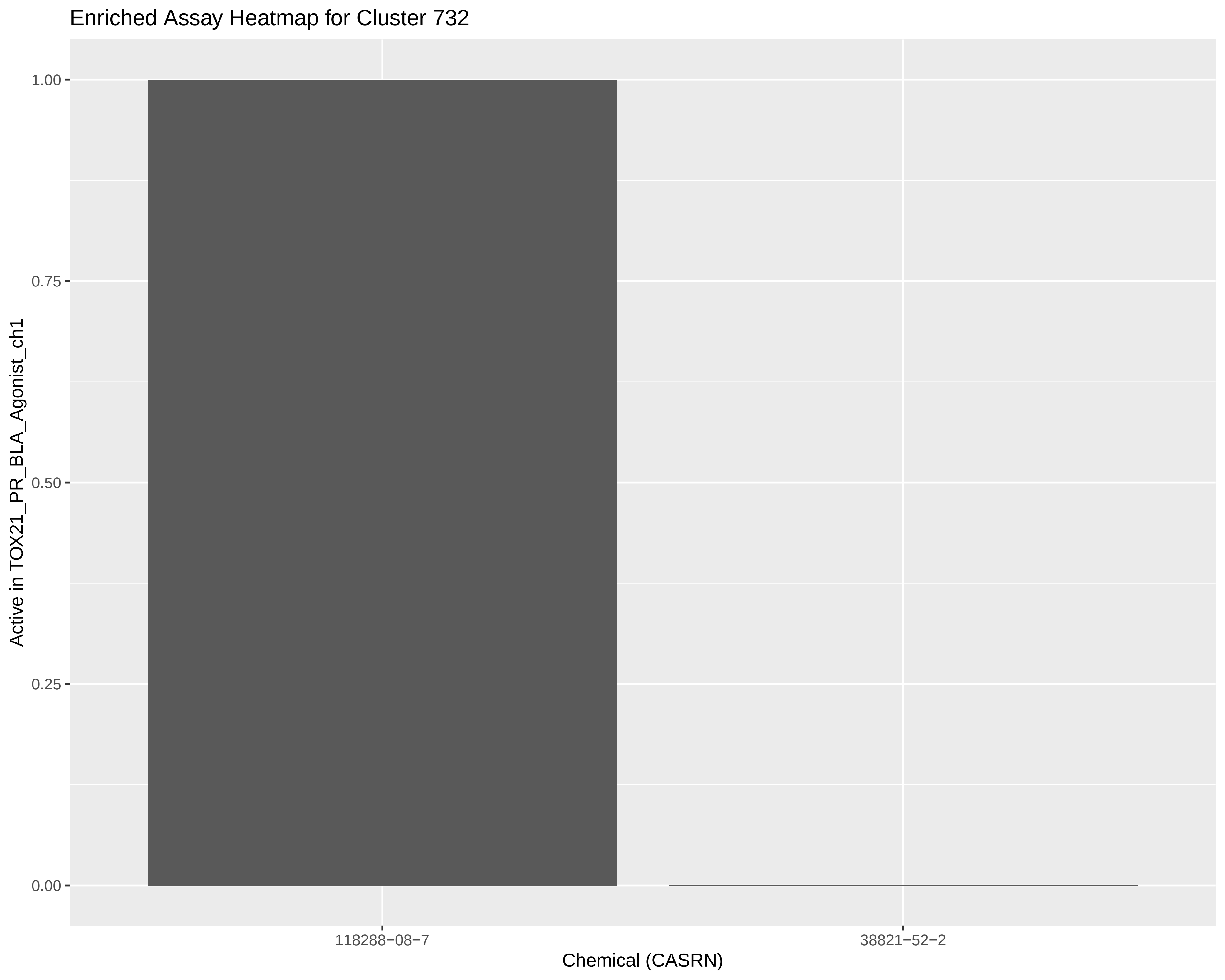

Enriched Assay Heatmap for Cluster 732
Active in TOX21_PR_BLA_Agonist_ch1
1.00
0.75
0.50
0.25
0.00
118288−08−7
38821−52−2
Chemical (CASRN)

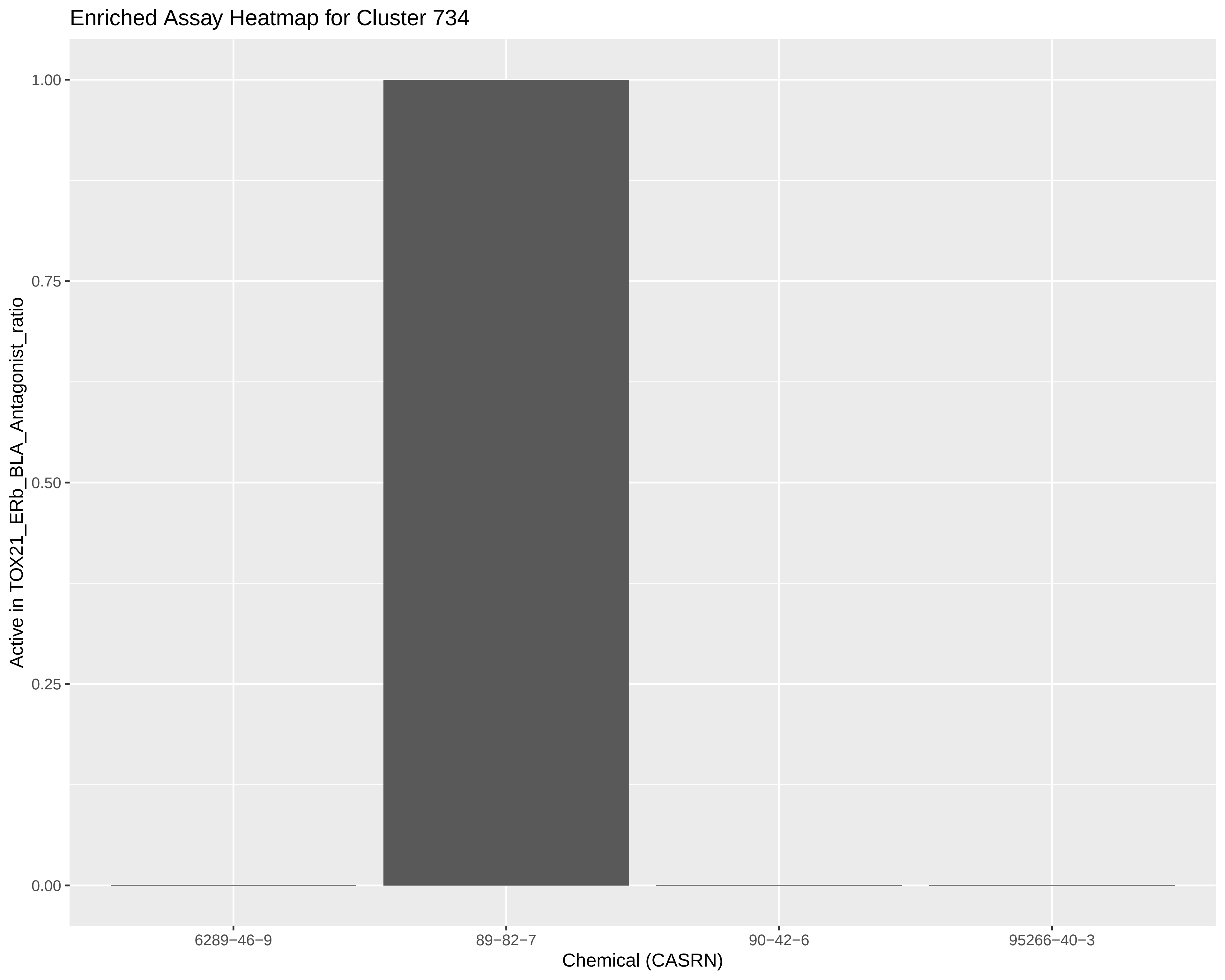
Enriched Assay Heatmap for Cluster 734
Active in TOX21_ERb_BLA_Antagonist_ratio
1.00
0.75
0.50
0.25
0.00
6289−46−9
89−82−7
90−42−6
95266−40−3
Chemical (CASRN)

Enriched Assay Heatmap for Cluster 740

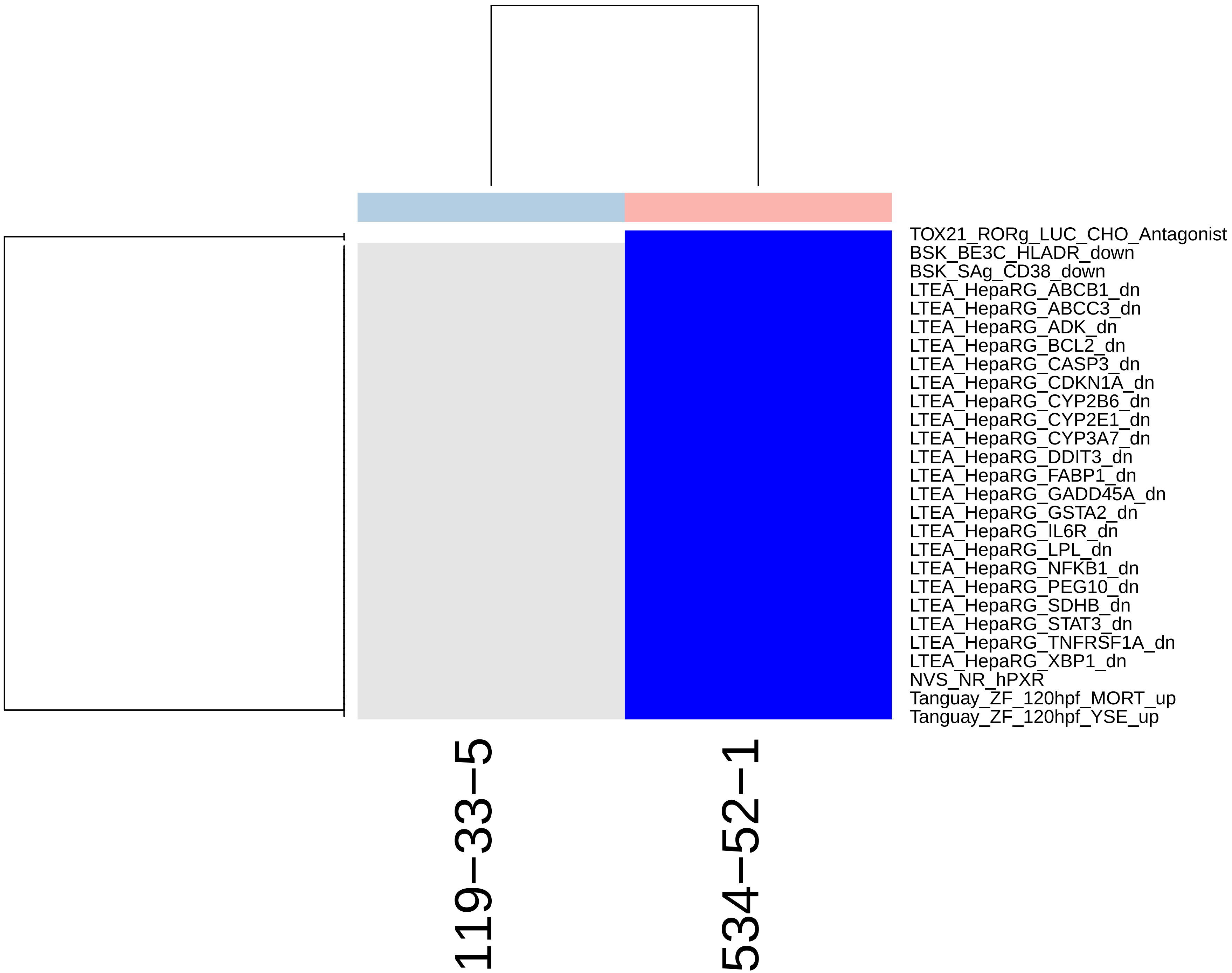

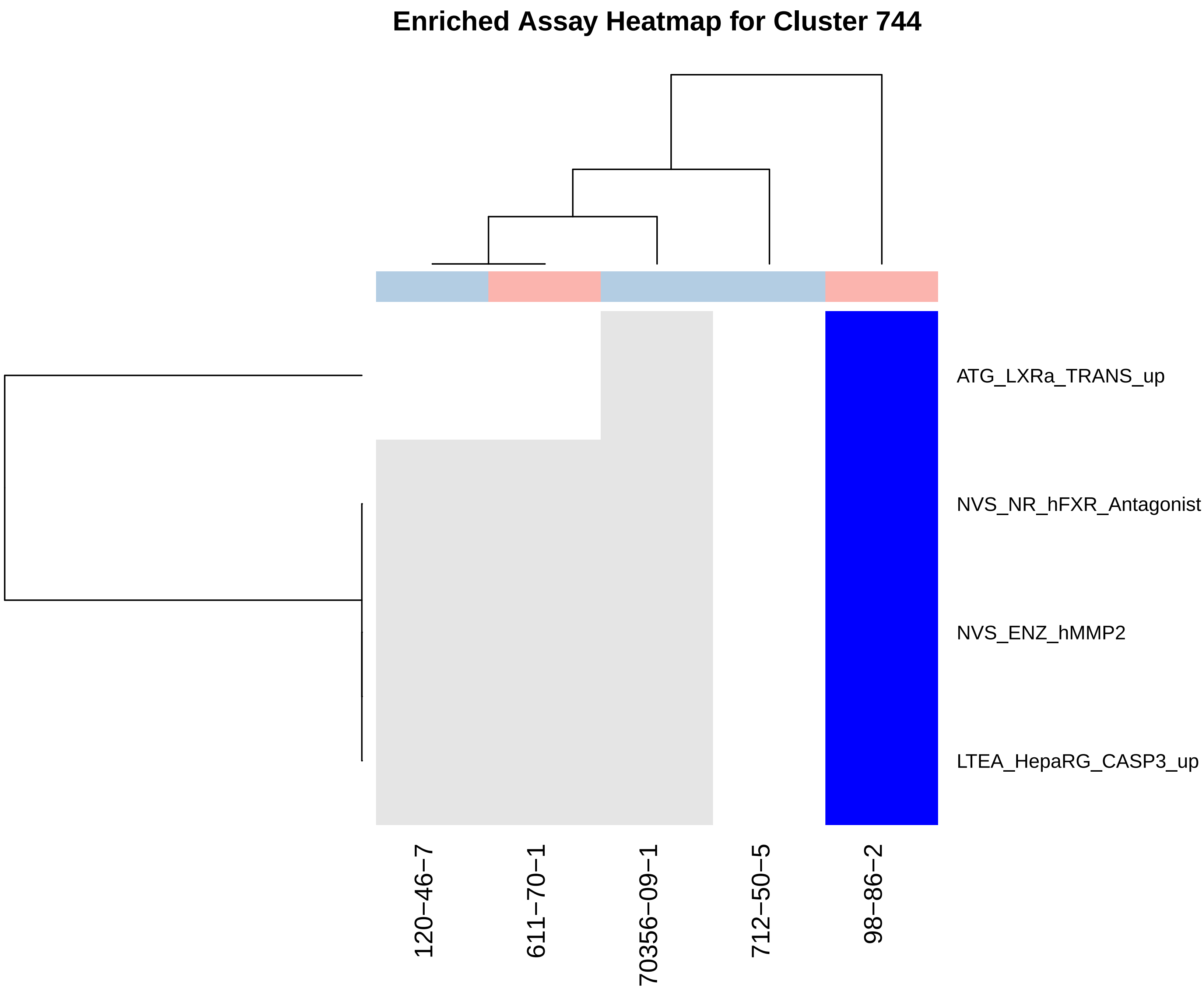
Enriched Assay Heatmap for Cluster 744
ATG_LXRa_TRANS_up
NVS_NR_hFXR_Antagonist
NVS_ENZ_hMMP2
LTEA_HepaRG_CASP3_up
120−46−7
611−70−1
70356−09−1
712−50−5
98−86−2

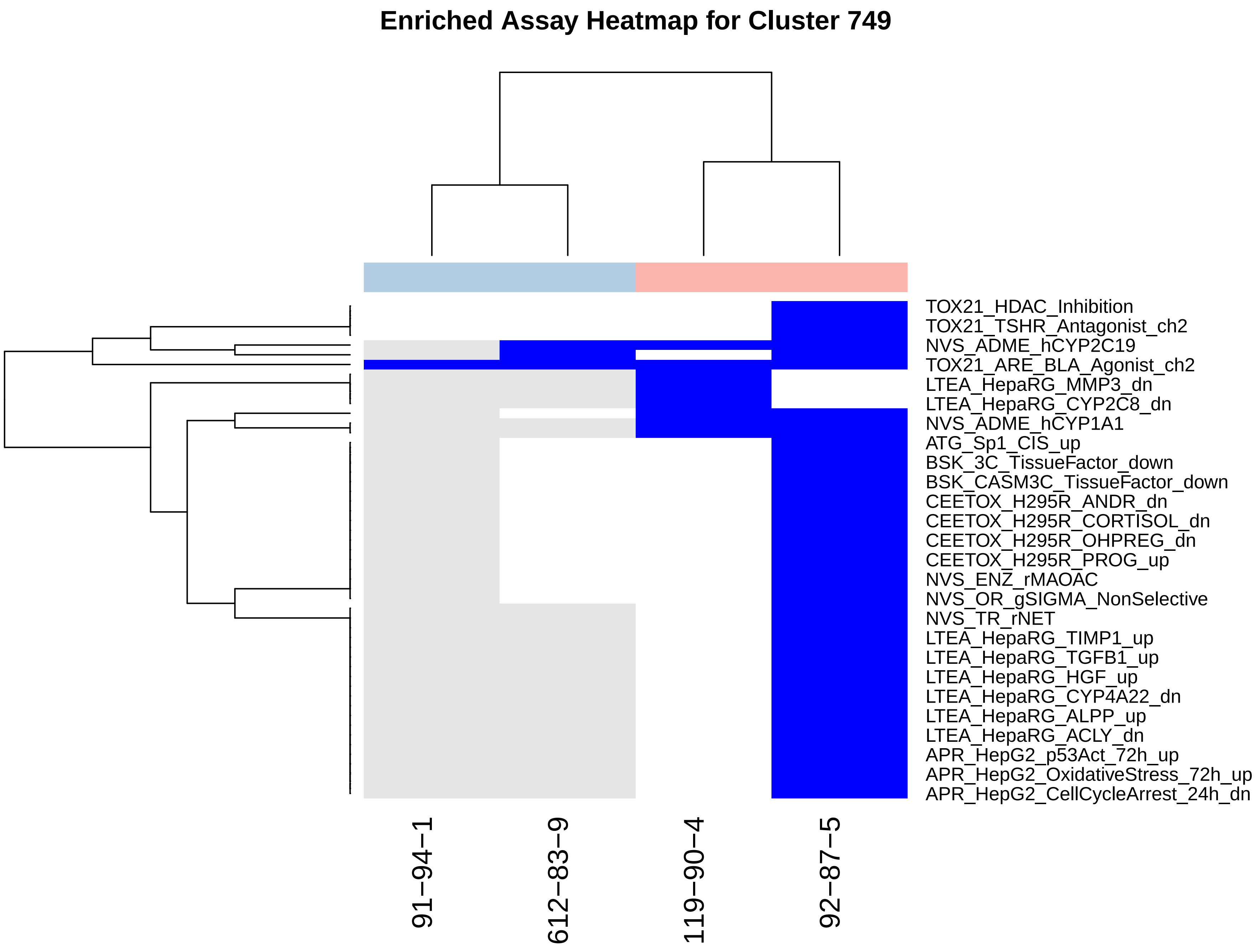
Enriched Assay Heatmap for Cluster 749
TOX21_HDAC_Inhibition
TOX21_TSHR_Antagonist_ch2
NVS_ADME_hCYP2C19
TOX21_ARE_BLA_Agonist_ch2
LTEA_HepaRG_MMP3_dn
LTEA_HepaRG_CYP2C8_dn
NVS_ADME_hCYP1A1
ATG_Sp1_CIS_up
BSK_3C_TissueFactor_down
BSK_CASM3C_TissueFactor_down
CEETOX_H295R_ANDR_dn
CEETOX_H295R_CORTISOL_dn
CEETOX_H295R_OHPREG_dn
CEETOX_H295R_PROG_up
NVS_ENZ_rMAOAC
NVS_OR_gSIGMA_NonSelective
NVS_TR_rNET
LTEA_HepaRG_TIMP1_up
LTEA_HepaRG_TGFB1_up
LTEA_HepaRG_HGF_up
LTEA_HepaRG_CYP4A22_dn
LTEA_HepaRG_ALPP_up
LTEA_HepaRG_ACLY_dn
APR_HepG2_p53Act_72h_up
APR_HepG2_OxidativeStress_72h_up
APR_HepG2_CellCycleArrest_24h_dn
91−94−1
612−83−9
119−90−4
92−87−5

Enriched Assay Heatmap for Cluster 750

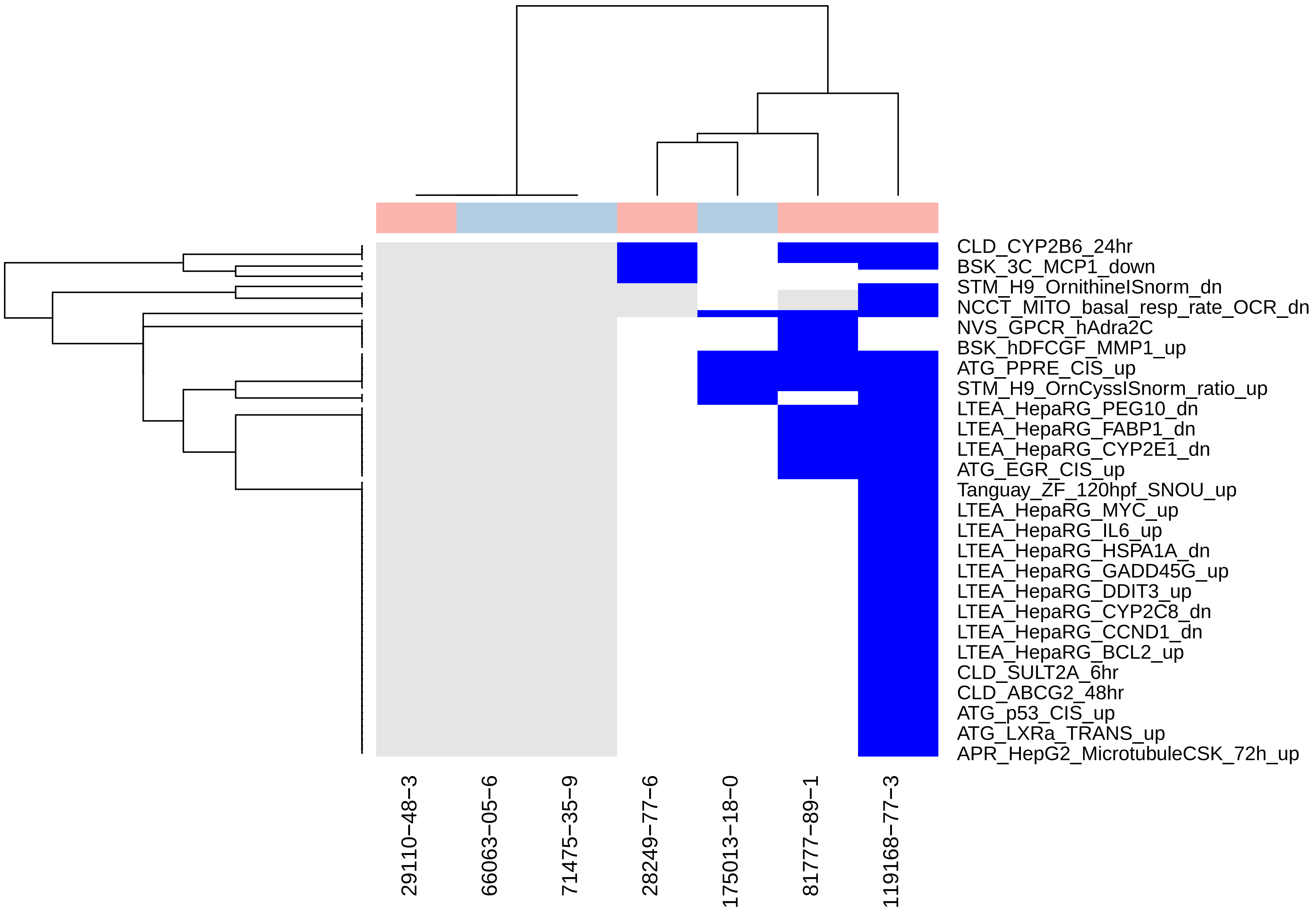

Enriched Assay Heatmap for Cluster 754

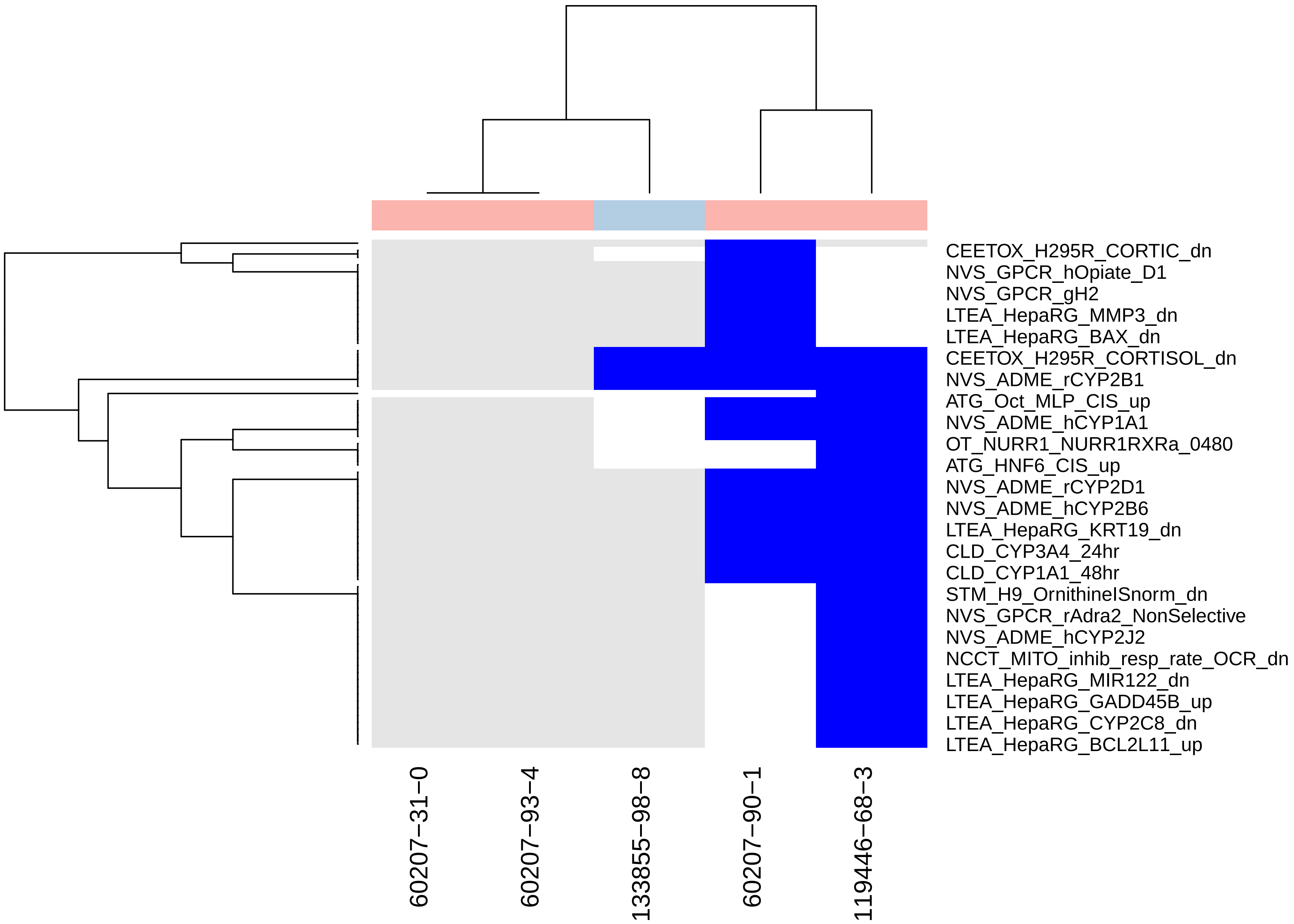

Enriched Assay Heatmap for Cluster 756

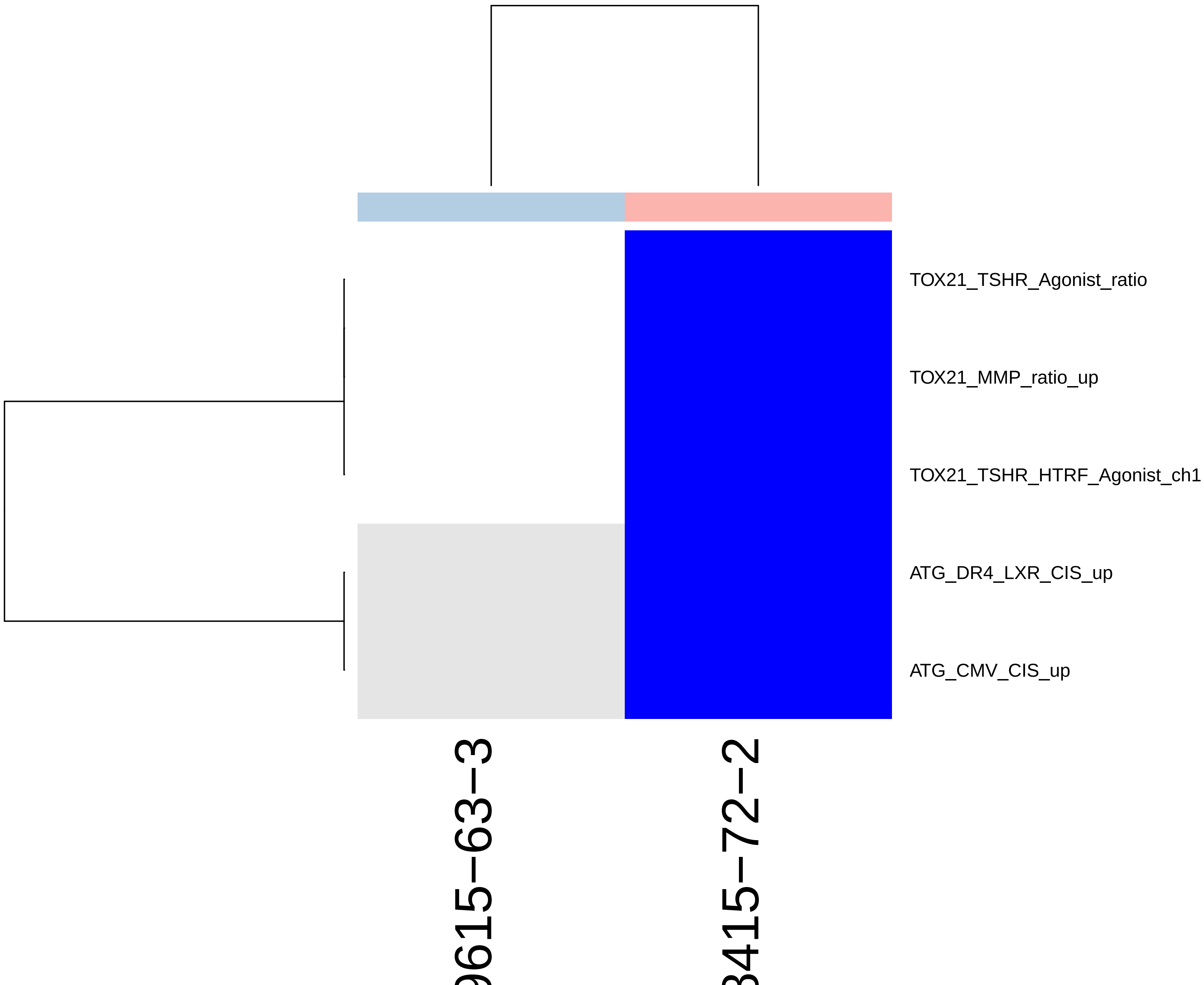

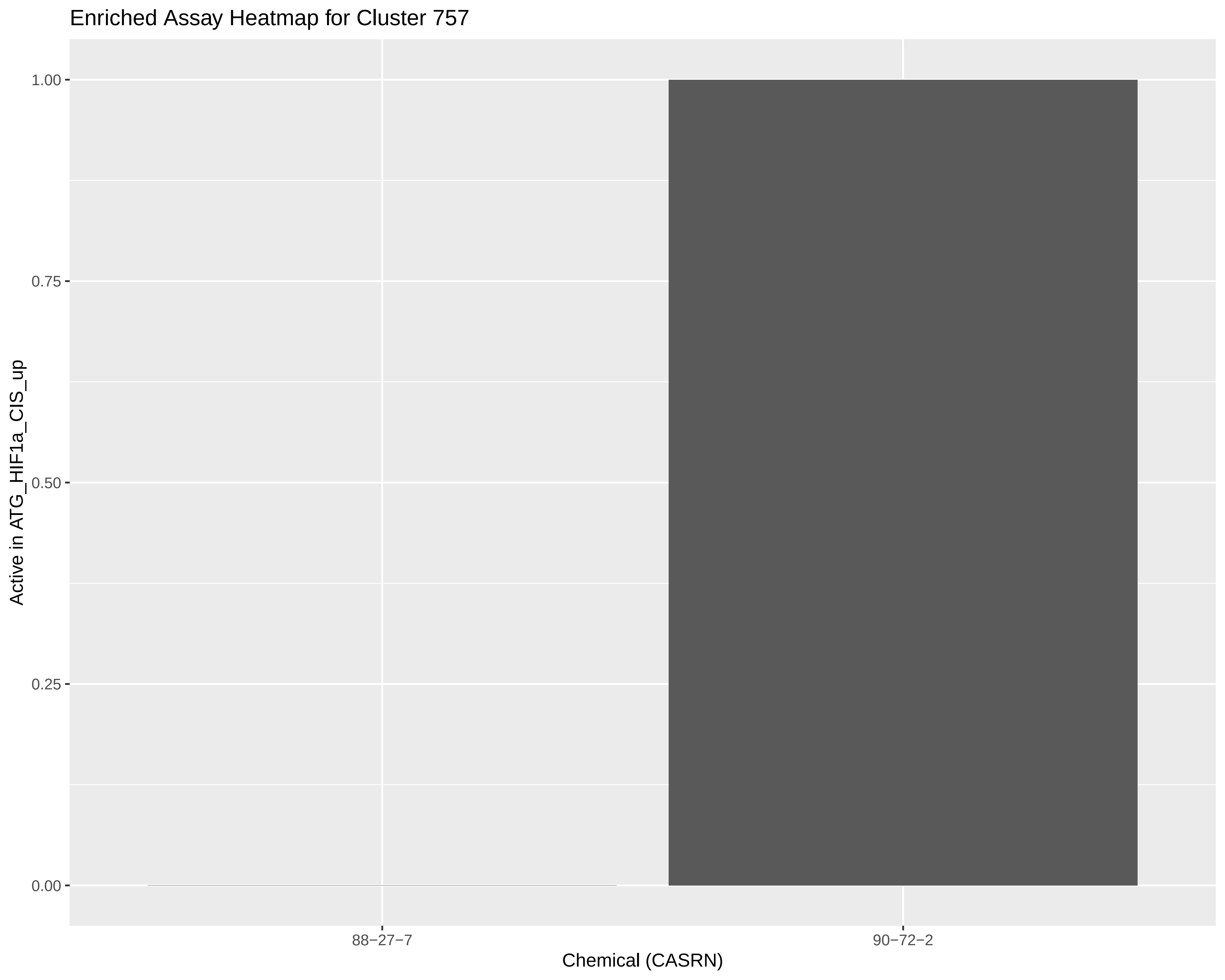
Enriched Assay Heatmap for Cluster 757
1.00
0.75
0.50
0.25
0.00
Active in ATG_HIF1a_CIS_up
88−27−7
90−72−2
Chemical (CASRN)

Enriched Assay Heatmap for Cluster 759

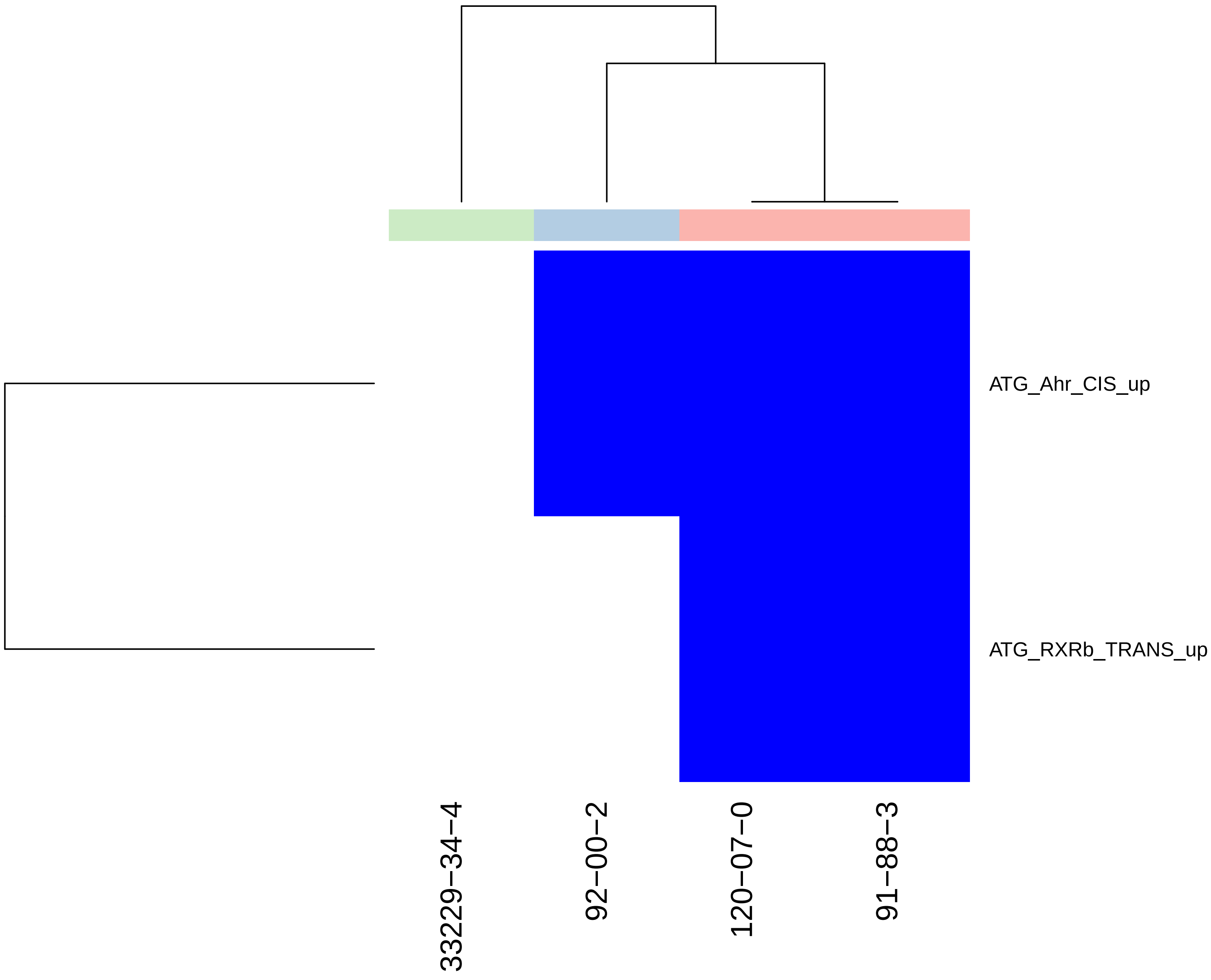

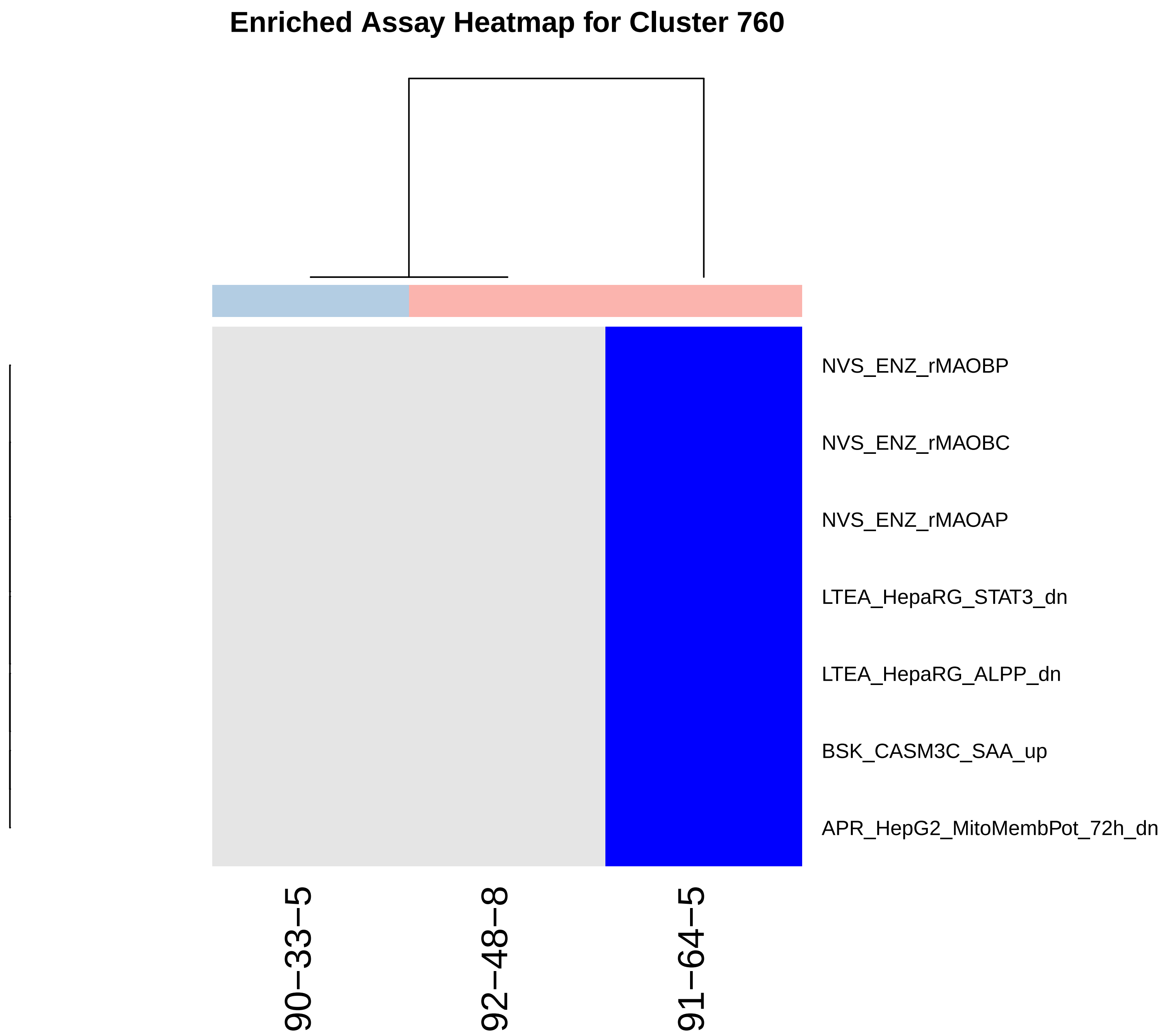

Enriched Assay Heatmap for Cluster 760
NVS_ENZ_rMAOBP
NVS_ENZ_rMAOBC
NVS_ENZ_rMAOAP
LTEA_HepaRG_STAT3_dn
LTEA_HepaRG_ALPP_dn
BSK_CASM3C_SAA_up
APR_HepG2_MitoMembPot_72h_dn
90−33−5
92−48−8
91−64−5

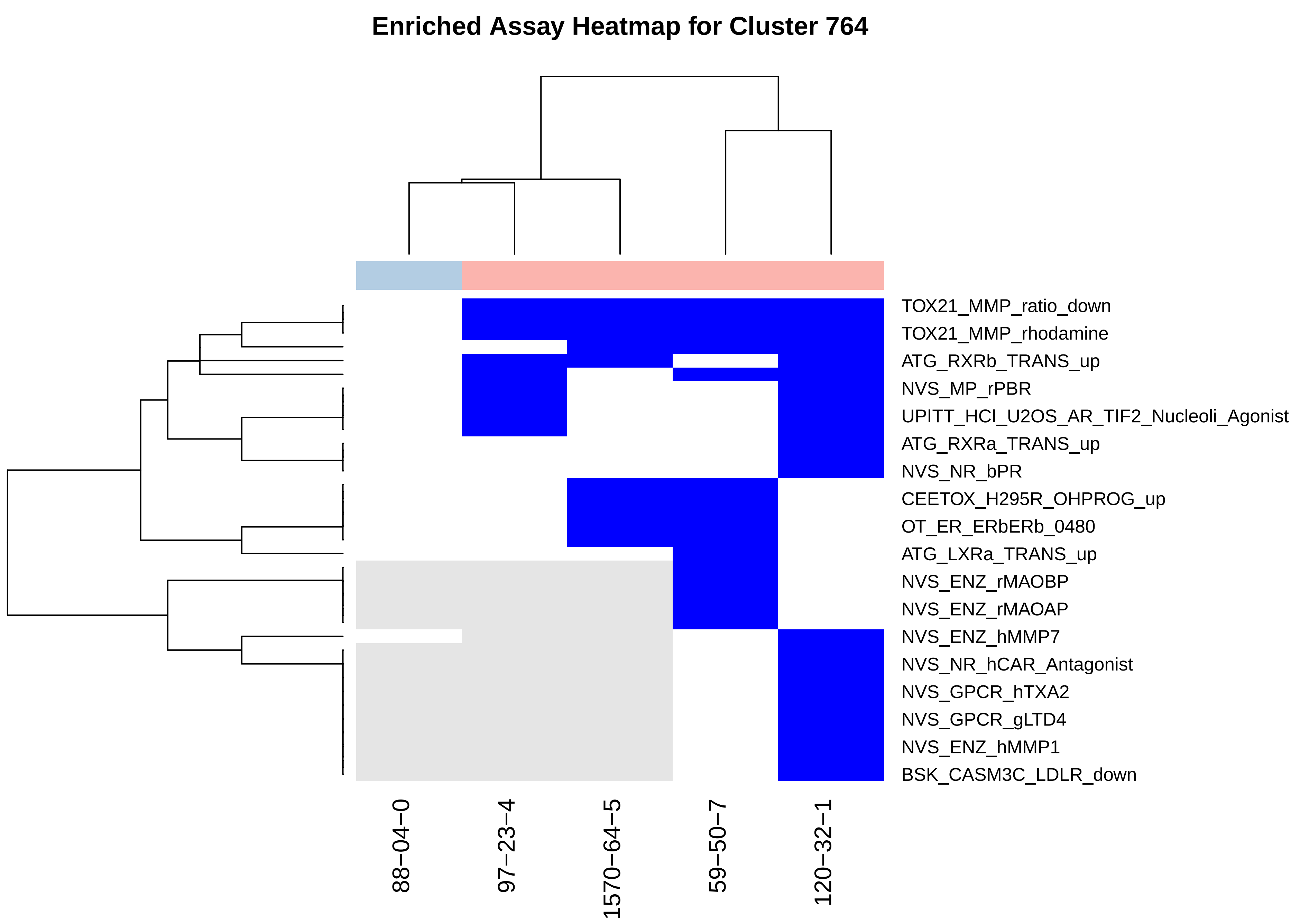
Enriched Assay Heatmap for Cluster 764
TOX21_MMP_ratio_down
TOX21_MMP_rhodamine
ATG_RXRb_TRANS_up
NVS_MP_rPBR
UPITT_HCI_U2OS_AR_TIF2_Nucleoli_Agonist
ATG_RXRa_TRANS_up
NVS_NR_bPR
CEETOX_H295R_OHPROG_up
OT_ER_ERbERb_0480
ATG_LXRa_TRANS_up
NVS_ENZ_rMAOBP
NVS_ENZ_rMAOAP
NVS_ENZ_hMMP7
NVS_NR_hCAR_Antagonist
NVS_GPCR_hTXA2
NVS_GPCR_gLTD4
NVS_ENZ_hMMP1
BSK_CASM3C_LDLR_down
88−04−0
97−23−4
1570−64−5
59−50−7
120−32−1

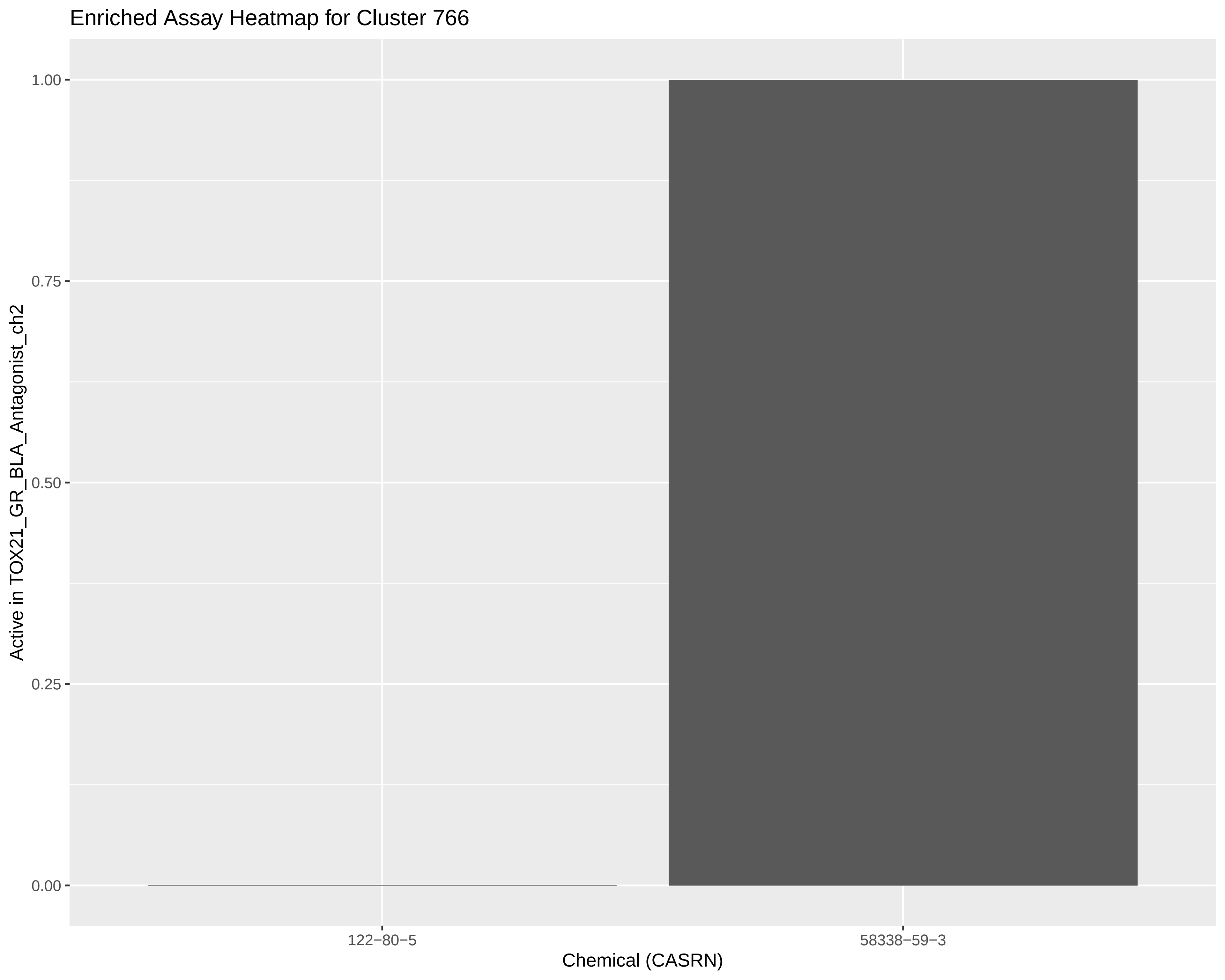

Enriched Assay Heatmap for Cluster 766
1.00
0.75
0.50
0.25
0.00
Active in TOX21_GR_BLA_Antagonist_ch2
122−80−5
58338−59−3
Chemical (CASRN)

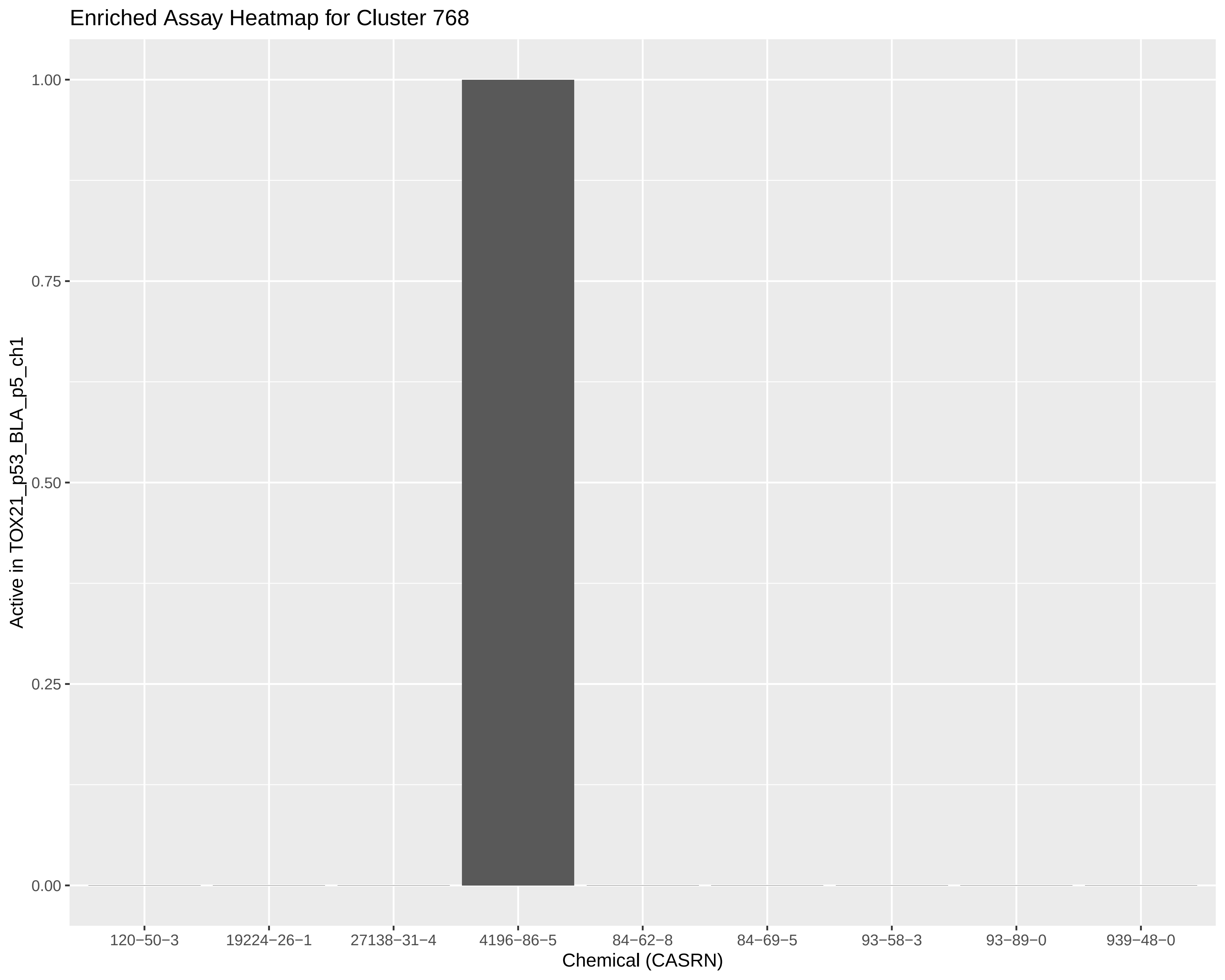
Enriched Assay Heatmap for Cluster 768
Active in TOX21_p53_BLA_p5_ch1
1.00
0.75
0.50
0.25
0.00
120−50−3
19224−26−1
27138−31−4
4196−86−5
84−62−8
84−69−5
93−58−3
93−89−0
939−48−0
Chemical (CASRN)

Enriched Assay Heatmap for Cluster 769

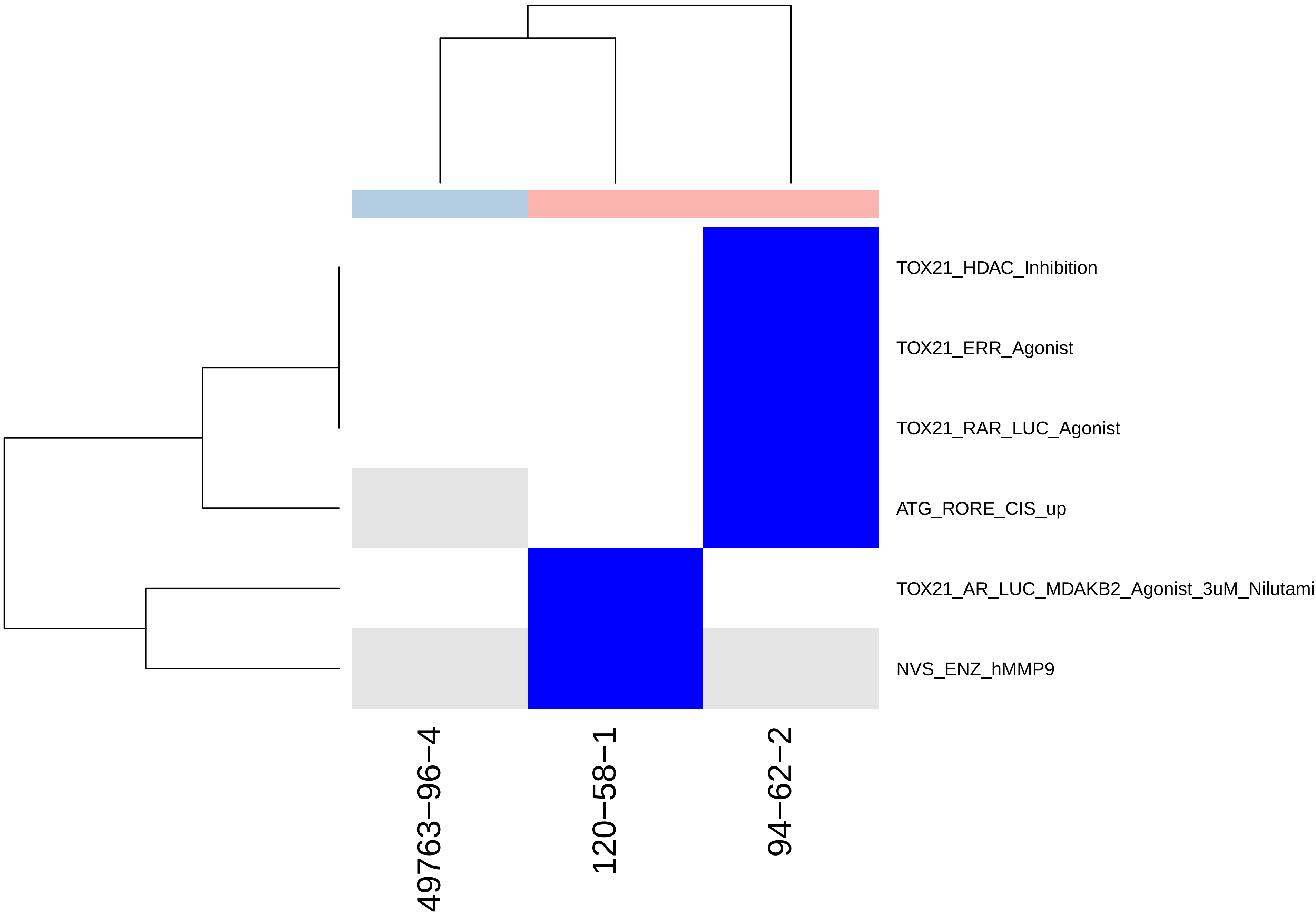

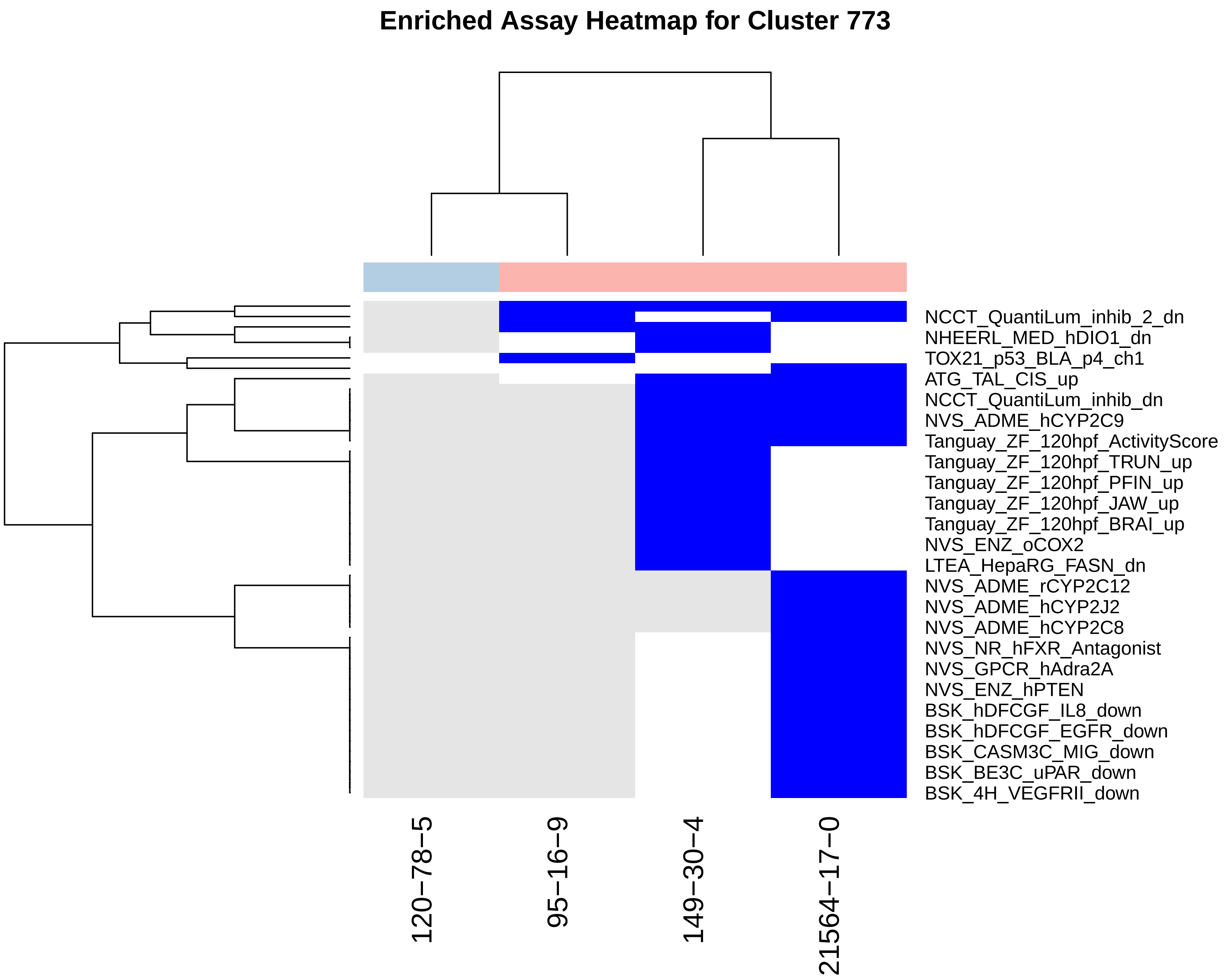
Enriched Assay Heatmap for Cluster 773
NCCT_QuantiLum_inhib_2_dn
NHEERL_MED_hDIO1_dn
TOX21_p53_BLA_p4_ch1
ATG_TAL_CIS_up
NCCT_QuantiLum_inhib_dn
NVS_ADME_hCYP2C9
Tanguay_ZF_120hpf_ActivityScore
Tanguay_ZF_120hpf_TRUN_up
Tanguay_ZF_120hpf_PFIN_up
Tanguay_ZF_120hpf_JAW_up
Tanguay_ZF_120hpf_BRAI_up
NVS_ENZ_oCOX2
LTEA_HepaRG_FASN_dn
NVS_ADME_rCYP2C12
NVS_ADME_hCYP2J2
NVS_ADME_hCYP2C8
NVS_NR_hFXR_Antagonist
NVS_GPCR_hAdra2A
NVS_ENZ_hPTEN
BSK_hDFCGF_IL8_down
BSK_hDFCGF_EGFR_down
BSK_CASM3C_MIG_down
BSK_BE3C_uPAR_down
BSK_4H_VEGFRII_down
120−78−5
95−16−9
149−30−4
21564−17−0

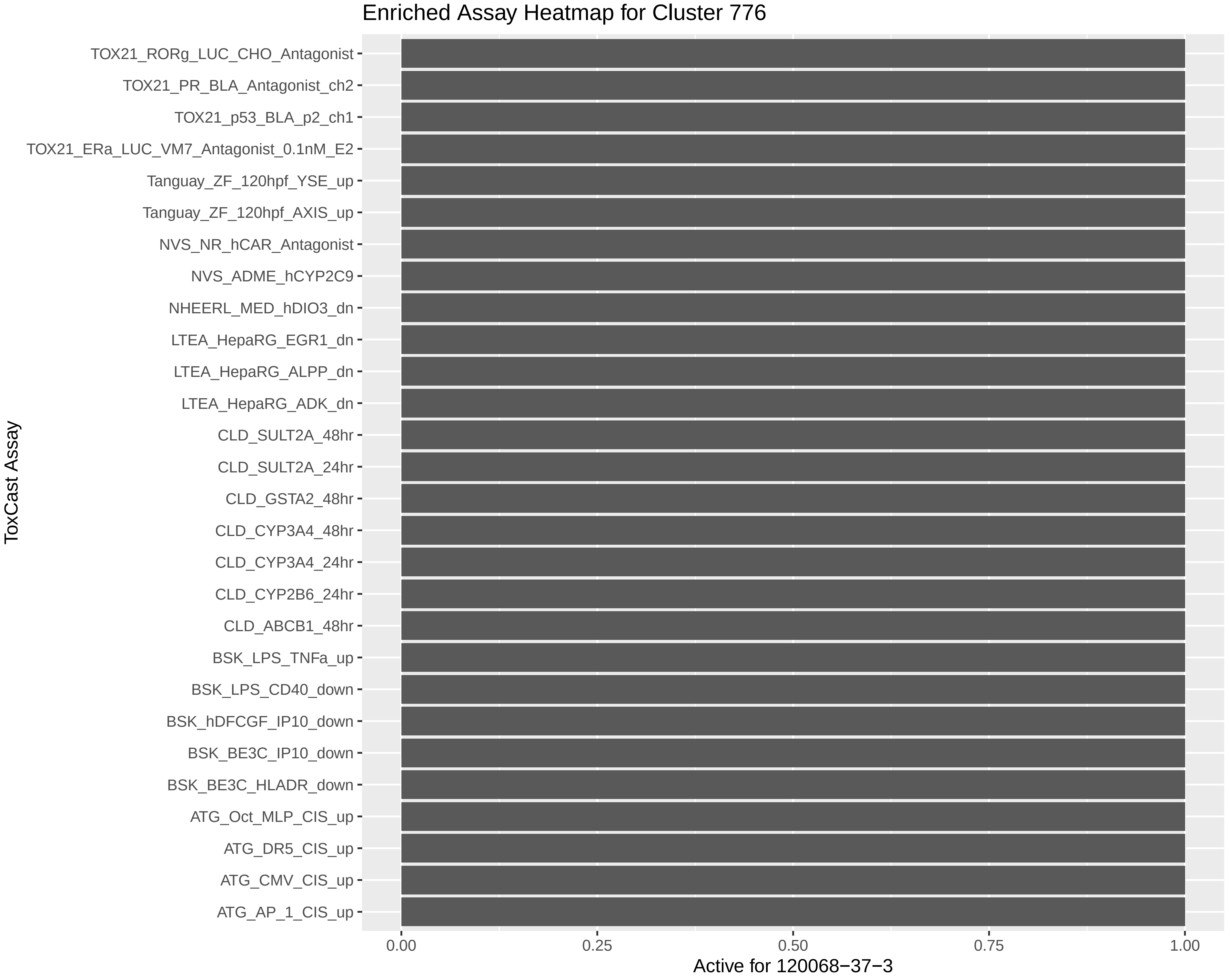

Enriched Assay Heatmap for Cluster 776
ToxCast Assay
TOX21_RORg_LUC_CHO_Antagonist
TOX21_PR_BLA_Antagonist_ch2
TOX21_p53_BLA_p2_ch1
TOX21_ERa_LUC_VM7_Antagonist_0.1nM_E2
Tanguay_ZF_120hpf_YSE_up
Tanguay_ZF_120hpf_AXIS_up
NVS_NR_hCAR_Antagonist
NVS_ADME_hCYP2C9
NHEERL_MED_hDIO3_dn
LTEA_HepaRG_EGR1_dn
LTEA_HepaRG_ALPP_dn
LTEA_HepaRG_ADK_dn
CLD_SULT2A_48hr
CLD_SULT2A_24hr
CLD_GSTA2_48hr
CLD_CYP3A4_48hr
CLD_CYP3A4_24hr
CLD_CYP2B6_24hr
CLD_ABCB1_48hr
BSK_LPS_TNFa_up
BSK_LPS_CD40_down
BSK_hDFCGF_IP10_down
BSK_BE3C_IP10_down
BSK_BE3C_HLADR_down
ATG_Oct_MLP_CIS_up
ATG_DR5_CIS_up
ATG_CMV_CIS_up
ATG_AP_1_CIS_up
0.00
0.25
0.50
0.75
1.00
Active for 120068−37−3

Enriched Assay Heatmap for Cluster 784

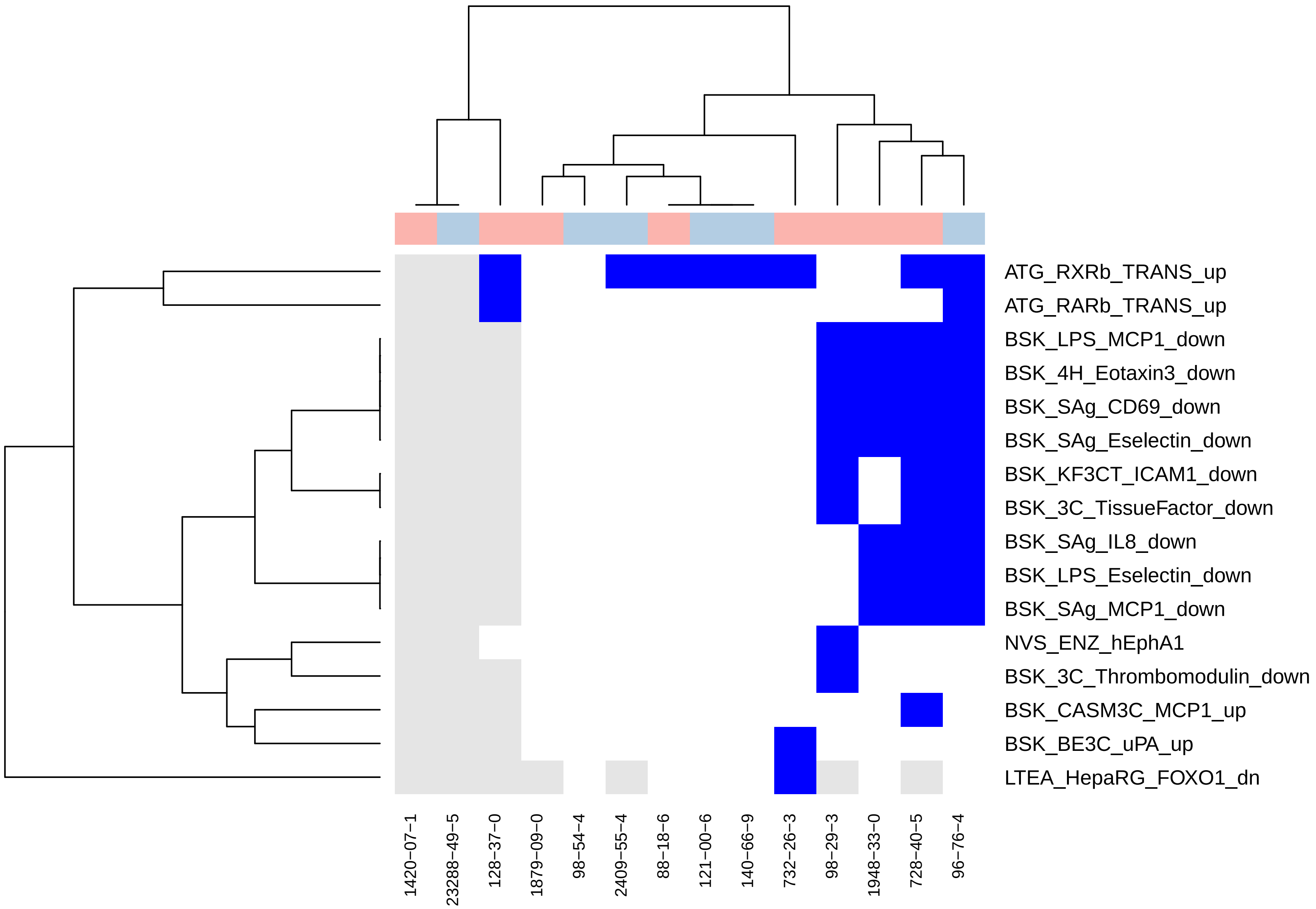

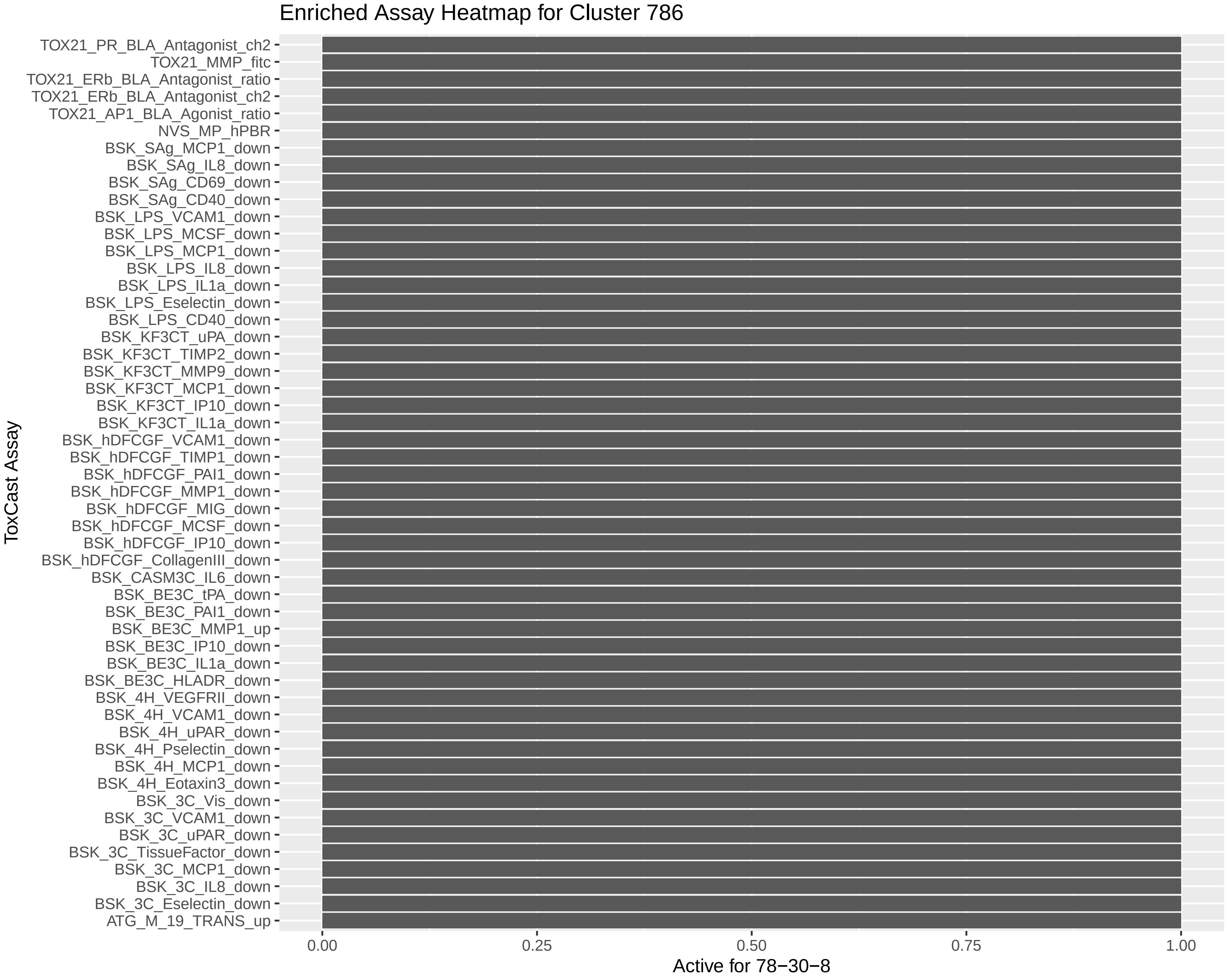

Enriched Assay Heatmap for Cluster 786
TOX21_PR_BLA_Antagonist_ch2
TOX21_MMP_fitc
TOX21_ERb_BLA_Antagonist_ratio
TOX21_ERb_BLA_Antagonist_ch2
TOX21_AP1_BLA_Agonist_ratio
NVS_MP_hPBR
BSK_SAg_MCP1_down
BSK_SAg_IL8_down
BSK_SAg_CD69_down
BSK_SAg_CD40_down
BSK_LPS_VCAM1_down
BSK_LPS_MCSF_down
BSK_LPS_MCP1_down
BSK_LPS_IL8_down
BSK_LPS_IL1a_down
BSK_LPS_Eselectin_down
BSK_LPS_CD40_down
BSK_KF3CT_uPA_down
BSK_KF3CT_TIMP2_down
BSK_KF3CT_MMP9_down
BSK_KF3CT_MCP1_down
BSK_KF3CT_IP10_down
BSK_KF3CT_IL1a_down
BSK_hDFCGF_VCAM1_down
BSK_hDFCGF_TIMP1_down
BSK_hDFCGF_PAI1_down
BSK_hDFCGF_MMP1_down
BSK_hDFCGF_MIG_down
BSK_hDFCGF_MCSF_down
BSK_hDFCGF_IP10_down
BSK_hDFCGF_CollagenIII_down
BSK_CASM3C_IL6_down
BSK_BE3C_tPA_down
BSK_BE3C_PAI1_down
BSK_BE3C_MMP1_up
BSK_BE3C_IP10_down
BSK_BE3C_IL1a_down
BSK_BE3C_HLADR_down
BSK_4H_VEGFRII_down
BSK_4H_VCAM1_down
BSK_4H_uPAR_down
BSK_4H_Pselectin_down
BSK_4H_MCP1_down
BSK_4H_Eotaxin3_down
BSK_3C_Vis_down
BSK_3C_VCAM1_down
BSK_3C_uPAR_down
BSK_3C_TissueFactor_down
BSK_3C_MCP1_down
BSK_3C_IL8_down
BSK_3C_Eselectin_down
ATG_M_19_TRANS_up
ToxCast Assay
0.00
0.25
0.50
0.75
1.00
Active for 78−30−8

Enriched Assay Heatmap for Cluster 787

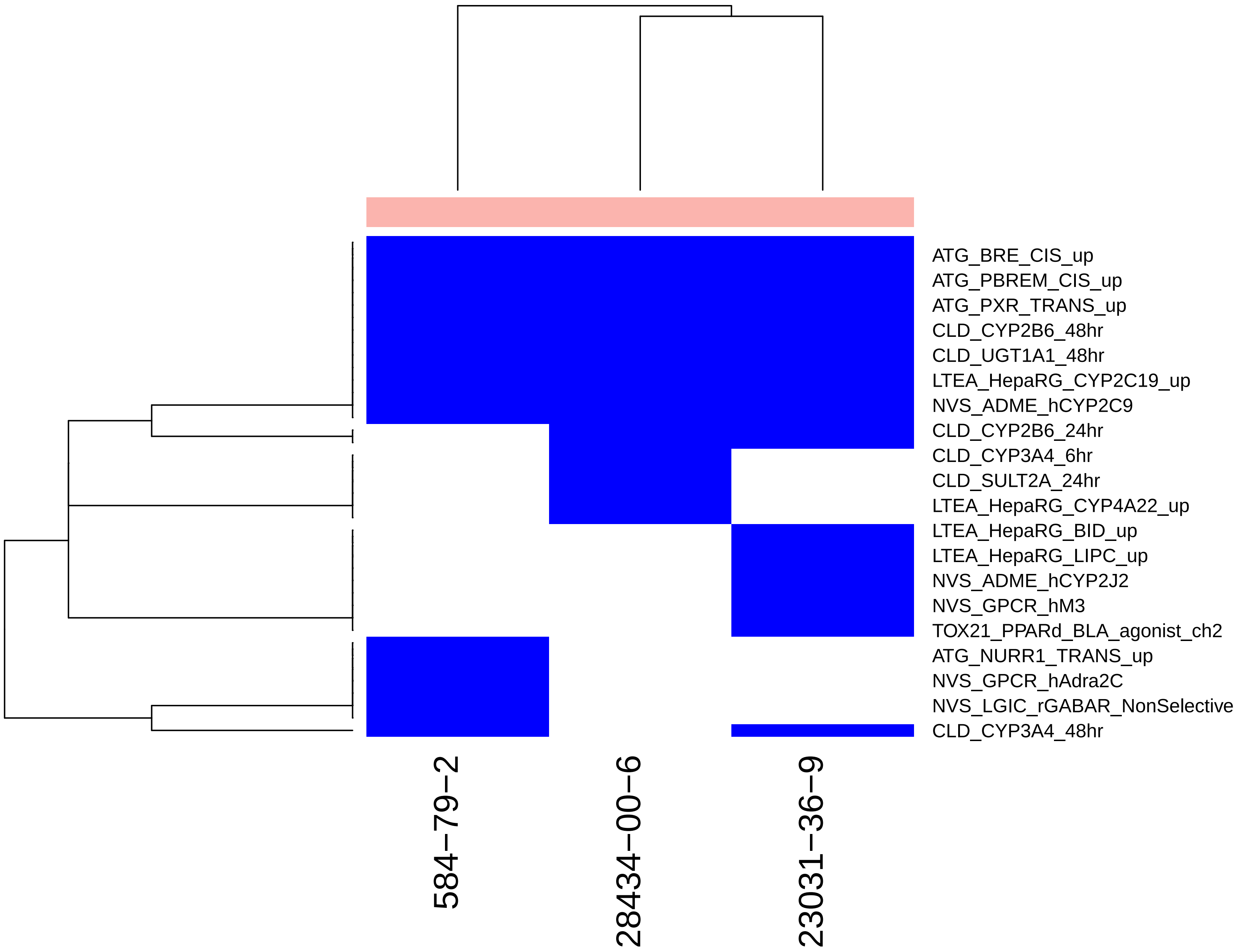

Enriched Assay Heatmap for Cluster 788

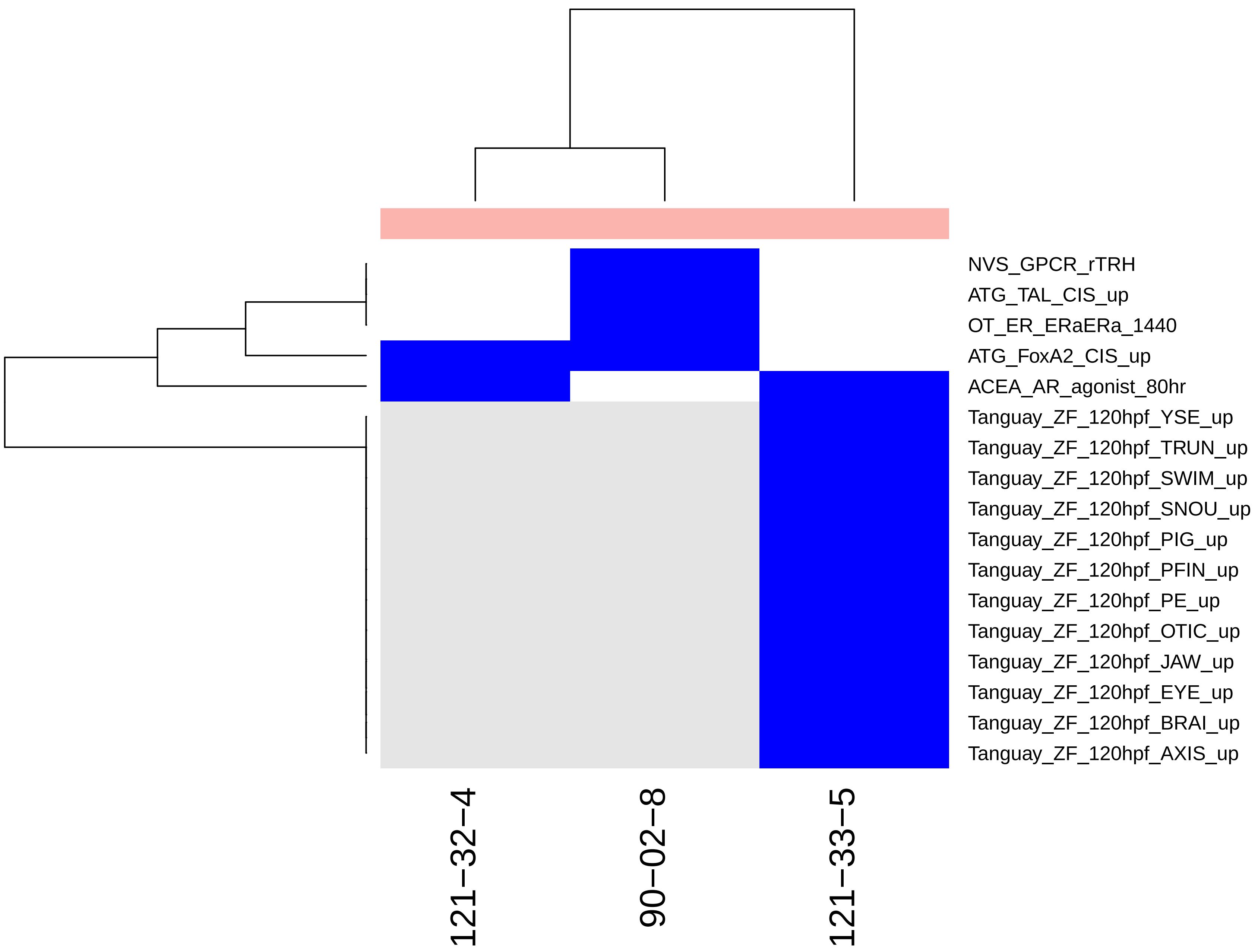

Enriched Assay Heatmap for Cluster 790

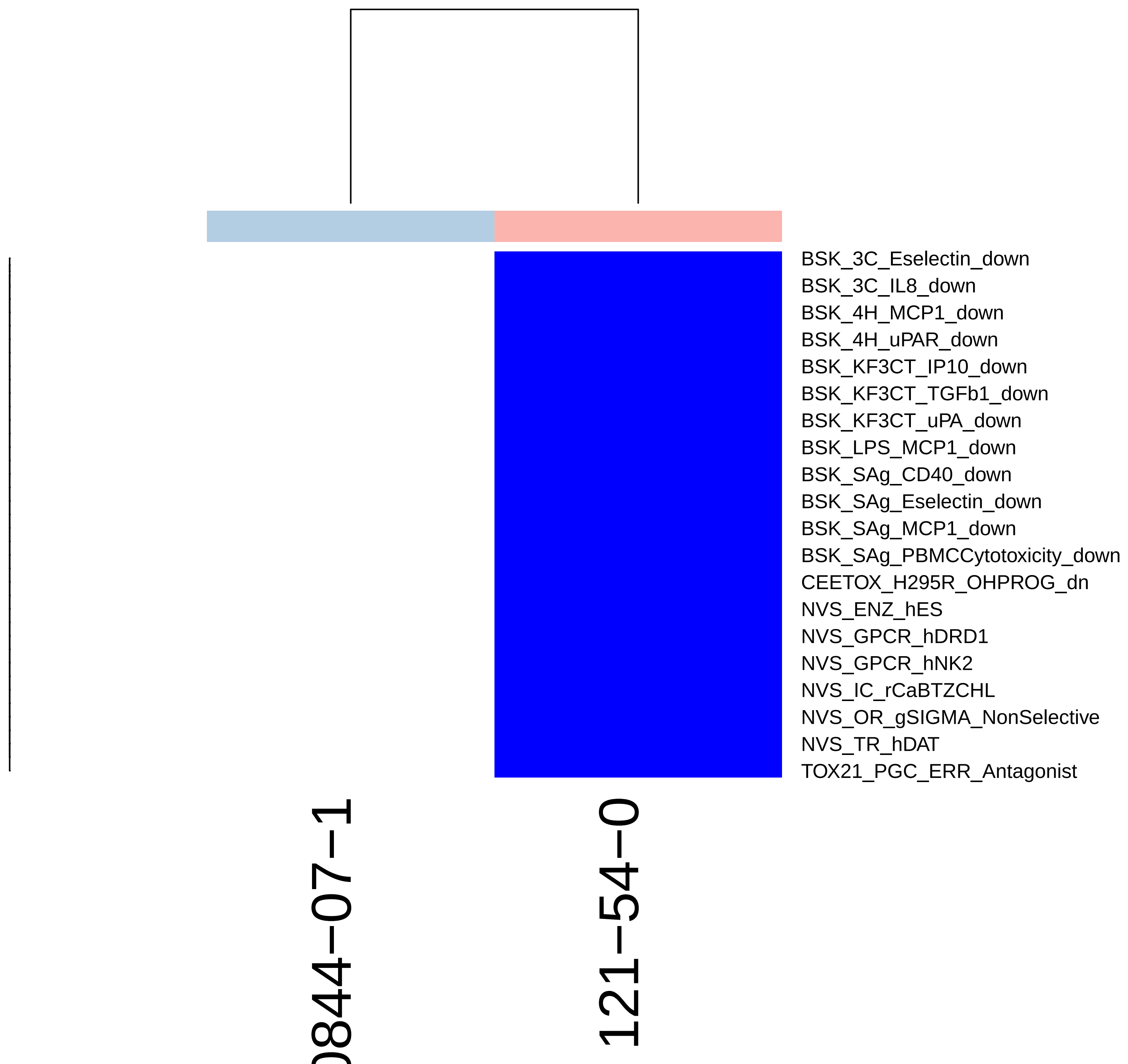

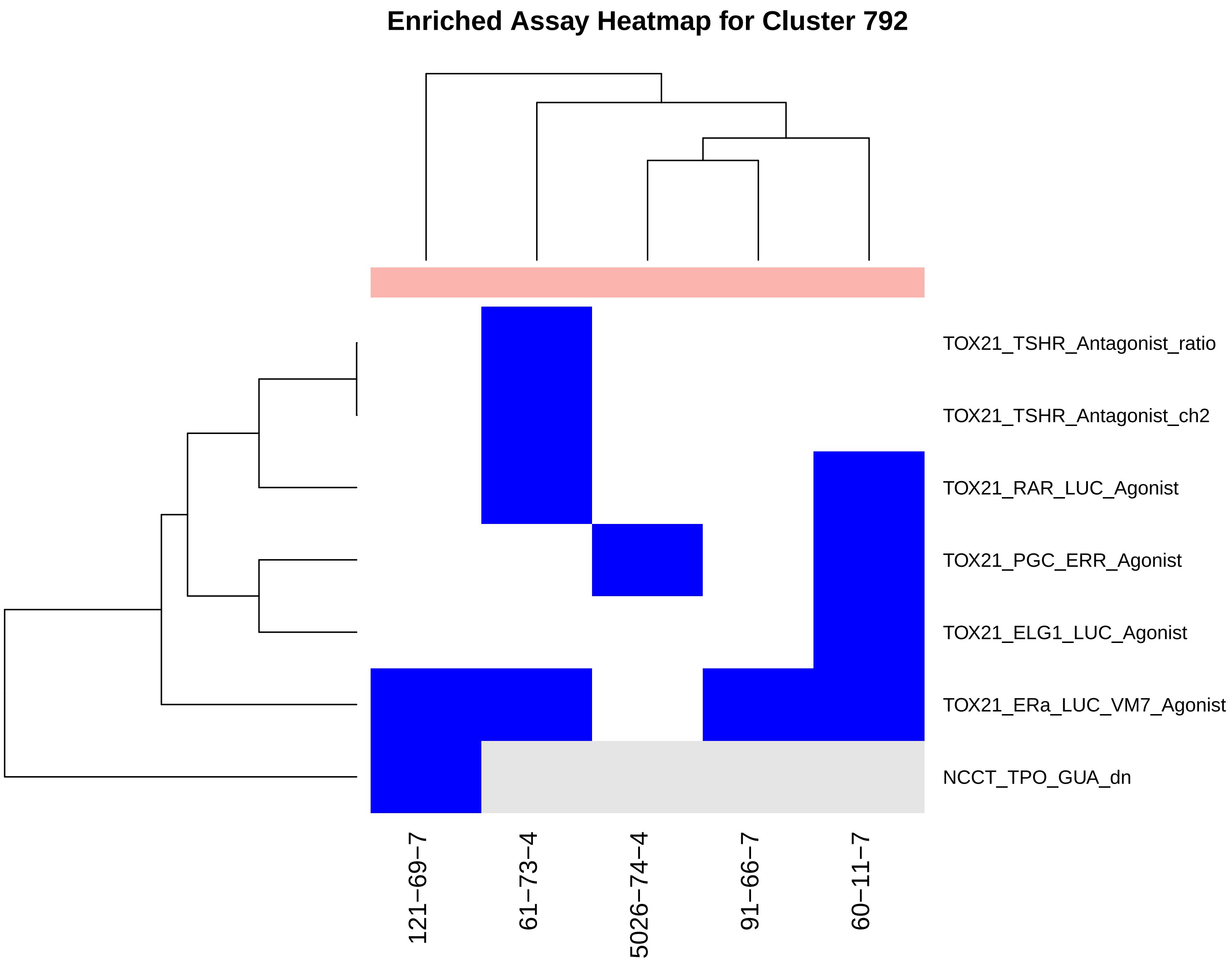

Enriched Assay Heatmap for Cluster 792
TOX21_TSHR_Antagonist_ratio
TOX21_TSHR_Antagonist_ch2
TOX21_RAR_LUC_Agonist
TOX21_PGC_ERR_Agonist
TOX21_ELG1_LUC_Agonist
TOX21_ERa_LUC_VM7_Agonist
NCCT_TPO_GUA_dn
121−69−7
61−73−4
5026−74−4
91−66−7
60−11−7

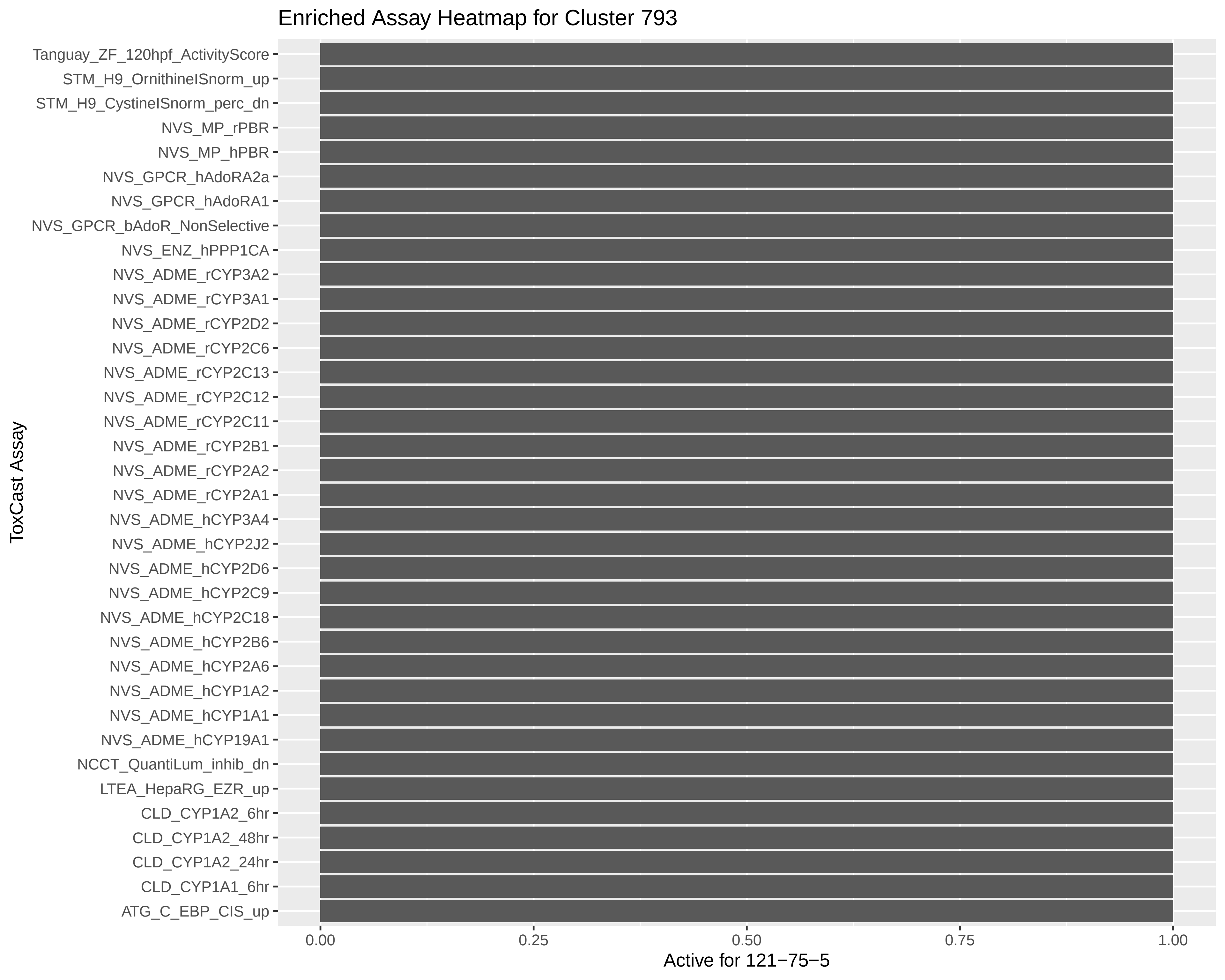

Enriched Assay Heatmap for Cluster 793
ToxCast Assay
Tanguay_ZF_120hpf_ActivityScore
STM_H9_OrnithineISnorm_up
STM_H9_CystineISnorm_perc_dn
NVS_MP_rPBR
NVS_MP_hPBR
NVS_GPCR_hAdoRA2a
NVS_GPCR_hAdoRA1
NVS_GPCR_bAdoR_NonSelective
NVS_ENZ_hPPP1CA
NVS_ADME_rCYP3A2
NVS_ADME_rCYP3A1
NVS_ADME_rCYP2D2
NVS_ADME_rCYP2C6
NVS_ADME_rCYP2C13
NVS_ADME_rCYP2C12
NVS_ADME_rCYP2C11
NVS_ADME_rCYP2B1
NVS_ADME_rCYP2A2
NVS_ADME_rCYP2A1
NVS_ADME_hCYP3A4
NVS_ADME_hCYP2J2
NVS_ADME_hCYP2D6
NVS_ADME_hCYP2C9
NVS_ADME_hCYP2C18
NVS_ADME_hCYP2B6
NVS_ADME_hCYP2A6
NVS_ADME_hCYP1A2
NVS_ADME_hCYP1A1
NVS_ADME_hCYP19A1
NCCT_QuantiLum_inhib_dn
LTEA_HepaRG_EZR_up
CLD_CYP1A2_6hr
CLD_CYP1A2_48hr
CLD_CYP1A2_24hr
CLD_CYP1A1_6hr
ATG_C_EBP_CIS_up
0.00
0.25
0.50
0.75
1.00
Active for 121−75−5

Enriched Assay Heatmap for Cluster 795

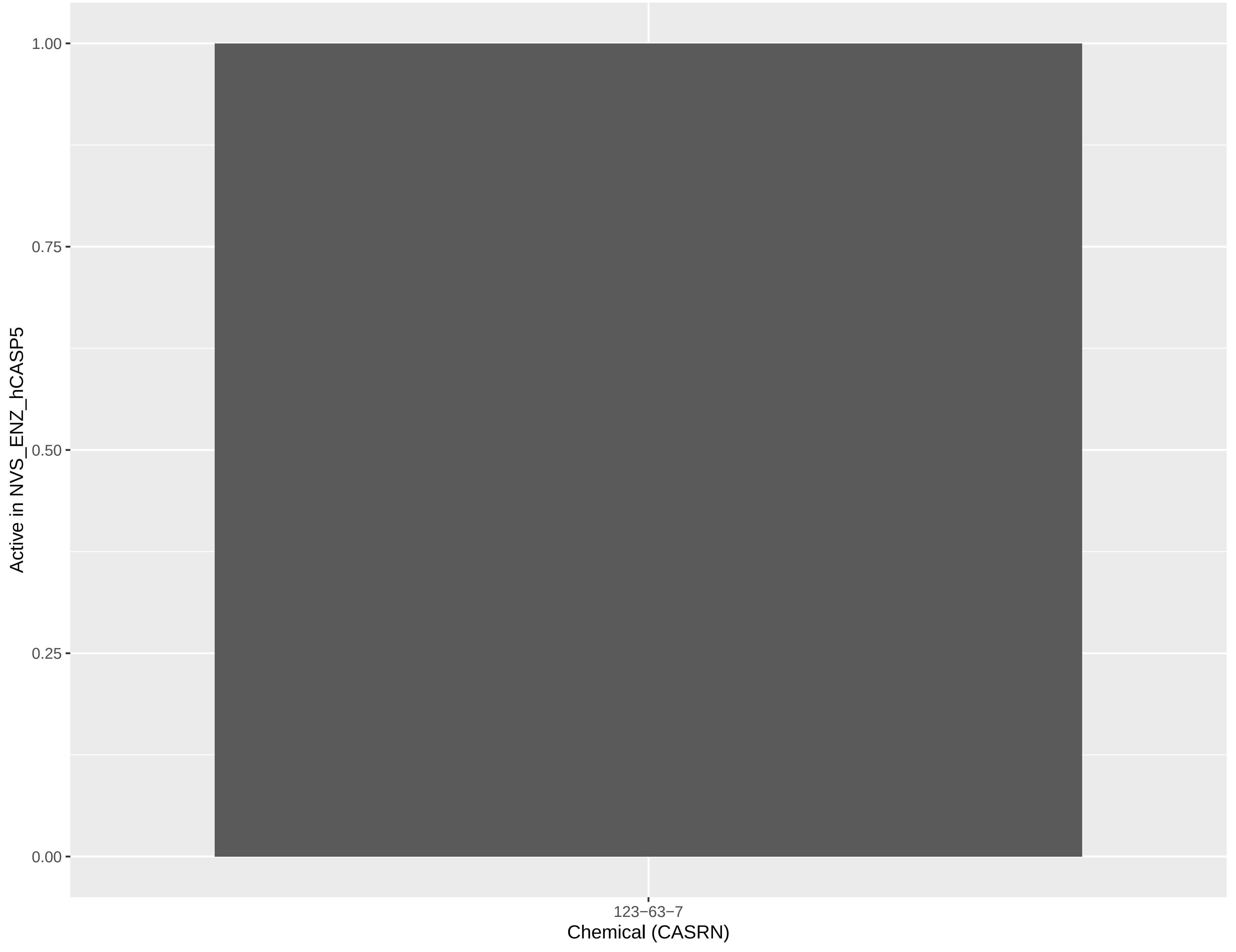

Enriched Assay Heatmap for Cluster 796

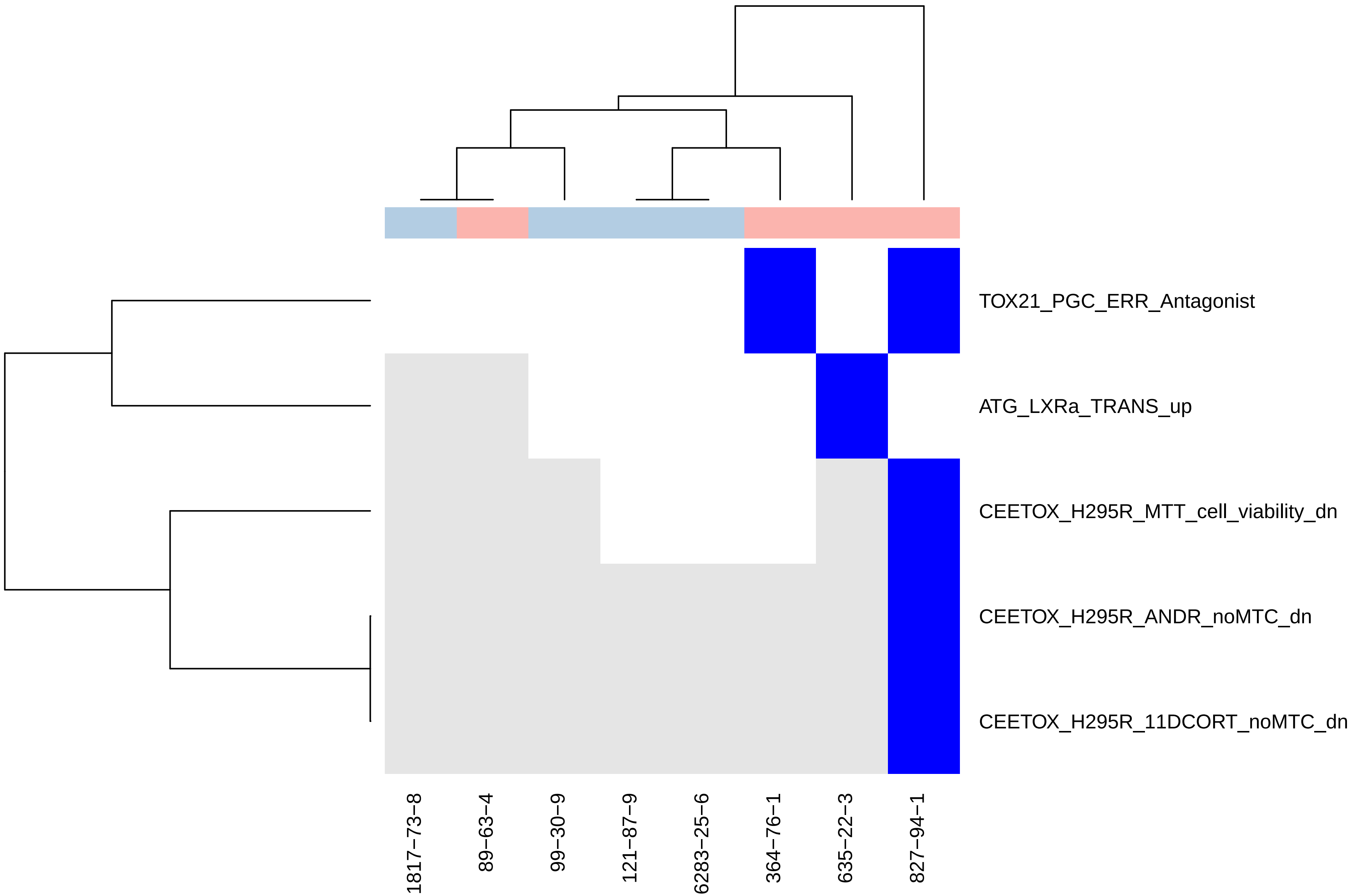

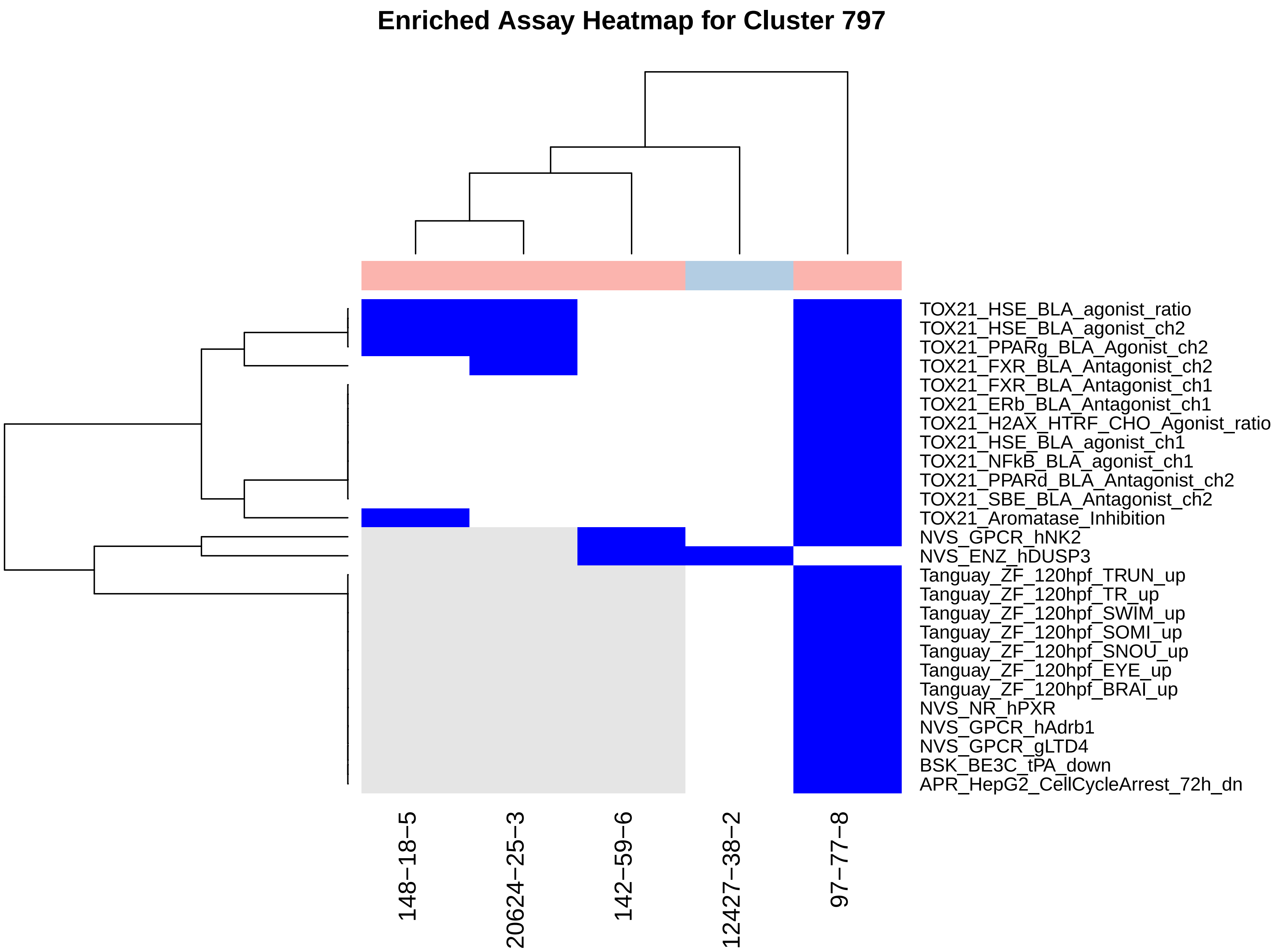

Enriched Assay Heatmap for Cluster 797
TOX21_HSE_BLA_agonist_ratio
TOX21_HSE_BLA_agonist_ch2
TOX21_PPARg_BLA_Agonist_ch2
TOX21_FXR_BLA_Antagonist_ch2
TOX21_FXR_BLA_Antagonist_ch1
TOX21_ERb_BLA_Antagonist_ch1
TOX21_H2AX_HTRF_CHO_Agonist_ratio
TOX21_HSE_BLA_agonist_ch1
TOX21_NFkB_BLA_agonist_ch1
TOX21_PPARd_BLA_Antagonist_ch2
TOX21_SBE_BLA_Antagonist_ch2
TOX21_Aromatase_Inhibition
NVS_GPCR_hNK2
NVS_ENZ_hDUSP3
Tanguay_ZF_120hpf_TRUN_up
Tanguay_ZF_120hpf_TR_up
Tanguay_ZF_120hpf_SWIM_up
Tanguay_ZF_120hpf_SOMI_up
Tanguay_ZF_120hpf_SNOU_up
Tanguay_ZF_120hpf_EYE_up
Tanguay_ZF_120hpf_BRAI_up
NVS_NR_hPXR
NVS_GPCR_hAdrb1
NVS_GPCR_gLTD4
BSK_BE3C_tPA_down
APR_HepG2_CellCycleArrest_72h_dn
148−18−5
20624−25−3
142−59−6
12427−38−2
97−77−8

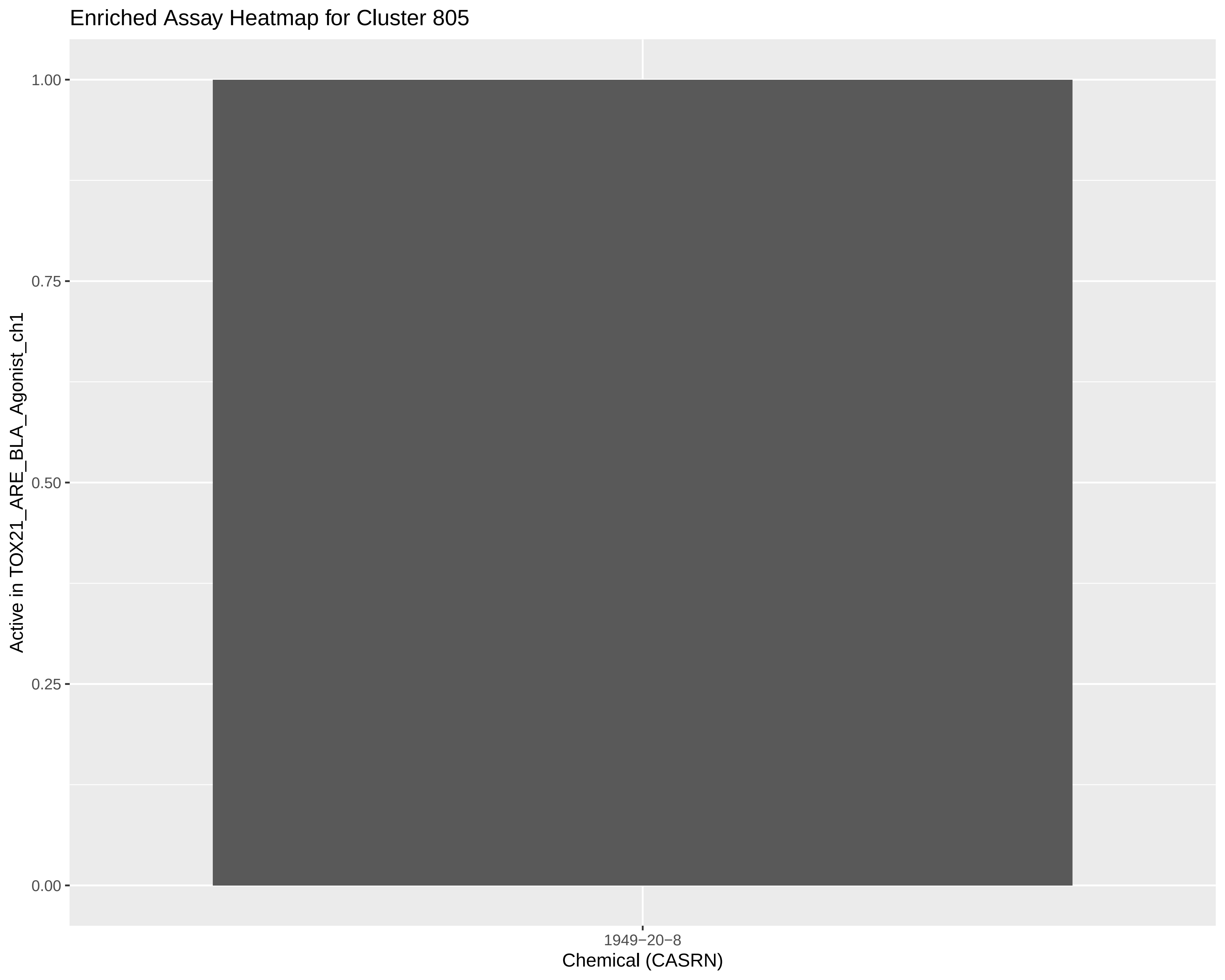

Enriched Assay Heatmap for Cluster 805
Active in TOX21_ARE_BLA_Agonist_ch1
1.00
0.75
0.50
0.25
0.00
1949−20−8
Chemical (CASRN)

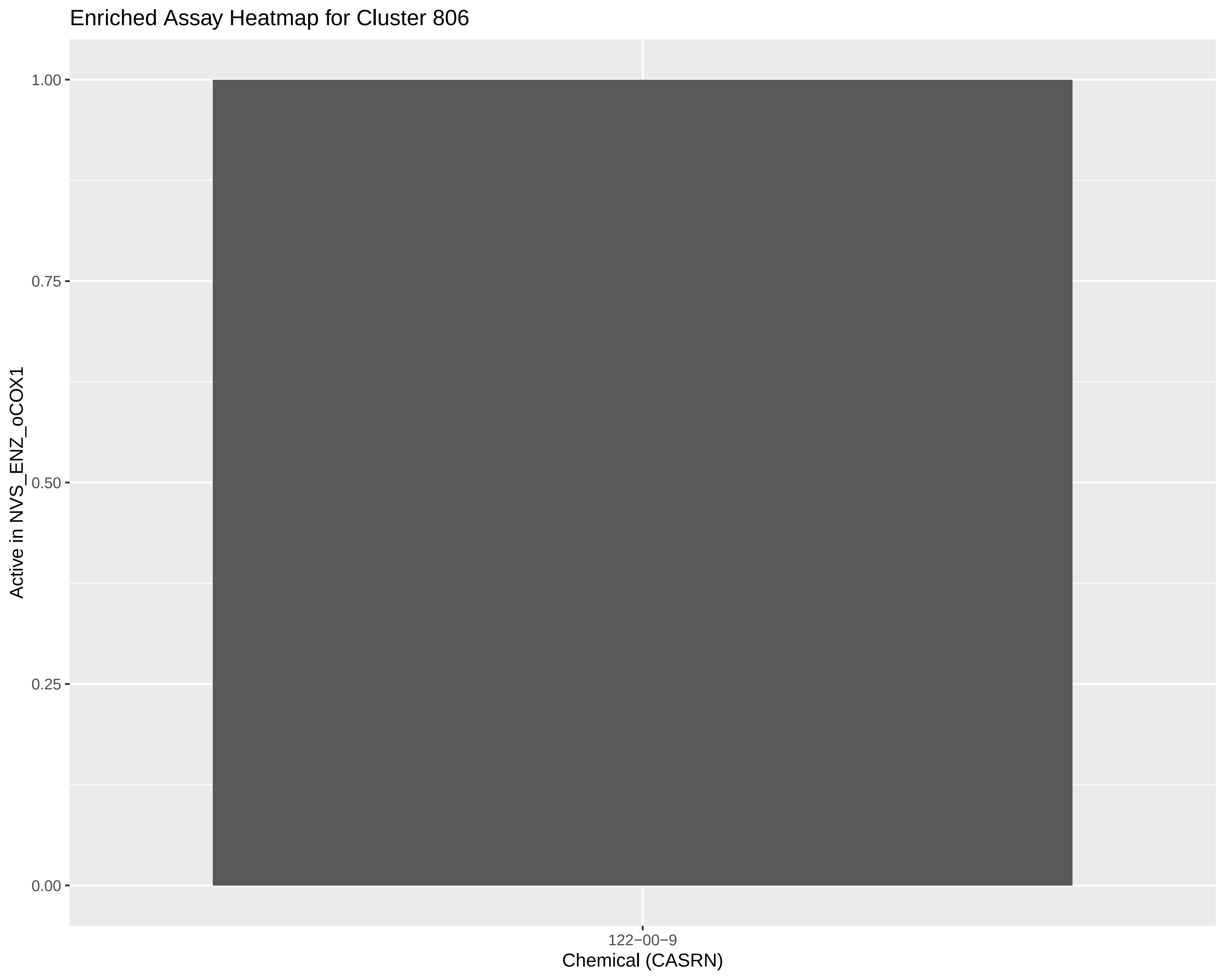
Enriched Assay Heatmap for Cluster 806
Active in NVS_ENZ_oCOX1
1.00
0.75
0.50
0.25
0.00
122−00−9
Chemical (CASRN)

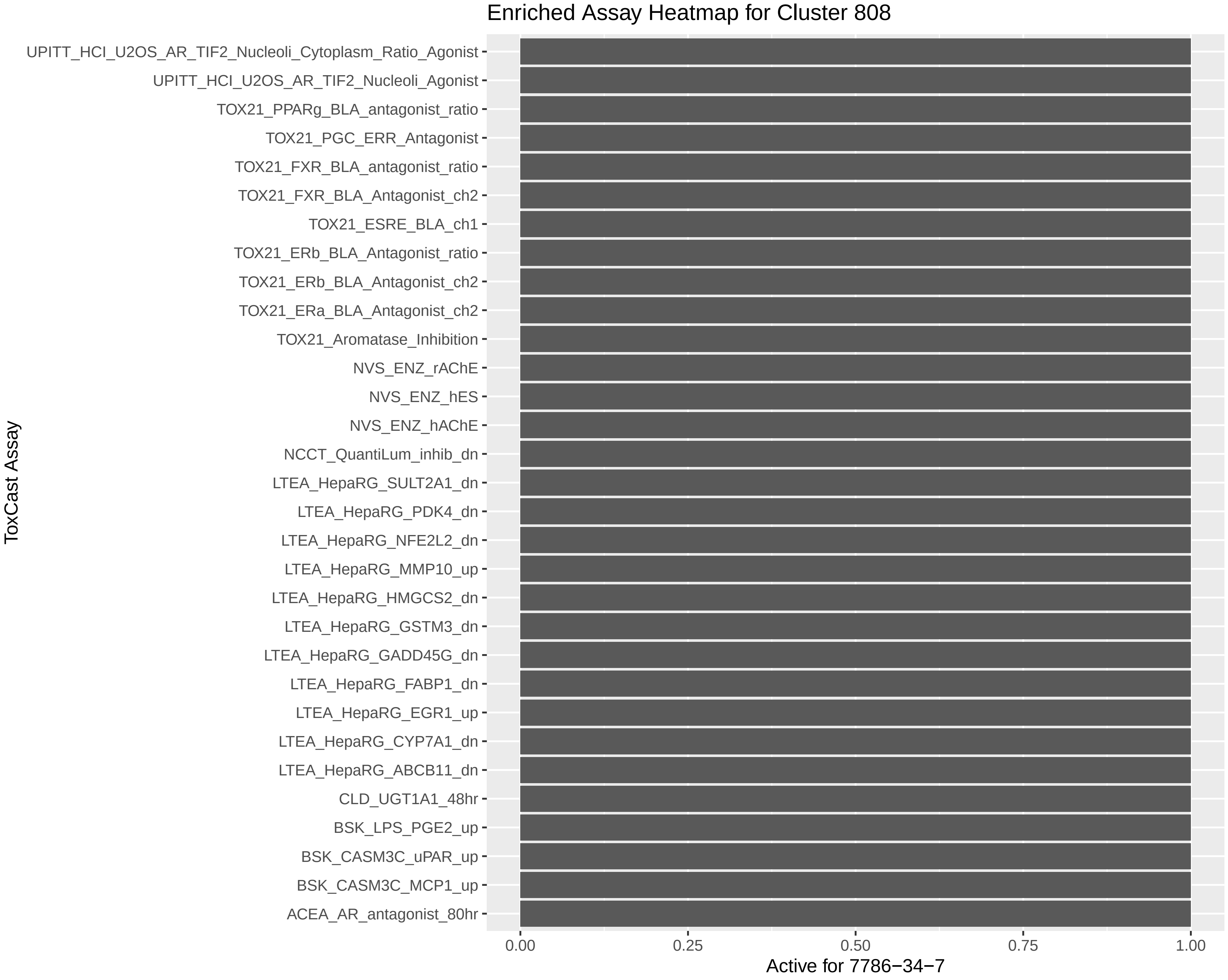

Enriched Assay Heatmap for Cluster 808
ToxCast Assay
UPITT_HCI_U2OS_AR_TIF2_Nucleoli_Cytoplasm_Ratio_Agonist
UPITT_HCI_U2OS_AR_TIF2_Nucleoli_Agonist
TOX21_PPARg_BLA_antagonist_ratio
TOX21_PGC_ERR_Antagonist
TOX21_FXR_BLA_antagonist_ratio
TOX21_FXR_BLA_Antagonist_ch2
TOX21_ESRE_BLA_ch1
TOX21_ERb_BLA_Antagonist_ratio
TOX21_ERb_BLA_Antagonist_ch2
TOX21_ERa_BLA_Antagonist_ch2
TOX21_Aromatase_Inhibition
NVS_ENZ_rAChE
NVS_ENZ_hES
NVS_ENZ_hAChE
NCCT_QuantiLum_inhib_dn
LTEA_HepaRG_SULT2A1_dn
LTEA_HepaRG_PDK4_dn
LTEA_HepaRG_NFE2L2_dn
LTEA_HepaRG_MMP10_up
LTEA_HepaRG_HMGCS2_dn
LTEA_HepaRG_GSTM3_dn
LTEA_HepaRG_GADD45G_dn
LTEA_HepaRG_FABP1_dn
LTEA_HepaRG_EGR1_up
LTEA_HepaRG_CYP7A1_dn
LTEA_HepaRG_ABCB11_dn
CLD_UGT1A1_48hr
BSK_LPS_PGE2_up
BSK_CASM3C_uPAR_up
BSK_CASM3C_MCP1_up
ACEA_AR_antagonist_80hr
0.00
0.25
0.50
0.75
1.00
Active for 7786−34−7

Enriched Assay Heatmap for Cluster 809

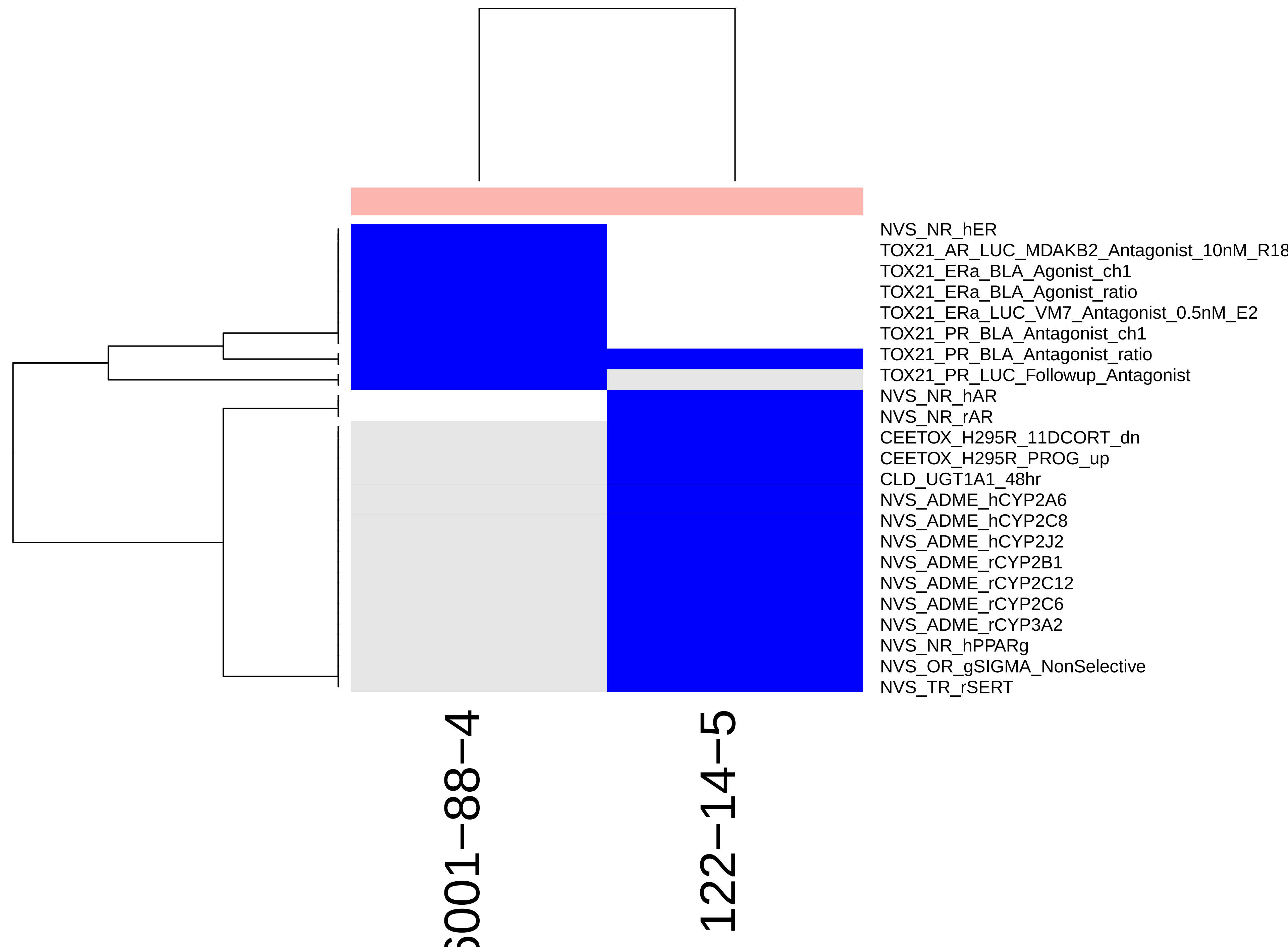

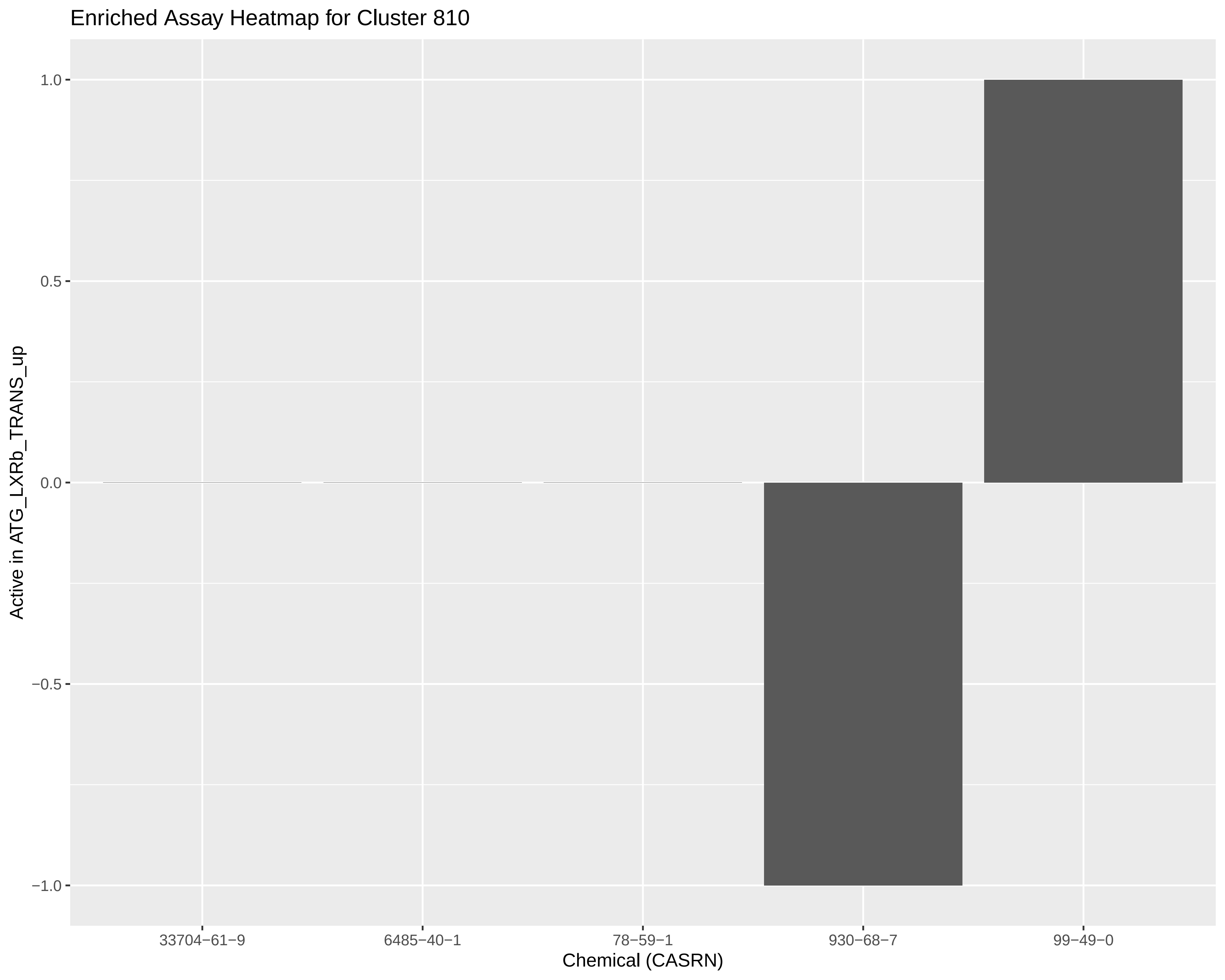

Enriched Assay Heatmap for Cluster 810
Active in ATG_LXRb_TRANS_up
1.0
0.5
0.0
−0.5
−1.0
33704−61−9
6485−40−1
78−59−1
930−68−7
99−49−0
Chemical (CASRN)

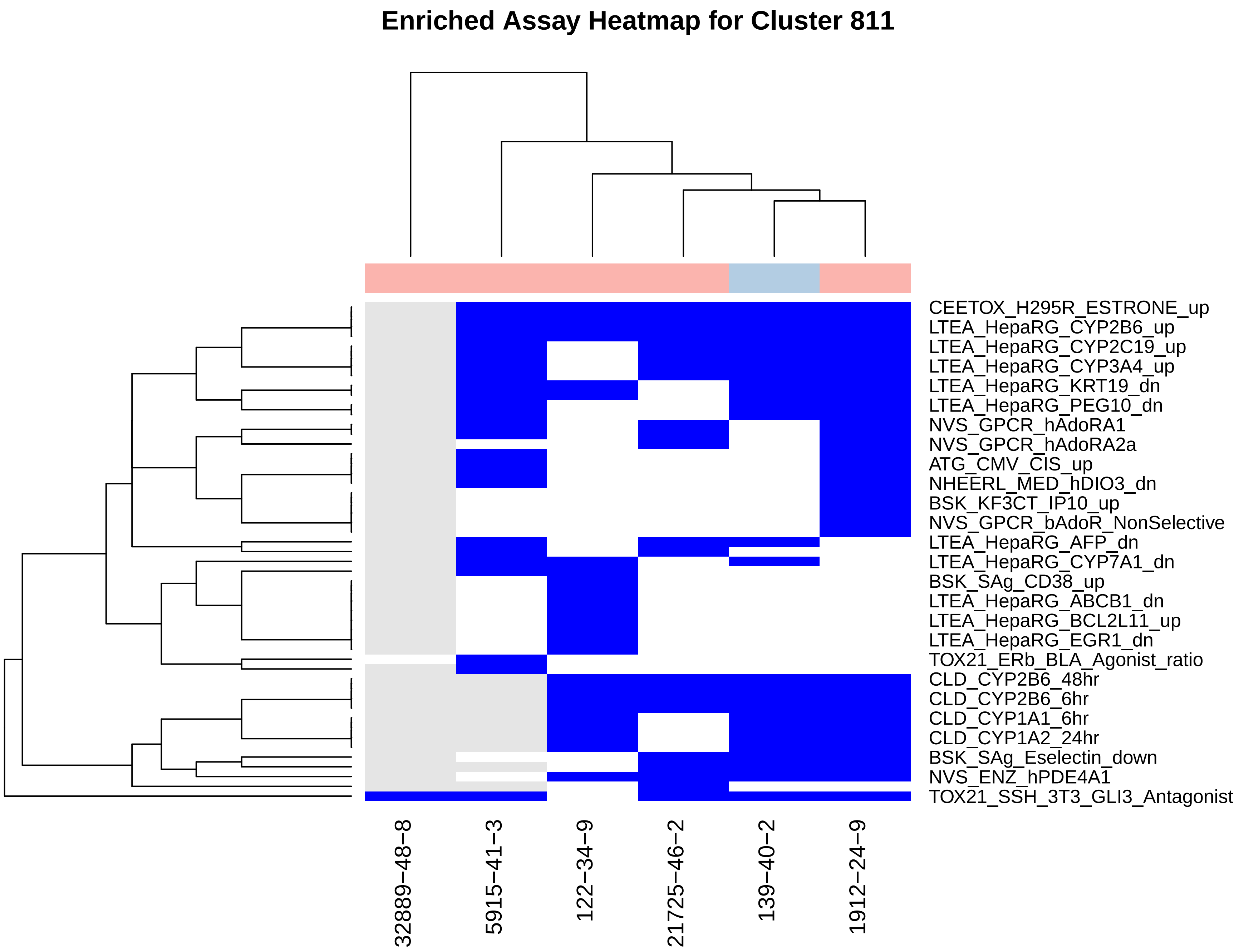
Enriched Assay Heatmap for Cluster 811
CEETOX_H295R_ESTRONE_up
LTEA_HepaRG_CYP2B6_up
LTEA_HepaRG_CYP2C19_up
LTEA_HepaRG_CYP3A4_up
LTEA_HepaRG_KRT19_dn
LTEA_HepaRG_PEG10_dn
NVS_GPCR_hAdoRA1
NVS_GPCR_hAdoRA2a
ATG_CMV_CIS_up
NHEERL_MED_hDIO3_dn
BSK_KF3CT_IP10_up
NVS_GPCR_bAdoR_NonSelective
LTEA_HepaRG_AFP_dn
LTEA_HepaRG_CYP7A1_dn
BSK_SAg_CD38_up
LTEA_HepaRG_ABCB1_dn
LTEA_HepaRG_BCL2L11_up
LTEA_HepaRG_EGR1_dn
TOX21_ERb_BLA_Agonist_ratio
CLD_CYP2B6_48hr
CLD_CYP2B6_6hr
CLD_CYP1A1_6hr
CLD_CYP1A2_24hr
BSK_SAg_Eselectin_down
NVS_ENZ_hPDE4A1
TOX21_SSH_3T3_GLI3_Antagonist
32889−48−8
5915−41−3
122−34−9
21725−46−2
139−40−2
1912−24−9

Enriched Assay Heatmap for Cluster 812

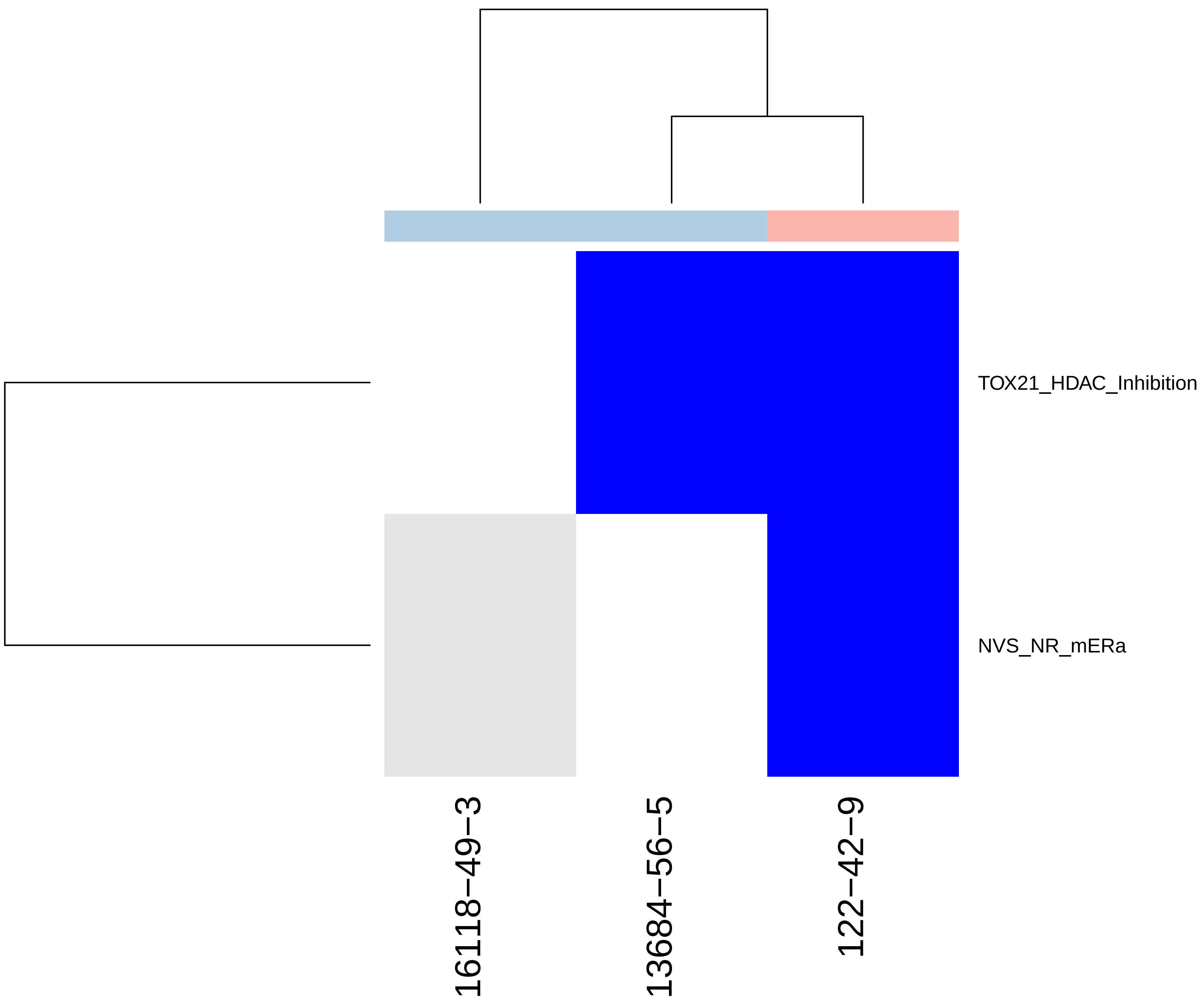

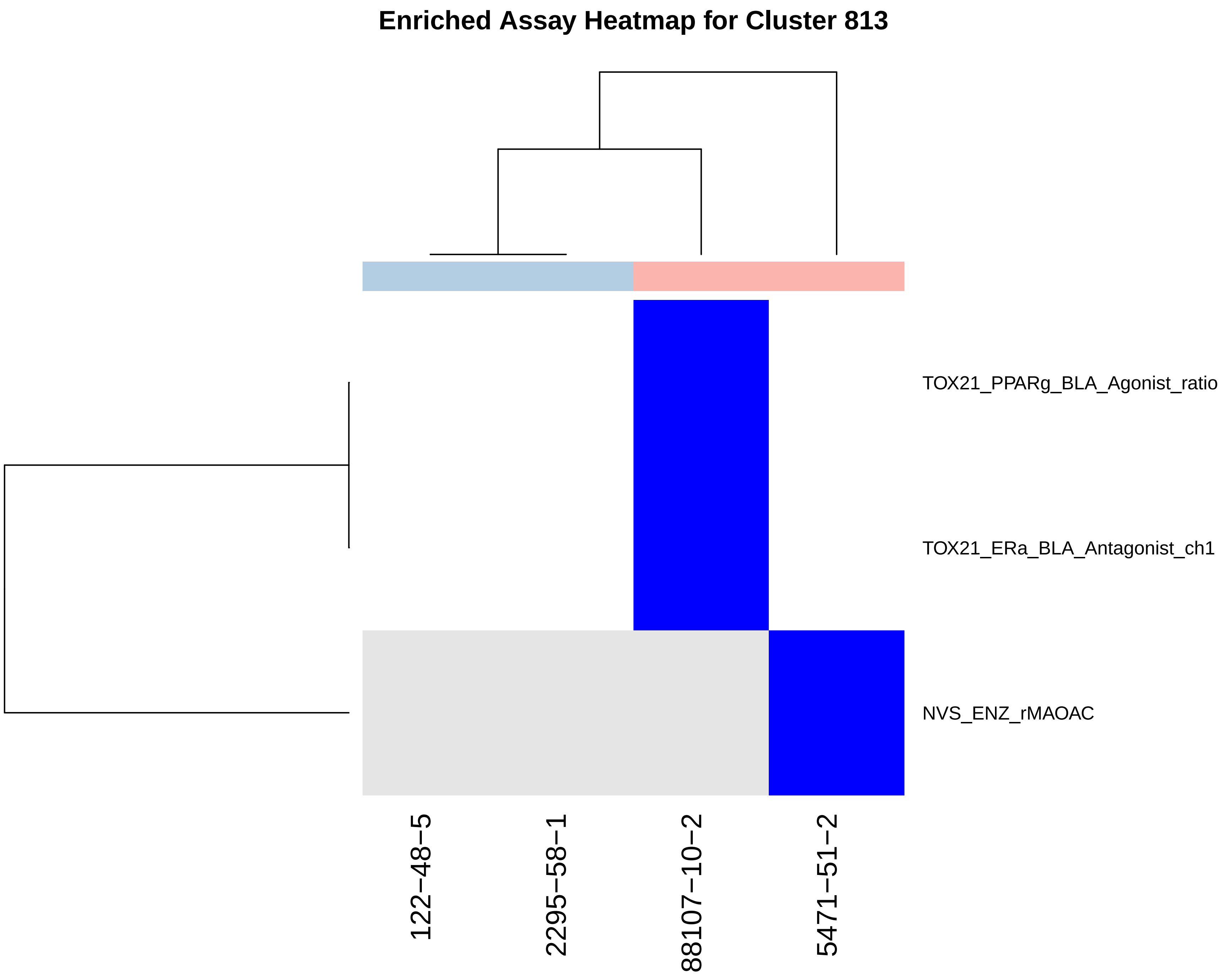
Enriched Assay Heatmap for Cluster 813
TOX21_PPARg_BLA_Agonist_ratio
TOX21_ERa_BLA_Antagonist_ch1
NVS_ENZ_rMAOAC
122−48−5
2295−58−1
88107−10−2
5471−51−2

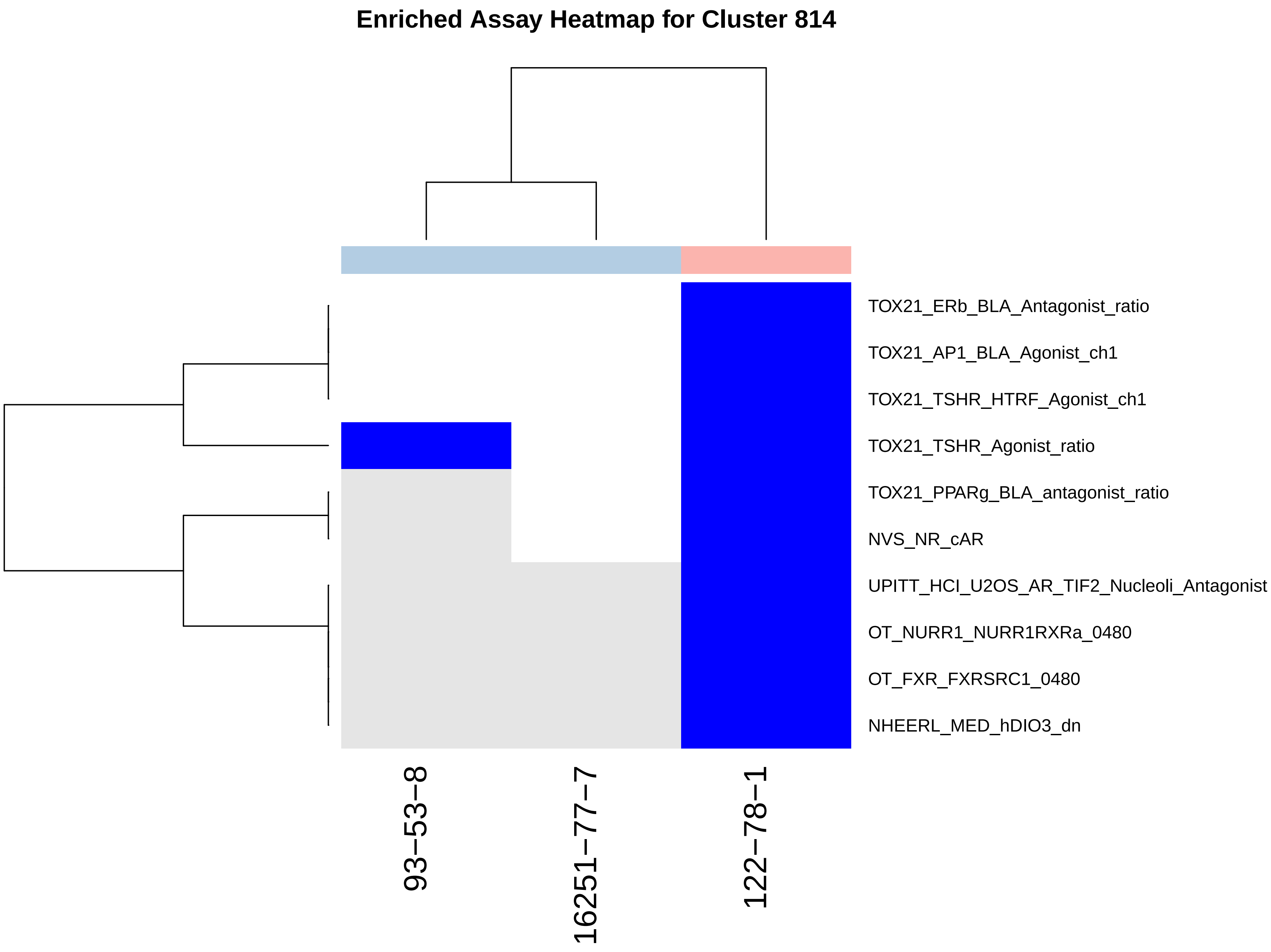
Enriched Assay Heatmap for Cluster 814
TOX21_ERb_BLA_Antagonist_ratio
TOX21_AP1_BLA_Agonist_ch1
TOX21_TSHR_HTRF_Agonist_ch1
TOX21_TSHR_Agonist_ratio
TOX21_PPARg_BLA_antagonist_ratio
NVS_NR_cAR
UPITT_HCI_U2OS_AR_TIF2_Nucleoli_Antagonist
OT_NURR1_NURR1RXRa_0480
OT_FXR_FXRSRC1_0480
NHEERL_MED_hDIO3_dn
93−53−8
16251−77−7
122−78−1

Enriched Assay Heatmap for Cluster 818

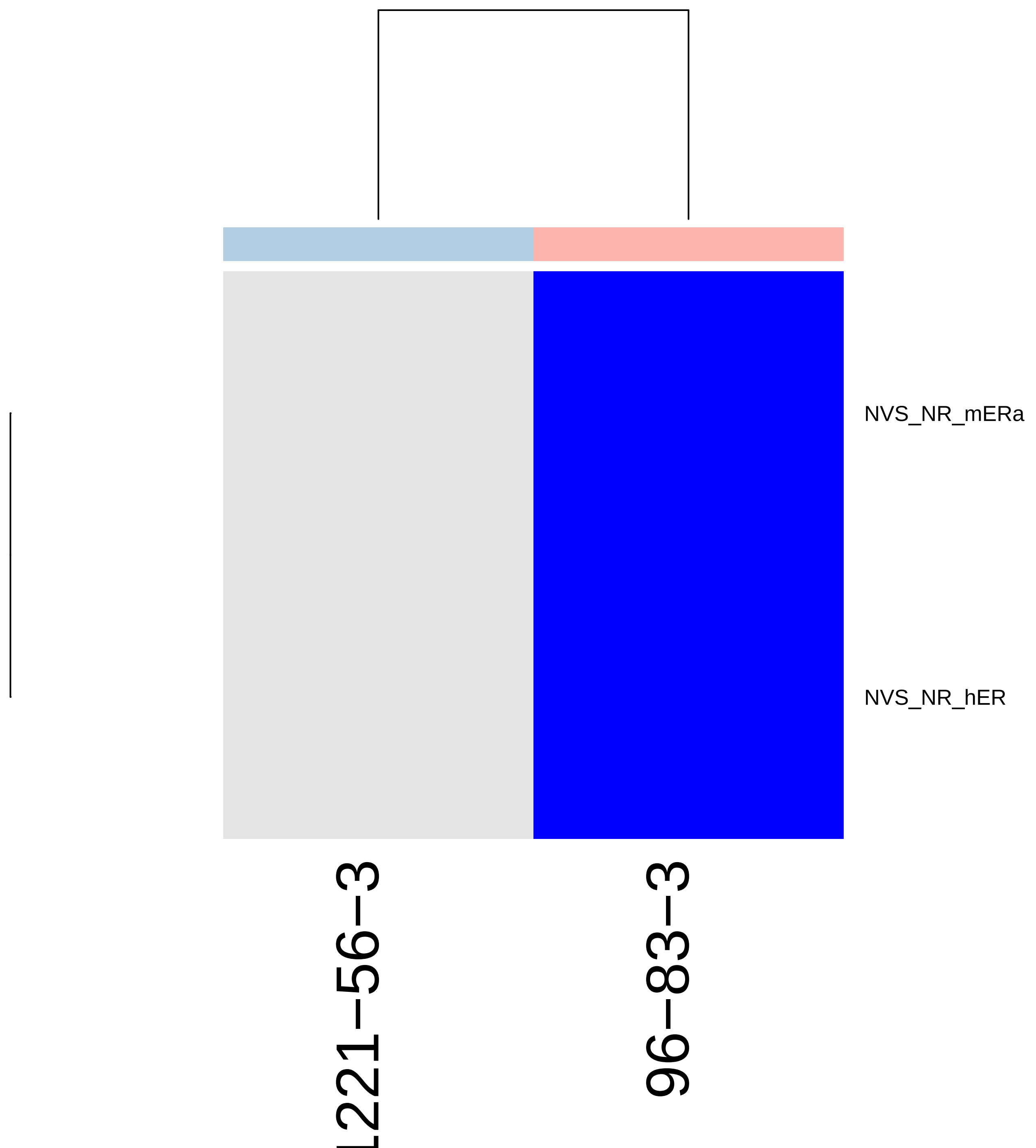

Enriched Assay Heatmap for Cluster 827

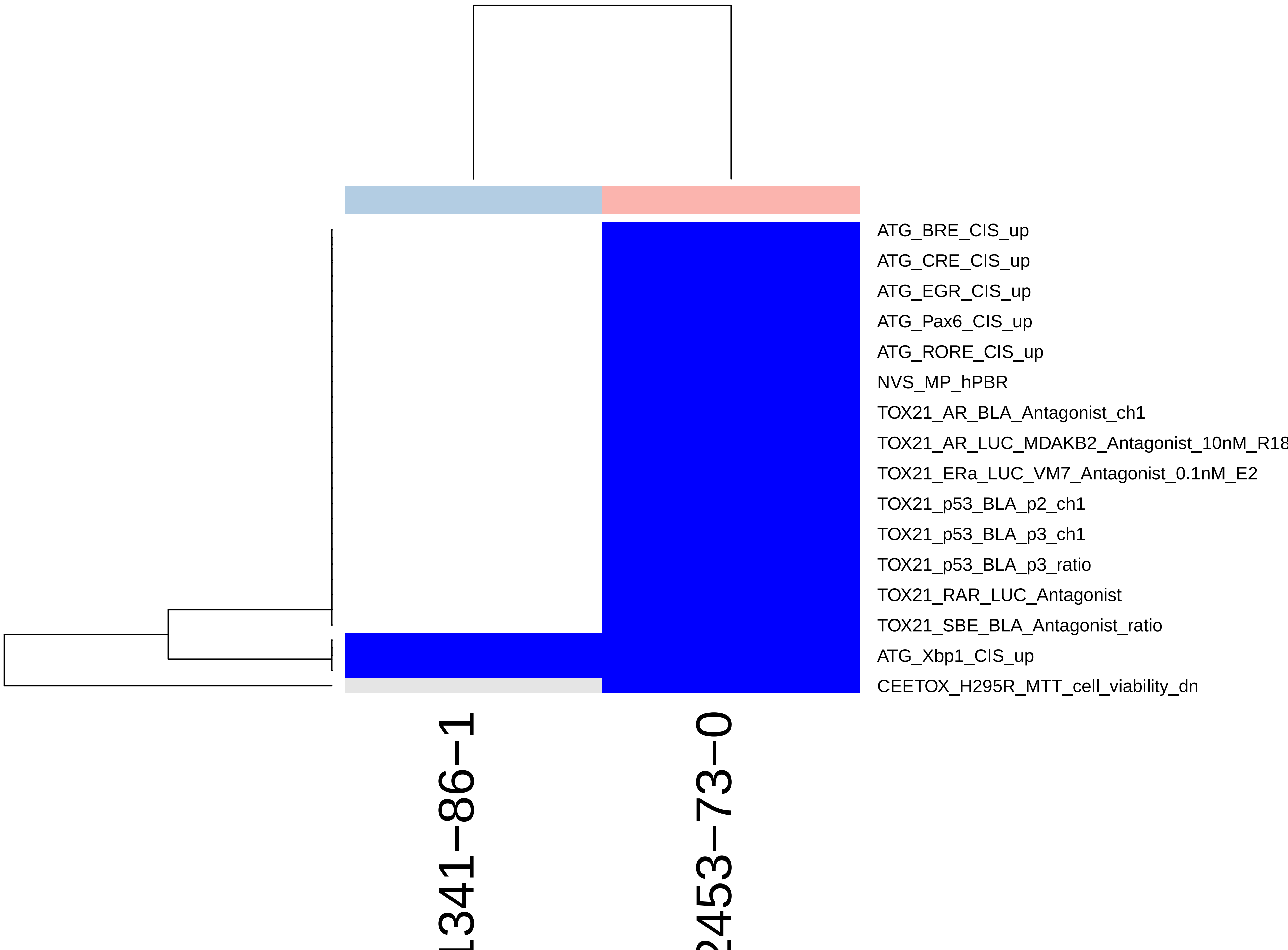

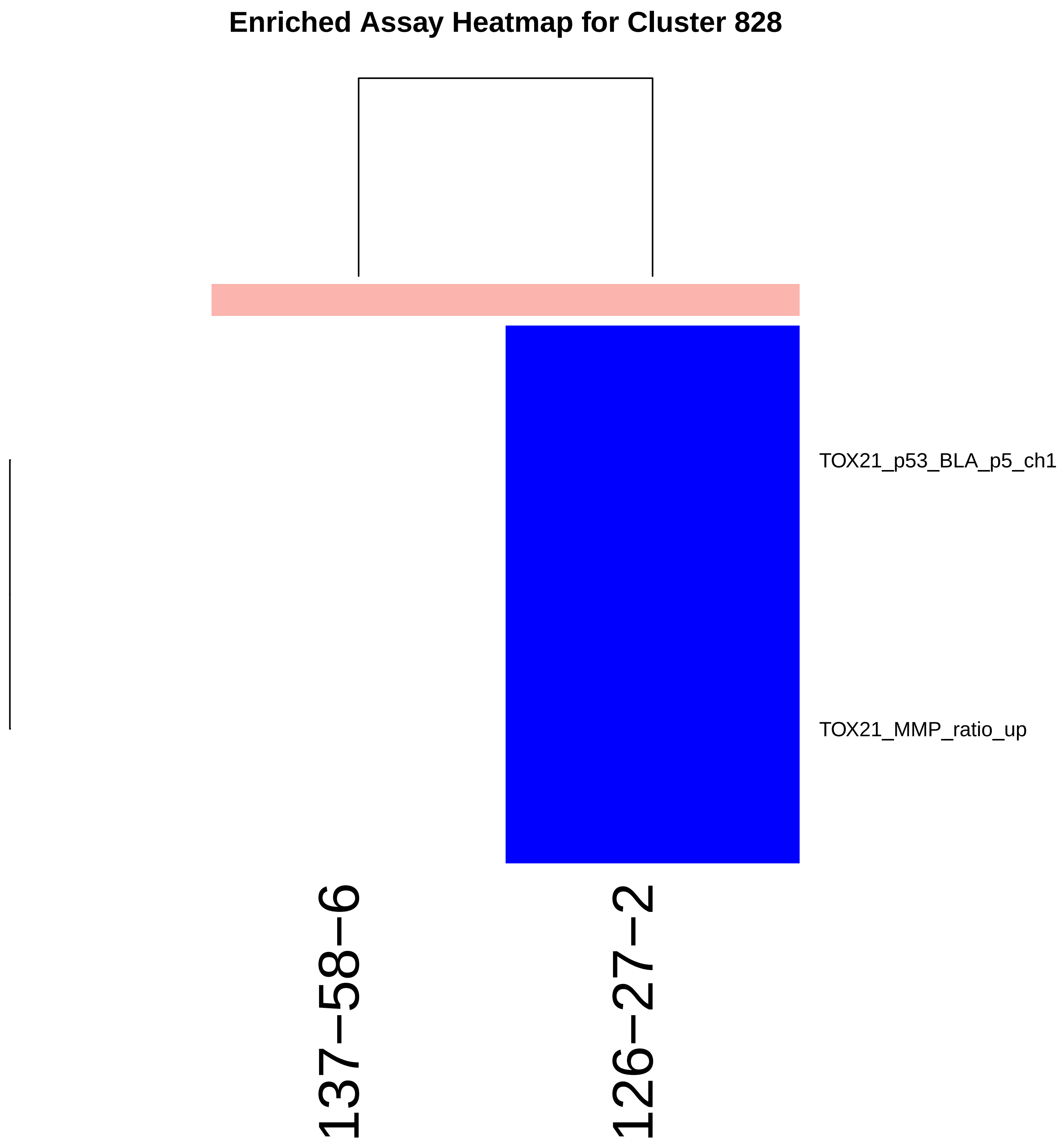
Enriched Assay Heatmap for Cluster 828
TOX21_p53_BLA_p5_ch1
TOX21_MMP_ratio_up
137−58−6
126−27−2

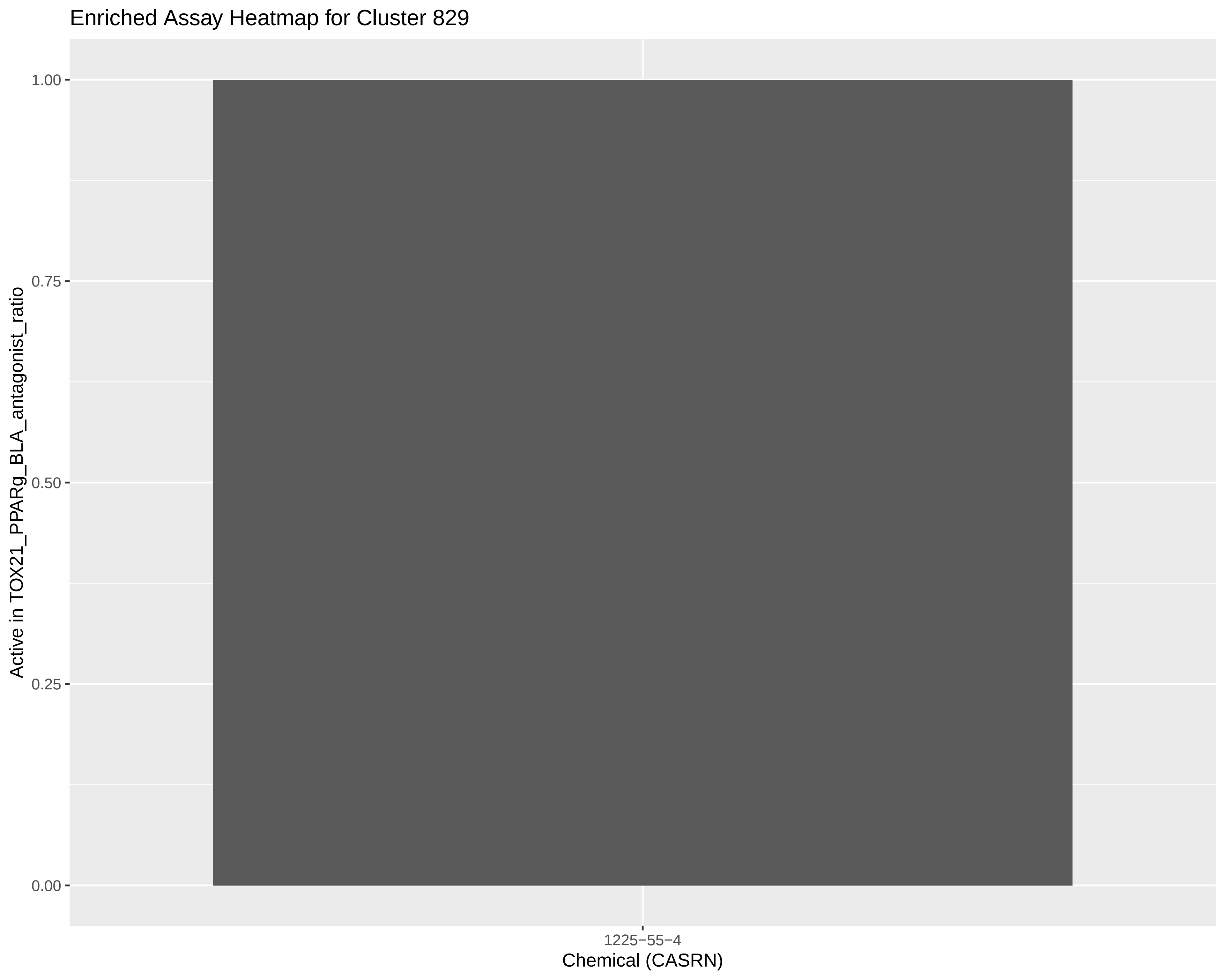

Enriched Assay Heatmap for Cluster 829
1.00
0.75
0.50
0.25
0.00
Active in TOX21_PPARg_BLA_antagonist_ratio
1225−55−4
Chemical (CASRN)

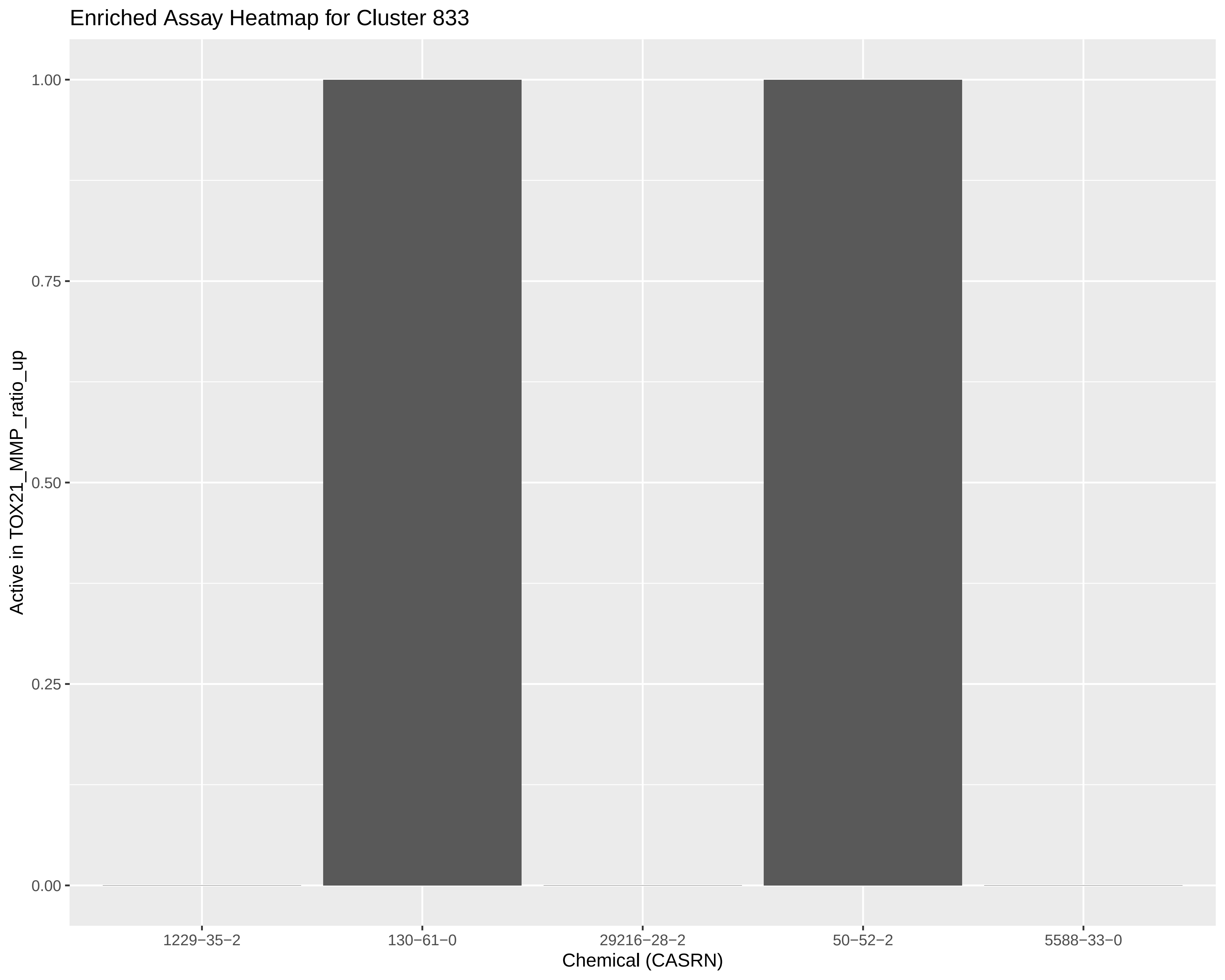

Enriched Assay Heatmap for Cluster 833
Active in TOX21_MMP_ratio_up
1.00
0.75
0.50
0.25
0.00
1229−35−2
130−61−0
29216−28−2
50−52−2
5588−33−0
Chemical (CASRN)

Enriched Assay Heatmap for Cluster 834

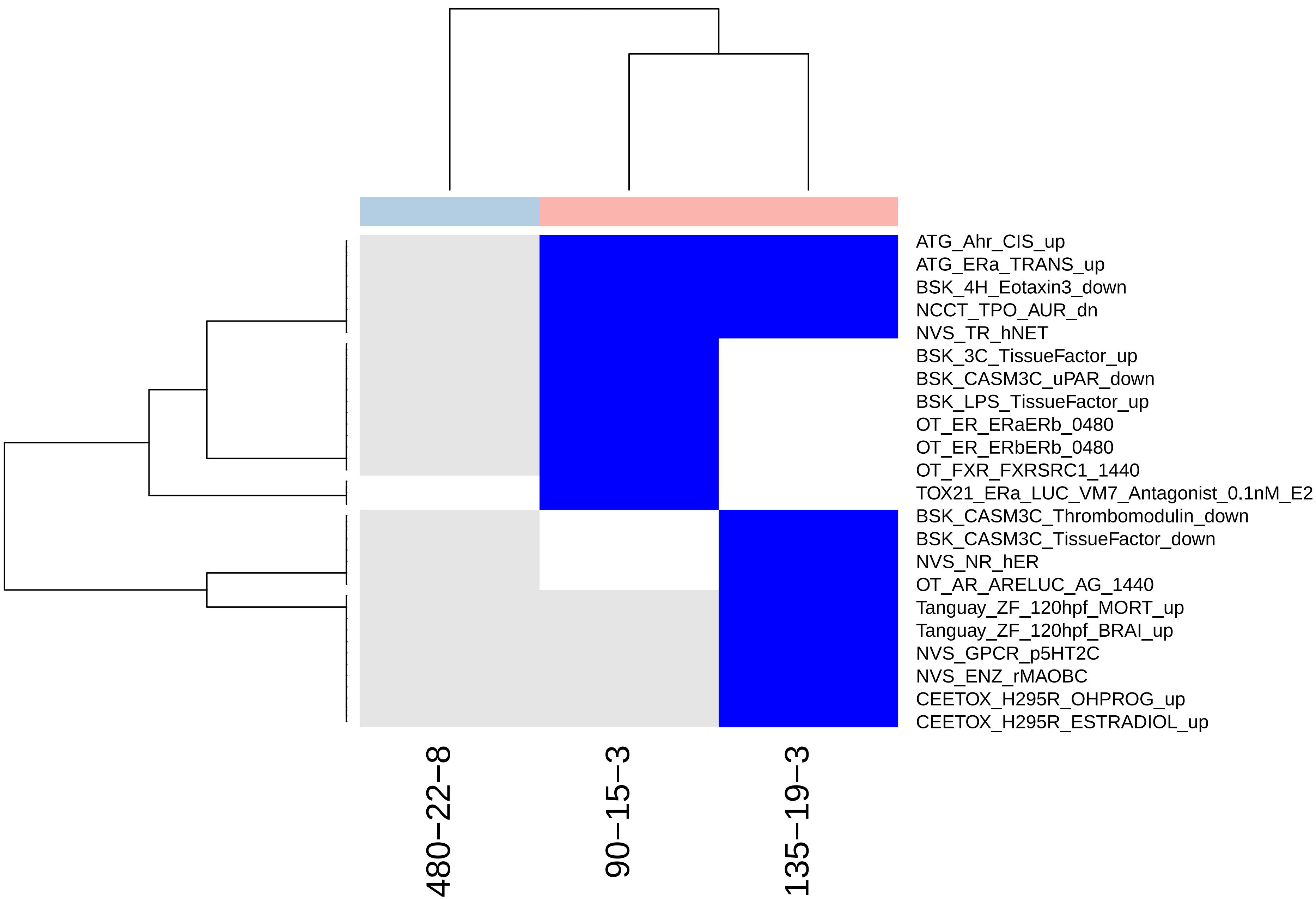

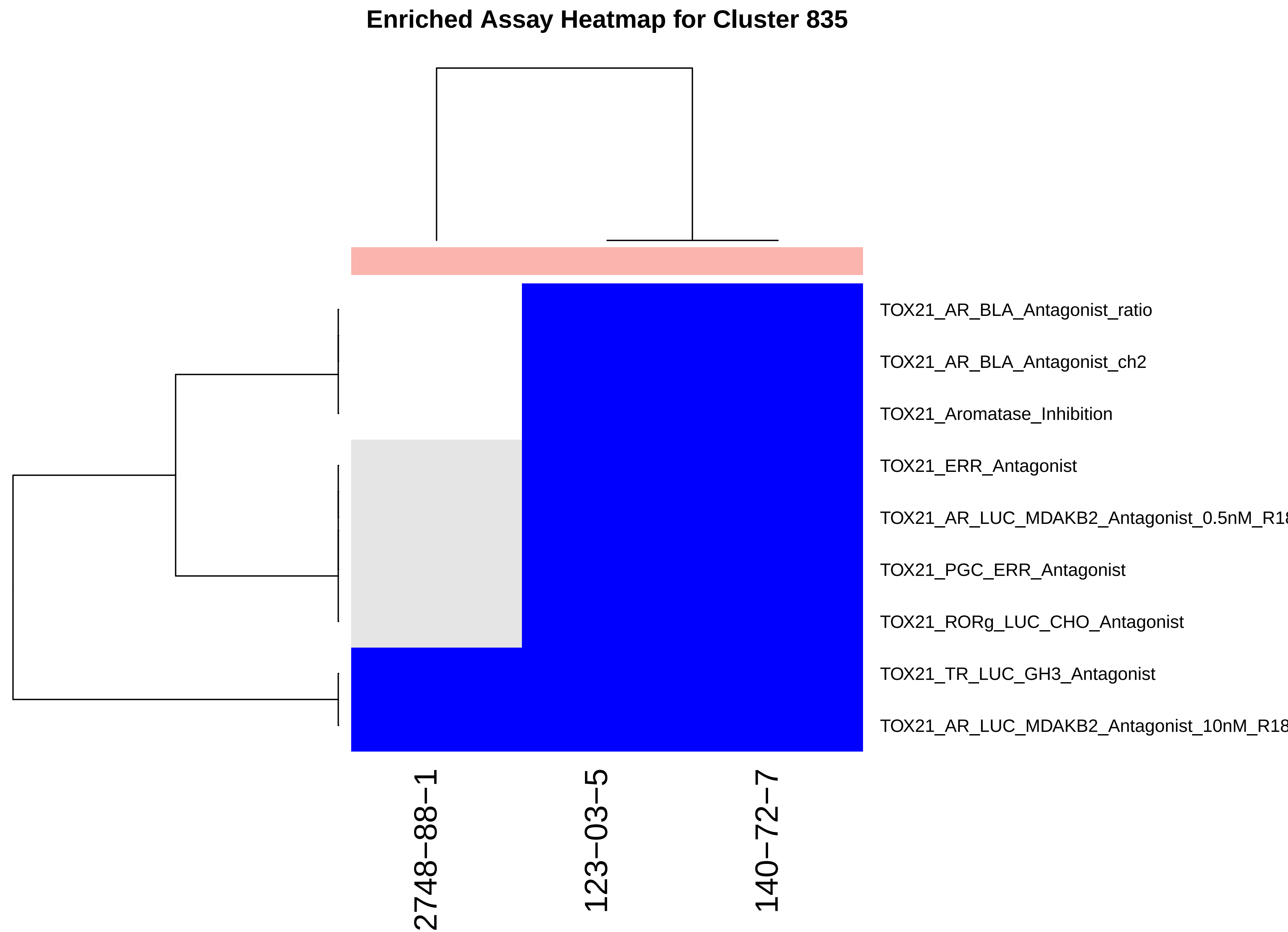
Enriched Assay Heatmap for Cluster 835
TOX21_AR_BLA_Antagonist_ratio
TOX21_AR_BLA_Antagonist_ch2
TOX21_Aromatase_Inhibition
TOX21_ERR_Antagonist
TOX21_AR_LUC_MDAKB2_Antagonist_0.5nM_R18
TOX21_PGC_ERR_Antagonist
TOX21_RORg_LUC_CHO_Antagonist
TOX21_TR_LUC_GH3_Antagonist
TOX21_AR_LUC_MDAKB2_Antagonist_10nM_R18
2748−88−1
123−03−5
140−72−7

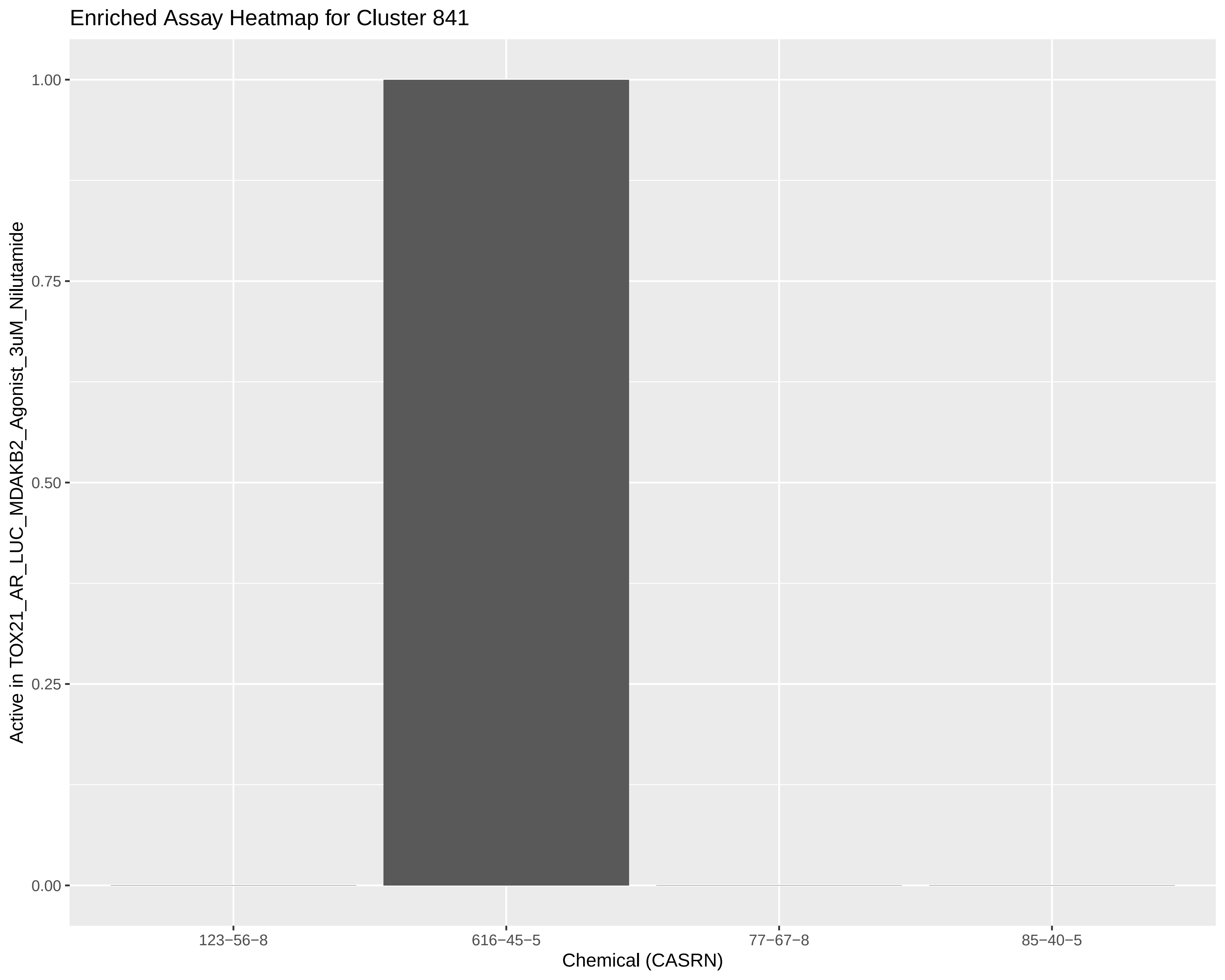

Enriched Assay Heatmap for Cluster 841
Active in TOX21_AR_LUC_MDAKB2_Agonist_3uM_Nilutamide
1.00
0.75
0.50
0.25
0.00
123−56−8
616−45−5
77−67−8
85−40−5
Chemical (CASRN)

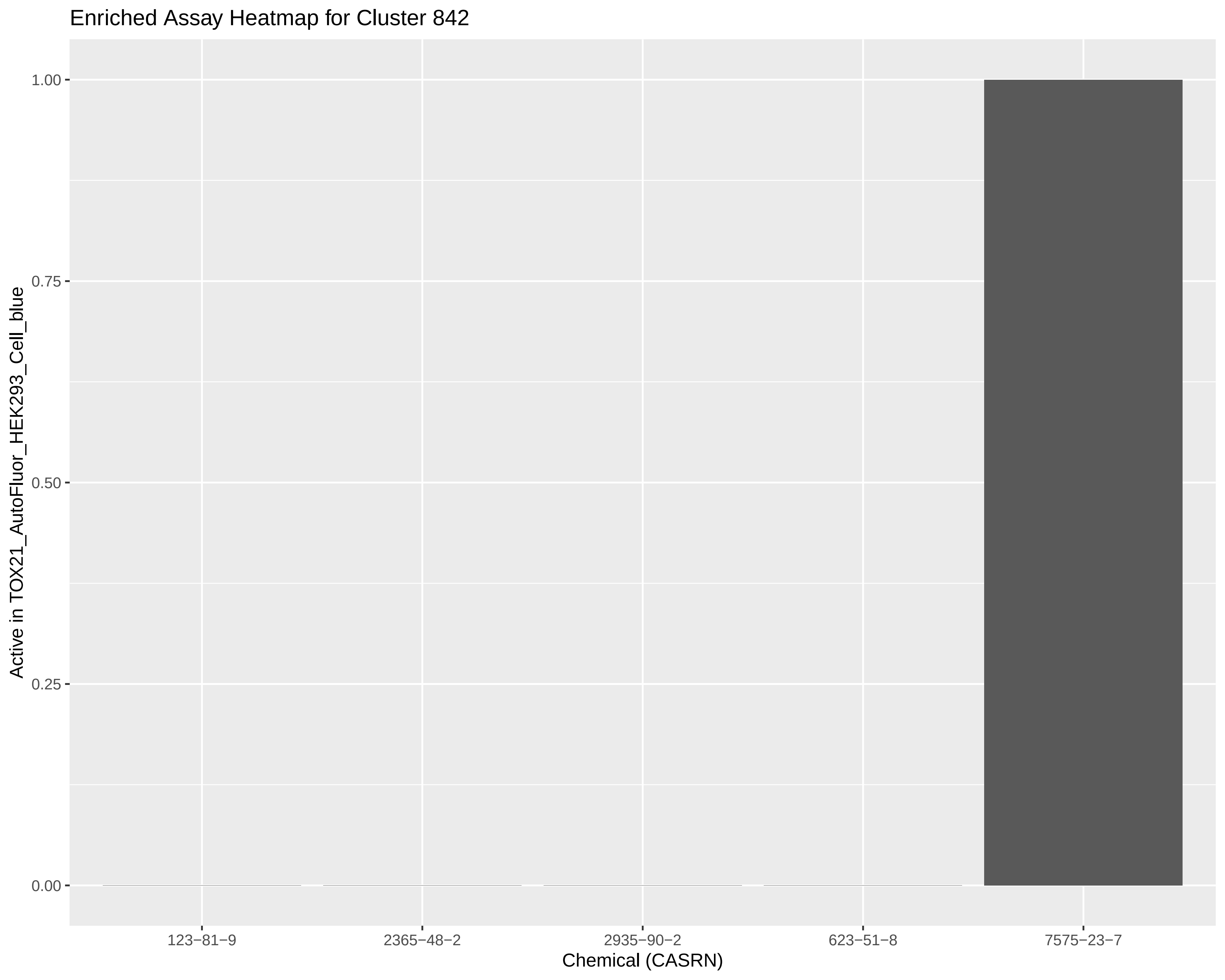

Enriched Assay Heatmap for Cluster 842
Active in TOX21_AutoFluor_HEK293_Cell_blue
1.00
0.75
0.50
0.25
0.00
123−81−9
2365−48−2
2935−90−2
623−51−8
7575−23−7
Chemical (CASRN)

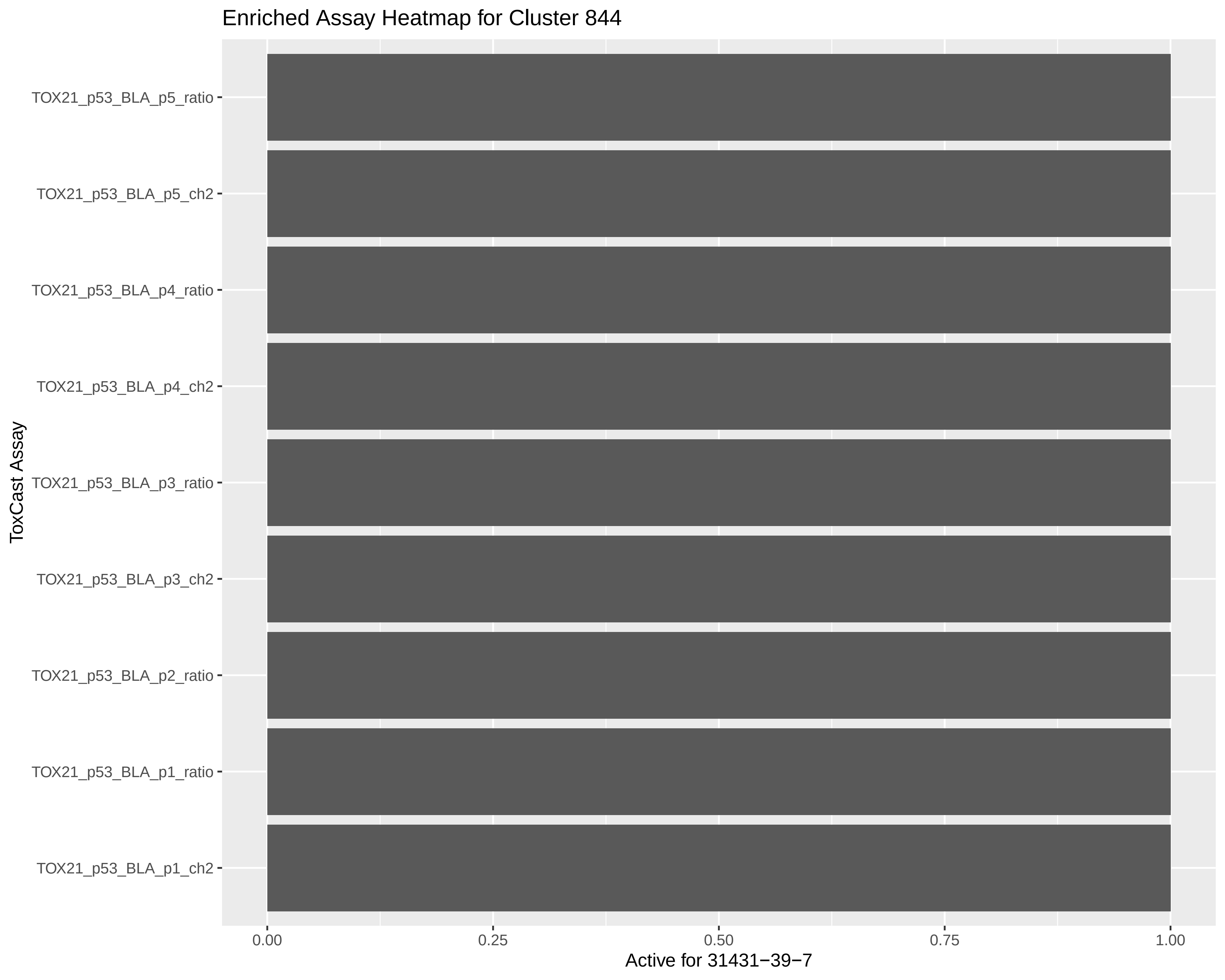
Enriched Assay Heatmap for Cluster 844
TOX21_p53_BLA_p5_ratio
TOX21_p53_BLA_p5_ch2
TOX21_p53_BLA_p4_ratio
TOX21_p53_BLA_p4_ch2
TOX21_p53_BLA_p3_ratio
TOX21_p53_BLA_p3_ch2
TOX21_p53_BLA_p2_ratio
TOX21_p53_BLA_p1_ratio
TOX21_p53_BLA_p1_ch2
ToxCast Assay
0.00
0.25
0.50
0.75
1.00
Active for 31431−39−7

Enriched Assay Heatmap for Cluster 850

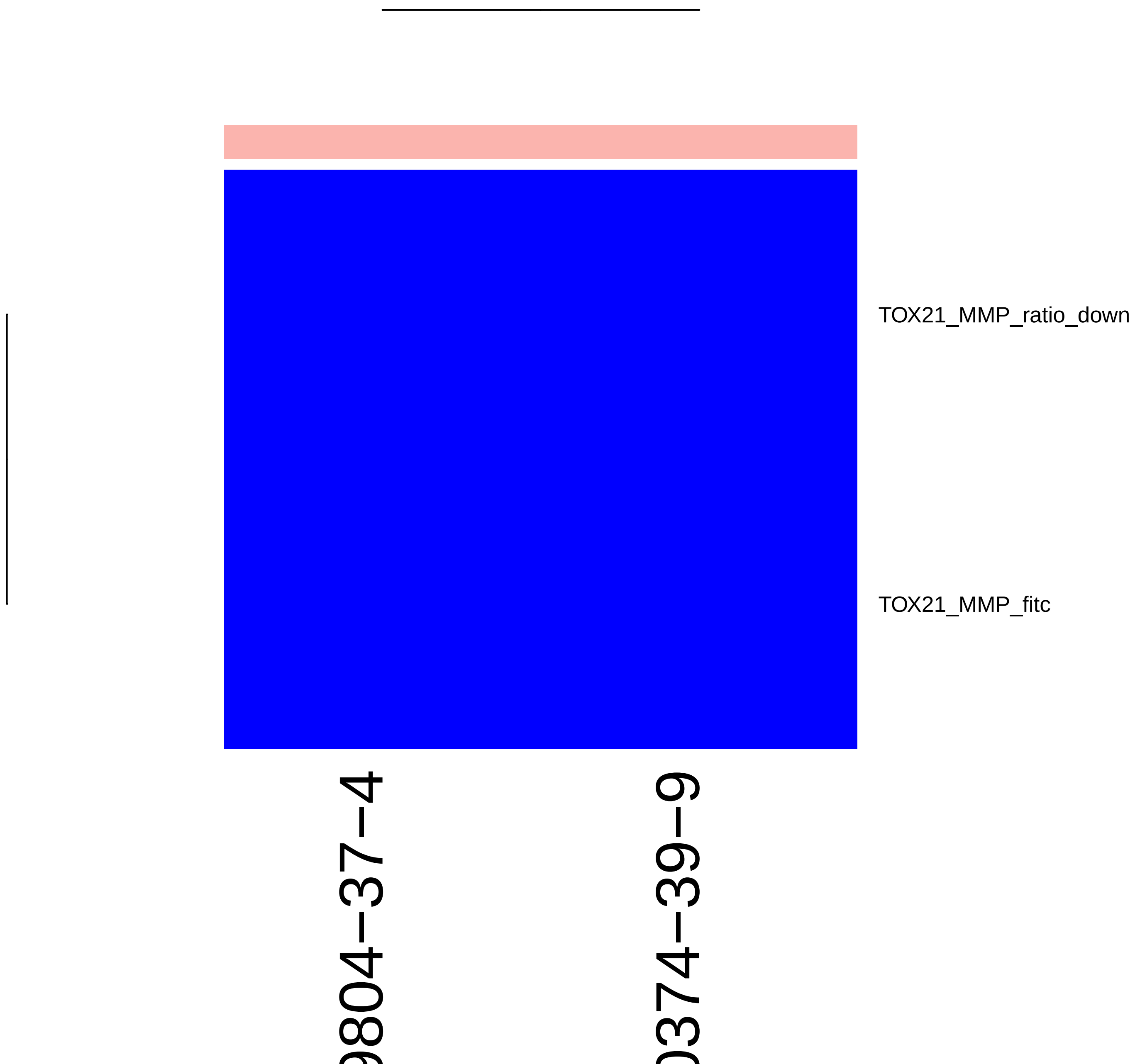

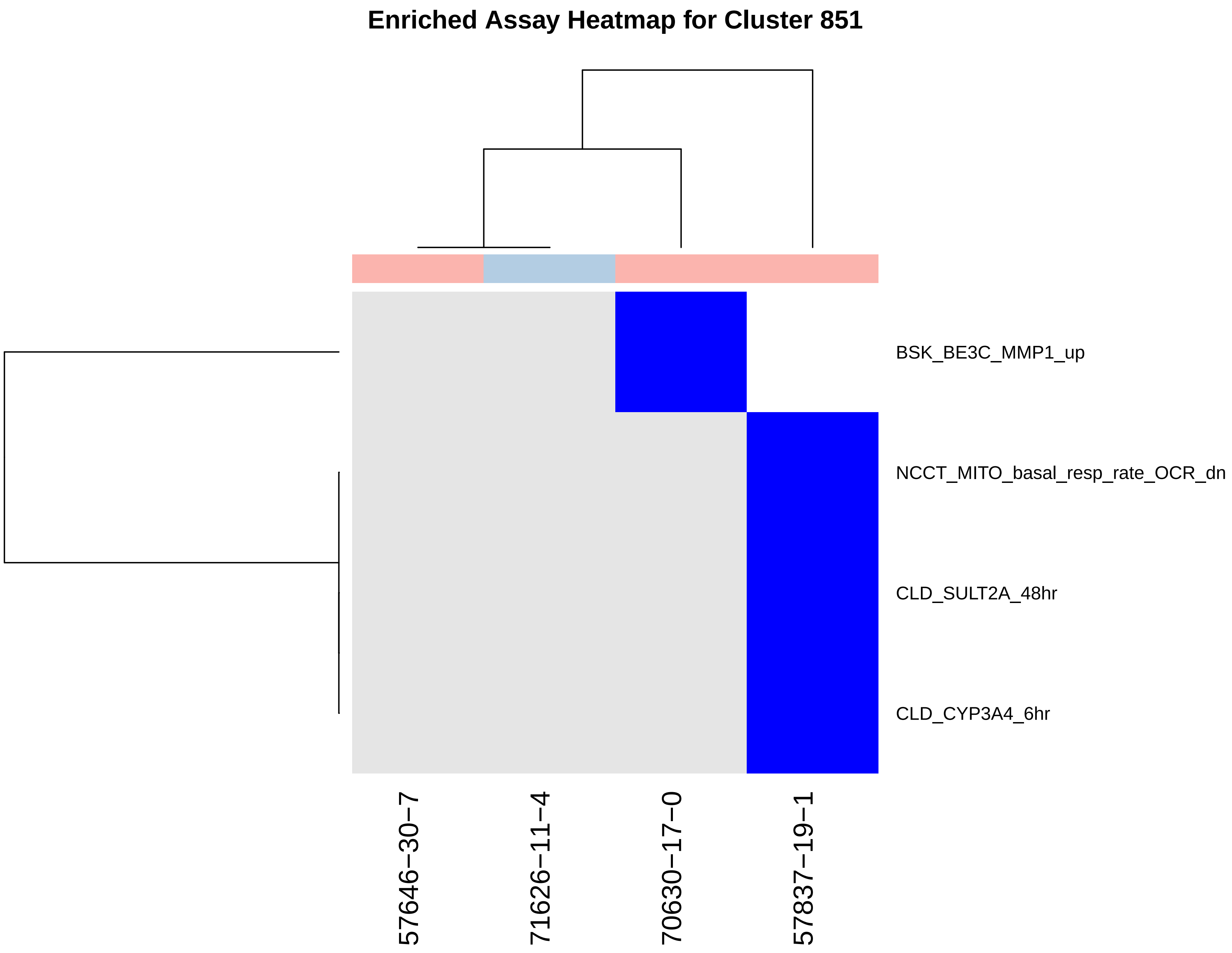

Enriched Assay Heatmap for Cluster 851
BSK_BE3C_MMP1_up
NCCT_MITO_basal_resp_rate_OCR_dn
CLD_SULT2A_48hr
CLD_CYP3A4_6hr
57646−30−7
71626−11−4
70630−17−0
57837−19−1

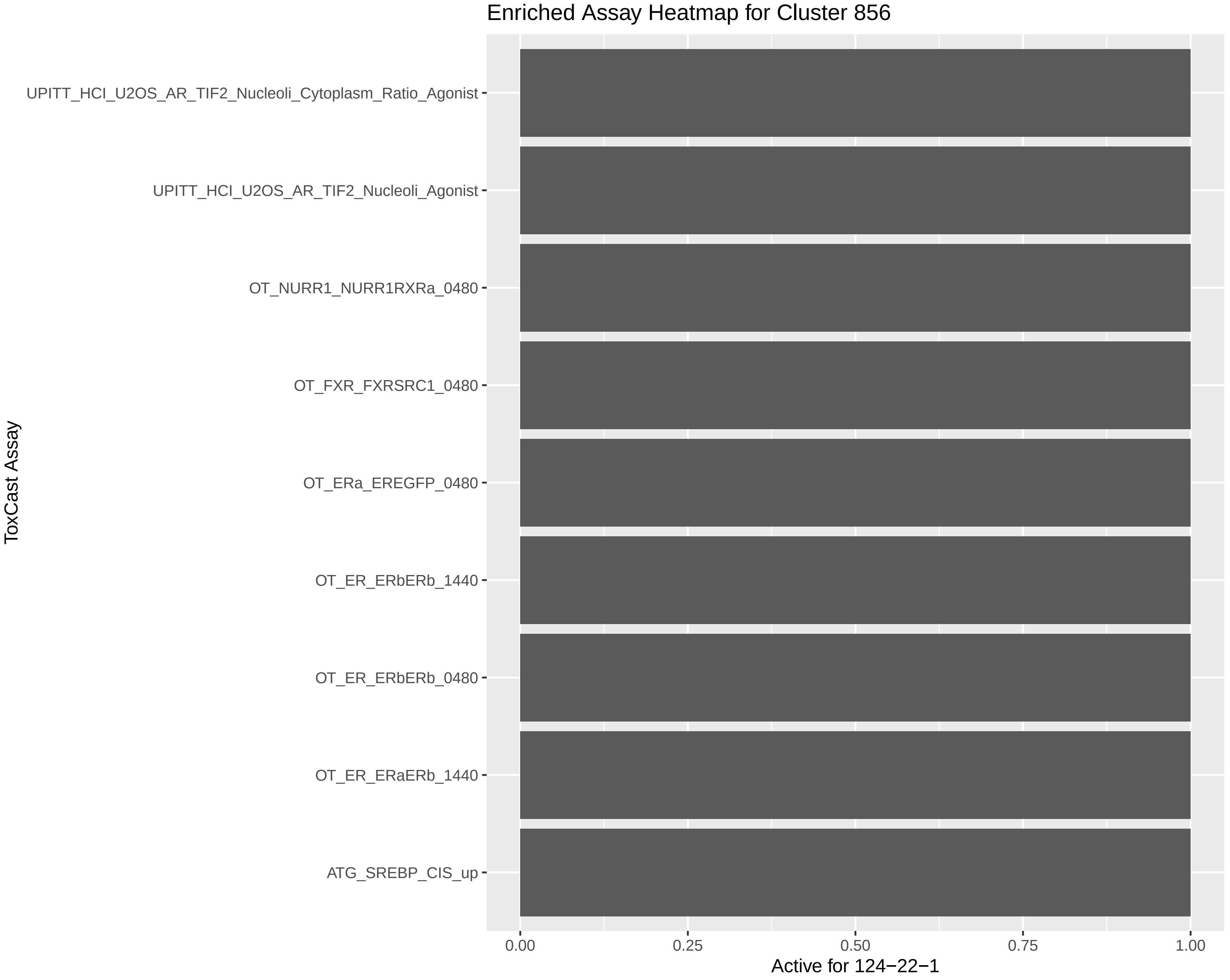
Enriched Assay Heatmap for Cluster 856
UPITT_HCI_U2OS_AR_TIF2_Nucleoli_Cytoplasm_Ratio_Agonist
UPITT_HCI_U2OS_AR_TIF2_Nucleoli_Agonist
OT_NURR1_NURR1RXRa_0480
OT_FXR_FXRSRC1_0480
OT_ERa_EREGFP_0480
OT_ER_ERbERb_1440
OT_ER_ERbERb_0480
OT_ER_ERaERb_1440
ATG_SREBP_CIS_up
ToxCast Assay
0.00
0.25
0.50
0.75
1.00
Active for 124−22−1

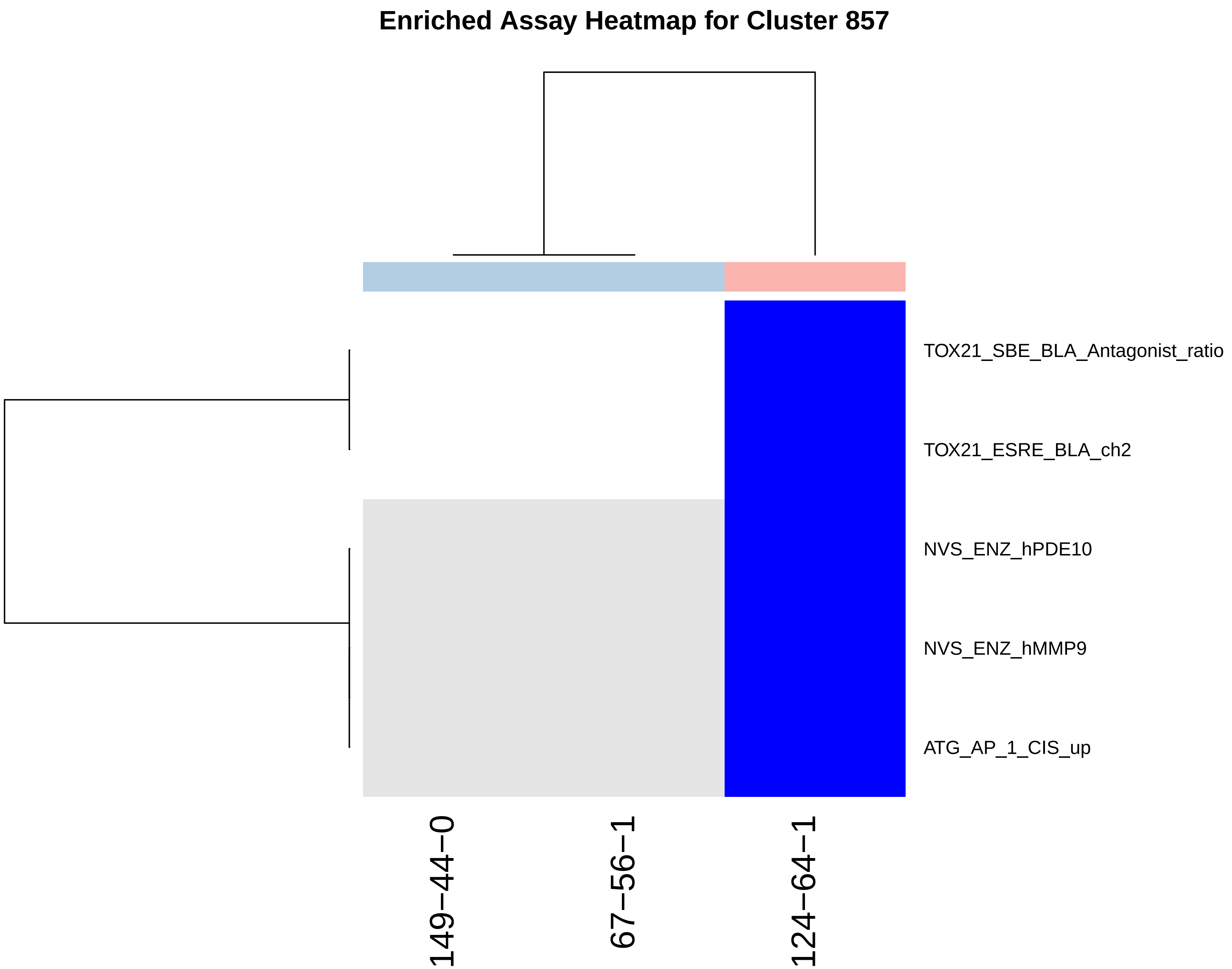
Enriched Assay Heatmap for Cluster 857
TOX21_SBE_BLA_Antagonist_ratio
TOX21_ESRE_BLA_ch2
NVS_ENZ_hPDE10
NVS_ENZ_hMMP9
ATG_AP_1_CIS_up
149−44−0
67−56−1
124−64−1

Enriched Assay Heatmap for Cluster 859

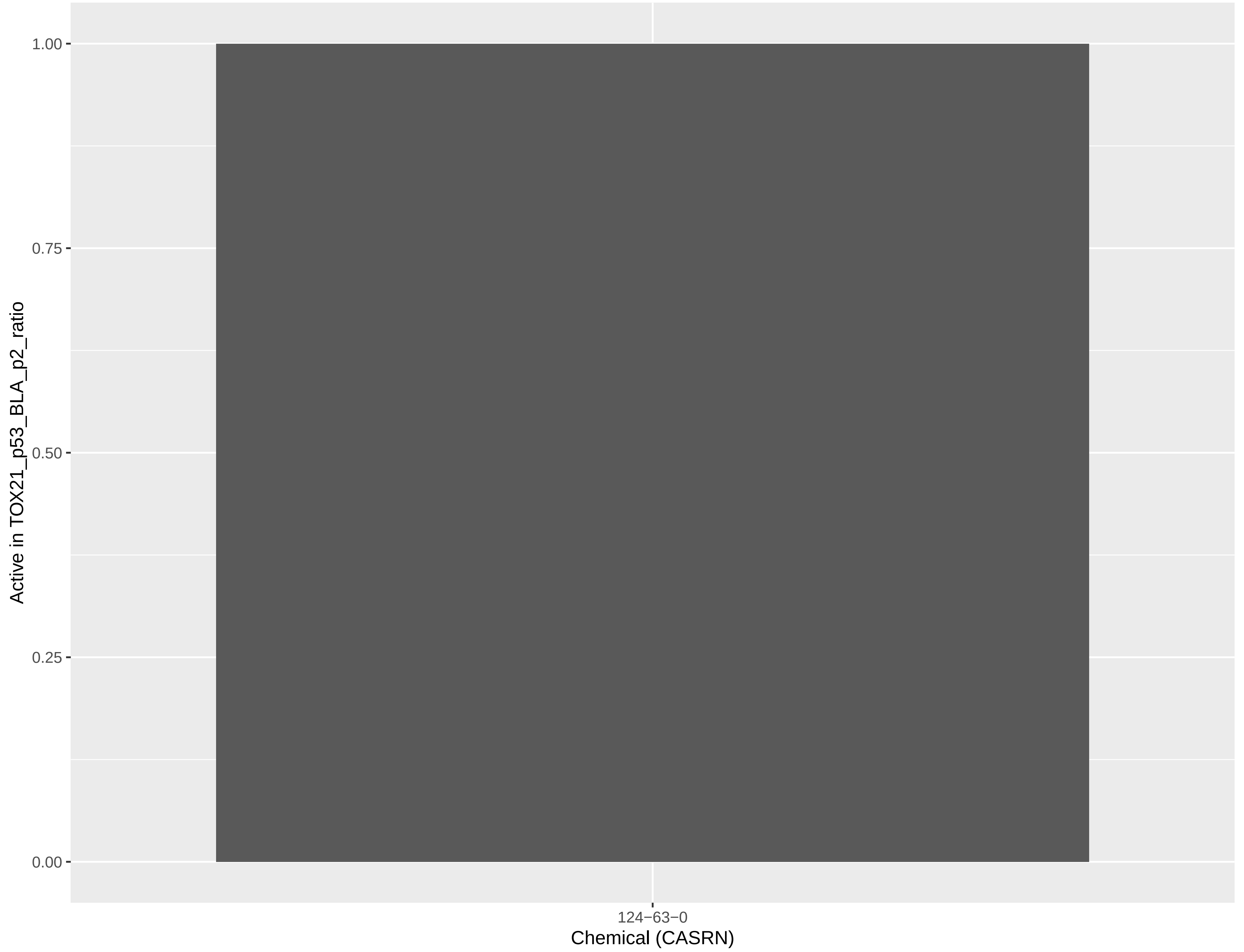

Enriched Assay Heatmap for Cluster 865

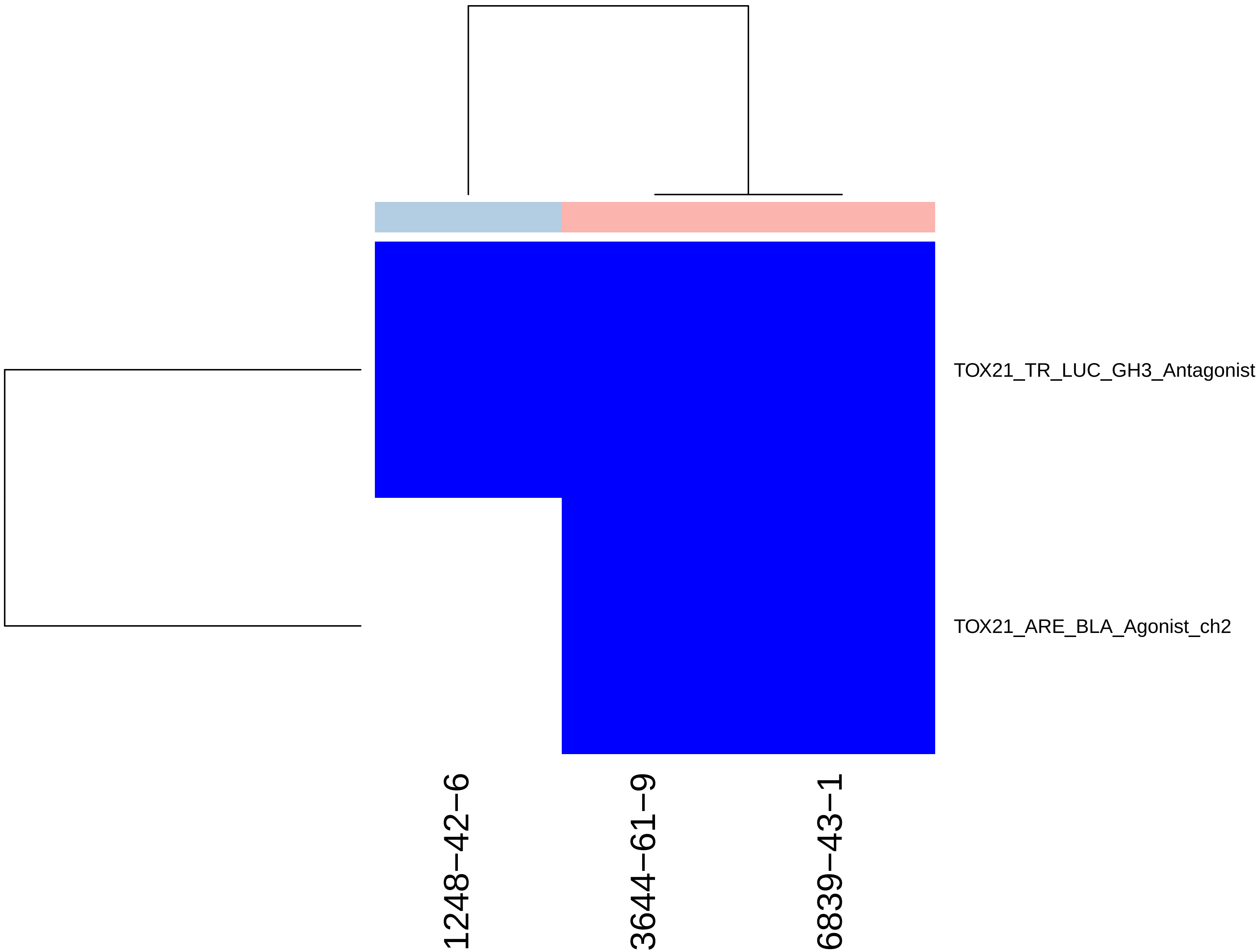

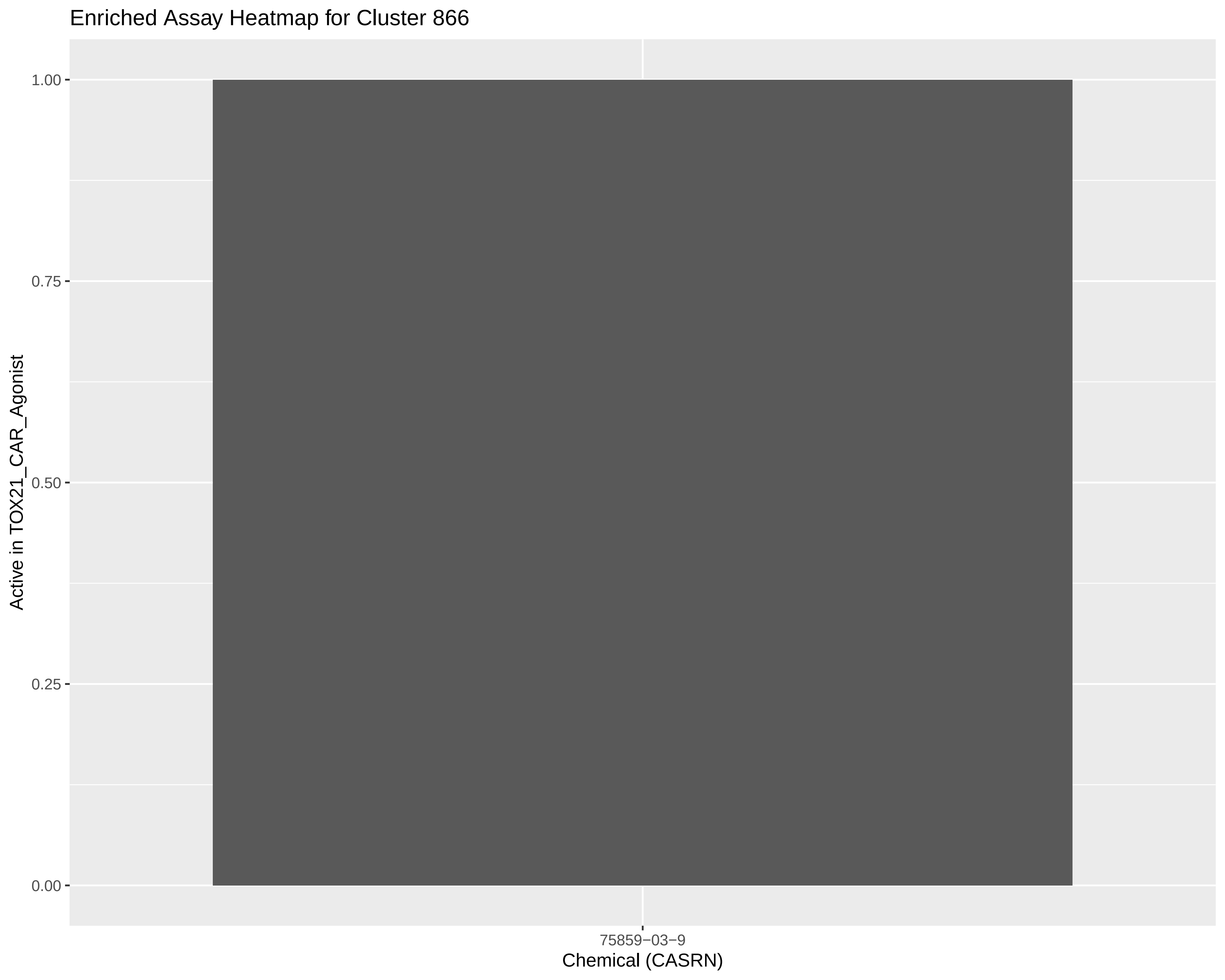

Enriched Assay Heatmap for Cluster 866
Active in TOX21_CAR_Agonist
1.00
0.75
0.50
0.25
0.00
75859−03−9
Chemical (CASRN)

Enriched Assay Heatmap for Cluster 870

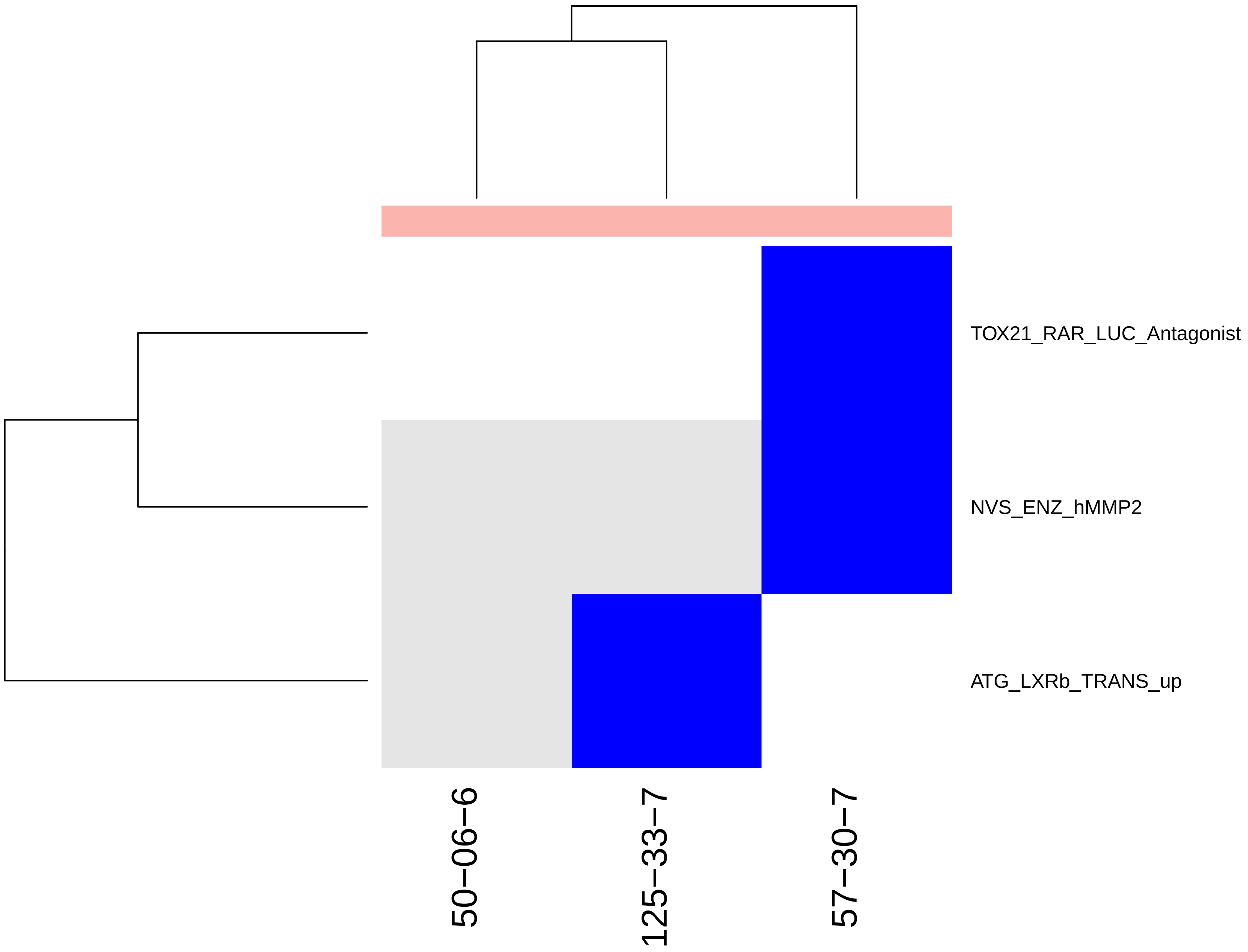

Enriched Assay Heatmap for Cluster 878

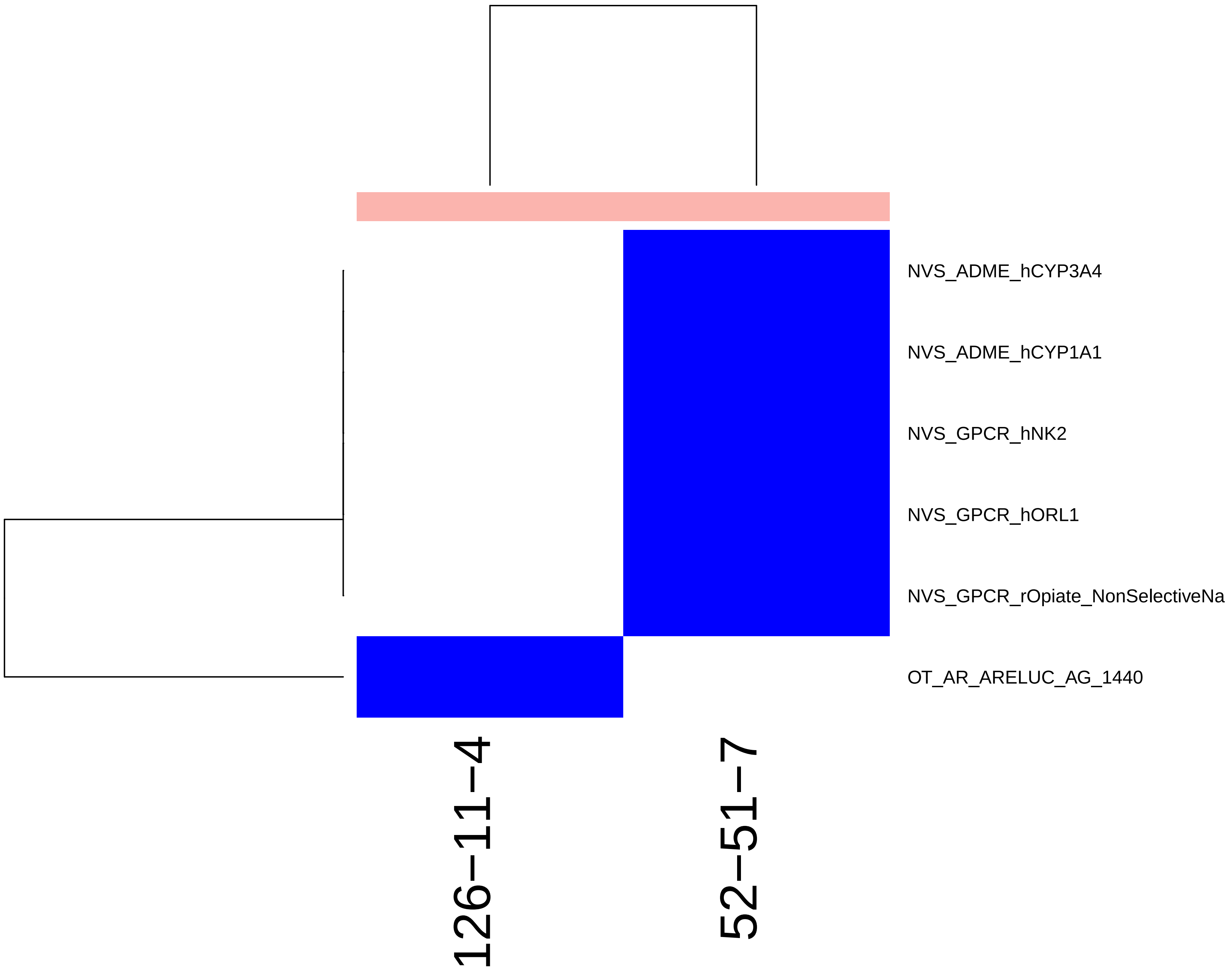

Enriched Assay Heatmap for Cluster 882

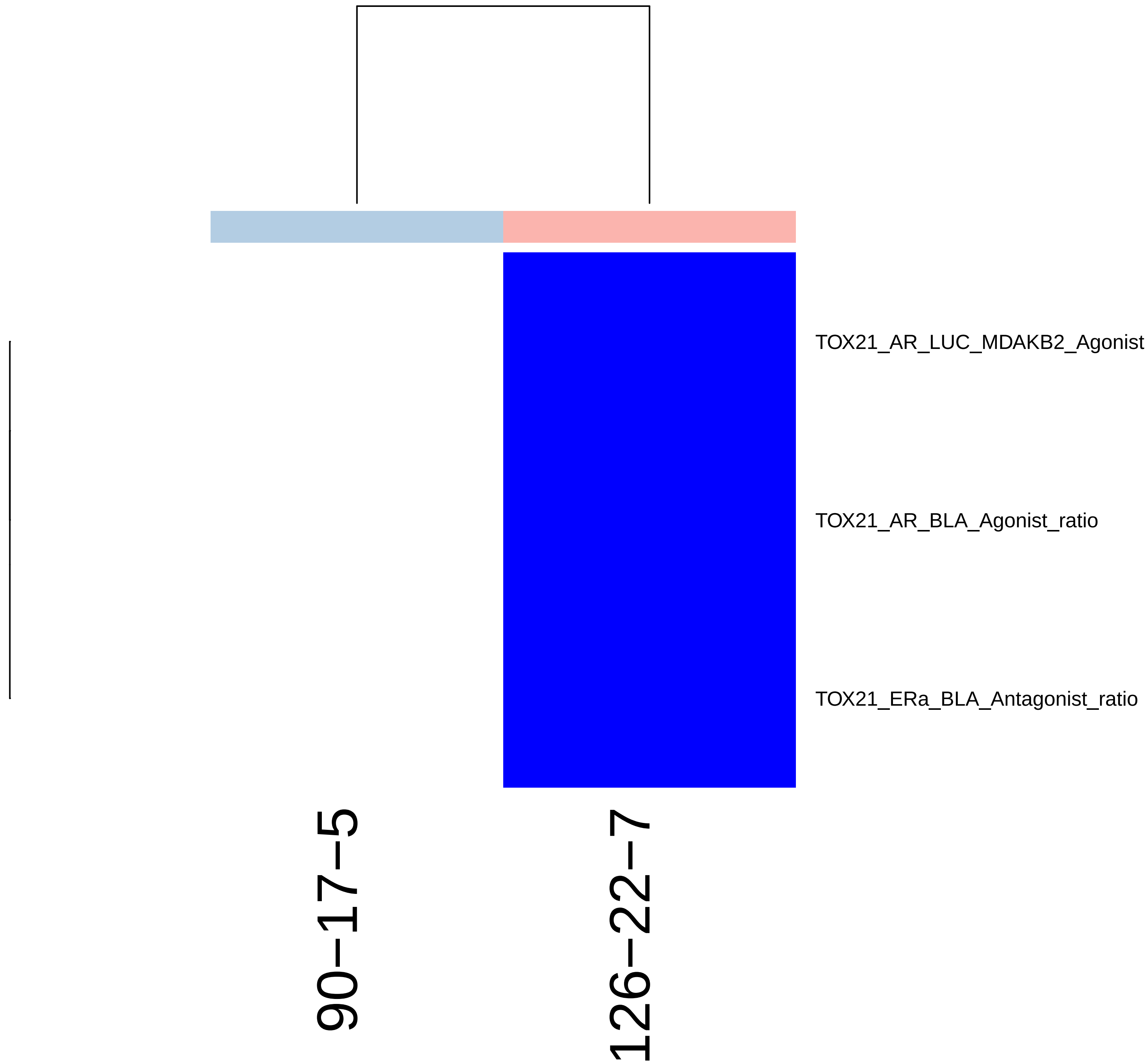

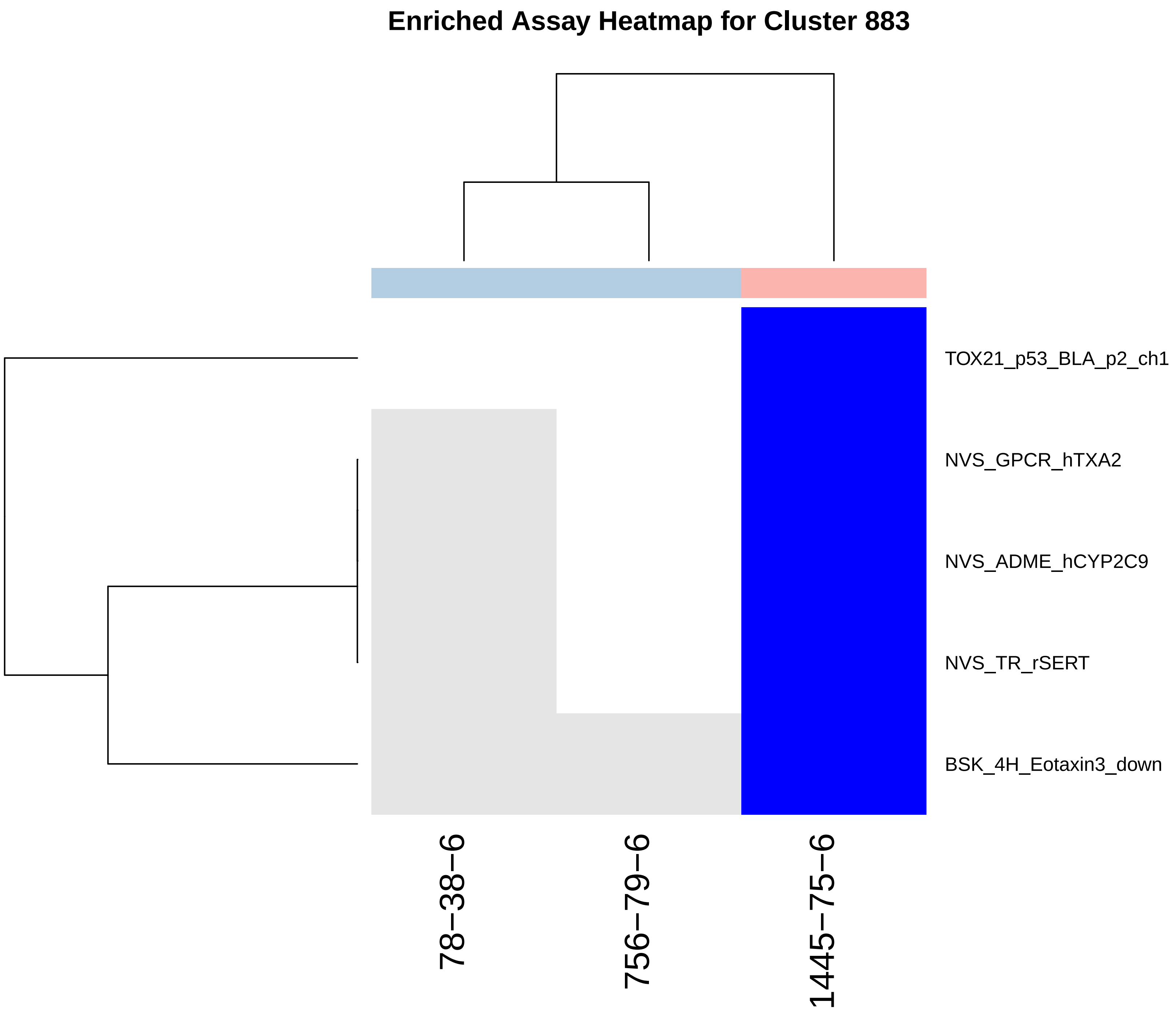
Enriched Assay Heatmap for Cluster 883
TOX21_p53_BLA_p2_ch1
NVS_GPCR_hTXA2
NVS_ADME_hCYP2C9
NVS_TR_rSERT
BSK_4H_Eotaxin3_down
78−38−6
756−79−6
1445−75−6

Enriched Assay Heatmap for Cluster 886

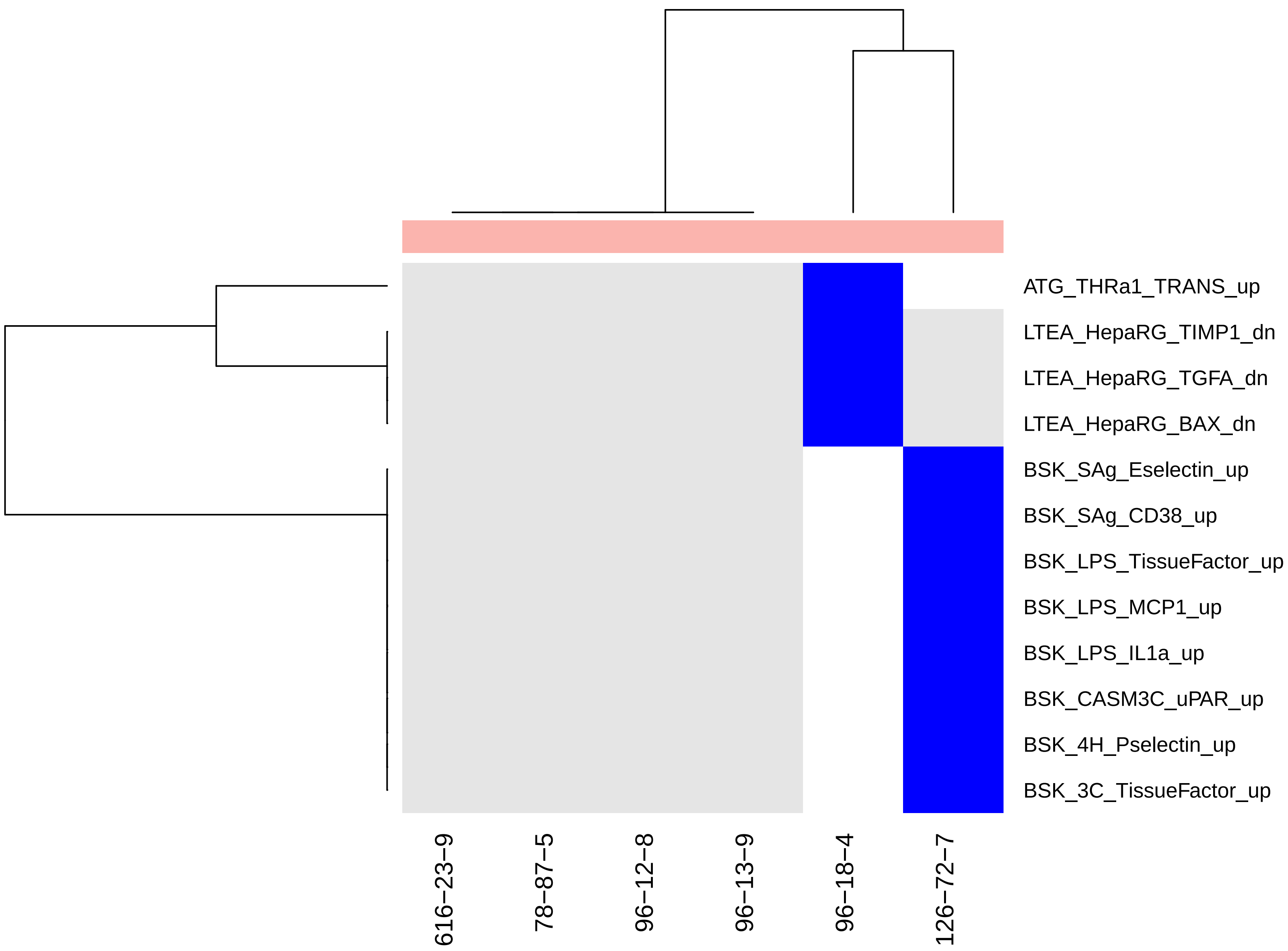

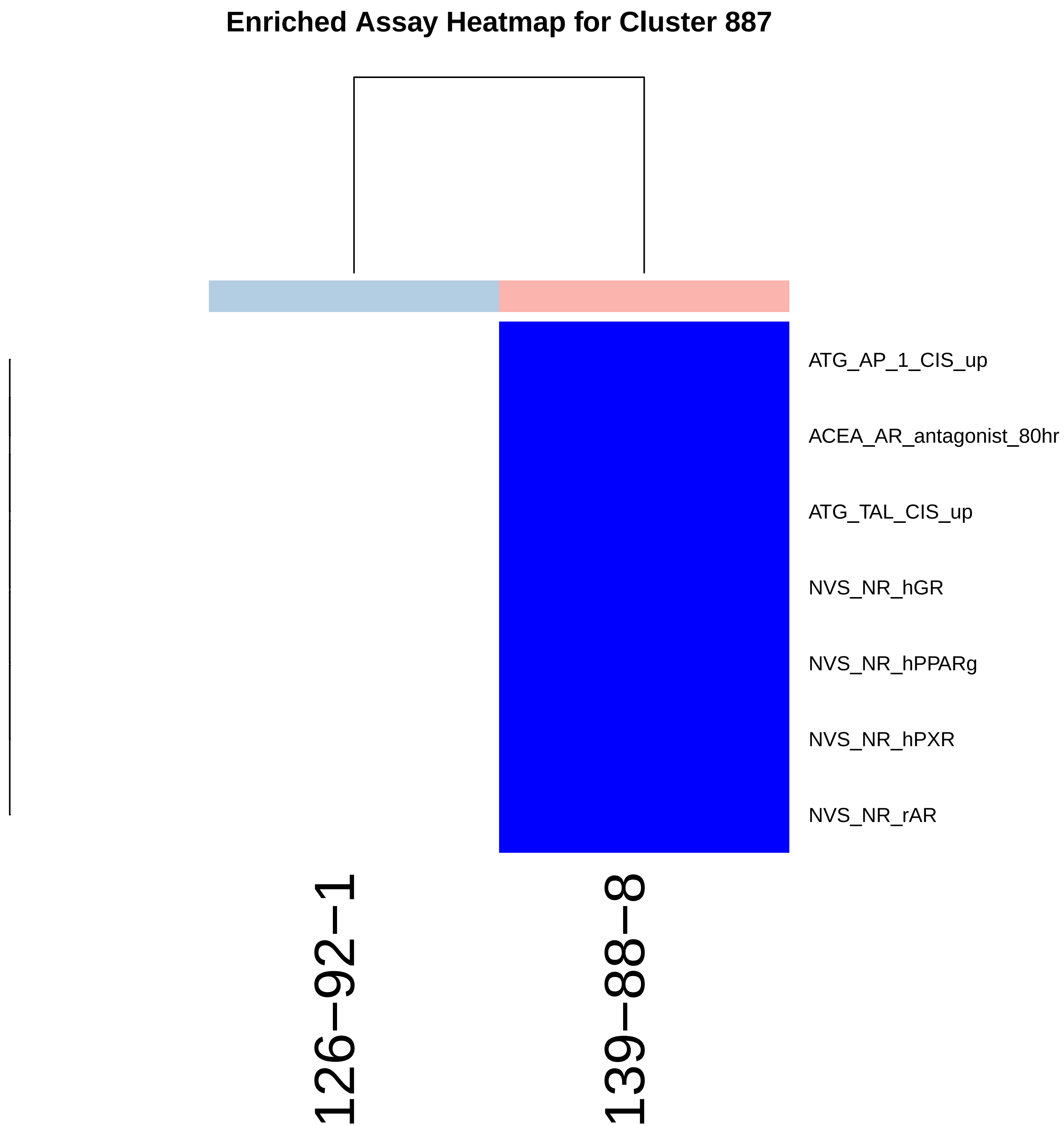

Enriched Assay Heatmap for Cluster 887
ATG_AP_1_CIS_up
ACEA_AR_antagonist_80hr
ATG_TAL_CIS_up
NVS_NR_hGR
NVS_NR_hPPARg
NVS_NR_hPXR
NVS_NR_rAR
126−92−1
139−88−8

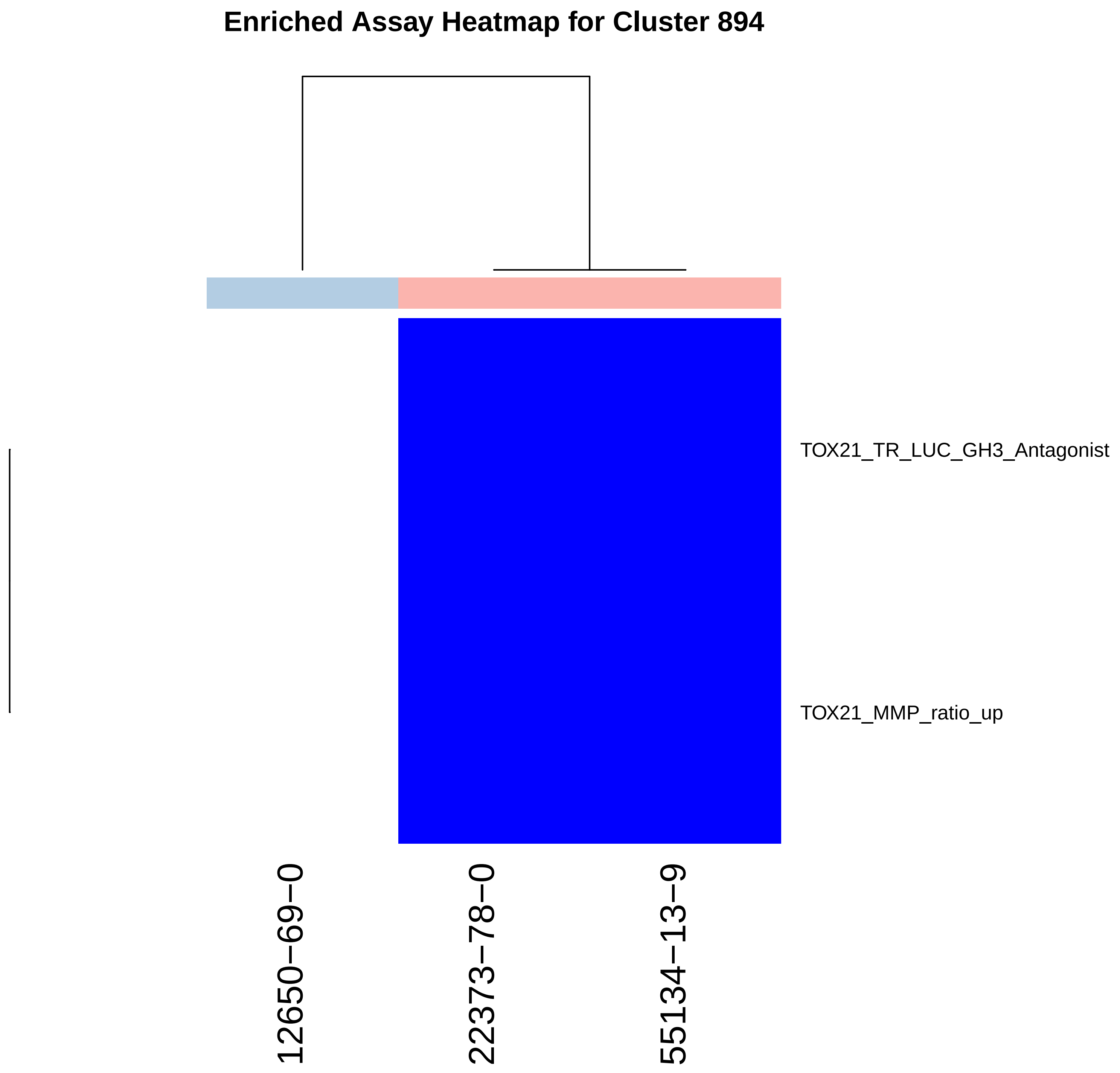
Enriched Assay Heatmap for Cluster 894
TOX21_TR_LUC_GH3_Antagonist
TOX21_MMP_ratio_up
12650−69−0
22373−78−0
55134−13−9

Enriched Assay Heatmap for Cluster 898

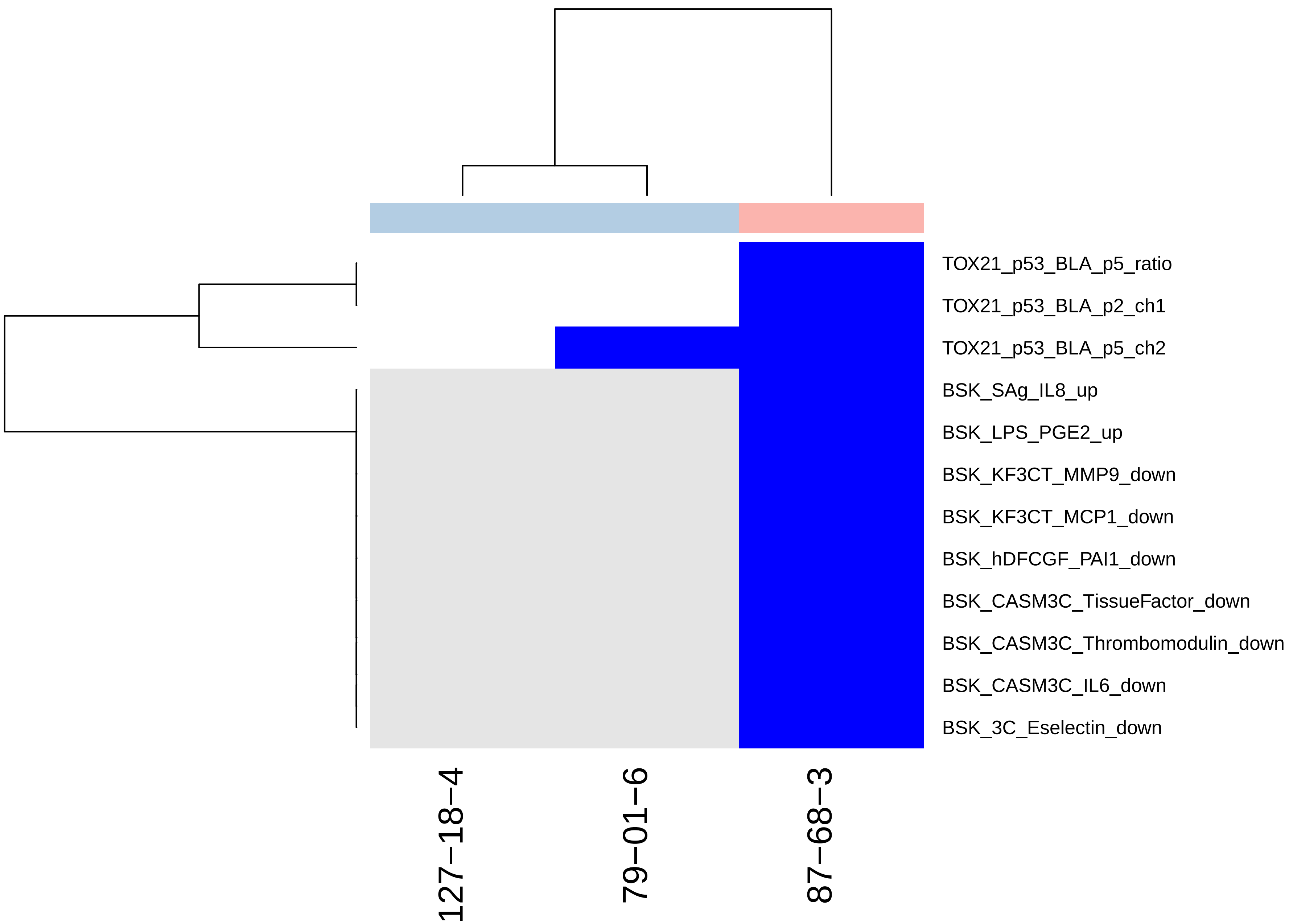

Enriched Assay Heatmap for Cluster 899

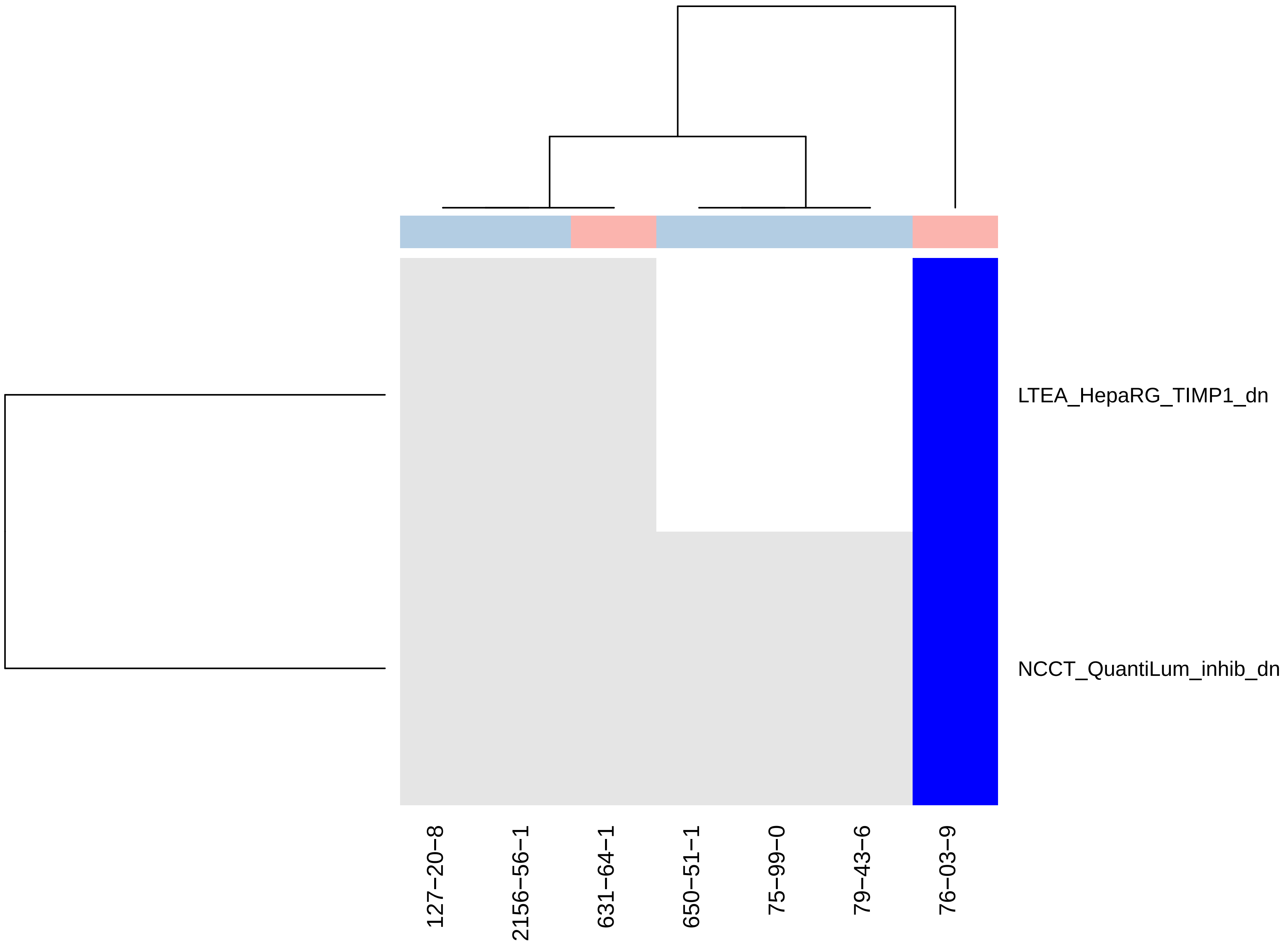

Enriched Assay Heatmap for Cluster 901

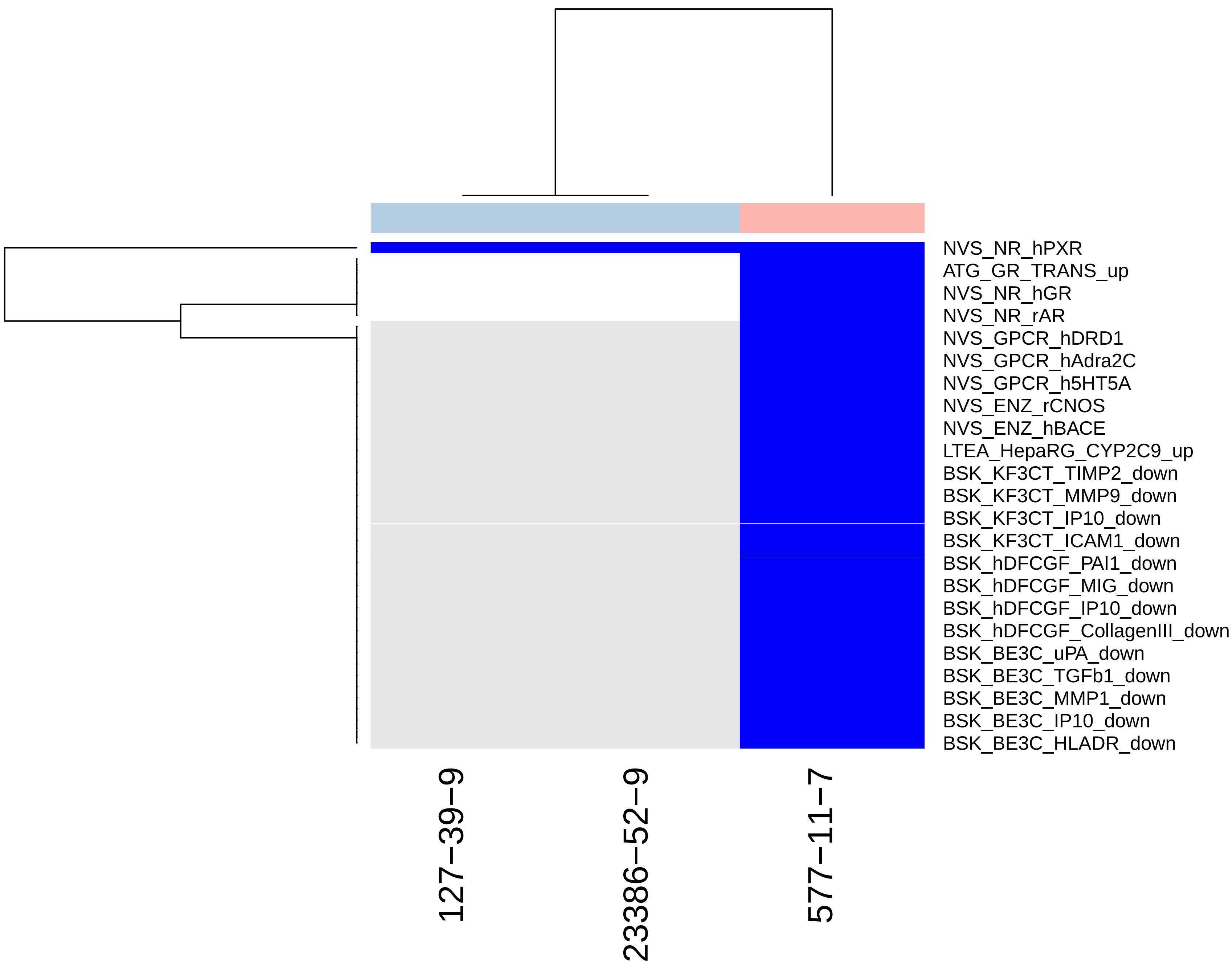

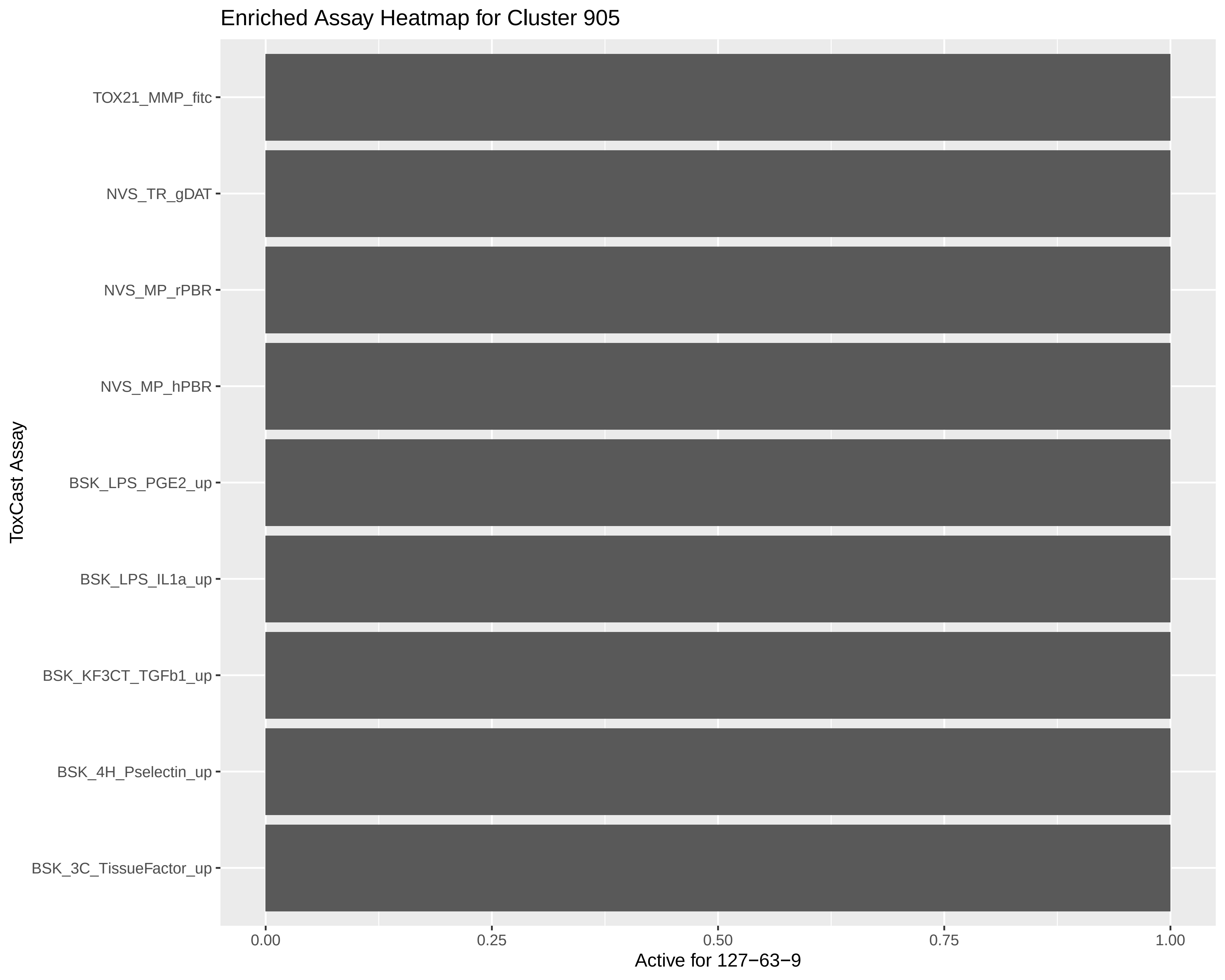

Enriched Assay Heatmap for Cluster 905
ToxCast Assay
TOX21_MMP_fitc
NVS_TR_gDAT
NVS_MP_rPBR
NVS_MP_hPBR
BSK_LPS_PGE2_up
BSK_LPS_IL1a_up
BSK_KF3CT_TGFb1_up
BSK_4H_Pselectin_up
BSK_3C_TissueFactor_up
0.00
0.25
0.50
0.75
1.00
Active for 127−63−9

Enriched Assay Heatmap for Cluster 906

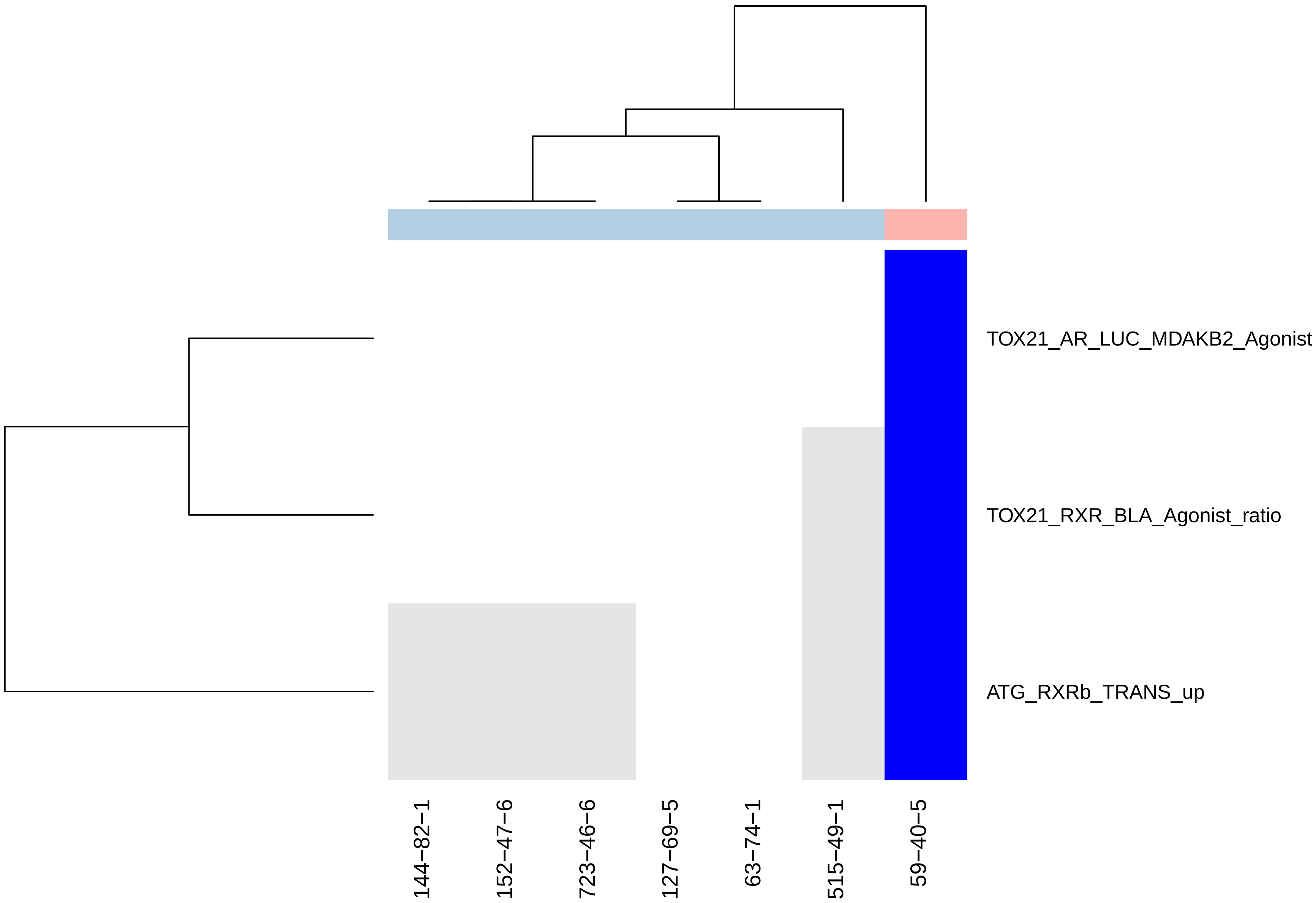

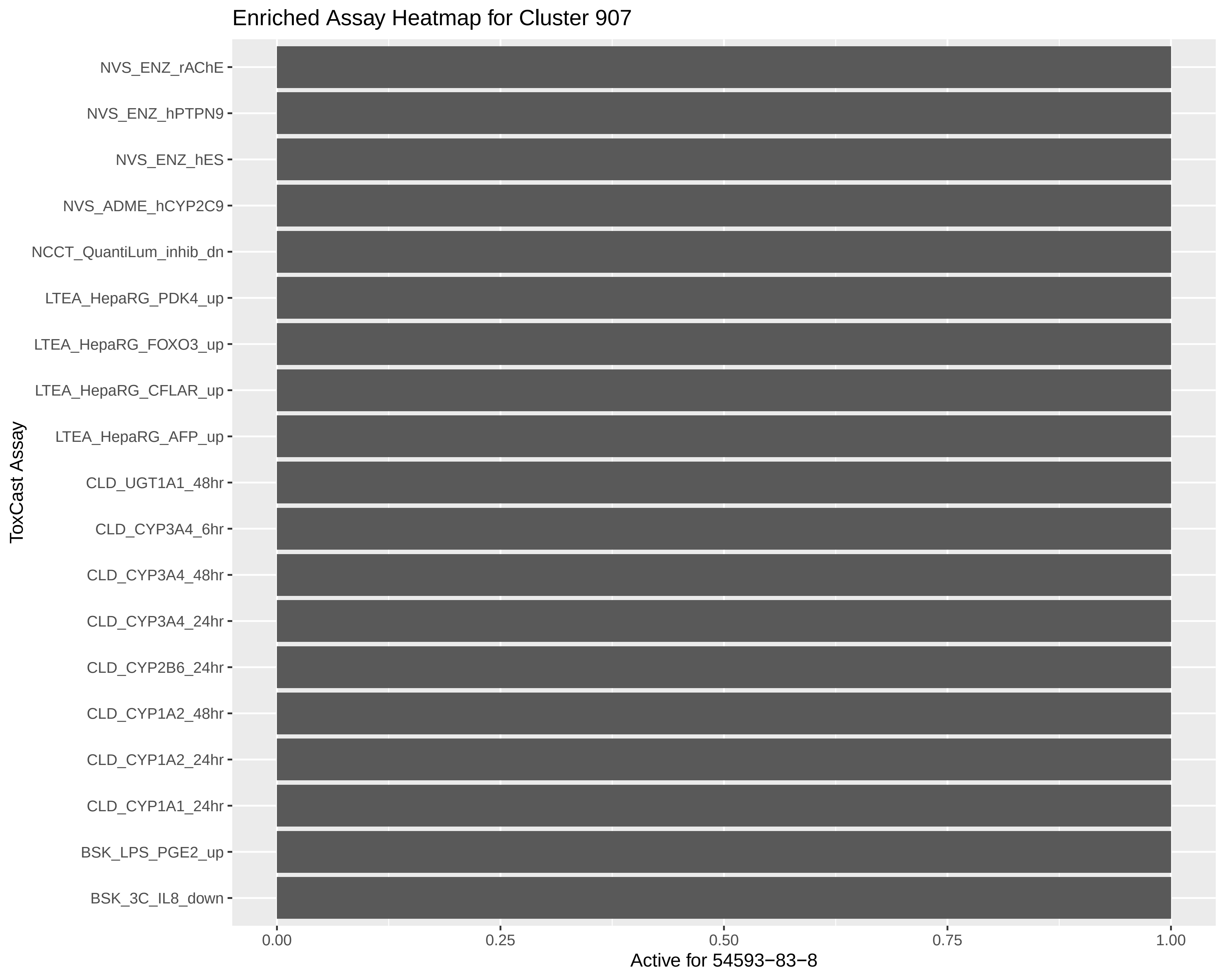

Enriched Assay Heatmap for Cluster 907
NVS_ENZ_rAChE
NVS_ENZ_hPTPN9
NVS_ENZ_hES
NVS_ADME_hCYP2C9
NCCT_QuantiLum_inhib_dn
LTEA_HepaRG_PDK4_up
LTEA_HepaRG_FOXO3_up
LTEA_HepaRG_CFLAR_up
LTEA_HepaRG_AFP_up
CLD_UGT1A1_48hr
CLD_CYP3A4_6hr
CLD_CYP3A4_48hr
CLD_CYP3A4_24hr
CLD_CYP2B6_24hr
CLD_CYP1A2_48hr
CLD_CYP1A2_24hr
CLD_CYP1A1_24hr
BSK_LPS_PGE2_up
BSK_3C_IL8_down
ToxCast Assay
0.00
0.25
0.50
0.75
1.00
Active for 54593−83−8

Enriched Assay Heatmap for Cluster 909

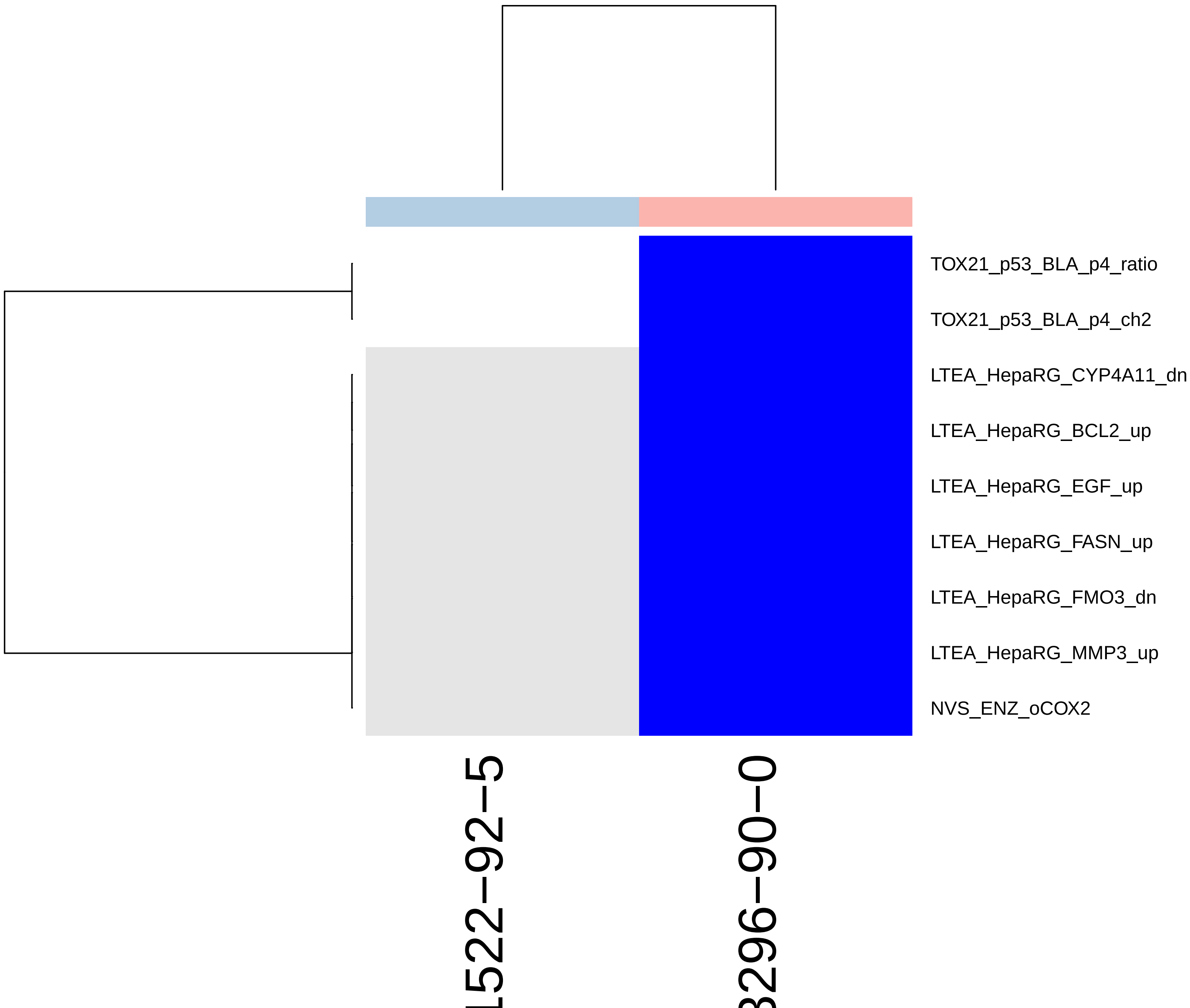

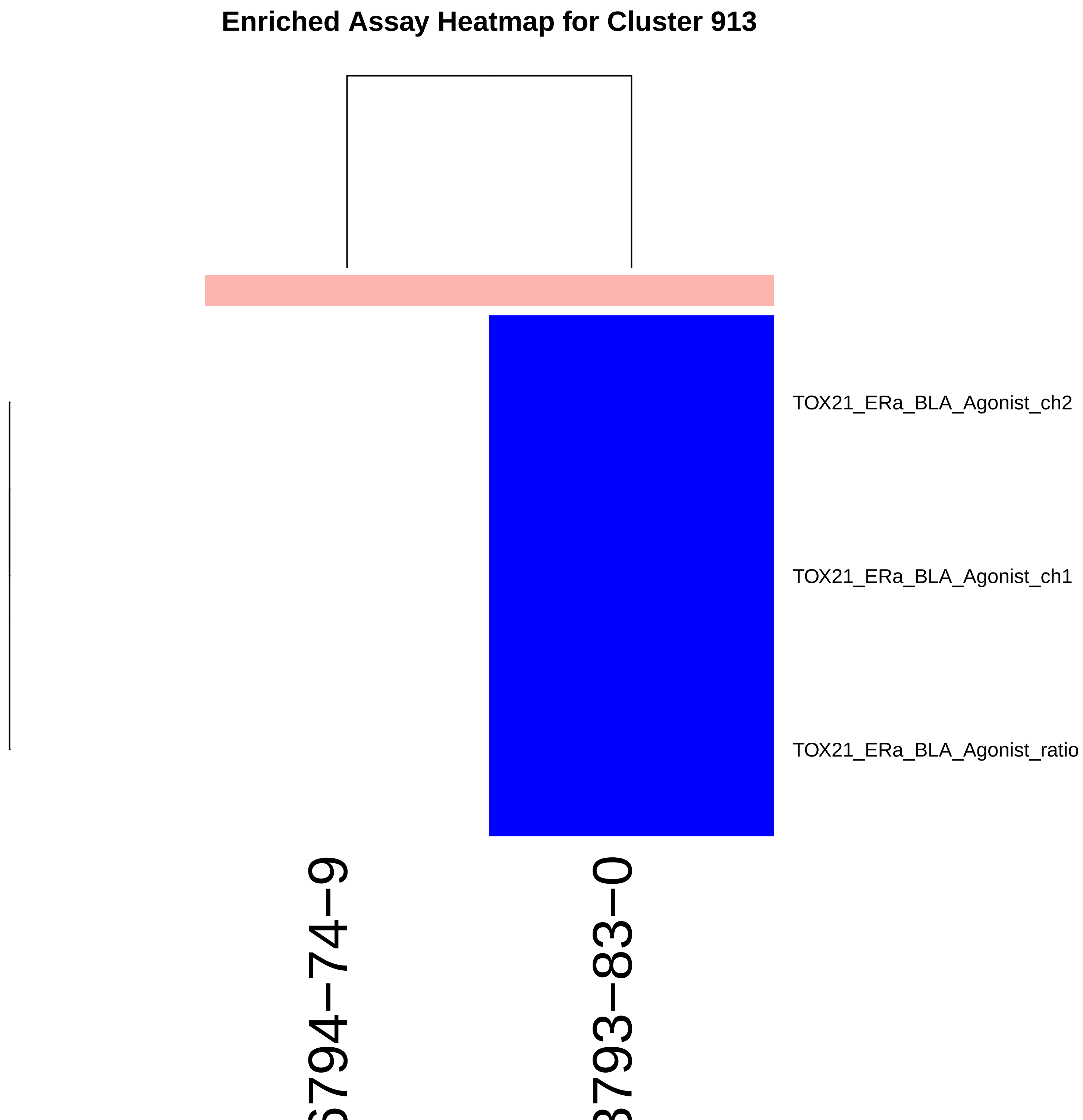

Enriched Assay Heatmap for Cluster 913
TOX21_ERa_BLA_Agonist_ch2
TOX21_ERa_BLA_Agonist_ch1
TOX21_ERa_BLA_Agonist_ratio

Enriched Assay Heatmap for Cluster 914

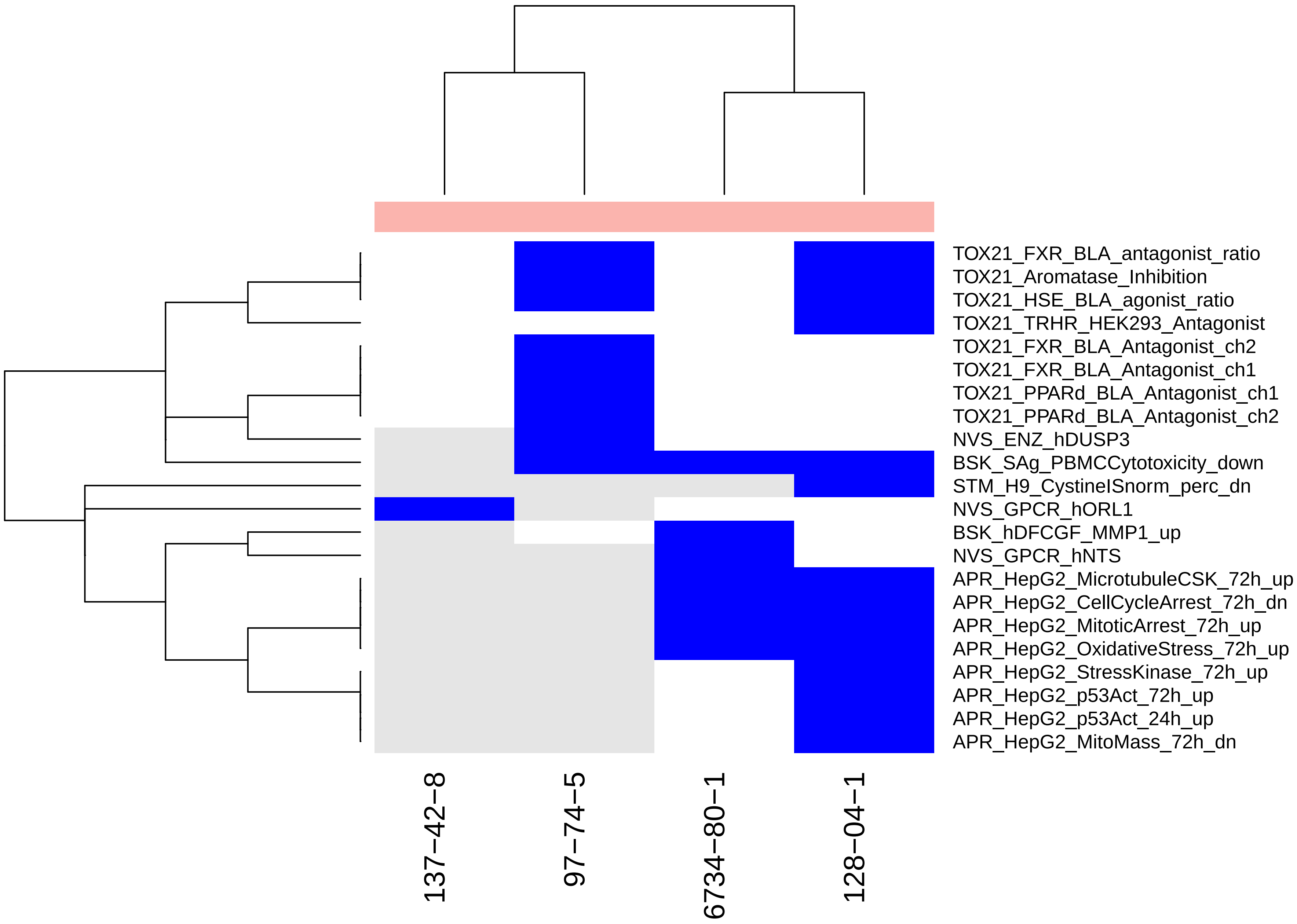

Enriched Assay Heatmap for Cluster 915

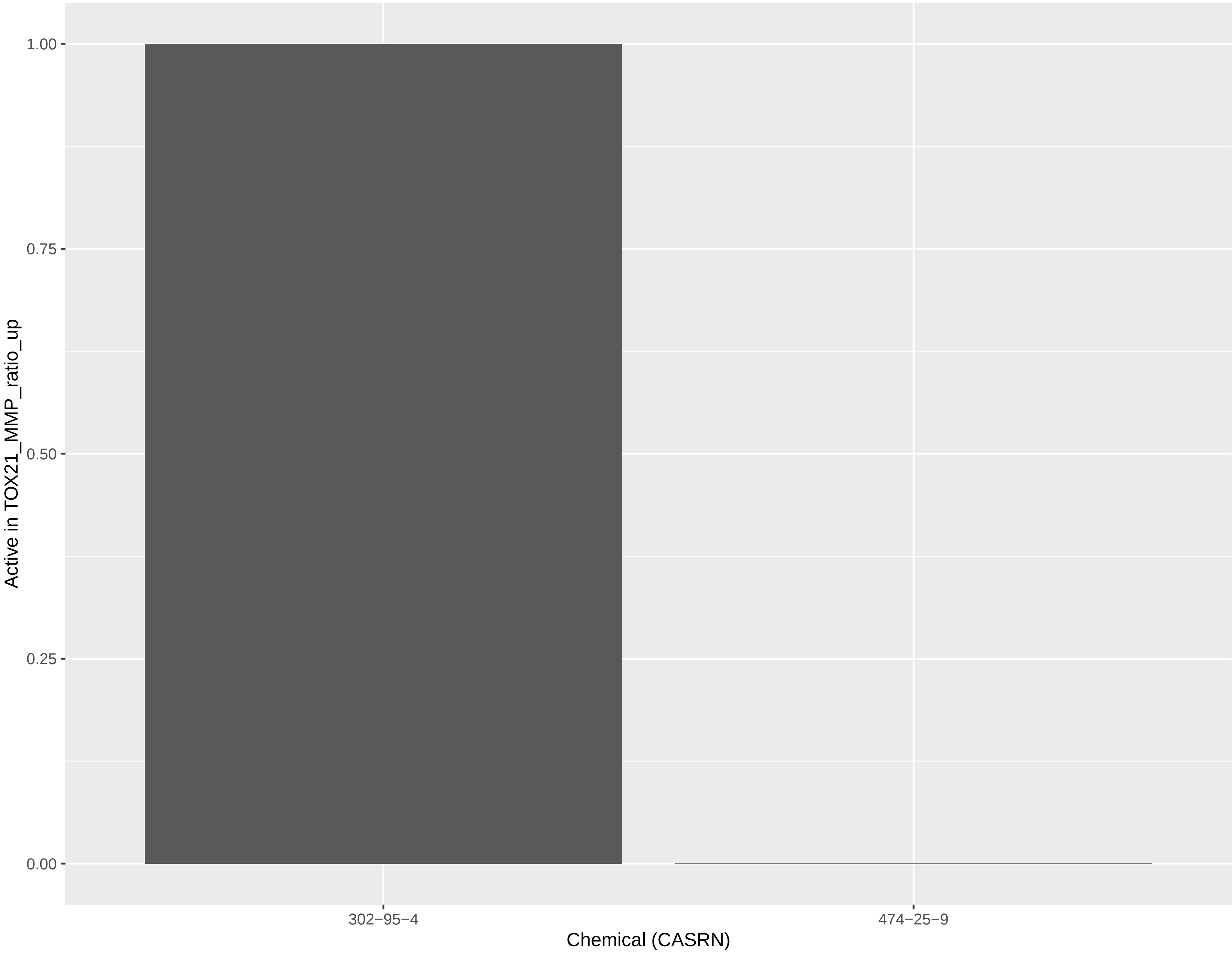

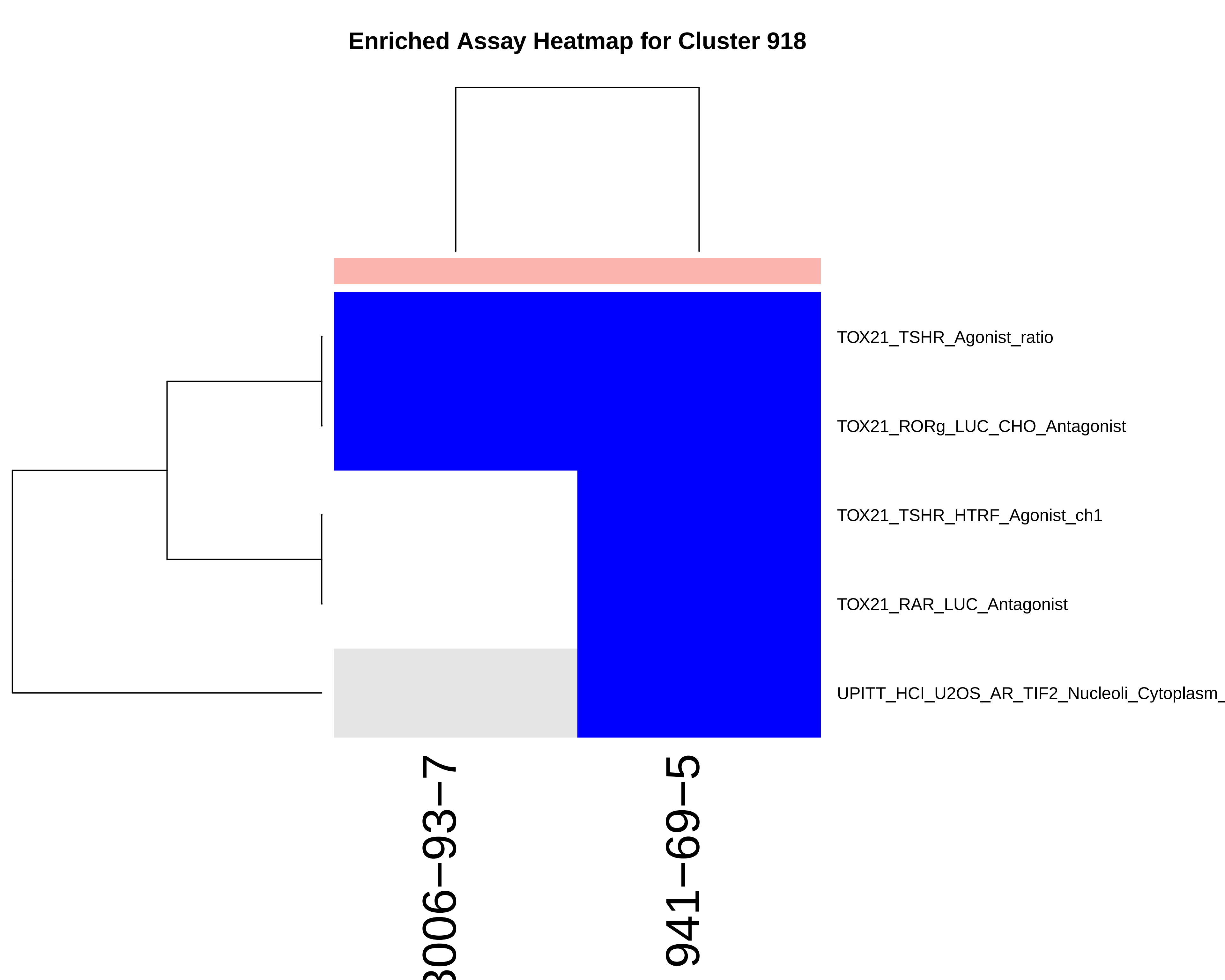
Enriched Assay Heatmap for Cluster 918
TOX21_TSHR_Agonist_ratio
TOX21_RORg_LUC_CHO_Antagonist
TOX21_TSHR_HTRF_Agonist_ch1
TOX21_RAR_LUC_Antagonist
UPITT_HCI_U2OS_AR_TIF2_Nucleoli_Cytoplasm_
306−93−7
941−69−5

Enriched Assay Heatmap for Cluster 921

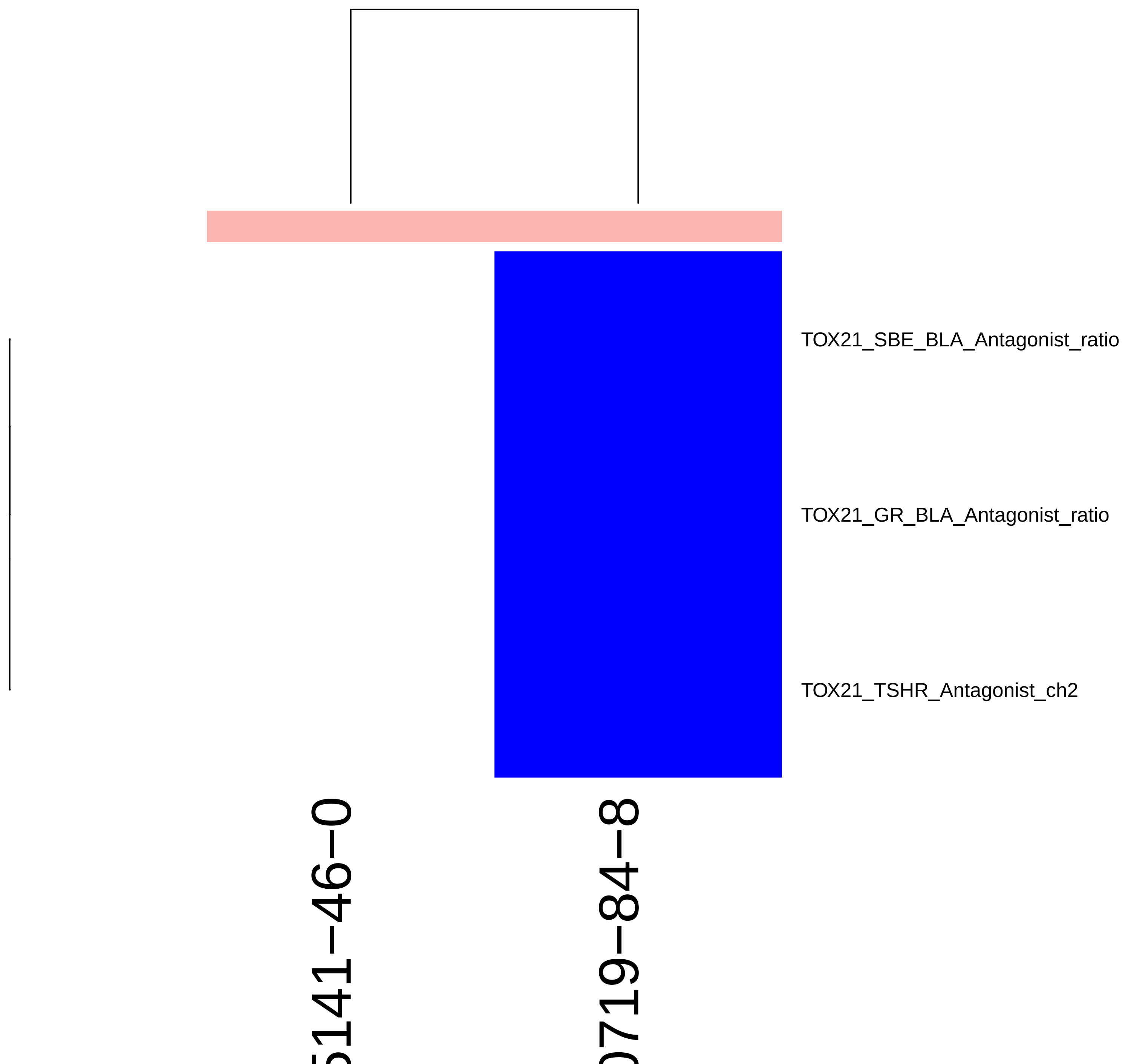

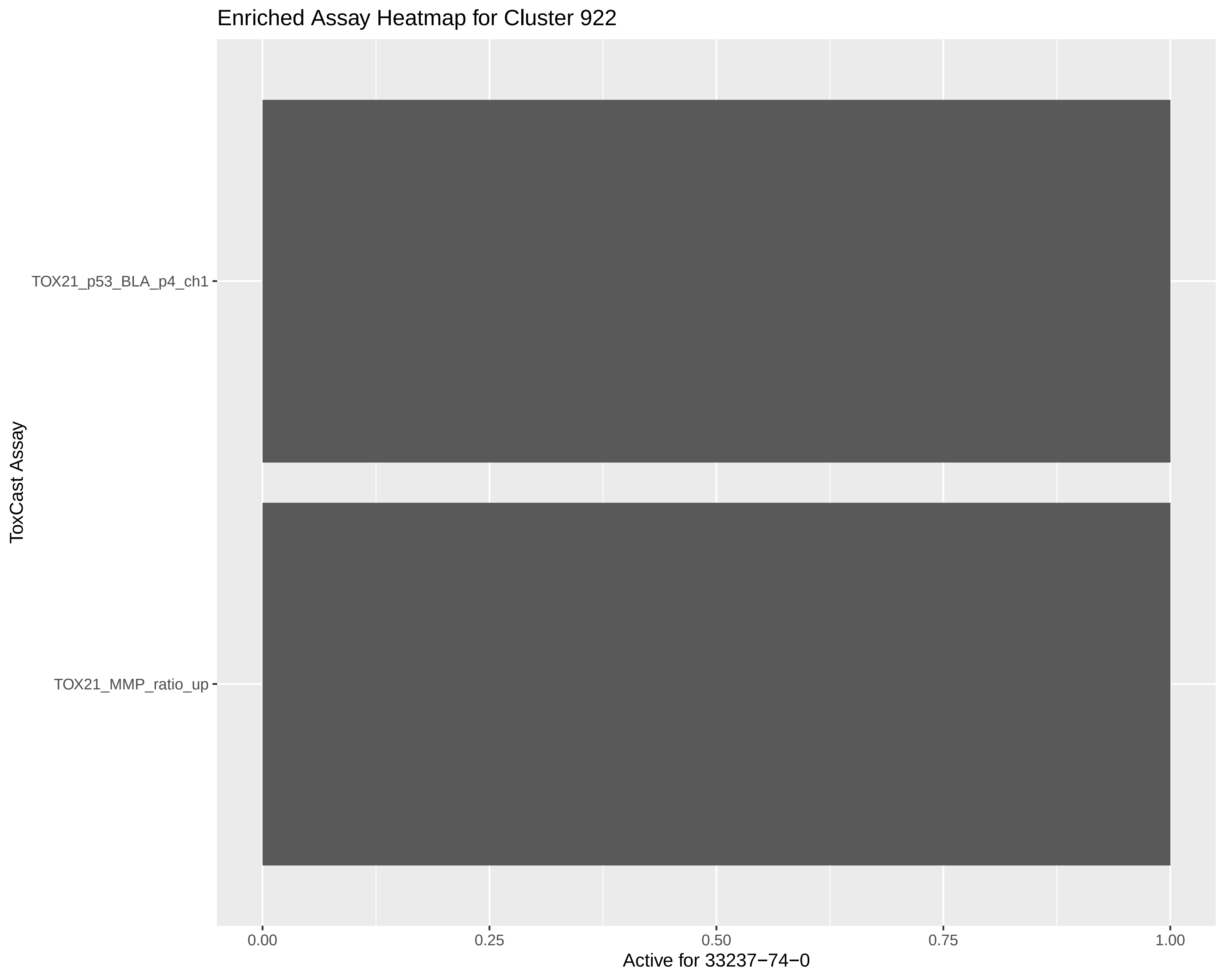
Enriched Assay Heatmap for Cluster 922
ToxCast Assay
TOX21_p53_BLA_p4_ch1
TOX21_MMP_ratio_up
0.00
0.25
0.50
0.75
1.00
Active for 33237−74−0

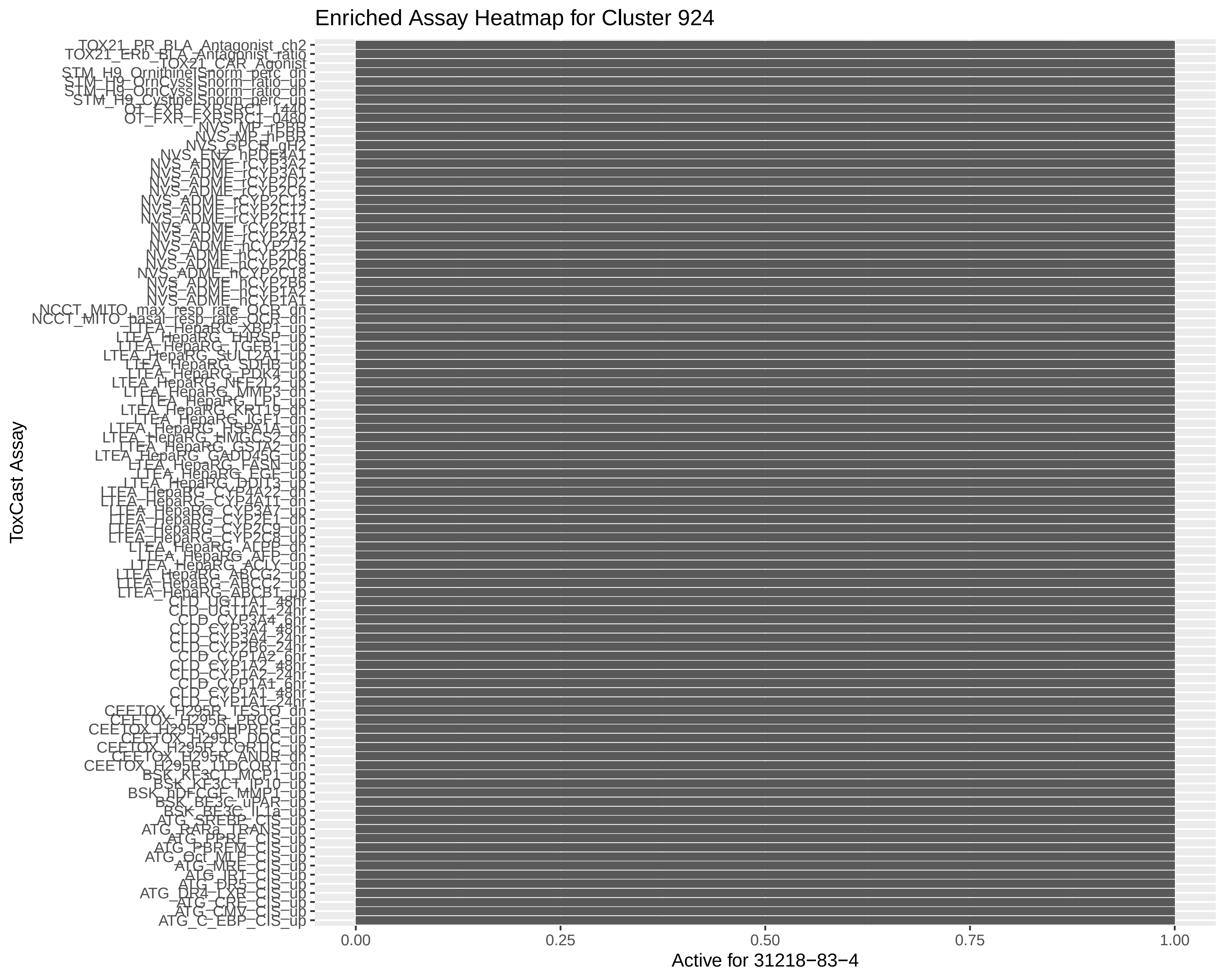

Enriched Assay Heatmap for Cluster 924
ToxCast Assay
Active for 31218−83−4
0.00
0.25
0.50
0.75
1.00

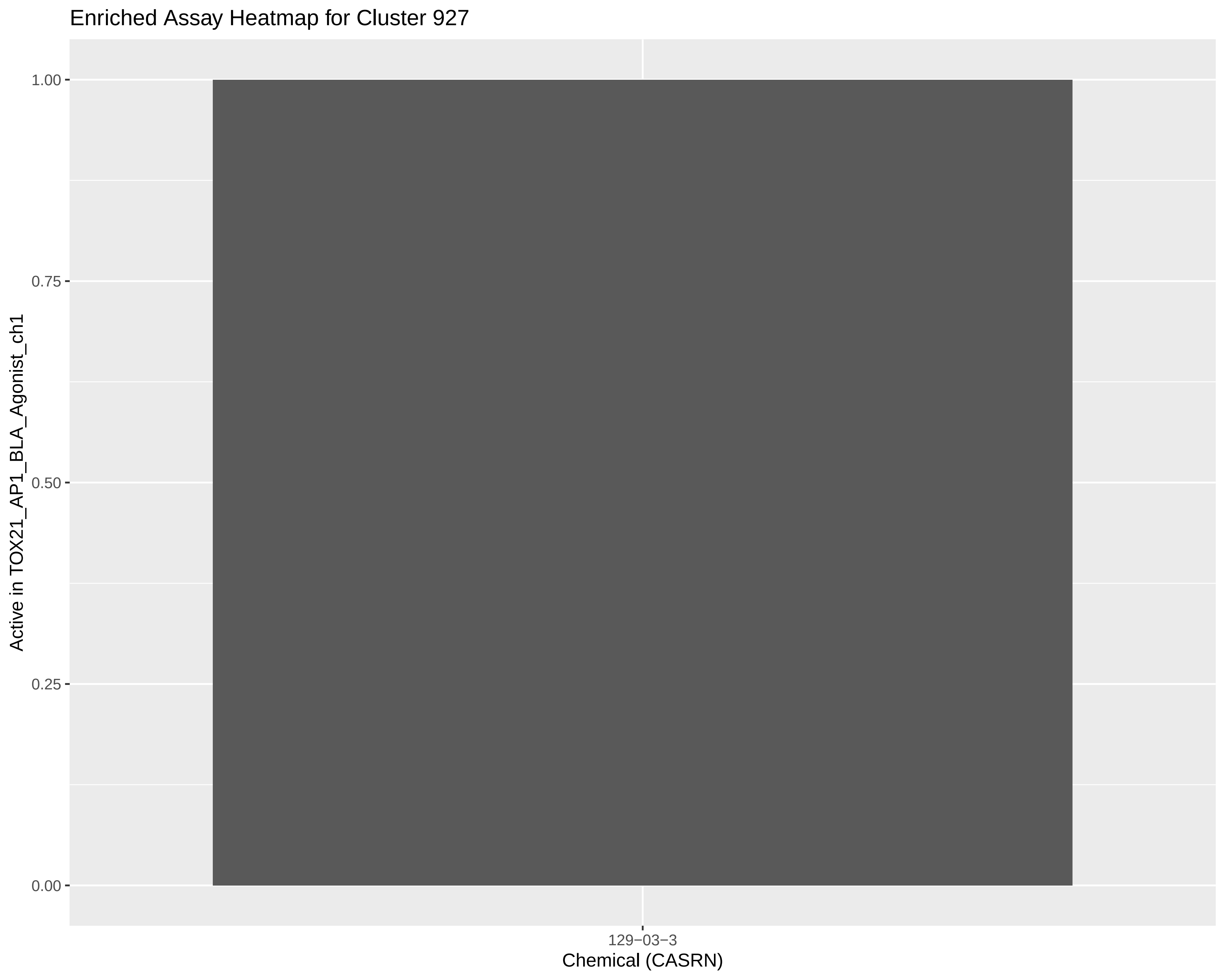
Enriched Assay Heatmap for Cluster 927
Active in TOX21_AP1_BLA_Agonist_ch1
1.00
0.75
0.50
0.25
0.00
129−03−3
Chemical (CASRN)

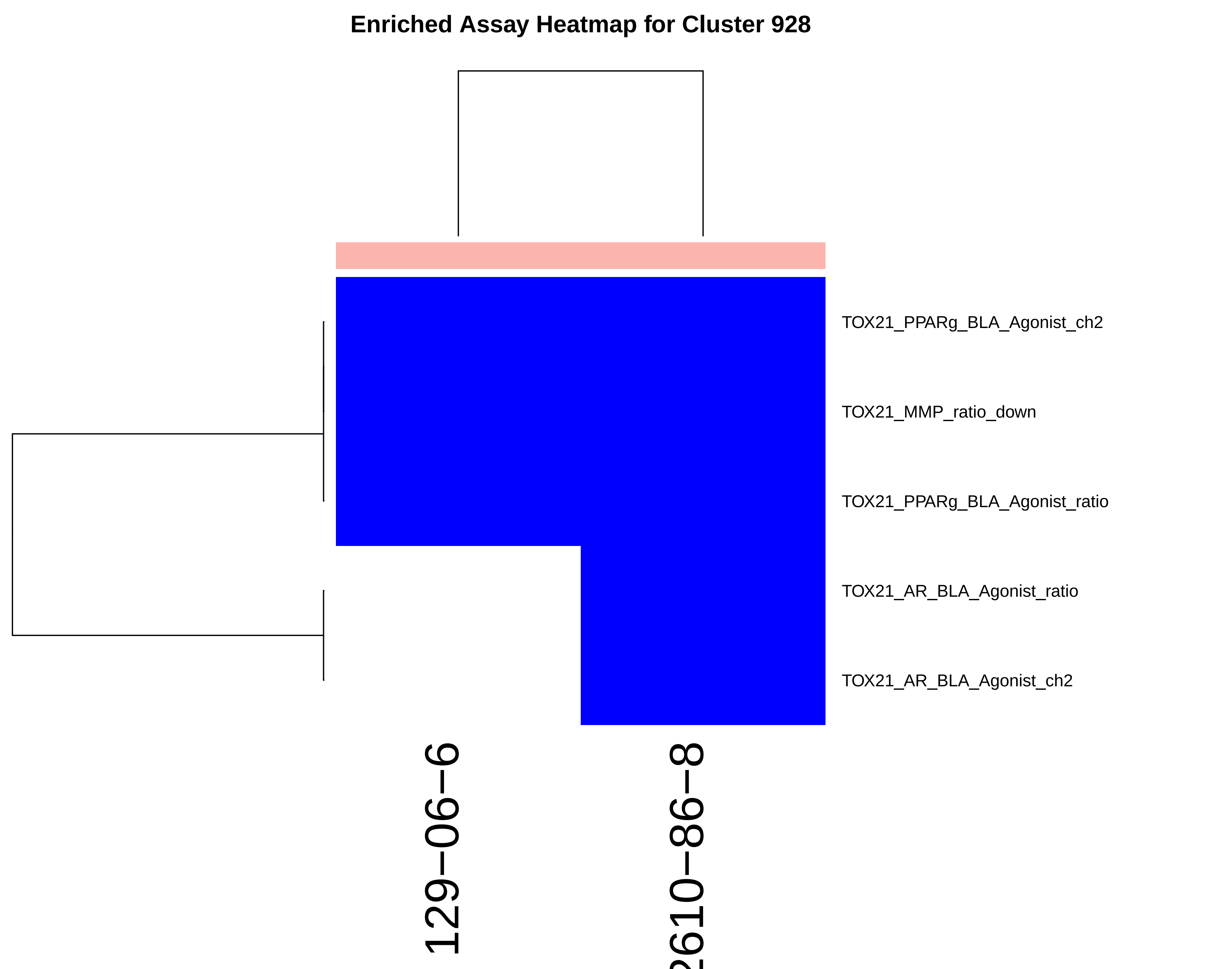
Enriched Assay Heatmap for Cluster 928
TOX21_PPARg_BLA_Agonist_ch2
TOX21_MMP_ratio_down
TOX21_PPARg_BLA_Agonist_ratio
TOX21_AR_BLA_Agonist_ratio
TOX21_AR_BLA_Agonist_ch2
129−06−6
2610−86−8

Enriched Assay Heatmap for Cluster 935

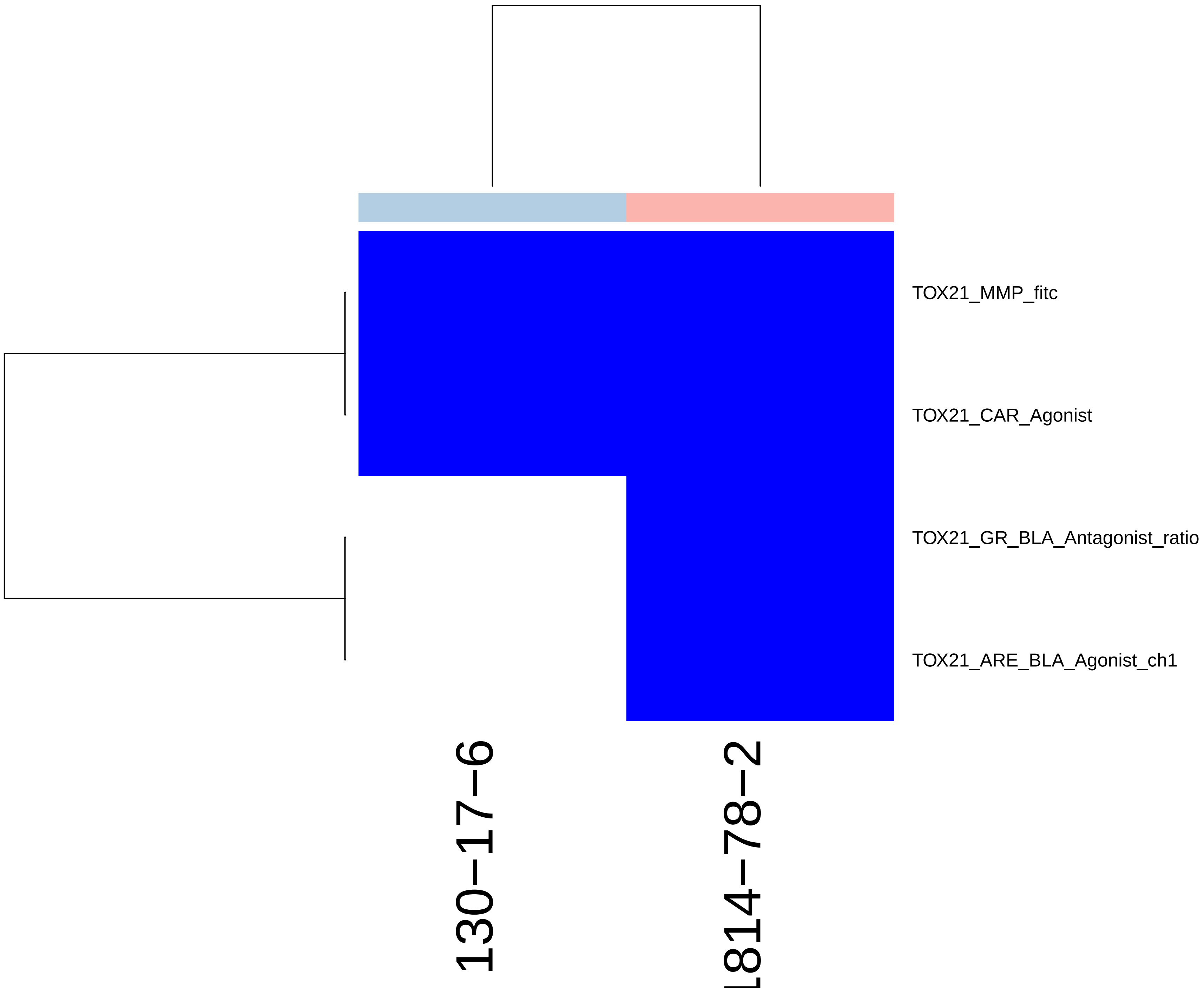

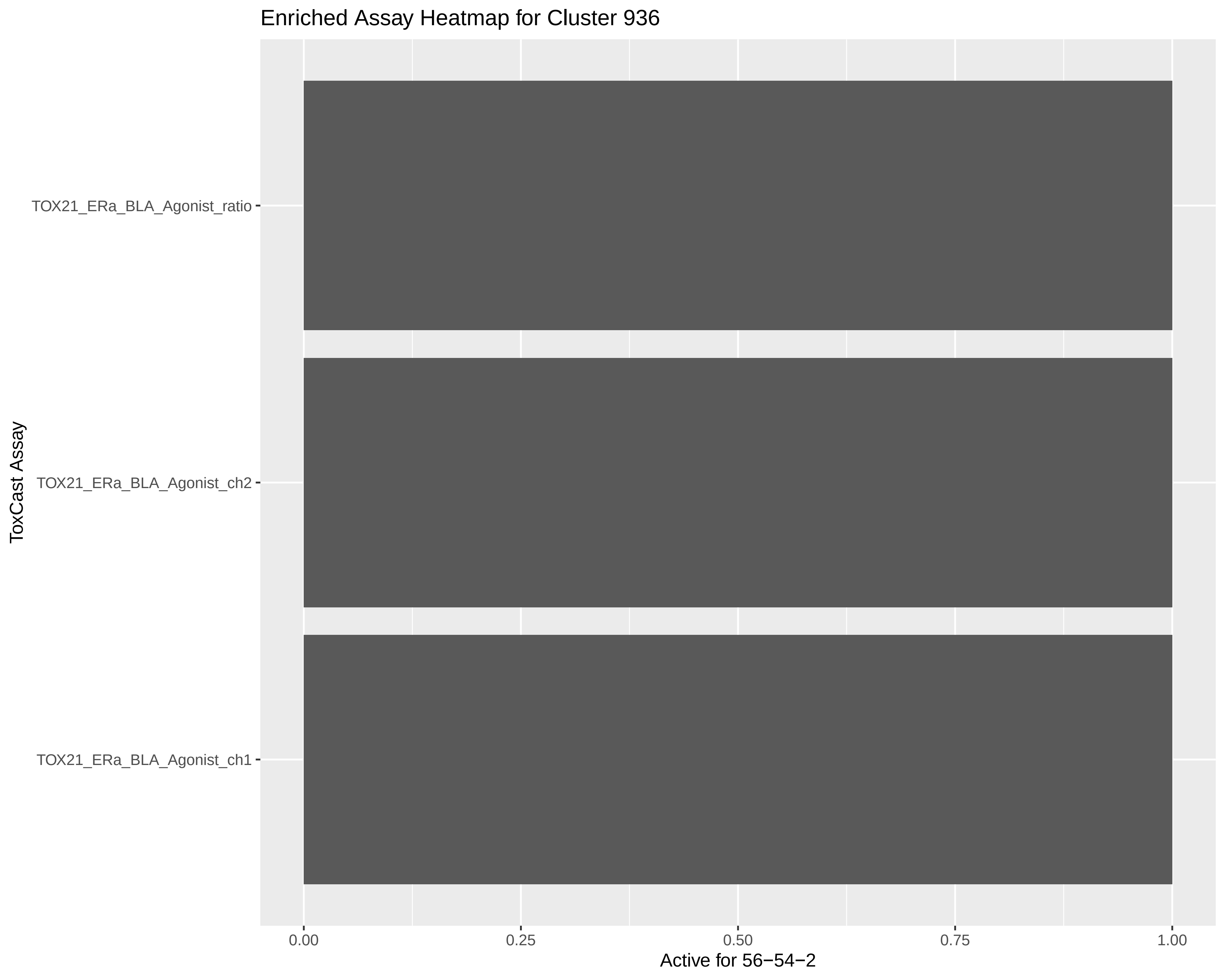

Enriched Assay Heatmap for Cluster 936
ToxCast Assay
TOX21_ERa_BLA_Agonist_ratio
TOX21_ERa_BLA_Agonist_ch2
TOX21_ERa_BLA_Agonist_ch1
0.00
0.25
0.50
0.75
1.00
Active for 56−54−2

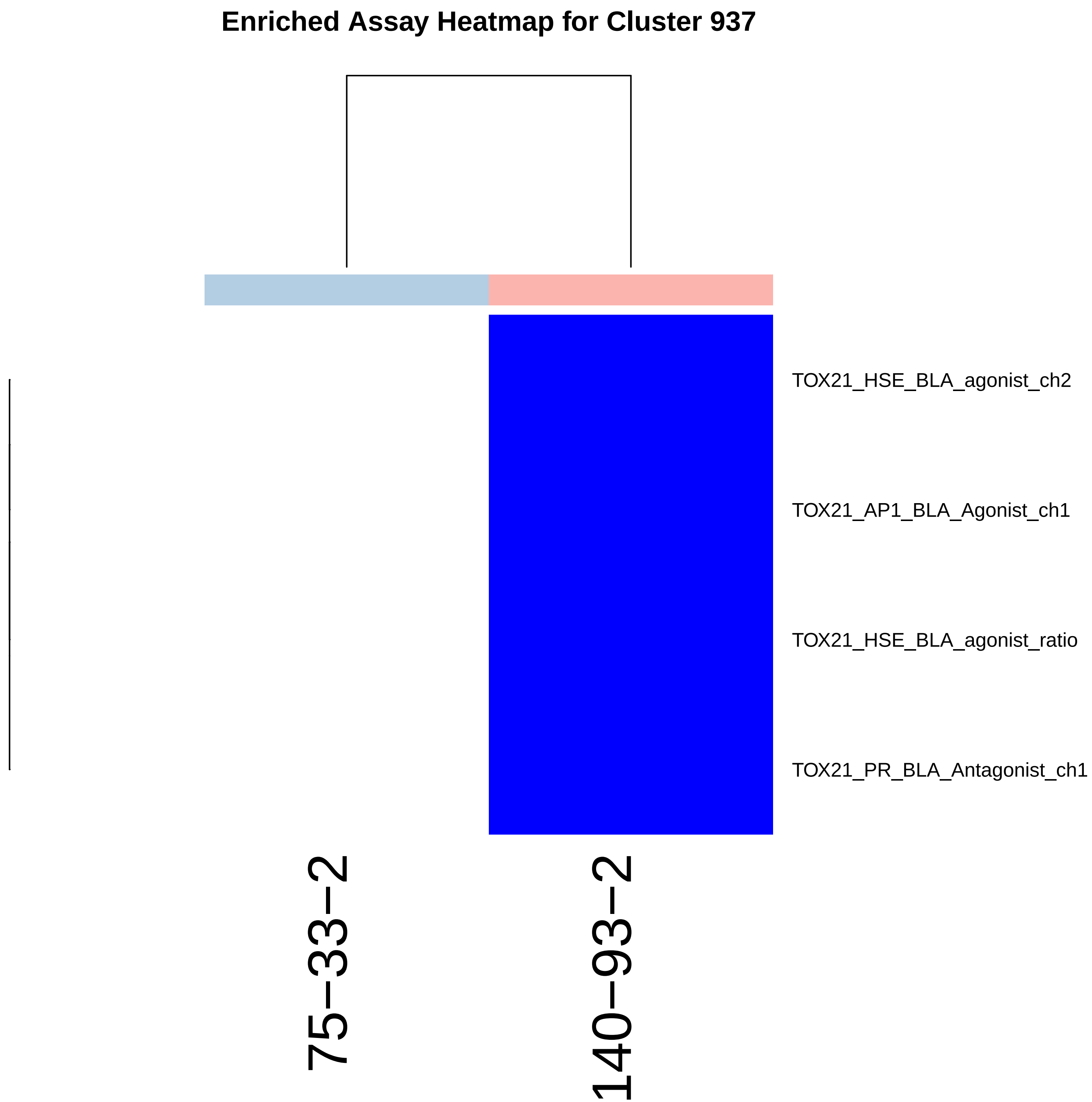
Enriched Assay Heatmap for Cluster 937
TOX21_HSE_BLA_agonist_ch2
TOX21_AP1_BLA_Agonist_ch1
TOX21_HSE_BLA_agonist_ratio
TOX21_PR_BLA_Antagonist_ch1
75−33−2
140−93−2

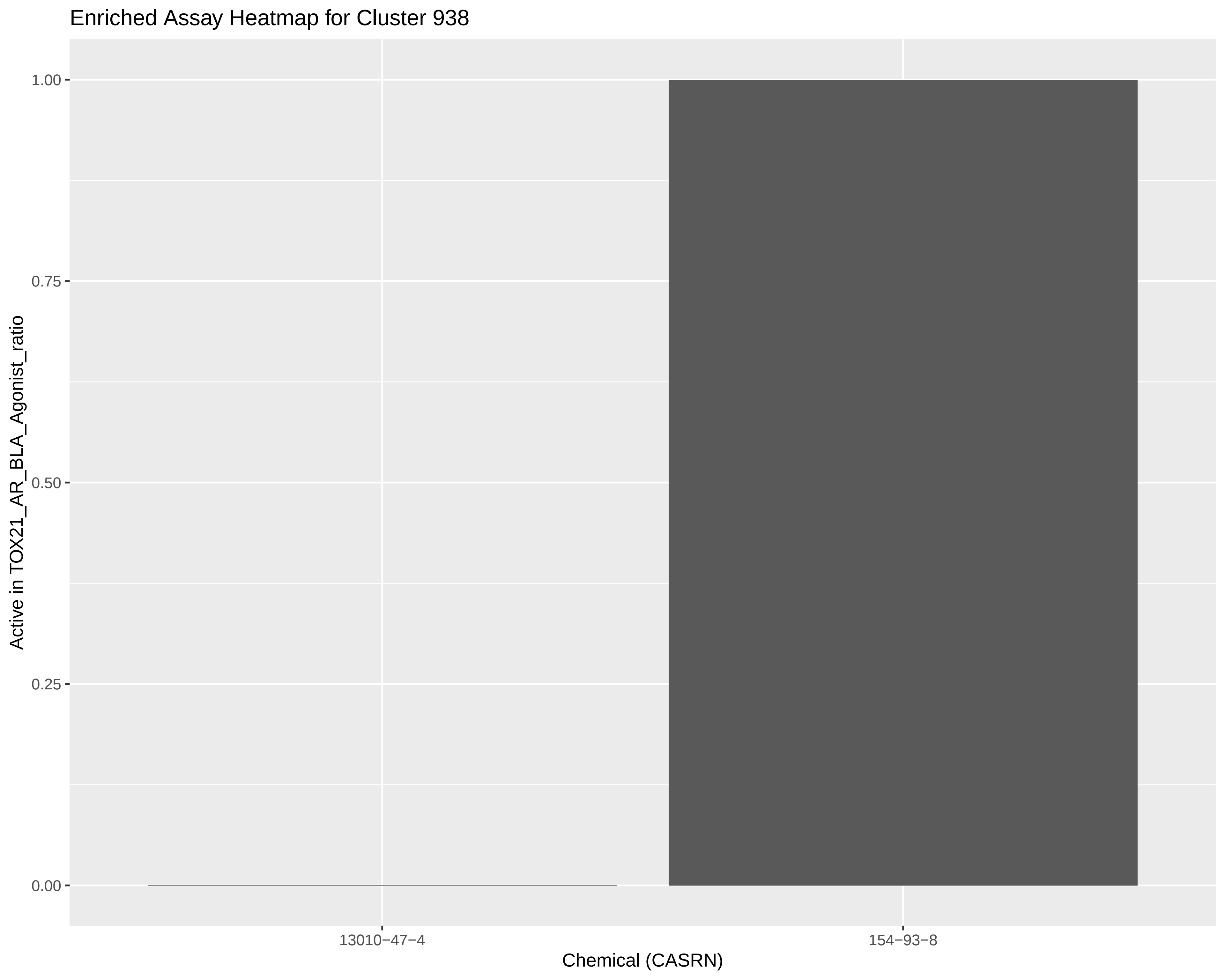
Enriched Assay Heatmap for Cluster 938
1.00
0.75
0.50
0.25
0.00
Active in TOX21_AR_BLA_Agonist_ratio
13010−47−4
154−93−8
Chemical (CASRN)

Enriched Assay Heatmap for Cluster 939

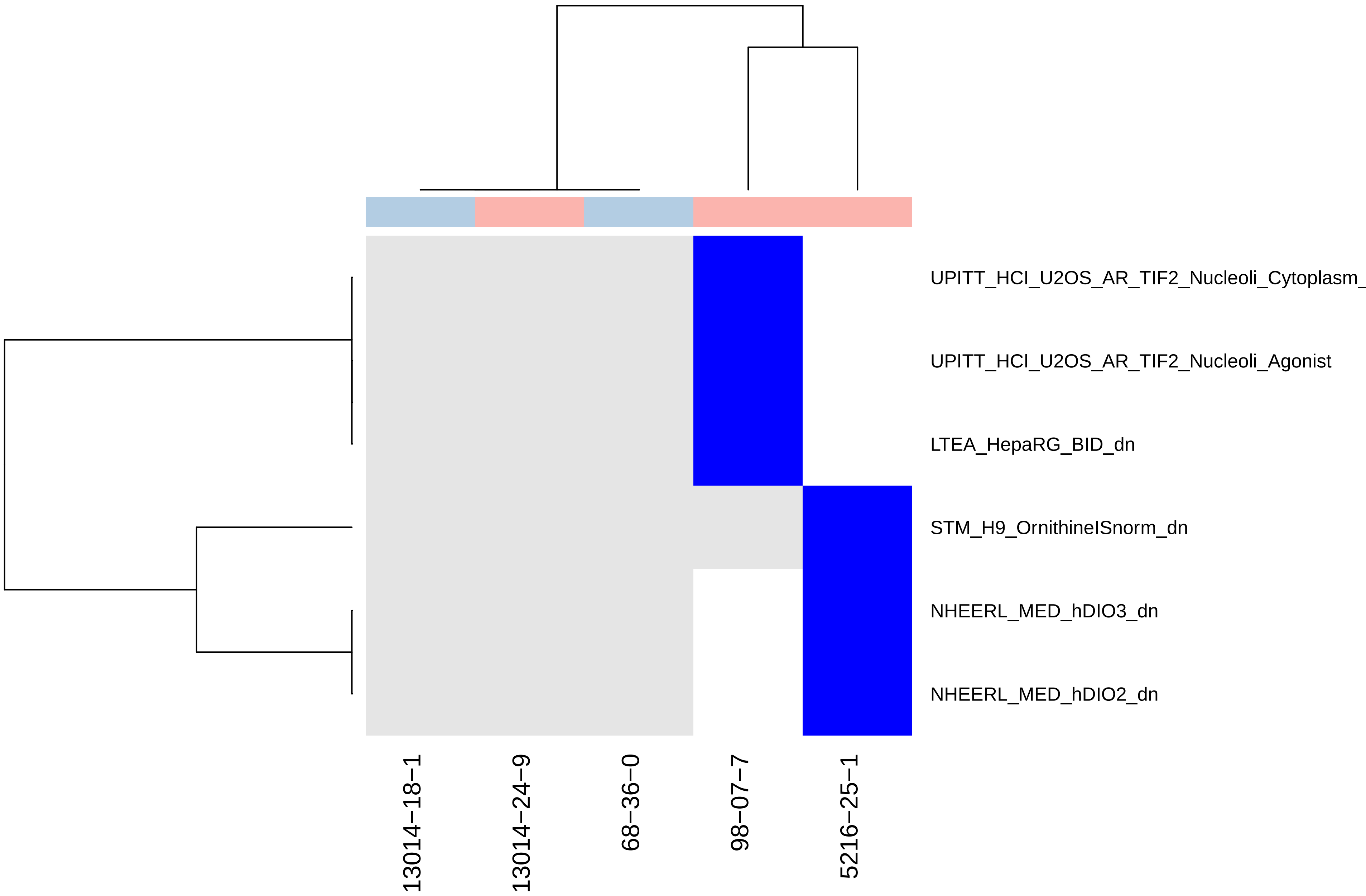

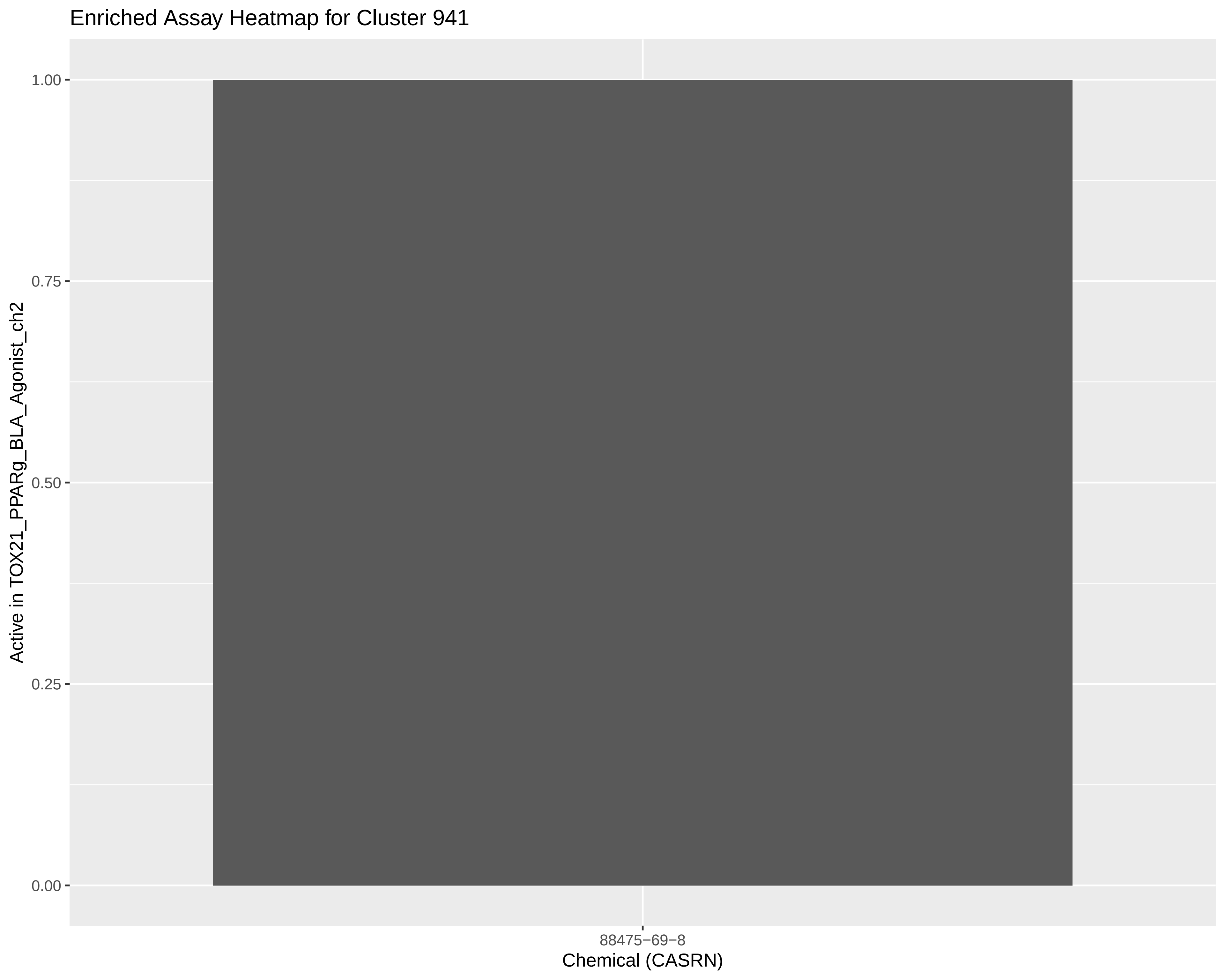

Enriched Assay Heatmap for Cluster 941
1.00
0.75
0.50
0.25
0.00
Active in TOX21_PPARg_BLA_Agonist_ch2
88475−69−8
Chemical (CASRN)

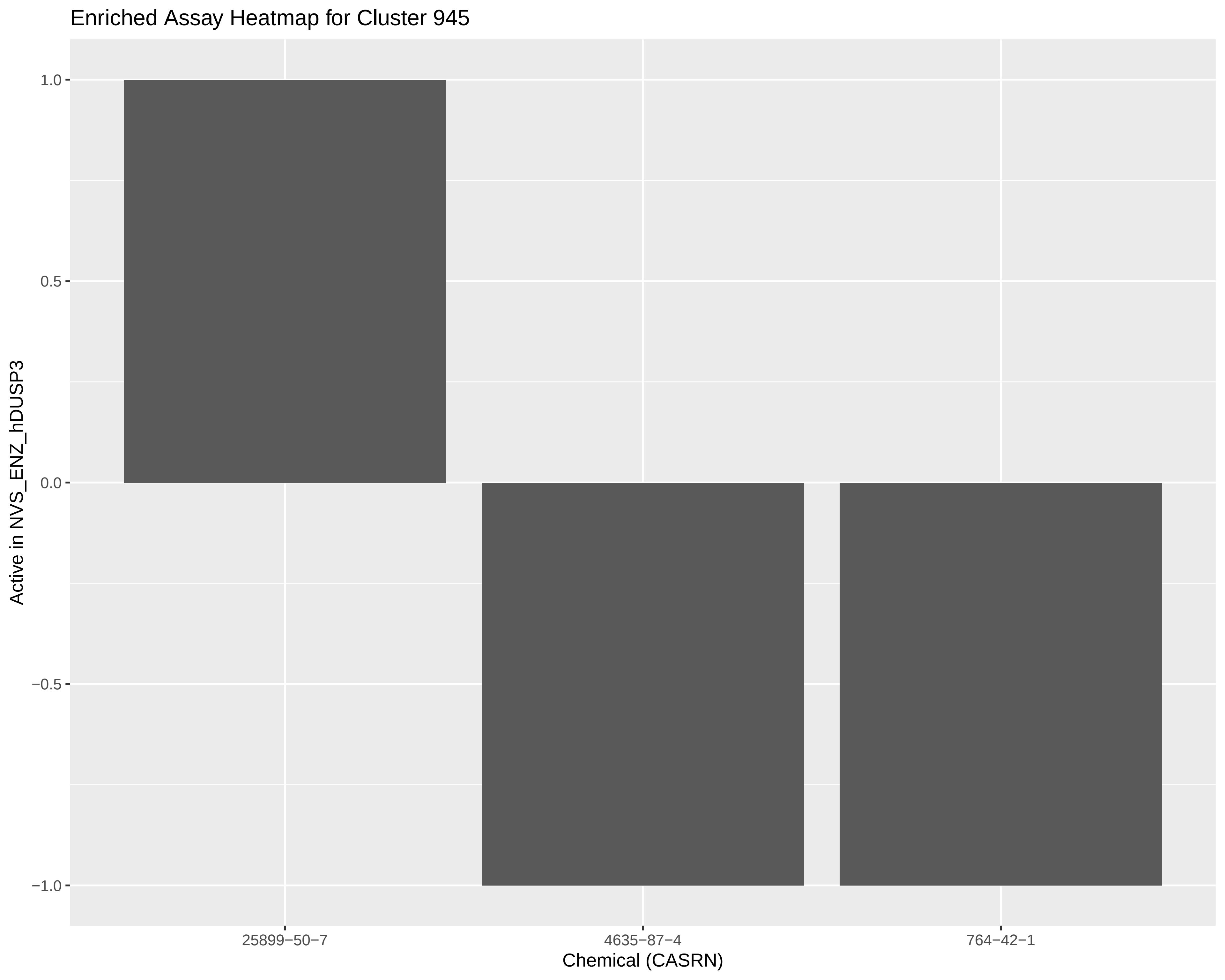

Enriched Assay Heatmap for Cluster 945
Active in NVS_ENZ_hDUSP3
1.0
0.5
0.0
−0.5
−1.0
25899−50−7
4635−87−4
764−42−1
Chemical (CASRN)

Enriched Assay Heatmap for Cluster 947

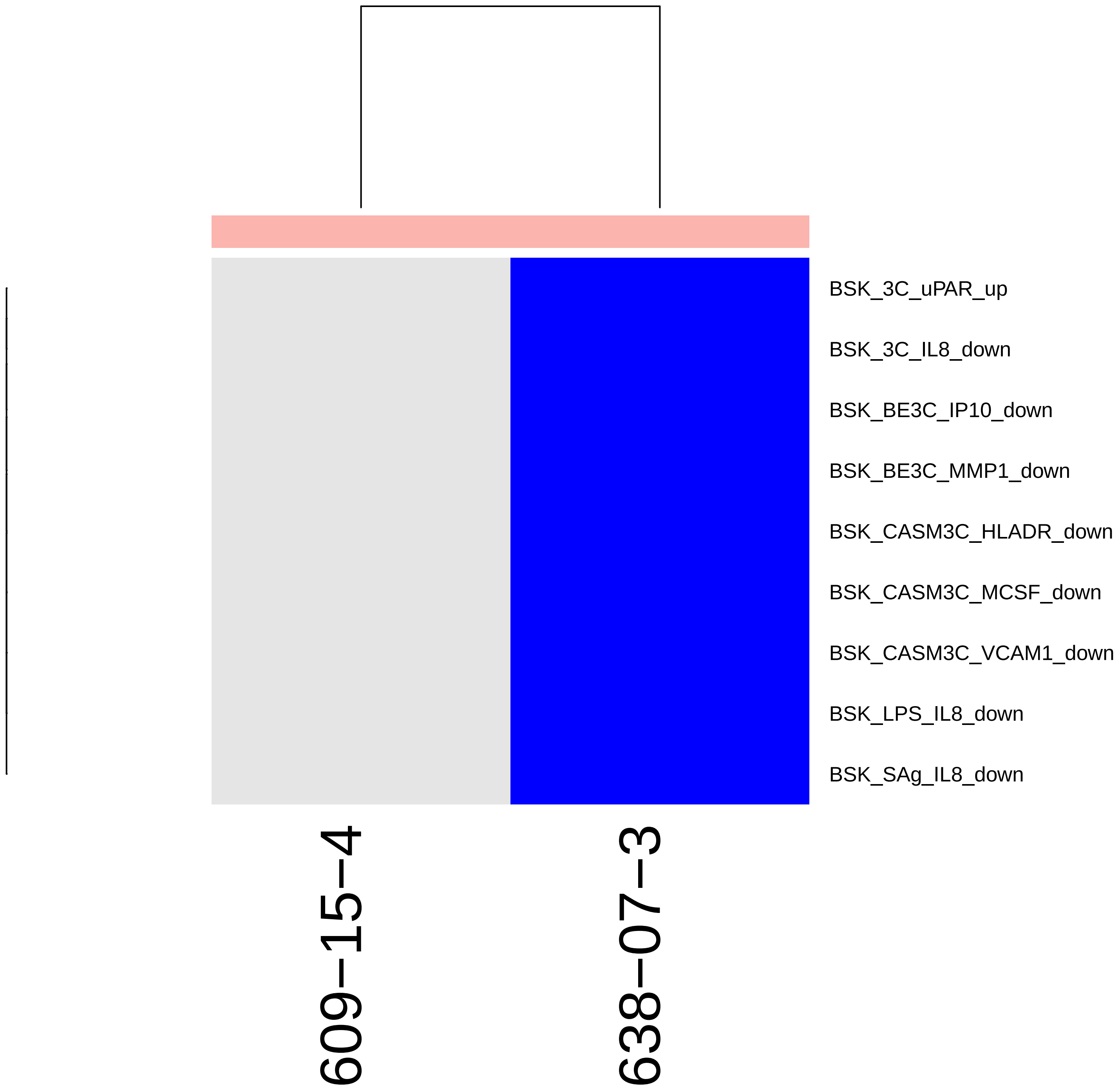

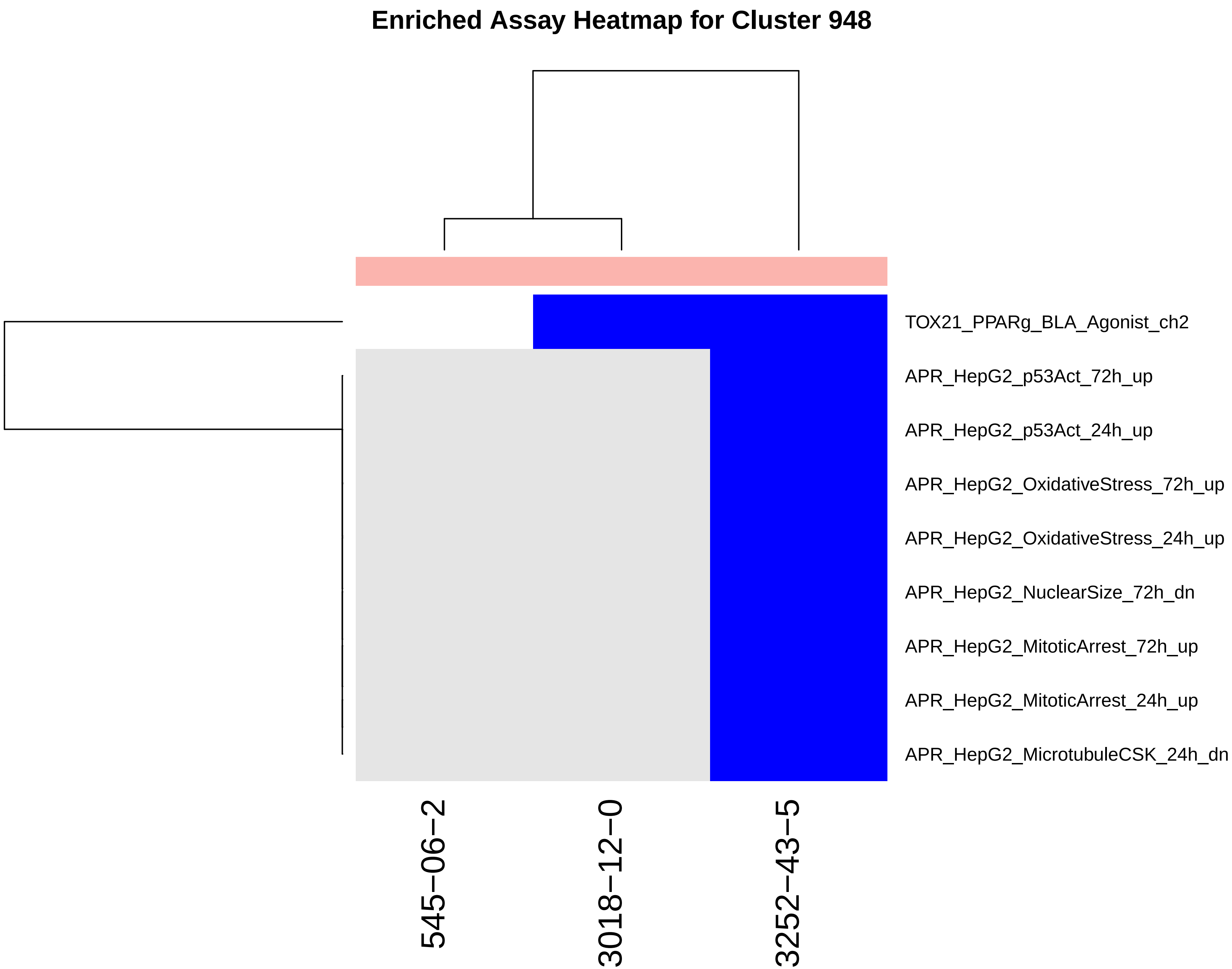

Enriched Assay Heatmap for Cluster 948
TOX21_PPARg_BLA_Agonist_ch2
APR_HepG2_p53Act_72h_up
APR_HepG2_p53Act_24h_up
APR_HepG2_OxidativeStress_72h_up
APR_HepG2_OxidativeStress_24h_up
APR_HepG2_NuclearSize_72h_dn
APR_HepG2_MitoticArrest_72h_up
APR_HepG2_MitoticArrest_24h_up
APR_HepG2_MicrotubuleCSK_24h_dn
545−06−2
3018−12−0
3252−43−5

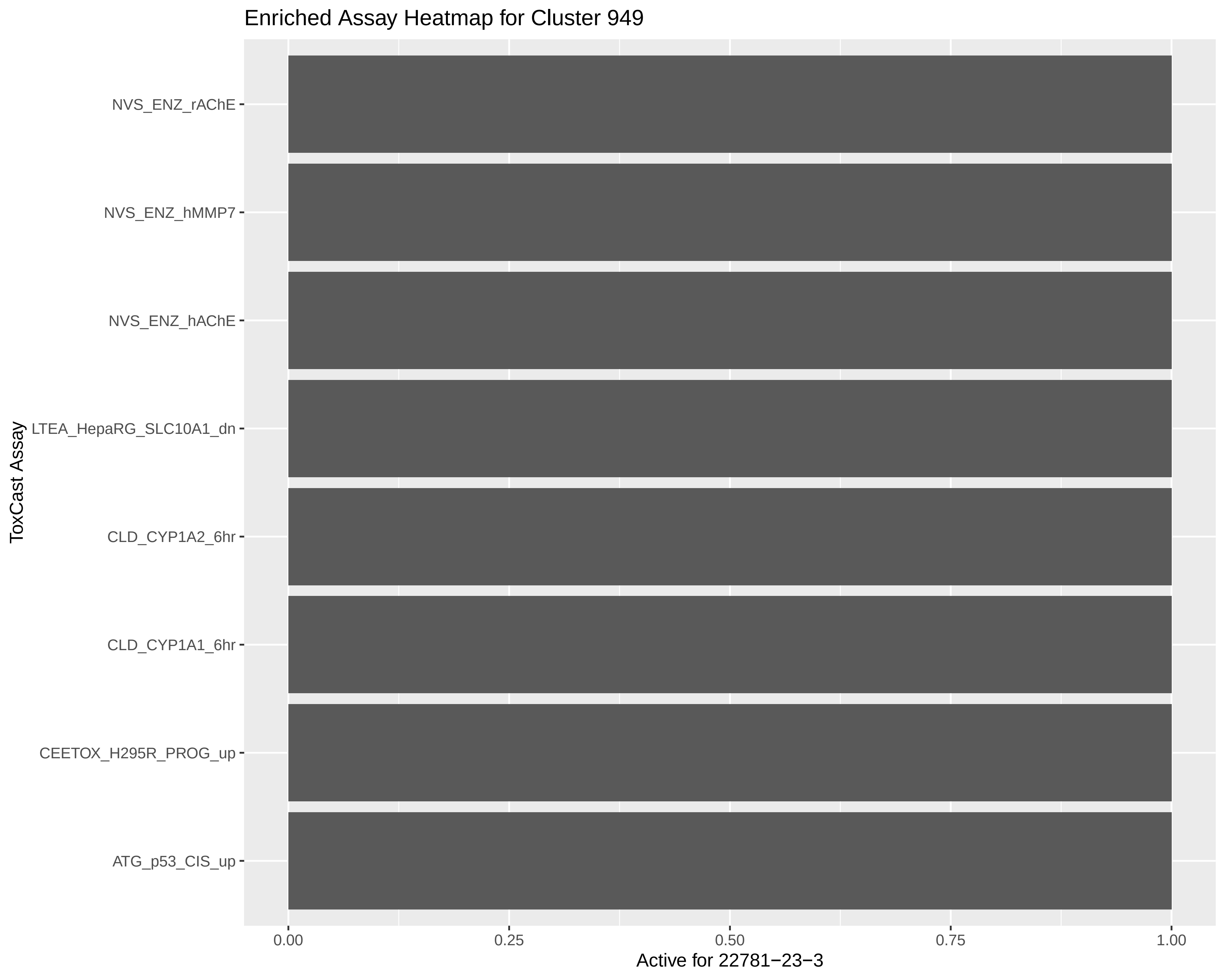
Enriched Assay Heatmap for Cluster 949
ToxCast Assay
NVS_ENZ_rAChE
NVS_ENZ_hMMP7
NVS_ENZ_hAChE
LTEA_HepaRG_SLC10A1_dn
CLD_CYP1A2_6hr
CLD_CYP1A1_6hr
CEETOX_H295R_PROG_up
ATG_p53_CIS_up
0.00
0.25
0.50
0.75
1.00
Active for 22781−23−3

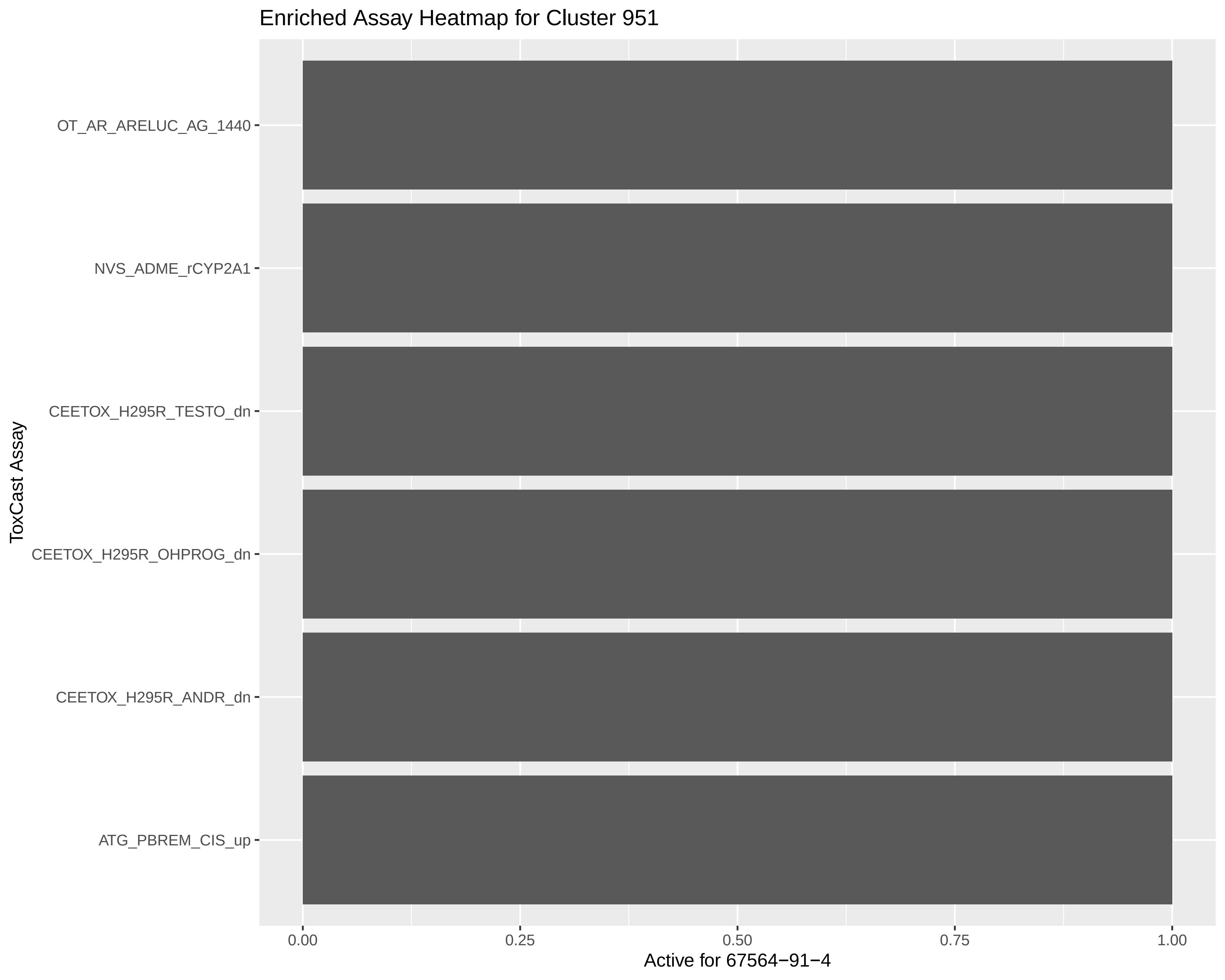
Enriched Assay Heatmap for Cluster 951
ToxCast Assay
OT_AR_ARELUC_AG_1440
NVS_ADME_rCYP2A1
CEETOX_H295R_TESTO_dn
CEETOX_H295R_OHPROG_dn
CEETOX_H295R_ANDR_dn
ATG_PBREM_CIS_up
0.00
0.25
0.50
0.75
1.00
Active for 67564−91−4

Enriched Assay Heatmap for Cluster 952

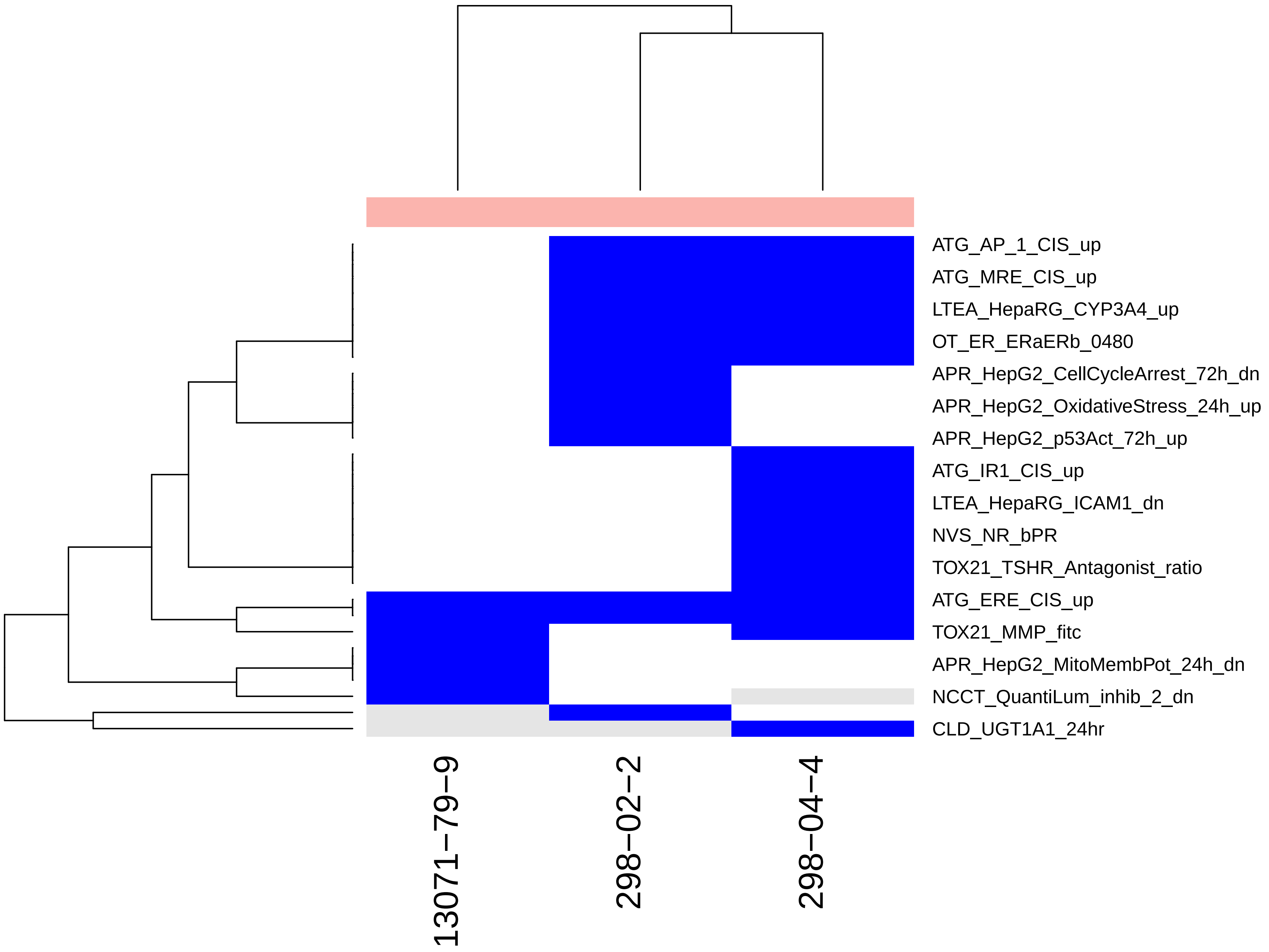

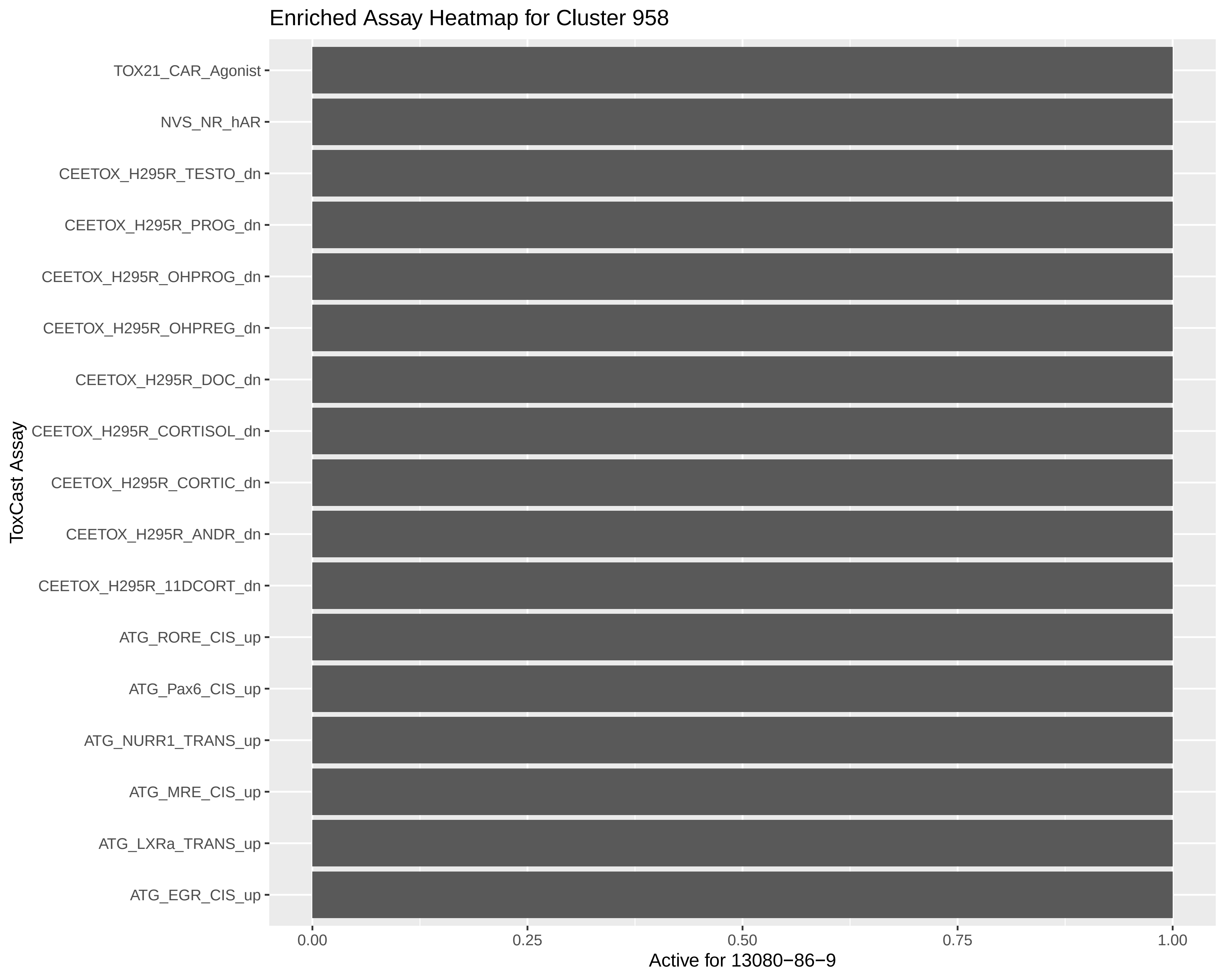

Enriched Assay Heatmap for Cluster 958
ToxCast Assay
TOX21_CAR_Agonist
NVS_NR_hAR
CEETOX_H295R_TESTO_dn
CEETOX_H295R_PROG_dn
CEETOX_H295R_OHPROG_dn
CEETOX_H295R_OHPREG_dn
CEETOX_H295R_DOC_dn
CEETOX_H295R_CORTISOL_dn
CEETOX_H295R_CORTIC_dn
CEETOX_H295R_ANDR_dn
CEETOX_H295R_11DCORT_dn
ATG_RORE_CIS_up
ATG_Pax6_CIS_up
ATG_NURR1_TRANS_up
ATG_MRE_CIS_up
ATG_LXRa_TRANS_up
ATG_EGR_CIS_up
0.00
0.25
0.50
0.75
1.00
Active for 13080−86−9

Enriched Assay Heatmap for Cluster 959

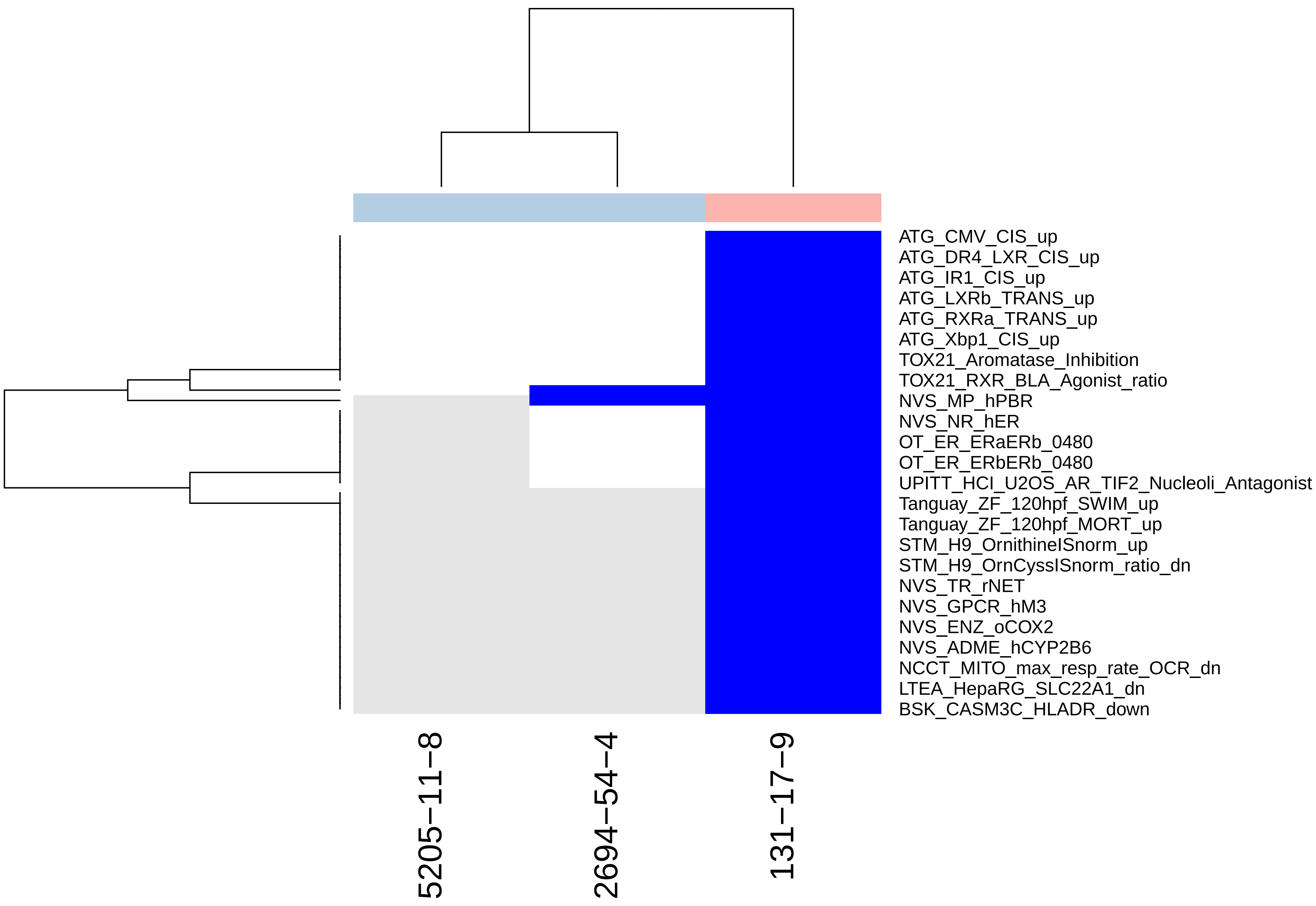

Enriched Assay Heatmap for Cluster 960

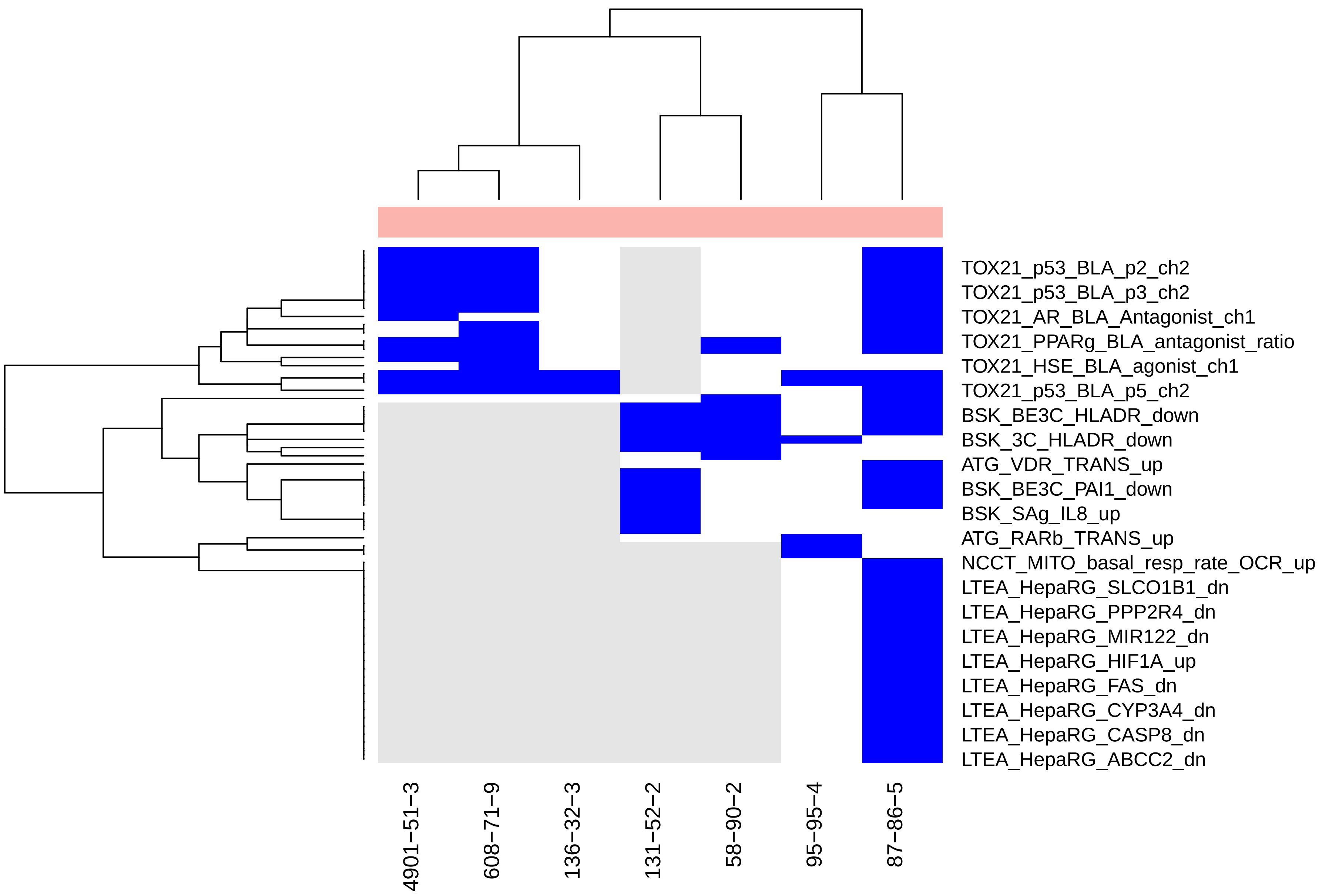

Enriched Assay Heatmap for Cluster 962

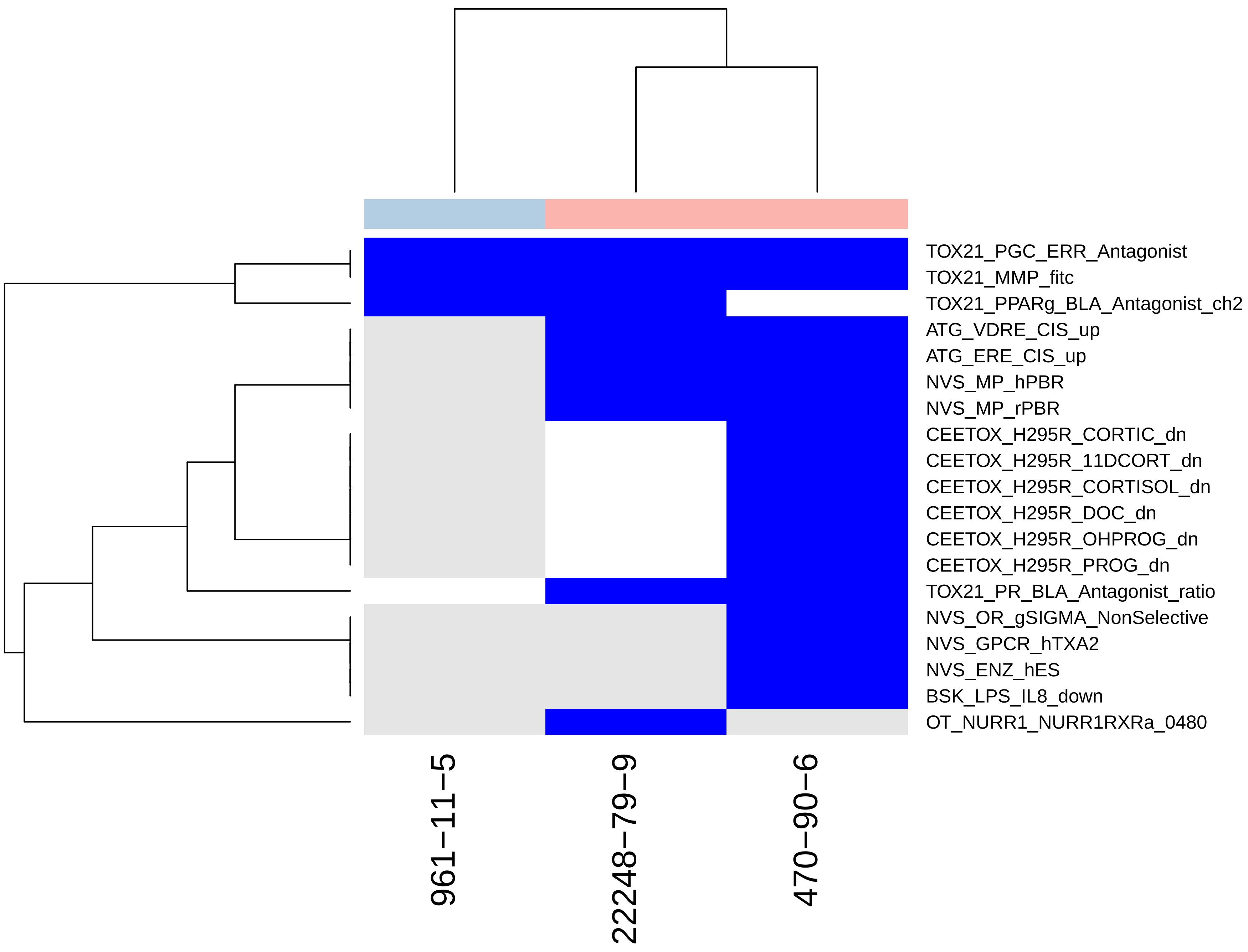

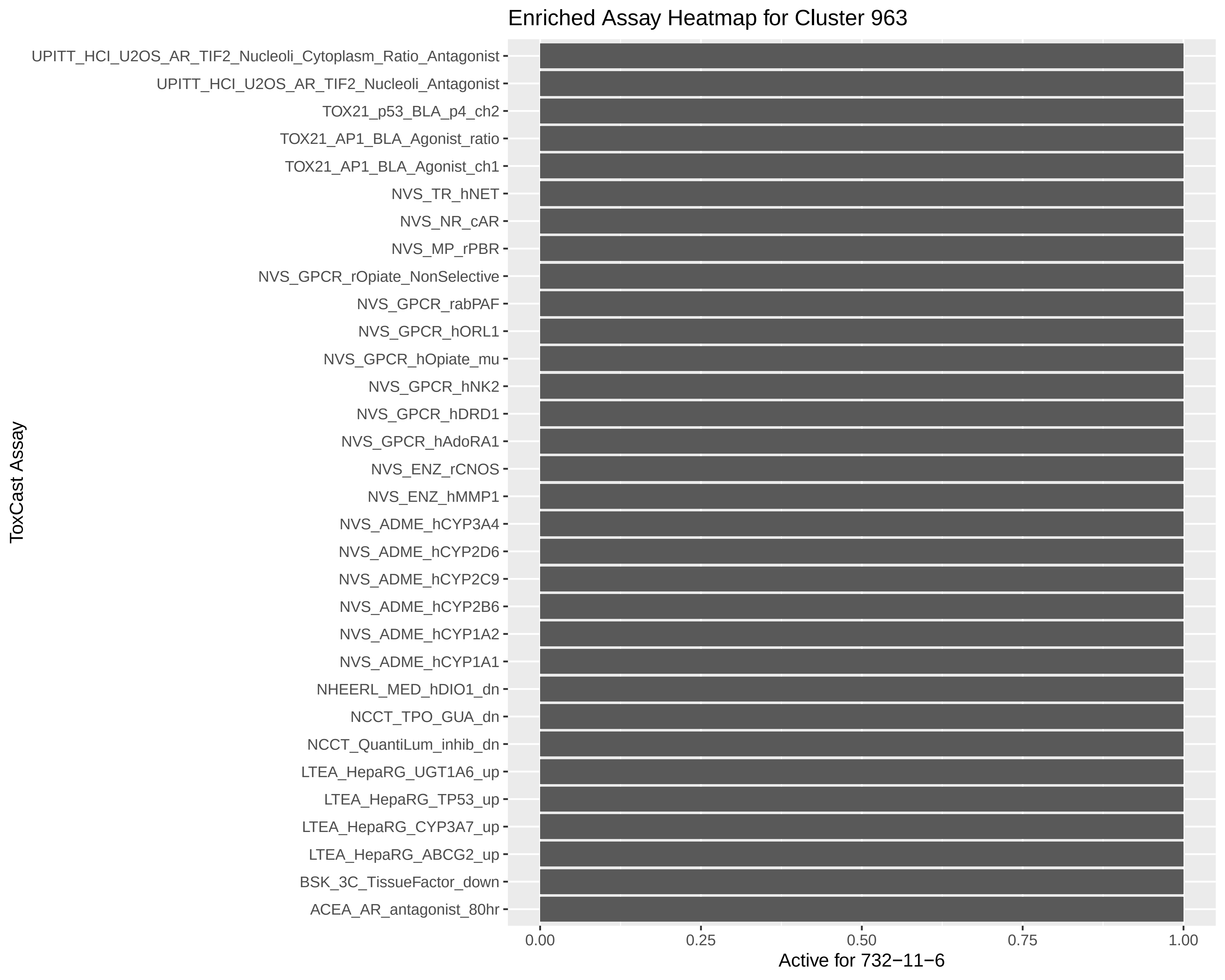
Enriched Assay Heatmap for Cluster 963
UPITT_HCI_U2OS_AR_TIF2_Nucleoli_Cytoplasm_Ratio_Antagonist
UPITT_HCI_U2OS_AR_TIF2_Nucleoli_Antagonist
TOX21_p53_BLA_p4_ch2
TOX21_AP1_BLA_Agonist_ratio
TOX21_AP1_BLA_Agonist_ch1
NVS_TR_hNET
NVS_NR_cAR
NVS_MP_rPBR
NVS_GPCR_rOpiate_NonSelective
NVS_GPCR_rabPAF
NVS_GPCR_hORL1
NVS_GPCR_hOpiate_mu
NVS_GPCR_hNK2
NVS_GPCR_hDRD1
NVS_GPCR_hAdoRA1
NVS_ENZ_rCNOS
NVS_ENZ_hMMP1
NVS_ADME_hCYP3A4
NVS_ADME_hCYP2D6
NVS_ADME_hCYP2C9
NVS_ADME_hCYP2B6
NVS_ADME_hCYP1A2
NVS_ADME_hCYP1A1
NHEERL_MED_hDIO1_dn
NCCT_TPO_GUA_dn
NCCT_QuantiLum_inhib_dn
LTEA_HepaRG_UGT1A6_up
LTEA_HepaRG_TP53_up
LTEA_HepaRG_CYP3A7_up
LTEA_HepaRG_ABCG2_up
BSK_3C_TissueFactor_down
ACEA_AR_antagonist_80hr
ToxCast Assay
0.00
0.25
0.50
0.75
1.00
Active for 732−11−6

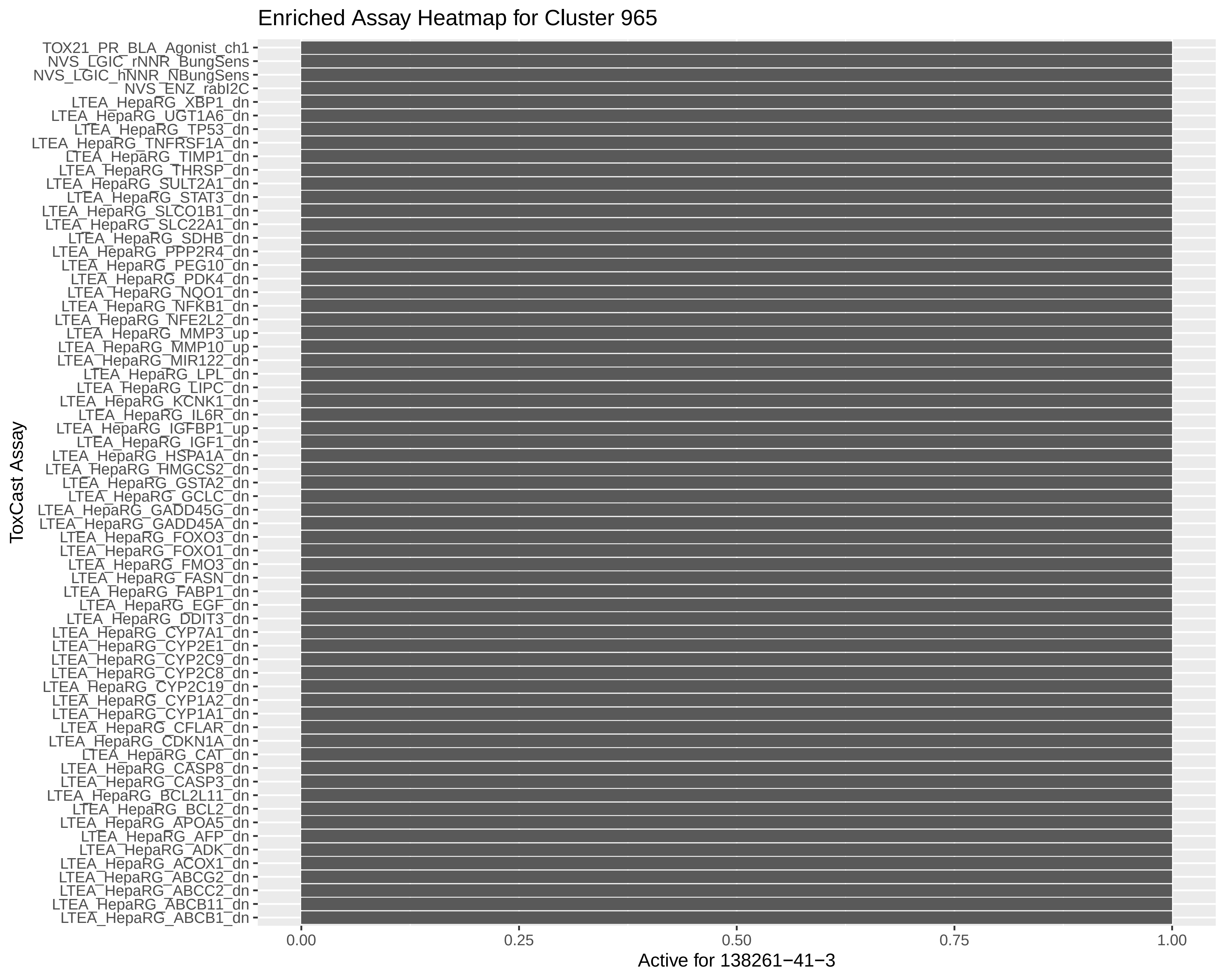
Enriched Assay Heatmap for Cluster 965
ToxCast Assay
TOX21_PR_BLA_Agonist_ch1
NVS_LGIC_rNNR_BungSens
NVS_LGIC_hNNR_NBungSens
NVS_ENZ_rabI2C
LTEA_HepaRG_XBP1_dn
LTEA_HepaRG_UGT1A6_dn
LTEA_HepaRG_TP53_dn
LTEA_HepaRG_TNFRSF1A_dn
LTEA_HepaRG_TIMP1_dn
LTEA_HepaRG_THRSP_dn
LTEA_HepaRG_SULT2A1_dn
LTEA_HepaRG_STAT3_dn
LTEA_HepaRG_SLCO1B1_dn
LTEA_HepaRG_SLC22A1_dn
LTEA_HepaRG_SDHB_dn
LTEA_HepaRG_PPP2R4_dn
LTEA_HepaRG_PEG10_dn
LTEA_HepaRG_PDK4_dn
LTEA_HepaRG_NQO1_dn
LTEA_HepaRG_NFKB1_dn
LTEA_HepaRG_NFE2L2_dn
LTEA_HepaRG_MMP3_up
LTEA_HepaRG_MMP10_up
LTEA_HepaRG_MIR122_dn
LTEA_HepaRG_LPL_dn
LTEA_HepaRG_LIPC_dn
LTEA_HepaRG_KCNK1_dn
LTEA_HepaRG_IL6R_dn
LTEA_HepaRG_IGFBP1_up
LTEA_HepaRG_IGF1_dn
LTEA_HepaRG_HSPA1A_dn
LTEA_HepaRG_HMGCS2_dn
LTEA_HepaRG_GSTA2_dn
LTEA_HepaRG_GCLC_dn
LTEA_HepaRG_GADD45G_dn
LTEA_HepaRG_GADD45A_dn
LTEA_HepaRG_FOXO3_dn
LTEA_HepaRG_FOXO1_dn
LTEA_HepaRG_FMO3_dn
LTEA_HepaRG_FASN_dn
LTEA_HepaRG_FABP1_dn
LTEA_HepaRG_EGF_dn
LTEA_HepaRG_DDIT3_dn
LTEA_HepaRG_CYP7A1_dn
LTEA_HepaRG_CYP2E1_dn
LTEA_HepaRG_CYP2C9_dn
LTEA_HepaRG_CYP2C8_dn
LTEA_HepaRG_CYP2C19_dn
LTEA_HepaRG_CYP1A2_dn
LTEA_HepaRG_CYP1A1_dn
LTEA_HepaRG_CFLAR_dn
LTEA_HepaRG_CDKN1A_dn
LTEA_HepaRG_CAT_dn
LTEA_HepaRG_CASP8_dn
LTEA_HepaRG_CASP3_dn
LTEA_HepaRG_BCL2L11_dn
LTEA_HepaRG_BCL2_dn
LTEA_HepaRG_APOA5_dn
LTEA_HepaRG_AFP_dn
LTEA_HepaRG_ADK_dn
LTEA_HepaRG_ACOX1_dn
LTEA_HepaRG_ABCG2_dn
LTEA_HepaRG_ABCC2_dn
LTEA_HepaRG_ABCB11_dn
LTEA_HepaRG_ABCB1_dn
0.00
0.25
0.50
0.75
1.00
Active for 138261−41−3

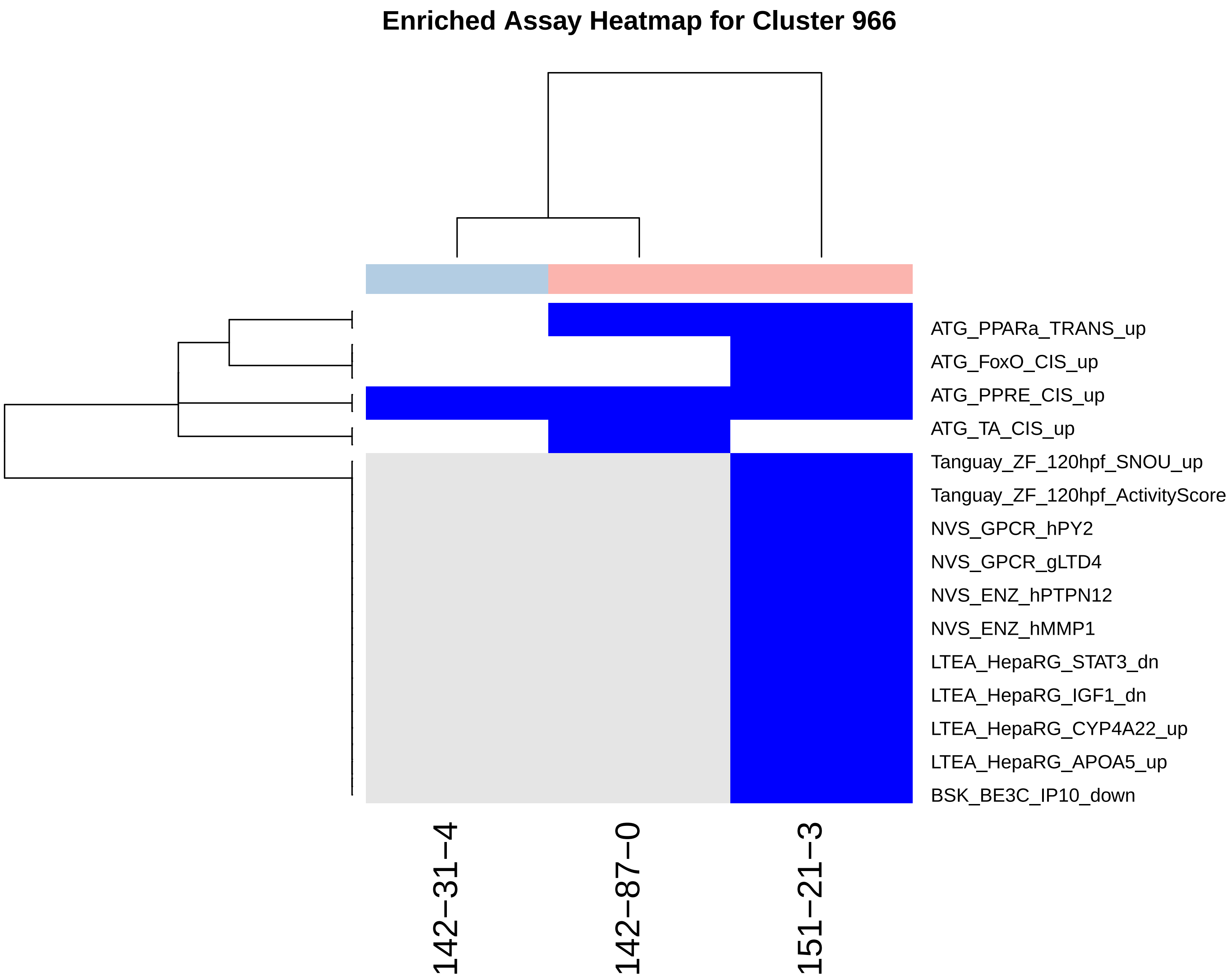
Enriched Assay Heatmap for Cluster 966
ATG_PPARa_TRANS_up
ATG_FoxO_CIS_up
ATG_PPRE_CIS_up
ATG_TA_CIS_up
Tanguay_ZF_120hpf_SNOU_up
Tanguay_ZF_120hpf_ActivityScore
NVS_GPCR_hPY2
NVS_GPCR_gLTD4
NVS_ENZ_hPTPN12
NVS_ENZ_hMMP1
LTEA_HepaRG_STAT3_dn
LTEA_HepaRG_IGF1_dn
LTEA_HepaRG_CYP4A22_up
LTEA_HepaRG_APOA5_up
BSK_BE3C_IP10_down
142−31−4
142−87−0
151−21−3

Enriched Assay Heatmap for Cluster 968

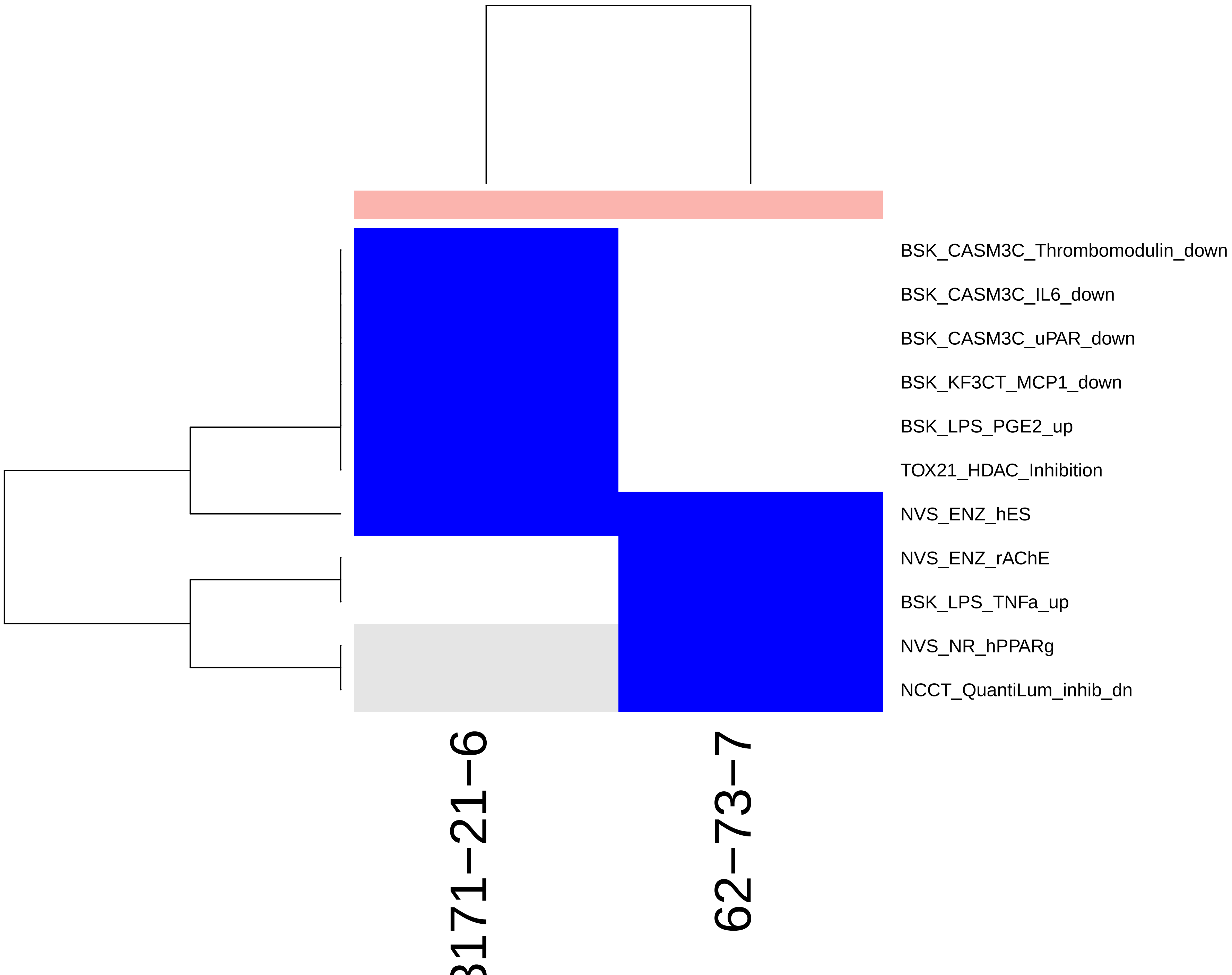

Enriched Assay Heatmap for Cluster 971

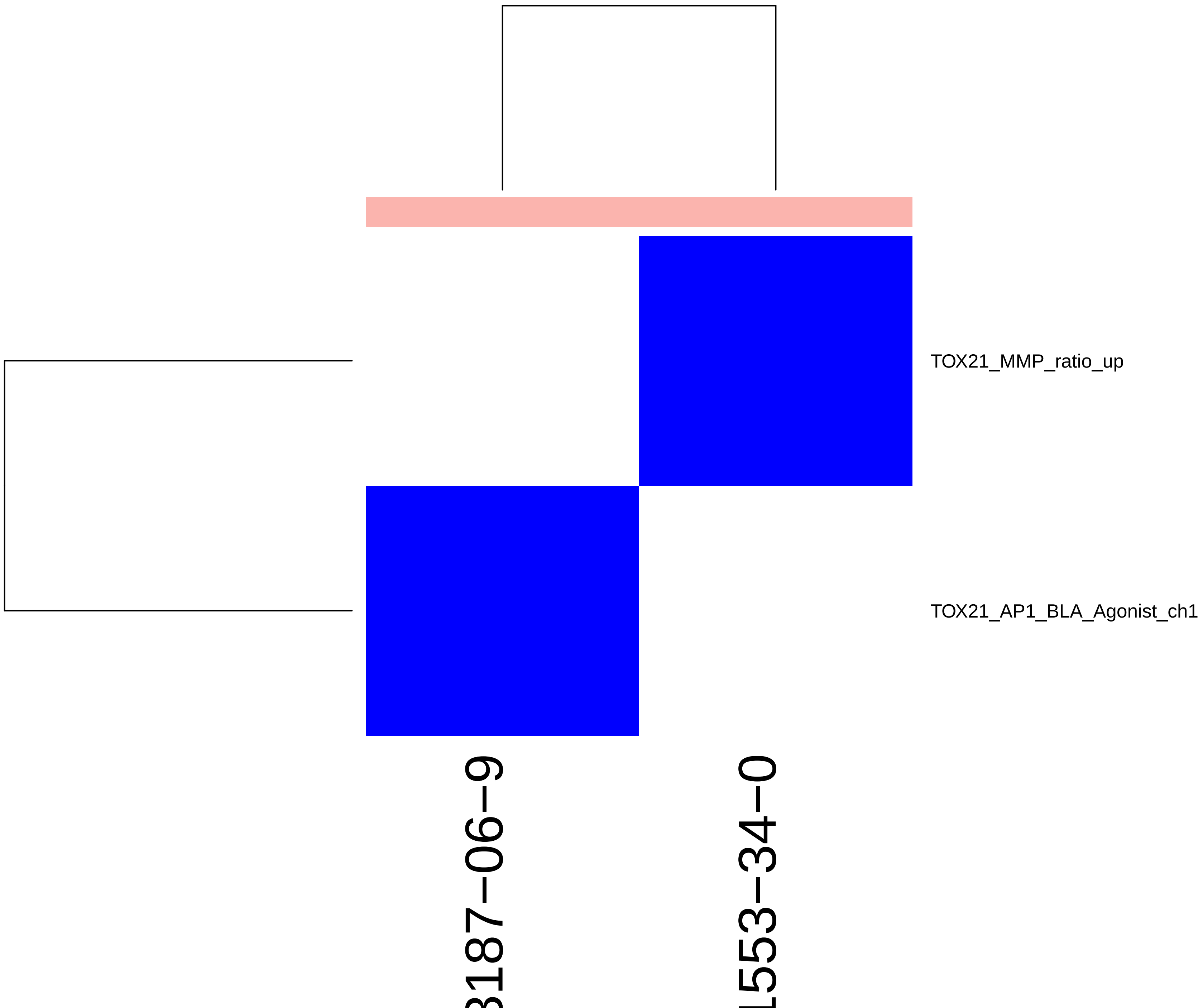

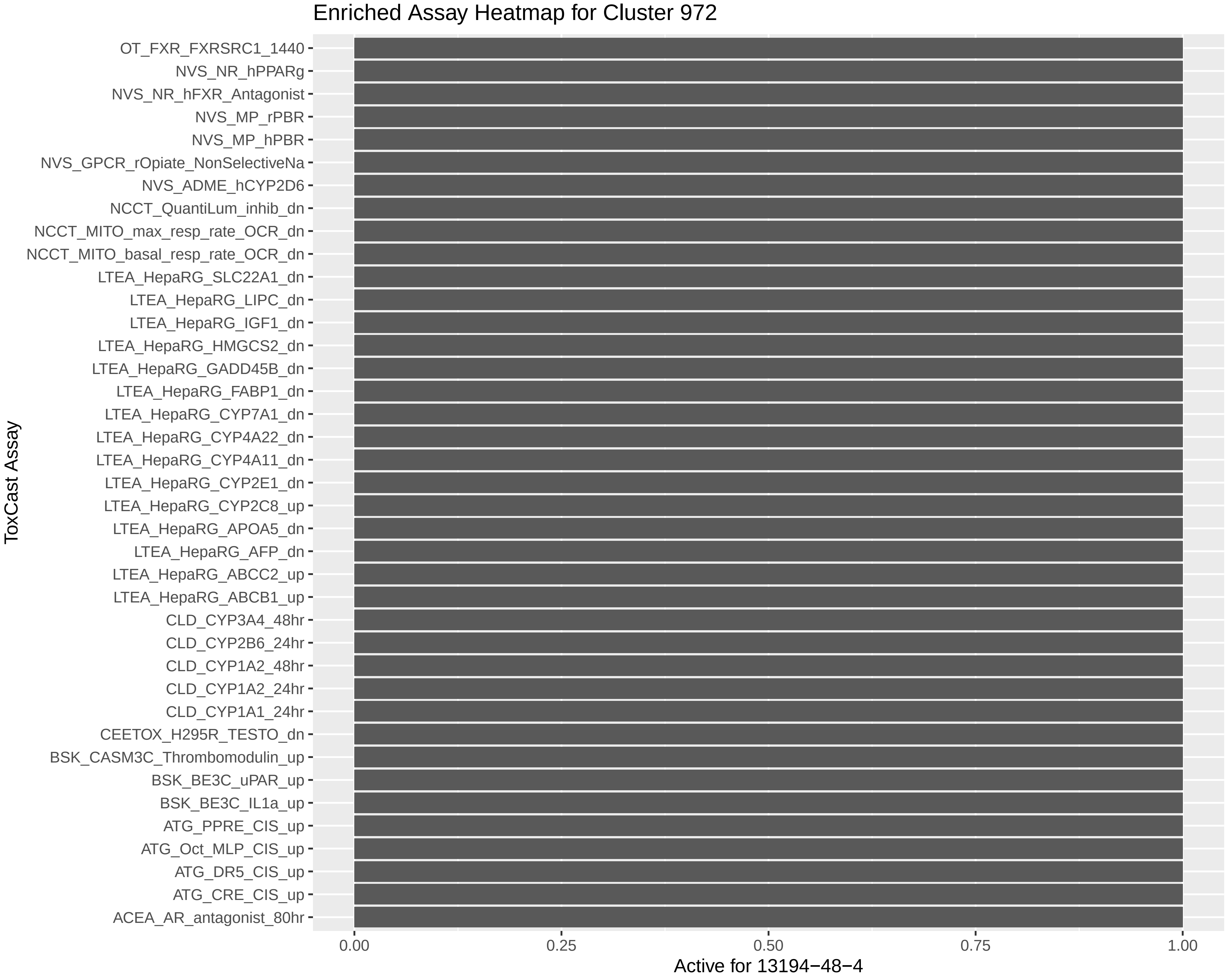
Enriched Assay Heatmap for Cluster 972
OT_FXR_FXRSRC1_1440
NVS_NR_hPPARg
NVS_NR_hFXR_Antagonist
NVS_MP_rPBR
NVS_MP_hPBR
NVS_GPCR_rOpiate_NonSelectiveNa
NVS_ADME_hCYP2D6
NCCT_QuantiLum_inhib_dn
NCCT_MITO_max_resp_rate_OCR_dn
NCCT_MITO_basal_resp_rate_OCR_dn
LTEA_HepaRG_SLC22A1_dn
LTEA_HepaRG_LIPC_dn
LTEA_HepaRG_IGF1_dn
LTEA_HepaRG_HMGCS2_dn
LTEA_HepaRG_GADD45B_dn
LTEA_HepaRG_FABP1_dn
LTEA_HepaRG_CYP7A1_dn
LTEA_HepaRG_CYP4A22_dn
LTEA_HepaRG_CYP4A11_dn
LTEA_HepaRG_CYP2E1_dn
LTEA_HepaRG_CYP2C8_up
LTEA_HepaRG_APOA5_dn
LTEA_HepaRG_AFP_dn
LTEA_HepaRG_ABCC2_up
LTEA_HepaRG_ABCB1_up
CLD_CYP3A4_48hr
CLD_CYP2B6_24hr
CLD_CYP1A2_48hr
CLD_CYP1A2_24hr
CLD_CYP1A1_24hr
CEETOX_H295R_TESTO_dn
BSK_CASM3C_Thrombomodulin_up
BSK_BE3C_uPAR_up
BSK_BE3C_IL1a_up
ATG_PPRE_CIS_up
ATG_Oct_MLP_CIS_up
ATG_DR5_CIS_up
ATG_CRE_CIS_up
ACEA_AR_antagonist_80hr
ToxCast Assay
0.00
0.25
0.50
0.75
1.00
Active for 13194−48−4

Enriched Assay Heatmap for Cluster 973

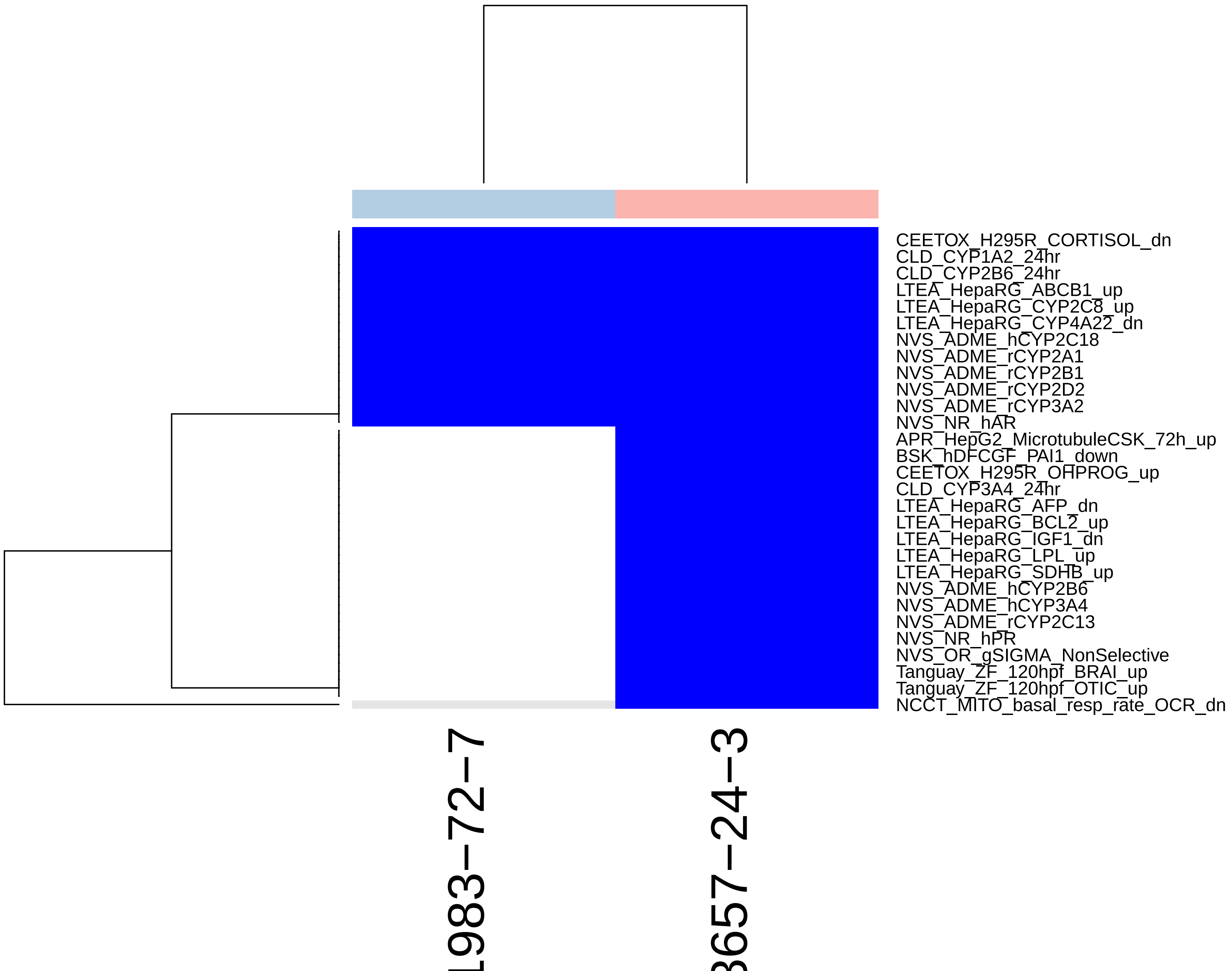

Enriched Assay Heatmap for Cluster 974

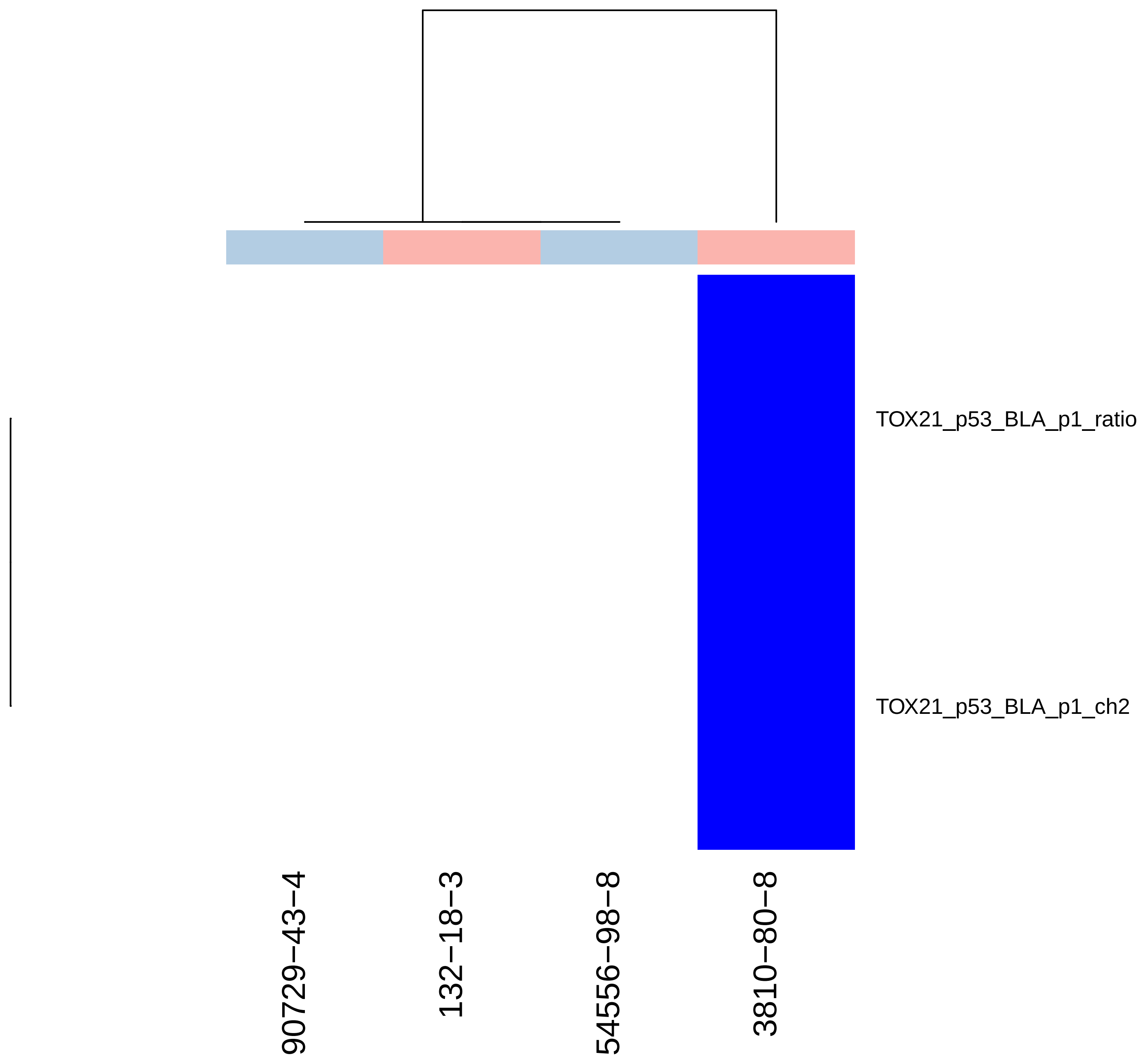

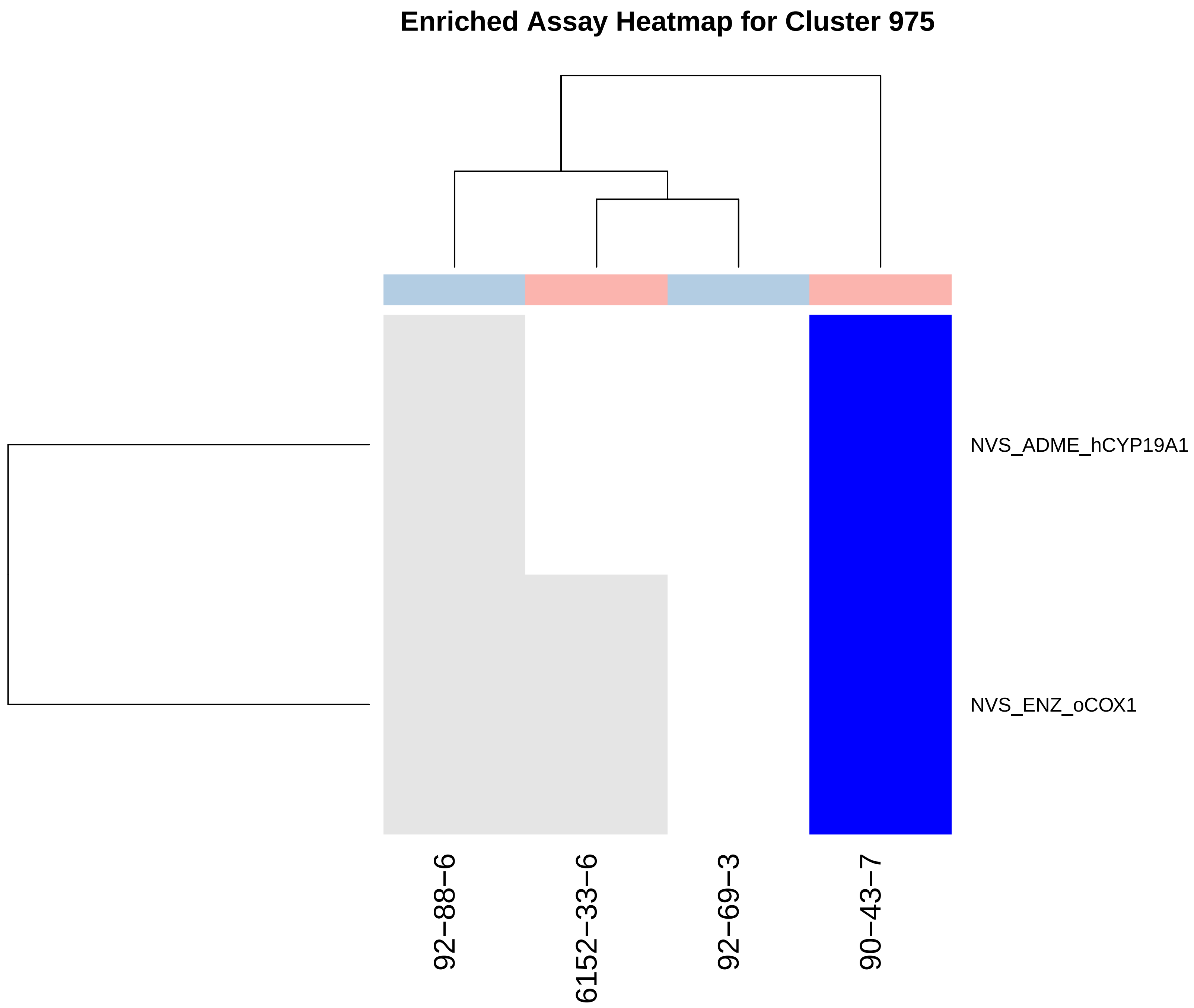
Enriched Assay Heatmap for Cluster 975
NVS_ADME_hCYP19A1
NVS_ENZ_oCOX1
92−88−6
6152−33−6
92−69−3
90−43−7

Enriched Assay Heatmap for Cluster 976

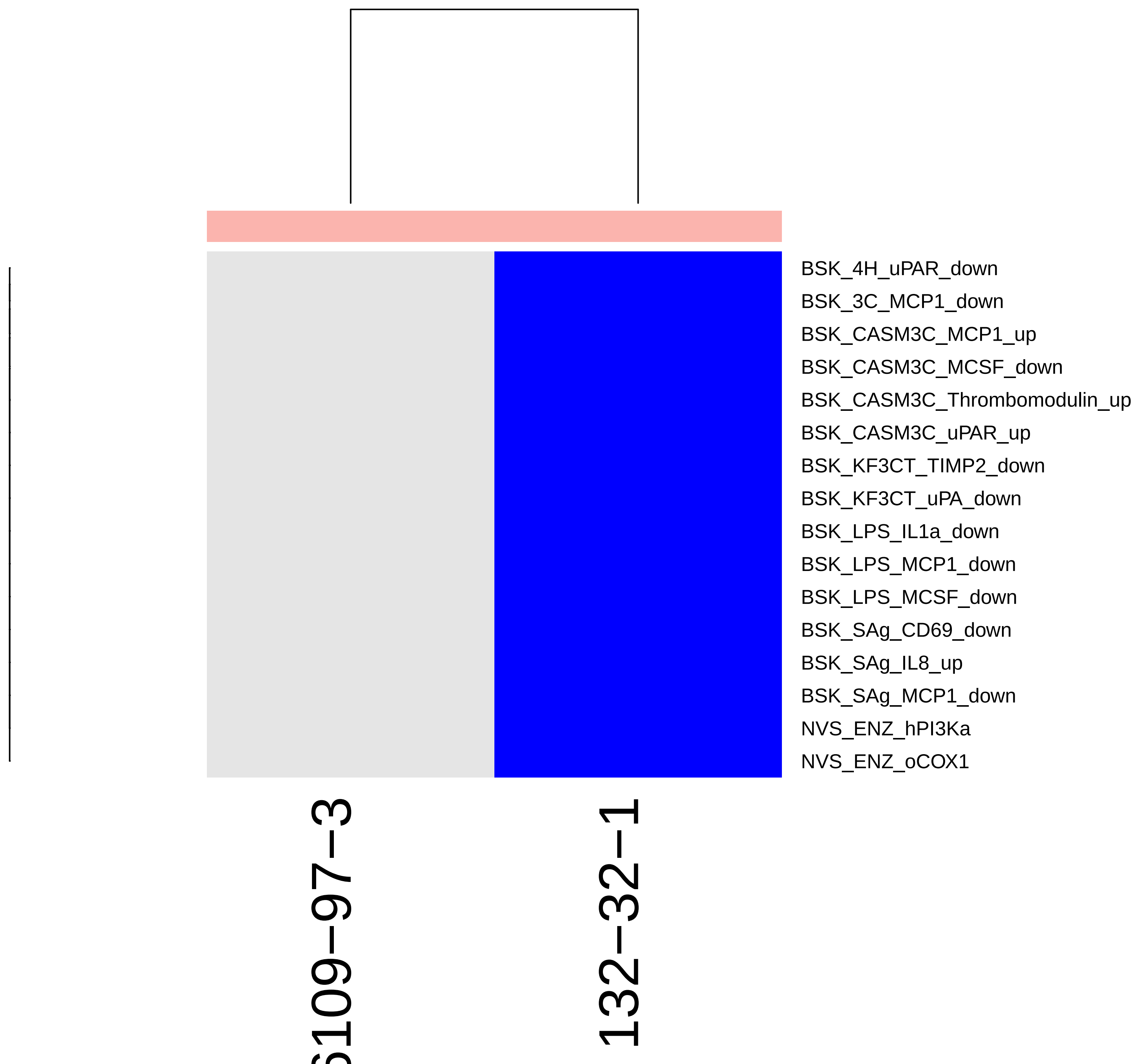

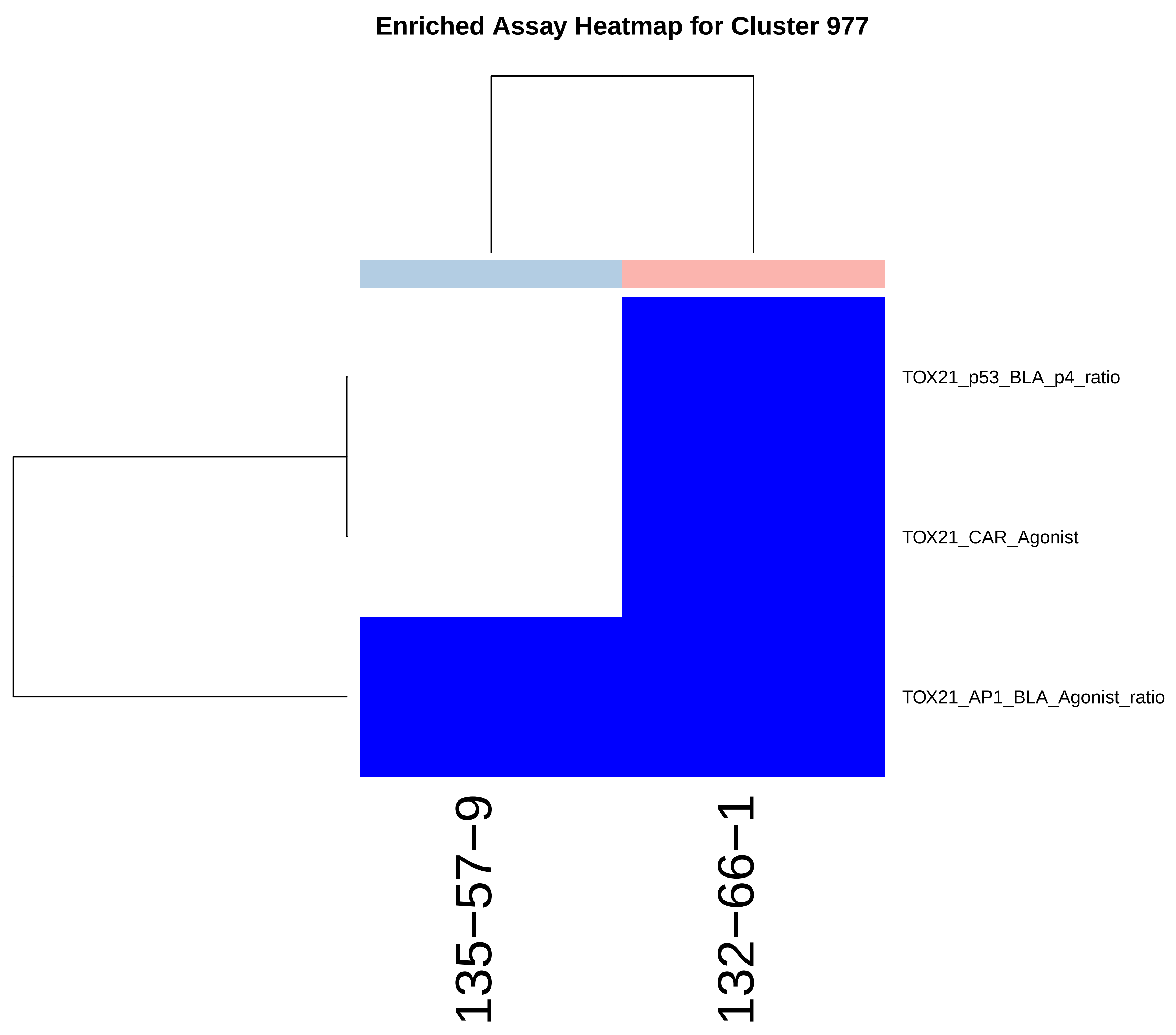
Enriched Assay Heatmap for Cluster 977
TOX21_p53_BLA_p4_ratio
TOX21_CAR_Agonist
TOX21_AP1_BLA_Agonist_ratio
135−57−9
132−66−1

Enriched Assay Heatmap for Cluster 981

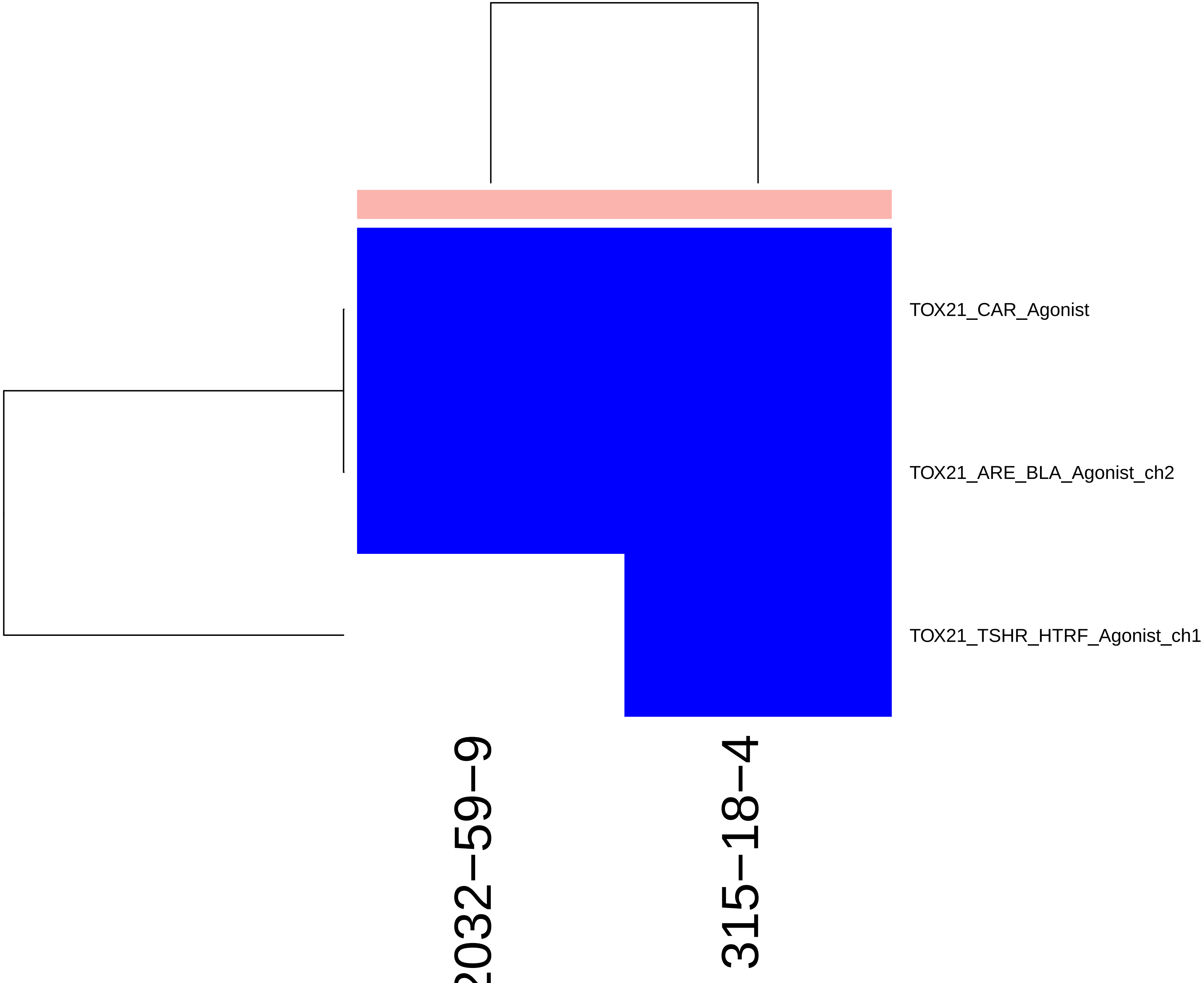

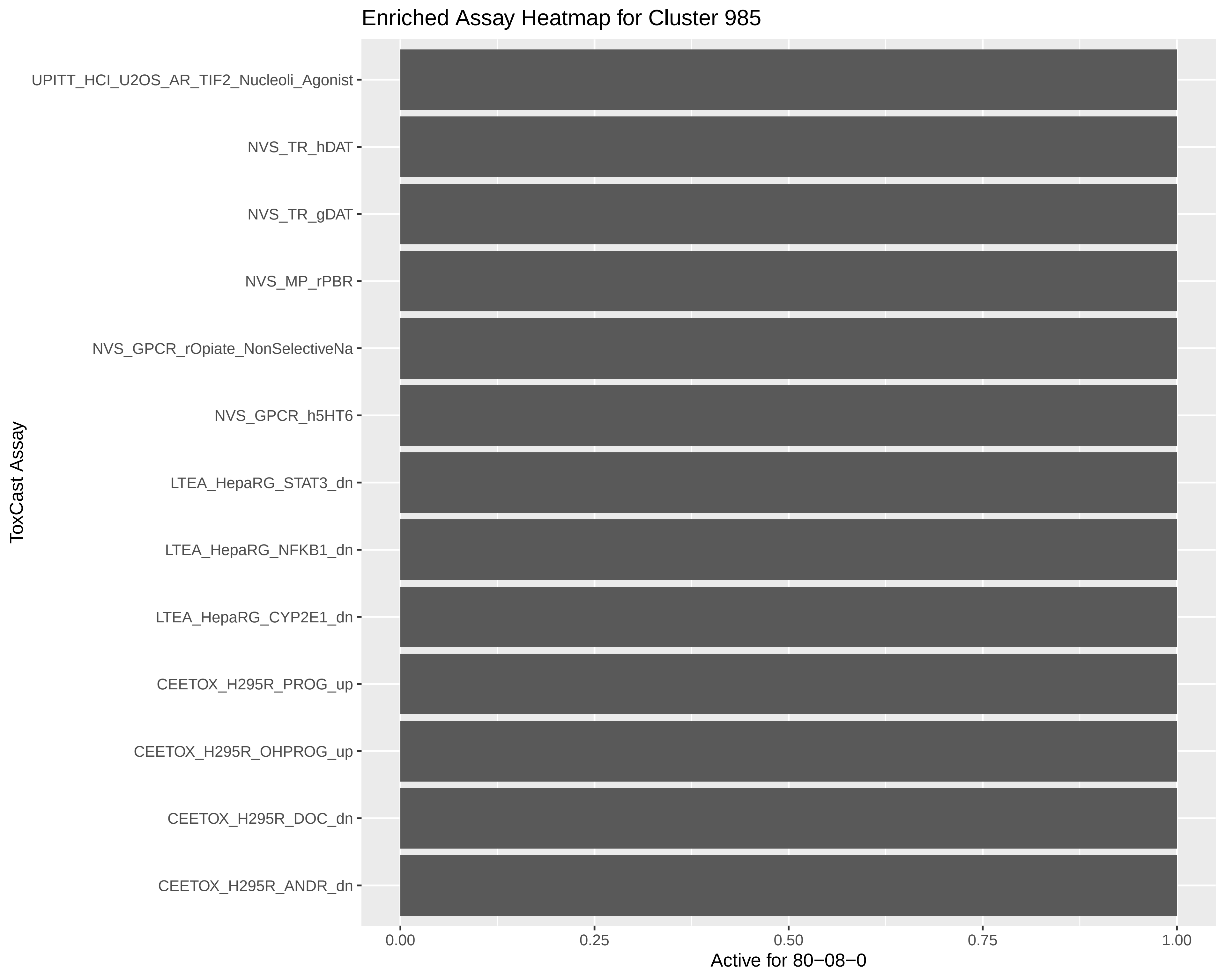

Enriched Assay Heatmap for Cluster 985
UPITT_HCI_U2OS_AR_TIF2_Nucleoli_Agonist
NVS_TR_hDAT
NVS_TR_gDAT
NVS_MP_rPBR
NVS_GPCR_rOpiate_NonSelectiveNa
NVS_GPCR_h5HT6
LTEA_HepaRG_STAT3_dn
LTEA_HepaRG_NFKB1_dn
LTEA_HepaRG_CYP2E1_dn
CEETOX_H295R_PROG_up
CEETOX_H295R_OHPROG_up
CEETOX_H295R_DOC_dn
CEETOX_H295R_ANDR_dn
ToxCast Assay
0.00
0.25
0.50
0.75
1.00
Active for 80−08−0

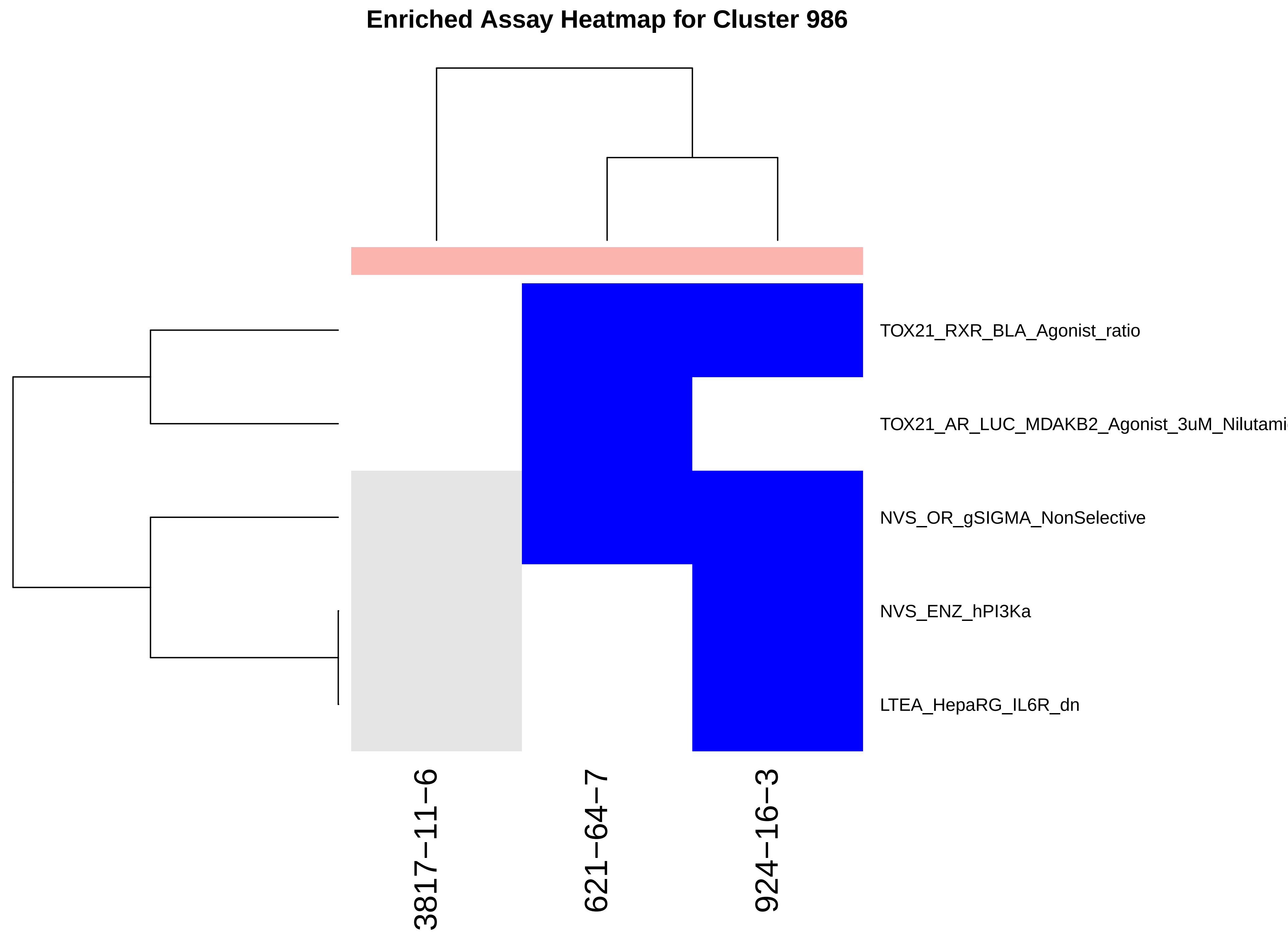
Enriched Assay Heatmap for Cluster 986
TOX21_RXR_BLA_Agonist_ratio
TOX21_AR_LUC_MDAKB2_Agonist_3uM_Nilutami
NVS_OR_gSIGMA_NonSelective
NVS_ENZ_hPI3Ka
LTEA_HepaRG_IL6R_dn
3817−11−6
621−64−7
924−16−3

Enriched Assay Heatmap for Cluster 992

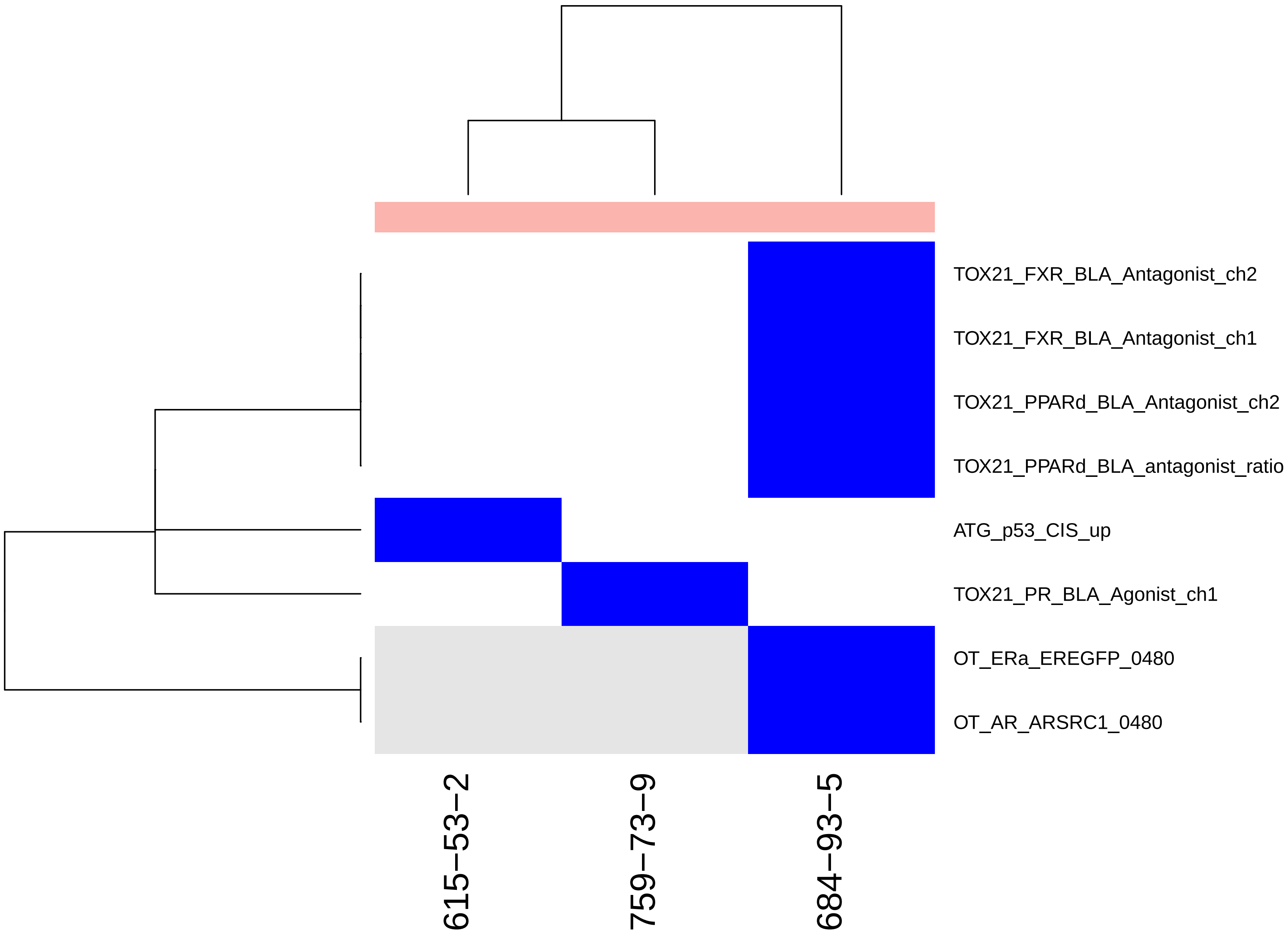

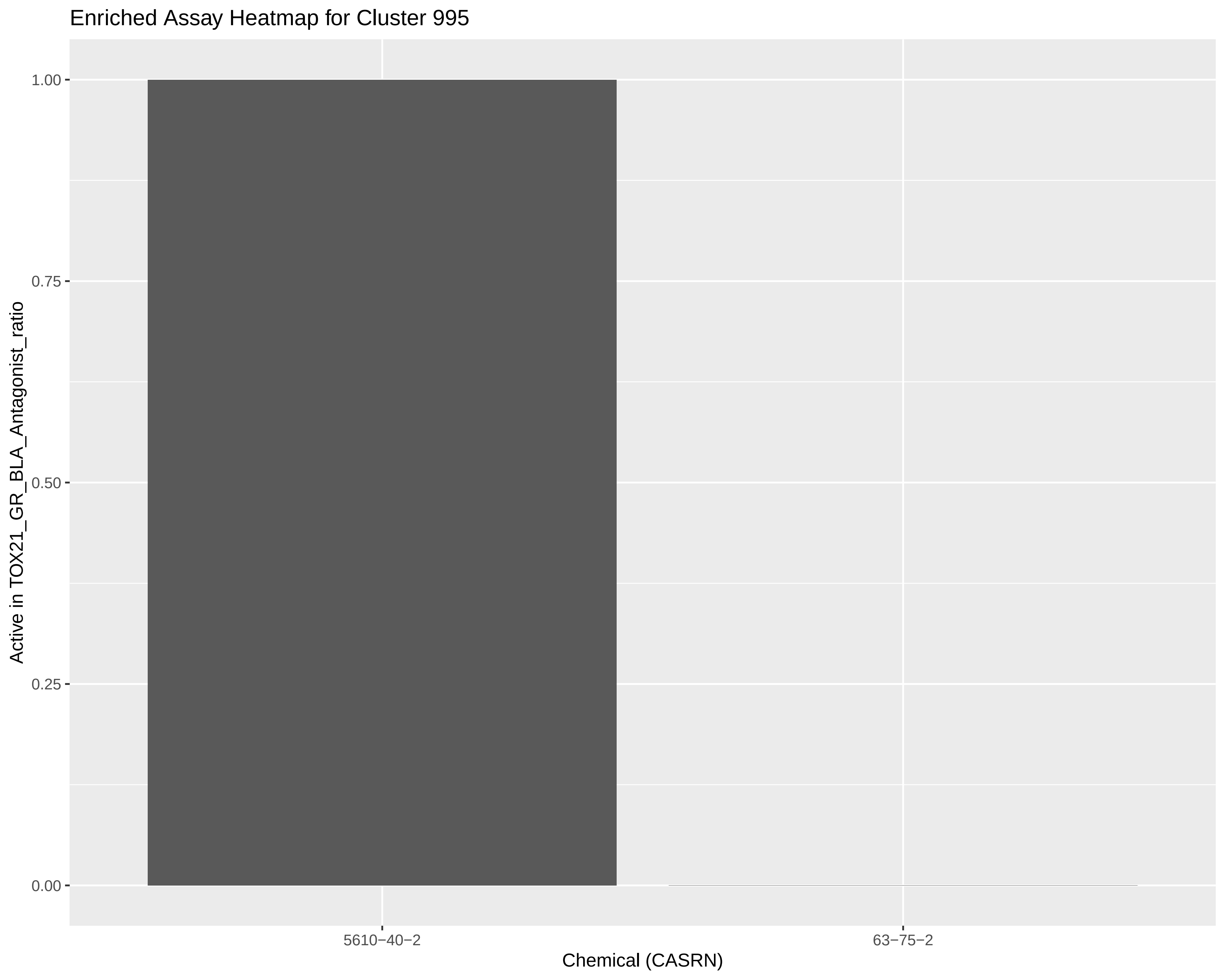
Enriched Assay Heatmap for Cluster 995
Active in TOX21_GR_BLA_Antagonist_ratio
1.00
0.75
0.50
0.25
0.00
5610−40−2
63−75−2
Chemical (CASRN)

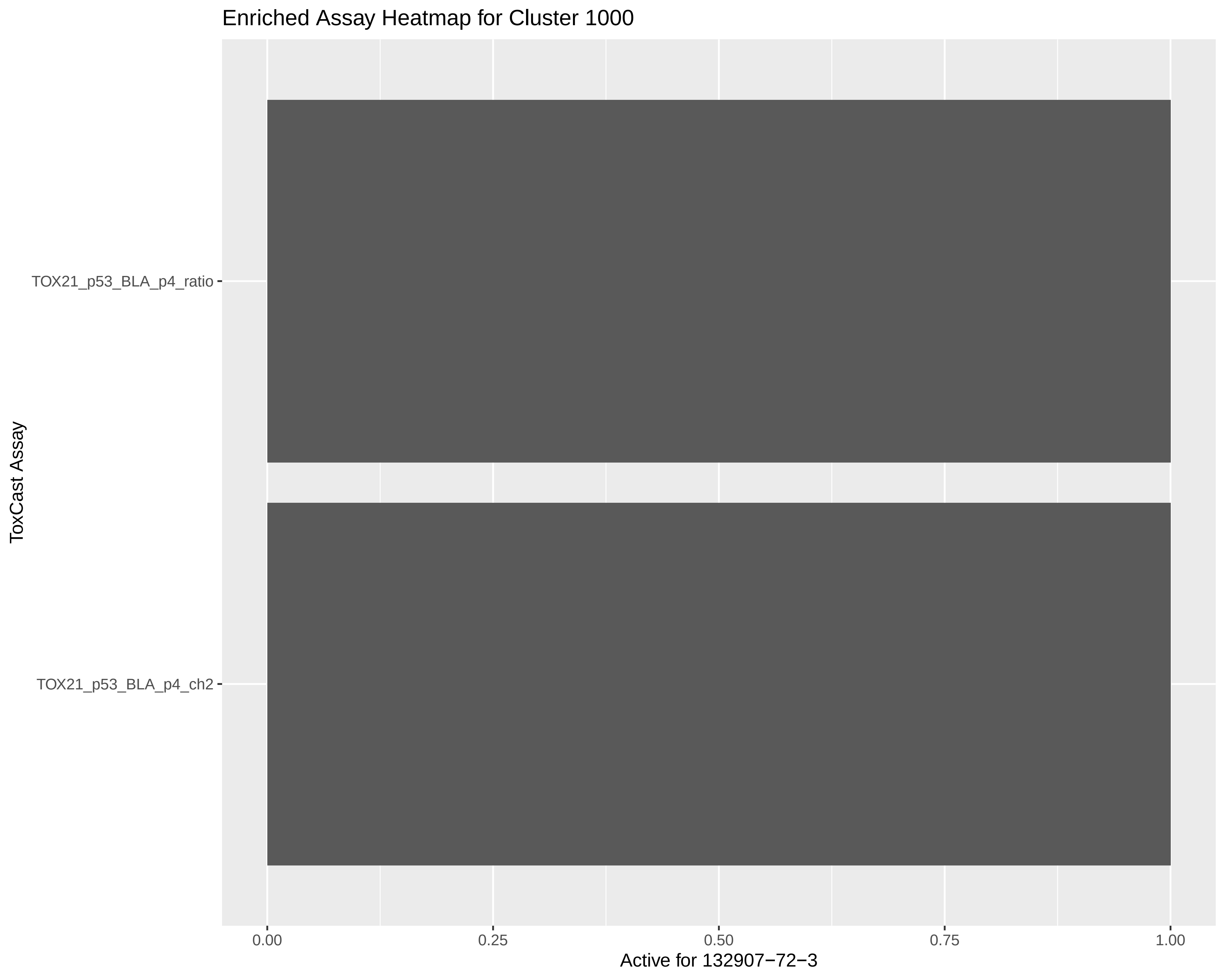

Enriched Assay Heatmap for Cluster 1000
ToxCast Assay
TOX21_p53_BLA_p4_ratio
TOX21_p53_BLA_p4_ch2
0.00
0.25
0.50
0.75
1.00
Active for 132907−72−3

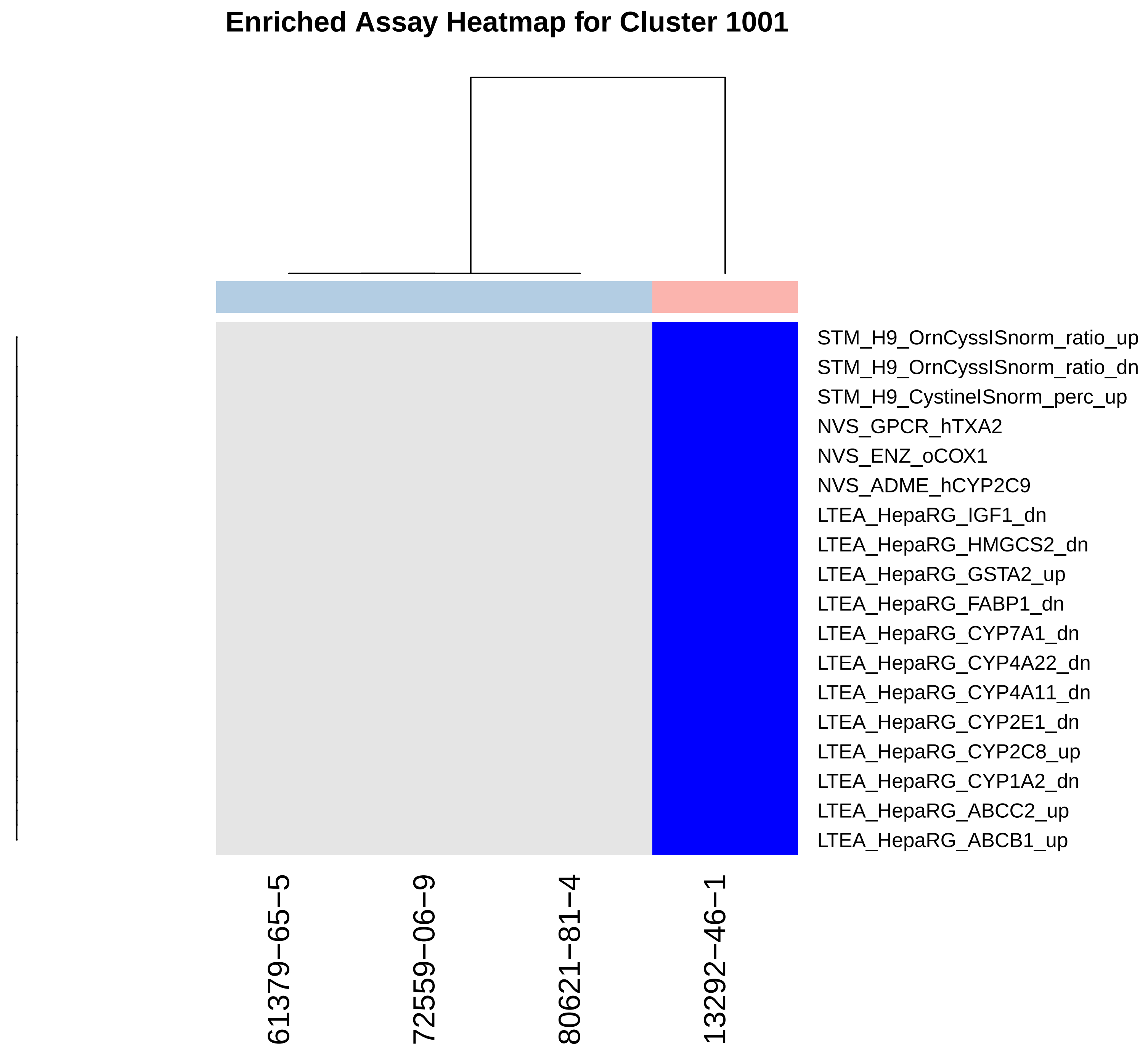
Enriched Assay Heatmap for Cluster 1001
STM_H9_OrnCyssISnorm_ratio_up
STM_H9_OrnCyssISnorm_ratio_dn
STM_H9_CystineISnorm_perc_up
NVS_GPCR_hTXA2
NVS_ENZ_oCOX1
NVS_ADME_hCYP2C9
LTEA_HepaRG_IGF1_dn
LTEA_HepaRG_HMGCS2_dn
LTEA_HepaRG_GSTA2_up
LTEA_HepaRG_FABP1_dn
LTEA_HepaRG_CYP7A1_dn
LTEA_HepaRG_CYP4A22_dn
LTEA_HepaRG_CYP4A11_dn
LTEA_HepaRG_CYP2E1_dn
LTEA_HepaRG_CYP2C8_up
LTEA_HepaRG_CYP1A2_dn
LTEA_HepaRG_ABCC2_up
LTEA_HepaRG_ABCB1_up
61379−65−5
72559−06−9
80621−81−4
13292−46−1

Enriched Assay Heatmap for Cluster 1010

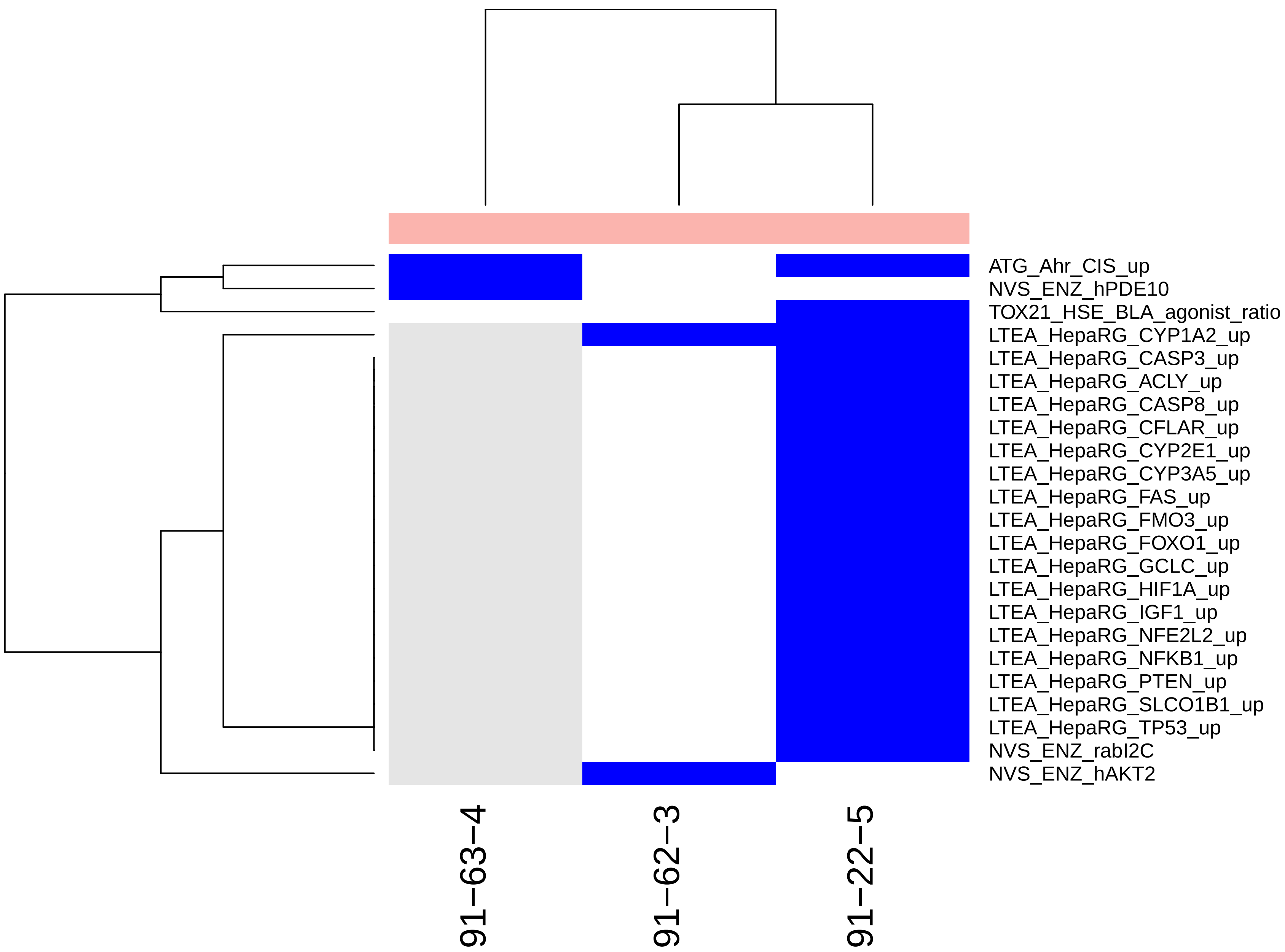

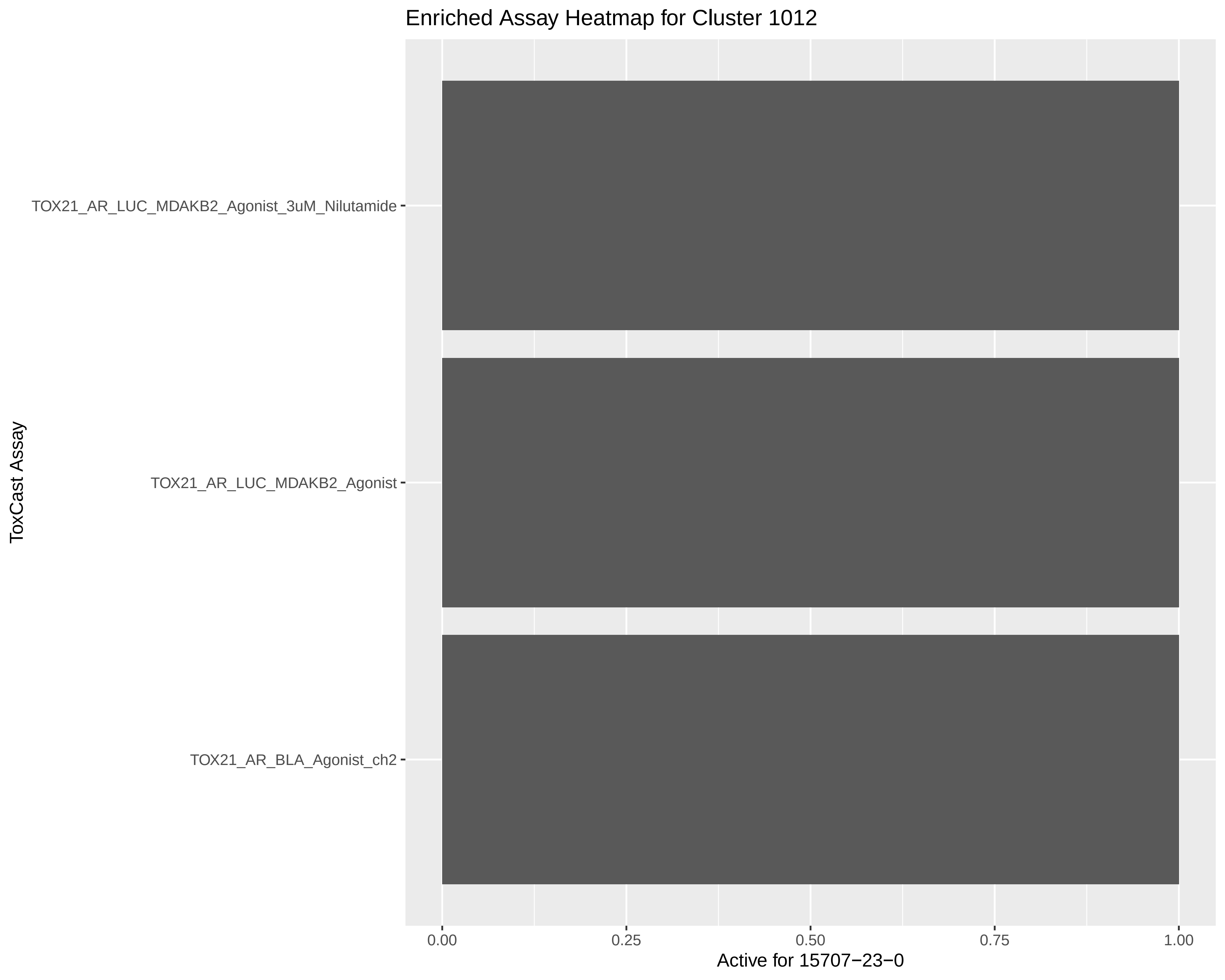

Enriched Assay Heatmap for Cluster 1012
ToxCast Assay
TOX21_AR_LUC_MDAKB2_Agonist_3uM_Nilutamide
TOX21_AR_LUC_MDAKB2_Agonist
TOX21_AR_BLA_Agonist_ch2
0.00
0.25
0.50
0.75
1.00
Active for 15707−23−0

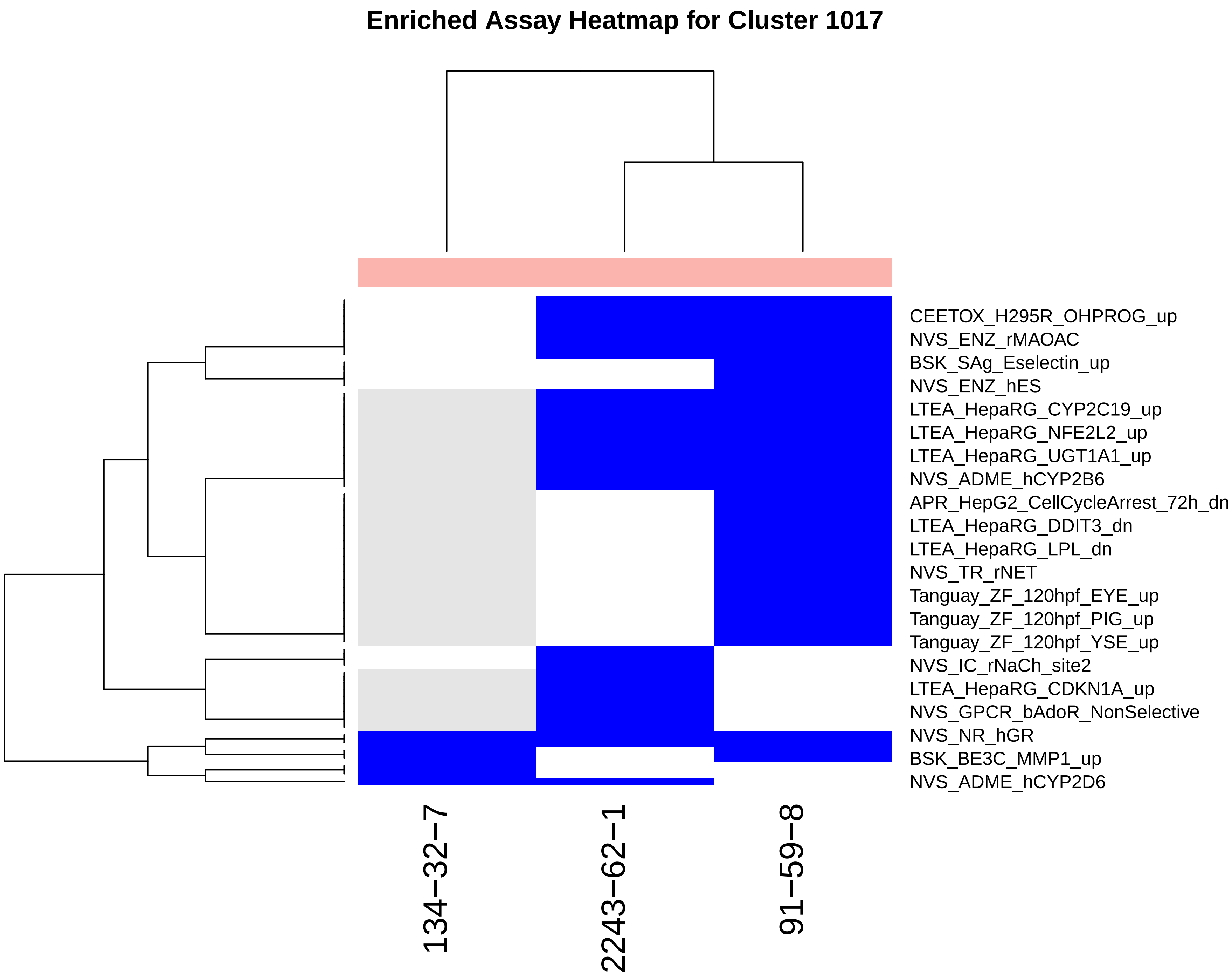
Enriched Assay Heatmap for Cluster 1017
CEETOX_H295R_OHPROG_up
NVS_ENZ_rMAOAC
BSK_SAg_Eselectin_up
NVS_ENZ_hES
LTEA_HepaRG_CYP2C19_up
LTEA_HepaRG_NFE2L2_up
LTEA_HepaRG_UGT1A1_up
NVS_ADME_hCYP2B6
APR_HepG2_CellCycleArrest_72h_dn
LTEA_HepaRG_DDIT3_dn
LTEA_HepaRG_LPL_dn
NVS_TR_rNET
Tanguay_ZF_120hpf_EYE_up
Tanguay_ZF_120hpf_PIG_up
Tanguay_ZF_120hpf_YSE_up
NVS_IC_rNaCh_site2
LTEA_HepaRG_CDKN1A_up
NVS_GPCR_bAdoR_NonSelective
NVS_NR_hGR
BSK_BE3C_MMP1_up
NVS_ADME_hCYP2D6
134−32−7
2243−62−1
91−59−8

Enriched Assay Heatmap for Cluster 1018

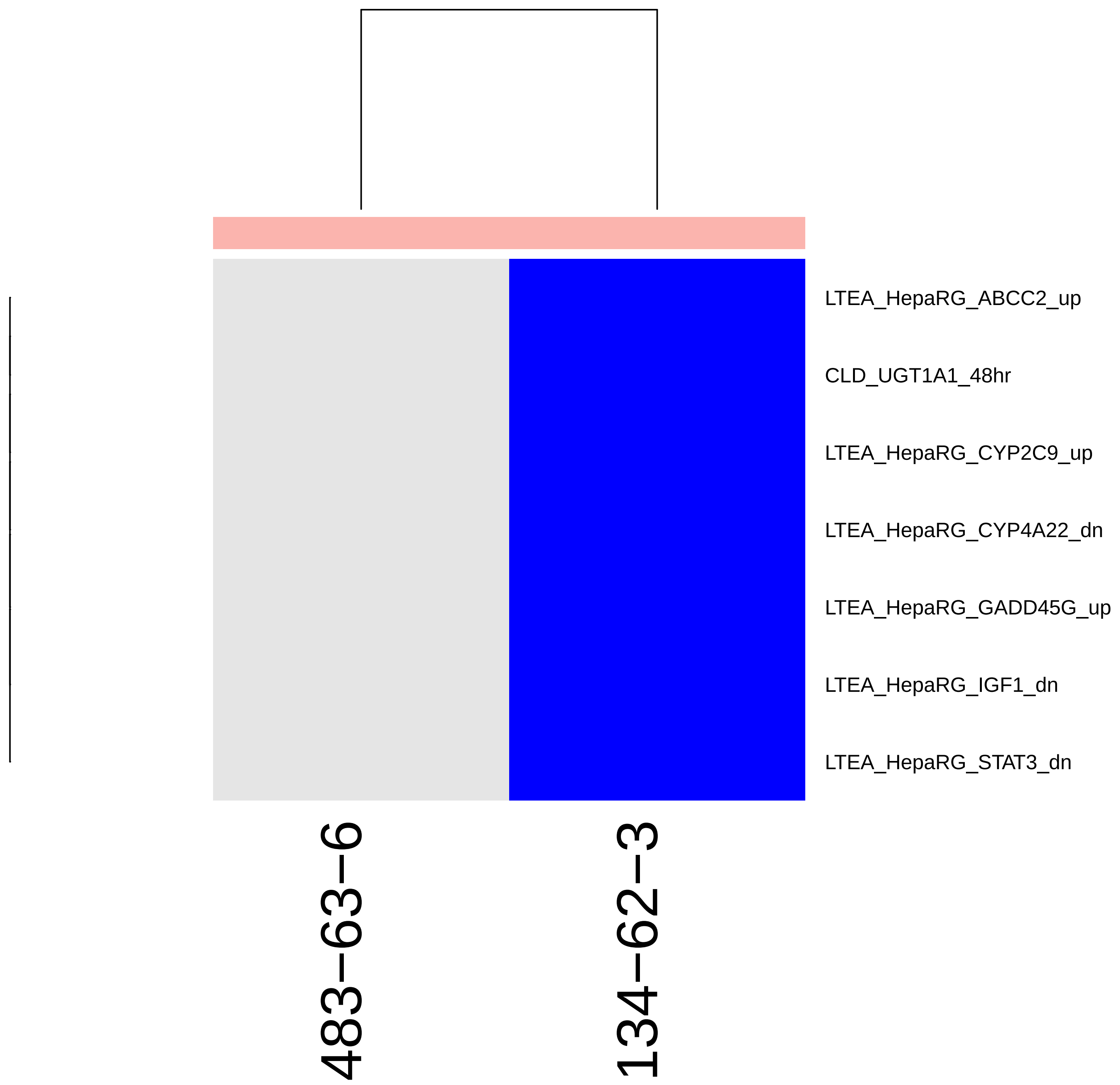

Enriched Assay Heatmap for Cluster 1020

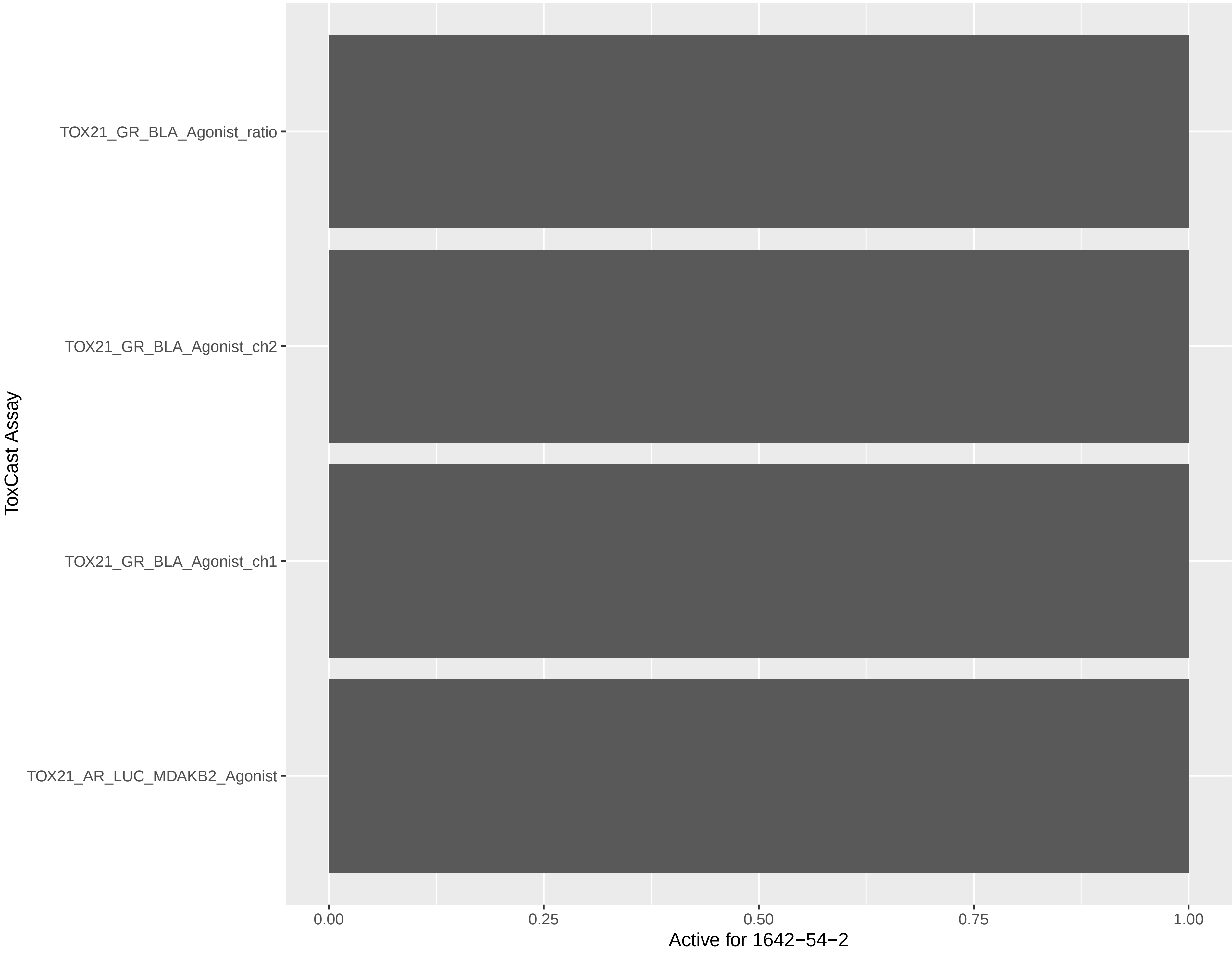

Enriched Assay Heatmap for Cluster 1026

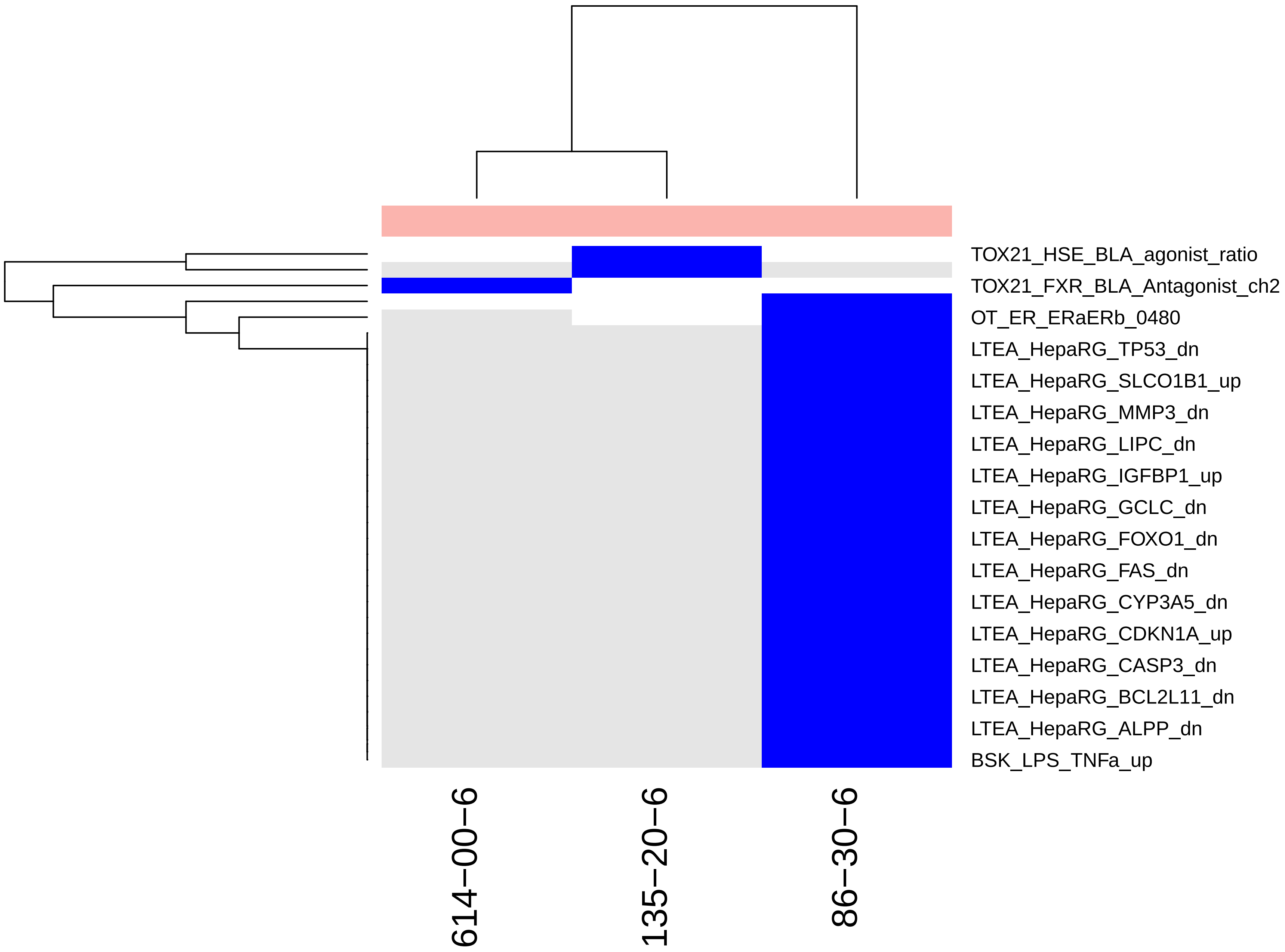

Enriched Assay Heatmap for Cluster 1035

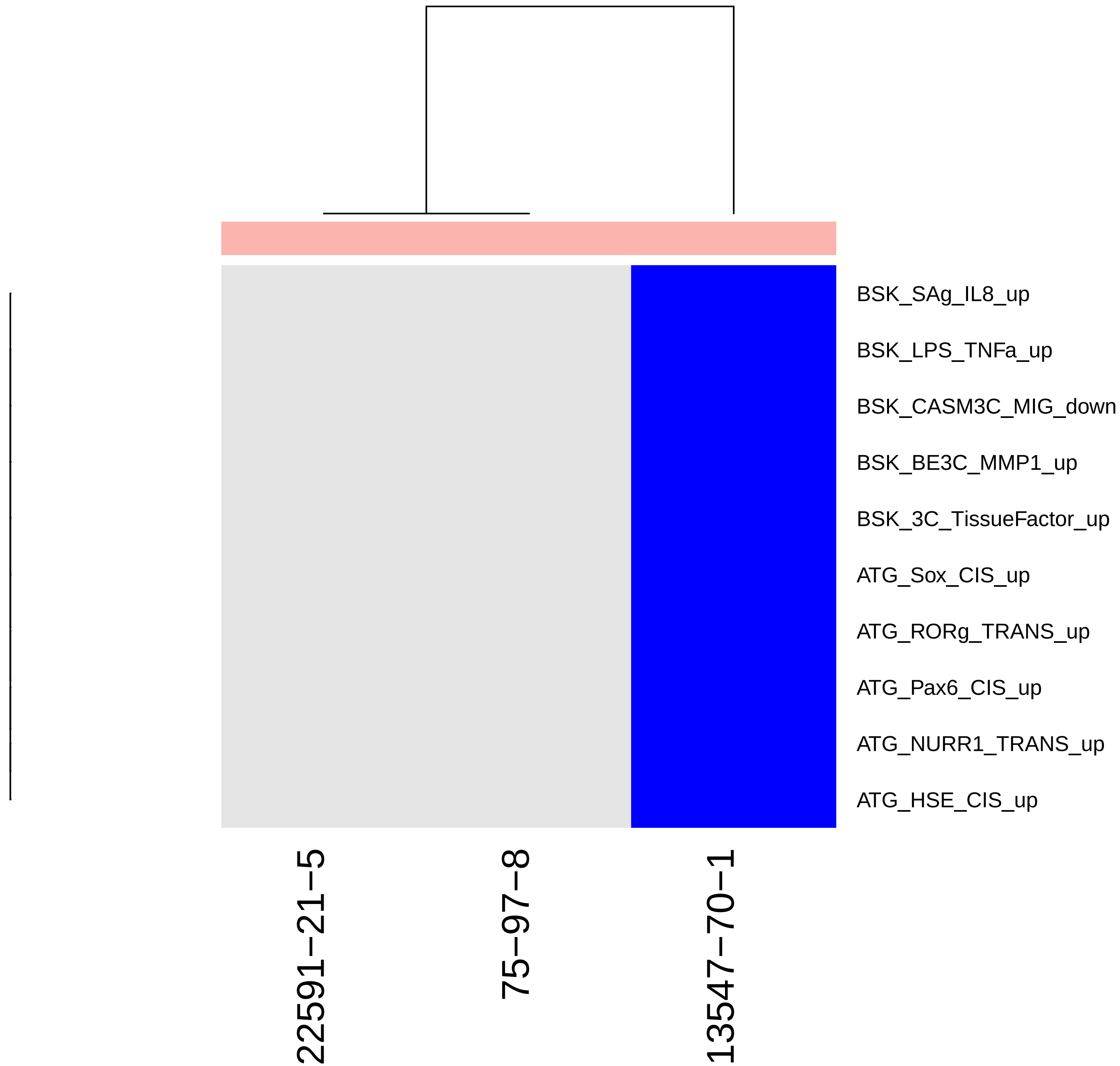

Enriched Assay Heatmap for Cluster 1041

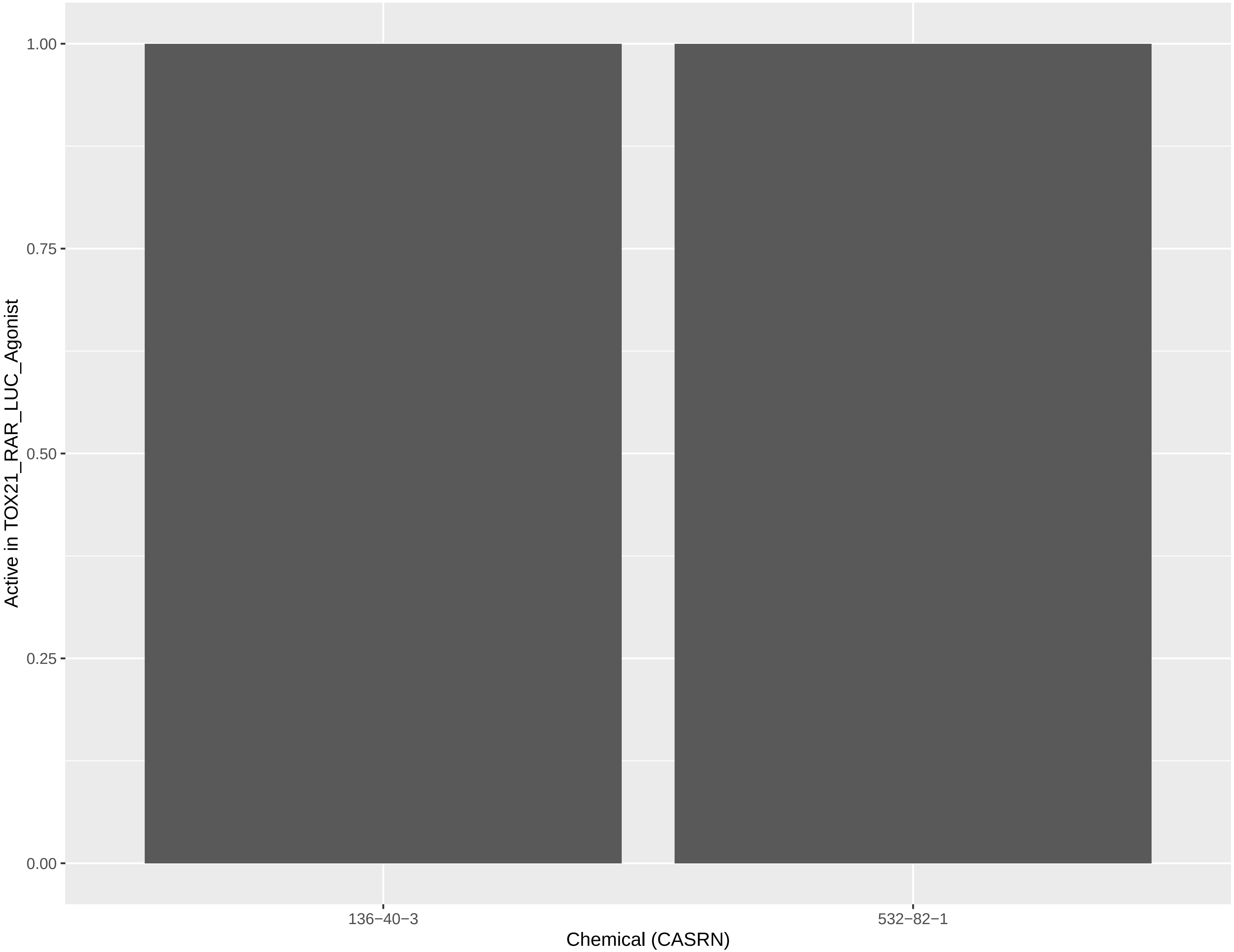

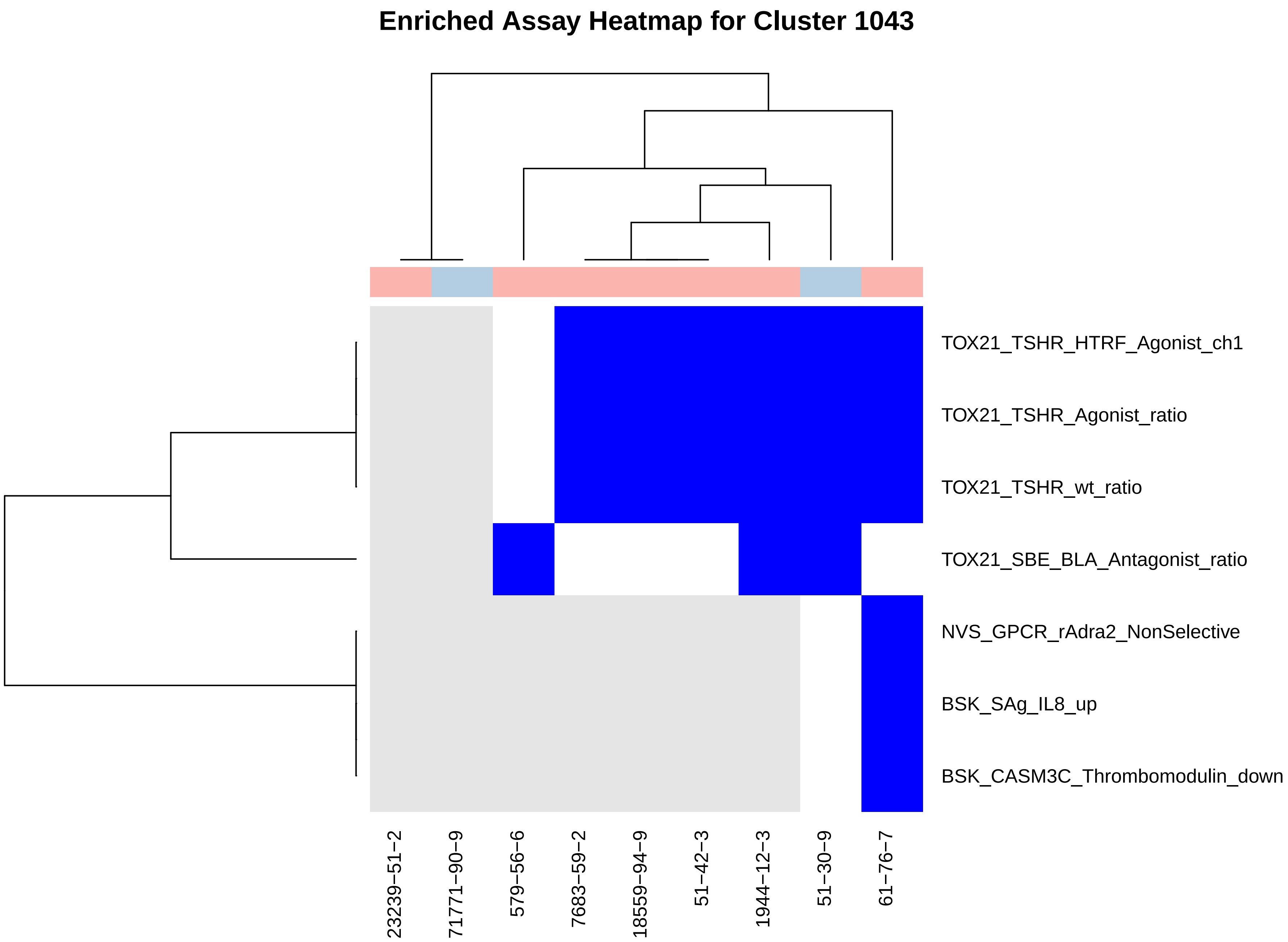

Enriched Assay Heatmap for Cluster 1043
TOX21_TSHR_HTRF_Agonist_ch1
TOX21_TSHR_Agonist_ratio
TOX21_TSHR_wt_ratio
TOX21_SBE_BLA_Antagonist_ratio
NVS_GPCR_rAdra2_NonSelective
BSK_SAg_IL8_up
BSK_CASM3C_Thrombomodulin_down
23239−51−2
71771−90−9
579−56−6
7683−59−2
18559−94−9
51−42−3
1944−12−3
51−30−9
61−76−7

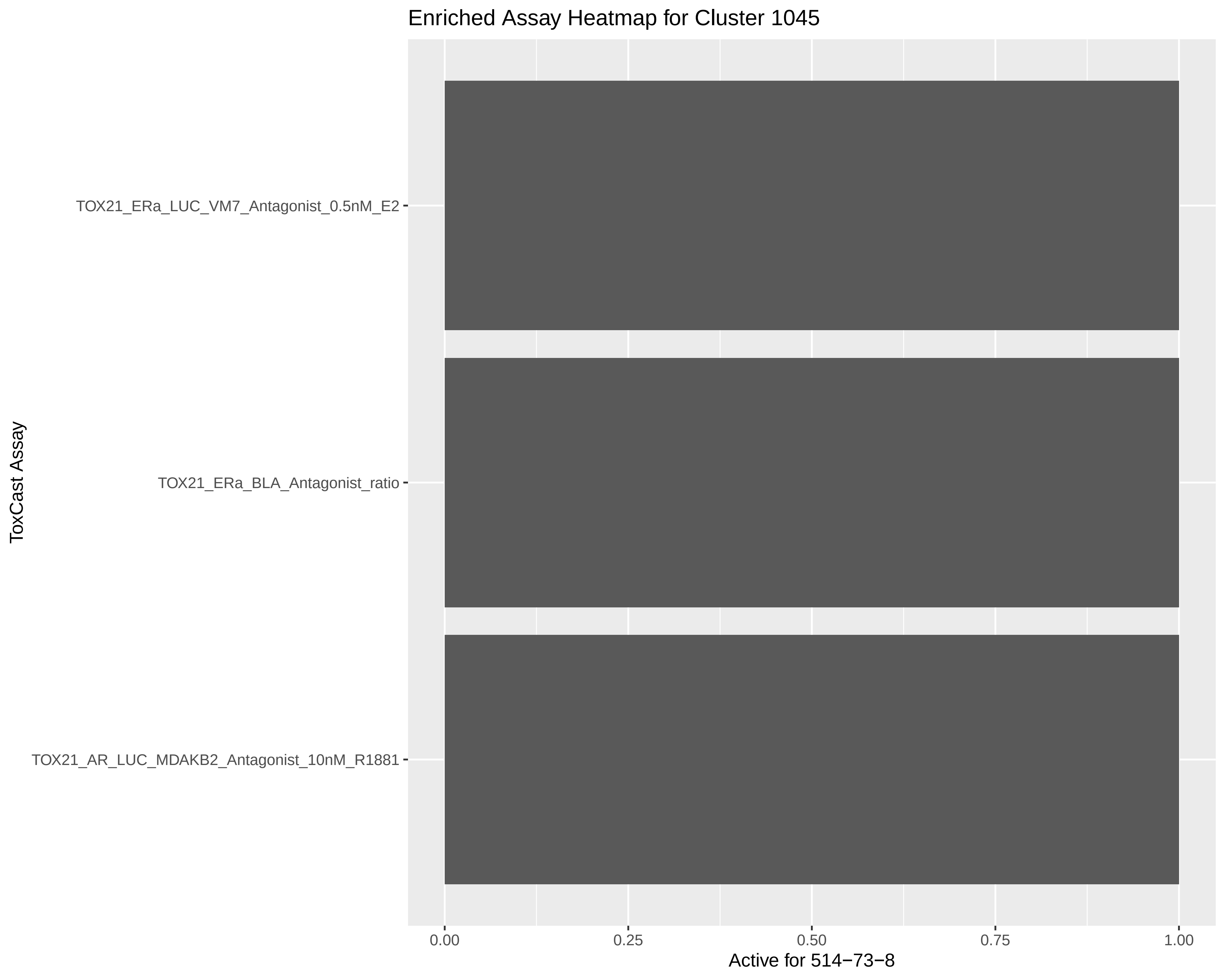

Enriched Assay Heatmap for Cluster 1045
ToxCast Assay
TOX21_ERa_LUC_VM7_Antagonist_0.5nM_E2
TOX21_ERa_BLA_Antagonist_ratio
TOX21_AR_LUC_MDAKB2_Antagonist_10nM_R1881
0.00
0.25
0.50
0.75
1.00
Active for 514−73−8

Enriched Assay Heatmap for Cluster 1046

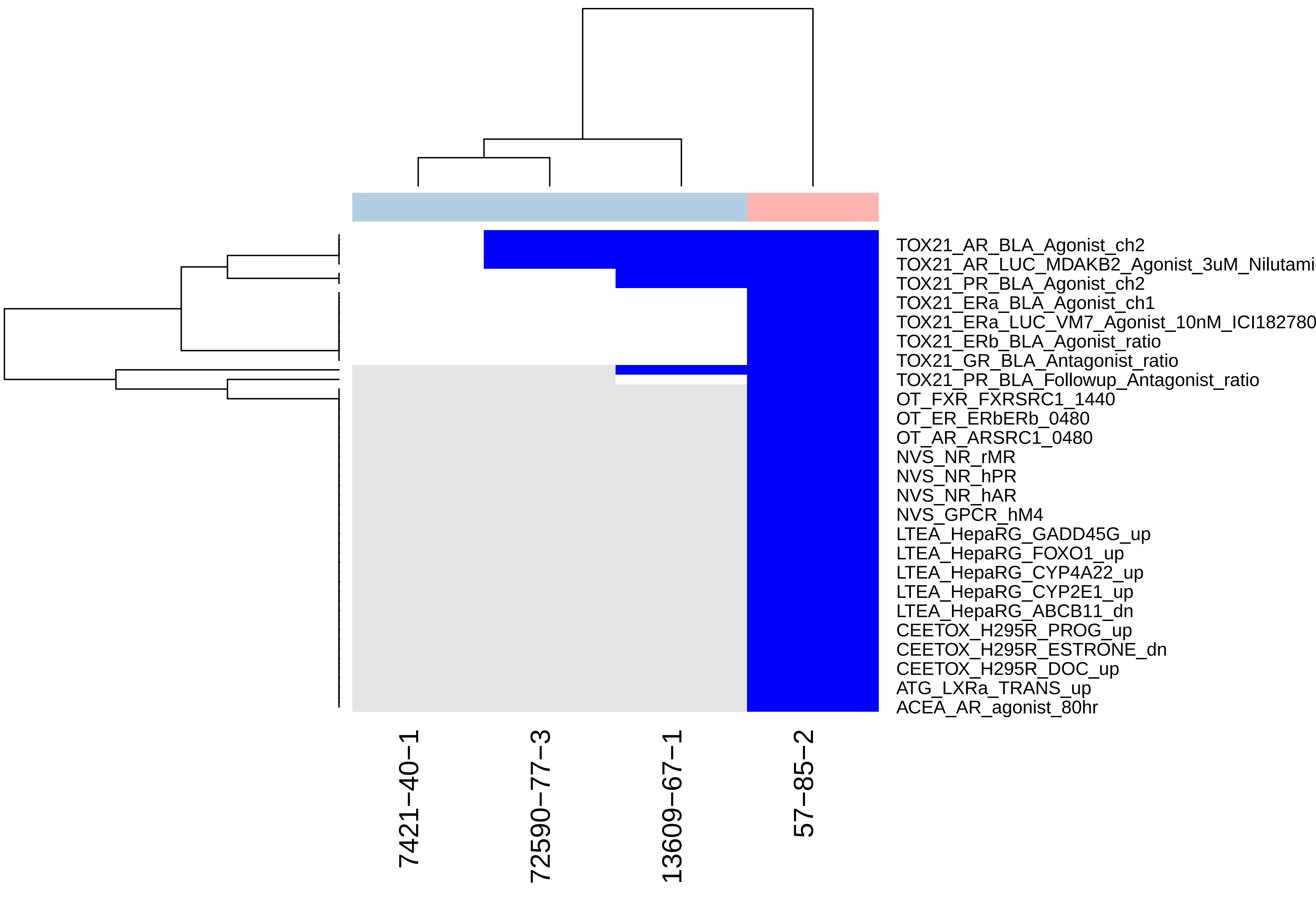

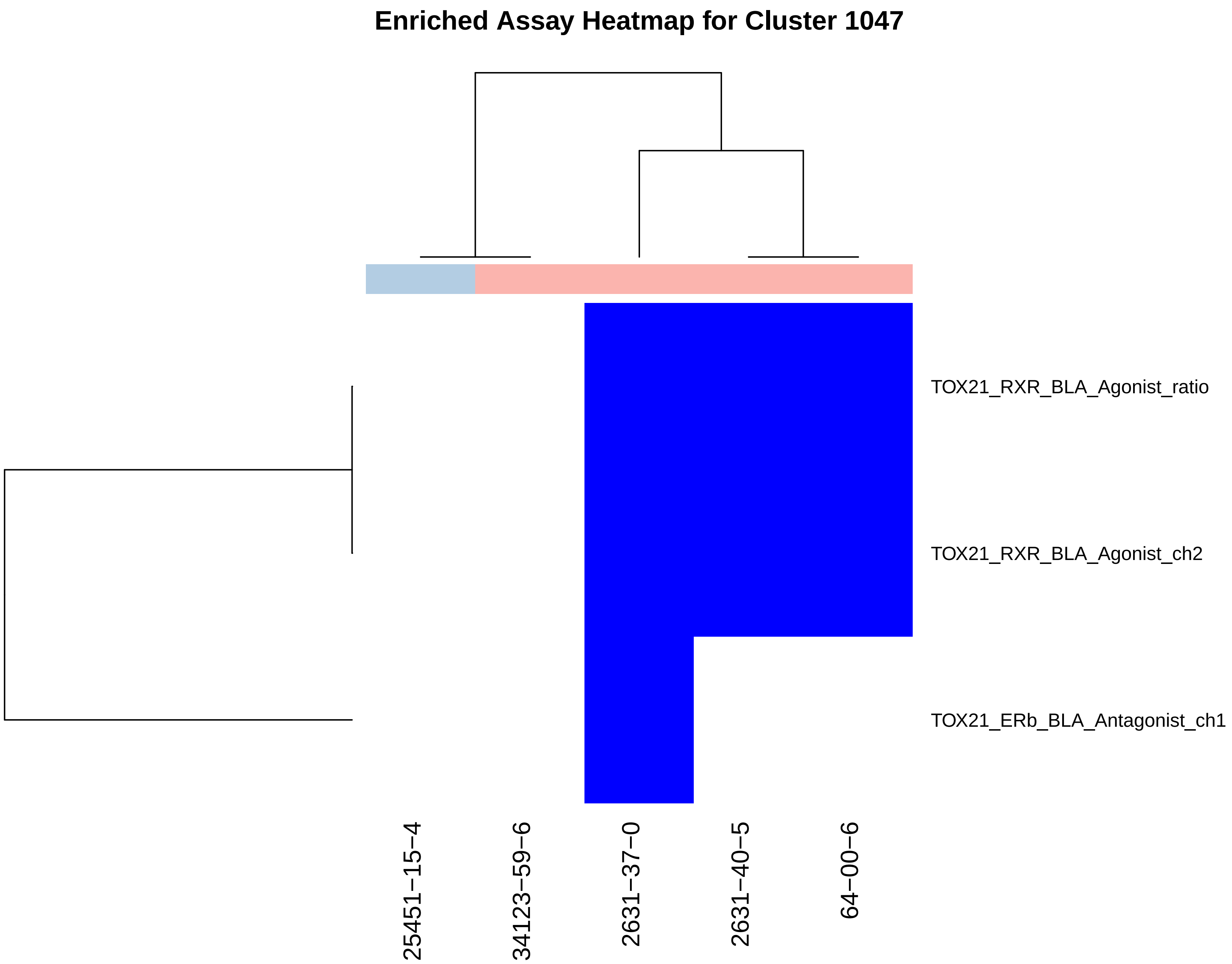

Enriched Assay Heatmap for Cluster 1047
TOX21_RXR_BLA_Agonist_ratio
TOX21_RXR_BLA_Agonist_ch2
TOX21_ERb_BLA_Antagonist_ch1
25451−15−4
34123−59−6
2631−37−0
2631−40−5
64−00−6

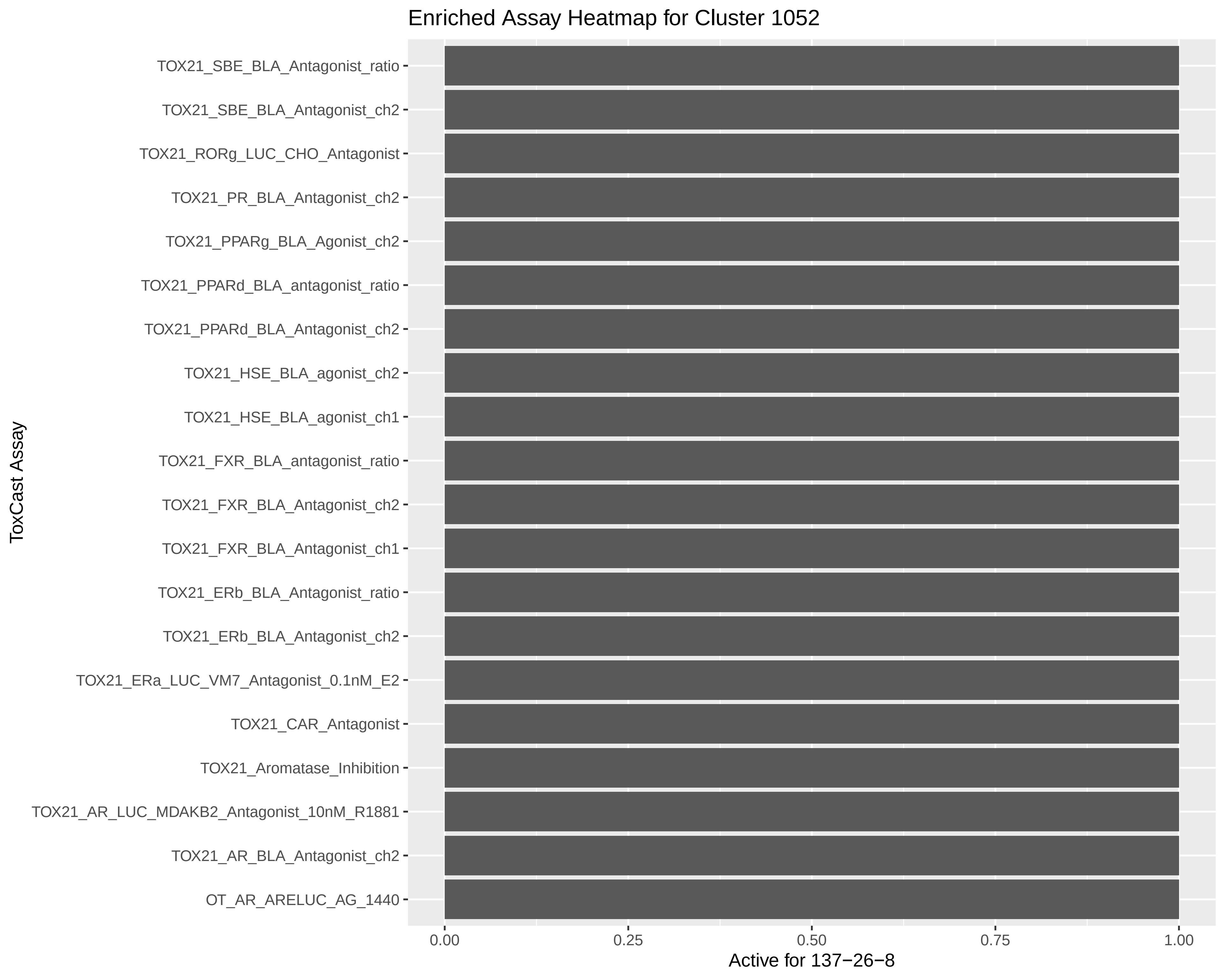

Enriched Assay Heatmap for Cluster 1052
TOX21_SBE_BLA_Antagonist_ratio
TOX21_SBE_BLA_Antagonist_ch2
TOX21_RORg_LUC_CHO_Antagonist
TOX21_PR_BLA_Antagonist_ch2
TOX21_PPARg_BLA_Agonist_ch2
TOX21_PPARd_BLA_antagonist_ratio
TOX21_PPARd_BLA_Antagonist_ch2
TOX21_HSE_BLA_agonist_ch2
TOX21_HSE_BLA_agonist_ch1
TOX21_FXR_BLA_antagonist_ratio
TOX21_FXR_BLA_Antagonist_ch2
TOX21_FXR_BLA_Antagonist_ch1
TOX21_ERb_BLA_Antagonist_ratio
TOX21_ERb_BLA_Antagonist_ch2
TOX21_ERa_LUC_VM7_Antagonist_0.1nM_E2
TOX21_CAR_Antagonist
TOX21_Aromatase_Inhibition
TOX21_AR_LUC_MDAKB2_Antagonist_10nM_R1881
TOX21_AR_BLA_Antagonist_ch2
OT_AR_ARELUC_AG_1440
ToxCast Assay
0.00
0.25
0.50
0.75
1.00
Active for 137−26−8

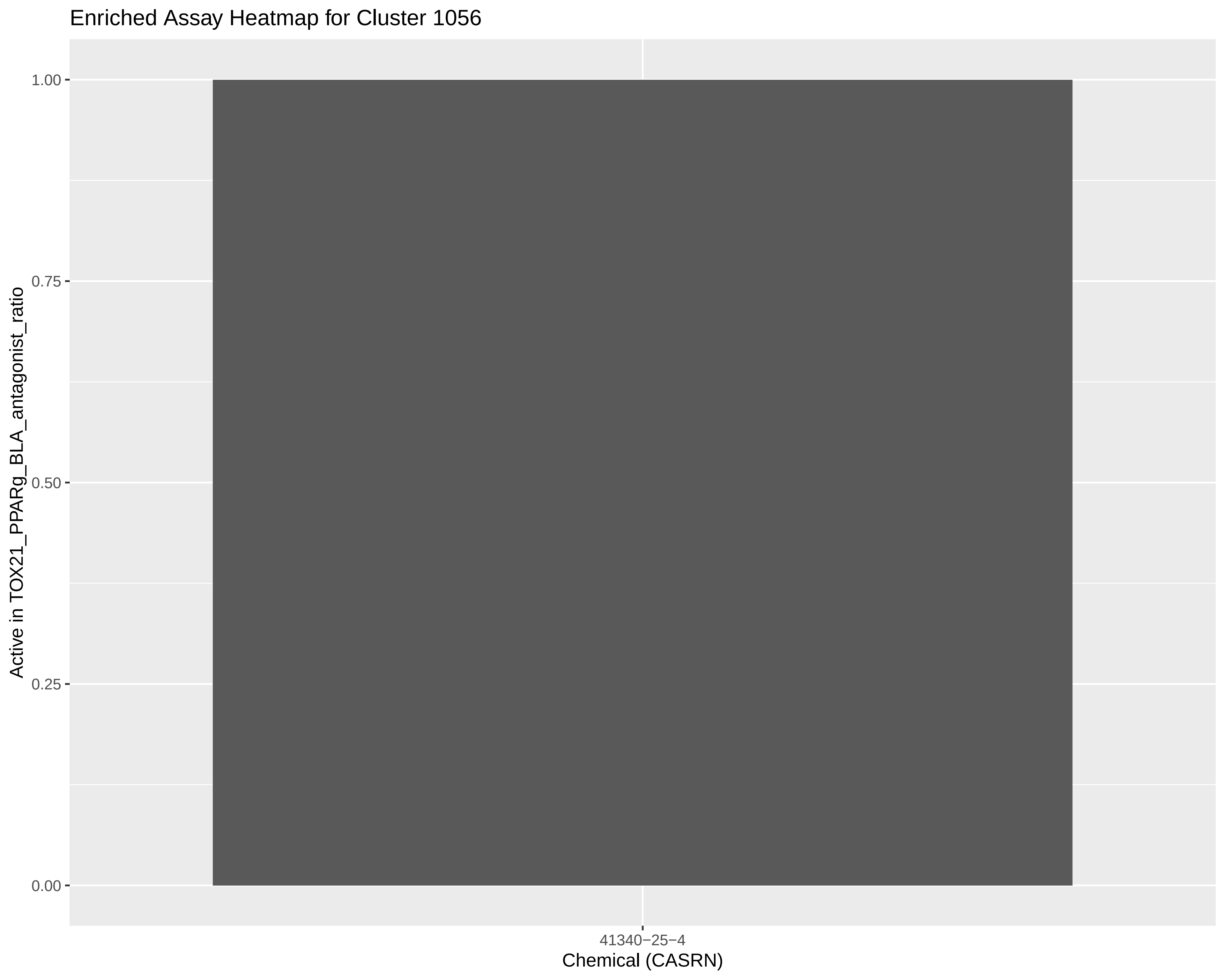
Enriched Assay Heatmap for Cluster 1056
Active in TOX21_PPARg_BLA_antagonist_ratio
1.00
0.75
0.50
0.25
0.00
41340−25−4
Chemical (CASRN)

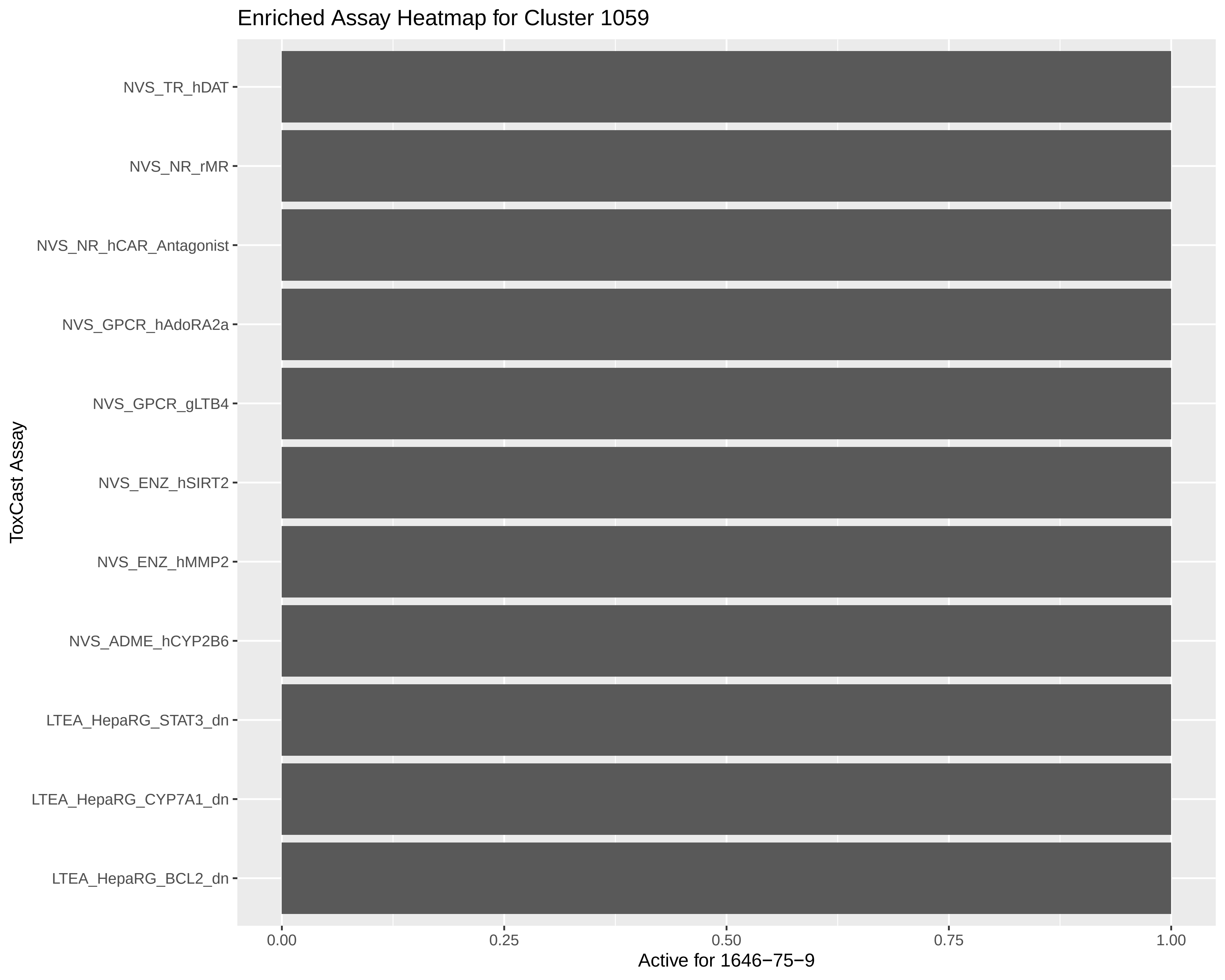
Enriched Assay Heatmap for Cluster 1059
ToxCast Assay
NVS_TR_hDAT
NVS_NR_rMR
NVS_NR_hCAR_Antagonist
NVS_GPCR_hAdoRA2a
NVS_GPCR_gLTB4
NVS_ENZ_hSIRT2
NVS_ENZ_hMMP2
NVS_ADME_hCYP2B6
LTEA_HepaRG_STAT3_dn
LTEA_HepaRG_CYP7A1_dn
LTEA_HepaRG_BCL2_dn
0.00
0.25
0.50
0.75
1.00
Active for 1646−75−9

Enriched Assay Heatmap for Cluster 1060

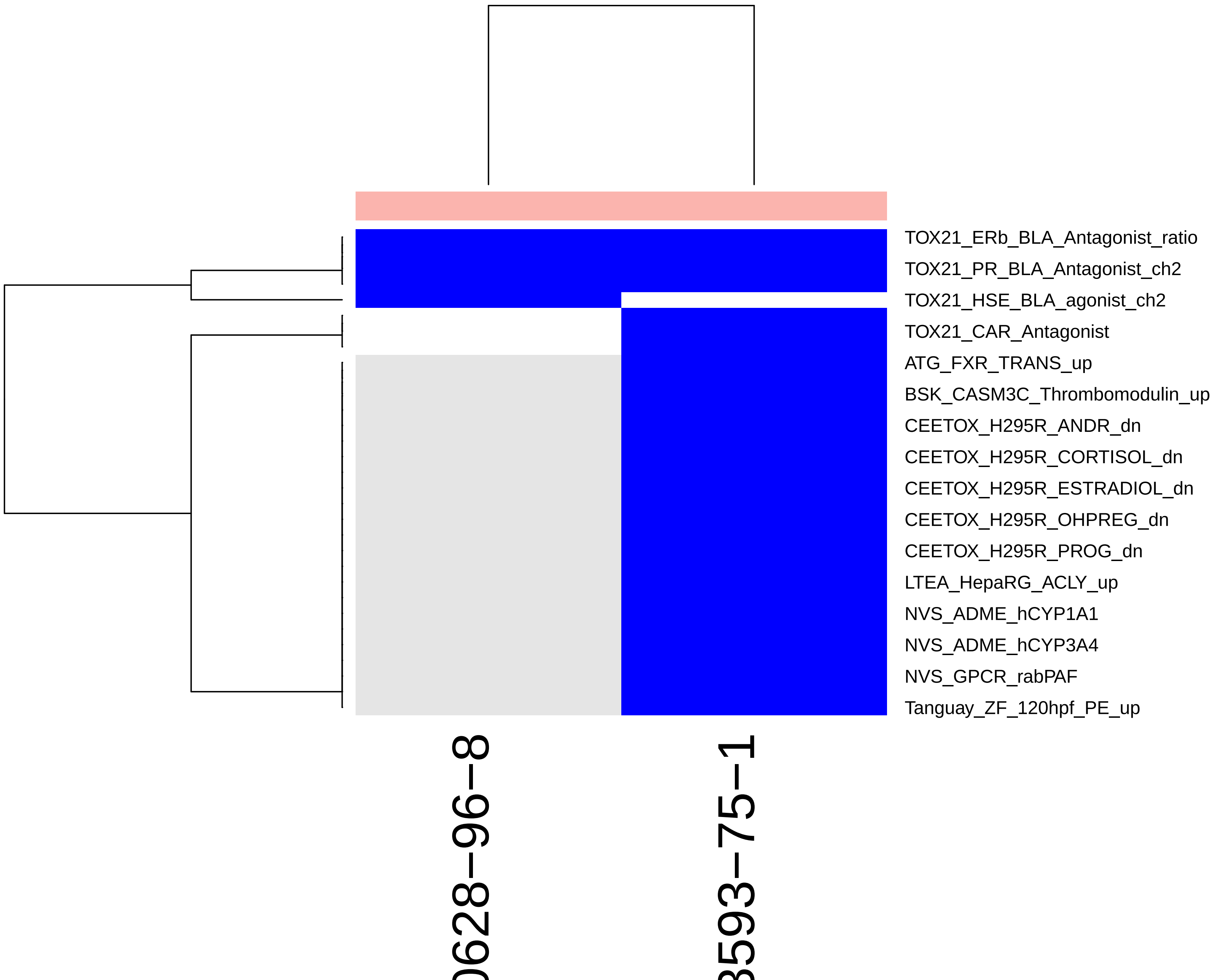

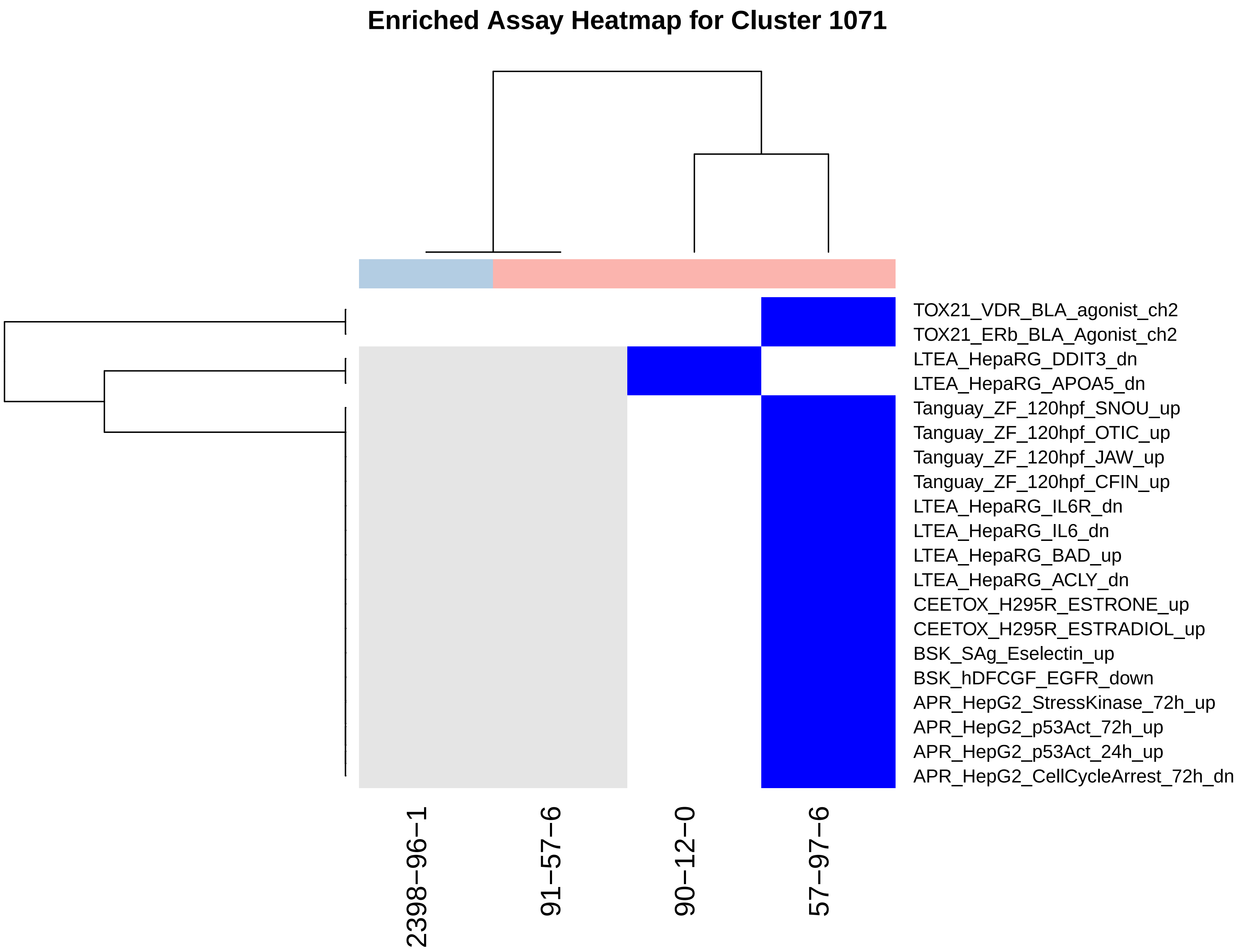
Enriched Assay Heatmap for Cluster 1071
TOX21_VDR_BLA_agonist_ch2
TOX21_ERb_BLA_Agonist_ch2
LTEA_HepaRG_DDIT3_dn
LTEA_HepaRG_APOA5_dn
Tanguay_ZF_120hpf_SNOU_up
Tanguay_ZF_120hpf_OTIC_up
Tanguay_ZF_120hpf_JAW_up
Tanguay_ZF_120hpf_CFIN_up
LTEA_HepaRG_IL6R_dn
LTEA_HepaRG_IL6_dn
LTEA_HepaRG_BAD_up
LTEA_HepaRG_ACLY_dn
CEETOX_H295R_ESTRONE_up
CEETOX_H295R_ESTRADIOL_up
BSK_SAg_Eselectin_up
BSK_hDFCGF_EGFR_down
APR_HepG2_StressKinase_72h_up
APR_HepG2_p53Act_72h_up
APR_HepG2_p53Act_24h_up
APR_HepG2_CellCycleArrest_72h_dn
2398−96−1
91−57−6
90−12−0
57−97−6

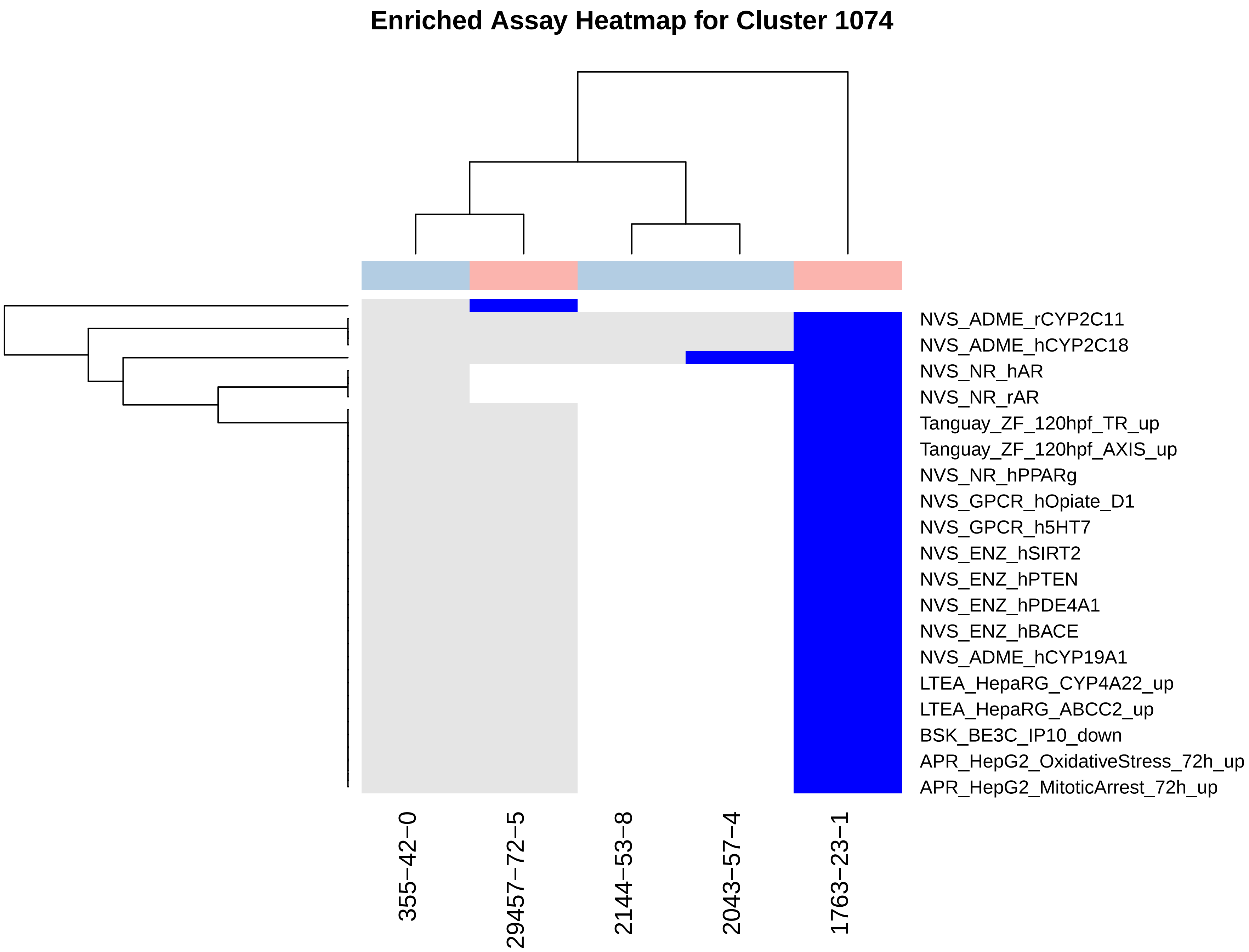

Enriched Assay Heatmap for Cluster 1074
NVS_ADME_rCYP2C11
NVS_ADME_hCYP2C18
NVS_NR_hAR
NVS_NR_rAR
Tanguay_ZF_120hpf_TR_up
Tanguay_ZF_120hpf_AXIS_up
NVS_NR_hPPARg
NVS_GPCR_hOpiate_D1
NVS_GPCR_h5HT7
NVS_ENZ_hSIRT2
NVS_ENZ_hPTEN
NVS_ENZ_hPDE4A1
NVS_ENZ_hBACE
NVS_ADME_hCYP19A1
LTEA_HepaRG_CYP4A22_up
LTEA_HepaRG_ABCC2_up
BSK_BE3C_IP10_down
APR_HepG2_OxidativeStress_72h_up
APR_HepG2_MitoticArrest_72h_up
355−42−0
29457−72−5
2144−53−8
2043−57−4
1763−23−1

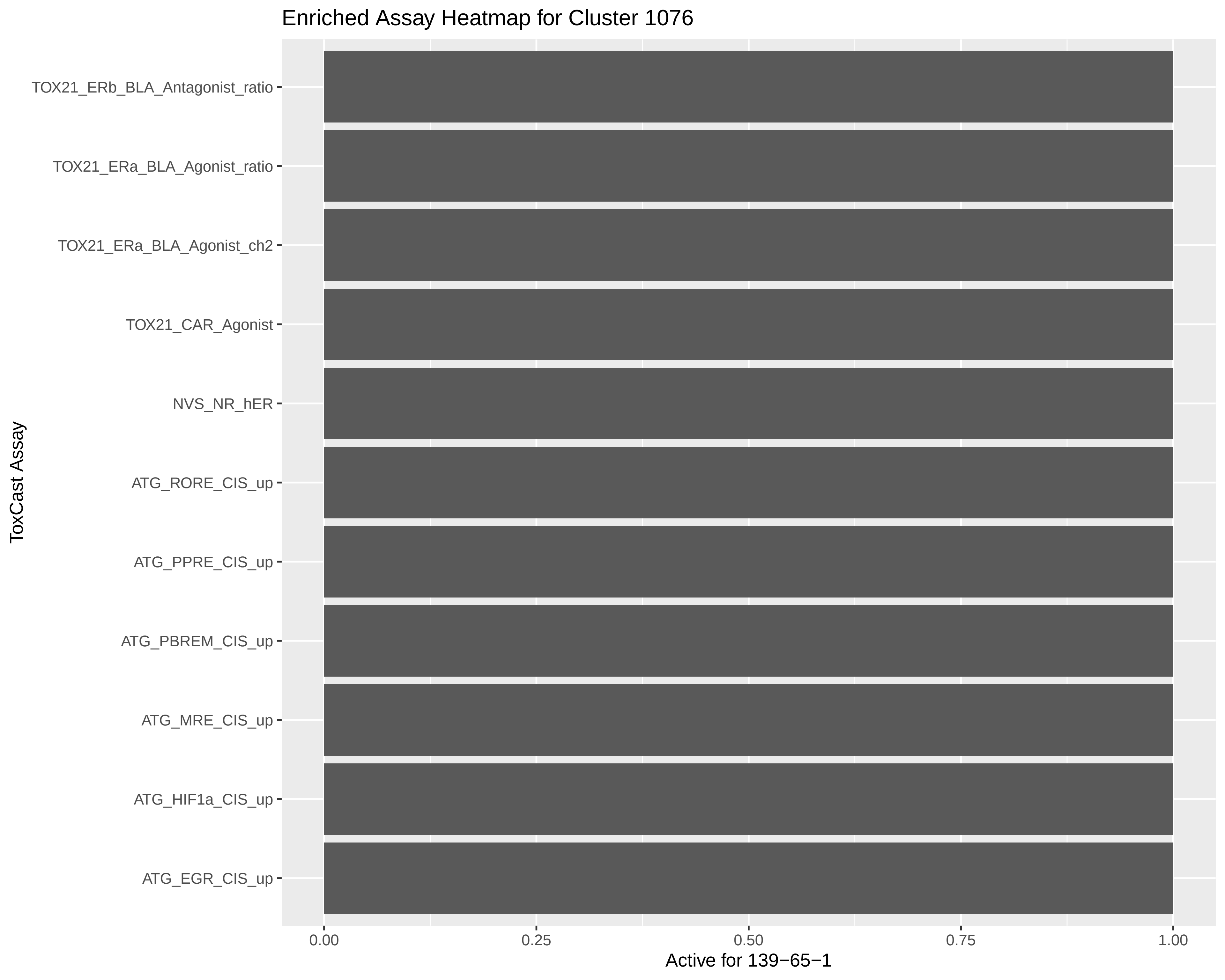

Enriched Assay Heatmap for Cluster 1076
TOX21_ERb_BLA_Antagonist_ratio
TOX21_ERa_BLA_Agonist_ratio
TOX21_ERa_BLA_Agonist_ch2
TOX21_CAR_Agonist
NVS_NR_hER
ATG_RORE_CIS_up
ATG_PPRE_CIS_up
ATG_PBREM_CIS_up
ATG_MRE_CIS_up
ATG_HIF1a_CIS_up
ATG_EGR_CIS_up
ToxCast Assay
0.00
0.25
0.50
0.75
1.00
Active for 139−65−1

Enriched Assay Heatmap for Cluster 1080

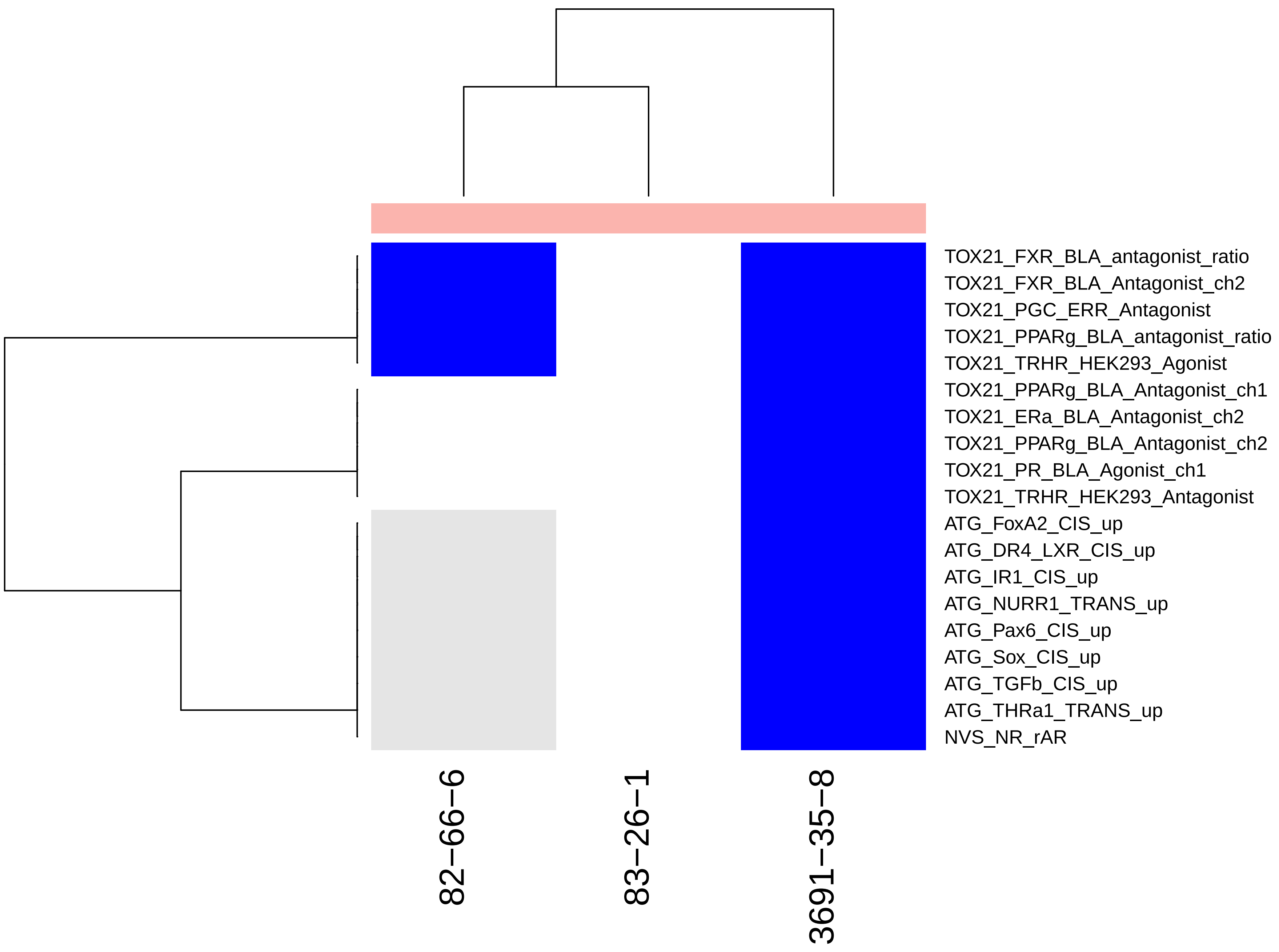

Enriched Assay Heatmap for Cluster 1083

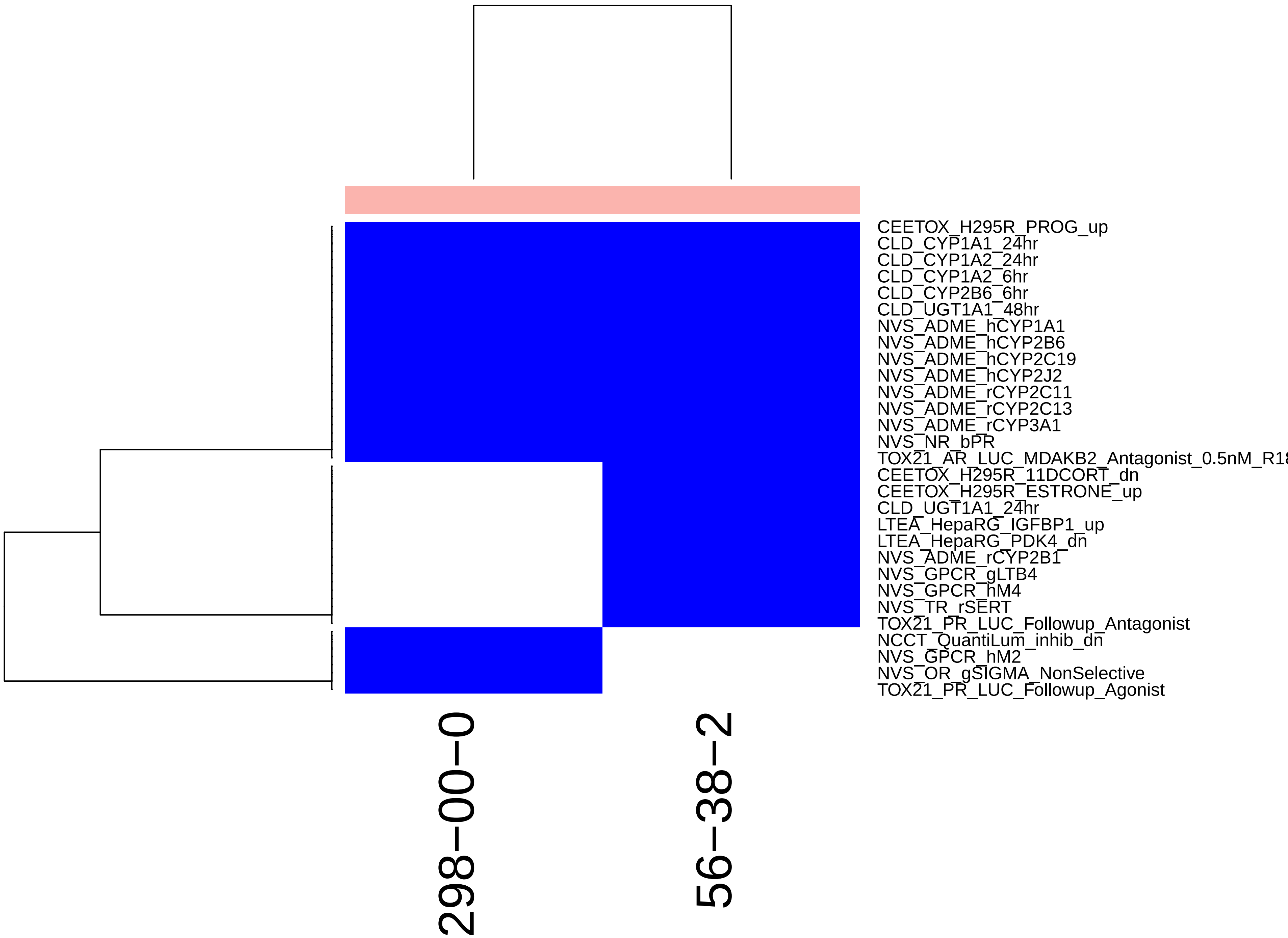

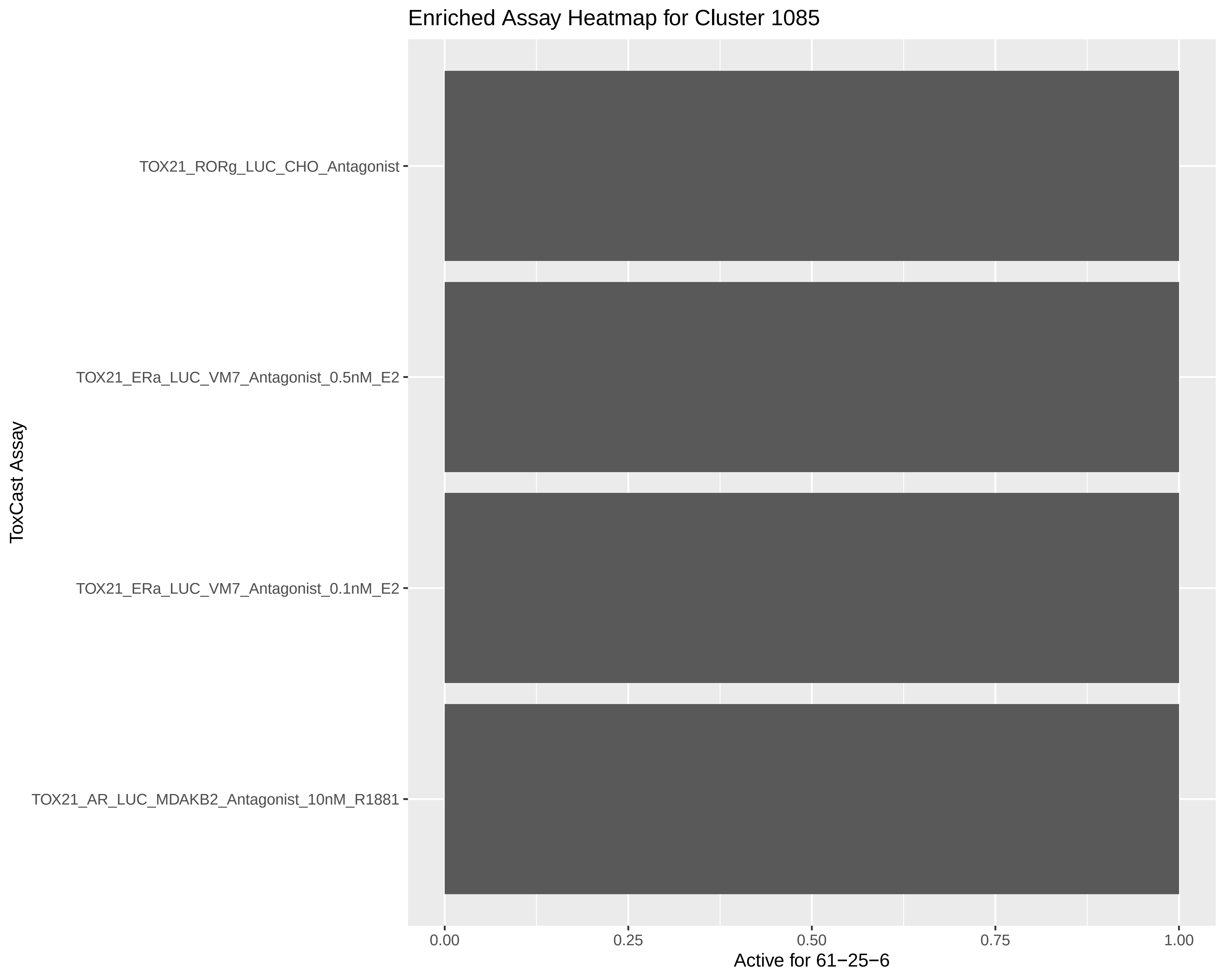

Enriched Assay Heatmap for Cluster 1085
ToxCast Assay
TOX21_RORg_LUC_CHO_Antagonist
TOX21_ERa_LUC_VM7_Antagonist_0.5nM_E2
TOX21_ERa_LUC_VM7_Antagonist_0.1nM_E2
TOX21_AR_LUC_MDAKB2_Antagonist_10nM_R1881
0.00
0.25
0.50
0.75
1.00
Active for 61−25−6

Enriched Assay Heatmap for Cluster 1086

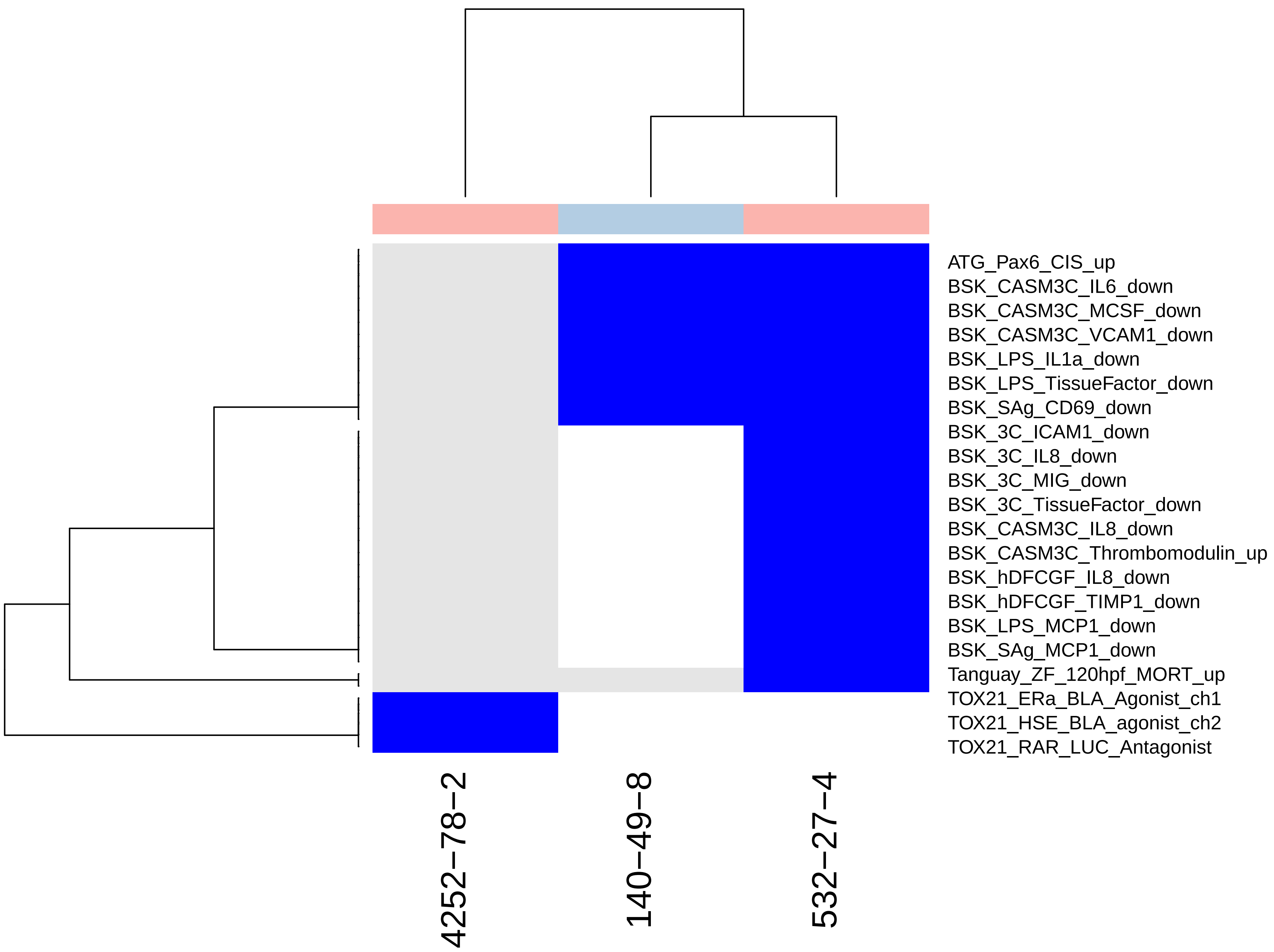

Enriched Assay Heatmap for Cluster 1087

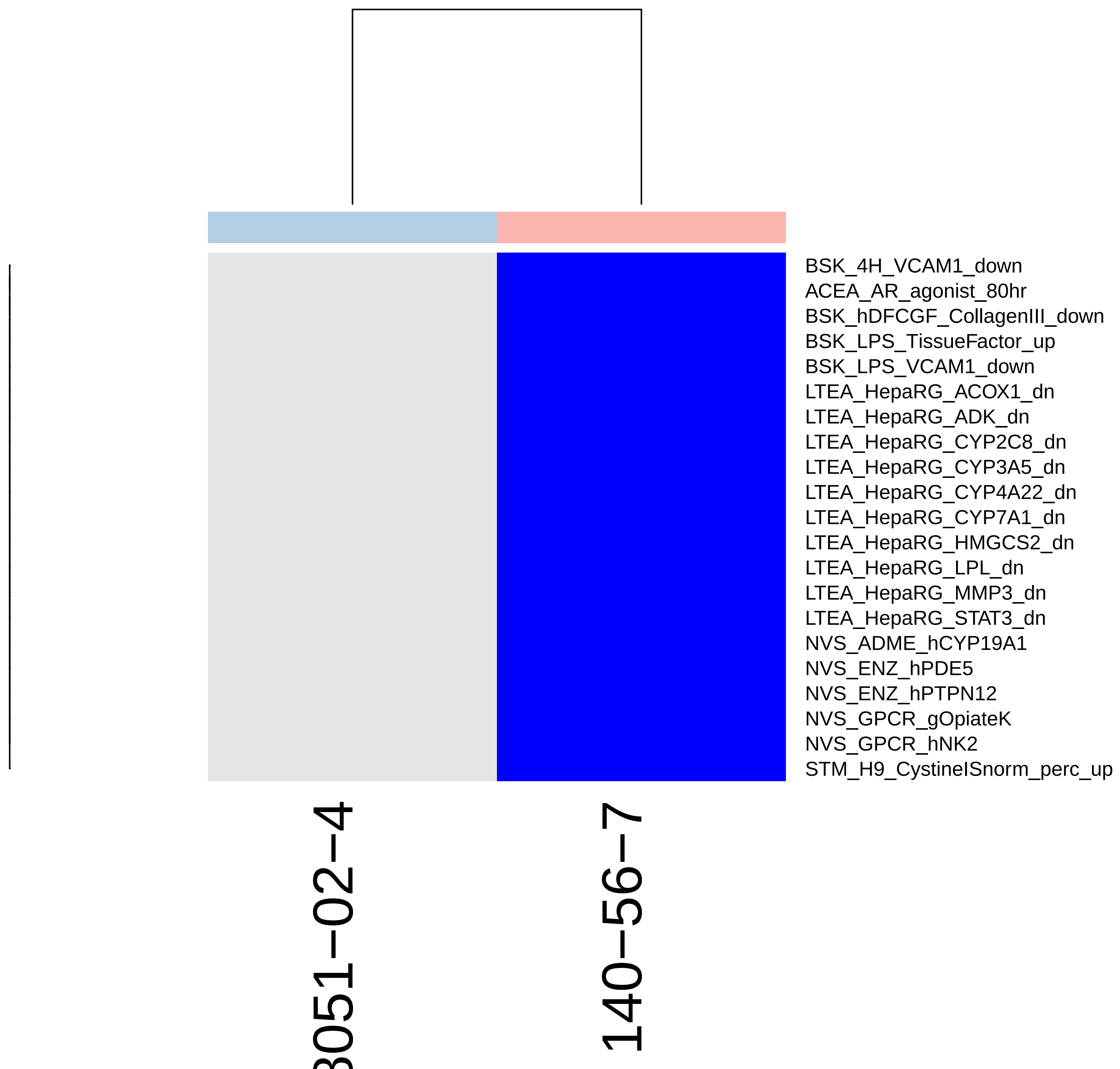

Enriched Assay Heatmap for Cluster 1090

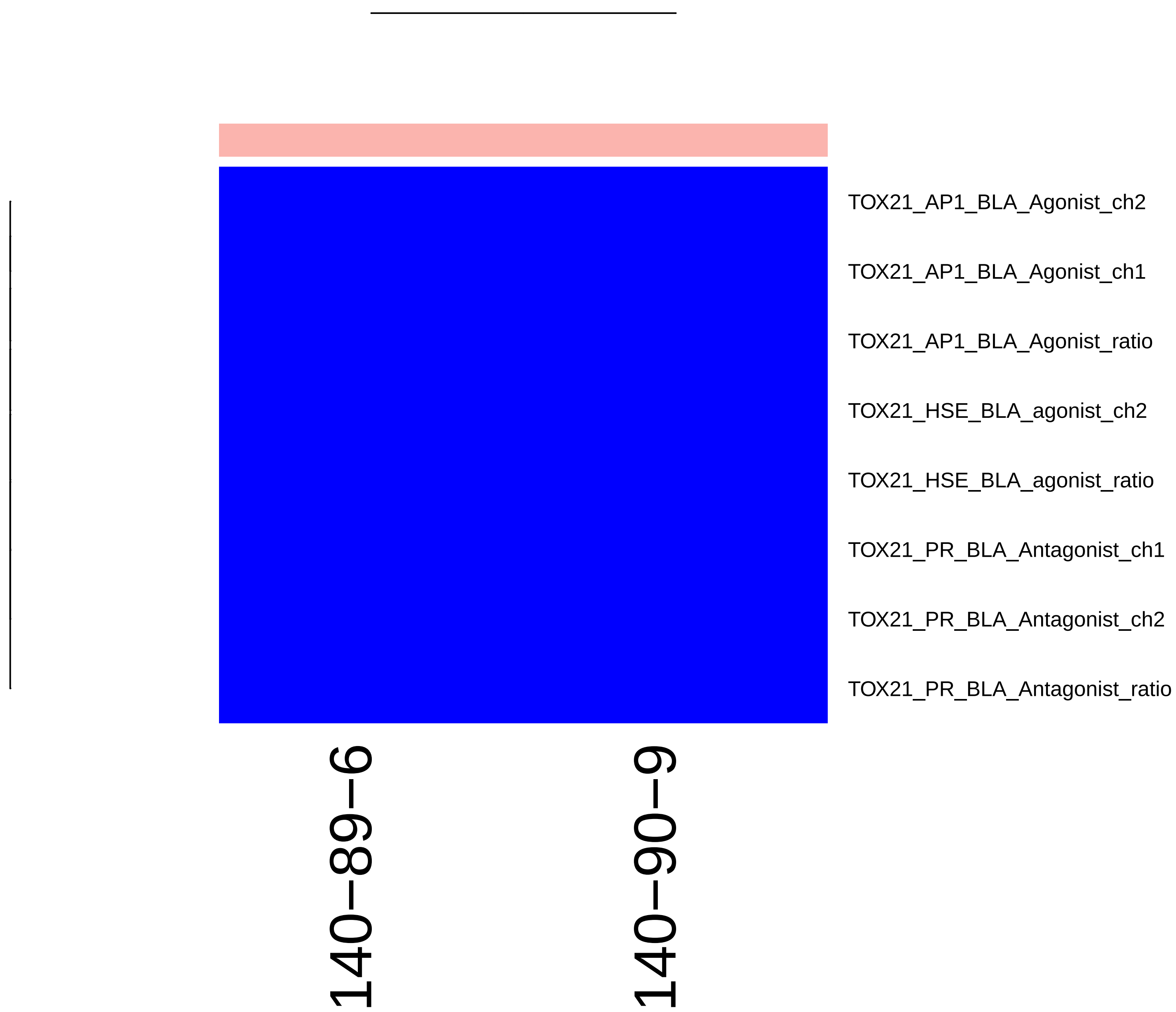

Enriched Assay Heatmap for Cluster 1091

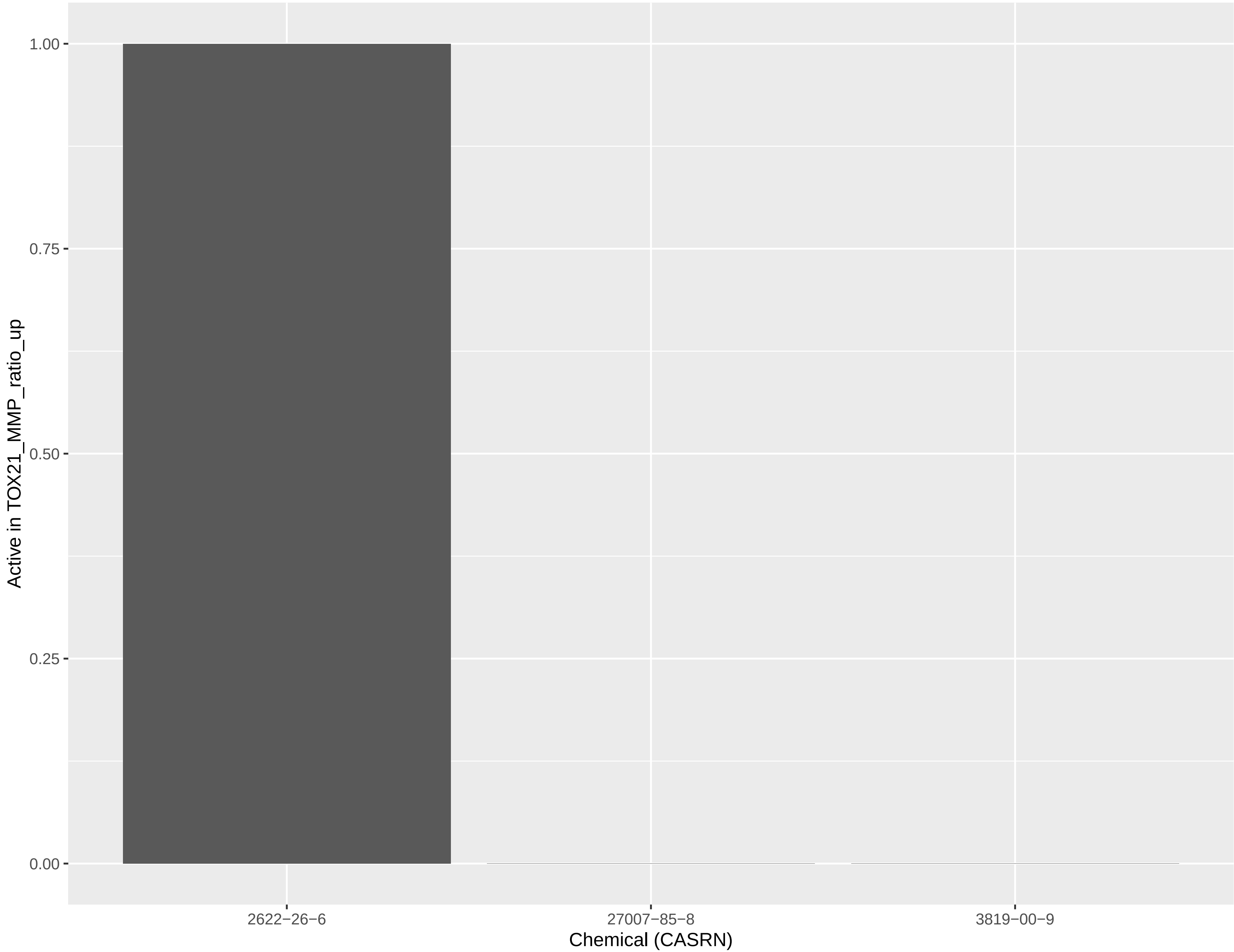

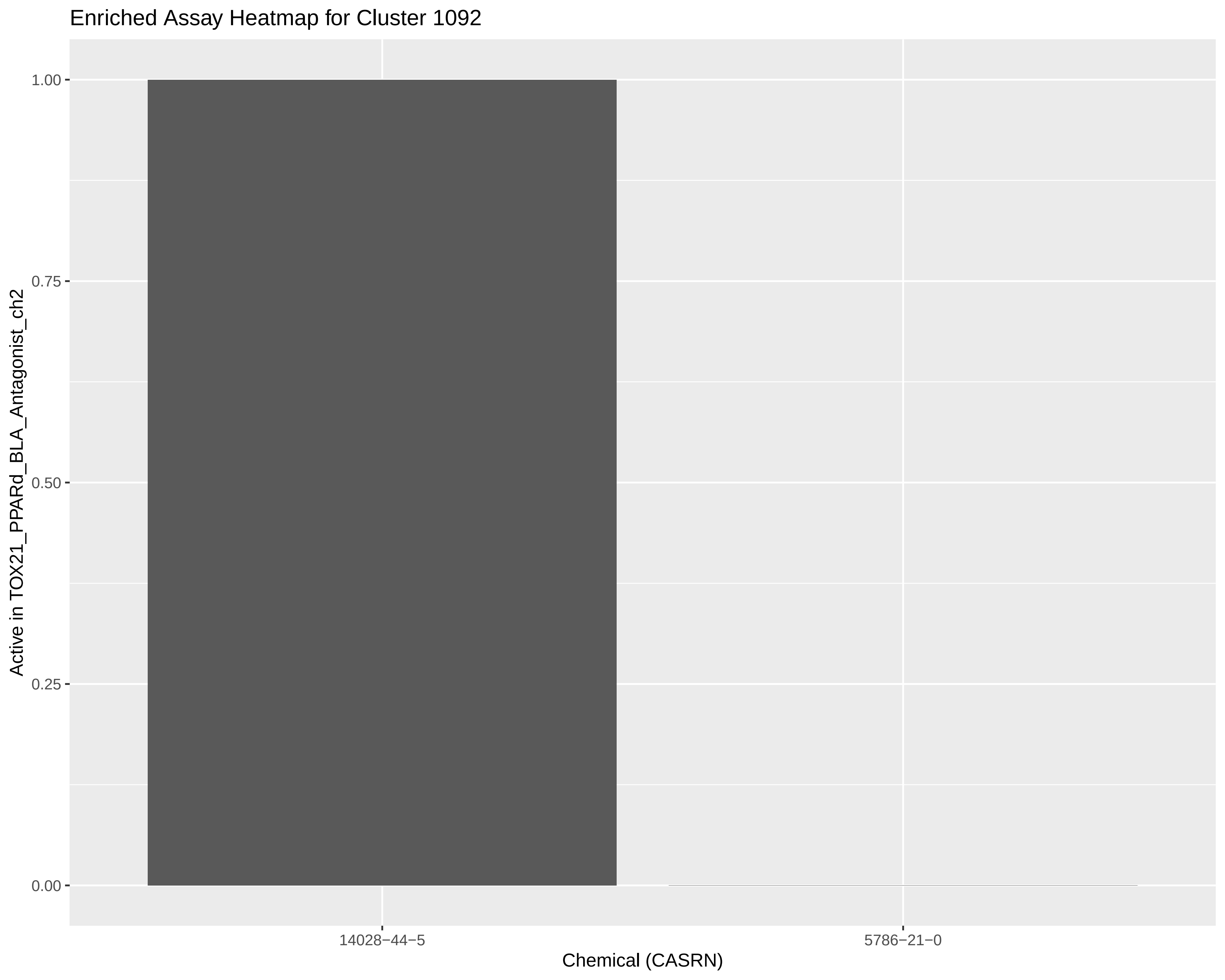

Enriched Assay Heatmap for Cluster 1092
Active in TOX21_PPARd_BLA_Antagonist_ch2
1.00
0.75
0.50
0.25
0.00
14028−44−5
5786−21−0
Chemical (CASRN)

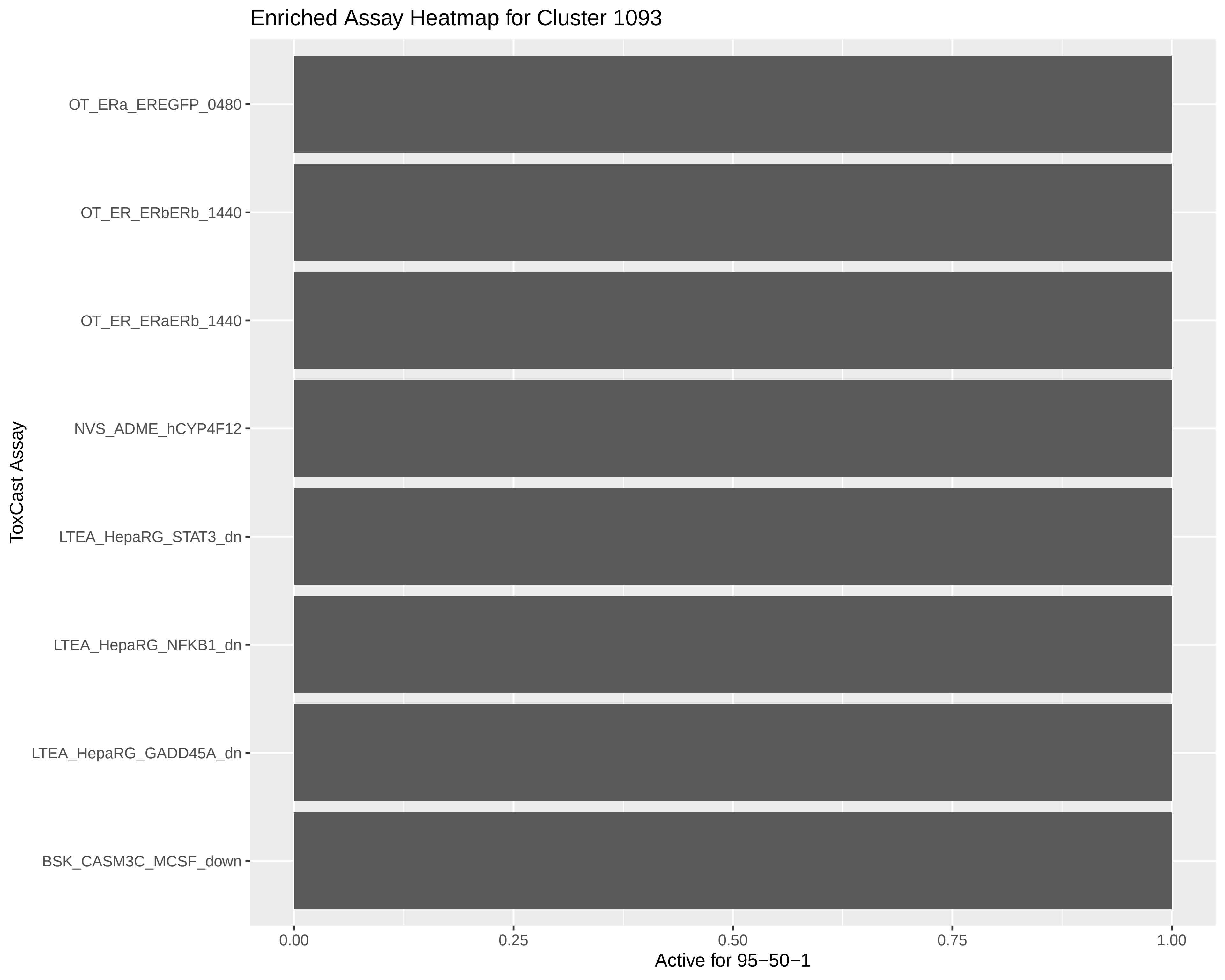
Enriched Assay Heatmap for Cluster 1093
ToxCast Assay
OT_ERa_EREGFP_0480
OT_ER_ERbERb_1440
OT_ER_ERaERb_1440
NVS_ADME_hCYP4F12
LTEA_HepaRG_STAT3_dn
LTEA_HepaRG_NFKB1_dn
LTEA_HepaRG_GADD45A_dn
BSK_CASM3C_MCSF_down
0.00
0.25
0.50
0.75
1.00
Active for 95−50−1

Enriched Assay Heatmap for Cluster 1099

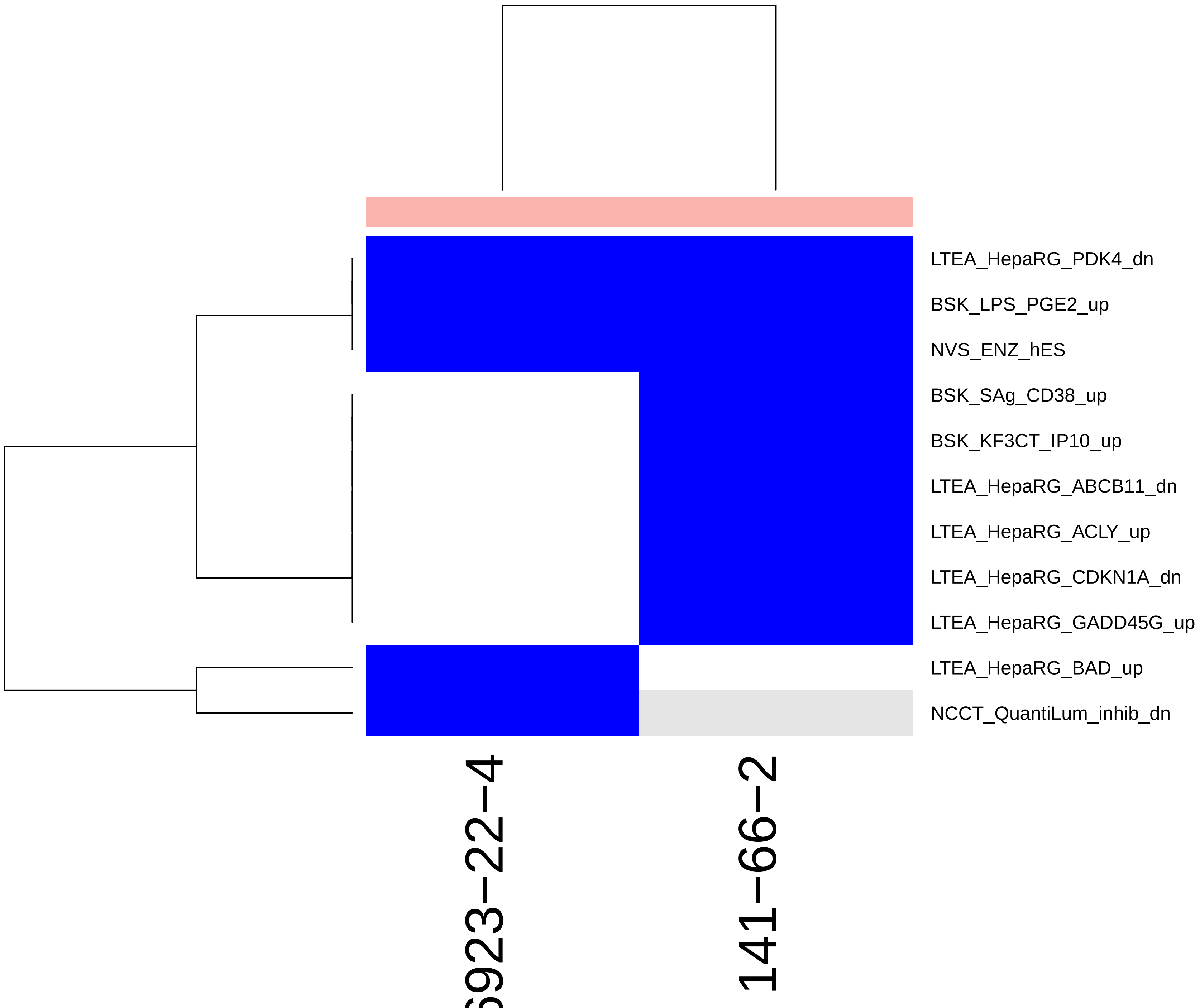

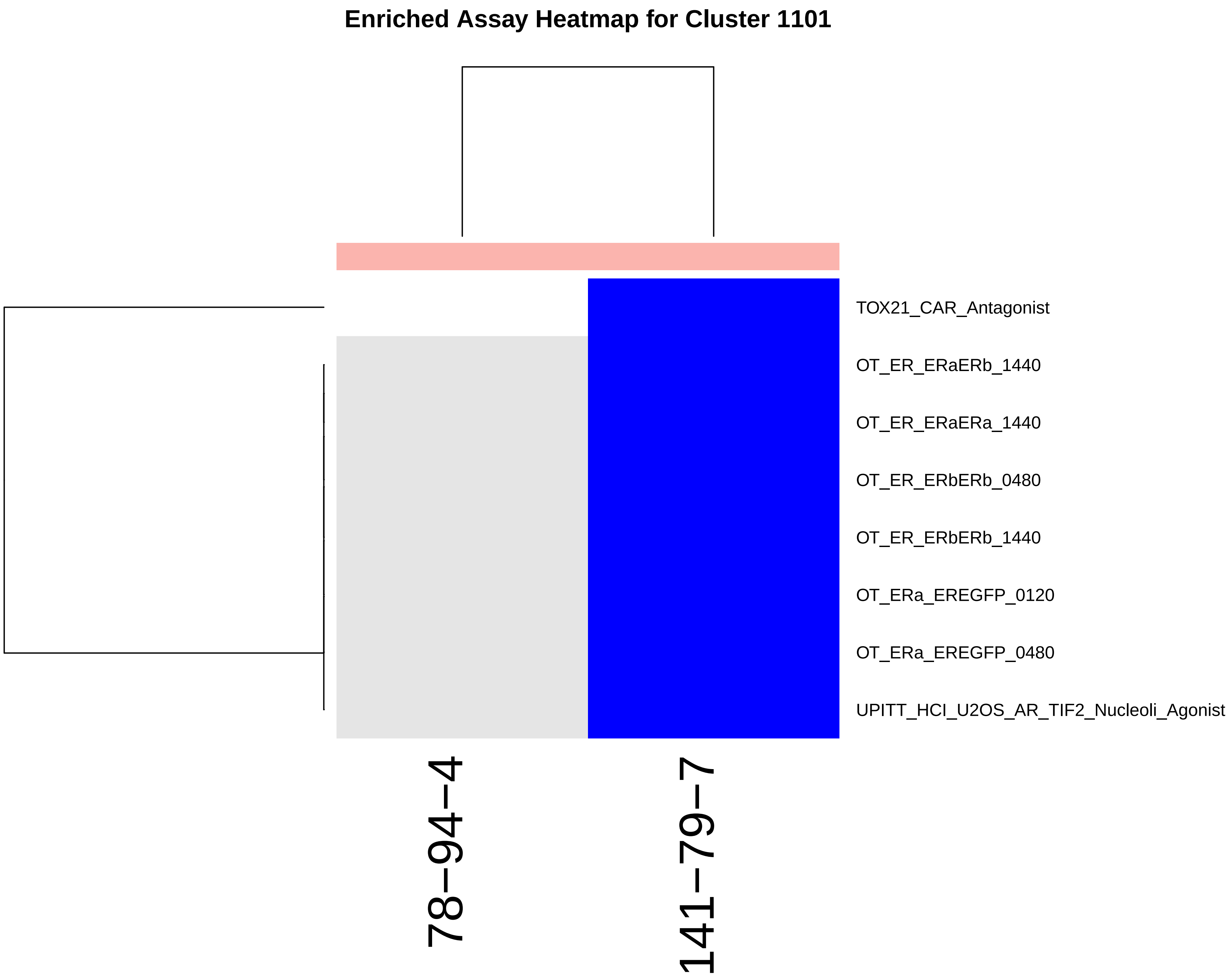

Enriched Assay Heatmap for Cluster 1101
TOX21_CAR_Antagonist
OT_ER_ERaERb_1440
OT_ER_ERaERa_1440
OT_ER_ERbERb_0480
OT_ER_ERbERb_1440
OT_ERa_EREGFP_0120
OT_ERa_EREGFP_0480
UPITT_HCI_U2OS_AR_TIF2_Nucleoli_Agonist
78−94−4
141−79−7

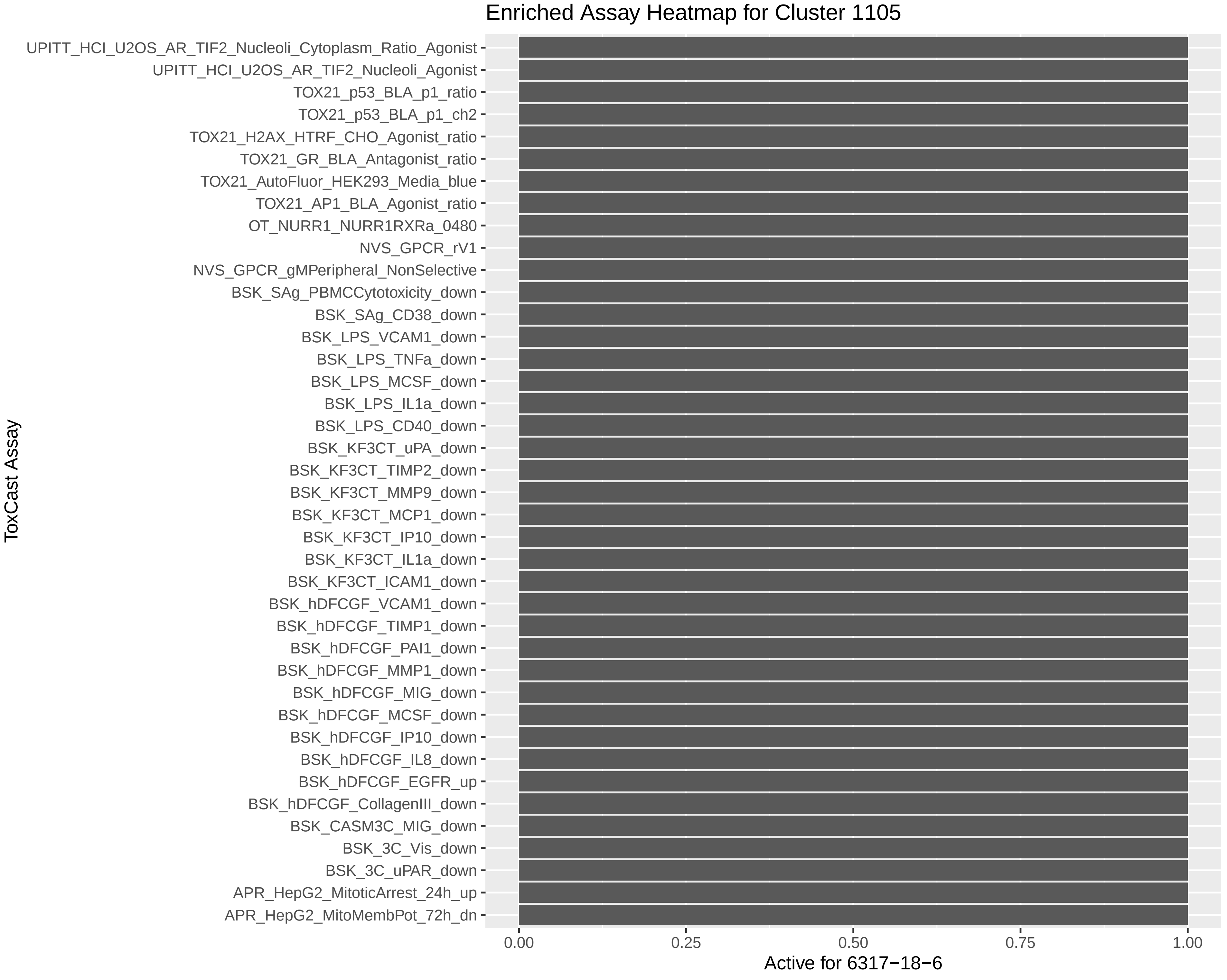
Enriched Assay Heatmap for Cluster 1105
UPITT_HCI_U2OS_AR_TIF2_Nucleoli_Cytoplasm_Ratio_Agonist
UPITT_HCI_U2OS_AR_TIF2_Nucleoli_Agonist
TOX21_p53_BLA_p1_ratio
TOX21_p53_BLA_p1_ch2
TOX21_H2AX_HTRF_CHO_Agonist_ratio
TOX21_GR_BLA_Antagonist_ratio
TOX21_AutoFluor_HEK293_Media_blue
TOX21_AP1_BLA_Agonist_ratio
OT_NURR1_NURR1RXRa_0480
NVS_GPCR_rV1
NVS_GPCR_gMPeripheral_NonSelective
BSK_SAg_PBMCCytotoxicity_down
BSK_SAg_CD38_down
BSK_LPS_VCAM1_down
BSK_LPS_TNFa_down
BSK_LPS_MCSF_down
BSK_LPS_IL1a_down
BSK_LPS_CD40_down
BSK_KF3CT_uPA_down
BSK_KF3CT_TIMP2_down
BSK_KF3CT_MMP9_down
BSK_KF3CT_MCP1_down
BSK_KF3CT_IP10_down
BSK_KF3CT_IL1a_down
BSK_KF3CT_ICAM1_down
BSK_hDFCGF_VCAM1_down
BSK_hDFCGF_TIMP1_down
BSK_hDFCGF_PAI1_down
BSK_hDFCGF_MMP1_down
BSK_hDFCGF_MIG_down
BSK_hDFCGF_MCSF_down
BSK_hDFCGF_IP10_down
BSK_hDFCGF_IL8_down
BSK_hDFCGF_EGFR_up
BSK_hDFCGF_CollagenIII_down
BSK_CASM3C_MIG_down
BSK_3C_Vis_down
BSK_3C_uPAR_down
APR_HepG2_MitoticArrest_24h_up
APR_HepG2_MitoMembPot_72h_dn
ToxCast Assay
0.00
0.25
0.50
0.75
1.00
Active for 6317−18−6

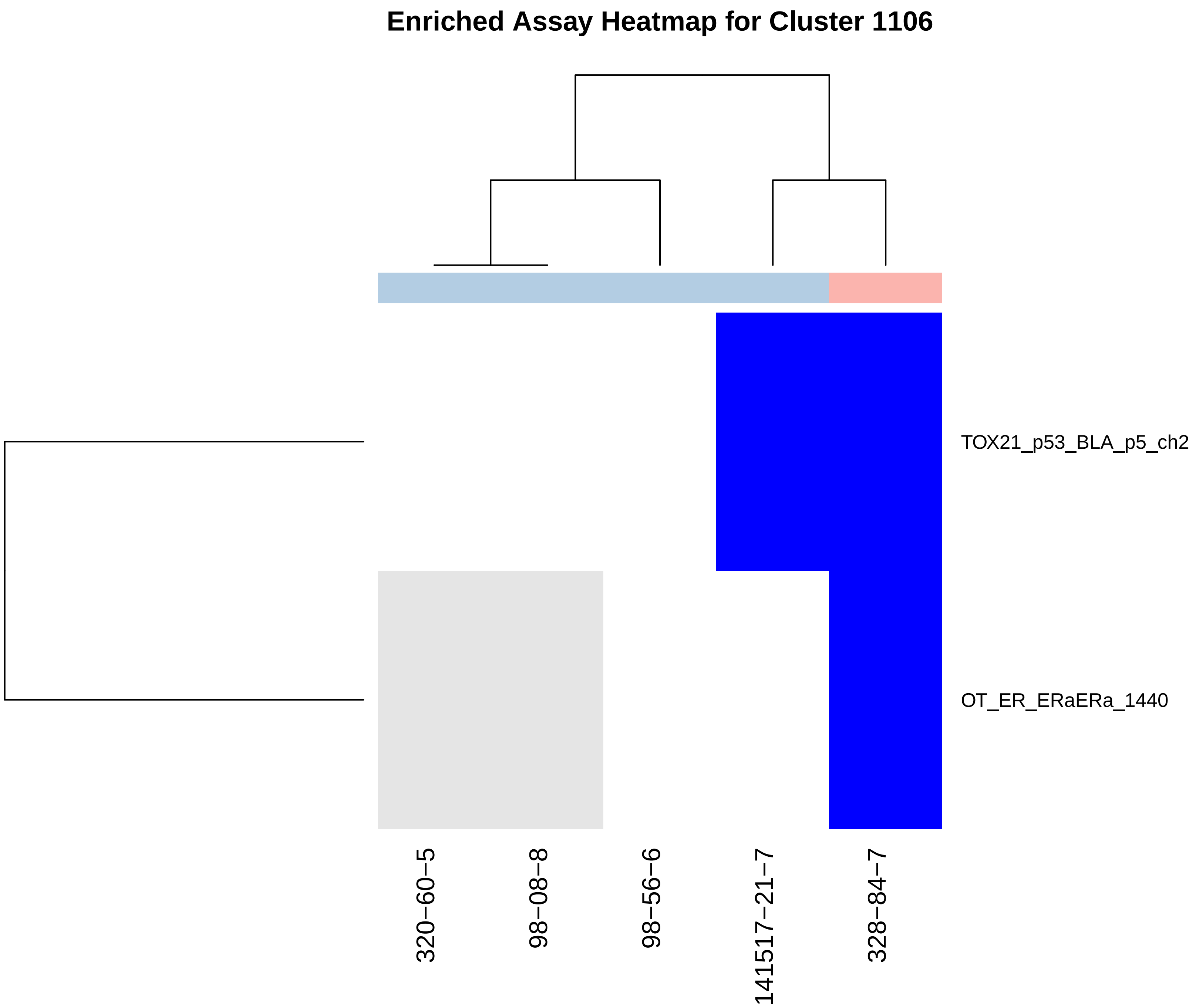
Enriched Assay Heatmap for Cluster 1106
TOX21_p53_BLA_p5_ch2
OT_ER_ERaERa_1440
320−60−5
98−08−8
98−56−6
141517−21−7
328−84−7

Enriched Assay Heatmap for Cluster 1108

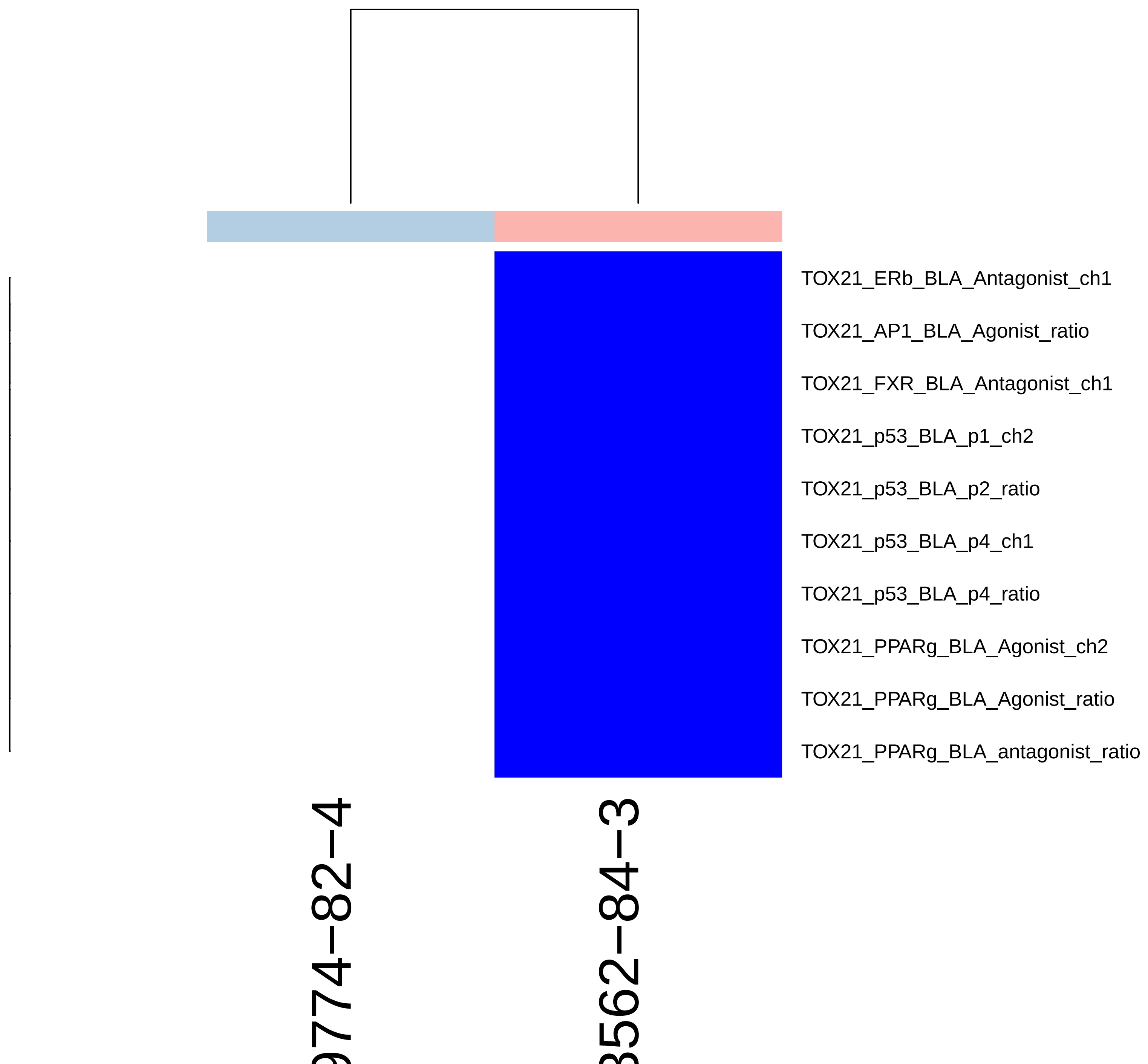

Enriched Assay Heatmap for Cluster 1111

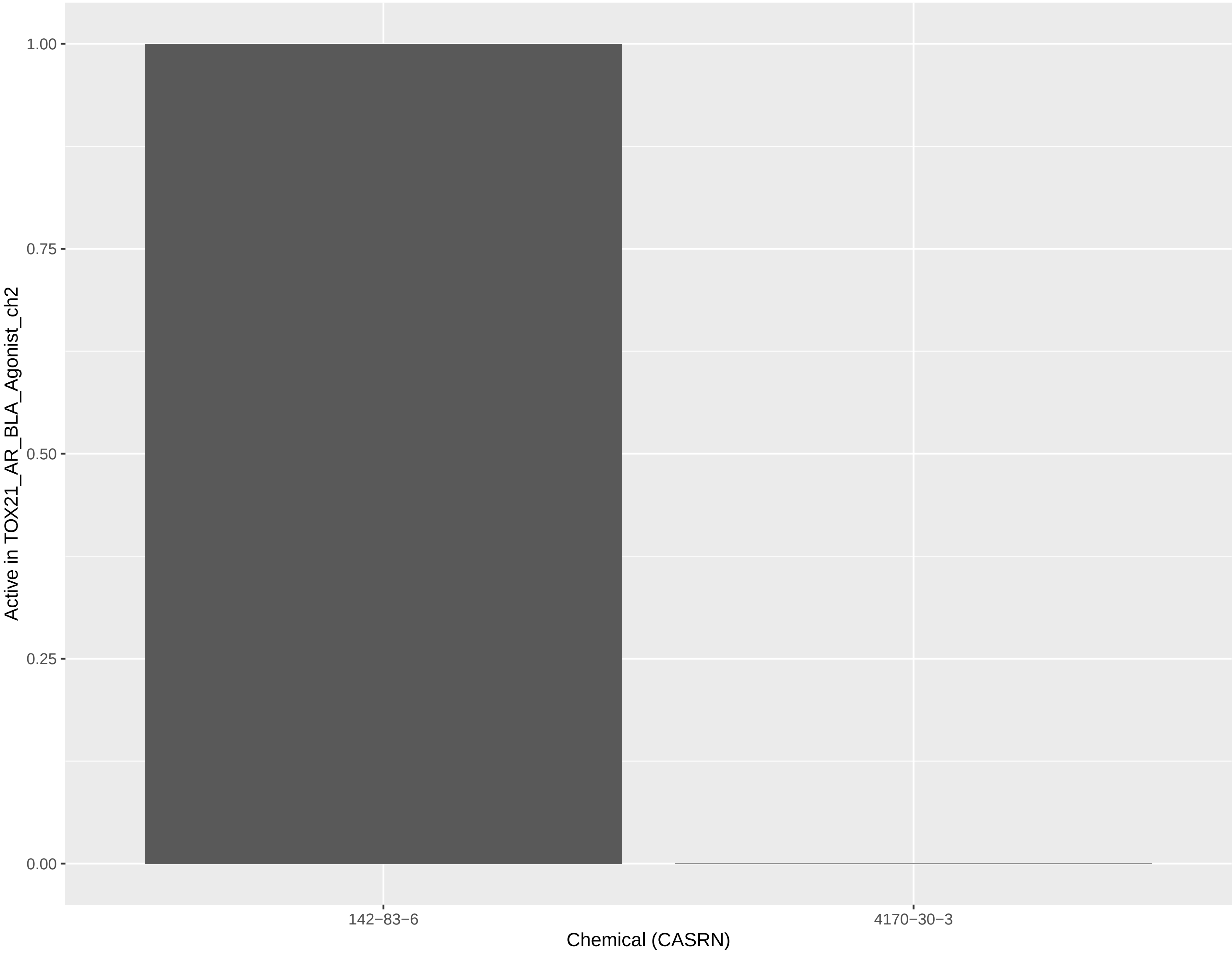

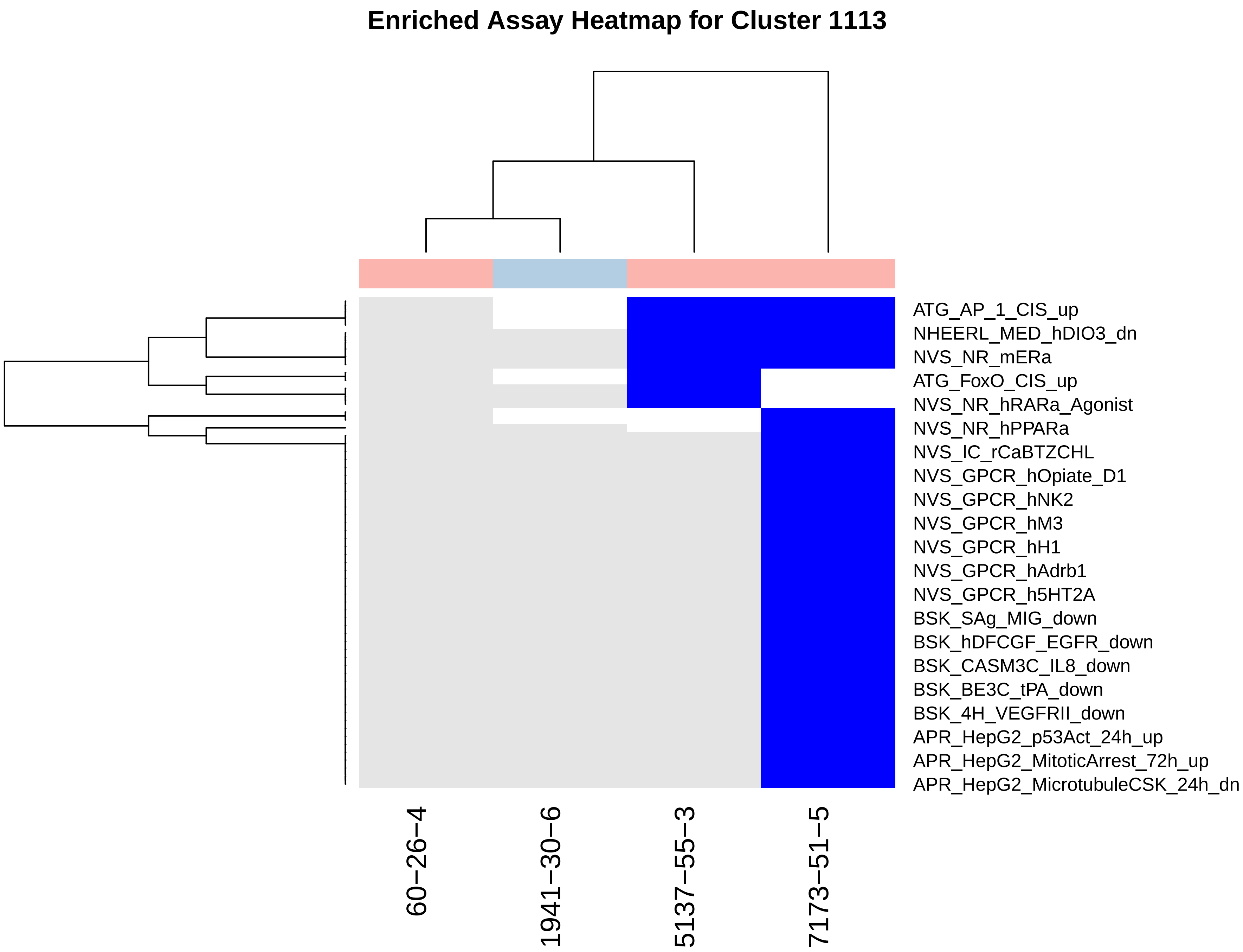
Enriched Assay Heatmap for Cluster 1113
ATG_AP_1_CIS_up
NHEERL_MED_hDIO3_dn
NVS_NR_mERa
ATG_FoxO_CIS_up
NVS_NR_hRARa_Agonist
NVS_NR_hPPARa
NVS_IC_rCaBTZCHL
NVS_GPCR_hOpiate_D1
NVS_GPCR_hNK2
NVS_GPCR_hM3
NVS_GPCR_hH1
NVS_GPCR_hAdrb1
NVS_GPCR_h5HT2A
BSK_SAg_MIG_down
BSK_hDFCGF_EGFR_down
BSK_CASM3C_IL8_down
BSK_BE3C_tPA_down
BSK_4H_VEGFRII_down
APR_HepG2_p53Act_24h_up
APR_HepG2_MitoticArrest_72h_up
APR_HepG2_MicrotubuleCSK_24h_dn
60−26−4
1941−30−6
5137−55−3
7173−51−5

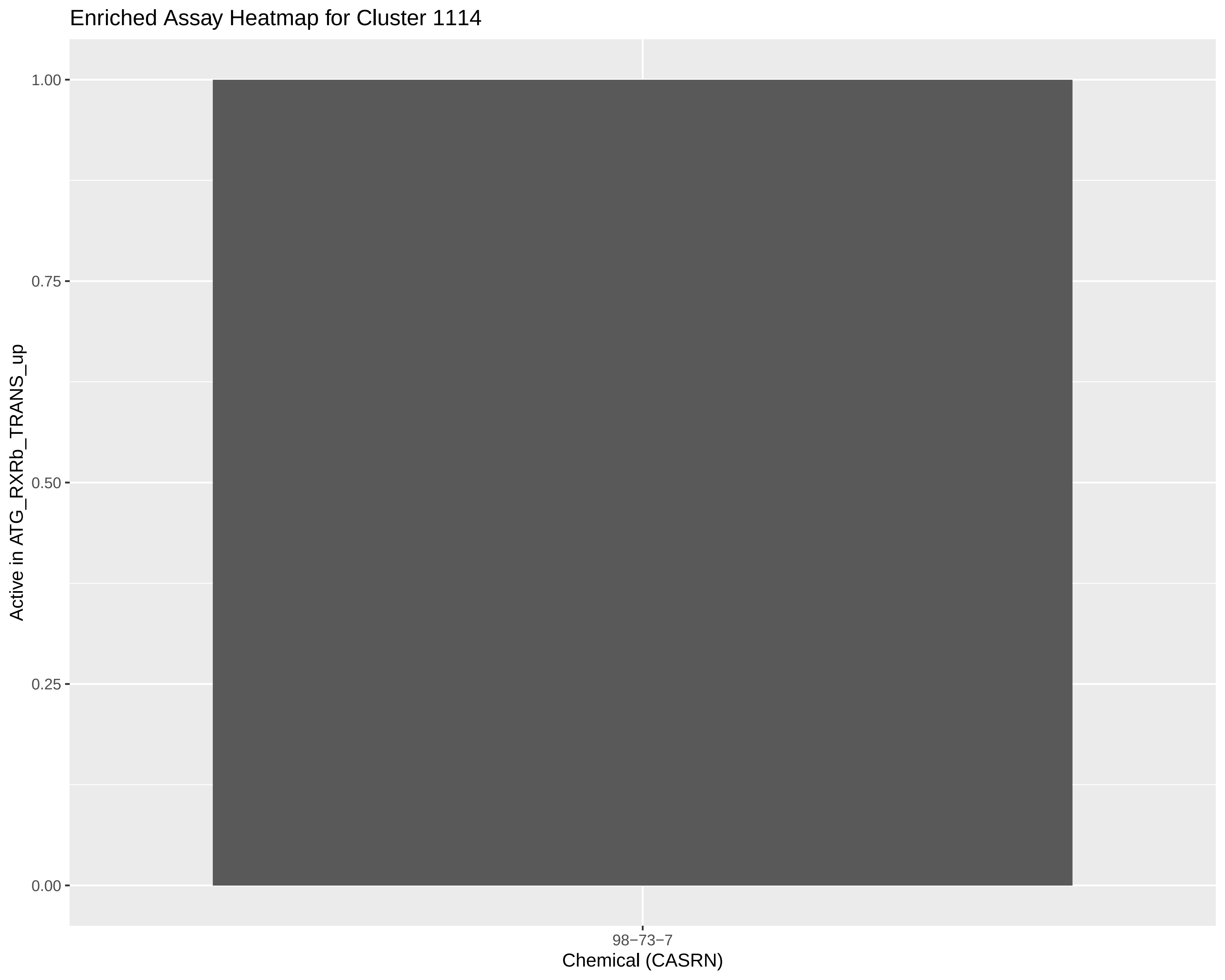

Enriched Assay Heatmap for Cluster 1114
1.00
0.75
0.50
0.25
0.00
Active in ATG_RXRb_TRANS_up
98−73−7
Chemical (CASRN)

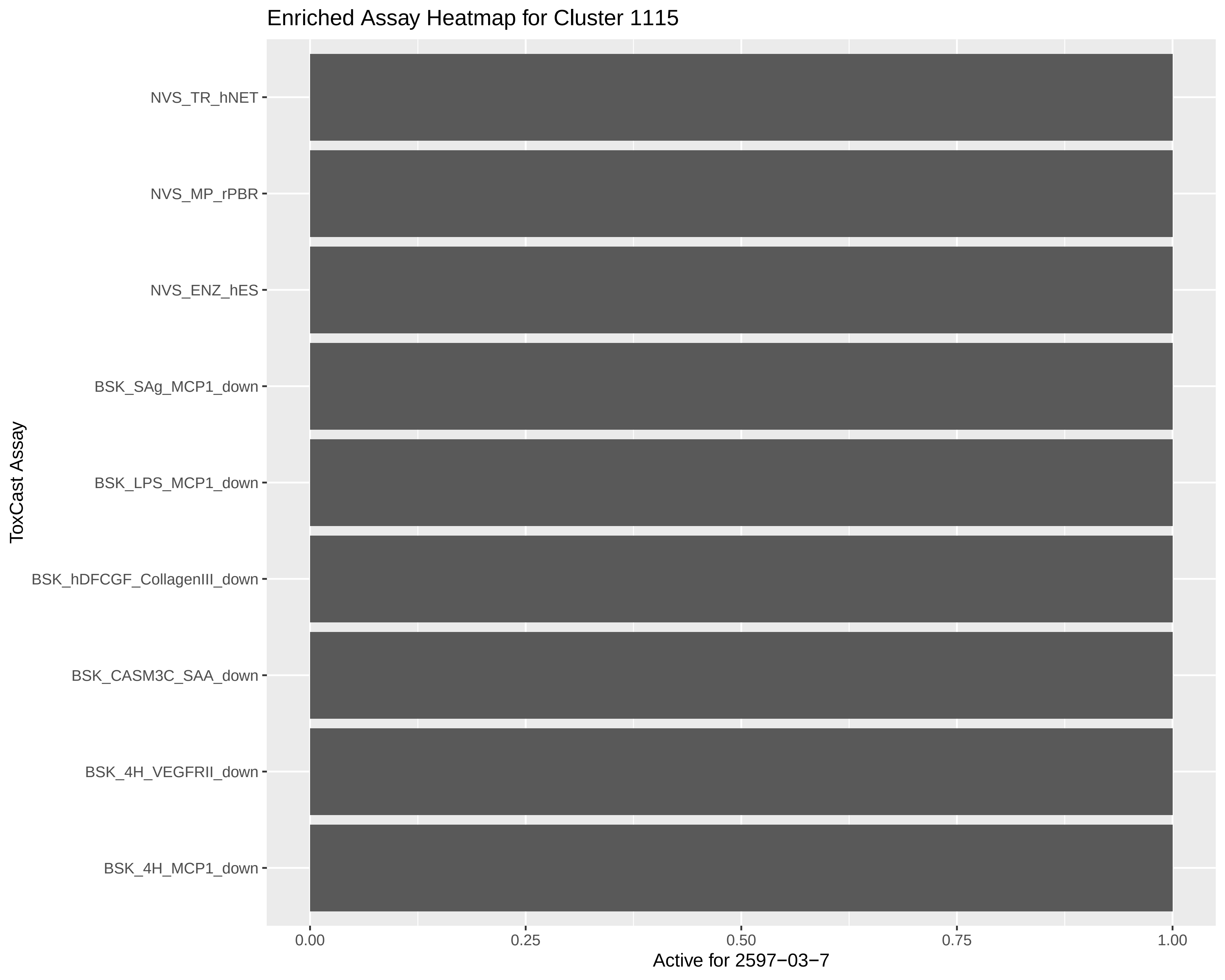

Enriched Assay Heatmap for Cluster 1115
ToxCast Assay
NVS_TR_hNET
NVS_MP_rPBR
NVS_ENZ_hES
BSK_SAg_MCP1_down
BSK_LPS_MCP1_down
BSK_hDFCGF_CollagenIII_down
BSK_CASM3C_SAA_down
BSK_4H_VEGFRII_down
BSK_4H_MCP1_down
0.00
0.25
0.50
0.75
1.00
Active for 2597−03−7

Enriched Assay Heatmap for Cluster 1118

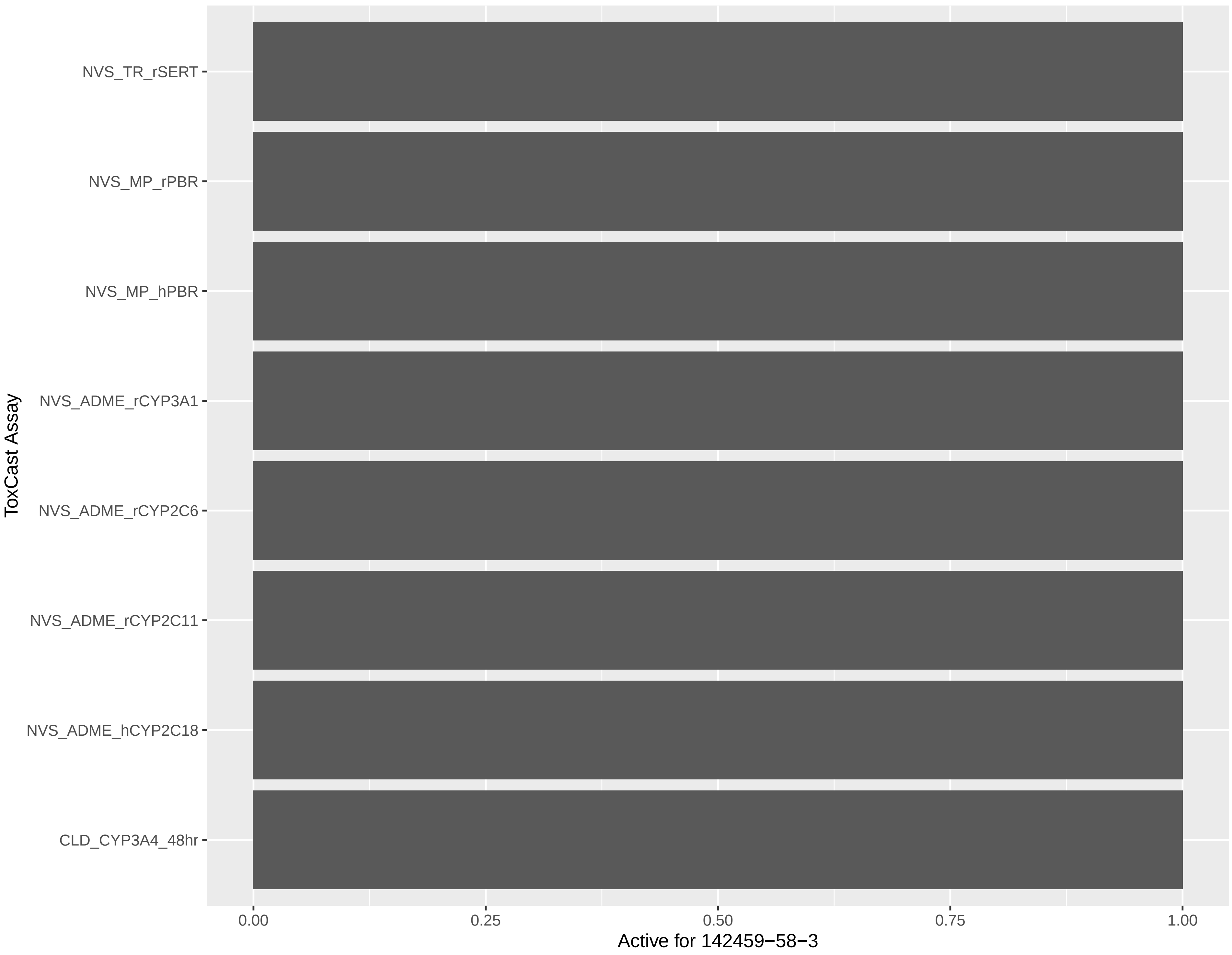

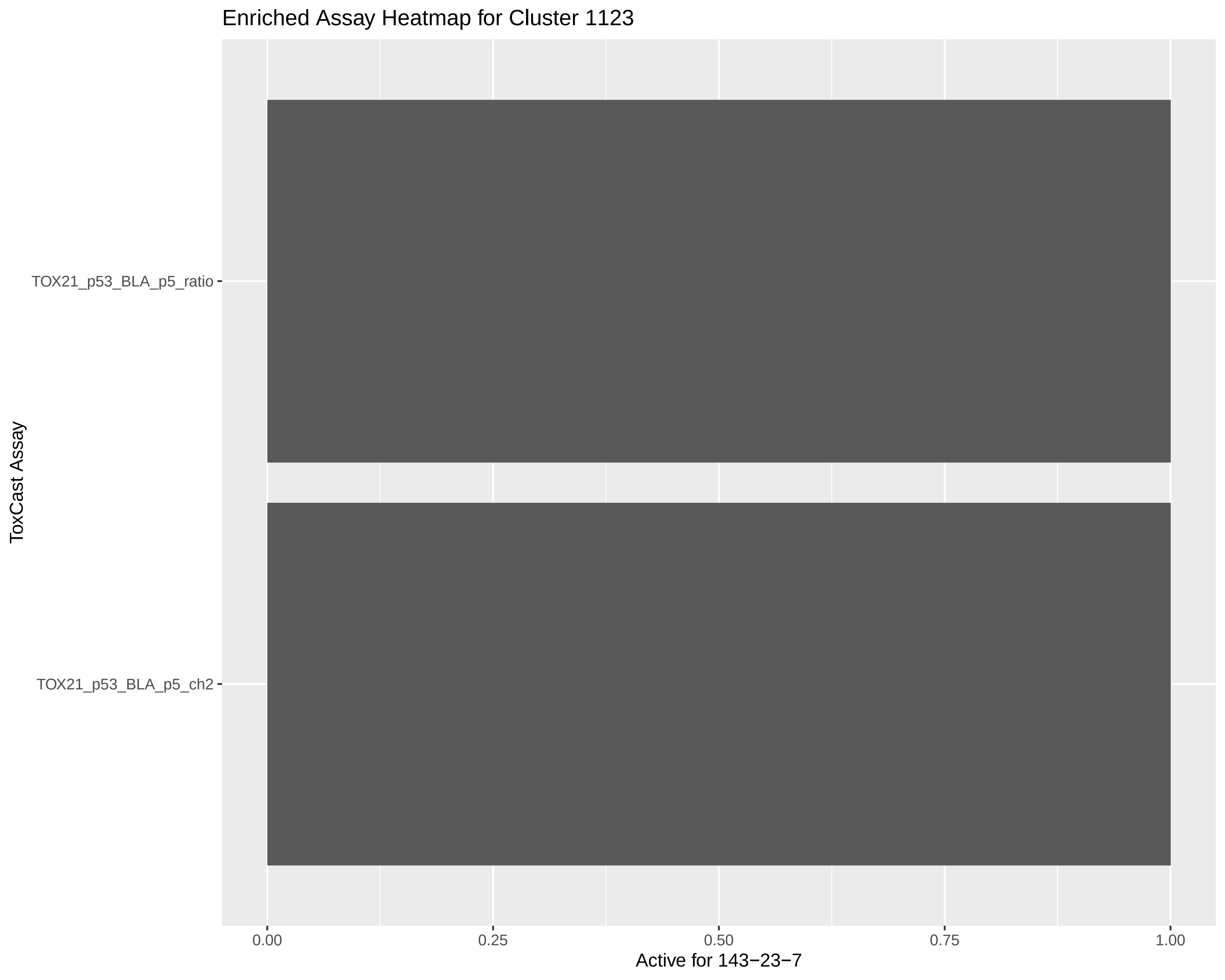
Enriched Assay Heatmap for Cluster 1123
ToxCast Assay
TOX21_p53_BLA_p5_ratio
TOX21_p53_BLA_p5_ch2
0.00
0.25
0.50
0.75
1.00
Active for 143−23−7

Enriched Assay Heatmap for Cluster 1124

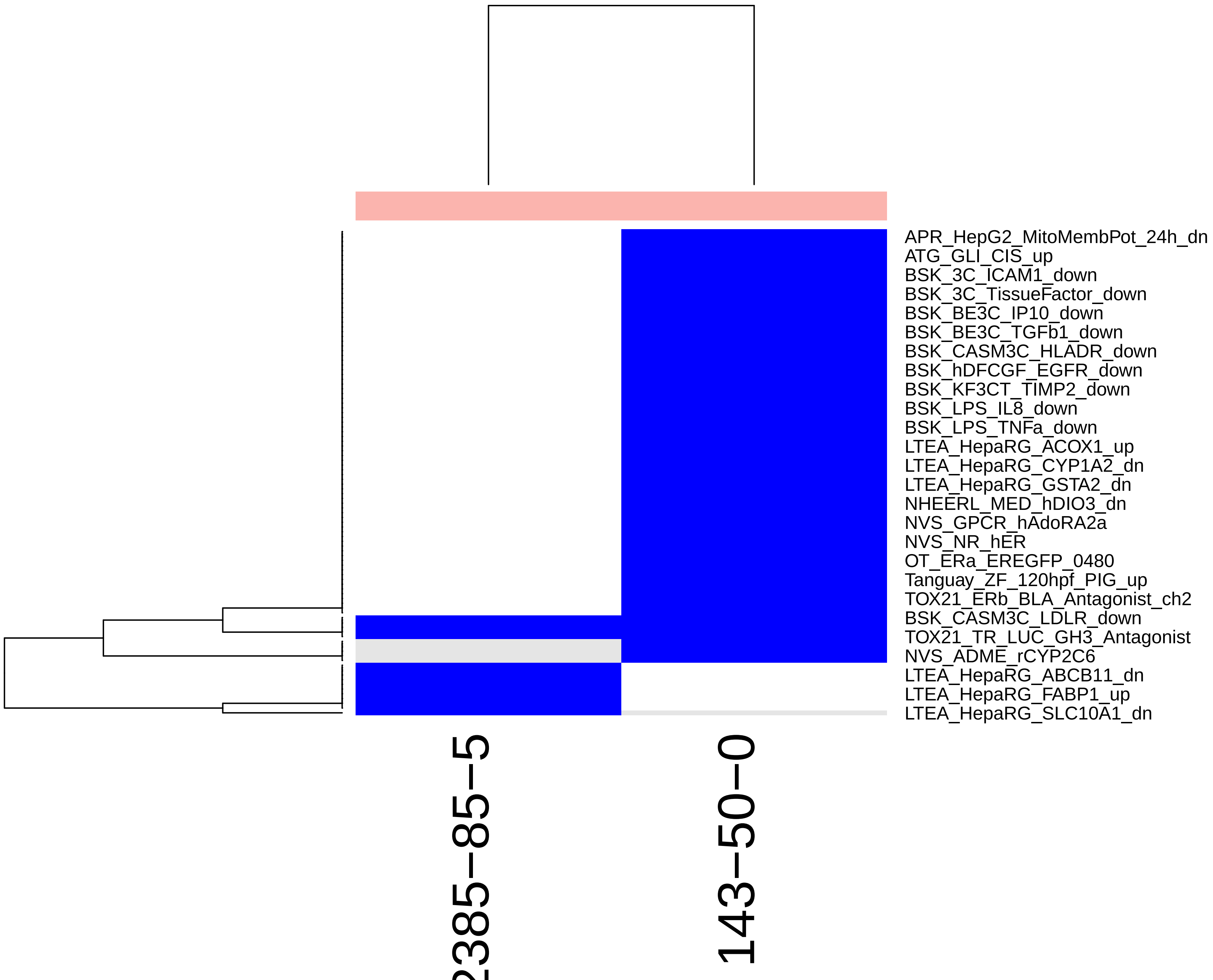

Enriched Assay Heatmap for Cluster 1125

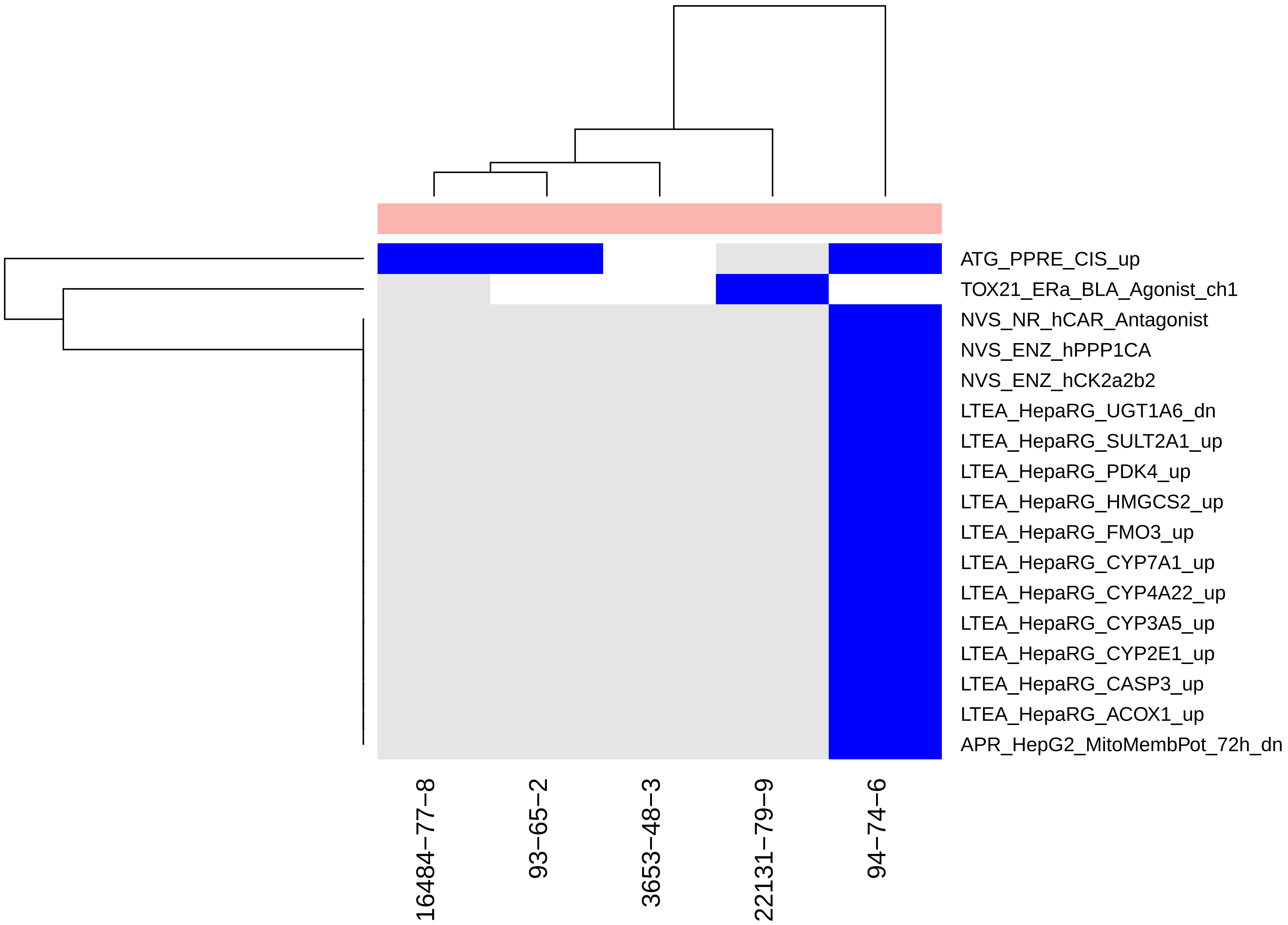

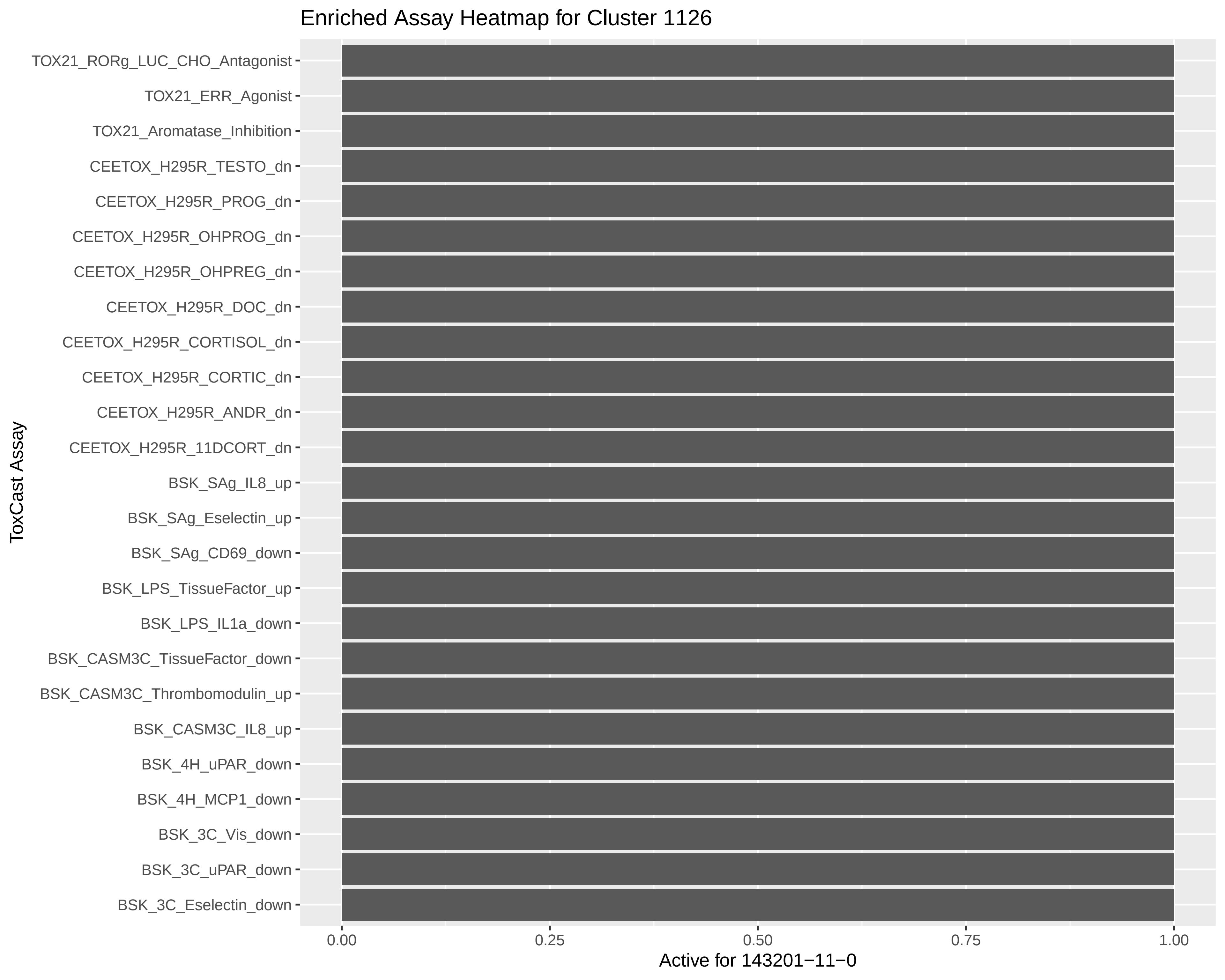
Enriched Assay Heatmap for Cluster 1126
ToxCast Assay
TOX21_RORg_LUC_CHO_Antagonist
TOX21_ERR_Agonist
TOX21_Aromatase_Inhibition
CEETOX_H295R_TESTO_dn
CEETOX_H295R_PROG_dn
CEETOX_H295R_OHPROG_dn
CEETOX_H295R_OHPREG_dn
CEETOX_H295R_DOC_dn
CEETOX_H295R_CORTISOL_dn
CEETOX_H295R_CORTIC_dn
CEETOX_H295R_ANDR_dn
CEETOX_H295R_11DCORT_dn
BSK_SAg_IL8_up
BSK_SAg_Eselectin_up
BSK_SAg_CD69_down
BSK_LPS_TissueFactor_up
BSK_LPS_IL1a_down
BSK_CASM3C_TissueFactor_down
BSK_CASM3C_Thrombomodulin_up
BSK_CASM3C_IL8_up
BSK_4H_uPAR_down
BSK_4H_MCP1_down
BSK_3C_Vis_down
BSK_3C_uPAR_down
BSK_3C_Eselectin_down
0.00
0.25
0.50
0.75
1.00
Active for 143201−11−0

Enriched Assay Heatmap for Cluster 1130

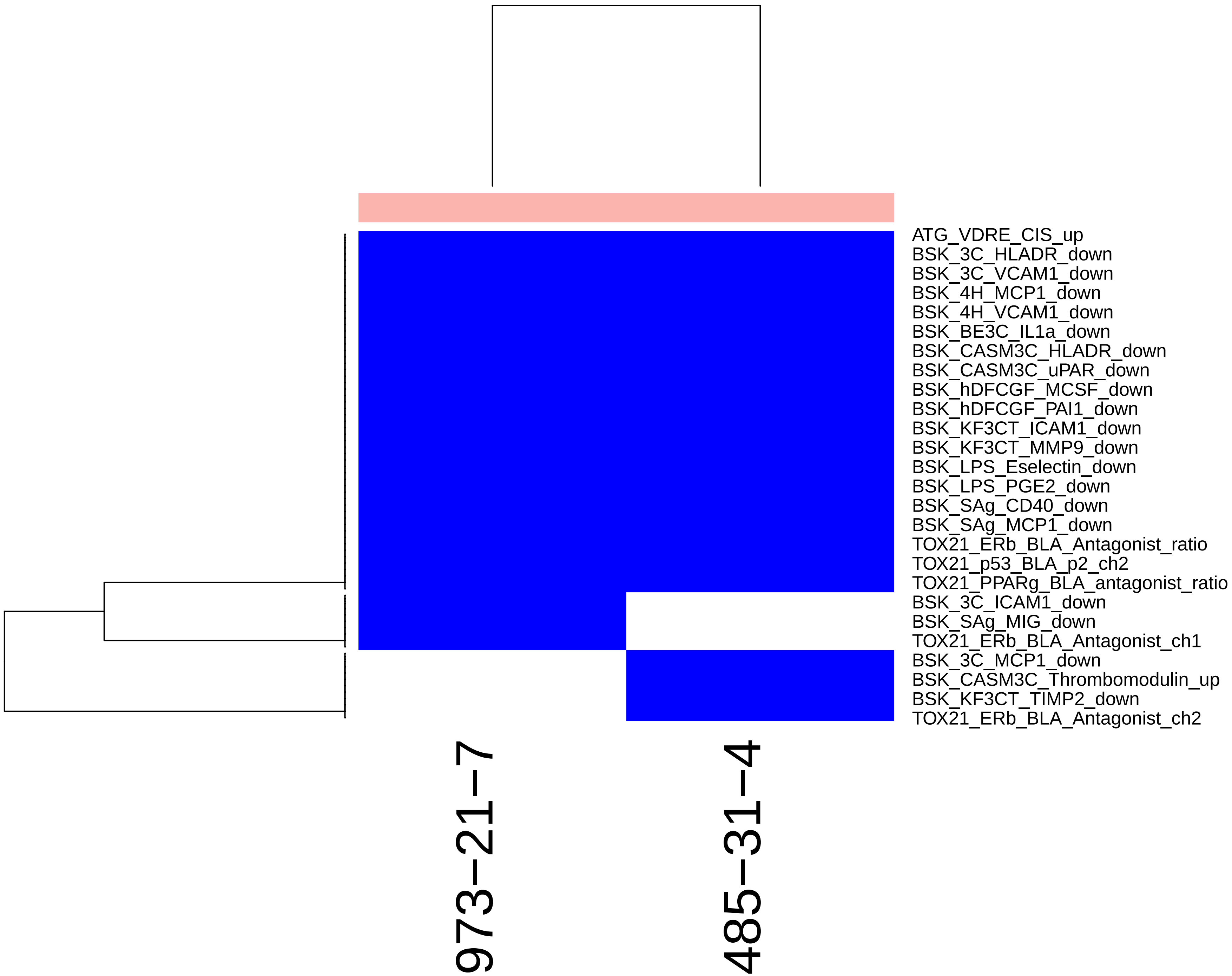

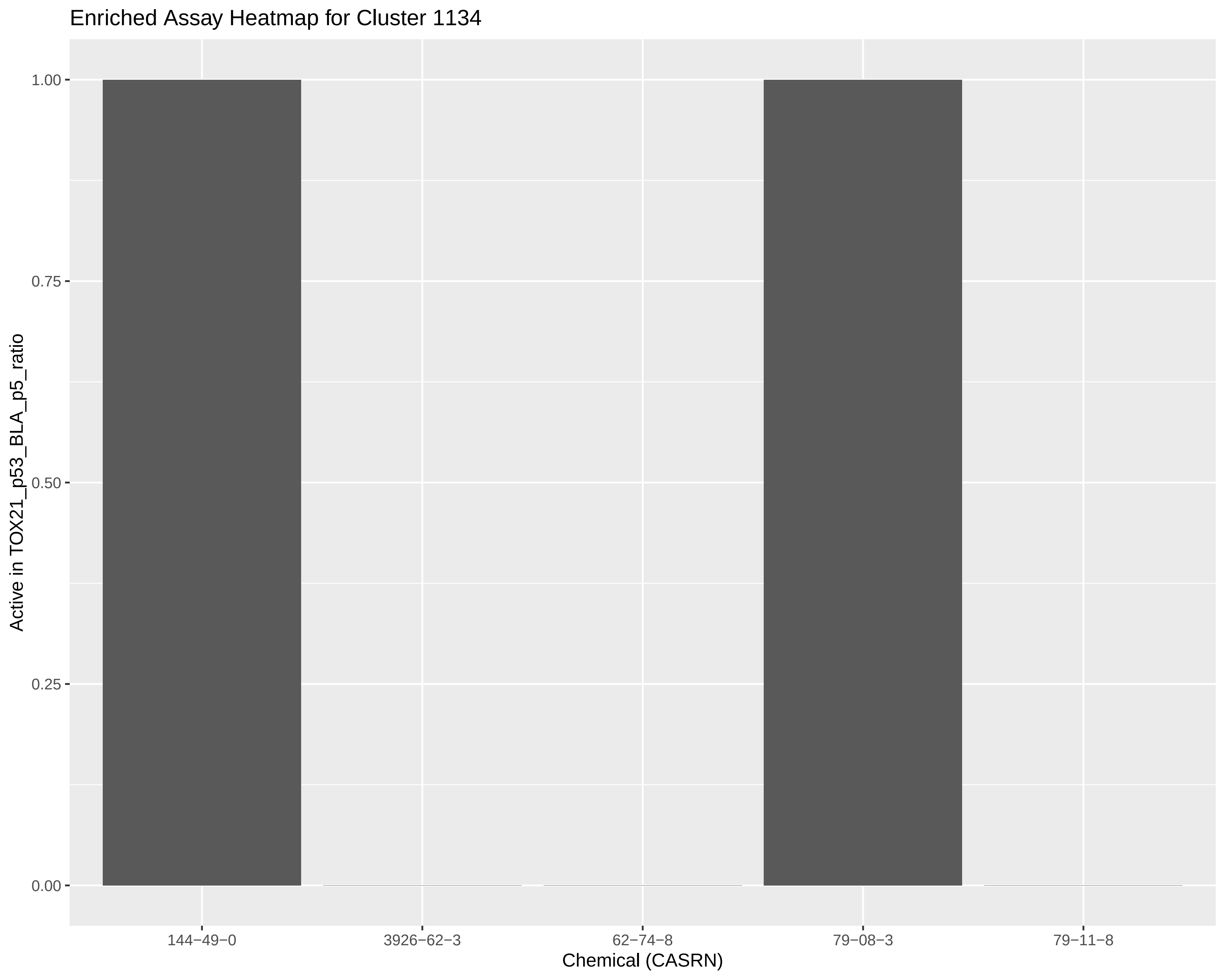

Enriched Assay Heatmap for Cluster 1134
1.00
0.75
0.50
0.25
0.00
Active in TOX21_p53_BLA_p5_ratio
144−49−0
3926−62−3
62−74−8
79−08−3
79−11−8
Chemical (CASRN)

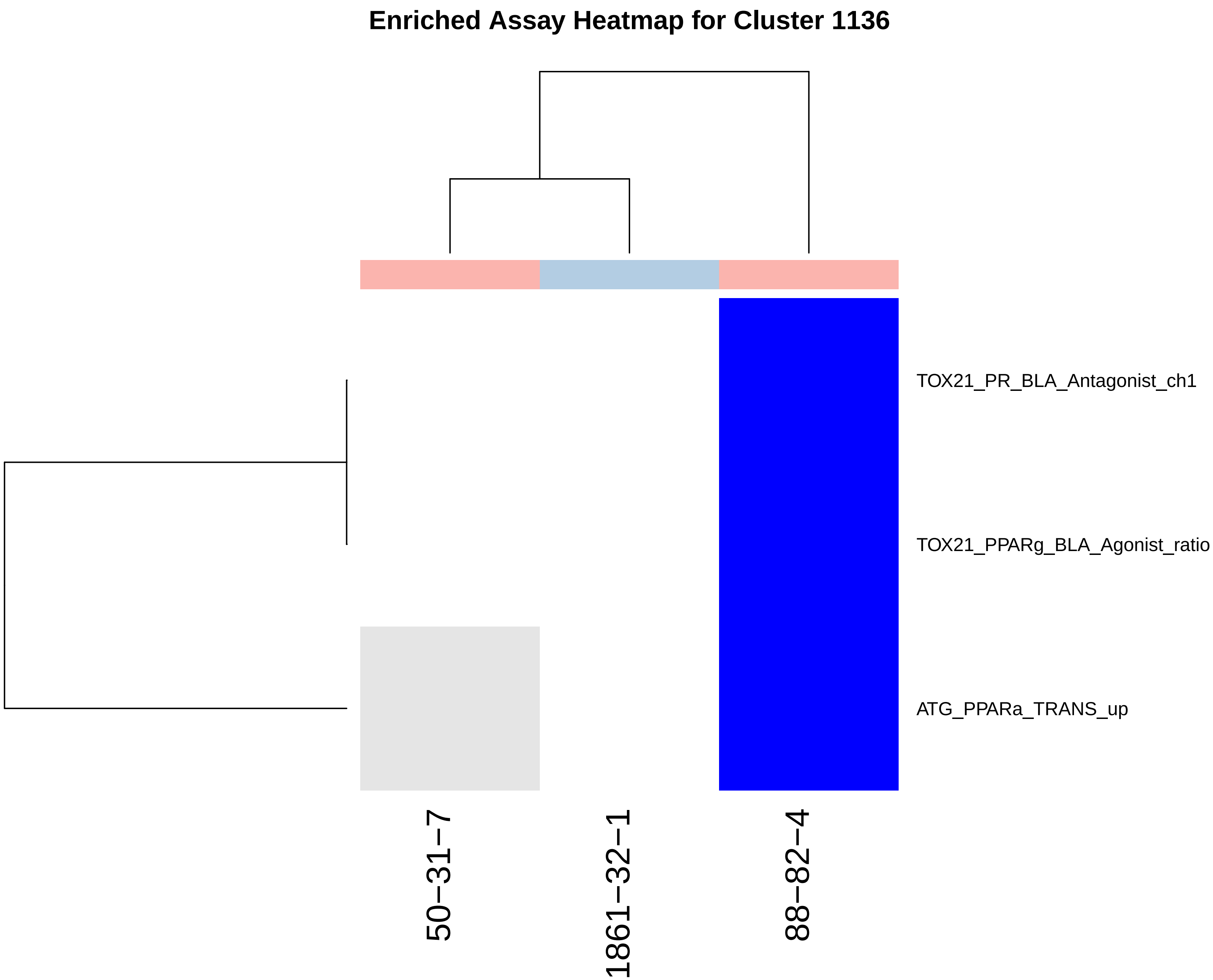
Enriched Assay Heatmap for Cluster 1136
TOX21_PR_BLA_Antagonist_ch1
TOX21_PPARg_BLA_Agonist_ratio
ATG_PPARa_TRANS_up
50−31−7
1861−32−1
88−82−4

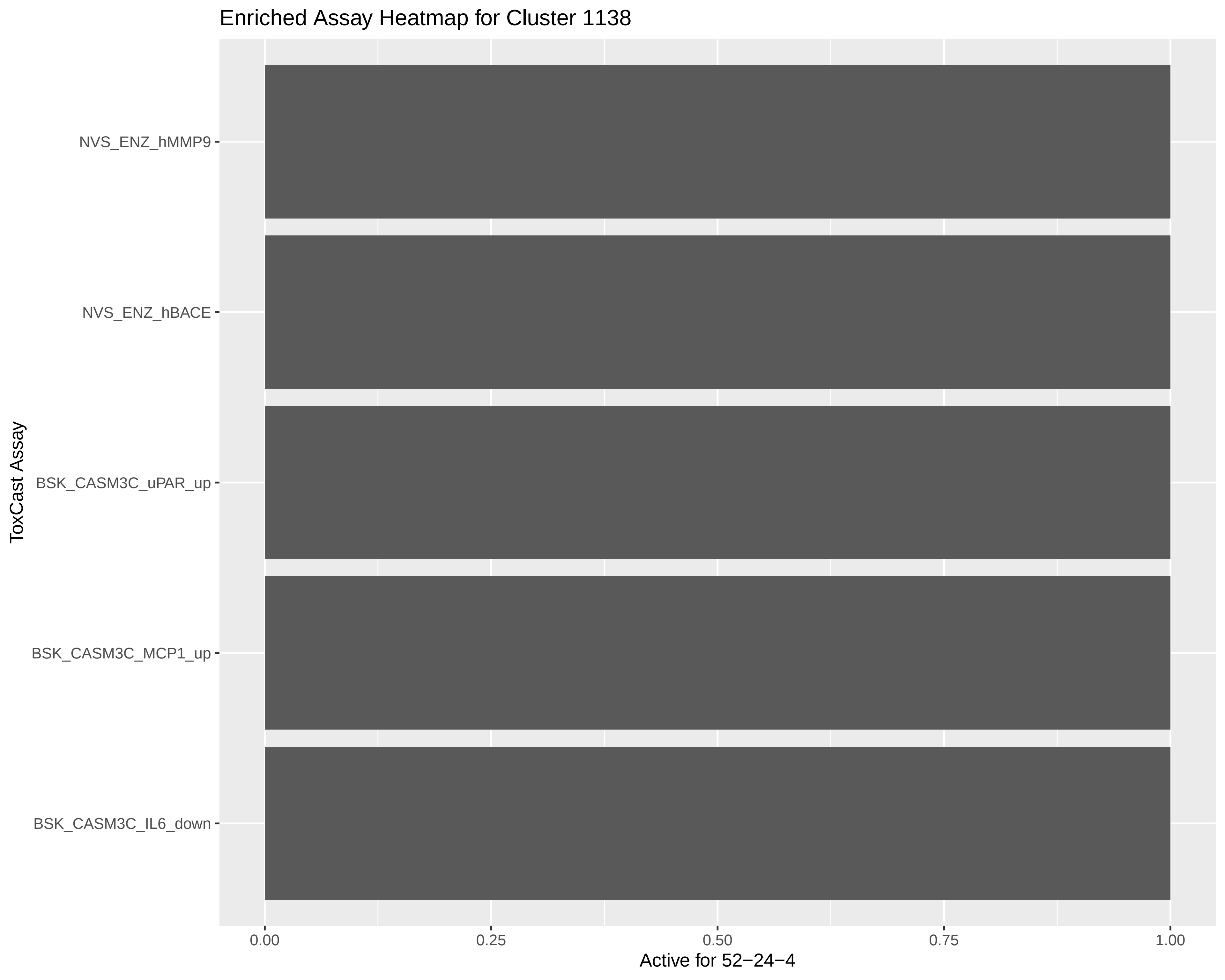

Enriched Assay Heatmap for Cluster 1138
NVS_ENZ_hMMP9
NVS_ENZ_hBACE
BSK_CASM3C_uPAR_up
BSK_CASM3C_MCP1_up
BSK_CASM3C_IL6_down
ToxCast Assay
0.00
0.25
0.50
0.75
1.00
Active for 52−24−4

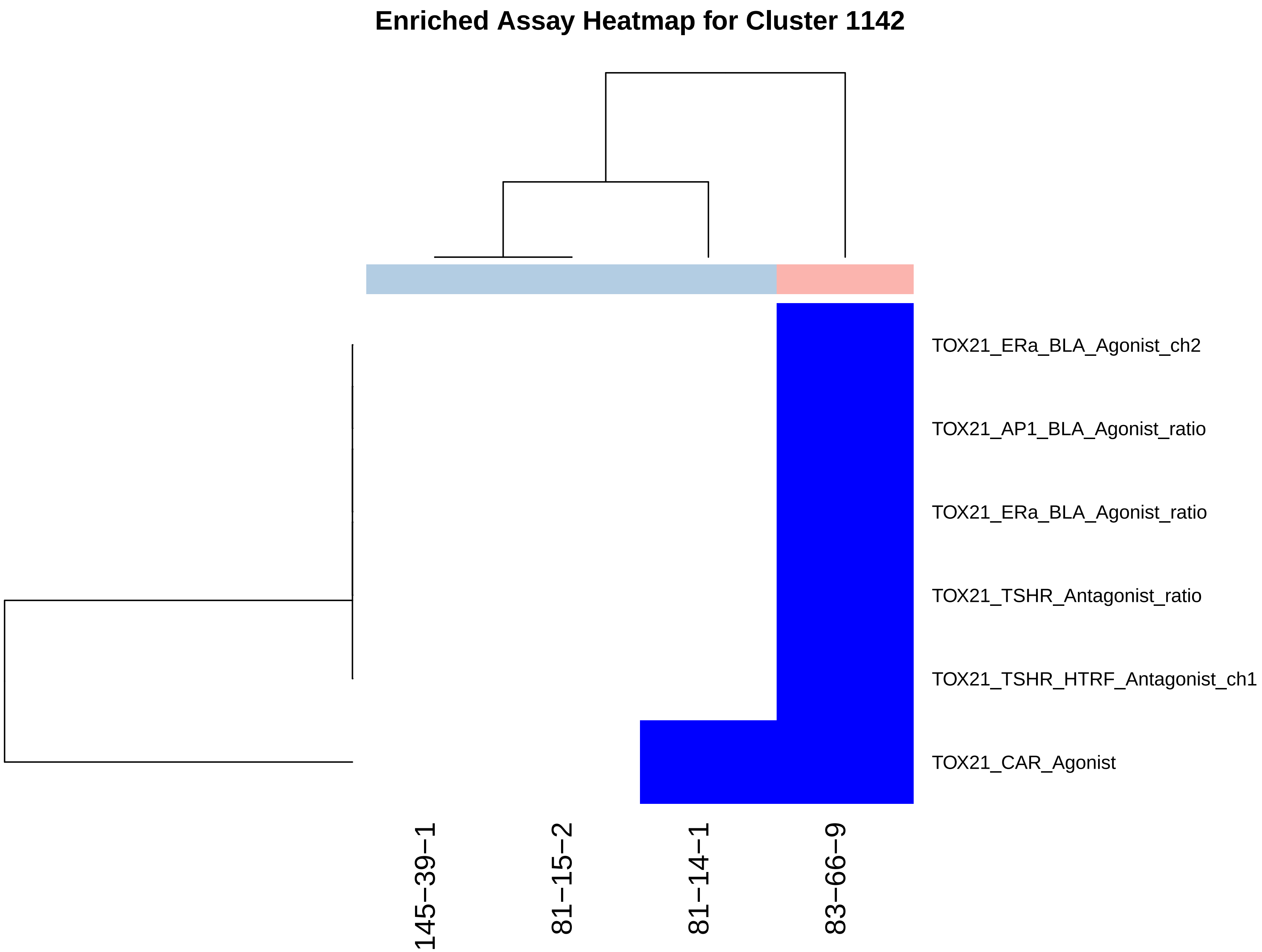
Enriched Assay Heatmap for Cluster 1142
TOX21_ERa_BLA_Agonist_ch2
TOX21_AP1_BLA_Agonist_ratio
TOX21_ERa_BLA_Agonist_ratio
TOX21_TSHR_Antagonist_ratio
TOX21_TSHR_HTRF_Antagonist_ch1
TOX21_CAR_Agonist
145−39−1
81−15−2
81−14−1
83−66−9

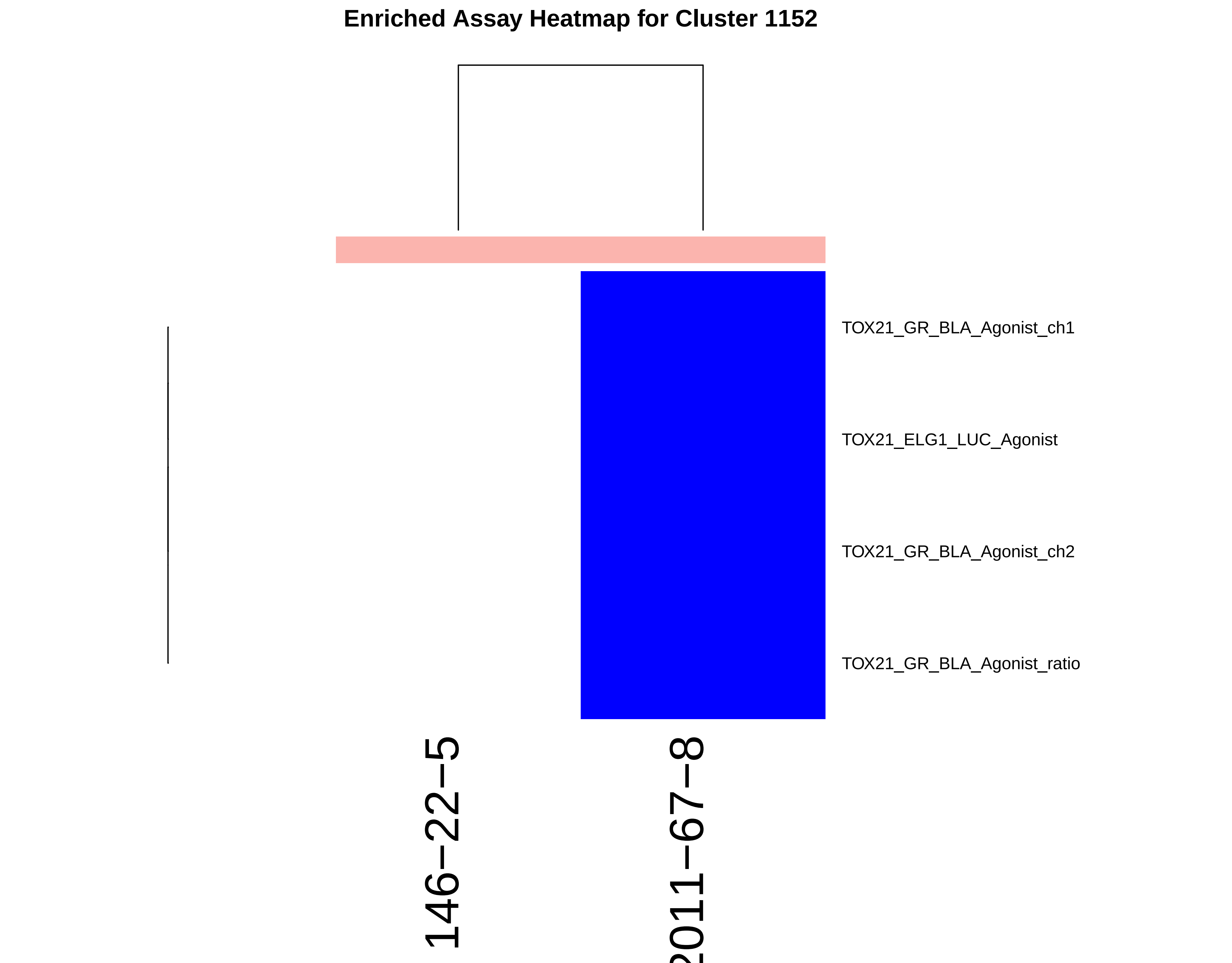
Enriched Assay Heatmap for Cluster 1152
TOX21_GR_BLA_Agonist_ch1
TOX21_ELG1_LUC_Agonist
TOX21_GR_BLA_Agonist_ch2
TOX21_GR_BLA_Agonist_ratio
146−22−5
2011−67−8

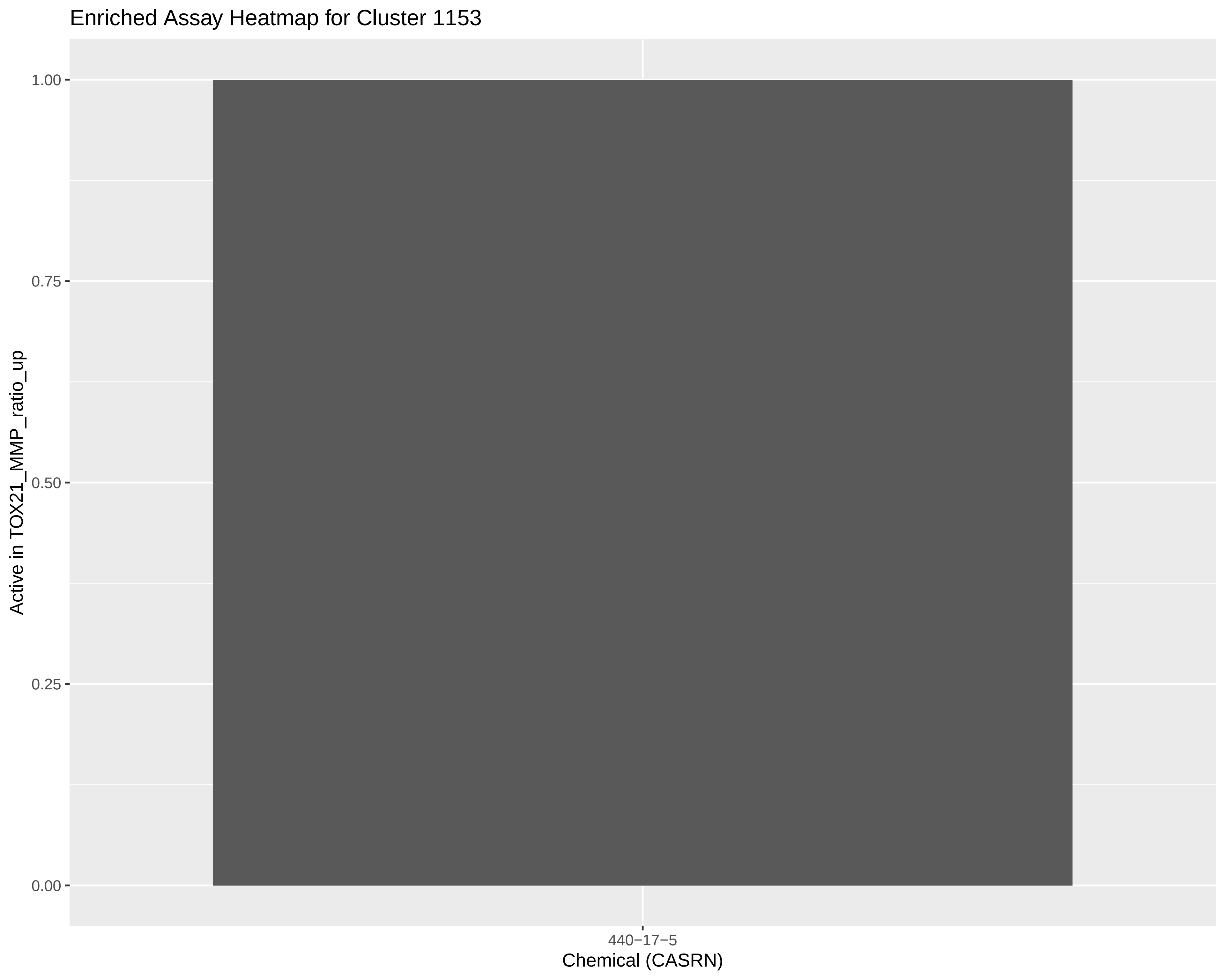

Enriched Assay Heatmap for Cluster 1153
Active in TOX21_MMP_ratio_up
1.00
0.75
0.50
0.25
0.00
440−17−5
Chemical (CASRN)

Enriched Assay Heatmap for Cluster 1157

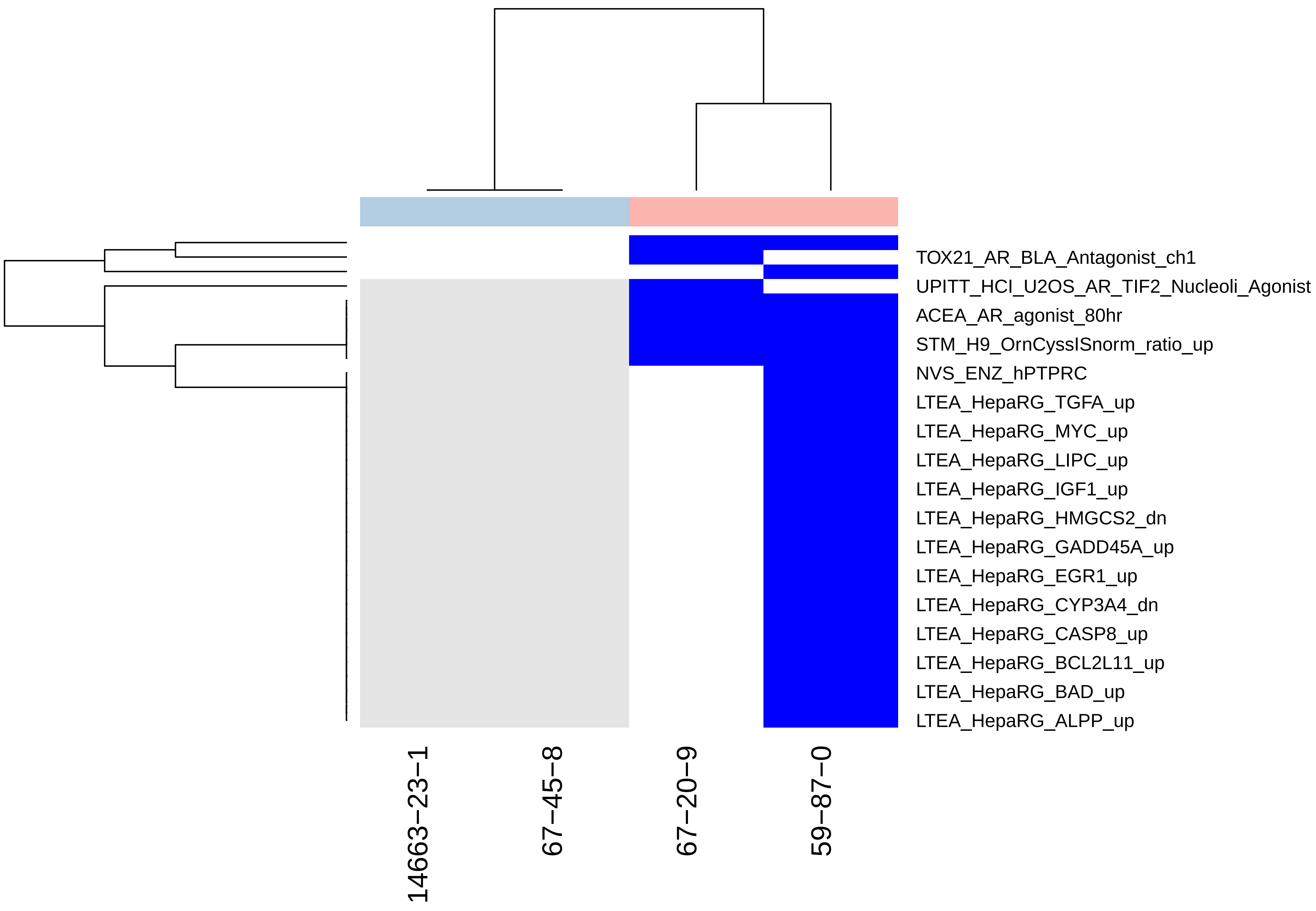

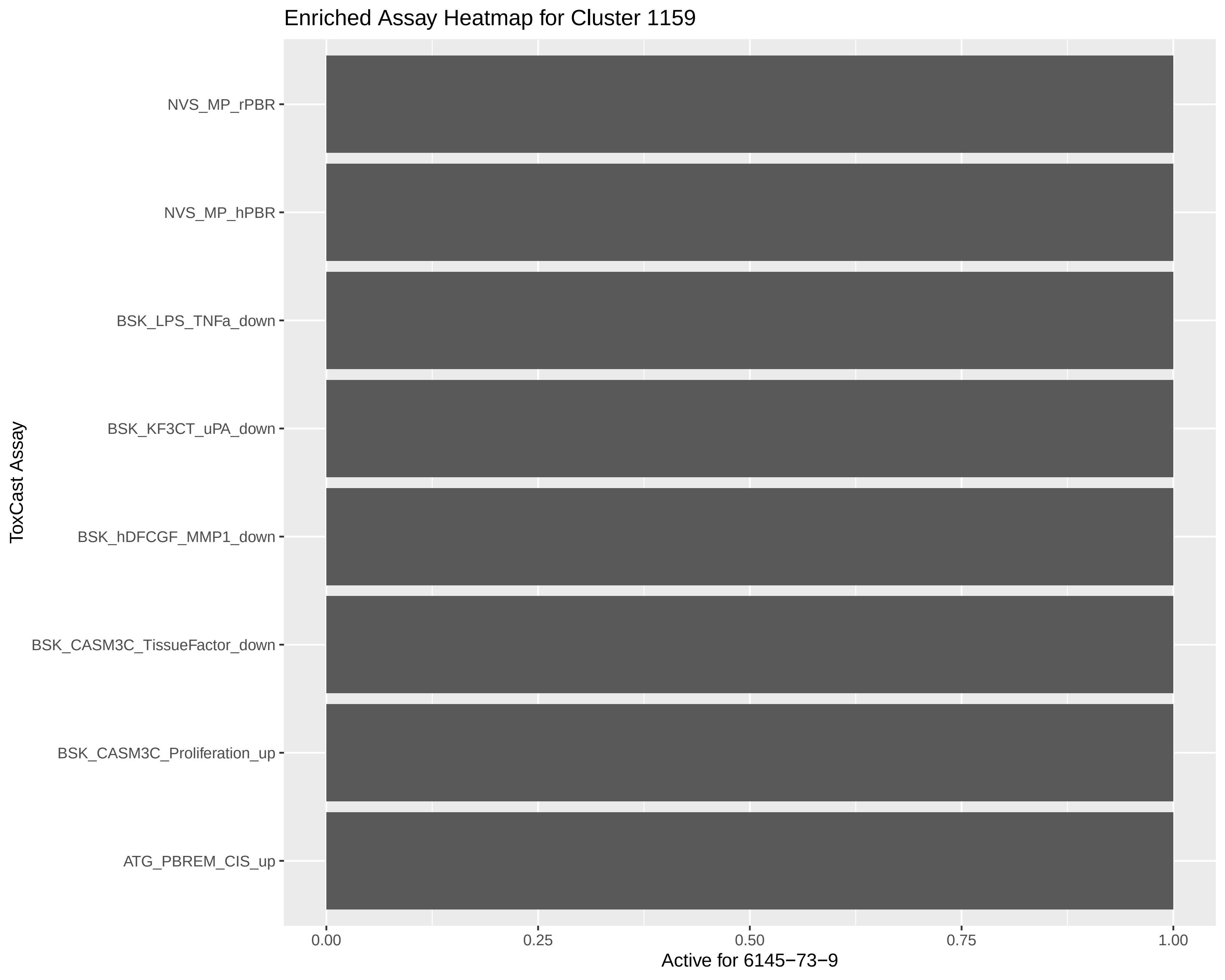

Enriched Assay Heatmap for Cluster 1159
ToxCast Assay
NVS_MP_rPBR
NVS_MP_hPBR
BSK_LPS_TNFa_down
BSK_KF3CT_uPA_down
BSK_hDFCGF_MMP1_down
BSK_CASM3C_TissueFactor_down
BSK_CASM3C_Proliferation_up
ATG_PBREM_CIS_up
0.00
0.25
0.50
0.75
1.00
Active for 6145−73−9

Enriched Assay Heatmap for Cluster 1167

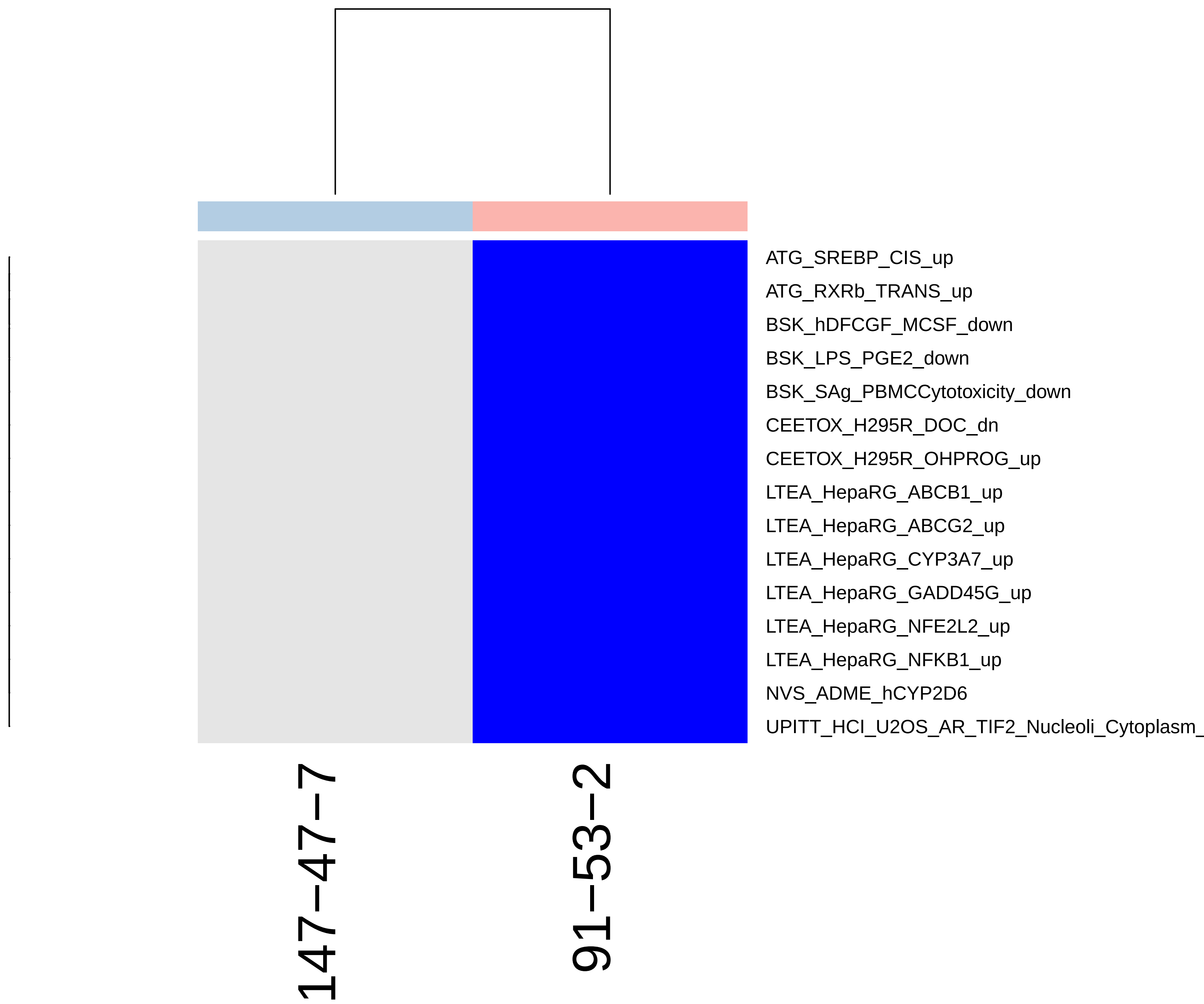

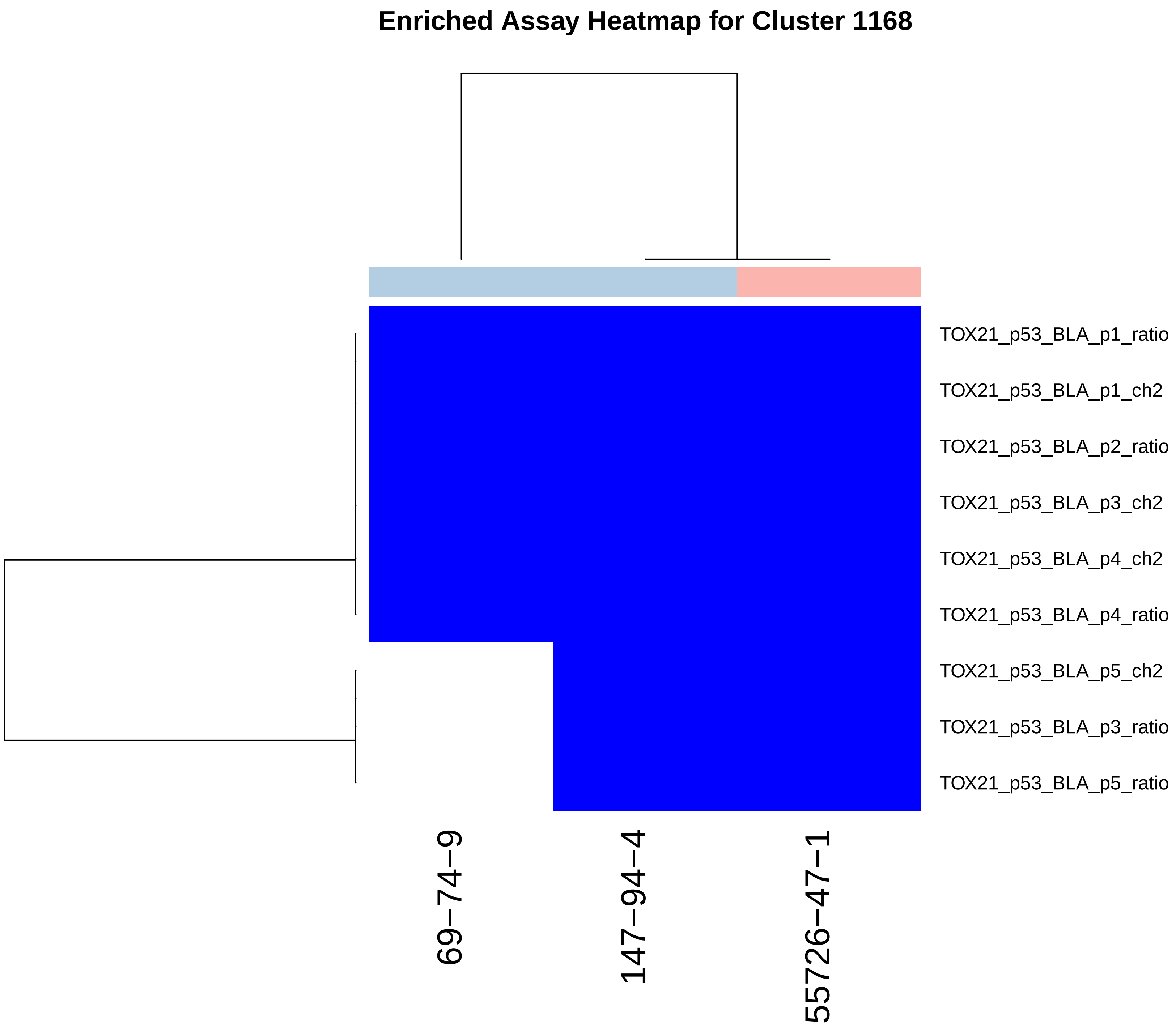
Enriched Assay Heatmap for Cluster 1168
TOX21_p53_BLA_p1_ratio
TOX21_p53_BLA_p1_ch2
TOX21_p53_BLA_p2_ratio
TOX21_p53_BLA_p3_ch2
TOX21_p53_BLA_p4_ch2
TOX21_p53_BLA_p4_ratio
TOX21_p53_BLA_p5_ch2
TOX21_p53_BLA_p3_ratio
TOX21_p53_BLA_p5_ratio
69−74−9
147−94−4
55726−47−1

Enriched Assay Heatmap for Cluster 1173

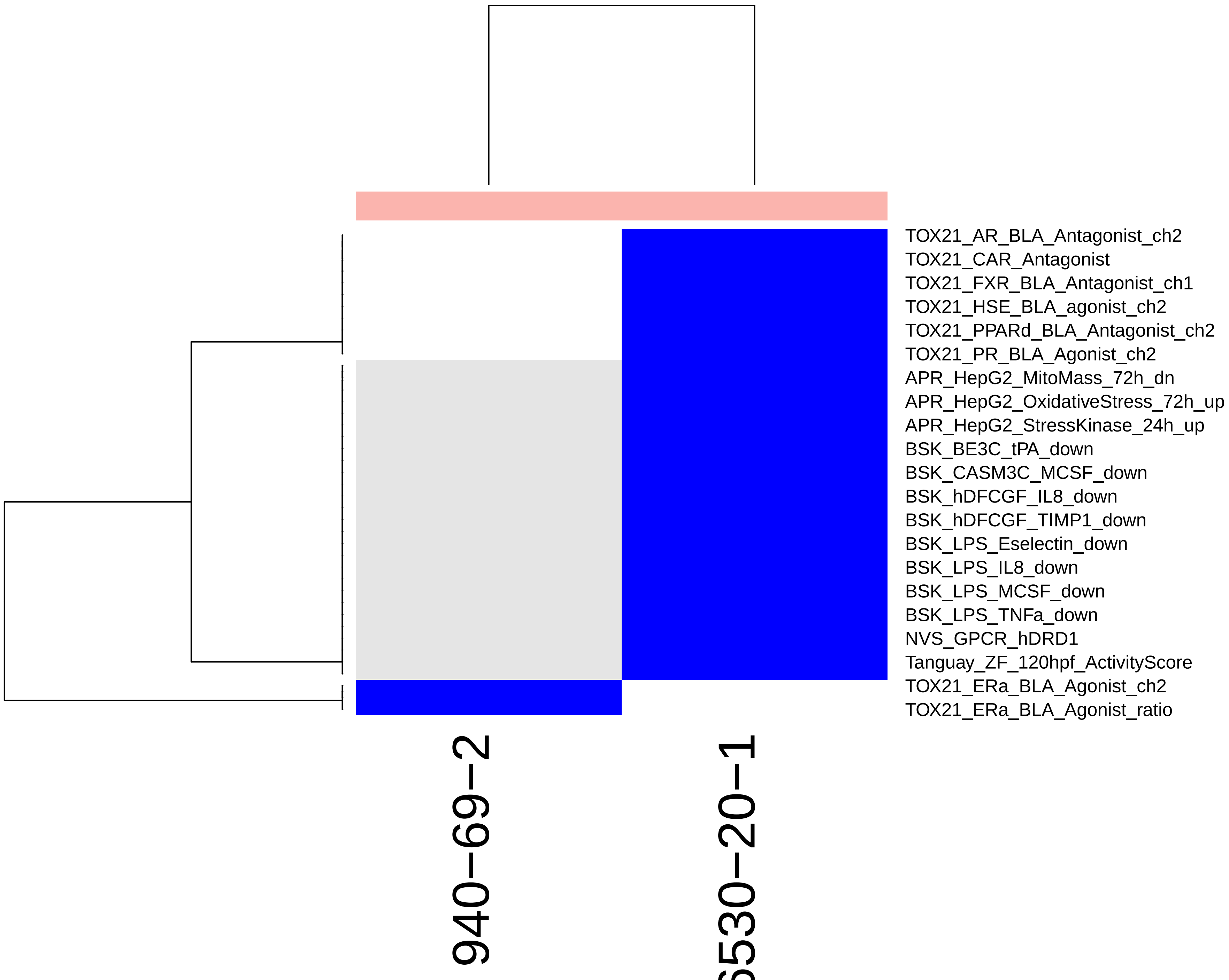

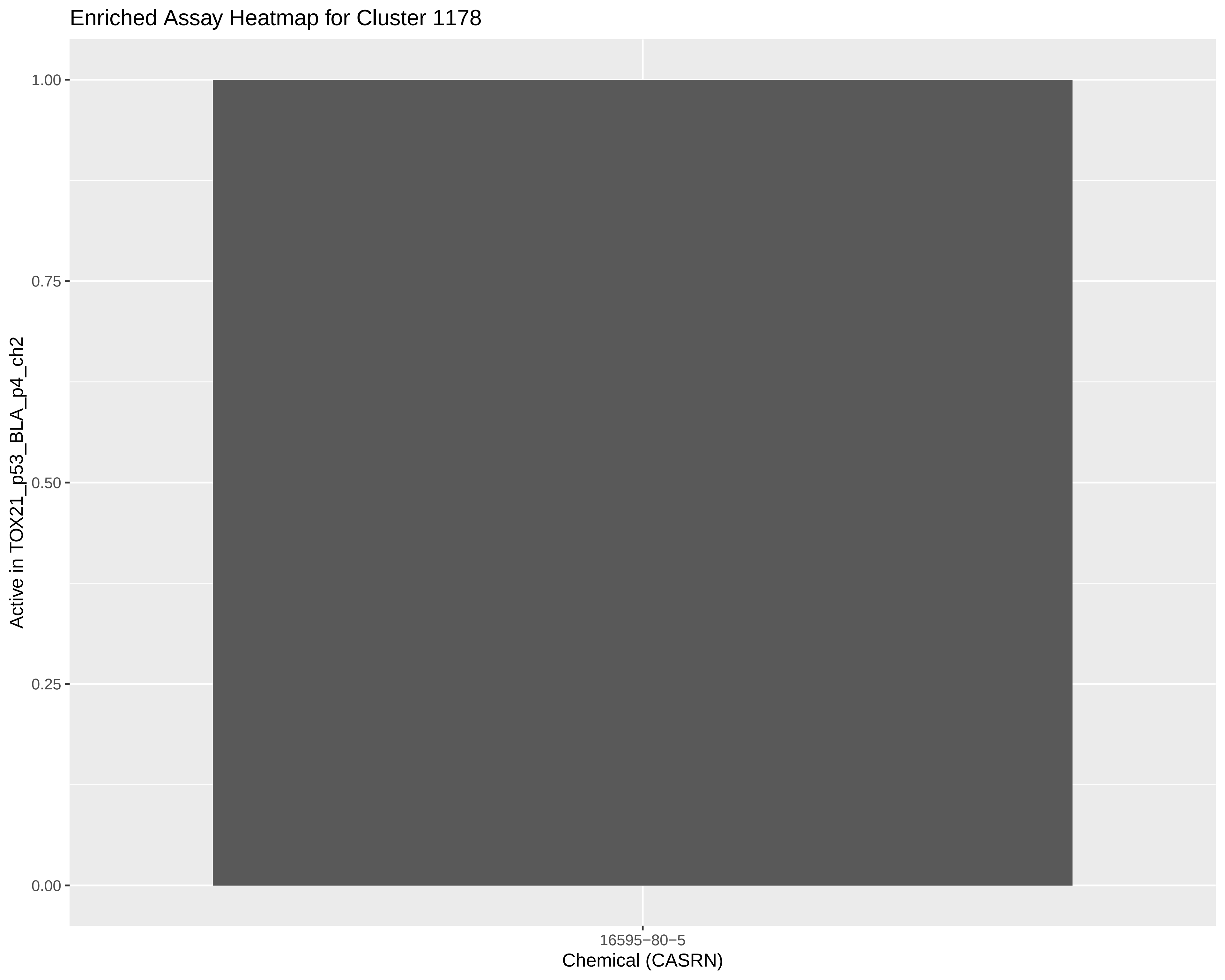
Enriched Assay Heatmap for Cluster 1178
Active in TOX21_p53_BLA_p4_ch2
1.00
0.75
0.50
0.25
0.00
16595−80−5
Chemical (CASRN)

Enriched Assay Heatmap for Cluster 1181

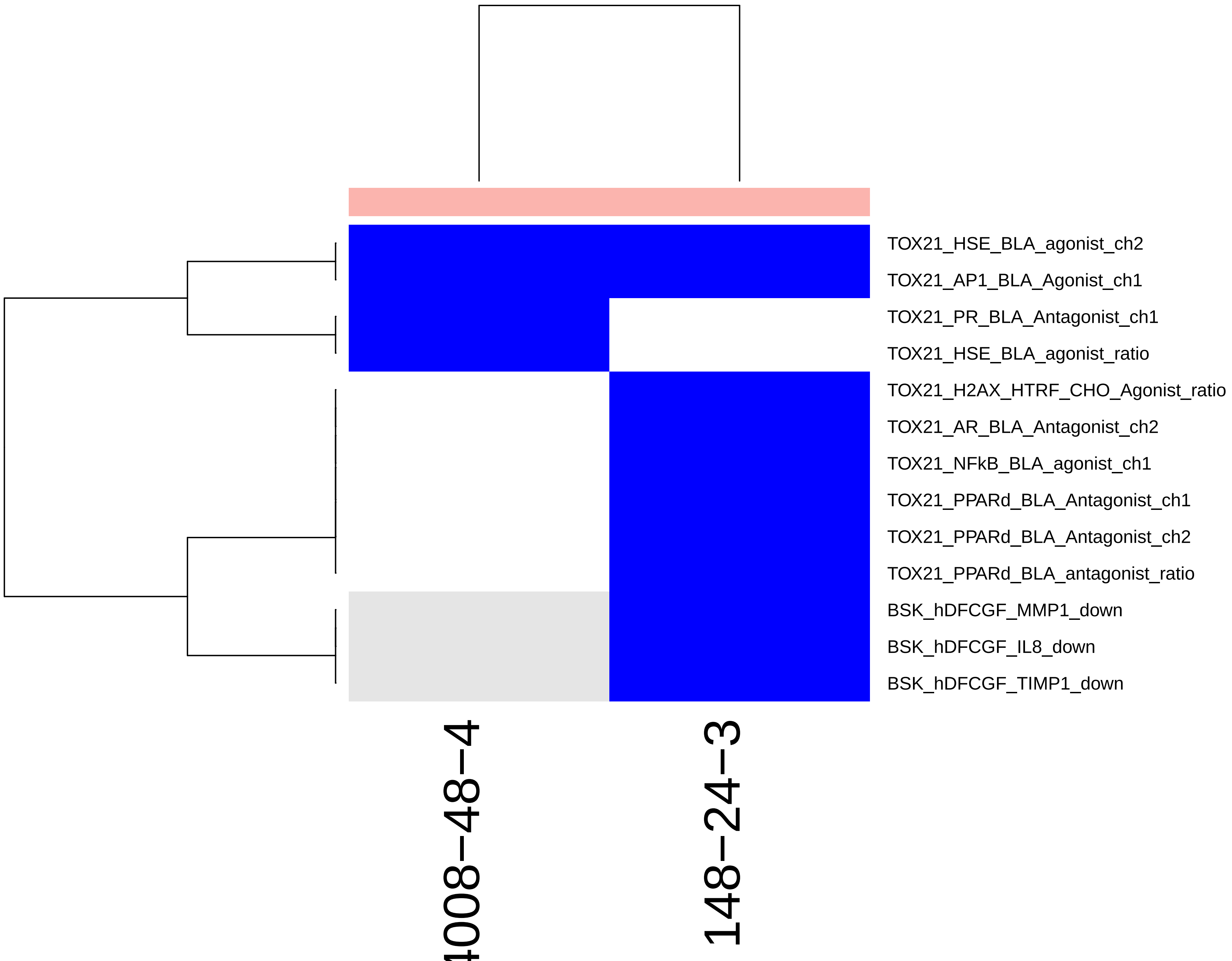

Enriched Assay Heatmap for Cluster 1184

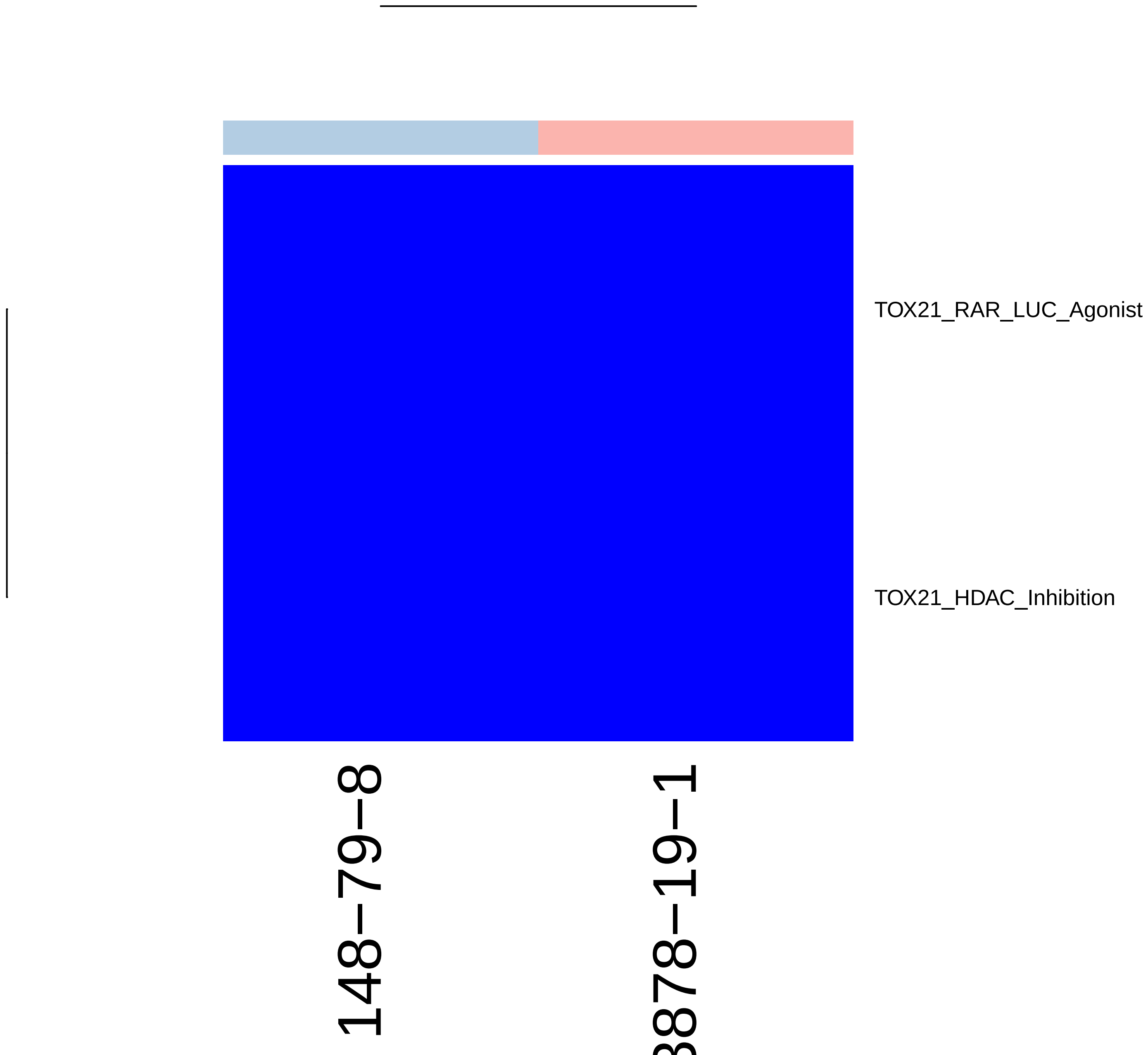

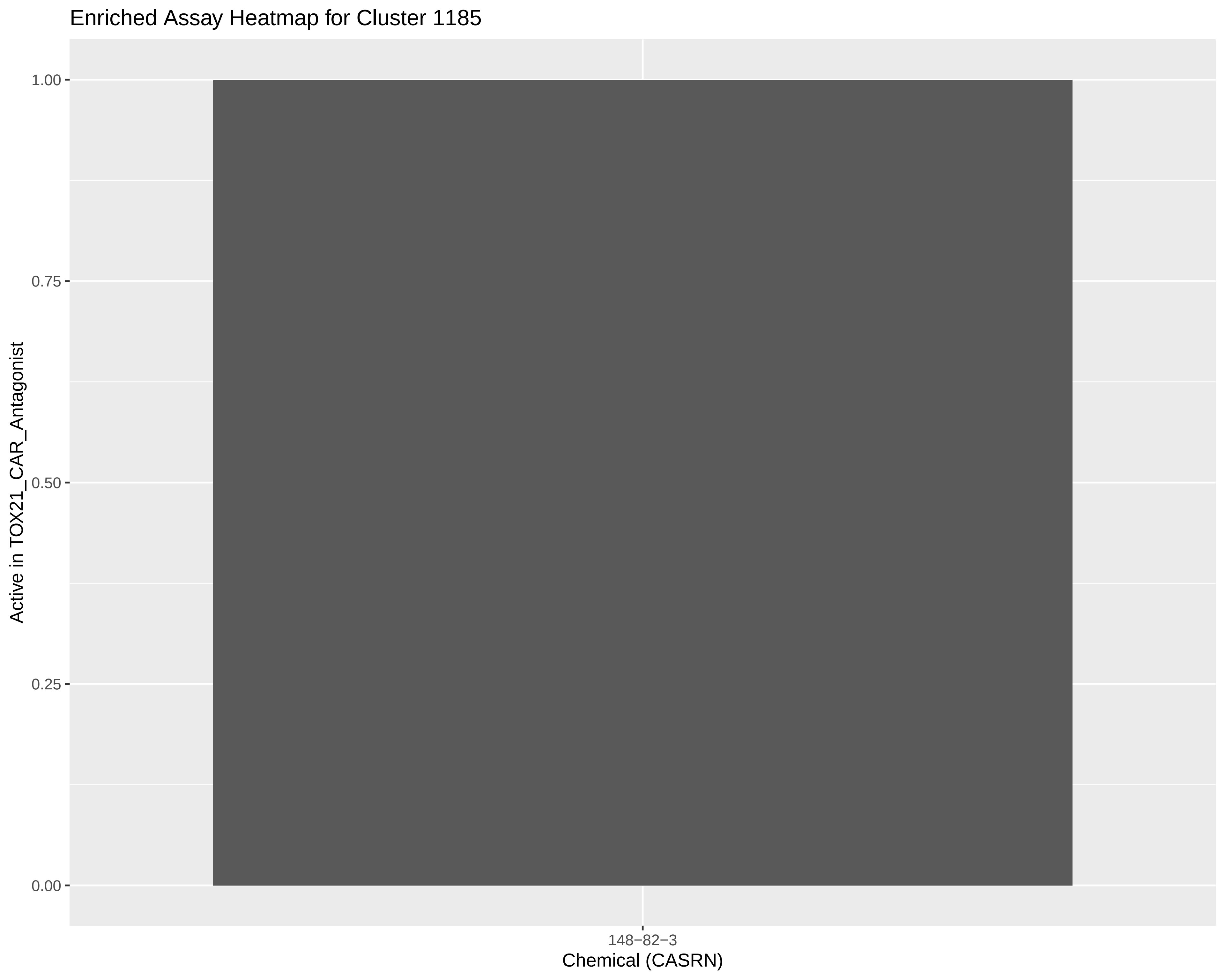
Enriched Assay Heatmap for Cluster 1185
1.00
0.75
0.50
0.25
0.00
Active in TOX21_CAR_Antagonist
148−82−3
Chemical (CASRN)

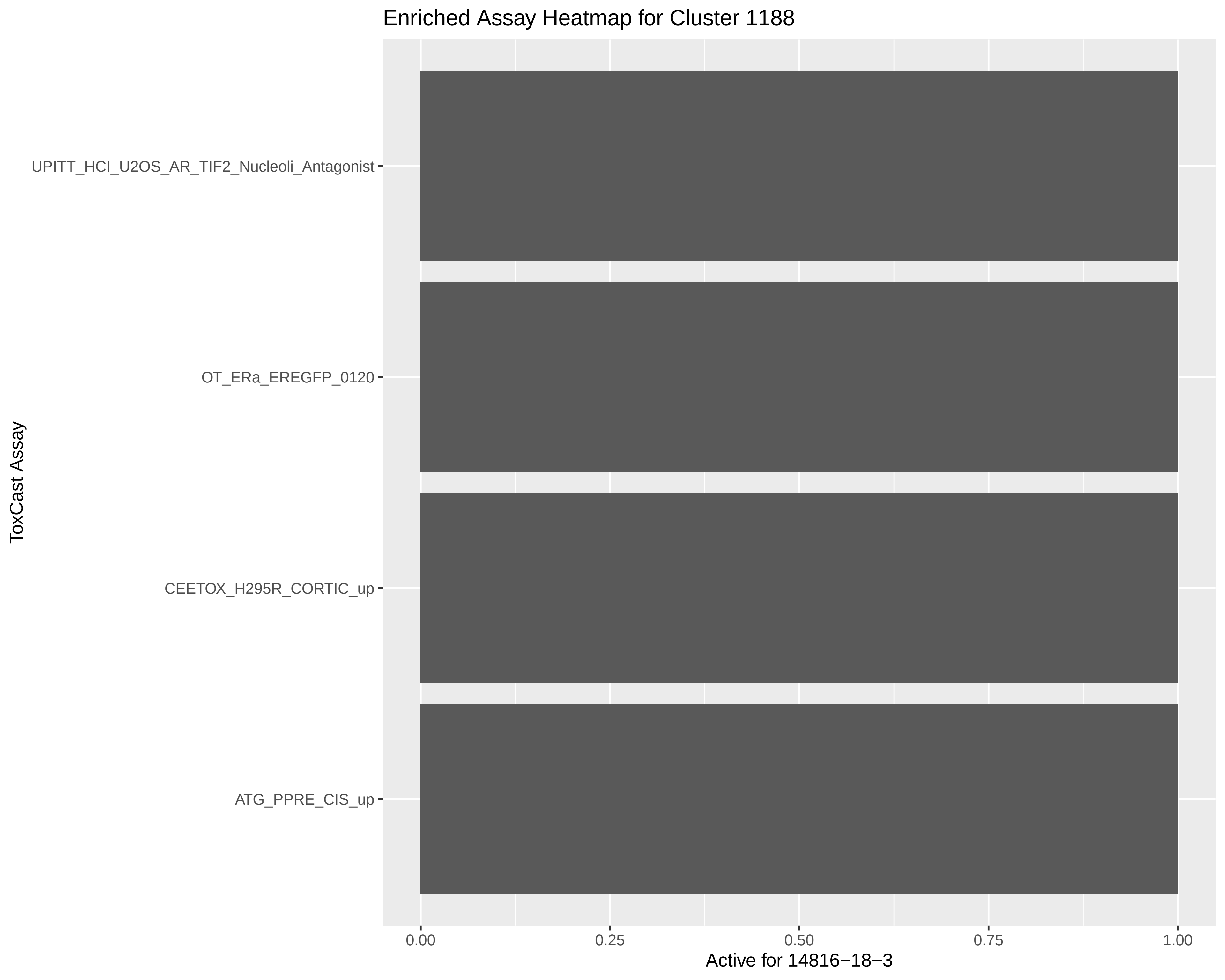
Enriched Assay Heatmap for Cluster 1188
ToxCast Assay
UPITT_HCI_U2OS_AR_TIF2_Nucleoli_Antagonist
OT_ERa_EREGFP_0120
CEETOX_H295R_CORTIC_up
ATG_PPRE_CIS_up
0.00
0.25
0.50
0.75
1.00
Active for 14816−18−3

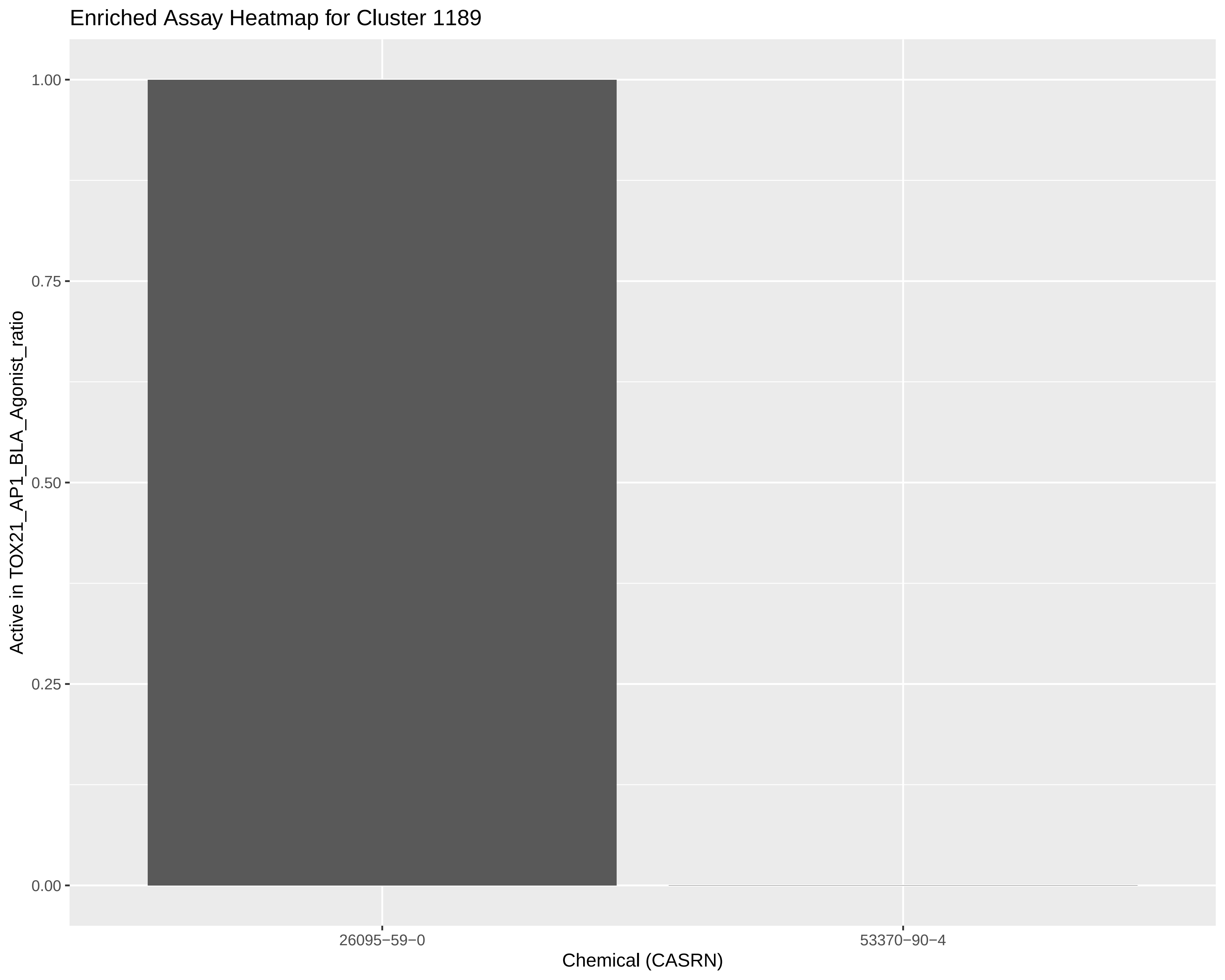
Enriched Assay Heatmap for Cluster 1189
Active in TOX21_AP1_BLA_Agonist_ratio
1.00
0.75
0.50
0.25
0.00
26095−59−0
53370−90−4
Chemical (CASRN)

Enriched Assay Heatmap for Cluster 1190

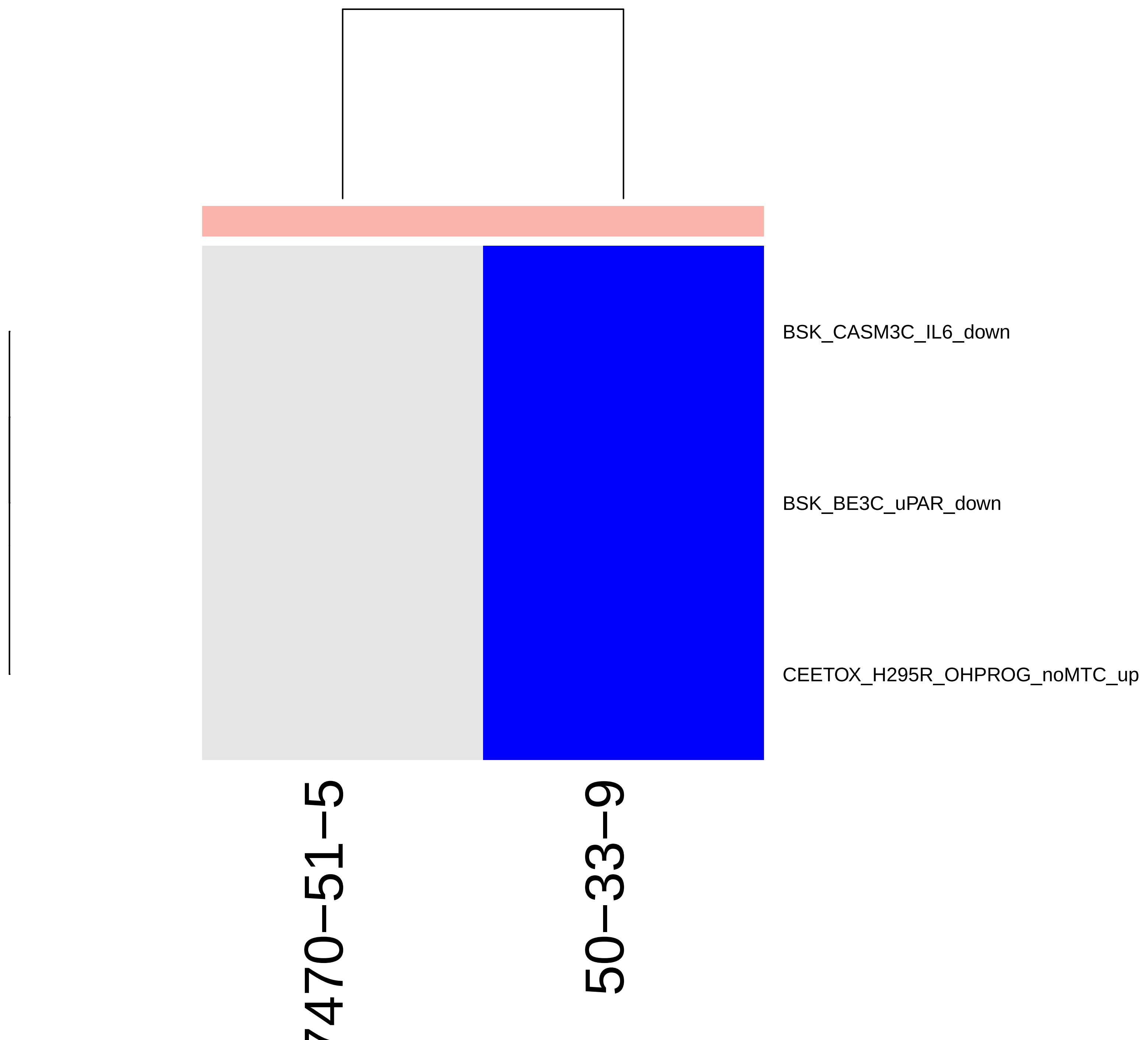

Enriched Assay Heatmap for Cluster 1204

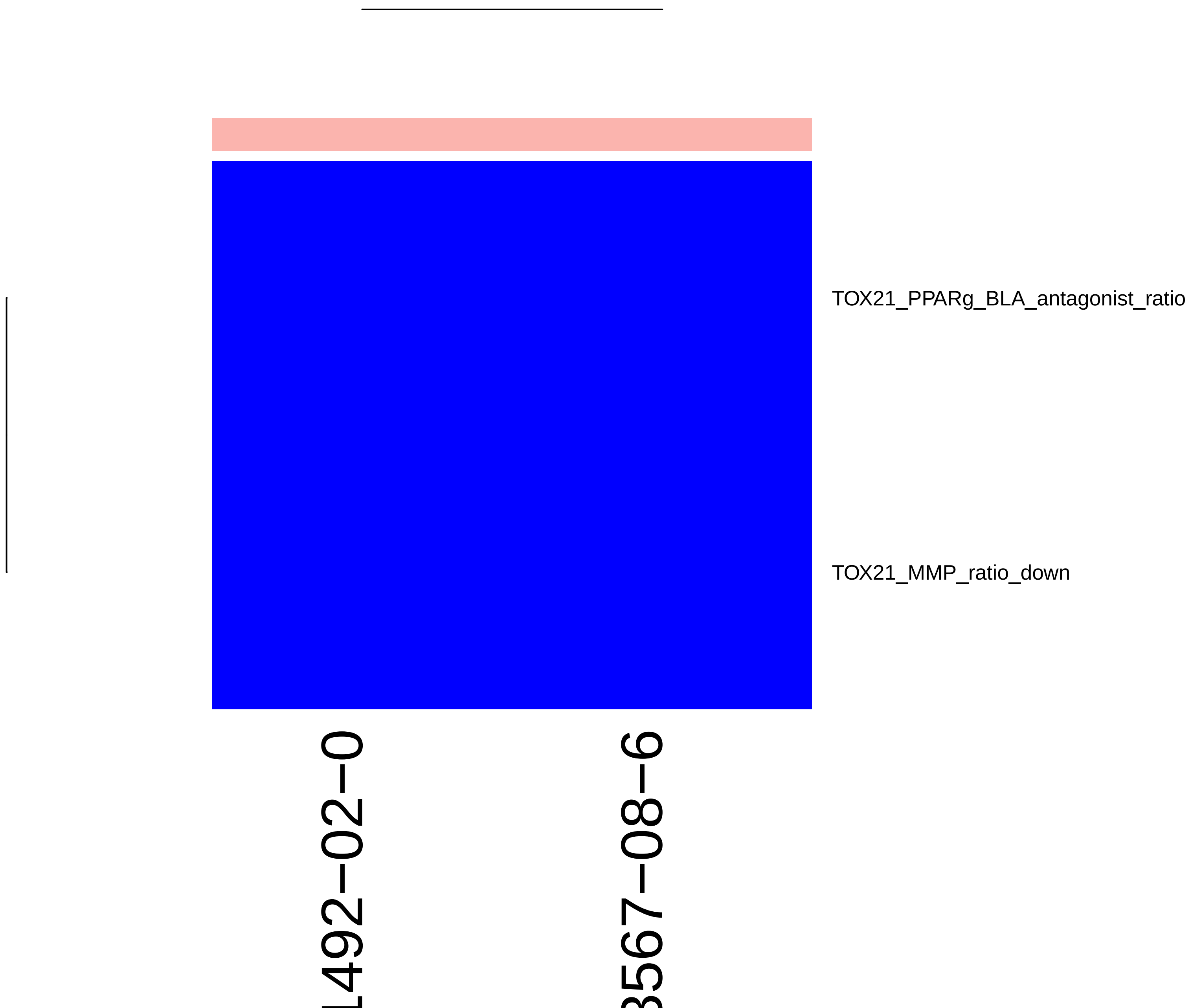

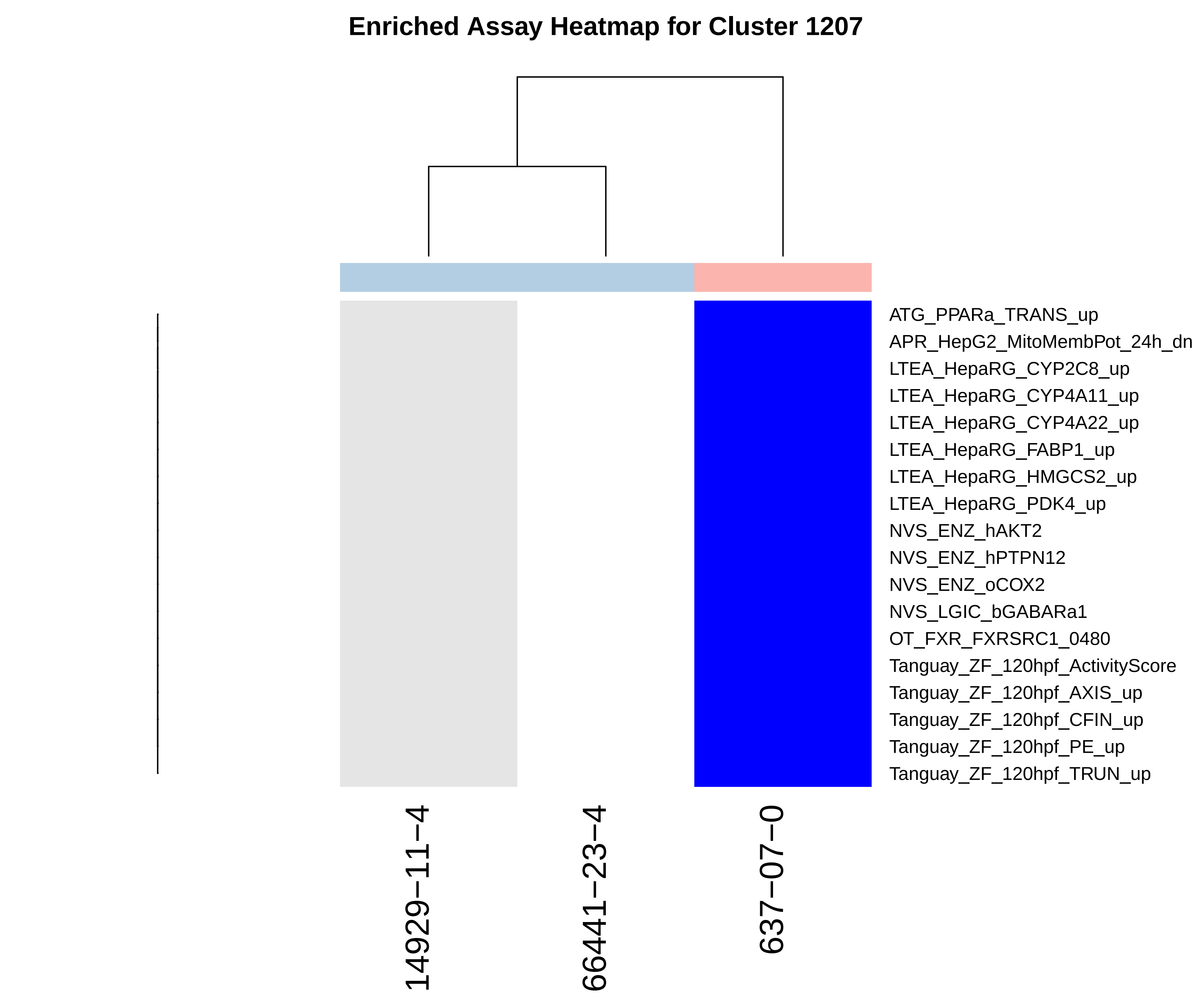

Enriched Assay Heatmap for Cluster 1207
ATG_PPARa_TRANS_up
APR_HepG2_MitoMembPot_24h_dn
LTEA_HepaRG_CYP2C8_up
LTEA_HepaRG_CYP4A11_up
LTEA_HepaRG_CYP4A22_up
LTEA_HepaRG_FABP1_up
LTEA_HepaRG_HMGCS2_up
LTEA_HepaRG_PDK4_up
NVS_ENZ_hAKT2
NVS_ENZ_hPTPN12
NVS_ENZ_oCOX2
NVS_LGIC_bGABARa1
OT_FXR_FXRSRC1_0480
Tanguay_ZF_120hpf_ActivityScore
Tanguay_ZF_120hpf_AXIS_up
Tanguay_ZF_120hpf_CFIN_up
Tanguay_ZF_120hpf_PE_up
Tanguay_ZF_120hpf_TRUN_up
14929−11−4
66441−23−4
637−07−0

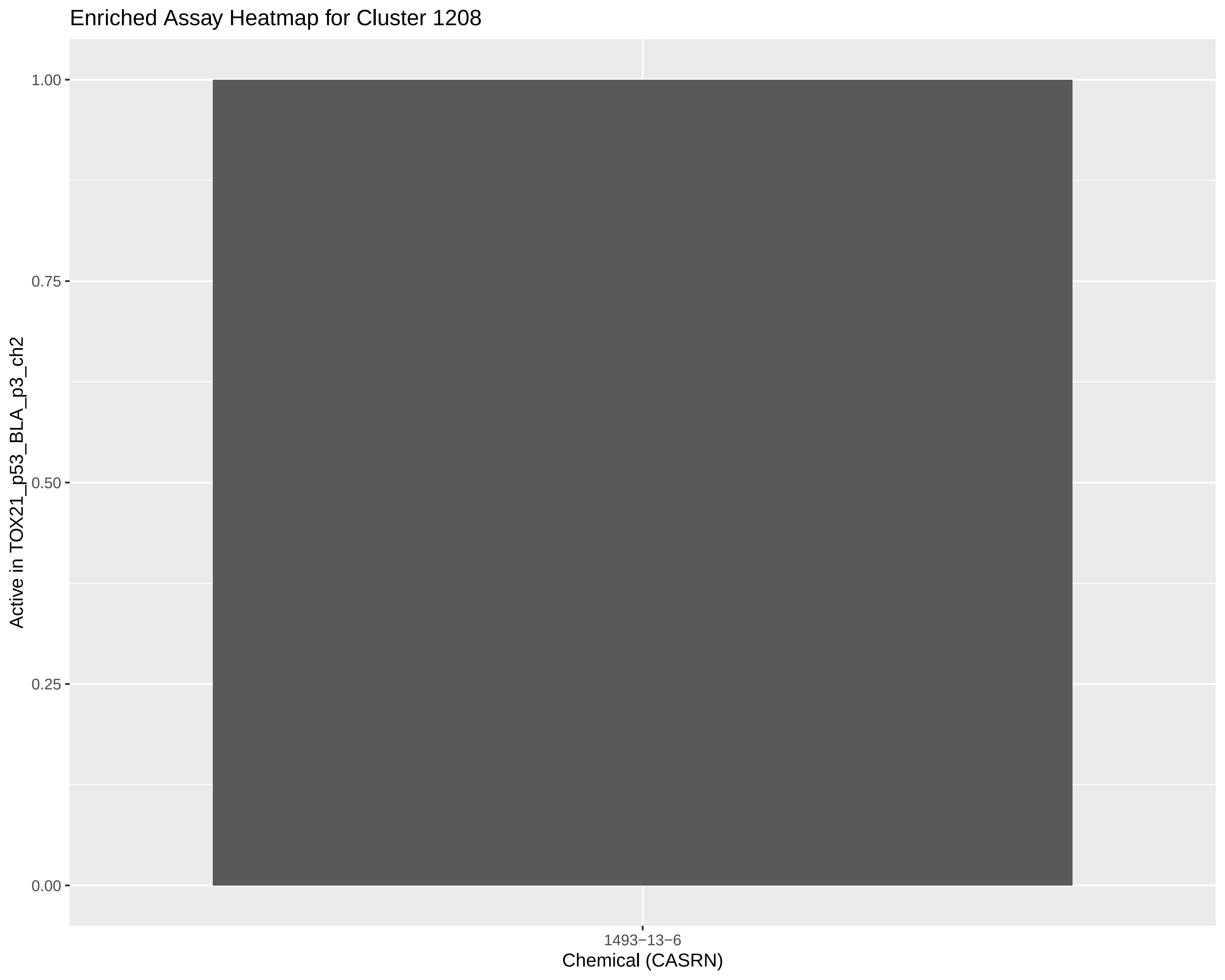

Enriched Assay Heatmap for Cluster 1208
1.00
0.75
0.50
0.25
0.00
Active in TOX21_p53_BLA_p3_ch2
1493−13−6
Chemical (CASRN)

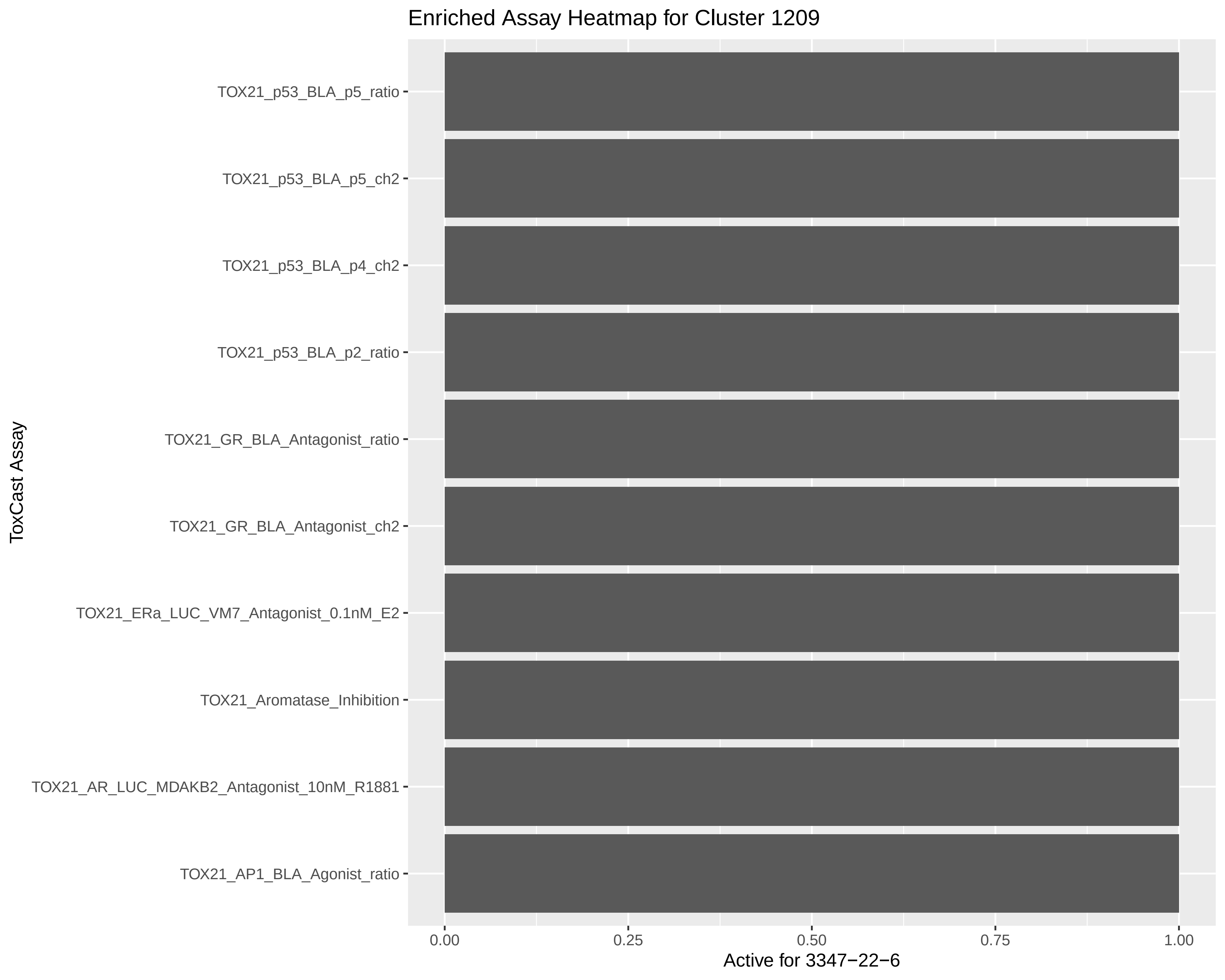
Enriched Assay Heatmap for Cluster 1209
TOX21_p53_BLA_p5_ratio
TOX21_p53_BLA_p5_ch2
TOX21_p53_BLA_p4_ch2
TOX21_p53_BLA_p2_ratio
TOX21_GR_BLA_Antagonist_ratio
TOX21_GR_BLA_Antagonist_ch2
TOX21_ERa_LUC_VM7_Antagonist_0.1nM_E2
TOX21_Aromatase_Inhibition
TOX21_AR_LUC_MDAKB2_Antagonist_10nM_R1881
TOX21_AP1_BLA_Agonist_ratio
ToxCast Assay
0.00
0.25
0.50
0.75
1.00
Active for 3347−22−6

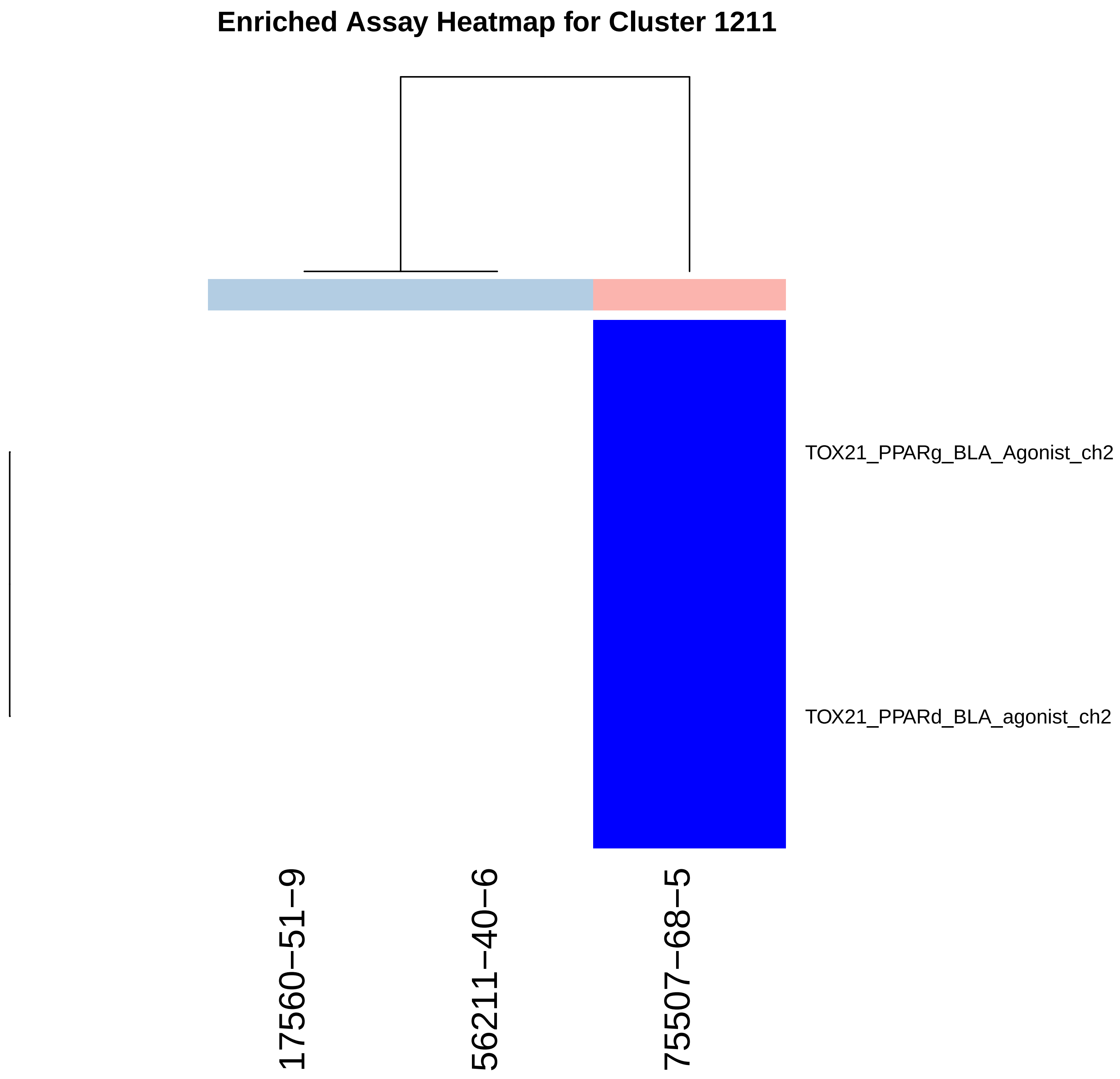
Enriched Assay Heatmap for Cluster 1211
TOX21_PPARg_BLA_Agonist_ch2
TOX21_PPARd_BLA_agonist_ch2
17560−51−9
56211−40−6
75507−68−5

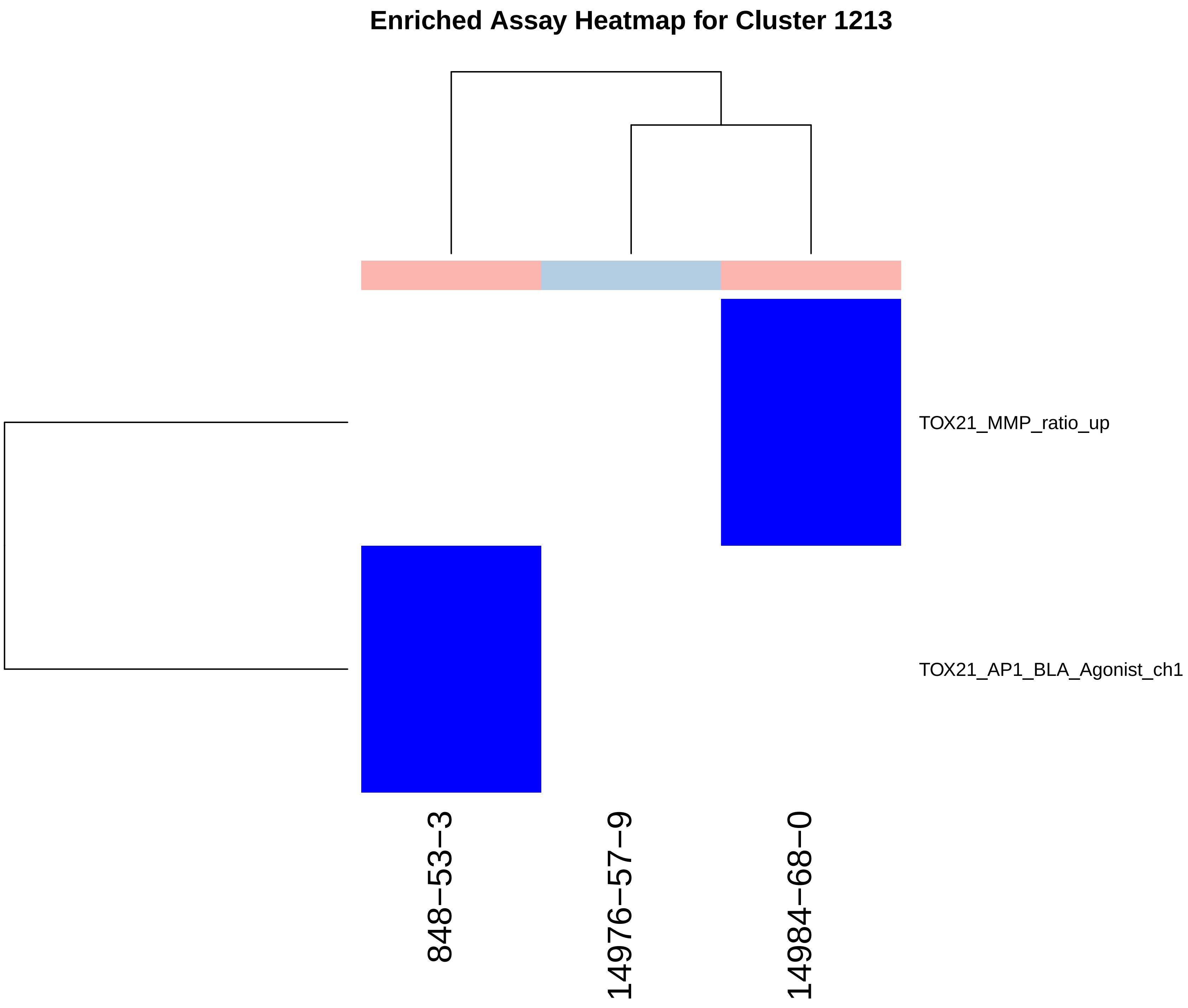
Enriched Assay Heatmap for Cluster 1213
TOX21_MMP_ratio_up
TOX21_AP1_BLA_Agonist_ch1
848−53−3
14976−57−9
14984−68−0

Enriched Assay Heatmap for Cluster 1215

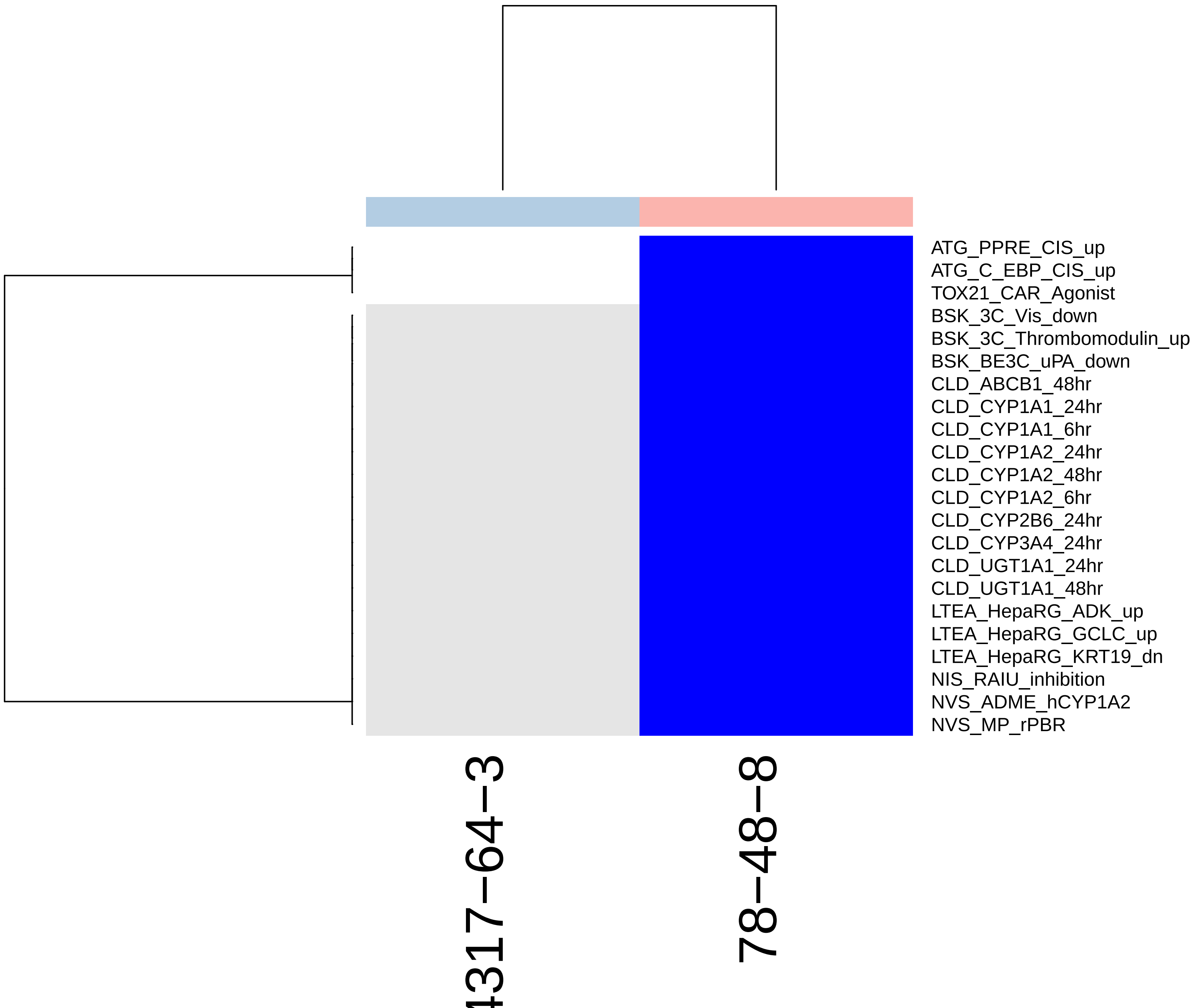

Enriched Assay Heatmap for Cluster 1216

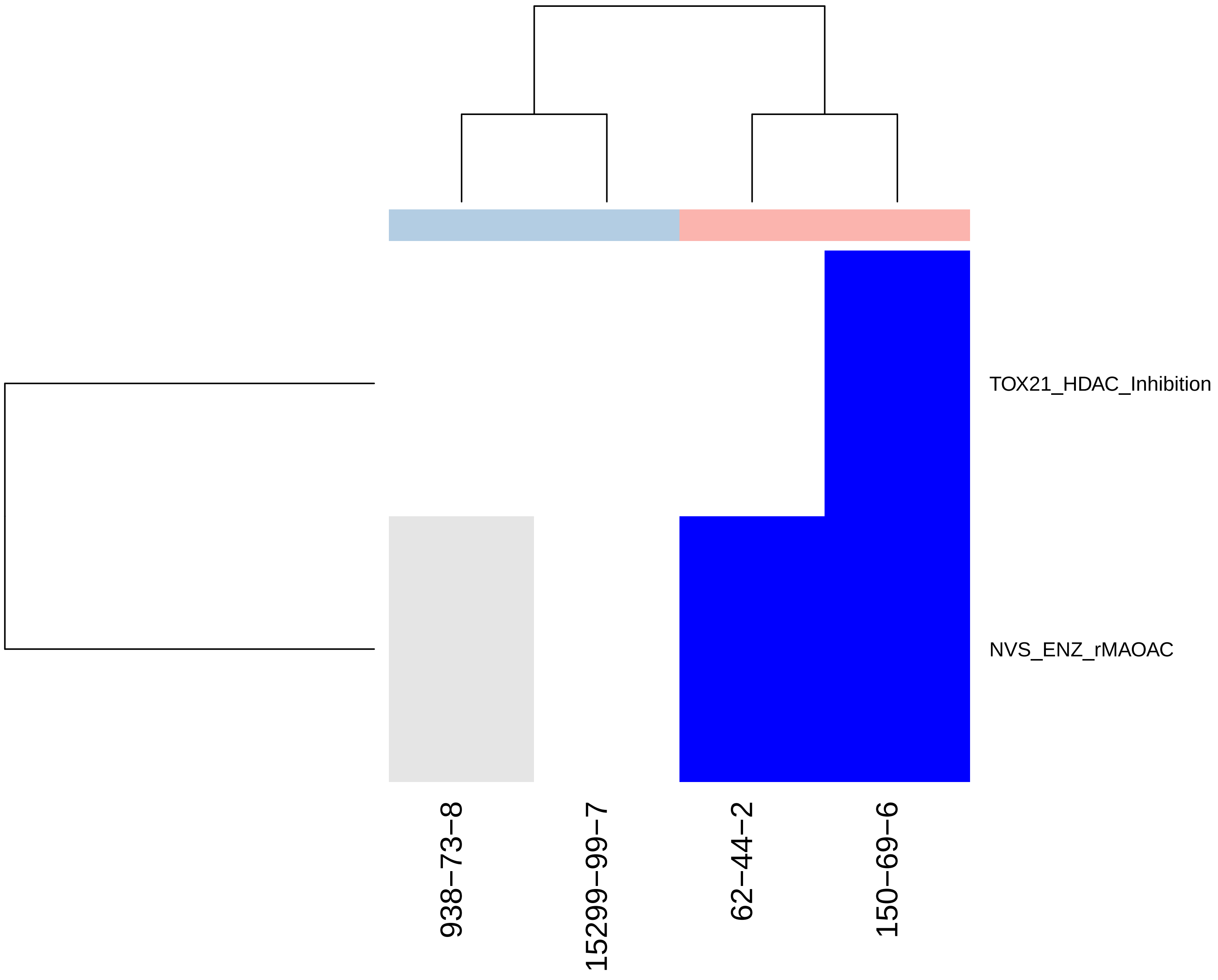

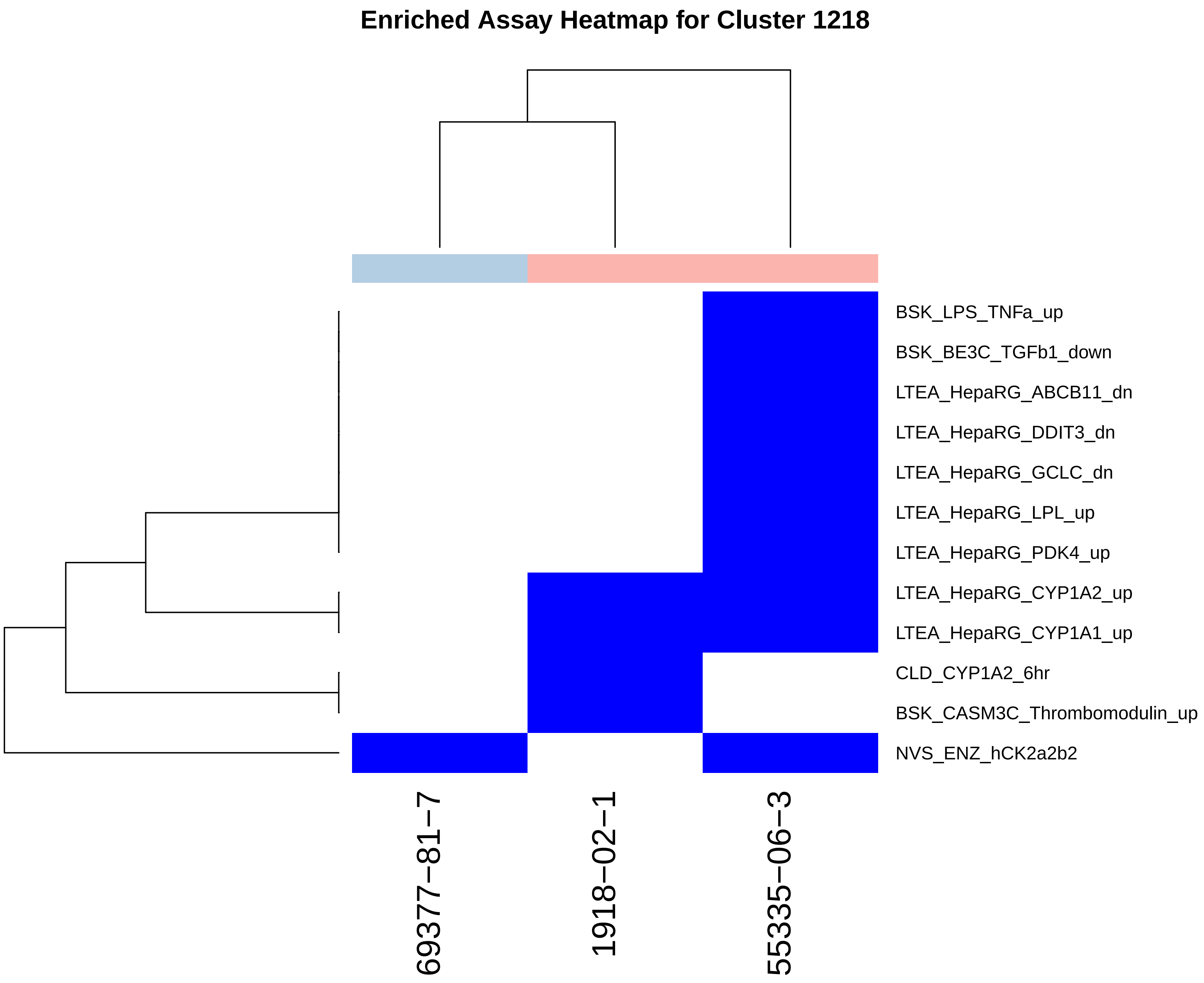
Enriched Assay Heatmap for Cluster 1218
BSK_LPS_TNFa_up
BSK_BE3C_TGFb1_down
LTEA_HepaRG_ABCB11_dn
LTEA_HepaRG_DDIT3_dn
LTEA_HepaRG_GCLC_dn
LTEA_HepaRG_LPL_up
LTEA_HepaRG_PDK4_up
LTEA_HepaRG_CYP1A2_up
LTEA_HepaRG_CYP1A1_up
CLD_CYP1A2_6hr
BSK_CASM3C_Thrombomodulin_up
NVS_ENZ_hCK2a2b2
69377−81−7
1918−02−1
55335−06−3

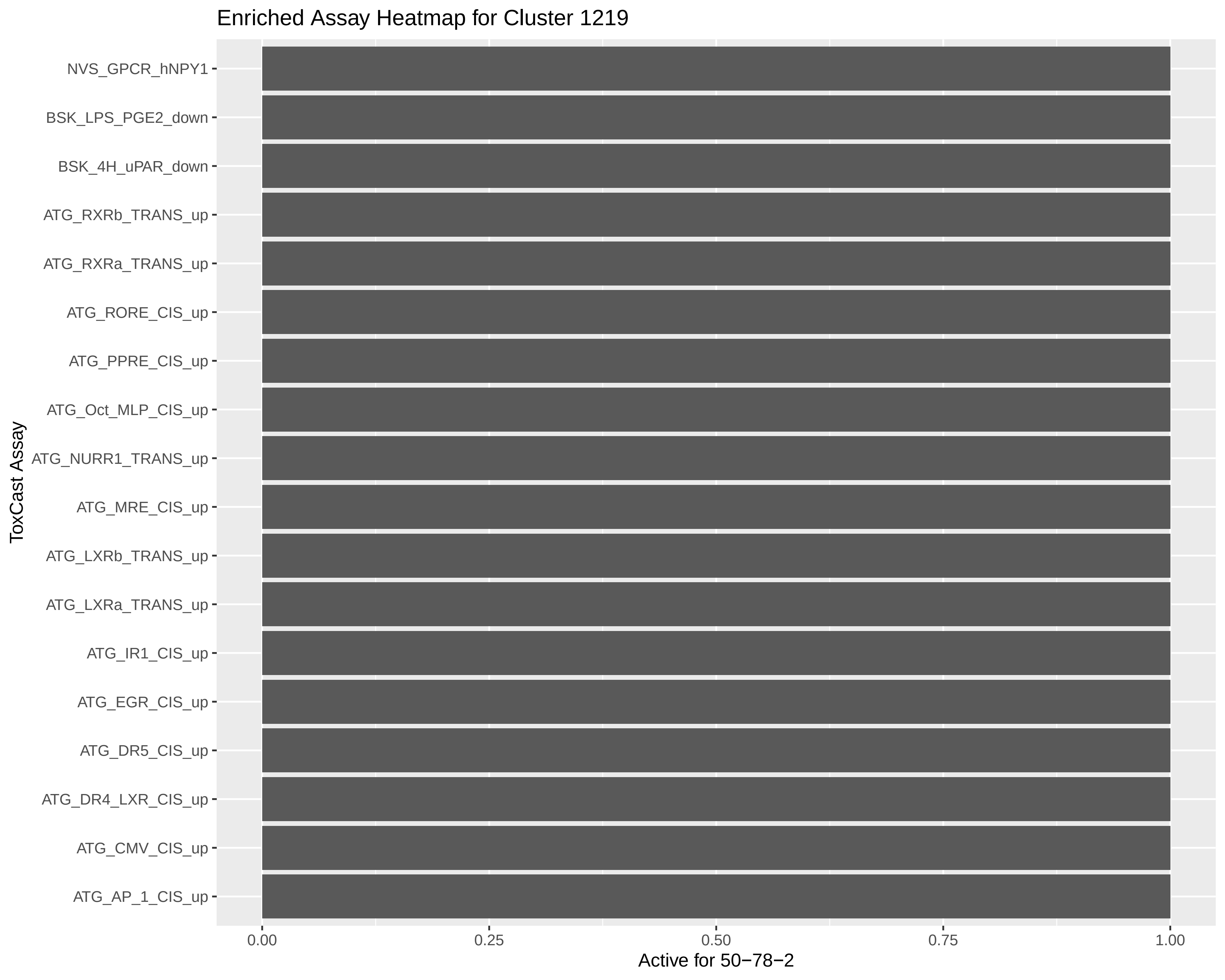

Enriched Assay Heatmap for Cluster 1219
NVS_GPCR_hNPY1
BSK_LPS_PGE2_down
BSK_4H_uPAR_down
ATG_RXRb_TRANS_up
ATG_RXRa_TRANS_up
ATG_RORE_CIS_up
ATG_PPRE_CIS_up
ATG_Oct_MLP_CIS_up
ATG_NURR1_TRANS_up
ATG_MRE_CIS_up
ATG_LXRb_TRANS_up
ATG_LXRa_TRANS_up
ATG_IR1_CIS_up
ATG_EGR_CIS_up
ATG_DR5_CIS_up
ATG_DR4_LXR_CIS_up
ATG_CMV_CIS_up
ATG_AP_1_CIS_up
ToxCast Assay
0.00
0.25
0.50
0.75
1.00
Active for 50−78−2

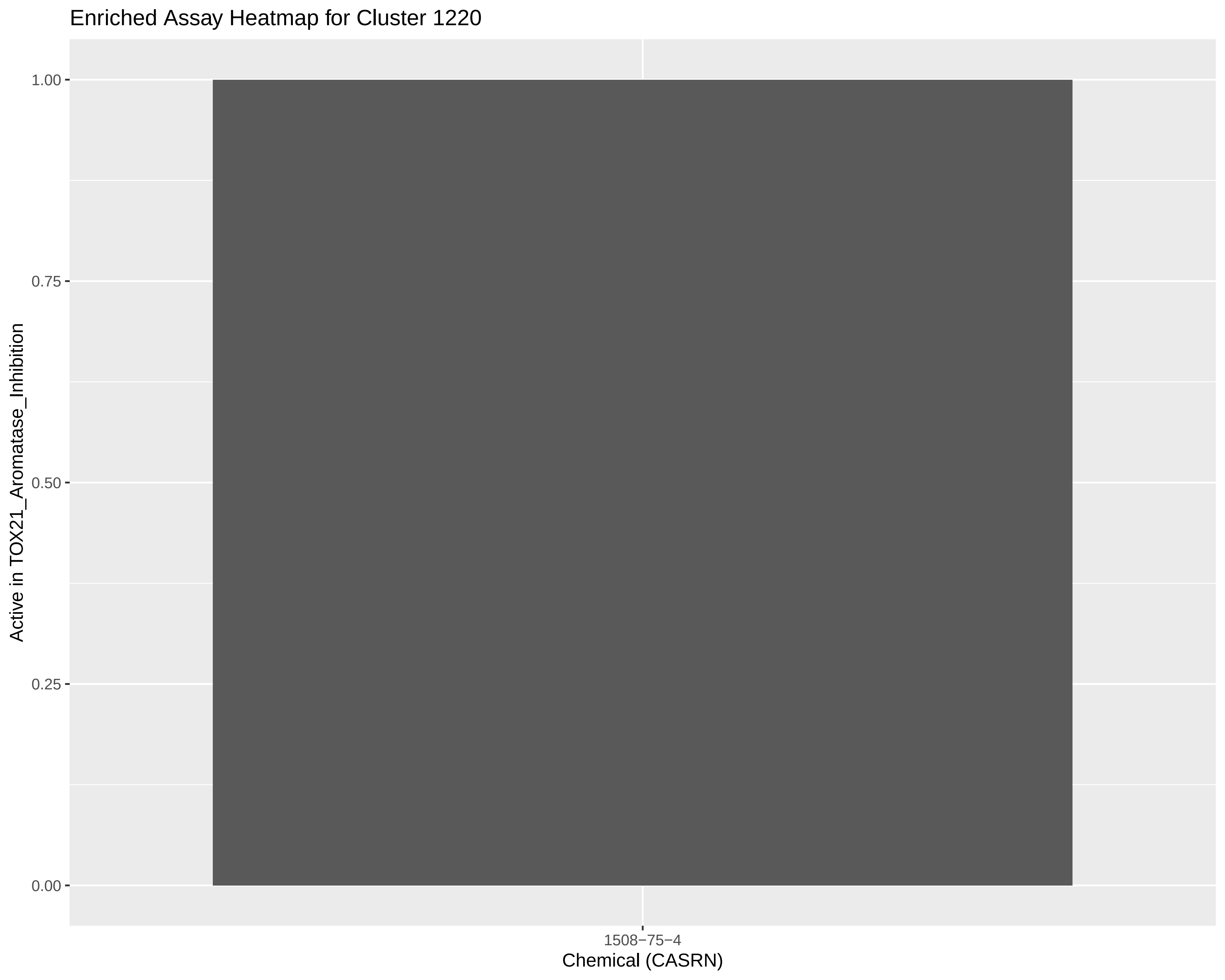
Enriched Assay Heatmap for Cluster 1220
Active in TOX21_Aromatase_Inhibition
1.00
0.75
0.50
0.25
0.00
1508−75−4
Chemical (CASRN)

Enriched Assay Heatmap for Cluster 1222

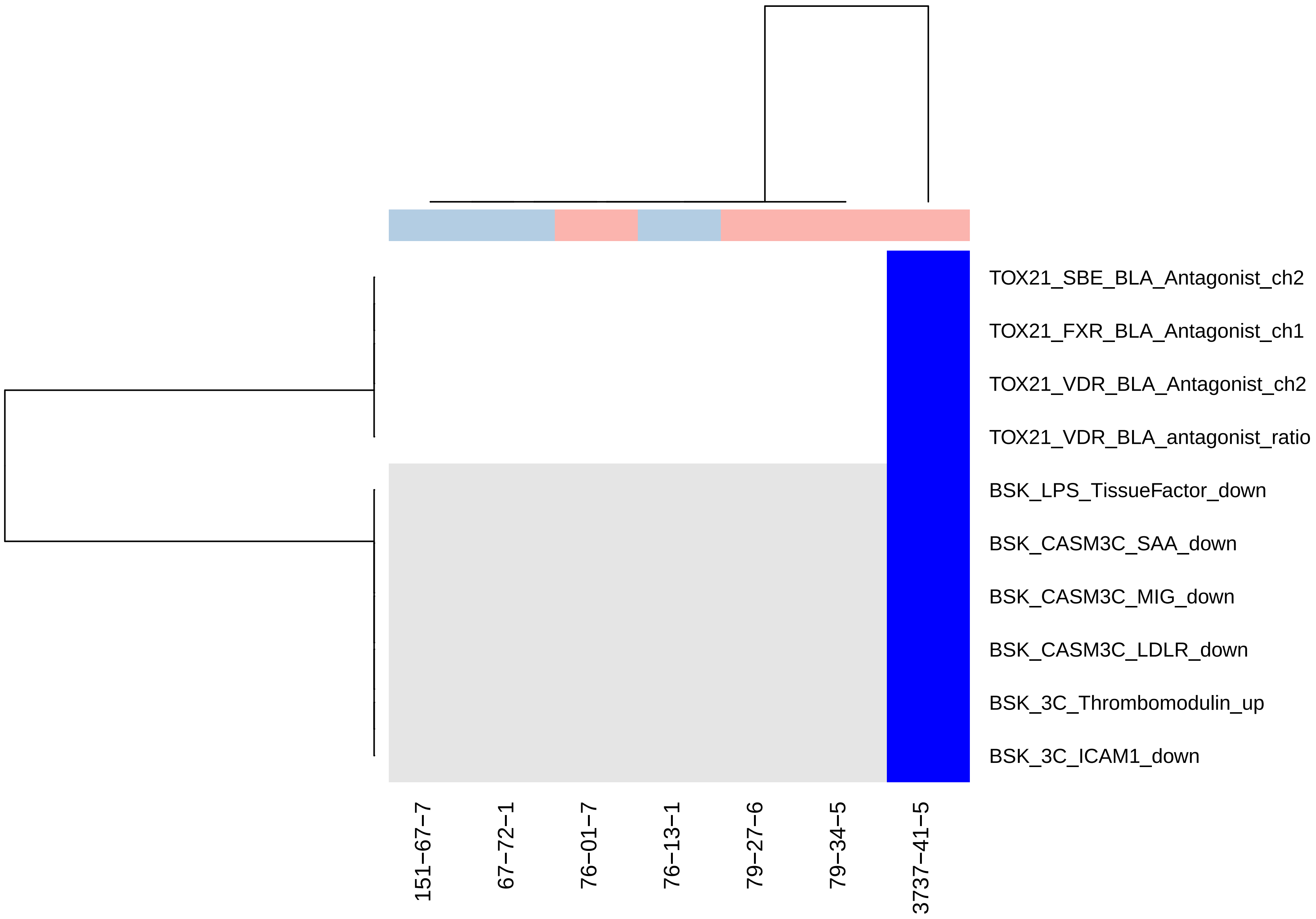

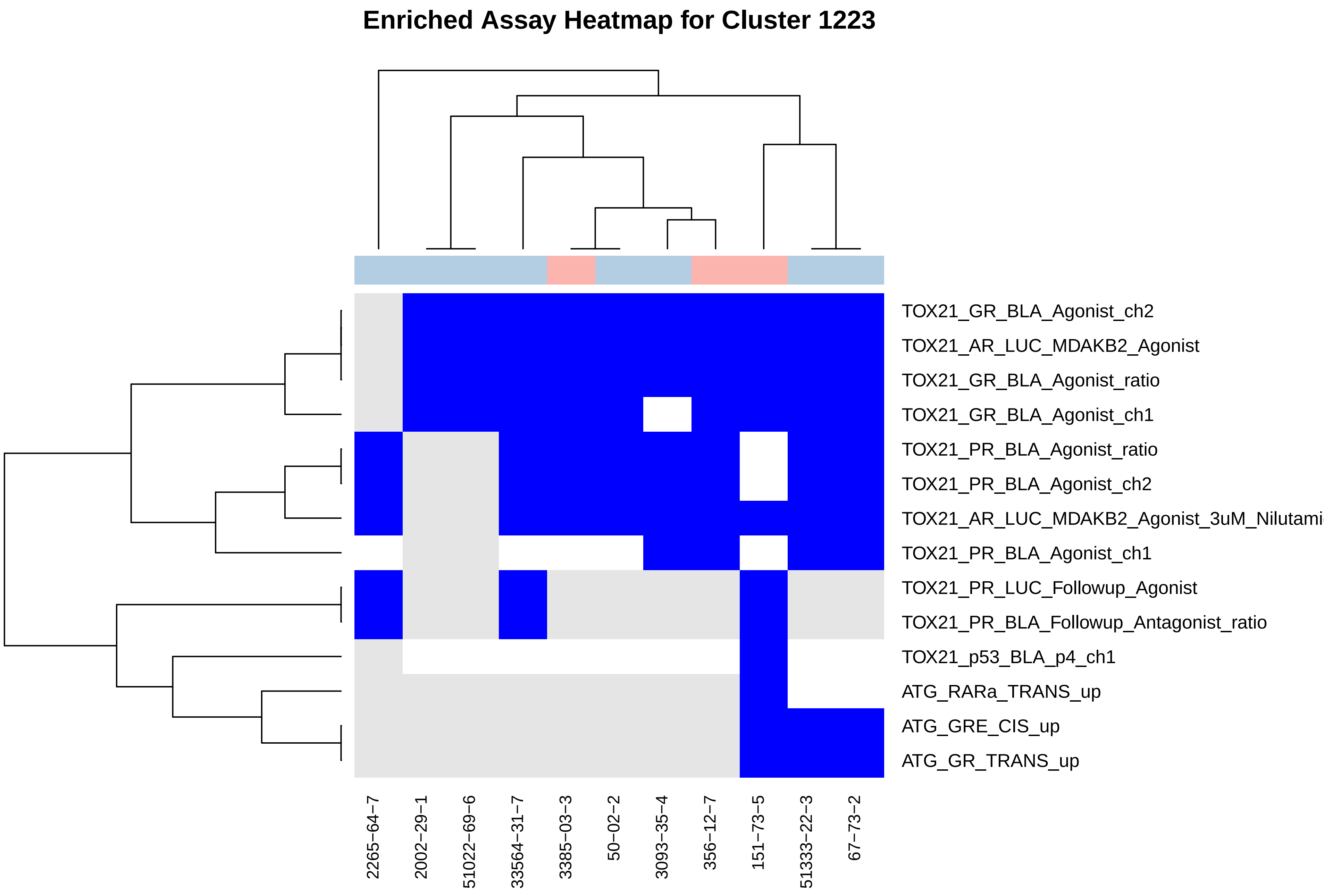
Enriched Assay Heatmap for Cluster 1223
TOX21_GR_BLA_Agonist_ch2
TOX21_AR_LUC_MDAKB2_Agonist
TOX21_GR_BLA_Agonist_ratio
TOX21_GR_BLA_Agonist_ch1
TOX21_PR_BLA_Agonist_ratio
TOX21_PR_BLA_Agonist_ch2
TOX21_AR_LUC_MDAKB2_Agonist_3uM_Nilutami
TOX21_PR_BLA_Agonist_ch1
TOX21_PR_LUC_Followup_Agonist
TOX21_PR_BLA_Followup_Antagonist_ratio
TOX21_p53_BLA_p4_ch1
ATG_RARa_TRANS_up
ATG_GRE_CIS_up
ATG_GR_TRANS_up
2265−64−7
2002−29−1
51022−69−6
33564−31−7
3385−03−3
50−02−2
3093−35−4
356−12−7
151−73−5
51333−22−3
67−73−2

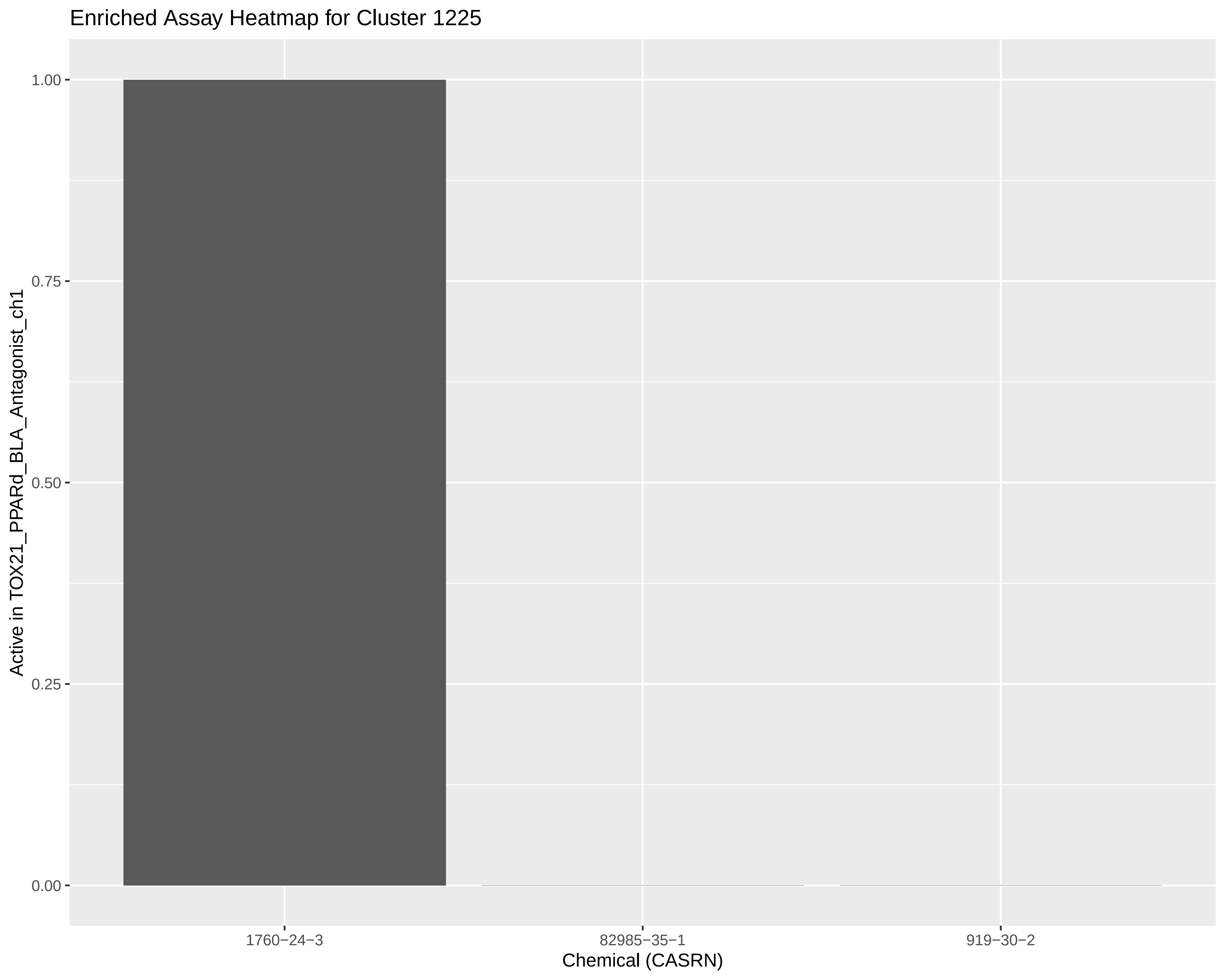
Enriched Assay Heatmap for Cluster 1225
Active in TOX21_PPARd_BLA_Antagonist_ch1
1.00
0.75
0.50
0.25
0.00
1760−24−3
82985−35−1
919−30−2
Chemical (CASRN)

Enriched Assay Heatmap for Cluster 1234

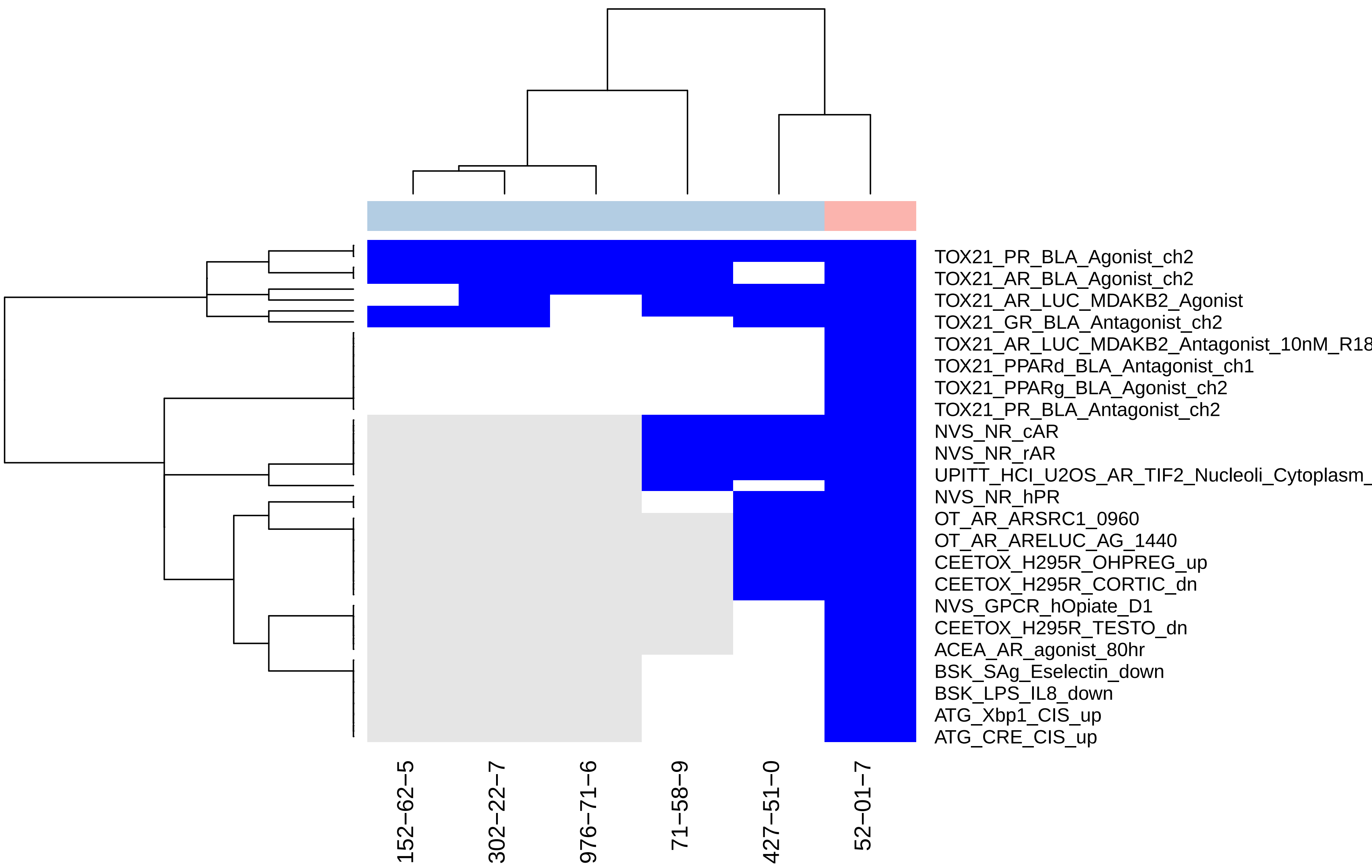

Enriched Assay Heatmap for Cluster 1235

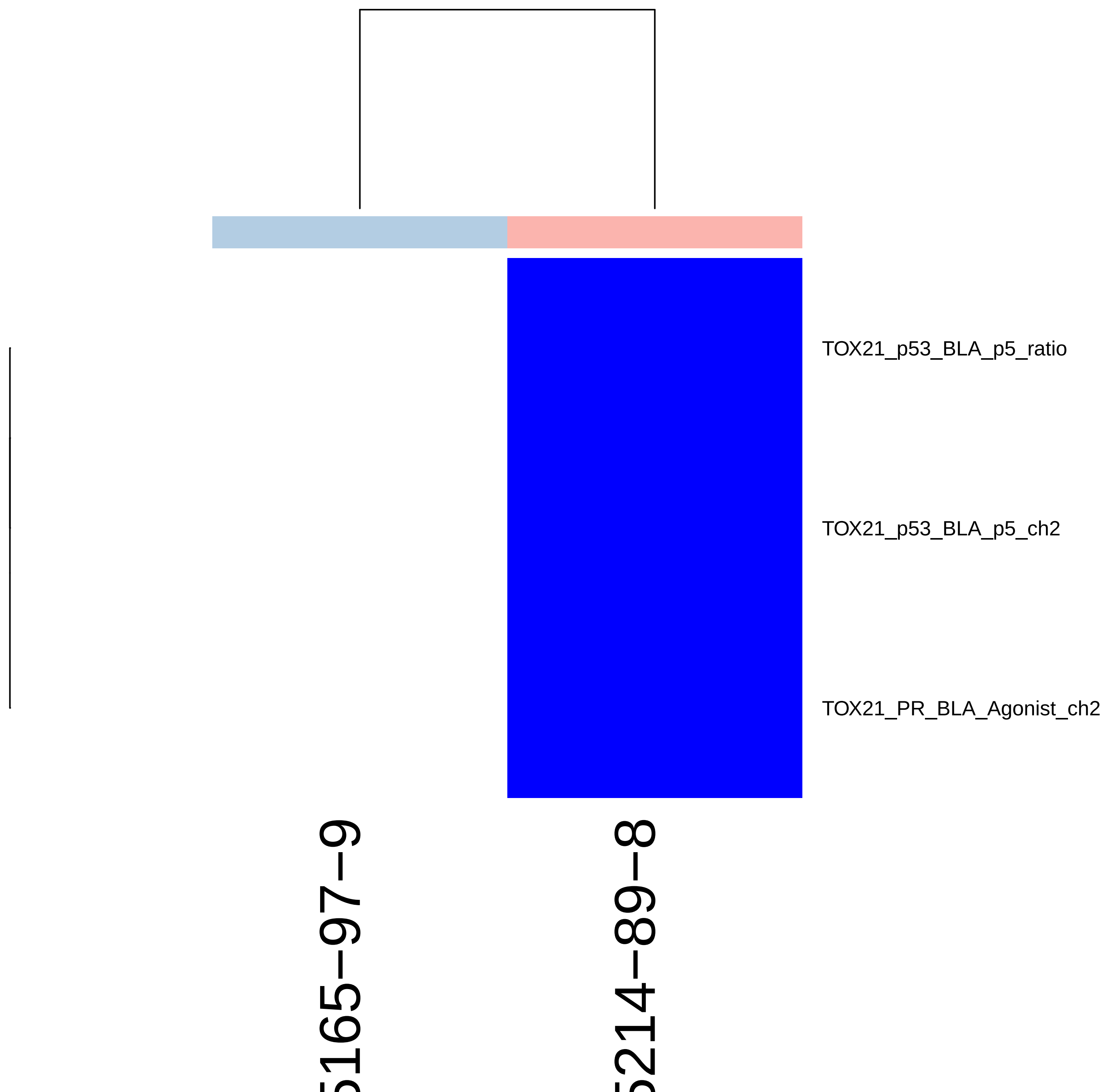

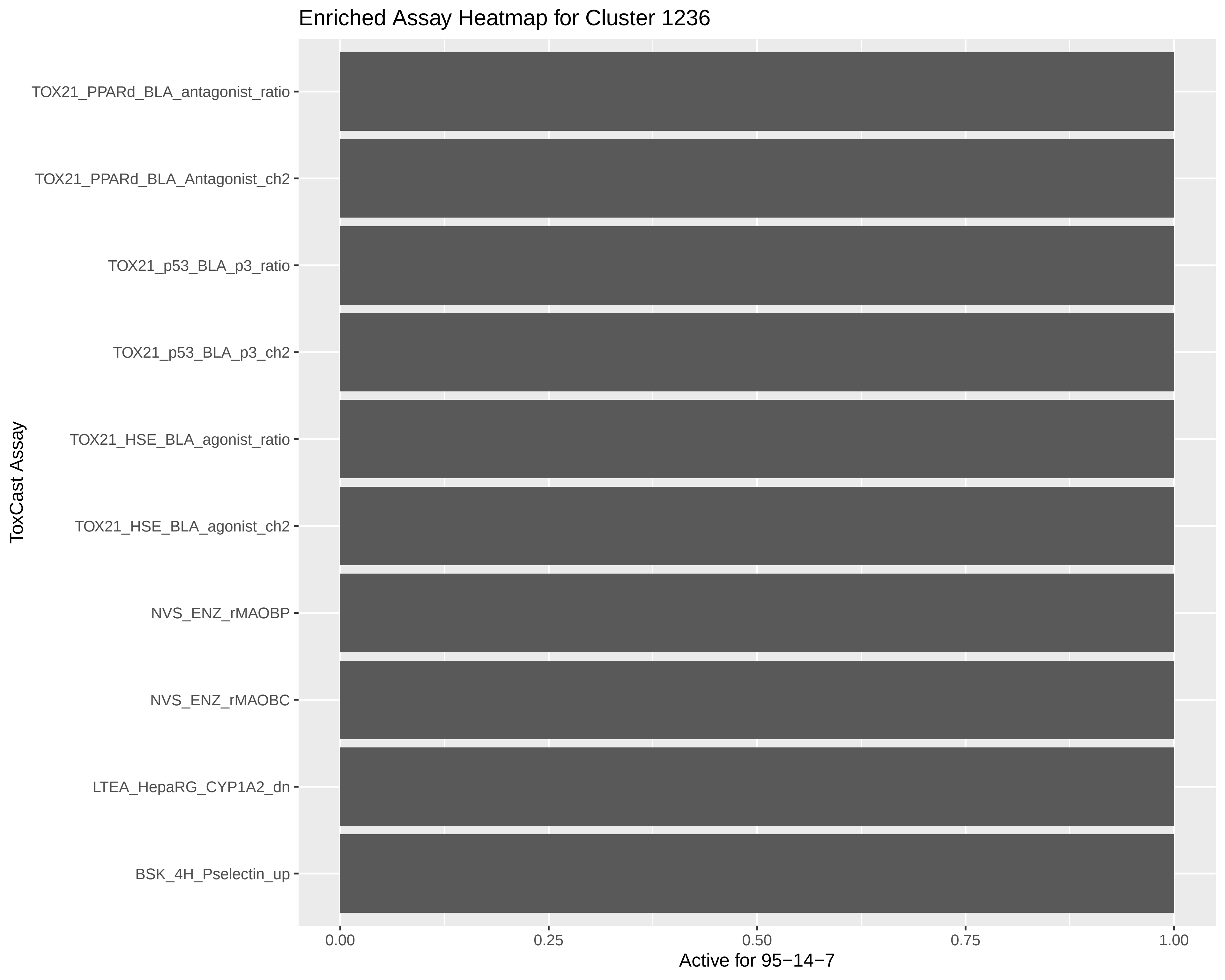
Enriched Assay Heatmap for Cluster 1236
ToxCast Assay
TOX21_PPARd_BLA_antagonist_ratio
TOX21_PPARd_BLA_Antagonist_ch2
TOX21_p53_BLA_p3_ratio
TOX21_p53_BLA_p3_ch2
TOX21_HSE_BLA_agonist_ratio
TOX21_HSE_BLA_agonist_ch2
NVS_ENZ_rMAOBP
NVS_ENZ_rMAOBC
LTEA_HepaRG_CYP1A2_dn
BSK_4H_Pselectin_up
0.00
0.25
0.50
0.75
1.00
Active for 95−14−7

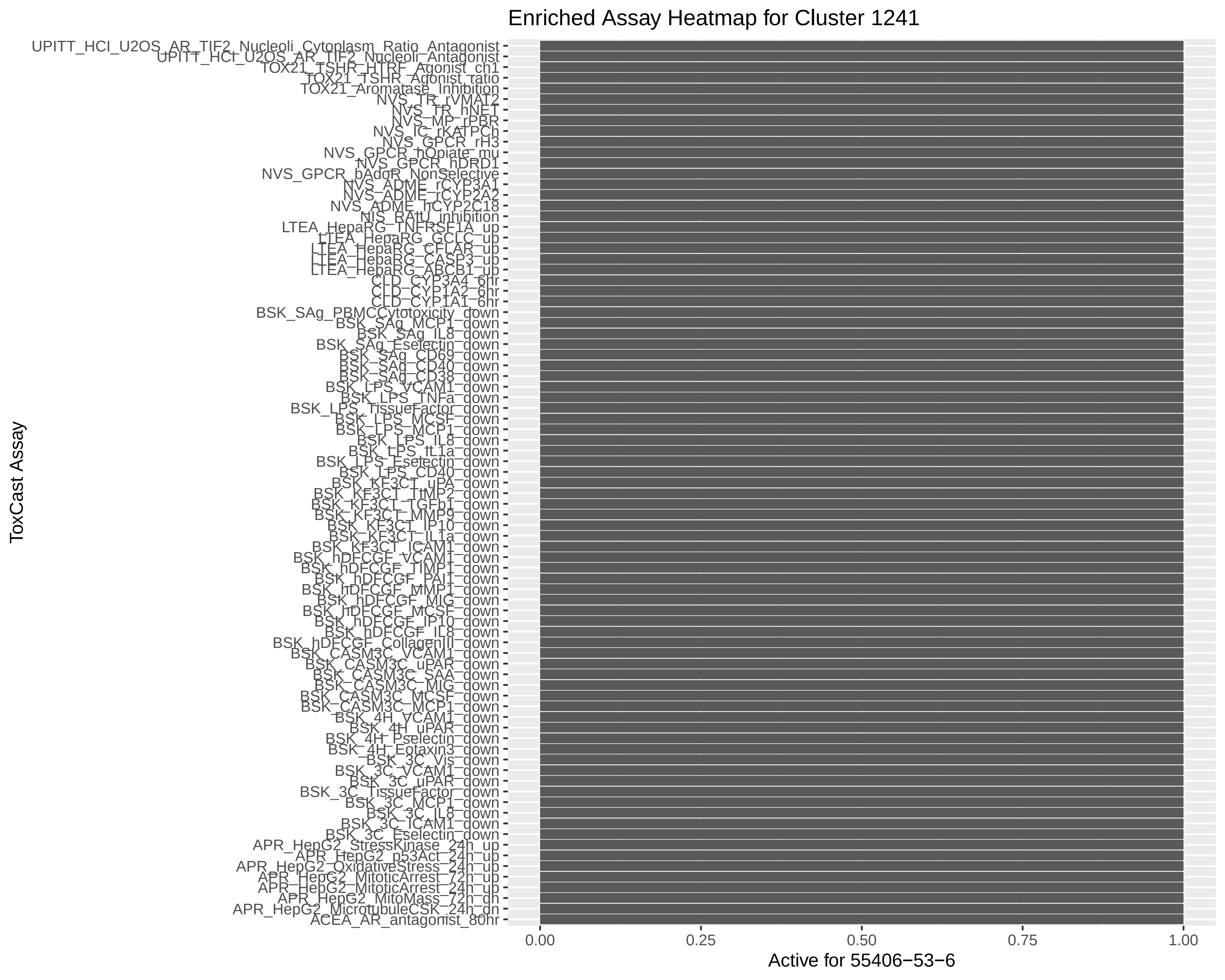

Enriched Assay Heatmap for Cluster 1241
ToxCast Assay
Active for 55406−53−6
0.00
0.25
0.50
0.75
1.00
UPITT_HCI_U2OS_AR_TIF2_Nucleoli_Cytoplasm_Ratio_Antagonist
UPITT_HCI_U2OS_AR_TIF2_Nucleoli_Antagonist
TOX21_TSHR_HTRF_Agonist_ch1
TOX21_TSHR_Agonist_ratio
TOX21_Aromatase_Inhibition
NVS_TR_rVMAT2
NVS_TR_hNET
NVS_MP_rPBR
NVS_IC_rKATPCh
NVS_GPCR_rH3
NVS_GPCR_hOpiate_mu
NVS_GPCR_hDRD1
NVS_GPCR_bAdoR_NonSelective
NVS_ADME_rCYP3A1
NVS_ADME_rCYP2A2
NVS_ADME_hCYP2C18
NIS_RAIU_inhibition
LTEA_HepaRG_TNFRSF1A_up
LTEA_HepaRG_GCLC_up
LTEA_HepaRG_CFLAR_up
LTEA_HepaRG_CASP3_up
LTEA_HepaRG_ABCB1_up
CLD_CYP3A4_6hr
CLD_CYP1A2_6hr
CLD_CYP1A1_6hr
BSK_SAg_PBMCCytotoxicity_down
BSK_SAg_MCP1_down
BSK_SAg_IL8_down
BSK_SAg_Eselectin_down
BSK_SAg_CD69_down
BSK_SAg_CD40_down
BSK_SAg_CD38_down
BSK_LPS_VCAM1_down
BSK_LPS_TNFa_down
BSK_LPS_TissueFactor_down
BSK_LPS_MCSF_down
BSK_LPS_MCP1_down
BSK_LPS_IL8_down
BSK_LPS_IL1a_down
BSK_LPS_Eselectin_down
BSK_LPS_CD40_down
BSK_KF3CT_uPA_down
BSK_KF3CT_TIMP2_down
BSK_KF3CT_TGFb1_down
BSK_KF3CT_MMP9_down
BSK_KF3CT_IP10_down
BSK_KF3CT_IL1a_down
BSK_KF3CT_ICAM1_down
BSK_hDFCGF_VCAM1_down
BSK_hDFCGF_TIMP1_down
BSK_hDFCGF_PAI1_down
BSK_hDFCGF_MMP1_down
BSK_hDFCGF_MIG_down
BSK_hDFCGF_MCSF_down
BSK_hDFCGF_IP10_down
BSK_hDFCGF_IL8_down
BSK_hDFCGF_CollagenIII_down
BSK_CASM3C_VCAM1_down
BSK_CASM3C_uPAR_down
BSK_CASM3C_SAA_down
BSK_CASM3C_MIG_down
BSK_CASM3C_MCSF_down
BSK_CASM3C_MCP1_down
BSK_4H_VCAM1_down
BSK_4H_uPAR_down
BSK_4H_Pselectin_down
BSK_4H_Eotaxin3_down
BSK_3C_Vis_down
BSK_3C_VCAM1_down
BSK_3C_uPAR_down
BSK_3C_TissueFactor_down
BSK_3C_MCP1_down
BSK_3C_IL8_down
BSK_3C_ICAM1_down
BSK_3C_Eselectin_down
APR_HepG2_StressKinase_24h_up
APR_HepG2_p53Act_24h_up
APR_HepG2_OxidativeStress_24h_up
APR_HepG2_MitoticArrest_72h_up
APR_HepG2_MitoticArrest_24h_up
APR_HepG2_MitoMass_72h_dn
APR_HepG2_MicrotubuleCSK_24h_dn
ACEA_AR_antagonist_80hr

Enriched Assay Heatmap for Cluster 1244

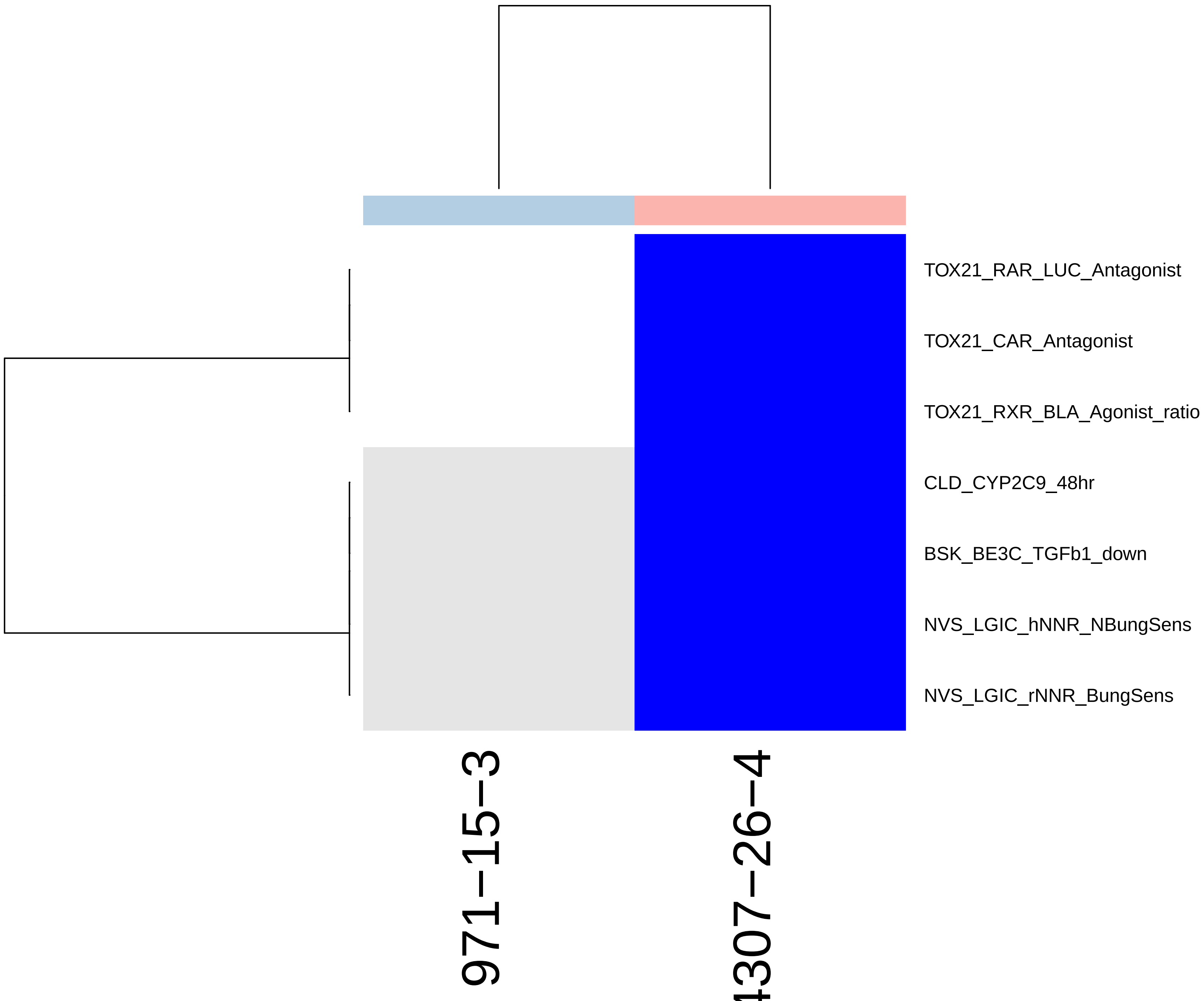

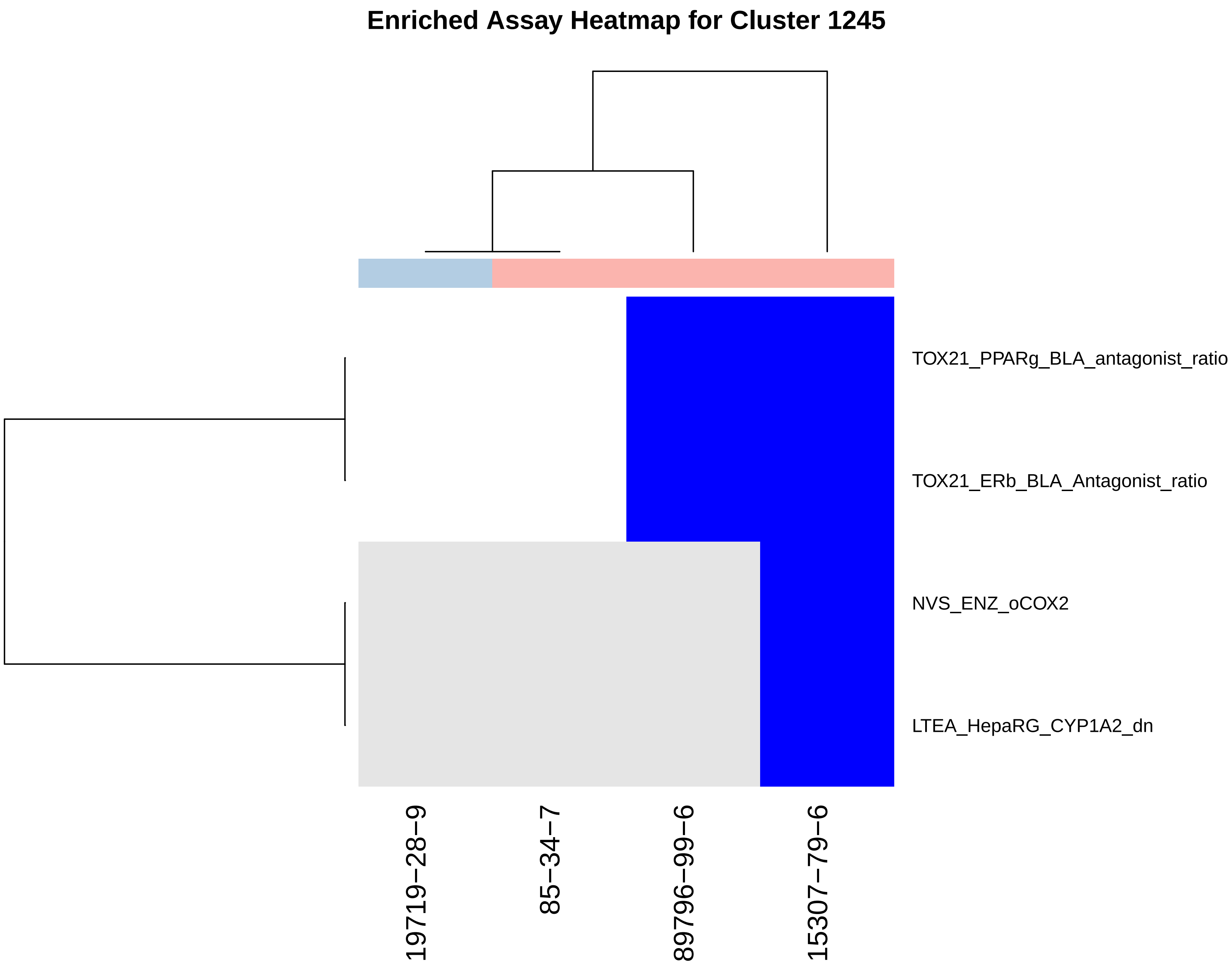
Enriched Assay Heatmap for Cluster 1245
TOX21_PPARg_BLA_antagonist_ratio
TOX21_ERb_BLA_Antagonist_ratio
NVS_ENZ_oCOX2
LTEA_HepaRG_CYP1A2_dn
19719−28−9
85−34−7
89796−99−6
15307−79−6

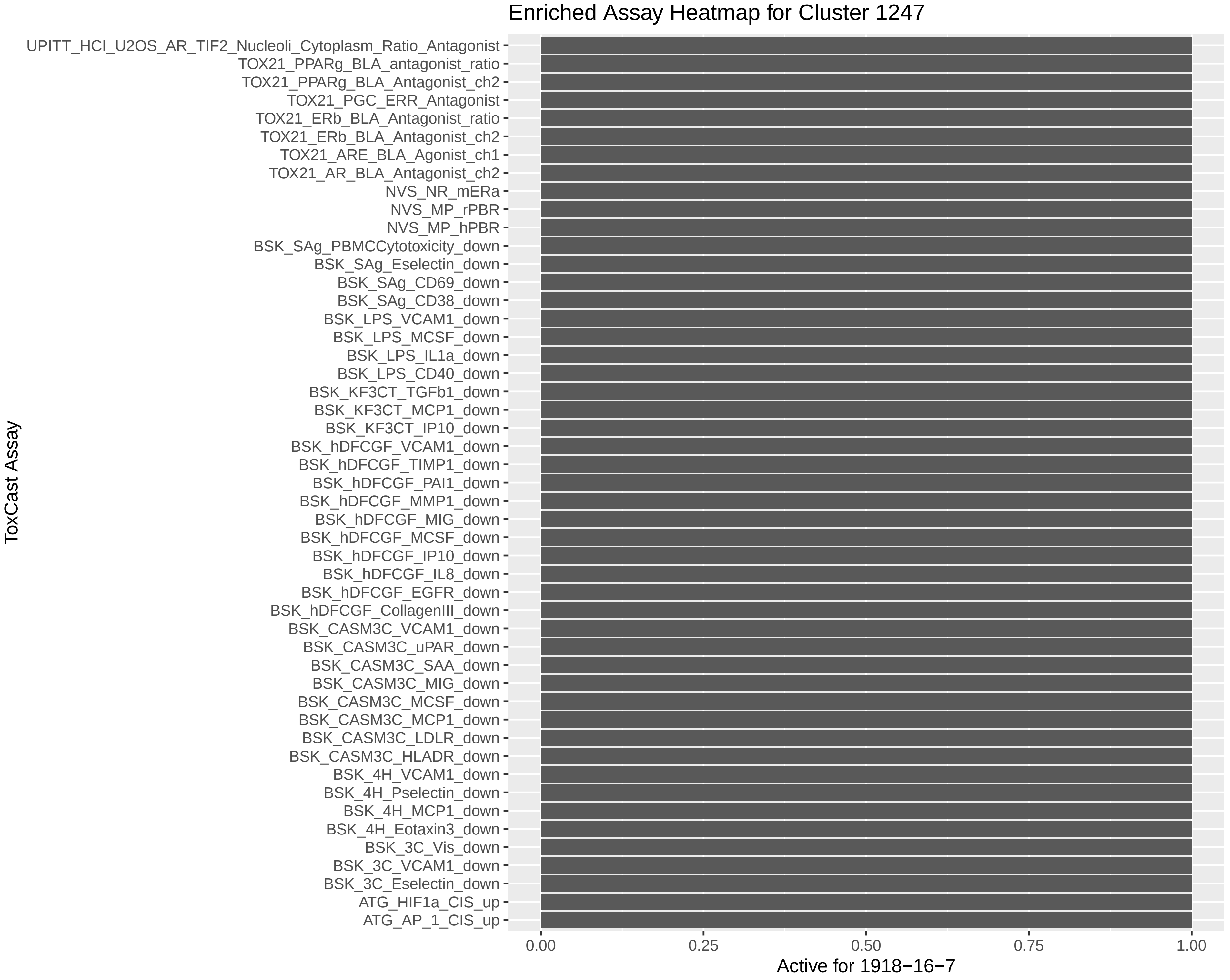
Enriched Assay Heatmap for Cluster 1247
UPITT_HCI_U2OS_AR_TIF2_Nucleoli_Cytoplasm_Ratio_Antagonist
TOX21_PPARg_BLA_antagonist_ratio
TOX21_PPARg_BLA_Antagonist_ch2
TOX21_PGC_ERR_Antagonist
TOX21_ERb_BLA_Antagonist_ratio
TOX21_ERb_BLA_Antagonist_ch2
TOX21_ARE_BLA_Agonist_ch1
TOX21_AR_BLA_Antagonist_ch2
NVS_NR_mERa
NVS_MP_rPBR
NVS_MP_hPBR
BSK_SAg_PBMCCytotoxicity_down
BSK_SAg_Eselectin_down
BSK_SAg_CD69_down
BSK_SAg_CD38_down
BSK_LPS_VCAM1_down
BSK_LPS_MCSF_down
BSK_LPS_IL1a_down
BSK_LPS_CD40_down
BSK_KF3CT_TGFb1_down
BSK_KF3CT_MCP1_down
BSK_KF3CT_IP10_down
BSK_hDFCGF_VCAM1_down
BSK_hDFCGF_TIMP1_down
BSK_hDFCGF_PAI1_down
BSK_hDFCGF_MMP1_down
BSK_hDFCGF_MIG_down
BSK_hDFCGF_MCSF_down
BSK_hDFCGF_IP10_down
BSK_hDFCGF_IL8_down
BSK_hDFCGF_EGFR_down
BSK_hDFCGF_CollagenIII_down
BSK_CASM3C_VCAM1_down
BSK_CASM3C_uPAR_down
BSK_CASM3C_SAA_down
BSK_CASM3C_MIG_down
BSK_CASM3C_MCSF_down
BSK_CASM3C_MCP1_down
BSK_CASM3C_LDLR_down
BSK_CASM3C_HLADR_down
BSK_4H_VCAM1_down
BSK_4H_Pselectin_down
BSK_4H_MCP1_down
BSK_4H_Eotaxin3_down
BSK_3C_Vis_down
BSK_3C_VCAM1_down
BSK_3C_Eselectin_down
ATG_HIF1a_CIS_up
ATG_AP_1_CIS_up
ToxCast Assay
0.00
0.25
0.50
0.75
1.00
Active for 1918−16−7

Enriched Assay Heatmap for Cluster 1253

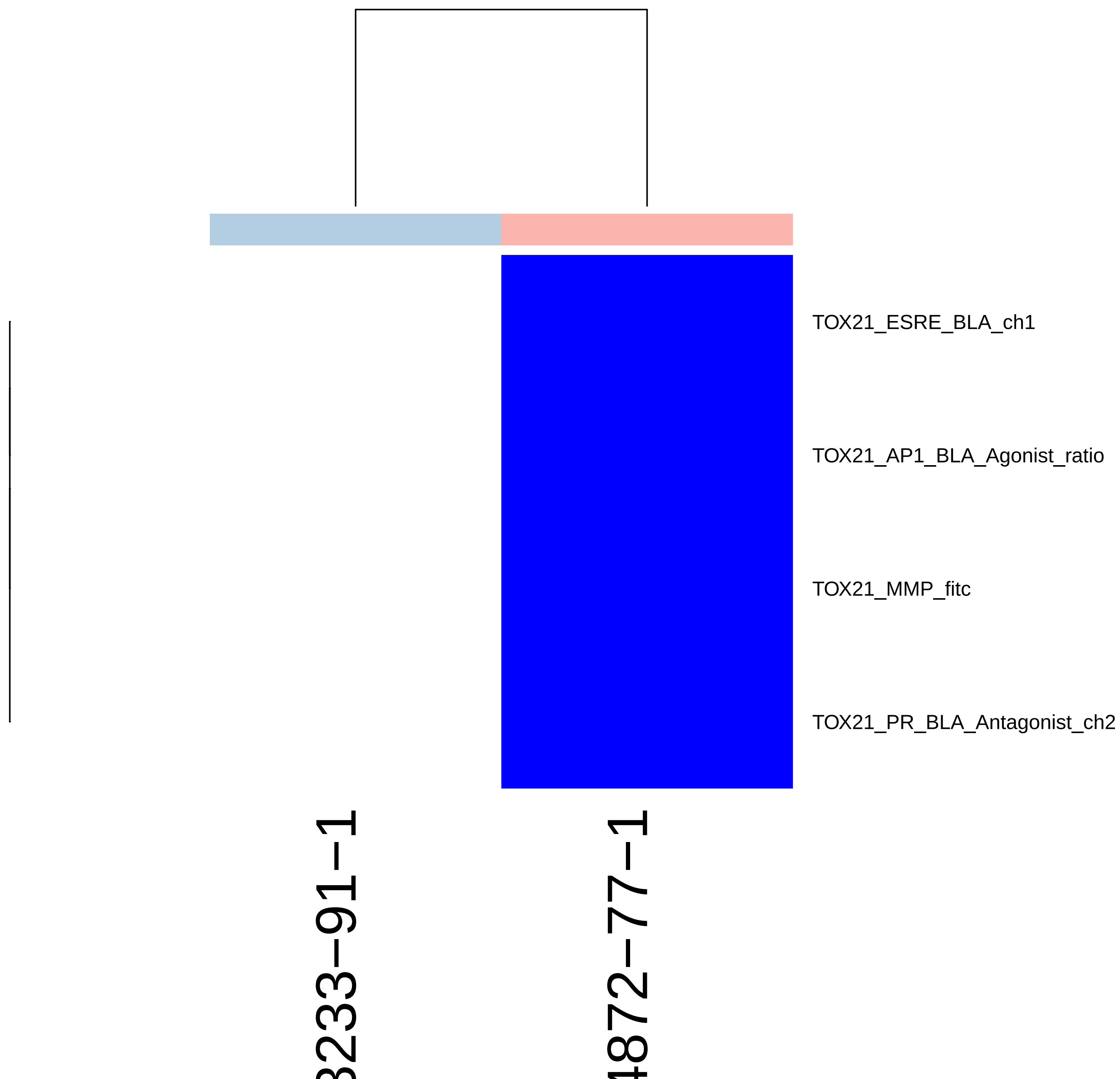

Enriched Assay Heatmap for Cluster 1256

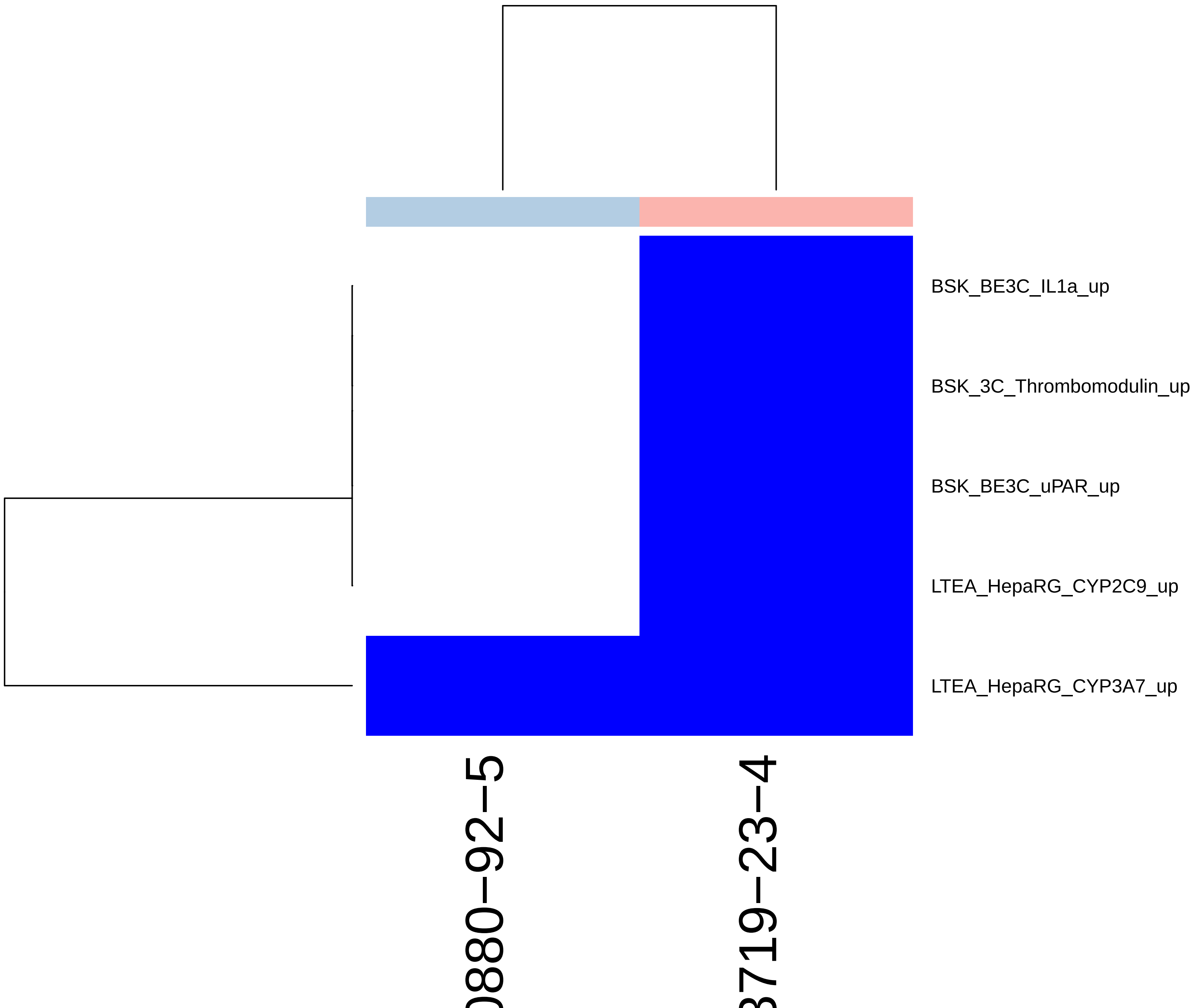

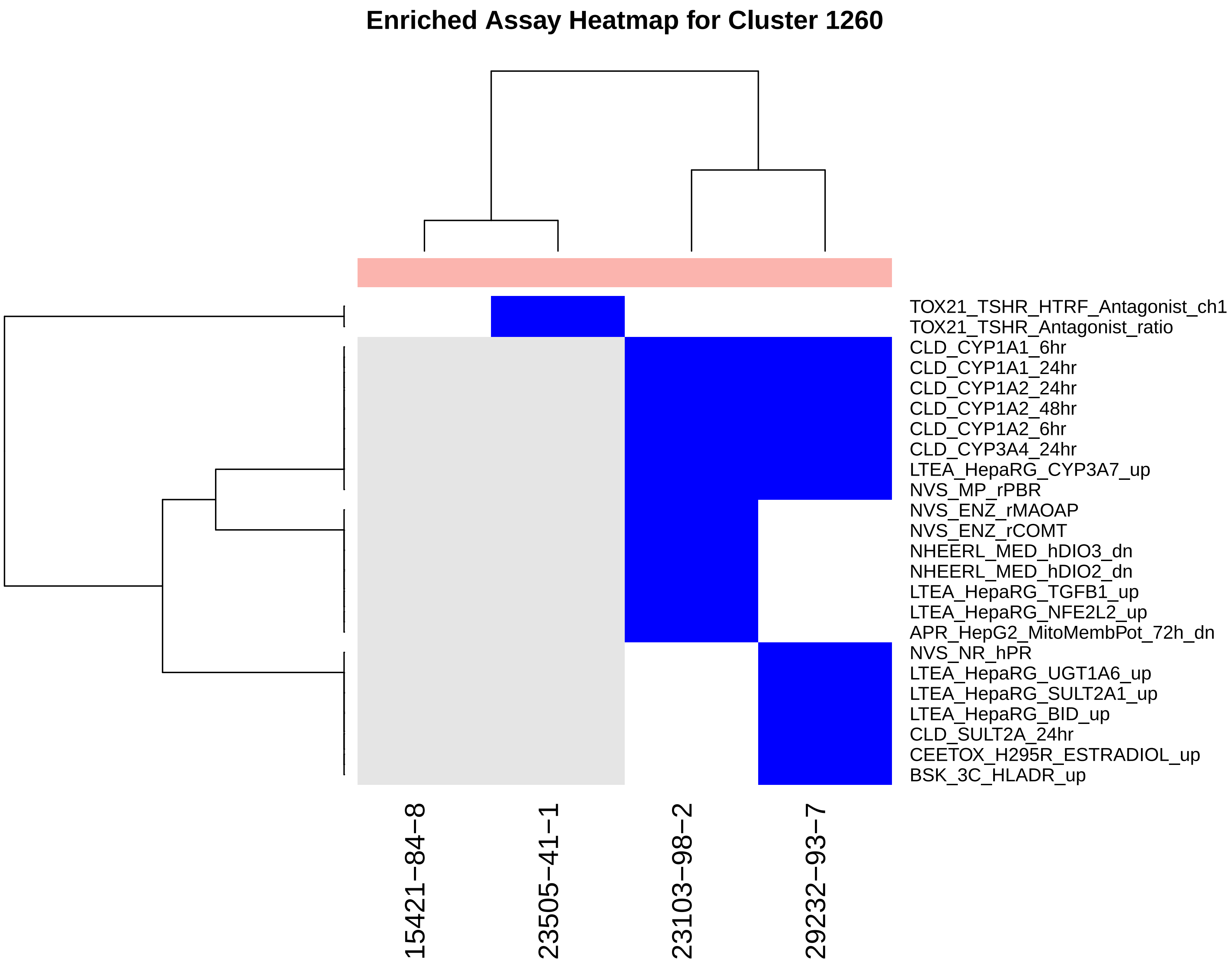

Enriched Assay Heatmap for Cluster 1260
TOX21_TSHR_HTRF_Antagonist_ch1
TOX21_TSHR_Antagonist_ratio
CLD_CYP1A1_6hr
CLD_CYP1A1_24hr
CLD_CYP1A2_24hr
CLD_CYP1A2_48hr
CLD_CYP1A2_6hr
CLD_CYP3A4_24hr
LTEA_HepaRG_CYP3A7_up
NVS_MP_rPBR
NVS_ENZ_rMAOAP
NVS_ENZ_rCOMT
NHEERL_MED_hDIO3_dn
NHEERL_MED_hDIO2_dn
LTEA_HepaRG_TGFB1_up
LTEA_HepaRG_NFE2L2_up
APR_HepG2_MitoMembPot_72h_dn
NVS_NR_hPR
LTEA_HepaRG_UGT1A6_up
LTEA_HepaRG_SULT2A1_up
LTEA_HepaRG_BID_up
CLD_SULT2A_24hr
CEETOX_H295R_ESTRADIOL_up
BSK_3C_HLADR_up
15421−84−8
23505−41−1
23103−98−2
29232−93−7

Enriched Assay Heatmap for Cluster 1263

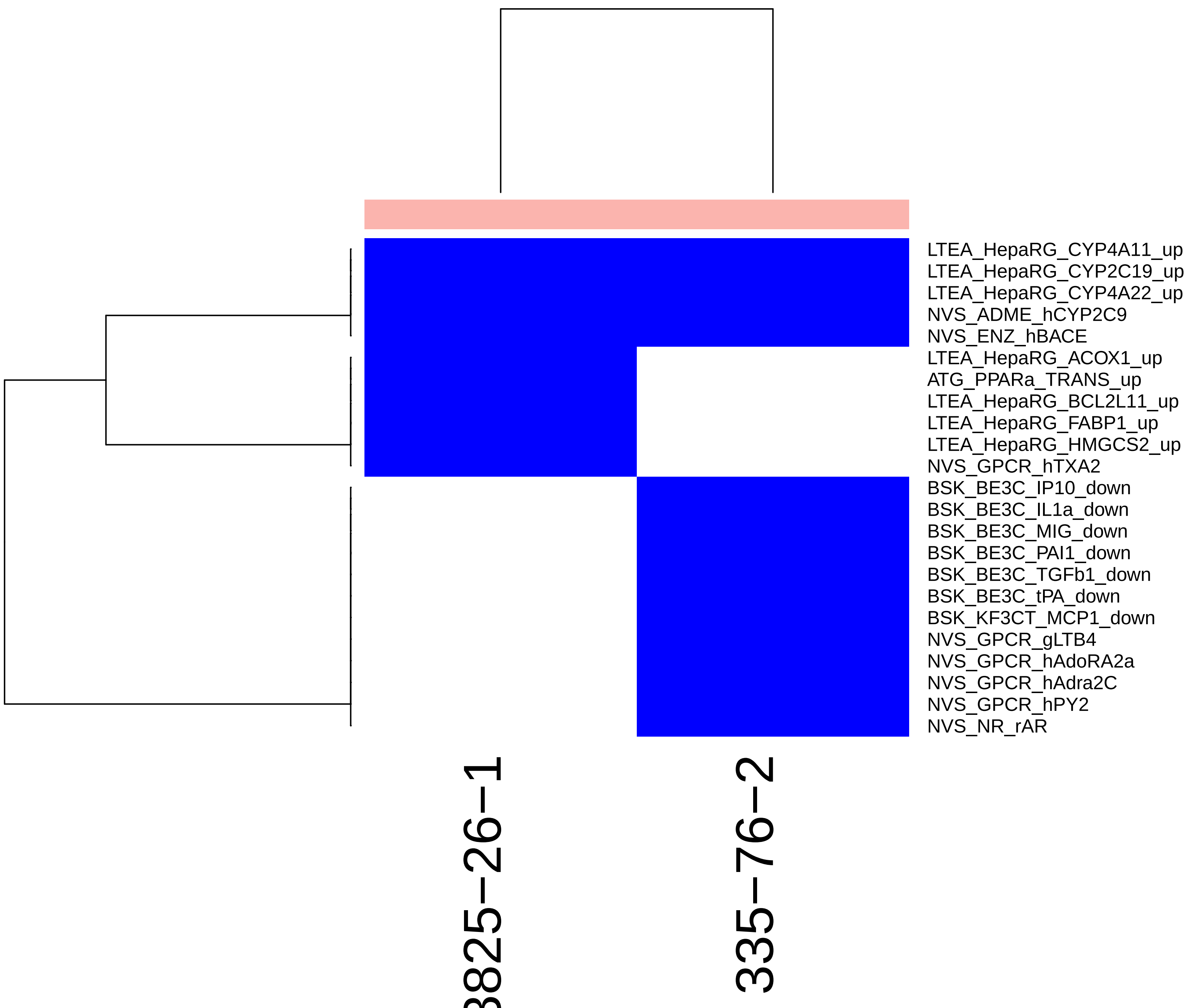

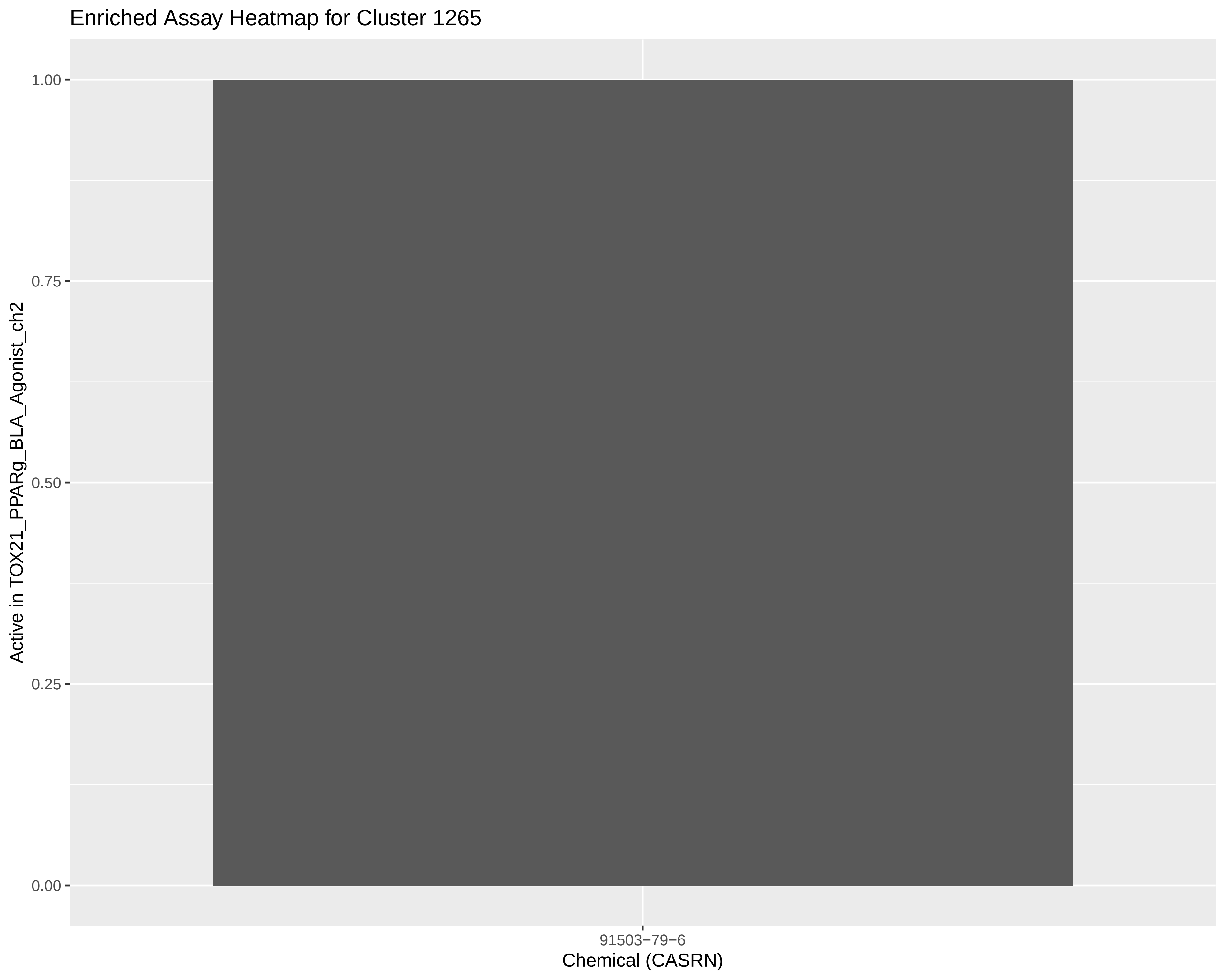
Enriched Assay Heatmap for Cluster 1265
Active in TOX21_PPARg_BLA_Agonist_ch2
1.00
0.75
0.50
0.25
0.00
91503−79−6
Chemical (CASRN)

Enriched Assay Heatmap for Cluster 1268

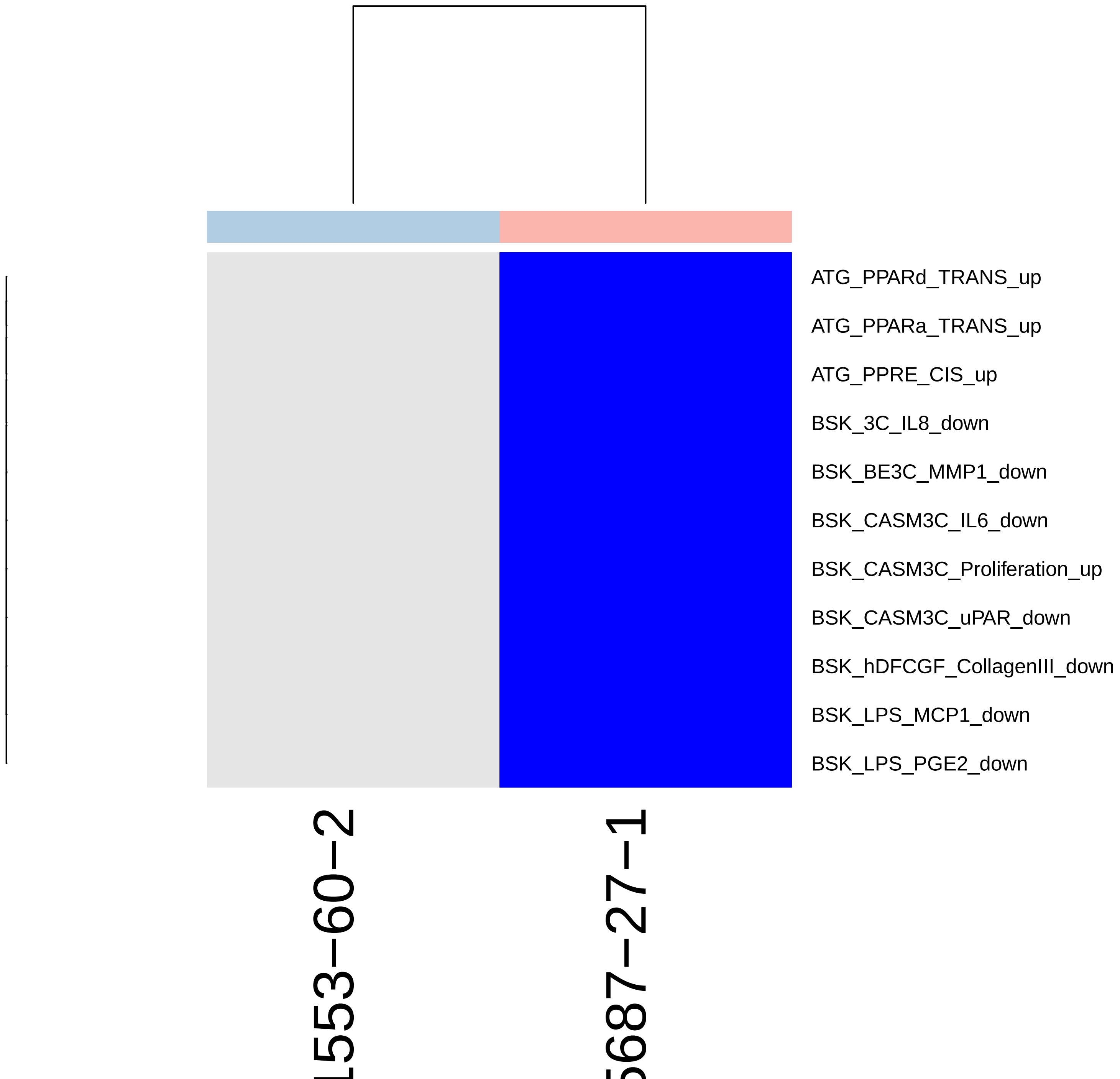

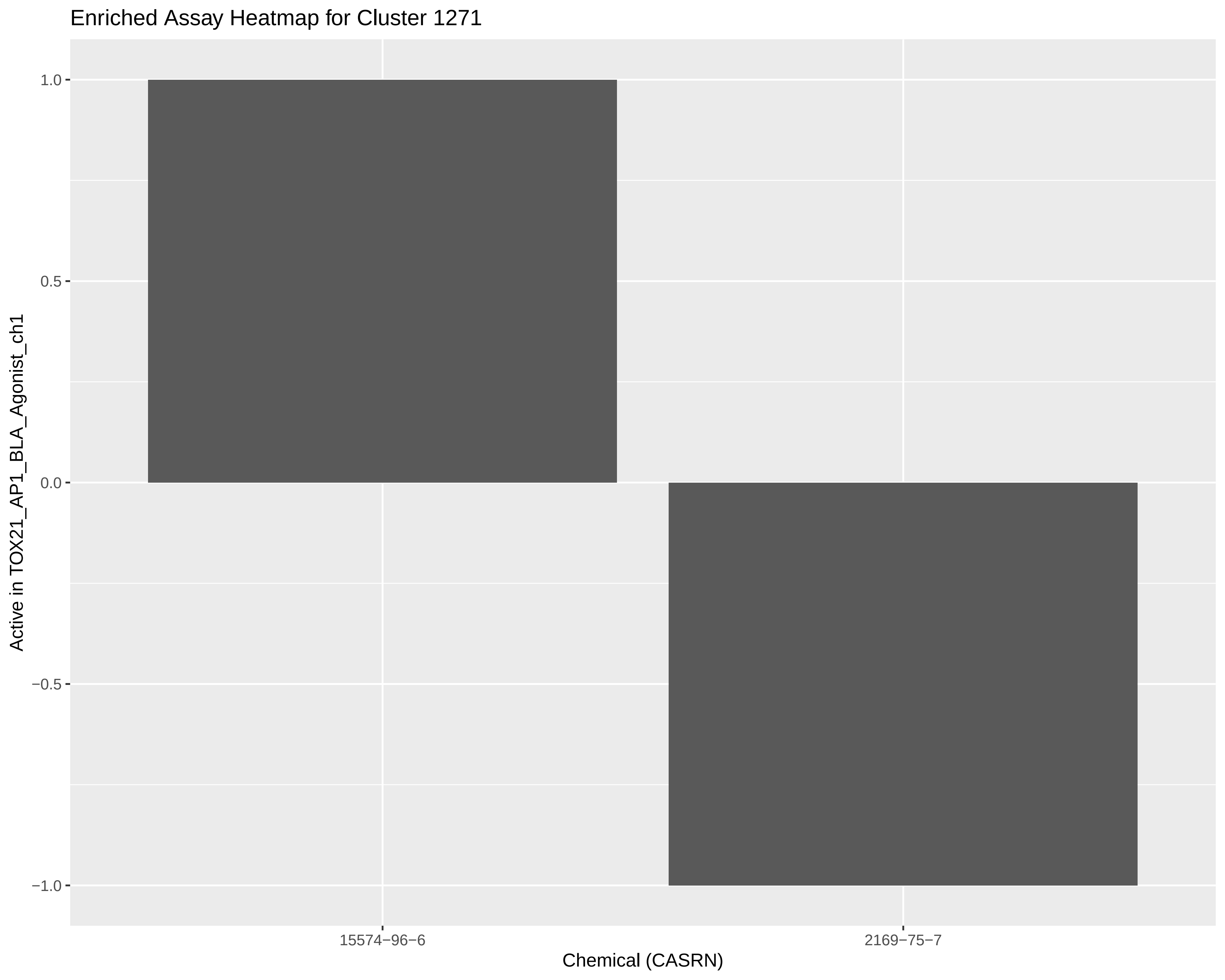
Enriched Assay Heatmap for Cluster 1271
1.0
0.5
0.0
−0.5
−1.0
Active in TOX21_AP1_BLA_Agonist_ch1
15574−96−6
2169−75−7
Chemical (CASRN)

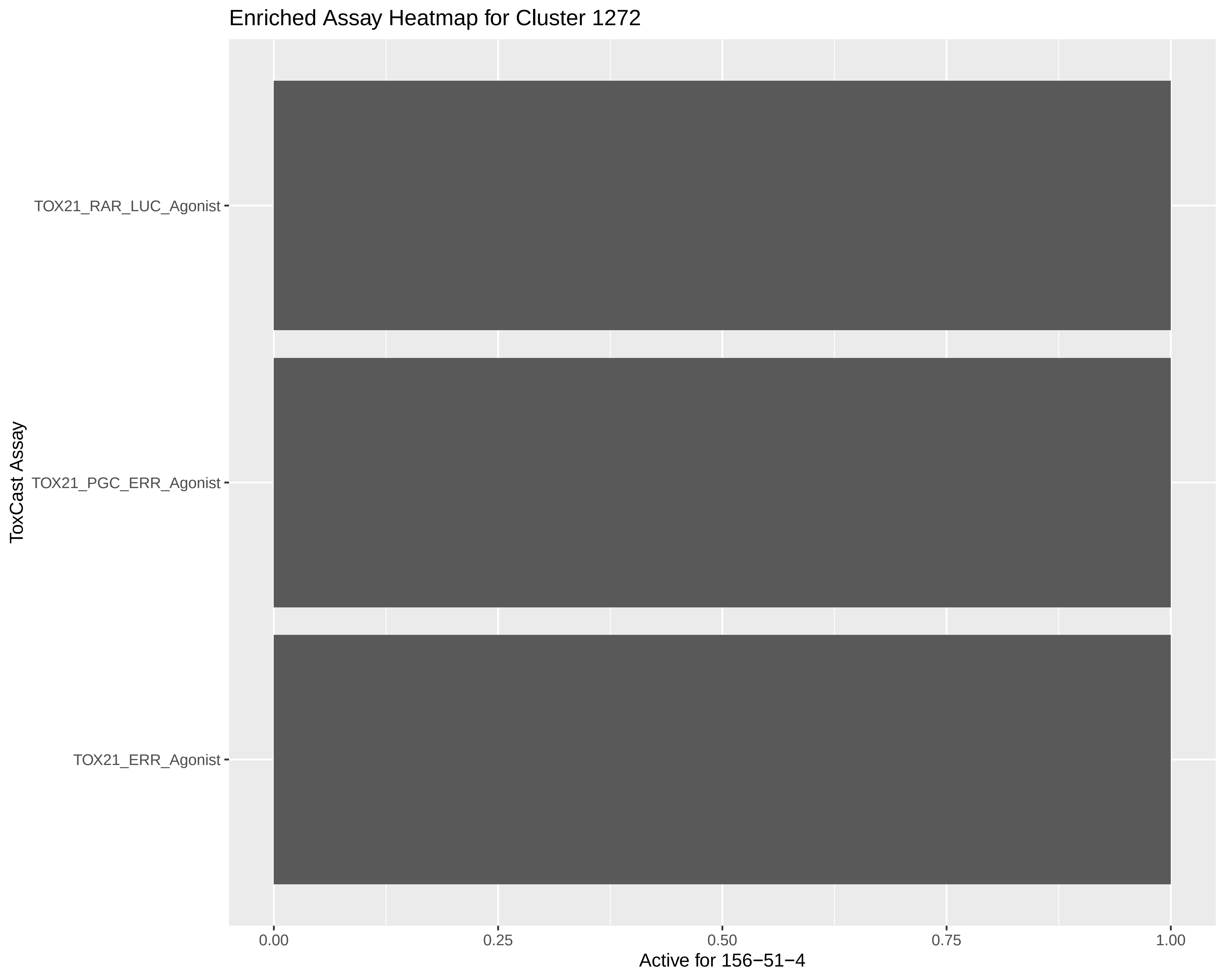
Enriched Assay Heatmap for Cluster 1272
ToxCast Assay
TOX21_RAR_LUC_Agonist
TOX21_PGC_ERR_Agonist
TOX21_ERR_Agonist
0.00
0.25
0.50
0.75
1.00
Active for 156−51−4

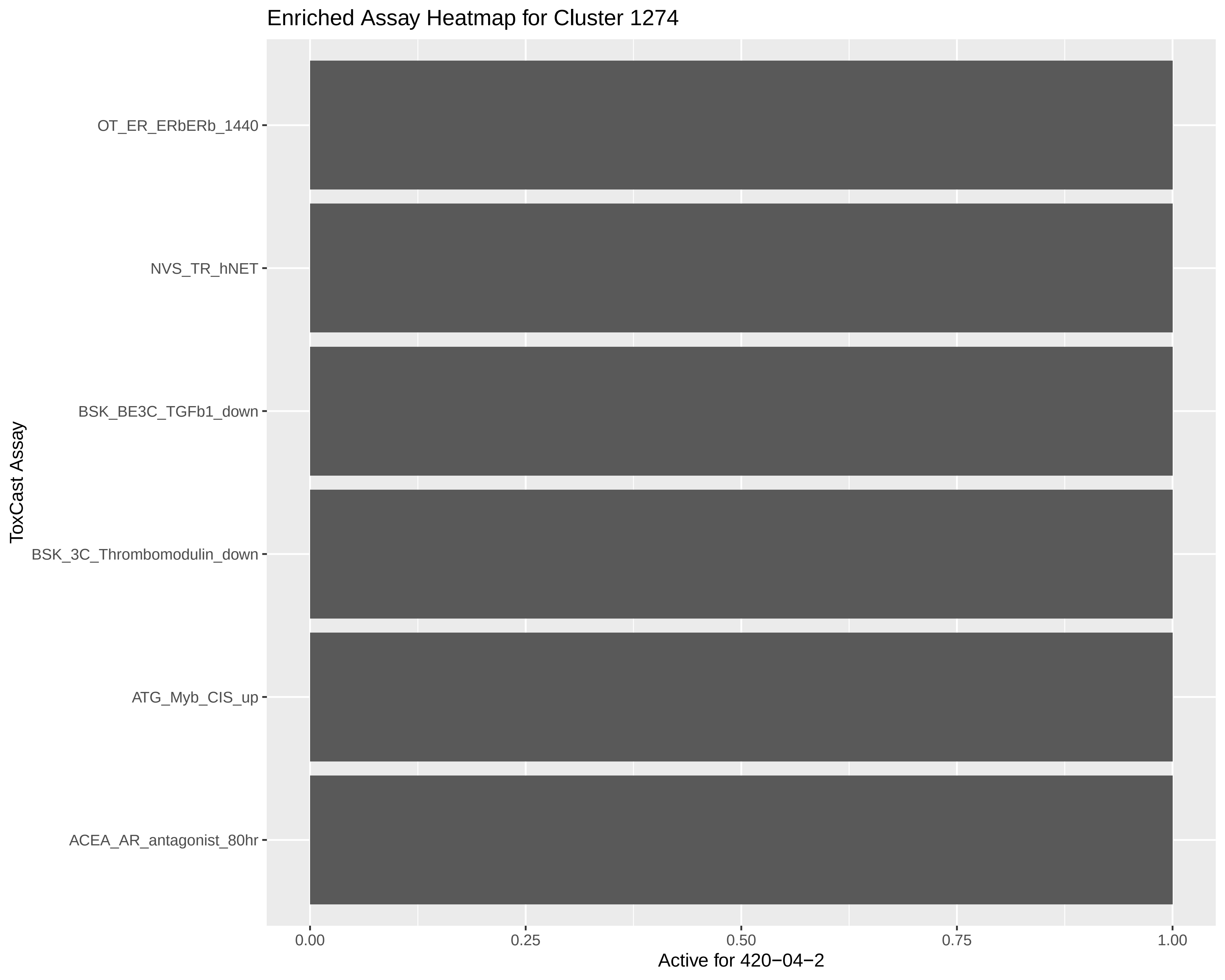

Enriched Assay Heatmap for Cluster 1274
OT_ER_ERbERb_1440
NVS_TR_hNET
BSK_BE3C_TGFb1_down
BSK_3C_Thrombomodulin_down
ATG_Myb_CIS_up
ACEA_AR_antagonist_80hr
ToxCast Assay
0.00
0.25
0.50
0.75
1.00
Active for 420−04−2

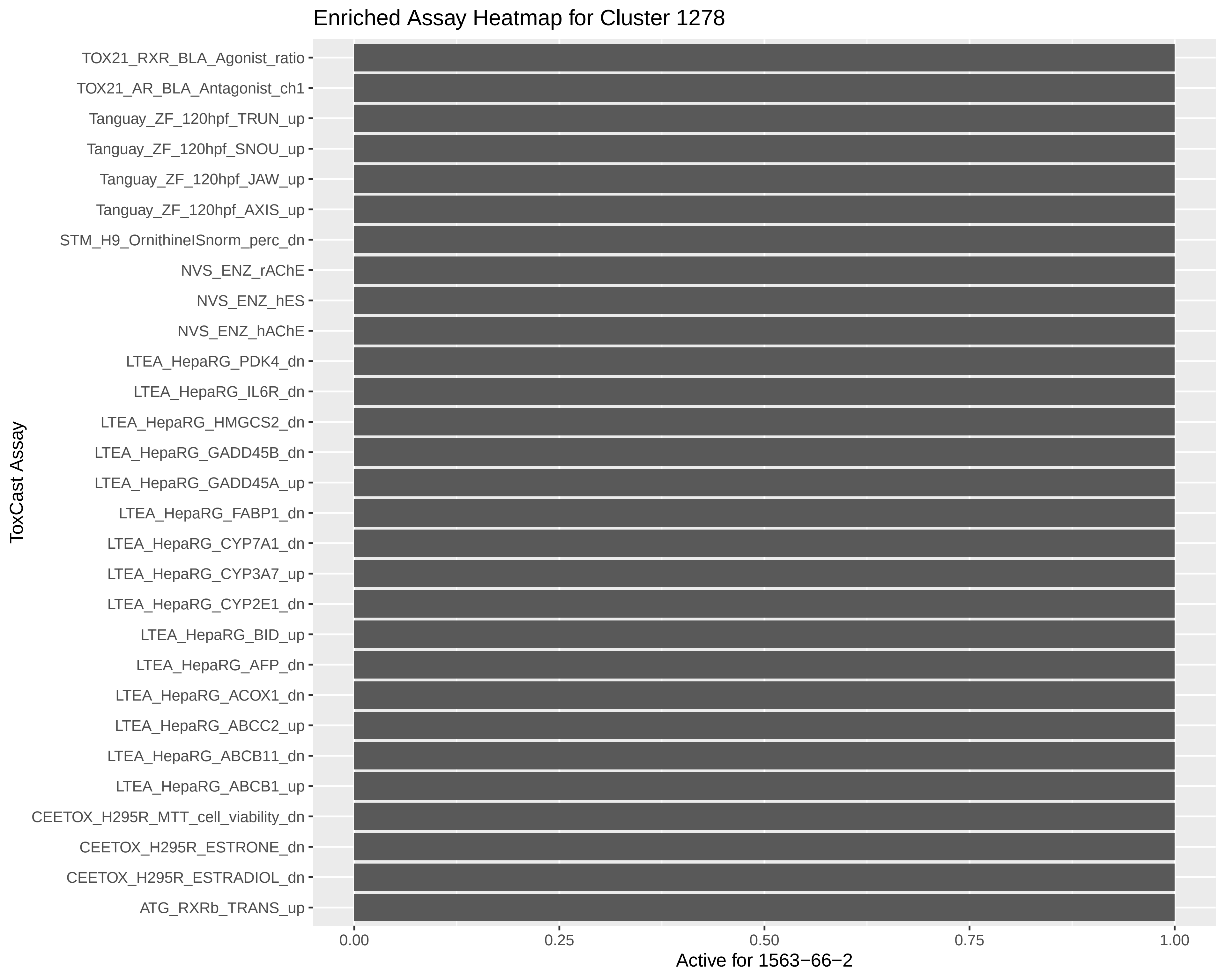
Enriched Assay Heatmap for Cluster 1278
ToxCast Assay
TOX21_RXR_BLA_Agonist_ratio
TOX21_AR_BLA_Antagonist_ch1
Tanguay_ZF_120hpf_TRUN_up
Tanguay_ZF_120hpf_SNOU_up
Tanguay_ZF_120hpf_JAW_up
Tanguay_ZF_120hpf_AXIS_up
STM_H9_OrnithineISnorm_perc_dn
NVS_ENZ_rAChE
NVS_ENZ_hES
NVS_ENZ_hAChE
LTEA_HepaRG_PDK4_dn
LTEA_HepaRG_IL6R_dn
LTEA_HepaRG_HMGCS2_dn
LTEA_HepaRG_GADD45B_dn
LTEA_HepaRG_GADD45A_up
LTEA_HepaRG_FABP1_dn
LTEA_HepaRG_CYP7A1_dn
LTEA_HepaRG_CYP3A7_up
LTEA_HepaRG_CYP2E1_dn
LTEA_HepaRG_BID_up
LTEA_HepaRG_AFP_dn
LTEA_HepaRG_ACOX1_dn
LTEA_HepaRG_ABCC2_up
LTEA_HepaRG_ABCB11_dn
LTEA_HepaRG_ABCB1_up
CEETOX_H295R_MTT_cell_viability_dn
CEETOX_H295R_ESTRONE_dn
CEETOX_H295R_ESTRADIOL_dn
ATG_RXRb_TRANS_up
0.00
0.25
0.50
0.75
1.00
Active for 1563−66−2

Enriched Assay Heatmap for Cluster 1282

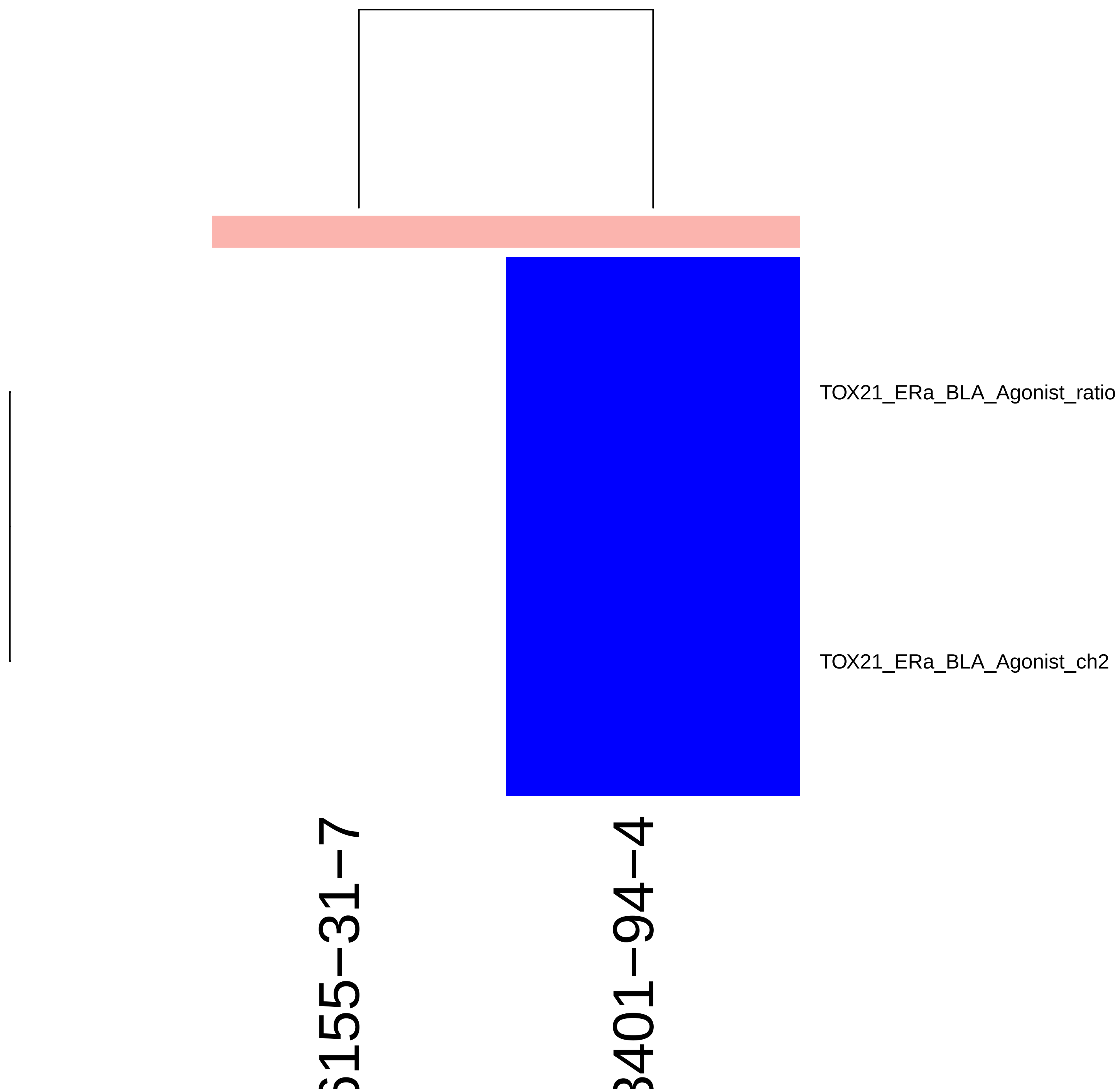

Enriched Assay Heatmap for Cluster 1283

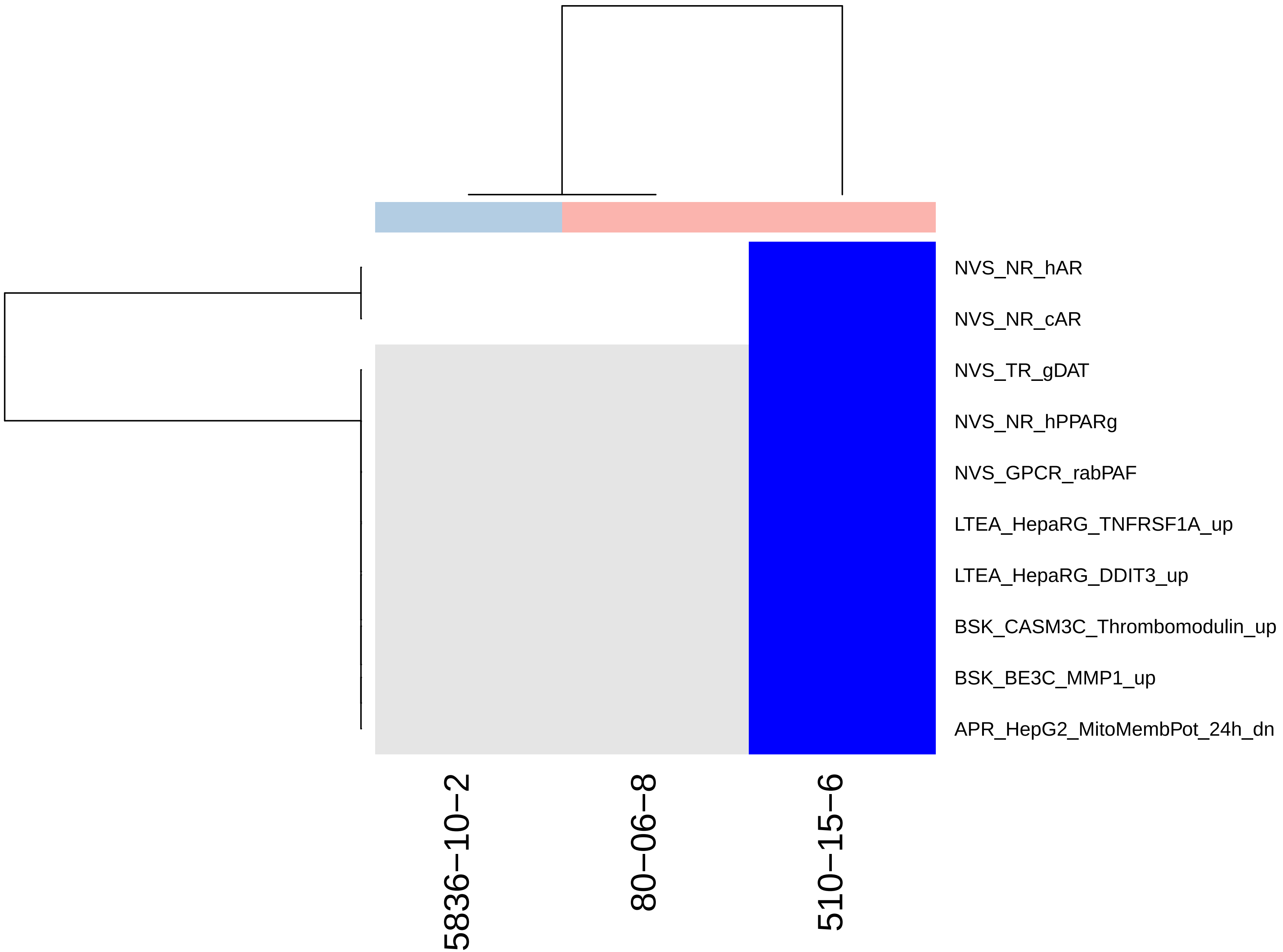

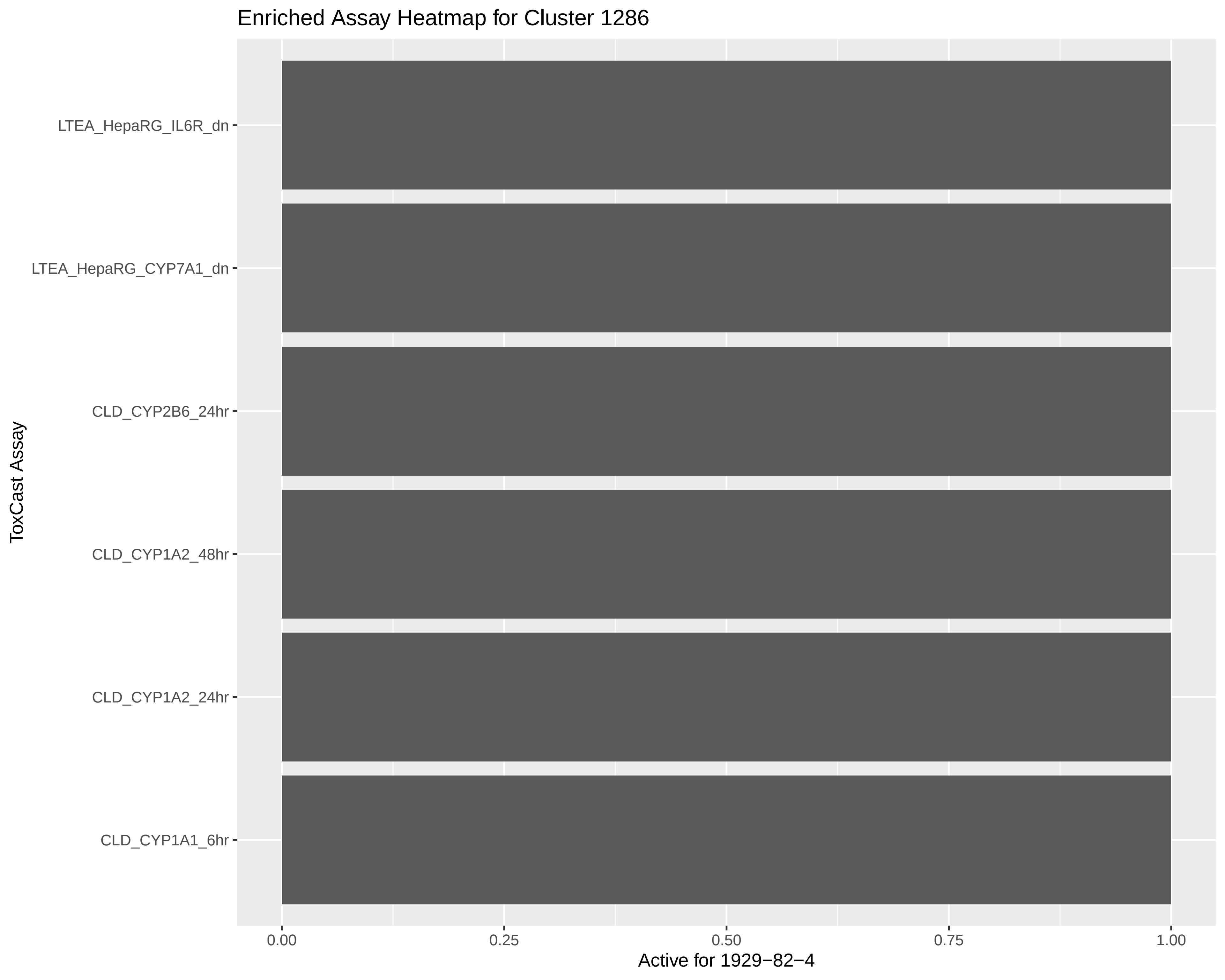

Enriched Assay Heatmap for Cluster 1286
ToxCast Assay
LTEA_HepaRG_IL6R_dn
LTEA_HepaRG_CYP7A1_dn
CLD_CYP2B6_24hr
CLD_CYP1A2_48hr
CLD_CYP1A2_24hr
CLD_CYP1A1_6hr
0.00
0.25
0.50
0.75
1.00
Active for 1929−82−4

Enriched Assay Heatmap for Cluster 1287

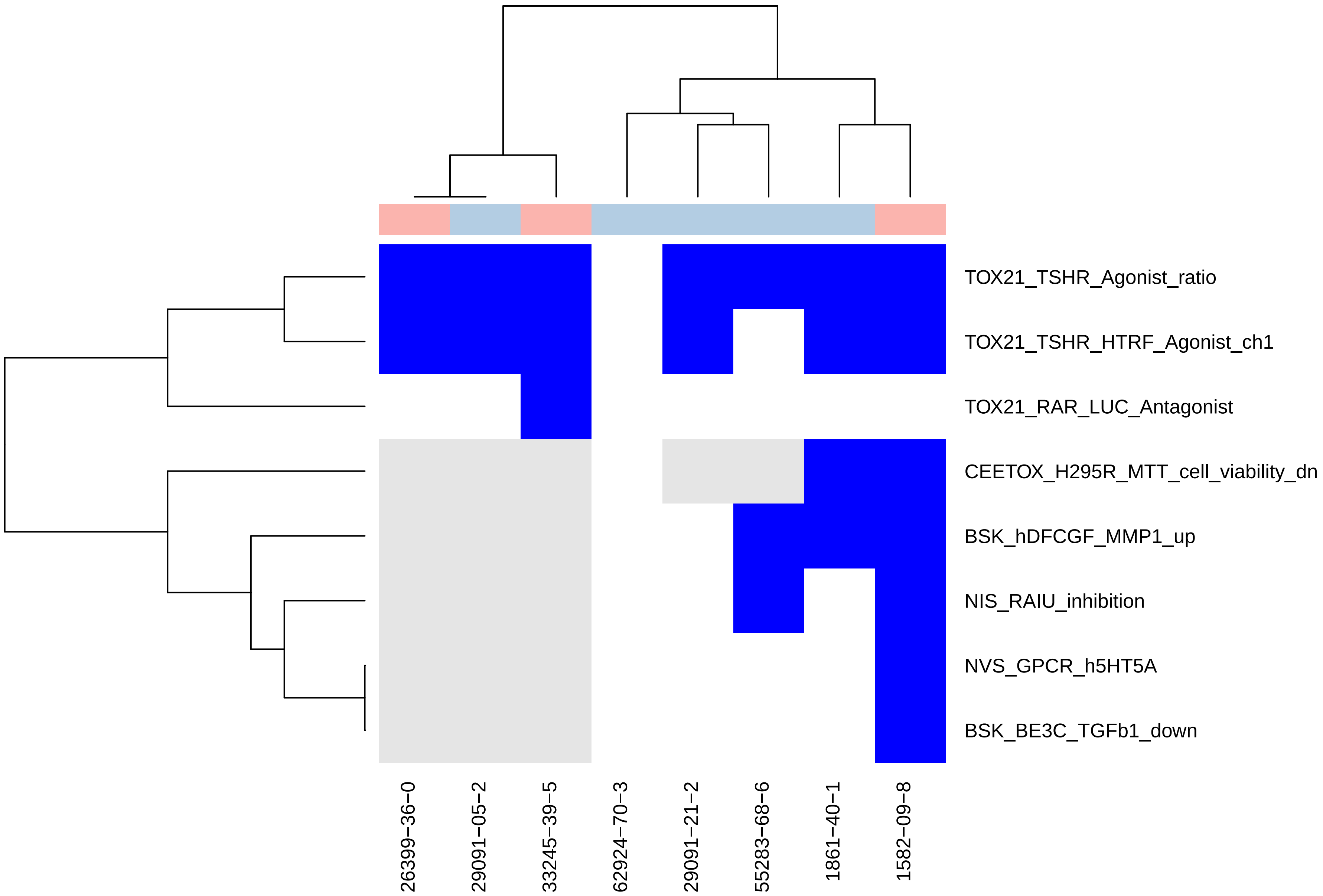

Enriched Assay Heatmap for Cluster 1295

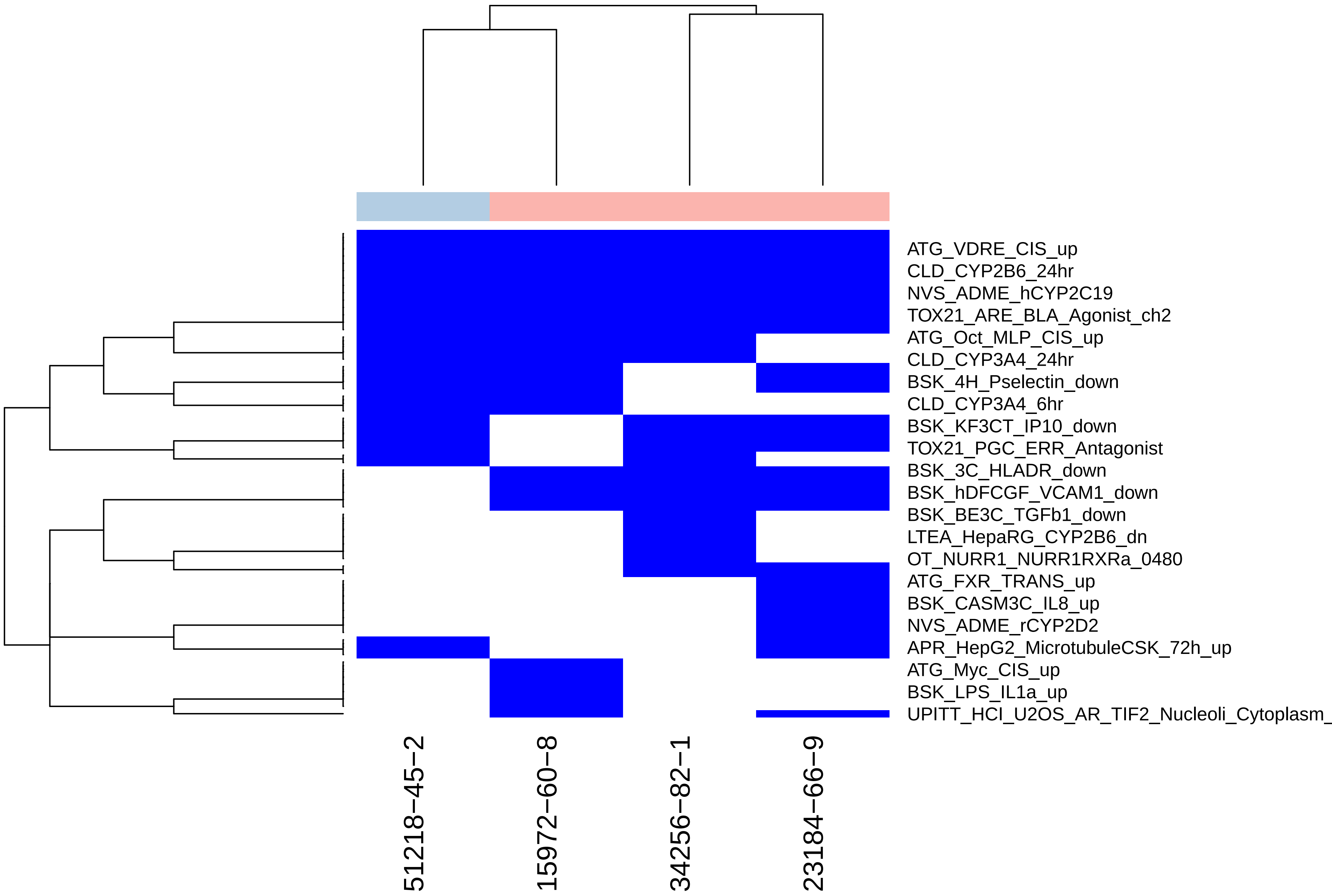

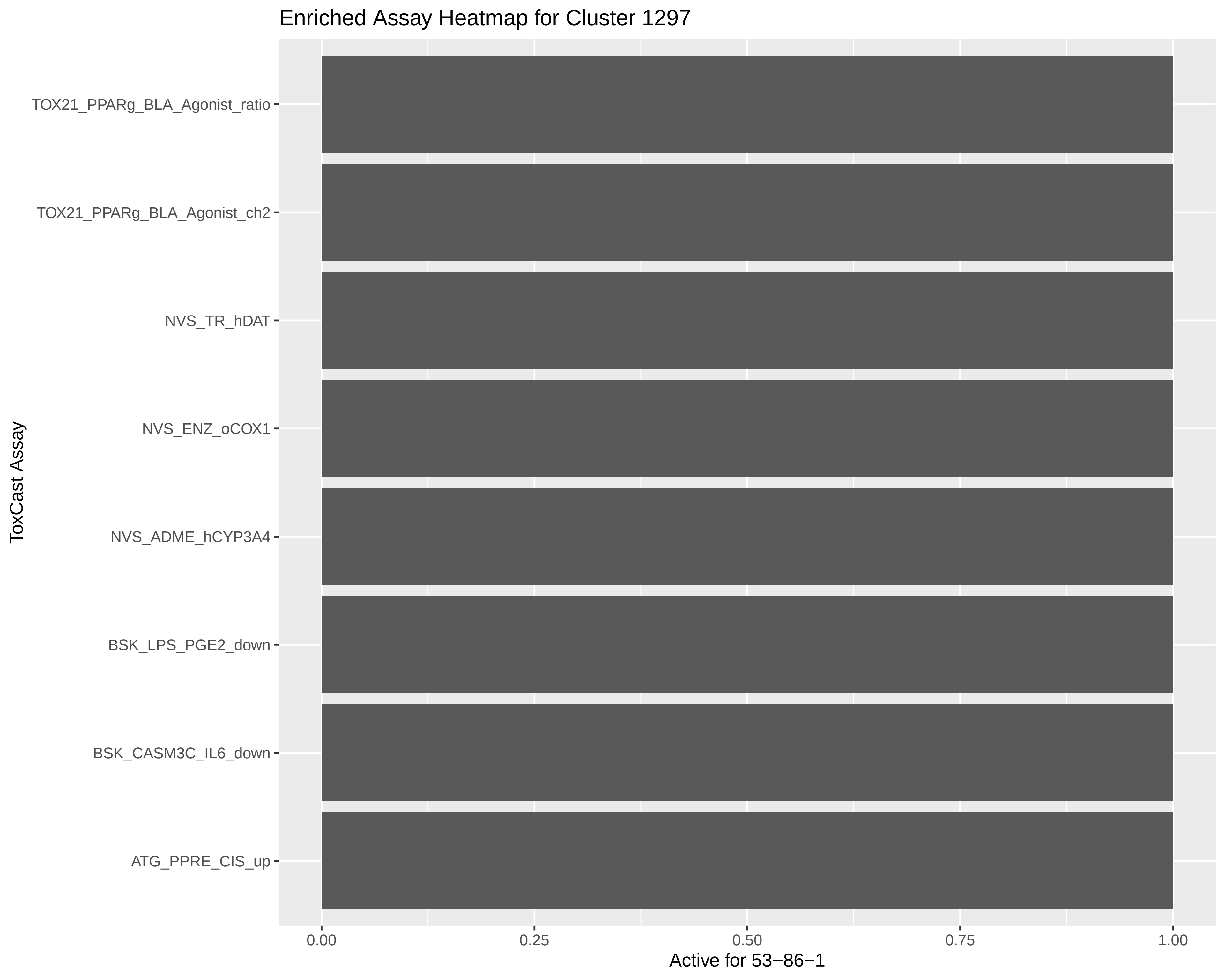

Enriched Assay Heatmap for Cluster 1297
TOX21_PPARg_BLA_Agonist_ratio
TOX21_PPARg_BLA_Agonist_ch2
NVS_TR_hDAT
NVS_ENZ_oCOX1
NVS_ADME_hCYP3A4
BSK_LPS_PGE2_down
BSK_CASM3C_IL6_down
ATG_PPRE_CIS_up
ToxCast Assay
0.00
0.25
0.50
0.75
1.00
Active for 53−86−1

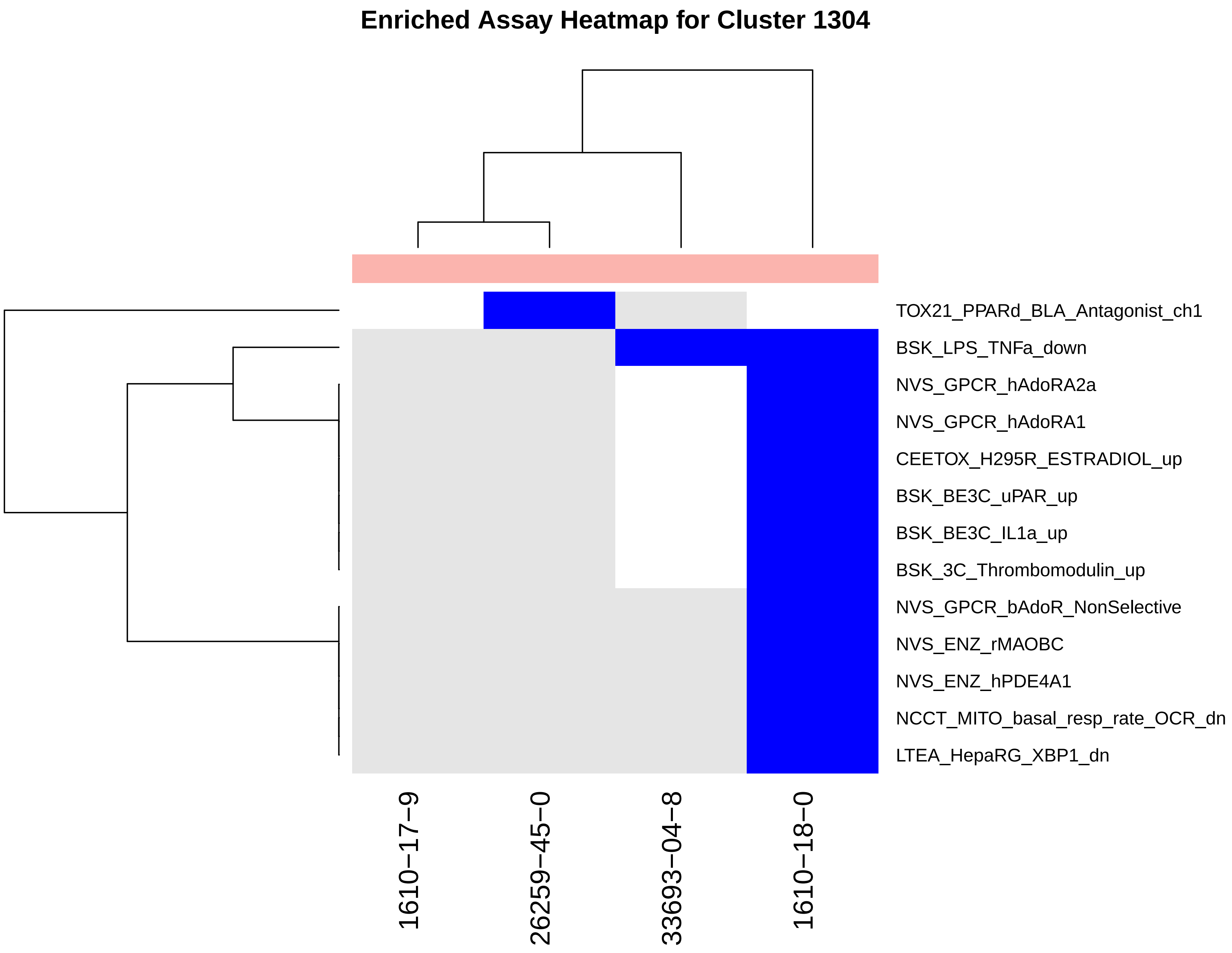

Enriched Assay Heatmap for Cluster 1304
TOX21_PPARd_BLA_Antagonist_ch1
BSK_LPS_TNFa_down
NVS_GPCR_hAdoRA2a
NVS_GPCR_hAdoRA1
CEETOX_H295R_ESTRADIOL_up
BSK_BE3C_uPAR_up
BSK_BE3C_IL1a_up
BSK_3C_Thrombomodulin_up
NVS_GPCR_bAdoR_NonSelective
NVS_ENZ_rMAOBC
NVS_ENZ_hPDE4A1
NCCT_MITO_basal_resp_rate_OCR_dn
LTEA_HepaRG_XBP1_dn
1610−17−9
26259−45−0
33693−04−8
1610−18−0

Enriched Assay Heatmap for Cluster 1311

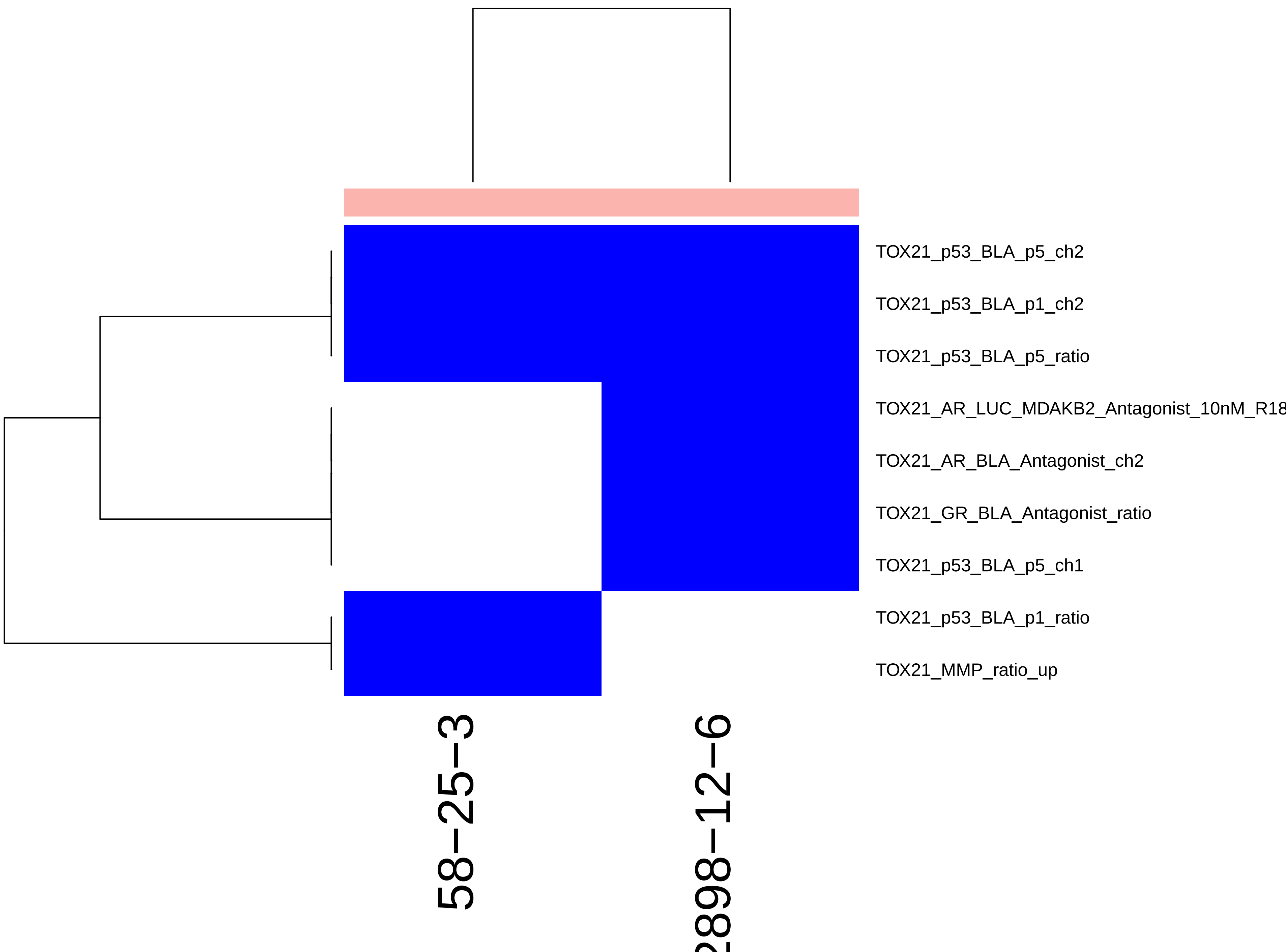

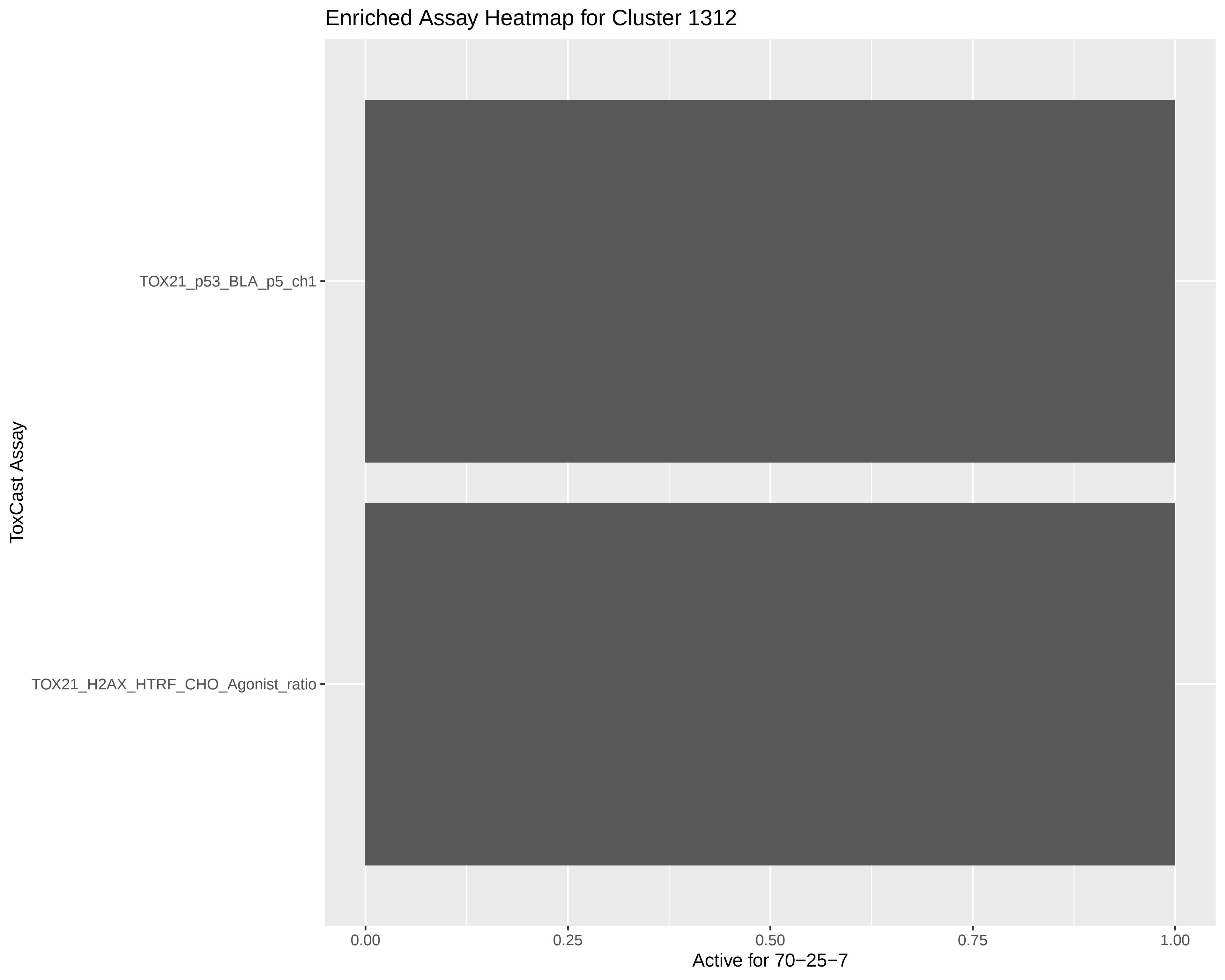
Enriched Assay Heatmap for Cluster 1312
TOX21_p53_BLA_p5_ch1
TOX21_H2AX_HTRF_CHO_Agonist_ratio
ToxCast Assay
0.00
0.25
0.50
0.75
1.00
Active for 70−25−7

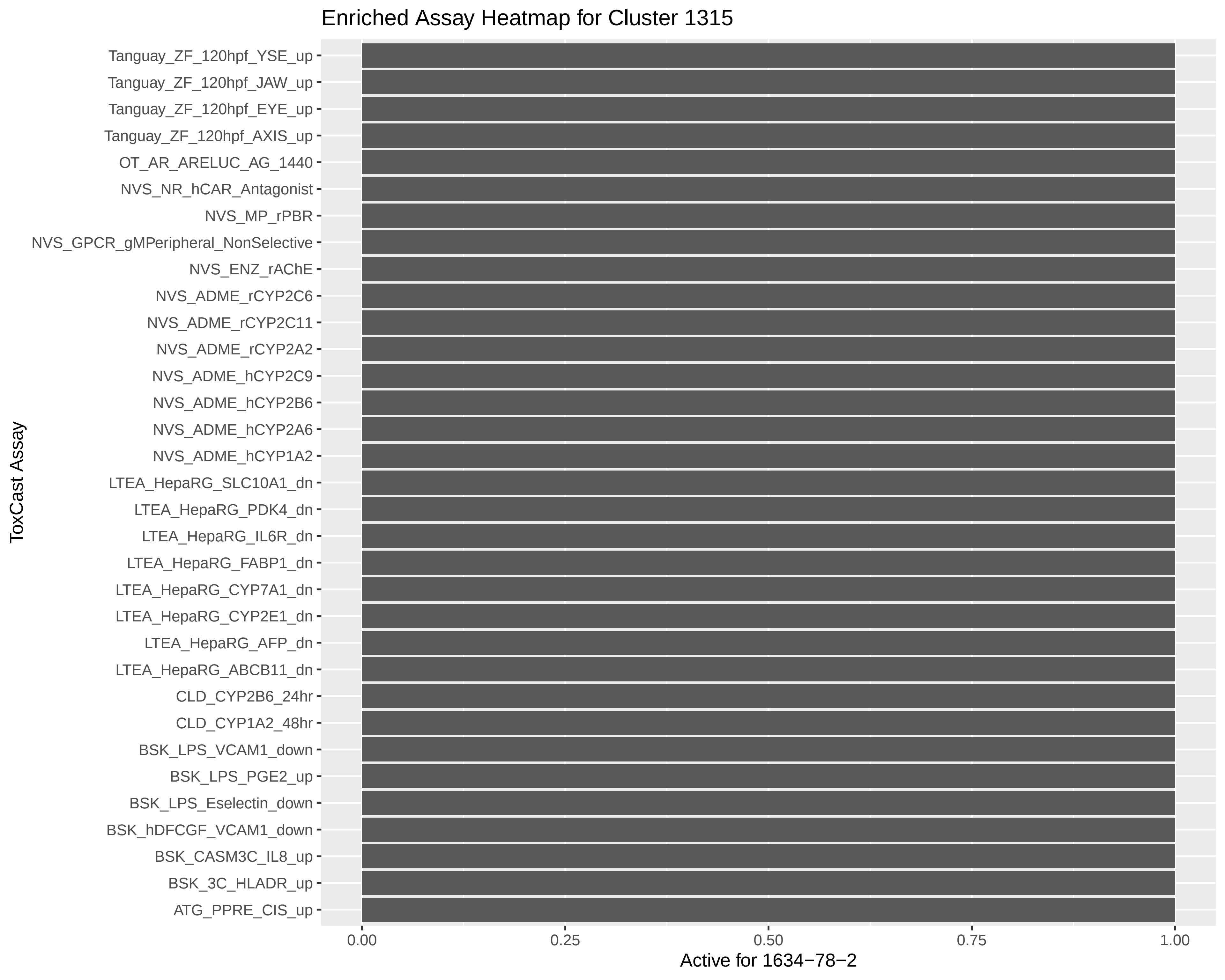

Enriched Assay Heatmap for Cluster 1315
ToxCast Assay
Tanguay_ZF_120hpf_YSE_up
Tanguay_ZF_120hpf_JAW_up
Tanguay_ZF_120hpf_EYE_up
Tanguay_ZF_120hpf_AXIS_up
OT_AR_ARELUC_AG_1440
NVS_NR_hCAR_Antagonist
NVS_MP_rPBR
NVS_GPCR_gMPeripheral_NonSelective
NVS_ENZ_rAChE
NVS_ADME_rCYP2C6
NVS_ADME_rCYP2C11
NVS_ADME_rCYP2A2
NVS_ADME_hCYP2C9
NVS_ADME_hCYP2B6
NVS_ADME_hCYP2A6
NVS_ADME_hCYP1A2
LTEA_HepaRG_SLC10A1_dn
LTEA_HepaRG_PDK4_dn
LTEA_HepaRG_IL6R_dn
LTEA_HepaRG_FABP1_dn
LTEA_HepaRG_CYP7A1_dn
LTEA_HepaRG_CYP2E1_dn
LTEA_HepaRG_AFP_dn
LTEA_HepaRG_ABCB11_dn
CLD_CYP2B6_24hr
CLD_CYP1A2_48hr
BSK_LPS_VCAM1_down
BSK_LPS_PGE2_up
BSK_LPS_Eselectin_down
BSK_hDFCGF_VCAM1_down
BSK_CASM3C_IL8_up
BSK_3C_HLADR_up
ATG_PPRE_CIS_up
0.00
0.25
0.50
0.75
1.00
Active for 1634−78−2

Enriched Assay Heatmap for Cluster 1319

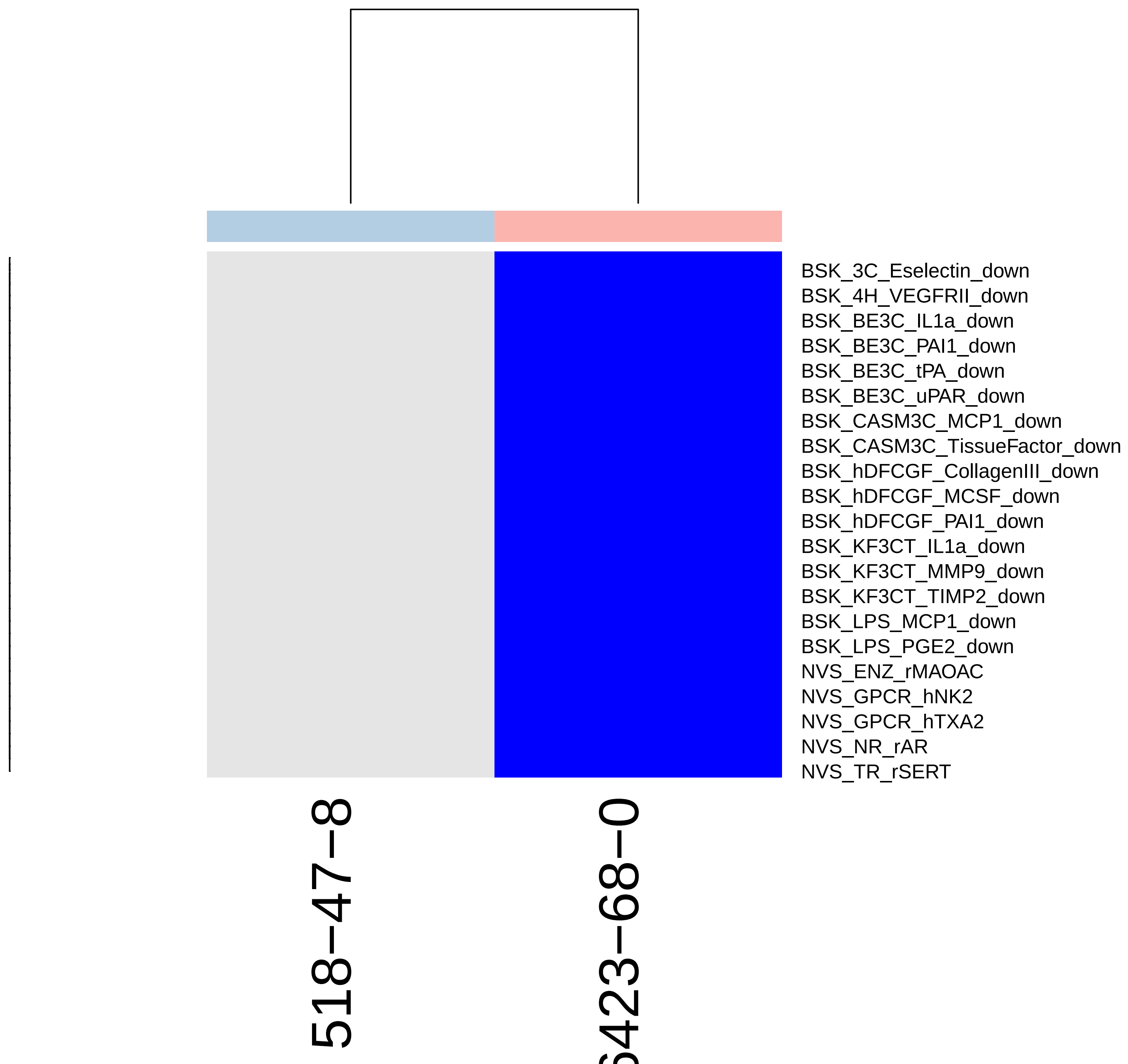

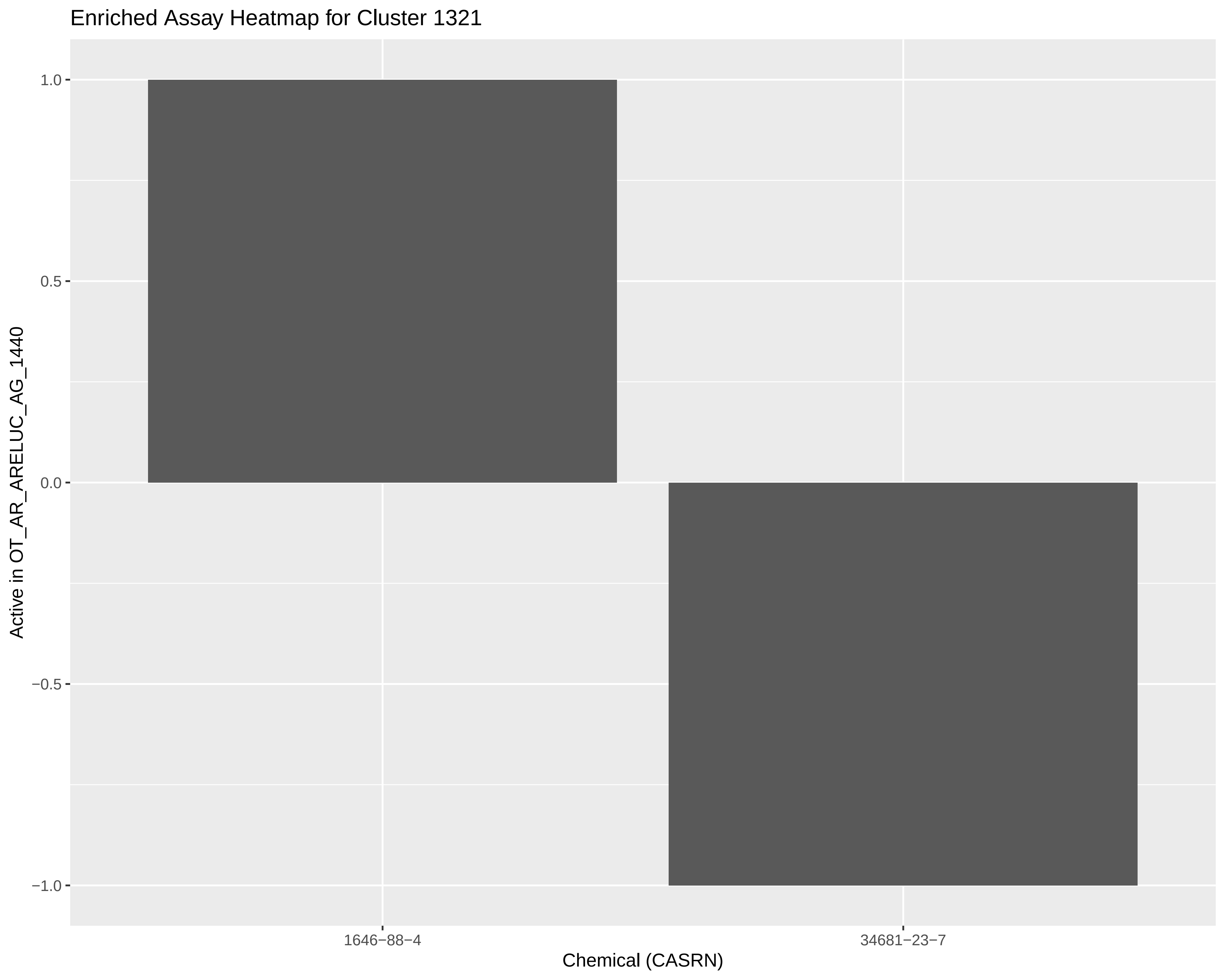
Enriched Assay Heatmap for Cluster 1321
Active in OT_AR_ARELUC_AG_1440
1.0
0.5
0.0
−0.5
−1.0
1646−88−4
34681−23−7
Chemical (CASRN)

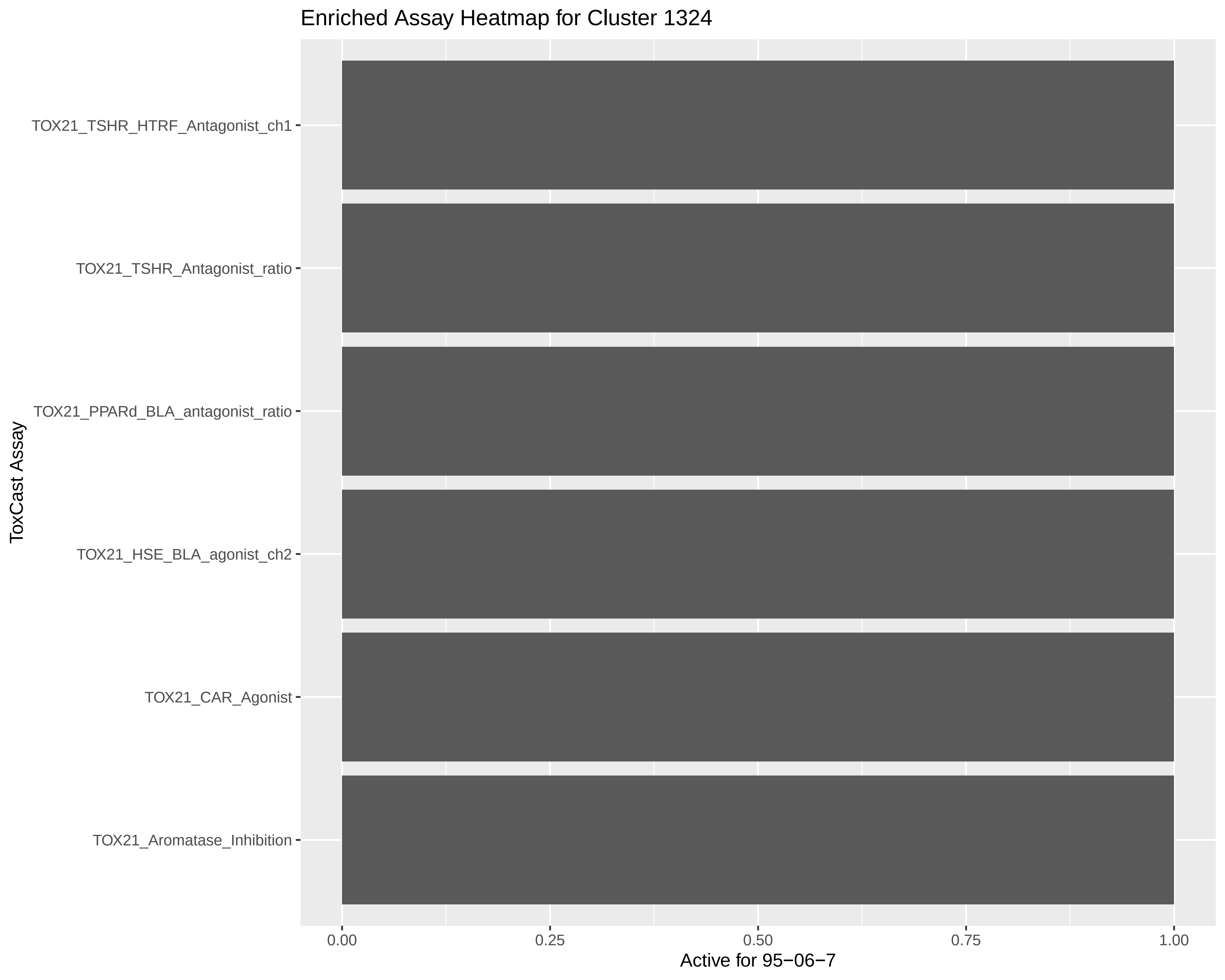
Enriched Assay Heatmap for Cluster 1324
TOX21_TSHR_HTRF_Antagonist_ch1
TOX21_TSHR_Antagonist_ratio
TOX21_PPARd_BLA_antagonist_ratio
TOX21_HSE_BLA_agonist_ch2
TOX21_CAR_Agonist
TOX21_Aromatase_Inhibition
ToxCast Assay
0.00
0.25
0.50
0.75
1.00
Active for 95−06−7

Enriched Assay Heatmap for Cluster 1325

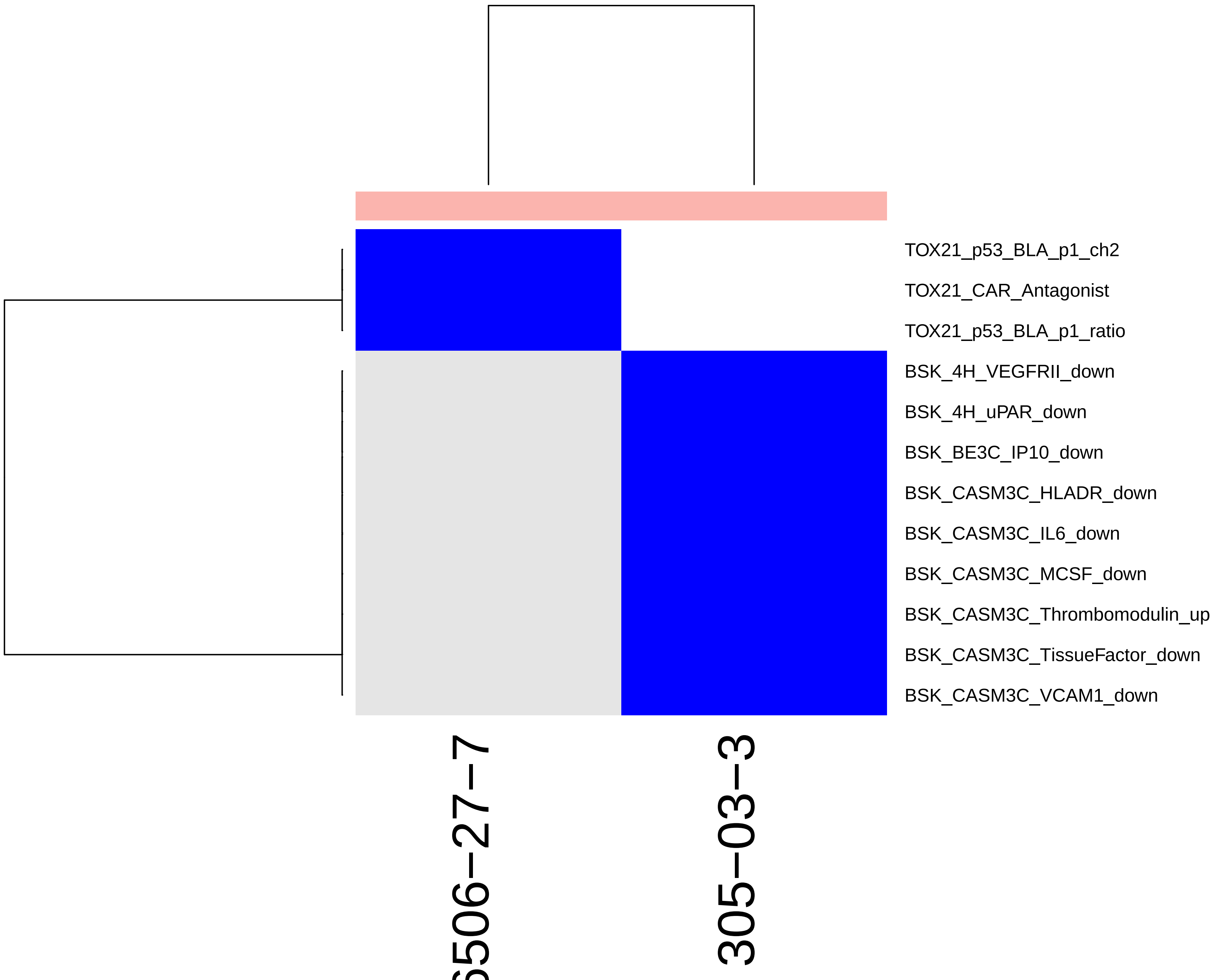

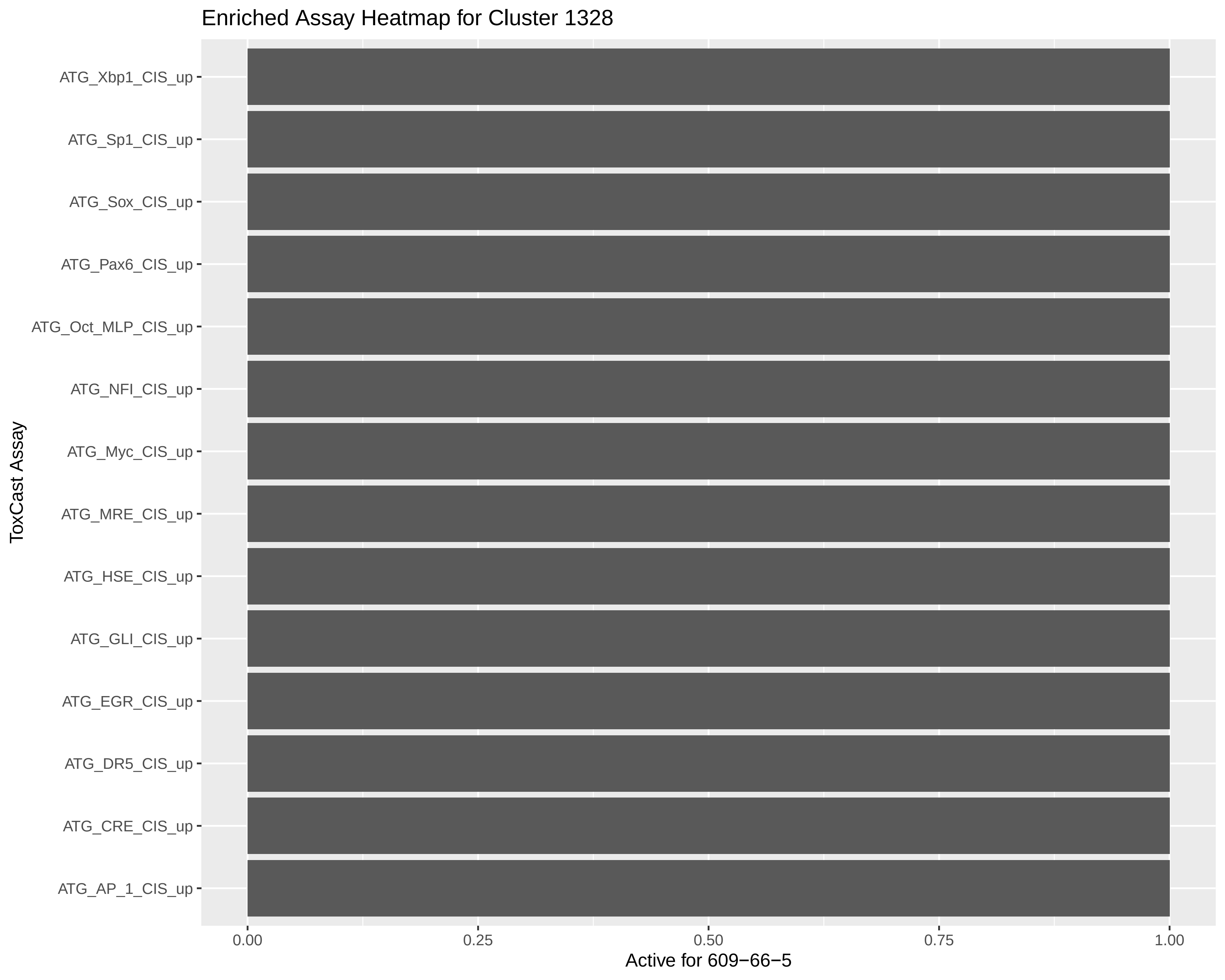

Enriched Assay Heatmap for Cluster 1328
ToxCast Assay
ATG_Xbp1_CIS_up
ATG_Sp1_CIS_up
ATG_Sox_CIS_up
ATG_Pax6_CIS_up
ATG_Oct_MLP_CIS_up
ATG_NFI_CIS_up
ATG_Myc_CIS_up
ATG_MRE_CIS_up
ATG_HSE_CIS_up
ATG_GLI_CIS_up
ATG_EGR_CIS_up
ATG_DR5_CIS_up
ATG_CRE_CIS_up
ATG_AP_1_CIS_up
0.00
0.25
0.50
0.75
1.00
Active for 609−66−5

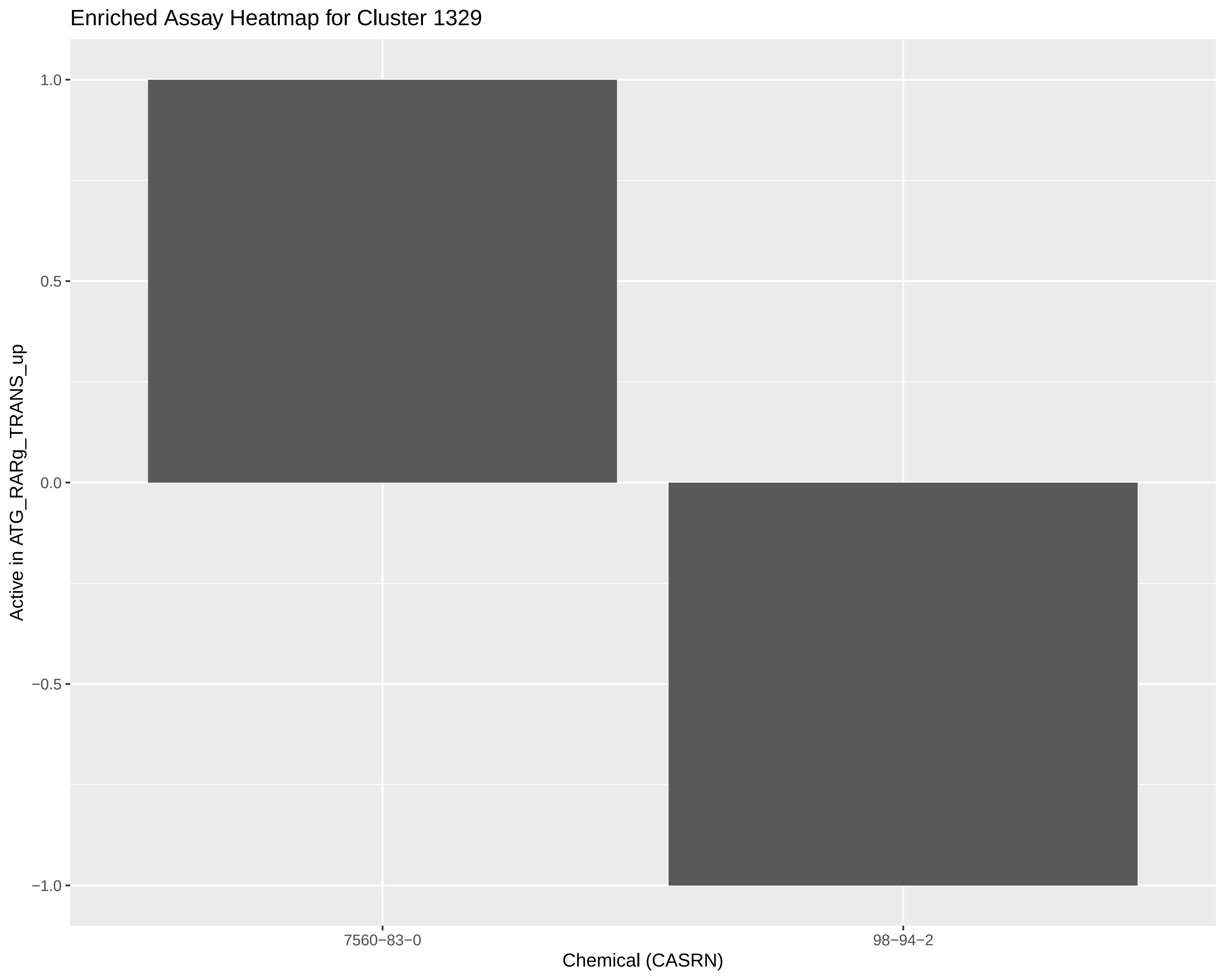

Enriched Assay Heatmap for Cluster 1329
Active in ATG_RARg_TRANS_up
1.0
0.5
0.0
−0.5
−1.0
7560−83−0
98−94−2
Chemical (CASRN)

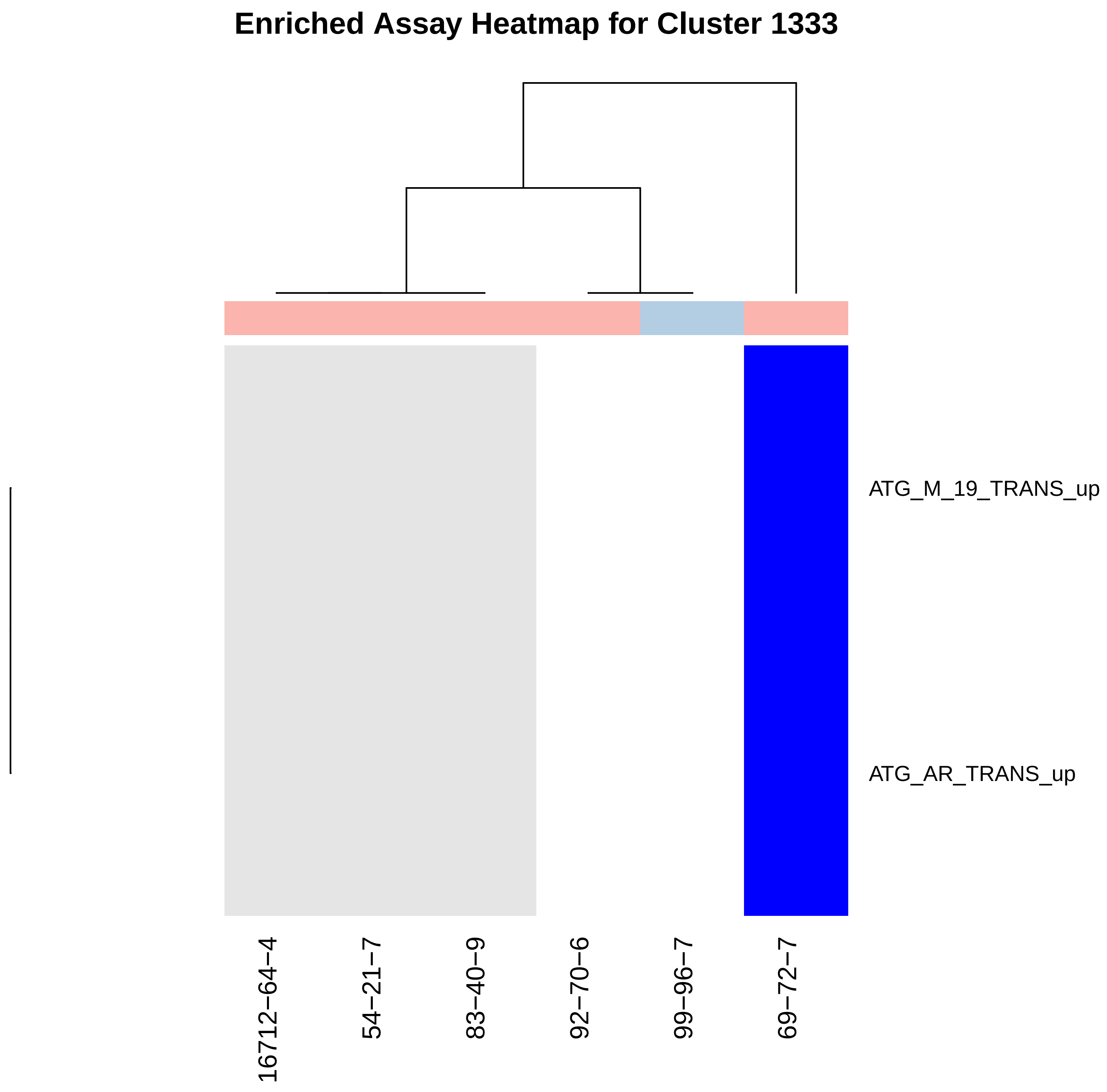
Enriched Assay Heatmap for Cluster 1333
ATG_M_19_TRANS_up
ATG_AR_TRANS_up
16712−64−4
54−21−7
83−40−9
92−70−6
99−96−7
69−72−7

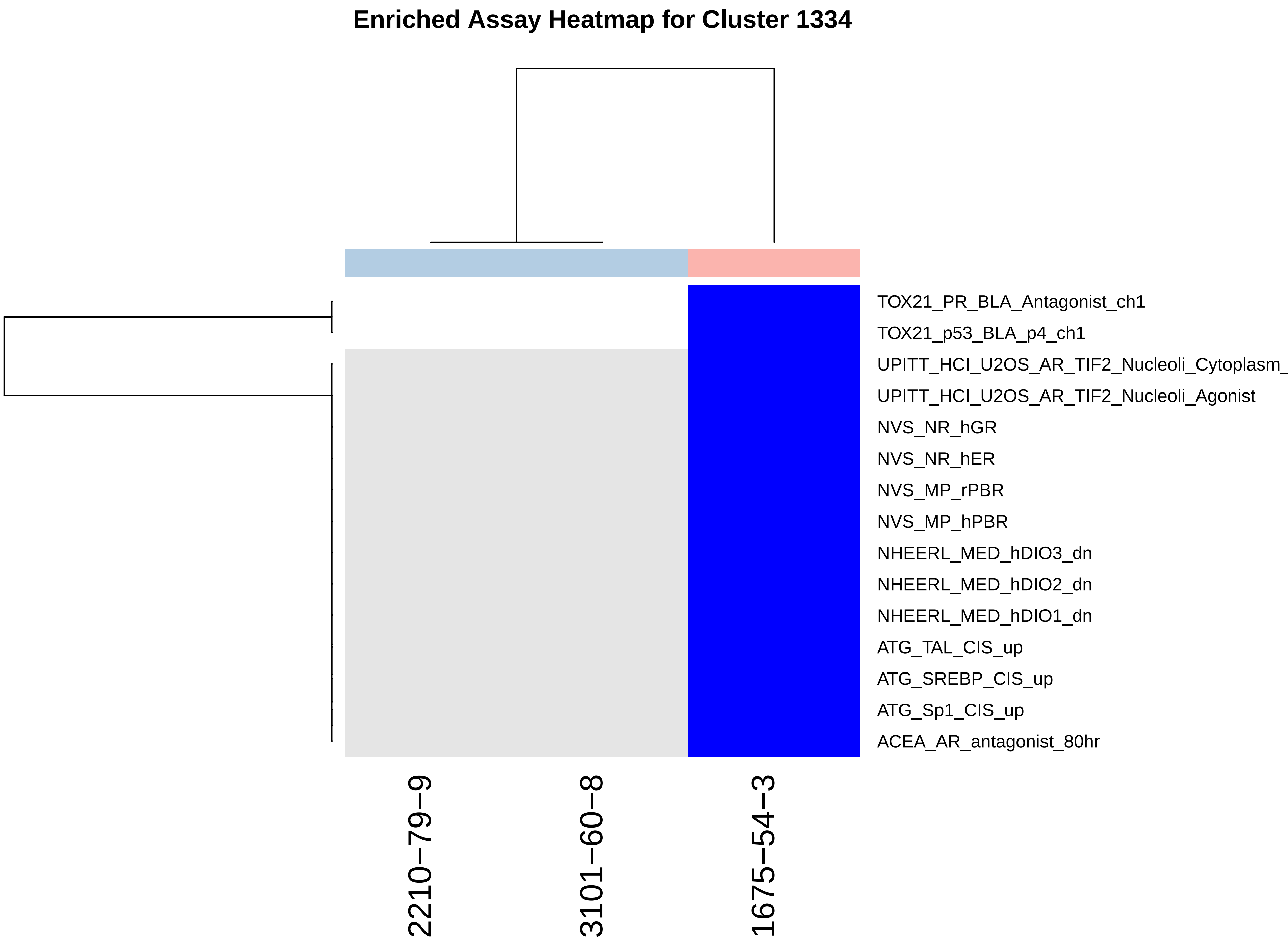
Enriched Assay Heatmap for Cluster 1334
TOX21_PR_BLA_Antagonist_ch1
TOX21_p53_BLA_p4_ch1
UPITT_HCI_U2OS_AR_TIF2_Nucleoli_Cytoplasm_
UPITT_HCI_U2OS_AR_TIF2_Nucleoli_Agonist
NVS_NR_hGR
NVS_NR_hER
NVS_MP_rPBR
NVS_MP_hPBR
NHEERL_MED_hDIO3_dn
NHEERL_MED_hDIO2_dn
NHEERL_MED_hDIO1_dn
ATG_TAL_CIS_up
ATG_SREBP_CIS_up
ATG_Sp1_CIS_up
ACEA_AR_antagonist_80hr
2210−79−9
3101−60−8
1675−54−3

Enriched Assay Heatmap for Cluster 1335

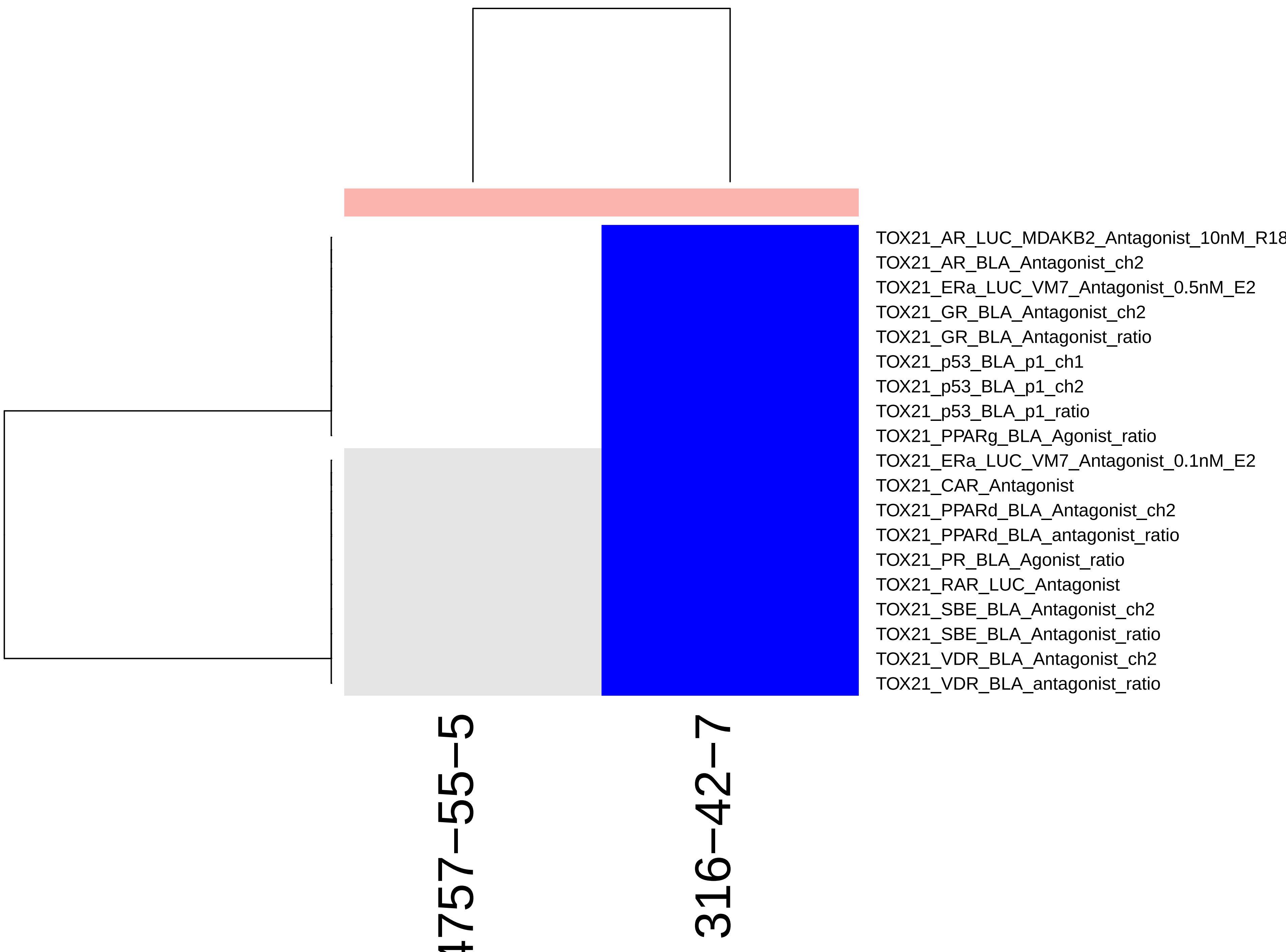

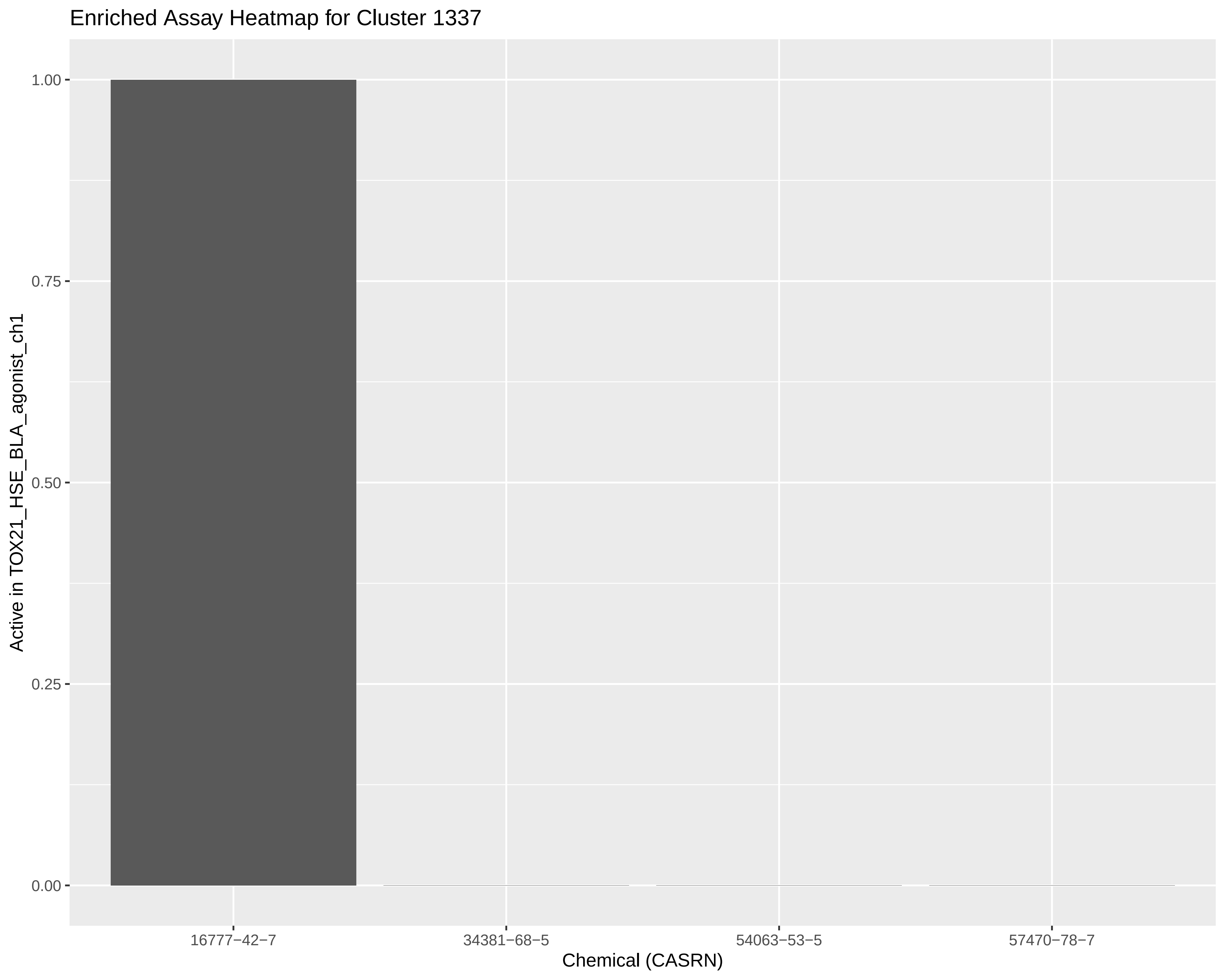

Enriched Assay Heatmap for Cluster 1337
Active in TOX21_HSE_BLA_agonist_ch1
1.00
0.75
0.50
0.25
0.00
16777−42−7
34381−68−5
54063−53−5
57470−78−7
Chemical (CASRN)

Enriched Assay Heatmap for Cluster 1345

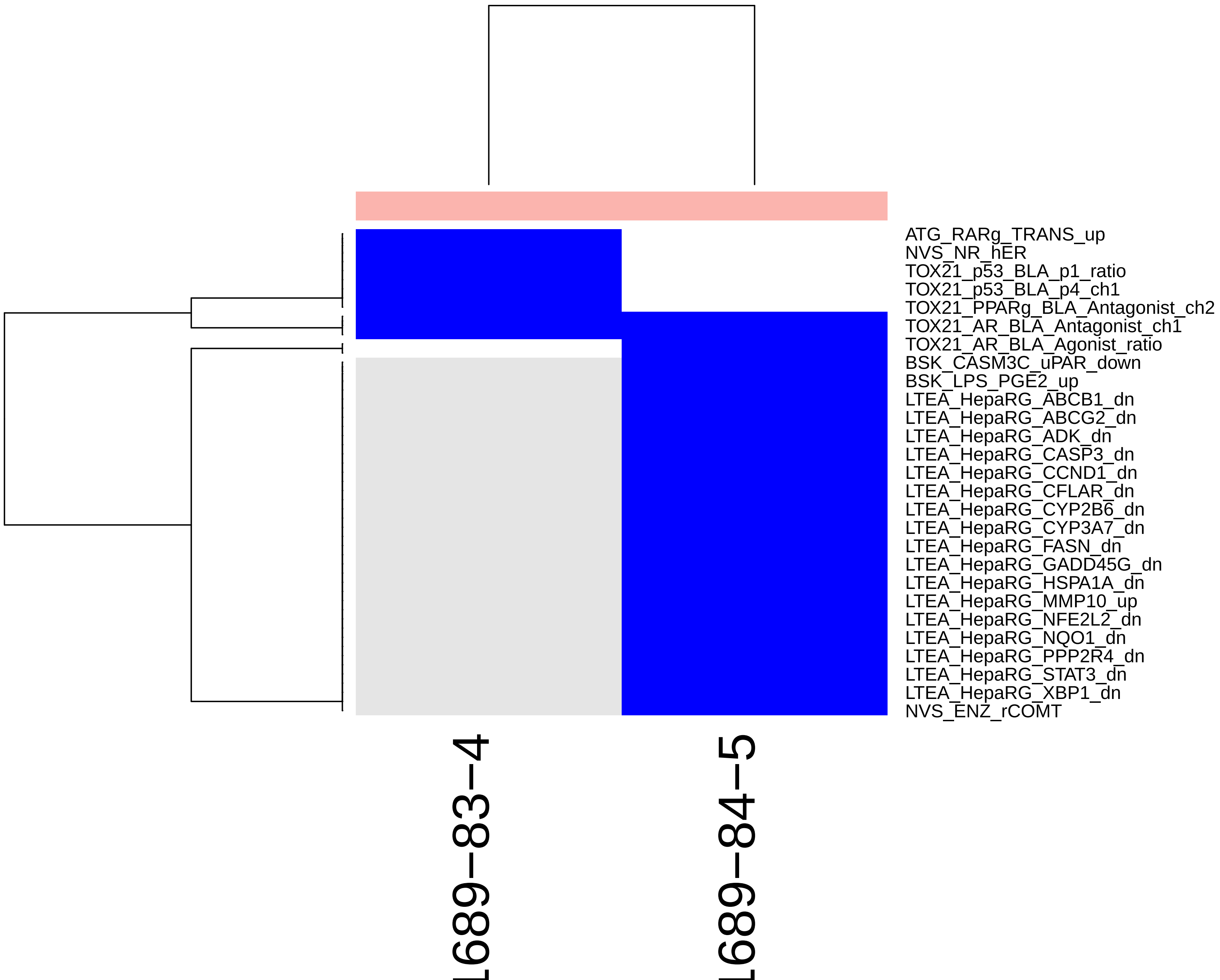

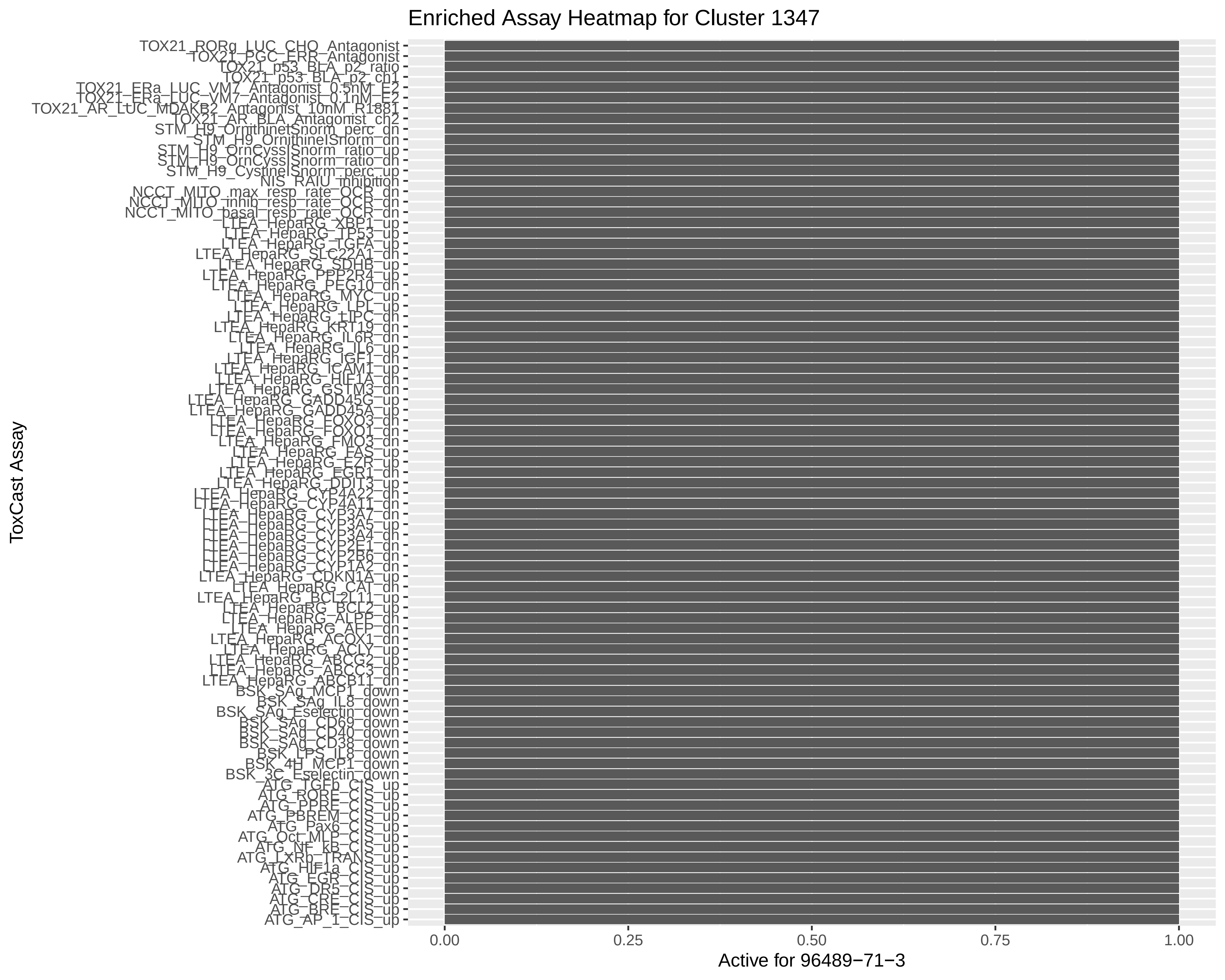
Enriched Assay Heatmap for Cluster 1347
ToxCast Assay
Active for 96489−71−3
0.00
0.25
0.50
0.75
1.00
TOX21_RORg_LUC_CHO_Antagonist
TOX21_PGC_ERR_Antagonist
TOX21_p53_BLA_p2_ratio
TOX21_p53_BLA_p2_ch1
TOX21_ERa_LUC_VM7_Antagonist_0.5nM_E2
TOX21_ERa_LUC_VM7_Antagonist_0.1nM_E2
TOX21_AR_LUC_MDAKB2_Antagonist_10nM_R1881
TOX21_AR_BLA_Antagonist_ch2
STM_H9_OrnithineISnorm_perc_dn
STM_H9_OrnithineISnorm_dn
STM_H9_OrnCyssISnorm_ratio_up
STM_H9_OrnCyssISnorm_ratio_dn
STM_H9_CystineISnorm_perc_up
NIS_RAIU_inhibition
NCCT_MITO_max_resp_rate_OCR_dn
NCCT_MITO_inhib_resp_rate_OCR_dn
NCCT_MITO_basal_resp_rate_OCR_dn
LTEA_HepaRG_XBP1_up
LTEA_HepaRG_TP53_up
LTEA_HepaRG_TGFA_up
LTEA_HepaRG_SLC22A1_dn
LTEA_HepaRG_SDHB_up
LTEA_HepaRG_PPP2R4_up
LTEA_HepaRG_PEG10_dn
LTEA_HepaRG_MYC_up
LTEA_HepaRG_LPL_up
LTEA_HepaRG_LIPC_dn
LTEA_HepaRG_KRT19_dn
LTEA_HepaRG_IL6R_dn
LTEA_HepaRG_IL6_up
LTEA_HepaRG_IGF1_dn
LTEA_HepaRG_ICAM1_up
LTEA_HepaRG_HIF1A_dn
LTEA_HepaRG_GSTM3_dn
LTEA_HepaRG_GADD45G_up
LTEA_HepaRG_GADD45A_up
LTEA_HepaRG_FOXO3_dn
LTEA_HepaRG_FOXO1_dn
LTEA_HepaRG_FMO3_dn
LTEA_HepaRG_FAS_up
LTEA_HepaRG_EZR_up
LTEA_HepaRG_EGR1_dn
LTEA_HepaRG_DDIT3_up
LTEA_HepaRG_CYP4A22_dn
LTEA_HepaRG_CYP4A11_dn
LTEA_HepaRG_CYP3A7_dn
LTEA_HepaRG_CYP3A5_up
LTEA_HepaRG_CYP3A4_dn
LTEA_HepaRG_CYP2E1_dn
LTEA_HepaRG_CYP2B6_dn
LTEA_HepaRG_CYP1A2_dn
LTEA_HepaRG_CDKN1A_up
LTEA_HepaRG_CAT_dn
LTEA_HepaRG_BCL2L11_up
LTEA_HepaRG_BCL2_up
LTEA_HepaRG_ALPP_dn
LTEA_HepaRG_AFP_dn
LTEA_HepaRG_ACOX1_dn
LTEA_HepaRG_ACLY_up
LTEA_HepaRG_ABCG2_up
LTEA_HepaRG_ABCC3_dn
LTEA_HepaRG_ABCB11_dn
BSK_SAg_MCP1_down
BSK_SAg_IL8_down
BSK_SAg_Eselectin_down
BSK_SAg_CD69_down
BSK_SAg_CD40_down
BSK_SAg_CD38_down
BSK_LPS_IL8_down
BSK_4H_MCP1_down
BSK_3C_Eselectin_down
ATG_TGFb_CIS_up
ATG_RORE_CIS_up
ATG_PPRE_CIS_up
ATG_PBREM_CIS_up
ATG_Pax6_CIS_up
ATG_Oct_MLP_CIS_up
ATG_NF_kB_CIS_up
ATG_LXRb_TRANS_up
ATG_HIF1a_CIS_up
ATG_EGR_CIS_up
ATG_DR5_CIS_up
ATG_CRE_CIS_up
ATG_BRE_CIS_up
ATG_AP_1_CIS_up

Enriched Assay Heatmap for Cluster 1349

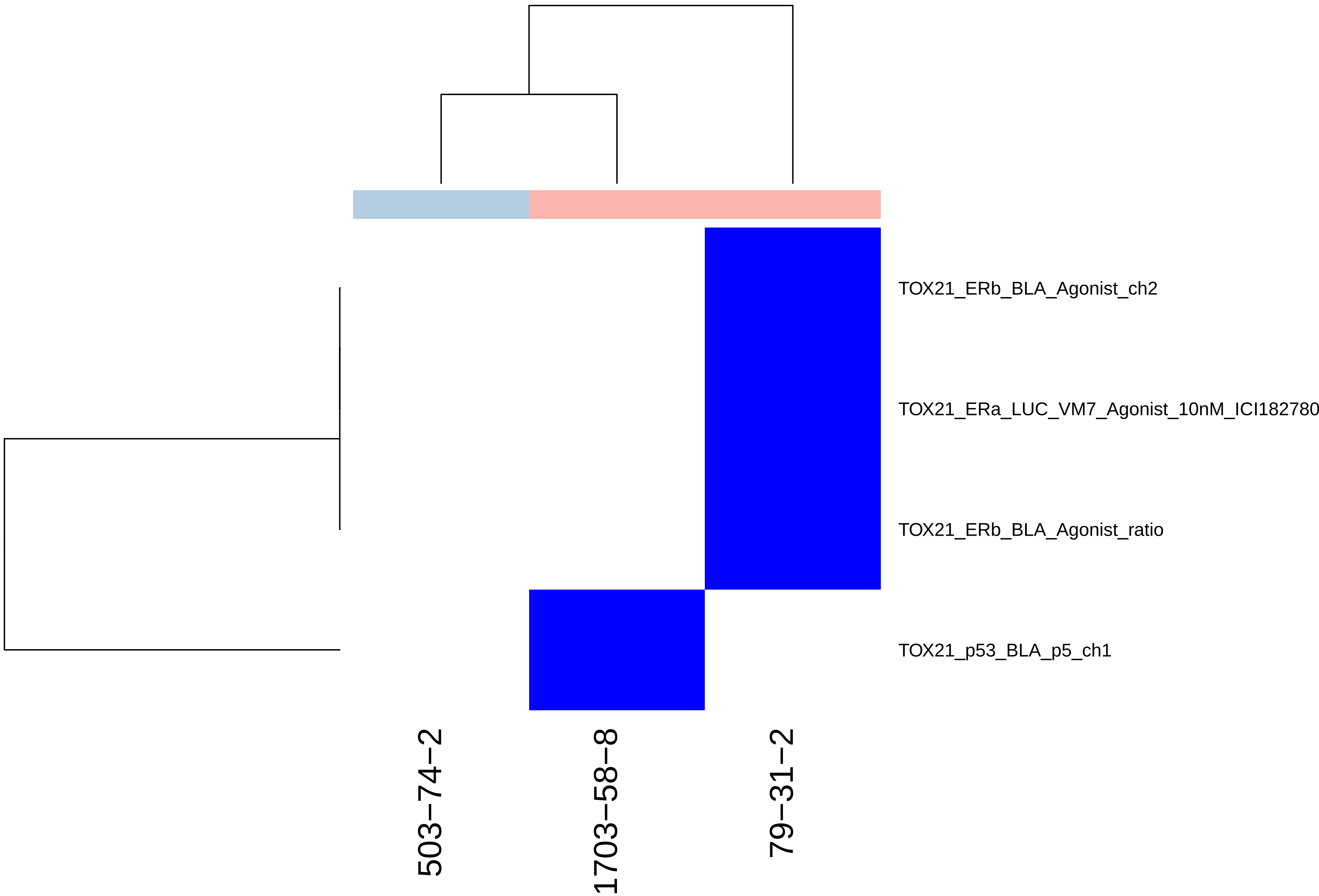

Enriched Assay Heatmap for Cluster 1350

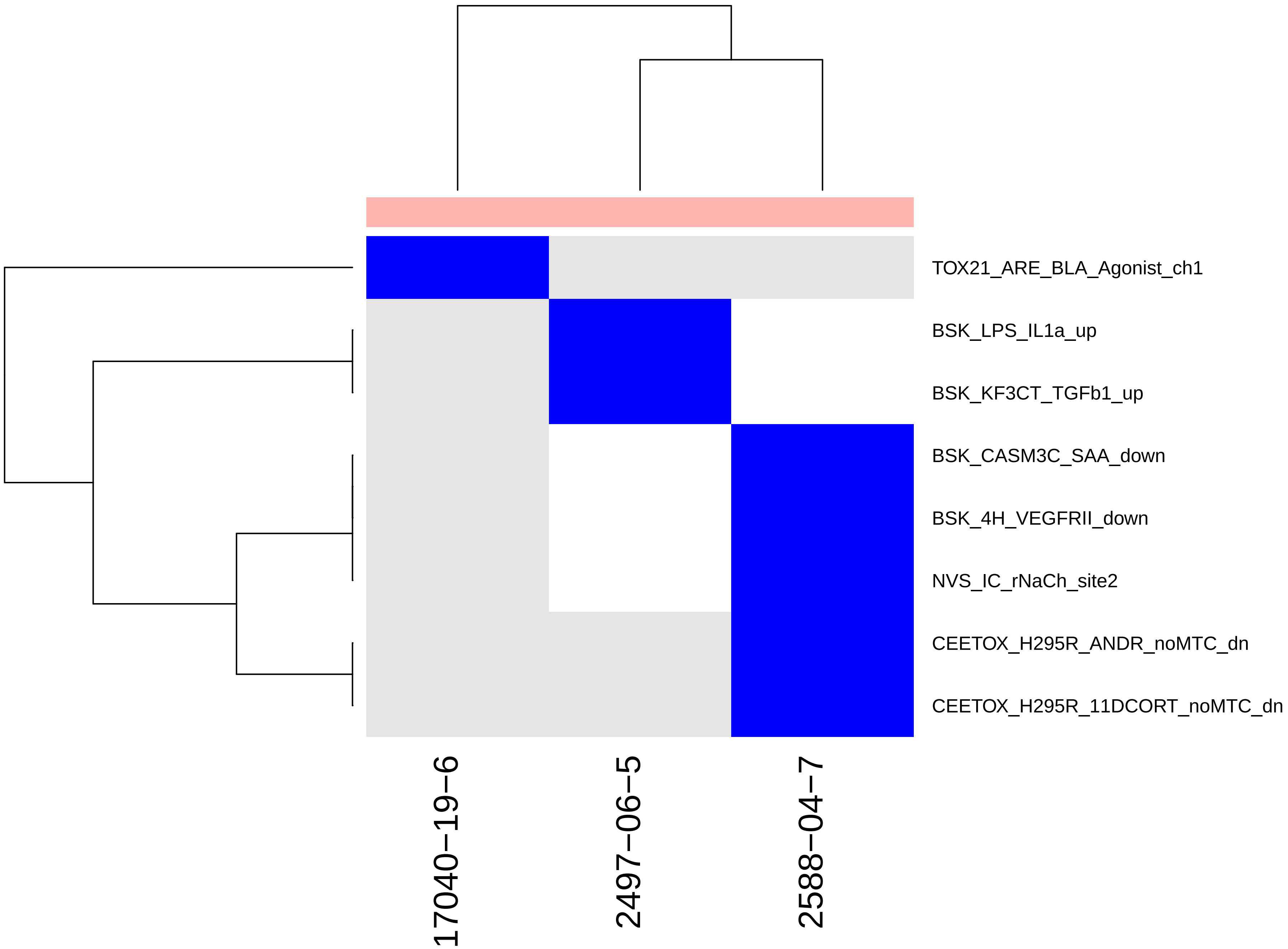

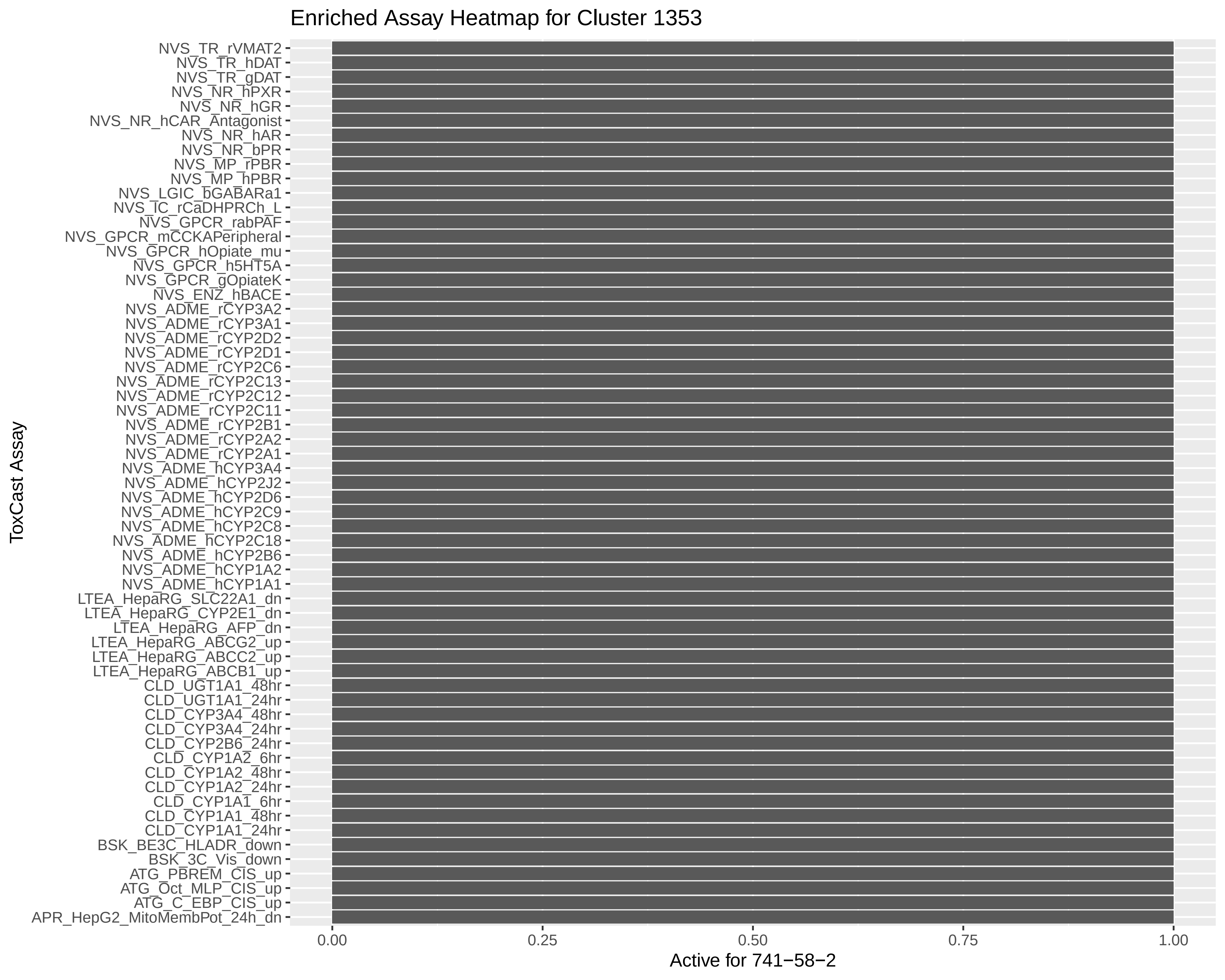

Enriched Assay Heatmap for Cluster 1353
ToxCast Assay
NVS_TR_rVMAT2
NVS_TR_hDAT
NVS_TR_gDAT
NVS_NR_hPXR
NVS_NR_hGR
NVS_NR_hCAR_Antagonist
NVS_NR_hAR
NVS_NR_bPR
NVS_MP_rPBR
NVS_MP_hPBR
NVS_LGIC_bGABARa1
NVS_IC_rCaDHPRCh_L
NVS_GPCR_rabPAF
NVS_GPCR_mCCKAPeripheral
NVS_GPCR_hOpiate_mu
NVS_GPCR_h5HT5A
NVS_GPCR_gOpiateK
NVS_ENZ_hBACE
NVS_ADME_rCYP3A2
NVS_ADME_rCYP3A1
NVS_ADME_rCYP2D2
NVS_ADME_rCYP2D1
NVS_ADME_rCYP2C6
NVS_ADME_rCYP2C13
NVS_ADME_rCYP2C12
NVS_ADME_rCYP2C11
NVS_ADME_rCYP2B1
NVS_ADME_rCYP2A2
NVS_ADME_rCYP2A1
NVS_ADME_hCYP3A4
NVS_ADME_hCYP2J2
NVS_ADME_hCYP2D6
NVS_ADME_hCYP2C9
NVS_ADME_hCYP2C8
NVS_ADME_hCYP2C18
NVS_ADME_hCYP2B6
NVS_ADME_hCYP1A2
NVS_ADME_hCYP1A1
LTEA_HepaRG_SLC22A1_dn
LTEA_HepaRG_CYP2E1_dn
LTEA_HepaRG_AFP_dn
LTEA_HepaRG_ABCG2_up
LTEA_HepaRG_ABCC2_up
LTEA_HepaRG_ABCB1_up
CLD_UGT1A1_48hr
CLD_UGT1A1_24hr
CLD_CYP3A4_48hr
CLD_CYP3A4_24hr
CLD_CYP2B6_24hr
CLD_CYP1A2_6hr
CLD_CYP1A2_48hr
CLD_CYP1A2_24hr
CLD_CYP1A1_6hr
CLD_CYP1A1_48hr
CLD_CYP1A1_24hr
BSK_BE3C_HLADR_down
BSK_3C_Vis_down
ATG_PBREM_CIS_up
ATG_Oct_MLP_CIS_up
ATG_C_EBP_CIS_up
APR_HepG2_MitoMembPot_24h_dn
0.00
0.25
0.50
0.75
1.00
Active for 741−58−2

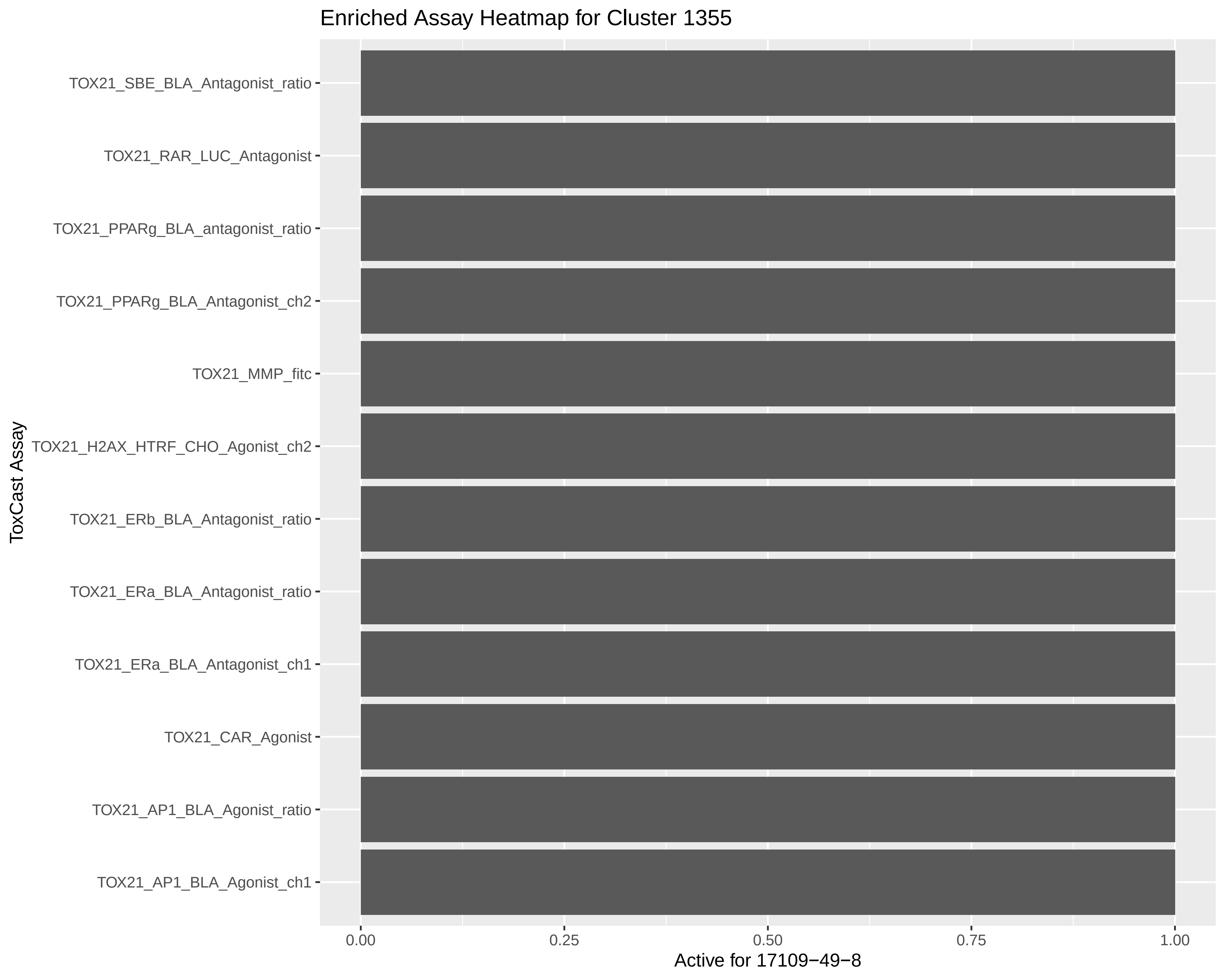
Enriched Assay Heatmap for Cluster 1355
TOX21_SBE_BLA_Antagonist_ratio
TOX21_RAR_LUC_Antagonist
TOX21_PPARg_BLA_antagonist_ratio
TOX21_PPARg_BLA_Antagonist_ch2
TOX21_MMP_fitc
TOX21_H2AX_HTRF_CHO_Agonist_ch2
TOX21_ERb_BLA_Antagonist_ratio
TOX21_ERa_BLA_Antagonist_ratio
TOX21_ERa_BLA_Antagonist_ch1
TOX21_CAR_Agonist
TOX21_AP1_BLA_Agonist_ratio
TOX21_AP1_BLA_Agonist_ch1
ToxCast Assay
0.00
0.25
0.50
0.75
1.00
Active for 17109−49−8

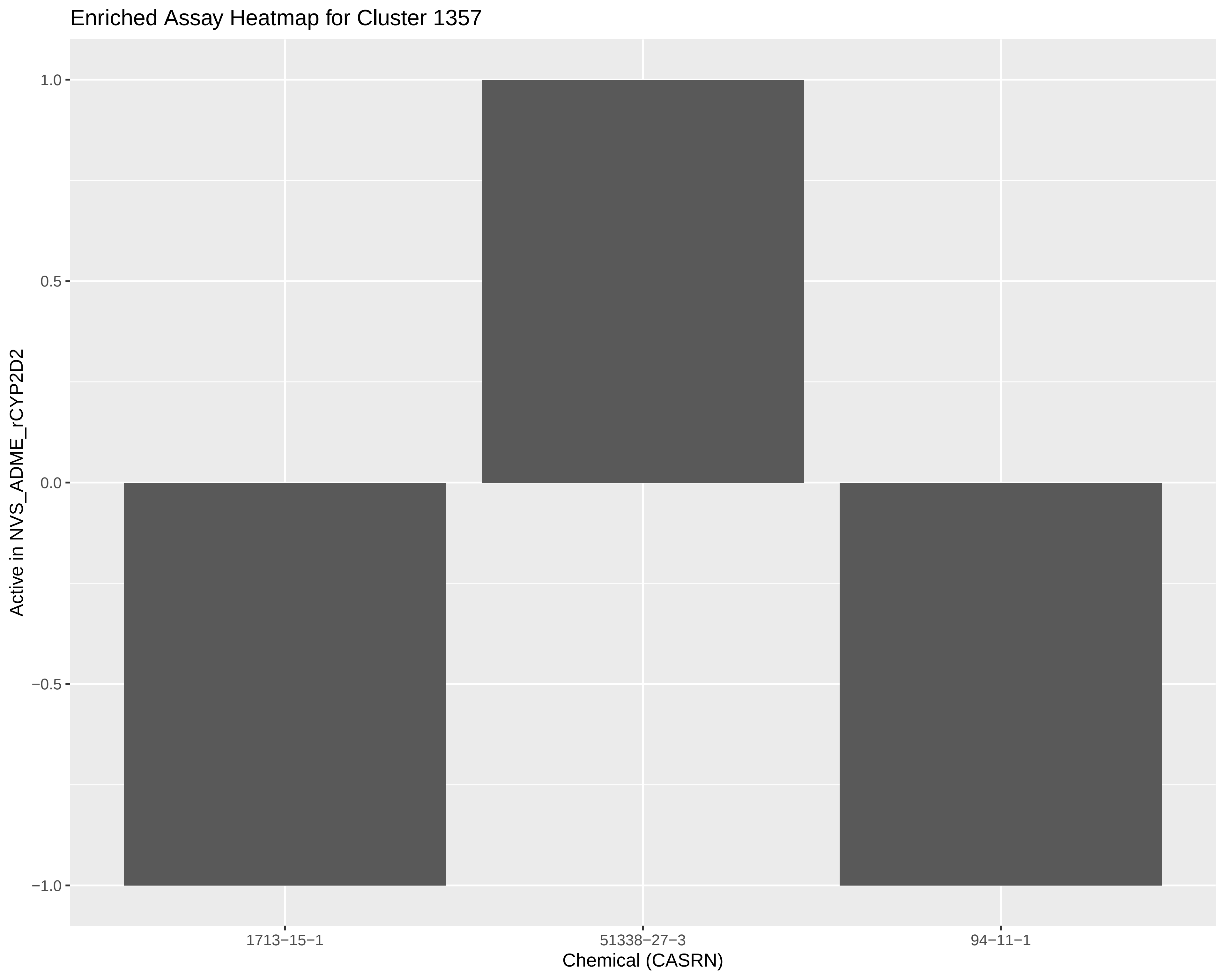
Enriched Assay Heatmap for Cluster 1357
Active in NVS_ADME_rCYP2D2
1.0
0.5
0.0
−0.5
−1.0
1713−15−1
51338−27−3
94−11−1
Chemical (CASRN)

Enriched Assay Heatmap for Cluster 1360

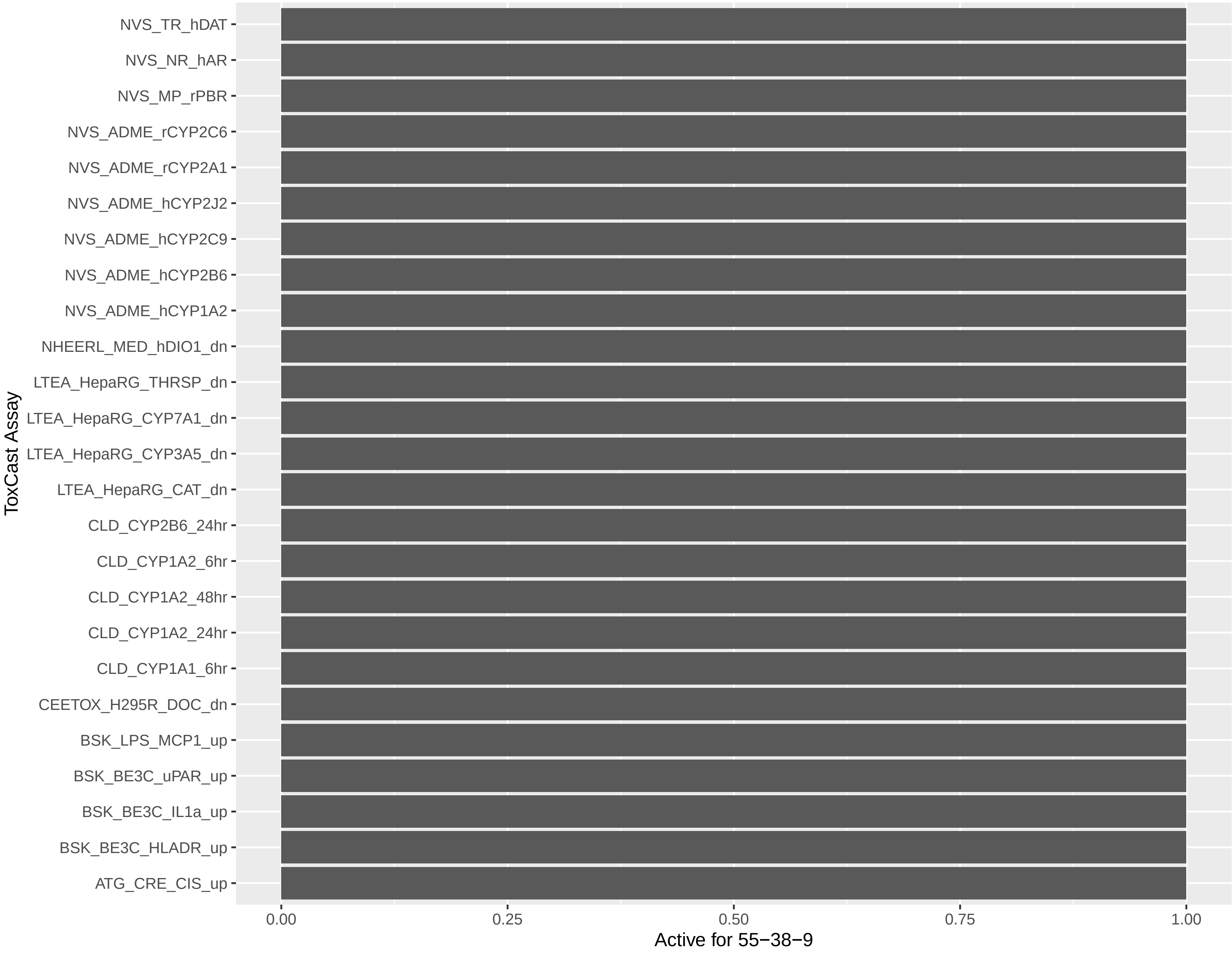

Enriched Assay Heatmap for Cluster 1363

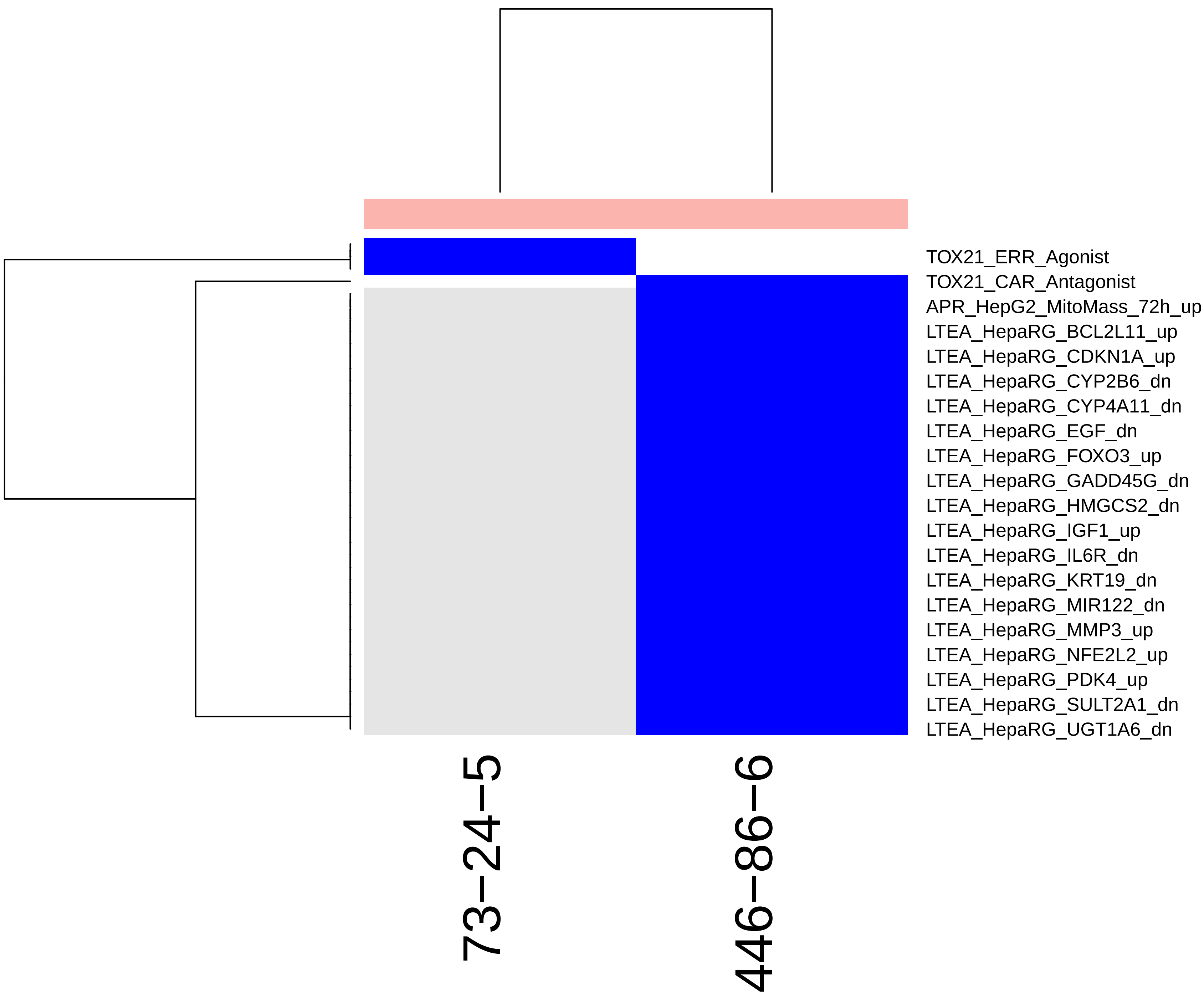

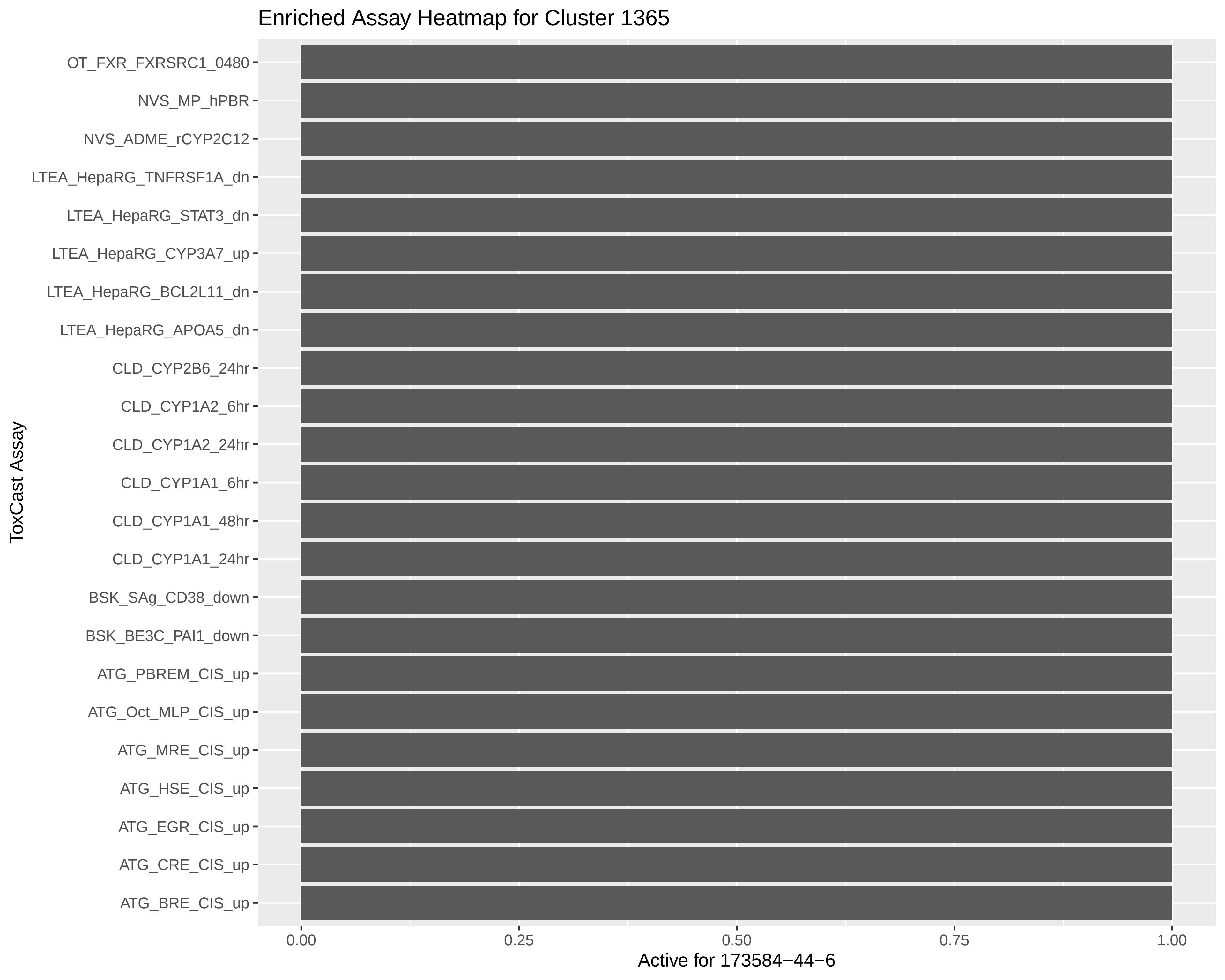

Enriched Assay Heatmap for Cluster 1365
OT_FXR_FXRSRC1_0480
NVS_MP_hPBR
NVS_ADME_rCYP2C12
LTEA_HepaRG_TNFRSF1A_dn
LTEA_HepaRG_STAT3_dn
LTEA_HepaRG_CYP3A7_up
LTEA_HepaRG_BCL2L11_dn
LTEA_HepaRG_APOA5_dn
CLD_CYP2B6_24hr
CLD_CYP1A2_6hr
CLD_CYP1A2_24hr
CLD_CYP1A1_6hr
CLD_CYP1A1_48hr
CLD_CYP1A1_24hr
BSK_SAg_CD38_down
BSK_BE3C_PAI1_down
ATG_PBREM_CIS_up
ATG_Oct_MLP_CIS_up
ATG_MRE_CIS_up
ATG_HSE_CIS_up
ATG_EGR_CIS_up
ATG_CRE_CIS_up
ATG_BRE_CIS_up
ToxCast Assay
0.00
0.25
0.50
0.75
1.00
Active for 173584−44−6

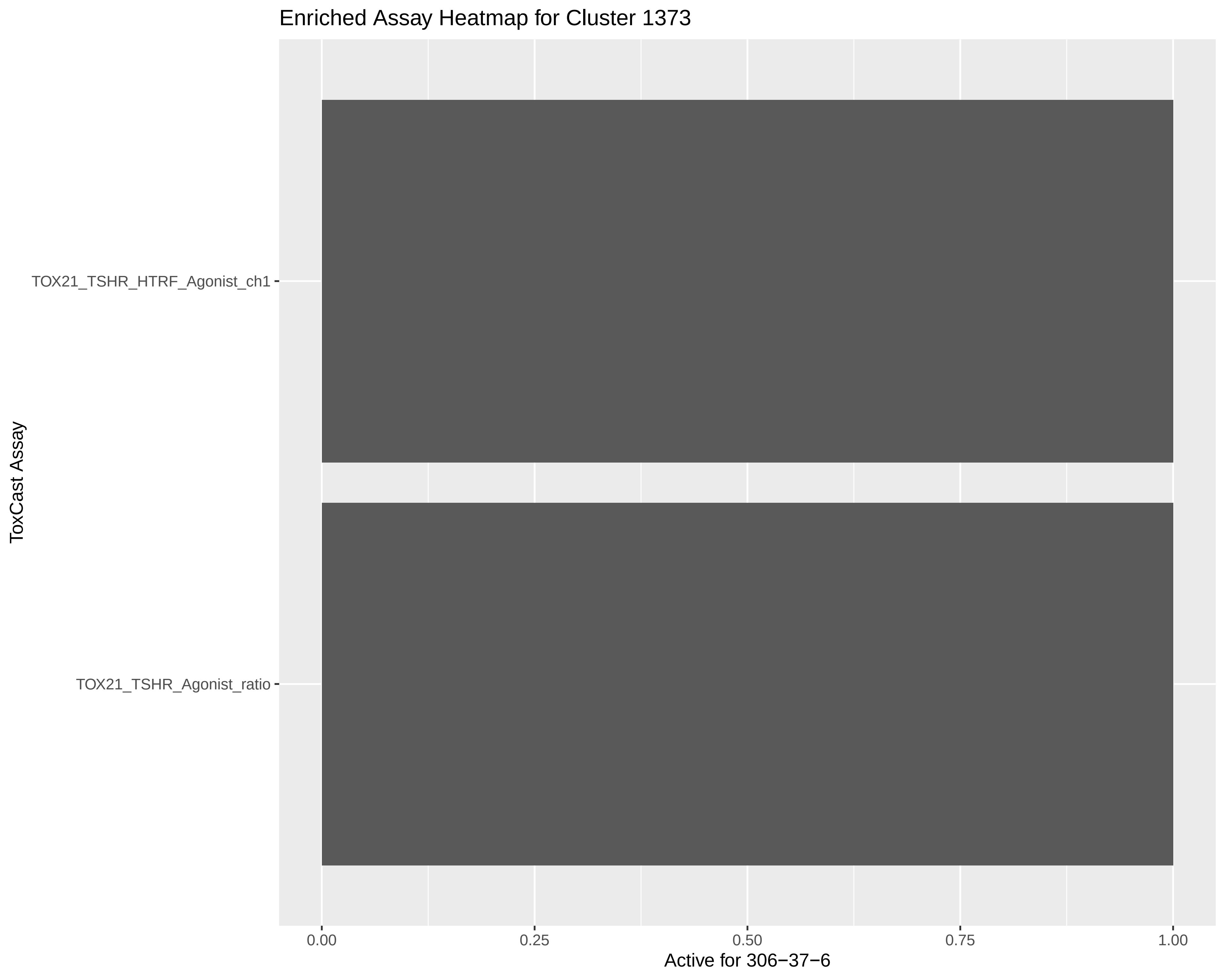

Enriched Assay Heatmap for Cluster 1373
ToxCast Assay
TOX21_TSHR_HTRF_Agonist_ch1
TOX21_TSHR_Agonist_ratio
0.00
0.25
0.50
0.75
1.00
Active for 306−37−6

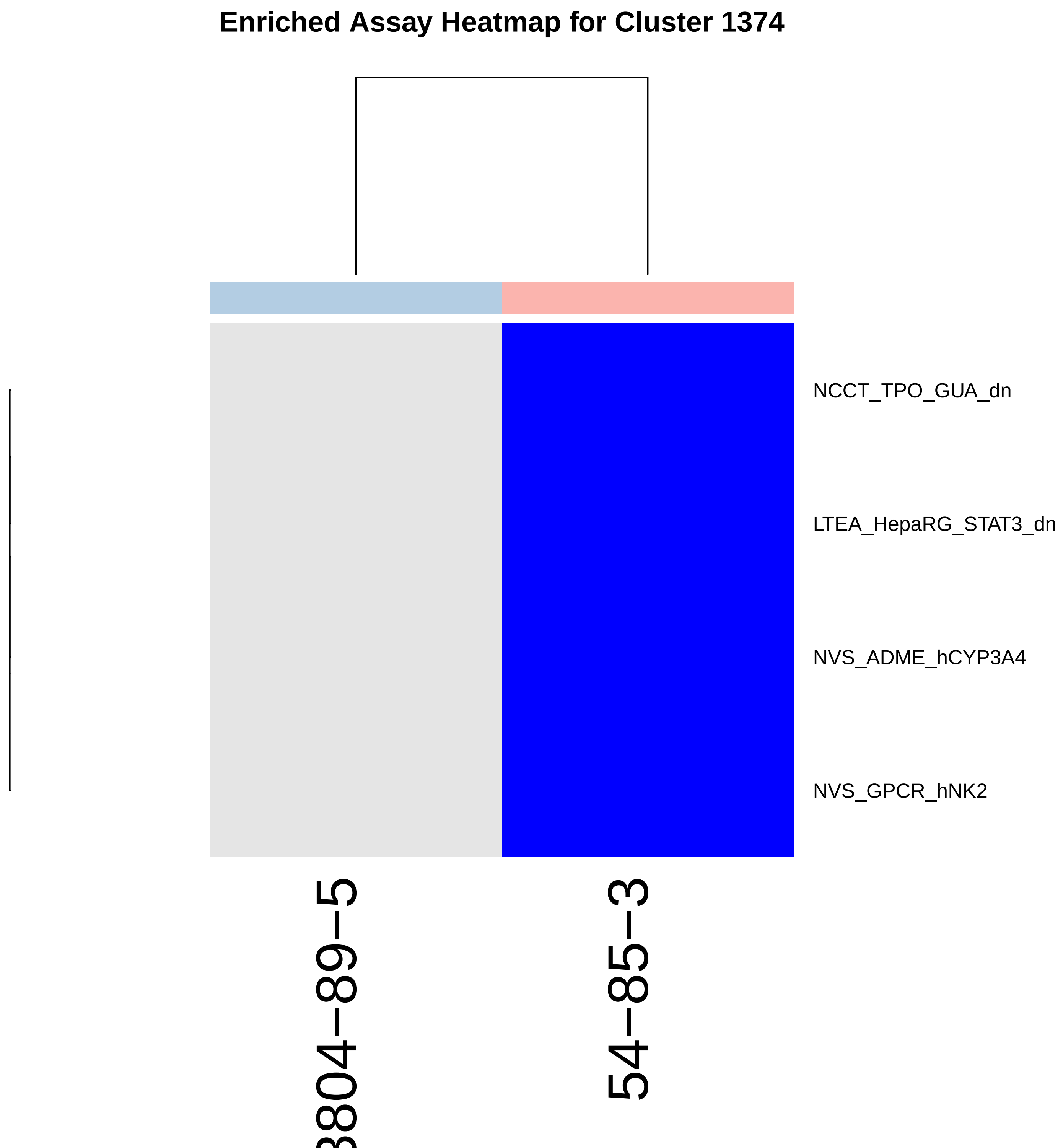
Enriched Assay Heatmap for Cluster 1374
NCCT_TPO_GUA_dn
LTEA_HepaRG_STAT3_dn
NVS_ADME_hCYP3A4
NVS_GPCR_hNK2
3804−89−5
54−85−3

Enriched Assay Heatmap for Cluster 1377

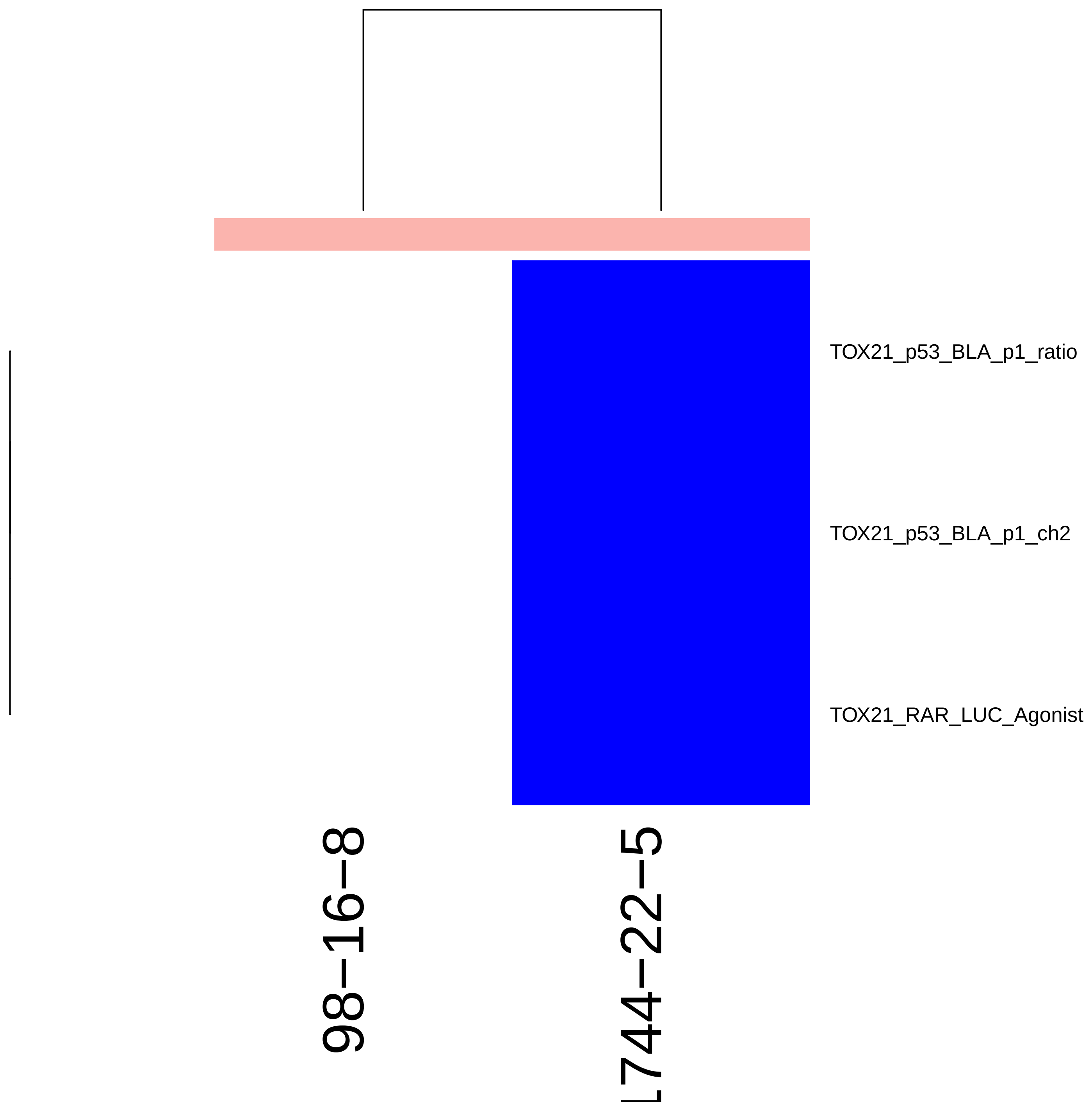

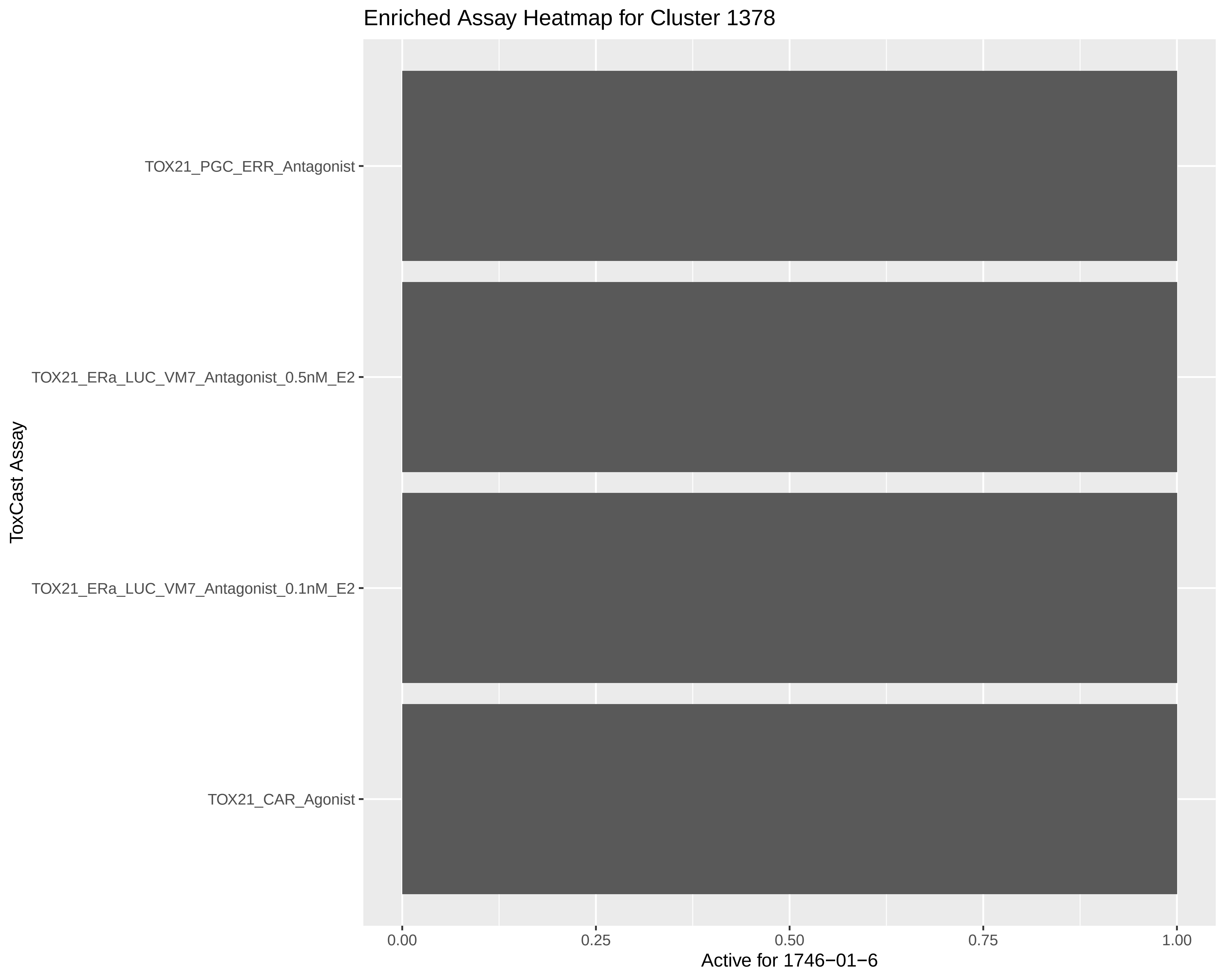

Enriched Assay Heatmap for Cluster 1378
ToxCast Assay
TOX21_PGC_ERR_Antagonist
TOX21_ERa_LUC_VM7_Antagonist_0.5nM_E2
TOX21_ERa_LUC_VM7_Antagonist_0.1nM_E2
TOX21_CAR_Agonist
0.00
0.25
0.50
0.75
1.00
Active for 1746−01−6

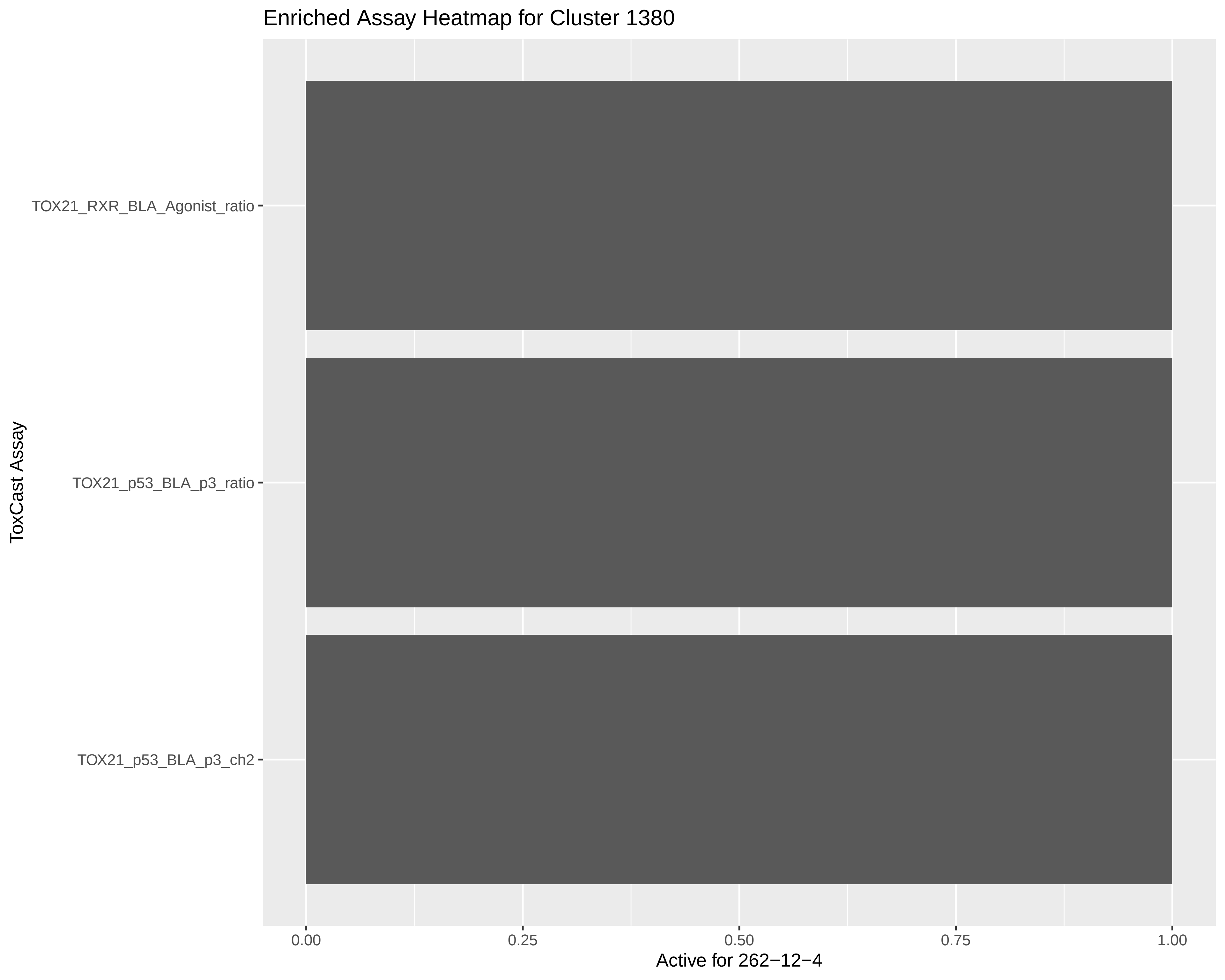
Enriched Assay Heatmap for Cluster 1380
ToxCast Assay
TOX21_RXR_BLA_Agonist_ratio
TOX21_p53_BLA_p3_ratio
TOX21_p53_BLA_p3_ch2
0.00
0.25
0.50
0.75
1.00
Active for 262−12−4

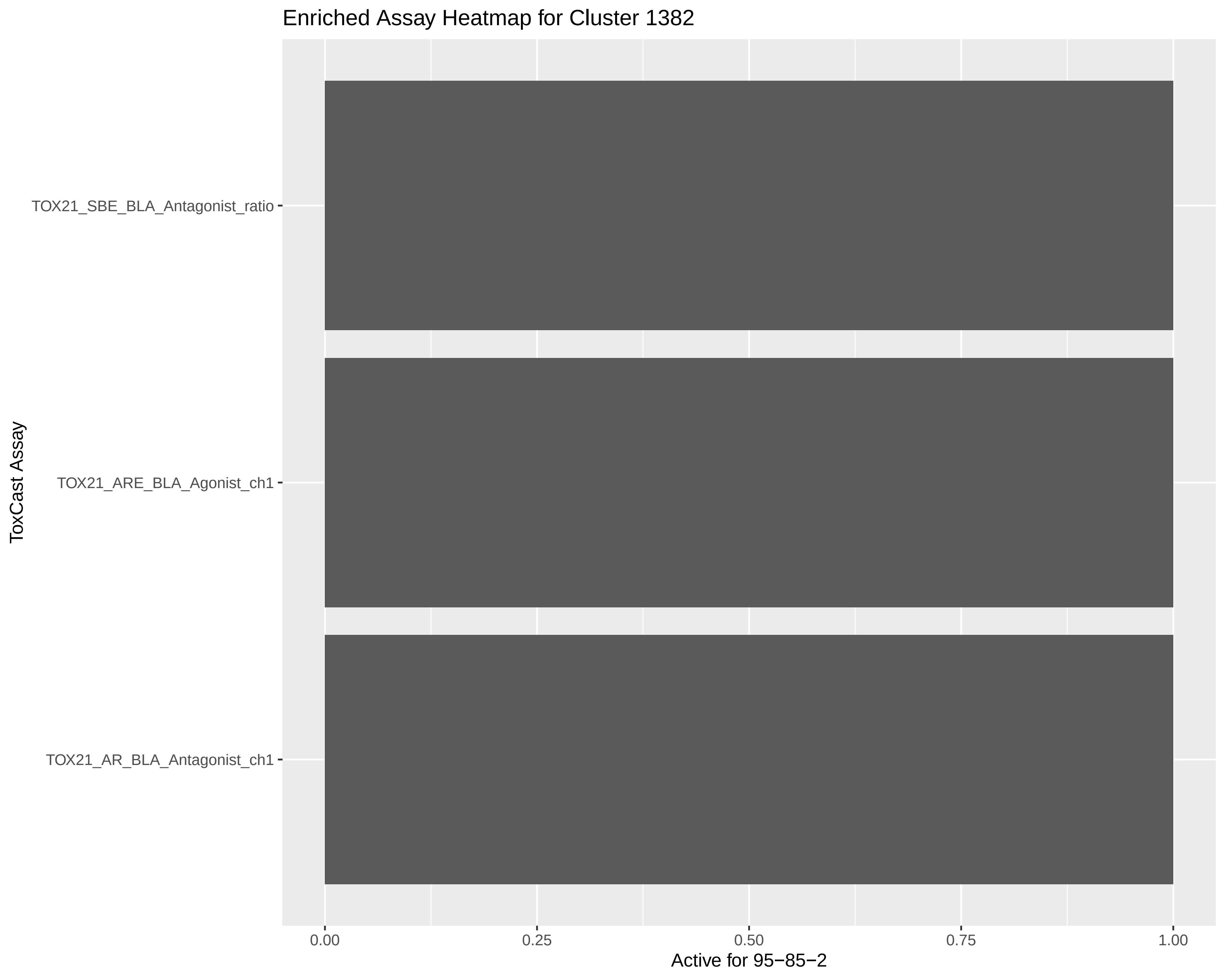

Enriched Assay Heatmap for Cluster 1382
TOX21_SBE_BLA_Antagonist_ratio
TOX21_ARE_BLA_Agonist_ch1
TOX21_AR_BLA_Antagonist_ch1
ToxCast Assay
0.00
0.25
0.50
0.75
1.00
Active for 95−85−2

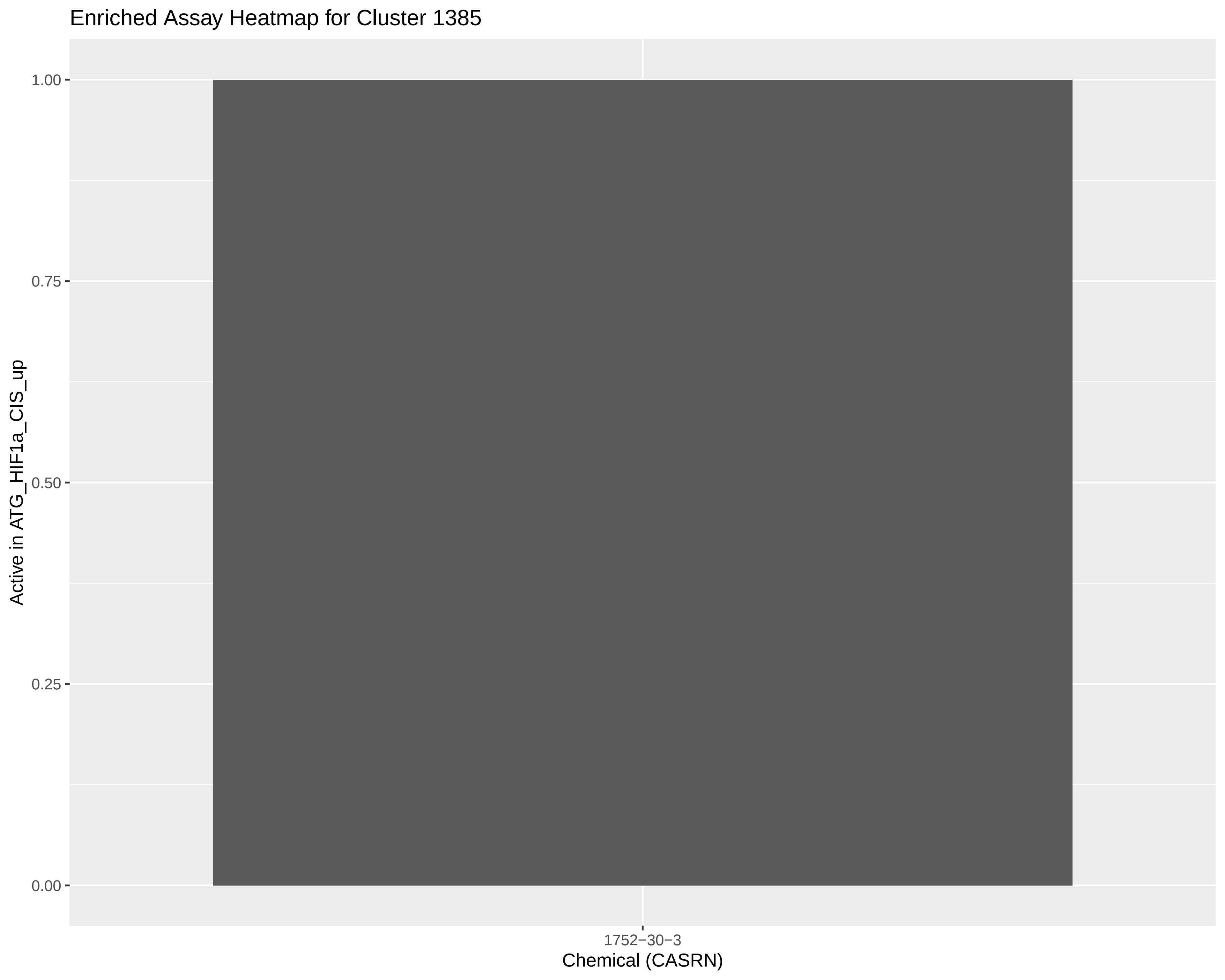

Enriched Assay Heatmap for Cluster 1385
1.00
0.75
0.50
0.25
0.00
Active in ATG_HIF1a_CIS_up
1752−30−3
Chemical (CASRN)

Enriched Assay Heatmap for Cluster 1394

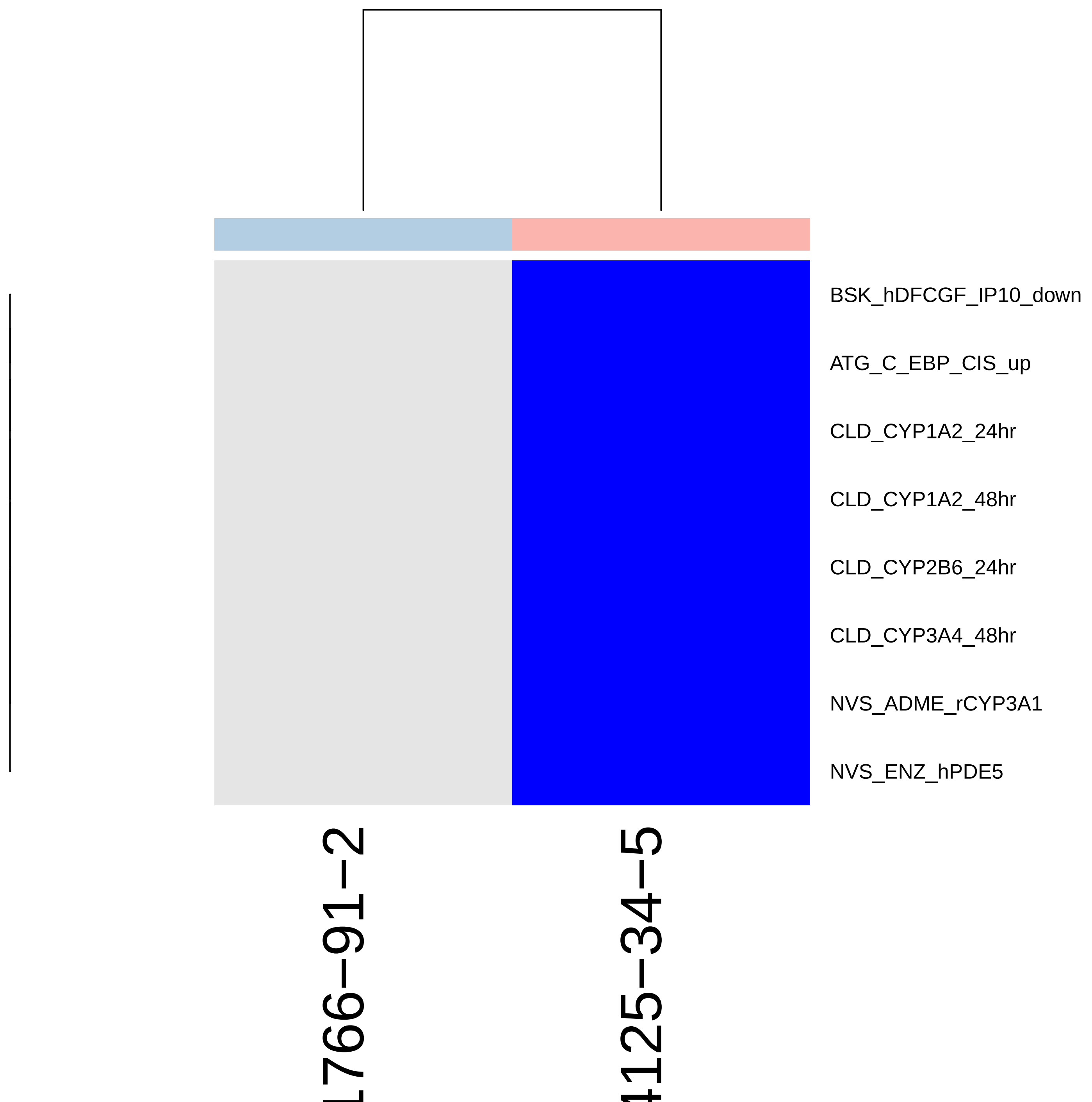

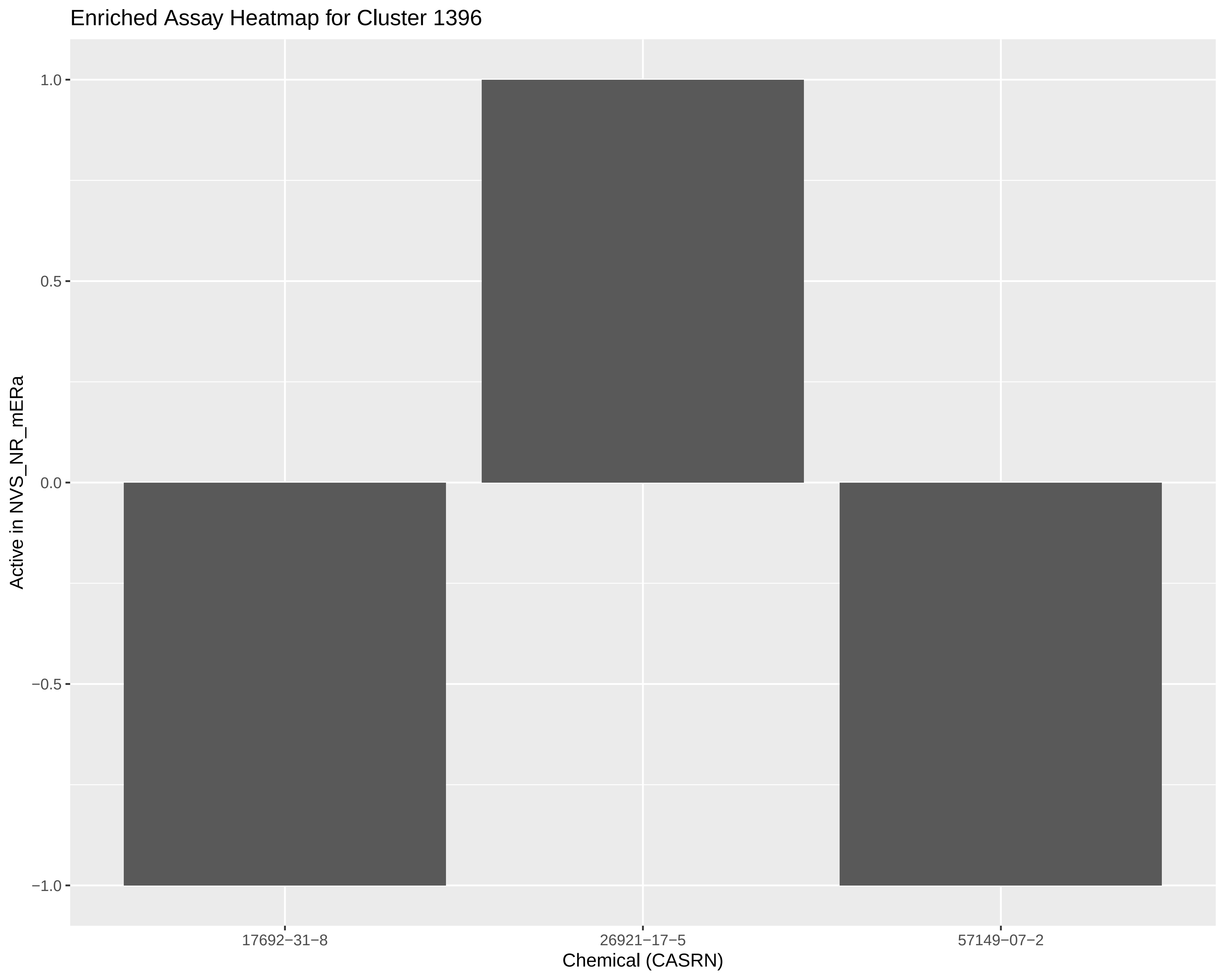

Enriched Assay Heatmap for Cluster 1396
1.0
0.5
0.0
−0.5
−1.0
Active in NVS_NR_mERa
17692−31−8
26921−17−5
57149−07−2
Chemical (CASRN)

Enriched Assay Heatmap for Cluster 1398

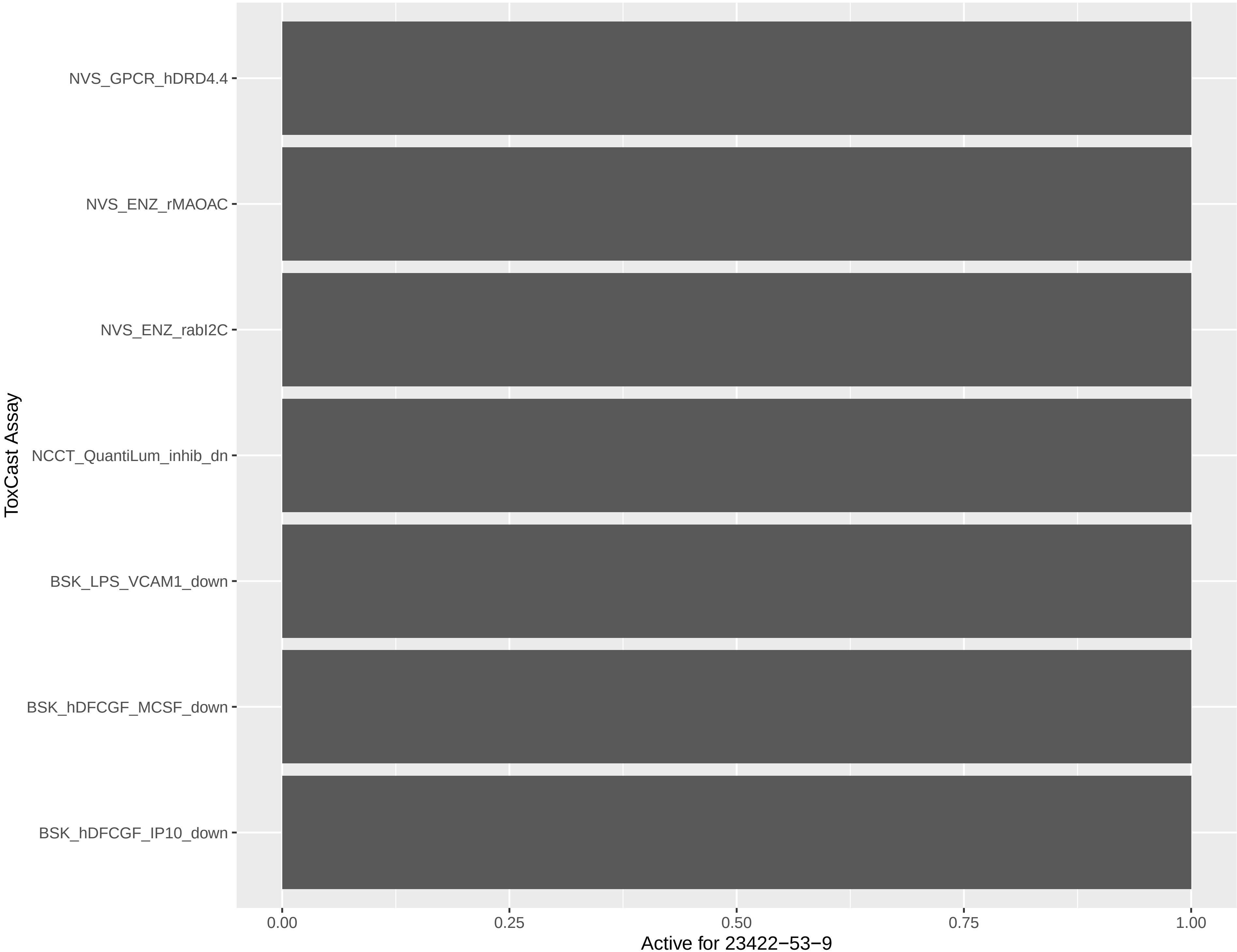

Enriched Assay Heatmap for Cluster 1410

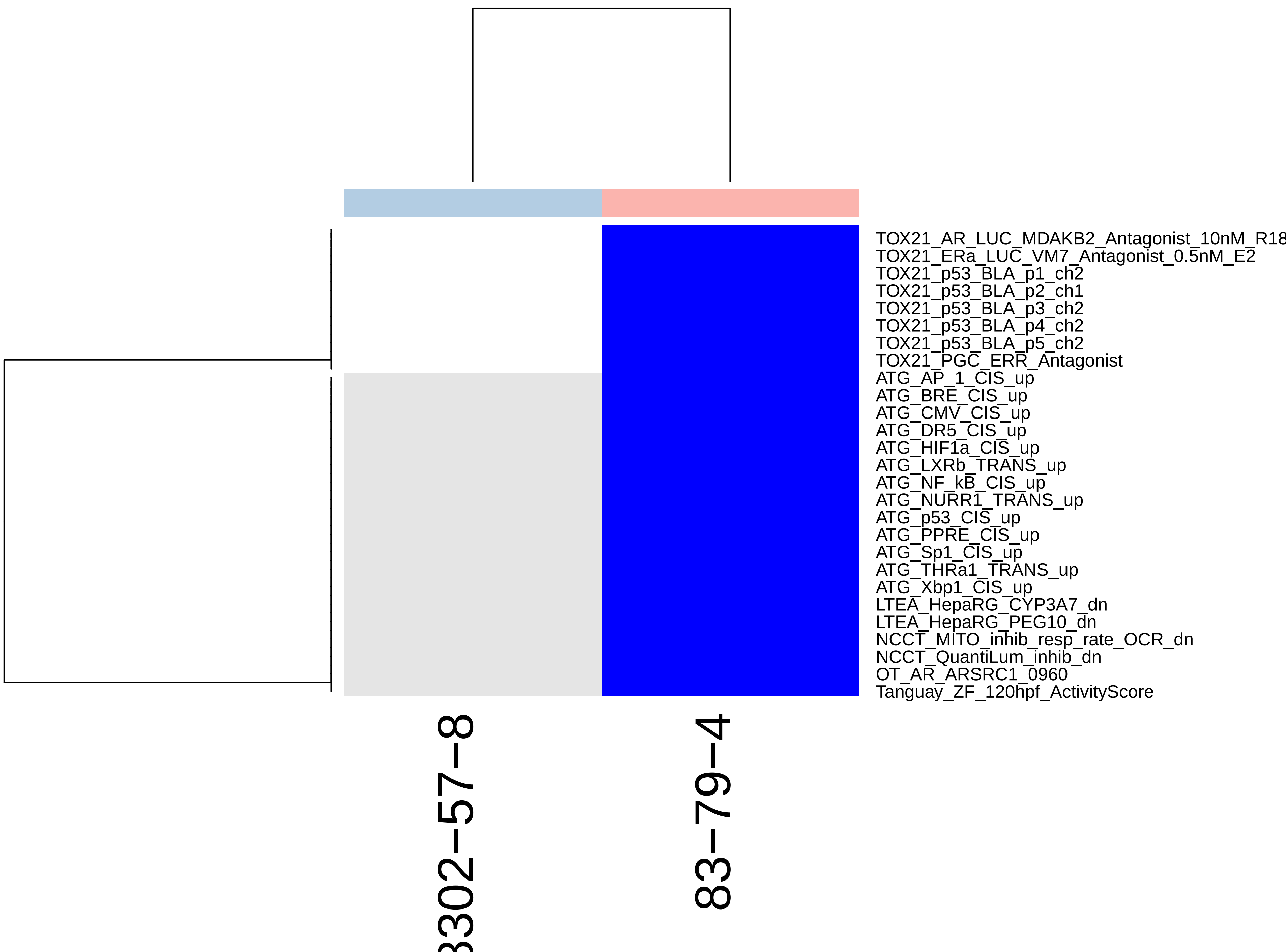

Enriched Assay Heatmap for Cluster 1415

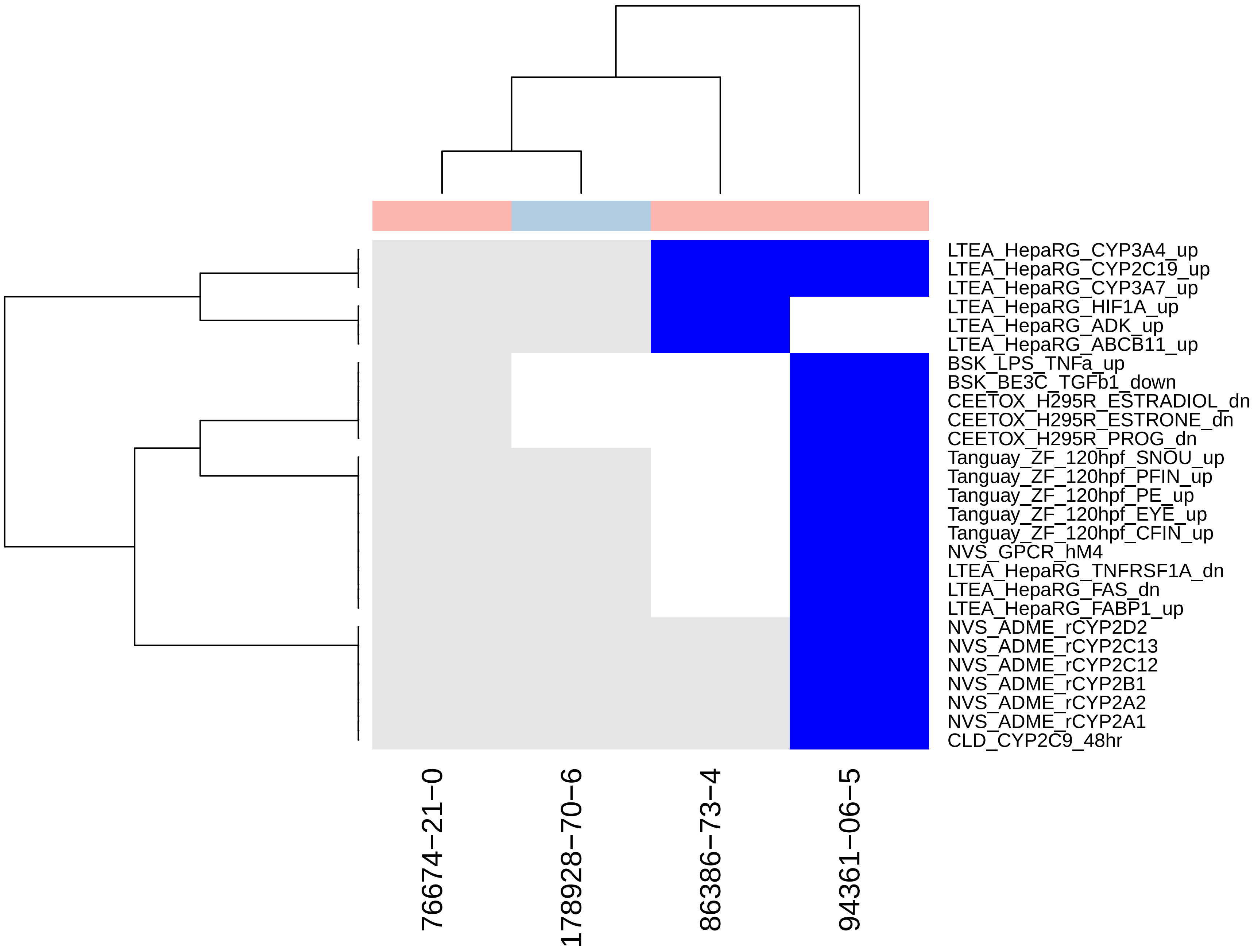

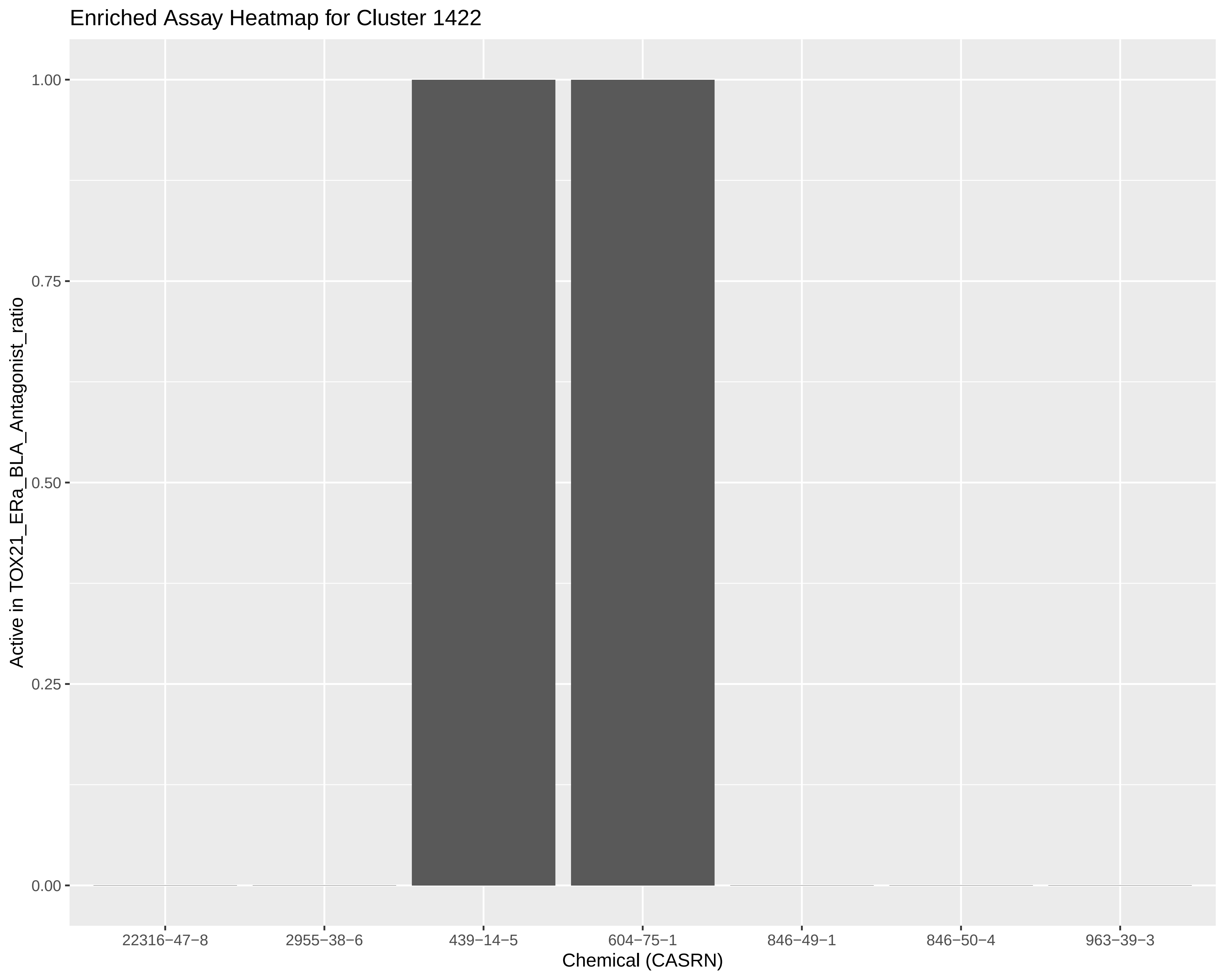
Enriched Assay Heatmap for Cluster 1422
Active in TOX21_ERa_BLA_Antagonist_ratio
1.00
0.75
0.50
0.25
0.00
22316−47−8
2955−38−6
439−14−5
604−75−1
846−49−1
846−50−4
963−39−3
Chemical (CASRN)

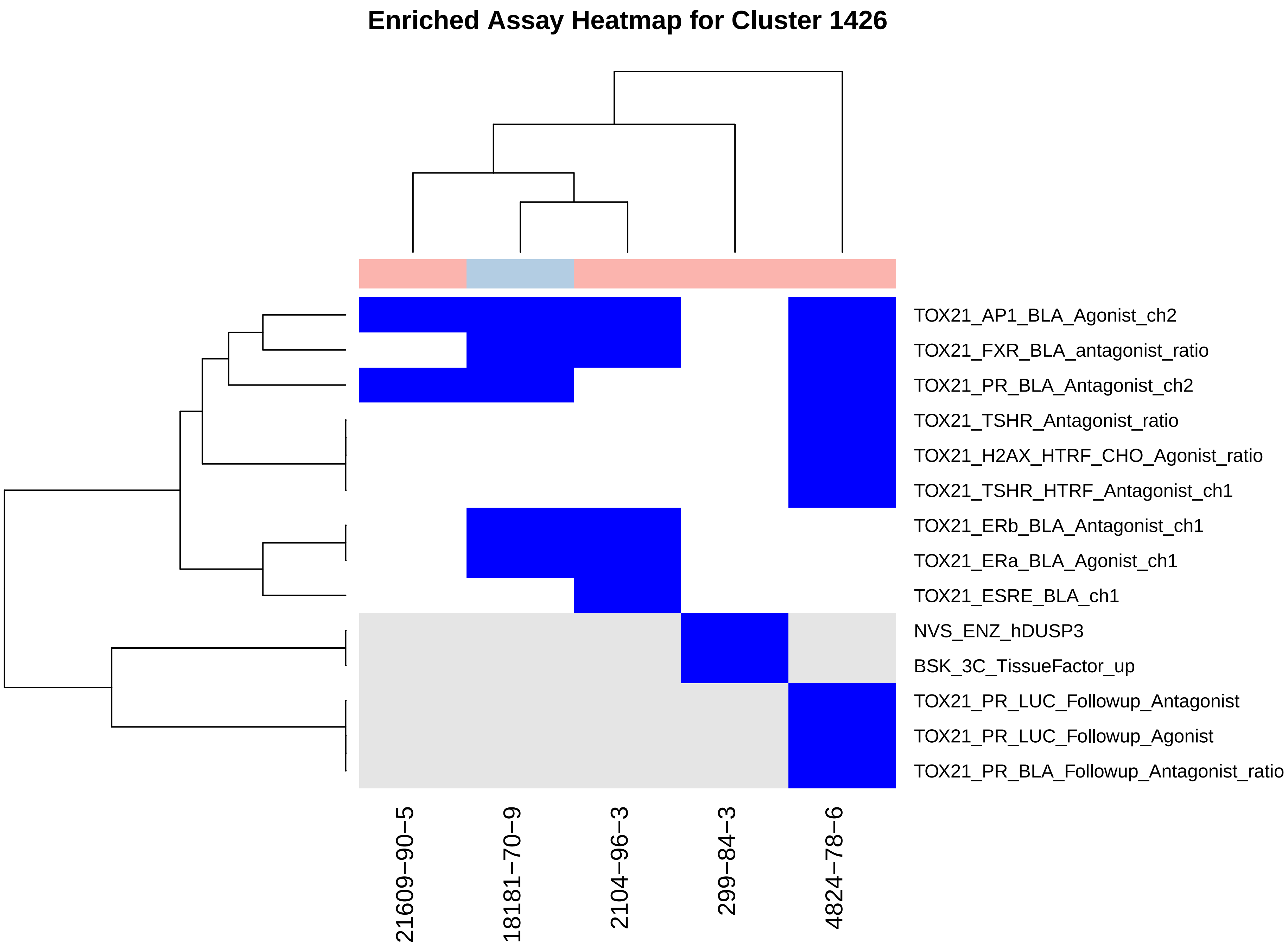

Enriched Assay Heatmap for Cluster 1426
TOX21_AP1_BLA_Agonist_ch2
TOX21_FXR_BLA_antagonist_ratio
TOX21_PR_BLA_Antagonist_ch2
TOX21_TSHR_Antagonist_ratio
TOX21_H2AX_HTRF_CHO_Agonist_ratio
TOX21_TSHR_HTRF_Antagonist_ch1
TOX21_ERb_BLA_Antagonist_ch1
TOX21_ERa_BLA_Agonist_ch1
TOX21_ESRE_BLA_ch1
NVS_ENZ_hDUSP3
BSK_3C_TissueFactor_up
TOX21_PR_LUC_Followup_Antagonist
TOX21_PR_LUC_Followup_Agonist
TOX21_PR_BLA_Followup_Antagonist_ratio
21609−90−5
18181−70−9
2104−96−3
299−84−3
4824−78−6

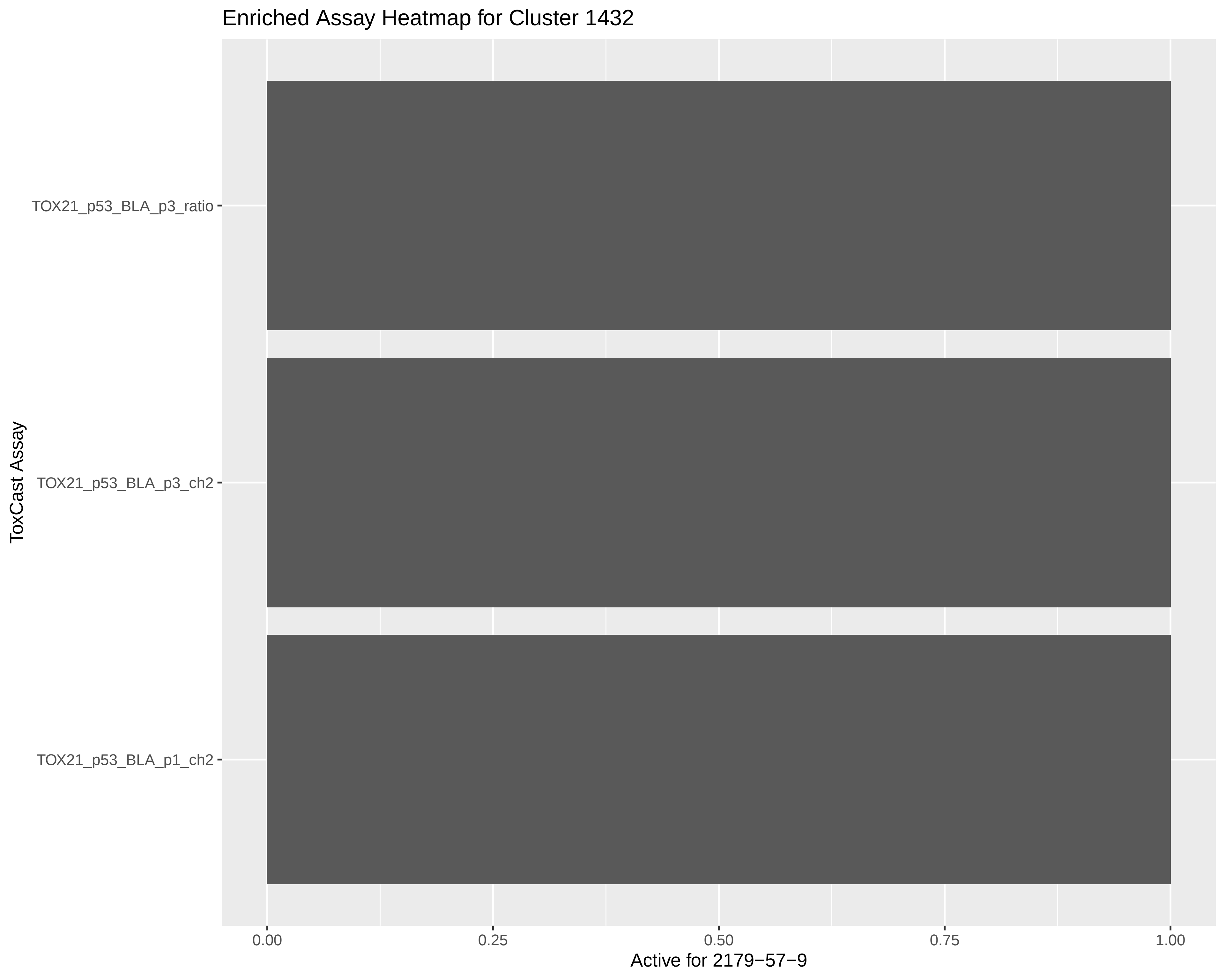

Enriched Assay Heatmap for Cluster 1432
ToxCast Assay
TOX21_p53_BLA_p3_ratio
TOX21_p53_BLA_p3_ch2
TOX21_p53_BLA_p1_ch2
0.00
0.25
0.50
0.75
1.00
Active for 2179−57−9

Enriched Assay Heatmap for Cluster 1438

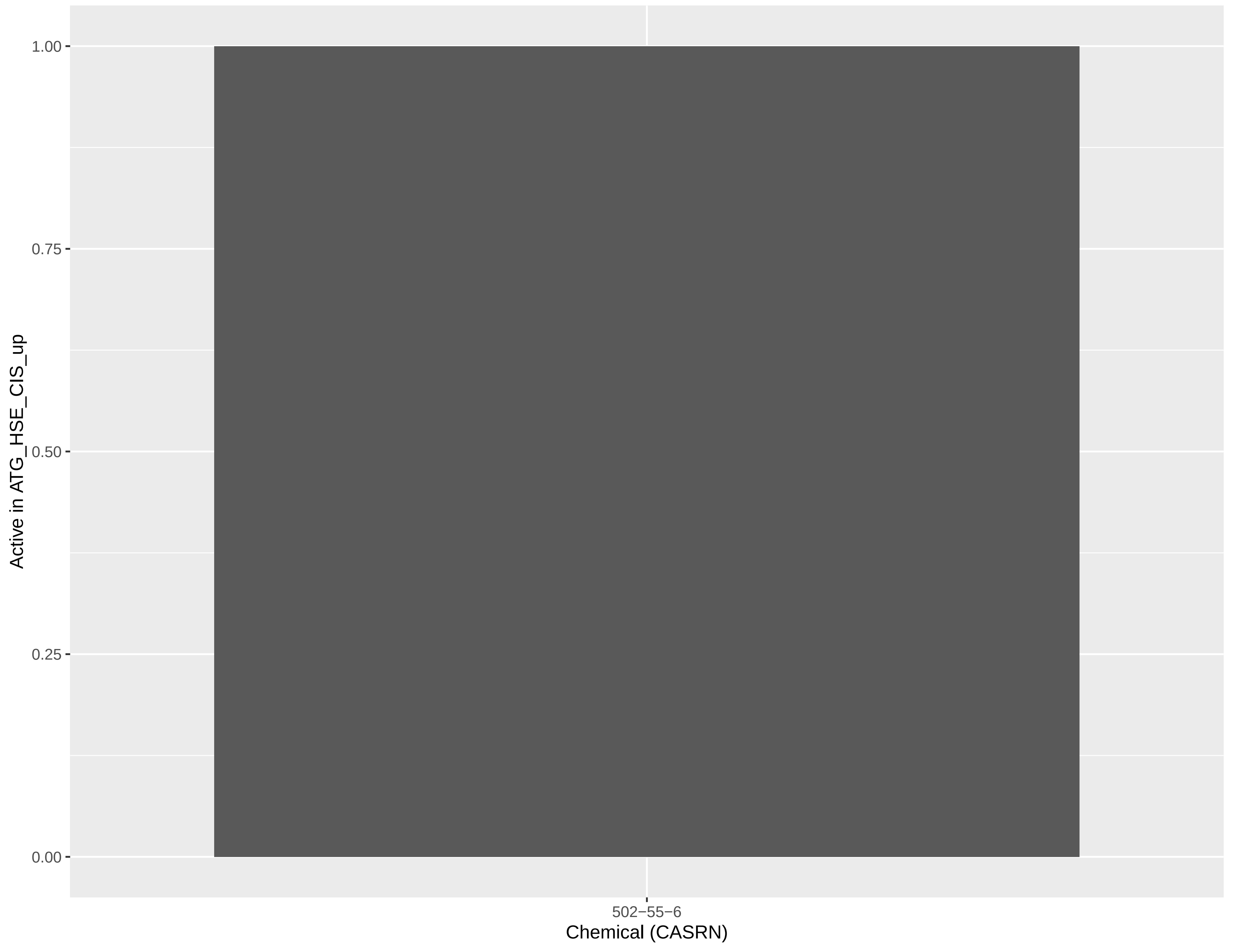

Enriched Assay Heatmap for Cluster 1442

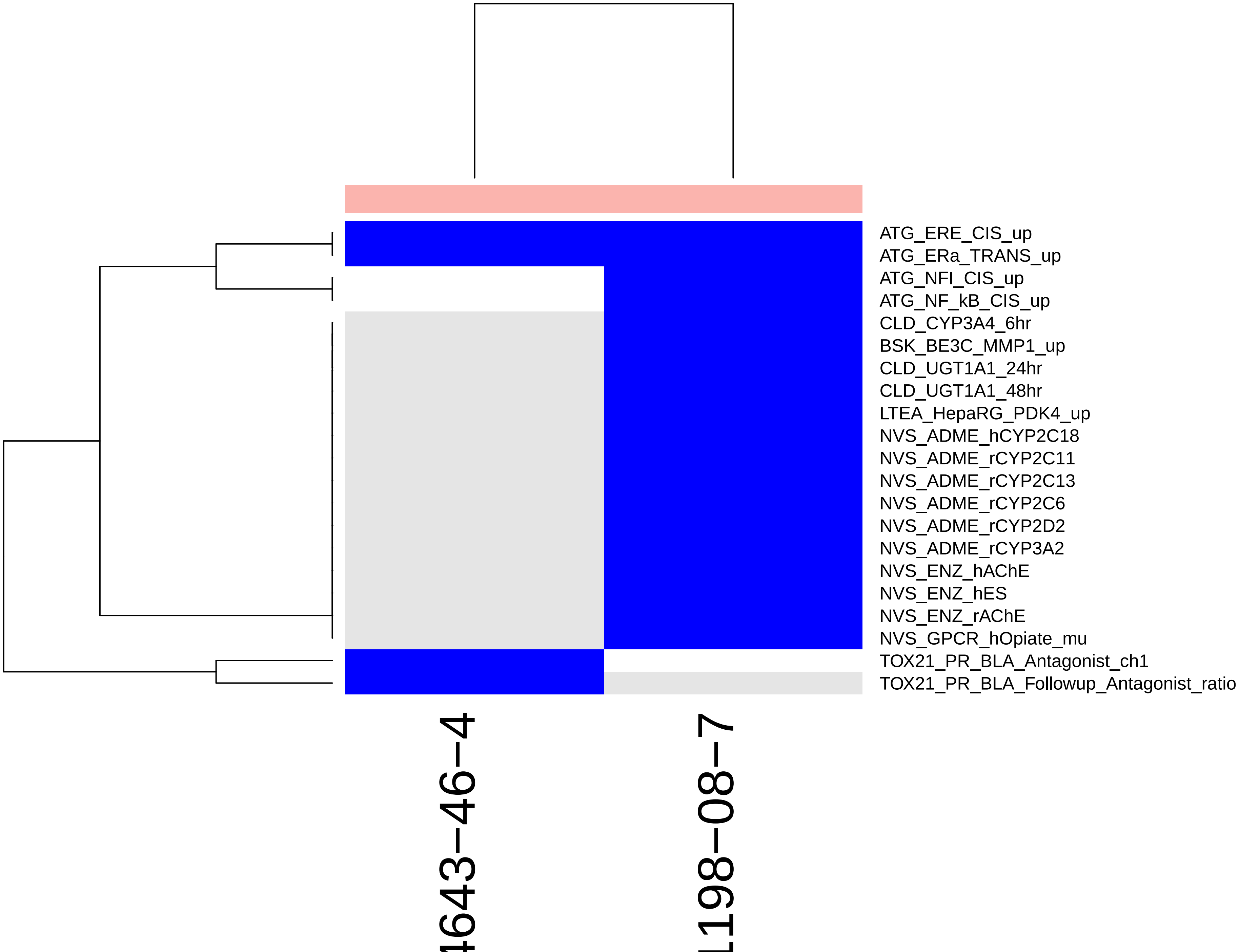

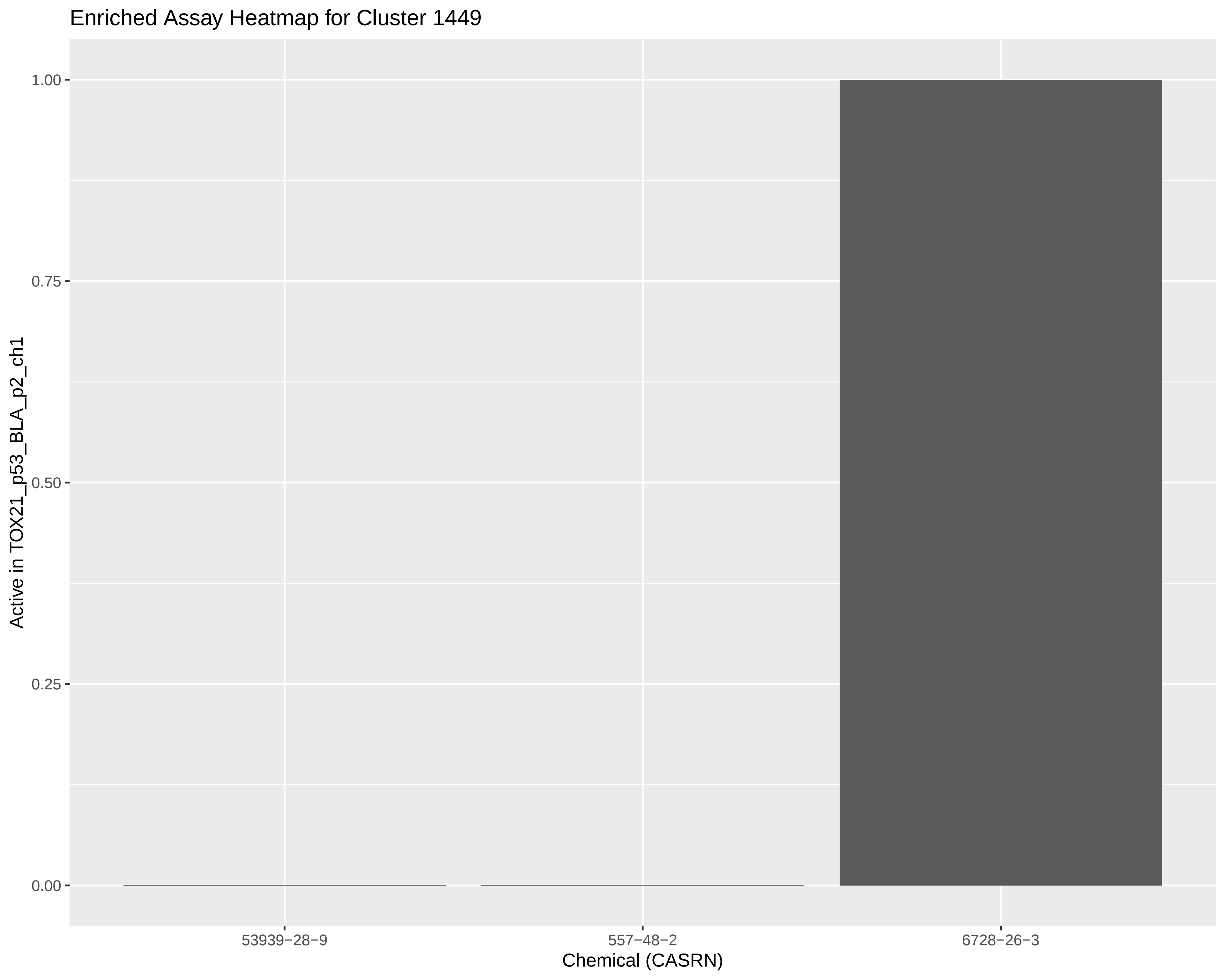
Enriched Assay Heatmap for Cluster 1449
Active in TOX21_p53_BLA_p2_ch1
1.00
0.75
0.50
0.25
0.00
53939−28−9
557−48−2
6728−26−3
Chemical (CASRN)

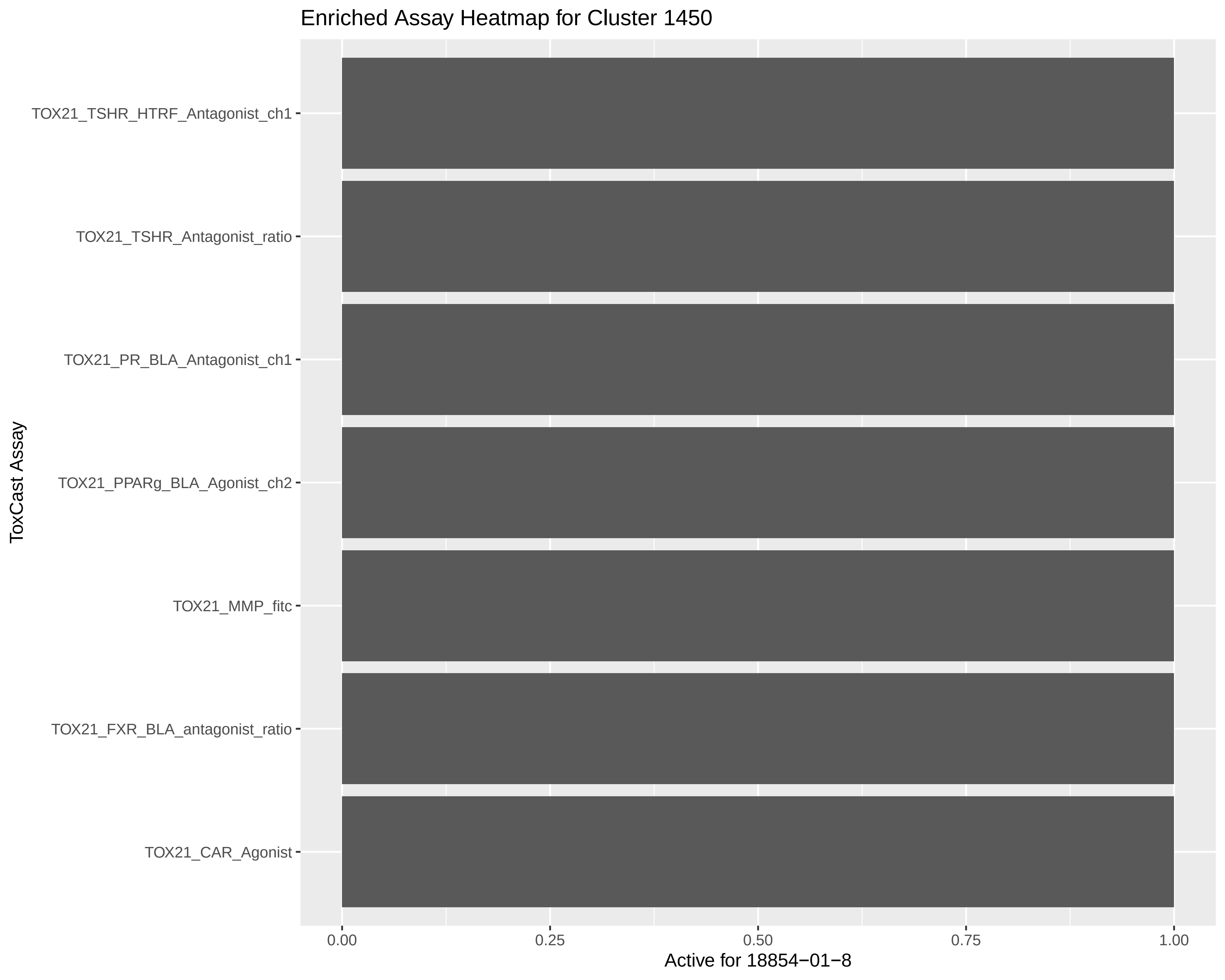
Enriched Assay Heatmap for Cluster 1450
ToxCast Assay
TOX21_TSHR_HTRF_Antagonist_ch1
TOX21_TSHR_Antagonist_ratio
TOX21_PR_BLA_Antagonist_ch1
TOX21_PPARg_BLA_Agonist_ch2
TOX21_MMP_fitc
TOX21_FXR_BLA_antagonist_ratio
TOX21_CAR_Agonist
0.00
0.25
0.50
0.75
1.00
Active for 18854−01−8

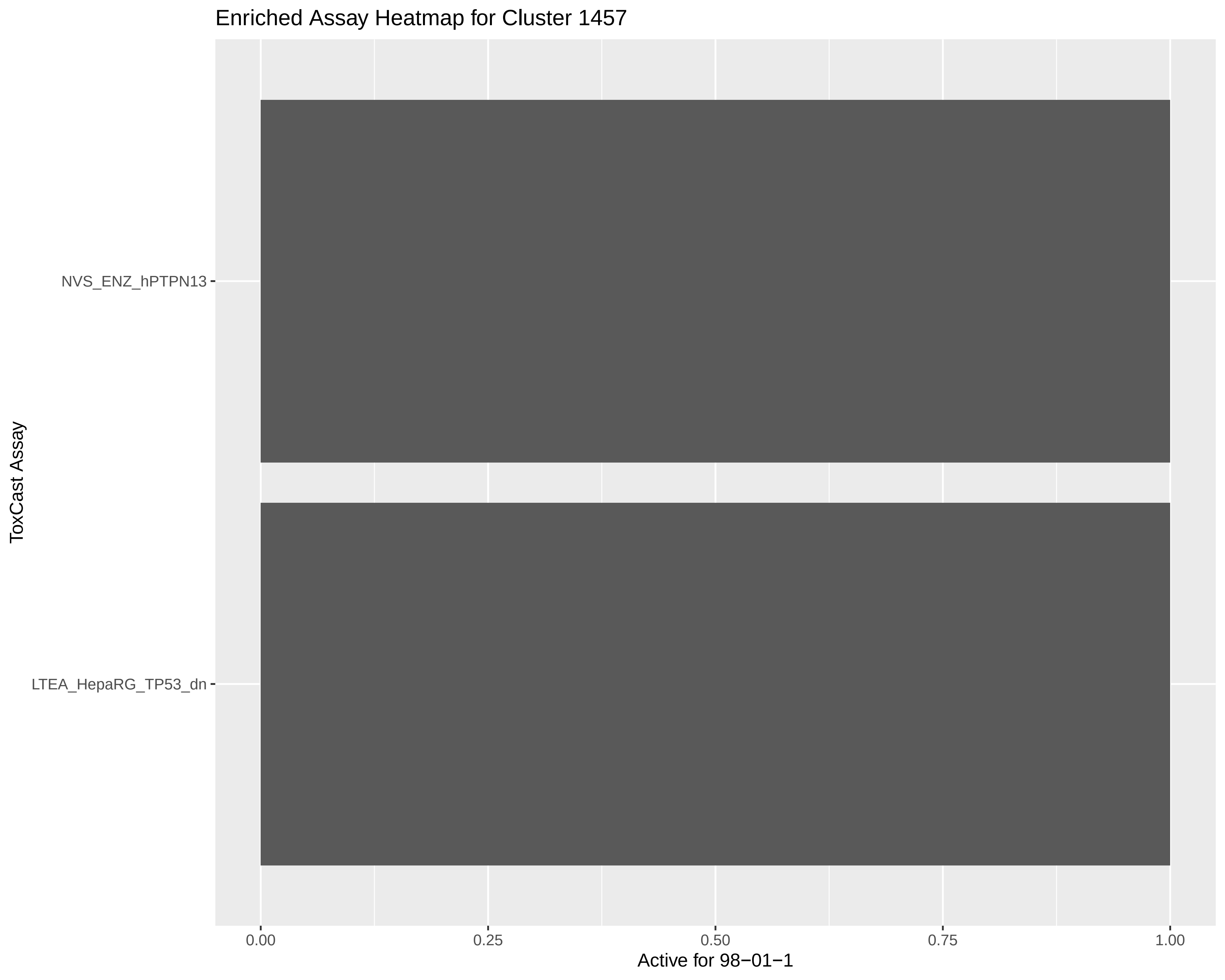
Enriched Assay Heatmap for Cluster 1457
ToxCast Assay
NVS_ENZ_hPTPN13
LTEA_HepaRG_TP53_dn
0.00
0.25
0.50
0.75
1.00
Active for 98−01−1

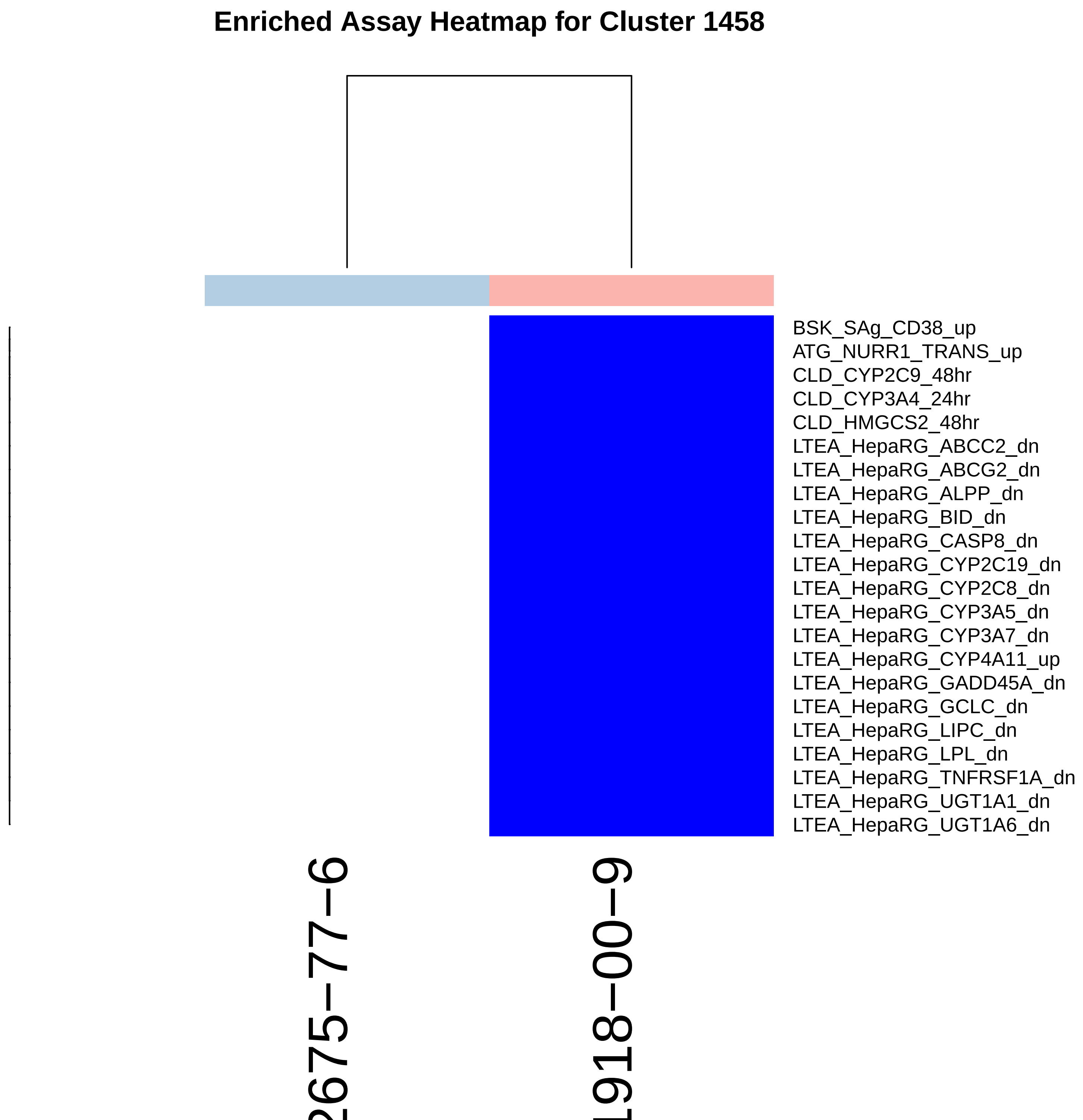
Enriched Assay Heatmap for Cluster 1458
BSK_SAg_CD38_up
ATG_NURR1_TRANS_up
CLD_CYP2C9_48hr
CLD_CYP3A4_24hr
CLD_HMGCS2_48hr
LTEA_HepaRG_ABCC2_dn
LTEA_HepaRG_ABCG2_dn
LTEA_HepaRG_ALPP_dn
LTEA_HepaRG_BID_dn
LTEA_HepaRG_CASP8_dn
LTEA_HepaRG_CYP2C19_dn
LTEA_HepaRG_CYP2C8_dn
LTEA_HepaRG_CYP3A5_dn
LTEA_HepaRG_CYP3A7_dn
LTEA_HepaRG_CYP4A11_up
LTEA_HepaRG_GADD45A_dn
LTEA_HepaRG_GCLC_dn
LTEA_HepaRG_LIPC_dn
LTEA_HepaRG_LPL_dn
LTEA_HepaRG_TNFRSF1A_dn
LTEA_HepaRG_UGT1A1_dn
LTEA_HepaRG_UGT1A6_dn
2675−77−6
918−00−9

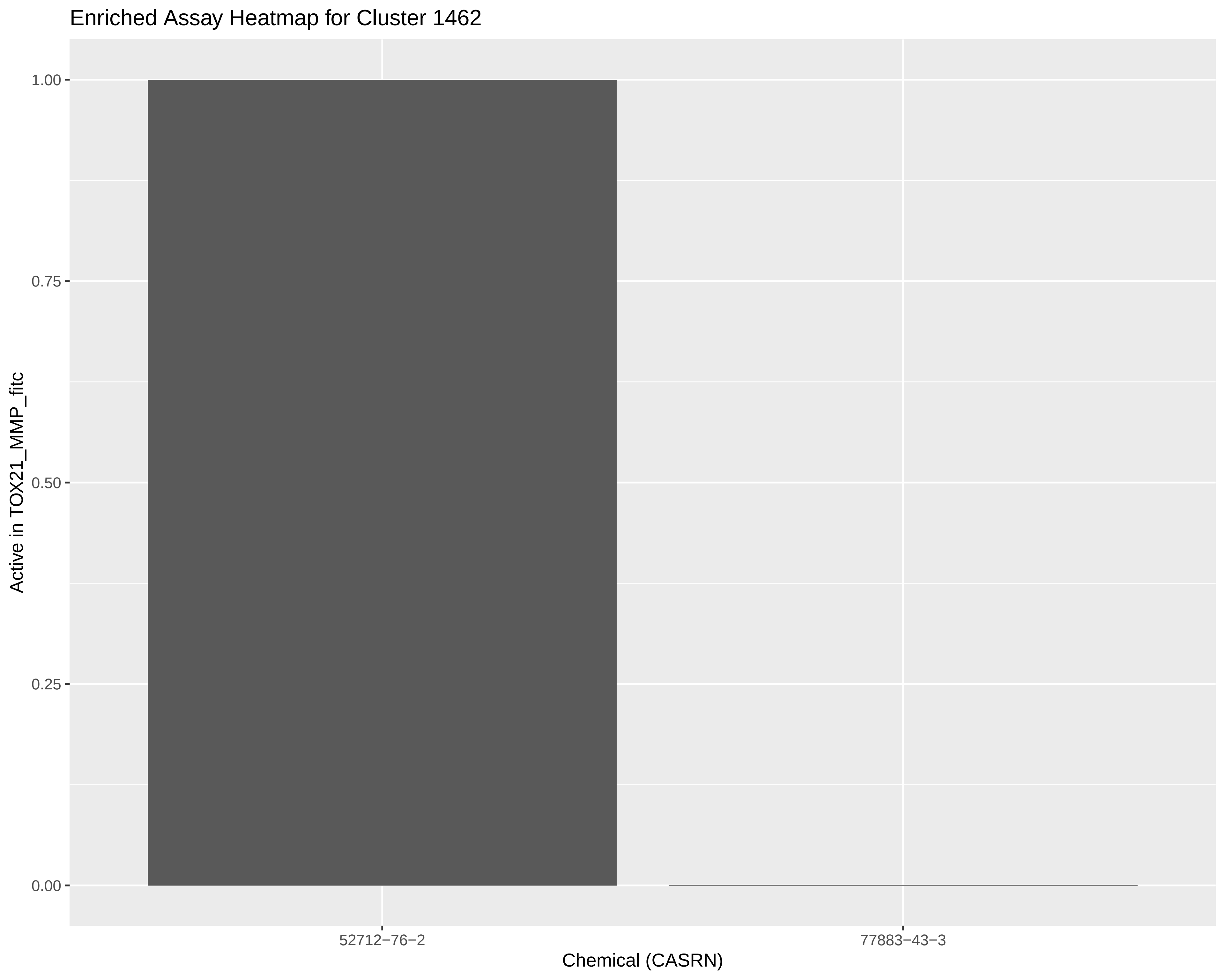

Enriched Assay Heatmap for Cluster 1462
Active in TOX21_MMP_fitc
1.00
0.75
0.50
0.25
0.00
52712−76−2
77883−43−3
Chemical (CASRN)

Enriched Assay Heatmap for Cluster 1464

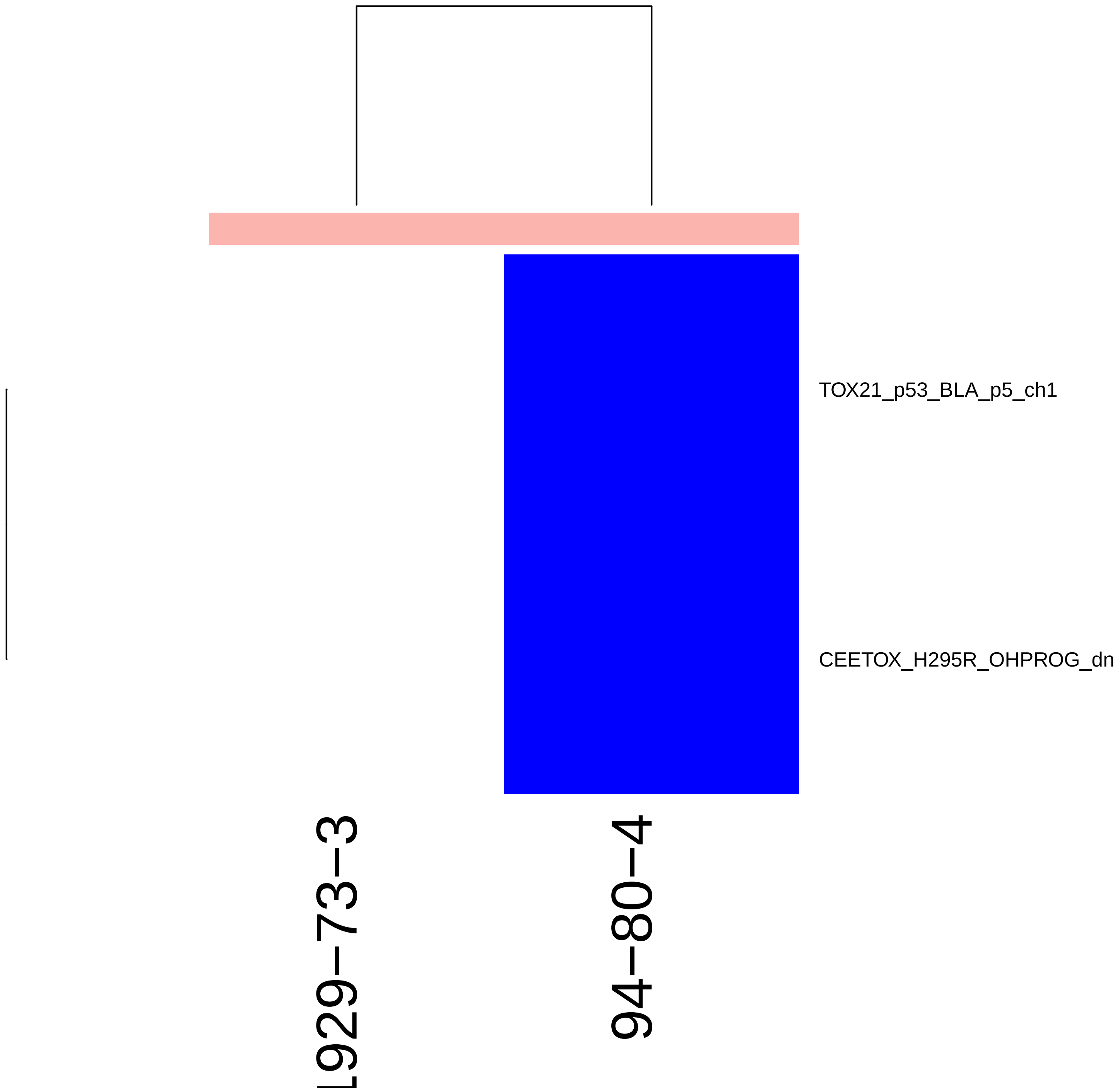

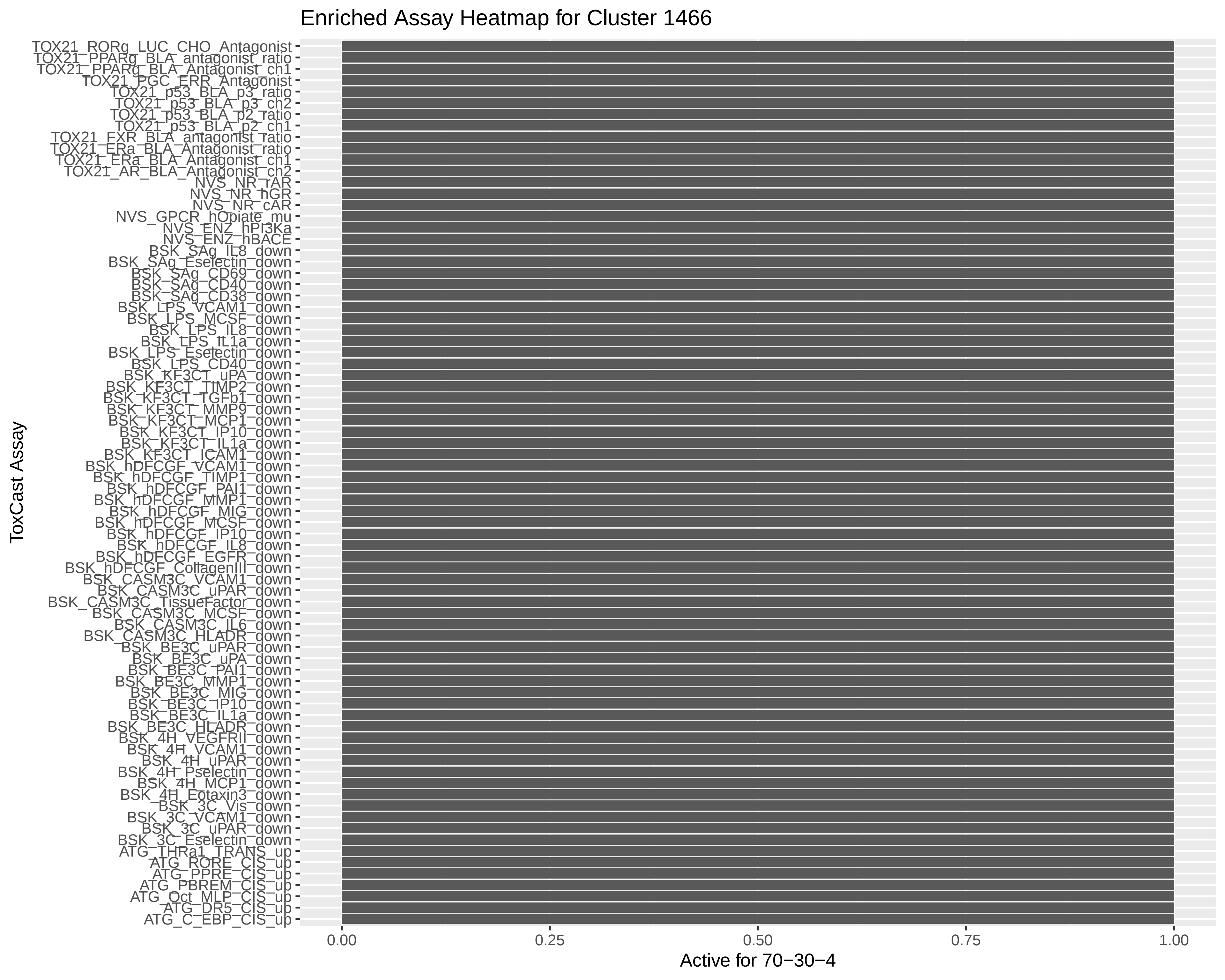

Enriched Assay Heatmap for Cluster 1466
ToxCast Assay
TOX21_RORg_LUC_CHO_Antagonist
TOX21_PPARg_BLA_antagonist_ratio
TOX21_PPARg_BLA_Antagonist_ch1
TOX21_PGC_ERR_Antagonist
TOX21_p53_BLA_p3_ratio
TOX21_p53_BLA_p3_ch2
TOX21_p53_BLA_p2_ratio
TOX21_p53_BLA_p2_ch1
TOX21_FXR_BLA_antagonist_ratio
TOX21_ERa_BLA_Antagonist_ratio
TOX21_ERa_BLA_Antagonist_ch1
TOX21_AR_BLA_Antagonist_ch2
NVS_NR_rAR
NVS_NR_hGR
NVS_NR_cAR
NVS_GPCR_hOpiate_mu
NVS_ENZ_hPI3Ka
NVS_ENZ_hBACE
BSK_SAg_IL8_down
BSK_SAg_Eselectin_down
BSK_SAg_CD69_down
BSK_SAg_CD40_down
BSK_SAg_CD38_down
BSK_LPS_VCAM1_down
BSK_LPS_MCSF_down
BSK_LPS_IL8_down
BSK_LPS_IL1a_down
BSK_LPS_Eselectin_down
BSK_LPS_CD40_down
BSK_KF3CT_uPA_down
BSK_KF3CT_TIMP2_down
BSK_KF3CT_TGFb1_down
BSK_KF3CT_MMP9_down
BSK_KF3CT_MCP1_down
BSK_KF3CT_IP10_down
BSK_KF3CT_IL1a_down
BSK_KF3CT_ICAM1_down
BSK_hDFCGF_VCAM1_down
BSK_hDFCGF_TIMP1_down
BSK_hDFCGF_PAI1_down
BSK_hDFCGF_MMP1_down
BSK_hDFCGF_MIG_down
BSK_hDFCGF_MCSF_down
BSK_hDFCGF_IP10_down
BSK_hDFCGF_IL8_down
BSK_hDFCGF_EGFR_down
BSK_hDFCGF_CollagenIII_down
BSK_CASM3C_VCAM1_down
BSK_CASM3C_uPAR_down
BSK_CASM3C_TissueFactor_down
BSK_CASM3C_MCSF_down
BSK_CASM3C_IL6_down
BSK_CASM3C_HLADR_down
BSK_BE3C_uPAR_down
BSK_BE3C_uPA_down
BSK_BE3C_PAI1_down
BSK_BE3C_MMP1_down
BSK_BE3C_MIG_down
BSK_BE3C_IP10_down
BSK_BE3C_IL1a_down
BSK_BE3C_HLADR_down
BSK_4H_VEGFRII_down
BSK_4H_VCAM1_down
BSK_4H_uPAR_down
BSK_4H_Pselectin_down
BSK_4H_MCP1_down
BSK_4H_Eotaxin3_down
BSK_3C_Vis_down
BSK_3C_VCAM1_down
BSK_3C_uPAR_down
BSK_3C_Eselectin_down
ATG_THRa1_TRANS_up
ATG_RORE_CIS_up
ATG_PPRE_CIS_up
ATG_PBREM_CIS_up
ATG_Oct_MLP_CIS_up
ATG_DR5_CIS_up
ATG_C_EBP_CIS_up
0.00
0.25
0.50
0.75
1.00
Active for 70−30−4

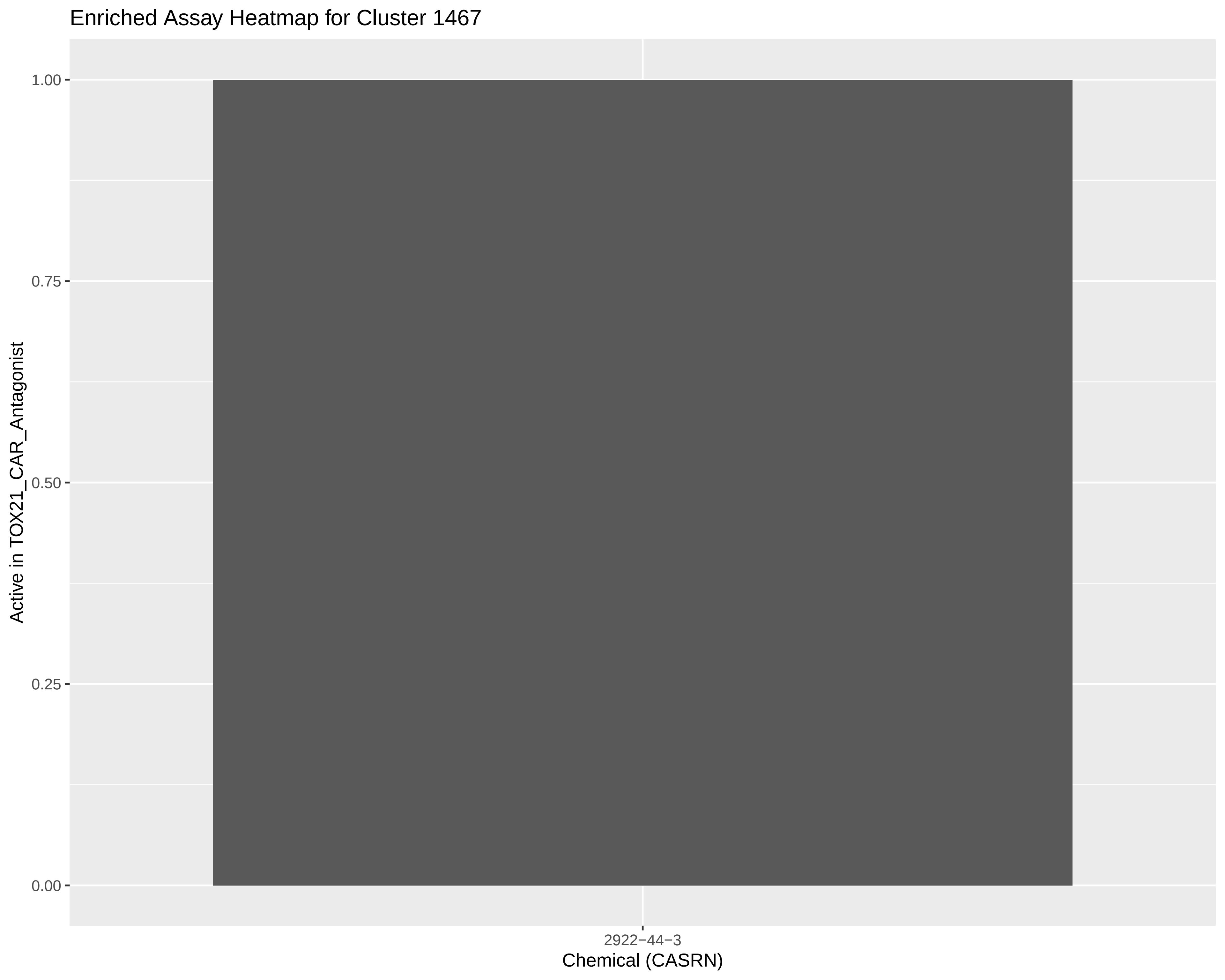
Enriched Assay Heatmap for Cluster 1467
1.00
0.75
0.50
0.25
0.00
Active in TOX21_CAR_Antagonist
2922−44−3
Chemical (CASRN)

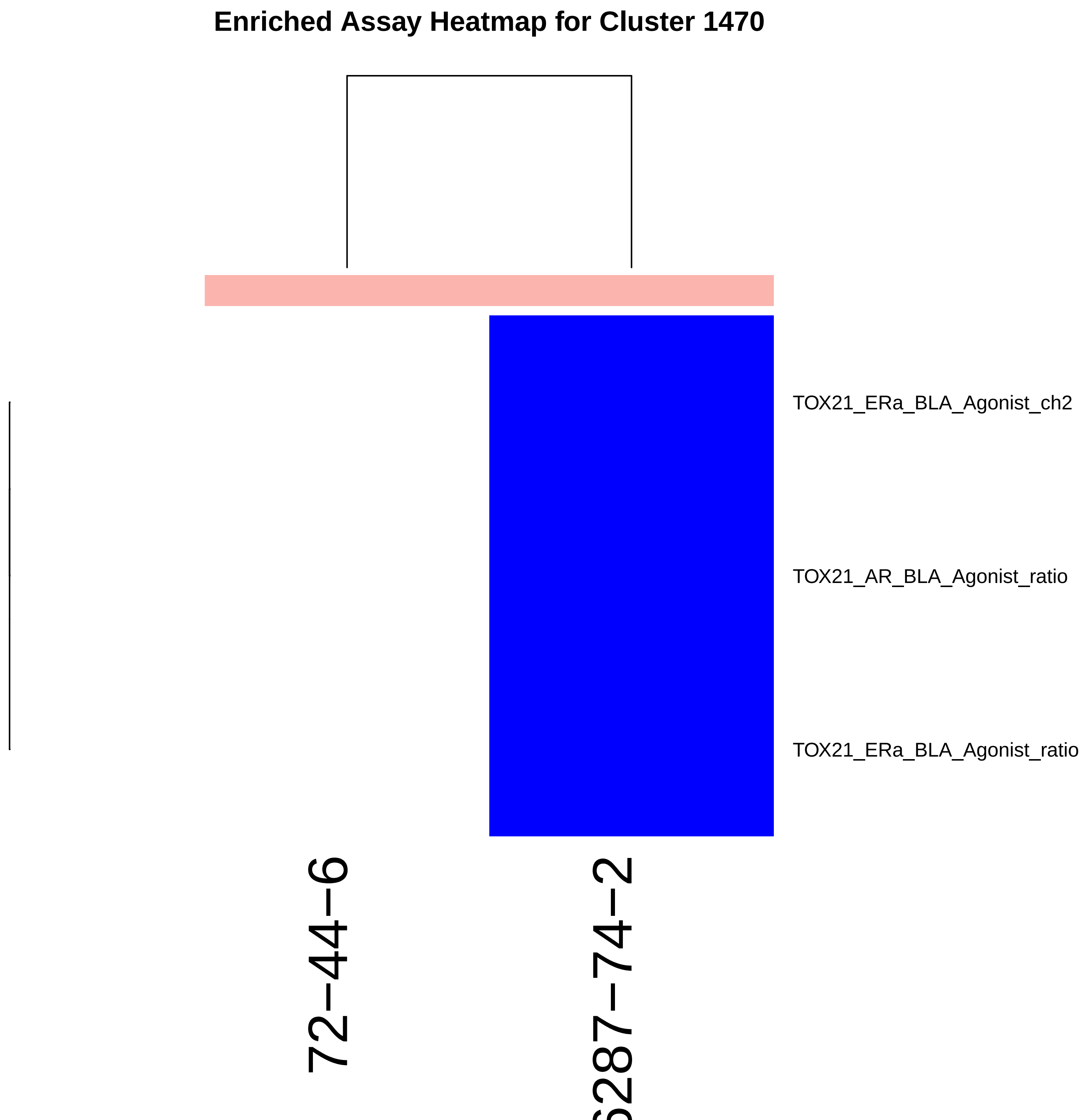
Enriched Assay Heatmap for Cluster 1470
TOX21_ERa_BLA_Agonist_ch2
TOX21_AR_BLA_Agonist_ratio
TOX21_ERa_BLA_Agonist_ratio
72−44−6

Enriched Assay Heatmap for Cluster 1478

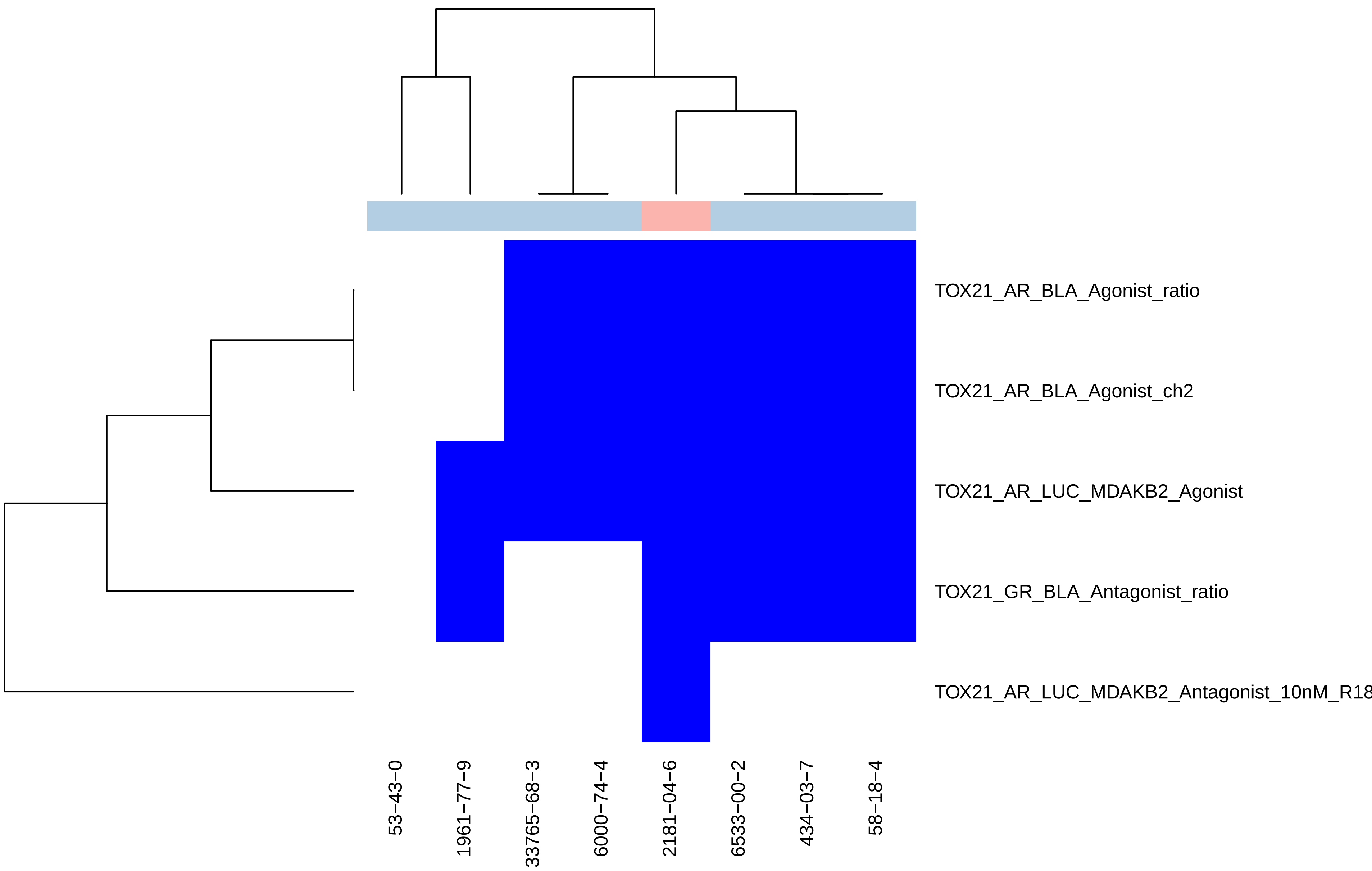

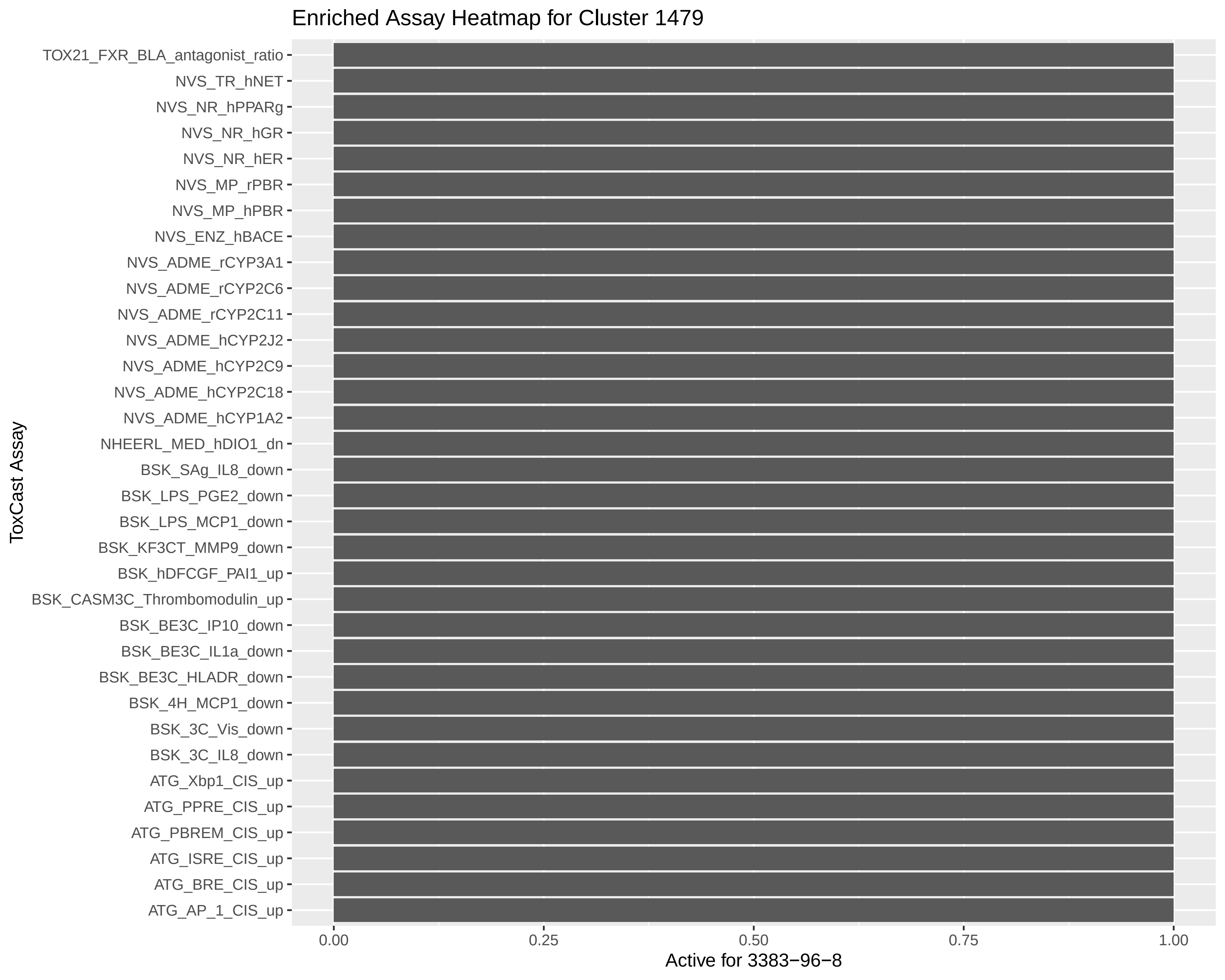

Enriched Assay Heatmap for Cluster 1479
ToxCast Assay
TOX21_FXR_BLA_antagonist_ratio
NVS_TR_hNET
NVS_NR_hPPARg
NVS_NR_hGR
NVS_NR_hER
NVS_MP_rPBR
NVS_MP_hPBR
NVS_ENZ_hBACE
NVS_ADME_rCYP3A1
NVS_ADME_rCYP2C6
NVS_ADME_rCYP2C11
NVS_ADME_hCYP2J2
NVS_ADME_hCYP2C9
NVS_ADME_hCYP2C18
NVS_ADME_hCYP1A2
NHEERL_MED_hDIO1_dn
BSK_SAg_IL8_down
BSK_LPS_PGE2_down
BSK_LPS_MCP1_down
BSK_KF3CT_MMP9_down
BSK_hDFCGF_PAI1_up
BSK_CASM3C_Thrombomodulin_up
BSK_BE3C_IP10_down
BSK_BE3C_IL1a_down
BSK_BE3C_HLADR_down
BSK_4H_MCP1_down
BSK_3C_Vis_down
BSK_3C_IL8_down
ATG_Xbp1_CIS_up
ATG_PPRE_CIS_up
ATG_PBREM_CIS_up
ATG_ISRE_CIS_up
ATG_BRE_CIS_up
ATG_AP_1_CIS_up
0.00
0.25
0.50
0.75
1.00
Active for 3383−96−8

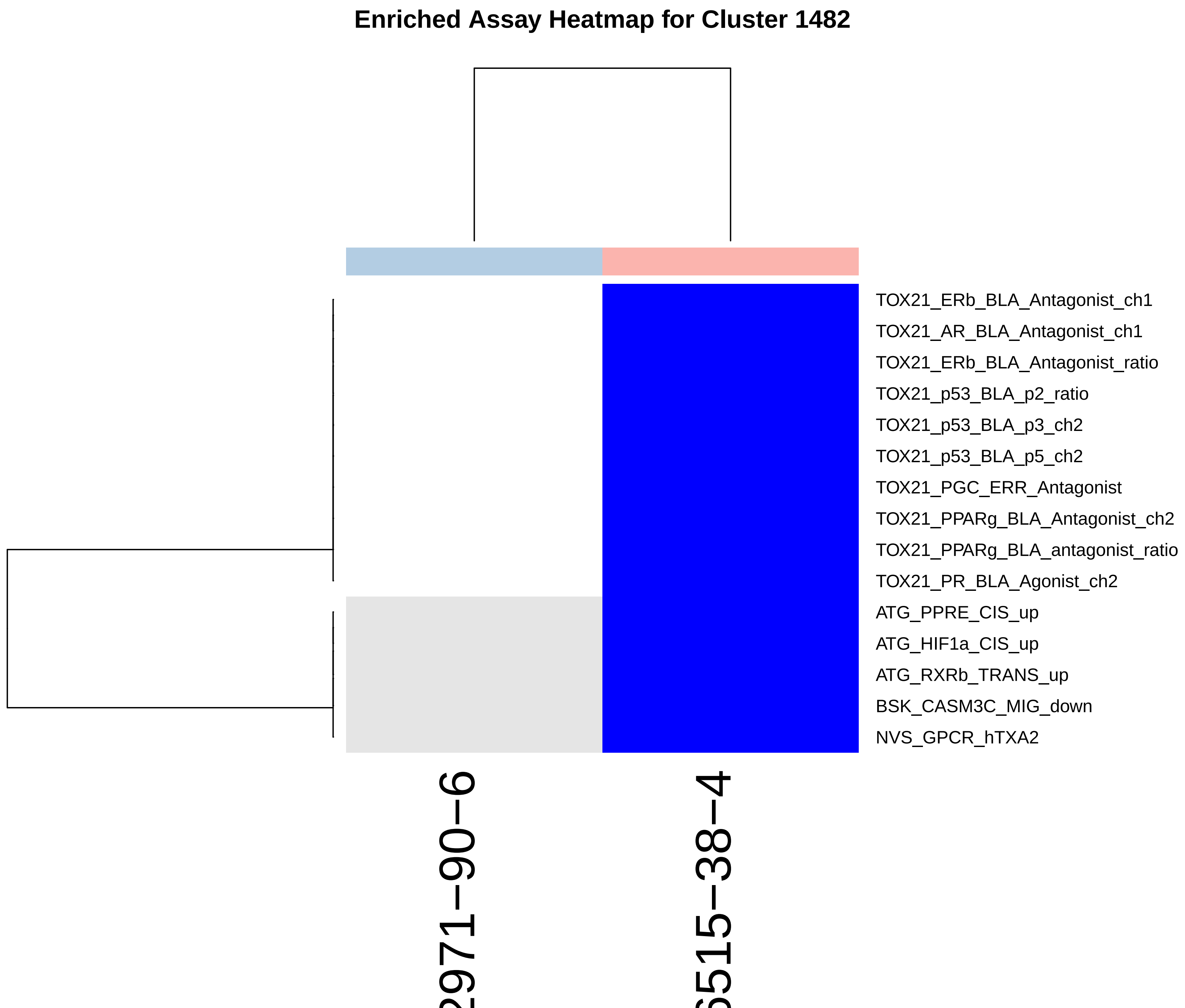
Enriched Assay Heatmap for Cluster 1482
TOX21_ERb_BLA_Antagonist_ch1
TOX21_AR_BLA_Antagonist_ch1
TOX21_ERb_BLA_Antagonist_ratio
TOX21_p53_BLA_p2_ratio
TOX21_p53_BLA_p3_ch2
TOX21_p53_BLA_p5_ch2
TOX21_PGC_ERR_Antagonist
TOX21_PPARg_BLA_Antagonist_ch2
TOX21_PPARg_BLA_antagonist_ratio
TOX21_PR_BLA_Agonist_ch2
ATG_PPRE_CIS_up
ATG_HIF1a_CIS_up
ATG_RXRb_TRANS_up
BSK_CASM3C_MIG_down
NVS_GPCR_hTXA2
971−90−6
515−38−4

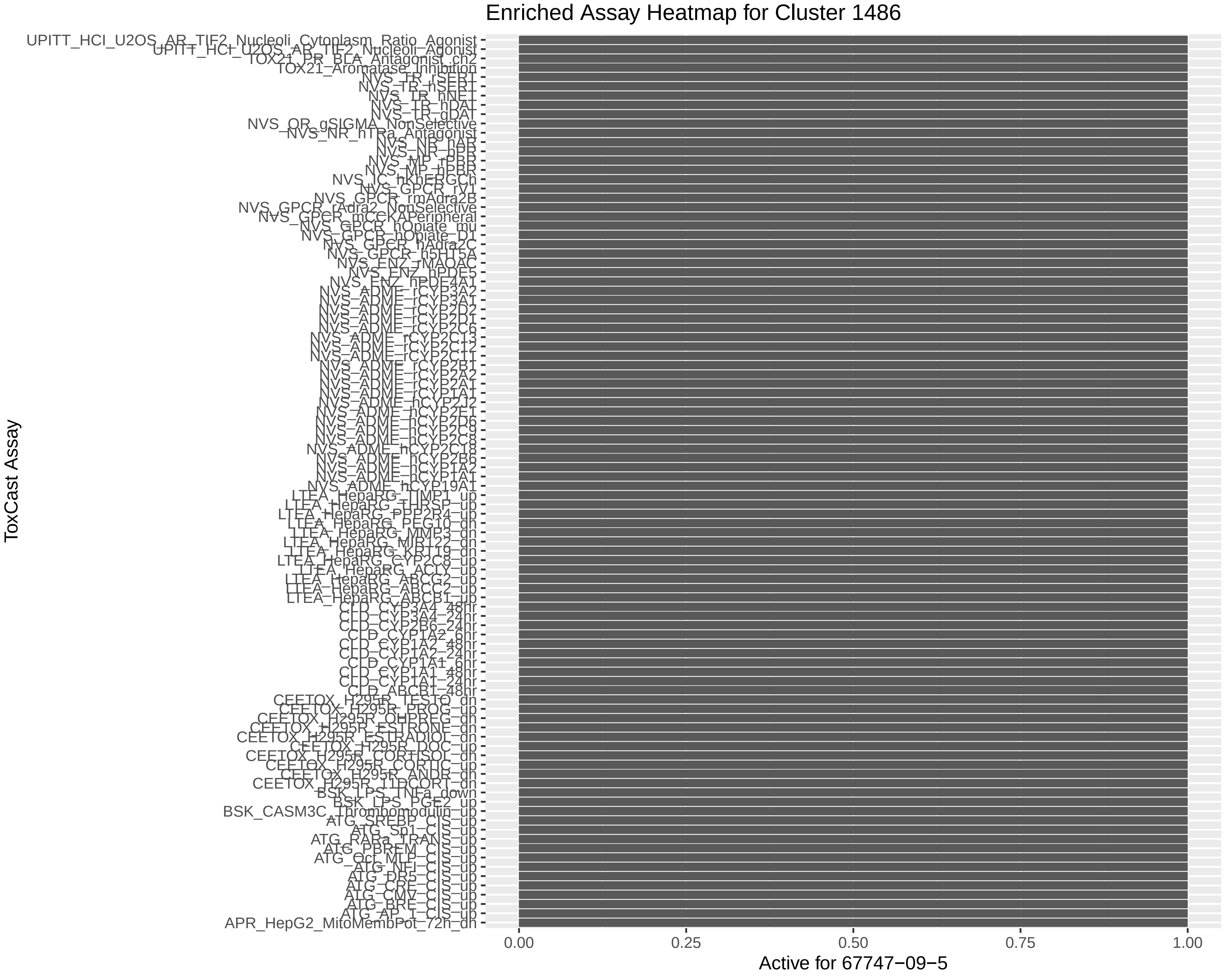
Enriched Assay Heatmap for Cluster 1486
ToxCast Assay
Active for 67747−09−5
0.00
0.25
0.50
0.75
1.00
UPITT_HCI_U2OS_AR_TIF2_Nucleoli_Cytoplasm_Ratio_Agonist
UPITT_HCI_U2OS_AR_TIF2_Nucleoli_Agonist
TOX21_PR_BLA_Antagonist_ch2
TOX21_Aromatase_Inhibition
APR_HepG2_MitoMembPot_72h_dn

Enriched Assay Heatmap for Cluster 1487

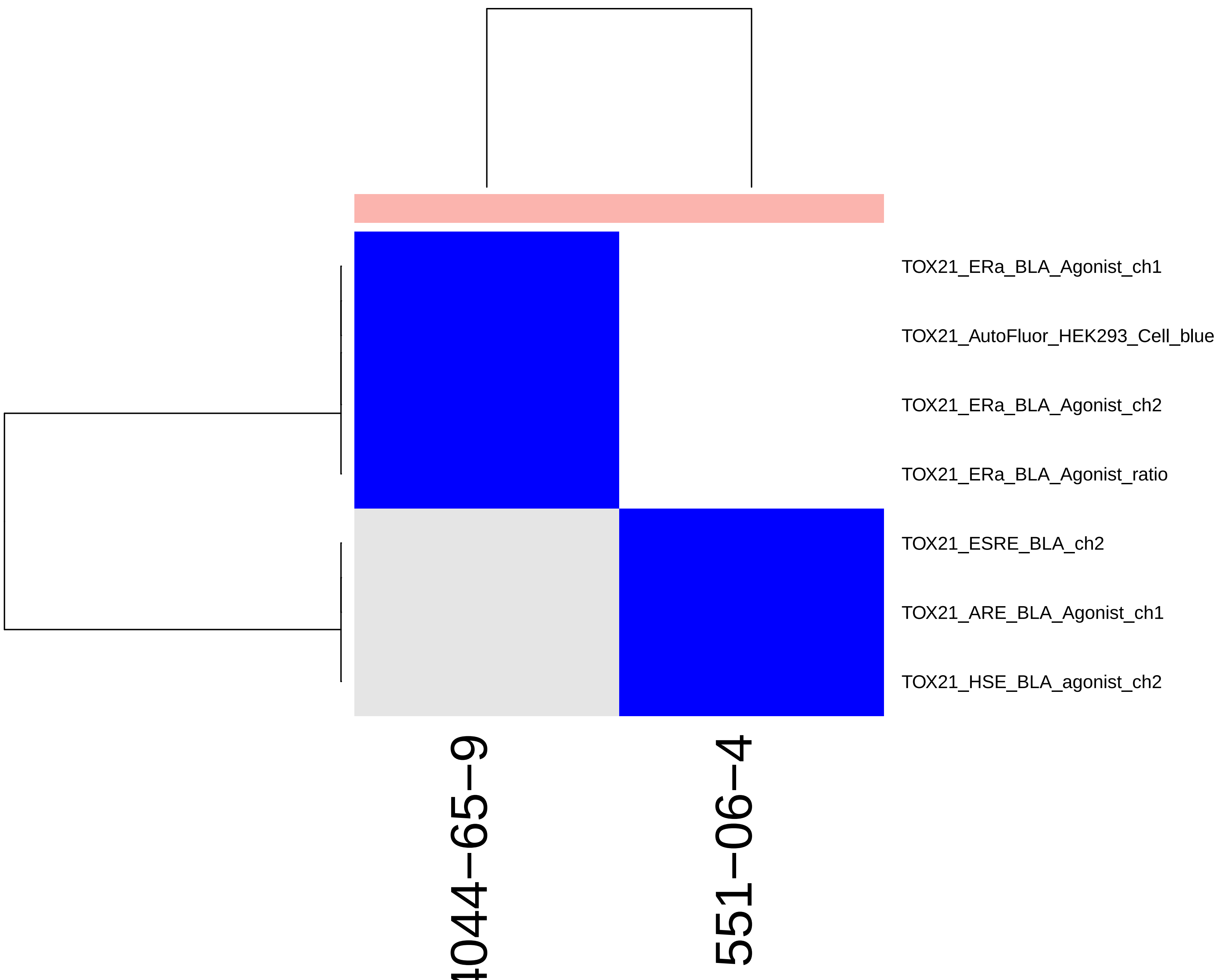

Enriched Assay Heatmap for Cluster 1488

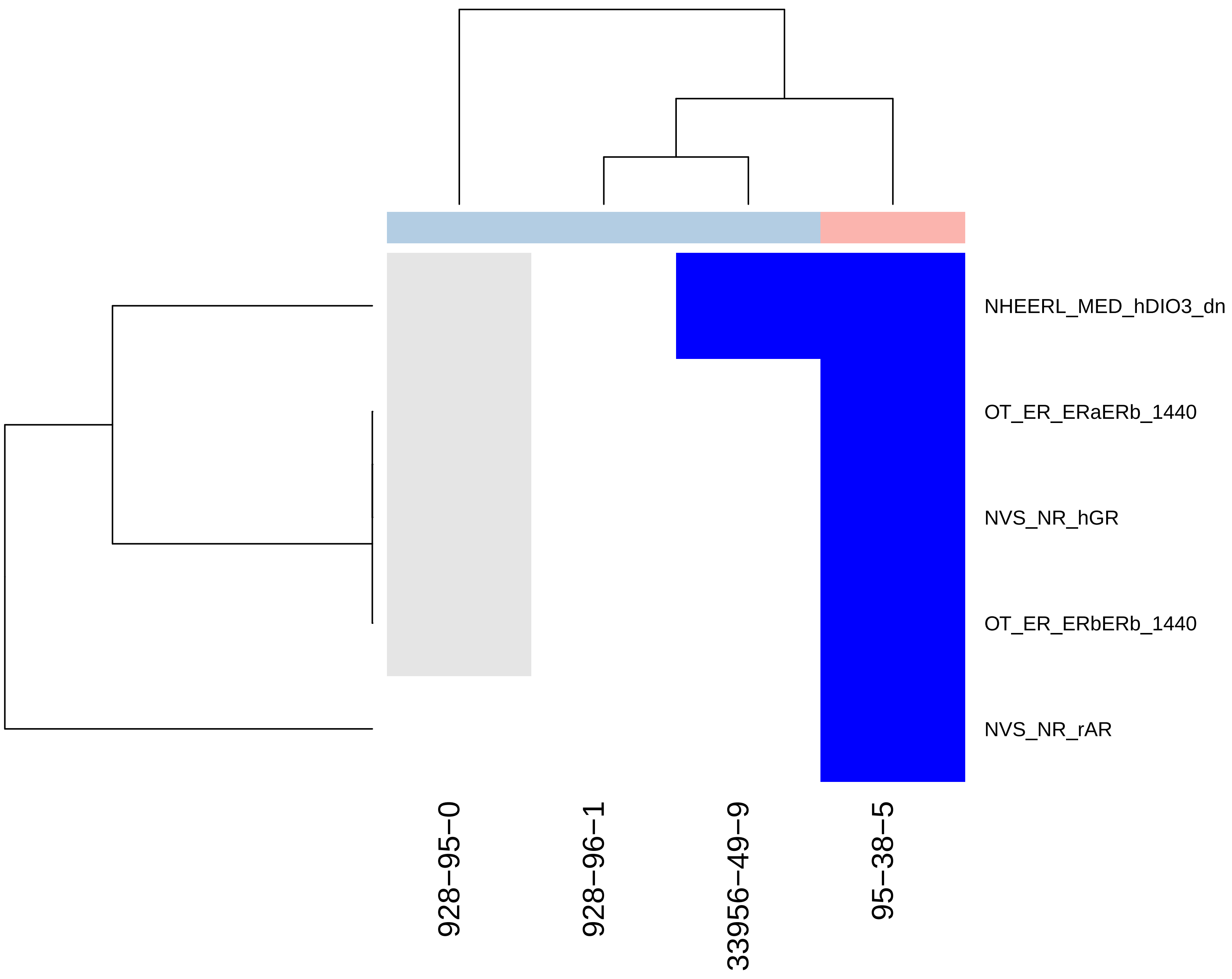

Enriched Assay Heatmap for Cluster 1489

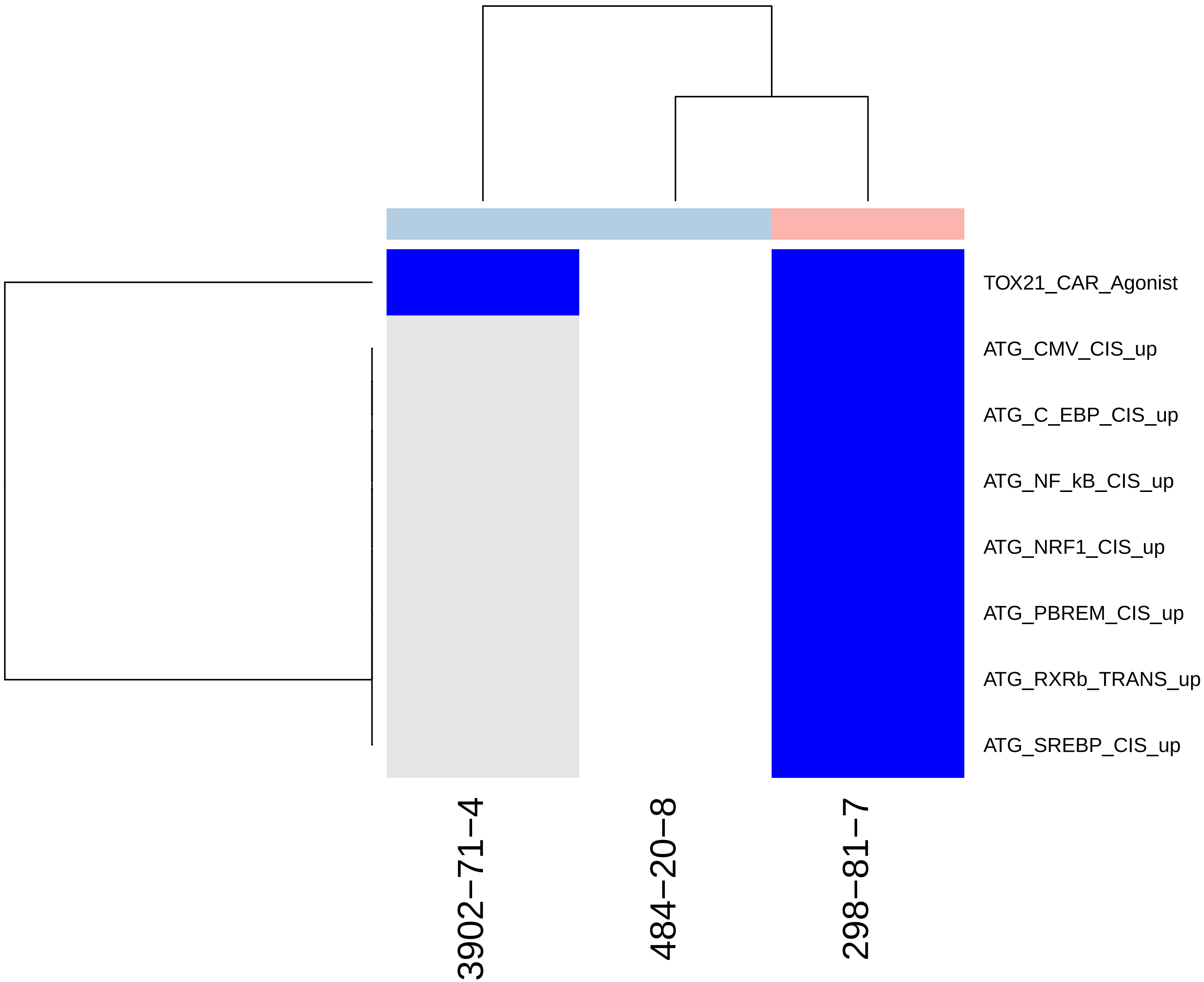

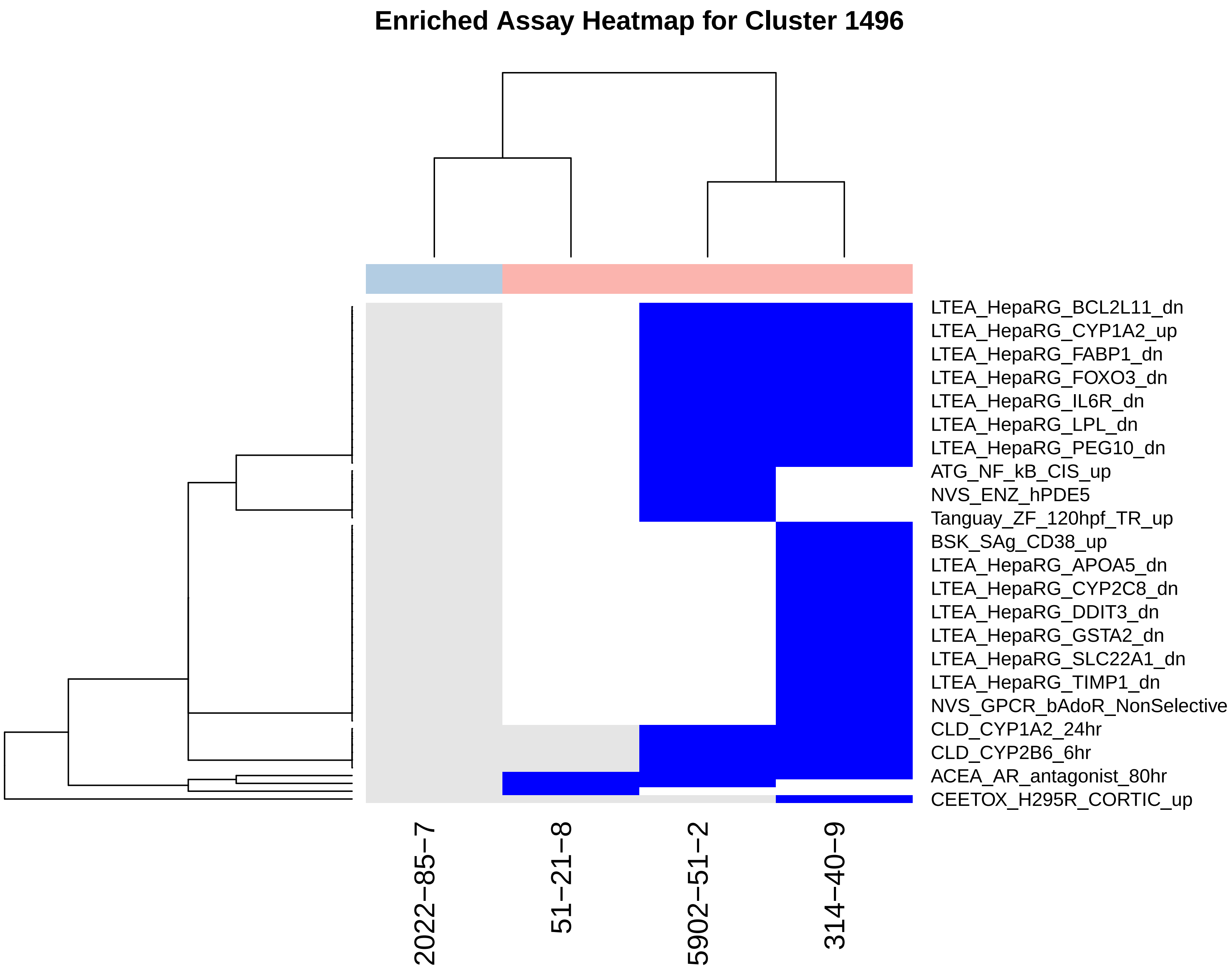

Enriched Assay Heatmap for Cluster 1496
LTEA_HepaRG_BCL2L11_dn
LTEA_HepaRG_CYP1A2_up
LTEA_HepaRG_FABP1_dn
LTEA_HepaRG_FOXO3_dn
LTEA_HepaRG_IL6R_dn
LTEA_HepaRG_LPL_dn
LTEA_HepaRG_PEG10_dn
ATG_NF_kB_CIS_up
NVS_ENZ_hPDE5
Tanguay_ZF_120hpf_TR_up
BSK_SAg_CD38_up
LTEA_HepaRG_APOA5_dn
LTEA_HepaRG_CYP2C8_dn
LTEA_HepaRG_DDIT3_dn
LTEA_HepaRG_GSTA2_dn
LTEA_HepaRG_SLC22A1_dn
LTEA_HepaRG_TIMP1_dn
NVS_GPCR_bAdoR_NonSelective
CLD_CYP1A2_24hr
CLD_CYP2B6_6hr
ACEA_AR_antagonist_80hr
CEETOX_H295R_CORTIC_up
2022−85−7
51−21−8
5902−51−2
314−40−9

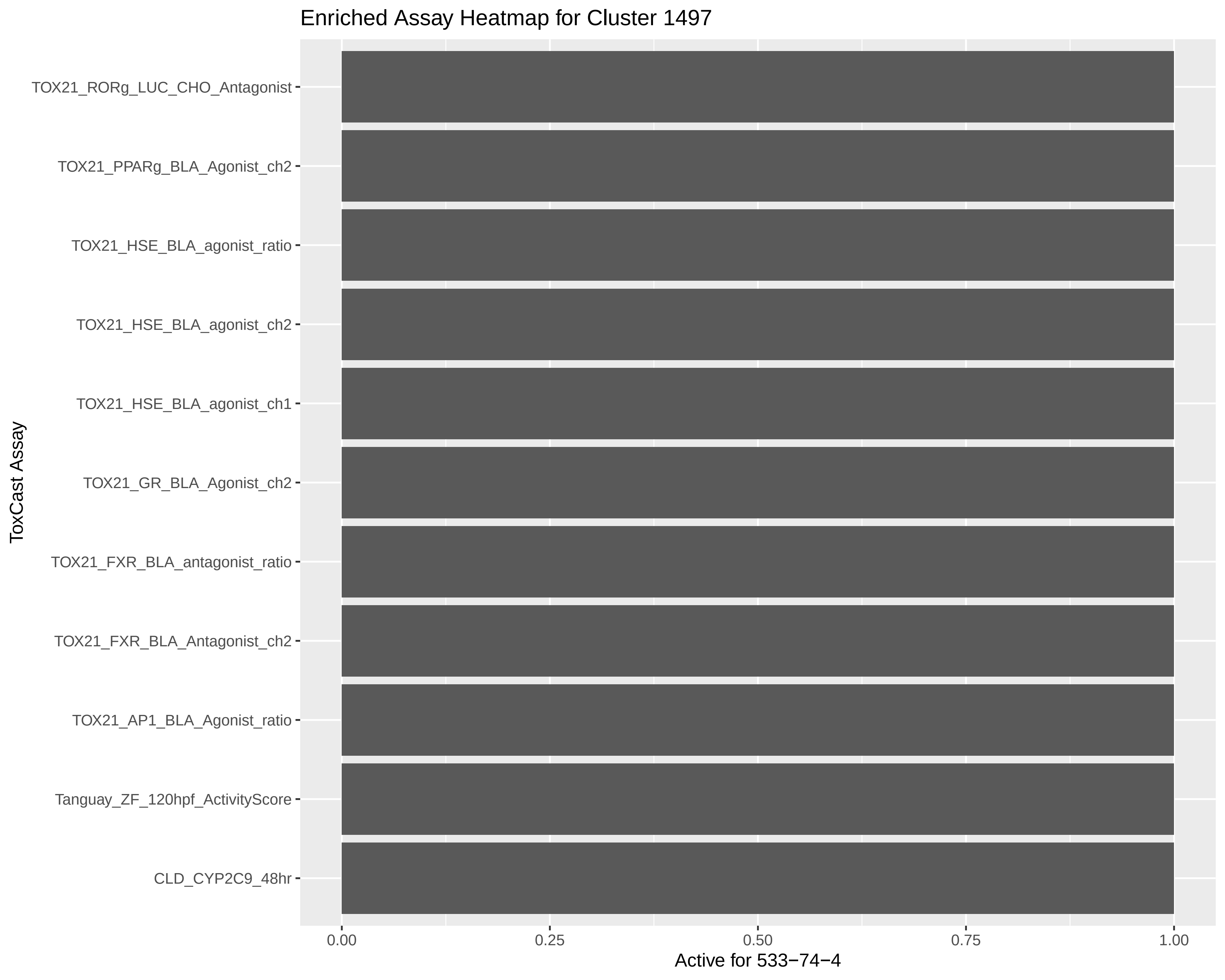

Enriched Assay Heatmap for Cluster 1497
TOX21_RORg_LUC_CHO_Antagonist
TOX21_PPARg_BLA_Agonist_ch2
TOX21_HSE_BLA_agonist_ratio
TOX21_HSE_BLA_agonist_ch2
TOX21_HSE_BLA_agonist_ch1
TOX21_GR_BLA_Agonist_ch2
TOX21_FXR_BLA_antagonist_ratio
TOX21_FXR_BLA_Antagonist_ch2
TOX21_AP1_BLA_Agonist_ratio
Tanguay_ZF_120hpf_ActivityScore
CLD_CYP2C9_48hr
ToxCast Assay
0.00
0.25
0.50
0.75
1.00
Active for 533−74−4

Enriched Assay Heatmap for Cluster 1499

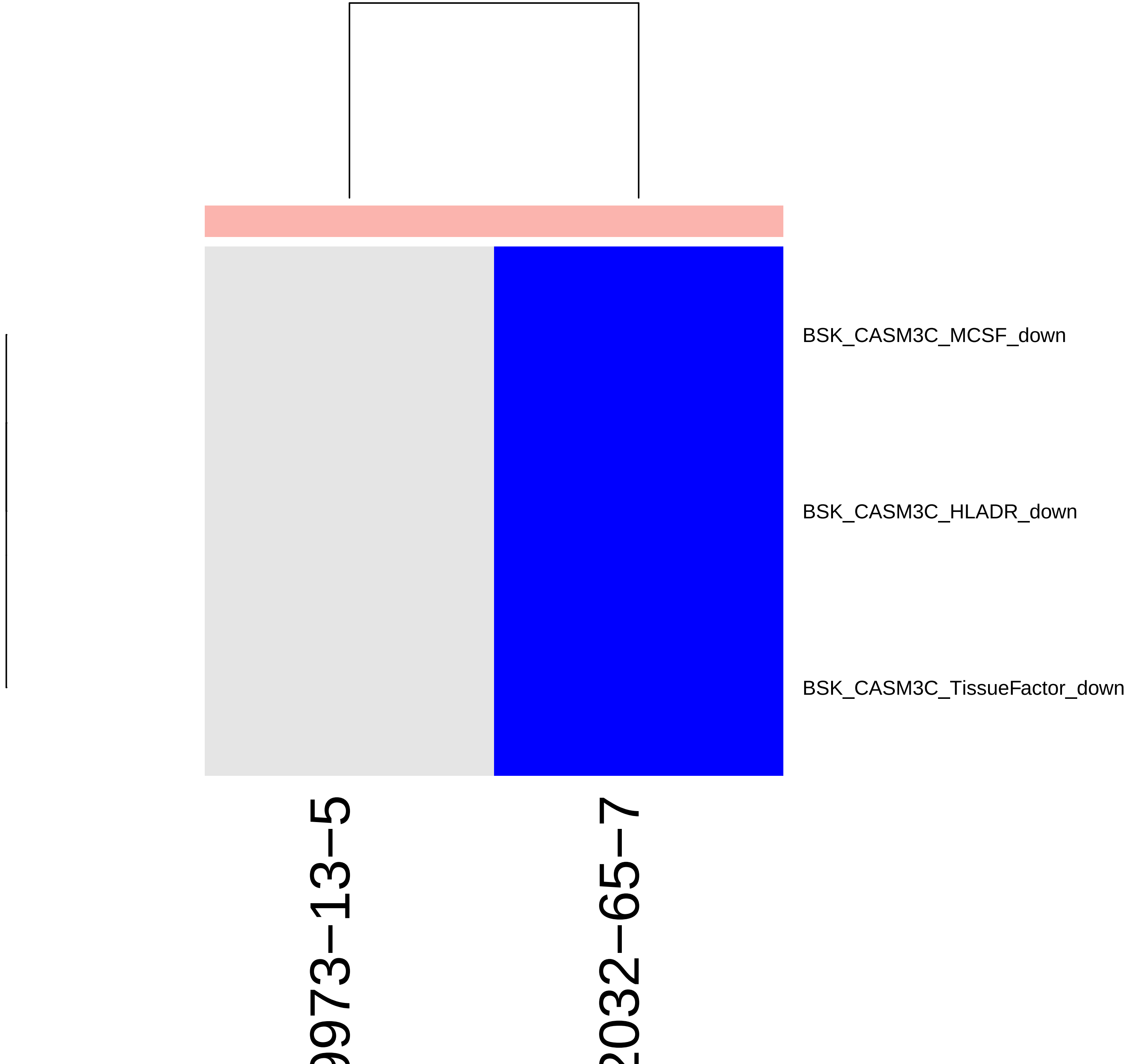

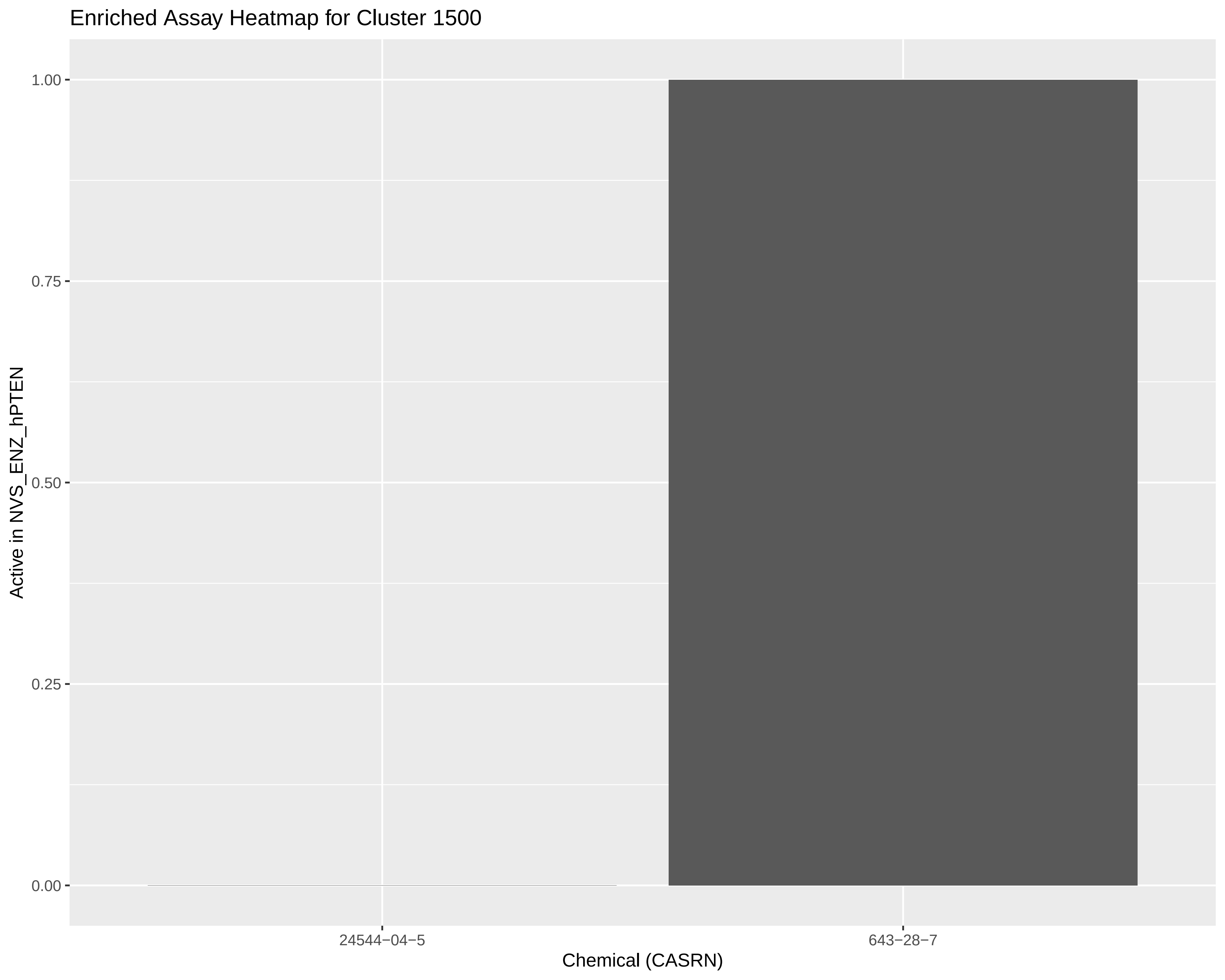
Enriched Assay Heatmap for Cluster 1500
Active in NVS_ENZ_hPTEN
1.00
0.75
0.50
0.25
0.00
24544−04−5
643−28−7
Chemical (CASRN)

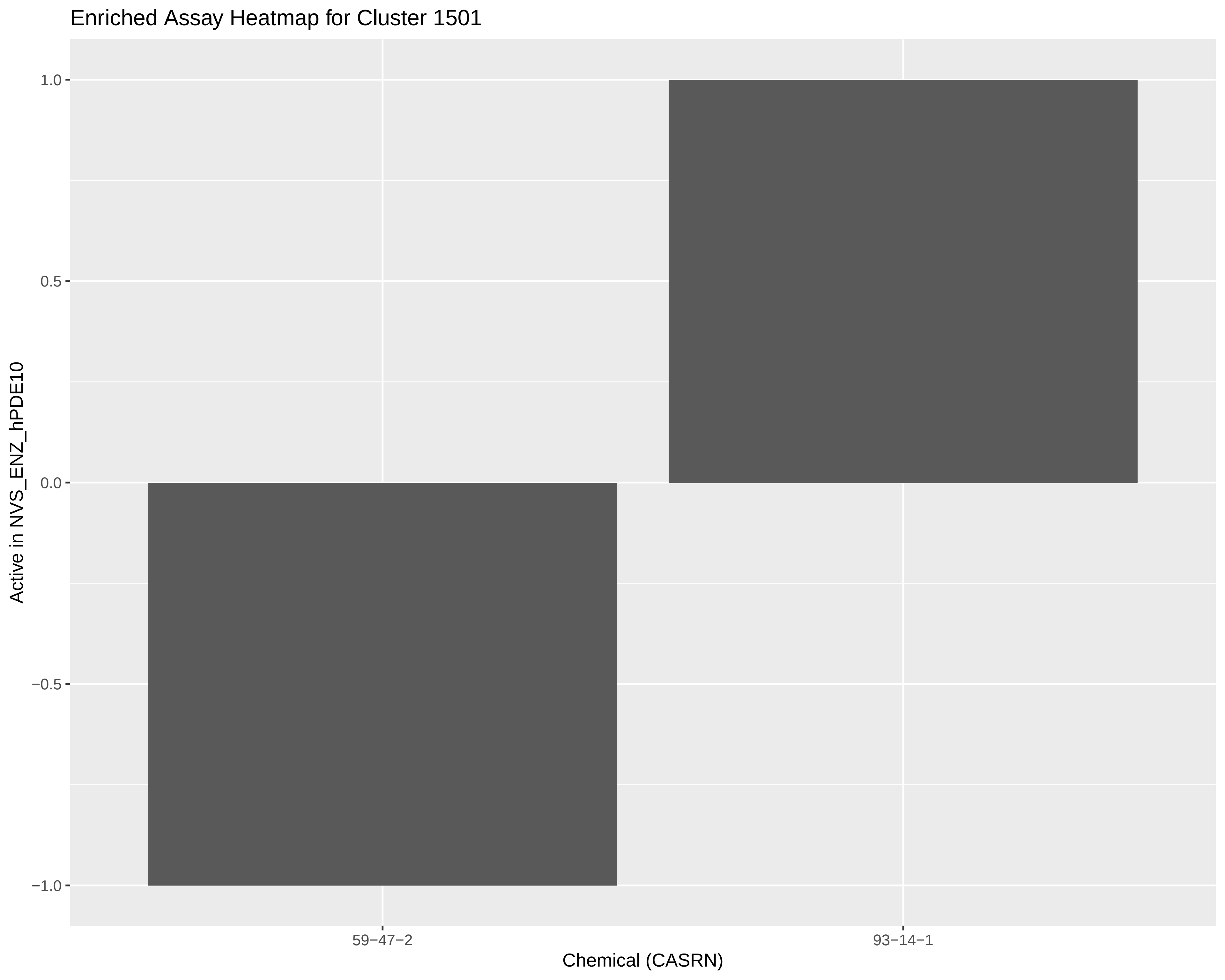
Enriched Assay Heatmap for Cluster 1501
1.0
0.5
0.0
−0.5
−1.0
Active in NVS_ENZ_hPDE10
59−47−2
93−14−1
Chemical (CASRN)

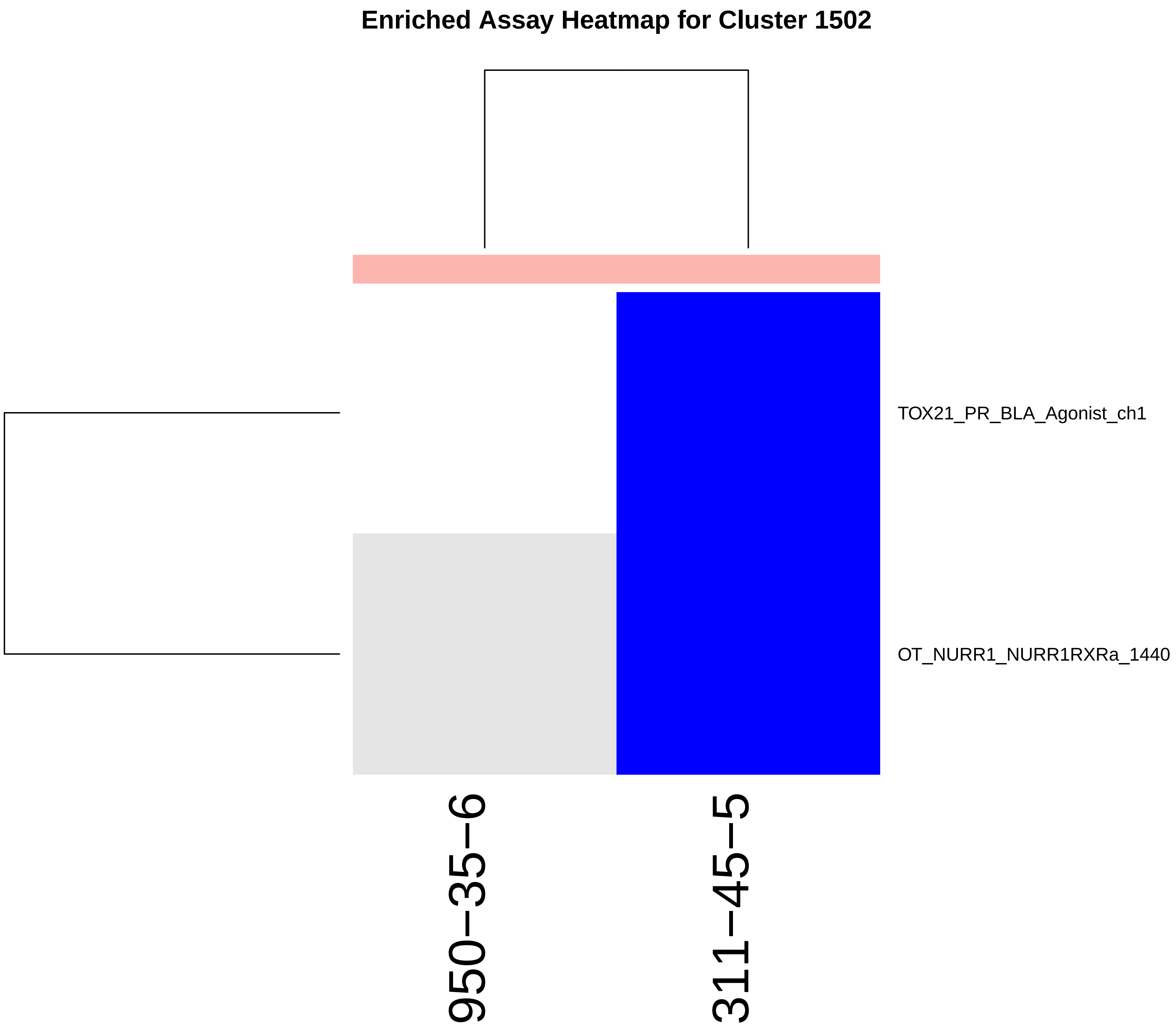
Enriched Assay Heatmap for Cluster 1502
TOX21_PR_BLA_Agonist_ch1
OT_NURR1_NURR1RXRa_1440
950−35−6
311−45−5

Enriched Assay Heatmap for Cluster 1504

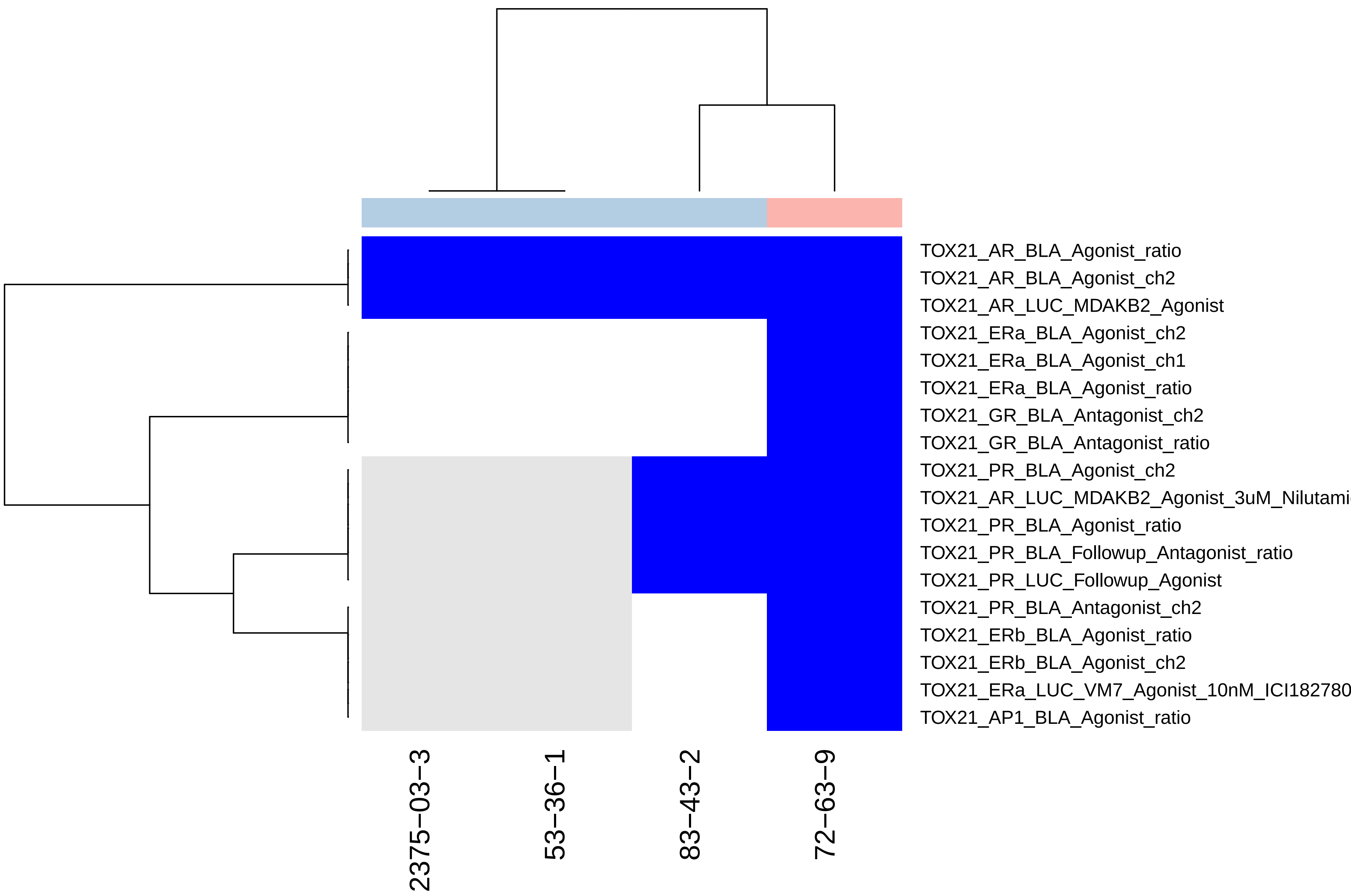

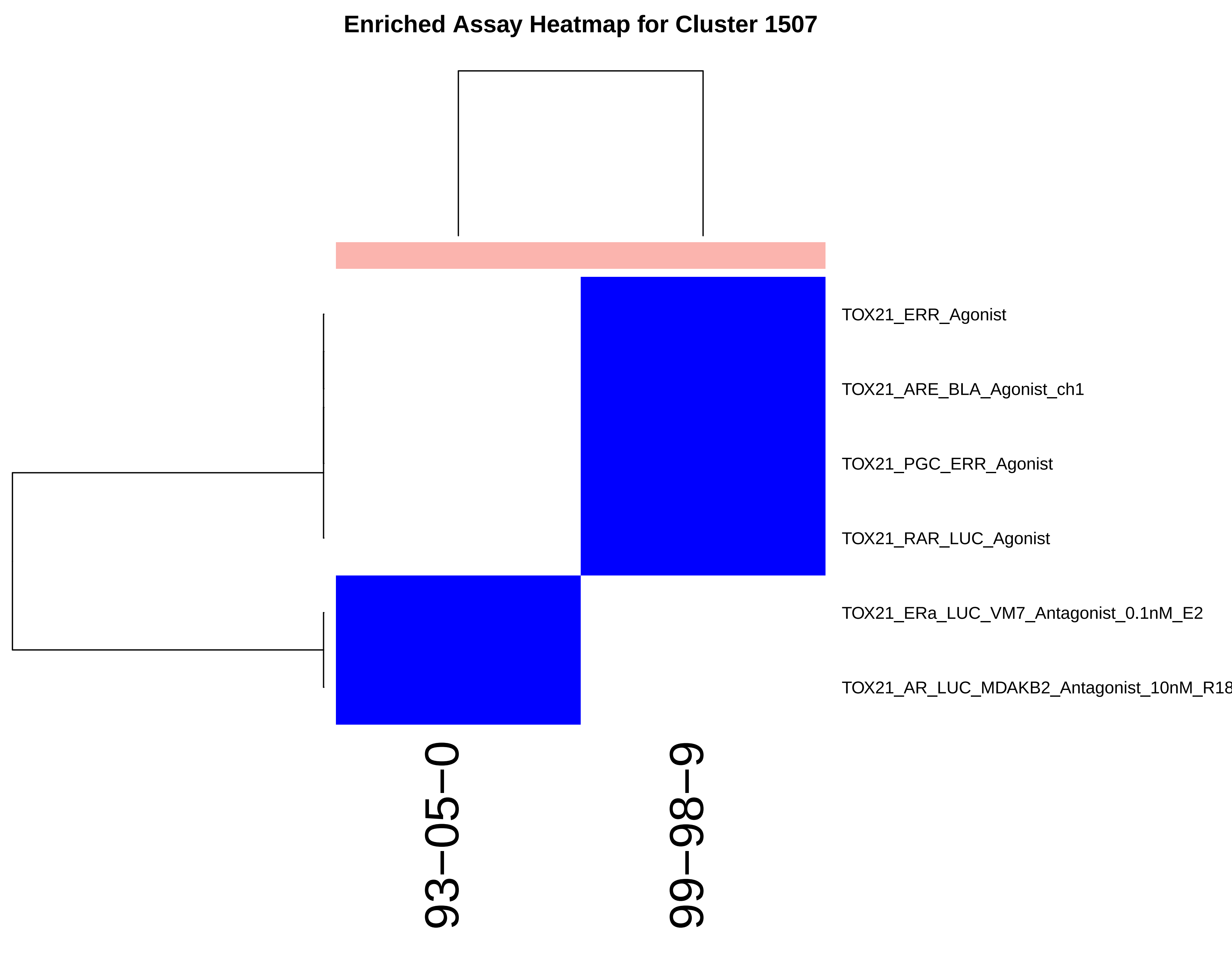
Enriched Assay Heatmap for Cluster 1507
TOX21_ERR_Agonist
TOX21_ARE_BLA_Agonist_ch1
TOX21_PGC_ERR_Agonist
TOX21_RAR_LUC_Agonist
TOX21_ERa_LUC_VM7_Antagonist_0.1nM_E2
TOX21_AR_LUC_MDAKB2_Antagonist_10nM_R18
93−05−0
99−98−9

Enriched Assay Heatmap for Cluster 1508

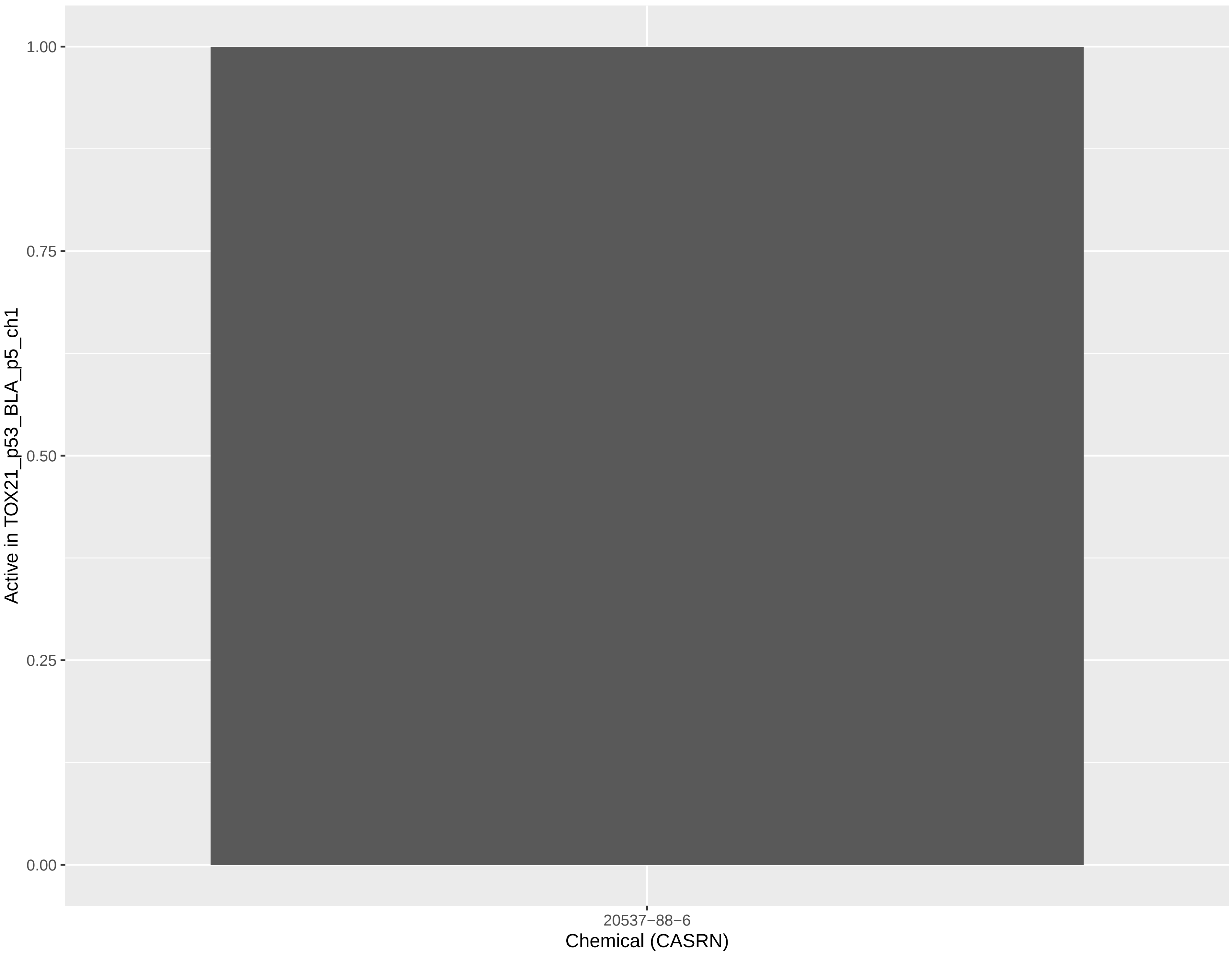

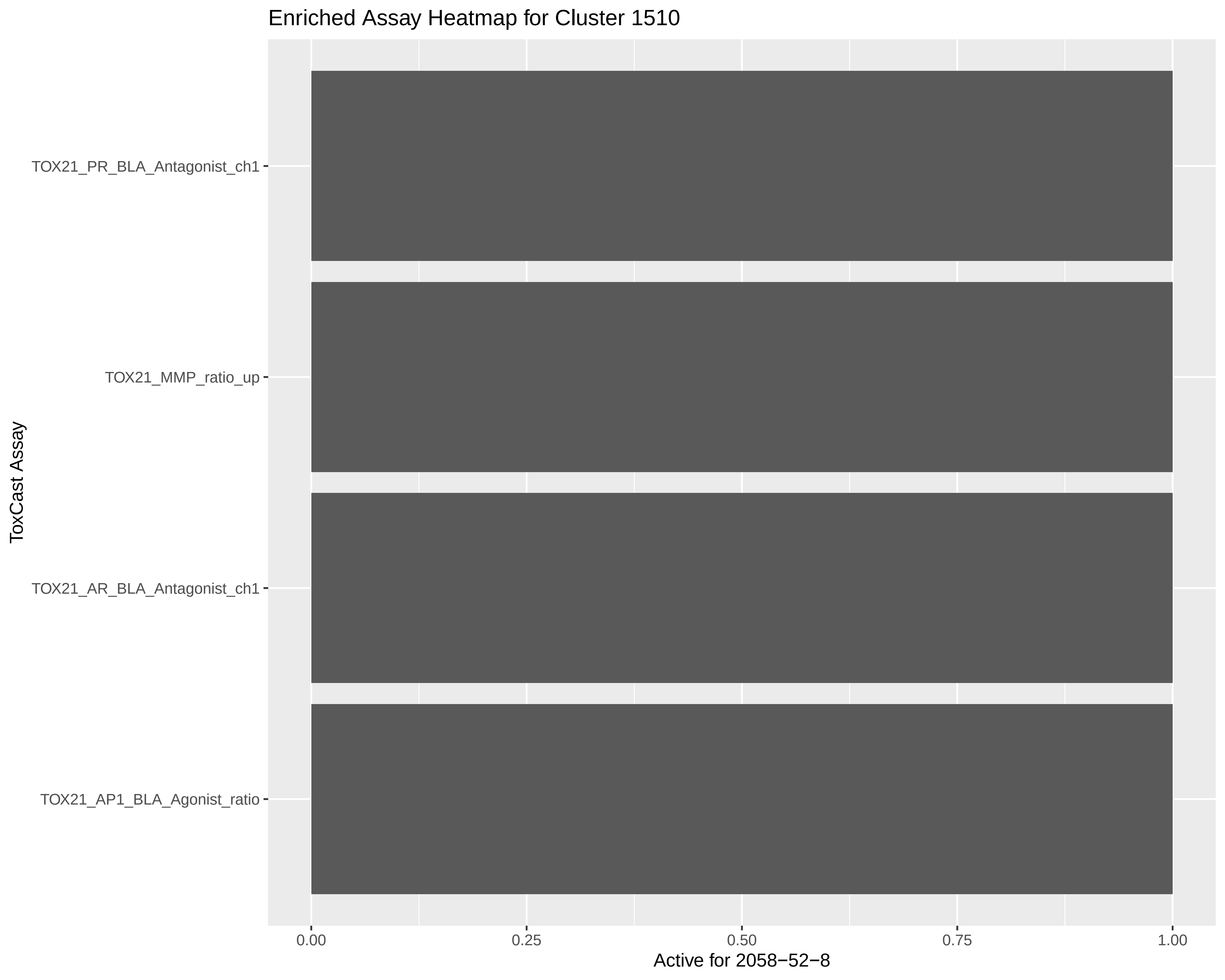

Enriched Assay Heatmap for Cluster 1510
ToxCast Assay
TOX21_PR_BLA_Antagonist_ch1
TOX21_MMP_ratio_up
TOX21_AR_BLA_Antagonist_ch1
TOX21_AP1_BLA_Agonist_ratio
0.00
0.25
0.50
0.75
1.00
Active for 2058−52−8

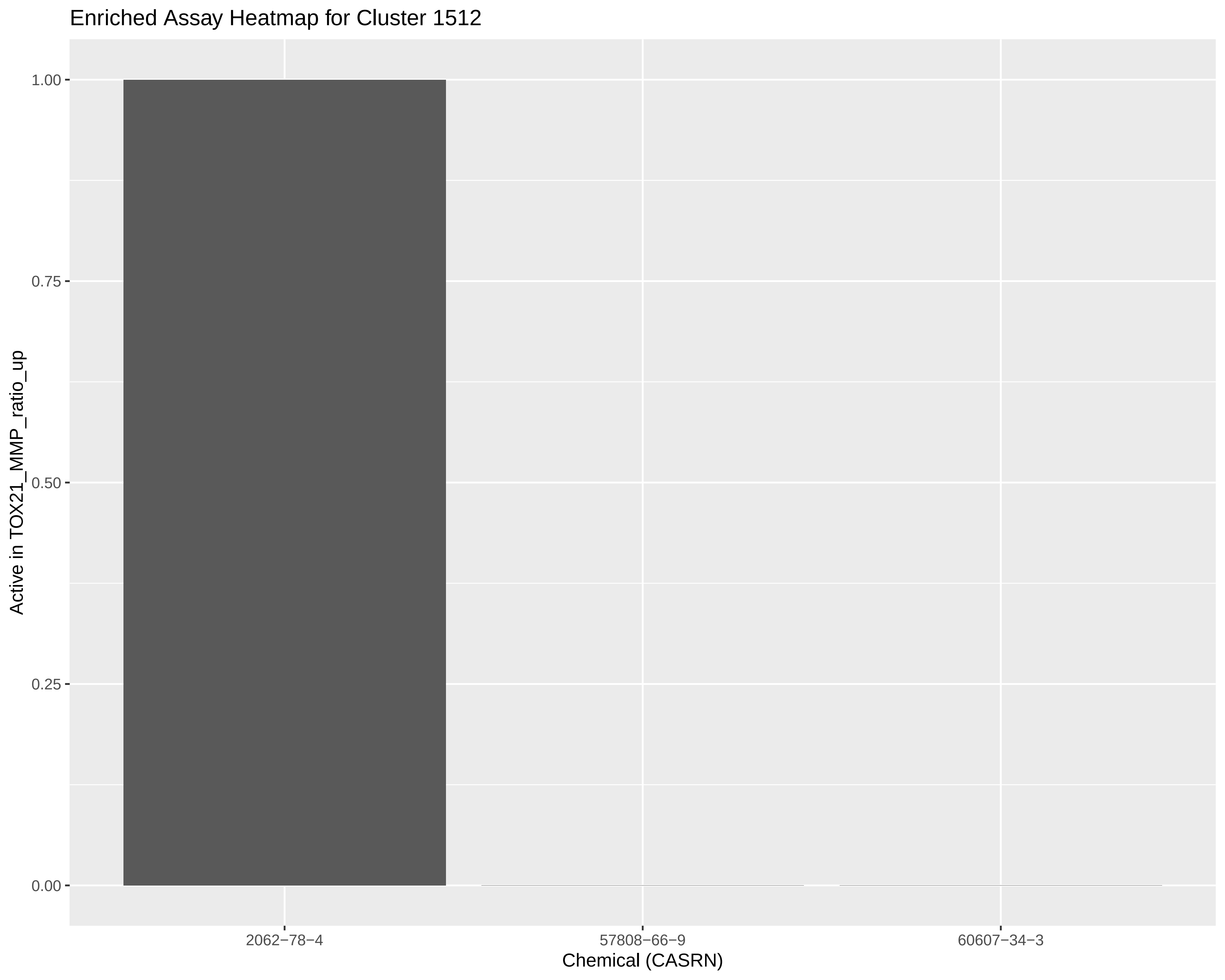

Enriched Assay Heatmap for Cluster 1512
Active in TOX21_MMP_ratio_up
1.00
0.75
0.50
0.25
0.00
2062−78−4
57808−66−9
60607−34−3
Chemical (CASRN)

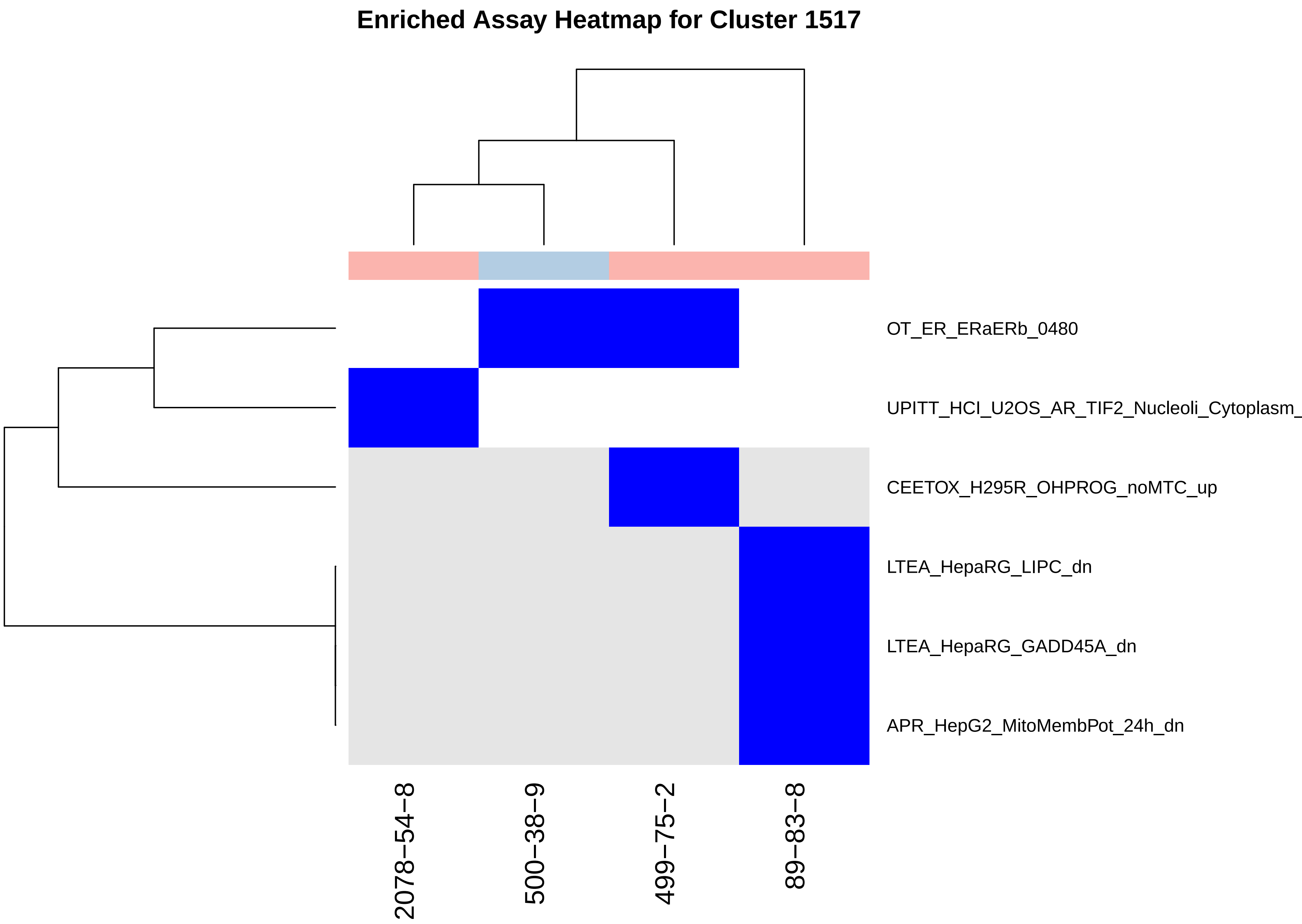
Enriched Assay Heatmap for Cluster 1517
OT_ER_ERaERb_0480
UPITT_HCI_U2OS_AR_TIF2_Nucleoli_Cytoplasm_
CEETOX_H295R_OHPROG_noMTC_up
LTEA_HepaRG_LIPC_dn
LTEA_HepaRG_GADD45A_dn
APR_HepG2_MitoMembPot_24h_dn
2078−54−8
500−38−9
499−75−2
89−83−8

Enriched Assay Heatmap for Cluster 1519

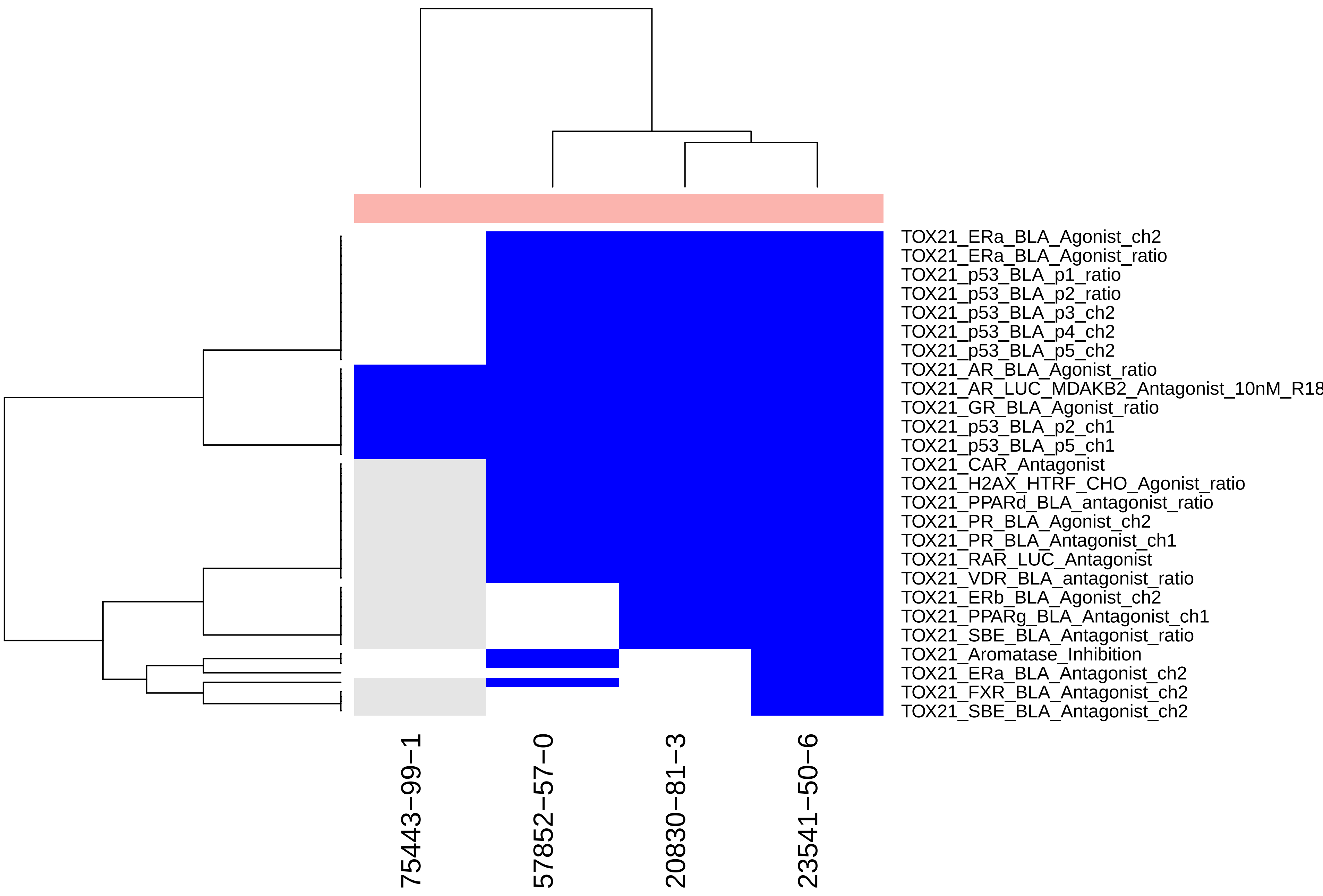

Enriched Assay Heatmap for Cluster 1521

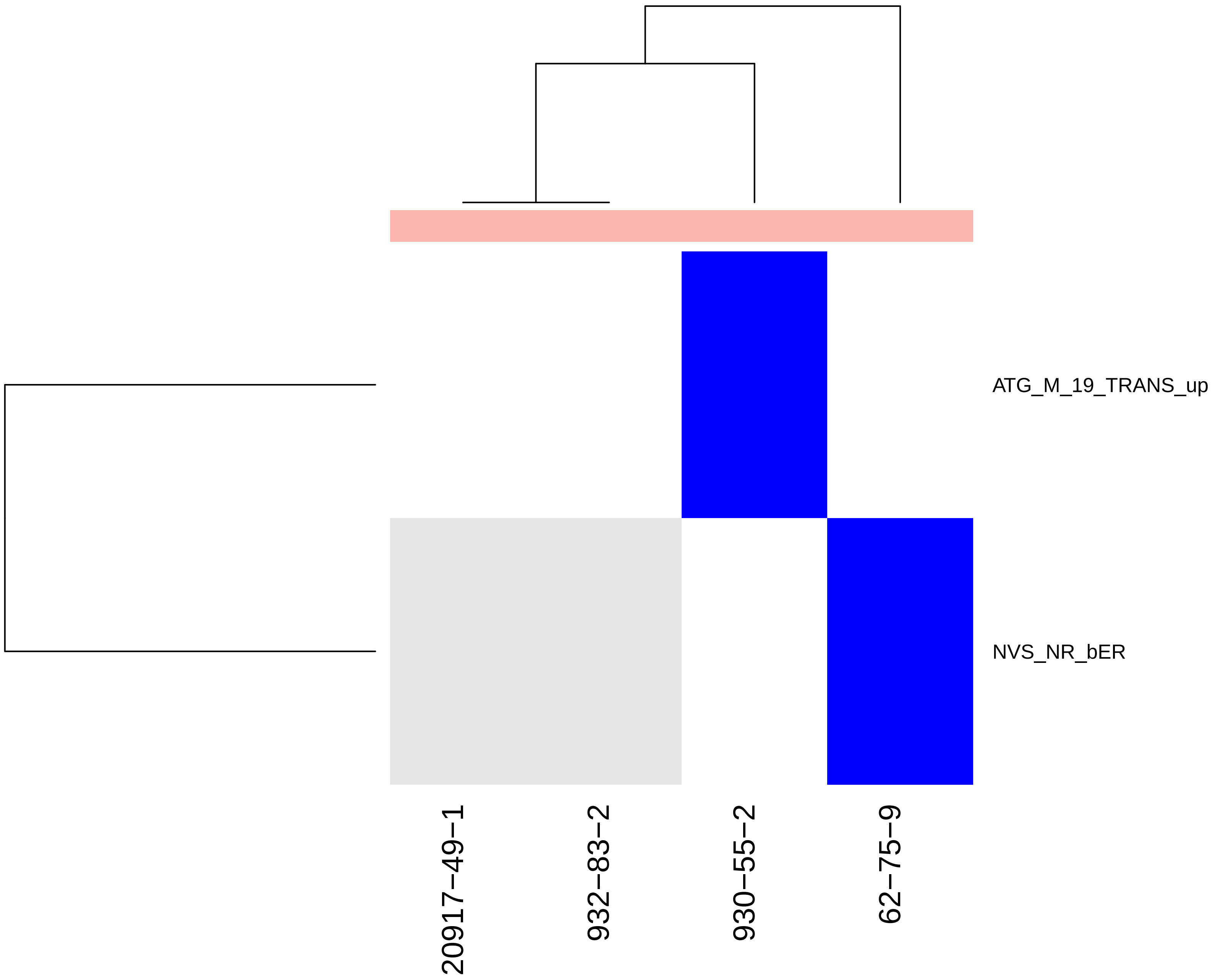

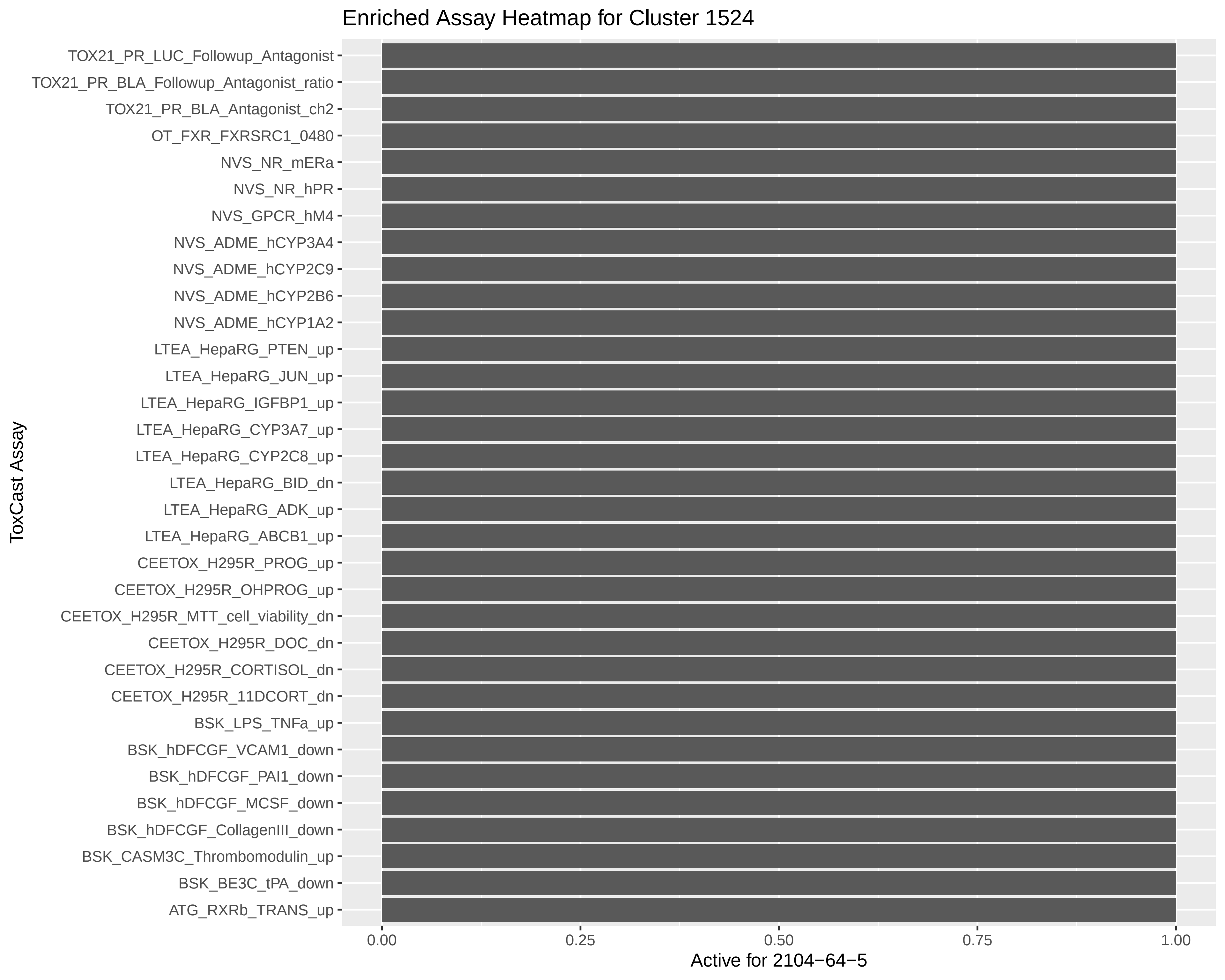

Enriched Assay Heatmap for Cluster 1524
ToxCast Assay
TOX21_PR_LUC_Followup_Antagonist
TOX21_PR_BLA_Followup_Antagonist_ratio
TOX21_PR_BLA_Antagonist_ch2
OT_FXR_FXRSRC1_0480
NVS_NR_mERa
NVS_NR_hPR
NVS_GPCR_hM4
NVS_ADME_hCYP3A4
NVS_ADME_hCYP2C9
NVS_ADME_hCYP2B6
NVS_ADME_hCYP1A2
LTEA_HepaRG_PTEN_up
LTEA_HepaRG_JUN_up
LTEA_HepaRG_IGFBP1_up
LTEA_HepaRG_CYP3A7_up
LTEA_HepaRG_CYP2C8_up
LTEA_HepaRG_BID_dn
LTEA_HepaRG_ADK_up
LTEA_HepaRG_ABCB1_up
CEETOX_H295R_PROG_up
CEETOX_H295R_OHPROG_up
CEETOX_H295R_MTT_cell_viability_dn
CEETOX_H295R_DOC_dn
CEETOX_H295R_CORTISOL_dn
CEETOX_H295R_11DCORT_dn
BSK_LPS_TNFa_up
BSK_hDFCGF_VCAM1_down
BSK_hDFCGF_PAI1_down
BSK_hDFCGF_MCSF_down
BSK_hDFCGF_CollagenIII_down
BSK_CASM3C_Thrombomodulin_up
BSK_BE3C_tPA_down
ATG_RXRb_TRANS_up
0.00
0.25
0.50
0.75
1.00
Active for 2104−64−5

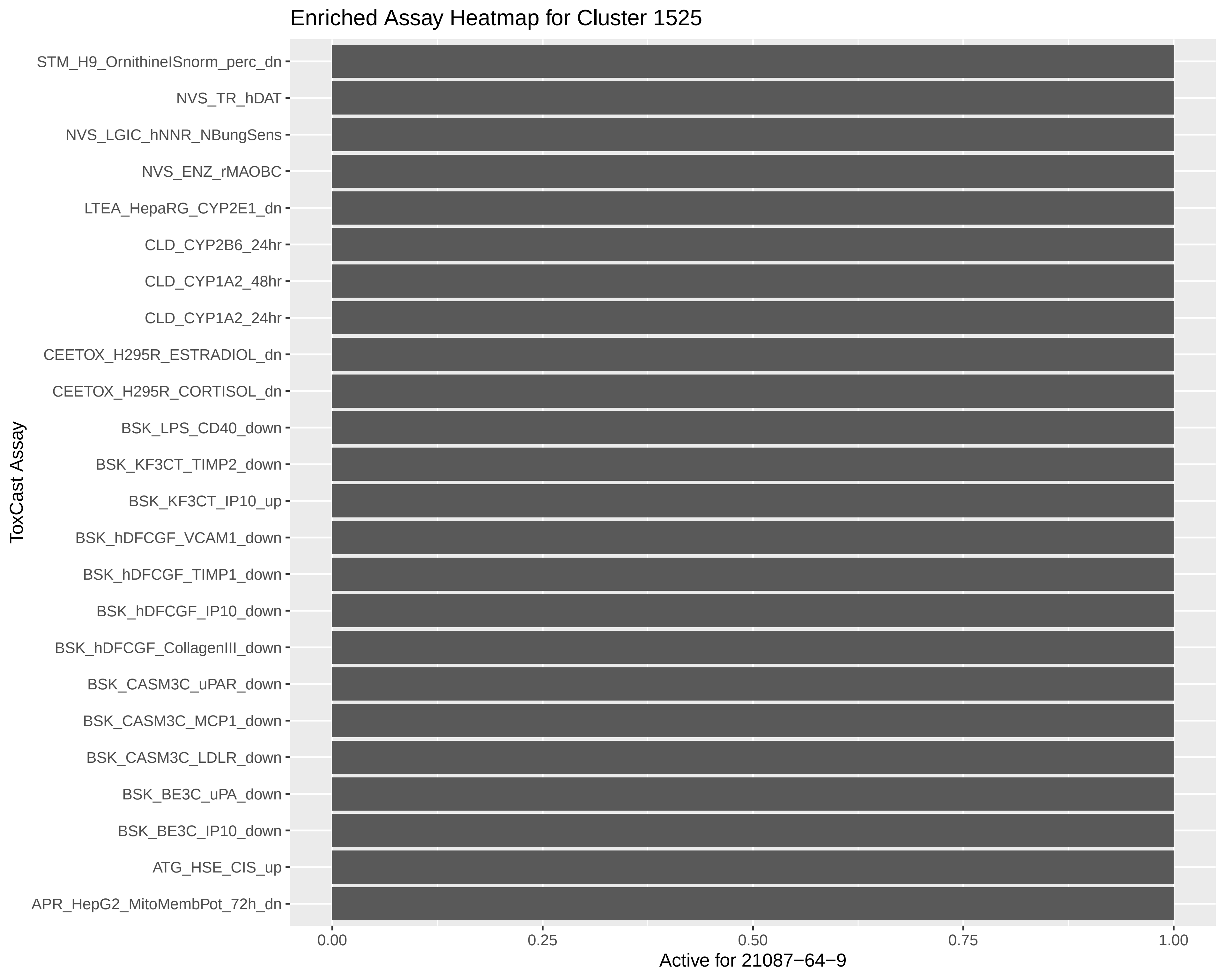

Enriched Assay Heatmap for Cluster 1525
STM_H9_OrnithineISnorm_perc_dn
NVS_TR_hDAT
NVS_LGIC_hNNR_NBungSens
NVS_ENZ_rMAOBC
LTEA_HepaRG_CYP2E1_dn
CLD_CYP2B6_24hr
CLD_CYP1A2_48hr
CLD_CYP1A2_24hr
CEETOX_H295R_ESTRADIOL_dn
CEETOX_H295R_CORTISOL_dn
BSK_LPS_CD40_down
BSK_KF3CT_TIMP2_down
BSK_KF3CT_IP10_up
BSK_hDFCGF_VCAM1_down
BSK_hDFCGF_TIMP1_down
BSK_hDFCGF_IP10_down
BSK_hDFCGF_CollagenIII_down
BSK_CASM3C_uPAR_down
BSK_CASM3C_MCP1_down
BSK_CASM3C_LDLR_down
BSK_BE3C_uPA_down
BSK_BE3C_IP10_down
ATG_HSE_CIS_up
APR_HepG2_MitoMembPot_72h_dn
ToxCast Assay
0.00
0.25
0.50
0.75
1.00
Active for 21087−64−9

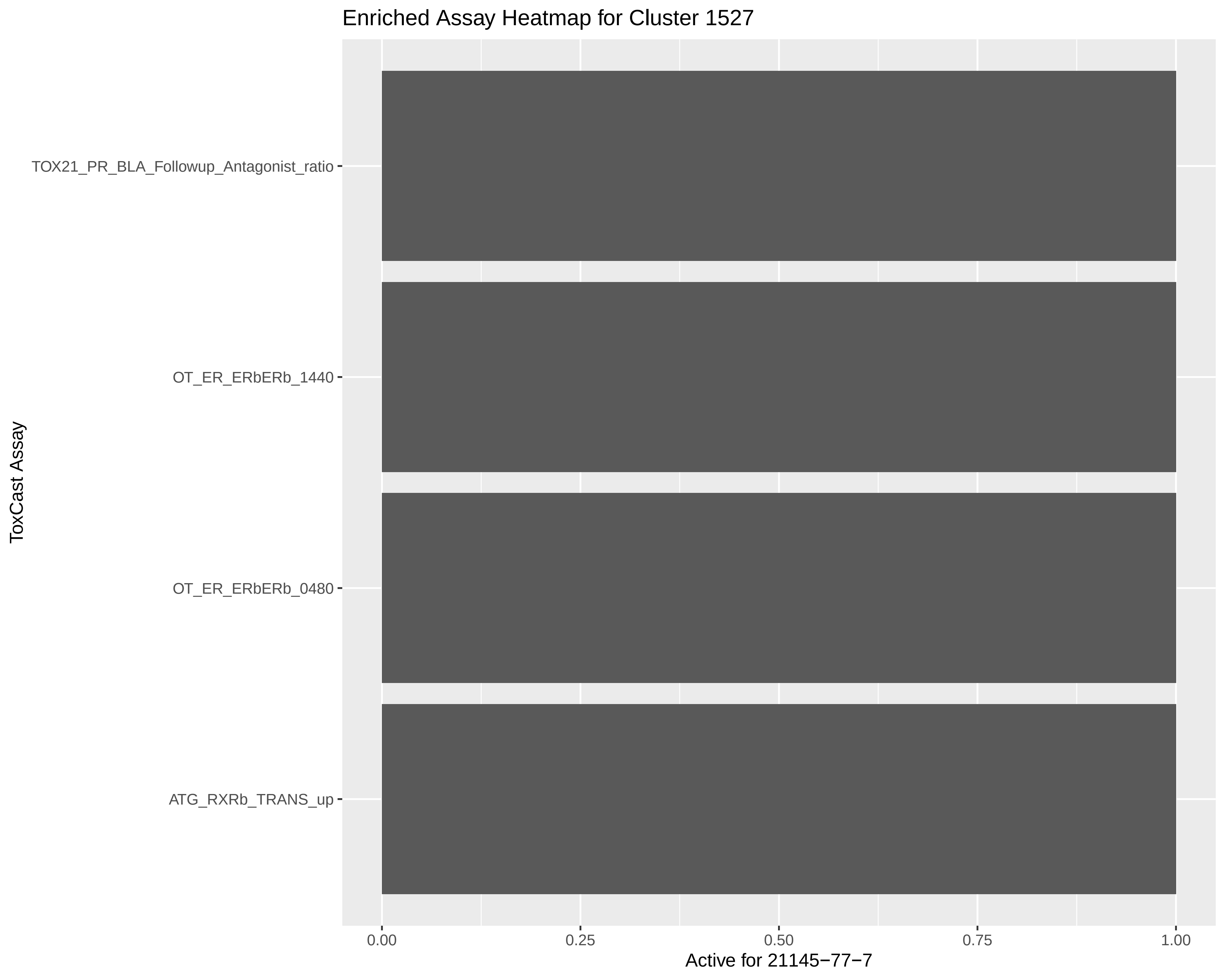

Enriched Assay Heatmap for Cluster 1527
TOX21_PR_BLA_Followup_Antagonist_ratio
OT_ER_ERbERb_1440
OT_ER_ERbERb_0480
ATG_RXRb_TRANS_up
ToxCast Assay
0.00
0.25
0.50
0.75
1.00
Active for 21145−77−7

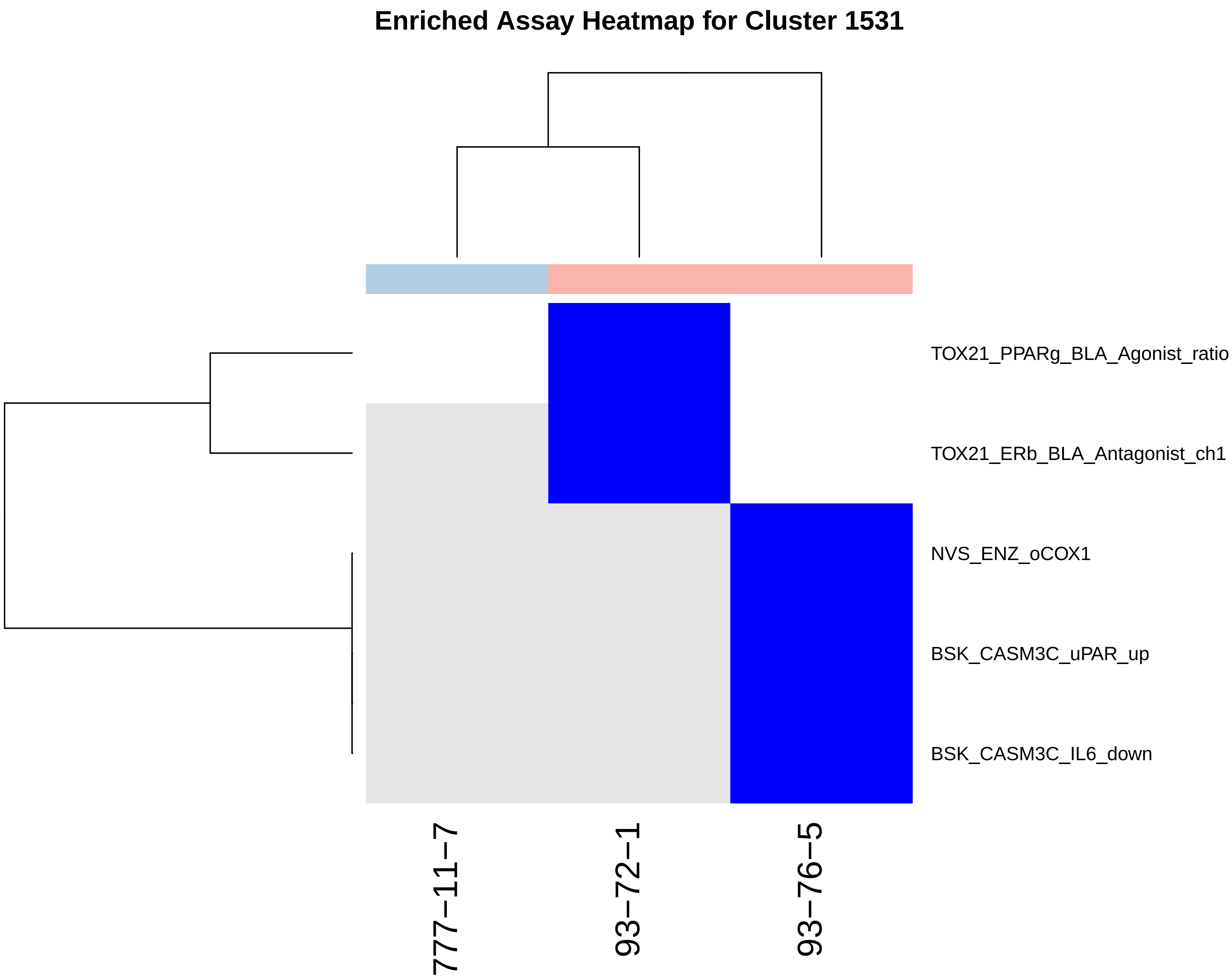

Enriched Assay Heatmap for Cluster 1531
TOX21_PPARg_BLA_Agonist_ratio
TOX21_ERb_BLA_Antagonist_ch1
NVS_ENZ_oCOX1
BSK_CASM3C_uPAR_up
BSK_CASM3C_IL6_down
777−11−7
93−72−1
93−76−5

Enriched Assay Heatmap for Cluster 1540

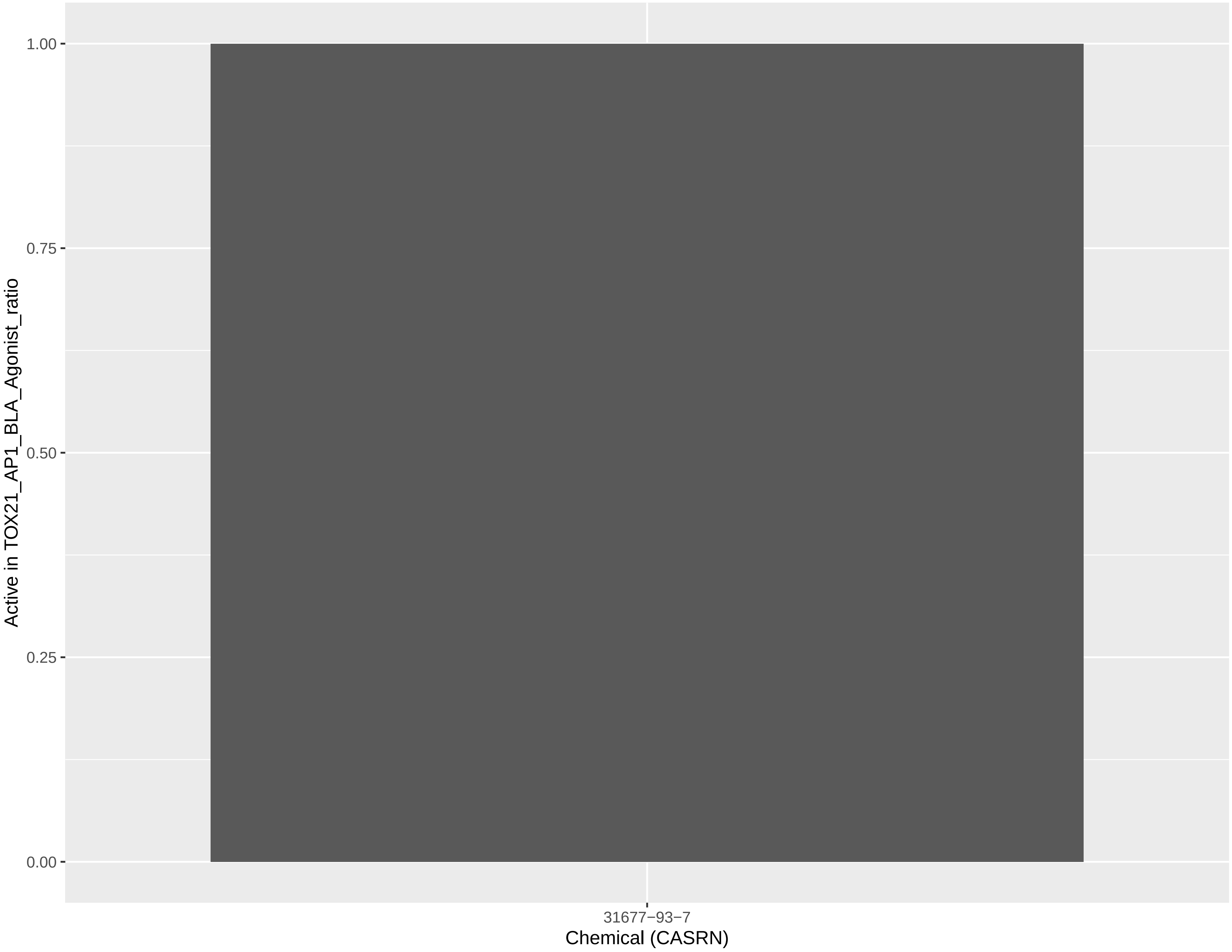

Enriched Assay Heatmap for Cluster 1541

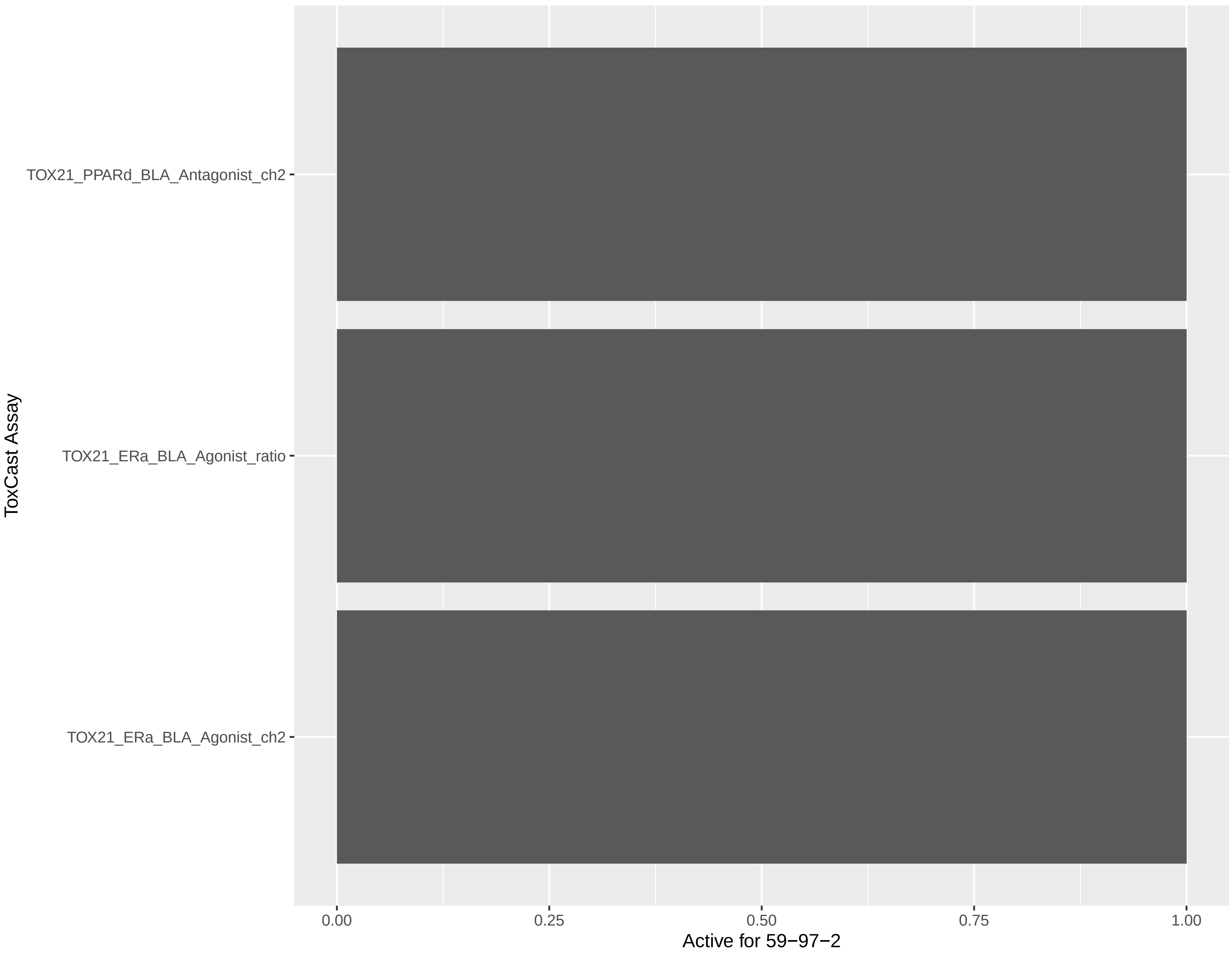

Enriched Assay Heatmap for Cluster 1553

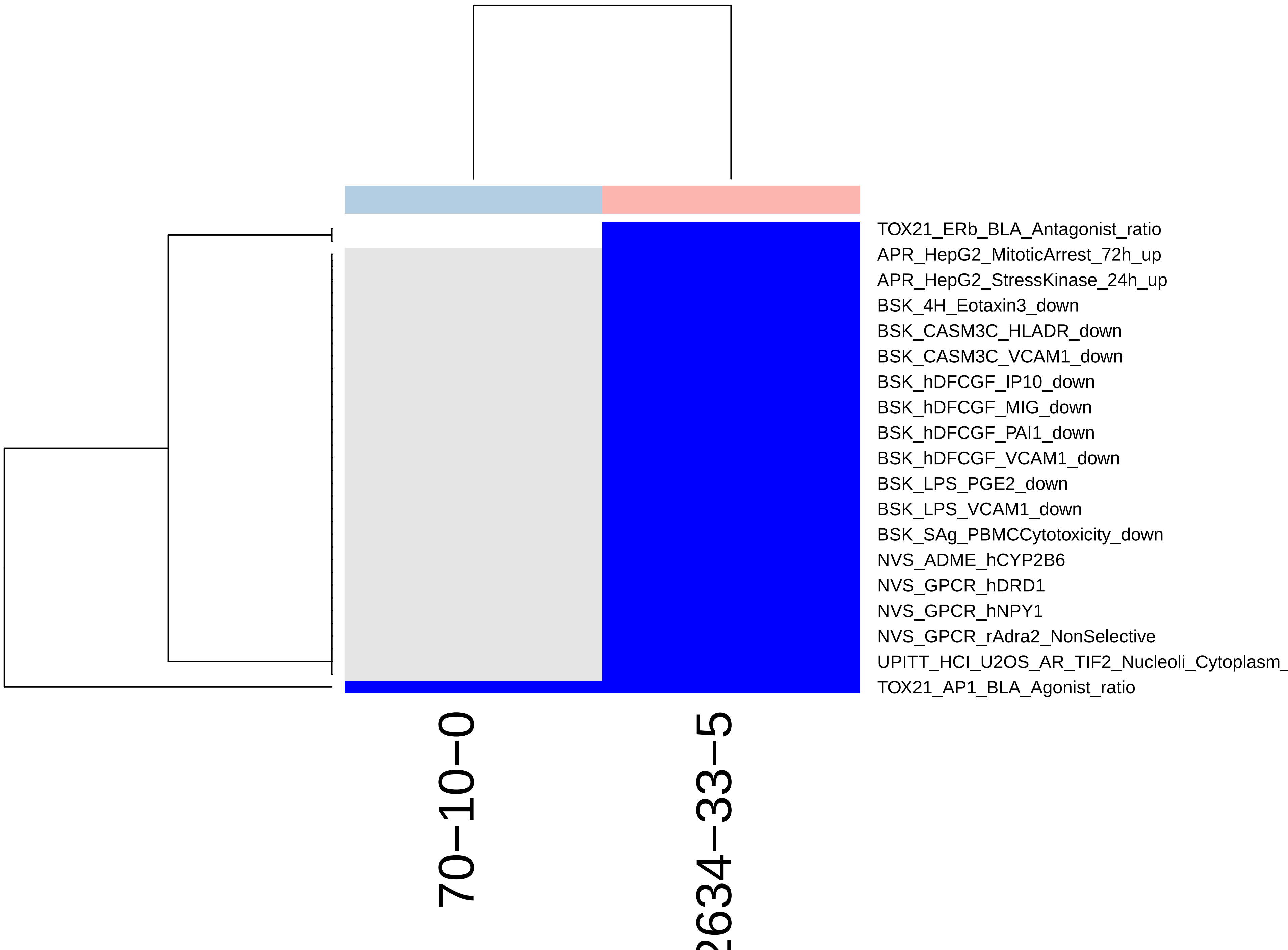

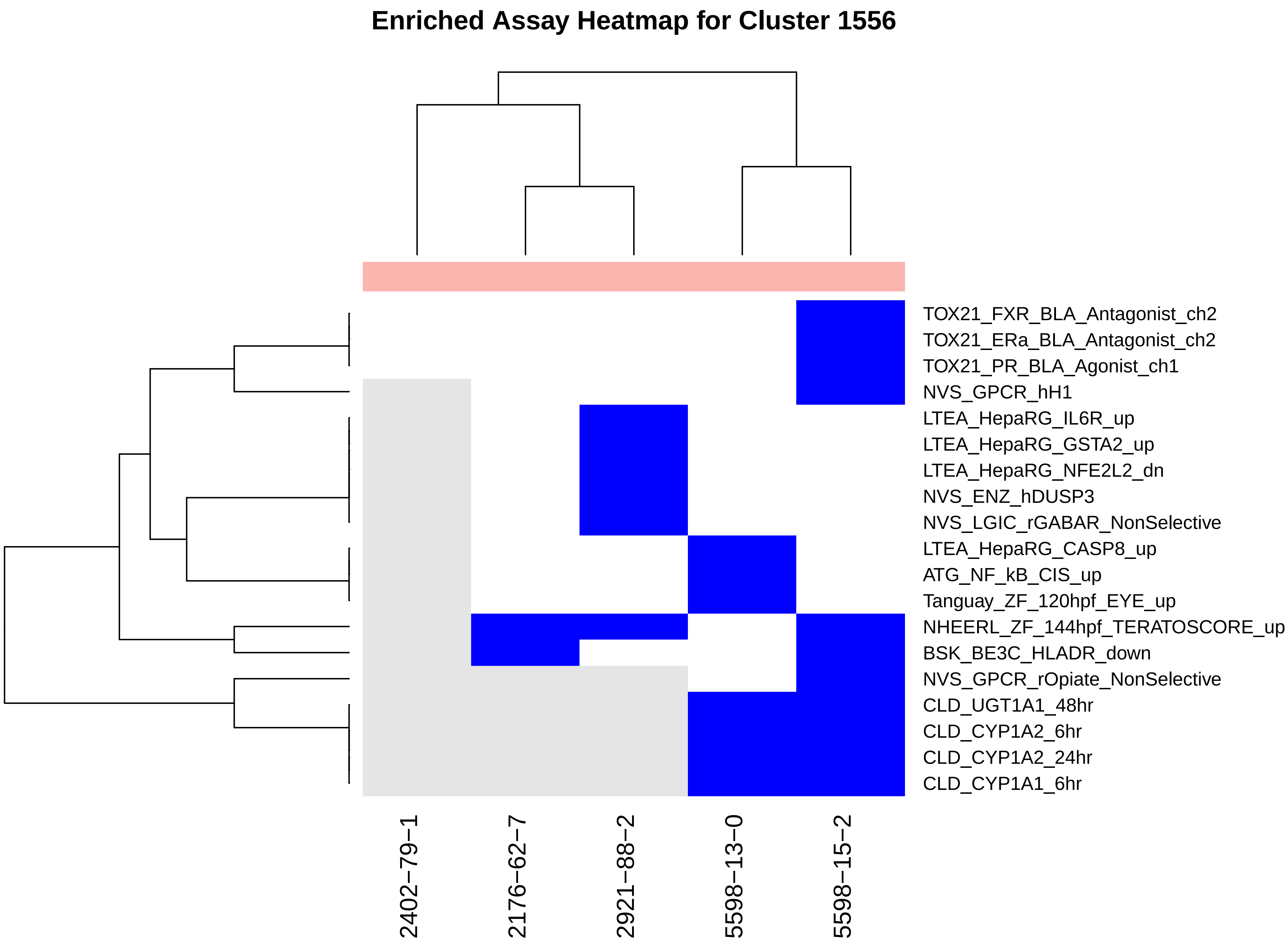
Enriched Assay Heatmap for Cluster 1556
TOX21_FXR_BLA_Antagonist_ch2
TOX21_ERa_BLA_Antagonist_ch2
TOX21_PR_BLA_Agonist_ch1
NVS_GPCR_hH1
LTEA_HepaRG_IL6R_up
LTEA_HepaRG_GSTA2_up
LTEA_HepaRG_NFE2L2_dn
NVS_ENZ_hDUSP3
NVS_LGIC_rGABAR_NonSelective
LTEA_HepaRG_CASP8_up
ATG_NF_kB_CIS_up
Tanguay_ZF_120hpf_EYE_up
NHEERL_ZF_144hpf_TERATOSCORE_up
BSK_BE3C_HLADR_down
NVS_GPCR_rOpiate_NonSelective
CLD_UGT1A1_48hr
CLD_CYP1A2_6hr
CLD_CYP1A2_24hr
CLD_CYP1A1_6hr
2402−79−1
2176−62−7
2921−88−2
5598−13−0
5598−15−2

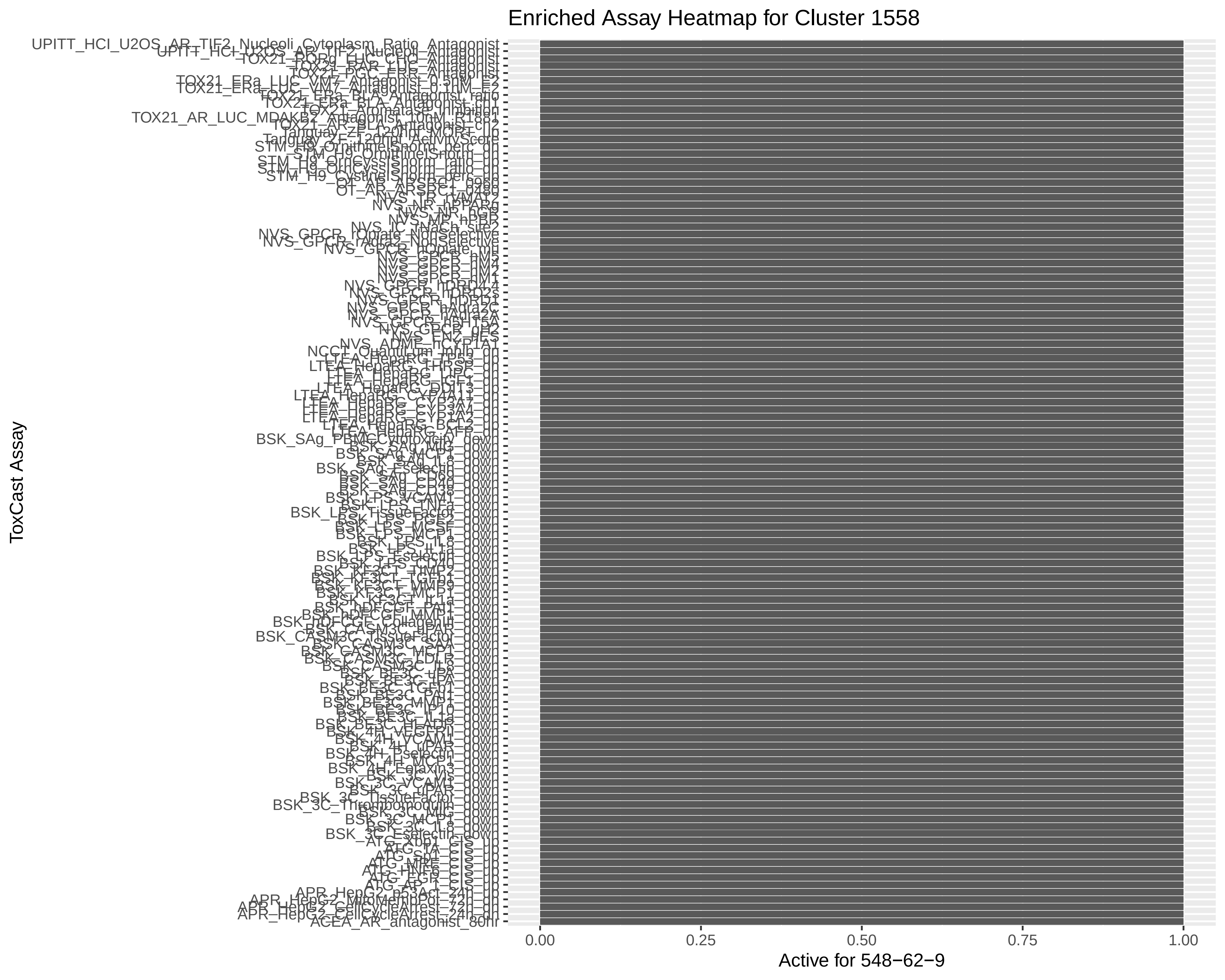

Enriched Assay Heatmap for Cluster 1558
ToxCast Assay
Active for 548−62−9
0.00
0.25
0.50
0.75
1.00
UPITT_HCI_U2OS_AR_TIF2_Nucleoli_Cytoplasm_Ratio_Antagonist
TOX21_AR_LUC_MDAKB2_Antagonist_0.5nM_R1881
NVS_GPCR_rOpiate_NonSelective
BSK_SAg_PBMCCytotoxicity_down
BSK_LPS_TissueFactor_down
BSK_CASM3C_TissueFactor_down
BSK_3C_Thrombomodulin_down
ACEA_AR_antagonist_80hr

Enriched Assay Heatmap for Cluster 1563

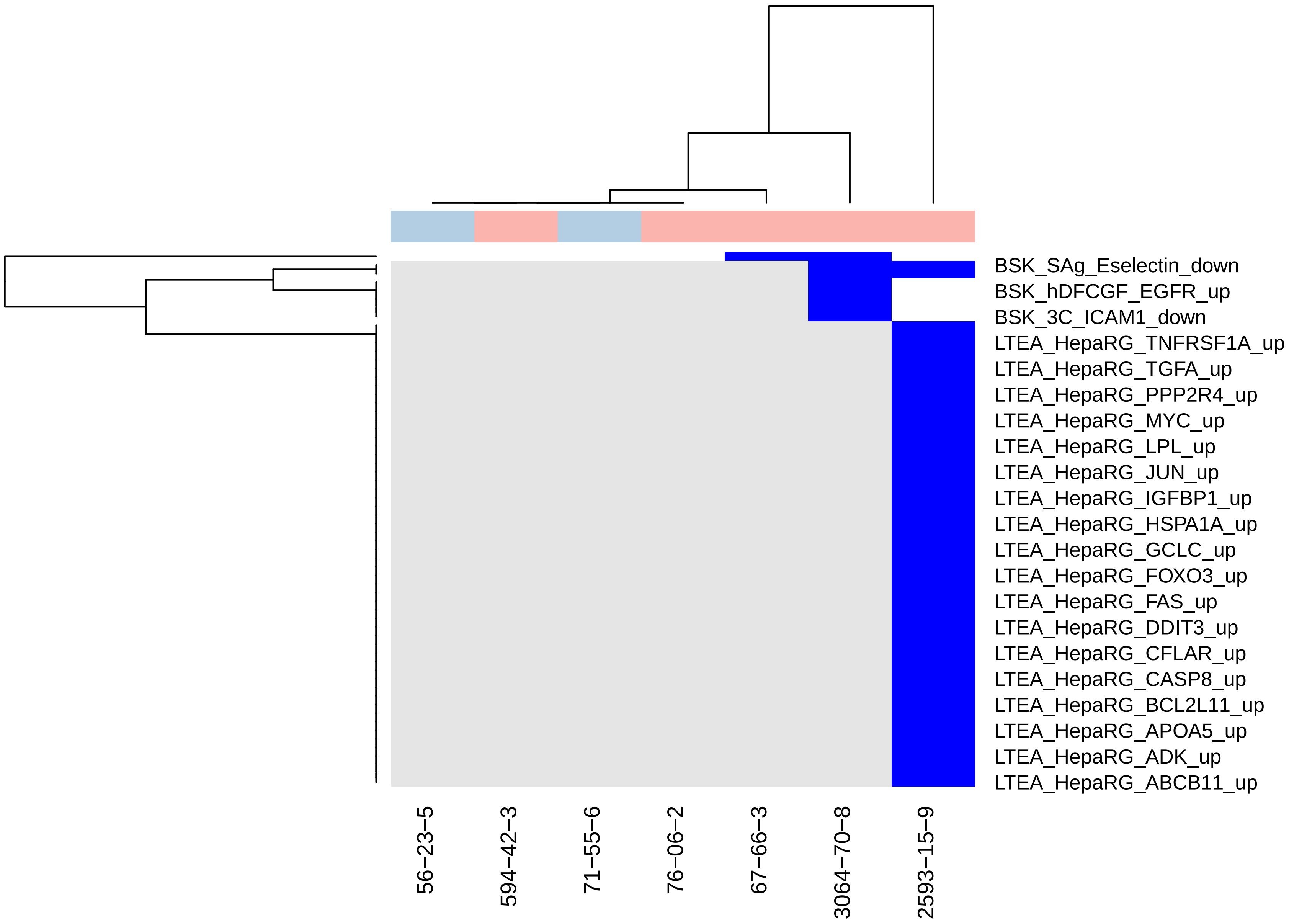

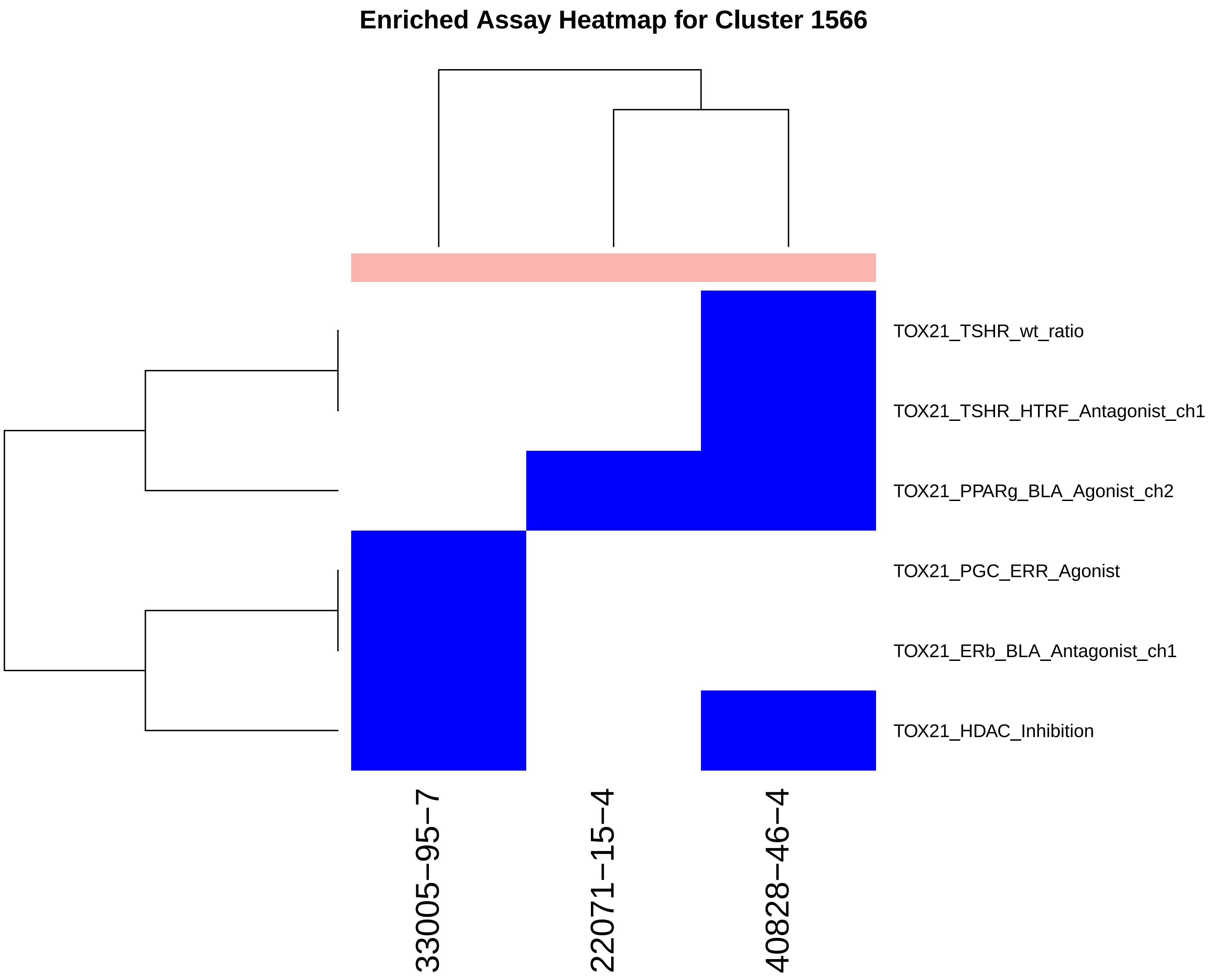

Enriched Assay Heatmap for Cluster 1566
TOX21_TSHR_wt_ratio
TOX21_TSHR_HTRF_Antagonist_ch1
TOX21_PPARg_BLA_Agonist_ch2
TOX21_PGC_ERR_Agonist
TOX21_ERb_BLA_Antagonist_ch1
TOX21_HDAC_Inhibition
33005−95−7
22071−15−4
40828−46−4

Enriched Assay Heatmap for Cluster 1567

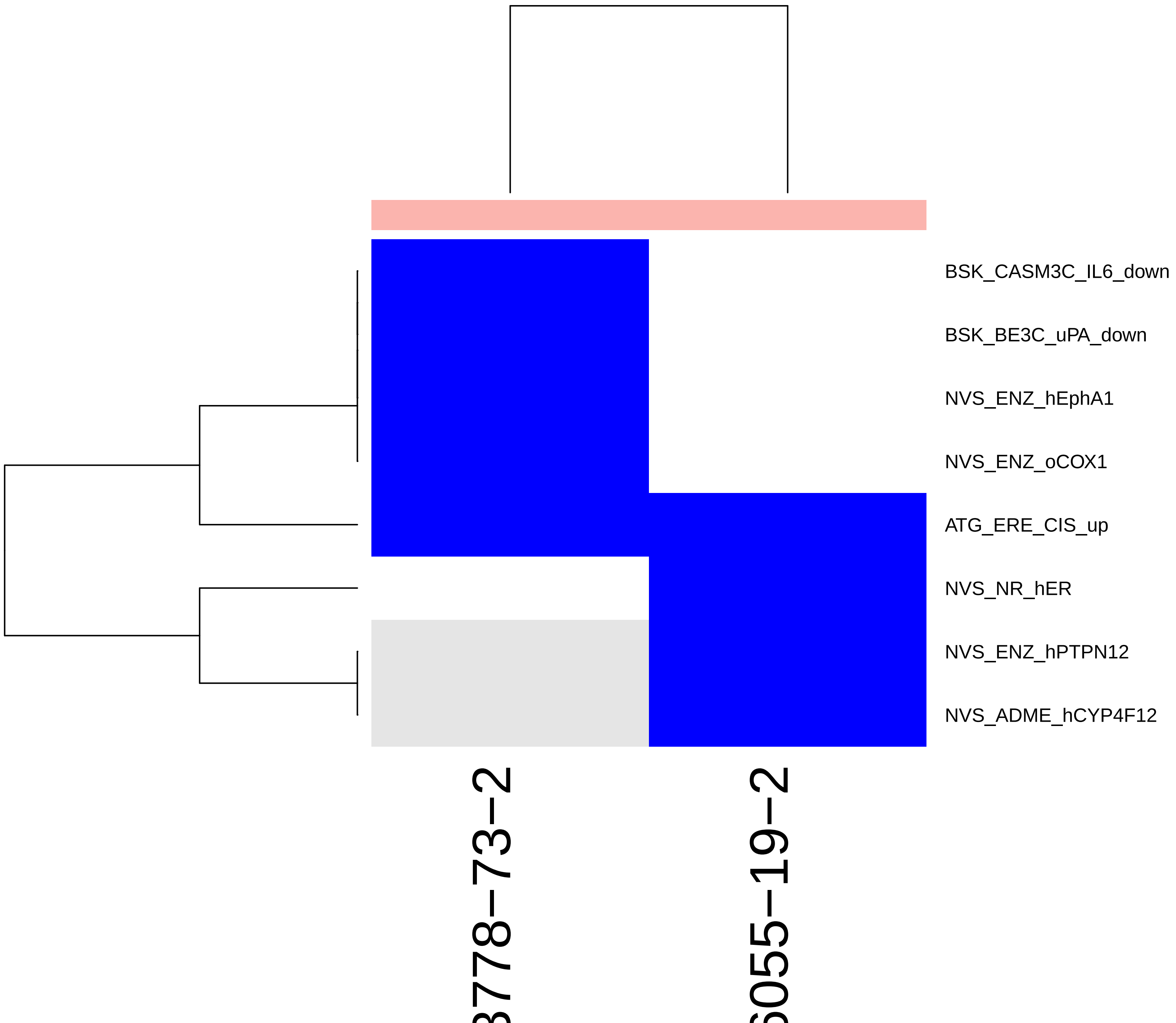

Enriched Assay Heatmap for Cluster 1568

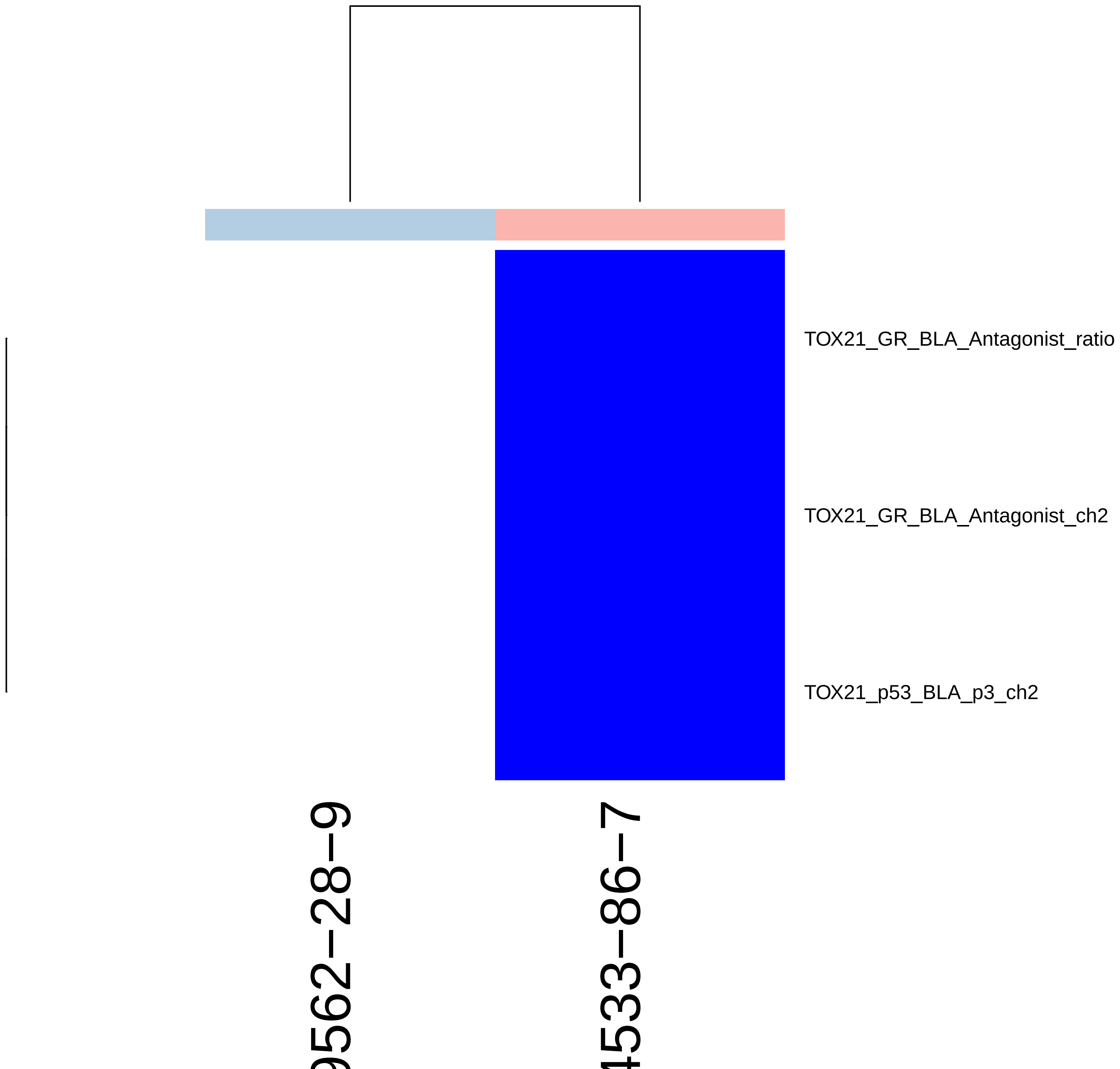

Enriched Assay Heatmap for Cluster 1571

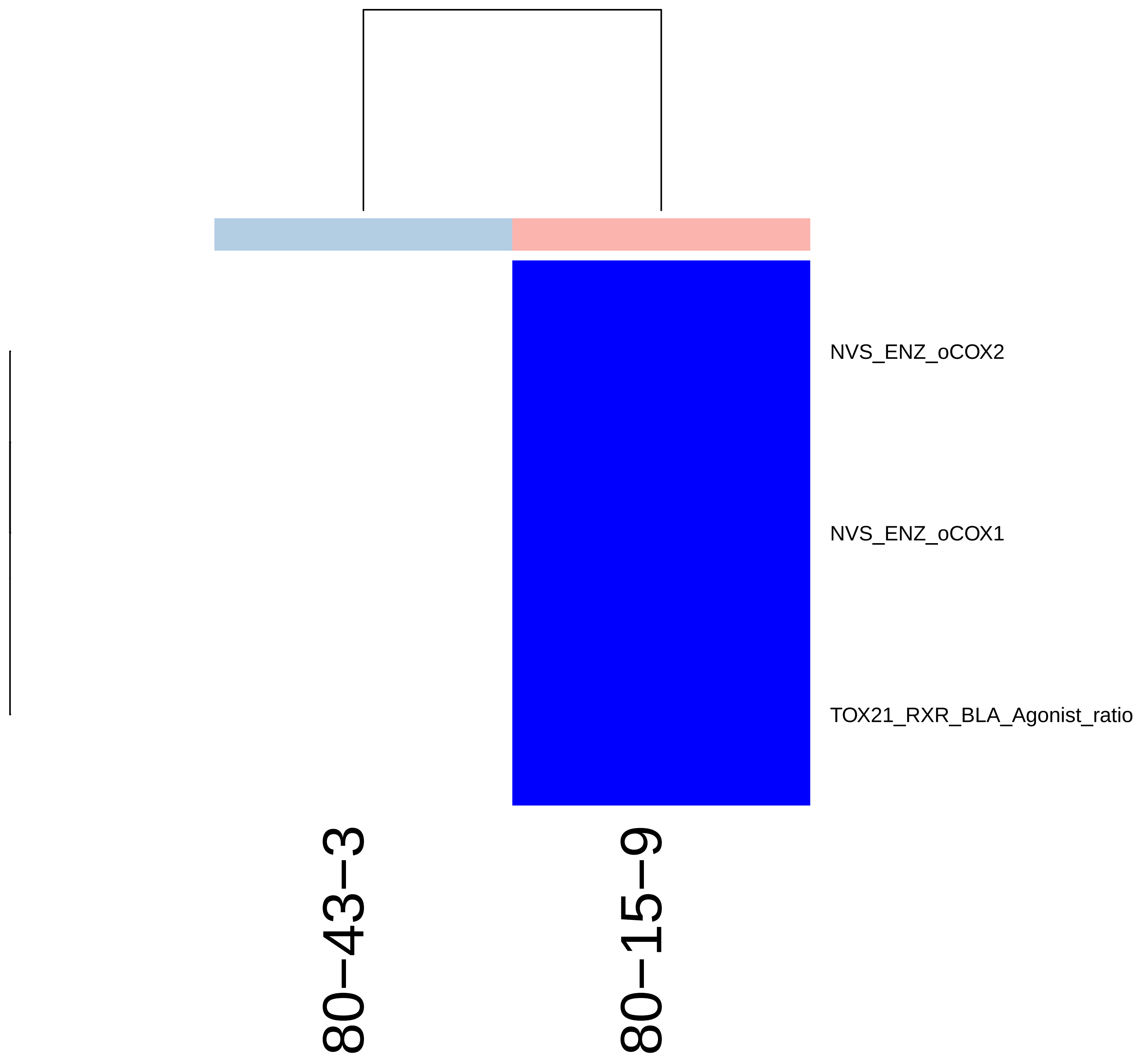

Enriched Assay Heatmap for Cluster 1573

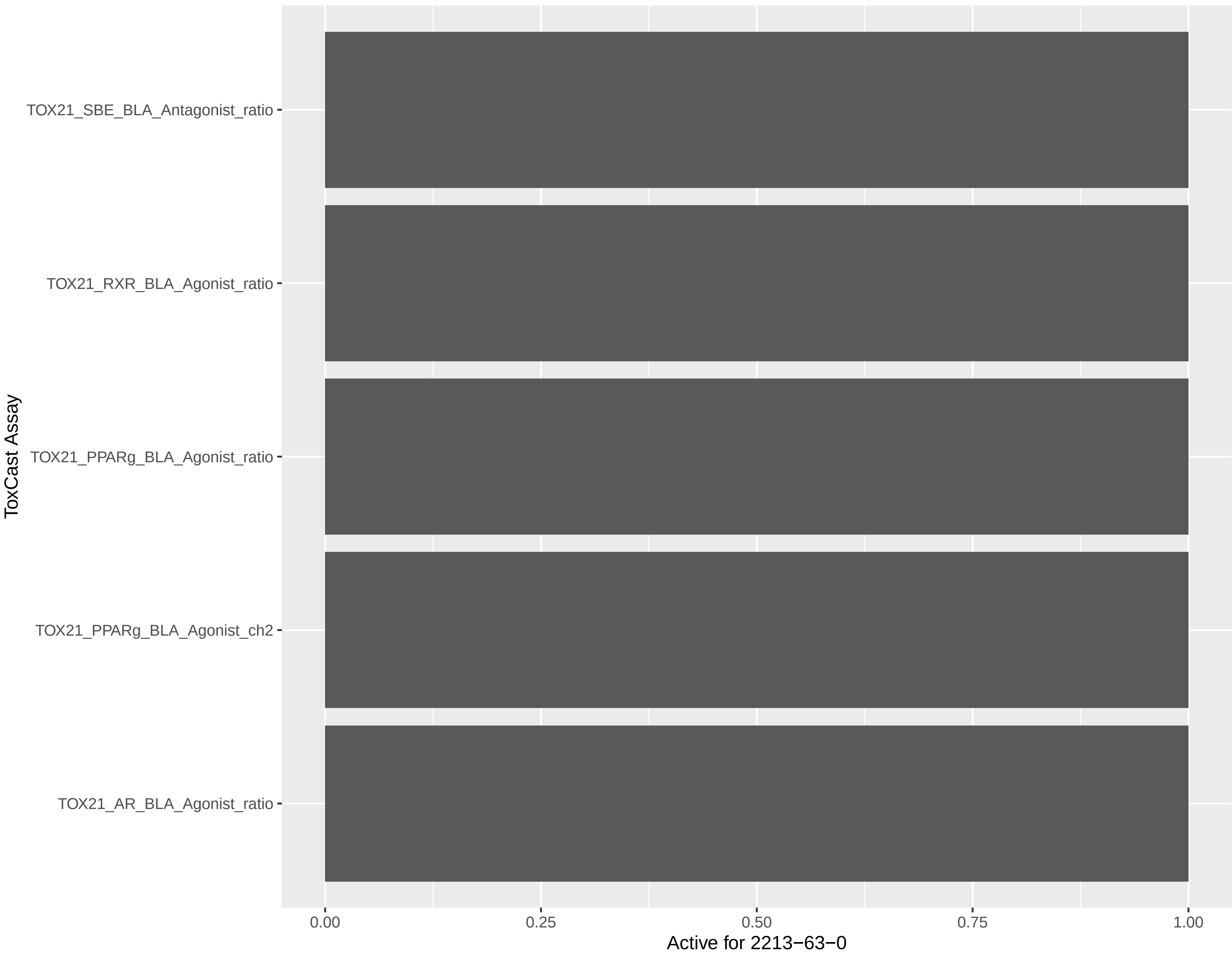

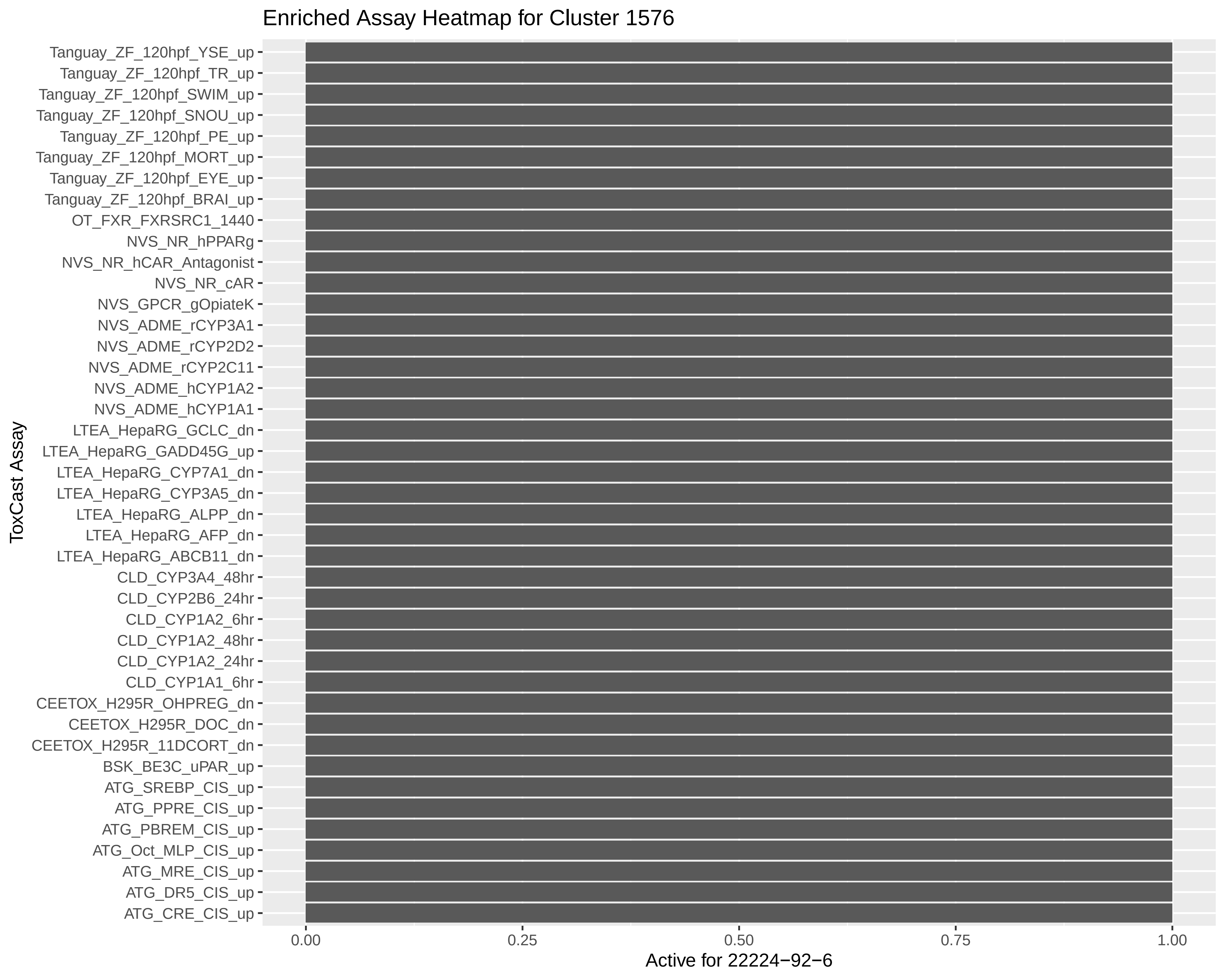

Enriched Assay Heatmap for Cluster 1576
ToxCast Assay
Tanguay_ZF_120hpf_YSE_up
Tanguay_ZF_120hpf_TR_up
Tanguay_ZF_120hpf_SWIM_up
Tanguay_ZF_120hpf_SNOU_up
Tanguay_ZF_120hpf_PE_up
Tanguay_ZF_120hpf_MORT_up
Tanguay_ZF_120hpf_EYE_up
Tanguay_ZF_120hpf_BRAI_up
OT_FXR_FXRSRC1_1440
NVS_NR_hPPARg
NVS_NR_hCAR_Antagonist
NVS_NR_cAR
NVS_GPCR_gOpiateK
NVS_ADME_rCYP3A1
NVS_ADME_rCYP2D2
NVS_ADME_rCYP2C11
NVS_ADME_hCYP1A2
NVS_ADME_hCYP1A1
LTEA_HepaRG_GCLC_dn
LTEA_HepaRG_GADD45G_up
LTEA_HepaRG_CYP7A1_dn
LTEA_HepaRG_CYP3A5_dn
LTEA_HepaRG_ALPP_dn
LTEA_HepaRG_AFP_dn
LTEA_HepaRG_ABCB11_dn
CLD_CYP3A4_48hr
CLD_CYP2B6_24hr
CLD_CYP1A2_6hr
CLD_CYP1A2_48hr
CLD_CYP1A2_24hr
CLD_CYP1A1_6hr
CEETOX_H295R_OHPREG_dn
CEETOX_H295R_DOC_dn
CEETOX_H295R_11DCORT_dn
BSK_BE3C_uPAR_up
ATG_SREBP_CIS_up
ATG_PPRE_CIS_up
ATG_PBREM_CIS_up
ATG_Oct_MLP_CIS_up
ATG_MRE_CIS_up
ATG_DR5_CIS_up
ATG_CRE_CIS_up
0.00
0.25
0.50
0.75
1.00
Active for 22224−92−6

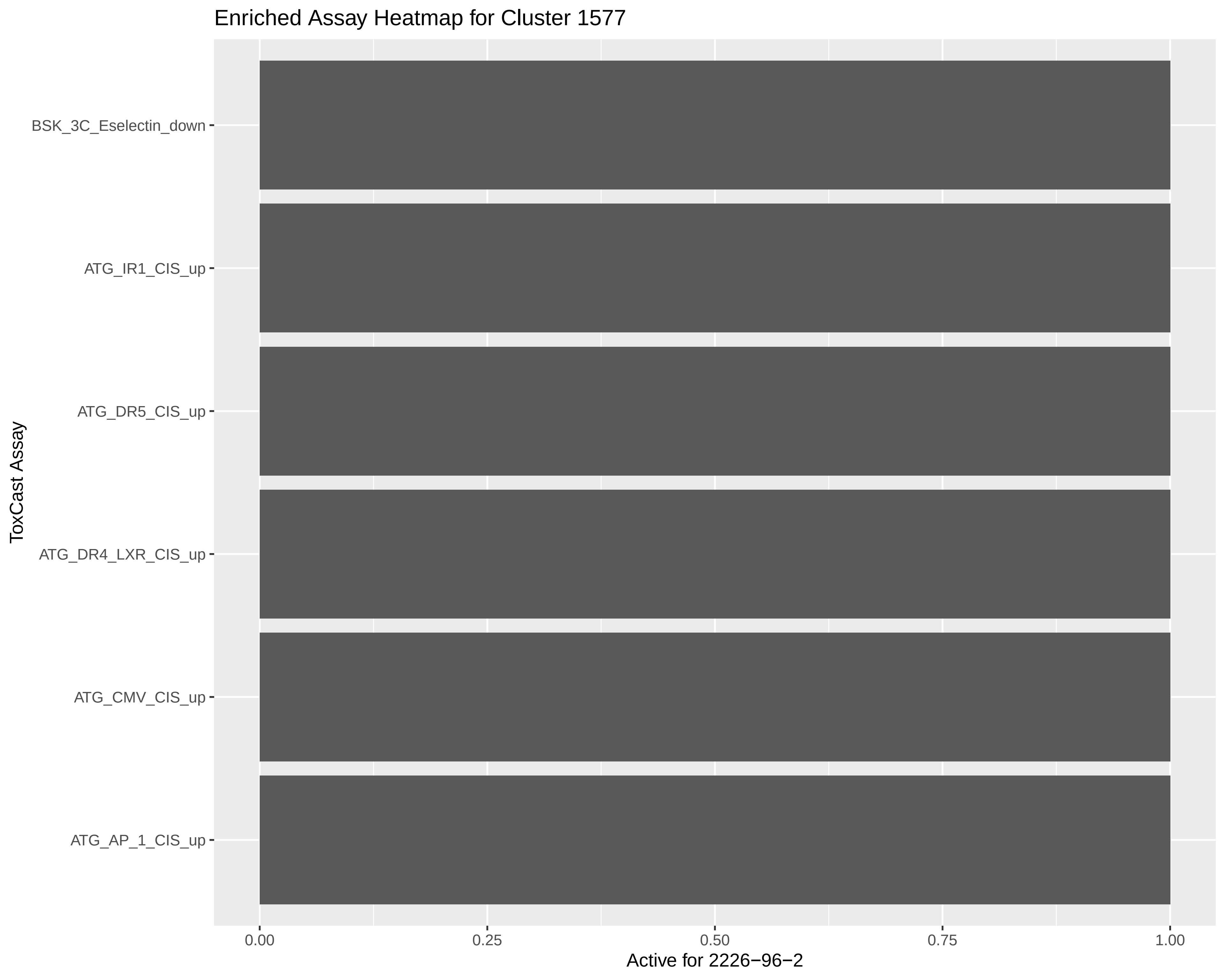

Enriched Assay Heatmap for Cluster 1577
ToxCast Assay
BSK_3C_Eselectin_down
ATG_IR1_CIS_up
ATG_DR5_CIS_up
ATG_DR4_LXR_CIS_up
ATG_CMV_CIS_up
ATG_AP_1_CIS_up
0.00
0.25
0.50
0.75
1.00
Active for 2226−96−2

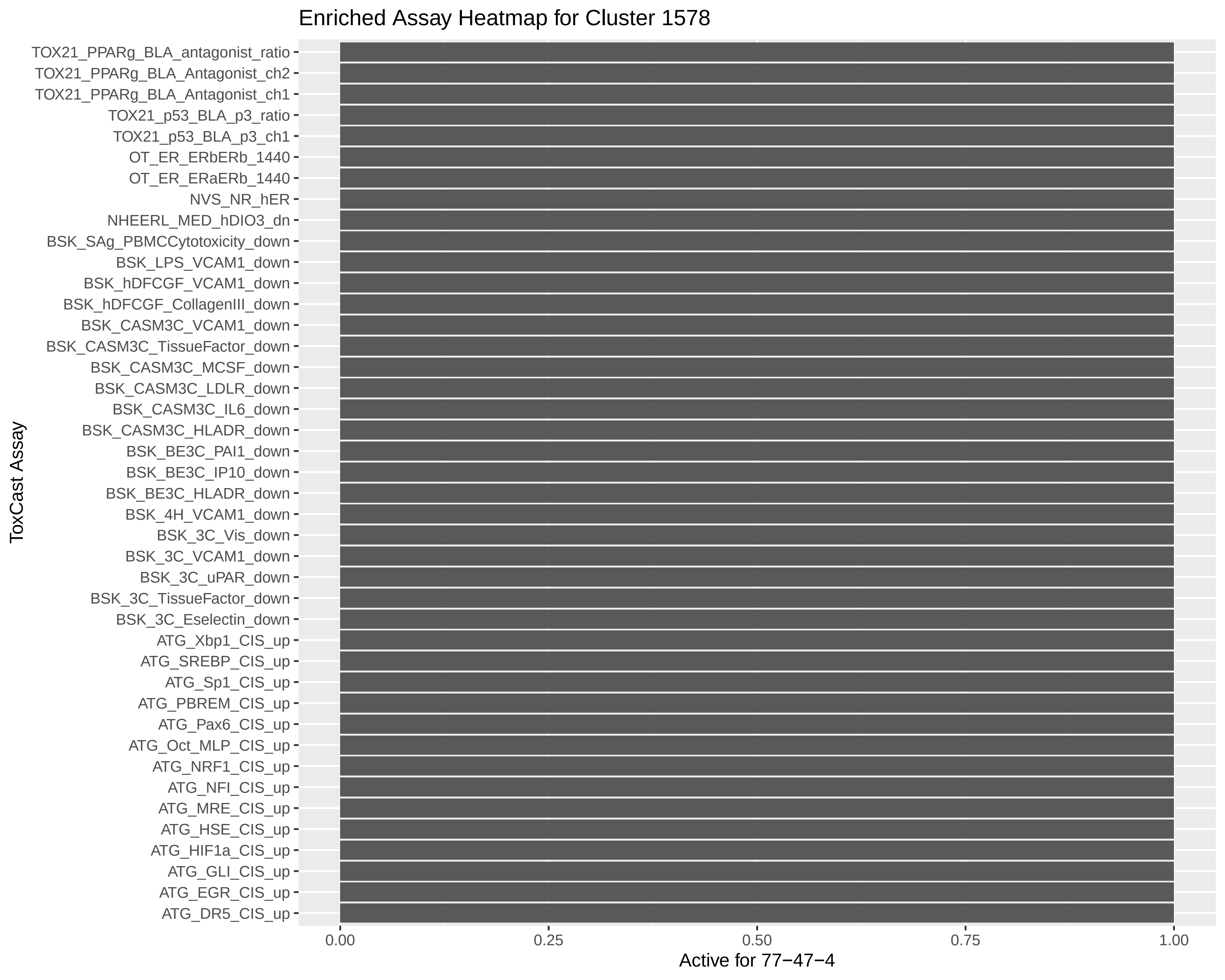

Enriched Assay Heatmap for Cluster 1578
ToxCast Assay
TOX21_PPARg_BLA_antagonist_ratio
TOX21_PPARg_BLA_Antagonist_ch2
TOX21_PPARg_BLA_Antagonist_ch1
TOX21_p53_BLA_p3_ratio
TOX21_p53_BLA_p3_ch1
OT_ER_ERbERb_1440
OT_ER_ERaERb_1440
NVS_NR_hER
NHEERL_MED_hDIO3_dn
BSK_SAg_PBMCCytotoxicity_down
BSK_LPS_VCAM1_down
BSK_hDFCGF_VCAM1_down
BSK_hDFCGF_CollagenIII_down
BSK_CASM3C_VCAM1_down
BSK_CASM3C_TissueFactor_down
BSK_CASM3C_MCSF_down
BSK_CASM3C_LDLR_down
BSK_CASM3C_IL6_down
BSK_CASM3C_HLADR_down
BSK_BE3C_PAI1_down
BSK_BE3C_IP10_down
BSK_BE3C_HLADR_down
BSK_4H_VCAM1_down
BSK_3C_Vis_down
BSK_3C_VCAM1_down
BSK_3C_uPAR_down
BSK_3C_TissueFactor_down
BSK_3C_Eselectin_down
ATG_Xbp1_CIS_up
ATG_SREBP_CIS_up
ATG_Sp1_CIS_up
ATG_PBREM_CIS_up
ATG_Pax6_CIS_up
ATG_Oct_MLP_CIS_up
ATG_NRF1_CIS_up
ATG_NFI_CIS_up
ATG_MRE_CIS_up
ATG_HSE_CIS_up
ATG_HIF1a_CIS_up
ATG_GLI_CIS_up
ATG_EGR_CIS_up
ATG_DR5_CIS_up
0.00
0.25
0.50
0.75
1.00
Active for 77−47−4

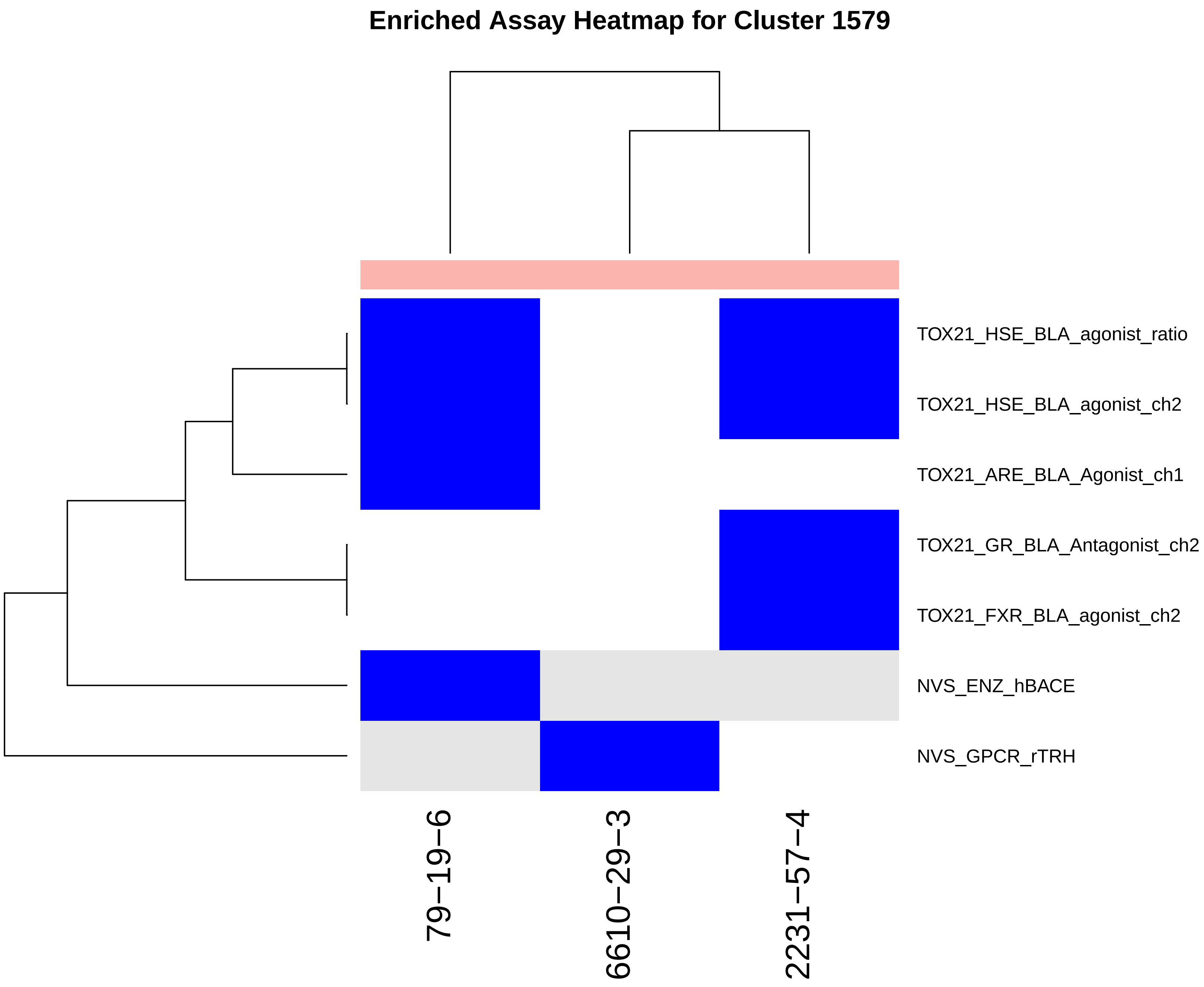

Enriched Assay Heatmap for Cluster 1579
TOX21_HSE_BLA_agonist_ratio
TOX21_HSE_BLA_agonist_ch2
TOX21_ARE_BLA_Agonist_ch1
TOX21_GR_BLA_Antagonist_ch2
TOX21_FXR_BLA_agonist_ch2
NVS_ENZ_hBACE
NVS_GPCR_rTRH
79−19−6
6610−29−3
2231−57−4

Enriched Assay Heatmap for Cluster 1584

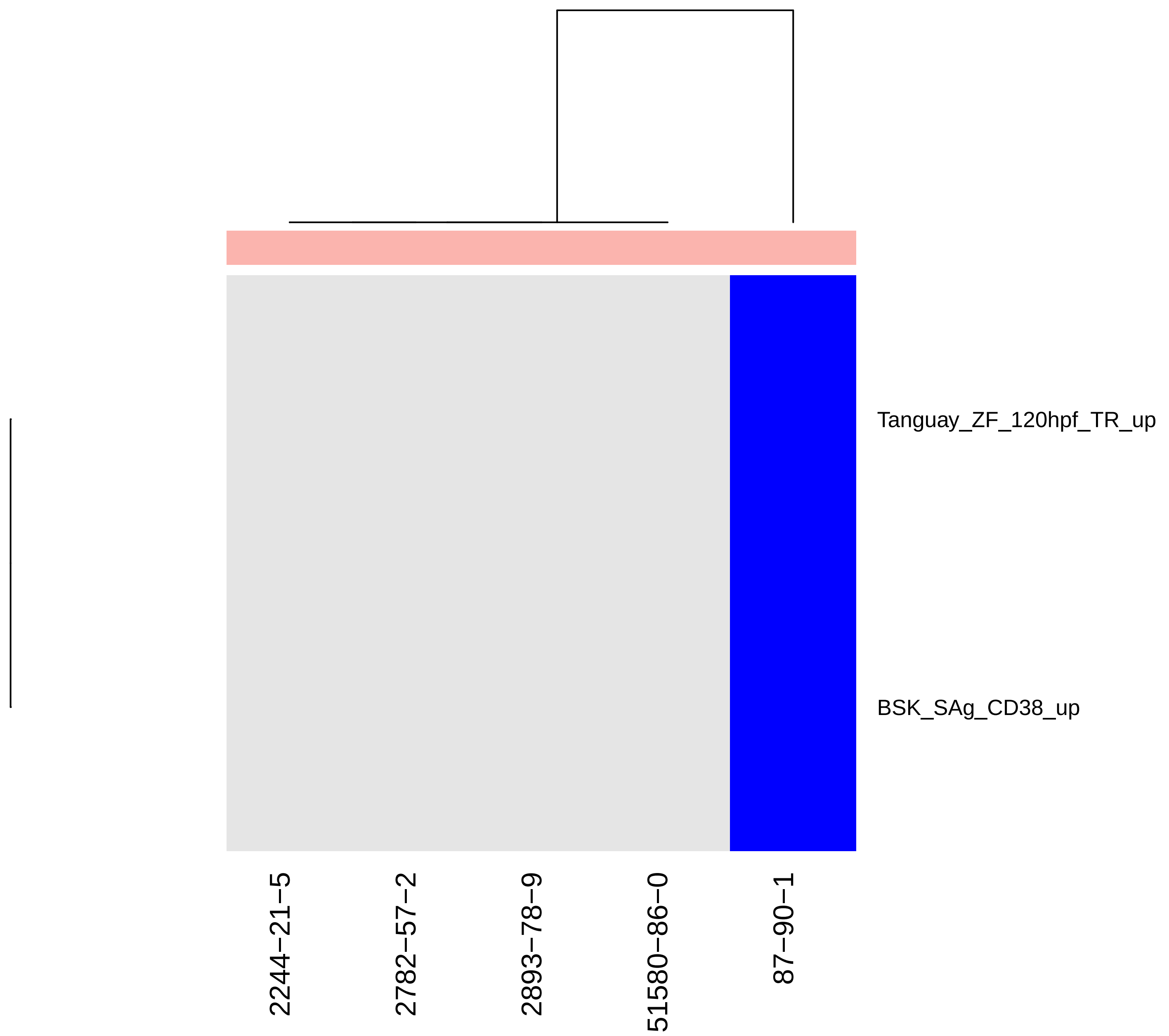

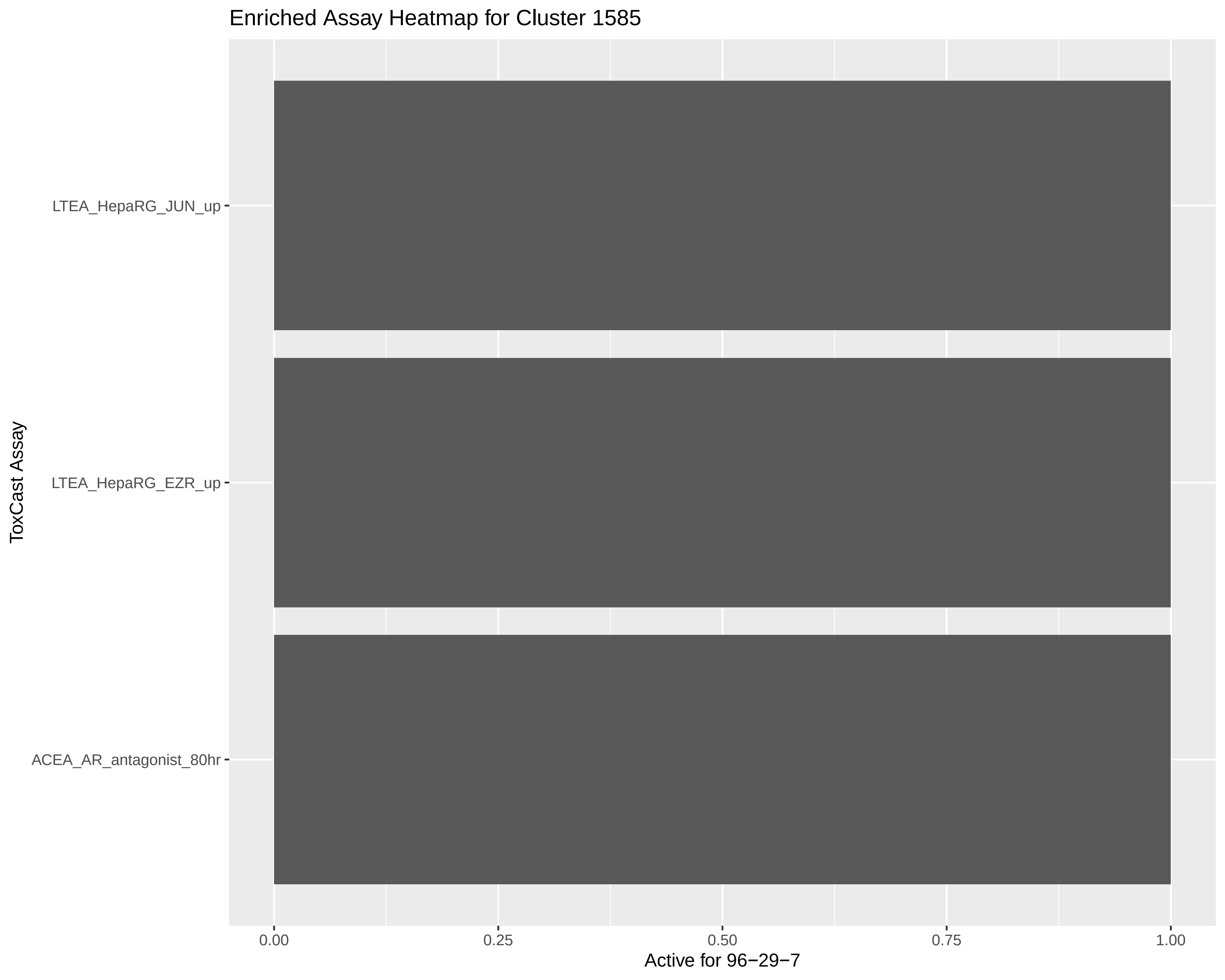

Enriched Assay Heatmap for Cluster 1585
ToxCast Assay
LTEA_HepaRG_JUN_up
LTEA_HepaRG_EZR_up
ACEA_AR_antagonist_80hr
0.00
0.25
0.50
0.75
1.00
Active for 96−29−7

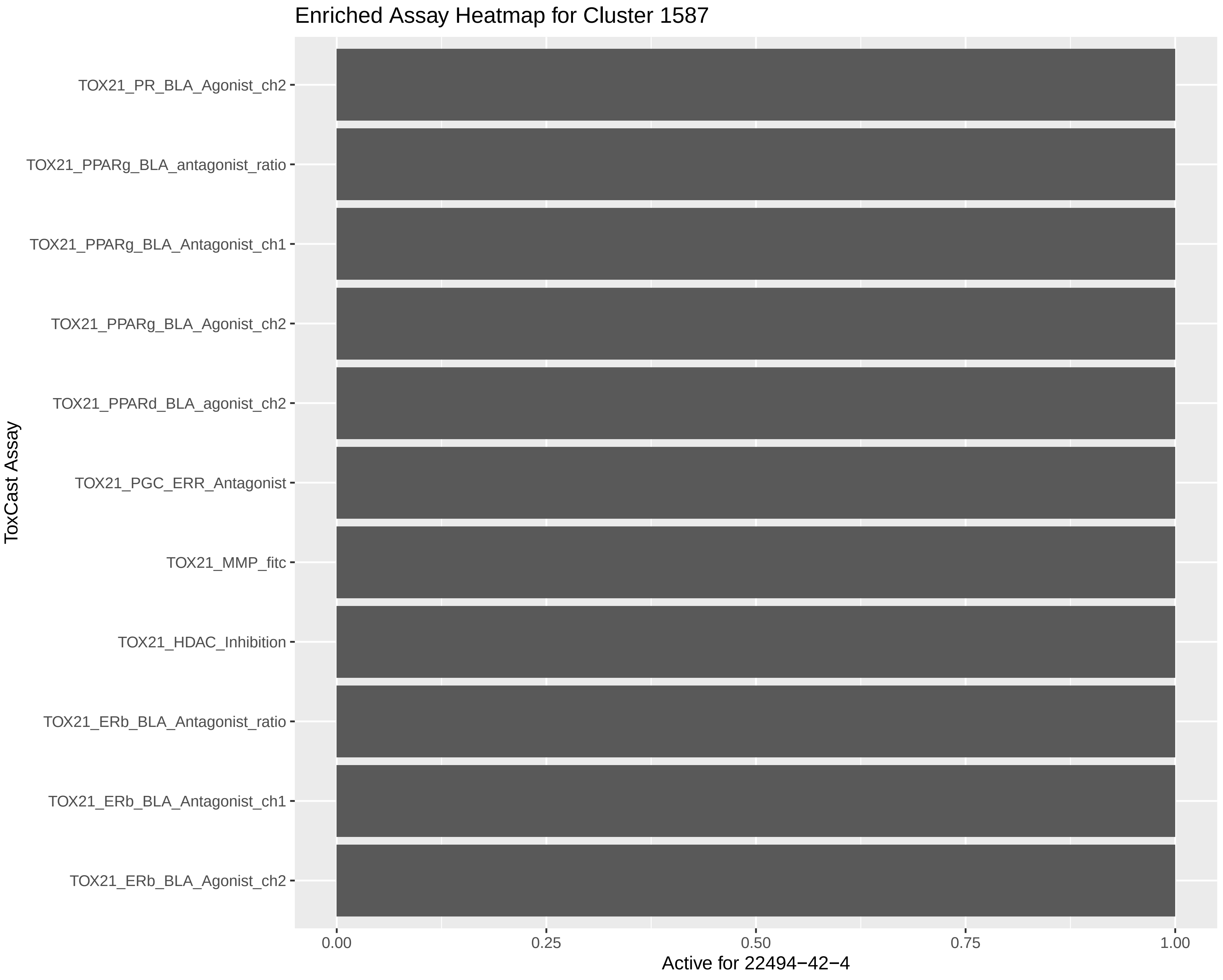

Enriched Assay Heatmap for Cluster 1587
ToxCast Assay
TOX21_PR_BLA_Agonist_ch2
TOX21_PPARg_BLA_antagonist_ratio
TOX21_PPARg_BLA_Antagonist_ch1
TOX21_PPARg_BLA_Agonist_ch2
TOX21_PPARd_BLA_agonist_ch2
TOX21_PGC_ERR_Antagonist
TOX21_MMP_fitc
TOX21_HDAC_Inhibition
TOX21_ERb_BLA_Antagonist_ratio
TOX21_ERb_BLA_Antagonist_ch1
TOX21_ERb_BLA_Agonist_ch2
0.00
0.25
0.50
0.75
1.00
Active for 22494−42−4

Enriched Assay Heatmap for Cluster 1589

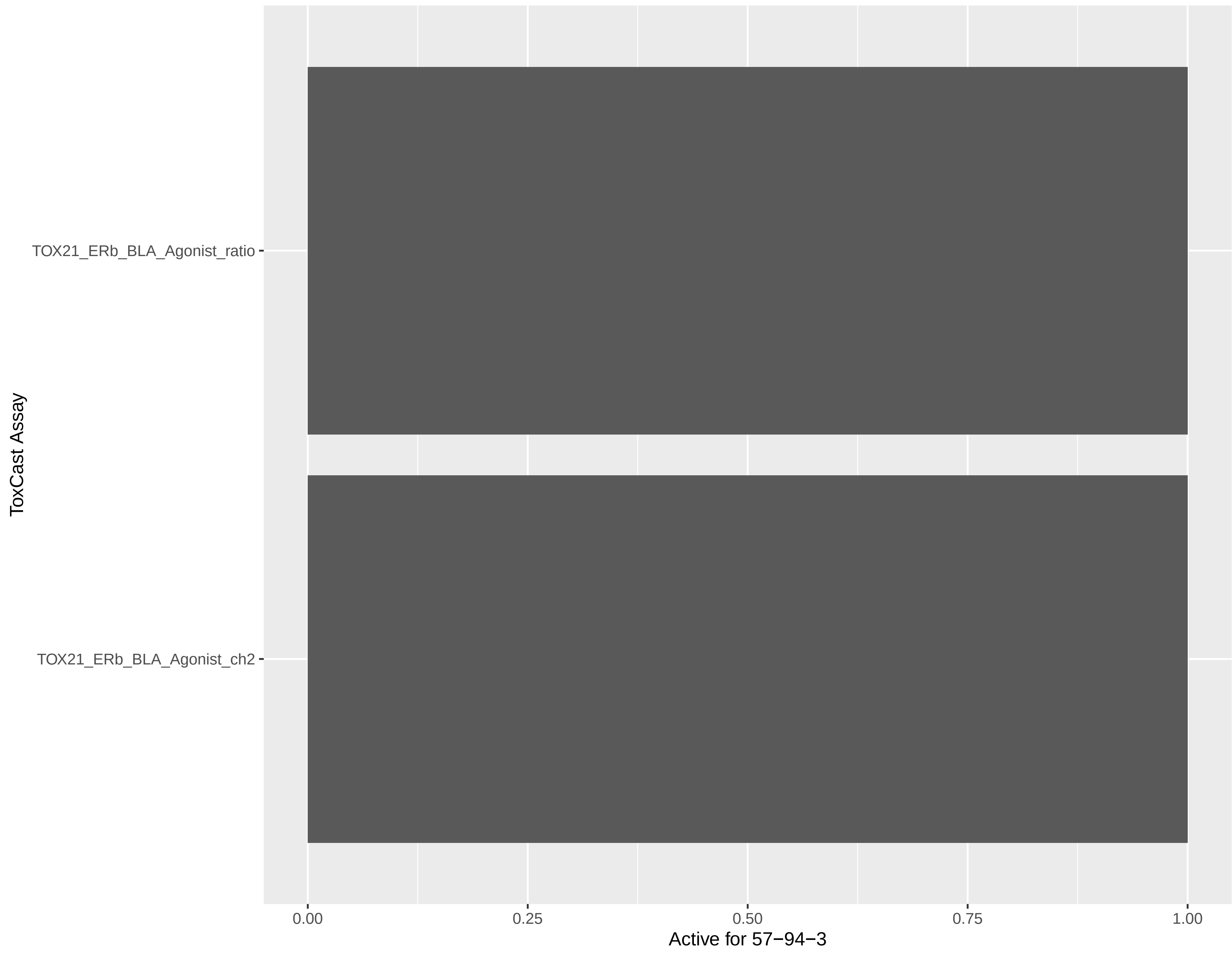

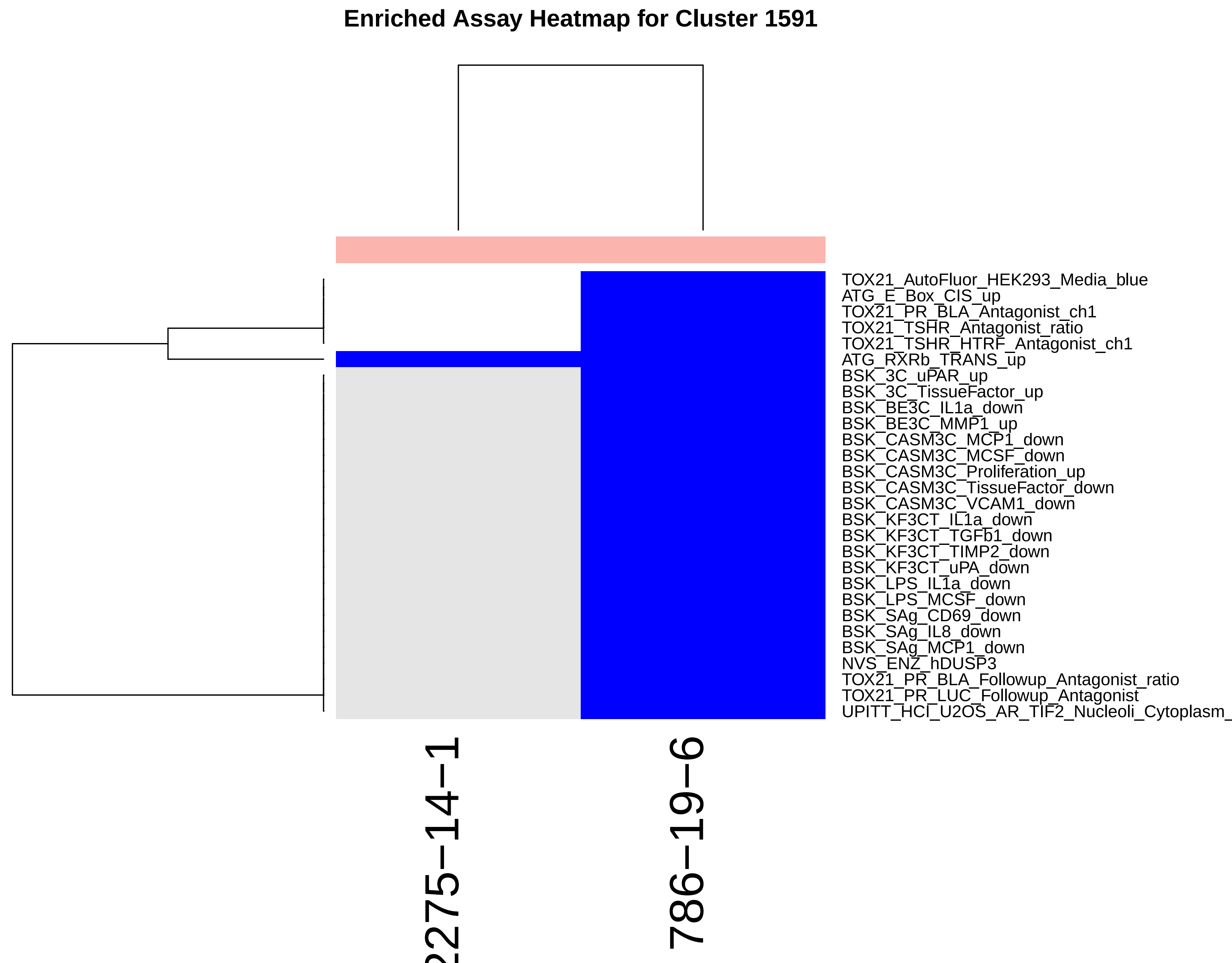

Enriched Assay Heatmap for Cluster 1591
TOX21_AutoFluor_HEK293_Media_blue
ATG_E_Box_CIS_up
TOX21_PR_BLA_Antagonist_ch1
TOX21_TSHR_Antagonist_ratio
TOX21_TSHR_HTRF_Antagonist_ch1
ATG_RXRb_TRANS_up
BSK_3C_uPAR_up
BSK_3C_TissueFactor_up
BSK_BE3C_IL1a_down
BSK_BE3C_MMP1_up
BSK_CASM3C_MCP1_down
BSK_CASM3C_MCSF_down
BSK_CASM3C_Proliferation_up
BSK_CASM3C_TissueFactor_down
BSK_CASM3C_VCAM1_down
BSK_KF3CT_IL1a_down
BSK_KF3CT_TGFb1_down
BSK_KF3CT_TIMP2_down
BSK_KF3CT_uPA_down
BSK_LPS_IL1a_down
BSK_LPS_MCSF_down
BSK_SAg_CD69_down
BSK_SAg_IL8_down
BSK_SAg_MCP1_down
NVS_ENZ_hDUSP3
TOX21_PR_BLA_Followup_Antagonist_ratio
TOX21_PR_LUC_Followup_Antagonist
UPITT_HCI_U2OS_AR_TIF2_Nucleoli_Cytoplasm_
2275−14−1
786−19−6

Enriched Assay Heatmap for Cluster 1593

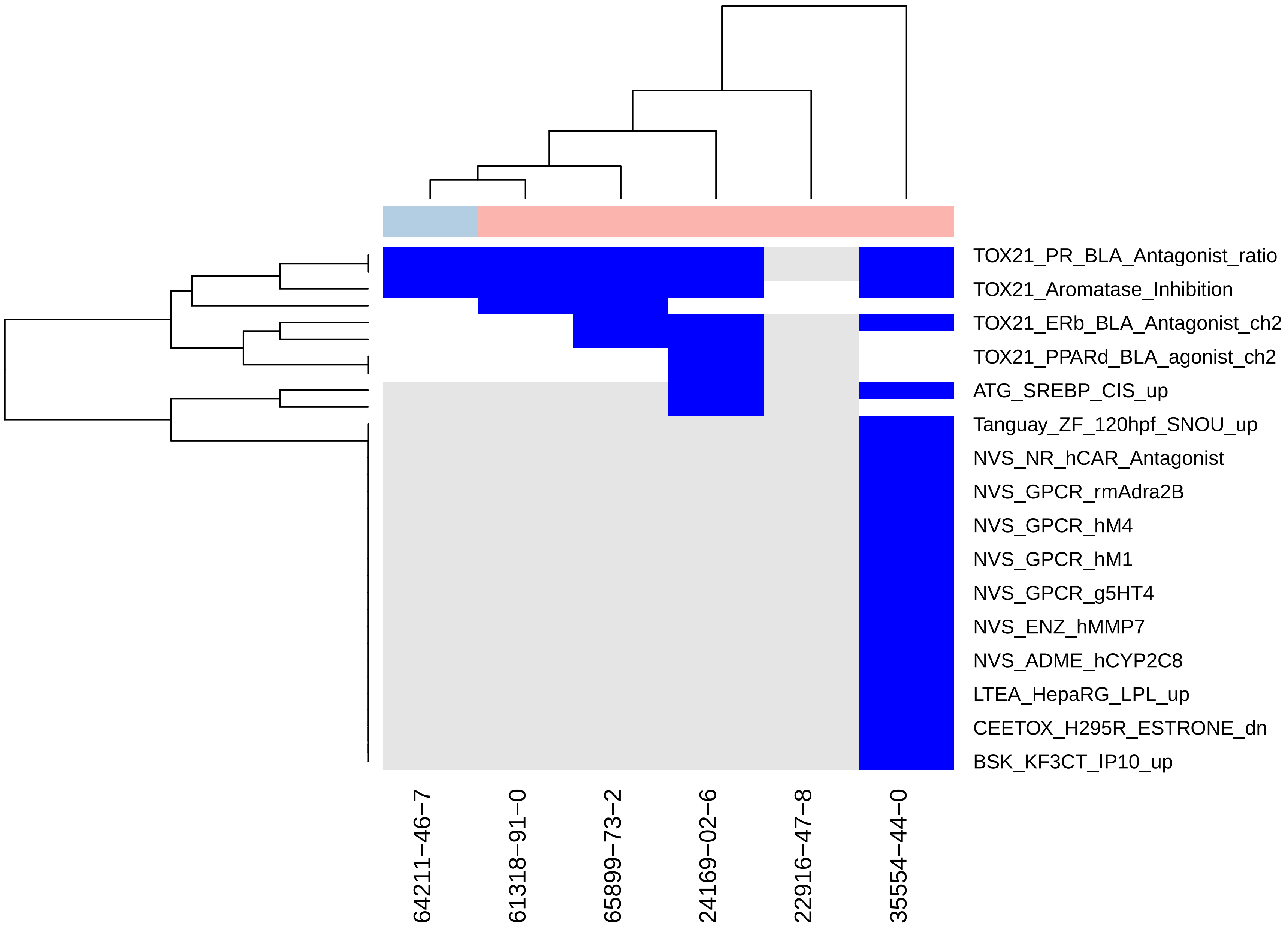

Enriched Assay Heatmap for Cluster 1594

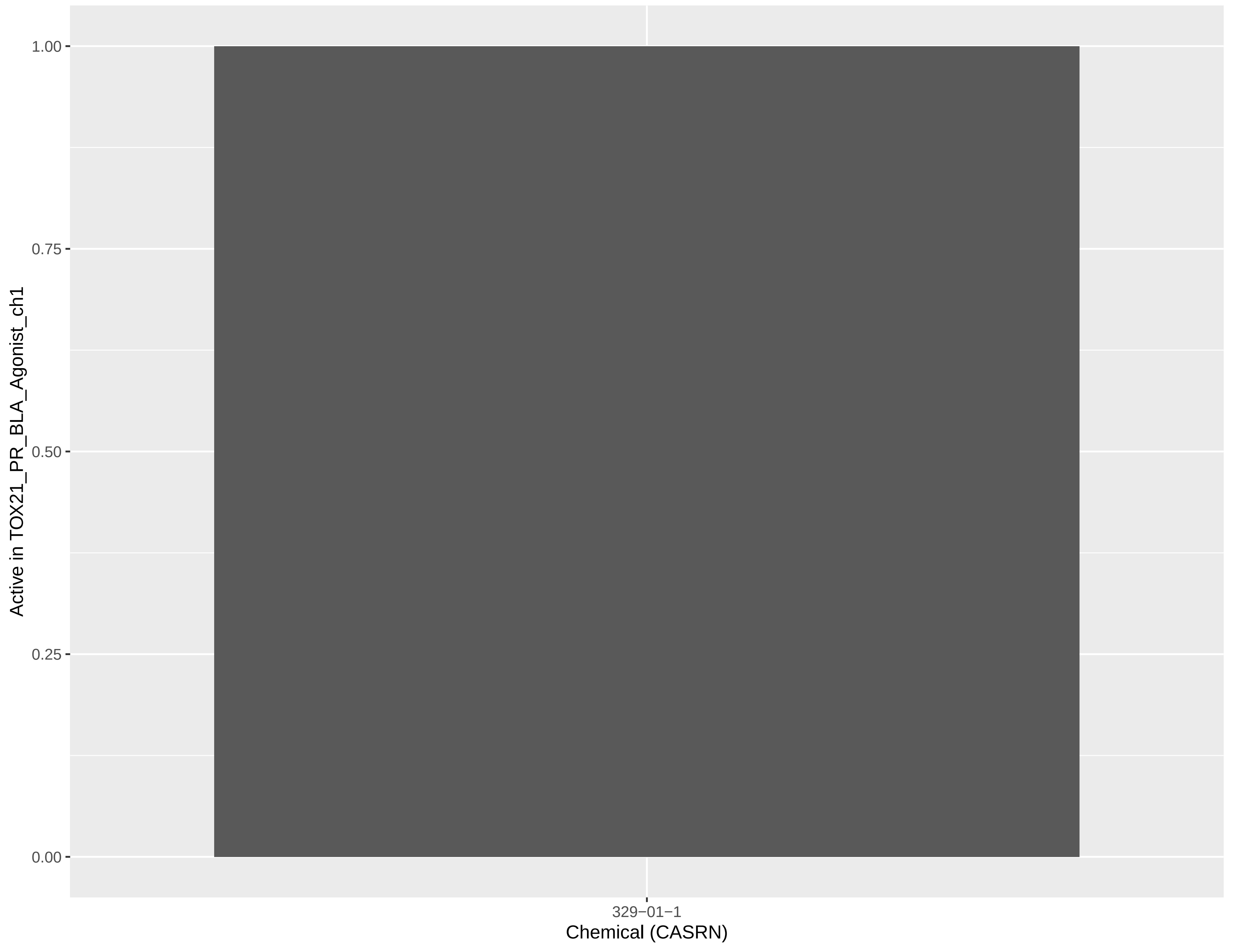

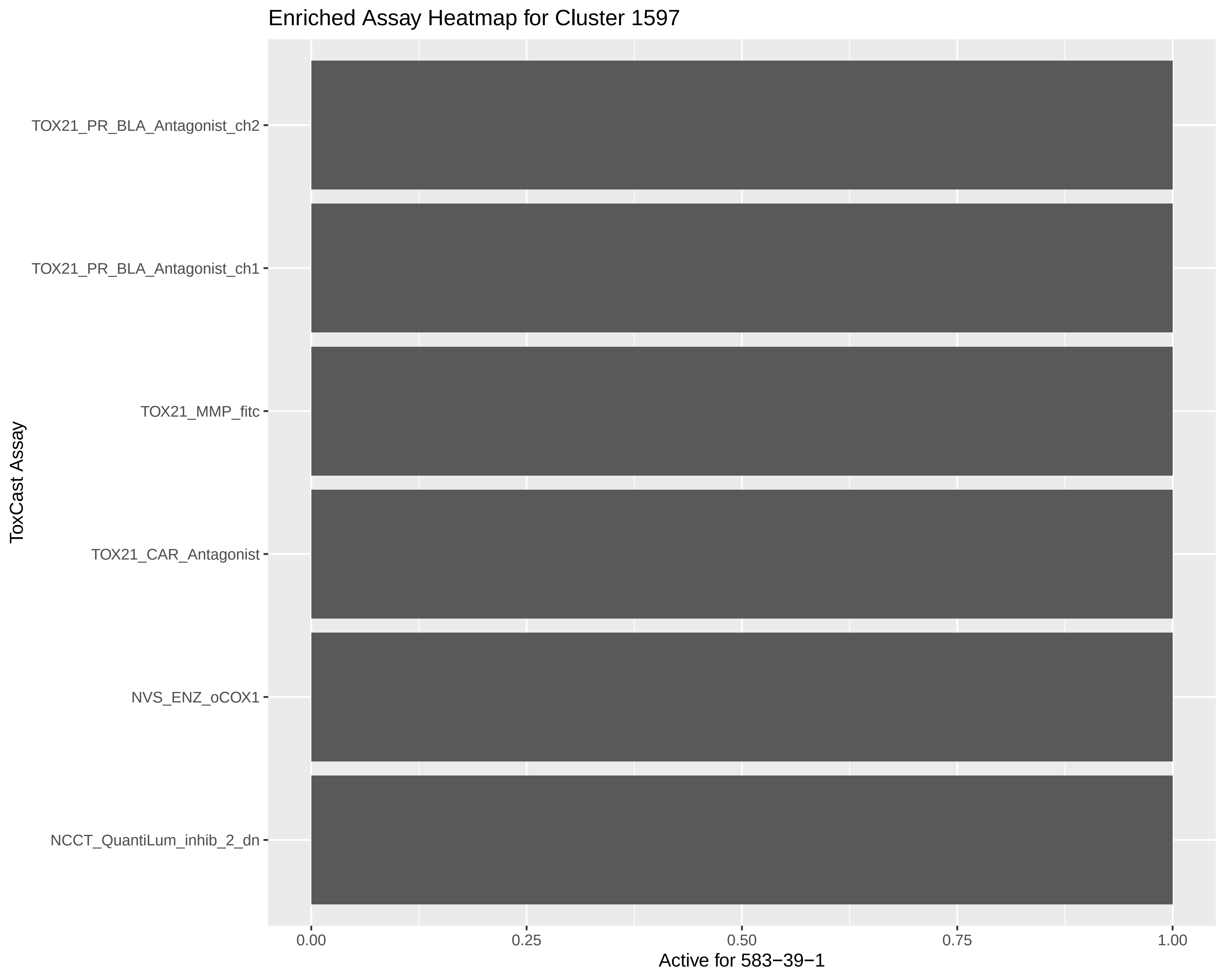
Enriched Assay Heatmap for Cluster 1597
ToxCast Assay
TOX21_PR_BLA_Antagonist_ch2
TOX21_PR_BLA_Antagonist_ch1
TOX21_MMP_fitc
TOX21_CAR_Antagonist
NVS_ENZ_oCOX1
NCCT_QuantiLum_inhib_2_dn
0.00
0.25
0.50
0.75
1.00
Active for 583−39−1

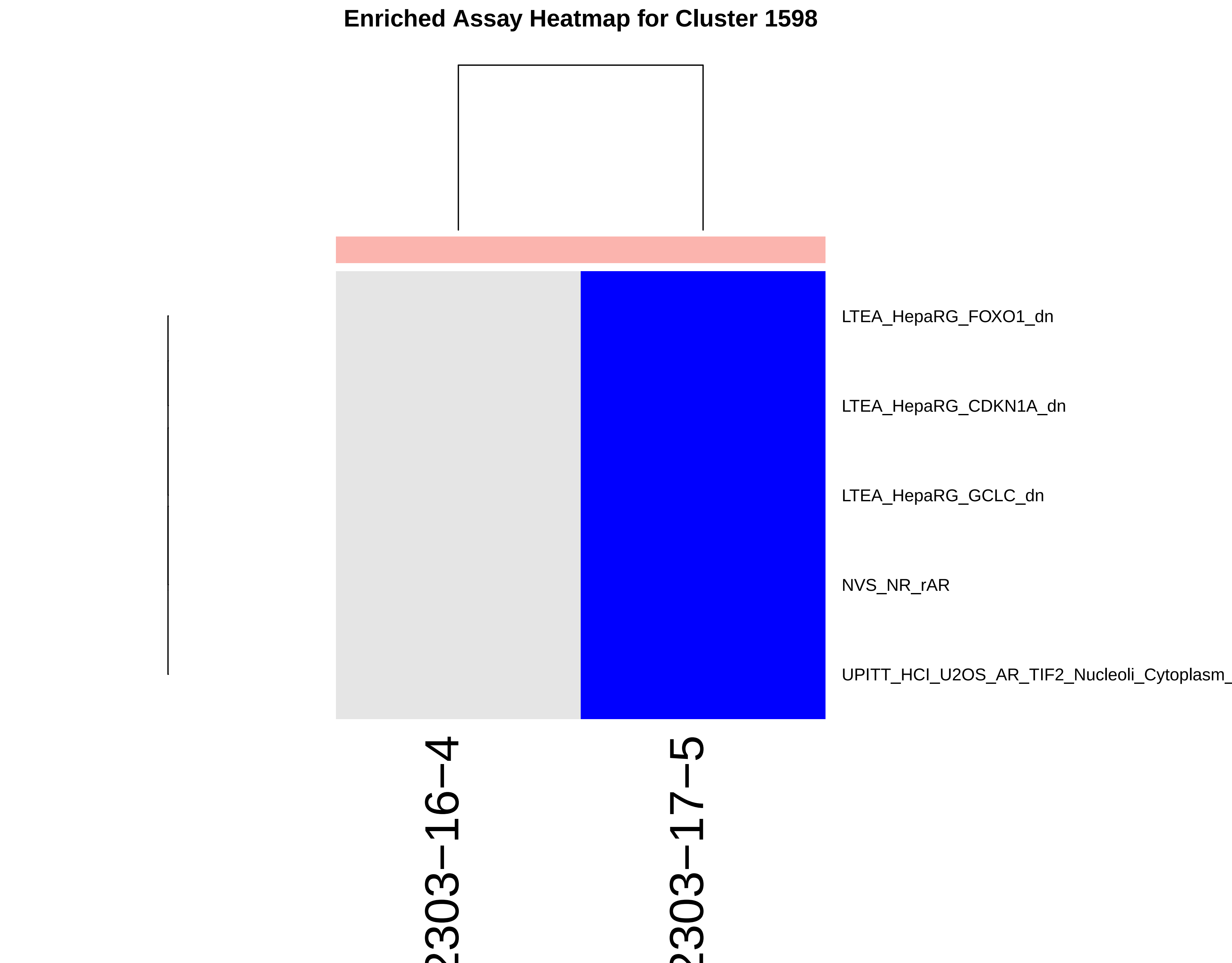
Enriched Assay Heatmap for Cluster 1598
LTEA_HepaRG_FOXO1_dn
LTEA_HepaRG_CDKN1A_dn
LTEA_HepaRG_GCLC_dn
NVS_NR_rAR
UPITT_HCI_U2OS_AR_TIF2_Nucleoli_Cytoplasm_
2303−16−4
2303−17−5

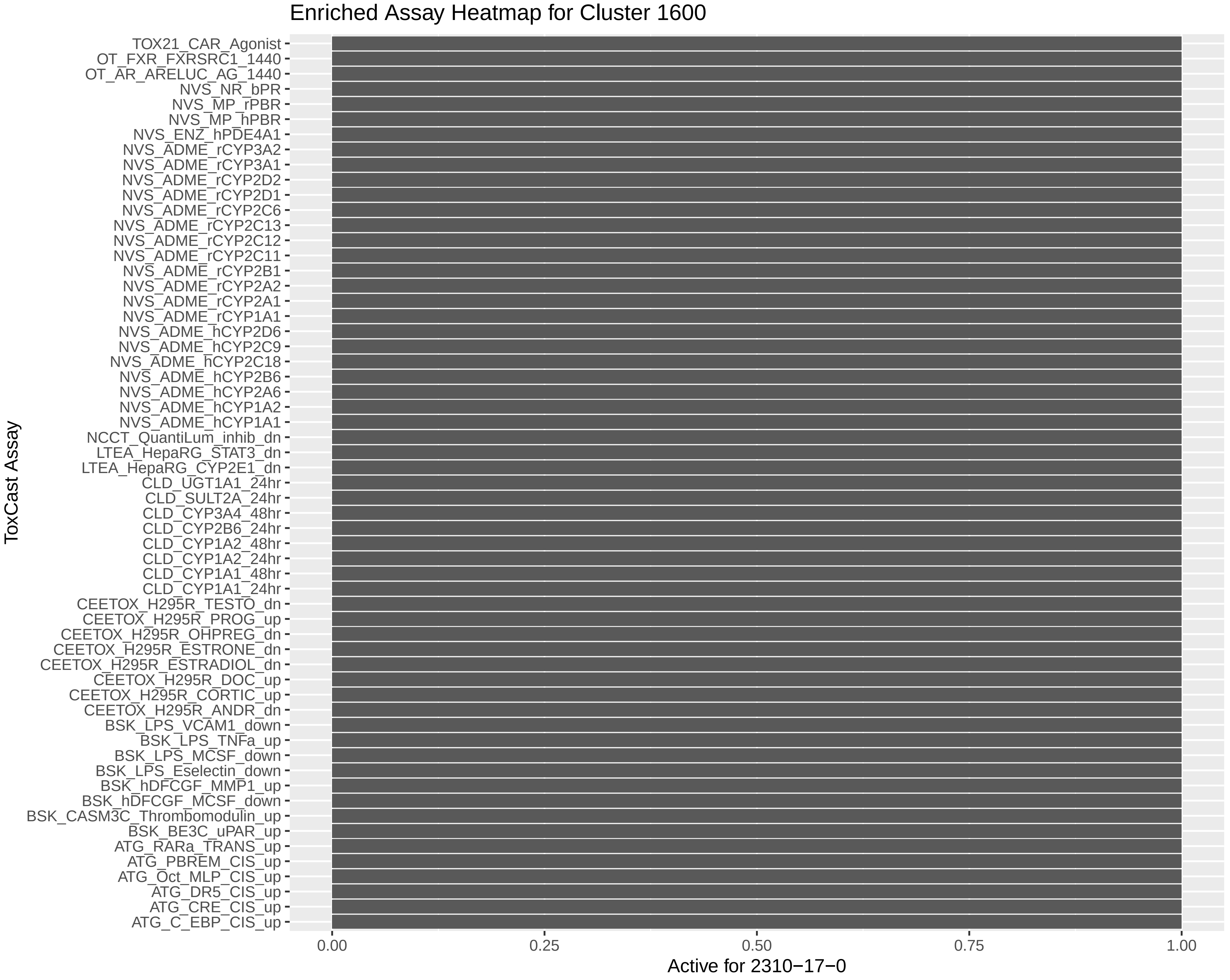
Enriched Assay Heatmap for Cluster 1600
ToxCast Assay
TOX21_CAR_Agonist
OT_FXR_FXRSRC1_1440
OT_AR_ARELUC_AG_1440
NVS_NR_bPR
NVS_MP_rPBR
NVS_MP_hPBR
NVS_ENZ_hPDE4A1
NVS_ADME_rCYP3A2
NVS_ADME_rCYP3A1
NVS_ADME_rCYP2D2
NVS_ADME_rCYP2D1
NVS_ADME_rCYP2C6
NVS_ADME_rCYP2C13
NVS_ADME_rCYP2C12
NVS_ADME_rCYP2C11
NVS_ADME_rCYP2B1
NVS_ADME_rCYP2A2
NVS_ADME_rCYP2A1
NVS_ADME_rCYP1A1
NVS_ADME_hCYP2D6
NVS_ADME_hCYP2C9
NVS_ADME_hCYP2C18
NVS_ADME_hCYP2B6
NVS_ADME_hCYP2A6
NVS_ADME_hCYP1A2
NVS_ADME_hCYP1A1
NCCT_QuantiLum_inhib_dn
LTEA_HepaRG_STAT3_dn
LTEA_HepaRG_CYP2E1_dn
CLD_UGT1A1_24hr
CLD_SULT2A_24hr
CLD_CYP3A4_48hr
CLD_CYP2B6_24hr
CLD_CYP1A2_48hr
CLD_CYP1A2_24hr
CLD_CYP1A1_48hr
CLD_CYP1A1_24hr
CEETOX_H295R_TESTO_dn
CEETOX_H295R_PROG_up
CEETOX_H295R_OHPREG_dn
CEETOX_H295R_ESTRONE_dn
CEETOX_H295R_ESTRADIOL_dn
CEETOX_H295R_DOC_up
CEETOX_H295R_CORTIC_up
CEETOX_H295R_ANDR_dn
BSK_LPS_VCAM1_down
BSK_LPS_TNFa_up
BSK_LPS_MCSF_down
BSK_LPS_Eselectin_down
BSK_hDFCGF_MMP1_up
BSK_hDFCGF_MCSF_down
BSK_CASM3C_Thrombomodulin_up
BSK_BE3C_uPAR_up
ATG_RARa_TRANS_up
ATG_PBREM_CIS_up
ATG_Oct_MLP_CIS_up
ATG_DR5_CIS_up
ATG_CRE_CIS_up
ATG_C_EBP_CIS_up
0.00
0.25
0.50
0.75
1.00
Active for 2310−17−0

Enriched Assay Heatmap for Cluster 1607

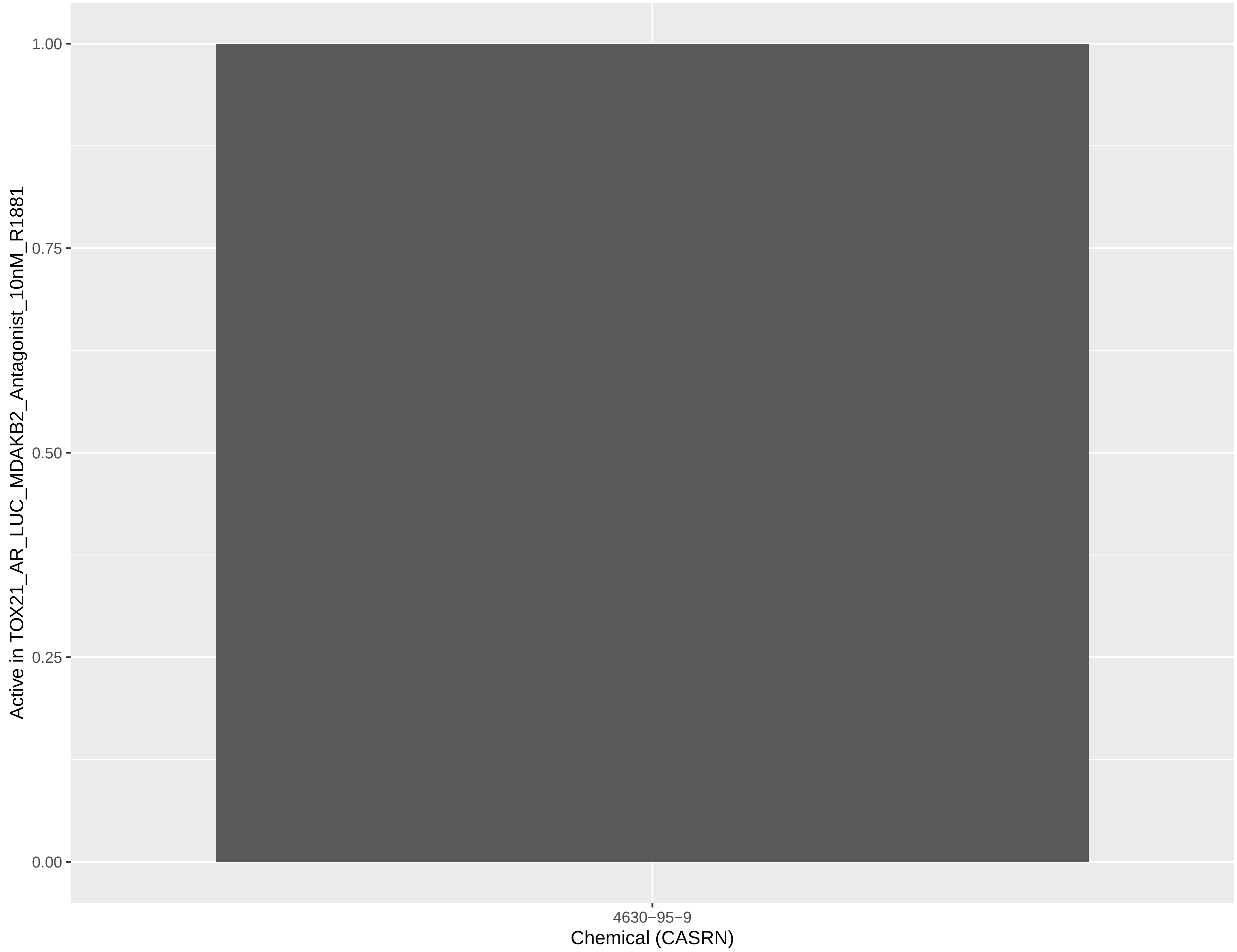

Enriched Assay Heatmap for Cluster 1609

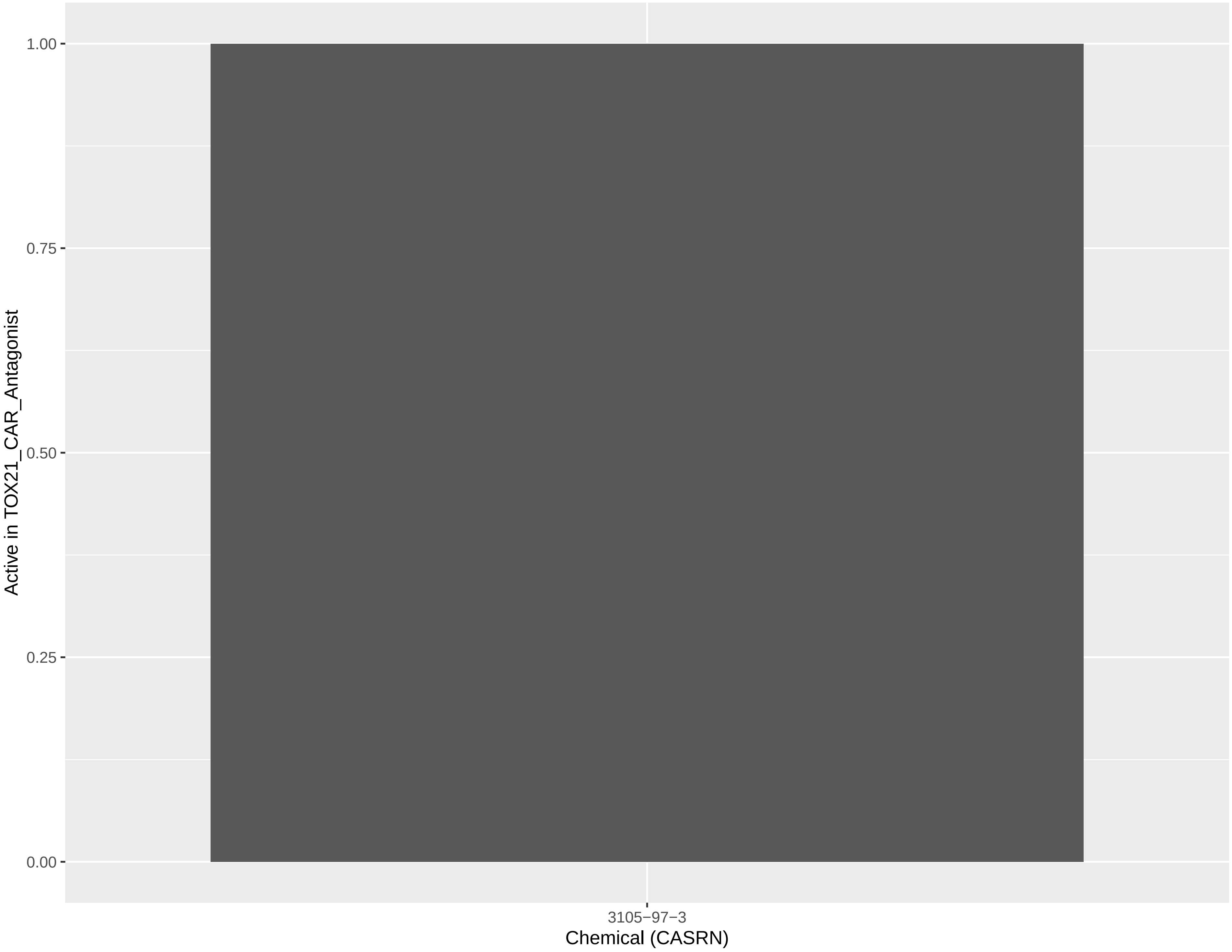

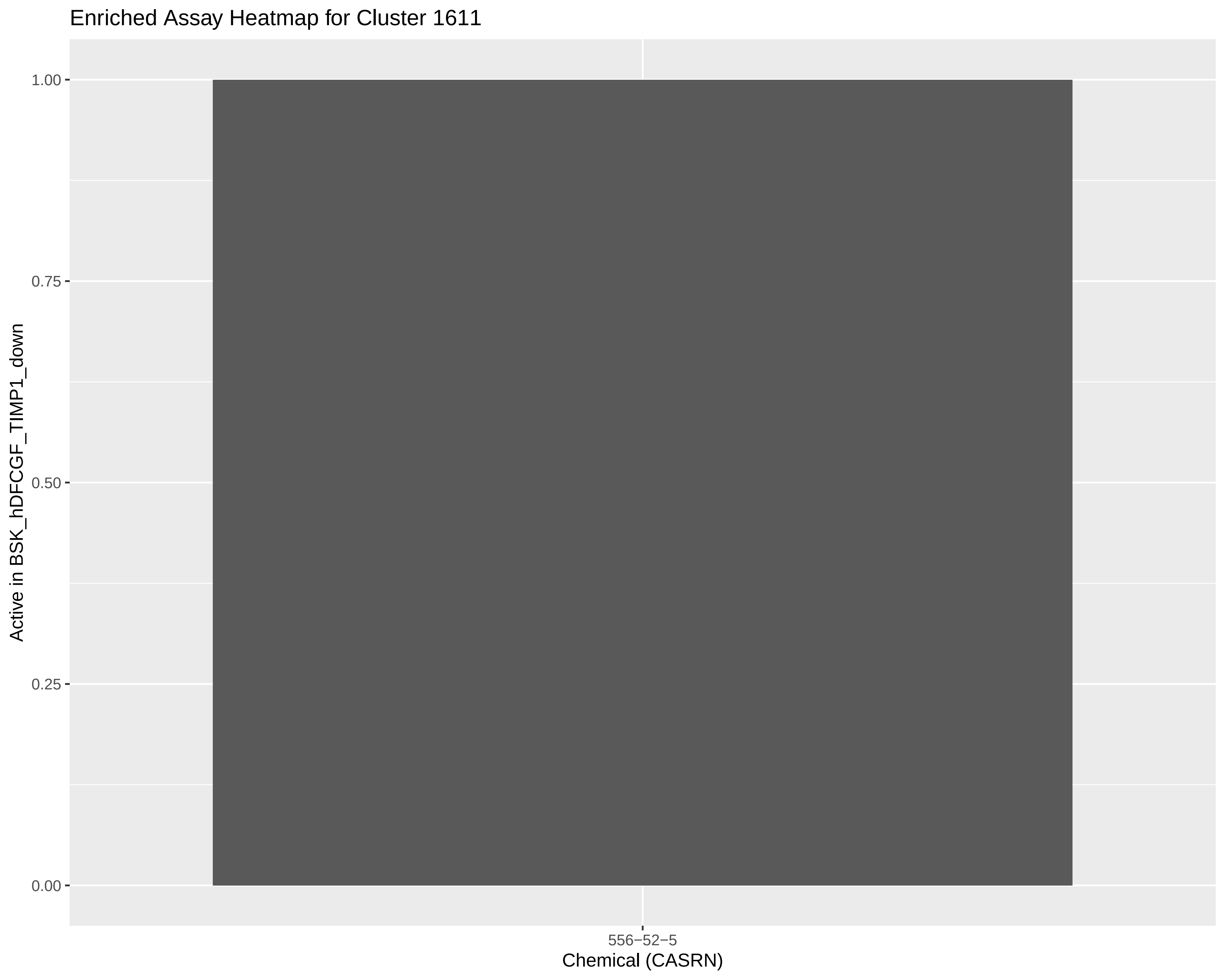

Enriched Assay Heatmap for Cluster 1611
1.00
0.75
0.50
0.25
0.00
Active in BSK_hDFCGF_TIMP1_down
556−52−5
Chemical (CASRN)

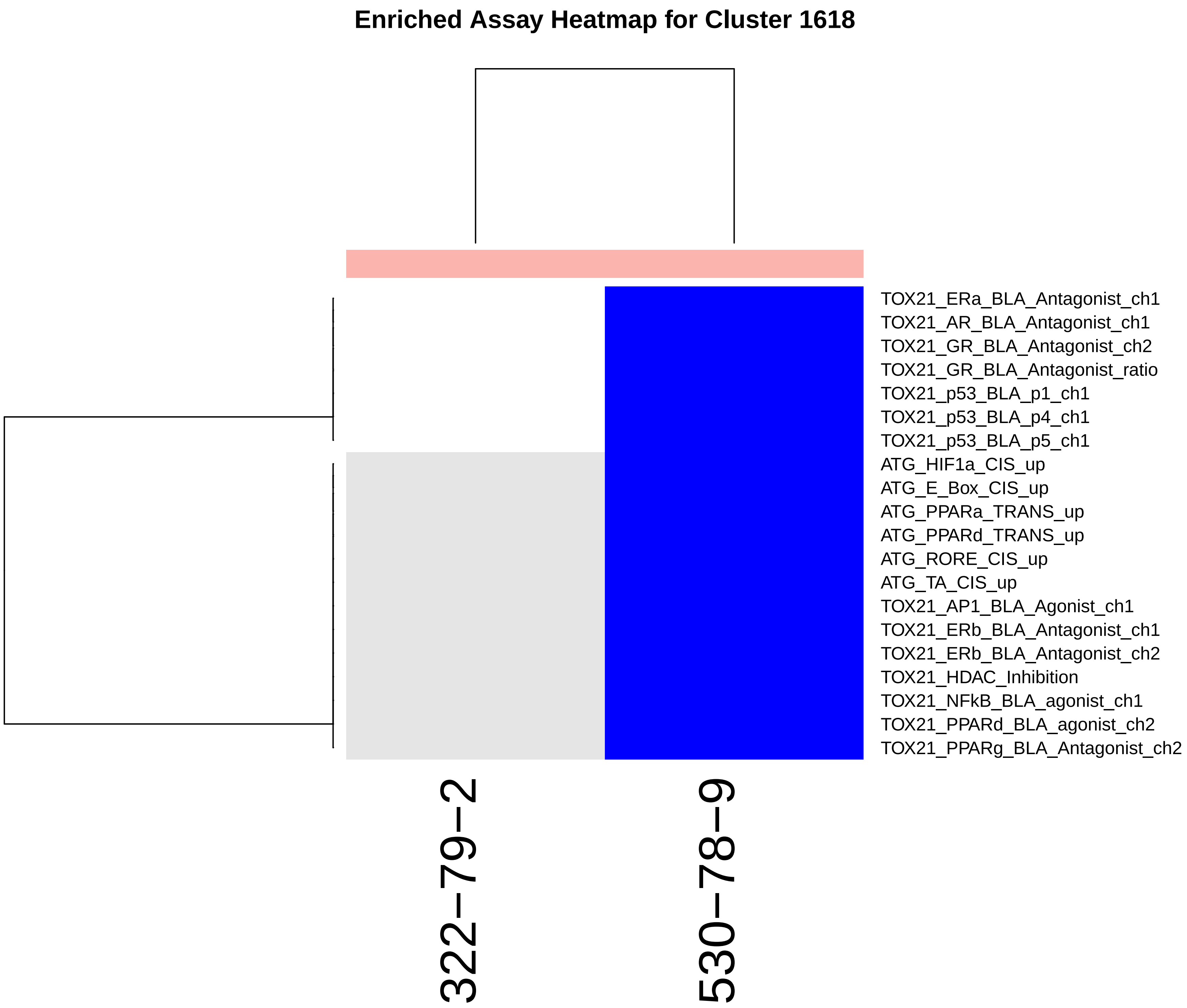
Enriched Assay Heatmap for Cluster 1618
TOX21_ERa_BLA_Antagonist_ch1
TOX21_AR_BLA_Antagonist_ch1
TOX21_GR_BLA_Antagonist_ch2
TOX21_GR_BLA_Antagonist_ratio
TOX21_p53_BLA_p1_ch1
TOX21_p53_BLA_p4_ch1
TOX21_p53_BLA_p5_ch1
ATG_HIF1a_CIS_up
ATG_E_Box_CIS_up
ATG_PPARa_TRANS_up
ATG_PPARd_TRANS_up
ATG_RORE_CIS_up
ATG_TA_CIS_up
TOX21_AP1_BLA_Agonist_ch1
TOX21_ERb_BLA_Antagonist_ch1
TOX21_ERb_BLA_Antagonist_ch2
TOX21_HDAC_Inhibition
TOX21_NFkB_BLA_agonist_ch1
TOX21_PPARd_BLA_agonist_ch2
TOX21_PPARg_BLA_Antagonist_ch2
322−79−2
530−78−9

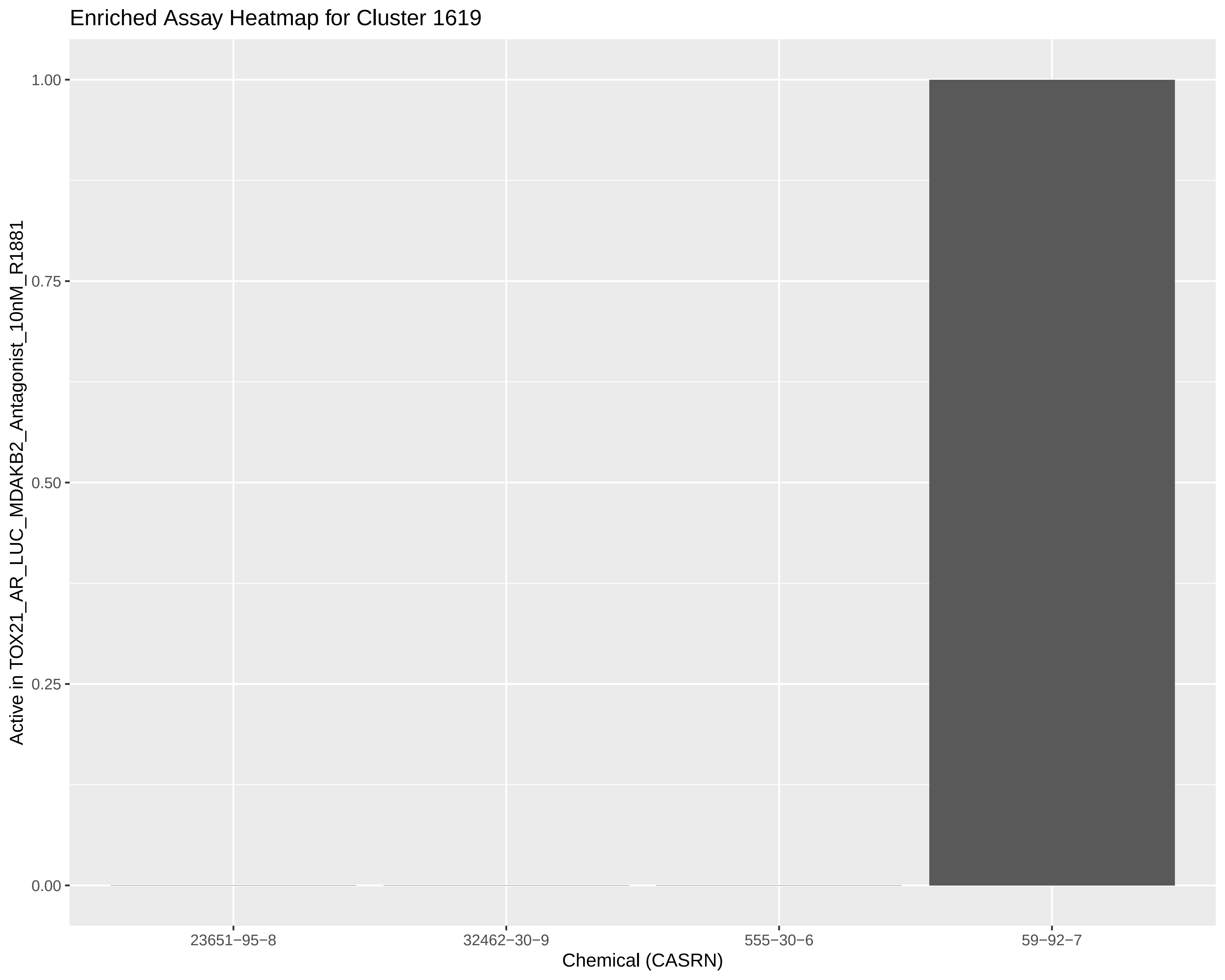
Enriched Assay Heatmap for Cluster 1619
Active in TOX21_AR_LUC_MDAKB2_Antagonist_10nM_R1881
1.00
0.75
0.50
0.25
0.00
23651−95−8
32462−30−9
555−30−6
59−92−7
Chemical (CASRN)

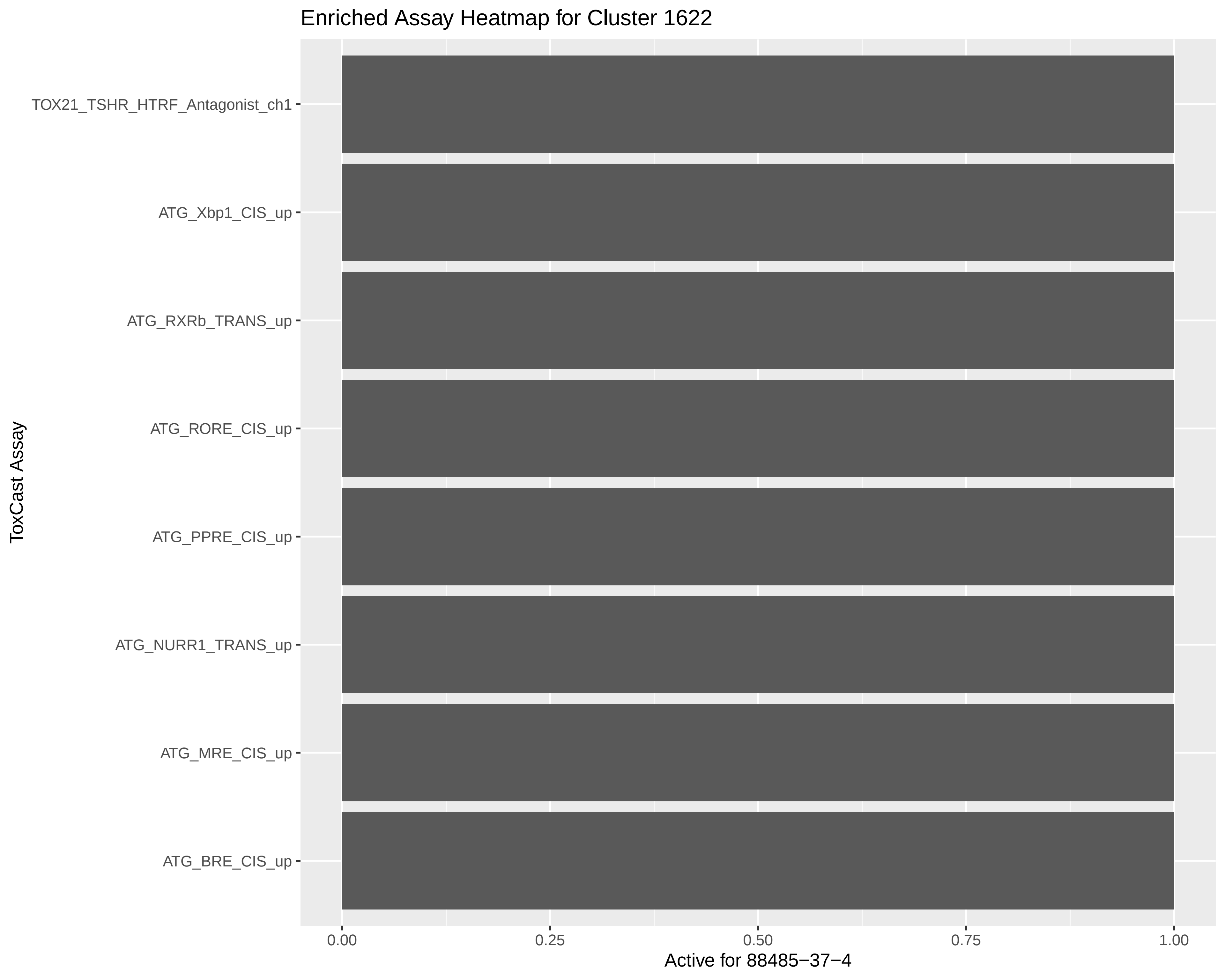

Enriched Assay Heatmap for Cluster 1622
TOX21_TSHR_HTRF_Antagonist_ch1
ATG_Xbp1_CIS_up
ATG_RXRb_TRANS_up
ATG_RORE_CIS_up
ATG_PPRE_CIS_up
ATG_NURR1_TRANS_up
ATG_MRE_CIS_up
ATG_BRE_CIS_up
ToxCast Assay
0.00
0.25
0.50
0.75
1.00
Active for 88485−37−4

Enriched Assay Heatmap for Cluster 1628

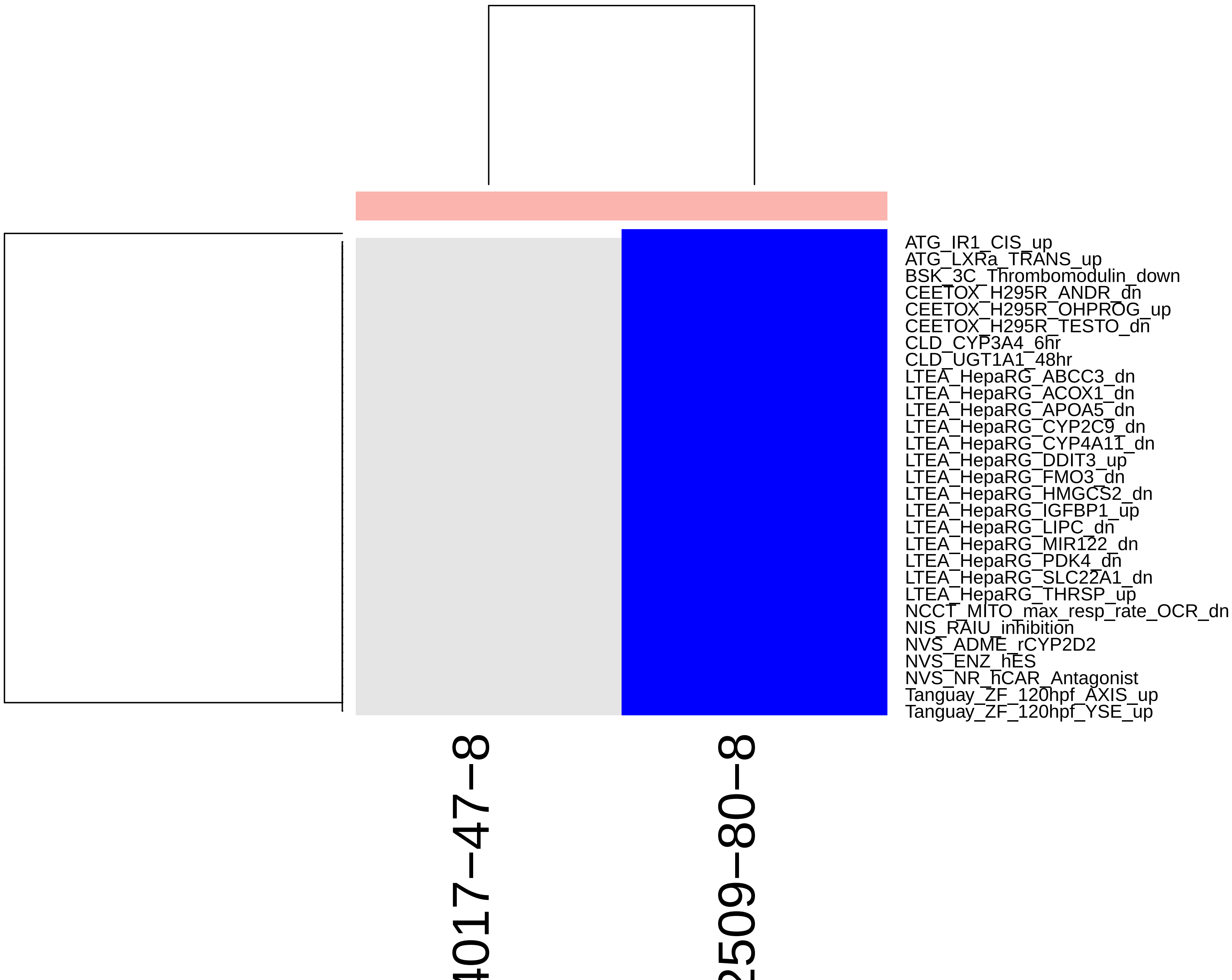

Enriched Assay Heatmap for Cluster 1629

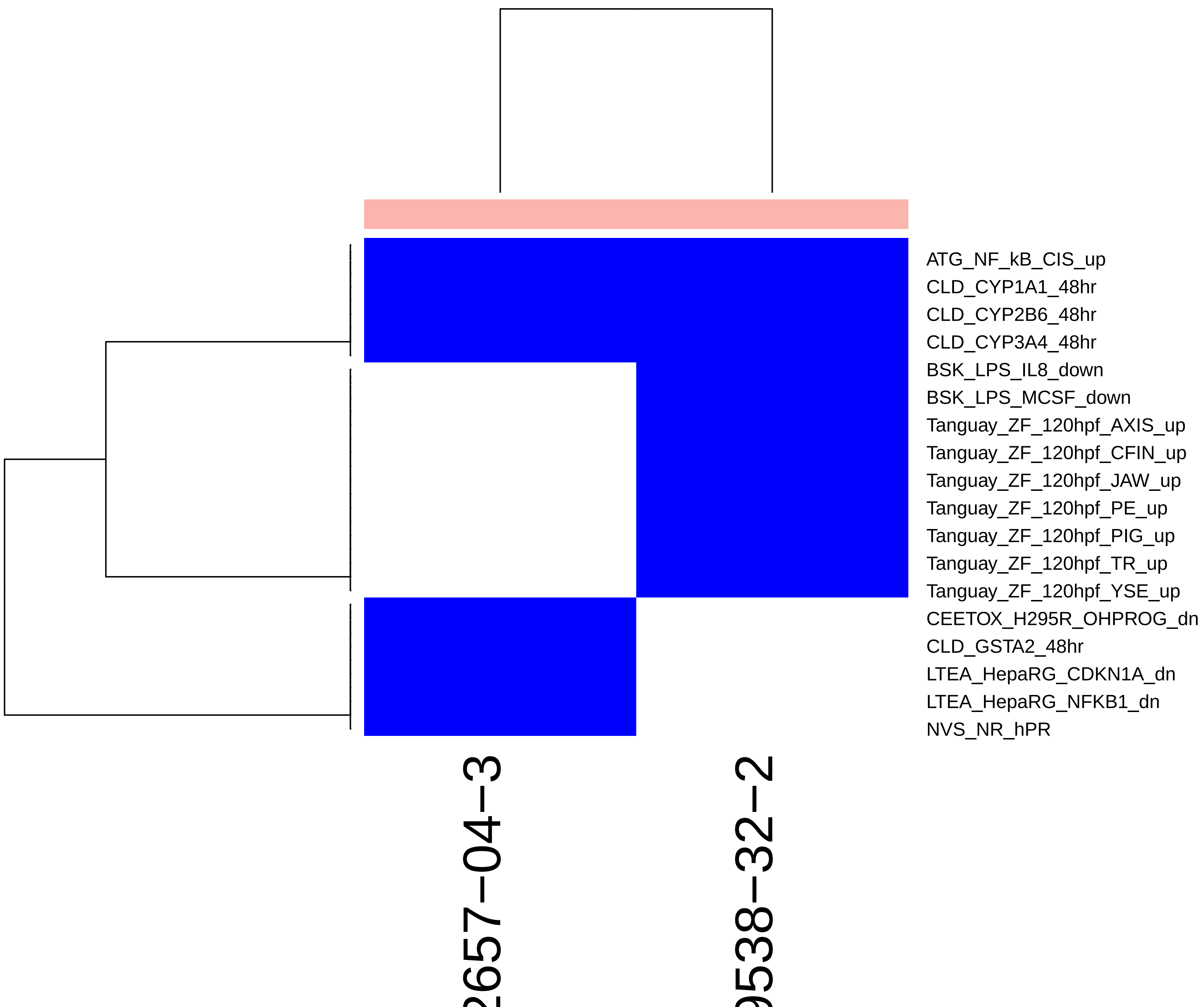

Enriched Assay Heatmap for Cluster 1631

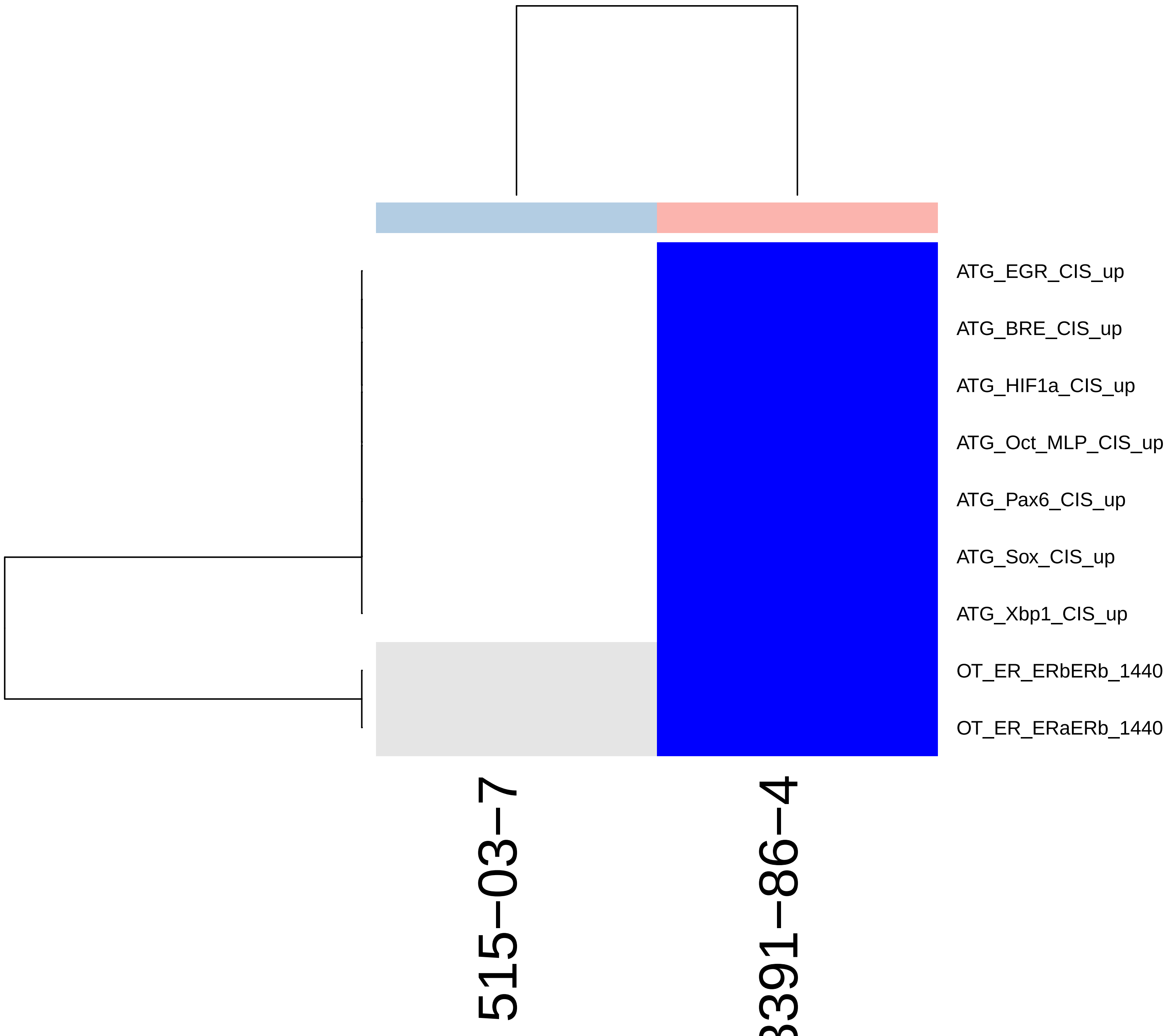

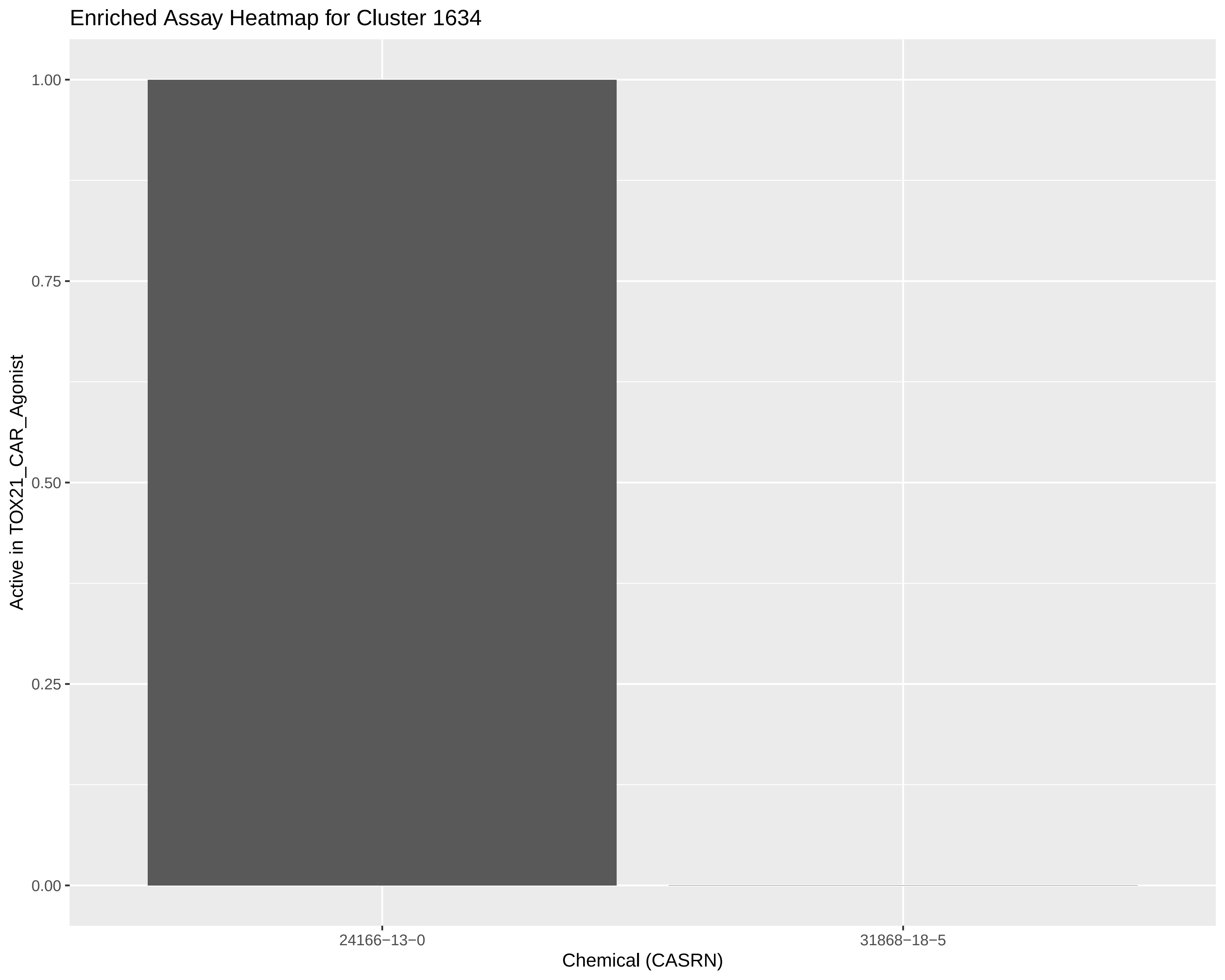

Enriched Assay Heatmap for Cluster 1634
Active in TOX21_CAR_Agonist
1.00
0.75
0.50
0.25
0.00
24166−13−0
31868−18−5
Chemical (CASRN)

Enriched Assay Heatmap for Cluster 1639

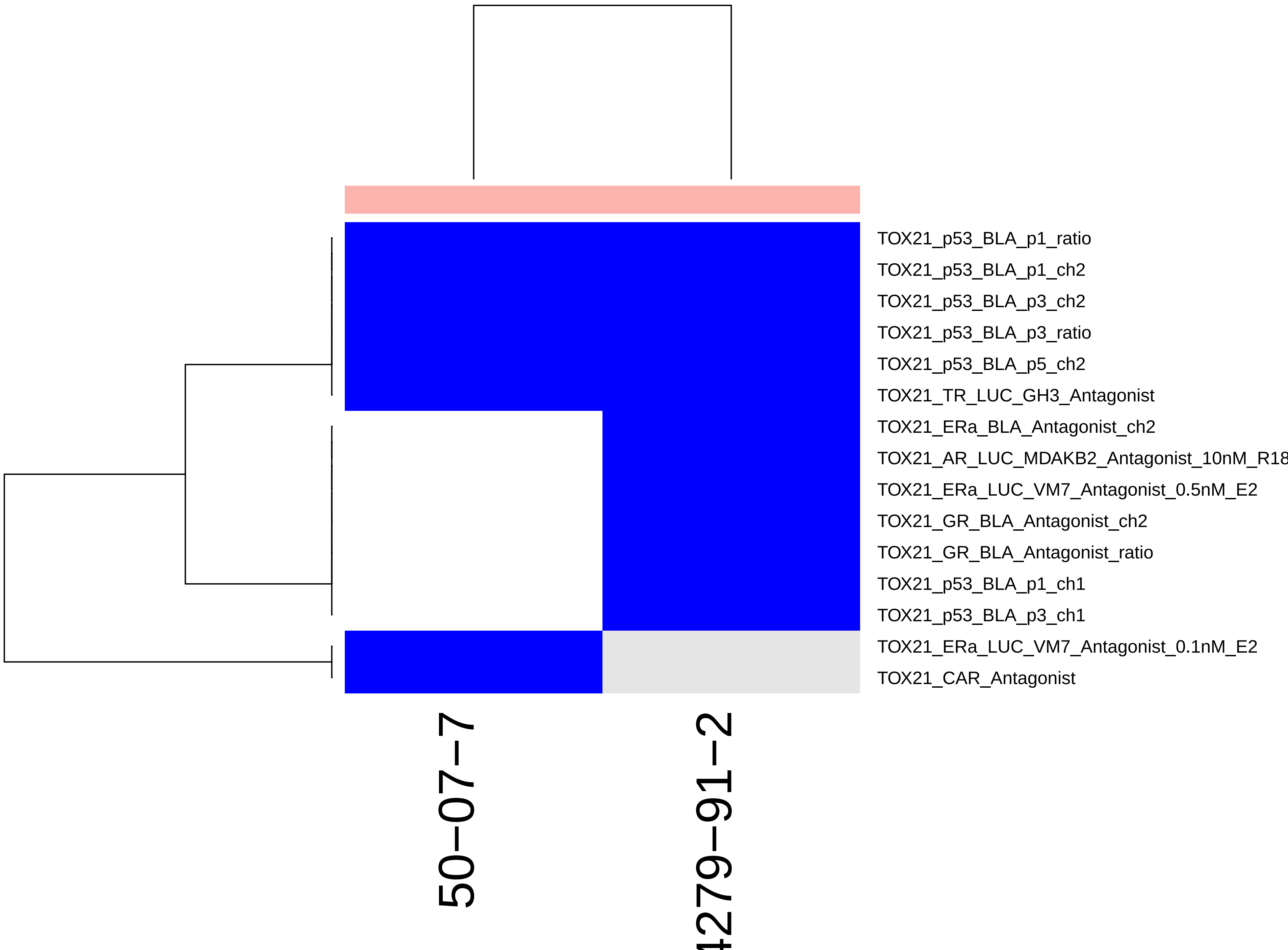

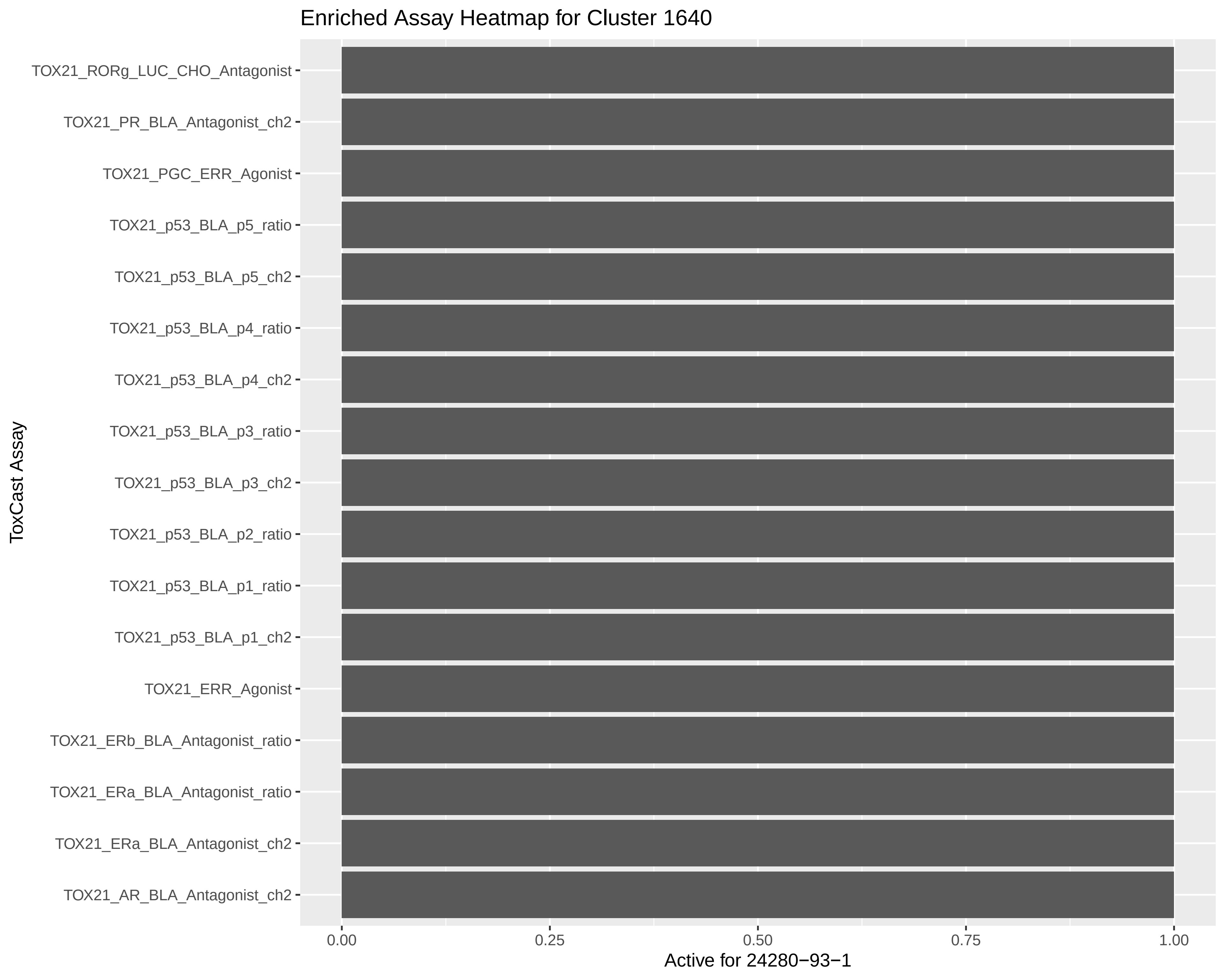

Enriched Assay Heatmap for Cluster 1640
TOX21_RORg_LUC_CHO_Antagonist
TOX21_PR_BLA_Antagonist_ch2
TOX21_PGC_ERR_Agonist
TOX21_p53_BLA_p5_ratio
TOX21_p53_BLA_p5_ch2
TOX21_p53_BLA_p4_ratio
TOX21_p53_BLA_p4_ch2
TOX21_p53_BLA_p3_ratio
TOX21_p53_BLA_p3_ch2
TOX21_p53_BLA_p2_ratio
TOX21_p53_BLA_p1_ratio
TOX21_p53_BLA_p1_ch2
TOX21_ERR_Agonist
TOX21_ERb_BLA_Antagonist_ratio
TOX21_ERa_BLA_Antagonist_ratio
TOX21_ERa_BLA_Antagonist_ch2
TOX21_AR_BLA_Antagonist_ch2
ToxCast Assay
0.00
0.25
0.50
0.75
1.00
Active for 24280−93−1

Enriched Assay Heatmap for Cluster 1642

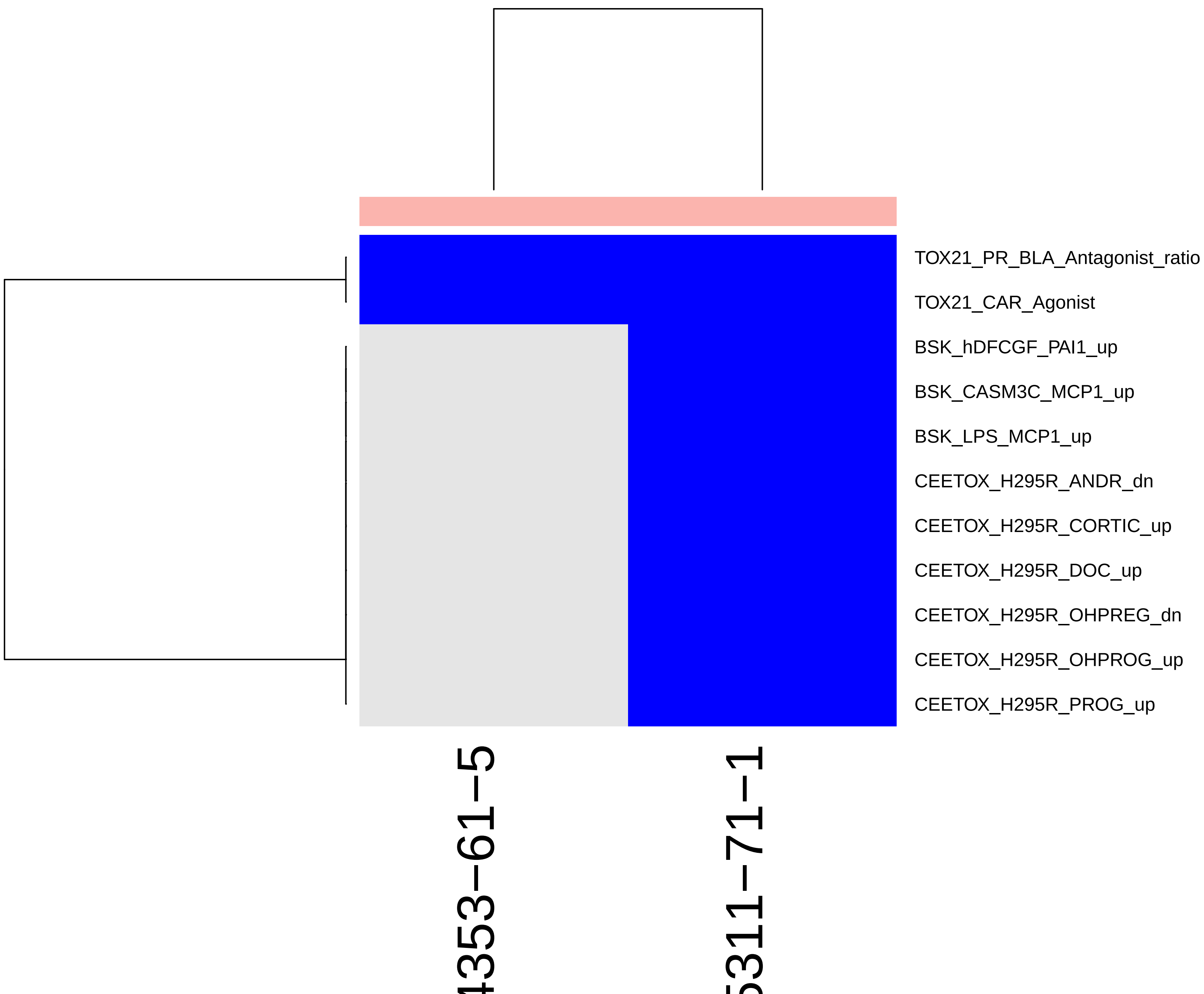

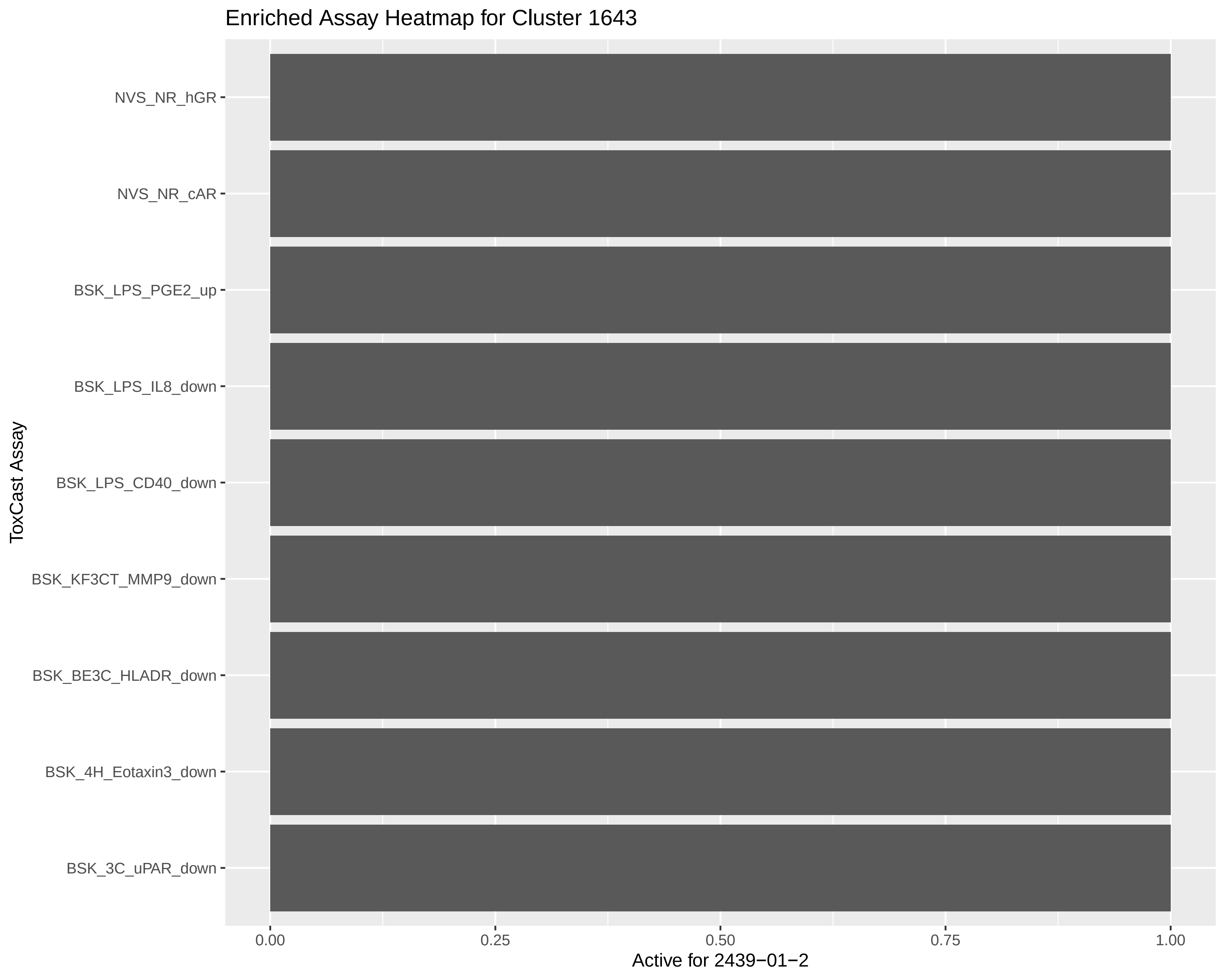

Enriched Assay Heatmap for Cluster 1643
ToxCast Assay
NVS_NR_hGR
NVS_NR_cAR
BSK_LPS_PGE2_up
BSK_LPS_IL8_down
BSK_LPS_CD40_down
BSK_KF3CT_MMP9_down
BSK_BE3C_HLADR_down
BSK_4H_Eotaxin3_down
BSK_3C_uPAR_down
0.00
0.25
0.50
0.75
1.00
Active for 2439−01−2

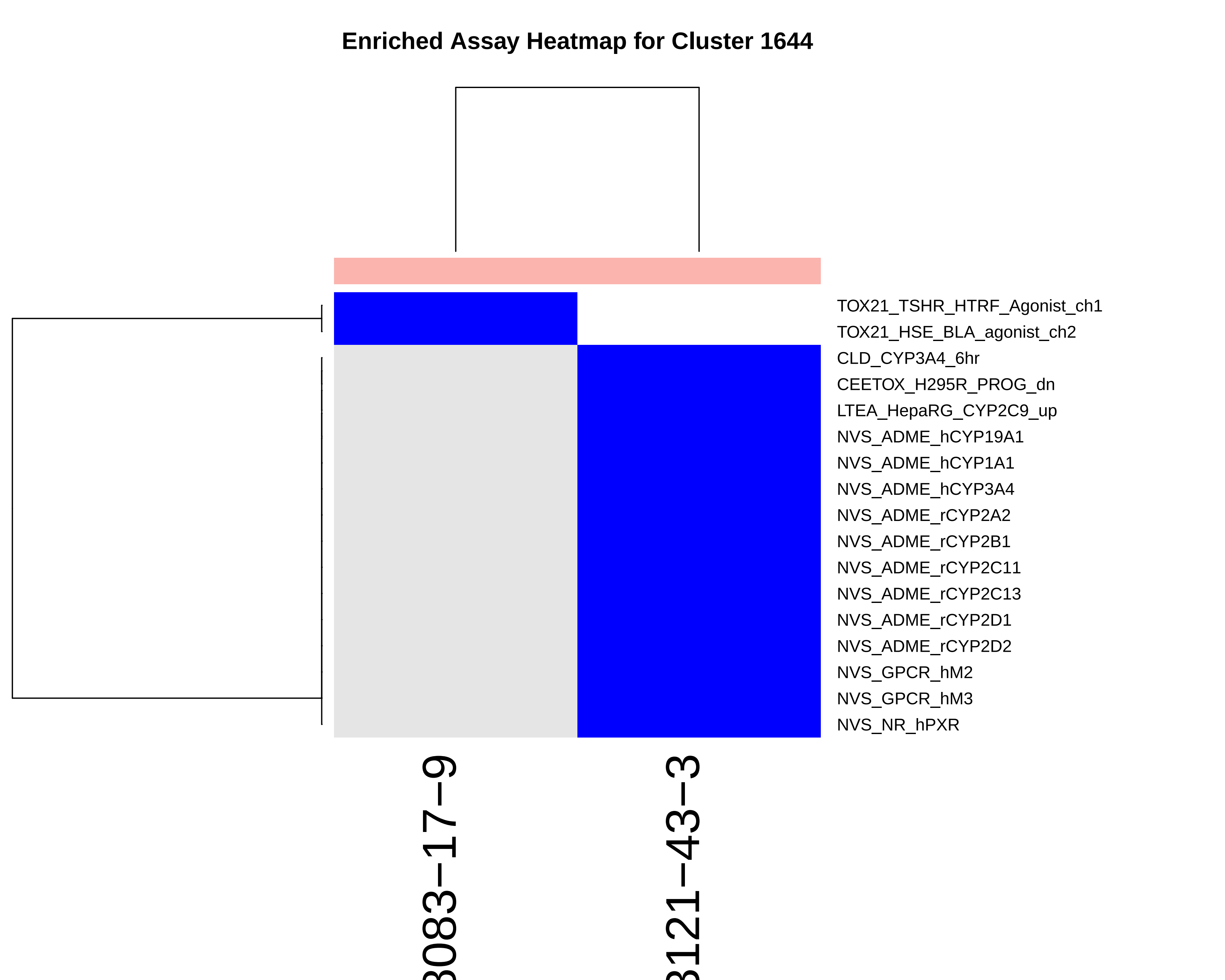

Enriched Assay Heatmap for Cluster 1644
TOX21_TSHR_HTRF_Agonist_ch1
TOX21_HSE_BLA_agonist_ch2
CLD_CYP3A4_6hr
CEETOX_H295R_PROG_dn
LTEA_HepaRG_CYP2C9_up
NVS_ADME_hCYP19A1
NVS_ADME_hCYP1A1
NVS_ADME_hCYP3A4
NVS_ADME_rCYP2A2
NVS_ADME_rCYP2B1
NVS_ADME_rCYP2C11
NVS_ADME_rCYP2C13
NVS_ADME_rCYP2D1
NVS_ADME_rCYP2D2
NVS_GPCR_hM2
NVS_GPCR_hM3
NVS_NR_hPXR

Enriched Assay Heatmap for Cluster 1647

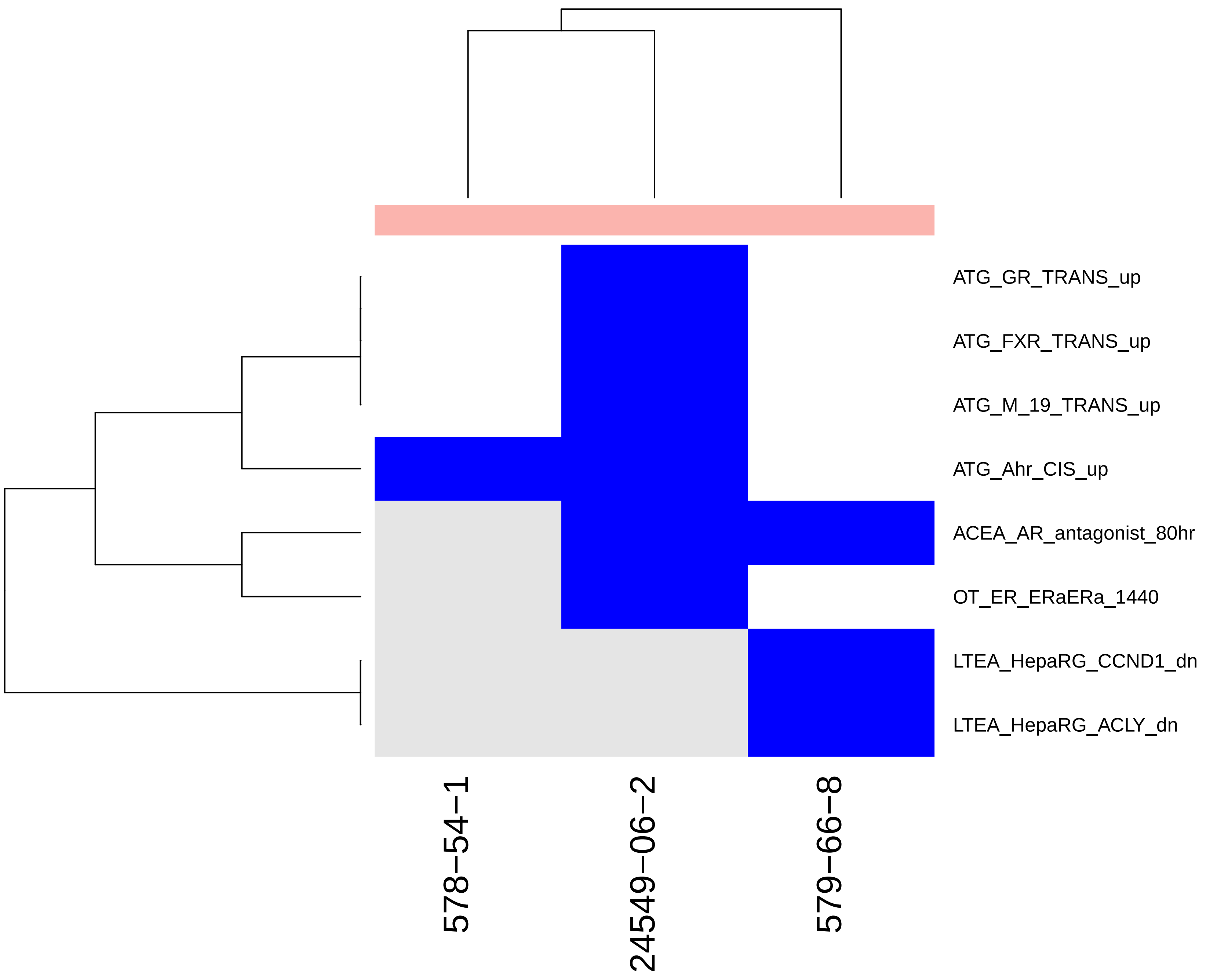

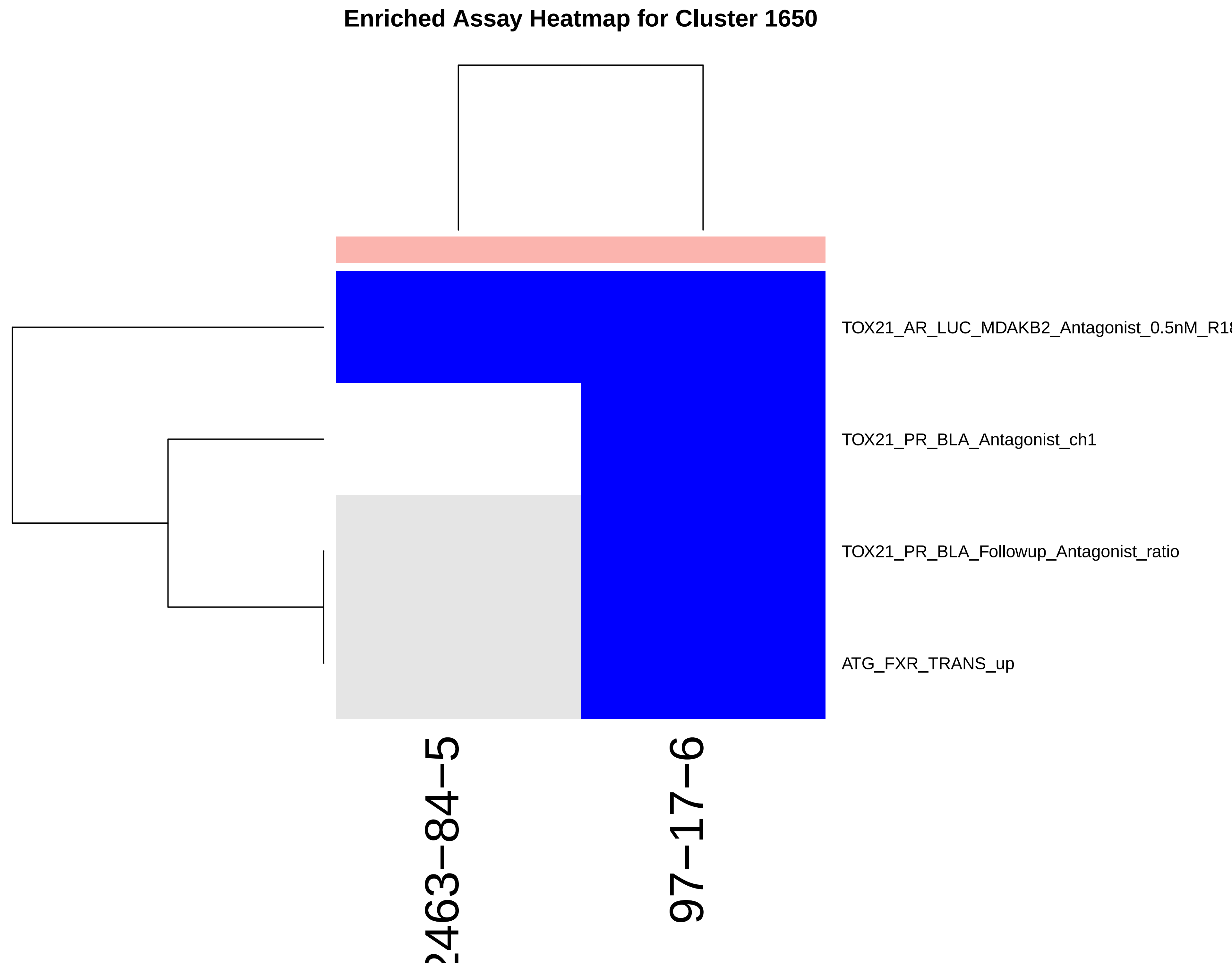

Enriched Assay Heatmap for Cluster 1650
TOX21_AR_LUC_MDAKB2_Antagonist_0.5nM_R18
TOX21_PR_BLA_Antagonist_ch1
TOX21_PR_BLA_Followup_Antagonist_ratio
ATG_FXR_TRANS_up
2463−84−5
97−17−6

Enriched Assay Heatmap for Cluster 1652

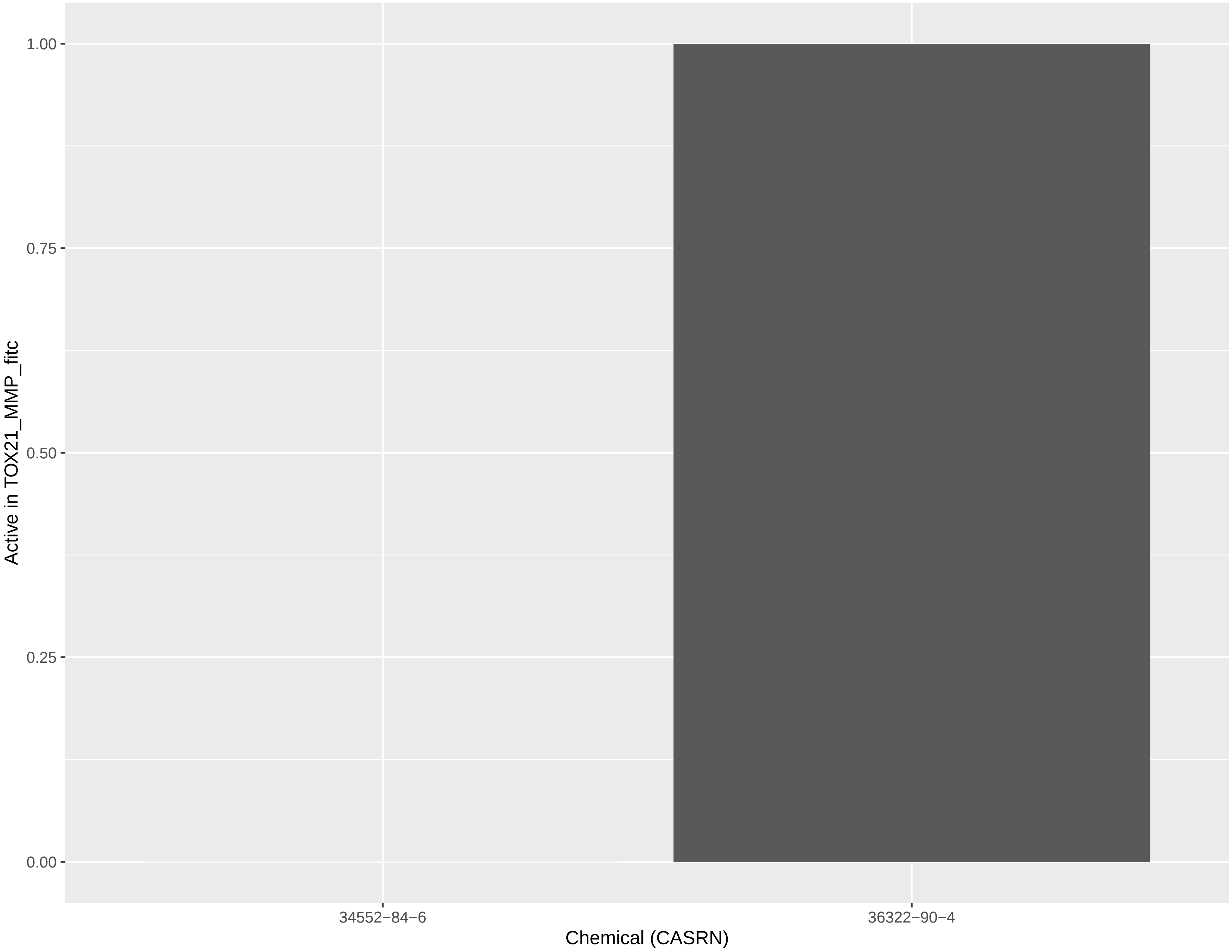

Enriched Assay Heatmap for Cluster 1653

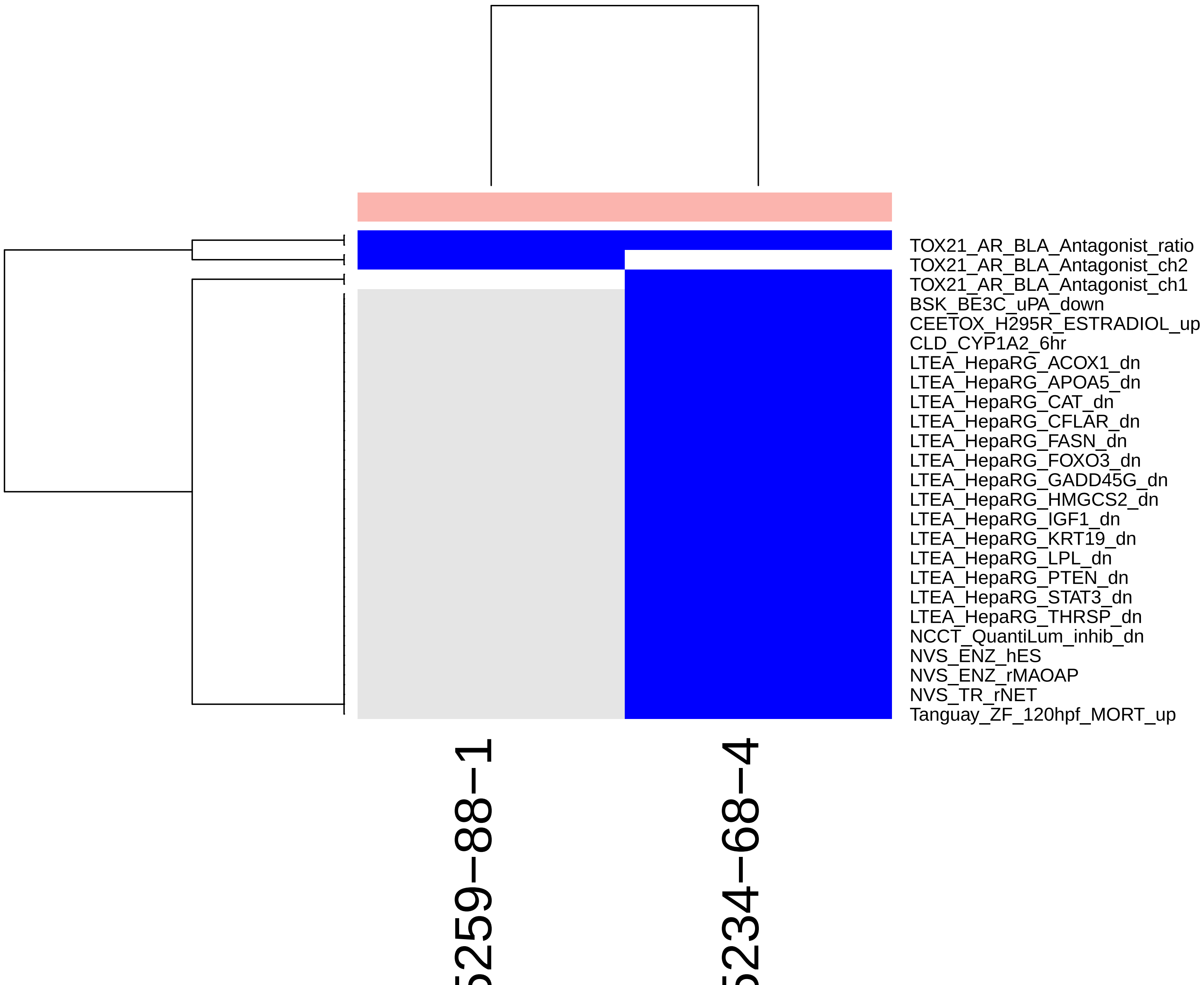

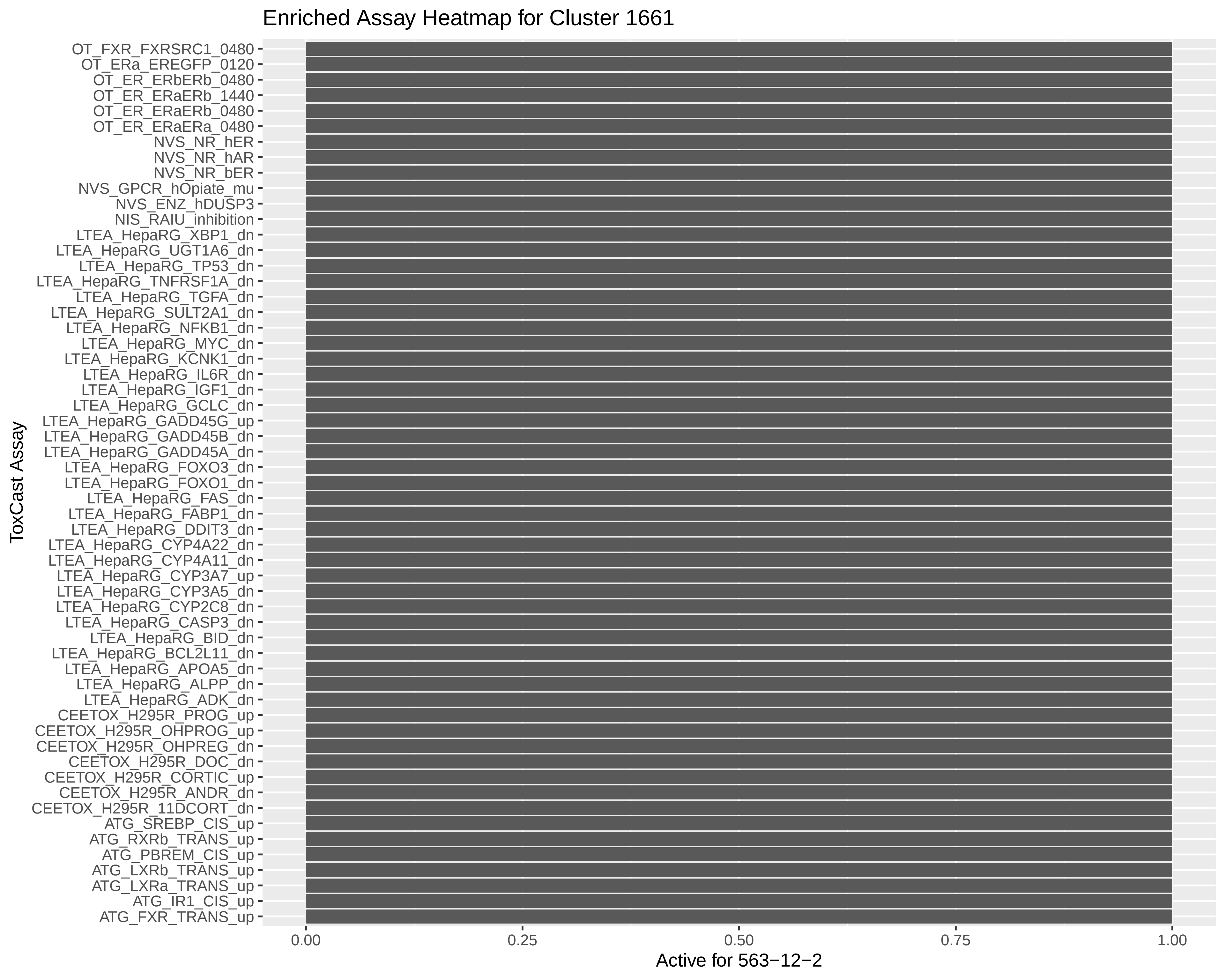

Enriched Assay Heatmap for Cluster 1661
ToxCast Assay
OT_FXR_FXRSRC1_0480
OT_ERa_EREGFP_0120
OT_ER_ERbERb_0480
OT_ER_ERaERb_1440
OT_ER_ERaERb_0480
OT_ER_ERaERa_0480
NVS_NR_hER
NVS_NR_hAR
NVS_NR_bER
NVS_GPCR_hOpiate_mu
NVS_ENZ_hDUSP3
NIS_RAIU_inhibition
LTEA_HepaRG_XBP1_dn
LTEA_HepaRG_UGT1A6_dn
LTEA_HepaRG_TP53_dn
LTEA_HepaRG_TNFRSF1A_dn
LTEA_HepaRG_TGFA_dn
LTEA_HepaRG_SULT2A1_dn
LTEA_HepaRG_NFKB1_dn
LTEA_HepaRG_MYC_dn
LTEA_HepaRG_KCNK1_dn
LTEA_HepaRG_IL6R_dn
LTEA_HepaRG_IGF1_dn
LTEA_HepaRG_GCLC_dn
LTEA_HepaRG_GADD45G_up
LTEA_HepaRG_GADD45B_dn
LTEA_HepaRG_GADD45A_dn
LTEA_HepaRG_FOXO3_dn
LTEA_HepaRG_FOXO1_dn
LTEA_HepaRG_FAS_dn
LTEA_HepaRG_FABP1_dn
LTEA_HepaRG_DDIT3_dn
LTEA_HepaRG_CYP4A22_dn
LTEA_HepaRG_CYP4A11_dn
LTEA_HepaRG_CYP3A7_up
LTEA_HepaRG_CYP3A5_dn
LTEA_HepaRG_CYP2C8_dn
LTEA_HepaRG_CASP3_dn
LTEA_HepaRG_BID_dn
LTEA_HepaRG_BCL2L11_dn
LTEA_HepaRG_APOA5_dn
LTEA_HepaRG_ALPP_dn
LTEA_HepaRG_ADK_dn
CEETOX_H295R_PROG_up
CEETOX_H295R_OHPROG_up
CEETOX_H295R_OHPREG_dn
CEETOX_H295R_DOC_dn
CEETOX_H295R_CORTIC_up
CEETOX_H295R_ANDR_dn
CEETOX_H295R_11DCORT_dn
ATG_SREBP_CIS_up
ATG_RXRb_TRANS_up
ATG_PBREM_CIS_up
ATG_LXRb_TRANS_up
ATG_LXRa_TRANS_up
ATG_IR1_CIS_up
ATG_FXR_TRANS_up
0.00
0.25
0.50
0.75
1.00
Active for 563−12−2

Enriched Assay Heatmap for Cluster 1662

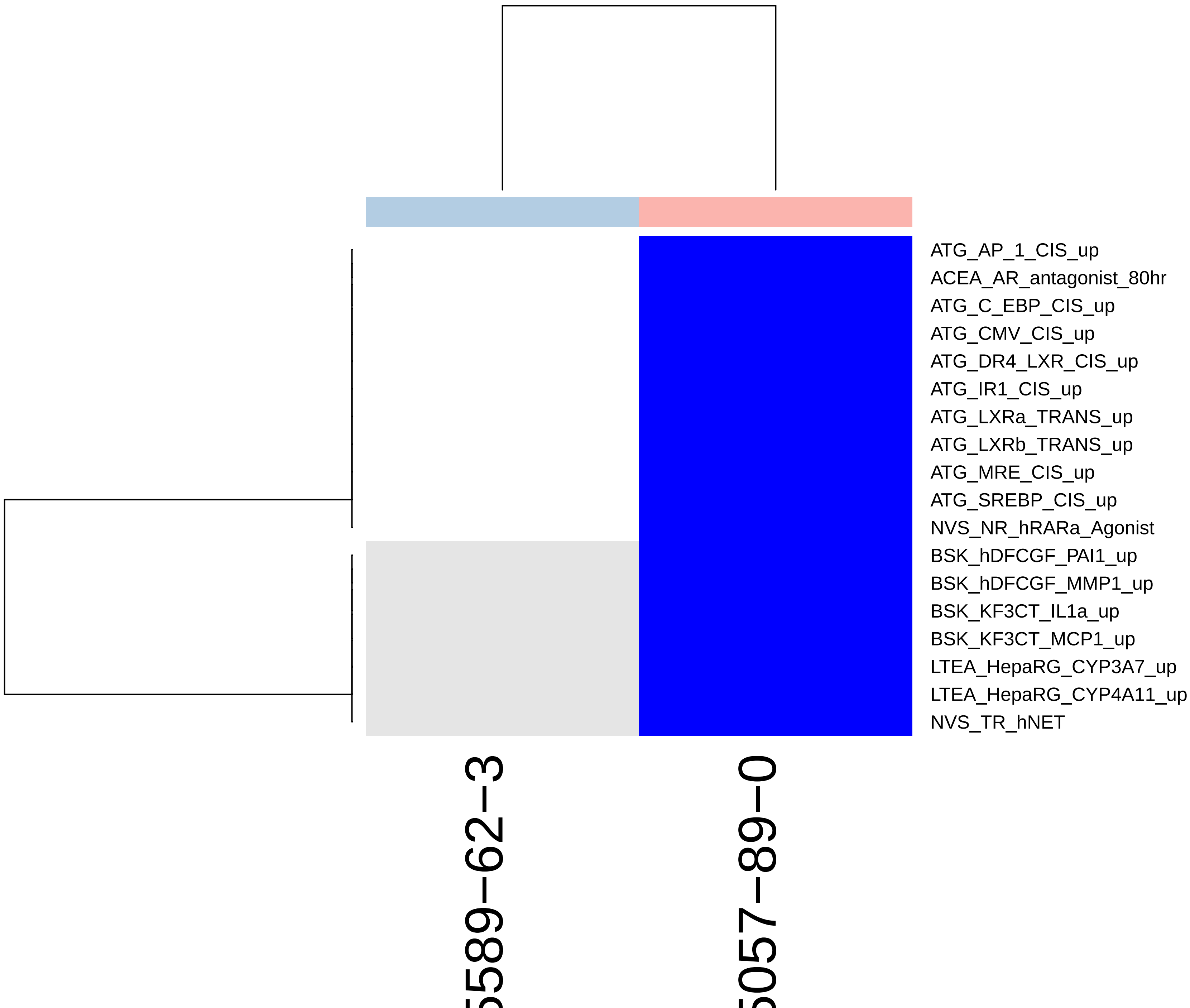

Enriched Assay Heatmap for Cluster 1667

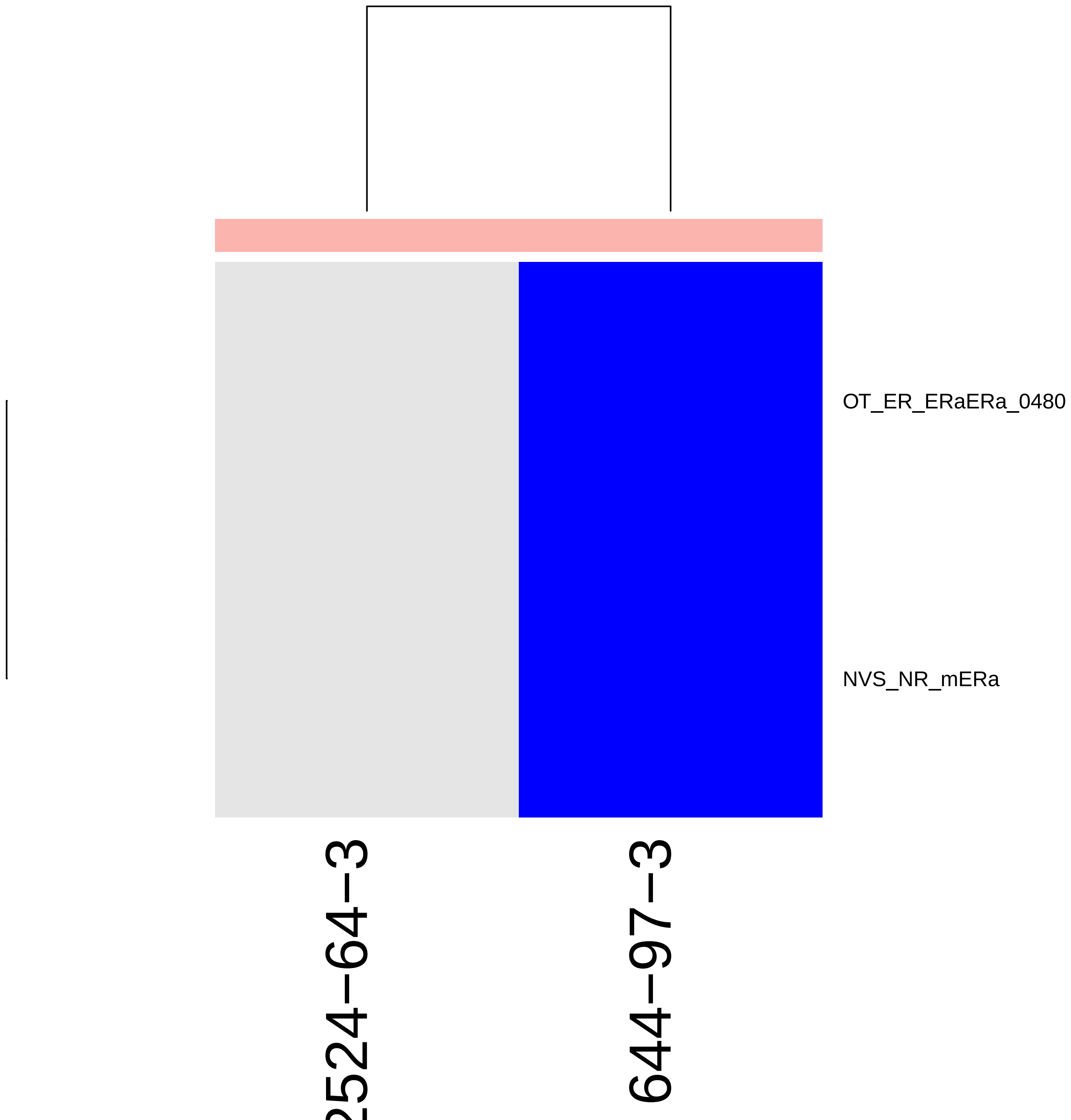

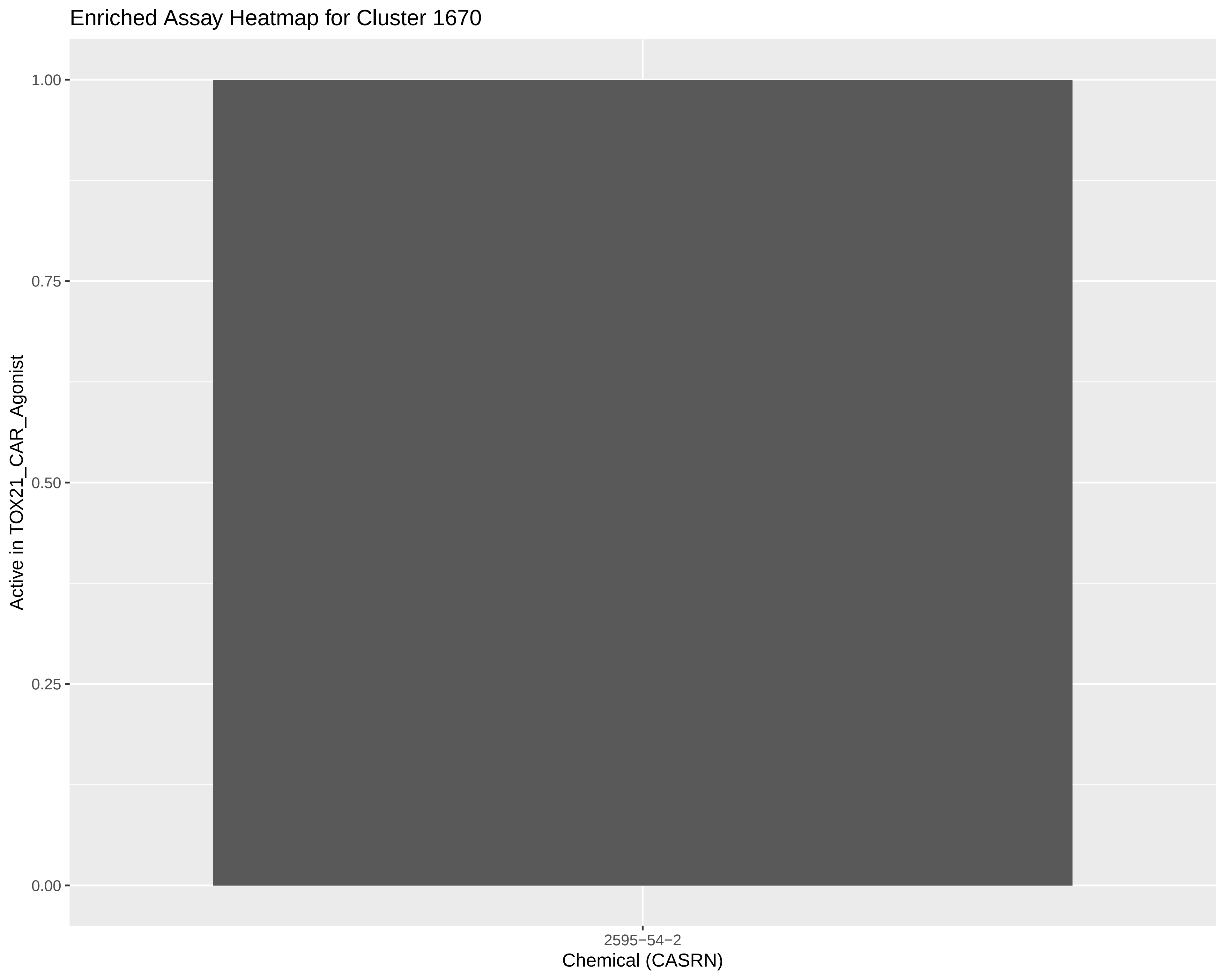

Enriched Assay Heatmap for Cluster 1670
1.00
0.75
0.50
0.25
0.00
Active in TOX21_CAR_Agonist
2595−54−2
Chemical (CASRN)

Enriched Assay Heatmap for Cluster 1677

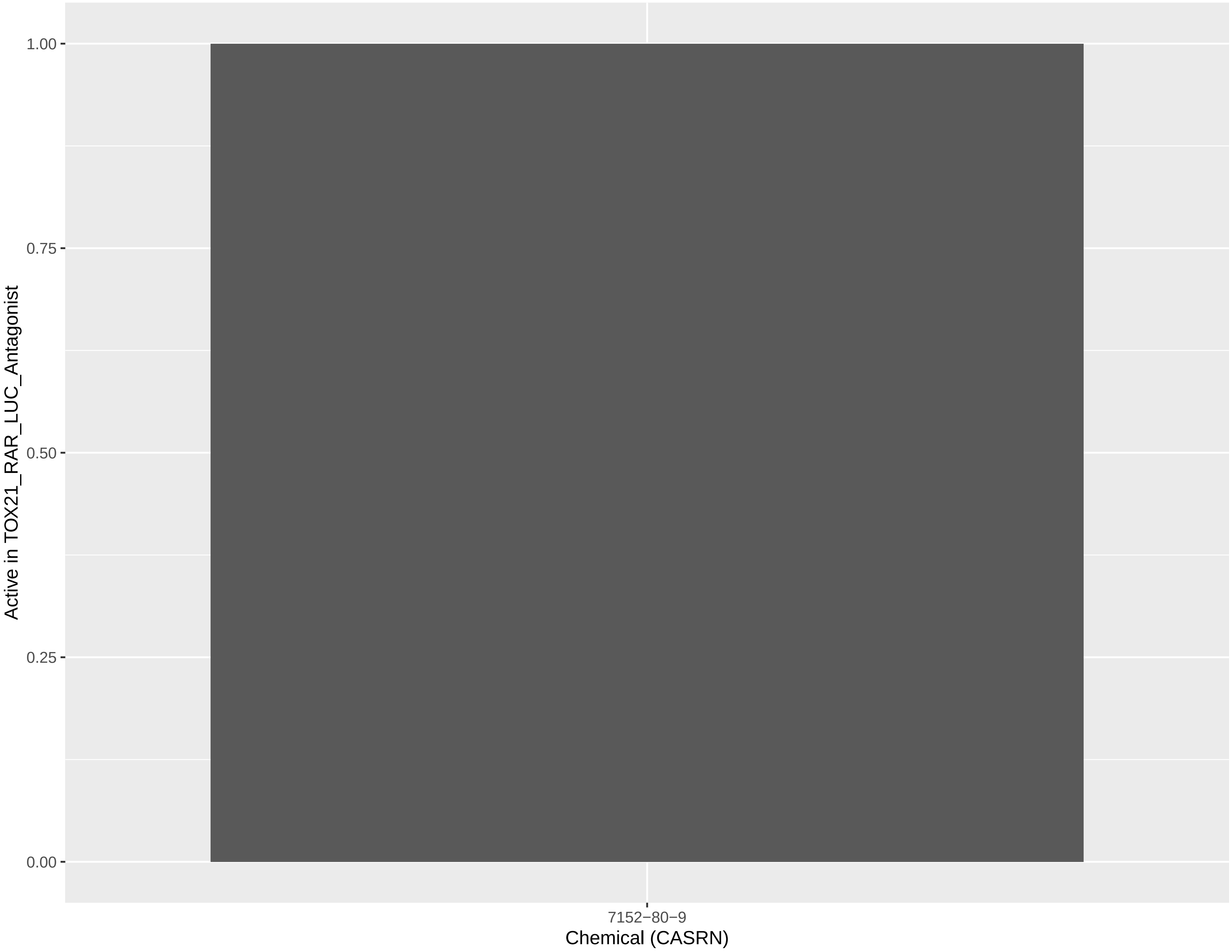

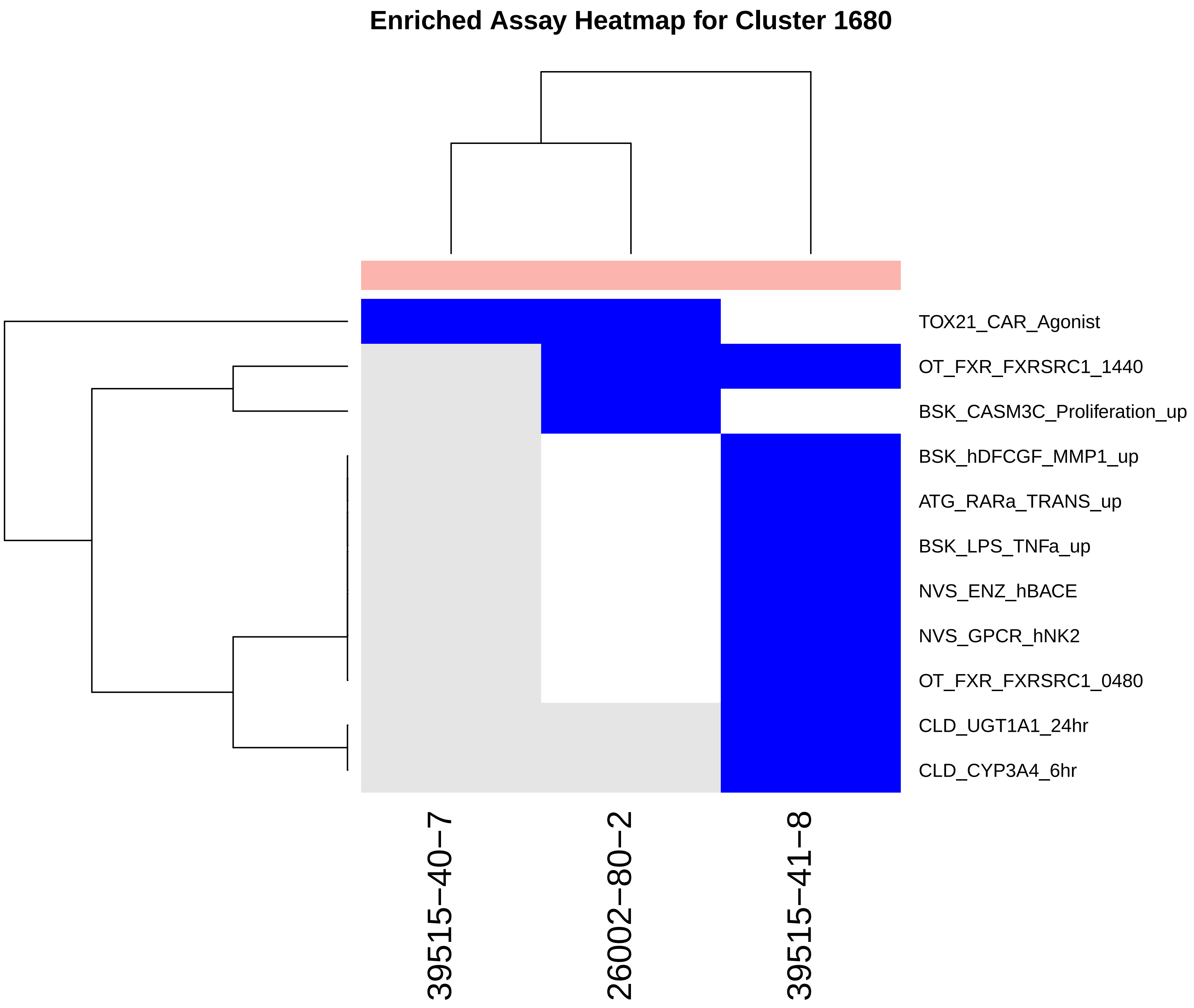
Enriched Assay Heatmap for Cluster 1680
TOX21_CAR_Agonist
OT_FXR_FXRSRC1_1440
BSK_CASM3C_Proliferation_up
BSK_hDFCGF_MMP1_up
ATG_RARa_TRANS_up
BSK_LPS_TNFa_up
NVS_ENZ_hBACE
NVS_GPCR_hNK2
OT_FXR_FXRSRC1_0480
CLD_UGT1A1_24hr
CLD_CYP3A4_6hr
39515−40−7
26002−80−2
39515−41−8

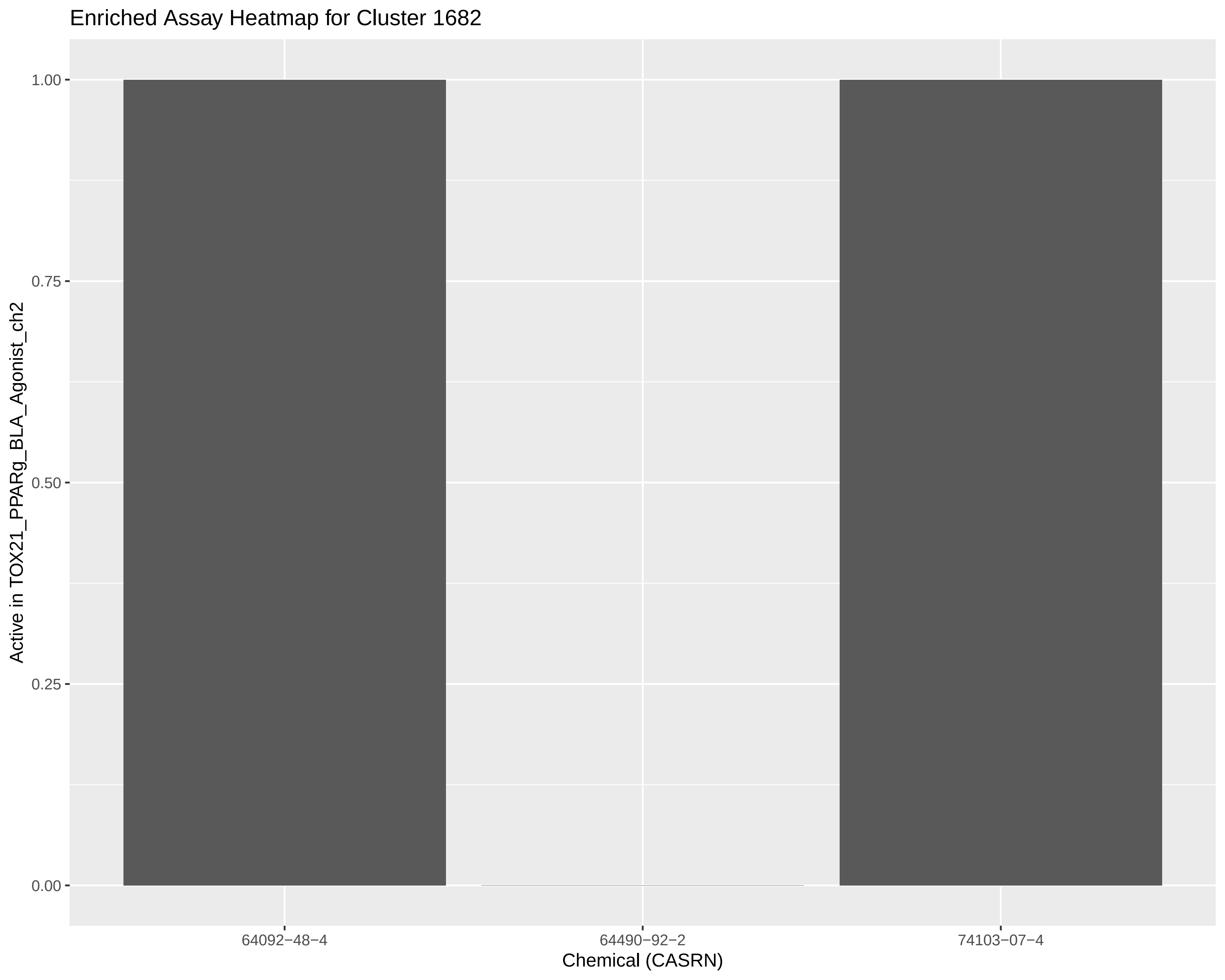

Enriched Assay Heatmap for Cluster 1682
Active in TOX21_PPARg_BLA_Agonist_ch2
1.00
0.75
0.50
0.25
0.00
64092−48−4
64490−92−2
74103−07−4
Chemical (CASRN)

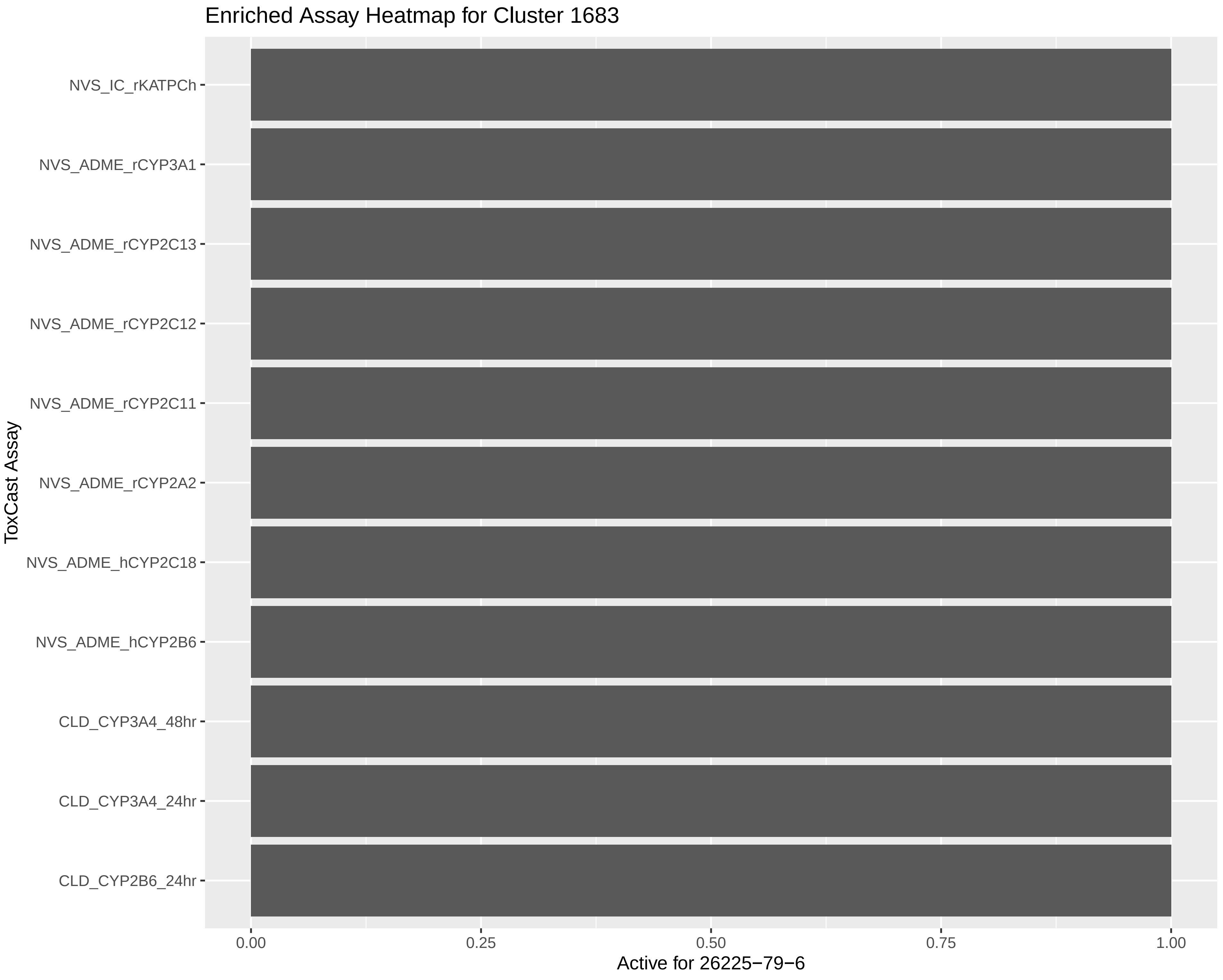
Enriched Assay Heatmap for Cluster 1683
NVS_IC_rKATPCh
NVS_ADME_rCYP3A1
NVS_ADME_rCYP2C13
NVS_ADME_rCYP2C12
NVS_ADME_rCYP2C11
NVS_ADME_rCYP2A2
NVS_ADME_hCYP2C18
NVS_ADME_hCYP2B6
CLD_CYP3A4_48hr
CLD_CYP3A4_24hr
CLD_CYP2B6_24hr
ToxCast Assay
0.00
0.25
0.50
0.75
1.00
Active for 26225−79−6

Enriched Assay Heatmap for Cluster 1686

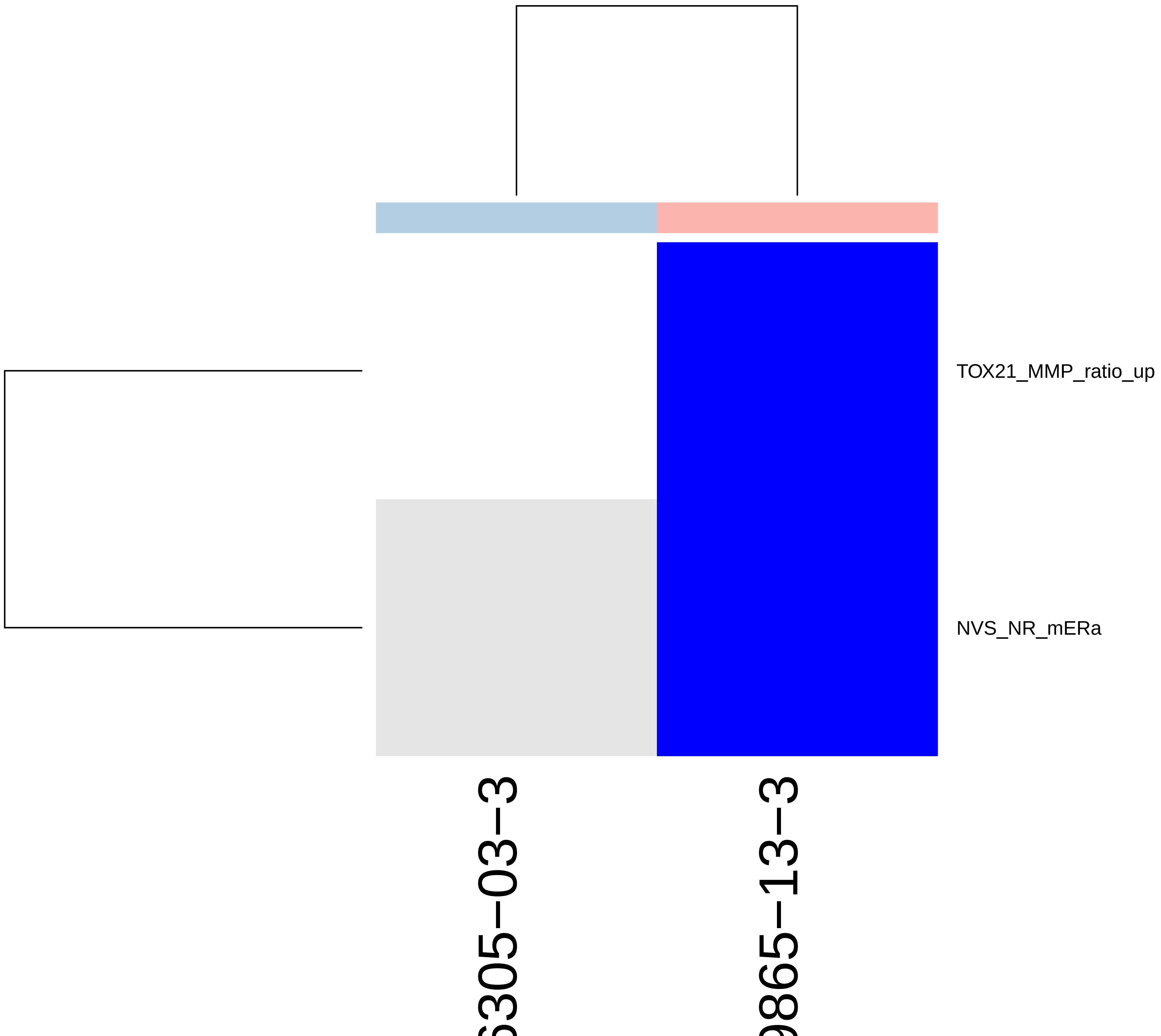

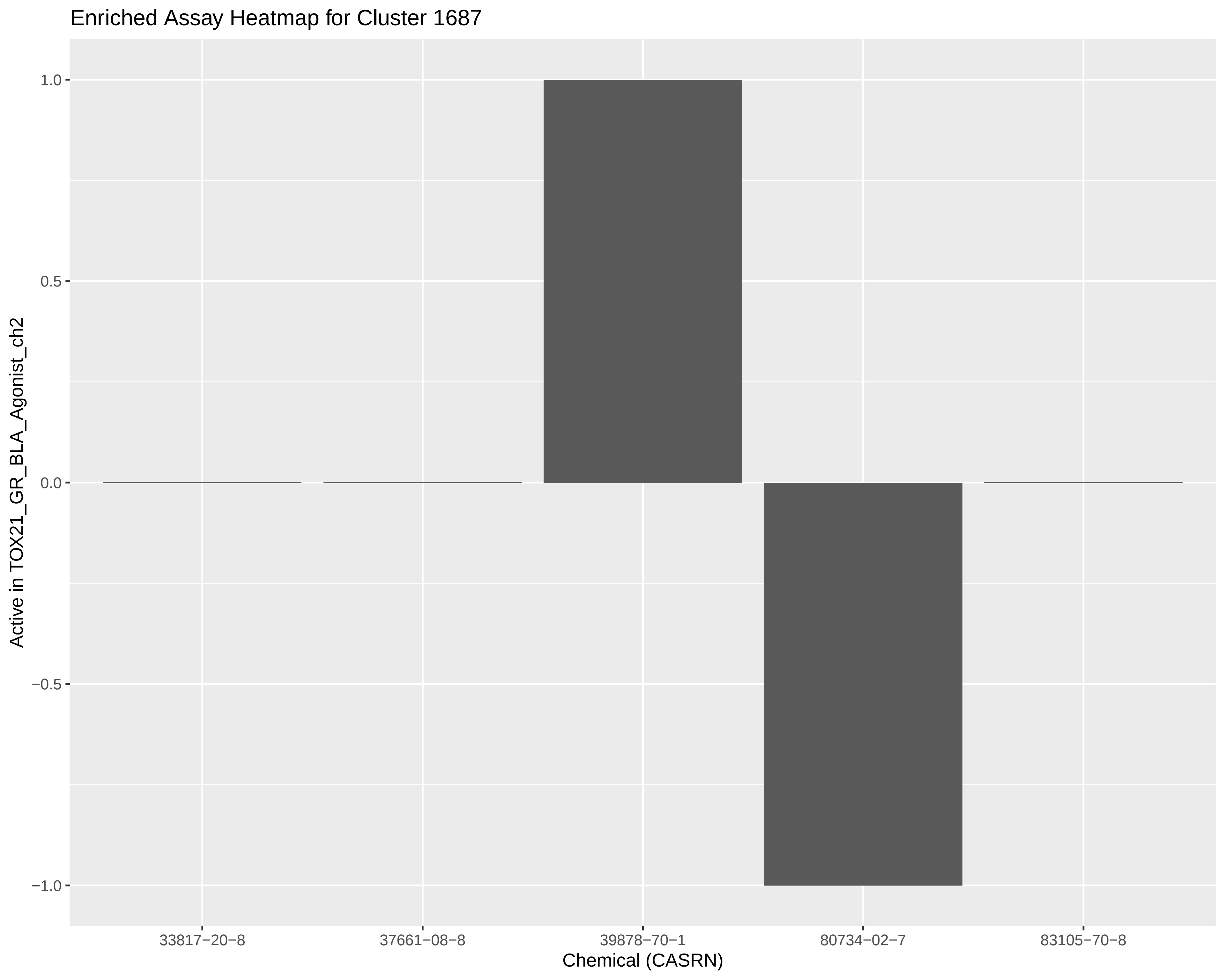
Enriched Assay Heatmap for Cluster 1687
Active in TOX21_GR_BLA_Agonist_ch2
1.0
0.5
0.0
−0.5
−1.0
33817−20−8
37661−08−8
39878−70−1
80734−02−7
83105−70−8
Chemical (CASRN)

Enriched Assay Heatmap for Cluster 1688

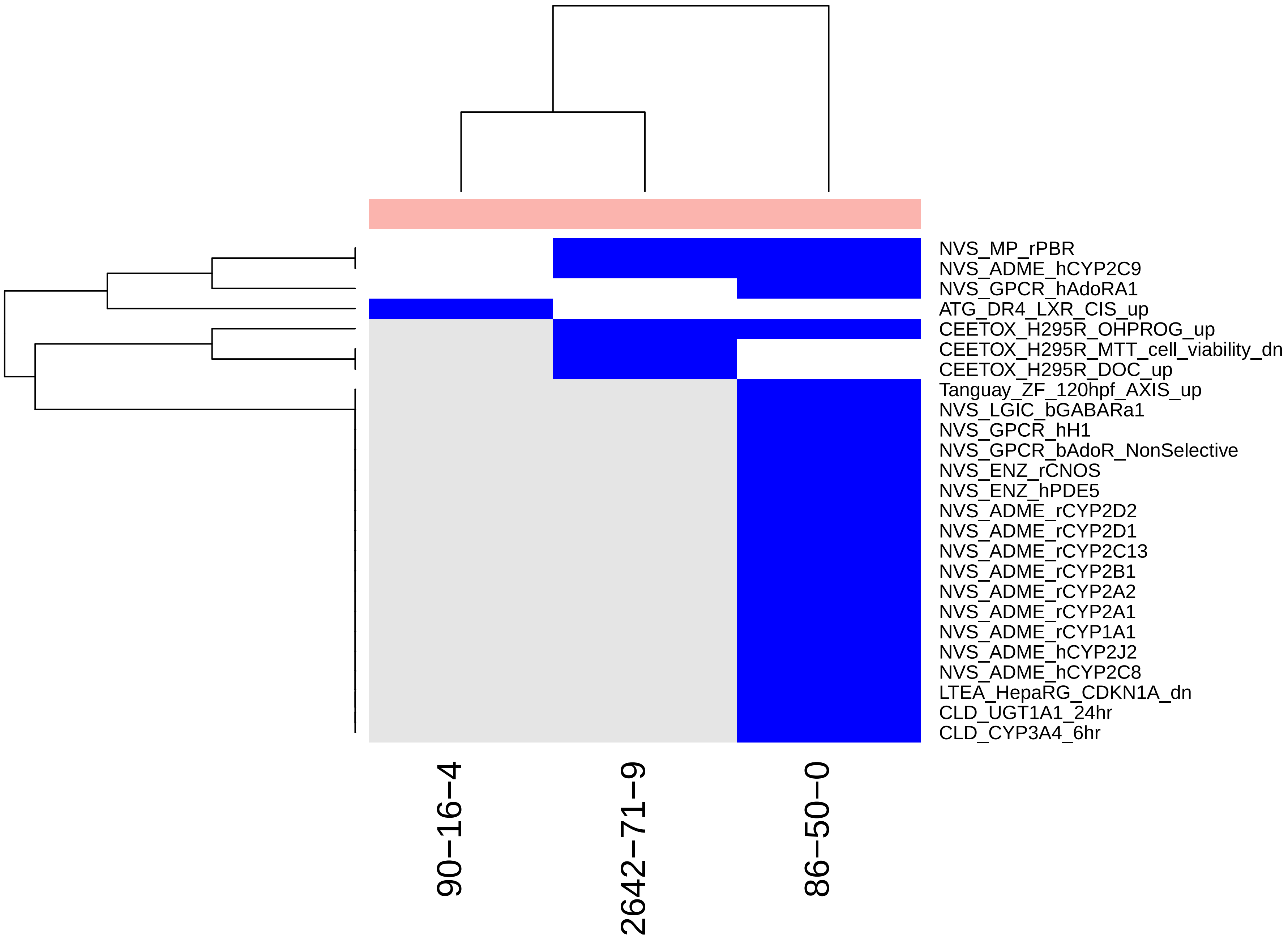

Enriched Assay Heatmap for Cluster 1689

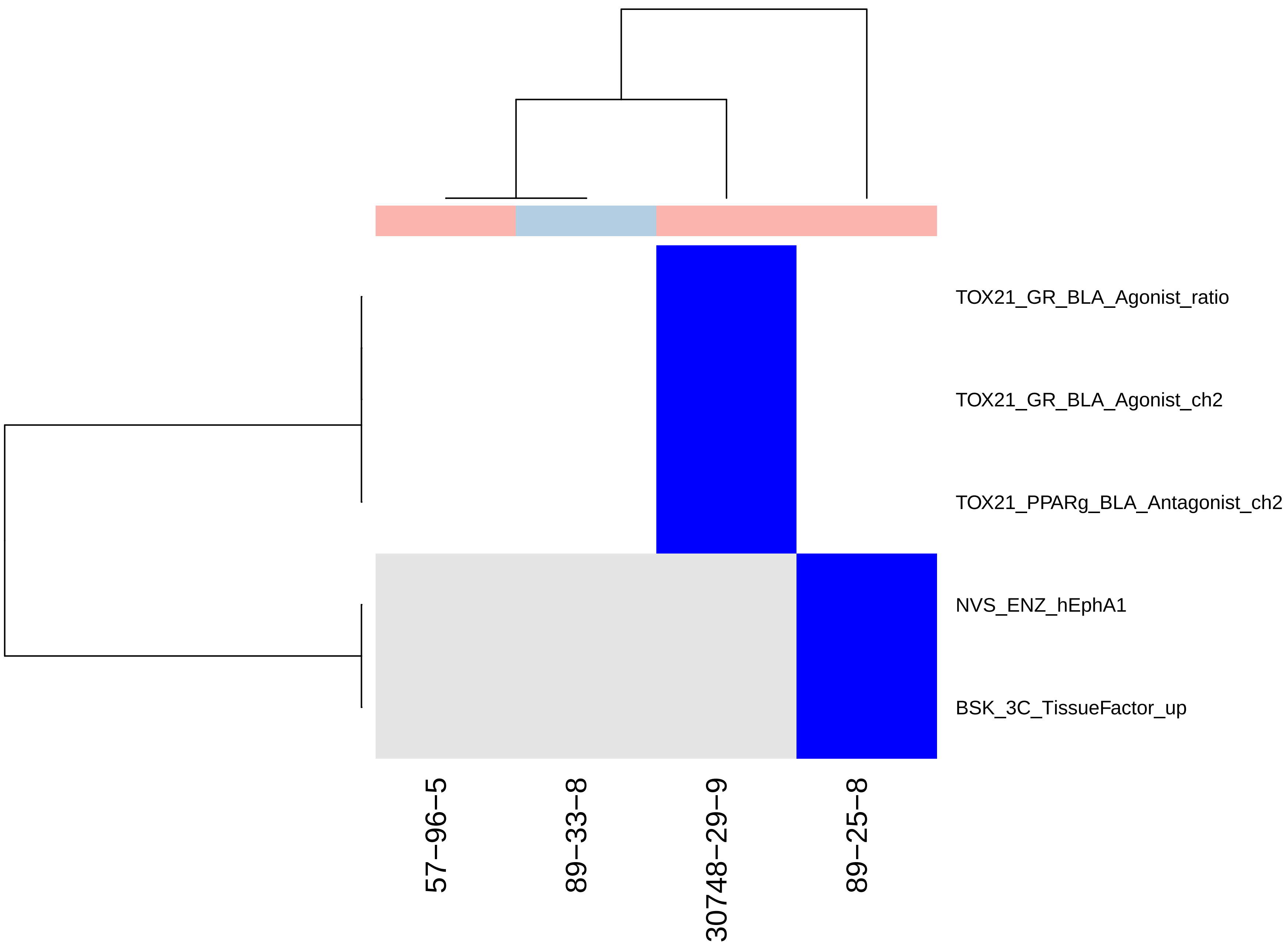

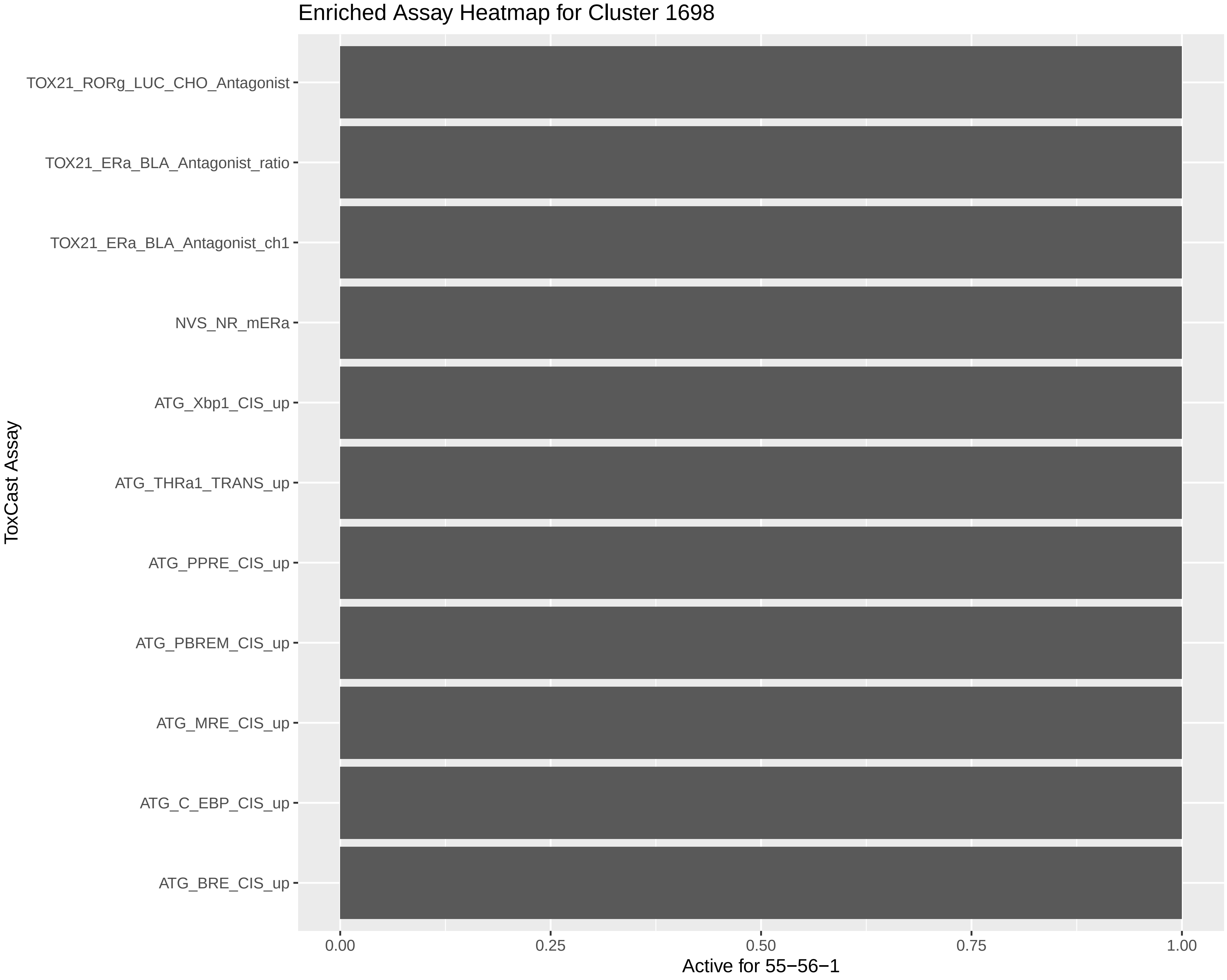

Enriched Assay Heatmap for Cluster 1698
ToxCast Assay
TOX21_RORg_LUC_CHO_Antagonist
TOX21_ERa_BLA_Antagonist_ratio
TOX21_ERa_BLA_Antagonist_ch1
NVS_NR_mERa
ATG_Xbp1_CIS_up
ATG_THRa1_TRANS_up
ATG_PPRE_CIS_up
ATG_PBREM_CIS_up
ATG_MRE_CIS_up
ATG_C_EBP_CIS_up
ATG_BRE_CIS_up
0.00
0.25
0.50
0.75
1.00
Active for 55−56−1

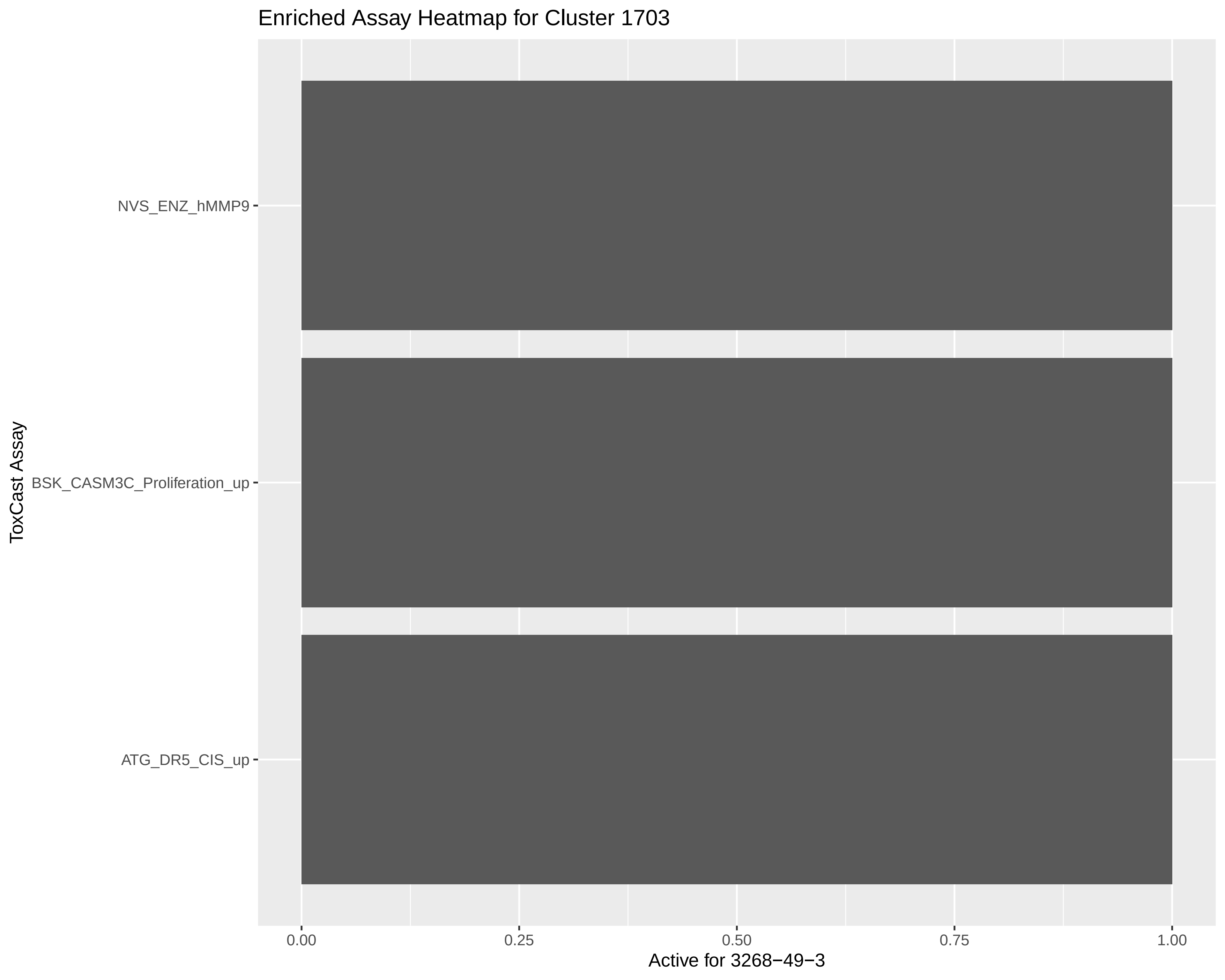

Enriched Assay Heatmap for Cluster 1703
ToxCast Assay
NVS_ENZ_hMMP9
BSK_CASM3C_Proliferation_up
ATG_DR5_CIS_up
0.00
0.25
0.50
0.75
1.00
Active for 3268−49−3

Enriched Assay Heatmap for Cluster 1706

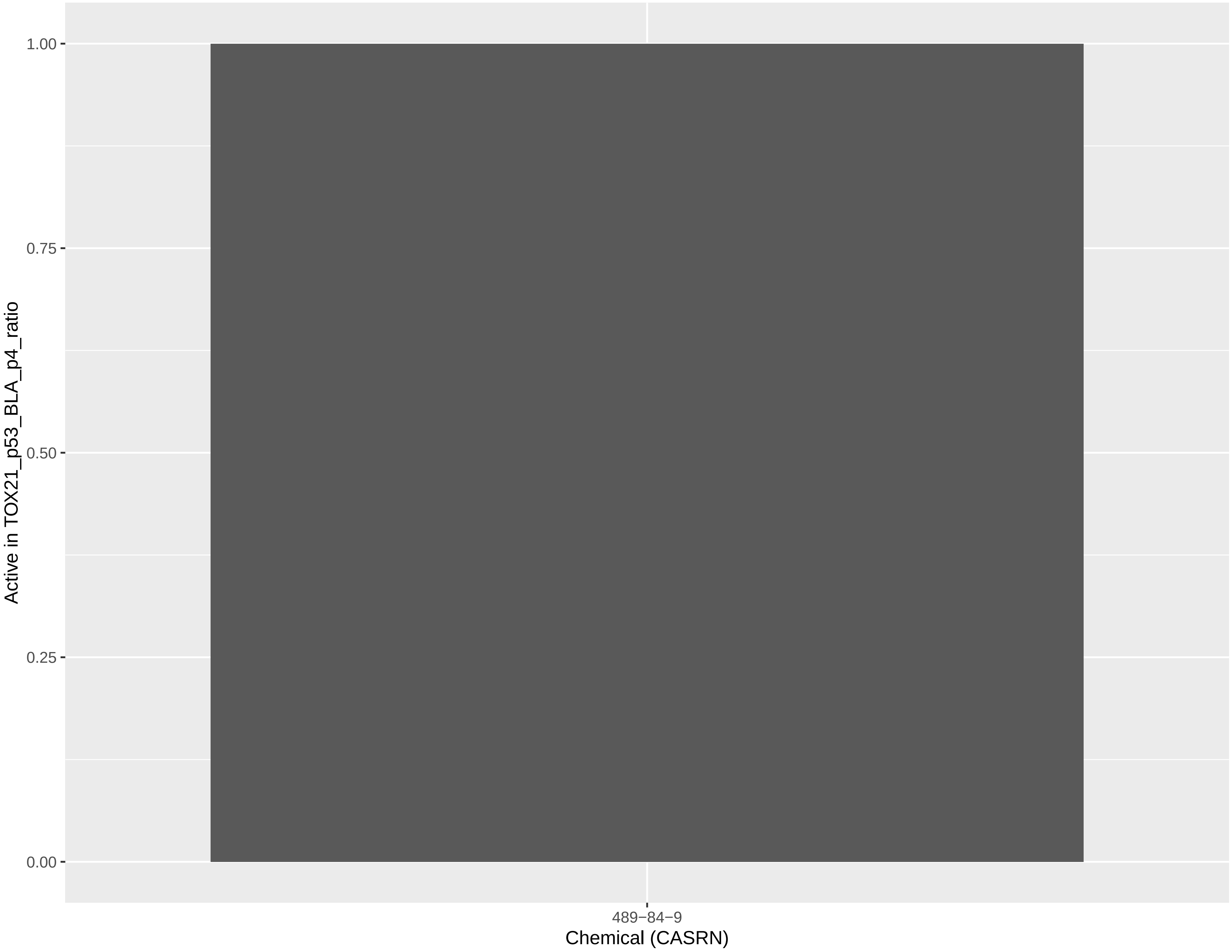

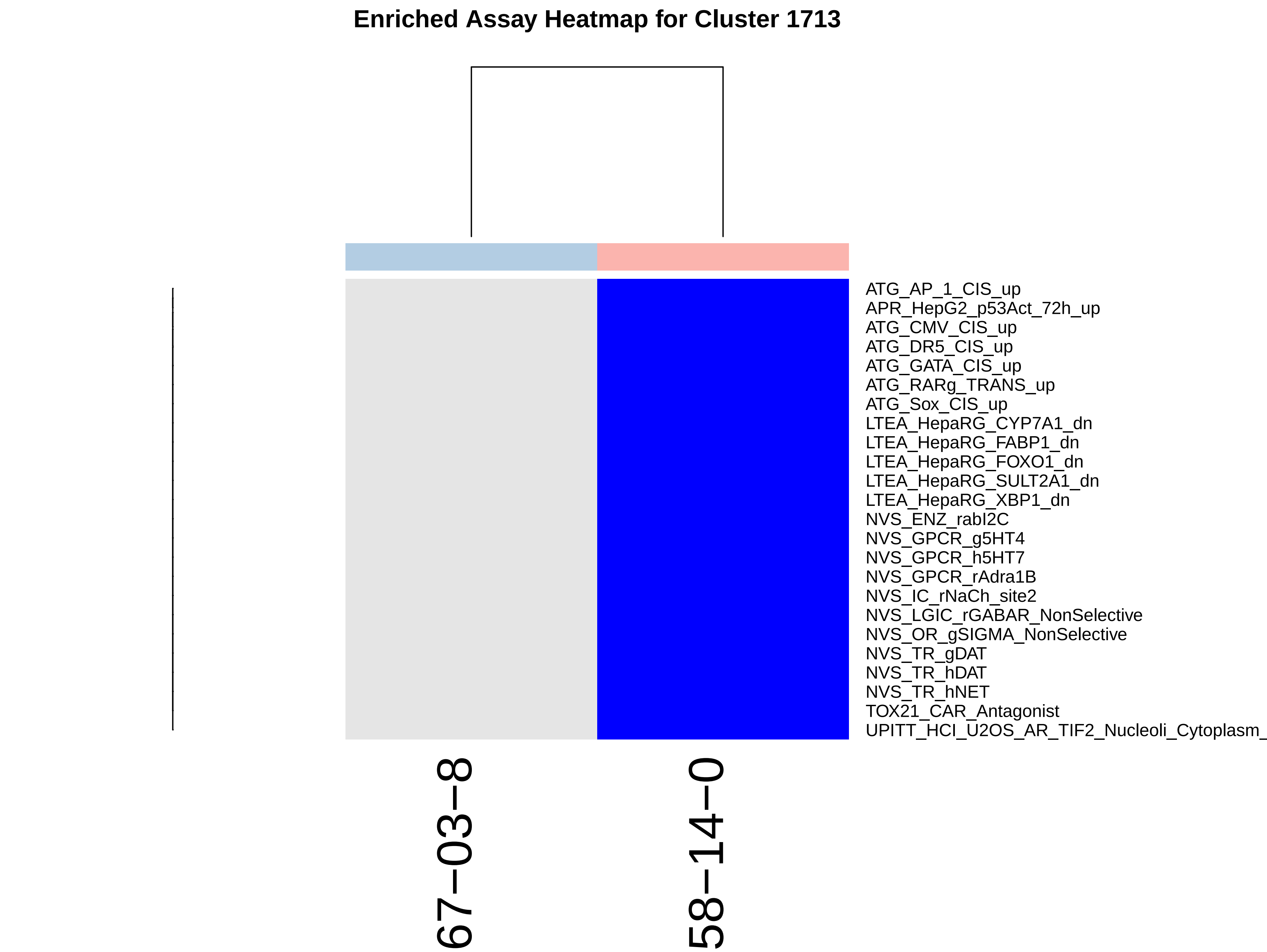
Enriched Assay Heatmap for Cluster 1713
ATG_AP_1_CIS_up
APR_HepG2_p53Act_72h_up
ATG_CMV_CIS_up
ATG_DR5_CIS_up
ATG_GATA_CIS_up
ATG_RARg_TRANS_up
ATG_Sox_CIS_up
LTEA_HepaRG_CYP7A1_dn
LTEA_HepaRG_FABP1_dn
LTEA_HepaRG_FOXO1_dn
LTEA_HepaRG_SULT2A1_dn
LTEA_HepaRG_XBP1_dn
NVS_ENZ_rabI2C
NVS_GPCR_g5HT4
NVS_GPCR_h5HT7
NVS_GPCR_rAdra1B
NVS_IC_rNaCh_site2
NVS_LGIC_rGABAR_NonSelective
NVS_OR_gSIGMA_NonSelective
NVS_TR_gDAT
NVS_TR_hDAT
NVS_TR_hNET
TOX21_CAR_Antagonist
UPITT_HCI_U2OS_AR_TIF2_Nucleoli_Cytoplasm_
67−03−8
58−14−0

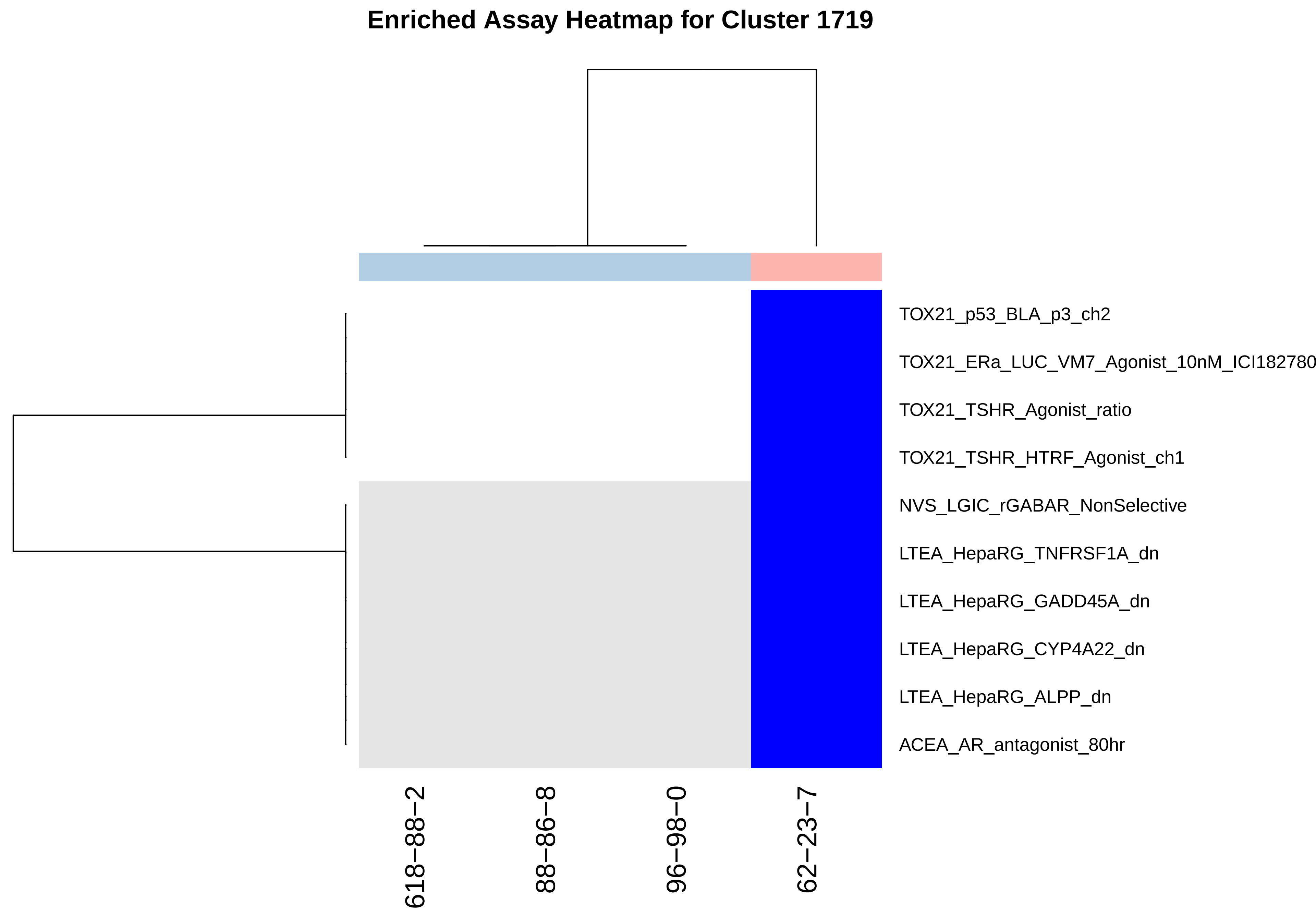

Enriched Assay Heatmap for Cluster 1719
TOX21_p53_BLA_p3_ch2
TOX21_ERa_LUC_VM7_Agonist_10nM_ICI182780
TOX21_TSHR_Agonist_ratio
TOX21_TSHR_HTRF_Agonist_ch1
NVS_LGIC_rGABAR_NonSelective
LTEA_HepaRG_TNFRSF1A_dn
LTEA_HepaRG_GADD45A_dn
LTEA_HepaRG_CYP4A22_dn
LTEA_HepaRG_ALPP_dn
ACEA_AR_antagonist_80hr
618−88−2
88−86−8
96−98−0
62−23−7

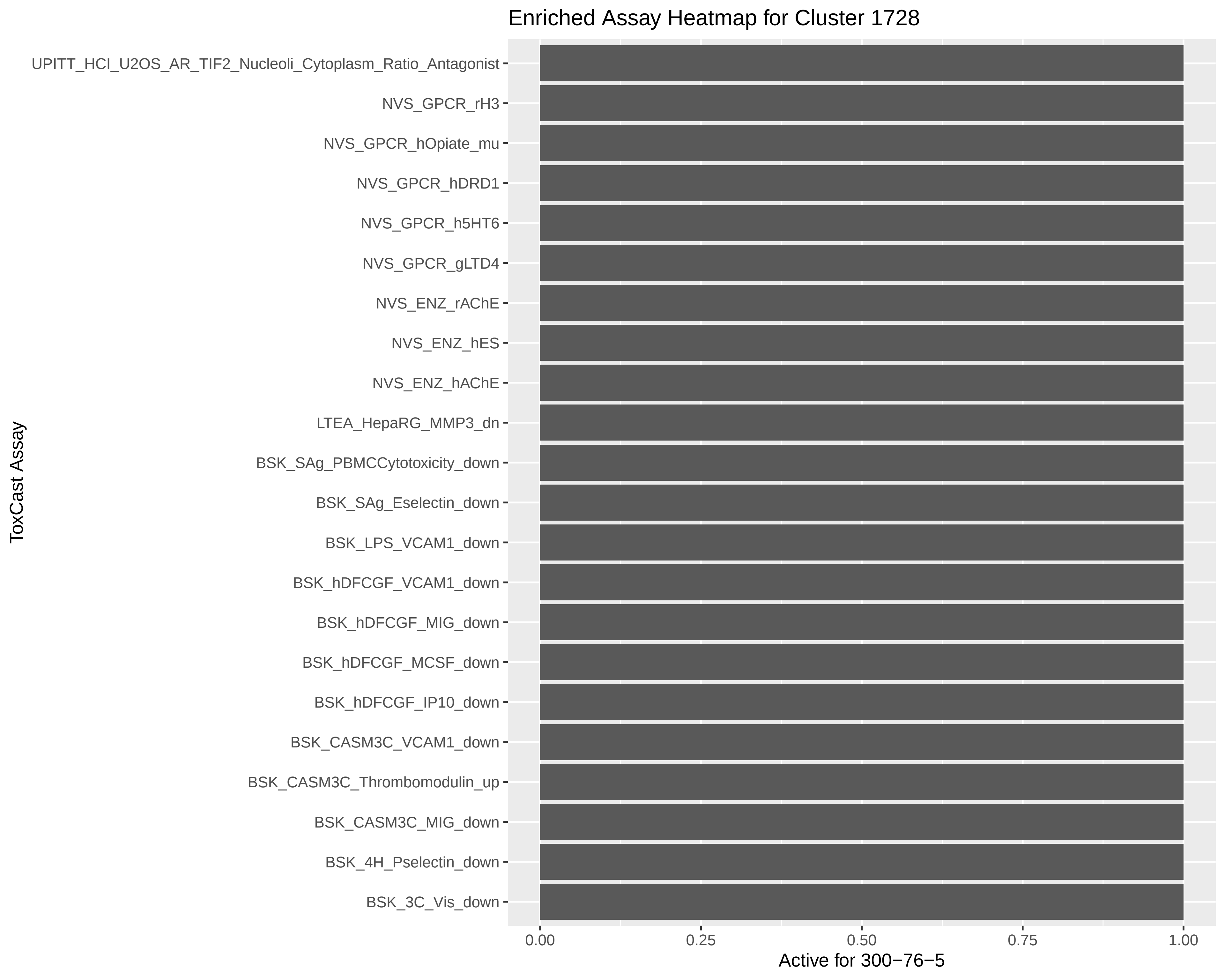

Enriched Assay Heatmap for Cluster 1728
ToxCast Assay
UPITT_HCI_U2OS_AR_TIF2_Nucleoli_Cytoplasm_Ratio_Antagonist
NVS_GPCR_rH3
NVS_GPCR_hOpiate_mu
NVS_GPCR_hDRD1
NVS_GPCR_h5HT6
NVS_GPCR_gLTD4
NVS_ENZ_rAChE
NVS_ENZ_hES
NVS_ENZ_hAChE
LTEA_HepaRG_MMP3_dn
BSK_SAg_PBMCCytotoxicity_down
BSK_SAg_Eselectin_down
BSK_LPS_VCAM1_down
BSK_hDFCGF_VCAM1_down
BSK_hDFCGF_MIG_down
BSK_hDFCGF_MCSF_down
BSK_hDFCGF_IP10_down
BSK_CASM3C_VCAM1_down
BSK_CASM3C_Thrombomodulin_up
BSK_CASM3C_MIG_down
BSK_4H_Pselectin_down
BSK_3C_Vis_down
0.00
0.25
0.50
0.75
1.00
Active for 300−76−5

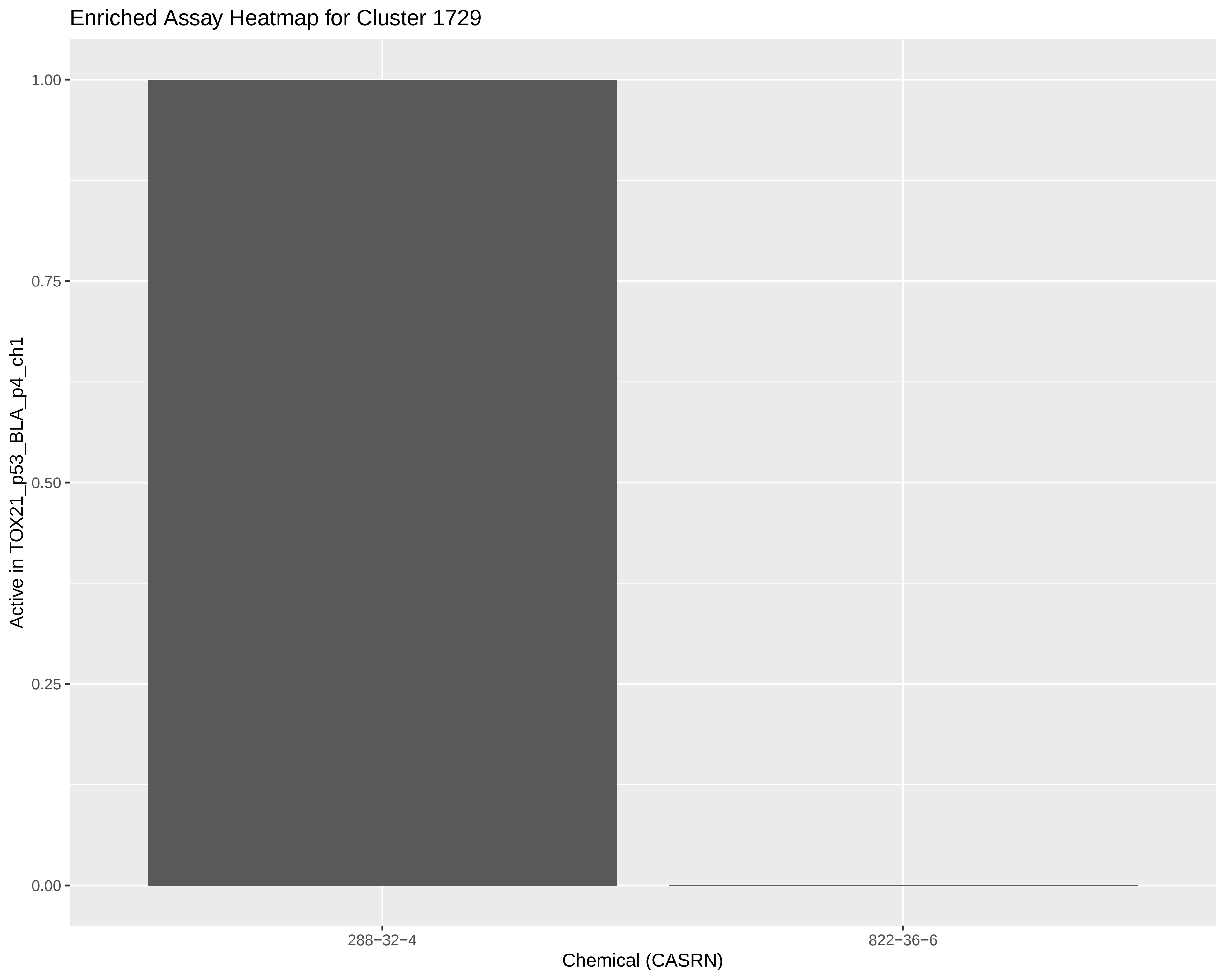

Enriched Assay Heatmap for Cluster 1729
Active in TOX21_p53_BLA_p4_ch1
1.00
0.75
0.50
0.25
0.00
288−32−4
822−36−6
Chemical (CASRN)

Enriched Assay Heatmap for Cluster 1730

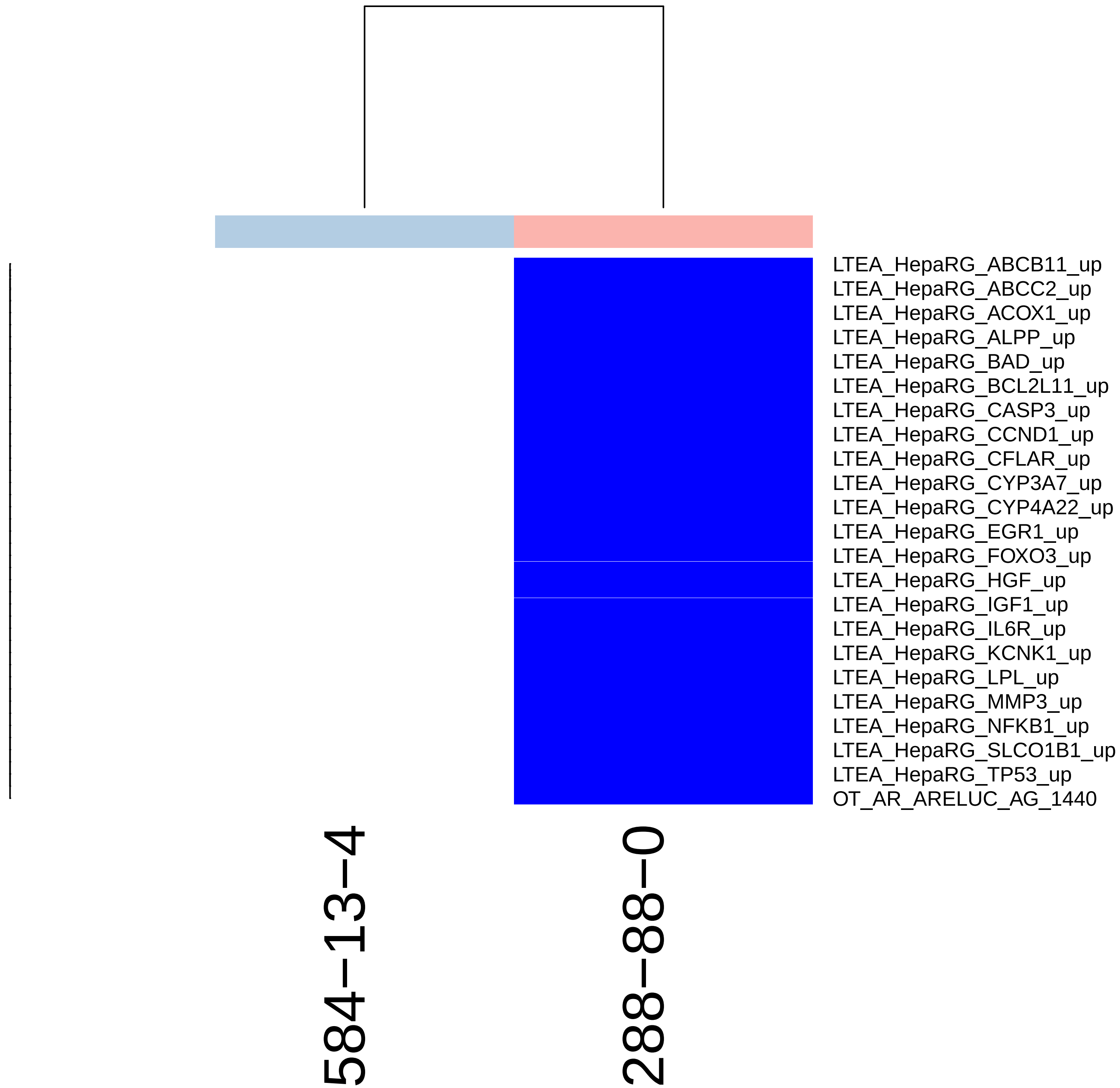

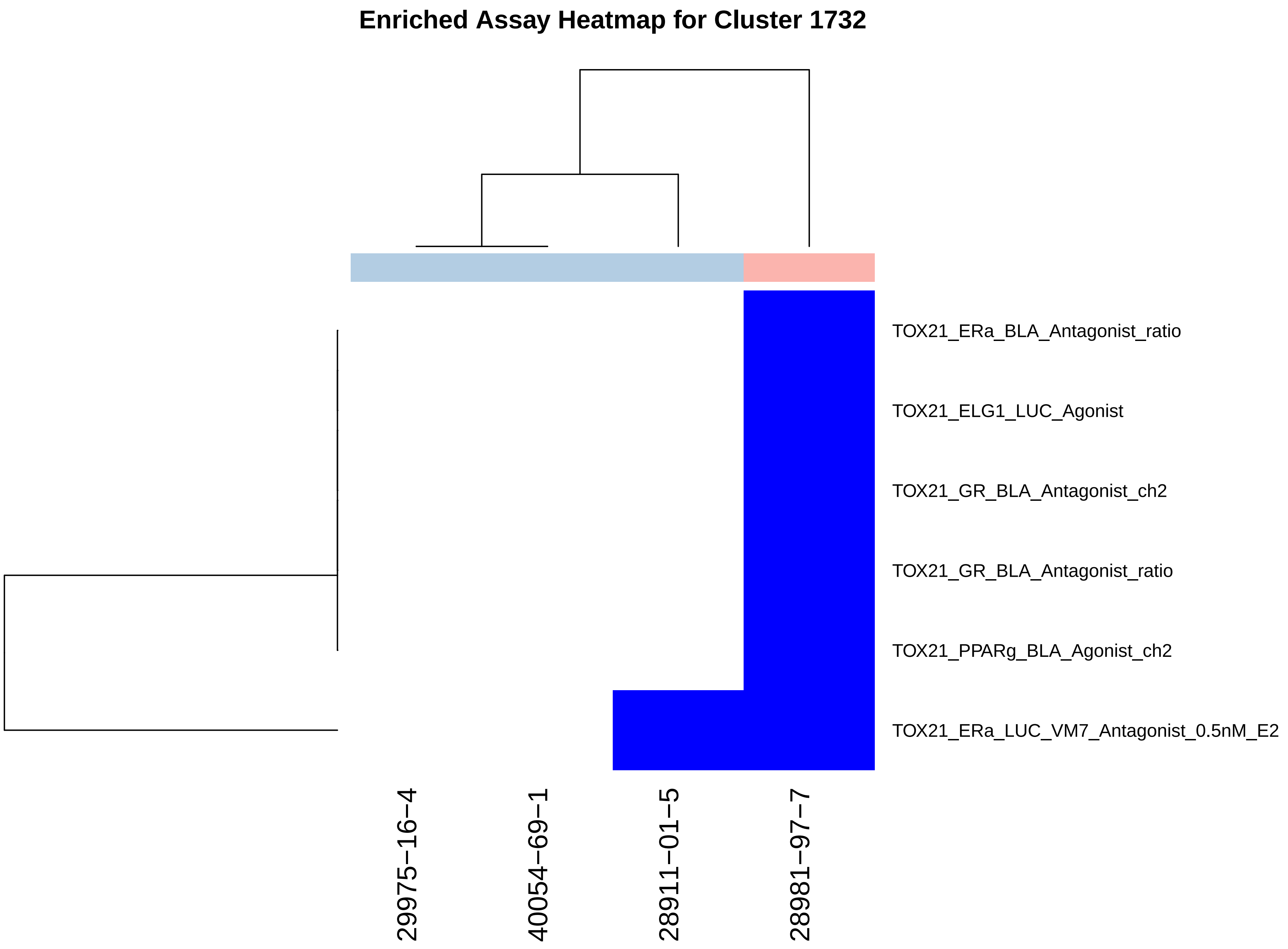
Enriched Assay Heatmap for Cluster 1732
TOX21_ERa_BLA_Antagonist_ratio
TOX21_ELG1_LUC_Agonist
TOX21_GR_BLA_Antagonist_ch2
TOX21_GR_BLA_Antagonist_ratio
TOX21_PPARg_BLA_Agonist_ch2
TOX21_ERa_LUC_VM7_Antagonist_0.5nM_E2
29975−16−4
40054−69−1
28911−01−5
28981−97−7

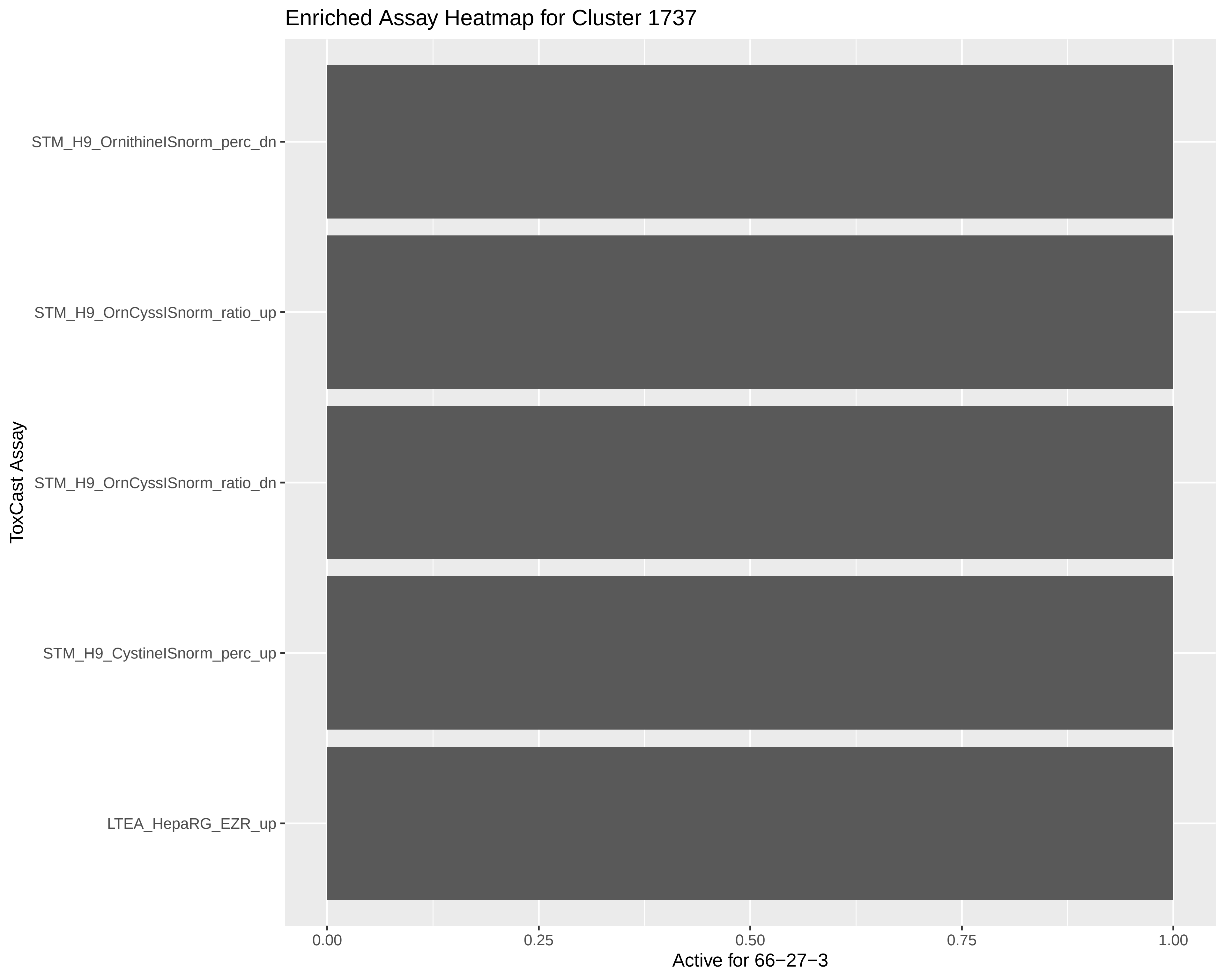

Enriched Assay Heatmap for Cluster 1737
ToxCast Assay
STM_H9_OrnithineISnorm_perc_dn
STM_H9_OrnCyssISnorm_ratio_up
STM_H9_OrnCyssISnorm_ratio_dn
STM_H9_CystineISnorm_perc_up
LTEA_HepaRG_EZR_up
0.00
0.25
0.50
0.75
1.00
Active for 66−27−3

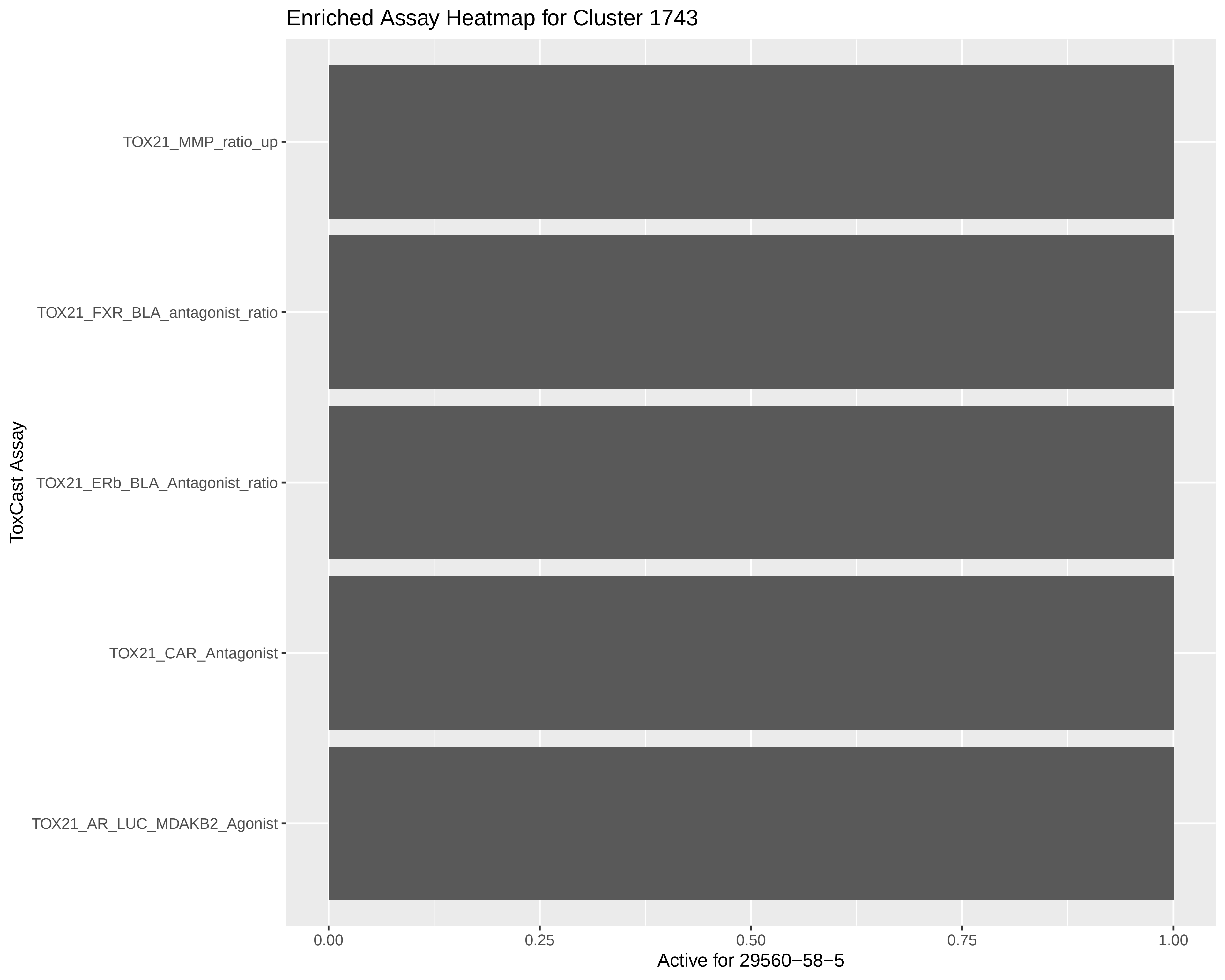
Enriched Assay Heatmap for Cluster 1743
ToxCast Assay
TOX21_MMP_ratio_up
TOX21_FXR_BLA_antagonist_ratio
TOX21_ERb_BLA_Antagonist_ratio
TOX21_CAR_Antagonist
TOX21_AR_LUC_MDAKB2_Agonist
0.00
0.25
0.50
0.75
1.00
Active for 29560−58−5

Enriched Assay Heatmap for Cluster 1745

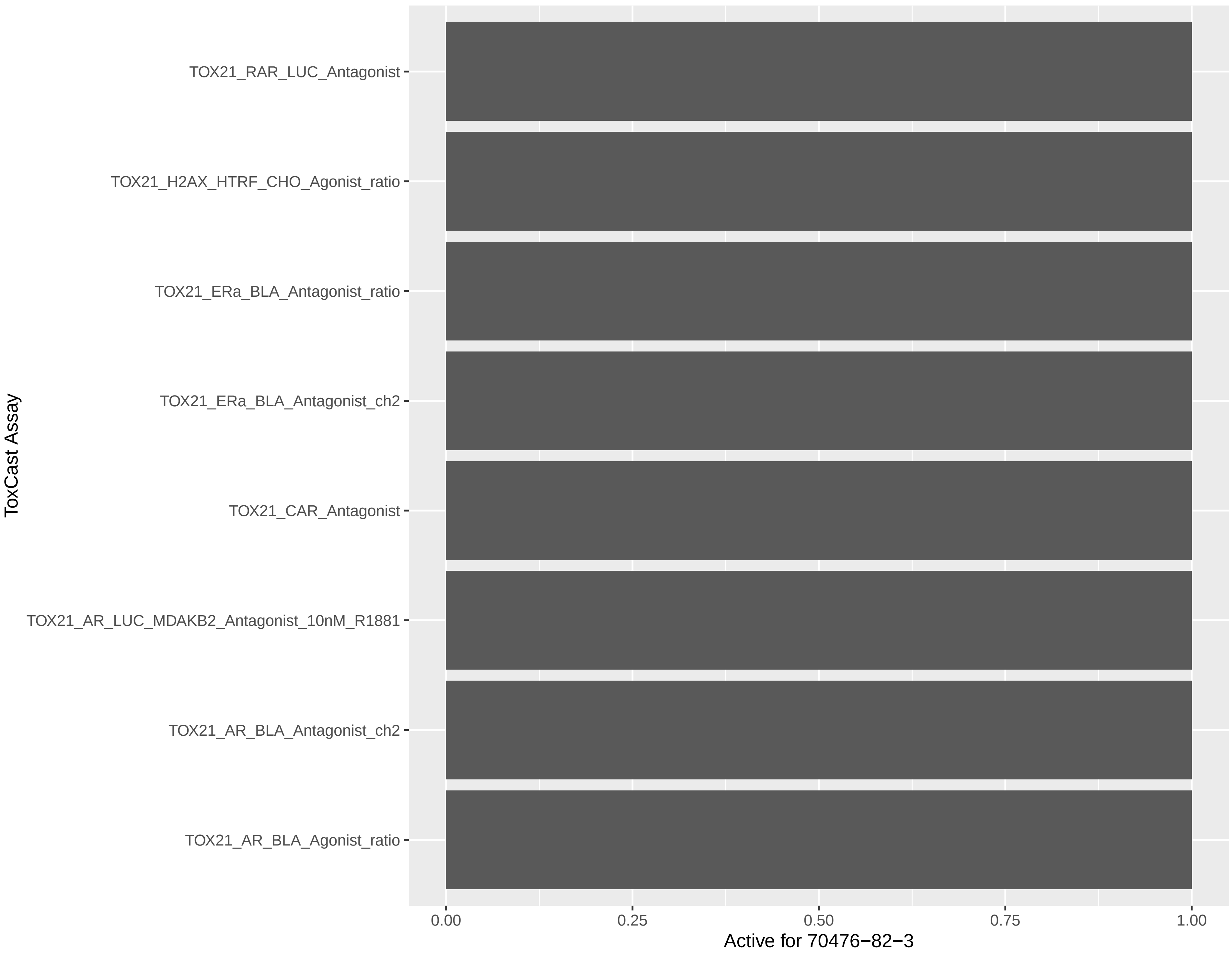

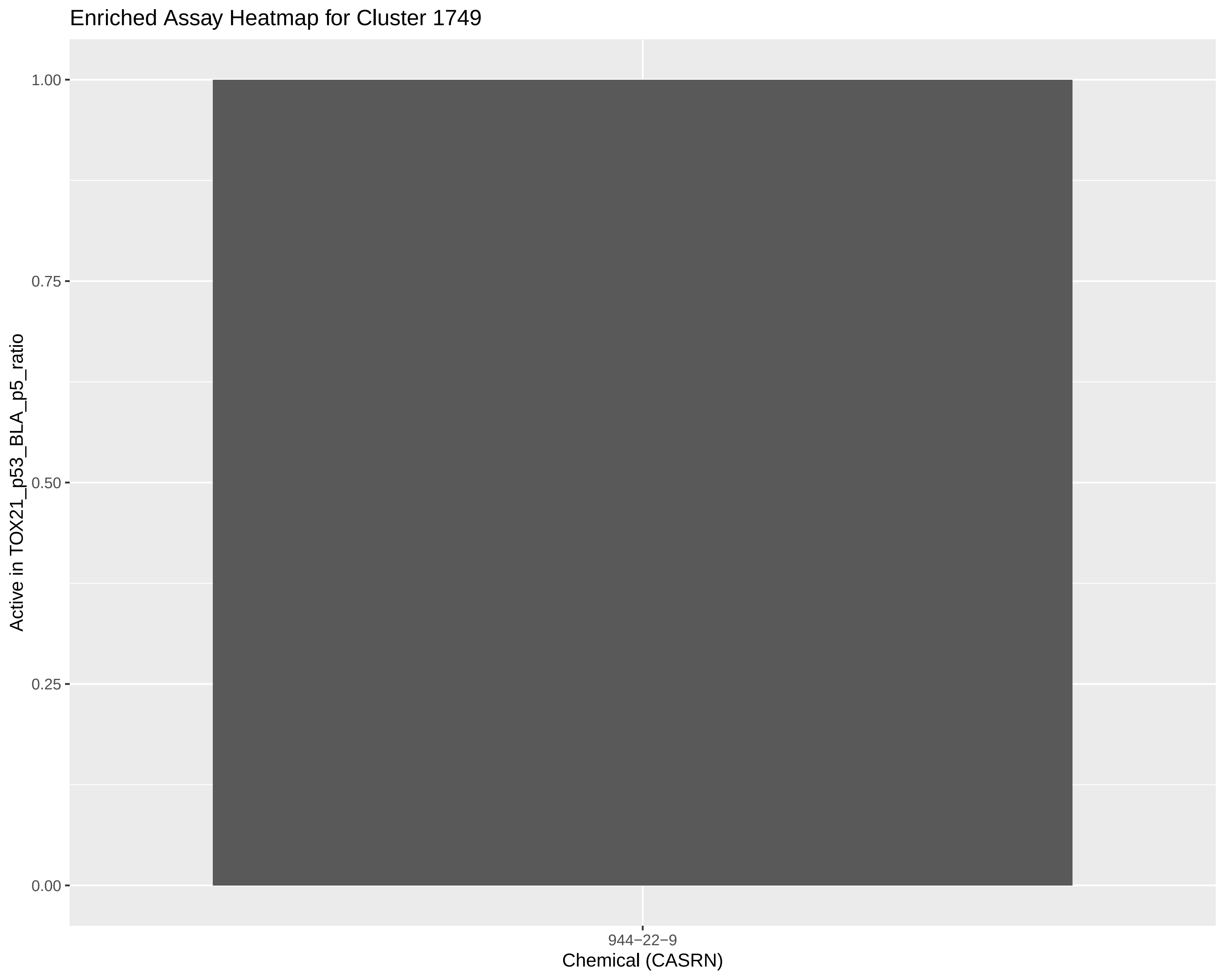
Enriched Assay Heatmap for Cluster 1749
1.00
0.75
0.50
0.25
0.00
Active in TOX21_p53_BLA_p5_ratio
944−22−9
Chemical (CASRN)

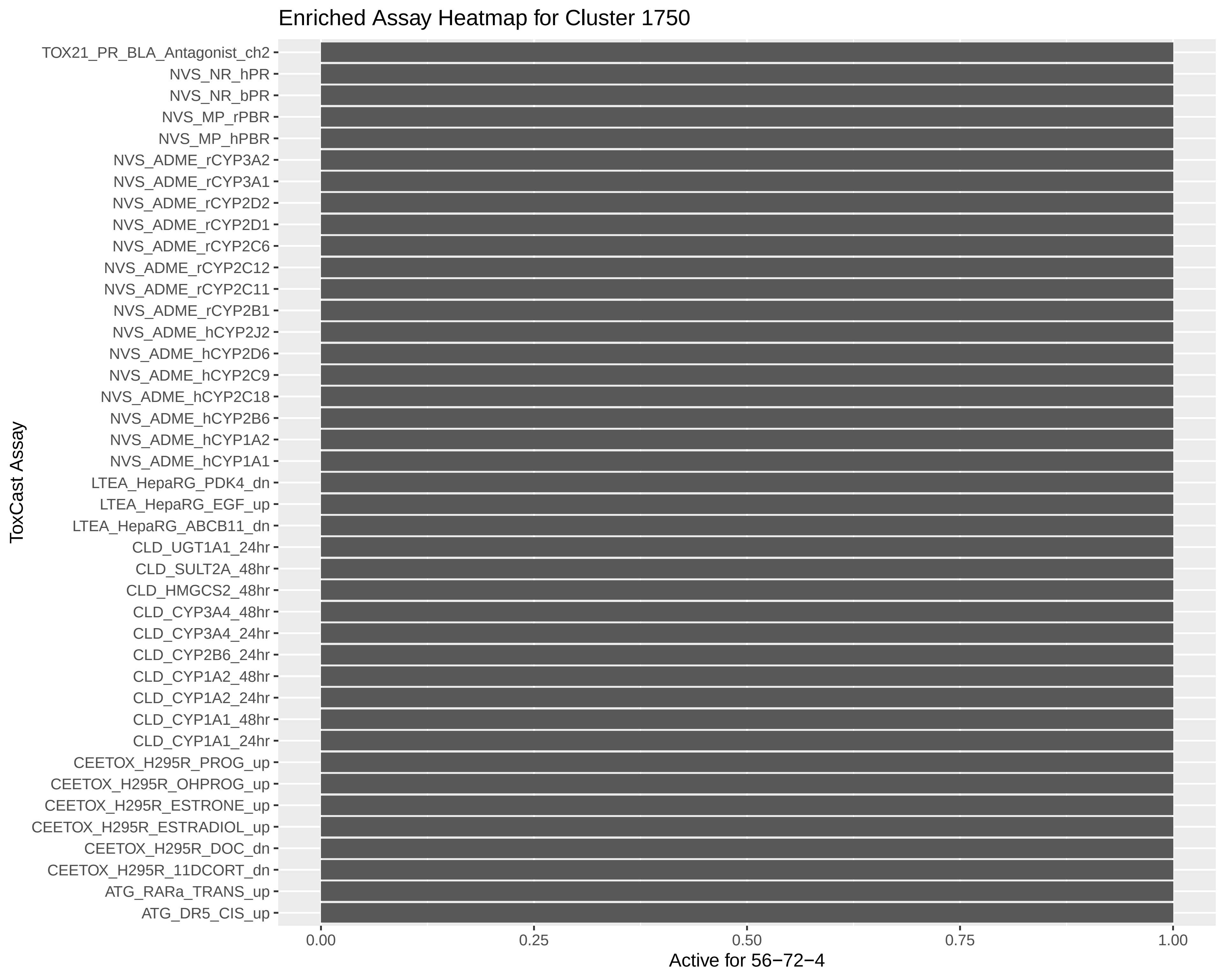
Enriched Assay Heatmap for Cluster 1750
ToxCast Assay
TOX21_PR_BLA_Antagonist_ch2
NVS_NR_hPR
NVS_NR_bPR
NVS_MP_rPBR
NVS_MP_hPBR
NVS_ADME_rCYP3A2
NVS_ADME_rCYP3A1
NVS_ADME_rCYP2D2
NVS_ADME_rCYP2D1
NVS_ADME_rCYP2C6
NVS_ADME_rCYP2C12
NVS_ADME_rCYP2C11
NVS_ADME_rCYP2B1
NVS_ADME_hCYP2J2
NVS_ADME_hCYP2D6
NVS_ADME_hCYP2C9
NVS_ADME_hCYP2C18
NVS_ADME_hCYP2B6
NVS_ADME_hCYP1A2
NVS_ADME_hCYP1A1
LTEA_HepaRG_PDK4_dn
LTEA_HepaRG_EGF_up
LTEA_HepaRG_ABCB11_dn
CLD_UGT1A1_24hr
CLD_SULT2A_48hr
CLD_HMGCS2_48hr
CLD_CYP3A4_48hr
CLD_CYP3A4_24hr
CLD_CYP2B6_24hr
CLD_CYP1A2_48hr
CLD_CYP1A2_24hr
CLD_CYP1A1_48hr
CLD_CYP1A1_24hr
CEETOX_H295R_PROG_up
CEETOX_H295R_OHPROG_up
CEETOX_H295R_ESTRONE_up
CEETOX_H295R_ESTRADIOL_up
CEETOX_H295R_DOC_dn
CEETOX_H295R_11DCORT_dn
ATG_RARa_TRANS_up
ATG_DR5_CIS_up
0.00
0.25
0.50
0.75
1.00
Active for 56−72−4

Enriched Assay Heatmap for Cluster 1752

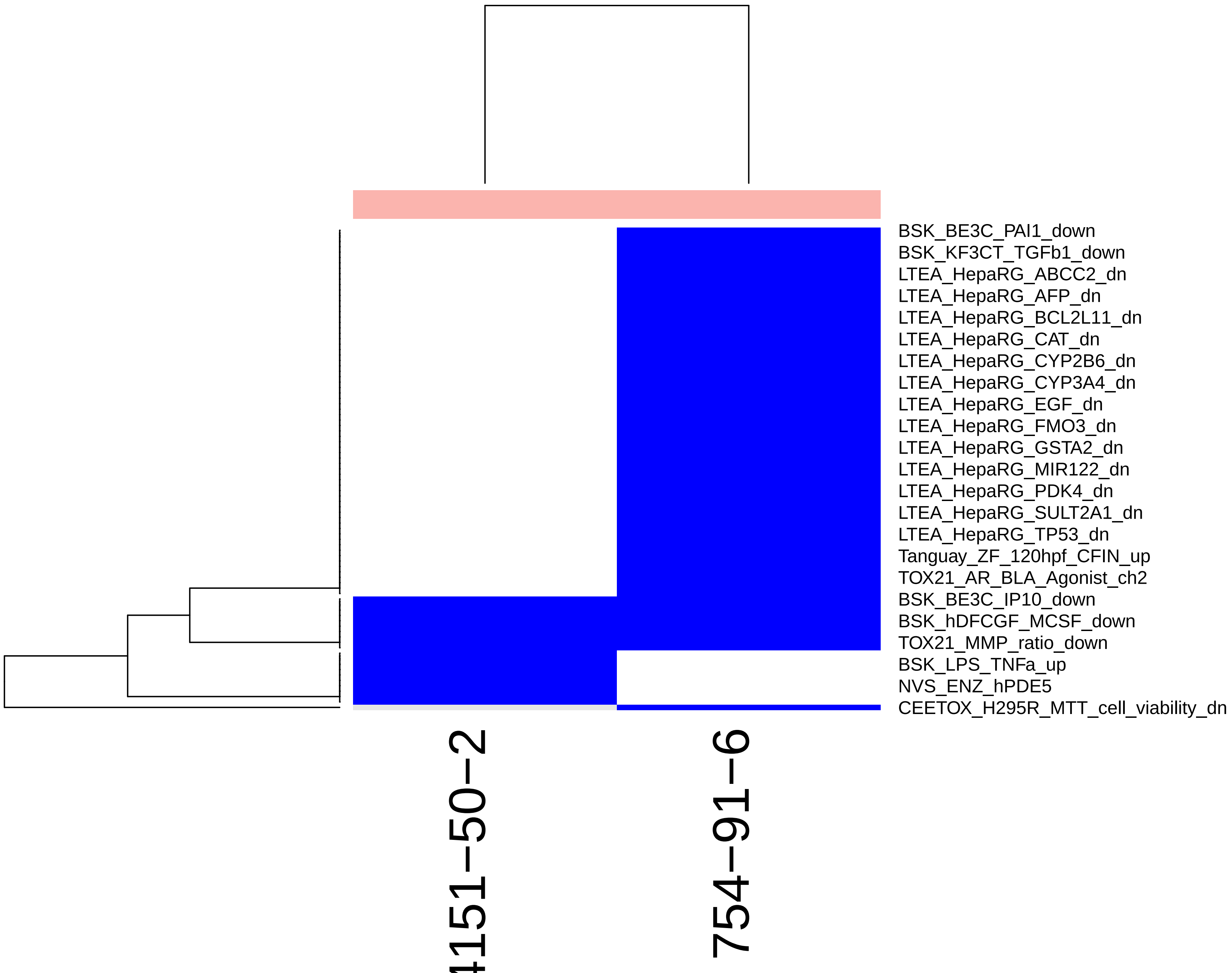

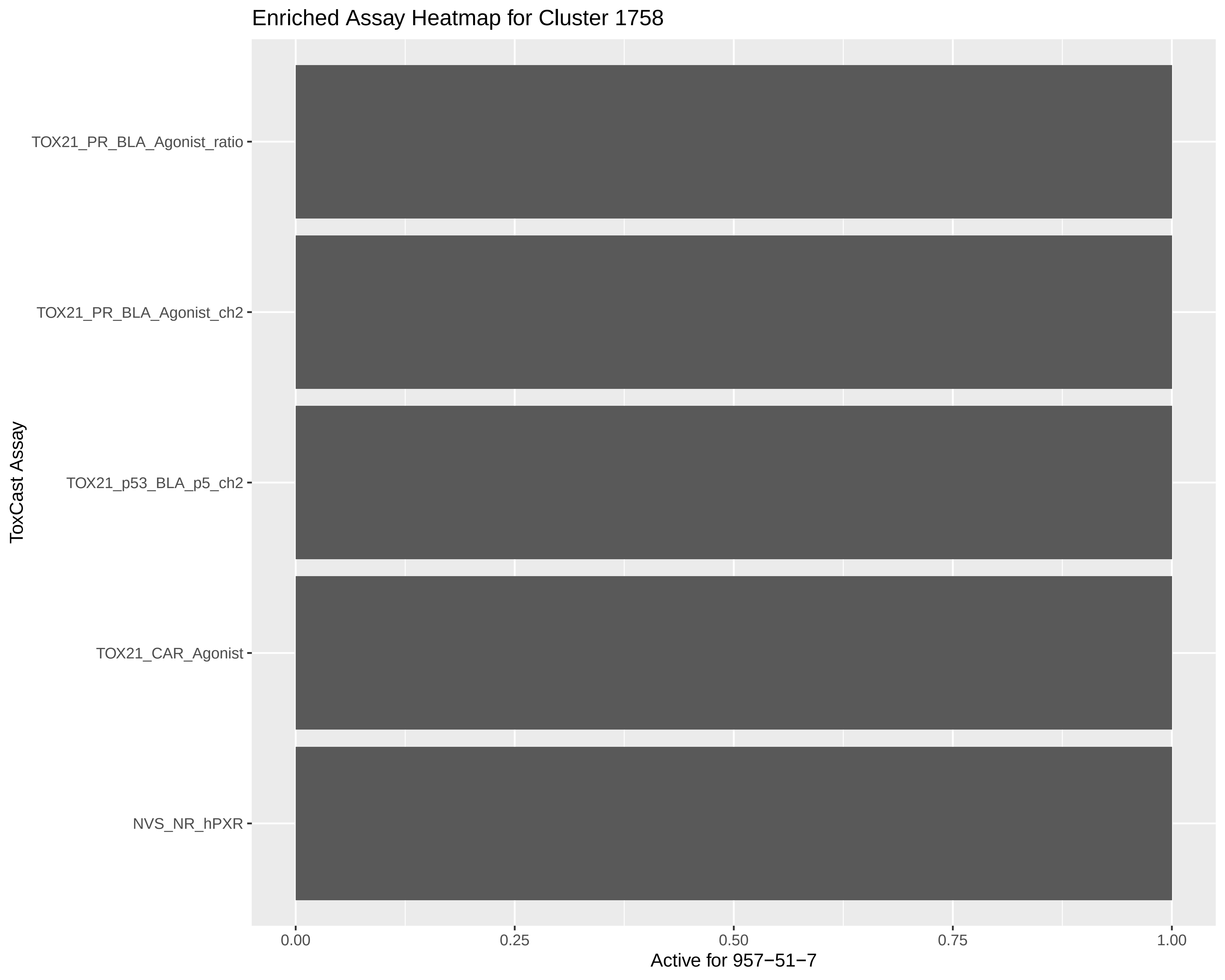

Enriched Assay Heatmap for Cluster 1758
ToxCast Assay
TOX21_PR_BLA_Agonist_ratio
TOX21_PR_BLA_Agonist_ch2
TOX21_p53_BLA_p5_ch2
TOX21_CAR_Agonist
NVS_NR_hPXR
0.00
0.25
0.50
0.75
1.00
Active for 957−51−7

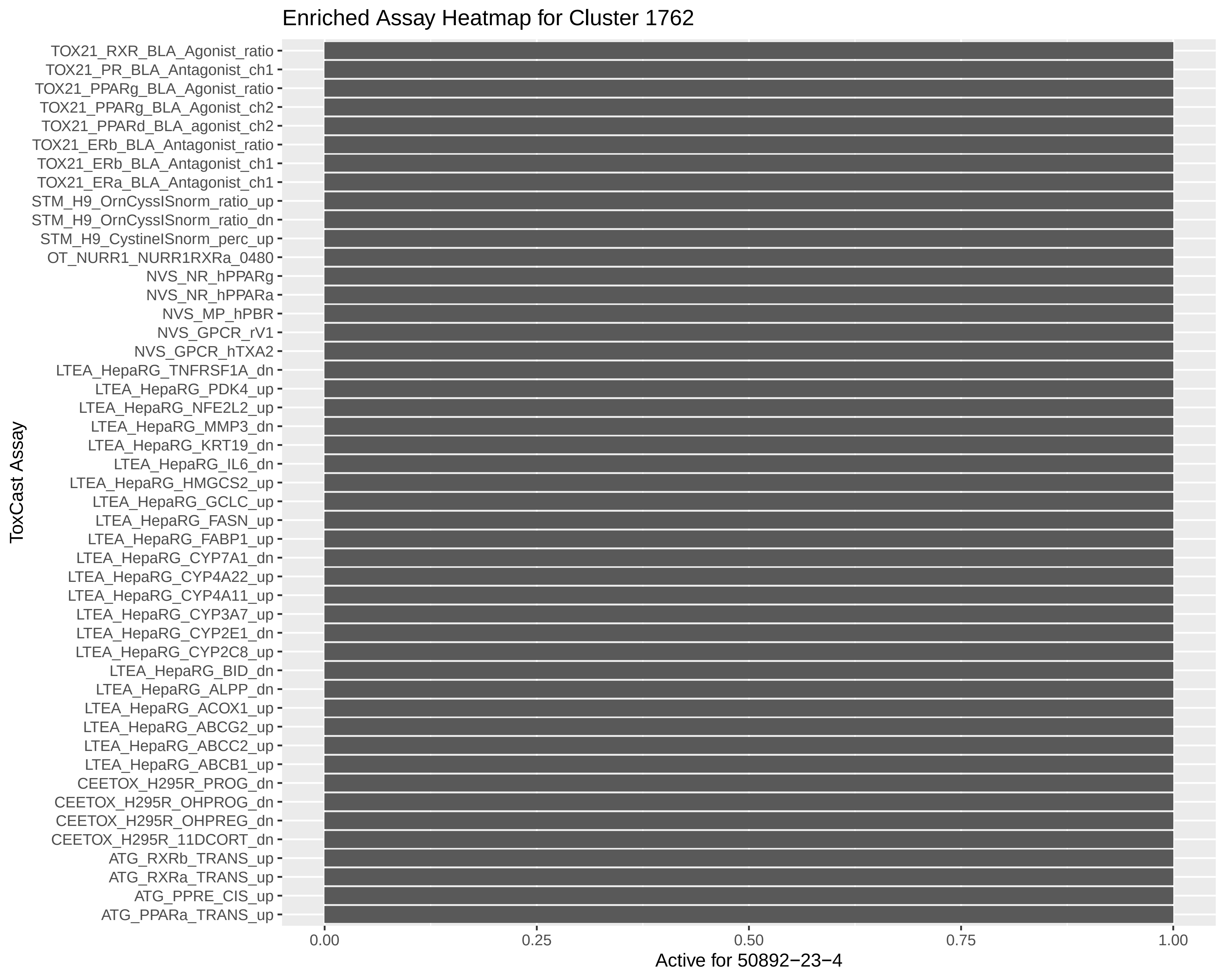

Enriched Assay Heatmap for Cluster 1762
ToxCast Assay
TOX21_RXR_BLA_Agonist_ratio
TOX21_PR_BLA_Antagonist_ch1
TOX21_PPARg_BLA_Agonist_ratio
TOX21_PPARg_BLA_Agonist_ch2
TOX21_PPARd_BLA_agonist_ch2
TOX21_ERb_BLA_Antagonist_ratio
TOX21_ERb_BLA_Antagonist_ch1
TOX21_ERa_BLA_Antagonist_ch1
STM_H9_OrnCyssISnorm_ratio_up
STM_H9_OrnCyssISnorm_ratio_dn
STM_H9_CystineISnorm_perc_up
OT_NURR1_NURR1RXRa_0480
NVS_NR_hPPARg
NVS_NR_hPPARa
NVS_MP_hPBR
NVS_GPCR_rV1
NVS_GPCR_hTXA2
LTEA_HepaRG_TNFRSF1A_dn
LTEA_HepaRG_PDK4_up
LTEA_HepaRG_NFE2L2_up
LTEA_HepaRG_MMP3_dn
LTEA_HepaRG_KRT19_dn
LTEA_HepaRG_IL6_dn
LTEA_HepaRG_HMGCS2_up
LTEA_HepaRG_GCLC_up
LTEA_HepaRG_FASN_up
LTEA_HepaRG_FABP1_up
LTEA_HepaRG_CYP7A1_dn
LTEA_HepaRG_CYP4A22_up
LTEA_HepaRG_CYP4A11_up
LTEA_HepaRG_CYP3A7_up
LTEA_HepaRG_CYP2E1_dn
LTEA_HepaRG_CYP2C8_up
LTEA_HepaRG_BID_dn
LTEA_HepaRG_ALPP_dn
LTEA_HepaRG_ACOX1_up
LTEA_HepaRG_ABCG2_up
LTEA_HepaRG_ABCC2_up
LTEA_HepaRG_ABCB1_up
CEETOX_H295R_PROG_dn
CEETOX_H295R_OHPROG_dn
CEETOX_H295R_OHPREG_dn
CEETOX_H295R_11DCORT_dn
ATG_RXRb_TRANS_up
ATG_RXRa_TRANS_up
ATG_PPRE_CIS_up
ATG_PPARa_TRANS_up
0.00
0.25
0.50
0.75
1.00
Active for 50892−23−4

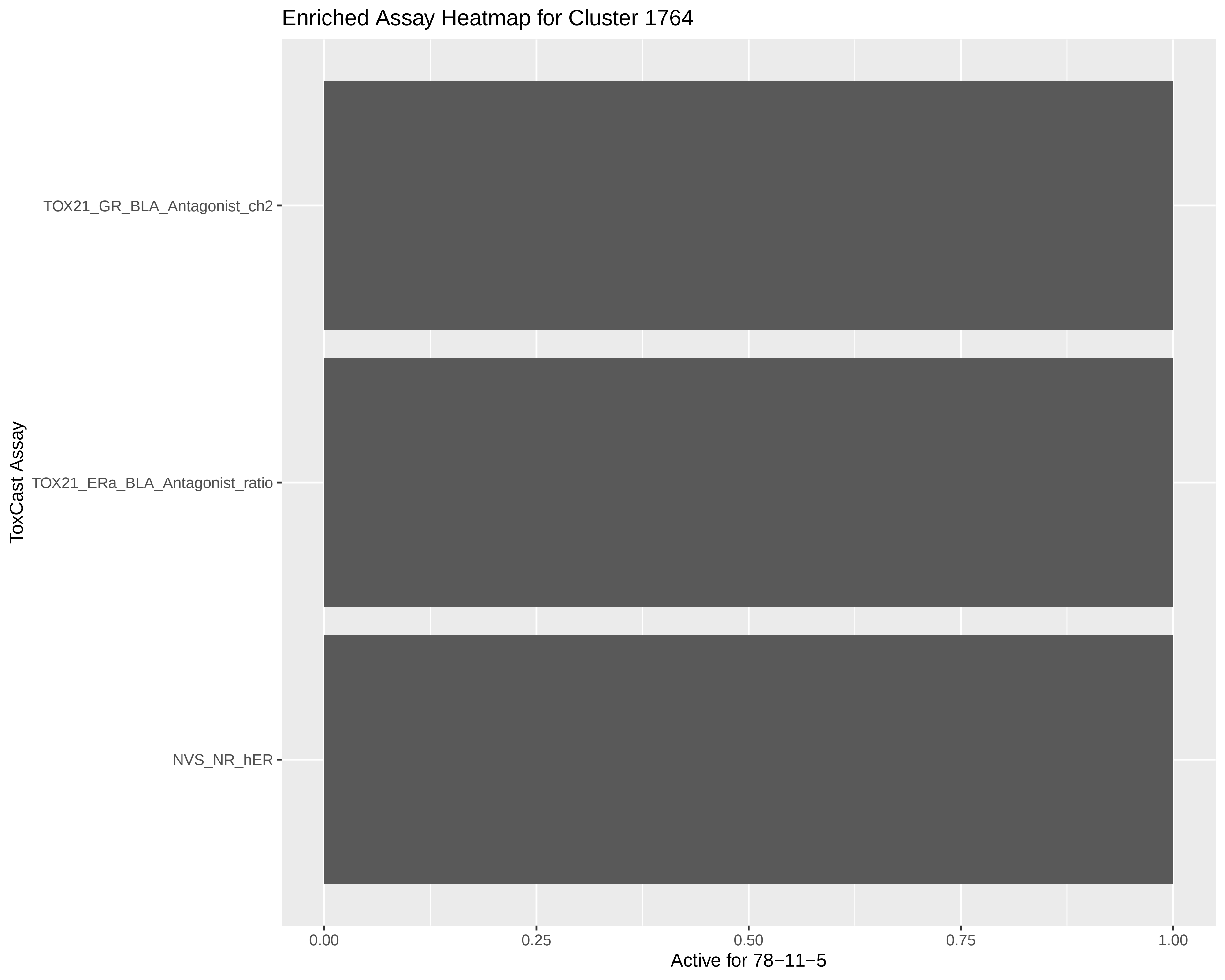

Enriched Assay Heatmap for Cluster 1764
TOX21_GR_BLA_Antagonist_ch2
TOX21_ERa_BLA_Antagonist_ratio
NVS_NR_hER
ToxCast Assay
0.00
0.25
0.50
0.75
1.00
Active for 78−11−5

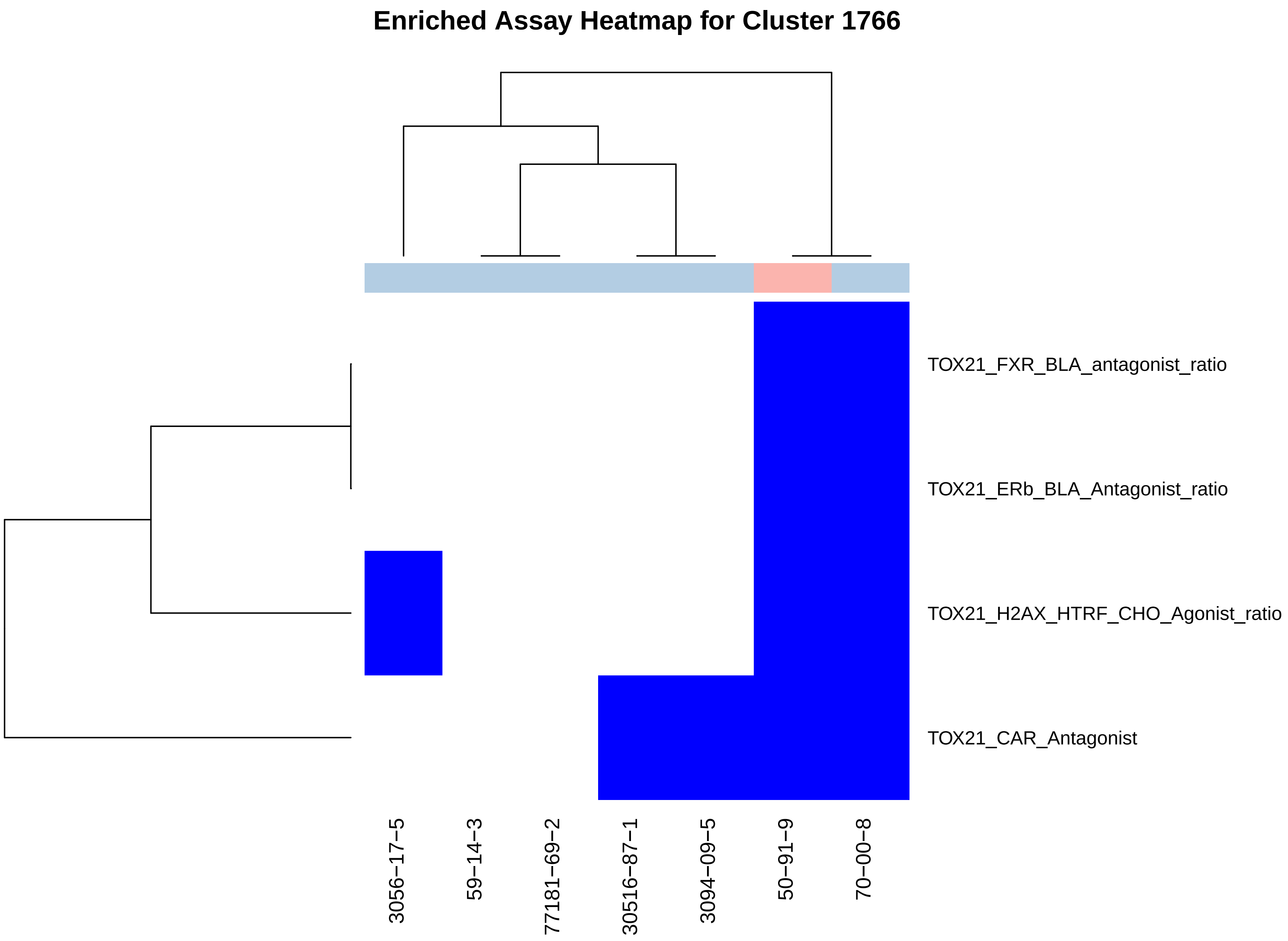
Enriched Assay Heatmap for Cluster 1766
TOX21_FXR_BLA_antagonist_ratio
TOX21_ERb_BLA_Antagonist_ratio
TOX21_H2AX_HTRF_CHO_Agonist_ratio
TOX21_CAR_Antagonist
3056−17−5
59−14−3
77181−69−2
30516−87−1
3094−09−5
50−91−9
70−00−8

Enriched Assay Heatmap for Cluster 1780

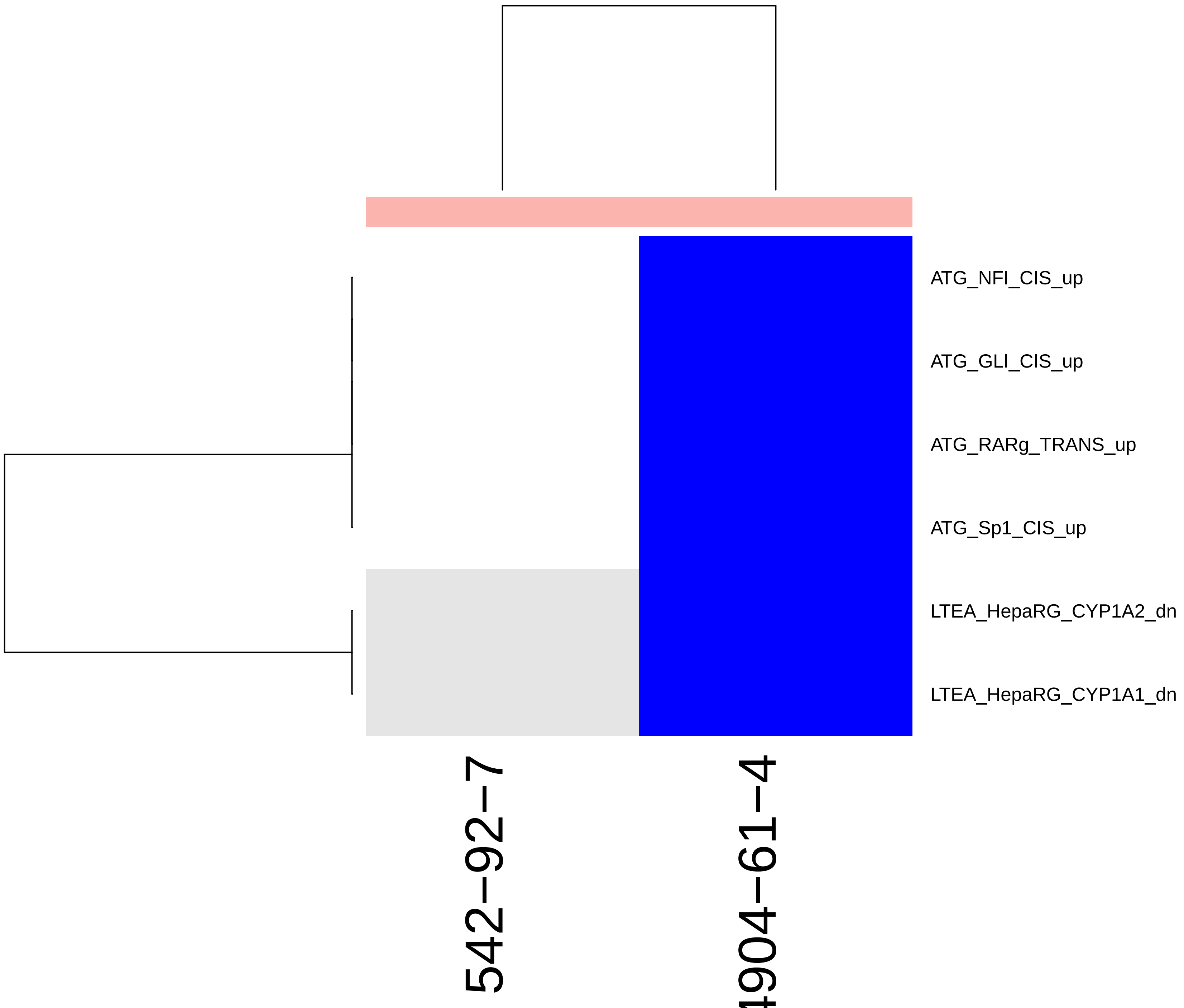

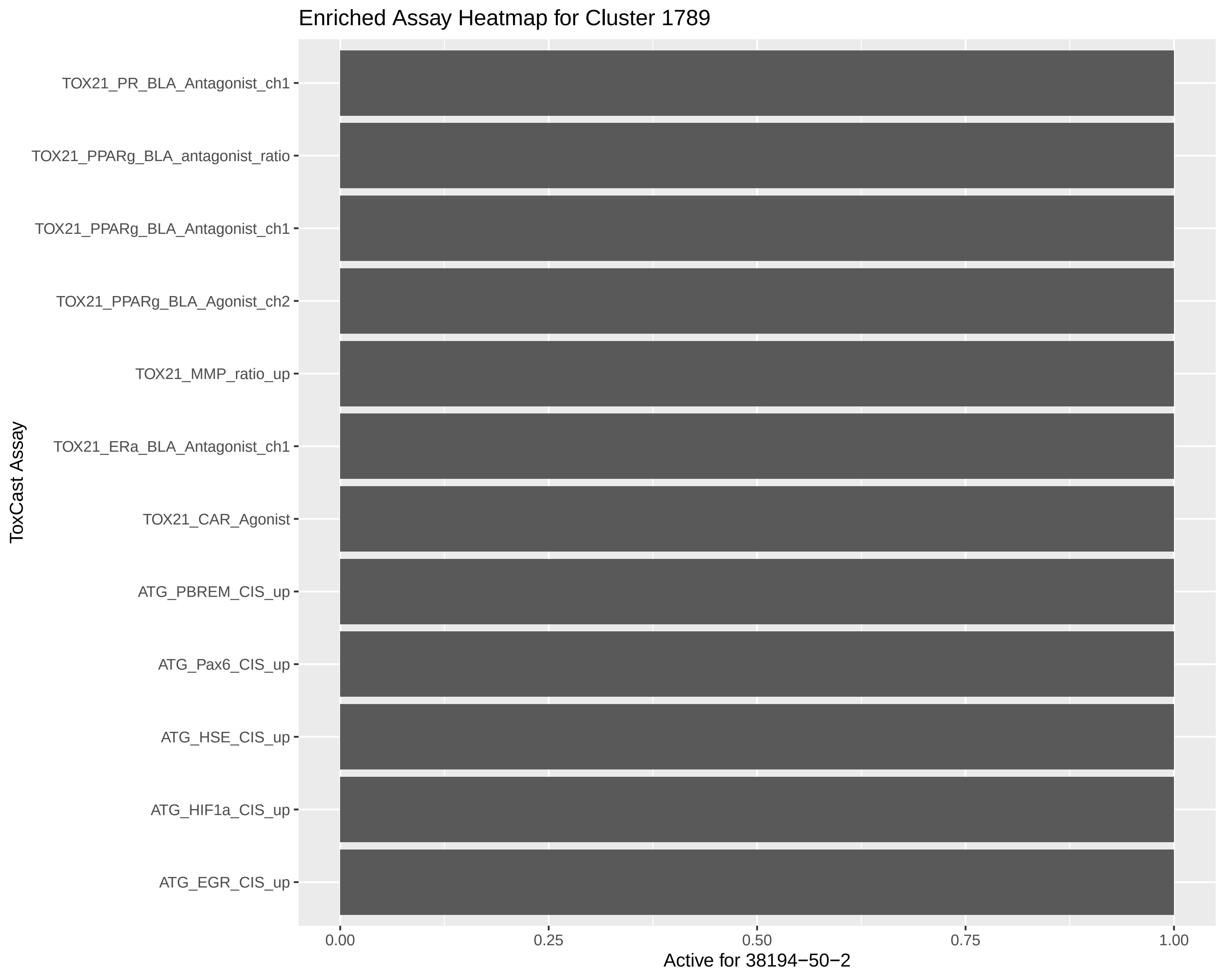
Enriched Assay Heatmap for Cluster 1789
TOX21_PR_BLA_Antagonist_ch1
TOX21_PPARg_BLA_antagonist_ratio
TOX21_PPARg_BLA_Antagonist_ch1
TOX21_PPARg_BLA_Agonist_ch2
TOX21_MMP_ratio_up
TOX21_ERa_BLA_Antagonist_ch1
TOX21_CAR_Agonist
ATG_PBREM_CIS_up
ATG_Pax6_CIS_up
ATG_HSE_CIS_up
ATG_HIF1a_CIS_up
ATG_EGR_CIS_up
ToxCast Assay
0.00
0.25
0.50
0.75
1.00
Active for 38194−50−2

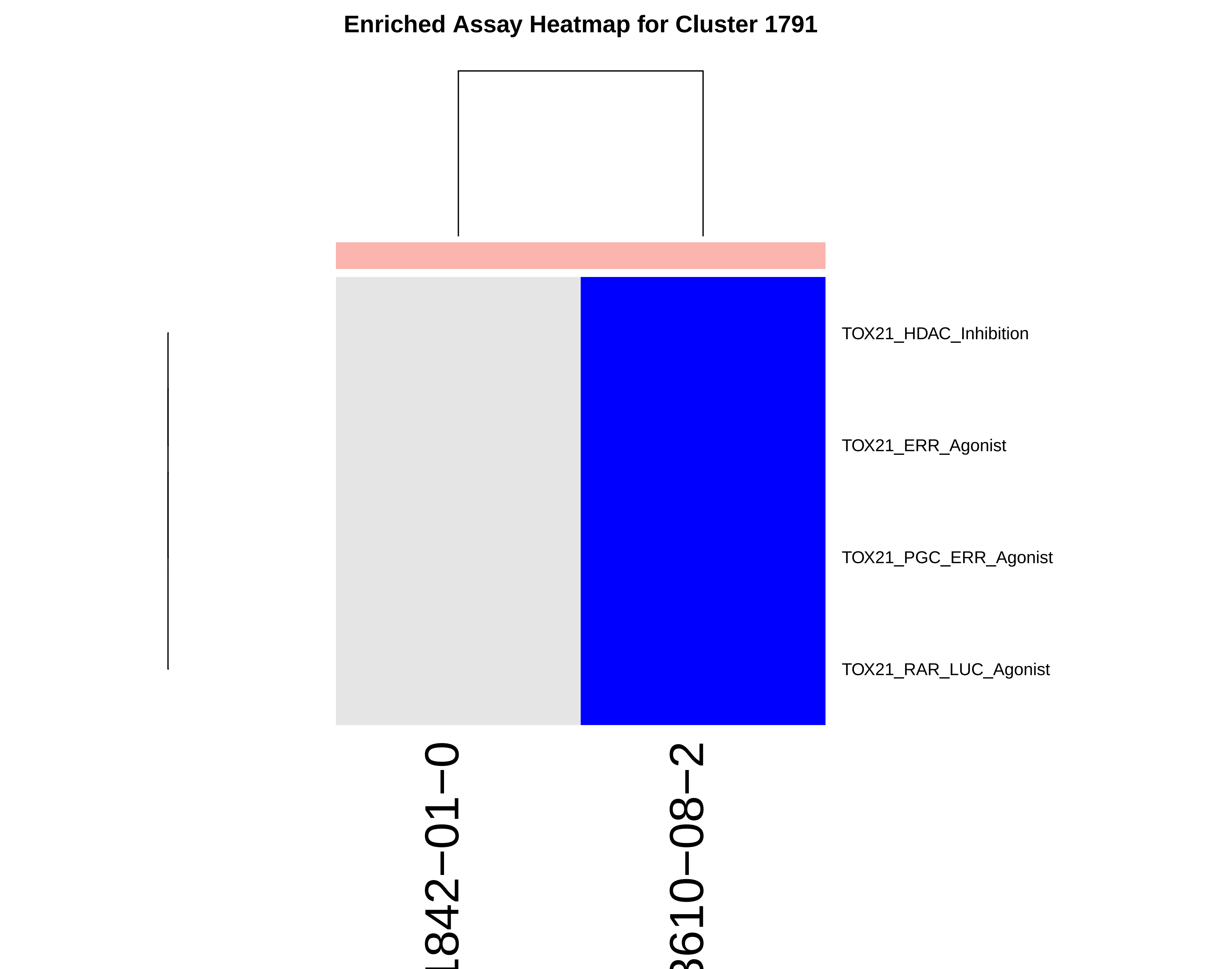

Enriched Assay Heatmap for Cluster 1791
TOX21_HDAC_Inhibition
TOX21_ERR_Agonist
TOX21_PGC_ERR_Agonist
TOX21_RAR_LUC_Agonist

Enriched Assay Heatmap for Cluster 1792

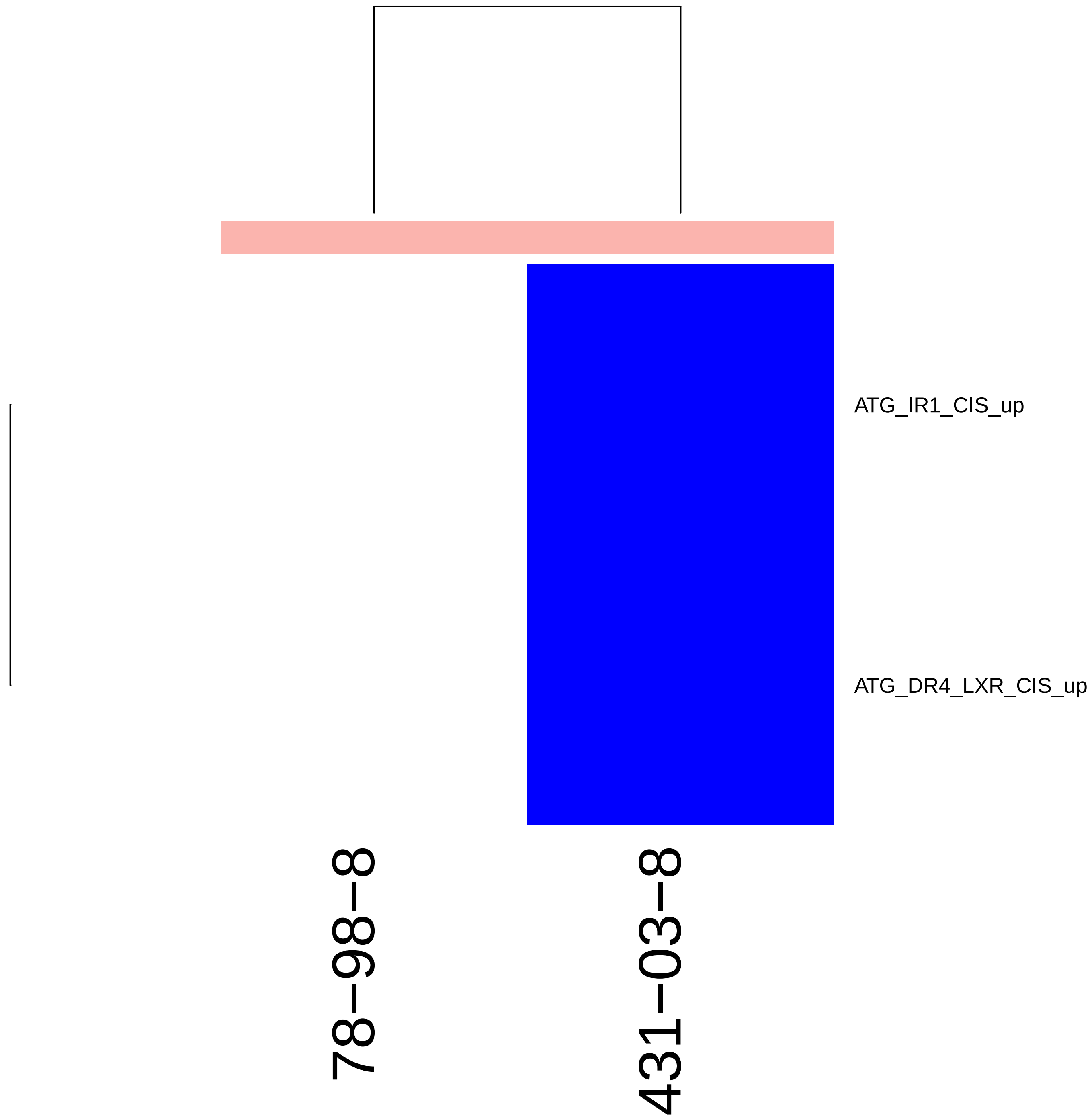

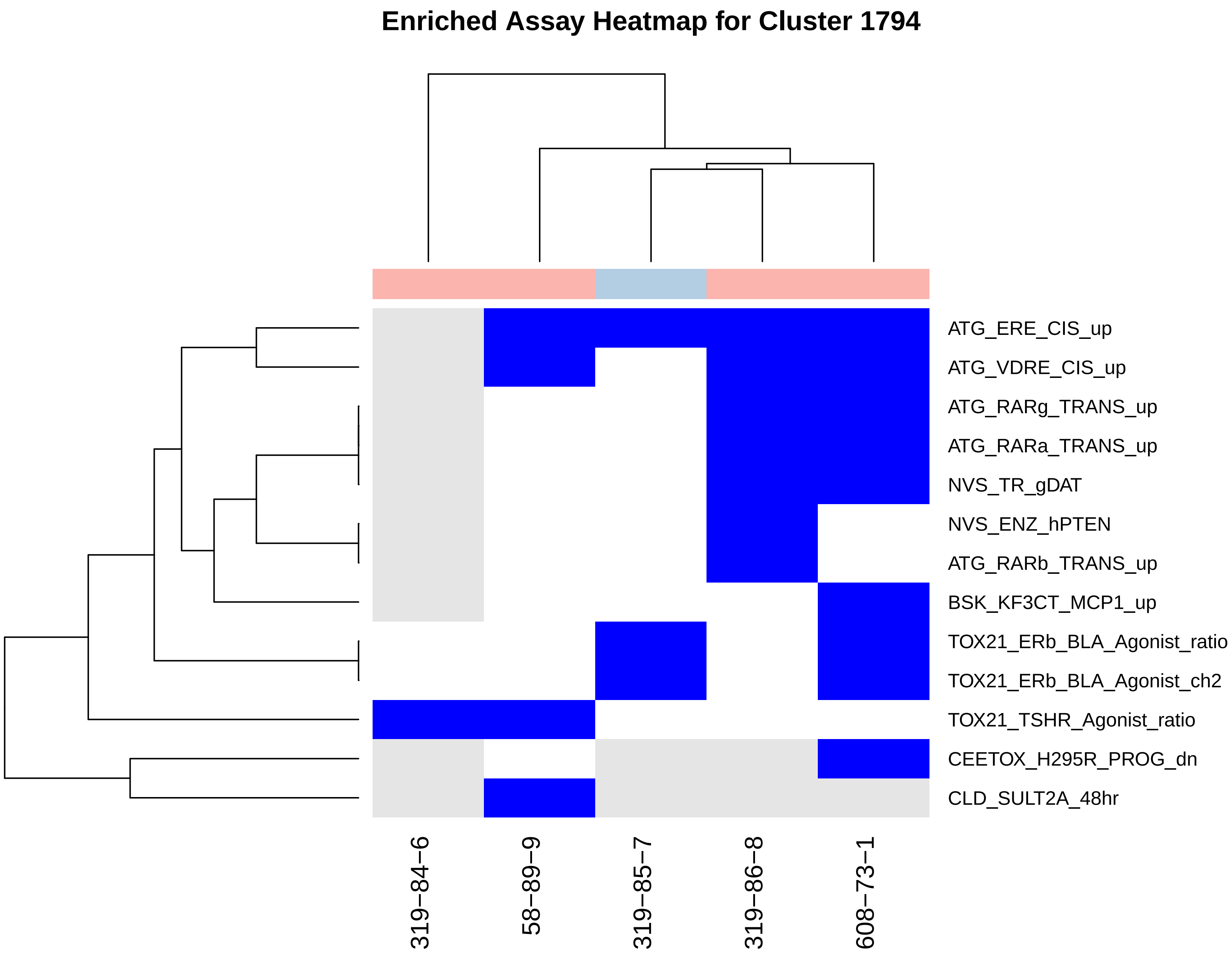
Enriched Assay Heatmap for Cluster 1794
ATG_ERE_CIS_up
ATG_VDRE_CIS_up
ATG_RARg_TRANS_up
ATG_RARa_TRANS_up
NVS_TR_gDAT
NVS_ENZ_hPTEN
ATG_RARb_TRANS_up
BSK_KF3CT_MCP1_up
TOX21_ERb_BLA_Agonist_ratio
TOX21_ERb_BLA_Agonist_ch2
TOX21_TSHR_Agonist_ratio
CEETOX_H295R_PROG_dn
CLD_SULT2A_48hr
319−84−6
58−89−9
319−85−7
319−86−8
608−73−1

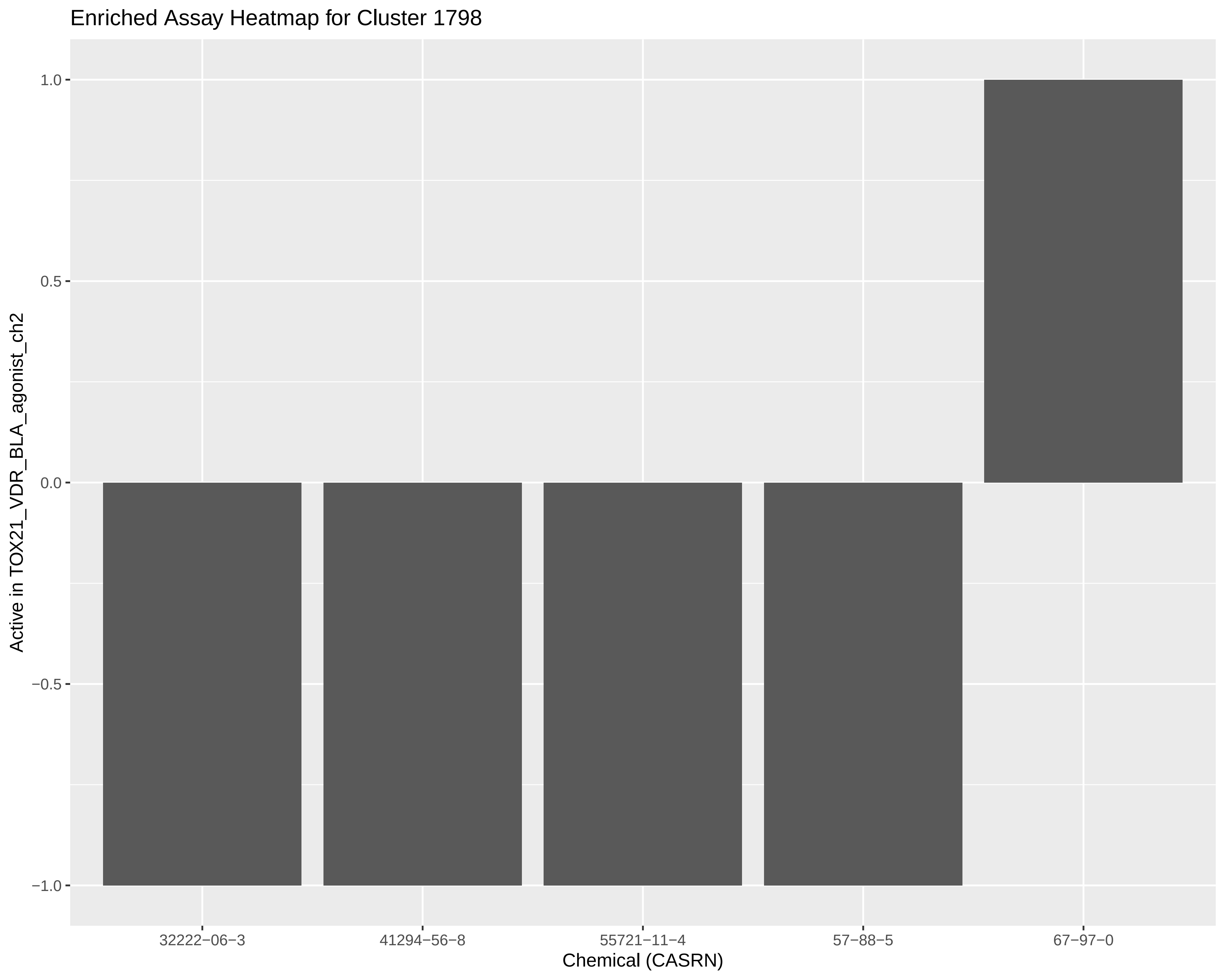
Enriched Assay Heatmap for Cluster 1798
Active in TOX21_VDR_BLA_agonist_ch2
1.0
0.5
0.0
−0.5
−1.0
32222−06−3
41294−56−8
55721−11−4
57−88−5
67−97−0
Chemical (CASRN)

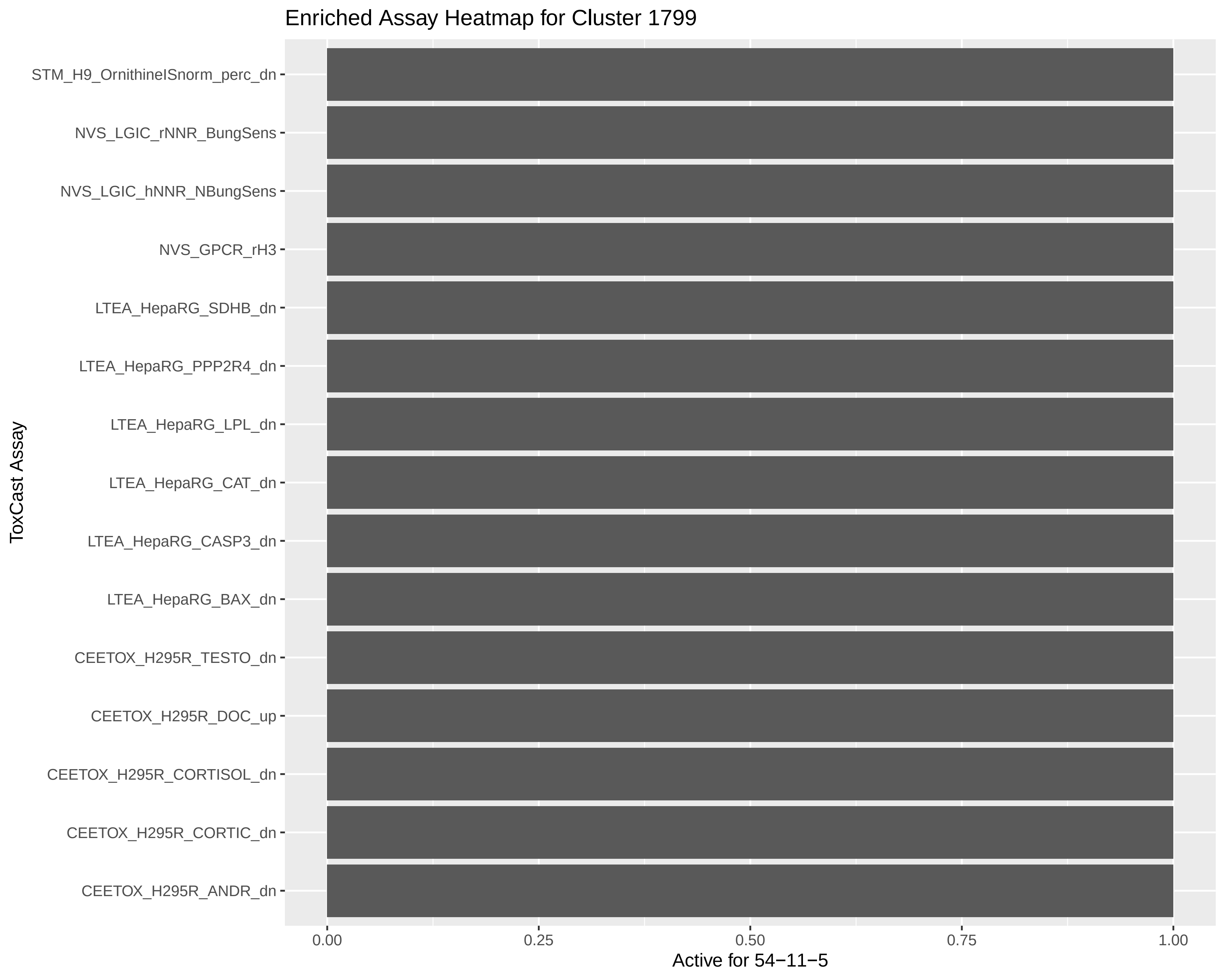

Enriched Assay Heatmap for Cluster 1799
ToxCast Assay
STM_H9_OrnithineISnorm_perc_dn
NVS_LGIC_rNNR_BungSens
NVS_LGIC_hNNR_NBungSens
NVS_GPCR_rH3
LTEA_HepaRG_SDHB_dn
LTEA_HepaRG_PPP2R4_dn
LTEA_HepaRG_LPL_dn
LTEA_HepaRG_CAT_dn
LTEA_HepaRG_CASP3_dn
LTEA_HepaRG_BAX_dn
CEETOX_H295R_TESTO_dn
CEETOX_H295R_DOC_up
CEETOX_H295R_CORTISOL_dn
CEETOX_H295R_CORTIC_dn
CEETOX_H295R_ANDR_dn
0.00
0.25
0.50
0.75
1.00
Active for 54−11−5

Enriched Assay Heatmap for Cluster 1800

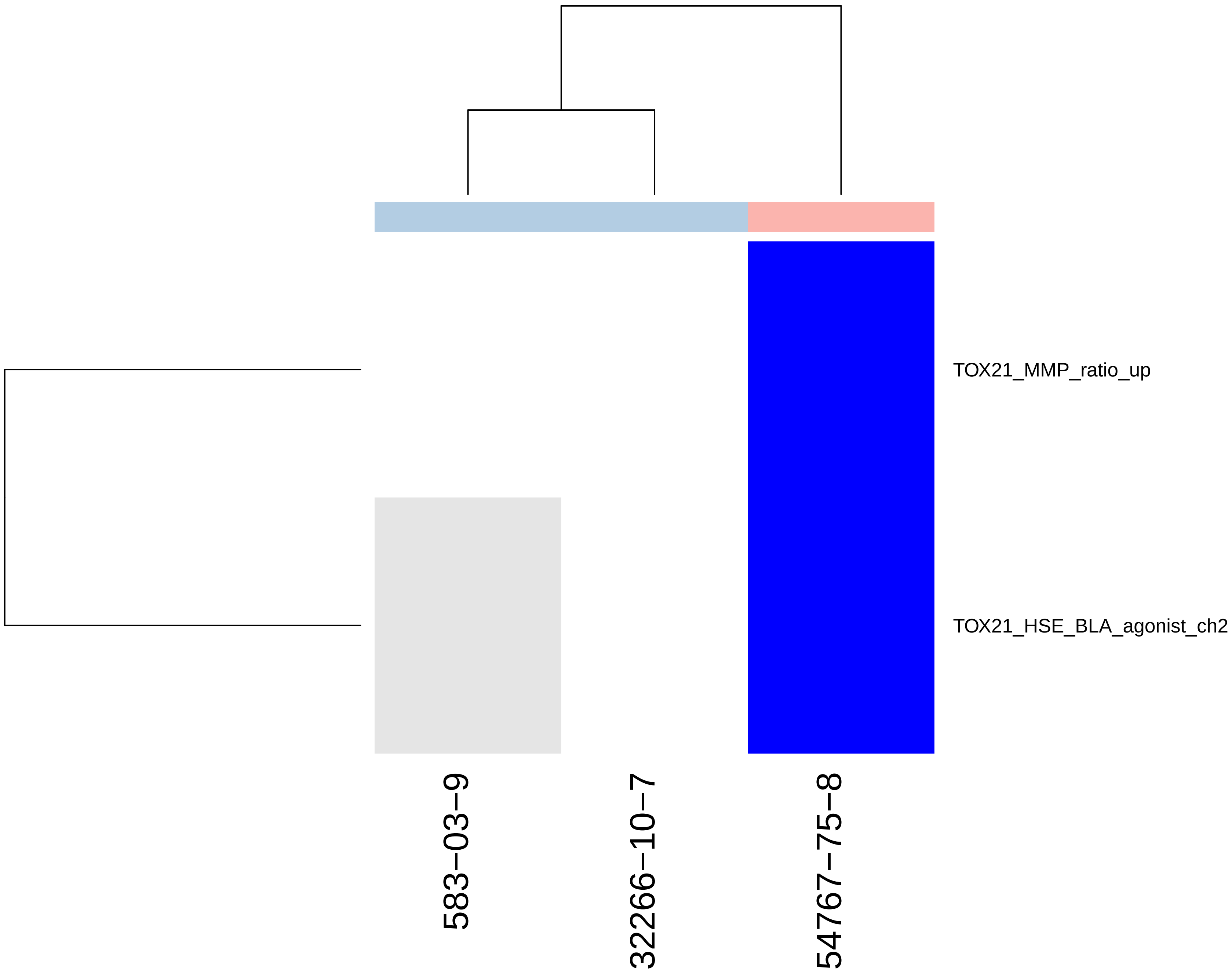

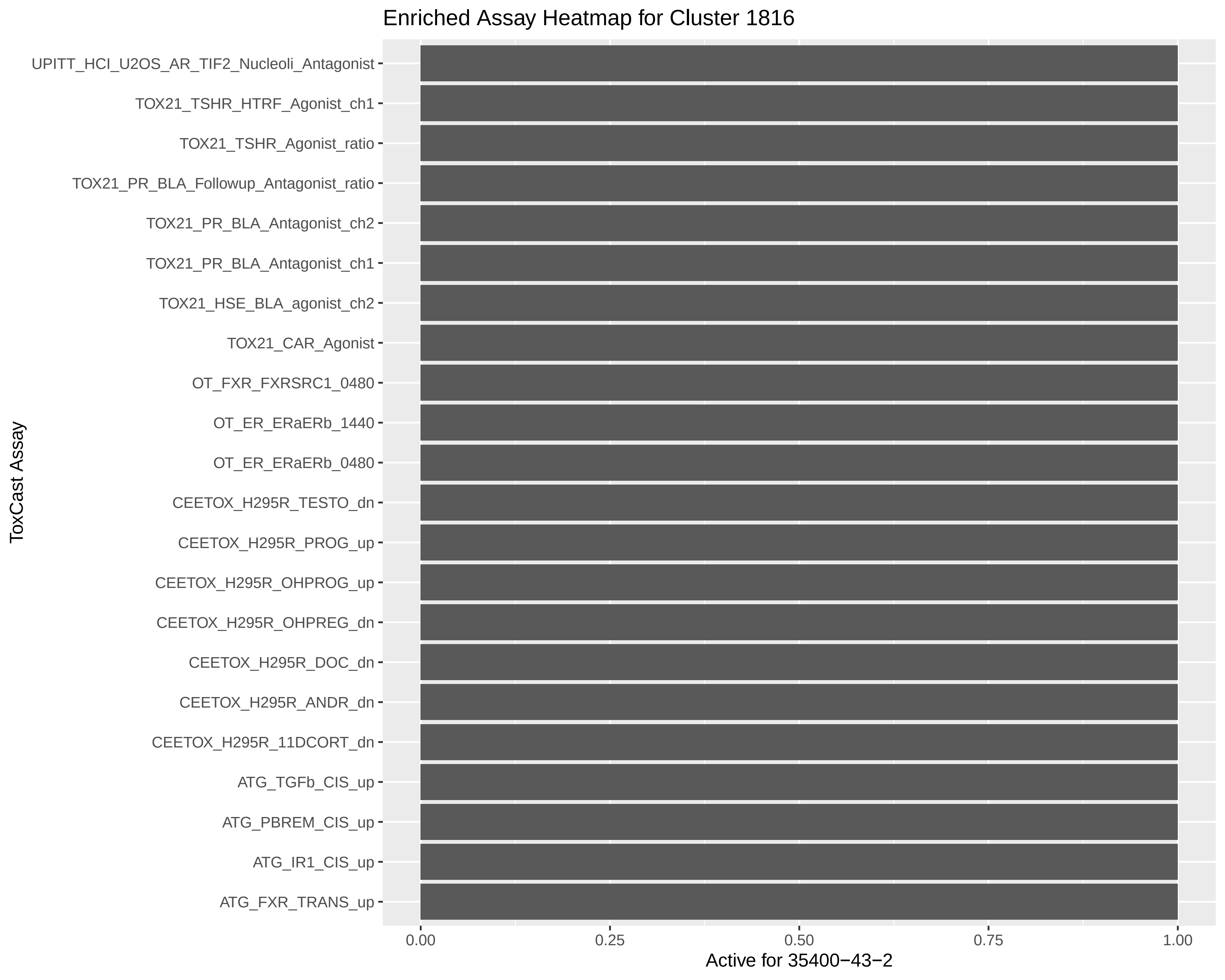

Enriched Assay Heatmap for Cluster 1816
UPITT_HCI_U2OS_AR_TIF2_Nucleoli_Antagonist
TOX21_TSHR_HTRF_Agonist_ch1
TOX21_TSHR_Agonist_ratio
TOX21_PR_BLA_Followup_Antagonist_ratio
TOX21_PR_BLA_Antagonist_ch2
TOX21_PR_BLA_Antagonist_ch1
TOX21_HSE_BLA_agonist_ch2
TOX21_CAR_Agonist
OT_FXR_FXRSRC1_0480
OT_ER_ERaERb_1440
OT_ER_ERaERb_0480
CEETOX_H295R_TESTO_dn
CEETOX_H295R_PROG_up
CEETOX_H295R_OHPROG_up
CEETOX_H295R_OHPREG_dn
CEETOX_H295R_DOC_dn
CEETOX_H295R_ANDR_dn
CEETOX_H295R_11DCORT_dn
ATG_TGFb_CIS_up
ATG_PBREM_CIS_up
ATG_IR1_CIS_up
ATG_FXR_TRANS_up
ToxCast Assay
0.00
0.25
0.50
0.75
1.00
Active for 35400−43−2

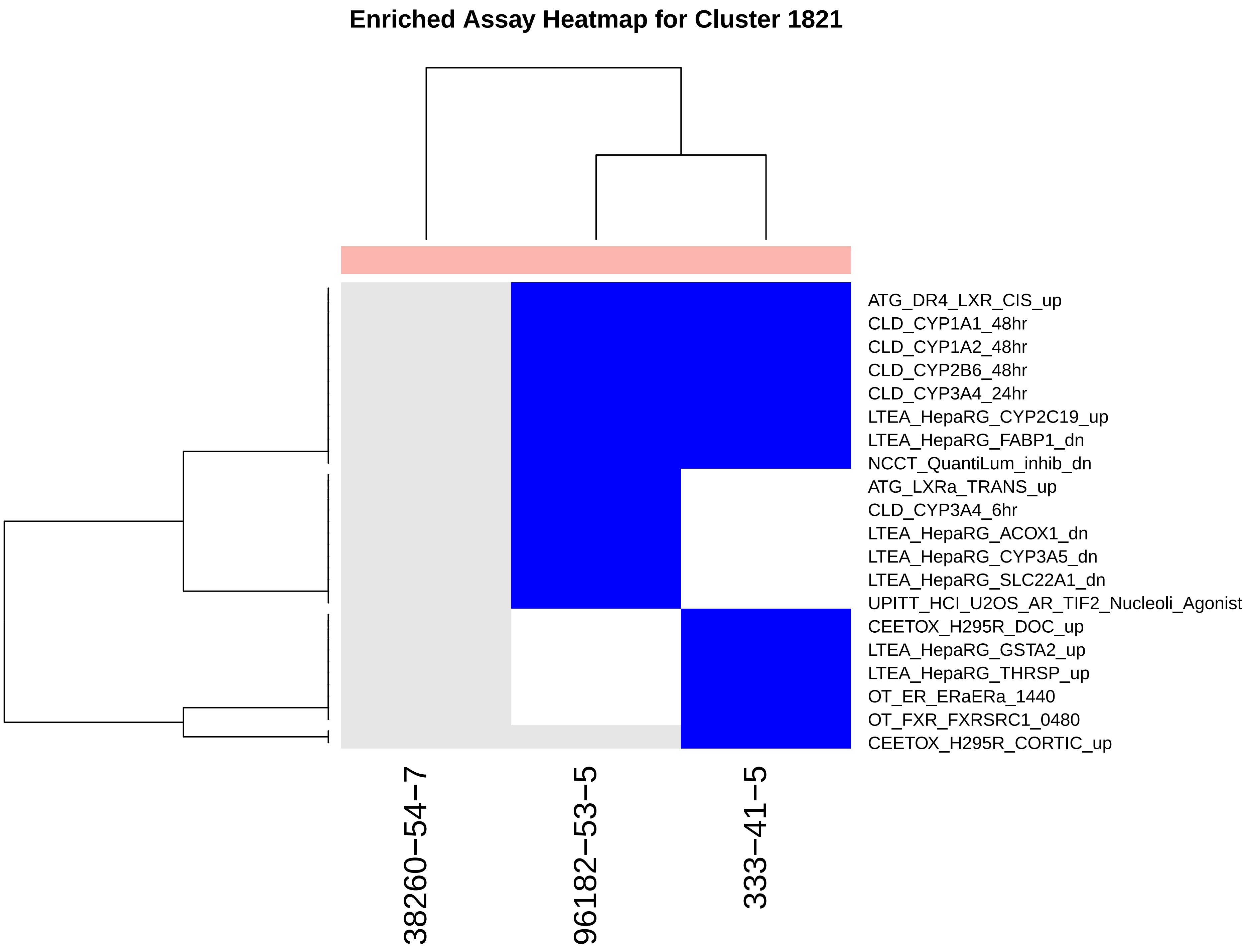
Enriched Assay Heatmap for Cluster 1821
ATG_DR4_LXR_CIS_up
CLD_CYP1A1_48hr
CLD_CYP1A2_48hr
CLD_CYP2B6_48hr
CLD_CYP3A4_24hr
LTEA_HepaRG_CYP2C19_up
LTEA_HepaRG_FABP1_dn
NCCT_QuantiLum_inhib_dn
ATG_LXRa_TRANS_up
CLD_CYP3A4_6hr
LTEA_HepaRG_ACOX1_dn
LTEA_HepaRG_CYP3A5_dn
LTEA_HepaRG_SLC22A1_dn
UPITT_HCI_U2OS_AR_TIF2_Nucleoli_Agonist
CEETOX_H295R_DOC_up
LTEA_HepaRG_GSTA2_up
LTEA_HepaRG_THRSP_up
OT_ER_ERaERa_1440
OT_FXR_FXRSRC1_0480
CEETOX_H295R_CORTIC_up
38260−54−7
96182−53−5
333−41−5

Enriched Assay Heatmap for Cluster 1822

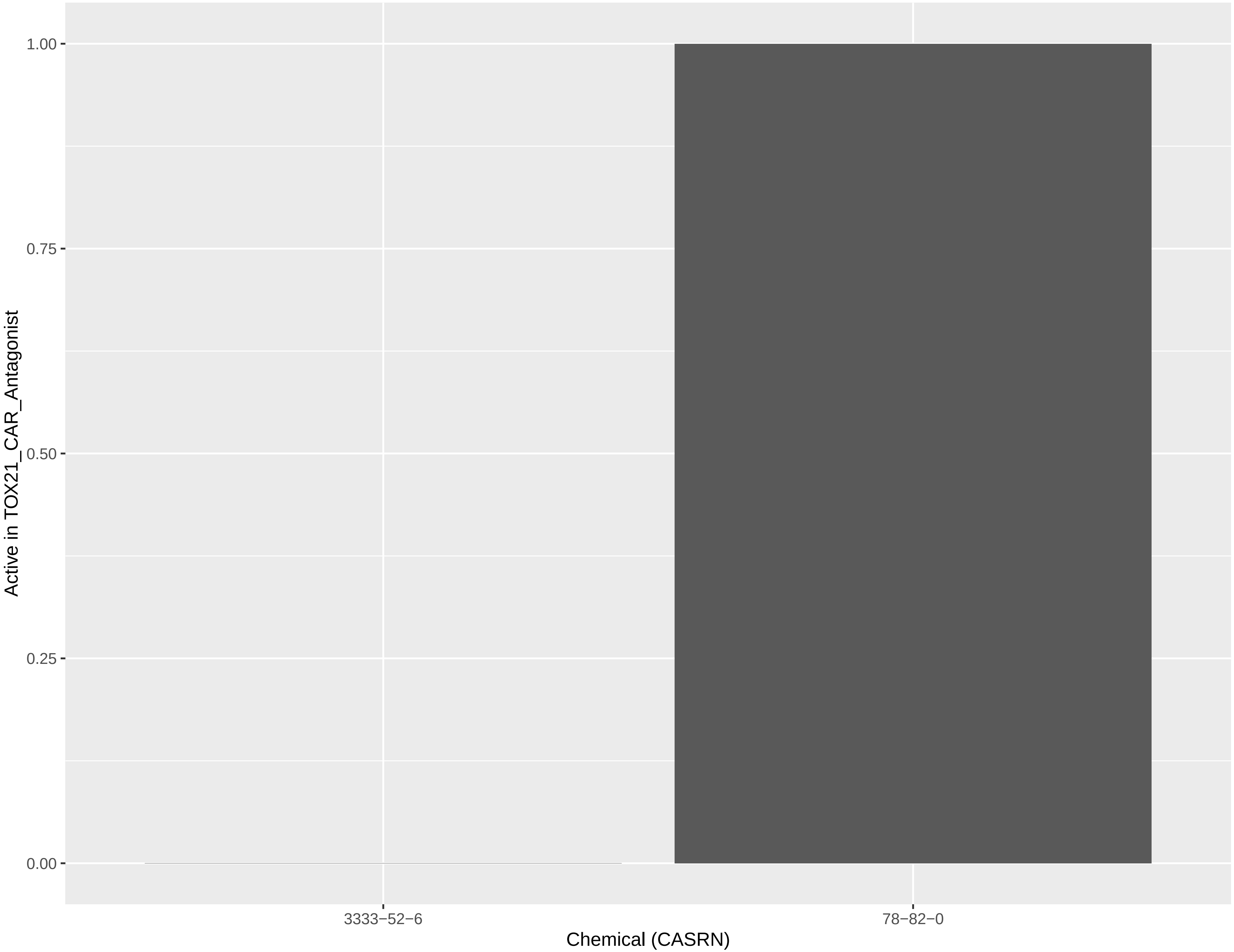

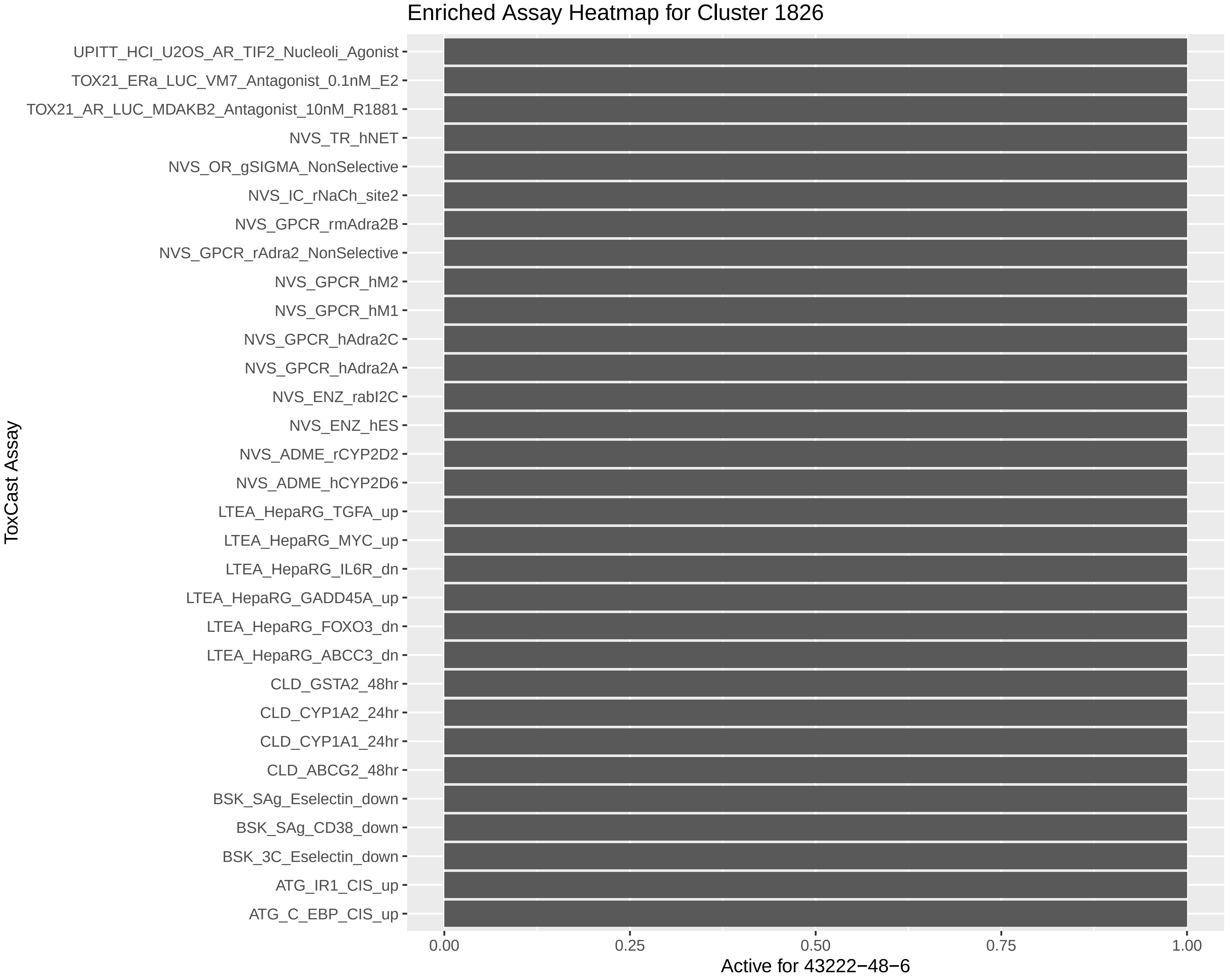

Enriched Assay Heatmap for Cluster 1826
UPITT_HCI_U2OS_AR_TIF2_Nucleoli_Agonist
TOX21_ERa_LUC_VM7_Antagonist_0.1nM_E2
TOX21_AR_LUC_MDAKB2_Antagonist_10nM_R1881
NVS_TR_hNET
NVS_OR_gSIGMA_NonSelective
NVS_IC_rNaCh_site2
NVS_GPCR_rmAdra2B
NVS_GPCR_rAdra2_NonSelective
NVS_GPCR_hM2
NVS_GPCR_hM1
NVS_GPCR_hAdra2C
NVS_GPCR_hAdra2A
NVS_ENZ_rabI2C
NVS_ENZ_hES
NVS_ADME_rCYP2D2
NVS_ADME_hCYP2D6
LTEA_HepaRG_TGFA_up
LTEA_HepaRG_MYC_up
LTEA_HepaRG_IL6R_dn
LTEA_HepaRG_GADD45A_up
LTEA_HepaRG_FOXO3_dn
LTEA_HepaRG_ABCC3_dn
CLD_GSTA2_48hr
CLD_CYP1A2_24hr
CLD_CYP1A1_24hr
CLD_ABCG2_48hr
BSK_SAg_Eselectin_down
BSK_SAg_CD38_down
BSK_3C_Eselectin_down
ATG_IR1_CIS_up
ATG_C_EBP_CIS_up
ToxCast Assay
0.00
0.25
0.50
0.75
1.00
Active for 43222−48−6

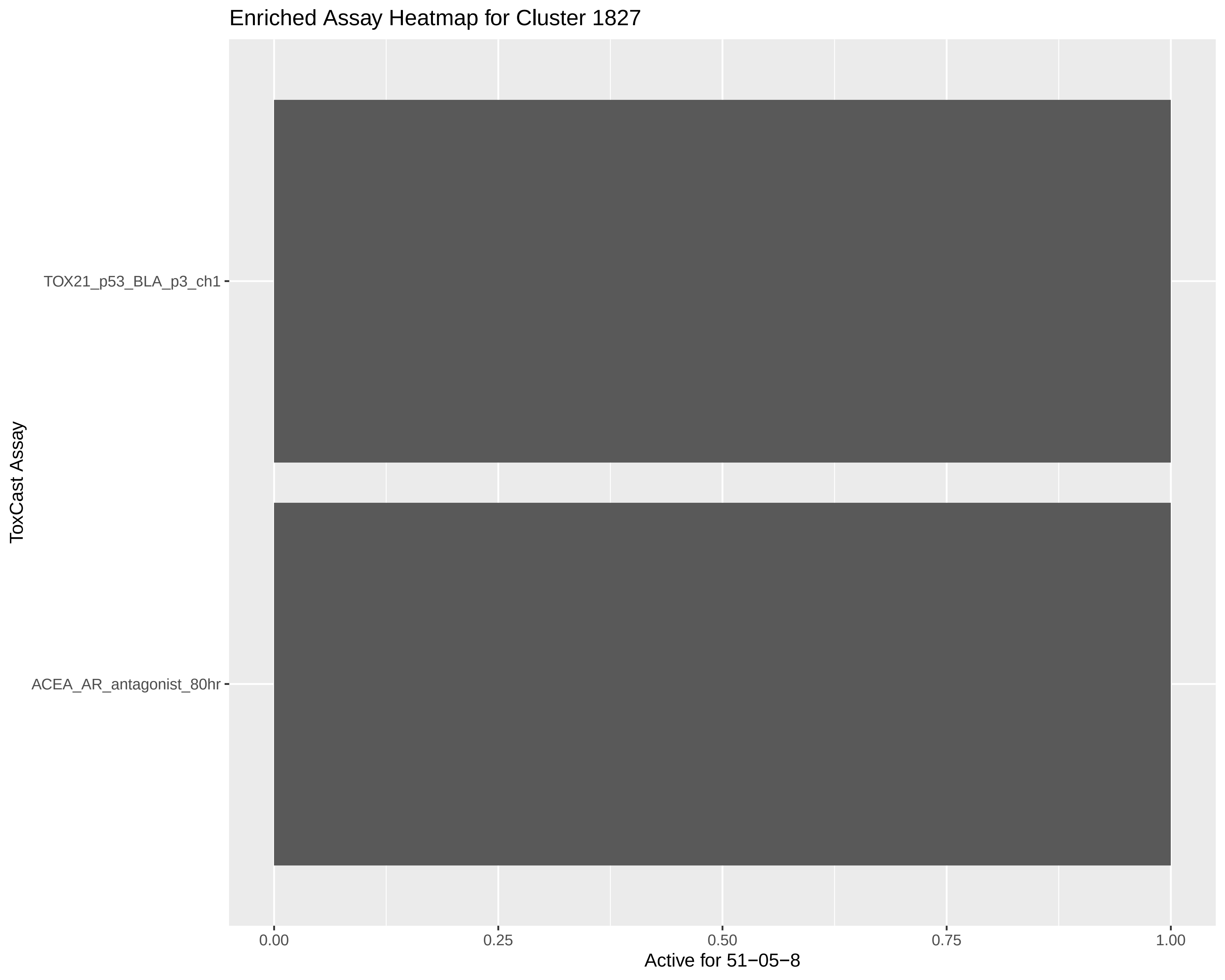
Enriched Assay Heatmap for Cluster 1827
ToxCast Assay
TOX21_p53_BLA_p3_ch1
ACEA_AR_antagonist_80hr
0.00
0.25
0.50
0.75
1.00
Active for 51−05−8

Enriched Assay Heatmap for Cluster 1834

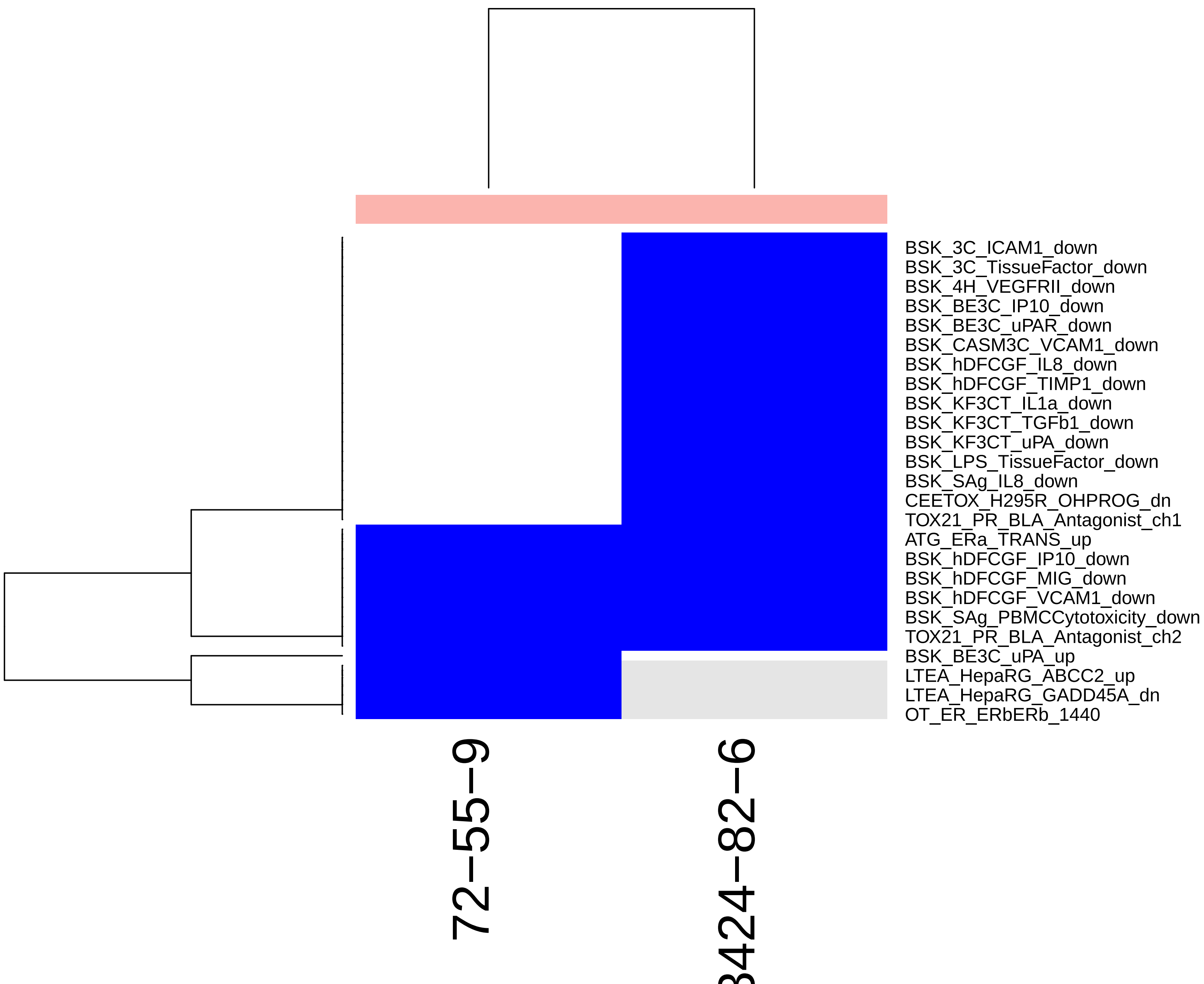

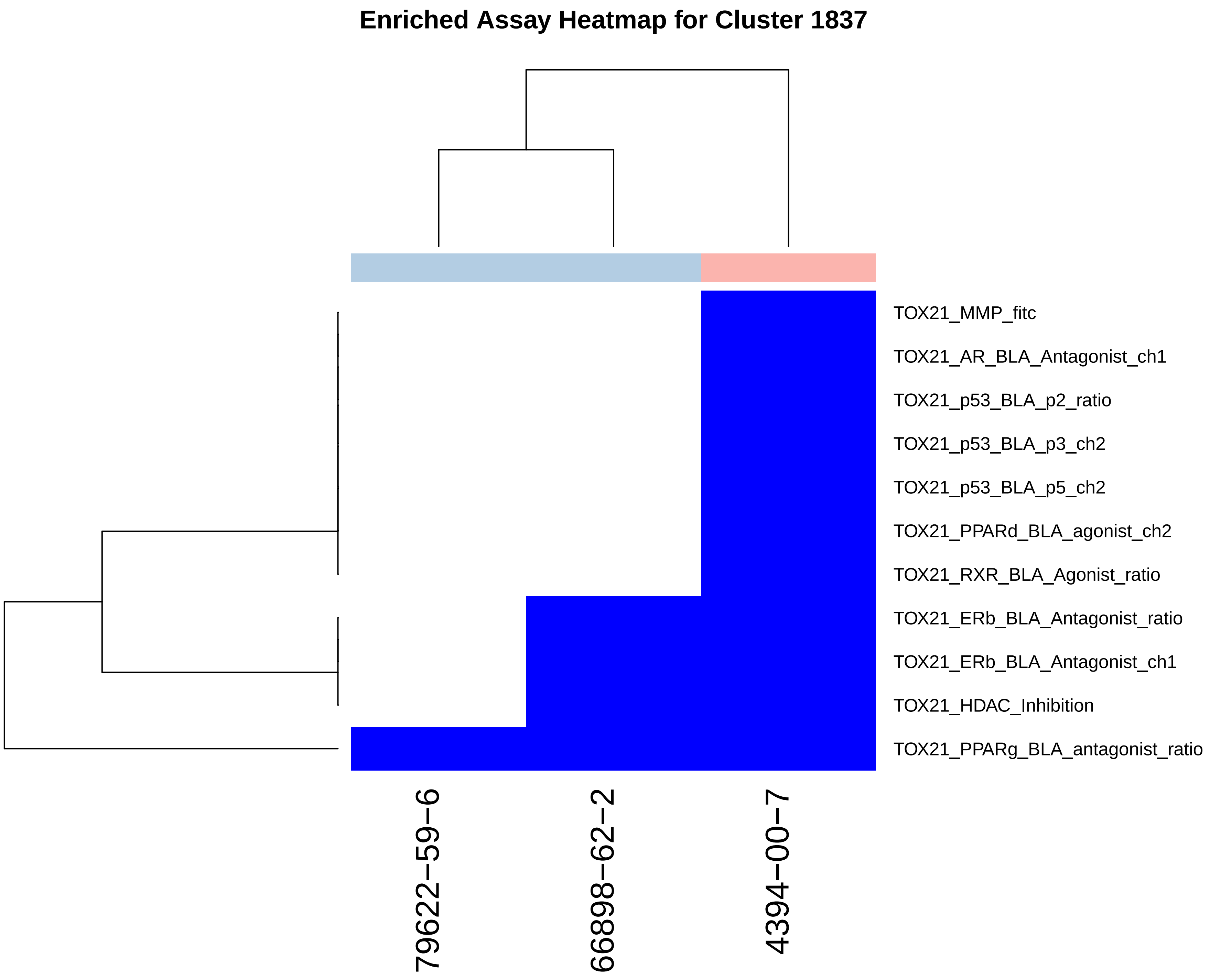
Enriched Assay Heatmap for Cluster 1837
TOX21_MMP_fitc
TOX21_AR_BLA_Antagonist_ch1
TOX21_p53_BLA_p2_ratio
TOX21_p53_BLA_p3_ch2
TOX21_p53_BLA_p5_ch2
TOX21_PPARd_BLA_agonist_ch2
TOX21_RXR_BLA_Agonist_ratio
TOX21_ERb_BLA_Antagonist_ratio
TOX21_ERb_BLA_Antagonist_ch1
TOX21_HDAC_Inhibition
TOX21_PPARg_BLA_antagonist_ratio
79622−59−6
66898−62−2
4394−00−7

Enriched Assay Heatmap for Cluster 1840

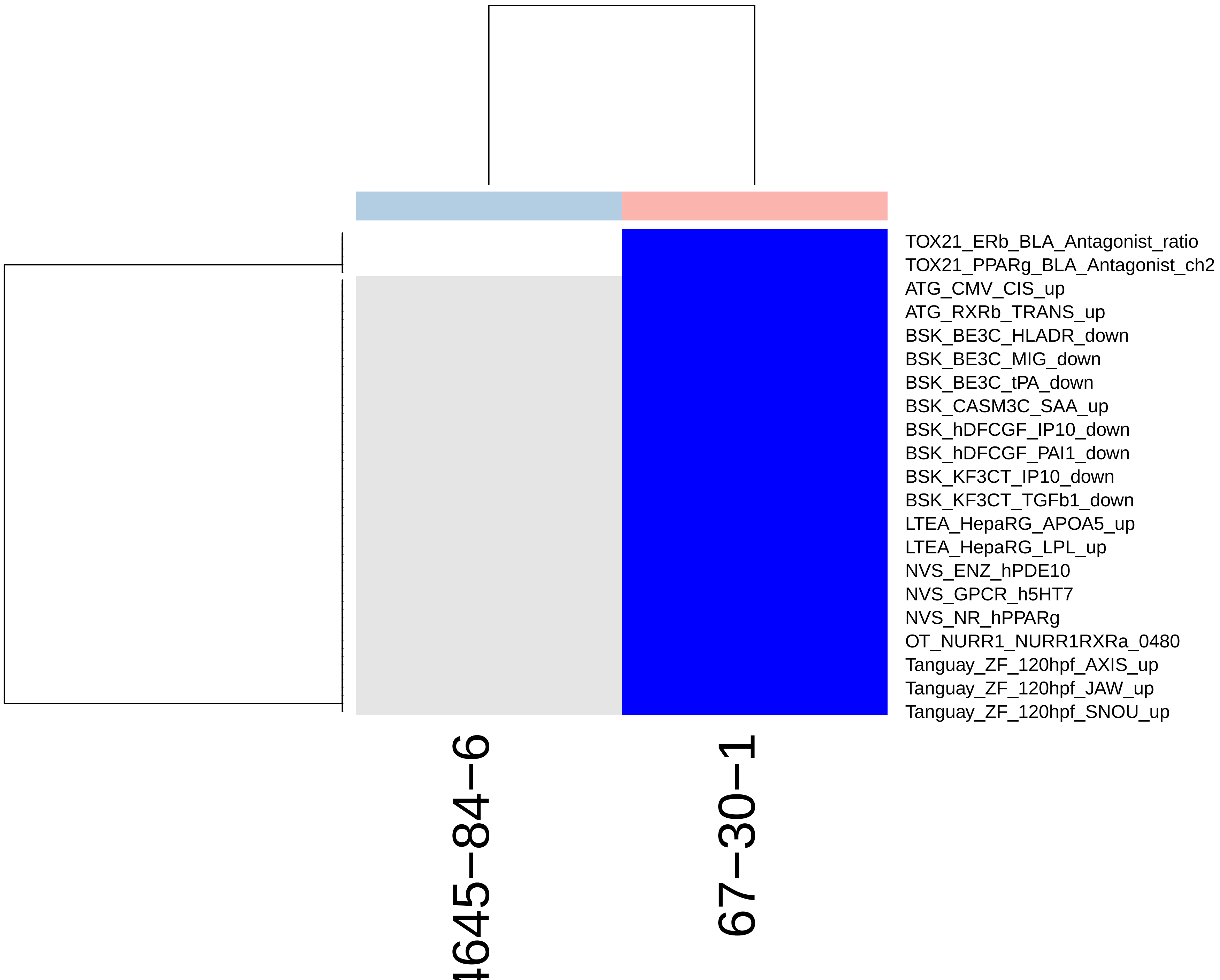

Enriched Assay Heatmap for Cluster 1841

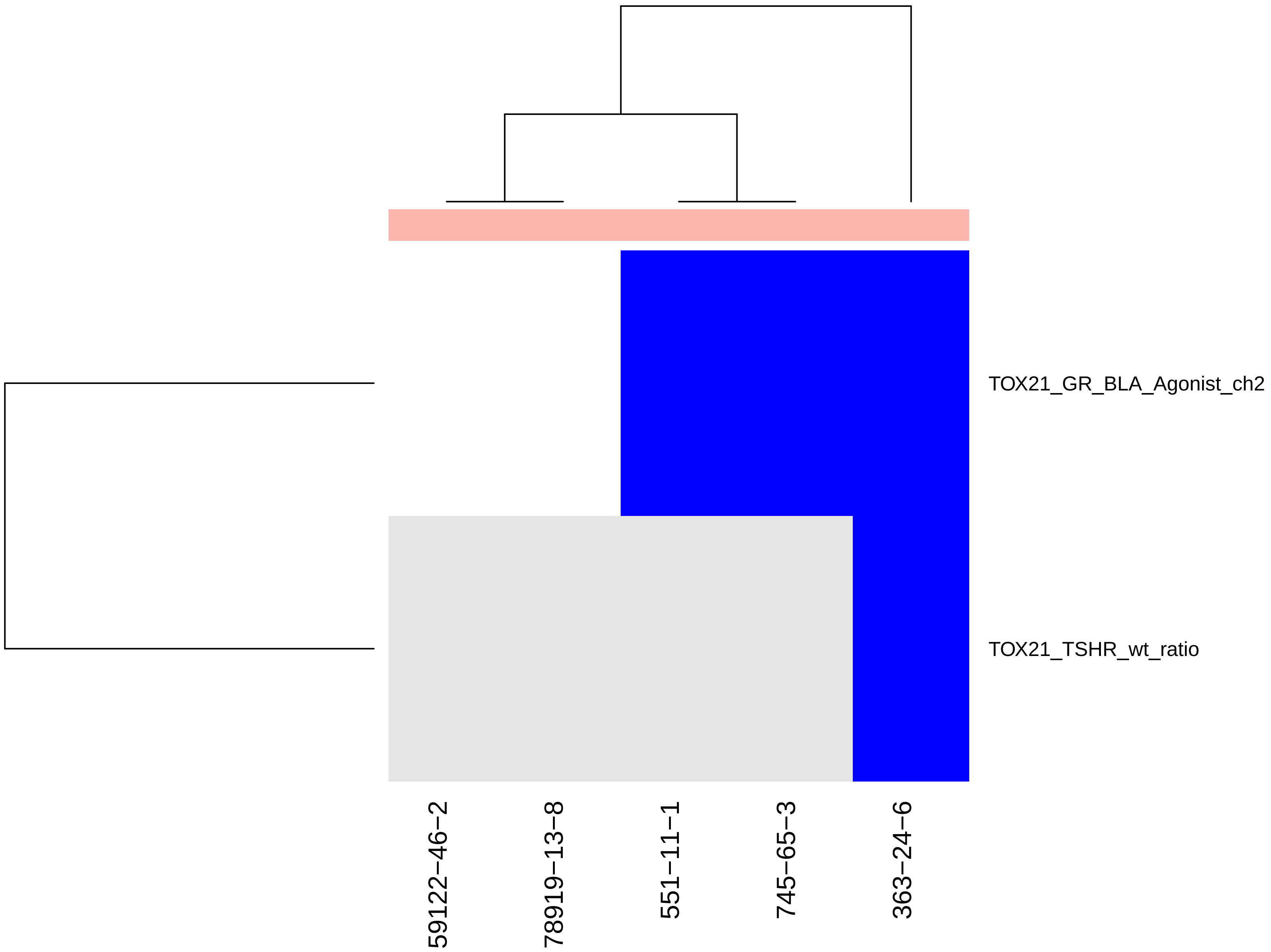

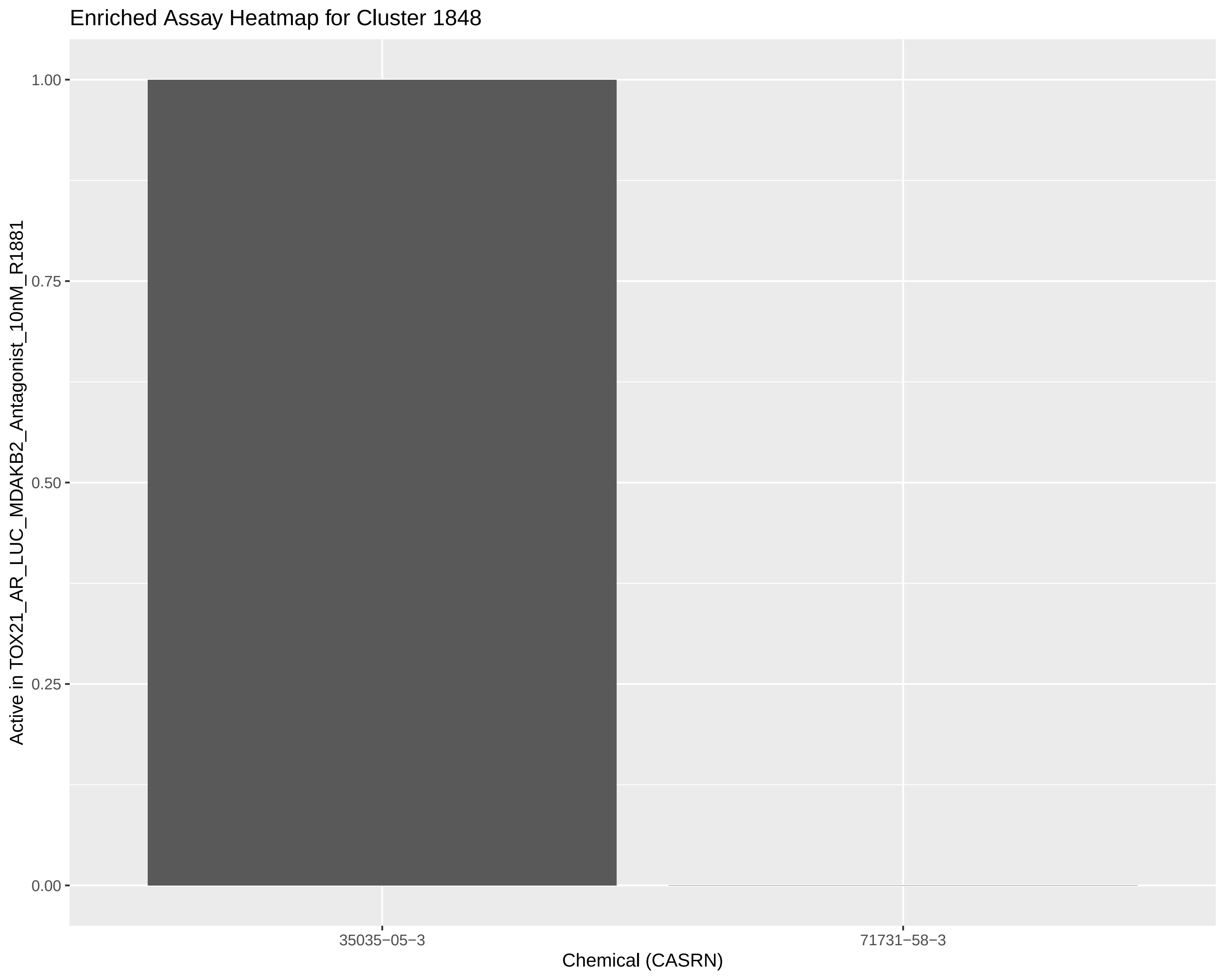
Enriched Assay Heatmap for Cluster 1848
Active in TOX21_AR_LUC_MDAKB2_Antagonist_10nM_R1881
1.00
0.75
0.50
0.25
0.00
35035−05−3
71731−58−3
Chemical (CASRN)

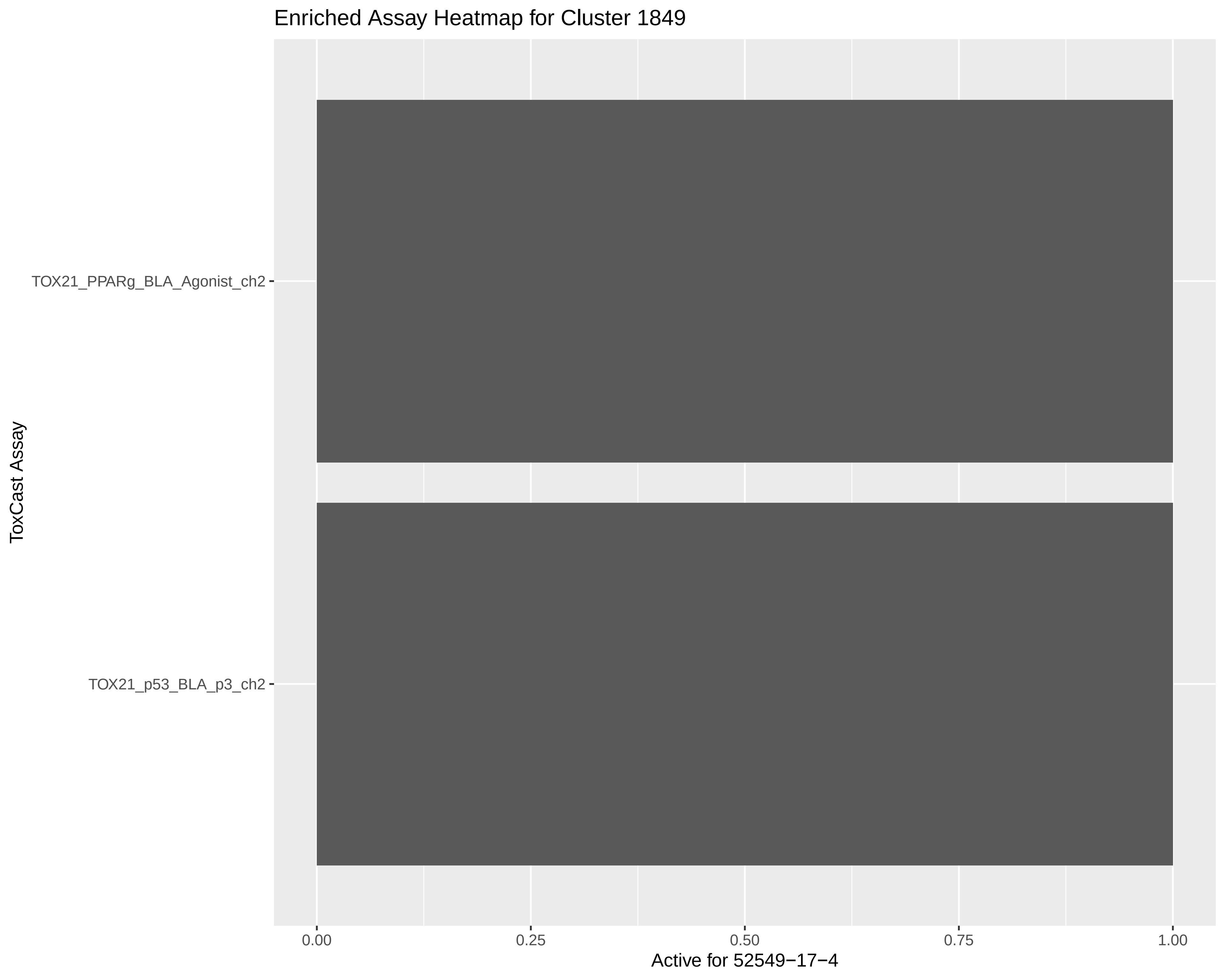
Enriched Assay Heatmap for Cluster 1849
ToxCast Assay
TOX21_PPARg_BLA_Agonist_ch2
TOX21_p53_BLA_p3_ch2
0.00
0.25
0.50
0.75
1.00
Active for 52549−17−4

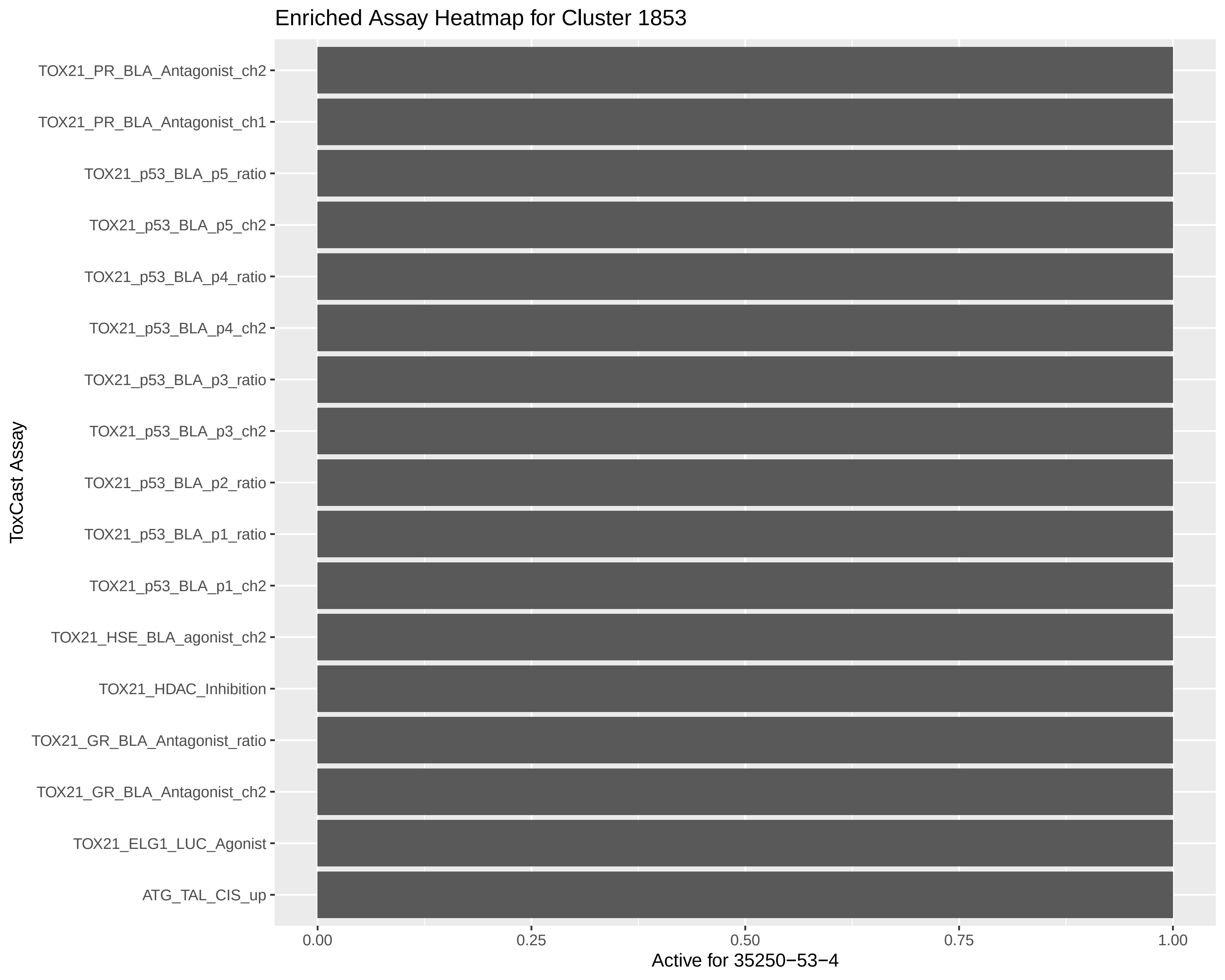
Enriched Assay Heatmap for Cluster 1853
ToxCast Assay
TOX21_PR_BLA_Antagonist_ch2
TOX21_PR_BLA_Antagonist_ch1
TOX21_p53_BLA_p5_ratio
TOX21_p53_BLA_p5_ch2
TOX21_p53_BLA_p4_ratio
TOX21_p53_BLA_p4_ch2
TOX21_p53_BLA_p3_ratio
TOX21_p53_BLA_p3_ch2
TOX21_p53_BLA_p2_ratio
TOX21_p53_BLA_p1_ratio
TOX21_p53_BLA_p1_ch2
TOX21_HSE_BLA_agonist_ch2
TOX21_HDAC_Inhibition
TOX21_GR_BLA_Antagonist_ratio
TOX21_GR_BLA_Antagonist_ch2
TOX21_ELG1_LUC_Agonist
ATG_TAL_CIS_up
0.00
0.25
0.50
0.75
1.00
Active for 35250−53−4

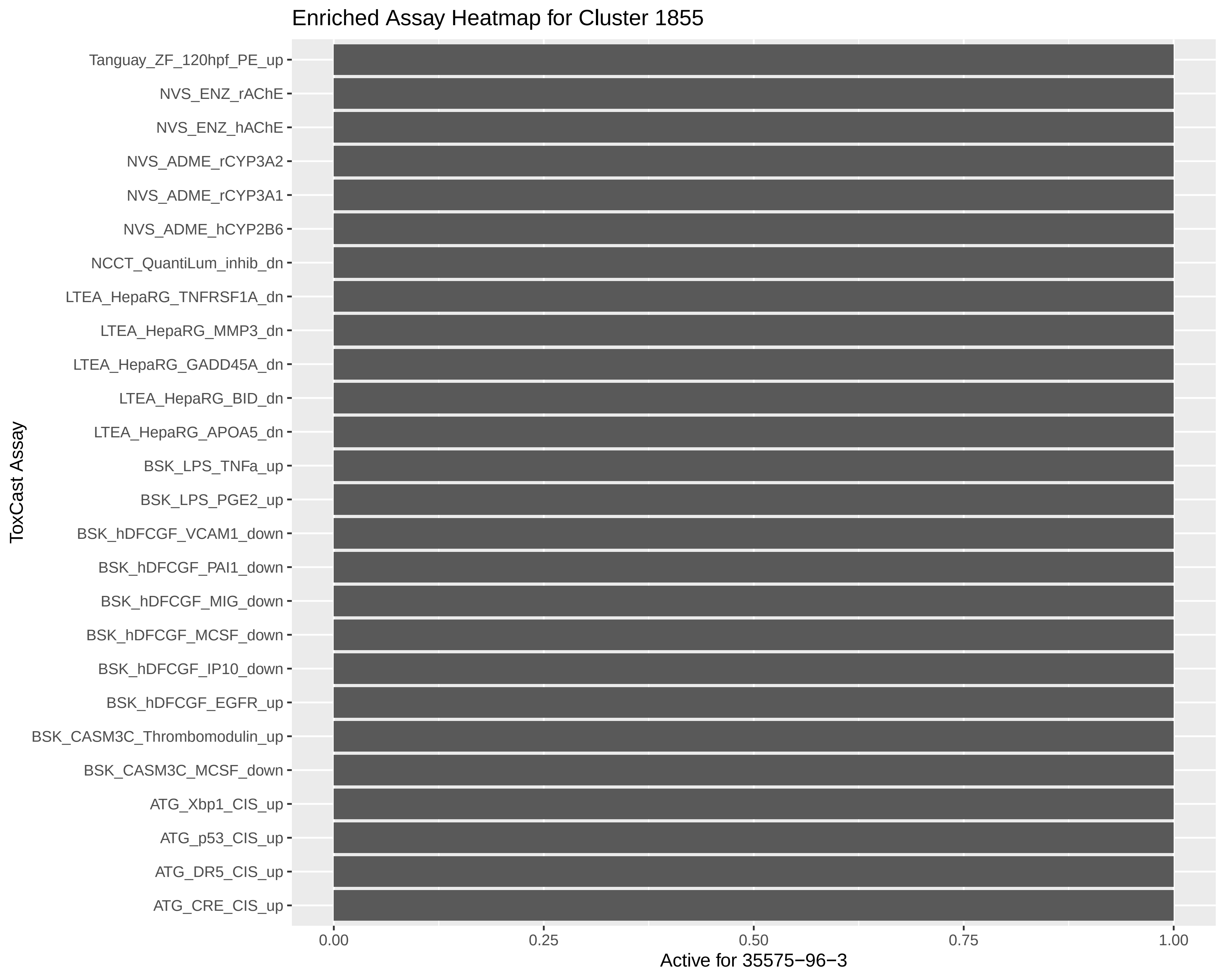
Enriched Assay Heatmap for Cluster 1855
ToxCast Assay
Tanguay_ZF_120hpf_PE_up
NVS_ENZ_rAChE
NVS_ENZ_hAChE
NVS_ADME_rCYP3A2
NVS_ADME_rCYP3A1
NVS_ADME_hCYP2B6
NCCT_QuantiLum_inhib_dn
LTEA_HepaRG_TNFRSF1A_dn
LTEA_HepaRG_MMP3_dn
LTEA_HepaRG_GADD45A_dn
LTEA_HepaRG_BID_dn
LTEA_HepaRG_APOA5_dn
BSK_LPS_TNFa_up
BSK_LPS_PGE2_up
BSK_hDFCGF_VCAM1_down
BSK_hDFCGF_PAI1_down
BSK_hDFCGF_MIG_down
BSK_hDFCGF_MCSF_down
BSK_hDFCGF_IP10_down
BSK_hDFCGF_EGFR_up
BSK_CASM3C_Thrombomodulin_up
BSK_CASM3C_MCSF_down
ATG_Xbp1_CIS_up
ATG_p53_CIS_up
ATG_DR5_CIS_up
ATG_CRE_CIS_up
0.00
0.25
0.50
0.75
1.00
Active for 35575−96−3

Enriched Assay Heatmap for Cluster 1856

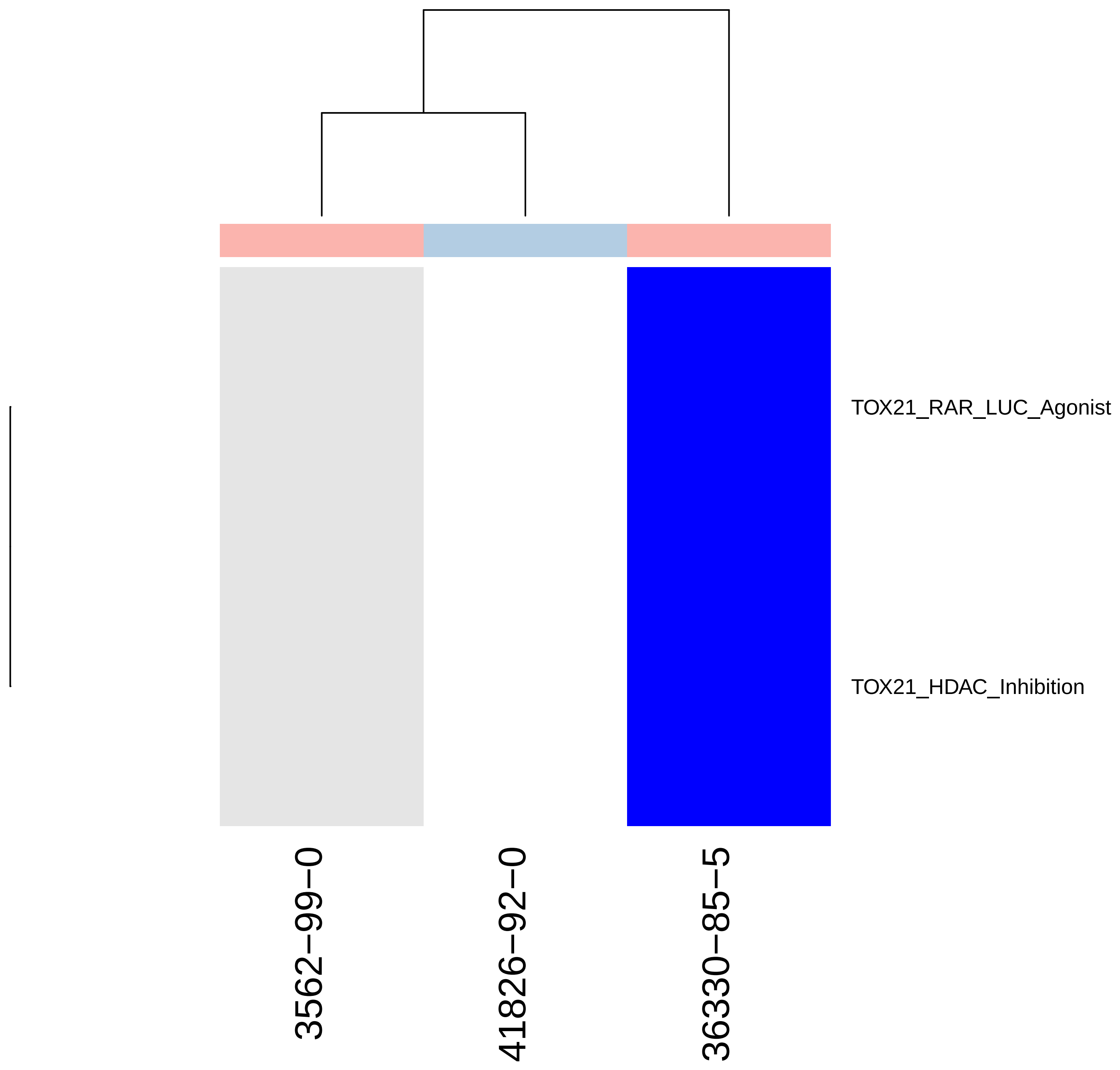

Enriched Assay Heatmap for Cluster 1857

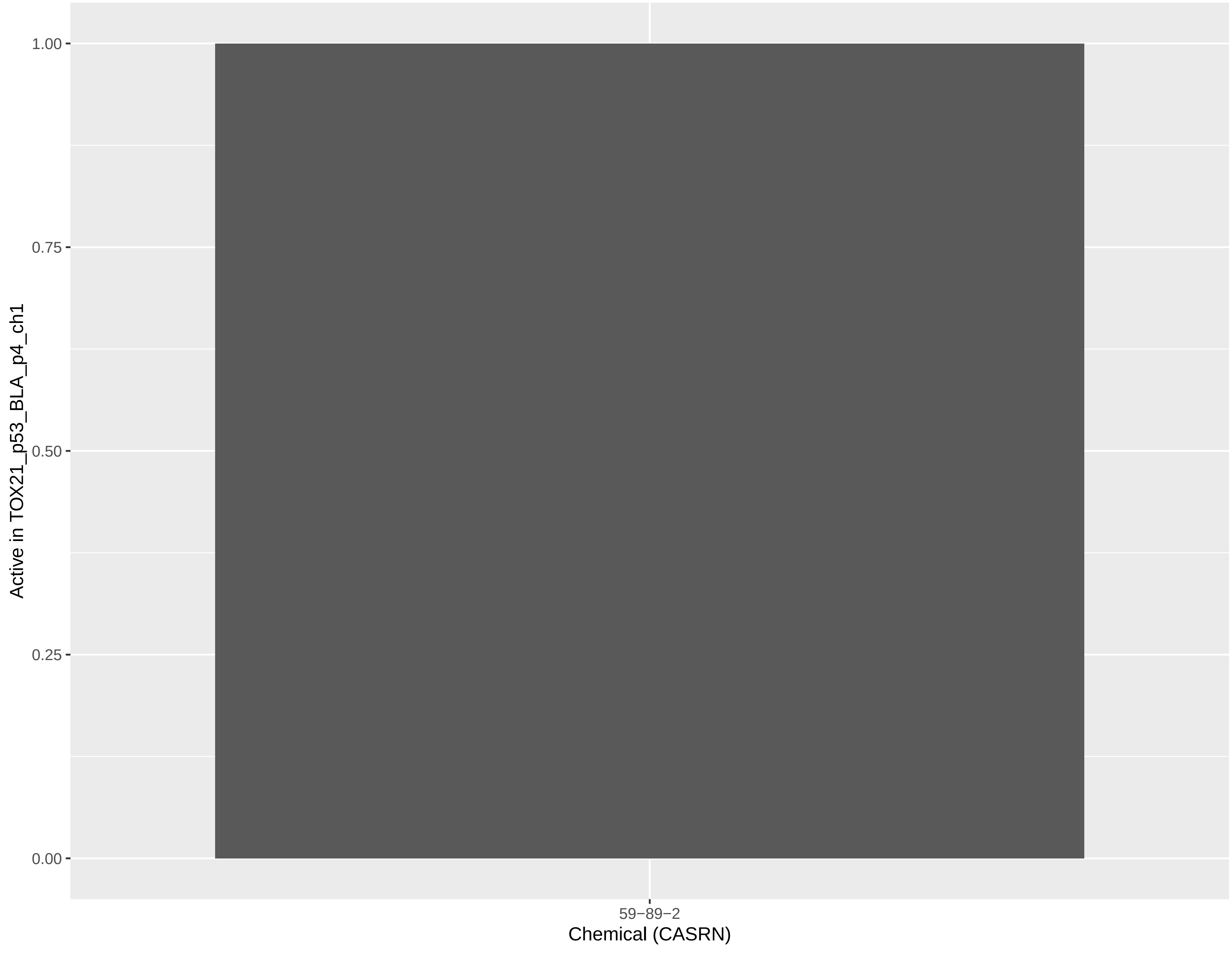

Enriched Assay Heatmap for Cluster 1860

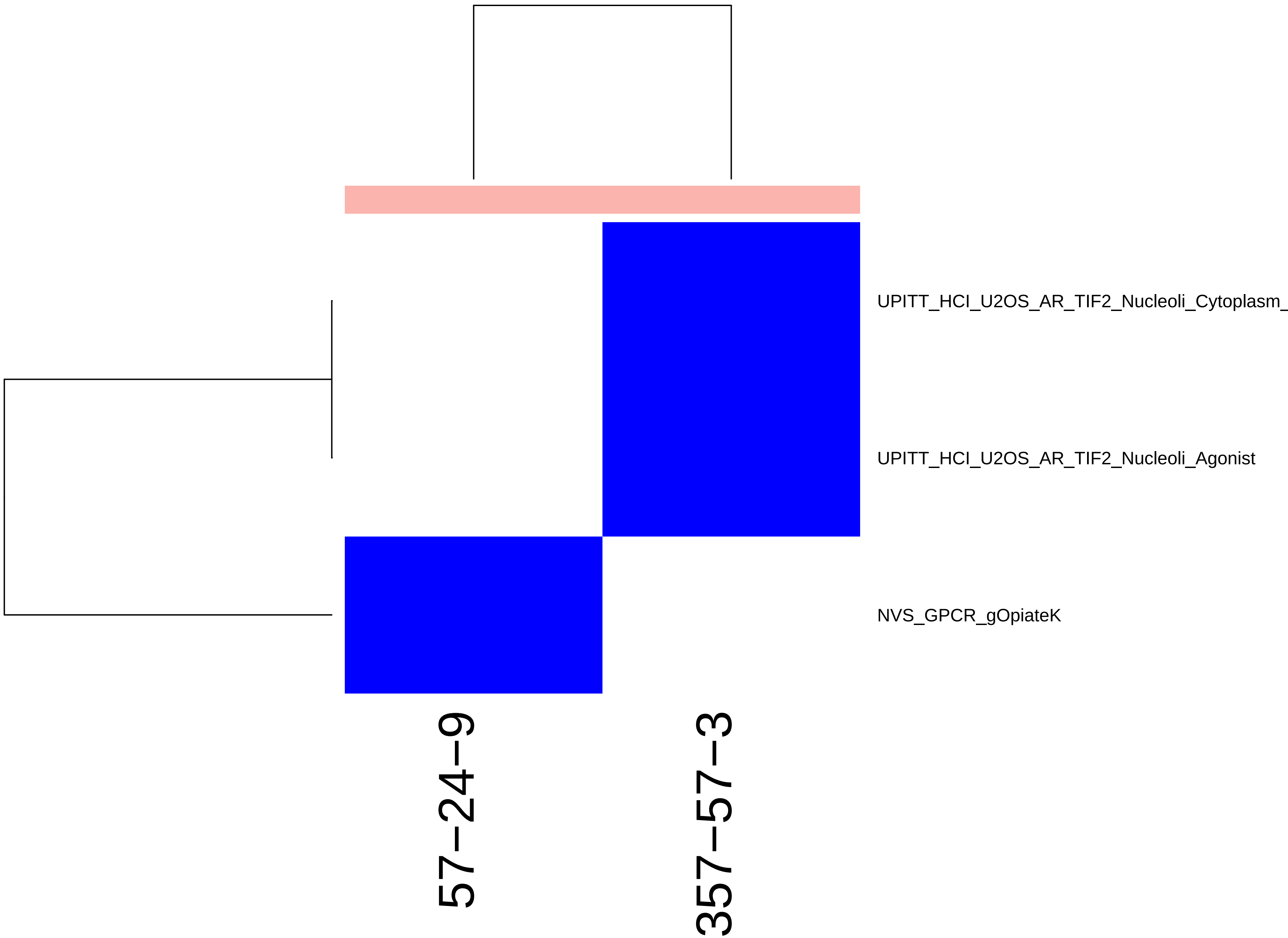

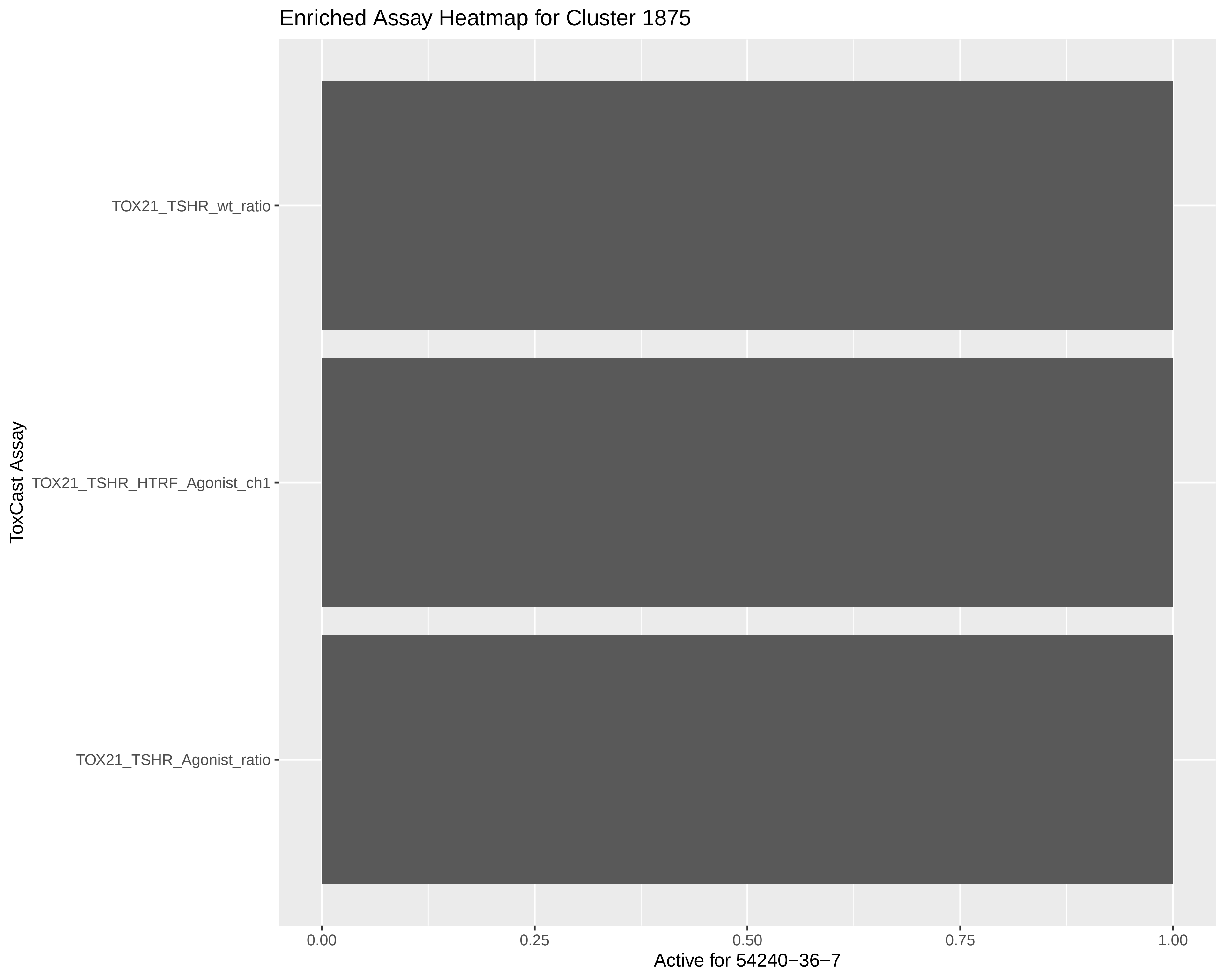
Enriched Assay Heatmap for Cluster 1875
ToxCast Assay
TOX21_TSHR_wt_ratio
TOX21_TSHR_HTRF_Agonist_ch1
TOX21_TSHR_Agonist_ratio
0.00
0.25
0.50
0.75
1.00
Active for 54240−36−7

Enriched Assay Heatmap for Cluster 1882

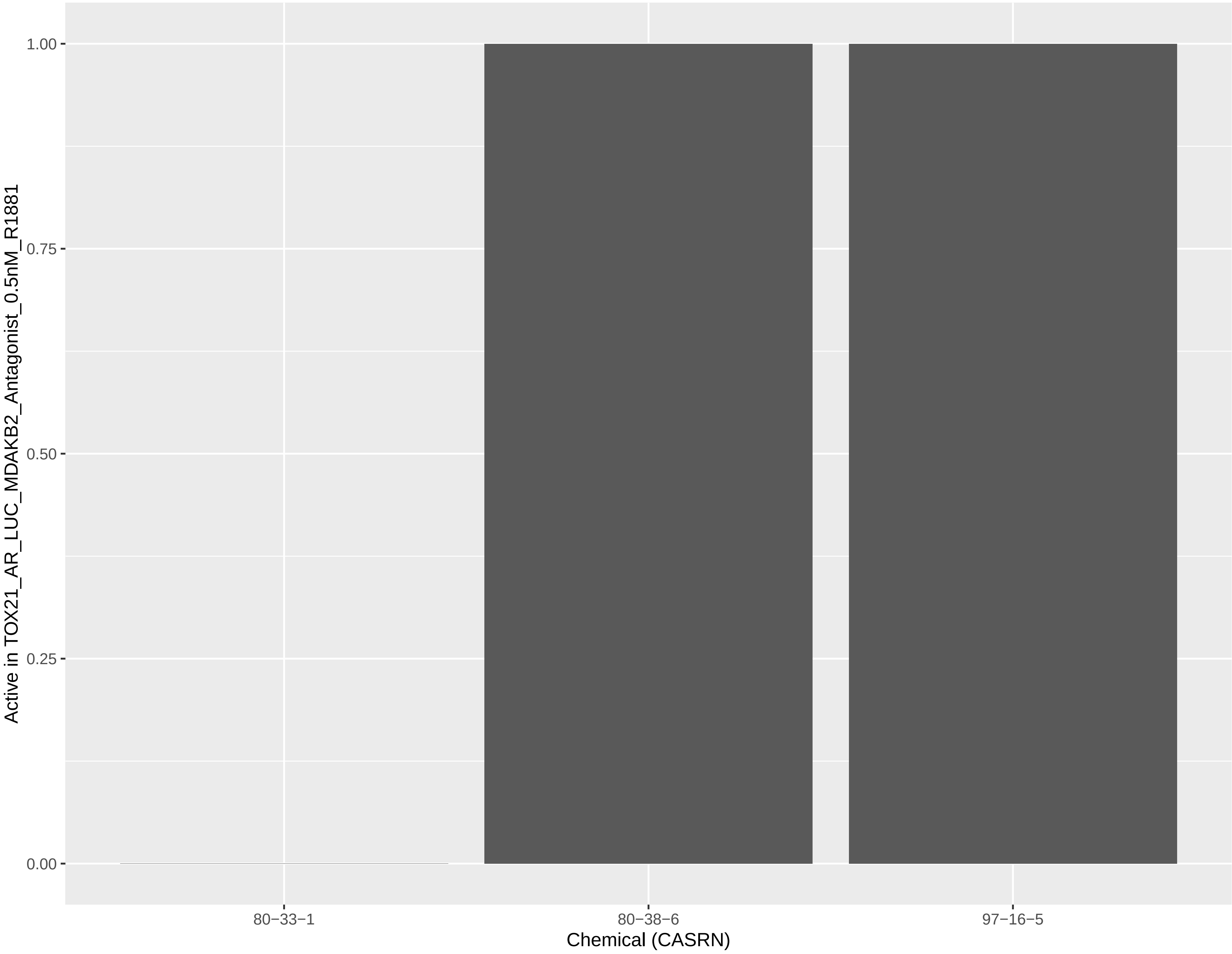

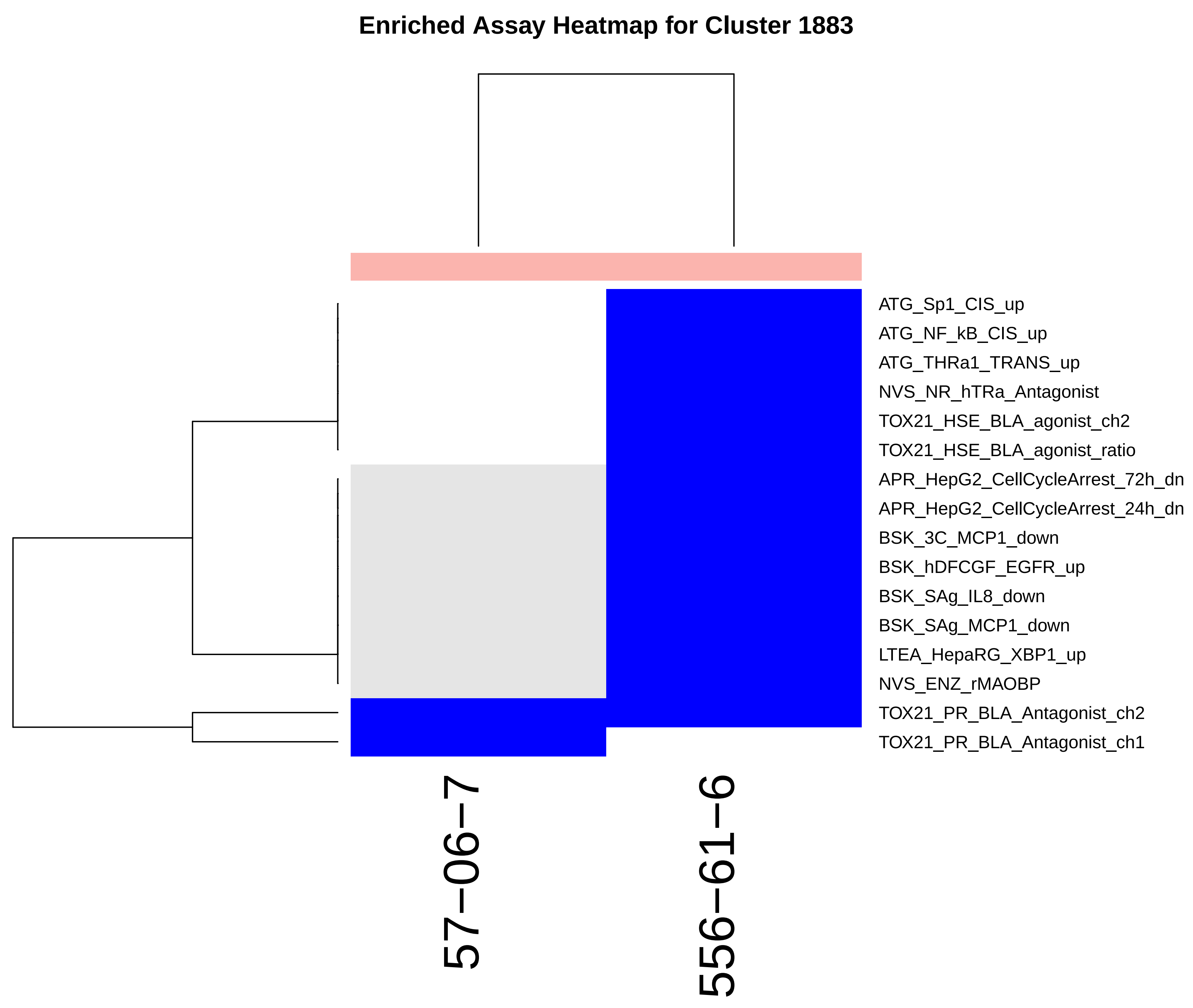
Enriched Assay Heatmap for Cluster 1883
ATG_Sp1_CIS_up
ATG_NF_kB_CIS_up
ATG_THRa1_TRANS_up
NVS_NR_hTRa_Antagonist
TOX21_HSE_BLA_agonist_ch2
TOX21_HSE_BLA_agonist_ratio
APR_HepG2_CellCycleArrest_72h_dn
APR_HepG2_CellCycleArrest_24h_dn
BSK_3C_MCP1_down
BSK_hDFCGF_EGFR_up
BSK_SAg_IL8_down
BSK_SAg_MCP1_down
LTEA_HepaRG_XBP1_up
NVS_ENZ_rMAOBP
TOX21_PR_BLA_Antagonist_ch2
TOX21_PR_BLA_Antagonist_ch1
57−06−7
556−61−6

Enriched Assay Heatmap for Cluster 1885

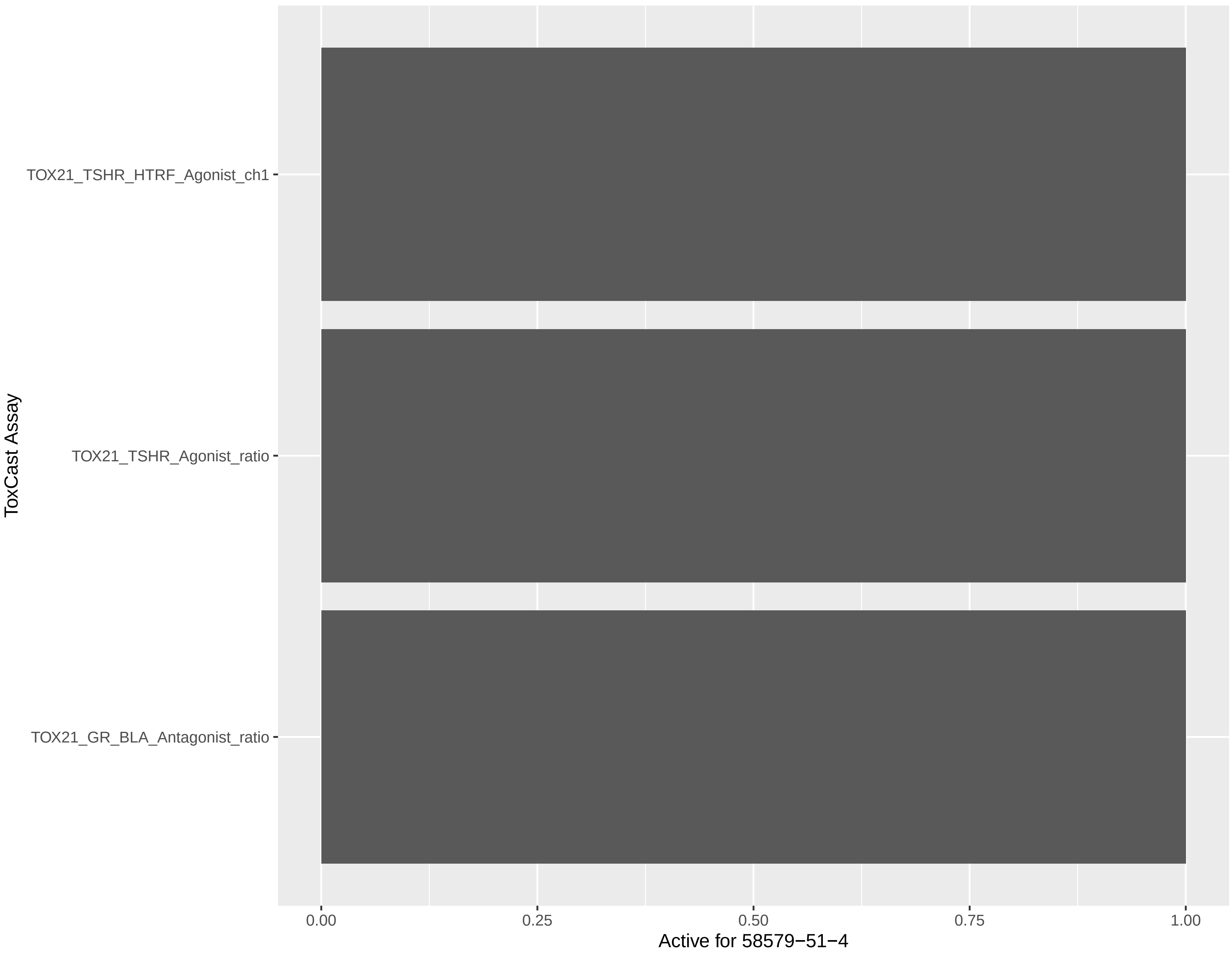

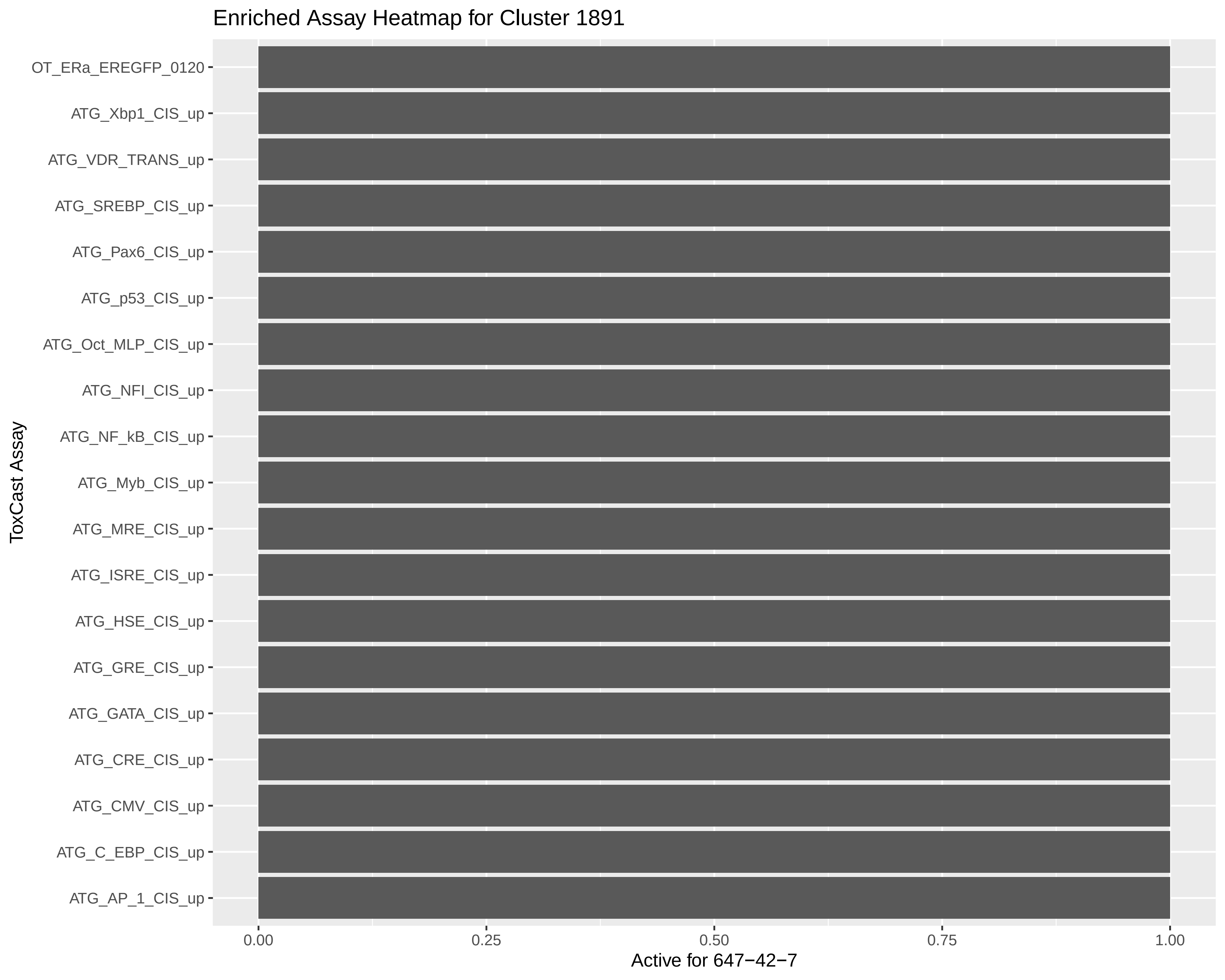
Enriched Assay Heatmap for Cluster 1891
ToxCast Assay
OT_ERa_EREGFP_0120
ATG_Xbp1_CIS_up
ATG_VDR_TRANS_up
ATG_SREBP_CIS_up
ATG_Pax6_CIS_up
ATG_p53_CIS_up
ATG_Oct_MLP_CIS_up
ATG_NFI_CIS_up
ATG_NF_kB_CIS_up
ATG_Myb_CIS_up
ATG_MRE_CIS_up
ATG_ISRE_CIS_up
ATG_HSE_CIS_up
ATG_GRE_CIS_up
ATG_GATA_CIS_up
ATG_CRE_CIS_up
ATG_CMV_CIS_up
ATG_C_EBP_CIS_up
ATG_AP_1_CIS_up
0.00
0.25
0.50
0.75
1.00
Active for 647−42−7

Enriched Assay Heatmap for Cluster 1893

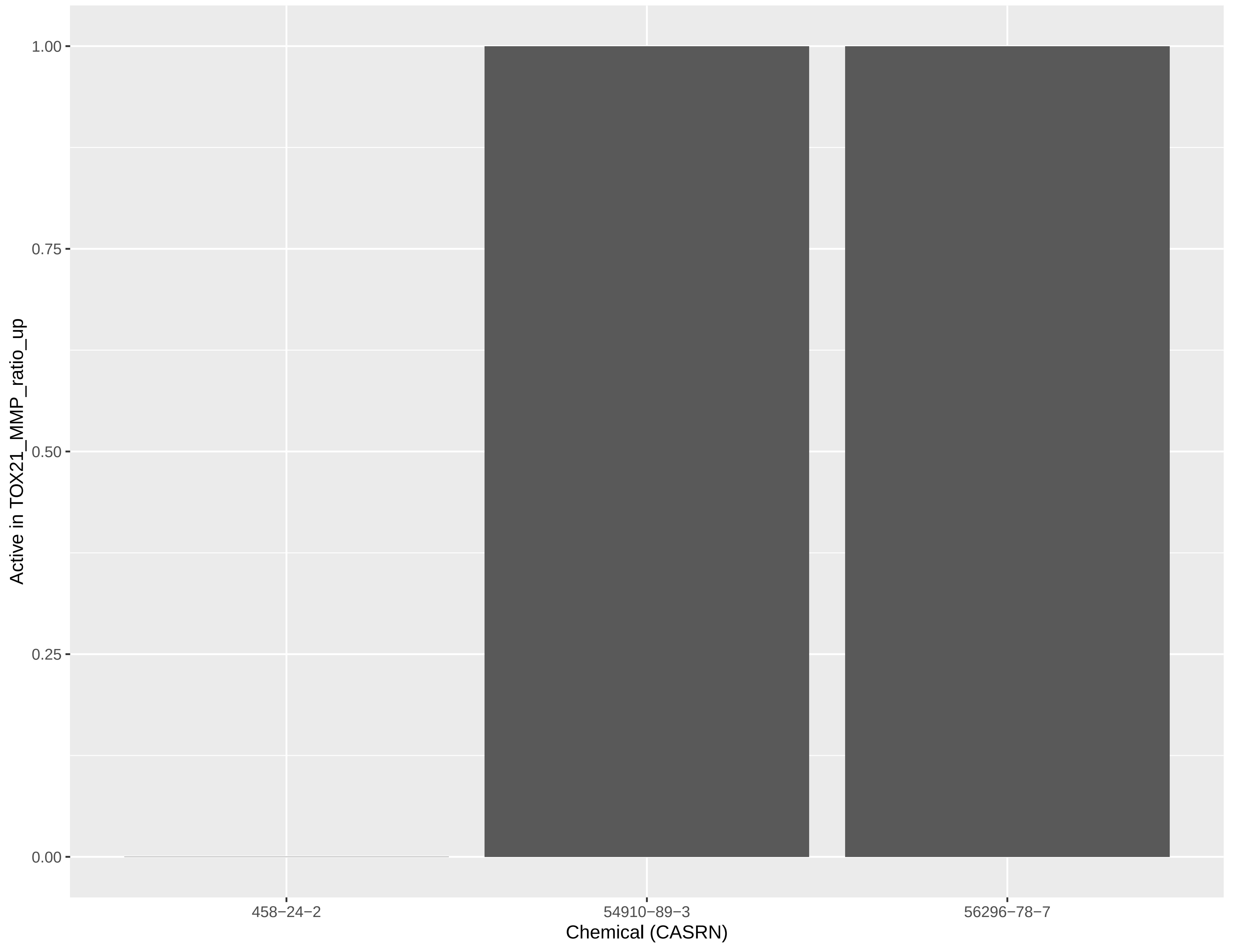

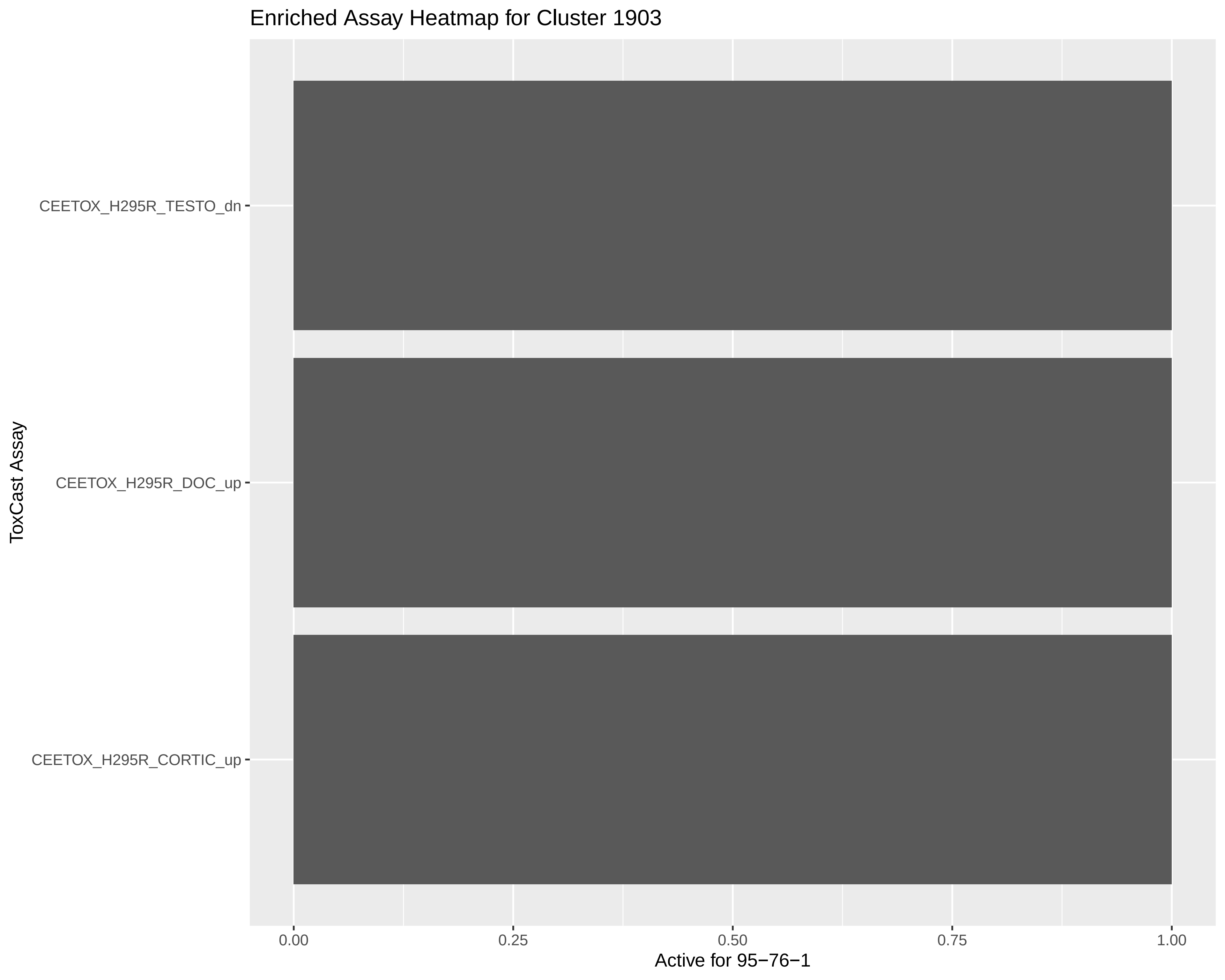

Enriched Assay Heatmap for Cluster 1903
ToxCast Assay
CEETOX_H295R_TESTO_dn
CEETOX_H295R_DOC_up
CEETOX_H295R_CORTIC_up
0.00
0.25
0.50
0.75
1.00
Active for 95−76−1

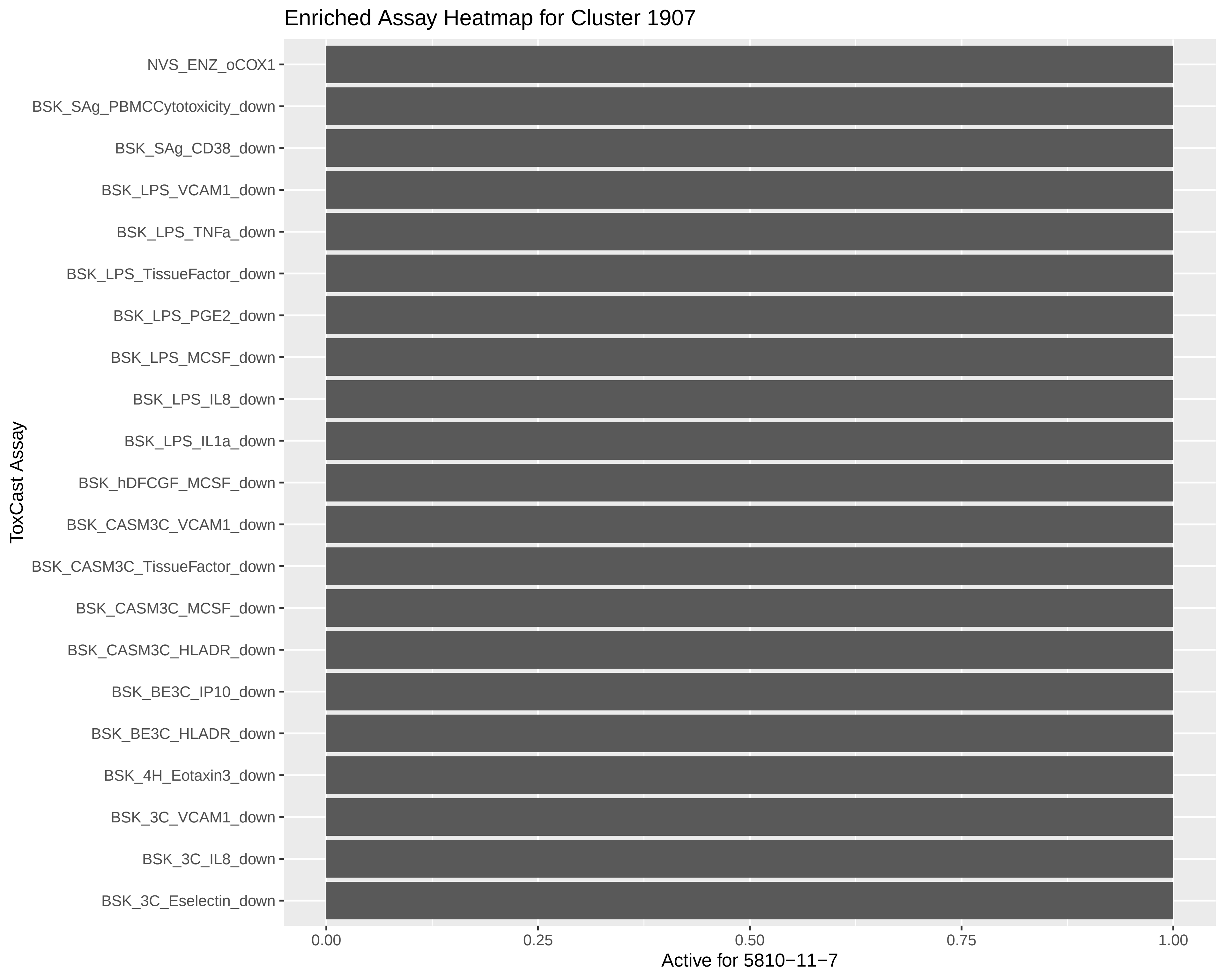

Enriched Assay Heatmap for Cluster 1907
NVS_ENZ_oCOX1
BSK_SAg_PBMCCytotoxicity_down
BSK_SAg_CD38_down
BSK_LPS_VCAM1_down
BSK_LPS_TNFa_down
BSK_LPS_TissueFactor_down
BSK_LPS_PGE2_down
BSK_LPS_MCSF_down
BSK_LPS_IL8_down
BSK_LPS_IL1a_down
BSK_hDFCGF_MCSF_down
BSK_CASM3C_VCAM1_down
BSK_CASM3C_TissueFactor_down
BSK_CASM3C_MCSF_down
BSK_CASM3C_HLADR_down
BSK_BE3C_IP10_down
BSK_BE3C_HLADR_down
BSK_4H_Eotaxin3_down
BSK_3C_VCAM1_down
BSK_3C_IL8_down
BSK_3C_Eselectin_down
ToxCast Assay
0.00
0.25
0.50
0.75
1.00
Active for 5810−11−7

Enriched Assay Heatmap for Cluster 1909

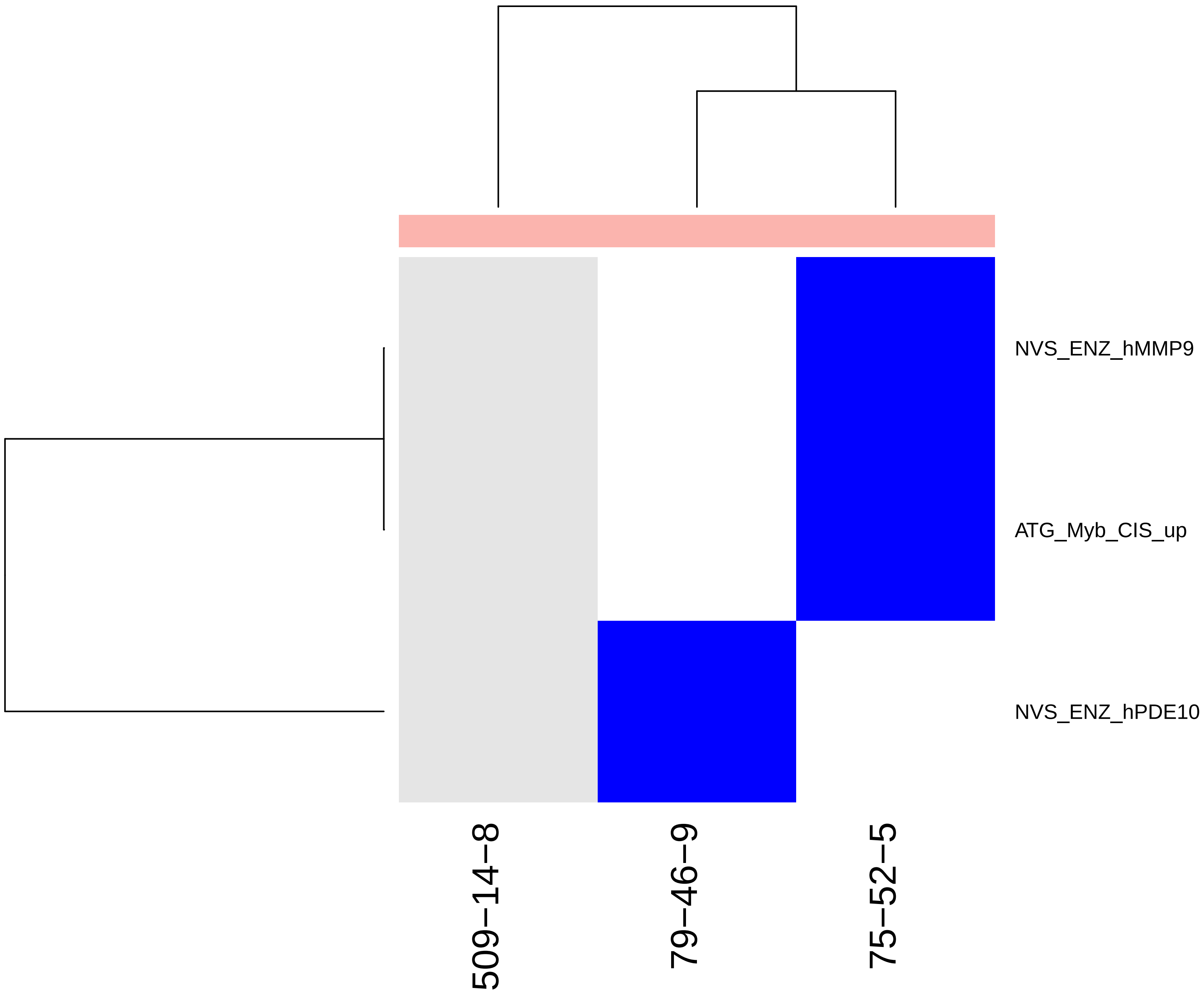

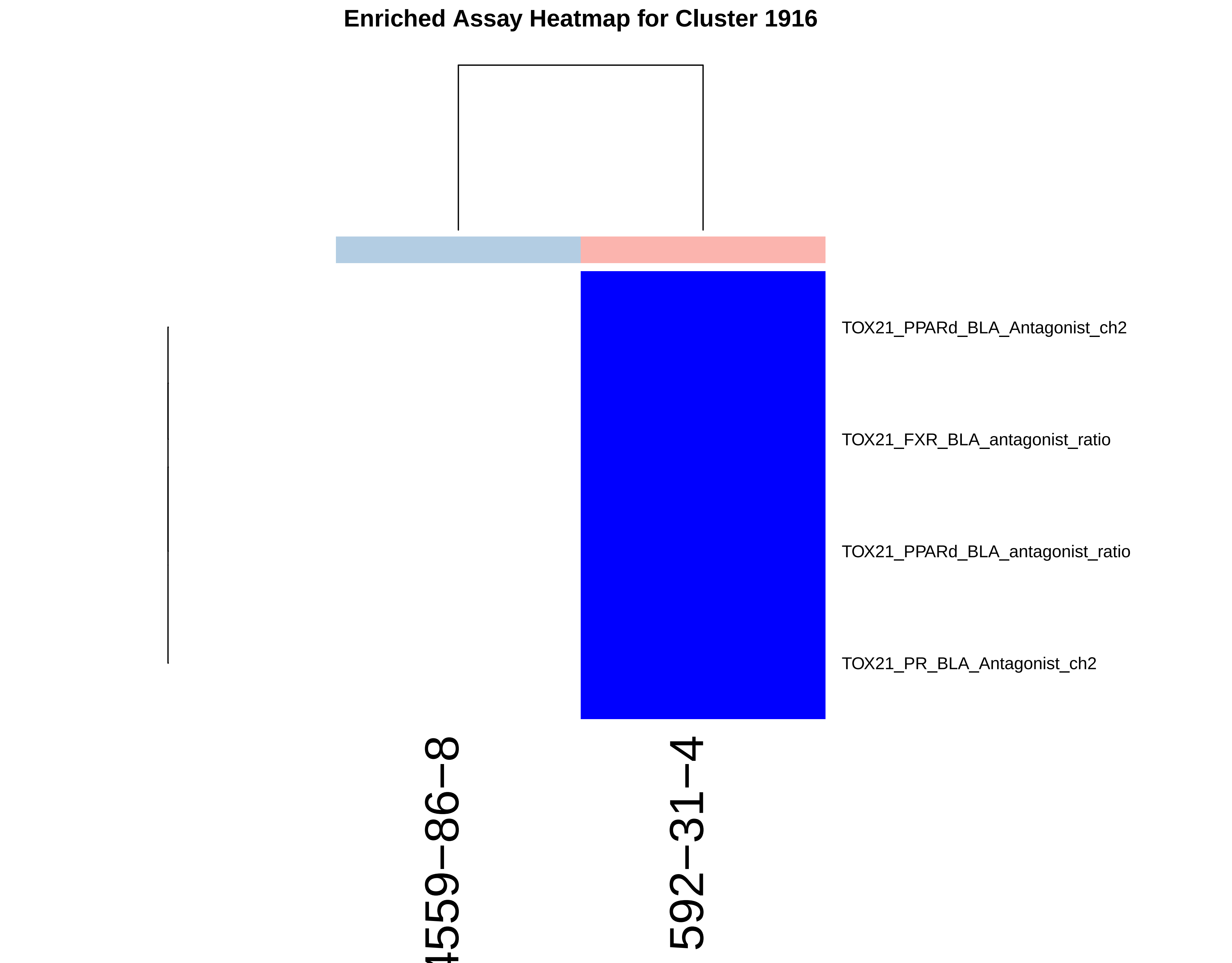

Enriched Assay Heatmap for Cluster 1916
TOX21_PPARd_BLA_Antagonist_ch2
TOX21_FXR_BLA_antagonist_ratio
TOX21_PPARd_BLA_antagonist_ratio
TOX21_PR_BLA_Antagonist_ch2
592−31−4

Enriched Assay Heatmap for Cluster 1919

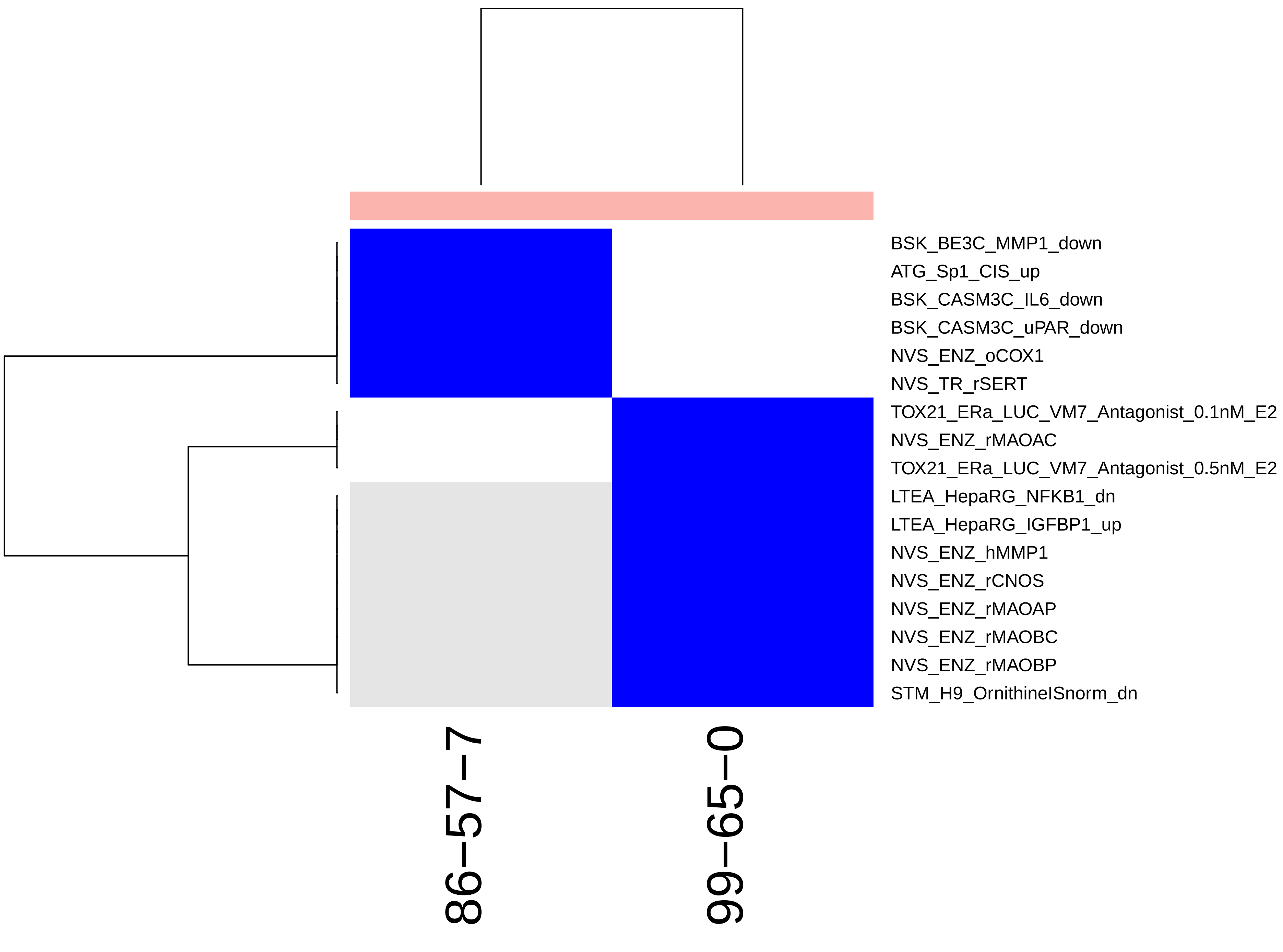

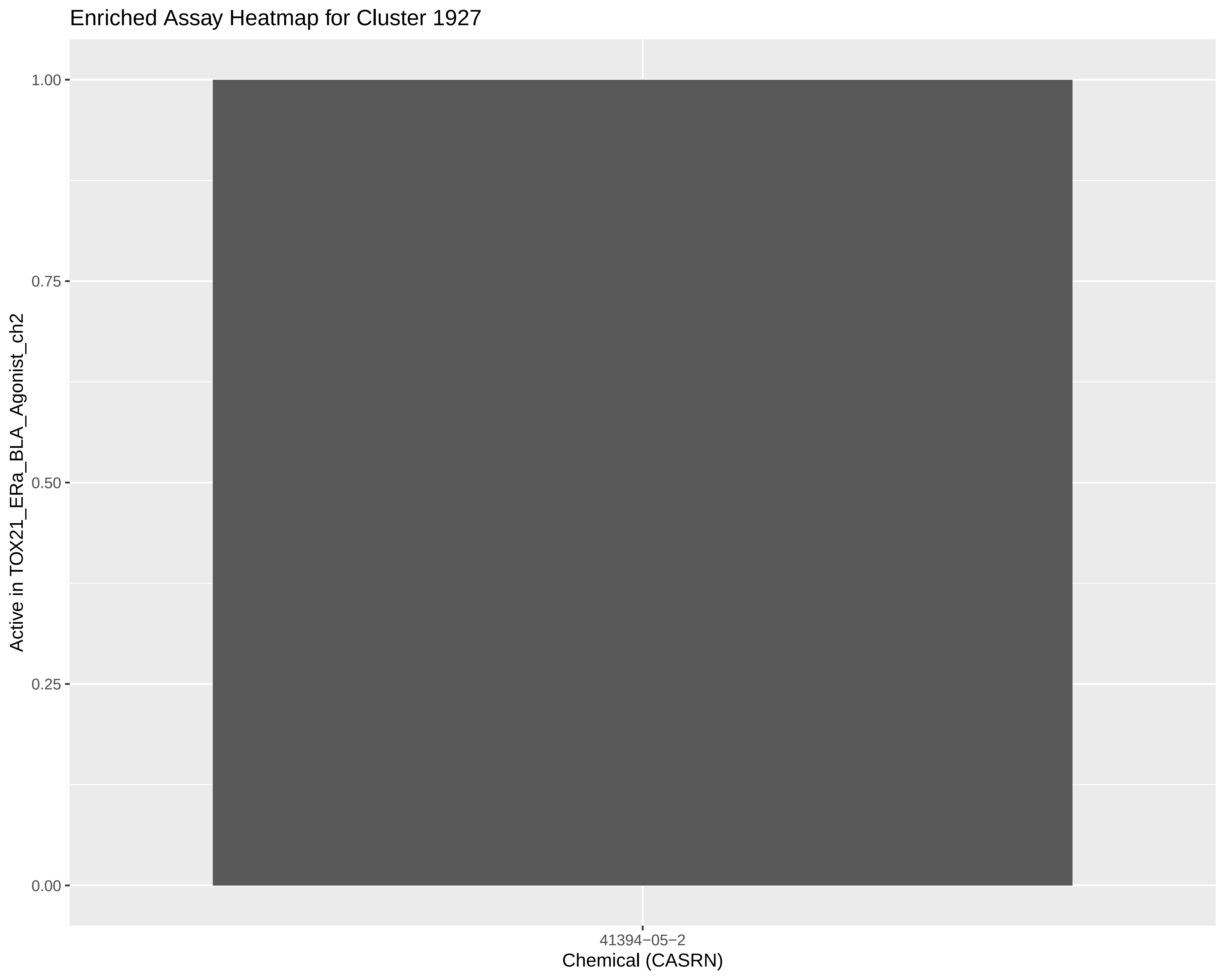
Enriched Assay Heatmap for Cluster 1927
1.00
0.75
0.50
0.25
0.00
Active in TOX21_ERa_BLA_Agonist_ch2
41394−05−2
Chemical (CASRN)

Enriched Assay Heatmap for Cluster 1939

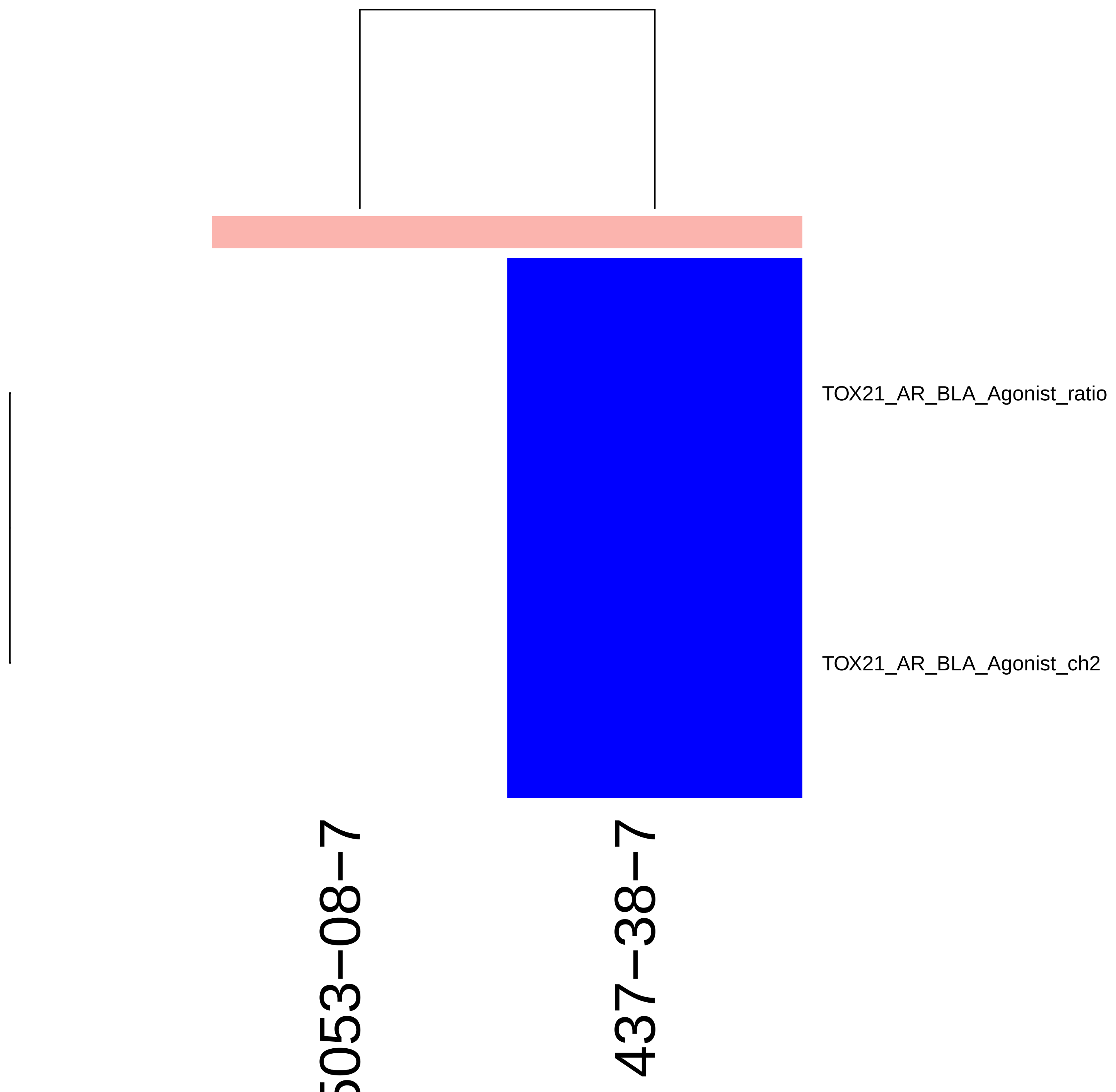

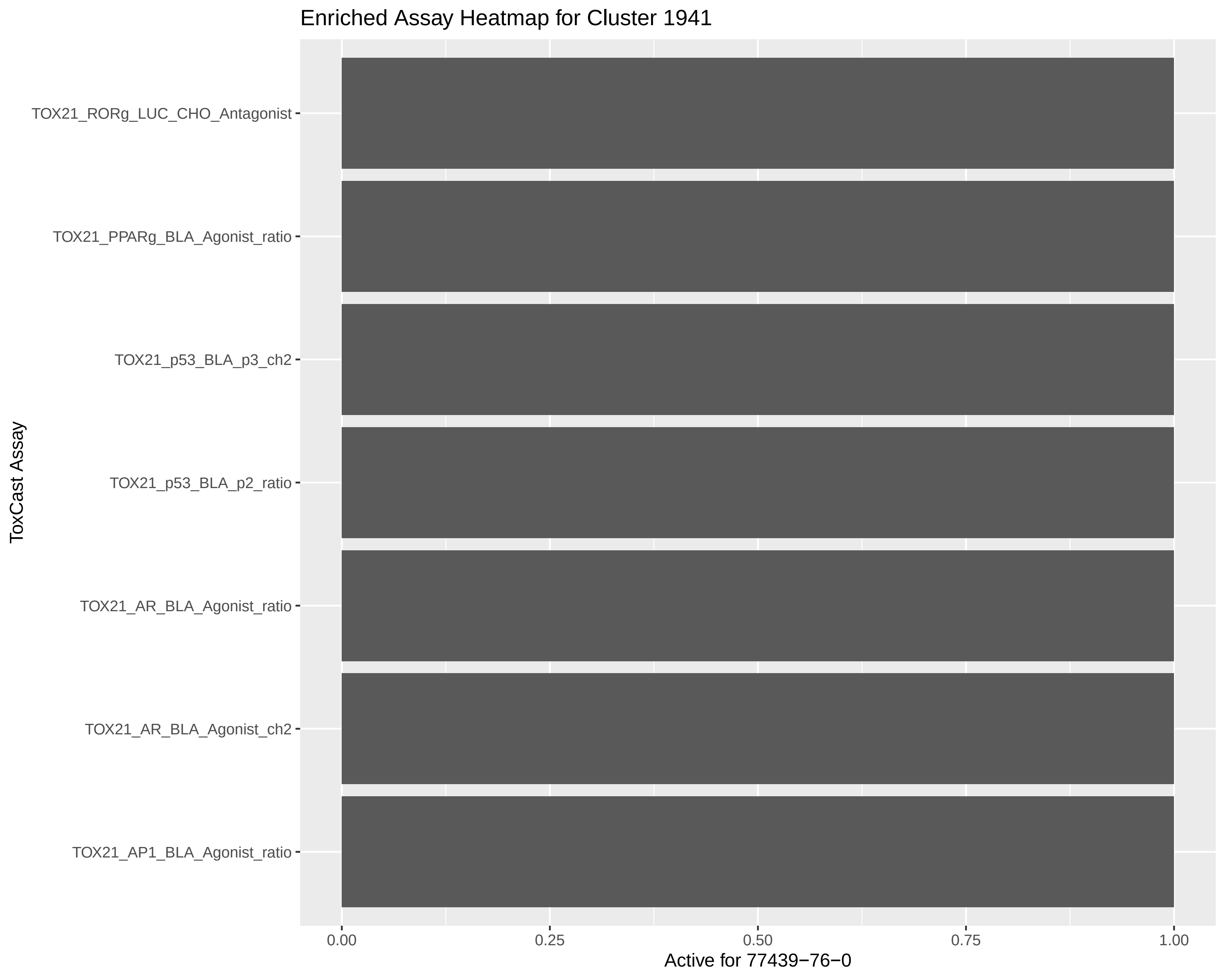

Enriched Assay Heatmap for Cluster 1941
ToxCast Assay
TOX21_RORg_LUC_CHO_Antagonist
TOX21_PPARg_BLA_Agonist_ratio
TOX21_p53_BLA_p3_ch2
TOX21_p53_BLA_p2_ratio
TOX21_AR_BLA_Agonist_ratio
TOX21_AR_BLA_Agonist_ch2
TOX21_AP1_BLA_Agonist_ratio
0.00
0.25
0.50
0.75
1.00
Active for 77439−76−0

Enriched Assay Heatmap for Cluster 1943

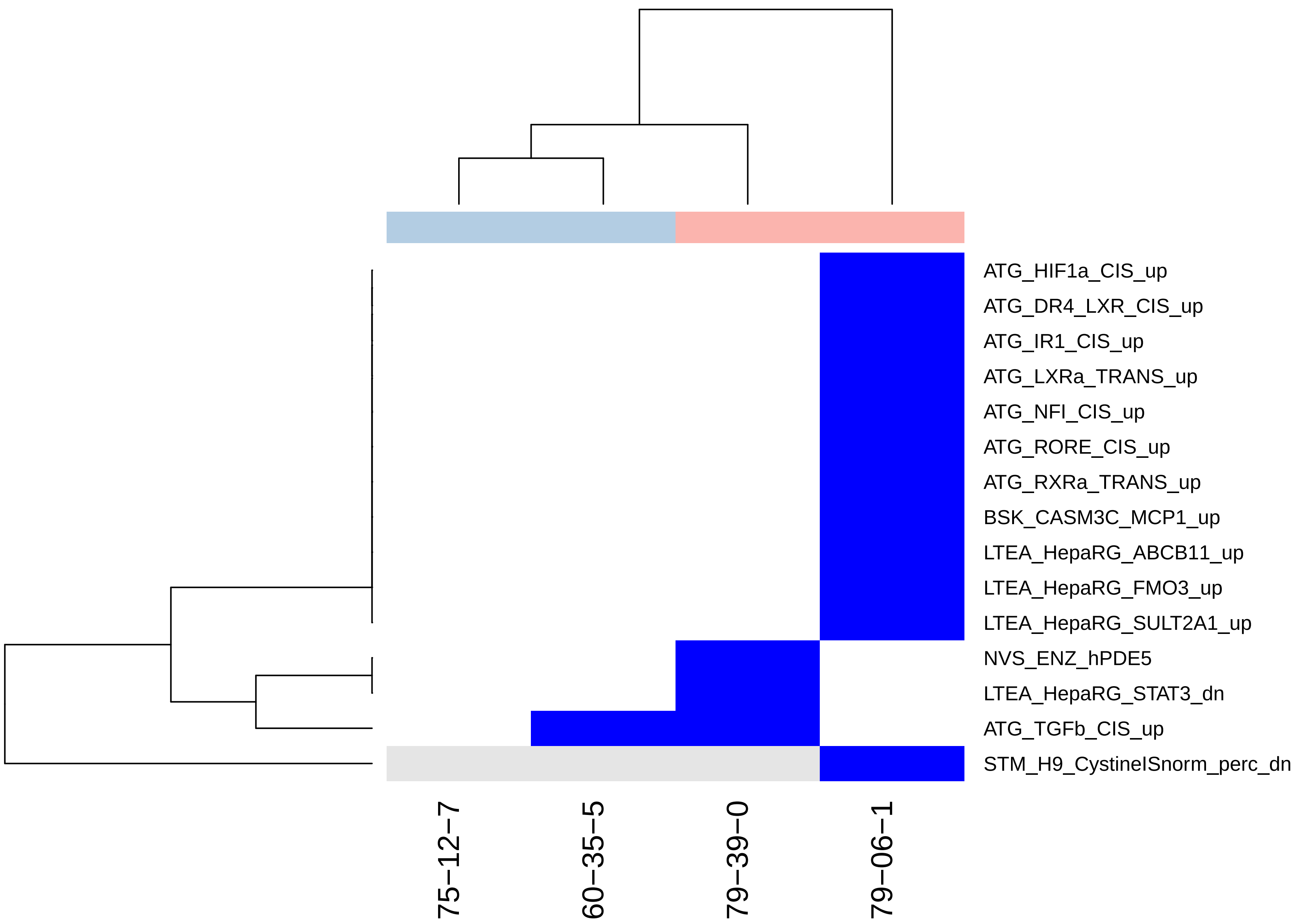

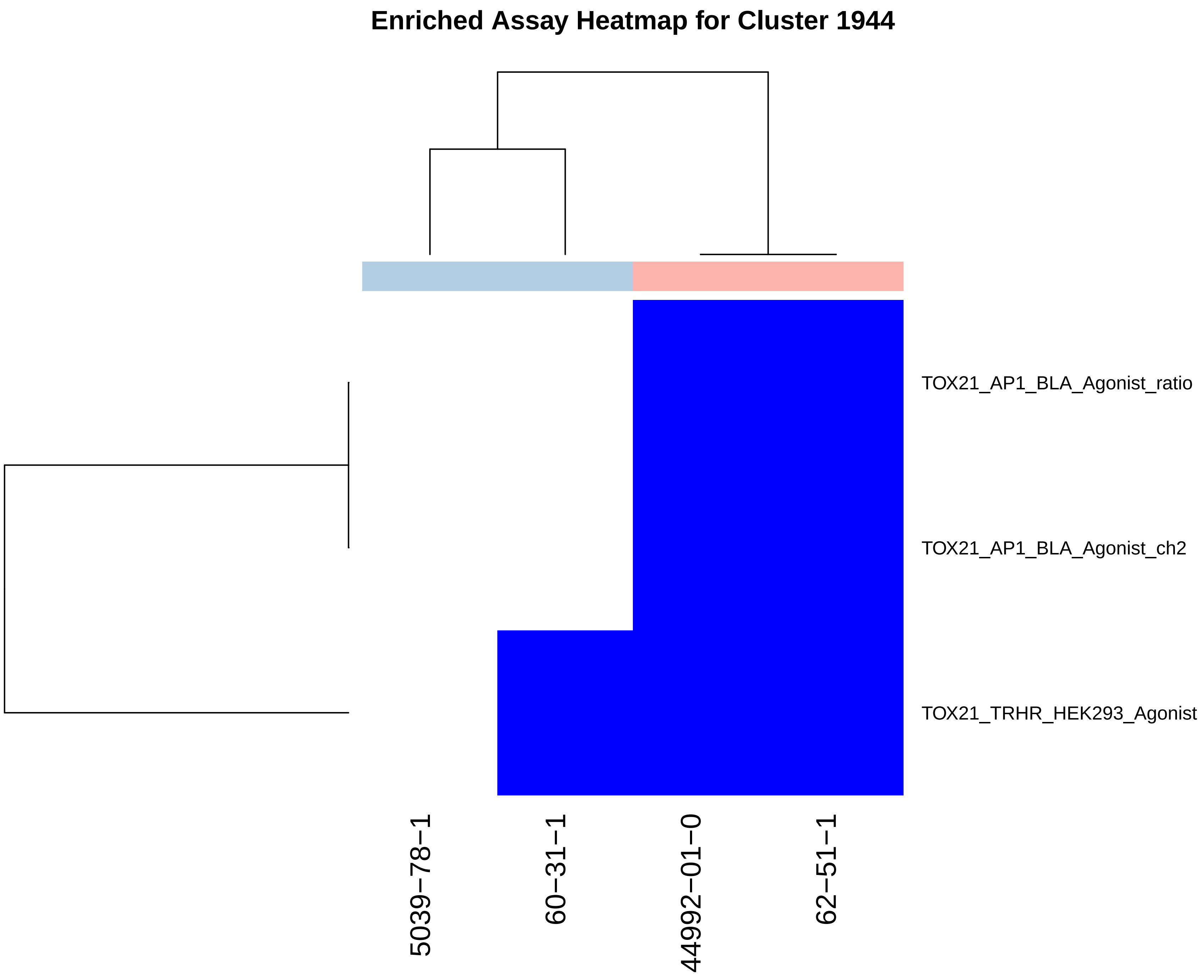
Enriched Assay Heatmap for Cluster 1944
TOX21_AP1_BLA_Agonist_ratio
TOX21_AP1_BLA_Agonist_ch2
TOX21_TRHR_HEK293_Agonist
5039−78−1
60−31−1
44992−01−0
62−51−1

Enriched Assay Heatmap for Cluster 1945

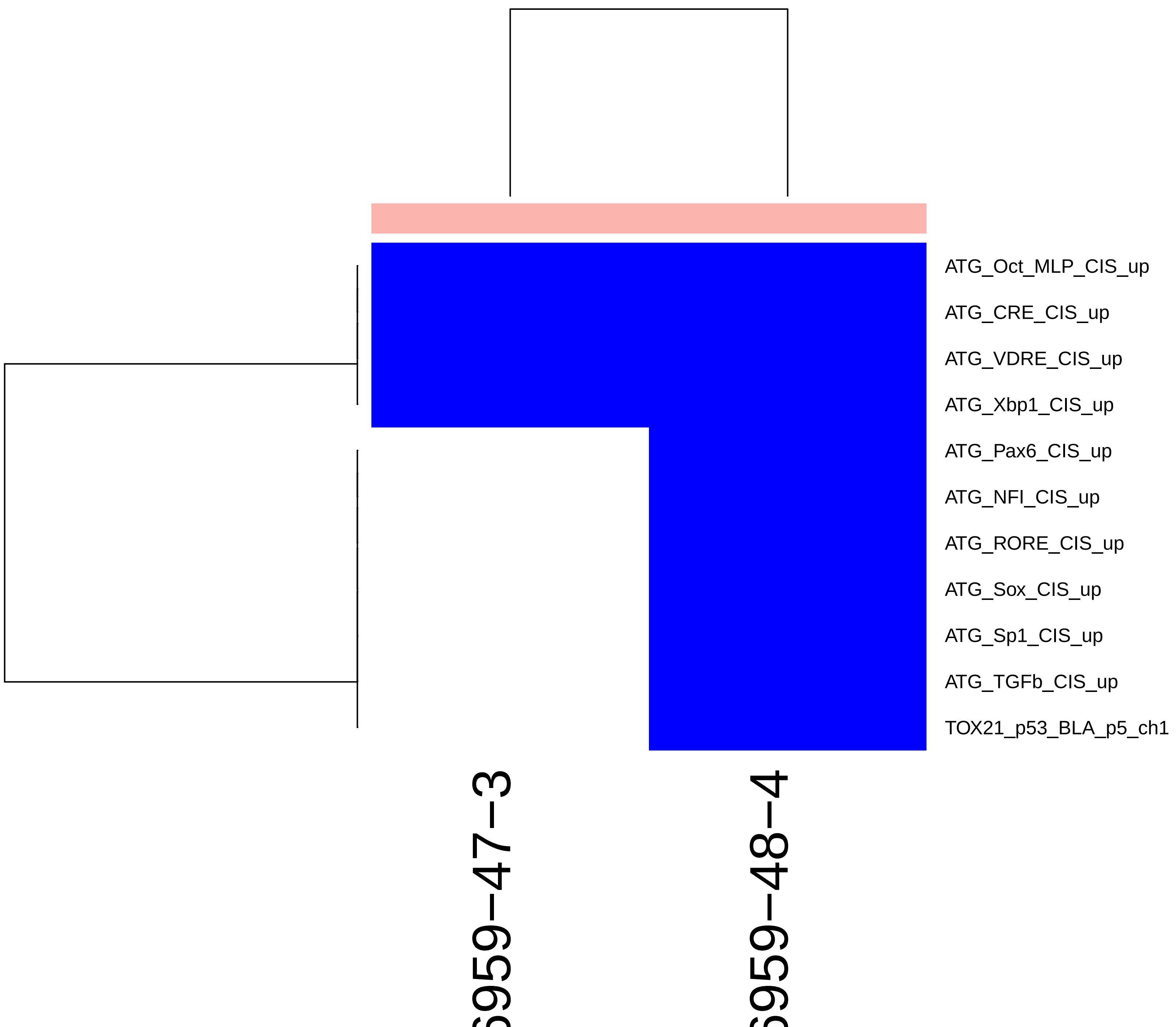

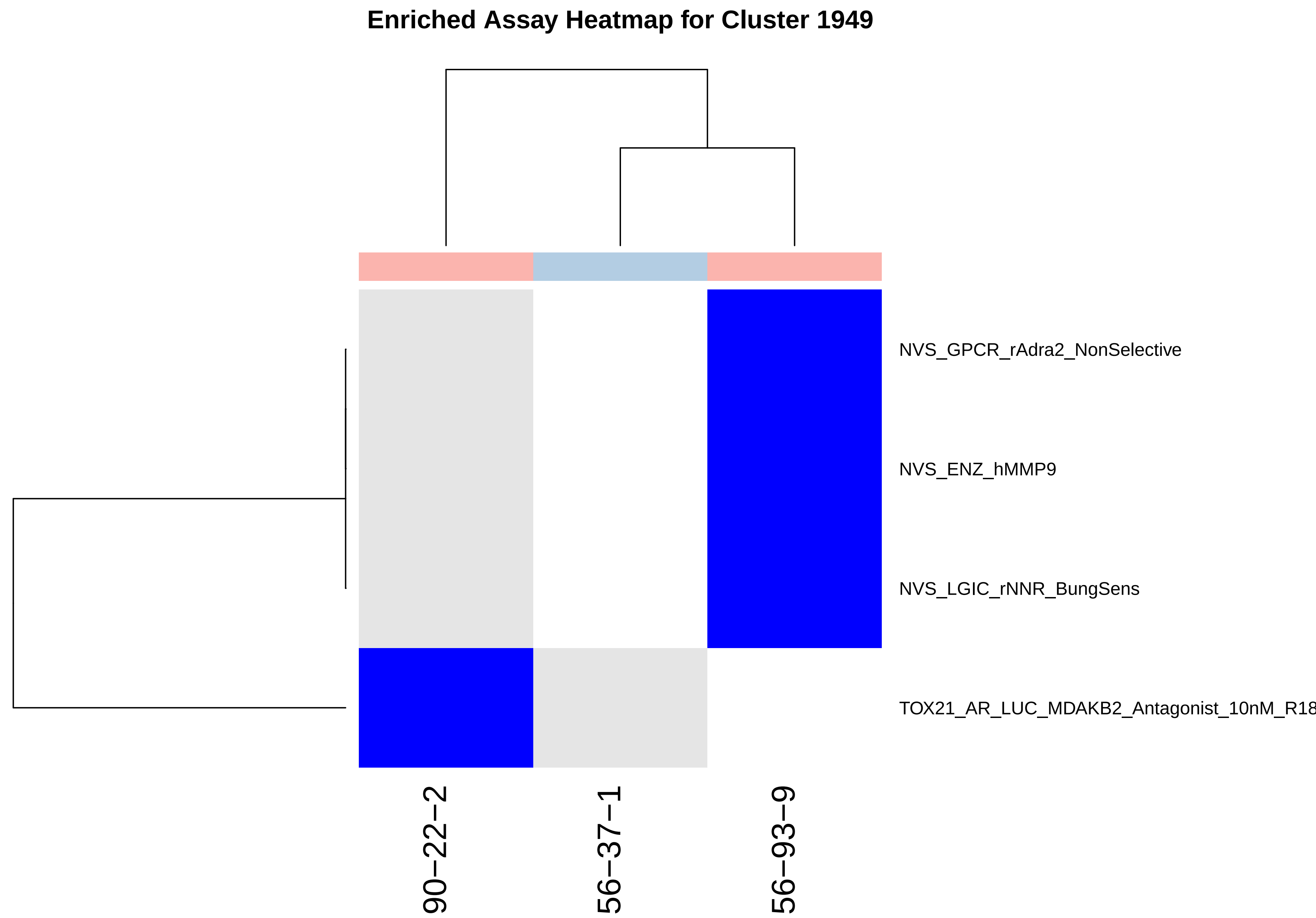
Enriched Assay Heatmap for Cluster 1949
NVS_GPCR_rAdra2_NonSelective
NVS_ENZ_hMMP9
NVS_LGIC_rNNR_BungSens
TOX21_AR_LUC_MDAKB2_Antagonist_10nM_R18
90−22−2
56−37−1
56−93−9

Enriched Assay Heatmap for Cluster 1964

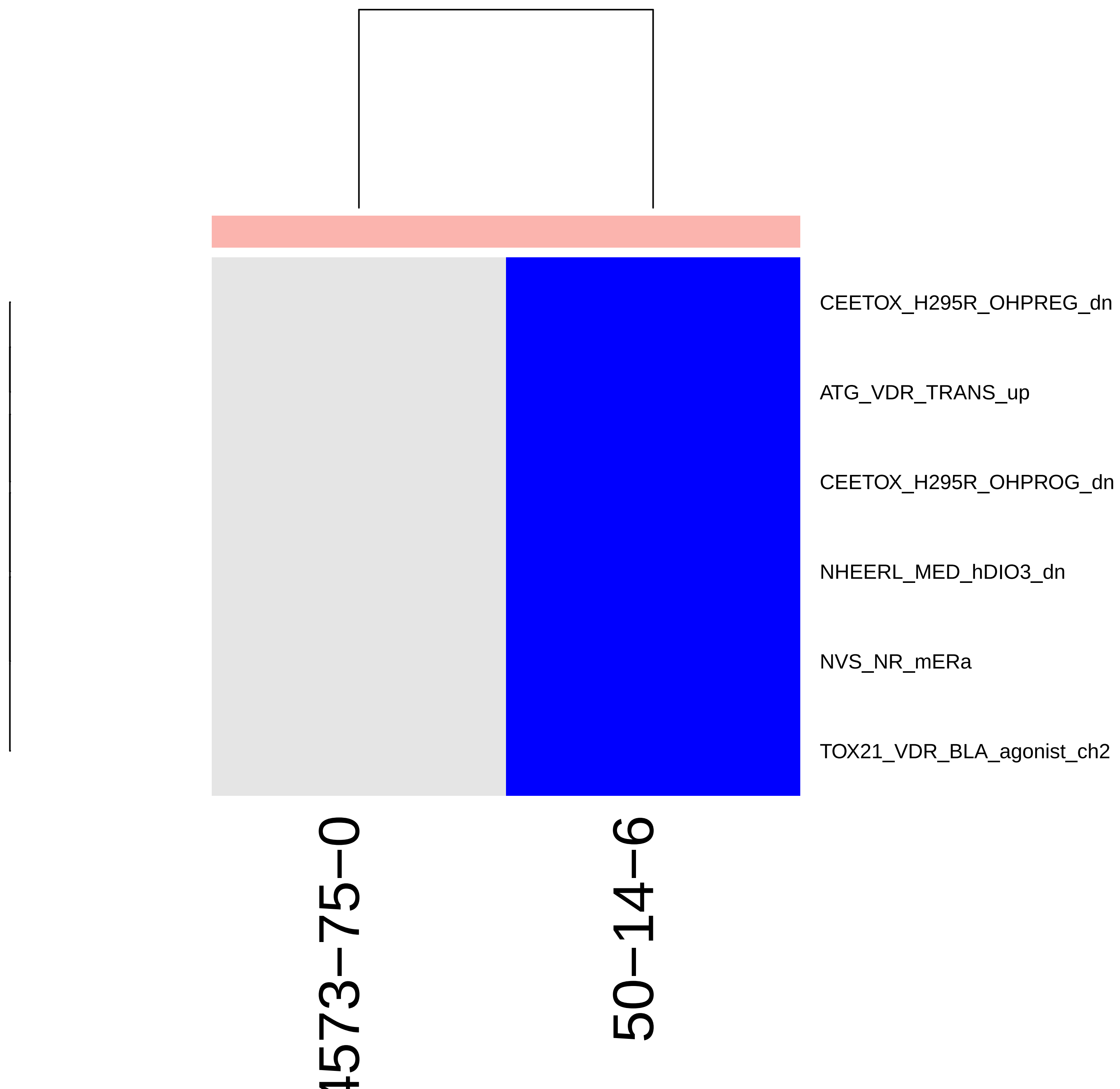

Enriched Assay Heatmap for Cluster 1965

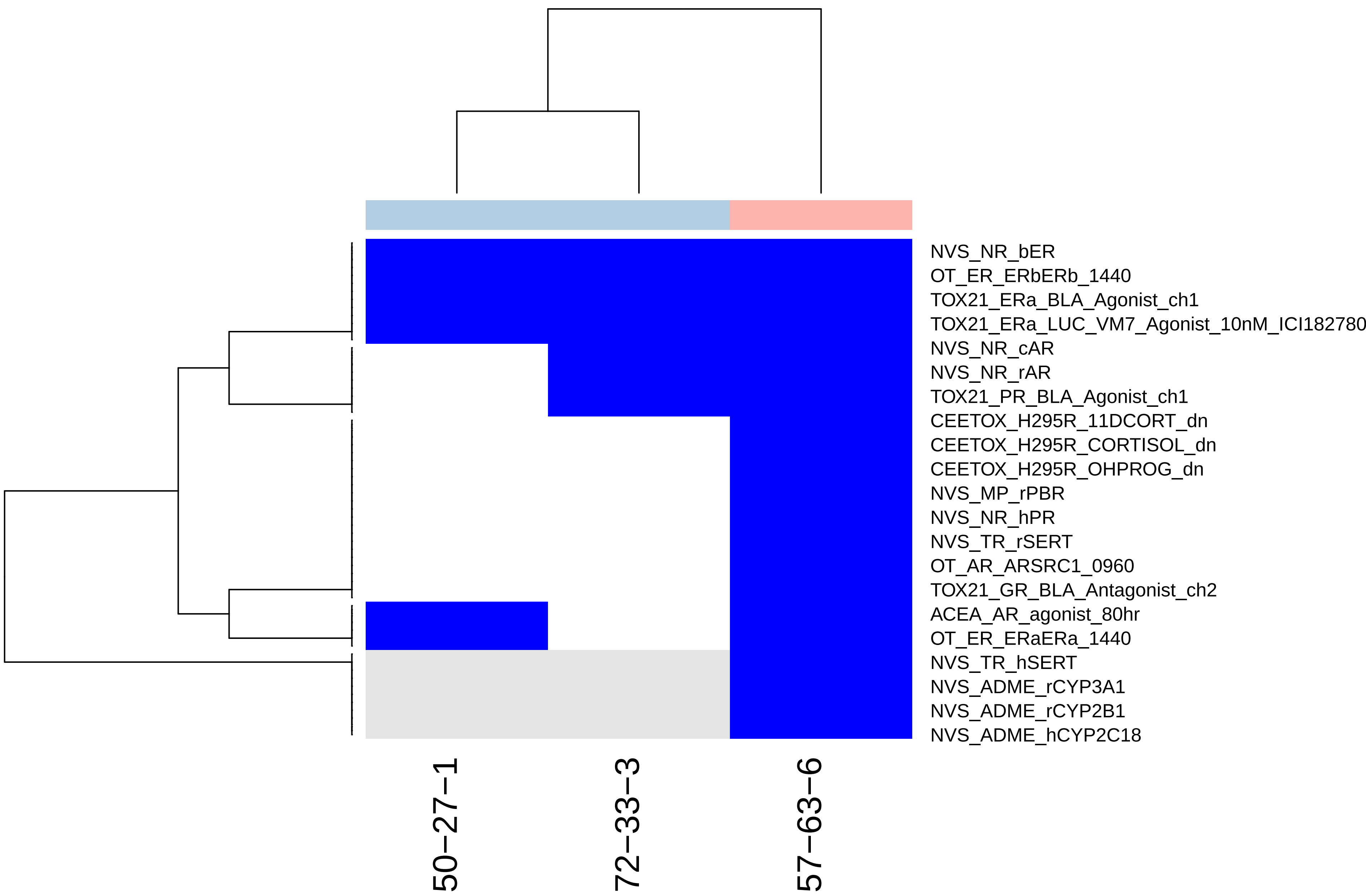

Enriched Assay Heatmap for Cluster 1966

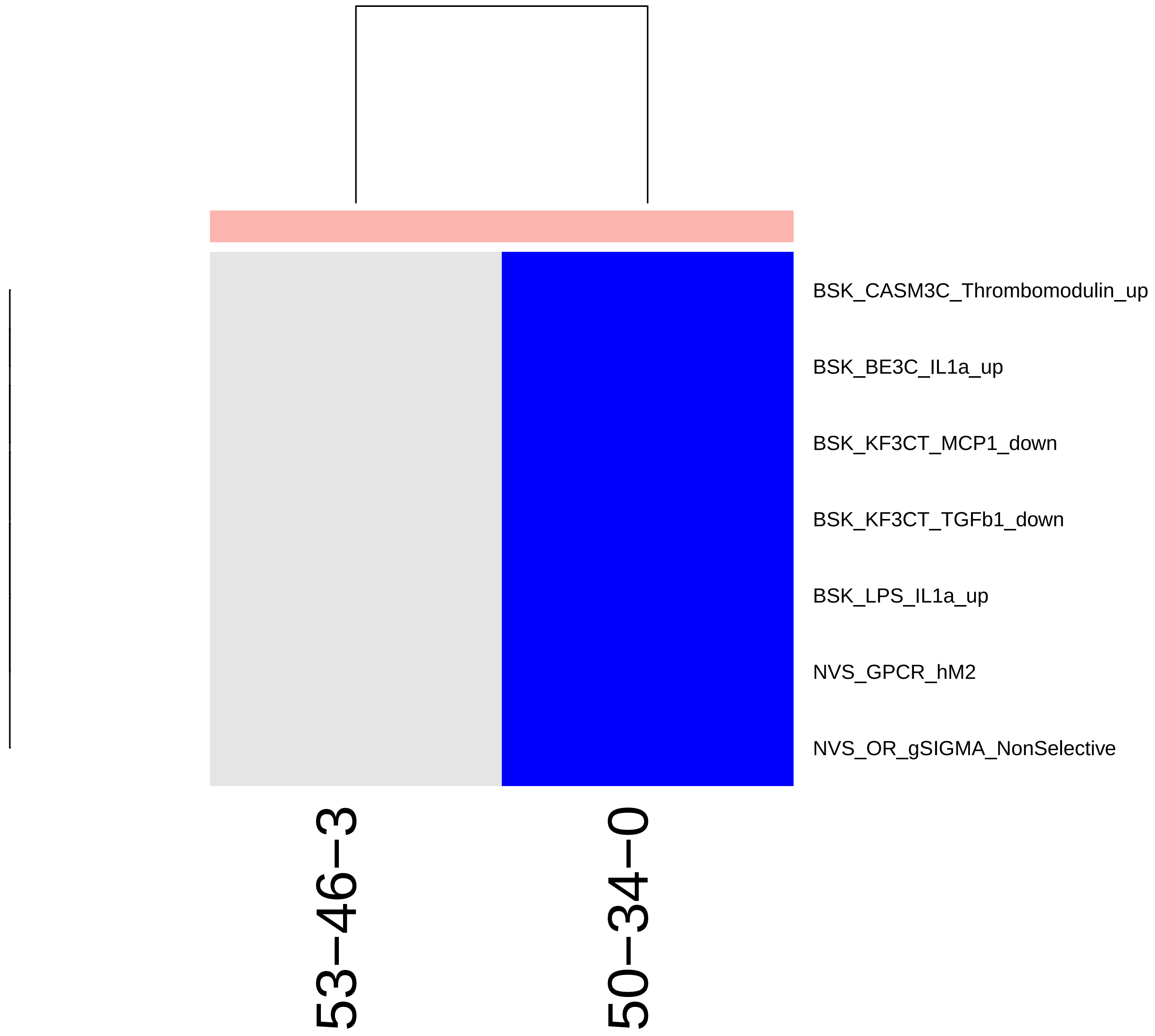

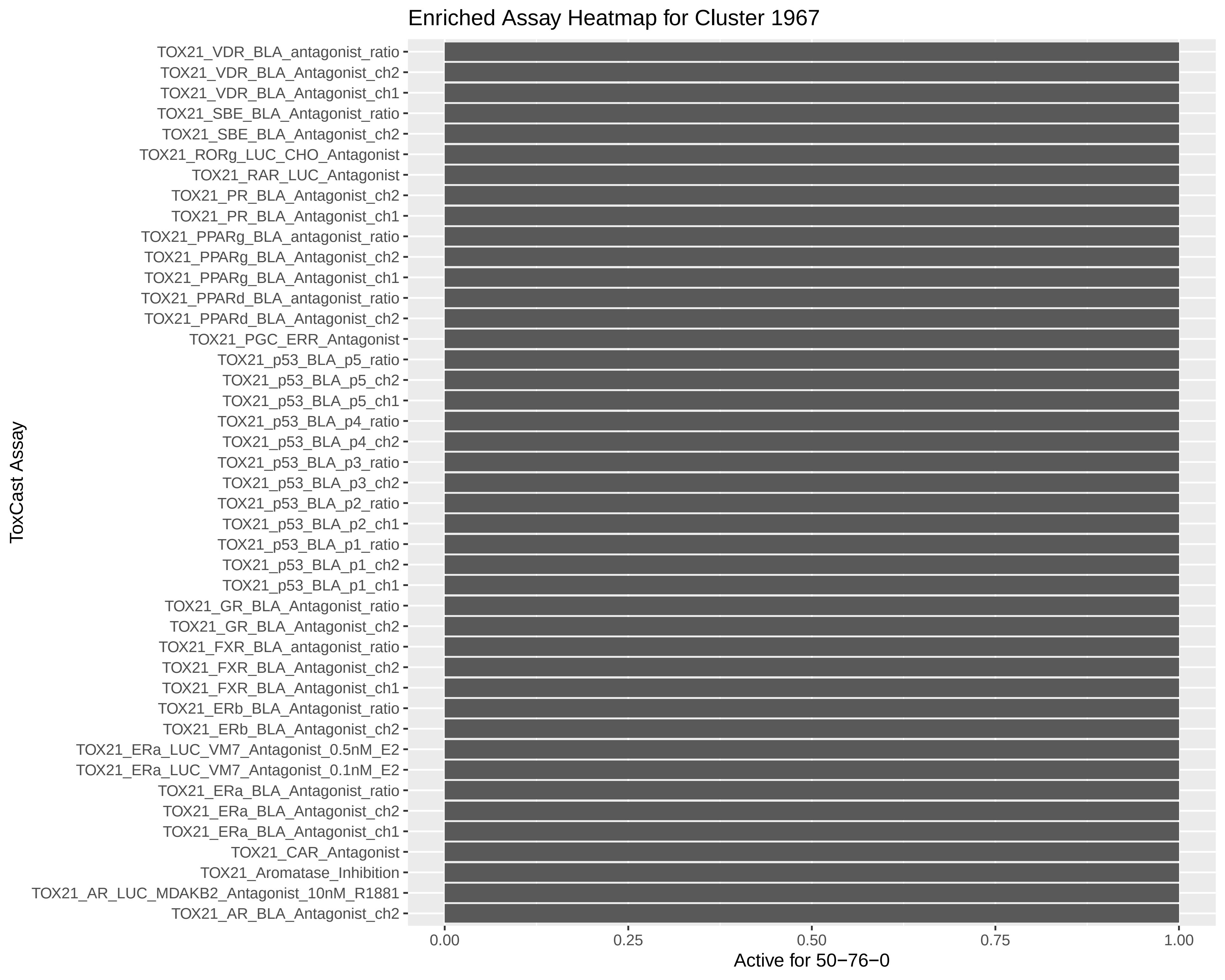

Enriched Assay Heatmap for Cluster 1967
ToxCast Assay
TOX21_VDR_BLA_antagonist_ratio
TOX21_VDR_BLA_Antagonist_ch2
TOX21_VDR_BLA_Antagonist_ch1
TOX21_SBE_BLA_Antagonist_ratio
TOX21_SBE_BLA_Antagonist_ch2
TOX21_RORg_LUC_CHO_Antagonist
TOX21_RAR_LUC_Antagonist
TOX21_PR_BLA_Antagonist_ch2
TOX21_PR_BLA_Antagonist_ch1
TOX21_PPARg_BLA_antagonist_ratio
TOX21_PPARg_BLA_Antagonist_ch2
TOX21_PPARg_BLA_Antagonist_ch1
TOX21_PPARd_BLA_antagonist_ratio
TOX21_PPARd_BLA_Antagonist_ch2
TOX21_PGC_ERR_Antagonist
TOX21_p53_BLA_p5_ratio
TOX21_p53_BLA_p5_ch2
TOX21_p53_BLA_p5_ch1
TOX21_p53_BLA_p4_ratio
TOX21_p53_BLA_p4_ch2
TOX21_p53_BLA_p3_ratio
TOX21_p53_BLA_p3_ch2
TOX21_p53_BLA_p2_ratio
TOX21_p53_BLA_p2_ch1
TOX21_p53_BLA_p1_ratio
TOX21_p53_BLA_p1_ch2
TOX21_p53_BLA_p1_ch1
TOX21_GR_BLA_Antagonist_ratio
TOX21_GR_BLA_Antagonist_ch2
TOX21_FXR_BLA_antagonist_ratio
TOX21_FXR_BLA_Antagonist_ch2
TOX21_FXR_BLA_Antagonist_ch1
TOX21_ERb_BLA_Antagonist_ratio
TOX21_ERb_BLA_Antagonist_ch2
TOX21_ERa_LUC_VM7_Antagonist_0.5nM_E2
TOX21_ERa_LUC_VM7_Antagonist_0.1nM_E2
TOX21_ERa_BLA_Antagonist_ratio
TOX21_ERa_BLA_Antagonist_ch2
TOX21_ERa_BLA_Antagonist_ch1
TOX21_CAR_Antagonist
TOX21_Aromatase_Inhibition
TOX21_AR_LUC_MDAKB2_Antagonist_10nM_R1881
TOX21_AR_BLA_Antagonist_ch2
0.00
0.25
0.50
0.75
1.00
Active for 50−76−0

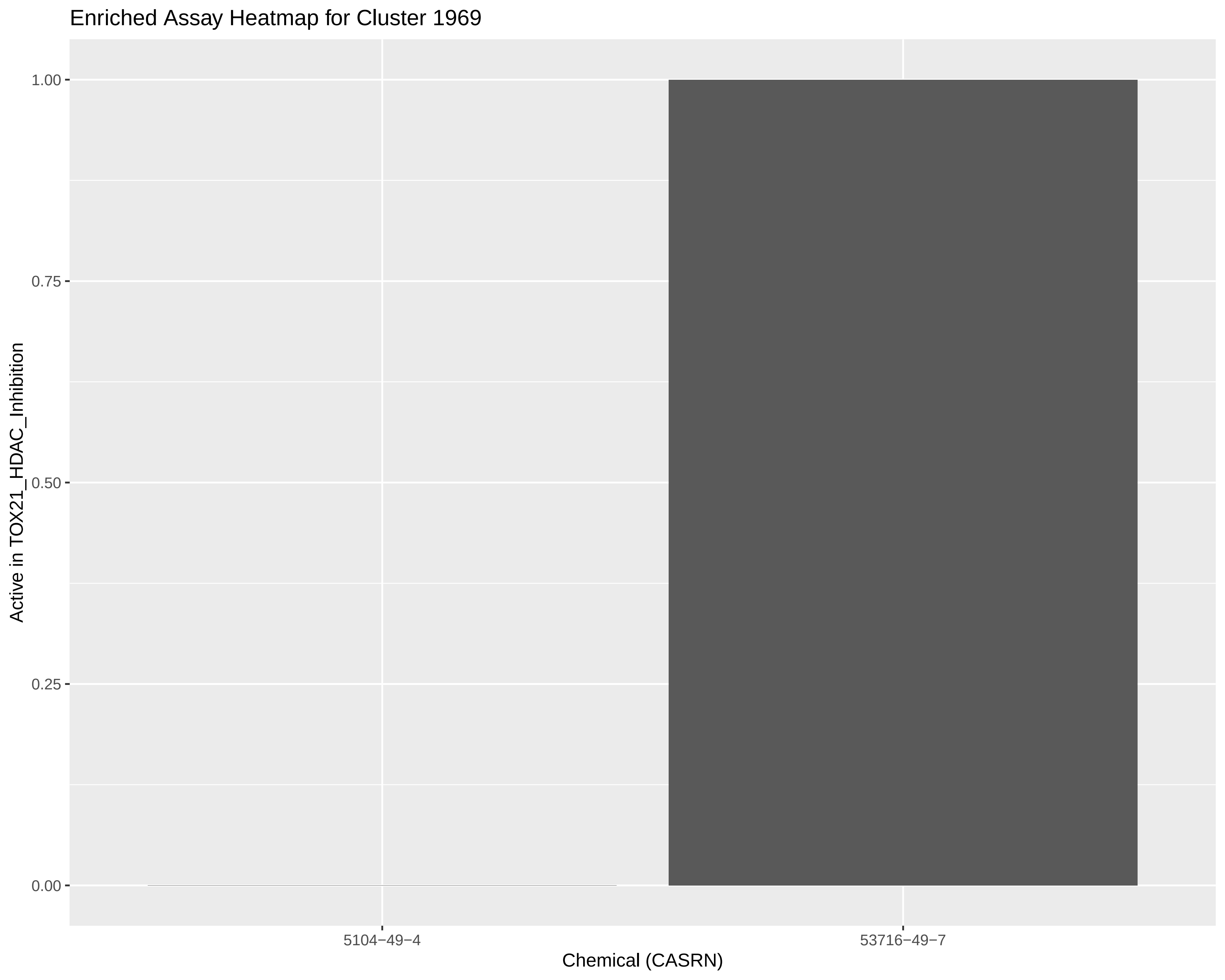
Enriched Assay Heatmap for Cluster 1969
Active in TOX21_HDAC_Inhibition
1.00
0.75
0.50
0.25
0.00
5104−49−4
53716−49−7
Chemical (CASRN)

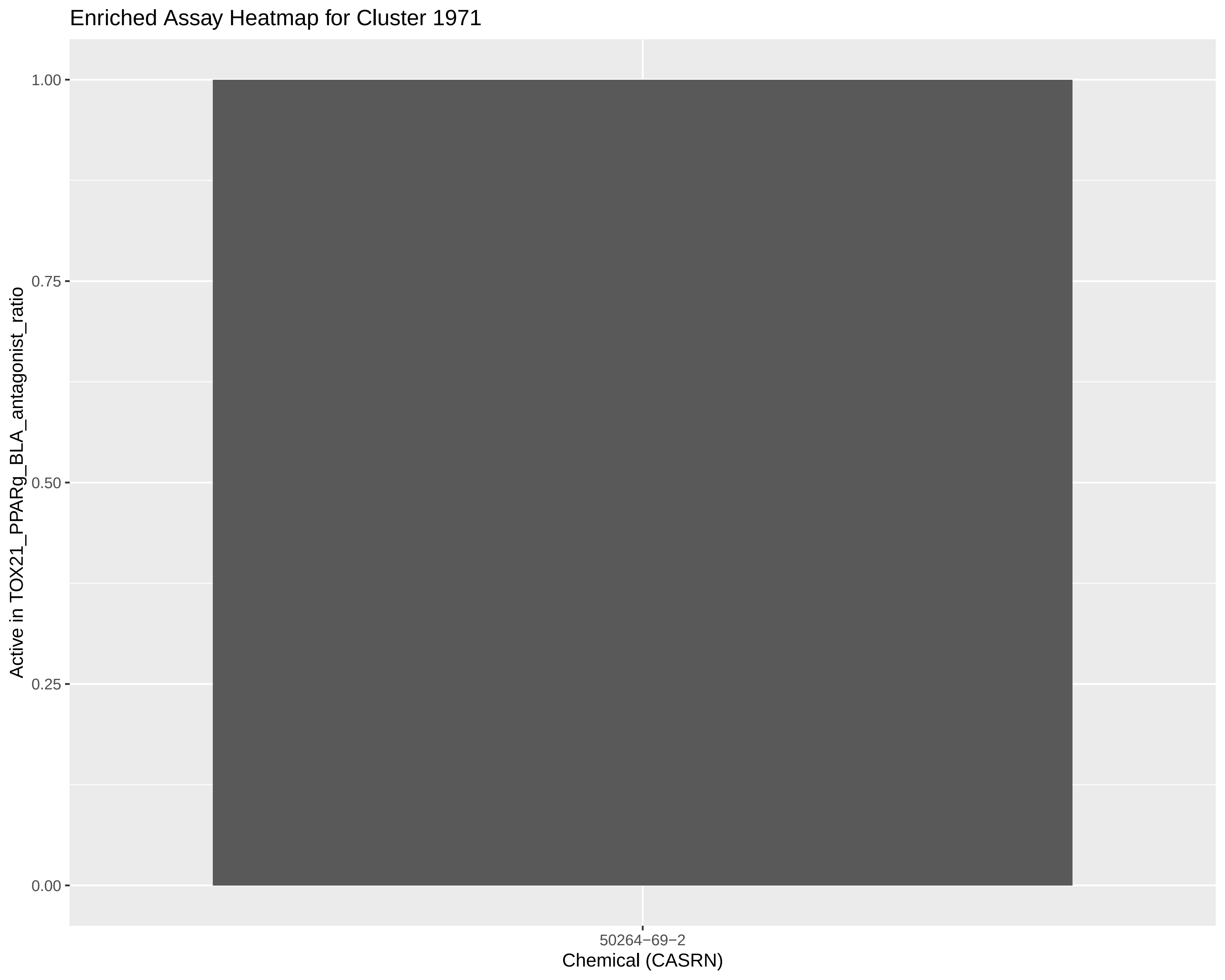
Enriched Assay Heatmap for Cluster 1971
Active in TOX21_PPARg_BLA_antagonist_ratio
1.00
0.75
0.50
0.25
0.00
50264−69−2
Chemical (CASRN)

Enriched Assay Heatmap for Cluster 1973

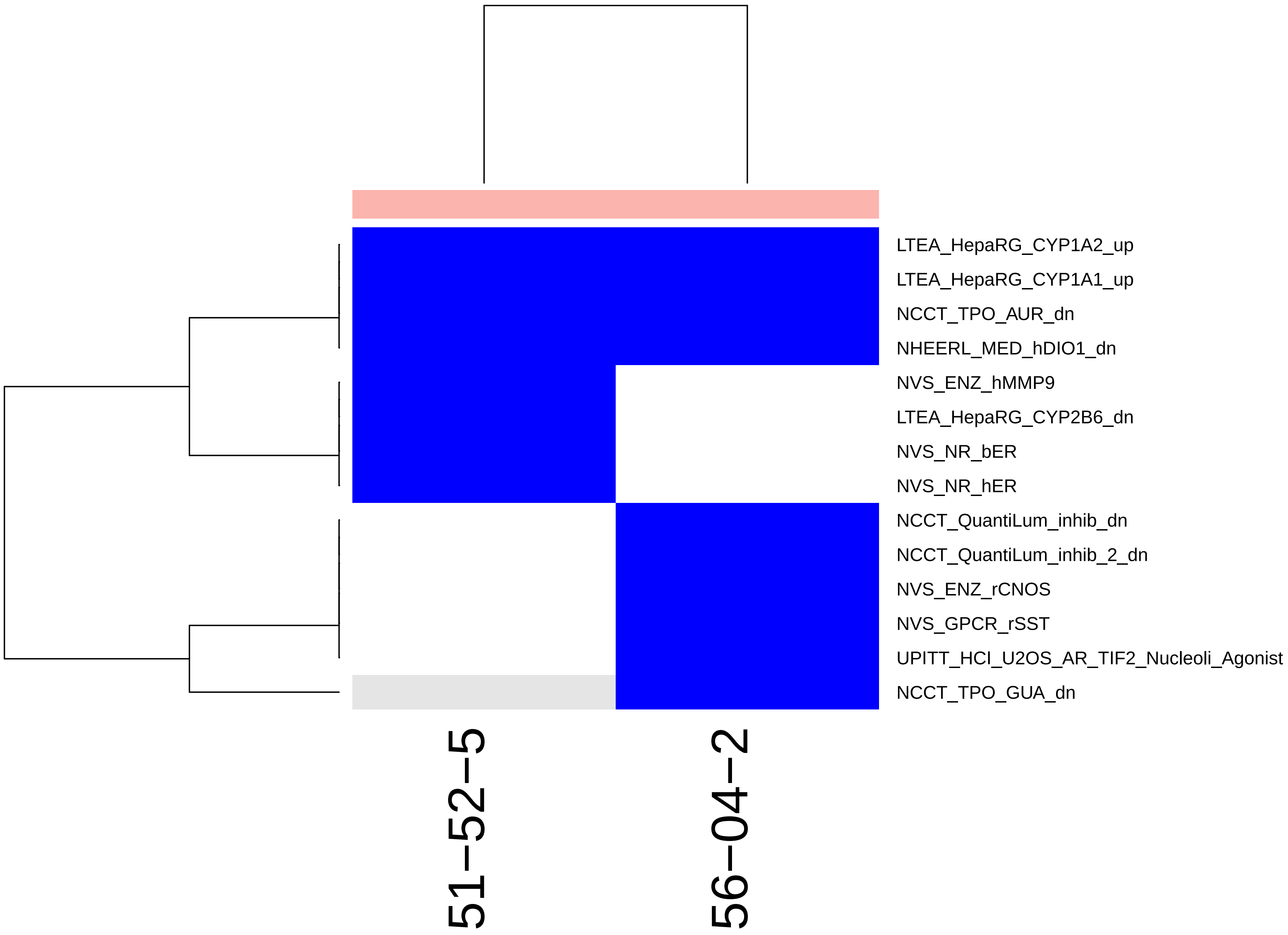

Enriched Assay Heatmap for Cluster 1976

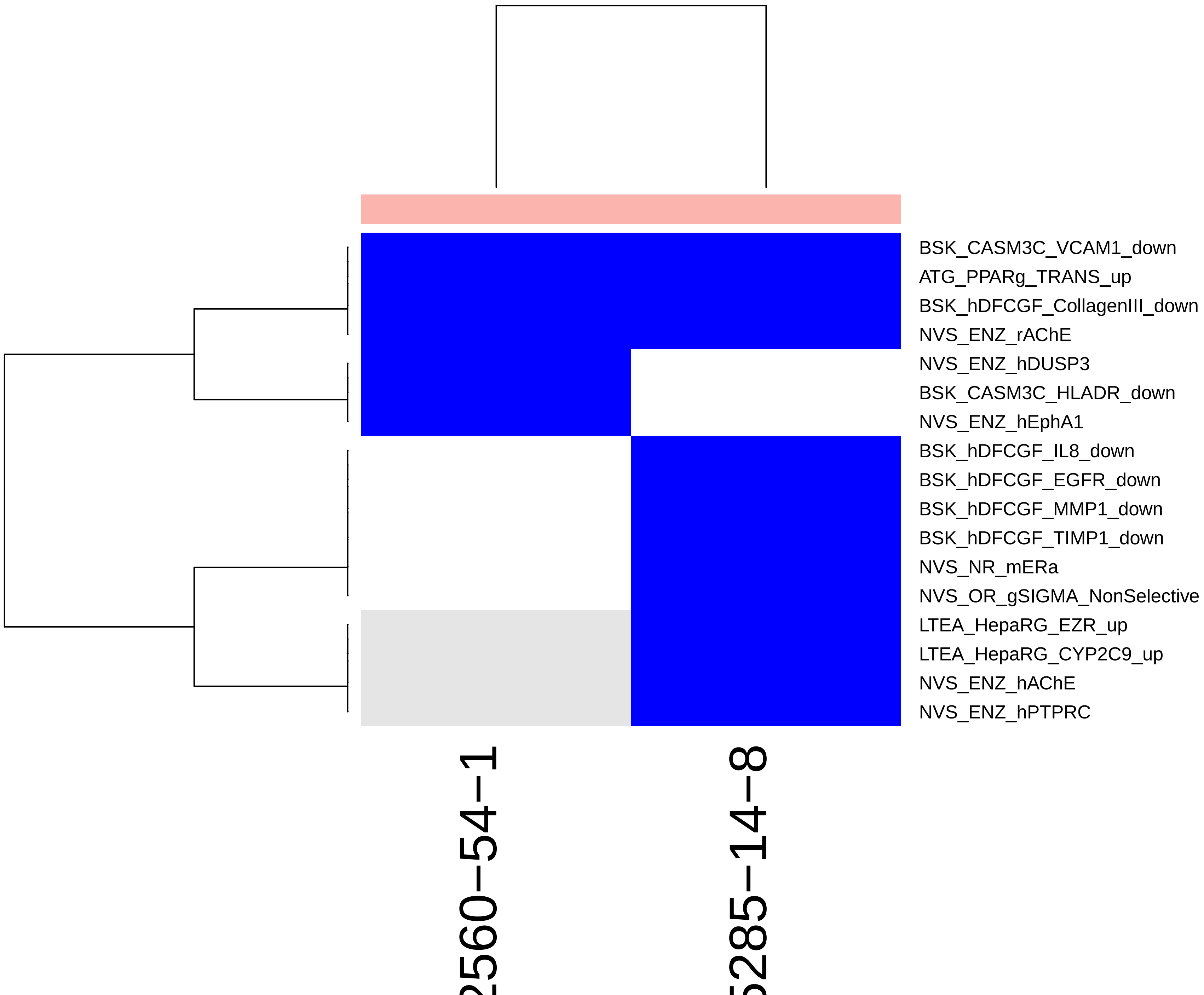

Enriched Assay Heatmap for Cluster 1983

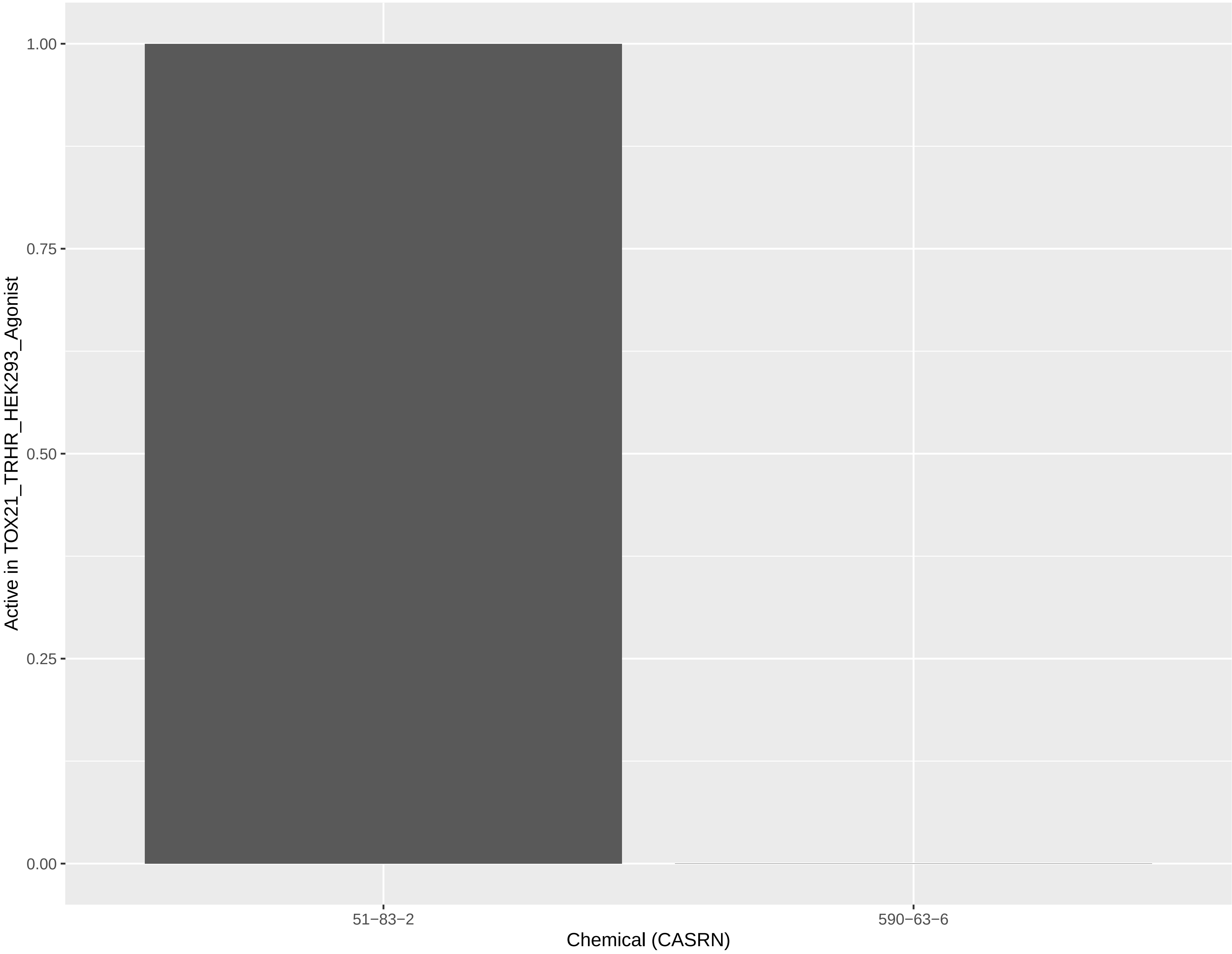

Enriched Assay Heatmap for Cluster 1987

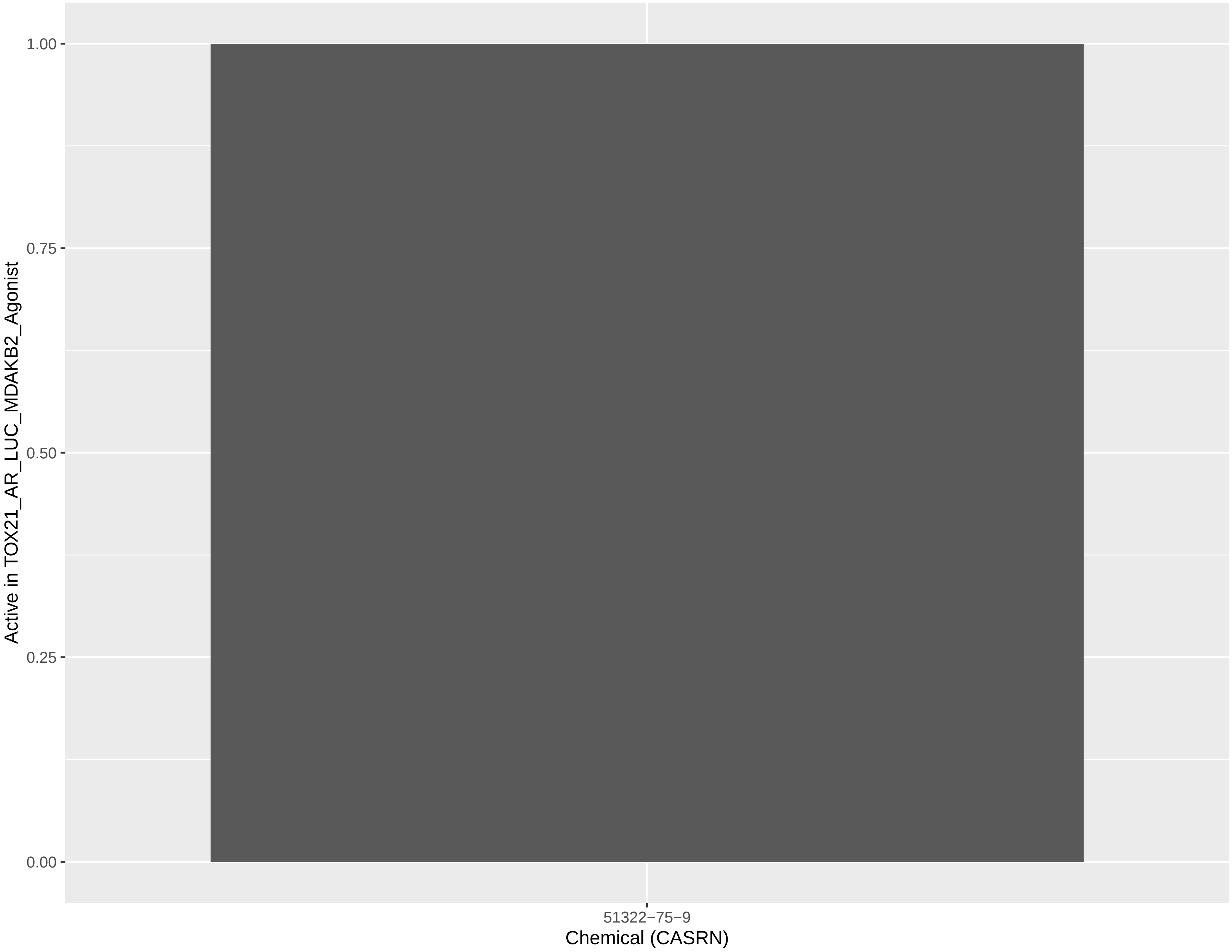

Enriched Assay Heatmap for Cluster 1994

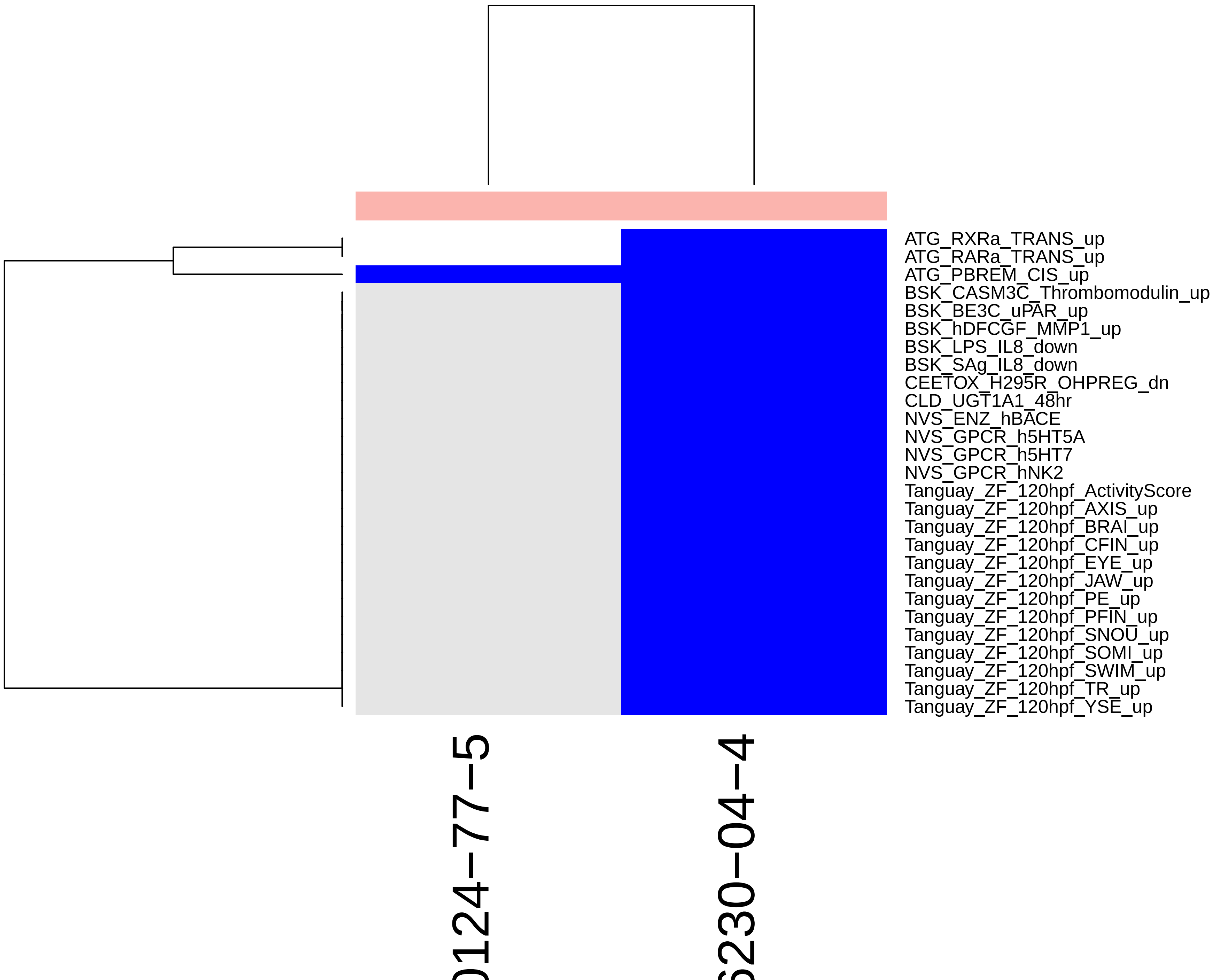

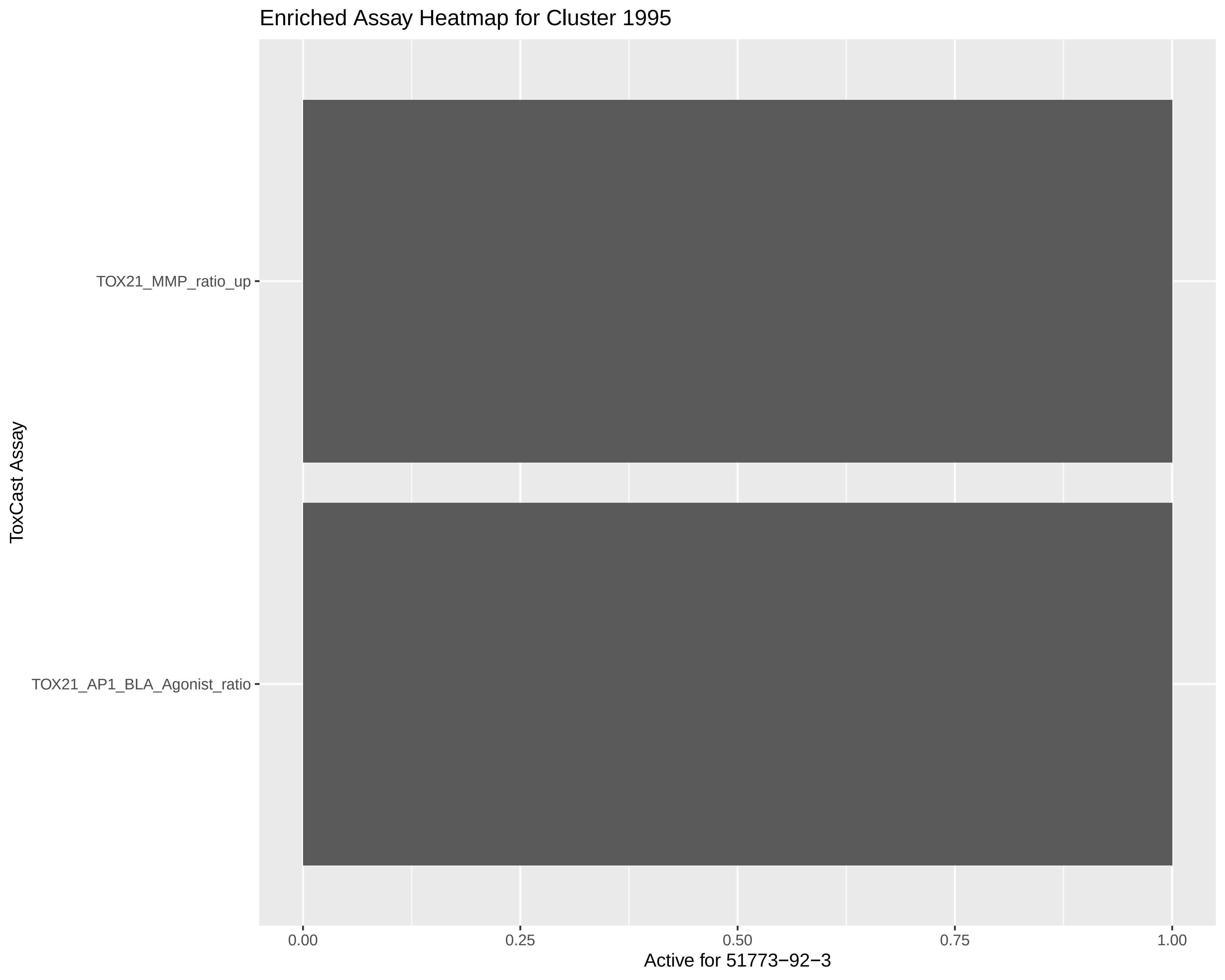

Enriched Assay Heatmap for Cluster 1995
ToxCast Assay
TOX21_MMP_ratio_up
TOX21_AP1_BLA_Agonist_ratio
0.00
0.25
0.50
0.75
1.00
Active for 51773−92−3

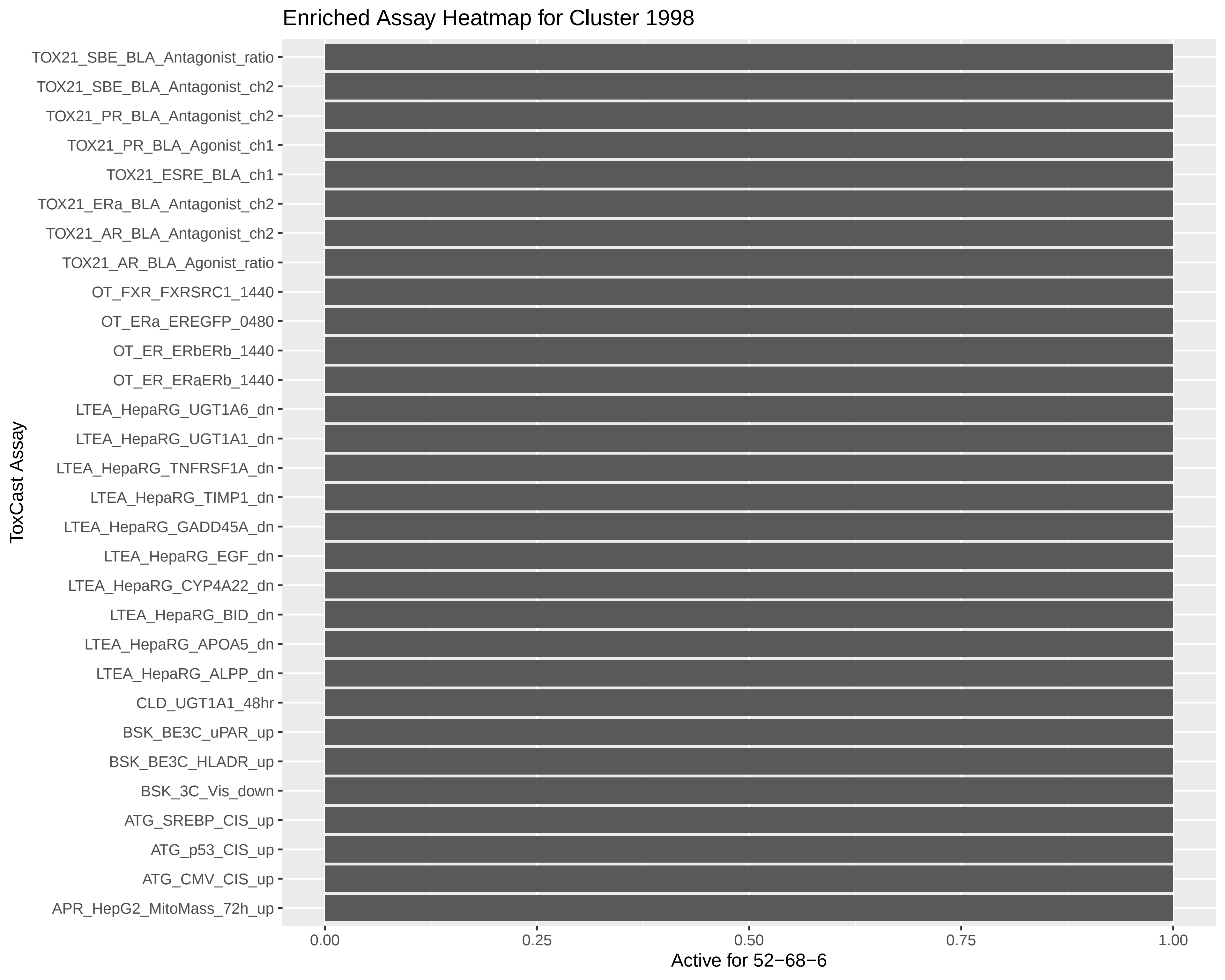
Enriched Assay Heatmap for Cluster 1998
ToxCast Assay
TOX21_SBE_BLA_Antagonist_ratio
TOX21_SBE_BLA_Antagonist_ch2
TOX21_PR_BLA_Antagonist_ch2
TOX21_PR_BLA_Agonist_ch1
TOX21_ESRE_BLA_ch1
TOX21_ERa_BLA_Antagonist_ch2
TOX21_AR_BLA_Antagonist_ch2
TOX21_AR_BLA_Agonist_ratio
OT_FXR_FXRSRC1_1440
OT_ERa_EREGFP_0480
OT_ER_ERbERb_1440
OT_ER_ERaERb_1440
LTEA_HepaRG_UGT1A6_dn
LTEA_HepaRG_UGT1A1_dn
LTEA_HepaRG_TNFRSF1A_dn
LTEA_HepaRG_TIMP1_dn
LTEA_HepaRG_GADD45A_dn
LTEA_HepaRG_EGF_dn
LTEA_HepaRG_CYP4A22_dn
LTEA_HepaRG_BID_dn
LTEA_HepaRG_APOA5_dn
LTEA_HepaRG_ALPP_dn
CLD_UGT1A1_48hr
BSK_BE3C_uPAR_up
BSK_BE3C_HLADR_up
BSK_3C_Vis_down
ATG_SREBP_CIS_up
ATG_p53_CIS_up
ATG_CMV_CIS_up
APR_HepG2_MitoMass_72h_up
0.00
0.25
0.50
0.75
1.00
Active for 52−68−6

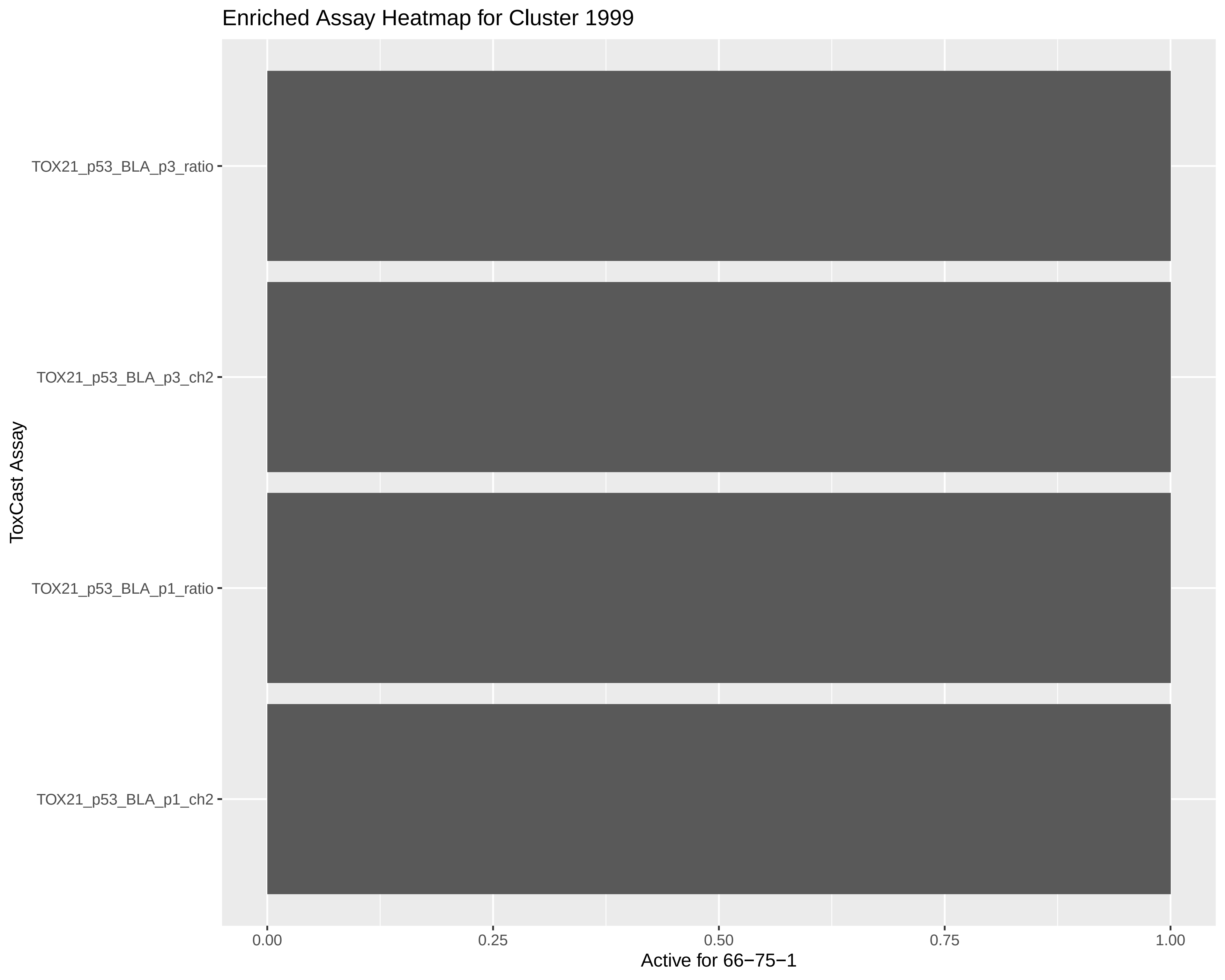
Enriched Assay Heatmap for Cluster 1999
ToxCast Assay
TOX21_p53_BLA_p3_ratio
TOX21_p53_BLA_p3_ch2
TOX21_p53_BLA_p1_ratio
TOX21_p53_BLA_p1_ch2
0.00
0.25
0.50
0.75
1.00
Active for 66−75−1

Enriched Assay Heatmap for Cluster 2001

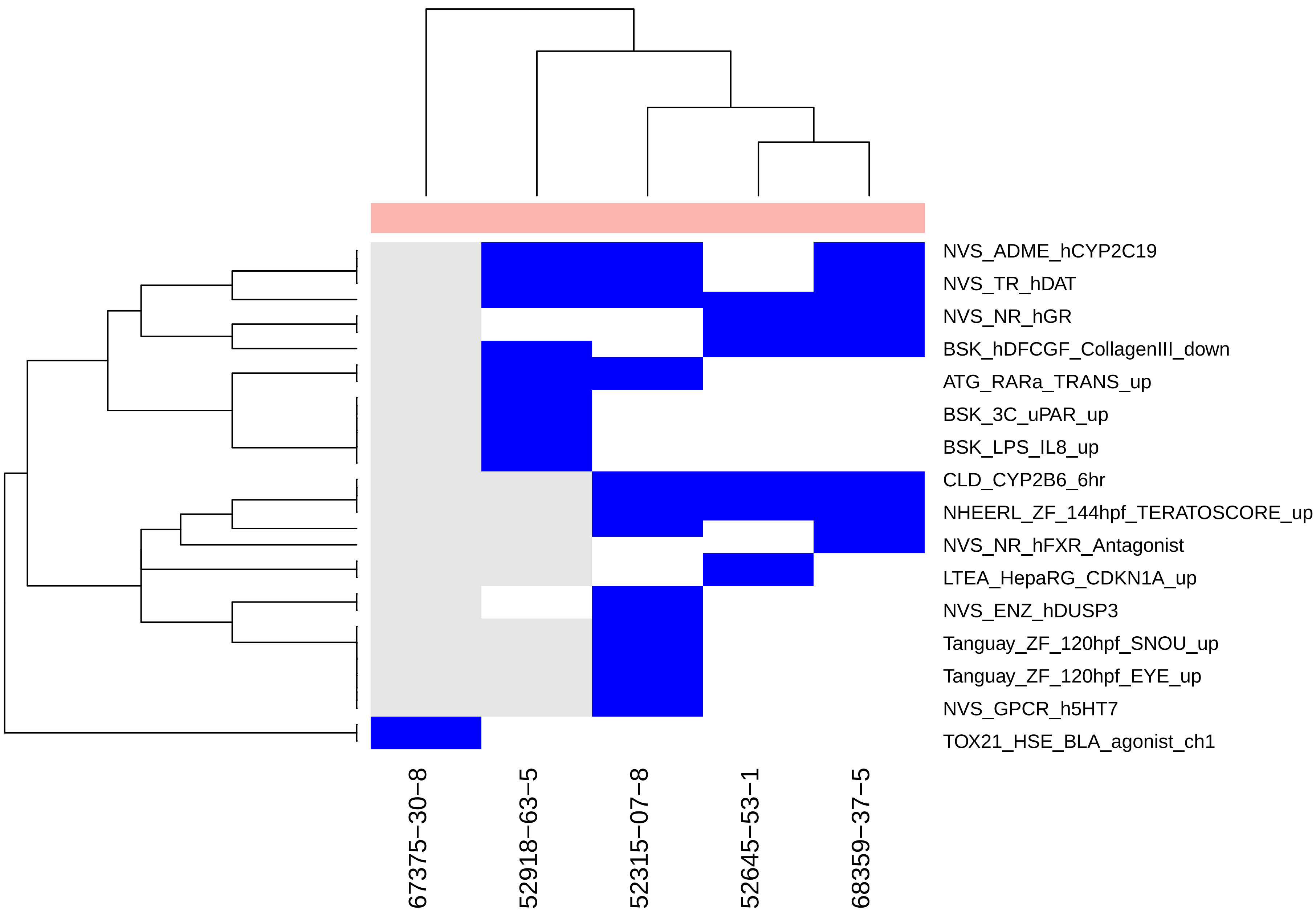

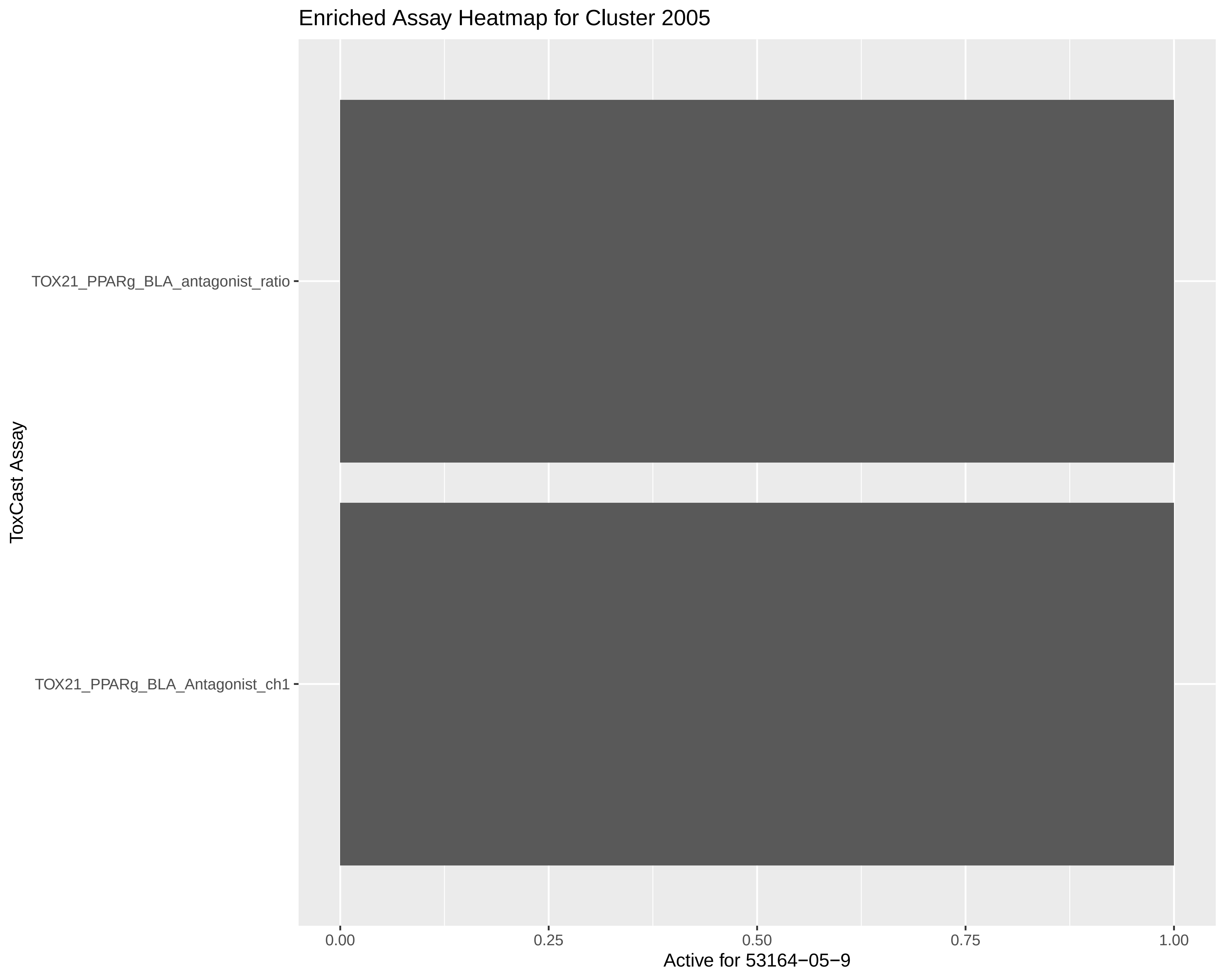
Enriched Assay Heatmap for Cluster 2005
ToxCast Assay
TOX21_PPARg_BLA_antagonist_ratio
TOX21_PPARg_BLA_Antagonist_ch1
0.00
0.25
0.50
0.75
1.00
Active for 53164−05−9

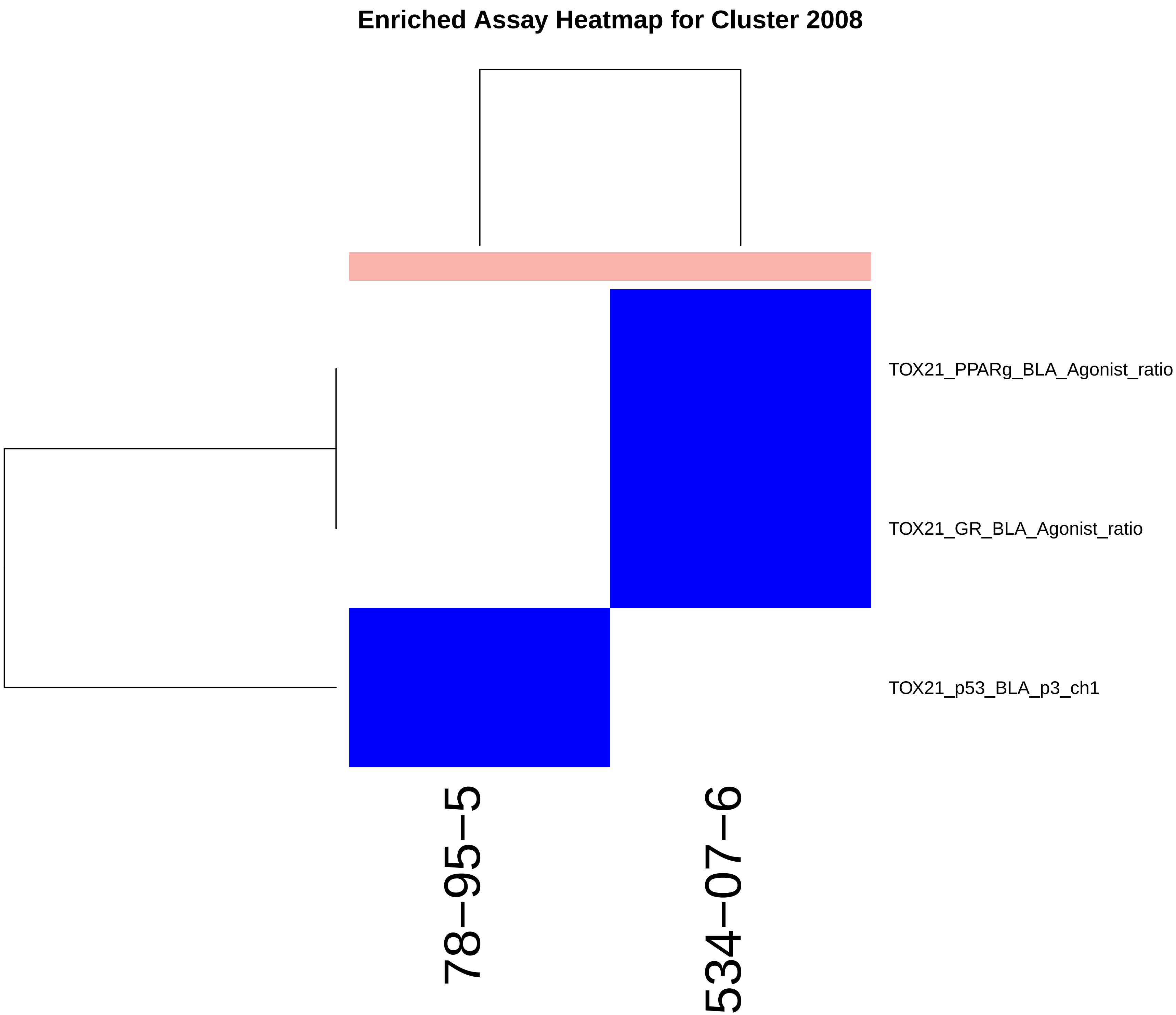

Enriched Assay Heatmap for Cluster 2008
TOX21_PPARg_BLA_Agonist_ratio
TOX21_GR_BLA_Agonist_ratio
TOX21_p53_BLA_p3_ch1
78−95−5
534−07−6

Enriched Assay Heatmap for Cluster 2009

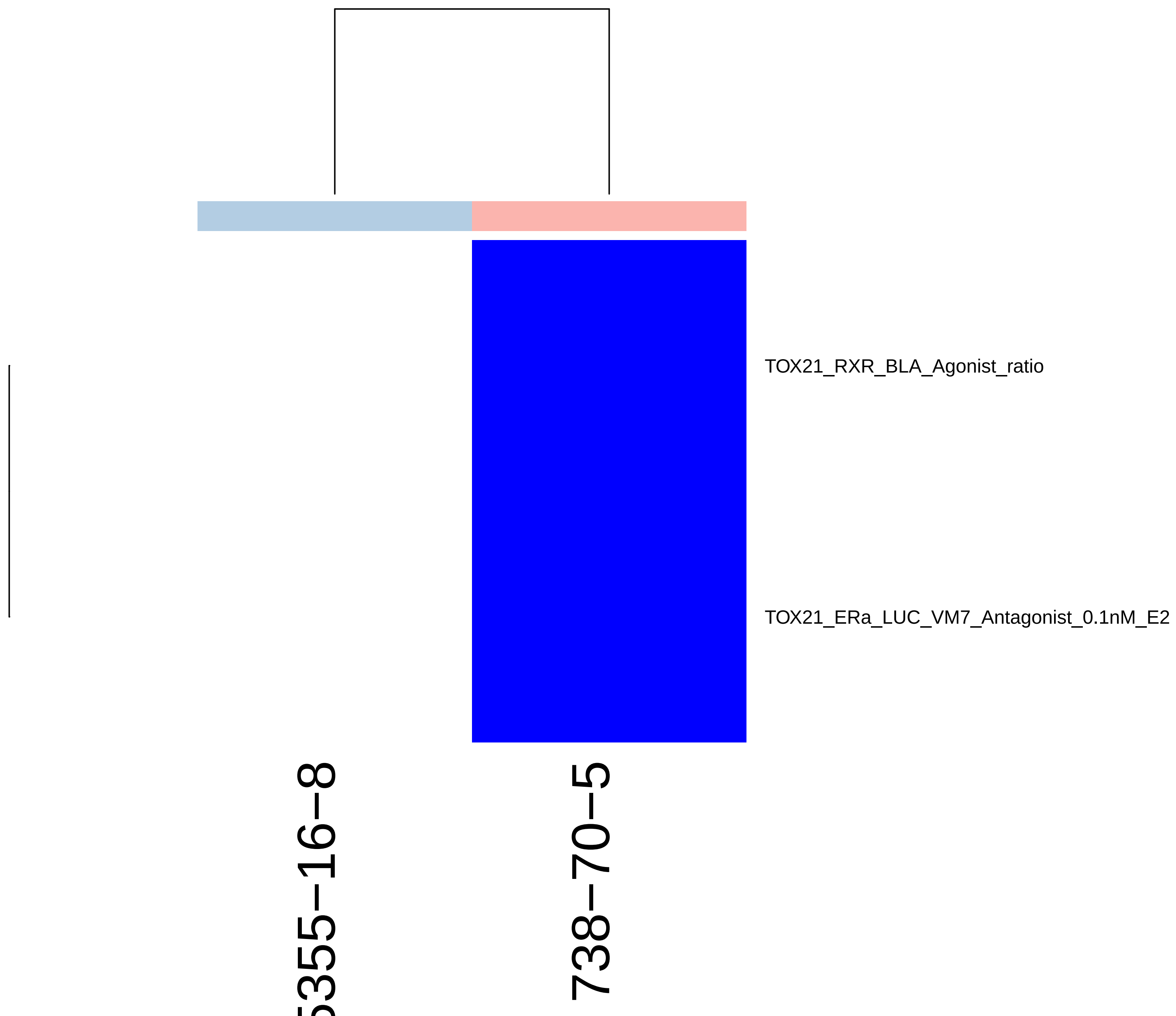

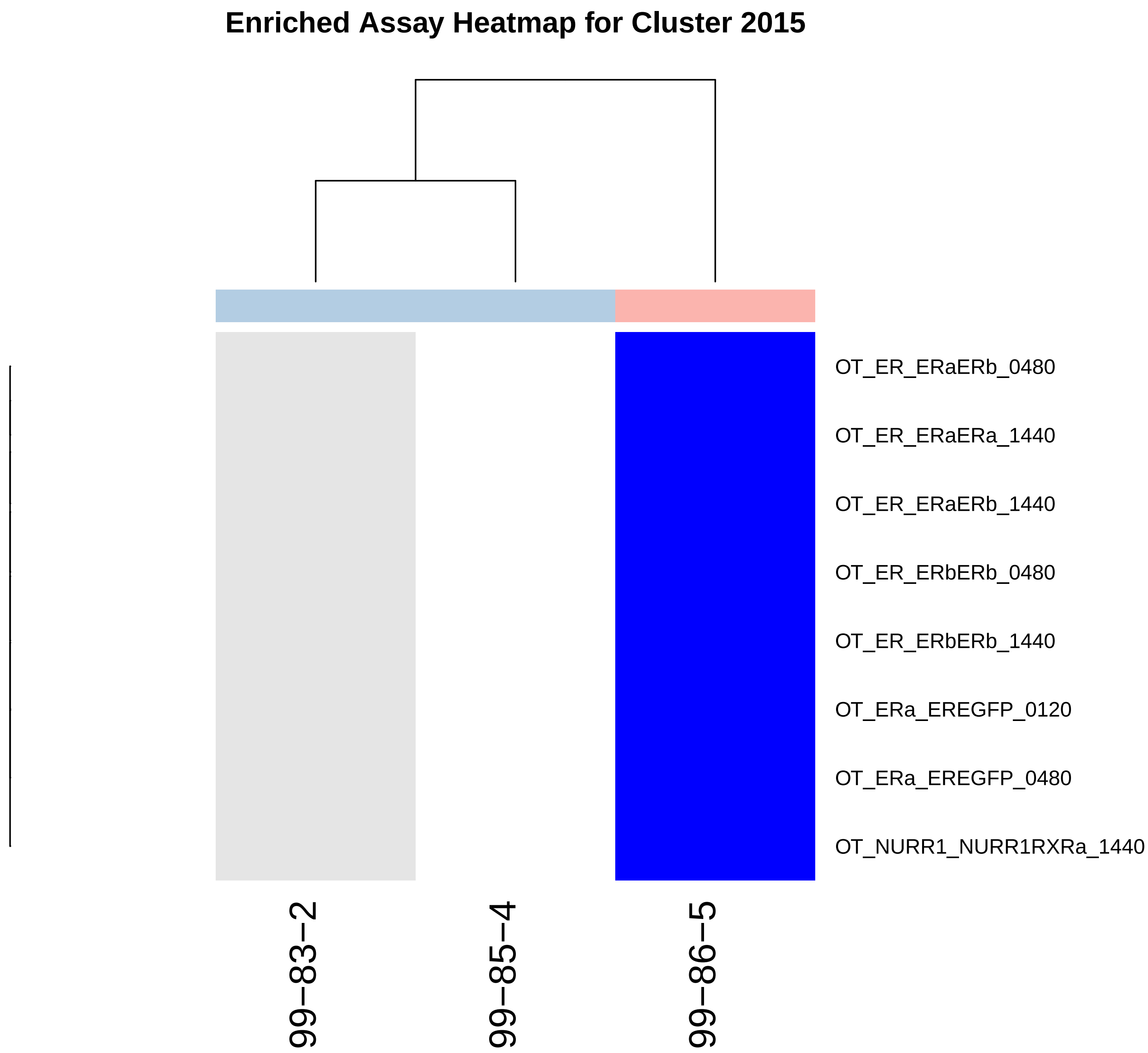
Enriched Assay Heatmap for Cluster 2015
OT_ER_ERaERb_0480
OT_ER_ERaERa_1440
OT_ER_ERaERb_1440
OT_ER_ERbERb_0480
OT_ER_ERbERb_1440
OT_ERa_EREGFP_0120
OT_ERa_EREGFP_0480
OT_NURR1_NURR1RXRa_1440
99−83−2
99−85−4
99−86−5

Enriched Assay Heatmap for Cluster 2017

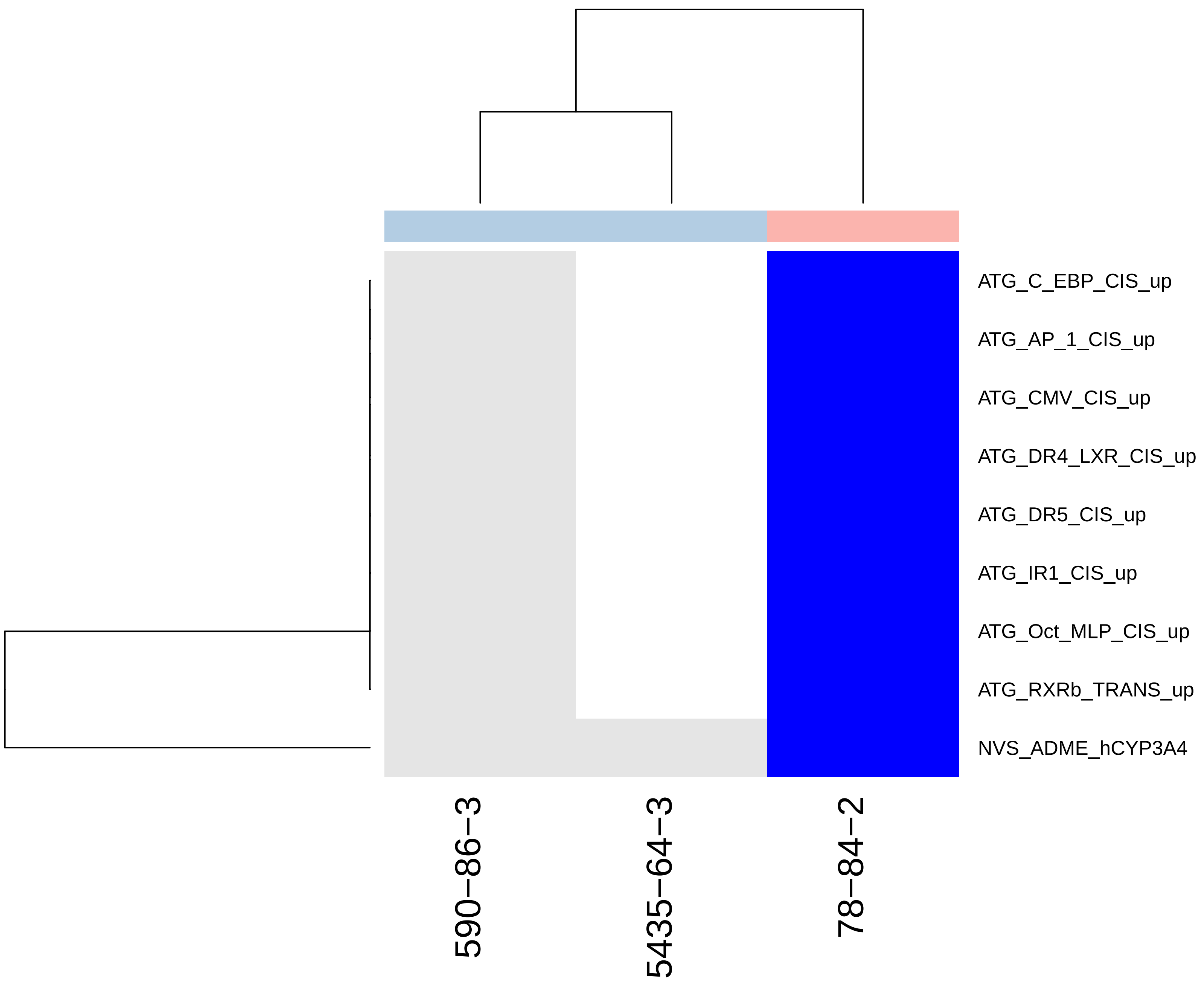

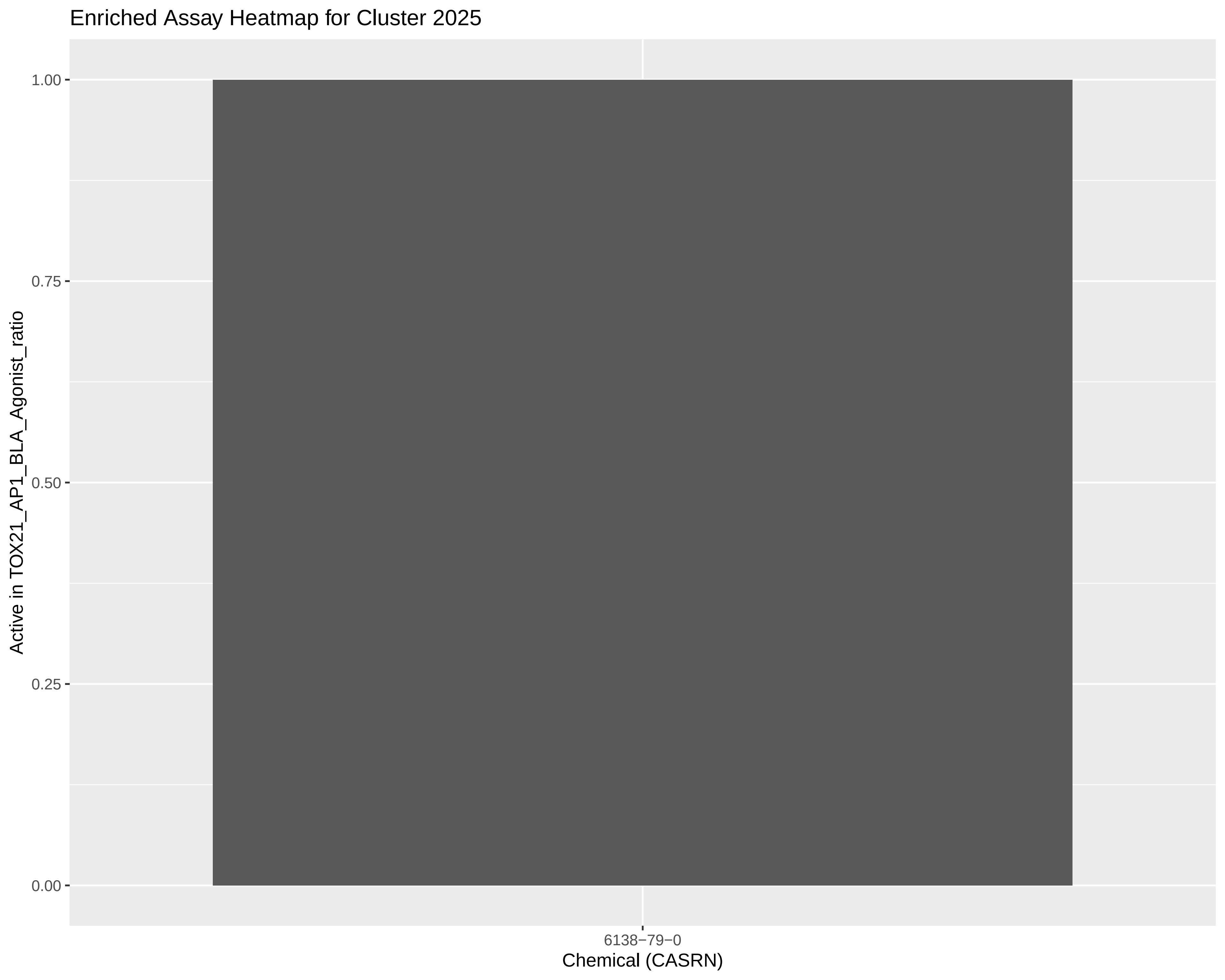

Enriched Assay Heatmap for Cluster 2025
Active in TOX21_AP1_BLA_Agonist_ratio
1.00
0.75
0.50
0.25
0.00
6138−79−0
Chemical (CASRN)

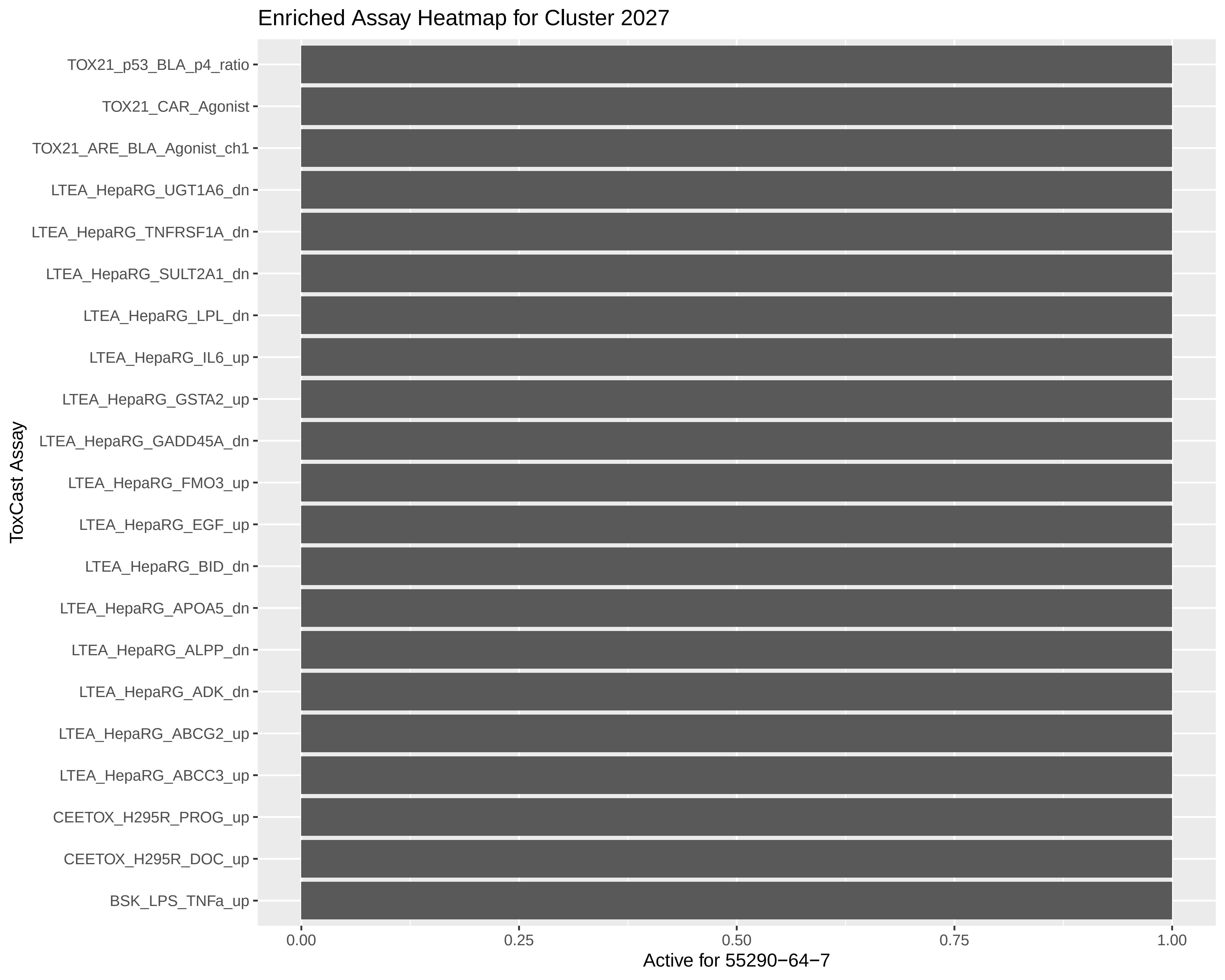

Enriched Assay Heatmap for Cluster 2027
TOX21_p53_BLA_p4_ratio
TOX21_CAR_Agonist
TOX21_ARE_BLA_Agonist_ch1
LTEA_HepaRG_UGT1A6_dn
LTEA_HepaRG_TNFRSF1A_dn
LTEA_HepaRG_SULT2A1_dn
LTEA_HepaRG_LPL_dn
LTEA_HepaRG_IL6_up
LTEA_HepaRG_GSTA2_up
LTEA_HepaRG_GADD45A_dn
LTEA_HepaRG_FMO3_up
LTEA_HepaRG_EGF_up
LTEA_HepaRG_BID_dn
LTEA_HepaRG_APOA5_dn
LTEA_HepaRG_ALPP_dn
LTEA_HepaRG_ADK_dn
LTEA_HepaRG_ABCG2_up
LTEA_HepaRG_ABCC3_up
CEETOX_H295R_PROG_up
CEETOX_H295R_DOC_up
BSK_LPS_TNFa_up
ToxCast Assay
0.00
0.25
0.50
0.75
1.00
Active for 55290−64−7

Enriched Assay Heatmap for Cluster 2031

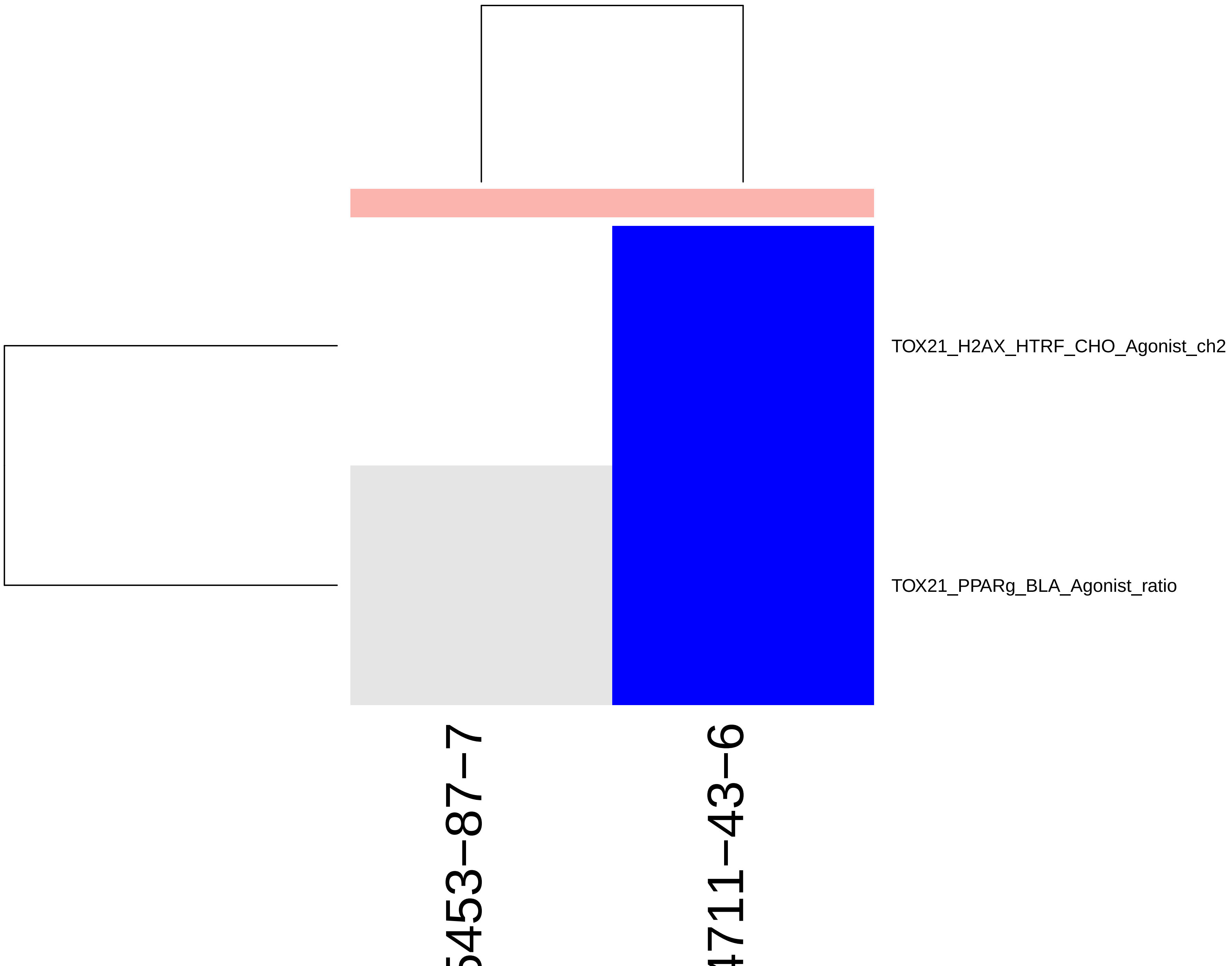

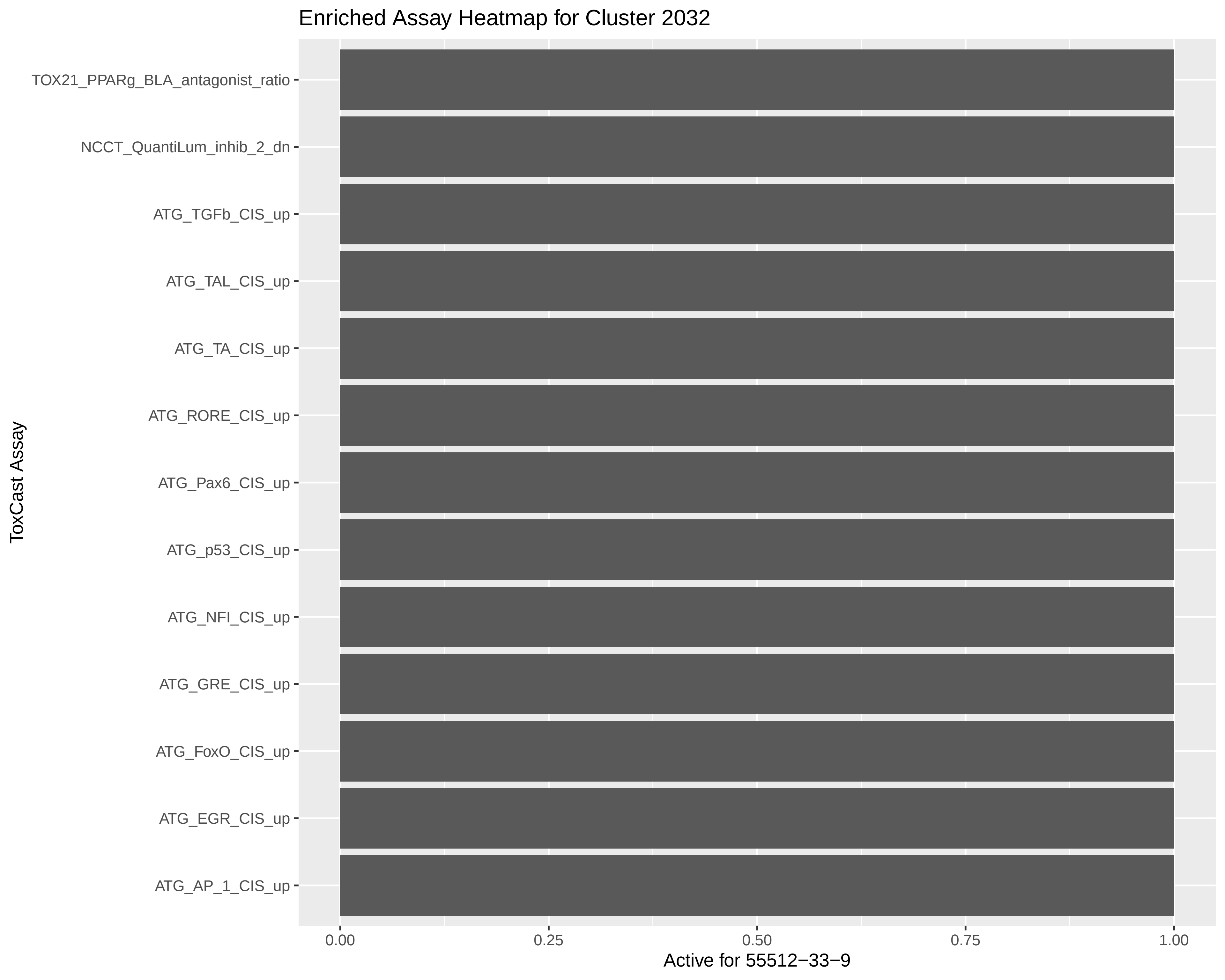
Enriched Assay Heatmap for Cluster 2032
ToxCast Assay
TOX21_PPARg_BLA_antagonist_ratio
NCCT_QuantiLum_inhib_2_dn
ATG_TGFb_CIS_up
ATG_TAL_CIS_up
ATG_TA_CIS_up
ATG_RORE_CIS_up
ATG_Pax6_CIS_up
ATG_p53_CIS_up
ATG_NFI_CIS_up
ATG_GRE_CIS_up
ATG_FoxO_CIS_up
ATG_EGR_CIS_up
ATG_AP_1_CIS_up
0.00
0.25
0.50
0.75
1.00
Active for 55512−33−9

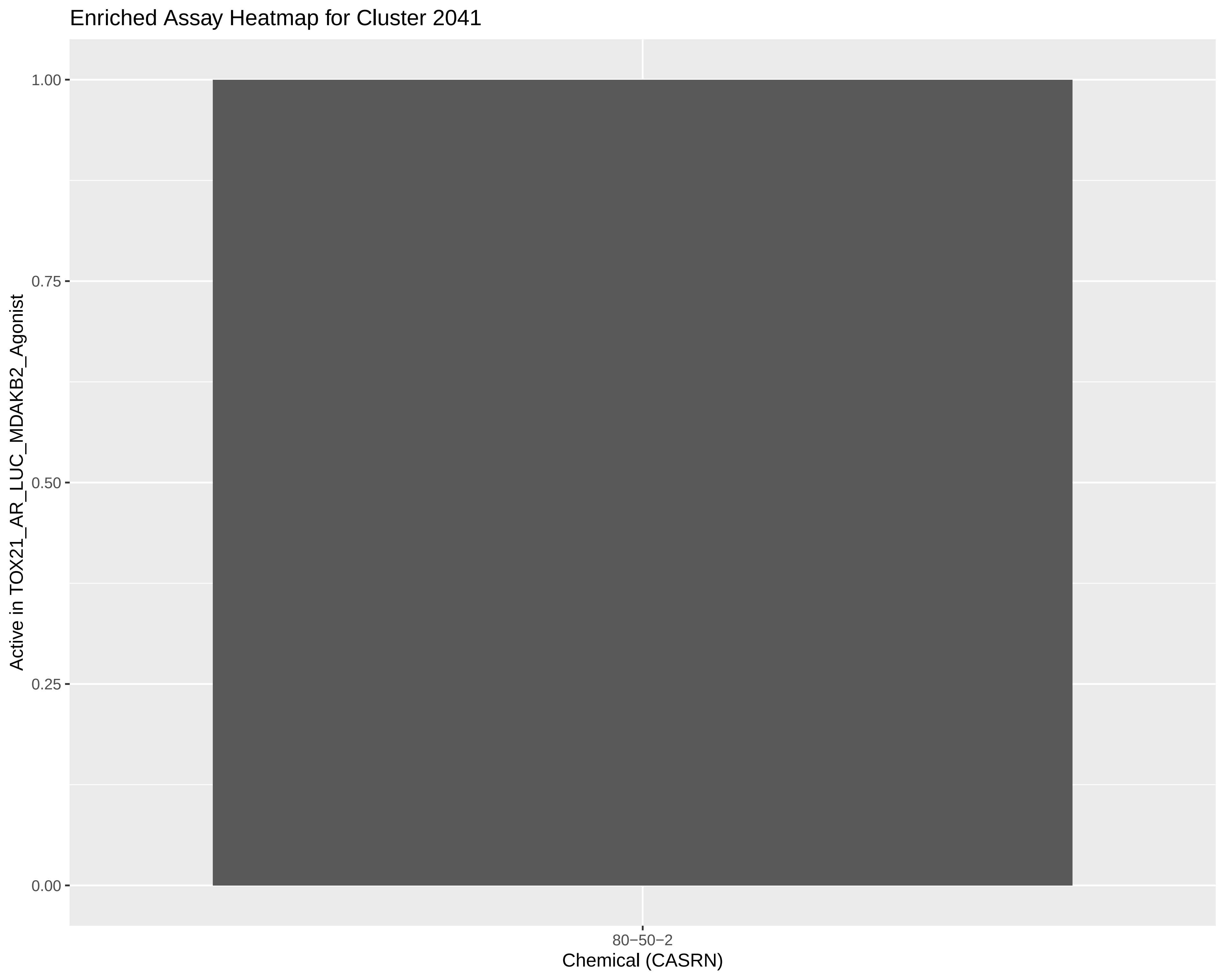

Enriched Assay Heatmap for Cluster 2041
Active in TOX21_AR_LUC_MDAKB2_Agonist
1.00
0.75
0.50
0.25
0.00
80−50−2
Chemical (CASRN)

Enriched Assay Heatmap for Cluster 2043

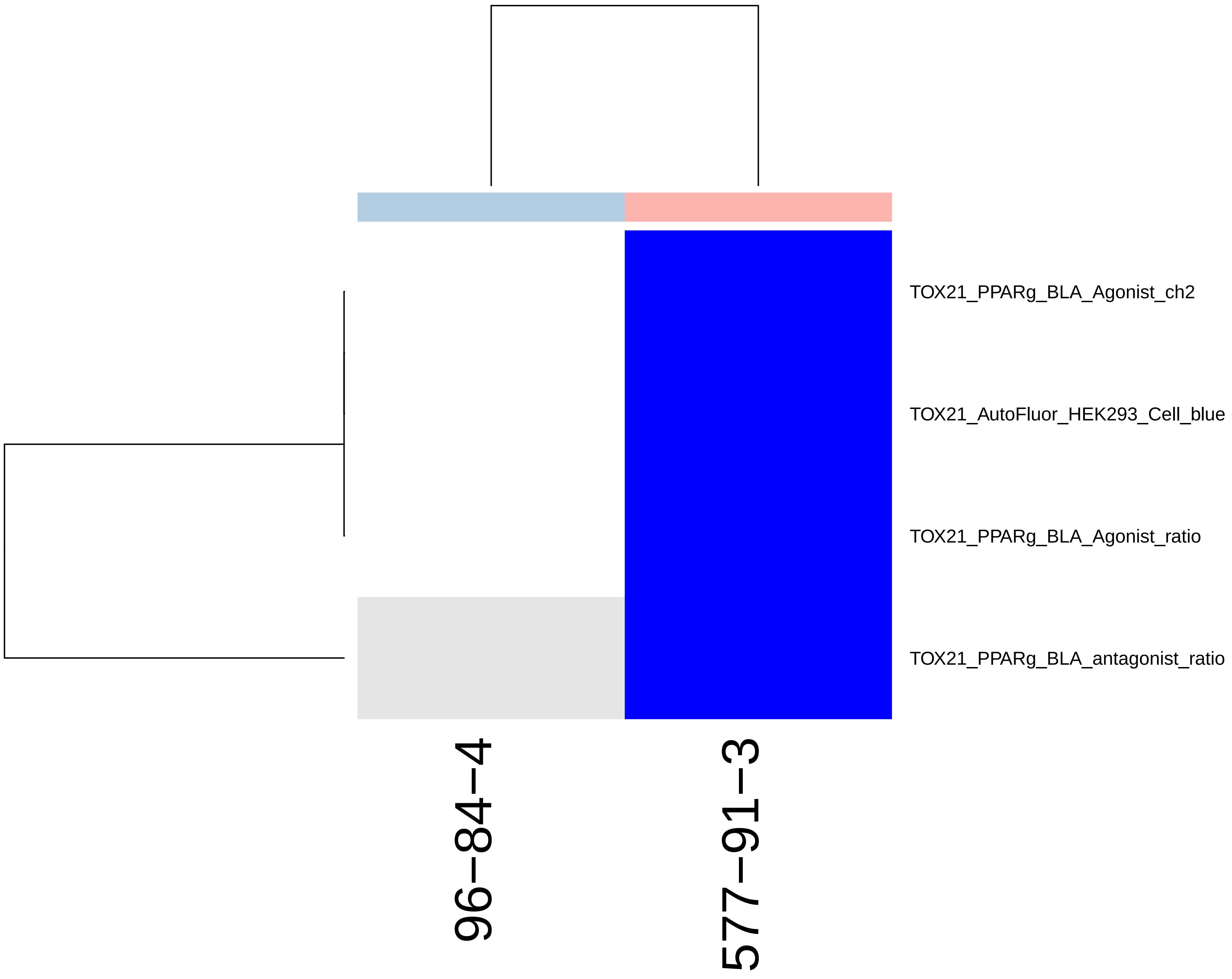

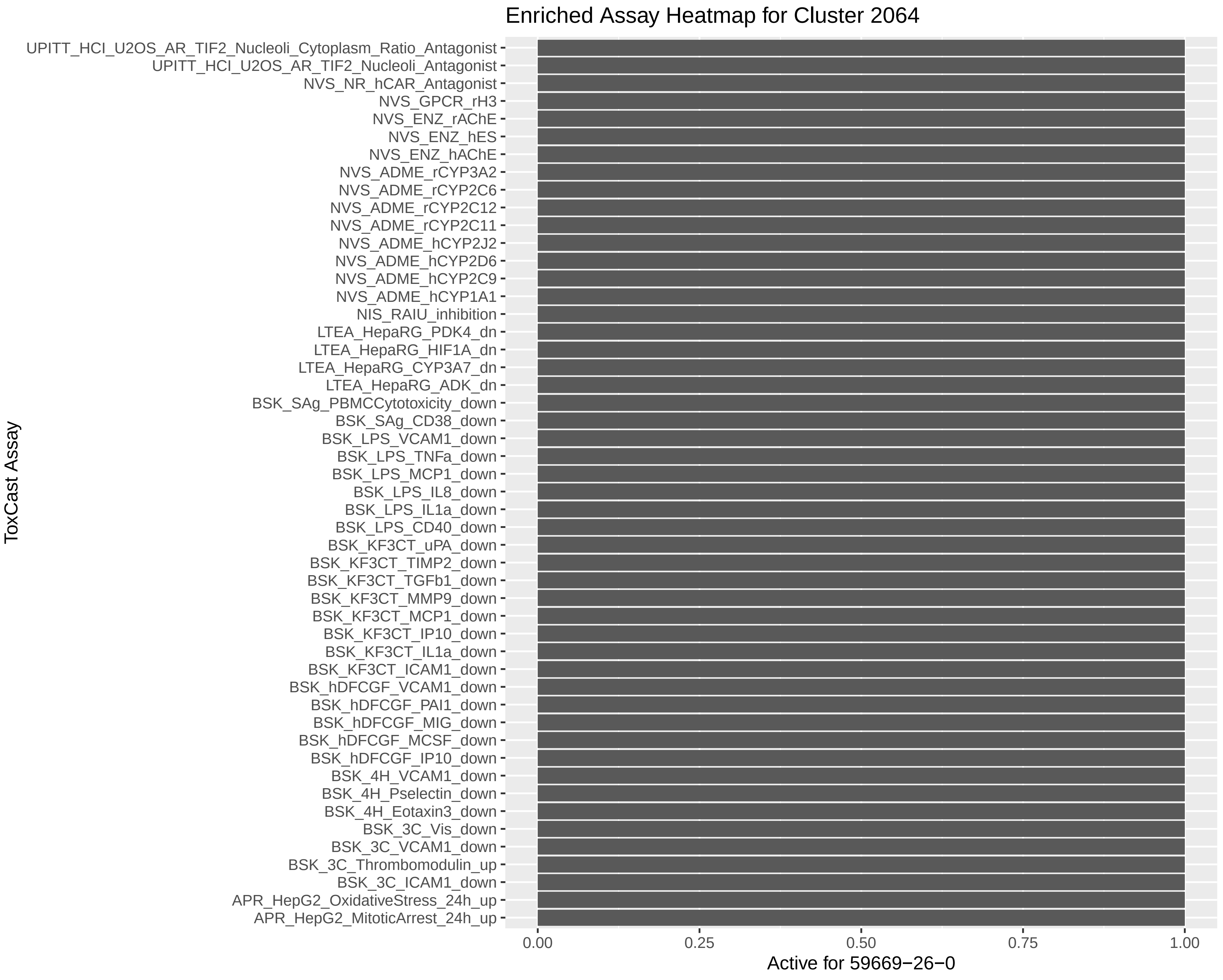

Enriched Assay Heatmap for Cluster 2064
ToxCast Assay
UPITT_HCI_U2OS_AR_TIF2_Nucleoli_Cytoplasm_Ratio_Antagonist
UPITT_HCI_U2OS_AR_TIF2_Nucleoli_Antagonist
NVS_NR_hCAR_Antagonist
NVS_GPCR_rH3
NVS_ENZ_rAChE
NVS_ENZ_hES
NVS_ENZ_hAChE
NVS_ADME_rCYP3A2
NVS_ADME_rCYP2C6
NVS_ADME_rCYP2C12
NVS_ADME_rCYP2C11
NVS_ADME_hCYP2J2
NVS_ADME_hCYP2D6
NVS_ADME_hCYP2C9
NVS_ADME_hCYP1A1
NIS_RAIU_inhibition
LTEA_HepaRG_PDK4_dn
LTEA_HepaRG_HIF1A_dn
LTEA_HepaRG_CYP3A7_dn
LTEA_HepaRG_ADK_dn
BSK_SAg_PBMCCytotoxicity_down
BSK_SAg_CD38_down
BSK_LPS_VCAM1_down
BSK_LPS_TNFa_down
BSK_LPS_MCP1_down
BSK_LPS_IL8_down
BSK_LPS_IL1a_down
BSK_LPS_CD40_down
BSK_KF3CT_uPA_down
BSK_KF3CT_TIMP2_down
BSK_KF3CT_TGFb1_down
BSK_KF3CT_MMP9_down
BSK_KF3CT_MCP1_down
BSK_KF3CT_IP10_down
BSK_KF3CT_IL1a_down
BSK_KF3CT_ICAM1_down
BSK_hDFCGF_VCAM1_down
BSK_hDFCGF_PAI1_down
BSK_hDFCGF_MIG_down
BSK_hDFCGF_MCSF_down
BSK_hDFCGF_IP10_down
BSK_4H_VCAM1_down
BSK_4H_Pselectin_down
BSK_4H_Eotaxin3_down
BSK_3C_Vis_down
BSK_3C_VCAM1_down
BSK_3C_Thrombomodulin_up
BSK_3C_ICAM1_down
APR_HepG2_OxidativeStress_24h_up
APR_HepG2_MitoticArrest_24h_up
0.00
0.25
0.50
0.75
1.00
Active for 59669−26−0

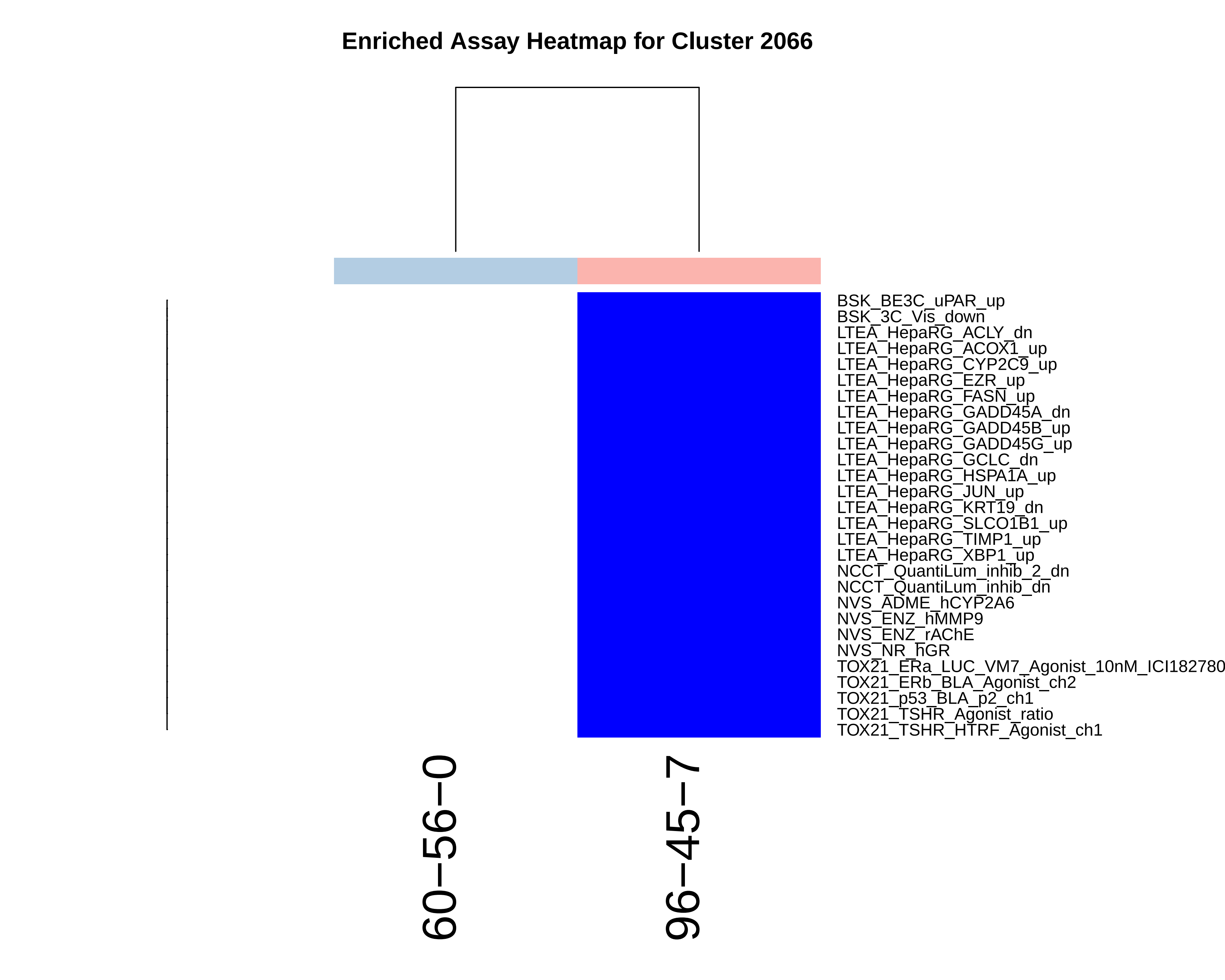
Enriched Assay Heatmap for Cluster 2066
BSK_BE3C_uPAR_up
BSK_3C_Vis_down
LTEA_HepaRG_ACLY_dn
LTEA_HepaRG_ACOX1_up
LTEA_HepaRG_CYP2C9_up
LTEA_HepaRG_EZR_up
LTEA_HepaRG_FASN_up
LTEA_HepaRG_GADD45A_dn
LTEA_HepaRG_GADD45B_up
LTEA_HepaRG_GADD45G_up
LTEA_HepaRG_GCLC_dn
LTEA_HepaRG_HSPA1A_up
LTEA_HepaRG_JUN_up
LTEA_HepaRG_KRT19_dn
LTEA_HepaRG_SLCO1B1_up
LTEA_HepaRG_TIMP1_up
LTEA_HepaRG_XBP1_up
NCCT_QuantiLum_inhib_2_dn
NCCT_QuantiLum_inhib_dn
NVS_ADME_hCYP2A6
NVS_ENZ_hMMP9
NVS_ENZ_rAChE
NVS_NR_hGR
TOX21_ERa_LUC_VM7_Agonist_10nM_ICI182780
TOX21_ERb_BLA_Agonist_ch2
TOX21_p53_BLA_p2_ch1
TOX21_TSHR_Agonist_ratio
TOX21_TSHR_HTRF_Agonist_ch1
60−56−0
96−45−7

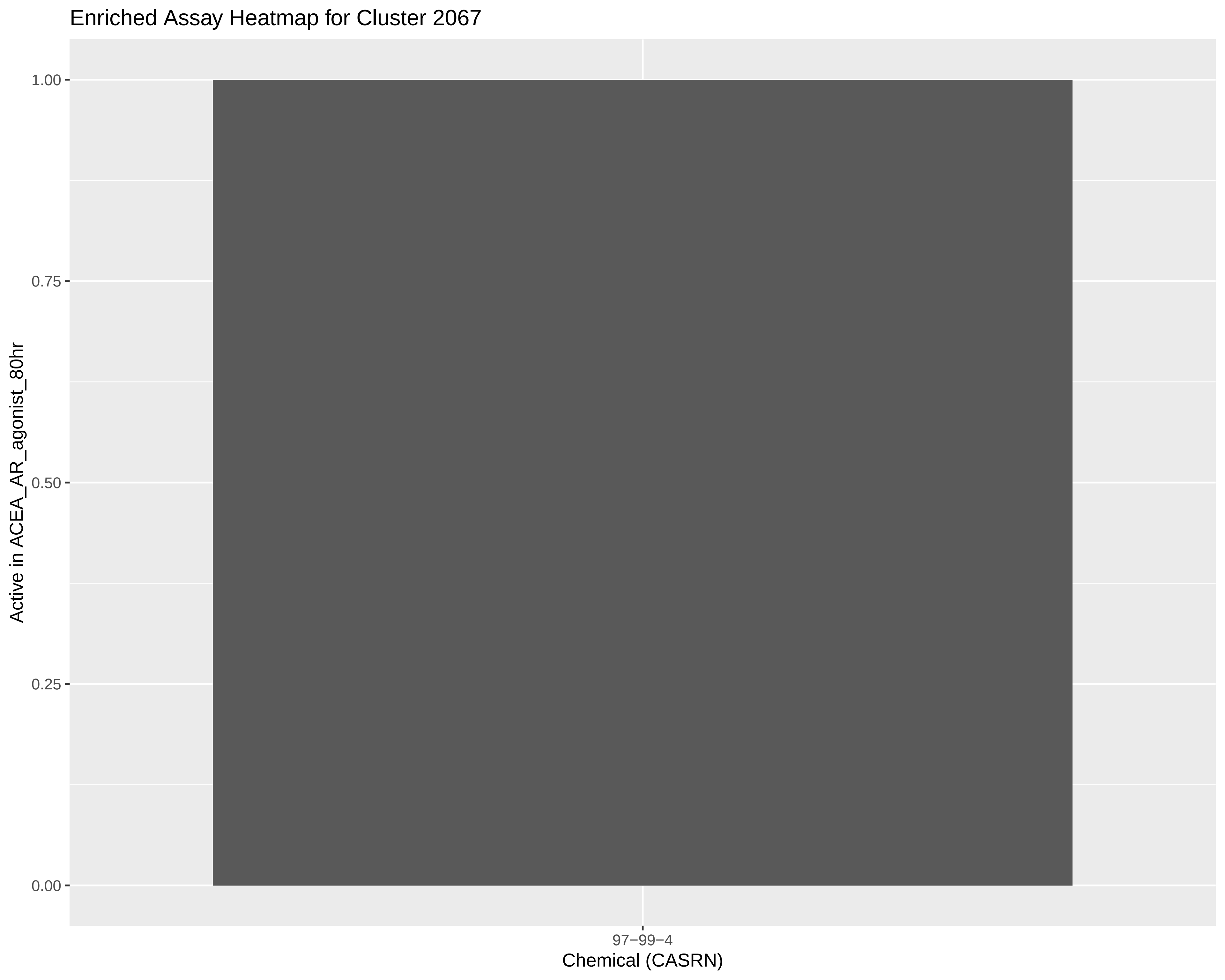

Enriched Assay Heatmap for Cluster 2067
Active in ACEA_AR_agonist_80hr
1.00
0.75
0.50
0.25
0.00
97−99−4
Chemical (CASRN)

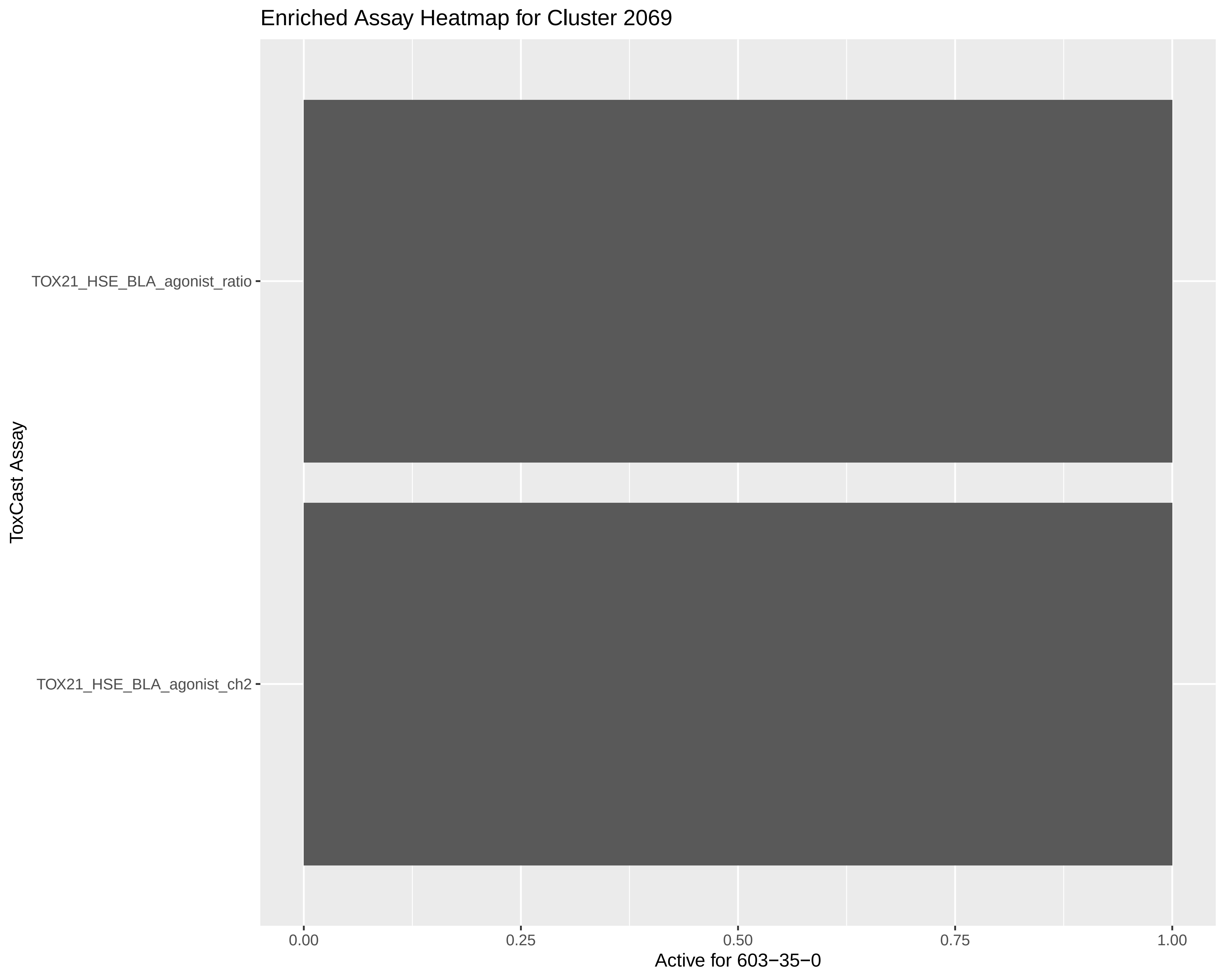
Enriched Assay Heatmap for Cluster 2069
ToxCast Assay
TOX21_HSE_BLA_agonist_ratio
TOX21_HSE_BLA_agonist_ch2
0.00
0.25
0.50
0.75
1.00
Active for 603−35−0

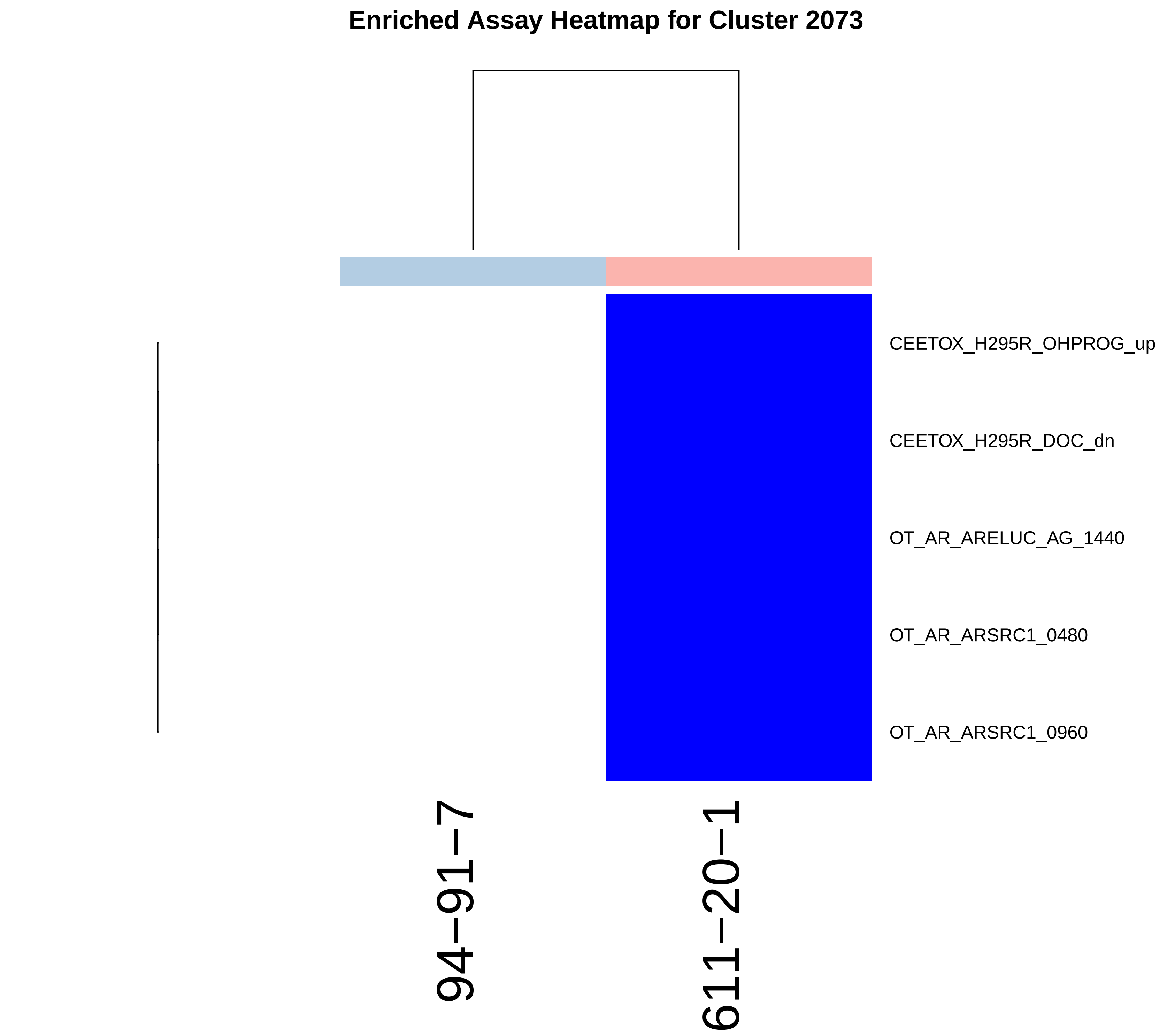

Enriched Assay Heatmap for Cluster 2073
CEETOX_H295R_OHPROG_up
CEETOX_H295R_DOC_dn
OT_AR_ARELUC_AG_1440
OT_AR_ARSRC1_0480
OT_AR_ARSRC1_0960
94−91−7
611−20−1

Enriched Assay Heatmap for Cluster 2083

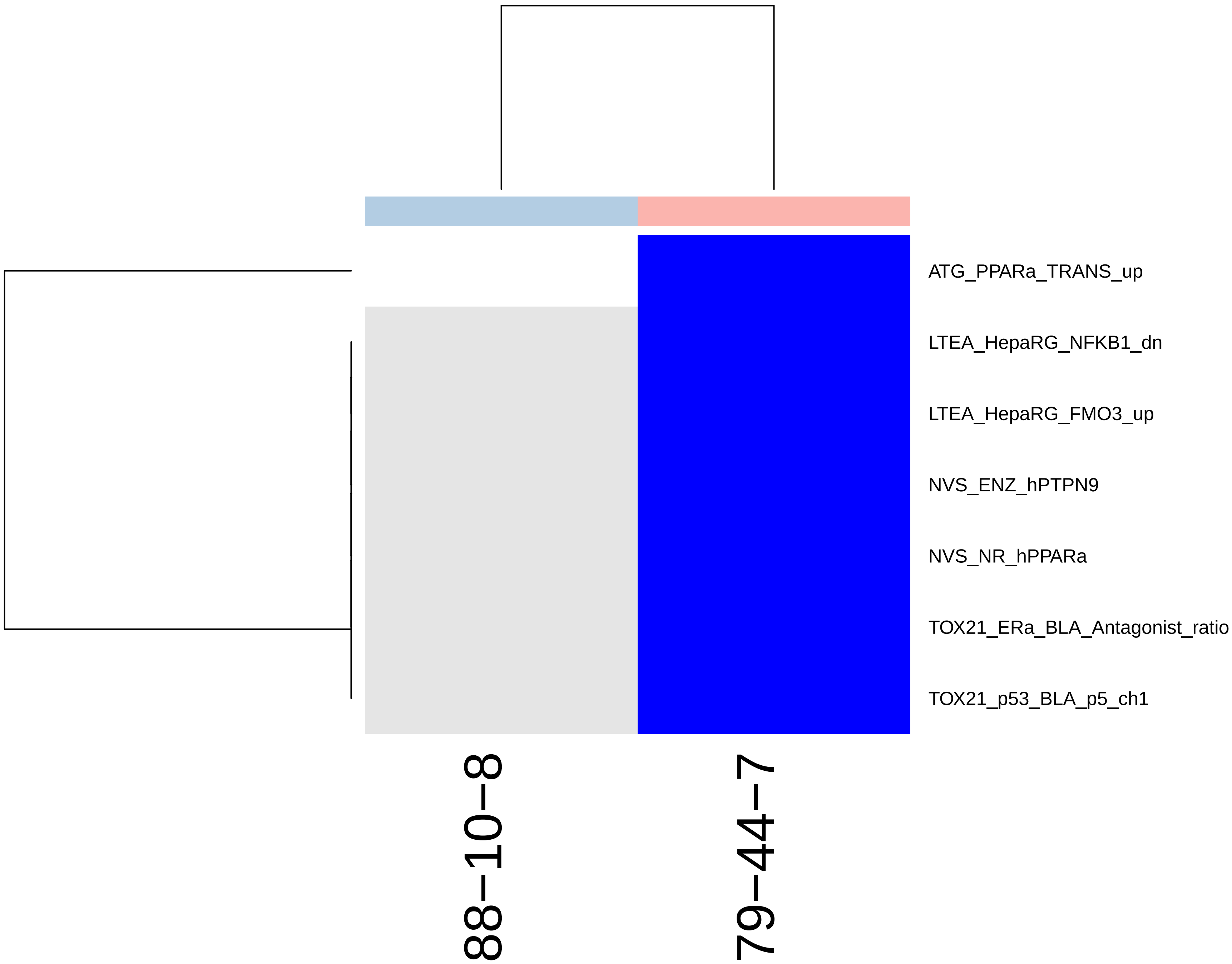

Enriched Assay Heatmap for Cluster 2101

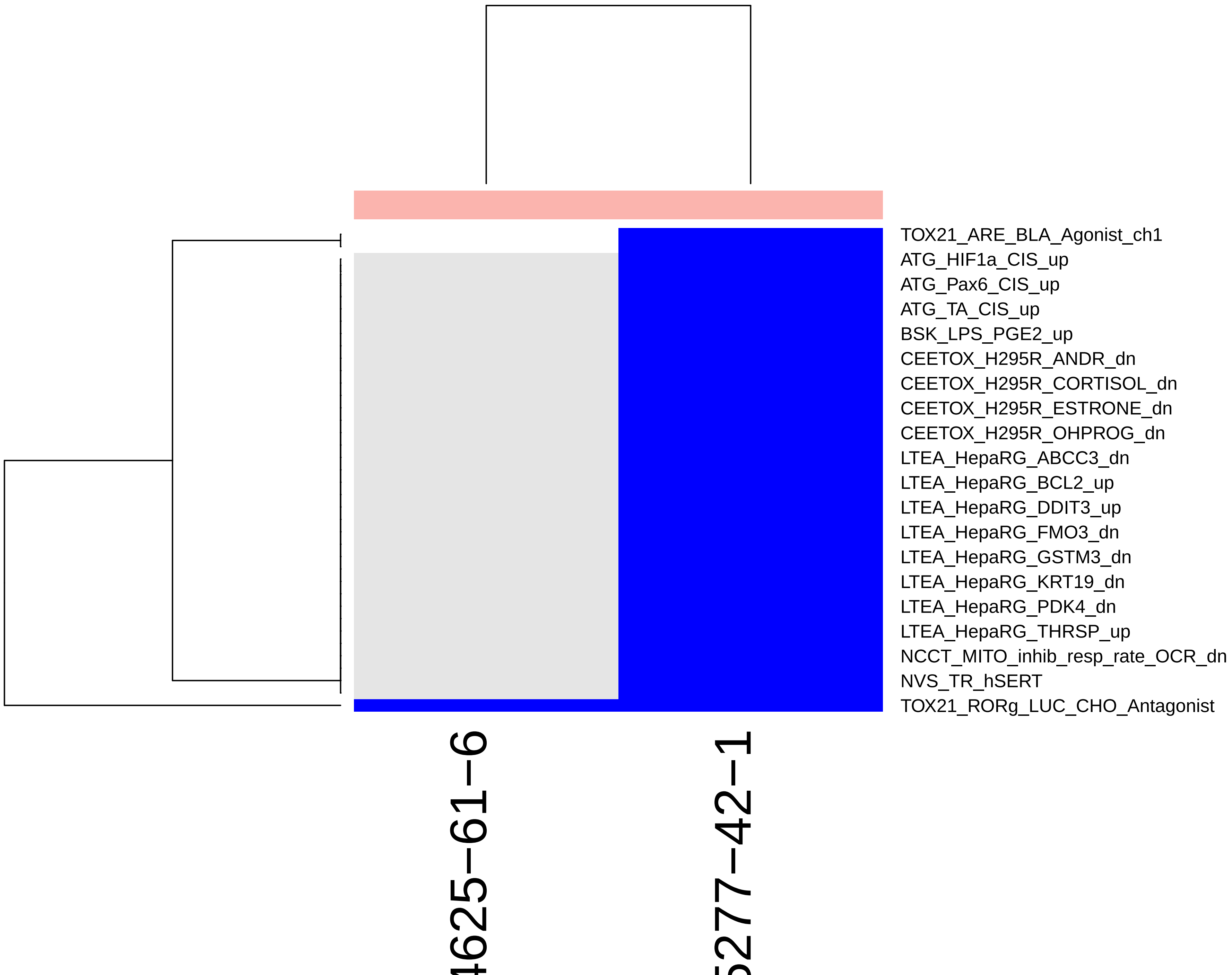

Enriched Assay Heatmap for Cluster 2108

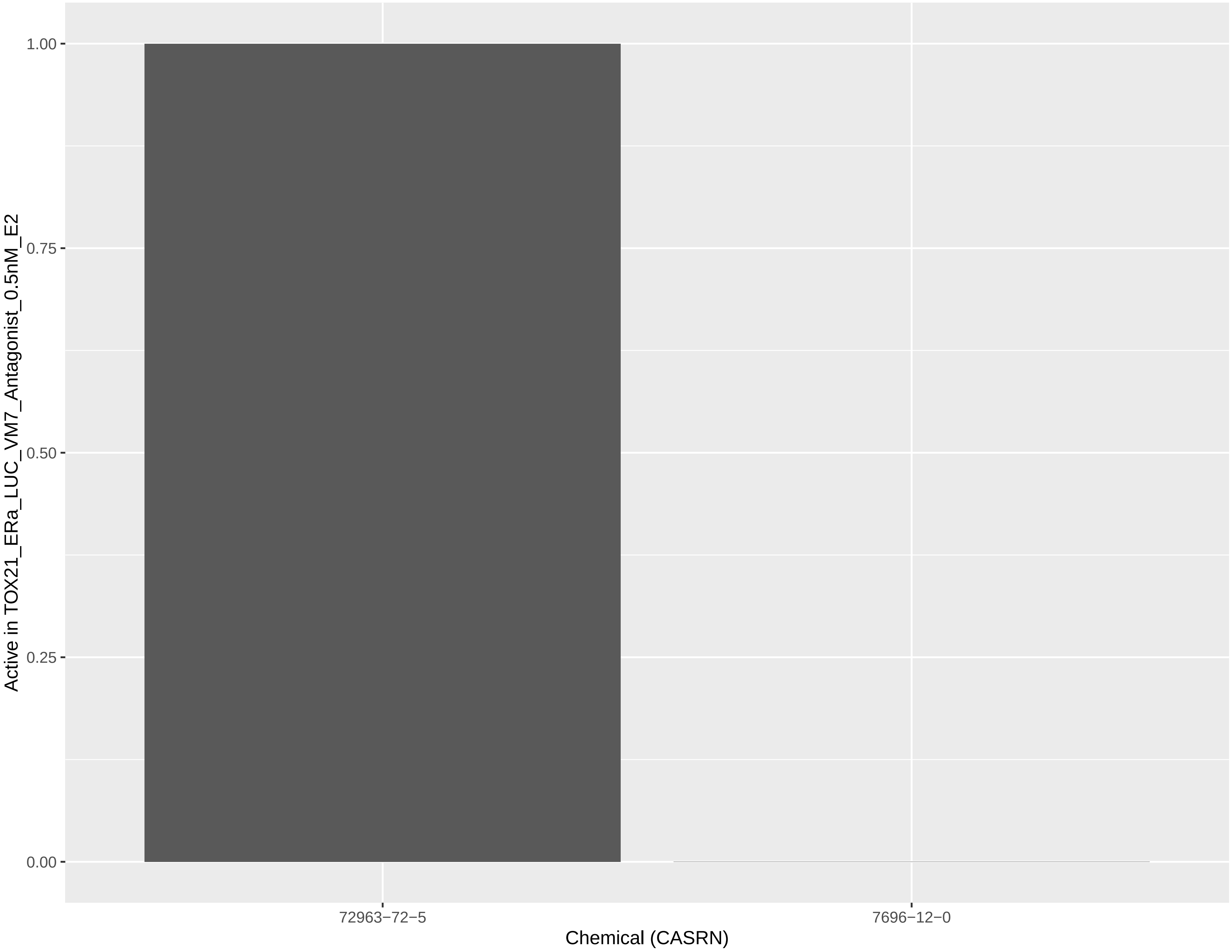

Enriched Assay Heatmap for Cluster 2111

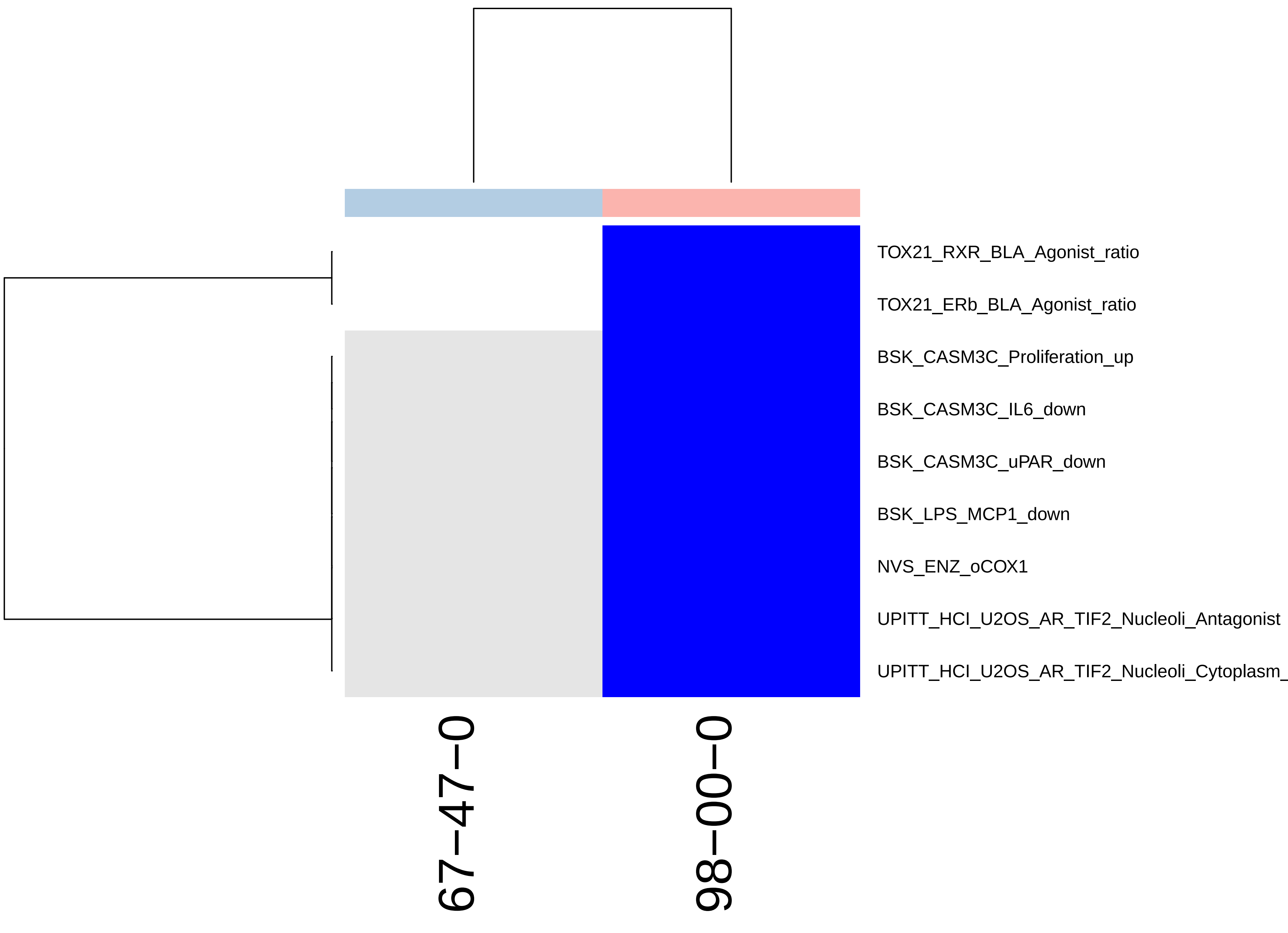

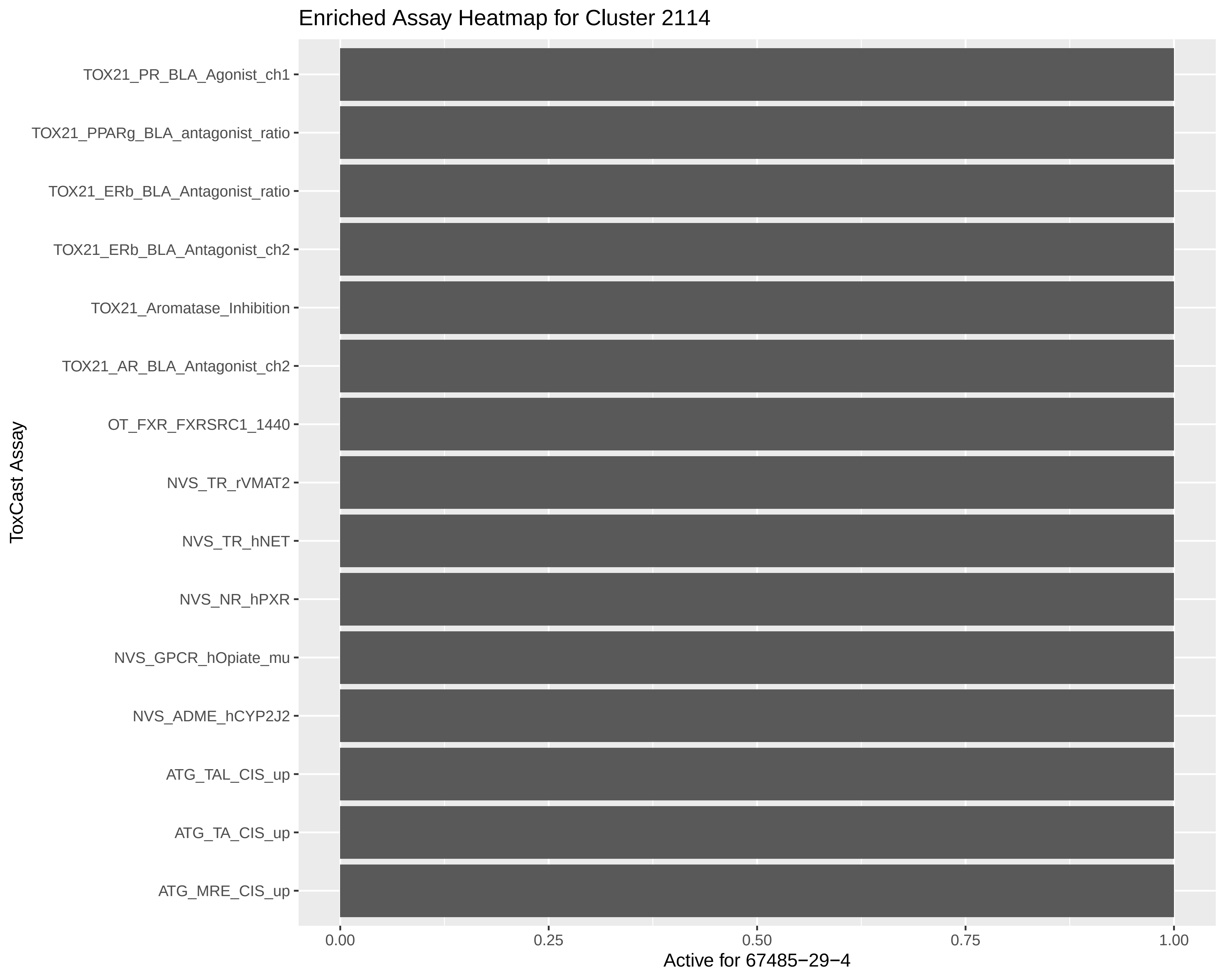

Enriched Assay Heatmap for Cluster 2114
ToxCast Assay
TOX21_PR_BLA_Agonist_ch1
TOX21_PPARg_BLA_antagonist_ratio
TOX21_ERb_BLA_Antagonist_ratio
TOX21_ERb_BLA_Antagonist_ch2
TOX21_Aromatase_Inhibition
TOX21_AR_BLA_Antagonist_ch2
OT_FXR_FXRSRC1_1440
NVS_TR_rVMAT2
NVS_TR_hNET
NVS_NR_hPXR
NVS_GPCR_hOpiate_mu
NVS_ADME_hCYP2J2
ATG_TAL_CIS_up
ATG_TA_CIS_up
ATG_MRE_CIS_up
0.00
0.25
0.50
0.75
1.00
Active for 67485−29−4

Enriched Assay Heatmap for Cluster 2121

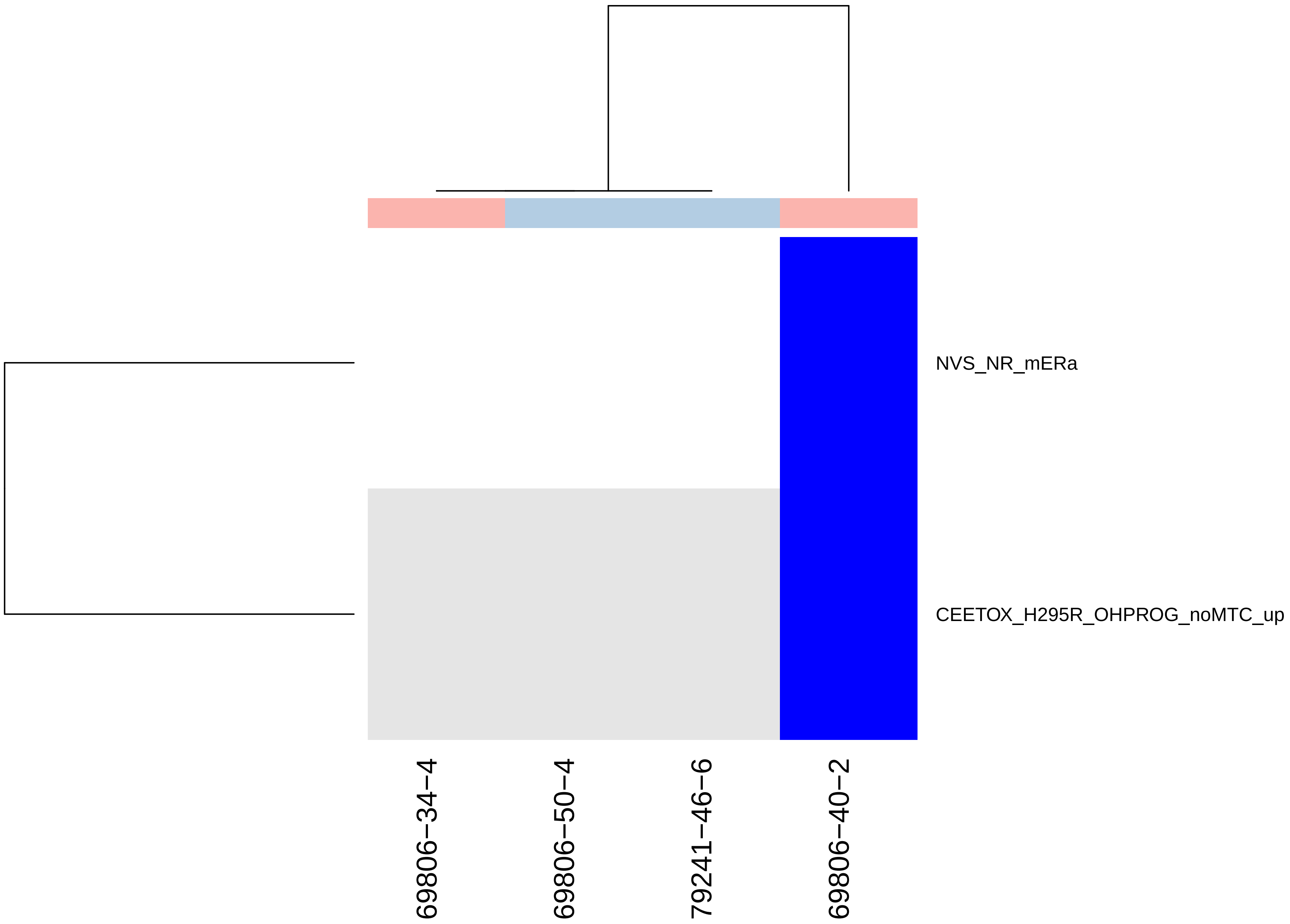

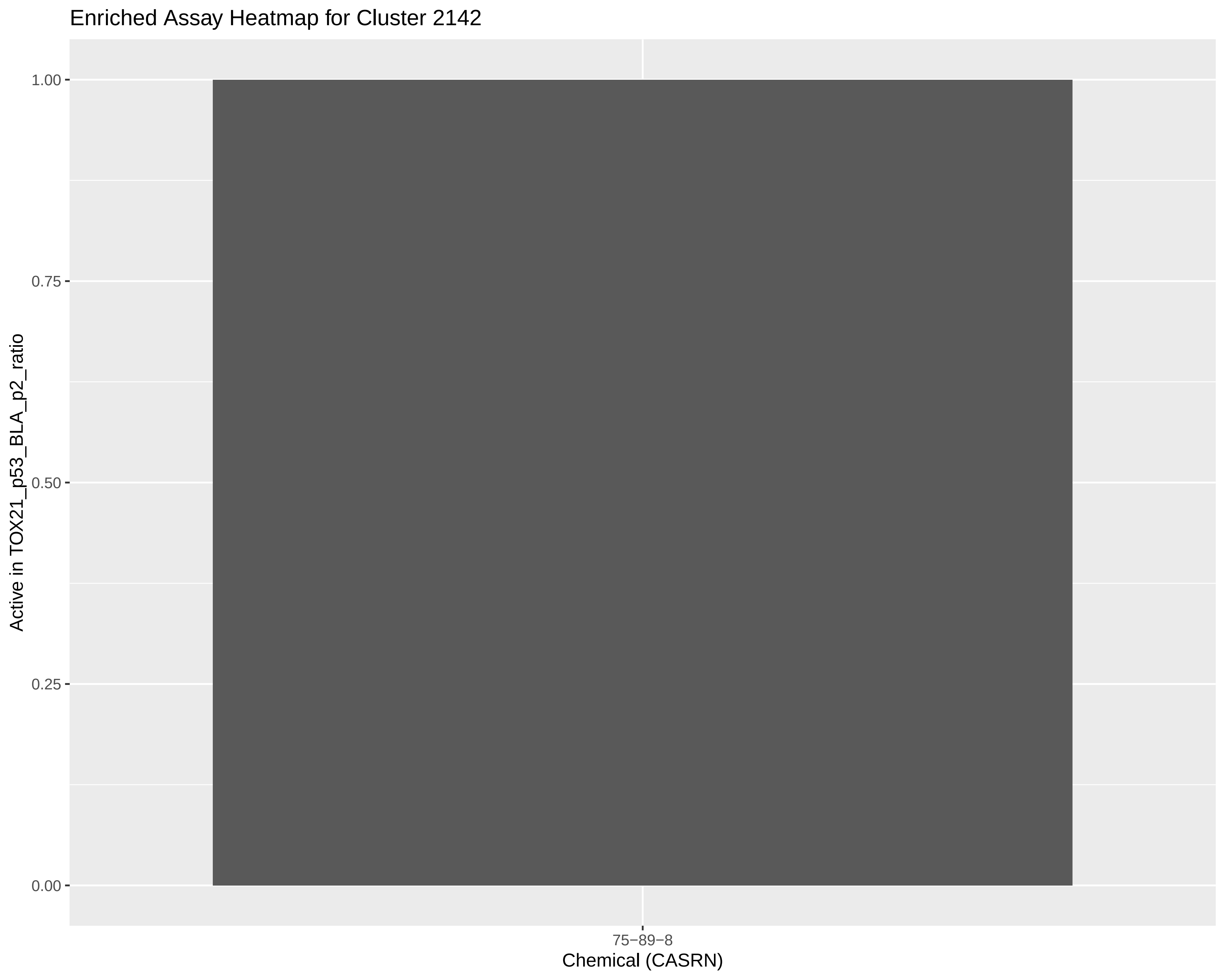
Enriched Assay Heatmap for Cluster 2142
1.00
0.75
0.50
0.25
0.00
Active in TOX21_p53_BLA_p2_ratio
75−89−8
Chemical (CASRN)

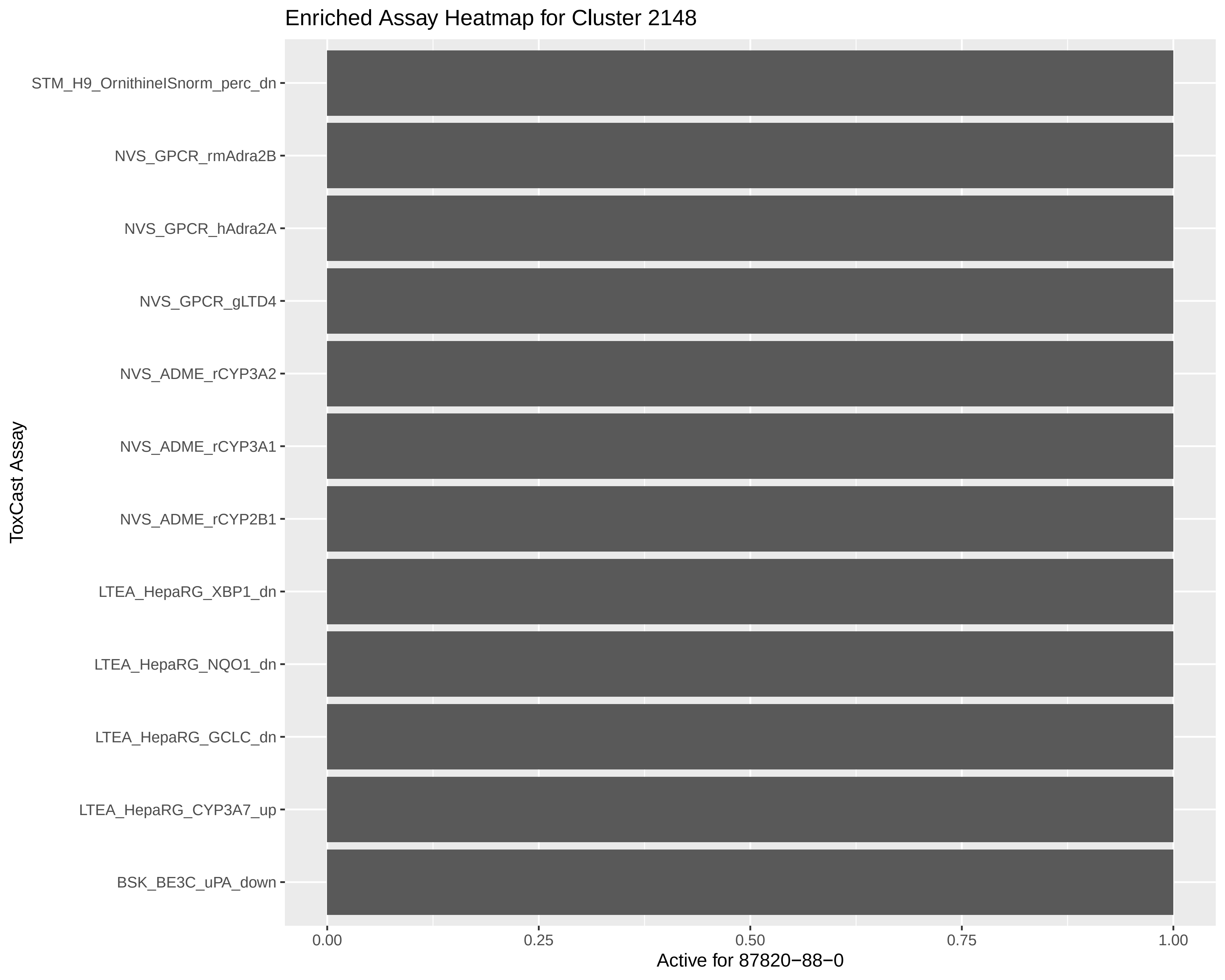

Enriched Assay Heatmap for Cluster 2148
STM_H9_OrnithineISnorm_perc_dn
NVS_GPCR_rmAdra2B
NVS_GPCR_hAdra2A
NVS_GPCR_gLTD4
NVS_ADME_rCYP3A2
NVS_ADME_rCYP3A1
NVS_ADME_rCYP2B1
LTEA_HepaRG_XBP1_dn
LTEA_HepaRG_NQO1_dn
LTEA_HepaRG_GCLC_dn
LTEA_HepaRG_CYP3A7_up
BSK_BE3C_uPA_down
ToxCast Assay
0.00
0.25
0.50
0.75
1.00
Active for 87820−88−0

Enriched Assay Heatmap for Cluster 2155

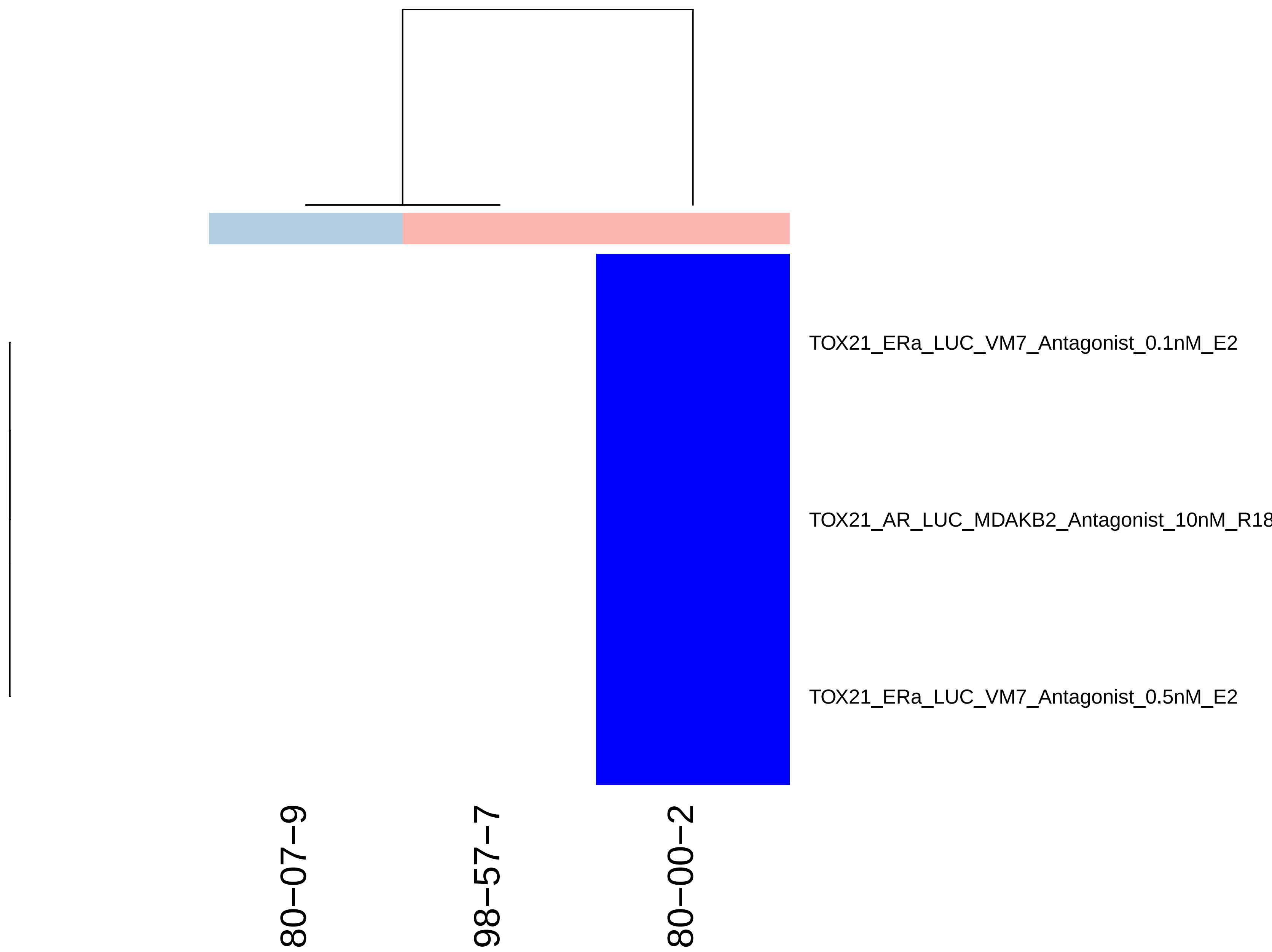

Enriched Assay Heatmap for Cluster 2160

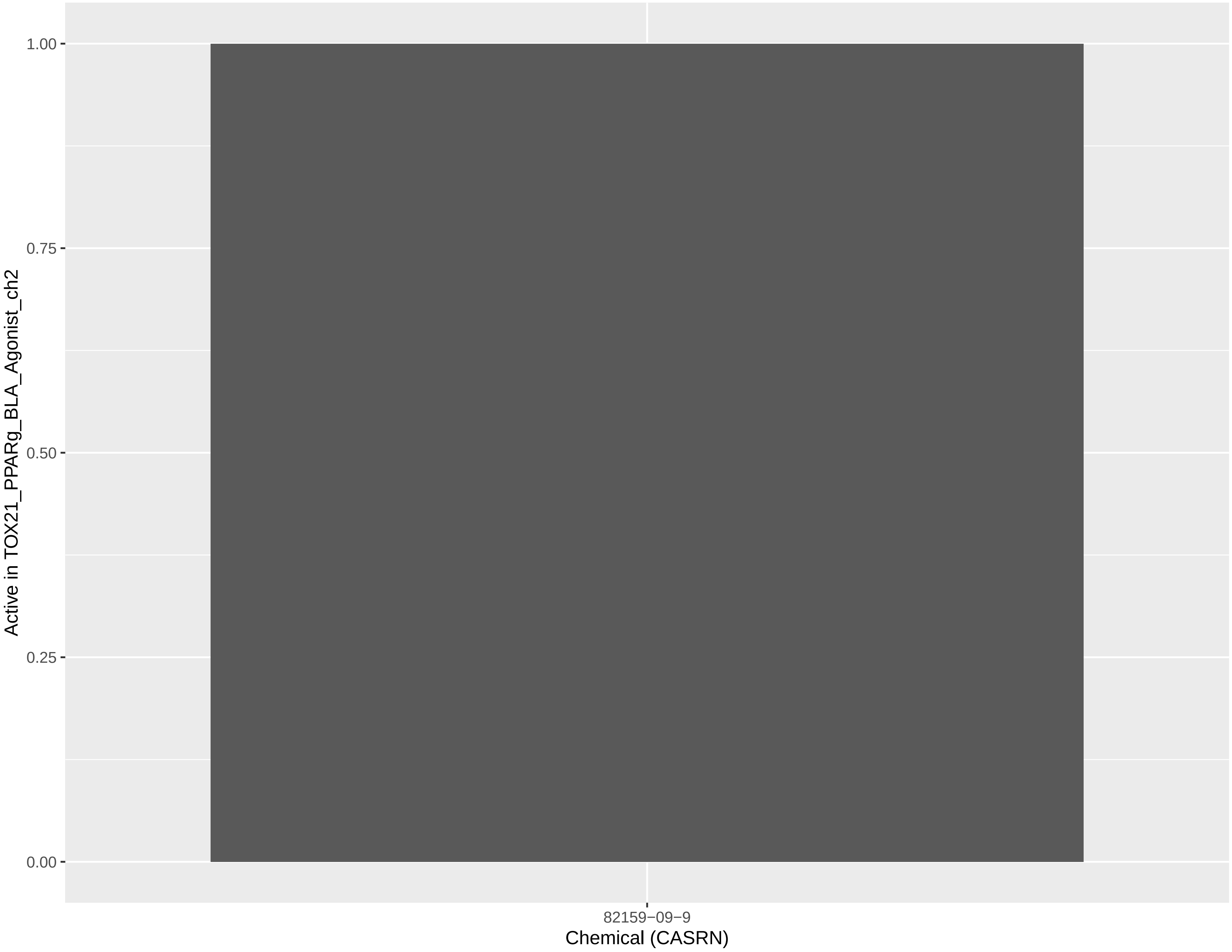

Enriched Assay Heatmap for Cluster 2162

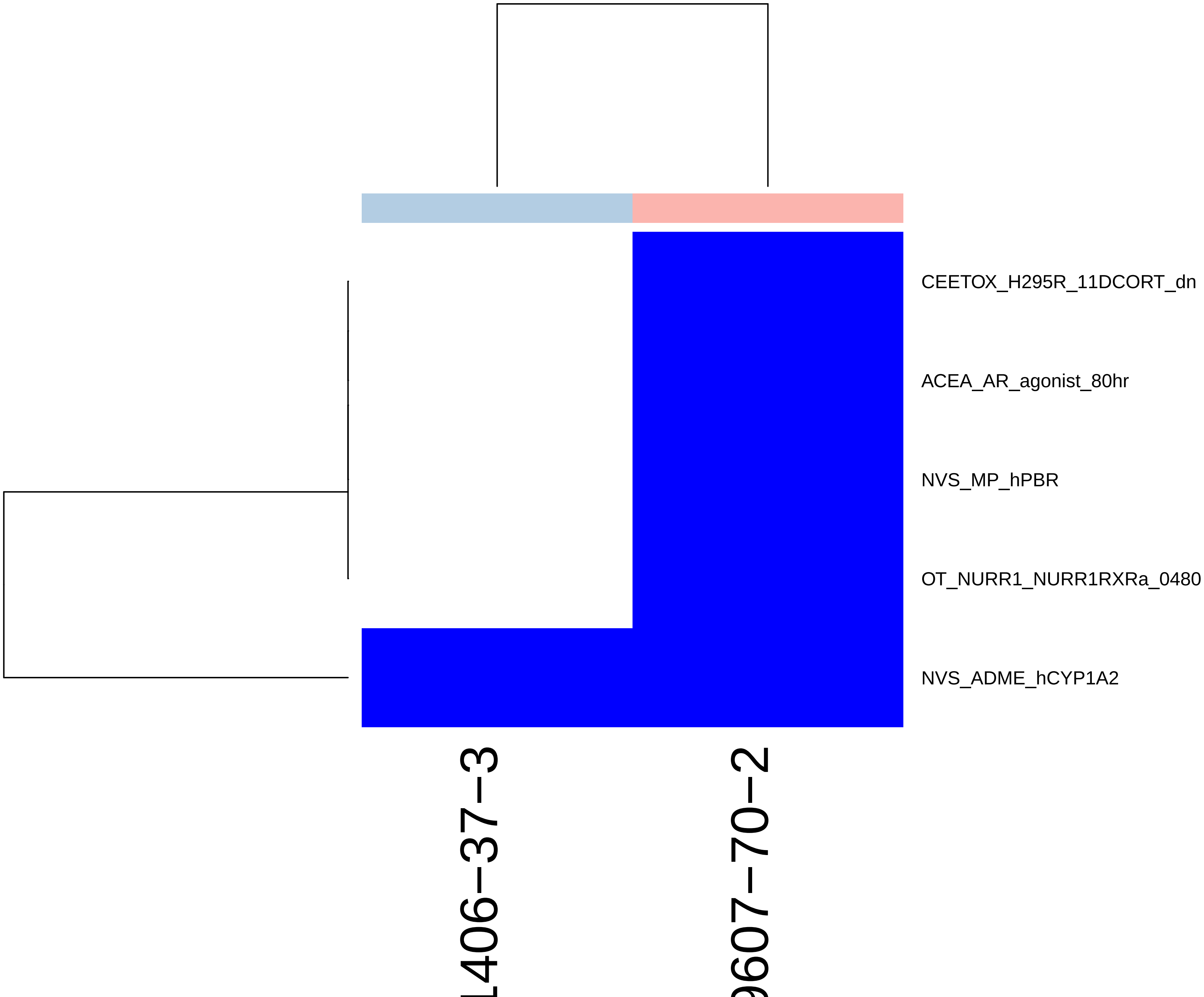

Enriched Assay Heatmap for Cluster 2166

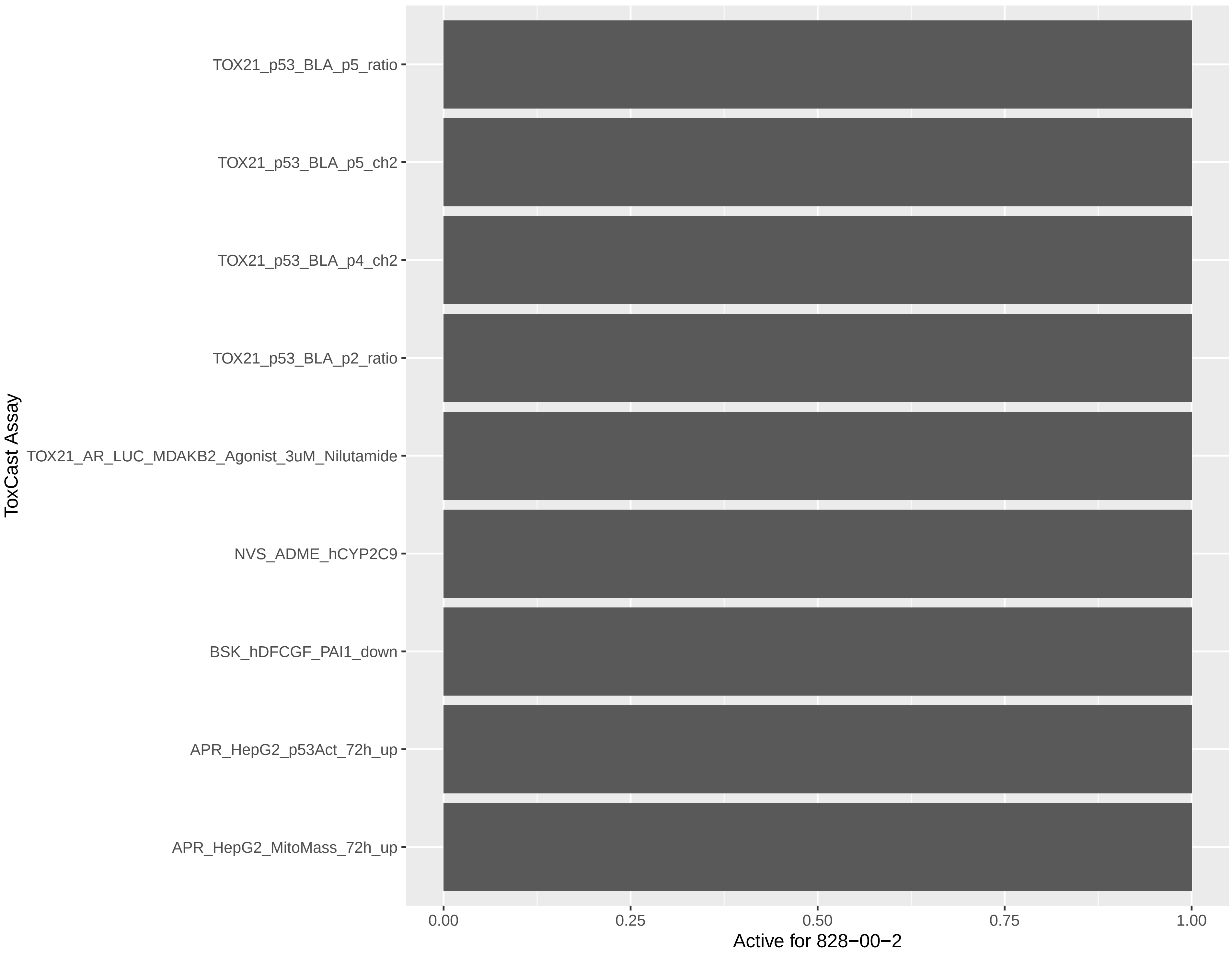

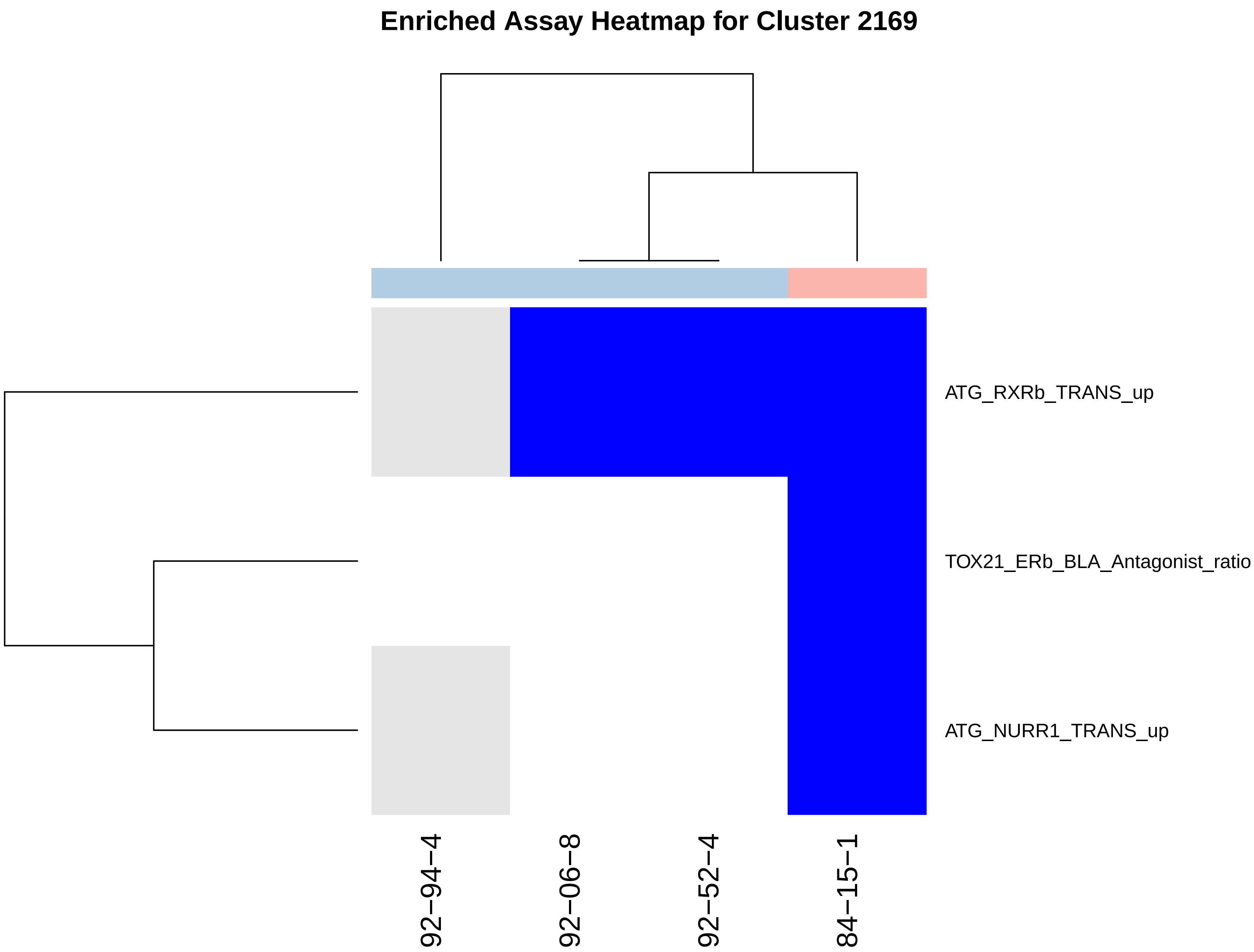
Enriched Assay Heatmap for Cluster 2169
ATG_RXRb_TRANS_up
TOX21_ERb_BLA_Antagonist_ratio
ATG_NURR1_TRANS_up
92−94−4
92−06−8
92−52−4
84−15−1

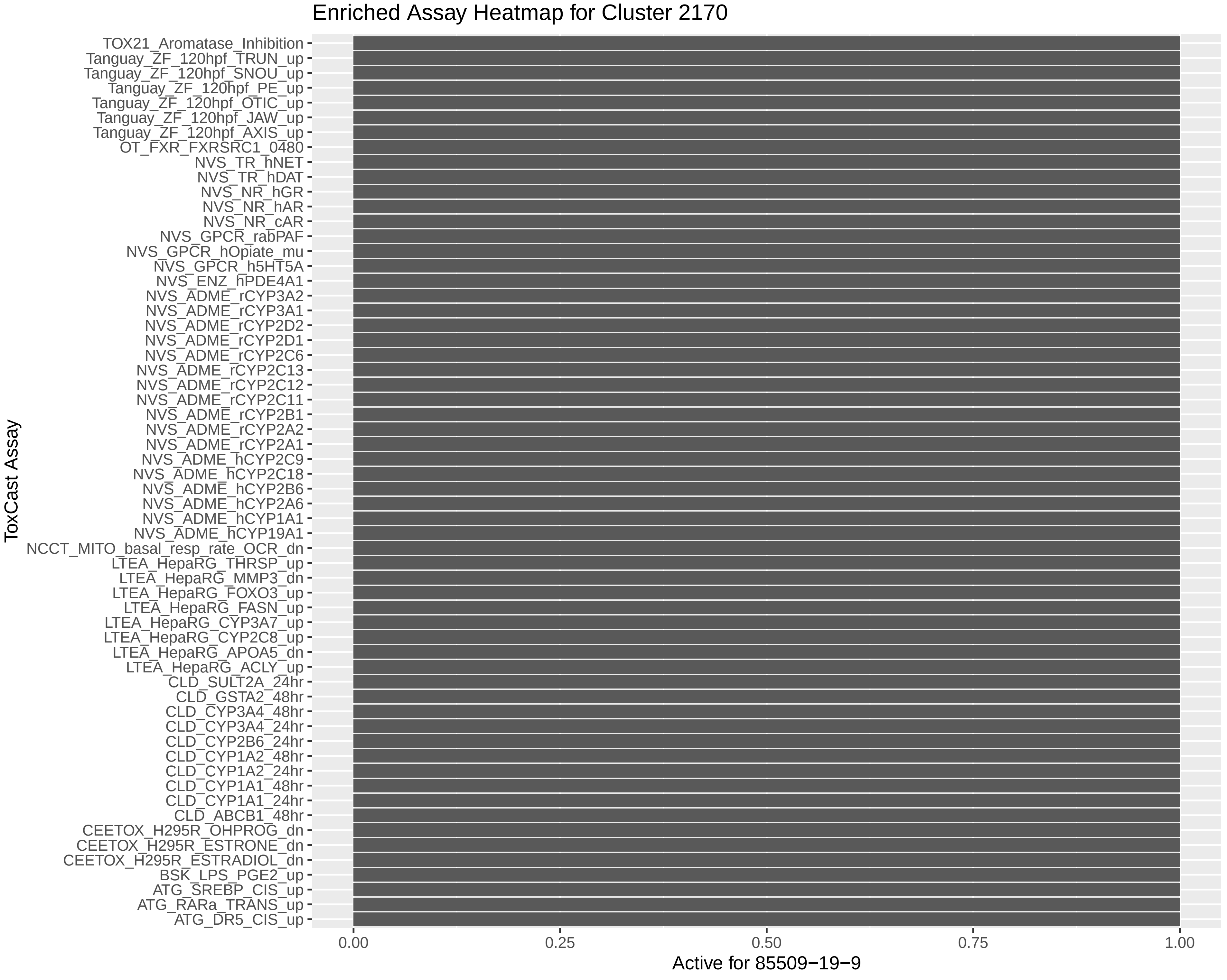

Enriched Assay Heatmap for Cluster 2170
ToxCast Assay
TOX21_Aromatase_Inhibition
Tanguay_ZF_120hpf_TRUN_up
Tanguay_ZF_120hpf_SNOU_up
Tanguay_ZF_120hpf_PE_up
Tanguay_ZF_120hpf_OTIC_up
Tanguay_ZF_120hpf_JAW_up
Tanguay_ZF_120hpf_AXIS_up
OT_FXR_FXRSRC1_0480
NVS_TR_hNET
NVS_TR_hDAT
NVS_NR_hGR
NVS_NR_hAR
NVS_NR_cAR
NVS_GPCR_rabPAF
NVS_GPCR_hOpiate_mu
NVS_GPCR_h5HT5A
NVS_ENZ_hPDE4A1
NVS_ADME_rCYP3A2
NVS_ADME_rCYP3A1
NVS_ADME_rCYP2D2
NVS_ADME_rCYP2D1
NVS_ADME_rCYP2C6
NVS_ADME_rCYP2C13
NVS_ADME_rCYP2C12
NVS_ADME_rCYP2C11
NVS_ADME_rCYP2B1
NVS_ADME_rCYP2A2
NVS_ADME_rCYP2A1
NVS_ADME_hCYP2C9
NVS_ADME_hCYP2C18
NVS_ADME_hCYP2B6
NVS_ADME_hCYP2A6
NVS_ADME_hCYP1A1
NVS_ADME_hCYP19A1
NCCT_MITO_basal_resp_rate_OCR_dn
LTEA_HepaRG_THRSP_up
LTEA_HepaRG_MMP3_dn
LTEA_HepaRG_FOXO3_up
LTEA_HepaRG_FASN_up
LTEA_HepaRG_CYP3A7_up
LTEA_HepaRG_CYP2C8_up
LTEA_HepaRG_APOA5_dn
LTEA_HepaRG_ACLY_up
CLD_SULT2A_24hr
CLD_GSTA2_48hr
CLD_CYP3A4_48hr
CLD_CYP3A4_24hr
CLD_CYP2B6_24hr
CLD_CYP1A2_48hr
CLD_CYP1A2_24hr
CLD_CYP1A1_48hr
CLD_CYP1A1_24hr
CLD_ABCB1_48hr
CEETOX_H295R_OHPROG_dn
CEETOX_H295R_ESTRONE_dn
CEETOX_H295R_ESTRADIOL_dn
BSK_LPS_PGE2_up
ATG_SREBP_CIS_up
ATG_RARa_TRANS_up
ATG_DR5_CIS_up
0.00
0.25
0.50
0.75
1.00
Active for 85509−19−9

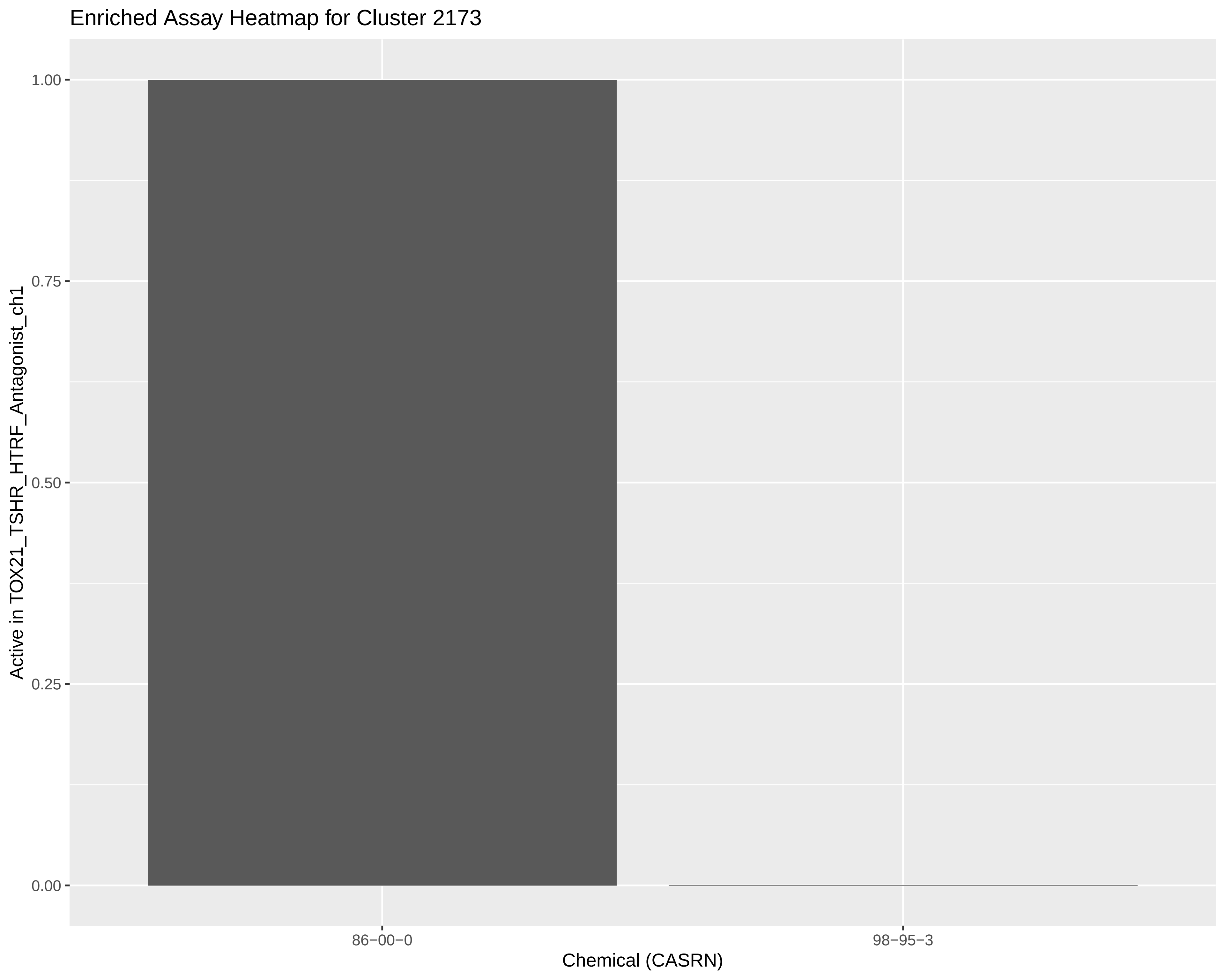
Enriched Assay Heatmap for Cluster 2173
Active in TOX21_TSHR_HTRF_Antagonist_ch1
1.00
0.75
0.50
0.25
0.00
86−00−0
98−95−3
Chemical (CASRN)

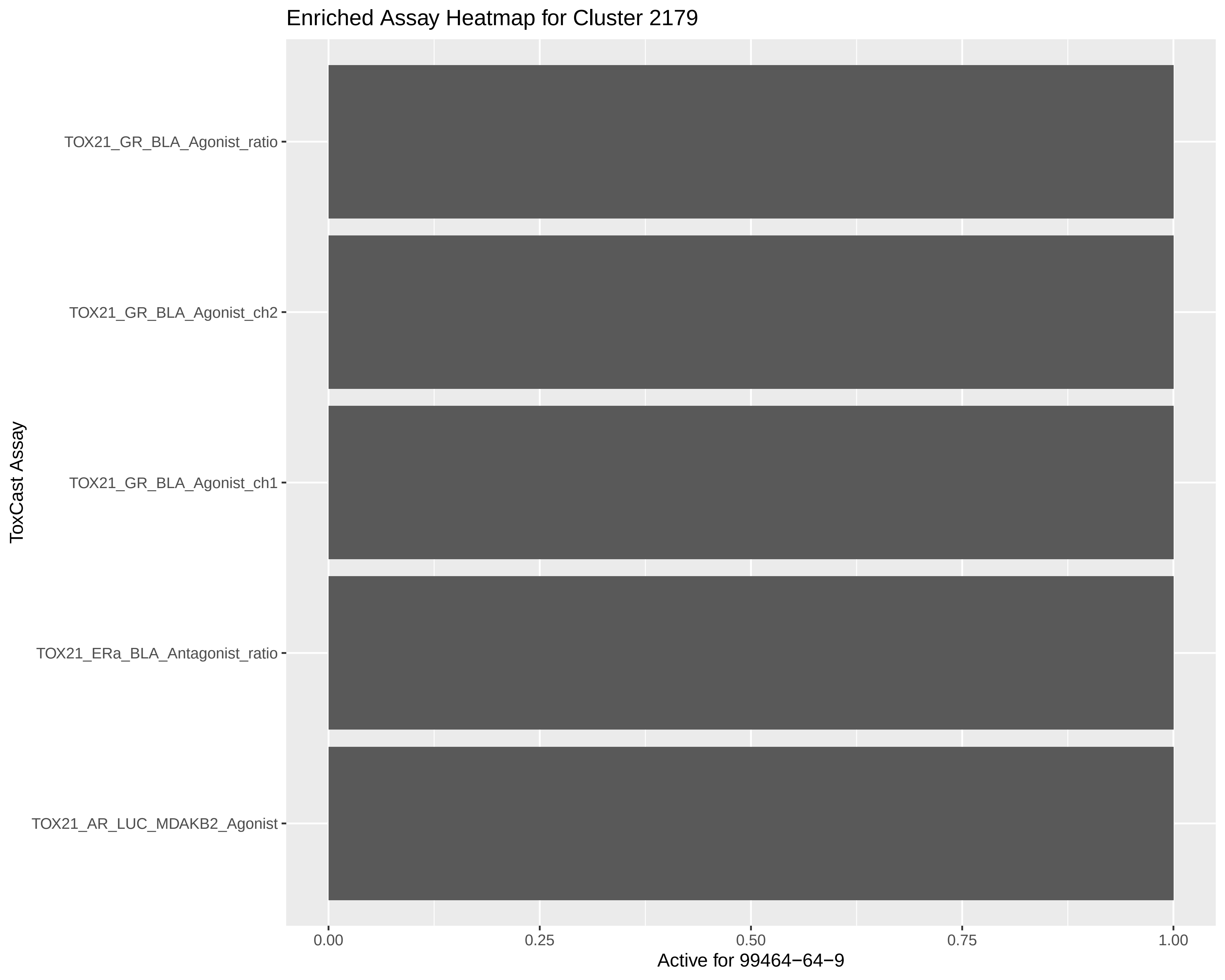
Enriched Assay Heatmap for Cluster 2179
ToxCast Assay
TOX21_GR_BLA_Agonist_ratio
TOX21_GR_BLA_Agonist_ch2
TOX21_GR_BLA_Agonist_ch1
TOX21_ERa_BLA_Antagonist_ratio
TOX21_AR_LUC_MDAKB2_Agonist
0.00
0.25
0.50
0.75
1.00
Active for 99464−64−9
